# Effect of implant abutment surface treatments on bacterial biofilm composition and structure

**DOI:** 10.1080/20002297.2025.2459922

**Published:** 2025-02-05

**Authors:** Eduardo Anitua, Alia Murias-Freijo, Roberto Tierno, Ricardo Tejero, Mohammad Hamdan Alkhraisat

**Affiliations:** a BTI-Biotechnology Institute, Vitoria, Spain; b University Institute for Regenerative Medicine & Oral Implantology, UIRMI (UPV/EHU-Eduardo Anitua Foundation), Vitoria, Spain; c Biomedical Research, Department of Cell Biology and Histology, Medicine and Nursing School, University of the Basque Country UPV/EHU, Leioa, Spain

**Keywords:** Biofilm, dental implant abutment, metagenomics, bacterial community, surface modifications, diversity

## Abstract

**Background:**

For the long-term success of dental implants, implant abutment surface should promote the attachment of oral epithelial cells and reduce bacterial adhesion. Titanium nitride (TiN) coatings show antimicrobial properties. Nevertheless, there is a lack of clinical trials that assess the biofilm formation on TiN abutments in the context of clinical practice. Thus, the objective of this study was to evaluate the effect of different abutment surfaces (machined, TiN and TiN oxidized) on bacterial biofilm composition and structure.

**Materials and methods:**

Implant abutments were connected to the dental implants. Bacterial communities were sampled at 1 and 60 days later. The relationship between surface, periodontal indices and bacterial community dynamics was assessed using 16S rRNA metagenomics. A total of 17 patients were involved in this study (14 included in final analyses: 15 machined, 16 TiN and 14 TiN oxidized abutments).

**Results:**

No significant differences between surfaces were found considering taxa abundance, most alpha diversity metrics or community structure. Time showed a significant effect on diversity and also on the abundance of several bacterial taxa.

**Conclusions:**

These results indicate that the effect of the three tested abutment surfaces on biofilm structure and composition was negligible, whereas patient and time exert strong influences on bacterial biofilm formation at different scales.

## KEY MESSAGES

The effect of three different implant abutment surfaces (machined, TiN and TiN oxidized) on biofilm structure and composition was negligible.

The variables patient and time exert a significant influence on bacterial community composition and structure at different scales

## Introduction

Peri-implant diseases are immune-mediated inflammatory condition that affects different tissues surrounding the dental implants. Two main forms have been identified: peri-implant mucositis and peri-implantitis. Peri-implant mucositis is a reversible oral reaction restricted to the peri-implant mucosa, while peri-implantitis is an inflammation of the peri-implant mucosa accompanied by the progressive loss of supporting bone and the destruction of soft tissues around implants, a process that compromises their stability, function and aesthetics [1]. As published by Lee et al. (2017) [2] weighted mean implant-based and subject-based peri-implant mucositis prevalences were 29.48% (95% Confidence Interval (CI): [22.65, 36.32]) and 46.83% (CI: [38.30, 55.36]), whereas those of peri-implantitis were 9.25% (95% (CI): [7.57, 10.93]) and 19.83% (CI: [15.38, 24.27]), respectively. The biofilm, which is a complex multicellular community of microorganisms embedded in a self-produced extracellular polymeric substances attached to biotic or abiotic surfaces, is the primary etiological factor in the development of peri-implant diseases [3].

As synthesized by Dib-Zaitum et al. (2022) [4], the capacity of peri-implant tissues for soft tissue sealing is relatively low. The biological space, which is a protective section of connective tissue and epithelium ranging from 2.2 to 3.5 mm in height, is correlated to the degree of bone remodeling that occurs after implant abutment connection. Thus, different morphological and compositional traits of implant abutments may influence epithelial-connective sealing and peri-implant marginal bone loss. The influence of surface chemistry and micro-topography on oral biofilm composition has been deeply investigated [5]. In this sense, Sterzenbach et al. (2020) [6] have highlighted the role of surface chemical composition on biofilm accumulation. Major biocompatibility-related characteristics affecting biofilm formation include biodegradability [7], nanoparticle functionalization [8,9], the corrosive release of metallic components [10], the residuals of unpolymerized fractions [11] and titanium ions released as a result of surface degradation [12,13]. As reviewed by Teughels et al. (2006) [14], rough surfaces usually harbour a more mature plaque, but most evidence points to modest effects and is limited to average heights (Ra) larger than 0.2 µm [15,16]. It has been hypothesized that a higher surface roughness increases surface area, offers shelter to colonizing microorganisms and difficults cleaning [6]. According to Bermejo et al. (2019) [17], certain pathogenic bacteria are more abundant in implants with moderate-roughness surfaces. However, since other studies have yielded contrasting results [18–20], more experiments comparing specific types of surface are required [21].

Soft and hard tissue characteristics, residual precipitants and surgical techniques, may also affect biofilm formation. In this sense, rough implant surfaces influence non-surgical treatment efficacy based on antiseptic and/or antibiotic agents, thus masking their direct effect on biofilm formation [22,23]. As stated by Kiremitci-Gumusderelioglu et al. (1996) [24] and Rzhepishevska et al. (2013) [25], the surface charge also modifies the adherence of microorganisms because of their tendency to adhere better to positively charged surfaces. Physicochemical surface properties of oral pellicles are also related with the physical and chemical nature of the primary hard surface [26]. In this context, surface free energy (SFE) of the underlying surface is also transferred through the pellicle layers and therefore shapes protein adsorption and bacterial adhesion [27]. As a result, hydrophobic surfaces tend to reduce biofilm formation [28].

Titanium (Ti) is typically classified as a bioinert material, inducing little or no detrimental effect on the surrounding tissues [29]. However, despite Ti and the majority of Ti alloys exhibit excellent biocompatibility, resistance to corrosion and mechanical properties and thus are considered the gold standard for endo-osseus dental implants production [30], without adequate surface treatment, may result in toxic reactions and poor integration with the bone and gingival tissue, which could lead to dental implant failures [31]. Therefore, the focus of Ti research as a biomaterial has shifted to the improvement of biocompatibility via structural modifications on the implant surface or surface bioactivation with molecules capable of improving the osseointegration process and preventing biofilm development [32]. For example, double acid etching (DAE) treatment of dental implants may be considered as a simple route to obtain key topographical features on surfaces to enhance osseointegration [33]. However, increased plaque accumulation has been observed in DAE healing abutments [34]. Physical Vapor Deposition (PVD) is a technique that is used to create a titanium nitride (TiN) ceramic layer on titanium machined abutment. Several reports have shown that TiN enhanced fibroblast proliferation, attachment and adhesion while reducing early bacterial colonization and biofilm formation [35]. However, most of these studies have investigated biofilm formation by using intraoral splints, which may not be representative of the use of implant abutments [36]. Therefore, further clinical research is required to assess the effect of TiN coated implant abutment on biofilm formation in the context of clinical practice (transgingival position and implant loading). The aim of this randomized clinical trial has been to evaluate the effect of implant abutment surface (machined, TiN and TiN-oxidized) on bacterial community characteristics and biofilm aging.

## Materials and methods

### Trial design

This research is included in a randomized clinical trial that was designed to assess the effect of implant abutment surface on biofilm formation. For that, different implant abutment surfaces were connected to dental implants. Bacterial communities were sampled and analyzed at 1 and 60 days after placement. The relationship between surface, plaque or inflammation related indices and bacterial community dynamics, with a particular emphasis on bacteria associated with peri-implant disease, was assessed at different stages of ecological succession. The trial was registered at Trial Registration ClinicalTrials.gov under the number NCT03554876. The study protocol and informed consent, in accordance with the ethical principles of the Declaration of Helsinki of 1975, as revisited in 2013, were approved by the Basque Country Ethics Committee (FIBEA-06-EC/17/Multi-Im). Eligible patients (nonsmokers with 18 years or more) needed for the placement of three or more dental implants. In addition, a complete mouth plaque index equal or lower than 20%, along with a bleeding index equal or lower than 30%, a pocket probing depth at the adjacent teeth lower than 4 mm and absence of active periodontal disease was required. They also had to not use antibiotics during the last 6 months. Moreover, it was necessary for the patients to sign an informed consent and be able to attend all the planned visits. Exclusion criteria include pregnant or breast-feeding women, patients suffering severe hematological diseases, temporomandibular joint disorders, metabolic osseous diseases, diabetes mellitus, oral mucosal diseases, disability to maintain a good oral hygiene or malignancies, patients receiving radiotherapy, chemotherapy, immunosuppressors, systemic corticoids, anticoagulants or bisphosphonates, or those participating in other studies.

### Study groups

**Figure 1.**
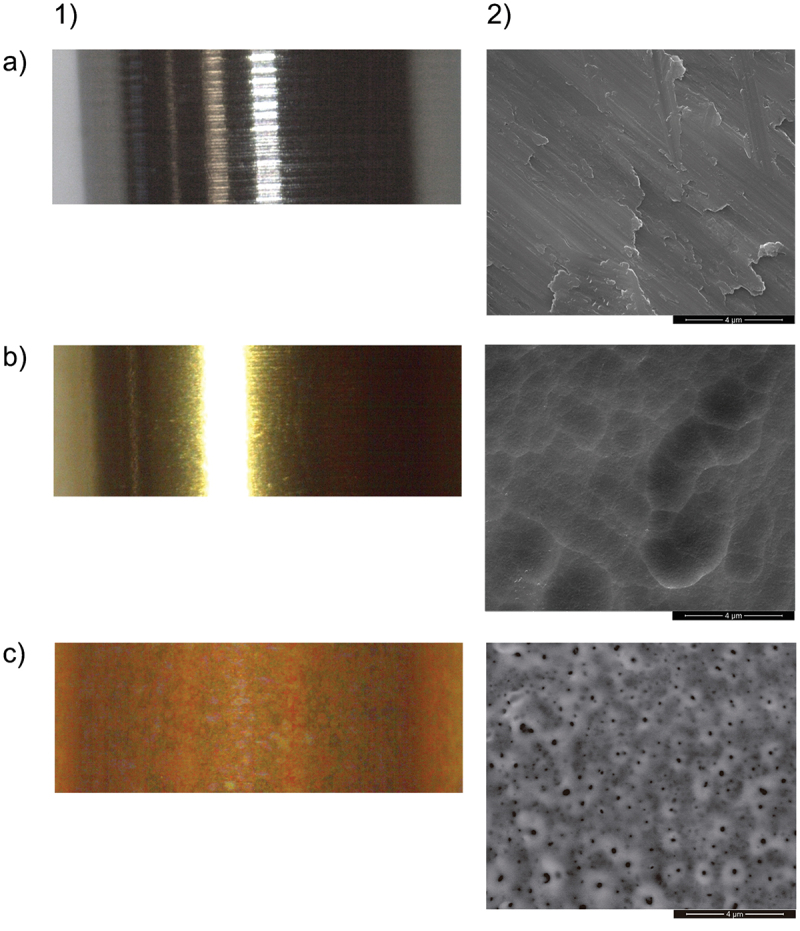
Macrophotographs (1) and scanning electron microscopy (SEM) microphotographs (2) representing the transepithelial abutment surfaces included in the present study: A) as machined (surface A), B) TiN (surface B) and C) Anodized TiN (surface C).

A total of three groups were included in the present study: implant abutment with machined surface as control group (A) and two experimental groups: (B) the abutment with the TiN surface (Ti-Golden®, BTI Biotechnology Institute S.L., Vitoria, Spain) and (C) the abutment with TiN oxidized surface (Figure 1). The implant abutments were 4.1 mm diameter and 2, 2.5 or 3 mm height. They were machined from a cylindrical commercially pure titanium grade IV bar (Multi-IM®, Universal® platform, BTI Biotechnology Institute S.L., Spain). All the abutments were straight and screw-retained. The as machined samples (machined; group A) were degreased and cleaned prior subsequent use. Physical Vapor Deposition (PVD) technique was used to produce the nitrided samples on as machined substrates in a nitrogen-rich atmosphere. Plasma sublimation permits the positive ionisation and an electric field imposed on the substrate allows the deposition of a 2–3 μm thick homogeneous layer of TiN. The process is maintained for 6 h at 480°C to assure an optimum adhesion of the coating. For the preparation of anodized TiN abutments (anodized TiN; group C), an additional electrochemical anodization step was used. Briefly, the samples were subjected to 1 min anodization at 90 ± 5 V in a titanium tinting electrolyte (WIELAND Edelmetalle GmbH, Germany).

A computer-generated randomization list was obtained to control potential sources of variation. An identification number was consecutively assigned to each implant. Each abutment type corresponds to an implant number according to the randomization list. A sealed envelope, identified only by the identification number, indicated the assignation of the intermediate abutment to the implant. Considering the characteristics of the treatments administered (placement of the implant abutment), the surgeon was not blinded as the type of the abutment was identifiable by its color (Figure 1). The evaluation of the patients, as well as the subsequent analyses of the variables were performed blindly. Likewise, the data collection notebook included the patient’s code as the only identifying data. The correspondence between the treatment and the patient’s number was kept in a document by the principal investigator and only disclosed after completing data processing and analyses.

### Study variables

The primary efficacy variable was the total abundance of selected bacterial taxa commonly associated with peri-implantitis. This set of top periimplant disease-related taxa was extracted from Leonhardt et al. (1999) [37], Belibasakis (2014) [38], Persson and Renvert (2014) [39] and Rakic et al. (2016) [40]. Secondary efficacy variables included the number of bacterial species (species richness), abundance of the top six most abundant bacterial species, abundance of selected peri-implantitis-related bacterial taxa in crevicular fluid samples, complete-mouth plaque index, gingival index, bleeding on probing and probing depth on the adjacent teeth. Other variables include demographic data (sex, age), clinical and dental history, state or preservation of teeth adjacent to dental implants, type of adjacent teeth, location of dental implants, date of surgery, diameter and length of the dental implants, diameter and length of the abutments and the time of placement of the implant-supported prosthesis. Moreover, as detailed below, a complete characterization of bacterial communities was performed in terms of alpha and beta diversity, differential abundance patterns and association network analyses.

### Sample size

A preliminary trial comparing the accumulation of bacterial taxa associated with peri-implantitis between the machined intermediate abutment (control group) and the Multi-Im® nano-Golden abutment (experimental group) was previously performed. The evaluation of the primary efficacy variable (Total abundance of the 25 most commonly associated with peri-implantitis) in the study group considering a control: experimental ratio of 1:1. Assuming a normal distribution and a standard deviation of 37,000. To detect a difference of 37,000 between the mean of the control group and the mean of the experimental group, 17 implants in each group will be needed to be able to reject the null hypothesis which assumes that the means of the control and experimental groups are equal with a statistical power of 0.8 and a type I error of 0.05. Similarly, 17 additional implants will be necessary for the third group.

### Implant surgery and prosthetic rehabilitation

All patients received antibiotic prophylaxis (2 g of amoxicillin 2 h before surgery). Implant sites were marked with an initial drill working at high speed with irrigation to the full length of the implant (UnicCa® BTI Biotechnology Institute, Vitoria, Spain). Diameter drills were used at low speed (125 rpm) without irrigation. Just before implant insertion, the implant site was treated with plasma-rich in growth factors (PRGF) (not activated fraction 2). To prepare the PRGF, commercial kits (KMU 15, BTI Biotechnology Institute, Vitoria, Spain) were used. Venous blood was extracted in tubes having sodium citrate as anticoagulant. After centrifugation (570 g for 8 min), the plasma column was separated into fraction 1 and fraction 2. The 2 ml of the plasma column just above the buffy coat was considered the Fraction 2, while the remaining plasma column was named as Fraction 1. All implants were placed at the bone level, and the final seating of the implant was performed with a calibrated wrench to identify the insertion torque. If adequate insertion torque (>30 Ncm) was obtained, one-stage surgery was conducted: abutments (Multi-Im®) were tightened (torque of 25 Ncm) immediately to the implant, and immediate loading was performed. Otherwise, two-stage surgery was carried out. In that scenario, after 3 months of healing period, the second surgical phase was implemented, with the simultaneous placement of the abutments and implant loading. During the prosthetic phase, impressions were captured with polyether (Impregum Penta Soft Pentamix 3 M España, S.A); impression copings were connected to the abutments in order to register dental implant positions, and screw-retained prosthesis was delivered. The design of the prosthesis in each and every case allowed the correct oral hygiene by the patient. Carefully polished acrylic resin was used to manufacture the screw-retained temporaries.

### Bacterial DNA extraction

Implant abutments with different surfaces were connected to the implants. Then, the abutments were then collected at two time points: 1 day after placement and 60 days later. Samples were stored at −80°C until processing. Bacterial DNA extraction from the biofilm matrix was performed via the DNeasy PowerBiofilm DNA isolation kit (Qiagen, Germany) according to the recommendations provided by the manufacturer. Additionally, sterile paper point size 30 (Maillefer, Ballaigues, Switzerland) were utilized to collect periodontal crevicular fluid from peri-implant crevicular fluid (TACF). The strips were homogenized (1 cycle at 6400 rpm for 30 s) with a Precellys 24 Tissue Homogenizer (Bertin Technologies, France) and the implant abutments were homogenized with an IKA MS 3 digital vortex (IKA, Germany) for 10 min at 2250 rpm. DNA quantification and quality control was performed using a Nanodrop 8000 (Thermo Fisher Scientific, MA, USA) and a Qubit fluorometer (Thermo Fisher, MA, USA). Extracted DNA was stored at −80°C until the extraction process.

### Library preparation and sequencing

16S rRNA library preparation workflow for MiSeq sequencing platform was performed as recommended by Illumina. 16S rRNA gene PCR primers (V3-V4 region) reported by Klindworth et al. (2013) [41] were combined with Illumina adapter overhang nucleotide sequences to generate a single amplicon of ~460 bp. After PCR product purification, dual-index barcodes and Illumina adapter sequences were ligated using the Nextera XT Index Kit (Illumina, CA, USA). Before sample pooling, libraries were quantified using a Qubit fluorimeter (Thermo Fisher, MA, USA) and diluted to an estimated sequencing depth of ~100.000 reads per sample. Eventually, pooled libraries were denatured with NaOH, diluted with hybridization buffer and then heat denatured before sequencing. As suggested by the manufacturer, 5% PhiX was included in each run to serve as an internal control. Paired-end sequencing (2 × 300 bp) was performed using MiSeq v3 reagent kits (600 cycles) (Illumina, CA, USA).

### Data processing

Secondary analysis was performed on BaseSpace using the 16S metagenomics application. After the assembly of full-length 16S rRNA amplicons, the Greengenes Consortium Database was used for taxonomic [41].

### Statistical analyses

[42] Since control and both treatments were represented in each patient, baseline demographic characteristics were described qualitatively (age and sex distribution). Distributional assumptions underlying parametric statistical procedures were checked by different statistical procedures, including visual inspection, Shapiro-Wilks for normality testing and Levene’s test for evaluating homoscedasticity. The effect of time on quantitative clinical variables (periodontal indices) was assessed via repeated-measures ANOVA. Significance level was set at 0.05 (*p* ≤ 0.05). The specific statistical and bioinformatic procedures performed to characterize the composition and structure of bacterial communities across times and transepithelial surfaces are detailed below. Statistical analyses were performed in R [43]. Graphical representation of data was performed via ggplot2 R package for data visualization [44].

#### Alpha diversity

The structure of bacterial biofilm communities in terms of OTU richness and dominance was explored using common alpha diversity metrics in metagenomic Next Generation Sequencing (mNGS) data using *vegan* [45], *phyloseq* [46] and *PMCMRplus* [47] R packages: observed richness, Chao index [48], Shannon index [49] and Simpson’s indices [50]. Generalized linear mixed-effects models were constructed, and ANOVA tests were computed using *lme4* to investigate the effect of time, surface, plaque and inflammation-related variables on alpha diversity indices [51]. Assumptions underlying parametric statistics were checked in model residuals through visual inspection (QQ Plots and density distributions) and significance tests (Shapiro-Wilk and Levene’s test for assessing normality and homoscedasticity). Multiple comparisons were performed using Bonferroni corrected post hoc tests in the *multcomp* package [52].

#### Data normalization

Libraries were normalized using different methods available in metagenomeSeq (Paulson et al. 2013) [53] and NetCoMi [54] R packages: Total Sum Scaling – TSS [55] and Centered Log-Ratio transformation – CLR [56].

#### Differential taxa abundance

Differential abundance testing was performed at different taxonomic ranks via differential expression analyses based on multivariate differential association using *MaAsLin2* R package [57].

#### Beta diversity

The effect of sampling method on beta diversity was assessed via Permutational Analyses of Variance (PERMANOVA) tests based on the Aitchison distance for the community composition at the OTU level [58]. For visual inspection, dissimilarity networks (Aitchison distance) were also constructed using *NetCoMi* R package [54].

#### Association networks

Differential network analyses based on SparCC (Sparse Correlations for Compositional data) correlation measure [59] were computed using the discordant method [60]. At the OTU level, differential plot networks were constructed and compared using the *NetCoMi* R package. A sparsification threshold of 0.5 was used to compare global network properties, centrality measures and hub taxa in the *NetCoMi* package via permutation (1000 permutations). Adjusted Rand Index (ARI) and Graphlet Correlation Distance (GCD) measures were computed to assess distance similarities [61,62].

## Results

### Demographic and clinical variables

**Figure 2.**
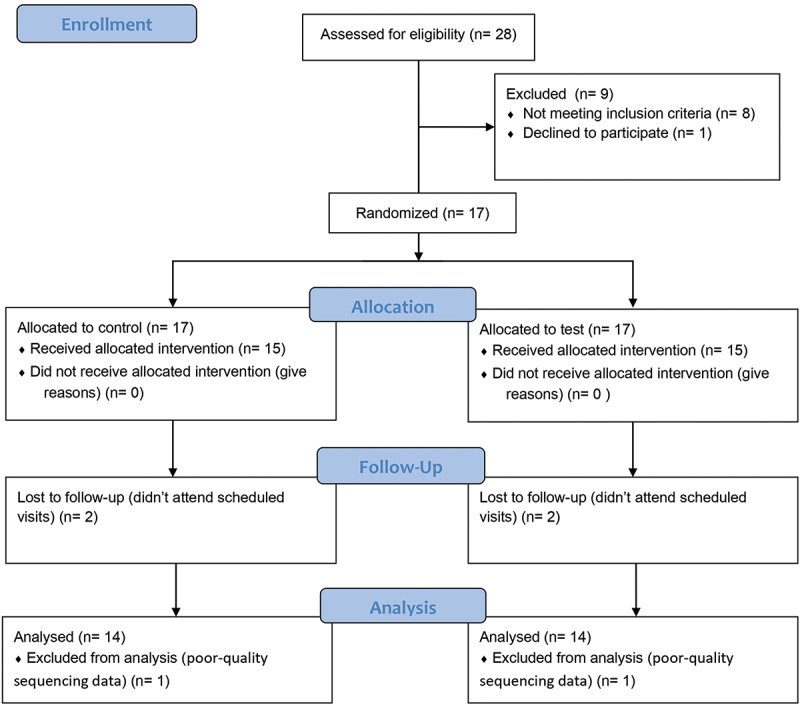
Flowchart of the randomized clinical trial.

As depicted in Figure 2, a total of 28 patients were screened for eligibility for the clinical trial. Seventeen patients were eligible to participate and signed the informed consent. Fifteen patients completed the study and a total of 2 were lost as they did not attend the scheduled visits. The mean age of study participants was 56.5 years, ranging from 38 to 73 years (IQR: 15.3), whereas the sex composition was 8 females (53%) and 7 males (47%) (Table 1). Thus, the study population dataset was composed of 15 patients comprising a total of 48 dental implants connected to 48 abutments, with at least two different surfaces represented in all the participants (n_A_ = 16, n_B_ = 17 and n_C_ = 15). Implant positions were, in descending order of frequency: 37 (6), 36 (6), 46 (6), 47 (5), 25 (4), 35 (5), 44 (4), 16 (2), 24 (2), 27 (2), 34 (2), 45 (2), 14 (1) and 15 (1). After evaluating the quality of metagenomic assemblies, data from another patient (6) were excluded from final analyses due to poor-quality sequencing results.

**Table 1.** Demographic characteristics of patients and periodontal indices at the time of implant placement.

Code	Sex	Implant positions	Age	Number of remaining teeth	Immediate loading	OLI	GI	BoP	SLI
1	Female	47/36/37	50	21	Yes	6.25	0.27	1.89	0.08
2	Male	44/46/47	69	9	No	0.00	1.90	7.40	0.25
3	Male	37/36/34/44/46/47	46	13	Yes	8.30	1.69	5.12	0.25
4	Female	14/24/25	61	6	Yes	8.33	0.42	8.33	0.25
5	Male	16/25/26	65	9	No	6.25	0.15	3.33	0.30
6	Male	44/35/36	73	15	Yes	11.6	1.46	11.1	0.25
7	Male	44/34/36	37	6	No	6.25	5.20	16.70	1.00
8	Female	16/25/27	70	6	No	8.30	1.50	2.70	0.25
9	Male	24/25/27	55	17	No	14.70	1.23	7.84	3.30
10	Female	47/46/37	59	17	Yes	15.60	0.82	10.80	1.75
11	Female	36/35/45	51	18	Yes	13.80	2.50	8.30	1.75
12	Female	37/35/46	58	15	Yes	5.00	1.50	7.14	0.5
14	Female	37/35/45	71	15	Yes	6.81	1.33	10.00	0.67
16	Male	47/46/15	51	20	No	7.50	0.45	5.80	0.33
17	Female	46/36/37	49	16	Yes	12.50	1.00	12.50	0.37

Abbreviations: O’Leary index (OLI), Gingival Index (GI), Bleeding on Probing (BoP) and Silness and Löe Index (SLI).

**Figure 3.**
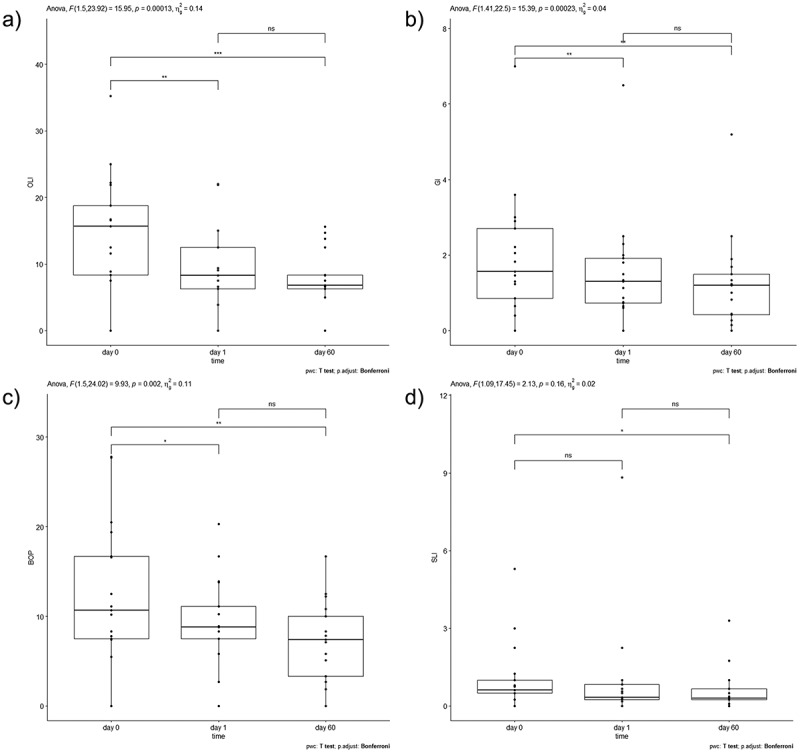
Boxplots of periodontal indices across time and *p* values obtained after computing repeated measures ANOVA and Bonferroni adjustment for multiple comparisons: A) OLI, B) GI, C) BoP and D) SLI.

The mean number of the remaining teeth was 14 teeth, ranging from 6 to 21 teeth (IQR: 8). Immediate loading was performed in nine patients and delayed loading was performed in six. A significant reduction in most periodontal indices was detected after comparing the abutment insertion time with subsequent time points (1 day and/or 60 days after abutment placement). Nevertheless, no changes were observed in OLI, GI, BoP or SLI between day 1 and day 60 after implant placement (Figure 3).

### Differential abundance

**Figure 4.**
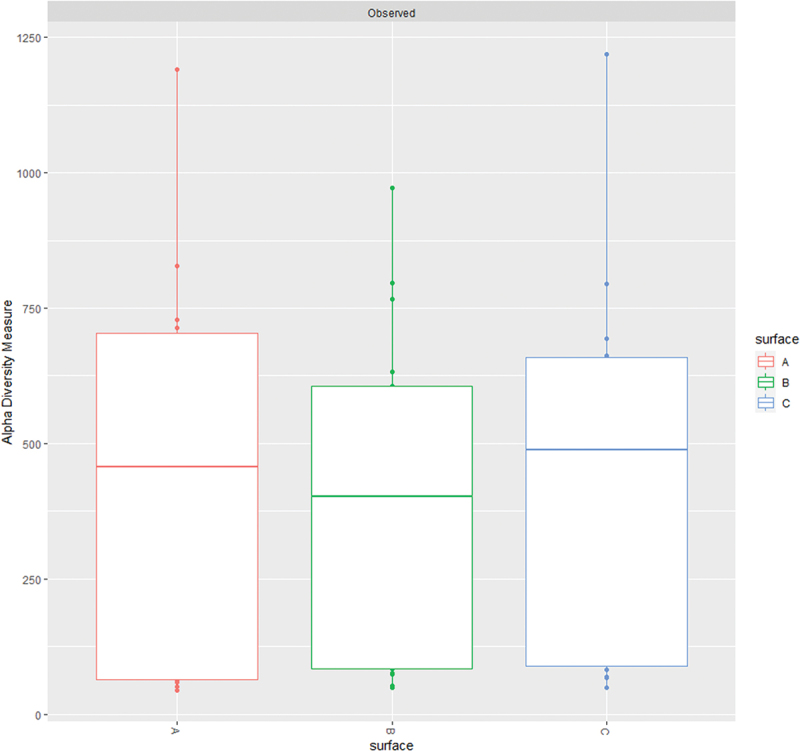
Mean bacterial species richness in the implant abutments collected 60 days after implant placement.

As shown in Figure 4, no significant differences were detected for bacterial richness between the abutment surfaces after 60 days.

**Figure 5.**
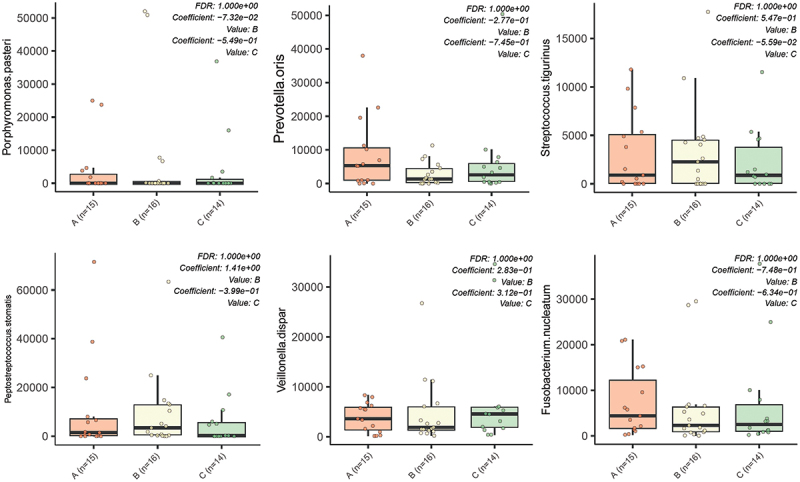
Absolute abundances of the top 6 most abundant bacterial species: *Porphyromonas pasteri, Prevotella oris, Streptococcus tigurinus, Peptostreptococcus stomatis, Veillonella dispar* and *Fusobacterium nucleatum* in the implant abutments collected 60 days after implant placement.

Accordingly, no significant differences were detected for the total abundance of the top six most abundant bacterial species in the abutments collected 60 days after implant placement between the three different surfaces (Figure 5).

**Figure 6.**
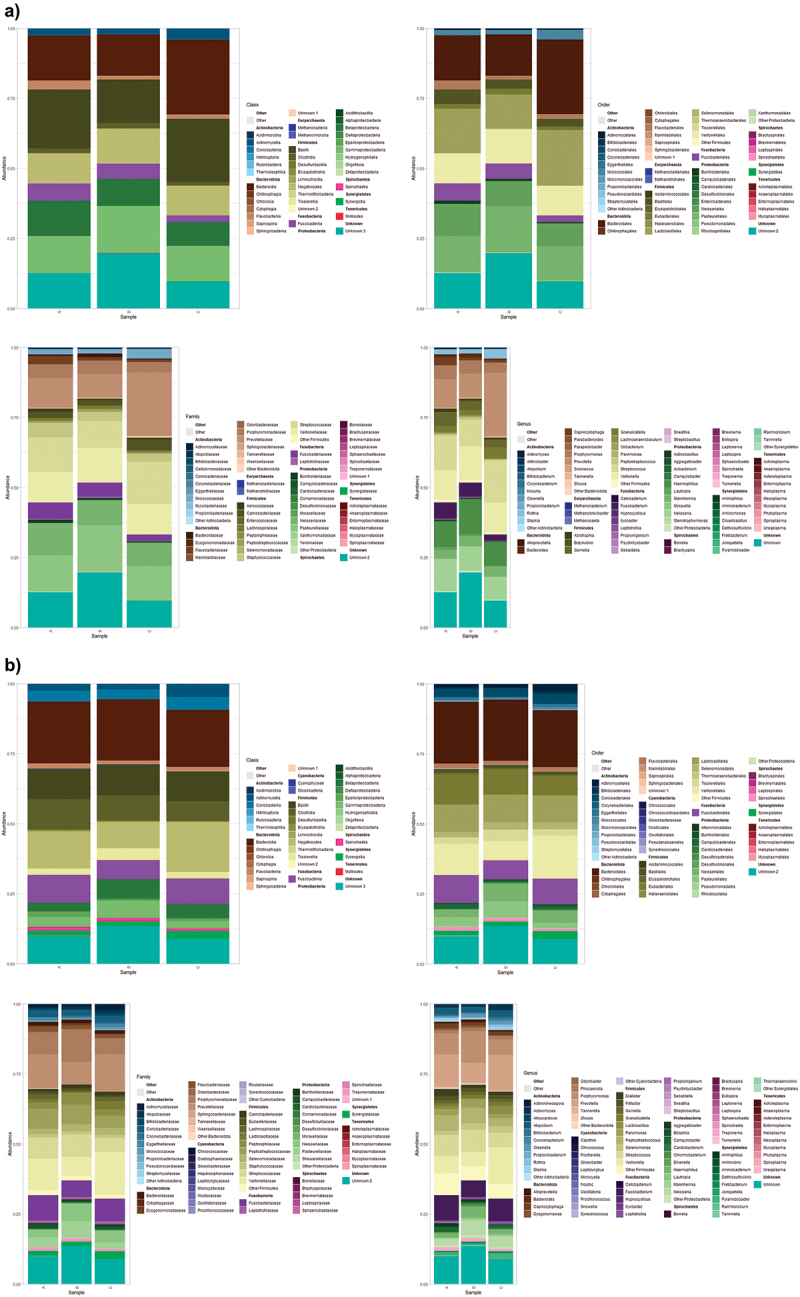
Stacked bar graphs representing cumulative abundances of taxa that represent up to 10 most abundant taxa nested by *Phylum* (only top 10 phyla were plotted) and grouped by sampling method (TSS normalized data) on the abutments collected: 1 (a) and 60 days (b) after abutment placement. The represented taxonomic ranks (*Class*, *Order*, *Family* and *Genus*) were nested by *Phylum*.

According to Figure 6a, *Firmicutes* (33%), *Proteobacteria* (25%), *Bacteroidota* (21%), *Fusobacteria* (4.6%) and *Actinobacteria* (2.9%) were the most common phyla 1 day after abutment placement. The most common classes were *Bacilli* (22%), *Bacteroidia* (17%), *Gammaproteobacteria* (16%), *Negativicutes* (11%) and *Betaproteobacteria* (10%). The orders *Bacteroidales* (19%), *Lactobacillales* (16%), *Pasteurellales* (14%), *Veillonellales* (11%) and *Neisseriales* (9.5%) reached higher frequencies. The families *Prevotellaceae* (14%), *Pasteurellaceae* (14%), *Streptococcaceae* (12%), *Veillonellaceae* (11%) and *Neisseriaceae* (9.5%) were also particularly prevalent taxa at this time point. Finally, the most abundant genera were *Prevotella* (14%), *Streptococcus* (12%), *Veillonella* (11%), *Haemophilus* (10%) and *Neisseria* (9.3%).

As detailed in Figure 6b, *Firmicutes* (42%), *Bacteroidota* (23%), *Proteobacteria* (14%), *Fusobacteria* (9.4%) and *Actinobacteria* (8.6%), were also the most abundant phyla in the abutments on day 60. At the class level, the most represented taxa were *Bacteroidia* (21%), *Bacilli* (14%), *Negativicutes* (14%), *Clostridia* (11%) and *Fusobacteriia* (9.4%), while the orders *Bacteroidales* (21%), *Lactobacillales* (13%), *Veillonellales* (11%), *Eubacteriales* (10%) and *Fusobacteriales* (9.4%) reached the highest relative frequencies. At lower taxonomic ranks, *Veillonellaceae* (11%), *Prevotellaceae* (11%), *Streptococcaceae* (9.1%), *Fusobacteriaceae* (8.1%) and *Porphyromonadaceae* (7.5%) were the most prevalent families, whereas *Prevotella* (10%), *Porphyromonas* (8.3%), *Fusobacterium* (7.8%), *Veillonella* (7.7%) and *Streptococcus* (6.9%) accounted a higher proportion of reads at the genus level.

The effect of surface and time on periodontal or peri-implant disease-related taxa are summarized in Table 2. This subset of potentially pathogenic taxa was extracted from different authors [36–39]. As revealed by multivariate linear models for microbiome data, no significant differential abundance between surfaces was identified regardless of the taxonomic rank. Nevertheless, the effect of time on the abundance of most periodontal and peri-implant disease-related taxa was significant (*p* ≤ 0.05). In the same line, significant associations between bacterial abundance and plaque indices or inflammation-related variables were detected. Boxplots and scatterplots resulting from multivariate linear models for testing the effect of time and plaque indices or inflammation-related variables on the abundance of bacteria are provided as supplementary material (supplementary material 2). Similarly, the effect of time on the abundance of taxa associated with peri-implant and periodontal disease was statistically significant (*p* ≤ 0.05) in the case of crevicular fluid samples (supplementary material 3). Boxplots and scatterplots resulting from multivariate linear models for testing the effect of time and periodontal indices on bacterial communities from the crevicular fluid around implant abutments are provided as supplementary material (supplementary material 4).

**Table 2.** Differential abundance analyses for periodontal or peri-implant disease-related taxa computed using Microbiome Multivariable Association with Linear Models (MaAslin) in transepithelial abutments.

Periodontal or peri-implant	Surface day 1 (B/C)*			Surface day 60 (B/C)*			Time (1 vs 60 days)		
disease related taxa	Coef (B/C)	SE	*p*	Coef (B/C)	SE	*p*	Coef	SE	*p*
*Actinomyces gerensceriae*	−0.32/-0.17	0.38/0.39	0.41/0.67	−0.86/-0.92	0.46/0.48	0.067/0.063	0.24	0.26	0.36
*Aggregatibacter actinomycetemcomitans*	0.51/-0.50	0.54/0.53	0.35/0.35	−0.17/-0.52	0.34/0.34	0.62/0.14	−0.69	0.29	0.020
*Campylobacter gracilis*	0.53/-0.43	0.58/0.56	0.37/0.45	−1.6/-0.50	1.13/1.12	0.16/0.66	6.4	0.65	2.6e-15
*Campylobacter rectus*	0/0	0/0	1/1	0.18/0.19	0.30/0.32	0.57/0.55	0.31	0.14	0.026
*Capnocytophaga sp.*	0.035/-0.55	0.85/0.83	0.97/0.52	−0.53/0.39	0.85/0.84	0.53/0.64	0.59	0.53	0.28
*Dialister invisus*	−1.17/-0.70	0.66/0.65	0.083/0.29	0.07/-0.19	1.2/1.3	0.95/0.88	6.8	0.65	2.5e-16
*Eikenella corrodens*	0.46/-0.85	1.0/1.0	0.65/0.42	−1.1/-0.56	0.73/0.71	0.16/0.44	3.6	0.60	7.5e-08
*Eubacterium infirmum*	0.35/-0.044	0.25/0.24	0.16/0.86	−0.23/-0.19	0.71/0.70	0.75/0.79	1.7	0.48	0.00073
*Eubacterium nodatum*	0.022/0.059	0.12/0.12	0.86/0.63	0.035/-0.14	0.25/0.25	0.89/0.59	0.094	0.11	0.39
*Filifactor alocis*	−0.66/-0.45	0.88/0.86	0.45/0.60	−1.2/-0.19	1.5/1.5	0.42/0.90	2.1	0.70	0.0046
*Fusobacterium nucleatum*	0.32/-0.98	0.71/0.69	0.64/0.17	−0.75/-0.63	0.73/0.76	0.31/0.41	3.9	0.48	4.1e-12
*Haemophilus influenzae*	0.072/-0.067	0.13/0.13	0.58/0.62	0.26/-0.19	0.31/0.31	0.42/0.55	0.33	0.18	0.064
*Mitsuokella sp.*	0/0	0/0	1/1	−0.067/-0.068	0.052/0.054	0.20/0.22	0.022	0.022	0.32
*Parvimonas micra*	−0.12/-0.47	0.39/0.37	0.75/0.22	0.33/-0.077	0.24/0.23	0.17/0.74	1.7	0.33	1.9e-06
*Peptostreptococcus stomatis*	1.2/0.81	1.1/1.1	0.27/0.46	1.4/0.40	1.5/1.5	0.35/0.79	3.4	0.79	6.4e-05
*Porphyromonas gingivalis*	−0.26/-0.047	0.91/0.89	0.78/0.96	−0.35/1.4	1.2/1.2	0.77/0.26	2.1	0.63	0.0014
*Prevotella intermedia*	0.27/-1.1	1.0/1.0	0.80/0.31	0.53/-0.75	1.2/1.2	0.67/0.54	0.85	0.64	0.19
*Prevotella nigrescens*	−0.58/0.77	1.0/1.0	0.58/0.48	0.46/-0.027	1.6/1.6	0.78/0.99	5.3	0.81	6.8e-09
*Pseudoramibacter alactolyticus*	−0.43/-0.11	0.41/0.43	0.31/0.80	1.0/2.5	1.4/1.4	0.47/0.074	2.9	0.64	3.17e-05
*Solobacterium moorei*	−0.065/-0.070	0.053/0.055	0.23/0.21	0.21/0.20	0.15/0.16	0.17/0.22	0.090	0.078	0.25
*Actinomyces* sp.	0.23/0.24	0.63/0.61	0.72/0.70	−0.64/-0.053	0.65/0.66	0.33/0.94	2.2	0.42	1.5e-06
*Campylobacter* sp.	−0.14/-0.14	0.73/0.71	0.85/0.85	−1.9/0.13	0.87/0.89	0.037/0.88	3.9	0.54	5.2e-10
*Chloroflexi*	0.27/0.15	0.27/0.26	0.33/0.55	0.50/-0.033	0.34/0.36	0.15/0.93	0.13	0.20	0.54

**Table 2.** Table continuation.

*Eubacterium* sp.	0.098/-0.12	0.45/0.45	0.83/0.79	-0.68/-0.77	0.50/0.53	0.18/0.15	1.2	0.34	00038
*Fusobacterium* sp	-0.10/-0.96	0.57/0.55	0.86/0.088	-0.83/-0.37	0.65/0.68	0.21/0.59	1.6	0.38	9.2e-05
*Leptotrichia* sp.	0.084/0.094	0.51/0.51	0.87/0.86	0.29/0.50	0.63/0.63	0.65/0.43	1.4	0.37	0.00038
*Mycoplasma* sp.	-0.59/0.34	0.41/0.40	0.16/0.41	0.093/-1.7	1.1/1.1	0.93/0.13	0.79	0.48	0.10
*Peptococcus* sp.	0.56/-0.64	0.55/0.54	0.31/0.24	0.32/0.037	0.38/0.37	0.40/0.92	1.8	0.40	4.0e-05
*Prevotella* sp.	0.20/0.99	0.50/0.48	0.70/0.050	-0.47/0.21	0.52/0.51	0.37/0.68	0.48	0.33	0.15
*Streptococcus* sp.	0.23/0.19	0.32/0.32	0.49/0.55	-0.045/-0.63	0.39/0.40	0.91/0.12	-0.63	0.21	0.0047
*Synergistetes*	0.31/-0.029	0.41/0.40	0.45/0.94	-0.57/-1.2	1.3/1.3	0.67/0.37	4.8	0.66	2.2e-10
*Tenericutes*	-0.20/0.21	0.34/0.34	0.57/0.55	-0.47/-1.4	0.75/0.78	0.53/0.085	1.1	0.36	0.0049

*The surface A was used as reference level for computing linear models.

### Alpha diversity metrics

**Figure 7.**
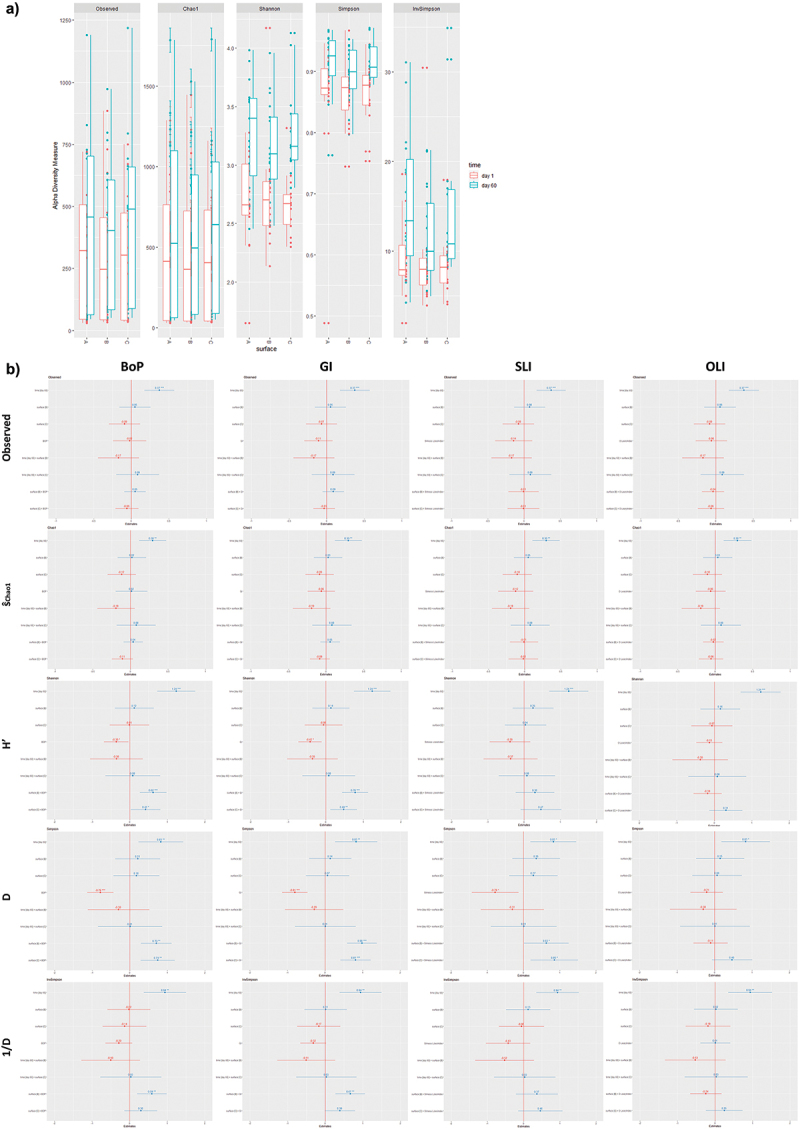
Alpha diversity metrics of biofilm samples: A) boxplots of alpha diversity indices comparing the abutments with different surfaces at 1 and 60 days after placement and B) forest plots of standardized beta values from linear mixed effects models constructed to estimate the effect of surface, time and plaque or inflammation related indices on alpha diversity metrics. Alpha diversity metrics were abbreviated as follows: Observed Richness (Observed), Chao1 Index (ŜChao1), Shannon Diversity Index (H’), Simpson’s Index (D) and Inverse Simpson’s Index (1/D).

Details of alpha diversity metrics of biofilm samples collected from different surfaces are shown in Figure 7a. After considering inflammation-related variables and plaque indices, the constructed linear mixed effects models showed a significantly positive marginal effect of time (*p* ≤ 0.01) on bacterial richness and diversity (Figure 7a). Thus, the impact of each unit of change in time on alpha diversity regardless of the metric used, while other variables are constant is significantly higher than zero. Fixed effects estimates also revealed a significant negative linear association between H’, D or 1/D and BoP (−0.05; *p* = 0.033 or − 0.01; *p* = 0.001) or GI (−0.38; *p* = 0.017, −0.07; *p* = 0.006 or − 5.15; *p* = 0.037) as depicted in Figure 2b. The effect of surface on H’ and D indices was also significant when BoP (*p* = 0.036 and *p* = 0.023) and GI (*p* = 0.040 and *p* = 0.013) were included as predictors. However, marginal effects extracted from these models also revealed significant interactions between surface and gingival inflammation-related variables (*p* ≤ 0.01). Nevertheless, H’ estimates increased with BoP and GI in the surfaces B (0.08; *p* = 0.001 and 0.31; *p* = 0.001) and C (0.05; *p* = 0.036 and 0.19; *p* = 0.009), whereas D increased with BoP, GI and SLI in the surfaces B (0.01; *p* = 0.001, 0.05; *p* ≤0.001 and 0.05; *p* = 0.045) and C (0.01; *p* = 0.002, 0.05; *p* ≤0.001 and 0.07; *p* = 0.011) and 1/D with BoP and GI in the surface B (1.03; *p* ≤ 0.004 and 3.67; *p* = 0.001).

No significant effect of variables related to inflammation, plaque indices or surfaces were observed on observed richness or Ŝ_Chao1_ after including BoP, GI SLI or OLI in the models. The proportion of variance explained by variations between different individuals and sequencing runs reached up to 95% (Observed and Ŝ_Chao1_), whereas the entire models (including random effects) explained about 38–59% of the variance of H’, D and 1/D when BoP or GI was included as predictors. However, fixed factors accounted for the 21–40% of the observed variation, indicating a strong effect of inter-individual and inter-run variability in the response of diversity to plaque indices and inflammation-related variables. Linear mixed effects model summaries are available as supplementary materials (supplementary materials 5).

### Beta diversity metrics and differential association

**Figure 8.**
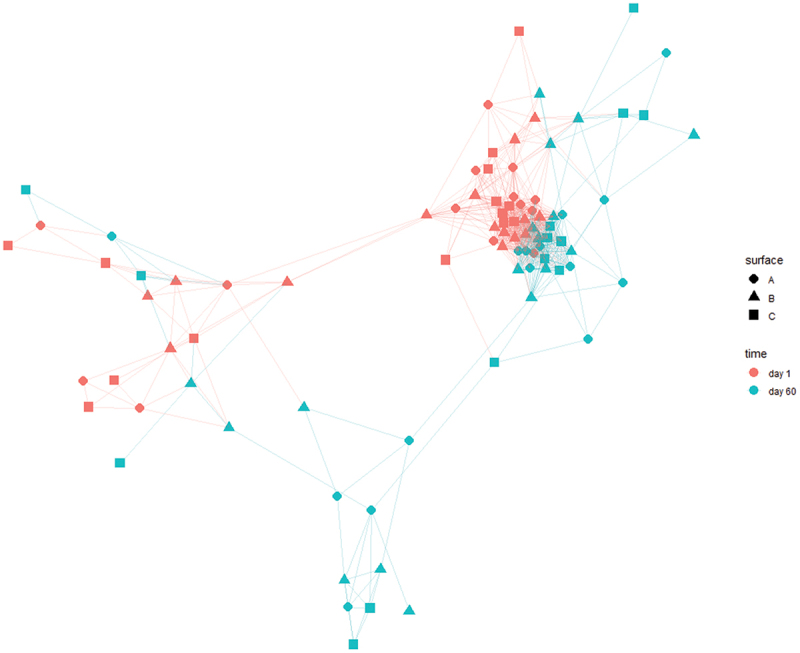
Dissimilarity network based on Aitchison distance matrix representing the beta-diversity at the OTU level (zeros were replaced via multiplicative simple replacement and k-nearest neighbor was used as sparsification method).

A compositional dissimilarity network based on Aitchison distances was constructed (Figure 8). Biofilm samples from the same time point clustered more tightly with each other. Nevertheless, the effect of surface assessed via visual inspection is negligible. Stratified PERMANOVA analyses concluded that the effect of time was significant on bacterial biofilm community (*p* ≤ 0.001), whereas surface was statistically non-significant at the OTU level (*p* = 0.92).

**Figure 9.**
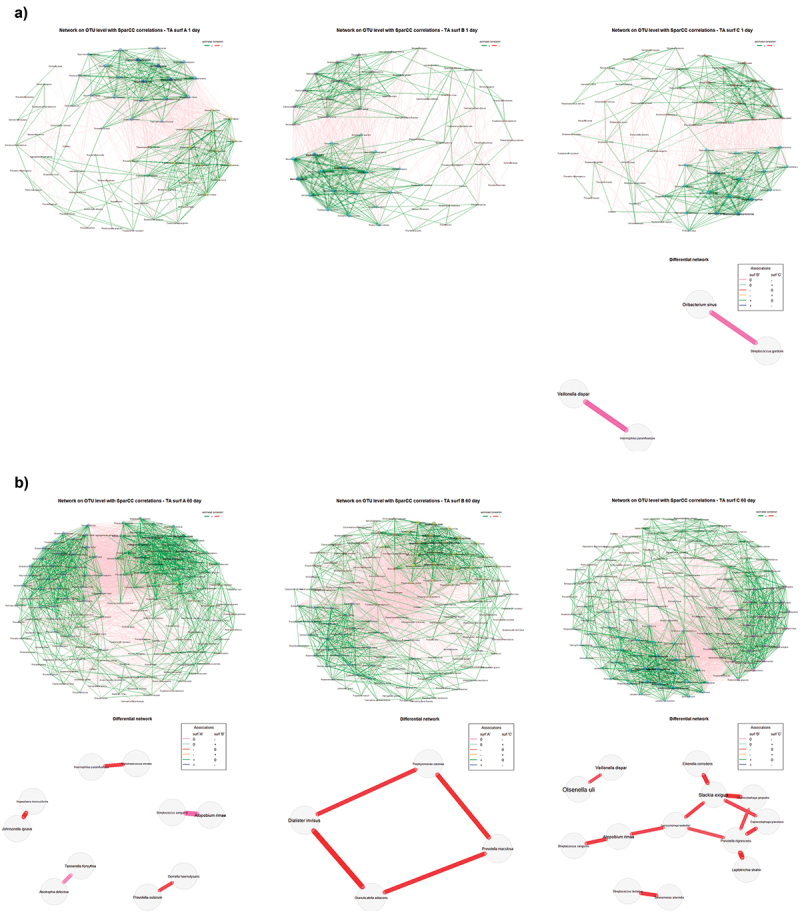
Bacterial association networks constructed using SparCC correlation coefficients for compositional data retaining OTUs with at least 0.1% of sequencing reads from all the studied surfaces at days 1 (A) and 60 (B). For network sparsification, only edges corresponding to an absolute association greater or equal than 0.7 were represented in order to improve network readability. Differential association networks based on association networks are also provided for all pairwise comparisons.

Bacterial association networks were computed for all the studied combinations of time points and surfaces (Figure 9). Differential network analyses revealed high clustering similarities in terms of Adjusted Rank Index (ARI) or Graphlet Correlation Distance (GCD) and minor changes in global network properties when comparing different surfaces within time points (Table 3). However, significant differences were detected in terms of hub taxa between A and B/C at 60 days (Jaccard Index: JI = 0.00, *p* = 0.0077), with a higher influence of *Eikenella corrodens*, *Prevotella tannerae* and *Neisseria lactamica* in B, and a higher interconnection density for *Streptococcus vestibularis* or *Veillonella denticariosi* in the surface C (Figure 9b). Significant differences were also identified in betweenness centrality after comparing B and C at both 1 and 60 days (Jaccard Index: JI = 0.087, *p* = 0.0068 and JI = 0.18, *p* = 0.027, respectively), with a higher influence of *Veillonella atypica*, *Gemella* spp., *Gemella cuniculi* and *Bifidobacterium subtile* in B and *Bacteroides denticanum* and *Streptococcus parasanguinis* in C (Figure 9a, B). As shown in Figure 4c, the effect of time on global network properties or on the similarity between the most central nodes was also limited. Nevertheless, significant differences were detected in betweenness centrality after comparing the studied time points within the A surface (Jaccard Index: JI = 0.16, *p* = 0.046), with a greater influence of *Streptococcus tigurinus* 1 day after transepithelial placement and a more prominent role of *Actinomyces odontolyticus* and *Streptococcus parasanguinis* 60 days later.

**Table 3.** Jaccard index values (*j*) corresponding to Figure 2 accounts for the similarity of the sets of most central nodes (centrality value above the empirical 70% quantile) and also of the sets of hub taxa between pairs of networks (*j* is 0 if the sets are completely different and 1 for exactly equal sets).

Jaccard Index (similarity between sets of most central nodes)									
Pairwisecomparisons by surface (1 day)									
Centrality measures	*j*_A-B_	P(J ≤ *j*)	P(J ≥ *j*)	*j*_A-C_	P(*J* ≤ *j*)	P(*J* ≥ *j*)	*j*_B-C_	P(*J* ≤ *j*)	P(*J* ≥ *j*)
Degree	0.29	0.48	0.18	0.24	0.25	0.88	0.29	0.48	0.72
Betweenness centrality	0.23	0.21	0.9	0.22	0.17	0.92	0.087	0.0068	1
Closeness centrality	0.75	1	0.00079***	0.56	0.99	0.043 *	0.53	0.97	0.075
Eigenvector centrality	0.75	1	0.00079***	0.87	1	0.000031 ***	0.63	1	0.016
Hub taxa	1	1	0.037 *	1	1	0.037 *	0.5	0.89	0.41
Pairwise comparisons by surface (60 days)									
Centrality measures	*j*_A-B_	P(J ≤ *j*)	P(J ≥ *j*)	*j*_A-C_	P(*J* ≤ *j*)	P(*J* ≥ *j*)	*j*_B-C_	P(*J* ≤ *j*)	P(*J* ≥ *j*)
Degree	0.57	1	0.0072**	0.47	0.97	0.058	0.57	1	0.0072**
Betweenness centrality	0.18	0.027*	0.99	0.29	0.32	0.79	0.18	0.027*	0.99
Closeness centrality	0.6	1	0.0025**	0.5	0.99	0.028*	0.6	1	0.0025**
Eigenvector centrality	0.71	1	0.000043***	0.29	0.32	0.79	0.71	1	0.000043***
Hub taxa	0.67	0.98	0.1	0	0.0077**	1	0.67	0.98	0.1
Pairwise comparisons by time point (1 vs day 60 within surface)									
Centrality measures	*j*_A 1–60 days_	P(J ≤ *j*)	P(J ≥ *j*)	*j*_B 1–60 days_	P(*J* ≤ *j*)	P(*J* ≥ *j*)	*j*_C 1–60 days_	P(*J* ≤ *j*)	P(*J* ≥ *j*)
Degree	0.25	0.3	0.85	0.44	0.87	0.26	0.34	0.76	0.45
Betweenness centrality	0.16	0.046 *	0.99	0.2	0.15	0.94	0.22	0.23	0.9
Closeness centrality	0.67	1	0.0039**	0.71	1	0.0040**	0.6	0.99	0.031*
Eigenvector centrality	0.88	1	0.000012***	1	1	0.0000020***	0.71	1	0.0040**
Hub taxa	0.2	0.46	0.87	0.5	0.89	0.41	0.5	0.89	0.41

Differential associations between OTUs 1 day after transepithelial placement were limited to the surfaces B and C particularly *Oribacterium sinus* and *Streptococcus gordonii* and *Veillonella dispar* and *Haemophilus parainfluenzae* were no correlated in the surface B but negatively correlated in C (Figure 8a). On the other hand, differential network analyses revealed significant correlation changes between certain OTUs 60 days after transepithelial placement (Figure 4b), including *Streptococcus sanguinis* and *Atopobium rimae*, and *Abiotrophia defectiva* and *Tannerella forsythia* (no correlated in A and inversely correlated in B), and *Megasphaera micronuciformis* and *Johnsonella ignava*, *Haemophilus parainfluenzae* and *Peptostreptococcus stomatis*, and *Prevotella oulorum* and *Gemella haemolysans* (no correlated in B and negatively correlated in A). After comparing the surfaces A and C, some negative correlations between taxa detected in A samples were not identified in C samples (*Dialister invisus* and *Granulicatella adiacens*, *Dialister invisus* and *Porphyromonas catoniae*, *Porphyromonas catoniae* and *Prevotella maculosa*, and *Prevotella maculosa* and *Granulicatella adjacens*). Moreover, pairwise comparisons between the surfaces B and C at the same time point also showed negative correlations in B samples that remained no significant in C samples, including *Olsenella uli* and *Veillonella dispar*, *Atopobium rimae* and *Streptococcus sanguinis*, *Leptotrichia shahii* and *Prevotella nigrescens*, or *Eikenella corrodens* and *Slackia exigua*. Finally, no significant differential associations were identified between samples collected 1 and 60 days after transepithelial placement regardless of the surface.

## Discussion

This randomized clinical trial would be the first to assess the biofilm formation on TiN-abutments in the context of its clinical use (transgingival position and implant loading). The characteristics of biofilm formation have been assessed at early (1 day) and late (60 days) stages. The multifactorial analysis indicated that bacterial biofilm properties are significantly affected by patient, time and periodontal indices at different scales, but the effect of abutment surface type (machined, TiN and TiN oxidized) at the community level was negligible. Despite the abundant literature that exists on oral microbiome acquisition and maturation [63–66], bacterial colonization dynamics on dental implants and restorative material surfaces is still poorly understood since most research is based on cross-sectional designs. According to Hu et al. (2022) [67], 12 hours (h) *in situ* biofilm on smooth restorative material surfaces was dominated by *Streptococcus*, *Neisseria*, *Gemella* and *Prevotella*. As summarized by Dhir et al. (2013) [68], bacterial cells colonize the tooth surface within 4 h of the pellicle formation, being the genus *Streptococcus* an important initial colonizer. After that, secondary colonizers, which are unable to adhere directly to the tooth surface, bind to the cell surfaces of the initial colonizing bacteria. Biofilm formation on implants surfaces shows similar microbial colonization and ecological succession patters [69]. Similar to previous reports, most bacterial taxa increased their abundance 60 days after abutment placement [70], including *Actinobacteria*, *Synergistetes*, *Spirochaetes*, *Fusobacteria*, *Actinomyces*, *Selenomonas*, *Eikenella*, *Megasphaera*, *Dialister*, *Filifactor*, *Treponema* or *Campylobacter*. Ramos de Freitas et al. (2018) [71] also reported higher abundances of *Fusobacterium* or *Selenomonas* 3 and 6 months after titanium implant placement in different sites. On the other hand, the abundance of *Proteobacteria*, *Haemophilus*, *Mannheimia*, *Neisseria*, *Streptococcus* spp., *Rothia* spp. and *Prevotella* spp. was significantly reduced 60 days after implant abutment placement.

Concerning periodontal or peri-implant disease-related taxa, a significant shift toward higher abundances has been detected in most cases (*Campylobacter* spp., *Dialister invisus*, *Eikenella corrodens*, *Eubacterium* spp., *Filifactor alocis*, *Porphyromonas gingivalis, Fusobacterium nucleatum* or *Peptostreptococcus stomatis*), whereas some of them rarefied over time (*Aggregatibacter actinomycetemcomitans* and *Streptococcus*). Accordingly, Wake et al. (2016) [72] reported a decrease in the relative proportion of *Streptococcus* that predominated until 16 h along with an increasing population of *Fusobacterium*, *Prevotella* and *Porphyromonas* that prevailed after 48 h. Despite the fact that very few studies assess the ecological succession in bacterial biofilms at long time periods [66,73–76] *Streptococcus*, *Haemophilus* spp., *Actinomyces* or *Veillonella* spp. are usually considered early colonizers, whereas *Porphyromonas gingivalis*, *Treponema denticola*, *Aggregatibacter actinomycetemcomitans*, *Prevotella* spp., *Selenomonas* spp., *Capnocytophaga* spp. and *Eubacterium* spp. are considered late colonizers [77–80] and *Fusobacterium nucleatum* is intentionally placed at the border between both categories since it coaggregates with all the early and late colonizers [81]. In the same line, Xu et al. (2022) [82] reported that *Streptococcus*, *Rothia* and *Haemophilus* constituted over 70% of total abundances at the initial stages of supragingival plaque formation. Our results confirm that biofilm formation is a continuous process consisting of a transition from the early aerobic environment characterized by Gram-positive aerobes or facultative anaerobes (*Bacilli*) to a highly oxygen depleted environment dominated by Gram-negative anaerobic taxa, including *Synergistetes* and *Fusobacteria* [83]. *Proteobacteria* populations (*Neisseria*, *Hemophilus* or *Mannheimia*), consisting mostly of aerobic or facultative anaerobic taxa, also declined over time.

As reported by Herrmann et al. (2020) [84], implant surface shows an effect on the abundance of certain periodontopathogens. This statement has been confirmed by several authors [85–89]. Surface chemical modifications, including antibacterial active metal and antibiotic coatings, also modify bacterial adhesion and biofilm formation patterns [90–92]. Focusing specifically on titanium, Xu et al. (2022) [93] summarized that bioactive coating methods, which are essential to synthetize a protective or biocompatible layer on the surface of titanium and titanium alloys, can deeply affect osseointegration, bacterial adhession and biofilm formation. Anodic oxidation is an electrochemical method used to oxidise the titanium surfaces into forming ceramic TiO_2_ layers of varying thicknesses (hundreds of nanometers to hundreds of micrometers), thus expanding the thin natural passive TiO_2_ film that occurs naturally under atmospheric conditions [94]. Modifications in this electrolytic oxidation process (affecting voltage, electrolyte composition and concentration, temperature or current density) lead to differential properties of the ceramic layer [95]. Surface characteristics of anodised titanium (higher surface energy, hydrophilicity and crystallinity) are recognised as possessing the ideal bioactive coating properties for osseointegration of implants [96].

According to Faveri et al. (2022) [97], anodization significantly reduced *in vitro* bacterial adhesion, but this reduction only affected around 30% of the studied taxa. In the same line, Fais et al. (2021) [98] detected significant changes in microbial adhesion, but no differences for biofilm formation after comparing anodized surfaces of titanium alloys. However, it should be considered that the results observed using *in vitro* approaches may not represent *in vivo* community dynamics [99]. In this sense, our results indicate that the effect of surface on alpha diversity metrics, beta diversity or bacterial abundance of the studied biofilm communities was negligible. However, after enriching the models with plaque and inflammation-related covariables, some interesting findings should be highlighted. The interaction term revealed that the effect of GI, BoP and SLI on certain diversity indices varied depending on the surface. For example, despite the fact that the overall association between BoP, GI or SLI and most diversity metrics was negative, higher values of BoP, GI and SLI were significantly associated with higher H’ and D in the surfaces Ti-Golden and nano-Golden, and with higher 1/D in the surface Ti-Golden. The positive response of bacterial diversity to inflammation and plaque indices detected in the surfaces Ti-Golden and nano-Golden was in agreement with findings of previous research in both animal [100,101] and human models [102–104]. The varying influence of richness, evenness and dominance in each alpha diversity index could explain the differences observed in relation to their interpretation and statistical significance [105].

Surprisingly, the opposite association was observed in the as machined surface. In this sense, Demmer et al. (2008) [106] detected a negative relationship between BoP and health-associated bacterial colonization levels in subgingival plaque. An inverse correlation between the presence of bleeding on probing and bacterial diversity was also confirmed by Daubert et al. (2018) [107], whereas positive correlations have been previously detected between BoP and bone resorption (ρ = 0.18; *p* = 0.001) and between PI and BOP (ρ = 0.13, *p* = 0.019) in patients with immediately loaded, implant-supported, full-arch prostheses [108]. Despite the fact that the relative abundance analyses did not reveal significant changes, bacterial adhesion and biofilm formation may still differ between surfaces. For example, Al-Ahmad et al. (2013) [109] and Pita et al. (2015) [110] reported that bacterial colonization in titanium machined surface has been significantly lower than that of other implant materials, including modified titanium, modified zirconia or alumina-toughened zirconia. Similar conclusions were drawn by Jordan et al. (2016) [19] and de Avila et al. (2017) [111]. Since D assigns much more weight to the most abundant bacteria, a reduction of the health-associated burden could lead to diminished D in surfaces with lower bacterial adhesion and biofilm formation [112]. Regarding the effect of time on bacterial abundance and alpha diversity, similar temporal dynamics of increasing bacterial diversity was reported by Esberg et al. (2022) [113] in different oral microbiota communities. In this sense, our results confirm that inter-individual variability is by far the most important factor that explains the observed differences in terms of bacterial diversity and composition [114–116]. In line with the present conclusions, previous findings suggest that success in implantology is less dependent on selecting specific implant systems and more on a deeper understanding of patient-specific risk factors [117]. However, the clinical significance of biofilm-related characteristics in terms for peri-implant tissue stability and oral health status could not be assessed during this investigation as it is out of the scope of the present clinical trial. To this effect, long-term follow-up studies are required.

## Conclusions

Despite minor variations in bacterial association patterns have been identified, changes in the properties of surface coatings on titanium abutment surfaces did not significantly affect bacterial alpha diversity or community composition. Nevertheless, the effect of patient and time on bacterial biofilm composition and diversity was statistically significant.

## Supplementary Material

Supplementary material.zip

## Data Availability

The datasets generated and/or analyzed during the current study are not publicly available due to commercial reasons but are available from the corresponding author upon reasonable request.

## Abbreviations

1/D: Inverse Simpson’s Index
ANOVA: Analysis of Variance
ARI: Adjusted Rand Index
BoP: Bleeding on Probing
CI: Confidence Interval
CLR: Centered Log-Ratio Transformation
D: Simpson’s Index
DNA: Deoxyribonucleic Acid
GCD: Graphlet Correlation Distance
GI: Gingival Index
H’: Shannon Diversity Index
j: Jaccard index values
mNGS: Next Generation Sequencing
OLI: O’Leary index
OUT: Operational Taxonomic Unit
p: p value
PERMANOVA: Permutational Analysis of Variance
rRNA: ribosomal RNA
TACF: Crevicular Fluid from Implant Abutment
Ti: Titanium
TiN: Titanium nitride
TSS: Total Sum Scaling
ŜChao: Chao Index
SE: Standard Error
SLI: Silness and Löe Index
SparCC: Sparse Correlations for Compositional data

## Author’s information

Eduardo Anitua: ORCID (0000–0002–8386–5303) email: eduardo@fundacioneduardoanitua.org

Alia Murias-Freijo: aliamurias@gmail.com

Roberto Tierno: roberto.tierno@bti-implant.es

Ricardo Tejero: ricardo.tejero@bti-implant.es

Mohammad Hamdan Alkhraisat: ORCID (0000–0003–4422–4527) email: mohammad.hamdan@bti-implant.es
