# Supplementary material for: Effect of implant abutment surface treatments on bacterial biofilm composition and structure
Source: J Oral Microbiol. 2025 Feb 5;17(1):2459922. doi: 10.1080/20002297.2025.2459922 (PMC11800344; doi:10.1080/20002297.2025.2459922)
Supplement: Supplementary material.zip [file ZJOM_A_2459922_SM4359.zip › Supplementary material 2.docx]

**Supplementary material 2.** Differential abundance analyses for periodontal or peri-implant disease related taxa computed using Microbiome Multivariable Association with Linear Models (MaAsLin) in crevicular fluid samples.

|  | **surface day 1 (B/C)*** | | | **surface day 60 (B/C)*** | | | **time (1 vs 60 days)** | | |
| --- | --- | --- | --- | --- | --- | --- | --- | --- | --- |
| **Periodontal or peri-implant**  **disease related taxa** | **Coef (B/C)** | **SE** | ***p*** | **Coef (B/C)** | **SE** | ***p*** | **Coef** | **SE** | ***p*** |
| *Actinomyces gerensceriae* | 0.067/4.7 | 0.059/0.064 | 0.26/1.0 | -0.79/-0.40 | 0.62/0.65 | 0.21/0.54 | 0.79 | 0.30 | 0.010 |
| *Aggregatibacter actinomycetemcomitans* | -0.52/-0.38 | 0.45/0.46 | 0.26/0.41 | -0.43/-0.36 | 0.34/0.33 | 0.21/0.29 | -0.51 | 0.30 | 0.093 |
| *Campylobacter gracilis* | -0.66/-0.21 | 0.33/0.34 | 0.057/0.54 | -0.66/1.2 | 1.3/1.4 | 0.62/0.40 | 5.6 | 0.67 | 3.9e-12 |
| *Campylobacter rectus* | 0/0 | 0/0 | 1/1 | 0.031/0.28 | 0.29/0.29 | 0.92/0.34 | 0.35 | 0.15 | 0.022 |
| *Capnocytophaga sp.* | -0.0043/-0.13 | 0.93/0.94 | 1.0/0.89 | -0.16/-0.012 | 0.73/0.72 | 0.83/0.99 | 0.66 | 0.49 | 0.18 |
| *Dialister invisus* | 0.15/0.43 | 0.40/0.42 | 0.70/0.31 | -1.4/-1.8 | 1.3/1.3 | 0.26/0.18 | 6.1 | 0.63 | 1.1e-14 |
| *Eikenella corrodens* | -0.51/-1.0 | 0.84/0.91 | 0.54/0.26 | -1.7/-0.84 | 0.95/0.97 | 0.087/0.39 | 2.3 | 0.54 | 8.2e-05 |
| *Eubacterium infirmum* | -0.071/-0.071 | 0.059/0.064 | 0.23/0.27 | -0.11/0.095 | 0.44/0.43 | 0.80/0.83 | 1.1 | 0.35 | 0.0016 |
| *Eubacterium nodatum* | 0.19/0.090 | 0.17/0.18 | 0.27/0.63 | 0.31/-0.17 | 0.43/0.42 | 0.47/0.68 | 0.38 | 0.22 | 0.094 |
| *Filifactor alocis* | -0.56/-0.38 | 0.37/0.37 | 0.14/0.32 | 0.30/-0.081 | 1.2/1.2 | 0.80/0.95 | 2.1 | 0.54 | 0.00017 |
| *Fusobacterium nucleatum* | 1.4/-0.69 | 0.97/1.0 | 0.16/0.51 | -0.51/-1.9 | 1.0/1.1 | 0.62/0.087 | 4.0 | 0.61 | 9.7e-09 |
| *Haemophilus influenzae* | 0/0 | 0/0 | 1/1 | -0.13/-0.23 | 0.52/0.53 | 0.81/0.67 | 0.65 | 0.24 | 0.0087 |
| *Mitsuokella sp.* | 0/0 | 0/0 | 1/1 | 0.059/0.093 | 0.086/0.090 | 0.50/0.31 | 0.050 | 0.038 | 0.19 |
| *Parvimonas micra* | -0.51/-0.26 | 0.39/0.39 | 0.20/0.52 | -0.25/0.74 | 0.56/0.55 | 0.65/0.19 | 1.6 | 0.36 | 3.6e-05 |
| *Peptostreptococcus stomatis* | 3.1/0.30 | 1.2/1.2 | 0.013/0.81 | -2.9/-1.6 | 1.2/1.2 | 0.026/0.20 | 0.29 | 0.76 | 0.71 |
| *Porphyromonas gingivalis* | 0.27/0.16 | 0.43/0.43 | 0.53/0.72 | 0.59/0.17 | 1.2/1.2 | 0.63/0.89 | 2.5 | 0.62 | 0.00017 |
| *Prevotella intermedia* | 1.4/-0.47 | 0.85/0.92 | 0.12/0.62 | -0.96/-0.40 | 0.99/0.98 | 0.34/0.69 | 1.4 | 0.64 | 0.037 |
| *Prevotella nigrescens* | 0.80/-0.14 | 0.74/0.80 | 0.29/0.87 | -1.7/-1.2 | 0.96/0.98 | 0.080/0.23 | 4.9 | 0.72 | 2.5e-09 |
| *Pseudoramibacter alactolyticus* | 0.24/-0.079 | 0.16/0.17 | 0.15/0.65 | -0.87/0.69 | 1.1/1.2 | 0.45/0.56 | 2.1 | 0.54 | 0.00027 |
| *Solobacterium moorei* | 0/0 | 0/0 | 1/1 | 0.059/-3.7 | 0.052/0.055 | 0.27/1.0 | 0.022 | 0.023 | 0.35 |
| *Actinomyces* sp. | -0.60/-1.0 | 0.78/0.80 | 0.45/0.21 | -0.67/0.34 | 0.47/0.47 | 0.16/0.47 | 2.9 | 0.42 | 2.5e-09 |
| *Campylobacter* sp. | 0.0081/-0.068 | 0.54/0.54 | 0.99/0.90 | 0.24/1.3 | 1.1/1.2 | 0.83/0.25 | 4.2 | 0.57 | 1.7e-10 |

**Supplementary material 2**. *Table continuation.*

|  | **surface day 1 (B/C)*** | | | **surface day 60 (B/C)*** | | | **time (1 vs 60 days)** | | |
| --- | --- | --- | --- | --- | --- | --- | --- | --- | --- |
| **Periodontal or peri-implant**  **disease related taxa** | **Coef (B/C)** | **SE** | ***p*** | **Coef (B/C)** | **SE** | ***p*** | **Coef** | **SE** | ***p*** |
| *Chloroflexi* | 0.37/-0.53 | 0.38/0.38 | 0.34/0.17 | -0.48/0.37 | 0.32/0.31 | 0.14/0.24 | 0.33 | 0.20 | 0.10 |
| *Eubacterium* sp. | -0.085/-0.20 | 0.38/0.39 | 0.83/0.61 | -0.45/0.16 | 0.43/0.44 | 0.30/0.71 | 1.1 | 0.32 | 0.00071 |
| *Fusobacterium* sp | 0.23/-1.5 | 1.0/1.1 | 0.83/0.17 | -0.62/-1.4 | 0.84/0.88 | 0.47/0.11 | 2.0 | 0.55 | 0.00049 |
| *Leptotrichia* sp. | 0.27/-0.11 | 0.66/0.68 | 0.69/0.88 | 0.26/0.67 | 0.78/0.78 | 0.74/0.39 | 2.2 | 0.49 | 3.2e-05 |
| *Mycoplasma* sp. | -0.55/-0.90 | 0.50/0.54 | 0.28/0.11 | -0.25/-0.92 | 0.78/0.78 | 0.75/0.25 | 1.2 | 0.42 | 0.0053 |
| *Peptococcus* sp. | -0.089/-0.82 | 0.49/0.53 | 0.86/0.13 | -0.90/-0.57 | 0.49/0.48 | 0.075/0.25 | 1.2 | 0.37 | 0.0014 |
| *Prevotella* sp. | -0.17/-0.58 | 0.62/0.63 | 0.79/0.37 | 0.17/0.41 | 0.63/0.65 | 0.79/0.54 | 0.19 | 0.42 | 0.66 |
| *Streptococcus* sp. | 0.33/0.98 | 0.55/0.56 | 0.55/0.091 | -0.25/0.40 | 0.52/0.54 | 0.63/0.46 | -0.78 | 0.33 | 0.023 |
| *Synergistetes* | -0.38/-0.18 | 0.31/0.34 | 0.23/0.60 | -0.39/-0.68 | 1.2/1.2 | 0.75/0.58 | 3.6 | 0.60 | 1.0e-07 |
| *Tenericutes* | -0.18/-0.60 | 0.39/0.42 | 0.65/0.16 | -0.40/-0.98 | 0.50/0.51 | 0.42/0.063 | 0.95 | 0.29 | 0.0020 |
