# Supplementary material for: Effect of implant abutment surface treatments on bacterial biofilm composition and structure
Source: J Oral Microbiol. 2025 Feb 5;17(1):2459922. doi: 10.1080/20002297.2025.2459922 (PMC11800344; doi:10.1080/20002297.2025.2459922)
Supplement: Supplementary material.zip [file ZJOM_A_2459922_SM4359.zip › Supplementary material 4.docx]

**Supplementary material 4.** Summary of linear mixed-model estimation results.

| **Observed** | | | | | | | | | | | | | | | |
| --- | --- | --- | --- | --- | --- | --- | --- | --- | --- | --- | --- | --- | --- | --- | --- |
| *Predictors* | *E* | *CI* | *p* | *Predictors* | *E* | *CI* | *p* | *Predictors* | *E* | *CI* | *p* | *Predictors* | *E* | *CI* | *p* |
| (Intercept) | 342.67 | -45.89 - 731.23 | **0.083** | (Intercept) | 369.19 | -10.36 - 748.74 | **0.056** | (Intercept) | 363.58 | -12.58 - 739.73 | **0.058** | (Intercept) | 362.56 | -25.02 - 750.14 | **0.066** |
| time [day 60] | 112.07 | 52.83 - 171.30 | **<0.001^***^** | time [day 60] | 112.07 | 52.90 - 171.24 | **<0.001^***^** | time [day 60] | 112.07 | 51.85 - 172.29 | **<0.001^***^** | time [day 60] | 112.07 | 52.01 - 172.13 | **<0.001^***^** |
| surface [B] | -15.56 | -123.20 - 92.08 | 0.774 | surface [B] | -16.26 | -97.56 - 65.05 | 0.691 | surface [B] | 24.78 | -54.88 - 104.45 | 0.537 | surface [B] | 37.36 | -64.28 - 139.00 | 0.466 |
| surface [C] | 6.97 | -98.48 - 112.43 | 0.895 | surface [C] | -8.45 | -91.28 - 74.38 | 0.839 | surface [C] | -20.93 | -96.78 - 54.93 | 0.584 | surface [C] | 11.10 | -105.47 - 127.67 | 0.850 |
| BOP | -1.60 | -18.97 - 15.77 | 0.842 | GI | -26-96 | -74.17 - 20.25 | 0.232 | SLI | -49.93 | -139.74 - 39.88 | 0.244 | OLI | -4.48 | -20.38 - 11.41 | 0.544 |
| time [day 60] × surface [B] | -50.78 | -132.72 - 31.16 | 0.221 | time [day 60] × surface [B] | -50.63 | -132.48 - 31.21 | 0.221 | time [day 60] × surface [B] | -51.32 | -34.61 - 31.97 | 0.223 | time [day 60] × surface [B] | -51.19 | -134.25 - 31.88 | 0.223 |
| time [day 60] × surface [C] | 25.86 | -59.39 - 111.12 | 0.547 | time [day 60] × surface [C] | 25.86 | -59.30 - 111.02 | 0.547 | time [day 60] × surface [C] | 25.86 | -60.81 - 112.54 | 0.554 | time [day 60] × surface [C] | 25.86 | -60.58 - 112.30 | 0.553 |
| surface [B] x BOP | 4.04 | -7.13 - 15.21 | 0.473 | surface [B] x GI | 22.05 | -13.40 - 57.51 | 0.219 | surface [B] x SLI | -2.07 | -75.81 - 71.67 | 0.956 | surface [B] x OLI | -2.72 | -13.91 - 8.46 | 0.629 |
| surface [C] x BOP | -4.48 | -16.64 - 7.68 | 0.465 | surface [C] x GI | -8.33 | -44.36 - 27.69 | 0.646 | surface [C] x SLI | -2.81 | -79.72 - 74.10 | 0.942 | surface [C] x OLI | -4.76 | -18.07 - 8.55 | 0.478 |
| N _seq_ | 3 |  |  | N _seq_ | 3 |  |  | N _seq_ | 3 |  |  | N _seq_ | 3 |  |  |
| N _patient_ | 14 |  |  | N _patient_ | 14 |  |  | N _patient_ | 14 |  |  | N _patient_ | 14 |  |  |
| Observations | 91 |  |  | Observations | 91 |  |  | Observations | 91 |  |  | Observations | 91 |  |  |
| R2m | 0.026 |  |  | R2m | 0.032 |  |  | R2m | 0.041 |  |  | R2m | 0.031 |  |  |
| R2c | 0.94 |  |  | R2c | 0.94 |  |  | R2c | 0.94 |  |  | R2c | 0.094 |  |  |

**Supplementary material 4.** *Table continuation.*

| **Ŝ_Chao1_** | | | | | | | | | | | | | | | |
| --- | --- | --- | --- | --- | --- | --- | --- | --- | --- | --- | --- | --- | --- | --- | --- |
| *Predictors* | *Estimates* | *CI* | *p* | *Predictors* | *Estimates* | *CI* | *p* | *Predictors* | *Estimates* | *CI* | *p* | *Predictors* | *Estimates* | *CI* | *p* |
| (Intercept) | 491.11 | -143.18 - 1125.40 | **0.127** | (Intercept) | 539.43 | -77.02 - 1155.88 | **0.085** | (Intercept) | 549.04 | -67.22 - 1165.30 | **0.080** | (Intercept) | 555.86 | -75.14 - 1186.86 | **0.083** |
| time [day 60] | 142.33 | 55.81 - 228.84 | **0.002^**^** | time [day 60] | 142.33 | 55.33 - 229.33 | **0.002^**^** | time [day 60] | 142.33 | 52.91 - 231.74 | **0.002^**^** | time [day 60] | 142.33 | 53.18 - 231.47 | **0.002^**^** |
| surface [B] | -25.31 | -182.73 - 132.10 | 0.749 | surface [B] | -18.14 | -137.94 - 101.65 | 0.764 | surface [B] | 27.98 | -90.53 - 146.48 | 0.639 | surface [B] | 39.02 | -111.90 - 189.94 | 0.608 |
| surface [C] | 43.78 | -110.25 - 197.82 | 0.573 | surface [C] | 6.46 | -115.44 - 128.36 | 0.916 | surface [C] | -41.67 | -154.37 - 71.04 | 0.463 | surface [C] | 1.82 | -171.30 - 174.94 | 0.983 |
| BOP | 1.92 | -24.72 - 28.57 | 0.875 | GI | -25.50 | -98.48 - 47.48 | 0.454 | SLI | -69.2 | -205.11 - 66.62 | 0.283 | OLI | -7.46 | -31.55 - 16.62 | 0.506 |
| time [day 60] × surface [B] | -91.79 | -211.46 - 27.87 | 0.131 | time [day 60] × surface [B] | -91.71 | -212.05 - 28.63 | 0.133 | time [day 60] × surface [B] | -92.42 | -216.08 - 31.25 | 0.141 | time [day 60] × surface [B] | -92.28 | -215.57 - 31.02 | 0.140 |
| time [day 60] × surface [C] | 37.29 | -87.23 - 161.80 | 0.552 | time [day 60] × surface [C] | 37.29 | -87.93 - 162.50 | 0.554 | time [day 60] × surface [C] | 37.29 | -91.40 - 165.98 | 0.565 | time [day 60] × surface [C] | 37.29 | -91.02 - 165.59 | 0.564 |
| surface [B] x BOP | 4.70 | -11.63 - 21.03 | 0.568 | surface [B] x GI | 21.82 | -30.35 - 74.00 | 0.407 | surface [B] x SLI | -4.74 | -114.47 - 104.99 | 0.932 | surface [B] x OLI | -3.00 | -19.61 - 13.61 | 0.720 |
| surface [C] x BOP | -13.31 | -31.08 - 4.46 | 0.140 | surface [C] x GI | -33.60 | -86.59 -19.39 | 0.210 | surface [C] x SLI | -9.16 | -123.55 - 105.24 | 0.874 | surface [C] x OLI | -6.82 | -26.59 - 12.95 | 0.494 |
| N _seq_ | 3 |  |  | N _seq_ | 3 |  |  | N _seq_ | 3 |  |  | N _seq_ | 3 |  |  |
| N _patient_ | 14 |  |  | N _patient_ | 14 |  |  | N _patient_ | 14 |  |  | N _patient_ | 14 |  |  |
| Observations | 91 |  |  | Observations | 91 |  |  | Observations | 91 |  |  | Observations | 91 |  |  |
| R2m | 0.017 |  |  | R2m | 0.021 |  |  | R2m | 0.028 |  |  | R2m | 0.021 |  |  |
| R2c | 0.95 |  |  | R2c | 0.95 |  |  | R2c | 0.95 |  |  | R2c | 0.95 |  |  |

**Supplementary material 4.** *Table continuation.*

| **H'** | | | | | | | | | | | | | | | |
| --- | --- | --- | --- | --- | --- | --- | --- | --- | --- | --- | --- | --- | --- | --- | --- |
| *Predictors* | *Estimates* | *CI* | *p* | *Predictors* | *Estimates* | *CI* | *p* | *Predictors* | *Estimates* | *CI* | *p* | *Predictors* | *Estimates* | *CI* | *p* |
| (Intercept) | 3.02 | 2.63 - 3.41 | **<0.001^***^** | (Intercept) | 2.94 | 2.61 - 3.26 | **<0.001^***^** | (Intercept) | 2.81 | 2.50 - 3.11 | **<0.001^***^** | (Intercept) | 2.82 | 2.43 - 3.21 | **<0.001^***^** |
| time [day 60] | 0.60 | 0.35 - 0.84 | **<0.001^***^** | time [day 60] | 0.60 | 0.36 - 0.83 | **<0.001^***^** | time [day 60] | 0.60 | 0.34 - 0.86 | **<0.001^***^** | time [day 60] | 0.60 | 0.34 - 0.85 | **<0.001^***^** |
| surface [B] | -0.53 | -0.96 - -0.10 | **0.016^*^** | surface [B] | -0.38 | -0.69 - 0.07 | **0.017^*^** | surface [B] | -0.01 | -0.34 - 0.86 | 0.963 | surface [B] | 0.24 | -0.18 - 0.67 | 0.259 |
| surface [C] | -0.40 | -0.84 - 0.03 | 0.067 | surface [C] | -0.30 | -0.63 - 0.02 | 0.067 | surface [C] | -0.18 | -0.50 - 0.15 | 0.276 | surface [C] | -0.31 | -0.79 - 0.16 | 0.195 |
| BOP | -0.05 | -0.09 - -0.00 | **0.033^*^** | GI | -0.17 | -0.29 - -0.04 | **0.013^*^** | SLI | -0.22 | -0.54 - 0.10 | 0.155 | OLI | -0.02 | -0.06 - 0.03 | 0.418 |
| time [day 60] × surface [B] | -0.17 | -0.51 - 0.17 | 0.317 | time [day 60] × surface [B] | -0.17 | -0.50 - 0.15 | 0.299 | time [day 60] × surface [B] | -0.18 | -0.54 - 0.18 | 0.322 | time [day 60] × surface [B] | -0.18 | -0.54 - 0.18 | 0.314 |
| time [day 60] × surface [C] | 0.04 | -0.31 - 0.39 | 0.827 | time [day 60] × surface [C] | 0.04 | -0.30 - 0.38 | 0.820 | time [day 60] × surface [C] | 0.04 | -0.34 - 0.41 | 0.837 | time [day 60] × surface [C] | 0.04 | -0.33 - 0.41 | 0.835 |
| surface [B] x BOP | 0.08 | 0.03 - 0.12 | **0.001^***^** | surface [B] x GI | 0.31 | 0.18 - 0.45 | **<0.001^***^** | surface [B] x SLI | 0.17 | -0.13 - 0.47 | 0.260 | surface [B] x OLI | -0.02 | -0.07 - 0.02 | 0.343 |
| surface [C] x BOP | 0.05 | 0.00 - 0.10 | **0.036^*^** | surface [C] x GI | 0.19 | 0.05 - 0.33 | **0.009^**^** | surface [C] x SLI | 0.26 | -0.05 - 0.58 | 0.102 | surface [C] x OLI | 0.04 | -0.02 - 0.09 | 0.170 |
| N _seq_ | 3 |  |  | N _seq_ | 3 |  |  | N _seq_ | 3 |  |  | N _seq_ | 3 |  |  |
| N _patient_ | 14 |  |  | N _patient_ | 14 |  |  | N _patient_ | 14 |  |  | N _patient_ | 14 |  |  |
| Observations | 91 |  |  | Observations | 91 |  |  | Observations | 91 |  |  | Observations | 91 |  |  |
| R2m | 0.36 |  |  | R2m | 0.40 |  |  | R2m | 0.33 |  |  | R2m | 0.36 |  |  |
| R2c | 0.56 |  |  | R2c | 0.59 |  |  | R2c | 0.49 |  |  | R2c | 0.50 |  |  |

**Supplementary material 4.** *Table continuation.*

| **D** | | | | | | | | | | | | | | | |
| --- | --- | --- | --- | --- | --- | --- | --- | --- | --- | --- | --- | --- | --- | --- | --- |
| *Predictors* | *Estimates* | *CI* | *p* | *Predictors* | *Estimates* | *CI* | *p* | *Predictors* | *Estimates* | *CI* | *p* | *Predictors* | *Estimates* | *CI* | *p* |
| (Intercept) | 0.95 | 0.90-1.00 | **<0.001^***^** | (Intercept) | 0.92 | 0.88-0.96 | **<0.001^***^** | (Intercept) | 0.89 | 0.85 - 0.93 | **<0.001^***^** | (Intercept) | 0.88 | 0.83 - 0.94 | **<0.001^***^** |
| time [day 60] | 0.05 | 0.02-0.09 | **0.007^**^** | time [day 60] | 0.05 | 0.02-0.09 | **0.004^**^** | time [day 60] | 0.05 | 0.01 - 0.10 | **0.011^*^** | time [day 60] | 0.05 | 0.01 - 0.10 | **0.013^*^** |
| surface [B] | -0.08 | -0.14 - -0.01 | **0.025^*^** | surface [B] | -0.07 | -0.12 - -0.02 | **0.006^**^** | surface [B] | -0.01 | -0.07 - 0.04 | 0.615 | surface [B] | 0.02 | -0.05 - 0.09 | 0.510 |
| surface [C] | -0.08 | -0.15 - -0.02 | **0.016*** | surface [C] | -0.06 | -0.11 - -0.01 | **0.022^*^** | surface [C] | -0.03 | -0.08 - 0.02 | 0.229 | surface [C] | -0.05 | -0.13 - 0.03 | 0.180 |
| BOP | -0.01 | -0.02 - -0.01 | **0.001^***^** | GI | -0.04 | -0.06 - -0.03 | **0.001^***^** | SLI | -0.06 | -0.11 - -0.01 | **0.023^*^** | OLI | -0.00 | -0.01 - 0.00 | 0.298 |
| time [day 60] × surface [B] | -0.02 | -0.07 - 0.03 | 0.469 | time [day 60] × surface [B] | -0.02 | -0.07 - 0.03 | 0.460 | time [day 60] × surface [B] | -0.02 | -0.08 - 0.04 | 0.486 | time [day 60] × surface [B] | -0.02 | -0.08 - 0.04 | 0.498 |
| time [day 60] × surface [C] | 0.00 | -0.06 - 0.06 | 0.983 | time [day 60] × surface [C] | 0.00 | -0.05 - 0.05 | 0.982 | time [day 60] × surface [C] | 0.00 | -0.06 - 0.06 | 0.984 | time [day 60] × surface [C] | 0.00 | -0.06 - 0.06 | 0.984 |
| surface [B] x BOP | 0.01 | 0.01 - 0.02 | **0.001^***^** | surface [B] x GI | 0.05 | 0.03 - 0.08 | **<0.001^***^** | surface [B] x SLI | 0.05 | 0.00 - 0.10 | **0.045^*^** | surface [B] x OLI | -0.00 | -0.01 - 0.01 | 0.642 |
| surface [C] x BOP | 0.01 | 0.00 - 0.02 | **0.002^**^** | surface [C] x GI | 0.04 | 0.02 - 0.07 | **<0.001^***^** | surface [C] x SLI | 0.07 | 0.02 - 0.12 | **0.011^*^** | surface [C] x OLI | 0.01 | -0.00 - 0.02 | 0.090 |
| N _seq_ | 3 |  |  | N _seq_ | 3 |  |  | N _seq_ | 3 |  |  | N _seq_ | 3 |  |  |
| N _patient_ | 14 |  |  | N _patient_ | 14 |  |  | N _patient_ | 14 |  |  | N _patient_ | 14 |  |  |
| Observations | 91 |  |  | Observations | 91 |  |  | Observations | 91 |  |  | Observations | 91 |  |  |
| R2m | 0.34 |  |  | R2m | 0.34 |  |  | R2m | 0.21 |  |  | R2m | 0.20 |  |  |
| R2c | 0.38 |  |  | R2c | 0.46 |  |  | R2c | 0.31 |  |  | R2c | 0.28 |  |  |

**Supplementary material 4.** *Table continuation.*

| **1/D** | | | | | | | | | | | | | | | |
| --- | --- | --- | --- | --- | --- | --- | --- | --- | --- | --- | --- | --- | --- | --- | --- |
| *Predictors* | *Estimates* | *CI* | *p* | *Predictors* | *Estimates* | *CI* | *p* | *Predictors* | *Estimates* | *CI* | *p* | *Predictors* | *Estimates* | *CI* | *p* |
| (Intercept) | 12.82 | 6.73 - 18.91 | **<0.001^***^** | (Intercept) | 11.82 | 6.76 - 16.88 | **<0.001^***^** | (Intercept) | 11.0 | 6.30 - 15.70 | **<0.001^***^** | (Intercept) | 9.31 | 3.12 - 15.51 | **0.004^**^** |
| time [day 60] | 6.20 | 2.47 - 9.92 | **0.001^***^** | time [day 60] | 6.20 | 2.51 - 9.88 | **0.001^***^** | time [day 60] | 6.20 | 2.32 - 10.07 | **0.002^**^** | time [day 60] | 6.20 | 2.34 - 10.05 | **0.002^**^** |
| surface [B] | -7.82 | -14.30 - -1.34 | **0.019^*^** | surface [B] | -5.15 | -10.00 - -0.31 | **0.037^*^** | surface [B] | -1.30 | -6.22 - 3.62 | 0.600 | surface [B] | 3.20 | -3.17 - 9.58 | 0.319 |
| surface [C] | -4.76 | -11.34 - 1.81 | 0.153 | surface [C] | -4.19 | -9.24 - 0.86 | 0.102 | surface [C] | -3.08 | -7.88 - 1.73 | 0.206 | surface [C] | -4.32 | -11.55 - 2.91 | 0.237 |
| BOP | -0.50 | -1.12 - 0.13 | 0.106 | GI | -1.75 | -3.64 - 0.14 | 0.066 | SLI | -3.31 | -8.04 - 1.43 | 0.151 | OLI | 0.01 | -0.65 - 0.68 | 0.962 |
| time [day 60] × surface [B] | -3.33 | -8.48 - 1.81 | 0.200 | time [day 60] × surface [B] | -3.34 | -8.43 - 1.76 | 0.196 | time [day 60] × surface [B] | -3.45 | -8.81 - 1.91 | 0.203 | time [day 60] × surface [B] | -3.48 | -8.81 - 1.84 | 0.196 |
| time [day 60] × surface [C] | 0.20 | -5.15- 5.56 | 0.940 | time [day 60] × surface [C] | 0.20 | -5.10 - 5.51 | 0.939 | time [day 60] × surface [C] | 0.20 | -5.38 - 5.78 | 0.942 | time [day 60] × surface [C] | 0.20 | -5.34 - 5.75 | 0.942 |
| surface [B] x BOP | 1.03 | 0.35 - 1.71 | **0.004^**^** | surface [B] x GI | 3.67 | -1.50 - 5.84 | **0.001^***^** | surface [B] x SLI | 2.85 | -1.65 - 7.35 | 0.211 | surface [B] x OLI | -0.41 | -1.10 - 0.29 | 0.245 |
| surface [C] x BOP | 0.52 | -0.24 - 1.27 | 0.177 | surface [C] x GI | 2.15 | -0.07 - 4.37 | 0.058 | surface [C] x SLI | 3.59 | -1.15 - 8.34 | 0.135 | surface [C] x OLI | 0.41 | -0.41 - 1.23 | 0.321 |
| N _seq_ | 3 |  |  | N _seq_ | 3 |  |  | N _seq_ | 3 |  |  | N _seq_ | 3 |  |  |
| N _patient_ | 14 |  |  | N _patient_ | 14 |  |  | N _patient_ | 14 |  |  | N _patient_ | 14 |  |  |
| Observations | 91 |  |  | Observations | 91 |  |  | Observations | 91 |  |  | Observations | 91 |  |  |
| R2m | 0.21 |  |  | R2m | 0.23 |  |  | R2m | 0.19 |  |  | R2m | 0.19 |  |  |
| R2c | 0.48 |  |  | R2c | 0.47 |  |  | R2c | 0.39 |  |  | R2c | 0.43 |  |  |
