# Supplementary material for: Effect of implant abutment surface treatments on bacterial biofilm composition and structure
Source: J Oral Microbiol. 2025 Feb 5;17(1):2459922. doi: 10.1080/20002297.2025.2459922 (PMC11800344; doi:10.1080/20002297.2025.2459922)
Supplement: Supplementary material.zip [file Supplementary material 1.pdf › Supplementary material 1.pdf]

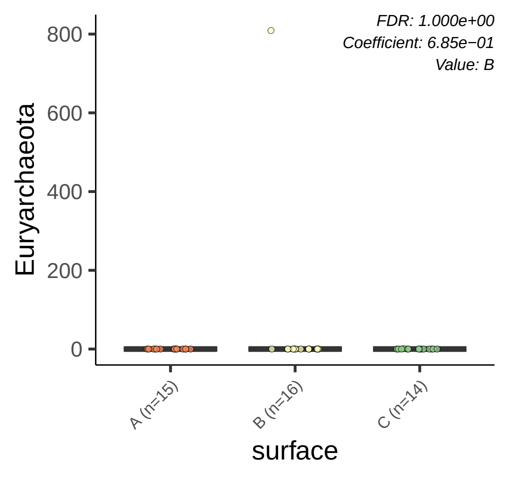

FDR: 1.000e+00
Coefficient: 6.85e−01
Value: B
Euryarchaeota
800
600
400
200
0
A (n=15)
B (n=16)
C (n=14)
surface

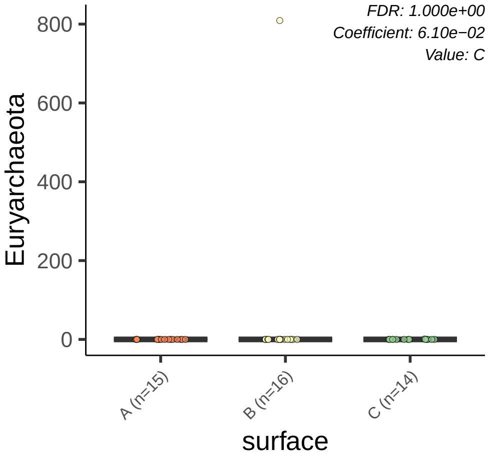
FDR: 1.000e+00
Coefficient: 6.10e−02
Value: C
Euryarchaeota
800
600
400
200
0
A (n=15)
B (n=16)
C (n=14)
surface

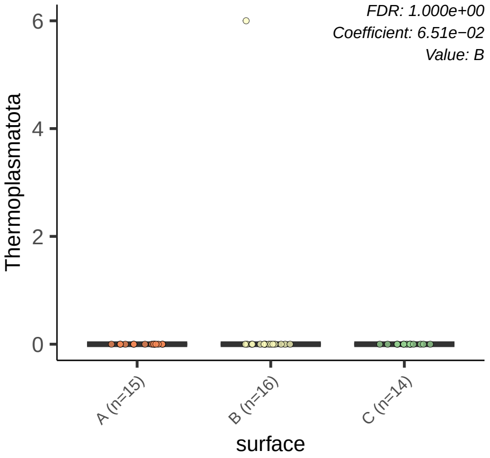
FDR: 1.000e+00
Coefficient: 6.51e−02
Value: B
Thermoplasmatota
6
4
2
0
A (n=15)
B (n=16)
C (n=14)
surface

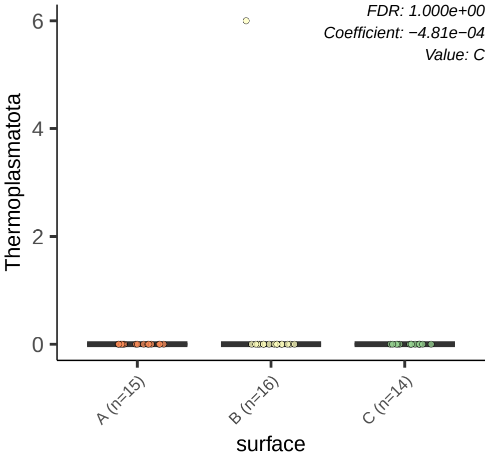
FDR: 1.000e+00
Coefficient: −4.81e−04
Value: C
Thermoplasmatota
6
4
2
0
A (n=15)
B (n=16)
C (n=14)
surface

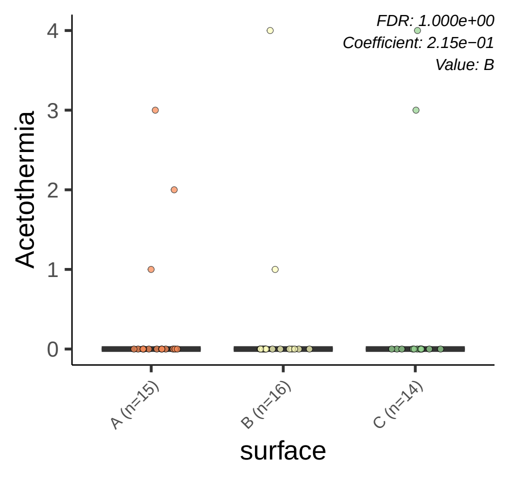

FDR: 1.000e+00
Coefficient: 2.15e−01
Value: B
Acetothermia
4
3
2
1
0
A (n=15)
B (n=16)
C (n=14)
surface

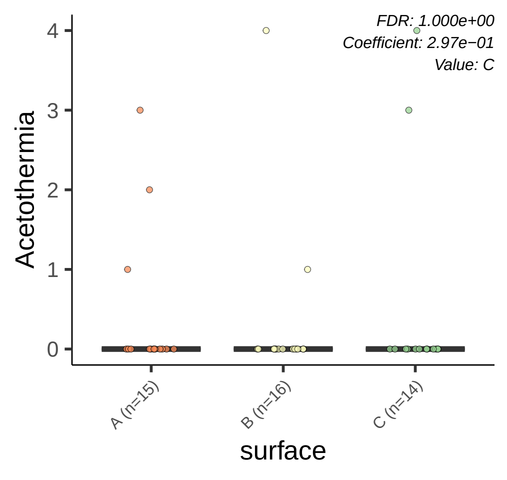

FDR: 1.000e+00
Coefficient: 2.97e−01
Value: C
Acetothermia
4
3
2
1
0
A (n=15)
B (n=16)
C (n=14)
surface

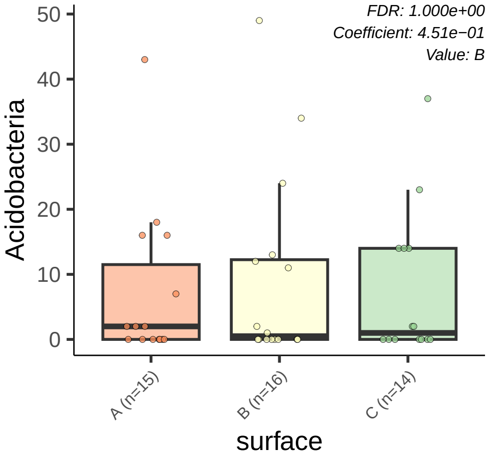

FDR: 1.000e+00
Coefficient: 4.51e−01
Value: B
Acidobacteria
0
10
20
30
40
50
A (n=15)
B (n=16)
C (n=14)
surface

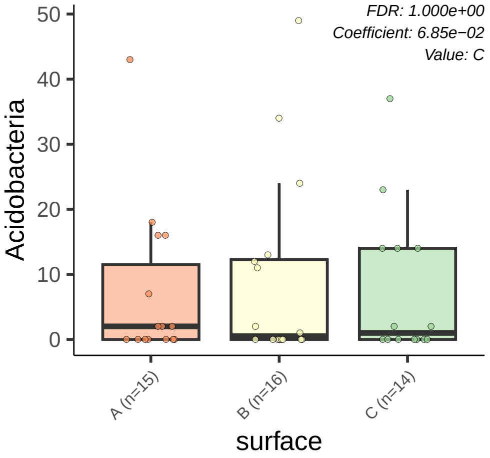

FDR: 1.000e+00
Coefficient: 6.85e−02
Value: C
Acidobacteria
50
40
30
20
10
0
A (n=15)
B (n=16)
C (n=14)
surface

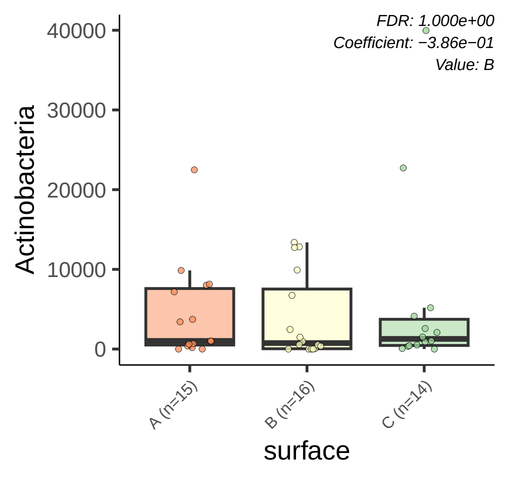
FDR: 1.000e+00
Coefficient: −3.86e−01
Value: B
Actinobacteria
40000
30000
20000
10000
0
A (n=15)
B (n=16)
C (n=14)
surface

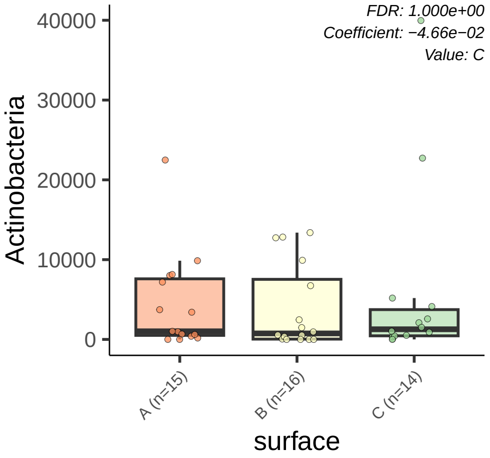
FDR: 1.000e+00
Coefficient: −4.66e−02
Value: C
Actinobacteria
40000
30000
20000
10000
0
A (n=15)
B (n=16)
C (n=14)
surface

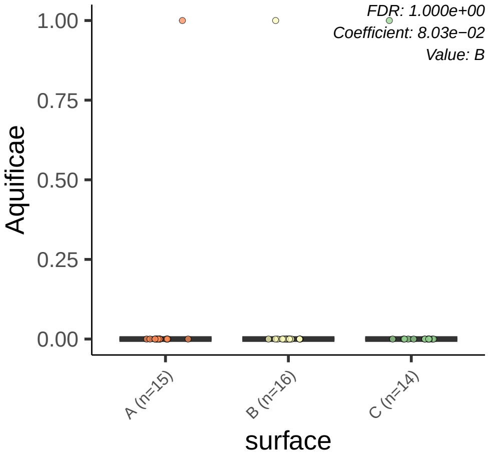

FDR: 1.000e+00
Coefficient: 8.03e−02
Value: B
Aquificae
1.00
0.75
0.50
0.25
0.00
A (n=15)
B (n=16)
C (n=14)
surface

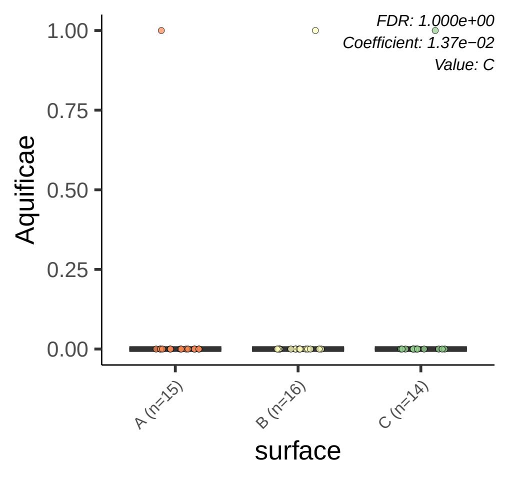

FDR: 1.000e+00
Coefficient: 1.37e−02
Value: C
Aquificae
1.00
0.75
0.50
0.25
0.00
A (n=15)
B (n=16)
C (n=14)
surface

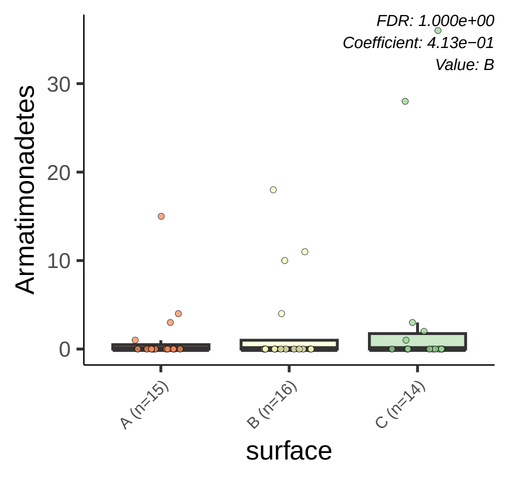
FDR: 1.000e+00
Coefficient: 4.13e−01
Value: B
Armatimonadetes
30
20
10
0
A (n=15)
B (n=16)
C (n=14)
surface

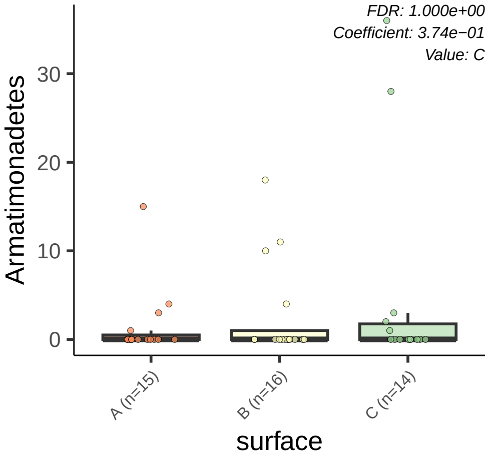

FDR: 1.000e+00
Coefficient: 3.74e−01
Value: C
Armatimonadetes
0
10
20
30
A (n=15)
B (n=16)
C (n=14)
surface

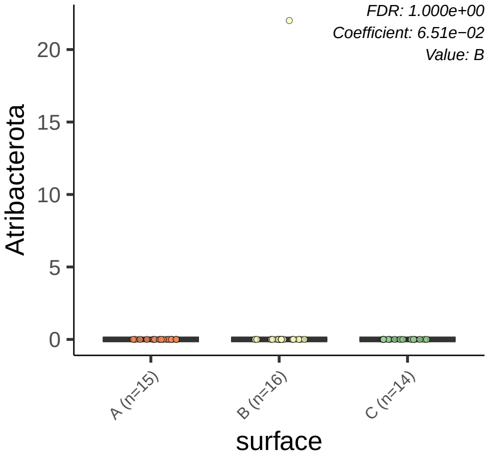

FDR: 1.000e+00
Coefficient: 6.51e−02
Value: B
Atribacterota
0
5
10
15
20
A (n=15)
B (n=16)
C (n=14)
surface

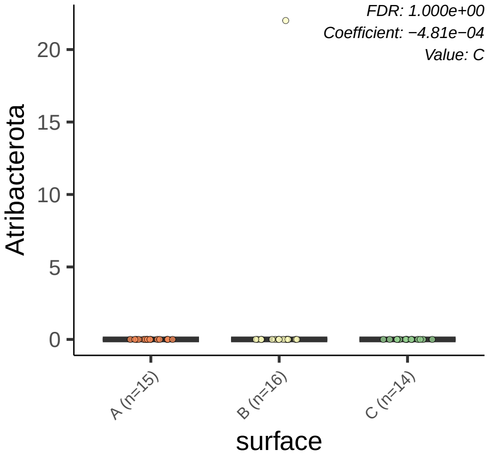
FDR: 1.000e+00
Coefficient: −4.81e−04
Value: C
Atribacterota
0
5
10
15
20
A (n=15)
B (n=16)
C (n=14)
surface

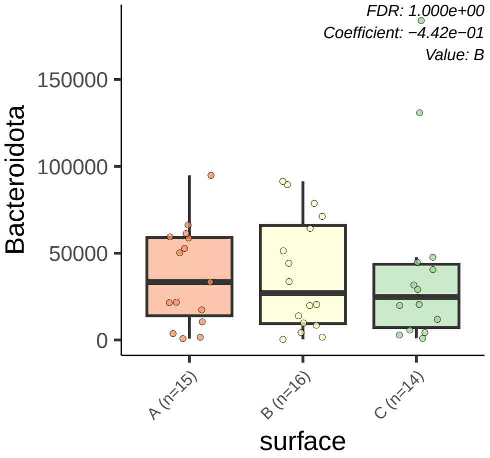
FDR: 1.000e+00
Coefficient: −4.42e−01
Value: B
Bacteroidota
150000
100000
50000
0
A (n=15)
B (n=16)
C (n=14)
surface

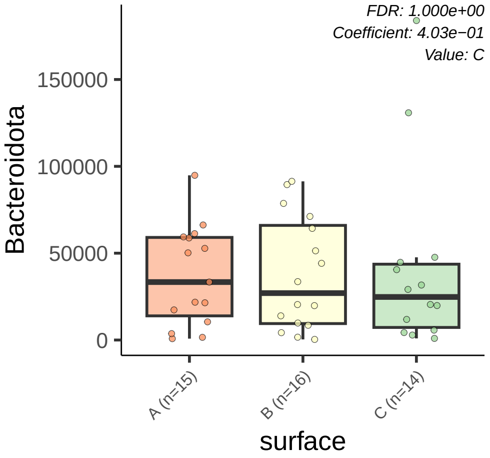
FDR: 1.000e+00
Coefficient: 4.03e−01
Value: C
Bacteroidota
150000
100000
50000
0
A (n=15)
B (n=16)
C (n=14)
surface

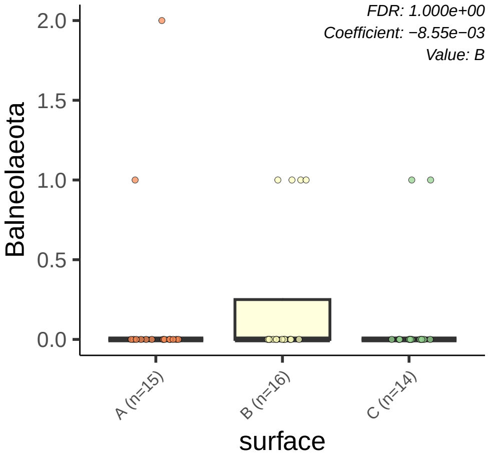

FDR: 1.000e+00
Coefficient: −8.55e−03
Value: B
Balneolaeota
2.0
1.5
1.0
0.5
0.0
A (n=15)
B (n=16)
C (n=14)
surface

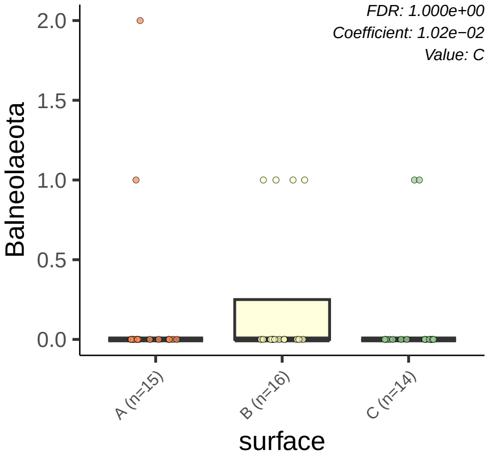
FDR: 1.000e+00
Coefficient: 1.02e−02
Value: C
Balneolaeota
2.0
1.5
1.0
0.5
0.0
A (n=15)
B (n=16)
C (n=14)
surface

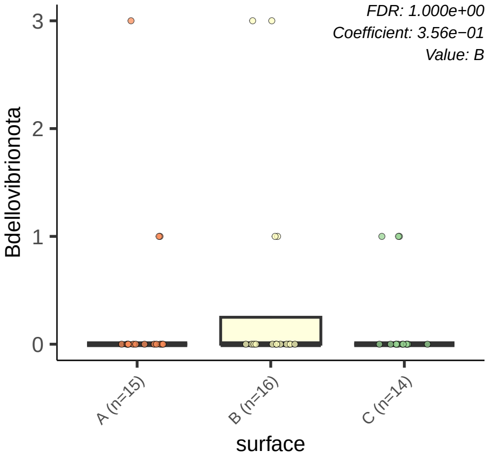
FDR: 1.000e+00
Coefficient: 3.56e−01
Value: B
Bdellovibrionota
3
2
1
0
A (n=15)
B (n=16)
C (n=14)
surface

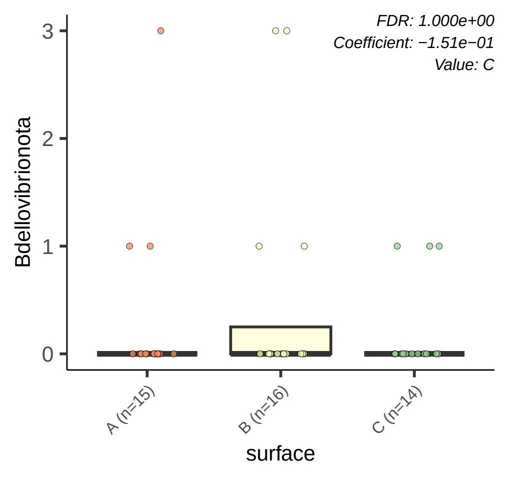
FDR: 1.000e+00
Coefficient: −1.51e−01
Value: C
Bdellovibrionota
3
2
1
0
A (n=15)
B (n=16)
C (n=14)
surface

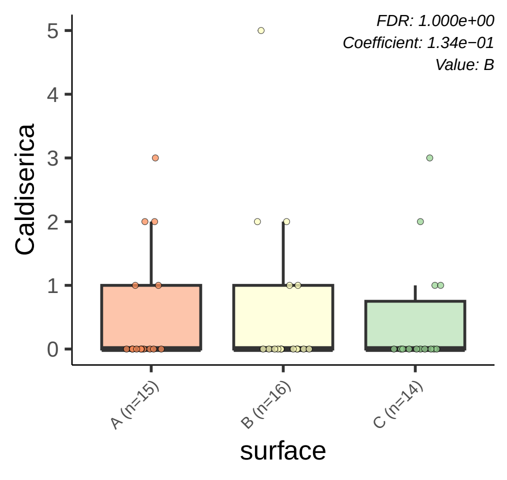
FDR: 1.000e+00
Coefficient: 1.34e−01
Value: B
Caldiserica
0
1
2
3
4
5
A (n=15)
B (n=16)
C (n=14)
surface

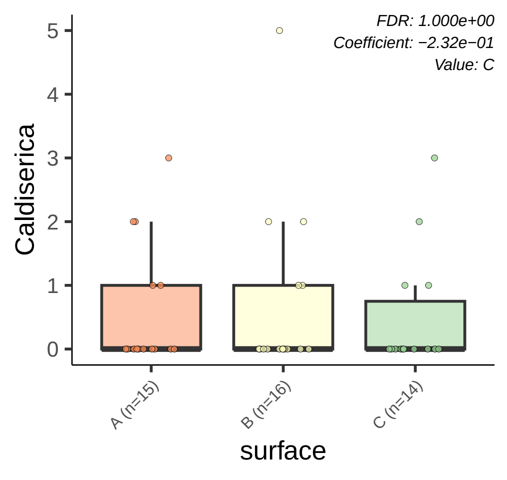
FDR: 1.000e+00
Coefficient: −2.32e−01
Value: C
Caldiserica
0
1
2
3
4
5
A (n=15)
B (n=16)
C (n=14)
surface

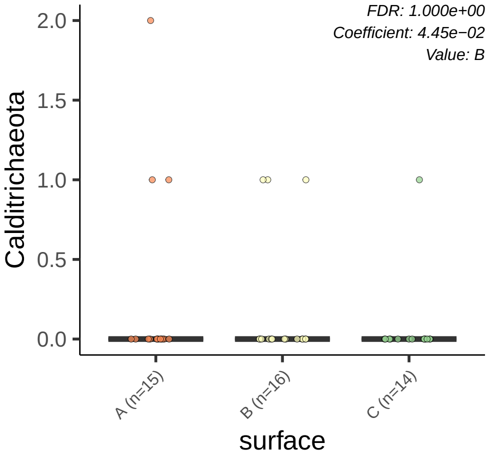
FDR: 1.000e+00
Coefficient: 4.45e−02
Value: B
Calditrichaeota
2.0
1.5
1.0
0.5
0.0
A (n=15)
B (n=16)
C (n=14)
surface

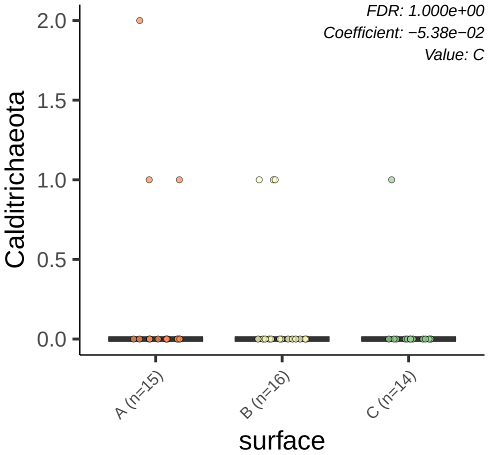

FDR: 1.000e+00
Coefficient: −5.38e−02
Value: C
Calditrichaeota
2.0
1.5
1.0
0.5
0.0
A (n=15)
B (n=16)
C (n=14)
surface

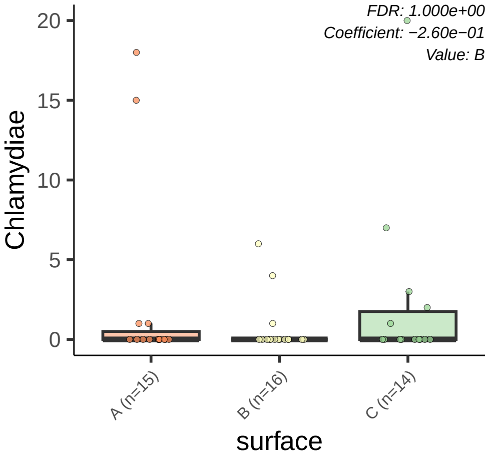
FDR: 1.000e+00
Coefficient: −2.60e−01
Value: B
Chlamydiae
0
5
10
15
20
A (n=15)
B (n=16)
C (n=14)
surface

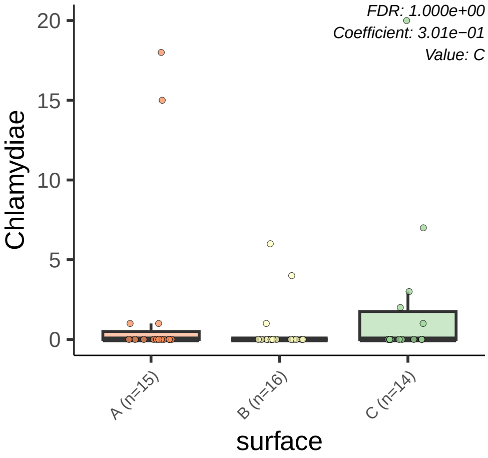

FDR: 1.000e+00
Coefficient: 3.01e−01
Value: C
Chlamydiae
20
15
10
5
0
A (n=15)
B (n=16)
C (n=14)
surface

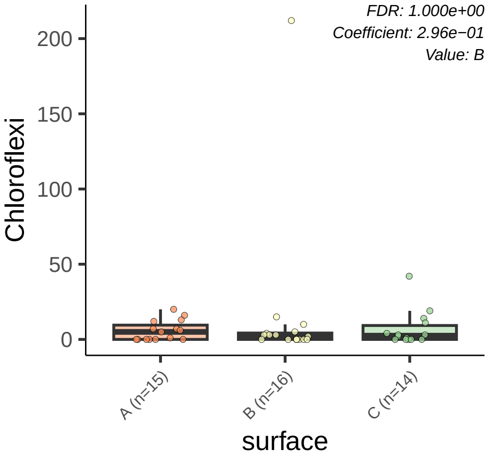

FDR: 1.000e+00
Coefficient: 2.96e−01
Value: B
Chloroflexi
200
150
100
50
0
A (n=15)
B (n=16)
C (n=14)
surface

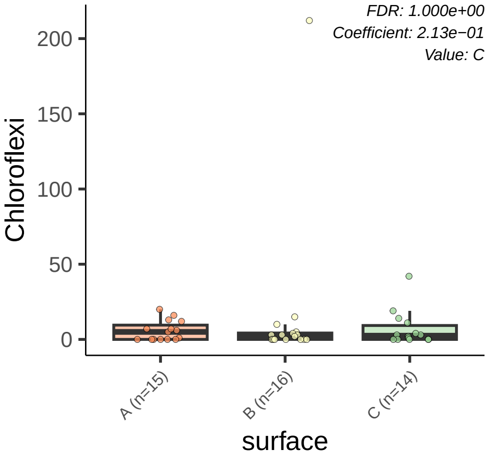
FDR: 1.000e+00
Coefficient: 2.13e−01
Value: C
Chloroflexi
200
150
100
50
0
A (n=15)
B (n=16)
C (n=14)
surface

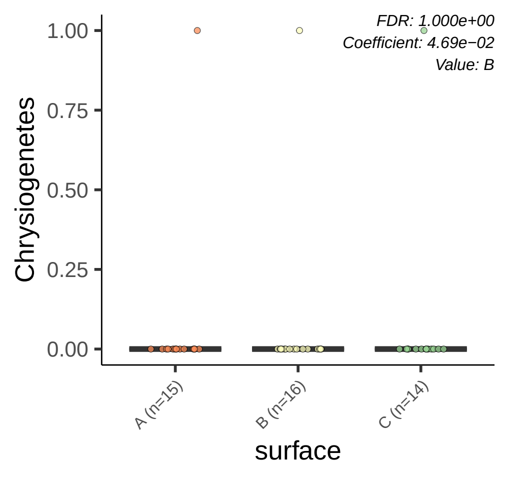

FDR: 1.000e+00
Coefficient: 4.69e−02
Value: B
Chrysiogenetes
1.00
0.75
0.50
0.25
0.00
A (n=15)
B (n=16)
C (n=14)
surface

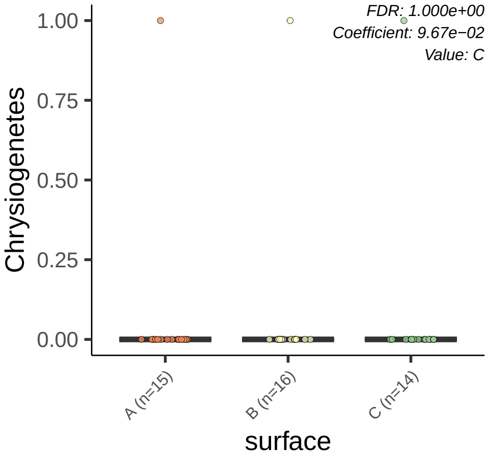
FDR: 1.000e+00
Coefficient: 9.67e−02
Value: C
Chrysiogenetes
1.00
0.75
0.50
0.25
0.00
A (n=15)
B (n=16)
C (n=14)
surface

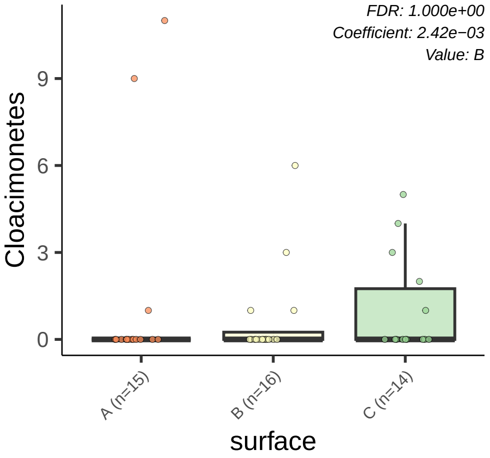
FDR: 1.000e+00
Coefficient: 2.42e−03
Value: B
Cloacimonetes
0
3
6
9
A (n=15)
B (n=16)
C (n=14)
surface

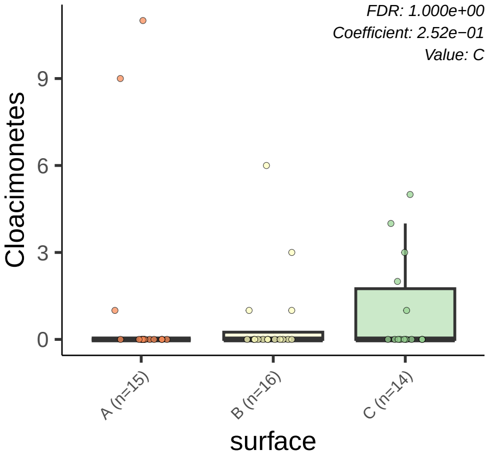
FDR: 1.000e+00
Coefficient: 2.52e−01
Value: C
Cloacimonetes
0
3
6
9
A (n=15)
B (n=16)
C (n=14)
surface

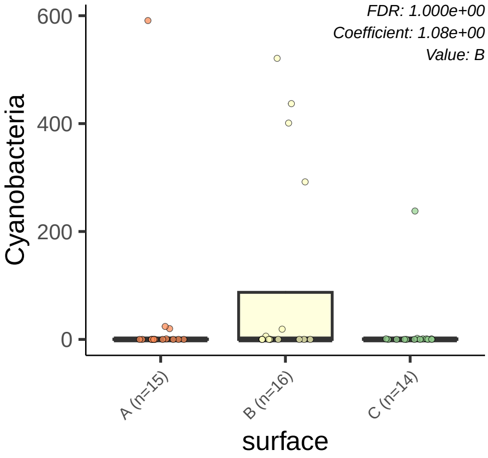
FDR: 1.000e+00
Coefficient: 1.08e+00
Value: B
Cyanobacteria
600
400
200
0
A (n=15)
B (n=16)
C (n=14)
surface

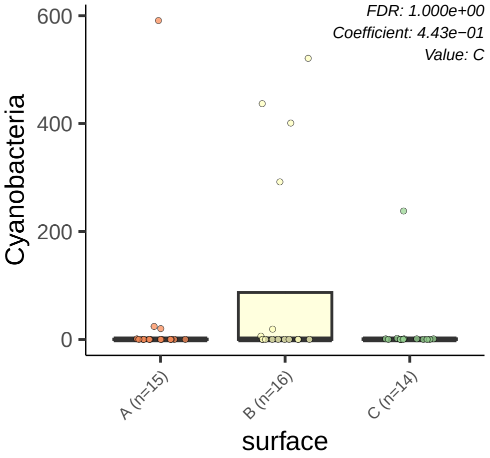

FDR: 1.000e+00
Coefficient: 4.43e−01
Value: C
Cyanobacteria
600
400
200
0
A (n=15)
B (n=16)
C (n=14)
surface

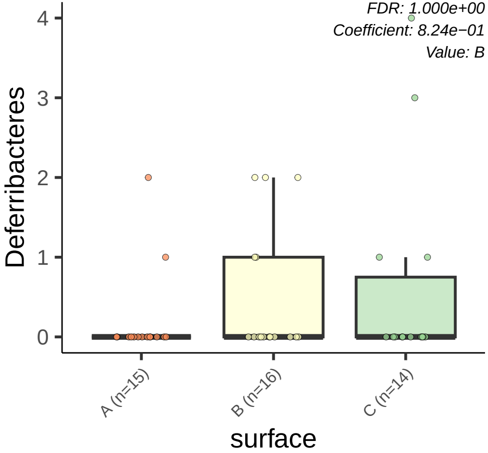
FDR: 1.000e+00
Coefficient: 8.24e−01
Value: B
Deferribacteres
0
1
2
3
4
A (n=15)
B (n=16)
C (n=14)
surface

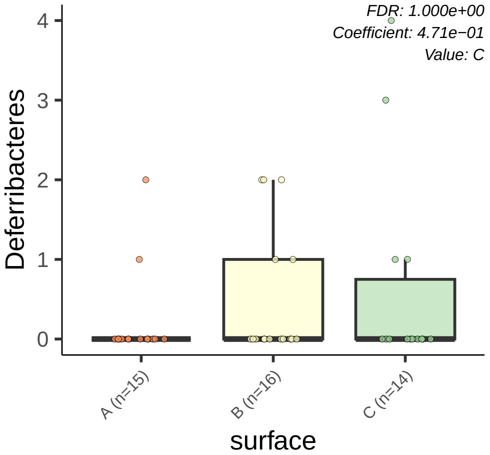
FDR: 1.000e+00
Coefficient: 4.71e−01
Value: C
Deferribacteres
0
1
2
3
4
A (n=15)
B (n=16)
C (n=14)
surface

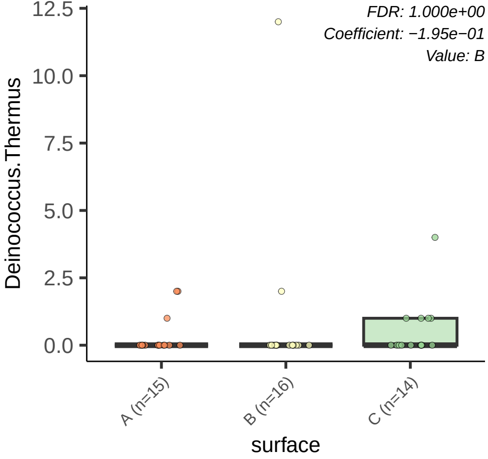
FDR: 1.000e+00
Coefficient: −1.95e−01
Value: B
Deinococcus.Thermus
12.5
10.0
7.5
5.0
2.5
0.0
A (n=15)
B (n=16)
C (n=14)
surface

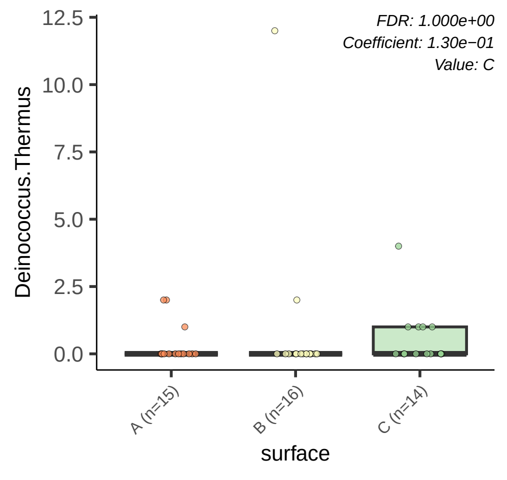

FDR: 1.000e+00
Coefficient: 1.30e−01
Value: C
12.5
10.0
7.5
5.0
2.5
0.0
Deinococcus.Thermus
A (n=15)
B (n=16)
C (n=14)
surface

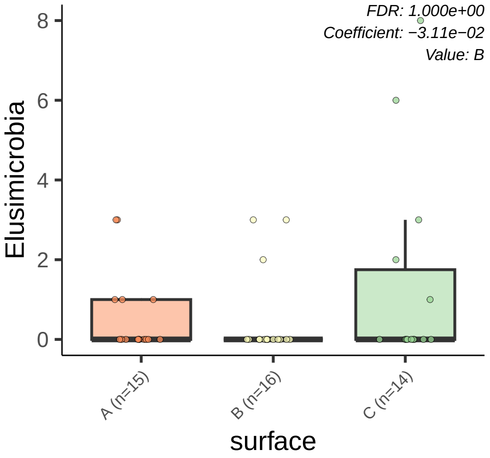

FDR: 1.000e+00
Coefficient: −3.11e−02
Value: B
Elusimicrobia
0
2
4
6
8
A (n=15)
B (n=16)
C (n=14)
surface

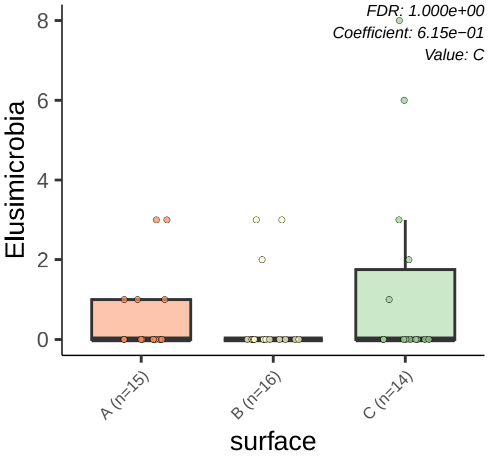
FDR: 1.000e+00
Coefficient: 6.15e−01
Value: C
Elusimicrobia
0
2
4
6
8
A (n=15)
B (n=16)
C (n=14)
surface

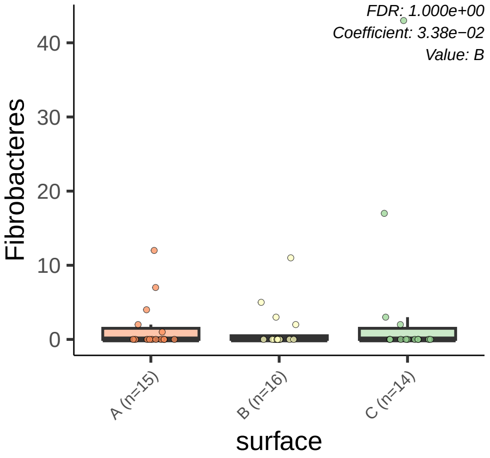
FDR: 1.000e+00
Coefficient: 3.38e−02
Value: B
Fibrobacteres
40
30
20
10
0
A (n=15)
B (n=16)
C (n=14)
surface

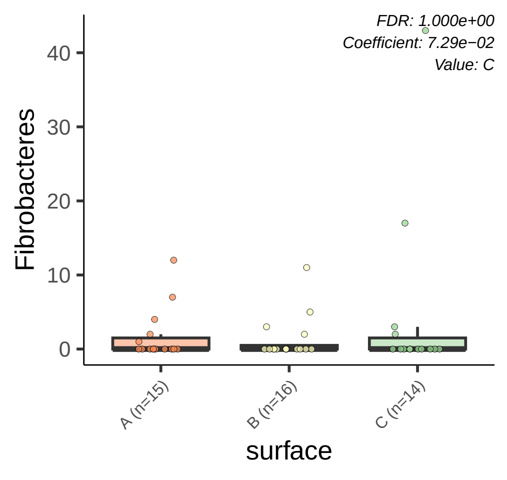

FDR: 1.000e+00
Coefficient: 7.29e−02
Value: C
Fibrobacteres
0
10
20
30
40
A (n=15)
B (n=16)
C (n=14)
surface

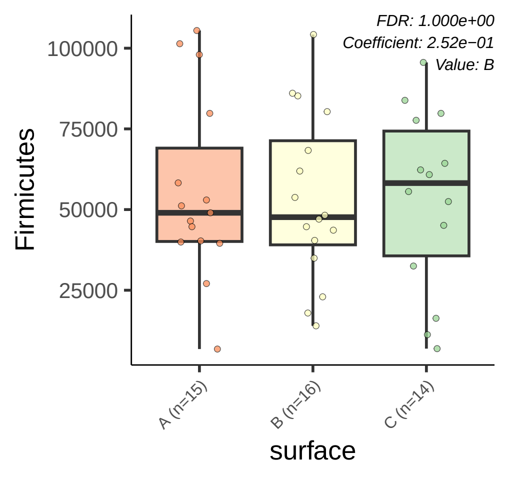
FDR: 1.000e+00
Coefficient: 2.52e−01
Value: B
Firmicutes
100000
75000
50000
25000
A (n=15)
B (n=16)
C (n=14)
surface

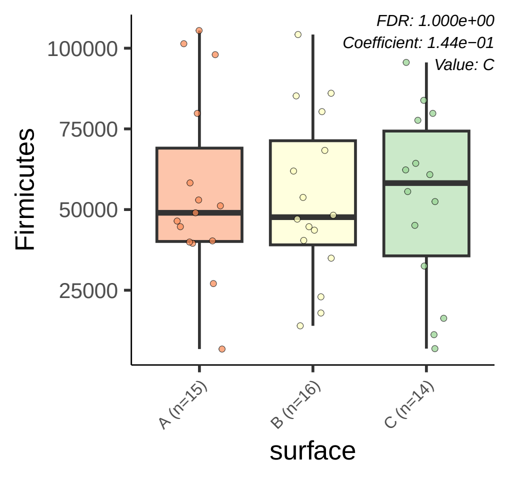
FDR: 1.000e+00
Coefficient: 1.44e−01
Value: C
Firmicutes
100000
75000
50000
25000
A (n=15)
B (n=16)
C (n=14)
surface

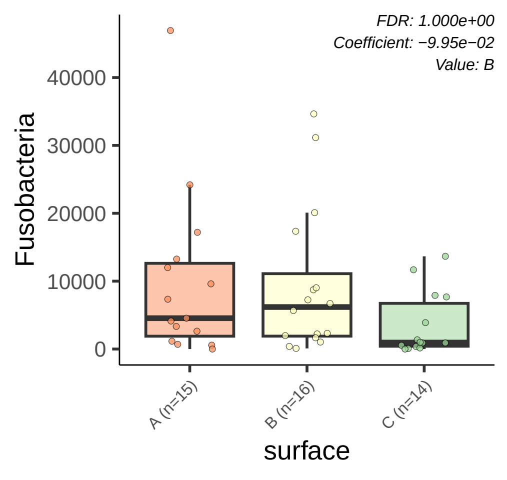

FDR: 1.000e+00
Coefficient: −9.95e−02
Value: B
Fusobacteria
40000
30000
20000
10000
0
A (n=15)
B (n=16)
C (n=14)
surface

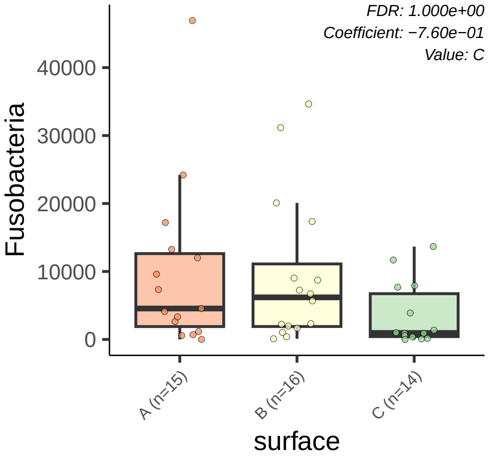

FDR: 1.000e+00
Coefficient: −7.60e−01
Value: C
Fusobacteria
40000
30000
20000
10000
0
A (n=15)
B (n=16)
C (n=14)
surface

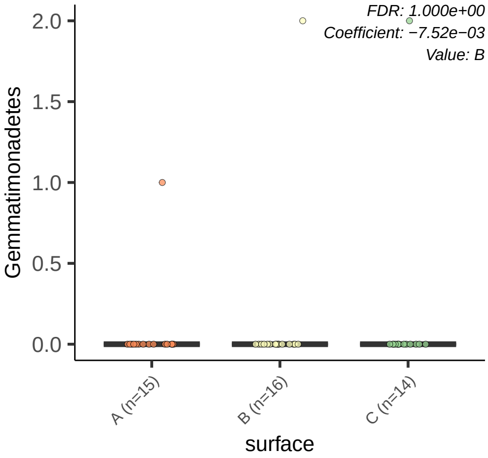
FDR: 1.000e+00
Coefficient: −7.52e−03
Value: B
Gemmatimonadetes
2.0
1.5
1.0
0.5
0.0
A (n=15)
B (n=16)
C (n=14)
surface

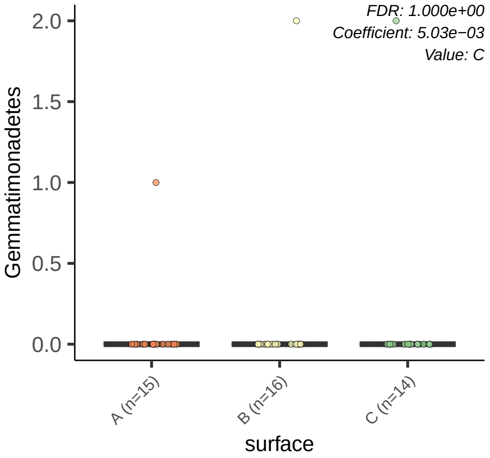

FDR: 1.000e+00
Coefficient: 5.03e−03
Value: C
2.0
1.5
1.0
0.5
0.0
Gemmatimonadetes
A (n=15)
B (n=16)
C (n=14)
surface

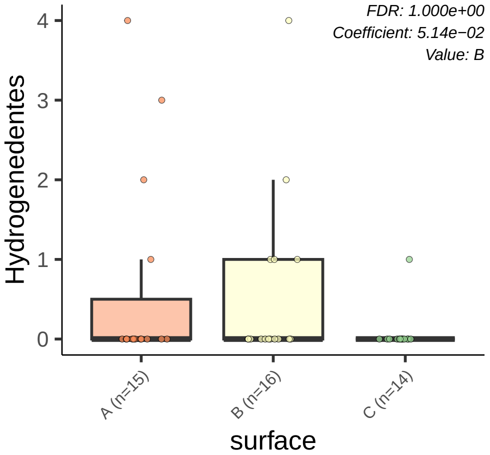
FDR: 1.000e+00
Coefficient: 5.14e−02
Value: B
Hydrogenedentes
0
1
2
3
4
A (n=15)
B (n=16)
C (n=14)
surface

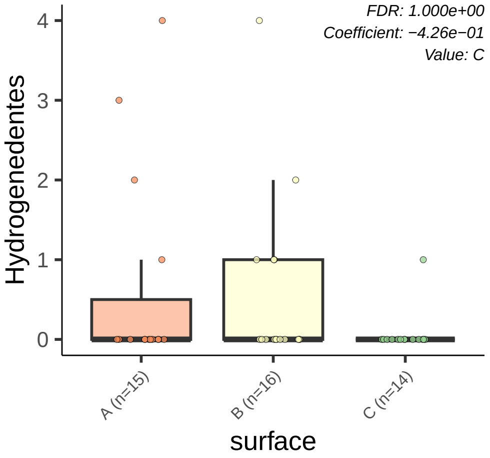
FDR: 1.000e+00
Coefficient: −4.26e−01
Value: C
Hydrogenedentes
0
1
2
3
4
A (n=15)
B (n=16)
C (n=14)
surface

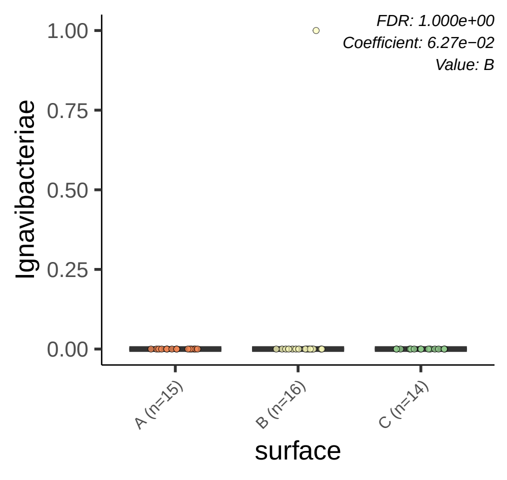

FDR: 1.000e+00
Coefficient: 6.27e−02
Value: B
Ignavibacteriae
1.00
0.75
0.50
0.25
0.00
A (n=15)
B (n=16)
C (n=14)
surface

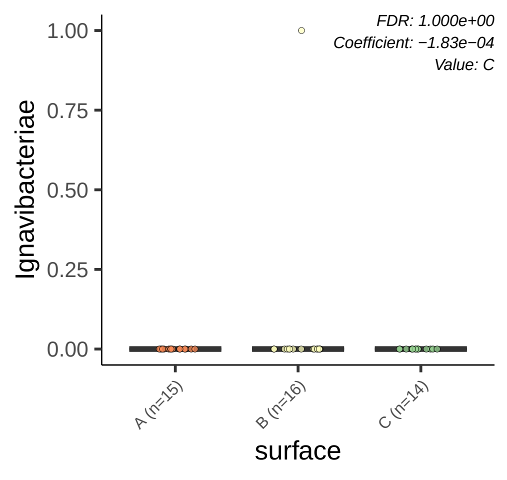
FDR: 1.000e+00
Coefficient: −1.83e−04
Value: C
Ignavibacteriae
1.00
0.75
0.50
0.25
0.00
A (n=15)
B (n=16)
C (n=14)
surface

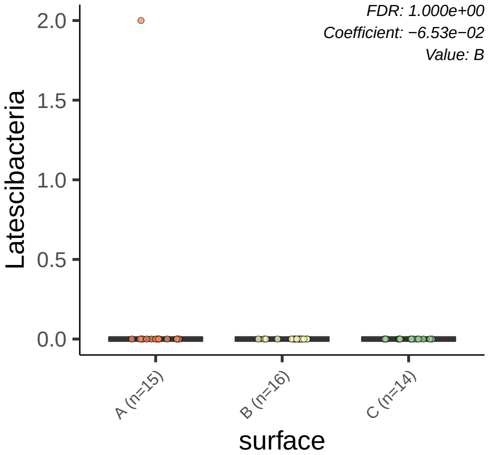
FDR: 1.000e+00
Coefficient: −6.53e−02
Value: B
Latescibacteria
2.0
1.5
1.0
0.5
0.0
A (n=15)
B (n=16)
C (n=14)
surface

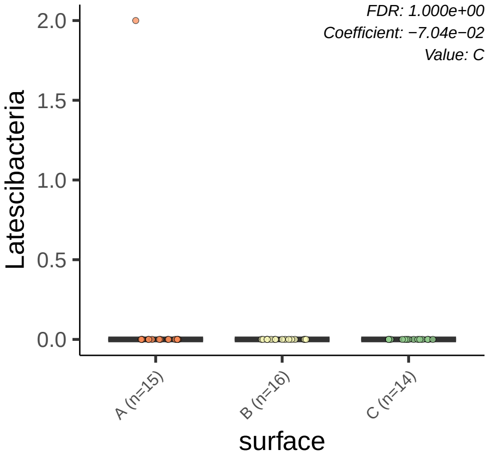

FDR: 1.000e+00
Coefficient: −7.04e−02
Value: C
Latescibacteria
2.0
1.5
1.0
0.5
0.0
A (n=15)
B (n=16)
C (n=14)
surface

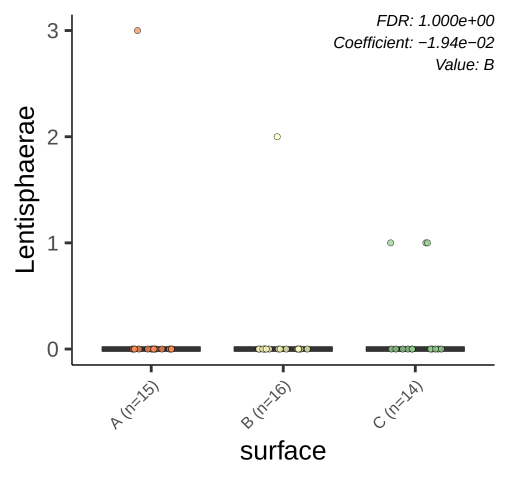
FDR: 1.000e+00
Coefficient: −1.94e−02
Value: B
Lentisphaerae
3
2
1
0
A (n=15)
B (n=16)
C (n=14)
surface

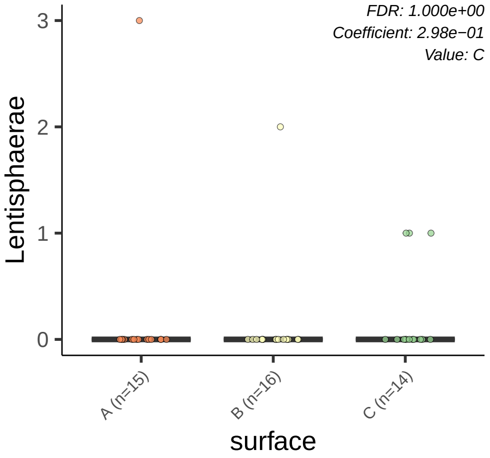

FDR: 1.000e+00
Coefficient: 2.98e−01
Value: C
Lentisphaerae
3
2
1
0
A (n=15)
B (n=16)
C (n=14)
surface

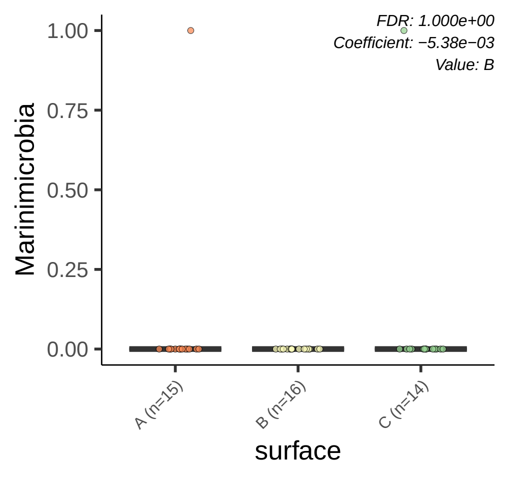
FDR: 1.000e+00
Coefficient: −5.38e−03
Value: B
Marinimicrobia
1.00
0.75
0.50
0.25
0.00
A (n=15)
B (n=16)
C (n=14)
surface

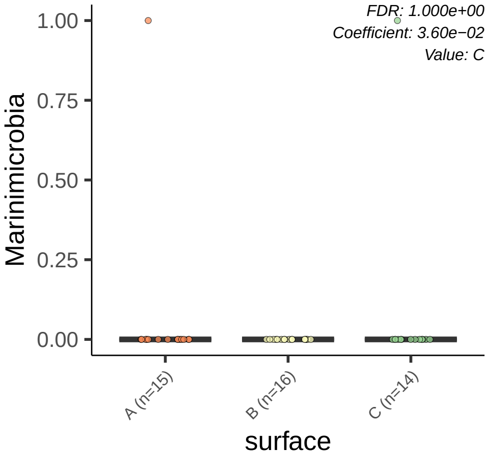

FDR: 1.000e+00
Coefficient: 3.60e−02
Value: C
Marinimicrobia
1.00
0.75
0.50
0.25
0.00
A (n=15)
B (n=16)
C (n=14)
surface

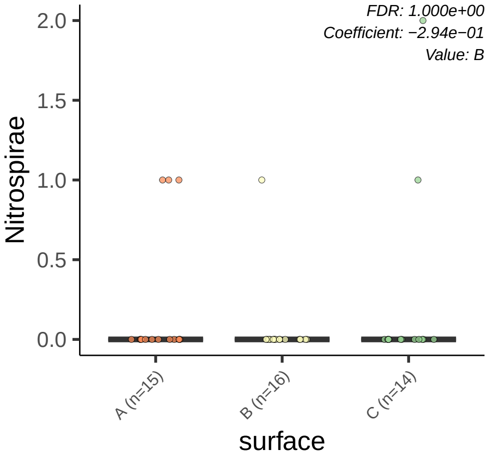
FDR: 1.000e+00
Coefficient: −2.94e−01
Value: B
Nitrospirae
2.0
1.5
1.0
0.5
0.0
A (n=15)
B (n=16)
C (n=14)
surface

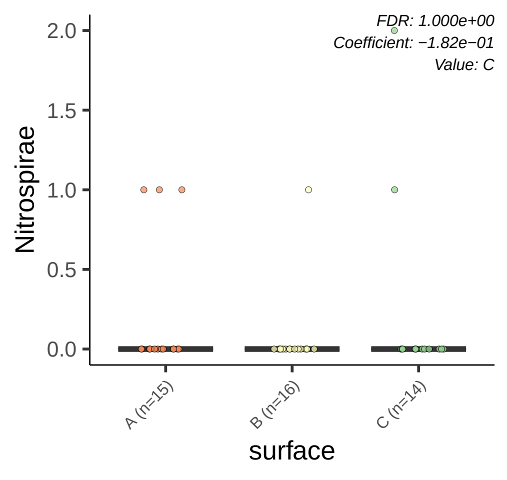
FDR: 1.000e+00
Coefficient: −1.82e−01
Value: C
Nitrospirae
2.0
1.5
1.0
0.5
0.0
A (n=15)
B (n=16)
C (n=14)
surface

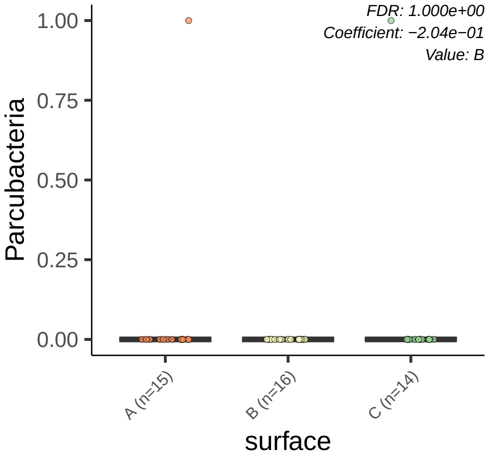
FDR: 1.000e+00
Coefficient: −2.04e−01
Value: B
Parcubacteria
1.00
0.75
0.50
0.25
0.00
A (n=15)
B (n=16)
C (n=14)
surface

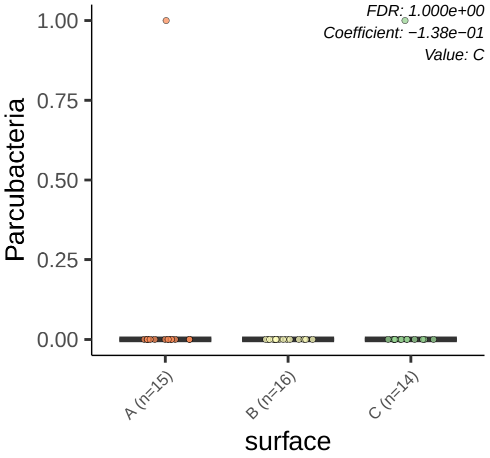

FDR: 1.000e+00
Coefficient: −1.38e−01
Value: C
Parcubacteria
1.00
0.75
0.50
0.25
0.00
A (n=15)
B (n=16)
C (n=14)
surface

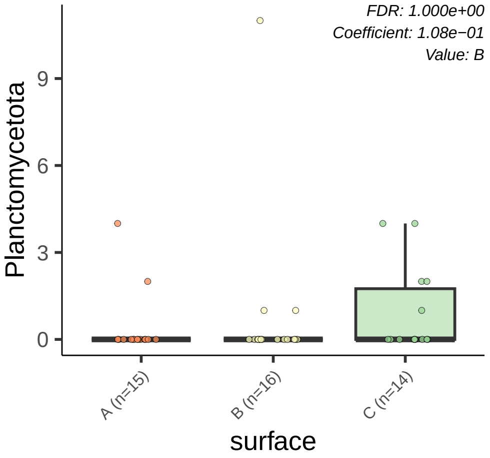
FDR: 1.000e+00
Coefficient: 1.08e−01
Value: B
Planctomycetota
9
6
3
0
A (n=15)
B (n=16)
C (n=14)
surface

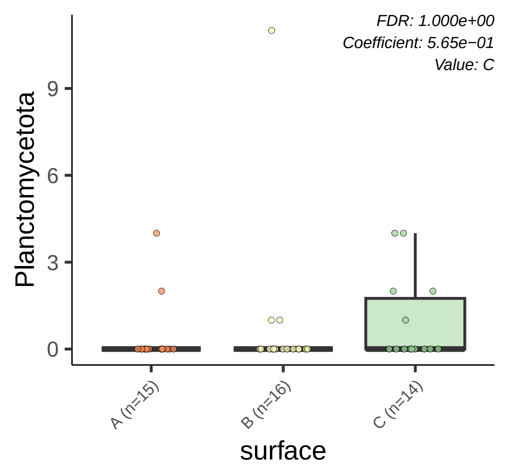

FDR: 1.000e+00
Coefficient: 5.65e−01
Value: C
Planctomycetota
0
3
6
9
A (n=15)
B (n=16)
C (n=14)
surface

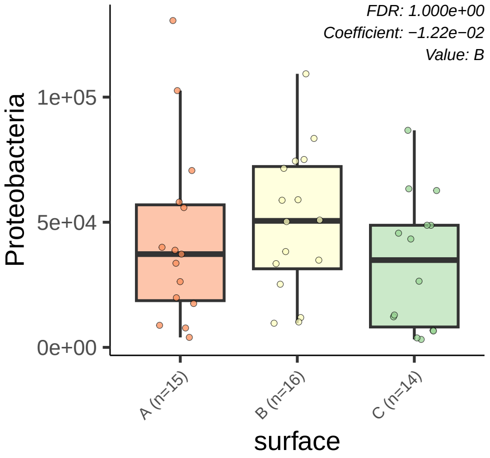

FDR: 1.000e+00
Coefficient: −1.22e−02
Value: B
Proteobacteria
1e+05
5e+04
0e+00
A (n=15)
B (n=16)
C (n=14)
surface

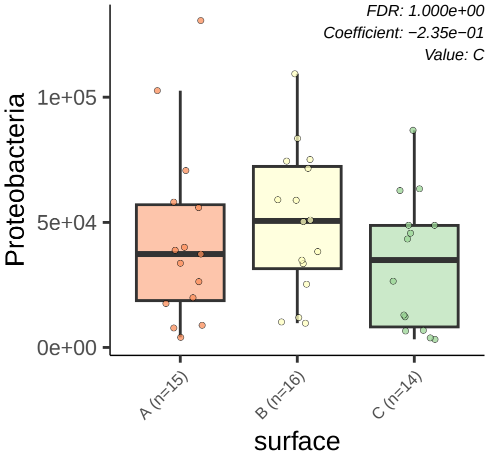

FDR: 1.000e+00
Coefficient: −2.35e−01
Value: C
Proteobacteria
1e+05
5e+04
0e+00
A (n=15)
B (n=16)
C (n=14)
surface

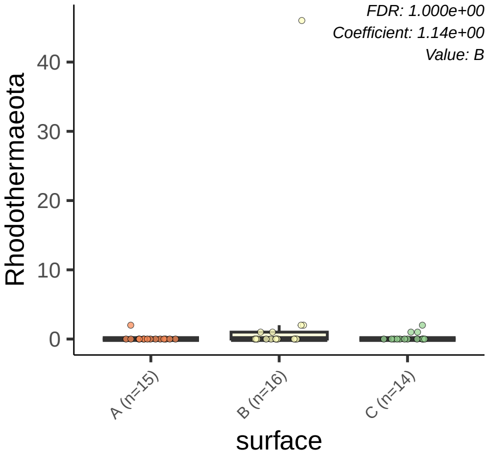

FDR: 1.000e+00
Coefficient: 1.14e+00
Value: B
Rhodothermaeota
0
10
20
30
40
A (n=15)
B (n=16)
C (n=14)
surface

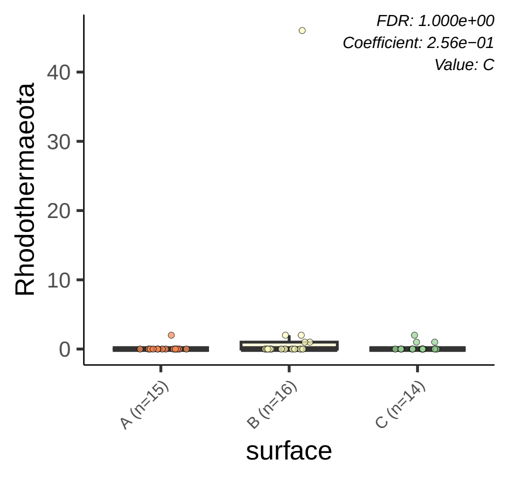

FDR: 1.000e+00
Coefficient: 2.56e−01
Value: C
Rhodothermaeota
0
10
20
30
40
A (n=15)
B (n=16)
C (n=14)
surface

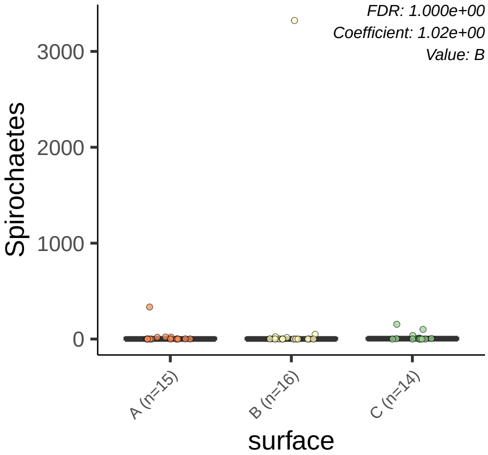
FDR: 1.000e+00
Coefficient: 1.02e+00
Value: B
Spirochaetes
3000
2000
1000
0
A (n=15)
B (n=16)
C (n=14)
surface

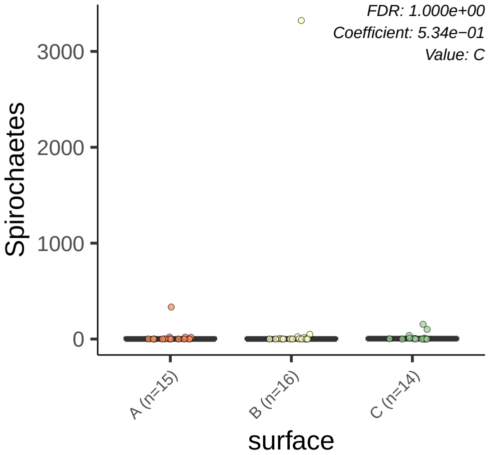
FDR: 1.000e+00
Coefficient: 5.34e−01
Value: C
Spirochaetes
3000
2000
1000
0
A (n=15)
B (n=16)
C (n=14)
surface

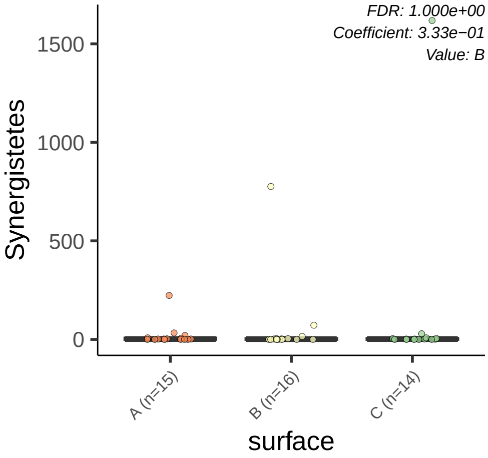
FDR: 1.000e+00
Coefficient: 3.33e−01
Value: B
Synergistetes
1500
1000
500
0
A (n=15)
B (n=16)
C (n=14)
surface

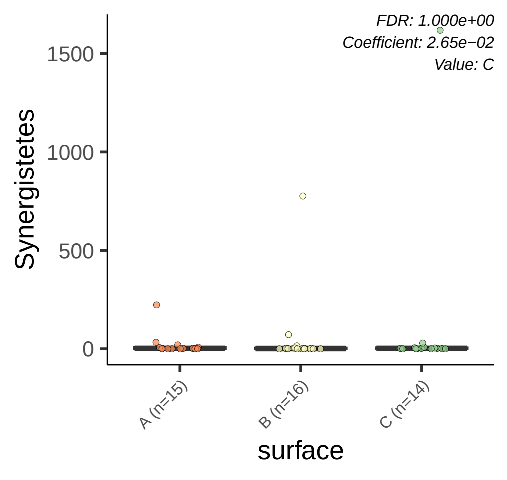

FDR: 1.000e+00
Coefficient: 2.65e−02
Value: C
Synergistetes
1500
1000
500
0
A (n=15)
B (n=16)
C (n=14)
surface

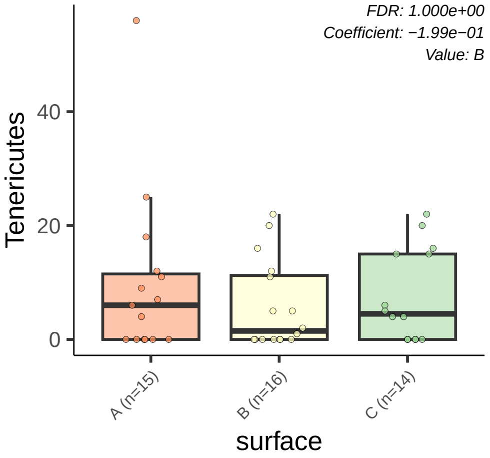

FDR: 1.000e+00
Coefficient: −1.99e−01
Value: B
Tenericutes
0
20
40
A (n=15)
B (n=16)
C (n=14)
surface

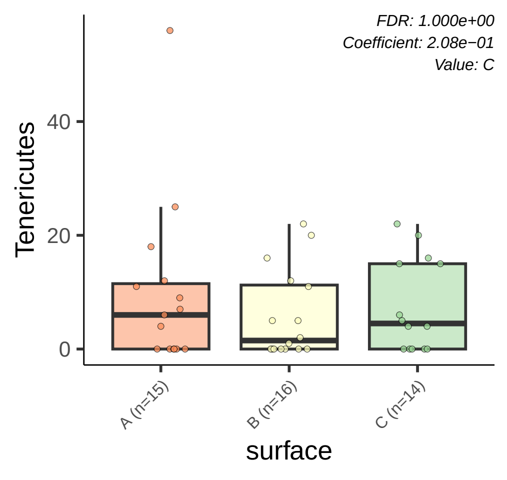
FDR: 1.000e+00
Coefficient: 2.08e−01
Value: C
Tenericutes
0
20
40
A (n=15)
B (n=16)
C (n=14)
surface

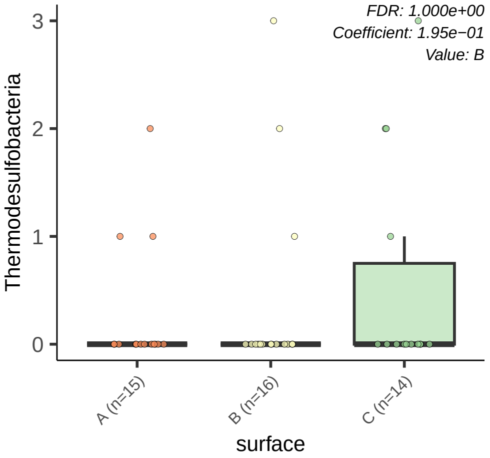
FDR: 1.000e+00
Coefficient: 1.95e−01
Value: B
Thermodesulfobacteria
3
2
1
0
A (n=15)
B (n=16)
C (n=14)
surface

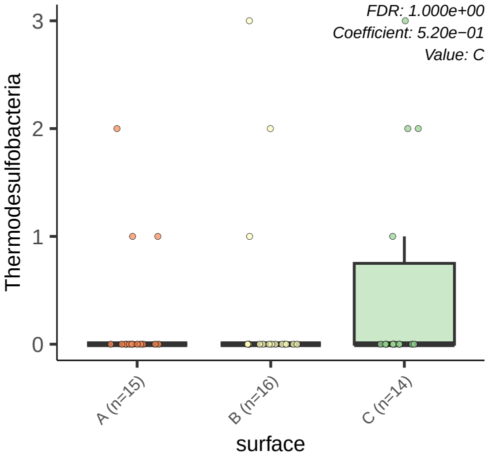

FDR: 1.000e+00
Coefficient: 5.20e−01
Value: C
Thermodesulfobacteria
3
2
1
0
A (n=15)
B (n=16)
C (n=14)
surface

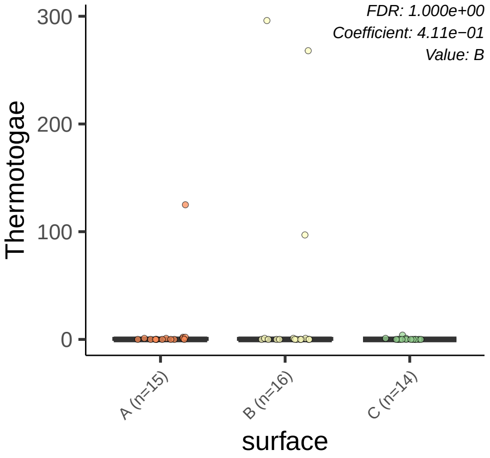
FDR: 1.000e+00
Coefficient: 4.11e−01
Value: B
Thermotogae
300
200
100
0
A (n=15)
B (n=16)
C (n=14)
surface

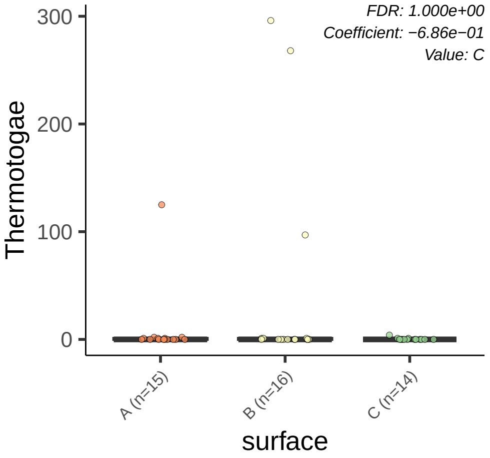
FDR: 1.000e+00
Coefficient: −6.86e−01
Value: C
300
200
100
0
Thermotogae
A (n=15)
B (n=16)
C (n=14)
surface

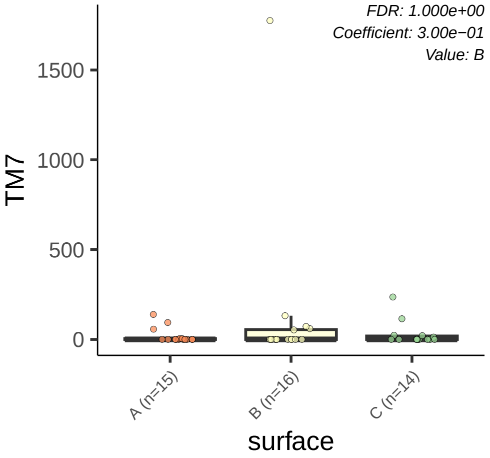

FDR: 1.000e+00
Coefficient: 3.00e−01
Value: B
TM7
1500
1000
500
0
A (n=15)
B (n=16)
C (n=14)
surface

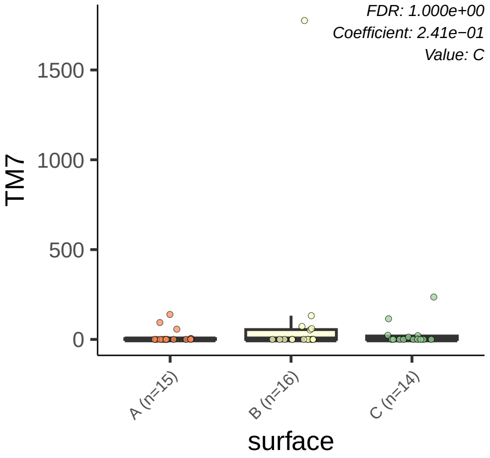

FDR: 1.000e+00
Coefficient: 2.41e−01
Value: C
TM7
1500
1000
500
0
A (n=15)
B (n=16)
C (n=14)
surface

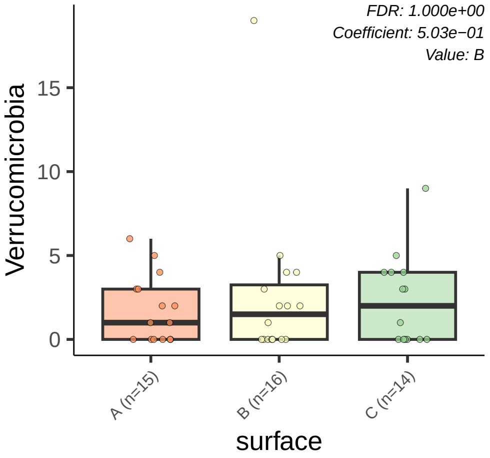
FDR: 1.000e+00
Coefficient: 5.03e−01
Value: B
Verrucomicrobia
15
10
5
0
A (n=15)
B (n=16)
C (n=14)
surface

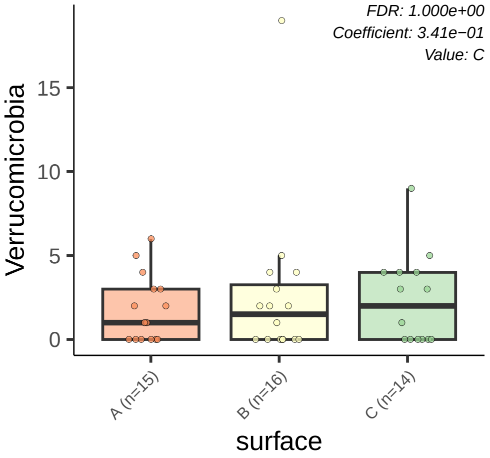

FDR: 1.000e+00
Coefficient: 3.41e−01
Value: C
Verrucomicrobia
15
10
5
0
A (n=15)
B (n=16)
C (n=14)
surface

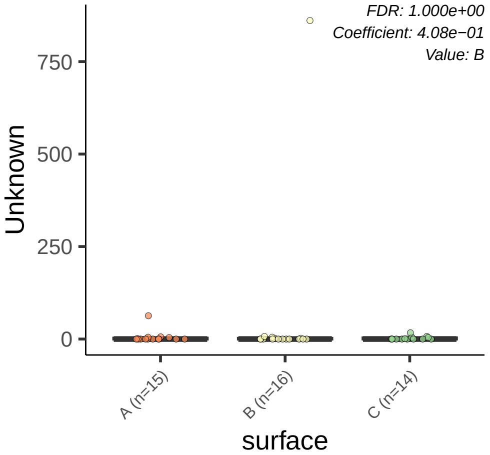

FDR: 1.000e+00
Coefficient: 4.08e−01
Value: B
Unknown
750
500
250
0
A (n=15)
B (n=16)
C (n=14)
surface

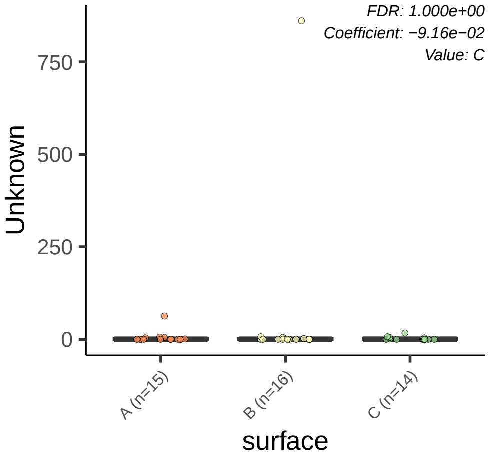

FDR: 1.000e+00
Coefficient: −9.16e−02
Value: C
Unknown
750
500
250
0
A (n=15)
B (n=16)
C (n=14)
surface

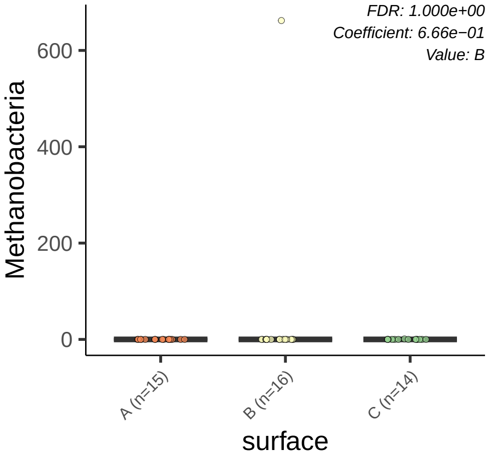
FDR: 1.000e+00
Coefficient: 6.66e−01
Value: B
Methanobacteria
600
400
200
0
A (n=15)
B (n=16)
C (n=14)
surface

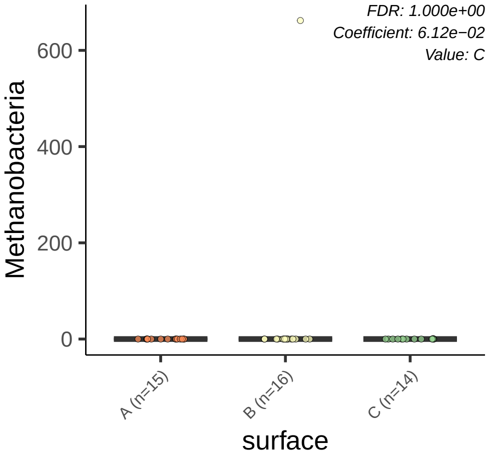
FDR: 1.000e+00
Coefficient: 6.12e−02
Value: C
Methanobacteria
600
400
200
0
A (n=15)
B (n=16)
C (n=14)
surface

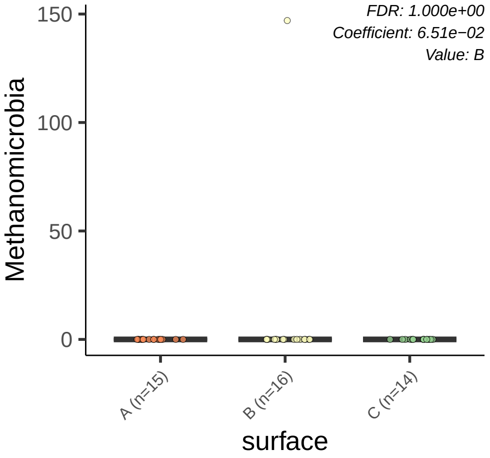

FDR: 1.000e+00
Coefficient: 6.51e−02
Value: B
Methanomicrobia
150
100
50
0
A (n=15)
B (n=16)
C (n=14)
surface

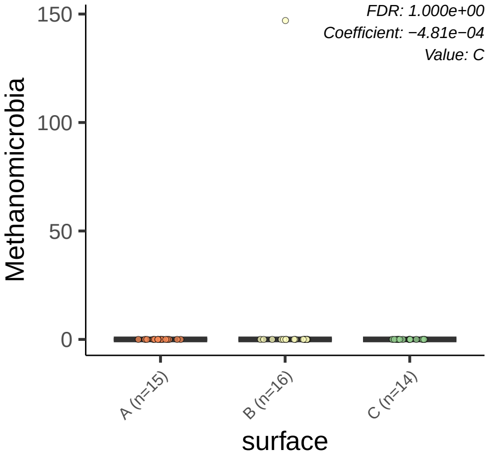

FDR: 1.000e+00
Coefficient: −4.81e−04
Value: C
Methanomicrobia
150
100
50
0
A (n=15)
B (n=16)
C (n=14)
surface

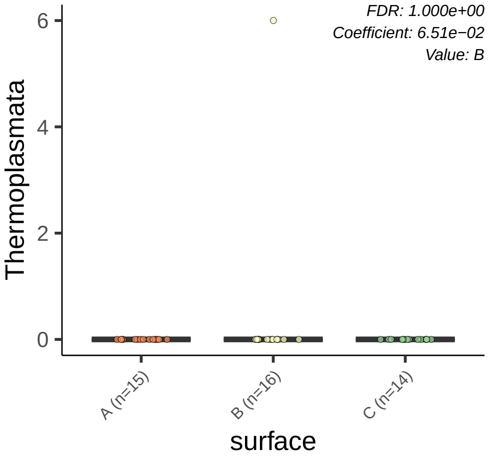

FDR: 1.000e+00
Coefficient: 6.51e−02
Value: B
Thermoplasmata
6
4
2
0
A (n=15)
B (n=16)
C (n=14)
surface

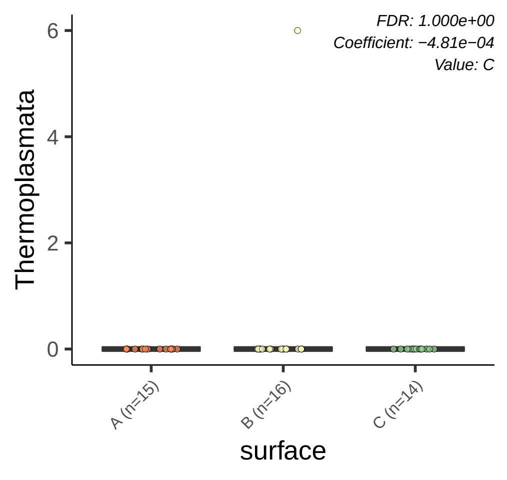
FDR: 1.000e+00
Coefficient: −4.81e−04
Value: C
Thermoplasmata
0
2
4
6
A (n=15)
B (n=16)
C (n=14)
surface

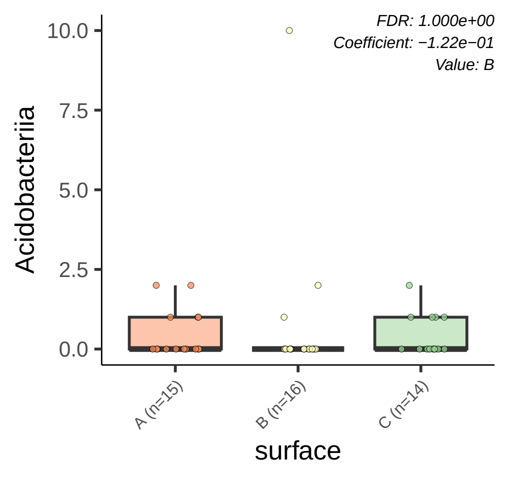

FDR: 1.000e+00
Coefficient: −1.22e−01
Value: B
Acidobacteriia
10.0
7.5
5.0
2.5
0.0
A (n=15)
B (n=16)
C (n=14)
surface

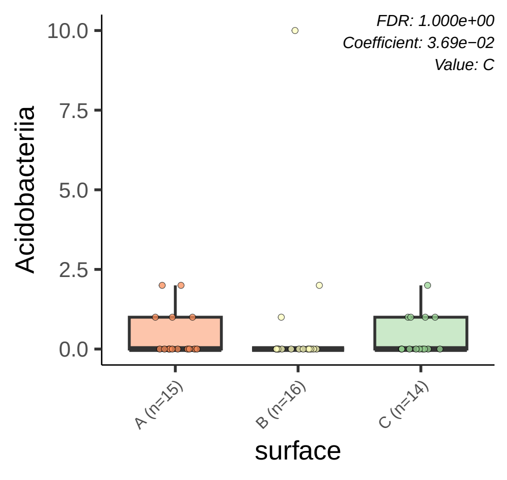

FDR: 1.000e+00
Coefficient: 3.69e−02
Value: C
Acidobacteriia
10.0
7.5
5.0
2.5
0.0
A (n=15)
B (n=16)
C (n=14)
surface

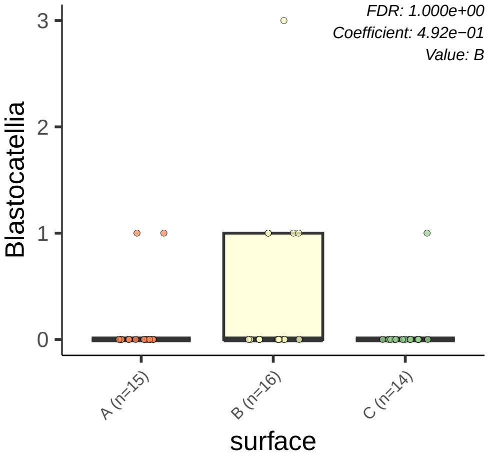
FDR: 1.000e+00
Coefficient: 4.92e−01
Value: B
Blastocatellia
0
1
2
3
A (n=15)
B (n=16)
C (n=14)
surface

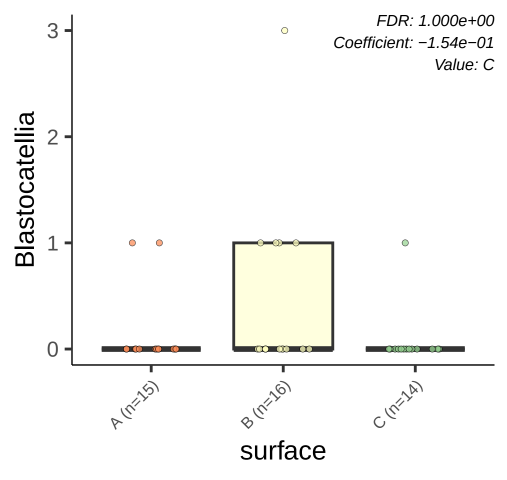
FDR: 1.000e+00
Coefficient: −1.54e−01
Value: C
Blastocatellia
0
1
2
3
A (n=15)
B (n=16)
C (n=14)
surface

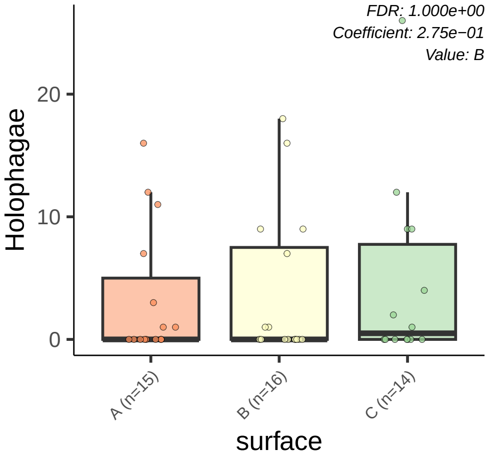

FDR: 1.000e+00
Coefficient: 2.75e−01
Value: B
Holophagae
20
10
0
A (n=15)
B (n=16)
C (n=14)
surface

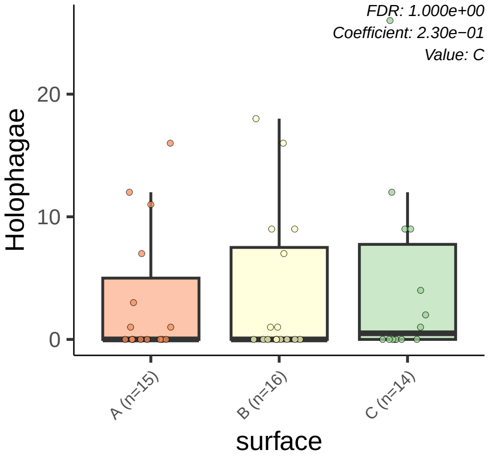

FDR: 1.000e+00
Coefficient: 2.30e−01
Value: C
Holophagae
20
10
0
A (n=15)
B (n=16)
C (n=14)
surface

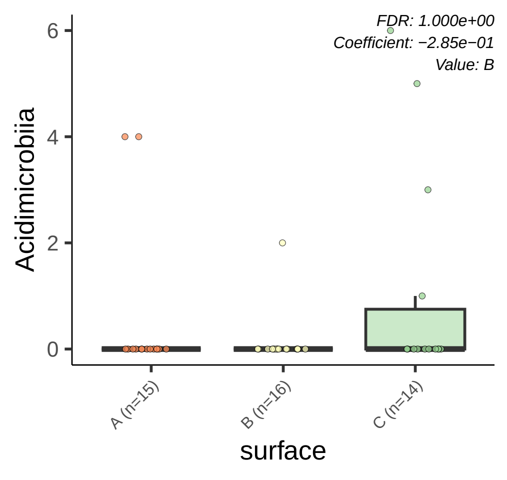

FDR: 1.000e+00
Coefficient: −2.85e−01
Value: B
Acidimicrobiia
6
4
2
0
A (n=15)
B (n=16)
C (n=14)
surface

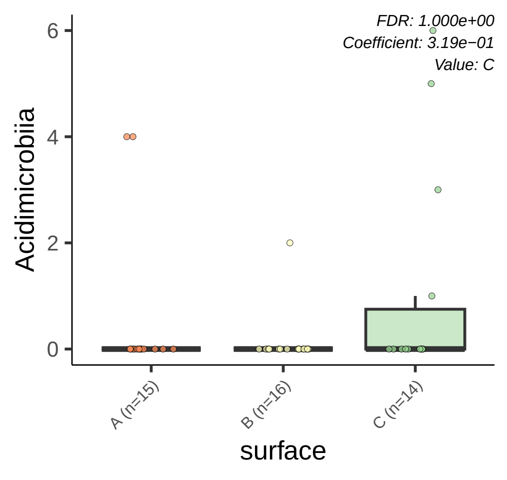

FDR: 1.000e+00
Coefficient: 3.19e−01
Value: C
Acidimicrobiia
0
2
4
6
A (n=15)
B (n=16)
C (n=14)
surface

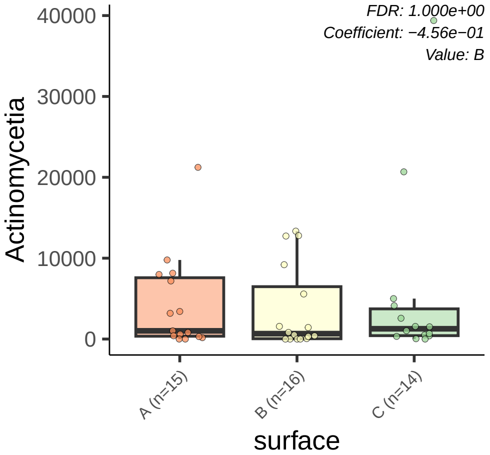
FDR: 1.000e+00
Coefficient: −4.56e−01
Value: B
Actinomycetia
40000
30000
20000
10000
0
A (n=15)
B (n=16)
C (n=14)
surface

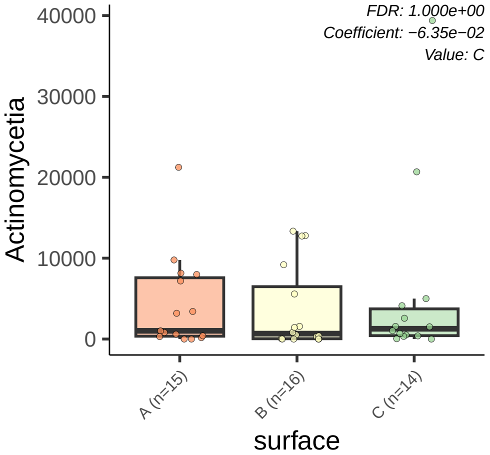
FDR: 1.000e+00
Coefficient: −6.35e−02
Value: C
Actinomycetia
40000
30000
20000
10000
0
A (n=15)
B (n=16)
C (n=14)
surface

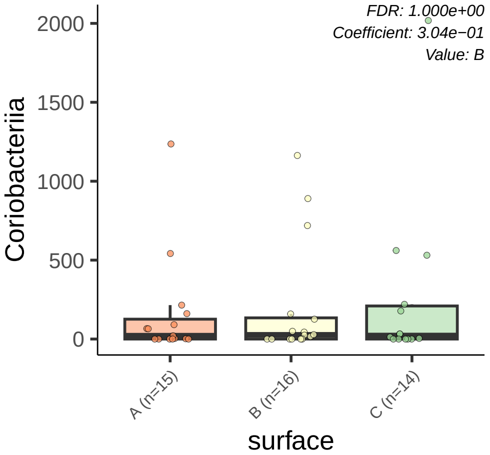

FDR: 1.000e+00
Coefficient: 3.04e−01
Value: B
Coriobacteriia
2000
1500
1000
500
0
A (n=15)
B (n=16)
C (n=14)
surface

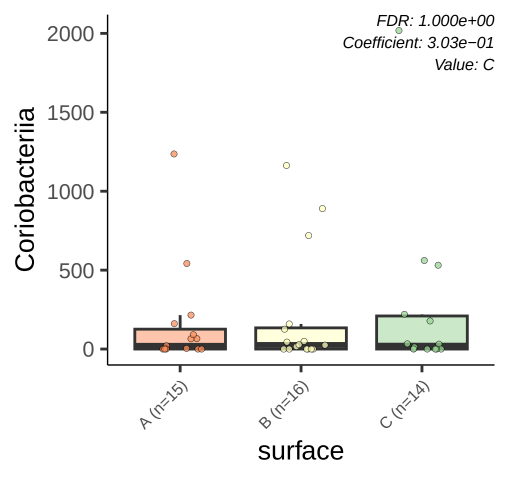
FDR: 1.000e+00
Coefficient: 3.03e−01
Value: C
Coriobacteriia
2000
1500
1000
500
0
A (n=15)
B (n=16)
C (n=14)
surface

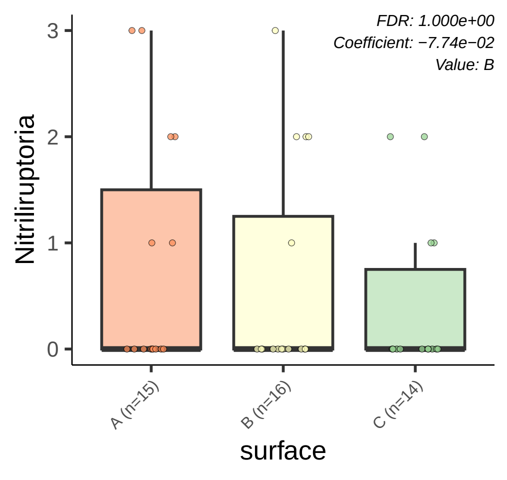

FDR: 1.000e+00
Coefficient: −7.74e−02
Value: B
Nitriliruptoria
0
1
2
3
A (n=15)
B (n=16)
C (n=14)
surface

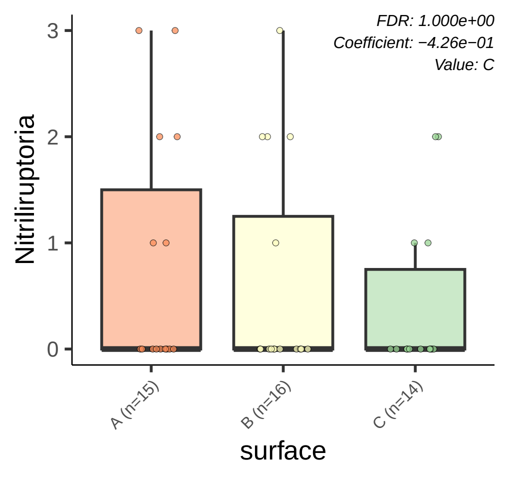

FDR: 1.000e+00
Coefficient: −4.26e−01
Value: C
Nitriliruptoria
0
1
2
3
A (n=15)
B (n=16)
C (n=14)
surface

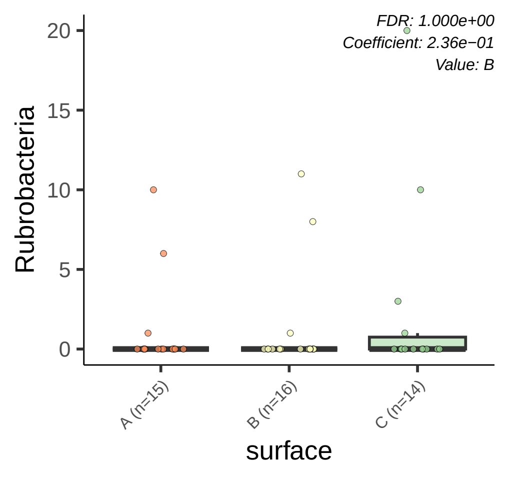
FDR: 1.000e+00
Coefficient: 2.36e−01
Value: B
Rubrobacteria
0
5
10
15
20
A (n=15)
B (n=16)
C (n=14)
surface

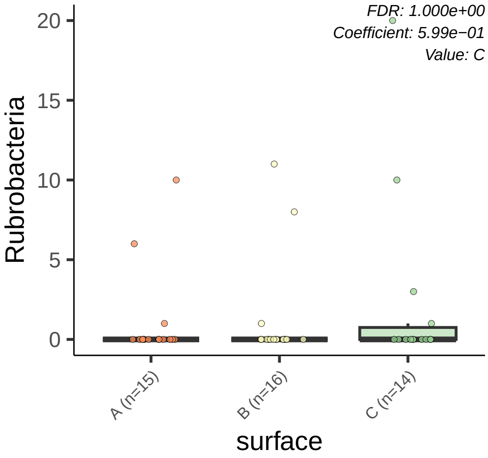
FDR: 1.000e+00
Coefficient: 5.99e−01
Value: C
Rubrobacteria
20
15
10
5
0
A (n=15)
B (n=16)
C (n=14)
surface

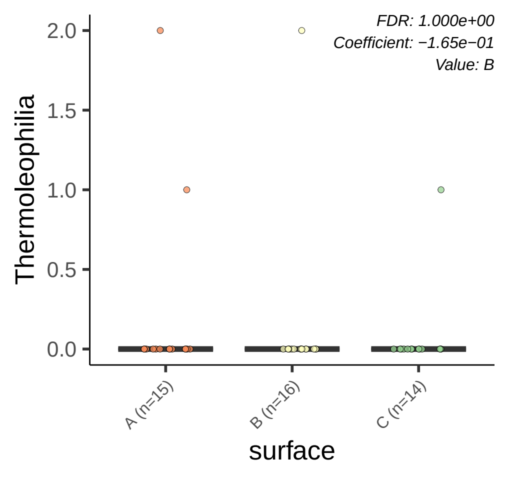
FDR: 1.000e+00
Coefficient: −1.65e−01
Value: B
Thermoleophilia
2.0
1.5
1.0
0.5
0.0
A (n=15)
B (n=16)
C (n=14)
surface

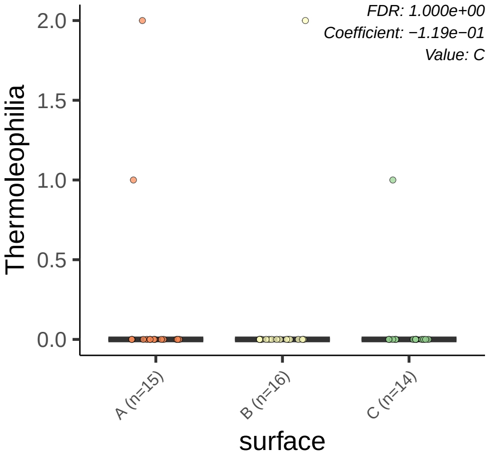

FDR: 1.000e+00
Coefficient: −1.19e−01
Value: C
Thermoleophilia
2.0
1.5
1.0
0.5
0.0
A (n=15)
B (n=16)
C (n=14)
surface

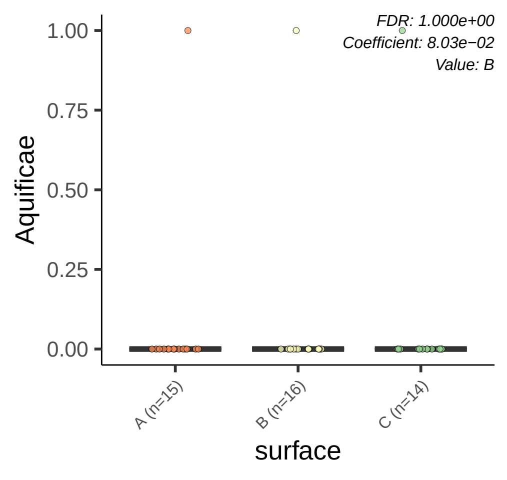

FDR: 1.000e+00
Coefficient: 8.03e−02
Value: B
Aquificae
1.00
0.75
0.50
0.25
0.00
A (n=15)
B (n=16)
C (n=14)
surface

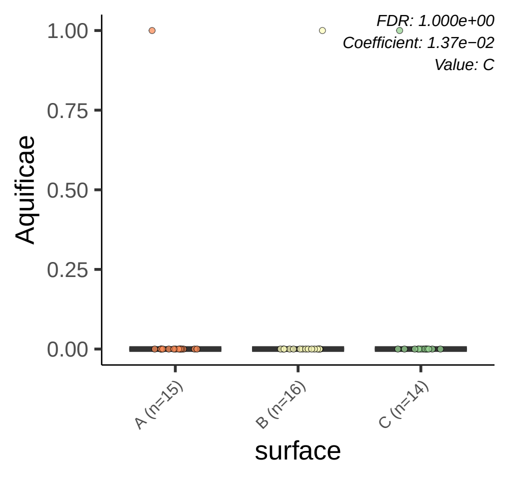
FDR: 1.000e+00
Coefficient: 1.37e−02
Value: C
Aquificae
1.00
0.75
0.50
0.25
0.00
A (n=15)
B (n=16)
C (n=14)
surface

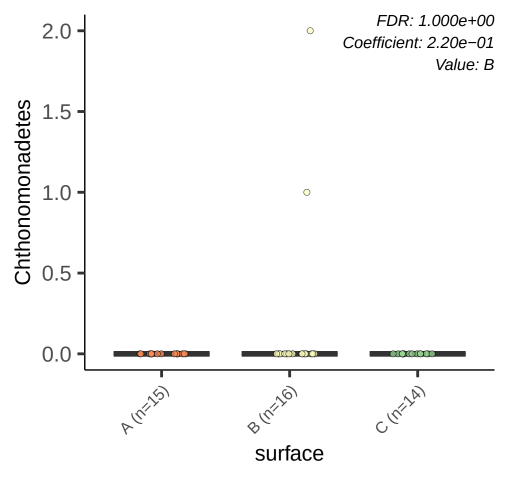
FDR: 1.000e+00
Coefficient: 2.20e−01
Value: B
Chthonomonadetes
2.0
1.5
1.0
0.5
0.0
A (n=15)
B (n=16)
C (n=14)
surface

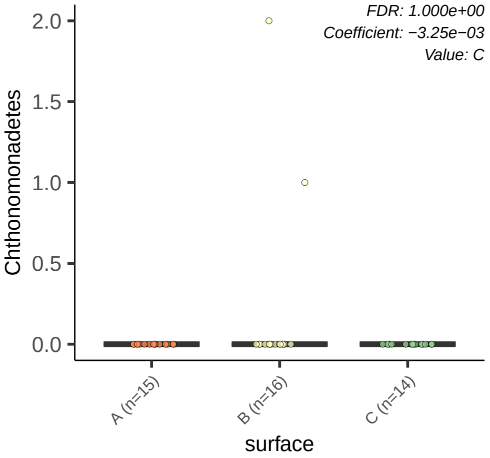
FDR: 1.000e+00
Coefficient: −3.25e−03
Value: C
Chthonomonadetes
2.0
1.5
1.0
0.5
0.0
A (n=15)
B (n=16)
C (n=14)
surface

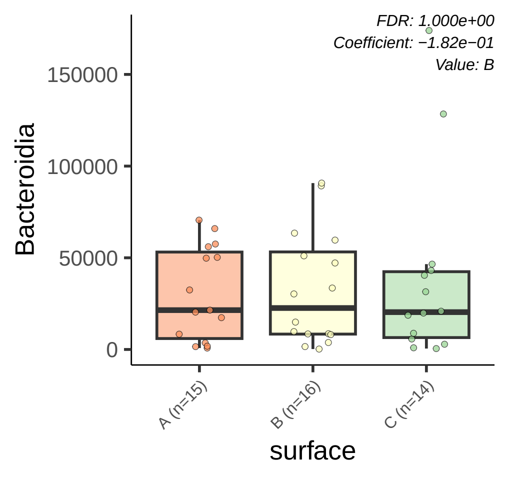
FDR: 1.000e+00
Coefficient: −1.82e−01
Value: B
Bacteroidia
150000
100000
50000
0
A (n=15)
B (n=16)
C (n=14)
surface

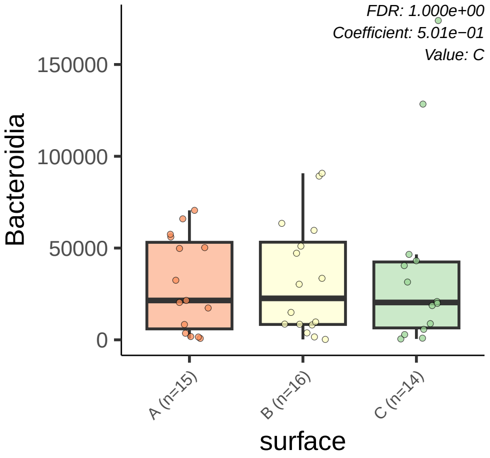

FDR: 1.000e+00
Coefficient: 5.01e−01
Value: C
Bacteroidia
150000
100000
50000
0
A (n=15)
B (n=16)
C (n=14)
surface

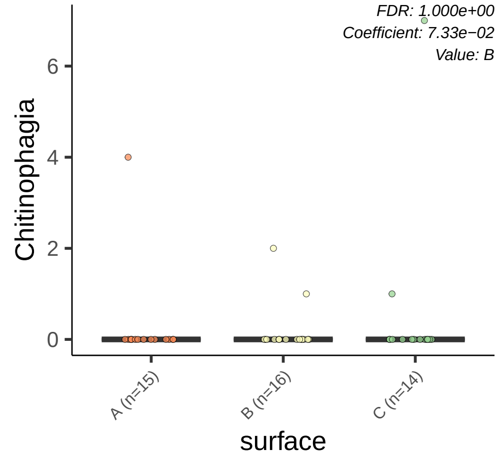

FDR: 1.000e+00
Coefficient: 7.33e−02
Value: B
Chitinophagia
0
2
4
6
A (n=15)
B (n=16)
C (n=14)
surface

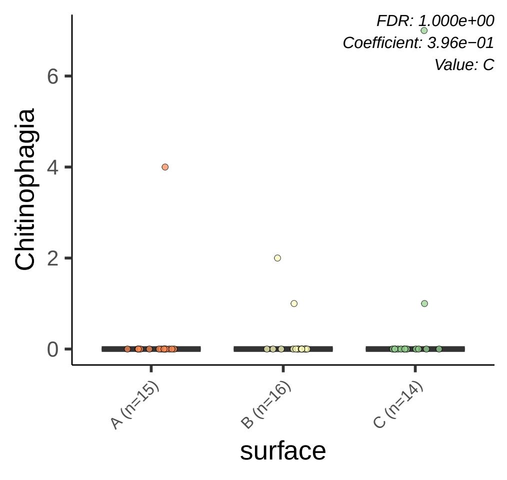
FDR: 1.000e+00
Coefficient: 3.96e−01
Value: C
Chitinophagia
0
2
4
6
A (n=15)
B (n=16)
C (n=14)
surface

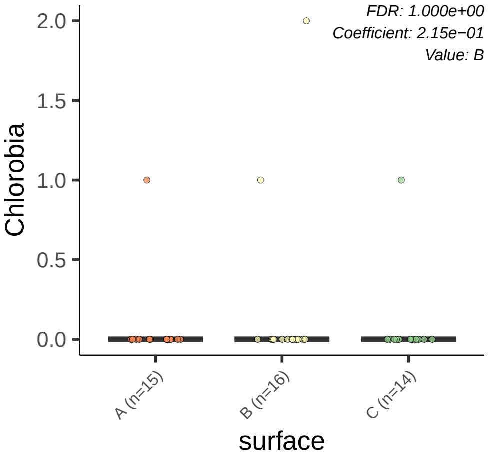
FDR: 1.000e+00
Coefficient: 2.15e−01
Value: B
Chlorobia
2.0
1.5
1.0
0.5
0.0
A (n=15)
B (n=16)
C (n=14)
surface

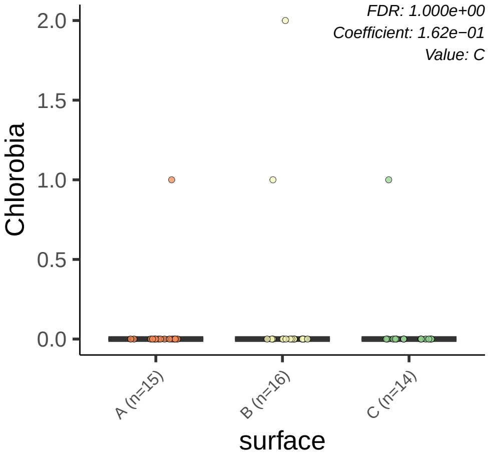

FDR: 1.000e+00
Coefficient: 1.62e−01
Value: C
2.0
1.5
1.0
0.5
0.0
Chlorobia
A (n=15)
B (n=16)
C (n=14)
surface

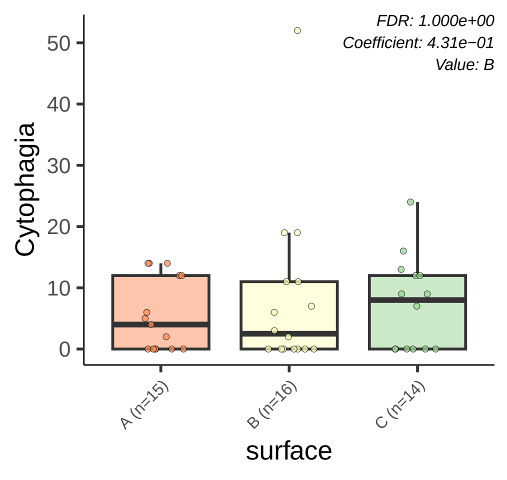

FDR: 1.000e+00
Coefficient: 4.31e−01
Value: B
Cytophagia
50
40
30
20
10
0
A (n=15)
B (n=16)
C (n=14)
surface

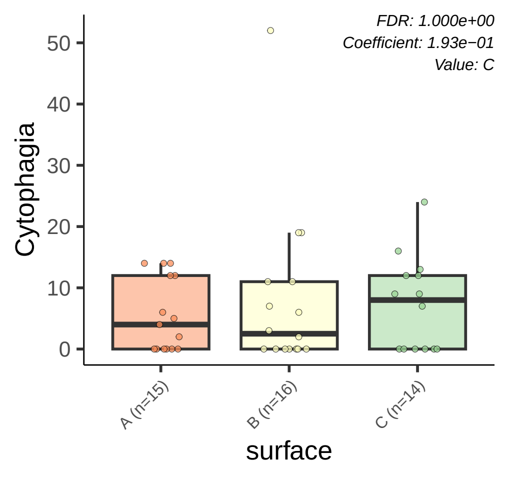
FDR: 1.000e+00
Coefficient: 1.93e−01
Value: C
Cytophagia
0
10
20
30
40
50
A (n=15)
B (n=16)
C (n=14)
surface

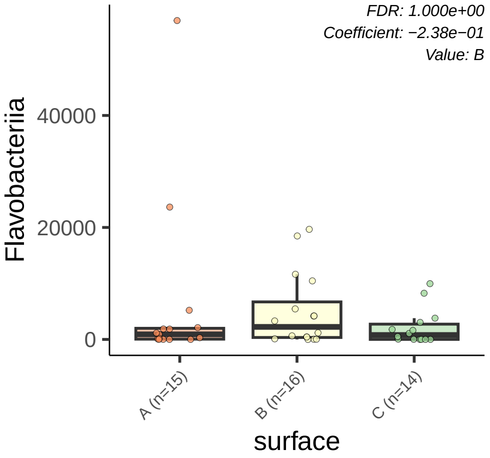

FDR: 1.000e+00
Coefficient: −2.38e−01
Value: B
Flavobacteriia
40000
20000
0
A (n=15)
B (n=16)
C (n=14)
surface

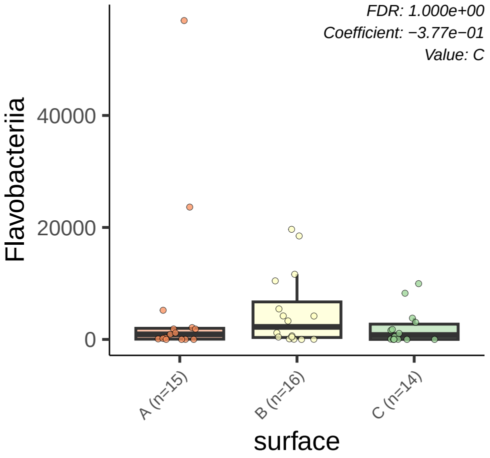

FDR: 1.000e+00
Coefficient: −3.77e−01
Value: C
Flavobacteriia
40000
20000
0
A (n=15)
B (n=16)
C (n=14)
surface

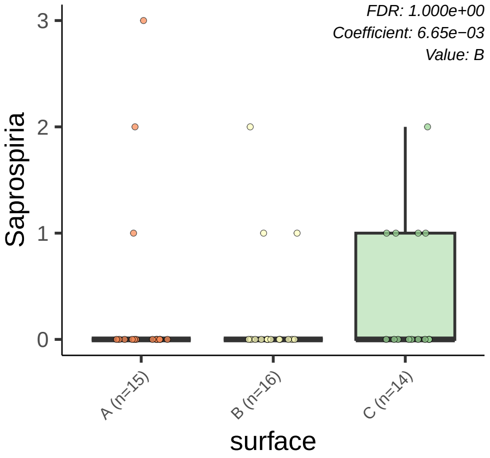

FDR: 1.000e+00
Coefficient: 6.65e−03
Value: B
Saprospiria
3
2
1
0
A (n=15)
B (n=16)
C (n=14)
surface

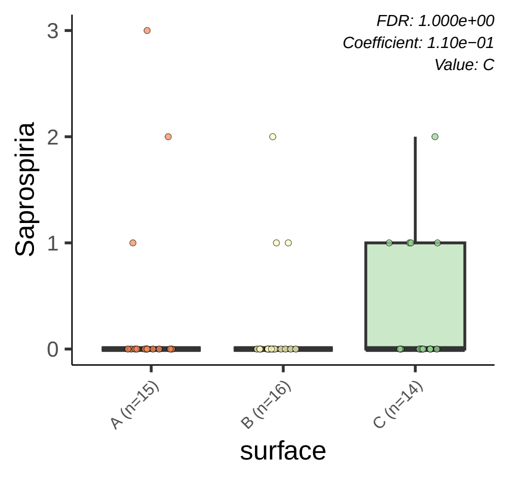
FDR: 1.000e+00
Coefficient: 1.10e−01
Value: C
Saprospiria
0
1
2
3
A (n=15)
B (n=16)
C (n=14)
surface

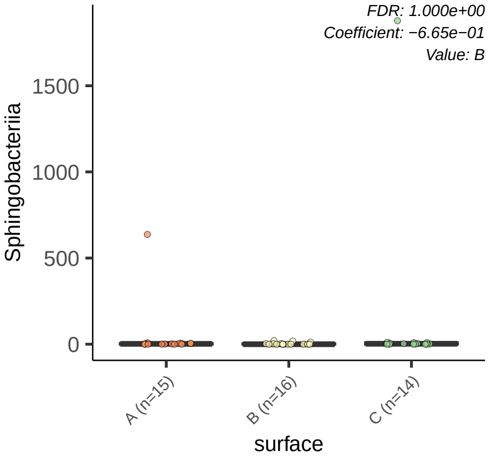
FDR: 1.000e+00
Coefficient: −6.65e−01
Value: B
Sphingobacteriia
1500
1000
500
0
A (n=15)
B (n=16)
C (n=14)
surface

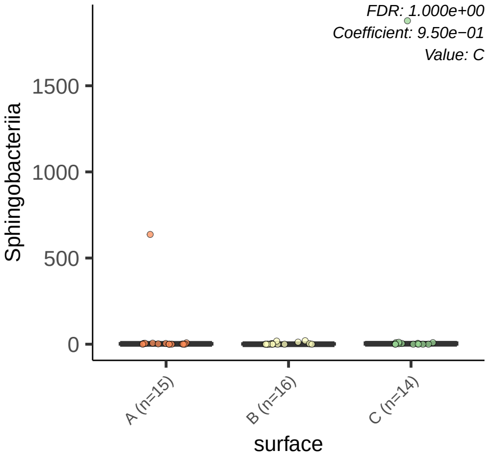

FDR: 1.000e+00
Coefficient: 9.50e−01
Value: C
Sphingobacteriia
1500
1000
500
0
A (n=15)
B (n=16)
C (n=14)
surface

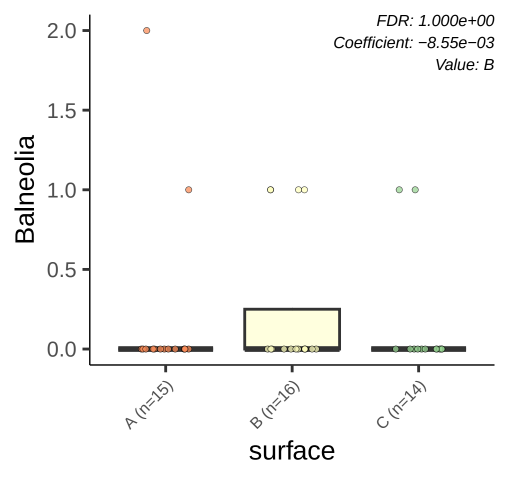
FDR: 1.000e+00
Coefficient: −8.55e−03
Value: B
Balneolia
2.0
1.5
1.0
0.5
0.0
A (n=15)
B (n=16)
C (n=14)
surface

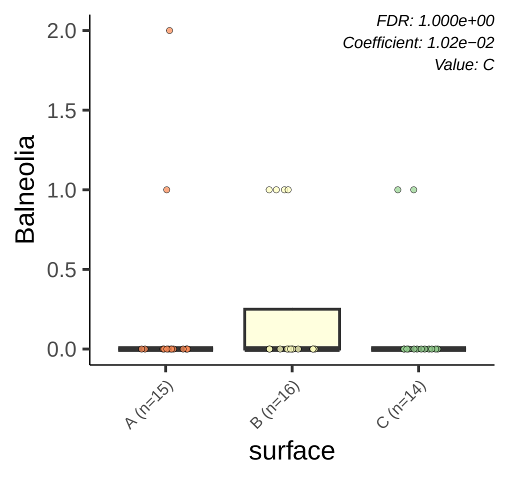
FDR: 1.000e+00
Coefficient: 1.02e−02
Value: C
Balneolia
2.0
1.5
1.0
0.5
0.0
A (n=15)
B (n=16)
C (n=14)
surface

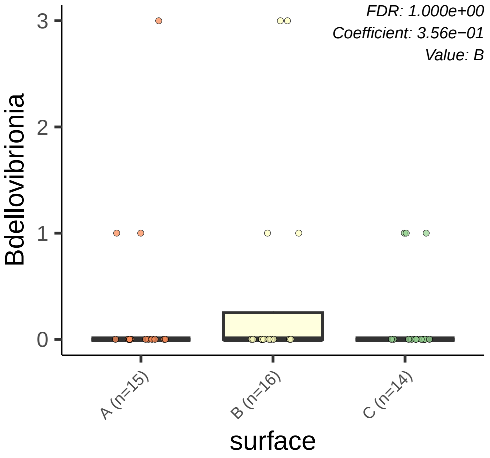

FDR: 1.000e+00
Coefficient: 3.56e−01
Value: B
Bdellovibrionia
3
2
1
0
A (n=15)
B (n=16)
C (n=14)
surface

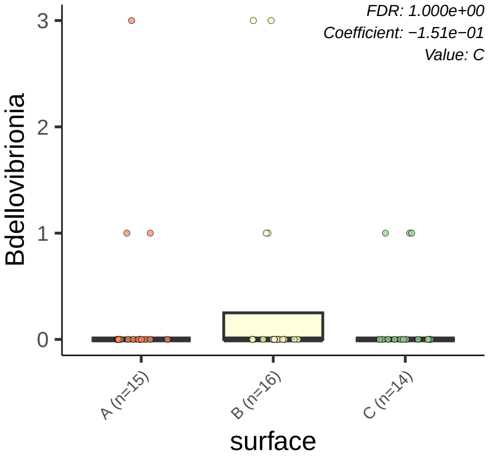

FDR: 1.000e+00
Coefficient: −1.51e−01
Value: C
Bdellovibrionia
3
2
1
0
A (n=15)
B (n=16)
C (n=14)
surface

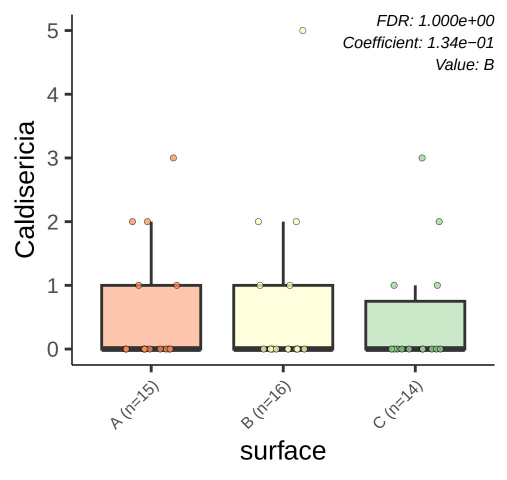

FDR: 1.000e+00
Coefficient: 1.34e−01
Value: B
Caldisericia
0
1
2
3
4
5
A (n=15)
B (n=16)
C (n=14)
surface

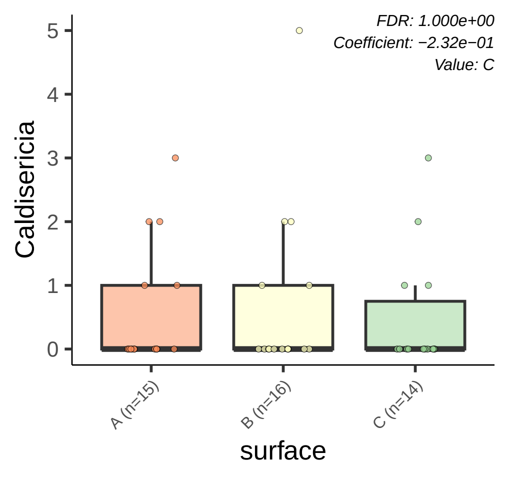
FDR: 1.000e+00
Coefficient: −2.32e−01
Value: C
Caldisericia
0
1
2
3
4
5
A (n=15)
B (n=16)
C (n=14)
surface

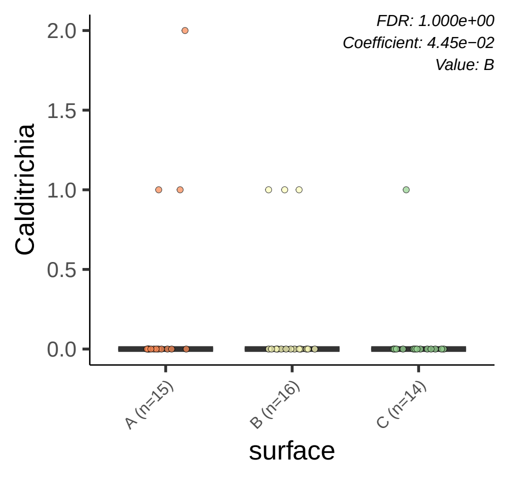
FDR: 1.000e+00
Coefficient: 4.45e−02
Value: B
Calditrichia
2.0
1.5
1.0
0.5
0.0
A (n=15)
B (n=16)
C (n=14)
surface

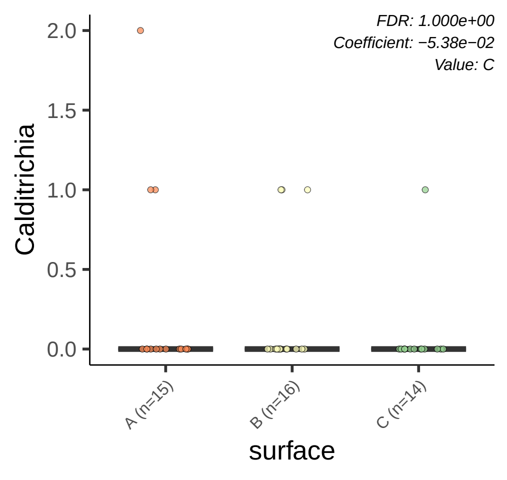
FDR: 1.000e+00
Coefficient: −5.38e−02
Value: C
Calditrichia
2.0
1.5
1.0
0.5
0.0
A (n=15)
B (n=16)
C (n=14)
surface

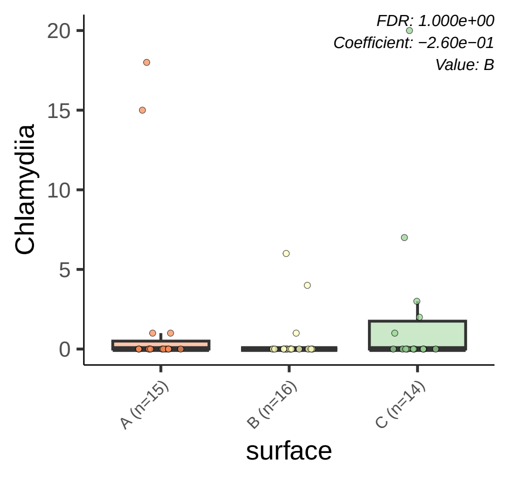
FDR: 1.000e+00
Coefficient: −2.60e−01
Value: B
Chlamydiia
20
15
10
5
0
A (n=15)
B (n=16)
C (n=14)
surface

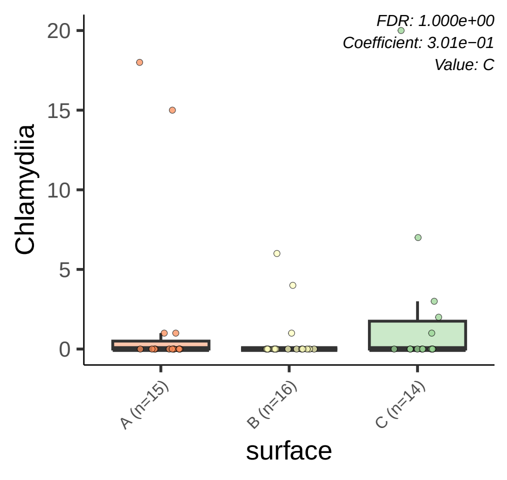

FDR: 1.000e+00
Coefficient: 3.01e−01
Value: C
Chlamydiia
20
15
10
5
0
A (n=15)
B (n=16)
C (n=14)
surface

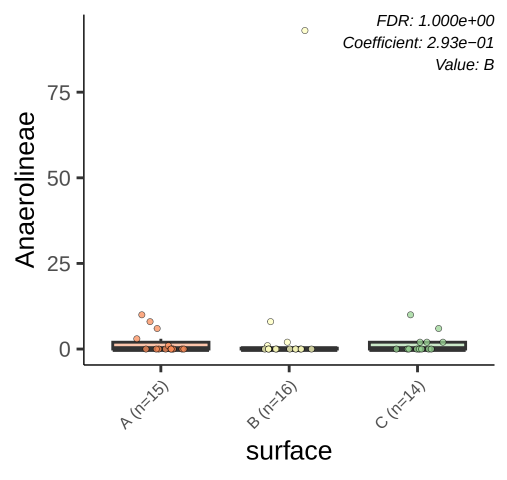

FDR: 1.000e+00
Coefficient: 2.93e−01
Value: B
Anaerolineae
75
50
25
0
A (n=15)
B (n=16)
C (n=14)
surface

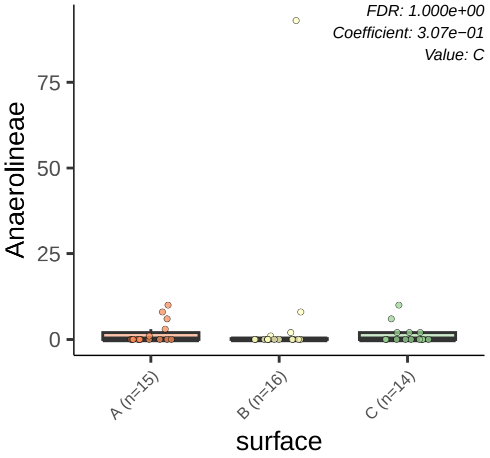

FDR: 1.000e+00
Coefficient: 3.07e−01
Value: C
Anaerolineae
75
50
25
0
A (n=15)
B (n=16)
C (n=14)
surface

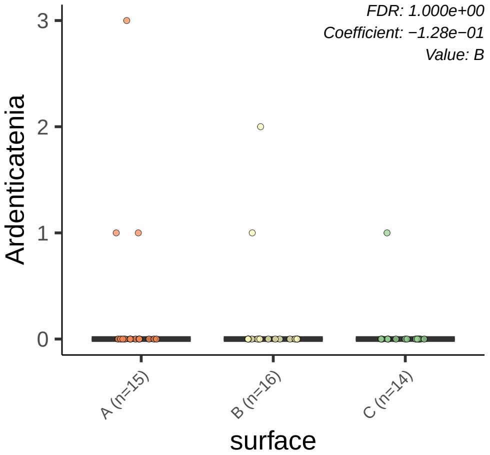

FDR: 1.000e+00
Coefficient: −1.28e−01
Value: B
Ardenticatenia
3
2
1
0
A (n=15)
B (n=16)
C (n=14)
surface

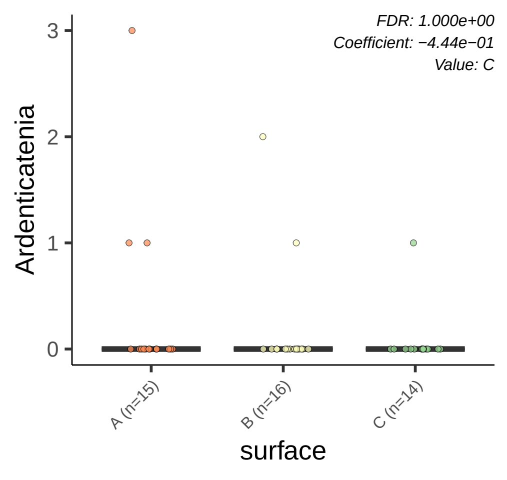

FDR: 1.000e+00
Coefficient: −4.44e−01
Value: C
Ardenticatenia
3
2
1
0
A (n=15)
B (n=16)
C (n=14)
surface

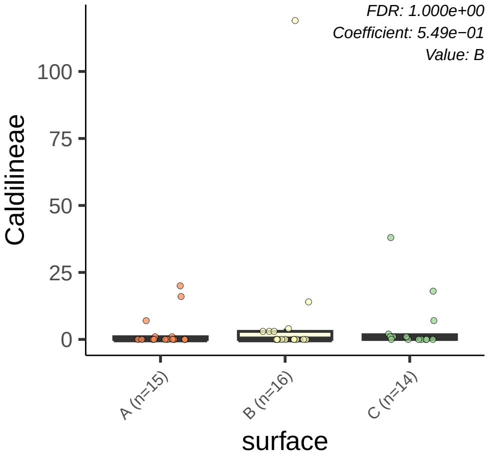
FDR: 1.000e+00
Coefficient: 5.49e−01
Value: B
Caldilineae
100
75
50
25
0
A (n=15)
B (n=16)
C (n=14)
surface

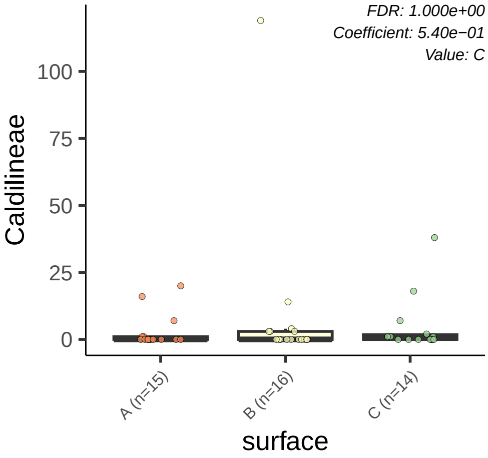
FDR: 1.000e+00
Coefficient: 5.40e−01
Value: C
Caldilineae
100
75
50
25
0
A (n=15)
B (n=16)
C (n=14)
surface

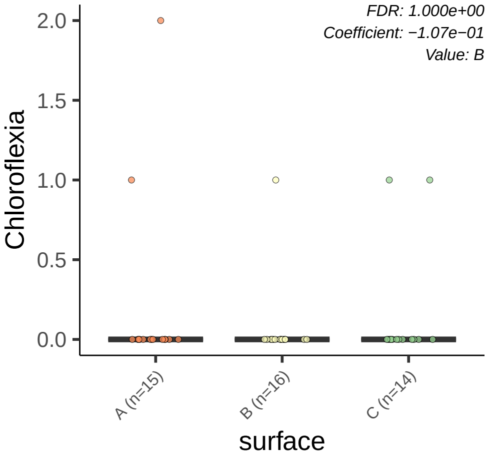

FDR: 1.000e+00
Coefficient: −1.07e−01
Value: B
Chloroflexia
2.0
1.5
1.0
0.5
0.0
A (n=15)
B (n=16)
C (n=14)
surface

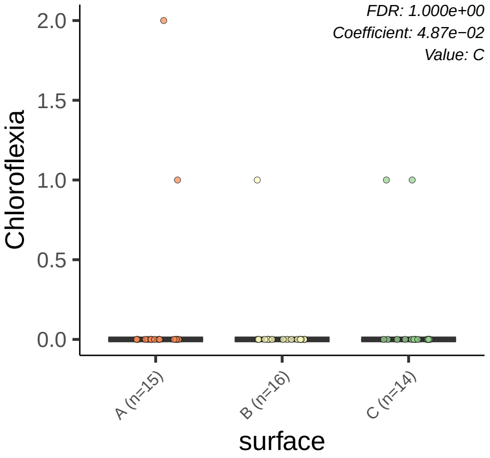

FDR: 1.000e+00
Coefficient: 4.87e−02
Value: C
Chloroflexia
2.0
1.5
1.0
0.5
0.0
A (n=15)
B (n=16)
C (n=14)
surface

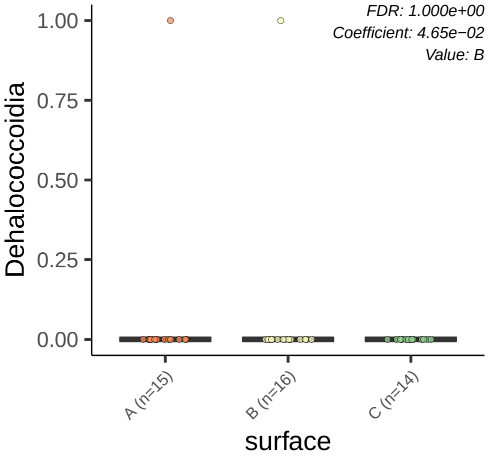
FDR: 1.000e+00
Coefficient: 4.65e−02
Value: B
Dehalococcoidia
1.00
0.75
0.50
0.25
0.00
A (n=15)
B (n=16)
C (n=14)
surface

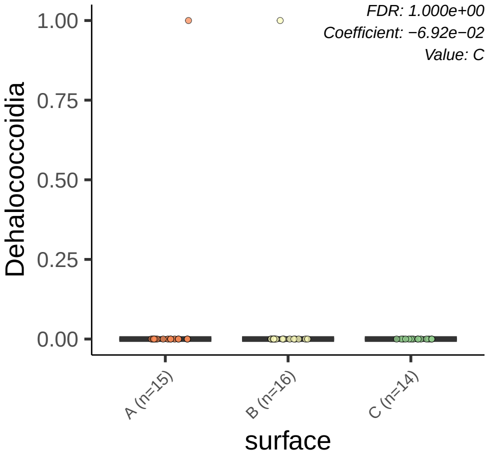

FDR: 1.000e+00
Coefficient: −6.92e−02
Value: C
Dehalococcoidia
1.00
0.75
0.50
0.25
0.00
A (n=15)
B (n=16)
C (n=14)
surface

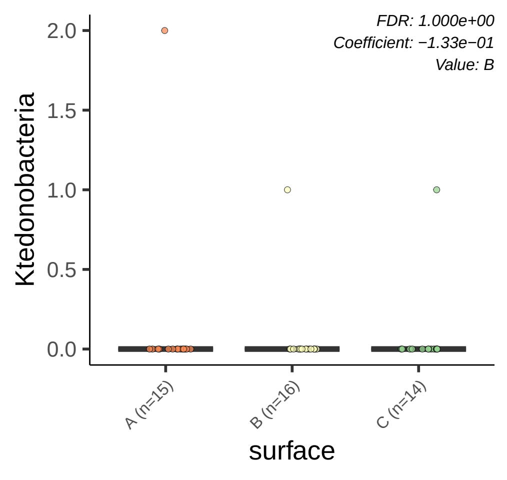

FDR: 1.000e+00
Coefficient: −1.33e−01
Value: B
Ktedonobacteria
2.0
1.5
1.0
0.5
0.0
A (n=15)
B (n=16)
C (n=14)
surface

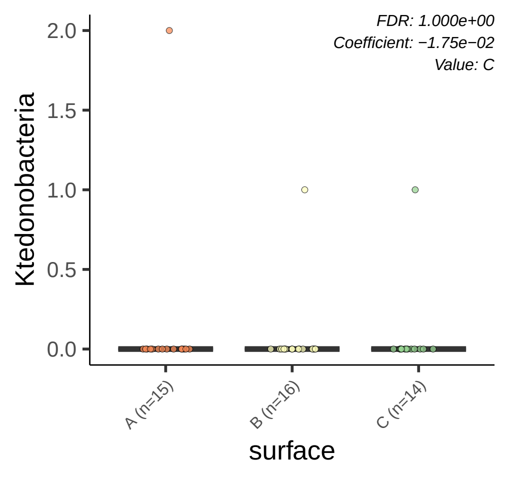
FDR: 1.000e+00
Coefficient: −1.75e−02
Value: C
Ktedonobacteria
2.0
1.5
1.0
0.5
0.0
A (n=15)
B (n=16)
C (n=14)
surface

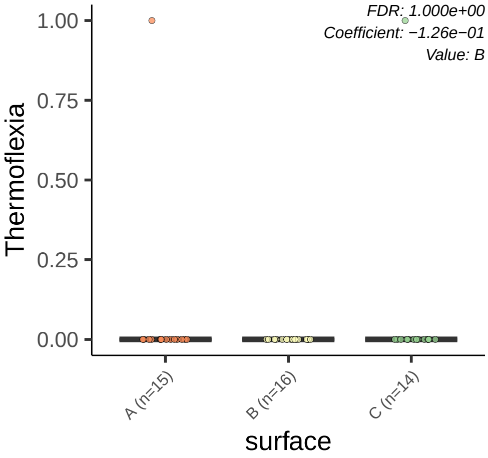
FDR: 1.000e+00
Coefficient: −1.26e−01
Value: B
Thermoflexia
1.00
0.75
0.50
0.25
0.00
A (n=15)
B (n=16)
C (n=14)
surface

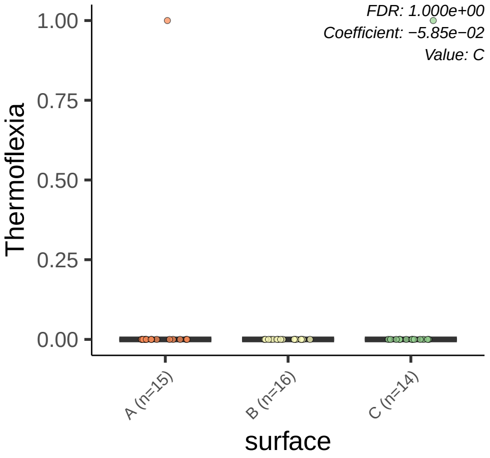
FDR: 1.000e+00
Coefficient: −5.85e−02
Value: C
Thermoflexia
1.00
0.75
0.50
0.25
0.00
A (n=15)
B (n=16)
C (n=14)
surface

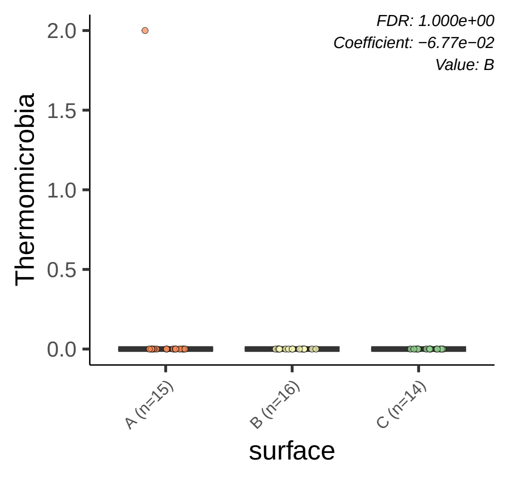
FDR: 1.000e+00
Coefficient: −6.77e−02
Value: B
Thermomicrobia
2.0
1.5
1.0
0.5
0.0
A (n=15)
B (n=16)
C (n=14)
surface

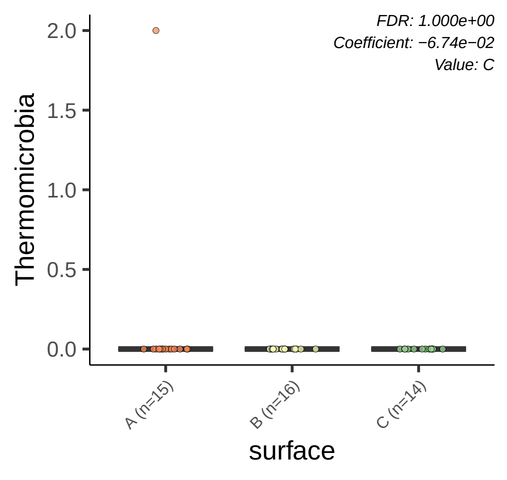
FDR: 1.000e+00
Coefficient: −6.74e−02
Value: C
Thermomicrobia
2.0
1.5
1.0
0.5
0.0
A (n=15)
B (n=16)
C (n=14)
surface

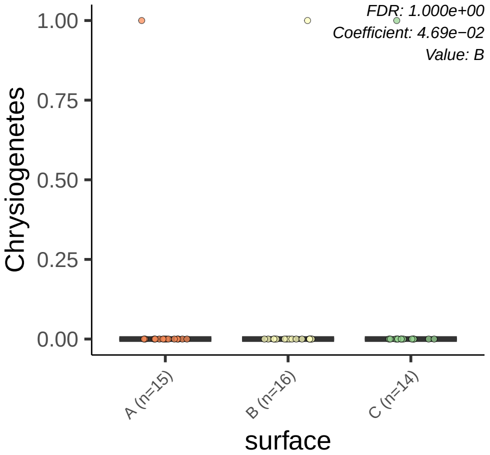
FDR: 1.000e+00
Coefficient: 4.69e−02
Value: B
Chrysiogenetes
1.00
0.75
0.50
0.25
0.00
A (n=15)
B (n=16)
C (n=14)
surface

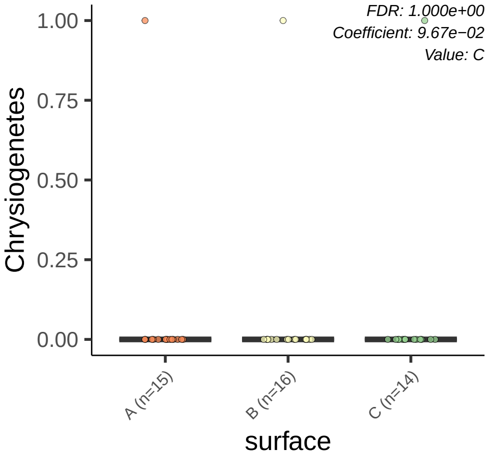
FDR: 1.000e+00
Coefficient: 9.67e−02
Value: C
Chrysiogenetes
1.00
0.75
0.50
0.25
0.00
A (n=15)
B (n=16)
C (n=14)
surface

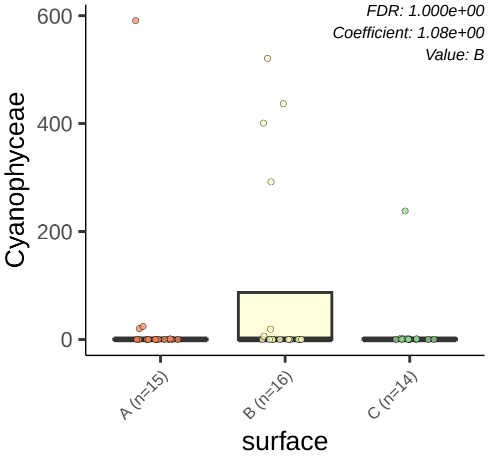

FDR: 1.000e+00
Coefficient: 1.08e+00
Value: B
Cyanophyceae
600
400
200
0
A (n=15)
B (n=16)
C (n=14)
surface

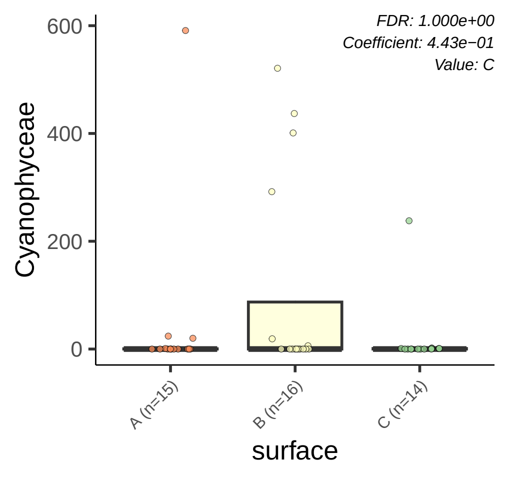

FDR: 1.000e+00
Coefficient: 4.43e−01
Value: C
Cyanophyceae
600
400
200
0
A (n=15)
B (n=16)
C (n=14)
surface

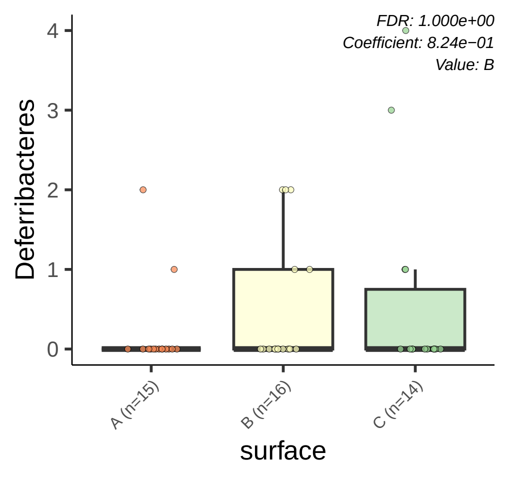

FDR: 1.000e+00
Coefficient: 8.24e−01
Value: B
Deferribacteres
4
3
2
1
0
A (n=15)
B (n=16)
C (n=14)
surface

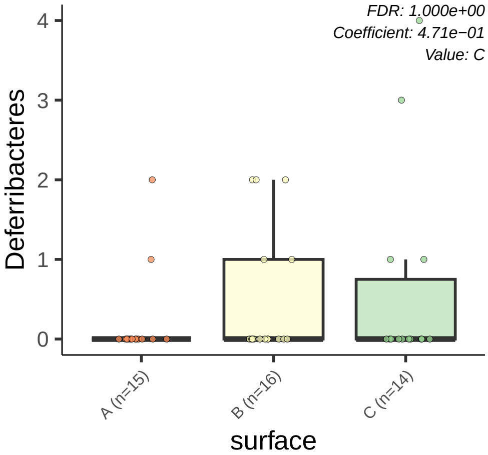
FDR: 1.000e+00
Coefficient: 4.71e−01
Value: C
Deferribacteres
4
3
2
1
0
A (n=15)
B (n=16)
C (n=14)
surface

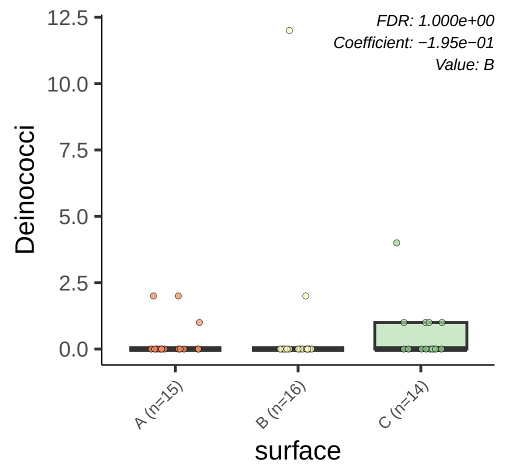

FDR: 1.000e+00
Coefficient: −1.95e−01
Value: B
Deinococci
12.5
10.0
7.5
5.0
2.5
0.0
A (n=15)
B (n=16)
C (n=14)
surface

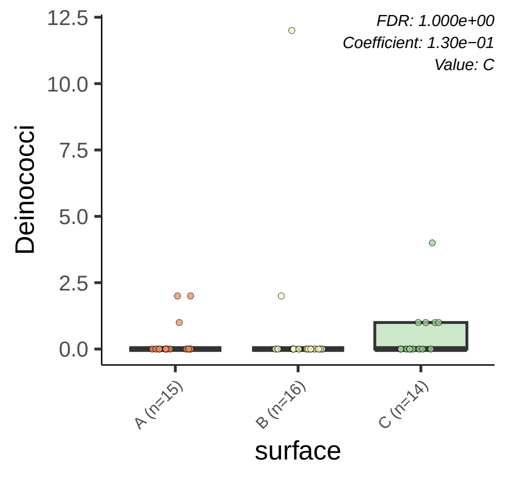

FDR: 1.000e+00
Coefficient: 1.30e−01
Value: C
Deinococci
12.5
10.0
7.5
5.0
2.5
0.0
A (n=15)
B (n=16)
C (n=14)
surface

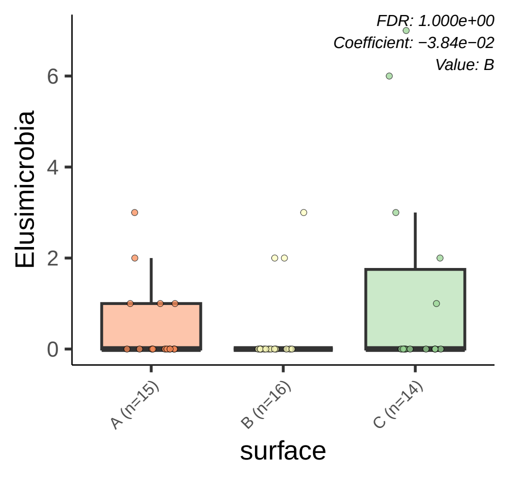
FDR: 1.000e+00
Coefficient: −3.84e−02
Value: B
Elusimicrobia
0
2
4
6
A (n=15)
B (n=16)
C (n=14)
surface

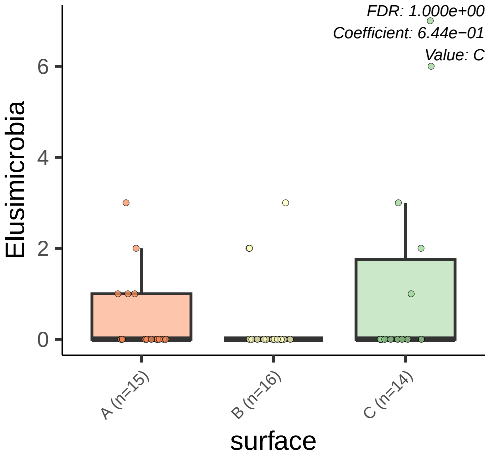
FDR: 1.000e+00
Coefficient: 6.44e−01
Value: C
Elusimicrobia
0
2
4
6
A (n=15)
B (n=16)
C (n=14)
surface

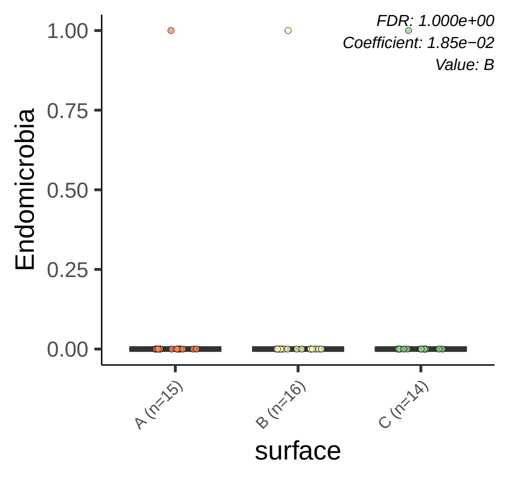
FDR: 1.000e+00
Coefficient: 1.85e−02
Value: B
Endomicrobia
1.00
0.75
0.50
0.25
0.00
A (n=15)
B (n=16)
C (n=14)
surface

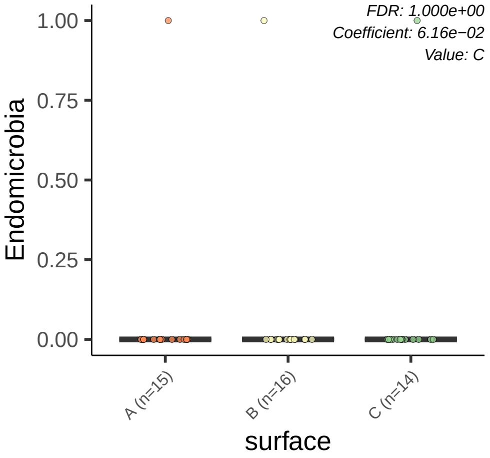

FDR: 1.000e+00
Coefficient: 6.16e−02
Value: C
Endomicrobia
1.00
0.75
0.50
0.25
0.00
A (n=15)
B (n=16)
C (n=14)
surface

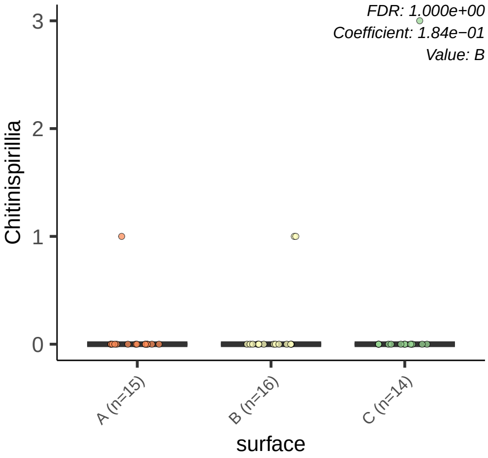
FDR: 1.000e+00
Coefficient: 1.84e−01
Value: B
Chitinispirillia
3
2
1
0
A (n=15)
B (n=16)
C (n=14)
surface

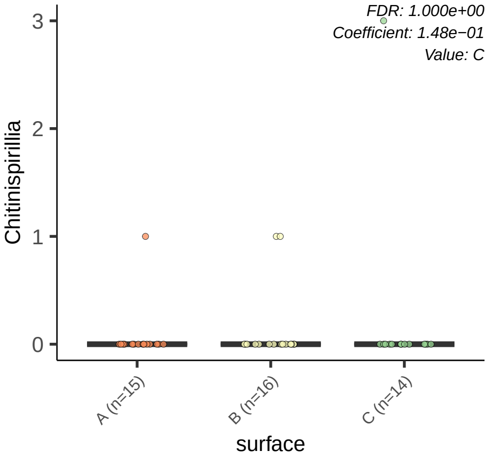
FDR: 1.000e+00
Coefficient: 1.48e−01
Value: C
Chitinispirillia
3
2
1
0
A (n=15)
B (n=16)
C (n=14)
surface

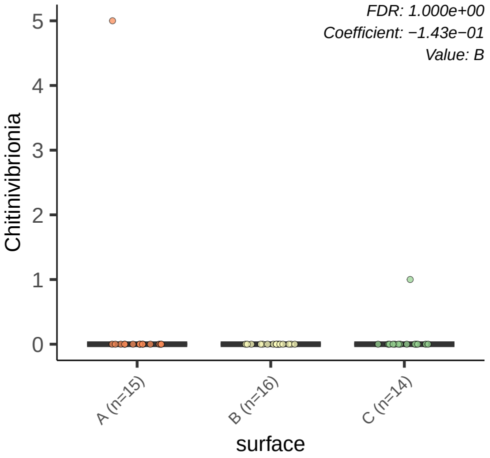
FDR: 1.000e+00
Coefficient: −1.43e−01
Value: B
Chitinivibrionia
5
4
3
2
1
0
A (n=15)
B (n=16)
C (n=14)
surface

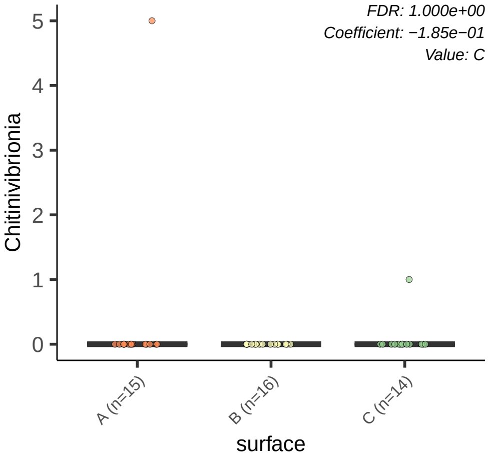
FDR: 1.000e+00
Coefficient: −1.85e−01
Value: C
Chitinivibrionia
0
1
2
3
4
5
A (n=15)
B (n=16)
C (n=14)
surface

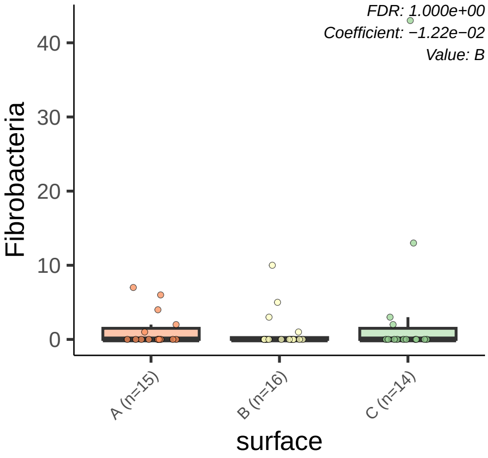

FDR: 1.000e+00
Coefficient: −1.22e−02
Value: B
Fibrobacteria
40
30
20
10
0
A (n=15)
B (n=16)
C (n=14)
surface

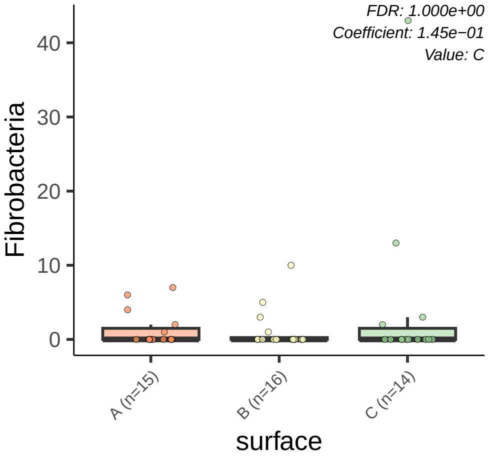
FDR: 1.000e+00
Coefficient: 1.45e−01
Value: C
Fibrobacteria
40
30
20
10
0
A (n=15)
B (n=16)
C (n=14)
surface

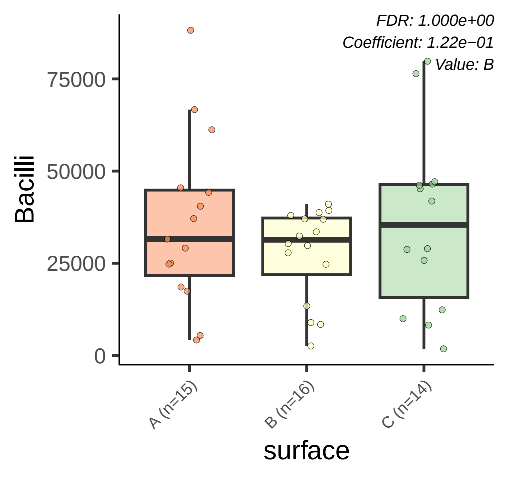

FDR: 1.000e+00
Coefficient: 1.22e−01
Value: B
Bacilli
75000
50000
25000
0
A (n=15)
B (n=16)
C (n=14)
surface

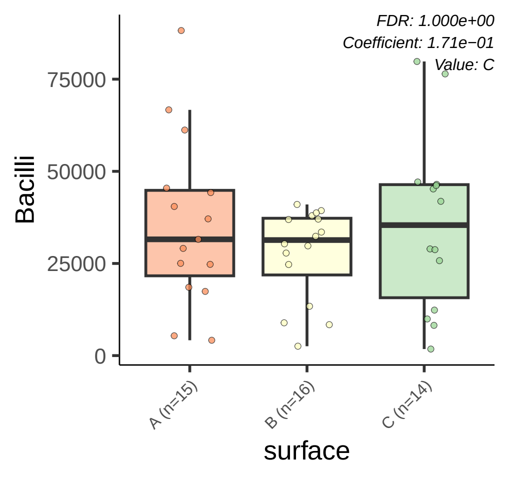
FDR: 1.000e+00
Coefficient: 1.71e−01
Value: C
Bacilli
75000
50000
25000
0
A (n=15)
B (n=16)
C (n=14)
surface

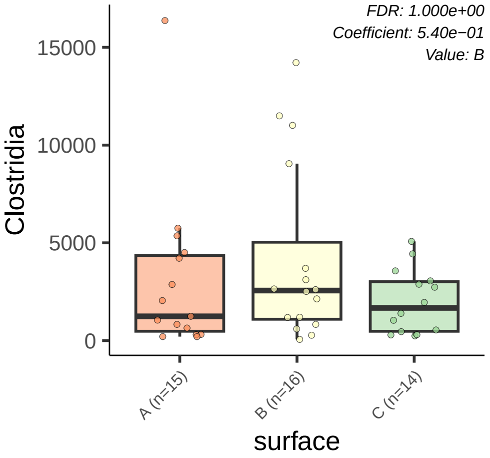
FDR: 1.000e+00
Coefficient: 5.40e−01
Value: B
15000
10000
5000
0
Clostridia
A (n=15)
B (n=16)
C (n=14)
surface

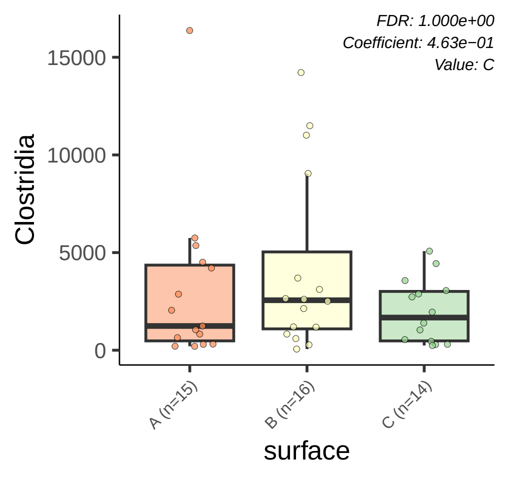

FDR: 1.000e+00
Coefficient: 4.63e−01
Value: C
Clostridia
15000
10000
5000
0
A (n=15)
B (n=16)
C (n=14)
surface

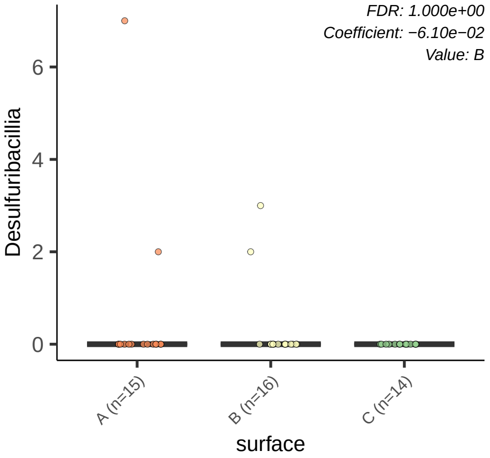

FDR: 1.000e+00
Coefficient: −6.10e−02
Value: B
Desulfuribacillia
0
2
4
6
A (n=15)
B (n=16)
C (n=14)
surface

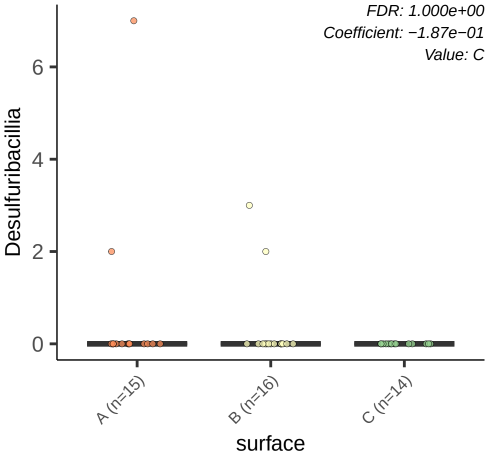
FDR: 1.000e+00
Coefficient: −1.87e−01
Value: C
Desulfuribacillia
0
2
4
6
A (n=15)
B (n=16)
C (n=14)
surface

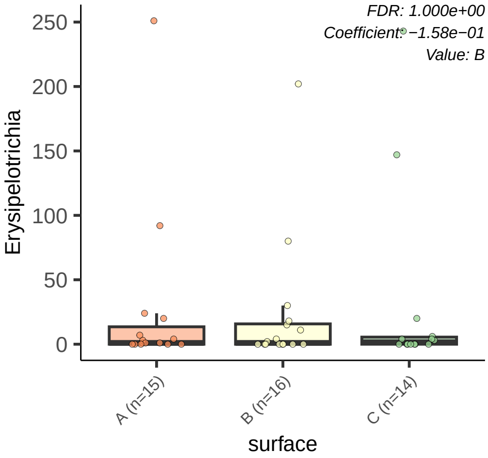

FDR: 1.000e+00
Coefficient: −1.58e−01
Value: B
Erysipelotrichia
250
200
150
100
50
0
A (n=15)
B (n=16)
C (n=14)
surface

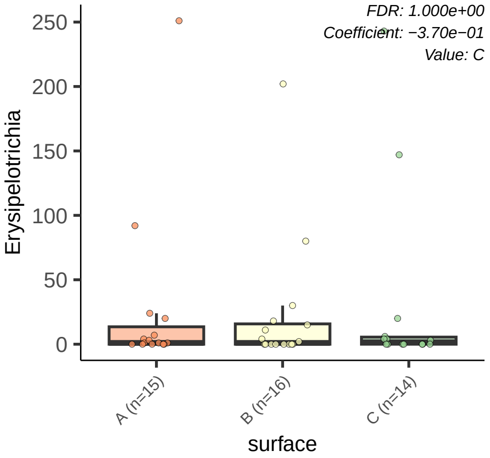

FDR: 1.000e+00
Coefficient: −3.70e−01
Value: C
Erysipelotrichia
250
200
150
100
50
0
A (n=15)
B (n=16)
C (n=14)
surface

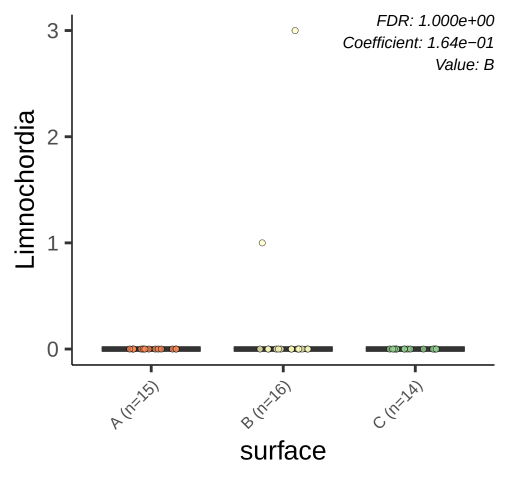

FDR: 1.000e+00
Coefficient: 1.64e−01
Value: B
Limnochordia
3
2
1
0
A (n=15)
B (n=16)
C (n=14)
surface

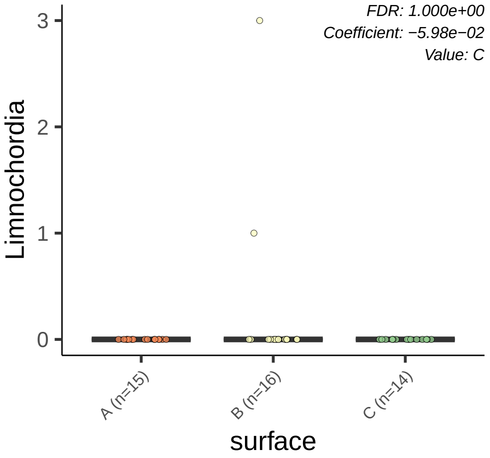
FDR: 1.000e+00
Coefficient: −5.98e−02
Value: C
Limnochordia
3
2
1
0
A (n=15)
B (n=16)
C (n=14)
surface

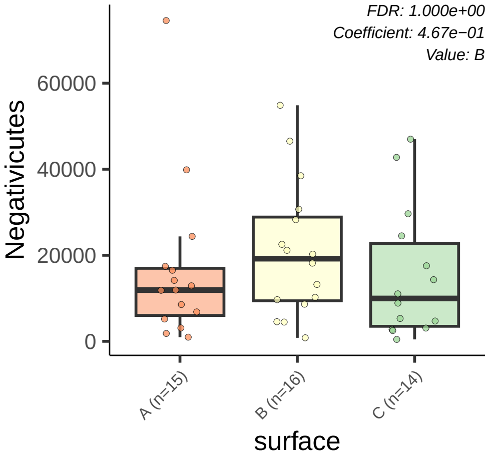

FDR: 1.000e+00
Coefficient: 4.67e−01
Value: B
Negativicutes
60000
40000
20000
0
A (n=15)
B (n=16)
C (n=14)
surface

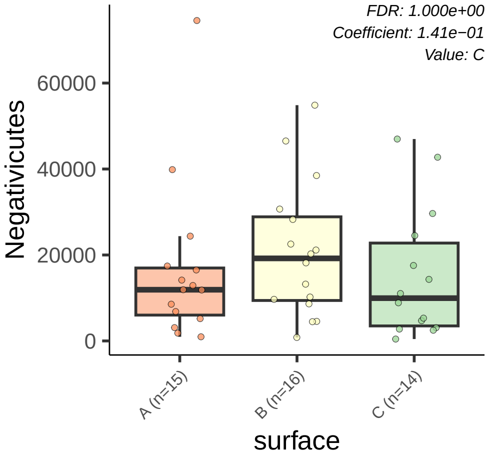
FDR: 1.000e+00
Coefficient: 1.41e−01
Value: C
Negativicutes
60000
40000
20000
0
A (n=15)
B (n=16)
C (n=14)
surface

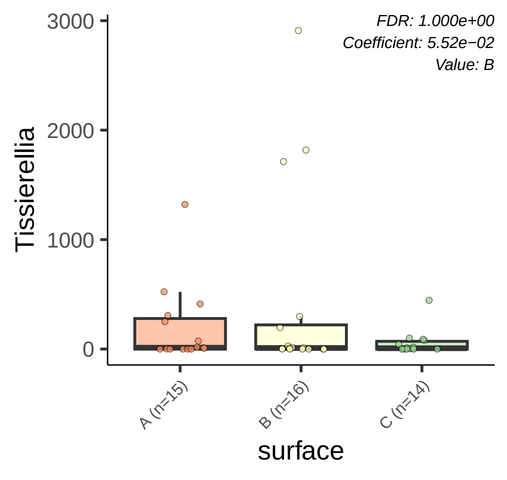

FDR: 1.000e+00
Coefficient: 5.52e−02
Value: B
Tissierellia
3000
2000
1000
0
A (n=15)
B (n=16)
C (n=14)
surface

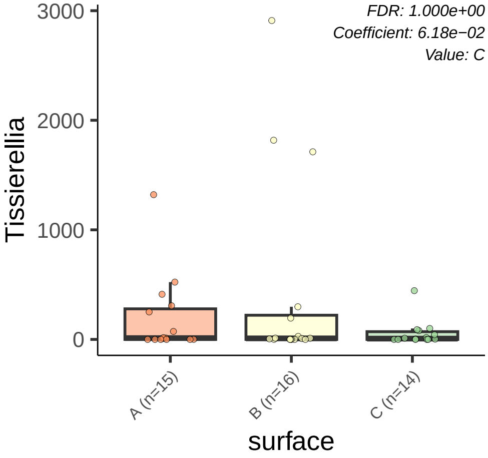
FDR: 1.000e+00
Coefficient: 6.18e−02
Value: C
Tissierellia
3000
2000
1000
0
A (n=15)
B (n=16)
C (n=14)
surface

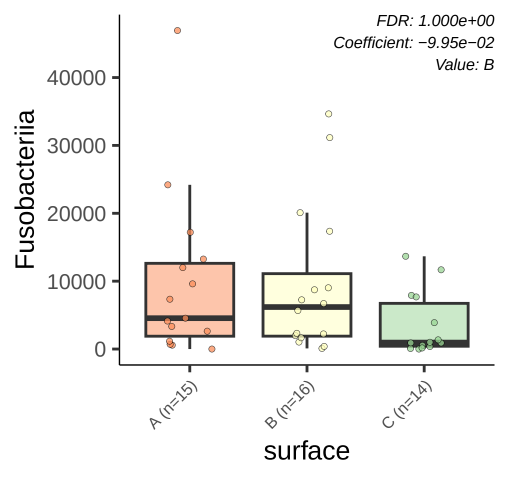
FDR: 1.000e+00
Coefficient: −9.95e−02
Value: B
Fusobacteriia
40000
30000
20000
10000
0
A (n=15)
B (n=16)
C (n=14)
surface

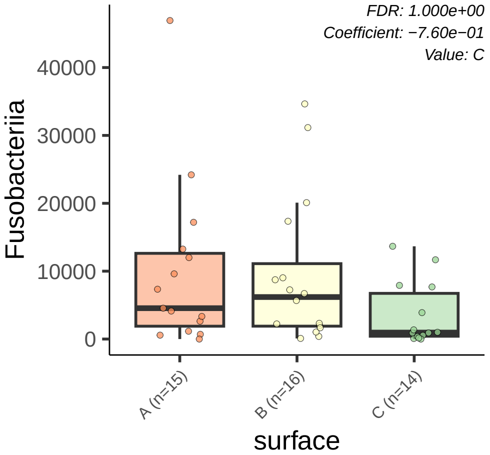

FDR: 1.000e+00
Coefficient: −7.60e−01
Value: C
Fusobacteriia
40000
30000
20000
10000
0
A (n=15)
B (n=16)
C (n=14)
surface

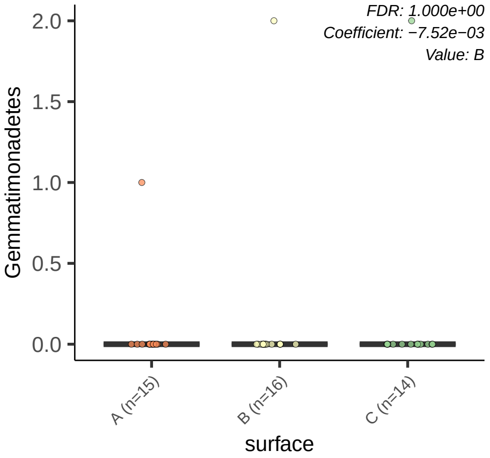
FDR: 1.000e+00
Coefficient: −7.52e−03
Value: B
Gemmatimonadetes
2.0
1.5
1.0
0.5
0.0
A (n=15)
B (n=16)
C (n=14)
surface

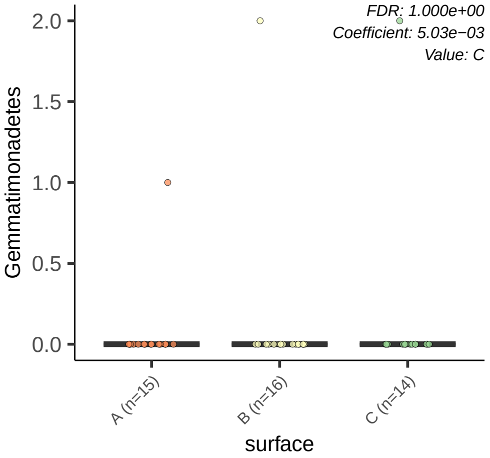
FDR: 1.000e+00
Coefficient: 5.03e−03
Value: C
Gemmatimonadetes
2.0
1.5
1.0
0.5
0.0
A (n=15)
B (n=16)
C (n=14)
surface

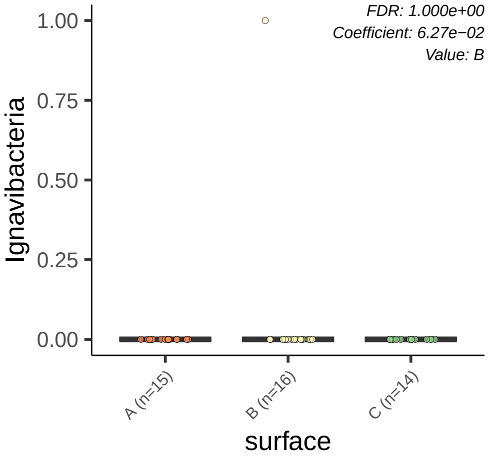

FDR: 1.000e+00
Coefficient: 6.27e−02
Value: B
Ignavibacteria
1.00
0.75
0.50
0.25
0.00
A (n=15)
B (n=16)
C (n=14)
surface

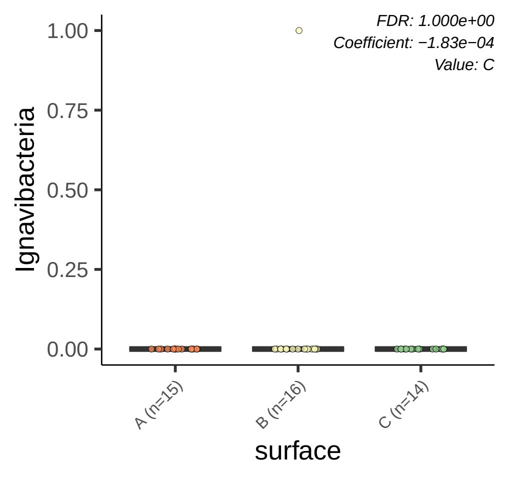
FDR: 1.000e+00
Coefficient: −1.83e−04
Value: C
Ignavibacteria
1.00
0.75
0.50
0.25
0.00
A (n=15)
B (n=16)
C (n=14)
surface

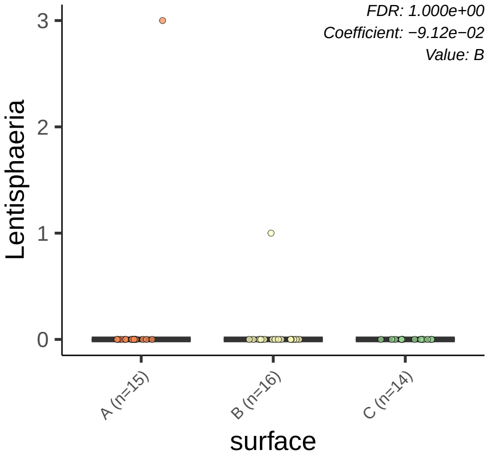
FDR: 1.000e+00
Coefficient: −9.12e−02
Value: B
Lentisphaeria
3
2
1
0
A (n=15)
B (n=16)
C (n=14)
surface

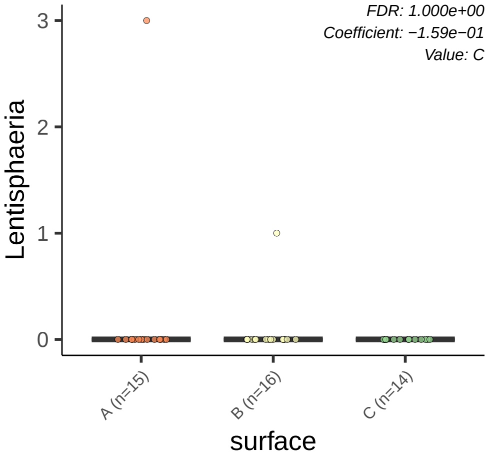
FDR: 1.000e+00
Coefficient: −1.59e−01
Value: C
Lentisphaeria
3
2
1
0
A (n=15)
B (n=16)
C (n=14)
surface

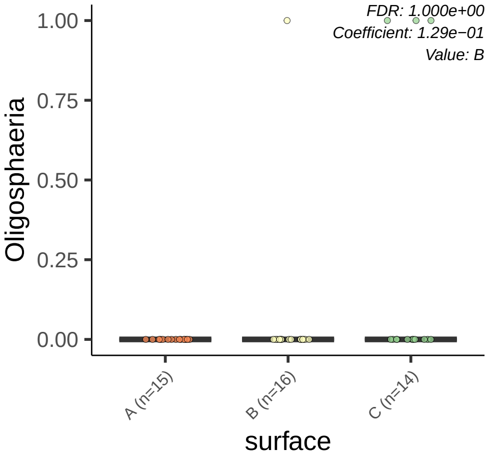
FDR: 1.000e+00
Coefficient: 1.29e−01
Value: B
Oligosphaeria
1.00
0.75
0.50
0.25
0.00
A (n=15)
B (n=16)
C (n=14)
surface

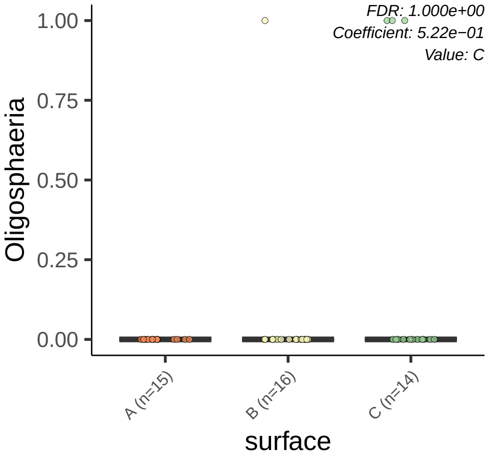

FDR: 1.000e+00
Coefficient: 5.22e−01
Value: C
Oligosphaeria
1.00
0.75
0.50
0.25
0.00
A (n=15)
B (n=16)
C (n=14)
surface

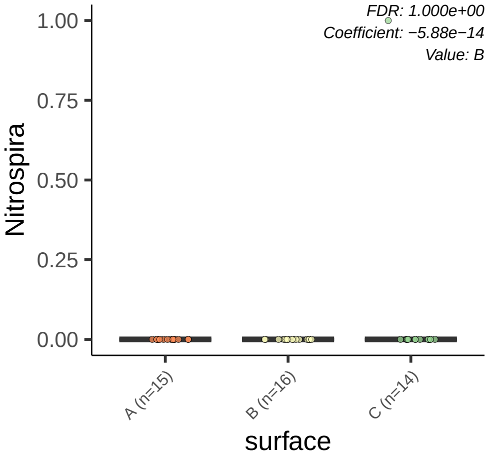

FDR: 1.000e+00
Coefficient: −5.88e−14
Value: B
1.00
0.75
0.50
0.25
0.00
Nitrospira
A (n=15)
B (n=16)
C (n=14)
surface

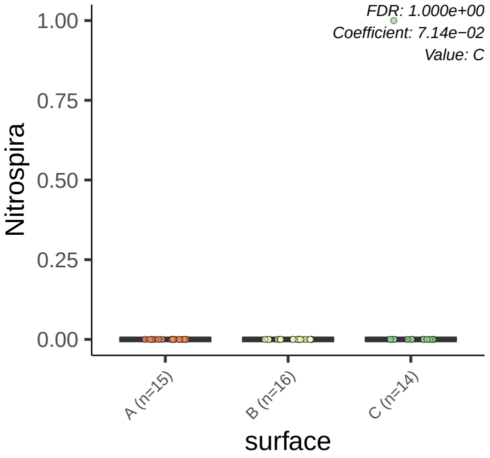

FDR: 1.000e+00
Coefficient: 7.14e−02
Value: C
Nitrospira
1.00
0.75
0.50
0.25
0.00
A (n=15)
B (n=16)
C (n=14)
surface

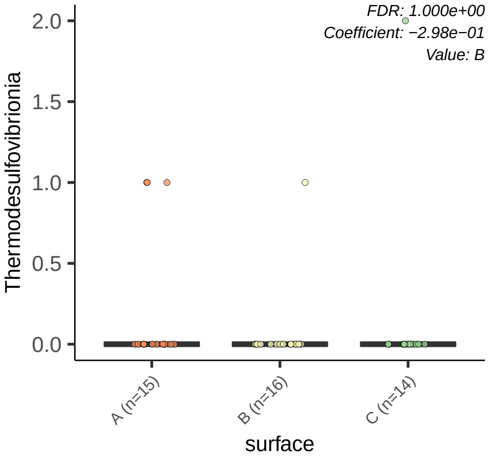
FDR: 1.000e+00
Coefficient: −2.98e−01
Value: B
Thermodesulfovibrionia
2.0
1.5
1.0
0.5
0.0
A (n=15)
B (n=16)
C (n=14)
surface

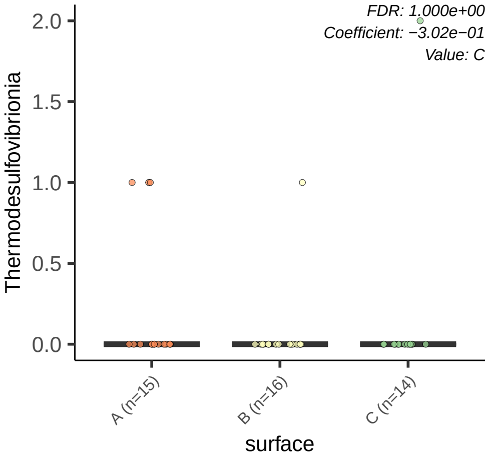

FDR: 1.000e+00
Coefficient: −3.02e−01
Value: C
Thermodesulfovibrionia
2.0
1.5
1.0
0.5
0.0
A (n=15)
B (n=16)
C (n=14)
surface

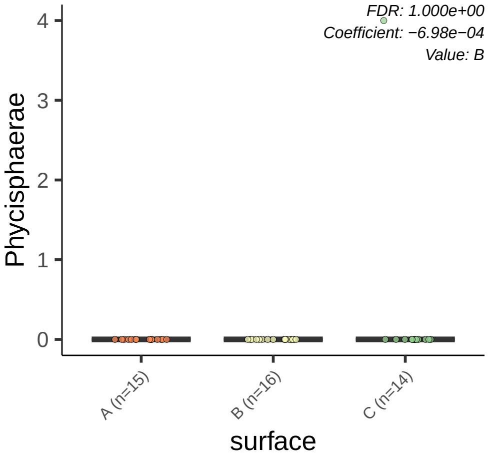
FDR: 1.000e+00
Coefficient: −6.98e−04
Value: B
Phycisphaerae
0
1
2
3
4
A (n=15)
B (n=16)
C (n=14)
surface

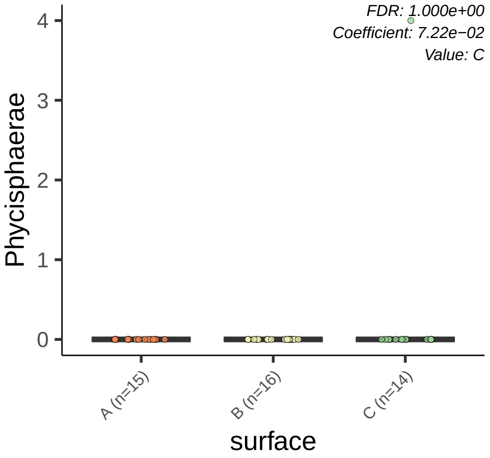
FDR: 1.000e+00
Coefficient: 7.22e−02
Value: C
Phycisphaerae
4
3
2
1
0
A (n=15)
B (n=16)
C (n=14)
surface

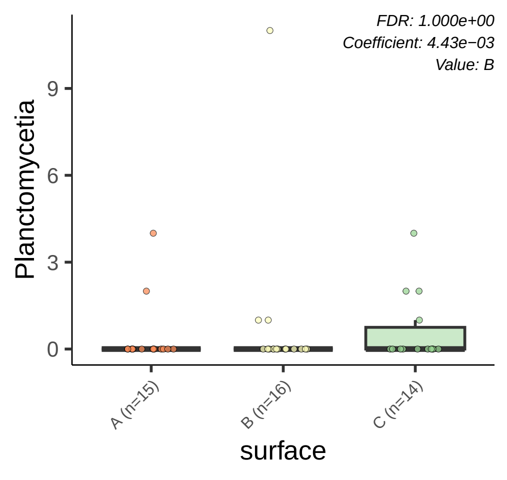
FDR: 1.000e+00
Coefficient: 4.43e−03
Value: B
Planctomycetia
9
6
3
0
A (n=15)
B (n=16)
C (n=14)
surface

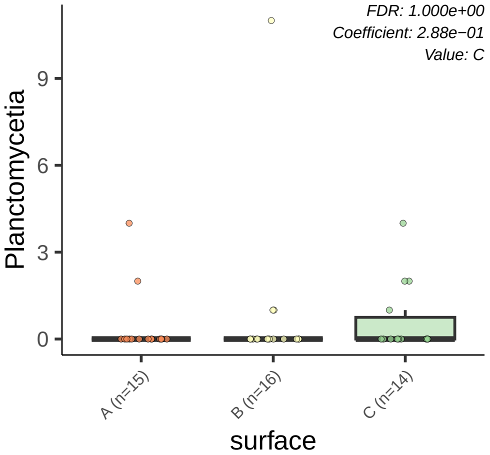

FDR: 1.000e+00
Coefficient: 2.88e−01
Value: C
Planctomycetia
9
6
3
0
A (n=15)
B (n=16)
C (n=14)
surface

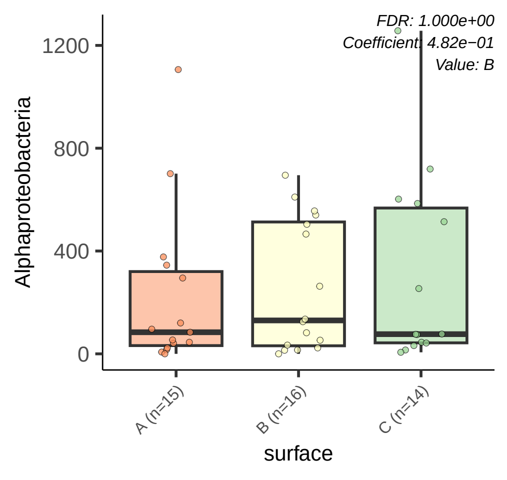

FDR: 1.000e+00
Coefficient: 4.82e−01
Value: B
1200
800
400
0
Alphaproteobacteria
A (n=15)
B (n=16)
C (n=14)
surface

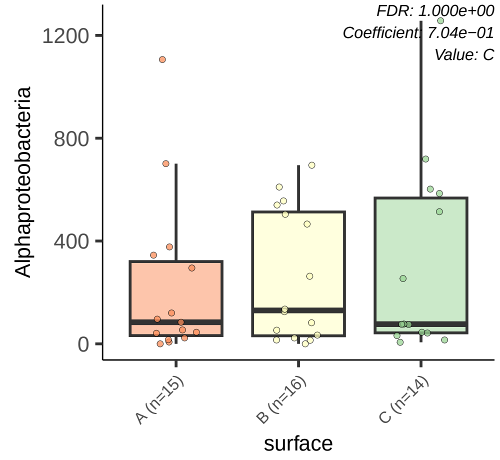

FDR: 1.000e+00
Coefficient: 7.04e−01
Value: C
Alphaproteobacteria
1200
800
400
0
A (n=15)
B (n=16)
C (n=14)
surface

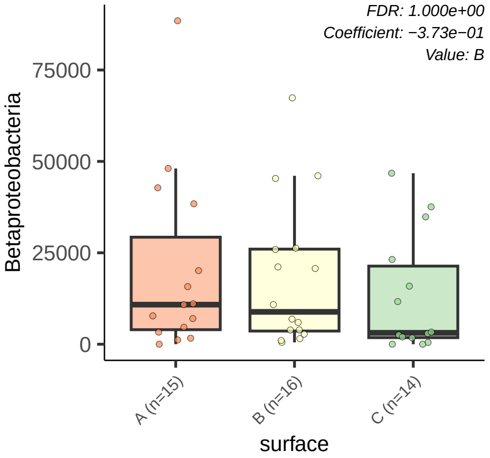
FDR: 1.000e+00
Coefficient: −3.73e−01
Value: B
Betaproteobacteria
75000
50000
25000
0
A (n=15)
B (n=16)
C (n=14)
surface

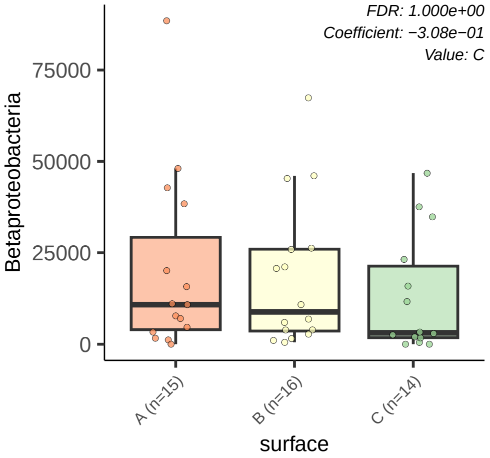
FDR: 1.000e+00
Coefficient: −3.08e−01
Value: C
Betaproteobacteria
75000
50000
25000
0
A (n=15)
B (n=16)
C (n=14)
surface

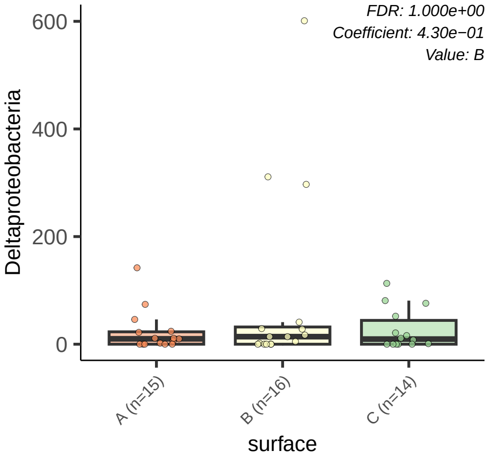
FDR: 1.000e+00
Coefficient: 4.30e−01
Value: B
Deltaproteobacteria
600
400
200
0
A (n=15)
B (n=16)
C (n=14)
surface

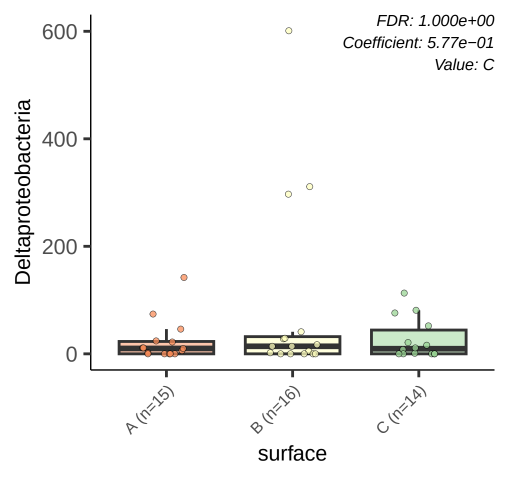

FDR: 1.000e+00
Coefficient: 5.77e−01
Value: C
600
400
200
0
Deltaproteobacteria
A (n=15)
B (n=16)
C (n=14)
surface

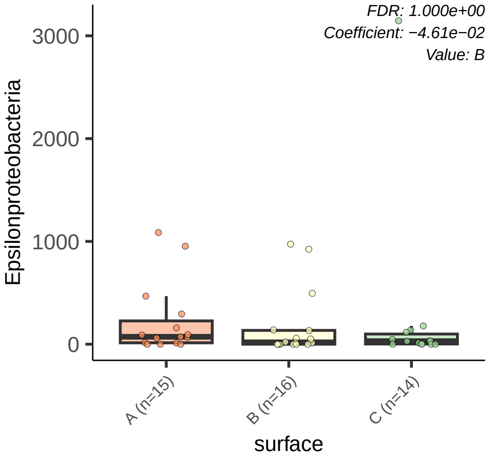
FDR: 1.000e+00
Coefficient: −4.61e−02
Value: B
Epsilonproteobacteria
3000
2000
1000
0
A (n=15)
B (n=16)
C (n=14)
surface

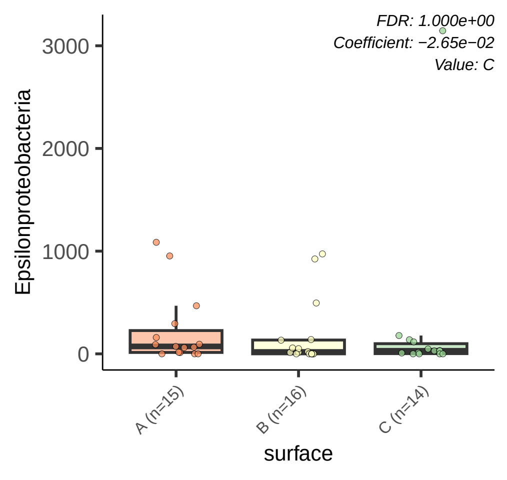

FDR: 1.000e+00
Coefficient: −2.65e−02
Value: C
Epsilonproteobacteria
3000
2000
1000
0
A (n=15)
B (n=16)
C (n=14)
surface

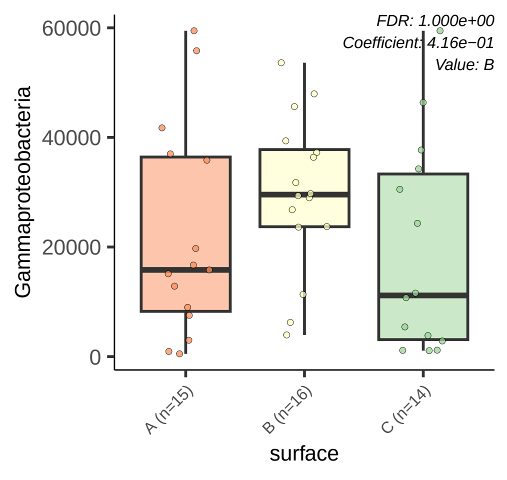

FDR: 1.000e+00
Coefficient: 4.16e−01
Value: B
Gammaproteobacteria
60000
40000
20000
0
A (n=15)
B (n=16)
C (n=14)
surface

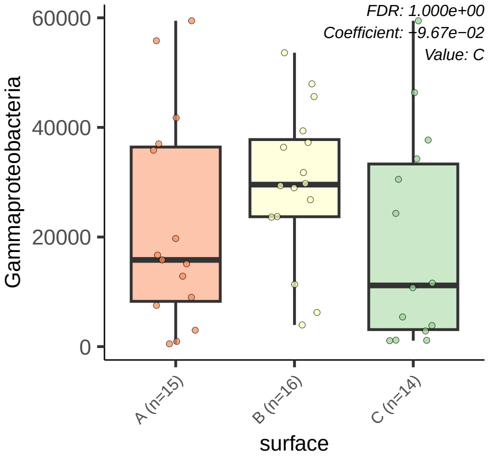
FDR: 1.000e+00
Coefficient: −9.67e−02
Value: C
Gammaproteobacteria
60000
40000
20000
0
A (n=15)
B (n=16)
C (n=14)
surface

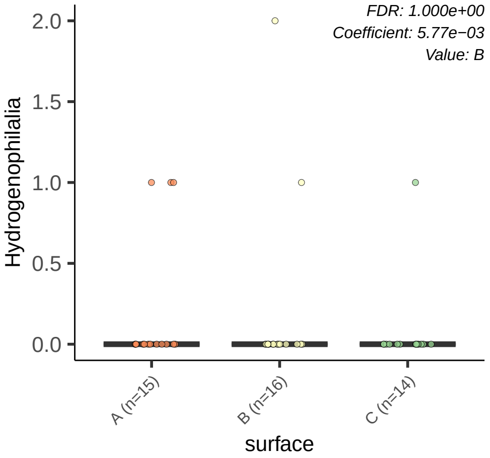
FDR: 1.000e+00
Coefficient: 5.77e−03
Value: B
Hydrogenophilalia
2.0
1.5
1.0
0.5
0.0
A (n=15)
B (n=16)
C (n=14)
surface

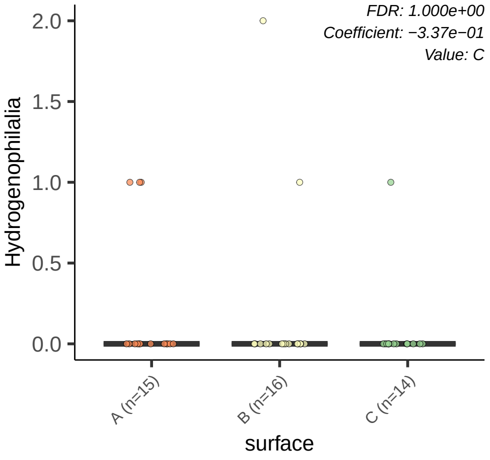

FDR: 1.000e+00
Coefficient: −3.37e−01
Value: C
Hydrogenophilalia
2.0
1.5
1.0
0.5
0.0
A (n=15)
B (n=16)
C (n=14)
surface

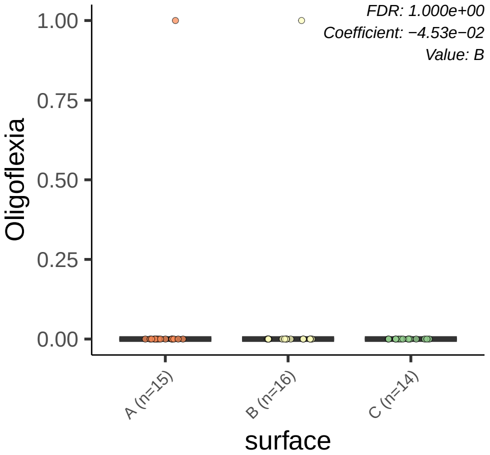
FDR: 1.000e+00
Coefficient: −4.53e−02
Value: B
Oligoflexia
1.00
0.75
0.50
0.25
0.00
A (n=15)
B (n=16)
C (n=14)
surface

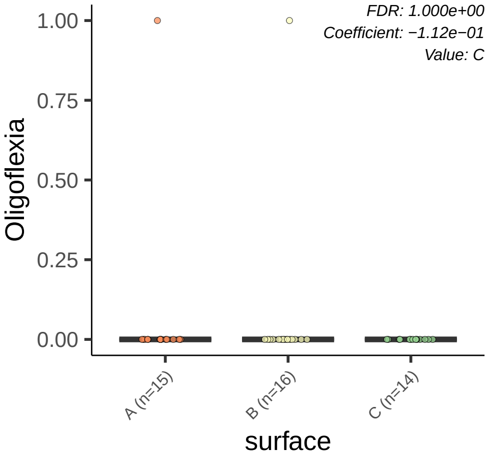

FDR: 1.000e+00
Coefficient: −1.12e−01
Value: C
Oligoflexia
1.00
0.75
0.50
0.25
0.00
A (n=15)
B (n=16)
C (n=14)
surface

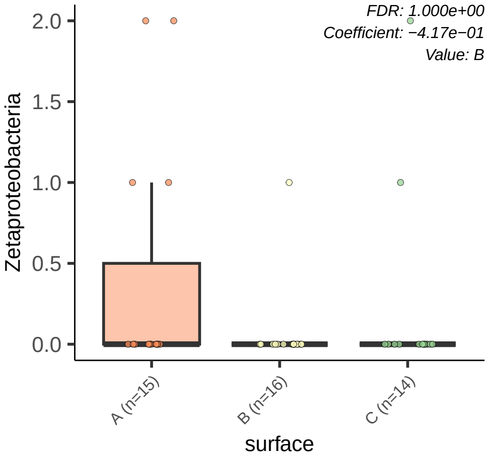
FDR: 1.000e+00
Coefficient: −4.17e−01
Value: B
Zetaproteobacteria
2.0
1.5
1.0
0.5
0.0
A (n=15)
B (n=16)
C (n=14)
surface

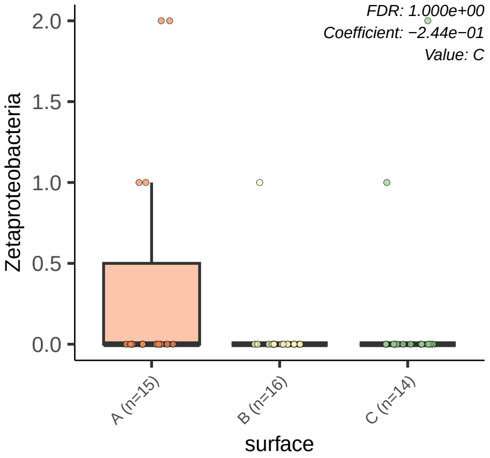

FDR: 1.000e+00
Coefficient: −2.44e−01
Value: C
Zetaproteobacteria
2.0
1.5
1.0
0.5
0.0
A (n=15)
B (n=16)
C (n=14)
surface

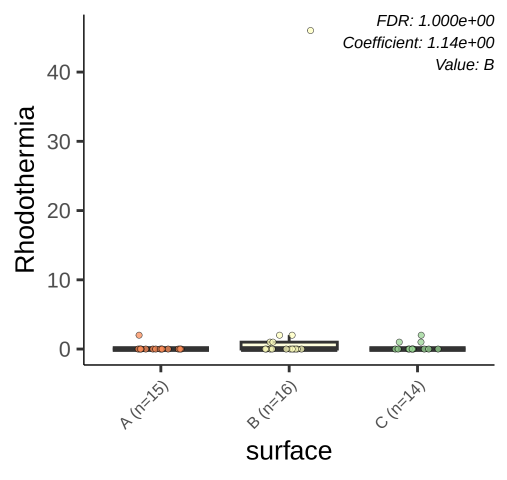
FDR: 1.000e+00
Coefficient: 1.14e+00
Value: B
Rhodothermia
40
30
20
10
0
A (n=15)
B (n=16)
C (n=14)
surface

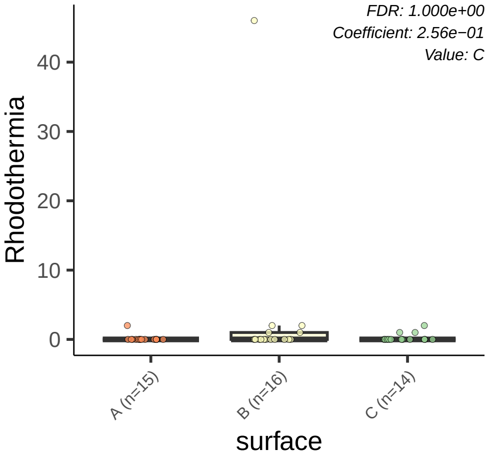

FDR: 1.000e+00
Coefficient: 2.56e−01
Value: C
Rhodothermia
0
10
20
30
40
A (n=15)
B (n=16)
C (n=14)
surface

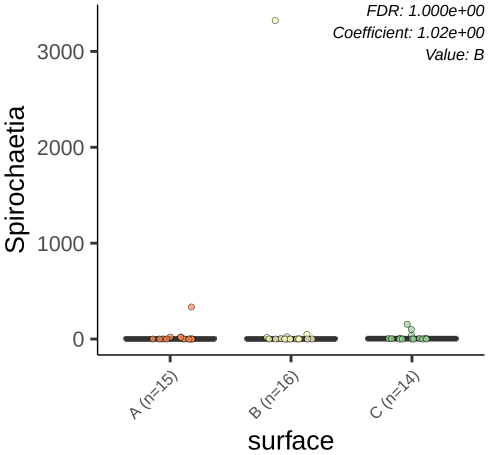
FDR: 1.000e+00
Coefficient: 1.02e+00
Value: B
Spirochaetia
3000
2000
1000
0
A (n=15)
B (n=16)
C (n=14)
surface

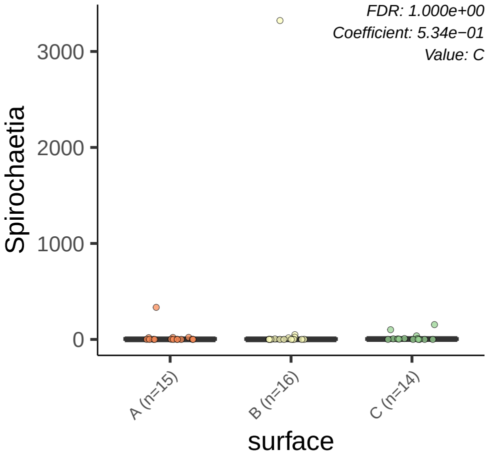

FDR: 1.000e+00
Coefficient: 5.34e−01
Value: C
Spirochaetia
3000
2000
1000
0
A (n=15)
B (n=16)
C (n=14)
surface

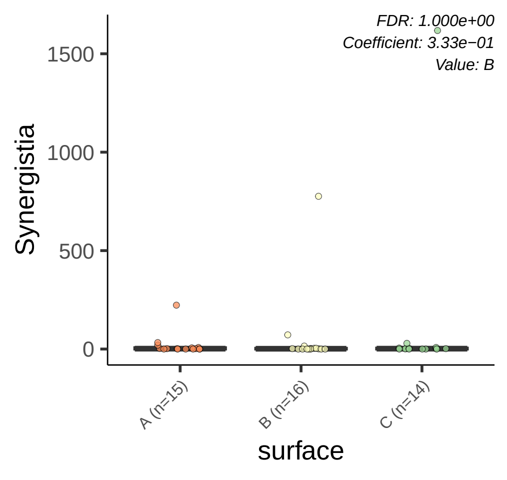
FDR: 1.000e+00
Coefficient: 3.33e−01
Value: B
Synergistia
1500
1000
500
0
A (n=15)
B (n=16)
C (n=14)
surface

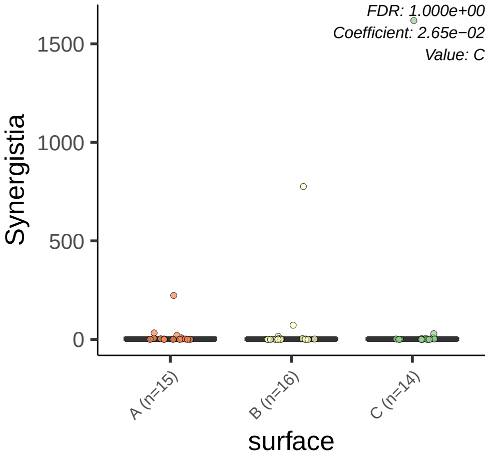
FDR: 1.000e+00
Coefficient: 2.65e−02
Value: C
Synergistia
1500
1000
500
0
A (n=15)
B (n=16)
C (n=14)
surface

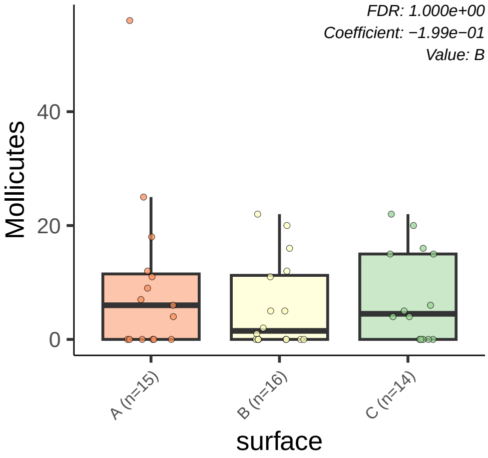
FDR: 1.000e+00
Coefficient: −1.99e−01
Value: B
Mollicutes
40
20
0
A (n=15)
B (n=16)
C (n=14)
surface

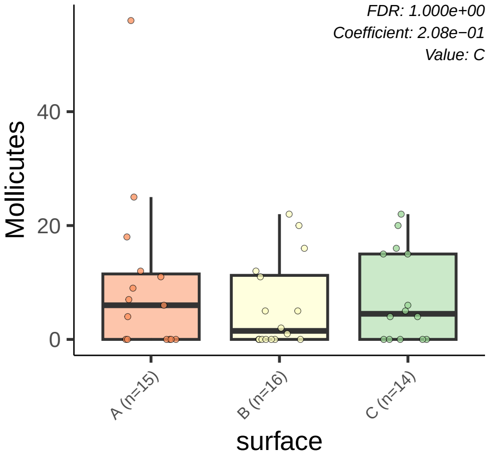
FDR: 1.000e+00
Coefficient: 2.08e−01
Value: C
Mollicutes
0
20
40
A (n=15)
B (n=16)
C (n=14)
surface

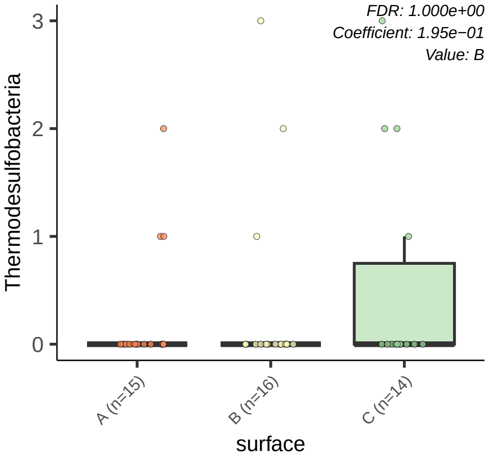

FDR: 1.000e+00
Coefficient: 1.95e−01
Value: B
Thermodesulfobacteria
3
2
1
0
A (n=15)
B (n=16)
C (n=14)
surface

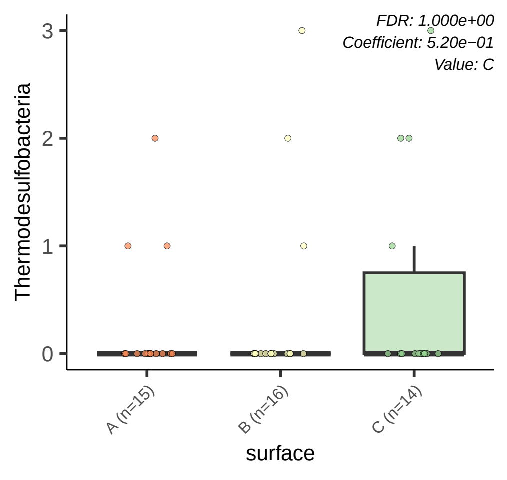
FDR: 1.000e+00
Coefficient: 5.20e−01
Value: C
Thermodesulfobacteria
0
1
2
3
A (n=15)
B (n=16)
C (n=14)
surface

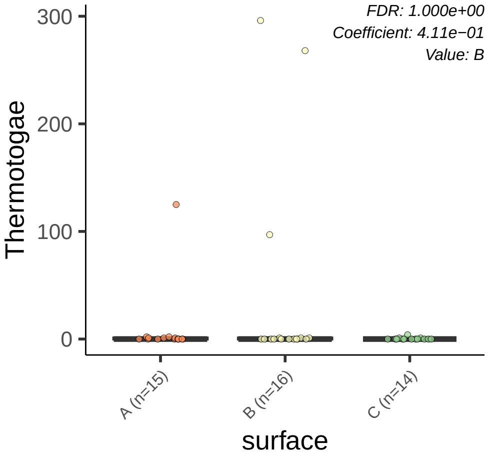
FDR: 1.000e+00
Coefficient: 4.11e−01
Value: B
300
200
100
0
Thermotogae
A (n=15)
B (n=16)
C (n=14)
surface

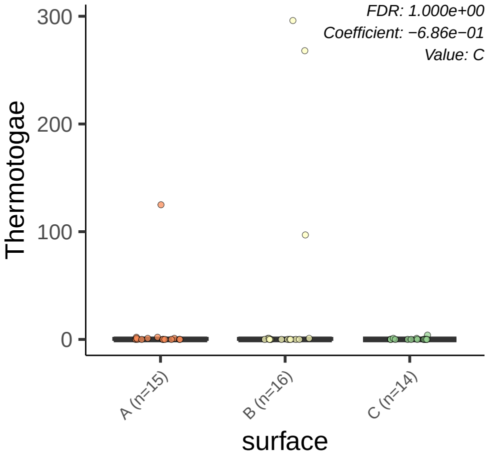

FDR: 1.000e+00
Coefficient: −6.86e−01
Value: C
300
200
100
0
Thermotogae
A (n=15)
B (n=16)
C (n=14)
surface

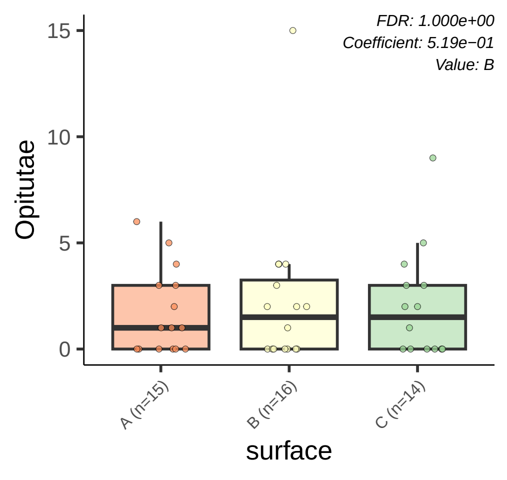
FDR: 1.000e+00
Coefficient: 5.19e−01
Value: B
Opitutae
15
10
5
0
A (n=15)
B (n=16)
C (n=14)
surface

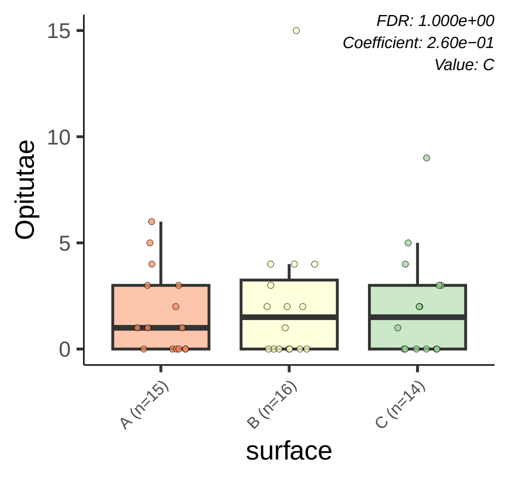
FDR: 1.000e+00
Coefficient: 2.60e−01
Value: C
Opitutae
15
10
5
0
A (n=15)
B (n=16)
C (n=14)
surface

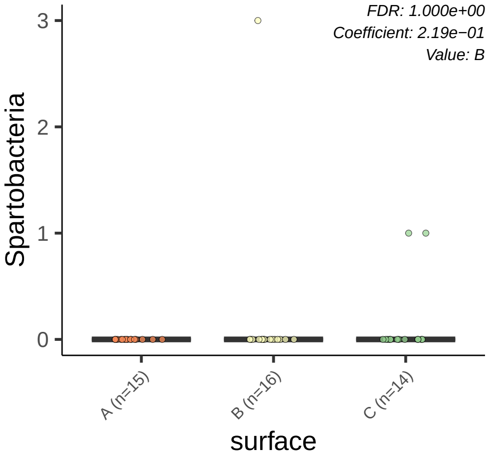
FDR: 1.000e+00
Coefficient: 2.19e−01
Value: B
Spartobacteria
3
2
1
0
A (n=15)
B (n=16)
C (n=14)
surface

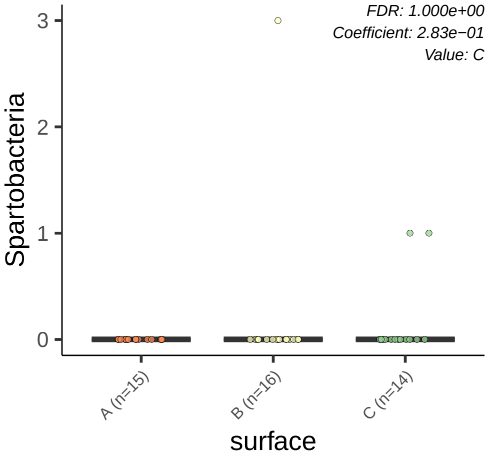
FDR: 1.000e+00
Coefficient: 2.83e−01
Value: C
Spartobacteria
0
1
2
3
A (n=15)
B (n=16)
C (n=14)
surface

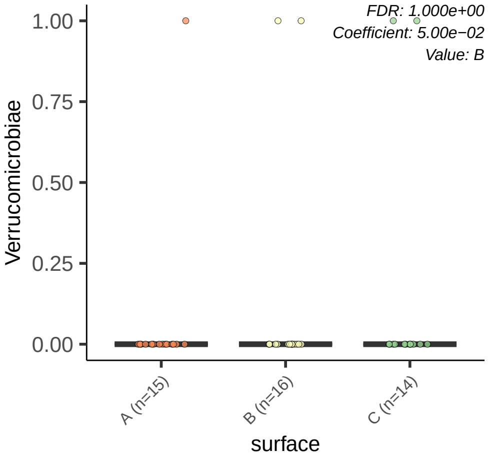

FDR: 1.000e+00
Coefficient: 5.00e−02
Value: B
Verrucomicrobiae
1.00
0.75
0.50
0.25
0.00
A (n=15)
B (n=16)
C (n=14)
surface

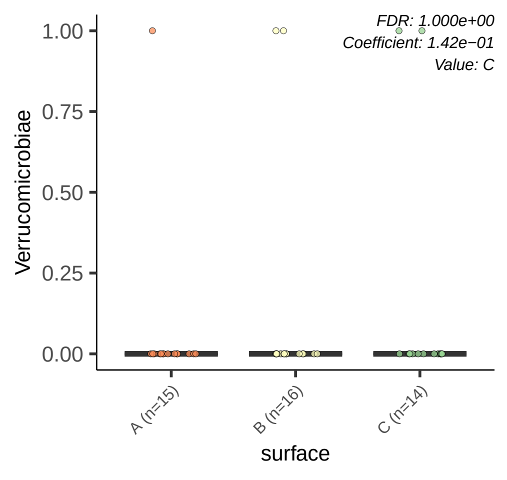
FDR: 1.000e+00
Coefficient: 1.42e−01
Value: C
Verrucomicrobiae
1.00
0.75
0.50
0.25
0.00
A (n=15)
B (n=16)
C (n=14)
surface

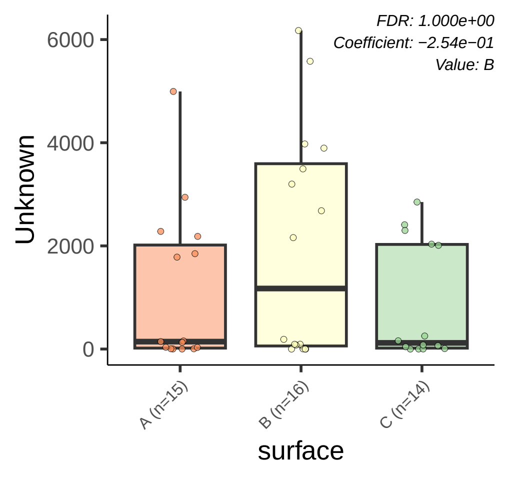

FDR: 1.000e+00
Coefficient: −2.54e−01
Value: B
Unknown
6000
4000
2000
0
A (n=15)
B (n=16)
C (n=14)
surface

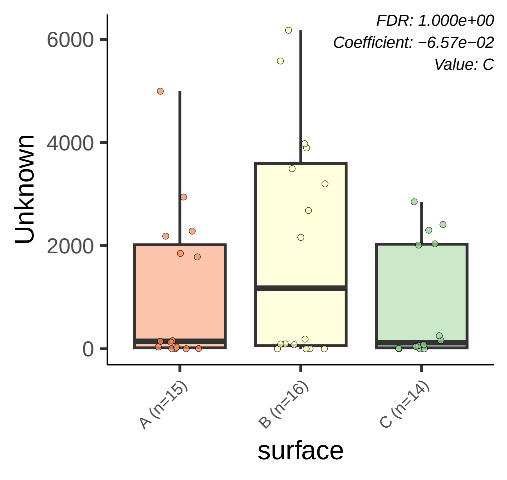

FDR: 1.000e+00
Coefficient: −6.57e−02
Value: C
Unknown
6000
4000
2000
0
A (n=15)
B (n=16)
C (n=14)
surface

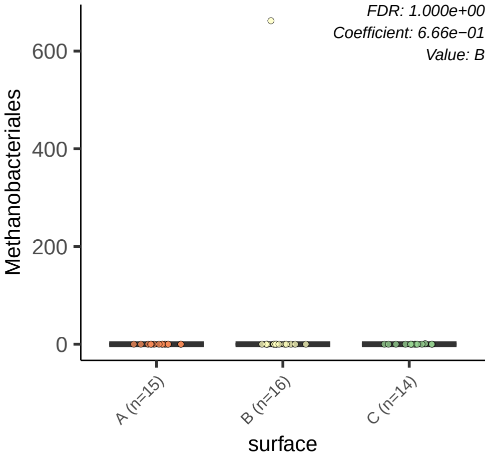
FDR: 1.000e+00
Coefficient: 6.66e−01
Value: B
Methanobacteriales
600
400
200
0
A (n=15)
B (n=16)
C (n=14)
surface

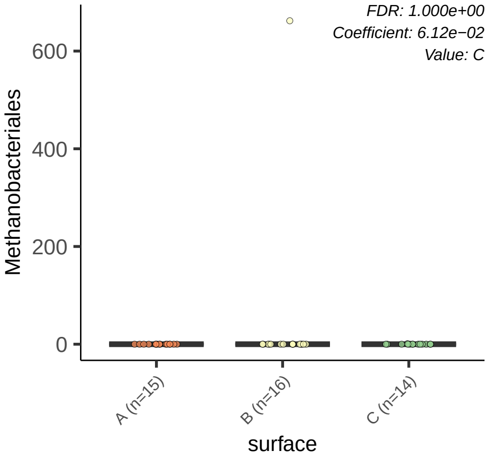
FDR: 1.000e+00
Coefficient: 6.12e−02
Value: C
Methanobacteriales
600
400
200
0
A (n=15)
B (n=16)
C (n=14)
surface

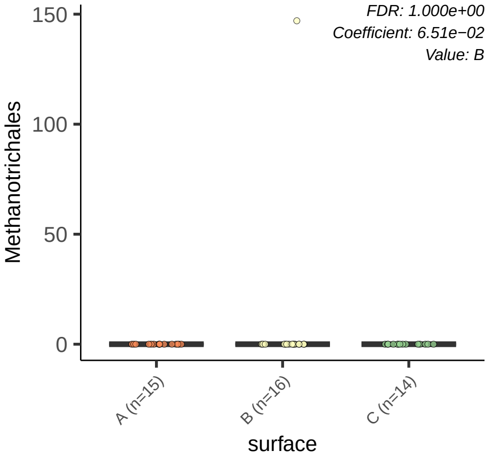

FDR: 1.000e+00
Coefficient: 6.51e−02
Value: B
Methanotrichales
150
100
50
0
A (n=15)
B (n=16)
C (n=14)
surface

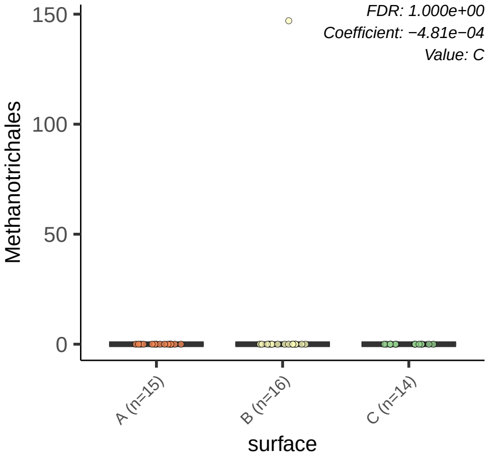
FDR: 1.000e+00
Coefficient: −4.81e−04
Value: C
Methanotrichales
150
100
50
0
A (n=15)
B (n=16)
C (n=14)
surface

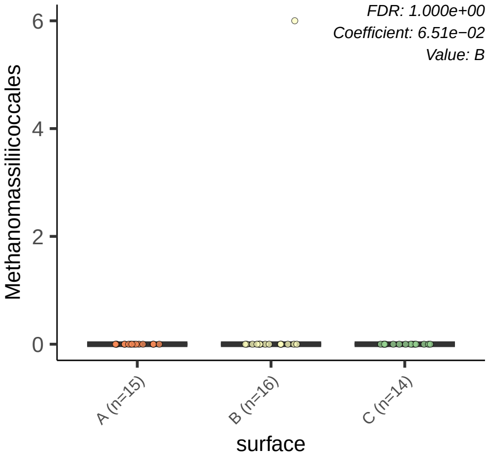

FDR: 1.000e+00
Coefficient: 6.51e−02
Value: B
Methanomassiliicoccales
6
4
2
0
A (n=15)
B (n=16)
C (n=14)
surface

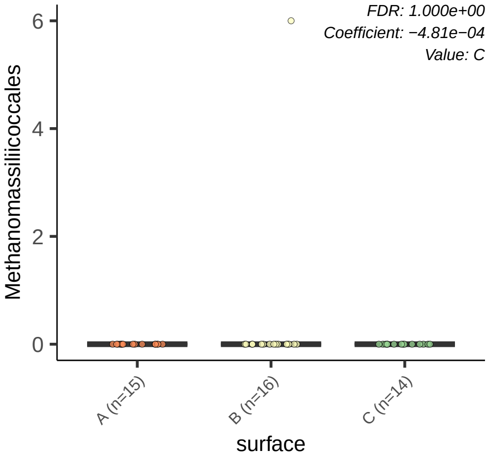
FDR: 1.000e+00
Coefficient: −4.81e−04
Value: C
Methanomassiliicoccales
6
4
2
0
A (n=15)
B (n=16)
C (n=14)
surface

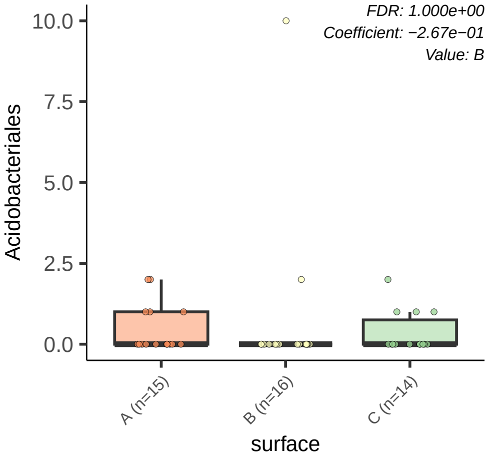

FDR: 1.000e+00
Coefficient: −2.67e−01
Value: B
Acidobacteriales
10.0
7.5
5.0
2.5
0.0
A (n=15)
B (n=16)
C (n=14)
surface

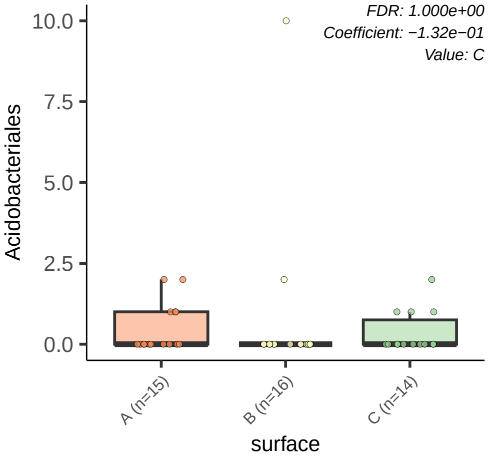
FDR: 1.000e+00
Coefficient: −1.32e−01
Value: C
Acidobacteriales
10.0
7.5
5.0
2.5
0.0
A (n=15)
B (n=16)
C (n=14)
surface

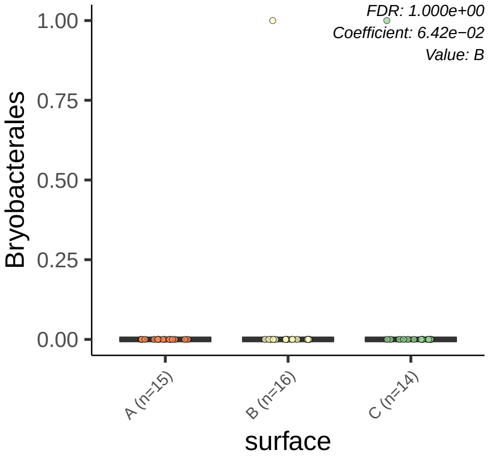

FDR: 1.000e+00
Coefficient: 6.42e−02
Value: B
1.00
0.75
0.50
0.25
0.00
Bryobacterales
A (n=15)
B (n=16)
C (n=14)
surface

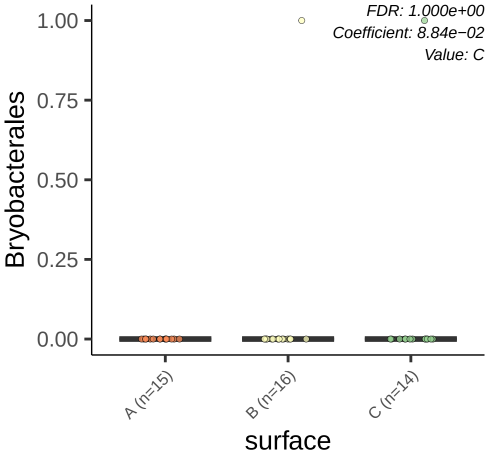

FDR: 1.000e+00
Coefficient: 8.84e−02
Value: C
Bryobacterales
1.00
0.75
0.50
0.25
0.00
A (n=15)
B (n=16)
C (n=14)
surface

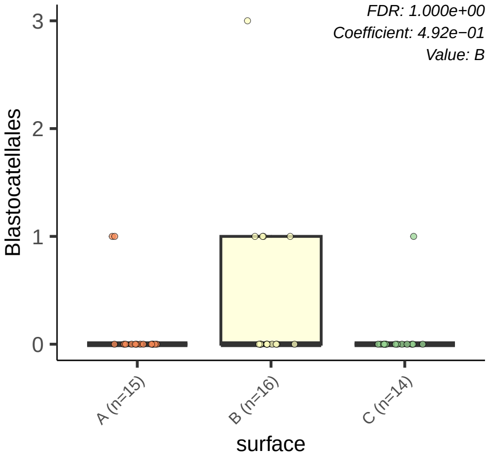

FDR: 1.000e+00
Coefficient: 4.92e−01
Value: B
Blastocatellales
3
2
1
0
A (n=15)
B (n=16)
C (n=14)
surface

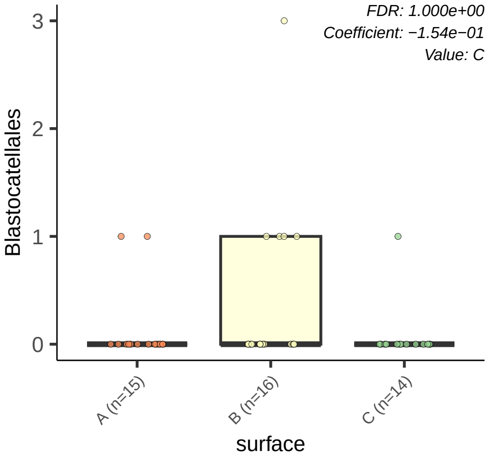
FDR: 1.000e+00
Coefficient: −1.54e−01
Value: C
Blastocatellales
3
2
1
0
A (n=15)
B (n=16)
C (n=14)
surface

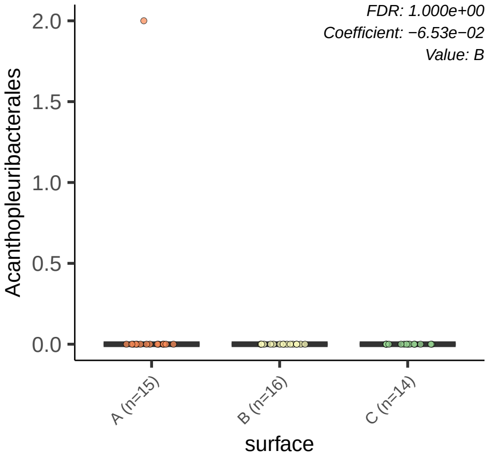

FDR: 1.000e+00
Coefficient: −6.53e−02
Value: B
Acanthopleuribacterales
2.0
1.5
1.0
0.5
0.0
A (n=15)
B (n=16)
C (n=14)
surface

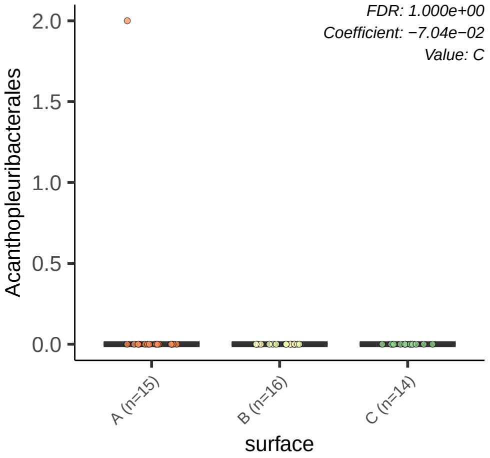
FDR: 1.000e+00
Coefficient: −7.04e−02
Value: C
Acanthopleuribacterales
2.0
1.5
1.0
0.5
0.0
A (n=15)
B (n=16)
C (n=14)
surface

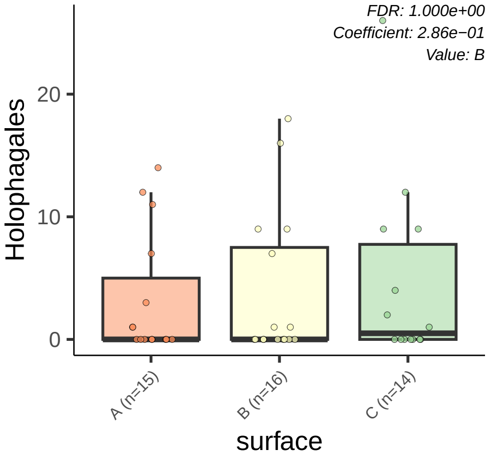
FDR: 1.000e+00
Coefficient: 2.86e−01
Value: B
Holophagales
20
10
0
A (n=15)
B (n=16)
C (n=14)
surface

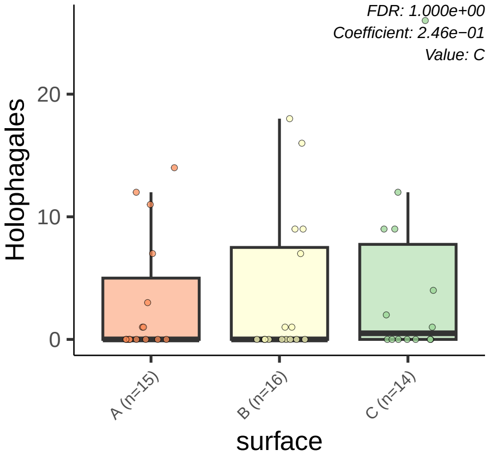
FDR: 1.000e+00
Coefficient: 2.46e−01
Value: C
Holophagales
20
10
0
A (n=15)
B (n=16)
C (n=14)
surface

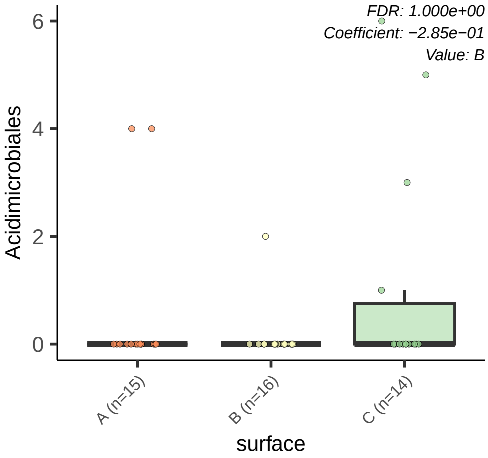

FDR: 1.000e+00
Coefficient: −2.85e−01
Value: B
Acidimicrobiales
6
4
2
0
A (n=15)
B (n=16)
C (n=14)
surface

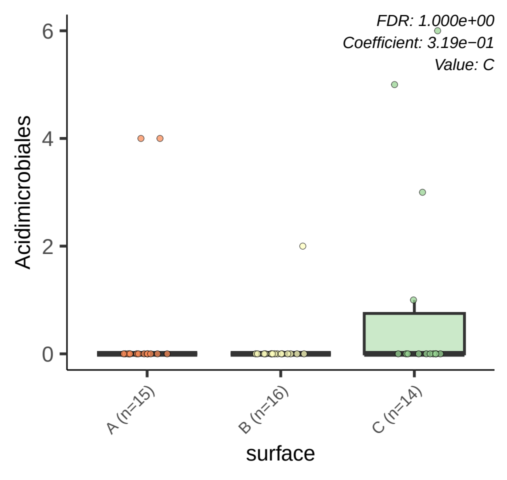
FDR: 1.000e+00
Coefficient: 3.19e−01
Value: C
Acidimicrobiales
6
4
2
0
A (n=15)
B (n=16)
C (n=14)
surface

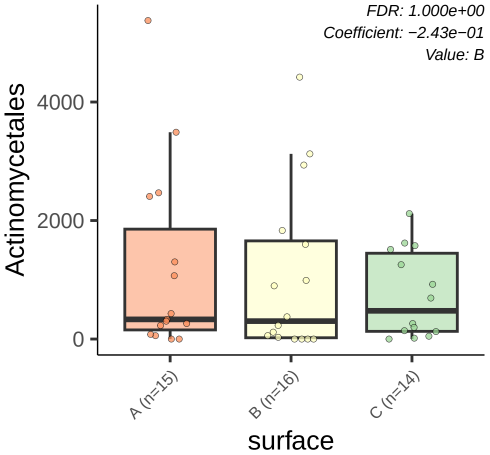

FDR: 1.000e+00
Coefficient: −2.43e−01
Value: B
Actinomycetales
4000
2000
0
A (n=15)
B (n=16)
C (n=14)
surface

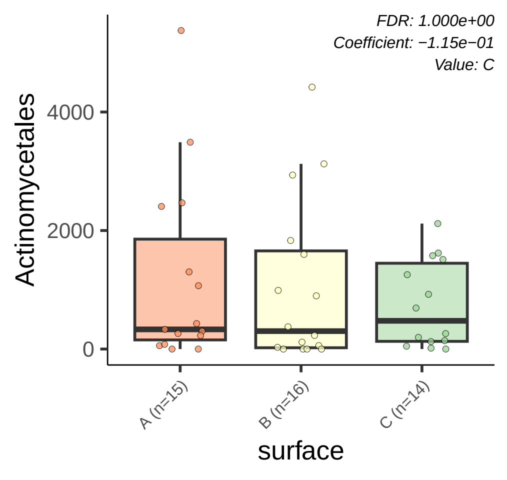
FDR: 1.000e+00
Coefficient: −1.15e−01
Value: C
Actinomycetales
4000
2000
0
A (n=15)
B (n=16)
C (n=14)
surface

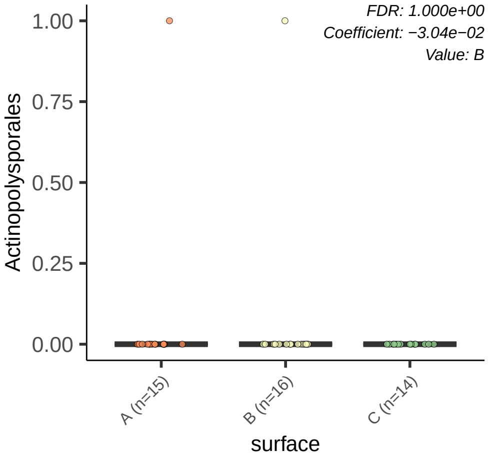
FDR: 1.000e+00
Coefficient: −3.04e−02
Value: B
Actinopolysporales
1.00
0.75
0.50
0.25
0.00
A (n=15)
B (n=16)
C (n=14)
surface

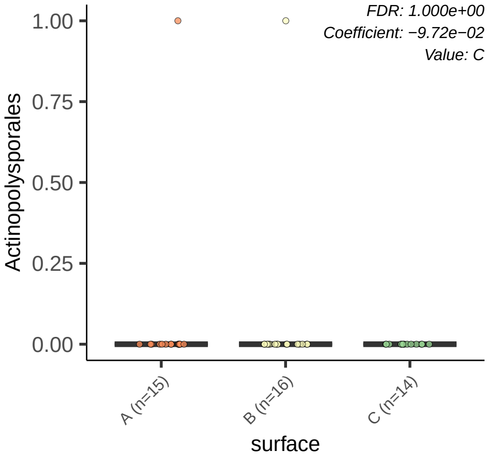
FDR: 1.000e+00
Coefficient: −9.72e−02
Value: C
Actinopolysporales
1.00
0.75
0.50
0.25
0.00
A (n=15)
B (n=16)
C (n=14)
surface

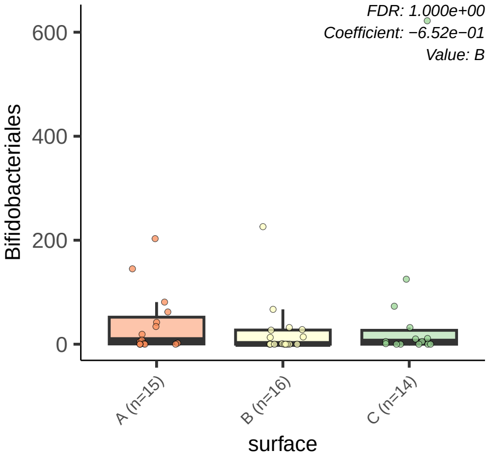

FDR: 1.000e+00
Coefficient: −6.52e−01
Value: B
Bifidobacteriales
600
400
200
0
A (n=15)
B (n=16)
C (n=14)
surface

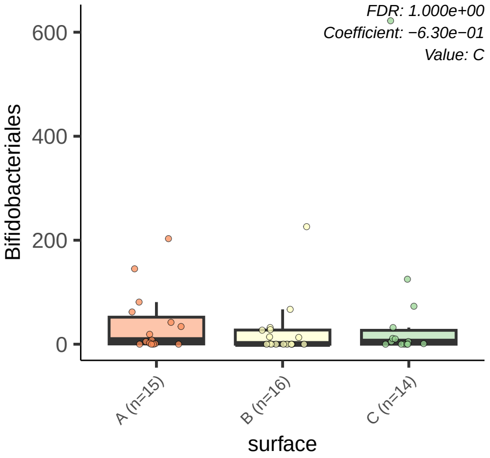

FDR: 1.000e+00
Coefficient: −6.30e−01
Value: C
Bifidobacteriales
600
400
200
0
A (n=15)
B (n=16)
C (n=14)
surface

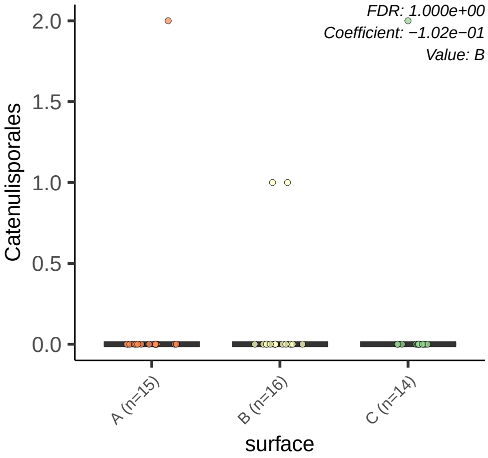

FDR: 1.000e+00
Coefficient: −1.02e−01
Value: B
Catenulisporales
2.0
1.5
1.0
0.5
0.0
A (n=15)
B (n=16)
C (n=14)
surface

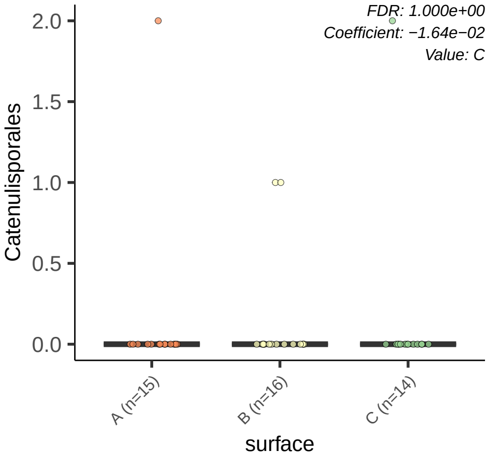

FDR: 1.000e+00
Coefficient: −1.64e−02
Value: C
Catenulisporales
2.0
1.5
1.0
0.5
0.0
A (n=15)
B (n=16)
C (n=14)
surface

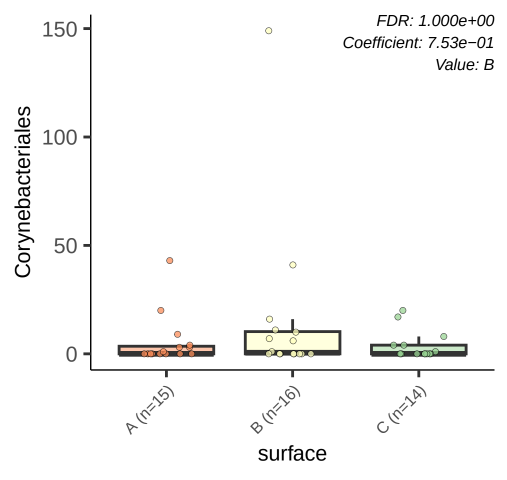

FDR: 1.000e+00
Coefficient: 7.53e−01
Value: B
Corynebacteriales
150
100
50
0
A (n=15)
B (n=16)
C (n=14)
surface

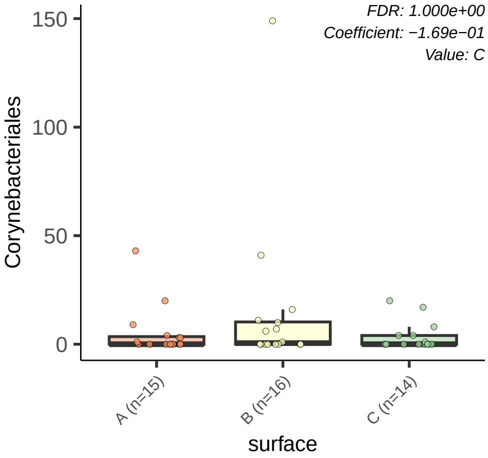

FDR: 1.000e+00
Coefficient: −1.69e−01
Value: C
Corynebacteriales
150
100
50
0
A (n=15)
B (n=16)
C (n=14)
surface

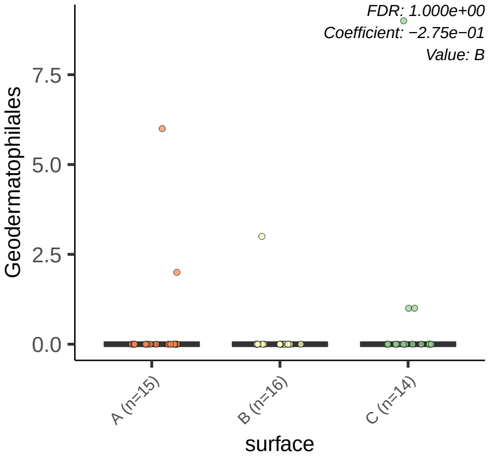
FDR: 1.000e+00
Coefficient: −2.75e−01
Value: B
Geodermatophilales
7.5
5.0
2.5
0.0
A (n=15)
B (n=16)
C (n=14)
surface

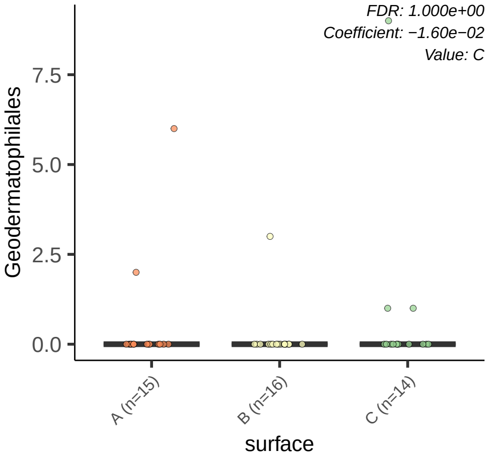
FDR: 1.000e+00
Coefficient: −1.60e−02
Value: C
Geodermatophilales
7.5
5.0
2.5
0.0
A (n=15)
B (n=16)
C (n=14)
surface

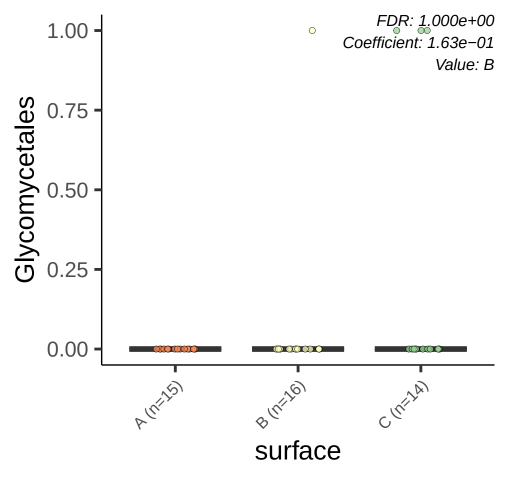

FDR: 1.000e+00
Coefficient: 1.63e−01
Value: B
Glycomycetales
1.00
0.75
0.50
0.25
0.00
A (n=15)
B (n=16)
C (n=14)
surface

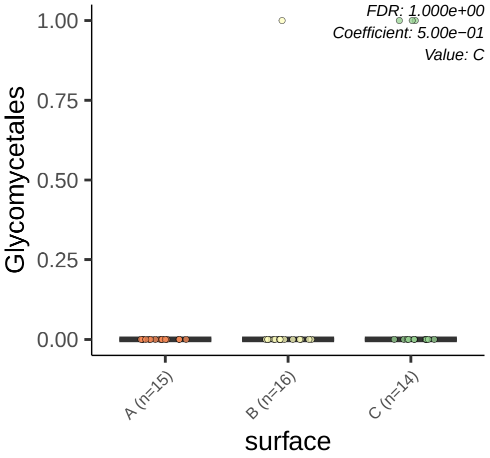
FDR: 1.000e+00
Coefficient: 5.00e−01
Value: C
Glycomycetales
1.00
0.75
0.50
0.25
0.00
A (n=15)
B (n=16)
C (n=14)
surface

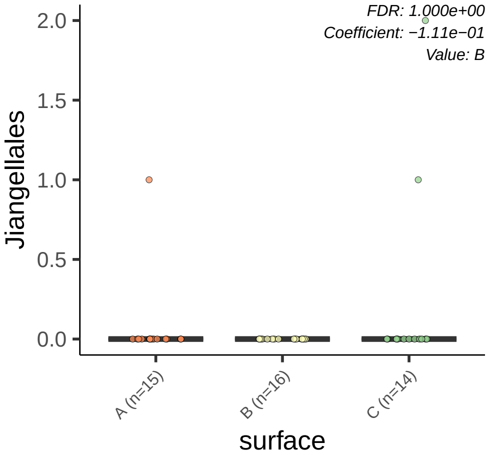

FDR: 1.000e+00
Coefficient: −1.11e−01
Value: B
Jiangellales
2.0
1.5
1.0
0.5
0.0
A (n=15)
B (n=16)
C (n=14)
surface

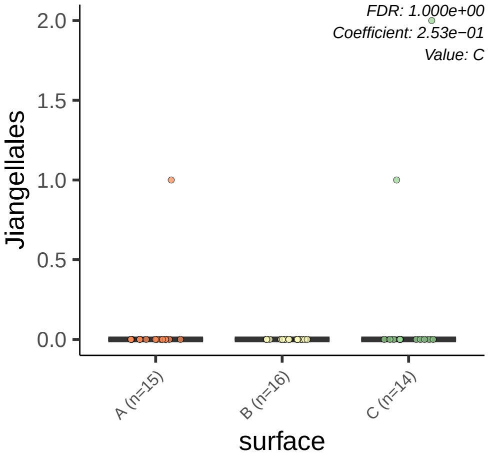
FDR: 1.000e+00
Coefficient: 2.53e−01
Value: C
Jiangellales
2.0
1.5
1.0
0.5
0.0
A (n=15)
B (n=16)
C (n=14)
surface

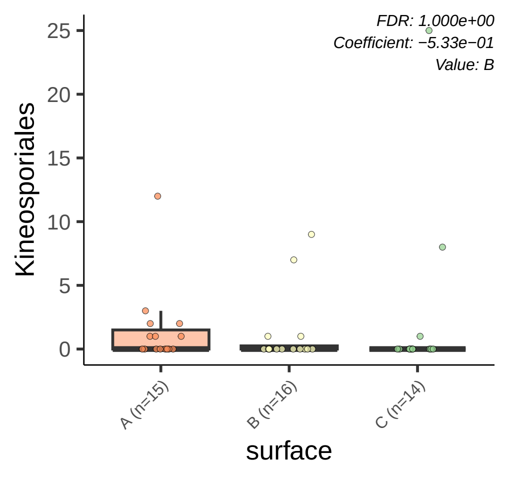

FDR: 1.000e+00
Coefficient: −5.33e−01
Value: B
Kineosporiales
25
20
15
10
5
0
A (n=15)
B (n=16)
C (n=14)
surface

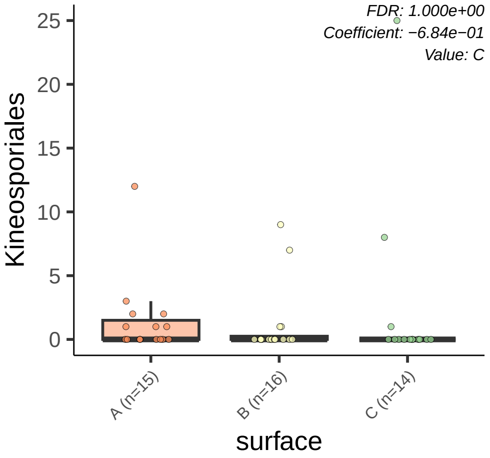

FDR: 1.000e+00
Coefficient: −6.84e−01
Value: C
Kineosporiales
25
20
15
10
5
0
A (n=15)
B (n=16)
C (n=14)
surface

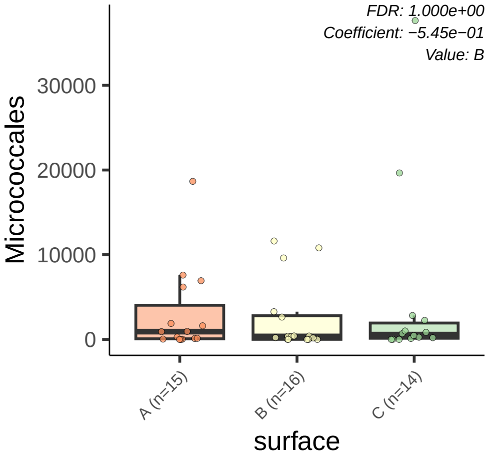
FDR: 1.000e+00
Coefficient: −5.45e−01
Value: B
Micrococcales
30000
20000
10000
0
A (n=15)
B (n=16)
C (n=14)
surface

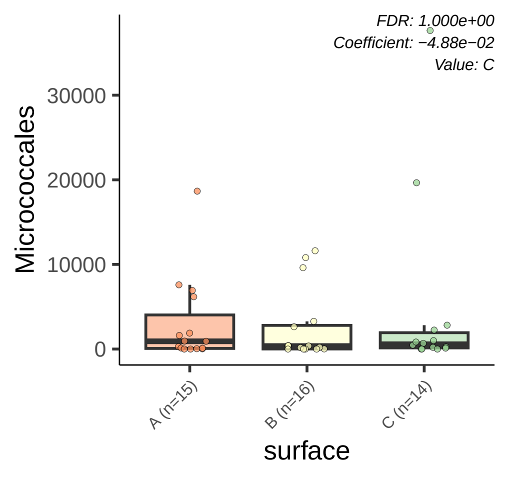

FDR: 1.000e+00
Coefficient: −4.88e−02
Value: C
Micrococcales
30000
20000
10000
0
A (n=15)
B (n=16)
C (n=14)
surface

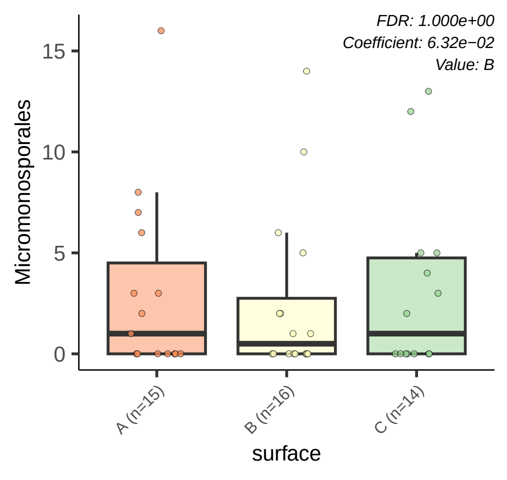
FDR: 1.000e+00
Coefficient: 6.32e−02
Value: B
Micromonosporales
0
5
10
15
A (n=15)
B (n=16)
C (n=14)
surface

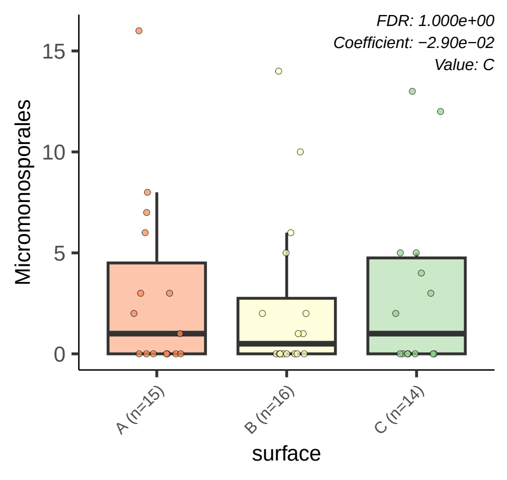
FDR: 1.000e+00
Coefficient: −2.90e−02
Value: C
Micromonosporales
0
5
10
15
A (n=15)
B (n=16)
C (n=14)
surface

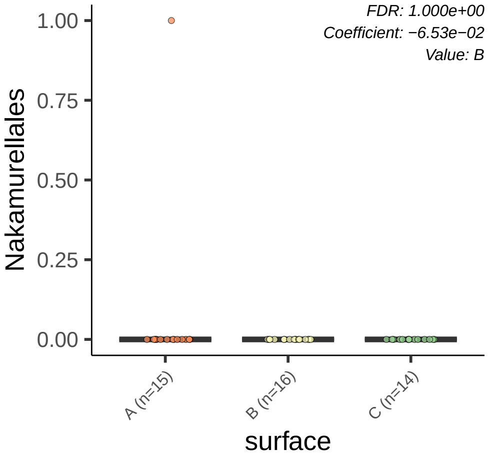
FDR: 1.000e+00
Coefficient: −6.53e−02
Value: B
Nakamurellales
1.00
0.75
0.50
0.25
0.00
A (n=15)
B (n=16)
C (n=14)
surface

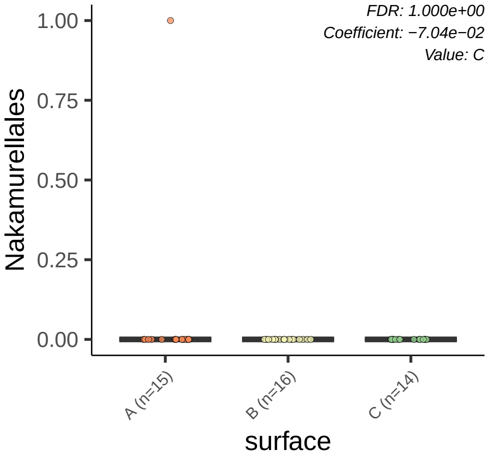
FDR: 1.000e+00
Coefficient: −7.04e−02
Value: C
Nakamurellales
1.00
0.75
0.50
0.25
0.00
A (n=15)
B (n=16)
C (n=14)
surface

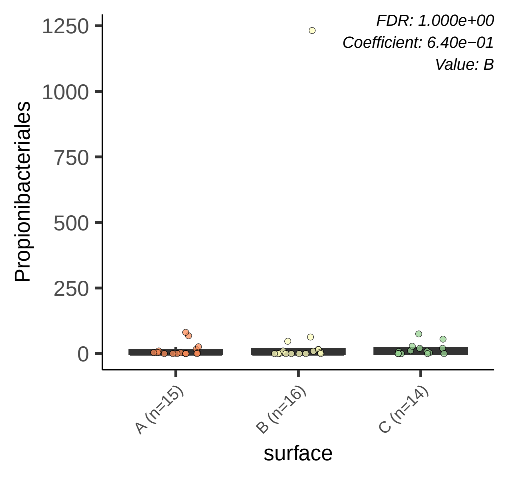

FDR: 1.000e+00
Coefficient: 6.40e−01
Value: B
Propionibacteriales
1250
1000
750
500
250
0
A (n=15)
B (n=16)
C (n=14)
surface

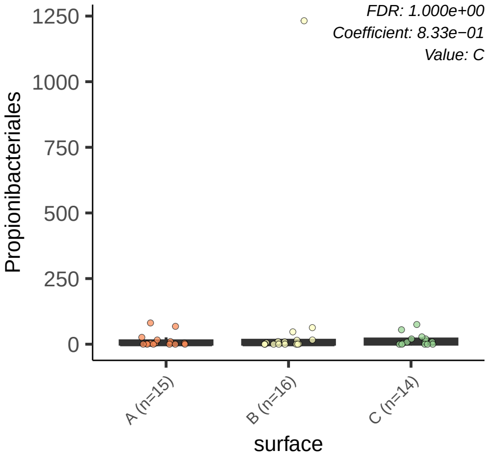

FDR: 1.000e+00
Coefficient: 8.33e−01
Value: C
Propionibacteriales
1250
1000
750
500
250
0
A (n=15)
B (n=16)
C (n=14)
surface

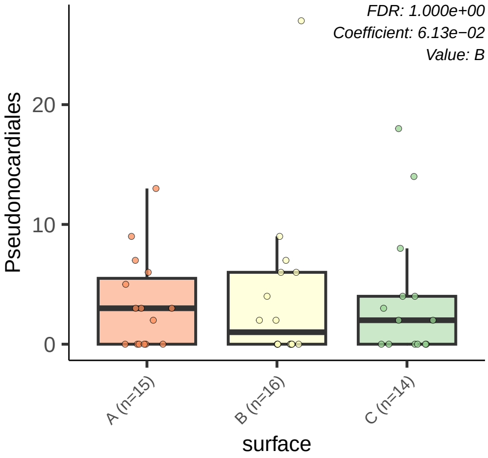
FDR: 1.000e+00
Coefficient: 6.13e−02
Value: B
Pseudonocardiales
0
10
20
A (n=15)
B (n=16)
C (n=14)
surface

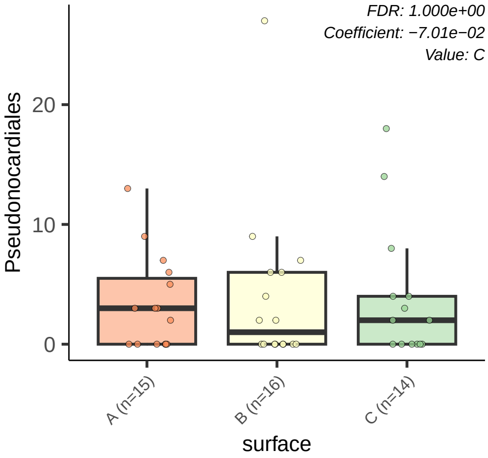

FDR: 1.000e+00
Coefficient: −7.01e−02
Value: C
Pseudonocardiales
20
10
0
A (n=15)
B (n=16)
C (n=14)
surface

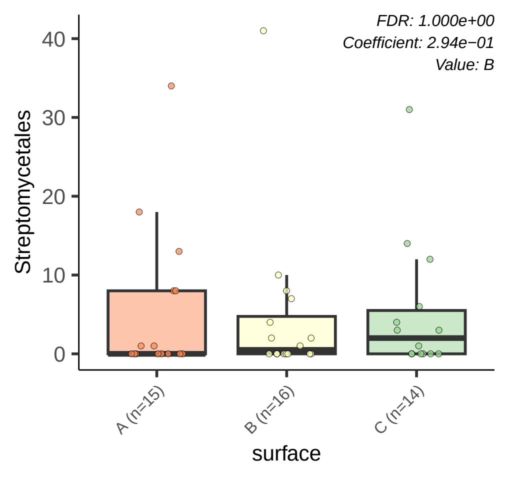

FDR: 1.000e+00
Coefficient: 2.94e−01
Value: B
Streptomycetales
0
10
20
30
40
A (n=15)
B (n=16)
C (n=14)
surface

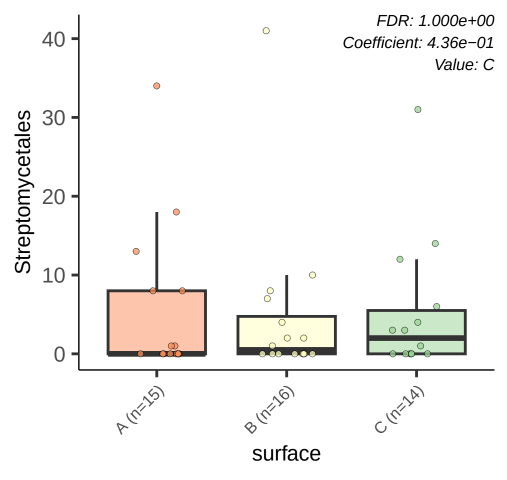

FDR: 1.000e+00
Coefficient: 4.36e−01
Value: C
Streptomycetales
40
30
20
10
0
A (n=15)
B (n=16)
C (n=14)
surface

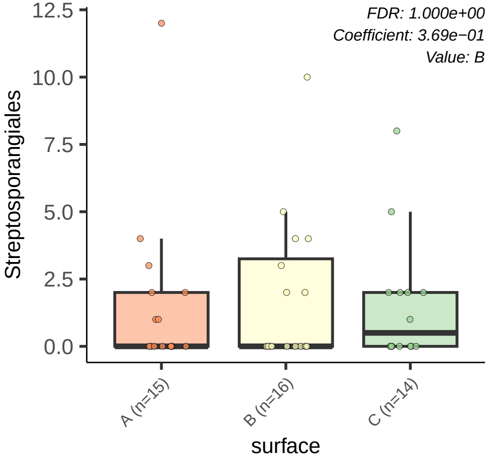

FDR: 1.000e+00
Coefficient: 3.69e−01
Value: B
12.5
10.0
7.5
5.0
2.5
0.0
Streptosporangiales
A (n=15)
B (n=16)
C (n=14)
surface

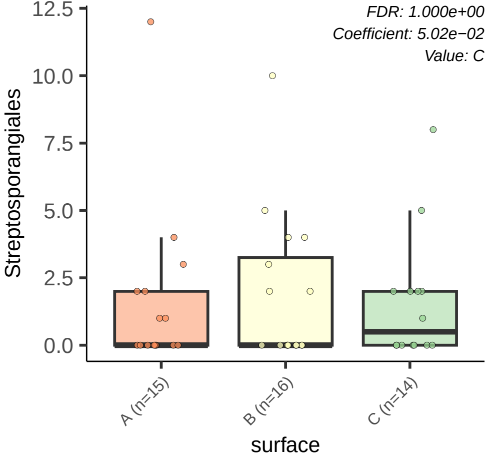

FDR: 1.000e+00
Coefficient: 5.02e−02
Value: C
Streptosporangiales
12.5
10.0
7.5
5.0
2.5
0.0
A (n=15)
B (n=16)
C (n=14)
surface

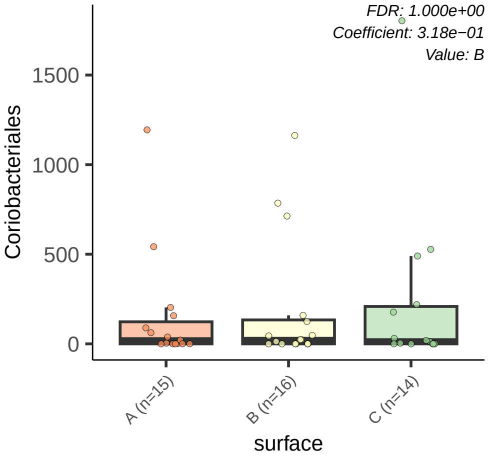

FDR: 1.000e+00
Coefficient: 3.18e−01
Value: B
Coriobacteriales
1500
1000
500
0
A (n=15)
B (n=16)
C (n=14)
surface

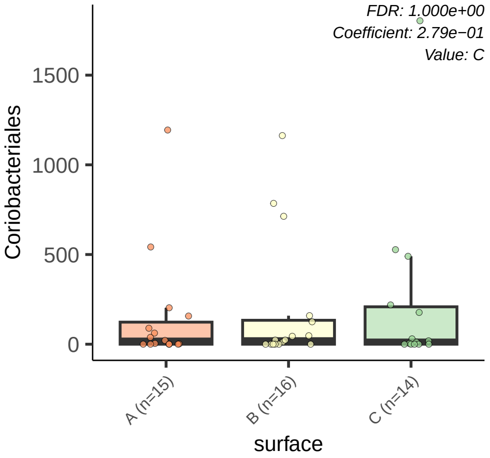
FDR: 1.000e+00
Coefficient: 2.79e−01
Value: C
Coriobacteriales
1500
1000
500
0
A (n=15)
B (n=16)
C (n=14)
surface

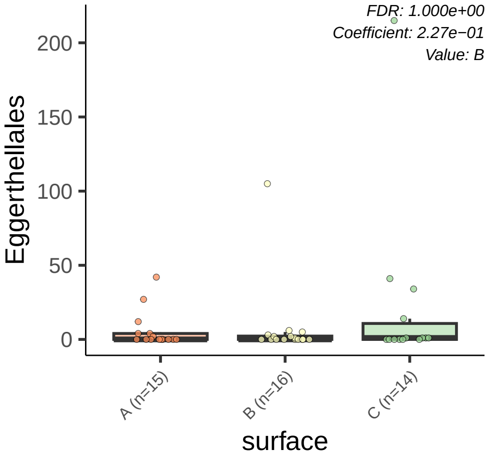
FDR: 1.000e+00
Coefficient: 2.27e−01
Value: B
Eggerthellales
200
150
100
50
0
A (n=15)
B (n=16)
C (n=14)
surface

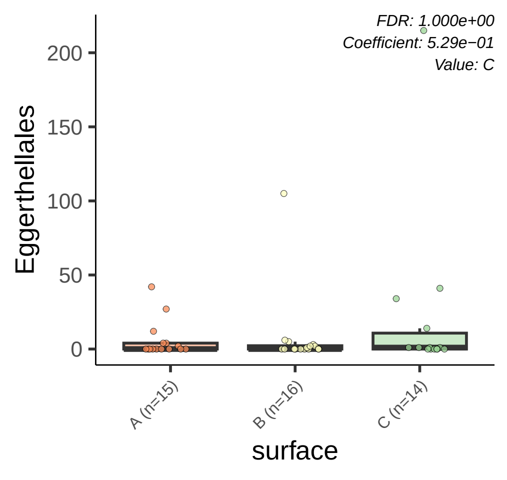
FDR: 1.000e+00
Coefficient: 5.29e−01
Value: C
Eggerthellales
200
150
100
50
0
A (n=15)
B (n=16)
C (n=14)
surface

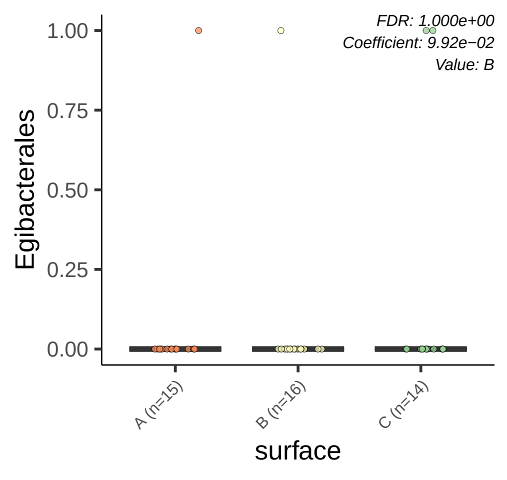
FDR: 1.000e+00
Coefficient: 9.92e−02
Value: B
Egibacterales
1.00
0.75
0.50
0.25
0.00
A (n=15)
B (n=16)
C (n=14)
surface

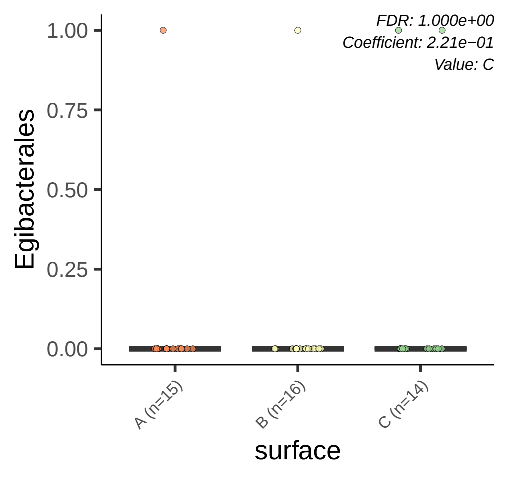
FDR: 1.000e+00
Coefficient: 2.21e−01
Value: C
Egibacterales
1.00
0.75
0.50
0.25
0.00
A (n=15)
B (n=16)
C (n=14)
surface

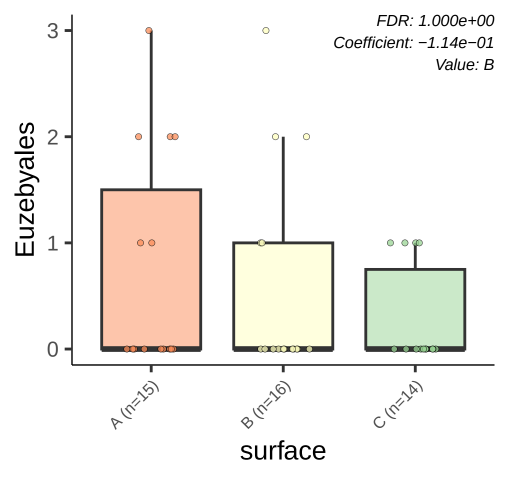

FDR: 1.000e+00
Coefficient: −1.14e−01
Value: B
Euzebyales
3
2
1
0
A (n=15)
B (n=16)
C (n=14)
surface

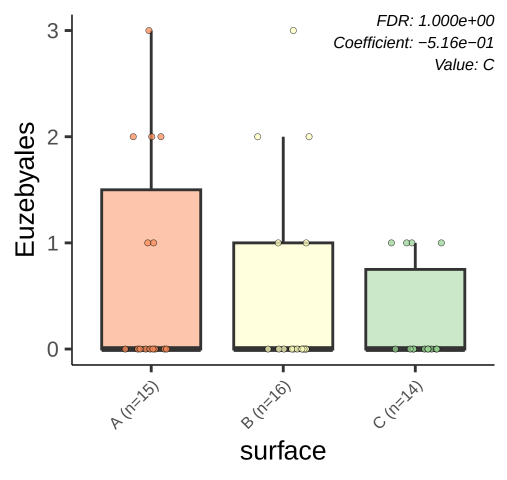

FDR: 1.000e+00
Coefficient: −5.16e−01
Value: C
Euzebyales
3
2
1
0
A (n=15)
B (n=16)
C (n=14)
surface

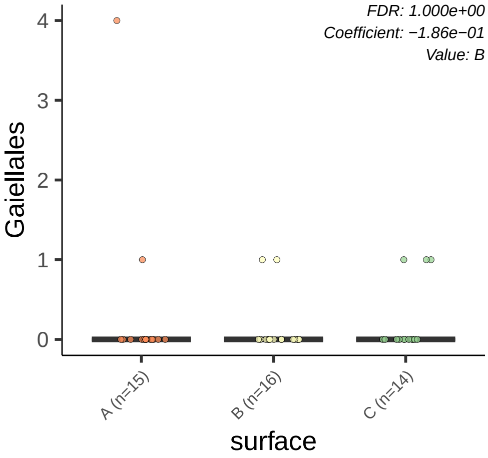

FDR: 1.000e+00
Coefficient: −1.86e−01
Value: B
Gaiellales
0
1
2
3
4
A (n=15)
B (n=16)
C (n=14)
surface

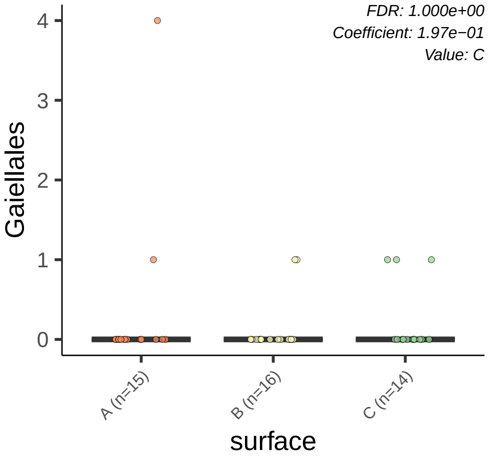
FDR: 1.000e+00
Coefficient: 1.97e−01
Value: C
Gaiellales
0
1
2
3
4
A (n=15)
B (n=16)
C (n=14)
surface

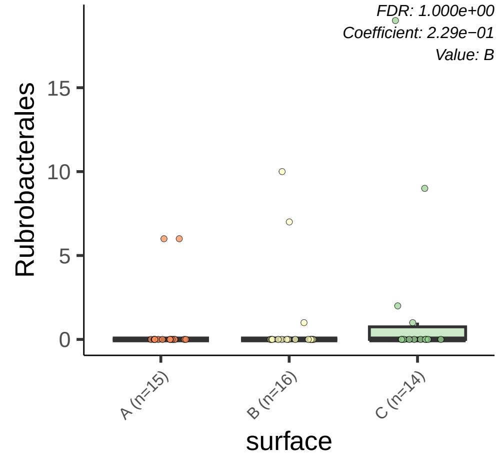
FDR: 1.000e+00
Coefficient: 2.29e−01
Value: B
Rubrobacterales
15
10
5
0
A (n=15)
B (n=16)
C (n=14)
surface

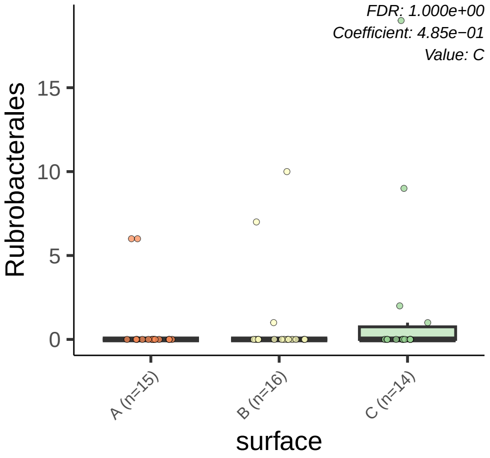
FDR: 1.000e+00
Coefficient: 4.85e−01
Value: C
Rubrobacterales
15
10
5
0
A (n=15)
B (n=16)
C (n=14)
surface

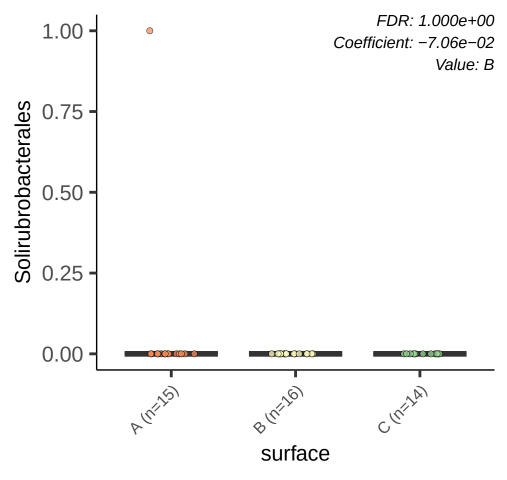
FDR: 1.000e+00
Coefficient: −7.06e−02
Value: B
Solirubrobacterales
1.00
0.75
0.50
0.25
0.00
A (n=15)
B (n=16)
C (n=14)
surface

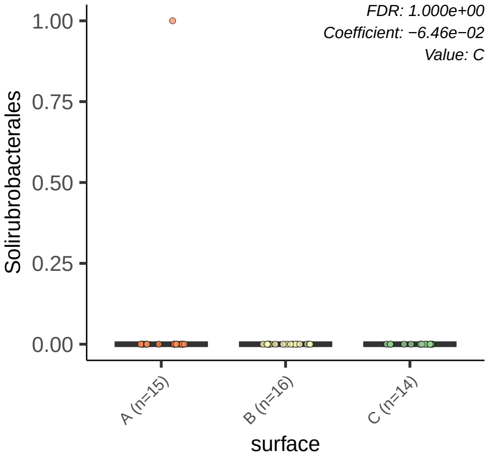
FDR: 1.000e+00
Coefficient: −6.46e−02
Value: C
Solirubrobacterales
1.00
0.75
0.50
0.25
0.00
A (n=15)
B (n=16)
C (n=14)
surface

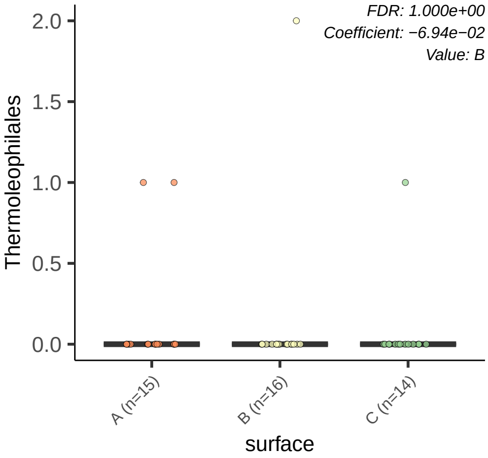
FDR: 1.000e+00
Coefficient: −6.94e−02
Value: B
Thermoleophilales
2.0
1.5
1.0
0.5
0.0
A (n=15)
B (n=16)
C (n=14)
surface

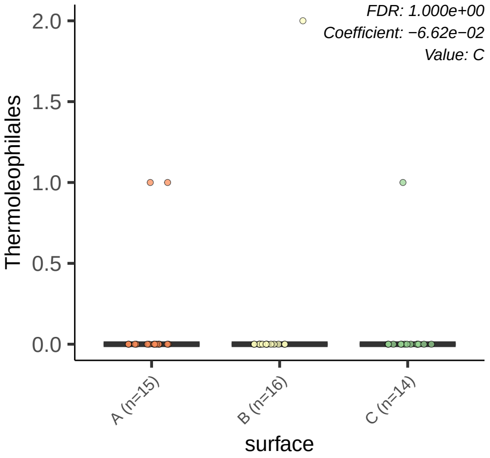
FDR: 1.000e+00
Coefficient: −6.62e−02
Value: C
Thermoleophilales
2.0
1.5
1.0
0.5
0.0
A (n=15)
B (n=16)
C (n=14)
surface

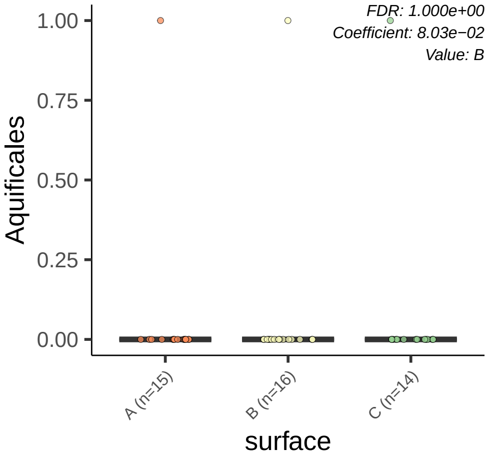
FDR: 1.000e+00
Coefficient: 8.03e−02
Value: B
Aquificales
1.00
0.75
0.50
0.25
0.00
A (n=15)
B (n=16)
C (n=14)
surface

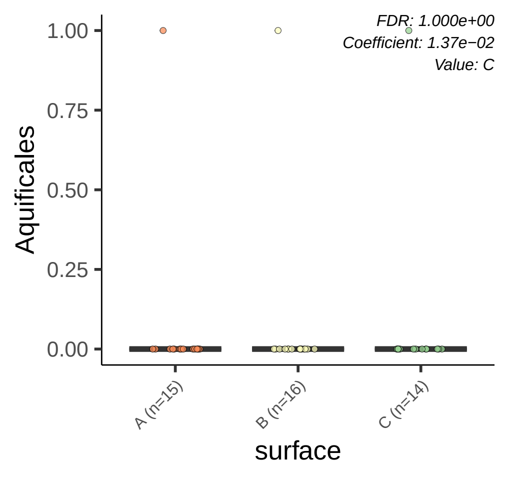

FDR: 1.000e+00
Coefficient: 1.37e−02
Value: C
Aquificales
1.00
0.75
0.50
0.25
0.00
A (n=15)
B (n=16)
C (n=14)
surface

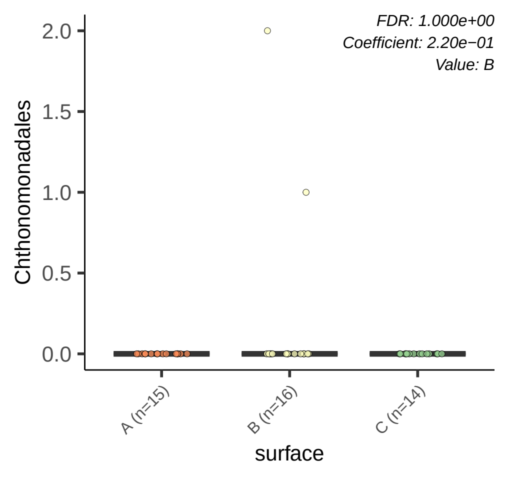
FDR: 1.000e+00
Coefficient: 2.20e−01
Value: B
Chthonomonadales
2.0
1.5
1.0
0.5
0.0
A (n=15)
B (n=16)
C (n=14)
surface

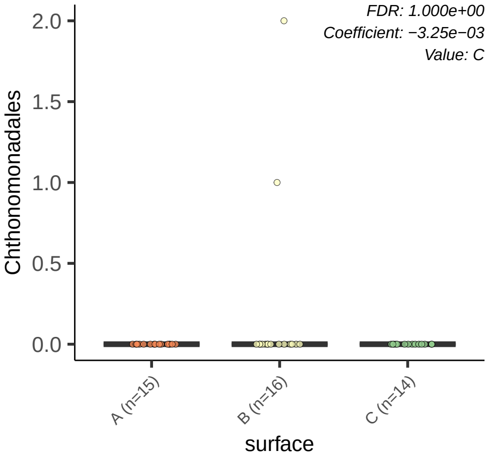

FDR: 1.000e+00
Coefficient: −3.25e−03
Value: C
Chthonomonadales
2.0
1.5
1.0
0.5
0.0
A (n=15)
B (n=16)
C (n=14)
surface

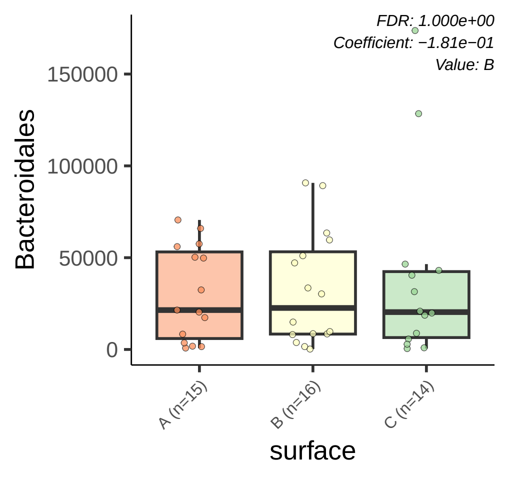
FDR: 1.000e+00
Coefficient: −1.81e−01
Value: B
Bacteroidales
150000
100000
50000
0
A (n=15)
B (n=16)
C (n=14)
surface

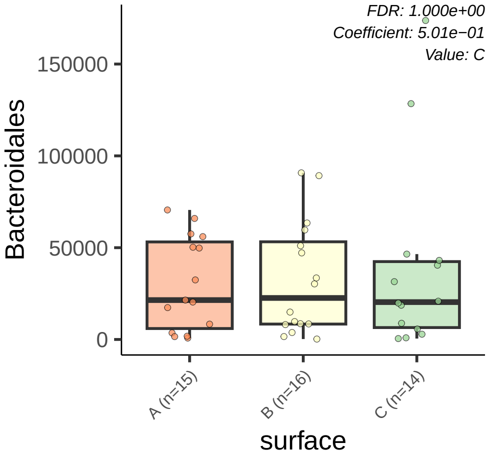
FDR: 1.000e+00
Coefficient: 5.01e−01
Value: C
Bacteroidales
150000
100000
50000
0
A (n=15)
B (n=16)
C (n=14)
surface

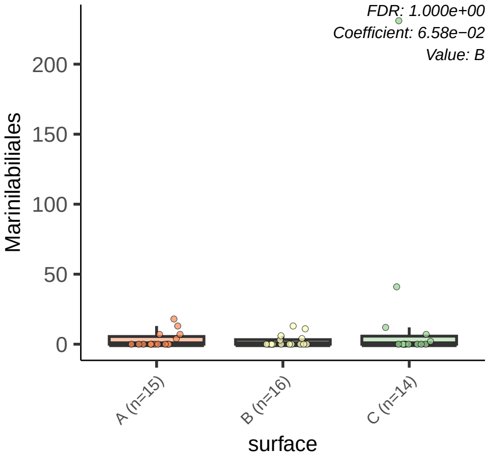
FDR: 1.000e+00
Coefficient: 6.58e−02
Value: B
Marinilabiliales
200
150
100
50
0
A (n=15)
B (n=16)
C (n=14)
surface

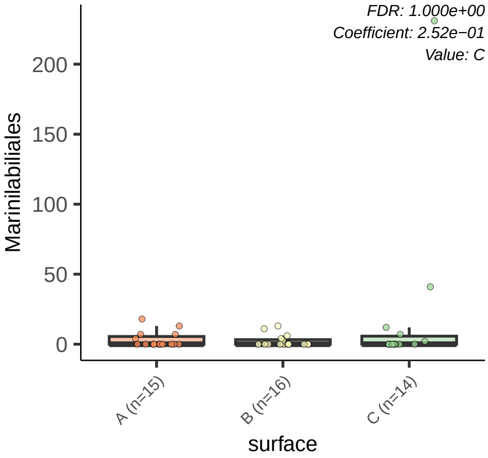

FDR: 1.000e+00
Coefficient: 2.52e−01
Value: C
Marinilabiliales
200
150
100
50
0
A (n=15)
B (n=16)
C (n=14)
surface

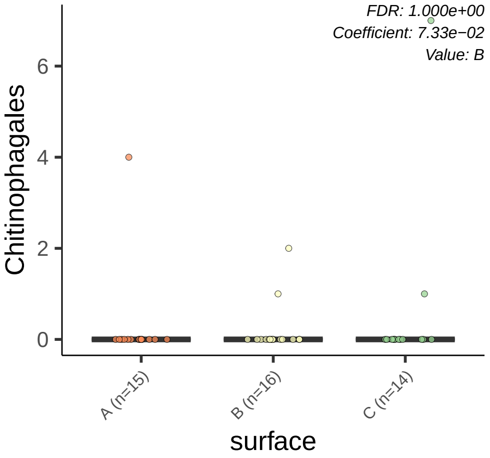

FDR: 1.000e+00
Coefficient: 7.33e−02
Value: B
Chitinophagales
0
2
4
6
A (n=15)
B (n=16)
C (n=14)
surface

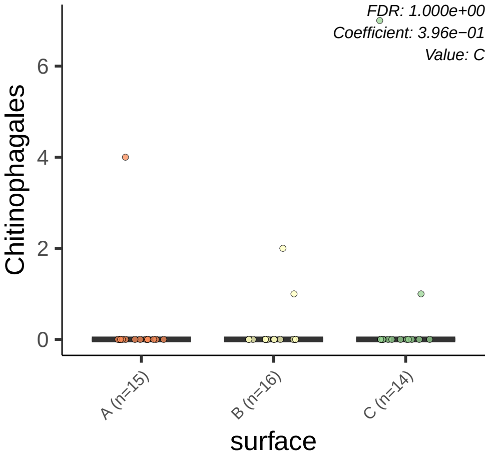
FDR: 1.000e+00
Coefficient: 3.96e−01
Value: C
Chitinophagales
0
2
4
6
A (n=15)
B (n=16)
C (n=14)
surface

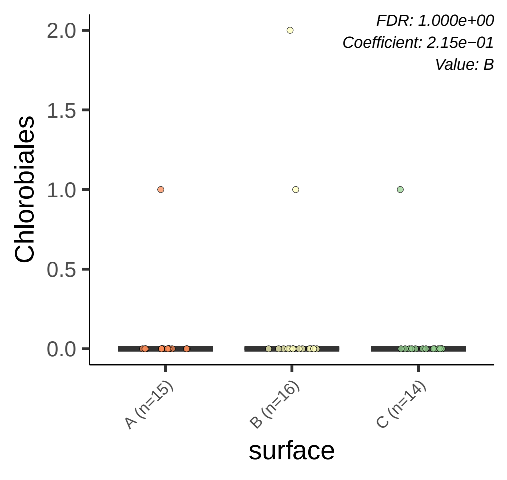

FDR: 1.000e+00
Coefficient: 2.15e−01
Value: B
Chlorobiales
2.0
1.5
1.0
0.5
0.0
A (n=15)
B (n=16)
C (n=14)
surface

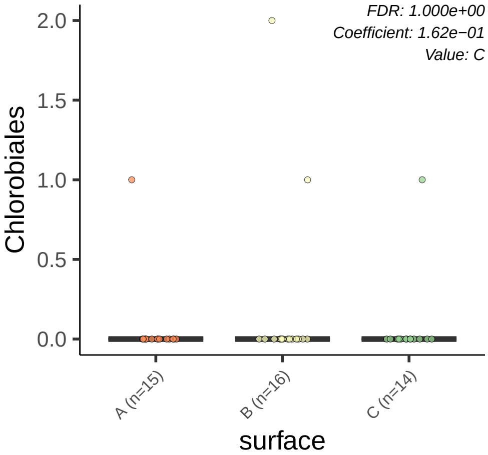

FDR: 1.000e+00
Coefficient: 1.62e−01
Value: C
Chlorobiales
2.0
1.5
1.0
0.5
0.0
A (n=15)
B (n=16)
C (n=14)
surface

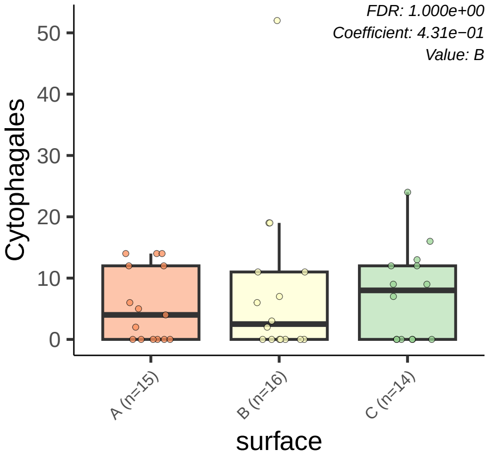
FDR: 1.000e+00
Coefficient: 4.31e−01
Value: B
Cytophagales
0
10
20
30
40
50
A (n=15)
B (n=16)
C (n=14)
surface

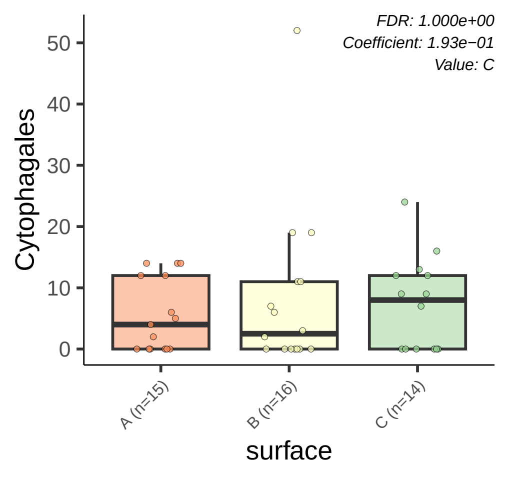

FDR: 1.000e+00
Coefficient: 1.93e−01
Value: C
Cytophagales
0
10
20
30
40
50
A (n=15)
B (n=16)
C (n=14)
surface

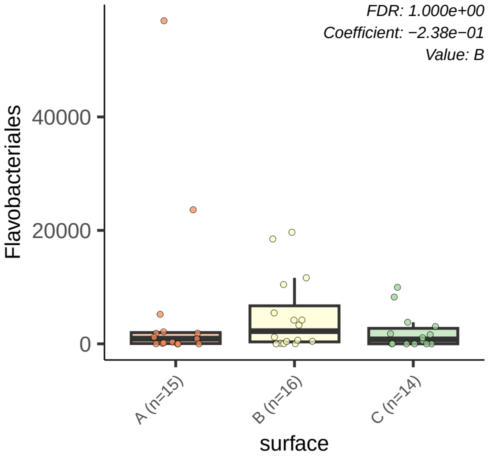

FDR: 1.000e+00
Coefficient: −2.38e−01
Value: B
Flavobacteriales
40000
20000
0
A (n=15)
B (n=16)
C (n=14)
surface

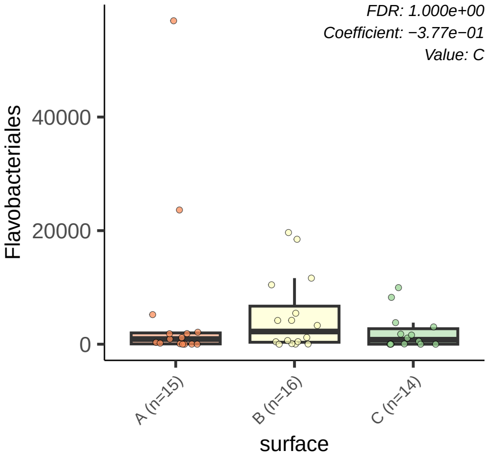
FDR: 1.000e+00
Coefficient: −3.77e−01
Value: C
Flavobacteriales
40000
20000
0
A (n=15)
B (n=16)
C (n=14)
surface

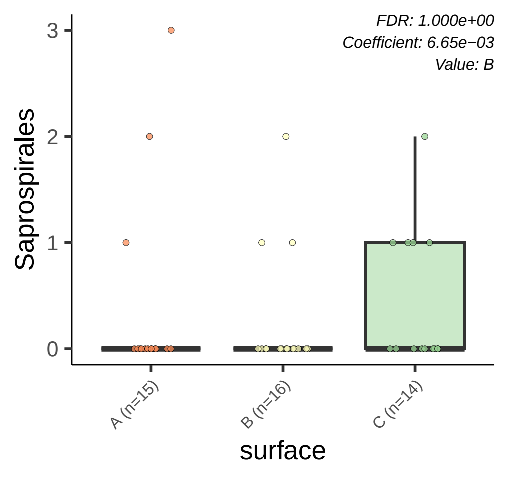
FDR: 1.000e+00
Coefficient: 6.65e−03
Value: B
Saprospirales
3
2
1
0
A (n=15)
B (n=16)
C (n=14)
surface

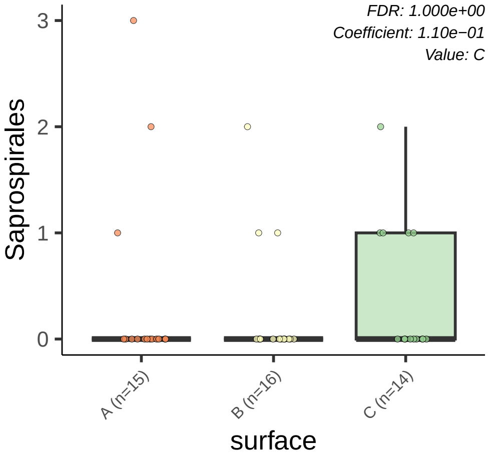
FDR: 1.000e+00
Coefficient: 1.10e−01
Value: C
Saprospirales
0
1
2
3
A (n=15)
B (n=16)
C (n=14)
surface

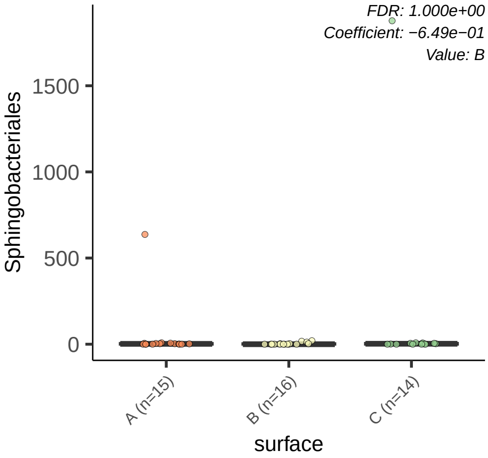

FDR: 1.000e+00
Coefficient: −6.49e−01
Value: B
Sphingobacteriales
1500
1000
500
0
A (n=15)
B (n=16)
C (n=14)
surface

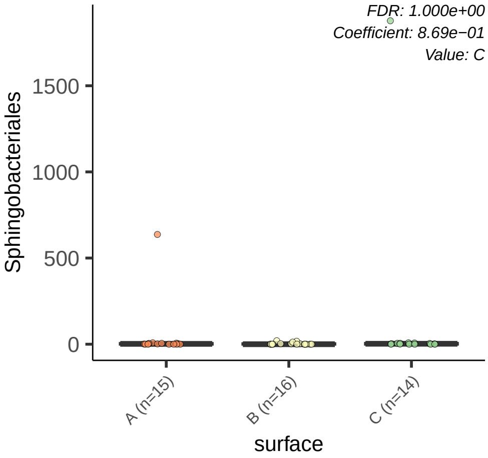
FDR: 1.000e+00
Coefficient: 8.69e−01
Value: C
Sphingobacteriales
1500
1000
500
0
A (n=15)
B (n=16)
C (n=14)
surface

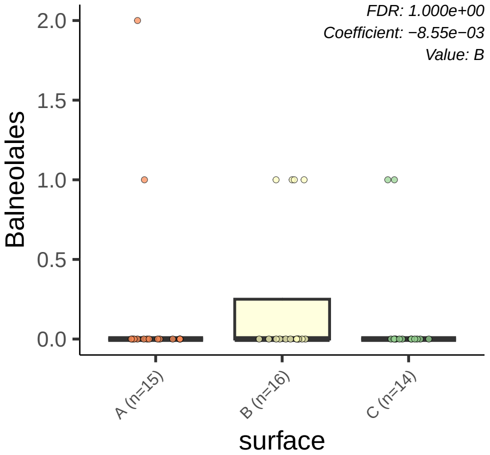
FDR: 1.000e+00
Coefficient: −8.55e−03
Value: B
Balneolales
2.0
1.5
1.0
0.5
0.0
A (n=15)
B (n=16)
C (n=14)
surface

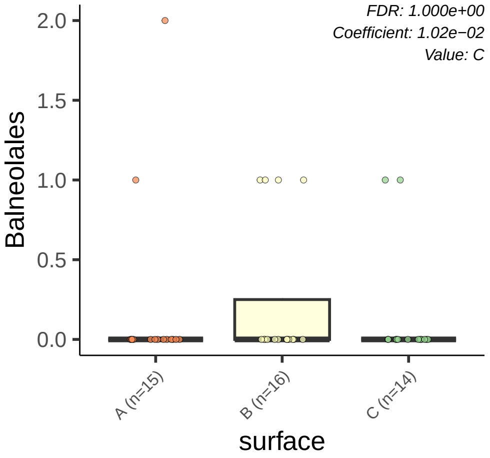

FDR: 1.000e+00
Coefficient: 1.02e−02
Value: C
Balneolales
2.0
1.5
1.0
0.5
0.0
A (n=15)
B (n=16)
C (n=14)
surface

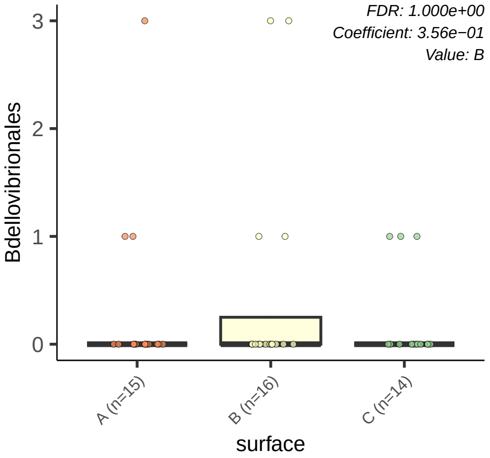
FDR: 1.000e+00
Coefficient: 3.56e−01
Value: B
Bdellovibrionales
3
2
1
0
A (n=15)
B (n=16)
C (n=14)
surface

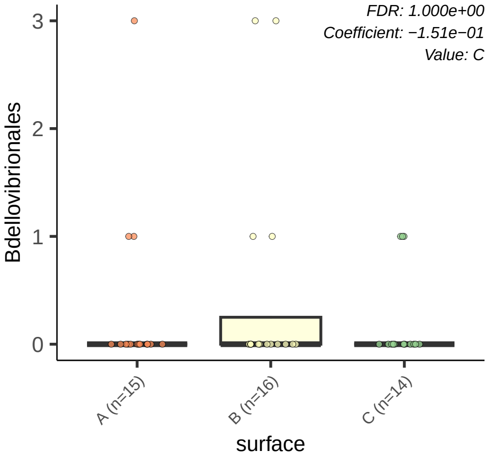

FDR: 1.000e+00
Coefficient: −1.51e−01
Value: C
Bdellovibrionales
3
2
1
0
A (n=15)
B (n=16)
C (n=14)
surface

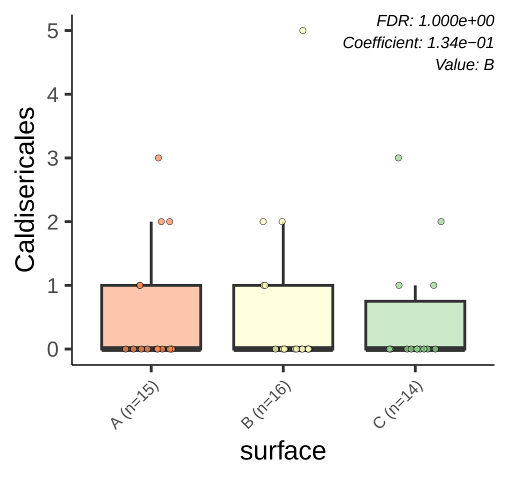
FDR: 1.000e+00
Coefficient: 1.34e−01
Value: B
Caldisericales
0
1
2
3
4
5
A (n=15)
B (n=16)
C (n=14)
surface

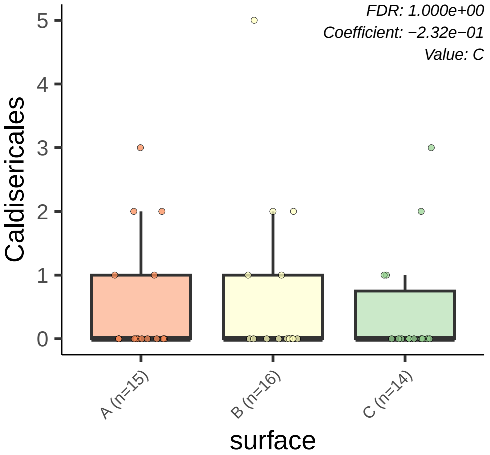

FDR: 1.000e+00
Coefficient: −2.32e−01
Value: C
Caldisericales
0
1
2
3
4
5
A (n=15)
B (n=16)
C (n=14)
surface

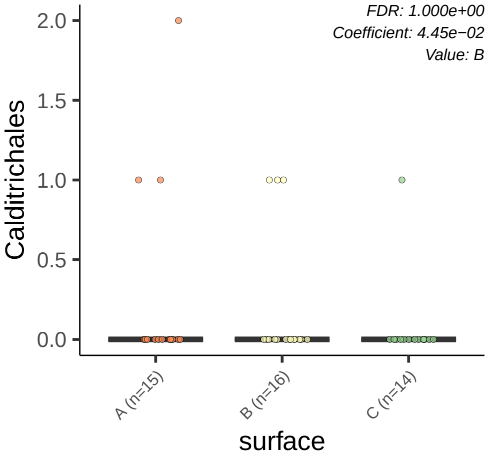
FDR: 1.000e+00
Coefficient: 4.45e−02
Value: B
Calditrichales
2.0
1.5
1.0
0.5
0.0
A (n=15)
B (n=16)
C (n=14)
surface

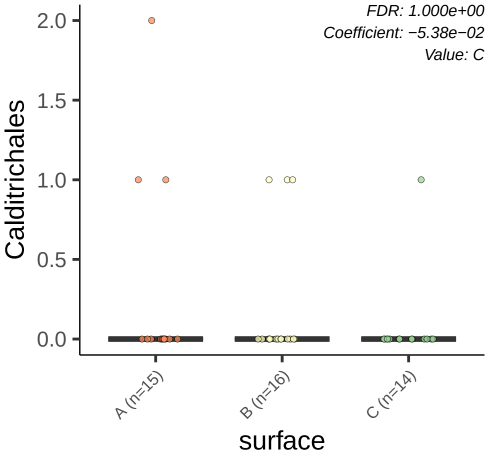
FDR: 1.000e+00
Coefficient: −5.38e−02
Value: C
Calditrichales
2.0
1.5
1.0
0.5
0.0
A (n=15)
B (n=16)
C (n=14)
surface

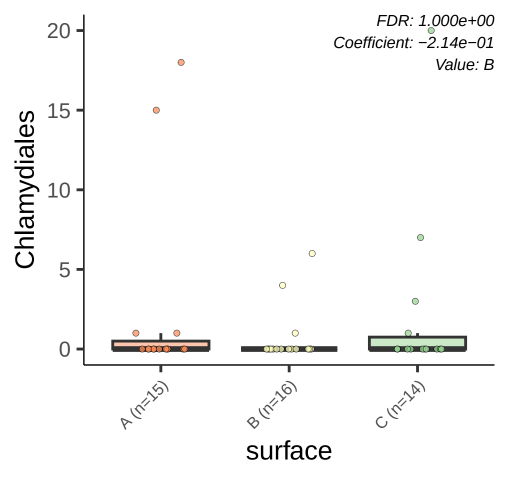
FDR: 1.000e+00
Coefficient: −2.14e−01
Value: B
Chlamydiales
20
15
10
5
0
A (n=15)
B (n=16)
C (n=14)
surface

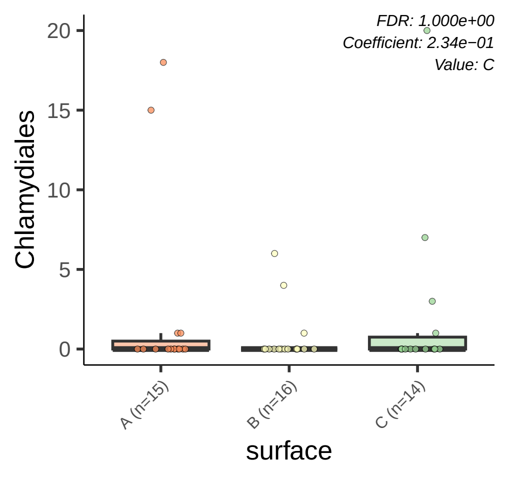

FDR: 1.000e+00
Coefficient: 2.34e−01
Value: C
Chlamydiales
0
5
10
15
20
A (n=15)
B (n=16)
C (n=14)
surface

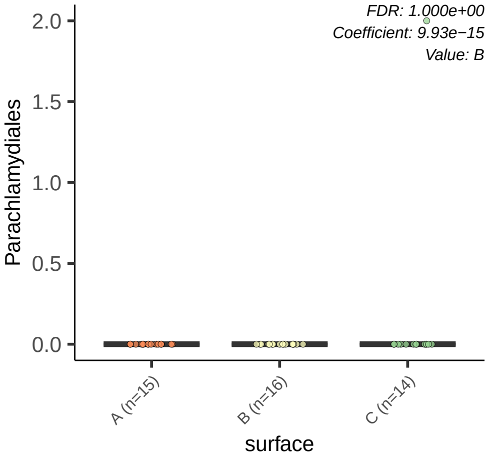
FDR: 1.000e+00
Coefficient: 9.93e−15
Value: B
Parachlamydiales
2.0
1.5
1.0
0.5
0.0
A (n=15)
B (n=16)
C (n=14)
surface

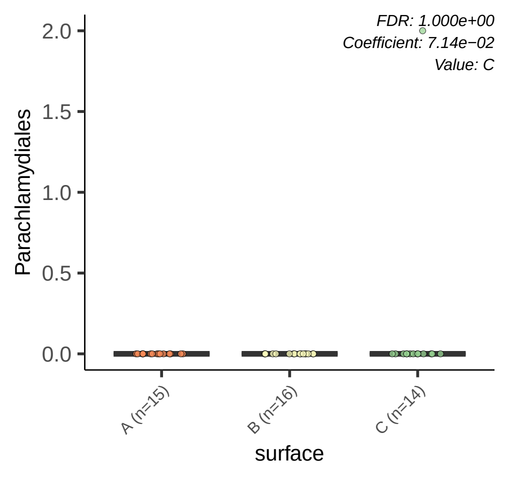
FDR: 1.000e+00
Coefficient: 7.14e−02
Value: C
2.0
1.5
1.0
0.5
0.0
Parachlamydiales
A (n=15)
B (n=16)
C (n=14)
surface

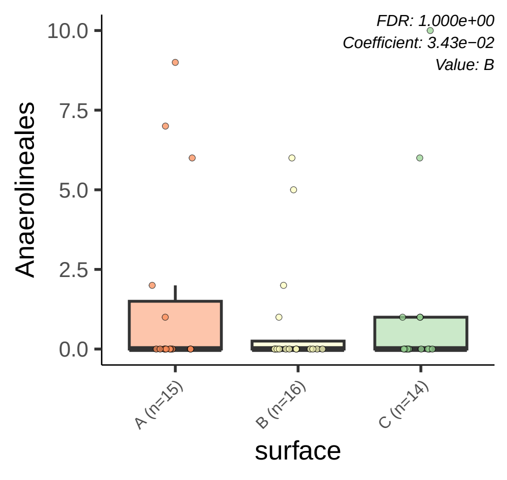

FDR: 1.000e+00
Coefficient: 3.43e−02
Value: B
Anaerolineales
10.0
7.5
5.0
2.5
0.0
A (n=15)
B (n=16)
C (n=14)
surface

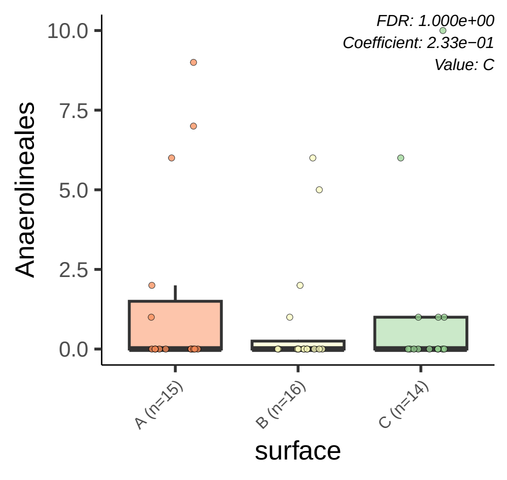
FDR: 1.000e+00
Coefficient: 2.33e−01
Value: C
Anaerolineales
10.0
7.5
5.0
2.5
0.0
A (n=15)
B (n=16)
C (n=14)
surface

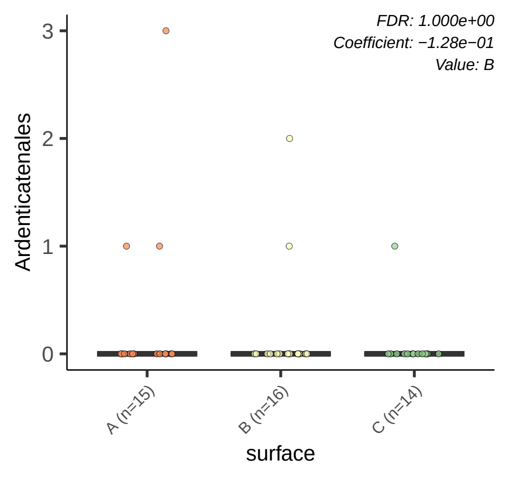
FDR: 1.000e+00
Coefficient: −1.28e−01
Value: B
Ardenticatenales
3
2
1
0
A (n=15)
B (n=16)
C (n=14)
surface

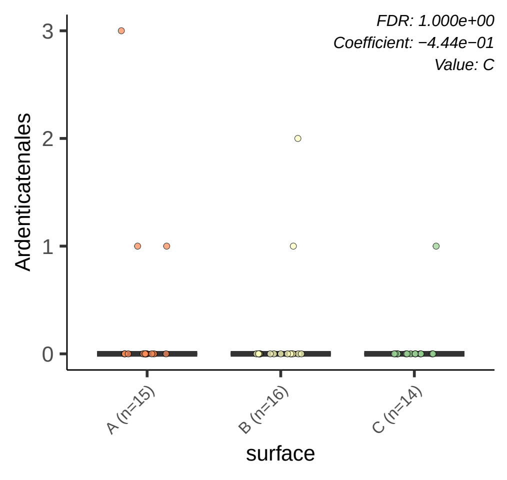

FDR: 1.000e+00
Coefficient: −4.44e−01
Value: C
Ardenticatenales
3
2
1
0
A (n=15)
B (n=16)
C (n=14)
surface

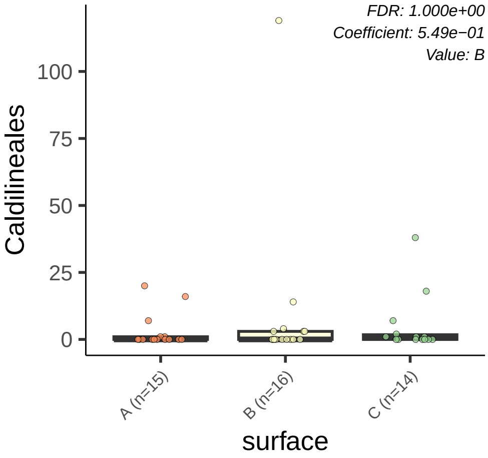
FDR: 1.000e+00
Coefficient: 5.49e−01
Value: B
Caldilineales
100
75
50
25
0
A (n=15)
B (n=16)
C (n=14)
surface

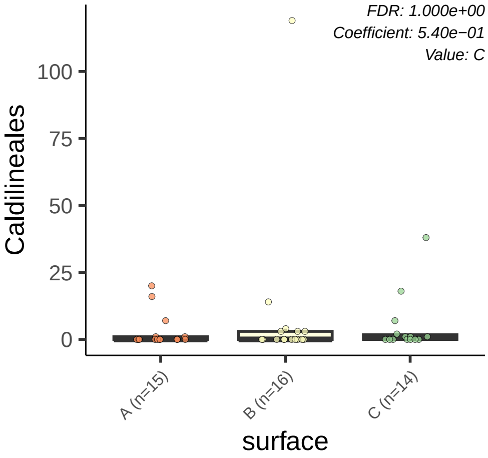

FDR: 1.000e+00
Coefficient: 5.40e−01
Value: C
Caldilineales
100
75
50
25
0
A (n=15)
B (n=16)
C (n=14)
surface

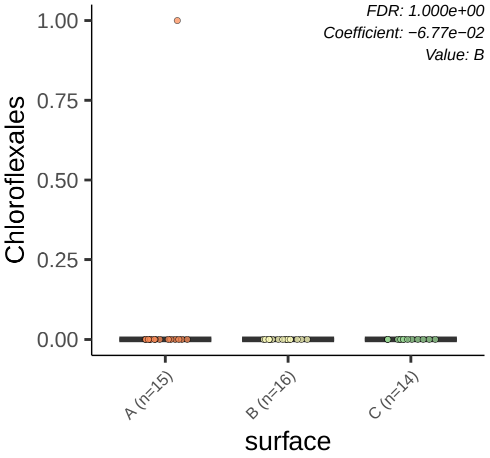
FDR: 1.000e+00
Coefficient: −6.77e−02
Value: B
Chloroflexales
1.00
0.75
0.50
0.25
0.00
A (n=15)
B (n=16)
C (n=14)
surface

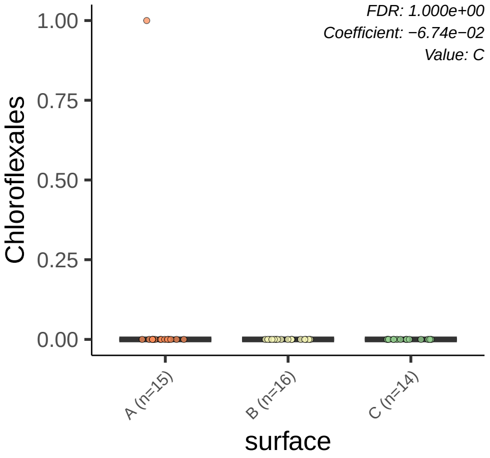

FDR: 1.000e+00
Coefficient: −6.74e−02
Value: C
Chloroflexales
1.00
0.75
0.50
0.25
0.00
A (n=15)
B (n=16)
C (n=14)
surface

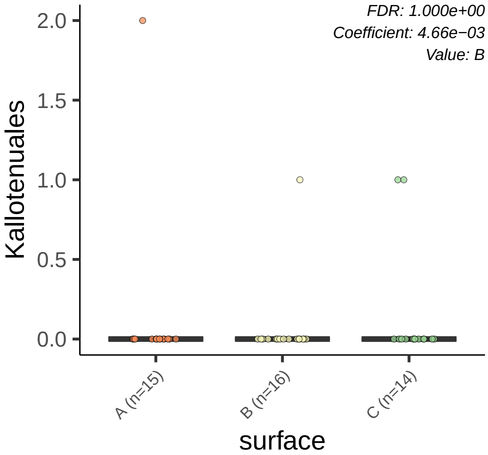
FDR: 1.000e+00
Coefficient: 4.66e−03
Value: B
Kallotenuales
2.0
1.5
1.0
0.5
0.0
A (n=15)
B (n=16)
C (n=14)
surface

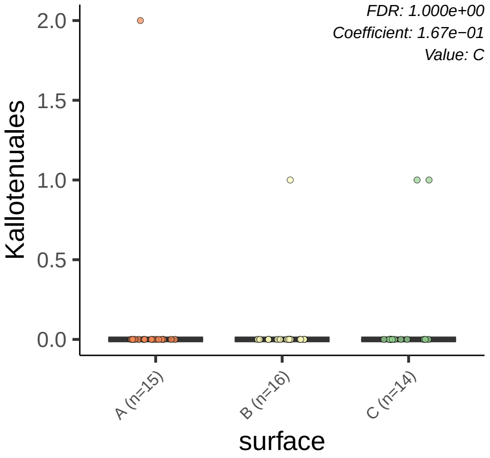
FDR: 1.000e+00
Coefficient: 1.67e−01
Value: C
Kallotenuales
2.0
1.5
1.0
0.5
0.0
A (n=15)
B (n=16)
C (n=14)
surface

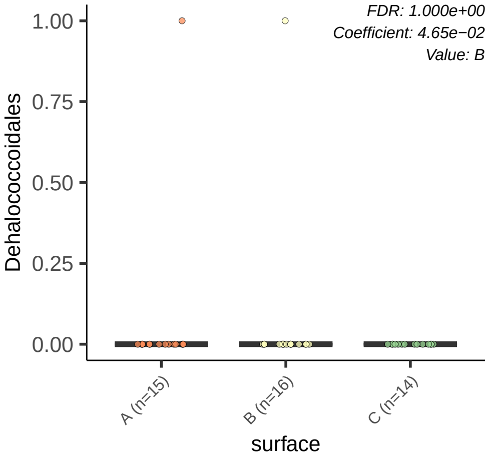
FDR: 1.000e+00
Coefficient: 4.65e−02
Value: B
Dehalococcoidales
1.00
0.75
0.50
0.25
0.00
A (n=15)
B (n=16)
C (n=14)
surface

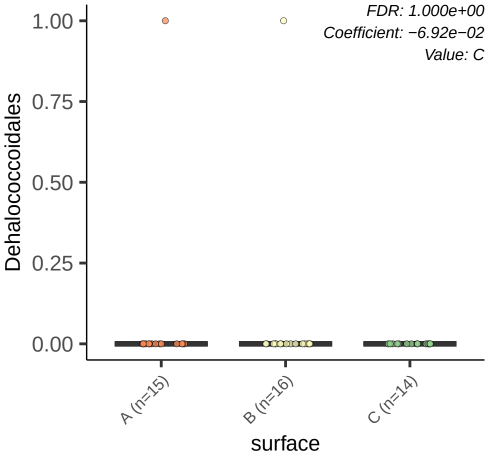
FDR: 1.000e+00
Coefficient: −6.92e−02
Value: C
Dehalococcoidales
1.00
0.75
0.50
0.25
0.00
A (n=15)
B (n=16)
C (n=14)
surface

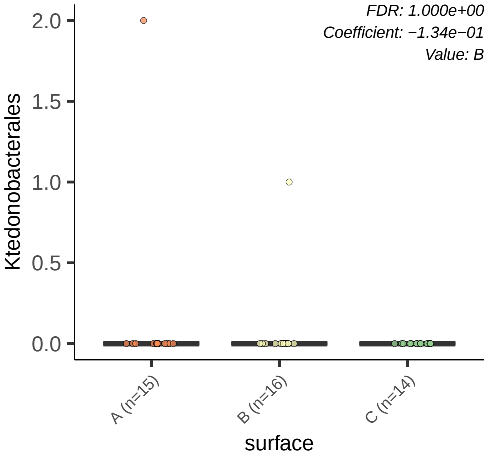
FDR: 1.000e+00
Coefficient: −1.34e−01
Value: B
Ktedonobacterales
2.0
1.5
1.0
0.5
0.0
A (n=15)
B (n=16)
C (n=14)
surface

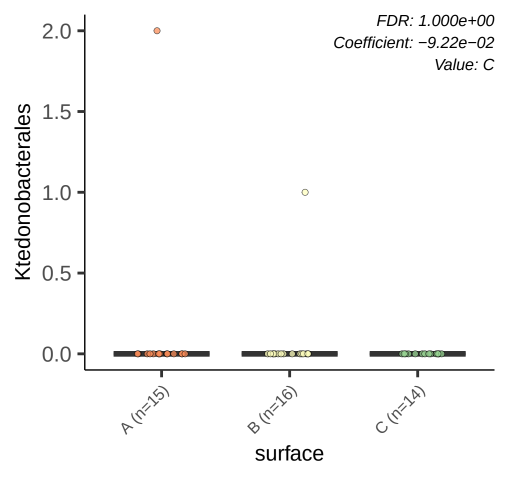
FDR: 1.000e+00
Coefficient: −9.22e−02
Value: C
Ktedonobacterales
2.0
1.5
1.0
0.5
0.0
A (n=15)
B (n=16)
C (n=14)
surface

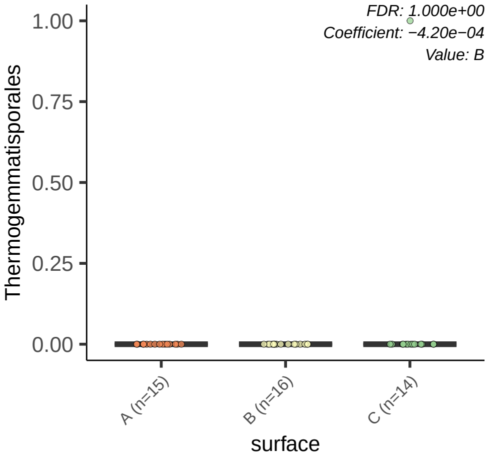
FDR: 1.000e+00
Coefficient: −4.20e−04
Value: B
Thermogemmatisporales
1.00
0.75
0.50
0.25
0.00
A (n=15)
B (n=16)
C (n=14)
surface

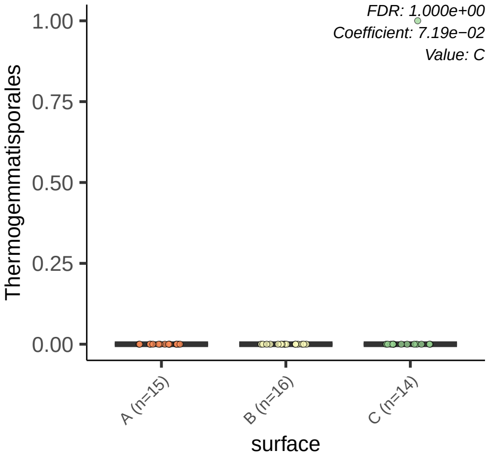

FDR: 1.000e+00
Coefficient: 7.19e−02
Value: C
Thermogemmatisporales
1.00
0.75
0.50
0.25
0.00
A (n=15)
B (n=16)
C (n=14)
surface

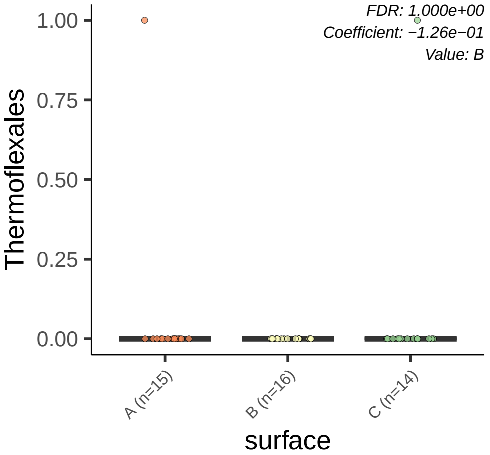
FDR: 1.000e+00
Coefficient: −1.26e−01
Value: B
Thermoflexales
1.00
0.75
0.50
0.25
0.00
A (n=15)
B (n=16)
C (n=14)
surface

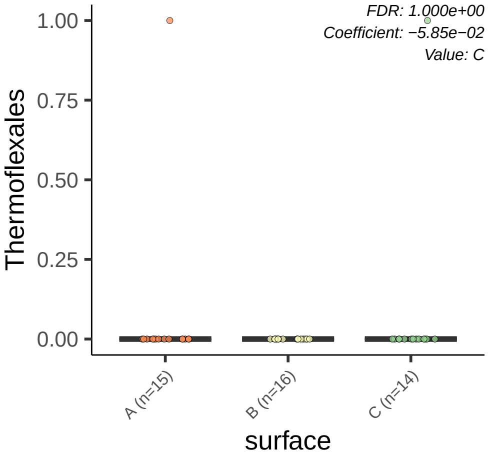

FDR: 1.000e+00
Coefficient: −5.85e−02
Value: C
Thermoflexales
1.00
0.75
0.50
0.25
0.00
A (n=15)
B (n=16)
C (n=14)
surface

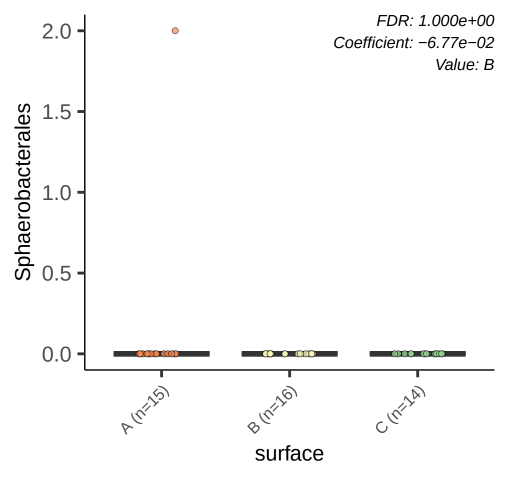
FDR: 1.000e+00
Coefficient: −6.77e−02
Value: B
Sphaerobacterales
2.0
1.5
1.0
0.5
0.0
A (n=15)
B (n=16)
C (n=14)
surface

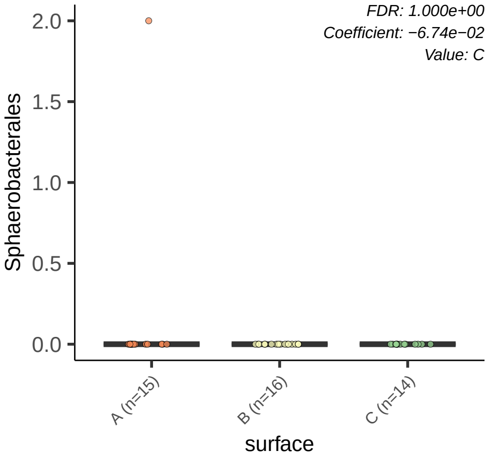
FDR: 1.000e+00
Coefficient: −6.74e−02
Value: C
Sphaerobacterales
2.0
1.5
1.0
0.5
0.0
A (n=15)
B (n=16)
C (n=14)
surface

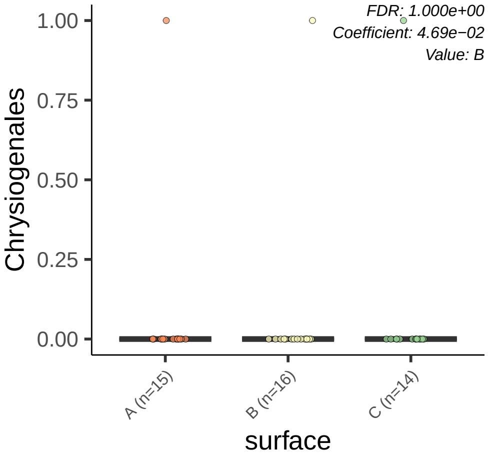
FDR: 1.000e+00
Coefficient: 4.69e−02
Value: B
Chrysiogenales
1.00
0.75
0.50
0.25
0.00
A (n=15)
B (n=16)
C (n=14)
surface

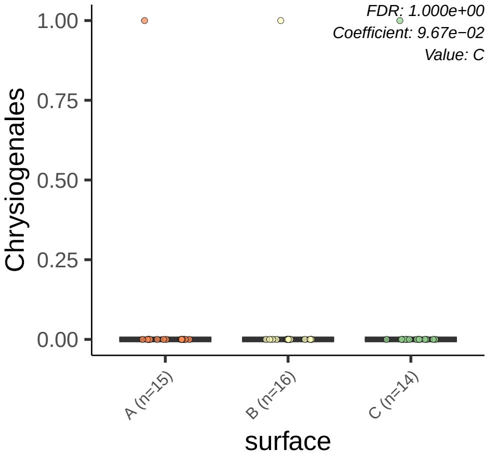
FDR: 1.000e+00
Coefficient: 9.67e−02
Value: C
Chrysiogenales
1.00
0.75
0.50
0.25
0.00
A (n=15)
B (n=16)
C (n=14)
surface

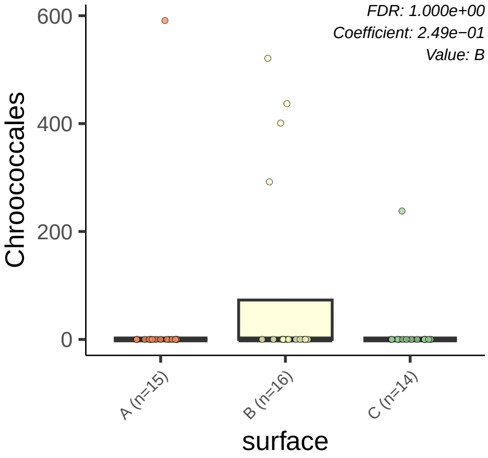

FDR: 1.000e+00
Coefficient: 2.49e−01
Value: B
600
400
200
0
Chroococcales
A (n=15)
B (n=16)
C (n=14)
surface

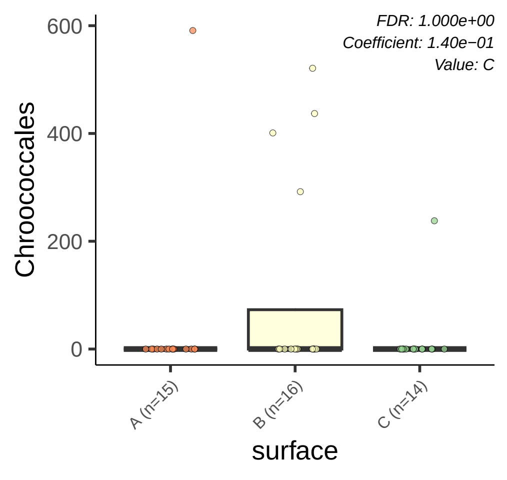
FDR: 1.000e+00
Coefficient: 1.40e−01
Value: C
600
400
200
0
Chroococcales
A (n=15)
B (n=16)
C (n=14)
surface

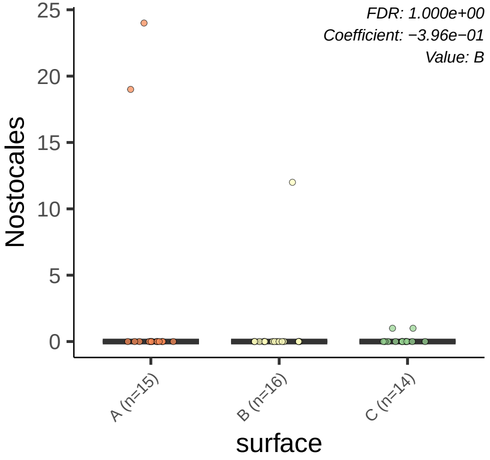

FDR: 1.000e+00
Coefficient: −3.96e−01
Value: B
Nostocales
25
20
15
10
5
0
A (n=15)
B (n=16)
C (n=14)
surface

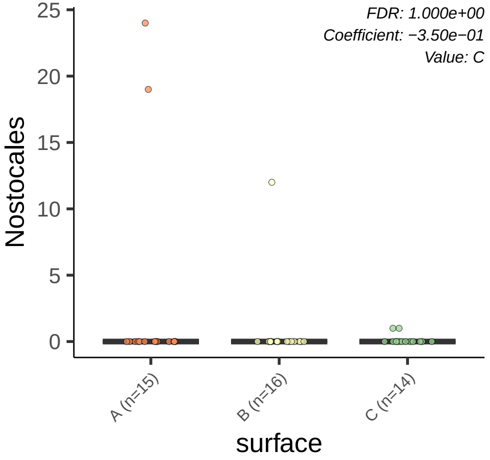
FDR: 1.000e+00
Coefficient: −3.50e−01
Value: C
Nostocales
25
20
15
10
5
0
A (n=15)
B (n=16)
C (n=14)
surface

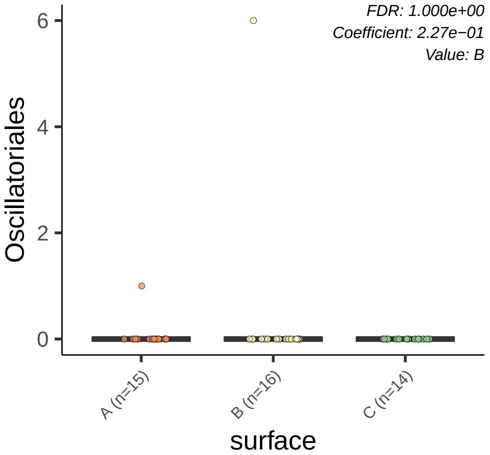
FDR: 1.000e+00
Coefficient: 2.27e−01
Value: B
Oscillatoriales
0
2
4
6
A (n=15)
B (n=16)
C (n=14)
surface

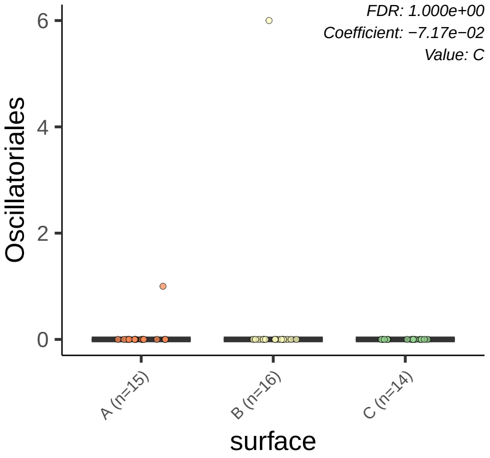
FDR: 1.000e+00
Coefficient: −7.17e−02
Value: C
Oscillatoriales
6
4
2
0
A (n=15)
B (n=16)
C (n=14)
surface

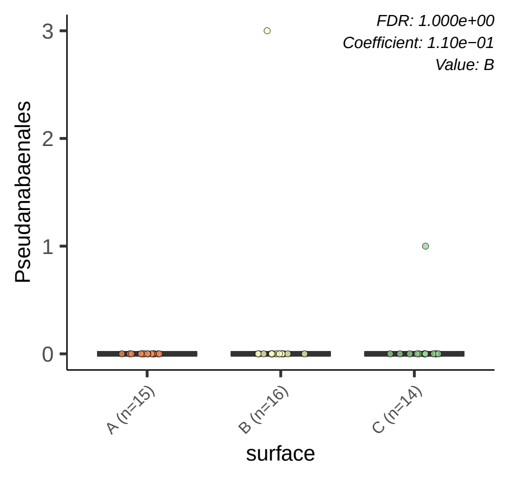

FDR: 1.000e+00
Coefficient: 1.10e−01
Value: B
Pseudanabaenales
0
1
2
3
A (n=15)
B (n=16)
C (n=14)
surface

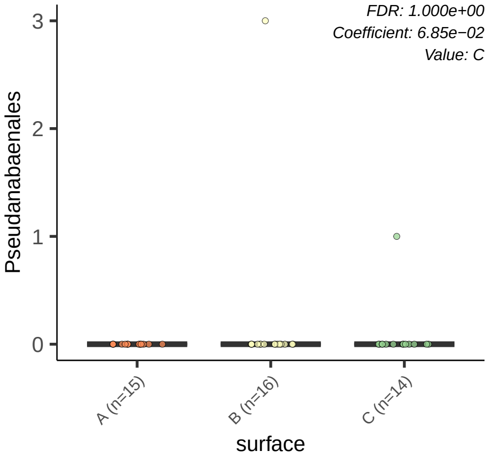

FDR: 1.000e+00
Coefficient: 6.85e−02
Value: C
Pseudanabaenales
3
2
1
0
A (n=15)
B (n=16)
C (n=14)
surface

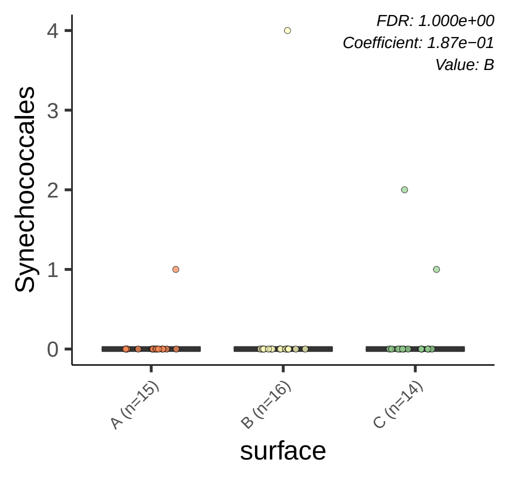

FDR: 1.000e+00
Coefficient: 1.87e−01
Value: B
Synechococcales
0
1
2
3
4
A (n=15)
B (n=16)
C (n=14)
surface

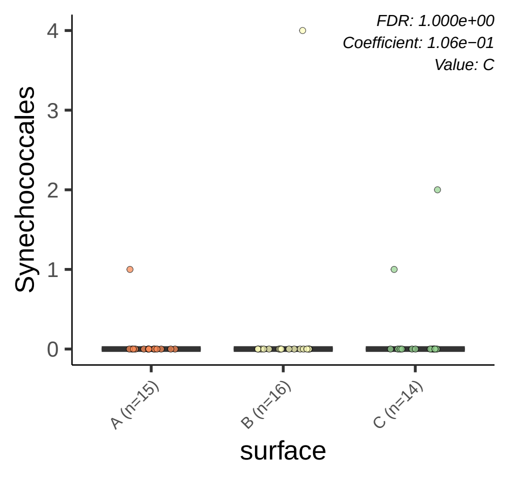

FDR: 1.000e+00
Coefficient: 1.06e−01
Value: C
Synechococcales
0
1
2
3
4
A (n=15)
B (n=16)
C (n=14)
surface

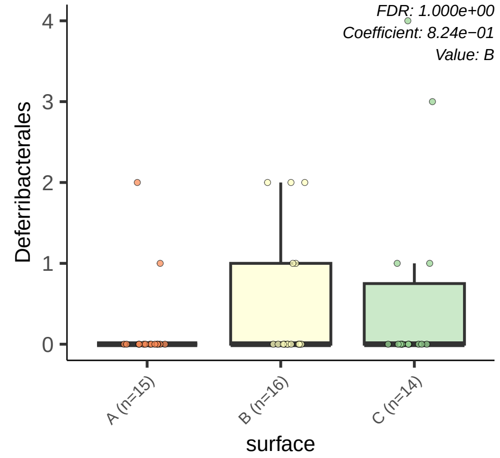
FDR: 1.000e+00
Coefficient: 8.24e−01
Value: B
Deferribacterales
4
3
2
1
0
A (n=15)
B (n=16)
C (n=14)
surface

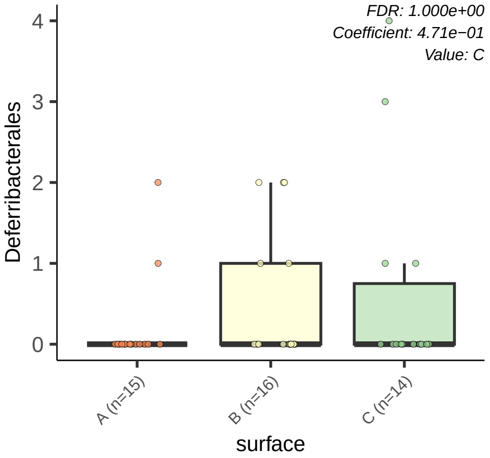

FDR: 1.000e+00
Coefficient: 4.71e−01
Value: C
Deferribacterales
0
1
2
3
4
A (n=15)
B (n=16)
C (n=14)
surface

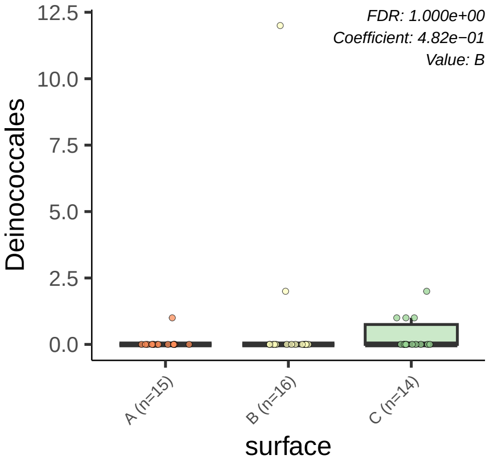

FDR: 1.000e+00
Coefficient: 4.82e−01
Value: B
Deinococcales
12.5
10.0
7.5
5.0
2.5
0.0
A (n=15)
B (n=16)
C (n=14)
surface

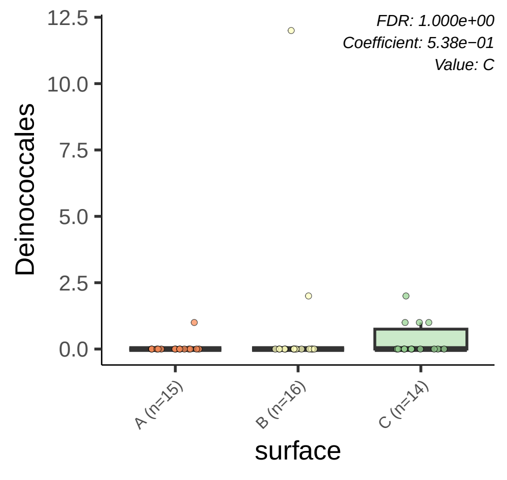

FDR: 1.000e+00
Coefficient: 5.38e−01
Value: C
Deinococcales
12.5
10.0
7.5
5.0
2.5
0.0
A (n=15)
B (n=16)
C (n=14)
surface

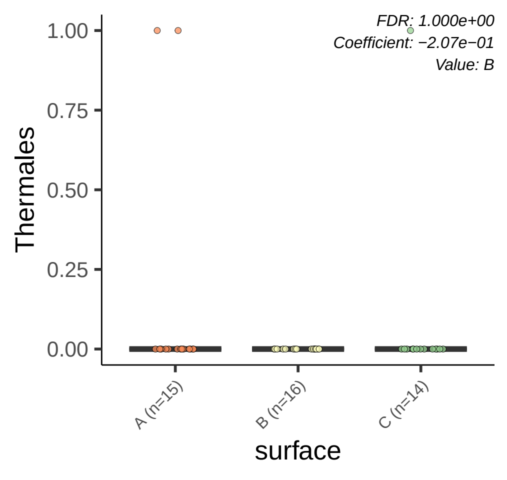

FDR: 1.000e+00
Coefficient: −2.07e−01
Value: B
Thermales
1.00
0.75
0.50
0.25
0.00
A (n=15)
B (n=16)
C (n=14)
surface

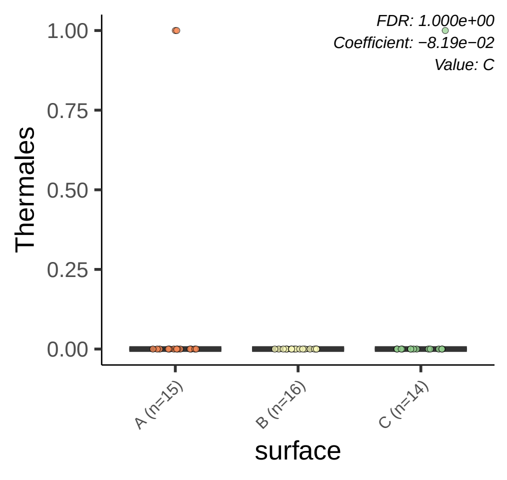
FDR: 1.000e+00
Coefficient: −8.19e−02
Value: C
Thermales
1.00
0.75
0.50
0.25
0.00
A (n=15)
B (n=16)
C (n=14)
surface

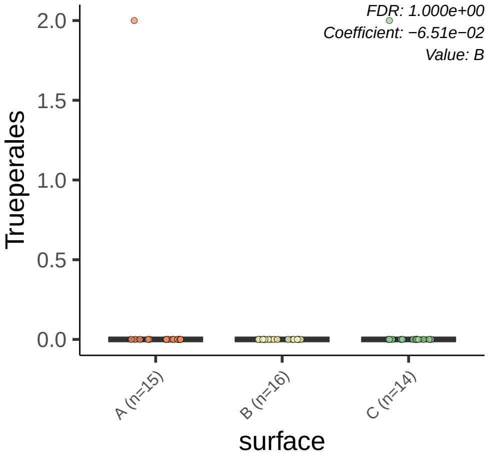
FDR: 1.000e+00
Coefficient: −6.51e−02
Value: B
Trueperales
2.0
1.5
1.0
0.5
0.0
A (n=15)
B (n=16)
C (n=14)
surface

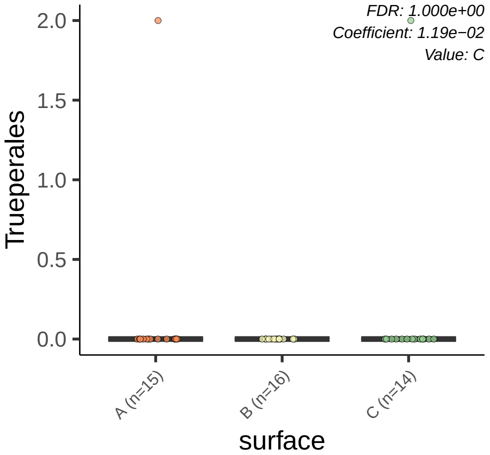
FDR: 1.000e+00
Coefficient: 1.19e−02
Value: C
Trueperales
2.0
1.5
1.0
0.5
0.0
A (n=15)
B (n=16)
C (n=14)
surface

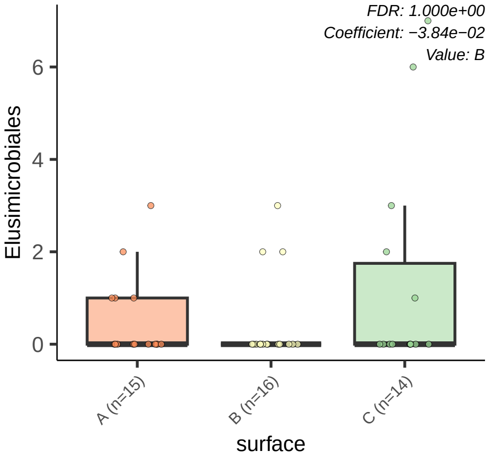
FDR: 1.000e+00
Coefficient: −3.84e−02
Value: B
Elusimicrobiales
0
2
4
6
A (n=15)
B (n=16)
C (n=14)
surface

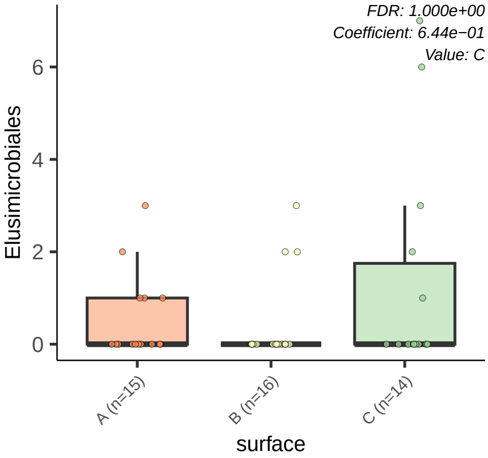
FDR: 1.000e+00
Coefficient: 6.44e−01
Value: C
Elusimicrobiales
0
2
4
6
A (n=15)
B (n=16)
C (n=14)
surface

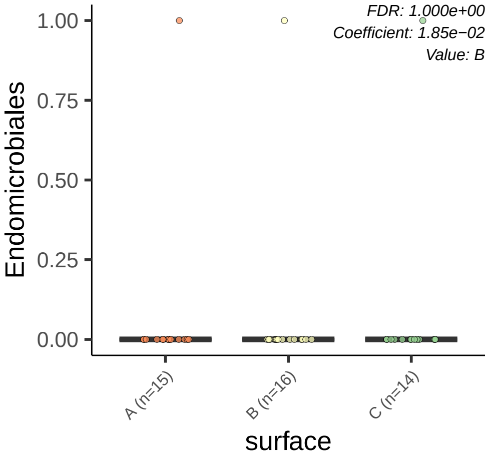
FDR: 1.000e+00
Coefficient: 1.85e−02
Value: B
Endomicrobiales
1.00
0.75
0.50
0.25
0.00
A (n=15)
B (n=16)
C (n=14)
surface

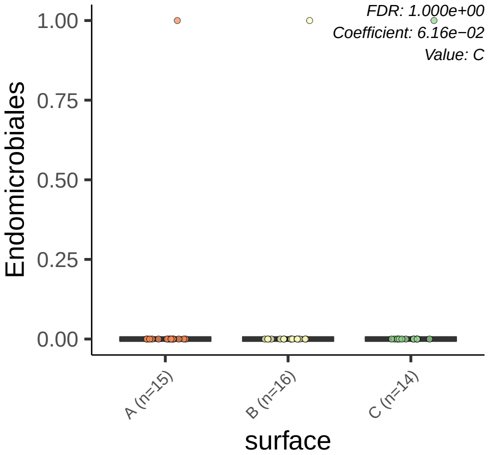

FDR: 1.000e+00
Coefficient: 6.16e−02
Value: C
Endomicrobiales
1.00
0.75
0.50
0.25
0.00
A (n=15)
B (n=16)
C (n=14)
surface

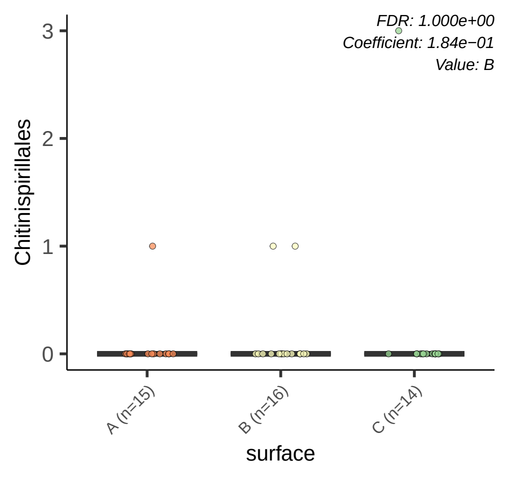

FDR: 1.000e+00
Coefficient: 1.84e−01
Value: B
Chitinispirillales
0
1
2
3
A (n=15)
B (n=16)
C (n=14)
surface

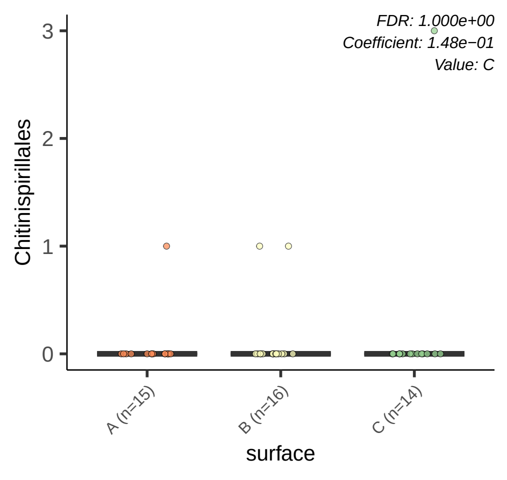

FDR: 1.000e+00
Coefficient: 1.48e−01
Value: C
Chitinispirillales
3
2
1
0
A (n=15)
B (n=16)
C (n=14)
surface

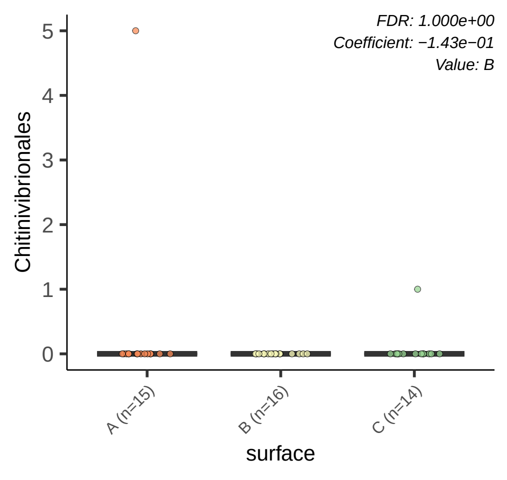
FDR: 1.000e+00
Coefficient: −1.43e−01
Value: B
Chitinivibrionales
0
1
2
3
4
5
A (n=15)
B (n=16)
C (n=14)
surface

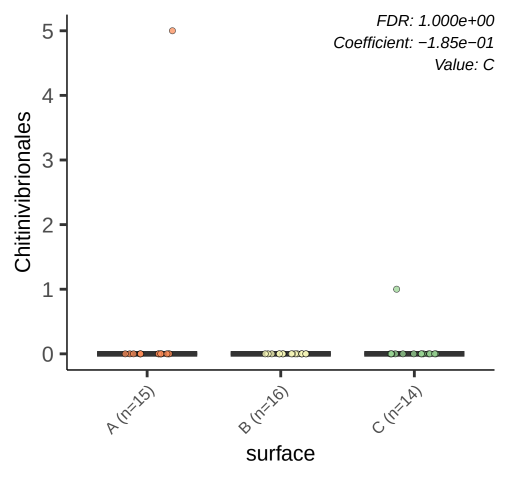
FDR: 1.000e+00
Coefficient: −1.85e−01
Value: C
Chitinivibrionales
5
4
3
2
1
0
A (n=15)
B (n=16)
C (n=14)
surface

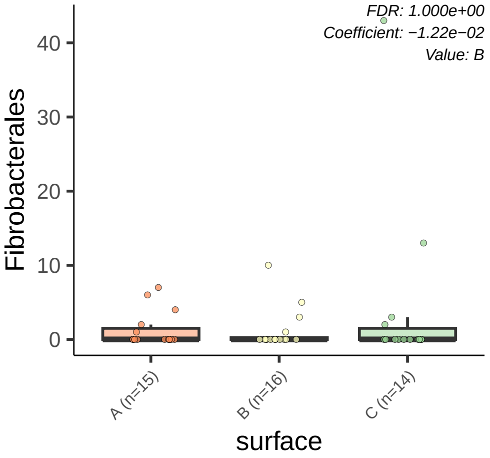

FDR: 1.000e+00
Coefficient: −1.22e−02
Value: B
Fibrobacterales
0
10
20
30
40
A (n=15)
B (n=16)
C (n=14)
surface

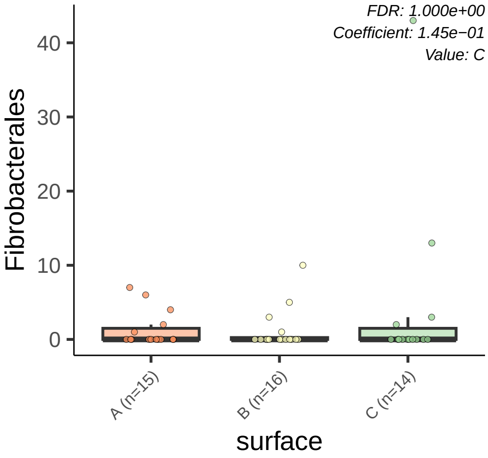

FDR: 1.000e+00
Coefficient: 1.45e−01
Value: C
Fibrobacterales
40
30
20
10
0
A (n=15)
B (n=16)
C (n=14)
surface

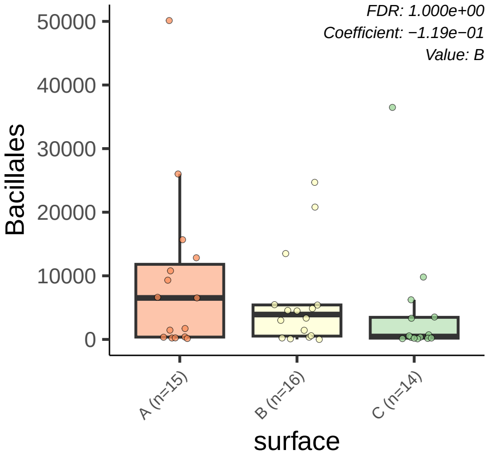
FDR: 1.000e+00
Coefficient: −1.19e−01
Value: B
Bacillales
50000
40000
30000
20000
10000
0
A (n=15)
B (n=16)
C (n=14)
surface

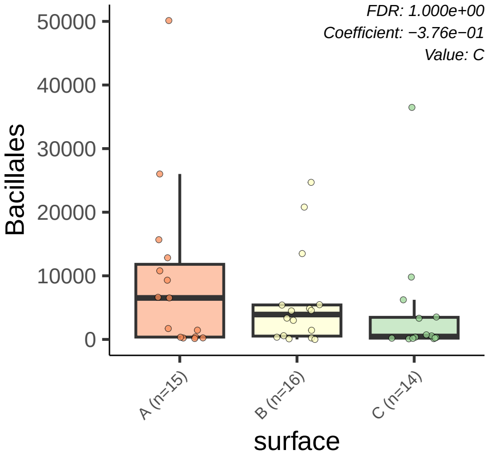
FDR: 1.000e+00
Coefficient: −3.76e−01
Value: C
Bacillales
50000
40000
30000
20000
10000
0
A (n=15)
B (n=16)
C (n=14)
surface

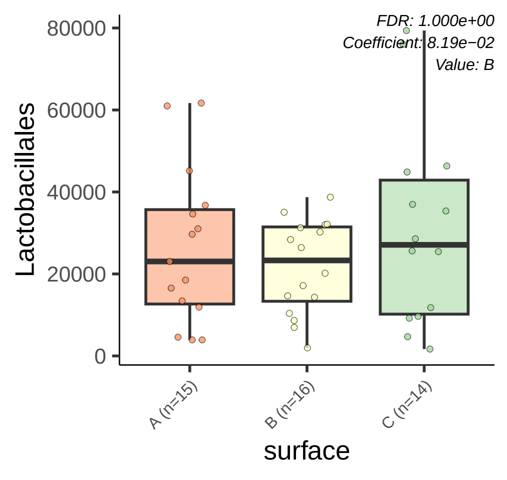
FDR: 1.000e+00
Coefficient: 8.19e−02
Value: B
Lactobacillales
80000
60000
40000
20000
0
A (n=15)
B (n=16)
C (n=14)
surface

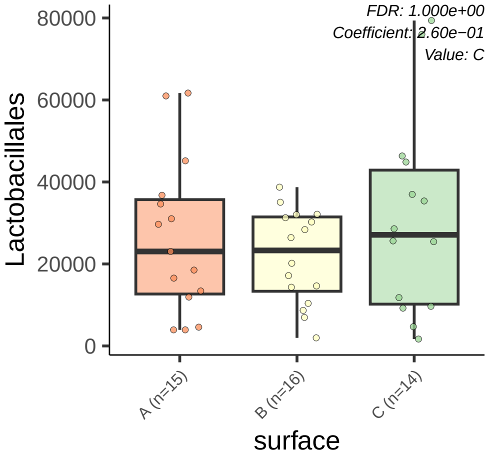

FDR: 1.000e+00
Coefficient: 2.60e−01
Value: C
Lactobacillales
80000
60000
40000
20000
0
A (n=15)
B (n=16)
C (n=14)
surface

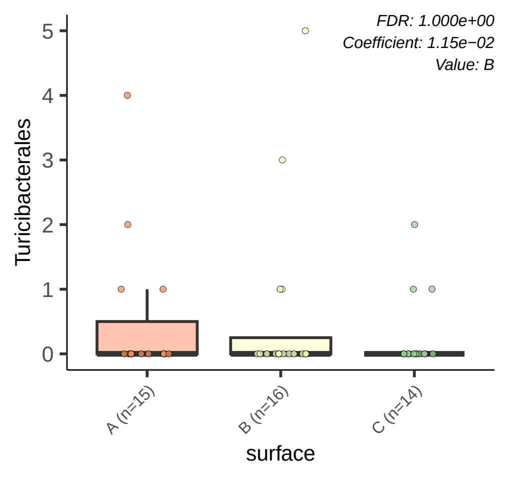

FDR: 1.000e+00
Coefficient: 1.15e−02
Value: B
Turicibacterales
0
1
2
3
4
5
A (n=15)
B (n=16)
C (n=14)
surface

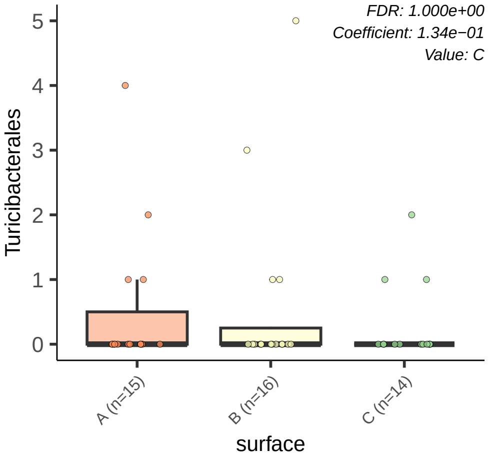

FDR: 1.000e+00
Coefficient: 1.34e−01
Value: C
Turicibacterales
0
1
2
3
4
5
A (n=15)
B (n=16)
C (n=14)
surface

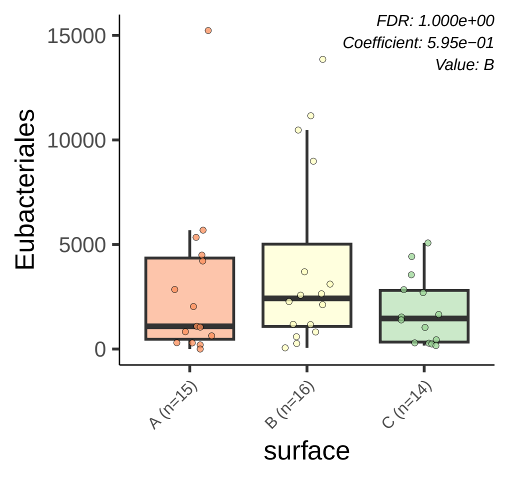

FDR: 1.000e+00
Coefficient: 5.95e−01
Value: B
15000
10000
5000
0
Eubacteriales
A (n=15)
B (n=16)
C (n=14)
surface

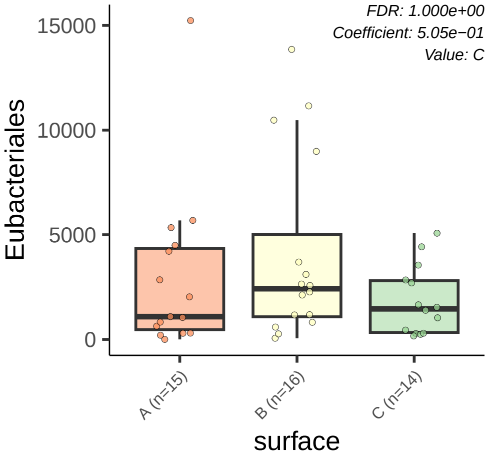

FDR: 1.000e+00
Coefficient: 5.05e−01
Value: C
Eubacteriales
15000
10000
5000
0
A (n=15)
B (n=16)
C (n=14)
surface

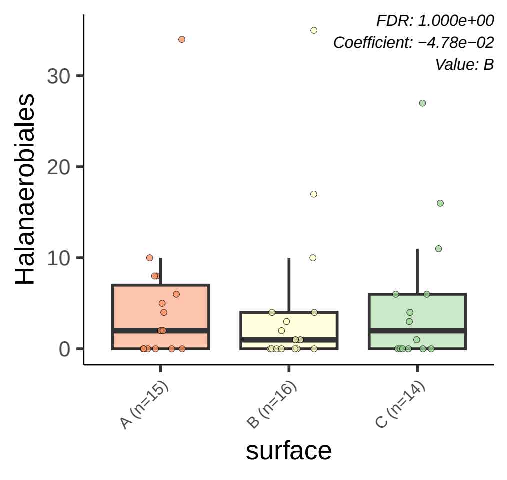

FDR: 1.000e+00
Coefficient: −4.78e−02
Value: B
Halanaerobiales
0
10
20
30
A (n=15)
B (n=16)
C (n=14)
surface

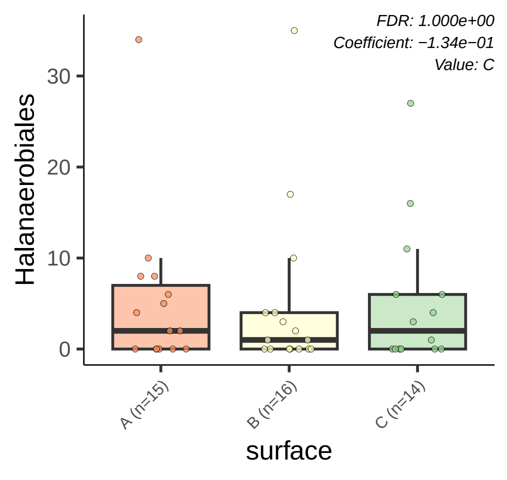

FDR: 1.000e+00
Coefficient: −1.34e−01
Value: C
Halanaerobiales
0
10
20
30
A (n=15)
B (n=16)
C (n=14)
surface

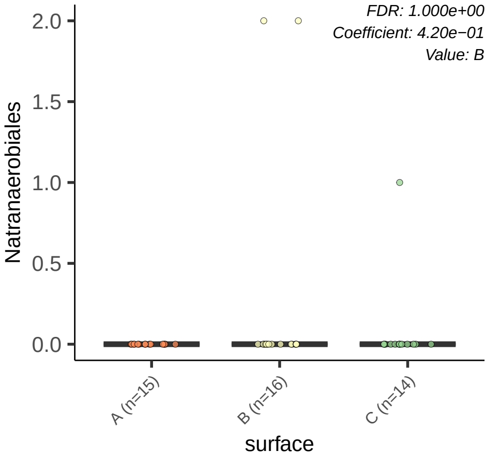

FDR: 1.000e+00
Coefficient: 4.20e−01
Value: B
Natranaerobiales
2.0
1.5
1.0
0.5
0.0
A (n=15)
B (n=16)
C (n=14)
surface

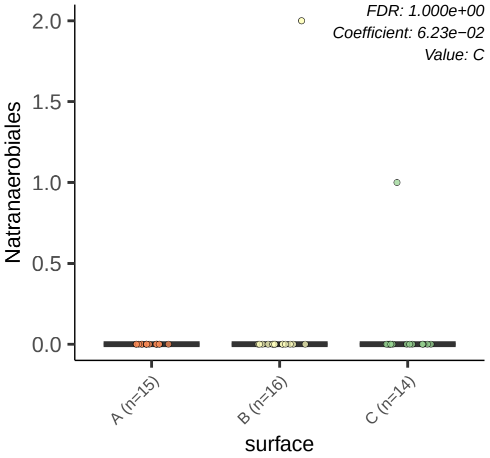

FDR: 1.000e+00
Coefficient: 6.23e−02
Value: C
Natranaerobiales
2.0
1.5
1.0
0.5
0.0
A (n=15)
B (n=16)
C (n=14)
surface

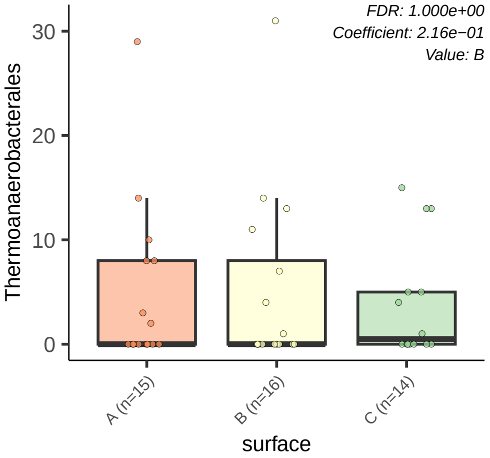
FDR: 1.000e+00
Coefficient: 2.16e−01
Value: B
Thermoanaerobacterales
30
20
10
0
A (n=15)
B (n=16)
C (n=14)
surface

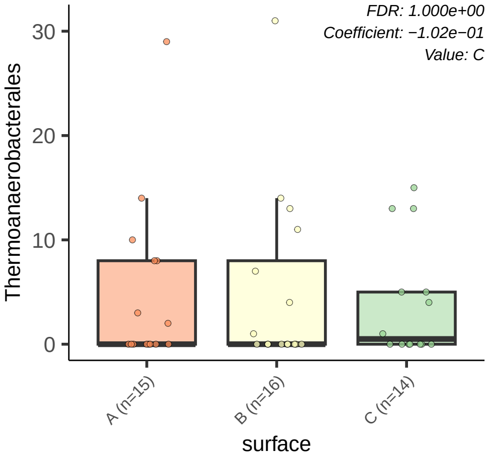
FDR: 1.000e+00
Coefficient: −1.02e−01
Value: C
Thermoanaerobacterales
30
20
10
0
A (n=15)
B (n=16)
C (n=14)
surface

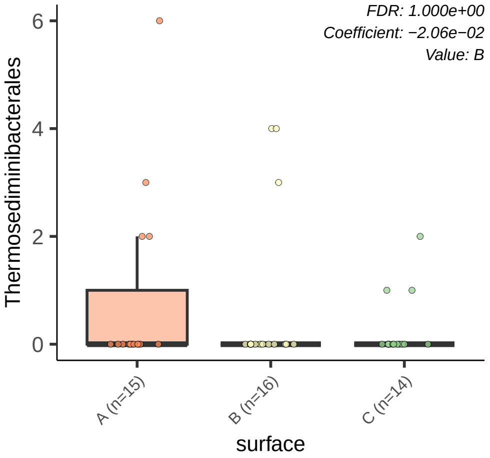

FDR: 1.000e+00
Coefficient: −2.06e−02
Value: B
Thermosediminibacterales
6
4
2
0
A (n=15)
B (n=16)
C (n=14)
surface

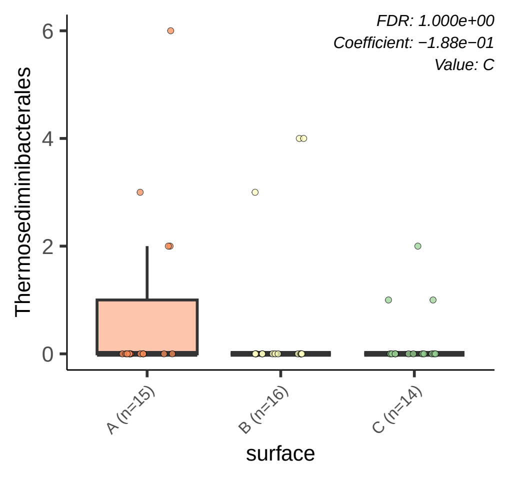
FDR: 1.000e+00
Coefficient: −1.88e−01
Value: C
Thermosediminibacterales
0
2
4
6
A (n=15)
B (n=16)
C (n=14)
surface

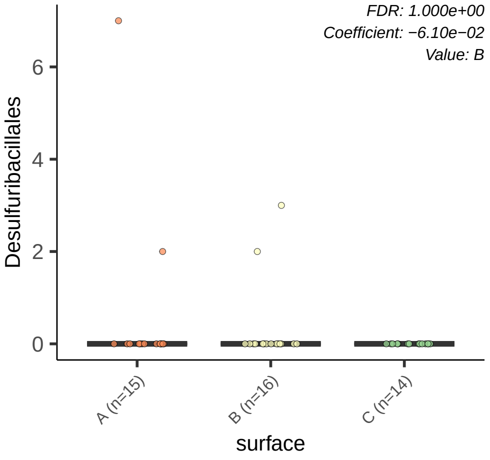

FDR: 1.000e+00
Coefficient: −6.10e−02
Value: B
Desulfuribacillales
0
2
4
6
A (n=15)
B (n=16)
C (n=14)
surface

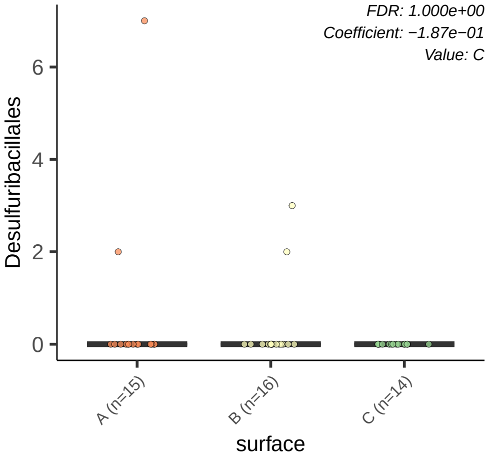

FDR: 1.000e+00
Coefficient: −1.87e−01
Value: C
Desulfuribacillales
0
2
4
6
A (n=15)
B (n=16)
C (n=14)
surface

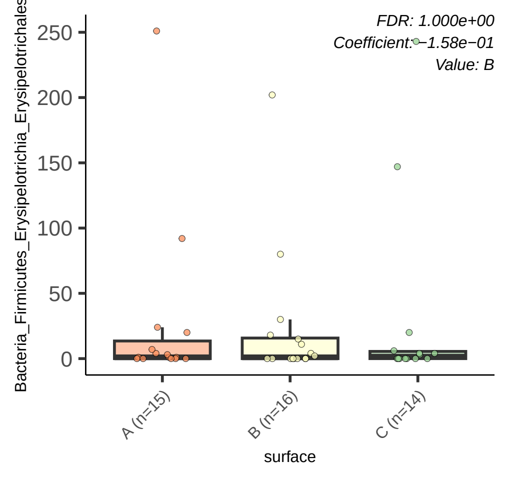
FDR: 1.000e+00
Coefficient: −1.58e−01
Value: B
Bacteria_Firmicutes_Erysipelotrichia_Erysipelotrichales
250
200
150
100
50
0
A (n=15)
B (n=16)
C (n=14)
surface

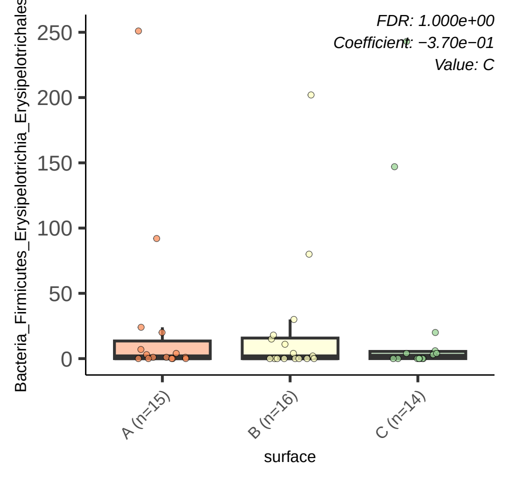

FDR: 1.000e+00
Coefficient: −3.70e−01
Value: C
Bacteria_Firmicutes_Erysipelotrichia_Erysipelotrichales
250
200
150
100
50
0
A (n=15)
B (n=16)
C (n=14)
surface

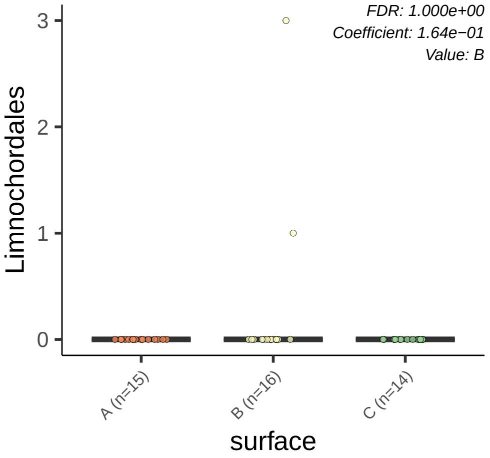
FDR: 1.000e+00
Coefficient: 1.64e−01
Value: B
Limnochordales
3
2
1
0
A (n=15)
B (n=16)
C (n=14)
surface

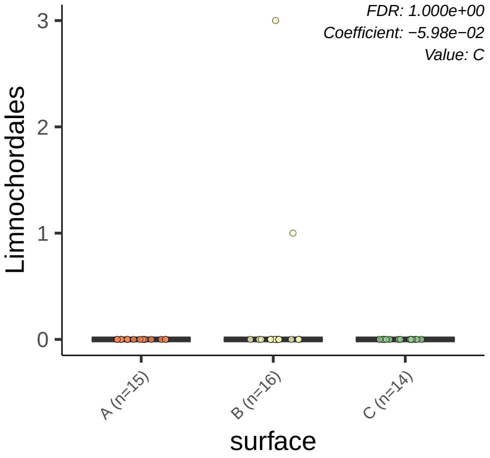
FDR: 1.000e+00
Coefficient: −5.98e−02
Value: C
Limnochordales
3
2
1
0
A (n=15)
B (n=16)
C (n=14)
surface

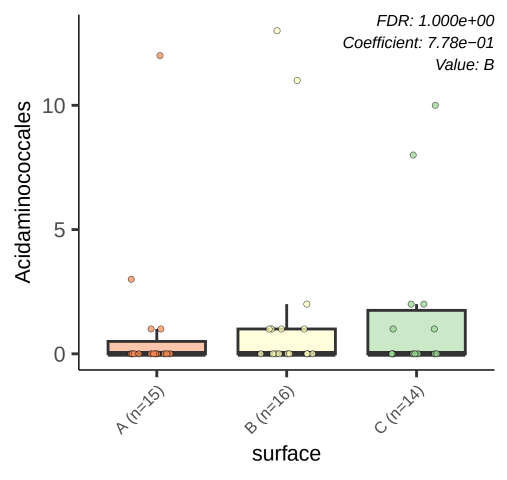
FDR: 1.000e+00
Coefficient: 7.78e−01
Value: B
Acidaminococcales
0
5
10
A (n=15)
B (n=16)
C (n=14)
surface

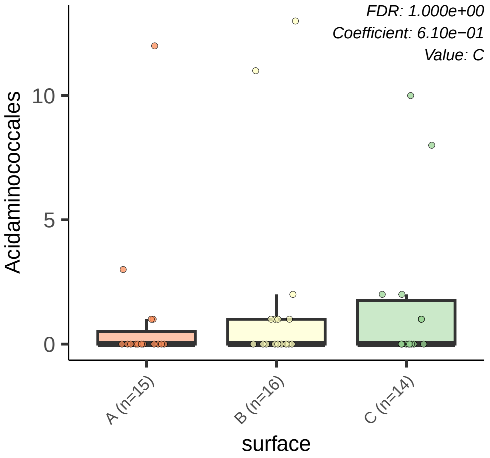

FDR: 1.000e+00
Coefficient: 6.10e−01
Value: C
Acidaminococcales
0
5
10
A (n=15)
B (n=16)
C (n=14)
surface

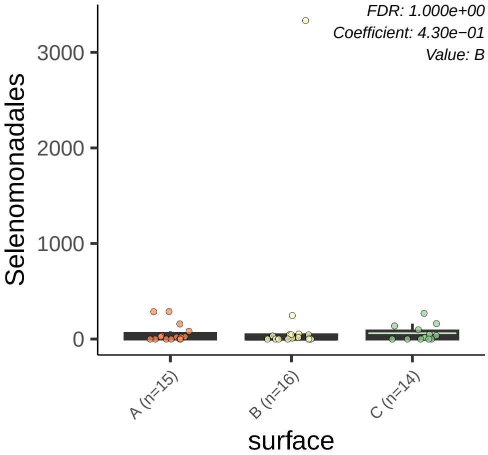
FDR: 1.000e+00
Coefficient: 4.30e−01
Value: B
Selenomonadales
3000
2000
1000
0
A (n=15)
B (n=16)
C (n=14)
surface

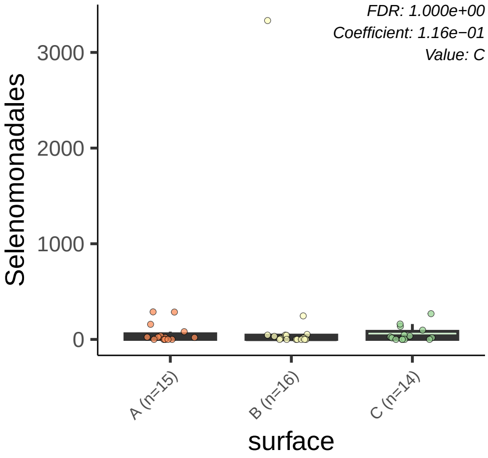

FDR: 1.000e+00
Coefficient: 1.16e−01
Value: C
Selenomonadales
3000
2000
1000
0
A (n=15)
B (n=16)
C (n=14)
surface

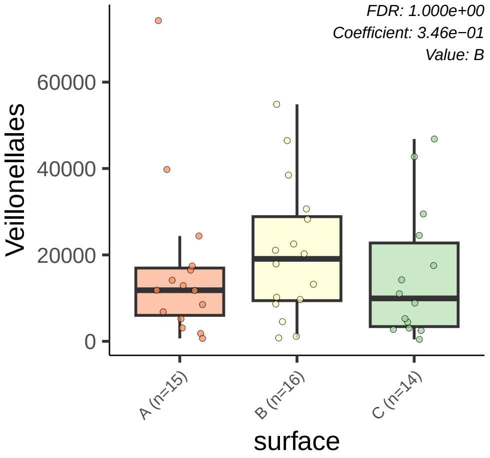

FDR: 1.000e+00
Coefficient: 3.46e−01
Value: B
Veillonellales
60000
40000
20000
0
A (n=15)
B (n=16)
C (n=14)
surface

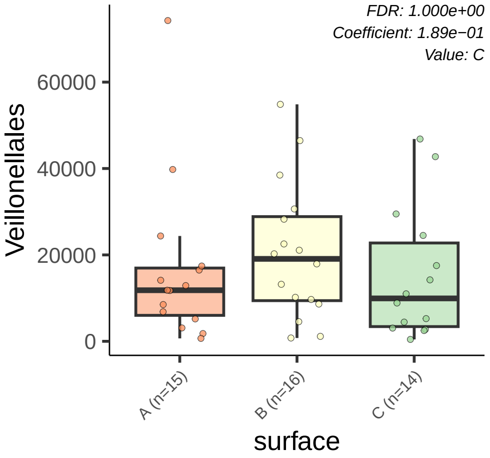
FDR: 1.000e+00
Coefficient: 1.89e−01
Value: C
Veillonellales
60000
40000
20000
0
A (n=15)
B (n=16)
C (n=14)
surface

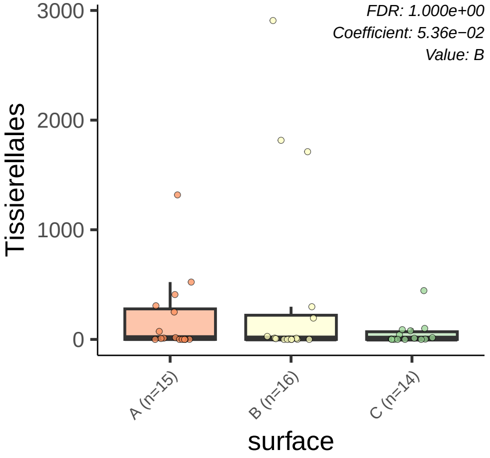
FDR: 1.000e+00
Coefficient: 5.36e−02
Value: B
Tissierellales
3000
2000
1000
0
A (n=15)
B (n=16)
C (n=14)
surface

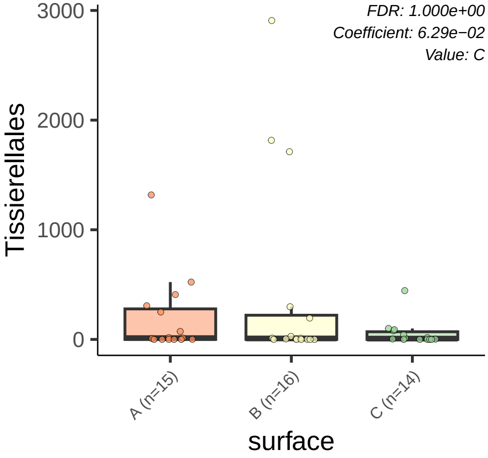

FDR: 1.000e+00
Coefficient: 6.29e−02
Value: C
Tissierellales
3000
2000
1000
0
A (n=15)
B (n=16)
C (n=14)
surface

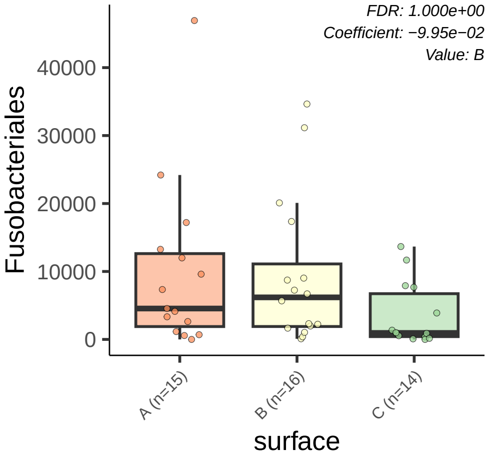

FDR: 1.000e+00
Coefficient: −9.95e−02
Value: B
Fusobacteriales
40000
30000
20000
10000
0
A (n=15)
B (n=16)
C (n=14)
surface

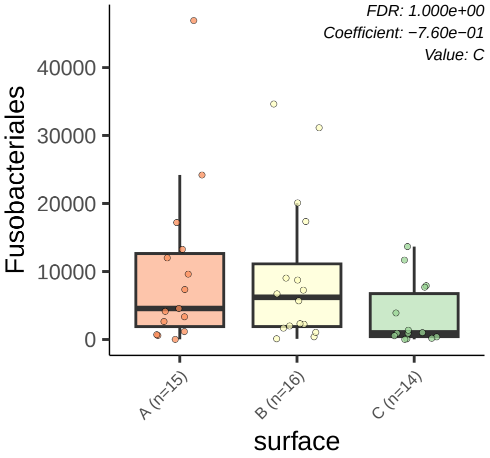
FDR: 1.000e+00
Coefficient: −7.60e−01
Value: C
Fusobacteriales
40000
30000
20000
10000
0
A (n=15)
B (n=16)
C (n=14)
surface

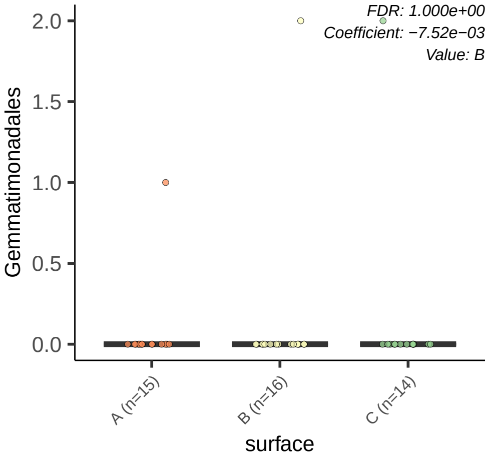
FDR: 1.000e+00
Coefficient: −7.52e−03
Value: B
Gemmatimonadales
2.0
1.5
1.0
0.5
0.0
A (n=15)
B (n=16)
C (n=14)
surface

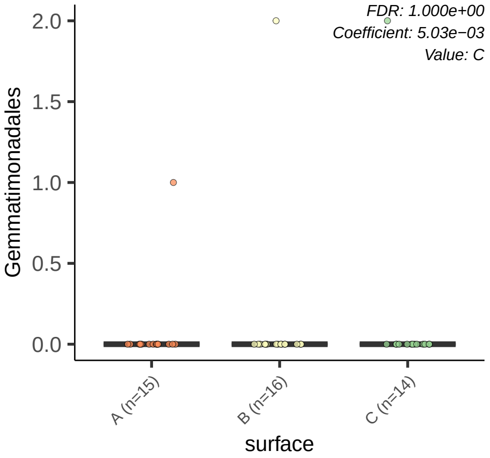

FDR: 1.000e+00
Coefficient: 5.03e−03
Value: C
Gemmatimonadales
2.0
1.5
1.0
0.5
0.0
A (n=15)
B (n=16)
C (n=14)
surface

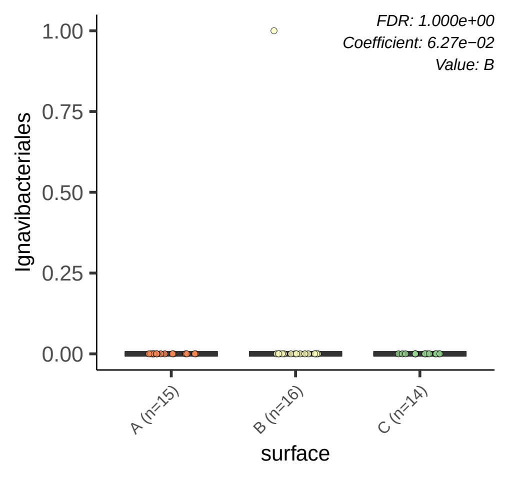

FDR: 1.000e+00
Coefficient: 6.27e−02
Value: B
Ignavibacteriales
1.00
0.75
0.50
0.25
0.00
A (n=15)
B (n=16)
C (n=14)
surface

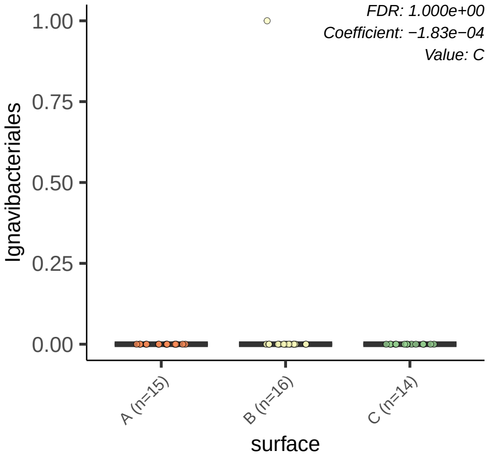

FDR: 1.000e+00
Coefficient: −1.83e−04
Value: C
Ignavibacteriales
1.00
0.75
0.50
0.25
0.00
A (n=15)
B (n=16)
C (n=14)
surface

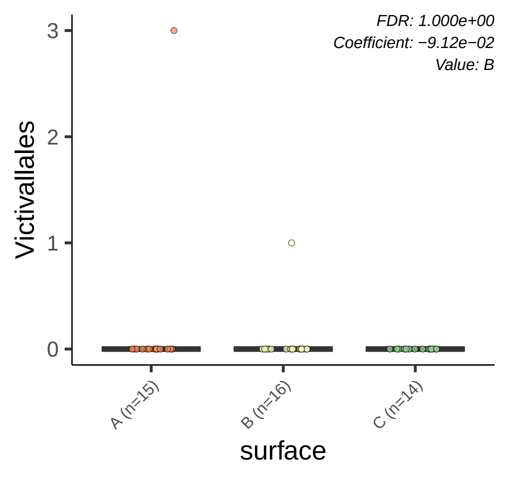
FDR: 1.000e+00
Coefficient: −9.12e−02
Value: B
Victivallales
0
1
2
3
A (n=15)
B (n=16)
C (n=14)
surface

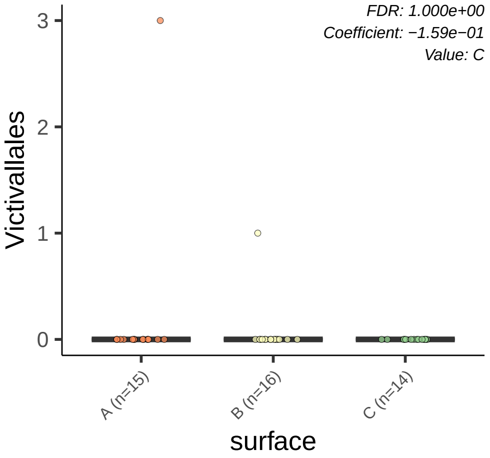
FDR: 1.000e+00
Coefficient: −1.59e−01
Value: C
Victivallales
0
1
2
3
A (n=15)
B (n=16)
C (n=14)
surface

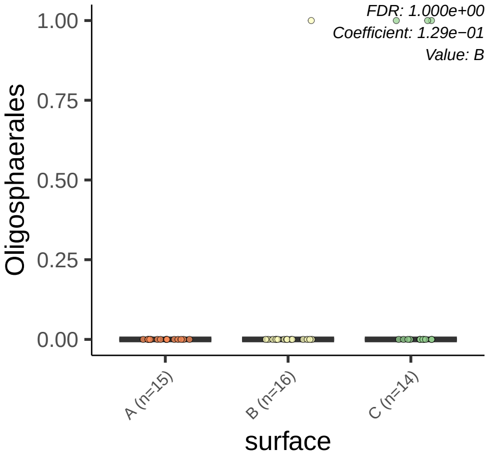

FDR: 1.000e+00
Coefficient: 1.29e−01
Value: B
1.00
0.75
0.50
0.25
0.00
Oligosphaerales
A (n=15)
B (n=16)
C (n=14)
surface

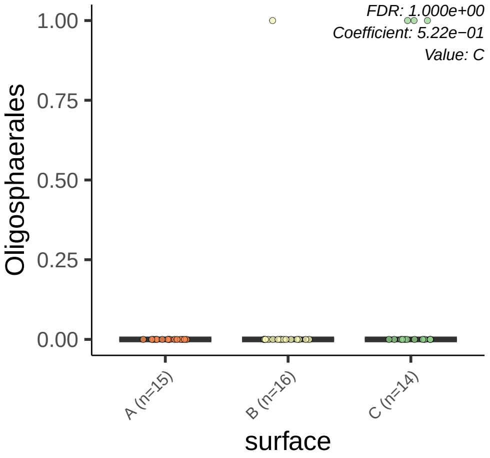
FDR: 1.000e+00
Coefficient: 5.22e−01
Value: C
Oligosphaerales
1.00
0.75
0.50
0.25
0.00
A (n=15)
B (n=16)
C (n=14)
surface

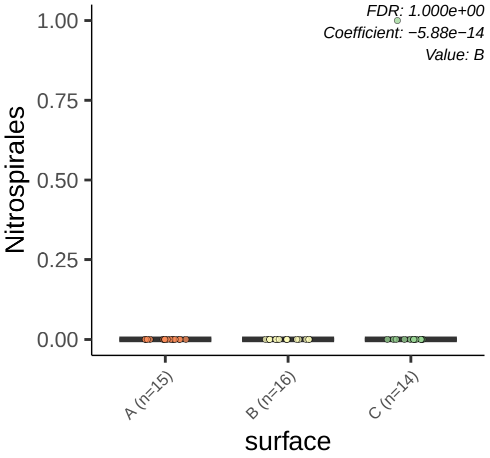
FDR: 1.000e+00
Coefficient: −5.88e−14
Value: B
Nitrospirales
1.00
0.75
0.50
0.25
0.00
A (n=15)
B (n=16)
C (n=14)
surface

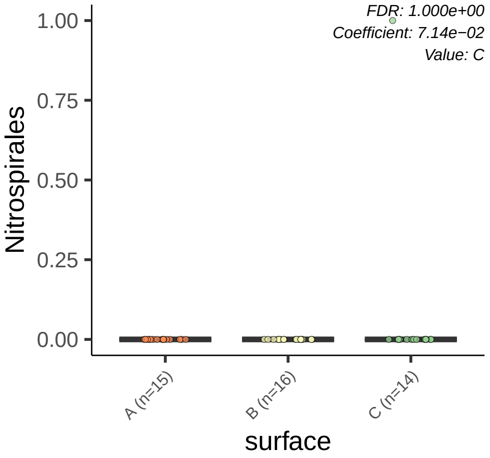
FDR: 1.000e+00
Coefficient: 7.14e−02
Value: C
Nitrospirales
1.00
0.75
0.50
0.25
0.00
A (n=15)
B (n=16)
C (n=14)
surface

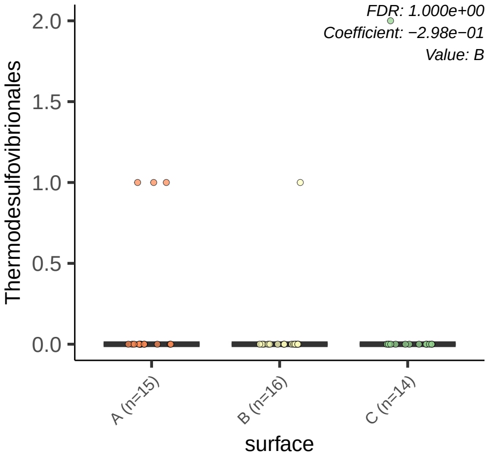
FDR: 1.000e+00
Coefficient: −2.98e−01
Value: B
Thermodesulfovibrionales
2.0
1.5
1.0
0.5
0.0
A (n=15)
B (n=16)
C (n=14)
surface

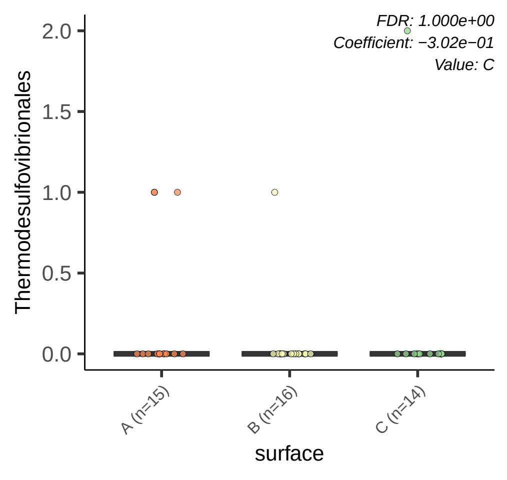

FDR: 1.000e+00
Coefficient: −3.02e−01
Value: C
Thermodesulfovibrionales
2.0
1.5
1.0
0.5
0.0
A (n=15)
B (n=16)
C (n=14)
surface

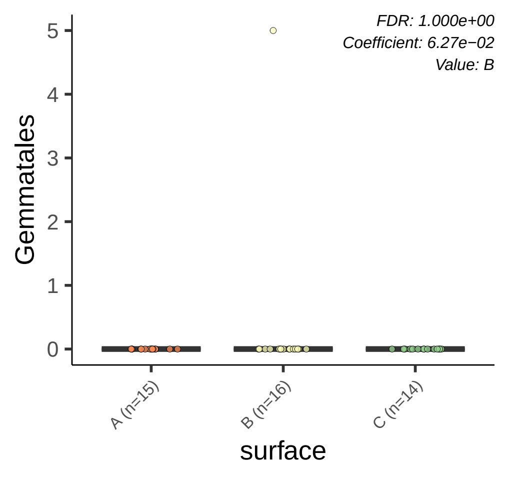
FDR: 1.000e+00
Coefficient: 6.27e−02
Value: B
Gemmatales
0
1
2
3
4
5
A (n=15)
B (n=16)
C (n=14)
surface

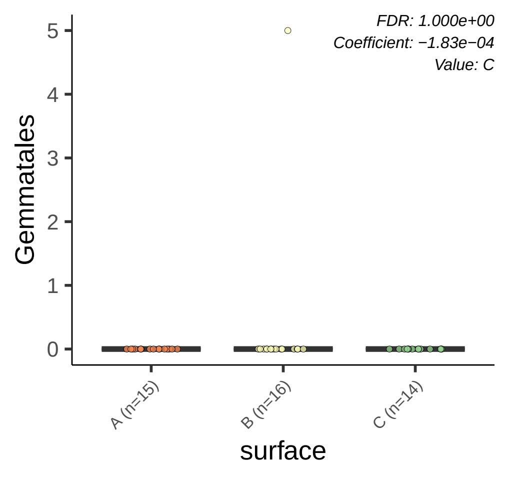

FDR: 1.000e+00
Coefficient: −1.83e−04
Value: C
Gemmatales
0
1
2
3
4
5
A (n=15)
B (n=16)
C (n=14)
surface

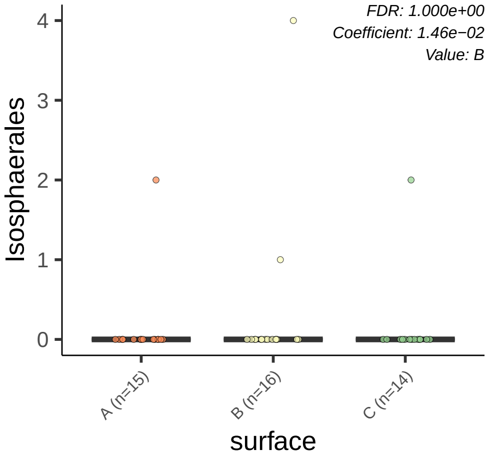
FDR: 1.000e+00
Coefficient: 1.46e−02
Value: B
Isosphaerales
4
3
2
1
0
A (n=15)
B (n=16)
C (n=14)
surface

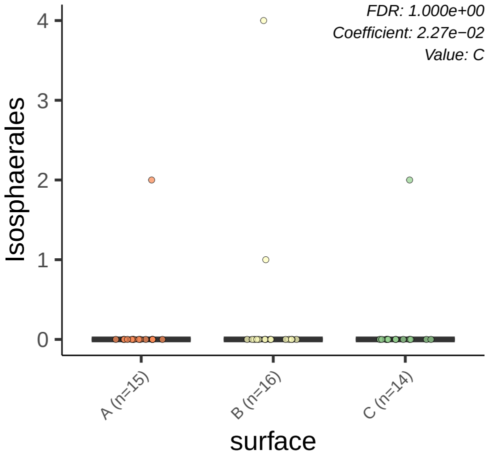
FDR: 1.000e+00
Coefficient: 2.27e−02
Value: C
Isosphaerales
4
3
2
1
0
A (n=15)
B (n=16)
C (n=14)
surface

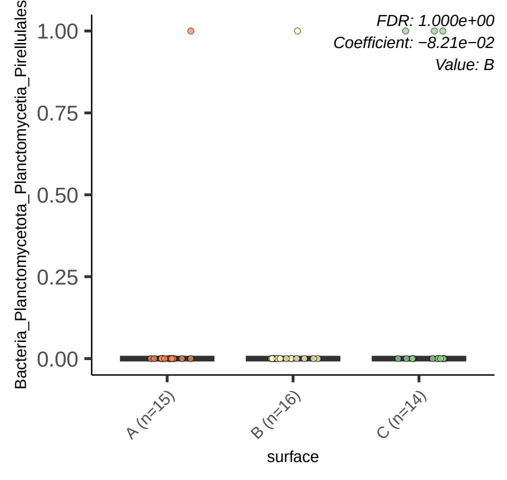
FDR: 1.000e+00
Coefficient: −8.21e−02
Value: B
Bacteria_Planctomycetota_Planctomycetia_Pirellulales
1.00
0.75
0.50
0.25
0.00
A (n=15)
B (n=16)
C (n=14)
surface

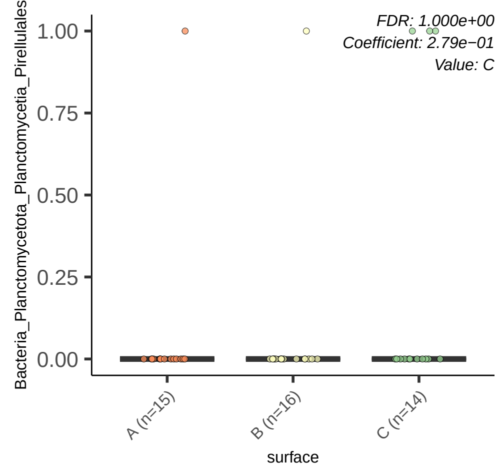
FDR: 1.000e+00
Coefficient: 2.79e−01
Value: C
Bacteria_Planctomycetota_Planctomycetia_Pirellulales
1.00
0.75
0.50
0.25
0.00
A (n=15)
B (n=16)
C (n=14)
surface

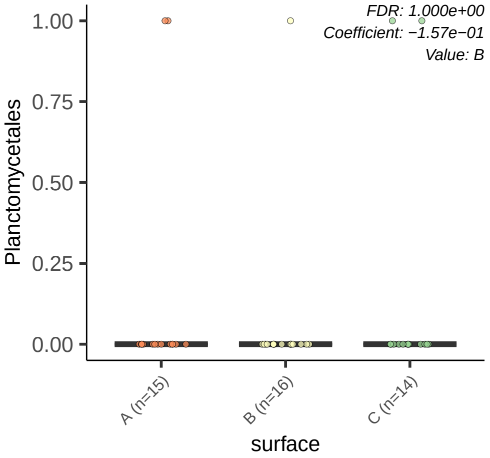
FDR: 1.000e+00
Coefficient: −1.57e−01
Value: B
Planctomycetales
1.00
0.75
0.50
0.25
0.00
A (n=15)
B (n=16)
C (n=14)
surface

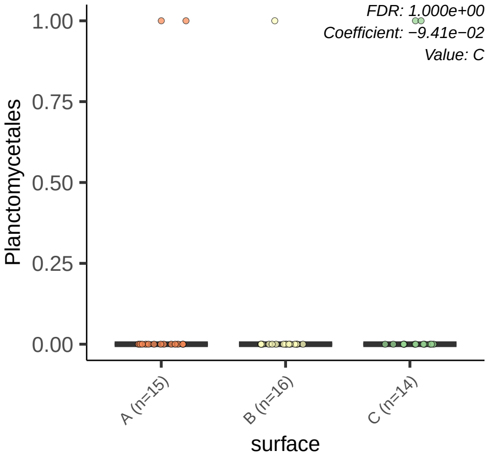

FDR: 1.000e+00
Coefficient: −9.41e−02
Value: C
Planctomycetales
1.00
0.75
0.50
0.25
0.00
A (n=15)
B (n=16)
C (n=14)
surface

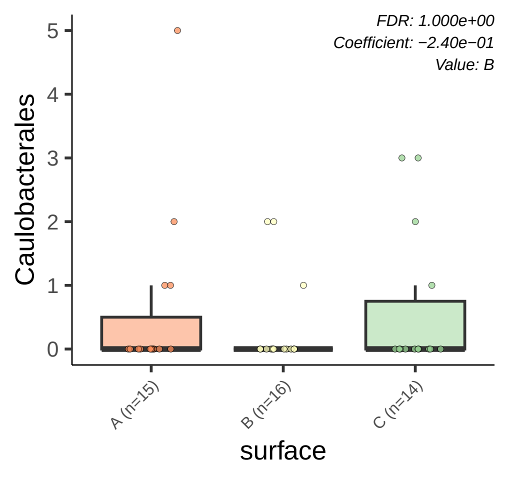
FDR: 1.000e+00
Coefficient: −2.40e−01
Value: B
Caulobacterales
5
4
3
2
1
0
A (n=15)
B (n=16)
C (n=14)
surface

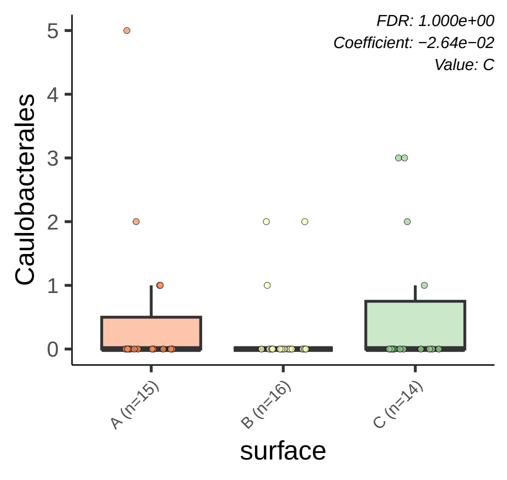

FDR: 1.000e+00
Coefficient: −2.64e−02
Value: C
Caulobacterales
0
1
2
3
4
5
A (n=15)
B (n=16)
C (n=14)
surface

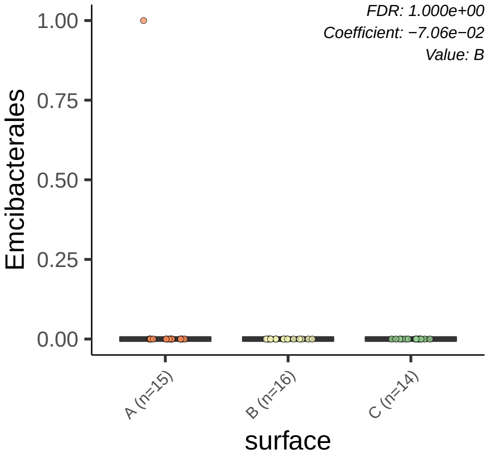
FDR: 1.000e+00
Coefficient: −7.06e−02
Value: B
Emcibacterales
1.00
0.75
0.50
0.25
0.00
A (n=15)
B (n=16)
C (n=14)
surface

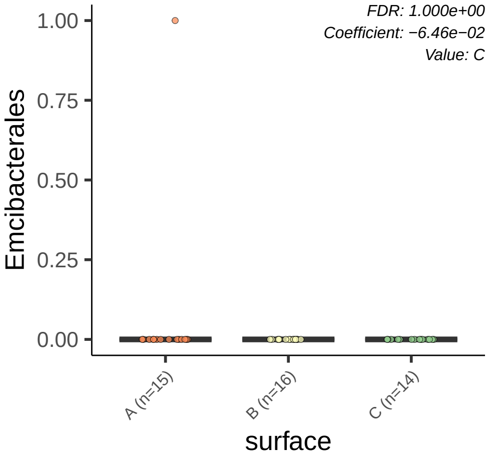
FDR: 1.000e+00
Coefficient: −6.46e−02
Value: C
Emcibacterales
1.00
0.75
0.50
0.25
0.00
A (n=15)
B (n=16)
C (n=14)
surface

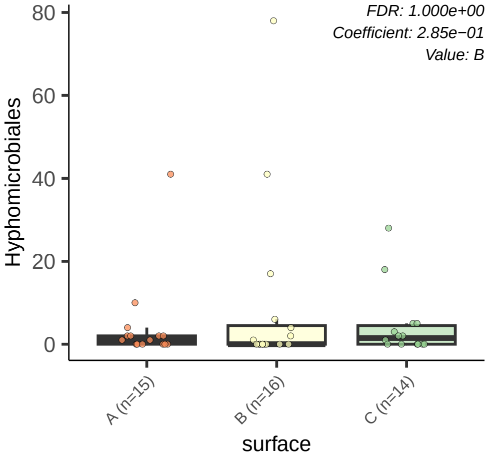
FDR: 1.000e+00
Coefficient: 2.85e−01
Value: B
Hyphomicrobiales
80
60
40
20
0
A (n=15)
B (n=16)
C (n=14)
surface

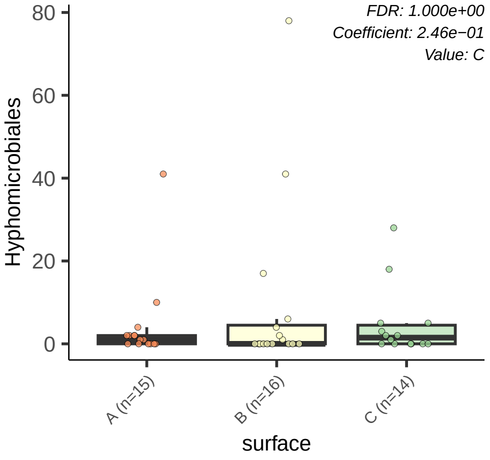

FDR: 1.000e+00
Coefficient: 2.46e−01
Value: C
Hyphomicrobiales
0
20
40
60
80
A (n=15)
B (n=16)
C (n=14)
surface

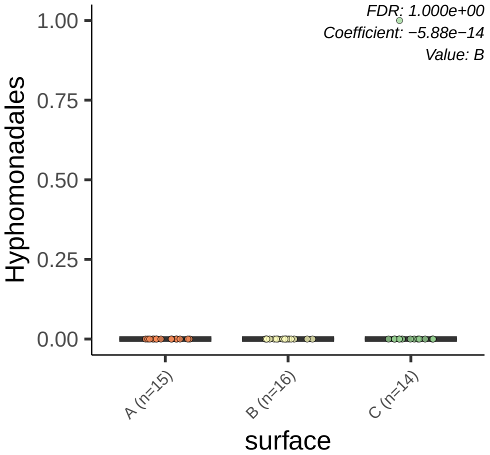
FDR: 1.000e+00
Coefficient: −5.88e−14
Value: B
Hyphomonadales
1.00
0.75
0.50
0.25
0.00
A (n=15)
B (n=16)
C (n=14)
surface

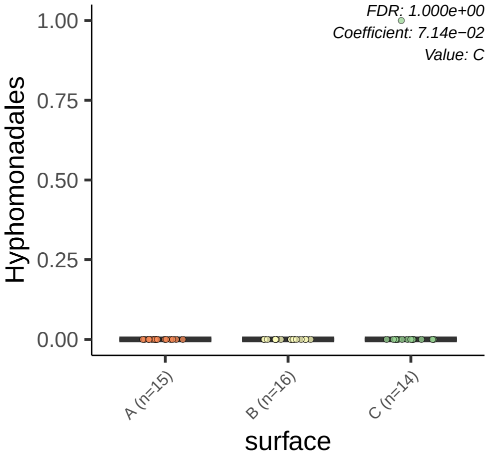
FDR: 1.000e+00
Coefficient: 7.14e−02
Value: C
Hyphomonadales
1.00
0.75
0.50
0.25
0.00
A (n=15)
B (n=16)
C (n=14)
surface

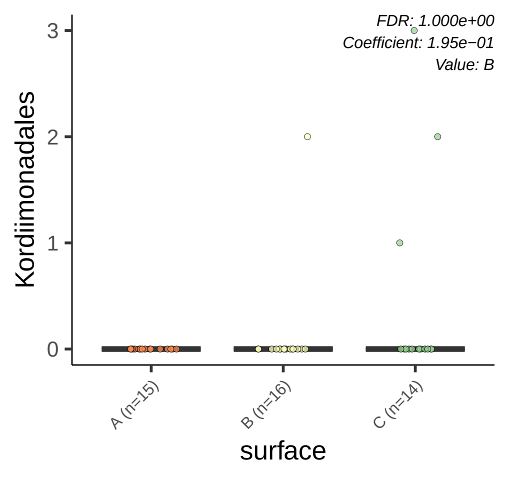
FDR: 1.000e+00
Coefficient: 1.95e−01
Value: B
Kordiimonadales
0
1
2
3
A (n=15)
B (n=16)
C (n=14)
surface

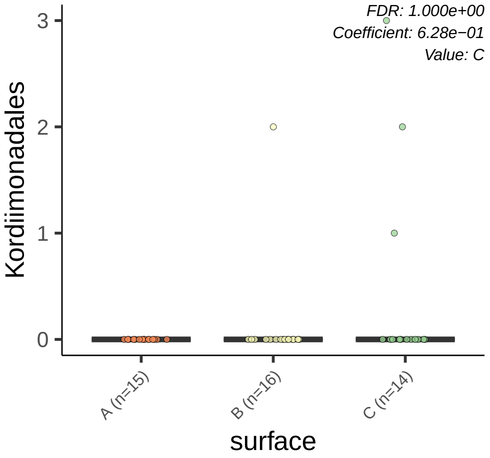

FDR: 1.000e+00
Coefficient: 6.28e−01
Value: C
Kordiimonadales
3
2
1
0
A (n=15)
B (n=16)
C (n=14)
surface

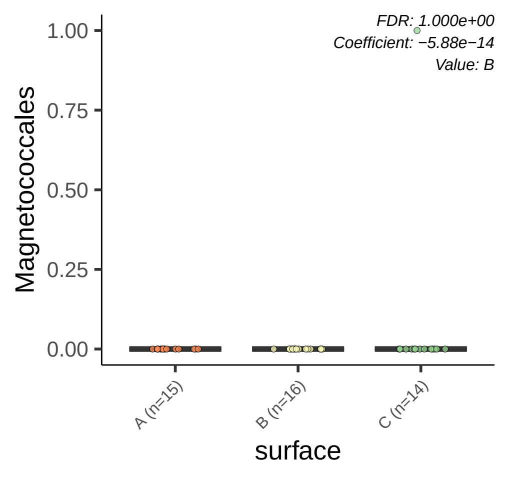
FDR: 1.000e+00
Coefficient: −5.88e−14
Value: B
Magnetococcales
1.00
0.75
0.50
0.25
0.00
A (n=15)
B (n=16)
C (n=14)
surface

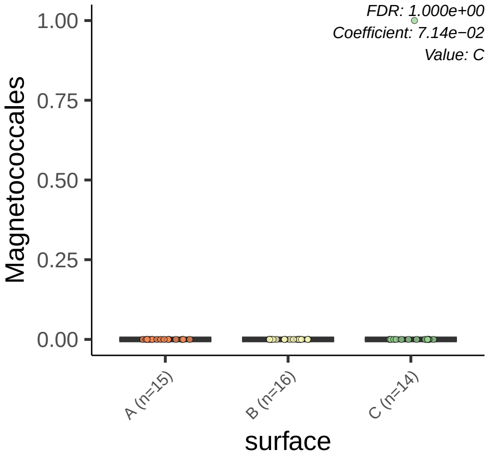

FDR: 1.000e+00
Coefficient: 7.14e−02
Value: C
Magnetococcales
1.00
0.75
0.50
0.25
0.00
A (n=15)
B (n=16)
C (n=14)
surface

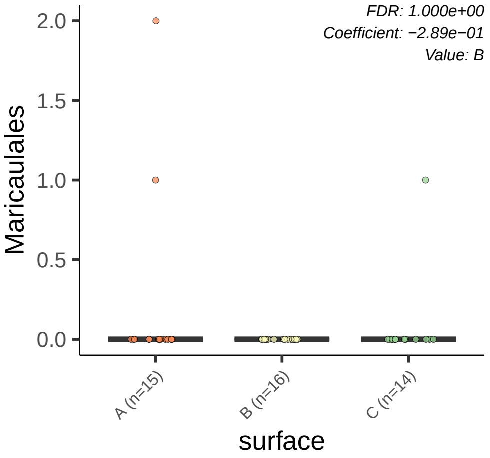
FDR: 1.000e+00
Coefficient: −2.89e−01
Value: B
Maricaulales
2.0
1.5
1.0
0.5
0.0
A (n=15)
B (n=16)
C (n=14)
surface

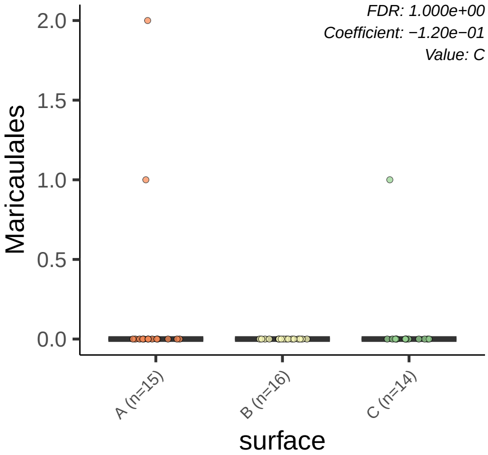

FDR: 1.000e+00
Coefficient: −1.20e−01
Value: C
Maricaulales
2.0
1.5
1.0
0.5
0.0
A (n=15)
B (n=16)
C (n=14)
surface

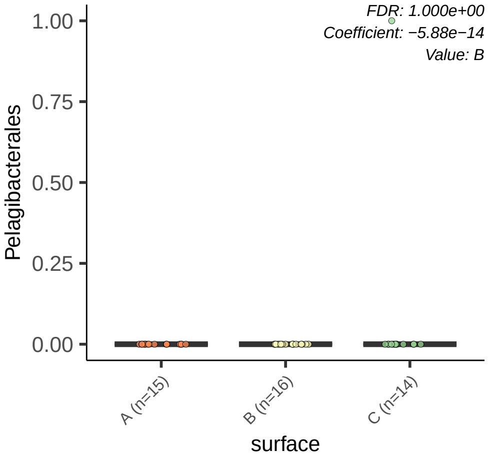

FDR: 1.000e+00
Coefficient: −5.88e−14
Value: B
Pelagibacterales
1.00
0.75
0.50
0.25
0.00
A (n=15)
B (n=16)
C (n=14)
surface

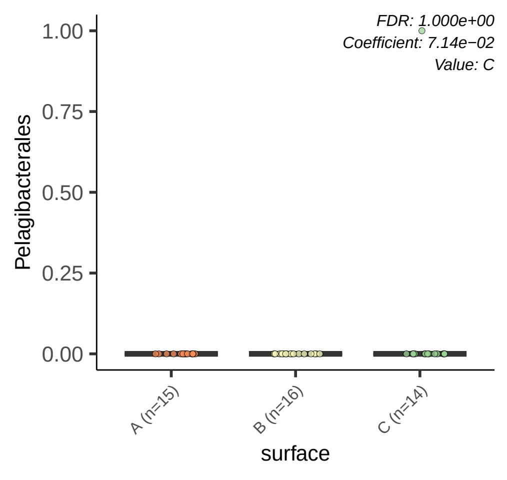
FDR: 1.000e+00
Coefficient: 7.14e−02
Value: C
1.00
0.75
0.50
0.25
0.00
Pelagibacterales
A (n=15)
B (n=16)
C (n=14)
surface

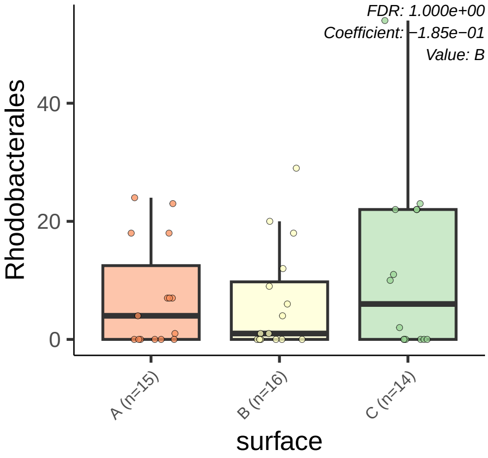

FDR: 1.000e+00
Coefficient: −1.85e−01
Value: B
Rhodobacterales
0
20
40
A (n=15)
B (n=16)
C (n=14)
surface

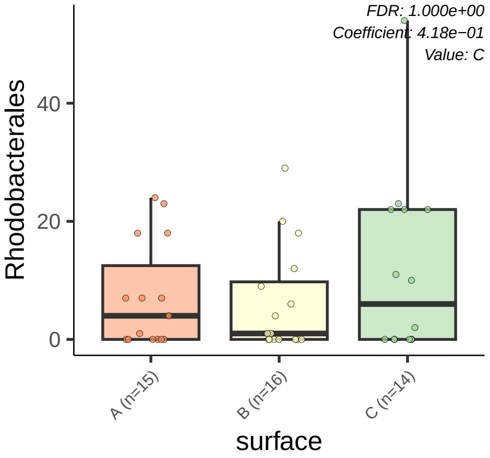
FDR: 1.000e+00
Coefficient: 4.18e−01
Value: C
Rhodobacterales
40
20
0
A (n=15)
B (n=16)
C (n=14)
surface

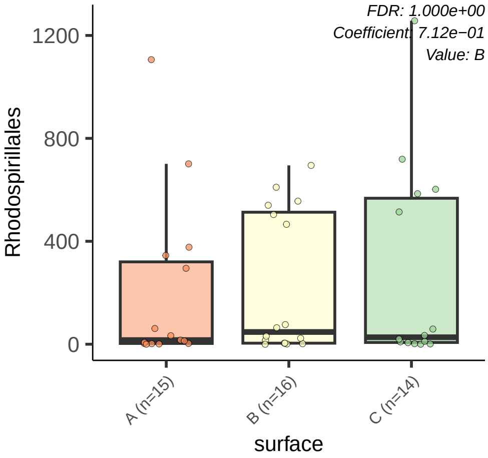

FDR: 1.000e+00
Coefficient: 7.12e−01
Value: B
Rhodospirillales
1200
800
400
0
A (n=15)
B (n=16)
C (n=14)
surface

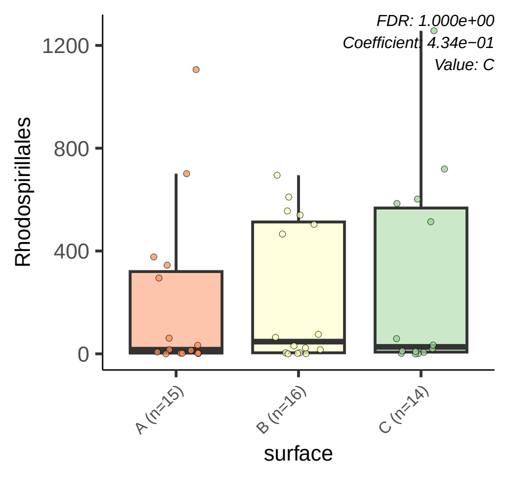

FDR: 1.000e+00
Coefficient: 4.34e−01
Value: C
Rhodospirillales
1200
800
400
0
A (n=15)
B (n=16)
C (n=14)
surface

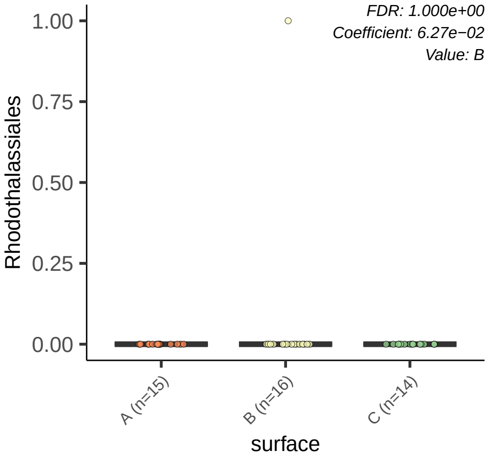
FDR: 1.000e+00
Coefficient: 6.27e−02
Value: B
Rhodothalassiales
1.00
0.75
0.50
0.25
0.00
A (n=15)
B (n=16)
C (n=14)
surface

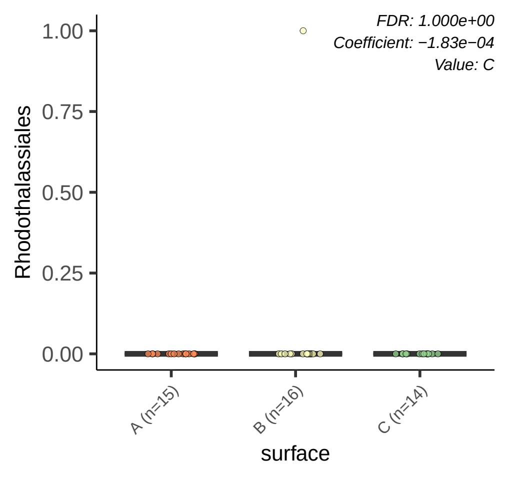

FDR: 1.000e+00
Coefficient: −1.83e−04
Value: C
1.00
0.75
0.50
0.25
0.00
Rhodothalassiales
A (n=15)
B (n=16)
C (n=14)
surface

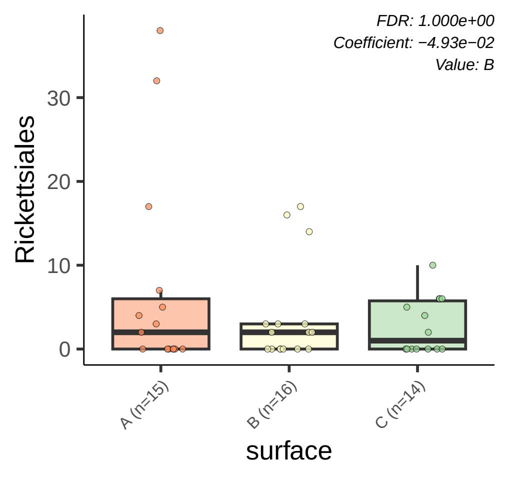
FDR: 1.000e+00
Coefficient: −4.93e−02
Value: B
Rickettsiales
0
10
20
30
A (n=15)
B (n=16)
C (n=14)
surface

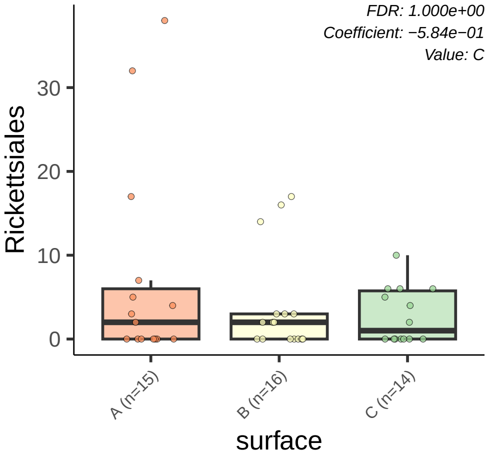

FDR: 1.000e+00
Coefficient: −5.84e−01
Value: C
Rickettsiales
30
20
10
0
A (n=15)
B (n=16)
C (n=14)
surface

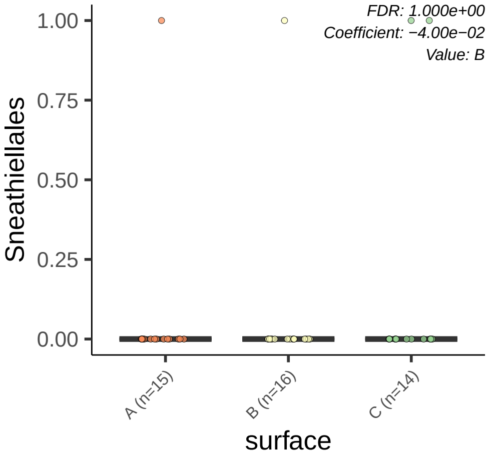
FDR: 1.000e+00
Coefficient: −4.00e−02
Value: B
Sneathiellales
1.00
0.75
0.50
0.25
0.00
A (n=15)
B (n=16)
C (n=14)
surface

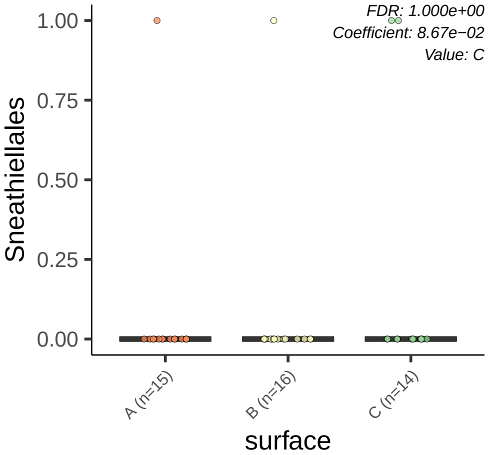

FDR: 1.000e+00
Coefficient: 8.67e−02
Value: C
Sneathiellales
1.00
0.75
0.50
0.25
0.00
A (n=15)
B (n=16)
C (n=14)
surface

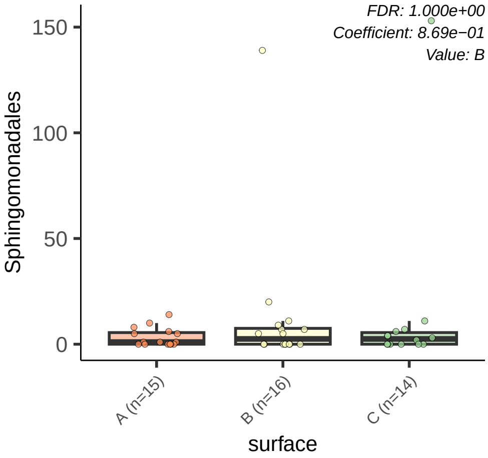

FDR: 1.000e+00
Coefficient: 8.69e−01
Value: B
Sphingomonadales
150
100
50
0
A (n=15)
B (n=16)
C (n=14)
surface

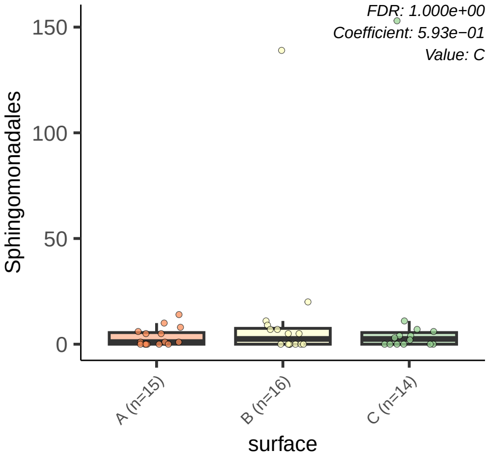
FDR: 1.000e+00
Coefficient: 5.93e−01
Value: C
Sphingomonadales
150
100
50
0
A (n=15)
B (n=16)
C (n=14)
surface

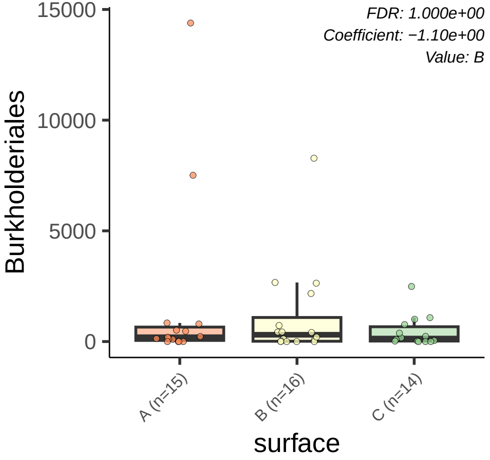

FDR: 1.000e+00
Coefficient: −1.10e+00
Value: B
15000
10000
5000
0
Burkholderiales
A (n=15)
B (n=16)
C (n=14)
surface

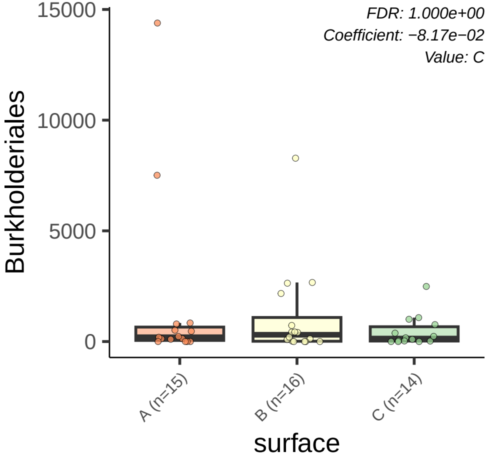
FDR: 1.000e+00
Coefficient: −8.17e−02
Value: C
Burkholderiales
15000
10000
5000
0
A (n=15)
B (n=16)
C (n=14)
surface

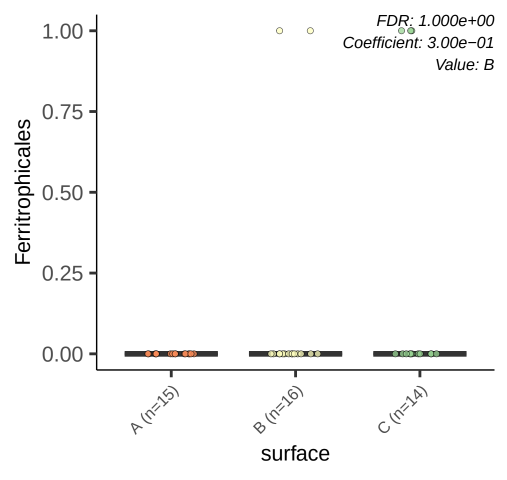

FDR: 1.000e+00
Coefficient: 3.00e−01
Value: B
Ferritrophicales
1.00
0.75
0.50
0.25
0.00
A (n=15)
B (n=16)
C (n=14)
surface

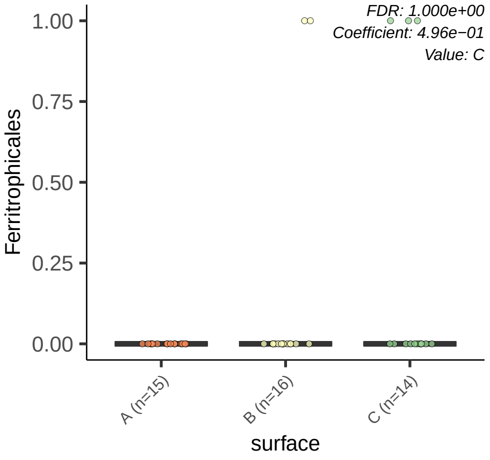
FDR: 1.000e+00
Coefficient: 4.96e−01
Value: C
Ferritrophicales
1.00
0.75
0.50
0.25
0.00
A (n=15)
B (n=16)
C (n=14)
surface

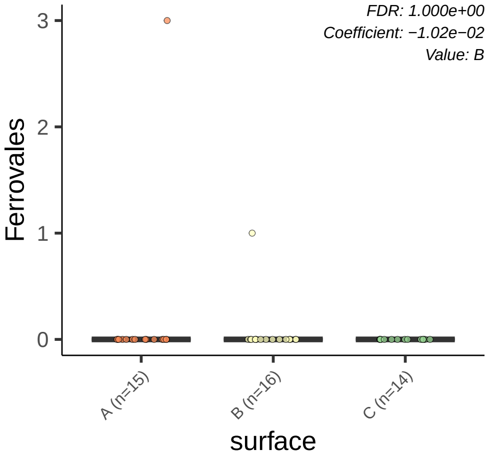
FDR: 1.000e+00
Coefficient: −1.02e−02
Value: B
Ferrovales
3
2
1
0
A (n=15)
B (n=16)
C (n=14)
surface

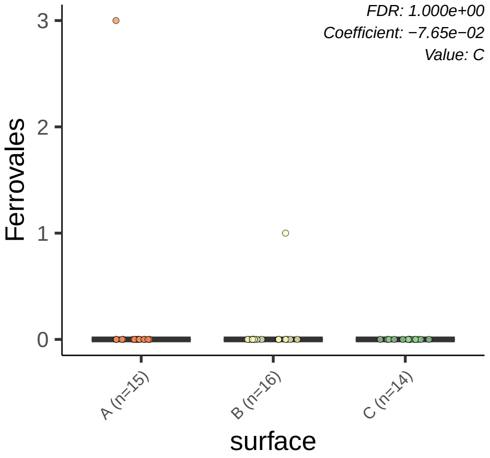

FDR: 1.000e+00
Coefficient: −7.65e−02
Value: C
Ferrovales
3
2
1
0
A (n=15)
B (n=16)
C (n=14)
surface

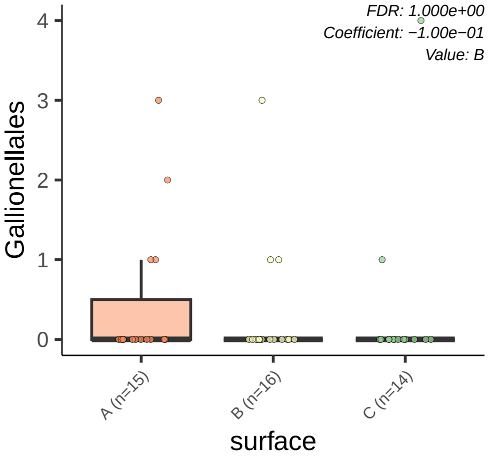
FDR: 1.000e+00
Coefficient: −1.00e−01
Value: B
Gallionellales
4
3
2
1
0
A (n=15)
B (n=16)
C (n=14)
surface

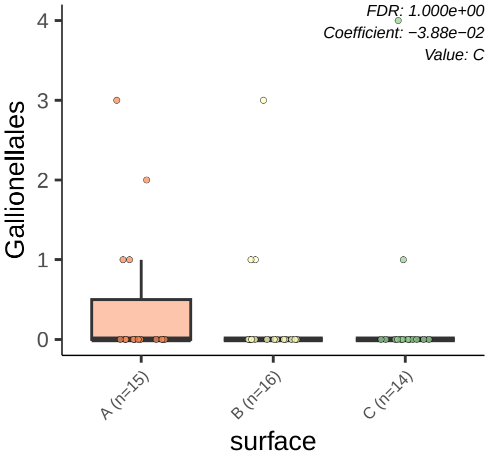
FDR: 1.000e+00
Coefficient: −3.88e−02
Value: C
Gallionellales
0
1
2
3
4
A (n=15)
B (n=16)
C (n=14)
surface

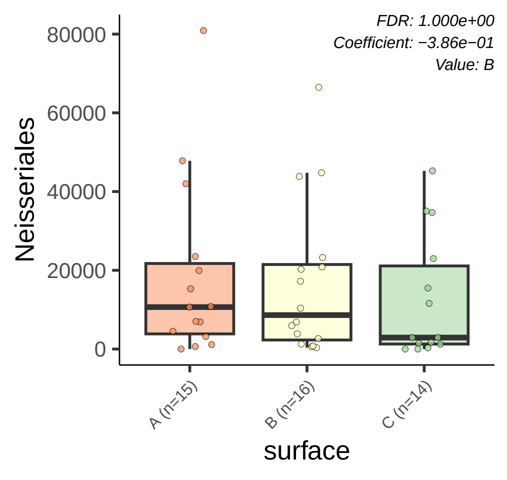

FDR: 1.000e+00
Coefficient: −3.86e−01
Value: B
Neisseriales
80000
60000
40000
20000
0
A (n=15)
B (n=16)
C (n=14)
surface

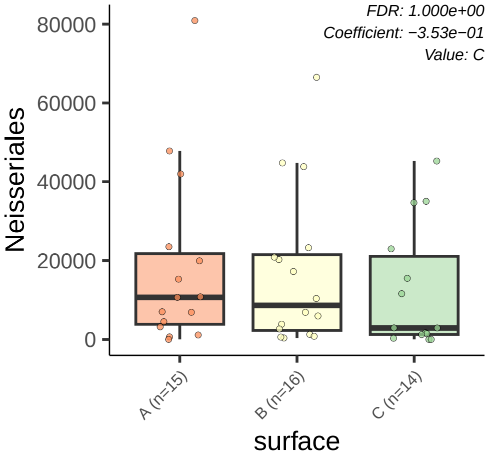

FDR: 1.000e+00
Coefficient: −3.53e−01
Value: C
Neisseriales
80000
60000
40000
20000
0
A (n=15)
B (n=16)
C (n=14)
surface

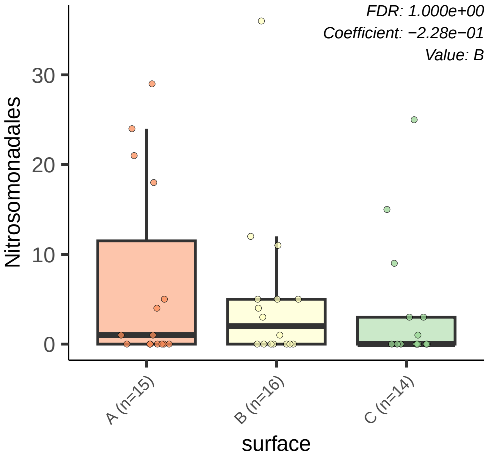
FDR: 1.000e+00
Coefficient: −2.28e−01
Value: B
Nitrosomonadales
0
10
20
30
A (n=15)
B (n=16)
C (n=14)
surface

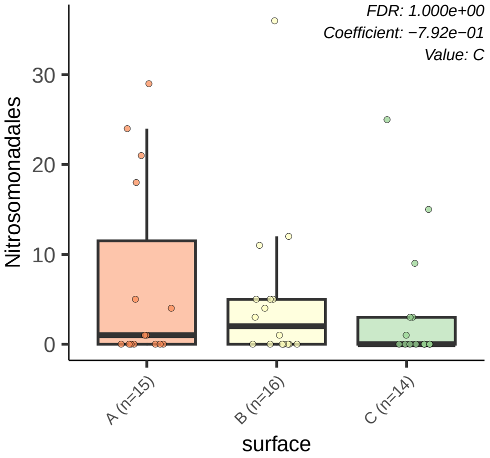
FDR: 1.000e+00
Coefficient: −7.92e−01
Value: C
Nitrosomonadales
30
20
10
0
A (n=15)
B (n=16)
C (n=14)
surface

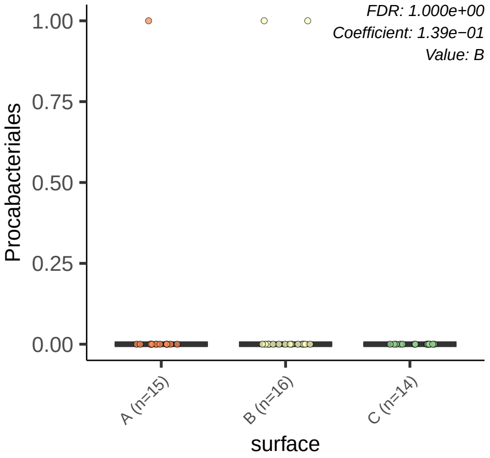

FDR: 1.000e+00
Coefficient: 1.39e−01
Value: B
Procabacteriales
1.00
0.75
0.50
0.25
0.00
A (n=15)
B (n=16)
C (n=14)
surface

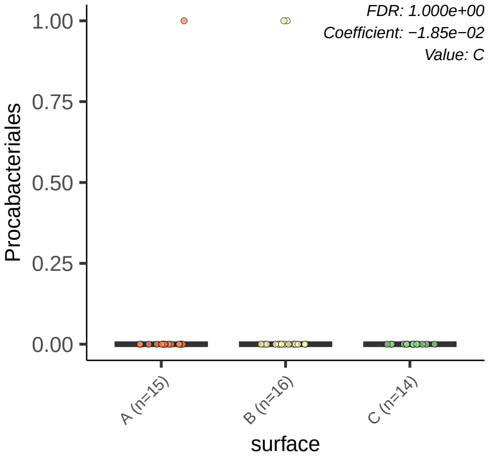
FDR: 1.000e+00
Coefficient: −1.85e−02
Value: C
Procabacteriales
1.00
0.75
0.50
0.25
0.00
A (n=15)
B (n=16)
C (n=14)
surface

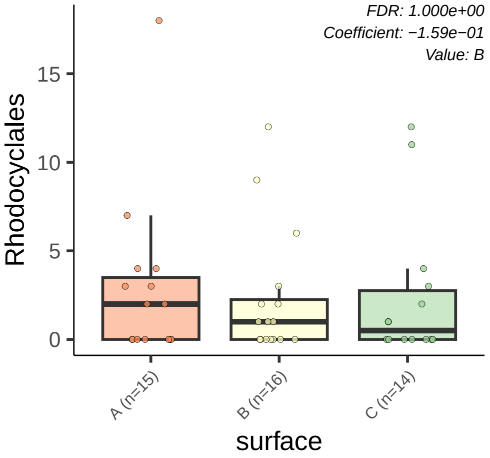

FDR: 1.000e+00
Coefficient: −1.59e−01
Value: B
Rhodocyclales
0
5
10
15
A (n=15)
B (n=16)
C (n=14)
surface

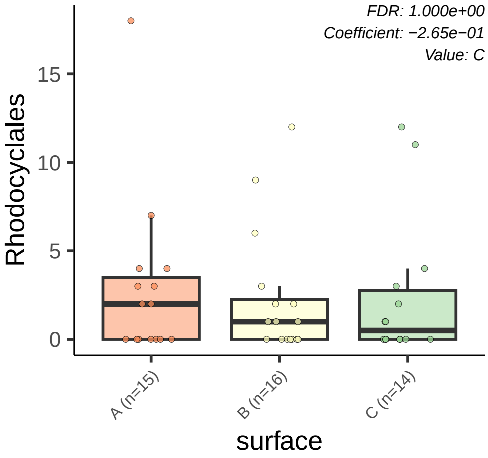

FDR: 1.000e+00
Coefficient: −2.65e−01
Value: C
Rhodocyclales
0
5
10
15
A (n=15)
B (n=16)
C (n=14)
surface

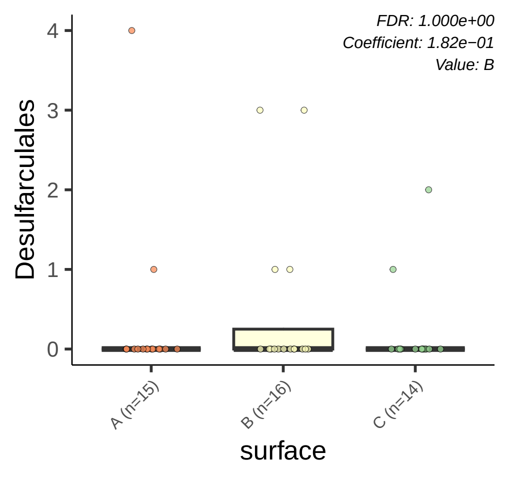
FDR: 1.000e+00
Coefficient: 1.82e−01
Value: B
Desulfarculales
0
1
2
3
4
A (n=15)
B (n=16)
C (n=14)
surface

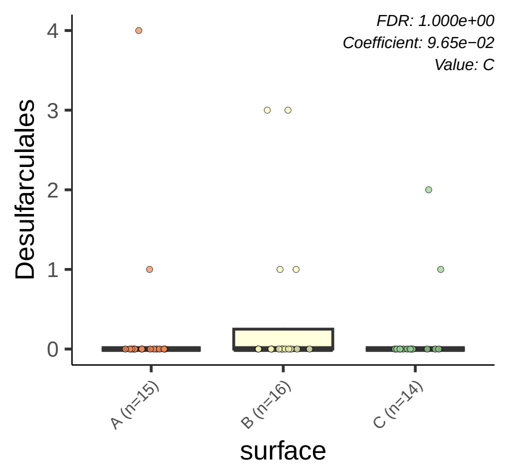
FDR: 1.000e+00
Coefficient: 9.65e−02
Value: C
Desulfarculales
4
3
2
1
0
A (n=15)
B (n=16)
C (n=14)
surface

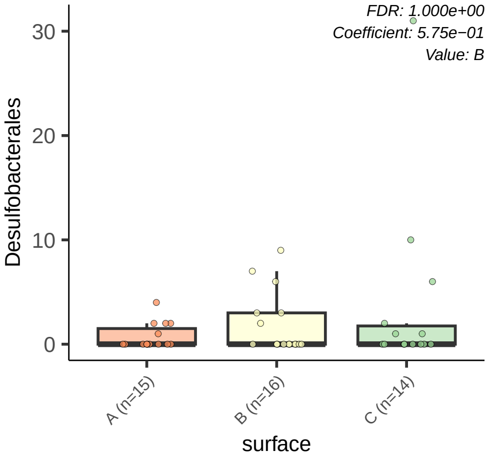
FDR: 1.000e+00
Coefficient: 5.75e−01
Value: B
Desulfobacterales
30
20
10
0
A (n=15)
B (n=16)
C (n=14)
surface

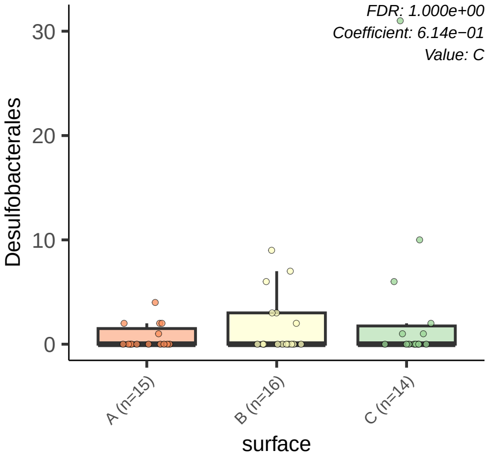

FDR: 1.000e+00
Coefficient: 6.14e−01
Value: C
Desulfobacterales
30
20
10
0
A (n=15)
B (n=16)
C (n=14)
surface

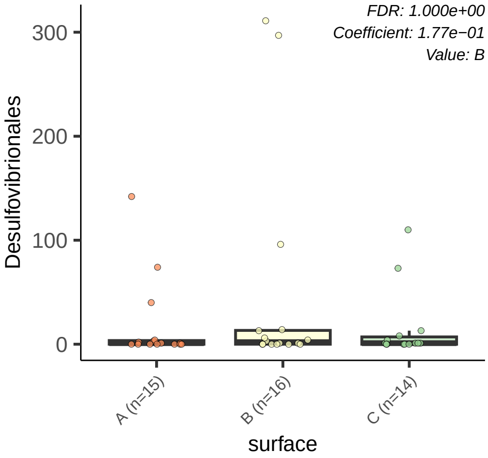
FDR: 1.000e+00
Coefficient: 1.77e−01
Value: B
Desulfovibrionales
300
200
100
0
A (n=15)
B (n=16)
C (n=14)
surface

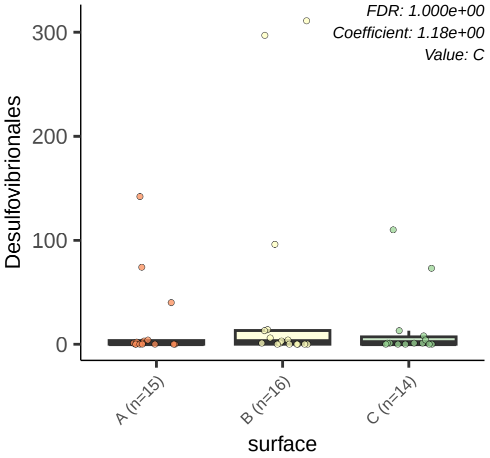
FDR: 1.000e+00
Coefficient: 1.18e+00
Value: C
Desulfovibrionales
300
200
100
0
A (n=15)
B (n=16)
C (n=14)
surface

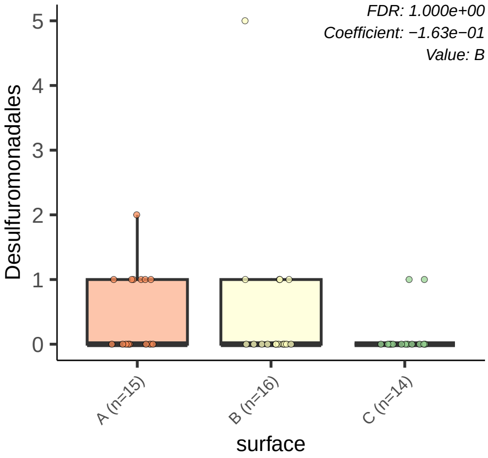

FDR: 1.000e+00
Coefficient: −1.63e−01
Value: B
Desulfuromonadales
0
1
2
3
4
5
A (n=15)
B (n=16)
C (n=14)
surface

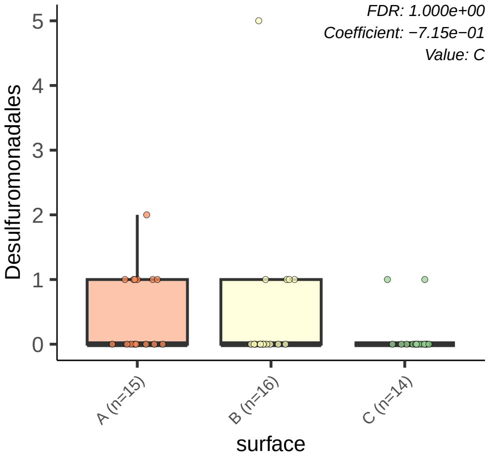
FDR: 1.000e+00
Coefficient: −7.15e−01
Value: C
Desulfuromonadales
0
1
2
3
4
5
A (n=15)
B (n=16)
C (n=14)
surface

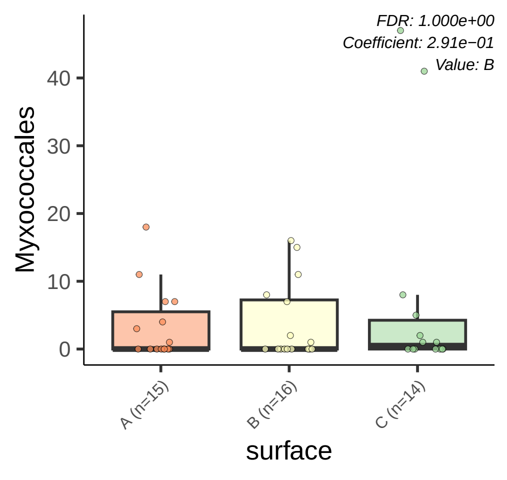

FDR: 1.000e+00
Coefficient: 2.91e−01
Value: B
Myxococcales
0
10
20
30
40
A (n=15)
B (n=16)
C (n=14)
surface

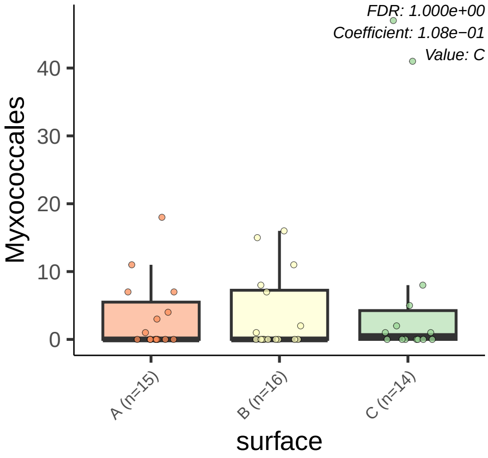

FDR: 1.000e+00
Coefficient: 1.08e−01
Value: C
Myxococcales
40
30
20
10
0
A (n=15)
B (n=16)
C (n=14)
surface

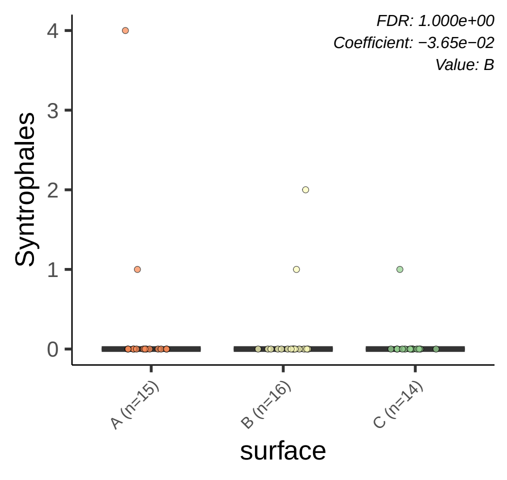

FDR: 1.000e+00
Coefficient: −3.65e−02
Value: B
Syntrophales
0
1
2
3
4
A (n=15)
B (n=16)
C (n=14)
surface

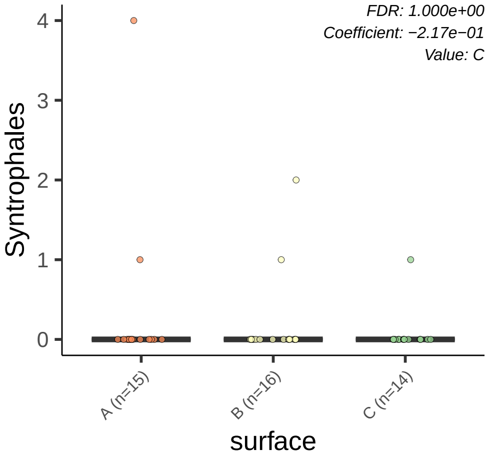
FDR: 1.000e+00
Coefficient: −2.17e−01
Value: C
Syntrophales
4
3
2
1
0
A (n=15)
B (n=16)
C (n=14)
surface

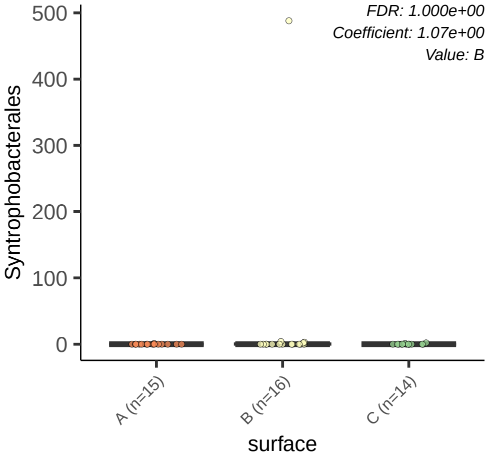
FDR: 1.000e+00
Coefficient: 1.07e+00
Value: B
Syntrophobacterales
0
100
200
300
400
500
A (n=15)
B (n=16)
C (n=14)
surface

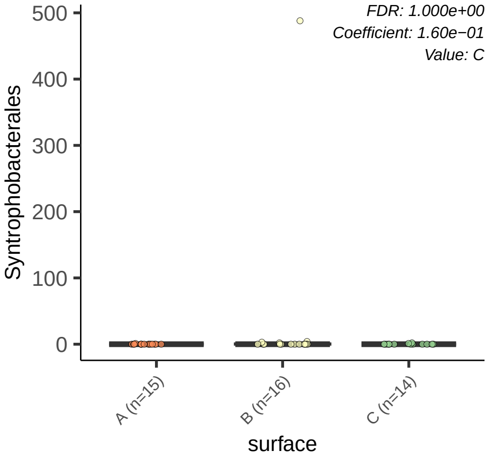
FDR: 1.000e+00
Coefficient: 1.60e−01
Value: C
Syntrophobacterales
500
400
300
200
100
0
A (n=15)
B (n=16)
C (n=14)
surface

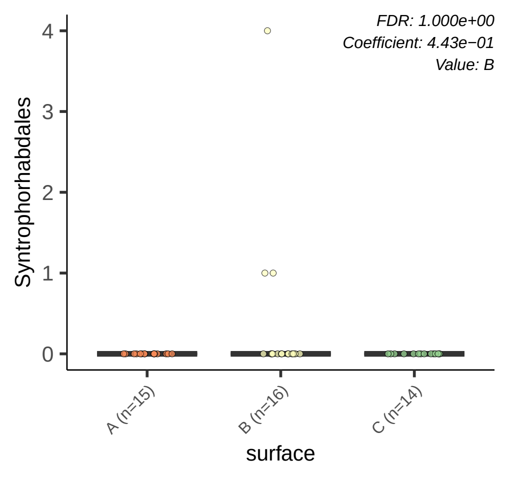
FDR: 1.000e+00
Coefficient: 4.43e−01
Value: B
Syntrophorhabdales
0
1
2
3
4
A (n=15)
B (n=16)
C (n=14)
surface

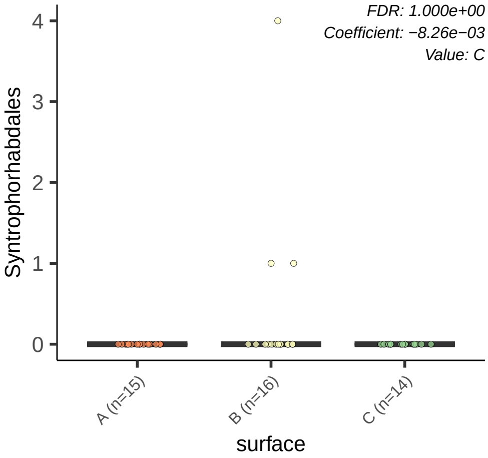
FDR: 1.000e+00
Coefficient: −8.26e−03
Value: C
Syntrophorhabdales
0
1
2
3
4
A (n=15)
B (n=16)
C (n=14)
surface

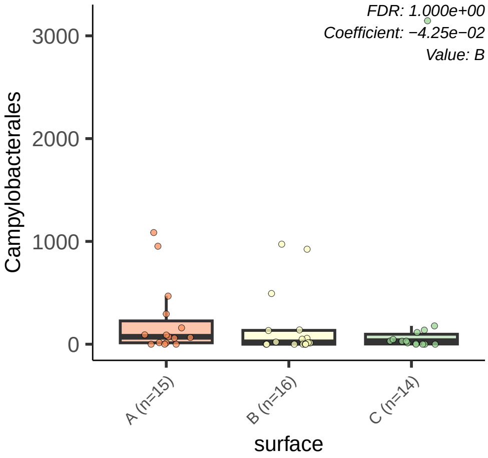
FDR: 1.000e+00
Coefficient: −4.25e−02
Value: B
Campylobacterales
3000
2000
1000
0
A (n=15)
B (n=16)
C (n=14)
surface

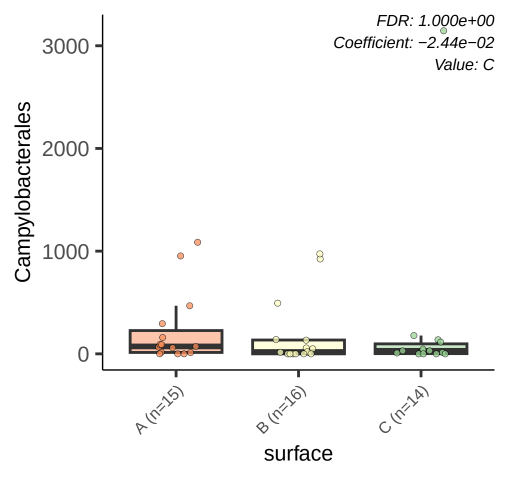

FDR: 1.000e+00
Coefficient: −2.44e−02
Value: C
Campylobacterales
3000
2000
1000
0
A (n=15)
B (n=16)
C (n=14)
surface

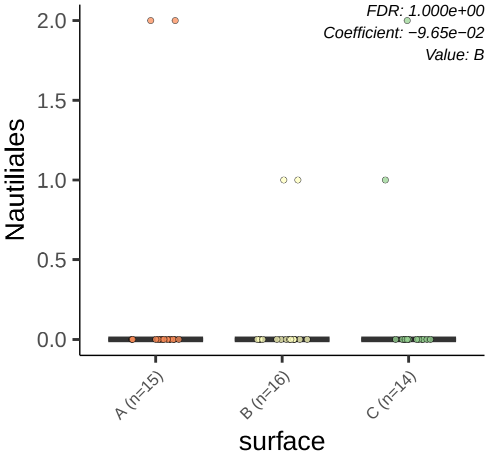
FDR: 1.000e+00
Coefficient: −9.65e−02
Value: B
Nautiliales
2.0
1.5
1.0
0.5
0.0
A (n=15)
B (n=16)
C (n=14)
surface

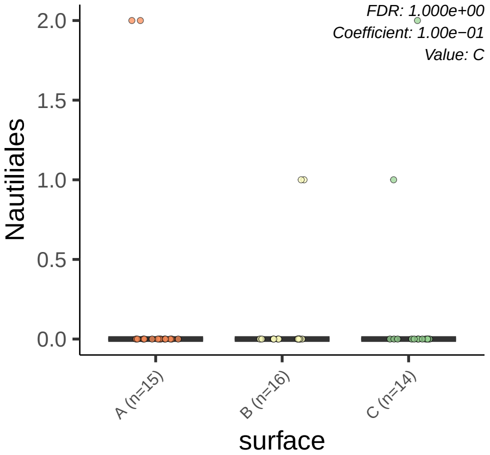

FDR: 1.000e+00
Coefficient: 1.00e−01
Value: C
Nautiliales
2.0
1.5
1.0
0.5
0.0
A (n=15)
B (n=16)
C (n=14)
surface

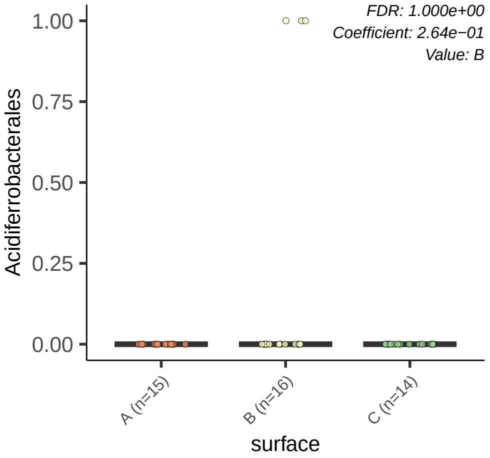

FDR: 1.000e+00
Coefficient: 2.64e−01
Value: B
Acidiferrobacterales
1.00
0.75
0.50
0.25
0.00
A (n=15)
B (n=16)
C (n=14)
surface

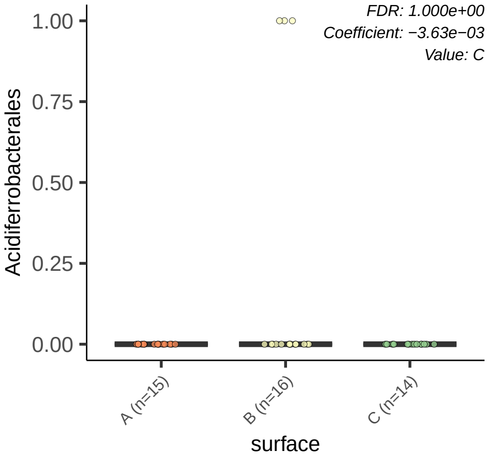
FDR: 1.000e+00
Coefficient: −3.63e−03
Value: C
Acidiferrobacterales
1.00
0.75
0.50
0.25
0.00
A (n=15)
B (n=16)
C (n=14)
surface

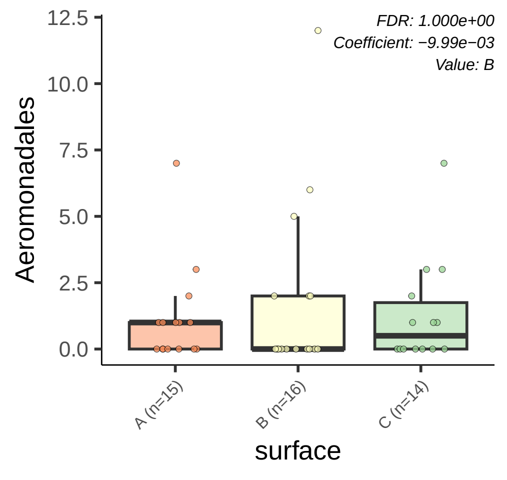
FDR: 1.000e+00
Coefficient: −9.99e−03
Value: B
Aeromonadales
12.5
10.0
7.5
5.0
2.5
0.0
A (n=15)
B (n=16)
C (n=14)
surface

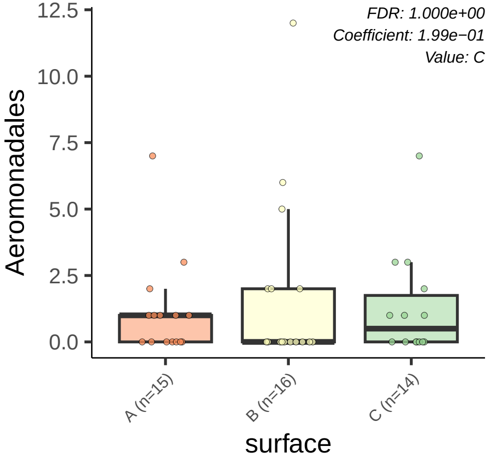

FDR: 1.000e+00
Coefficient: 1.99e−01
Value: C
Aeromonadales
12.5
10.0
7.5
5.0
2.5
0.0
A (n=15)
B (n=16)
C (n=14)
surface

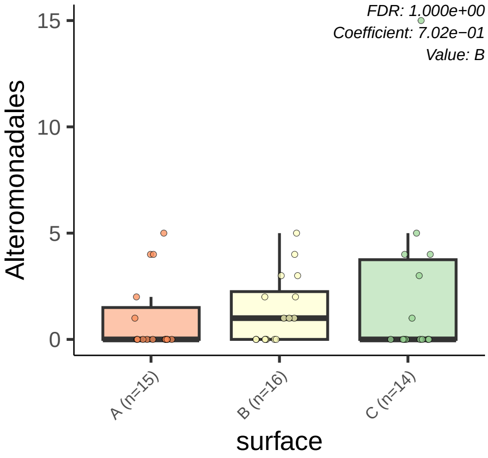

FDR: 1.000e+00
Coefficient: 7.02e−01
Value: B
Alteromonadales
15
10
5
0
A (n=15)
B (n=16)
C (n=14)
surface

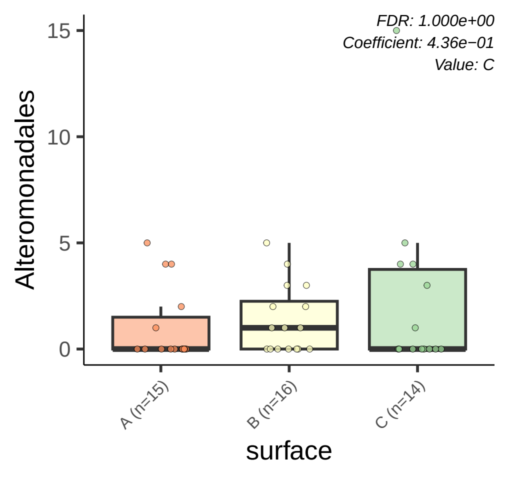
FDR: 1.000e+00
Coefficient: 4.36e−01
Value: C
Alteromonadales
15
10
5
0
A (n=15)
B (n=16)
C (n=14)
surface

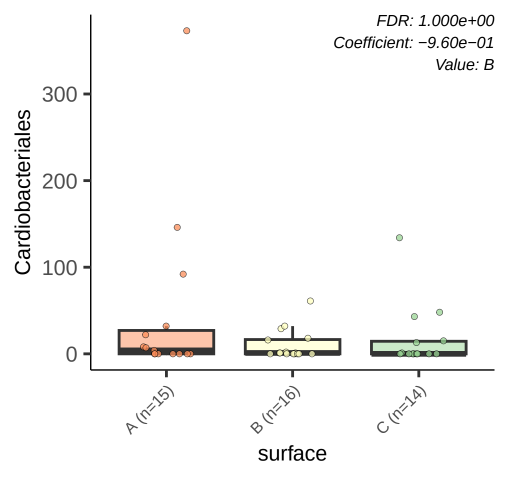
FDR: 1.000e+00
Coefficient: −9.60e−01
Value: B
Cardiobacteriales
300
200
100
0
A (n=15)
B (n=16)
C (n=14)
surface

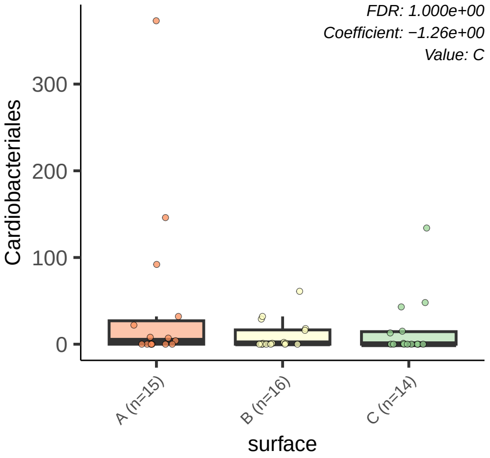

FDR: 1.000e+00
Coefficient: −1.26e+00
Value: C
Cardiobacteriales
300
200
100
0
A (n=15)
B (n=16)
C (n=14)
surface

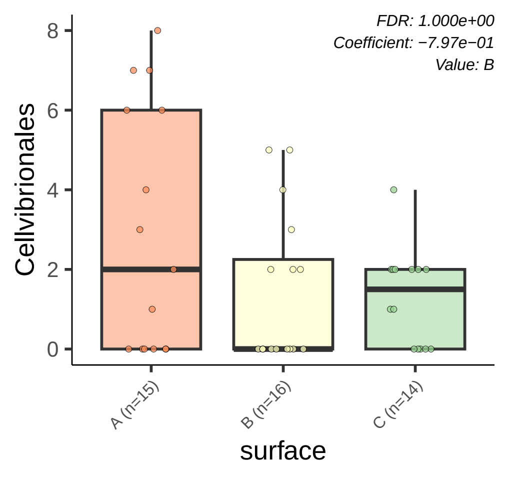

FDR: 1.000e+00
Coefficient: −7.97e−01
Value: B
Cellvibrionales
0
2
4
6
8
A (n=15)
B (n=16)
C (n=14)
surface

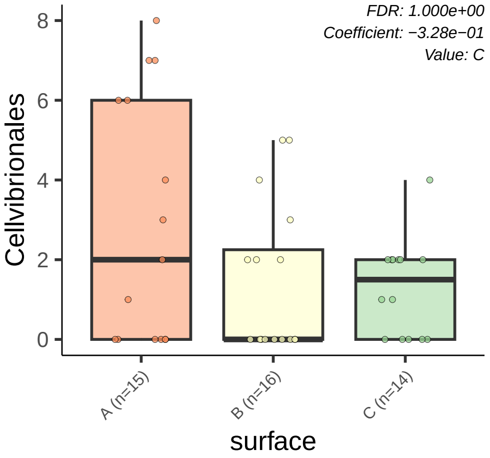

FDR: 1.000e+00
Coefficient: −3.28e−01
Value: C
Cellvibrionales
0
2
4
6
8
A (n=15)
B (n=16)
C (n=14)
surface

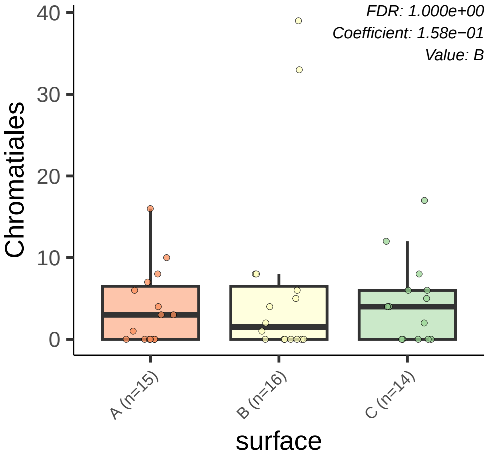

FDR: 1.000e+00
Coefficient: 1.58e−01
Value: B
Chromatiales
40
30
20
10
0
A (n=15)
B (n=16)
C (n=14)
surface

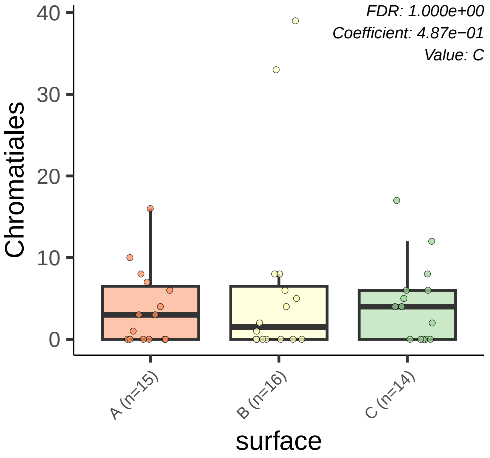
FDR: 1.000e+00
Coefficient: 4.87e−01
Value: C
Chromatiales
0
10
20
30
40
A (n=15)
B (n=16)
C (n=14)
surface

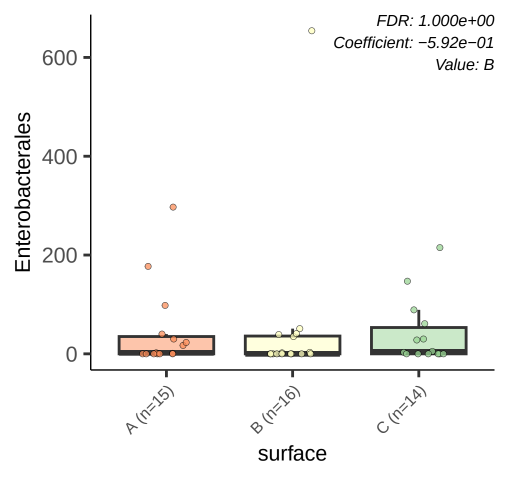
FDR: 1.000e+00
Coefficient: −5.92e−01
Value: B
Enterobacterales
0
200
400
600
A (n=15)
B (n=16)
C (n=14)
surface

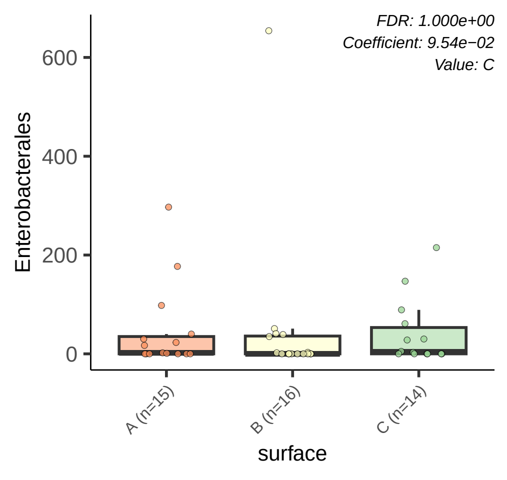
FDR: 1.000e+00
Coefficient: 9.54e−02
Value: C
Enterobacterales
0
200
400
600
A (n=15)
B (n=16)
C (n=14)
surface

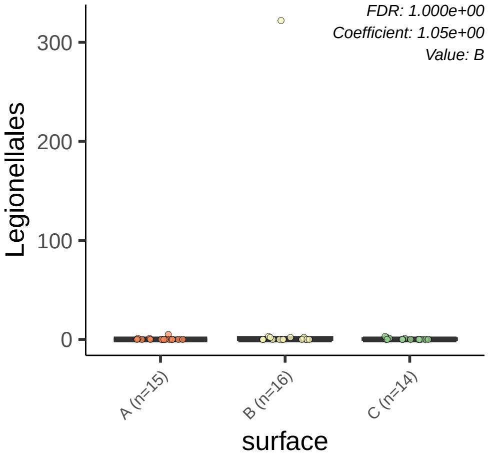
FDR: 1.000e+00
Coefficient: 1.05e+00
Value: B
Legionellales
300
200
100
0
A (n=15)
B (n=16)
C (n=14)
surface

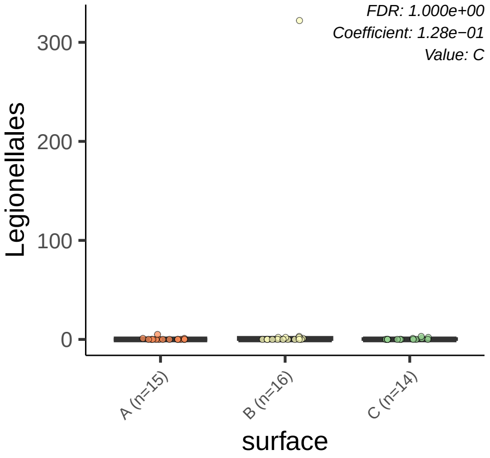
FDR: 1.000e+00
Coefficient: 1.28e−01
Value: C
Legionellales
300
200
100
0
A (n=15)
B (n=16)
C (n=14)
surface

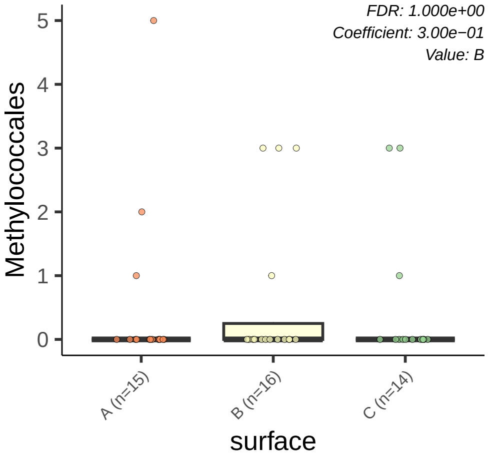
FDR: 1.000e+00
Coefficient: 3.00e−01
Value: B
Methylococcales
0
1
2
3
4
5
A (n=15)
B (n=16)
C (n=14)
surface

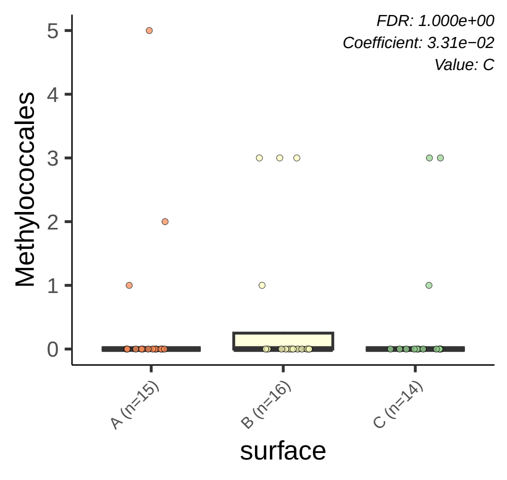

FDR: 1.000e+00
Coefficient: 3.31e−02
Value: C
Methylococcales
5
4
3
2
1
0
A (n=15)
B (n=16)
C (n=14)
surface

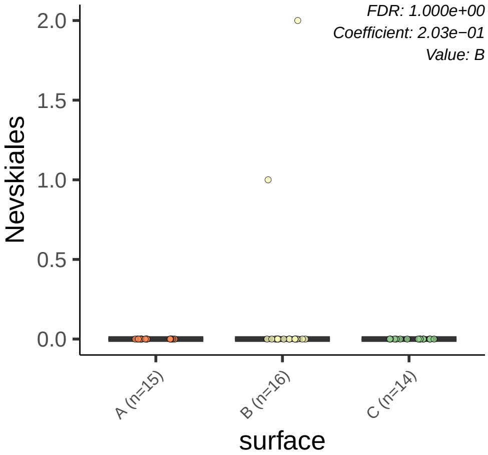

FDR: 1.000e+00
Coefficient: 2.03e−01
Value: B
Nevskiales
2.0
1.5
1.0
0.5
0.0
A (n=15)
B (n=16)
C (n=14)
surface

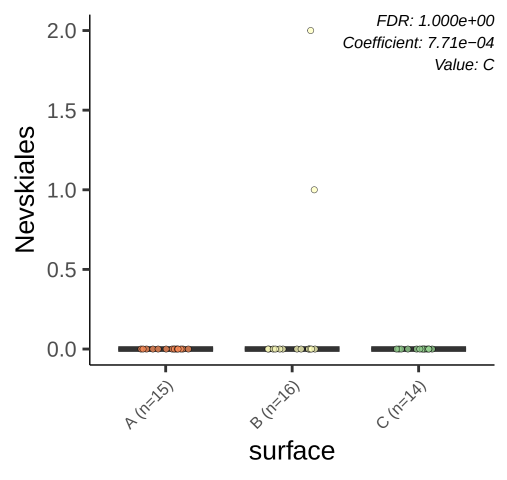

FDR: 1.000e+00
Coefficient: 7.71e−04
Value: C
Nevskiales
2.0
1.5
1.0
0.5
0.0
A (n=15)
B (n=16)
C (n=14)
surface

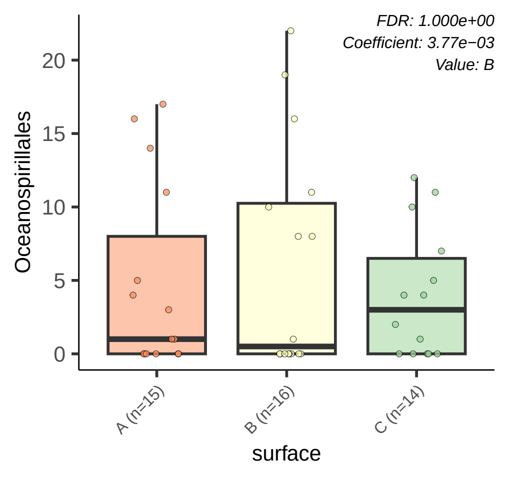
FDR: 1.000e+00
Coefficient: 3.77e−03
Value: B
Oceanospirillales
0
5
10
15
20
A (n=15)
B (n=16)
C (n=14)
surface

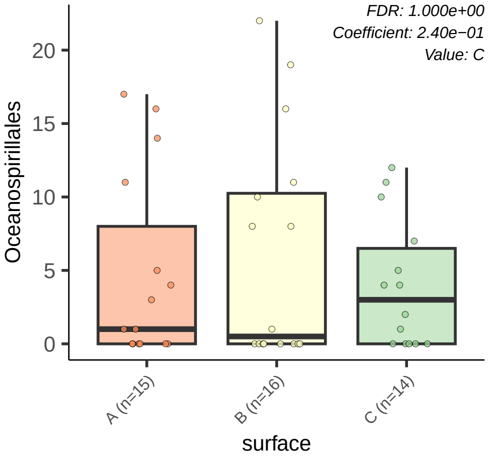

FDR: 1.000e+00
Coefficient: 2.40e−01
Value: C
Oceanospirillales
0
5
10
15
20
A (n=15)
B (n=16)
C (n=14)
surface

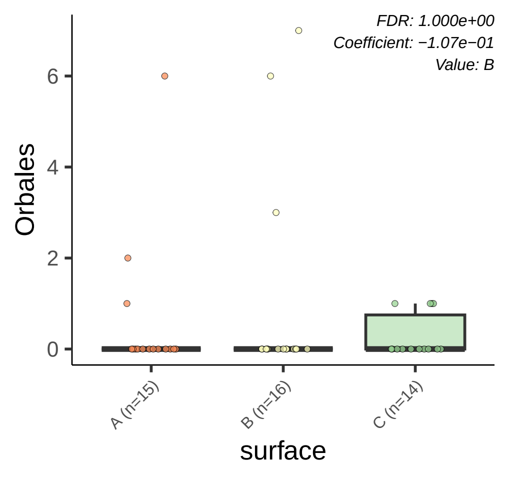

FDR: 1.000e+00
Coefficient: −1.07e−01
Value: B
Orbales
0
2
4
6
A (n=15)
B (n=16)
C (n=14)
surface

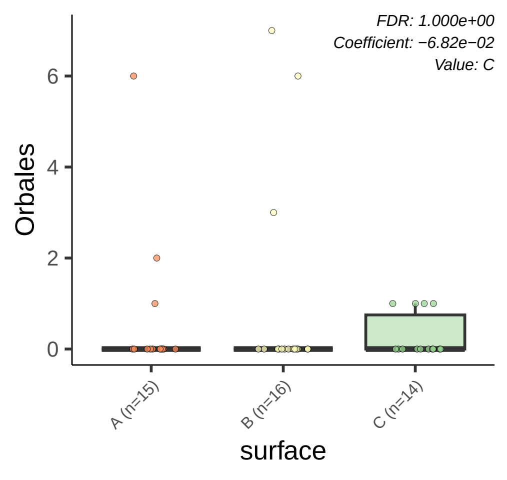

FDR: 1.000e+00
Coefficient: −6.82e−02
Value: C
Orbales
0
2
4
6
A (n=15)
B (n=16)
C (n=14)
surface

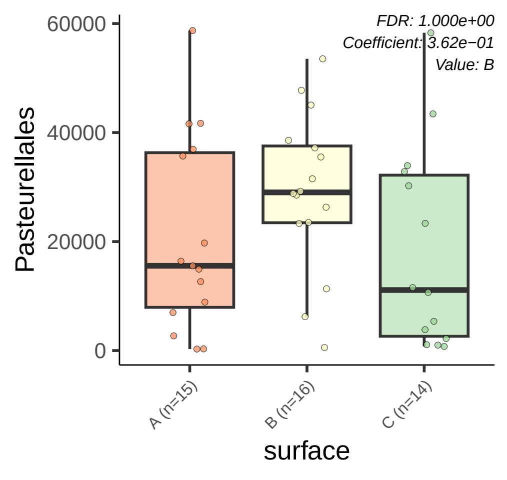

FDR: 1.000e+00
Coefficient: 3.62e−01
Value: B
Pasteurellales
60000
40000
20000
0
A (n=15)
B (n=16)
C (n=14)
surface

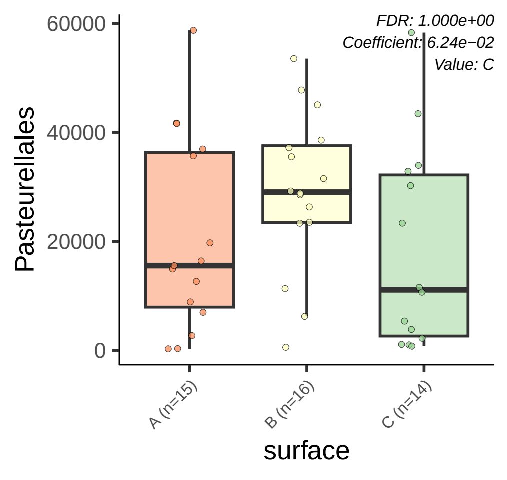

FDR: 1.000e+00
Coefficient: 6.24e−02
Value: C
Pasteurellales
60000
40000
20000
0
A (n=15)
B (n=16)
C (n=14)
surface

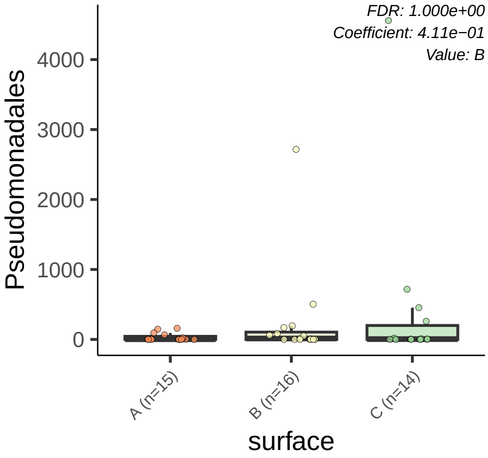

FDR: 1.000e+00
Coefficient: 4.11e−01
Value: B
Pseudomonadales
4000
3000
2000
1000
0
A (n=15)
B (n=16)
C (n=14)
surface

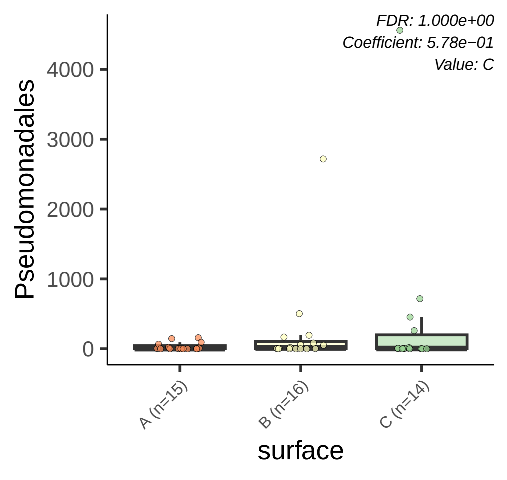
FDR: 1.000e+00
Coefficient: 5.78e−01
Value: C
Pseudomonadales
4000
3000
2000
1000
0
A (n=15)
B (n=16)
C (n=14)
surface

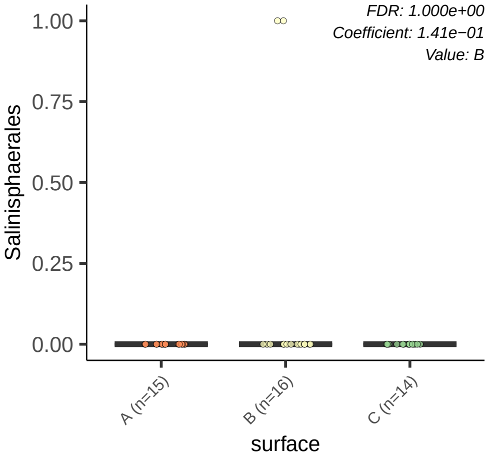

FDR: 1.000e+00
Coefficient: 1.41e−01
Value: B
Salinisphaerales
1.00
0.75
0.50
0.25
0.00
A (n=15)
B (n=16)
C (n=14)
surface

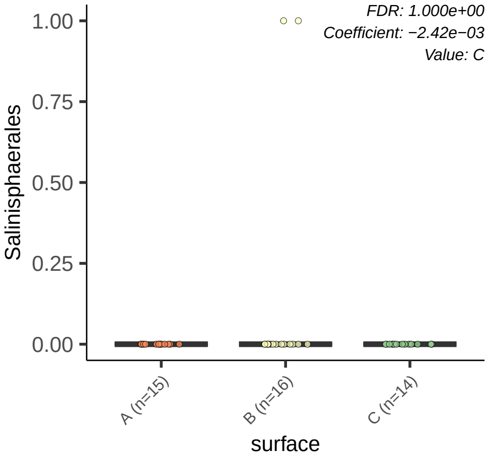
FDR: 1.000e+00
Coefficient: −2.42e−03
Value: C
1.00
0.75
0.50
0.25
0.00
Salinisphaerales
A (n=15)
B (n=16)
C (n=14)
surface

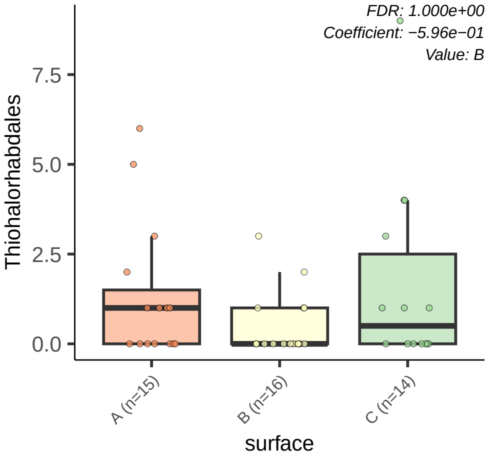

FDR: 1.000e+00
Coefficient: −5.96e−01
Value: B
Thiohalorhabdales
7.5
5.0
2.5
0.0
A (n=15)
B (n=16)
C (n=14)
surface

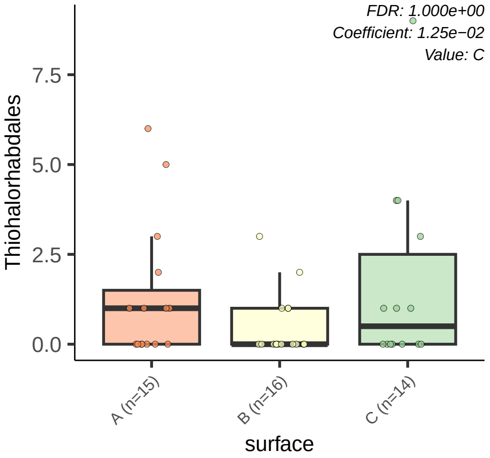

FDR: 1.000e+00
Coefficient: 1.25e−02
Value: C
Thiohalorhabdales
7.5
5.0
2.5
0.0
A (n=15)
B (n=16)
C (n=14)
surface

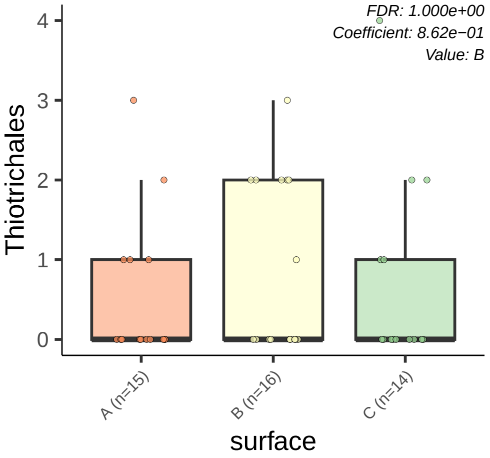
FDR: 1.000e+00
Coefficient: 8.62e−01
Value: B
Thiotrichales
0
1
2
3
4
A (n=15)
B (n=16)
C (n=14)
surface

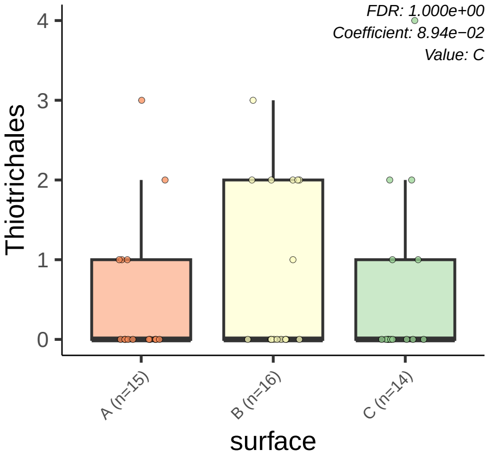

FDR: 1.000e+00
Coefficient: 8.94e−02
Value: C
Thiotrichales
0
1
2
3
4
A (n=15)
B (n=16)
C (n=14)
surface

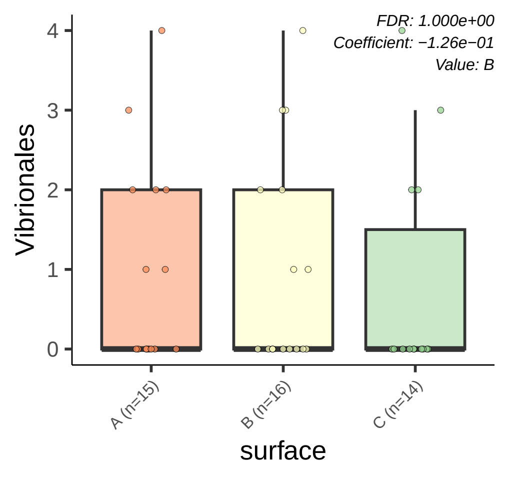

FDR: 1.000e+00
Coefficient: −1.26e−01
Value: B
Vibrionales
0
1
2
3
4
A (n=15)
B (n=16)
C (n=14)
surface

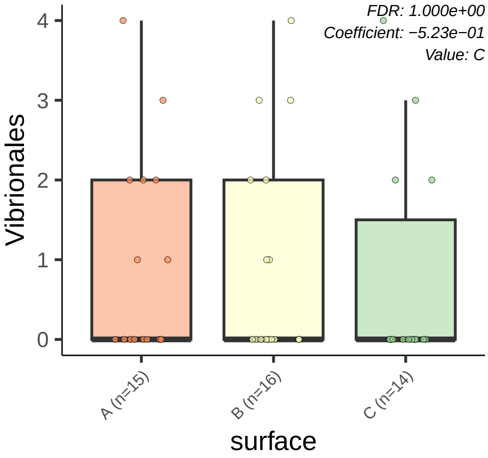

FDR: 1.000e+00
Coefficient: −5.23e−01
Value: C
Vibrionales
4
3
2
1
0
A (n=15)
B (n=16)
C (n=14)
surface

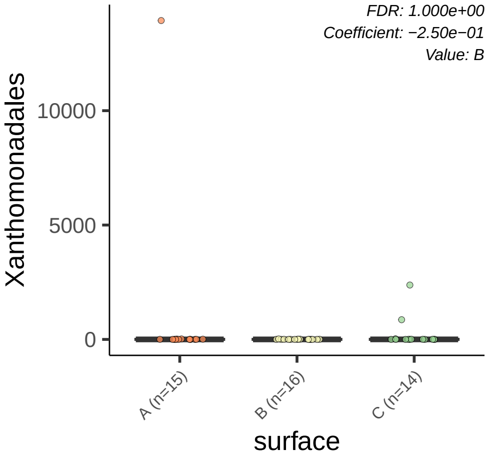
FDR: 1.000e+00
Coefficient: −2.50e−01
Value: B
Xanthomonadales
10000
5000
0
A (n=15)
B (n=16)
C (n=14)
surface

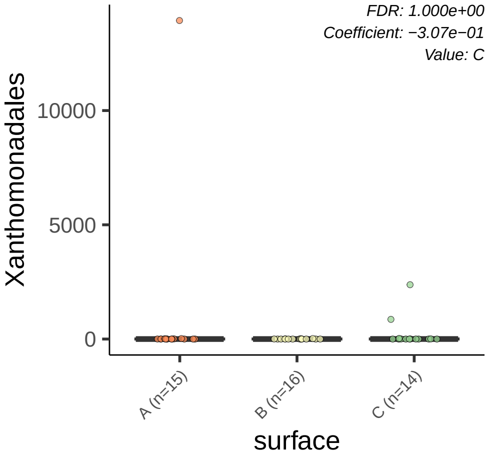

FDR: 1.000e+00
Coefficient: −3.07e−01
Value: C
Xanthomonadales
10000
5000
0
A (n=15)
B (n=16)
C (n=14)
surface

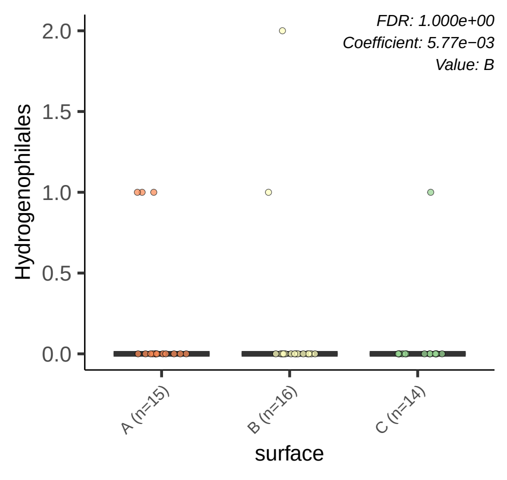
FDR: 1.000e+00
Coefficient: 5.77e−03
Value: B
Hydrogenophilales
2.0
1.5
1.0
0.5
0.0
A (n=15)
B (n=16)
C (n=14)
surface

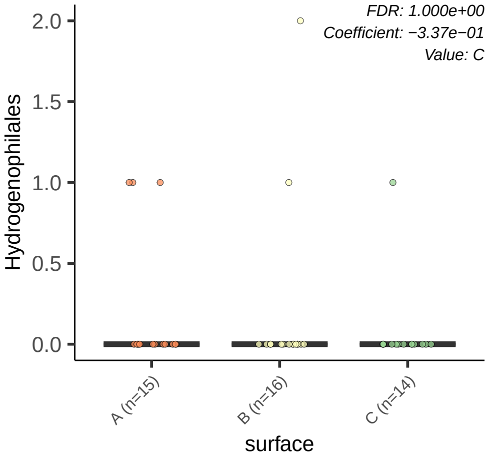

FDR: 1.000e+00
Coefficient: −3.37e−01
Value: C
Hydrogenophilales
2.0
1.5
1.0
0.5
0.0
A (n=15)
B (n=16)
C (n=14)
surface

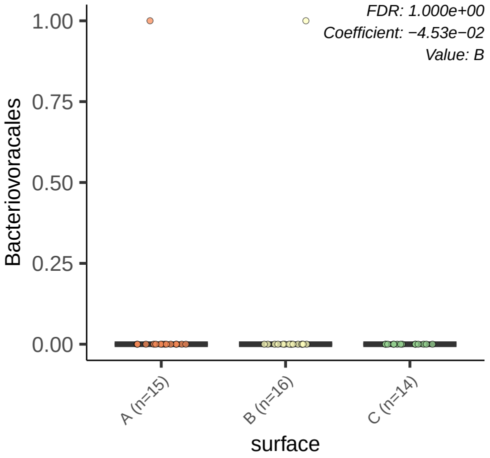
FDR: 1.000e+00
Coefficient: −4.53e−02
Value: B
Bacteriovoracales
1.00
0.75
0.50
0.25
0.00
A (n=15)
B (n=16)
C (n=14)
surface

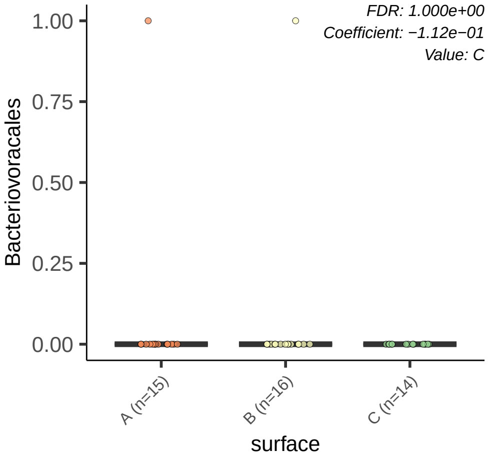

FDR: 1.000e+00
Coefficient: −1.12e−01
Value: C
Bacteriovoracales
1.00
0.75
0.50
0.25
0.00
A (n=15)
B (n=16)
C (n=14)
surface

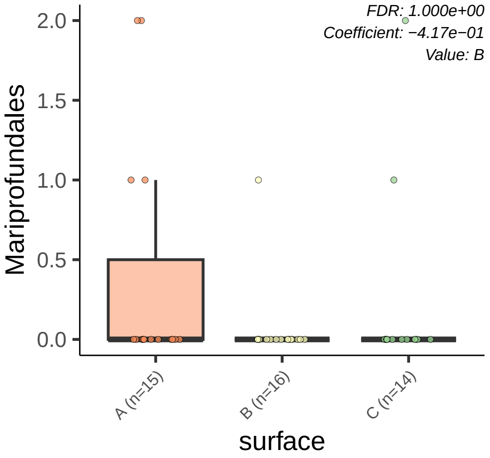
FDR: 1.000e+00
Coefficient: −4.17e−01
Value: B
Mariprofundales
2.0
1.5
1.0
0.5
0.0
A (n=15)
B (n=16)
C (n=14)
surface

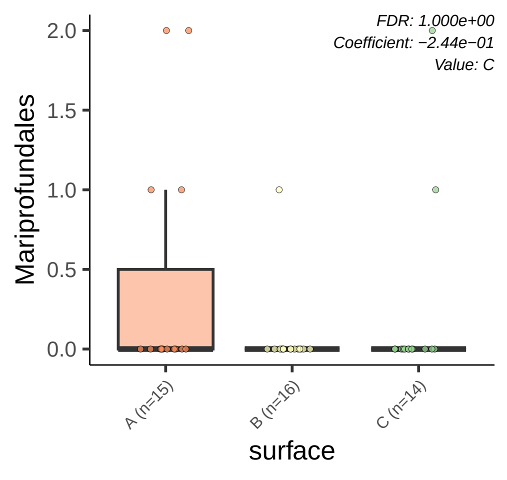

FDR: 1.000e+00
Coefficient: −2.44e−01
Value: C
Mariprofundales
2.0
1.5
1.0
0.5
0.0
A (n=15)
B (n=16)
C (n=14)
surface

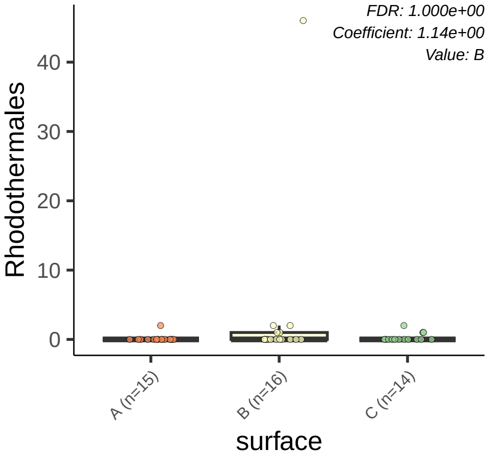

FDR: 1.000e+00
Coefficient: 1.14e+00
Value: B
Rhodothermales
0
10
20
30
40
A (n=15)
B (n=16)
C (n=14)
surface

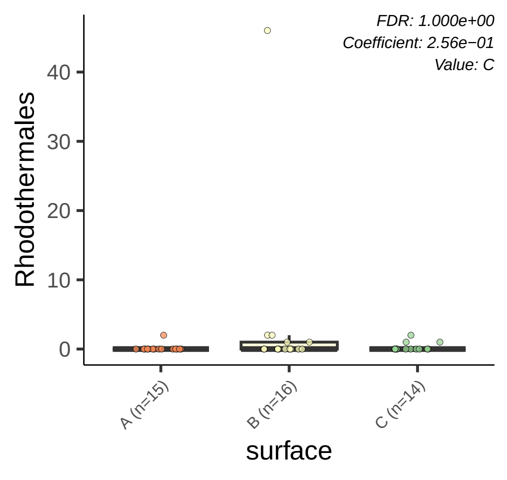

FDR: 1.000e+00
Coefficient: 2.56e−01
Value: C
Rhodothermales
0
10
20
30
40
A (n=15)
B (n=16)
C (n=14)
surface

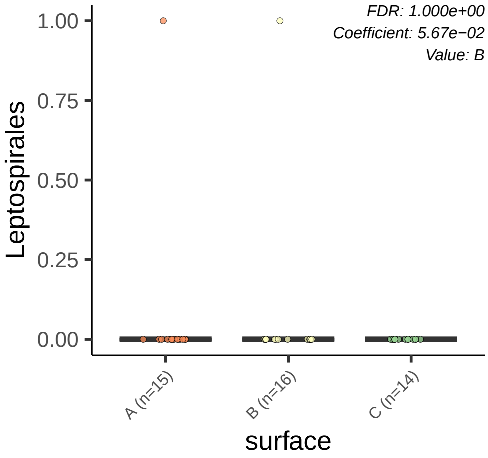
FDR: 1.000e+00
Coefficient: 5.67e−02
Value: B
Leptospirales
1.00
0.75
0.50
0.25
0.00
A (n=15)
B (n=16)
C (n=14)
surface

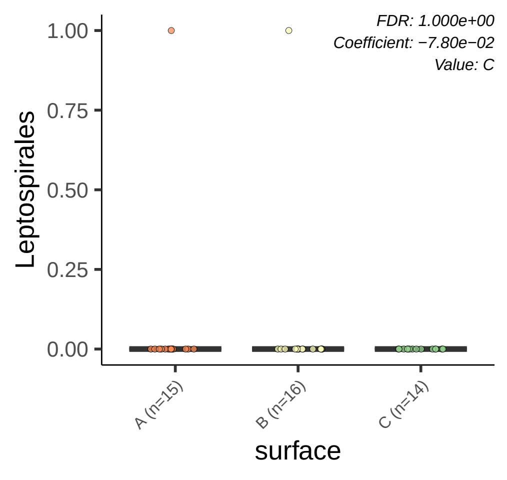
FDR: 1.000e+00
Coefficient: −7.80e−02
Value: C
1.00
0.75
0.50
0.25
0.00
Leptospirales
A (n=15)
B (n=16)
C (n=14)
surface

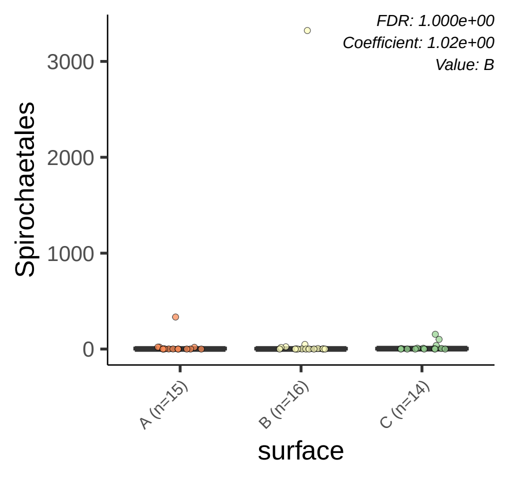
FDR: 1.000e+00
Coefficient: 1.02e+00
Value: B
Spirochaetales
3000
2000
1000
0
A (n=15)
B (n=16)
C (n=14)
surface

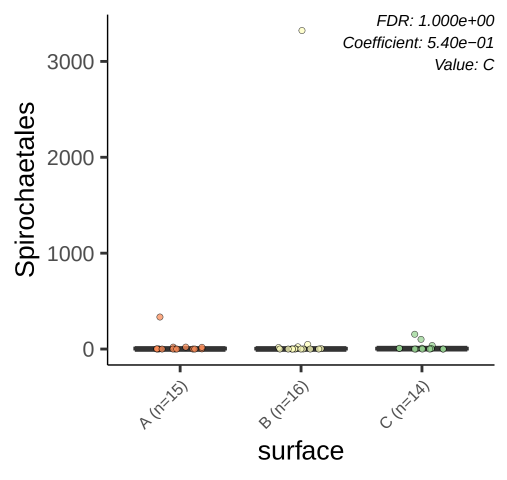

FDR: 1.000e+00
Coefficient: 5.40e−01
Value: C
Spirochaetales
3000
2000
1000
0
A (n=15)
B (n=16)
C (n=14)
surface

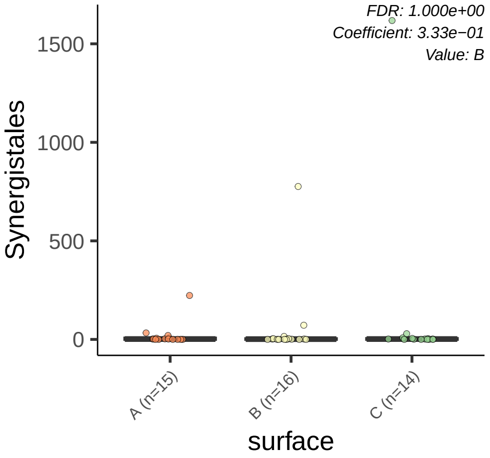

FDR: 1.000e+00
Coefficient: 3.33e−01
Value: B
Synergistales
1500
1000
500
0
A (n=15)
B (n=16)
C (n=14)
surface

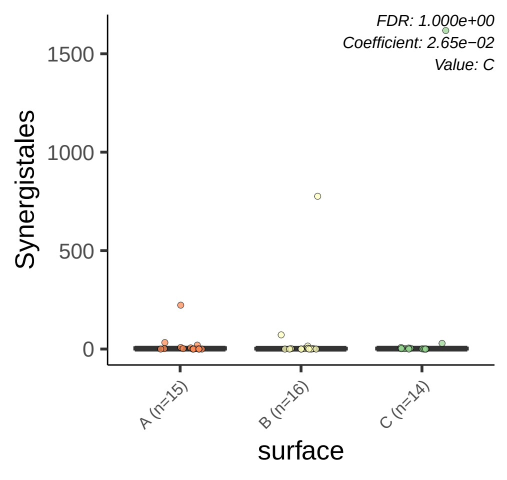
FDR: 1.000e+00
Coefficient: 2.65e−02
Value: C
Synergistales
1500
1000
500
0
A (n=15)
B (n=16)
C (n=14)
surface

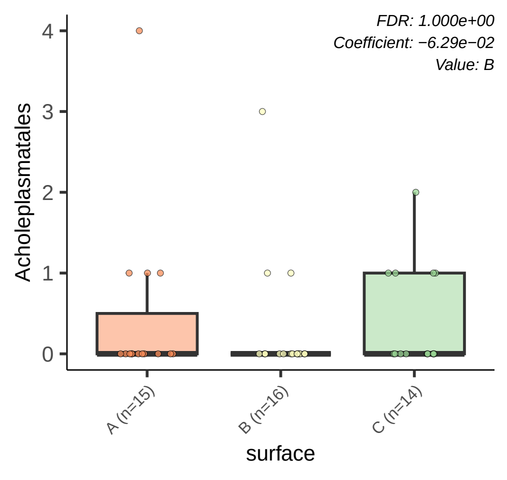

FDR: 1.000e+00
Coefficient: −6.29e−02
Value: B
Acholeplasmatales
0
1
2
3
4
A (n=15)
B (n=16)
C (n=14)
surface

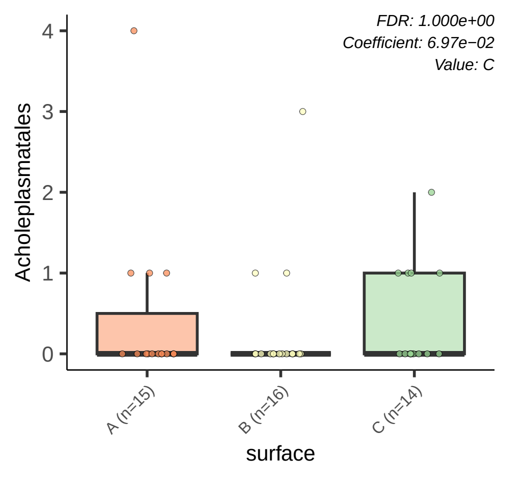
FDR: 1.000e+00
Coefficient: 6.97e−02
Value: C
Acholeplasmatales
0
1
2
3
4
A (n=15)
B (n=16)
C (n=14)
surface

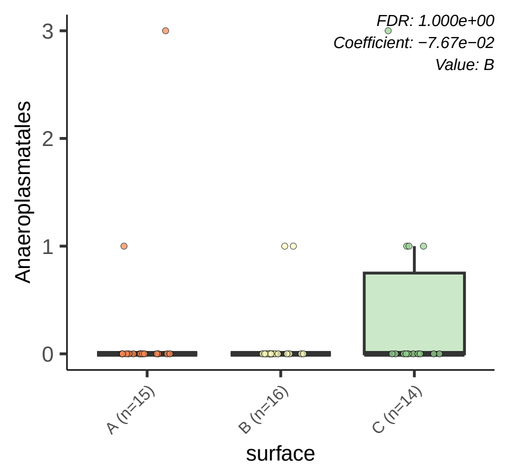

FDR: 1.000e+00
Coefficient: −7.67e−02
Value: B
Anaeroplasmatales
3
2
1
0
A (n=15)
B (n=16)
C (n=14)
surface

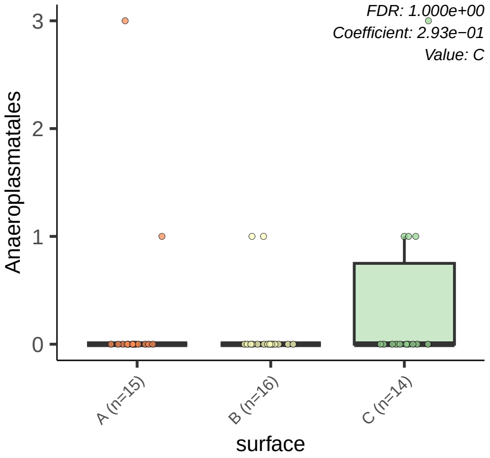

FDR: 1.000e+00
Coefficient: 2.93e−01
Value: C
Anaeroplasmatales
0
1
2
3
A (n=15)
B (n=16)
C (n=14)
surface

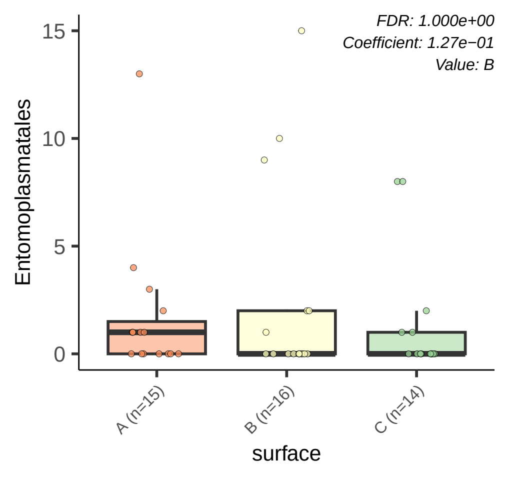
FDR: 1.000e+00
Coefficient: 1.27e−01
Value: B
Entomoplasmatales
15
10
5
0
A (n=15)
B (n=16)
C (n=14)
surface

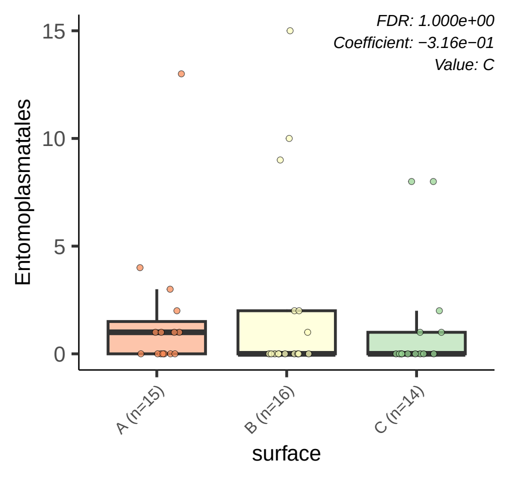
FDR: 1.000e+00
Coefficient: −3.16e−01
Value: C
Entomoplasmatales
15
10
5
0
A (n=15)
B (n=16)
C (n=14)
surface

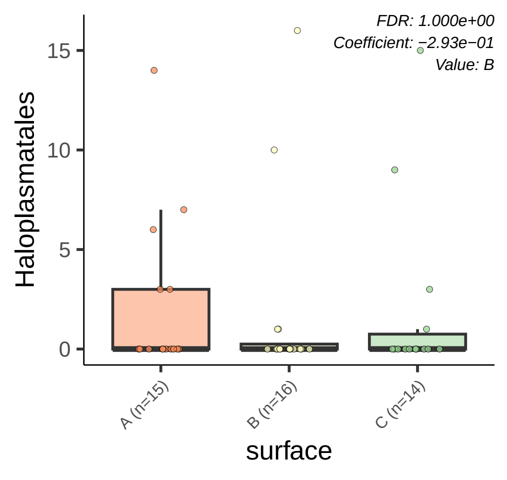
FDR: 1.000e+00
Coefficient: −2.93e−01
Value: B
Haloplasmatales
15
10
5
0
A (n=15)
B (n=16)
C (n=14)
surface

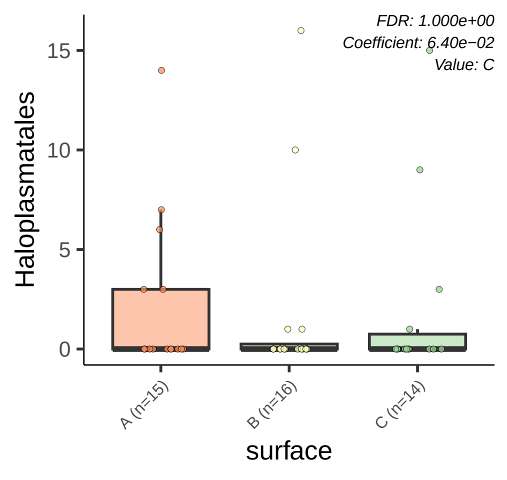

FDR: 1.000e+00
Coefficient: 6.40e−02
Value: C
Haloplasmatales
0
5
10
15
A (n=15)
B (n=16)
C (n=14)
surface

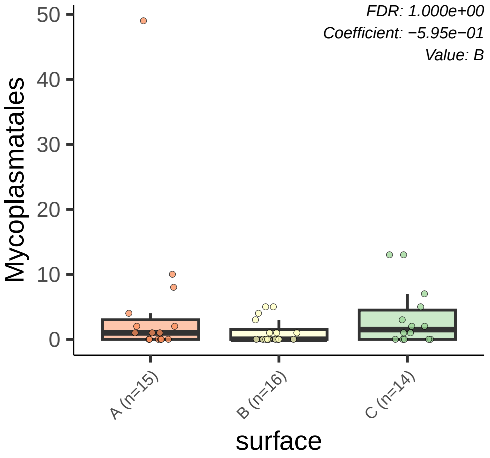
FDR: 1.000e+00
Coefficient: −5.95e−01
Value: B
Mycoplasmatales
50
40
30
20
10
0
A (n=15)
B (n=16)
C (n=14)
surface

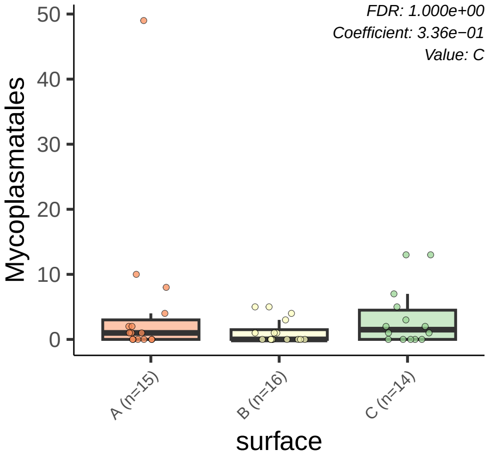
FDR: 1.000e+00
Coefficient: 3.36e−01
Value: C
Mycoplasmatales
50
40
30
20
10
0
A (n=15)
B (n=16)
C (n=14)
surface

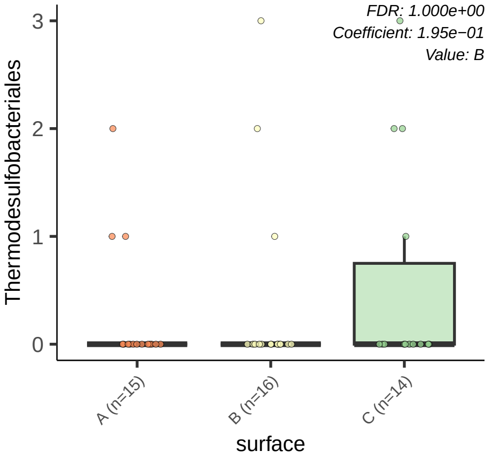

FDR: 1.000e+00
Coefficient: 1.95e−01
Value: B
Thermodesulfobacteriales
0
1
2
3
A (n=15)
B (n=16)
C (n=14)
surface

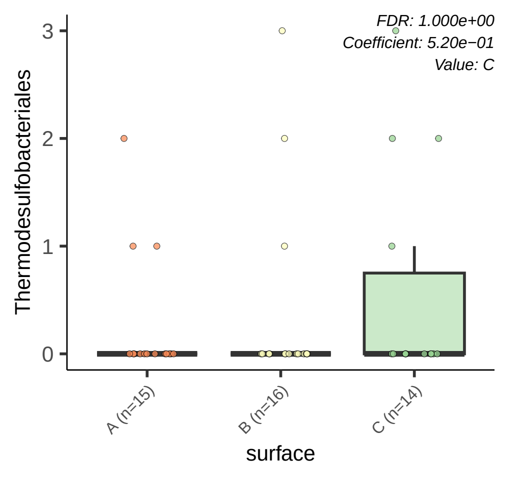

FDR: 1.000e+00
Coefficient: 5.20e−01
Value: C
Thermodesulfobacteriales
3
2
1
0
A (n=15)
B (n=16)
C (n=14)
surface

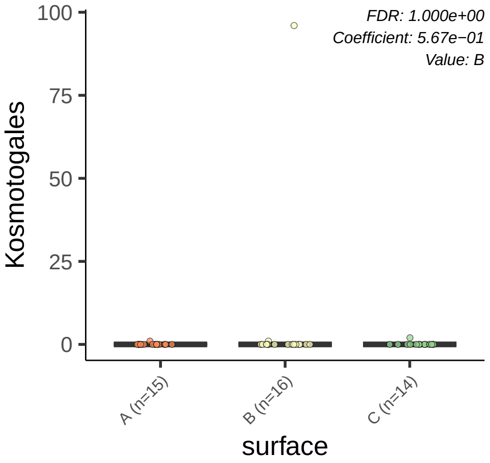

FDR: 1.000e+00
Coefficient: 5.67e−01
Value: B
100
75
50
25
0
Kosmotogales
A (n=15)
B (n=16)
C (n=14)
surface

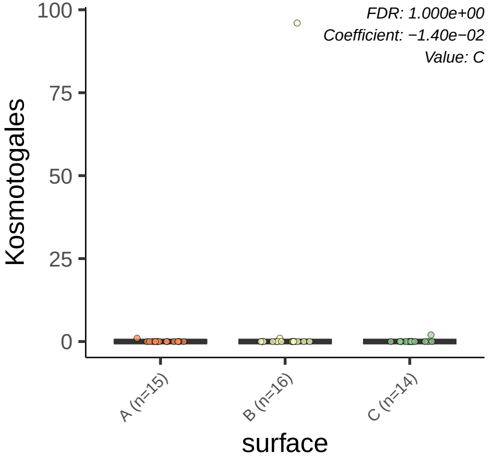
FDR: 1.000e+00
Coefficient: −1.40e−02
Value: C
Kosmotogales
100
75
50
25
0
A (n=15)
B (n=16)
C (n=14)
surface

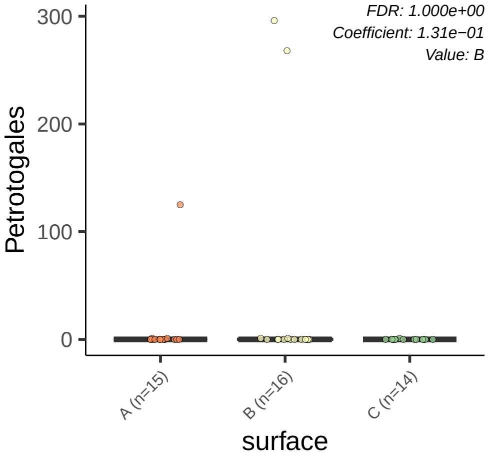

FDR: 1.000e+00
Coefficient: 1.31e−01
Value: B
Petrotogales
300
200
100
0
A (n=15)
B (n=16)
C (n=14)
surface

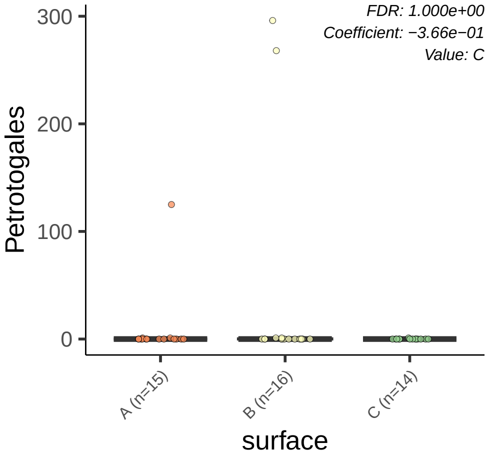
FDR: 1.000e+00
Coefficient: −3.66e−01
Value: C
Petrotogales
300
200
100
0
A (n=15)
B (n=16)
C (n=14)
surface

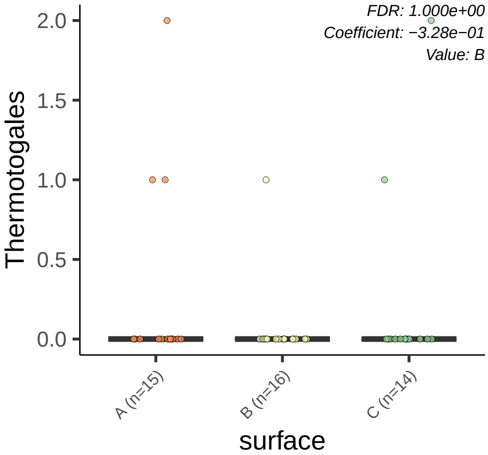
FDR: 1.000e+00
Coefficient: −3.28e−01
Value: B
Thermotogales
2.0
1.5
1.0
0.5
0.0
A (n=15)
B (n=16)
C (n=14)
surface

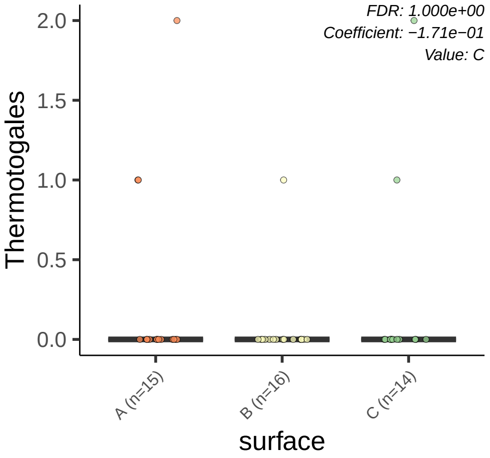

FDR: 1.000e+00
Coefficient: −1.71e−01
Value: C
Thermotogales
2.0
1.5
1.0
0.5
0.0
A (n=15)
B (n=16)
C (n=14)
surface

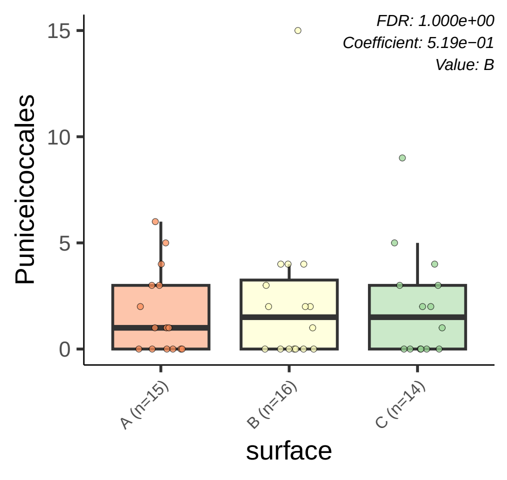

FDR: 1.000e+00
Coefficient: 5.19e−01
Value: B
Puniceicoccales
15
10
5
0
A (n=15)
B (n=16)
C (n=14)
surface

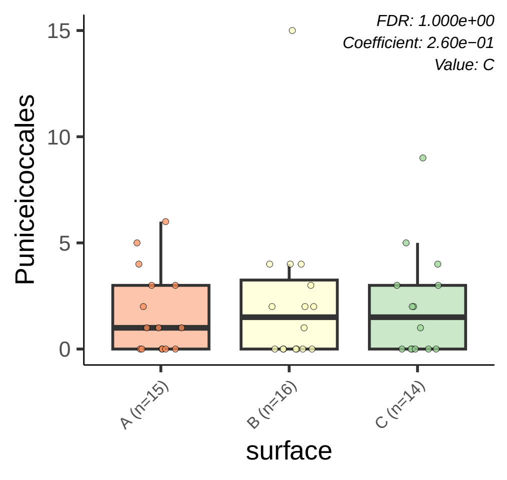

FDR: 1.000e+00
Coefficient: 2.60e−01
Value: C
Puniceicoccales
15
10
5
0
A (n=15)
B (n=16)
C (n=14)
surface

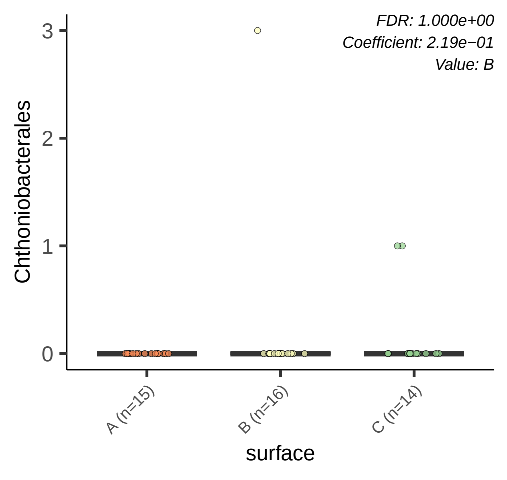

FDR: 1.000e+00
Coefficient: 2.19e−01
Value: B
Chthoniobacterales
3
2
1
0
A (n=15)
B (n=16)
C (n=14)
surface

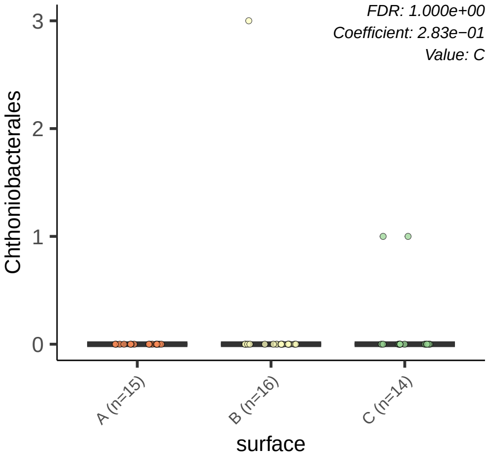

FDR: 1.000e+00
Coefficient: 2.83e−01
Value: C
Chthoniobacterales
3
2
1
0
A (n=15)
B (n=16)
C (n=14)
surface

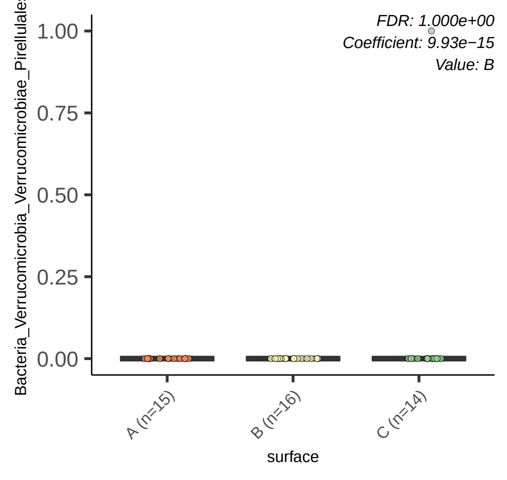

FDR: 1.000e+00
Coefficient: 9.93e−15
Value: B
1.00
0.75
0.50
0.25
0.00
Bacteria_Verrucomicrobia_Verrucomicrobiae_Pirellulales
A (n=15)
B (n=16)
C (n=14)
surface

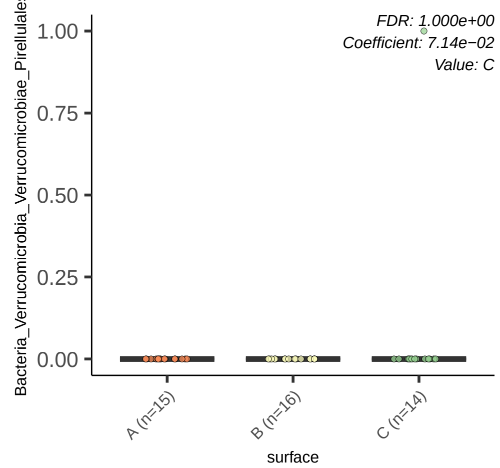

FDR: 1.000e+00
Coefficient: 7.14e−02
Value: C
Bacteria_Verrucomicrobia_Verrucomicrobiae_Pirellulales
1.00
0.75
0.50
0.25
0.00
A (n=15)
B (n=16)
C (n=14)
surface

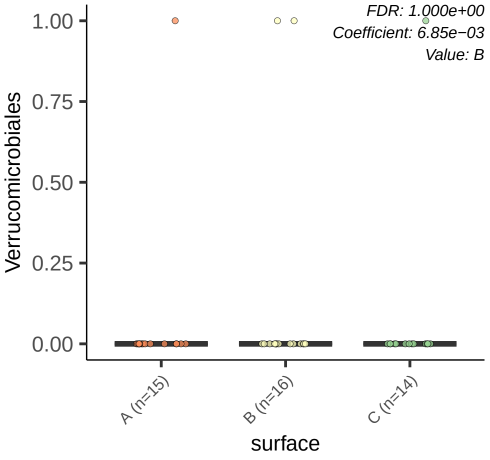
FDR: 1.000e+00
Coefficient: 6.85e−03
Value: B
Verrucomicrobiales
1.00
0.75
0.50
0.25
0.00
A (n=15)
B (n=16)
C (n=14)
surface

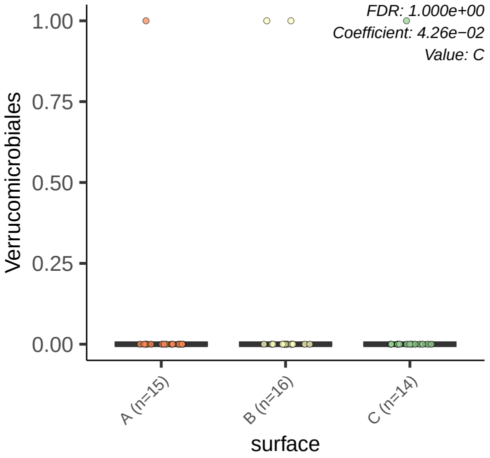
FDR: 1.000e+00
Coefficient: 4.26e−02
Value: C
Verrucomicrobiales
1.00
0.75
0.50
0.25
0.00
A (n=15)
B (n=16)
C (n=14)
surface

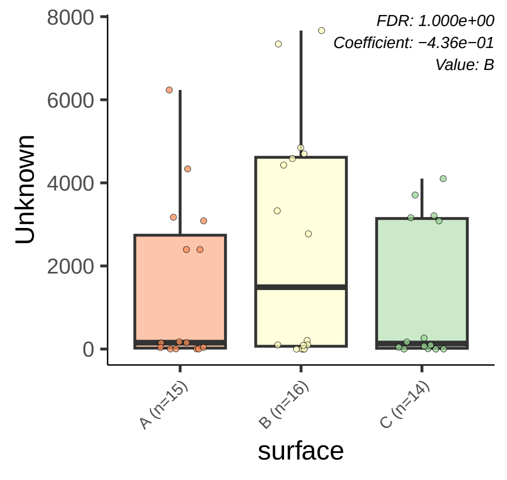
FDR: 1.000e+00
Coefficient: −4.36e−01
Value: B
Unknown
8000
6000
4000
2000
0
A (n=15)
B (n=16)
C (n=14)
surface

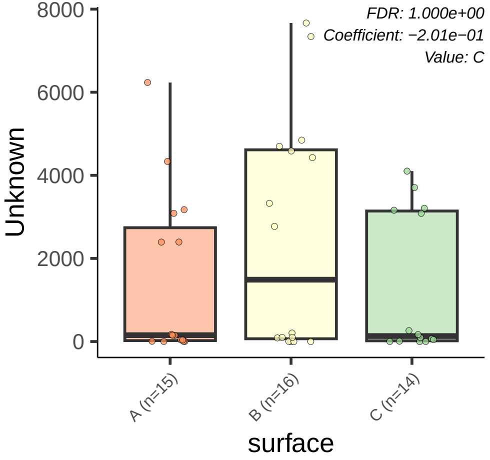

FDR: 1.000e+00
Coefficient: −2.01e−01
Value: C
Unknown
8000
6000
4000
2000
0
A (n=15)
B (n=16)
C (n=14)
surface

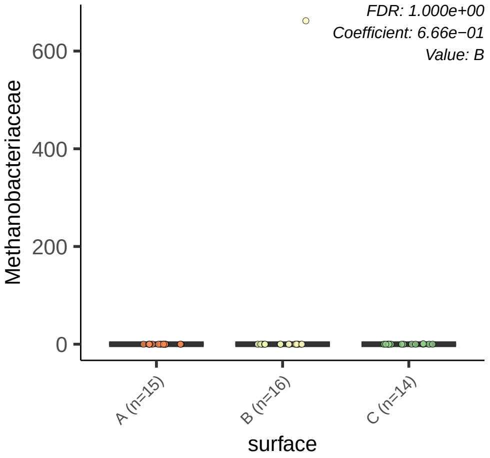

FDR: 1.000e+00
Coefficient: 6.66e−01
Value: B
Methanobacteriaceae
600
400
200
0
A (n=15)
B (n=16)
C (n=14)
surface

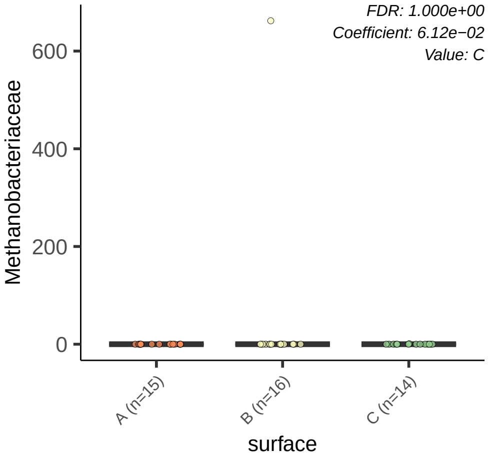
FDR: 1.000e+00
Coefficient: 6.12e−02
Value: C
Methanobacteriaceae
600
400
200
0
A (n=15)
B (n=16)
C (n=14)
surface

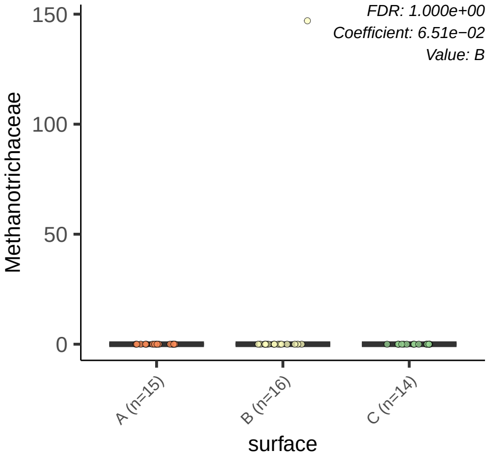
FDR: 1.000e+00
Coefficient: 6.51e−02
Value: B
Methanotrichaceae
150
100
50
0
A (n=15)
B (n=16)
C (n=14)
surface

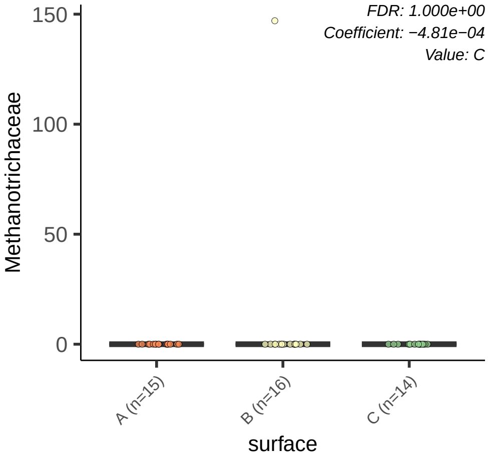

FDR: 1.000e+00
Coefficient: −4.81e−04
Value: C
150
100
50
0
Methanotrichaceae
A (n=15)
B (n=16)
C (n=14)
surface

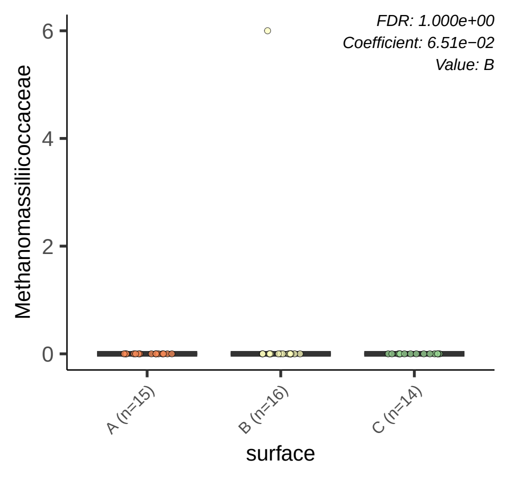
FDR: 1.000e+00
Coefficient: 6.51e−02
Value: B
Methanomassiliicoccaceae
0
2
4
6
A (n=15)
B (n=16)
C (n=14)
surface

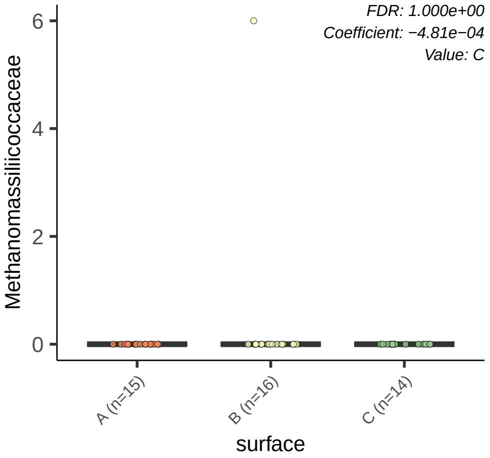
FDR: 1.000e+00
Coefficient: −4.81e−04
Value: C
Methanomassiliicoccaceae
0
2
4
6
A (n=15)
B (n=16)
C (n=14)
surface

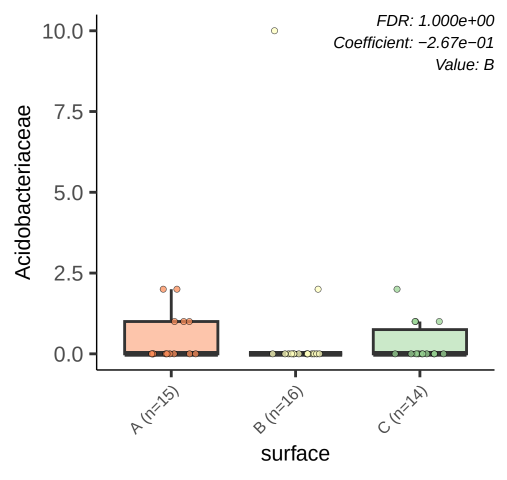
FDR: 1.000e+00
Coefficient: −2.67e−01
Value: B
10.0
7.5
5.0
2.5
0.0
Acidobacteriaceae
A (n=15)
B (n=16)
C (n=14)
surface

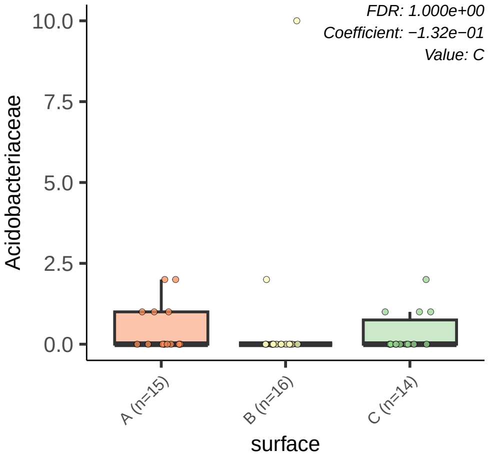
FDR: 1.000e+00
Coefficient: −1.32e−01
Value: C
Acidobacteriaceae
10.0
7.5
5.0
2.5
0.0
A (n=15)
B (n=16)
C (n=14)
surface

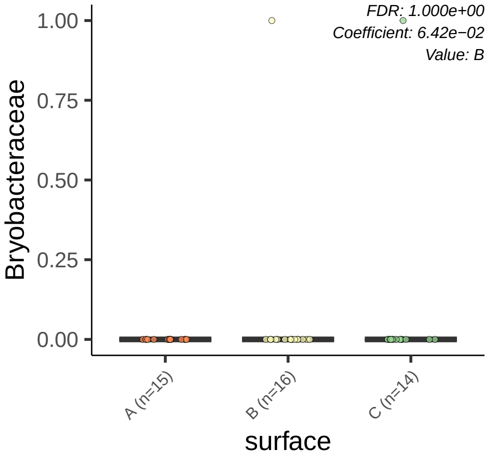
FDR: 1.000e+00
Coefficient: 6.42e−02
Value: B
Bryobacteraceae
1.00
0.75
0.50
0.25
0.00
A (n=15)
B (n=16)
C (n=14)
surface

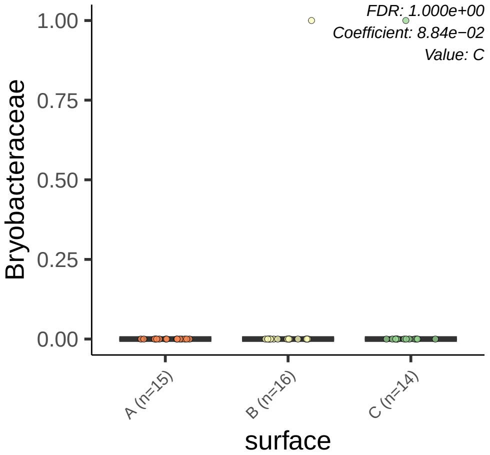
FDR: 1.000e+00
Coefficient: 8.84e−02
Value: C
Bryobacteraceae
1.00
0.75
0.50
0.25
0.00
A (n=15)
B (n=16)
C (n=14)
surface

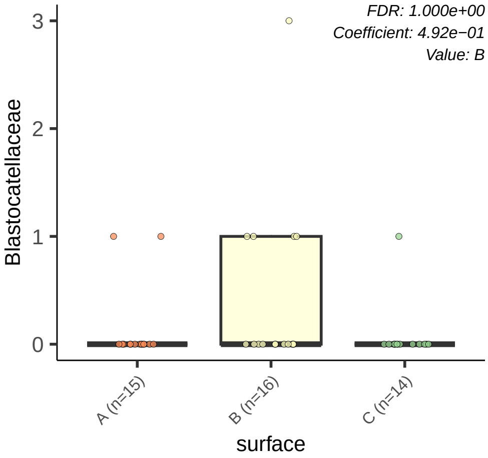

FDR: 1.000e+00
Coefficient: 4.92e−01
Value: B
Blastocatellaceae
3
2
1
0
A (n=15)
B (n=16)
C (n=14)
surface

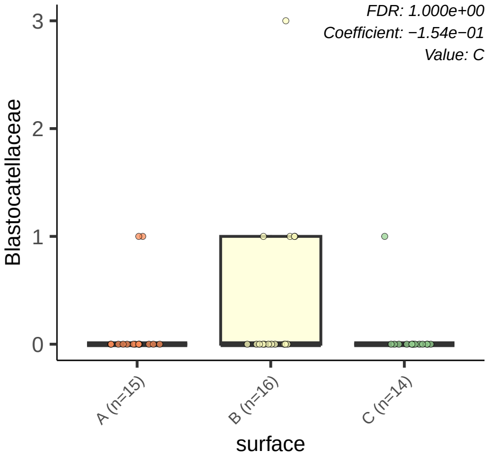
FDR: 1.000e+00
Coefficient: −1.54e−01
Value: C
Blastocatellaceae
3
2
1
0
A (n=15)
B (n=16)
C (n=14)
surface

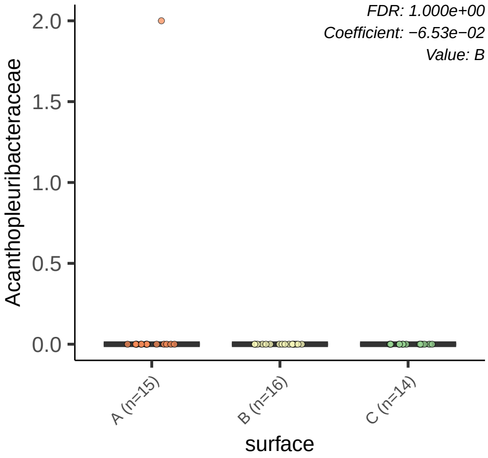

FDR: 1.000e+00
Coefficient: −6.53e−02
Value: B
Acanthopleuribacteraceae
2.0
1.5
1.0
0.5
0.0
A (n=15)
B (n=16)
C (n=14)
surface

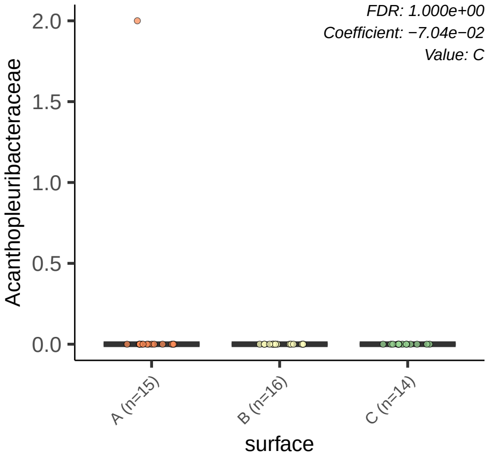
FDR: 1.000e+00
Coefficient: −7.04e−02
Value: C
Acanthopleuribacteraceae
2.0
1.5
1.0
0.5
0.0
A (n=15)
B (n=16)
C (n=14)
surface

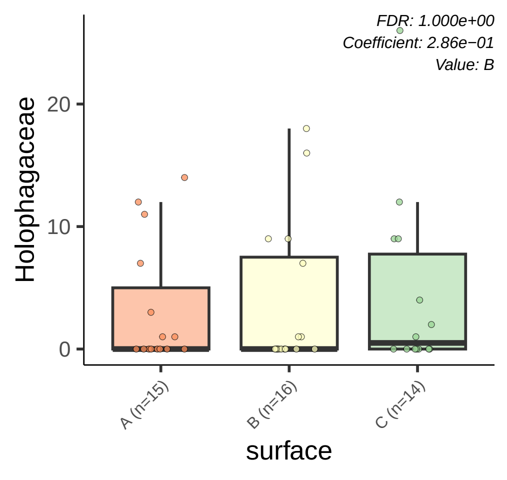
FDR: 1.000e+00
Coefficient: 2.86e−01
Value: B
Holophagaceae
20
10
0
A (n=15)
B (n=16)
C (n=14)
surface

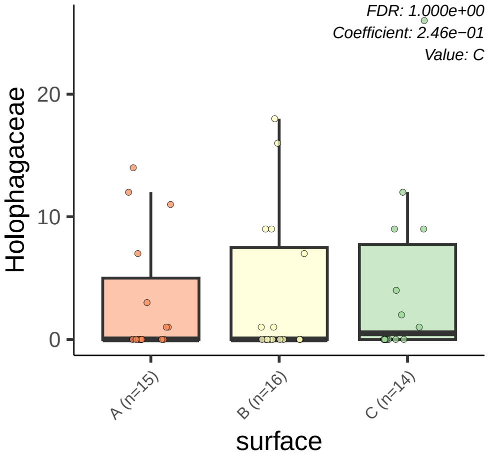
FDR: 1.000e+00
Coefficient: 2.46e−01
Value: C
Holophagaceae
20
10
0
A (n=15)
B (n=16)
C (n=14)
surface

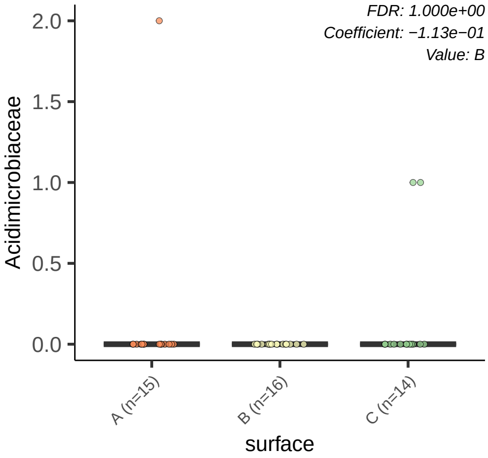

FDR: 1.000e+00
Coefficient: −1.13e−01
Value: B
Acidimicrobiaceae
2.0
1.5
1.0
0.5
0.0
A (n=15)
B (n=16)
C (n=14)
surface

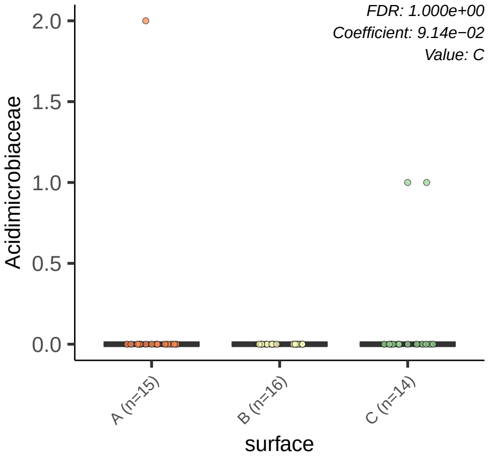

FDR: 1.000e+00
Coefficient: 9.14e−02
Value: C
Acidimicrobiaceae
2.0
1.5
1.0
0.5
0.0
A (n=15)
B (n=16)
C (n=14)
surface

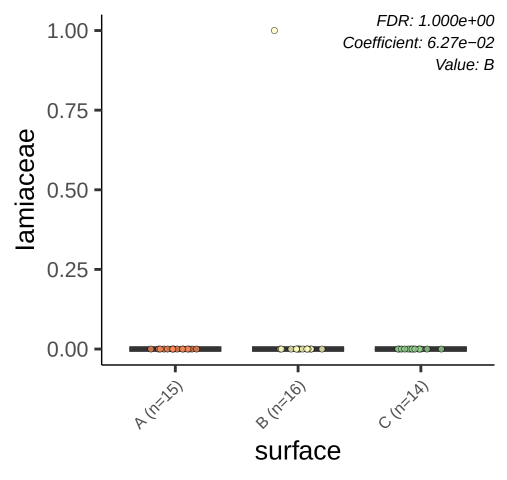
FDR: 1.000e+00
Coefficient: 6.27e−02
Value: B
1.00
0.75
0.50
0.25
0.00
lamiaceae
A (n=15)
B (n=16)
C (n=14)
surface

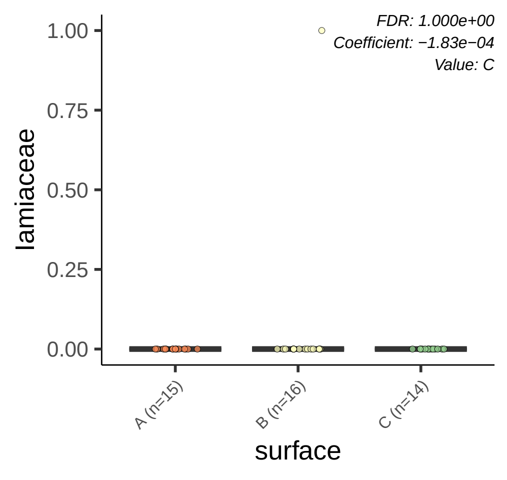
FDR: 1.000e+00
Coefficient: −1.83e−04
Value: C
lamiaceae
1.00
0.75
0.50
0.25
0.00
A (n=15)
B (n=16)
C (n=14)
surface

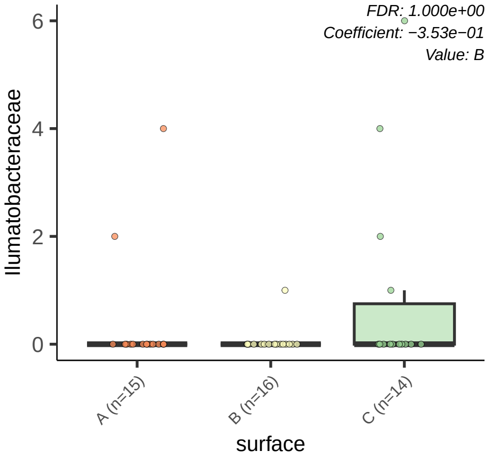

FDR: 1.000e+00
Coefficient: −3.53e−01
Value: B
Ilumatobacteraceae
0
2
4
6
A (n=15)
B (n=16)
C (n=14)
surface

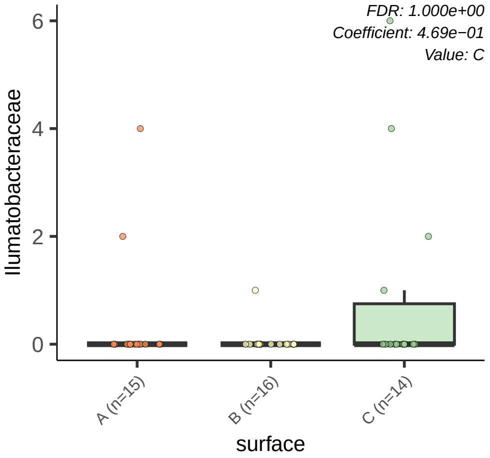

FDR: 1.000e+00
Coefficient: 4.69e−01
Value: C
Ilumatobacteraceae
0
2
4
6
A (n=15)
B (n=16)
C (n=14)
surface

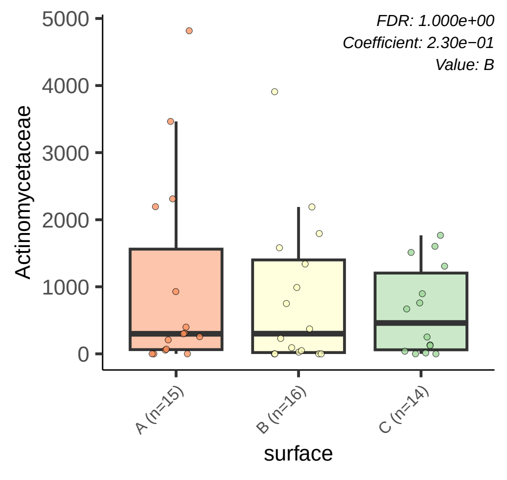
FDR: 1.000e+00
Coefficient: 2.30e−01
Value: B
5000
4000
3000
2000
1000
0
Actinomycetaceae
A (n=15)
B (n=16)
C (n=14)
surface

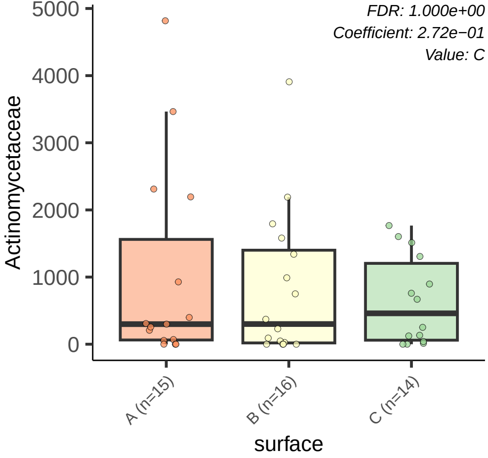
FDR: 1.000e+00
Coefficient: 2.72e−01
Value: C
Actinomycetaceae
5000
4000
3000
2000
1000
0
A (n=15)
B (n=16)
C (n=14)
surface

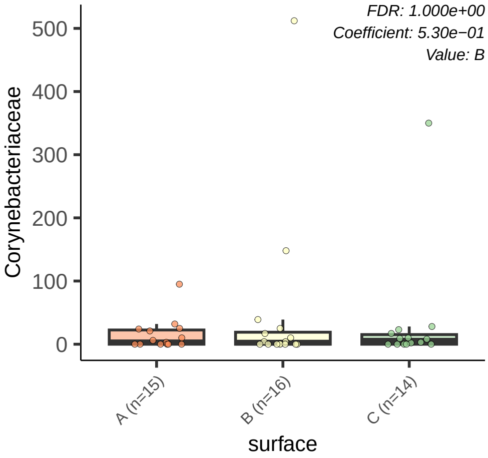

FDR: 1.000e+00
Coefficient: 5.30e−01
Value: B
Corynebacteriaceae
500
400
300
200
100
0
A (n=15)
B (n=16)
C (n=14)
surface

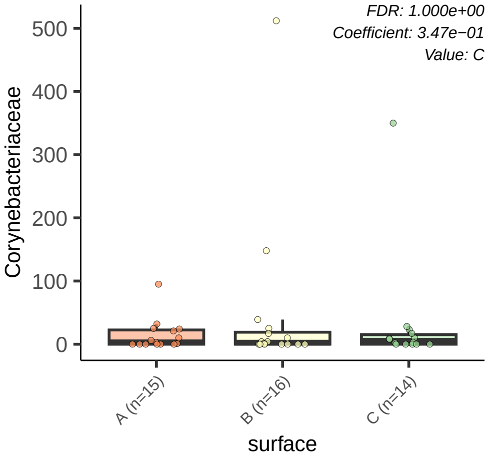

FDR: 1.000e+00
Coefficient: 3.47e−01
Value: C
Corynebacteriaceae
0
100
200
300
400
500
A (n=15)
B (n=16)
C (n=14)
surface

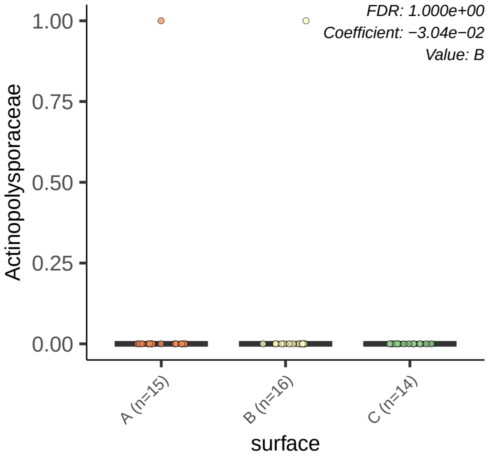
FDR: 1.000e+00
Coefficient: −3.04e−02
Value: B
Actinopolysporaceae
1.00
0.75
0.50
0.25
0.00
A (n=15)
B (n=16)
C (n=14)
surface

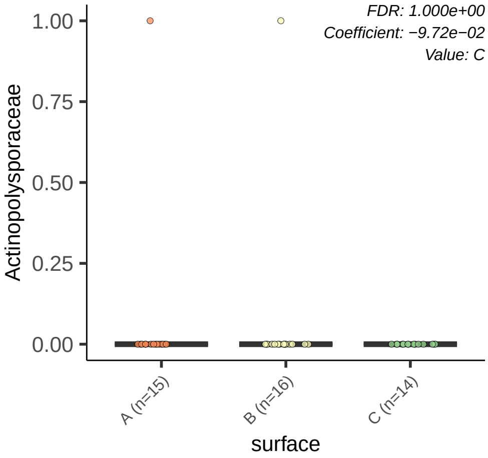

FDR: 1.000e+00
Coefficient: −9.72e−02
Value: C
Actinopolysporaceae
1.00
0.75
0.50
0.25
0.00
A (n=15)
B (n=16)
C (n=14)
surface

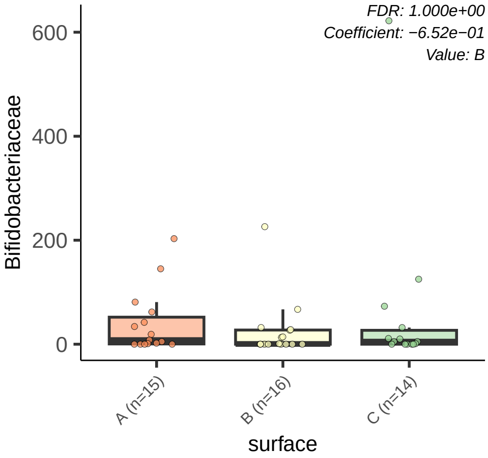

FDR: 1.000e+00
Coefficient: −6.52e−01
Value: B
Bifidobacteriaceae
600
400
200
0
A (n=15)
B (n=16)
C (n=14)
surface

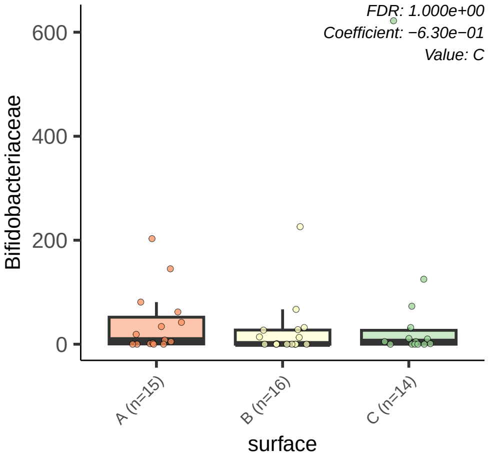
FDR: 1.000e+00
Coefficient: −6.30e−01
Value: C
Bifidobacteriaceae
0
200
400
600
A (n=15)
B (n=16)
C (n=14)
surface

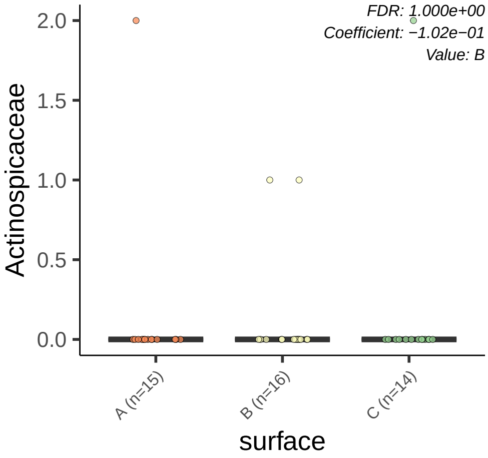
FDR: 1.000e+00
Coefficient: −1.02e−01
Value: B
Actinospicaceae
2.0
1.5
1.0
0.5
0.0
A (n=15)
B (n=16)
C (n=14)
surface

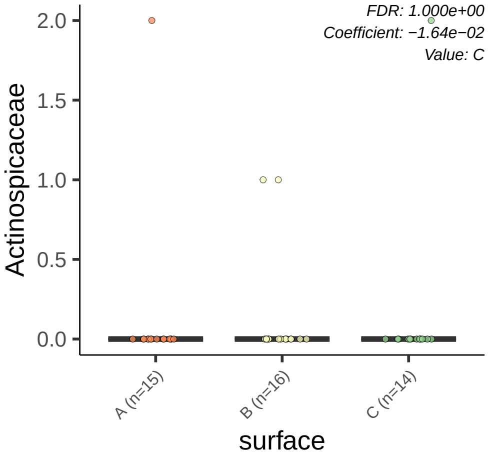
FDR: 1.000e+00
Coefficient: −1.64e−02
Value: C
Actinospicaceae
2.0
1.5
1.0
0.5
0.0
A (n=15)
B (n=16)
C (n=14)
surface

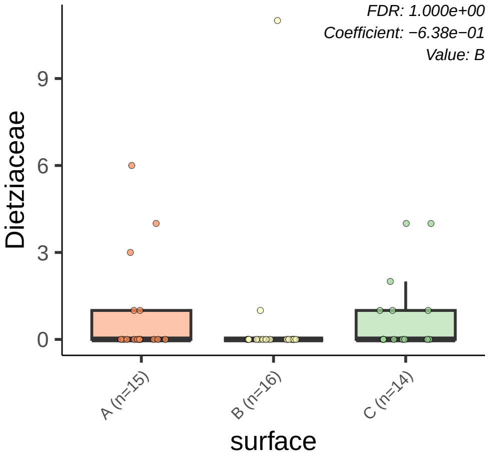
FDR: 1.000e+00
Coefficient: −6.38e−01
Value: B
Dietziaceae
9
6
3
0
A (n=15)
B (n=16)
C (n=14)
surface

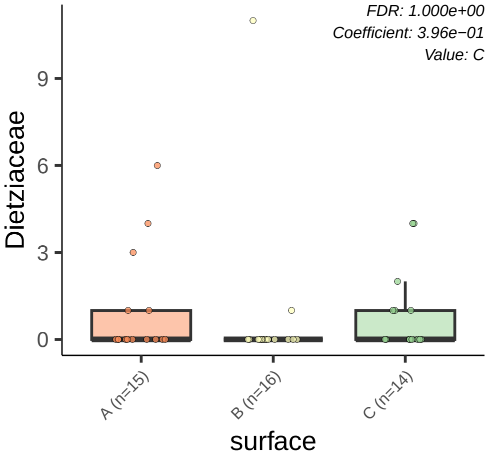

FDR: 1.000e+00
Coefficient: 3.96e−01
Value: C
Dietziaceae
9
6
3
0
A (n=15)
B (n=16)
C (n=14)
surface

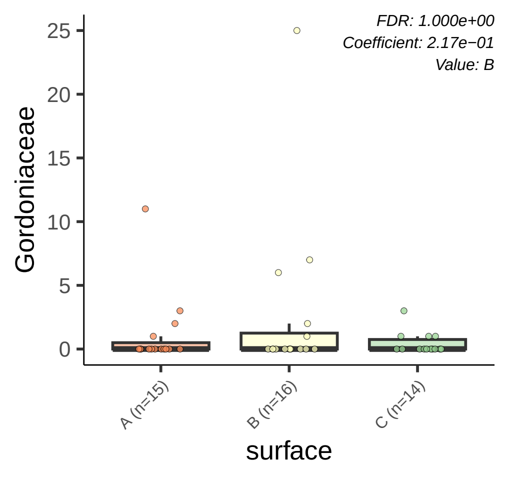
FDR: 1.000e+00
Coefficient: 2.17e−01
Value: B
Gordoniaceae
25
20
15
10
5
0
A (n=15)
B (n=16)
C (n=14)
surface

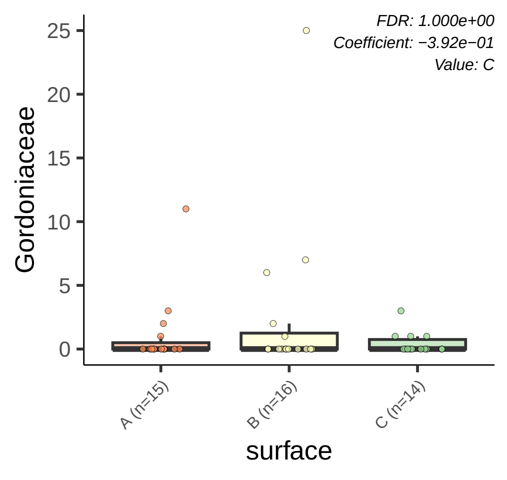
FDR: 1.000e+00
Coefficient: −3.92e−01
Value: C
Gordoniaceae
0
5
10
15
20
25
A (n=15)
B (n=16)
C (n=14)
surface

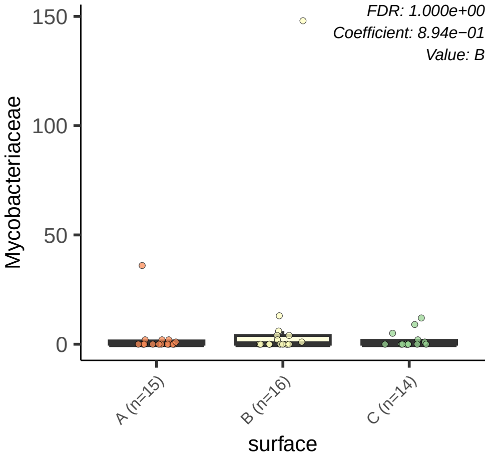

FDR: 1.000e+00
Coefficient: 8.94e−01
Value: B
Mycobacteriaceae
150
100
50
0
A (n=15)
B (n=16)
C (n=14)
surface

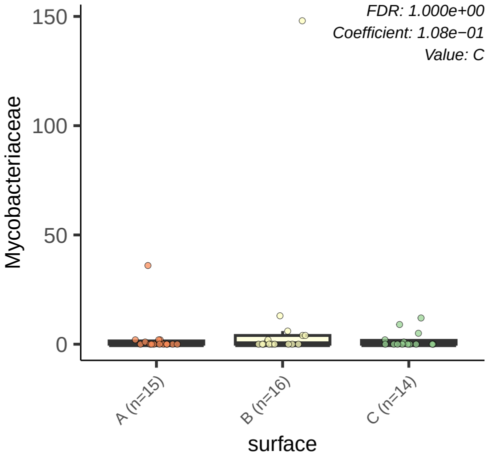

FDR: 1.000e+00
Coefficient: 1.08e−01
Value: C
Mycobacteriaceae
150
100
50
0
A (n=15)
B (n=16)
C (n=14)
surface

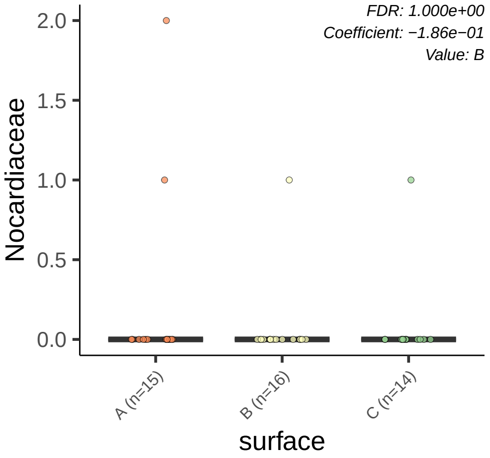
FDR: 1.000e+00
Coefficient: −1.86e−01
Value: B
Nocardiaceae
2.0
1.5
1.0
0.5
0.0
A (n=15)
B (n=16)
C (n=14)
surface

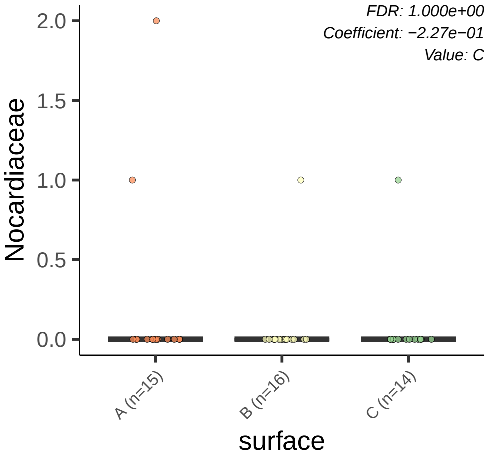
FDR: 1.000e+00
Coefficient: −2.27e−01
Value: C
Nocardiaceae
2.0
1.5
1.0
0.5
0.0
A (n=15)
B (n=16)
C (n=14)
surface

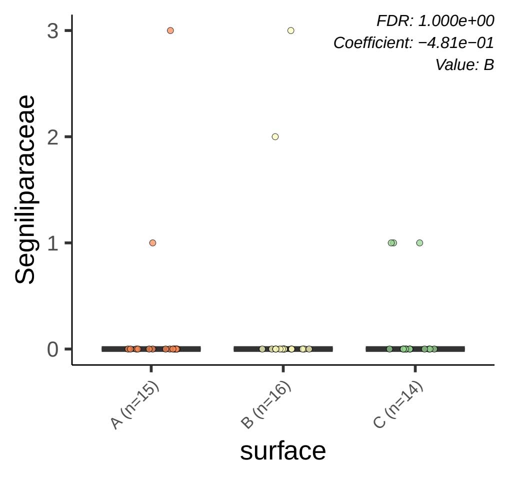

FDR: 1.000e+00
Coefficient: −4.81e−01
Value: B
Segniliparaceae
3
2
1
0
A (n=15)
B (n=16)
C (n=14)
surface

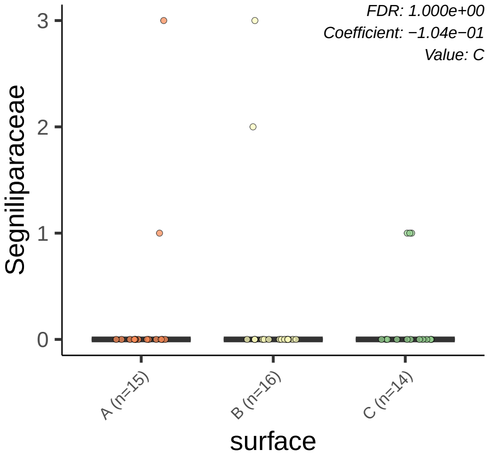

FDR: 1.000e+00
Coefficient: −1.04e−01
Value: C
Segniliparaceae
3
2
1
0
A (n=15)
B (n=16)
C (n=14)
surface

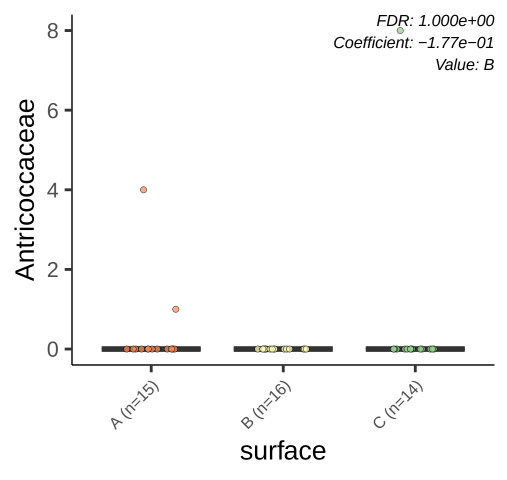
FDR: 1.000e+00
Coefficient: −1.77e−01
Value: B
Antricoccaceae
8
6
4
2
0
A (n=15)
B (n=16)
C (n=14)
surface

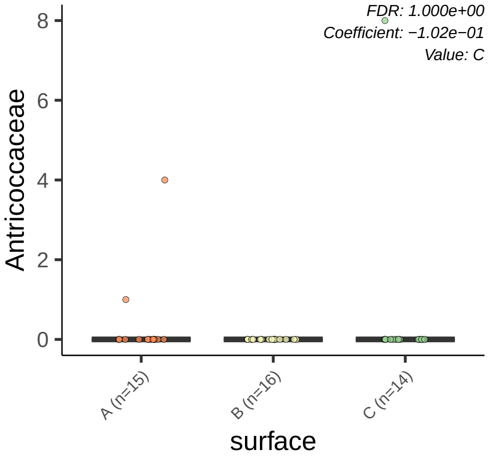

FDR: 1.000e+00
Coefficient: −1.02e−01
Value: C
Antricoccaceae
8
6
4
2
0
A (n=15)
B (n=16)
C (n=14)
surface

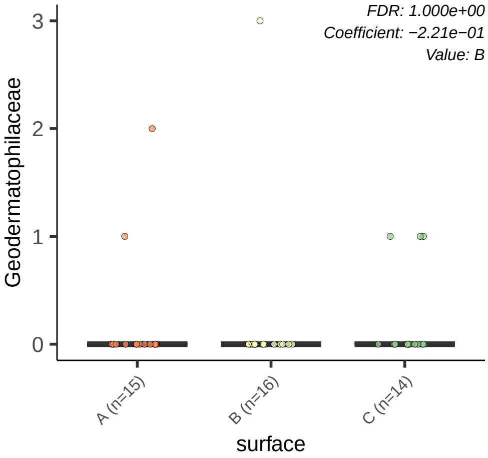
FDR: 1.000e+00
Coefficient: −2.21e−01
Value: B
Geodermatophilaceae
0
1
2
3
A (n=15)
B (n=16)
C (n=14)
surface

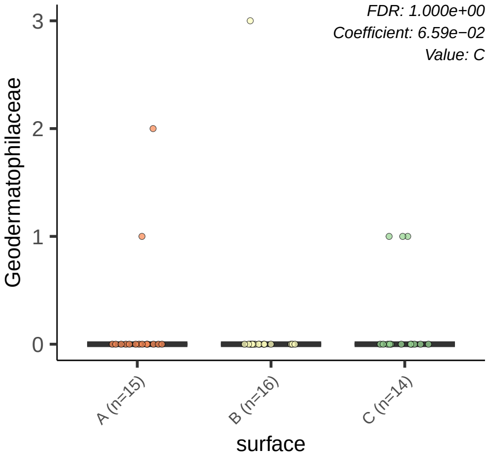

FDR: 1.000e+00
Coefficient: 6.59e−02
Value: C
Geodermatophilaceae
3
2
1
0
A (n=15)
B (n=16)
C (n=14)
surface

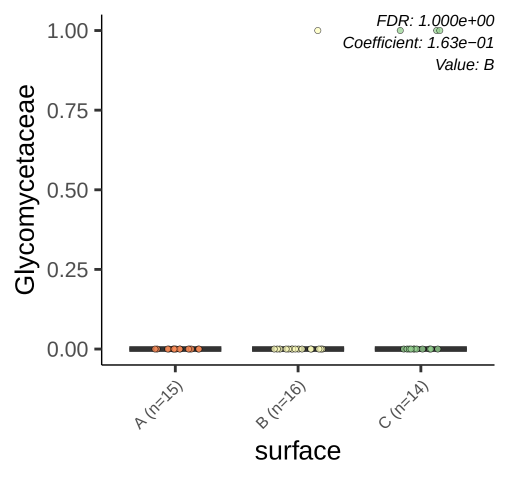
FDR: 1.000e+00
Coefficient: 1.63e−01
Value: B
Glycomycetaceae
1.00
0.75
0.50
0.25
0.00
A (n=15)
B (n=16)
C (n=14)
surface

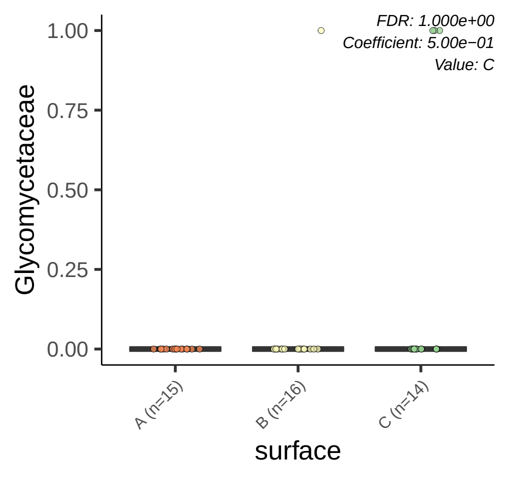
FDR: 1.000e+00
Coefficient: 5.00e−01
Value: C
Glycomycetaceae
1.00
0.75
0.50
0.25
0.00
A (n=15)
B (n=16)
C (n=14)
surface

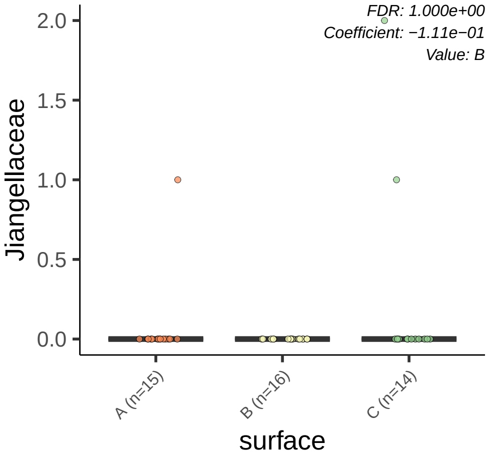
FDR: 1.000e+00
Coefficient: −1.11e−01
Value: B
Jiangellaceae
2.0
1.5
1.0
0.5
0.0
A (n=15)
B (n=16)
C (n=14)
surface

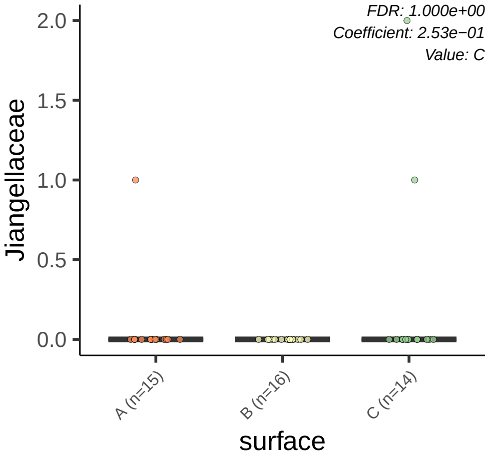

FDR: 1.000e+00
Coefficient: 2.53e−01
Value: C
2.0
1.5
1.0
0.5
0.0
Jiangellaceae
A (n=15)
B (n=16)
C (n=14)
surface

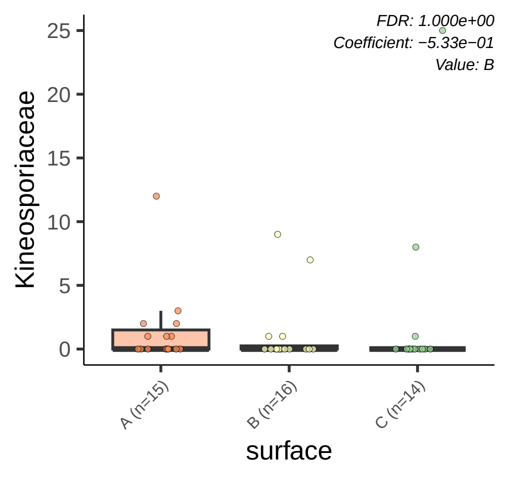

FDR: 1.000e+00
Coefficient: −5.33e−01
Value: B
Kineosporiaceae
0
5
10
15
20
25
A (n=15)
B (n=16)
C (n=14)
surface

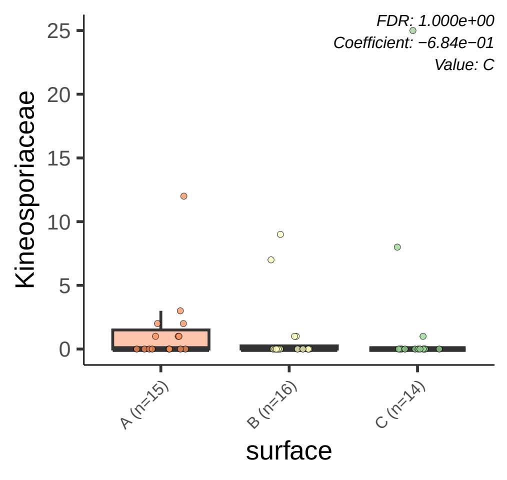

FDR: 1.000e+00
Coefficient: −6.84e−01
Value: C
Kineosporiaceae
0
5
10
15
20
25
A (n=15)
B (n=16)
C (n=14)
surface

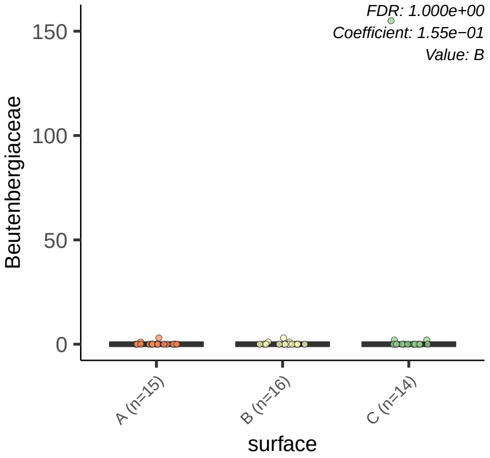

FDR: 1.000e+00
Coefficient: 1.55e−01
Value: B
Beutenbergiaceae
150
100
50
0
A (n=15)
B (n=16)
C (n=14)
surface

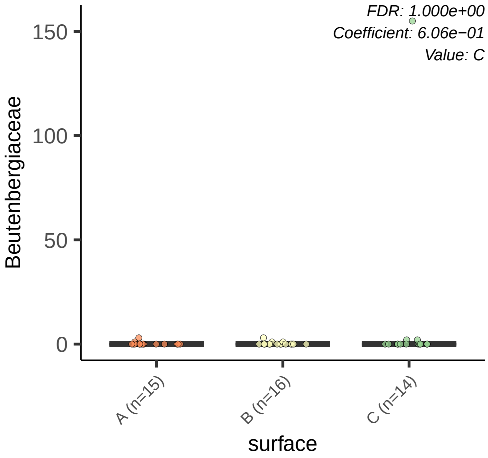
FDR: 1.000e+00
Coefficient: 6.06e−01
Value: C
Beutenbergiaceae
150
100
50
0
A (n=15)
B (n=16)
C (n=14)
surface

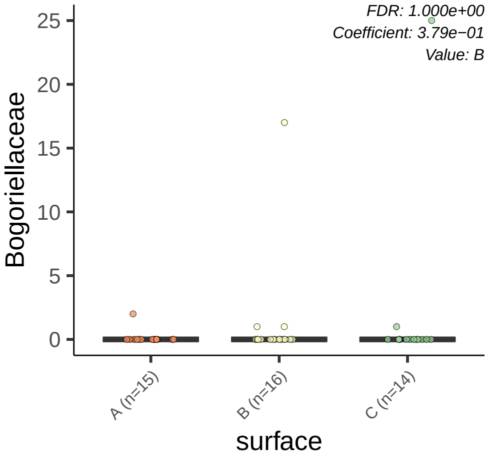

FDR: 1.000e+00
Coefficient: 3.79e−01
Value: B
Bogoriellaceae
25
20
15
10
5
0
A (n=15)
B (n=16)
C (n=14)
surface

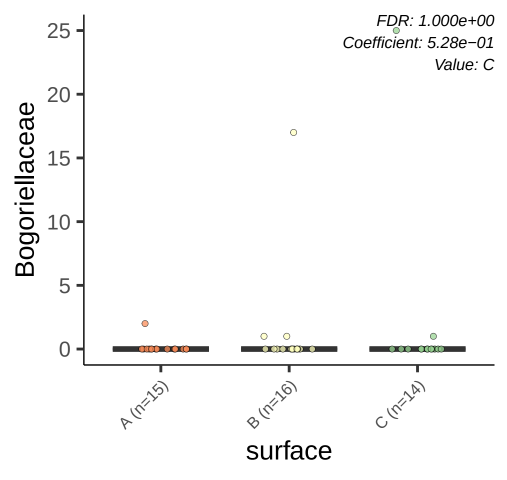

FDR: 1.000e+00
Coefficient: 5.28e−01
Value: C
Bogoriellaceae
25
20
15
10
5
0
A (n=15)
B (n=16)
C (n=14)
surface

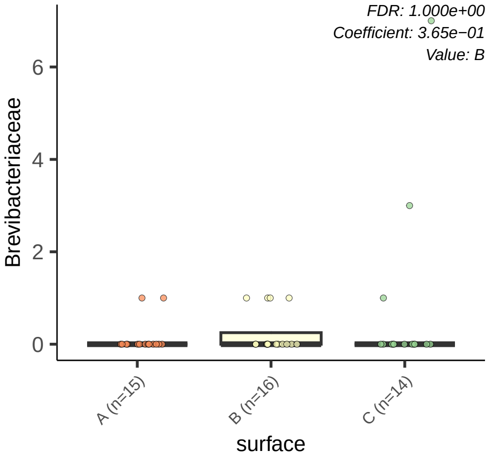
FDR: 1.000e+00
Coefficient: 3.65e−01
Value: B
Brevibacteriaceae
0
2
4
6
A (n=15)
B (n=16)
C (n=14)
surface

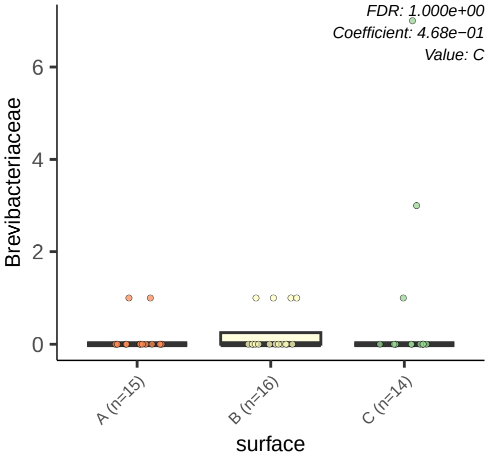
FDR: 1.000e+00
Coefficient: 4.68e−01
Value: C
Brevibacteriaceae
0
2
4
6
A (n=15)
B (n=16)
C (n=14)
surface

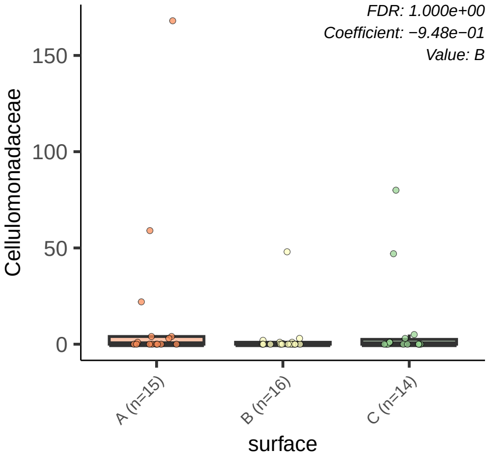
FDR: 1.000e+00
Coefficient: −9.48e−01
Value: B
Cellulomonadaceae
150
100
50
0
A (n=15)
B (n=16)
C (n=14)
surface

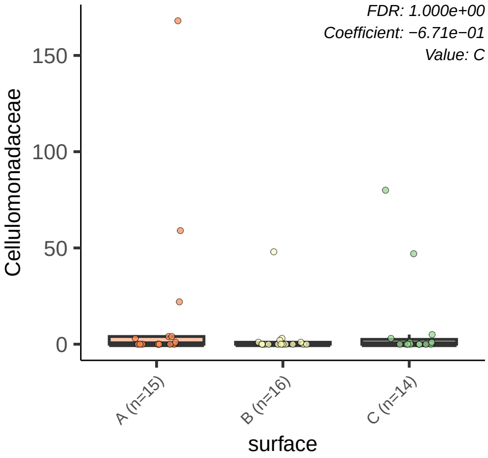
FDR: 1.000e+00
Coefficient: −6.71e−01
Value: C
Cellulomonadaceae
150
100
50
0
A (n=15)
B (n=16)
C (n=14)
surface

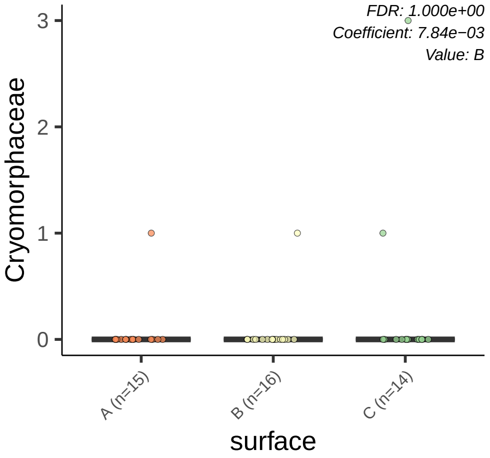
FDR: 1.000e+00
Coefficient: 7.84e−03
Value: B
Cryomorphaceae
3
2
1
0
A (n=15)
B (n=16)
C (n=14)
surface

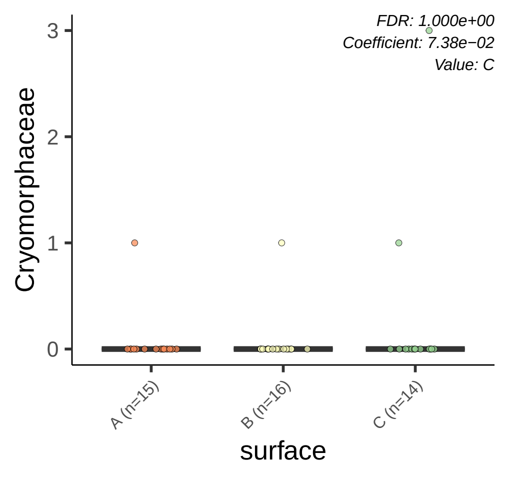

FDR: 1.000e+00
Coefficient: 7.38e−02
Value: C
Cryomorphaceae
3
2
1
0
A (n=15)
B (n=16)
C (n=14)
surface

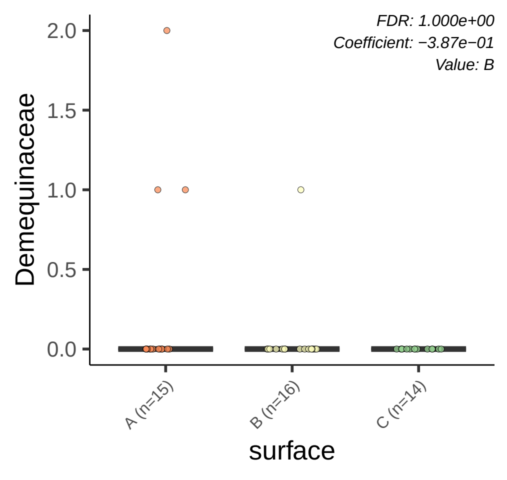

FDR: 1.000e+00
Coefficient: −3.87e−01
Value: B
Demequinaceae
2.0
1.5
1.0
0.5
0.0
A (n=15)
B (n=16)
C (n=14)
surface

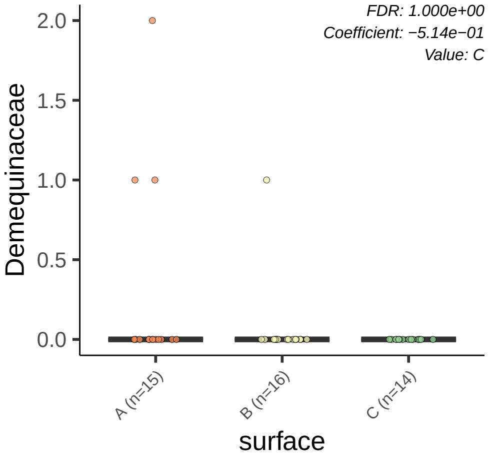

FDR: 1.000e+00
Coefficient: −5.14e−01
Value: C
Demequinaceae
2.0
1.5
1.0
0.5
0.0
A (n=15)
B (n=16)
C (n=14)
surface

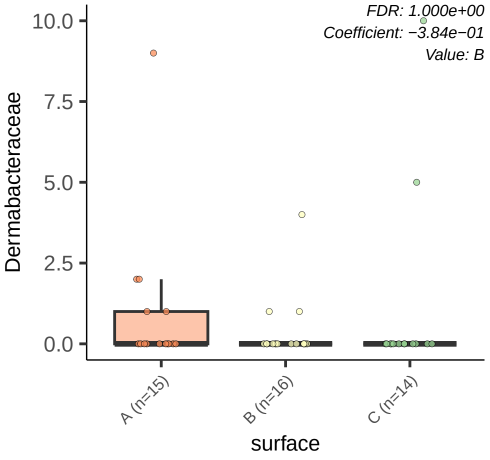
FDR: 1.000e+00
Coefficient: −3.84e−01
Value: B
Dermabacteraceae
10.0
7.5
5.0
2.5
0.0
A (n=15)
B (n=16)
C (n=14)
surface

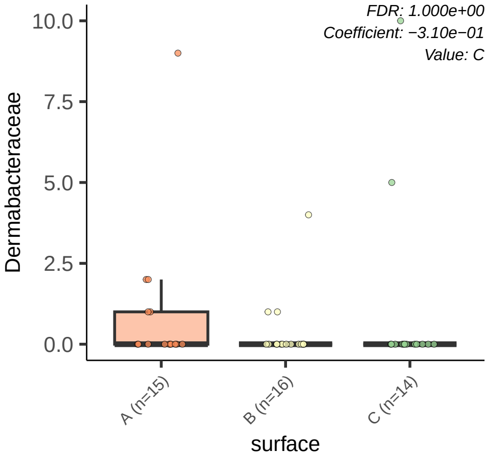

FDR: 1.000e+00
Coefficient: −3.10e−01
Value: C
Dermabacteraceae
10.0
7.5
5.0
2.5
0.0
A (n=15)
B (n=16)
C (n=14)
surface

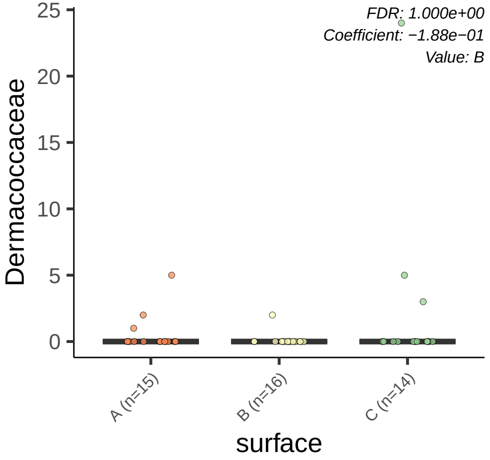

FDR: 1.000e+00
Coefficient: −1.88e−01
Value: B
Dermacoccaceae
25
20
15
10
5
0
A (n=15)
B (n=16)
C (n=14)
surface

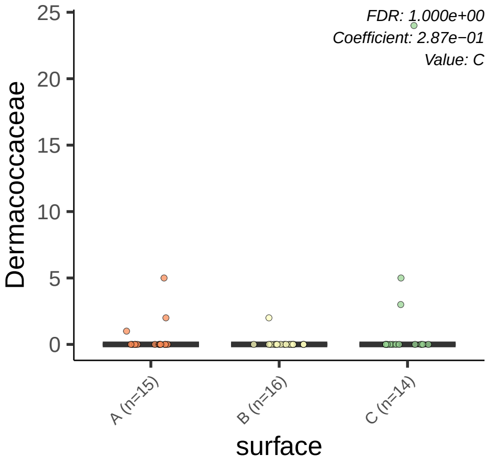
FDR: 1.000e+00
Coefficient: 2.87e−01
Value: C
Dermacoccaceae
25
20
15
10
5
0
A (n=15)
B (n=16)
C (n=14)
surface

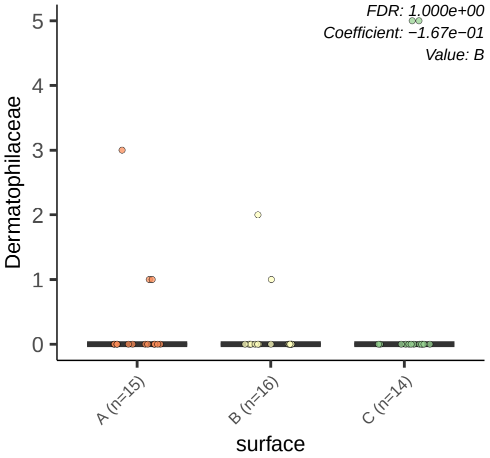
FDR: 1.000e+00
Coefficient: −1.67e−01
Value: B
Dermatophilaceae
0
1
2
3
4
5
A (n=15)
B (n=16)
C (n=14)
surface

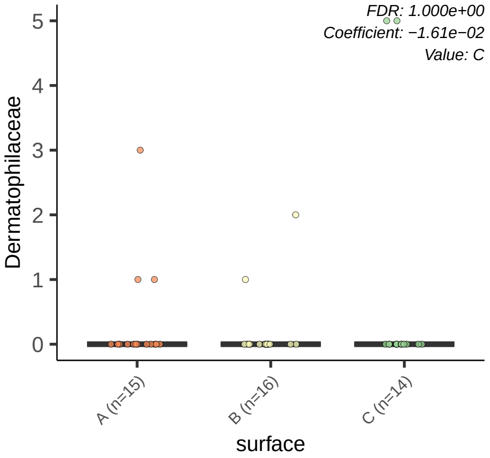

FDR: 1.000e+00
Coefficient: −1.61e−02
Value: C
Dermatophilaceae
0
1
2
3
4
5
A (n=15)
B (n=16)
C (n=14)
surface

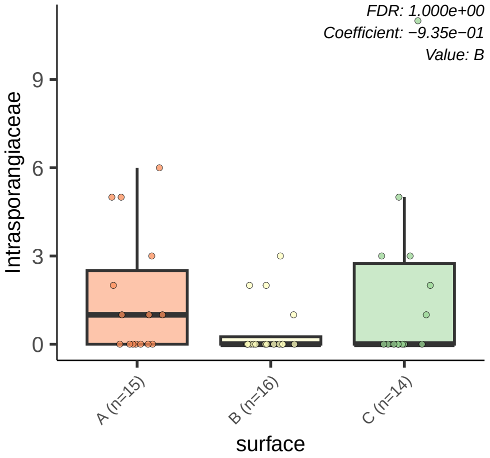

FDR: 1.000e+00
Coefficient: −9.35e−01
Value: B
Intrasporangiaceae
0
3
6
9
A (n=15)
B (n=16)
C (n=14)
surface

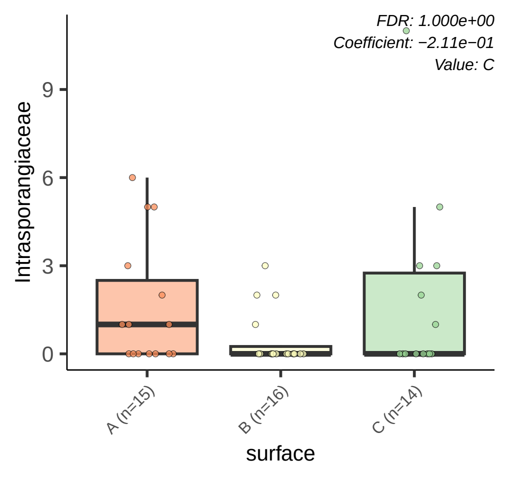
FDR: 1.000e+00
Coefficient: −2.11e−01
Value: C
Intrasporangiaceae
9
6
3
0
A (n=15)
B (n=16)
C (n=14)
surface

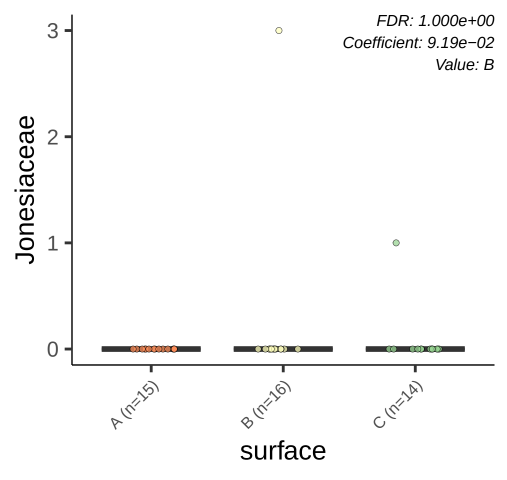

FDR: 1.000e+00
Coefficient: 9.19e−02
Value: B
Jonesiaceae
3
2
1
0
A (n=15)
B (n=16)
C (n=14)
surface

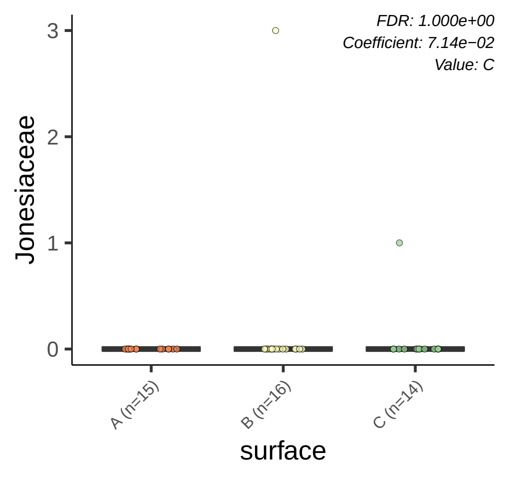
FDR: 1.000e+00
Coefficient: 7.14e−02
Value: C
Jonesiaceae
0
1
2
3
A (n=15)
B (n=16)
C (n=14)
surface

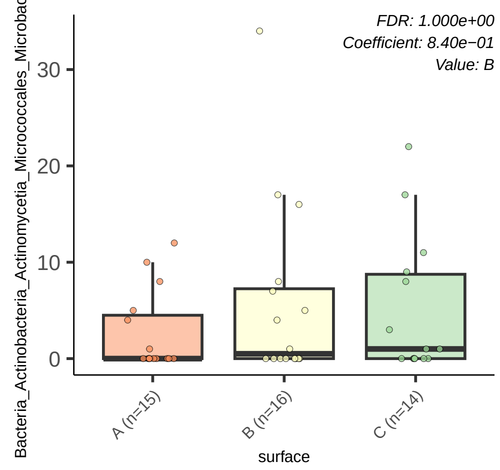

FDR: 1.000e+00
Coefficient: 8.40e−01
Value: B
30
20
10
0
Bacteria_Actinobacteria_Actinomycetia_Micrococcales_Microbac
A (n=15)
B (n=16)
C (n=14)
surface

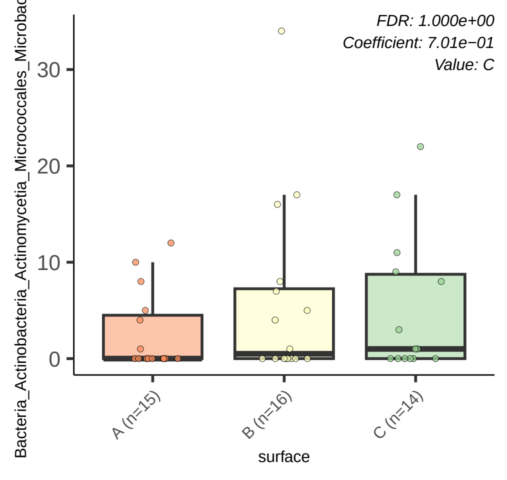

FDR: 1.000e+00
Coefficient: 7.01e−01
Value: C
Bacteria_Actinobacteria_Actinomycetia_Micrococcales_Microbac
30
20
10
0
A (n=15)
B (n=16)
C (n=14)
surface

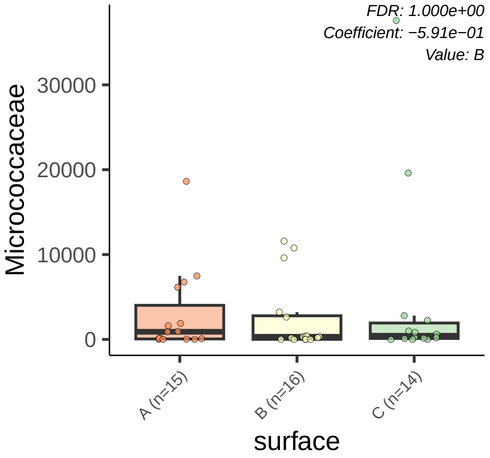
FDR: 1.000e+00
Coefficient: −5.91e−01
Value: B
Micrococcaceae
30000
20000
10000
0
A (n=15)
B (n=16)
C (n=14)
surface

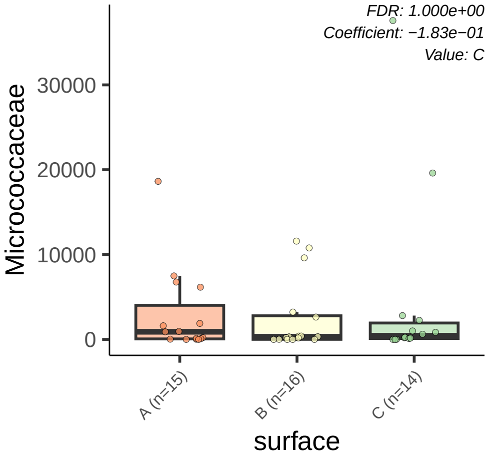
FDR: 1.000e+00
Coefficient: −1.83e−01
Value: C
Micrococcaceae
30000
20000
10000
0
A (n=15)
B (n=16)
C (n=14)
surface

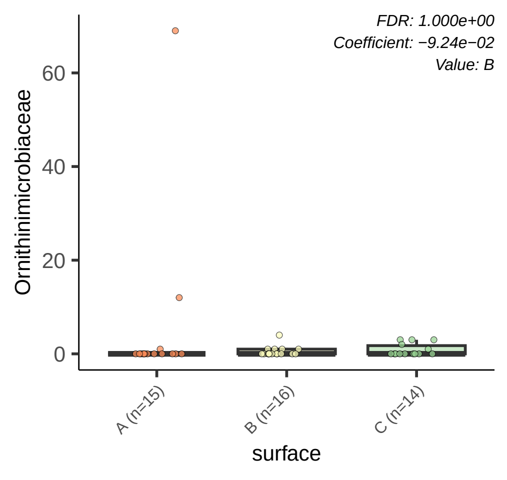

FDR: 1.000e+00
Coefficient: −9.24e−02
Value: B
Ornithinimicrobiaceae
0
20
40
60
A (n=15)
B (n=16)
C (n=14)
surface

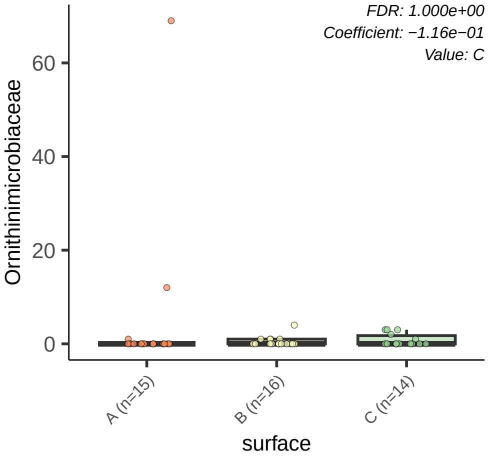

FDR: 1.000e+00
Coefficient: −1.16e−01
Value: C
Ornithinimicrobiaceae
0
20
40
60
A (n=15)
B (n=16)
C (n=14)
surface

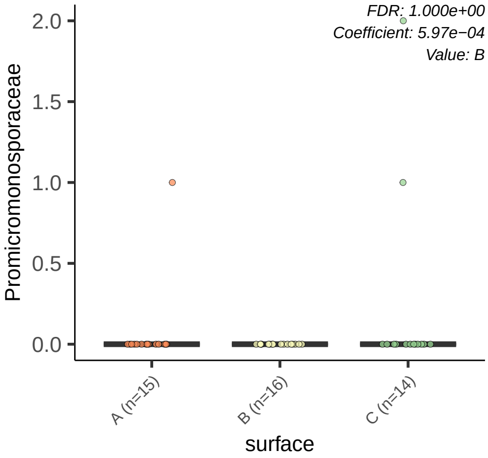
FDR: 1.000e+00
Coefficient: 5.97e−04
Value: B
Promicromonosporaceae
2.0
1.5
1.0
0.5
0.0
A (n=15)
B (n=16)
C (n=14)
surface

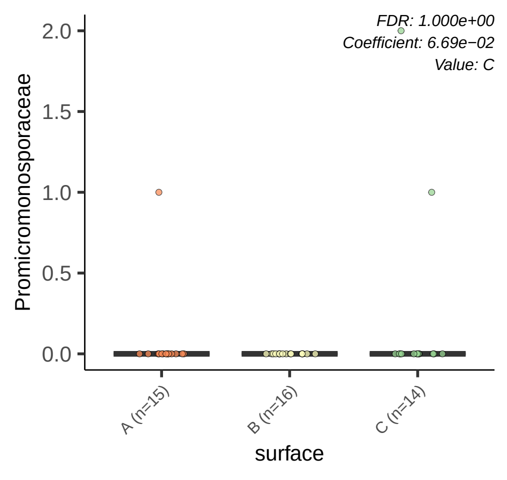
FDR: 1.000e+00
Coefficient: 6.69e−02
Value: C
Promicromonosporaceae
2.0
1.5
1.0
0.5
0.0
A (n=15)
B (n=16)
C (n=14)
surface

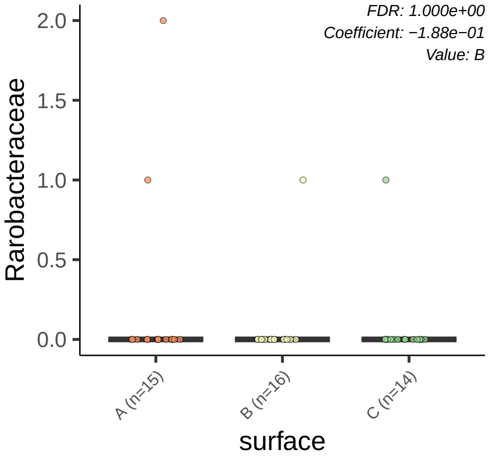
FDR: 1.000e+00
Coefficient: −1.88e−01
Value: B
Rarobacteraceae
2.0
1.5
1.0
0.5
0.0
A (n=15)
B (n=16)
C (n=14)
surface

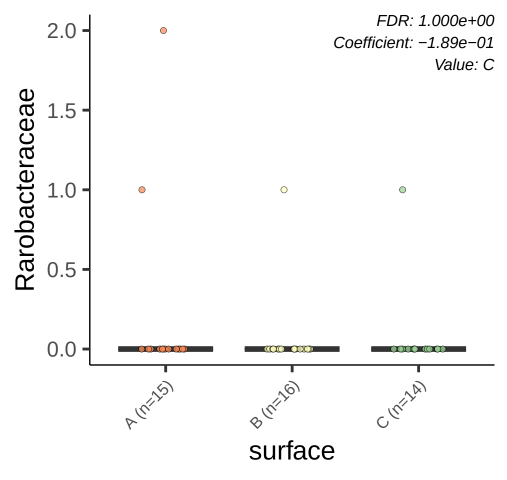
FDR: 1.000e+00
Coefficient: −1.89e−01
Value: C
Rarobacteraceae
2.0
1.5
1.0
0.5
0.0
A (n=15)
B (n=16)
C (n=14)
surface

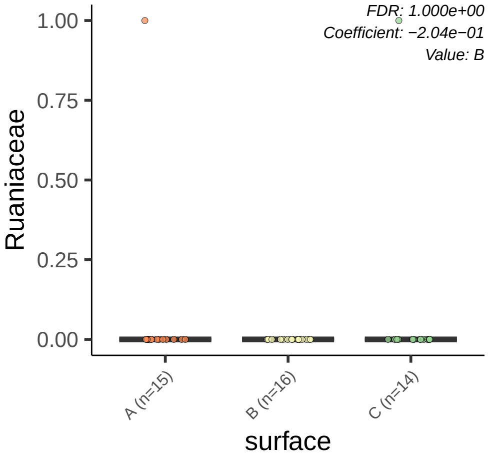

FDR: 1.000e+00
Coefficient: −2.04e−01
Value: B
Ruaniaceae
1.00
0.75
0.50
0.25
0.00
A (n=15)
B (n=16)
C (n=14)
surface

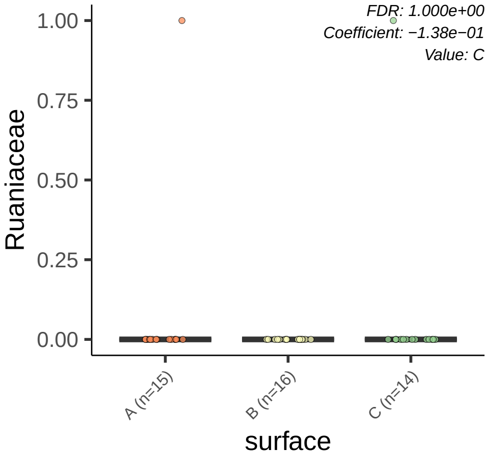

FDR: 1.000e+00
Coefficient: −1.38e−01
Value: C
Ruaniaceae
1.00
0.75
0.50
0.25
0.00
A (n=15)
B (n=16)
C (n=14)
surface

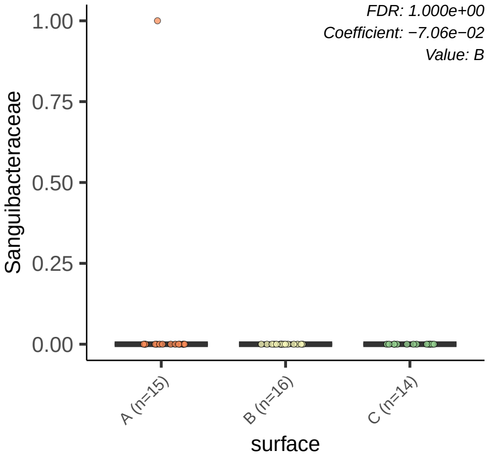
FDR: 1.000e+00
Coefficient: −7.06e−02
Value: B
Sanguibacteraceae
1.00
0.75
0.50
0.25
0.00
A (n=15)
B (n=16)
C (n=14)
surface

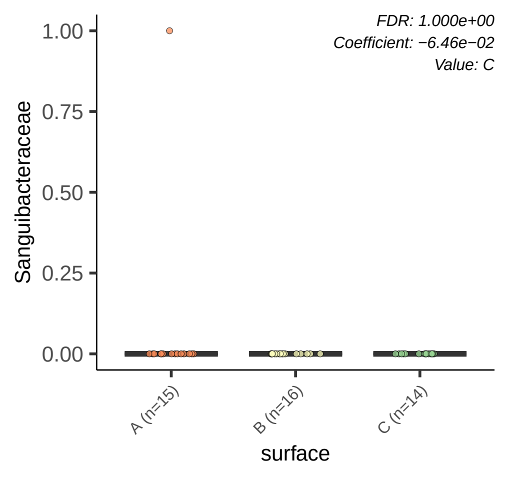
FDR: 1.000e+00
Coefficient: −6.46e−02
Value: C
Sanguibacteraceae
1.00
0.75
0.50
0.25
0.00
A (n=15)
B (n=16)
C (n=14)
surface

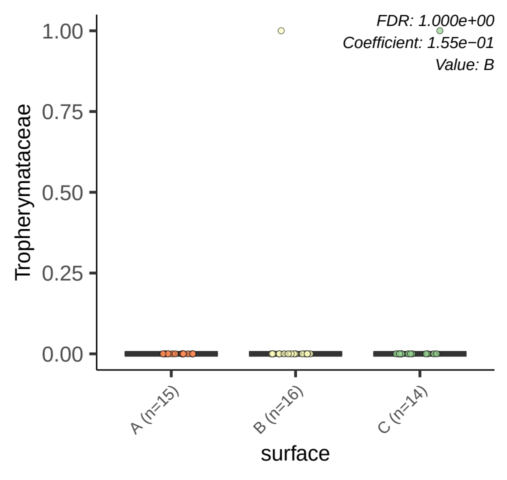
FDR: 1.000e+00
Coefficient: 1.55e−01
Value: B
Tropherymataceae
1.00
0.75
0.50
0.25
0.00
A (n=15)
B (n=16)
C (n=14)
surface

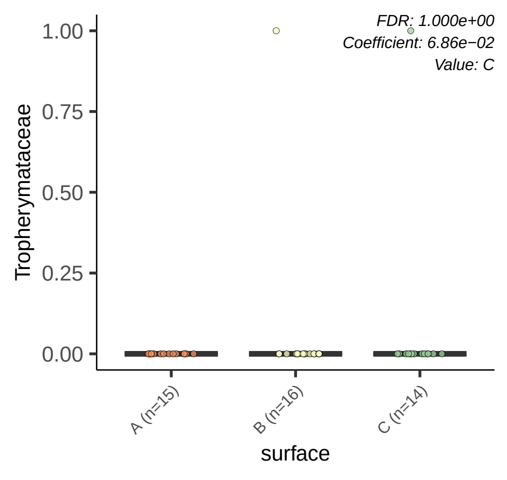
FDR: 1.000e+00
Coefficient: 6.86e−02
Value: C
Tropherymataceae
1.00
0.75
0.50
0.25
0.00
A (n=15)
B (n=16)
C (n=14)
surface

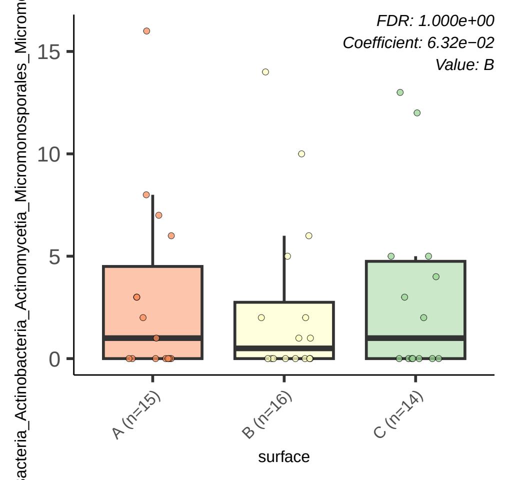

FDR: 1.000e+00
Coefficient: 6.32e−02
Value: B
0
5
10
15
A (n=15)
B (n=16)
C (n=14)
surface
acteria_Actinobacteria_Actinomycetia_Micromonosporales_Microm

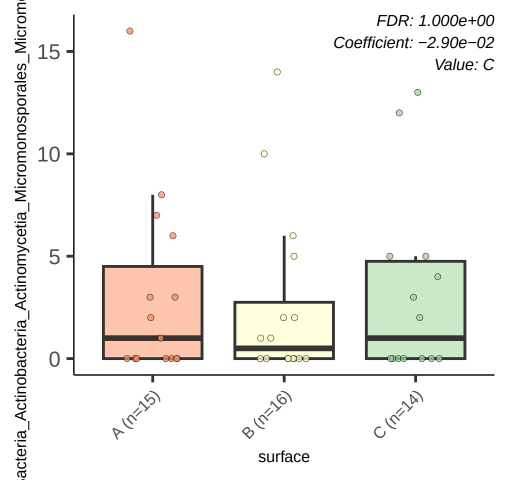
FDR: 1.000e+00
Coefficient: −2.90e−02
Value: C
0
5
10
15
A (n=15)
B (n=16)
C (n=14)
surface
acteria_Actinobacteria_Actinomycetia_Micromonosporales_Microm

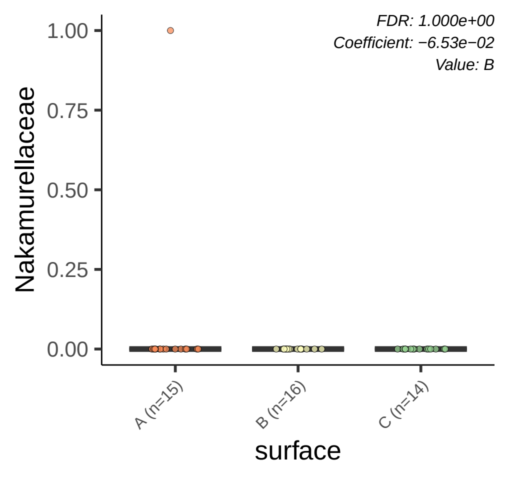
FDR: 1.000e+00
Coefficient: −6.53e−02
Value: B
Nakamurellaceae
1.00
0.75
0.50
0.25
0.00
A (n=15)
B (n=16)
C (n=14)
surface

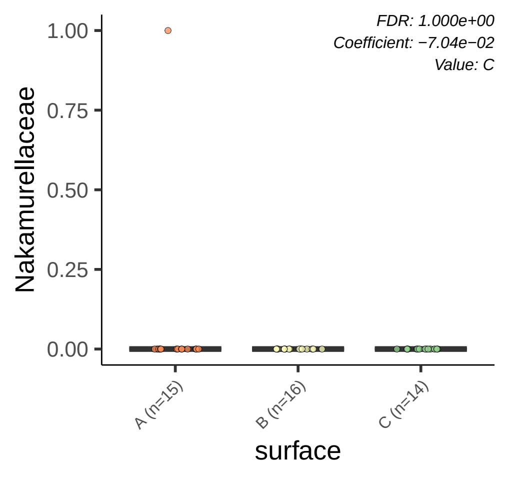
FDR: 1.000e+00
Coefficient: −7.04e−02
Value: C
Nakamurellaceae
1.00
0.75
0.50
0.25
0.00
A (n=15)
B (n=16)
C (n=14)
surface

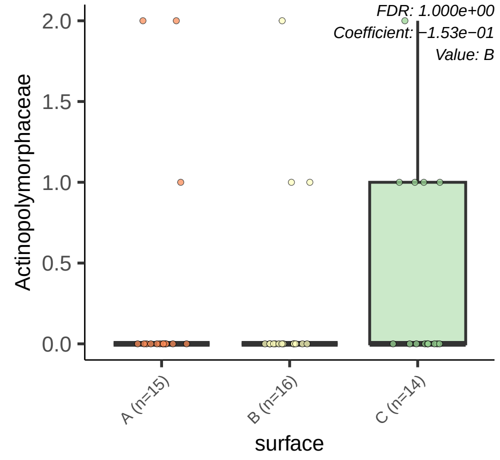
FDR: 1.000e+00
Coefficient: −1.53e−01
Value: B
Actinopolymorphaceae
2.0
1.5
1.0
0.5
0.0
A (n=15)
B (n=16)
C (n=14)
surface

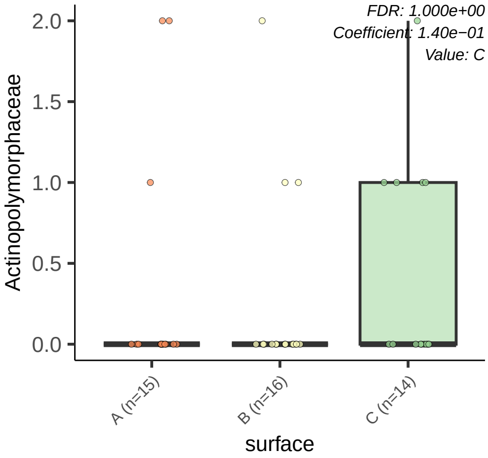

FDR: 1.000e+00
Coefficient: 1.40e−01
Value: C
Actinopolymorphaceae
2.0
1.5
1.0
0.5
0.0
A (n=15)
B (n=16)
C (n=14)
surface

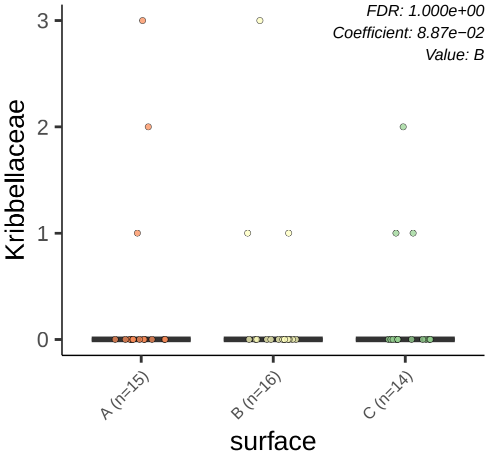

FDR: 1.000e+00
Coefficient: 8.87e−02
Value: B
Kribbellaceae
3
2
1
0
A (n=15)
B (n=16)
C (n=14)
surface

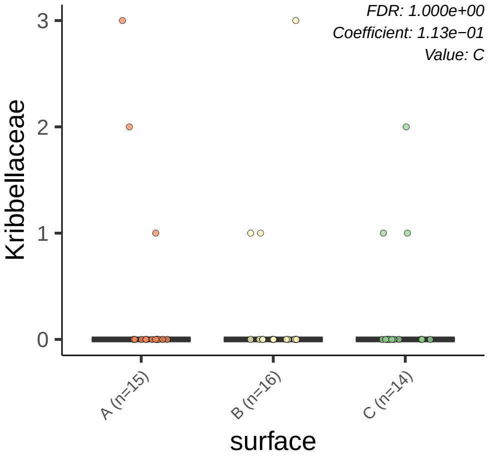
FDR: 1.000e+00
Coefficient: 1.13e−01
Value: C
Kribbellaceae
3
2
1
0
A (n=15)
B (n=16)
C (n=14)
surface

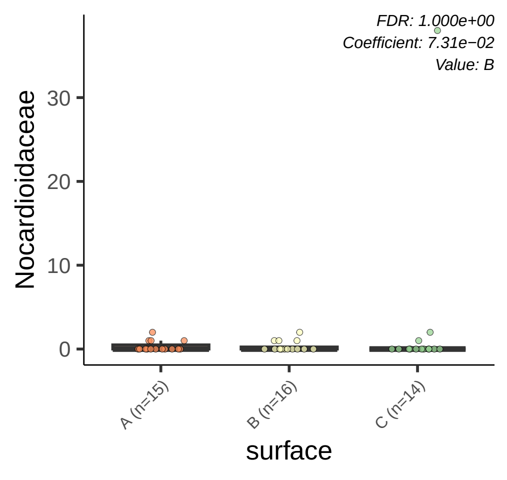

FDR: 1.000e+00
Coefficient: 7.31e−02
Value: B
Nocardioidaceae
30
20
10
0
A (n=15)
B (n=16)
C (n=14)
surface

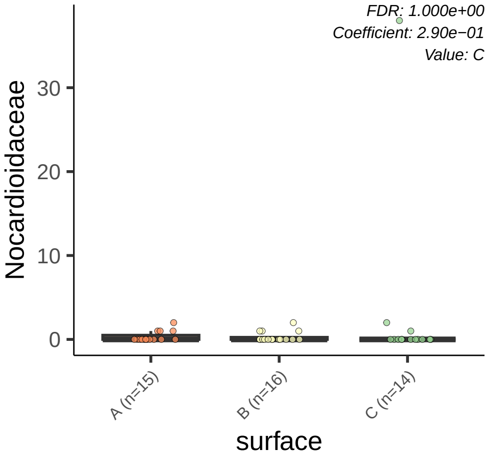
FDR: 1.000e+00
Coefficient: 2.90e−01
Value: C
Nocardioidaceae
30
20
10
0
A (n=15)
B (n=16)
C (n=14)
surface

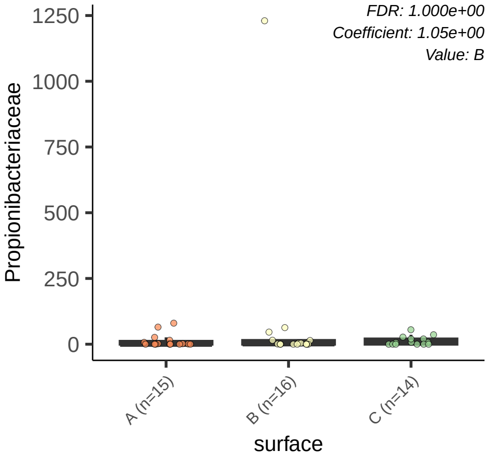
FDR: 1.000e+00
Coefficient: 1.05e+00
Value: B
Propionibacteriaceae
1250
1000
750
500
250
0
A (n=15)
B (n=16)
C (n=14)
surface

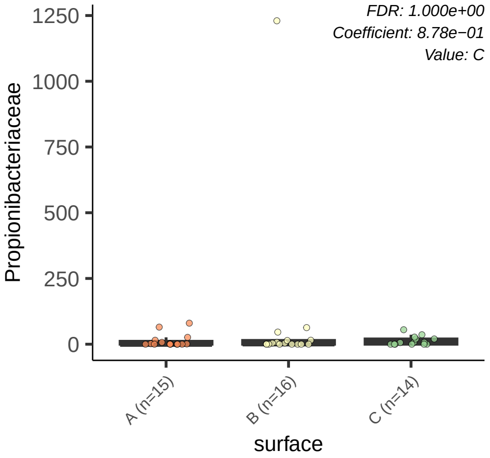
FDR: 1.000e+00
Coefficient: 8.78e−01
Value: C
Propionibacteriaceae
1250
1000
750
500
250
0
A (n=15)
B (n=16)
C (n=14)
surface

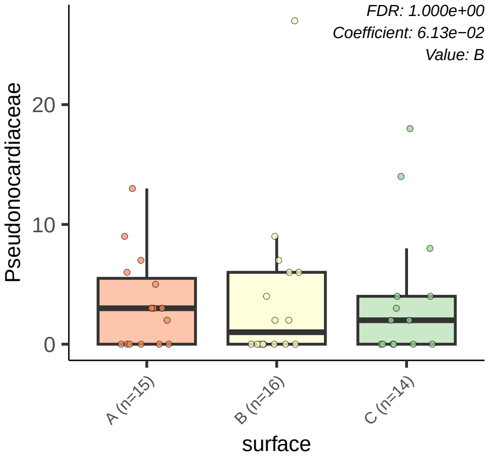

FDR: 1.000e+00
Coefficient: 6.13e−02
Value: B
Pseudonocardiaceae
0
10
20
A (n=15)
B (n=16)
C (n=14)
surface

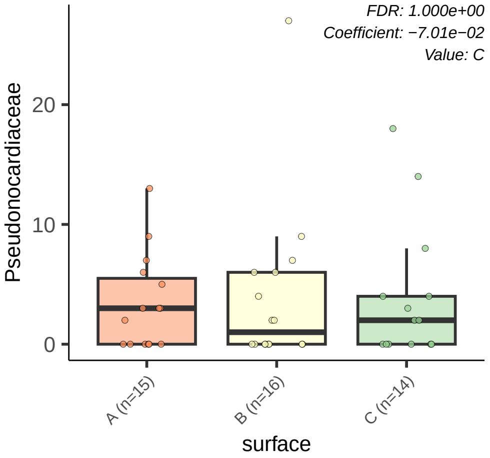

FDR: 1.000e+00
Coefficient: −7.01e−02
Value: C
Pseudonocardiaceae
20
10
0
A (n=15)
B (n=16)
C (n=14)
surface

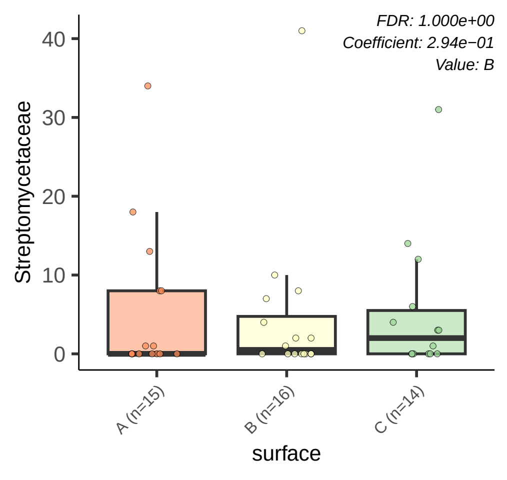

FDR: 1.000e+00
Coefficient: 2.94e−01
Value: B
Streptomycetaceae
40
30
20
10
0
A (n=15)
B (n=16)
C (n=14)
surface

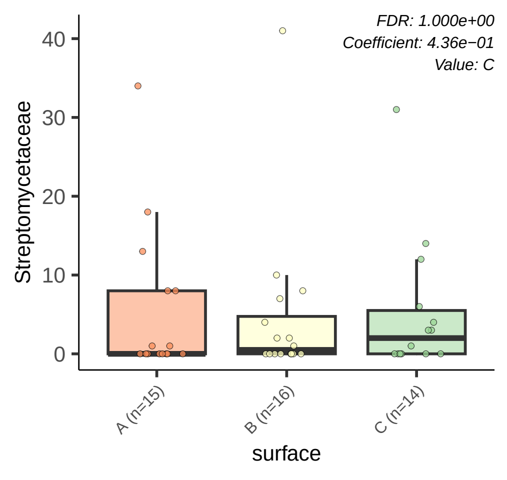

FDR: 1.000e+00
Coefficient: 4.36e−01
Value: C
Streptomycetaceae
40
30
20
10
0
A (n=15)
B (n=16)
C (n=14)
surface

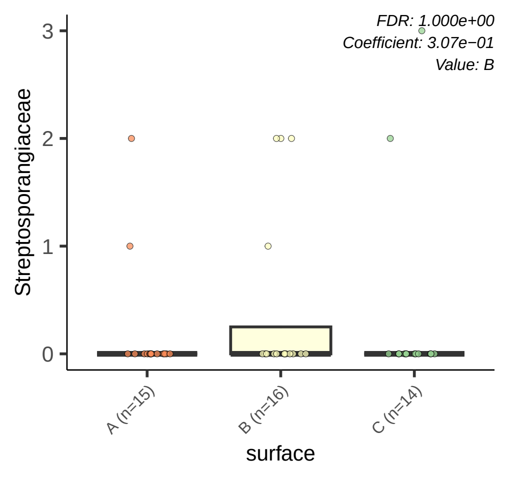
FDR: 1.000e+00
Coefficient: 3.07e−01
Value: B
Streptosporangiaceae
0
1
2
3
A (n=15)
B (n=16)
C (n=14)
surface

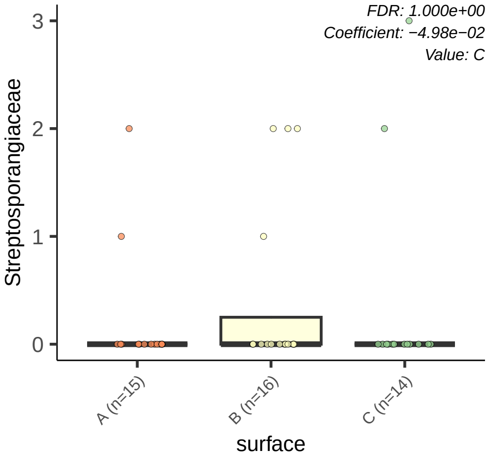
FDR: 1.000e+00
Coefficient: −4.98e−02
Value: C
Streptosporangiaceae
0
1
2
3
A (n=15)
B (n=16)
C (n=14)
surface

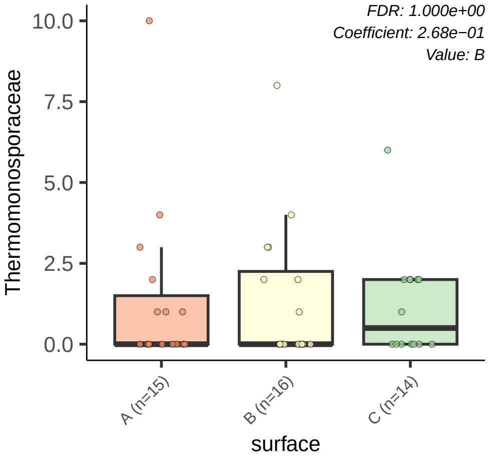

FDR: 1.000e+00
Coefficient: 2.68e−01
Value: B
Thermomonosporaceae
10.0
7.5
5.0
2.5
0.0
A (n=15)
B (n=16)
C (n=14)
surface

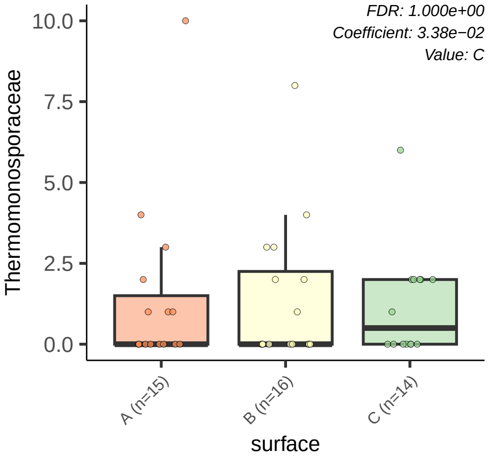
FDR: 1.000e+00
Coefficient: 3.38e−02
Value: C
Thermomonosporaceae
10.0
7.5
5.0
2.5
0.0
A (n=15)
B (n=16)
C (n=14)
surface

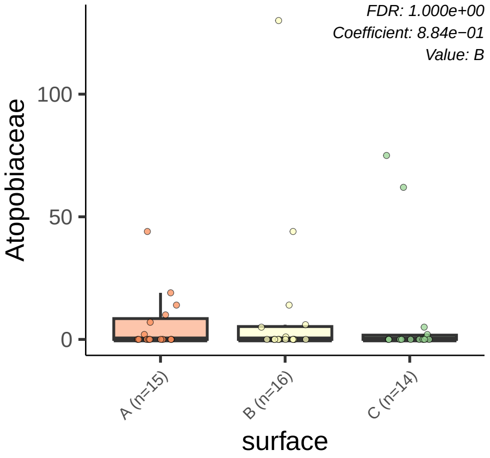
FDR: 1.000e+00
Coefficient: 8.84e−01
Value: B
Atopobiaceae
100
50
0
A (n=15)
B (n=16)
C (n=14)
surface

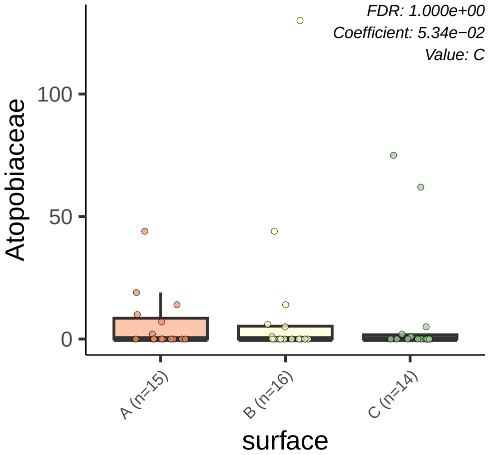

FDR: 1.000e+00
Coefficient: 5.34e−02
Value: C
Atopobiaceae
100
50
0
A (n=15)
B (n=16)
C (n=14)
surface

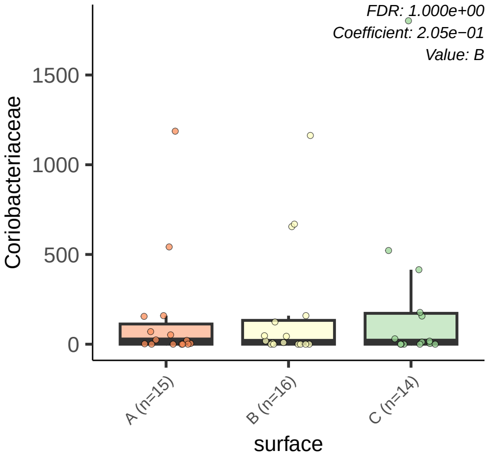
FDR: 1.000e+00
Coefficient: 2.05e−01
Value: B
Coriobacteriaceae
1500
1000
500
0
A (n=15)
B (n=16)
C (n=14)
surface

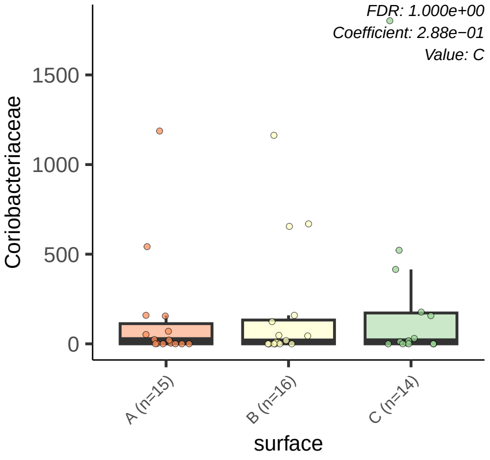
FDR: 1.000e+00
Coefficient: 2.88e−01
Value: C
Coriobacteriaceae
1500
1000
500
0
A (n=15)
B (n=16)
C (n=14)
surface

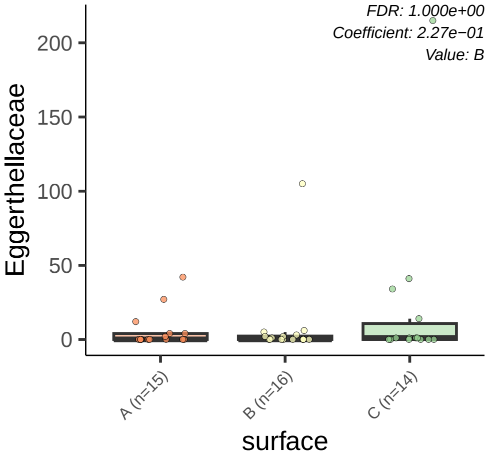
FDR: 1.000e+00
Coefficient: 2.27e−01
Value: B
Eggerthellaceae
200
150
100
50
0
A (n=15)
B (n=16)
C (n=14)
surface

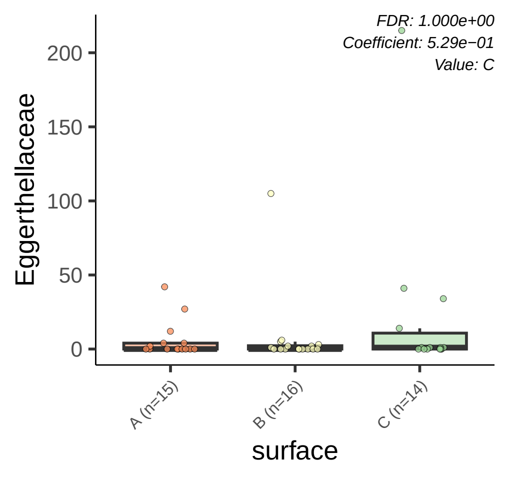

FDR: 1.000e+00
Coefficient: 5.29e−01
Value: C
Eggerthellaceae
200
150
100
50
0
A (n=15)
B (n=16)
C (n=14)
surface

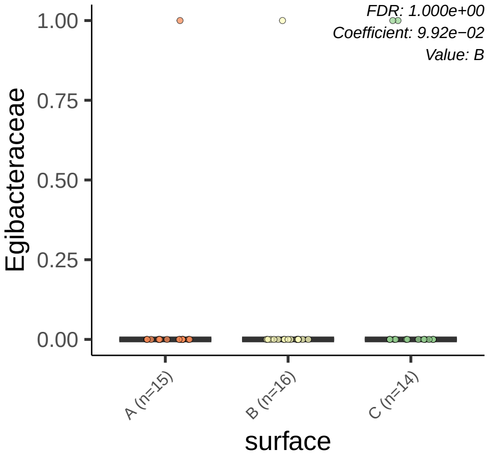
FDR: 1.000e+00
Coefficient: 9.92e−02
Value: B
Egibacteraceae
1.00
0.75
0.50
0.25
0.00
A (n=15)
B (n=16)
C (n=14)
surface

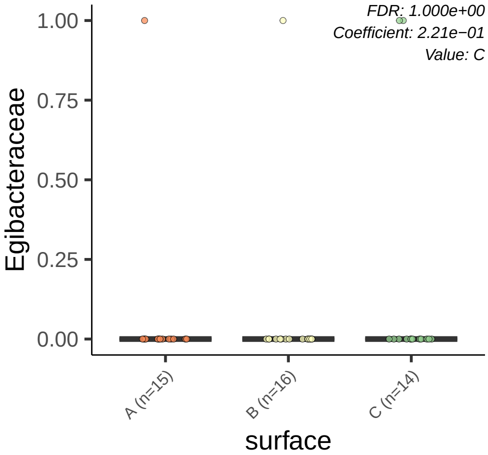
FDR: 1.000e+00
Coefficient: 2.21e−01
Value: C
Egibacteraceae
1.00
0.75
0.50
0.25
0.00
A (n=15)
B (n=16)
C (n=14)
surface

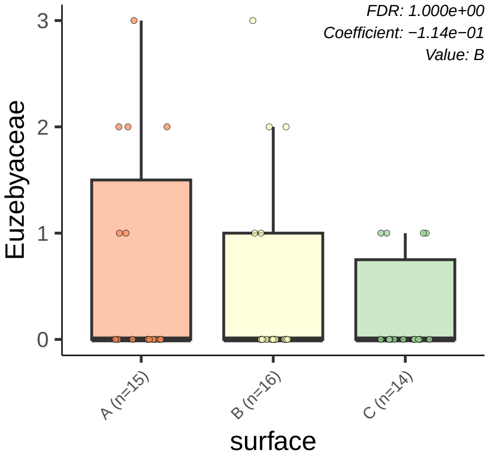
FDR: 1.000e+00
Coefficient: −1.14e−01
Value: B
Euzebyaceae
3
2
1
0
A (n=15)
B (n=16)
C (n=14)
surface

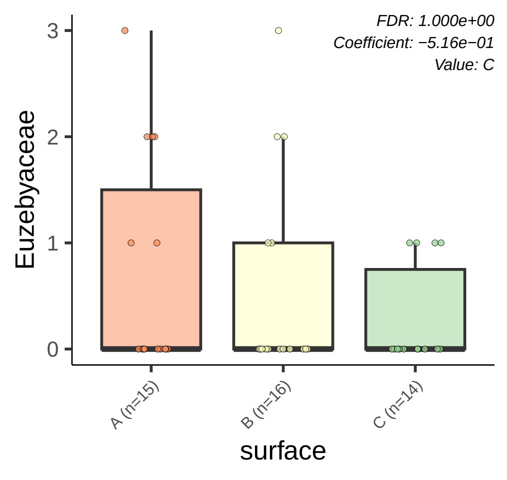
FDR: 1.000e+00
Coefficient: −5.16e−01
Value: C
Euzebyaceae
0
1
2
3
A (n=15)
B (n=16)
C (n=14)
surface

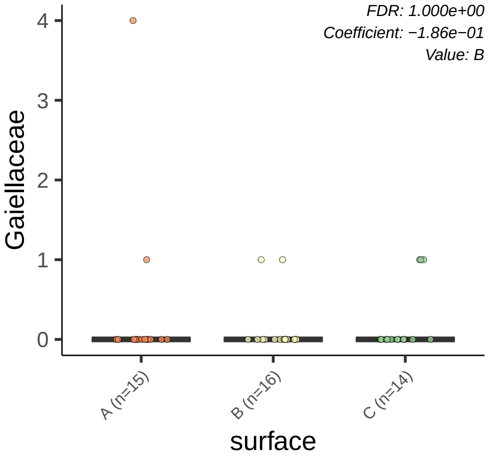

FDR: 1.000e+00
Coefficient: −1.86e−01
Value: B
Gaiellaceae
0
1
2
3
4
A (n=15)
B (n=16)
C (n=14)
surface

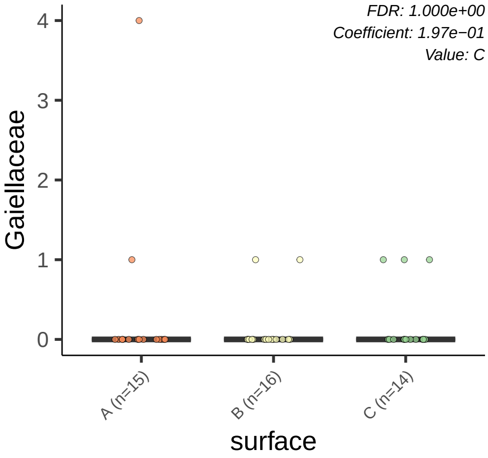

FDR: 1.000e+00
Coefficient: 1.97e−01
Value: C
Gaiellaceae
4
3
2
1
0
A (n=15)
B (n=16)
C (n=14)
surface

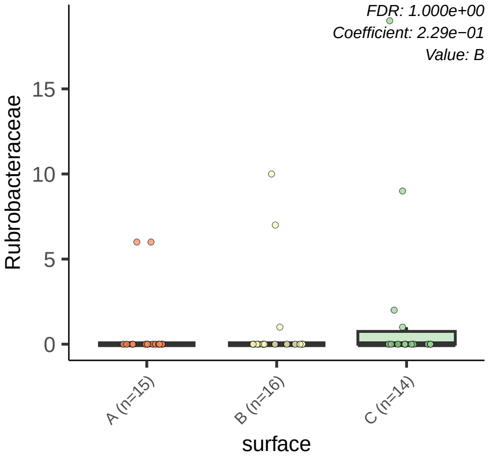
FDR: 1.000e+00
Coefficient: 2.29e−01
Value: B
Rubrobacteraceae
0
5
10
15
A (n=15)
B (n=16)
C (n=14)
surface

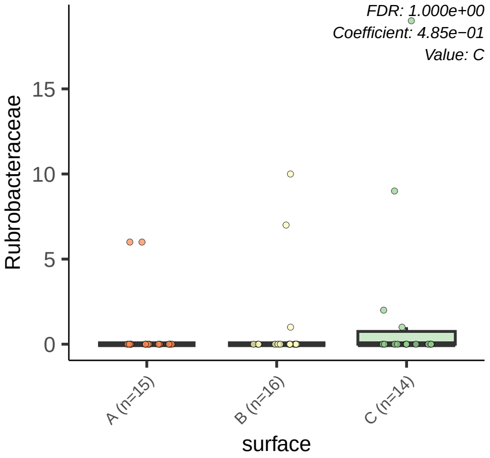

FDR: 1.000e+00
Coefficient: 4.85e−01
Value: C
Rubrobacteraceae
15
10
5
0
A (n=15)
B (n=16)
C (n=14)
surface

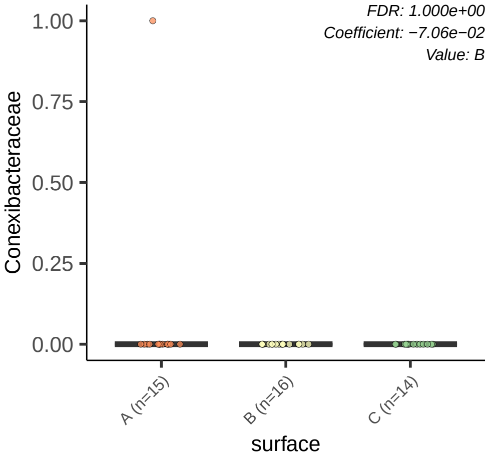

FDR: 1.000e+00
Coefficient: −7.06e−02
Value: B
Conexibacteraceae
1.00
0.75
0.50
0.25
0.00
A (n=15)
B (n=16)
C (n=14)
surface

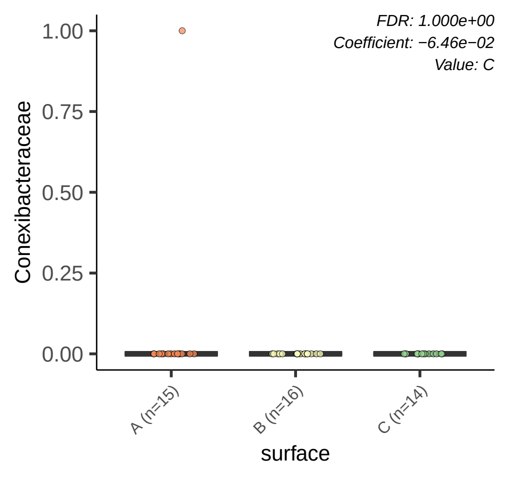

FDR: 1.000e+00
Coefficient: −6.46e−02
Value: C
Conexibacteraceae
1.00
0.75
0.50
0.25
0.00
A (n=15)
B (n=16)
C (n=14)
surface

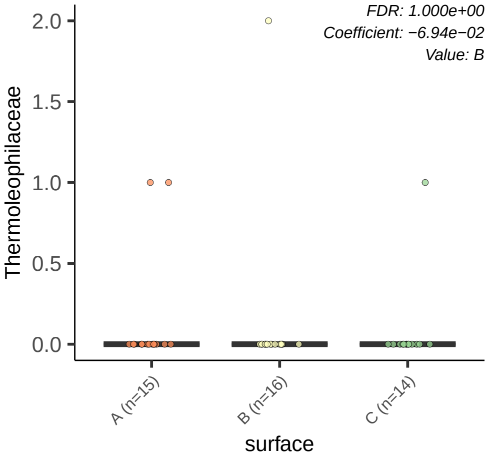
FDR: 1.000e+00
Coefficient: −6.94e−02
Value: B
Thermoleophilaceae
2.0
1.5
1.0
0.5
0.0
A (n=15)
B (n=16)
C (n=14)
surface

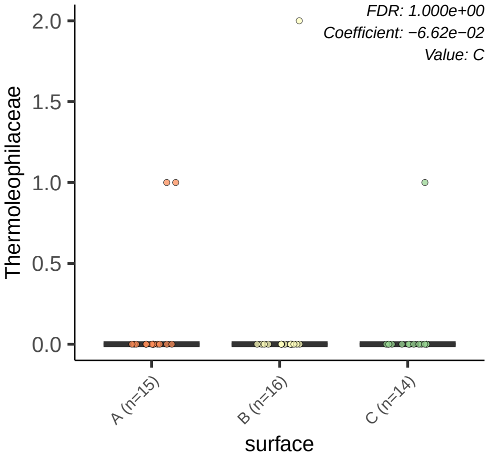
FDR: 1.000e+00
Coefficient: −6.62e−02
Value: C
Thermoleophilaceae
2.0
1.5
1.0
0.5
0.0
A (n=15)
B (n=16)
C (n=14)
surface

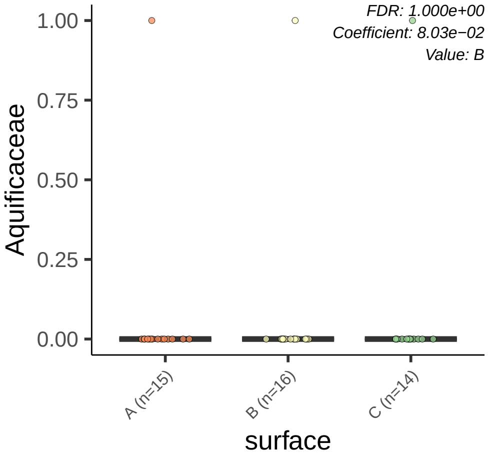
FDR: 1.000e+00
Coefficient: 8.03e−02
Value: B
Aquificaceae
1.00
0.75
0.50
0.25
0.00
A (n=15)
B (n=16)
C (n=14)
surface

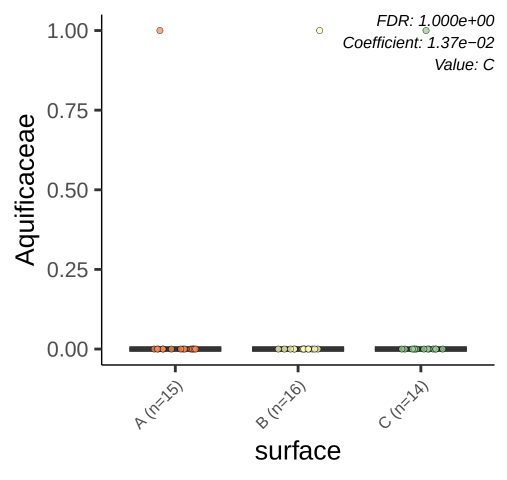

FDR: 1.000e+00
Coefficient: 1.37e−02
Value: C
Aquificaceae
1.00
0.75
0.50
0.25
0.00
A (n=15)
B (n=16)
C (n=14)
surface

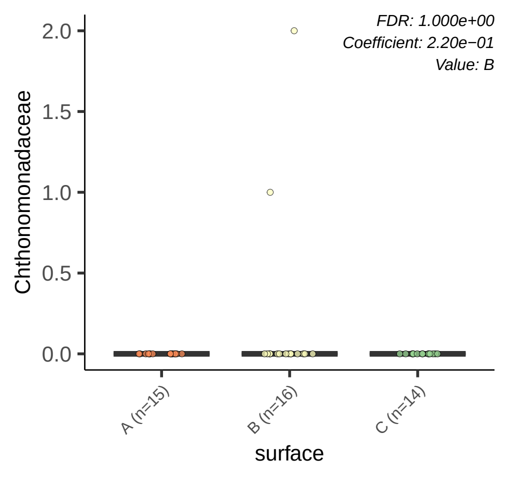

FDR: 1.000e+00
Coefficient: 2.20e−01
Value: B
Chthonomonadaceae
2.0
1.5
1.0
0.5
0.0
A (n=15)
B (n=16)
C (n=14)
surface

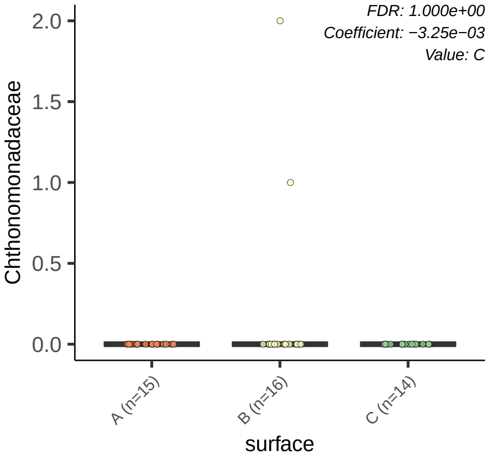
FDR: 1.000e+00
Coefficient: −3.25e−03
Value: C
Chthonomonadaceae
2.0
1.5
1.0
0.5
0.0
A (n=15)
B (n=16)
C (n=14)
surface

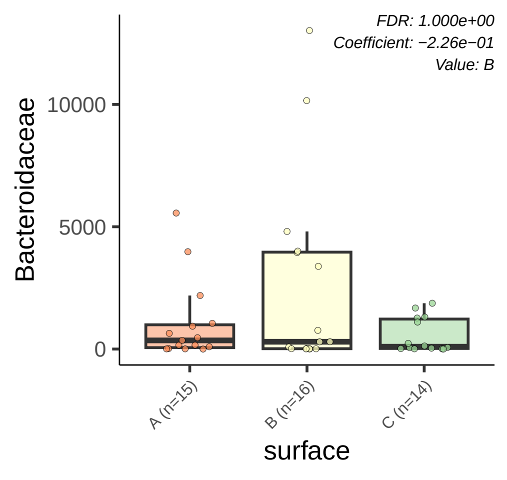

FDR: 1.000e+00
Coefficient: −2.26e−01
Value: B
Bacteroidaceae
10000
5000
0
A (n=15)
B (n=16)
C (n=14)
surface

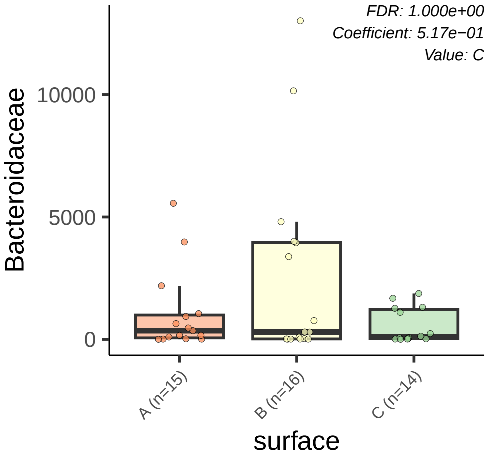
FDR: 1.000e+00
Coefficient: 5.17e−01
Value: C
Bacteroidaceae
10000
5000
0
A (n=15)
B (n=16)
C (n=14)
surface

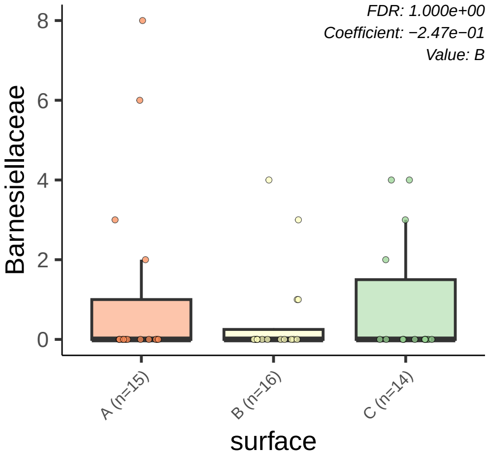
FDR: 1.000e+00
Coefficient: −2.47e−01
Value: B
Barnesiellaceae
0
2
4
6
8
A (n=15)
B (n=16)
C (n=14)
surface

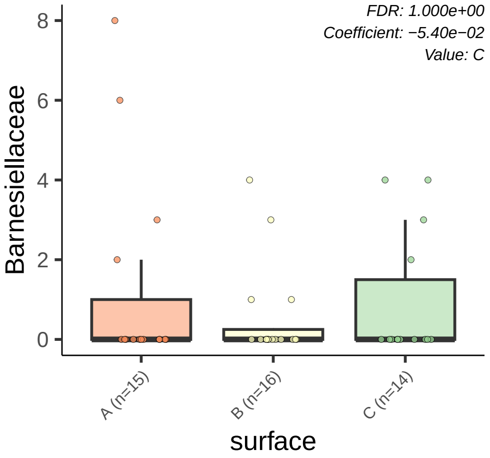
FDR: 1.000e+00
Coefficient: −5.40e−02
Value: C
Barnesiellaceae
0
2
4
6
8
A (n=15)
B (n=16)
C (n=14)
surface

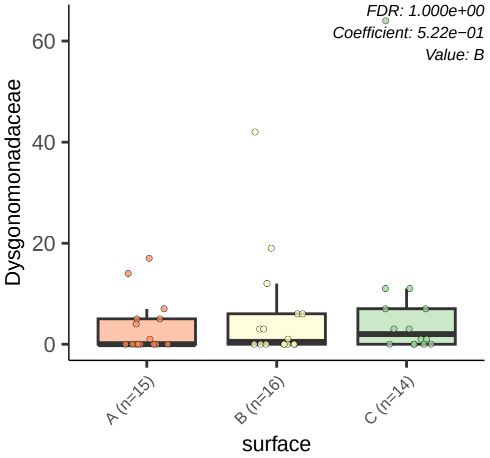

FDR: 1.000e+00
Coefficient: 5.22e−01
Value: B
Dysgonomonadaceae
0
20
40
60
A (n=15)
B (n=16)
C (n=14)
surface

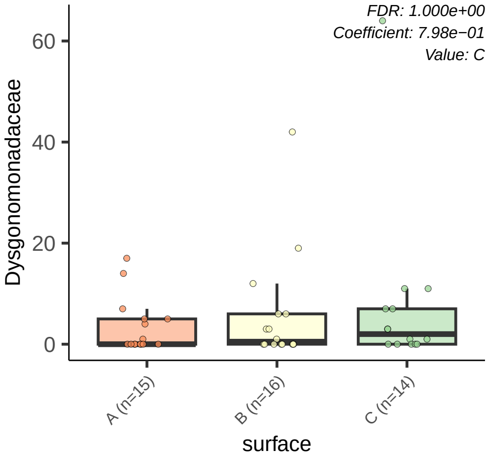
FDR: 1.000e+00
Coefficient: 7.98e−01
Value: C
Dysgonomonadaceae
0
20
40
60
A (n=15)
B (n=16)
C (n=14)
surface

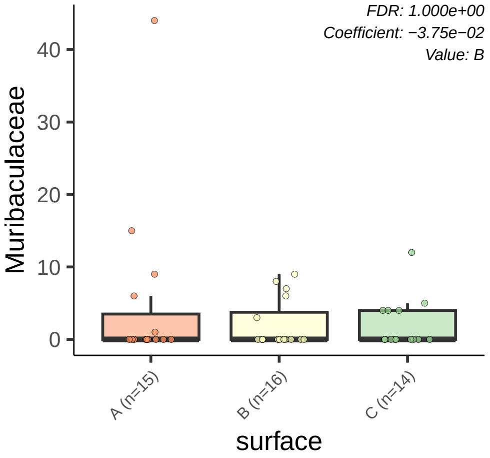
FDR: 1.000e+00
Coefficient: −3.75e−02
Value: B
Muribaculaceae
0
10
20
30
40
A (n=15)
B (n=16)
C (n=14)
surface

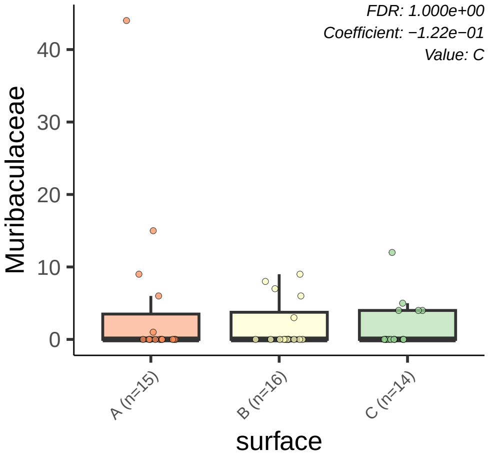
FDR: 1.000e+00
Coefficient: −1.22e−01
Value: C
Muribaculaceae
40
30
20
10
0
A (n=15)
B (n=16)
C (n=14)
surface

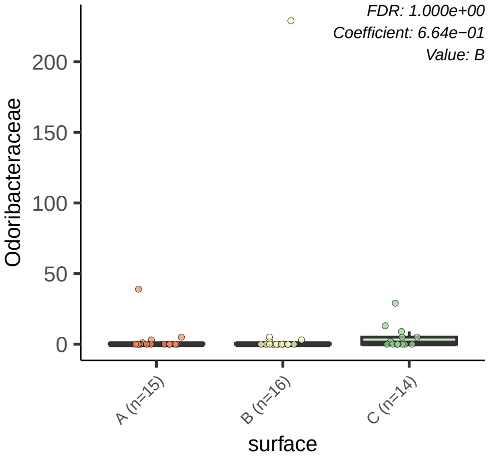
FDR: 1.000e+00
Coefficient: 6.64e−01
Value: B
Odoribacteraceae
0
50
100
150
200
A (n=15)
B (n=16)
C (n=14)
surface

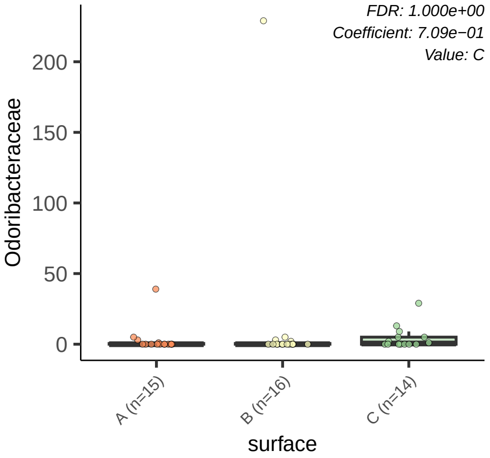
FDR: 1.000e+00
Coefficient: 7.09e−01
Value: C
Odoribacteraceae
200
150
100
50
0
A (n=15)
B (n=16)
C (n=14)
surface

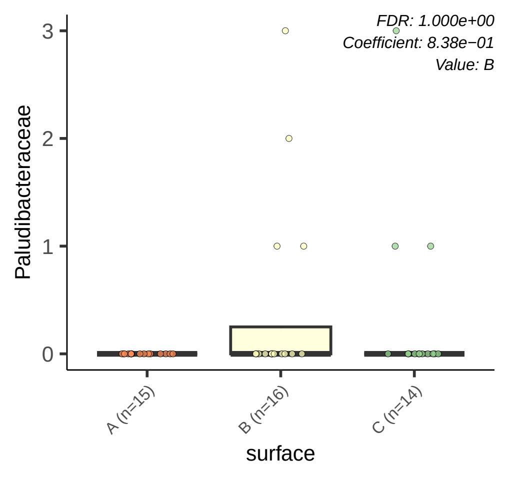
FDR: 1.000e+00
Coefficient: 8.38e−01
Value: B
Paludibacteraceae
3
2
1
0
A (n=15)
B (n=16)
C (n=14)
surface

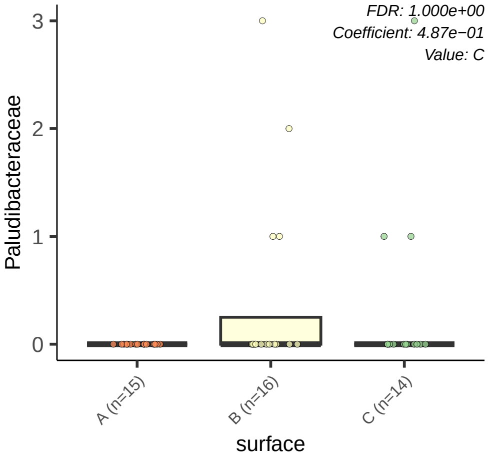

FDR: 1.000e+00
Coefficient: 4.87e−01
Value: C
Paludibacteraceae
3
2
1
0
A (n=15)
B (n=16)
C (n=14)
surface

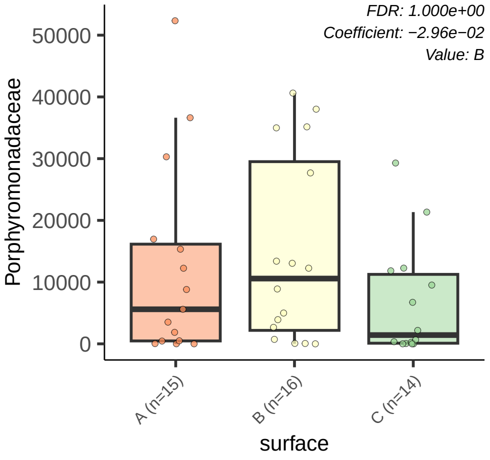

FDR: 1.000e+00
Coefficient: −2.96e−02
Value: B
Porphyromonadaceae
50000
40000
30000
20000
10000
0
A (n=15)
B (n=16)
C (n=14)
surface

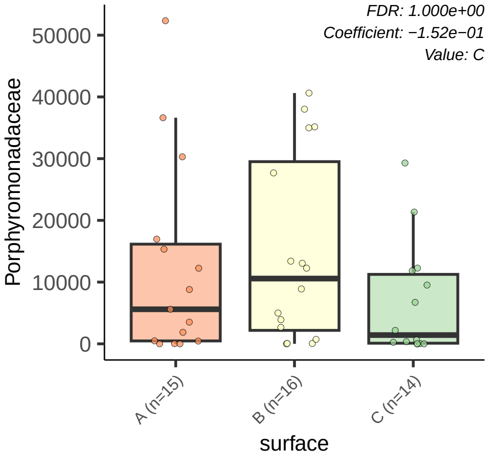

FDR: 1.000e+00
Coefficient: −1.52e−01
Value: C
Porphyromonadaceae
50000
40000
30000
20000
10000
0
A (n=15)
B (n=16)
C (n=14)
surface

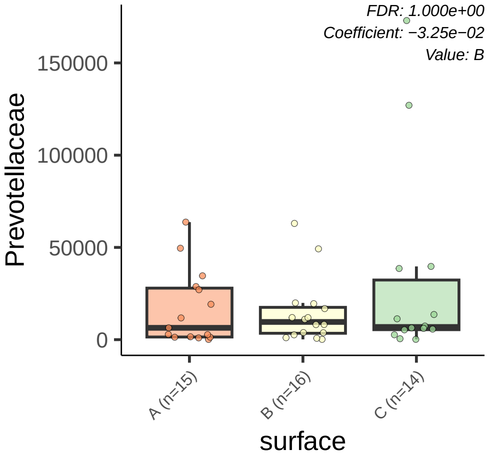

FDR: 1.000e+00
Coefficient: −3.25e−02
Value: B
Prevotellaceae
150000
100000
50000
0
A (n=15)
B (n=16)
C (n=14)
surface

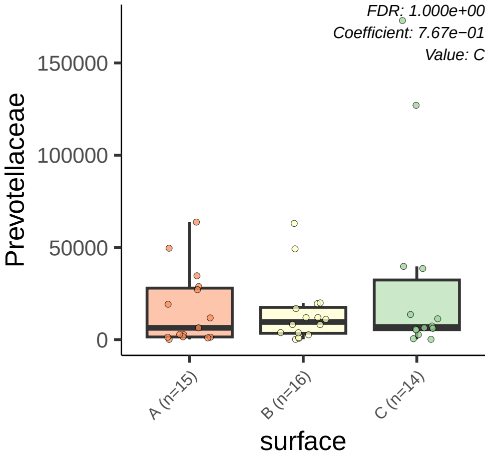

FDR: 1.000e+00
Coefficient: 7.67e−01
Value: C
Prevotellaceae
150000
100000
50000
0
A (n=15)
B (n=16)
C (n=14)
surface

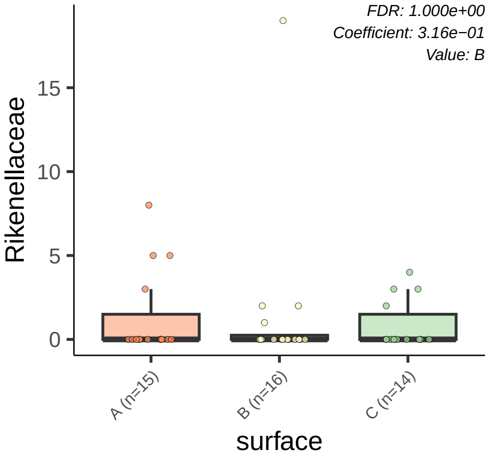

FDR: 1.000e+00
Coefficient: 3.16e−01
Value: B
Rikenellaceae
0
5
10
15
A (n=15)
B (n=16)
C (n=14)
surface

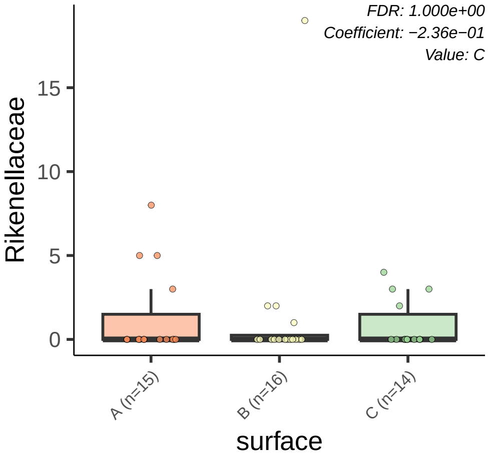

FDR: 1.000e+00
Coefficient: −2.36e−01
Value: C
Rikenellaceae
0
5
10
15
A (n=15)
B (n=16)
C (n=14)
surface

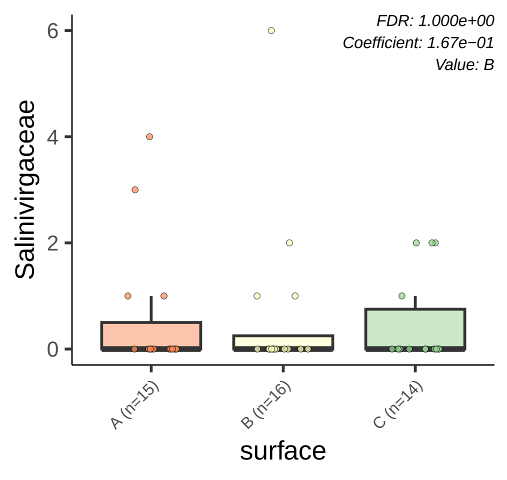
FDR: 1.000e+00
Coefficient: 1.67e−01
Value: B
Salinivirgaceae
0
2
4
6
A (n=15)
B (n=16)
C (n=14)
surface

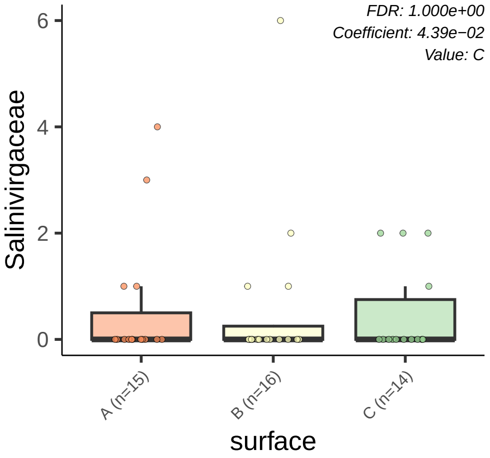
FDR: 1.000e+00
Coefficient: 4.39e−02
Value: C
Salinivirgaceae
0
2
4
6
A (n=15)
B (n=16)
C (n=14)
surface

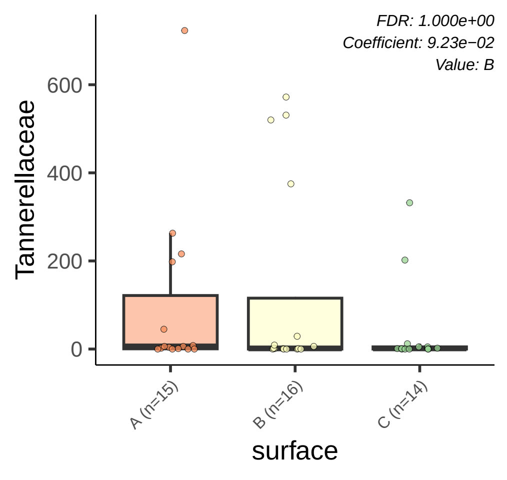
FDR: 1.000e+00
Coefficient: 9.23e−02
Value: B
Tannerellaceae
0
200
400
600
A (n=15)
B (n=16)
C (n=14)
surface

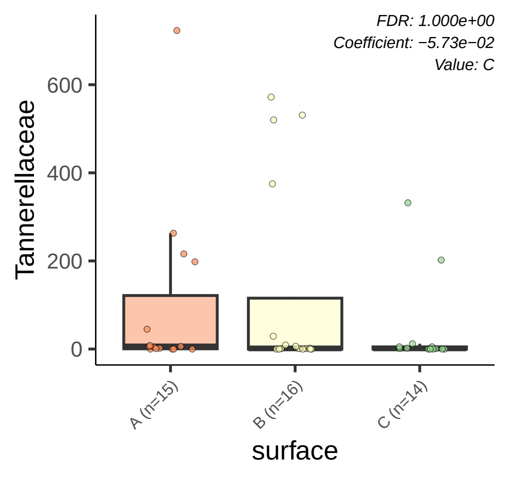

FDR: 1.000e+00
Coefficient: −5.73e−02
Value: C
Tannerellaceae
600
400
200
0
A (n=15)
B (n=16)
C (n=14)
surface

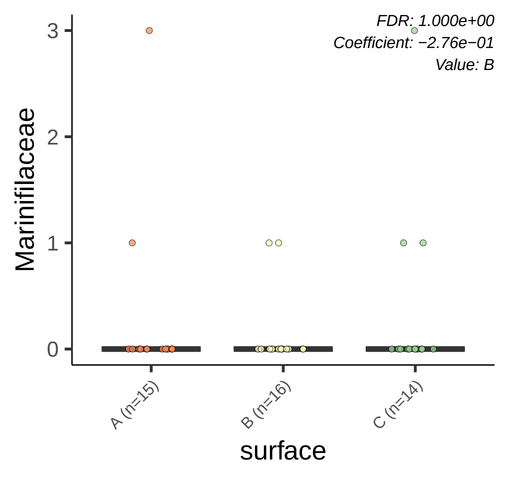
FDR: 1.000e+00
Coefficient: −2.76e−01
Value: B
Marinifilaceae
3
2
1
0
A (n=15)
B (n=16)
C (n=14)
surface

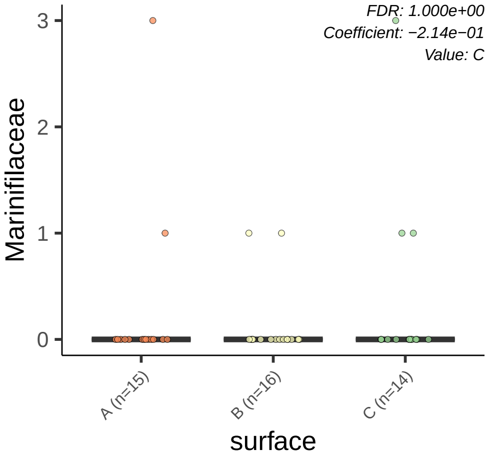
FDR: 1.000e+00
Coefficient: −2.14e−01
Value: C
Marinifilaceae
3
2
1
0
A (n=15)
B (n=16)
C (n=14)
surface

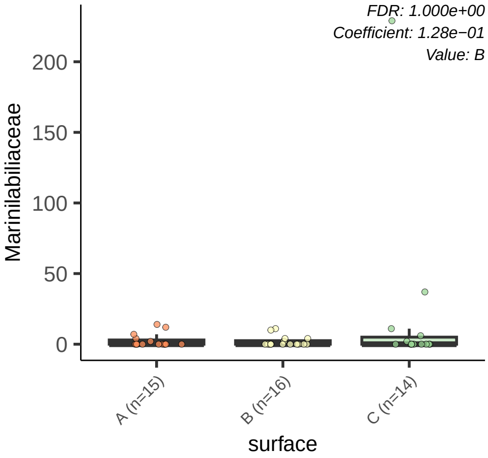
FDR: 1.000e+00
Coefficient: 1.28e−01
Value: B
Marinilabiliaceae
200
150
100
50
0
A (n=15)
B (n=16)
C (n=14)
surface

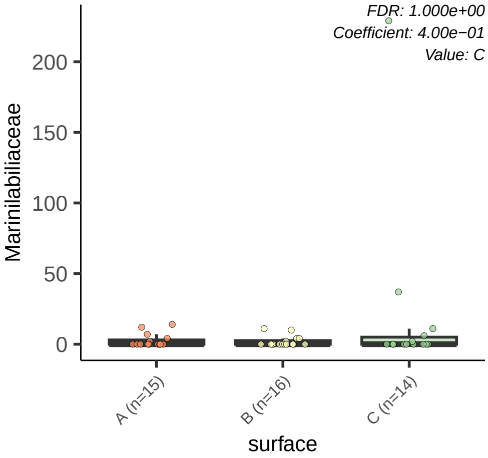
FDR: 1.000e+00
Coefficient: 4.00e−01
Value: C
Marinilabiliaceae
200
150
100
50
0
A (n=15)
B (n=16)
C (n=14)
surface

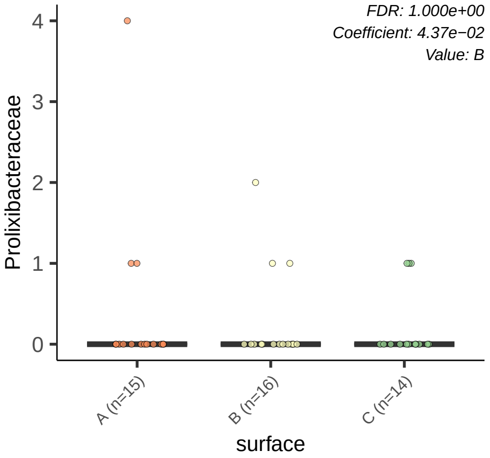
FDR: 1.000e+00
Coefficient: 4.37e−02
Value: B
Prolixibacteraceae
4
3
2
1
0
A (n=15)
B (n=16)
C (n=14)
surface

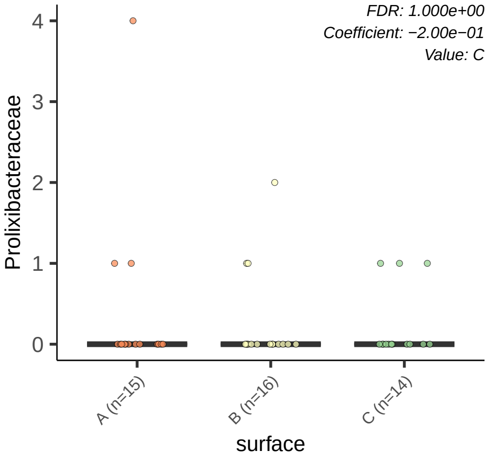
FDR: 1.000e+00
Coefficient: −2.00e−01
Value: C
Prolixibacteraceae
4
3
2
1
0
A (n=15)
B (n=16)
C (n=14)
surface

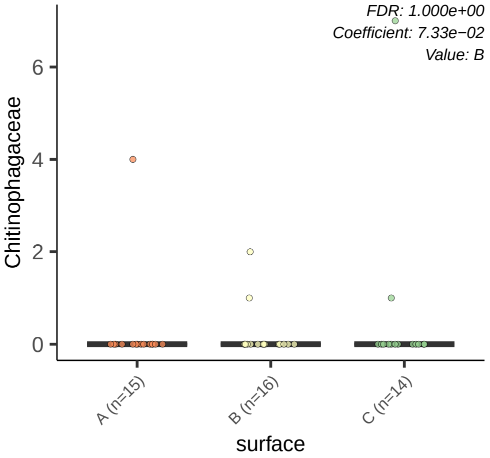

FDR: 1.000e+00
Coefficient: 7.33e−02
Value: B
Chitinophagaceae
0
2
4
6
A (n=15)
B (n=16)
C (n=14)
surface

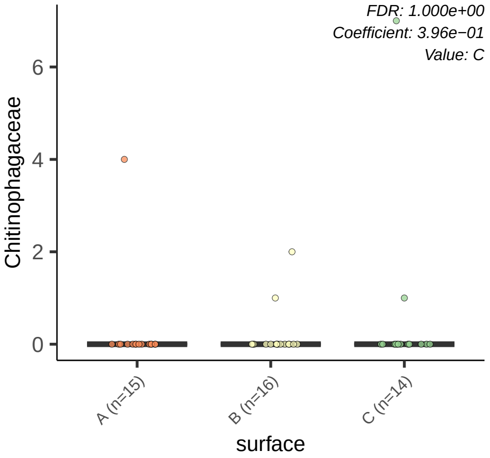

FDR: 1.000e+00
Coefficient: 3.96e−01
Value: C
Chitinophagaceae
0
2
4
6
A (n=15)
B (n=16)
C (n=14)
surface

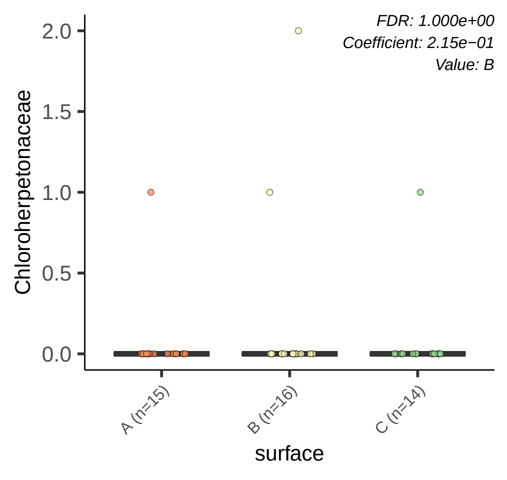
FDR: 1.000e+00
Coefficient: 2.15e−01
Value: B
Chloroherpetonaceae
2.0
1.5
1.0
0.5
0.0
A (n=15)
B (n=16)
C (n=14)
surface

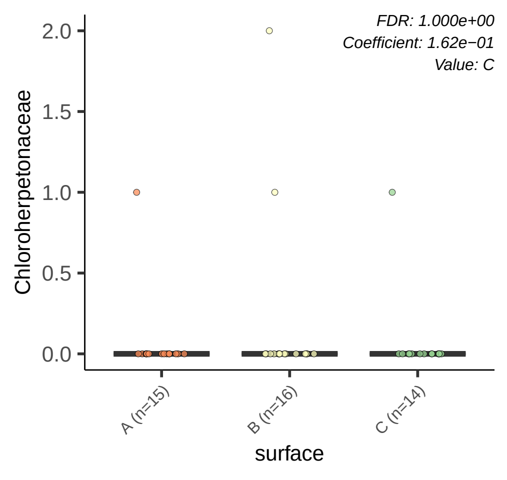

FDR: 1.000e+00
Coefficient: 1.62e−01
Value: C
Chloroherpetonaceae
2.0
1.5
1.0
0.5
0.0
A (n=15)
B (n=16)
C (n=14)
surface

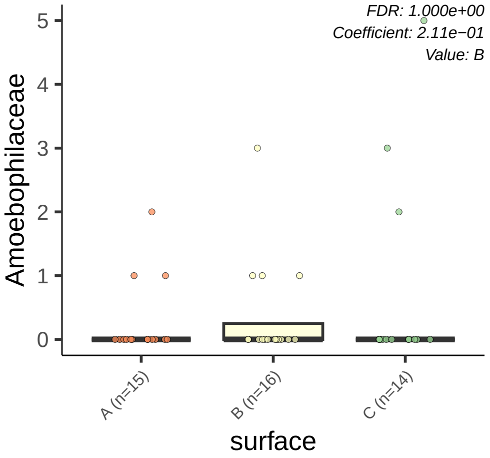

FDR: 1.000e+00
Coefficient: 2.11e−01
Value: B
Amoebophilaceae
0
1
2
3
4
5
A (n=15)
B (n=16)
C (n=14)
surface

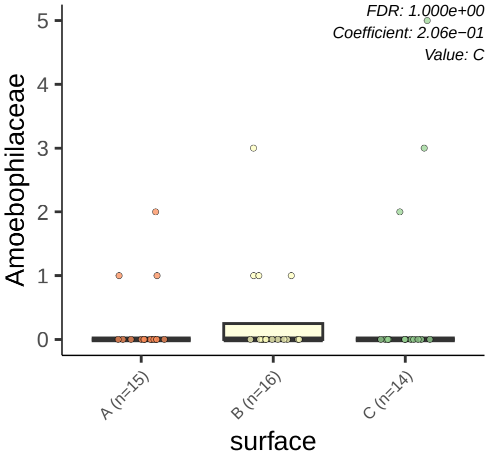
FDR: 1.000e+00
Coefficient: 2.06e−01
Value: C
Amoebophilaceae
0
1
2
3
4
5
A (n=15)
B (n=16)
C (n=14)
surface

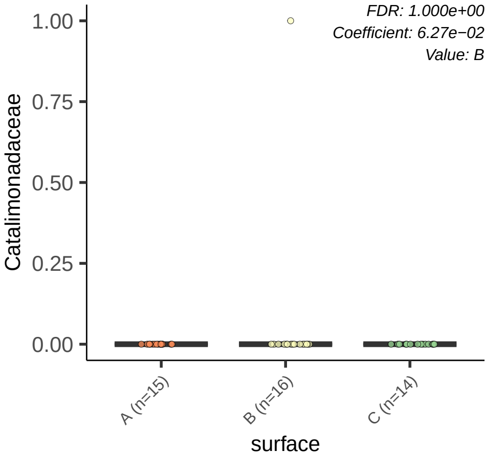
FDR: 1.000e+00
Coefficient: 6.27e−02
Value: B
Catalimonadaceae
1.00
0.75
0.50
0.25
0.00
A (n=15)
B (n=16)
C (n=14)
surface

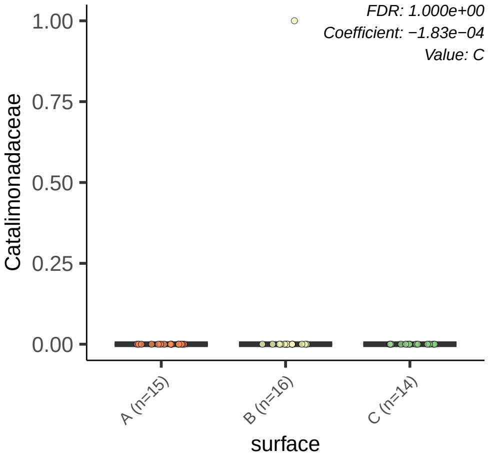
FDR: 1.000e+00
Coefficient: −1.83e−04
Value: C
Catalimonadaceae
1.00
0.75
0.50
0.25
0.00
A (n=15)
B (n=16)
C (n=14)
surface

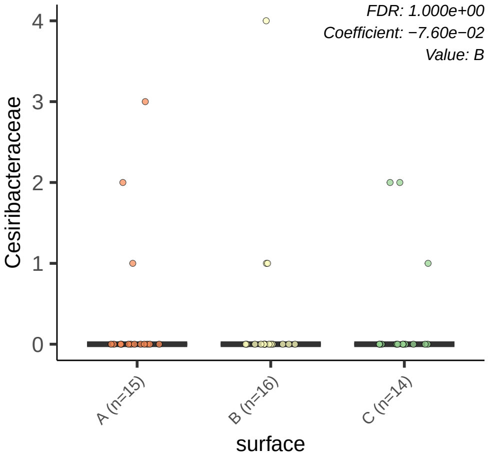
FDR: 1.000e+00
Coefficient: −7.60e−02
Value: B
Cesiribacteraceae
0
1
2
3
4
A (n=15)
B (n=16)
C (n=14)
surface

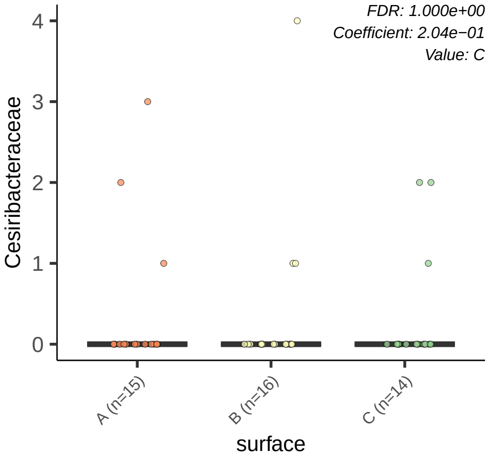

FDR: 1.000e+00
Coefficient: 2.04e−01
Value: C
Cesiribacteraceae
0
1
2
3
4
A (n=15)
B (n=16)
C (n=14)
surface

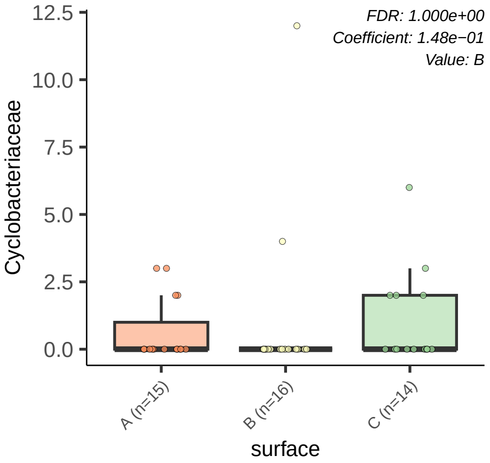

FDR: 1.000e+00
Coefficient: 1.48e−01
Value: B
Cyclobacteriaceae
12.5
10.0
7.5
5.0
2.5
0.0
A (n=15)
B (n=16)
C (n=14)
surface

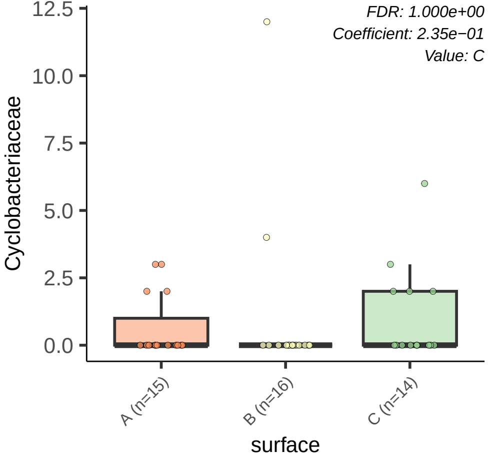

FDR: 1.000e+00
Coefficient: 2.35e−01
Value: C
12.5
10.0
7.5
5.0
2.5
0.0
Cyclobacteriaceae
A (n=15)
B (n=16)
C (n=14)
surface

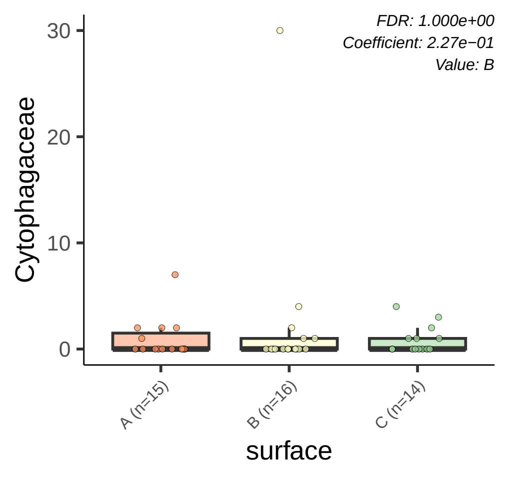
FDR: 1.000e+00
Coefficient: 2.27e−01
Value: B
Cytophagaceae
30
20
10
0
A (n=15)
B (n=16)
C (n=14)
surface

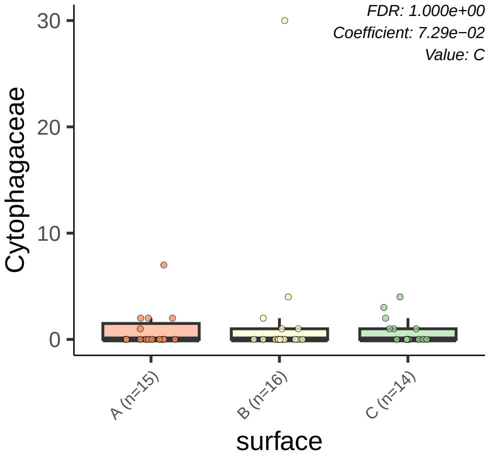
FDR: 1.000e+00
Coefficient: 7.29e−02
Value: C
Cytophagaceae
30
20
10
0
A (n=15)
B (n=16)
C (n=14)
surface

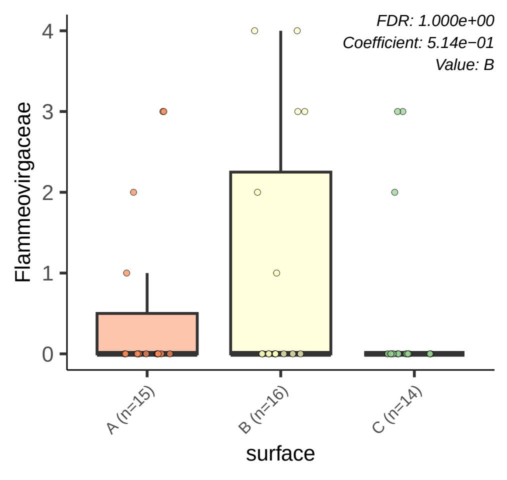

FDR: 1.000e+00
Coefficient: 5.14e−01
Value: B
Flammeovirgaceae
4
3
2
1
0
A (n=15)
B (n=16)
C (n=14)
surface

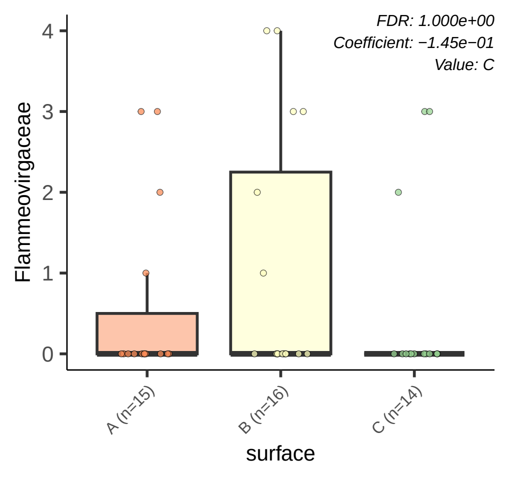
FDR: 1.000e+00
Coefficient: −1.45e−01
Value: C
Flammeovirgaceae
0
1
2
3
4
A (n=15)
B (n=16)
C (n=14)
surface

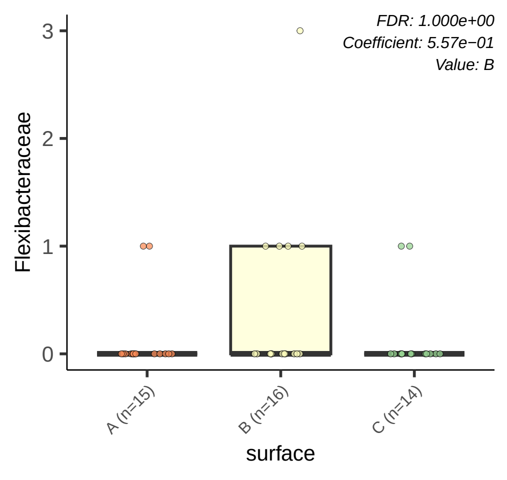
FDR: 1.000e+00
Coefficient: 5.57e−01
Value: B
Flexibacteraceae
3
2
1
0
A (n=15)
B (n=16)
C (n=14)
surface

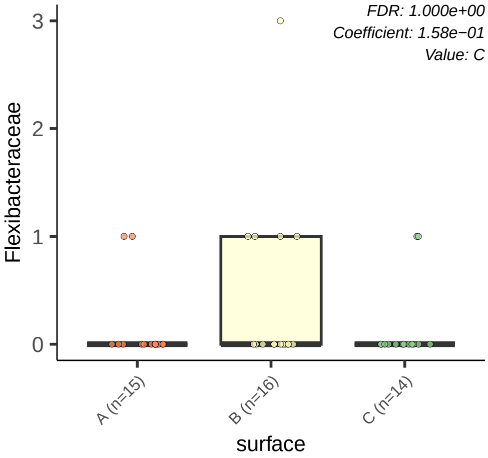

FDR: 1.000e+00
Coefficient: 1.58e−01
Value: C
Flexibacteraceae
3
2
1
0
A (n=15)
B (n=16)
C (n=14)
surface

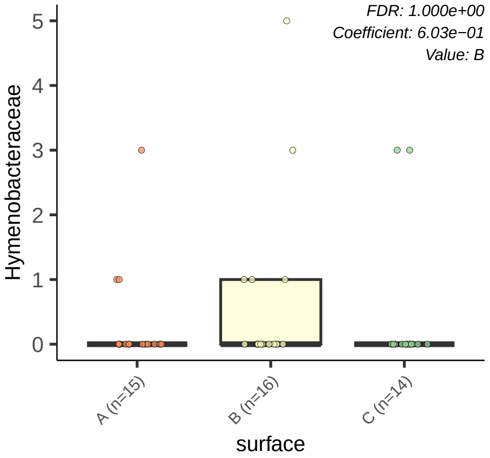
FDR: 1.000e+00
Coefficient: 6.03e−01
Value: B
Hymenobacteraceae
0
1
2
3
4
5
A (n=15)
B (n=16)
C (n=14)
surface

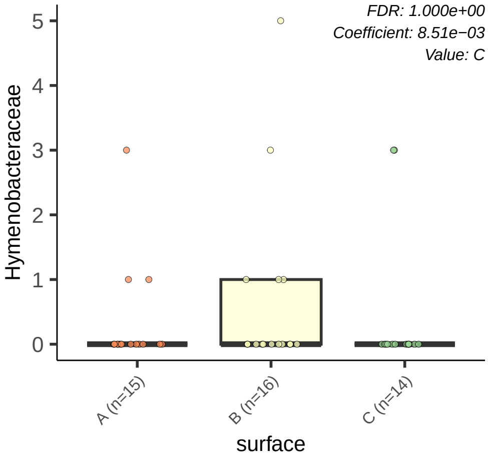

FDR: 1.000e+00
Coefficient: 8.51e−03
Value: C
Hymenobacteraceae
0
1
2
3
4
5
A (n=15)
B (n=16)
C (n=14)
surface

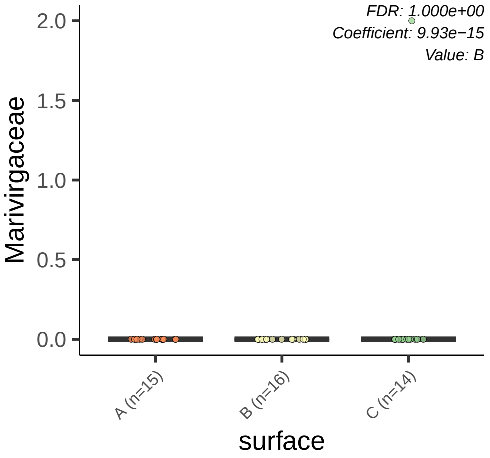
FDR: 1.000e+00
Coefficient: 9.93e−15
Value: B
Marivirgaceae
2.0
1.5
1.0
0.5
0.0
A (n=15)
B (n=16)
C (n=14)
surface

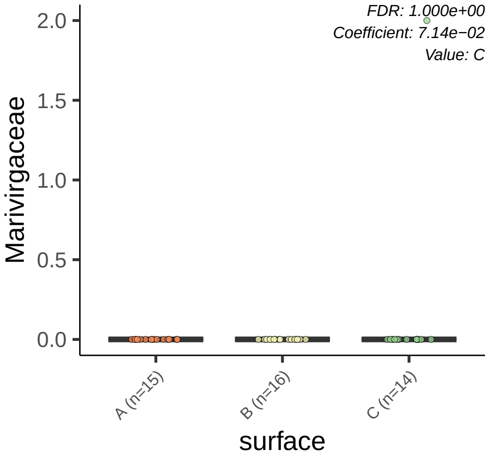

FDR: 1.000e+00
Coefficient: 7.14e−02
Value: C
Marivirgaceae
2.0
1.5
1.0
0.5
0.0
A (n=15)
B (n=16)
C (n=14)
surface

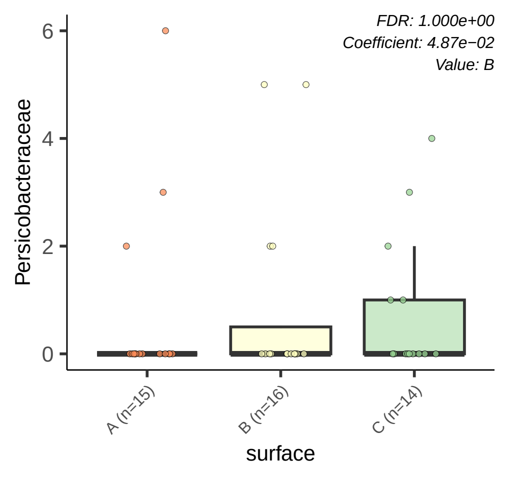
FDR: 1.000e+00
Coefficient: 4.87e−02
Value: B
Persicobacteraceae
6
4
2
0
A (n=15)
B (n=16)
C (n=14)
surface

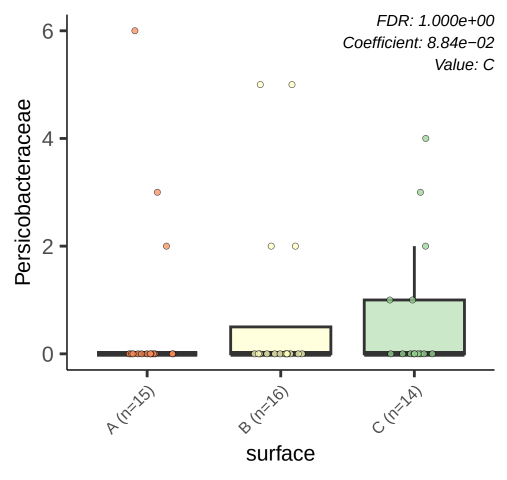

FDR: 1.000e+00
Coefficient: 8.84e−02
Value: C
Persicobacteraceae
6
4
2
0
A (n=15)
B (n=16)
C (n=14)
surface

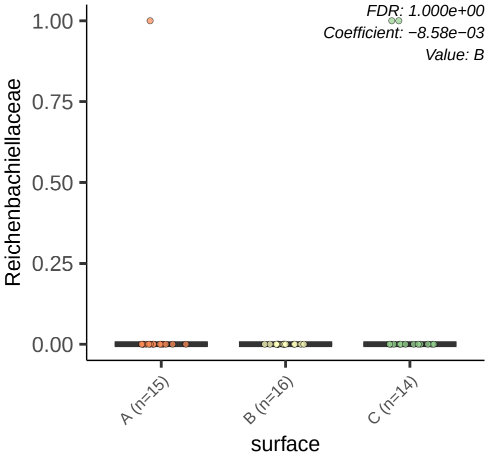
FDR: 1.000e+00
Coefficient: −8.58e−03
Value: B
Reichenbachiellaceae
1.00
0.75
0.50
0.25
0.00
A (n=15)
B (n=16)
C (n=14)
surface

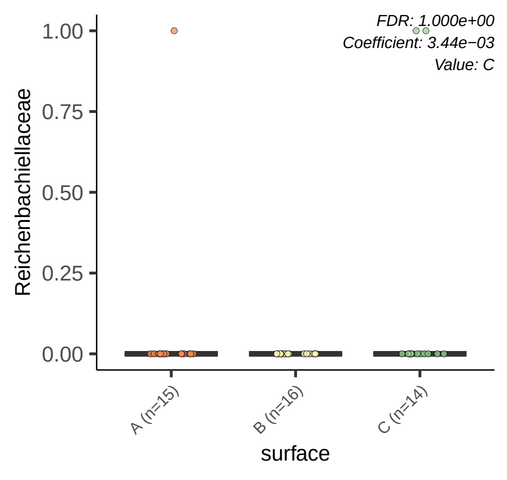
FDR: 1.000e+00
Coefficient: 3.44e−03
Value: C
Reichenbachiellaceae
1.00
0.75
0.50
0.25
0.00
A (n=15)
B (n=16)
C (n=14)
surface

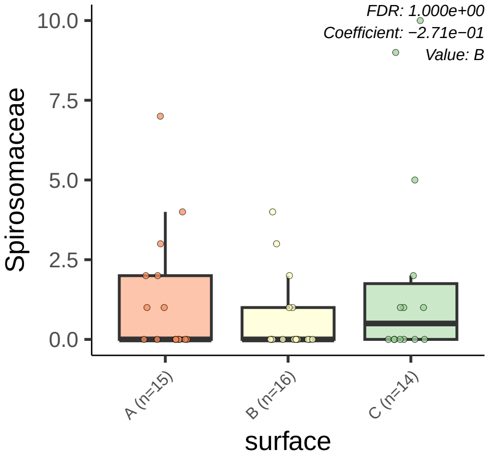

FDR: 1.000e+00
Coefficient: −2.71e−01
Value: B
10.0
7.5
5.0
2.5
0.0
Spirosomaceae
A (n=15)
B (n=16)
C (n=14)
surface

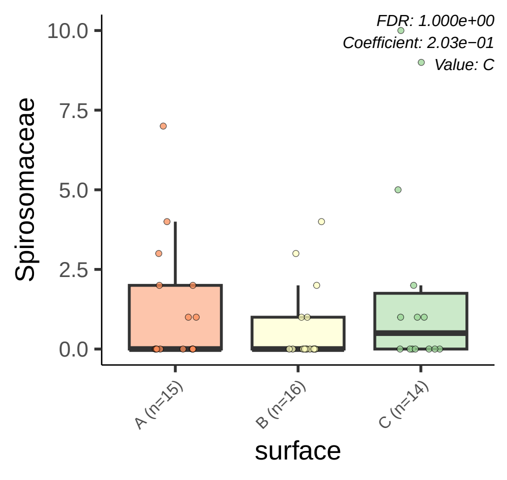
FDR: 1.000e+00
Coefficient: 2.03e−01
Value: C
10.0
7.5
5.0
2.5
0.0
Spirosomaceae
A (n=15)
B (n=16)
C (n=14)
surface

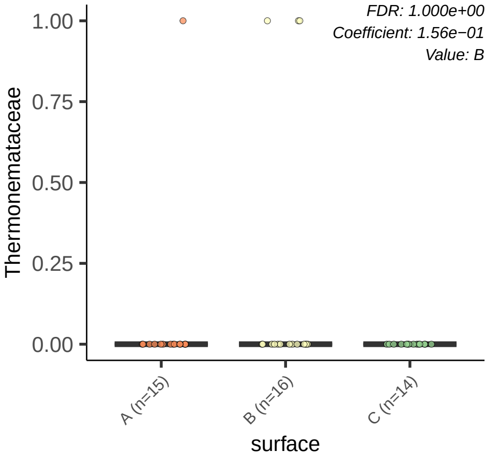FDR: 1.000e+00
Coefficient: 1.56e−01
Value: B
Thermonemataceae
1.00
0.75
0.50
0.25
0.00
A (n=15)
B (n=16)
C (n=14)
surface

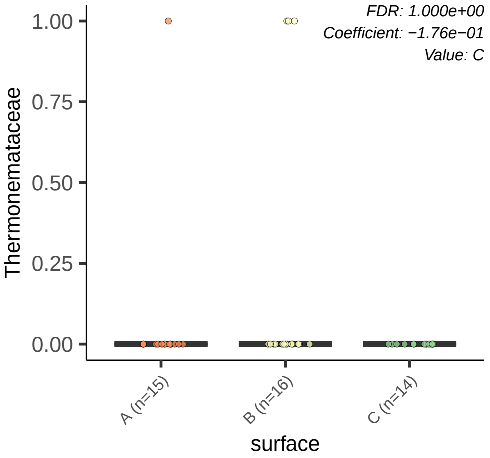
FDR: 1.000e+00
Coefficient: −1.76e−01
Value: C
Thermonemataceae
1.00
0.75
0.50
0.25
0.00
A (n=15)
B (n=16)
C (n=14)
surface

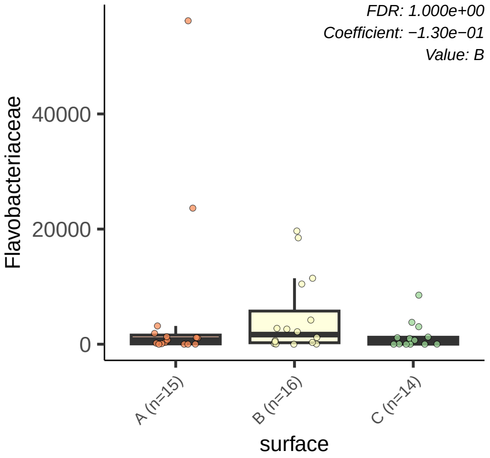

FDR: 1.000e+00
Coefficient: −1.30e−01
Value: B
Flavobacteriaceae
40000
20000
0
A (n=15)
B (n=16)
C (n=14)
surface

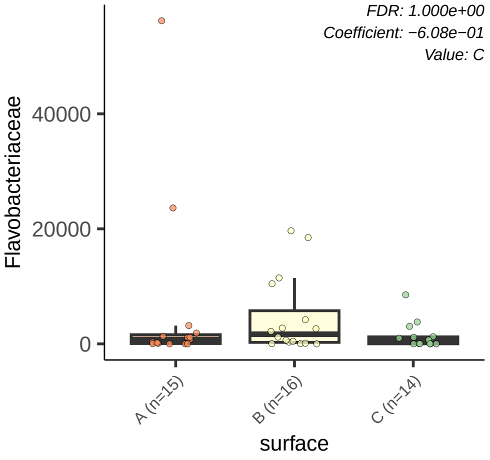

FDR: 1.000e+00
Coefficient: −6.08e−01
Value: C
Flavobacteriaceae
40000
20000
0
A (n=15)
B (n=16)
C (n=14)
surface

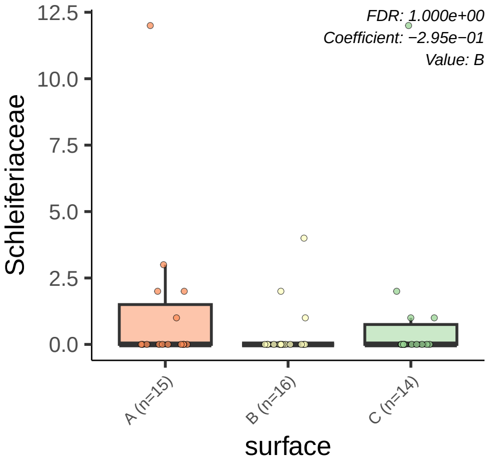

FDR: 1.000e+00
Coefficient: −2.95e−01
Value: B
Schleiferiaceae
12.5
10.0
7.5
5.0
2.5
0.0
A (n=15)
B (n=16)
C (n=14)
surface

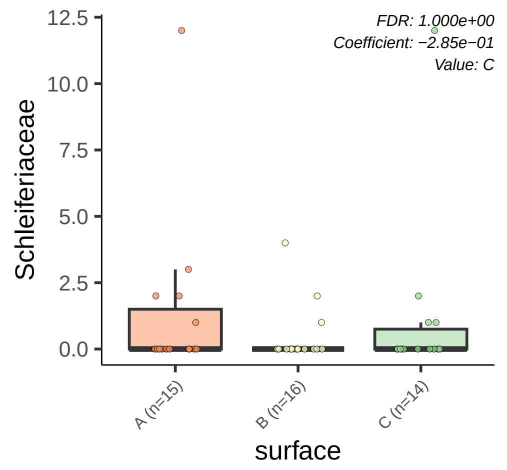
FDR: 1.000e+00
Coefficient: −2.85e−01
Value: C
Schleiferiaceae
12.5
10.0
7.5
5.0
2.5
0.0
A (n=15)
B (n=16)
C (n=14)
surface

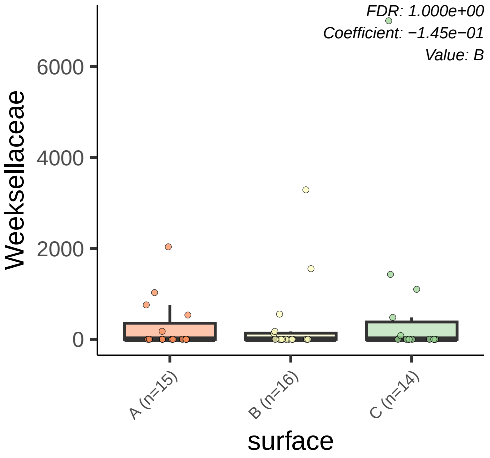
FDR: 1.000e+00
Coefficient: −1.45e−01
Value: B
Weeksellaceae
6000
4000
2000
0
A (n=15)
B (n=16)
C (n=14)
surface

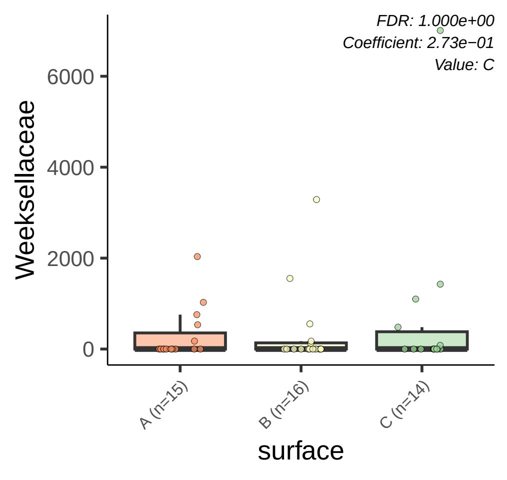

FDR: 1.000e+00
Coefficient: 2.73e−01
Value: C
6000
4000
2000
0
Weeksellaceae
A (n=15)
B (n=16)
C (n=14)
surface

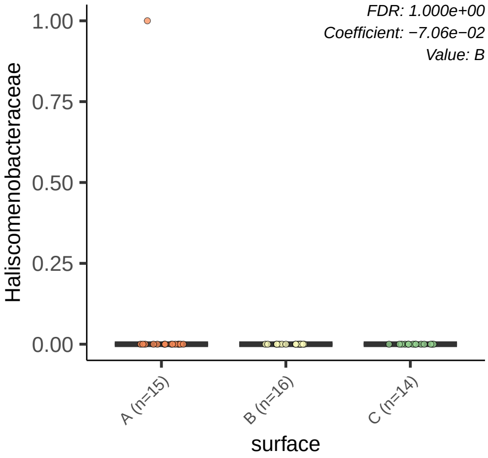
FDR: 1.000e+00
Coefficient: −7.06e−02
Value: B
Haliscomenobacteraceae
1.00
0.75
0.50
0.25
0.00
A (n=15)
B (n=16)
C (n=14)
surface

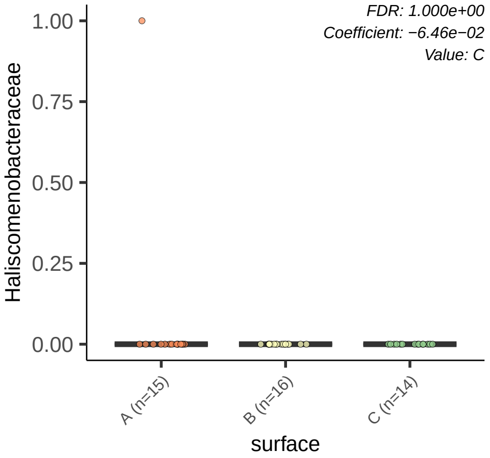

FDR: 1.000e+00
Coefficient: −6.46e−02
Value: C
Haliscomenobacteraceae
1.00
0.75
0.50
0.25
0.00
A (n=15)
B (n=16)
C (n=14)
surface

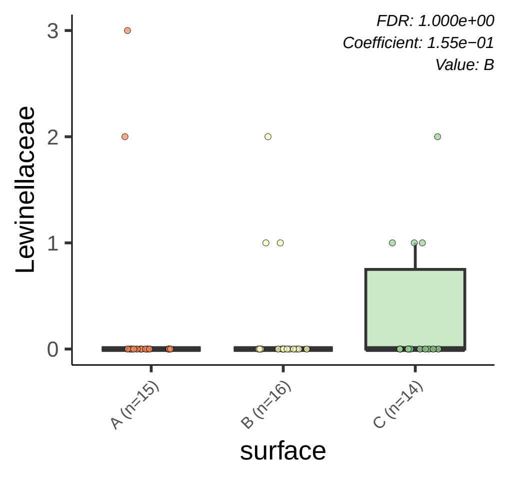
FDR: 1.000e+00
Coefficient: 1.55e−01
Value: B
Lewinellaceae
3
2
1
0
A (n=15)
B (n=16)
C (n=14)
surface

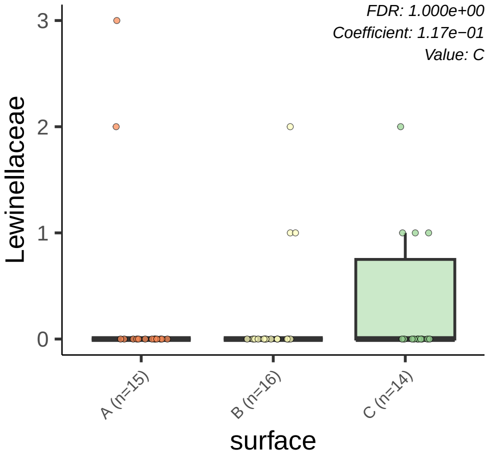
FDR: 1.000e+00
Coefficient: 1.17e−01
Value: C
Lewinellaceae
3
2
1
0
A (n=15)
B (n=16)
C (n=14)
surface

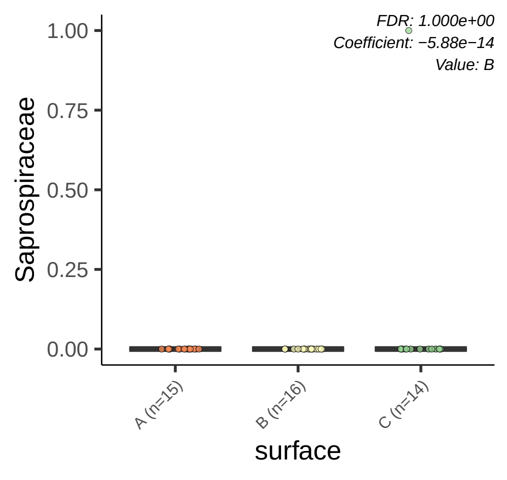
FDR: 1.000e+00
Coefficient: −5.88e−14
Value: B
Saprospiraceae
1.00
0.75
0.50
0.25
0.00
A (n=15)
B (n=16)
C (n=14)
surface

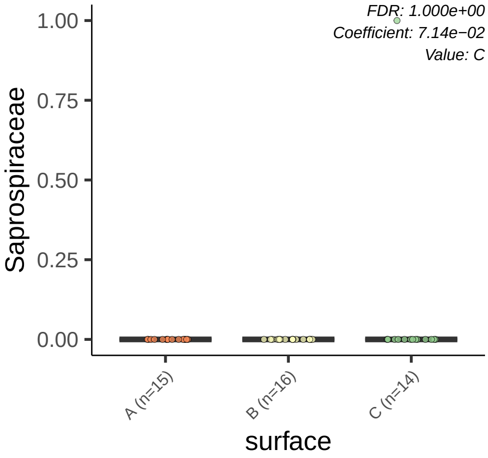

FDR: 1.000e+00
Coefficient: 7.14e−02
Value: C
Saprospiraceae
1.00
0.75
0.50
0.25
0.00
A (n=15)
B (n=16)
C (n=14)
surface

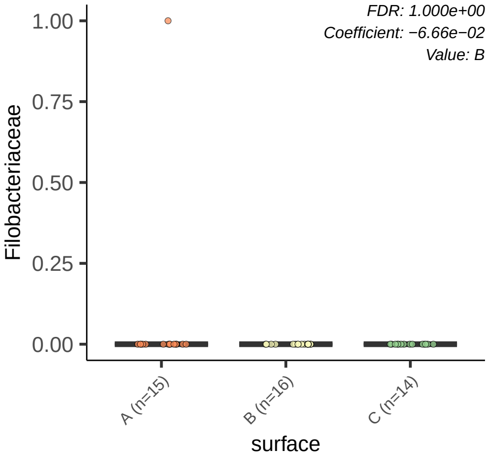
FDR: 1.000e+00
Coefficient: −6.66e−02
Value: B
Filobacteriaceae
1.00
0.75
0.50
0.25
0.00
A (n=15)
B (n=16)
C (n=14)
surface

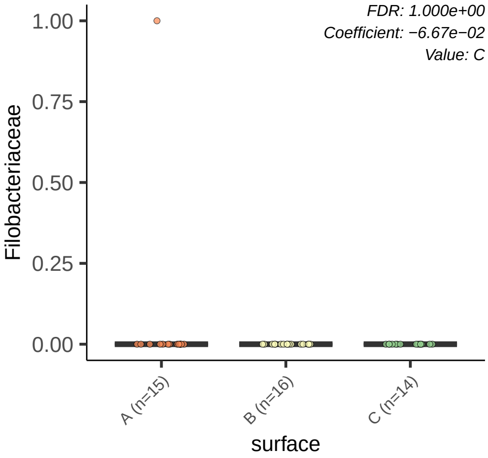
FDR: 1.000e+00
Coefficient: −6.67e−02
Value: C
Filobacteriaceae
1.00
0.75
0.50
0.25
0.00
A (n=15)
B (n=16)
C (n=14)
surface

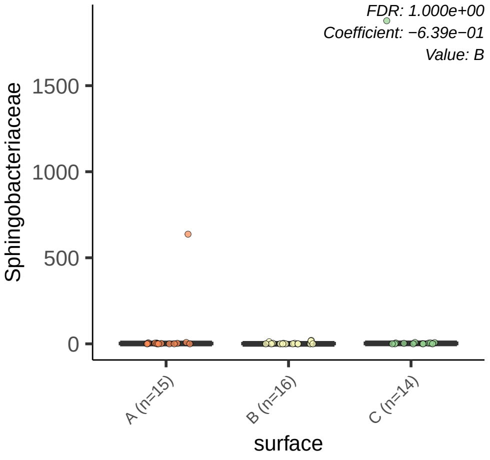

FDR: 1.000e+00
Coefficient: −6.39e−01
Value: B
Sphingobacteriaceae
1500
1000
500
0
A (n=15)
B (n=16)
C (n=14)
surface

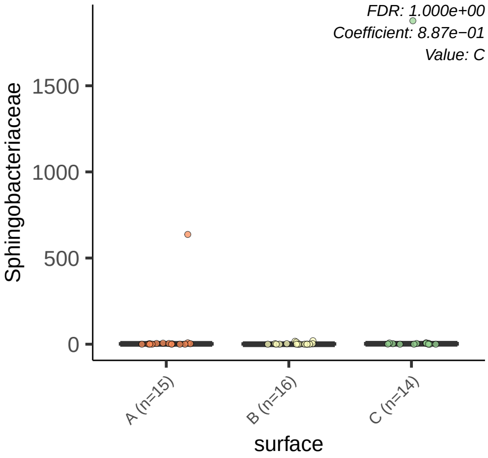
FDR: 1.000e+00
Coefficient: 8.87e−01
Value: C
Sphingobacteriaceae
1500
1000
500
0
A (n=15)
B (n=16)
C (n=14)
surface

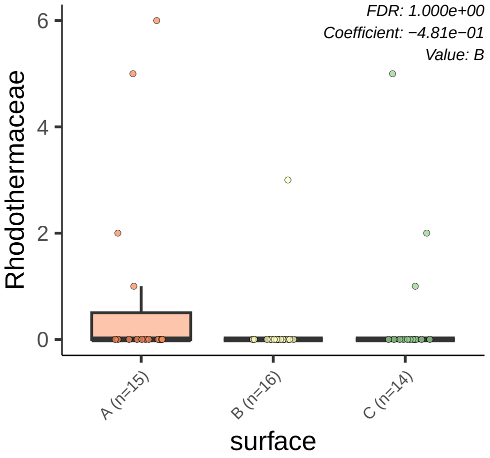

FDR: 1.000e+00
Coefficient: −4.81e−01
Value: B
Rhodothermaceae
6
4
2
0
A (n=15)
B (n=16)
C (n=14)
surface

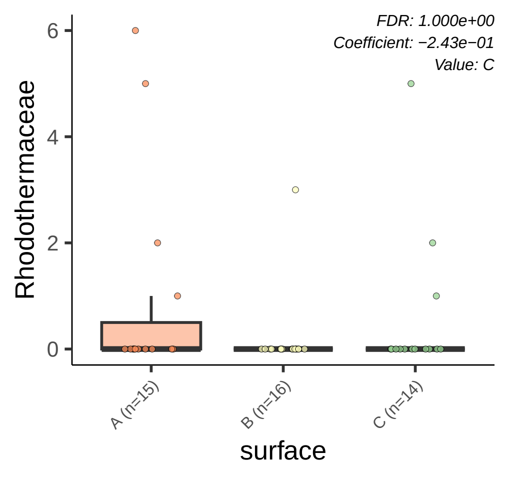

FDR: 1.000e+00
Coefficient: −2.43e−01
Value: C
Rhodothermaceae
0
2
4
6
A (n=15)
B (n=16)
C (n=14)
surface

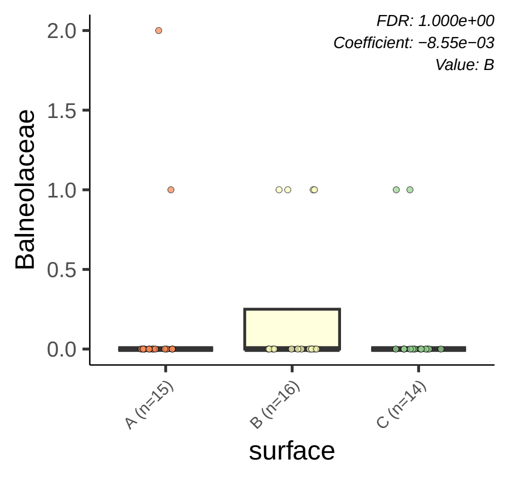
FDR: 1.000e+00
Coefficient: −8.55e−03
Value: B
Balneolaceae
2.0
1.5
1.0
0.5
0.0
A (n=15)
B (n=16)
C (n=14)
surface

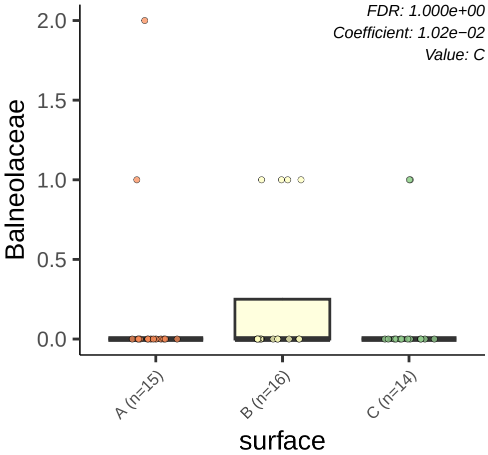
FDR: 1.000e+00
Coefficient: 1.02e−02
Value: C
Balneolaceae
2.0
1.5
1.0
0.5
0.0
A (n=15)
B (n=16)
C (n=14)
surface

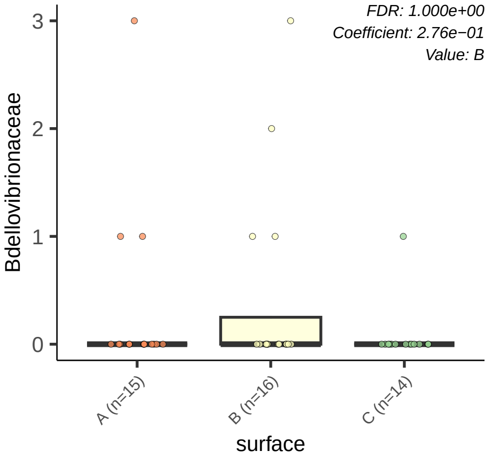
FDR: 1.000e+00
Coefficient: 2.76e−01
Value: B
Bdellovibrionaceae
3
2
1
0
A (n=15)
B (n=16)
C (n=14)
surface

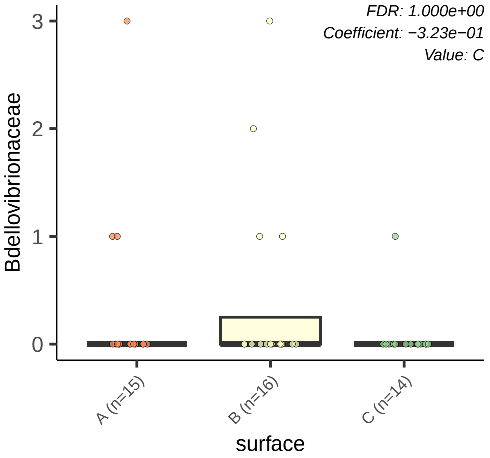

FDR: 1.000e+00
Coefficient: −3.23e−01
Value: C
Bdellovibrionaceae
3
2
1
0
A (n=15)
B (n=16)
C (n=14)
surface

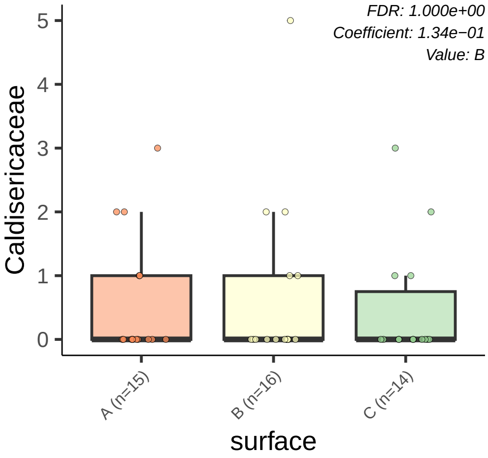

FDR: 1.000e+00
Coefficient: 1.34e−01
Value: B
Caldisericaceae
0
1
2
3
4
5
A (n=15)
B (n=16)
C (n=14)
surface

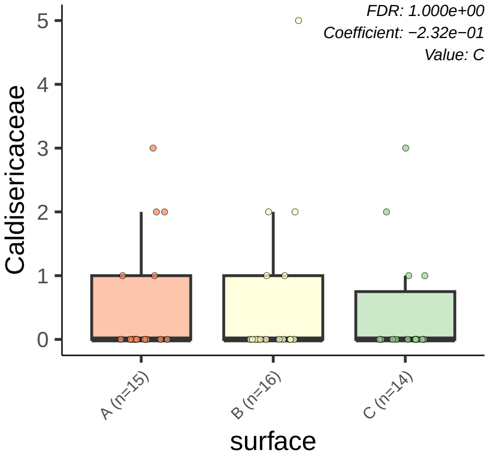

FDR: 1.000e+00
Coefficient: −2.32e−01
Value: C
Caldisericaceae
0
1
2
3
4
5
A (n=15)
B (n=16)
C (n=14)
surface

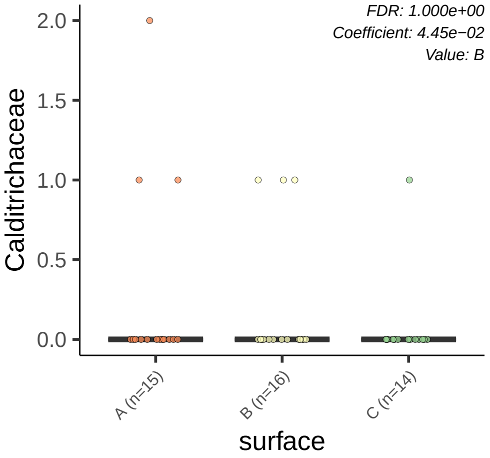
FDR: 1.000e+00
Coefficient: 4.45e−02
Value: B
Calditrichaceae
2.0
1.5
1.0
0.5
0.0
A (n=15)
B (n=16)
C (n=14)
surface

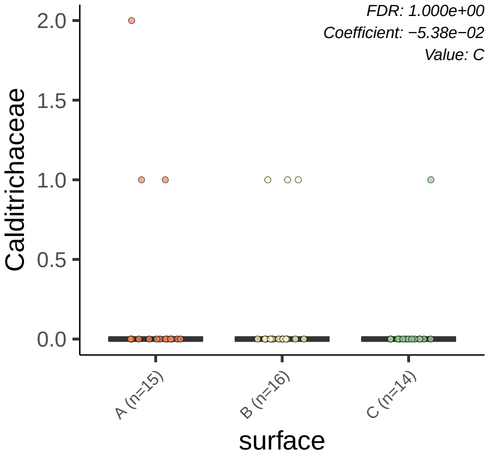
FDR: 1.000e+00
Coefficient: −5.38e−02
Value: C
Calditrichaceae
2.0
1.5
1.0
0.5
0.0
A (n=15)
B (n=16)
C (n=14)
surface

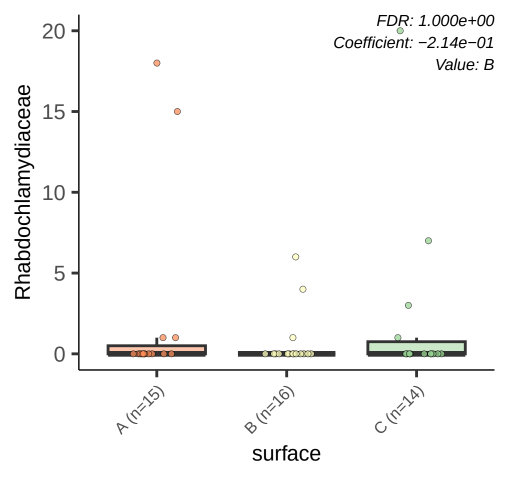
FDR: 1.000e+00
Coefficient: −2.14e−01
Value: B
Rhabdochlamydiaceae
20
15
10
5
0
A (n=15)
B (n=16)
C (n=14)
surface

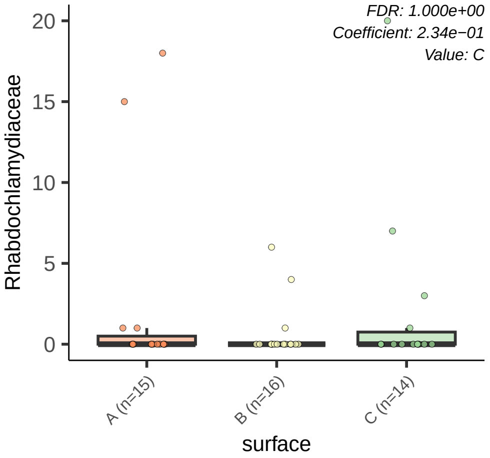
FDR: 1.000e+00
Coefficient: 2.34e−01
Value: C
Rhabdochlamydiaceae
20
15
10
5
0
A (n=15)
B (n=16)
C (n=14)
surface

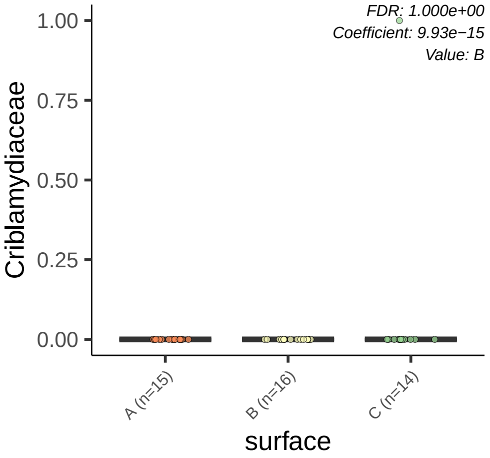
FDR: 1.000e+00
Coefficient: 9.93e−15
Value: B
Criblamydiaceae
1.00
0.75
0.50
0.25
0.00
A (n=15)
B (n=16)
C (n=14)
surface

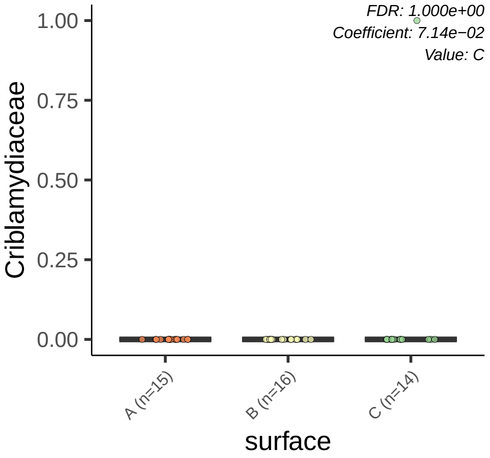
FDR: 1.000e+00
Coefficient: 7.14e−02
Value: C
Criblamydiaceae
1.00
0.75
0.50
0.25
0.00
A (n=15)
B (n=16)
C (n=14)
surface

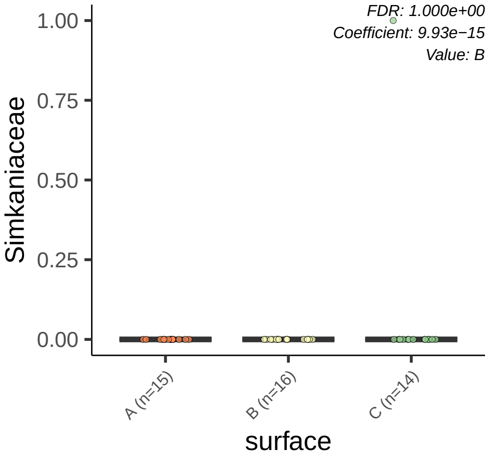

FDR: 1.000e+00
Coefficient: 9.93e−15
Value: B
Simkaniaceae
1.00
0.75
0.50
0.25
0.00
A (n=15)
B (n=16)
C (n=14)
surface

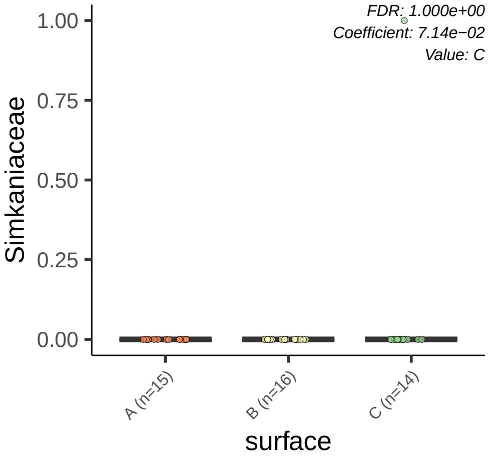
FDR: 1.000e+00
Coefficient: 7.14e−02
Value: C
Simkaniaceae
1.00
0.75
0.50
0.25
0.00
A (n=15)
B (n=16)
C (n=14)
surface

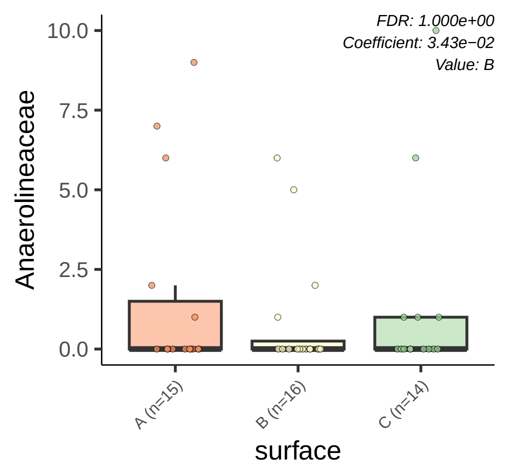

FDR: 1.000e+00
Coefficient: 3.43e−02
Value: B
Anaerolineaceae
10.0
7.5
5.0
2.5
0.0
A (n=15)
B (n=16)
C (n=14)
surface

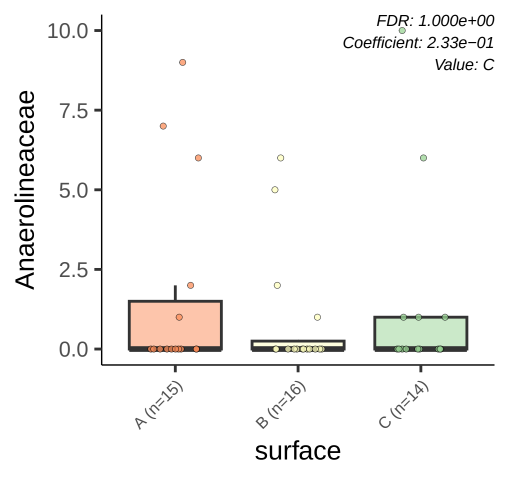
FDR: 1.000e+00
Coefficient: 2.33e−01
Value: C
Anaerolineaceae
10.0
7.5
5.0
2.5
0.0
A (n=15)
B (n=16)
C (n=14)
surface

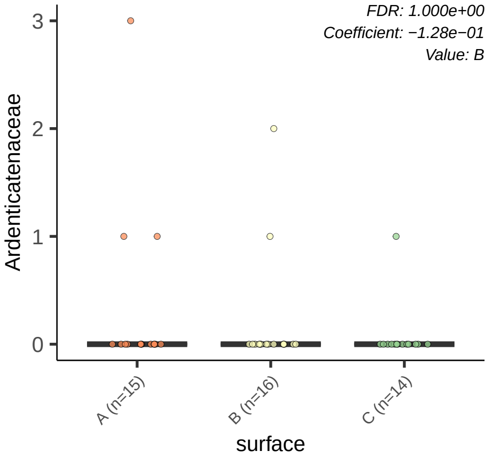

FDR: 1.000e+00
Coefficient: −1.28e−01
Value: B
Ardenticatenaceae
0
1
2
3
A (n=15)
B (n=16)
C (n=14)
surface

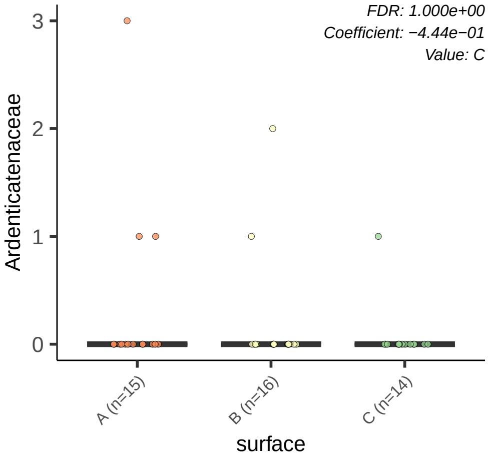
FDR: 1.000e+00
Coefficient: −4.44e−01
Value: C
Ardenticatenaceae
3
2
1
0
A (n=15)
B (n=16)
C (n=14)
surface

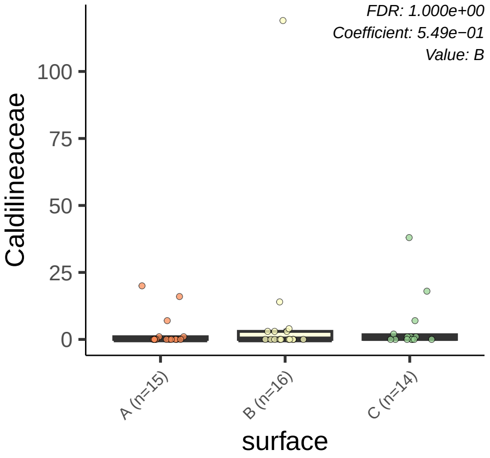
FDR: 1.000e+00
Coefficient: 5.49e−01
Value: B
Caldilineaceae
0
25
50
75
100
A (n=15)
B (n=16)
C (n=14)
surface

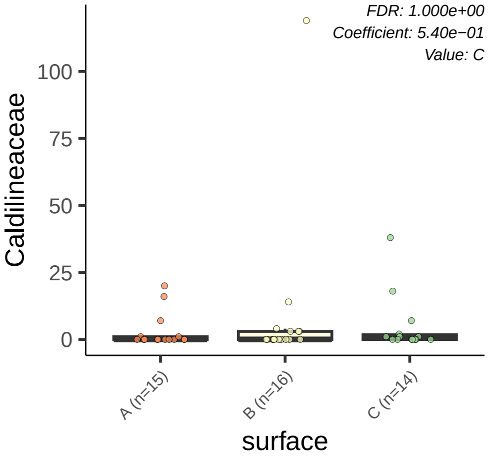
FDR: 1.000e+00
Coefficient: 5.40e−01
Value: C
Caldilineaceae
100
75
50
25
0
A (n=15)
B (n=16)
C (n=14)
surface

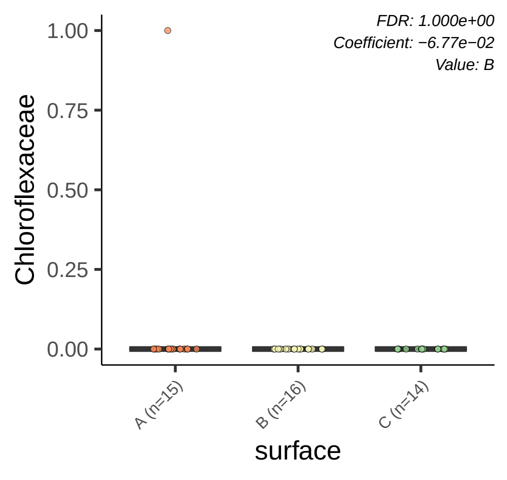
FDR: 1.000e+00
Coefficient: −6.77e−02
Value: B
Chloroflexaceae
1.00
0.75
0.50
0.25
0.00
A (n=15)
B (n=16)
C (n=14)
surface

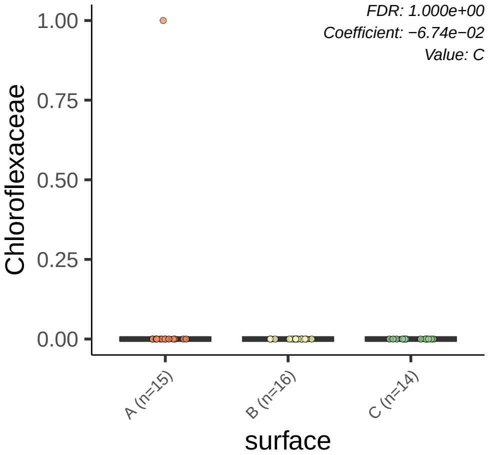

FDR: 1.000e+00
Coefficient: −6.74e−02
Value: C
Chloroflexaceae
1.00
0.75
0.50
0.25
0.00
A (n=15)
B (n=16)
C (n=14)
surface

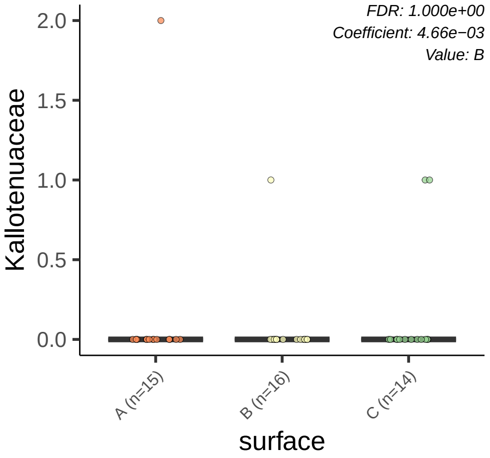

FDR: 1.000e+00
Coefficient: 4.66e−03
Value: B
Kallotenuaceae
2.0
1.5
1.0
0.5
0.0
A (n=15)
B (n=16)
C (n=14)
surface

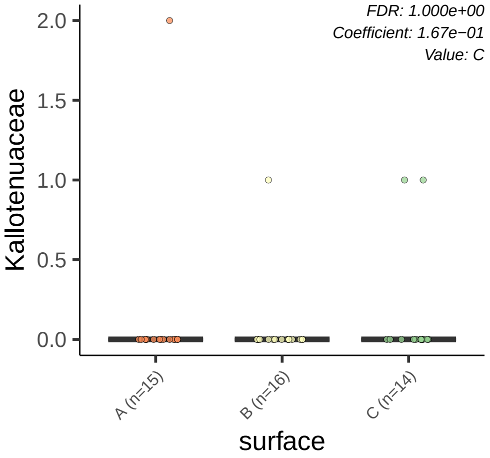

FDR: 1.000e+00
Coefficient: 1.67e−01
Value: C
Kallotenuaceae
2.0
1.5
1.0
0.5
0.0
A (n=15)
B (n=16)
C (n=14)
surface

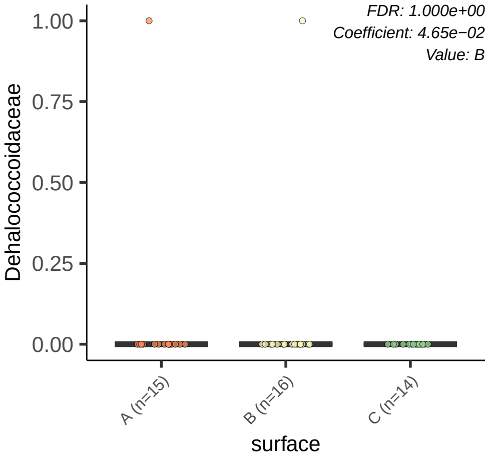

FDR: 1.000e+00
Coefficient: 4.65e−02
Value: B
Dehalococcoidaceae
1.00
0.75
0.50
0.25
0.00
A (n=15)
B (n=16)
C (n=14)
surface

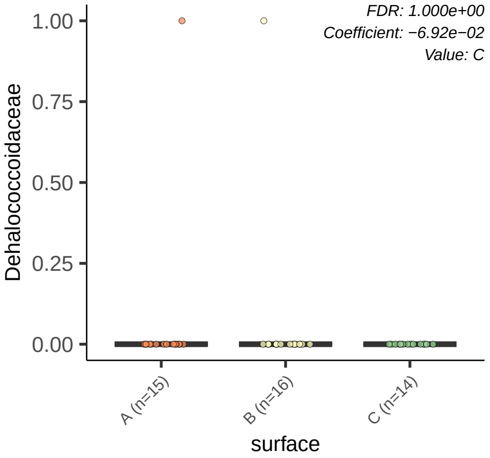

FDR: 1.000e+00
Coefficient: −6.92e−02
Value: C
Dehalococcoidaceae
1.00
0.75
0.50
0.25
0.00
A (n=15)
B (n=16)
C (n=14)
surface

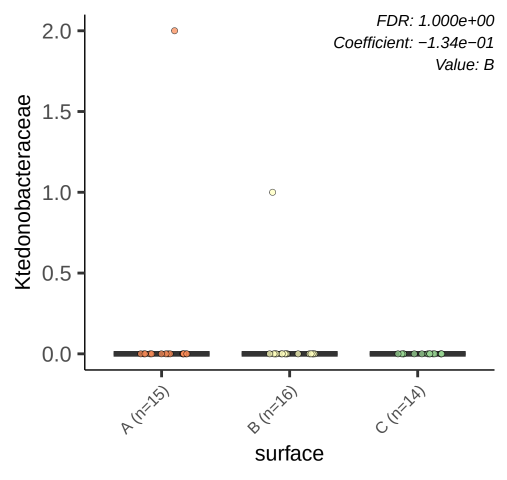
FDR: 1.000e+00
Coefficient: −1.34e−01
Value: B
Ktedonobacteraceae
2.0
1.5
1.0
0.5
0.0
A (n=15)
B (n=16)
C (n=14)
surface

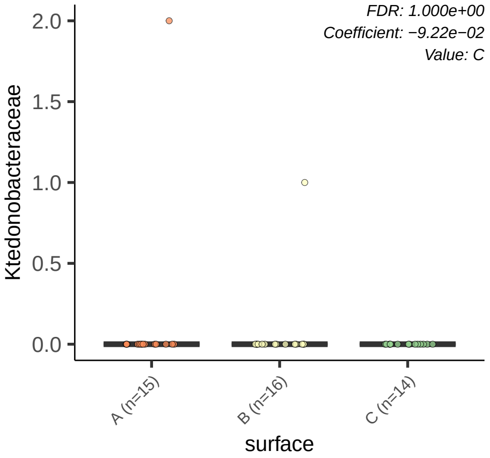
FDR: 1.000e+00
Coefficient: −9.22e−02
Value: C
Ktedonobacteraceae
2.0
1.5
1.0
0.5
0.0
A (n=15)
B (n=16)
C (n=14)
surface

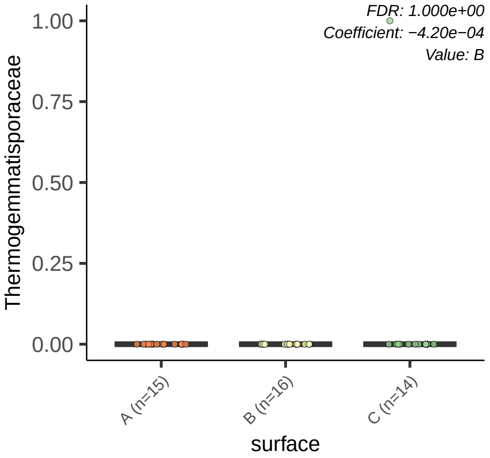
FDR: 1.000e+00
Coefficient: −4.20e−04
Value: B
Thermogemmatisporaceae
1.00
0.75
0.50
0.25
0.00
A (n=15)
B (n=16)
C (n=14)
surface

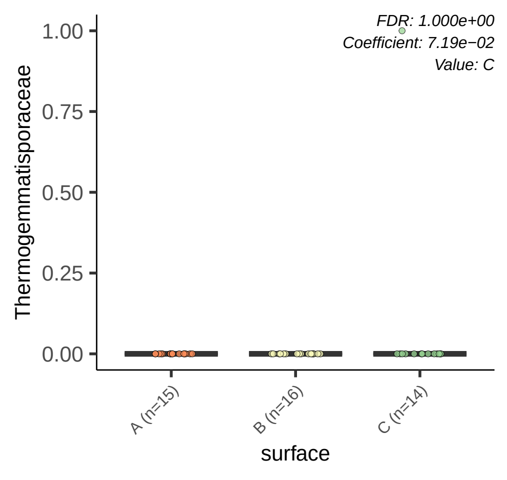
FDR: 1.000e+00
Coefficient: 7.19e−02
Value: C
Thermogemmatisporaceae
1.00
0.75
0.50
0.25
0.00
A (n=15)
B (n=16)
C (n=14)
surface

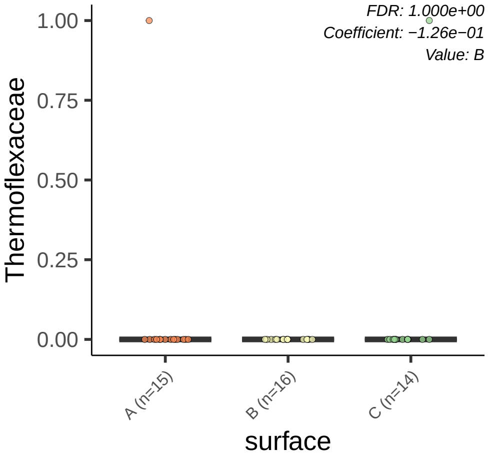
FDR: 1.000e+00
Coefficient: −1.26e−01
Value: B
Thermoflexaceae
1.00
0.75
0.50
0.25
0.00
A (n=15)
B (n=16)
C (n=14)
surface

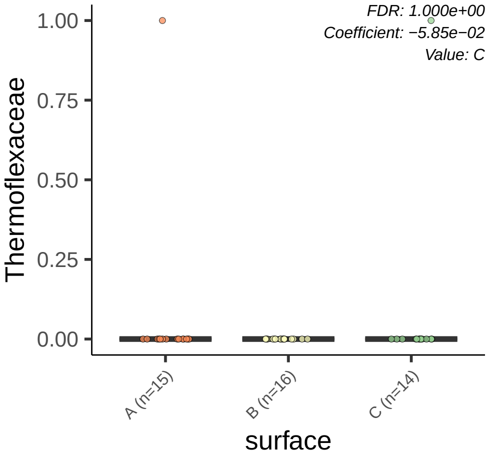
FDR: 1.000e+00
Coefficient: −5.85e−02
Value: C
Thermoflexaceae
1.00
0.75
0.50
0.25
0.00
A (n=15)
B (n=16)
C (n=14)
surface

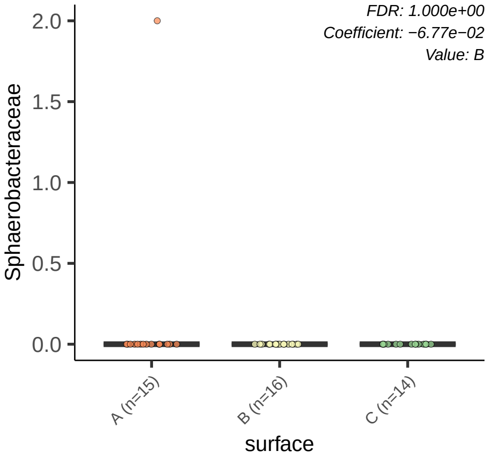

FDR: 1.000e+00
Coefficient: −6.77e−02
Value: B
Sphaerobacteraceae
2.0
1.5
1.0
0.5
0.0
A (n=15)
B (n=16)
C (n=14)
surface

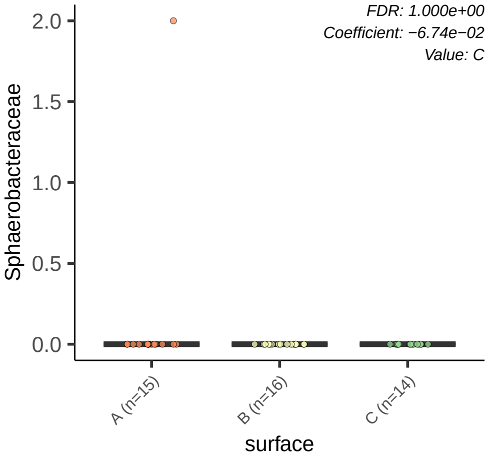
FDR: 1.000e+00
Coefficient: −6.74e−02
Value: C
Sphaerobacteraceae
2.0
1.5
1.0
0.5
0.0
A (n=15)
B (n=16)
C (n=14)
surface

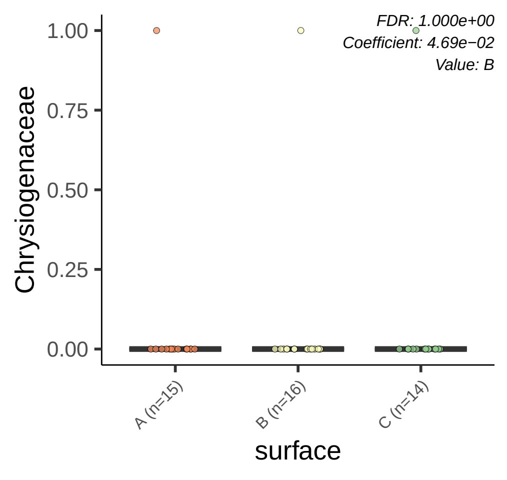

FDR: 1.000e+00
Coefficient: 4.69e−02
Value: B
Chrysiogenaceae
1.00
0.75
0.50
0.25
0.00
A (n=15)
B (n=16)
C (n=14)
surface

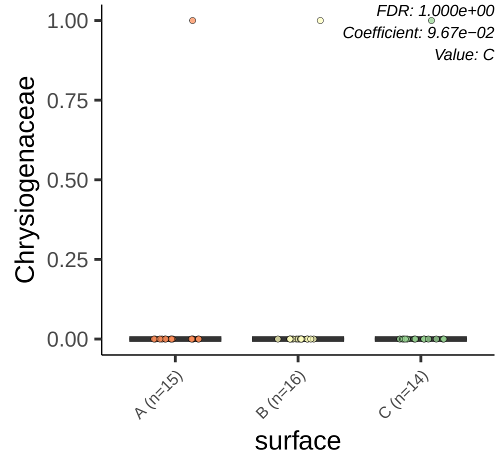
FDR: 1.000e+00
Coefficient: 9.67e−02
Value: C
Chrysiogenaceae
1.00
0.75
0.50
0.25
0.00
A (n=15)
B (n=16)
C (n=14)
surface

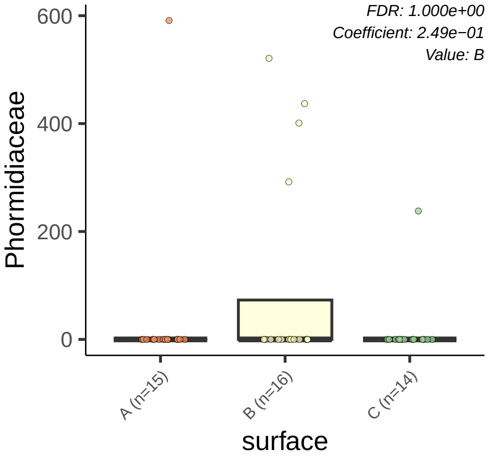
FDR: 1.000e+00
Coefficient: 2.49e−01
Value: B
Phormidiaceae
600
400
200
0
A (n=15)
B (n=16)
C (n=14)
surface

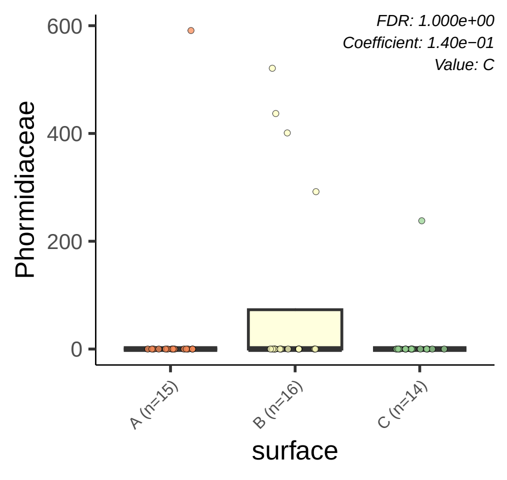
FDR: 1.000e+00
Coefficient: 1.40e−01
Value: C
Phormidiaceae
600
400
200
0
A (n=15)
B (n=16)
C (n=14)
surface

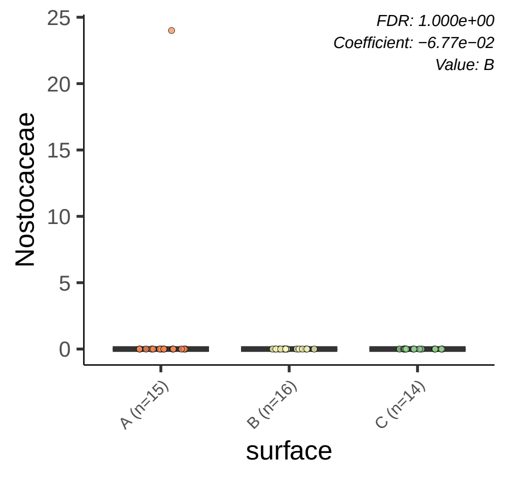

FDR: 1.000e+00
Coefficient: −6.77e−02
Value: B
Nostocaceae
25
20
15
10
5
0
A (n=15)
B (n=16)
C (n=14)
surface

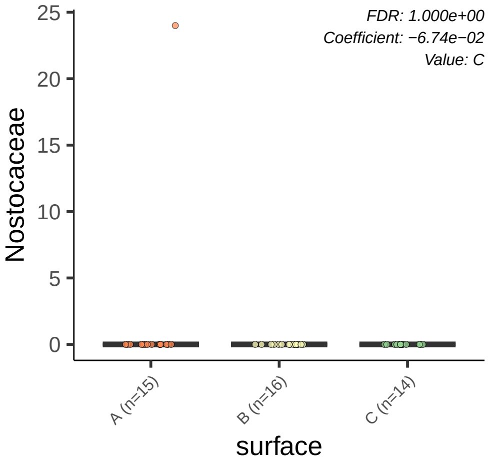

FDR: 1.000e+00
Coefficient: −6.74e−02
Value: C
Nostocaceae
25
20
15
10
5
0
A (n=15)
B (n=16)
C (n=14)
surface

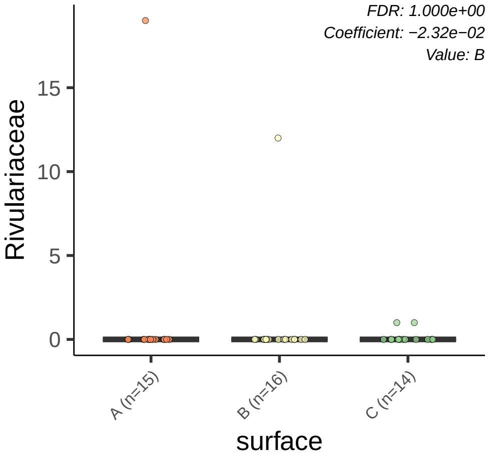
FDR: 1.000e+00
Coefficient: −2.32e−02
Value: B
Rivulariaceae
15
10
5
0
A (n=15)
B (n=16)
C (n=14)
surface

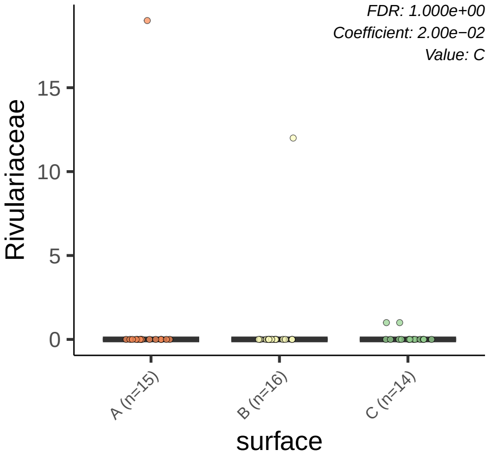

FDR: 1.000e+00
Coefficient: 2.00e−02
Value: C
Rivulariaceae
15
10
5
0
A (n=15)
B (n=16)
C (n=14)
surface

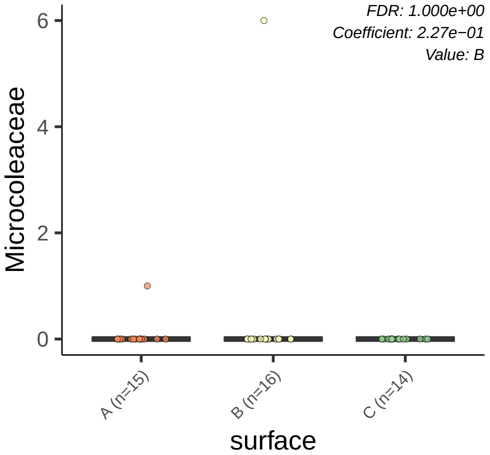
FDR: 1.000e+00
Coefficient: 2.27e−01
Value: B
Microcoleaceae
6
4
2
0
A (n=15)
B (n=16)
C (n=14)
surface

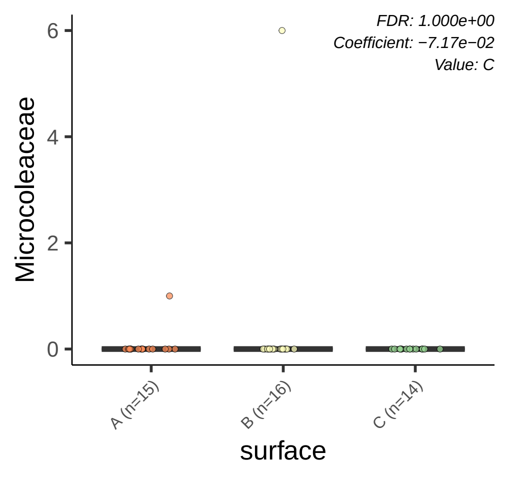
FDR: 1.000e+00
Coefficient: −7.17e−02
Value: C
Microcoleaceae
6
4
2
0
A (n=15)
B (n=16)
C (n=14)
surface

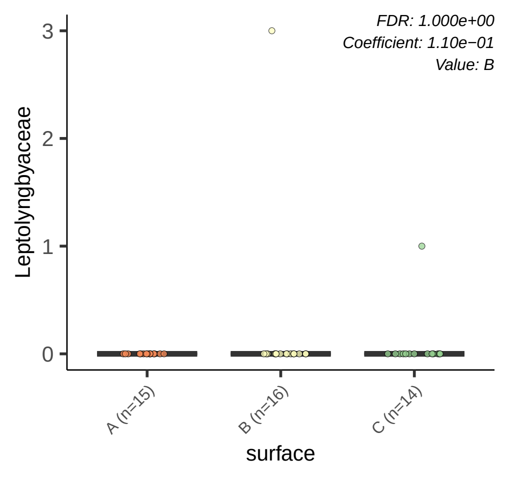
FDR: 1.000e+00
Coefficient: 1.10e−01
Value: B
Leptolyngbyaceae
3
2
1
0
A (n=15)
B (n=16)
C (n=14)
surface

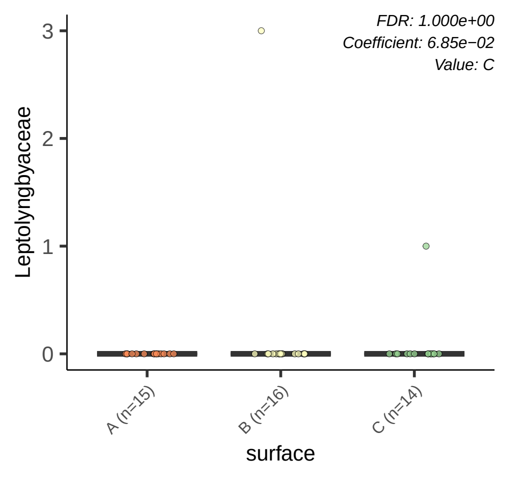

FDR: 1.000e+00
Coefficient: 6.85e−02
Value: C
Leptolyngbyaceae
3
2
1
0
A (n=15)
B (n=16)
C (n=14)
surface

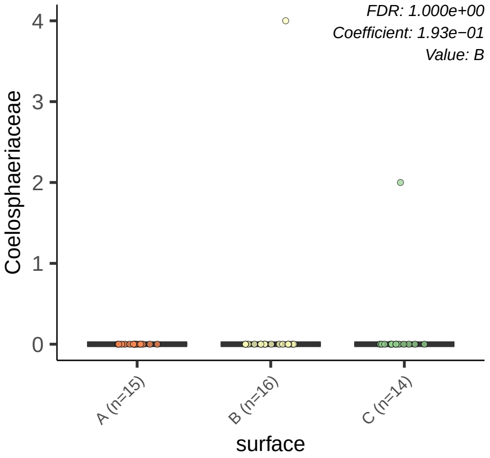

FDR: 1.000e+00
Coefficient: 1.93e−01
Value: B
Coelosphaeriaceae
0
1
2
3
4
A (n=15)
B (n=16)
C (n=14)
surface

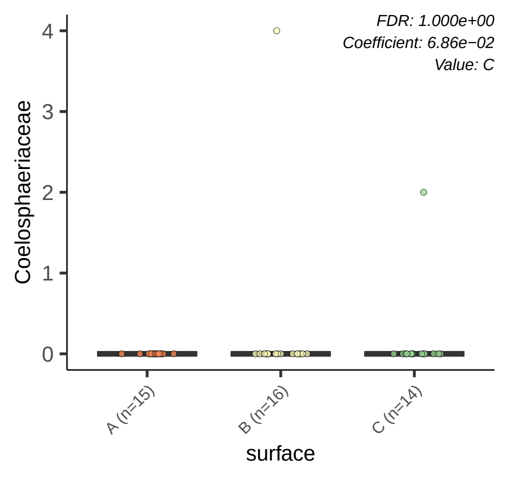
FDR: 1.000e+00
Coefficient: 6.86e−02
Value: C
Coelosphaeriaceae
4
3
2
1
0
A (n=15)
B (n=16)
C (n=14)
surface

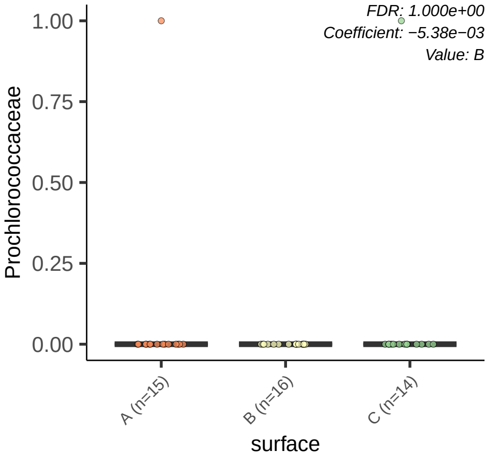

FDR: 1.000e+00
Coefficient: −5.38e−03
Value: B
Prochlorococcaceae
1.00
0.75
0.50
0.25
0.00
A (n=15)
B (n=16)
C (n=14)
surface

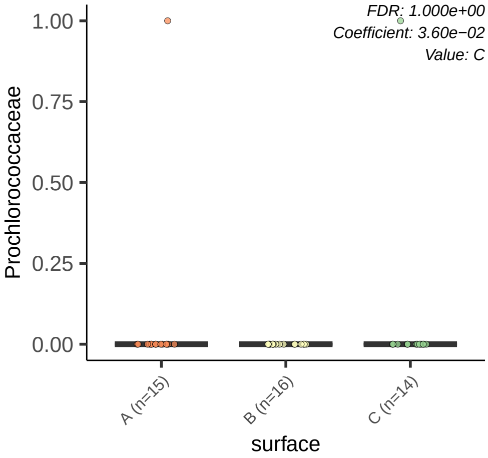
FDR: 1.000e+00
Coefficient: 3.60e−02
Value: C
Prochlorococcaceae
1.00
0.75
0.50
0.25
0.00
A (n=15)
B (n=16)
C (n=14)
surface

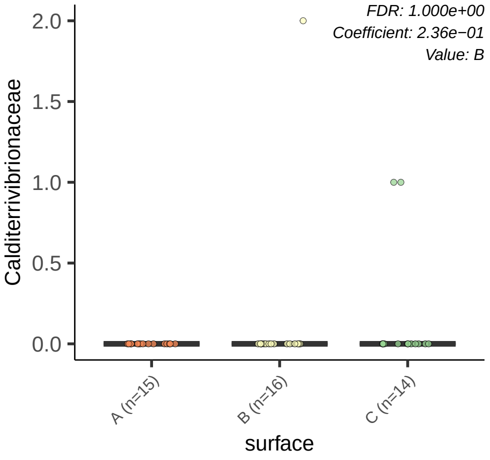
FDR: 1.000e+00
Coefficient: 2.36e−01
Value: B
Calditerrivibrionaceae
2.0
1.5
1.0
0.5
0.0
A (n=15)
B (n=16)
C (n=14)
surface

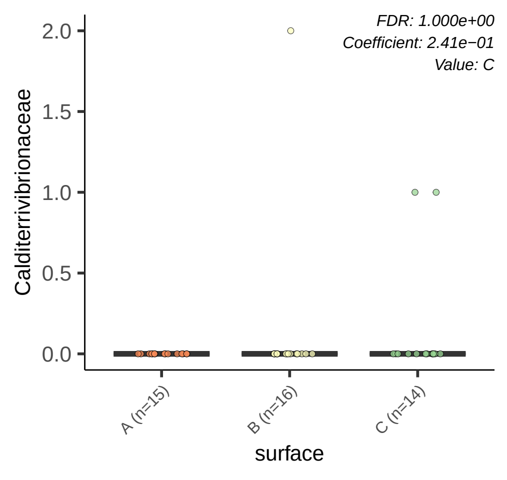
FDR: 1.000e+00
Coefficient: 2.41e−01
Value: C
Calditerrivibrionaceae
2.0
1.5
1.0
0.5
0.0
A (n=15)
B (n=16)
C (n=14)
surface

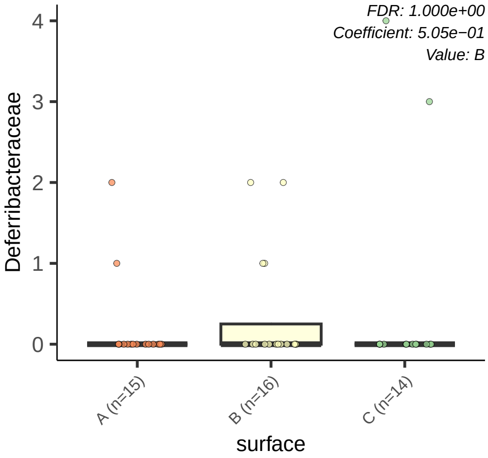
FDR: 1.000e+00
Coefficient: 5.05e−01
Value: B
Deferribacteraceae
0
1
2
3
4
A (n=15)
B (n=16)
C (n=14)
surface

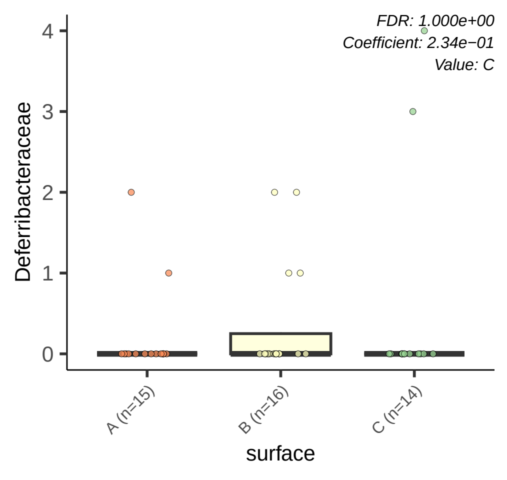
FDR: 1.000e+00
Coefficient: 2.34e−01
Value: C
Deferribacteraceae
0
1
2
3
4
A (n=15)
B (n=16)
C (n=14)
surface

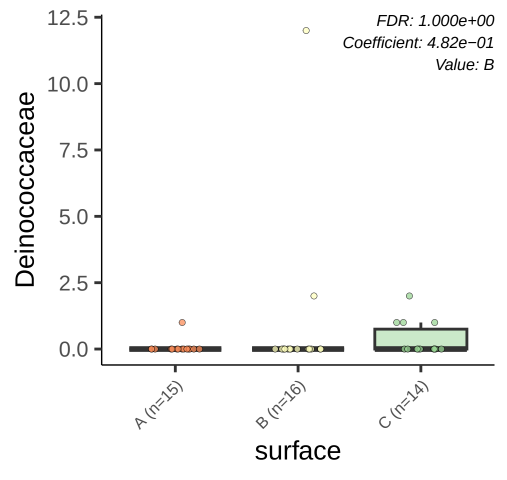
FDR: 1.000e+00
Coefficient: 4.82e−01
Value: B
Deinococcaceae
12.5
10.0
7.5
5.0
2.5
0.0
A (n=15)
B (n=16)
C (n=14)
surface

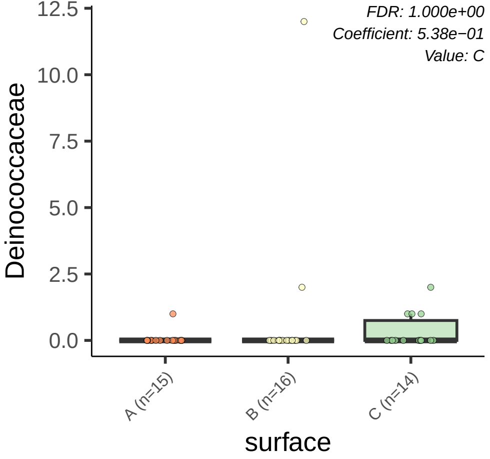
FDR: 1.000e+00
Coefficient: 5.38e−01
Value: C
Deinococcaceae
12.5
10.0
7.5
5.0
2.5
0.0
A (n=15)
B (n=16)
C (n=14)
surface

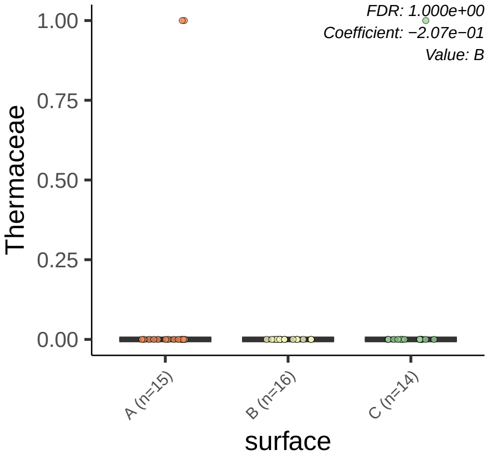

FDR: 1.000e+00
Coefficient: −2.07e−01
Value: B
Thermaceae
1.00
0.75
0.50
0.25
0.00
A (n=15)
B (n=16)
C (n=14)
surface

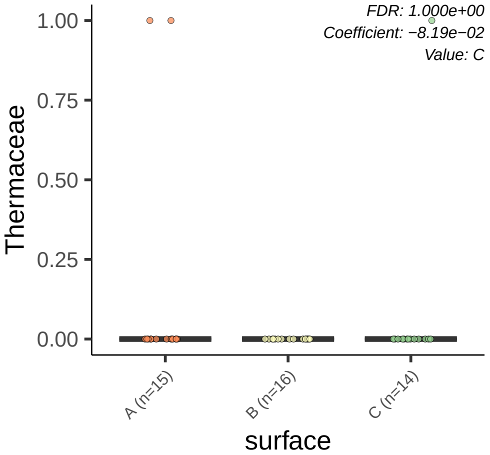
FDR: 1.000e+00
Coefficient: −8.19e−02
Value: C
Thermaceae
1.00
0.75
0.50
0.25
0.00
A (n=15)
B (n=16)
C (n=14)
surface

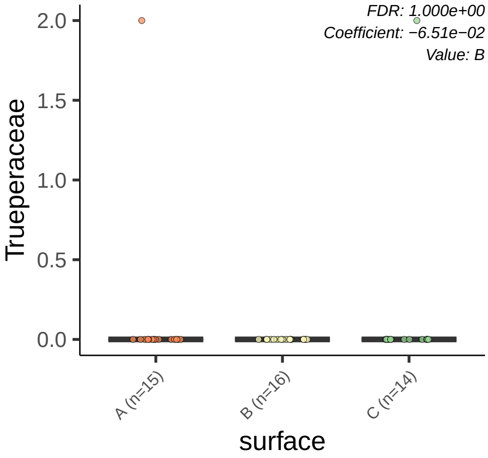
FDR: 1.000e+00
Coefficient: −6.51e−02
Value: B
Trueperaceae
2.0
1.5
1.0
0.5
0.0
A (n=15)
B (n=16)
C (n=14)
surface

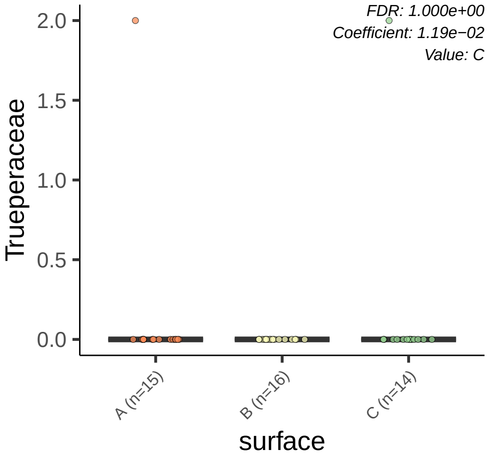
FDR: 1.000e+00
Coefficient: 1.19e−02
Value: C
Trueperaceae
2.0
1.5
1.0
0.5
0.0
A (n=15)
B (n=16)
C (n=14)
surface

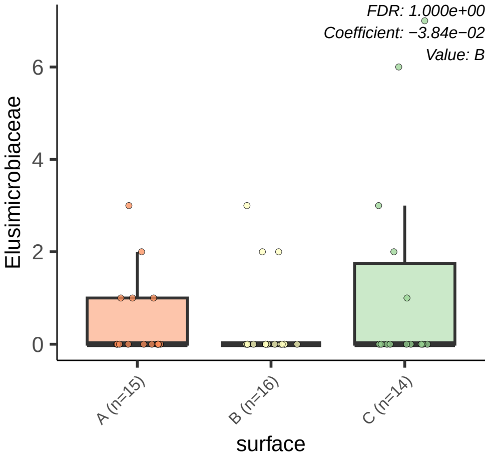
FDR: 1.000e+00
Coefficient: −3.84e−02
Value: B
Elusimicrobiaceae
0
2
4
6
A (n=15)
B (n=16)
C (n=14)
surface

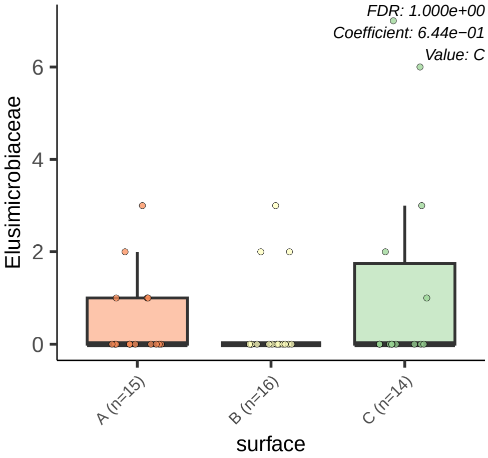
FDR: 1.000e+00
Coefficient: 6.44e−01
Value: C
Elusimicrobiaceae
0
2
4
6
A (n=15)
B (n=16)
C (n=14)
surface

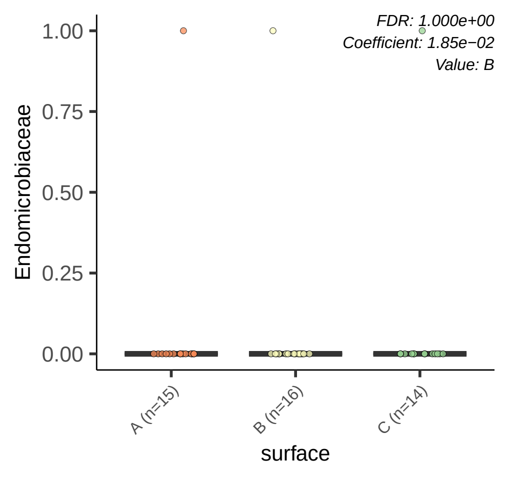
FDR: 1.000e+00
Coefficient: 1.85e−02
Value: B
Endomicrobiaceae
1.00
0.75
0.50
0.25
0.00
A (n=15)
B (n=16)
C (n=14)
surface

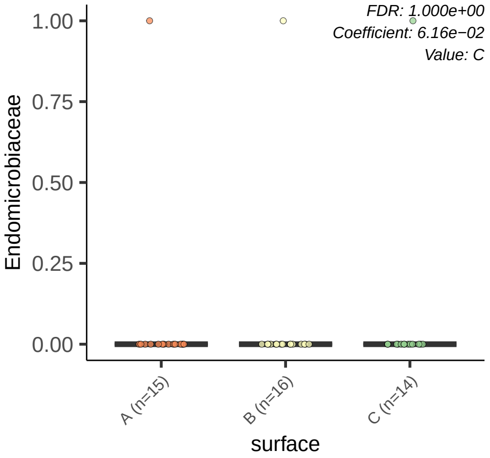

FDR: 1.000e+00
Coefficient: 6.16e−02
Value: C
Endomicrobiaceae
1.00
0.75
0.50
0.25
0.00
A (n=15)
B (n=16)
C (n=14)
surface

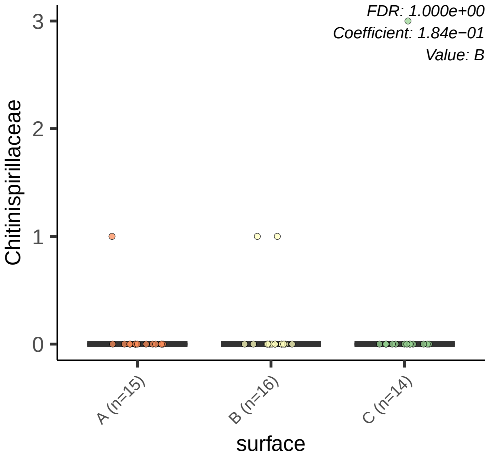
FDR: 1.000e+00
Coefficient: 1.84e−01
Value: B
Chitinispirillaceae
3
2
1
0
A (n=15)
B (n=16)
C (n=14)
surface

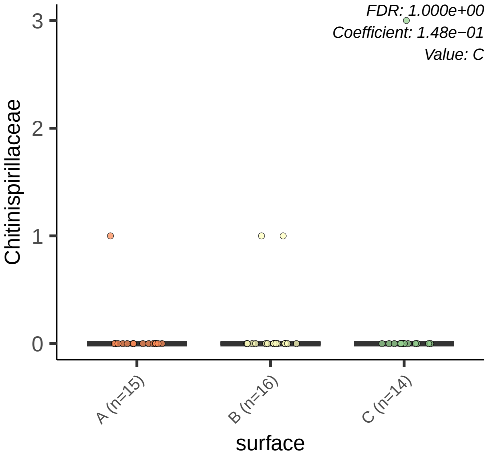
FDR: 1.000e+00
Coefficient: 1.48e−01
Value: C
Chitinispirillaceae
0
1
2
3
A (n=15)
B (n=16)
C (n=14)
surface

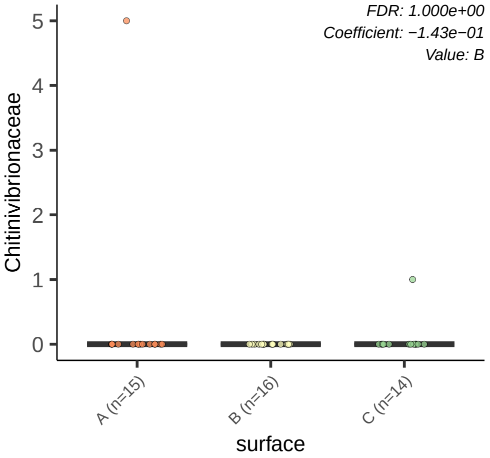

FDR: 1.000e+00
Coefficient: −1.43e−01
Value: B
Chitinivibrionaceae
0
1
2
3
4
5
A (n=15)
B (n=16)
C (n=14)
surface

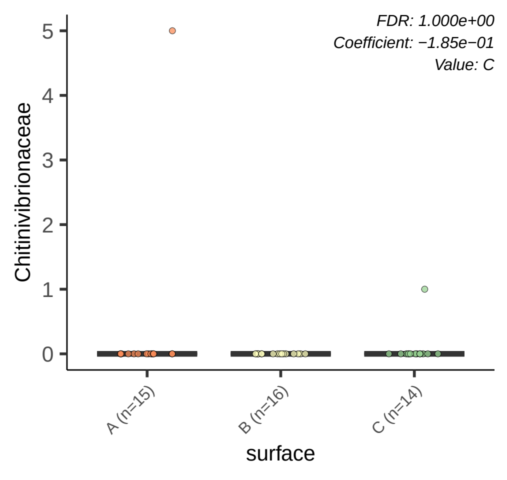
FDR: 1.000e+00
Coefficient: −1.85e−01
Value: C
Chitinivibrionaceae
0
1
2
3
4
5
A (n=15)
B (n=16)
C (n=14)
surface

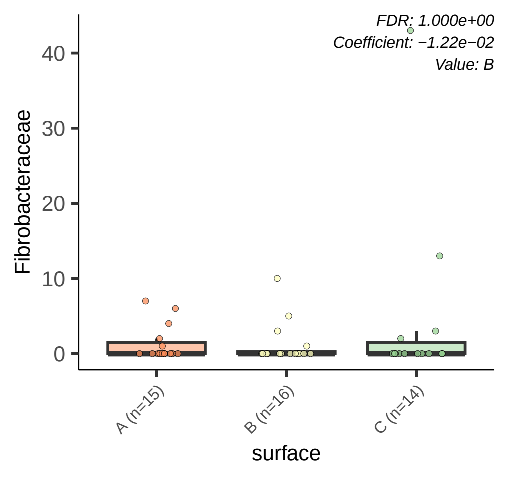
FDR: 1.000e+00
Coefficient: −1.22e−02
Value: B
Fibrobacteraceae
0
10
20
30
40
A (n=15)
B (n=16)
C (n=14)
surface

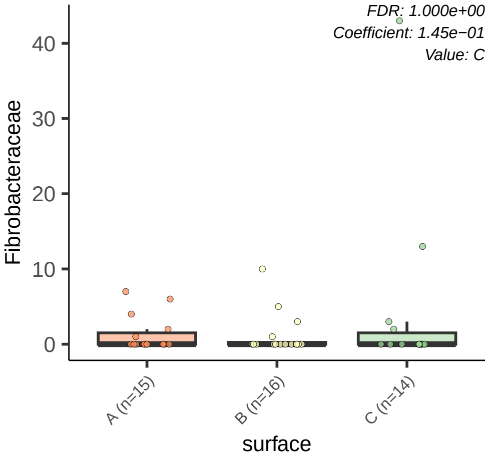

FDR: 1.000e+00
Coefficient: 1.45e−01
Value: C
Fibrobacteraceae
0
10
20
30
40
A (n=15)
B (n=16)
C (n=14)
surface

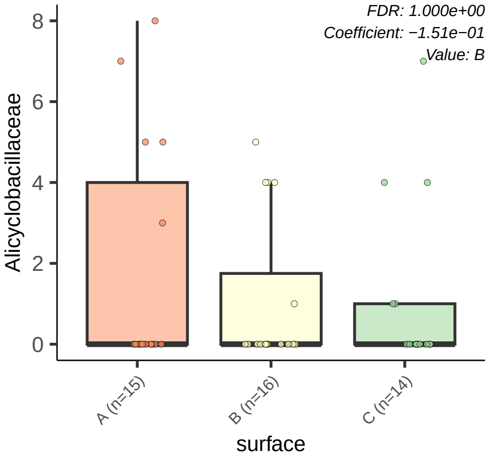
FDR: 1.000e+00
Coefficient: −1.51e−01
Value: B
Alicyclobacillaceae
8
6
4
2
0
A (n=15)
B (n=16)
C (n=14)
surface

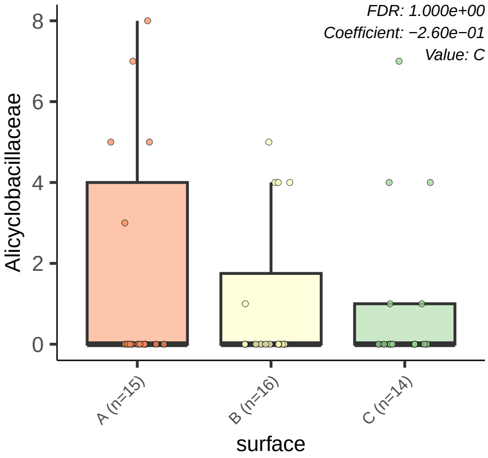
FDR: 1.000e+00
Coefficient: −2.60e−01
Value: C
Alicyclobacillaceae
8
6
4
2
0
A (n=15)
B (n=16)
C (n=14)
surface

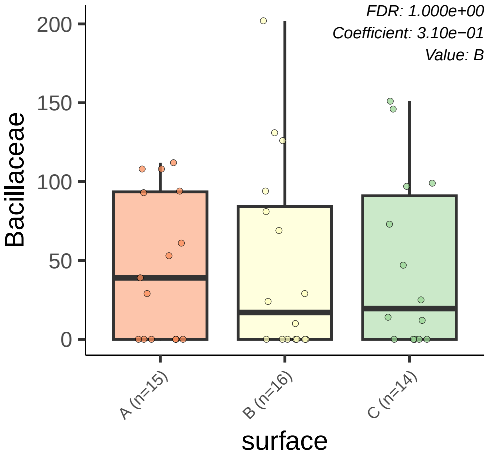
FDR: 1.000e+00
Coefficient: 3.10e−01
Value: B
Bacillaceae
200
150
100
50
0
A (n=15)
B (n=16)
C (n=14)
surface

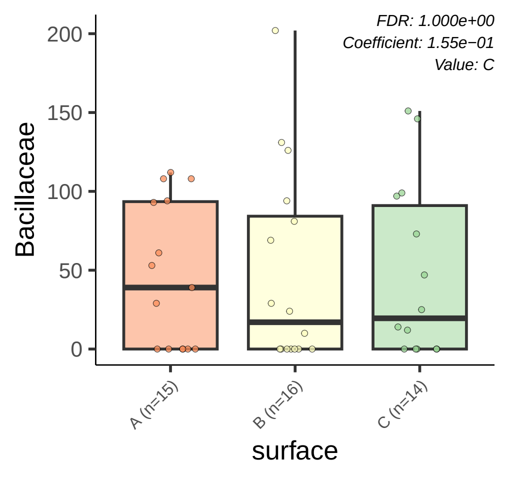

FDR: 1.000e+00
Coefficient: 1.55e−01
Value: C
Bacillaceae
200
150
100
50
0
A (n=15)
B (n=16)
C (n=14)
surface

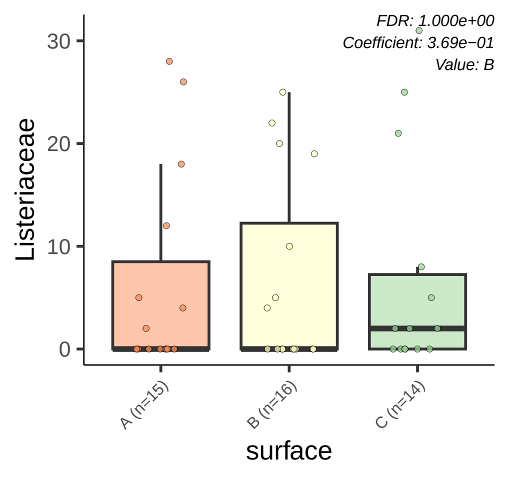

FDR: 1.000e+00
Coefficient: 3.69e−01
Value: B
Listeriaceae
30
20
10
0
A (n=15)
B (n=16)
C (n=14)
surface

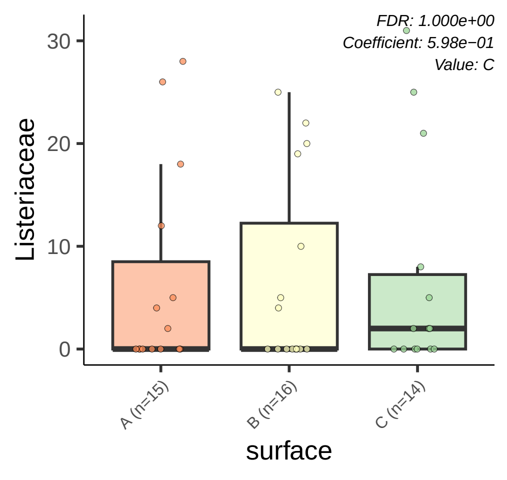
FDR: 1.000e+00
Coefficient: 5.98e−01
Value: C
Listeriaceae
30
20
10
0
A (n=15)
B (n=16)
C (n=14)
surface

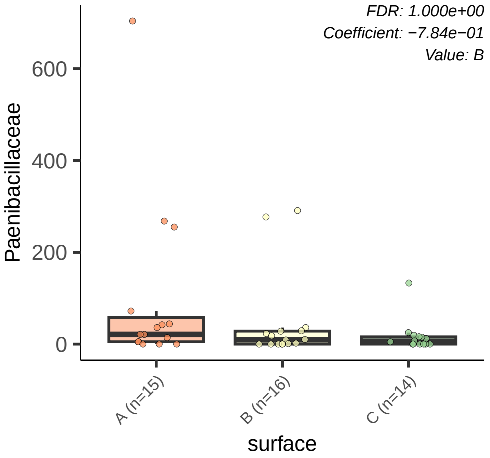
FDR: 1.000e+00
Coefficient: −7.84e−01
Value: B
Paenibacillaceae
600
400
200
0
A (n=15)
B (n=16)
C (n=14)
surface

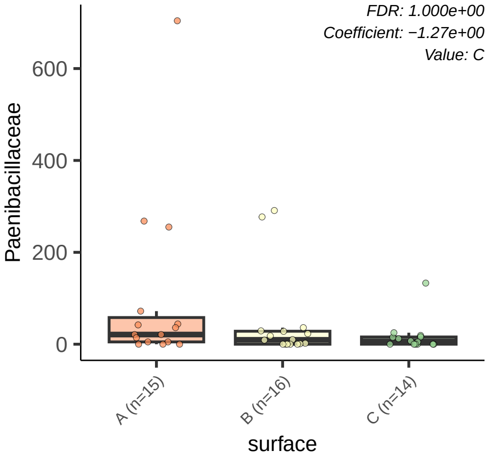

FDR: 1.000e+00
Coefficient: −1.27e+00
Value: C
Paenibacillaceae
600
400
200
0
A (n=15)
B (n=16)
C (n=14)
surface

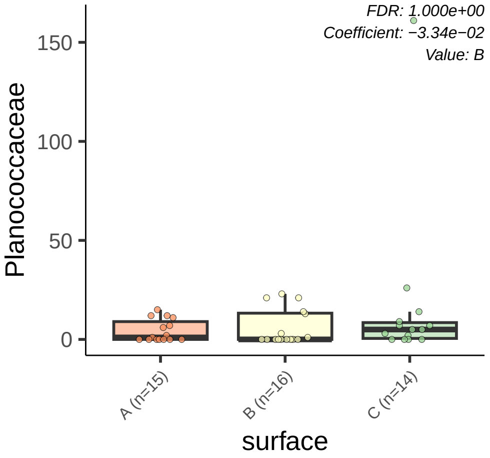
FDR: 1.000e+00
Coefficient: −3.34e−02
Value: B
Planococcaceae
150
100
50
0
A (n=15)
B (n=16)
C (n=14)
surface

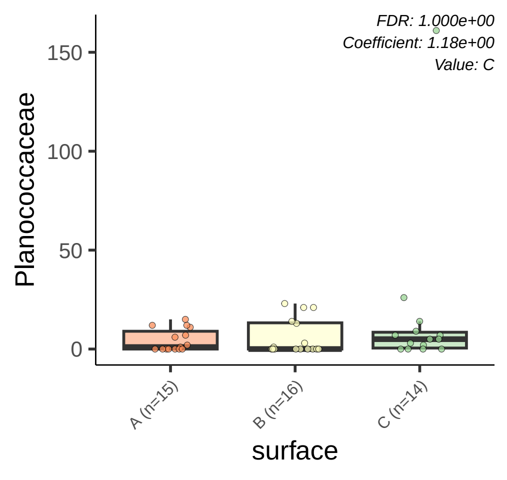

FDR: 1.000e+00
Coefficient: 1.18e+00
Value: C
Planococcaceae
150
100
50
0
A (n=15)
B (n=16)
C (n=14)
surface

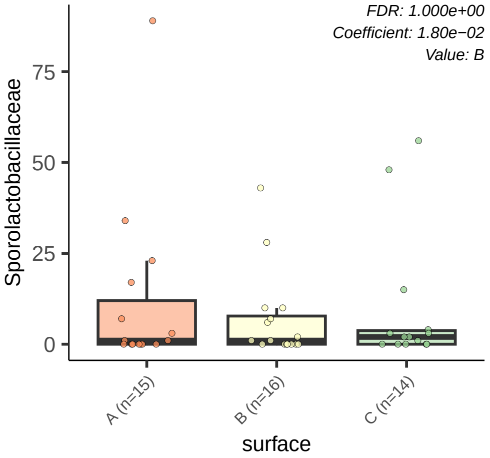
FDR: 1.000e+00
Coefficient: 1.80e−02
Value: B
Sporolactobacillaceae
75
50
25
0
A (n=15)
B (n=16)
C (n=14)
surface

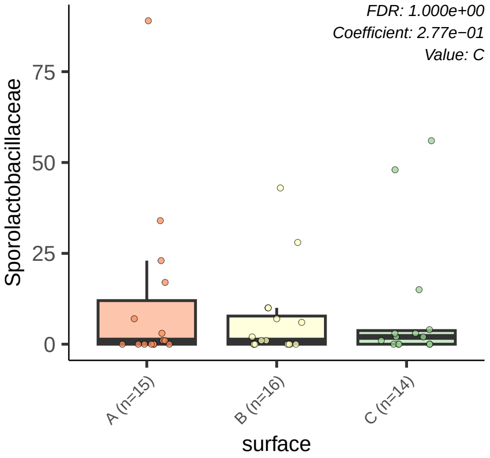
FDR: 1.000e+00
Coefficient: 2.77e−01
Value: C
Sporolactobacillaceae
75
50
25
0
A (n=15)
B (n=16)
C (n=14)
surface

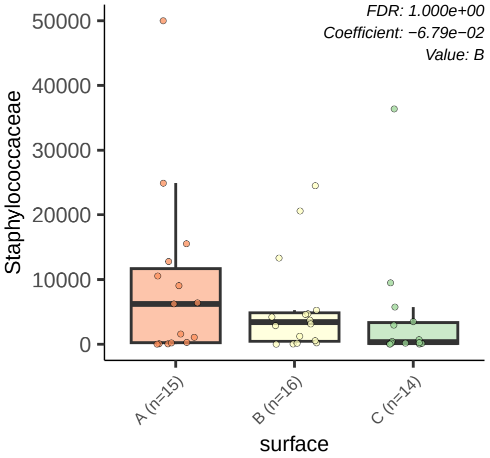
FDR: 1.000e+00
Coefficient: −6.79e−02
Value: B
Staphylococcaceae
50000
40000
30000
20000
10000
0
A (n=15)
B (n=16)
C (n=14)
surface

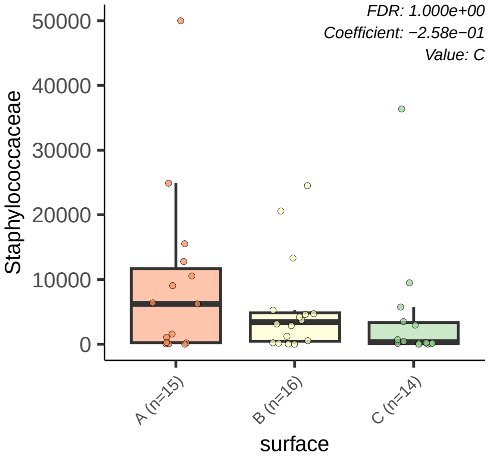

FDR: 1.000e+00
Coefficient: −2.58e−01
Value: C
Staphylococcaceae
50000
40000
30000
20000
10000
0
A (n=15)
B (n=16)
C (n=14)
surface

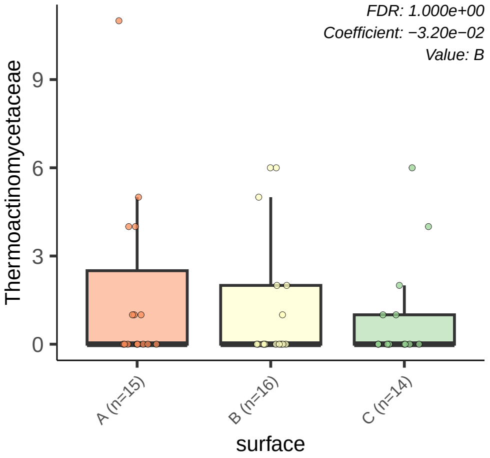
FDR: 1.000e+00
Coefficient: −3.20e−02
Value: B
Thermoactinomycetaceae
0
3
6
9
A (n=15)
B (n=16)
C (n=14)
surface

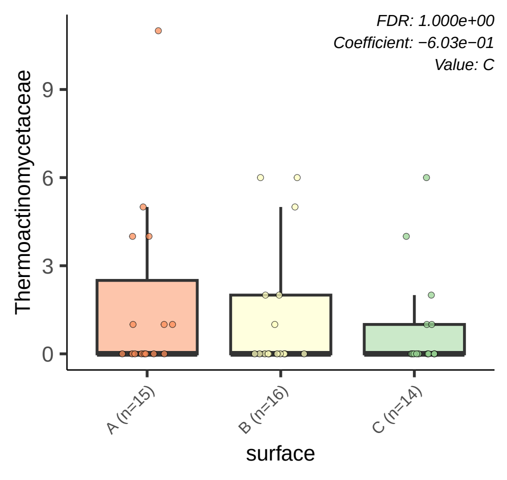

FDR: 1.000e+00
Coefficient: −6.03e−01
Value: C
Thermoactinomycetaceae
0
3
6
9
A (n=15)
B (n=16)
C (n=14)
surface

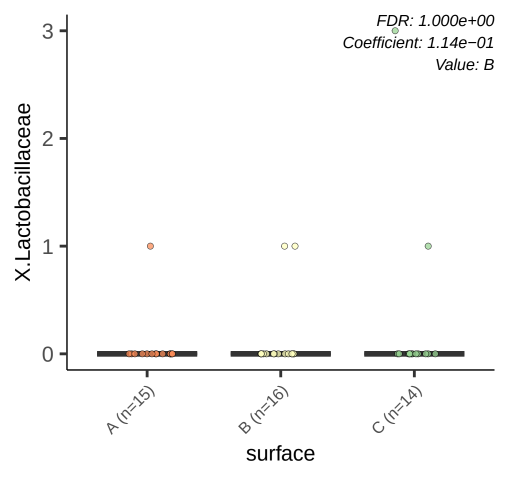
FDR: 1.000e+00
Coefficient: 1.14e−01
Value: B
X.Lactobacillaceae
3
2
1
0
A (n=15)
B (n=16)
C (n=14)
surface

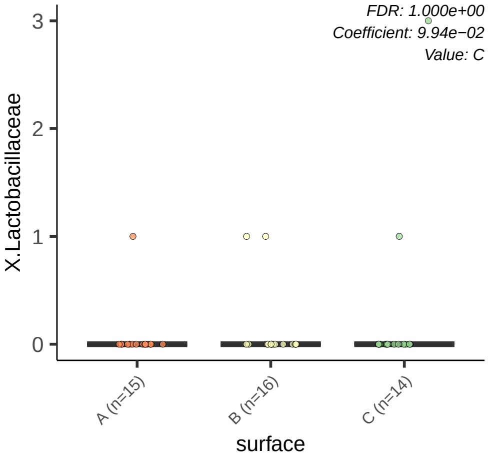

FDR: 1.000e+00
Coefficient: 9.94e−02
Value: C
X.Lactobacillaceae
3
2
1
0
A (n=15)
B (n=16)
C (n=14)
surface

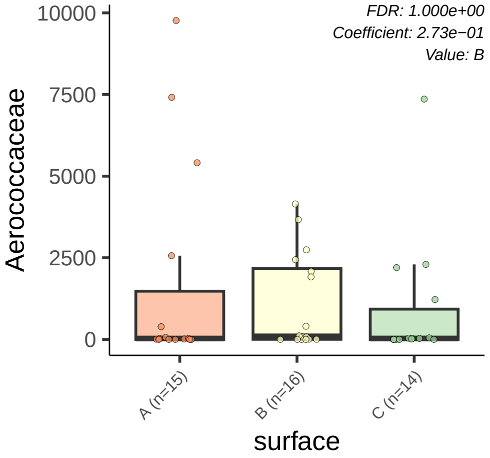

FDR: 1.000e+00
Coefficient: 2.73e−01
Value: B
Aerococcaceae
10000
7500
5000
2500
0
A (n=15)
B (n=16)
C (n=14)
surface

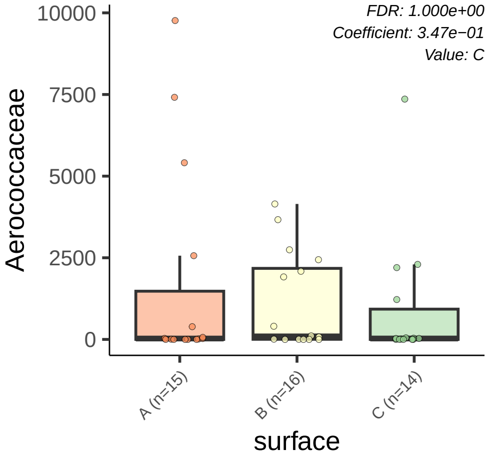

FDR: 1.000e+00
Coefficient: 3.47e−01
Value: C
Aerococcaceae
10000
7500
5000
2500
0
A (n=15)
B (n=16)
C (n=14)
surface

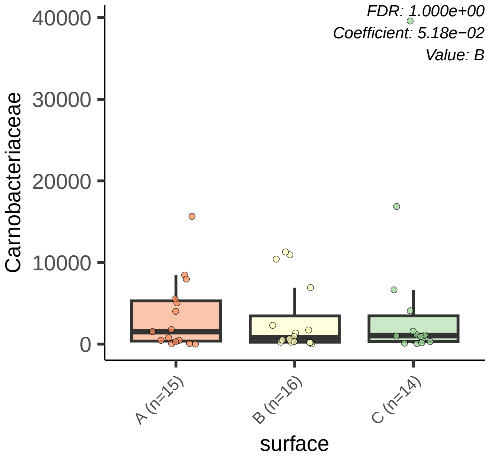

FDR: 1.000e+00
Coefficient: 5.18e−02
Value: B
40000
30000
20000
10000
0
Carnobacteriaceae
A (n=15)
B (n=16)
C (n=14)
surface

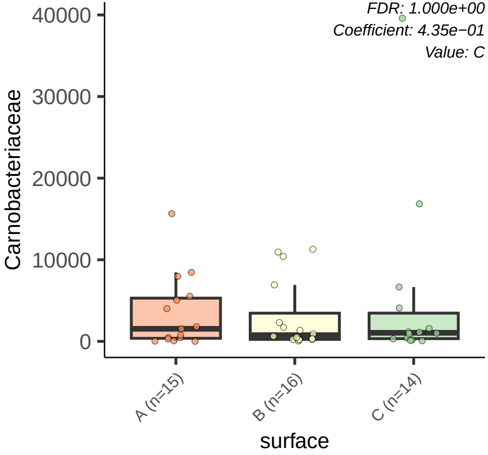
FDR: 1.000e+00
Coefficient: 4.35e−01
Value: C
Carnobacteriaceae
40000
30000
20000
10000
0
A (n=15)
B (n=16)
C (n=14)
surface

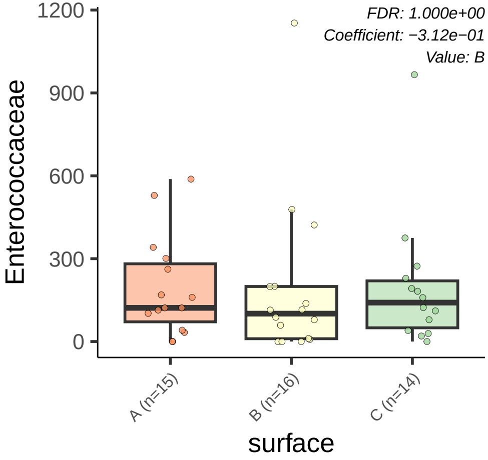
FDR: 1.000e+00
Coefficient: −3.12e−01
Value: B
Enterococcaceae
1200
900
600
300
0
A (n=15)
B (n=16)
C (n=14)
surface

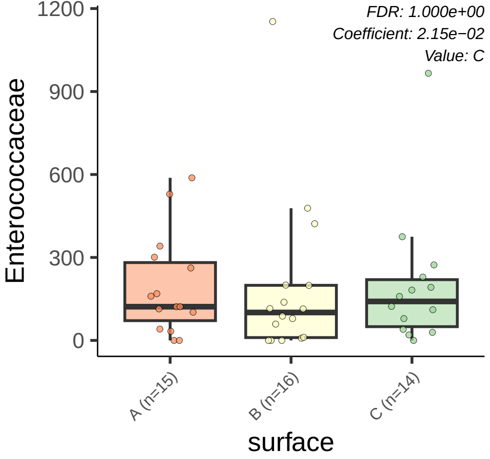

FDR: 1.000e+00
Coefficient: 2.15e−02
Value: C
Enterococcaceae
1200
900
600
300
0
A (n=15)
B (n=16)
C (n=14)
surface

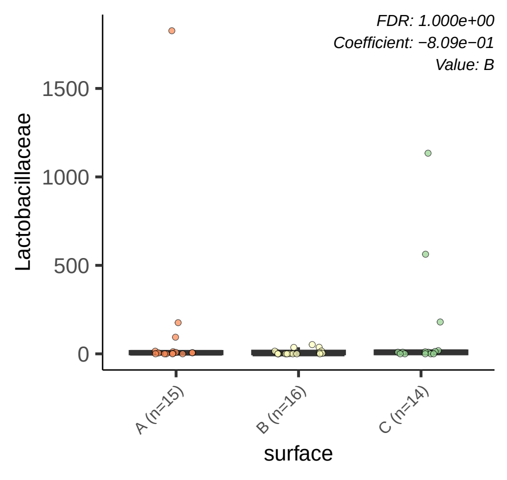
FDR: 1.000e+00
Coefficient: −8.09e−01
Value: B
Lactobacillaceae
1500
1000
500
0
A (n=15)
B (n=16)
C (n=14)
surface

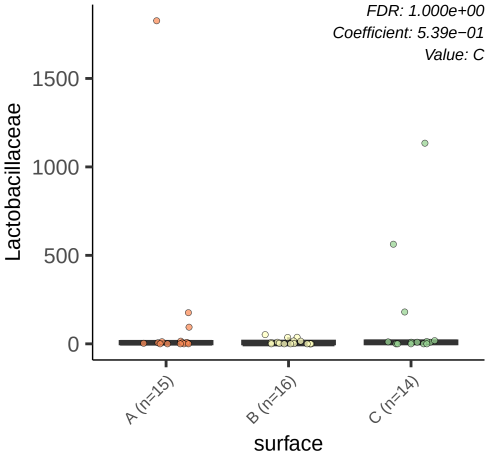
FDR: 1.000e+00
Coefficient: 5.39e−01
Value: C
Lactobacillaceae
1500
1000
500
0
A (n=15)
B (n=16)
C (n=14)
surface

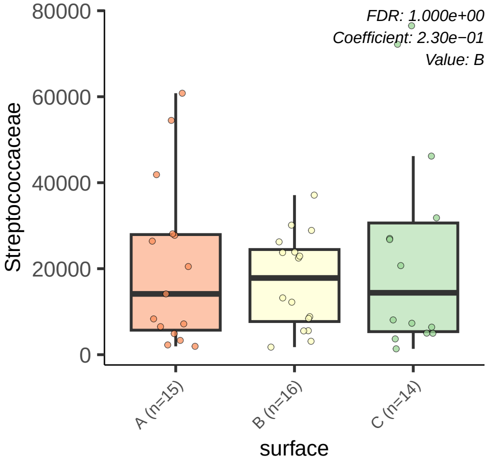
FDR: 1.000e+00
Coefficient: 2.30e−01
Value: B
Streptococcaceae
80000
60000
40000
20000
0
A (n=15)
B (n=16)
C (n=14)
surface

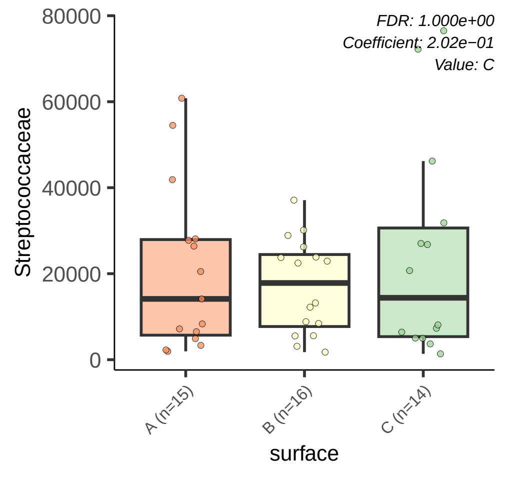
FDR: 1.000e+00
Coefficient: 2.02e−01
Value: C
Streptococcaceae
80000
60000
40000
20000
0
A (n=15)
B (n=16)
C (n=14)
surface

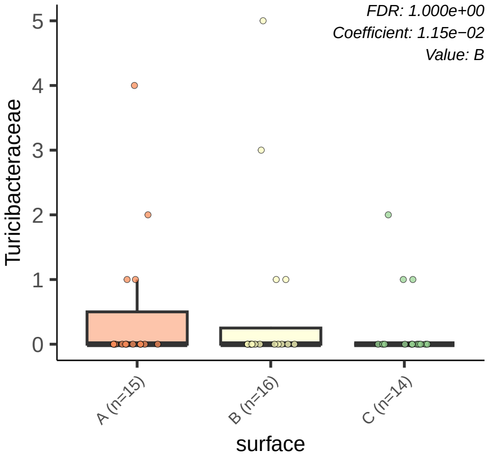

FDR: 1.000e+00
Coefficient: 1.15e−02
Value: B
Turicibacteraceae
0
1
2
3
4
5
A (n=15)
B (n=16)
C (n=14)
surface

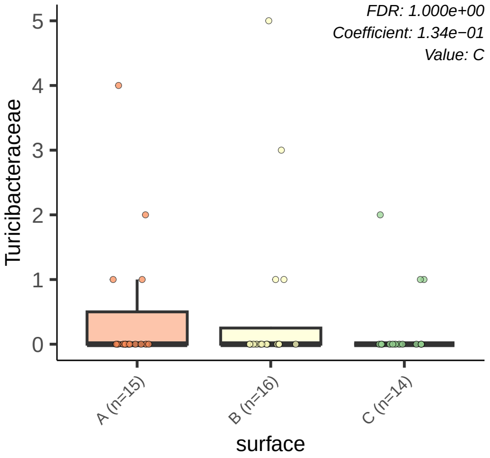

FDR: 1.000e+00
Coefficient: 1.34e−01
Value: C
Turicibacteraceae
0
1
2
3
4
5
A (n=15)
B (n=16)
C (n=14)
surface

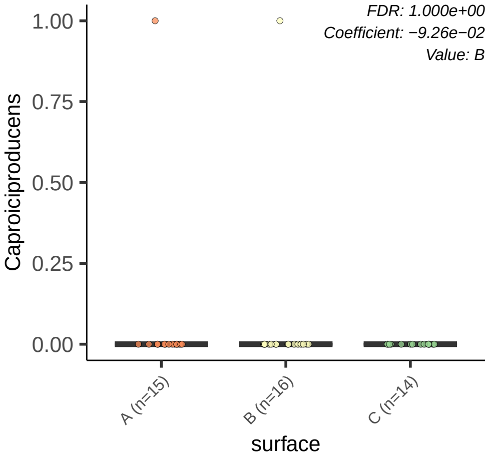
FDR: 1.000e+00
Coefficient: −9.26e−02
Value: B
Caproiciproducens
1.00
0.75
0.50
0.25
0.00
A (n=15)
B (n=16)
C (n=14)
surface

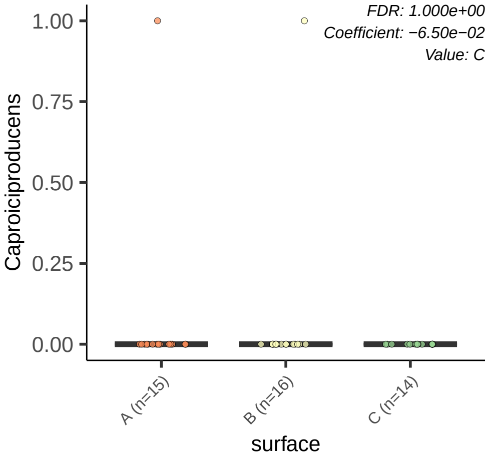

FDR: 1.000e+00
Coefficient: −6.50e−02
Value: C
Caproiciproducens
1.00
0.75
0.50
0.25
0.00
A (n=15)
B (n=16)
C (n=14)
surface

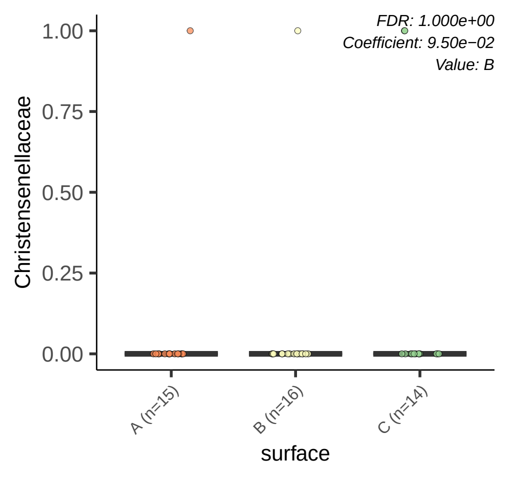
FDR: 1.000e+00
Coefficient: 9.50e−02
Value: B
Christensenellaceae
1.00
0.75
0.50
0.25
0.00
A (n=15)
B (n=16)
C (n=14)
surface

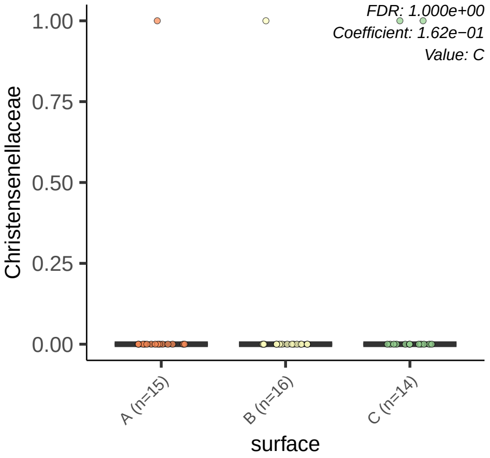
FDR: 1.000e+00
Coefficient: 1.62e−01
Value: C
Christensenellaceae
1.00
0.75
0.50
0.25
0.00
A (n=15)
B (n=16)
C (n=14)
surface

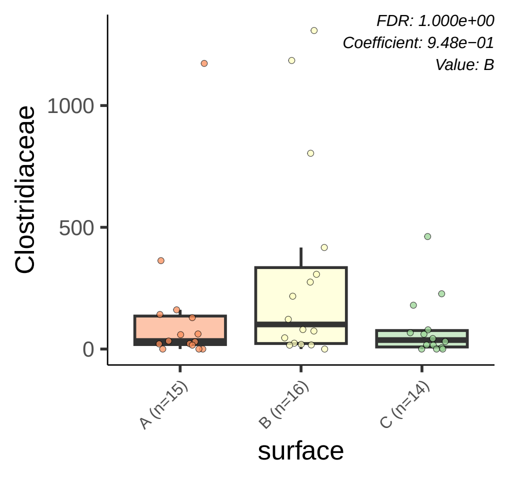
FDR: 1.000e+00
Coefficient: 9.48e−01
Value: B
Clostridiaceae
1000
500
0
A (n=15)
B (n=16)
C (n=14)
surface

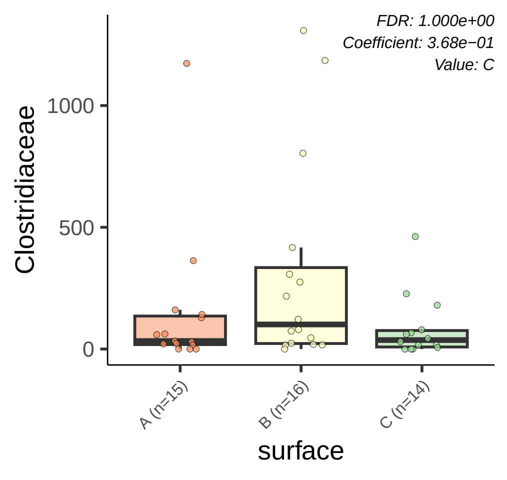
FDR: 1.000e+00
Coefficient: 3.68e−01
Value: C
Clostridiaceae
1000
500
0
A (n=15)
B (n=16)
C (n=14)
surface

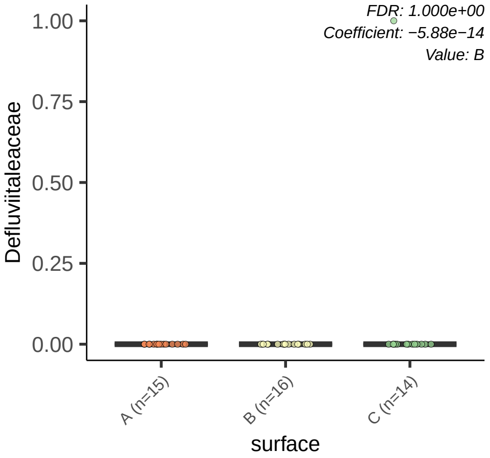
FDR: 1.000e+00
Coefficient: −5.88e−14
Value: B
Defluviitaleaceae
1.00
0.75
0.50
0.25
0.00
A (n=15)
B (n=16)
C (n=14)
surface

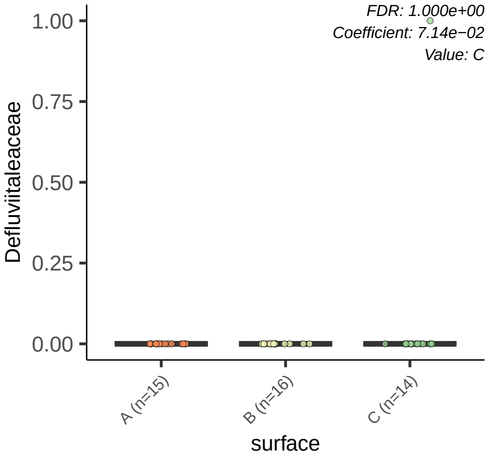
FDR: 1.000e+00
Coefficient: 7.14e−02
Value: C
Defluviitaleaceae
1.00
0.75
0.50
0.25
0.00
A (n=15)
B (n=16)
C (n=14)
surface

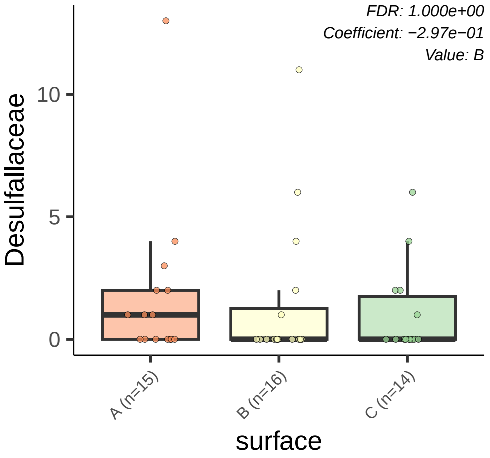
FDR: 1.000e+00
Coefficient: −2.97e−01
Value: B
Desulfallaceae
10
5
0
A (n=15)
B (n=16)
C (n=14)
surface

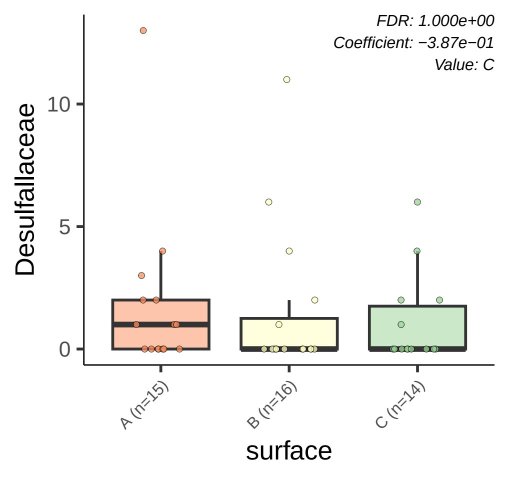

FDR: 1.000e+00
Coefficient: −3.87e−01
Value: C
Desulfallaceae
10
5
0
A (n=15)
B (n=16)
C (n=14)
surface

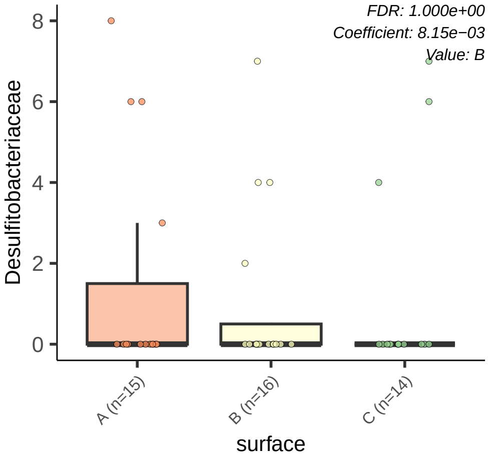
FDR: 1.000e+00
Coefficient: 8.15e−03
Value: B
Desulfitobacteriaceae
0
2
4
6
8
A (n=15)
B (n=16)
C (n=14)
surface

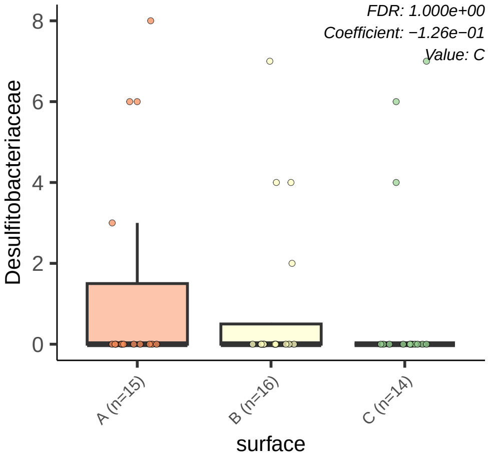
FDR: 1.000e+00
Coefficient: −1.26e−01
Value: C
Desulfitobacteriaceae
8
6
4
2
0
A (n=15)
B (n=16)
C (n=14)
surface

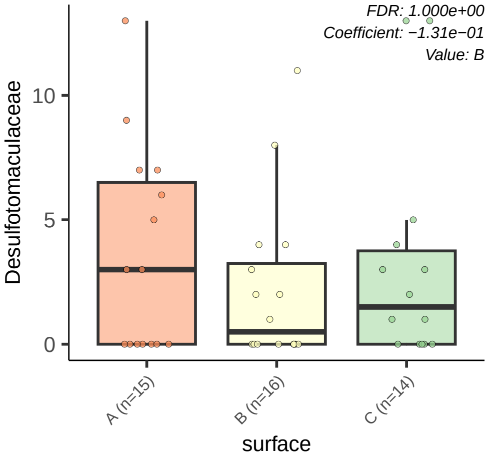
FDR: 1.000e+00
Coefficient: −1.31e−01
Value: B
Desulfotomaculaceae
10
5
0
A (n=15)
B (n=16)
C (n=14)
surface

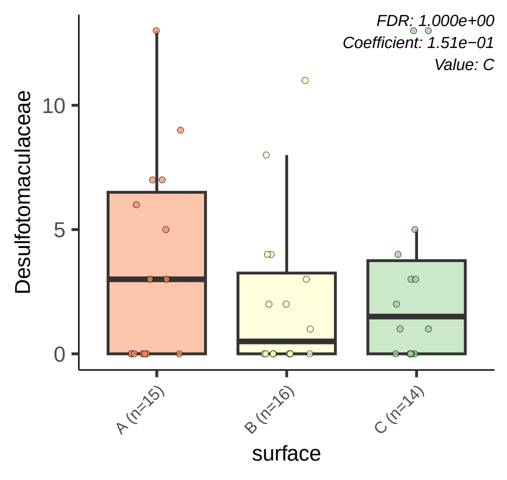
FDR: 1.000e+00
Coefficient: 1.51e−01
Value: C
Desulfotomaculaceae
0
5
10
A (n=15)
B (n=16)
C (n=14)
surface

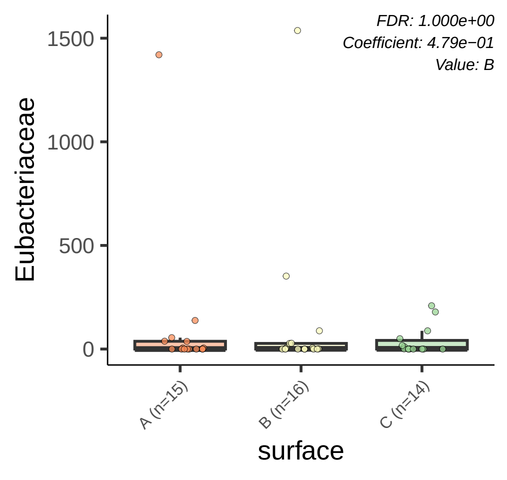
FDR: 1.000e+00
Coefficient: 4.79e−01
Value: B
Eubacteriaceae
1500
1000
500
0
A (n=15)
B (n=16)
C (n=14)
surface

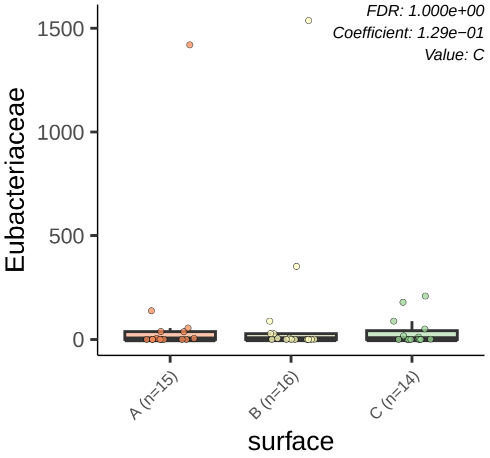

FDR: 1.000e+00
Coefficient: 1.29e−01
Value: C
Eubacteriaceae
1500
1000
500
0
A (n=15)
B (n=16)
C (n=14)
surface

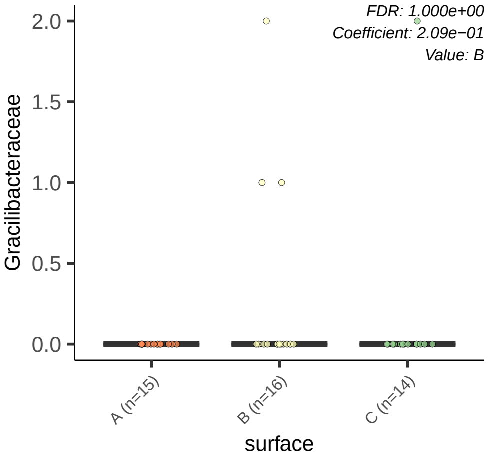

FDR: 1.000e+00
Coefficient: 2.09e−01
Value: B
Gracilibacteraceae
2.0
1.5
1.0
0.5
0.0
A (n=15)
B (n=16)
C (n=14)
surface

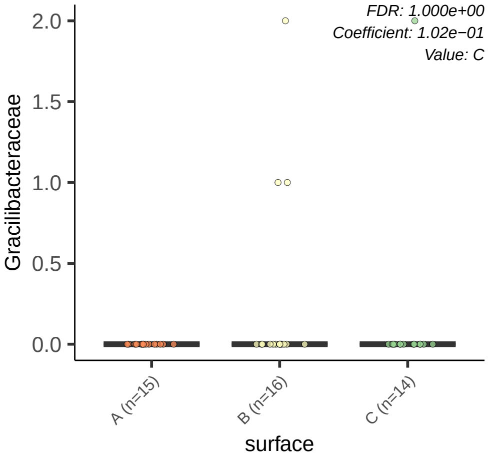
FDR: 1.000e+00
Coefficient: 1.02e−01
Value: C
Gracilibacteraceae
2.0
1.5
1.0
0.5
0.0
A (n=15)
B (n=16)
C (n=14)
surface

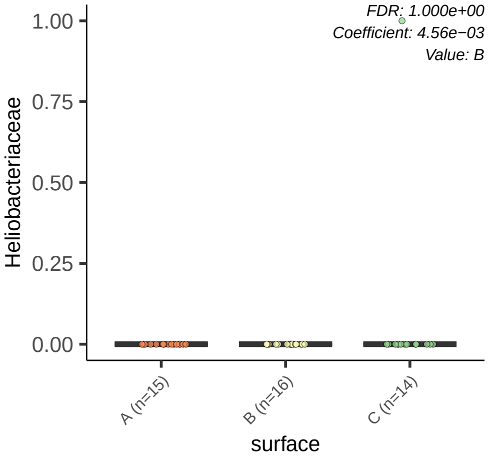
FDR: 1.000e+00
Coefficient: 4.56e−03
Value: B
Heliobacteriaceae
1.00
0.75
0.50
0.25
0.00
A (n=15)
B (n=16)
C (n=14)
surface

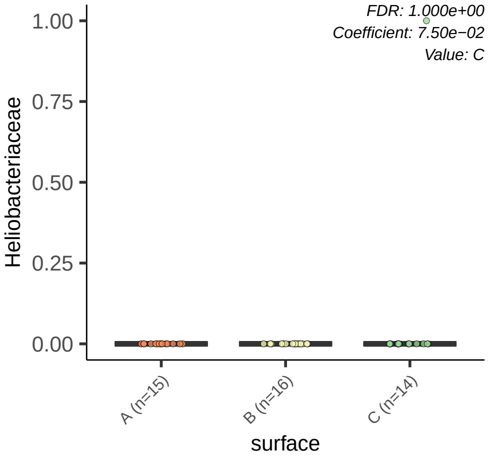
FDR: 1.000e+00
Coefficient: 7.50e−02
Value: C
1.00
0.75
0.50
0.25
0.00
Heliobacteriaceae
A (n=15)
B (n=16)
C (n=14)
surface

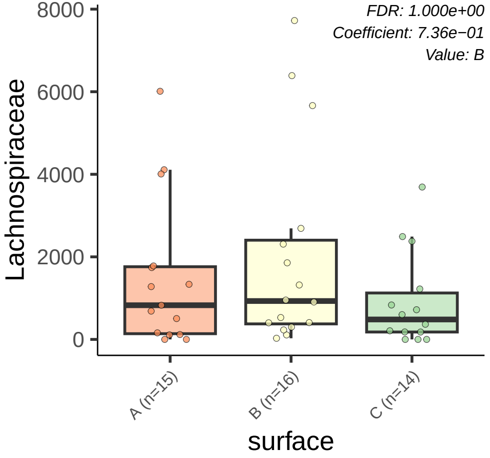
FDR: 1.000e+00
Coefficient: 7.36e−01
Value: B
Lachnospiraceae
8000
6000
4000
2000
0
A (n=15)
B (n=16)
C (n=14)
surface

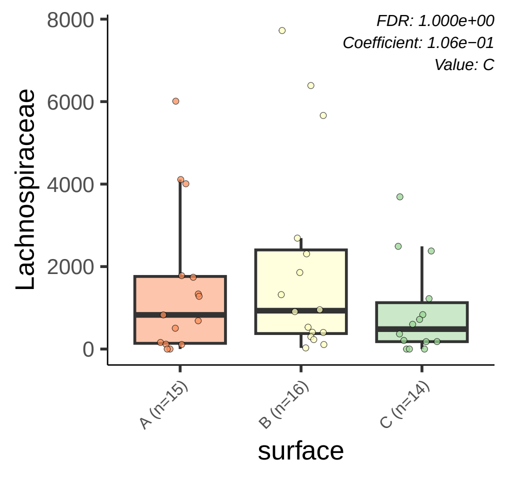

FDR: 1.000e+00
Coefficient: 1.06e−01
Value: C
8000
6000
4000
2000
0
Lachnospiraceae
A (n=15)
B (n=16)
C (n=14)
surface

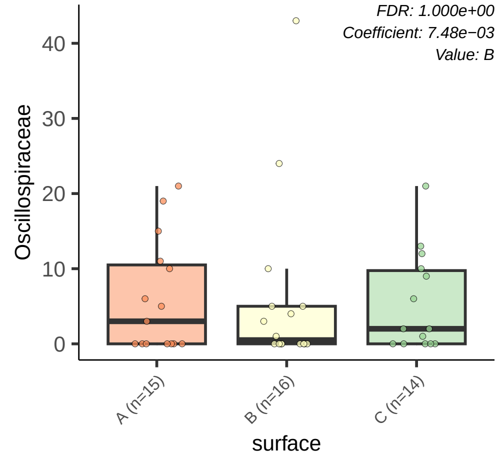
FDR: 1.000e+00
Coefficient: 7.48e−03
Value: B
Oscillospiraceae
40
30
20
10
0
A (n=15)
B (n=16)
C (n=14)
surface

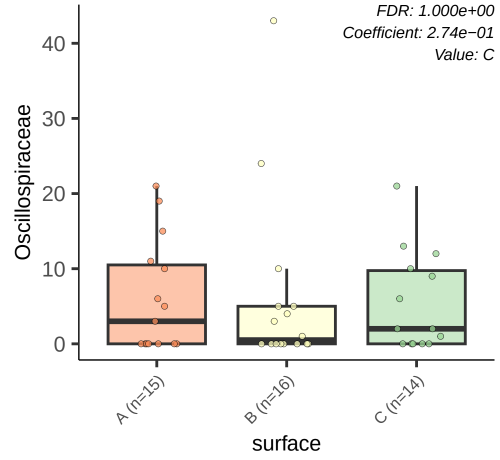
FDR: 1.000e+00
Coefficient: 2.74e−01
Value: C
Oscillospiraceae
0
10
20
30
40
A (n=15)
B (n=16)
C (n=14)
surface

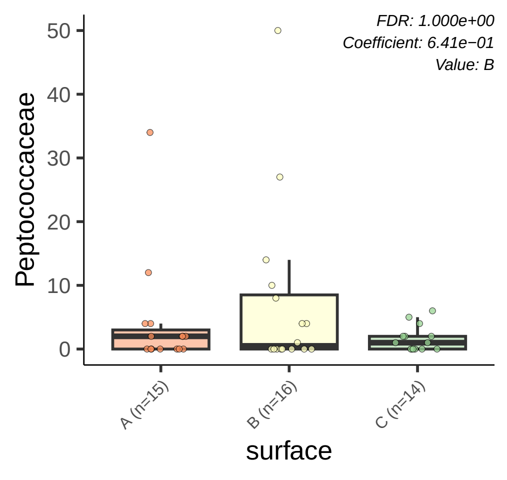

FDR: 1.000e+00
Coefficient: 6.41e−01
Value: B
Peptococcaceae
50
40
30
20
10
0
A (n=15)
B (n=16)
C (n=14)
surface

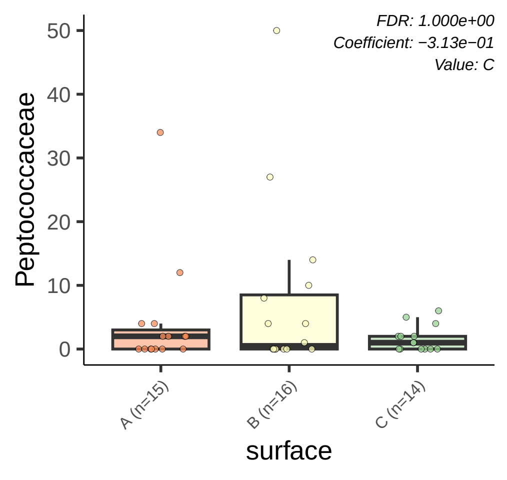
FDR: 1.000e+00
Coefficient: −3.13e−01
Value: C
Peptococcaceae
0
10
20
30
40
50
A (n=15)
B (n=16)
C (n=14)
surface

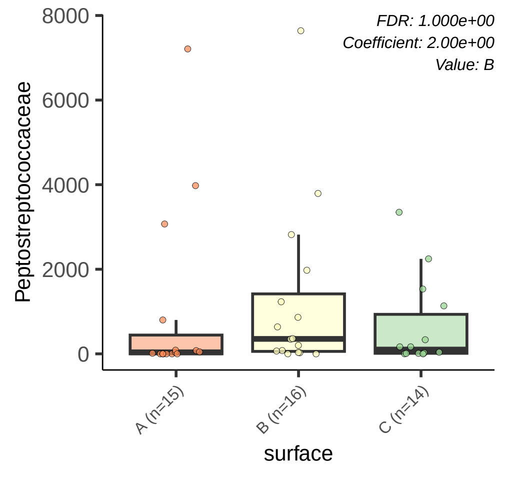
FDR: 1.000e+00
Coefficient: 2.00e+00
Value: B
Peptostreptococcaceae
8000
6000
4000
2000
0
A (n=15)
B (n=16)
C (n=14)
surface

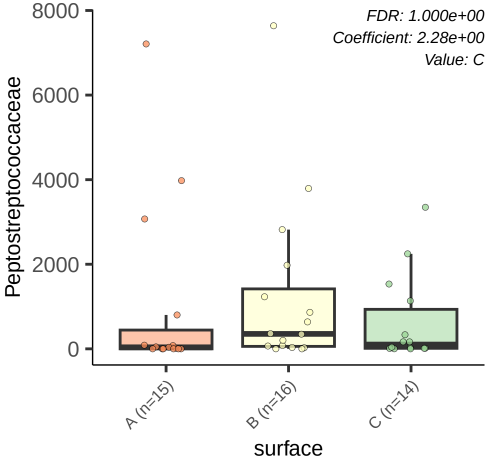
FDR: 1.000e+00
Coefficient: 2.28e+00
Value: C
8000
6000
4000
2000
0
Peptostreptococcaceae
A (n=15)
B (n=16)
C (n=14)
surface

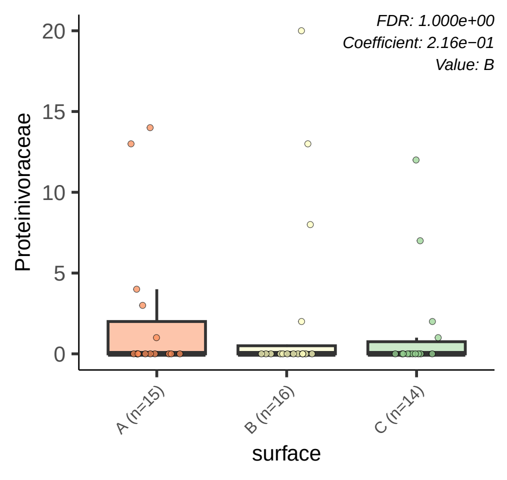
FDR: 1.000e+00
Coefficient: 2.16e−01
Value: B
Proteinivoraceae
20
15
10
5
0
A (n=15)
B (n=16)
C (n=14)
surface

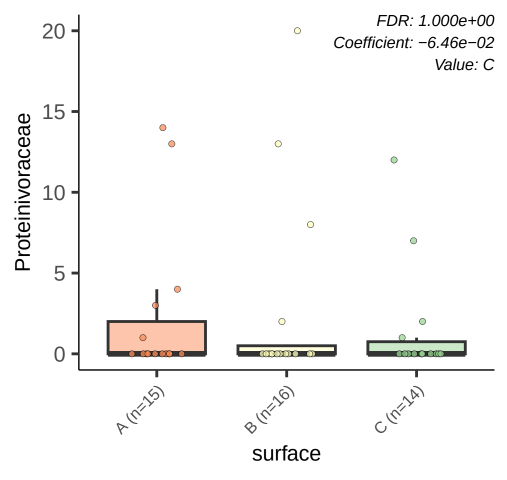

FDR: 1.000e+00
Coefficient: −6.46e−02
Value: C
Proteinivoraceae
20
15
10
5
0
A (n=15)
B (n=16)
C (n=14)
surface

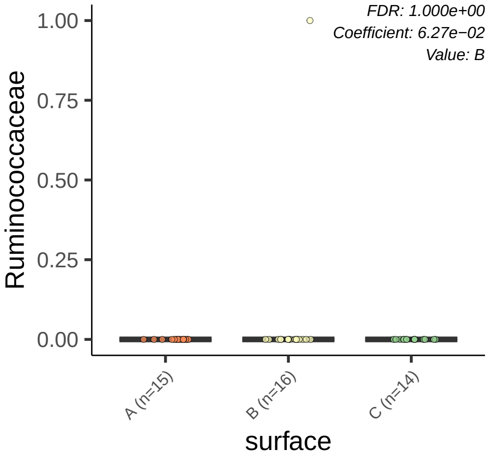

FDR: 1.000e+00
Coefficient: 6.27e−02
Value: B
Ruminococcaceae
1.00
0.75
0.50
0.25
0.00
A (n=15)
B (n=16)
C (n=14)
surface

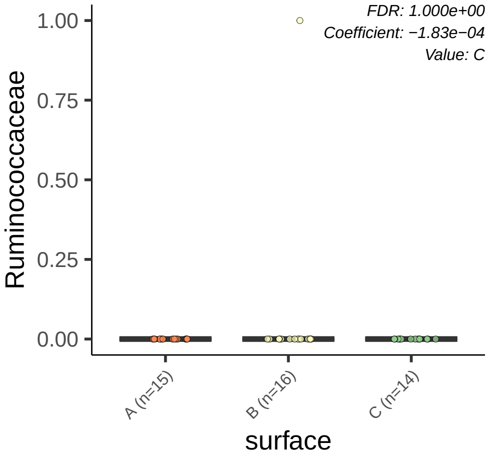
FDR: 1.000e+00
Coefficient: −1.83e−04
Value: C
1.00
0.75
0.50
0.25
0.00
Ruminococcaceae
A (n=15)
B (n=16)
C (n=14)
surface

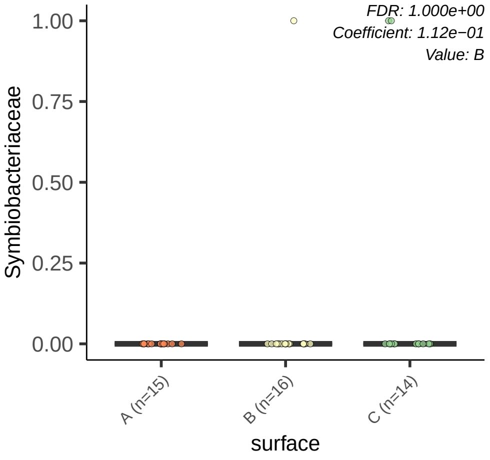
FDR: 1.000e+00
Coefficient: 1.12e−01
Value: B
Symbiobacteriaceae
1.00
0.75
0.50
0.25
0.00
A (n=15)
B (n=16)
C (n=14)
surface

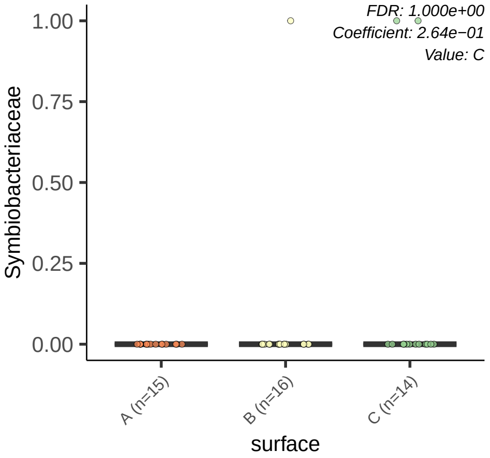
FDR: 1.000e+00
Coefficient: 2.64e−01
Value: C
Symbiobacteriaceae
1.00
0.75
0.50
0.25
0.00
A (n=15)
B (n=16)
C (n=14)
surface

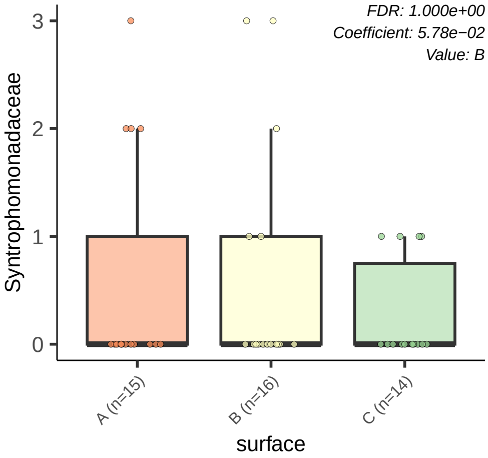
FDR: 1.000e+00
Coefficient: 5.78e−02
Value: B
Syntrophomonadaceae
3
2
1
0
A (n=15)
B (n=16)
C (n=14)
surface

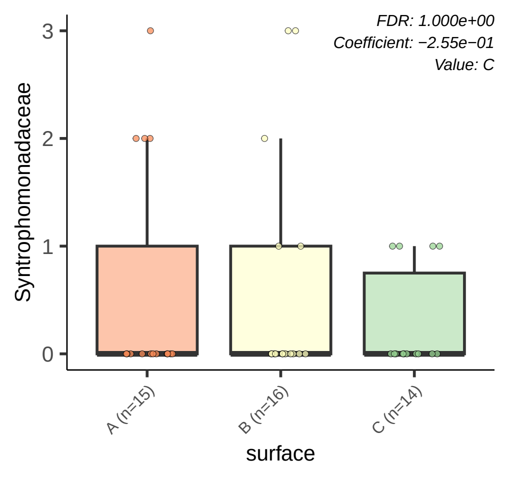

FDR: 1.000e+00
Coefficient: −2.55e−01
Value: C
Syntrophomonadaceae
3
2
1
0
A (n=15)
B (n=16)
C (n=14)
surface

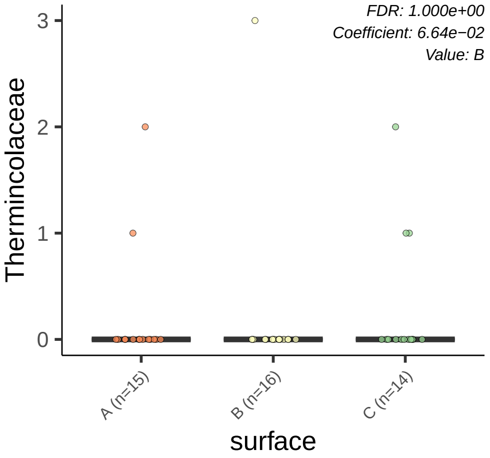
FDR: 1.000e+00
Coefficient: 6.64e−02
Value: B
Thermincolaceae
0
1
2
3
A (n=15)
B (n=16)
C (n=14)
surface

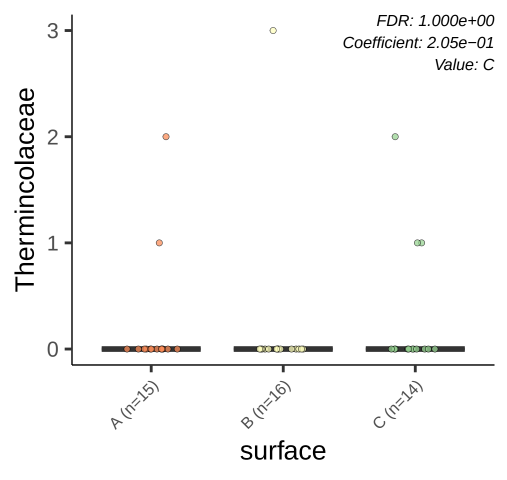

FDR: 1.000e+00
Coefficient: 2.05e−01
Value: C
Thermincolaceae
3
2
1
0
A (n=15)
B (n=16)
C (n=14)
surface

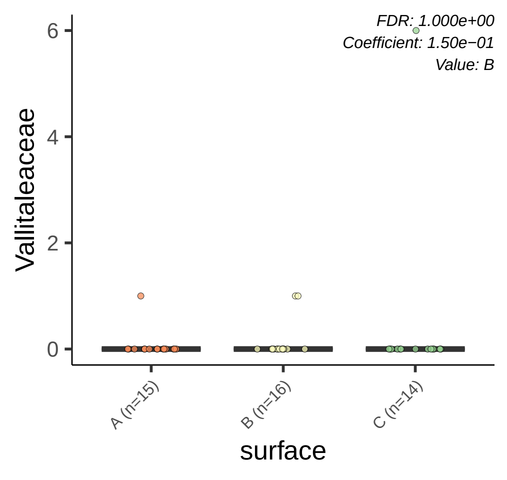

FDR: 1.000e+00
Coefficient: 1.50e−01
Value: B
Vallitaleaceae
6
4
2
0
A (n=15)
B (n=16)
C (n=14)
surface

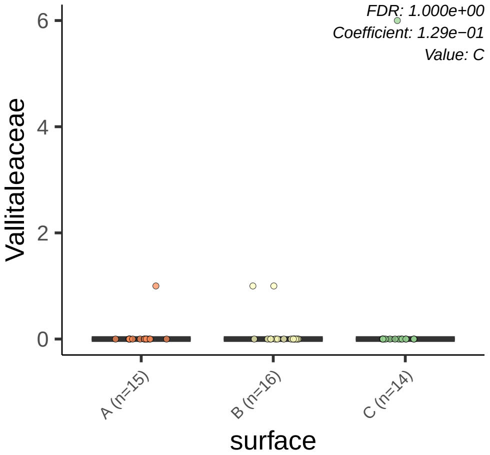
FDR: 1.000e+00
Coefficient: 1.29e−01
Value: C
Vallitaleaceae
0
2
4
6
A (n=15)
B (n=16)
C (n=14)
surface

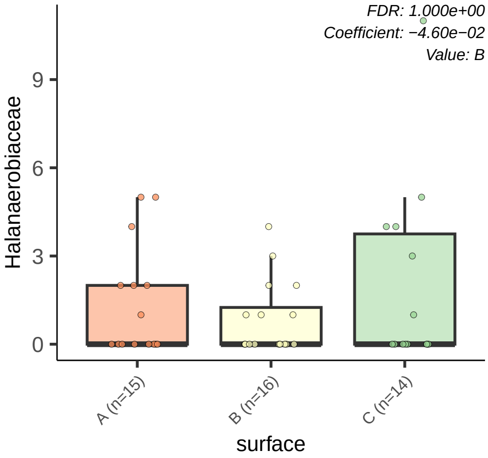
FDR: 1.000e+00
Coefficient: −4.60e−02
Value: B
Halanaerobiaceae
0
3
6
9
A (n=15)
B (n=16)
C (n=14)
surface

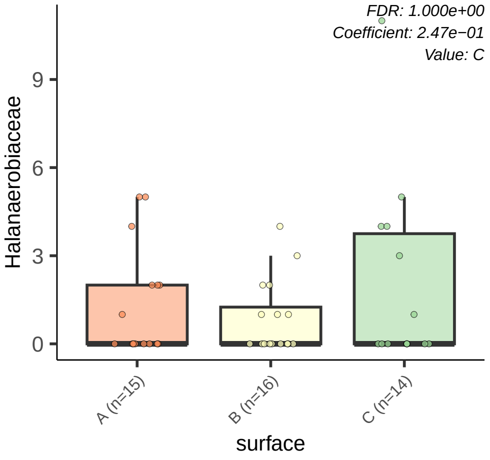
FDR: 1.000e+00
Coefficient: 2.47e−01
Value: C
Halanaerobiaceae
0
3
6
9
A (n=15)
B (n=16)
C (n=14)
surface

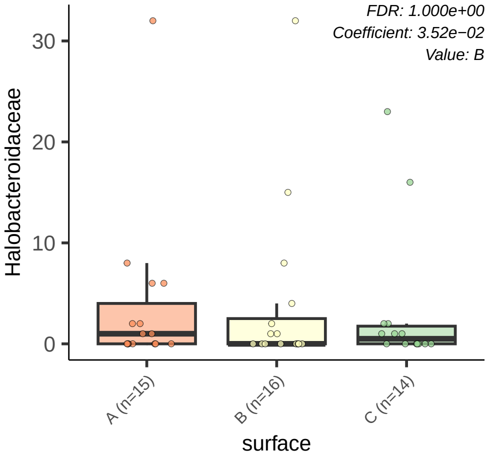
FDR: 1.000e+00
Coefficient: 3.52e−02
Value: B
Halobacteroidaceae
0
10
20
30
A (n=15)
B (n=16)
C (n=14)
surface

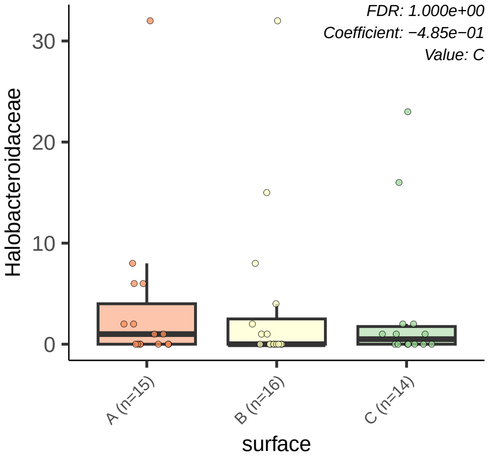

FDR: 1.000e+00
Coefficient: −4.85e−01
Value: C
Halobacteroidaceae
0
10
20
30
A (n=15)
B (n=16)
C (n=14)
surface

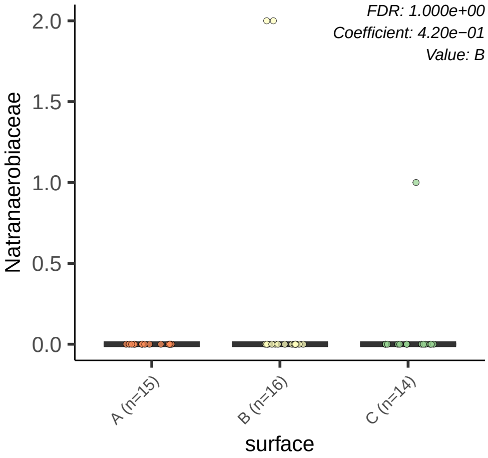

FDR: 1.000e+00
Coefficient: 4.20e−01
Value: B
Natranaerobiaceae
2.0
1.5
1.0
0.5
0.0
A (n=15)
B (n=16)
C (n=14)
surface

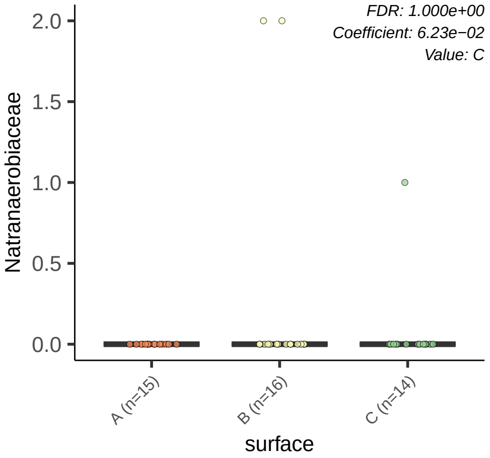

FDR: 1.000e+00
Coefficient: 6.23e−02
Value: C
Natranaerobiaceae
2.0
1.5
1.0
0.5
0.0
A (n=15)
B (n=16)
C (n=14)
surface

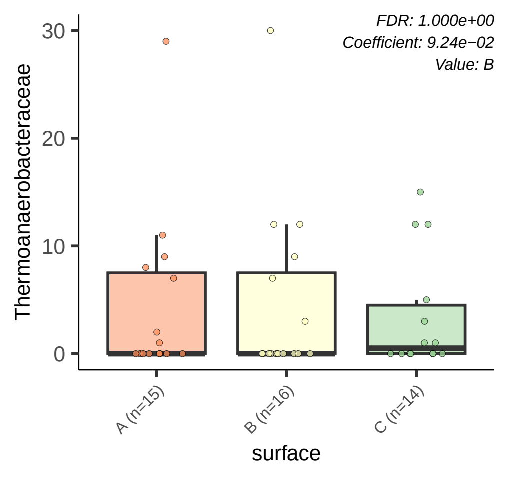
FDR: 1.000e+00
Coefficient: 9.24e−02
Value: B
Thermoanaerobacteraceae
30
20
10
0
A (n=15)
B (n=16)
C (n=14)
surface

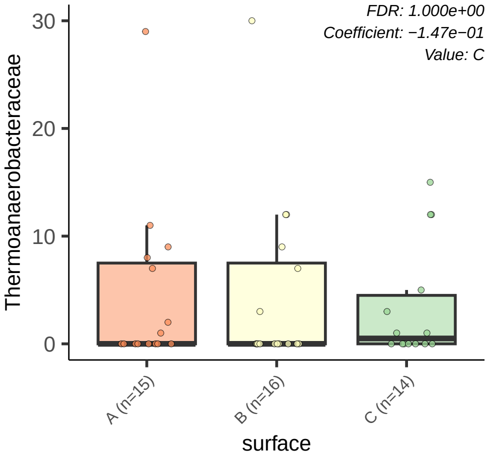

FDR: 1.000e+00
Coefficient: −1.47e−01
Value: C
Thermoanaerobacteraceae
30
20
10
0
A (n=15)
B (n=16)
C (n=14)
surface

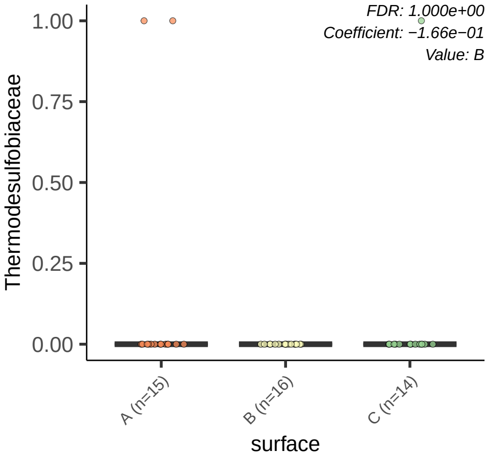
FDR: 1.000e+00
Coefficient: −1.66e−01
Value: B
Thermodesulfobiaceae
1.00
0.75
0.50
0.25
0.00
A (n=15)
B (n=16)
C (n=14)
surface

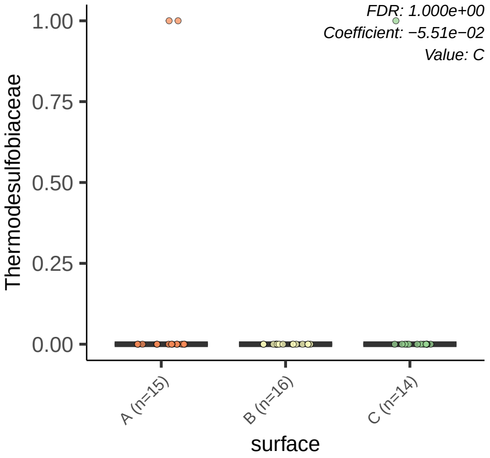
FDR: 1.000e+00
Coefficient: −5.51e−02
Value: C
Thermodesulfobiaceae
1.00
0.75
0.50
0.25
0.00
A (n=15)
B (n=16)
C (n=14)
surface

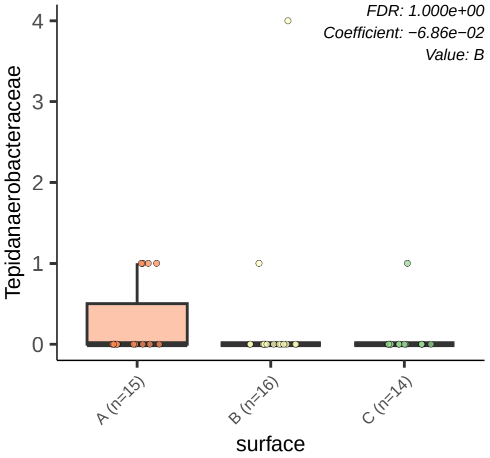
FDR: 1.000e+00
Coefficient: −6.86e−02
Value: B
Tepidanaerobacteraceae
4
3
2
1
0
A (n=15)
B (n=16)
C (n=14)
surface

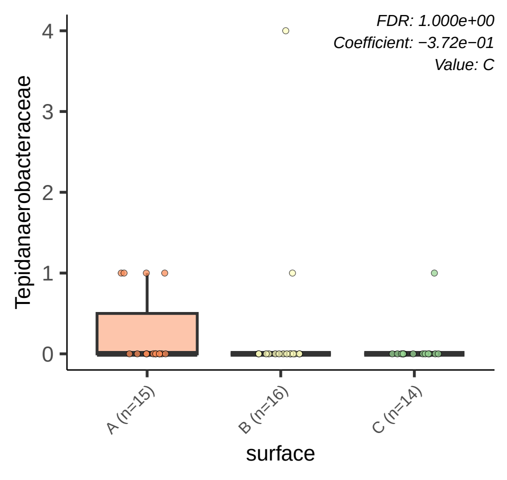

FDR: 1.000e+00
Coefficient: −3.72e−01
Value: C
Tepidanaerobacteraceae
0
1
2
3
4
A (n=15)
B (n=16)
C (n=14)
surface

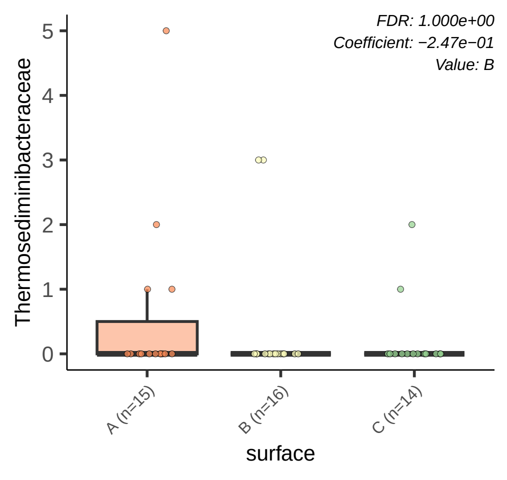

FDR: 1.000e+00
Coefficient: −2.47e−01
Value: B
Thermosediminibacteraceae
0
1
2
3
4
5
A (n=15)
B (n=16)
C (n=14)
surface

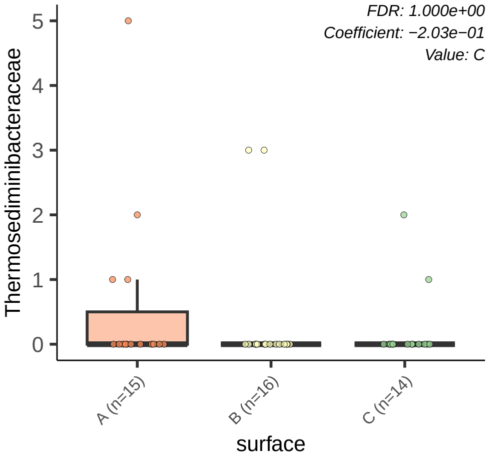
FDR: 1.000e+00
Coefficient: −2.03e−01
Value: C
Thermosediminibacteraceae
0
1
2
3
4
5
A (n=15)
B (n=16)
C (n=14)
surface

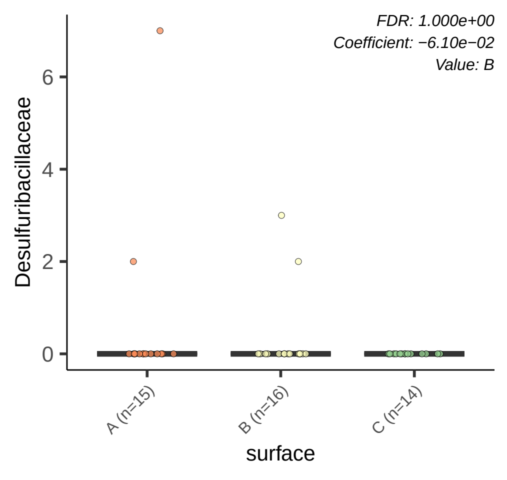

FDR: 1.000e+00
Coefficient: −6.10e−02
Value: B
Desulfuribacillaceae
0
2
4
6
A (n=15)
B (n=16)
C (n=14)
surface

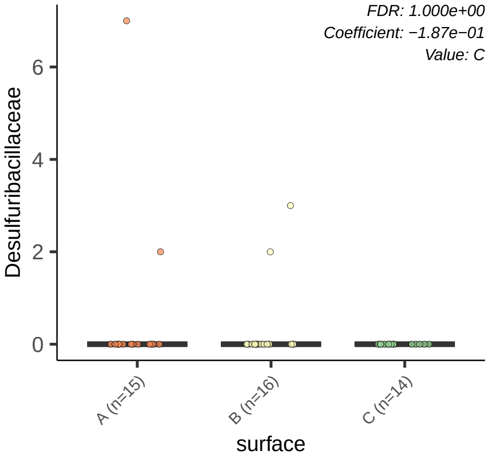

FDR: 1.000e+00
Coefficient: −1.87e−01
Value: C
Desulfuribacillaceae
6
4
2
0
A (n=15)
B (n=16)
C (n=14)
surface

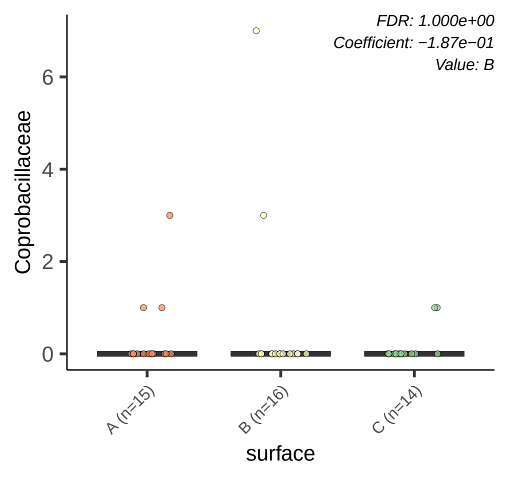
FDR: 1.000e+00
Coefficient: −1.87e−01
Value: B
Coprobacillaceae
0
2
4
6
A (n=15)
B (n=16)
C (n=14)
surface

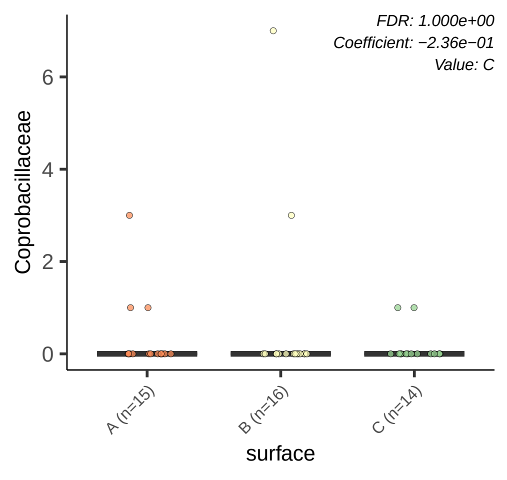
FDR: 1.000e+00
Coefficient: −2.36e−01
Value: C
Coprobacillaceae
0
2
4
6
A (n=15)
B (n=16)
C (n=14)
surface

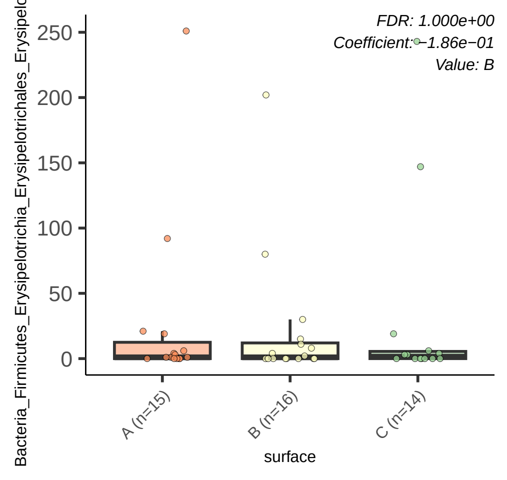
FDR: 1.000e+00
Coefficient: −1.86e−01
Value: B
Bacteria_Firmicutes_Erysipelotrichia_Erysipelotrichales_Erysipelo
250
200
150
100
50
0
A (n=15)
B (n=16)
C (n=14)
surface

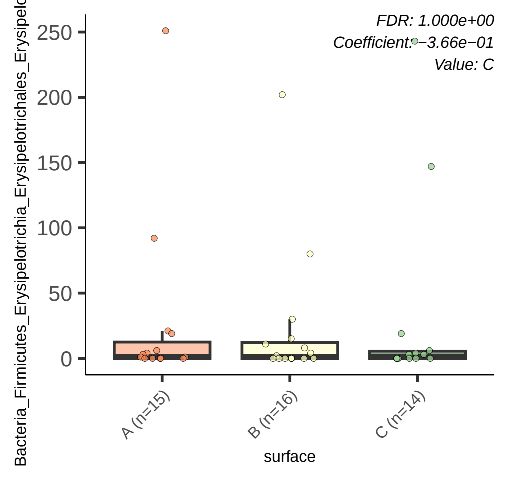

FDR: 1.000e+00
Coefficient: −3.66e−01
Value: C
Bacteria_Firmicutes_Erysipelotrichia_Erysipelotrichales_Erysipelo
250
200
150
100
50
0
A (n=15)
B (n=16)
C (n=14)
surface

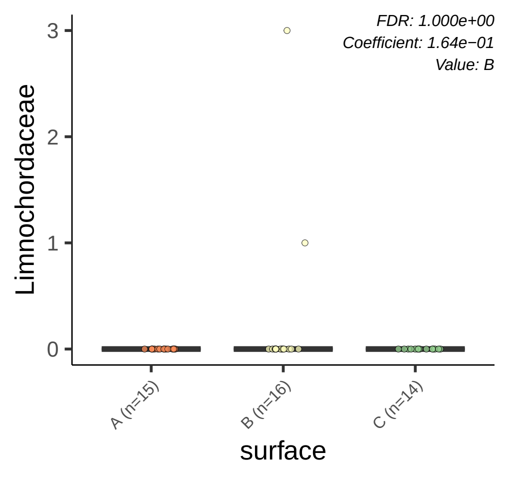

FDR: 1.000e+00
Coefficient: 1.64e−01
Value: B
Limnochordaceae
3
2
1
0
A (n=15)
B (n=16)
C (n=14)
surface

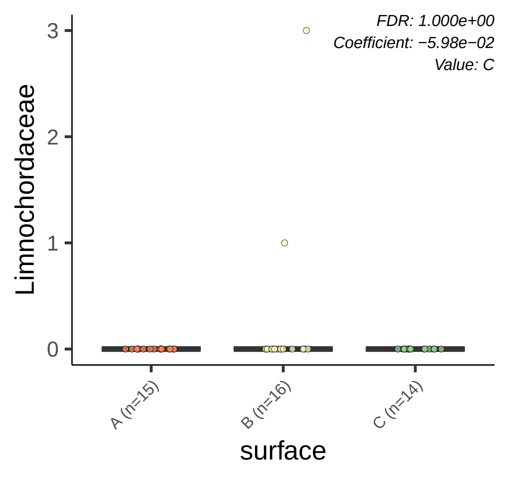

FDR: 1.000e+00
Coefficient: −5.98e−02
Value: C
Limnochordaceae
3
2
1
0
A (n=15)
B (n=16)
C (n=14)
surface

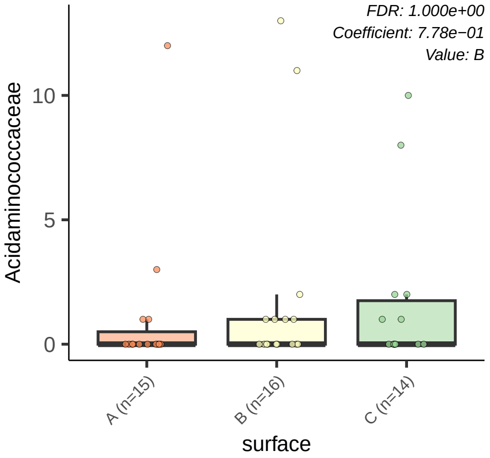
FDR: 1.000e+00
Coefficient: 7.78e−01
Value: B
Acidaminococcaceae
0
5
10
A (n=15)
B (n=16)
C (n=14)
surface

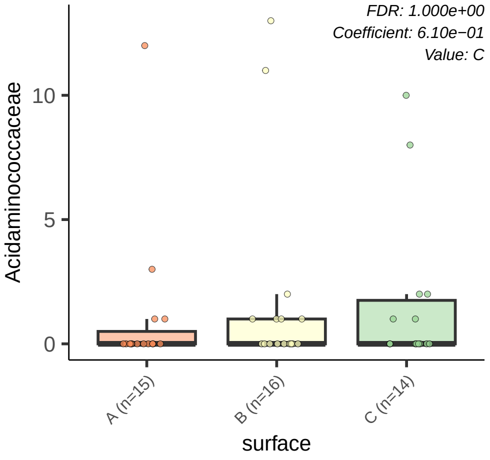
FDR: 1.000e+00
Coefficient: 6.10e−01
Value: C
Acidaminococcaceae
0
5
10
A (n=15)
B (n=16)
C (n=14)
surface

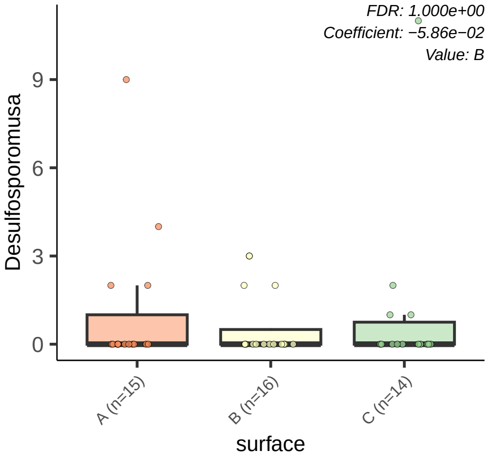
FDR: 1.000e+00
Coefficient: −5.86e−02
Value: B
Desulfosporomusa
9
6
3
0
A (n=15)
B (n=16)
C (n=14)
surface

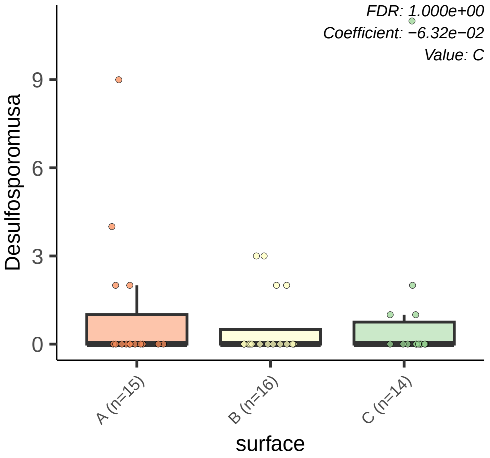
FDR: 1.000e+00
Coefficient: −6.32e−02
Value: C
Desulfosporomusa
0
3
6
9
A (n=15)
B (n=16)
C (n=14)
surface

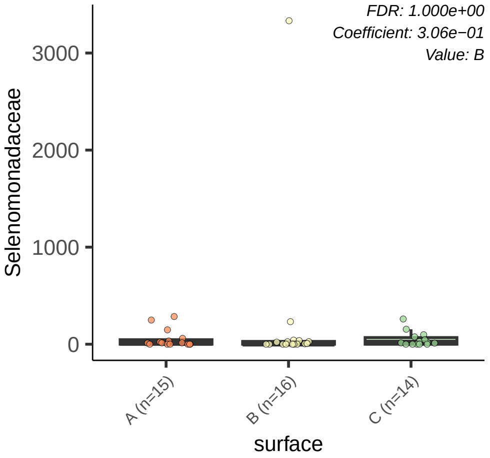
FDR: 1.000e+00
Coefficient: 3.06e−01
Value: B
Selenomonadaceae
3000
2000
1000
0
A (n=15)
B (n=16)
C (n=14)
surface

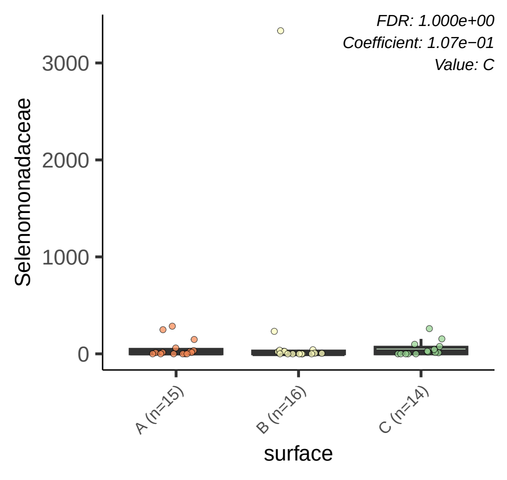

FDR: 1.000e+00
Coefficient: 1.07e−01
Value: C
Selenomonadaceae
3000
2000
1000
0
A (n=15)
B (n=16)
C (n=14)
surface

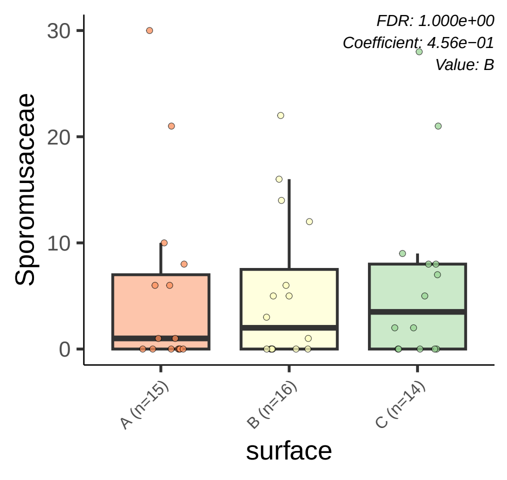
FDR: 1.000e+00
Coefficient: 4.56e−01
Value: B
Sporomusaceae
30
20
10
0
A (n=15)
B (n=16)
C (n=14)
surface

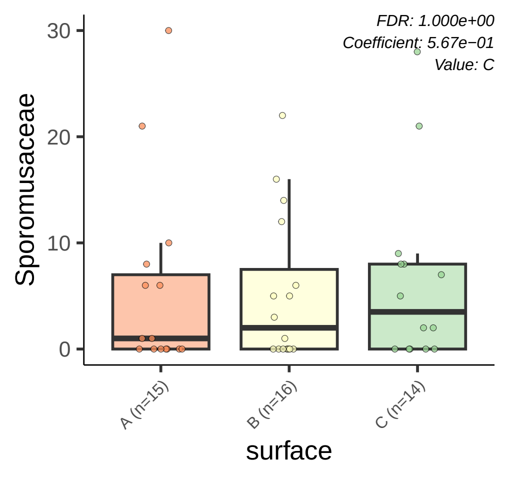

FDR: 1.000e+00
Coefficient: 5.67e−01
Value: C
Sporomusaceae
0
10
20
30
A (n=15)
B (n=16)
C (n=14)
surface

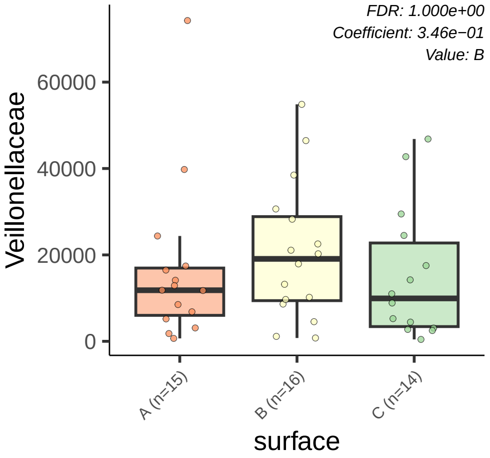
FDR: 1.000e+00
Coefficient: 3.46e−01
Value: B
Veillonellaceae
60000
40000
20000
0
A (n=15)
B (n=16)
C (n=14)
surface

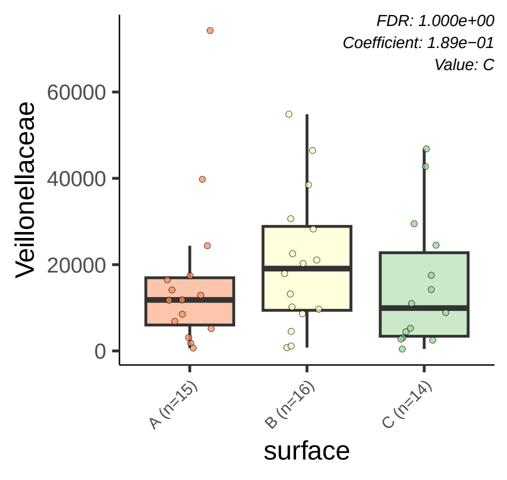
FDR: 1.000e+00
Coefficient: 1.89e−01
Value: C
Veillonellaceae
60000
40000
20000
0
A (n=15)
B (n=16)
C (n=14)
surface

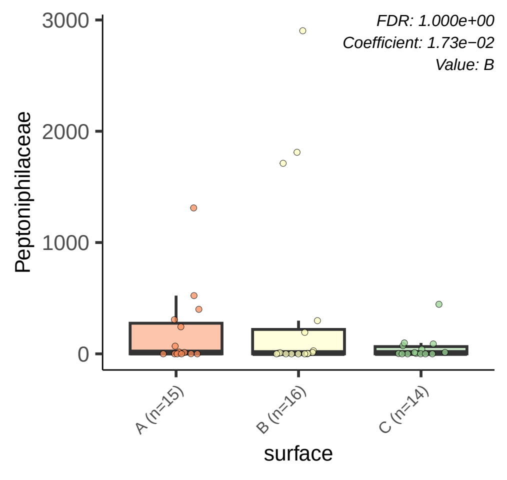

FDR: 1.000e+00
Coefficient: 1.73e−02
Value: B
Peptoniphilaceae
3000
2000
1000
0
A (n=15)
B (n=16)
C (n=14)
surface

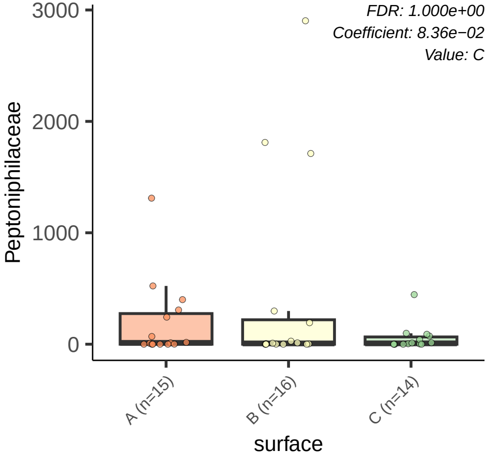
FDR: 1.000e+00
Coefficient: 8.36e−02
Value: C
3000
2000
1000
0
Peptoniphilaceae
A (n=15)
B (n=16)
C (n=14)
surface

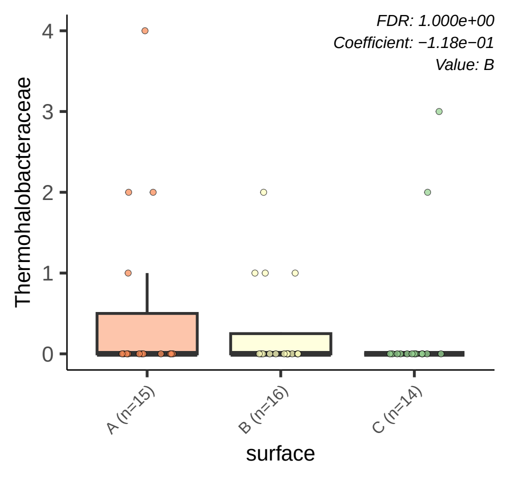
FDR: 1.000e+00
Coefficient: −1.18e−01
Value: B
Thermohalobacteraceae
0
1
2
3
4
A (n=15)
B (n=16)
C (n=14)
surface

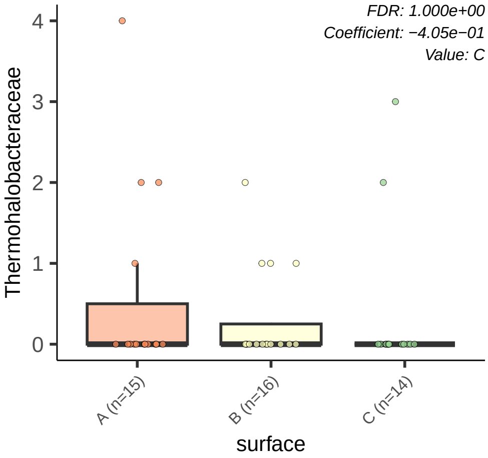

FDR: 1.000e+00
Coefficient: −4.05e−01
Value: C
Thermohalobacteraceae
4
3
2
1
0
A (n=15)
B (n=16)
C (n=14)
surface

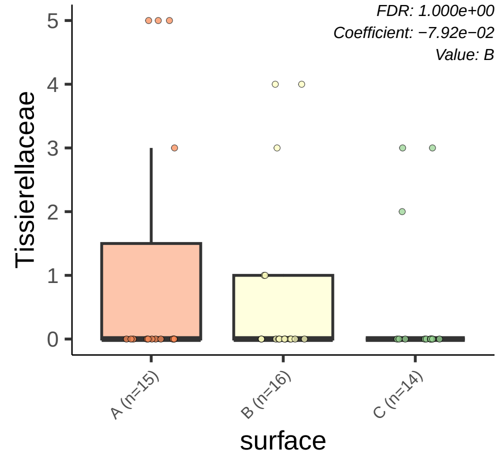
FDR: 1.000e+00
Coefficient: −7.92e−02
Value: B
Tissierellaceae
5
4
3
2
1
0
A (n=15)
B (n=16)
C (n=14)
surface

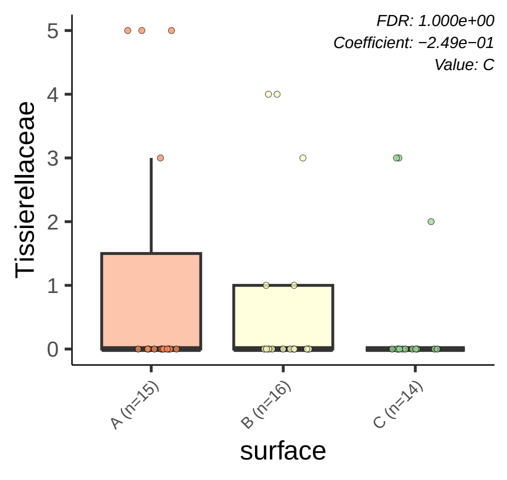
FDR: 1.000e+00
Coefficient: −2.49e−01
Value: C
Tissierellaceae
5
4
3
2
1
0
A (n=15)
B (n=16)
C (n=14)
surface

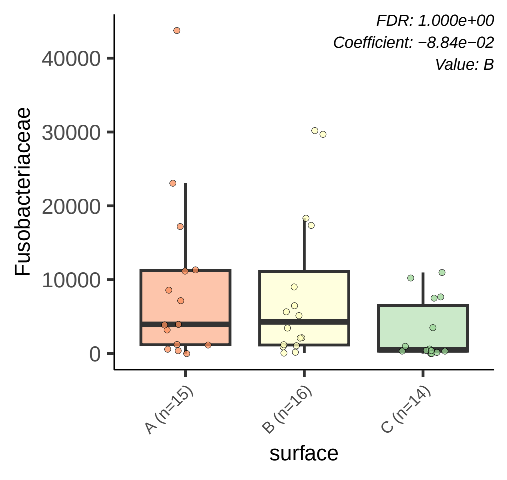
FDR: 1.000e+00
Coefficient: −8.84e−02
Value: B
Fusobacteriaceae
40000
30000
20000
10000
0
A (n=15)
B (n=16)
C (n=14)
surface

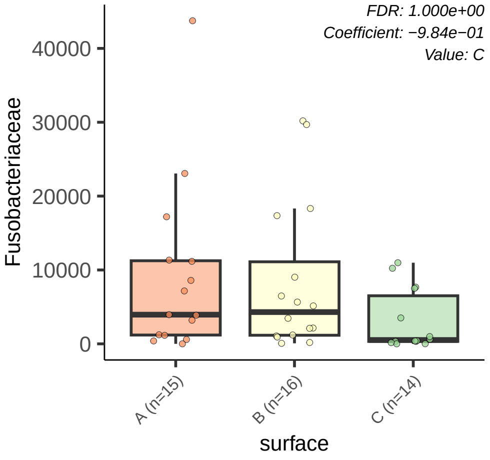
FDR: 1.000e+00
Coefficient: −9.84e−01
Value: C
Fusobacteriaceae
40000
30000
20000
10000
0
A (n=15)
B (n=16)
C (n=14)
surface

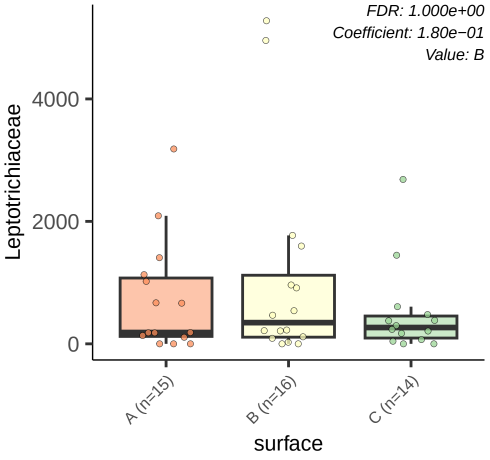

FDR: 1.000e+00
Coefficient: 1.80e−01
Value: B
Leptotrichiaceae
4000
2000
0
A (n=15)
B (n=16)
C (n=14)
surface

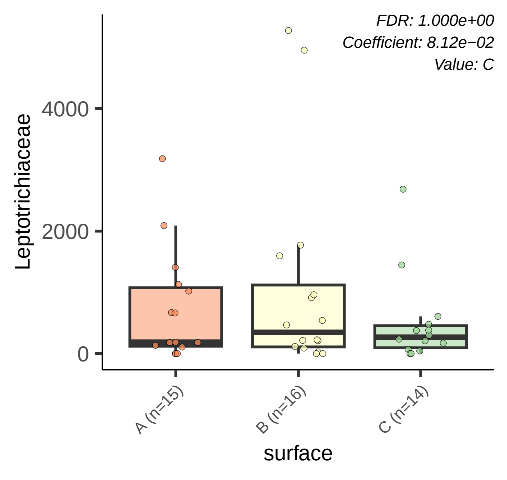
FDR: 1.000e+00
Coefficient: 8.12e−02
Value: C
Leptotrichiaceae
4000
2000
0
A (n=15)
B (n=16)
C (n=14)
surface

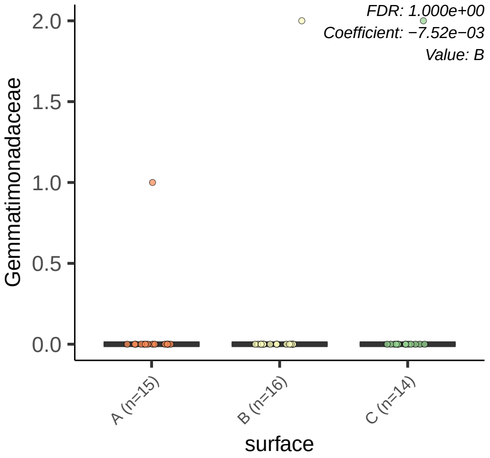

FDR: 1.000e+00
Coefficient: −7.52e−03
Value: B
Gemmatimonadaceae
2.0
1.5
1.0
0.5
0.0
A (n=15)
B (n=16)
C (n=14)
surface

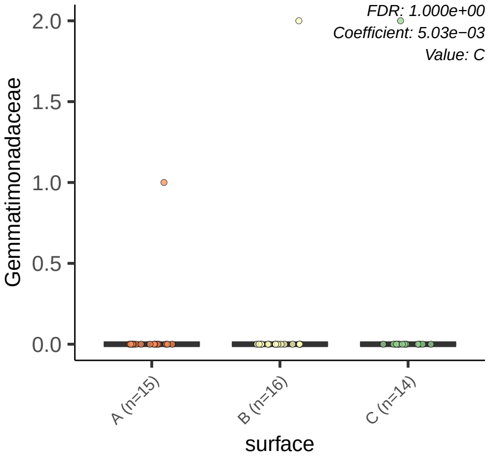
FDR: 1.000e+00
Coefficient: 5.03e−03
Value: C
Gemmatimonadaceae
2.0
1.5
1.0
0.5
0.0
A (n=15)
B (n=16)
C (n=14)
surface

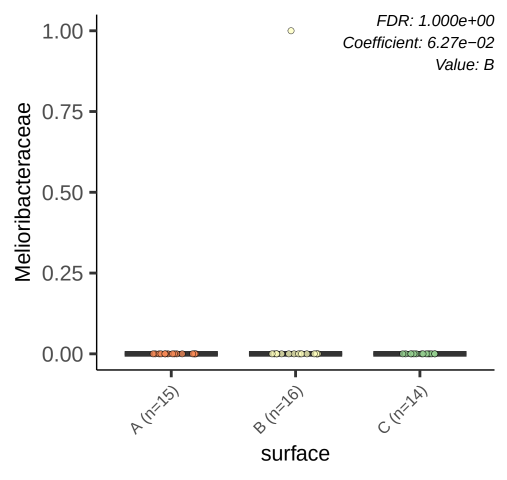
FDR: 1.000e+00
Coefficient: 6.27e−02
Value: B
Melioribacteraceae
1.00
0.75
0.50
0.25
0.00
A (n=15)
B (n=16)
C (n=14)
surface

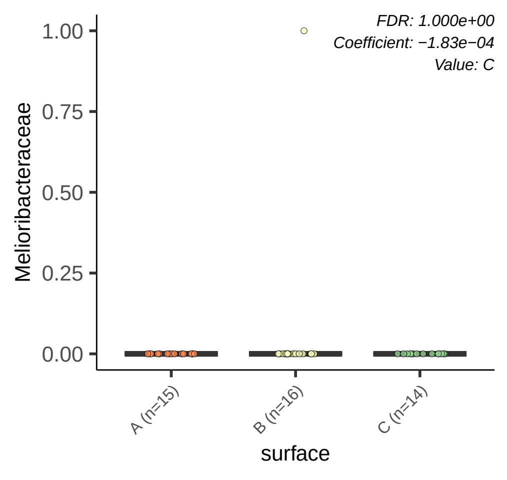
FDR: 1.000e+00
Coefficient: −1.83e−04
Value: C
Melioribacteraceae
1.00
0.75
0.50
0.25
0.00
A (n=15)
B (n=16)
C (n=14)
surface

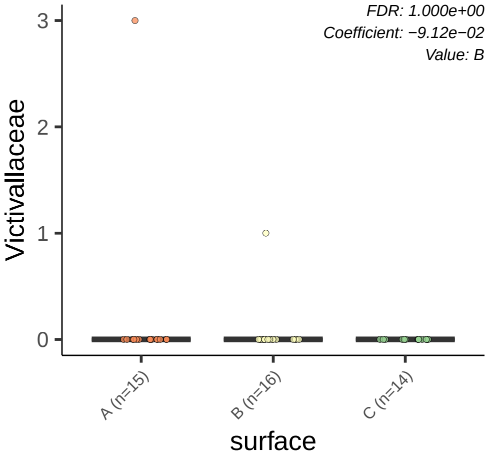

FDR: 1.000e+00
Coefficient: −9.12e−02
Value: B
Victivallaceae
3
2
1
0
A (n=15)
B (n=16)
C (n=14)
surface

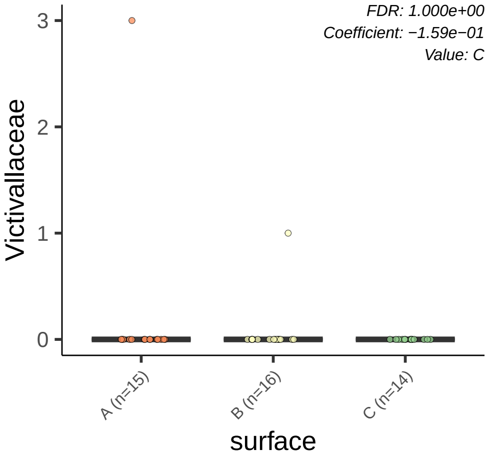

FDR: 1.000e+00
Coefficient: −1.59e−01
Value: C
Victivallaceae
3
2
1
0
A (n=15)
B (n=16)
C (n=14)
surface

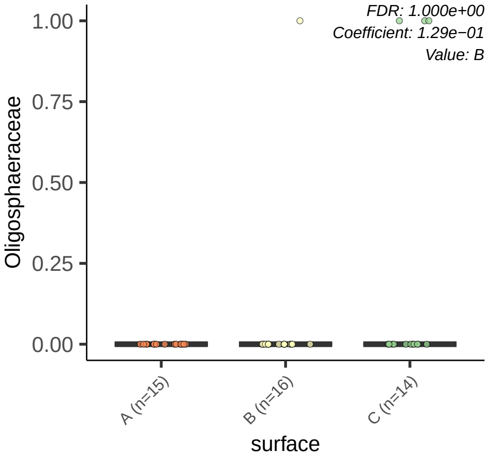

FDR: 1.000e+00
Coefficient: 1.29e−01
Value: B
Oligosphaeraceae
1.00
0.75
0.50
0.25
0.00
A (n=15)
B (n=16)
C (n=14)
surface

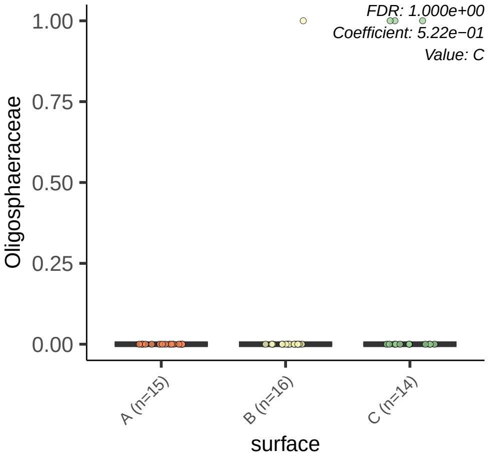

FDR: 1.000e+00
Coefficient: 5.22e−01
Value: C
1.00
0.75
0.50
0.25
0.00
Oligosphaeraceae
A (n=15)
B (n=16)
C (n=14)
surface

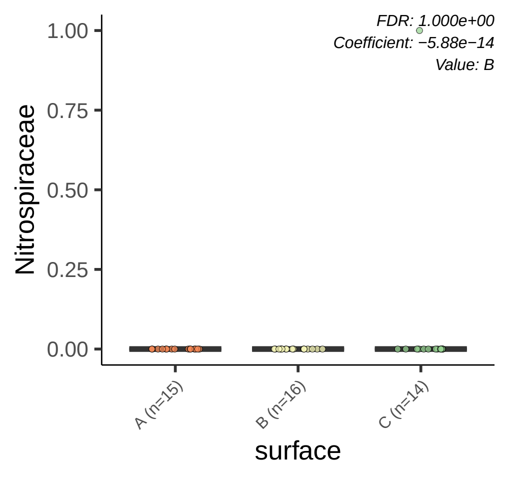
FDR: 1.000e+00
Coefficient: −5.88e−14
Value: B
Nitrospiraceae
1.00
0.75
0.50
0.25
0.00
A (n=15)
B (n=16)
C (n=14)
surface

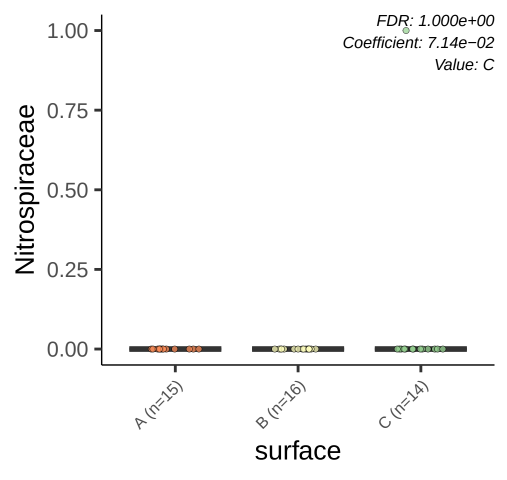
FDR: 1.000e+00
Coefficient: 7.14e−02
Value: C
Nitrospiraceae
1.00
0.75
0.50
0.25
0.00
A (n=15)
B (n=16)
C (n=14)
surface

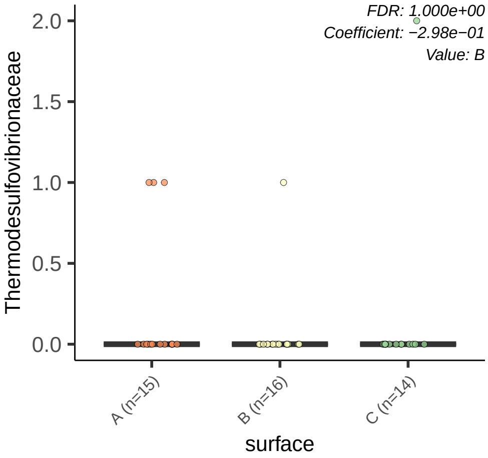

FDR: 1.000e+00
Coefficient: −2.98e−01
Value: B
Thermodesulfovibrionaceae
2.0
1.5
1.0
0.5
0.0
A (n=15)
B (n=16)
C (n=14)
surface

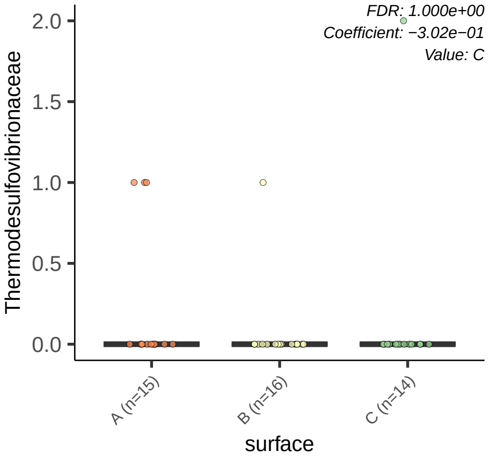

FDR: 1.000e+00
Coefficient: −3.02e−01
Value: C
Thermodesulfovibrionaceae
2.0
1.5
1.0
0.5
0.0
A (n=15)
B (n=16)
C (n=14)
surface

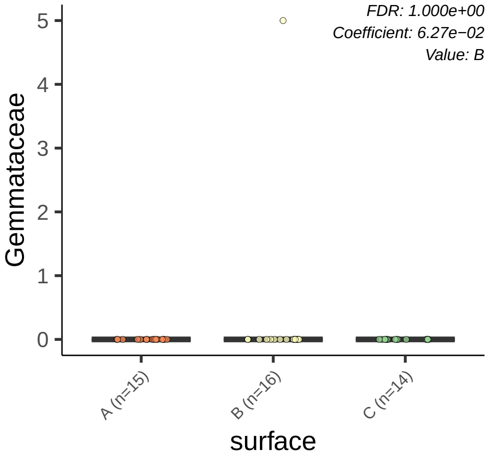
FDR: 1.000e+00
Coefficient: 6.27e−02
Value: B
Gemmataceae
0
1
2
3
4
5
A (n=15)
B (n=16)
C (n=14)
surface

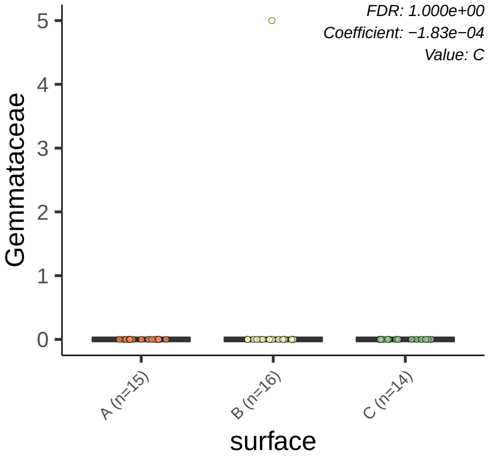

FDR: 1.000e+00
Coefficient: −1.83e−04
Value: C
Gemmataceae
5
4
3
2
1
0
A (n=15)
B (n=16)
C (n=14)
surface

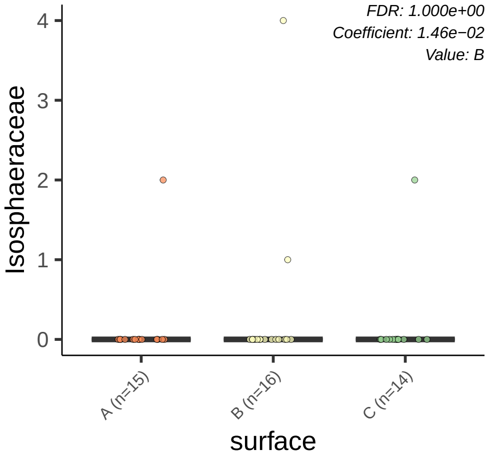

FDR: 1.000e+00
Coefficient: 1.46e−02
Value: B
Isosphaeraceae
0
1
2
3
4
A (n=15)
B (n=16)
C (n=14)
surface

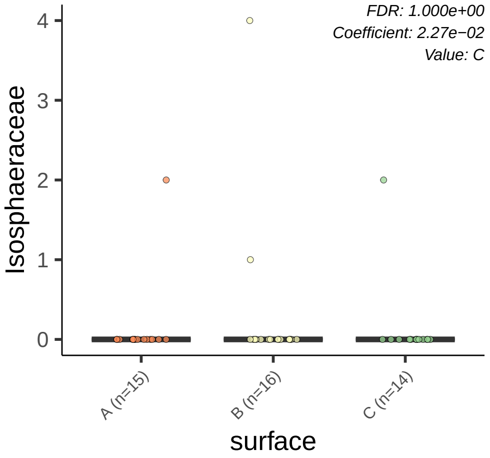

FDR: 1.000e+00
Coefficient: 2.27e−02
Value: C
Isosphaeraceae
0
1
2
3
4
A (n=15)
B (n=16)
C (n=14)
surface

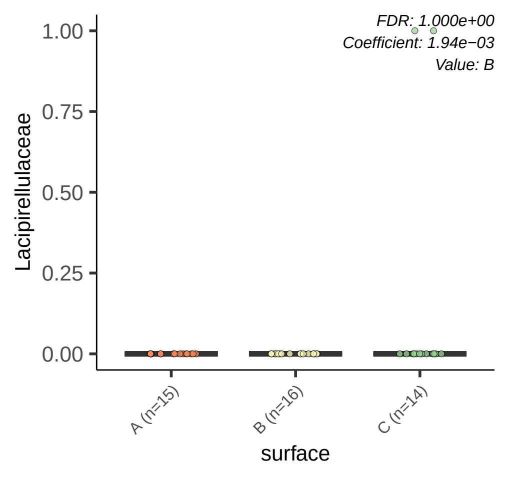
FDR: 1.000e+00
Coefficient: 1.94e−03
Value: B
Lacipirellulaceae
1.00
0.75
0.50
0.25
0.00
A (n=15)
B (n=16)
C (n=14)
surface

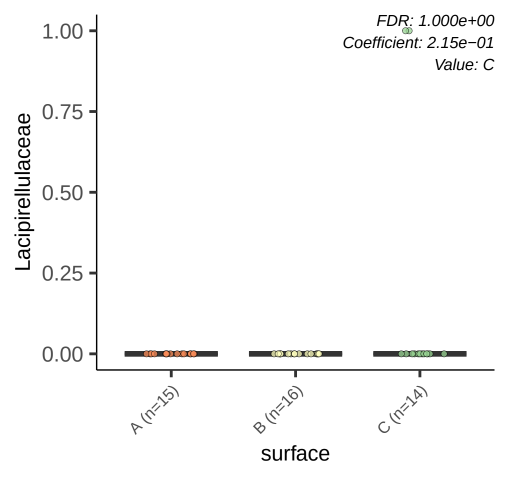

FDR: 1.000e+00
Coefficient: 2.15e−01
Value: C
Lacipirellulaceae
1.00
0.75
0.50
0.25
0.00
A (n=15)
B (n=16)
C (n=14)
surface

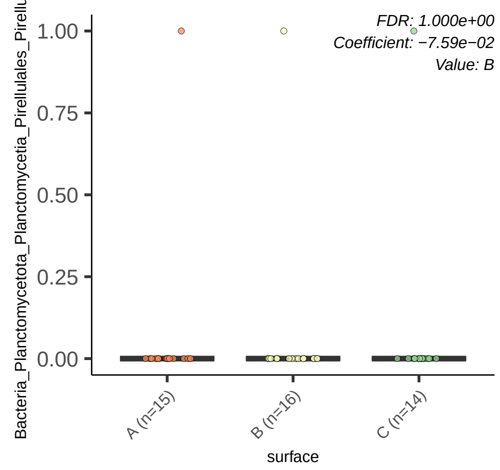

FDR: 1.000e+00
Coefficient: −7.59e−02
Value: B
1.00
0.75
0.50
0.25
0.00
Bacteria_Planctomycetota_Planctomycetia_Pirellulales_Pirellu
A (n=15)
B (n=16)
C (n=14)
surface

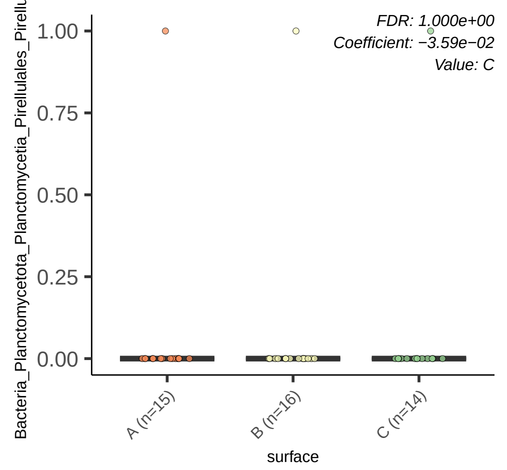

FDR: 1.000e+00
Coefficient: −3.59e−02
Value: C
Bacteria_Planctomycetota_Planctomycetia_Pirellulales_Pirellu
1.00
0.75
0.50
0.25
0.00
A (n=15)
B (n=16)
C (n=14)
surface

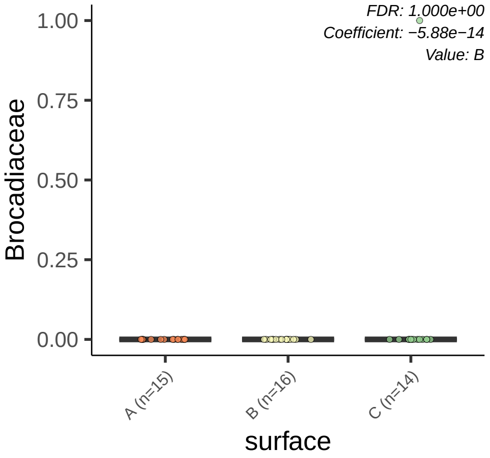
FDR: 1.000e+00
Coefficient: −5.88e−14
Value: B
Brocadiaceae
1.00
0.75
0.50
0.25
0.00
A (n=15)
B (n=16)
C (n=14)
surface

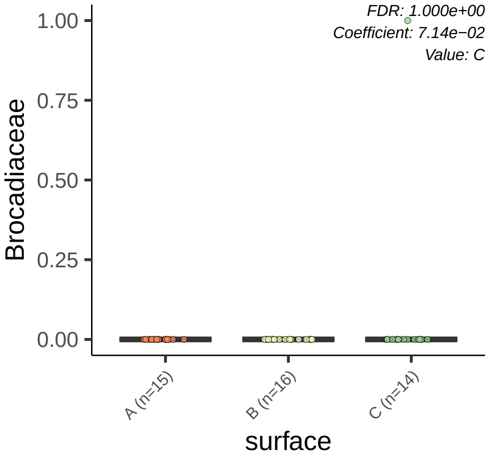
FDR: 1.000e+00
Coefficient: 7.14e−02
Value: C
1.00
0.75
0.50
0.25
0.00
Brocadiaceae
A (n=15)
B (n=16)
C (n=14)
surface

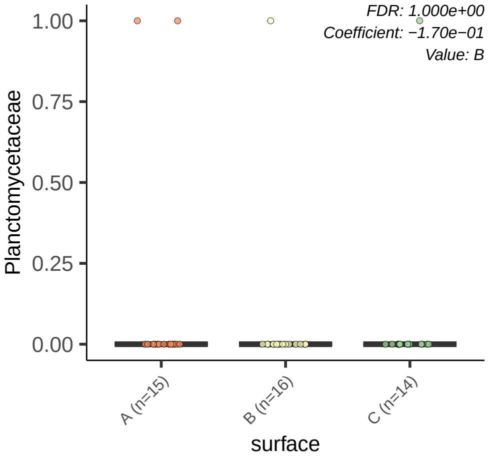
FDR: 1.000e+00
Coefficient: −1.70e−01
Value: B
Planctomycetaceae
1.00
0.75
0.50
0.25
0.00
A (n=15)
B (n=16)
C (n=14)
surface

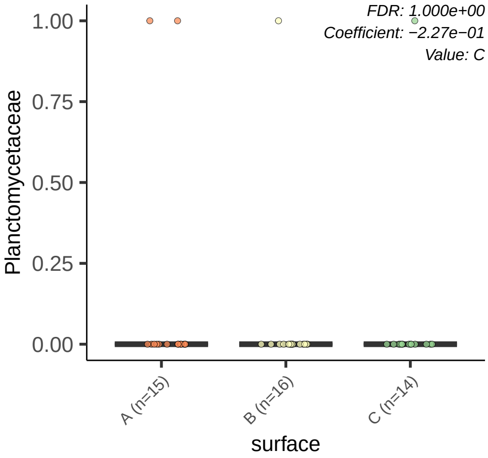

FDR: 1.000e+00
Coefficient: −2.27e−01
Value: C
Planctomycetaceae
1.00
0.75
0.50
0.25
0.00
A (n=15)
B (n=16)
C (n=14)
surface

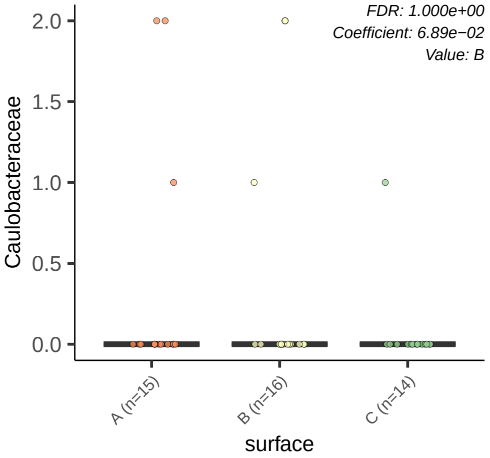
FDR: 1.000e+00
Coefficient: 6.89e−02
Value: B
Caulobacteraceae
2.0
1.5
1.0
0.5
0.0
A (n=15)
B (n=16)
C (n=14)
surface

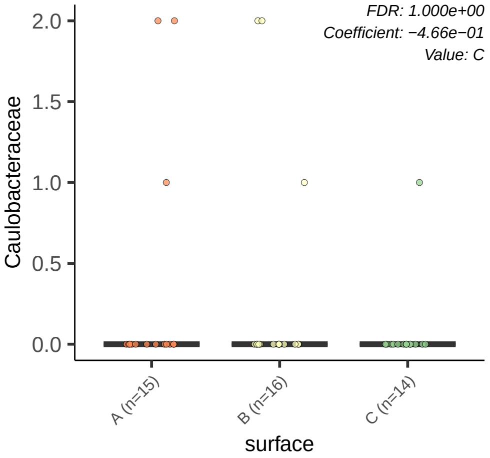
FDR: 1.000e+00
Coefficient: −4.66e−01
Value: C
Caulobacteraceae
2.0
1.5
1.0
0.5
0.0
A (n=15)
B (n=16)
C (n=14)
surface

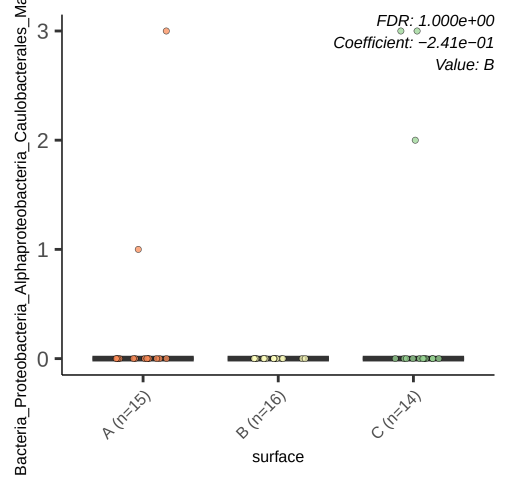
FDR: 1.000e+00
Coefficient: −2.41e−01
Value: B
Bacteria_Proteobacteria_Alphaproteobacteria_Caulobacterales_Ma
3
2
1
0
A (n=15)
B (n=16)
C (n=14)
surface

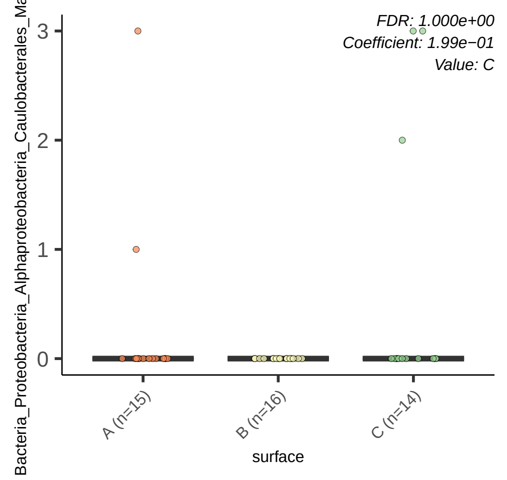

FDR: 1.000e+00
Coefficient: 1.99e−01
Value: C
Bacteria_Proteobacteria_Alphaproteobacteria_Caulobacterales_Ma
0
1
2
3
A (n=15)
B (n=16)
C (n=14)
surface

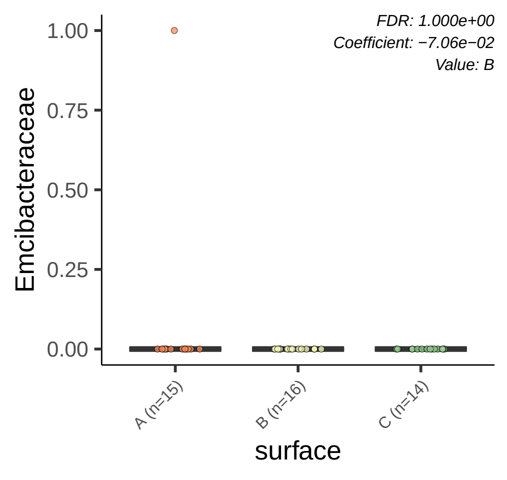
FDR: 1.000e+00
Coefficient: −7.06e−02
Value: B
Emcibacteraceae
1.00
0.75
0.50
0.25
0.00
A (n=15)
B (n=16)
C (n=14)
surface

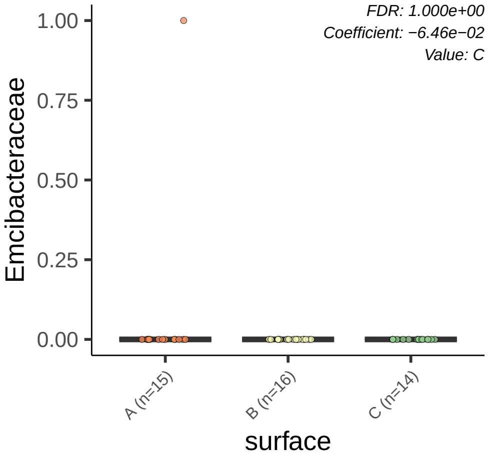

FDR: 1.000e+00
Coefficient: −6.46e−02
Value: C
Emcibacteraceae
1.00
0.75
0.50
0.25
0.00
A (n=15)
B (n=16)
C (n=14)
surface

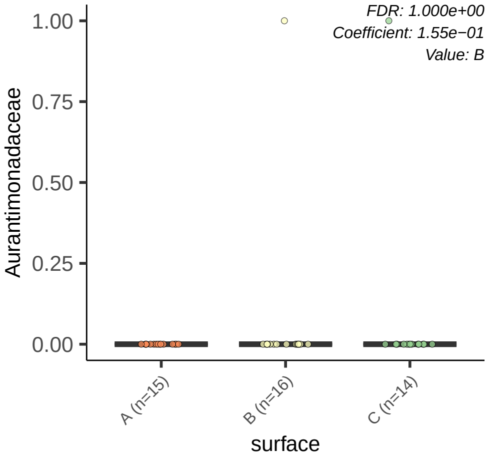

FDR: 1.000e+00
Coefficient: 1.55e−01
Value: B
Aurantimonadaceae
1.00
0.75
0.50
0.25
0.00
A (n=15)
B (n=16)
C (n=14)
surface

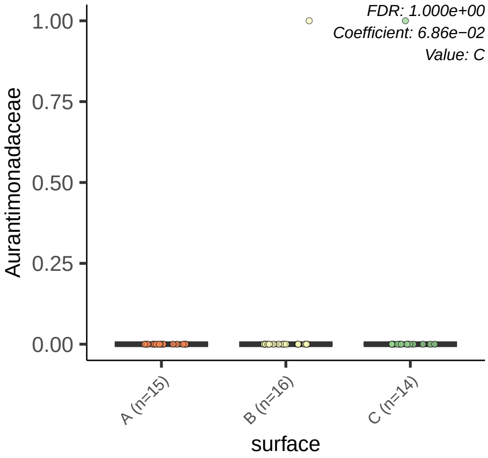
FDR: 1.000e+00
Coefficient: 6.86e−02
Value: C
Aurantimonadaceae
1.00
0.75
0.50
0.25
0.00
A (n=15)
B (n=16)
C (n=14)
surface

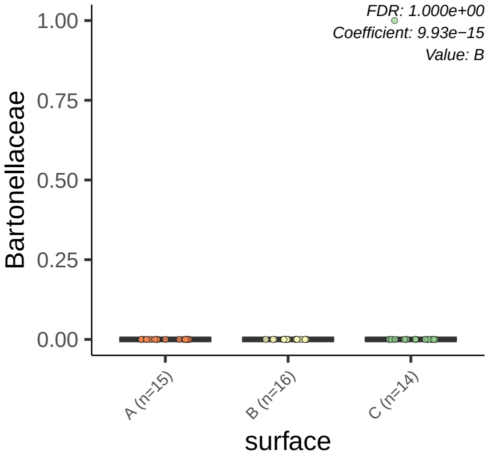

FDR: 1.000e+00
Coefficient: 9.93e−15
Value: B
1.00
0.75
0.50
0.25
0.00
Bartonellaceae
A (n=15)
B (n=16)
C (n=14)
surface

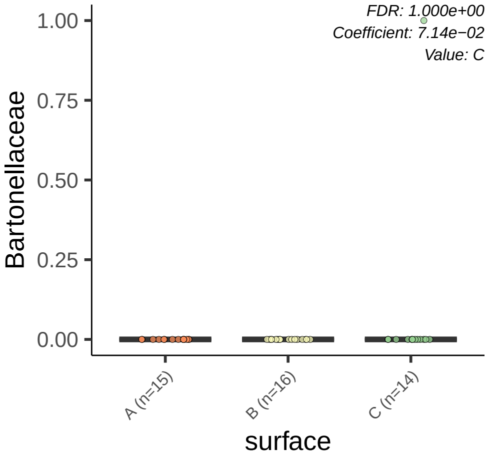

FDR: 1.000e+00
Coefficient: 7.14e−02
Value: C
1.00
0.75
0.50
0.25
0.00
Bartonellaceae
A (n=15)
B (n=16)
C (n=14)
surface

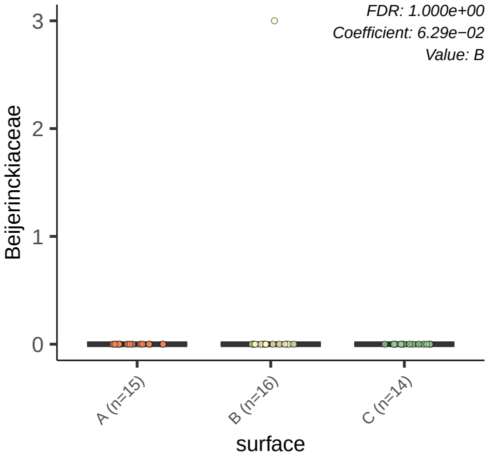
FDR: 1.000e+00
Coefficient: 6.29e−02
Value: B
Beijerinckiaceae
0
1
2
3
A (n=15)
B (n=16)
C (n=14)
surface

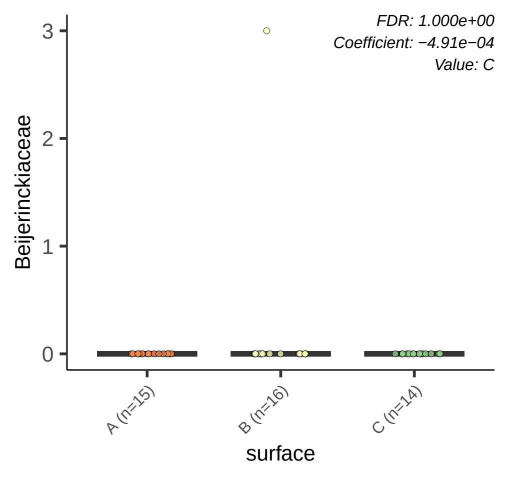
FDR: 1.000e+00
Coefficient: −4.91e−04
Value: C
Beijerinckiaceae
3
2
1
0
A (n=15)
B (n=16)
C (n=14)
surface

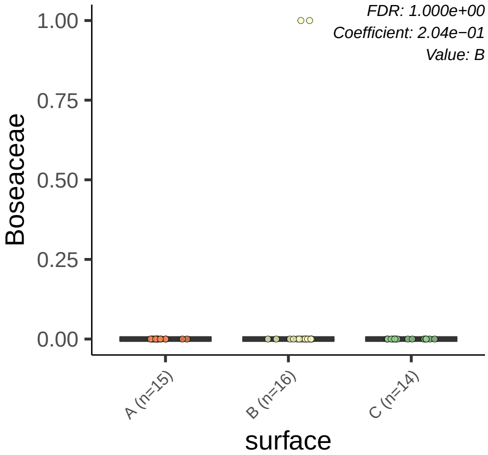
FDR: 1.000e+00
Coefficient: 2.04e−01
Value: B
Boseaceae
1.00
0.75
0.50
0.25
0.00
A (n=15)
B (n=16)
C (n=14)
surface

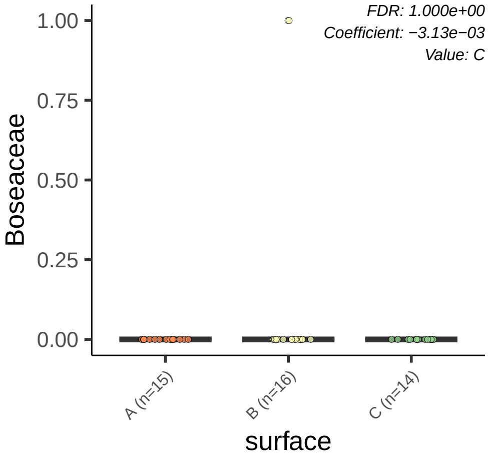

FDR: 1.000e+00
Coefficient: −3.13e−03
Value: C
1.00
0.75
0.50
0.25
0.00
Boseaceae
A (n=15)
B (n=16)
C (n=14)
surface

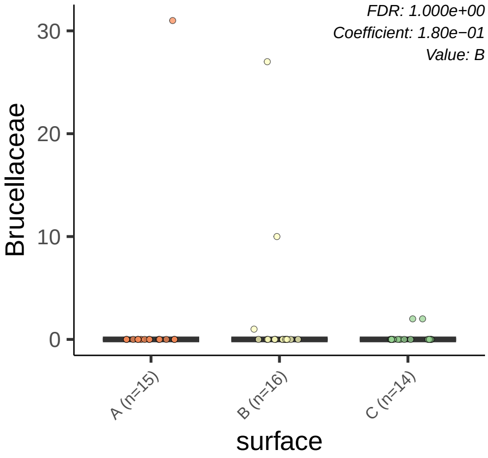

FDR: 1.000e+00
Coefficient: 1.80e−01
Value: B
Brucellaceae
30
20
10
0
A (n=15)
B (n=16)
C (n=14)
surface

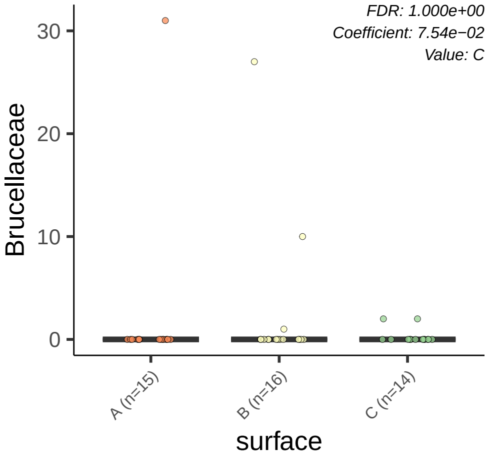
FDR: 1.000e+00
Coefficient: 7.54e−02
Value: C
Brucellaceae
30
20
10
0
A (n=15)
B (n=16)
C (n=14)
surface

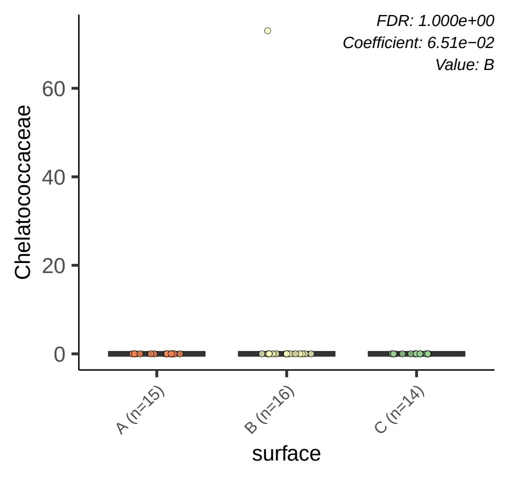
FDR: 1.000e+00
Coefficient: 6.51e−02
Value: B
Chelatococcaceae
60
40
20
0
A (n=15)
B (n=16)
C (n=14)
surface

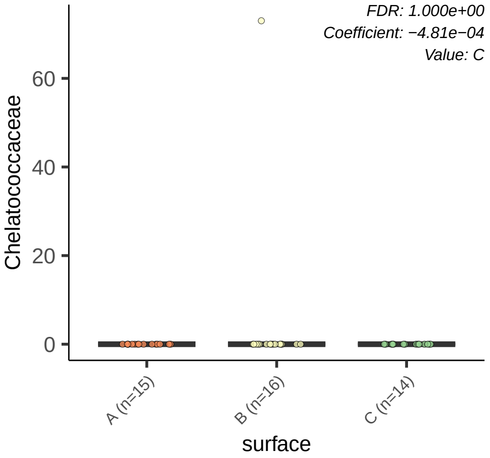

FDR: 1.000e+00
Coefficient: −4.81e−04
Value: C
Chelatococcaceae
0
20
40
60
A (n=15)
B (n=16)
C (n=14)
surface

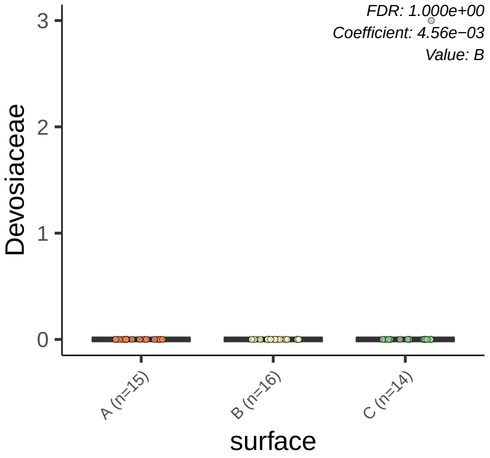

FDR: 1.000e+00
Coefficient: 4.56e−03
Value: B
Devosiaceae
3
2
1
0
A (n=15)
B (n=16)
C (n=14)
surface

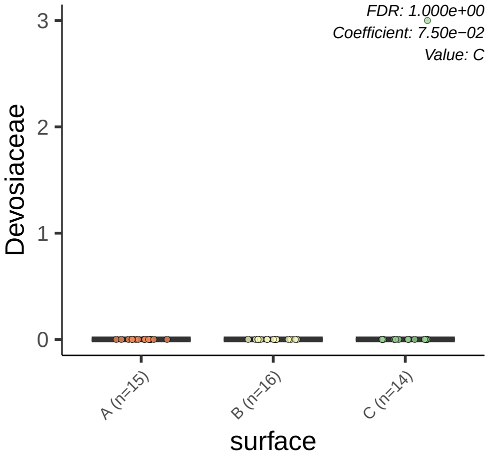
FDR: 1.000e+00
Coefficient: 7.50e−02
Value: C
Devosiaceae
0
1
2
3
A (n=15)
B (n=16)
C (n=14)
surface

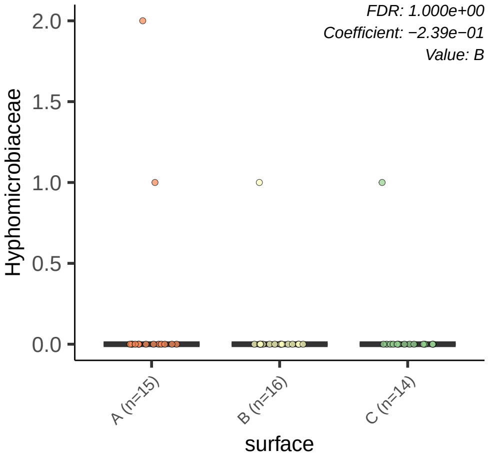
FDR: 1.000e+00
Coefficient: −2.39e−01
Value: B
Hyphomicrobiaceae
2.0
1.5
1.0
0.5
0.0
A (n=15)
B (n=16)
C (n=14)
surface

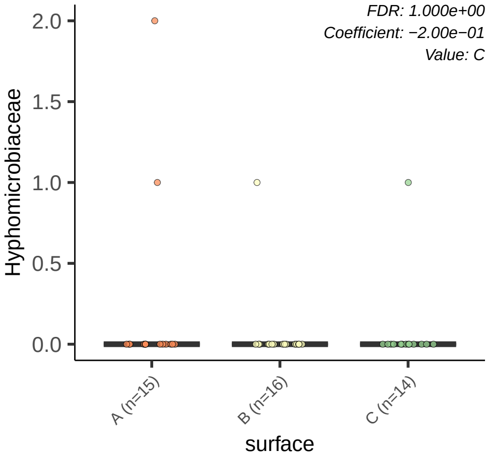

FDR: 1.000e+00
Coefficient: −2.00e−01
Value: C
Hyphomicrobiaceae
2.0
1.5
1.0
0.5
0.0
A (n=15)
B (n=16)
C (n=14)
surface

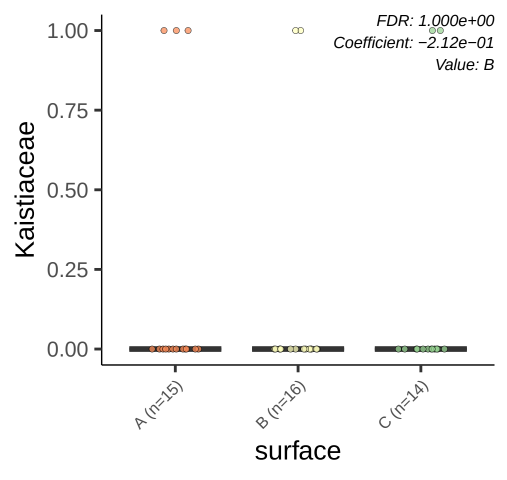

FDR: 1.000e+00
Coefficient: −2.12e−01
Value: B
Kaistiaceae
1.00
0.75
0.50
0.25
0.00
A (n=15)
B (n=16)
C (n=14)
surface

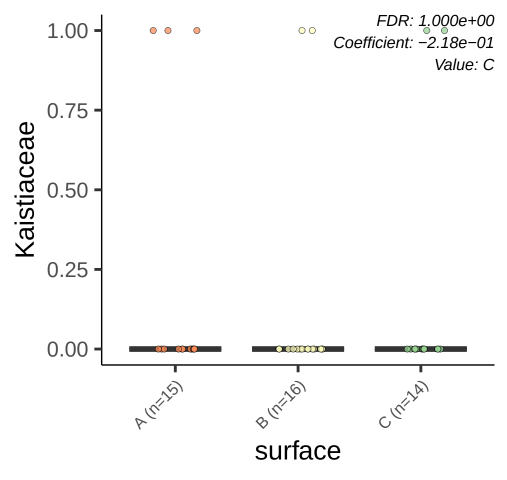
FDR: 1.000e+00
Coefficient: −2.18e−01
Value: C
Kaistiaceae
1.00
0.75
0.50
0.25
0.00
A (n=15)
B (n=16)
C (n=14)
surface

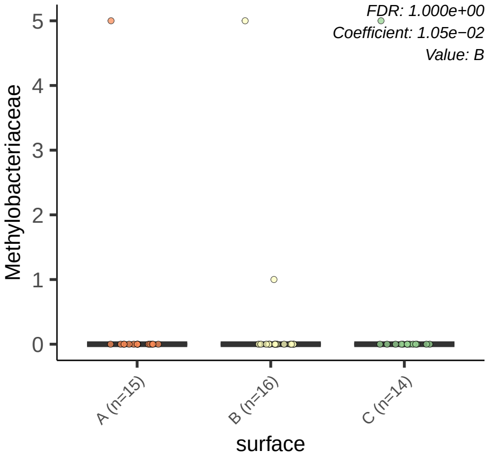

FDR: 1.000e+00
Coefficient: 1.05e−02
Value: B
Methylobacteriaceae
0
1
2
3
4
5
A (n=15)
B (n=16)
C (n=14)
surface

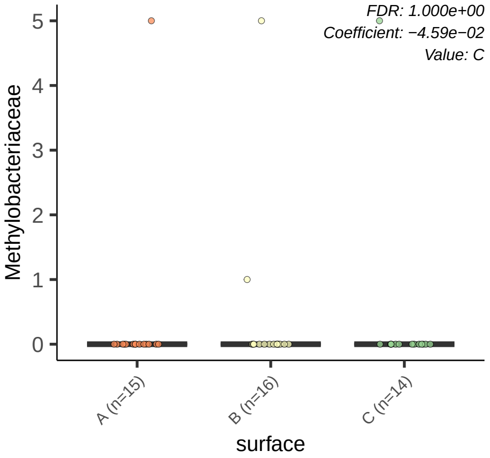

FDR: 1.000e+00
Coefficient: −4.59e−02
Value: C
Methylobacteriaceae
0
1
2
3
4
5
A (n=15)
B (n=16)
C (n=14)
surface

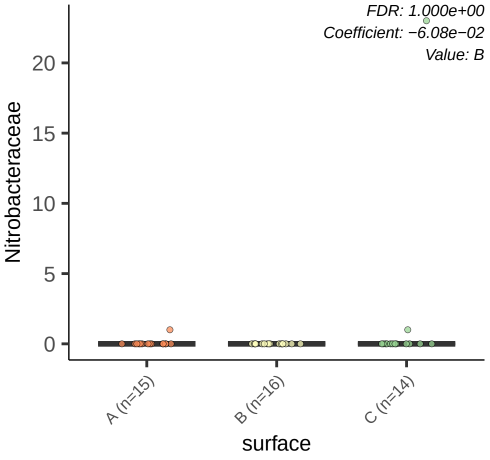

FDR: 1.000e+00
Coefficient: −6.08e−02
Value: B
Nitrobacteraceae
20
15
10
5
0
A (n=15)
B (n=16)
C (n=14)
surface

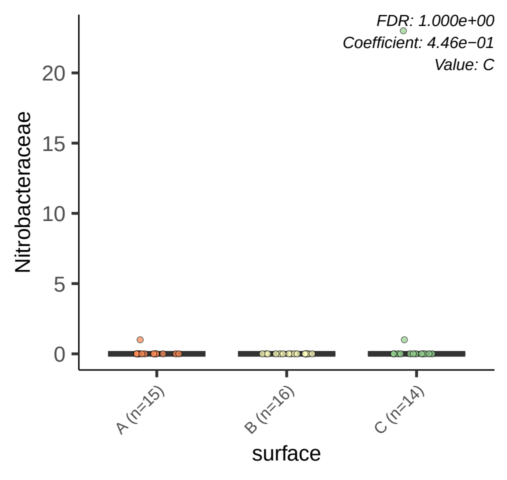

FDR: 1.000e+00
Coefficient: 4.46e−01
Value: C
Nitrobacteraceae
20
15
10
5
0
A (n=15)
B (n=16)
C (n=14)
surface

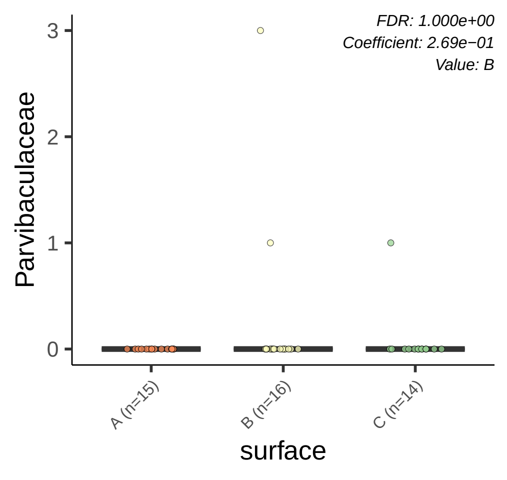

FDR: 1.000e+00
Coefficient: 2.69e−01
Value: B
Parvibaculaceae
3
2
1
0
A (n=15)
B (n=16)
C (n=14)
surface

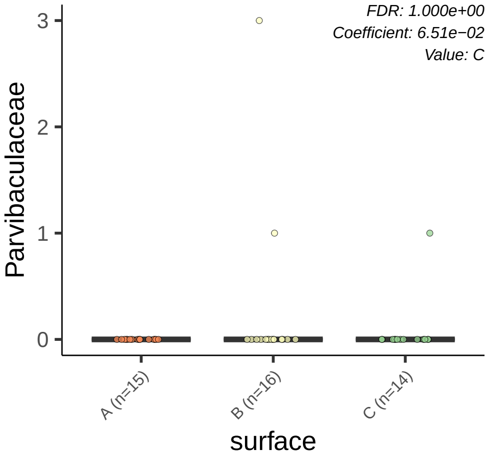
FDR: 1.000e+00
Coefficient: 6.51e−02
Value: C
Parvibaculaceae
3
2
1
0
A (n=15)
B (n=16)
C (n=14)
surface

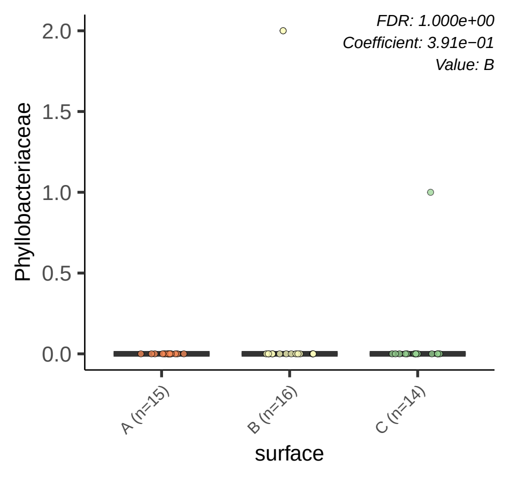
FDR: 1.000e+00
Coefficient: 3.91e−01
Value: B
Phyllobacteriaceae
2.0
1.5
1.0
0.5
0.0
A (n=15)
B (n=16)
C (n=14)
surface

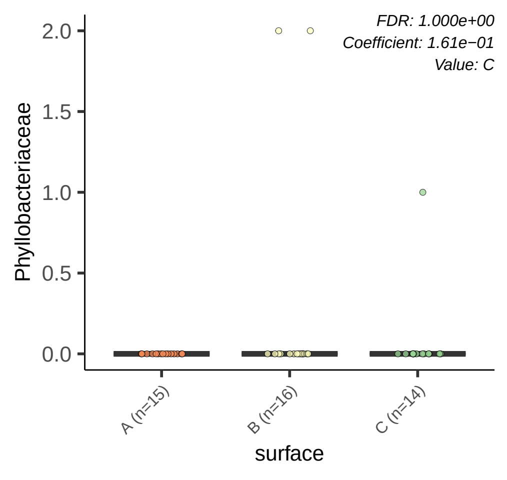
FDR: 1.000e+00
Coefficient: 1.61e−01
Value: C
Phyllobacteriaceae
2.0
1.5
1.0
0.5
0.0
A (n=15)
B (n=16)
C (n=14)
surface

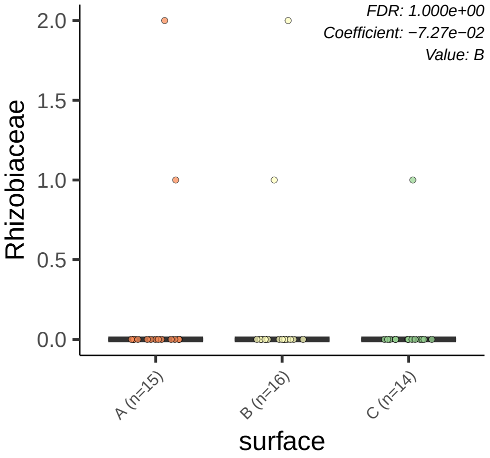

FDR: 1.000e+00
Coefficient: −7.27e−02
Value: B
Rhizobiaceae
2.0
1.5
1.0
0.5
0.0
A (n=15)
B (n=16)
C (n=14)
surface

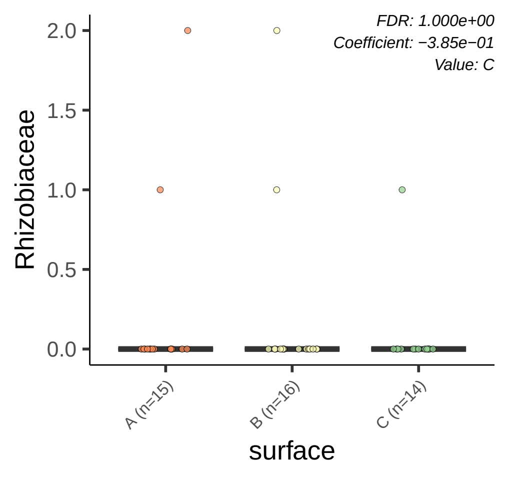
FDR: 1.000e+00
Coefficient: −3.85e−01
Value: C
Rhizobiaceae
2.0
1.5
1.0
0.5
0.0
A (n=15)
B (n=16)
C (n=14)
surface

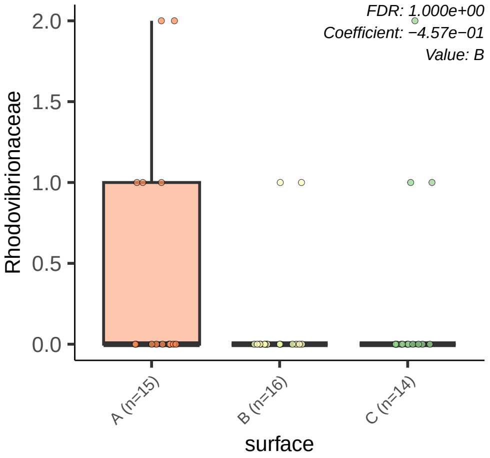

FDR: 1.000e+00
Coefficient: −4.57e−01
Value: B
Rhodovibrionaceae
2.0
1.5
1.0
0.5
0.0
A (n=15)
B (n=16)
C (n=14)
surface

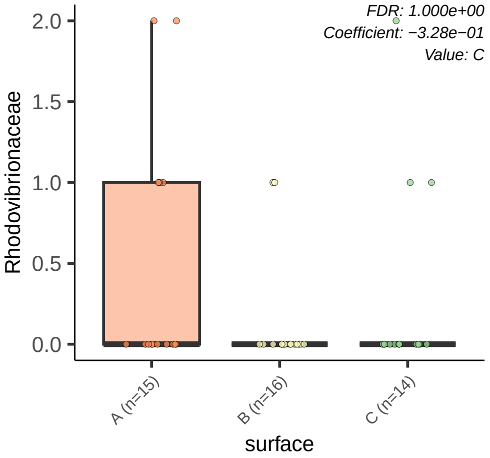

FDR: 1.000e+00
Coefficient: −3.28e−01
Value: C
Rhodovibrionaceae
2.0
1.5
1.0
0.5
0.0
A (n=15)
B (n=16)
C (n=14)
surface

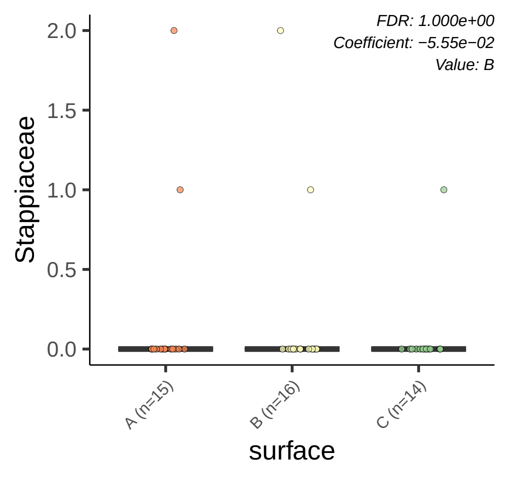

FDR: 1.000e+00
Coefficient: −5.55e−02
Value: B
Stappiaceae
2.0
1.5
1.0
0.5
0.0
A (n=15)
B (n=16)
C (n=14)
surface

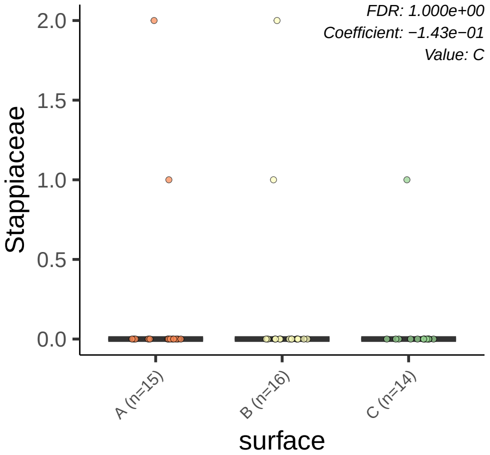
FDR: 1.000e+00
Coefficient: −1.43e−01
Value: C
Stappiaceae
2.0
1.5
1.0
0.5
0.0
A (n=15)
B (n=16)
C (n=14)
surface

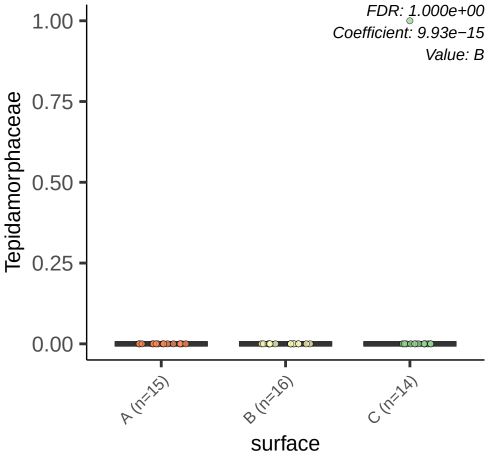

FDR: 1.000e+00
Coefficient: 9.93e−15
Value: B
Tepidamorphaceae
1.00
0.75
0.50
0.25
0.00
A (n=15)
B (n=16)
C (n=14)
surface

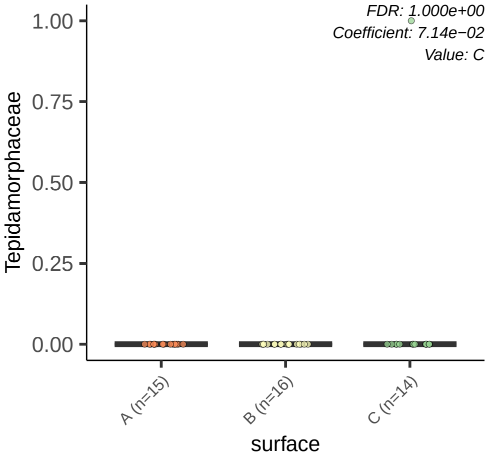
FDR: 1.000e+00
Coefficient: 7.14e−02
Value: C
Tepidamorphaceae
1.00
0.75
0.50
0.25
0.00
A (n=15)
B (n=16)
C (n=14)
surface

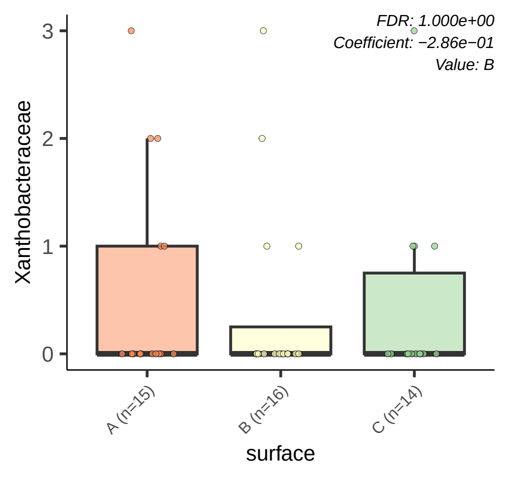

FDR: 1.000e+00
Coefficient: −2.86e−01
Value: B
Xanthobacteraceae
3
2
1
0
A (n=15)
B (n=16)
C (n=14)
surface

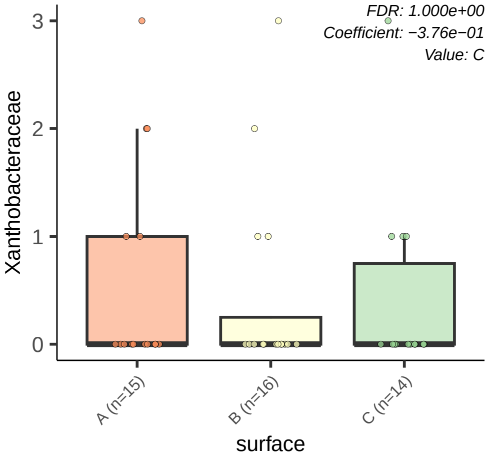

FDR: 1.000e+00
Coefficient: −3.76e−01
Value: C
Xanthobacteraceae
0
1
2
3
A (n=15)
B (n=16)
C (n=14)
surface

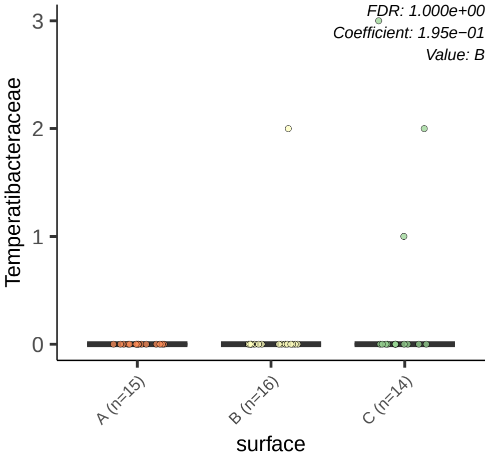

FDR: 1.000e+00
Coefficient: 1.95e−01
Value: B
Temperatibacteraceae
3
2
1
0
A (n=15)
B (n=16)
C (n=14)
surface

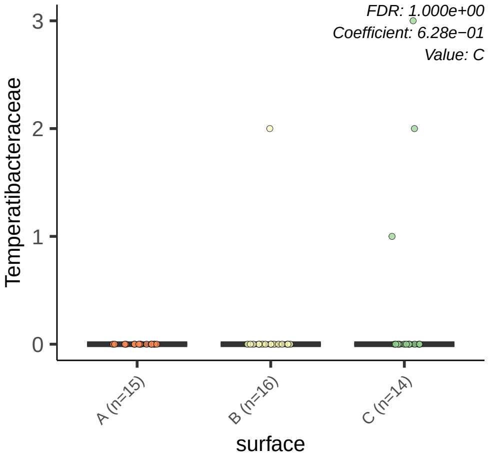

FDR: 1.000e+00
Coefficient: 6.28e−01
Value: C
Temperatibacteraceae
0
1
2
3
A (n=15)
B (n=16)
C (n=14)
surface

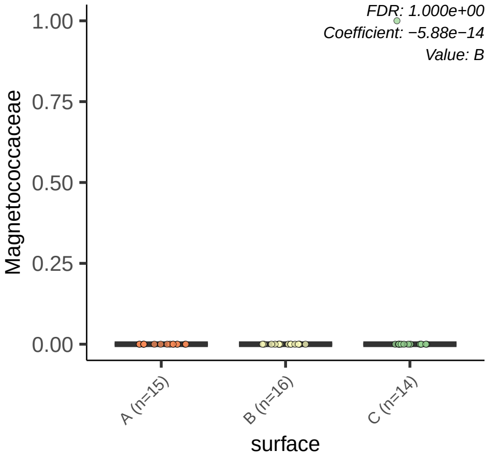
FDR: 1.000e+00
Coefficient: −5.88e−14
Value: B
Magnetococcaceae
1.00
0.75
0.50
0.25
0.00
A (n=15)
B (n=16)
C (n=14)
surface

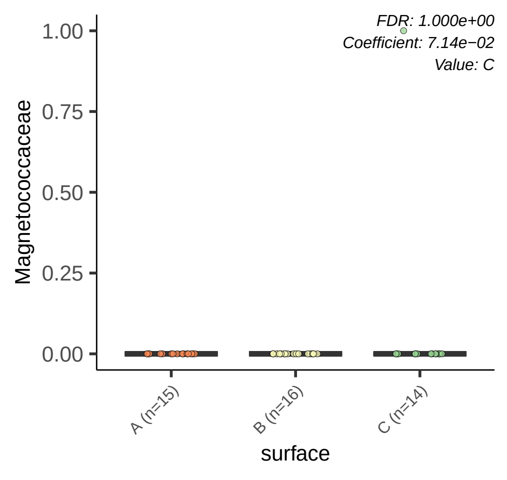
FDR: 1.000e+00
Coefficient: 7.14e−02
Value: C
Magnetococcaceae
1.00
0.75
0.50
0.25
0.00
A (n=15)
B (n=16)
C (n=14)
surface

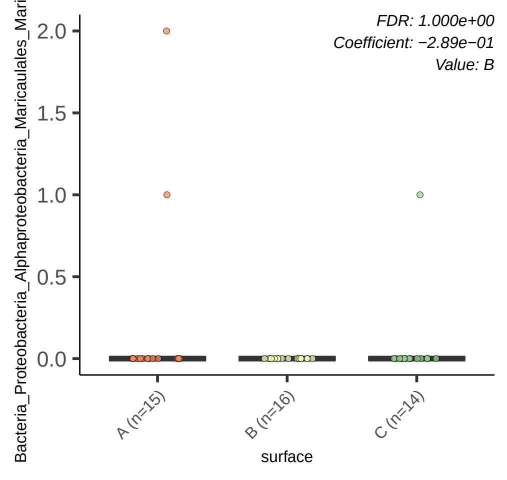

FDR: 1.000e+00
Coefficient: −2.89e−01
Value: B
Bacteria_Proteobacteria_Alphaproteobacteria_Maricaulales_Mari
2.0
1.5
1.0
0.5
0.0
A (n=15)
B (n=16)
C (n=14)
surface

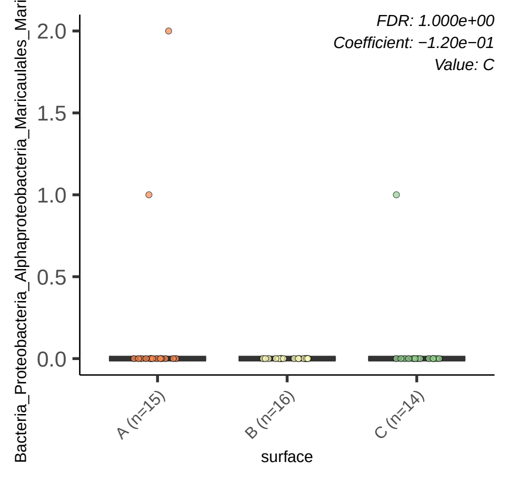

FDR: 1.000e+00
Coefficient: −1.20e−01
Value: C
Bacteria_Proteobacteria_Alphaproteobacteria_Maricaulales_Mari
2.0
1.5
1.0
0.5
0.0
A (n=15)
B (n=16)
C (n=14)
surface

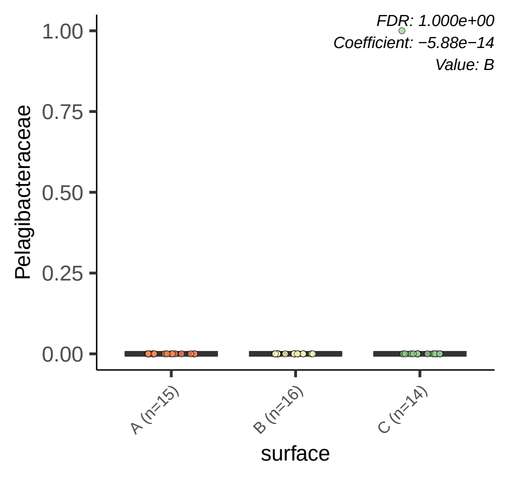
FDR: 1.000e+00
Coefficient: −5.88e−14
Value: B
Pelagibacteraceae
1.00
0.75
0.50
0.25
0.00
A (n=15)
B (n=16)
C (n=14)
surface

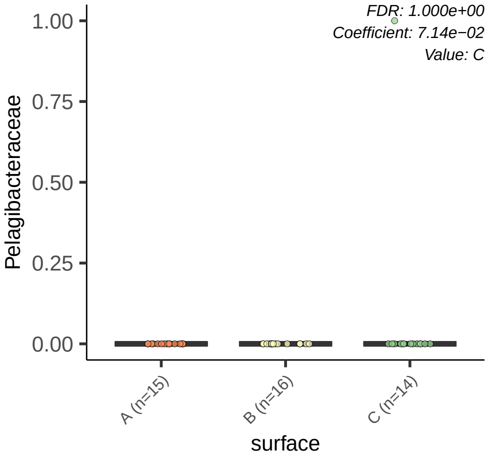

FDR: 1.000e+00
Coefficient: 7.14e−02
Value: C
Pelagibacteraceae
1.00
0.75
0.50
0.25
0.00
A (n=15)
B (n=16)
C (n=14)
surface

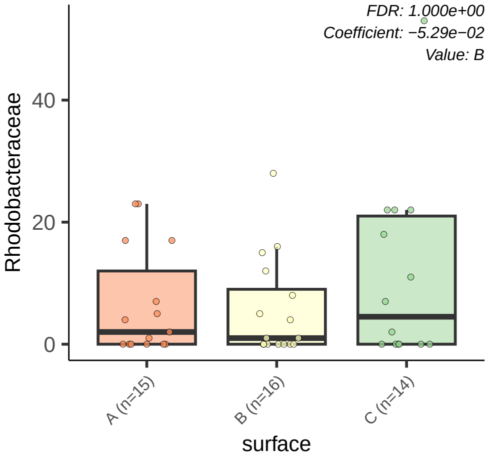

FDR: 1.000e+00
Coefficient: −5.29e−02
Value: B
Rhodobacteraceae
0
20
40
A (n=15)
B (n=16)
C (n=14)
surface

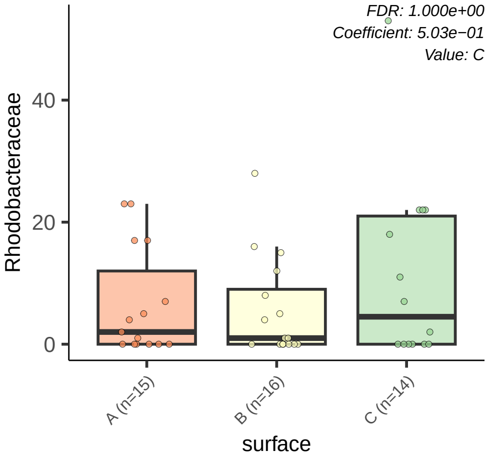

FDR: 1.000e+00
Coefficient: 5.03e−01
Value: C
Rhodobacteraceae
40
20
0
A (n=15)
B (n=16)
C (n=14)
surface

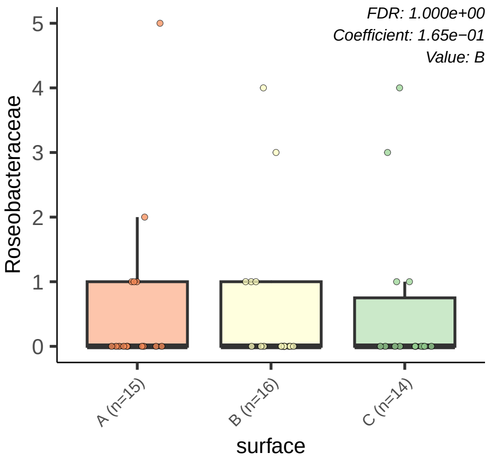
FDR: 1.000e+00
Coefficient: 1.65e−01
Value: B
Roseobacteraceae
0
1
2
3
4
5
A (n=15)
B (n=16)
C (n=14)
surface

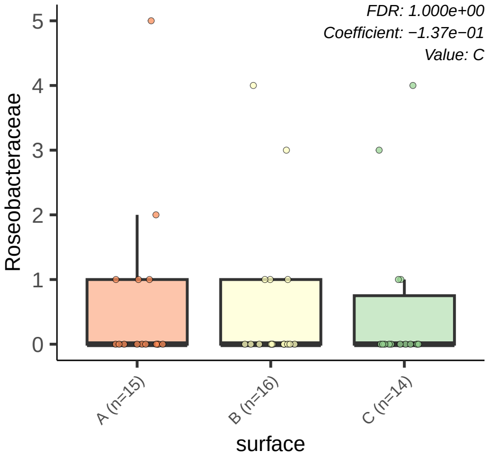

FDR: 1.000e+00
Coefficient: −1.37e−01
Value: C
Roseobacteraceae
0
1
2
3
4
5
A (n=15)
B (n=16)
C (n=14)
surface

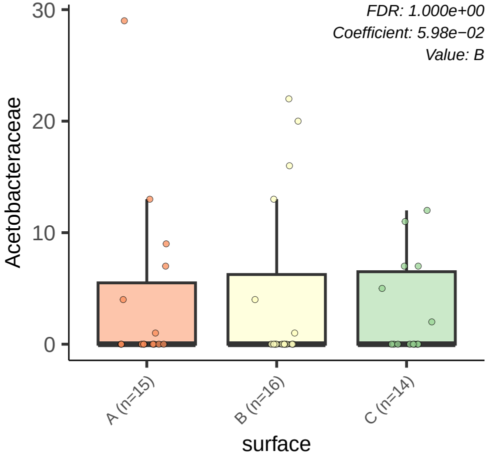
FDR: 1.000e+00
Coefficient: 5.98e−02
Value: B
Acetobacteraceae
30
20
10
0
A (n=15)
B (n=16)
C (n=14)
surface

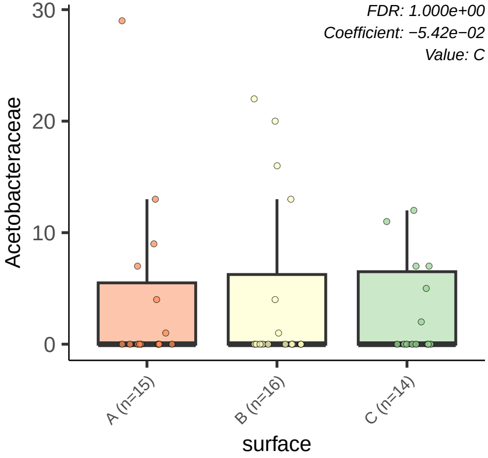
FDR: 1.000e+00
Coefficient: −5.42e−02
Value: C
Acetobacteraceae
30
20
10
0
A (n=15)
B (n=16)
C (n=14)
surface

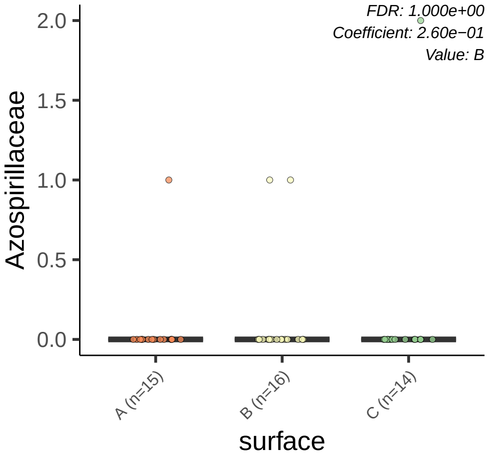

FDR: 1.000e+00
Coefficient: 2.60e−01
Value: B
Azospirillaceae
2.0
1.5
1.0
0.5
0.0
A (n=15)
B (n=16)
C (n=14)
surface

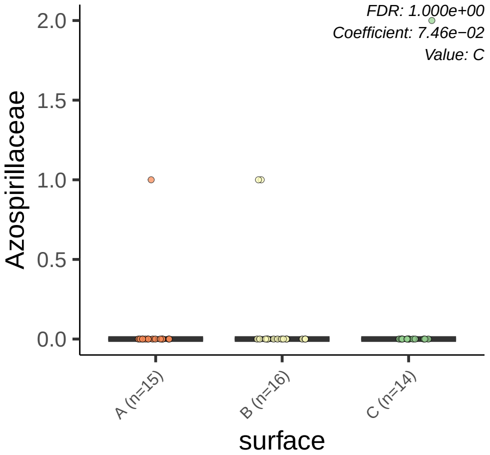
FDR: 1.000e+00
Coefficient: 7.46e−02
Value: C
Azospirillaceae
2.0
1.5
1.0
0.5
0.0
A (n=15)
B (n=16)
C (n=14)
surface

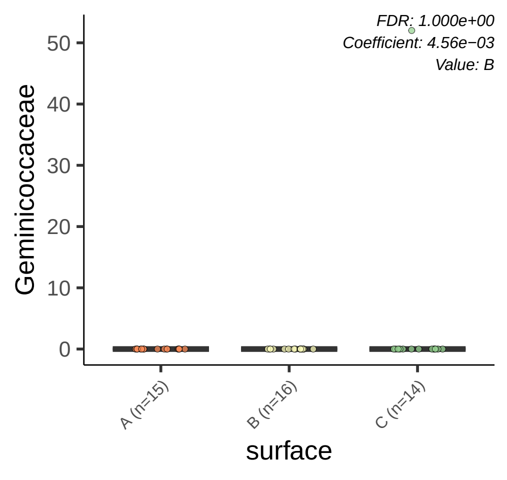
FDR: 1.000e+00
Coefficient: 4.56e−03
Value: B
Geminicoccaceae
50
40
30
20
10
0
A (n=15)
B (n=16)
C (n=14)
surface

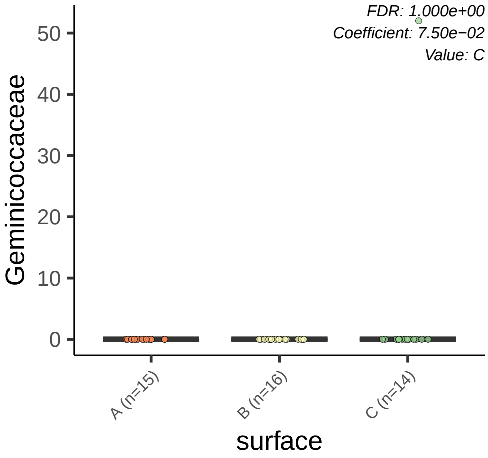
FDR: 1.000e+00
Coefficient: 7.50e−02
Value: C
Geminicoccaceae
50
40
30
20
10
0
A (n=15)
B (n=16)
C (n=14)
surface

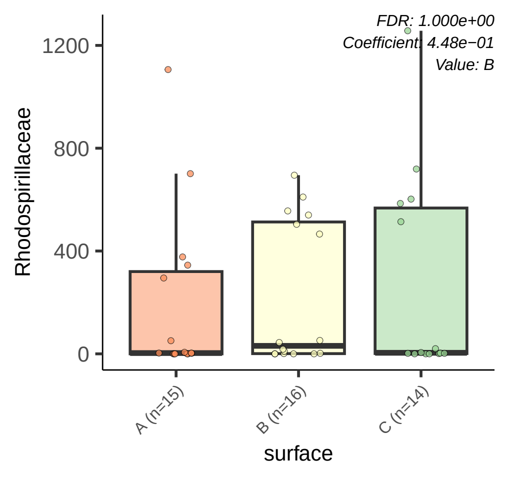

FDR: 1.000e+00
Coefficient: 4.48e−01
Value: B
Rhodospirillaceae
1200
800
400
0
A (n=15)
B (n=16)
C (n=14)
surface

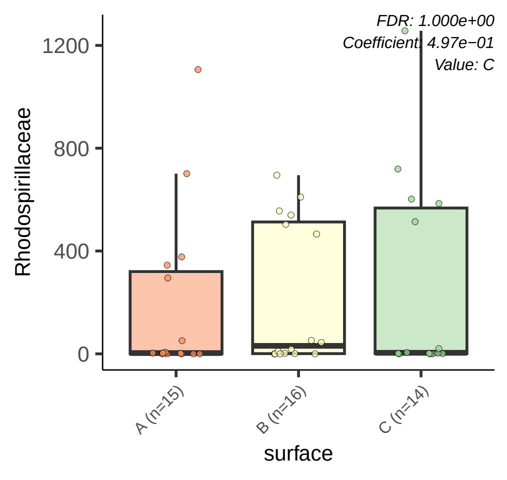
FDR: 1.000e+00
Coefficient: 4.97e−01
Value: C
Rhodospirillaceae
1200
800
400
0
A (n=15)
B (n=16)
C (n=14)
surface

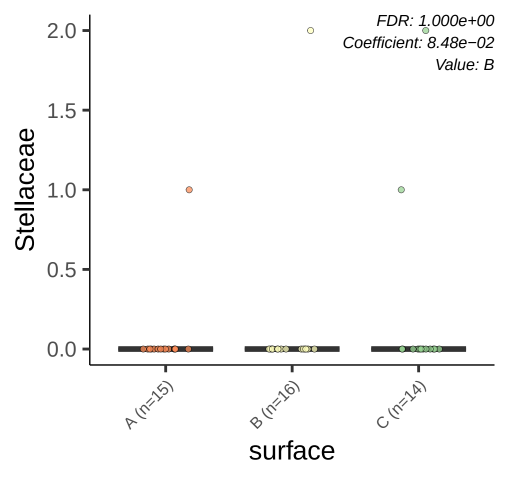
FDR: 1.000e+00
Coefficient: 8.48e−02
Value: B
Stellaceae
2.0
1.5
1.0
0.5
0.0
A (n=15)
B (n=16)
C (n=14)
surface

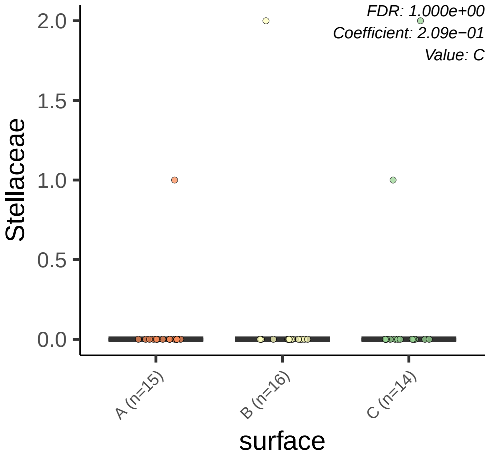
FDR: 1.000e+00
Coefficient: 2.09e−01
Value: C
Stellaceae
2.0
1.5
1.0
0.5
0.0
A (n=15)
B (n=16)
C (n=14)
surface

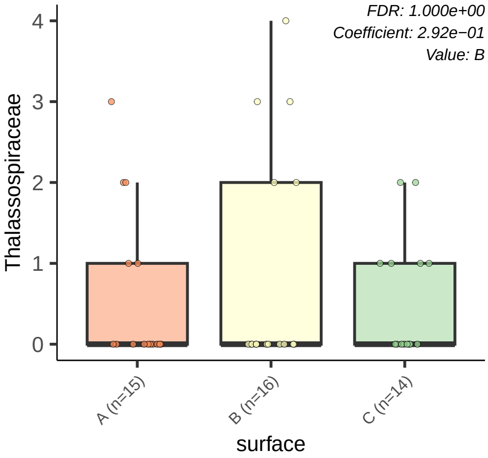

FDR: 1.000e+00
Coefficient: 2.92e−01
Value: B
Thalassospiraceae
0
1
2
3
4
A (n=15)
B (n=16)
C (n=14)
surface

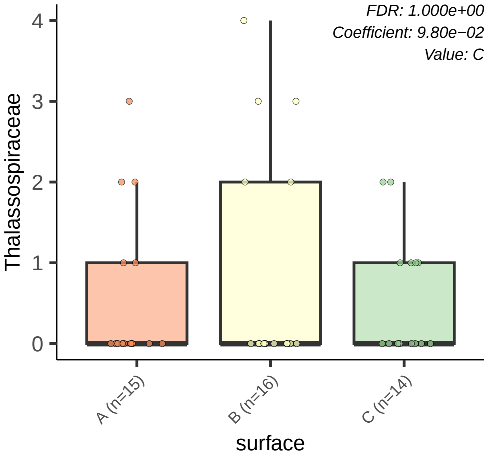

FDR: 1.000e+00
Coefficient: 9.80e−02
Value: C
Thalassospiraceae
0
1
2
3
4
A (n=15)
B (n=16)
C (n=14)
surface

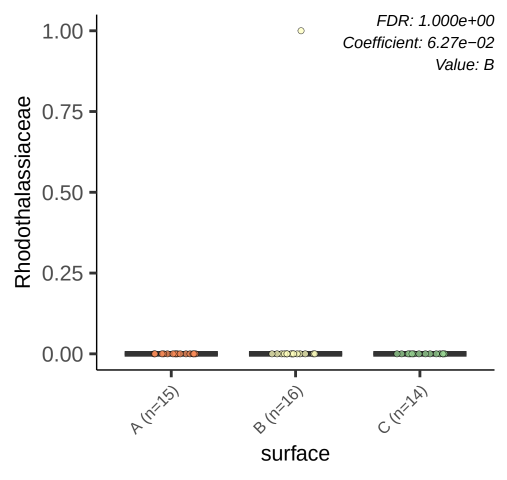
FDR: 1.000e+00
Coefficient: 6.27e−02
Value: B
Rhodothalassiaceae
1.00
0.75
0.50
0.25
0.00
A (n=15)
B (n=16)
C (n=14)
surface

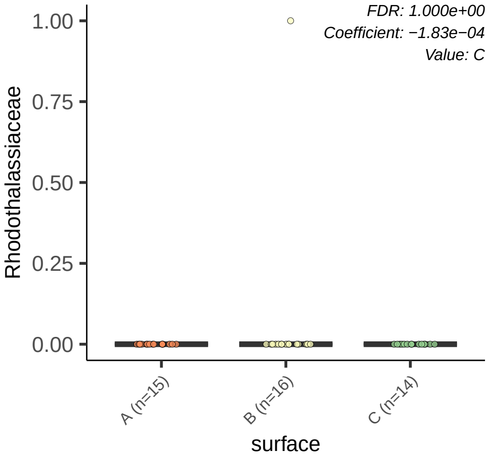
FDR: 1.000e+00
Coefficient: −1.83e−04
Value: C
Rhodothalassiaceae
1.00
0.75
0.50
0.25
0.00
A (n=15)
B (n=16)
C (n=14)
surface

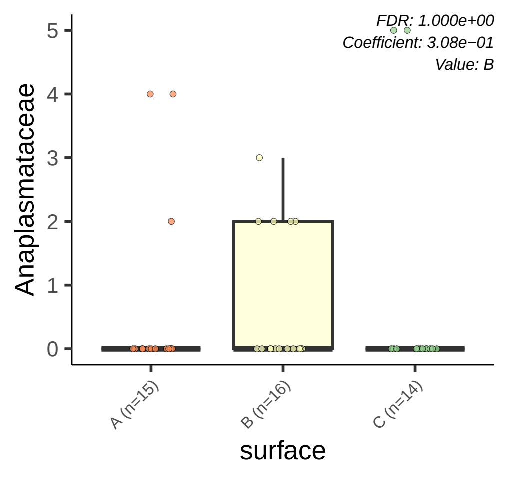

FDR: 1.000e+00
Coefficient: 3.08e−01
Value: B
Anaplasmataceae
5
4
3
2
1
0
A (n=15)
B (n=16)
C (n=14)
surface

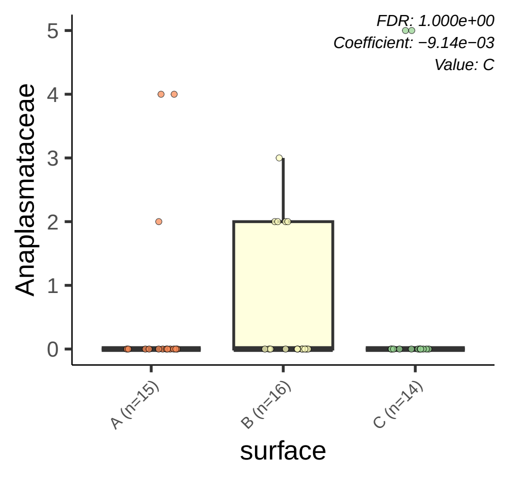

FDR: 1.000e+00
Coefficient: −9.14e−03
Value: C
Anaplasmataceae
0
1
2
3
4
5
A (n=15)
B (n=16)
C (n=14)
surface

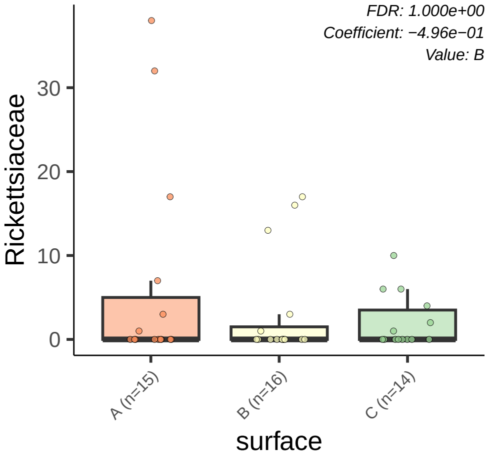

FDR: 1.000e+00
Coefficient: −4.96e−01
Value: B
Rickettsiaceae
0
10
20
30
A (n=15)
B (n=16)
C (n=14)
surface

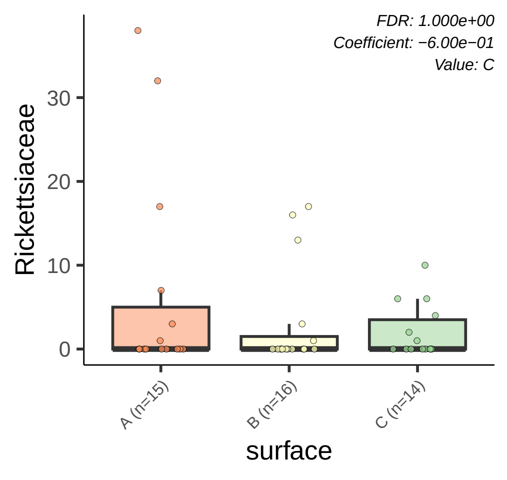
FDR: 1.000e+00
Coefficient: −6.00e−01
Value: C
Rickettsiaceae
0
10
20
30
A (n=15)
B (n=16)
C (n=14)
surface

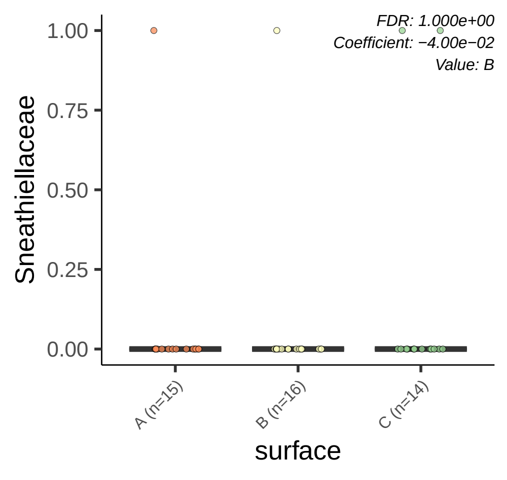
FDR: 1.000e+00
Coefficient: −4.00e−02
Value: B
Sneathiellaceae
1.00
0.75
0.50
0.25
0.00
A (n=15)
B (n=16)
C (n=14)
surface

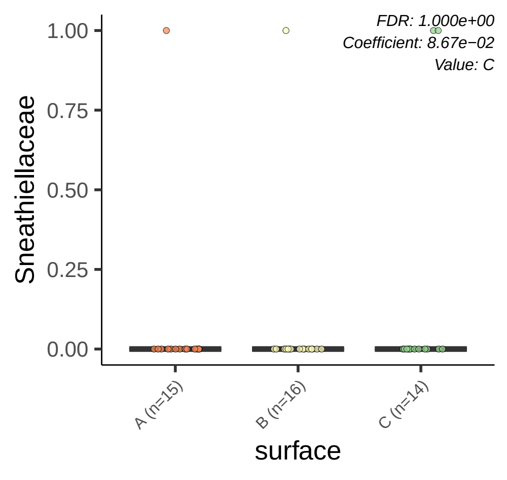

FDR: 1.000e+00
Coefficient: 8.67e−02
Value: C
Sneathiellaceae
1.00
0.75
0.50
0.25
0.00
A (n=15)
B (n=16)
C (n=14)
surface

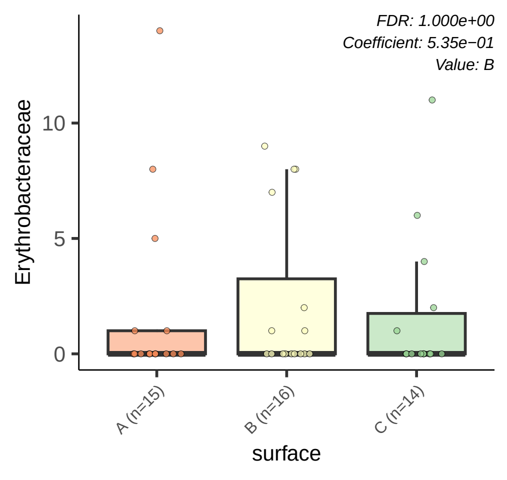
FDR: 1.000e+00
Coefficient: 5.35e−01
Value: B
Erythrobacteraceae
10
5
0
A (n=15)
B (n=16)
C (n=14)
surface

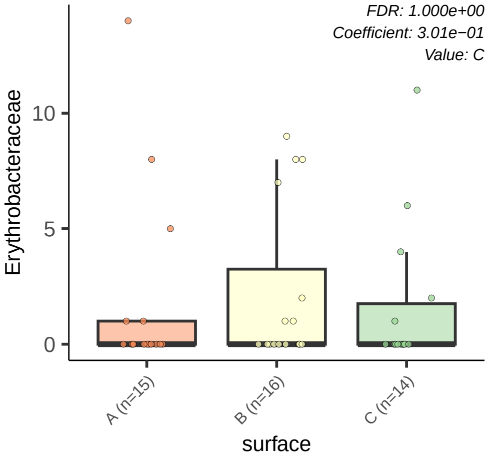

FDR: 1.000e+00
Coefficient: 3.01e−01
Value: C
Erythrobacteraceae
10
5
0
A (n=15)
B (n=16)
C (n=14)
surface

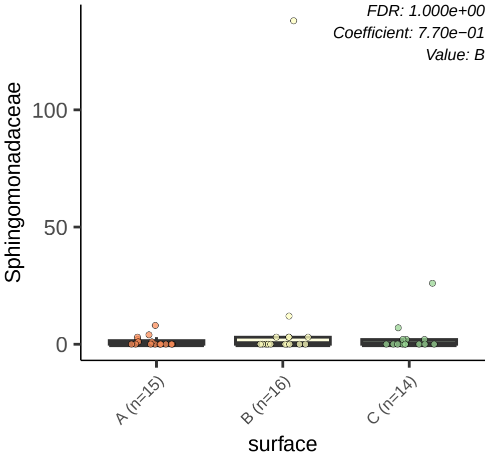

FDR: 1.000e+00
Coefficient: 7.70e−01
Value: B
Sphingomonadaceae
100
50
0
A (n=15)
B (n=16)
C (n=14)
surface

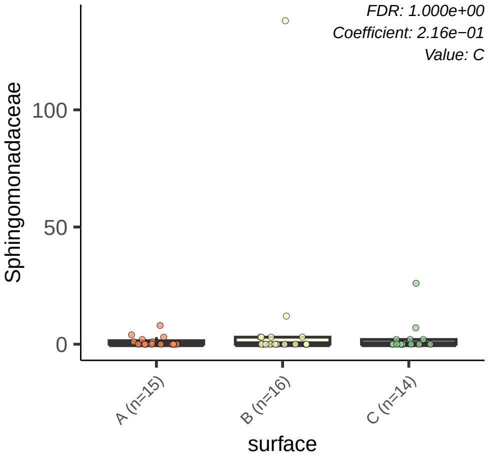
FDR: 1.000e+00
Coefficient: 2.16e−01
Value: C
Sphingomonadaceae
100
50
0
A (n=15)
B (n=16)
C (n=14)
surface

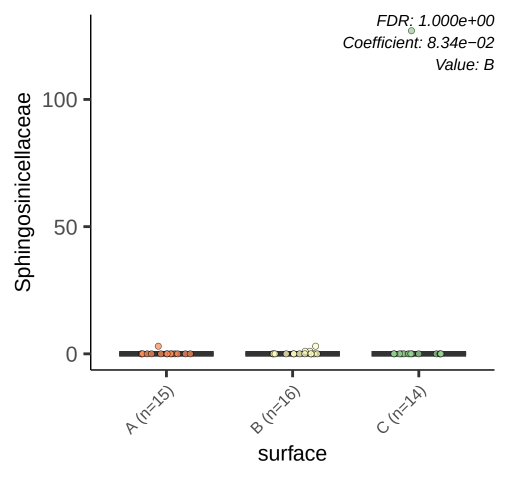
FDR: 1.000e+00
Coefficient: 8.34e−02
Value: B
Sphingosinicellaceae
100
50
0
A (n=15)
B (n=16)
C (n=14)
surface

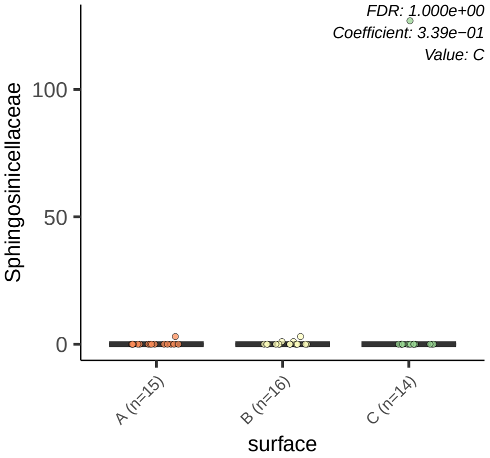
FDR: 1.000e+00
Coefficient: 3.39e−01
Value: C
100
50
0
Sphingosinicellaceae
A (n=15)
B (n=16)
C (n=14)
surface

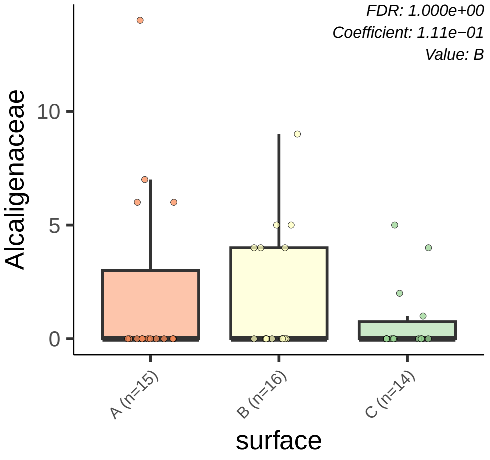
FDR: 1.000e+00
Coefficient: 1.11e−01
Value: B
Alcaligenaceae
10
5
0
A (n=15)
B (n=16)
C (n=14)
surface

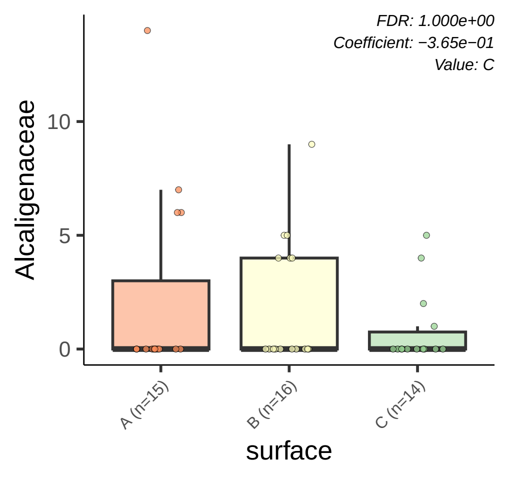
FDR: 1.000e+00
Coefficient: −3.65e−01
Value: C
Alcaligenaceae
10
5
0
A (n=15)
B (n=16)
C (n=14)
surface

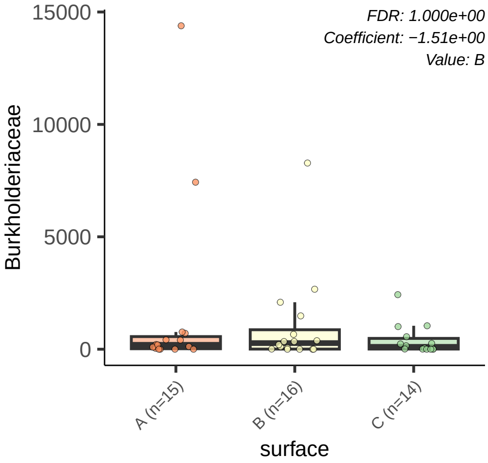

FDR: 1.000e+00
Coefficient: −1.51e+00
Value: B
15000
10000
5000
0
Burkholderiaceae
A (n=15)
B (n=16)
C (n=14)
surface

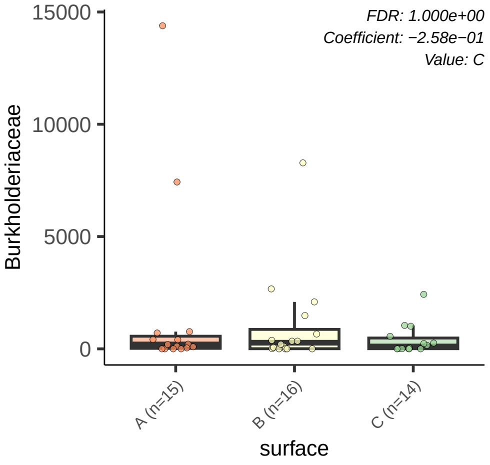

FDR: 1.000e+00
Coefficient: −2.58e−01
Value: C
Burkholderiaceae
15000
10000
5000
0
A (n=15)
B (n=16)
C (n=14)
surface

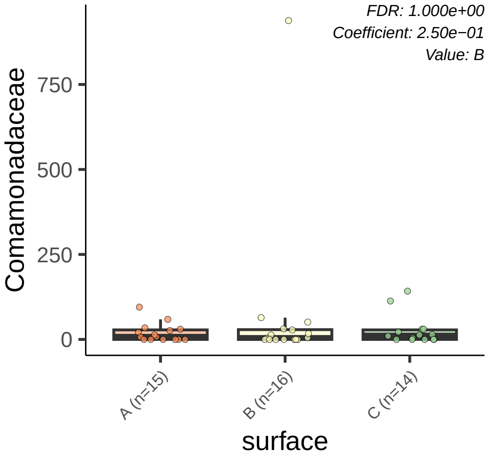

FDR: 1.000e+00
Coefficient: 2.50e−01
Value: B
Comamonadaceae
750
500
250
0
A (n=15)
B (n=16)
C (n=14)
surface

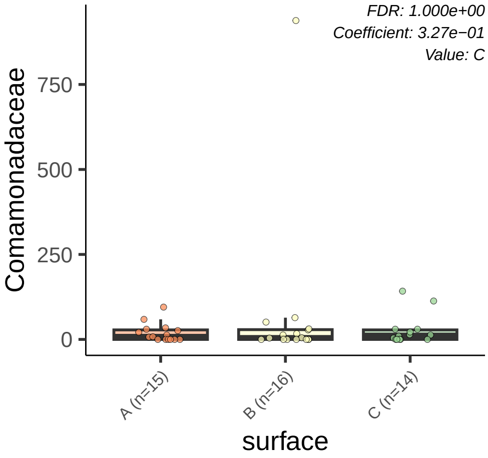

FDR: 1.000e+00
Coefficient: 3.27e−01
Value: C
Comamonadaceae
750
500
250
0
A (n=15)
B (n=16)
C (n=14)
surface

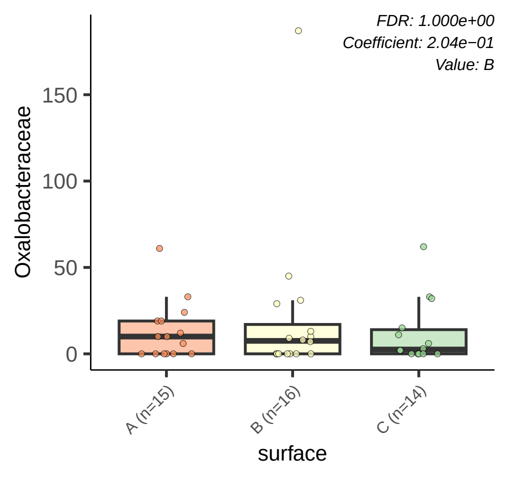
FDR: 1.000e+00
Coefficient: 2.04e−01
Value: B
Oxalobacteraceae
150
100
50
0
A (n=15)
B (n=16)
C (n=14)
surface

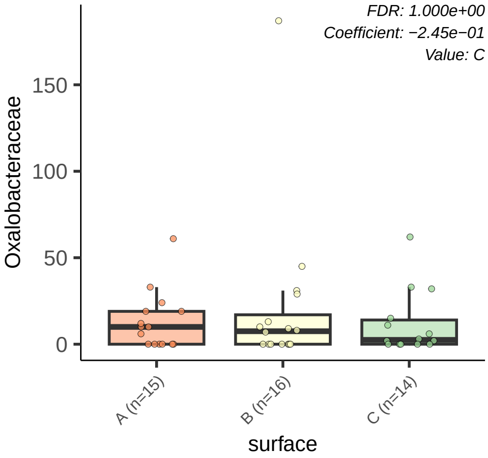

FDR: 1.000e+00
Coefficient: −2.45e−01
Value: C
Oxalobacteraceae
150
100
50
0
A (n=15)
B (n=16)
C (n=14)
surface

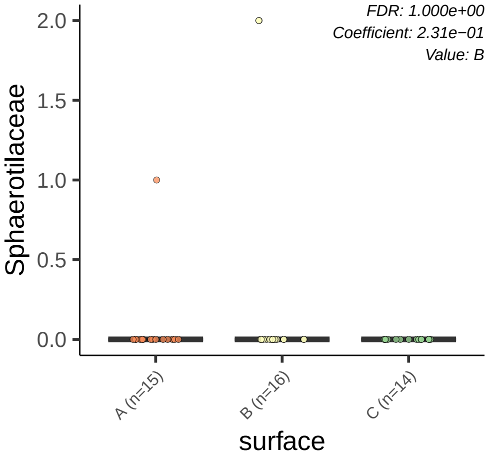
FDR: 1.000e+00
Coefficient: 2.31e−01
Value: B
Sphaerotilaceae
2.0
1.5
1.0
0.5
0.0
A (n=15)
B (n=16)
C (n=14)
surface

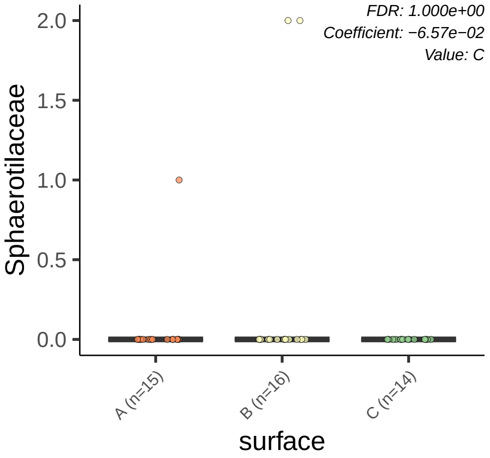
FDR: 1.000e+00
Coefficient: −6.57e−02
Value: C
Sphaerotilaceae
2.0
1.5
1.0
0.5
0.0
A (n=15)
B (n=16)
C (n=14)
surface

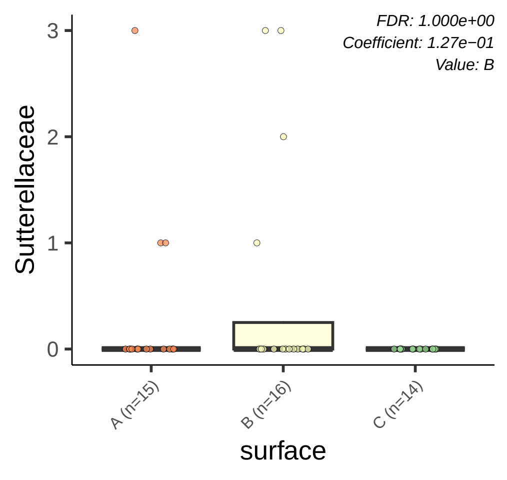

FDR: 1.000e+00
Coefficient: 1.27e−01
Value: B
Sutterellaceae
0
1
2
3
A (n=15)
B (n=16)
C (n=14)
surface

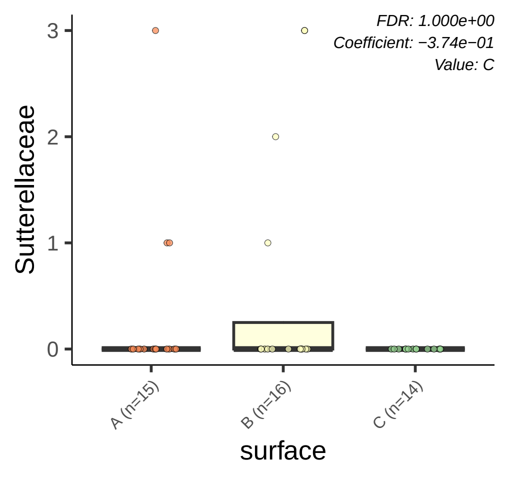
FDR: 1.000e+00
Coefficient: −3.74e−01
Value: C
Sutterellaceae
0
1
2
3
A (n=15)
B (n=16)
C (n=14)
surface

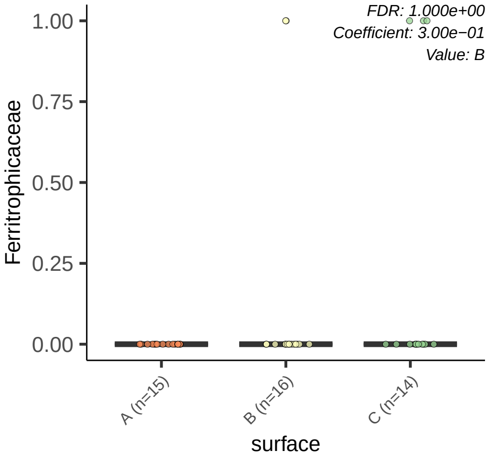

FDR: 1.000e+00
Coefficient: 3.00e−01
Value: B
Ferritrophicaceae
1.00
0.75
0.50
0.25
0.00
A (n=15)
B (n=16)
C (n=14)
surface

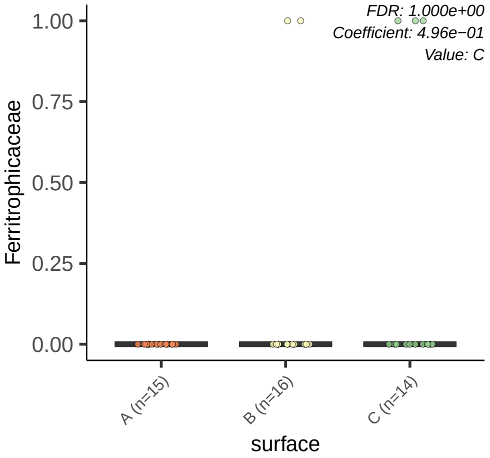
FDR: 1.000e+00
Coefficient: 4.96e−01
Value: C
Ferritrophicaceae
1.00
0.75
0.50
0.25
0.00
A (n=15)
B (n=16)
C (n=14)
surface

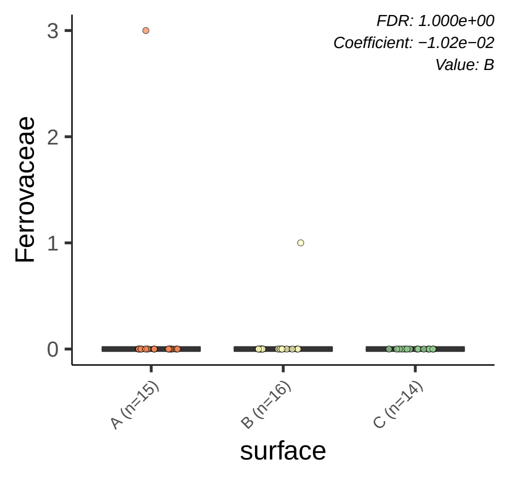
FDR: 1.000e+00
Coefficient: −1.02e−02
Value: B
Ferrovaceae
3
2
1
0
A (n=15)
B (n=16)
C (n=14)
surface

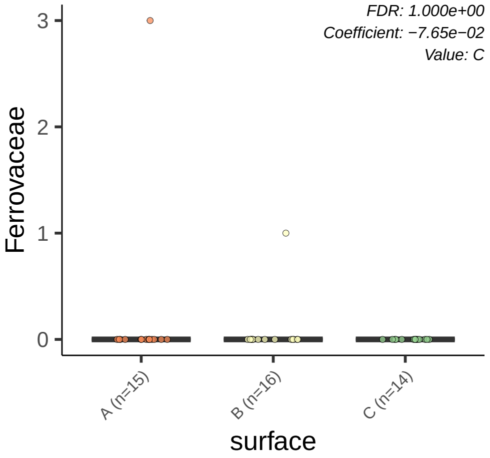
FDR: 1.000e+00
Coefficient: −7.65e−02
Value: C
Ferrovaceae
3
2
1
0
A (n=15)
B (n=16)
C (n=14)
surface

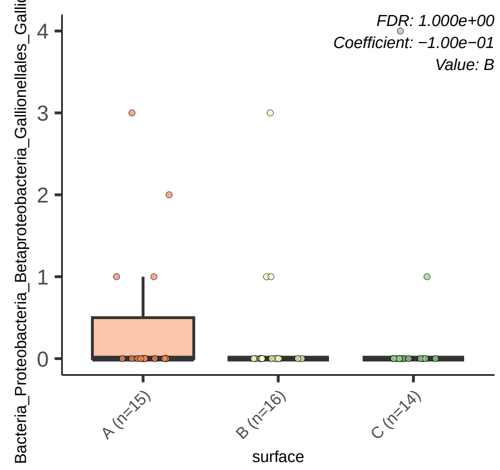
FDR: 1.000e+00
Coefficient: −1.00e−01
Value: B
Bacteria_Proteobacteria_Betaproteobacteria_Gallionellales_Gallio
4
3
2
1
0
A (n=15)
B (n=16)
C (n=14)
surface

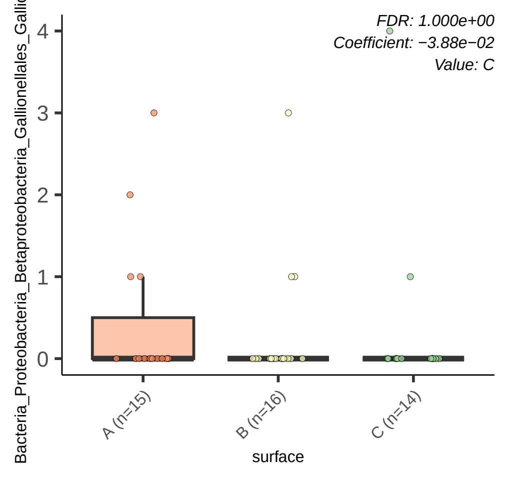

FDR: 1.000e+00
Coefficient: −3.88e−02
Value: C
Bacteria_Proteobacteria_Betaproteobacteria_Gallionellales_Gallio
0
1
2
3
4
A (n=15)
B (n=16)
C (n=14)
surface

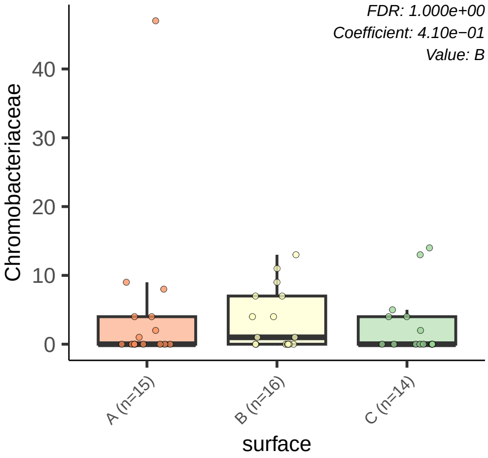
FDR: 1.000e+00
Coefficient: 4.10e−01
Value: B
Chromobacteriaceae
0
10
20
30
40
A (n=15)
B (n=16)
C (n=14)
surface

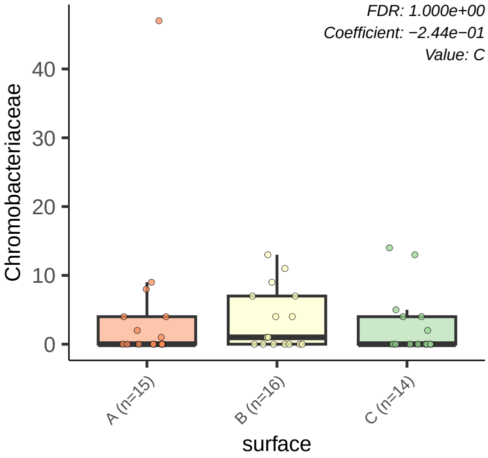

FDR: 1.000e+00
Coefficient: −2.44e−01
Value: C
Chromobacteriaceae
0
10
20
30
40
A (n=15)
B (n=16)
C (n=14)
surface

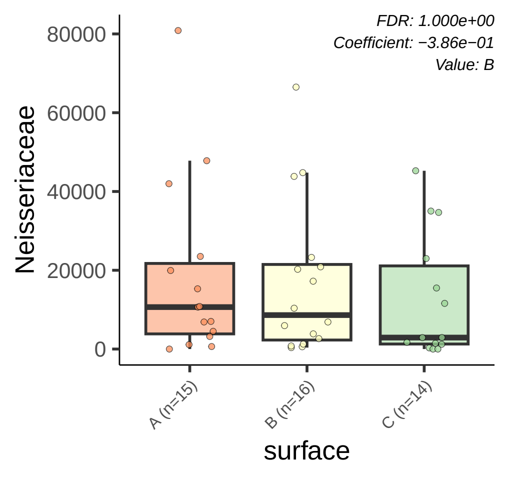
FDR: 1.000e+00
Coefficient: −3.86e−01
Value: B
Neisseriaceae
80000
60000
40000
20000
0
A (n=15)
B (n=16)
C (n=14)
surface

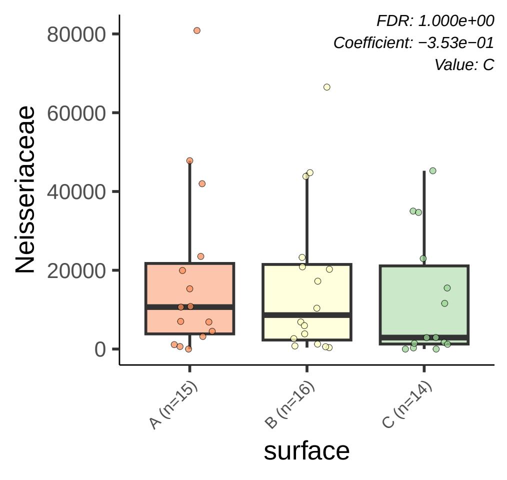
FDR: 1.000e+00
Coefficient: −3.53e−01
Value: C
Neisseriaceae
80000
60000
40000
20000
0
A (n=15)
B (n=16)
C (n=14)
surface

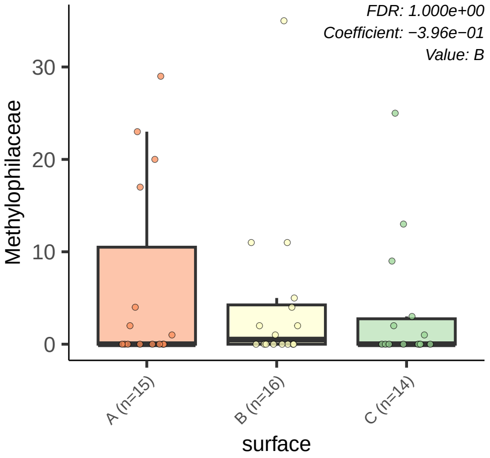

FDR: 1.000e+00
Coefficient: −3.96e−01
Value: B
Methylophilaceae
0
10
20
30
A (n=15)
B (n=16)
C (n=14)
surface

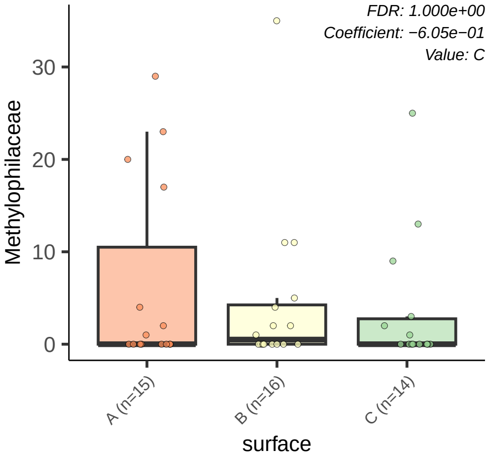
FDR: 1.000e+00
Coefficient: −6.05e−01
Value: C
Methylophilaceae
0
10
20
30
A (n=15)
B (n=16)
C (n=14)
surface

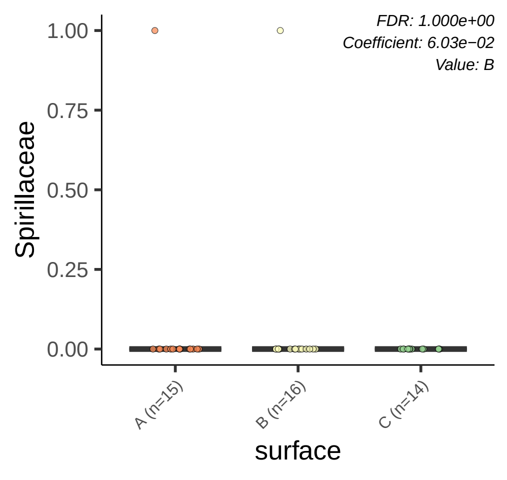
FDR: 1.000e+00
Coefficient: 6.03e−02
Value: B
Spirillaceae
1.00
0.75
0.50
0.25
0.00
A (n=15)
B (n=16)
C (n=14)
surface

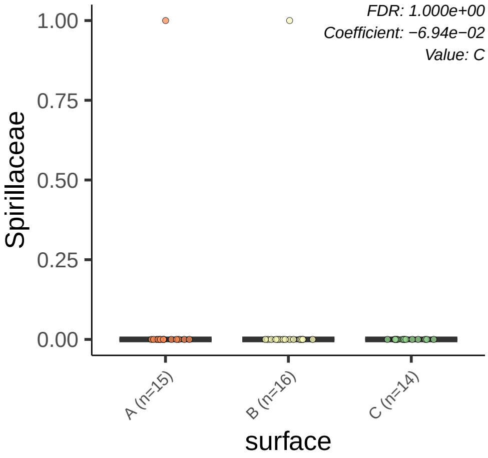

FDR: 1.000e+00
Coefficient: −6.94e−02
Value: C
Spirillaceae
1.00
0.75
0.50
0.25
0.00
A (n=15)
B (n=16)
C (n=14)
surface

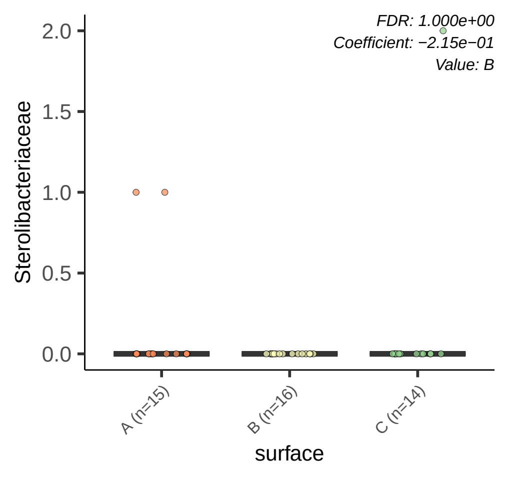
FDR: 1.000e+00
Coefficient: −2.15e−01
Value: B
Sterolibacteriaceae
2.0
1.5
1.0
0.5
0.0
A (n=15)
B (n=16)
C (n=14)
surface

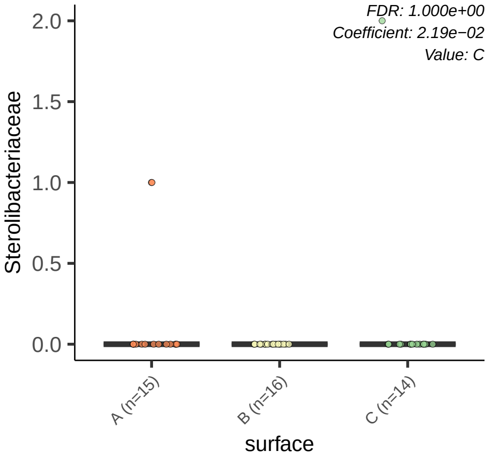
FDR: 1.000e+00
Coefficient: 2.19e−02
Value: C
Sterolibacteriaceae
2.0
1.5
1.0
0.5
0.0
A (n=15)
B (n=16)
C (n=14)
surface

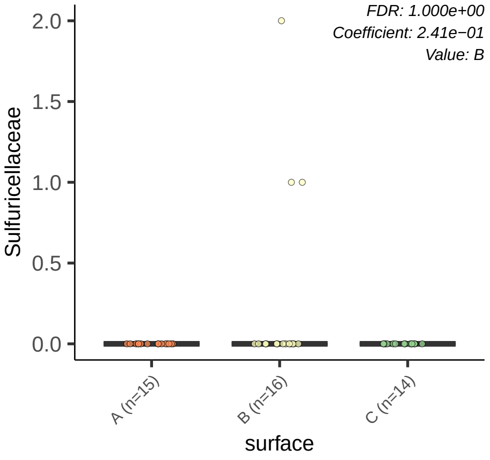

FDR: 1.000e+00
Coefficient: 2.41e−01
Value: B
Sulfuricellaceae
2.0
1.5
1.0
0.5
0.0
A (n=15)
B (n=16)
C (n=14)
surface

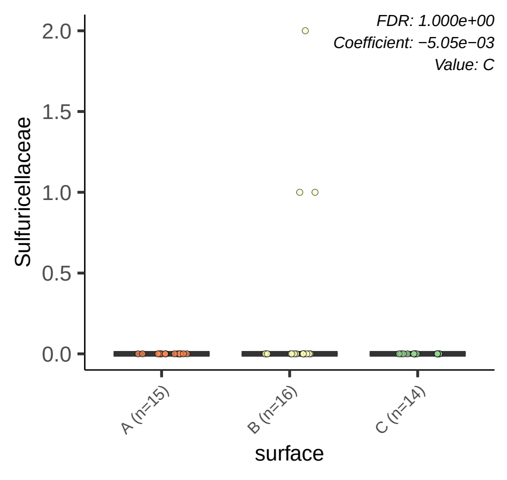
FDR: 1.000e+00
Coefficient: −5.05e−03
Value: C
Sulfuricellaceae
2.0
1.5
1.0
0.5
0.0
A (n=15)
B (n=16)
C (n=14)
surface

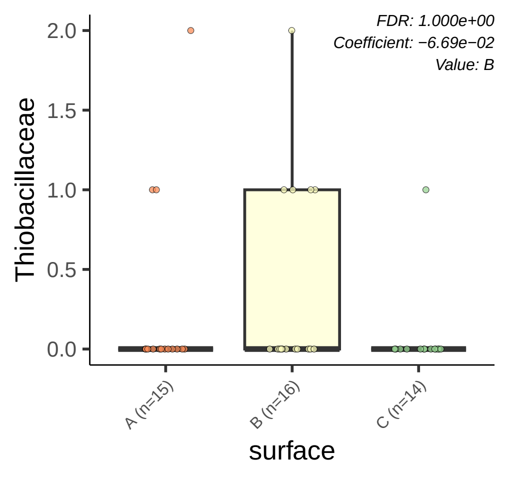
FDR: 1.000e+00
Coefficient: −6.69e−02
Value: B
Thiobacillaceae
2.0
1.5
1.0
0.5
0.0
A (n=15)
B (n=16)
C (n=14)
surface

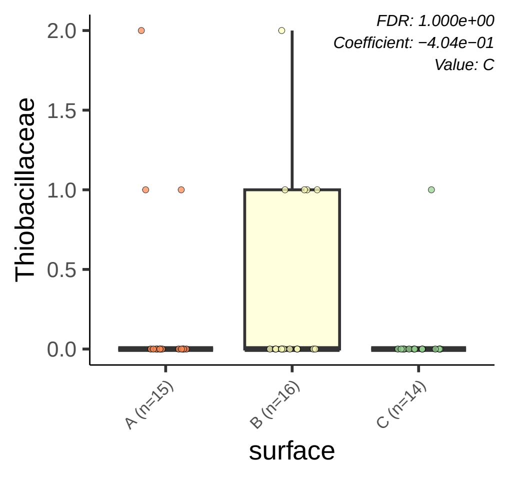
FDR: 1.000e+00
Coefficient: −4.04e−01
Value: C
Thiobacillaceae
2.0
1.5
1.0
0.5
0.0
A (n=15)
B (n=16)
C (n=14)
surface

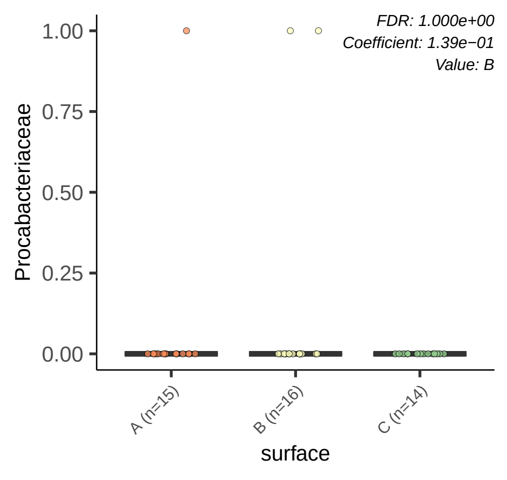
FDR: 1.000e+00
Coefficient: 1.39e−01
Value: B
Procabacteriaceae
1.00
0.75
0.50
0.25
0.00
A (n=15)
B (n=16)
C (n=14)
surface

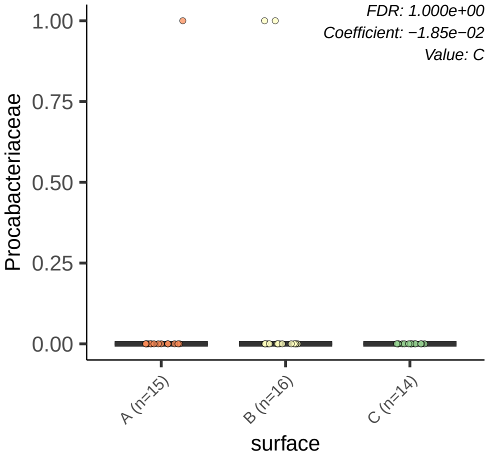
FDR: 1.000e+00
Coefficient: −1.85e−02
Value: C
Procabacteriaceae
1.00
0.75
0.50
0.25
0.00
A (n=15)
B (n=16)
C (n=14)
surface

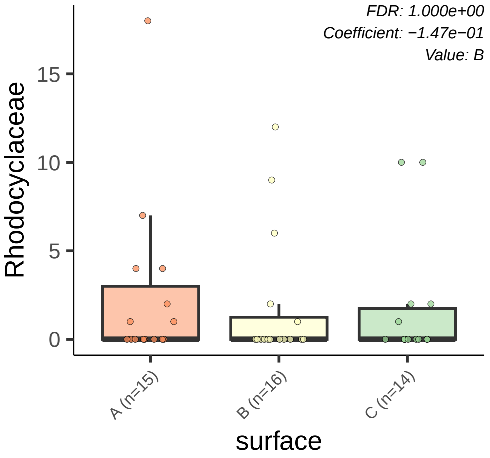

FDR: 1.000e+00
Coefficient: −1.47e−01
Value: B
Rhodocyclaceae
15
10
5
0
A (n=15)
B (n=16)
C (n=14)
surface

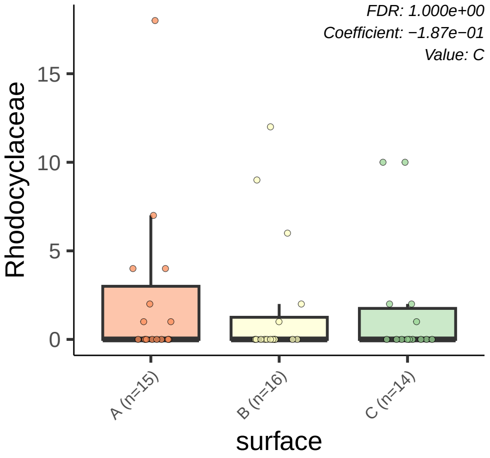
FDR: 1.000e+00
Coefficient: −1.87e−01
Value: C
Rhodocyclaceae
15
10
5
0
A (n=15)
B (n=16)
C (n=14)
surface

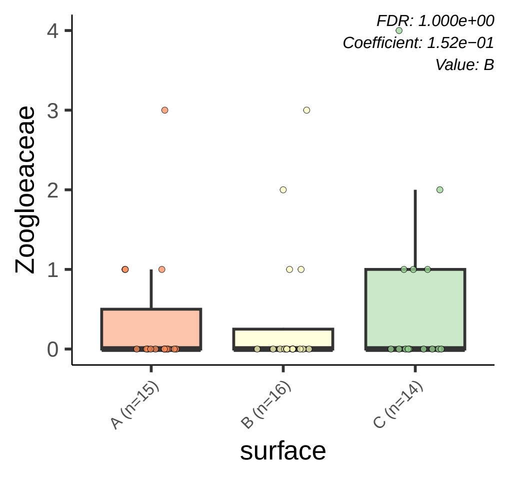
FDR: 1.000e+00
Coefficient: 1.52e−01
Value: B
Zoogloeaceae
0
1
2
3
4
A (n=15)
B (n=16)
C (n=14)
surface

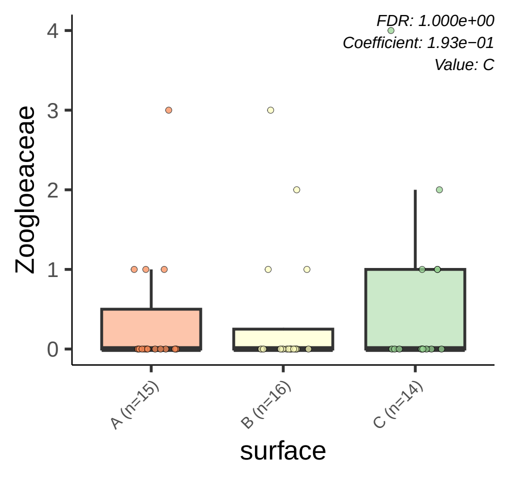
FDR: 1.000e+00
Coefficient: 1.93e−01
Value: C
Zoogloeaceae
0
1
2
3
4
A (n=15)
B (n=16)
C (n=14)
surface

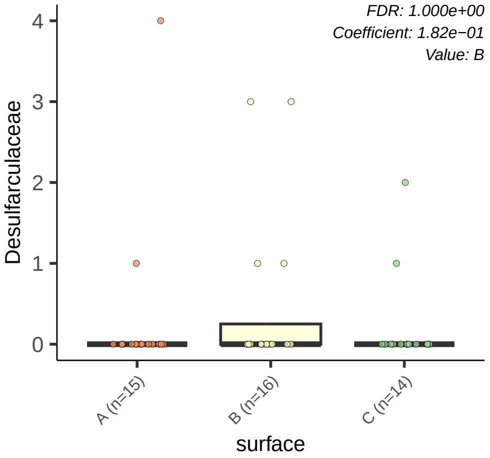

FDR: 1.000e+00
Coefficient: 1.82e−01
Value: B
Desulfarculaceae
0
1
2
3
4
A (n=15)
B (n=16)
C (n=14)
surface

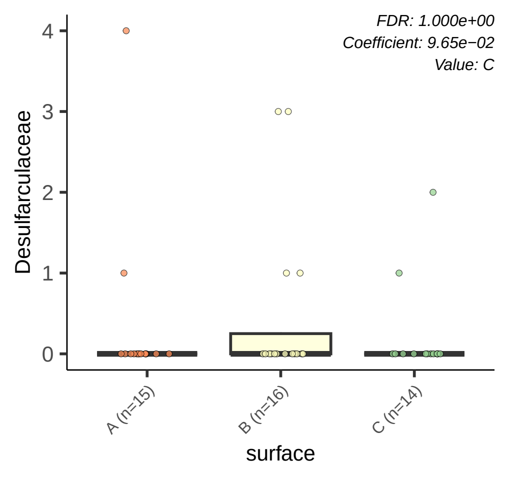
FDR: 1.000e+00
Coefficient: 9.65e−02
Value: C
Desulfarculaceae
0
1
2
3
4
A (n=15)
B (n=16)
C (n=14)
surface

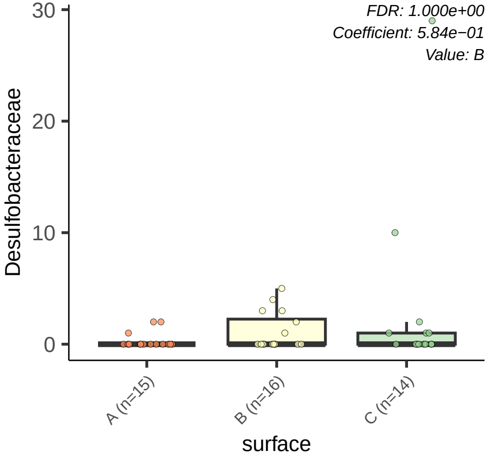
FDR: 1.000e+00
Coefficient: 5.84e−01
Value: B
Desulfobacteraceae
30
20
10
0
A (n=15)
B (n=16)
C (n=14)
surface

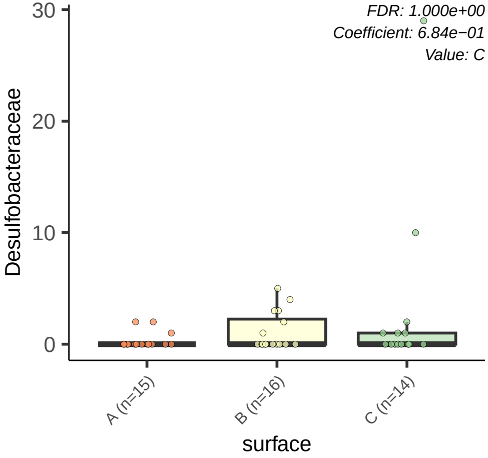

FDR: 1.000e+00
Coefficient: 6.84e−01
Value: C
Desulfobacteraceae
30
20
10
0
A (n=15)
B (n=16)
C (n=14)
surface

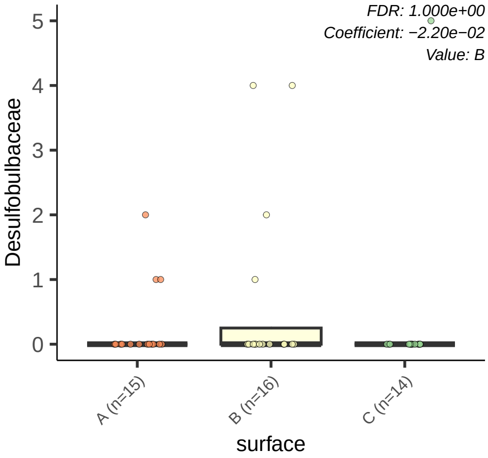

FDR: 1.000e+00
Coefficient: −2.20e−02
Value: B
Desulfobulbaceae
0
1
2
3
4
5
A (n=15)
B (n=16)
C (n=14)
surface

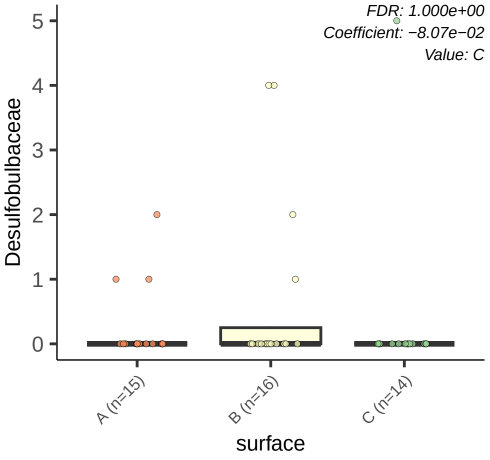

FDR: 1.000e+00
Coefficient: −8.07e−02
Value: C
Desulfobulbaceae
0
1
2
3
4
5
A (n=15)
B (n=16)
C (n=14)
surface

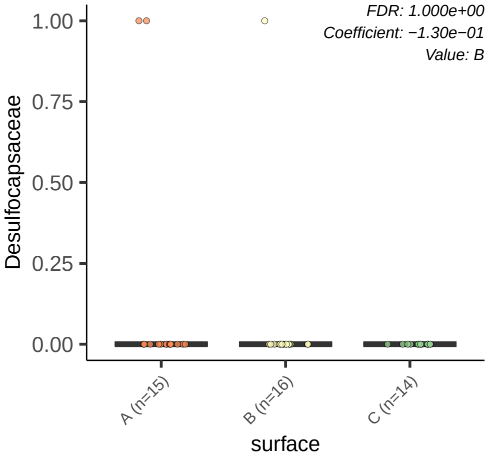
FDR: 1.000e+00
Coefficient: −1.30e−01
Value: B
Desulfocapsaceae
1.00
0.75
0.50
0.25
0.00
A (n=15)
B (n=16)
C (n=14)
surface

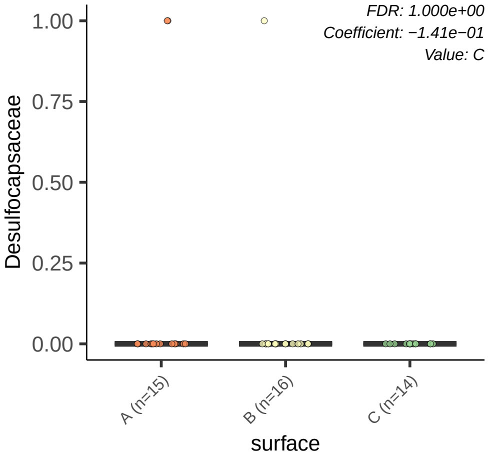
FDR: 1.000e+00
Coefficient: −1.41e−01
Value: C
1.00
0.75
0.50
0.25
0.00
Desulfocapsaceae
A (n=15)
B (n=16)
C (n=14)
surface

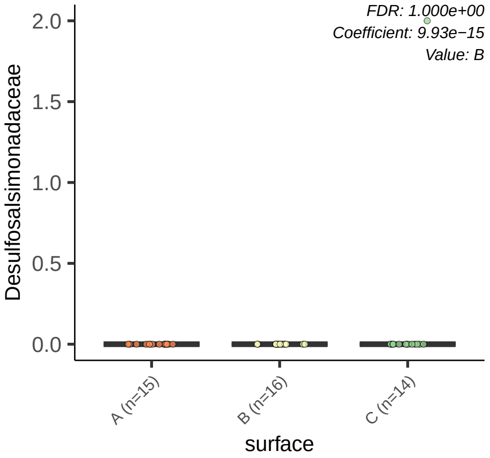
FDR: 1.000e+00
Coefficient: 9.93e−15
Value: B
Desulfosalsimonadaceae
2.0
1.5
1.0
0.5
0.0
A (n=15)
B (n=16)
C (n=14)
surface

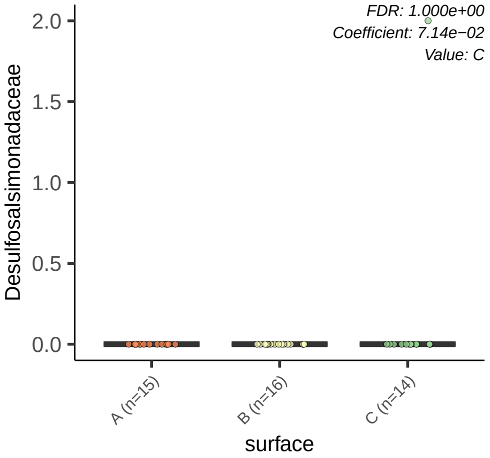

FDR: 1.000e+00
Coefficient: 7.14e−02
Value: C
Desulfosalsimonadaceae
2.0
1.5
1.0
0.5
0.0
A (n=15)
B (n=16)
C (n=14)
surface

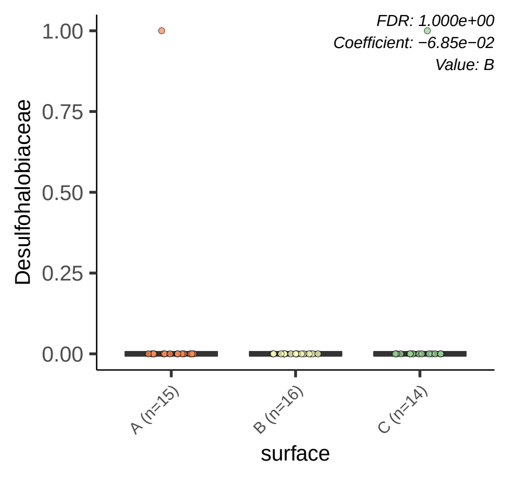
FDR: 1.000e+00
Coefficient: −6.85e−02
Value: B
Desulfohalobiaceae
1.00
0.75
0.50
0.25
0.00
A (n=15)
B (n=16)
C (n=14)
surface

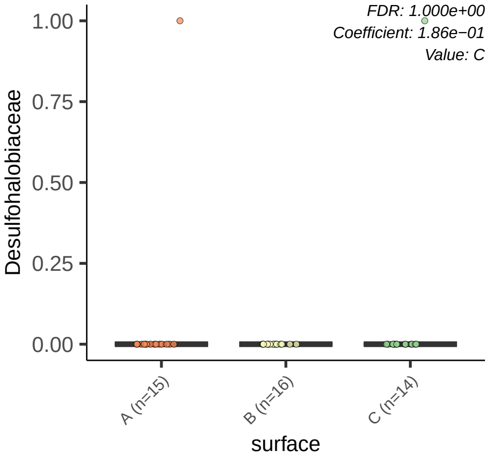
FDR: 1.000e+00
Coefficient: 1.86e−01
Value: C
Desulfohalobiaceae
1.00
0.75
0.50
0.25
0.00
A (n=15)
B (n=16)
C (n=14)
surface

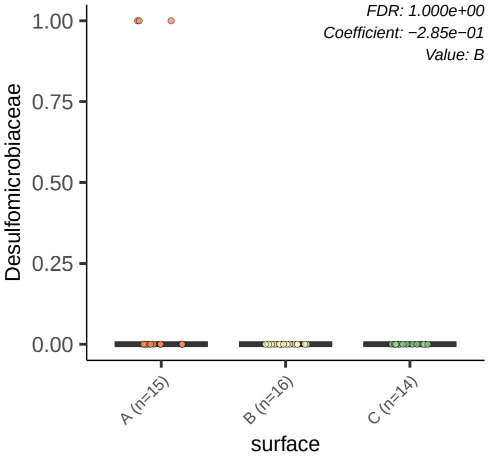

FDR: 1.000e+00
Coefficient: −2.85e−01
Value: B
Desulfomicrobiaceae
1.00
0.75
0.50
0.25
0.00
A (n=15)
B (n=16)
C (n=14)
surface

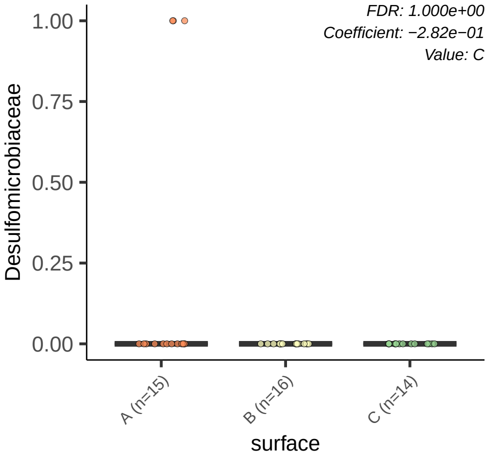
FDR: 1.000e+00
Coefficient: −2.82e−01
Value: C
Desulfomicrobiaceae
1.00
0.75
0.50
0.25
0.00
A (n=15)
B (n=16)
C (n=14)
surface

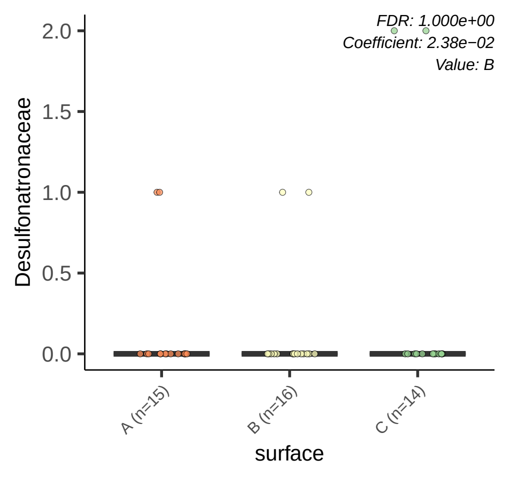
FDR: 1.000e+00
Coefficient: 2.38e−02
Value: B
Desulfonatronaceae
2.0
1.5
1.0
0.5
0.0
A (n=15)
B (n=16)
C (n=14)
surface

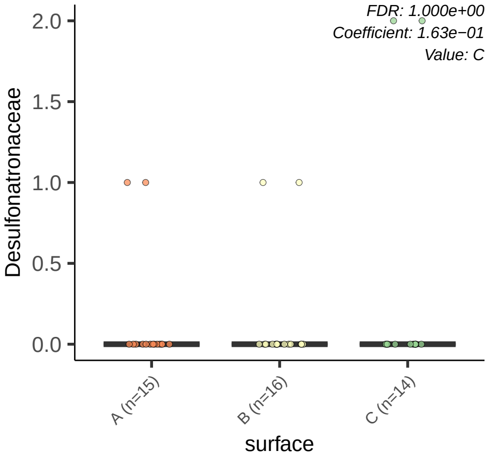

FDR: 1.000e+00
Coefficient: 1.63e−01
Value: C
Desulfonatronaceae
2.0
1.5
1.0
0.5
0.0
A (n=15)
B (n=16)
C (n=14)
surface

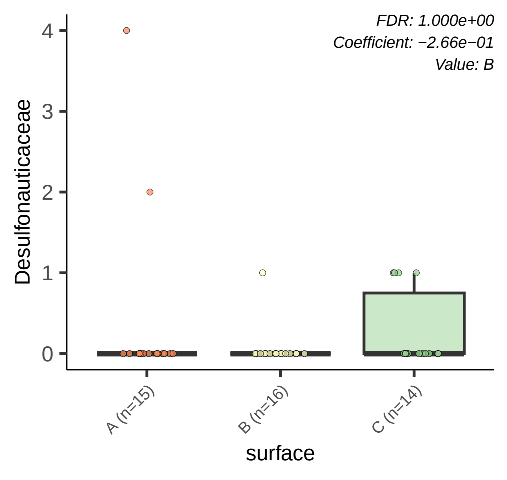
FDR: 1.000e+00
Coefficient: −2.66e−01
Value: B
Desulfonauticaceae
0
1
2
3
4
A (n=15)
B (n=16)
C (n=14)
surface

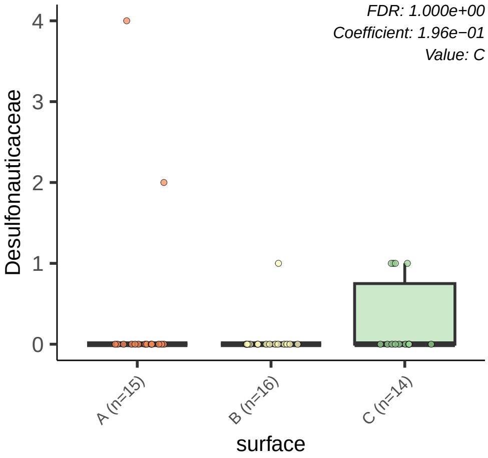
FDR: 1.000e+00
Coefficient: 1.96e−01
Value: C
Desulfonauticaceae
4
3
2
1
0
A (n=15)
B (n=16)
C (n=14)
surface

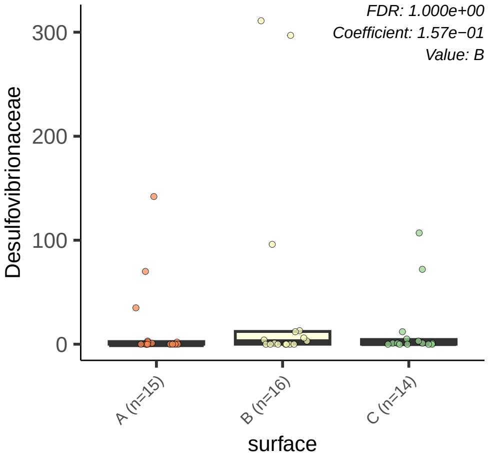
FDR: 1.000e+00
Coefficient: 1.57e−01
Value: B
300
200
100
0
Desulfovibrionaceae
A (n=15)
B (n=16)
C (n=14)
surface

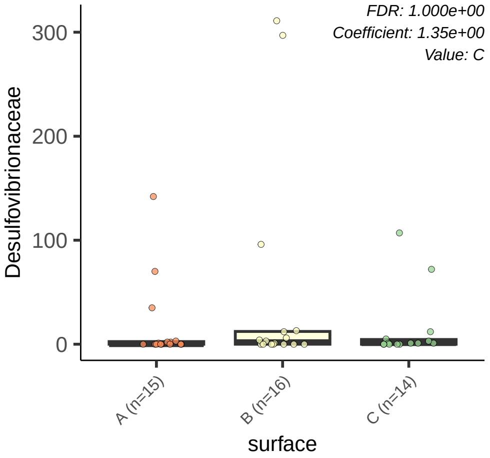
FDR: 1.000e+00
Coefficient: 1.35e+00
Value: C
Desulfovibrionaceae
300
200
100
0
A (n=15)
B (n=16)
C (n=14)
surface

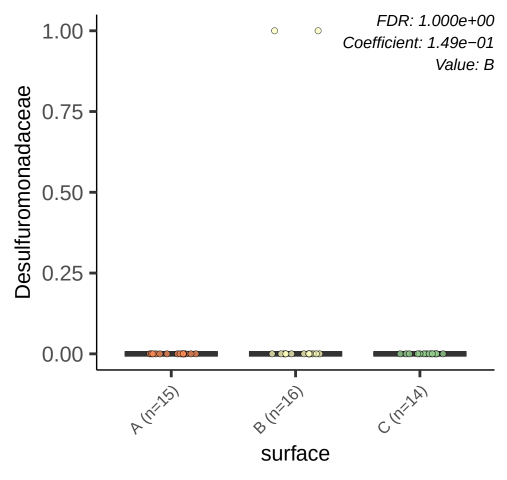
FDR: 1.000e+00
Coefficient: 1.49e−01
Value: B
Desulfuromonadaceae
1.00
0.75
0.50
0.25
0.00
A (n=15)
B (n=16)
C (n=14)
surface

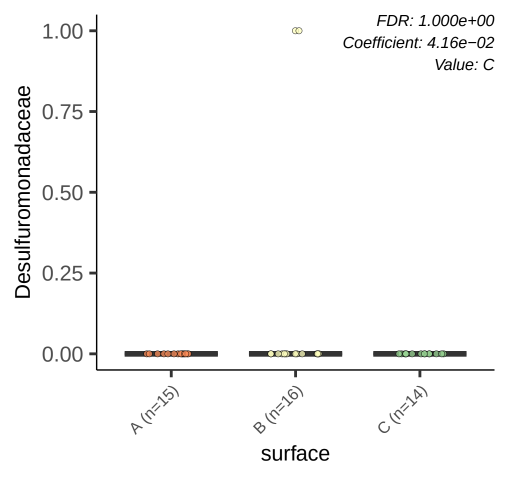

FDR: 1.000e+00
Coefficient: 4.16e−02
Value: C
Desulfuromonadaceae
1.00
0.75
0.50
0.25
0.00
A (n=15)
B (n=16)
C (n=14)
surface

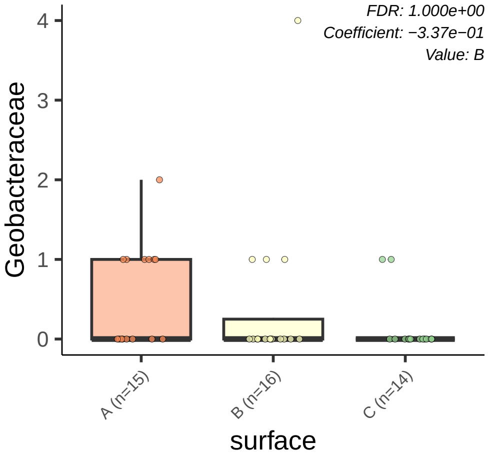

FDR: 1.000e+00
Coefficient: −3.37e−01
Value: B
Geobacteraceae
0
1
2
3
4
A (n=15)
B (n=16)
C (n=14)
surface

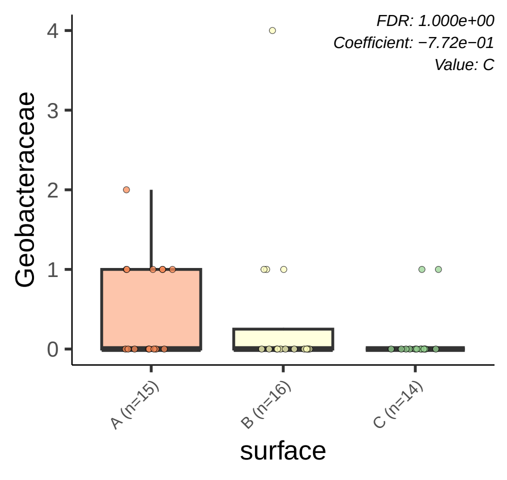
FDR: 1.000e+00
Coefficient: −7.72e−01
Value: C
Geobacteraceae
0
1
2
3
4
A (n=15)
B (n=16)
C (n=14)
surface

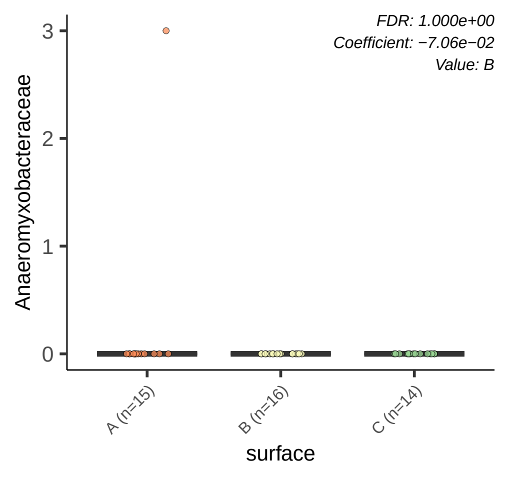

FDR: 1.000e+00
Coefficient: −7.06e−02
Value: B
Anaeromyxobacteraceae
0
1
2
3
A (n=15)
B (n=16)
C (n=14)
surface

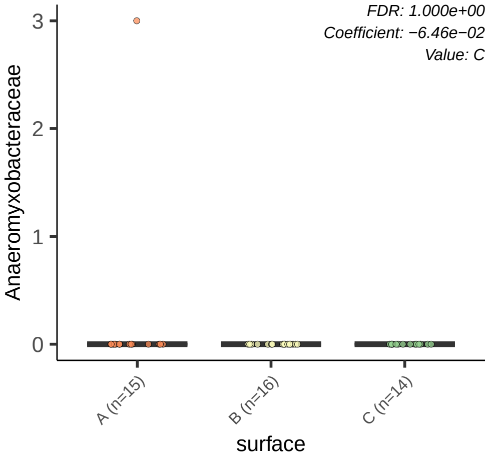

FDR: 1.000e+00
Coefficient: −6.46e−02
Value: C
Anaeromyxobacteraceae
0
1
2
3
A (n=15)
B (n=16)
C (n=14)
surface

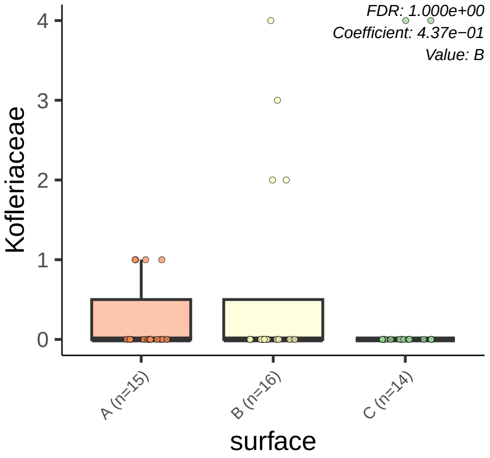
FDR: 1.000e+00
Coefficient: 4.37e−01
Value: B
Kofleriaceae
0
1
2
3
4
A (n=15)
B (n=16)
C (n=14)
surface

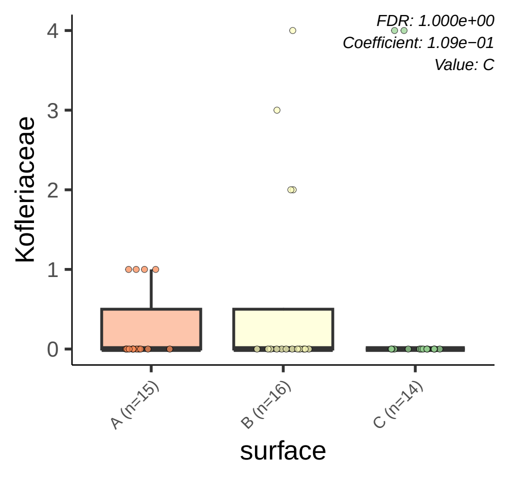
FDR: 1.000e+00
Coefficient: 1.09e−01
Value: C
Kofleriaceae
0
1
2
3
4
A (n=15)
B (n=16)
C (n=14)
surface

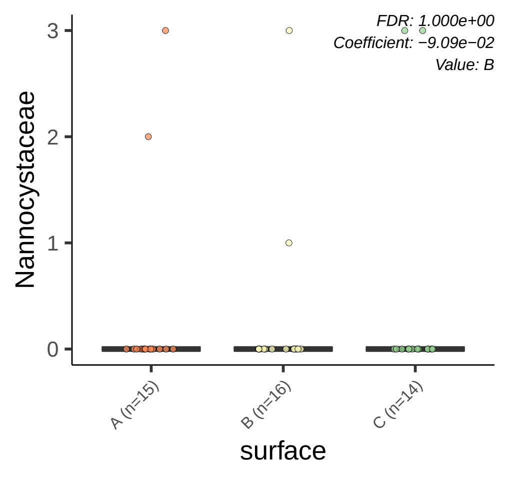
FDR: 1.000e+00
Coefficient: −9.09e−02
Value: B
Nannocystaceae
3
2
1
0
A (n=15)
B (n=16)
C (n=14)
surface

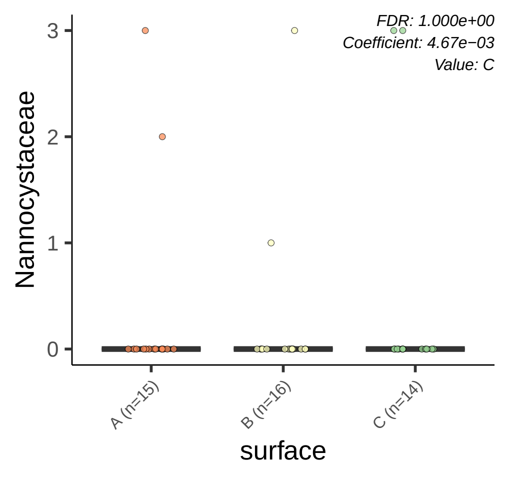

FDR: 1.000e+00
Coefficient: 4.67e−03
Value: C
Nannocystaceae
3
2
1
0
A (n=15)
B (n=16)
C (n=14)
surface

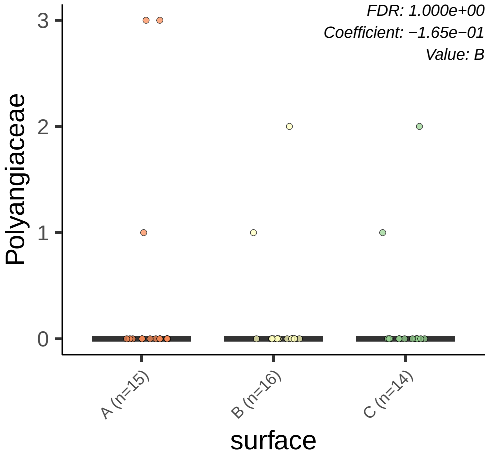

FDR: 1.000e+00
Coefficient: −1.65e−01
Value: B
Polyangiaceae
3
2
1
0
A (n=15)
B (n=16)
C (n=14)
surface

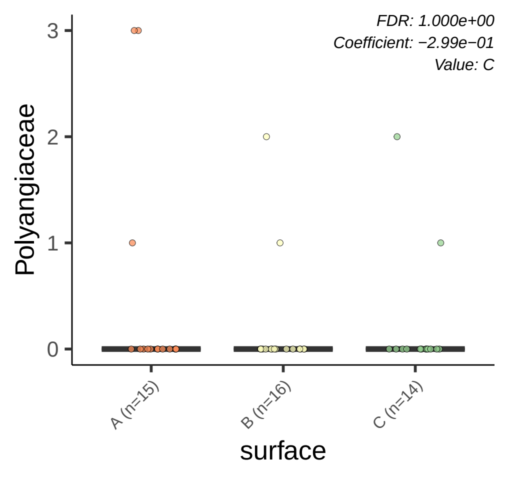
FDR: 1.000e+00
Coefficient: −2.99e−01
Value: C
Polyangiaceae
0
1
2
3
A (n=15)
B (n=16)
C (n=14)
surface

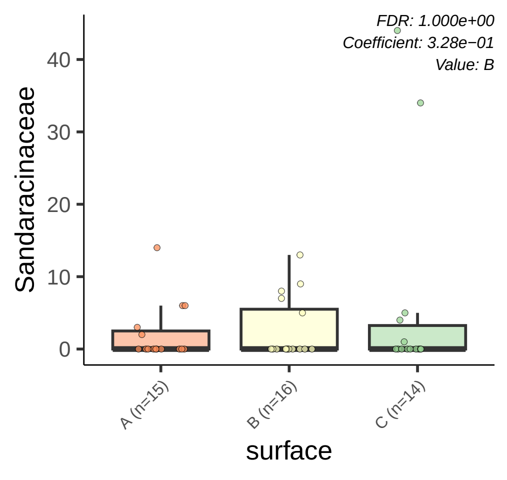

FDR: 1.000e+00
Coefficient: 3.28e−01
Value: B
Sandaracinaceae
0
10
20
30
40
A (n=15)
B (n=16)
C (n=14)
surface

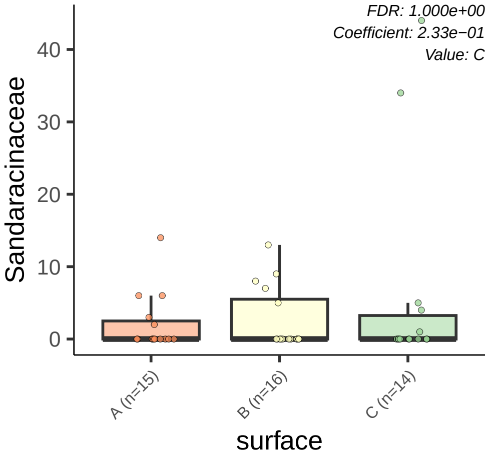
FDR: 1.000e+00
Coefficient: 2.33e−01
Value: C
Sandaracinaceae
0
10
20
30
40
A (n=15)
B (n=16)
C (n=14)
surface

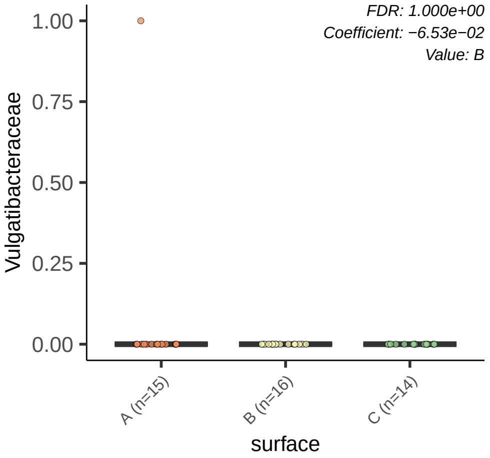
FDR: 1.000e+00
Coefficient: −6.53e−02
Value: B
Vulgatibacteraceae
1.00
0.75
0.50
0.25
0.00
A (n=15)
B (n=16)
C (n=14)
surface

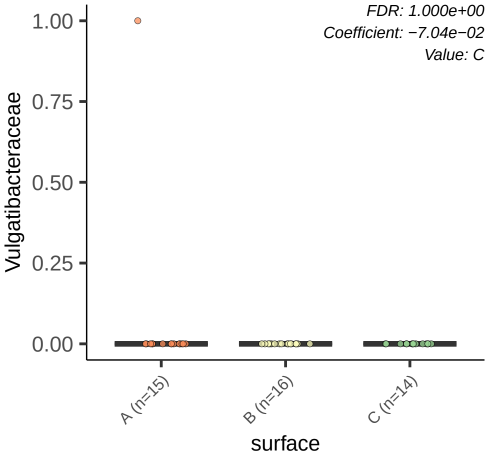

FDR: 1.000e+00
Coefficient: −7.04e−02
Value: C
Vulgatibacteraceae
1.00
0.75
0.50
0.25
0.00
A (n=15)
B (n=16)
C (n=14)
surface

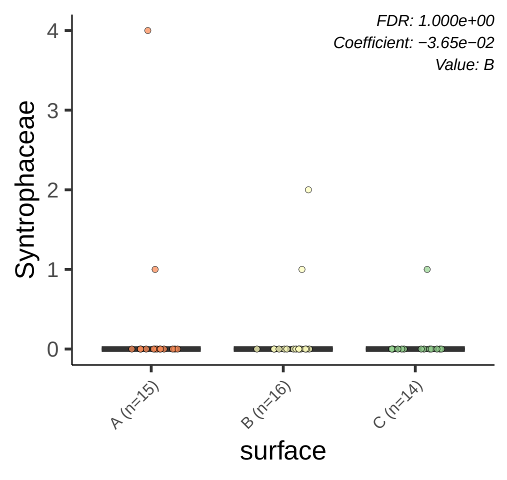
FDR: 1.000e+00
Coefficient: −3.65e−02
Value: B
Syntrophaceae
4
3
2
1
0
A (n=15)
B (n=16)
C (n=14)
surface

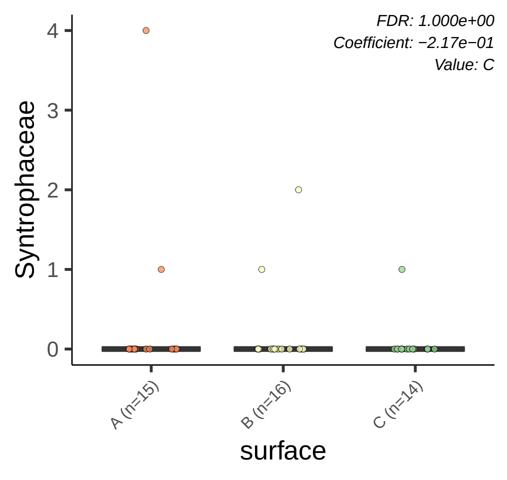
FDR: 1.000e+00
Coefficient: −2.17e−01
Value: C
Syntrophaceae
0
1
2
3
4
A (n=15)
B (n=16)
C (n=14)
surface

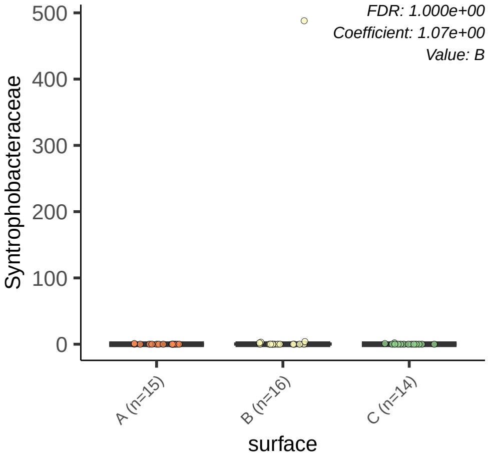
FDR: 1.000e+00
Coefficient: 1.07e+00
Value: B
Syntrophobacteraceae
500
400
300
200
100
0
A (n=15)
B (n=16)
C (n=14)
surface

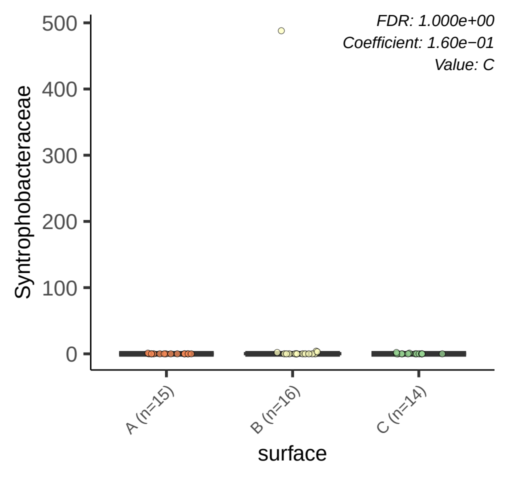

FDR: 1.000e+00
Coefficient: 1.60e−01
Value: C
Syntrophobacteraceae
500
400
300
200
100
0
A (n=15)
B (n=16)
C (n=14)
surface

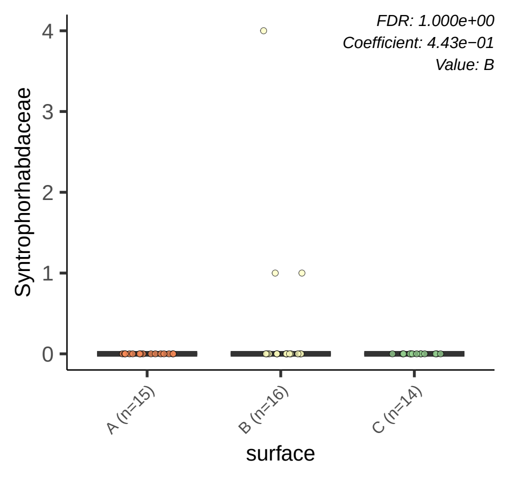
FDR: 1.000e+00
Coefficient: 4.43e−01
Value: B
Syntrophorhabdaceae
0
1
2
3
4
A (n=15)
B (n=16)
C (n=14)
surface

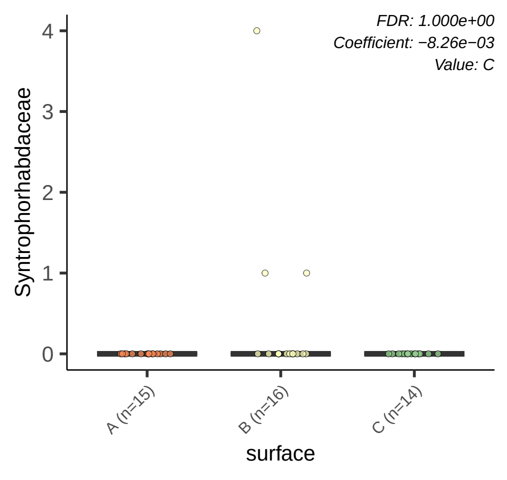

FDR: 1.000e+00
Coefficient: −8.26e−03
Value: C
Syntrophorhabdaceae
0
1
2
3
4
A (n=15)
B (n=16)
C (n=14)
surface

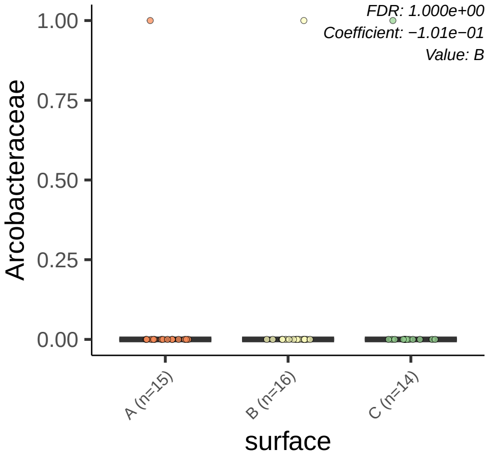
FDR: 1.000e+00
Coefficient: −1.01e−01
Value: B
Arcobacteraceae
1.00
0.75
0.50
0.25
0.00
A (n=15)
B (n=16)
C (n=14)
surface

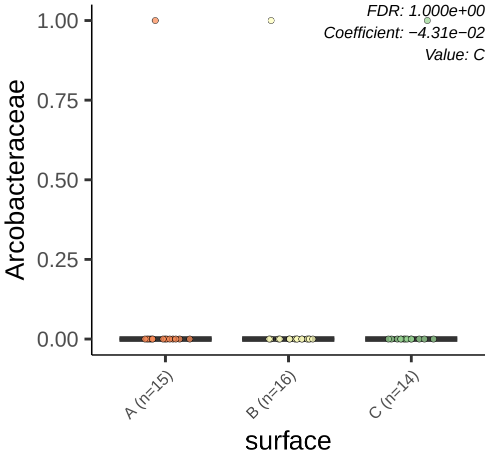
FDR: 1.000e+00
Coefficient: −4.31e−02
Value: C
Arcobacteraceae
1.00
0.75
0.50
0.25
0.00
A (n=15)
B (n=16)
C (n=14)
surface

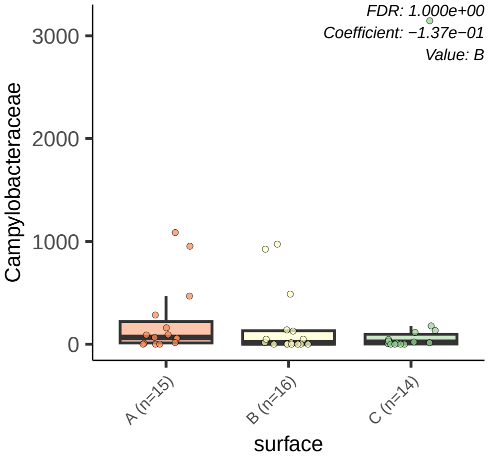
FDR: 1.000e+00
Coefficient: −1.37e−01
Value: B
Campylobacteraceae
3000
2000
1000
0
A (n=15)
B (n=16)
C (n=14)
surface

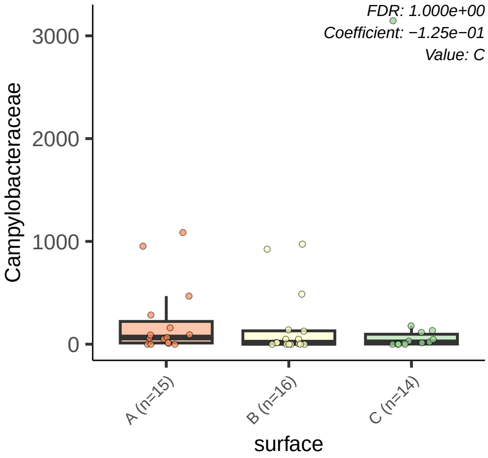
FDR: 1.000e+00
Coefficient: −1.25e−01
Value: C
3000
2000
1000
0
Campylobacteraceae
A (n=15)
B (n=16)
C (n=14)
surface

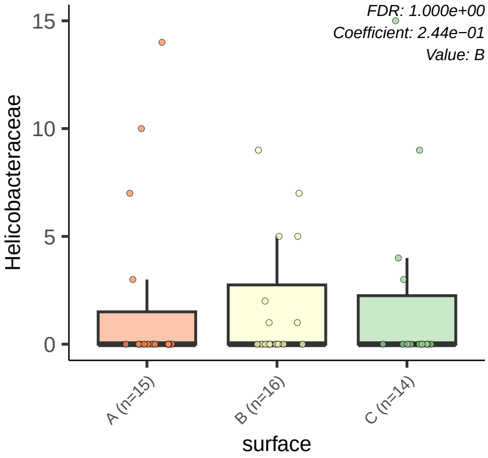

FDR: 1.000e+00
Coefficient: 2.44e−01
Value: B
Helicobacteraceae
15
10
5
0
A (n=15)
B (n=16)
C (n=14)
surface

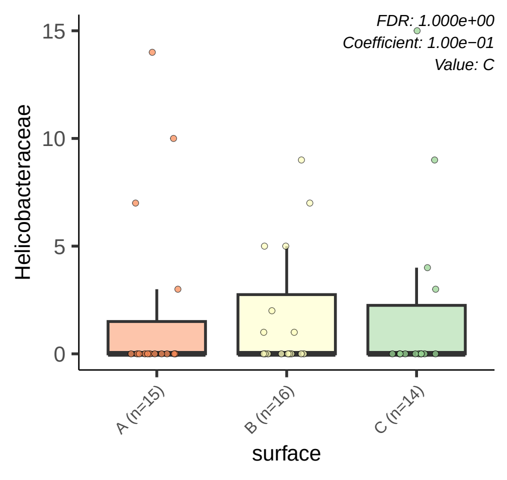
FDR: 1.000e+00
Coefficient: 1.00e−01
Value: C
Helicobacteraceae
15
10
5
0
A (n=15)
B (n=16)
C (n=14)
surface

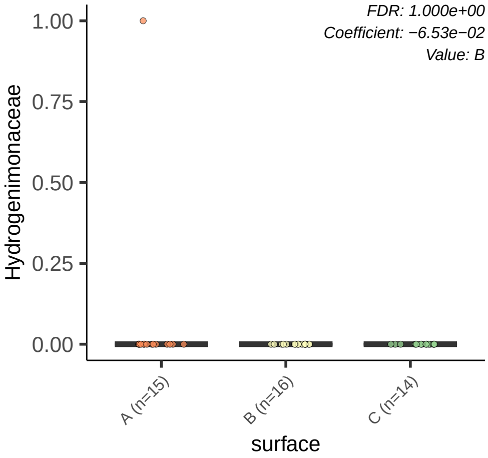
FDR: 1.000e+00
Coefficient: −6.53e−02
Value: B
Hydrogenimonaceae
1.00
0.75
0.50
0.25
0.00
A (n=15)
B (n=16)
C (n=14)
surface

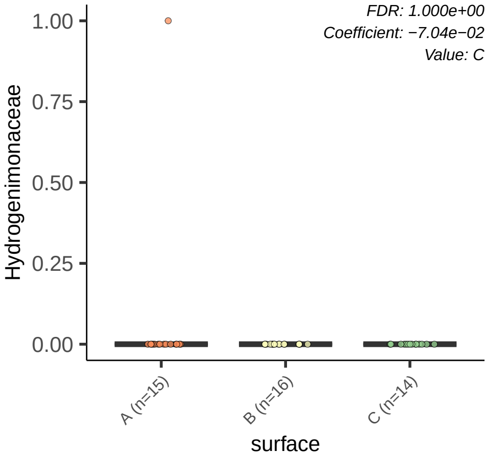

FDR: 1.000e+00
Coefficient: −7.04e−02
Value: C
Hydrogenimonaceae
1.00
0.75
0.50
0.25
0.00
A (n=15)
B (n=16)
C (n=14)
surface

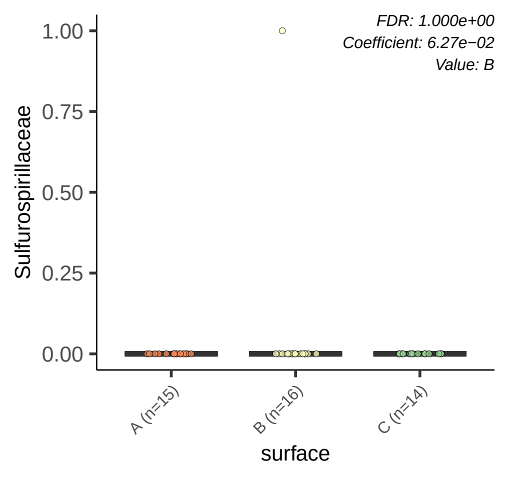

FDR: 1.000e+00
Coefficient: 6.27e−02
Value: B
Sulfurospirillaceae
1.00
0.75
0.50
0.25
0.00
A (n=15)
B (n=16)
C (n=14)
surface

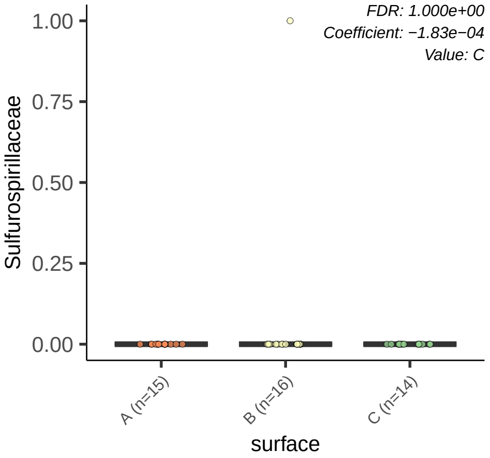

FDR: 1.000e+00
Coefficient: −1.83e−04
Value: C
Sulfurospirillaceae
1.00
0.75
0.50
0.25
0.00
A (n=15)
B (n=16)
C (n=14)
surface

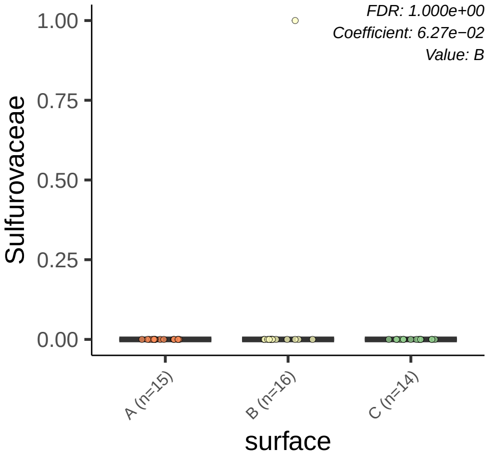

FDR: 1.000e+00
Coefficient: 6.27e−02
Value: B
Sulfurovaceae
1.00
0.75
0.50
0.25
0.00
A (n=15)
B (n=16)
C (n=14)
surface

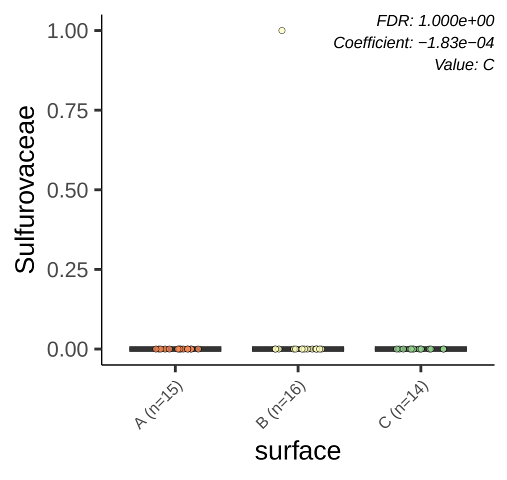
FDR: 1.000e+00
Coefficient: −1.83e−04
Value: C
Sulfurovaceae
1.00
0.75
0.50
0.25
0.00
A (n=15)
B (n=16)
C (n=14)
surface

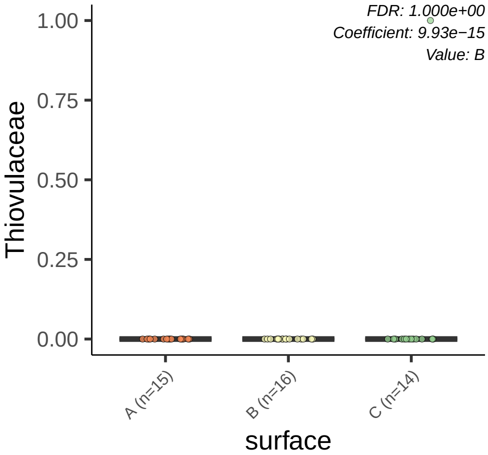

FDR: 1.000e+00
Coefficient: 9.93e−15
Value: B
Thiovulaceae
1.00
0.75
0.50
0.25
0.00
A (n=15)
B (n=16)
C (n=14)
surface

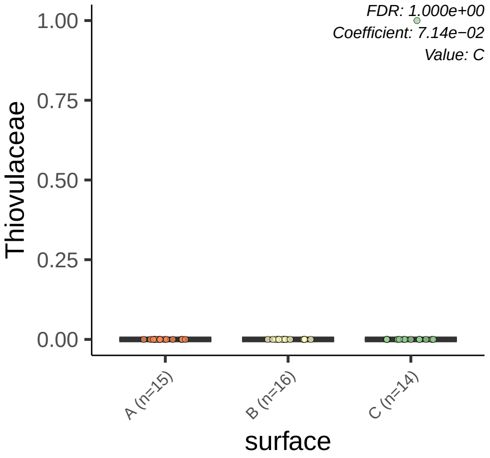

FDR: 1.000e+00
Coefficient: 7.14e−02
Value: C
Thiovulaceae
1.00
0.75
0.50
0.25
0.00
A (n=15)
B (n=16)
C (n=14)
surface

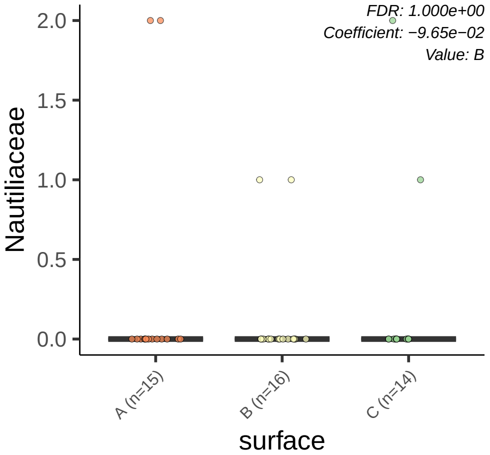
FDR: 1.000e+00
Coefficient: −9.65e−02
Value: B
Nautiliaceae
2.0
1.5
1.0
0.5
0.0
A (n=15)
B (n=16)
C (n=14)
surface

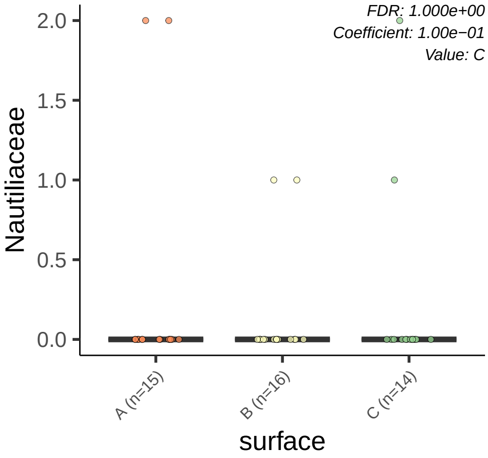

FDR: 1.000e+00
Coefficient: 1.00e−01
Value: C
Nautiliaceae
2.0
1.5
1.0
0.5
0.0
A (n=15)
B (n=16)
C (n=14)
surface

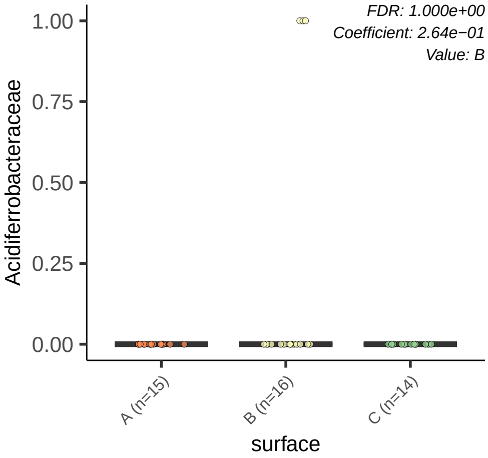
FDR: 1.000e+00
Coefficient: 2.64e−01
Value: B
Acidiferrobacteraceae
1.00
0.75
0.50
0.25
0.00
A (n=15)
B (n=16)
C (n=14)
surface

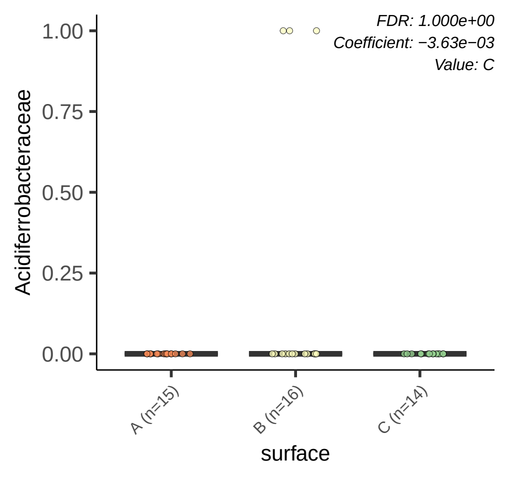
FDR: 1.000e+00
Coefficient: −3.63e−03
Value: C
Acidiferrobacteraceae
1.00
0.75
0.50
0.25
0.00
A (n=15)
B (n=16)
C (n=14)
surface

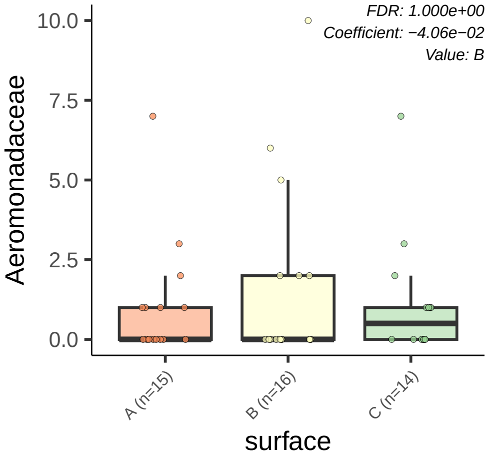
FDR: 1.000e+00
Coefficient: −4.06e−02
Value: B
Aeromonadaceae
10.0
7.5
5.0
2.5
0.0
A (n=15)
B (n=16)
C (n=14)
surface

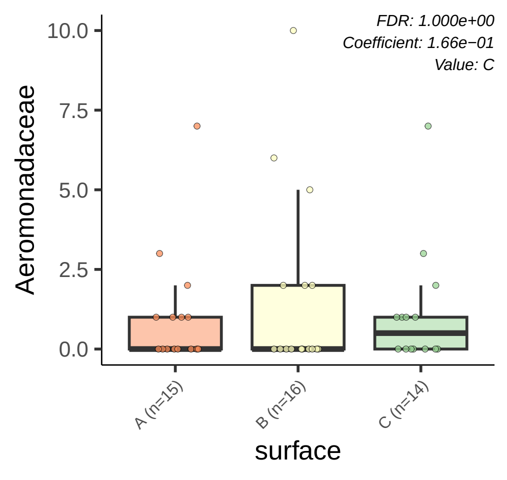
FDR: 1.000e+00
Coefficient: 1.66e−01
Value: C
Aeromonadaceae
10.0
7.5
5.0
2.5
0.0
A (n=15)
B (n=16)
C (n=14)
surface

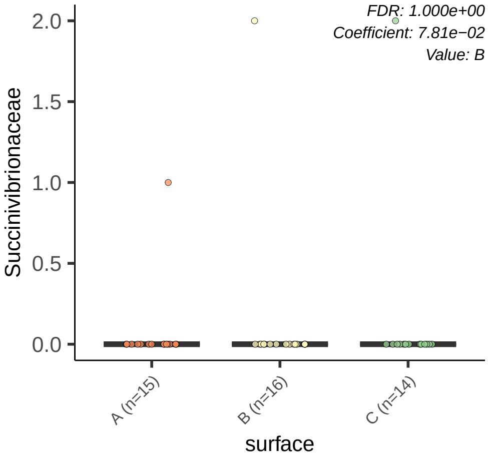
FDR: 1.000e+00
Coefficient: 7.81e−02
Value: B
Succinivibrionaceae
2.0
1.5
1.0
0.5
0.0
A (n=15)
B (n=16)
C (n=14)
surface

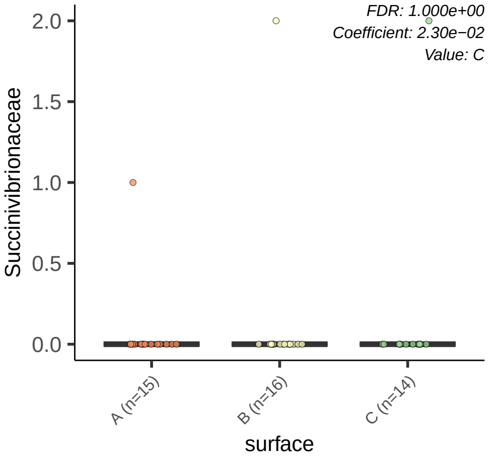

FDR: 1.000e+00
Coefficient: 2.30e−02
Value: C
Succinivibrionaceae
2.0
1.5
1.0
0.5
0.0
A (n=15)
B (n=16)
C (n=14)
surface

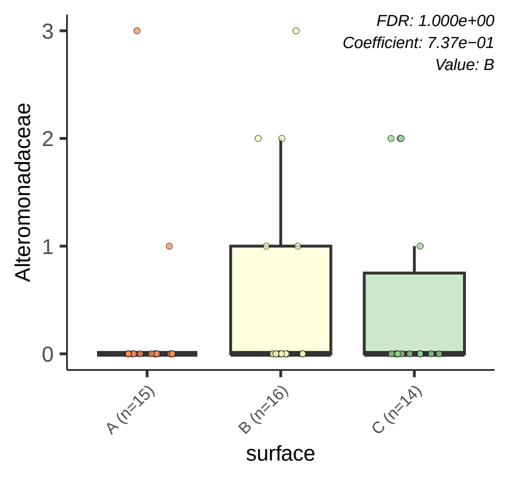
FDR: 1.000e+00
Coefficient: 7.37e−01
Value: B
Alteromonadaceae
0
1
2
3
A (n=15)
B (n=16)
C (n=14)
surface

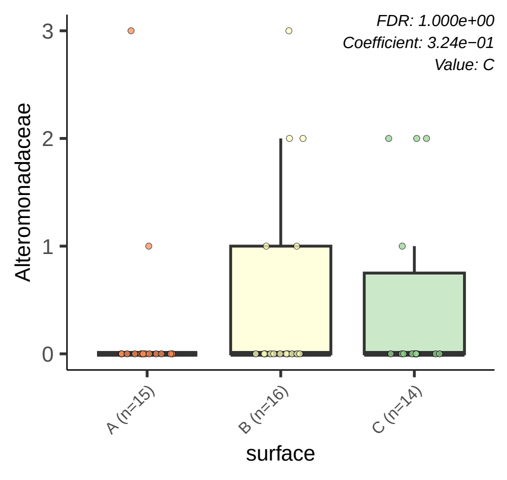
FDR: 1.000e+00
Coefficient: 3.24e−01
Value: C
Alteromonadaceae
0
1
2
3
A (n=15)
B (n=16)
C (n=14)
surface

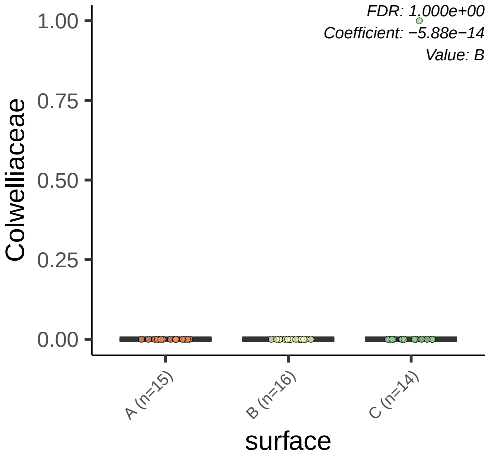

FDR: 1.000e+00
Coefficient: −5.88e−14
Value: B
Colwelliaceae
1.00
0.75
0.50
0.25
0.00
A (n=15)
B (n=16)
C (n=14)
surface

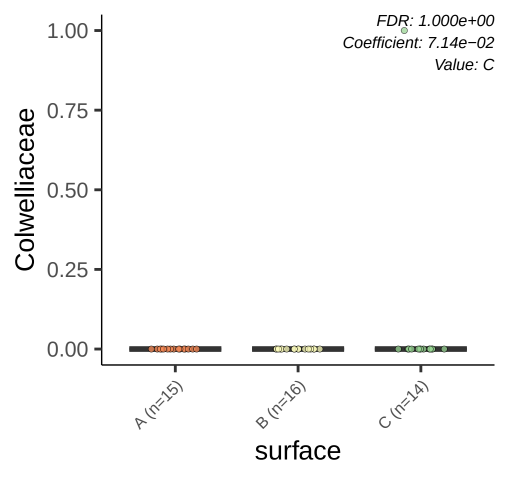
FDR: 1.000e+00
Coefficient: 7.14e−02
Value: C
Colwelliaceae
1.00
0.75
0.50
0.25
0.00
A (n=15)
B (n=16)
C (n=14)
surface

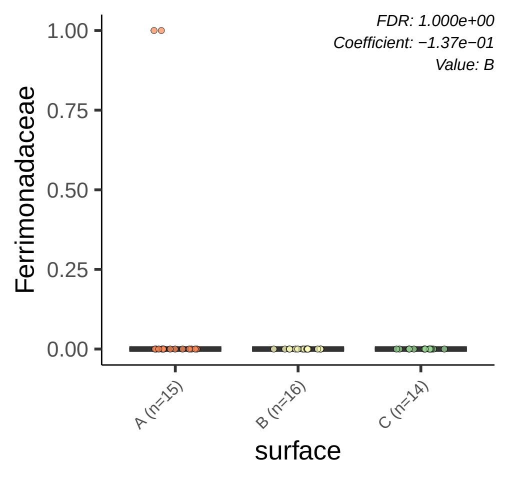

FDR: 1.000e+00
Coefficient: −1.37e−01
Value: B
Ferrimonadaceae
1.00
0.75
0.50
0.25
0.00
A (n=15)
B (n=16)
C (n=14)
surface

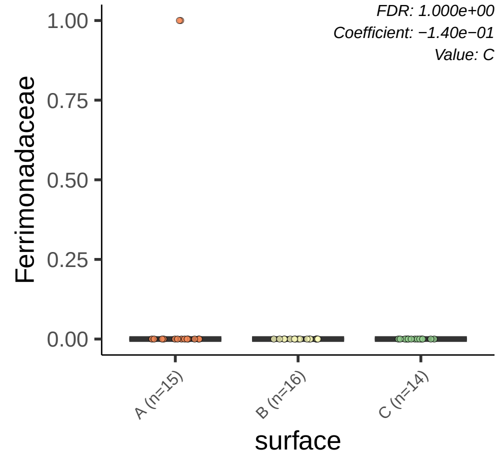
FDR: 1.000e+00
Coefficient: −1.40e−01
Value: C
Ferrimonadaceae
1.00
0.75
0.50
0.25
0.00
A (n=15)
B (n=16)
C (n=14)
surface

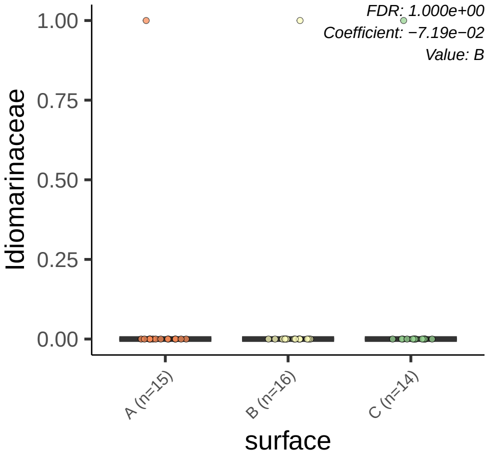

FDR: 1.000e+00
Coefficient: −7.19e−02
Value: B
Idiomarinaceae
1.00
0.75
0.50
0.25
0.00
A (n=15)
B (n=16)
C (n=14)
surface

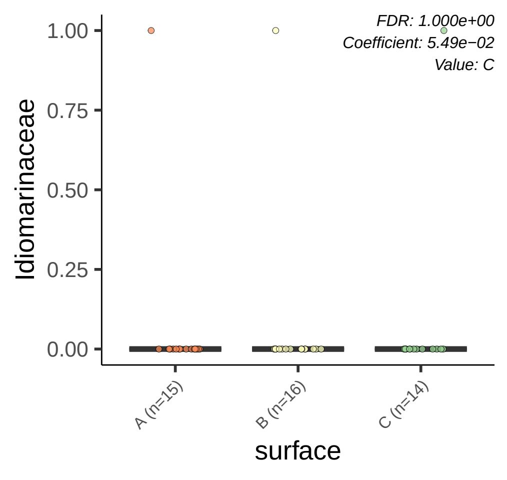
FDR: 1.000e+00
Coefficient: 5.49e−02
Value: C
Idiomarinaceae
1.00
0.75
0.50
0.25
0.00
A (n=15)
B (n=16)
C (n=14)
surface

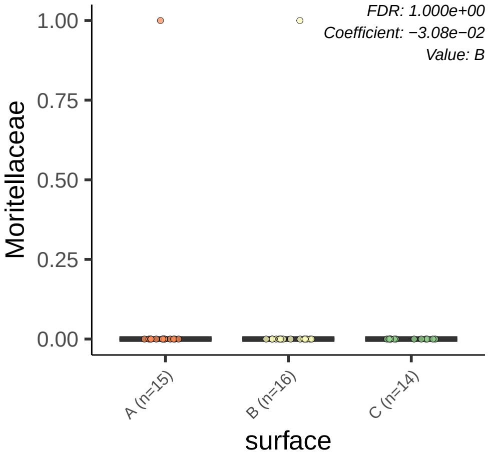
FDR: 1.000e+00
Coefficient: −3.08e−02
Value: B
Moritellaceae
1.00
0.75
0.50
0.25
0.00
A (n=15)
B (n=16)
C (n=14)
surface

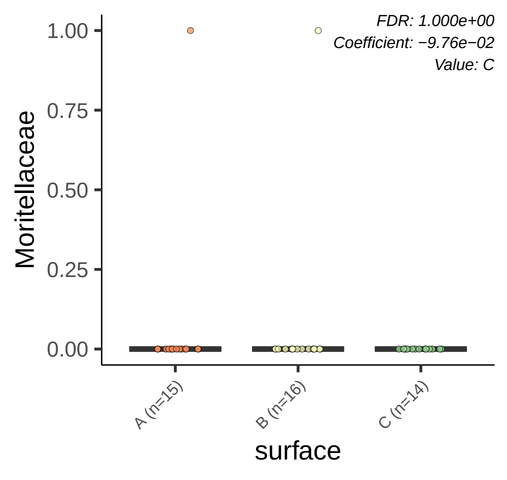
FDR: 1.000e+00
Coefficient: −9.76e−02
Value: C
Moritellaceae
1.00
0.75
0.50
0.25
0.00
A (n=15)
B (n=16)
C (n=14)
surface

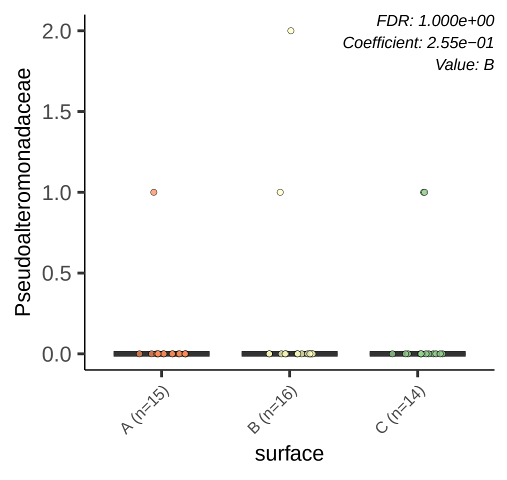
FDR: 1.000e+00
Coefficient: 2.55e−01
Value: B
Pseudoalteromonadaceae
2.0
1.5
1.0
0.5
0.0
A (n=15)
B (n=16)
C (n=14)
surface

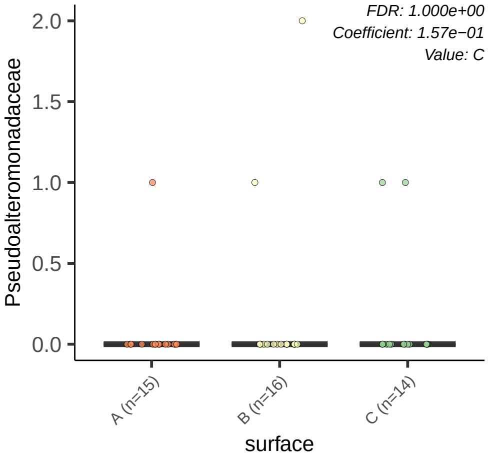

FDR: 1.000e+00
Coefficient: 1.57e−01
Value: C
Pseudoalteromonadaceae
2.0
1.5
1.0
0.5
0.0
A (n=15)
B (n=16)
C (n=14)
surface

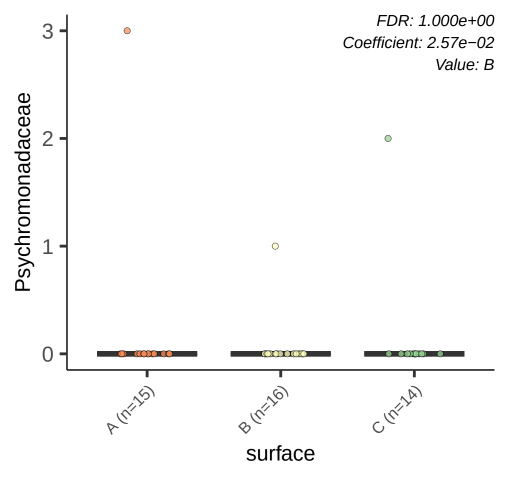

FDR: 1.000e+00
Coefficient: 2.57e−02
Value: B
Psychromonadaceae
3
2
1
0
A (n=15)
B (n=16)
C (n=14)
surface

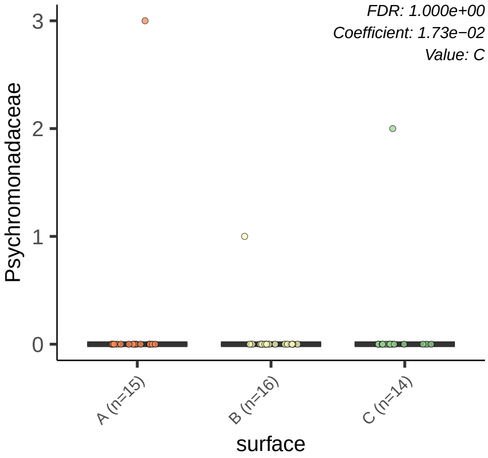

FDR: 1.000e+00
Coefficient: 1.73e−02
Value: C
Psychromonadaceae
3
2
1
0
A (n=15)
B (n=16)
C (n=14)
surface

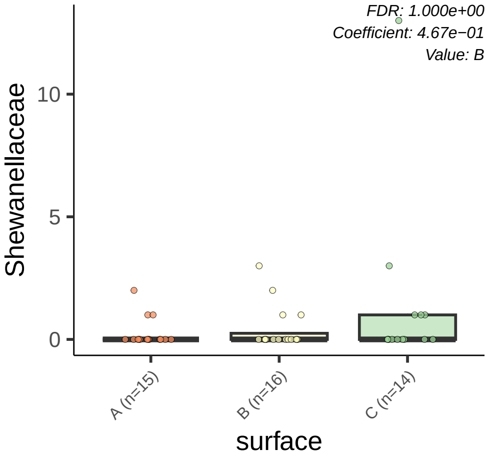
FDR: 1.000e+00
Coefficient: 4.67e−01
Value: B
Shewanellaceae
10
5
0
A (n=15)
B (n=16)
C (n=14)
surface

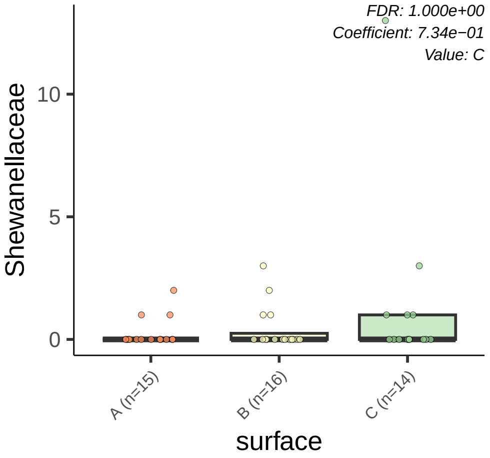
FDR: 1.000e+00
Coefficient: 7.34e−01
Value: C
Shewanellaceae
10
5
0
A (n=15)
B (n=16)
C (n=14)
surface

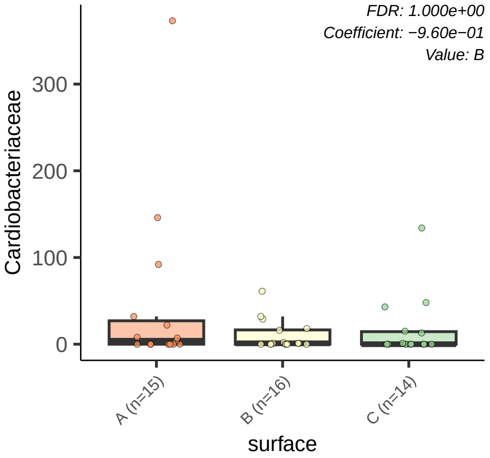
FDR: 1.000e+00
Coefficient: −9.60e−01
Value: B
Cardiobacteriaceae
300
200
100
0
A (n=15)
B (n=16)
C (n=14)
surface

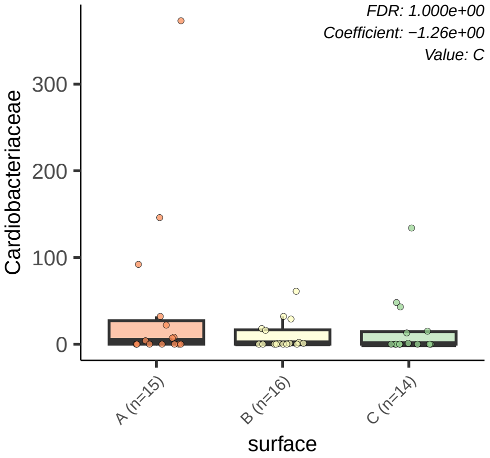
FDR: 1.000e+00
Coefficient: −1.26e+00
Value: C
Cardiobacteriaceae
300
200
100
0
A (n=15)
B (n=16)
C (n=14)
surface

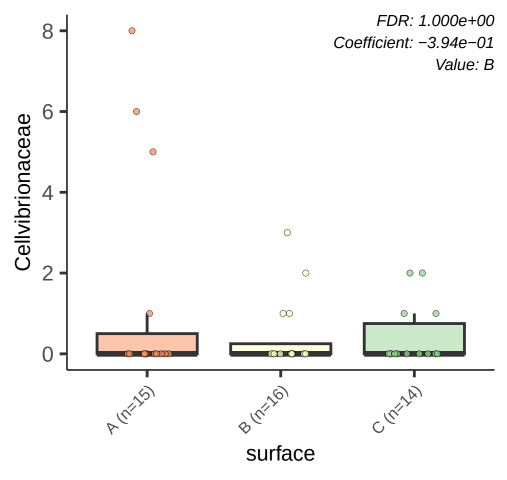
FDR: 1.000e+00
Coefficient: −3.94e−01
Value: B
Cellvibrionaceae
0
2
4
6
8
A (n=15)
B (n=16)
C (n=14)
surface

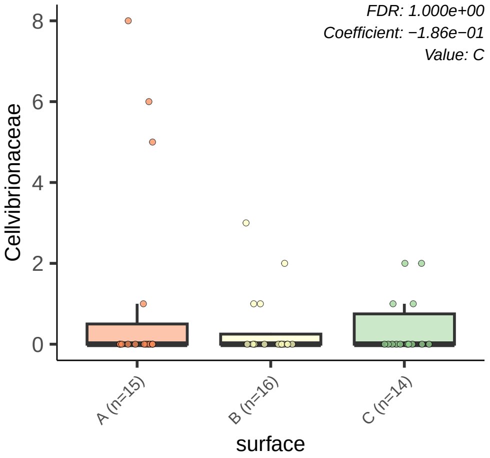
FDR: 1.000e+00
Coefficient: −1.86e−01
Value: C
Cellvibrionaceae
0
2
4
6
8
A (n=15)
B (n=16)
C (n=14)
surface

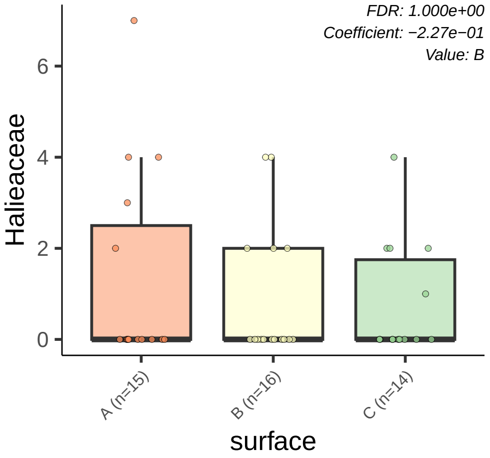
FDR: 1.000e+00
Coefficient: −2.27e−01
Value: B
Halieaceae
0
2
4
6
A (n=15)
B (n=16)
C (n=14)
surface

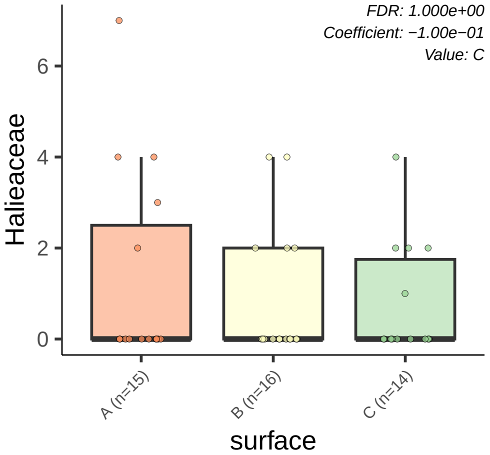
FDR: 1.000e+00
Coefficient: −1.00e−01
Value: C
Halieaceae
0
2
4
6
A (n=15)
B (n=16)
C (n=14)
surface

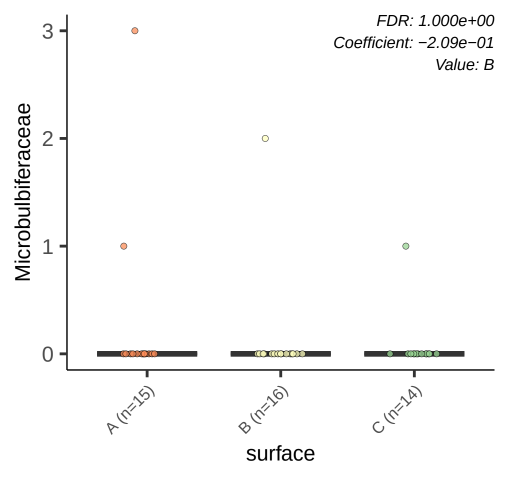
FDR: 1.000e+00
Coefficient: −2.09e−01
Value: B
Microbulbiferaceae
3
2
1
0
A (n=15)
B (n=16)
C (n=14)
surface

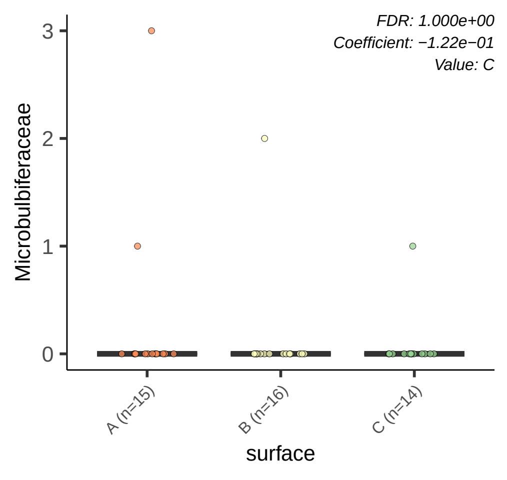

FDR: 1.000e+00
Coefficient: −1.22e−01
Value: C
Microbulbiferaceae
3
2
1
0
A (n=15)
B (n=16)
C (n=14)
surface

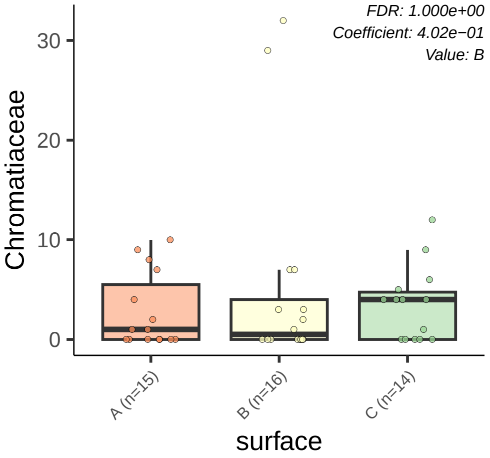

FDR: 1.000e+00
Coefficient: 4.02e−01
Value: B
Chromatiaceae
0
10
20
30
A (n=15)
B (n=16)
C (n=14)
surface

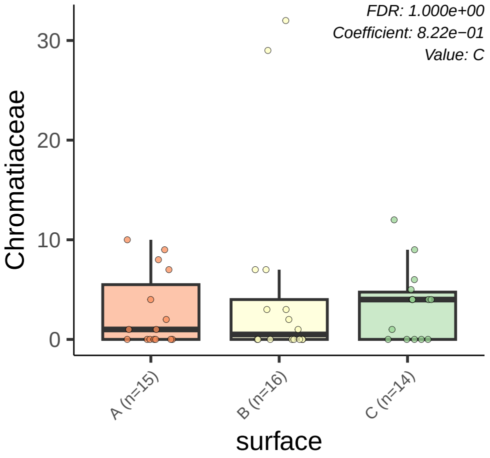
FDR: 1.000e+00
Coefficient: 8.22e−01
Value: C
Chromatiaceae
0
10
20
30
A (n=15)
B (n=16)
C (n=14)
surface

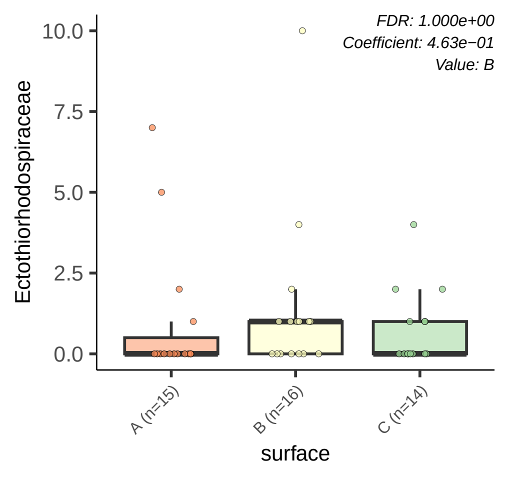

FDR: 1.000e+00
Coefficient: 4.63e−01
Value: B
Ectothiorhodospiraceae
10.0
7.5
5.0
2.5
0.0
A (n=15)
B (n=16)
C (n=14)
surface

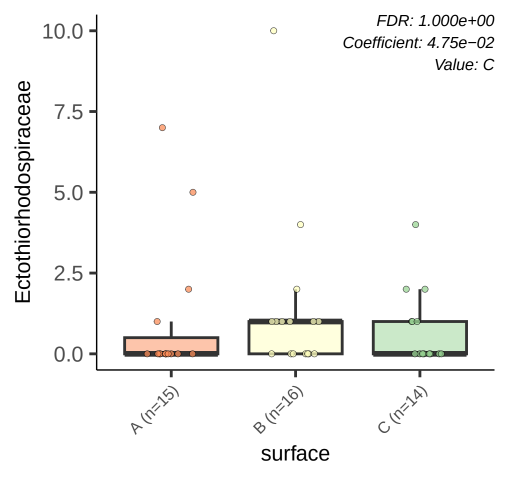

FDR: 1.000e+00
Coefficient: 4.75e−02
Value: C
Ectothiorhodospiraceae
10.0
7.5
5.0
2.5
0.0
A (n=15)
B (n=16)
C (n=14)
surface

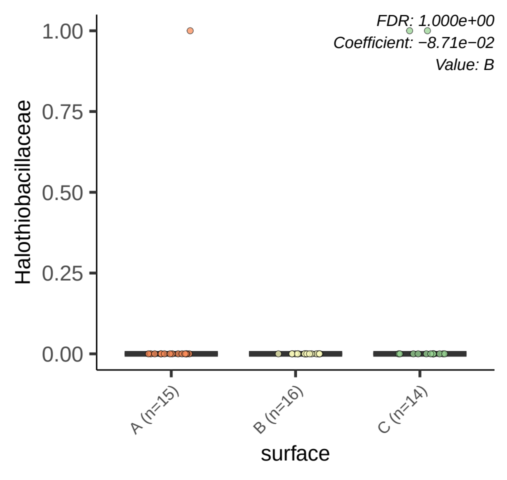
FDR: 1.000e+00
Coefficient: −8.71e−02
Value: B
Halothiobacillaceae
1.00
0.75
0.50
0.25
0.00
A (n=15)
B (n=16)
C (n=14)
surface

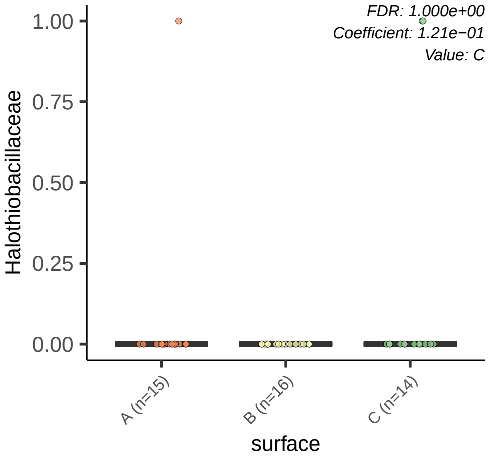

FDR: 1.000e+00
Coefficient: 1.21e−01
Value: C
Halothiobacillaceae
1.00
0.75
0.50
0.25
0.00
A (n=15)
B (n=16)
C (n=14)
surface

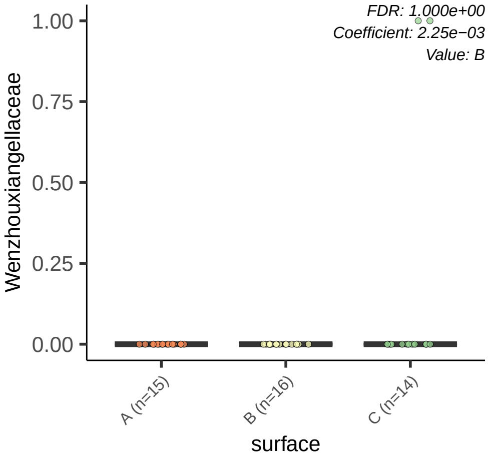
FDR: 1.000e+00
Coefficient: 2.25e−03
Value: B
Wenzhouxiangellaceae
1.00
0.75
0.50
0.25
0.00
A (n=15)
B (n=16)
C (n=14)
surface

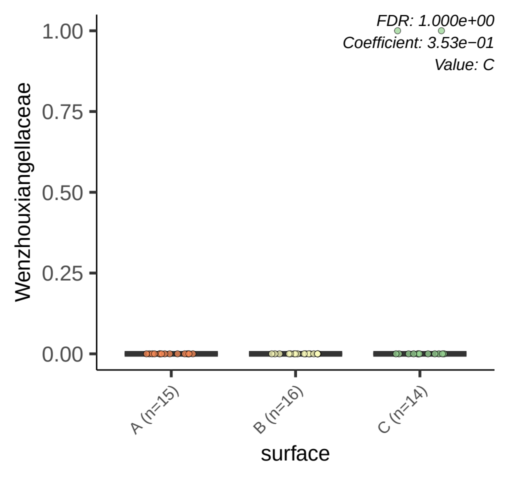
FDR: 1.000e+00
Coefficient: 3.53e−01
Value: C
Wenzhouxiangellaceae
1.00
0.75
0.50
0.25
0.00
A (n=15)
B (n=16)
C (n=14)
surface

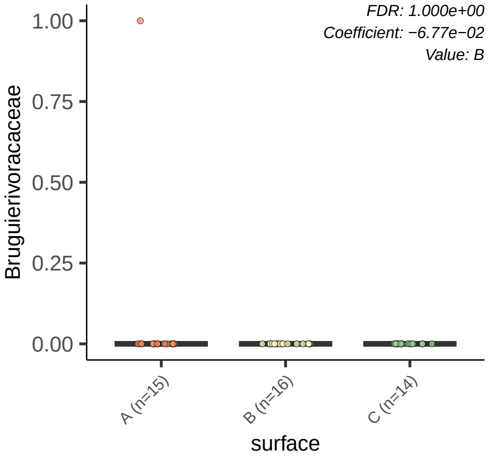
FDR: 1.000e+00
Coefficient: −6.77e−02
Value: B
Bruguierivoracaceae
1.00
0.75
0.50
0.25
0.00
A (n=15)
B (n=16)
C (n=14)
surface

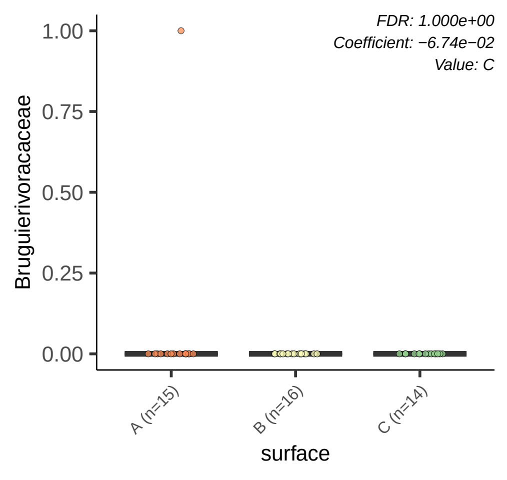

FDR: 1.000e+00
Coefficient: −6.74e−02
Value: C
Bruguierivoracaceae
1.00
0.75
0.50
0.25
0.00
A (n=15)
B (n=16)
C (n=14)
surface

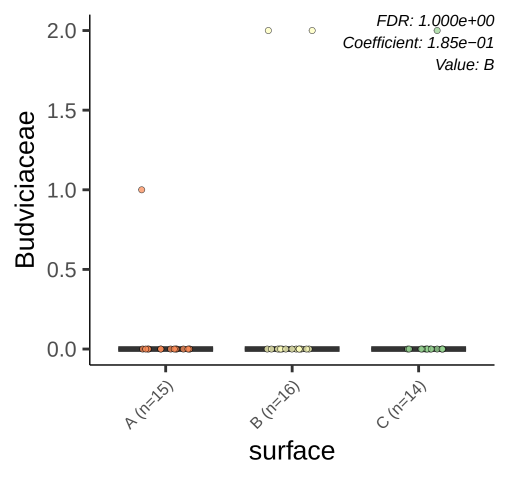
FDR: 1.000e+00
Coefficient: 1.85e−01
Value: B
Budviciaceae
2.0
1.5
1.0
0.5
0.0
A (n=15)
B (n=16)
C (n=14)
surface

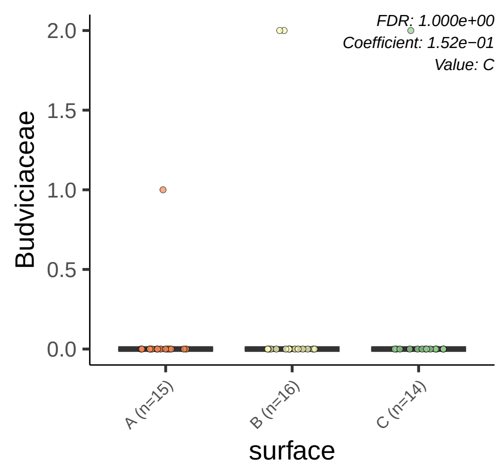
FDR: 1.000e+00
Coefficient: 1.52e−01
Value: C
Budviciaceae
2.0
1.5
1.0
0.5
0.0
A (n=15)
B (n=16)
C (n=14)
surface

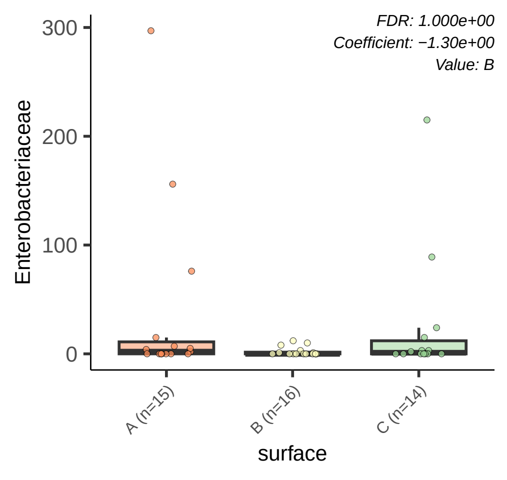

FDR: 1.000e+00
Coefficient: −1.30e+00
Value: B
Enterobacteriaceae
300
200
100
0
A (n=15)
B (n=16)
C (n=14)
surface

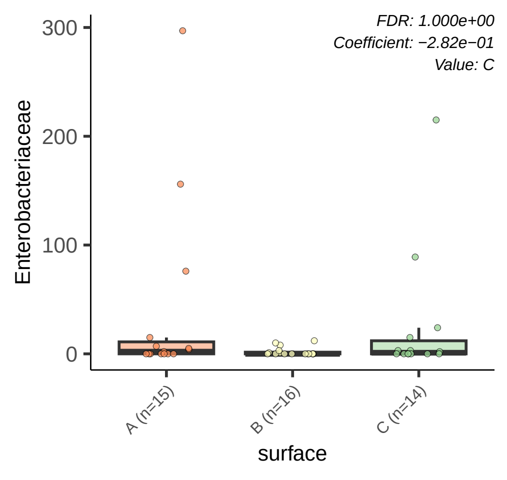
FDR: 1.000e+00
Coefficient: −2.82e−01
Value: C
Enterobacteriaceae
300
200
100
0
A (n=15)
B (n=16)
C (n=14)
surface

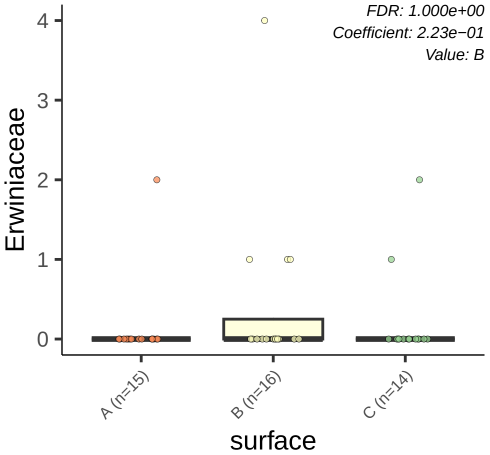
FDR: 1.000e+00
Coefficient: 2.23e−01
Value: B
Erwiniaceae
4
3
2
1
0
A (n=15)
B (n=16)
C (n=14)
surface

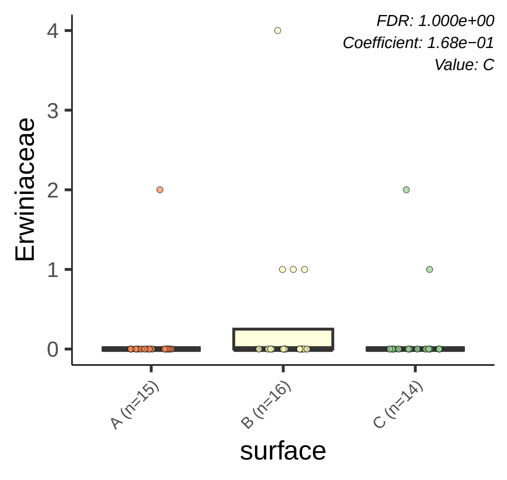
FDR: 1.000e+00
Coefficient: 1.68e−01
Value: C
Erwiniaceae
0
1
2
3
4
A (n=15)
B (n=16)
C (n=14)
surface

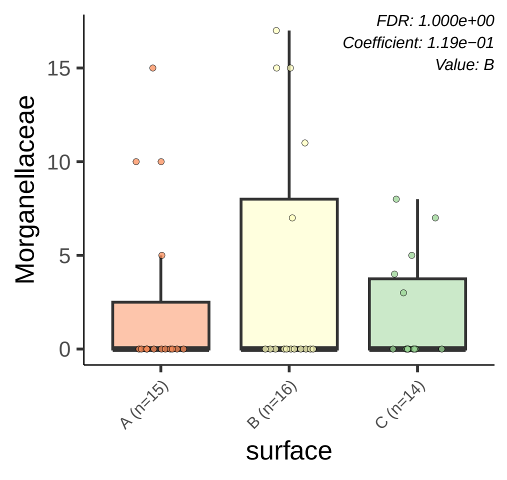

FDR: 1.000e+00
Coefficient: 1.19e−01
Value: B
Morganellaceae
0
5
10
15
A (n=15)
B (n=16)
C (n=14)
surface

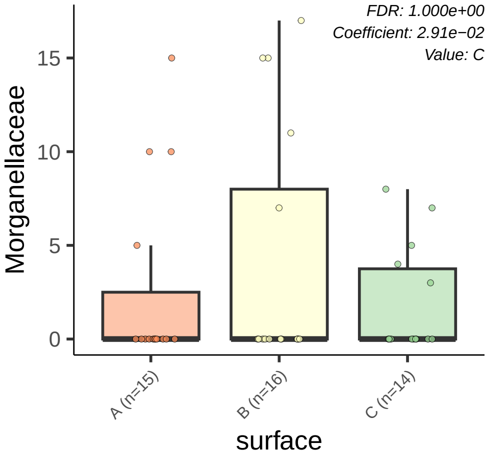
FDR: 1.000e+00
Coefficient: 2.91e−02
Value: C
Morganellaceae
0
5
10
15
A (n=15)
B (n=16)
C (n=14)
surface

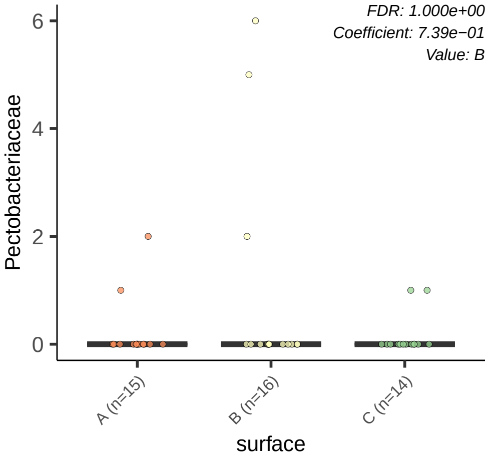
FDR: 1.000e+00
Coefficient: 7.39e−01
Value: B
Pectobacteriaceae
0
2
4
6
A (n=15)
B (n=16)
C (n=14)
surface

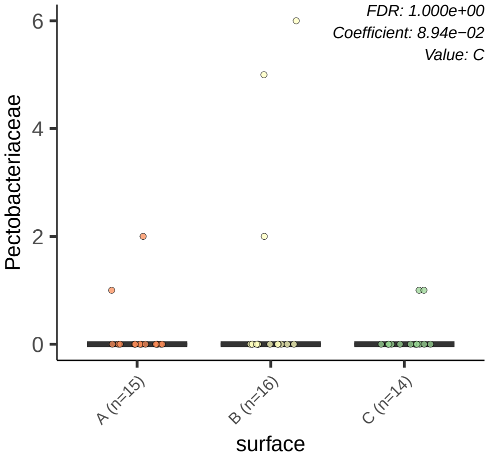
FDR: 1.000e+00
Coefficient: 8.94e−02
Value: C
Pectobacteriaceae
0
2
4
6
A (n=15)
B (n=16)
C (n=14)
surface

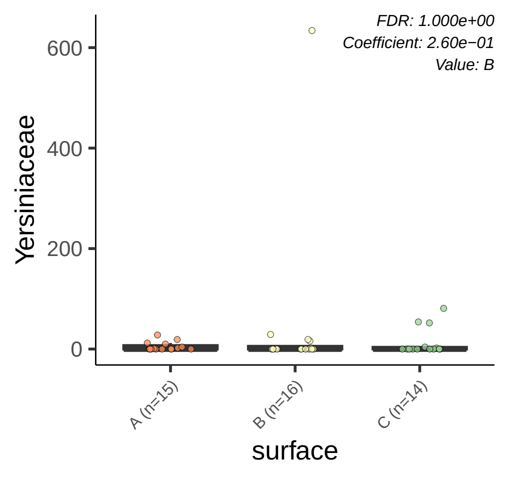
FDR: 1.000e+00
Coefficient: 2.60e−01
Value: B
Yersiniaceae
600
400
200
0
A (n=15)
B (n=16)
C (n=14)
surface

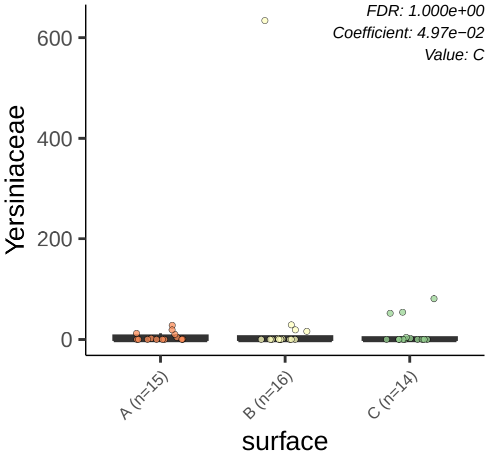

FDR: 1.000e+00
Coefficient: 4.97e−02
Value: C
Yersiniaceae
600
400
200
0
A (n=15)
B (n=16)
C (n=14)
surface

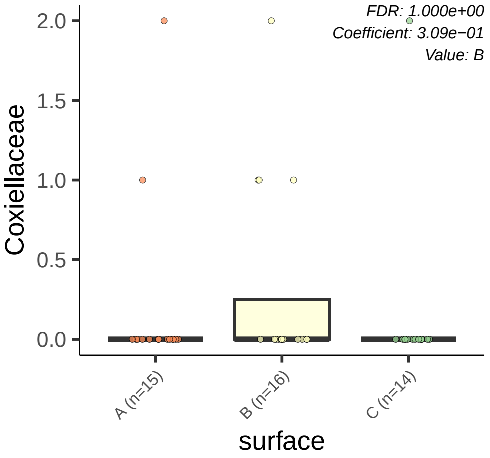

FDR: 1.000e+00
Coefficient: 3.09e−01
Value: B
Coxiellaceae
2.0
1.5
1.0
0.5
0.0
A (n=15)
B (n=16)
C (n=14)
surface

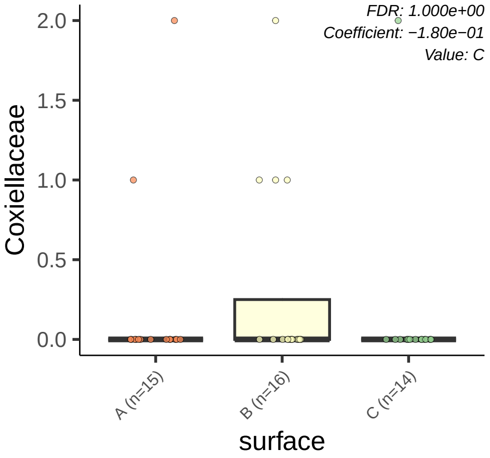
FDR: 1.000e+00
Coefficient: −1.80e−01
Value: C
Coxiellaceae
2.0
1.5
1.0
0.5
0.0
A (n=15)
B (n=16)
C (n=14)
surface

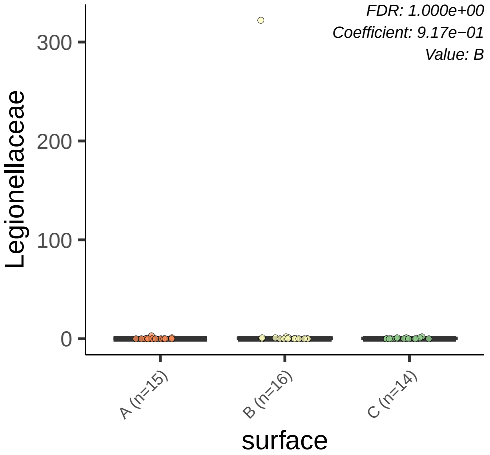

FDR: 1.000e+00
Coefficient: 9.17e−01
Value: B
Legionellaceae
300
200
100
0
A (n=15)
B (n=16)
C (n=14)
surface

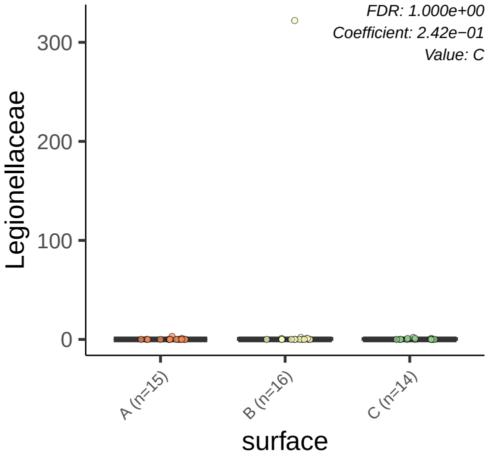

FDR: 1.000e+00
Coefficient: 2.42e−01
Value: C
300
200
100
0
Legionellaceae
A (n=15)
B (n=16)
C (n=14)
surface

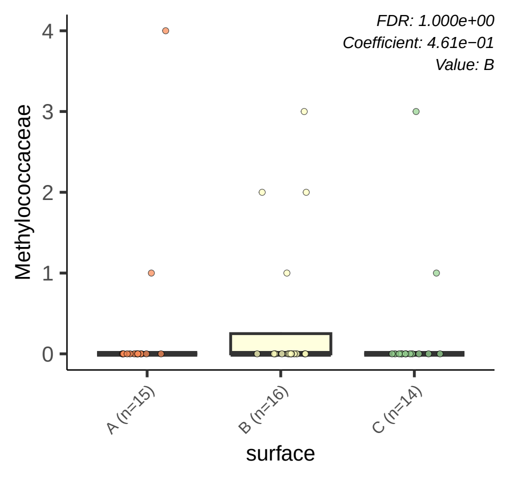
FDR: 1.000e+00
Coefficient: 4.61e−01
Value: B
Methylococcaceae
0
1
2
3
4
A (n=15)
B (n=16)
C (n=14)
surface

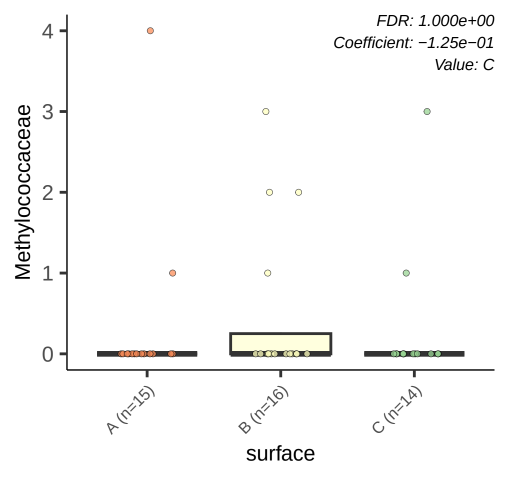
FDR: 1.000e+00
Coefficient: −1.25e−01
Value: C
Methylococcaceae
0
1
2
3
4
A (n=15)
B (n=16)
C (n=14)
surface

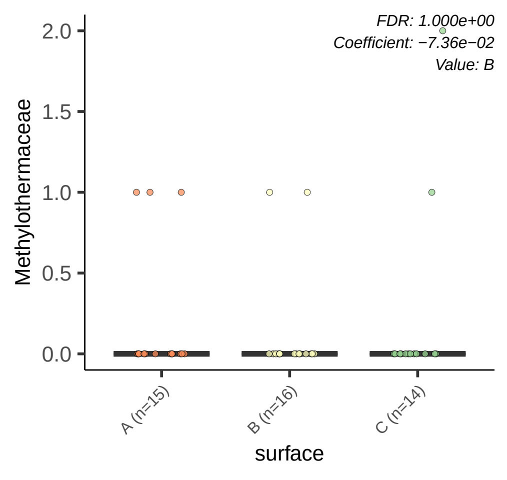

FDR: 1.000e+00
Coefficient: −7.36e−02
Value: B
Methylothermaceae
2.0
1.5
1.0
0.5
0.0
A (n=15)
B (n=16)
C (n=14)
surface

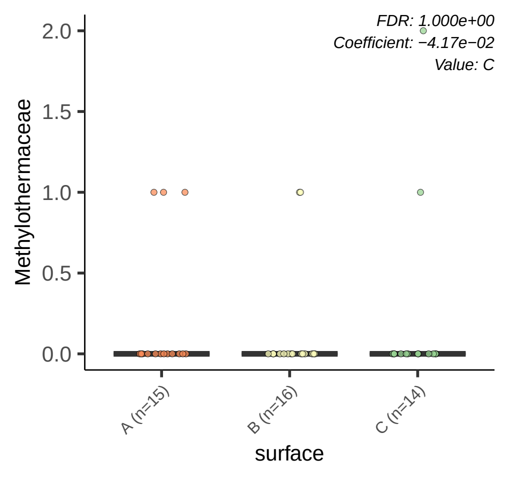

FDR: 1.000e+00
Coefficient: −4.17e−02
Value: C
Methylothermaceae
2.0
1.5
1.0
0.5
0.0
A (n=15)
B (n=16)
C (n=14)
surface

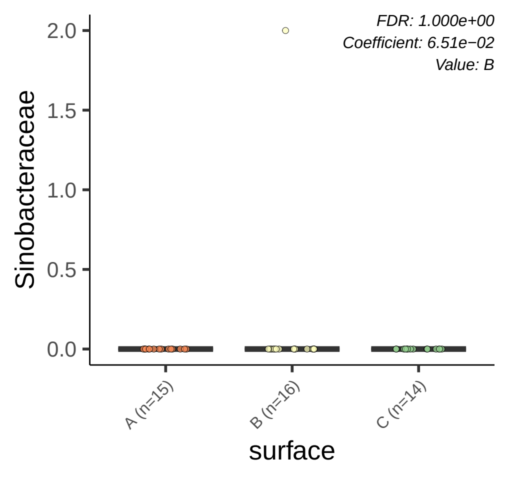
FDR: 1.000e+00
Coefficient: 6.51e−02
Value: B
Sinobacteraceae
2.0
1.5
1.0
0.5
0.0
A (n=15)
B (n=16)
C (n=14)
surface

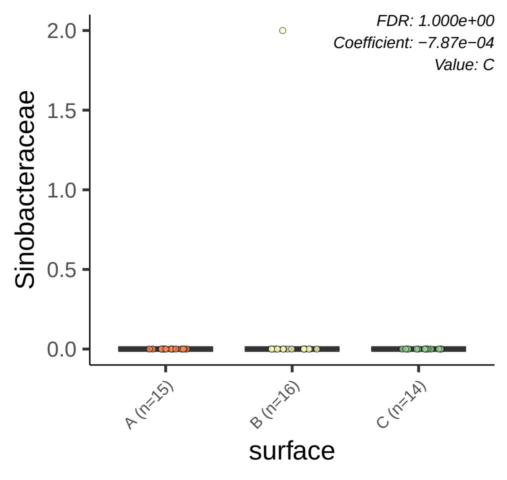
FDR: 1.000e+00
Coefficient: −7.87e−04
Value: C
Sinobacteraceae
2.0
1.5
1.0
0.5
0.0
A (n=15)
B (n=16)
C (n=14)
surface

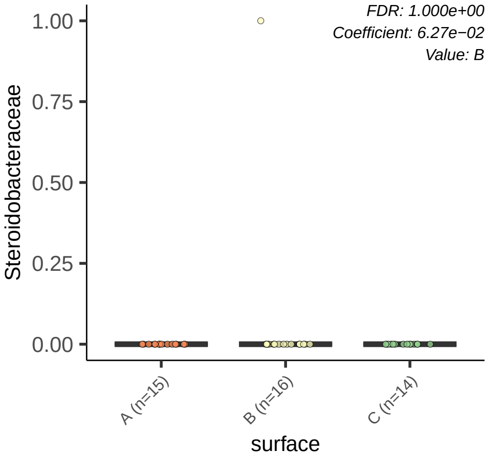

FDR: 1.000e+00
Coefficient: 6.27e−02
Value: B
Steroidobacteraceae
1.00
0.75
0.50
0.25
0.00
A (n=15)
B (n=16)
C (n=14)
surface

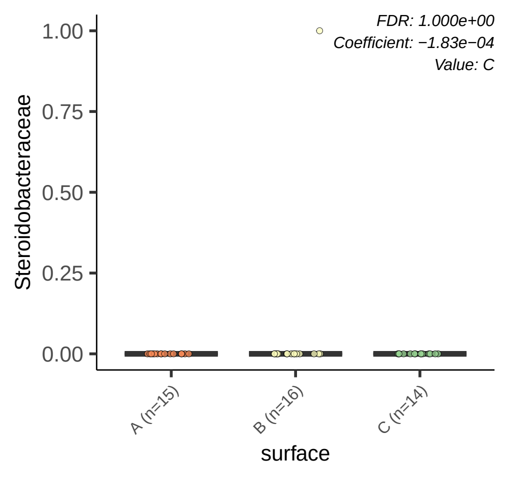
FDR: 1.000e+00
Coefficient: −1.83e−04
Value: C
Steroidobacteraceae
1.00
0.75
0.50
0.25
0.00
A (n=15)
B (n=16)
C (n=14)
surface

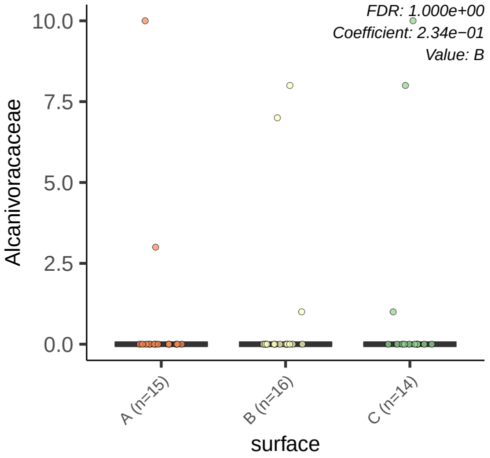
FDR: 1.000e+00
Coefficient: 2.34e−01
Value: B
Alcanivoracaceae
10.0
7.5
5.0
2.5
0.0
A (n=15)
B (n=16)
C (n=14)
surface

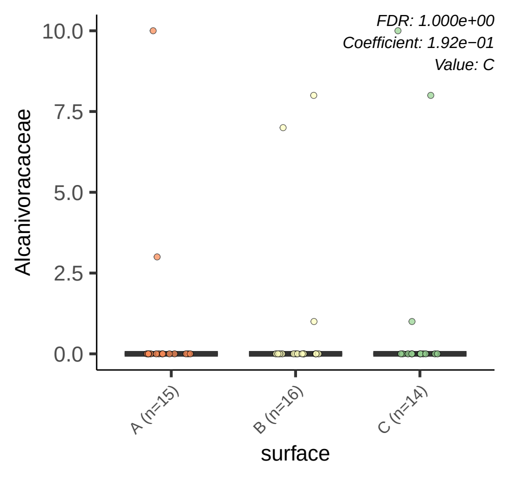
FDR: 1.000e+00
Coefficient: 1.92e−01
Value: C
Alcanivoracaceae
10.0
7.5
5.0
2.5
0.0
A (n=15)
B (n=16)
C (n=14)
surface

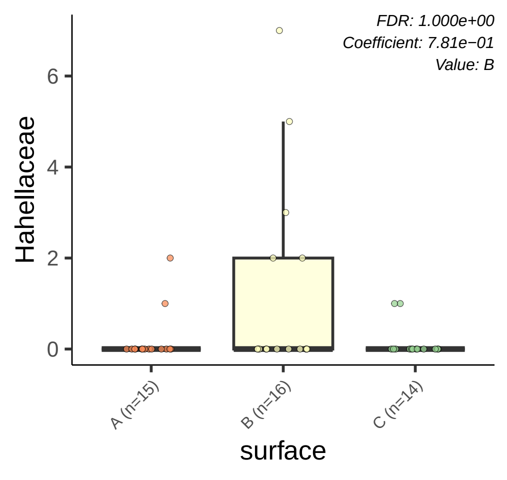
FDR: 1.000e+00
Coefficient: 7.81e−01
Value: B
Hahellaceae
6
4
2
0
A (n=15)
B (n=16)
C (n=14)
surface

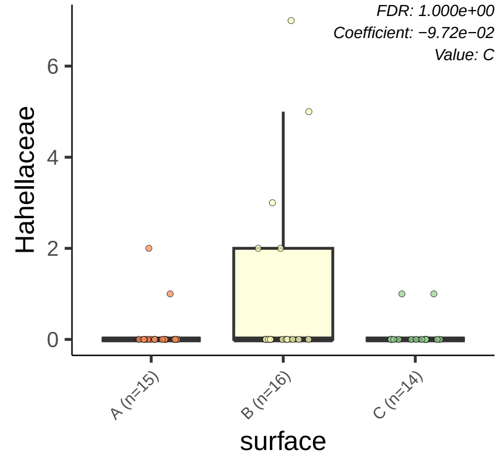
FDR: 1.000e+00
Coefficient: −9.72e−02
Value: C
Hahellaceae
0
2
4
6
A (n=15)
B (n=16)
C (n=14)
surface

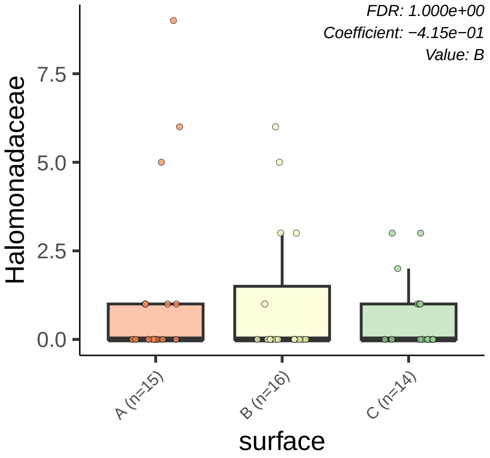

FDR: 1.000e+00
Coefficient: −4.15e−01
Value: B
Halomonadaceae
7.5
5.0
2.5
0.0
A (n=15)
B (n=16)
C (n=14)
surface

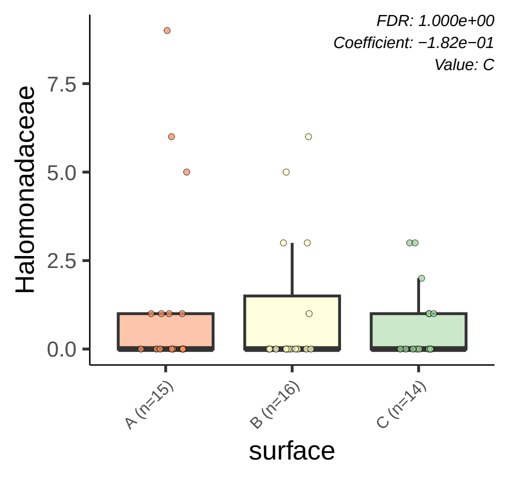
FDR: 1.000e+00
Coefficient: −1.82e−01
Value: C
Halomonadaceae
7.5
5.0
2.5
0.0
A (n=15)
B (n=16)
C (n=14)
surface

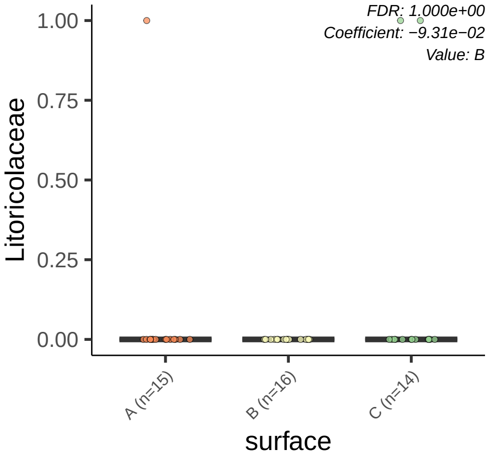

FDR: 1.000e+00
Coefficient: −9.31e−02
Value: B
Litoricolaceae
1.00
0.75
0.50
0.25
0.00
A (n=15)
B (n=16)
C (n=14)
surface

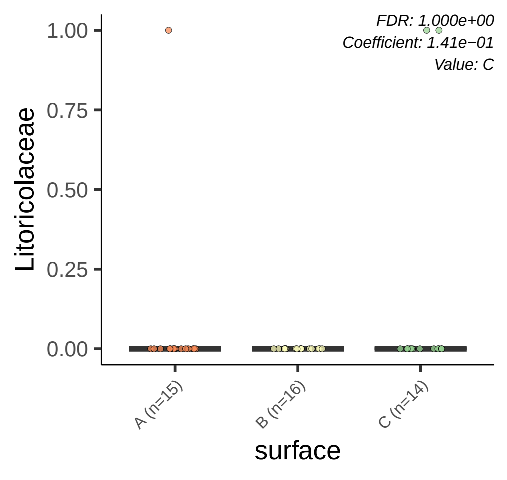

FDR: 1.000e+00
Coefficient: 1.41e−01
Value: C
Litoricolaceae
1.00
0.75
0.50
0.25
0.00
A (n=15)
B (n=16)
C (n=14)
surface

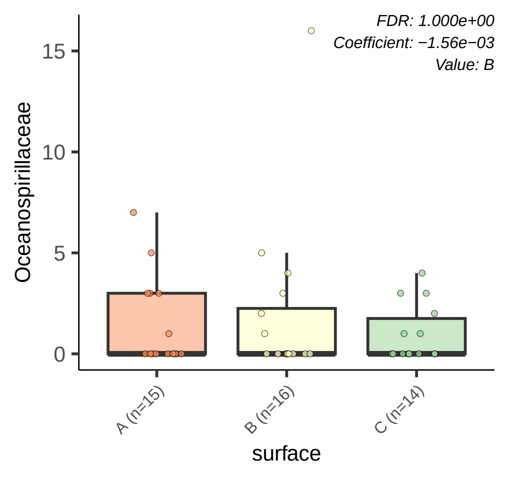

FDR: 1.000e+00
Coefficient: −1.56e−03
Value: B
Oceanospirillaceae
0
5
10
15
A (n=15)
B (n=16)
C (n=14)
surface

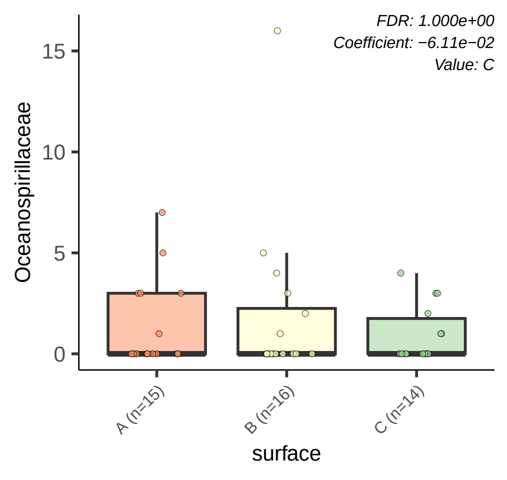

FDR: 1.000e+00
Coefficient: −6.11e−02
Value: C
Oceanospirillaceae
15
10
5
0
A (n=15)
B (n=16)
C (n=14)
surface

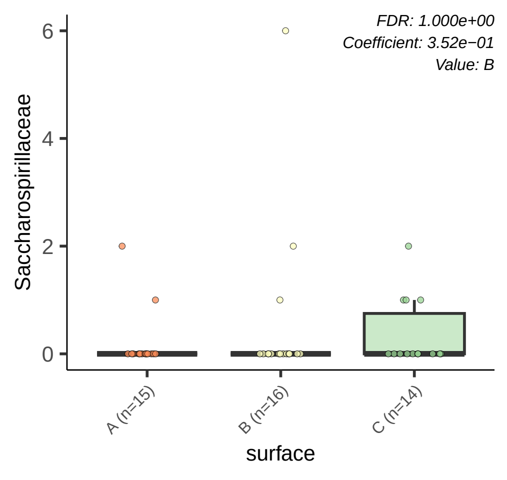

FDR: 1.000e+00
Coefficient: 3.52e−01
Value: B
Saccharospirillaceae
0
2
4
6
A (n=15)
B (n=16)
C (n=14)
surface

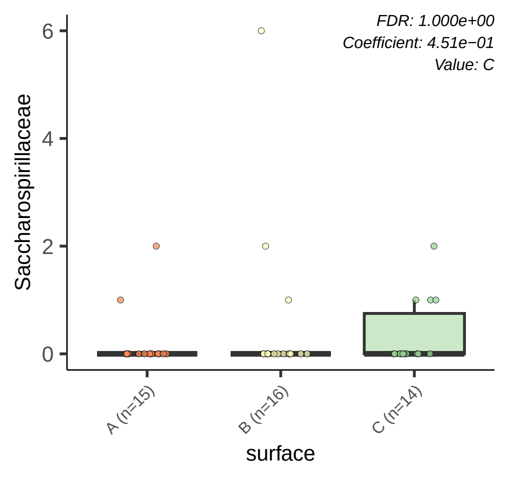

FDR: 1.000e+00
Coefficient: 4.51e−01
Value: C
Saccharospirillaceae
0
2
4
6
A (n=15)
B (n=16)
C (n=14)
surface

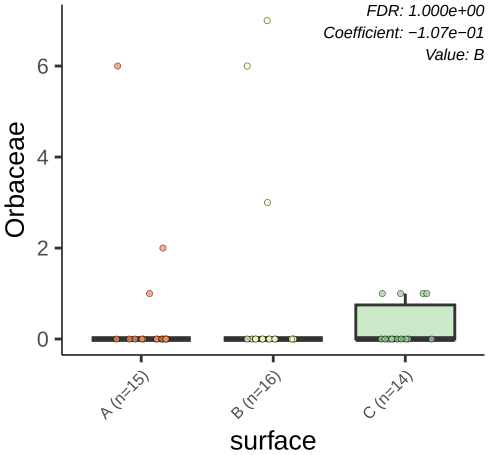
FDR: 1.000e+00
Coefficient: −1.07e−01
Value: B
Orbaceae
0
2
4
6
A (n=15)
B (n=16)
C (n=14)
surface

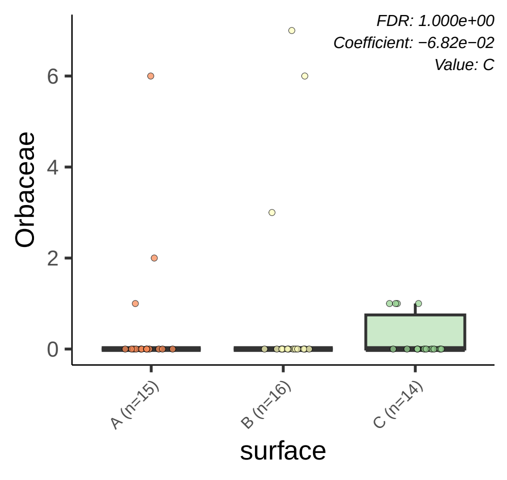
FDR: 1.000e+00
Coefficient: −6.82e−02
Value: C
Orbaceae
0
2
4
6
A (n=15)
B (n=16)
C (n=14)
surface

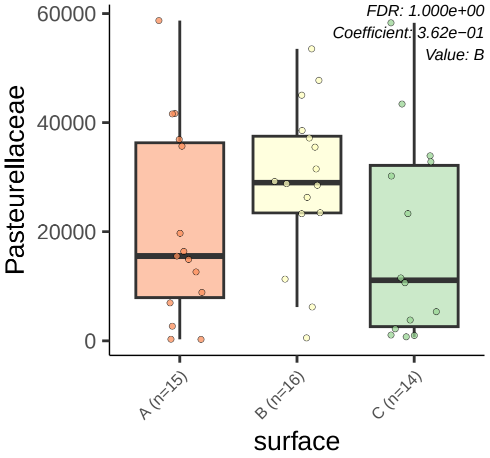
FDR: 1.000e+00
Coefficient: 3.62e−01
Value: B
Pasteurellaceae
60000
40000
20000
0
A (n=15)
B (n=16)
C (n=14)
surface

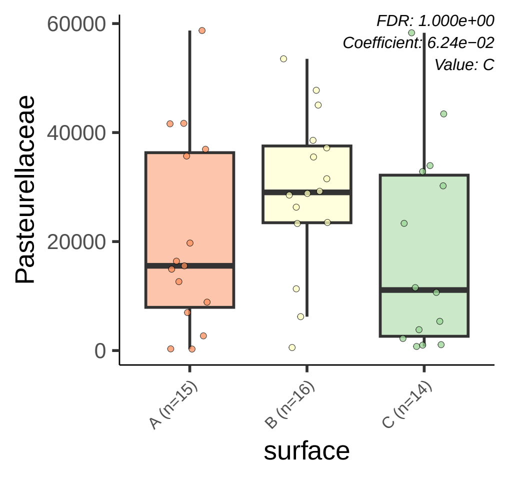
FDR: 1.000e+00
Coefficient: 6.24e−02
Value: C
60000
40000
20000
0
Pasteurellaceae
A (n=15)
B (n=16)
C (n=14)
surface

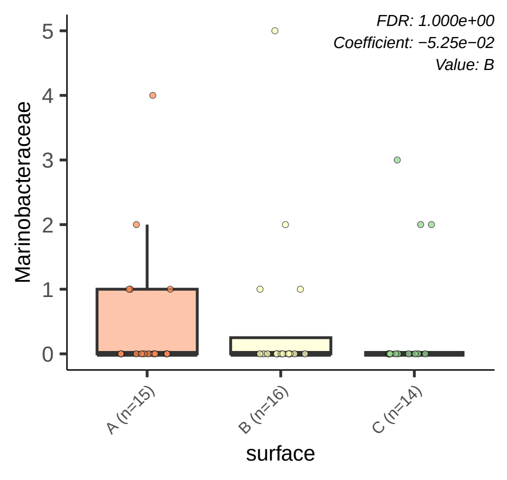
FDR: 1.000e+00
Coefficient: −5.25e−02
Value: B
Marinobacteraceae
0
1
2
3
4
5
A (n=15)
B (n=16)
C (n=14)
surface

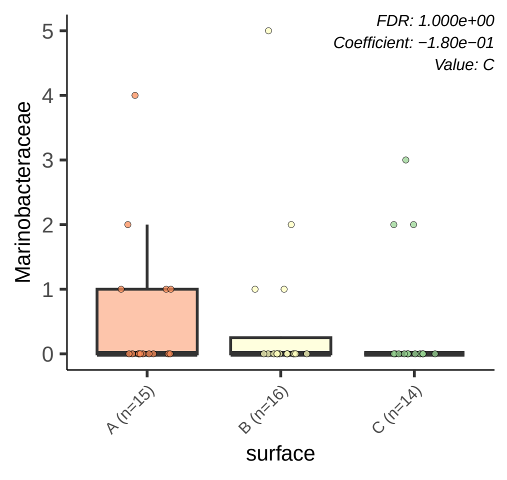

FDR: 1.000e+00
Coefficient: −1.80e−01
Value: C
Marinobacteraceae
5
4
3
2
1
0
A (n=15)
B (n=16)
C (n=14)
surface

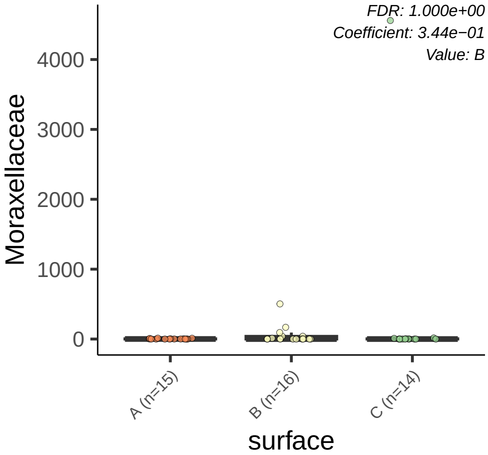
FDR: 1.000e+00
Coefficient: 3.44e−01
Value: B
Moraxellaceae
4000
3000
2000
1000
0
A (n=15)
B (n=16)
C (n=14)
surface

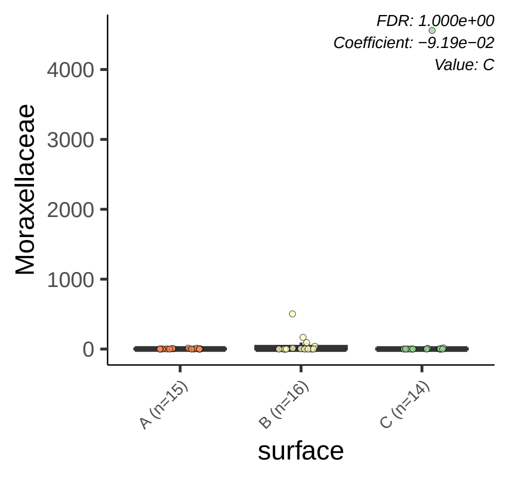
FDR: 1.000e+00
Coefficient: −9.19e−02
Value: C
Moraxellaceae
4000
3000
2000
1000
0
A (n=15)
B (n=16)
C (n=14)
surface

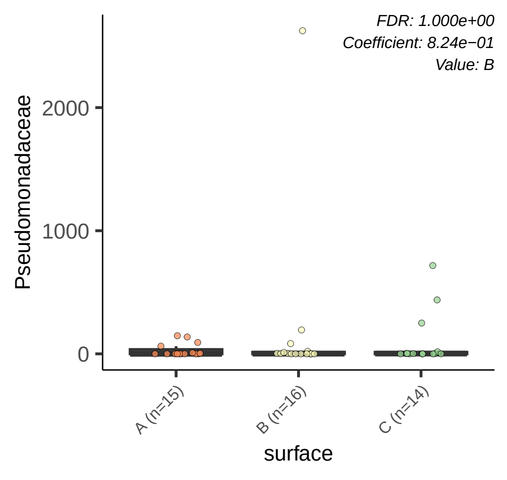
FDR: 1.000e+00
Coefficient: 8.24e−01
Value: B
Pseudomonadaceae
2000
1000
0
A (n=15)
B (n=16)
C (n=14)
surface

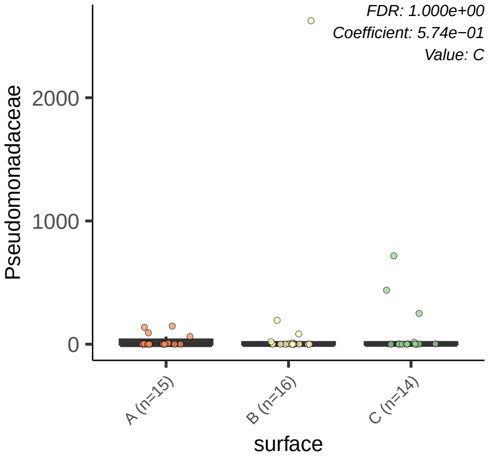

FDR: 1.000e+00
Coefficient: 5.74e−01
Value: C
Pseudomonadaceae
2000
1000
0
A (n=15)
B (n=16)
C (n=14)
surface

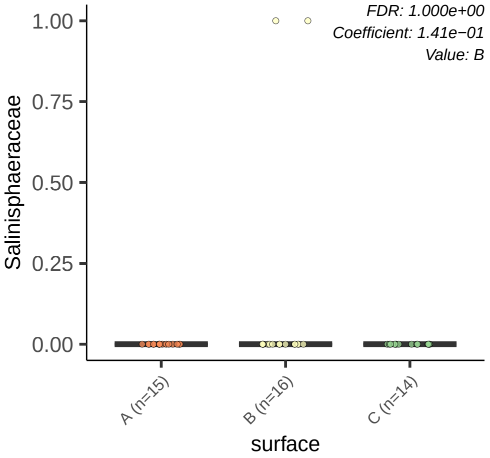

FDR: 1.000e+00
Coefficient: 1.41e−01
Value: B
Salinisphaeraceae
1.00
0.75
0.50
0.25
0.00
A (n=15)
B (n=16)
C (n=14)
surface

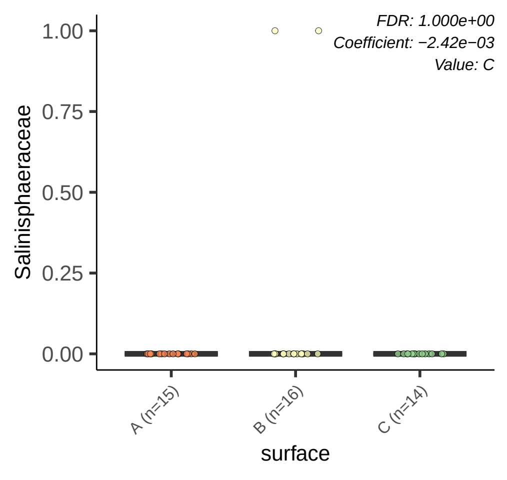

FDR: 1.000e+00
Coefficient: −2.42e−03
Value: C
Salinisphaeraceae
1.00
0.75
0.50
0.25
0.00
A (n=15)
B (n=16)
C (n=14)
surface

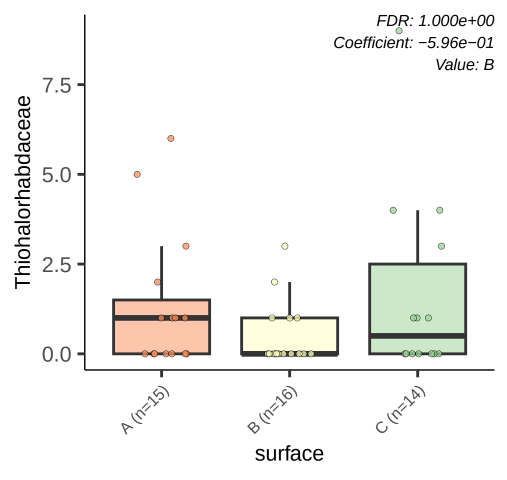
FDR: 1.000e+00
Coefficient: −5.96e−01
Value: B
Thiohalorhabdaceae
7.5
5.0
2.5
0.0
A (n=15)
B (n=16)
C (n=14)
surface

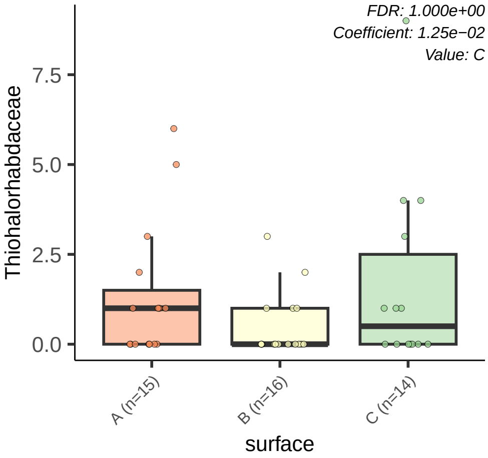

FDR: 1.000e+00
Coefficient: 1.25e−02
Value: C
Thiohalorhabdaceae
7.5
5.0
2.5
0.0
A (n=15)
B (n=16)
C (n=14)
surface

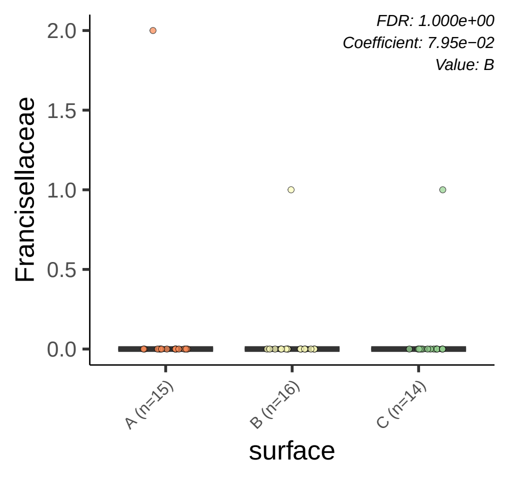

FDR: 1.000e+00
Coefficient: 7.95e−02
Value: B
Francisellaceae
2.0
1.5
1.0
0.5
0.0
A (n=15)
B (n=16)
C (n=14)
surface

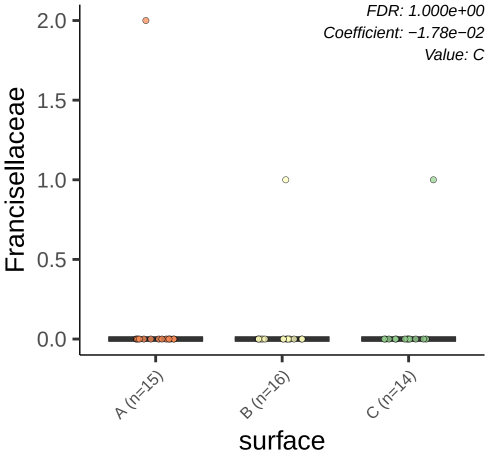
FDR: 1.000e+00
Coefficient: −1.78e−02
Value: C
Francisellaceae
2.0
1.5
1.0
0.5
0.0
A (n=15)
B (n=16)
C (n=14)
surface

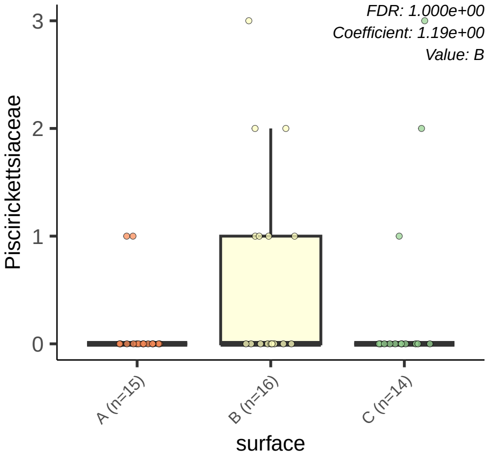
FDR: 1.000e+00
Coefficient: 1.19e+00
Value: B
Piscirickettsiaceae
0
1
2
3
A (n=15)
B (n=16)
C (n=14)
surface

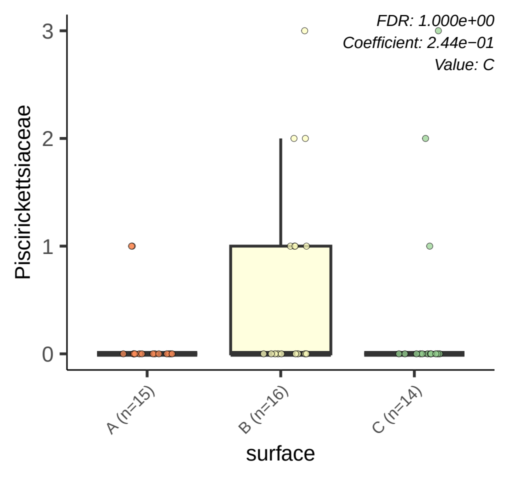

FDR: 1.000e+00
Coefficient: 2.44e−01
Value: C
Piscirickettsiaceae
0
1
2
3
A (n=15)
B (n=16)
C (n=14)
surface

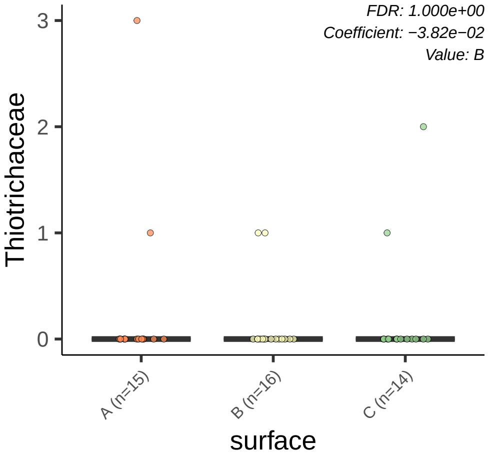
FDR: 1.000e+00
Coefficient: −3.82e−02
Value: B
Thiotrichaceae
3
2
1
0
A (n=15)
B (n=16)
C (n=14)
surface

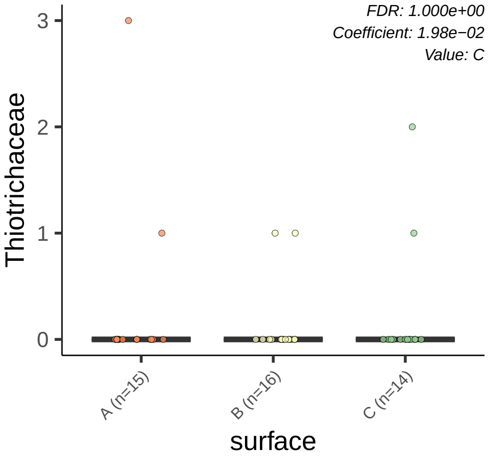
FDR: 1.000e+00
Coefficient: 1.98e−02
Value: C
Thiotrichaceae
3
2
1
0
A (n=15)
B (n=16)
C (n=14)
surface

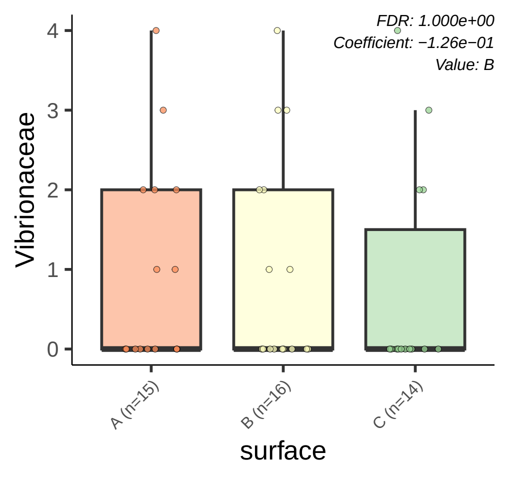
FDR: 1.000e+00
Coefficient: −1.26e−01
Value: B
Vibrionaceae
0
1
2
3
4
A (n=15)
B (n=16)
C (n=14)
surface

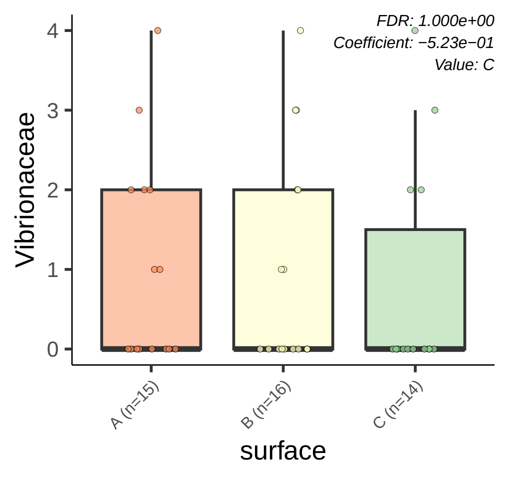
FDR: 1.000e+00
Coefficient: −5.23e−01
Value: C
Vibrionaceae
0
1
2
3
4
A (n=15)
B (n=16)
C (n=14)
surface

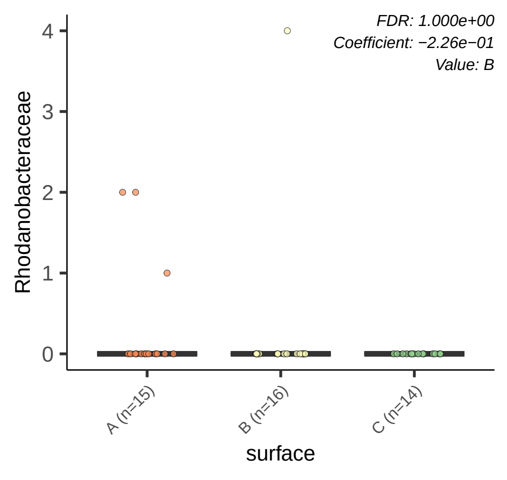
FDR: 1.000e+00
Coefficient: −2.26e−01
Value: B
Rhodanobacteraceae
0
1
2
3
4
A (n=15)
B (n=16)
C (n=14)
surface

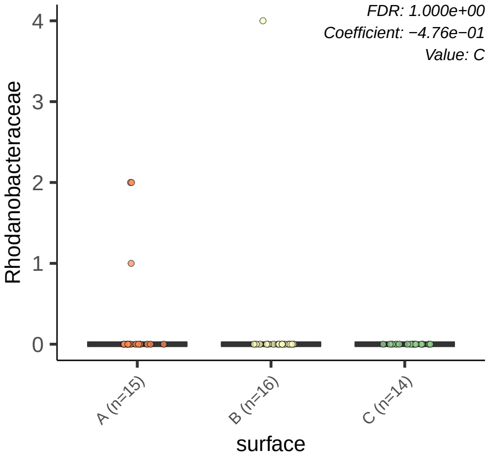
FDR: 1.000e+00
Coefficient: −4.76e−01
Value: C
Rhodanobacteraceae
0
1
2
3
4
A (n=15)
B (n=16)
C (n=14)
surface

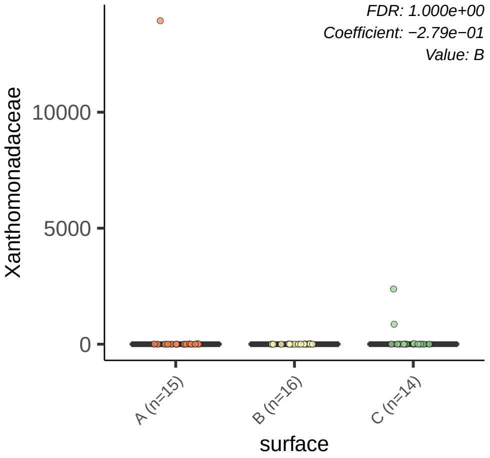
FDR: 1.000e+00
Coefficient: −2.79e−01
Value: B
Xanthomonadaceae
10000
5000
0
A (n=15)
B (n=16)
C (n=14)
surface

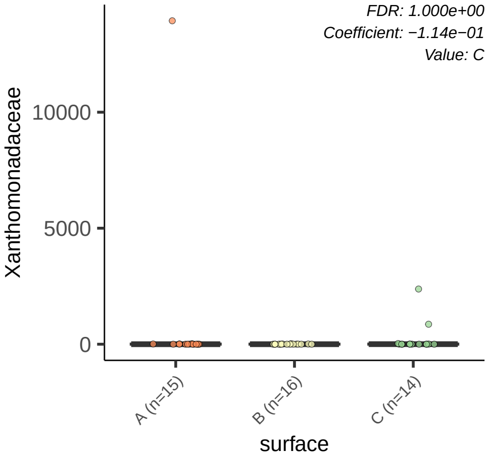
FDR: 1.000e+00
Coefficient: −1.14e−01
Value: C
Xanthomonadaceae
10000
5000
0
A (n=15)
B (n=16)
C (n=14)
surface

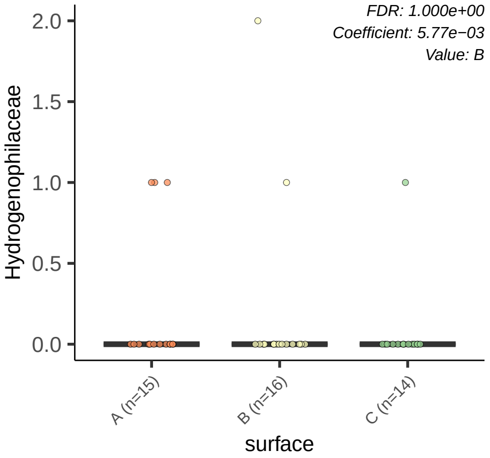
FDR: 1.000e+00
Coefficient: 5.77e−03
Value: B
Hydrogenophilaceae
2.0
1.5
1.0
0.5
0.0
A (n=15)
B (n=16)
C (n=14)
surface

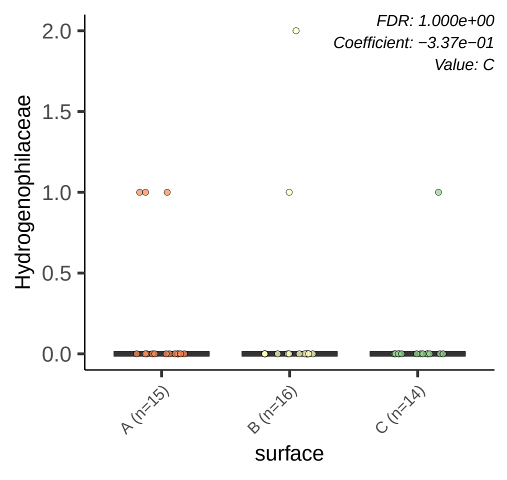

FDR: 1.000e+00
Coefficient: −3.37e−01
Value: C
Hydrogenophilaceae
2.0
1.5
1.0
0.5
0.0
A (n=15)
B (n=16)
C (n=14)
surface

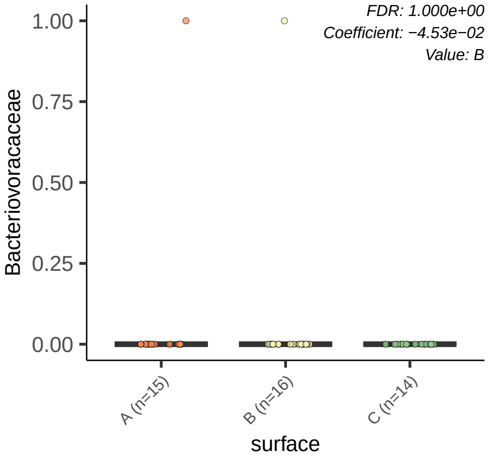

FDR: 1.000e+00
Coefficient: −4.53e−02
Value: B
Bacteriovoracaceae
1.00
0.75
0.50
0.25
0.00
A (n=15)
B (n=16)
C (n=14)
surface

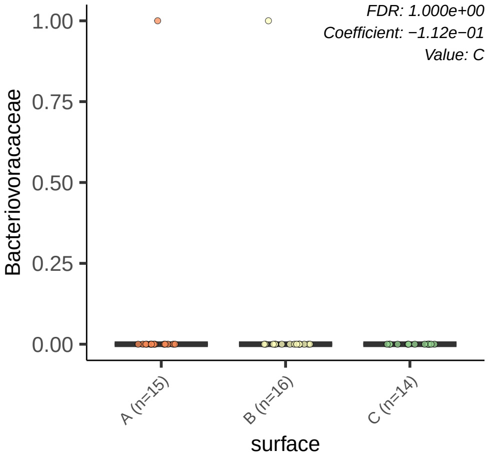
FDR: 1.000e+00
Coefficient: −1.12e−01
Value: C
Bacteriovoracaceae
1.00
0.75
0.50
0.25
0.00
A (n=15)
B (n=16)
C (n=14)
surface

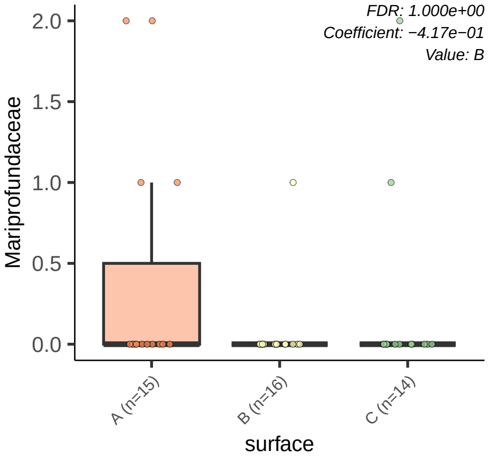
FDR: 1.000e+00
Coefficient: −4.17e−01
Value: B
Mariprofundaceae
2.0
1.5
1.0
0.5
0.0
A (n=15)
B (n=16)
C (n=14)
surface

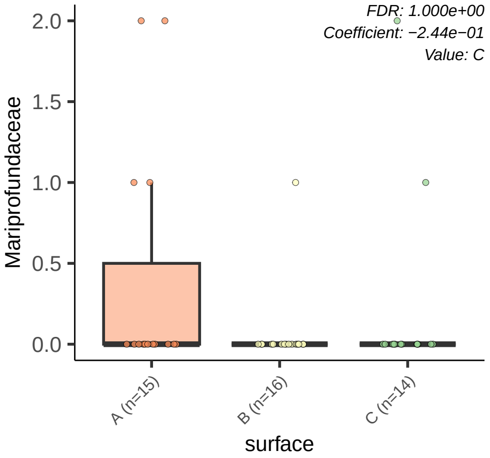
FDR: 1.000e+00
Coefficient: −2.44e−01
Value: C
Mariprofundaceae
2.0
1.5
1.0
0.5
0.0
A (n=15)
B (n=16)
C (n=14)
surface

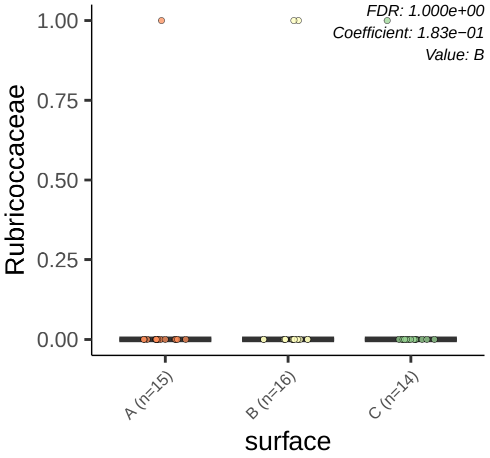
FDR: 1.000e+00
Coefficient: 1.83e−01
Value: B
Rubricoccaceae
1.00
0.75
0.50
0.25
0.00
A (n=15)
B (n=16)
C (n=14)
surface

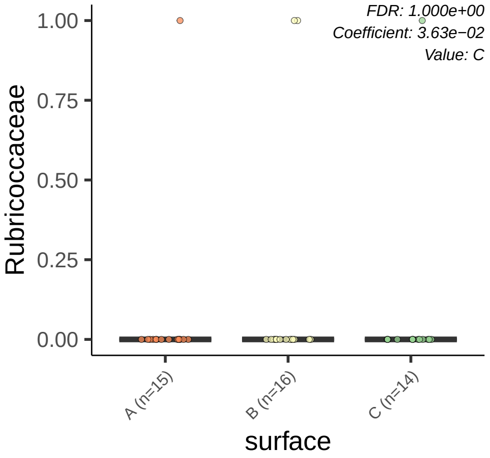
FDR: 1.000e+00
Coefficient: 3.63e−02
Value: C
Rubricoccaceae
1.00
0.75
0.50
0.25
0.00
A (n=15)
B (n=16)
C (n=14)
surface

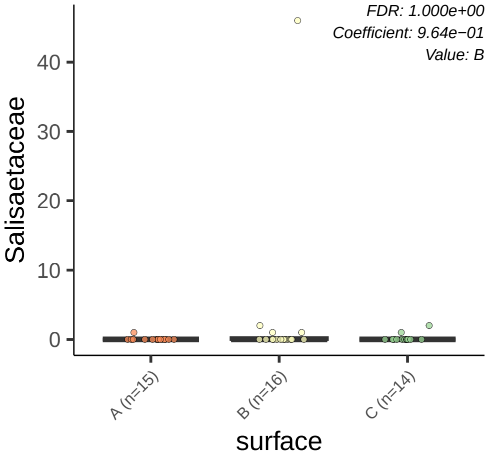
FDR: 1.000e+00
Coefficient: 9.64e−01
Value: B
Salisaetaceae
0
10
20
30
40
A (n=15)
B (n=16)
C (n=14)
surface

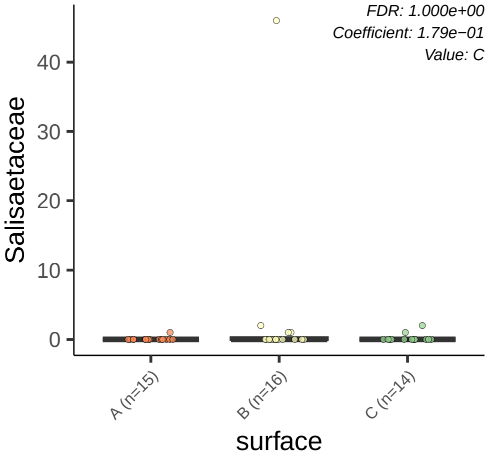

FDR: 1.000e+00
Coefficient: 1.79e−01
Value: C
Salisaetaceae
0
10
20
30
40
A (n=15)
B (n=16)
C (n=14)
surface

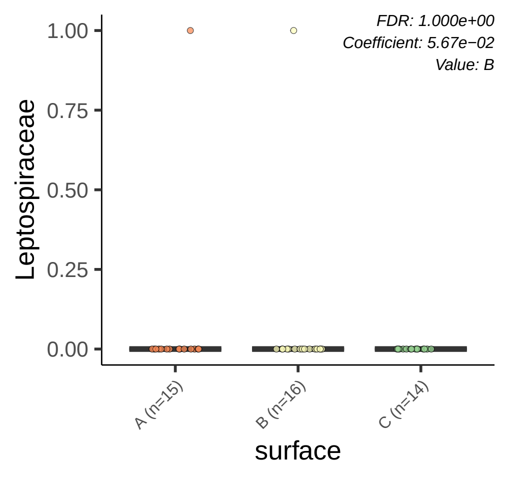
FDR: 1.000e+00
Coefficient: 5.67e−02
Value: B
Leptospiraceae
1.00
0.75
0.50
0.25
0.00
A (n=15)
B (n=16)
C (n=14)
surface

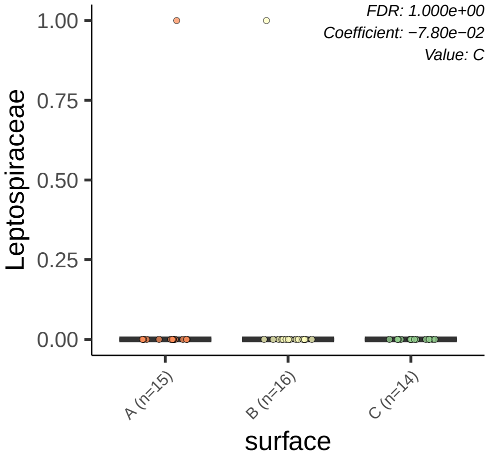

FDR: 1.000e+00
Coefficient: −7.80e−02
Value: C
Leptospiraceae
1.00
0.75
0.50
0.25
0.00
A (n=15)
B (n=16)
C (n=14)
surface

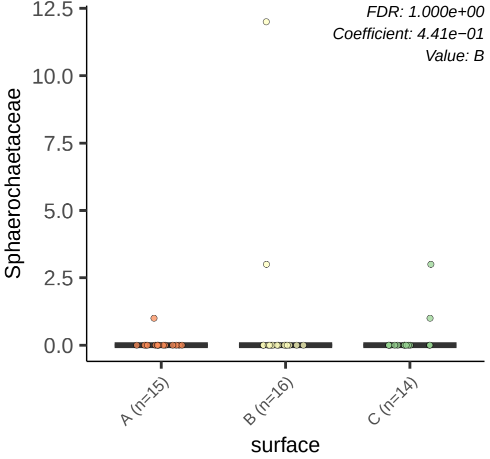

FDR: 1.000e+00
Coefficient: 4.41e−01
Value: B
Sphaerochaetaceae
12.5
10.0
7.5
5.0
2.5
0.0
A (n=15)
B (n=16)
C (n=14)
surface

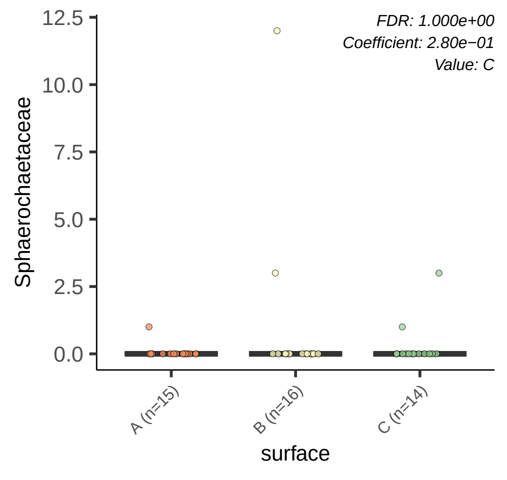
FDR: 1.000e+00
Coefficient: 2.80e−01
Value: C
Sphaerochaetaceae
12.5
10.0
7.5
5.0
2.5
0.0
A (n=15)
B (n=16)
C (n=14)
surface

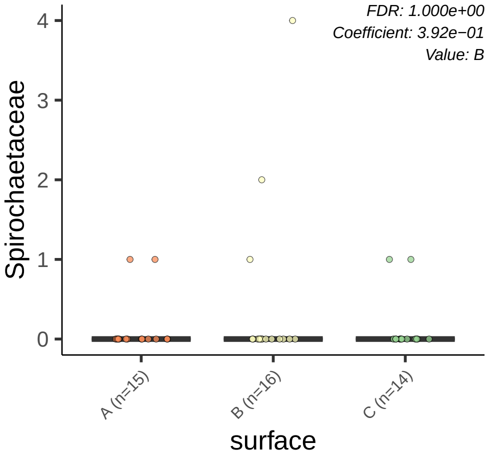
FDR: 1.000e+00
Coefficient: 3.92e−01
Value: B
Spirochaetaceae
0
1
2
3
4
A (n=15)
B (n=16)
C (n=14)
surface

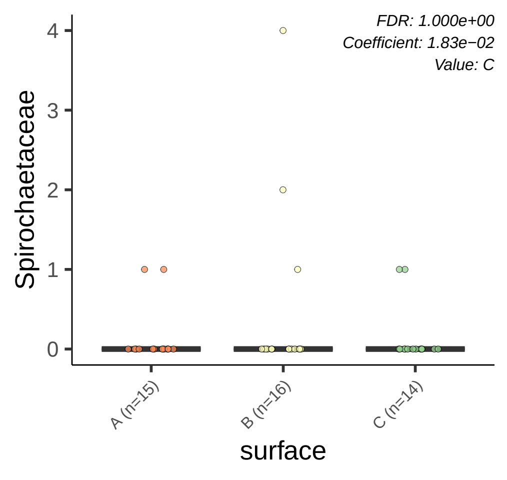
FDR: 1.000e+00
Coefficient: 1.83e−02
Value: C
Spirochaetaceae
0
1
2
3
4
A (n=15)
B (n=16)
C (n=14)
surface

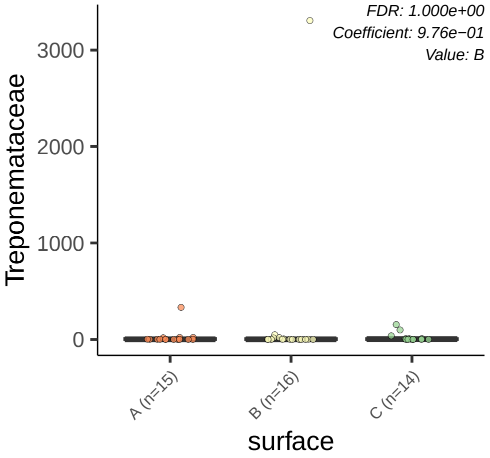
FDR: 1.000e+00
Coefficient: 9.76e−01
Value: B
Treponemataceae
3000
2000
1000
0
A (n=15)
B (n=16)
C (n=14)
surface

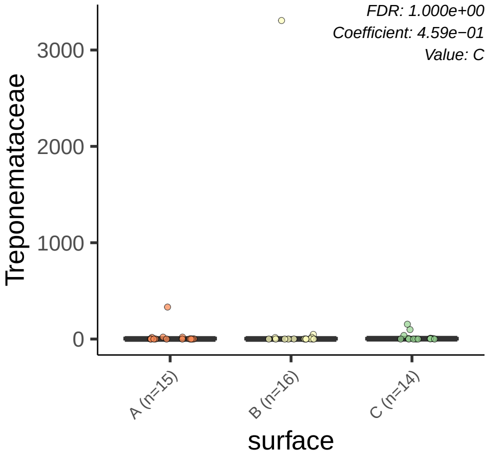
FDR: 1.000e+00
Coefficient: 4.59e−01
Value: C
Treponemataceae
3000
2000
1000
0
A (n=15)
B (n=16)
C (n=14)
surface

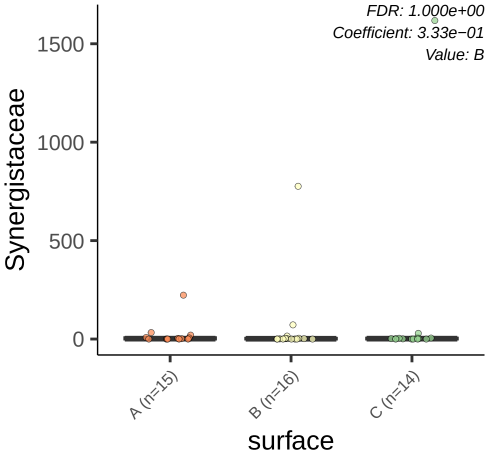

FDR: 1.000e+00
Coefficient: 3.33e−01
Value: B
Synergistaceae
1500
1000
500
0
A (n=15)
B (n=16)
C (n=14)
surface

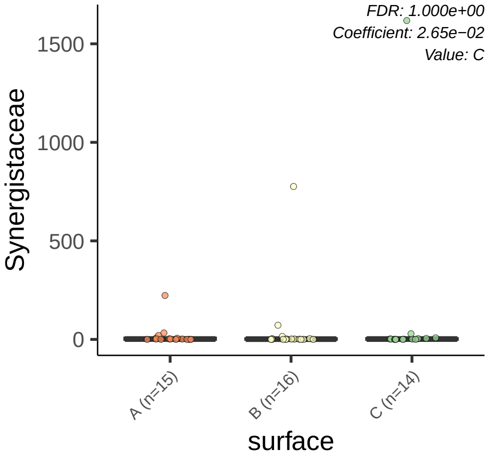

FDR: 1.000e+00
Coefficient: 2.65e−02
Value: C
Synergistaceae
1500
1000
500
0
A (n=15)
B (n=16)
C (n=14)
surface

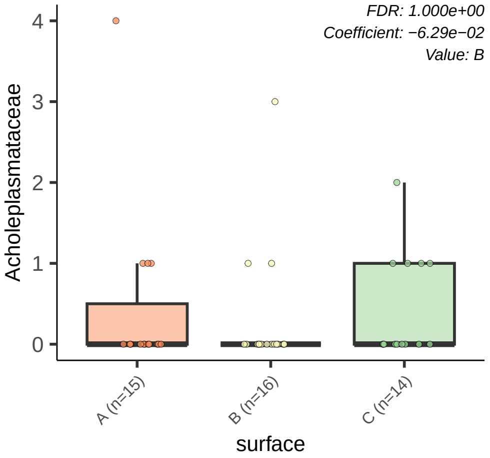
FDR: 1.000e+00
Coefficient: −6.29e−02
Value: B
Acholeplasmataceae
0
1
2
3
4
A (n=15)
B (n=16)
C (n=14)
surface

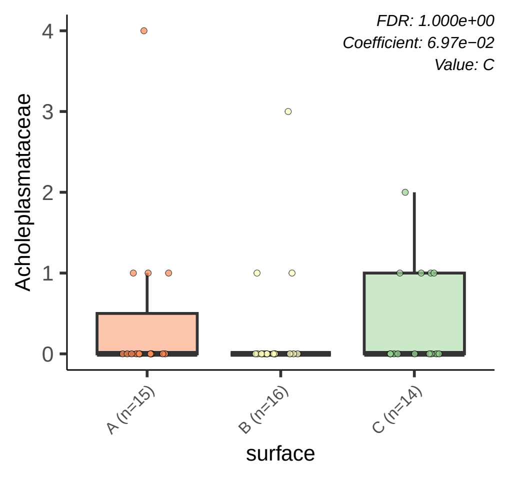
FDR: 1.000e+00
Coefficient: 6.97e−02
Value: C
Acholeplasmataceae
0
1
2
3
4
A (n=15)
B (n=16)
C (n=14)
surface

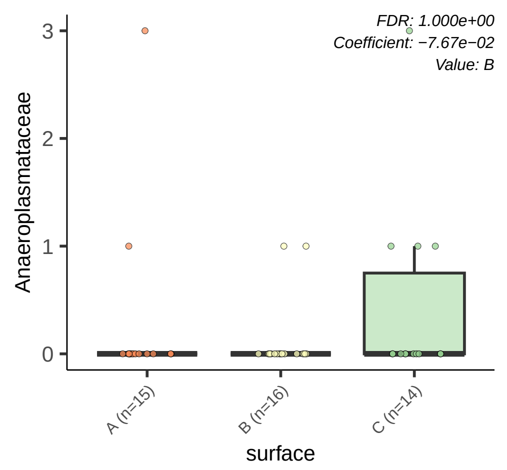

FDR: 1.000e+00
Coefficient: −7.67e−02
Value: B
Anaeroplasmataceae
3
2
1
0
A (n=15)
B (n=16)
C (n=14)
surface

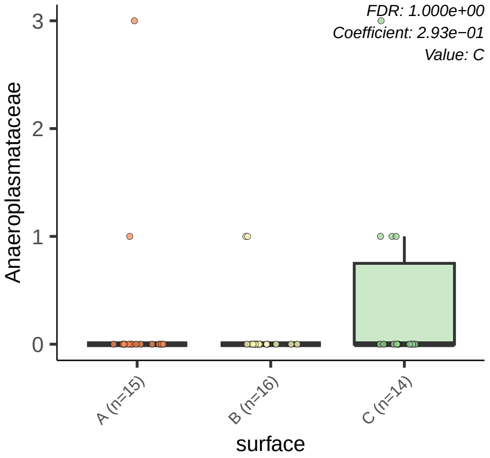

FDR: 1.000e+00
Coefficient: 2.93e−01
Value: C
Anaeroplasmataceae
0
1
2
3
A (n=15)
B (n=16)
C (n=14)
surface

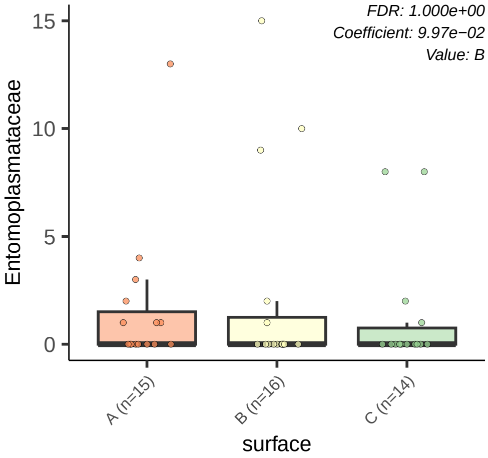

FDR: 1.000e+00
Coefficient: 9.97e−02
Value: B
Entomoplasmataceae
15
10
5
0
A (n=15)
B (n=16)
C (n=14)
surface

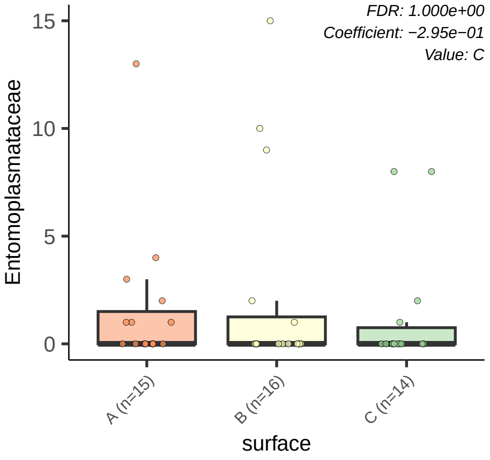

FDR: 1.000e+00
Coefficient: −2.95e−01
Value: C
Entomoplasmataceae
15
10
5
0
A (n=15)
B (n=16)
C (n=14)
surface

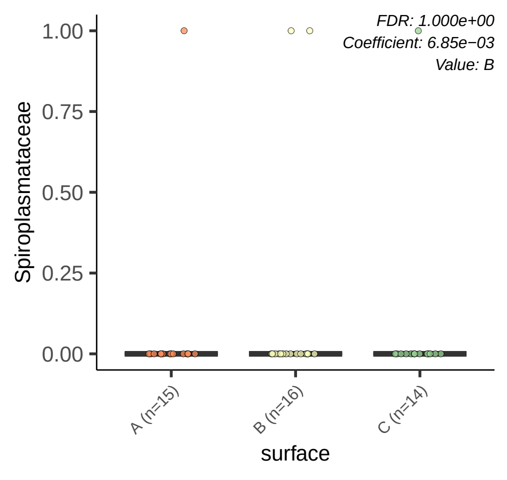
FDR: 1.000e+00
Coefficient: 6.85e−03
Value: B
Spiroplasmataceae
1.00
0.75
0.50
0.25
0.00
A (n=15)
B (n=16)
C (n=14)
surface

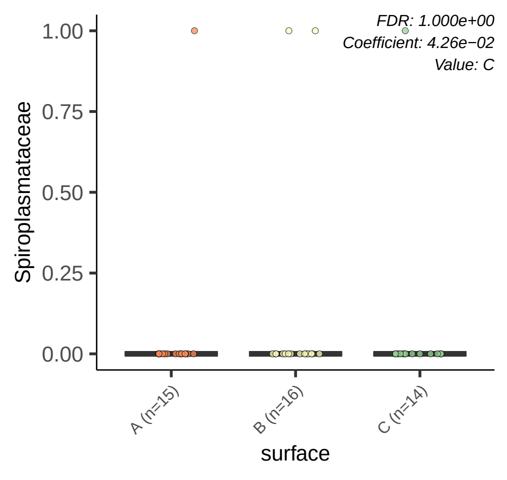

FDR: 1.000e+00
Coefficient: 4.26e−02
Value: C
Spiroplasmataceae
1.00
0.75
0.50
0.25
0.00
A (n=15)
B (n=16)
C (n=14)
surface

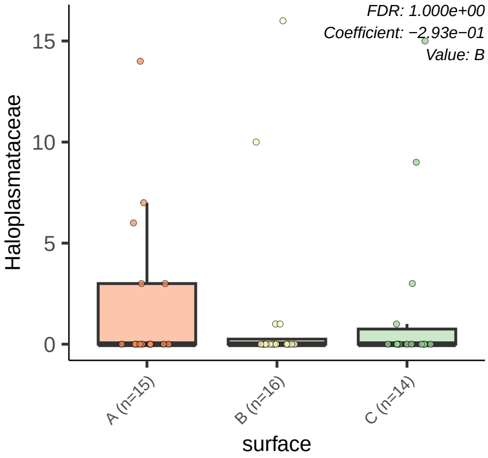

FDR: 1.000e+00
Coefficient: −2.93e−01
Value: B
Haloplasmataceae
15
10
5
0
A (n=15)
B (n=16)
C (n=14)
surface

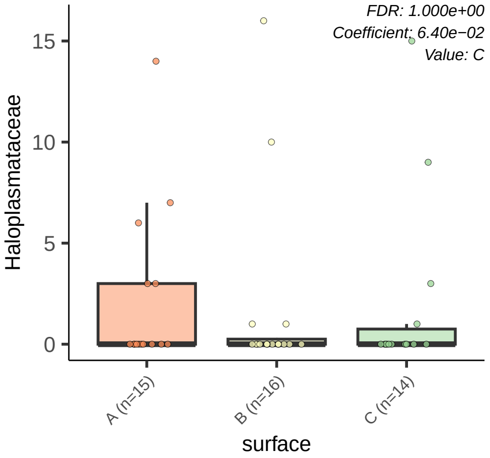
FDR: 1.000e+00
Coefficient: 6.40e−02
Value: C
Haloplasmataceae
15
10
5
0
A (n=15)
B (n=16)
C (n=14)
surface

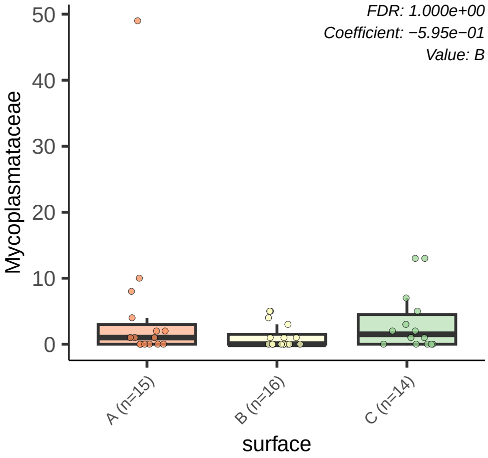
FDR: 1.000e+00
Coefficient: −5.95e−01
Value: B
Mycoplasmataceae
50
40
30
20
10
0
A (n=15)
B (n=16)
C (n=14)
surface

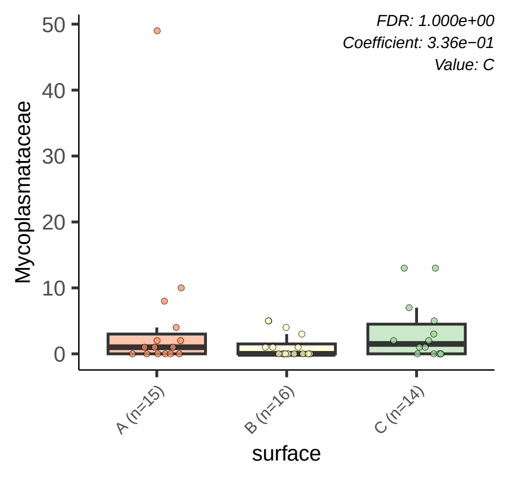
FDR: 1.000e+00
Coefficient: 3.36e−01
Value: C
Mycoplasmataceae
0
10
20
30
40
50
A (n=15)
B (n=16)
C (n=14)
surface

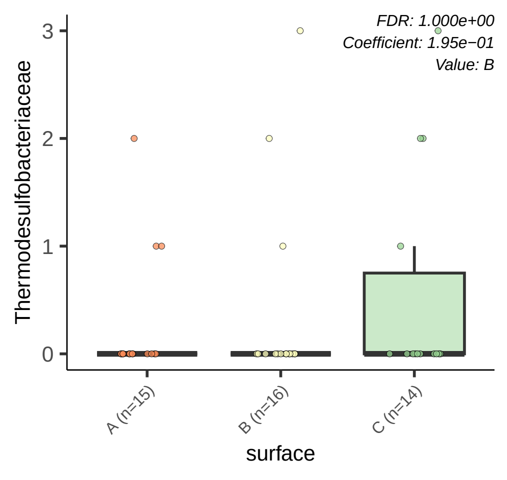
FDR: 1.000e+00
Coefficient: 1.95e−01
Value: B
Thermodesulfobacteriaceae
3
2
1
0
A (n=15)
B (n=16)
C (n=14)
surface

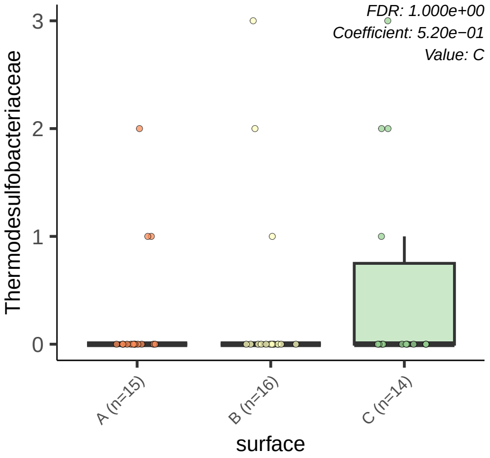
FDR: 1.000e+00
Coefficient: 5.20e−01
Value: C
Thermodesulfobacteriaceae
0
1
2
3
A (n=15)
B (n=16)
C (n=14)
surface

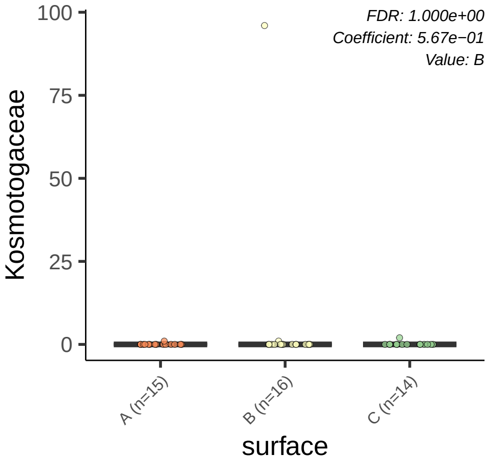
FDR: 1.000e+00
Coefficient: 5.67e−01
Value: B
Kosmotogaceae
100
75
50
25
0
A (n=15)
B (n=16)
C (n=14)
surface

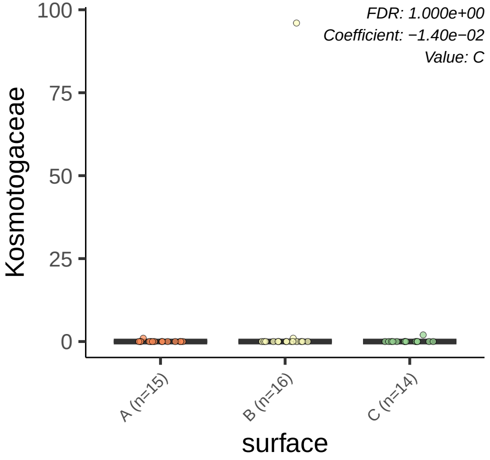

FDR: 1.000e+00
Coefficient: −1.40e−02
Value: C
Kosmotogaceae
100
75
50
25
0
A (n=15)
B (n=16)
C (n=14)
surface

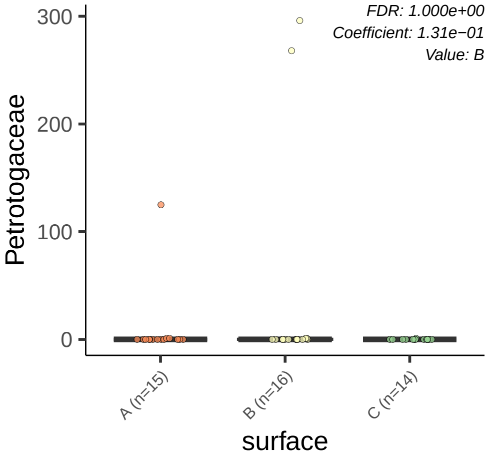

FDR: 1.000e+00
Coefficient: 1.31e−01
Value: B
Petrotogaceae
300
200
100
0
A (n=15)
B (n=16)
C (n=14)
surface

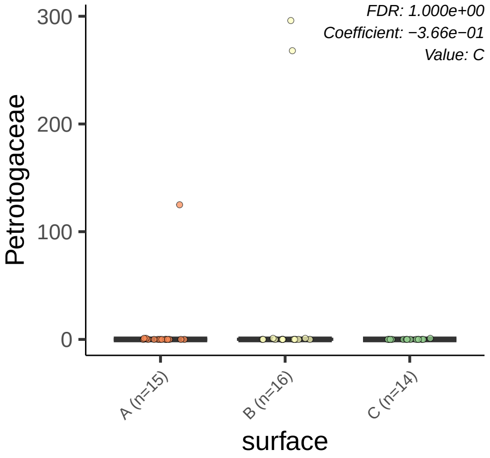
FDR: 1.000e+00
Coefficient: −3.66e−01
Value: C
Petrotogaceae
300
200
100
0
A (n=15)
B (n=16)
C (n=14)
surface

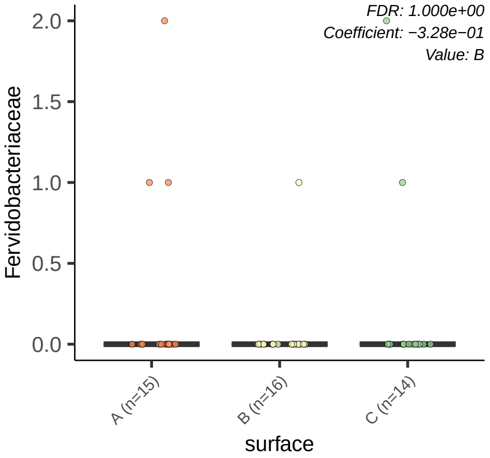

FDR: 1.000e+00
Coefficient: −3.28e−01
Value: B
Fervidobacteriaceae
2.0
1.5
1.0
0.5
0.0
A (n=15)
B (n=16)
C (n=14)
surface

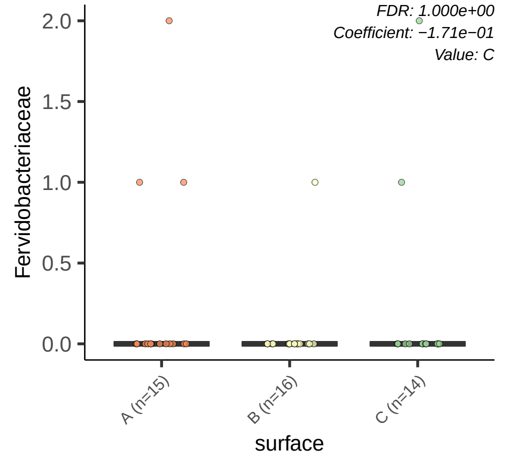

FDR: 1.000e+00
Coefficient: −1.71e−01
Value: C
Fervidobacteriaceae
2.0
1.5
1.0
0.5
0.0
A (n=15)
B (n=16)
C (n=14)
surface

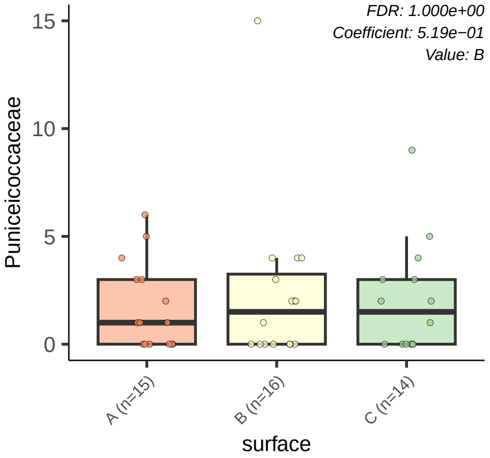

FDR: 1.000e+00
Coefficient: 5.19e−01
Value: B
Puniceicoccaceae
15
10
5
0
A (n=15)
B (n=16)
C (n=14)
surface

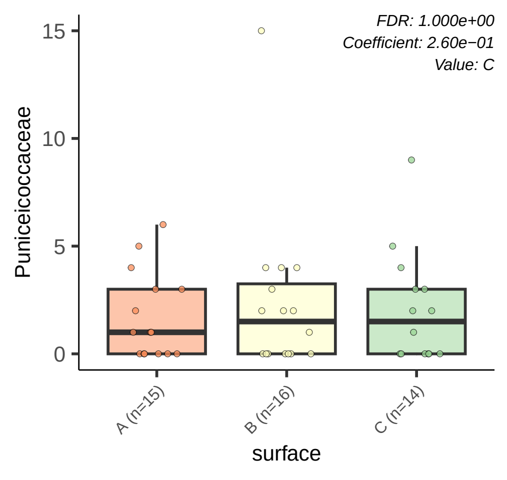
FDR: 1.000e+00
Coefficient: 2.60e−01
Value: C
Puniceicoccaceae
15
10
5
0
A (n=15)
B (n=16)
C (n=14)
surface

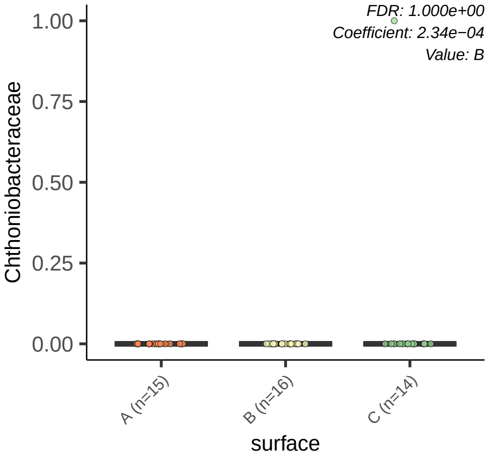
FDR: 1.000e+00
Coefficient: 2.34e−04
Value: B
Chthoniobacteraceae
1.00
0.75
0.50
0.25
0.00
A (n=15)
B (n=16)
C (n=14)
surface

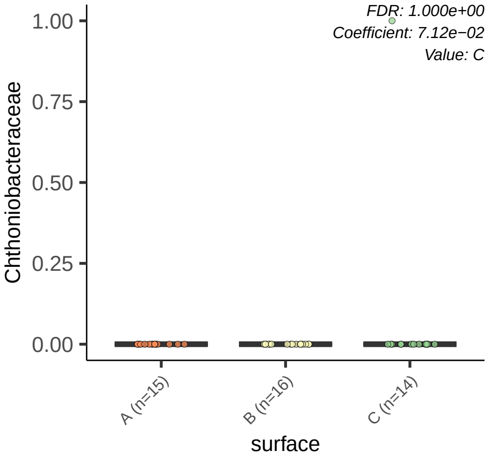

FDR: 1.000e+00
Coefficient: 7.12e−02
Value: C
Chthoniobacteraceae
1.00
0.75
0.50
0.25
0.00
A (n=15)
B (n=16)
C (n=14)
surface

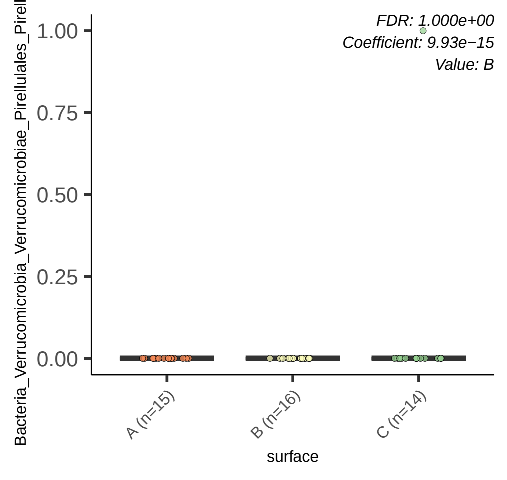
FDR: 1.000e+00
Coefficient: 9.93e−15
Value: B
Bacteria_Verrucomicrobia_Verrucomicrobiae_Pirellulales_Pirell
1.00
0.75
0.50
0.25
0.00
A (n=15)
B (n=16)
C (n=14)
surface

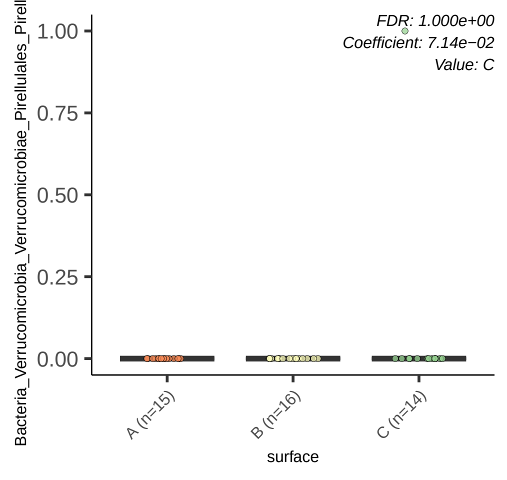
FDR: 1.000e+00
Coefficient: 7.14e−02
Value: C
Bacteria_Verrucomicrobia_Verrucomicrobiae_Pirellulales_Pirell
1.00
0.75
0.50
0.25
0.00
A (n=15)
B (n=16)
C (n=14)
surface

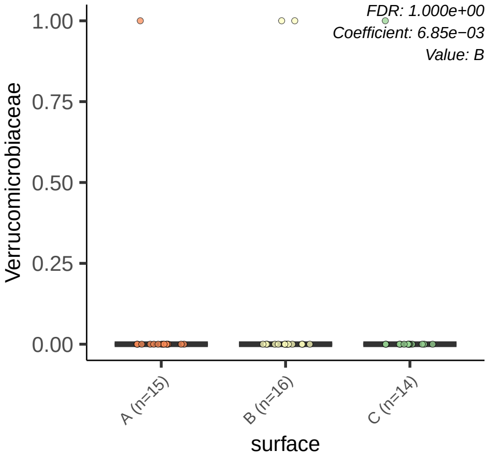

FDR: 1.000e+00
Coefficient: 6.85e−03
Value: B
Verrucomicrobiaceae
1.00
0.75
0.50
0.25
0.00
A (n=15)
B (n=16)
C (n=14)
surface

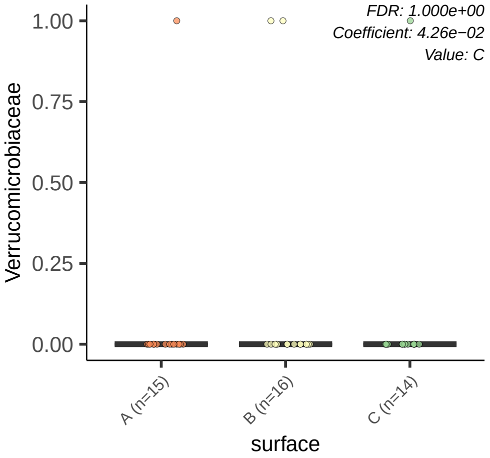

FDR: 1.000e+00
Coefficient: 4.26e−02
Value: C
Verrucomicrobiaceae
1.00
0.75
0.50
0.25
0.00
A (n=15)
B (n=16)
C (n=14)
surface

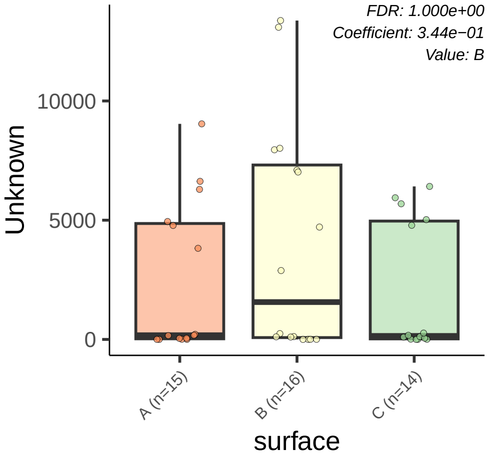
FDR: 1.000e+00
Coefficient: 3.44e−01
Value: B
Unknown
10000
5000
0
A (n=15)
B (n=16)
C (n=14)
surface

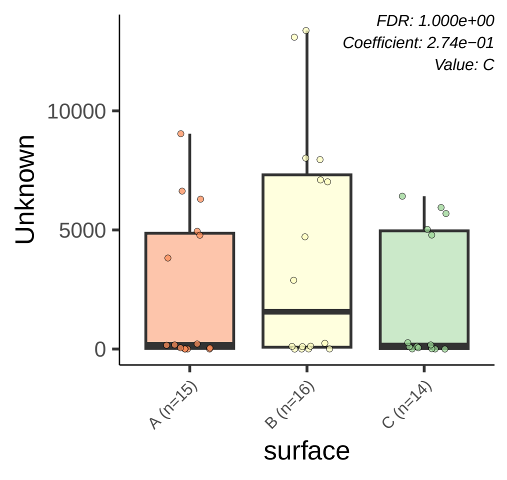
FDR: 1.000e+00
Coefficient: 2.74e−01
Value: C
Unknown
10000
5000
0
A (n=15)
B (n=16)
C (n=14)
surface

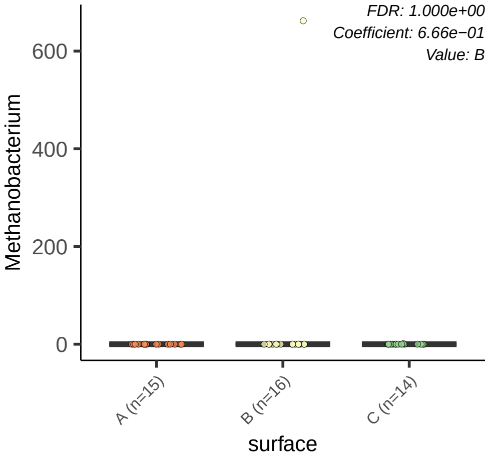

FDR: 1.000e+00
Coefficient: 6.66e−01
Value: B
Methanobacterium
600
400
200
0
A (n=15)
B (n=16)
C (n=14)
surface

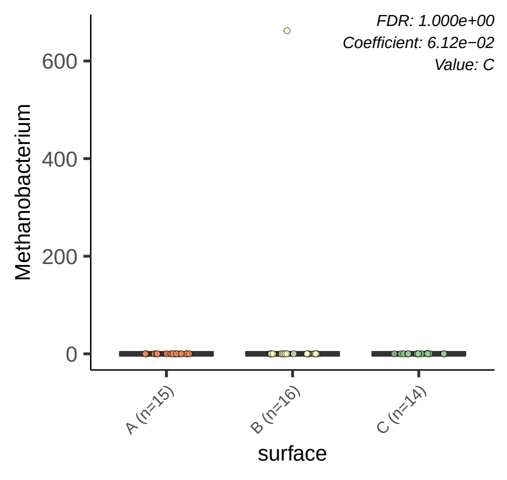
FDR: 1.000e+00
Coefficient: 6.12e−02
Value: C
Methanobacterium
600
400
200
0
A (n=15)
B (n=16)
C (n=14)
surface

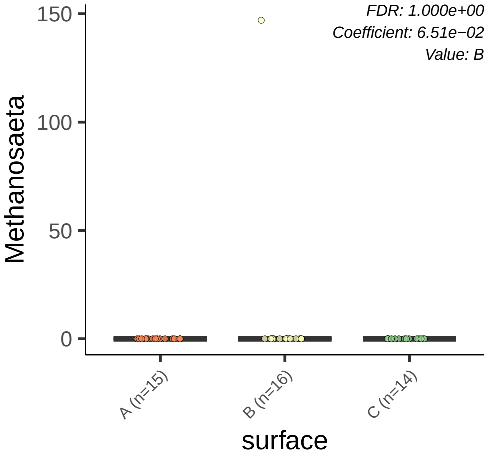

FDR: 1.000e+00
Coefficient: 6.51e−02
Value: B
150
100
50
0
Methanosaeta
A (n=15)
B (n=16)
C (n=14)
surface

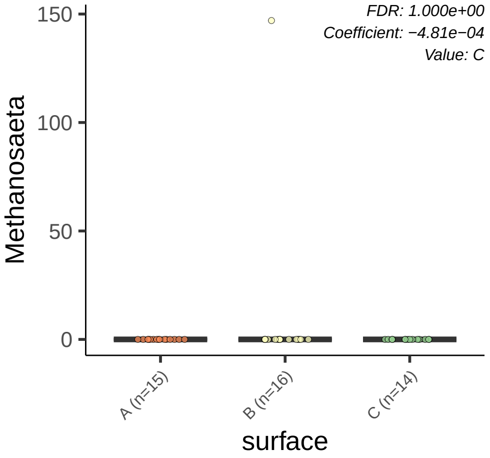
FDR: 1.000e+00
Coefficient: −4.81e−04
Value: C
Methanosaeta
150
100
50
0
A (n=15)
B (n=16)
C (n=14)
surface

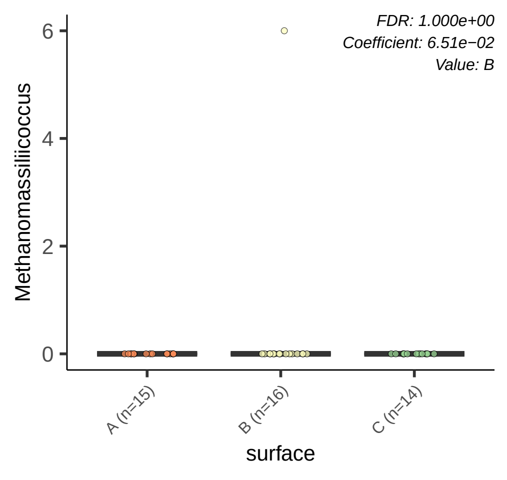
FDR: 1.000e+00
Coefficient: 6.51e−02
Value: B
Methanomassiliicoccus
0
2
4
6
A (n=15)
B (n=16)
C (n=14)
surface

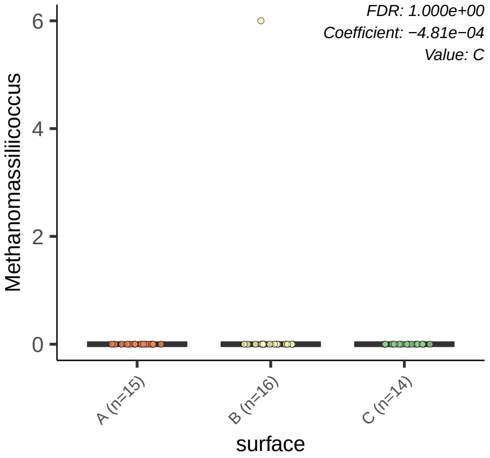

FDR: 1.000e+00
Coefficient: −4.81e−04
Value: C
Methanomassiliicoccus
6
4
2
0
A (n=15)
B (n=16)
C (n=14)
surface

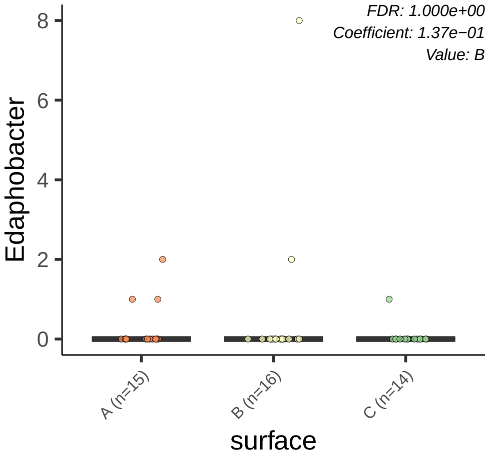
FDR: 1.000e+00
Coefficient: 1.37e−01
Value: B
Edaphobacter
8
6
4
2
0
A (n=15)
B (n=16)
C (n=14)
surface

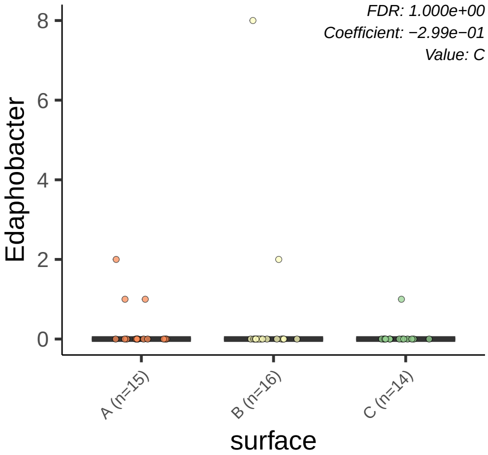

FDR: 1.000e+00
Coefficient: −2.99e−01
Value: C
Edaphobacter
0
2
4
6
8
A (n=15)
B (n=16)
C (n=14)
surface

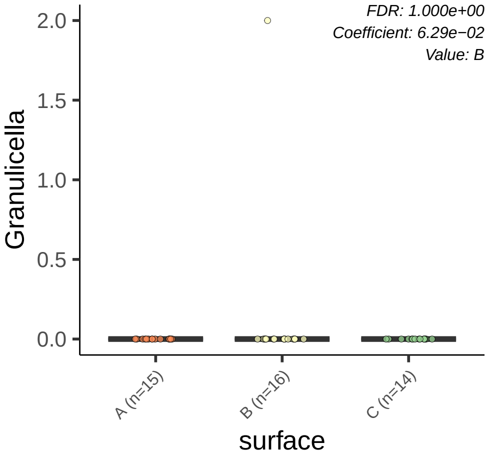

FDR: 1.000e+00
Coefficient: 6.29e−02
Value: B
Granulicella
2.0
1.5
1.0
0.5
0.0
A (n=15)
B (n=16)
C (n=14)
surface

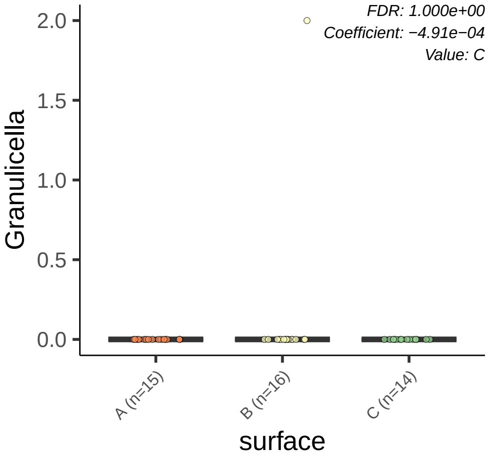
FDR: 1.000e+00
Coefficient: −4.91e−04
Value: C
2.0
1.5
1.0
0.5
0.0
Granulicella
A (n=15)
B (n=16)
C (n=14)
surface

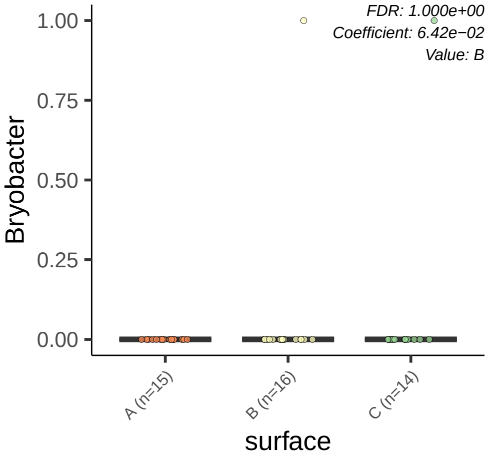
FDR: 1.000e+00
Coefficient: 6.42e−02
Value: B
Bryobacter
1.00
0.75
0.50
0.25
0.00
A (n=15)
B (n=16)
C (n=14)
surface

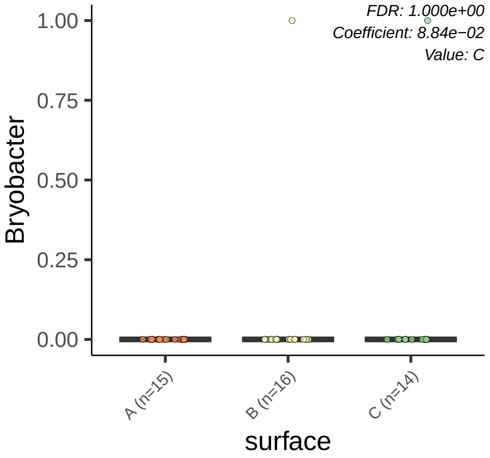
FDR: 1.000e+00
Coefficient: 8.84e−02
Value: C
Bryobacter
1.00
0.75
0.50
0.25
0.00
A (n=15)
B (n=16)
C (n=14)
surface

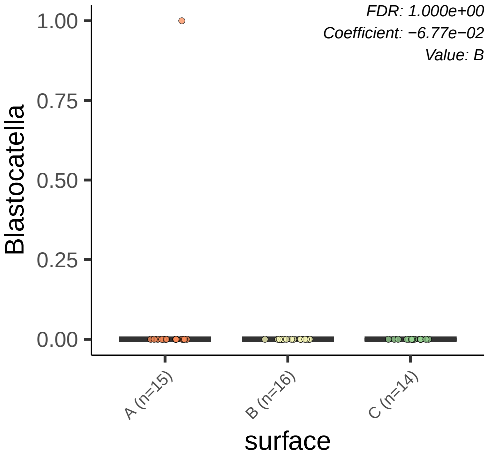

FDR: 1.000e+00
Coefficient: −6.77e−02
Value: B
Blastocatella
1.00
0.75
0.50
0.25
0.00
A (n=15)
B (n=16)
C (n=14)
surface

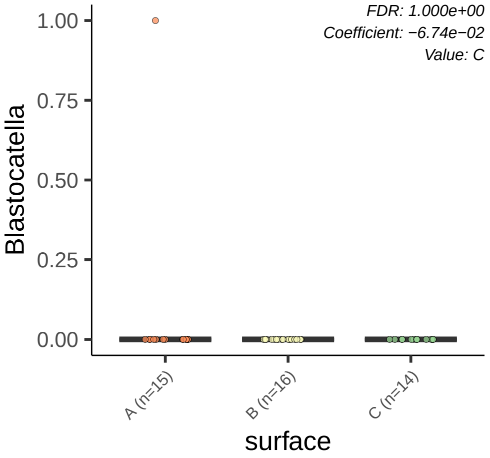
FDR: 1.000e+00
Coefficient: −6.74e−02
Value: C
Blastocatella
1.00
0.75
0.50
0.25
0.00
A (n=15)
B (n=16)
C (n=14)
surface

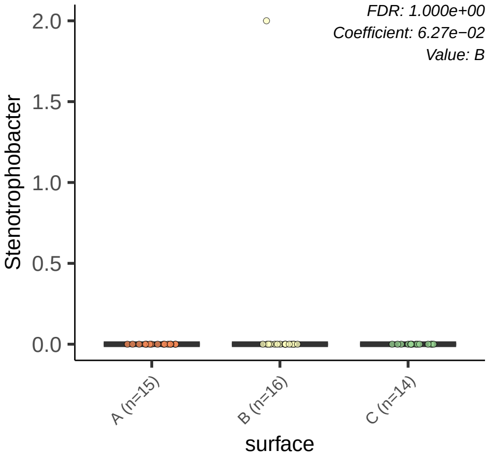
FDR: 1.000e+00
Coefficient: 6.27e−02
Value: B
Stenotrophobacter
2.0
1.5
1.0
0.5
0.0
A (n=15)
B (n=16)
C (n=14)
surface

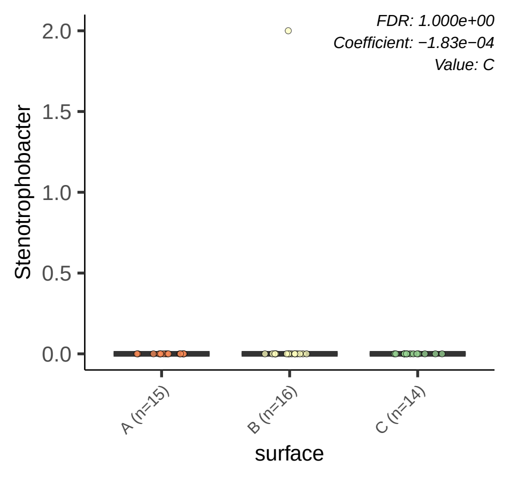

FDR: 1.000e+00
Coefficient: −1.83e−04
Value: C
Stenotrophobacter
2.0
1.5
1.0
0.5
0.0
A (n=15)
B (n=16)
C (n=14)
surface

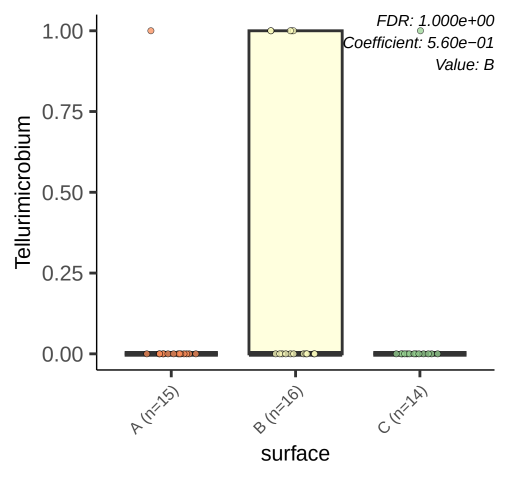

FDR: 1.000e+00
Coefficient: 5.60e−01
Value: B
Tellurimicrobium
1.00
0.75
0.50
0.25
0.00
A (n=15)
B (n=16)
C (n=14)
surface

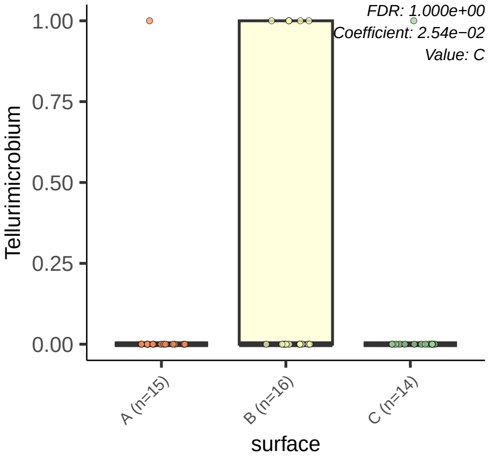
FDR: 1.000e+00
Coefficient: 2.54e−02
Value: C
1.00
0.75
0.50
0.25
0.00
Tellurimicrobium
A (n=15)
B (n=16)
C (n=14)
surface

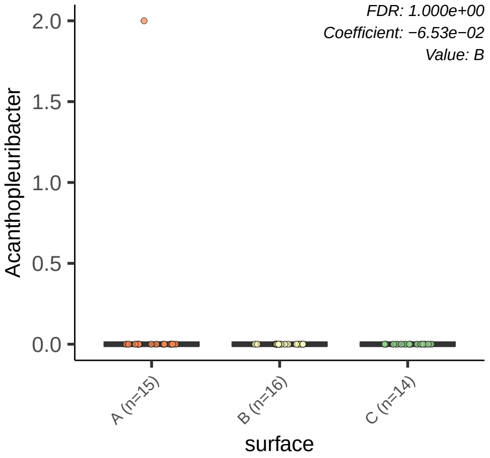
FDR: 1.000e+00
Coefficient: −6.53e−02
Value: B
Acanthopleuribacter
2.0
1.5
1.0
0.5
0.0
A (n=15)
B (n=16)
C (n=14)
surface

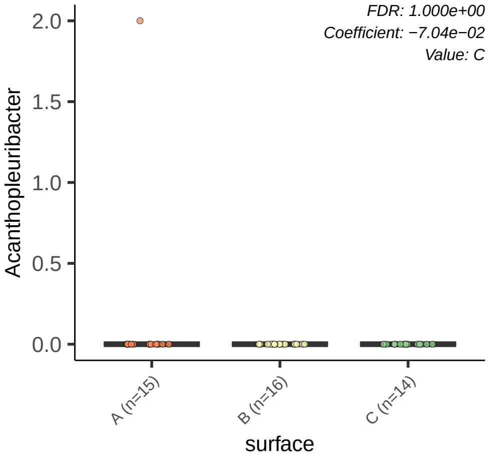
FDR: 1.000e+00
Coefficient: −7.04e−02
Value: C
Acanthopleuribacter
2.0
1.5
1.0
0.5
0.0
A (n=15)
B (n=16)
C (n=14)
surface

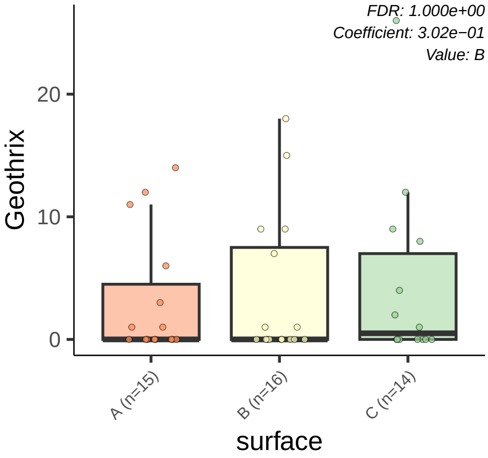

FDR: 1.000e+00
Coefficient: 3.02e−01
Value: B
Geothrix
20
10
0
A (n=15)
B (n=16)
C (n=14)
surface

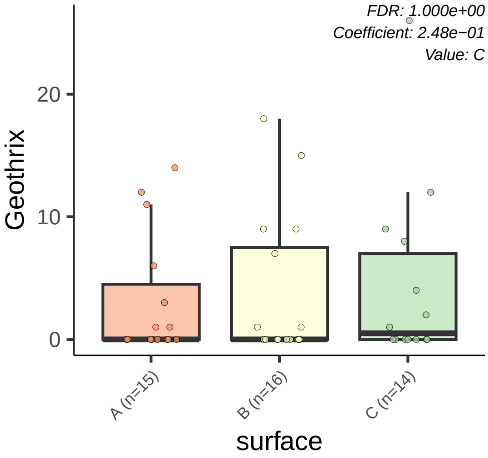

FDR: 1.000e+00
Coefficient: 2.48e−01
Value: C
Geothrix
0
10
20
A (n=15)
B (n=16)
C (n=14)
surface

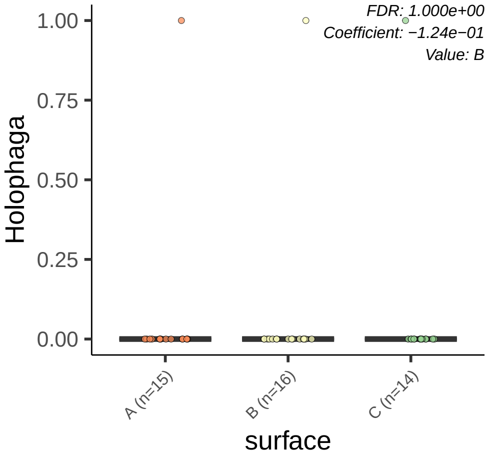

FDR: 1.000e+00
Coefficient: −1.24e−01
Value: B
Holophaga
1.00
0.75
0.50
0.25
0.00
A (n=15)
B (n=16)
C (n=14)
surface

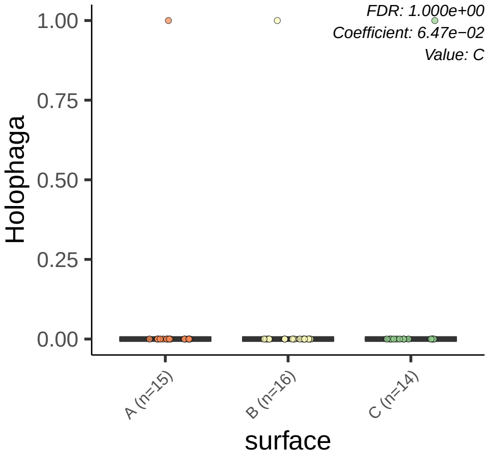

FDR: 1.000e+00
Coefficient: 6.47e−02
Value: C
Holophaga
1.00
0.75
0.50
0.25
0.00
A (n=15)
B (n=16)
C (n=14)
surface

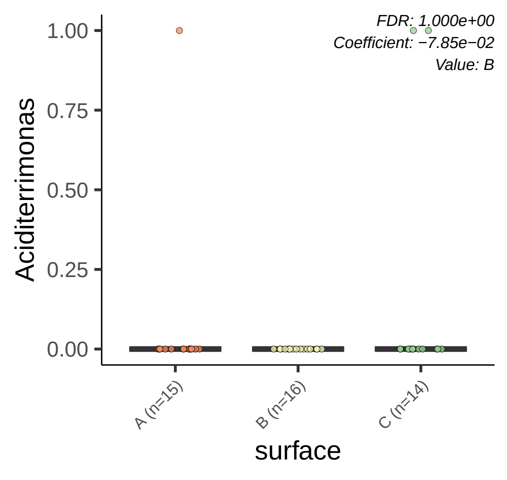
FDR: 1.000e+00
Coefficient: −7.85e−02
Value: B
Aciditerrimonas
1.00
0.75
0.50
0.25
0.00
A (n=15)
B (n=16)
C (n=14)
surface

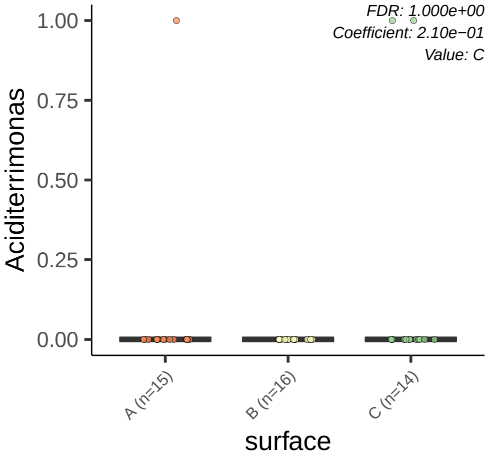

FDR: 1.000e+00
Coefficient: 2.10e−01
Value: C
Aciditerrimonas
1.00
0.75
0.50
0.25
0.00
A (n=15)
B (n=16)
C (n=14)
surface

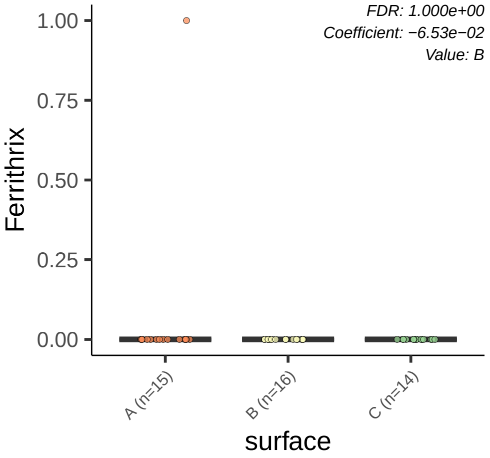
FDR: 1.000e+00
Coefficient: −6.53e−02
Value: B
Ferrithrix
1.00
0.75
0.50
0.25
0.00
A (n=15)
B (n=16)
C (n=14)
surface

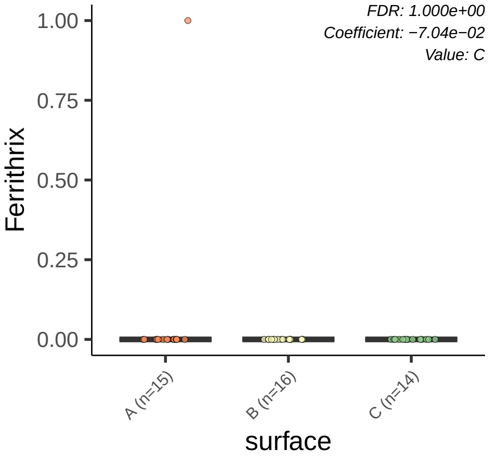
FDR: 1.000e+00
Coefficient: −7.04e−02
Value: C
Ferrithrix
1.00
0.75
0.50
0.25
0.00
A (n=15)
B (n=16)
C (n=14)
surface

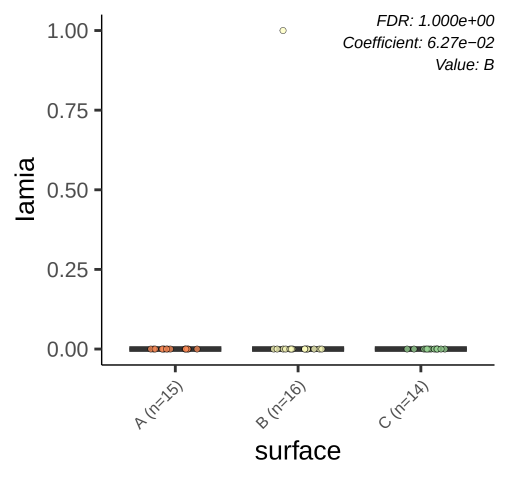

FDR: 1.000e+00
Coefficient: 6.27e−02
Value: B
1.00
0.75
0.50
0.25
0.00
lamia
A (n=15)
B (n=16)
C (n=14)
surface

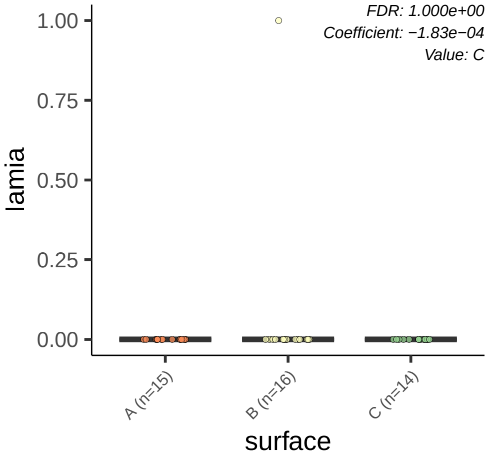

FDR: 1.000e+00
Coefficient: −1.83e−04
Value: C
lamia
1.00
0.75
0.50
0.25
0.00
A (n=15)
B (n=16)
C (n=14)
surface

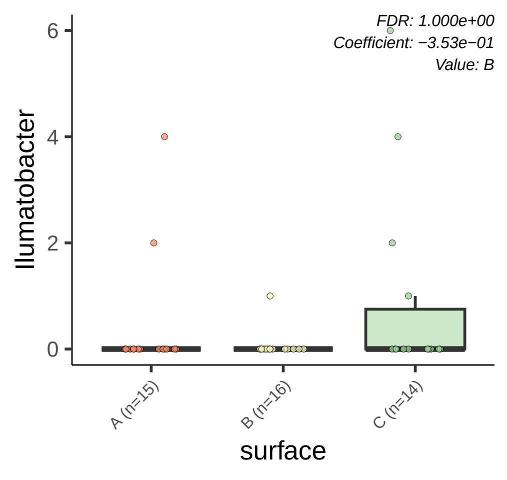
FDR: 1.000e+00
Coefficient: −3.53e−01
Value: B
Ilumatobacter
6
4
2
0
A (n=15)
B (n=16)
C (n=14)
surface

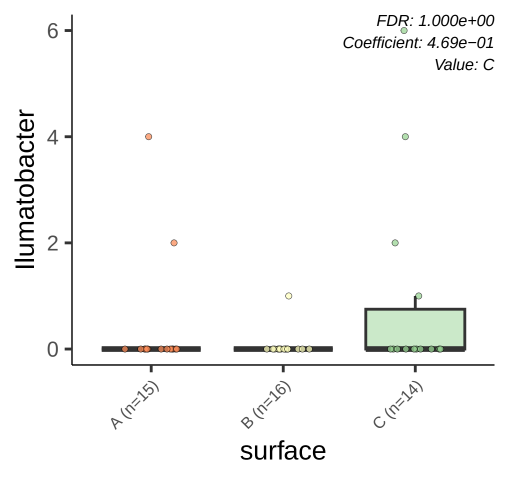
FDR: 1.000e+00
Coefficient: 4.69e−01
Value: C
Ilumatobacter
0
2
4
6
A (n=15)
B (n=16)
C (n=14)
surface

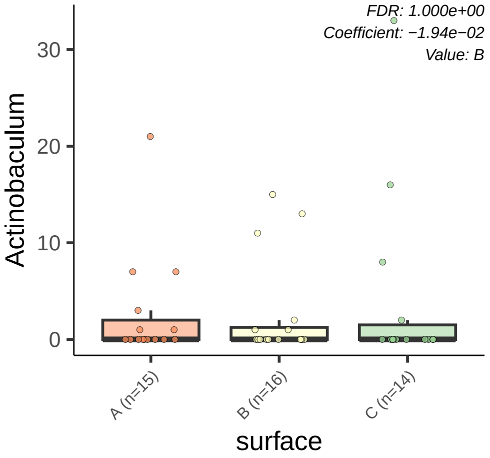

FDR: 1.000e+00
Coefficient: −1.94e−02
Value: B
Actinobaculum
30
20
10
0
A (n=15)
B (n=16)
C (n=14)
surface

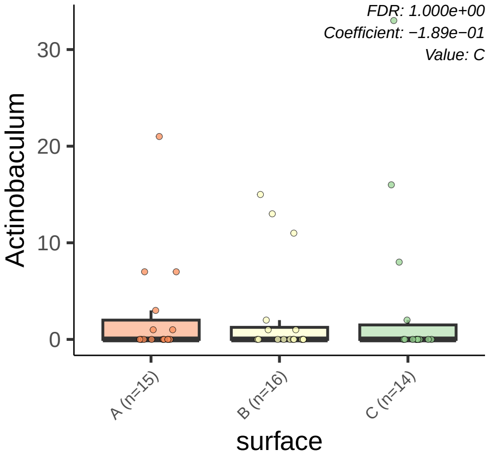
FDR: 1.000e+00
Coefficient: −1.89e−01
Value: C
Actinobaculum
30
20
10
0
A (n=15)
B (n=16)
C (n=14)
surface

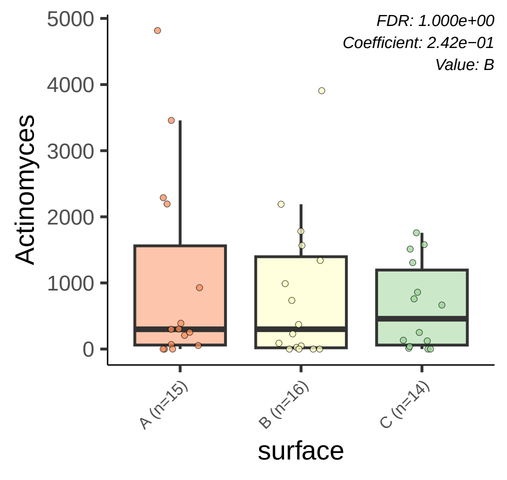
FDR: 1.000e+00
Coefficient: 2.42e−01
Value: B
Actinomyces
5000
4000
3000
2000
1000
0
A (n=15)
B (n=16)
C (n=14)
surface

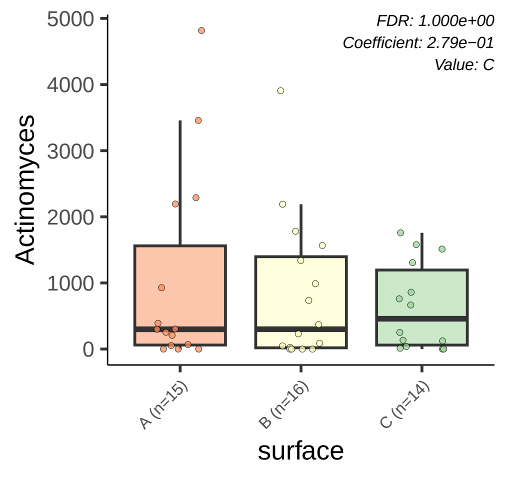
FDR: 1.000e+00
Coefficient: 2.79e−01
Value: C
Actinomyces
5000
4000
3000
2000
1000
0
A (n=15)
B (n=16)
C (n=14)
surface

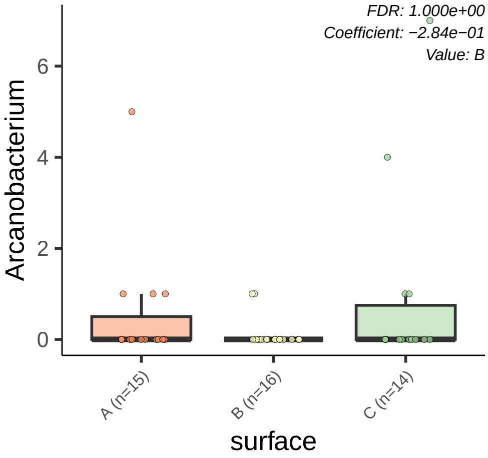

FDR: 1.000e+00
Coefficient: −2.84e−01
Value: B
Arcanobacterium
0
2
4
6
A (n=15)
B (n=16)
C (n=14)
surface

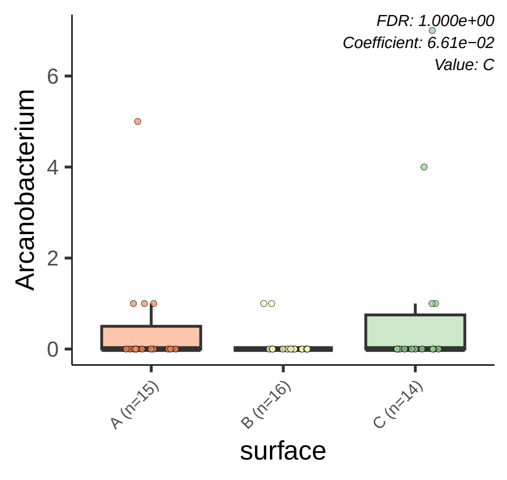
FDR: 1.000e+00
Coefficient: 6.61e−02
Value: C
Arcanobacterium
0
2
4
6
A (n=15)
B (n=16)
C (n=14)
surface

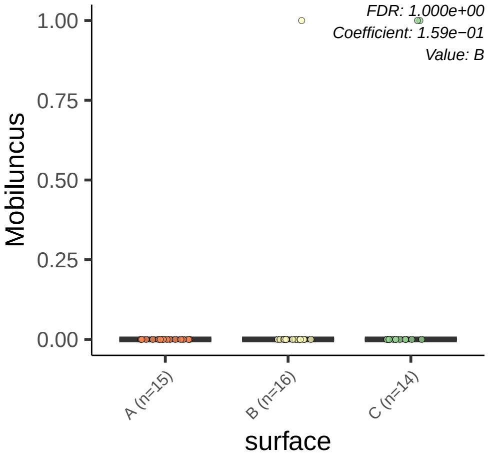

FDR: 1.000e+00
Coefficient: 1.59e−01
Value: B
Mobiluncus
1.00
0.75
0.50
0.25
0.00
A (n=15)
B (n=16)
C (n=14)
surface

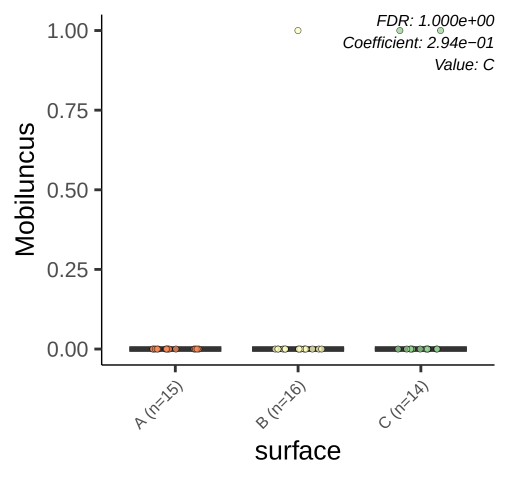
FDR: 1.000e+00
Coefficient: 2.94e−01
Value: C
Mobiluncus
1.00
0.75
0.50
0.25
0.00
A (n=15)
B (n=16)
C (n=14)
surface

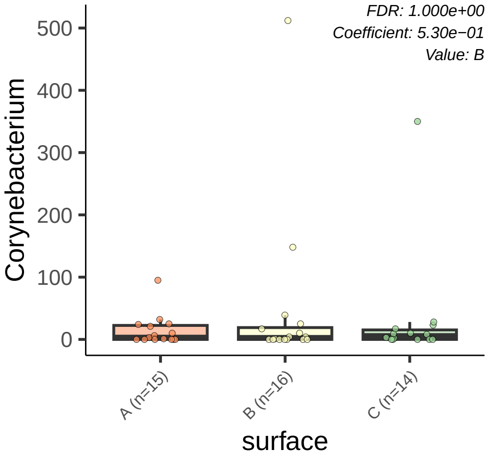

FDR: 1.000e+00
Coefficient: 5.30e−01
Value: B
Corynebacterium
500
400
300
200
100
0
A (n=15)
B (n=16)
C (n=14)
surface

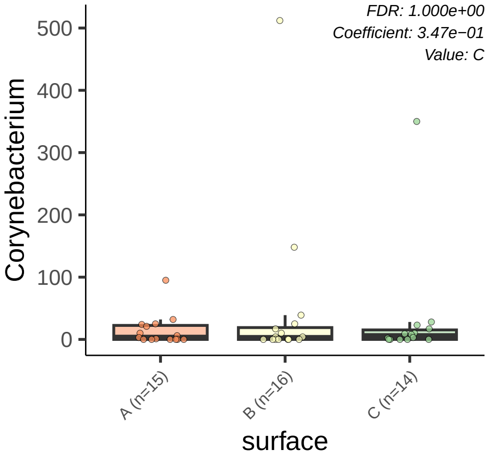
FDR: 1.000e+00
Coefficient: 3.47e−01
Value: C
Corynebacterium
0
100
200
300
400
500
A (n=15)
B (n=16)
C (n=14)
surface

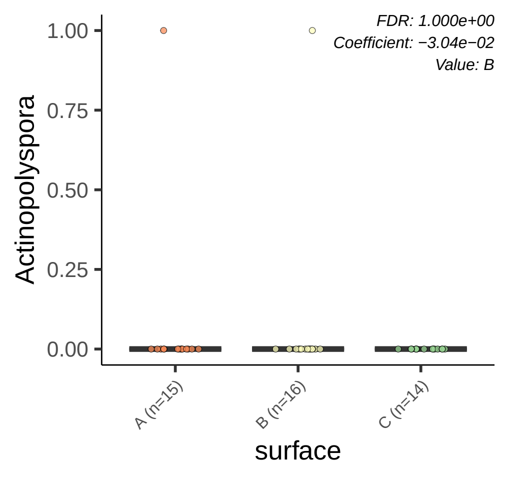
FDR: 1.000e+00
Coefficient: −3.04e−02
Value: B
Actinopolyspora
1.00
0.75
0.50
0.25
0.00
A (n=15)
B (n=16)
C (n=14)
surface

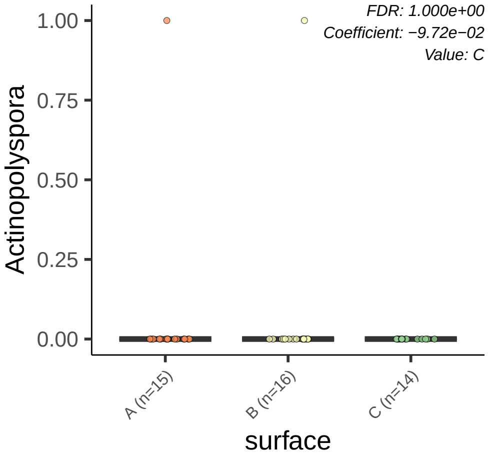

FDR: 1.000e+00
Coefficient: −9.72e−02
Value: C
Actinopolyspora
1.00
0.75
0.50
0.25
0.00
A (n=15)
B (n=16)
C (n=14)
surface

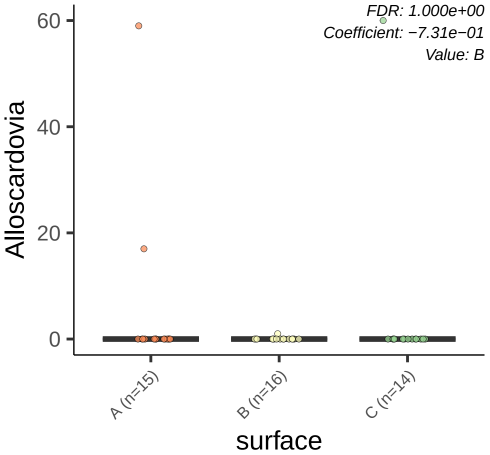

FDR: 1.000e+00
Coefficient: −7.31e−01
Value: B
Alloscardovia
60
40
20
0
A (n=15)
B (n=16)
C (n=14)
surface

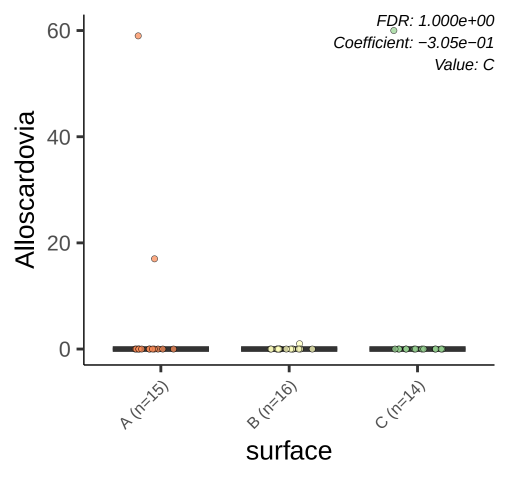
FDR: 1.000e+00
Coefficient: −3.05e−01
Value: C
Alloscardovia
60
40
20
0
A (n=15)
B (n=16)
C (n=14)
surface

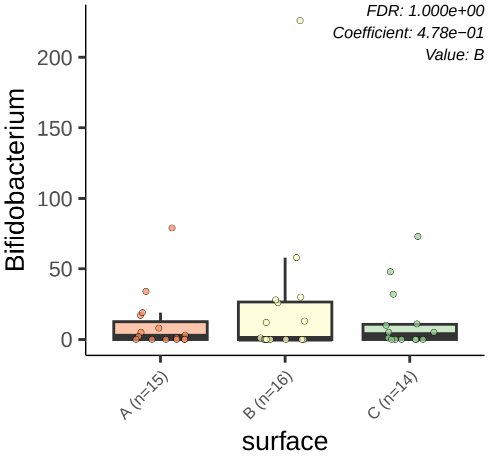
FDR: 1.000e+00
Coefficient: 4.78e−01
Value: B
Bifidobacterium
200
150
100
50
0
A (n=15)
B (n=16)
C (n=14)
surface

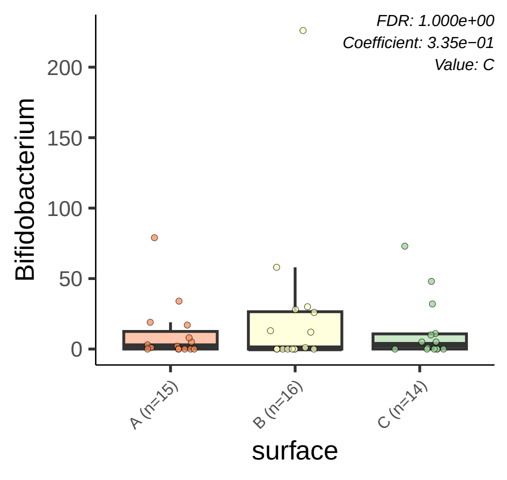

FDR: 1.000e+00
Coefficient: 3.35e−01
Value: C
Bifidobacterium
200
150
100
50
0
A (n=15)
B (n=16)
C (n=14)
surface

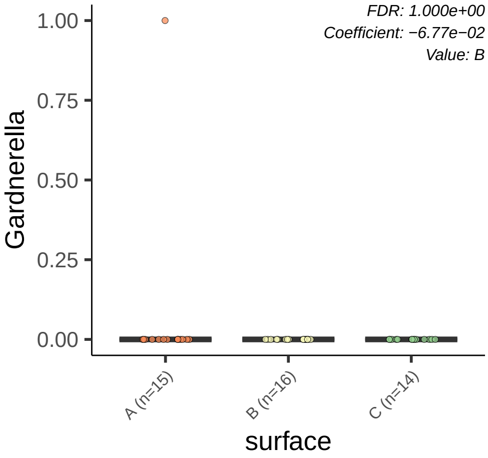
FDR: 1.000e+00
Coefficient: −6.77e−02
Value: B
Gardnerella
1.00
0.75
0.50
0.25
0.00
A (n=15)
B (n=16)
C (n=14)
surface

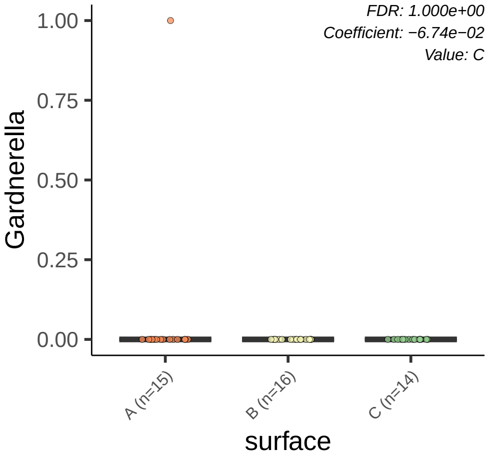
FDR: 1.000e+00
Coefficient: −6.74e−02
Value: C
Gardnerella
1.00
0.75
0.50
0.25
0.00
A (n=15)
B (n=16)
C (n=14)
surface

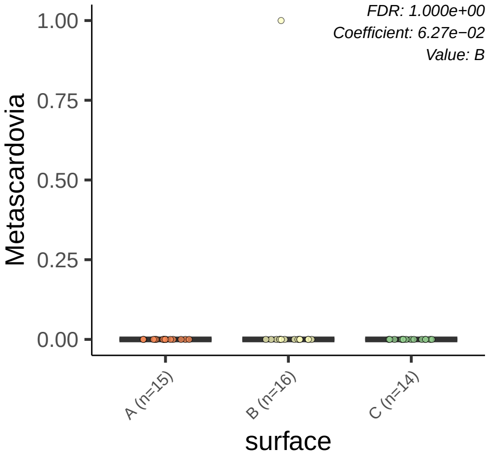
FDR: 1.000e+00
Coefficient: 6.27e−02
Value: B
Metascardovia
1.00
0.75
0.50
0.25
0.00
A (n=15)
B (n=16)
C (n=14)
surface

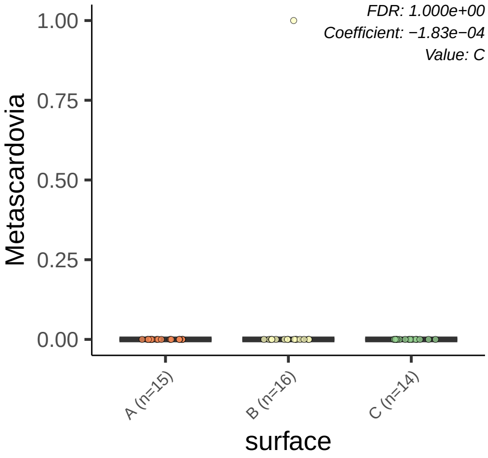
FDR: 1.000e+00
Coefficient: −1.83e−04
Value: C
Metascardovia
1.00
0.75
0.50
0.25
0.00
A (n=15)
B (n=16)
C (n=14)
surface

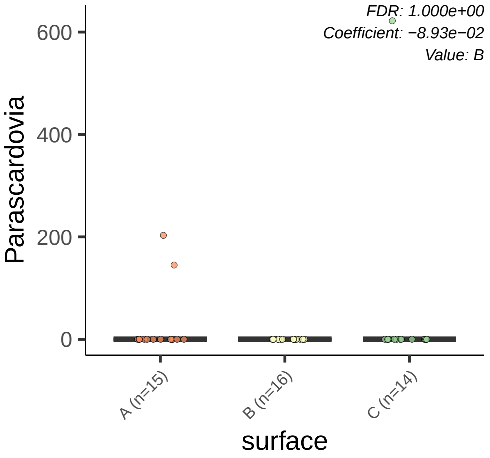

FDR: 1.000e+00
Coefficient: −8.93e−02
Value: B
600
400
200
0
Parascardovia
A (n=15)
B (n=16)
C (n=14)
surface

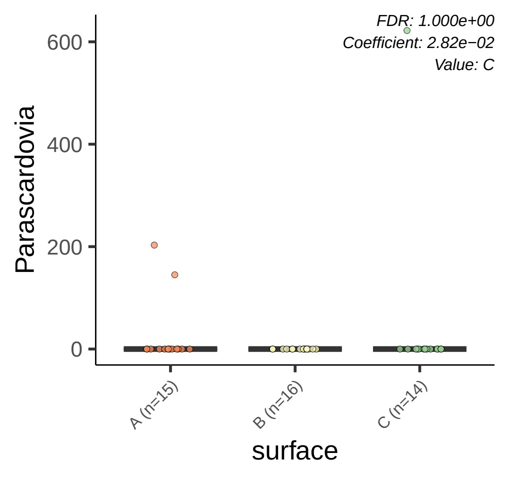

FDR: 1.000e+00
Coefficient: 2.82e−02
Value: C
600
400
200
0
Parascardovia
A (n=15)
B (n=16)
C (n=14)
surface

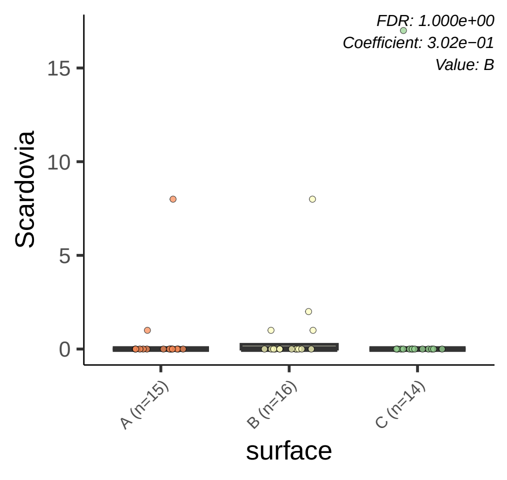
FDR: 1.000e+00
Coefficient: 3.02e−01
Value: B
Scardovia
15
10
5
0
A (n=15)
B (n=16)
C (n=14)
surface

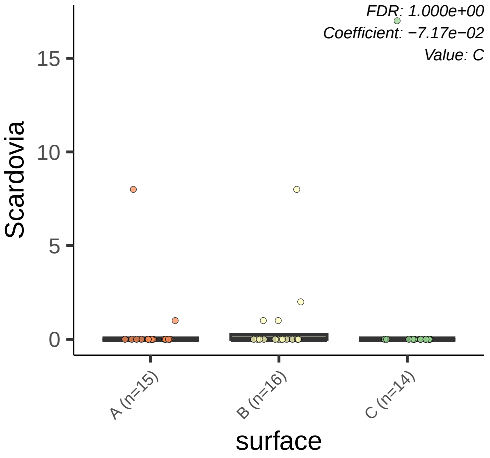

FDR: 1.000e+00
Coefficient: −7.17e−02
Value: C
Scardovia
15
10
5
0
A (n=15)
B (n=16)
C (n=14)
surface

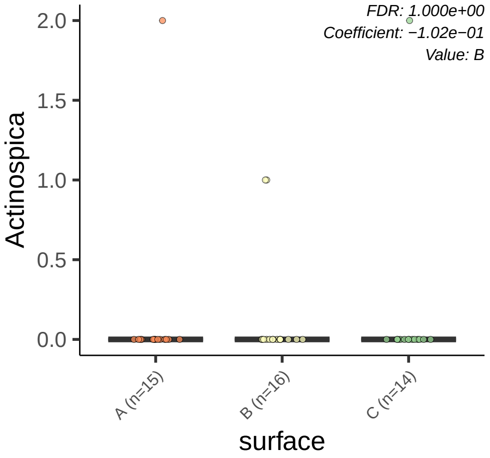
FDR: 1.000e+00
Coefficient: −1.02e−01
Value: B
Actinospica
2.0
1.5
1.0
0.5
0.0
A (n=15)
B (n=16)
C (n=14)
surface

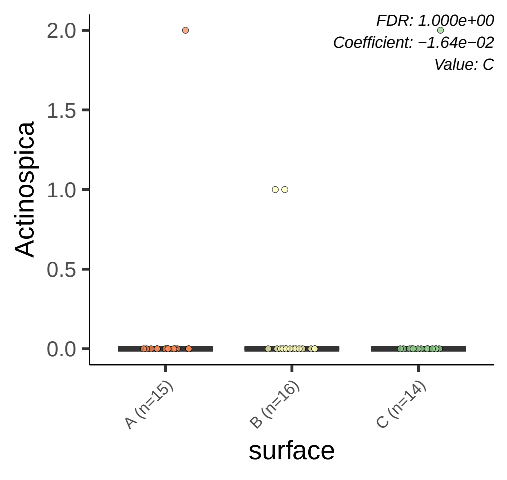
FDR: 1.000e+00
Coefficient: −1.64e−02
Value: C
Actinospica
2.0
1.5
1.0
0.5
0.0
A (n=15)
B (n=16)
C (n=14)
surface

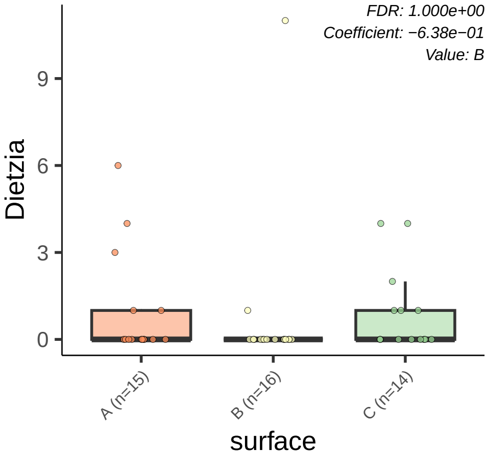
FDR: 1.000e+00
Coefficient: −6.38e−01
Value: B
Dietzia
0
3
6
9
A (n=15)
B (n=16)
C (n=14)
surface

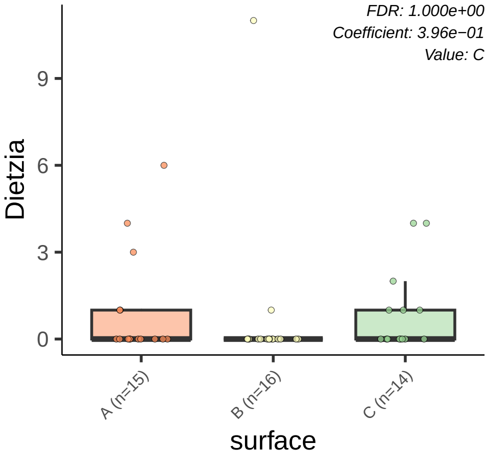

FDR: 1.000e+00
Coefficient: 3.96e−01
Value: C
Dietzia
9
6
3
0
A (n=15)
B (n=16)
C (n=14)
surface

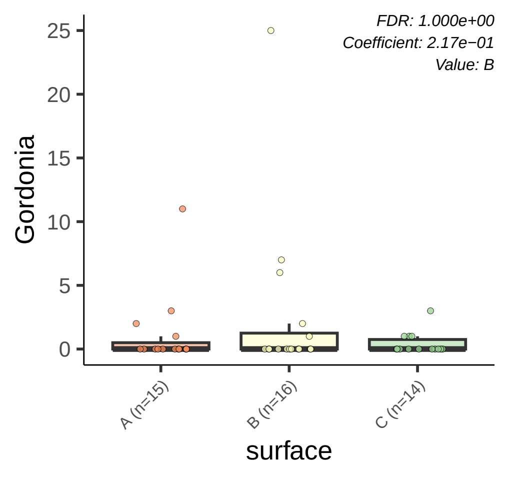
FDR: 1.000e+00
Coefficient: 2.17e−01
Value: B
Gordonia
25
20
15
10
5
0
A (n=15)
B (n=16)
C (n=14)
surface

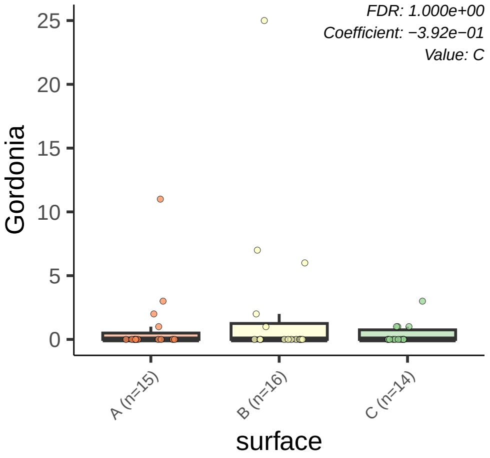
FDR: 1.000e+00
Coefficient: −3.92e−01
Value: C
Gordonia
0
5
10
15
20
25
A (n=15)
B (n=16)
C (n=14)
surface

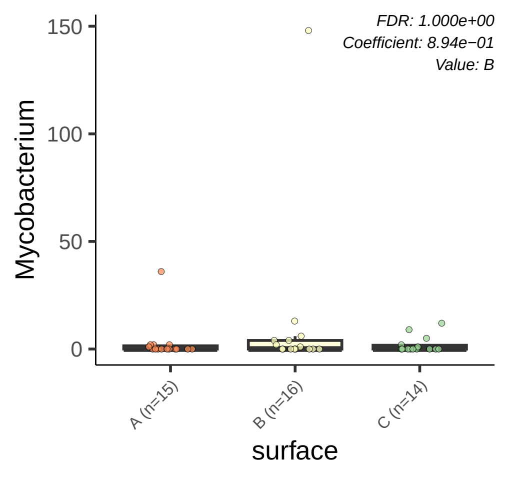
FDR: 1.000e+00
Coefficient: 8.94e−01
Value: B
Mycobacterium
150
100
50
0
A (n=15)
B (n=16)
C (n=14)
surface

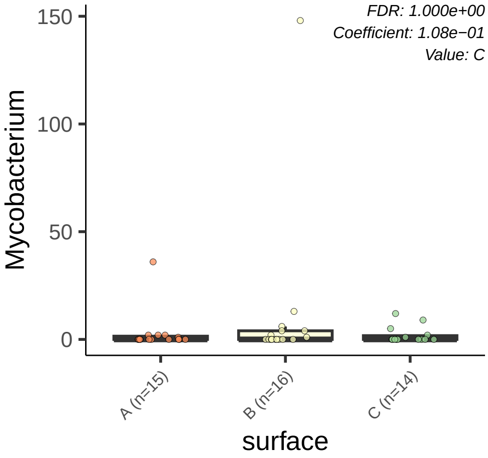
FDR: 1.000e+00
Coefficient: 1.08e−01
Value: C
Mycobacterium
150
100
50
0
A (n=15)
B (n=16)
C (n=14)
surface

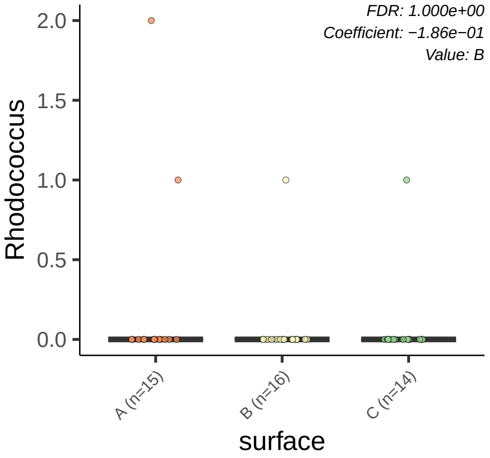
FDR: 1.000e+00
Coefficient: −1.86e−01
Value: B
Rhodococcus
2.0
1.5
1.0
0.5
0.0
A (n=15)
B (n=16)
C (n=14)
surface

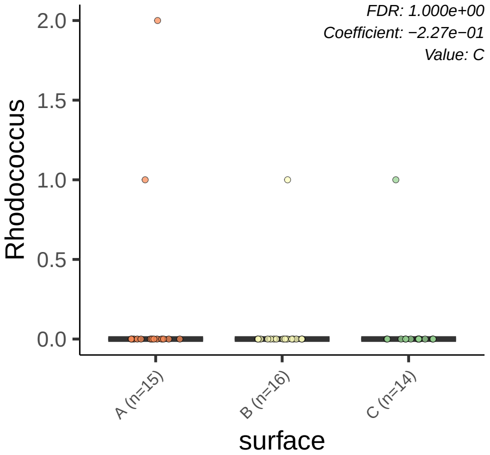
FDR: 1.000e+00
Coefficient: −2.27e−01
Value: C
Rhodococcus
2.0
1.5
1.0
0.5
0.0
A (n=15)
B (n=16)
C (n=14)
surface

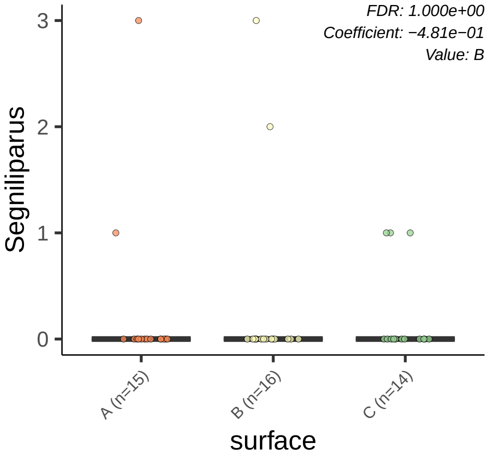

FDR: 1.000e+00
Coefficient: −4.81e−01
Value: B
Segniliparus
3
2
1
0
A (n=15)
B (n=16)
C (n=14)
surface

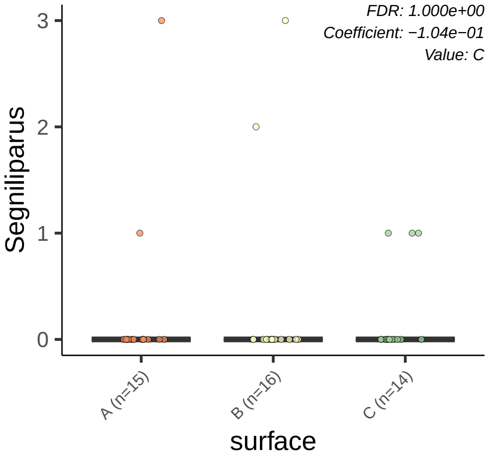

FDR: 1.000e+00
Coefficient: −1.04e−01
Value: C
Segniliparus
0
1
2
3
A (n=15)
B (n=16)
C (n=14)
surface

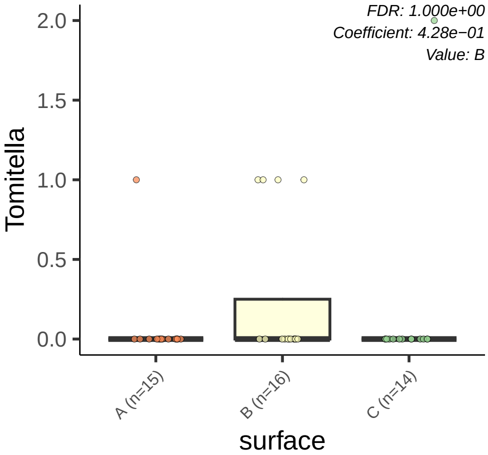
FDR: 1.000e+00
Coefficient: 4.28e−01
Value: B
Tomitella
2.0
1.5
1.0
0.5
0.0
A (n=15)
B (n=16)
C (n=14)
surface

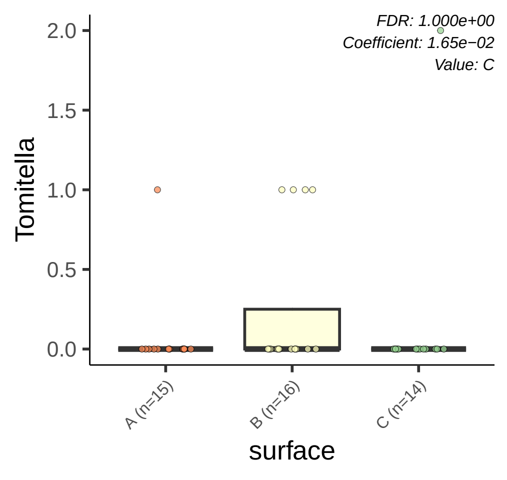
FDR: 1.000e+00
Coefficient: 1.65e−02
Value: C
Tomitella
2.0
1.5
1.0
0.5
0.0
A (n=15)
B (n=16)
C (n=14)
surface

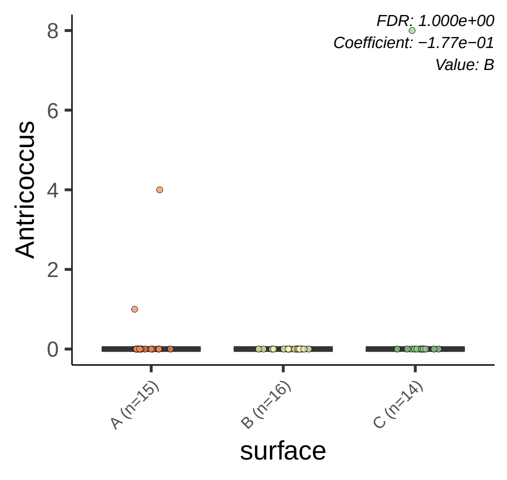

FDR: 1.000e+00
Coefficient: −1.77e−01
Value: B
Antricoccus
8
6
4
2
0
A (n=15)
B (n=16)
C (n=14)
surface

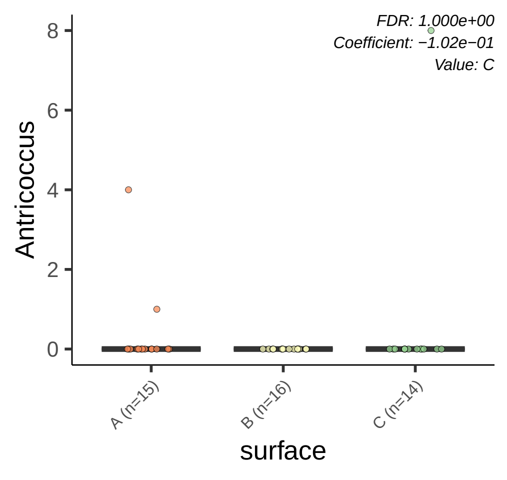
FDR: 1.000e+00
Coefficient: −1.02e−01
Value: C
Antricoccus
8
6
4
2
0
A (n=15)
B (n=16)
C (n=14)
surface

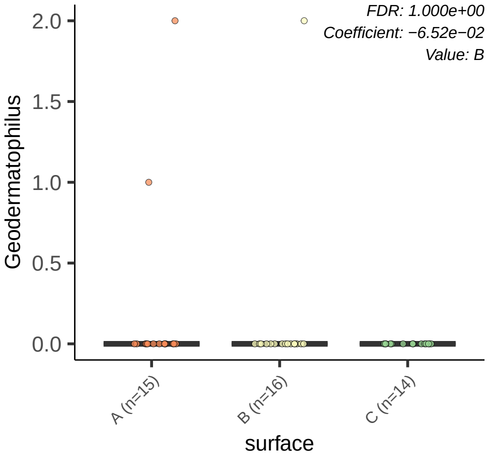
FDR: 1.000e+00
Coefficient: −6.52e−02
Value: B
Geodermatophilus
2.0
1.5
1.0
0.5
0.0
A (n=15)
B (n=16)
C (n=14)
surface

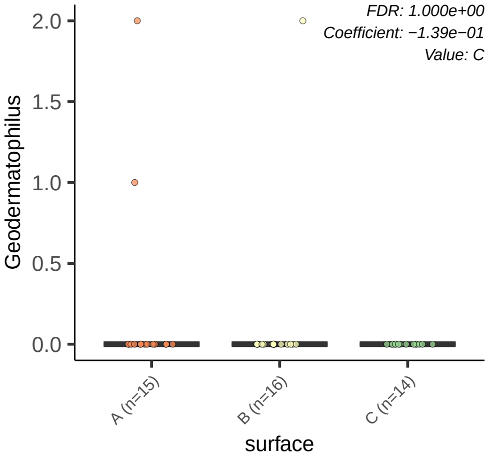
FDR: 1.000e+00
Coefficient: −1.39e−01
Value: C
Geodermatophilus
2.0
1.5
1.0
0.5
0.0
A (n=15)
B (n=16)
C (n=14)
surface

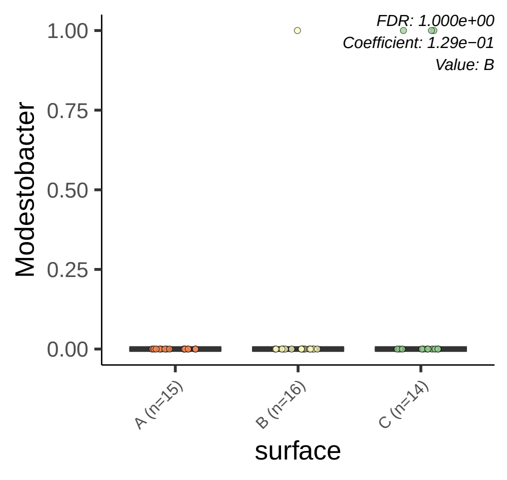
FDR: 1.000e+00
Coefficient: 1.29e−01
Value: B
Modestobacter
1.00
0.75
0.50
0.25
0.00
A (n=15)
B (n=16)
C (n=14)
surface

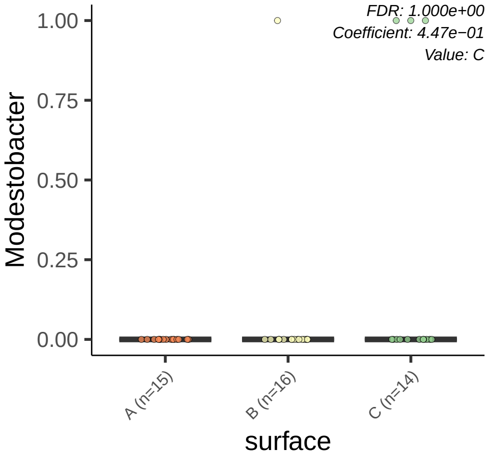
FDR: 1.000e+00
Coefficient: 4.47e−01
Value: C
Modestobacter
1.00
0.75
0.50
0.25
0.00
A (n=15)
B (n=16)
C (n=14)
surface

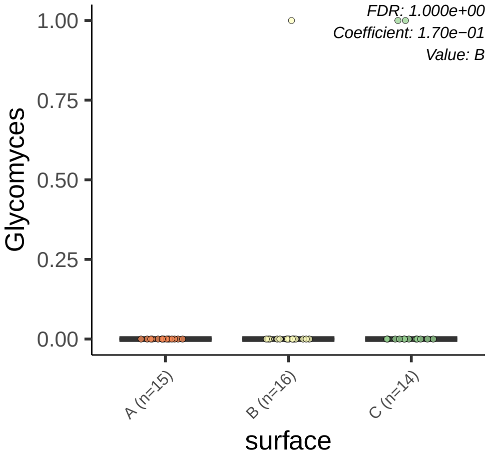
FDR: 1.000e+00
Coefficient: 1.70e−01
Value: B
Glycomyces
1.00
0.75
0.50
0.25
0.00
A (n=15)
B (n=16)
C (n=14)
surface

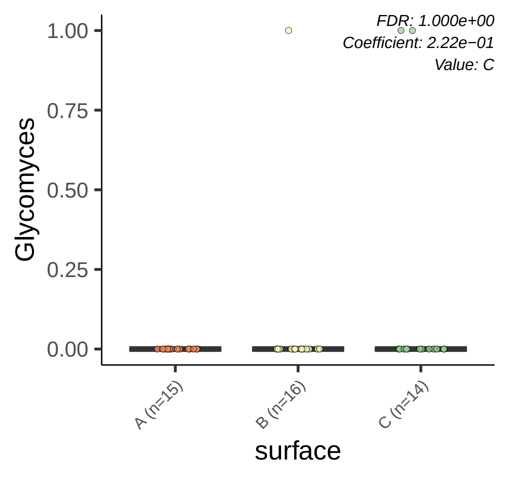

FDR: 1.000e+00
Coefficient: 2.22e−01
Value: C
Glycomyces
1.00
0.75
0.50
0.25
0.00
A (n=15)
B (n=16)
C (n=14)
surface

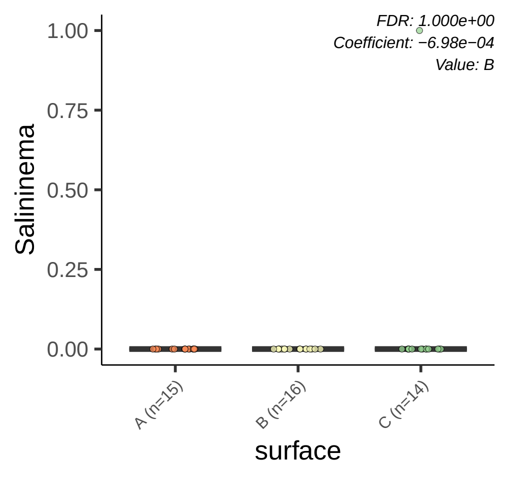
FDR: 1.000e+00
Coefficient: −6.98e−04
Value: B
Salininema
1.00
0.75
0.50
0.25
0.00
A (n=15)
B (n=16)
C (n=14)
surface

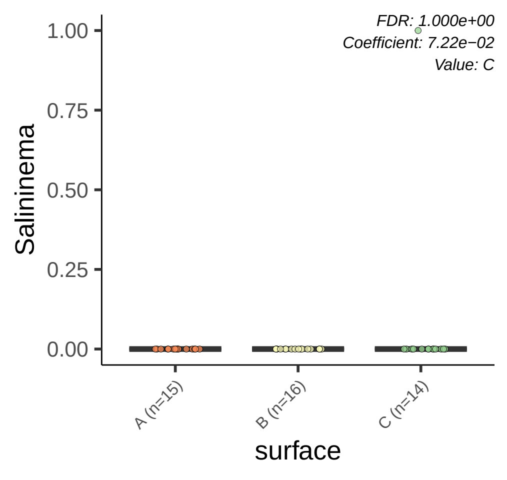
FDR: 1.000e+00
Coefficient: 7.22e−02
Value: C
Salininema
1.00
0.75
0.50
0.25
0.00
A (n=15)
B (n=16)
C (n=14)
surface

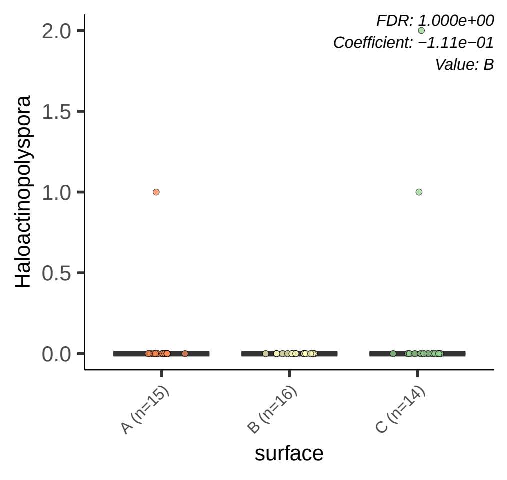
FDR: 1.000e+00
Coefficient: −1.11e−01
Value: B
Haloactinopolyspora
2.0
1.5
1.0
0.5
0.0
A (n=15)
B (n=16)
C (n=14)
surface

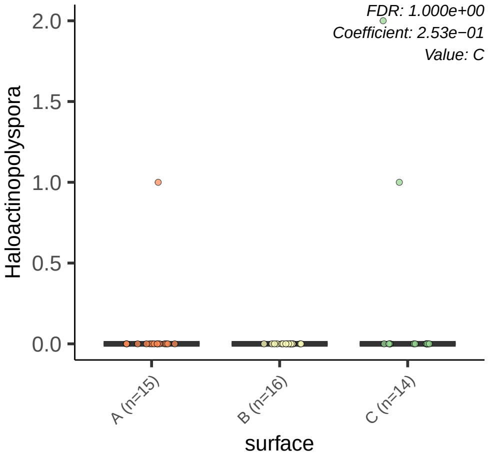
FDR: 1.000e+00
Coefficient: 2.53e−01
Value: C
Haloactinopolyspora
2.0
1.5
1.0
0.5
0.0
A (n=15)
B (n=16)
C (n=14)
surface

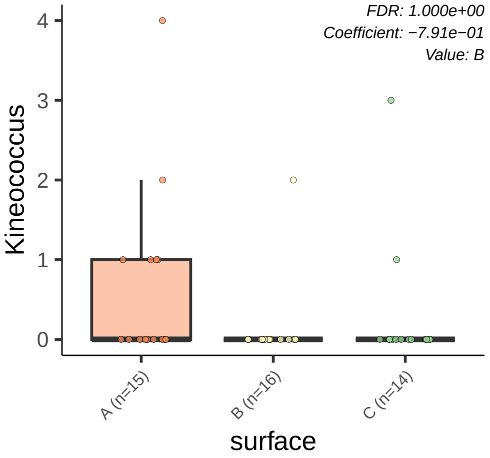
FDR: 1.000e+00
Coefficient: −7.91e−01
Value: B
Kineococcus
0
1
2
3
4
A (n=15)
B (n=16)
C (n=14)
surface

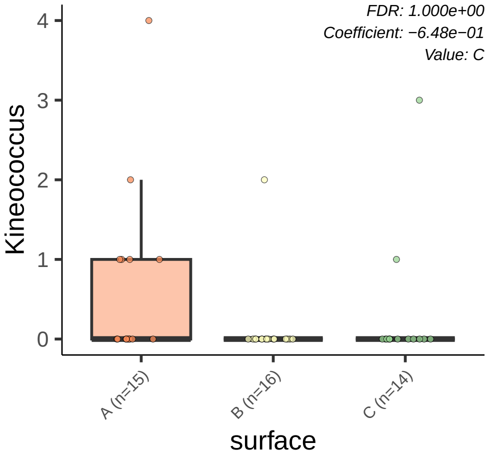
FDR: 1.000e+00
Coefficient: −6.48e−01
Value: C
Kineococcus
0
1
2
3
4
A (n=15)
B (n=16)
C (n=14)
surface

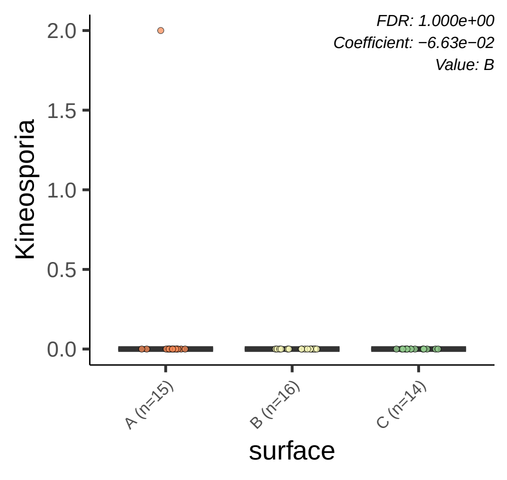
FDR: 1.000e+00
Coefficient: −6.63e−02
Value: B
Kineosporia
2.0
1.5
1.0
0.5
0.0
A (n=15)
B (n=16)
C (n=14)
surface

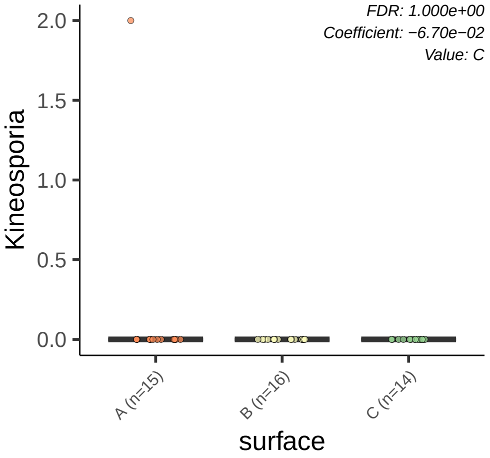

FDR: 1.000e+00
Coefficient: −6.70e−02
Value: C
Kineosporia
2.0
1.5
1.0
0.5
0.0
A (n=15)
B (n=16)
C (n=14)
surface

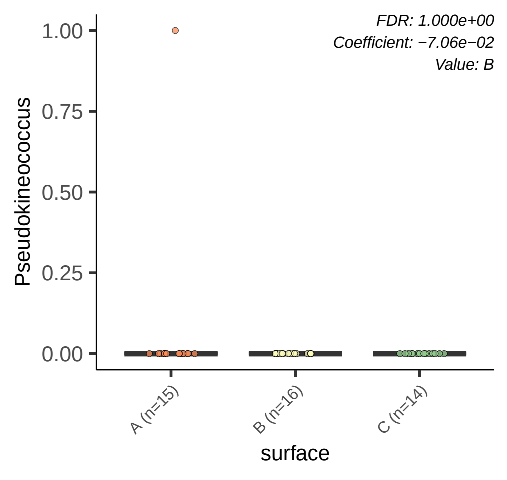
FDR: 1.000e+00
Coefficient: −7.06e−02
Value: B
Pseudokineococcus
1.00
0.75
0.50
0.25
0.00
A (n=15)
B (n=16)
C (n=14)
surface

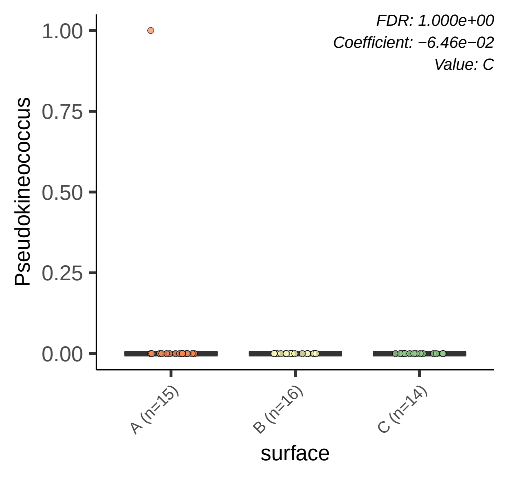

FDR: 1.000e+00
Coefficient: −6.46e−02
Value: C
Pseudokineococcus
1.00
0.75
0.50
0.25
0.00
A (n=15)
B (n=16)
C (n=14)
surface

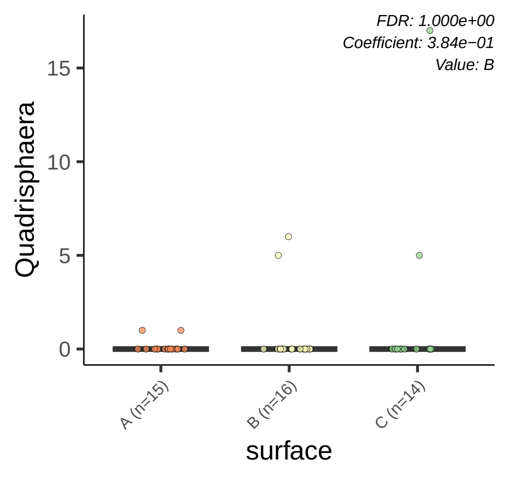

FDR: 1.000e+00
Coefficient: 3.84e−01
Value: B
Quadrisphaera
15
10
5
0
A (n=15)
B (n=16)
C (n=14)
surface

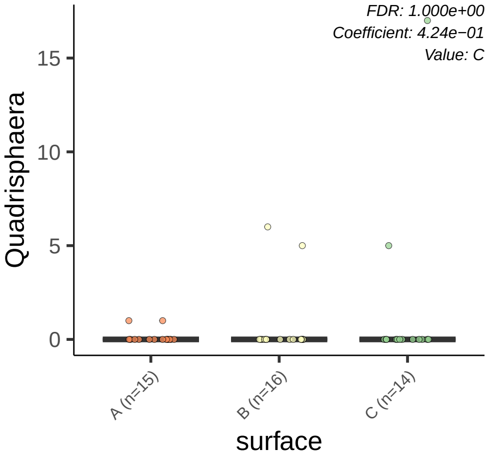

FDR: 1.000e+00
Coefficient: 4.24e−01
Value: C
Quadrisphaera
15
10
5
0
A (n=15)
B (n=16)
C (n=14)
surface

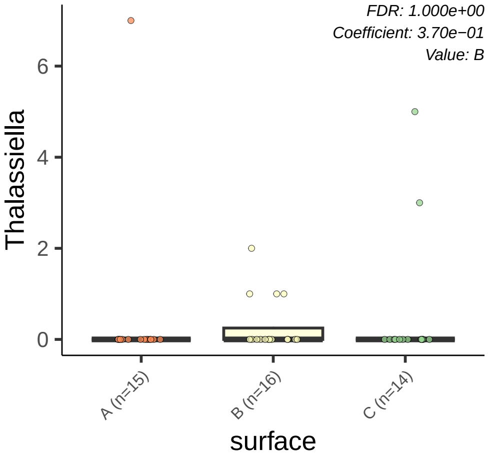

FDR: 1.000e+00
Coefficient: 3.70e−01
Value: B
Thalassiella
0
2
4
6
A (n=15)
B (n=16)
C (n=14)
surface

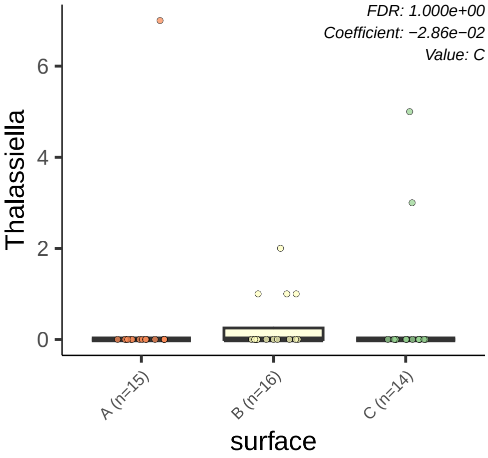
FDR: 1.000e+00
Coefficient: −2.86e−02
Value: C
Thalassiella
0
2
4
6
A (n=15)
B (n=16)
C (n=14)
surface

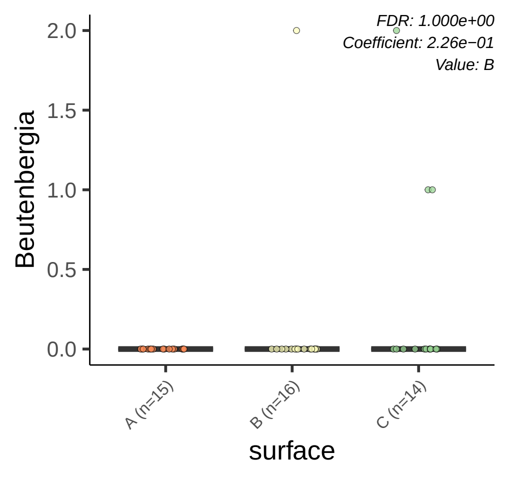
FDR: 1.000e+00
Coefficient: 2.26e−01
Value: B
Beutenbergia
2.0
1.5
1.0
0.5
0.0
A (n=15)
B (n=16)
C (n=14)
surface

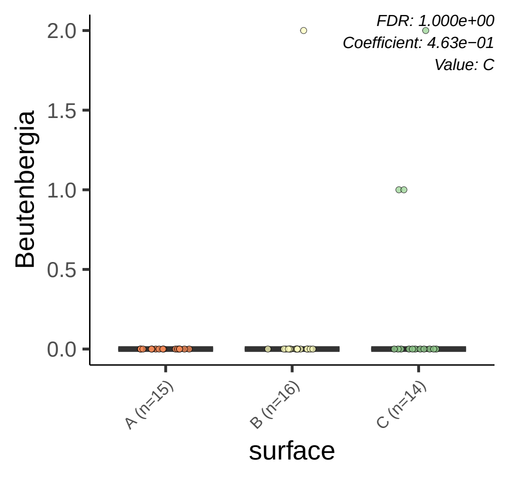

FDR: 1.000e+00
Coefficient: 4.63e−01
Value: C
Beutenbergia
2.0
1.5
1.0
0.5
0.0
A (n=15)
B (n=16)
C (n=14)
surface

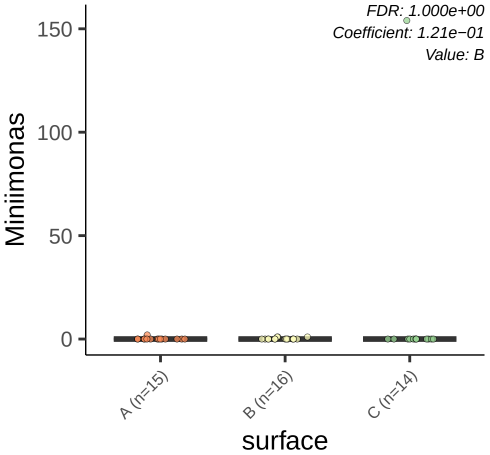
FDR: 1.000e+00
Coefficient: 1.21e−01
Value: B
Miniimonas
150
100
50
0
A (n=15)
B (n=16)
C (n=14)
surface

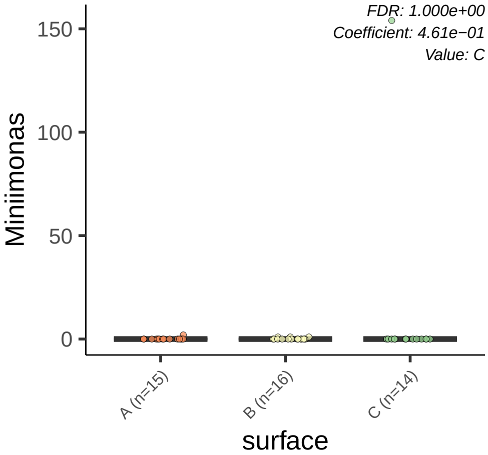

FDR: 1.000e+00
Coefficient: 4.61e−01
Value: C
Miniimonas
150
100
50
0
A (n=15)
B (n=16)
C (n=14)
surface

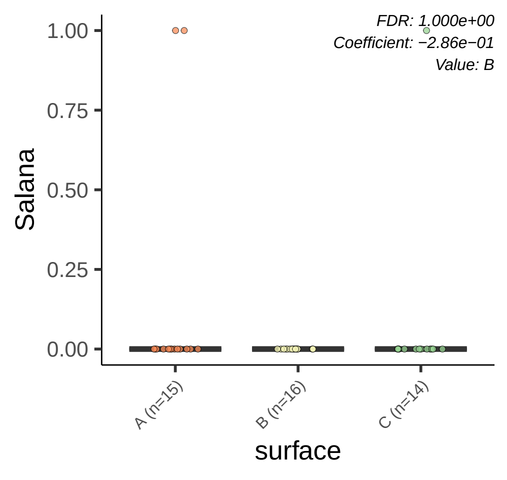
FDR: 1.000e+00
Coefficient: −2.86e−01
Value: B
Salana
1.00
0.75
0.50
0.25
0.00
A (n=15)
B (n=16)
C (n=14)
surface

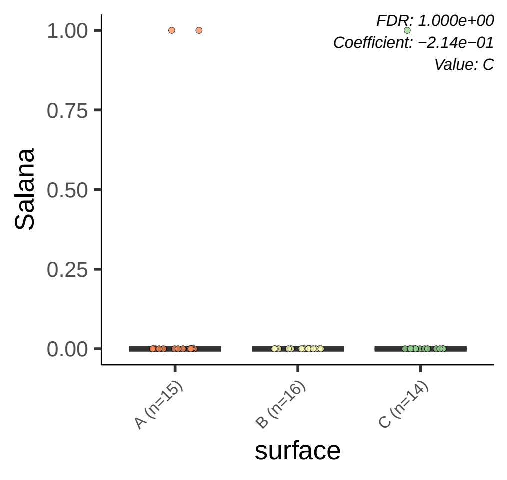
FDR: 1.000e+00
Coefficient: −2.14e−01
Value: C
Salana
1.00
0.75
0.50
0.25
0.00
A (n=15)
B (n=16)
C (n=14)
surface

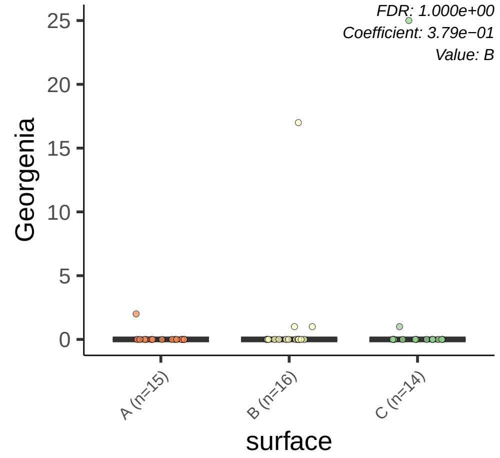

FDR: 1.000e+00
Coefficient: 3.79e−01
Value: B
Georgenia
25
20
15
10
5
0
A (n=15)
B (n=16)
C (n=14)
surface

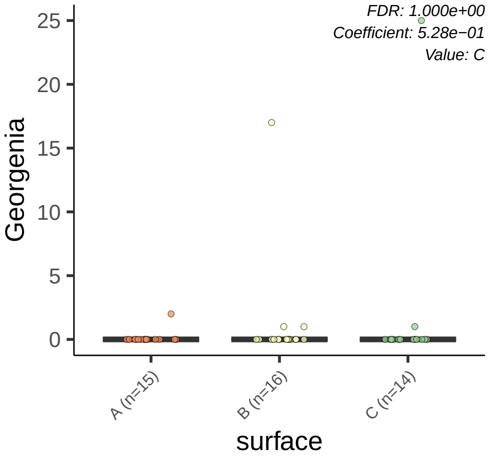

FDR: 1.000e+00
Coefficient: 5.28e−01
Value: C
Georgenia
25
20
15
10
5
0
A (n=15)
B (n=16)
C (n=14)
surface

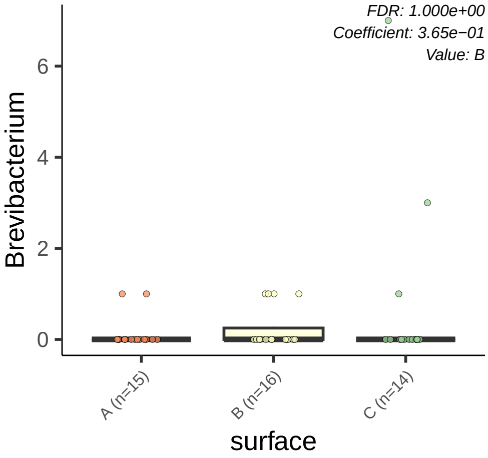

FDR: 1.000e+00
Coefficient: 3.65e−01
Value: B
Brevibacterium
0
2
4
6
A (n=15)
B (n=16)
C (n=14)
surface

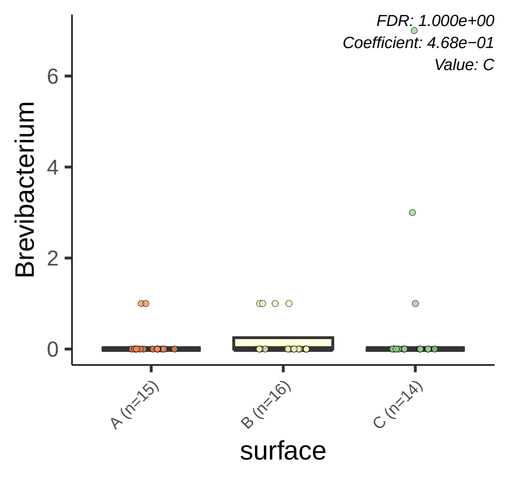
FDR: 1.000e+00
Coefficient: 4.68e−01
Value: C
Brevibacterium
0
2
4
6
A (n=15)
B (n=16)
C (n=14)
surface

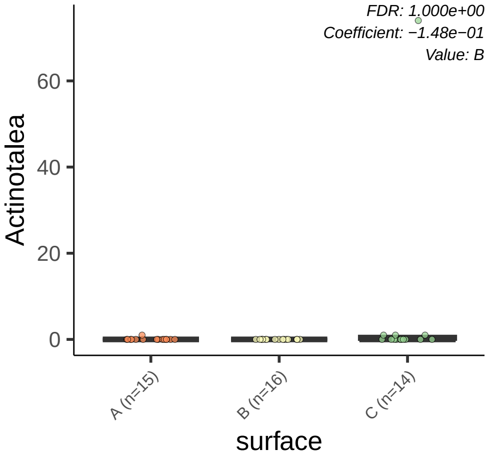
FDR: 1.000e+00
Coefficient: −1.48e−01
Value: B
Actinotalea
60
40
20
0
A (n=15)
B (n=16)
C (n=14)
surface

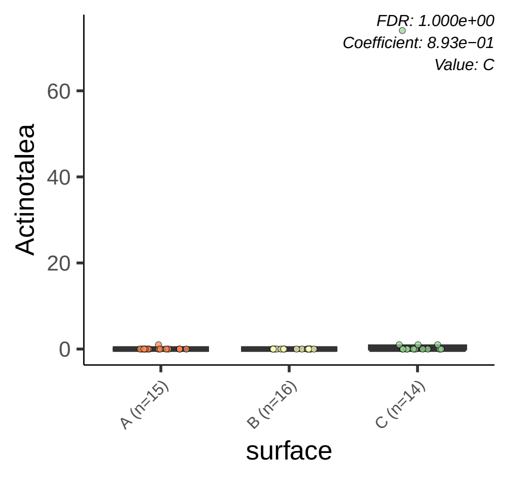
FDR: 1.000e+00
Coefficient: 8.93e−01
Value: C
60
40
20
0
Actinotalea
A (n=15)
B (n=16)
C (n=14)
surface

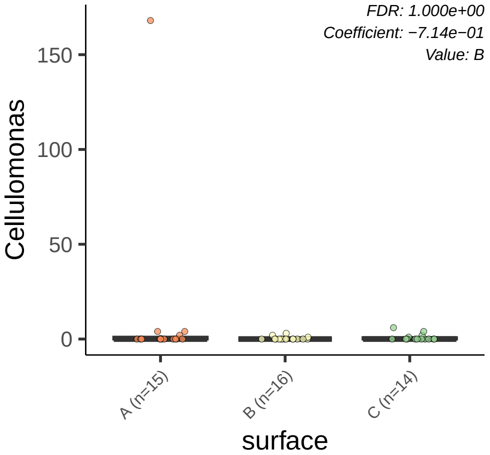
FDR: 1.000e+00
Coefficient: −7.14e−01
Value: B
Cellulomonas
150
100
50
0
A (n=15)
B (n=16)
C (n=14)
surface

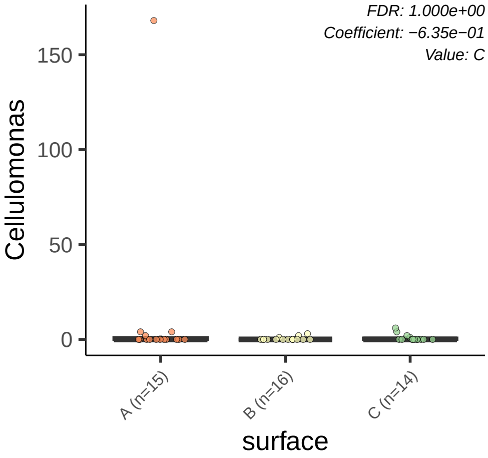
FDR: 1.000e+00
Coefficient: −6.35e−01
Value: C
Cellulomonas
150
100
50
0
A (n=15)
B (n=16)
C (n=14)
surface

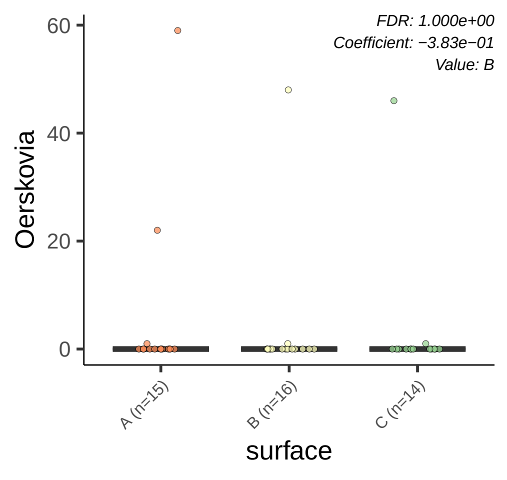
FDR: 1.000e+00
Coefficient: −3.83e−01
Value: B
Oerskovia
60
40
20
0
A (n=15)
B (n=16)
C (n=14)
surface

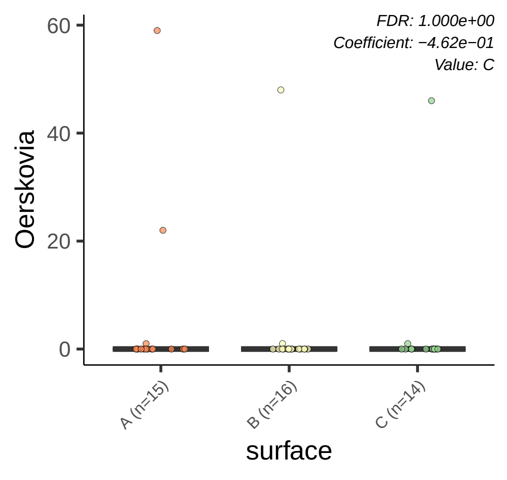
FDR: 1.000e+00
Coefficient: −4.62e−01
Value: C
Oerskovia
60
40
20
0
A (n=15)
B (n=16)
C (n=14)
surface

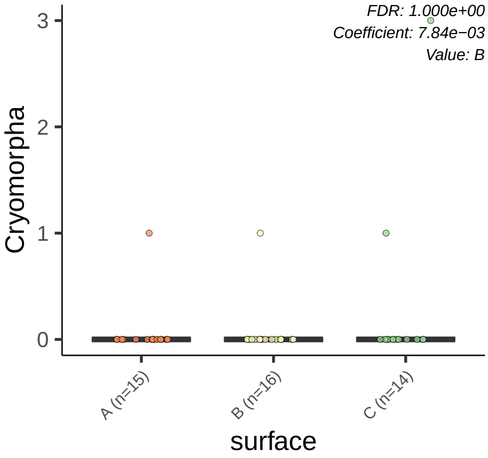
FDR: 1.000e+00
Coefficient: 7.84e−03
Value: B
Cryomorpha
0
1
2
3
A (n=15)
B (n=16)
C (n=14)
surface

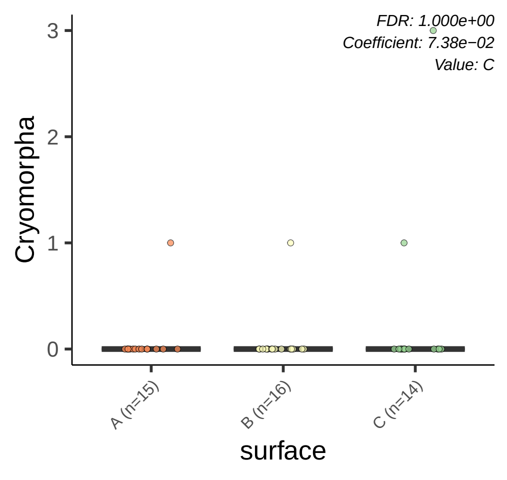
FDR: 1.000e+00
Coefficient: 7.38e−02
Value: C
Cryomorpha
3
2
1
0
A (n=15)
B (n=16)
C (n=14)
surface

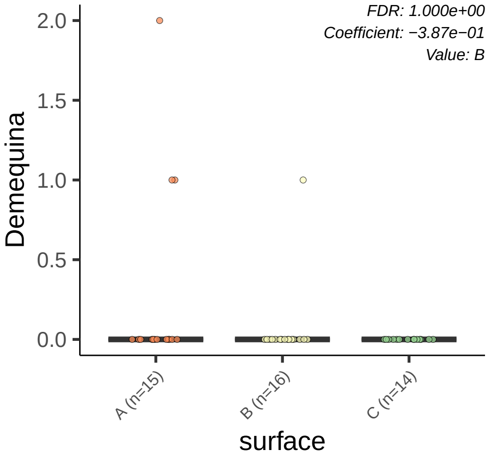
FDR: 1.000e+00
Coefficient: −3.87e−01
Value: B
Demequina
2.0
1.5
1.0
0.5
0.0
A (n=15)
B (n=16)
C (n=14)
surface

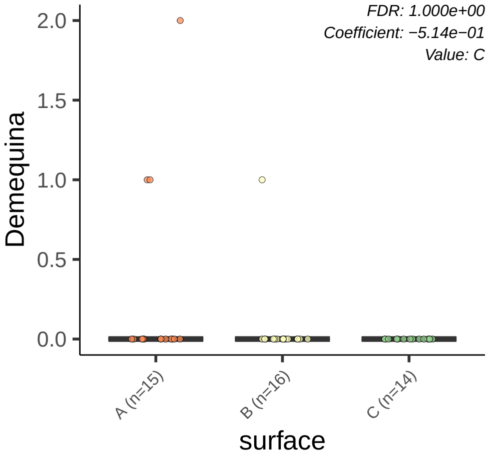

FDR: 1.000e+00
Coefficient: −5.14e−01
Value: C
Demequina
2.0
1.5
1.0
0.5
0.0
A (n=15)
B (n=16)
C (n=14)
surface

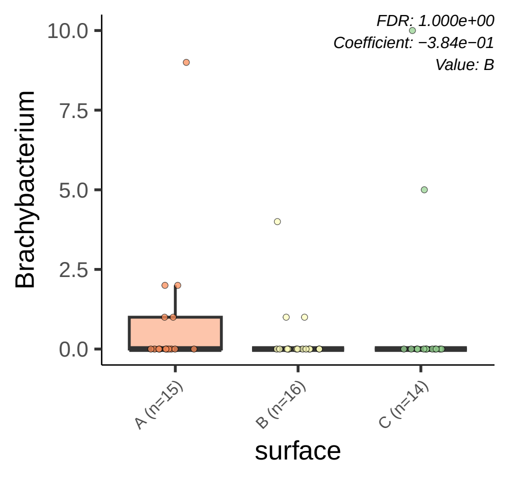
FDR: 1.000e+00
Coefficient: −3.84e−01
Value: B
Brachybacterium
10.0
7.5
5.0
2.5
0.0
A (n=15)
B (n=16)
C (n=14)
surface

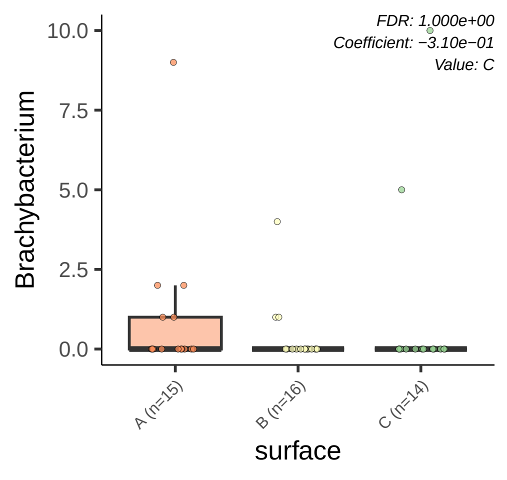

FDR: 1.000e+00
Coefficient: −3.10e−01
Value: C
10.0
7.5
5.0
2.5
0.0
Brachybacterium
A (n=15)
B (n=16)
C (n=14)
surface

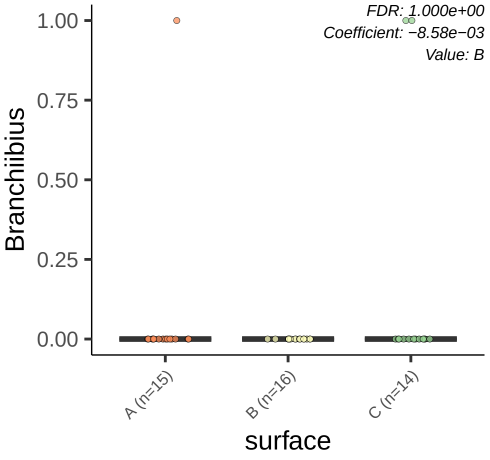

FDR: 1.000e+00
Coefficient: −8.58e−03
Value: B
Branchiibius
1.00
0.75
0.50
0.25
0.00
A (n=15)
B (n=16)
C (n=14)
surface

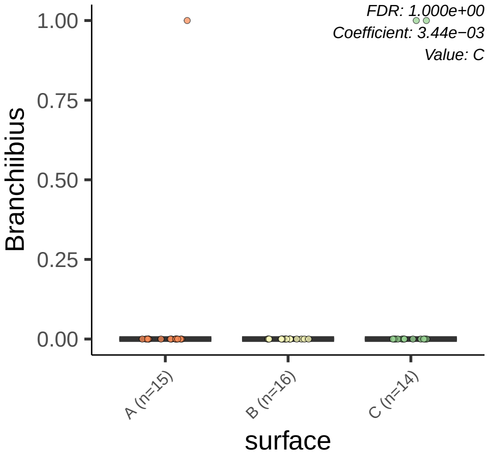
FDR: 1.000e+00
Coefficient: 3.44e−03
Value: C
Branchiibius
1.00
0.75
0.50
0.25
0.00
A (n=15)
B (n=16)
C (n=14)
surface

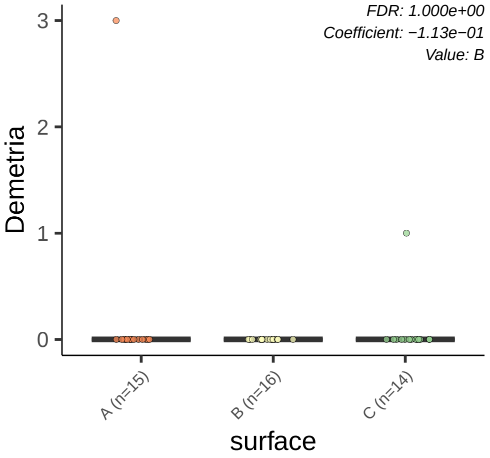
FDR: 1.000e+00
Coefficient: −1.13e−01
Value: B
Demetria
3
2
1
0
A (n=15)
B (n=16)
C (n=14)
surface

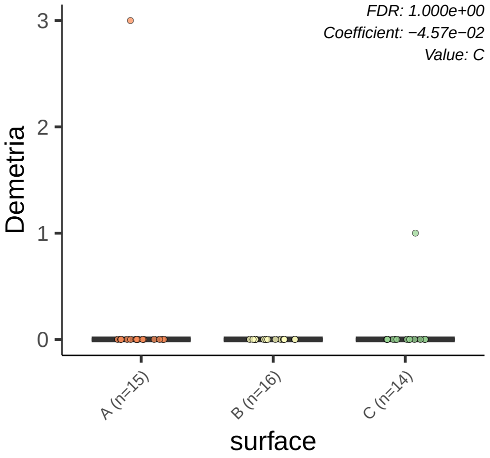
FDR: 1.000e+00
Coefficient: −4.57e−02
Value: C
Demetria
3
2
1
0
A (n=15)
B (n=16)
C (n=14)
surface

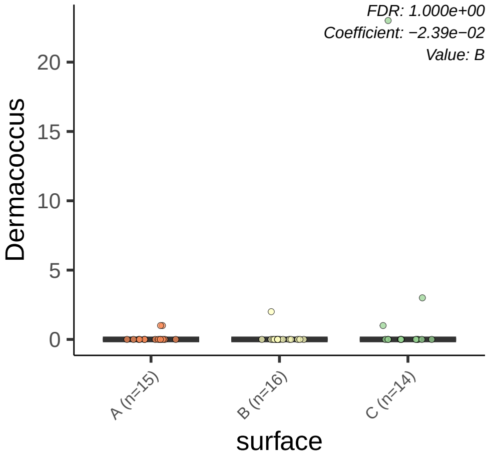

FDR: 1.000e+00
Coefficient: −2.39e−02
Value: B
Dermacoccus
20
15
10
5
0
A (n=15)
B (n=16)
C (n=14)
surface

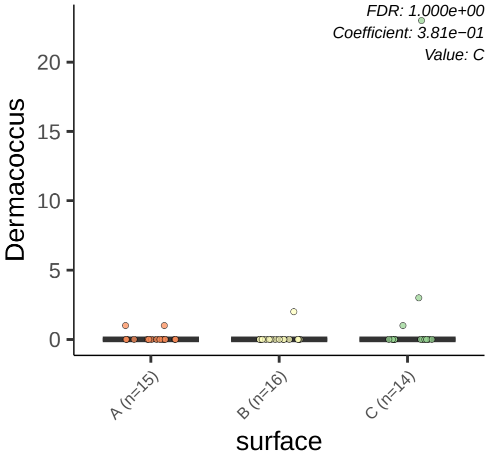
FDR: 1.000e+00
Coefficient: 3.81e−01
Value: C
Dermacoccus
20
15
10
5
0
A (n=15)
B (n=16)
C (n=14)
surface

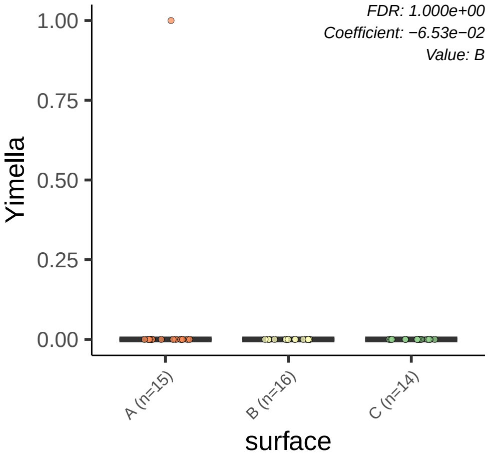
FDR: 1.000e+00
Coefficient: −6.53e−02
Value: B
1.00
0.75
0.50
0.25
0.00
Yimella
A (n=15)
B (n=16)
C (n=14)
surface

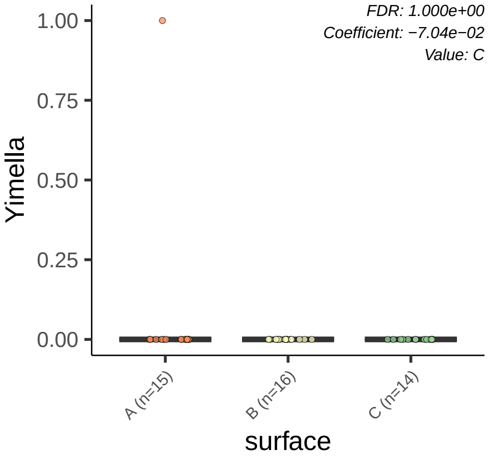

FDR: 1.000e+00
Coefficient: −7.04e−02
Value: C
Yimella
1.00
0.75
0.50
0.25
0.00
A (n=15)
B (n=16)
C (n=14)
surface

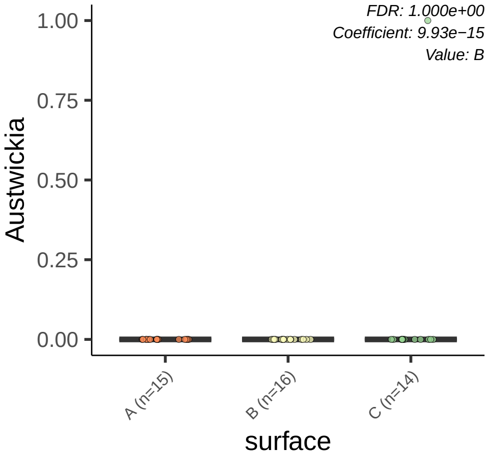
FDR: 1.000e+00
Coefficient: 9.93e−15
Value: B
Austwickia
1.00
0.75
0.50
0.25
0.00
A (n=15)
B (n=16)
C (n=14)
surface

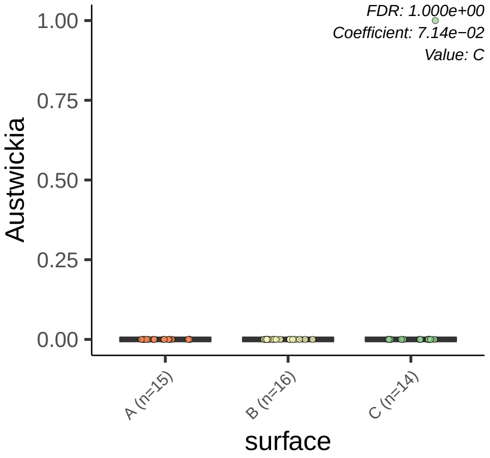

FDR: 1.000e+00
Coefficient: 7.14e−02
Value: C
Austwickia
1.00
0.75
0.50
0.25
0.00
A (n=15)
B (n=16)
C (n=14)
surface

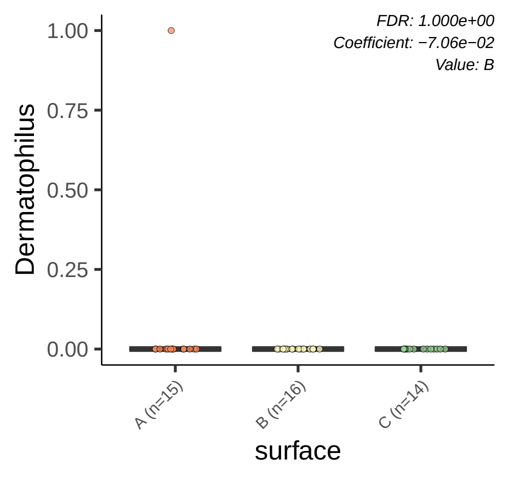

FDR: 1.000e+00
Coefficient: −7.06e−02
Value: B
Dermatophilus
1.00
0.75
0.50
0.25
0.00
A (n=15)
B (n=16)
C (n=14)
surface

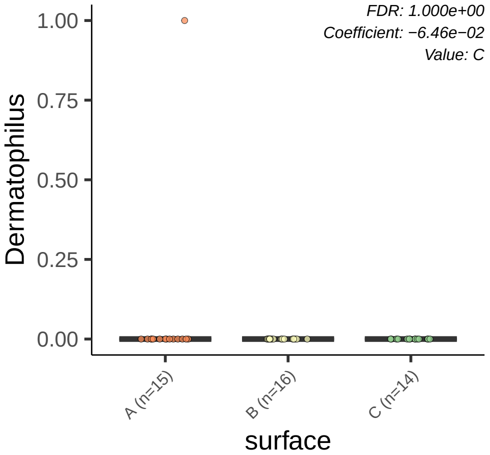
FDR: 1.000e+00
Coefficient: −6.46e−02
Value: C
Dermatophilus
1.00
0.75
0.50
0.25
0.00
A (n=15)
B (n=16)
C (n=14)
surface

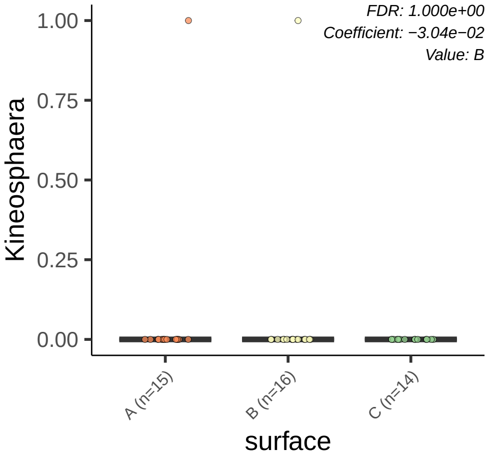
FDR: 1.000e+00
Coefficient: −3.04e−02
Value: B
Kineosphaera
1.00
0.75
0.50
0.25
0.00
A (n=15)
B (n=16)
C (n=14)
surface

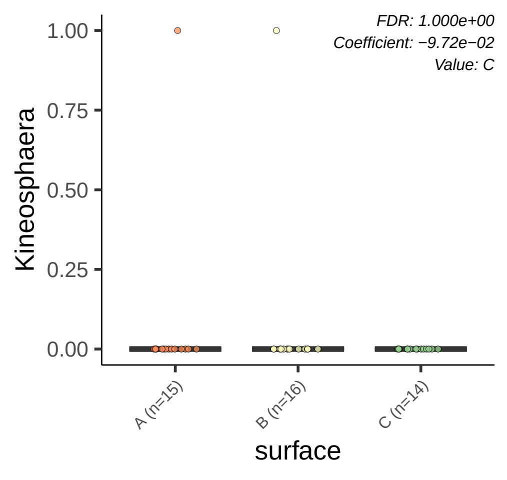

FDR: 1.000e+00
Coefficient: −9.72e−02
Value: C
Kineosphaera
1.00
0.75
0.50
0.25
0.00
A (n=15)
B (n=16)
C (n=14)
surface

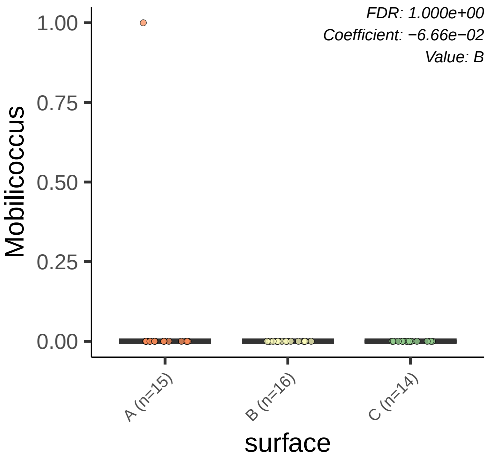
FDR: 1.000e+00
Coefficient: −6.66e−02
Value: B
Mobilicoccus
1.00
0.75
0.50
0.25
0.00
A (n=15)
B (n=16)
C (n=14)
surface

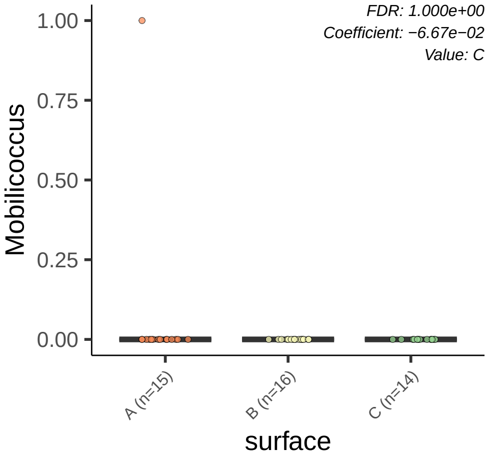
FDR: 1.000e+00
Coefficient: −6.67e−02
Value: C
Mobilicoccus
1.00
0.75
0.50
0.25
0.00
A (n=15)
B (n=16)
C (n=14)
surface

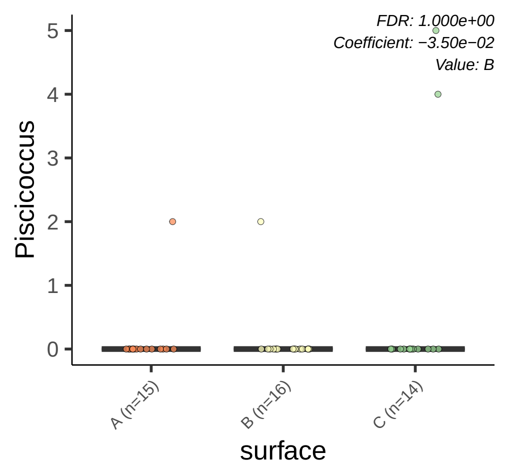
FDR: 1.000e+00
Coefficient: −3.50e−02
Value: B
Piscicoccus
0
1
2
3
4
5
A (n=15)
B (n=16)
C (n=14)
surface

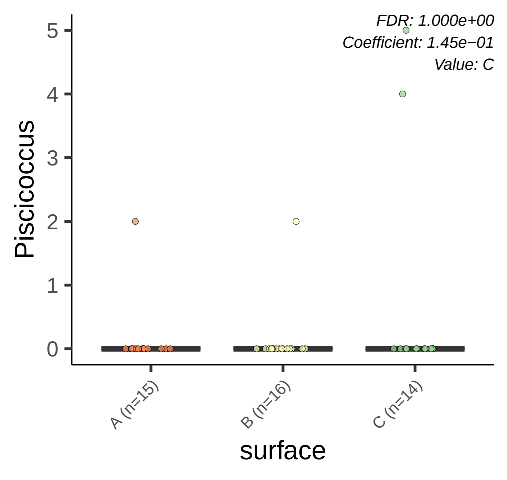
FDR: 1.000e+00
Coefficient: 1.45e−01
Value: C
Piscicoccus
0
1
2
3
4
5
A (n=15)
B (n=16)
C (n=14)
surface

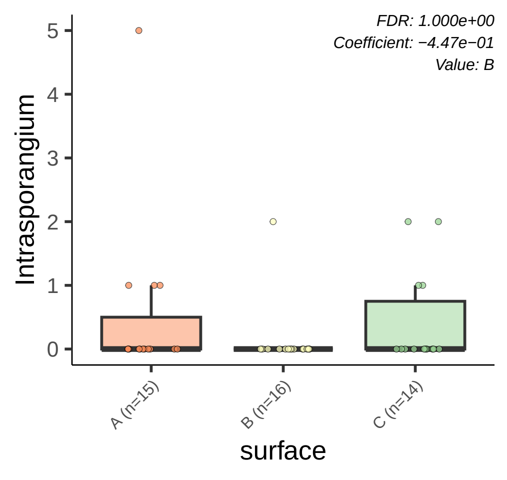
FDR: 1.000e+00
Coefficient: −4.47e−01
Value: B
Intrasporangium
5
4
3
2
1
0
A (n=15)
B (n=16)
C (n=14)
surface

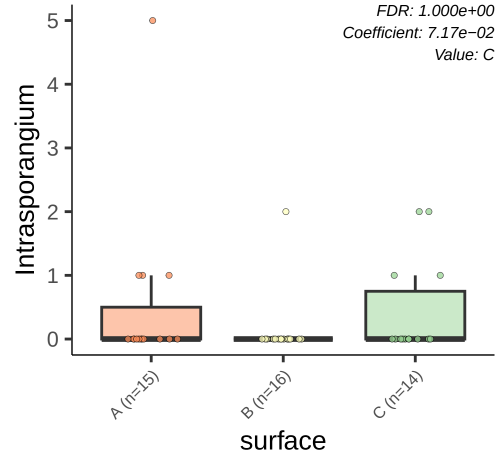
FDR: 1.000e+00
Coefficient: 7.17e−02
Value: C
Intrasporangium
5
4
3
2
1
0
A (n=15)
B (n=16)
C (n=14)
surface

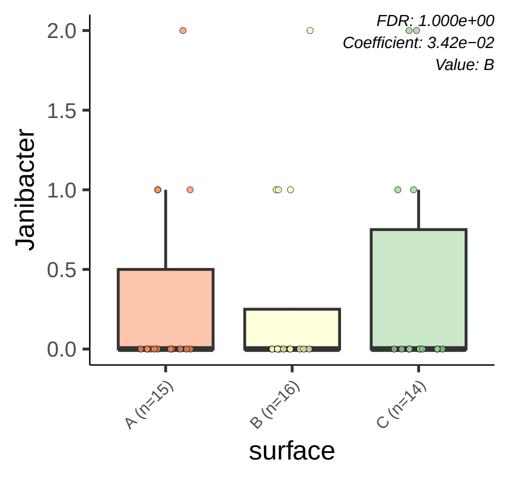
FDR: 1.000e+00
Coefficient: 3.42e−02
Value: B
2.0
1.5
1.0
0.5
0.0
Janibacter
A (n=15)
B (n=16)
C (n=14)
surface

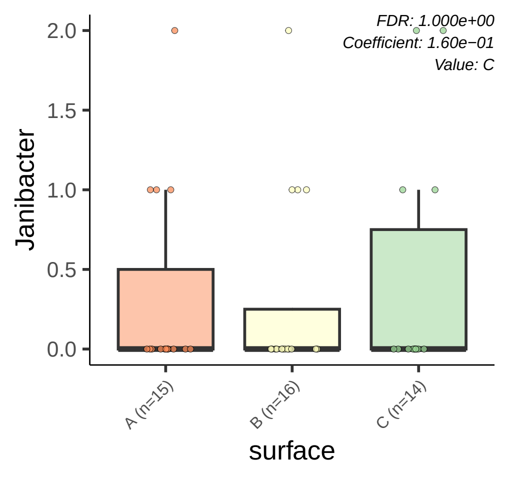

FDR: 1.000e+00
Coefficient: 1.60e−01
Value: C
Janibacter
2.0
1.5
1.0
0.5
0.0
A (n=15)
B (n=16)
C (n=14)
surface

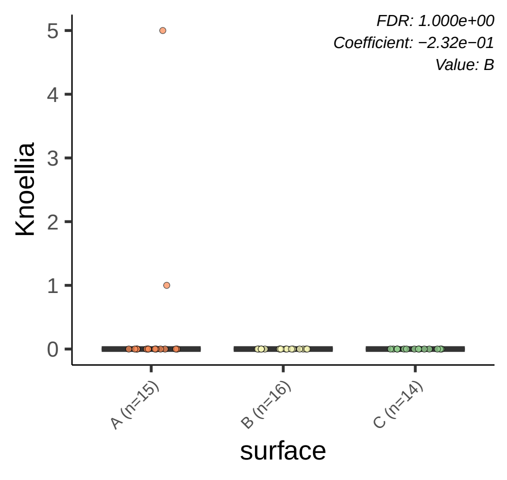
FDR: 1.000e+00
Coefficient: −2.32e−01
Value: B
Knoellia
5
4
3
2
1
0
A (n=15)
B (n=16)
C (n=14)
surface

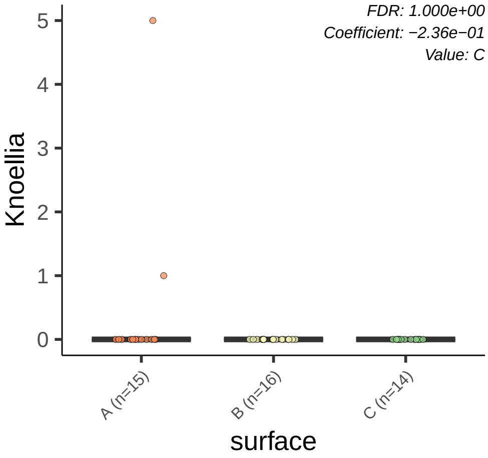
FDR: 1.000e+00
Coefficient: −2.36e−01
Value: C
Knoellia
0
1
2
3
4
5
A (n=15)
B (n=16)
C (n=14)
surface

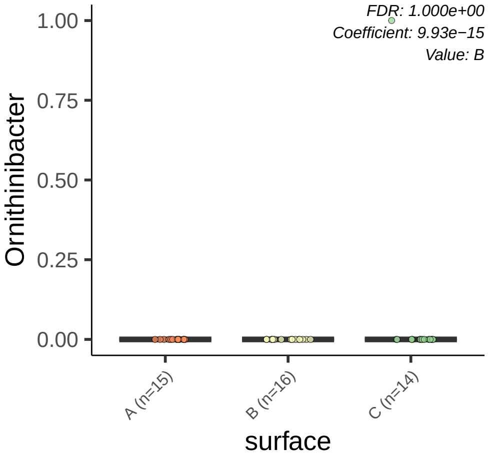
FDR: 1.000e+00
Coefficient: 9.93e−15
Value: B
Ornithinibacter
1.00
0.75
0.50
0.25
0.00
A (n=15)
B (n=16)
C (n=14)
surface

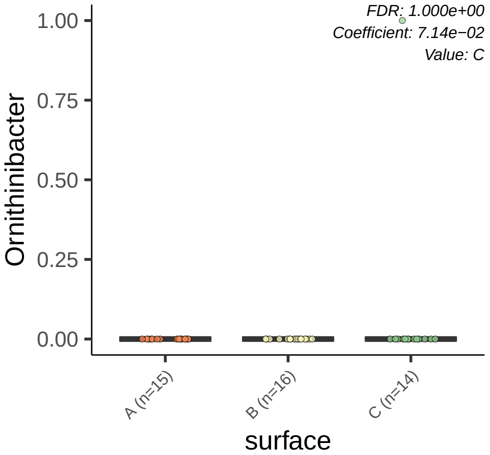
FDR: 1.000e+00
Coefficient: 7.14e−02
Value: C
Ornithinibacter
1.00
0.75
0.50
0.25
0.00
A (n=15)
B (n=16)
C (n=14)
surface

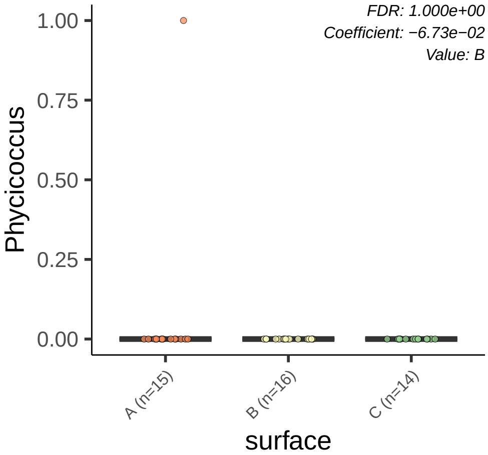
FDR: 1.000e+00
Coefficient: −6.73e−02
Value: B
Phycicoccus
1.00
0.75
0.50
0.25
0.00
A (n=15)
B (n=16)
C (n=14)
surface

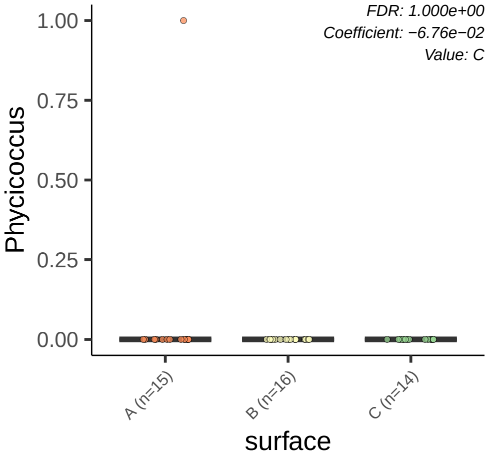

FDR: 1.000e+00
Coefficient: −6.76e−02
Value: C
Phycicoccus
1.00
0.75
0.50
0.25
0.00
A (n=15)
B (n=16)
C (n=14)
surface

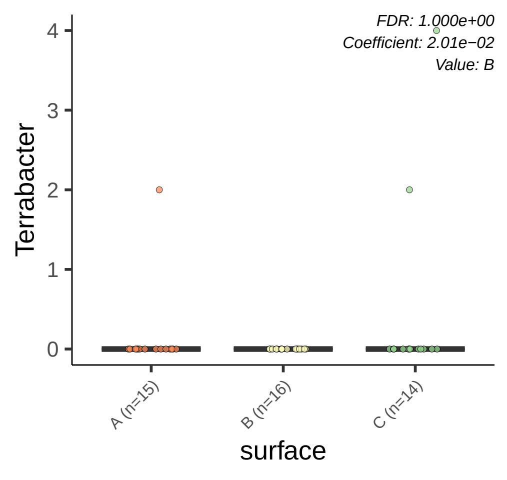
FDR: 1.000e+00
Coefficient: 2.01e−02
Value: B
Terrabacter
0
1
2
3
4
A (n=15)
B (n=16)
C (n=14)
surface

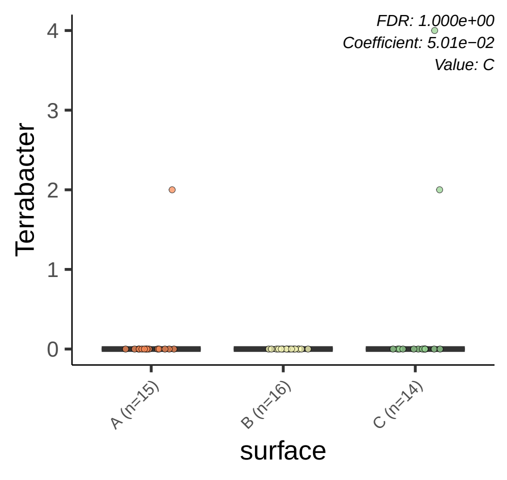
FDR: 1.000e+00
Coefficient: 5.01e−02
Value: C
Terrabacter
0
1
2
3
4
A (n=15)
B (n=16)
C (n=14)
surface

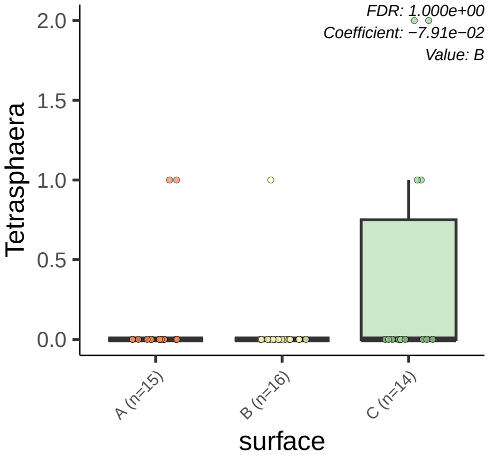

FDR: 1.000e+00
Coefficient: −7.91e−02
Value: B
Tetrasphaera
2.0
1.5
1.0
0.5
0.0
A (n=15)
B (n=16)
C (n=14)
surface

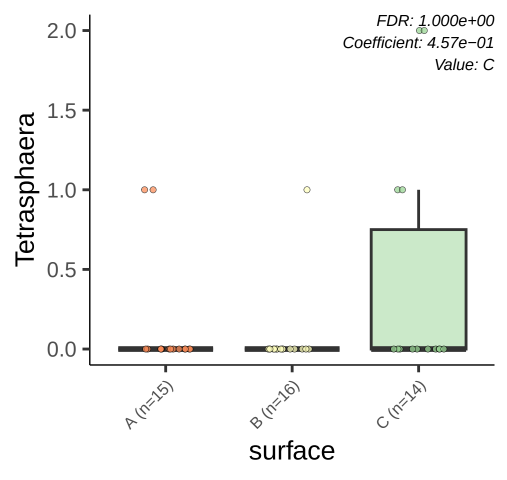

FDR: 1.000e+00
Coefficient: 4.57e−01
Value: C
Tetrasphaera
2.0
1.5
1.0
0.5
0.0
A (n=15)
B (n=16)
C (n=14)
surface

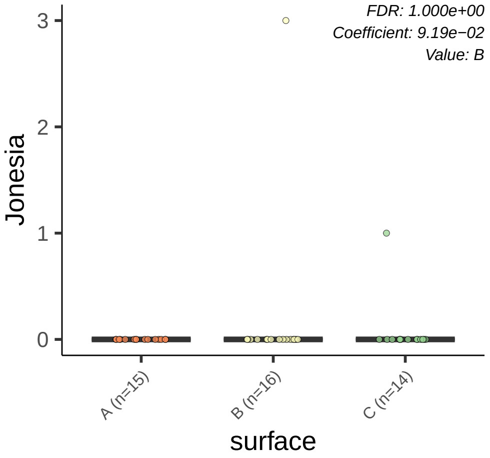
FDR: 1.000e+00
Coefficient: 9.19e−02
Value: B
Jonesia
3
2
1
0
A (n=15)
B (n=16)
C (n=14)
surface

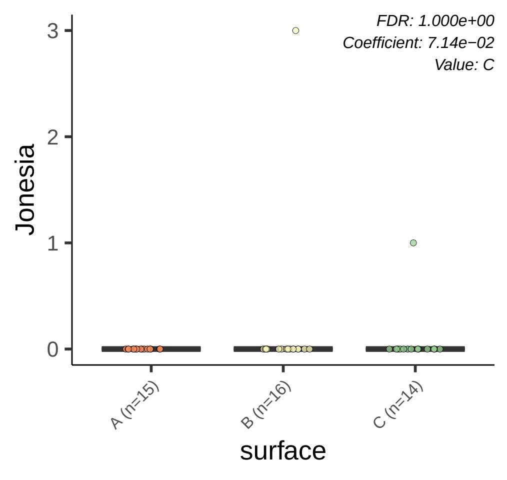
FDR: 1.000e+00
Coefficient: 7.14e−02
Value: C
Jonesia
3
2
1
0
A (n=15)
B (n=16)
C (n=14)
surface

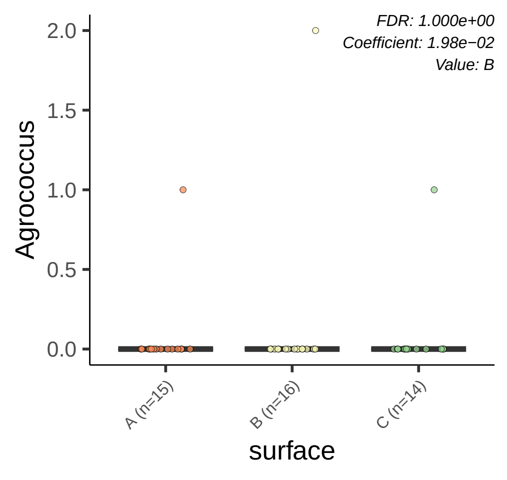

FDR: 1.000e+00
Coefficient: 1.98e−02
Value: B
2.0
1.5
1.0
0.5
0.0
Agrococcus
A (n=15)
B (n=16)
C (n=14)
surface

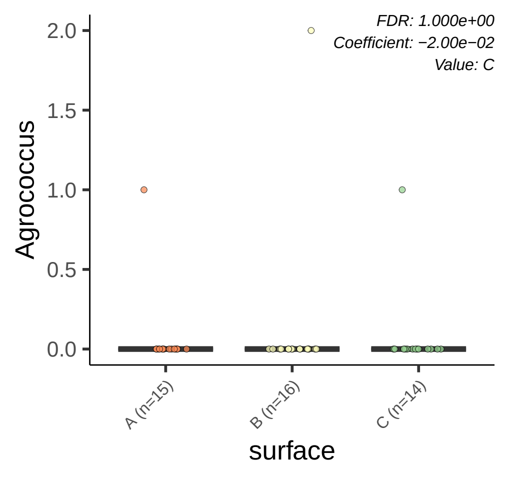
FDR: 1.000e+00
Coefficient: −2.00e−02
Value: C
Agrococcus
2.0
1.5
1.0
0.5
0.0
A (n=15)
B (n=16)
C (n=14)
surface

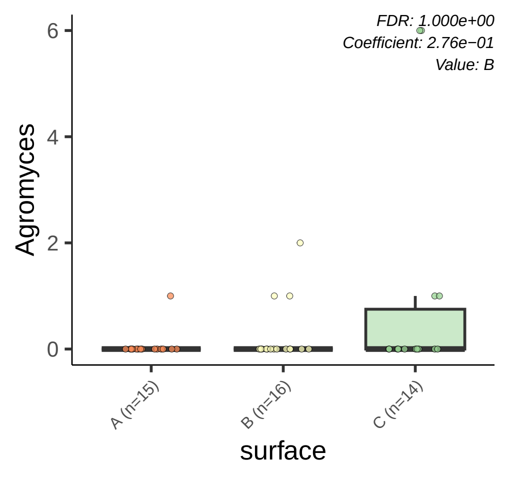
FDR: 1.000e+00
Coefficient: 2.76e−01
Value: B
Agromyces
6
4
2
0
A (n=15)
B (n=16)
C (n=14)
surface

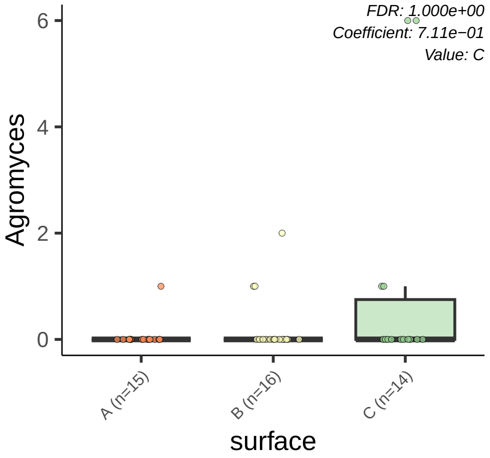
FDR: 1.000e+00
Coefficient: 7.11e−01
Value: C
Agromyces
6
4
2
0
A (n=15)
B (n=16)
C (n=14)
surface

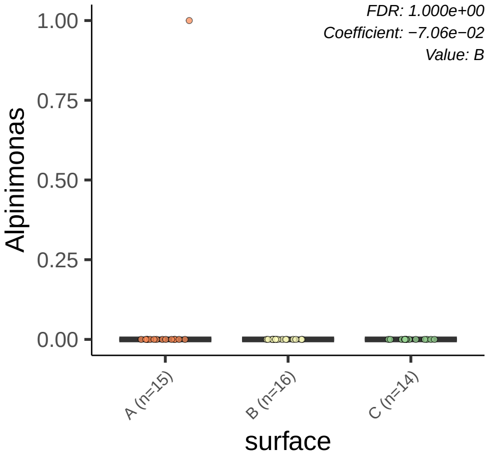
FDR: 1.000e+00
Coefficient: −7.06e−02
Value: B
Alpinimonas
1.00
0.75
0.50
0.25
0.00
A (n=15)
B (n=16)
C (n=14)
surface

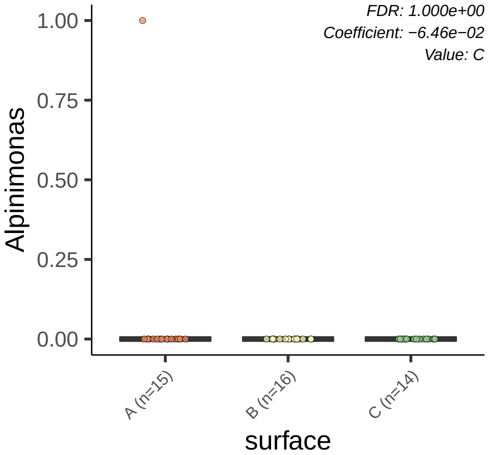
FDR: 1.000e+00
Coefficient: −6.46e−02
Value: C
Alpinimonas
1.00
0.75
0.50
0.25
0.00
A (n=15)
B (n=16)
C (n=14)
surface

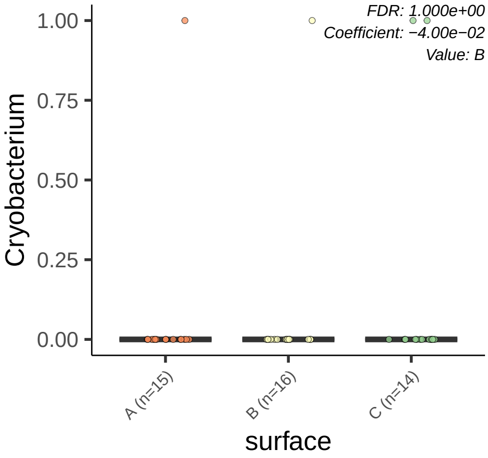
FDR: 1.000e+00
Coefficient: −4.00e−02
Value: B
Cryobacterium
1.00
0.75
0.50
0.25
0.00
A (n=15)
B (n=16)
C (n=14)
surface

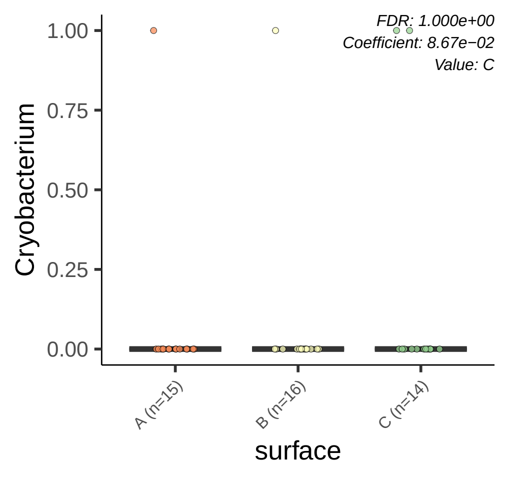

FDR: 1.000e+00
Coefficient: 8.67e−02
Value: C
Cryobacterium
1.00
0.75
0.50
0.25
0.00
A (n=15)
B (n=16)
C (n=14)
surface

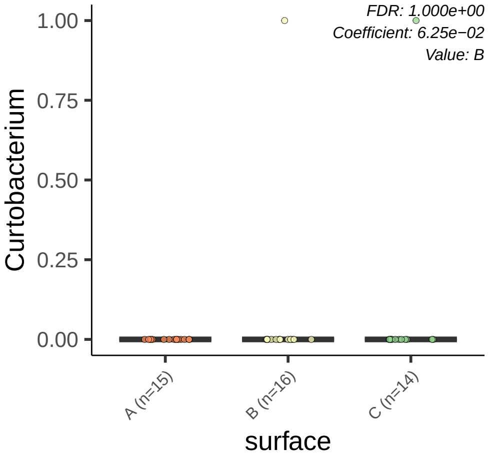

FDR: 1.000e+00
Coefficient: 6.25e−02
Value: B
1.00
0.75
0.50
0.25
0.00
Curtobacterium
A (n=15)
B (n=16)
C (n=14)
surface

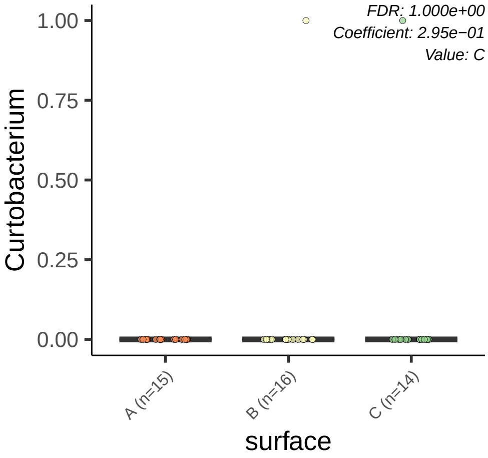

FDR: 1.000e+00
Coefficient: 2.95e−01
Value: C
Curtobacterium
1.00
0.75
0.50
0.25
0.00
A (n=15)
B (n=16)
C (n=14)
surface

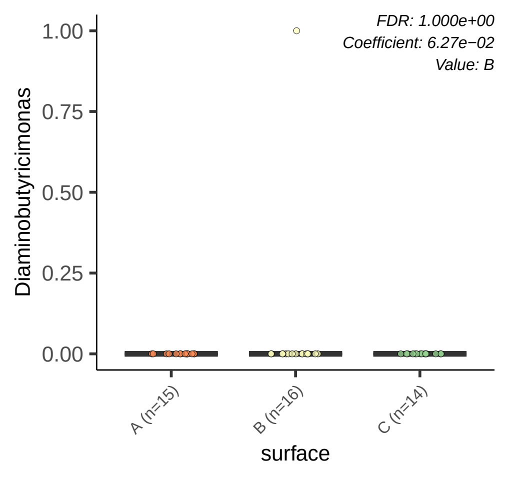

FDR: 1.000e+00
Coefficient: 6.27e−02
Value: B
Diaminobutyricimonas
1.00
0.75
0.50
0.25
0.00
A (n=15)
B (n=16)
C (n=14)
surface

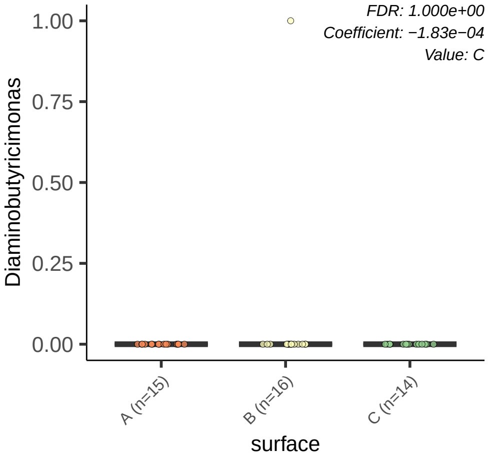

FDR: 1.000e+00
Coefficient: −1.83e−04
Value: C
Diaminobutyricimonas
1.00
0.75
0.50
0.25
0.00
A (n=15)
B (n=16)
C (n=14)
surface

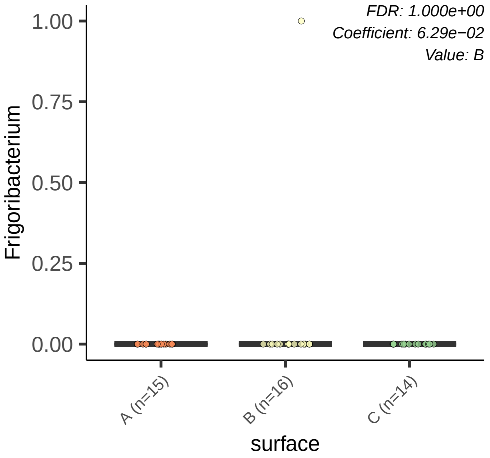

FDR: 1.000e+00
Coefficient: 6.29e−02
Value: B
Frigoribacterium
1.00
0.75
0.50
0.25
0.00
A (n=15)
B (n=16)
C (n=14)
surface

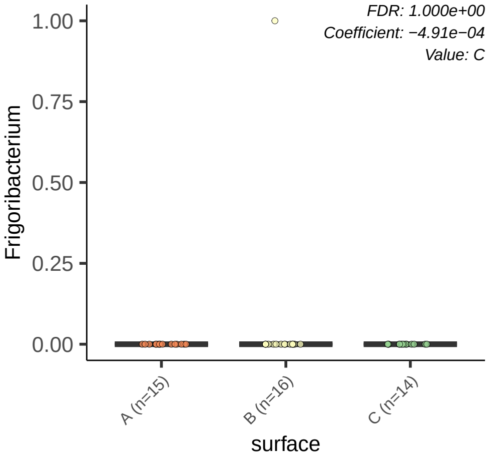

FDR: 1.000e+00
Coefficient: −4.91e−04
Value: C
Frigoribacterium
1.00
0.75
0.50
0.25
0.00
A (n=15)
B (n=16)
C (n=14)
surface

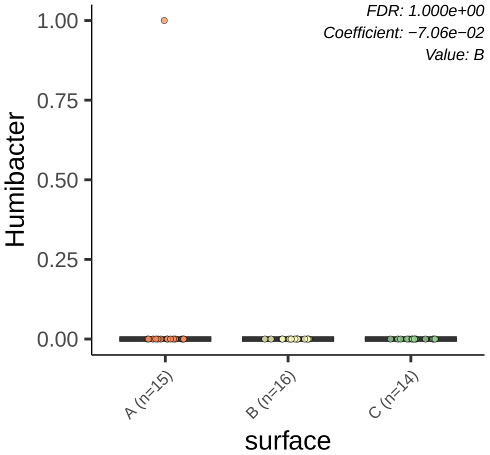
FDR: 1.000e+00
Coefficient: −7.06e−02
Value: B
Humibacter
1.00
0.75
0.50
0.25
0.00
A (n=15)
B (n=16)
C (n=14)
surface

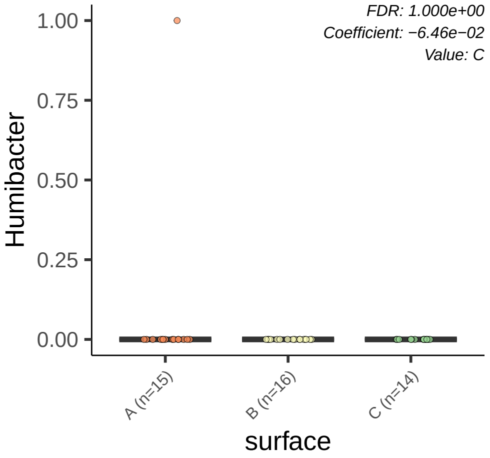
FDR: 1.000e+00
Coefficient: −6.46e−02
Value: C
Humibacter
1.00
0.75
0.50
0.25
0.00
A (n=15)
B (n=16)
C (n=14)
surface

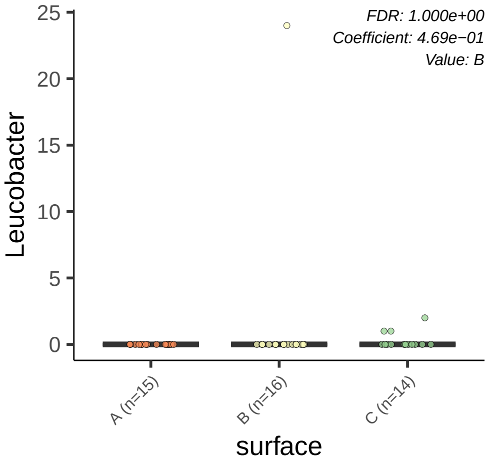
FDR: 1.000e+00
Coefficient: 4.69e−01
Value: B
Leucobacter
25
20
15
10
5
0
A (n=15)
B (n=16)
C (n=14)
surface

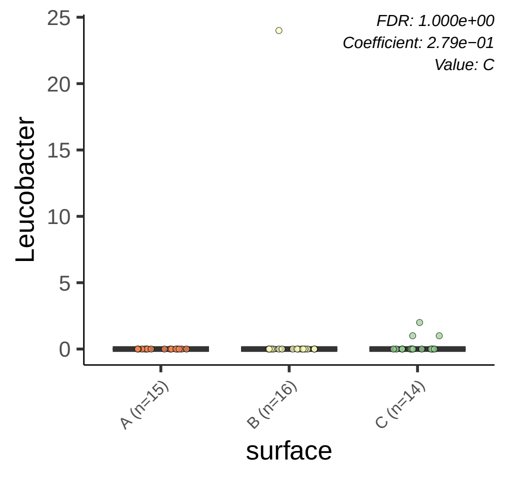
FDR: 1.000e+00
Coefficient: 2.79e−01
Value: C
Leucobacter
25
20
15
10
5
0
A (n=15)
B (n=16)
C (n=14)
surface

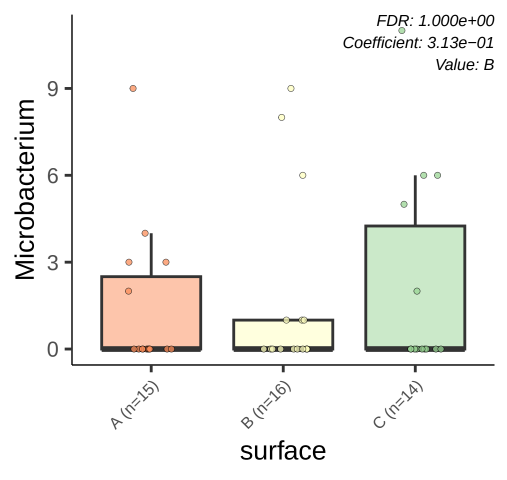
FDR: 1.000e+00
Coefficient: 3.13e−01
Value: B
Microbacterium
9
6
3
0
A (n=15)
B (n=16)
C (n=14)
surface

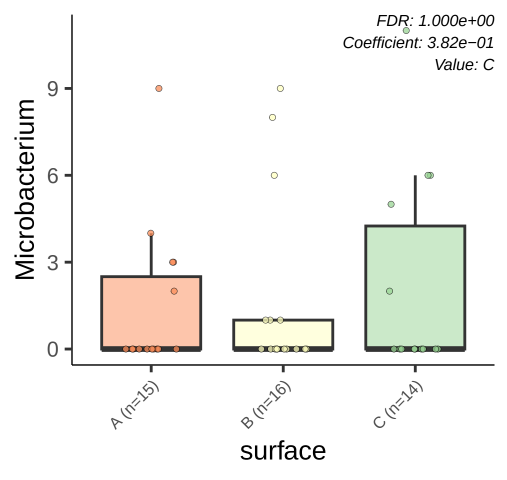

FDR: 1.000e+00
Coefficient: 3.82e−01
Value: C
Microbacterium
9
6
3
0
A (n=15)
B (n=16)
C (n=14)
surface

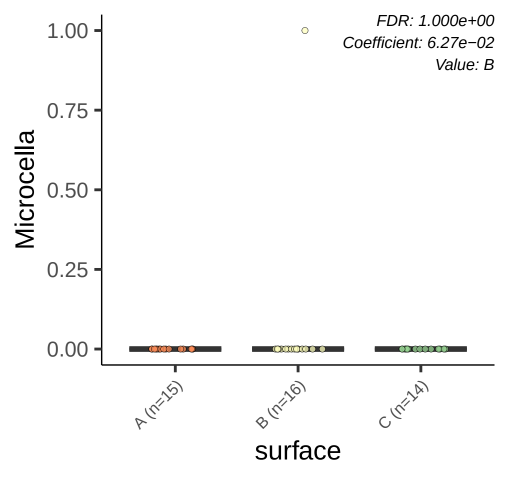
FDR: 1.000e+00
Coefficient: 6.27e−02
Value: B
Microcella
1.00
0.75
0.50
0.25
0.00
A (n=15)
B (n=16)
C (n=14)
surface

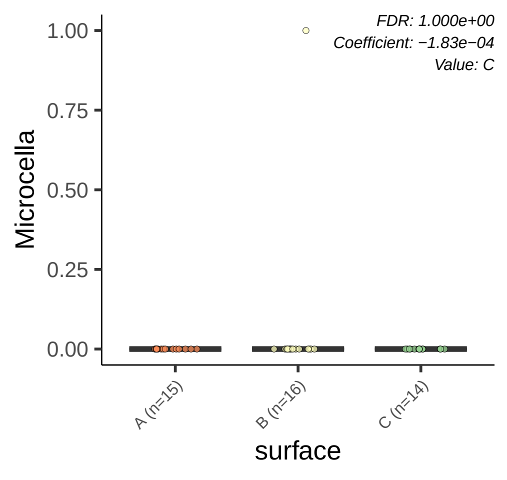

FDR: 1.000e+00
Coefficient: −1.83e−04
Value: C
Microcella
1.00
0.75
0.50
0.25
0.00
A (n=15)
B (n=16)
C (n=14)
surface

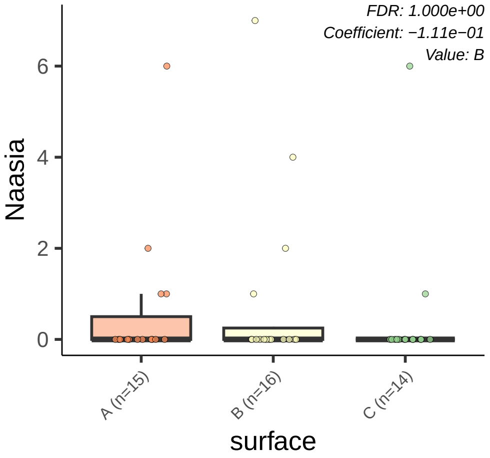

FDR: 1.000e+00
Coefficient: −1.11e−01
Value: B
Naasia
0
2
4
6
A (n=15)
B (n=16)
C (n=14)
surface

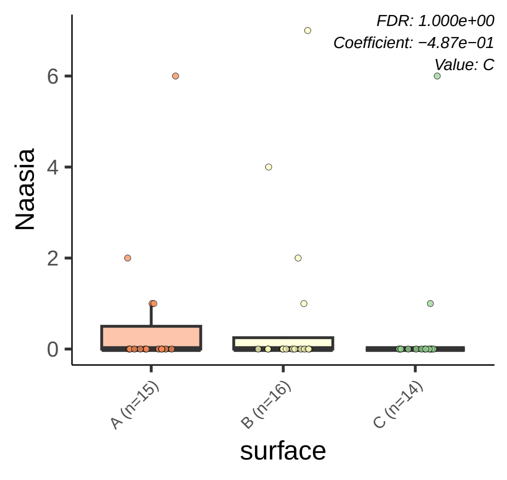

FDR: 1.000e+00
Coefficient: −4.87e−01
Value: C
Naasia
0
2
4
6
A (n=15)
B (n=16)
C (n=14)
surface

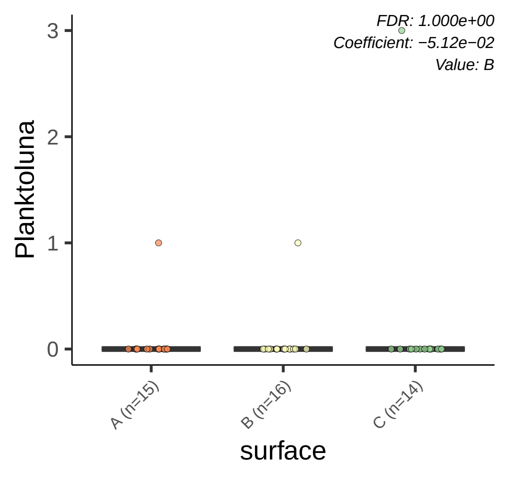
FDR: 1.000e+00
Coefficient: −5.12e−02
Value: B
Planktoluna
3
2
1
0
A (n=15)
B (n=16)
C (n=14)
surface

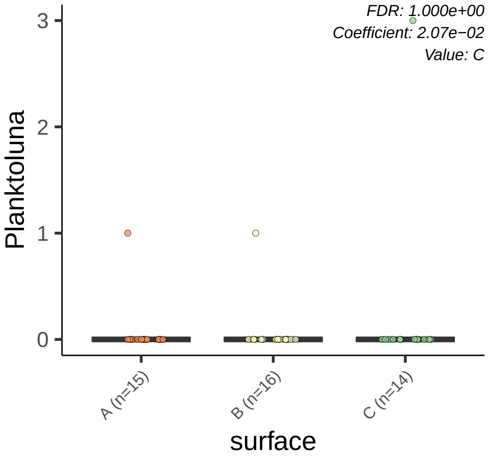

FDR: 1.000e+00
Coefficient: 2.07e−02
Value: C
Planktoluna
3
2
1
0
A (n=15)
B (n=16)
C (n=14)
surface

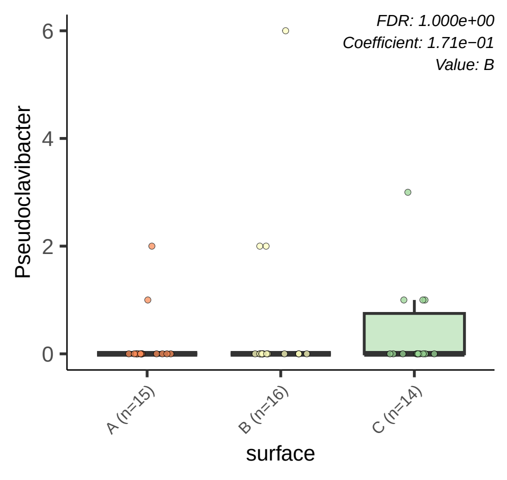
FDR: 1.000e+00
Coefficient: 1.71e−01
Value: B
Pseudoclavibacter
0
2
4
6
A (n=15)
B (n=16)
C (n=14)
surface

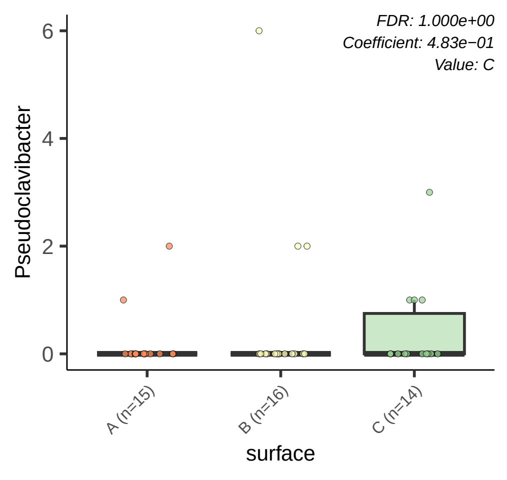
FDR: 1.000e+00
Coefficient: 4.83e−01
Value: C
Pseudoclavibacter
6
4
2
0
A (n=15)
B (n=16)
C (n=14)
surface

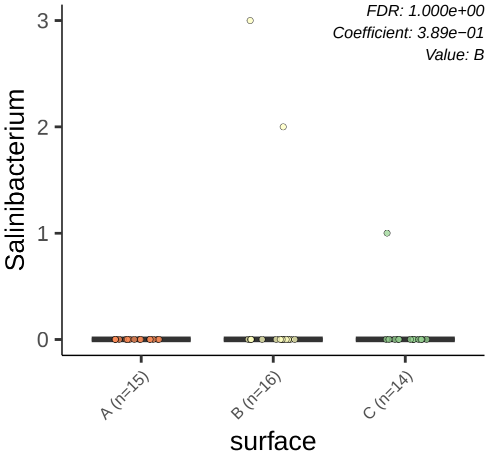

FDR: 1.000e+00
Coefficient: 3.89e−01
Value: B
Salinibacterium
3
2
1
0
A (n=15)
B (n=16)
C (n=14)
surface

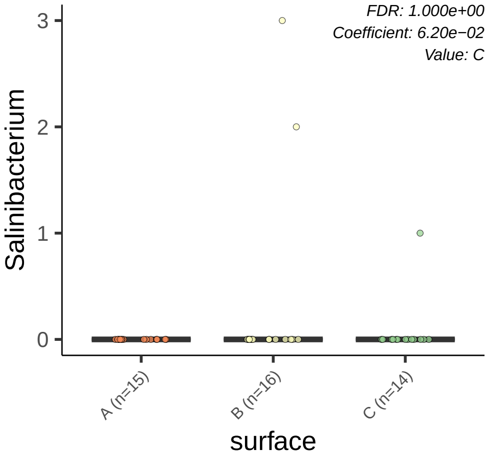
FDR: 1.000e+00
Coefficient: 6.20e−02
Value: C
Salinibacterium
3
2
1
0
A (n=15)
B (n=16)
C (n=14)
surface

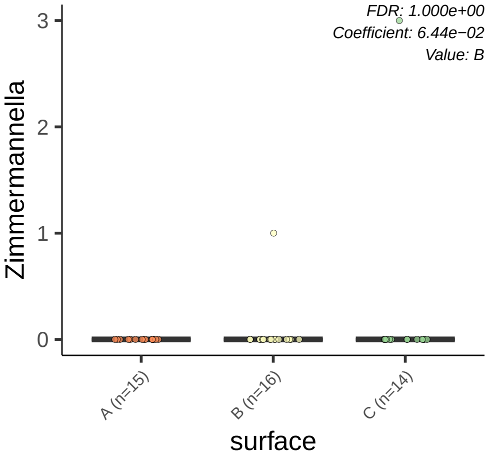
FDR: 1.000e+00
Coefficient: 6.44e−02
Value: B
Zimmermannella
3
2
1
0
A (n=15)
B (n=16)
C (n=14)
surface

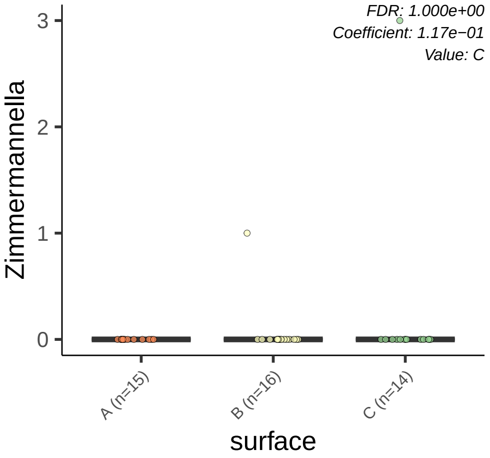

FDR: 1.000e+00
Coefficient: 1.17e−01
Value: C
Zimmermannella
0
1
2
3
A (n=15)
B (n=16)
C (n=14)
surface

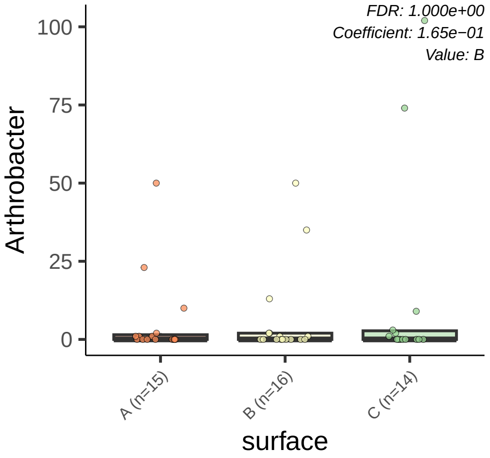
FDR: 1.000e+00
Coefficient: 1.65e−01
Value: B
Arthrobacter
100
75
50
25
0
A (n=15)
B (n=16)
C (n=14)
surface

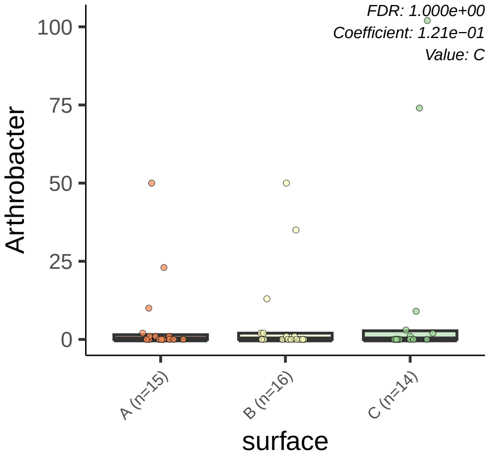
FDR: 1.000e+00
Coefficient: 1.21e−01
Value: C
Arthrobacter
100
75
50
25
0
A (n=15)
B (n=16)
C (n=14)
surface

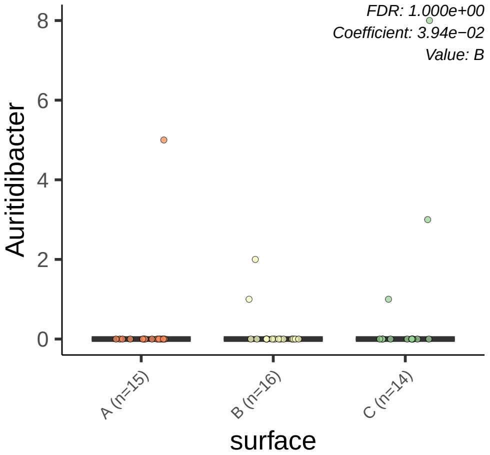

FDR: 1.000e+00
Coefficient: 3.94e−02
Value: B
Auritidibacter
0
2
4
6
8
A (n=15)
B (n=16)
C (n=14)
surface

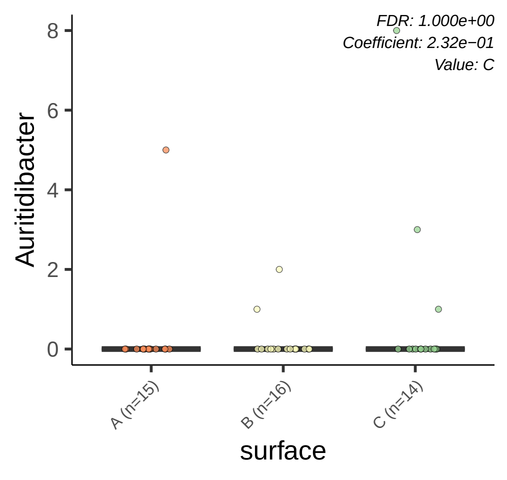

FDR: 1.000e+00
Coefficient: 2.32e−01
Value: C
Auritidibacter
8
6
4
2
0
A (n=15)
B (n=16)
C (n=14)
surface

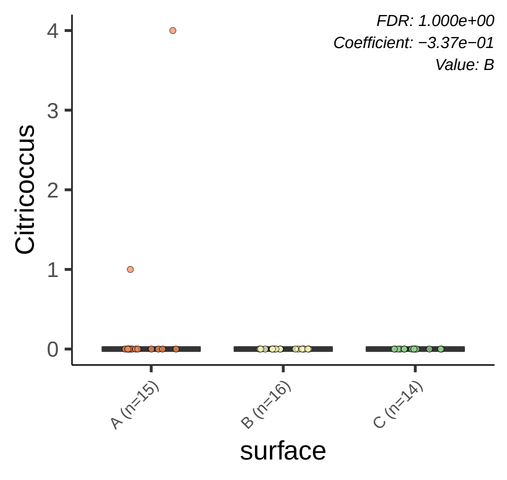

FDR: 1.000e+00
Coefficient: −3.37e−01
Value: B
Citricoccus
0
1
2
3
4
A (n=15)
B (n=16)
C (n=14)
surface

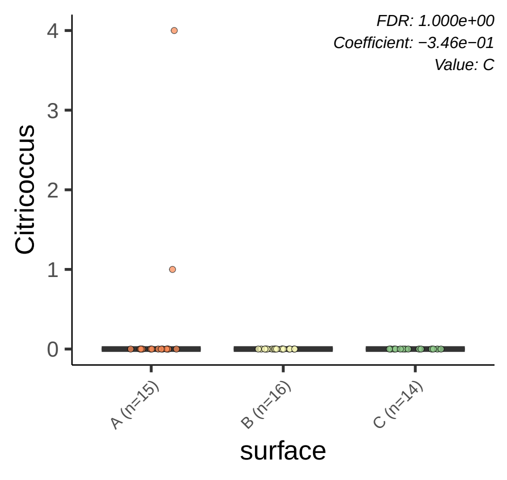
FDR: 1.000e+00
Coefficient: −3.46e−01
Value: C
Citricoccus
0
1
2
3
4
A (n=15)
B (n=16)
C (n=14)
surface

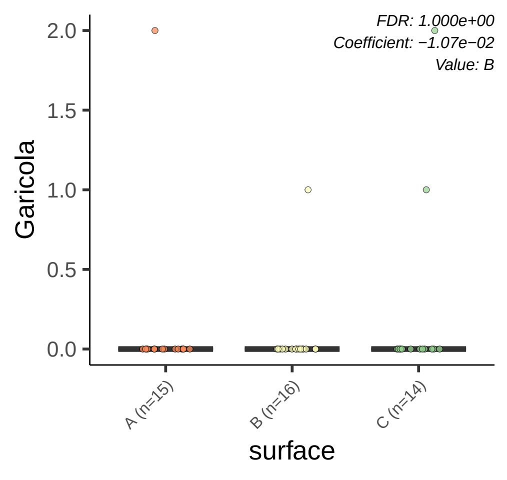
FDR: 1.000e+00
Coefficient: −1.07e−02
Value: B
Garicola
2.0
1.5
1.0
0.5
0.0
A (n=15)
B (n=16)
C (n=14)
surface

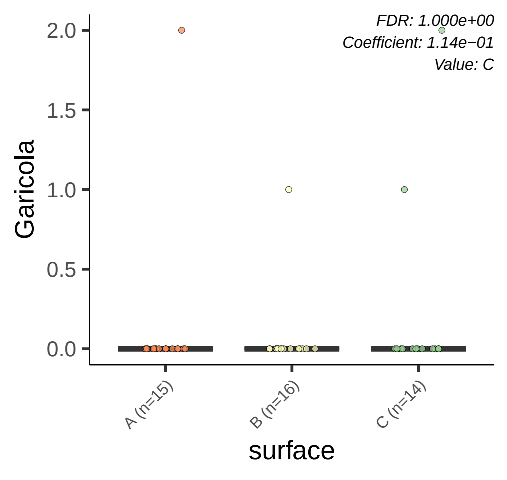

FDR: 1.000e+00
Coefficient: 1.14e−01
Value: C
2.0
1.5
1.0
0.5
0.0
Garicola
A (n=15)
B (n=16)
C (n=14)
surface

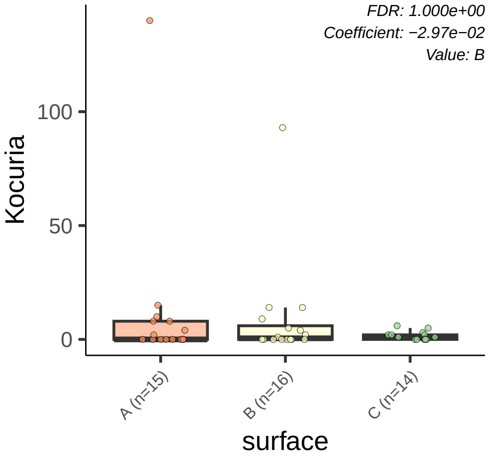
FDR: 1.000e+00
Coefficient: −2.97e−02
Value: B
Kocuria
100
50
0
A (n=15)
B (n=16)
C (n=14)
surface

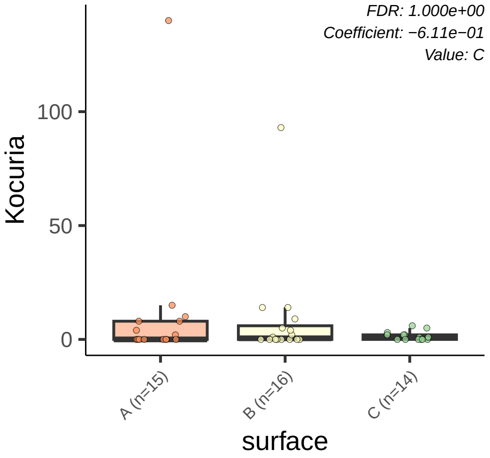
FDR: 1.000e+00
Coefficient: −6.11e−01
Value: C
Kocuria
100
50
0
A (n=15)
B (n=16)
C (n=14)
surface

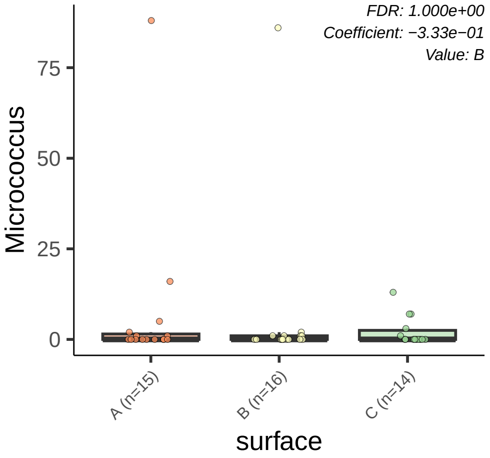
FDR: 1.000e+00
Coefficient: −3.33e−01
Value: B
Micrococcus
0
25
50
75
A (n=15)
B (n=16)
C (n=14)
surface

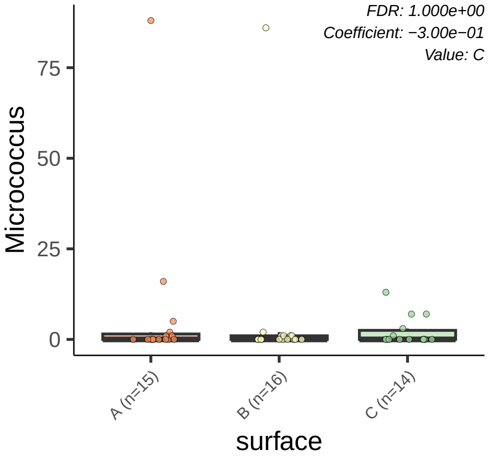
FDR: 1.000e+00
Coefficient: −3.00e−01
Value: C
Micrococcus
75
50
25
0
A (n=15)
B (n=16)
C (n=14)
surface

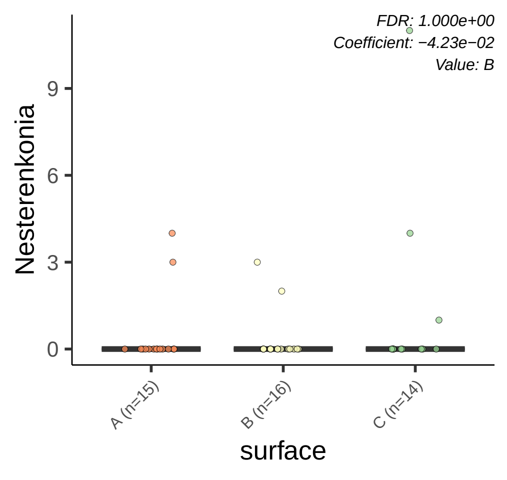
FDR: 1.000e+00
Coefficient: −4.23e−02
Value: B
Nesterenkonia
0
3
6
9
A (n=15)
B (n=16)
C (n=14)
surface

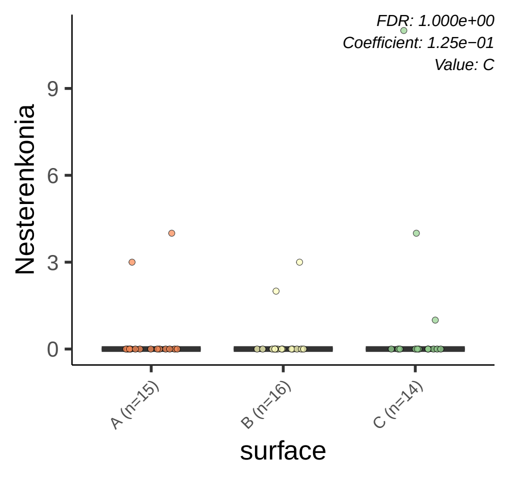
FDR: 1.000e+00
Coefficient: 1.25e−01
Value: C
Nesterenkonia
0
3
6
9
A (n=15)
B (n=16)
C (n=14)
surface

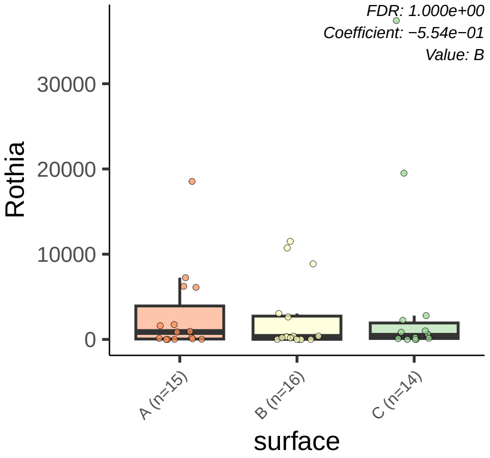
FDR: 1.000e+00
Coefficient: −5.54e−01
Value: B
Rothia
30000
20000
10000
0
A (n=15)
B (n=16)
C (n=14)
surface

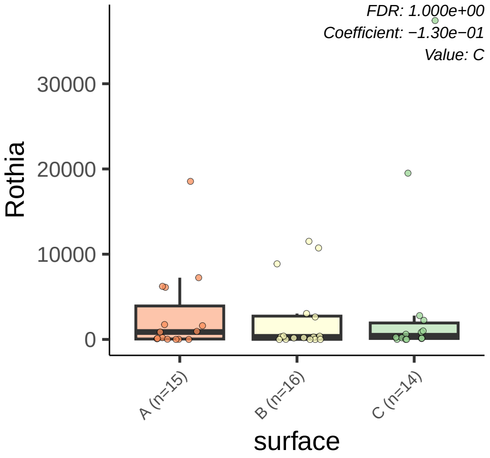
FDR: 1.000e+00
Coefficient: −1.30e−01
Value: C
Rothia
30000
20000
10000
0
A (n=15)
B (n=16)
C (n=14)
surface

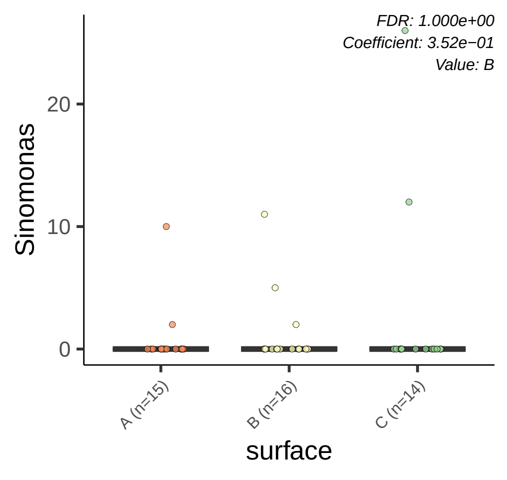

FDR: 1.000e+00
Coefficient: 3.52e−01
Value: B
Sinomonas
20
10
0
A (n=15)
B (n=16)
C (n=14)
surface

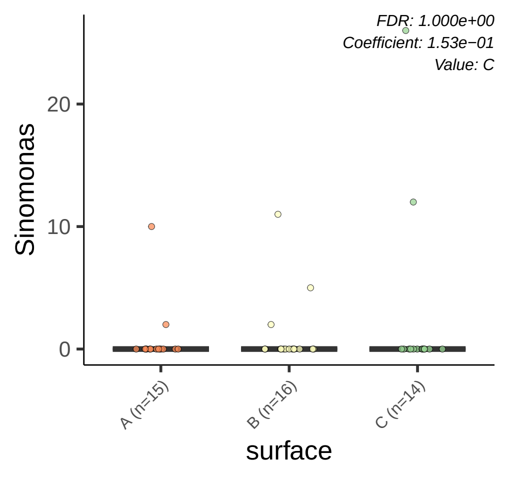
FDR: 1.000e+00
Coefficient: 1.53e−01
Value: C
Sinomonas
0
10
20
A (n=15)
B (n=16)
C (n=14)
surface

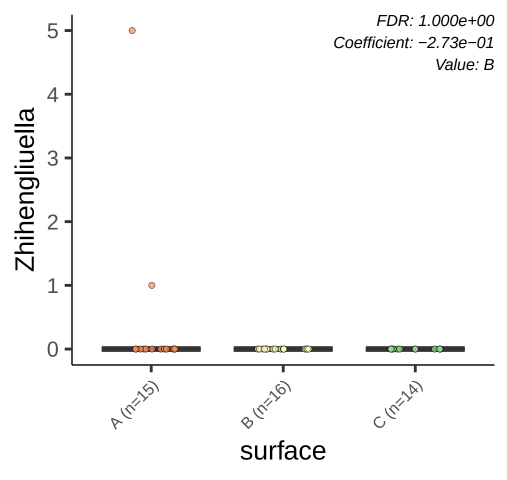
FDR: 1.000e+00
Coefficient: −2.73e−01
Value: B
Zhihengliuella
5
4
3
2
1
0
A (n=15)
B (n=16)
C (n=14)
surface

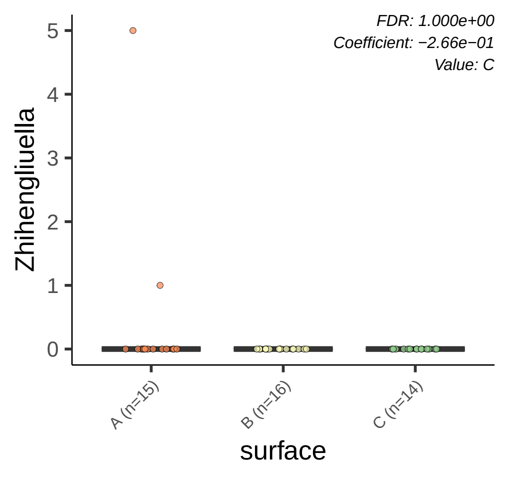

FDR: 1.000e+00
Coefficient: −2.66e−01
Value: C
Zhihengliuella
5
4
3
2
1
0
A (n=15)
B (n=16)
C (n=14)
surface

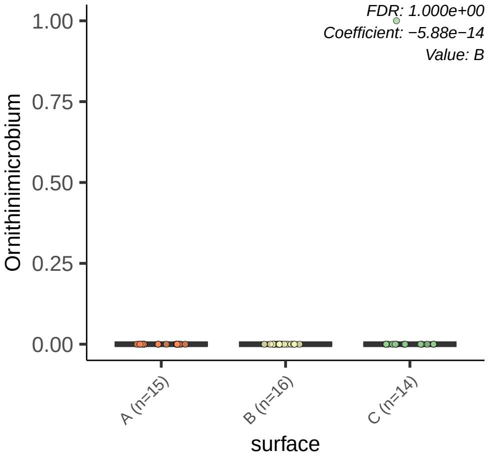
FDR: 1.000e+00
Coefficient: −5.88e−14
Value: B
Ornithinimicrobium
1.00
0.75
0.50
0.25
0.00
A (n=15)
B (n=16)
C (n=14)
surface

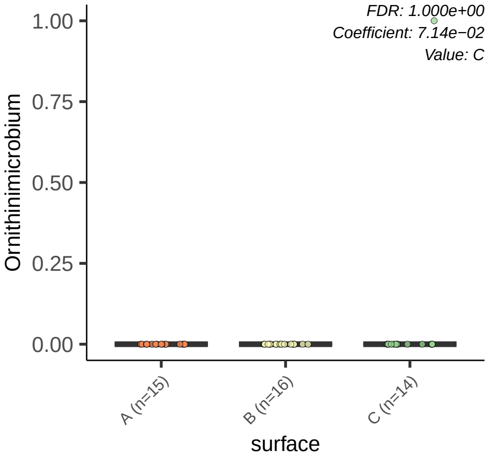
FDR: 1.000e+00
Coefficient: 7.14e−02
Value: C
Ornithinimicrobium
1.00
0.75
0.50
0.25
0.00
A (n=15)
B (n=16)
C (n=14)
surface

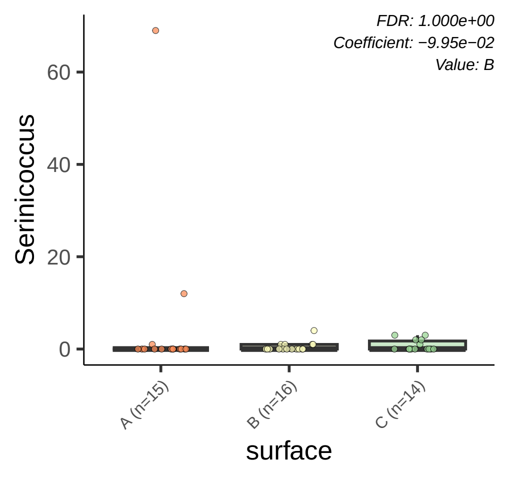
FDR: 1.000e+00
Coefficient: −9.95e−02
Value: B
Serinicoccus
60
40
20
0
A (n=15)
B (n=16)
C (n=14)
surface

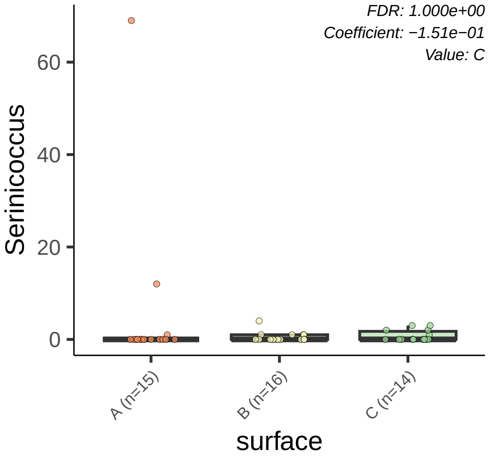

FDR: 1.000e+00
Coefficient: −1.51e−01
Value: C
Serinicoccus
60
40
20
0
A (n=15)
B (n=16)
C (n=14)
surface

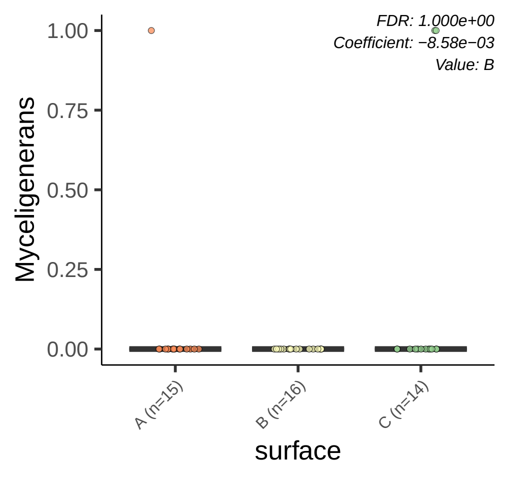

FDR: 1.000e+00
Coefficient: −8.58e−03
Value: B
Myceligenerans
1.00
0.75
0.50
0.25
0.00
A (n=15)
B (n=16)
C (n=14)
surface

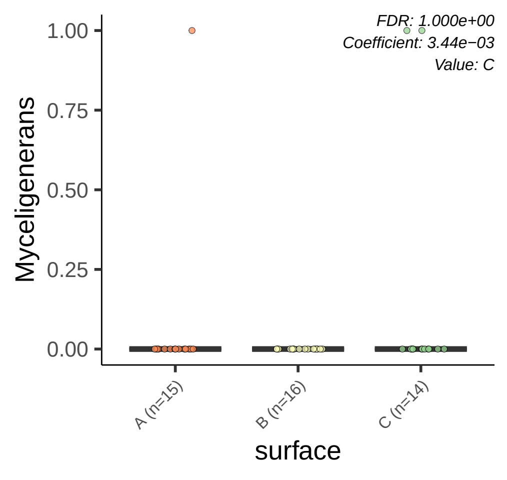

FDR: 1.000e+00
Coefficient: 3.44e−03
Value: C
Myceligenerans
1.00
0.75
0.50
0.25
0.00
A (n=15)
B (n=16)
C (n=14)
surface

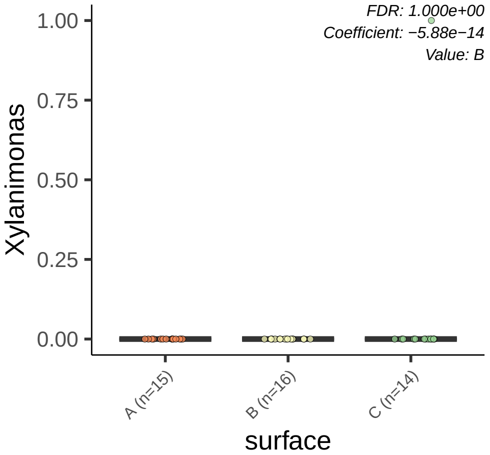

FDR: 1.000e+00
Coefficient: −5.88e−14
Value: B
Xylanimonas
1.00
0.75
0.50
0.25
0.00
A (n=15)
B (n=16)
C (n=14)
surface

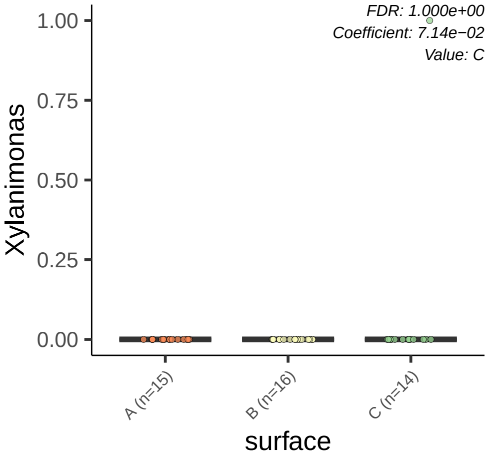
FDR: 1.000e+00
Coefficient: 7.14e−02
Value: C
Xylanimonas
1.00
0.75
0.50
0.25
0.00
A (n=15)
B (n=16)
C (n=14)
surface

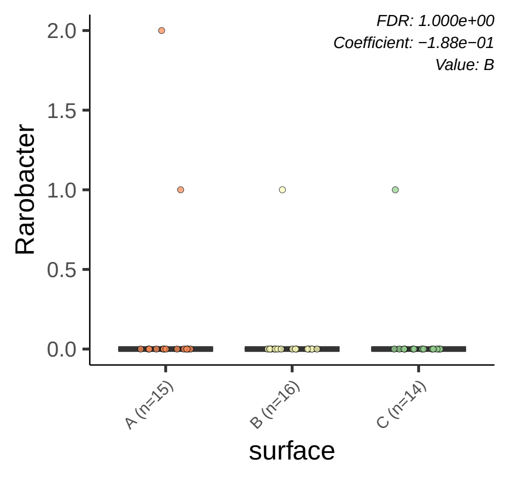

FDR: 1.000e+00
Coefficient: −1.88e−01
Value: B
Rarobacter
2.0
1.5
1.0
0.5
0.0
A (n=15)
B (n=16)
C (n=14)
surface

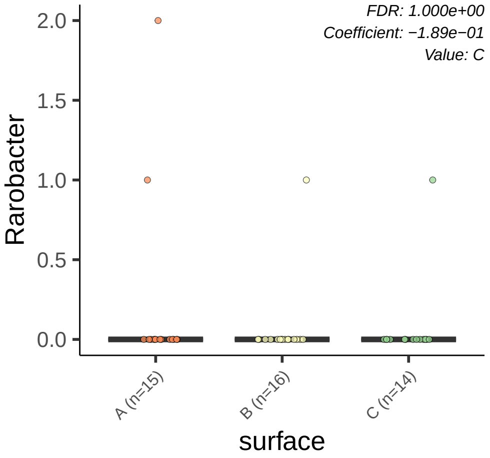
FDR: 1.000e+00
Coefficient: −1.89e−01
Value: C
Rarobacter
2.0
1.5
1.0
0.5
0.0
A (n=15)
B (n=16)
C (n=14)
surface

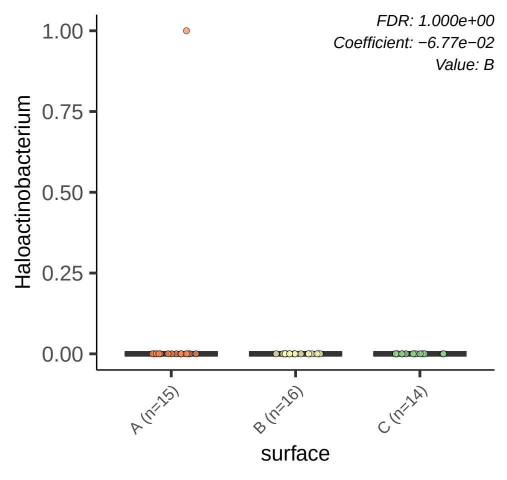

FDR: 1.000e+00
Coefficient: −6.77e−02
Value: B
Haloactinobacterium
1.00
0.75
0.50
0.25
0.00
A (n=15)
B (n=16)
C (n=14)
surface

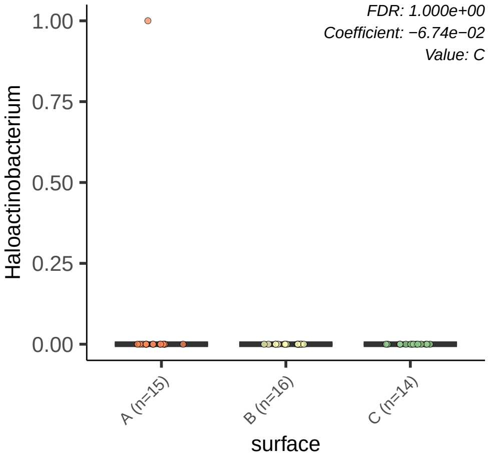
FDR: 1.000e+00
Coefficient: −6.74e−02
Value: C
Haloactinobacterium
1.00
0.75
0.50
0.25
0.00
A (n=15)
B (n=16)
C (n=14)
surface

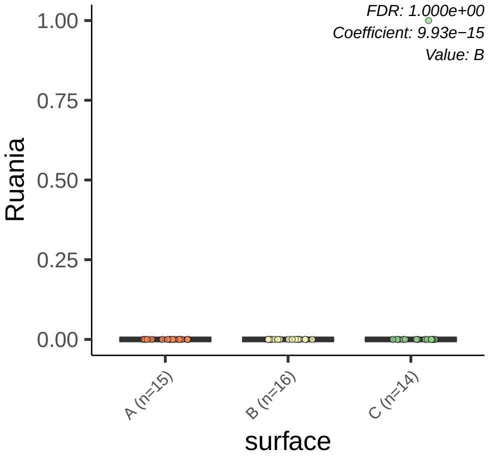
FDR: 1.000e+00
Coefficient: 9.93e−15
Value: B
Ruania
1.00
0.75
0.50
0.25
0.00
A (n=15)
B (n=16)
C (n=14)
surface

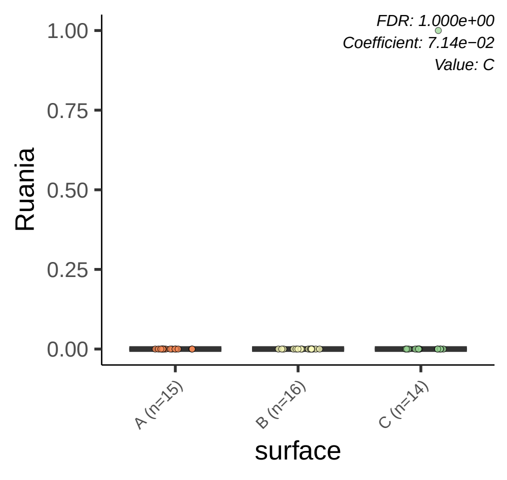
FDR: 1.000e+00
Coefficient: 7.14e−02
Value: C
Ruania
1.00
0.75
0.50
0.25
0.00
A (n=15)
B (n=16)
C (n=14)
surface

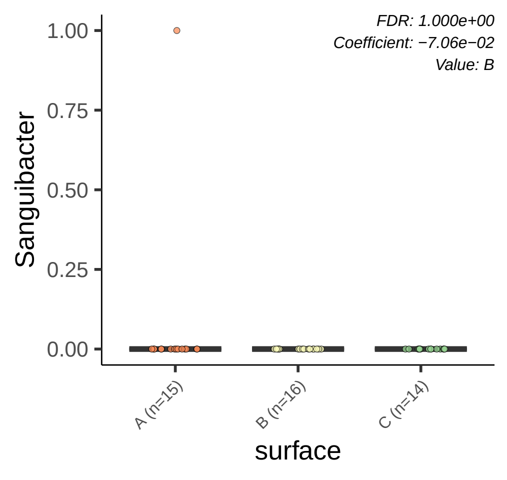

FDR: 1.000e+00
Coefficient: −7.06e−02
Value: B
Sanguibacter
1.00
0.75
0.50
0.25
0.00
A (n=15)
B (n=16)
C (n=14)
surface

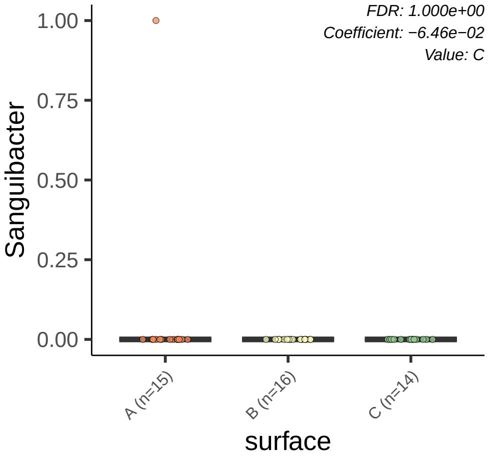
FDR: 1.000e+00
Coefficient: −6.46e−02
Value: C
Sanguibacter
1.00
0.75
0.50
0.25
0.00
A (n=15)
B (n=16)
C (n=14)
surface

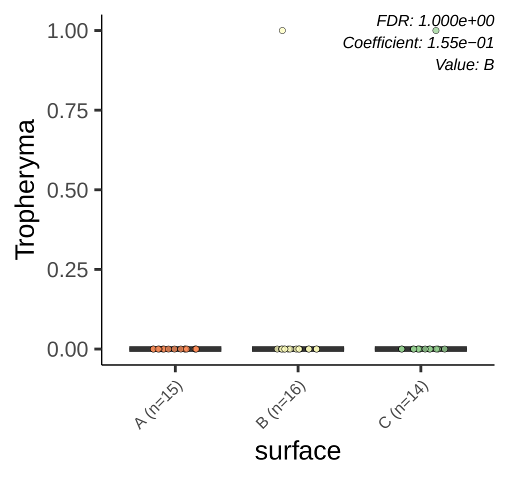
FDR: 1.000e+00
Coefficient: 1.55e−01
Value: B
Tropheryma
1.00
0.75
0.50
0.25
0.00
A (n=15)
B (n=16)
C (n=14)
surface

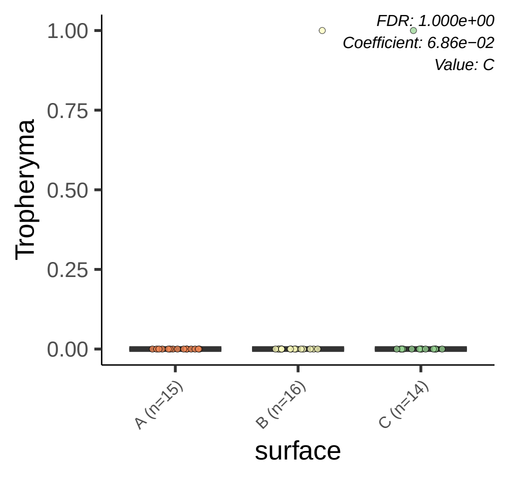
FDR: 1.000e+00
Coefficient: 6.86e−02
Value: C
1.00
0.75
0.50
0.25
0.00
Tropheryma
A (n=15)
B (n=16)
C (n=14)
surface

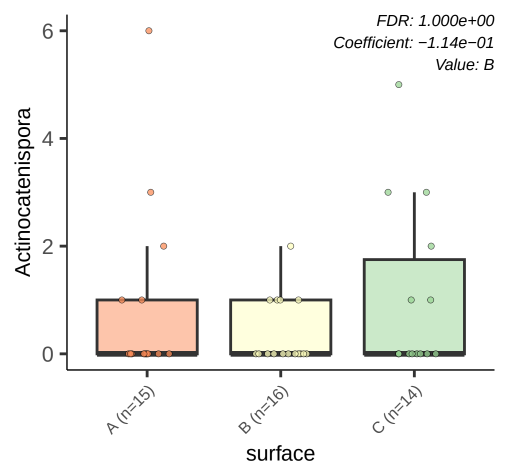

FDR: 1.000e+00
Coefficient: −1.14e−01
Value: B
Actinocatenispora
0
2
4
6
A (n=15)
B (n=16)
C (n=14)
surface

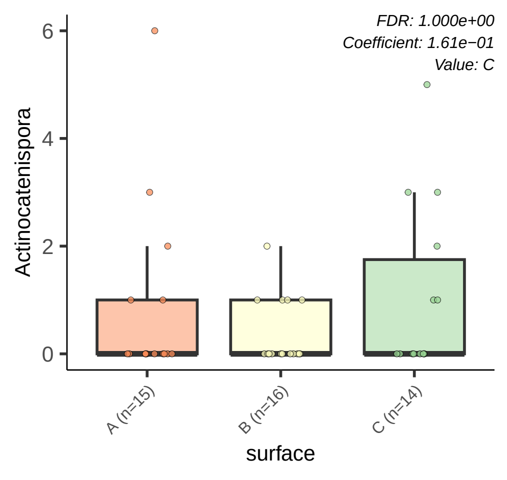

FDR: 1.000e+00
Coefficient: 1.61e−01
Value: C
Actinocatenispora
0
2
4
6
A (n=15)
B (n=16)
C (n=14)
surface

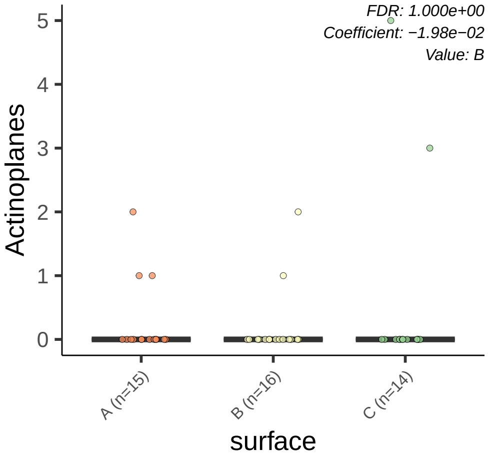
FDR: 1.000e+00
Coefficient: −1.98e−02
Value: B
Actinoplanes
0
1
2
3
4
5
A (n=15)
B (n=16)
C (n=14)
surface

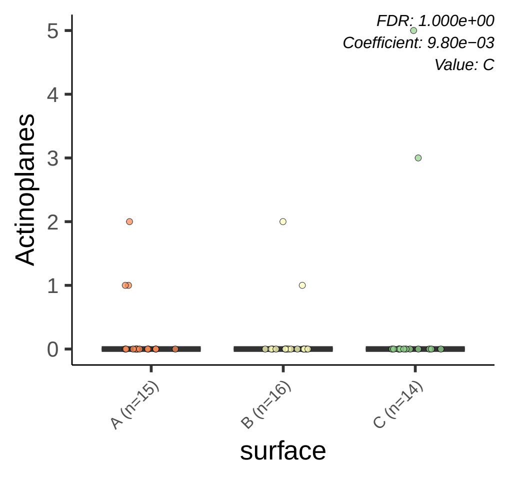
FDR: 1.000e+00
Coefficient: 9.80e−03
Value: C
Actinoplanes
0
1
2
3
4
5
A (n=15)
B (n=16)
C (n=14)
surface

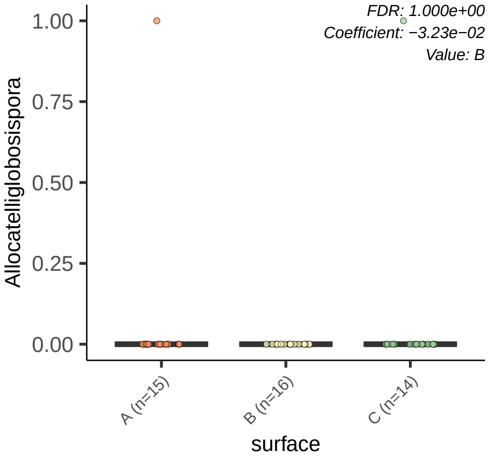

FDR: 1.000e+00
Coefficient: −3.23e−02
Value: B
Allocatelliglobosispora
1.00
0.75
0.50
0.25
0.00
A (n=15)
B (n=16)
C (n=14)
surface

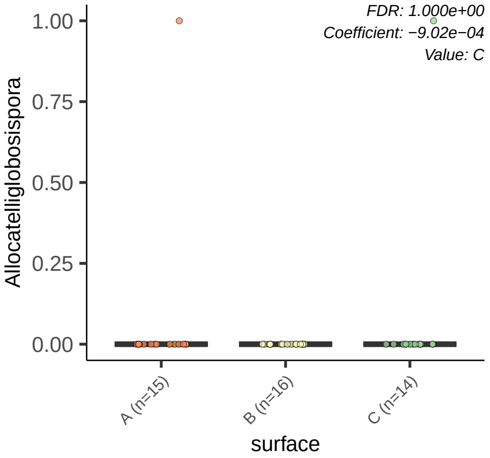

FDR: 1.000e+00
Coefficient: −9.02e−04
Value: C
Allocatelliglobosispora
1.00
0.75
0.50
0.25
0.00
A (n=15)
B (n=16)
C (n=14)
surface

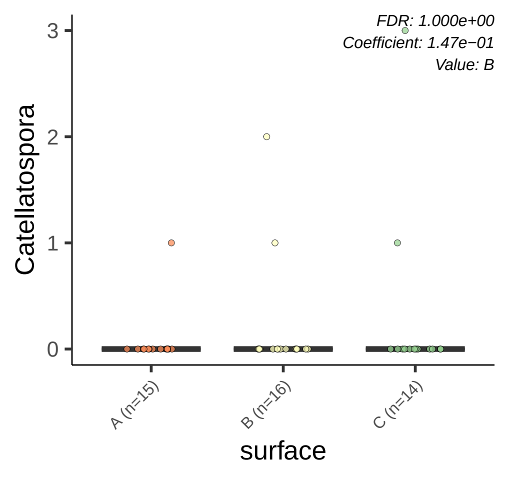
FDR: 1.000e+00
Coefficient: 1.47e−01
Value: B
Catellatospora
3
2
1
0
A (n=15)
B (n=16)
C (n=14)
surface

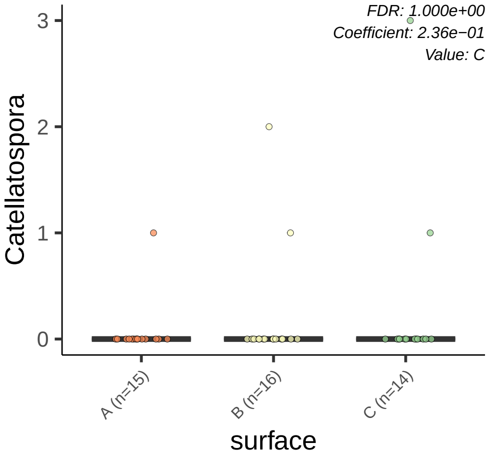

FDR: 1.000e+00
Coefficient: 2.36e−01
Value: C
Catellatospora
0
1
2
3
A (n=15)
B (n=16)
C (n=14)
surface

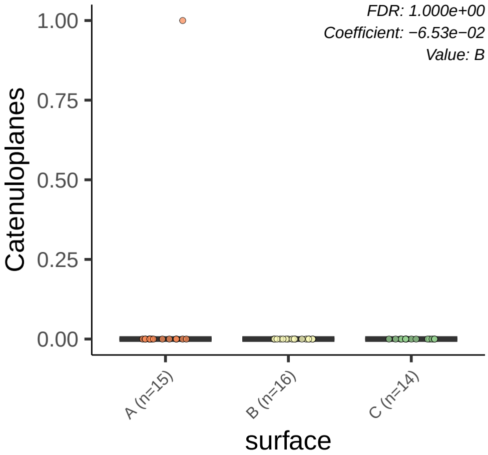

FDR: 1.000e+00
Coefficient: −6.53e−02
Value: B
Catenuloplanes
1.00
0.75
0.50
0.25
0.00
A (n=15)
B (n=16)
C (n=14)
surface

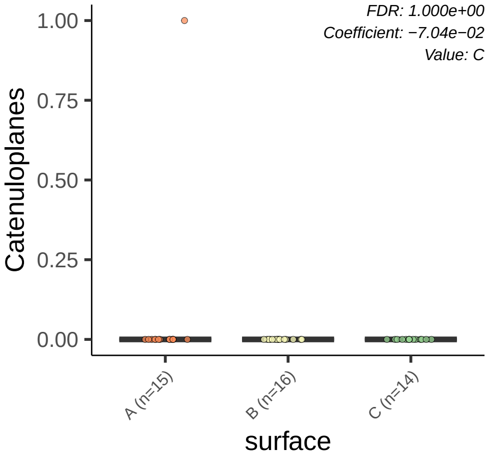
FDR: 1.000e+00
Coefficient: −7.04e−02
Value: C
Catenuloplanes
1.00
0.75
0.50
0.25
0.00
A (n=15)
B (n=16)
C (n=14)
surface

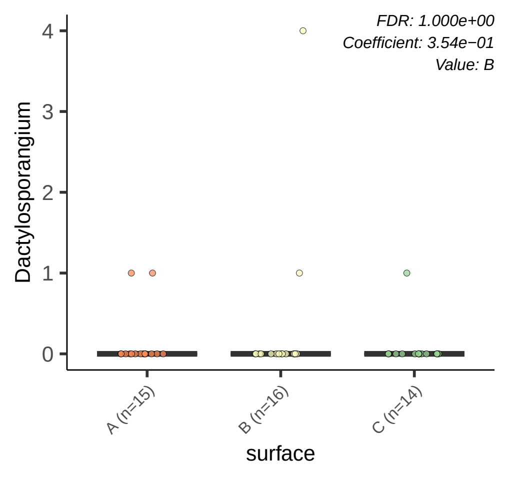
FDR: 1.000e+00
Coefficient: 3.54e−01
Value: B
Dactylosporangium
4
3
2
1
0
A (n=15)
B (n=16)
C (n=14)
surface

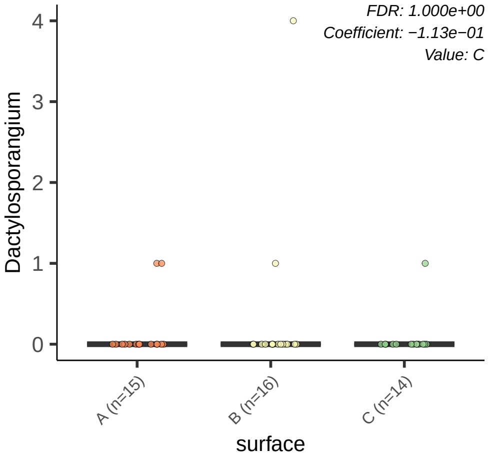

FDR: 1.000e+00
Coefficient: −1.13e−01
Value: C
Dactylosporangium
0
1
2
3
4
A (n=15)
B (n=16)
C (n=14)
surface

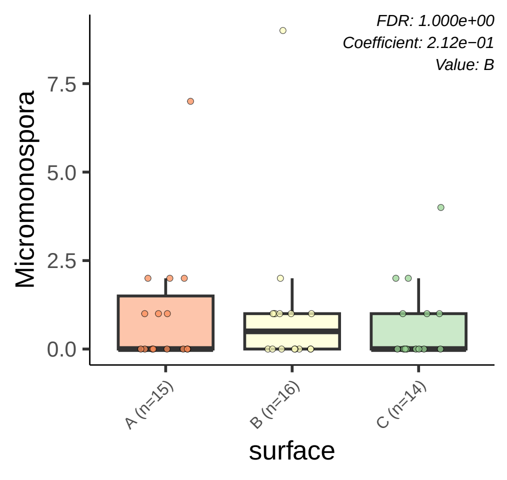

FDR: 1.000e+00
Coefficient: 2.12e−01
Value: B
Micromonospora
7.5
5.0
2.5
0.0
A (n=15)
B (n=16)
C (n=14)
surface

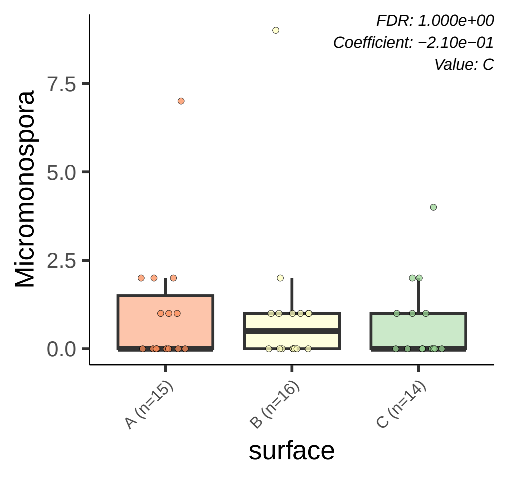

FDR: 1.000e+00
Coefficient: −2.10e−01
Value: C
Micromonospora
7.5
5.0
2.5
0.0
A (n=15)
B (n=16)
C (n=14)
surface

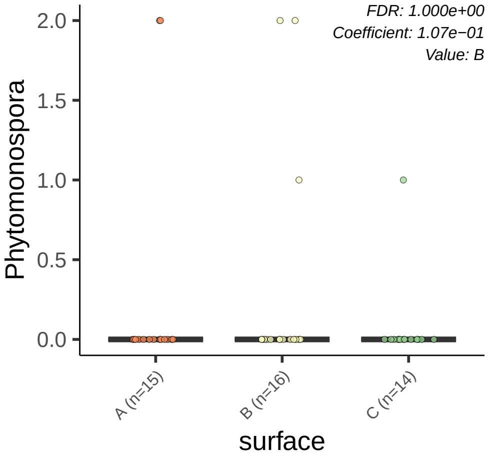
FDR: 1.000e+00
Coefficient: 1.07e−01
Value: B
Phytomonospora
2.0
1.5
1.0
0.5
0.0
A (n=15)
B (n=16)
C (n=14)
surface

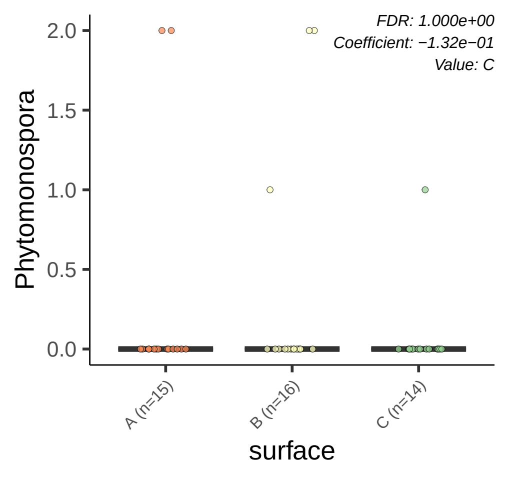
FDR: 1.000e+00
Coefficient: −1.32e−01
Value: C
Phytomonospora
2.0
1.5
1.0
0.5
0.0
A (n=15)
B (n=16)
C (n=14)
surface

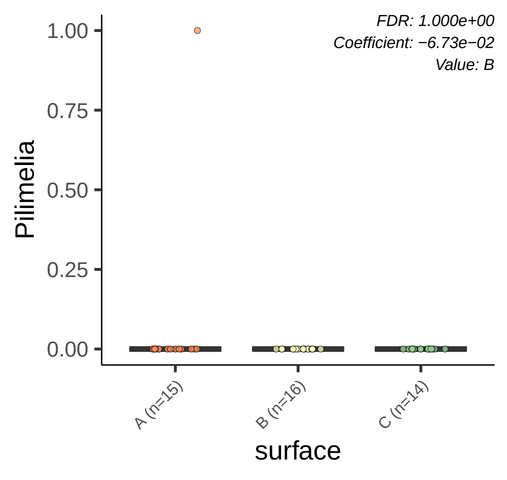
FDR: 1.000e+00
Coefficient: −6.73e−02
Value: B
Pilimelia
1.00
0.75
0.50
0.25
0.00
A (n=15)
B (n=16)
C (n=14)
surface

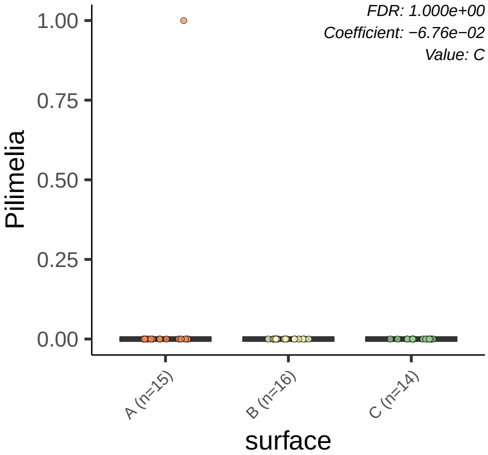

FDR: 1.000e+00
Coefficient: −6.76e−02
Value: C
Pilimelia
1.00
0.75
0.50
0.25
0.00
A (n=15)
B (n=16)
C (n=14)
surface

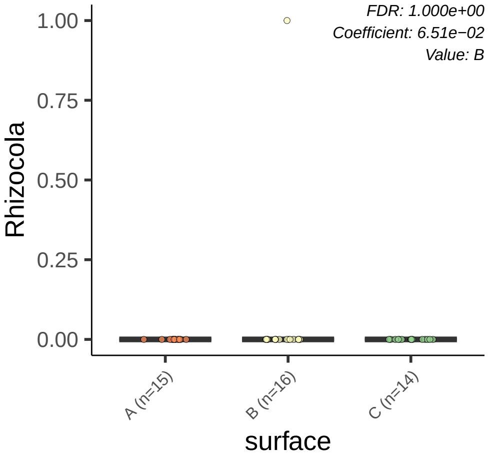

FDR: 1.000e+00
Coefficient: 6.51e−02
Value: B
Rhizocola
1.00
0.75
0.50
0.25
0.00
A (n=15)
B (n=16)
C (n=14)
surface

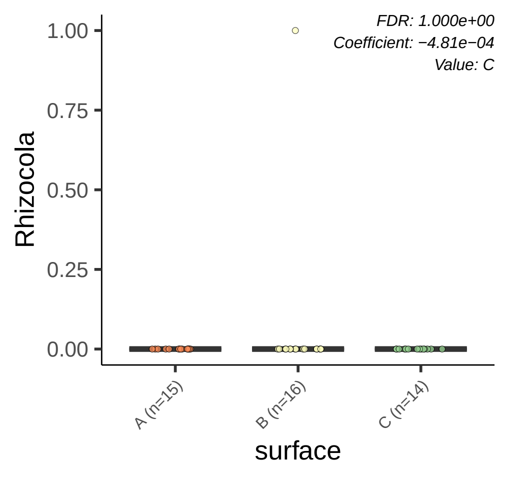

FDR: 1.000e+00
Coefficient: −4.81e−04
Value: C
Rhizocola
1.00
0.75
0.50
0.25
0.00
A (n=15)
B (n=16)
C (n=14)
surface

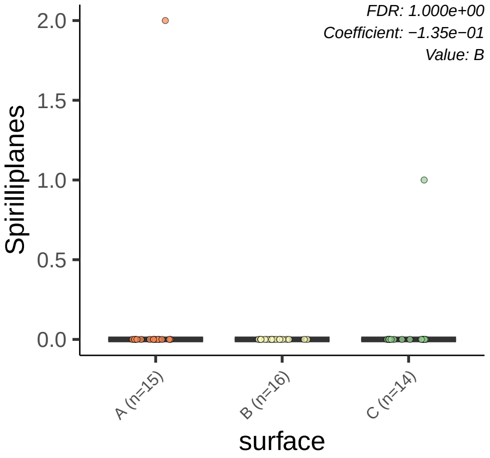
FDR: 1.000e+00
Coefficient: −1.35e−01
Value: B
Spirilliplanes
2.0
1.5
1.0
0.5
0.0
A (n=15)
B (n=16)
C (n=14)
surface

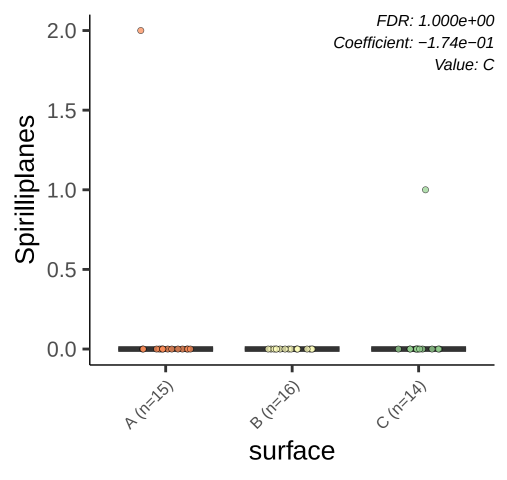
FDR: 1.000e+00
Coefficient: −1.74e−01
Value: C
Spirilliplanes
2.0
1.5
1.0
0.5
0.0
A (n=15)
B (n=16)
C (n=14)
surface

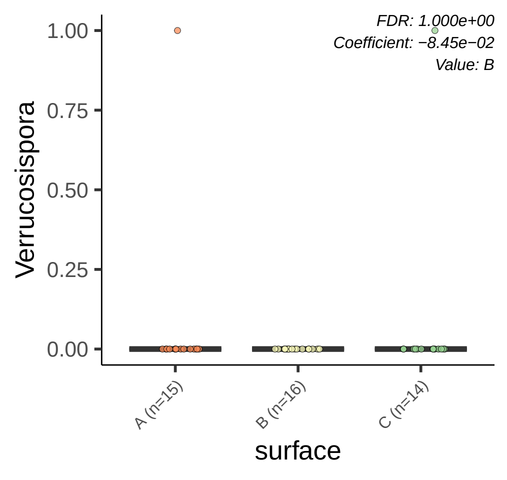

FDR: 1.000e+00
Coefficient: −8.45e−02
Value: B
Verrucosispora
1.00
0.75
0.50
0.25
0.00
A (n=15)
B (n=16)
C (n=14)
surface

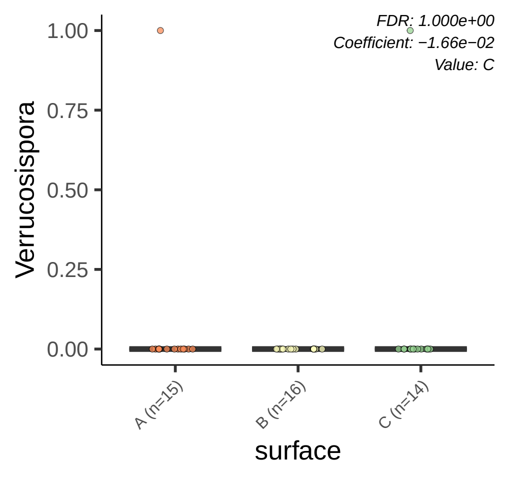
FDR: 1.000e+00
Coefficient: −1.66e−02
Value: C
Verrucosispora
1.00
0.75
0.50
0.25
0.00
A (n=15)
B (n=16)
C (n=14)
surface

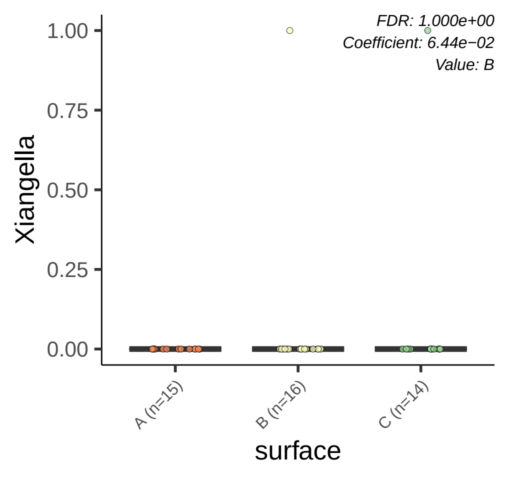

FDR: 1.000e+00
Coefficient: 6.44e−02
Value: B
Xiangella
1.00
0.75
0.50
0.25
0.00
A (n=15)
B (n=16)
C (n=14)
surface

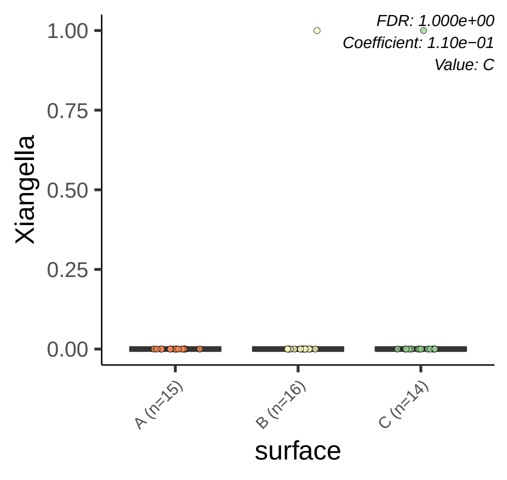
FDR: 1.000e+00
Coefficient: 1.10e−01
Value: C
Xiangella
1.00
0.75
0.50
0.25
0.00
A (n=15)
B (n=16)
C (n=14)
surface

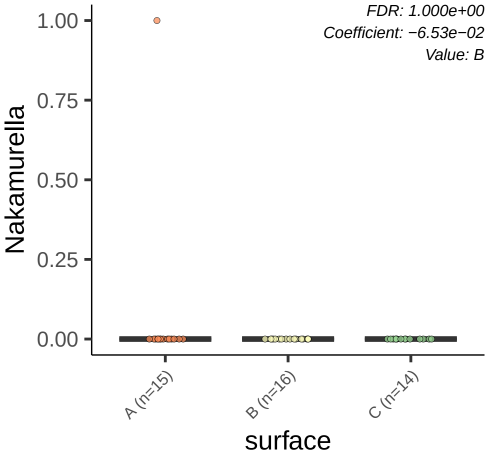
FDR: 1.000e+00
Coefficient: −6.53e−02
Value: B
Nakamurella
1.00
0.75
0.50
0.25
0.00
A (n=15)
B (n=16)
C (n=14)
surface

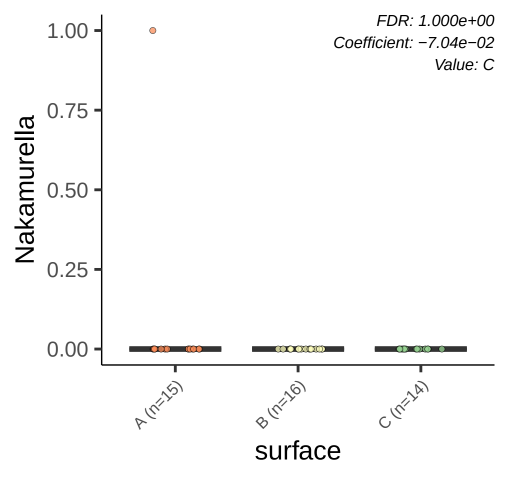
FDR: 1.000e+00
Coefficient: −7.04e−02
Value: C
Nakamurella
1.00
0.75
0.50
0.25
0.00
A (n=15)
B (n=16)
C (n=14)
surface

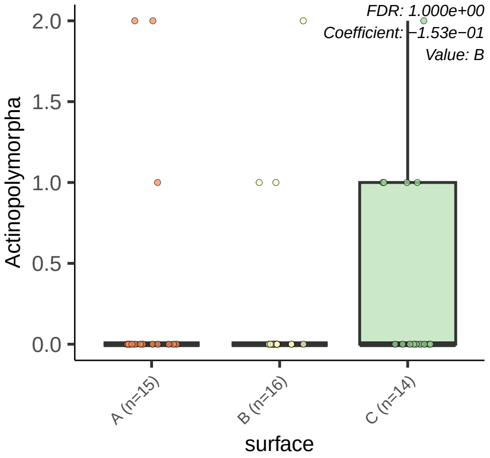

FDR: 1.000e+00
Coefficient: −1.53e−01
Value: B
Actinopolymorpha
2.0
1.5
1.0
0.5
0.0
A (n=15)
B (n=16)
C (n=14)
surface

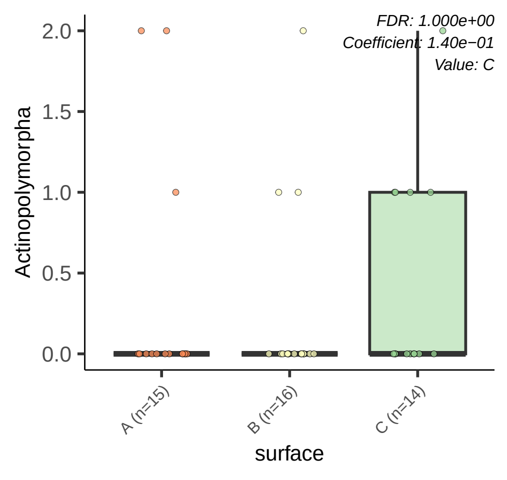

FDR: 1.000e+00
Coefficient: 1.40e−01
Value: C
Actinopolymorpha
2.0
1.5
1.0
0.5
0.0
A (n=15)
B (n=16)
C (n=14)
surface

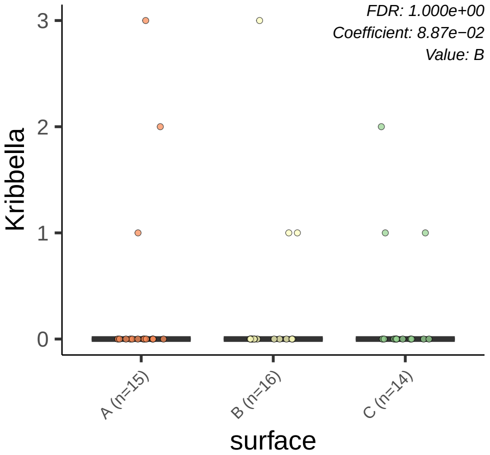

FDR: 1.000e+00
Coefficient: 8.87e−02
Value: B
Kribbella
3
2
1
0
A (n=15)
B (n=16)
C (n=14)
surface

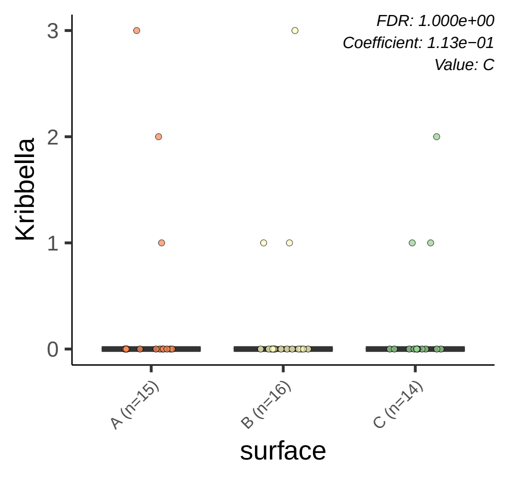

FDR: 1.000e+00
Coefficient: 1.13e−01
Value: C
Kribbella
3
2
1
0
A (n=15)
B (n=16)
C (n=14)
surface

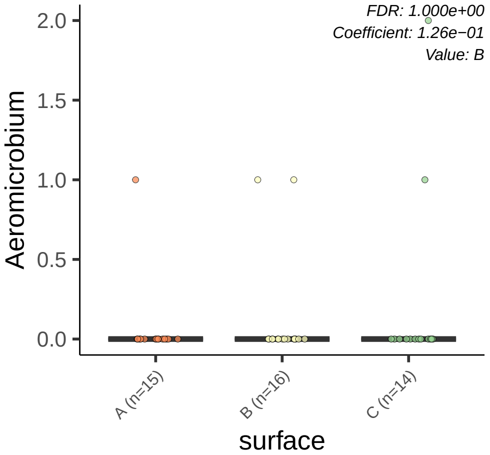
FDR: 1.000e+00
Coefficient: 1.26e−01
Value: B
Aeromicrobium
2.0
1.5
1.0
0.5
0.0
A (n=15)
B (n=16)
C (n=14)
surface

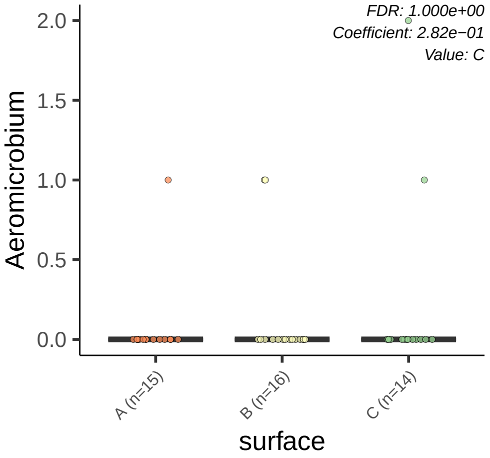
FDR: 1.000e+00
Coefficient: 2.82e−01
Value: C
Aeromicrobium
2.0
1.5
1.0
0.5
0.0
A (n=15)
B (n=16)
C (n=14)
surface

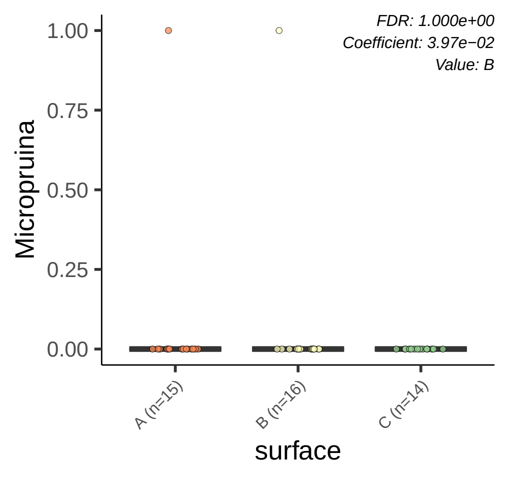

FDR: 1.000e+00
Coefficient: 3.97e−02
Value: B
Micropruina
1.00
0.75
0.50
0.25
0.00
A (n=15)
B (n=16)
C (n=14)
surface

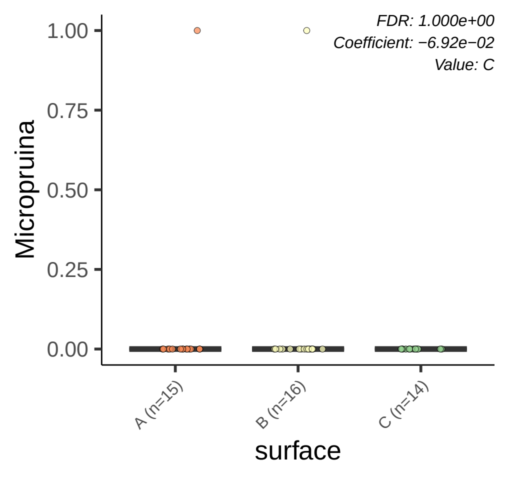
FDR: 1.000e+00
Coefficient: −6.92e−02
Value: C
Micropruina
1.00
0.75
0.50
0.25
0.00
A (n=15)
B (n=16)
C (n=14)
surface

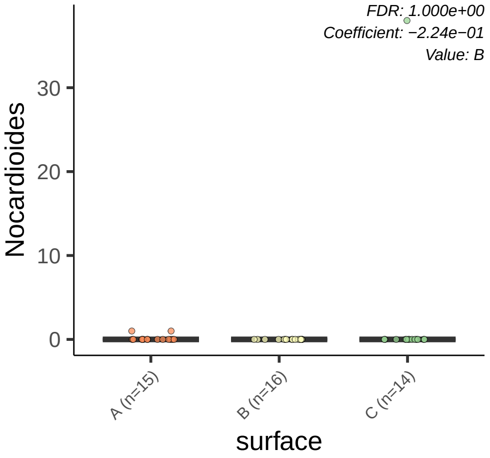
FDR: 1.000e+00
Coefficient: −2.24e−01
Value: B
Nocardioides
30
20
10
0
A (n=15)
B (n=16)
C (n=14)
surface

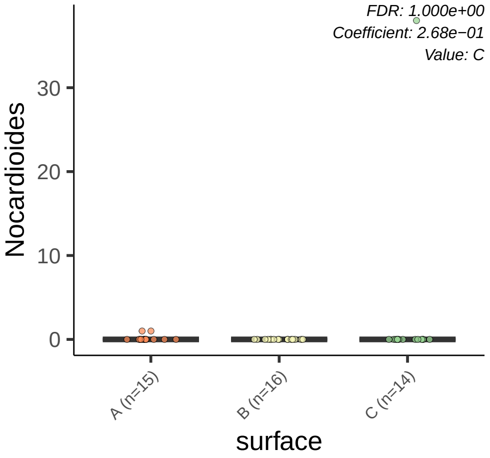

FDR: 1.000e+00
Coefficient: 2.68e−01
Value: C
Nocardioides
30
20
10
0
A (n=15)
B (n=16)
C (n=14)
surface

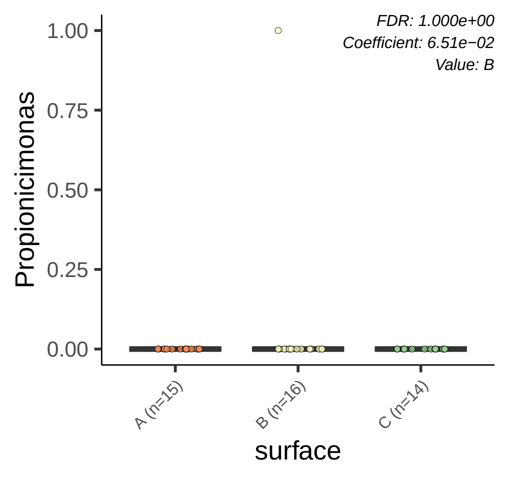
FDR: 1.000e+00
Coefficient: 6.51e−02
Value: B
Propionicimonas
1.00
0.75
0.50
0.25
0.00
A (n=15)
B (n=16)
C (n=14)
surface

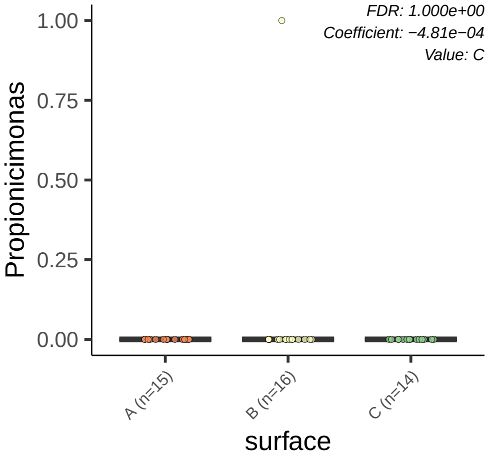

FDR: 1.000e+00
Coefficient: −4.81e−04
Value: C
Propionicimonas
1.00
0.75
0.50
0.25
0.00
A (n=15)
B (n=16)
C (n=14)
surface

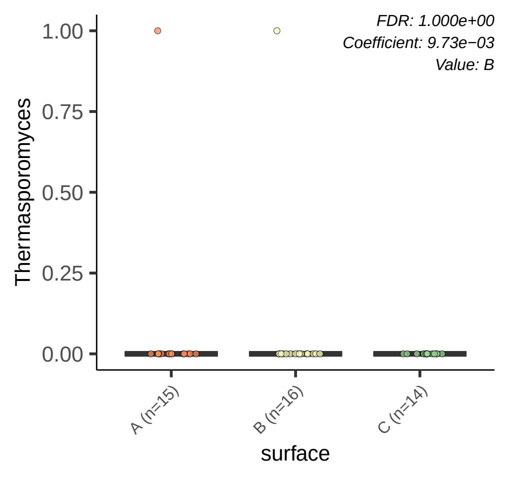
FDR: 1.000e+00
Coefficient: 9.73e−03
Value: B
Thermasporomyces
1.00
0.75
0.50
0.25
0.00
A (n=15)
B (n=16)
C (n=14)
surface

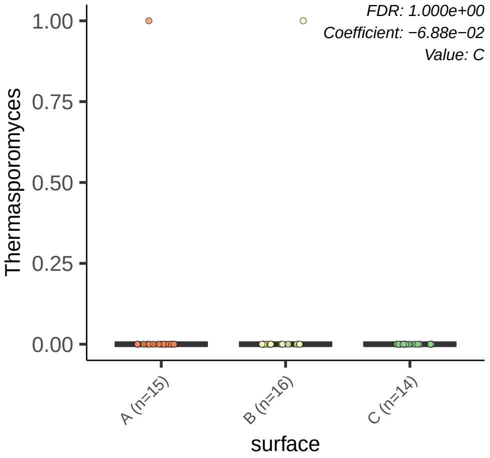
FDR: 1.000e+00
Coefficient: −6.88e−02
Value: C
Thermasporomyces
1.00
0.75
0.50
0.25
0.00
A (n=15)
B (n=16)
C (n=14)
surface

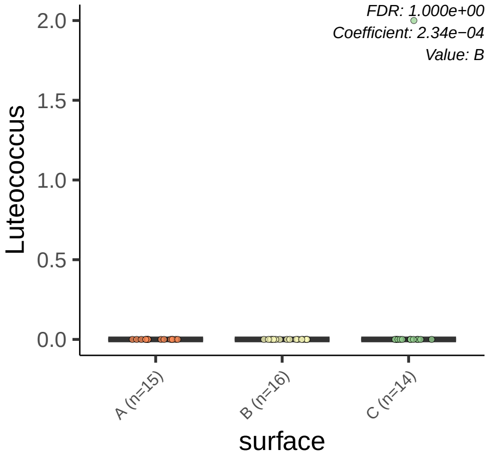

FDR: 1.000e+00
Coefficient: 2.34e−04
Value: B
2.0
1.5
1.0
0.5
0.0
Luteococcus
A (n=15)
B (n=16)
C (n=14)
surface

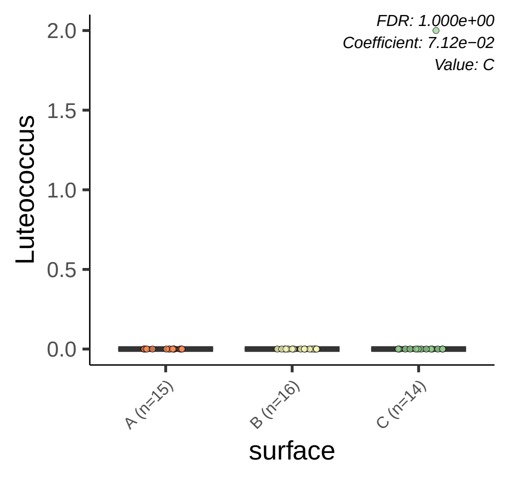

FDR: 1.000e+00
Coefficient: 7.12e−02
Value: C
Luteococcus
2.0
1.5
1.0
0.5
0.0
A (n=15)
B (n=16)
C (n=14)
surface

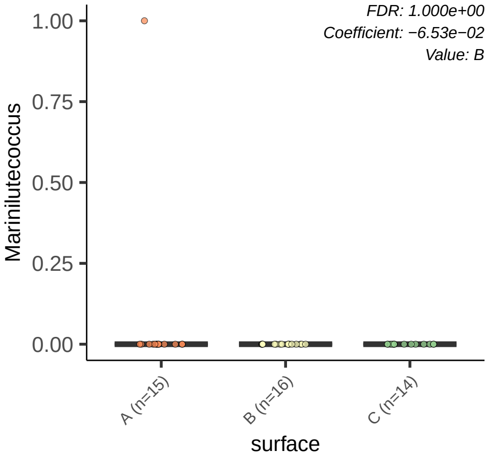
FDR: 1.000e+00
Coefficient: −6.53e−02
Value: B
Marinilutecoccus
1.00
0.75
0.50
0.25
0.00
A (n=15)
B (n=16)
C (n=14)
surface

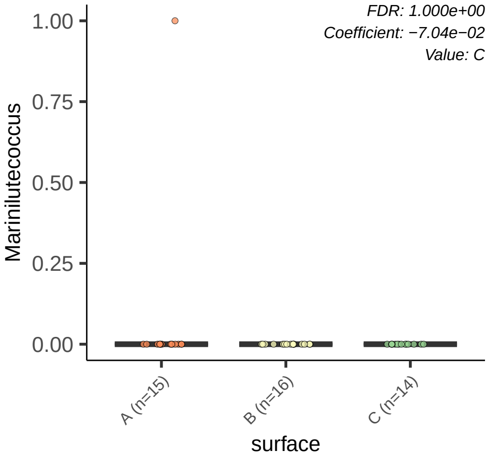

FDR: 1.000e+00
Coefficient: −7.04e−02
Value: C
Marinilutecoccus
1.00
0.75
0.50
0.25
0.00
A (n=15)
B (n=16)
C (n=14)
surface

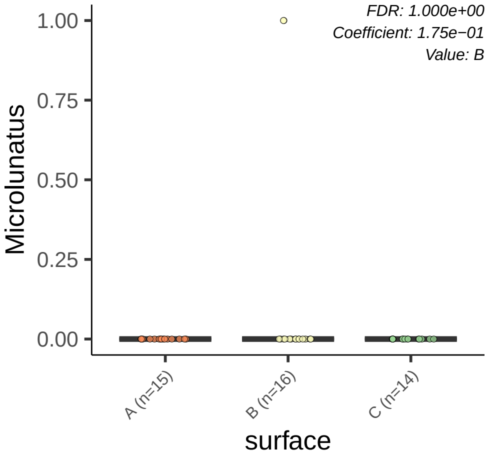

FDR: 1.000e+00
Coefficient: 1.75e−01
Value: B
Microlunatus
1.00
0.75
0.50
0.25
0.00
A (n=15)
B (n=16)
C (n=14)
surface

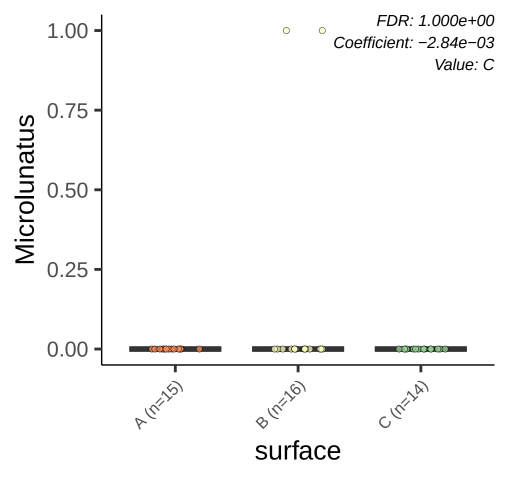

FDR: 1.000e+00
Coefficient: −2.84e−03
Value: C
Microlunatus
1.00
0.75
0.50
0.25
0.00
A (n=15)
B (n=16)
C (n=14)
surface

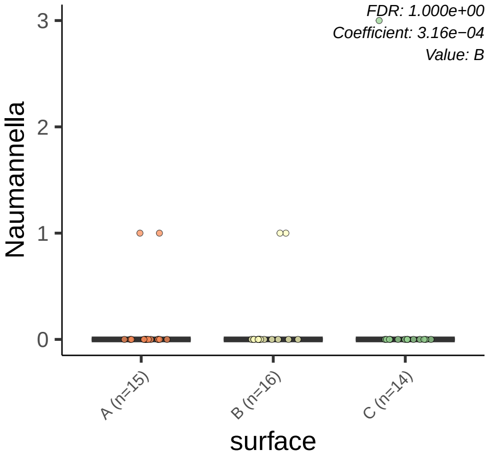
FDR: 1.000e+00
Coefficient: 3.16e−04
Value: B
Naumannella
3
2
1
0
A (n=15)
B (n=16)
C (n=14)
surface

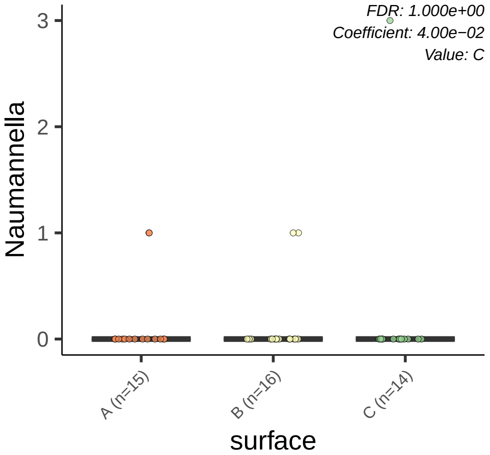
FDR: 1.000e+00
Coefficient: 4.00e−02
Value: C
Naumannella
0
1
2
3
A (n=15)
B (n=16)
C (n=14)
surface

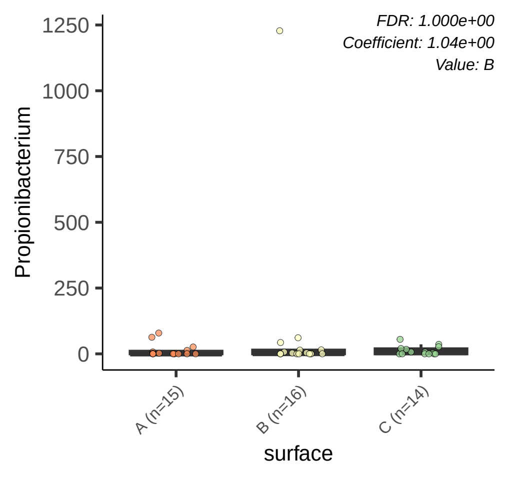
FDR: 1.000e+00
Coefficient: 1.04e+00
Value: B
Propionibacterium
1250
1000
750
500
250
0
A (n=15)
B (n=16)
C (n=14)
surface

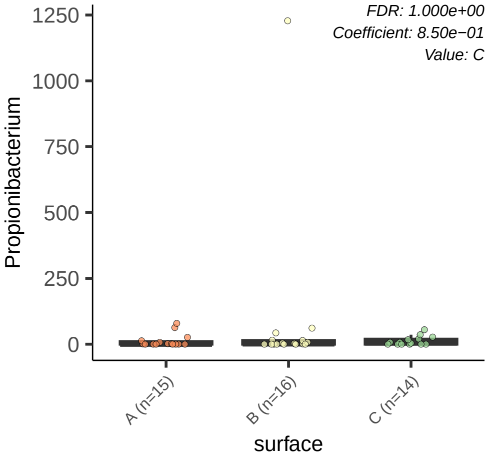

FDR: 1.000e+00
Coefficient: 8.50e−01
Value: C
Propionibacterium
1250
1000
750
500
250
0
A (n=15)
B (n=16)
C (n=14)
surface

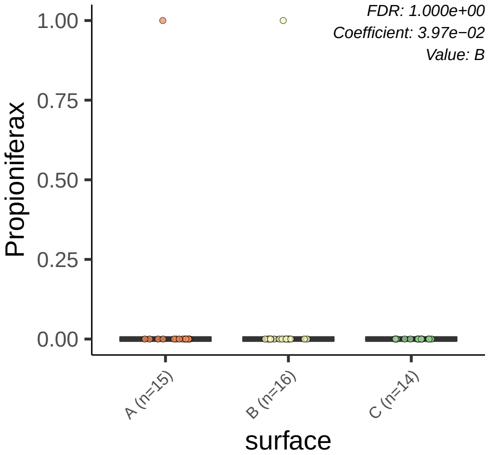

FDR: 1.000e+00
Coefficient: 3.97e−02
Value: B
Propioniferax
1.00
0.75
0.50
0.25
0.00
A (n=15)
B (n=16)
C (n=14)
surface

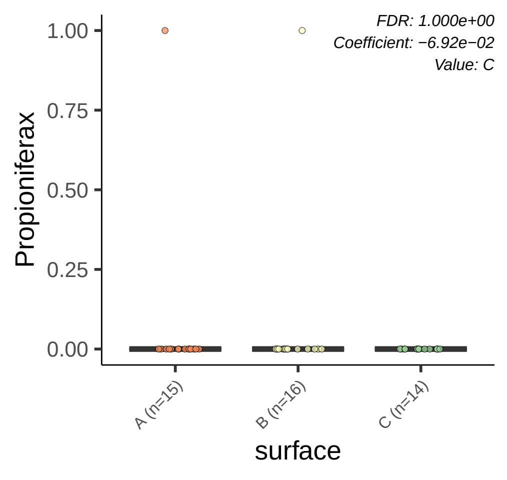
FDR: 1.000e+00
Coefficient: −6.92e−02
Value: C
Propioniferax
1.00
0.75
0.50
0.25
0.00
A (n=15)
B (n=16)
C (n=14)
surface

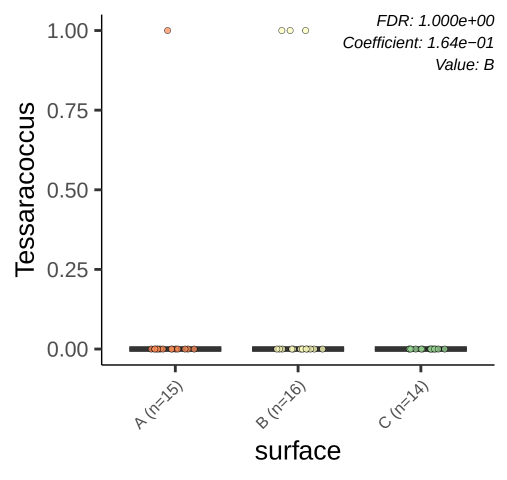

FDR: 1.000e+00
Coefficient: 1.64e−01
Value: B
Tessaracoccus
1.00
0.75
0.50
0.25
0.00
A (n=15)
B (n=16)
C (n=14)
surface

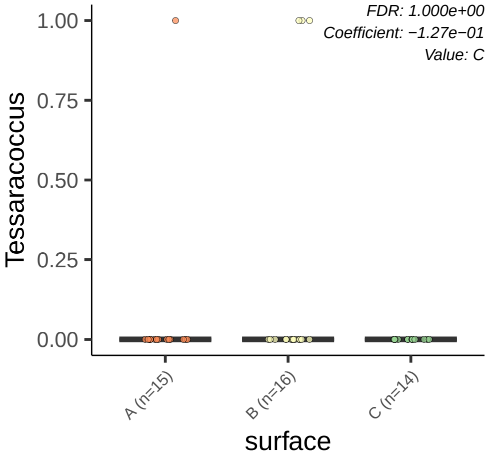
FDR: 1.000e+00
Coefficient: −1.27e−01
Value: C
Tessaracoccus
1.00
0.75
0.50
0.25
0.00
A (n=15)
B (n=16)
C (n=14)
surface

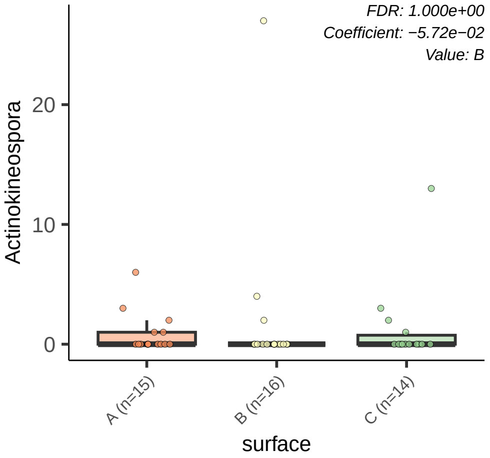

FDR: 1.000e+00
Coefficient: −5.72e−02
Value: B
Actinokineospora
0
10
20
A (n=15)
B (n=16)
C (n=14)
surface

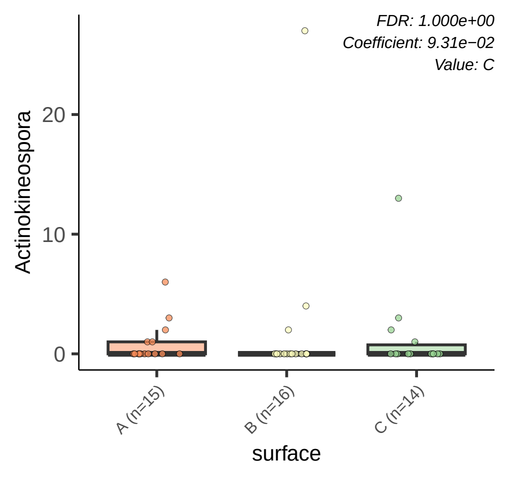
FDR: 1.000e+00
Coefficient: 9.31e−02
Value: C
Actinokineospora
0
10
20
A (n=15)
B (n=16)
C (n=14)
surface

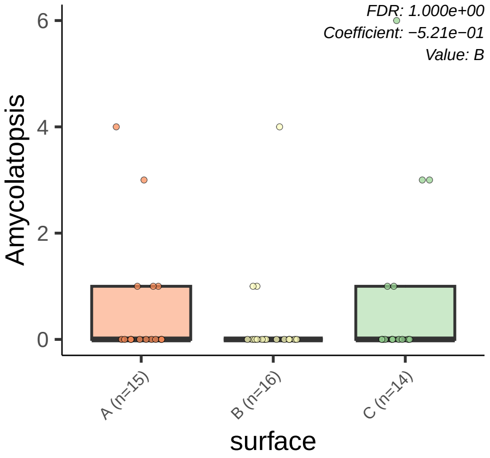

FDR: 1.000e+00
Coefficient: −5.21e−01
Value: B
Amycolatopsis
0
2
4
6
A (n=15)
B (n=16)
C (n=14)
surface

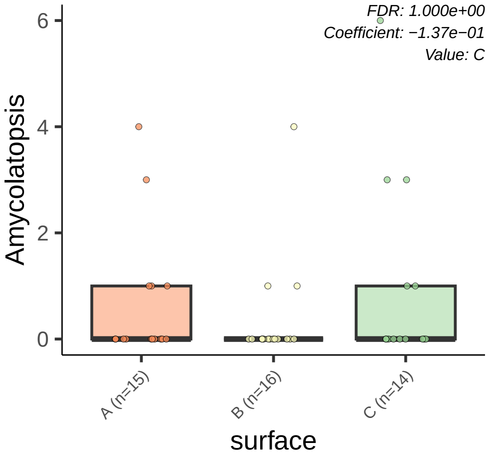
FDR: 1.000e+00
Coefficient: −1.37e−01
Value: C
Amycolatopsis
6
4
2
0
A (n=15)
B (n=16)
C (n=14)
surface

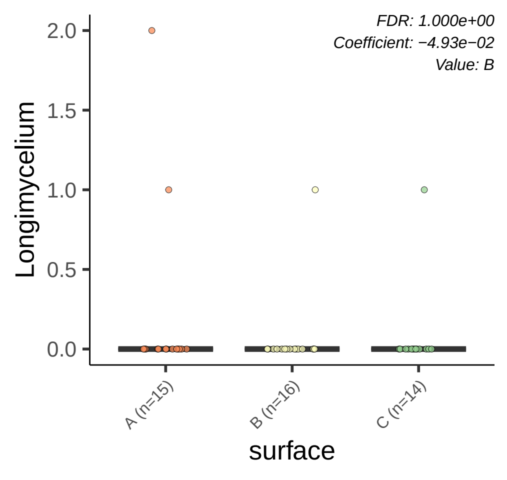

FDR: 1.000e+00
Coefficient: −4.93e−02
Value: B
Longimycelium
2.0
1.5
1.0
0.5
0.0
A (n=15)
B (n=16)
C (n=14)
surface

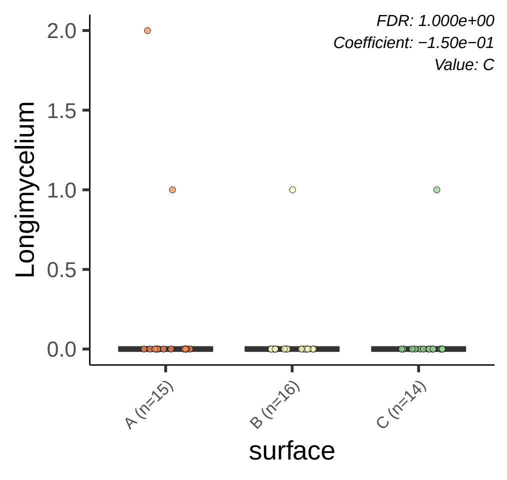
FDR: 1.000e+00
Coefficient: −1.50e−01
Value: C
2.0
1.5
1.0
0.5
0.0
Longimycelium
A (n=15)
B (n=16)
C (n=14)
surface

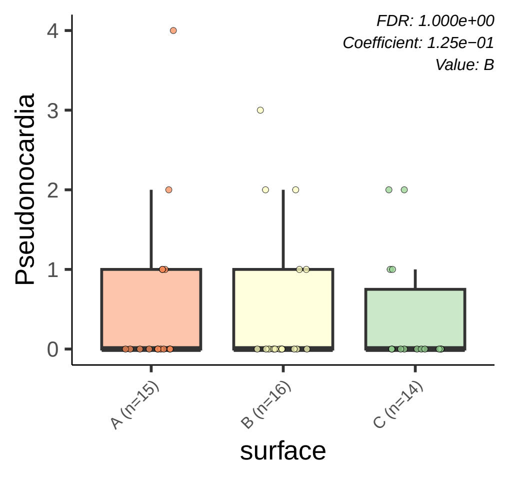
FDR: 1.000e+00
Coefficient: 1.25e−01
Value: B
Pseudonocardia
4
3
2
1
0
A (n=15)
B (n=16)
C (n=14)
surface

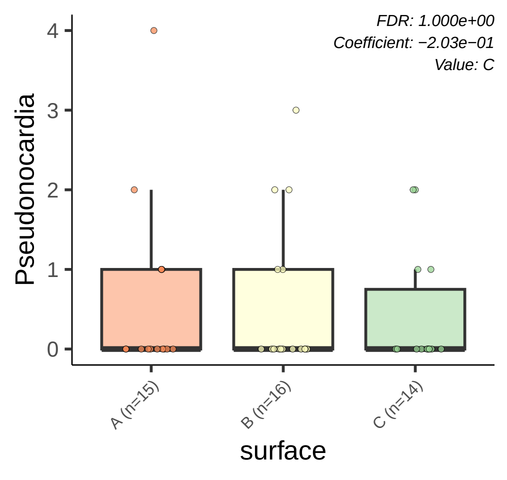

FDR: 1.000e+00
Coefficient: −2.03e−01
Value: C
Pseudonocardia
0
1
2
3
4
A (n=15)
B (n=16)
C (n=14)
surface

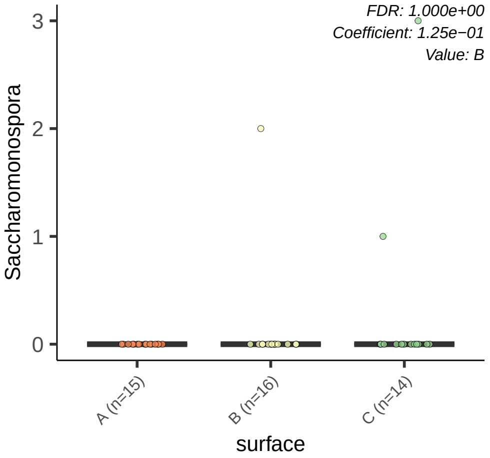
FDR: 1.000e+00
Coefficient: 1.25e−01
Value: B
Saccharomonospora
3
2
1
0
A (n=15)
B (n=16)
C (n=14)
surface

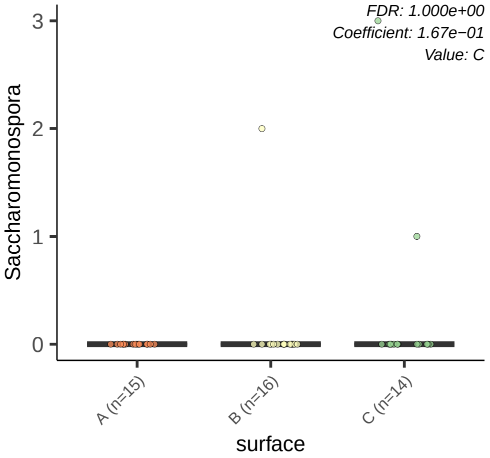
FDR: 1.000e+00
Coefficient: 1.67e−01
Value: C
Saccharomonospora
3
2
1
0
A (n=15)
B (n=16)
C (n=14)
surface

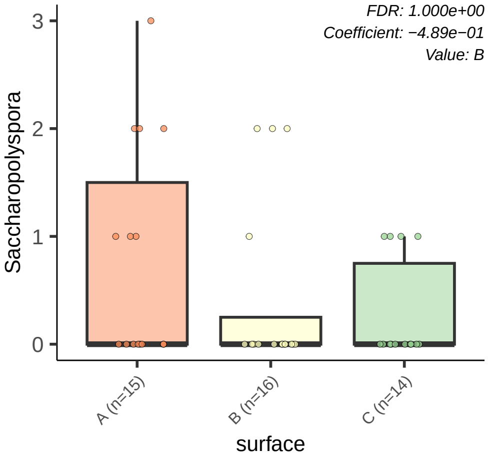

FDR: 1.000e+00
Coefficient: −4.89e−01
Value: B
Saccharopolyspora
3
2
1
0
A (n=15)
B (n=16)
C (n=14)
surface

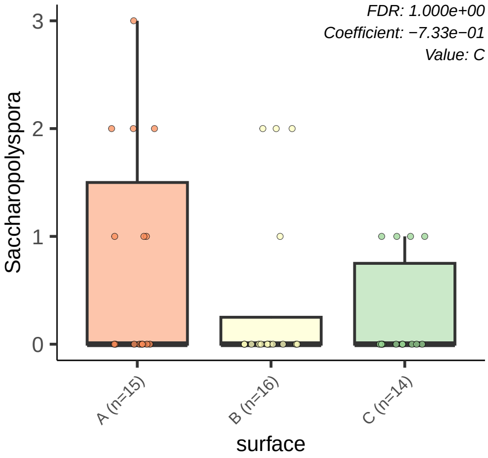
FDR: 1.000e+00
Coefficient: −7.33e−01
Value: C
Saccharopolyspora
3
2
1
0
A (n=15)
B (n=16)
C (n=14)
surface

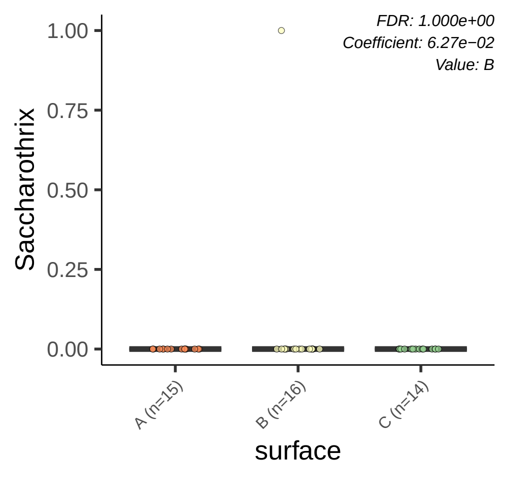

FDR: 1.000e+00
Coefficient: 6.27e−02
Value: B
Saccharothrix
1.00
0.75
0.50
0.25
0.00
A (n=15)
B (n=16)
C (n=14)
surface

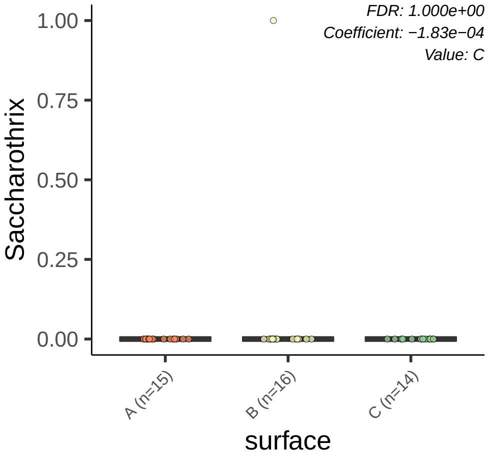
FDR: 1.000e+00
Coefficient: −1.83e−04
Value: C
Saccharothrix
1.00
0.75
0.50
0.25
0.00
A (n=15)
B (n=16)
C (n=14)
surface

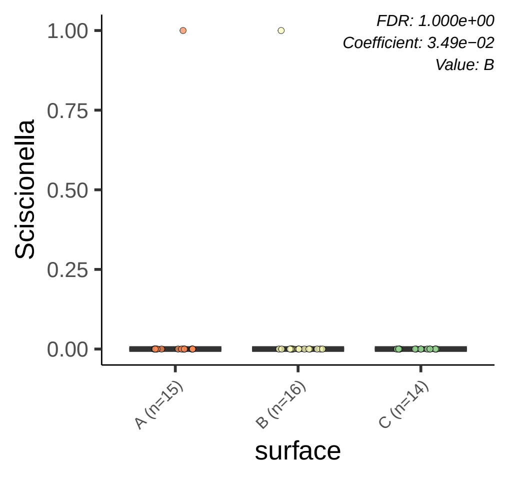
FDR: 1.000e+00
Coefficient: 3.49e−02
Value: B
Sciscionella
1.00
0.75
0.50
0.25
0.00
A (n=15)
B (n=16)
C (n=14)
surface

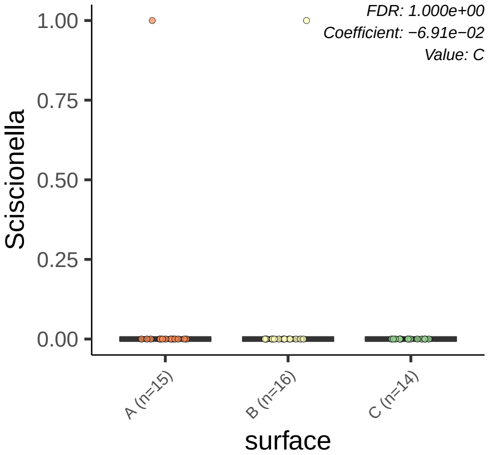

FDR: 1.000e+00
Coefficient: −6.91e−02
Value: C
Sciscionella
1.00
0.75
0.50
0.25
0.00
A (n=15)
B (n=16)
C (n=14)
surface

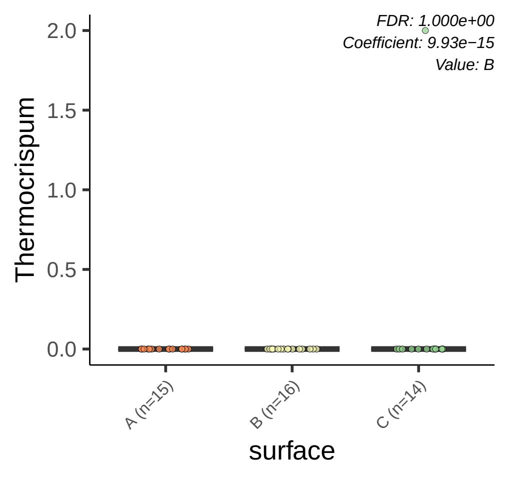
FDR: 1.000e+00
Coefficient: 9.93e−15
Value: B
Thermocrispum
2.0
1.5
1.0
0.5
0.0
A (n=15)
B (n=16)
C (n=14)
surface

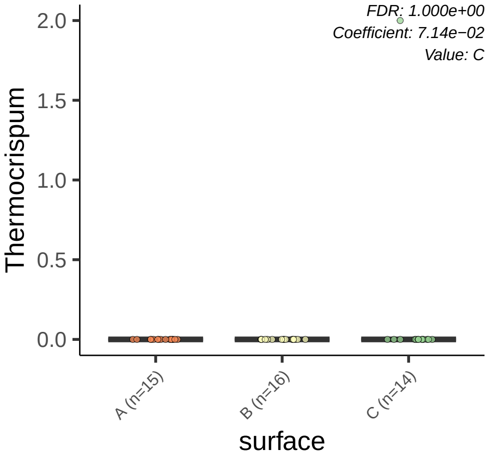
FDR: 1.000e+00
Coefficient: 7.14e−02
Value: C
Thermocrispum
2.0
1.5
1.0
0.5
0.0
A (n=15)
B (n=16)
C (n=14)
surface

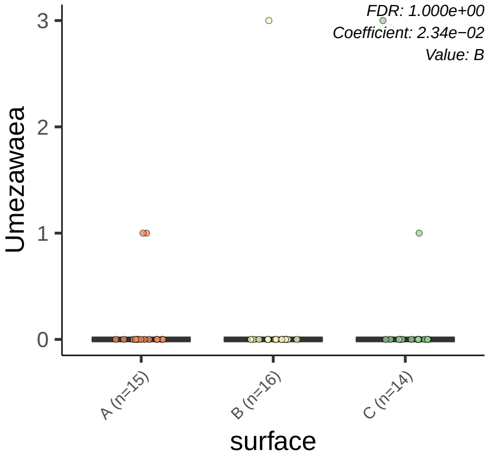
FDR: 1.000e+00
Coefficient: 2.34e−02
Value: B
Umezawaea
3
2
1
0
A (n=15)
B (n=16)
C (n=14)
surface

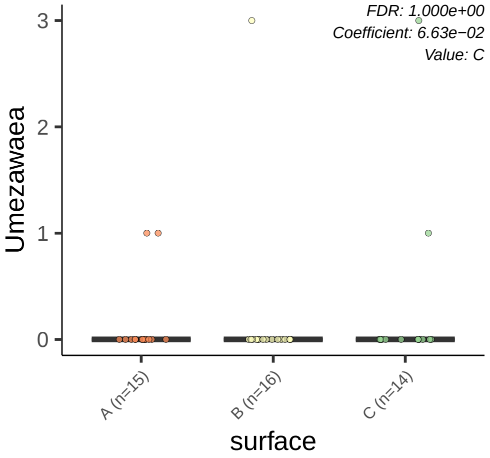

FDR: 1.000e+00
Coefficient: 6.63e−02
Value: C
Umezawaea
3
2
1
0
A (n=15)
B (n=16)
C (n=14)
surface

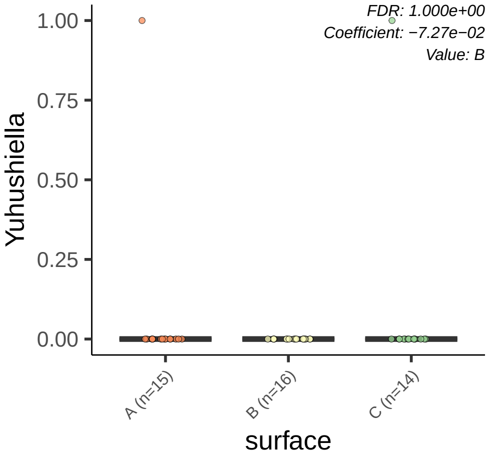

FDR: 1.000e+00
Coefficient: −7.27e−02
Value: B
Yuhushiella
1.00
0.75
0.50
0.25
0.00
A (n=15)
B (n=16)
C (n=14)
surface

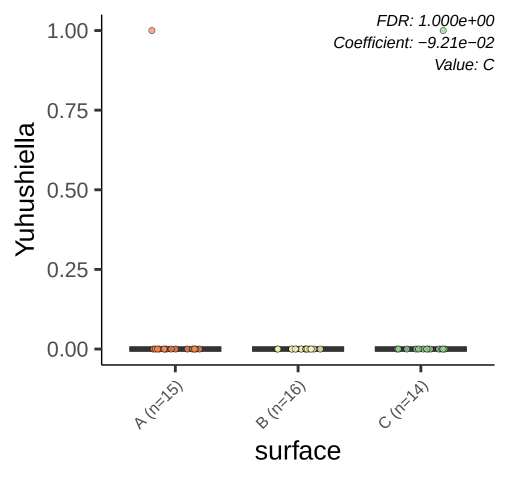
FDR: 1.000e+00
Coefficient: −9.21e−02
Value: C
1.00
0.75
0.50
0.25
0.00
Yuhushiella
A (n=15)
B (n=16)
C (n=14)
surface

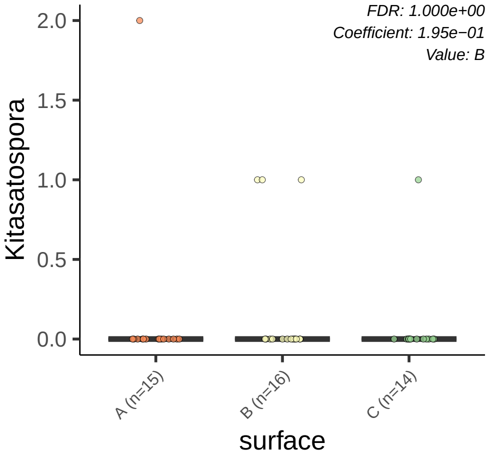
FDR: 1.000e+00
Coefficient: 1.95e−01
Value: B
Kitasatospora
2.0
1.5
1.0
0.5
0.0
A (n=15)
B (n=16)
C (n=14)
surface

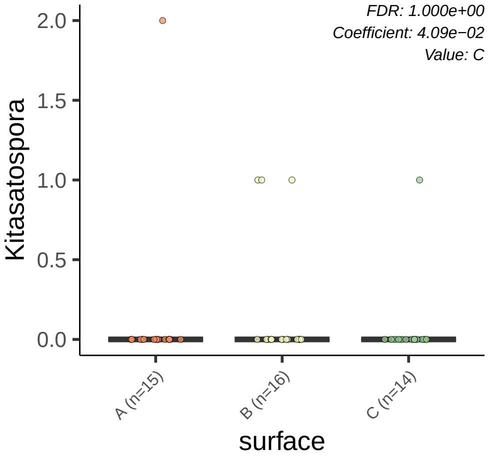
FDR: 1.000e+00
Coefficient: 4.09e−02
Value: C
Kitasatospora
2.0
1.5
1.0
0.5
0.0
A (n=15)
B (n=16)
C (n=14)
surface

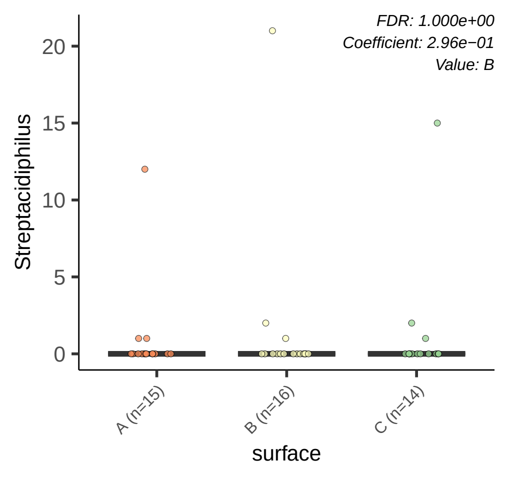
FDR: 1.000e+00
Coefficient: 2.96e−01
Value: B
Streptacidiphilus
20
15
10
5
0
A (n=15)
B (n=16)
C (n=14)
surface

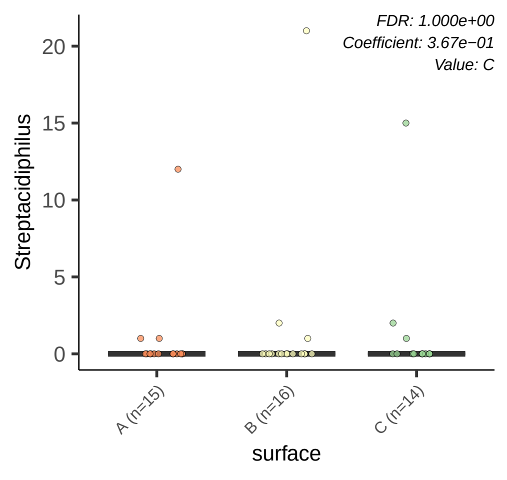

FDR: 1.000e+00
Coefficient: 3.67e−01
Value: C
Streptacidiphilus
20
15
10
5
0
A (n=15)
B (n=16)
C (n=14)
surface

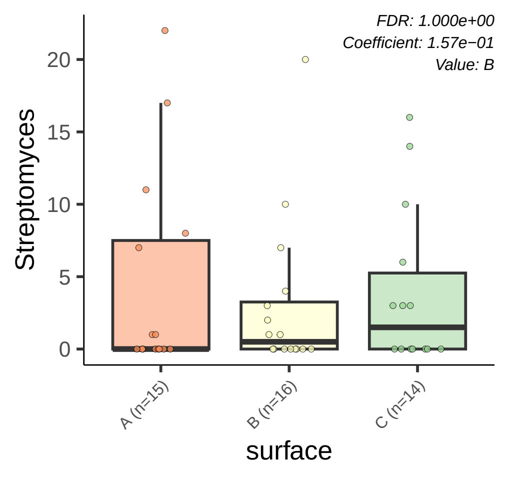
FDR: 1.000e+00
Coefficient: 1.57e−01
Value: B
Streptomyces
0
5
10
15
20
A (n=15)
B (n=16)
C (n=14)
surface

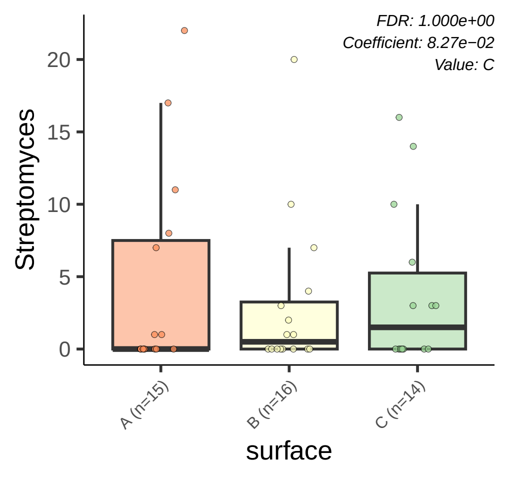
FDR: 1.000e+00
Coefficient: 8.27e−02
Value: C
Streptomyces
0
5
10
15
20
A (n=15)
B (n=16)
C (n=14)
surface

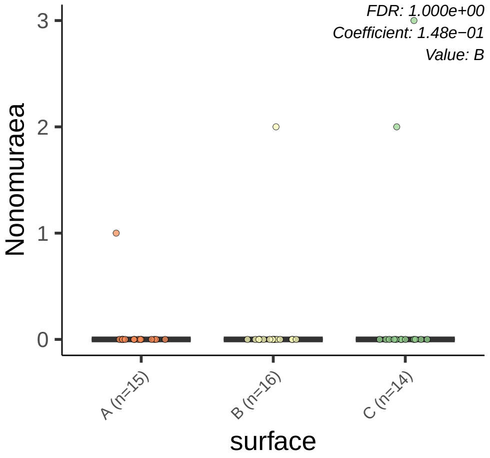

FDR: 1.000e+00
Coefficient: 1.48e−01
Value: B
Nonomuraea
0
1
2
3
A (n=15)
B (n=16)
C (n=14)
surface

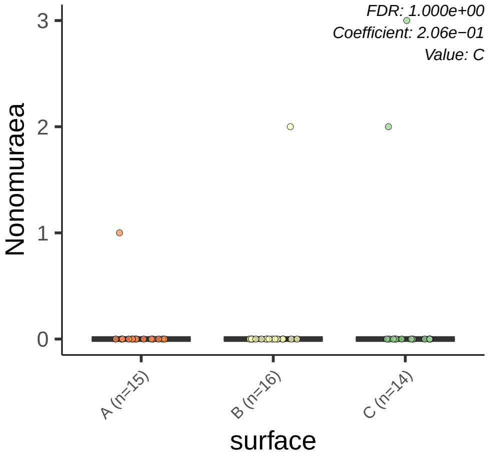

FDR: 1.000e+00
Coefficient: 2.06e−01
Value: C
Nonomuraea
3
2
1
0
A (n=15)
B (n=16)
C (n=14)
surface

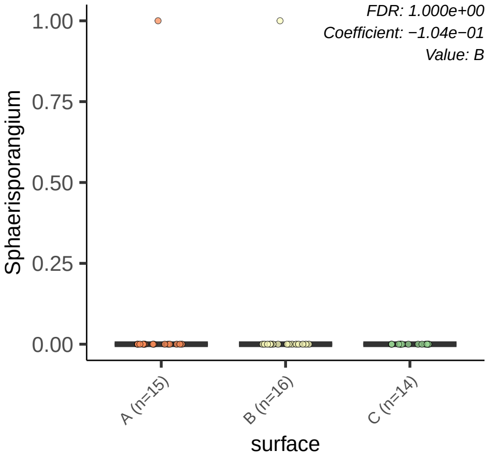
FDR: 1.000e+00
Coefficient: −1.04e−01
Value: B
Sphaerisporangium
1.00
0.75
0.50
0.25
0.00
A (n=15)
B (n=16)
C (n=14)
surface

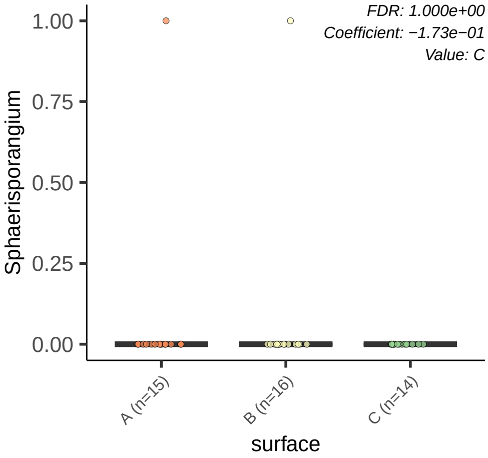
FDR: 1.000e+00
Coefficient: −1.73e−01
Value: C
Sphaerisporangium
1.00
0.75
0.50
0.25
0.00
A (n=15)
B (n=16)
C (n=14)
surface

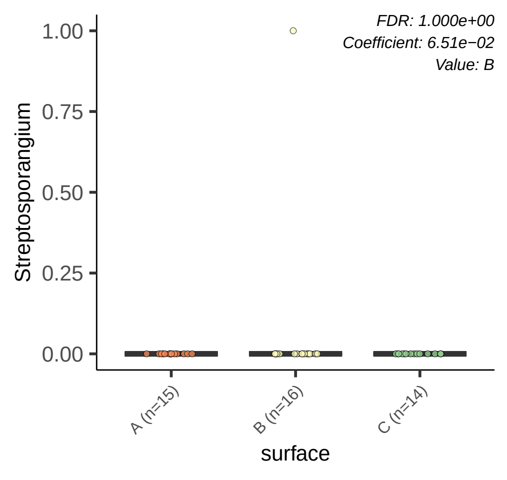
FDR: 1.000e+00
Coefficient: 6.51e−02
Value: B
Streptosporangium
1.00
0.75
0.50
0.25
0.00
A (n=15)
B (n=16)
C (n=14)
surface

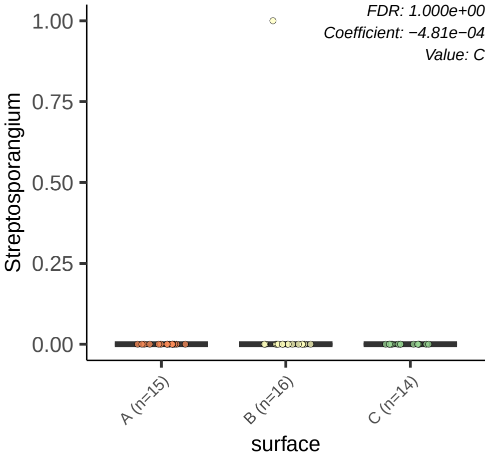
FDR: 1.000e+00
Coefficient: −4.81e−04
Value: C
Streptosporangium
1.00
0.75
0.50
0.25
0.00
A (n=15)
B (n=16)
C (n=14)
surface

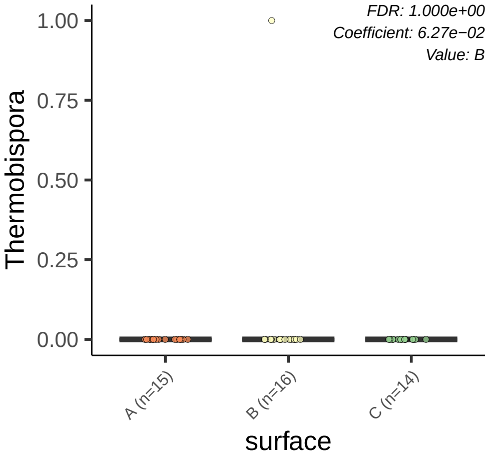

FDR: 1.000e+00
Coefficient: 6.27e−02
Value: B
Thermobispora
1.00
0.75
0.50
0.25
0.00
A (n=15)
B (n=16)
C (n=14)
surface

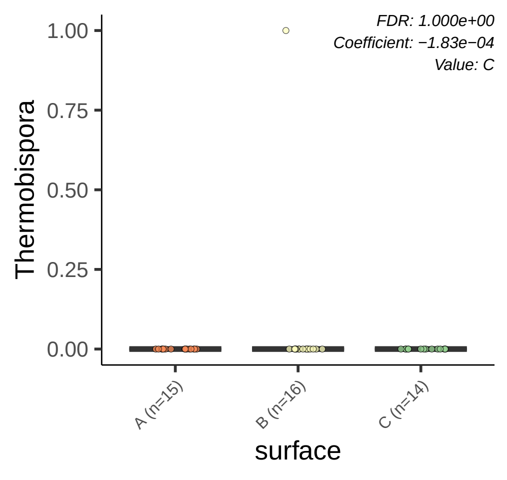
FDR: 1.000e+00
Coefficient: −1.83e−04
Value: C
Thermobispora
1.00
0.75
0.50
0.25
0.00
A (n=15)
B (n=16)
C (n=14)
surface

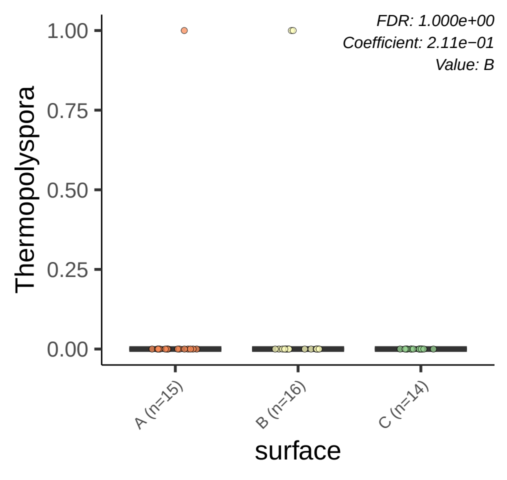
FDR: 1.000e+00
Coefficient: 2.11e−01
Value: B
Thermopolyspora
1.00
0.75
0.50
0.25
0.00
A (n=15)
B (n=16)
C (n=14)
surface

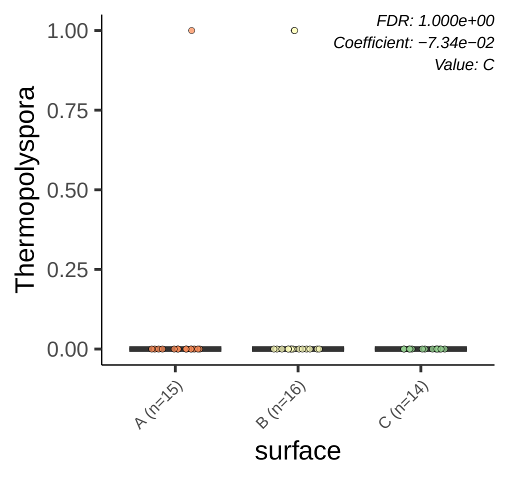
FDR: 1.000e+00
Coefficient: −7.34e−02
Value: C
Thermopolyspora
1.00
0.75
0.50
0.25
0.00
A (n=15)
B (n=16)
C (n=14)
surface

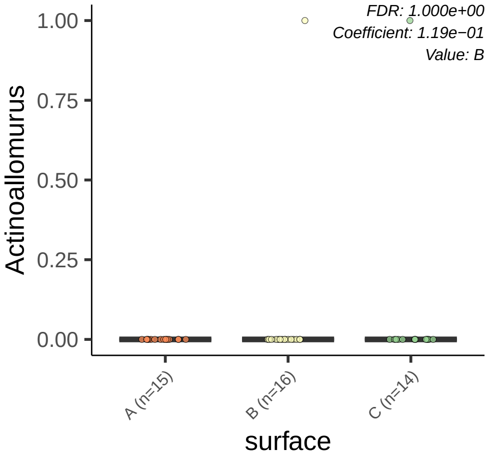
FDR: 1.000e+00
Coefficient: 1.19e−01
Value: B
Actinoallomurus
1.00
0.75
0.50
0.25
0.00
A (n=15)
B (n=16)
C (n=14)
surface

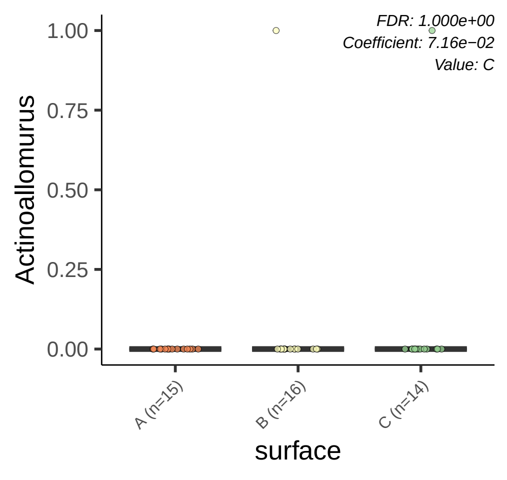

FDR: 1.000e+00
Coefficient: 7.16e−02
Value: C
Actinoallomurus
1.00
0.75
0.50
0.25
0.00
A (n=15)
B (n=16)
C (n=14)
surface

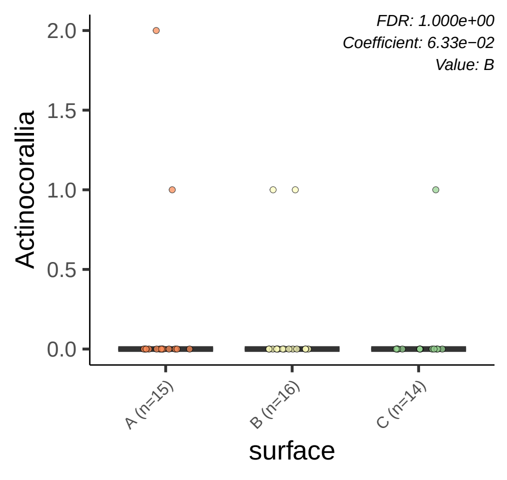
FDR: 1.000e+00
Coefficient: 6.33e−02
Value: B
Actinocorallia
2.0
1.5
1.0
0.5
0.0
A (n=15)
B (n=16)
C (n=14)
surface

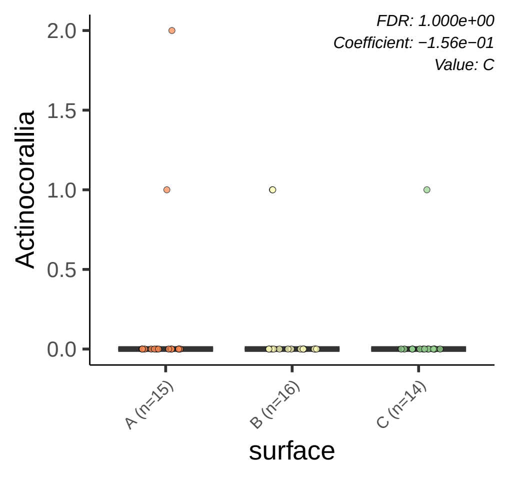

FDR: 1.000e+00
Coefficient: −1.56e−01
Value: C
Actinocorallia
2.0
1.5
1.0
0.5
0.0
A (n=15)
B (n=16)
C (n=14)
surface

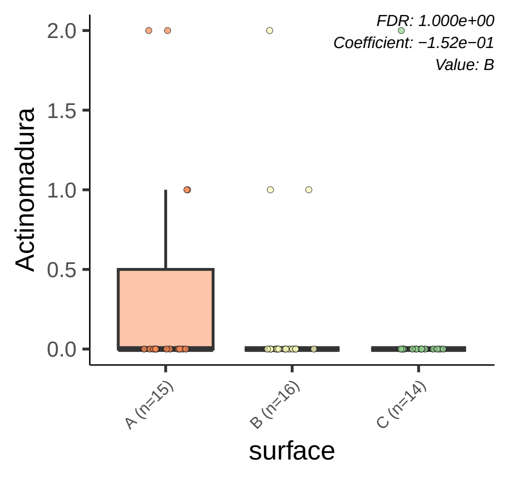
FDR: 1.000e+00
Coefficient: −1.52e−01
Value: B
Actinomadura
2.0
1.5
1.0
0.5
0.0
A (n=15)
B (n=16)
C (n=14)
surface

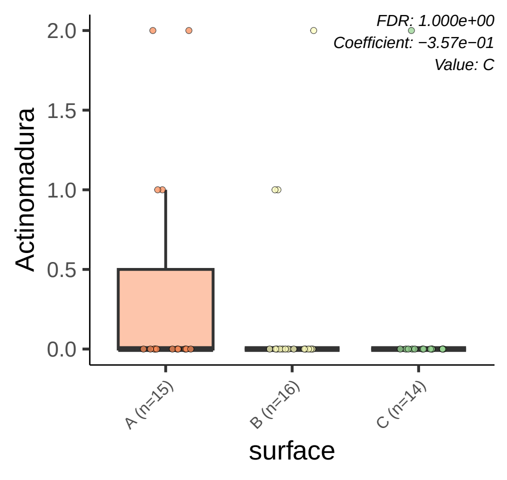

FDR: 1.000e+00
Coefficient: −3.57e−01
Value: C
Actinomadura
2.0
1.5
1.0
0.5
0.0
A (n=15)
B (n=16)
C (n=14)
surface

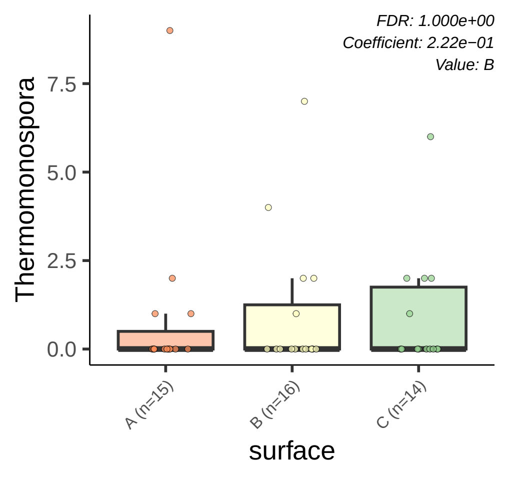

FDR: 1.000e+00
Coefficient: 2.22e−01
Value: B
Thermomonospora
7.5
5.0
2.5
0.0
A (n=15)
B (n=16)
C (n=14)
surface

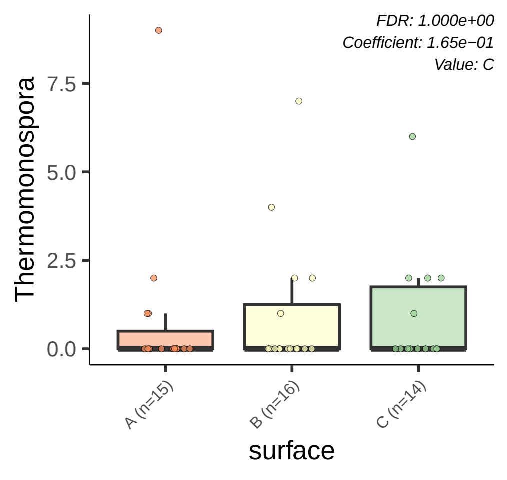
FDR: 1.000e+00
Coefficient: 1.65e−01
Value: C
Thermomonospora
7.5
5.0
2.5
0.0
A (n=15)
B (n=16)
C (n=14)
surface

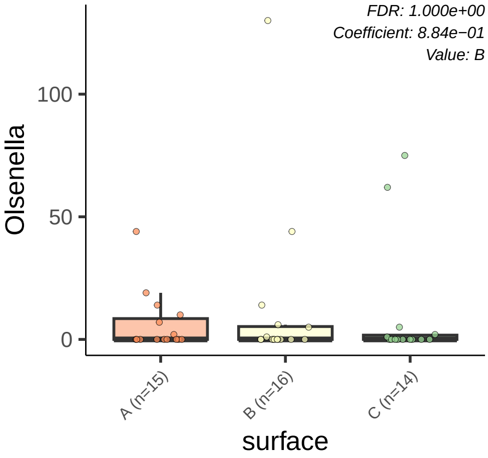

FDR: 1.000e+00
Coefficient: 8.84e−01
Value: B
Olsenella
100
50
0
A (n=15)
B (n=16)
C (n=14)
surface

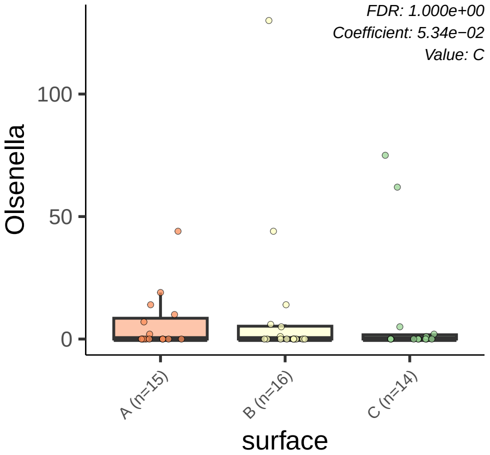

FDR: 1.000e+00
Coefficient: 5.34e−02
Value: C
Olsenella
100
50
0
A (n=15)
B (n=16)
C (n=14)
surface

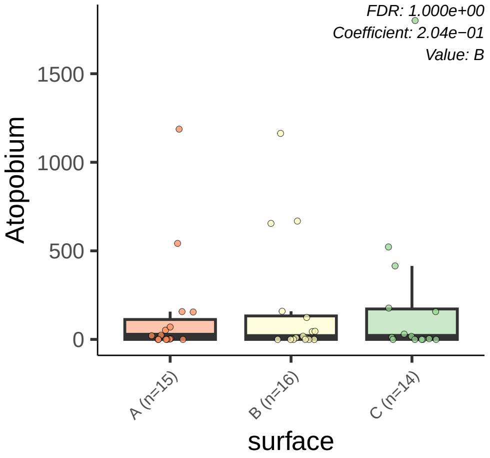

FDR: 1.000e+00
Coefficient: 2.04e−01
Value: B
Atopobium
1500
1000
500
0
A (n=15)
B (n=16)
C (n=14)
surface

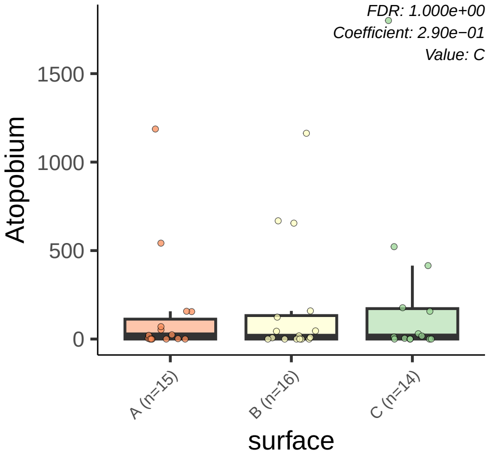

FDR: 1.000e+00
Coefficient: 2.90e−01
Value: C
Atopobium
1500
1000
500
0
A (n=15)
B (n=16)
C (n=14)
surface

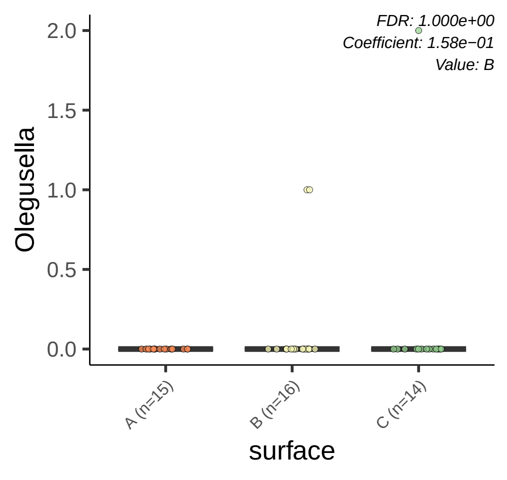

FDR: 1.000e+00
Coefficient: 1.58e−01
Value: B
Olegusella
2.0
1.5
1.0
0.5
0.0
A (n=15)
B (n=16)
C (n=14)
surface

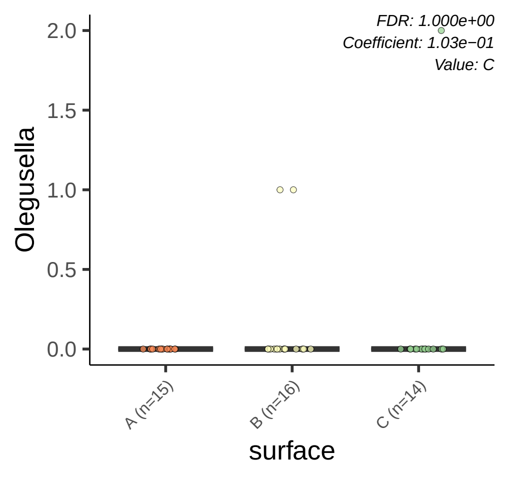

FDR: 1.000e+00
Coefficient: 1.03e−01
Value: C
Olegusella
2.0
1.5
1.0
0.5
0.0
A (n=15)
B (n=16)
C (n=14)
surface

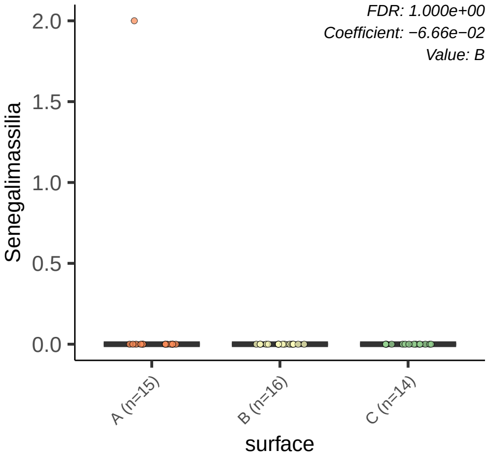

FDR: 1.000e+00
Coefficient: −6.66e−02
Value: B
Senegalimassilia
2.0
1.5
1.0
0.5
0.0
A (n=15)
B (n=16)
C (n=14)
surface

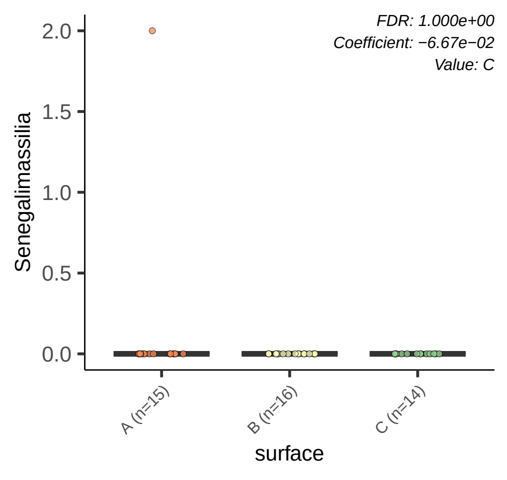

FDR: 1.000e+00
Coefficient: −6.67e−02
Value: C
Senegalimassilia
2.0
1.5
1.0
0.5
0.0
A (n=15)
B (n=16)
C (n=14)
surface

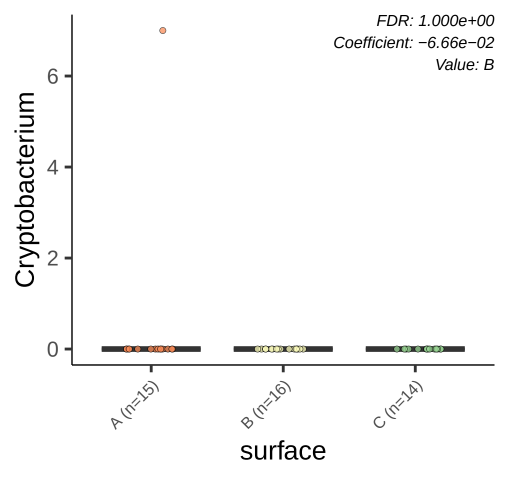

FDR: 1.000e+00
Coefficient: −6.66e−02
Value: B
Cryptobacterium
6
4
2
0
A (n=15)
B (n=16)
C (n=14)
surface

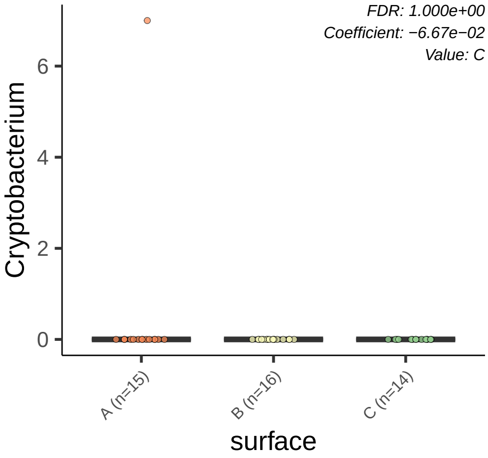
FDR: 1.000e+00
Coefficient: −6.67e−02
Value: C
Cryptobacterium
0
2
4
6
A (n=15)
B (n=16)
C (n=14)
surface

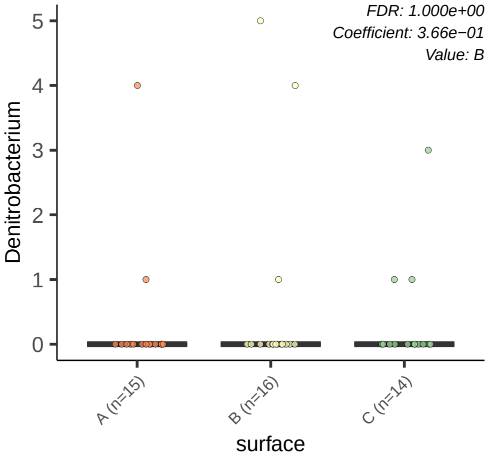

FDR: 1.000e+00
Coefficient: 3.66e−01
Value: B
Denitrobacterium
0
1
2
3
4
5
A (n=15)
B (n=16)
C (n=14)
surface

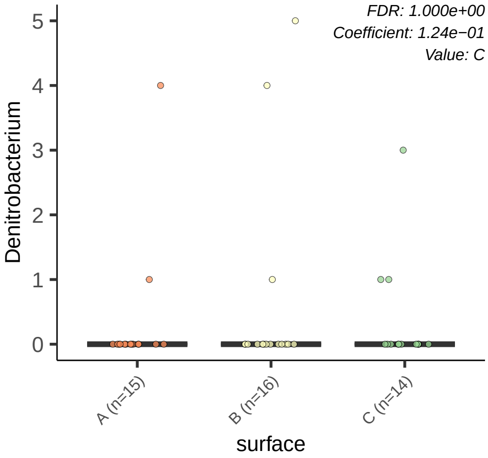

FDR: 1.000e+00
Coefficient: 1.24e−01
Value: C
Denitrobacterium
0
1
2
3
4
5
A (n=15)
B (n=16)
C (n=14)
surface

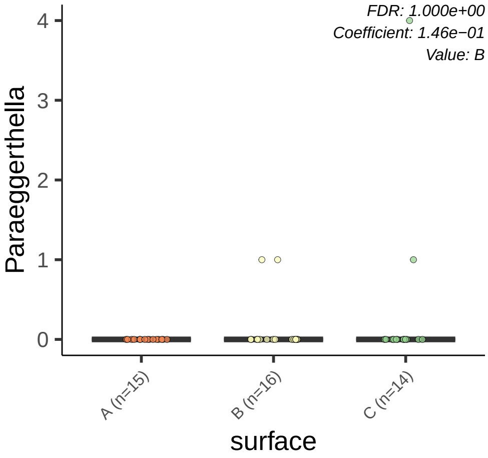

FDR: 1.000e+00
Coefficient: 1.46e−01
Value: B
Paraeggerthella
0
1
2
3
4
A (n=15)
B (n=16)
C (n=14)
surface

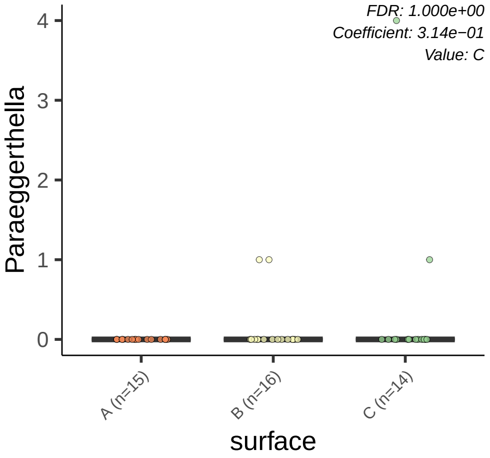

FDR: 1.000e+00
Coefficient: 3.14e−01
Value: C
Paraeggerthella
4
3
2
1
0
A (n=15)
B (n=16)
C (n=14)
surface

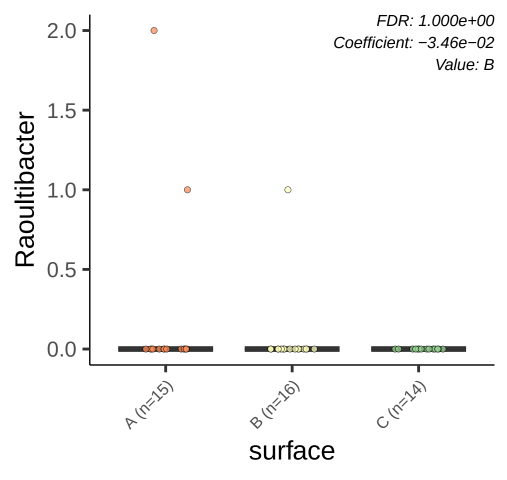

FDR: 1.000e+00
Coefficient: −3.46e−02
Value: B
Raoultibacter
2.0
1.5
1.0
0.5
0.0
A (n=15)
B (n=16)
C (n=14)
surface

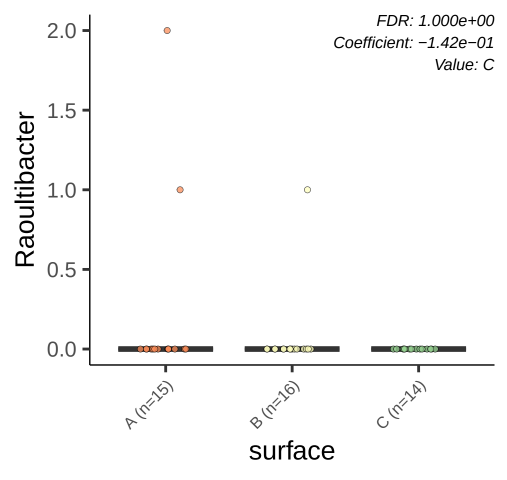
FDR: 1.000e+00
Coefficient: −1.42e−01
Value: C
Raoultibacter
2.0
1.5
1.0
0.5
0.0
A (n=15)
B (n=16)
C (n=14)
surface

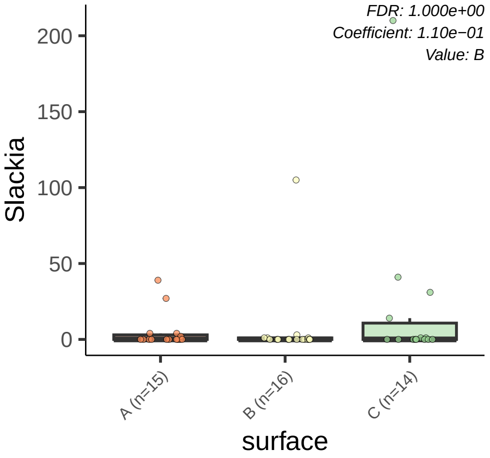
FDR: 1.000e+00
Coefficient: 1.10e−01
Value: B
Slackia
200
150
100
50
0
A (n=15)
B (n=16)
C (n=14)
surface

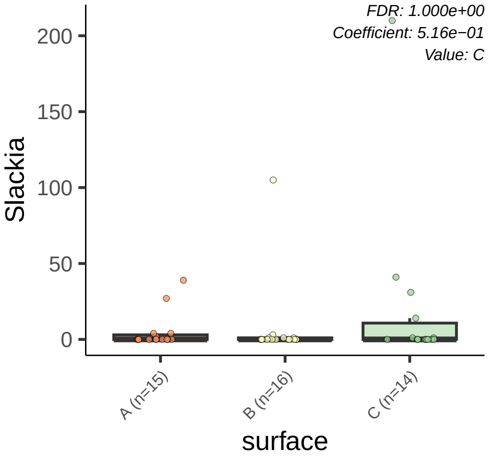
FDR: 1.000e+00
Coefficient: 5.16e−01
Value: C
Slackia
200
150
100
50
0
A (n=15)
B (n=16)
C (n=14)
surface

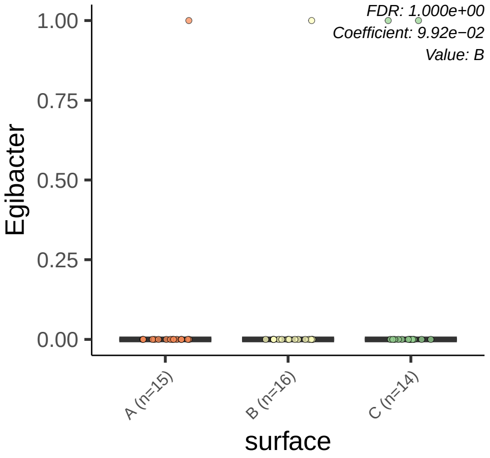

FDR: 1.000e+00
Coefficient: 9.92e−02
Value: B
Egibacter
1.00
0.75
0.50
0.25
0.00
A (n=15)
B (n=16)
C (n=14)
surface

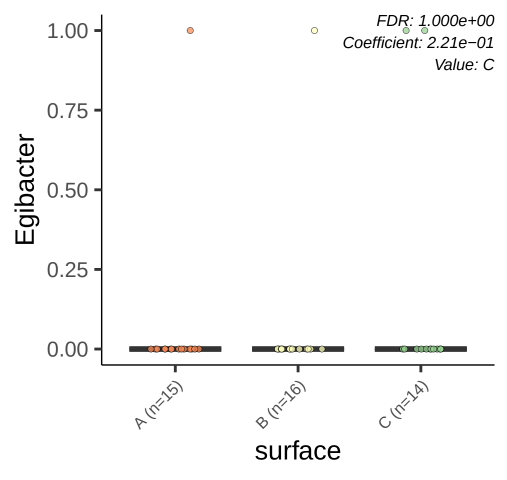
FDR: 1.000e+00
Coefficient: 2.21e−01
Value: C
Egibacter
1.00
0.75
0.50
0.25
0.00
A (n=15)
B (n=16)
C (n=14)
surface

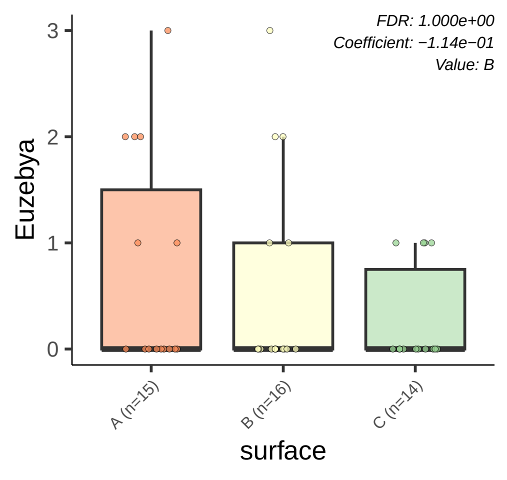

FDR: 1.000e+00
Coefficient: −1.14e−01
Value: B
Euzebya
3
2
1
0
A (n=15)
B (n=16)
C (n=14)
surface

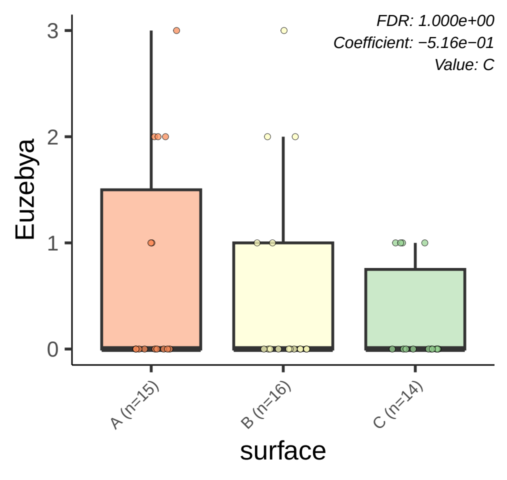

FDR: 1.000e+00
Coefficient: −5.16e−01
Value: C
Euzebya
3
2
1
0
A (n=15)
B (n=16)
C (n=14)
surface

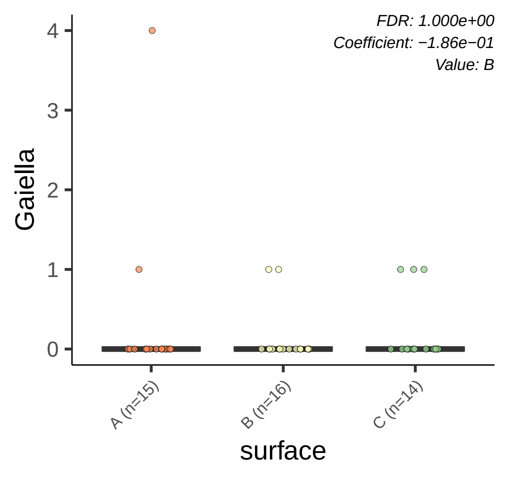

FDR: 1.000e+00
Coefficient: −1.86e−01
Value: B
Gaiella
4
3
2
1
0
A (n=15)
B (n=16)
C (n=14)
surface

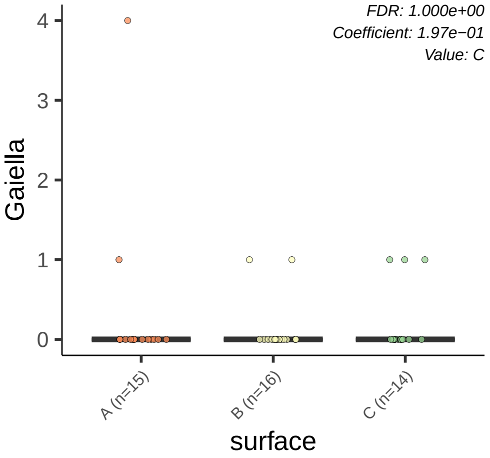
FDR: 1.000e+00
Coefficient: 1.97e−01
Value: C
Gaiella
4
3
2
1
0
A (n=15)
B (n=16)
C (n=14)
surface

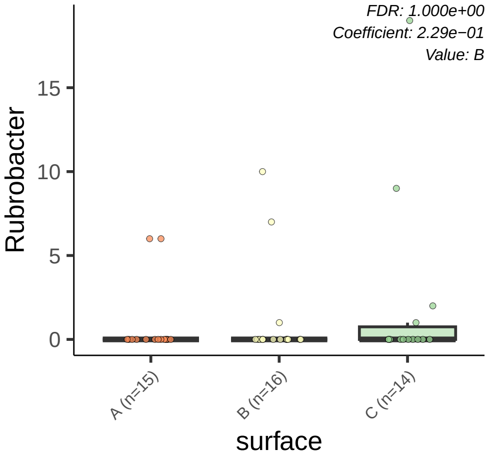
FDR: 1.000e+00
Coefficient: 2.29e−01
Value: B
Rubrobacter
15
10
5
0
A (n=15)
B (n=16)
C (n=14)
surface

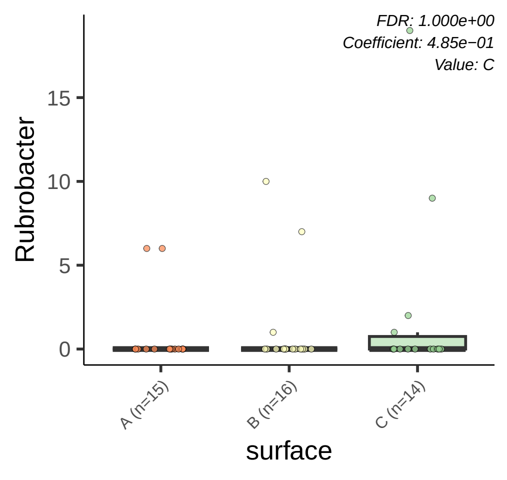

FDR: 1.000e+00
Coefficient: 4.85e−01
Value: C
Rubrobacter
15
10
5
0
A (n=15)
B (n=16)
C (n=14)
surface

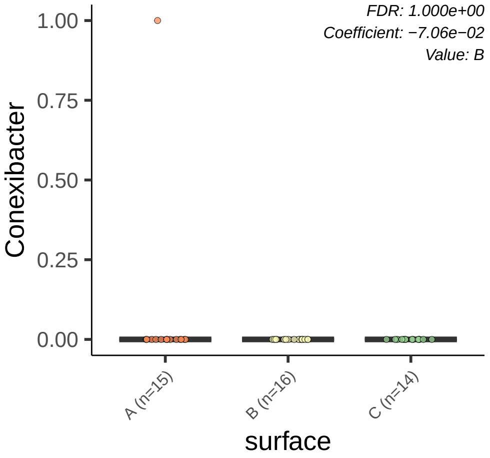

FDR: 1.000e+00
Coefficient: −7.06e−02
Value: B
Conexibacter
1.00
0.75
0.50
0.25
0.00
A (n=15)
B (n=16)
C (n=14)
surface

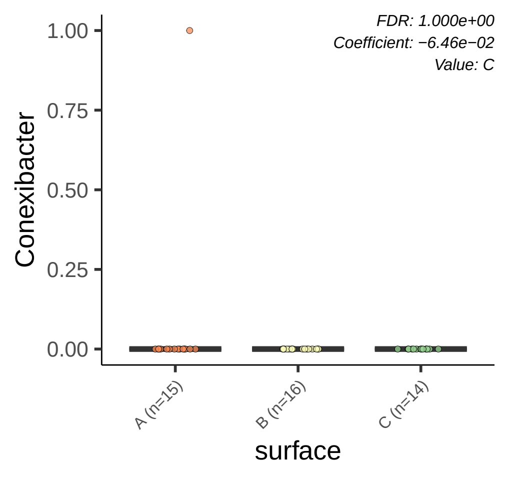

FDR: 1.000e+00
Coefficient: −6.46e−02
Value: C
Conexibacter
1.00
0.75
0.50
0.25
0.00
A (n=15)
B (n=16)
C (n=14)
surface

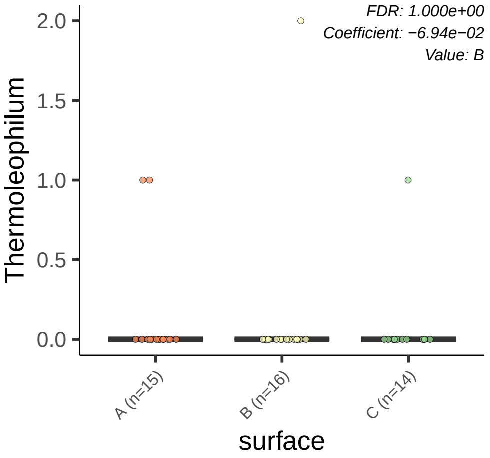

FDR: 1.000e+00
Coefficient: −6.94e−02
Value: B
Thermoleophilum
2.0
1.5
1.0
0.5
0.0
A (n=15)
B (n=16)
C (n=14)
surface

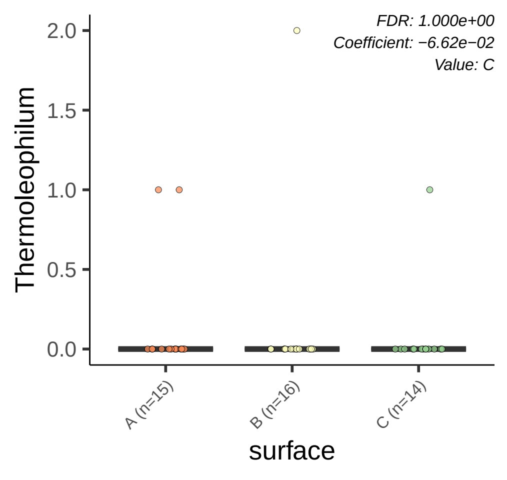
FDR: 1.000e+00
Coefficient: −6.62e−02
Value: C
Thermoleophilum
2.0
1.5
1.0
0.5
0.0
A (n=15)
B (n=16)
C (n=14)
surface

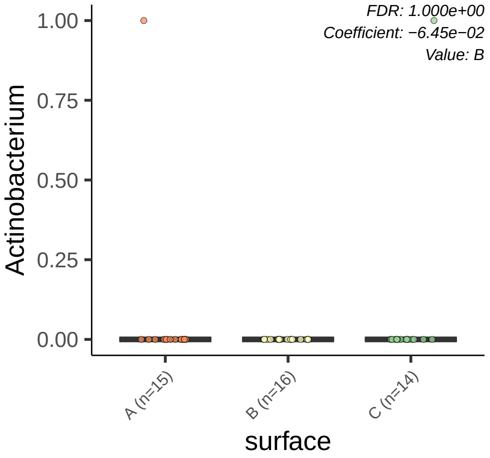
FDR: 1.000e+00
Coefficient: −6.45e−02
Value: B
Actinobacterium
1.00
0.75
0.50
0.25
0.00
A (n=15)
B (n=16)
C (n=14)
surface

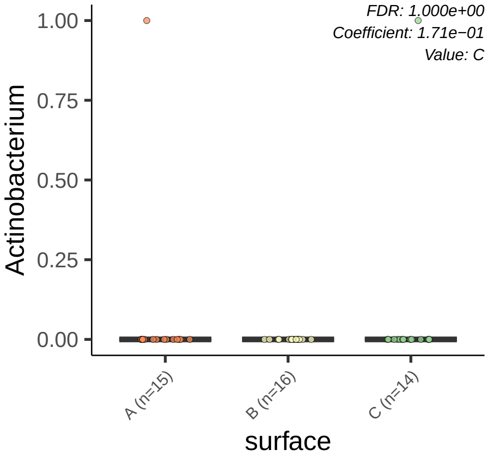
FDR: 1.000e+00
Coefficient: 1.71e−01
Value: C
Actinobacterium
1.00
0.75
0.50
0.25
0.00
A (n=15)
B (n=16)
C (n=14)
surface

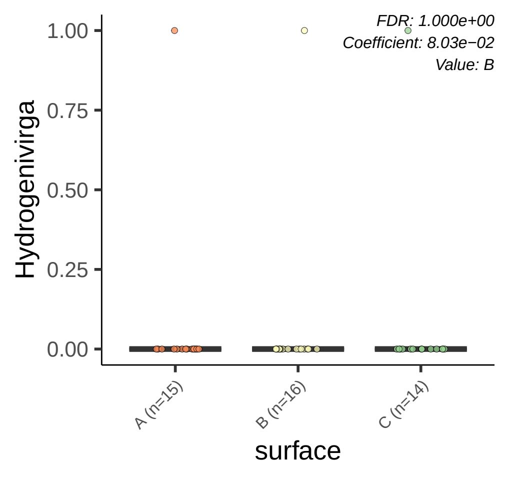
FDR: 1.000e+00
Coefficient: 8.03e−02
Value: B
Hydrogenivirga
1.00
0.75
0.50
0.25
0.00
A (n=15)
B (n=16)
C (n=14)
surface

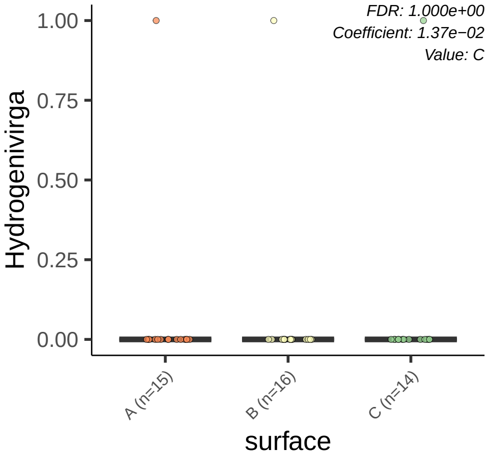
FDR: 1.000e+00
Coefficient: 1.37e−02
Value: C
Hydrogenivirga
1.00
0.75
0.50
0.25
0.00
A (n=15)
B (n=16)
C (n=14)
surface

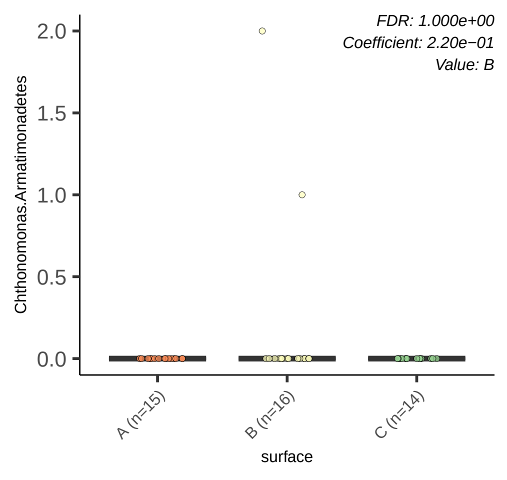

FDR: 1.000e+00
Coefficient: 2.20e−01
Value: B
Chthonomonas.Armatimonadetes
2.0
1.5
1.0
0.5
0.0
A (n=15)
B (n=16)
C (n=14)
surface

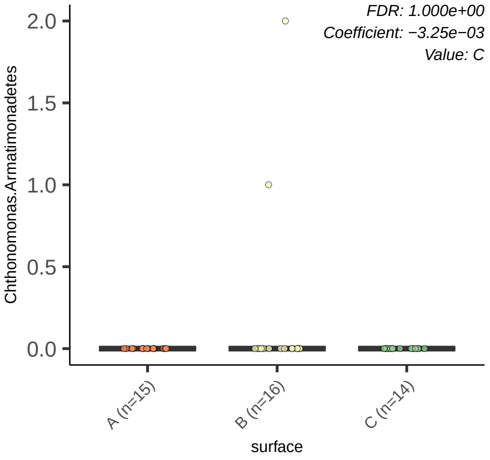

FDR: 1.000e+00
Coefficient: −3.25e−03
Value: C
2.0
1.5
1.0
0.5
0.0
Chthonomonas.Armatimonadetes
A (n=15)
B (n=16)
C (n=14)
surface

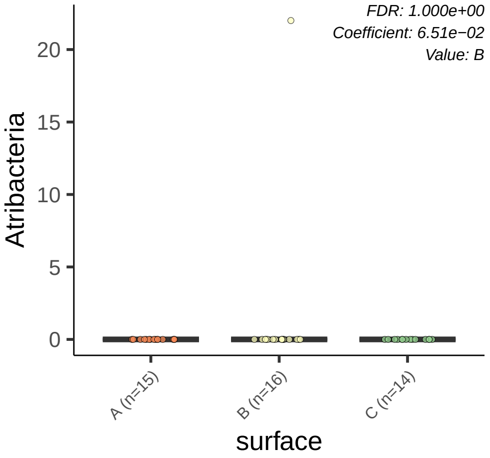
FDR: 1.000e+00
Coefficient: 6.51e−02
Value: B
Atribacteria
20
15
10
5
0
A (n=15)
B (n=16)
C (n=14)
surface

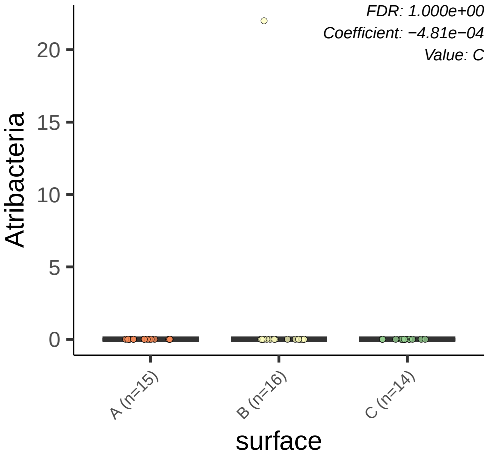
FDR: 1.000e+00
Coefficient: −4.81e−04
Value: C
Atribacteria
20
15
10
5
0
A (n=15)
B (n=16)
C (n=14)
surface

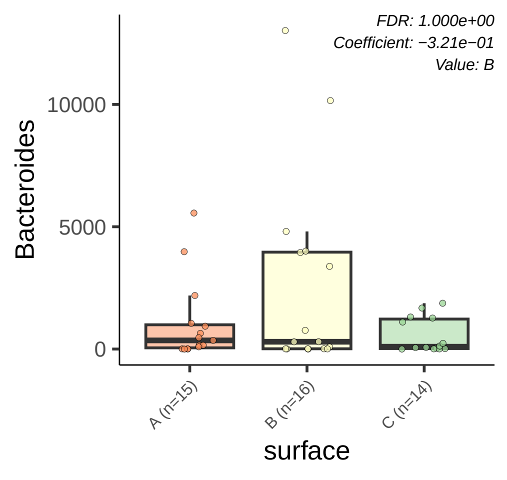

FDR: 1.000e+00
Coefficient: −3.21e−01
Value: B
Bacteroides
10000
5000
0
A (n=15)
B (n=16)
C (n=14)
surface

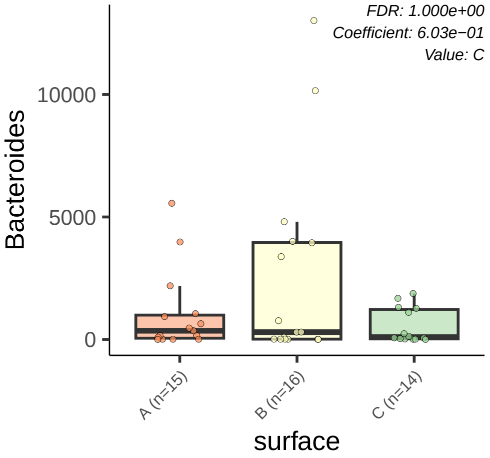

FDR: 1.000e+00
Coefficient: 6.03e−01
Value: C
Bacteroides
10000
5000
0
A (n=15)
B (n=16)
C (n=14)
surface

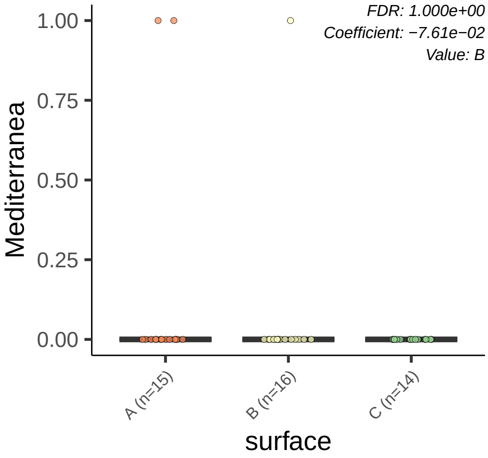
FDR: 1.000e+00
Coefficient: −7.61e−02
Value: B
Mediterranea
1.00
0.75
0.50
0.25
0.00
A (n=15)
B (n=16)
C (n=14)
surface

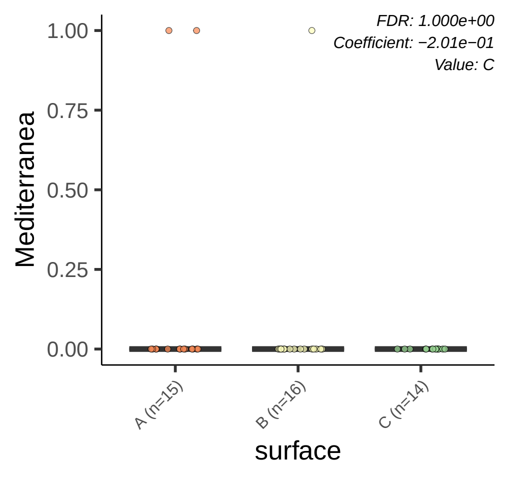
FDR: 1.000e+00
Coefficient: −2.01e−01
Value: C
Mediterranea
1.00
0.75
0.50
0.25
0.00
A (n=15)
B (n=16)
C (n=14)
surface

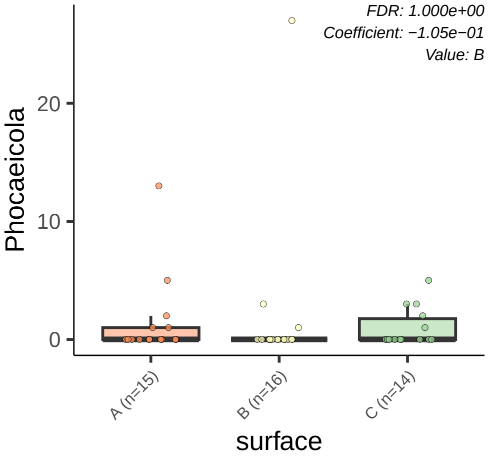
FDR: 1.000e+00
Coefficient: −1.05e−01
Value: B
Phocaeicola
0
10
20
A (n=15)
B (n=16)
C (n=14)
surface

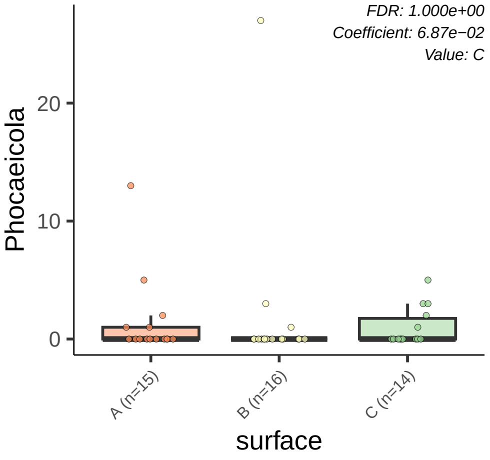
FDR: 1.000e+00
Coefficient: 6.87e−02
Value: C
Phocaeicola
20
10
0
A (n=15)
B (n=16)
C (n=14)
surface

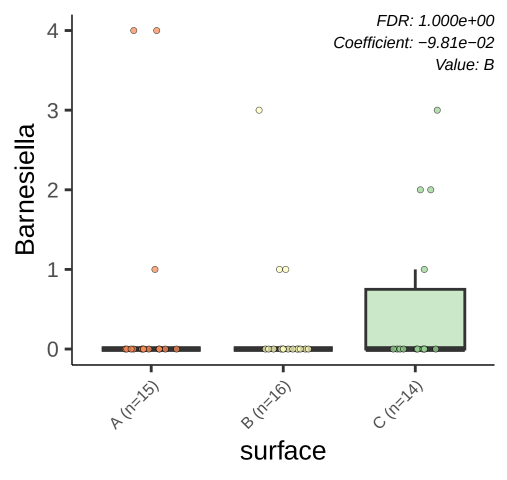

FDR: 1.000e+00
Coefficient: −9.81e−02
Value: B
Barnesiella
0
1
2
3
4
A (n=15)
B (n=16)
C (n=14)
surface

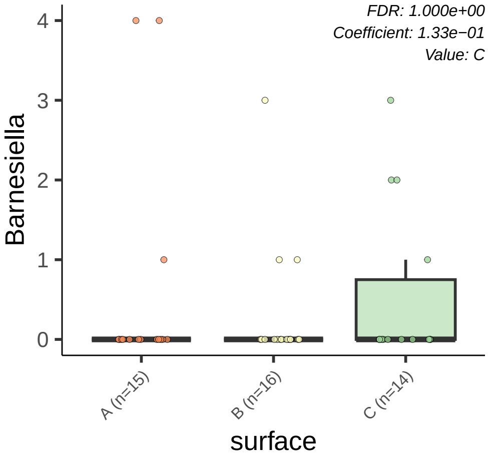
FDR: 1.000e+00
Coefficient: 1.33e−01
Value: C
Barnesiella
0
1
2
3
4
A (n=15)
B (n=16)
C (n=14)
surface

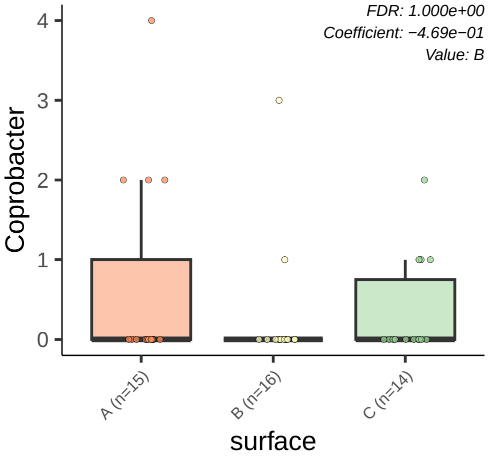

FDR: 1.000e+00
Coefficient: −4.69e−01
Value: B
Coprobacter
0
1
2
3
4
A (n=15)
B (n=16)
C (n=14)
surface

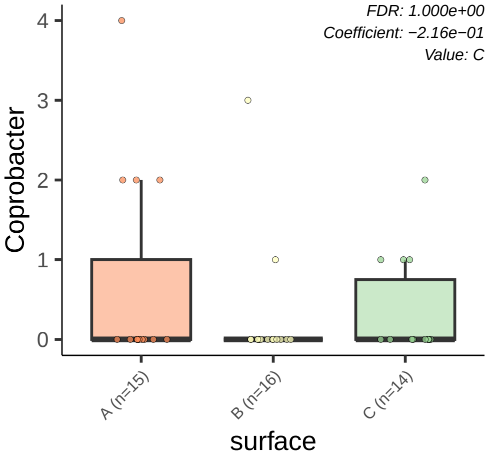
FDR: 1.000e+00
Coefficient: −2.16e−01
Value: C
Coprobacter
0
1
2
3
4
A (n=15)
B (n=16)
C (n=14)
surface

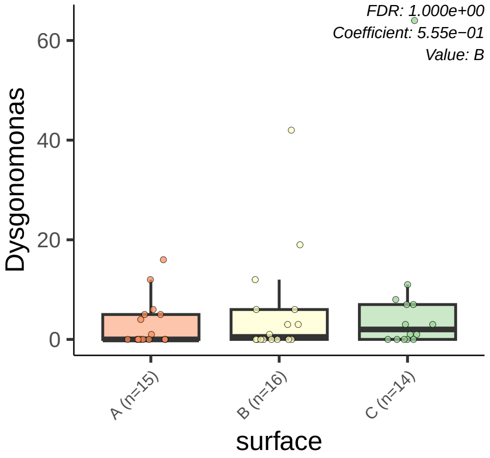

FDR: 1.000e+00
Coefficient: 5.55e−01
Value: B
Dysgonomonas
60
40
20
0
A (n=15)
B (n=16)
C (n=14)
surface

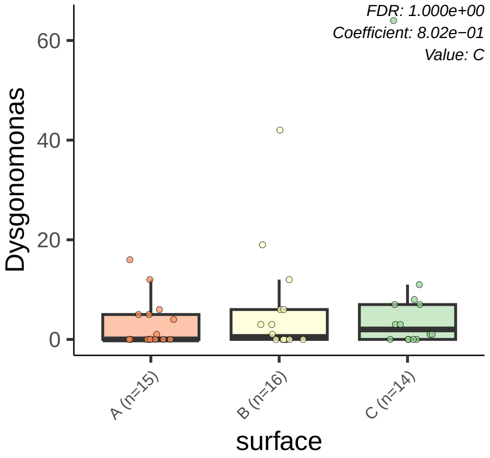
FDR: 1.000e+00
Coefficient: 8.02e−01
Value: C
60
40
20
0
Dysgonomonas
A (n=15)
B (n=16)
C (n=14)
surface

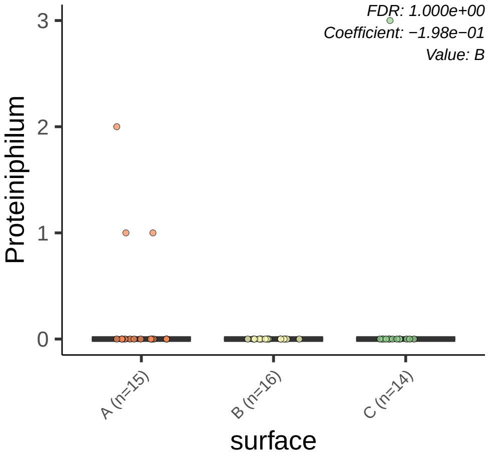
FDR: 1.000e+00
Coefficient: −1.98e−01
Value: B
Proteiniphilum
3
2
1
0
A (n=15)
B (n=16)
C (n=14)
surface

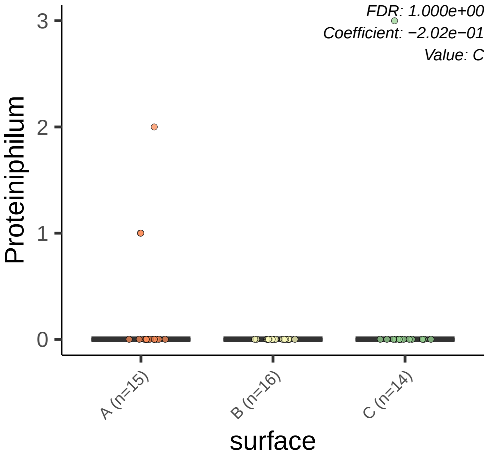
FDR: 1.000e+00
Coefficient: −2.02e−01
Value: C
Proteiniphilum
3
2
1
0
A (n=15)
B (n=16)
C (n=14)
surface

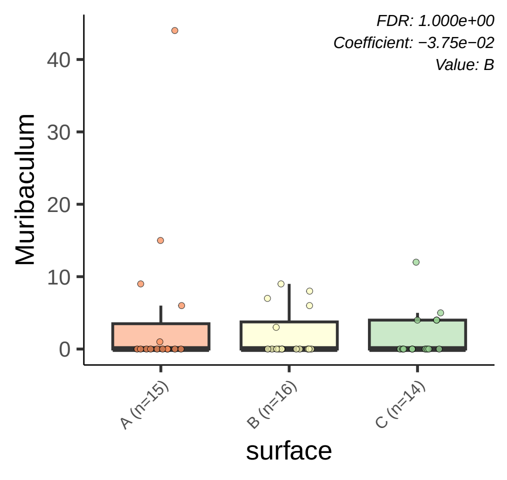
FDR: 1.000e+00
Coefficient: −3.75e−02
Value: B
Muribaculum
0
10
20
30
40
A (n=15)
B (n=16)
C (n=14)
surface

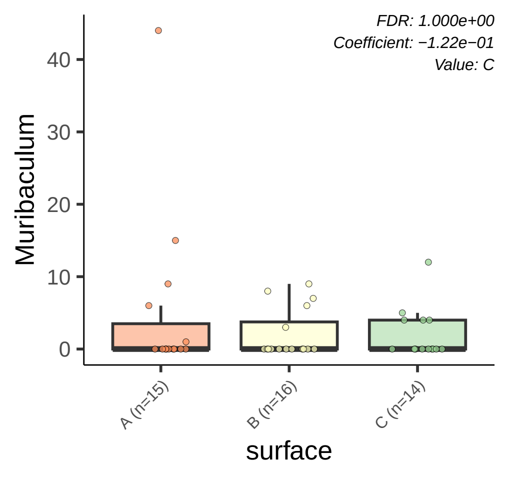
FDR: 1.000e+00
Coefficient: −1.22e−01
Value: C
Muribaculum
0
10
20
30
40
A (n=15)
B (n=16)
C (n=14)
surface

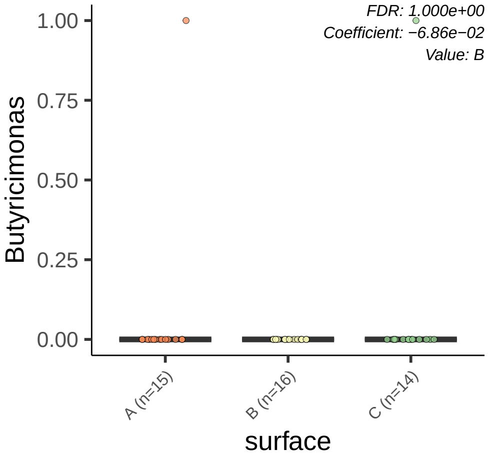
FDR: 1.000e+00
Coefficient: −6.86e−02
Value: B
Butyricimonas
1.00
0.75
0.50
0.25
0.00
A (n=15)
B (n=16)
C (n=14)
surface

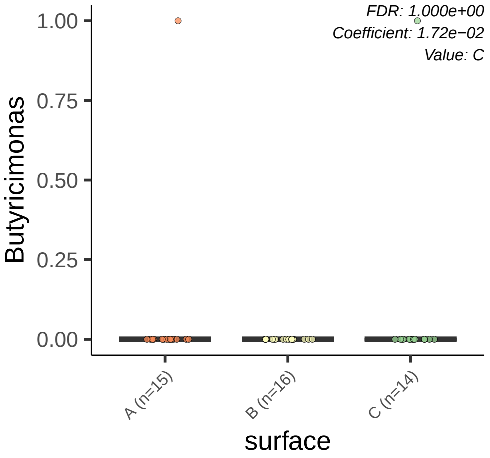

FDR: 1.000e+00
Coefficient: 1.72e−02
Value: C
Butyricimonas
1.00
0.75
0.50
0.25
0.00
A (n=15)
B (n=16)
C (n=14)
surface

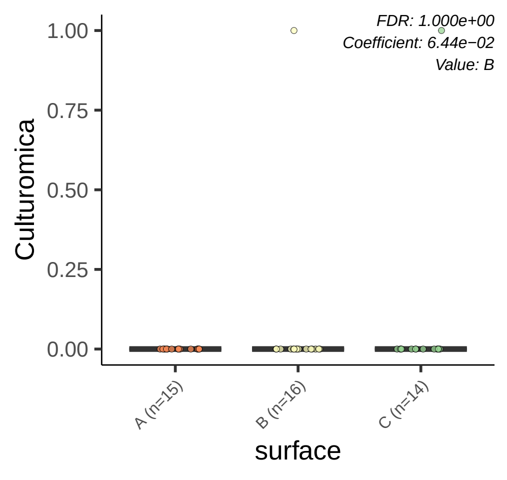

FDR: 1.000e+00
Coefficient: 6.44e−02
Value: B
Culturomica
1.00
0.75
0.50
0.25
0.00
A (n=15)
B (n=16)
C (n=14)
surface

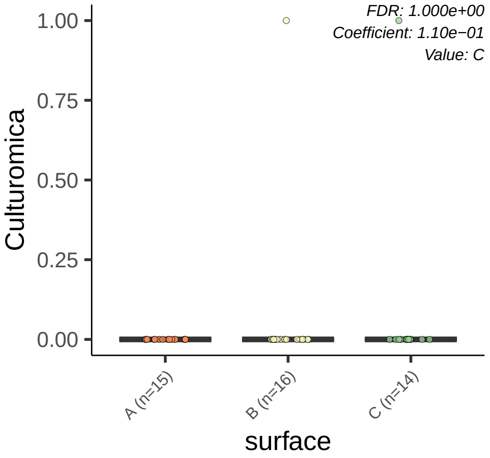
FDR: 1.000e+00
Coefficient: 1.10e−01
Value: C
Culturomica
1.00
0.75
0.50
0.25
0.00
A (n=15)
B (n=16)
C (n=14)
surface

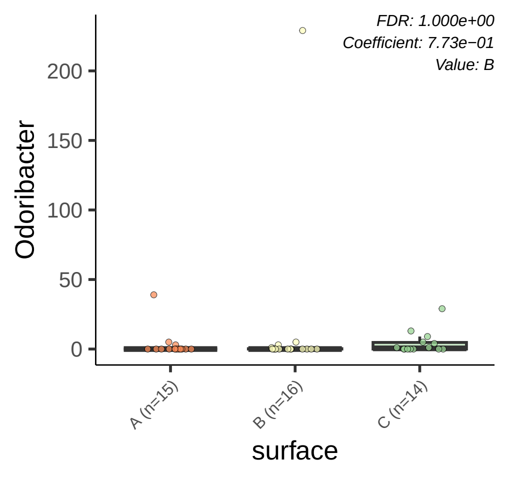

FDR: 1.000e+00
Coefficient: 7.73e−01
Value: B
Odoribacter
200
150
100
50
0
A (n=15)
B (n=16)
C (n=14)
surface

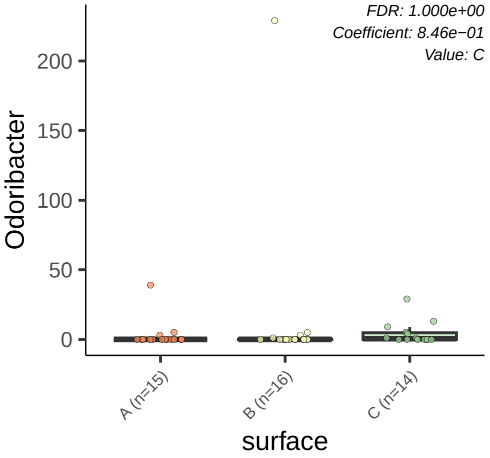
FDR: 1.000e+00
Coefficient: 8.46e−01
Value: C
Odoribacter
200
150
100
50
0
A (n=15)
B (n=16)
C (n=14)
surface

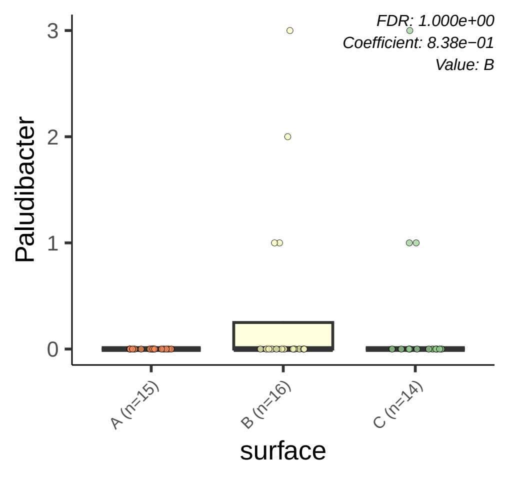
FDR: 1.000e+00
Coefficient: 8.38e−01
Value: B
Paludibacter
0
1
2
3
A (n=15)
B (n=16)
C (n=14)
surface

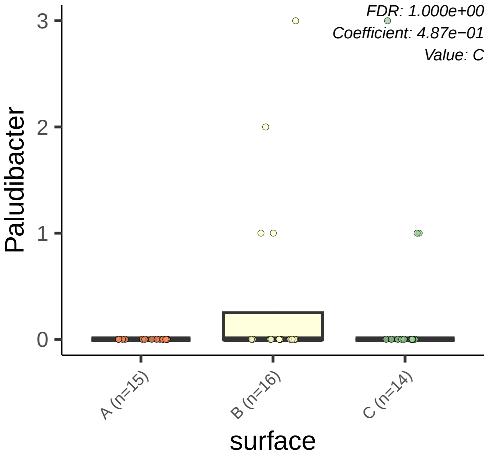

FDR: 1.000e+00
Coefficient: 4.87e−01
Value: C
Paludibacter
3
2
1
0
A (n=15)
B (n=16)
C (n=14)
surface

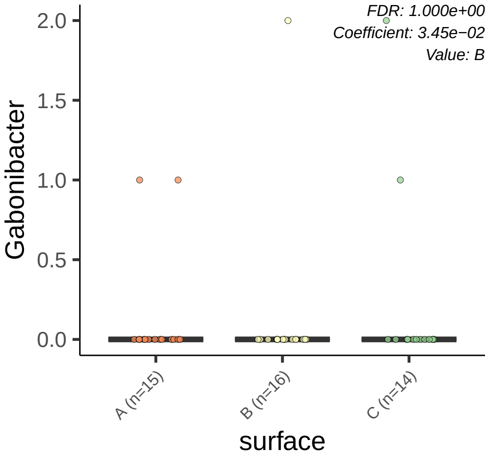

FDR: 1.000e+00
Coefficient: 3.45e−02
Value: B
Gabonibacter
2.0
1.5
1.0
0.5
0.0
A (n=15)
B (n=16)
C (n=14)
surface

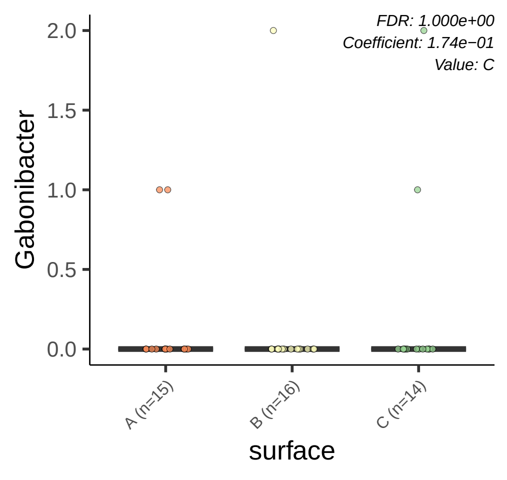

FDR: 1.000e+00
Coefficient: 1.74e−01
Value: C
Gabonibacter
2.0
1.5
1.0
0.5
0.0
A (n=15)
B (n=16)
C (n=14)
surface

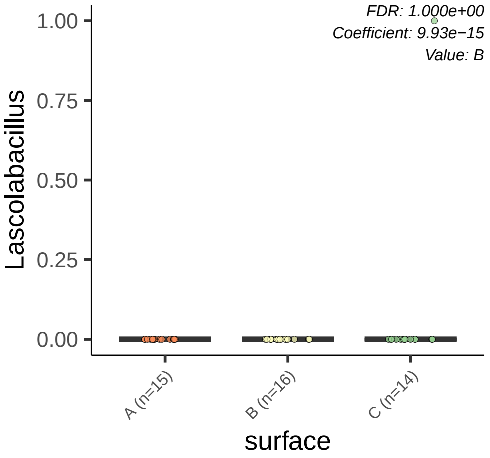

FDR: 1.000e+00
Coefficient: 9.93e−15
Value: B
Lascolabacillus
1.00
0.75
0.50
0.25
0.00
A (n=15)
B (n=16)
C (n=14)
surface

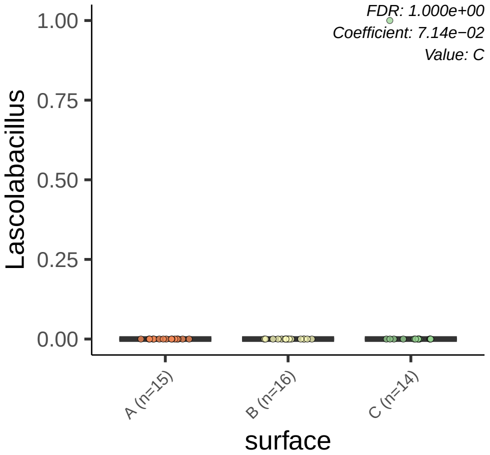
FDR: 1.000e+00
Coefficient: 7.14e−02
Value: C
Lascolabacillus
1.00
0.75
0.50
0.25
0.00
A (n=15)
B (n=16)
C (n=14)
surface

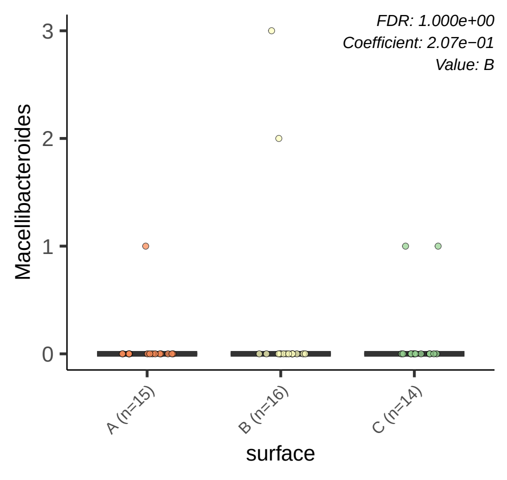

FDR: 1.000e+00
Coefficient: 2.07e−01
Value: B
Macellibacteroides
3
2
1
0
A (n=15)
B (n=16)
C (n=14)
surface

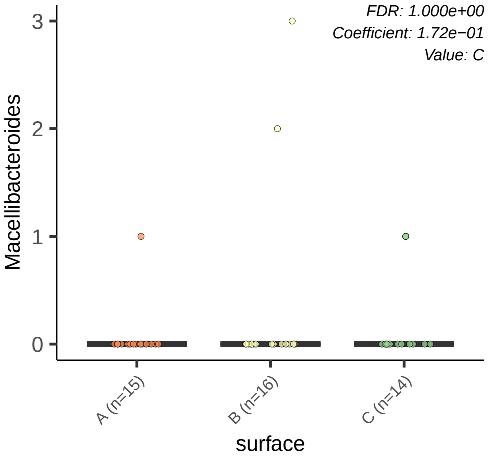
FDR: 1.000e+00
Coefficient: 1.72e−01
Value: C
Macellibacteroides
3
2
1
0
A (n=15)
B (n=16)
C (n=14)
surface

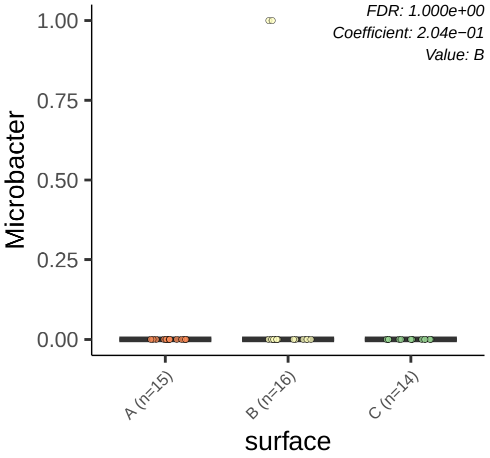
FDR: 1.000e+00
Coefficient: 2.04e−01
Value: B
Microbacter
1.00
0.75
0.50
0.25
0.00
A (n=15)
B (n=16)
C (n=14)
surface

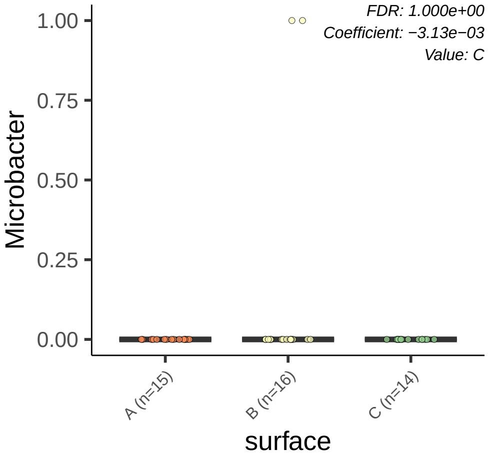
FDR: 1.000e+00
Coefficient: −3.13e−03
Value: C
1.00
0.75
0.50
0.25
0.00
Microbacter
A (n=15)
B (n=16)
C (n=14)
surface

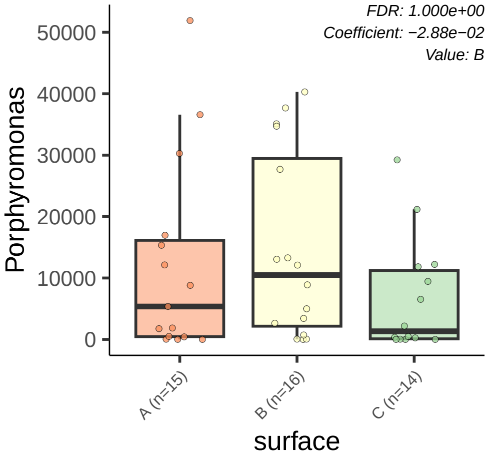

FDR: 1.000e+00
Coefficient: −2.88e−02
Value: B
Porphyromonas
50000
40000
30000
20000
10000
0
A (n=15)
B (n=16)
C (n=14)
surface

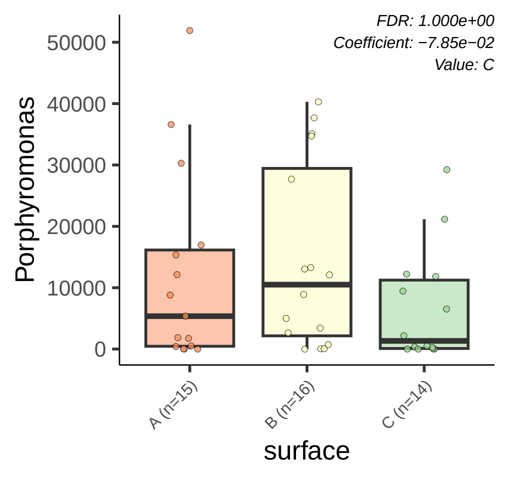
FDR: 1.000e+00
Coefficient: −7.85e−02
Value: C
Porphyromonas
50000
40000
30000
20000
10000
0
A (n=15)
B (n=16)
C (n=14)
surface

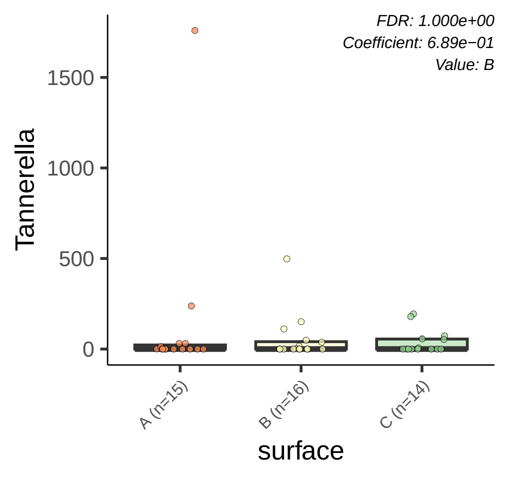
FDR: 1.000e+00
Coefficient: 6.89e−01
Value: B
Tannerella
1500
1000
500
0
A (n=15)
B (n=16)
C (n=14)
surface

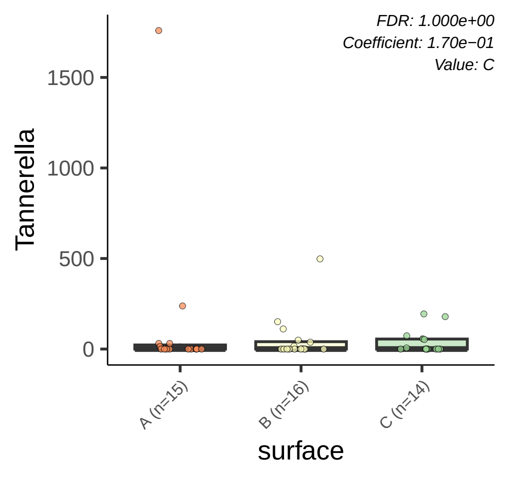

FDR: 1.000e+00
Coefficient: 1.70e−01
Value: C
Tannerella
1500
1000
500
0
A (n=15)
B (n=16)
C (n=14)
surface

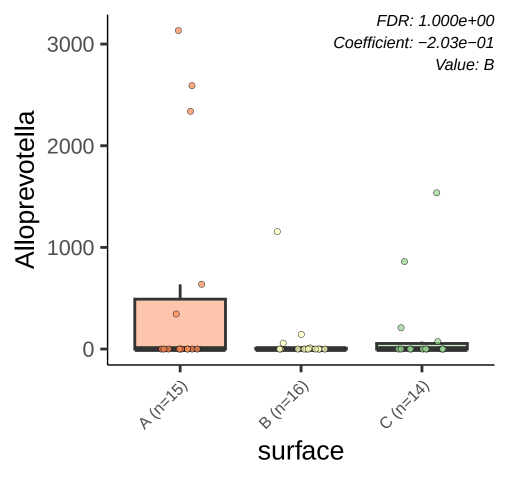
FDR: 1.000e+00
Coefficient: −2.03e−01
Value: B
Alloprevotella
3000
2000
1000
0
A (n=15)
B (n=16)
C (n=14)
surface

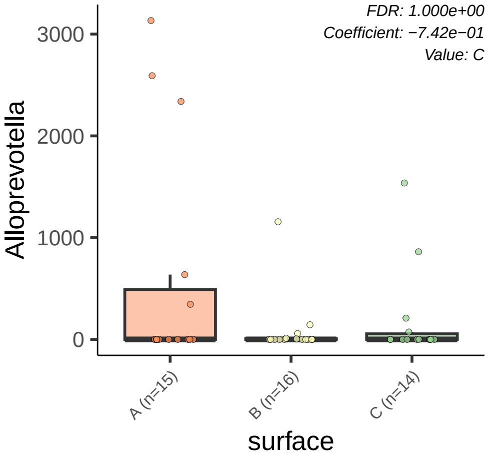
FDR: 1.000e+00
Coefficient: −7.42e−01
Value: C
Alloprevotella
3000
2000
1000
0
A (n=15)
B (n=16)
C (n=14)
surface

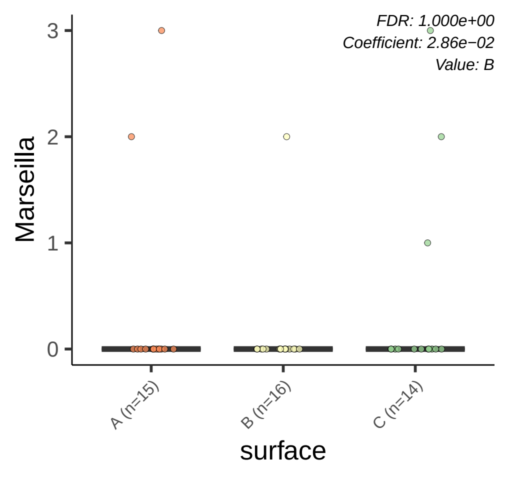

FDR: 1.000e+00
Coefficient: 2.86e−02
Value: B
Marseilla
3
2
1
0
A (n=15)
B (n=16)
C (n=14)
surface

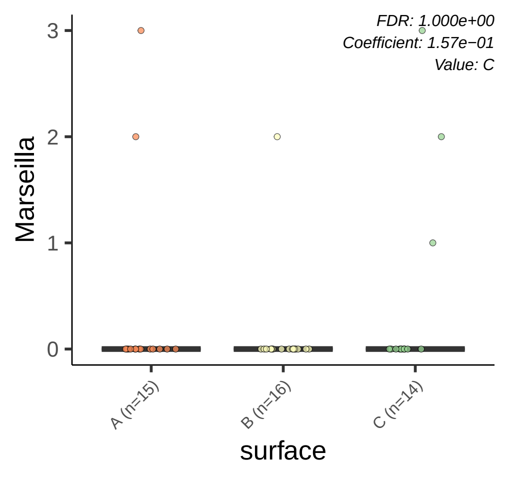
FDR: 1.000e+00
Coefficient: 1.57e−01
Value: C
Marseilla
3
2
1
0
A (n=15)
B (n=16)
C (n=14)
surface

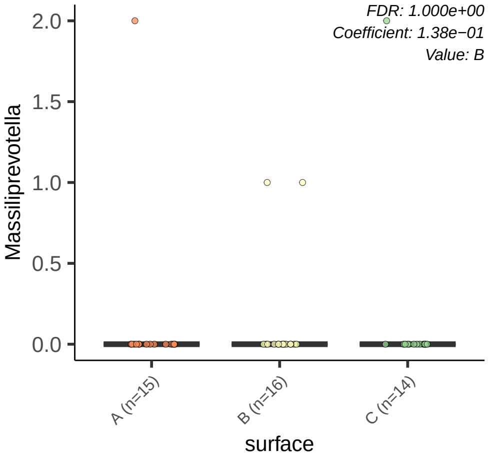
FDR: 1.000e+00
Coefficient: 1.38e−01
Value: B
Massiliprevotella
2.0
1.5
1.0
0.5
0.0
A (n=15)
B (n=16)
C (n=14)
surface

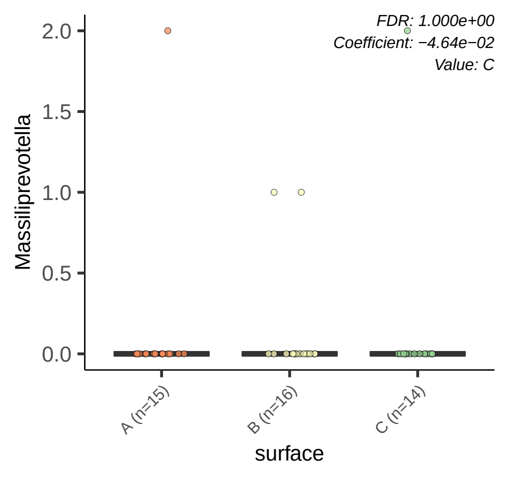
FDR: 1.000e+00
Coefficient: −4.64e−02
Value: C
Massiliprevotella
2.0
1.5
1.0
0.5
0.0
A (n=15)
B (n=16)
C (n=14)
surface

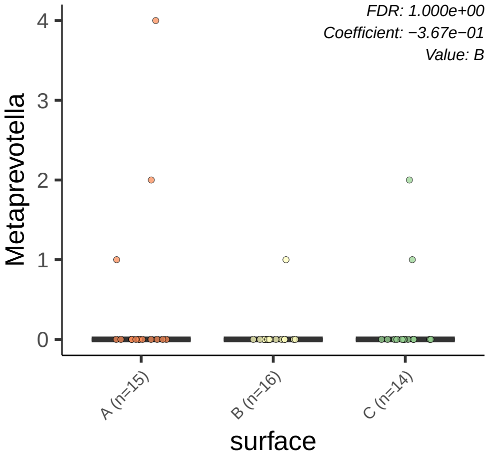

FDR: 1.000e+00
Coefficient: −3.67e−01
Value: B
Metaprevotella
0
1
2
3
4
A (n=15)
B (n=16)
C (n=14)
surface

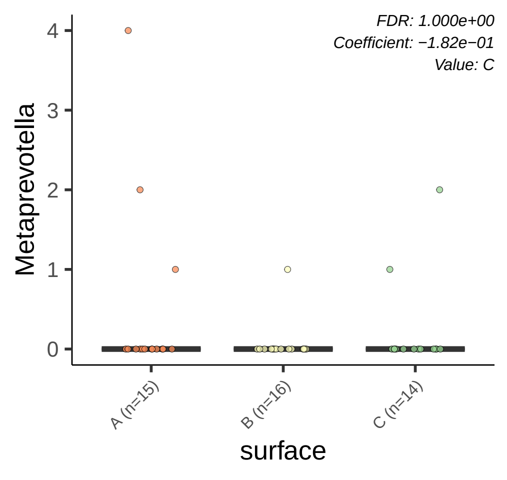

FDR: 1.000e+00
Coefficient: −1.82e−01
Value: C
Metaprevotella
4
3
2
1
0
A (n=15)
B (n=16)
C (n=14)
surface

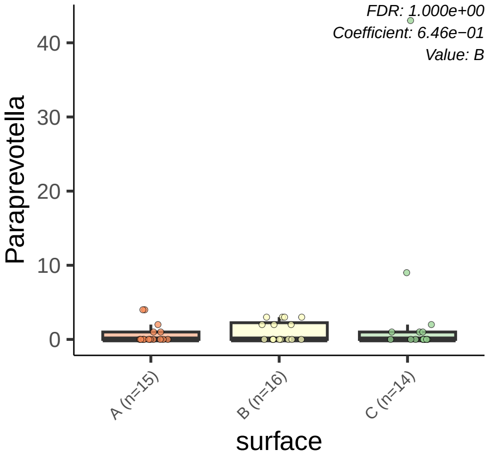

FDR: 1.000e+00
Coefficient: 6.46e−01
Value: B
40
30
20
10
0
Paraprevotella
A (n=15)
B (n=16)
C (n=14)
surface

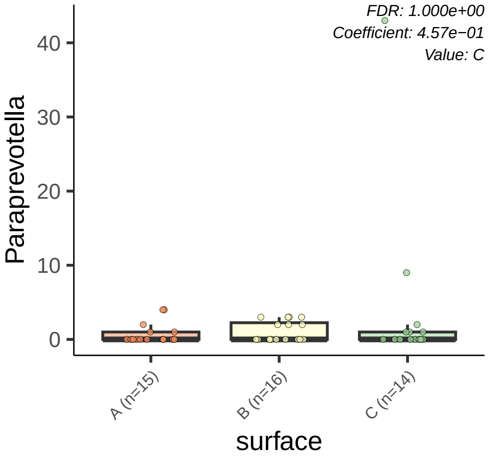
FDR: 1.000e+00
Coefficient: 4.57e−01
Value: C
Paraprevotella
0
10
20
30
40
A (n=15)
B (n=16)
C (n=14)
surface

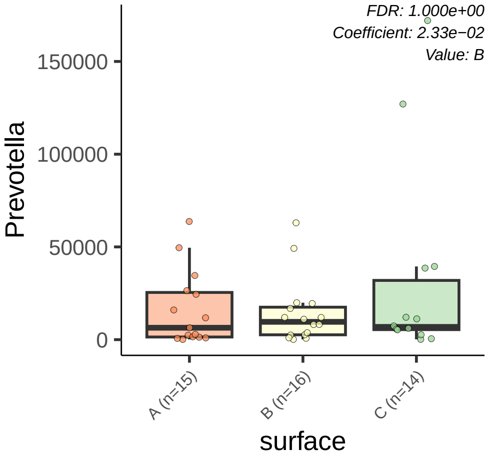

FDR: 1.000e+00
Coefficient: 2.33e−02
Value: B
Prevotella
150000
100000
50000
0
A (n=15)
B (n=16)
C (n=14)
surface

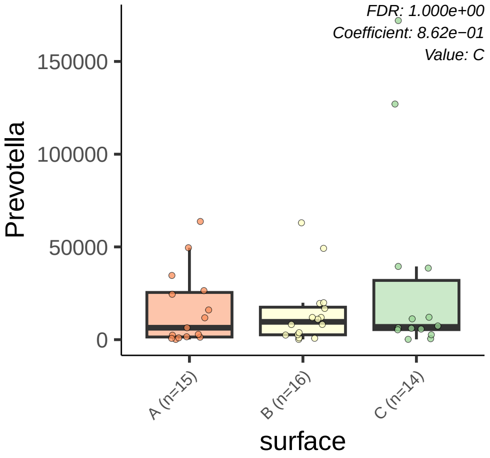

FDR: 1.000e+00
Coefficient: 8.62e−01
Value: C
Prevotella
150000
100000
50000
0
A (n=15)
B (n=16)
C (n=14)
surface

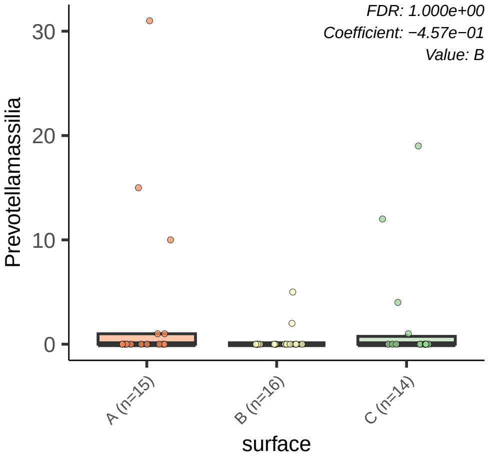

FDR: 1.000e+00
Coefficient: −4.57e−01
Value: B
Prevotellamassilia
30
20
10
0
A (n=15)
B (n=16)
C (n=14)
surface

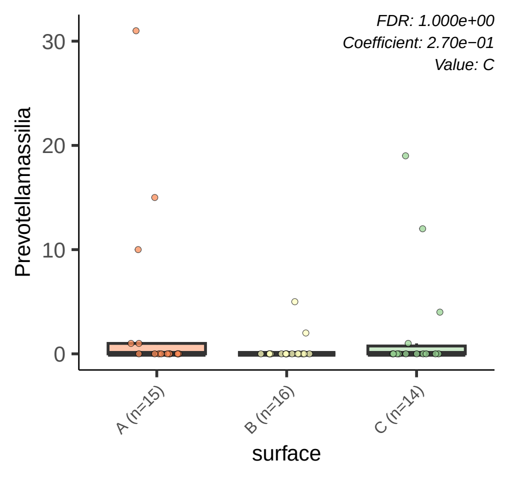
FDR: 1.000e+00
Coefficient: 2.70e−01
Value: C
Prevotellamassilia
30
20
10
0
A (n=15)
B (n=16)
C (n=14)
surface

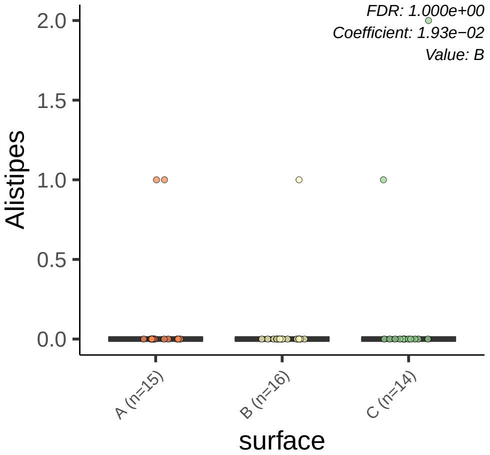

FDR: 1.000e+00
Coefficient: 1.93e−02
Value: B
2.0
1.5
1.0
0.5
0.0
Alistipes
A (n=15)
B (n=16)
C (n=14)
surface

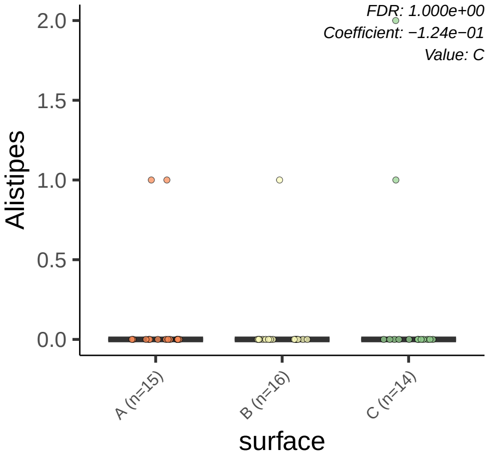
FDR: 1.000e+00
Coefficient: −1.24e−01
Value: C
Alistipes
2.0
1.5
1.0
0.5
0.0
A (n=15)
B (n=16)
C (n=14)
surface

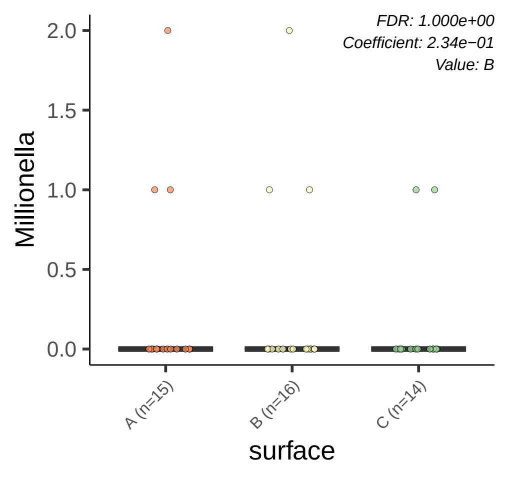

FDR: 1.000e+00
Coefficient: 2.34e−01
Value: B
Millionella
2.0
1.5
1.0
0.5
0.0
A (n=15)
B (n=16)
C (n=14)
surface

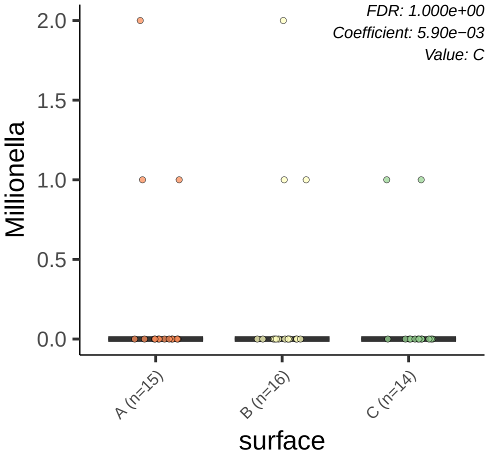
FDR: 1.000e+00
Coefficient: 5.90e−03
Value: C
Millionella
2.0
1.5
1.0
0.5
0.0
A (n=15)
B (n=16)
C (n=14)
surface

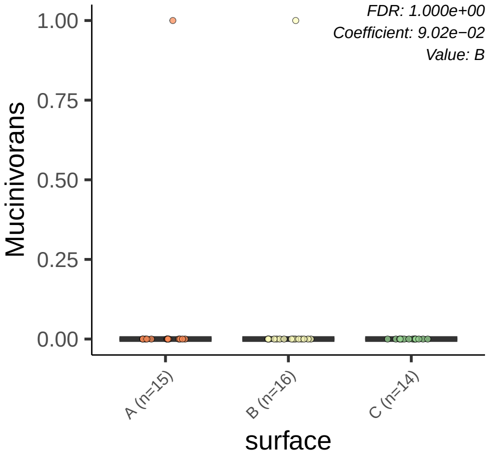
FDR: 1.000e+00
Coefficient: 9.02e−02
Value: B
Mucinivorans
1.00
0.75
0.50
0.25
0.00
A (n=15)
B (n=16)
C (n=14)
surface

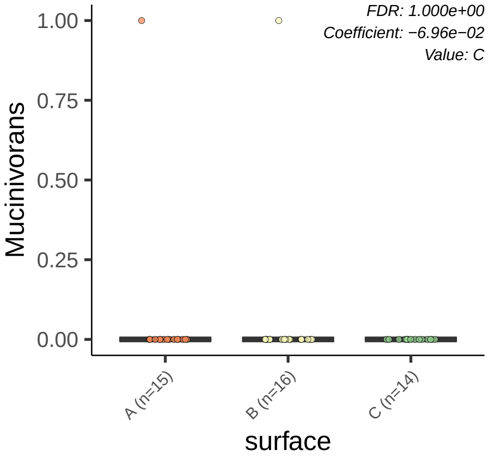

FDR: 1.000e+00
Coefficient: −6.96e−02
Value: C
Mucinivorans
1.00
0.75
0.50
0.25
0.00
A (n=15)
B (n=16)
C (n=14)
surface

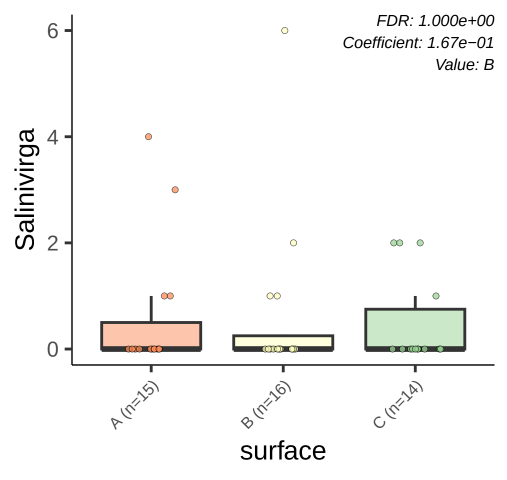

FDR: 1.000e+00
Coefficient: 1.67e−01
Value: B
Salinivirga
0
2
4
6
A (n=15)
B (n=16)
C (n=14)
surface

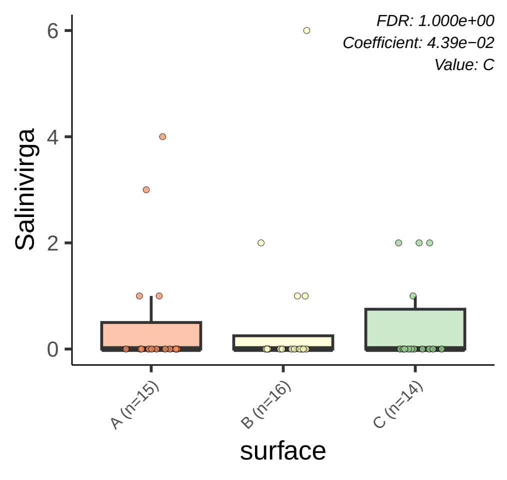
FDR: 1.000e+00
Coefficient: 4.39e−02
Value: C
Salinivirga
0
2
4
6
A (n=15)
B (n=16)
C (n=14)
surface

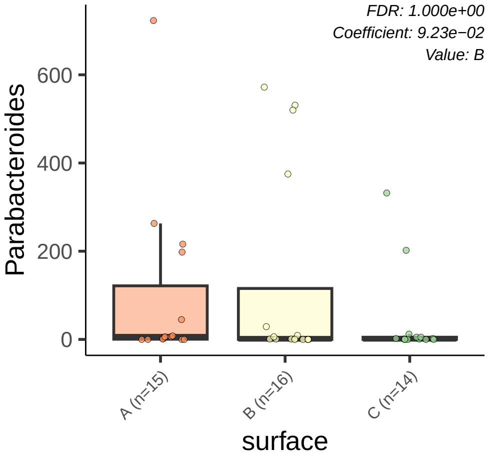

FDR: 1.000e+00
Coefficient: 9.23e−02
Value: B
Parabacteroides
0
200
400
600
A (n=15)
B (n=16)
C (n=14)
surface

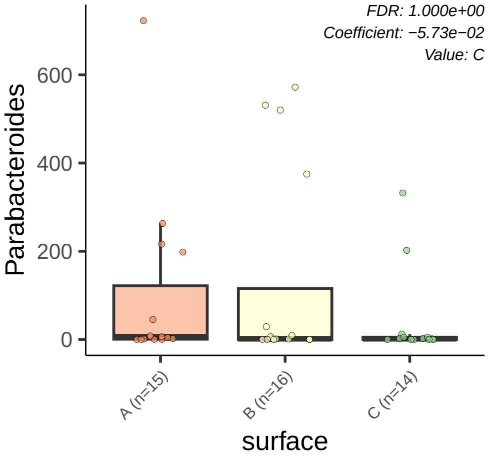

FDR: 1.000e+00
Coefficient: −5.73e−02
Value: C
Parabacteroides
600
400
200
0
A (n=15)
B (n=16)
C (n=14)
surface

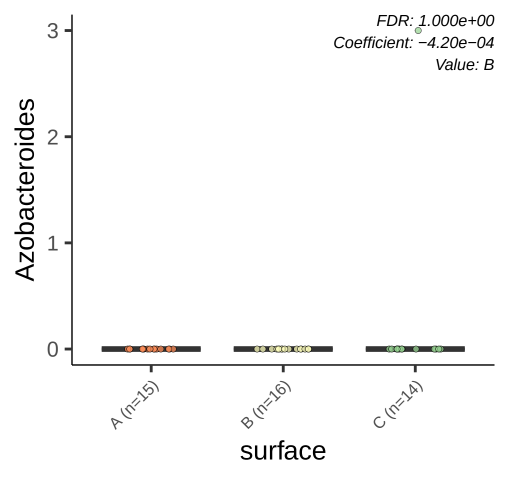
FDR: 1.000e+00
Coefficient: −4.20e−04
Value: B
Azobacteroides
0
1
2
3
A (n=15)
B (n=16)
C (n=14)
surface

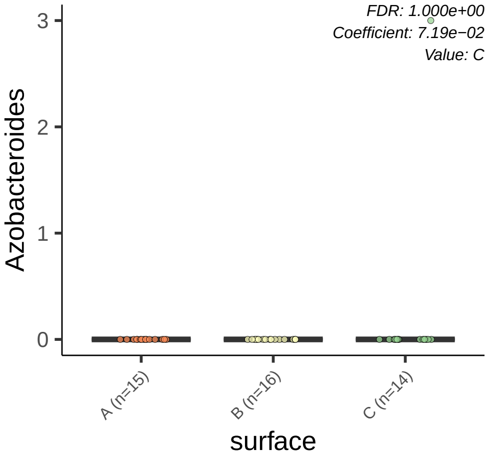

FDR: 1.000e+00
Coefficient: 7.19e−02
Value: C
Azobacteroides
3
2
1
0
A (n=15)
B (n=16)
C (n=14)
surface

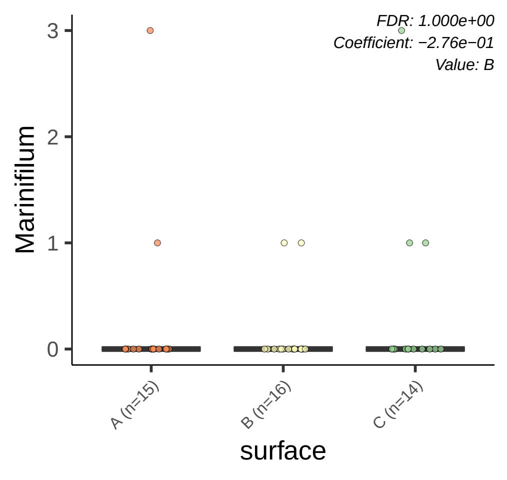
FDR: 1.000e+00
Coefficient: −2.76e−01
Value: B
Marinifilum
3
2
1
0
A (n=15)
B (n=16)
C (n=14)
surface

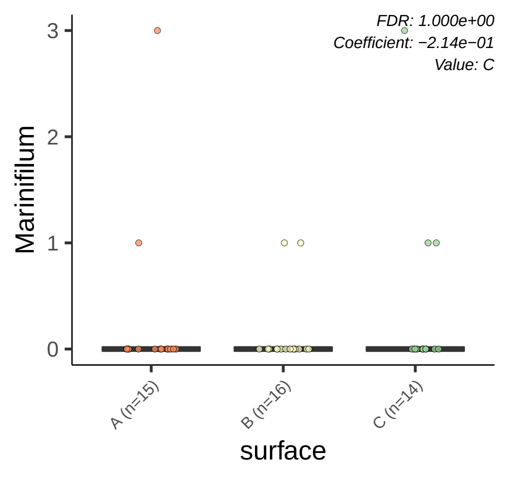
FDR: 1.000e+00
Coefficient: −2.14e−01
Value: C
Marinifilum
3
2
1
0
A (n=15)
B (n=16)
C (n=14)
surface

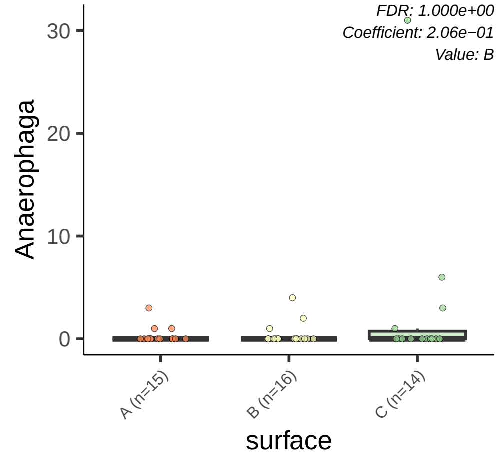
FDR: 1.000e+00
Coefficient: 2.06e−01
Value: B
Anaerophaga
30
20
10
0
A (n=15)
B (n=16)
C (n=14)
surface

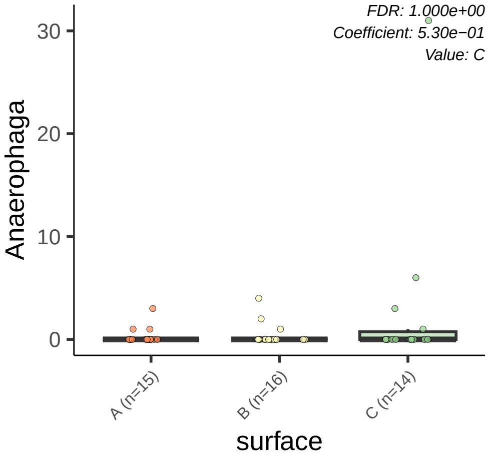

FDR: 1.000e+00
Coefficient: 5.30e−01
Value: C
Anaerophaga
30
20
10
0
A (n=15)
B (n=16)
C (n=14)
surface

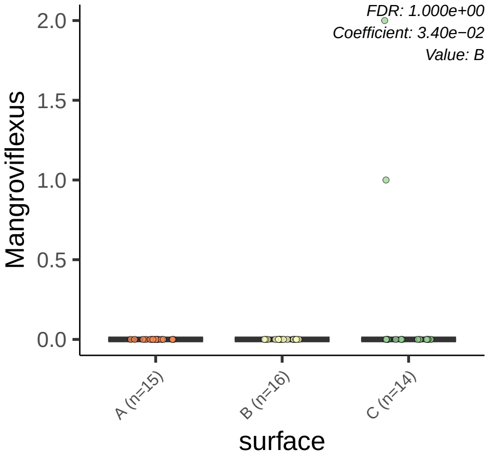

FDR: 1.000e+00
Coefficient: 3.40e−02
Value: B
Mangroviflexus
2.0
1.5
1.0
0.5
0.0
A (n=15)
B (n=16)
C (n=14)
surface

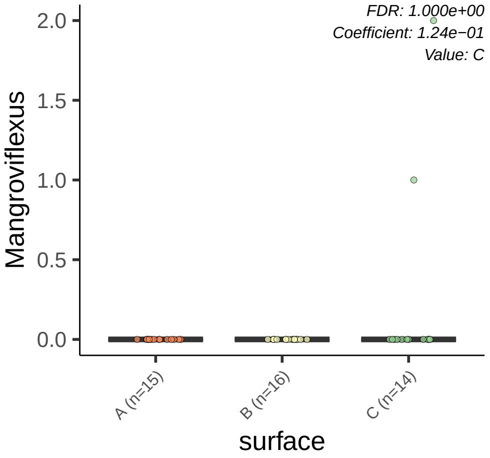
FDR: 1.000e+00
Coefficient: 1.24e−01
Value: C
Mangroviflexus
2.0
1.5
1.0
0.5
0.0
A (n=15)
B (n=16)
C (n=14)
surface

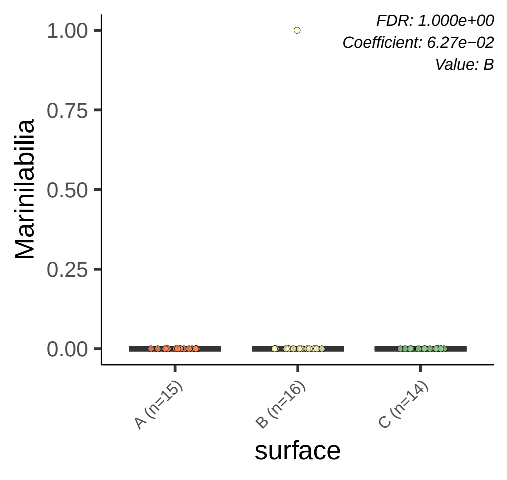

FDR: 1.000e+00
Coefficient: 6.27e−02
Value: B
1.00
0.75
0.50
0.25
0.00
Marinilabilia
A (n=15)
B (n=16)
C (n=14)
surface

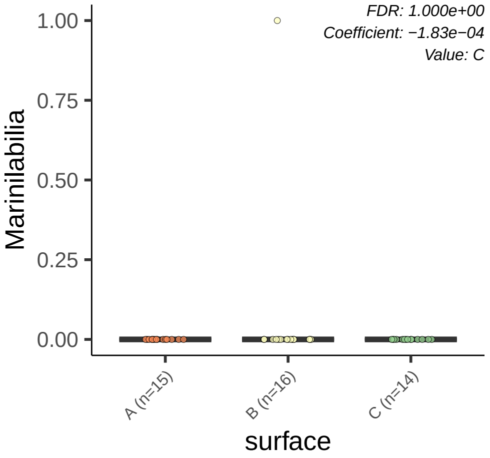
FDR: 1.000e+00
Coefficient: −1.83e−04
Value: C
Marinilabilia
1.00
0.75
0.50
0.25
0.00
A (n=15)
B (n=16)
C (n=14)
surface

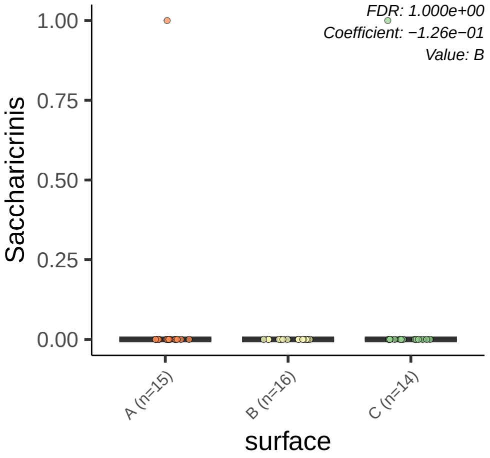

FDR: 1.000e+00
Coefficient: −1.26e−01
Value: B
Saccharicrinis
1.00
0.75
0.50
0.25
0.00
A (n=15)
B (n=16)
C (n=14)
surface

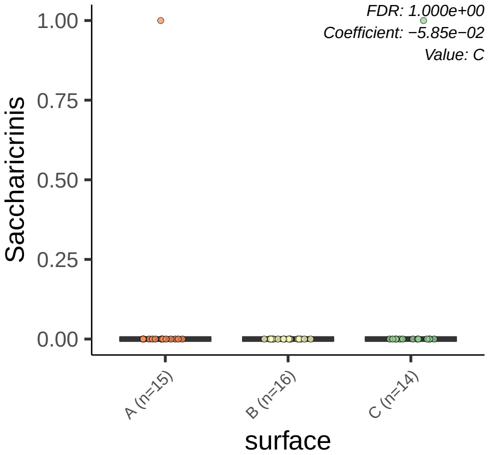

FDR: 1.000e+00
Coefficient: −5.85e−02
Value: C
Saccharicrinis
1.00
0.75
0.50
0.25
0.00
A (n=15)
B (n=16)
C (n=14)
surface

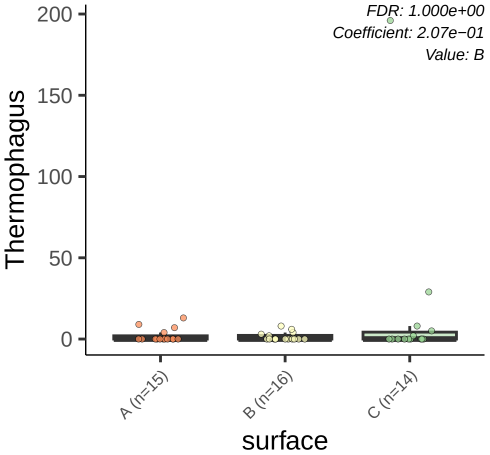
FDR: 1.000e+00
Coefficient: 2.07e−01
Value: B
Thermophagus
200
150
100
50
0
A (n=15)
B (n=16)
C (n=14)
surface

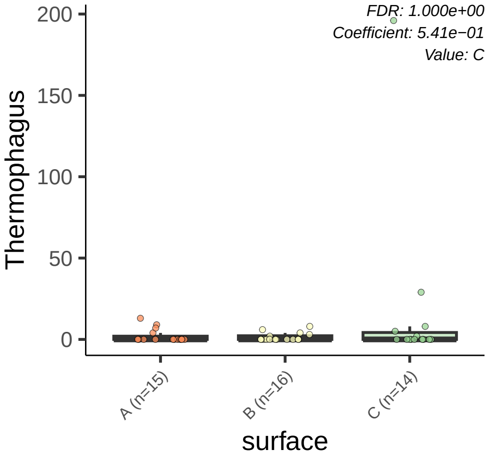

FDR: 1.000e+00
Coefficient: 5.41e−01
Value: C
Thermophagus
200
150
100
50
0
A (n=15)
B (n=16)
C (n=14)
surface

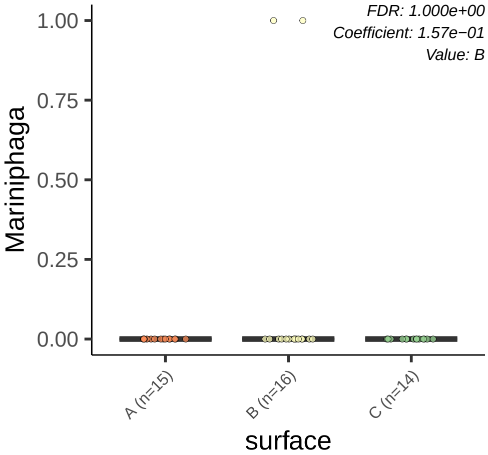

FDR: 1.000e+00
Coefficient: 1.57e−01
Value: B
1.00
0.75
0.50
0.25
0.00
Mariniphaga
A (n=15)
B (n=16)
C (n=14)
surface

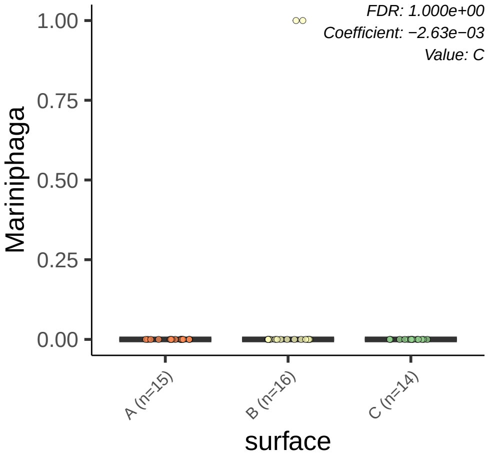
FDR: 1.000e+00
Coefficient: −2.63e−03
Value: C
Mariniphaga
1.00
0.75
0.50
0.25
0.00
A (n=15)
B (n=16)
C (n=14)
surface

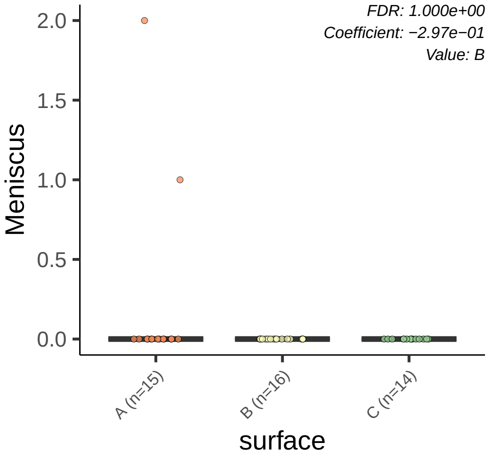

FDR: 1.000e+00
Coefficient: −2.97e−01
Value: B
Meniscus
2.0
1.5
1.0
0.5
0.0
A (n=15)
B (n=16)
C (n=14)
surface

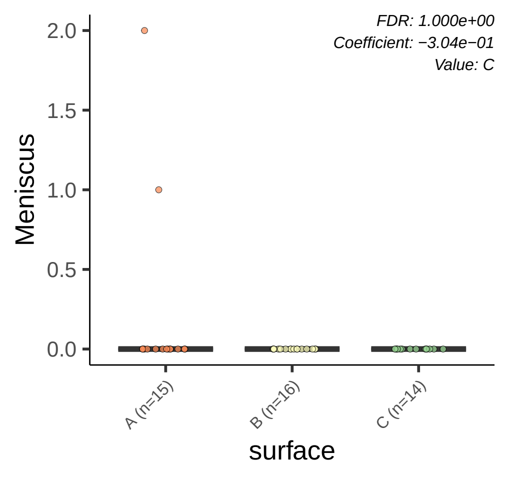

FDR: 1.000e+00
Coefficient: −3.04e−01
Value: C
Meniscus
2.0
1.5
1.0
0.5
0.0
A (n=15)
B (n=16)
C (n=14)
surface

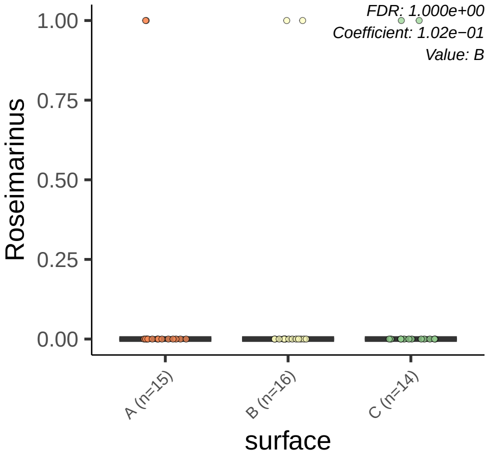
FDR: 1.000e+00
Coefficient: 1.02e−01
Value: B
1.00
0.75
0.50
0.25
0.00
Roseimarinus
A (n=15)
B (n=16)
C (n=14)
surface

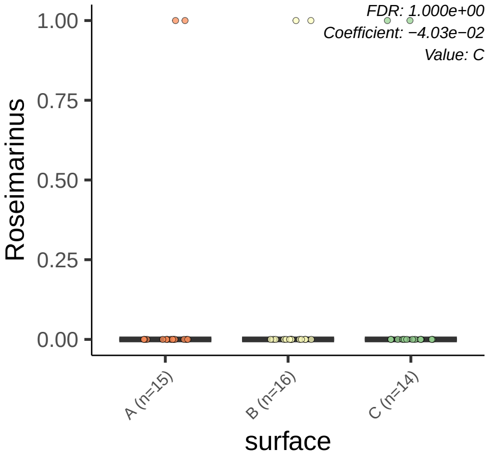
FDR: 1.000e+00
Coefficient: −4.03e−02
Value: C
Roseimarinus
1.00
0.75
0.50
0.25
0.00
A (n=15)
B (n=16)
C (n=14)
surface

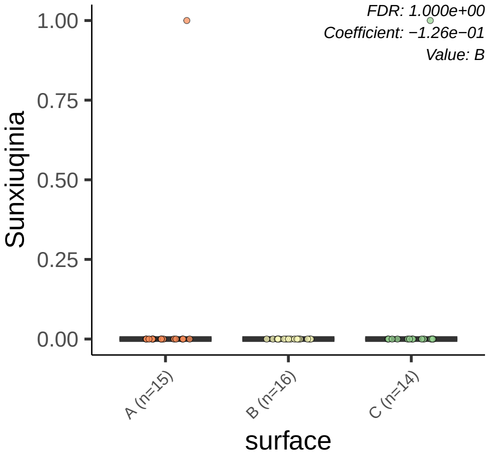
FDR: 1.000e+00
Coefficient: −1.26e−01
Value: B
Sunxiuqinia
1.00
0.75
0.50
0.25
0.00
A (n=15)
B (n=16)
C (n=14)
surface

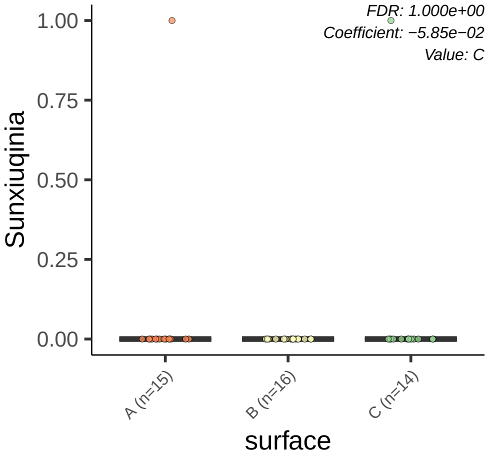

FDR: 1.000e+00
Coefficient: −5.85e−02
Value: C
Sunxiuqinia
1.00
0.75
0.50
0.25
0.00
A (n=15)
B (n=16)
C (n=14)
surface

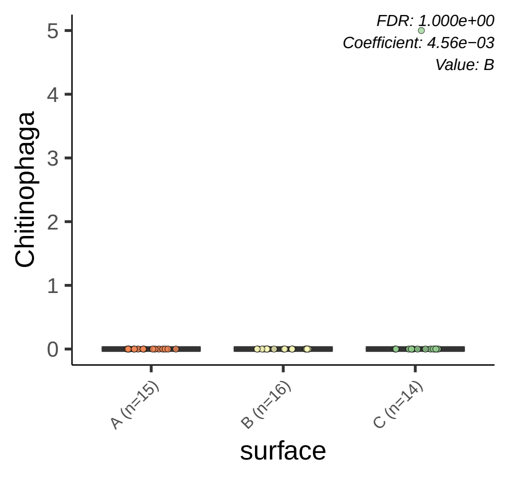

FDR: 1.000e+00
Coefficient: 4.56e−03
Value: B
Chitinophaga
5
4
3
2
1
0
A (n=15)
B (n=16)
C (n=14)
surface

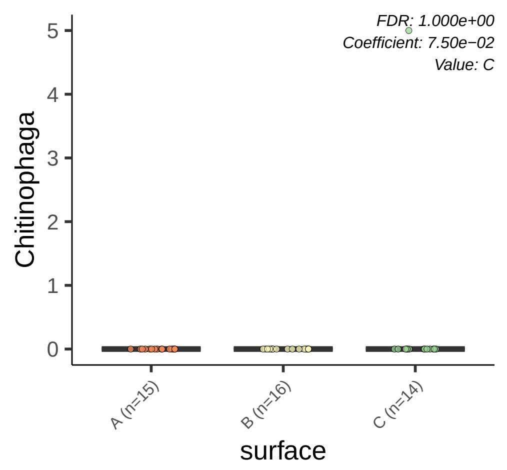

FDR: 1.000e+00
Coefficient: 7.50e−02
Value: C
Chitinophaga
5
4
3
2
1
0
A (n=15)
B (n=16)
C (n=14)
surface

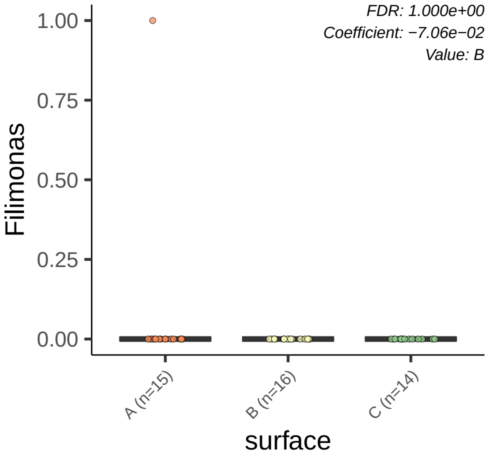
FDR: 1.000e+00
Coefficient: −7.06e−02
Value: B
Filimonas
1.00
0.75
0.50
0.25
0.00
A (n=15)
B (n=16)
C (n=14)
surface

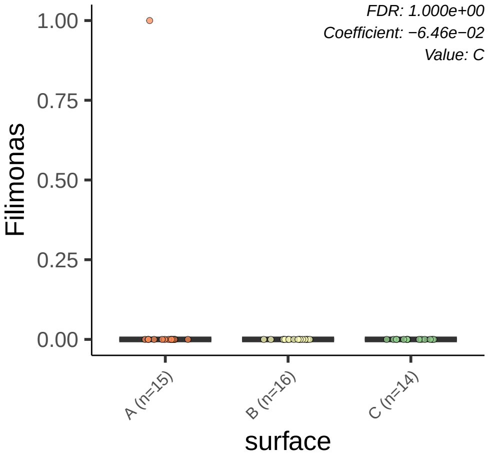
FDR: 1.000e+00
Coefficient: −6.46e−02
Value: C
Filimonas
1.00
0.75
0.50
0.25
0.00
A (n=15)
B (n=16)
C (n=14)
surface

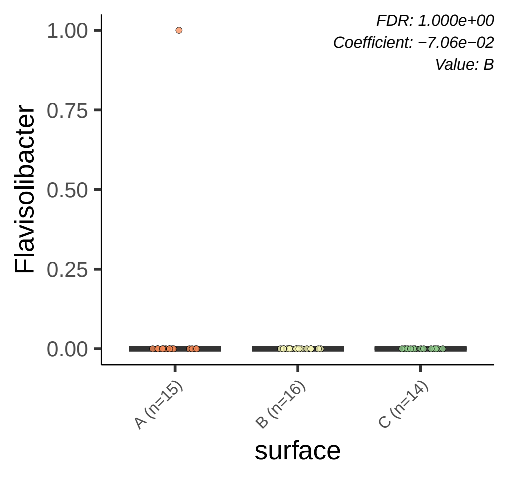
FDR: 1.000e+00
Coefficient: −7.06e−02
Value: B
Flavisolibacter
1.00
0.75
0.50
0.25
0.00
A (n=15)
B (n=16)
C (n=14)
surface

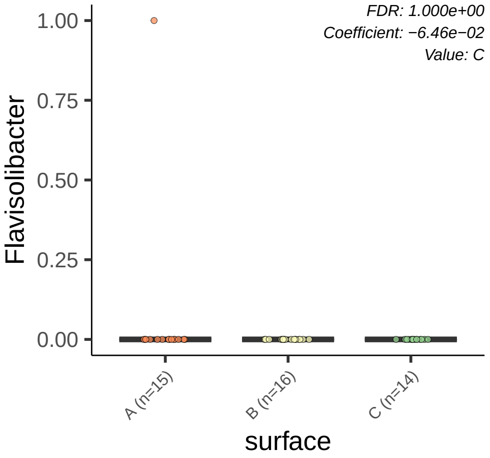
FDR: 1.000e+00
Coefficient: −6.46e−02
Value: C
Flavisolibacter
1.00
0.75
0.50
0.25
0.00
A (n=15)
B (n=16)
C (n=14)
surface

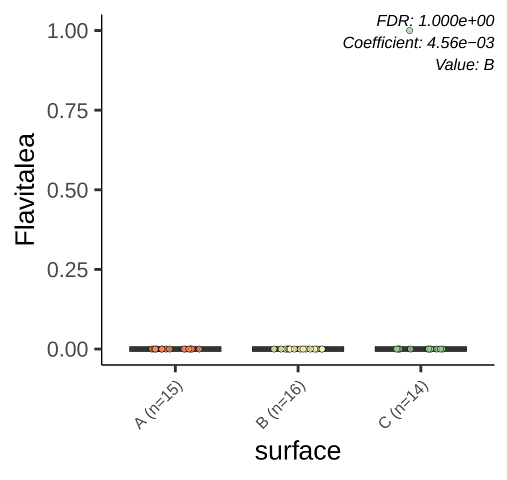

FDR: 1.000e+00
Coefficient: 4.56e−03
Value: B
Flavitalea
1.00
0.75
0.50
0.25
0.00
A (n=15)
B (n=16)
C (n=14)
surface

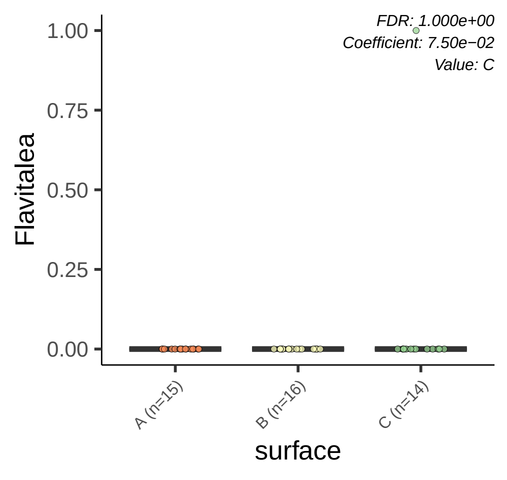
FDR: 1.000e+00
Coefficient: 7.50e−02
Value: C
Flavitalea
1.00
0.75
0.50
0.25
0.00
A (n=15)
B (n=16)
C (n=14)
surface

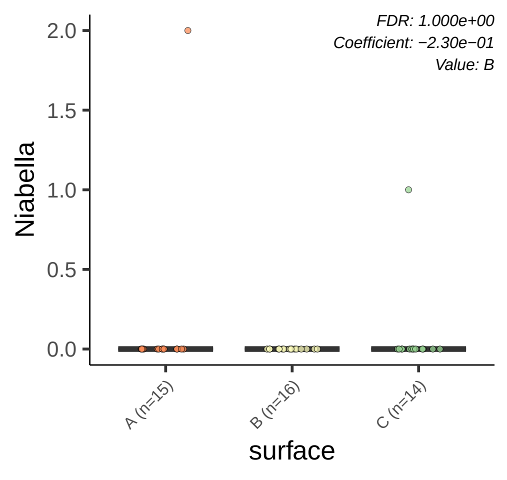

FDR: 1.000e+00
Coefficient: −2.30e−01
Value: B
Niabella
2.0
1.5
1.0
0.5
0.0
A (n=15)
B (n=16)
C (n=14)
surface

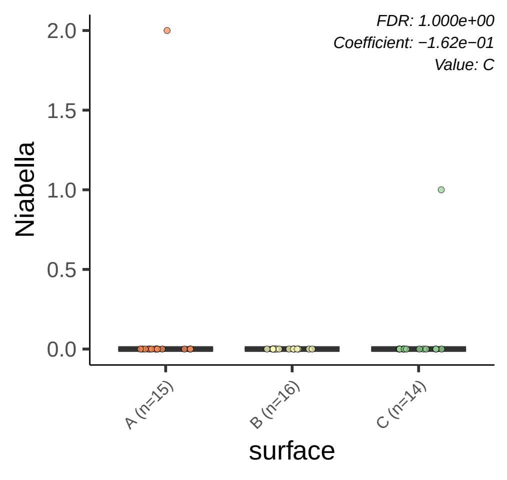

FDR: 1.000e+00
Coefficient: −1.62e−01
Value: C
Niabella
2.0
1.5
1.0
0.5
0.0
A (n=15)
B (n=16)
C (n=14)
surface

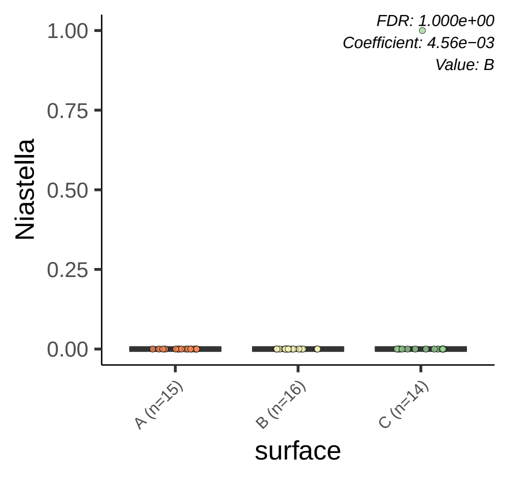

FDR: 1.000e+00
Coefficient: 4.56e−03
Value: B
Niastella
1.00
0.75
0.50
0.25
0.00
A (n=15)
B (n=16)
C (n=14)
surface

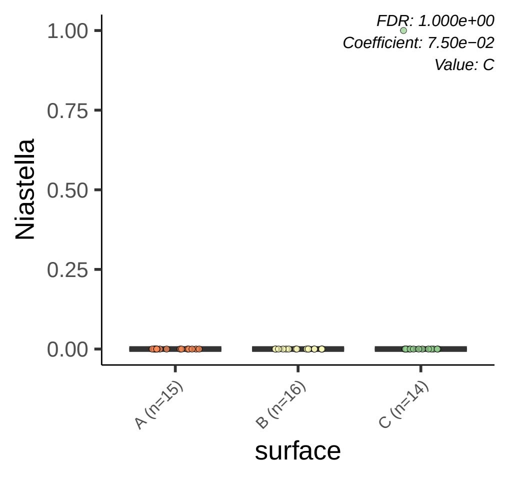

FDR: 1.000e+00
Coefficient: 7.50e−02
Value: C
1.00
0.75
0.50
0.25
0.00
Niastella
A (n=15)
B (n=16)
C (n=14)
surface

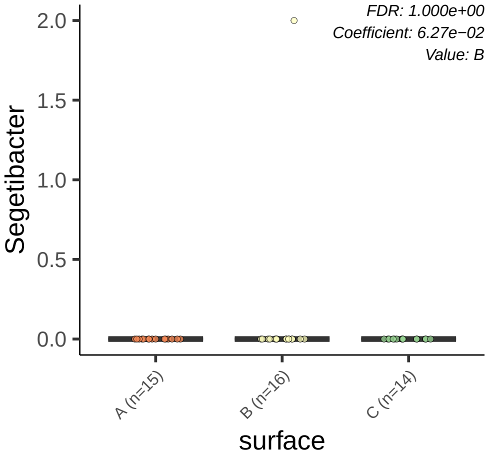

FDR: 1.000e+00
Coefficient: 6.27e−02
Value: B
Segetibacter
2.0
1.5
1.0
0.5
0.0
A (n=15)
B (n=16)
C (n=14)
surface

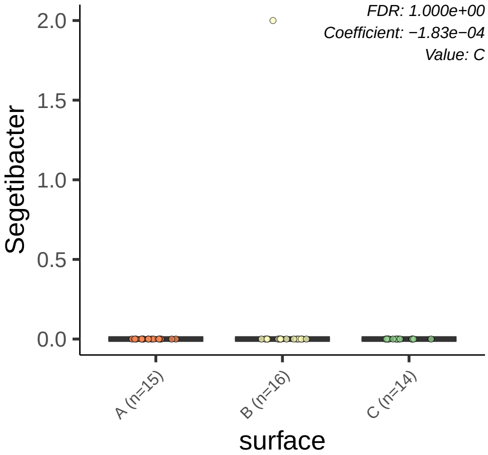
FDR: 1.000e+00
Coefficient: −1.83e−04
Value: C
Segetibacter
2.0
1.5
1.0
0.5
0.0
A (n=15)
B (n=16)
C (n=14)
surface

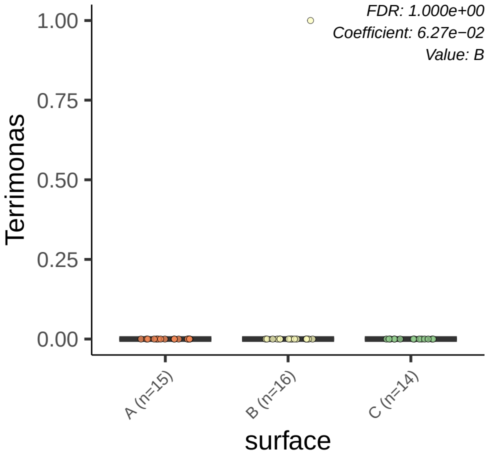
FDR: 1.000e+00
Coefficient: 6.27e−02
Value: B
Terrimonas
1.00
0.75
0.50
0.25
0.00
A (n=15)
B (n=16)
C (n=14)
surface

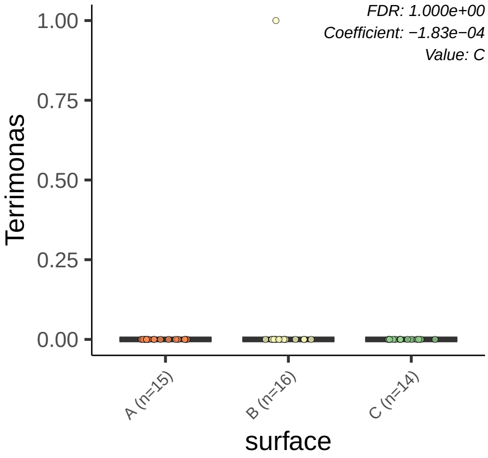
FDR: 1.000e+00
Coefficient: −1.83e−04
Value: C
Terrimonas
1.00
0.75
0.50
0.25
0.00
A (n=15)
B (n=16)
C (n=14)
surface

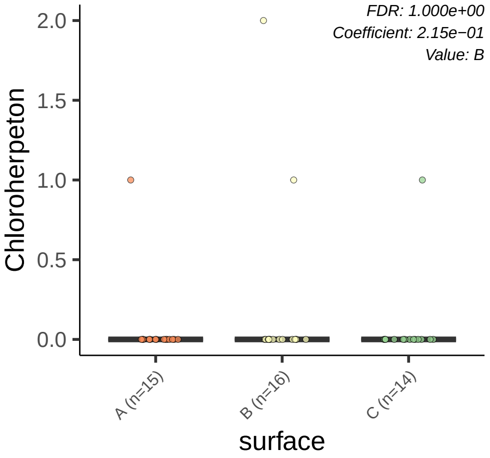
FDR: 1.000e+00
Coefficient: 2.15e−01
Value: B
Chloroherpeton
2.0
1.5
1.0
0.5
0.0
A (n=15)
B (n=16)
C (n=14)
surface

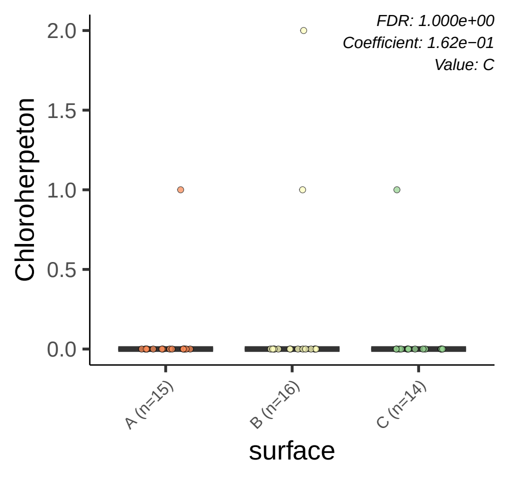
FDR: 1.000e+00
Coefficient: 1.62e−01
Value: C
Chloroherpeton
2.0
1.5
1.0
0.5
0.0
A (n=15)
B (n=16)
C (n=14)
surface

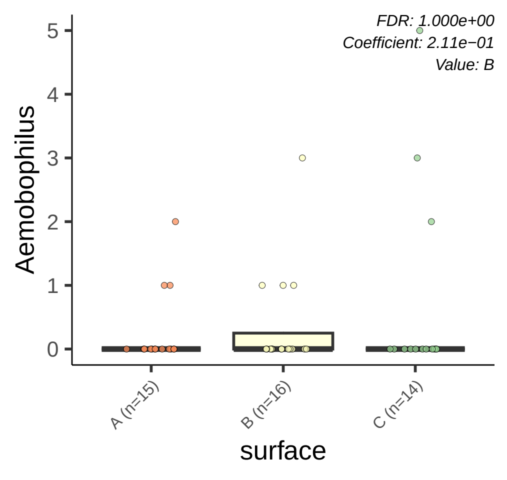
FDR: 1.000e+00
Coefficient: 2.11e−01
Value: B
Aemobophilus
0
1
2
3
4
5
A (n=15)
B (n=16)
C (n=14)
surface

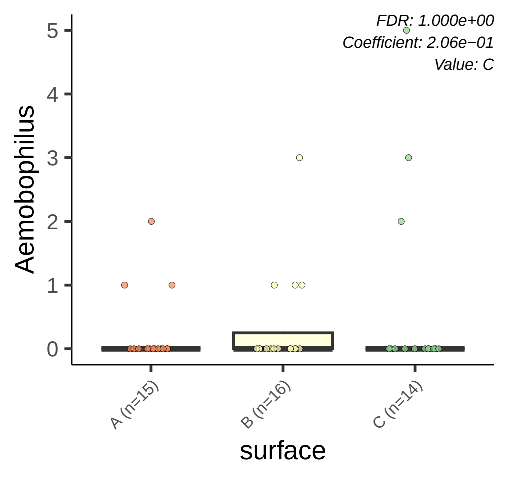

FDR: 1.000e+00
Coefficient: 2.06e−01
Value: C
Aemobophilus
0
1
2
3
4
5
A (n=15)
B (n=16)
C (n=14)
surface

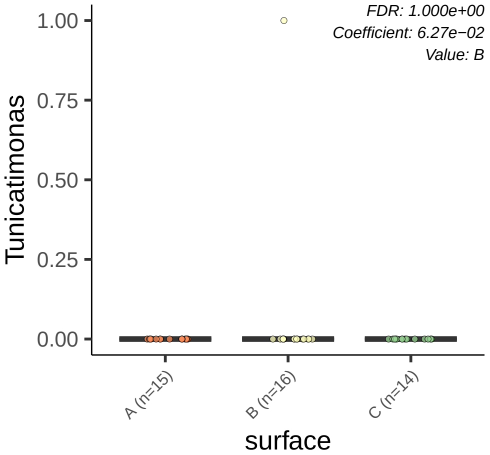

FDR: 1.000e+00
Coefficient: 6.27e−02
Value: B
Tunicatimonas
1.00
0.75
0.50
0.25
0.00
A (n=15)
B (n=16)
C (n=14)
surface

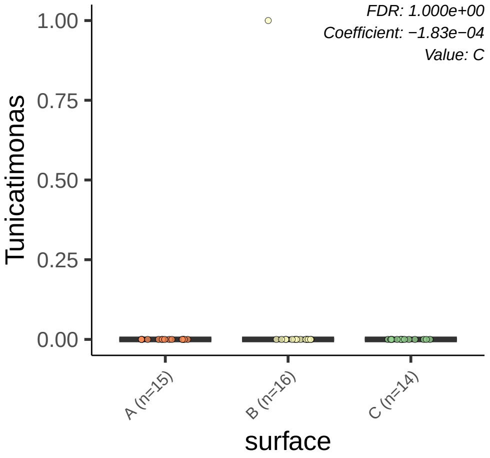

FDR: 1.000e+00
Coefficient: −1.83e−04
Value: C
1.00
0.75
0.50
0.25
0.00
Tunicatimonas
A (n=15)
B (n=16)
C (n=14)
surface

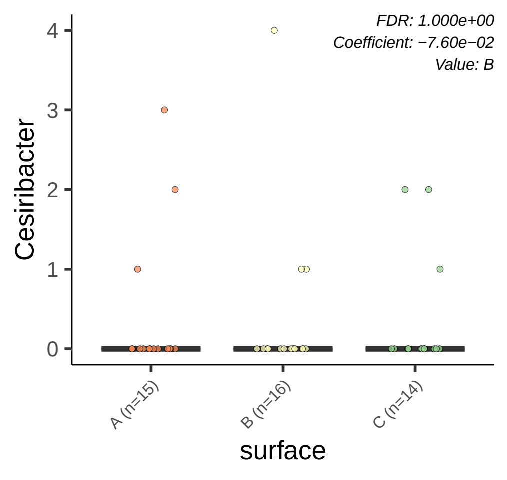
FDR: 1.000e+00
Coefficient: −7.60e−02
Value: B
Cesiribacter
0
1
2
3
4
A (n=15)
B (n=16)
C (n=14)
surface

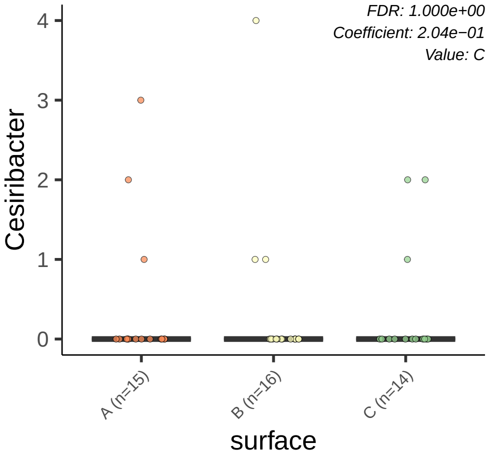
FDR: 1.000e+00
Coefficient: 2.04e−01
Value: C
Cesiribacter
4
3
2
1
0
A (n=15)
B (n=16)
C (n=14)
surface

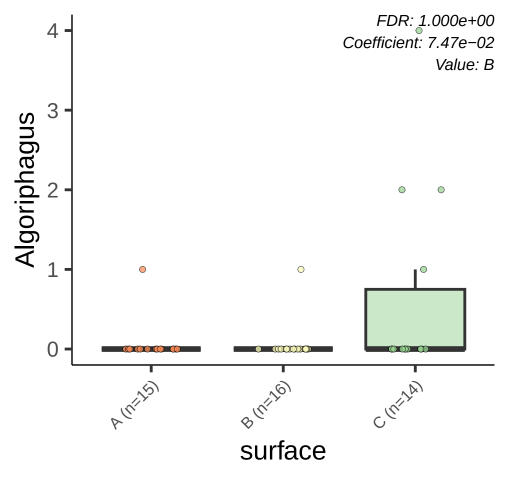
FDR: 1.000e+00
Coefficient: 7.47e−02
Value: B
Algoriphagus
0
1
2
3
4
A (n=15)
B (n=16)
C (n=14)
surface

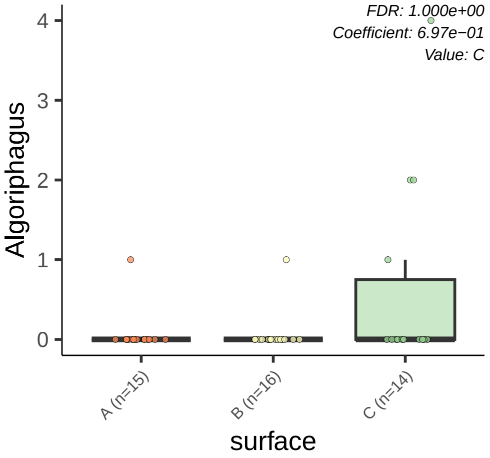

FDR: 1.000e+00
Coefficient: 6.97e−01
Value: C
Algoriphagus
4
3
2
1
0
A (n=15)
B (n=16)
C (n=14)
surface

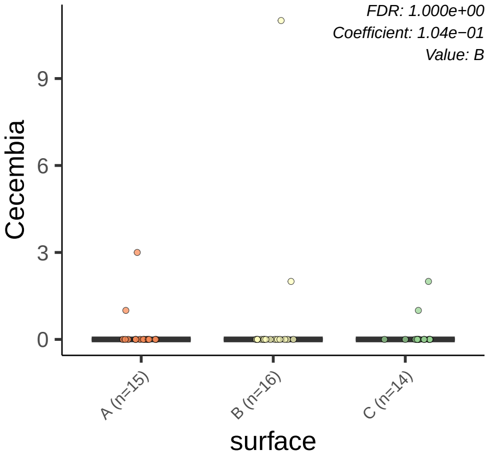
FDR: 1.000e+00
Coefficient: 1.04e−01
Value: B
Cecembia
0
3
6
9
A (n=15)
B (n=16)
C (n=14)
surface

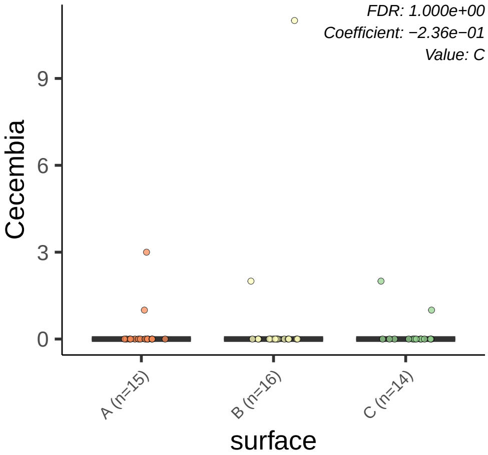

FDR: 1.000e+00
Coefficient: −2.36e−01
Value: C
Cecembia
0
3
6
9
A (n=15)
B (n=16)
C (n=14)
surface

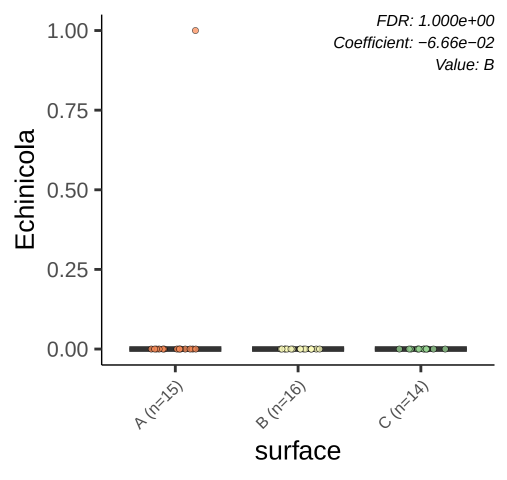

FDR: 1.000e+00
Coefficient: −6.66e−02
Value: B
Echinicola
1.00
0.75
0.50
0.25
0.00
A (n=15)
B (n=16)
C (n=14)
surface

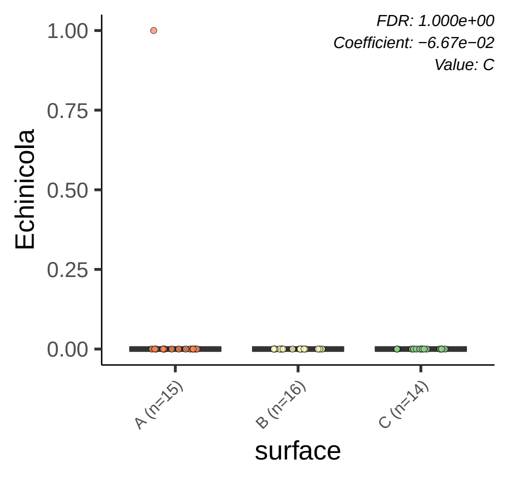

FDR: 1.000e+00
Coefficient: −6.67e−02
Value: C
1.00
0.75
0.50
0.25
0.00
Echinicola
A (n=15)
B (n=16)
C (n=14)
surface

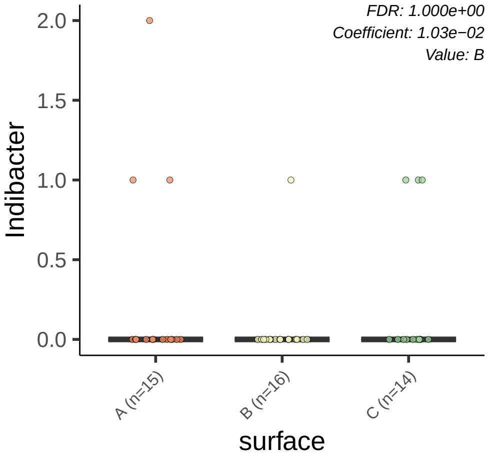

FDR: 1.000e+00
Coefficient: 1.03e−02
Value: B
Indibacter
2.0
1.5
1.0
0.5
0.0
A (n=15)
B (n=16)
C (n=14)
surface

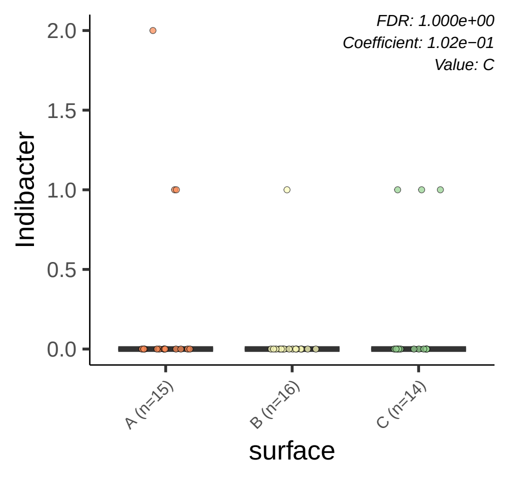

FDR: 1.000e+00
Coefficient: 1.02e−01
Value: C
Indibacter
2.0
1.5
1.0
0.5
0.0
A (n=15)
B (n=16)
C (n=14)
surface

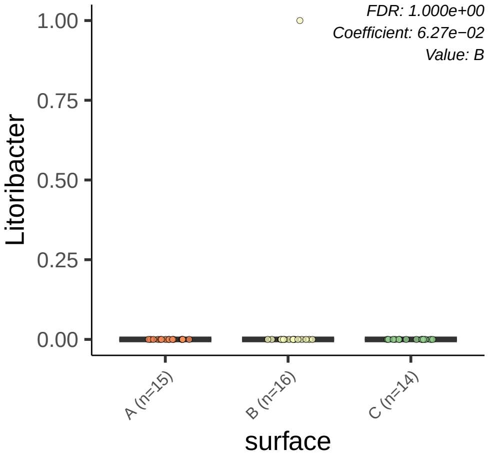
FDR: 1.000e+00
Coefficient: 6.27e−02
Value: B
Litoribacter
1.00
0.75
0.50
0.25
0.00
A (n=15)
B (n=16)
C (n=14)
surface

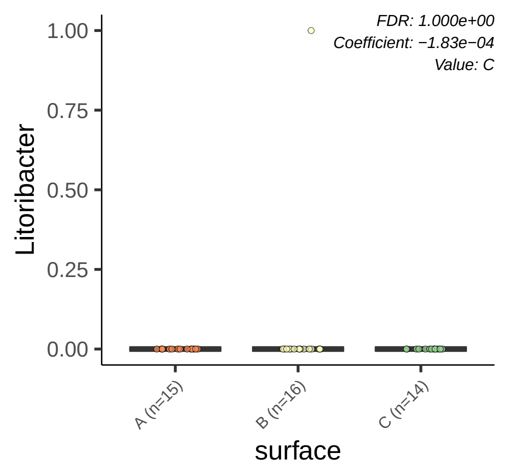
FDR: 1.000e+00
Coefficient: −1.83e−04
Value: C
Litoribacter
1.00
0.75
0.50
0.25
0.00
A (n=15)
B (n=16)
C (n=14)
surface

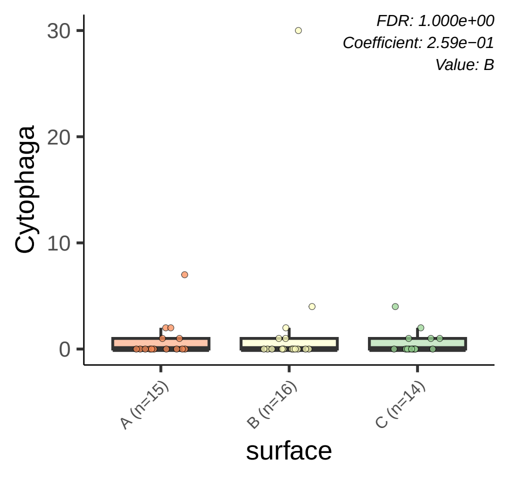

FDR: 1.000e+00
Coefficient: 2.59e−01
Value: B
Cytophaga
30
20
10
0
A (n=15)
B (n=16)
C (n=14)
surface

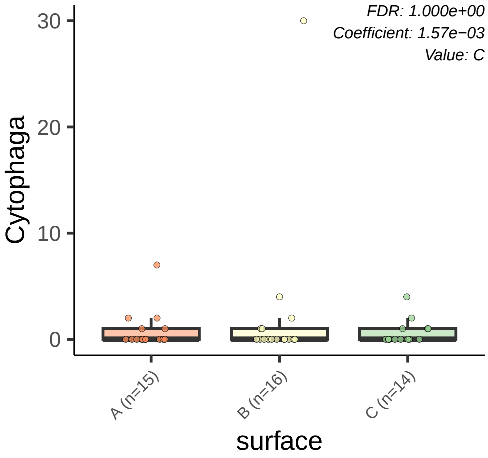
FDR: 1.000e+00
Coefficient: 1.57e−03
Value: C
Cytophaga
30
20
10
0
A (n=15)
B (n=16)
C (n=14)
surface

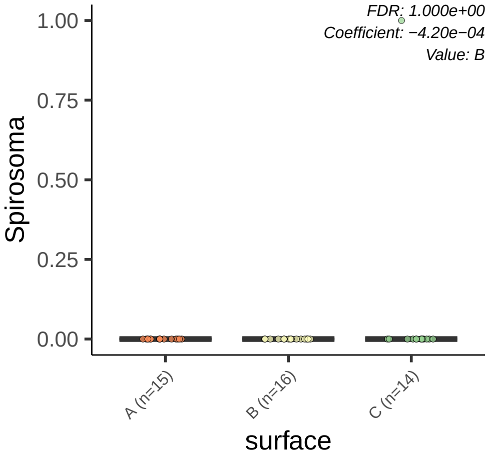
FDR: 1.000e+00
Coefficient: −4.20e−04
Value: B
Spirosoma
1.00
0.75
0.50
0.25
0.00
A (n=15)
B (n=16)
C (n=14)
surface

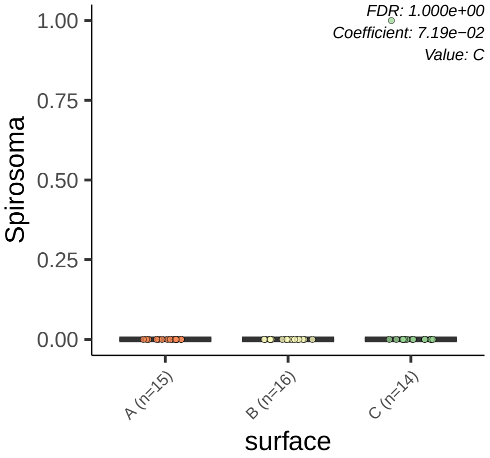

FDR: 1.000e+00
Coefficient: 7.19e−02
Value: C
Spirosoma
1.00
0.75
0.50
0.25
0.00
A (n=15)
B (n=16)
C (n=14)
surface

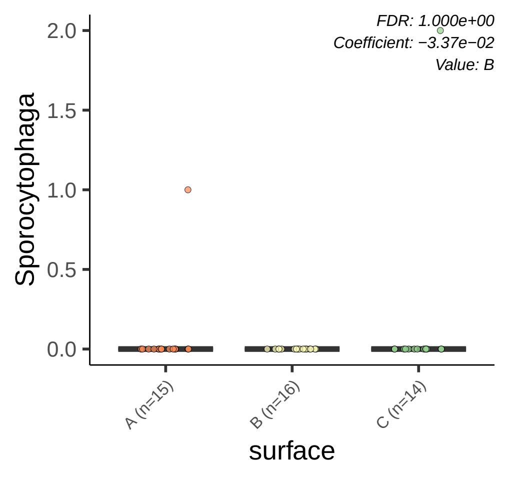

FDR: 1.000e+00
Coefficient: −3.37e−02
Value: B
Sporocytophaga
2.0
1.5
1.0
0.5
0.0
A (n=15)
B (n=16)
C (n=14)
surface

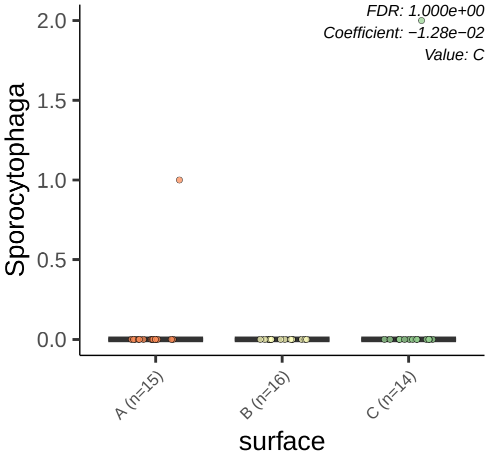
FDR: 1.000e+00
Coefficient: −1.28e−02
Value: C
Sporocytophaga
2.0
1.5
1.0
0.5
0.0
A (n=15)
B (n=16)
C (n=14)
surface

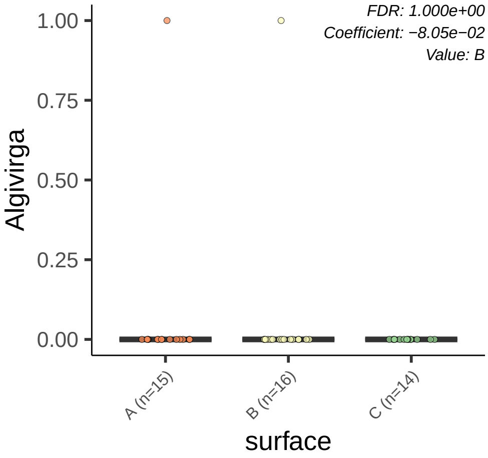
FDR: 1.000e+00
Coefficient: −8.05e−02
Value: B
Algivirga
1.00
0.75
0.50
0.25
0.00
A (n=15)
B (n=16)
C (n=14)
surface

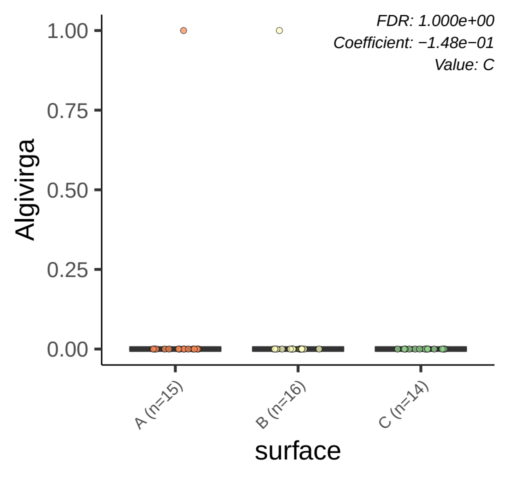
FDR: 1.000e+00
Coefficient: −1.48e−01
Value: C
Algivirga
1.00
0.75
0.50
0.25
0.00
A (n=15)
B (n=16)
C (n=14)
surface

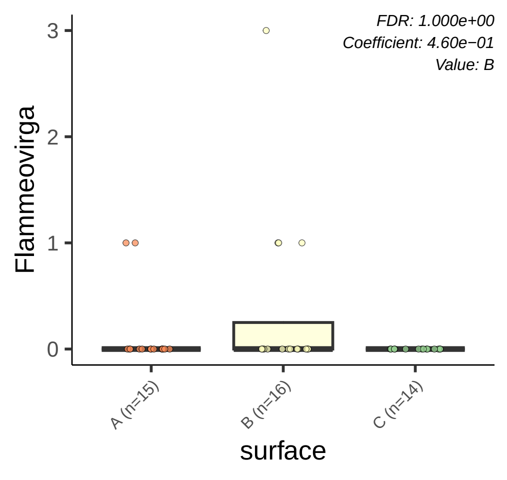

FDR: 1.000e+00
Coefficient: 4.60e−01
Value: B
Flammeovirga
3
2
1
0
A (n=15)
B (n=16)
C (n=14)
surface

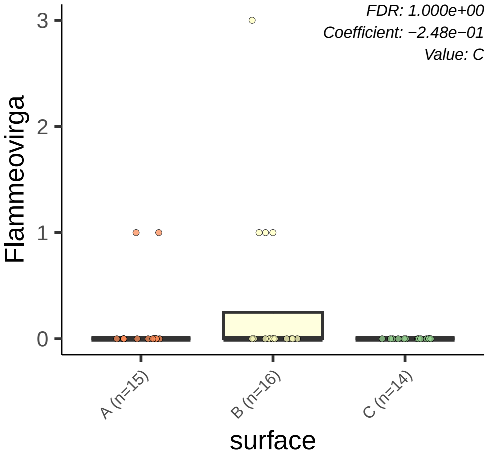

FDR: 1.000e+00
Coefficient: −2.48e−01
Value: C
Flammeovirga
0
1
2
3
A (n=15)
B (n=16)
C (n=14)
surface

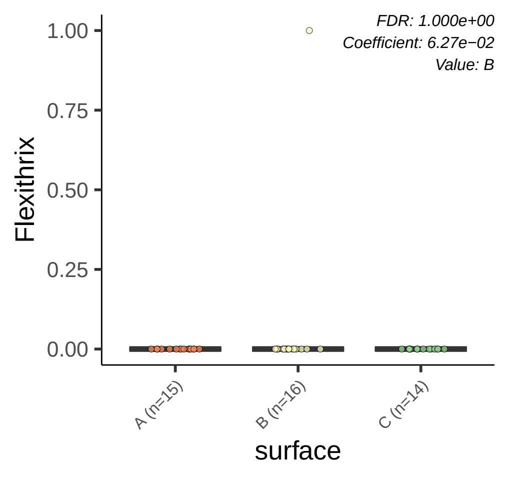

FDR: 1.000e+00
Coefficient: 6.27e−02
Value: B
Flexithrix
1.00
0.75
0.50
0.25
0.00
A (n=15)
B (n=16)
C (n=14)
surface

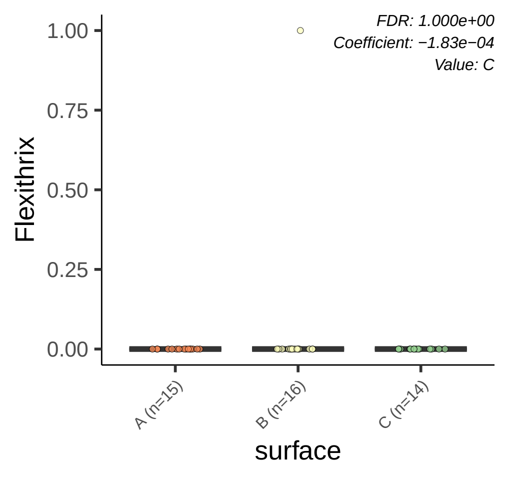
FDR: 1.000e+00
Coefficient: −1.83e−04
Value: C
Flexithrix
1.00
0.75
0.50
0.25
0.00
A (n=15)
B (n=16)
C (n=14)
surface

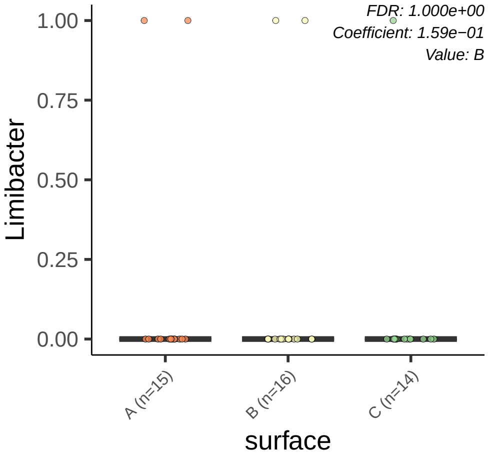

FDR: 1.000e+00
Coefficient: 1.59e−01
Value: B
Limibacter
1.00
0.75
0.50
0.25
0.00
A (n=15)
B (n=16)
C (n=14)
surface

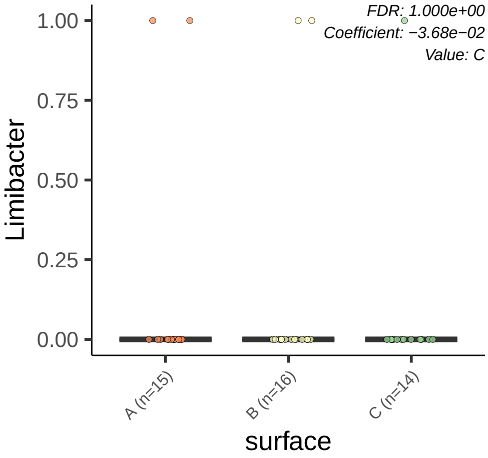

FDR: 1.000e+00
Coefficient: −3.68e−02
Value: C
Limibacter
1.00
0.75
0.50
0.25
0.00
A (n=15)
B (n=16)
C (n=14)
surface

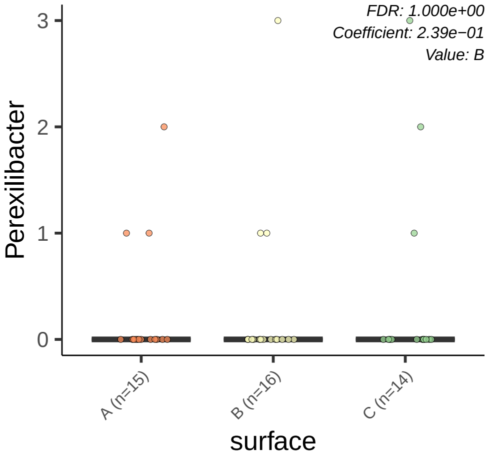
FDR: 1.000e+00
Coefficient: 2.39e−01
Value: B
Perexilibacter
3
2
1
0
A (n=15)
B (n=16)
C (n=14)
surface

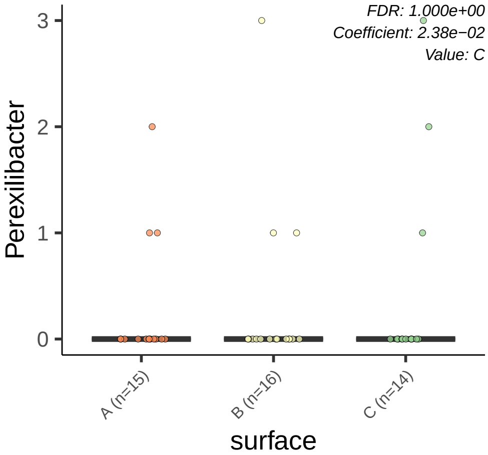

FDR: 1.000e+00
Coefficient: 2.38e−02
Value: C
Perexilibacter
3
2
1
0
A (n=15)
B (n=16)
C (n=14)
surface

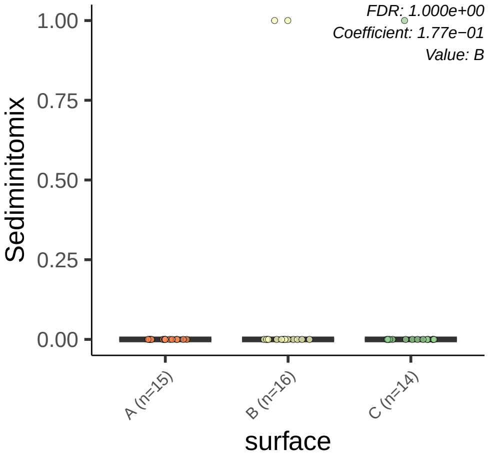

FDR: 1.000e+00
Coefficient: 1.77e−01
Value: B
1.00
0.75
0.50
0.25
0.00
Sedimінitomix
A (n=15)
B (n=16)
C (n=14)
surface

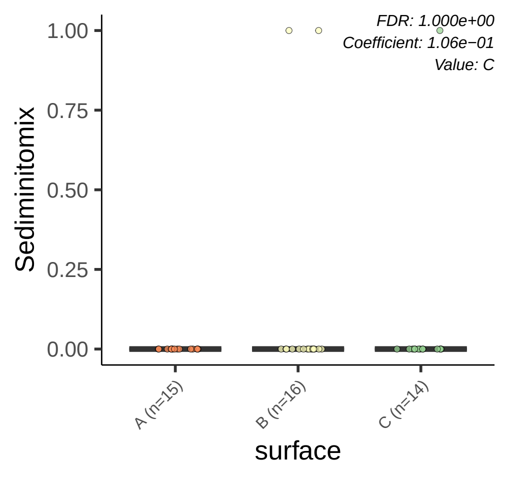

FDR: 1.000e+00
Coefficient: 1.06e−01
Value: C
Sedimintomix
1.00
0.75
0.50
0.25
0.00
A (n=15)
B (n=16)
C (n=14)
surface

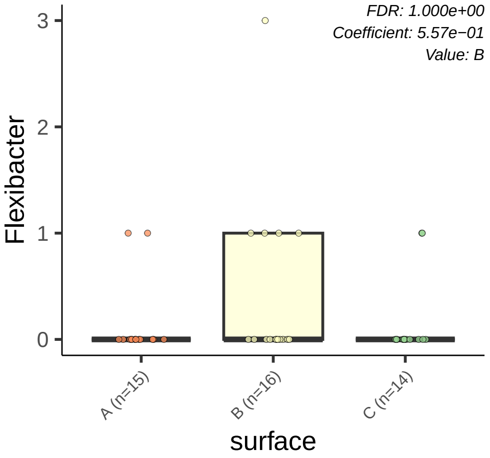
FDR: 1.000e+00
Coefficient: 5.57e−01
Value: B
Flexibacter
3
2
1
0
A (n=15)
B (n=16)
C (n=14)
surface

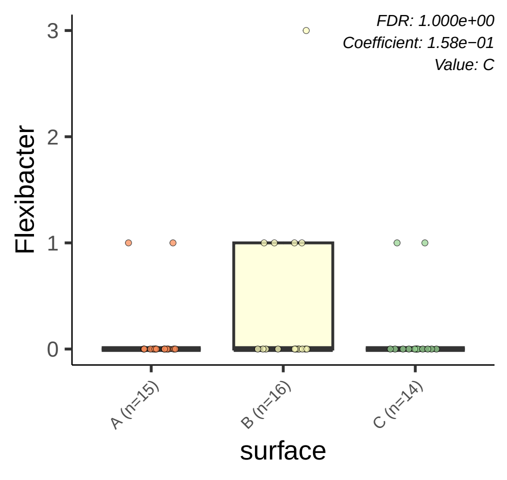
FDR: 1.000e+00
Coefficient: 1.58e−01
Value: C
Flexibacter
0
1
2
3
A (n=15)
B (n=16)
C (n=14)
surface

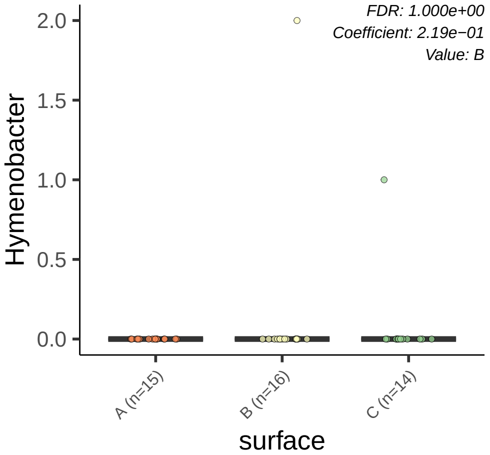
FDR: 1.000e+00
Coefficient: 2.19e−01
Value: B
Hymenobacter
2.0
1.5
1.0
0.5
0.0
A (n=15)
B (n=16)
C (n=14)
surface

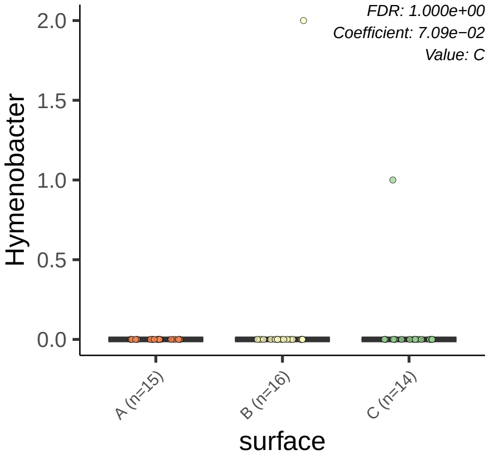
FDR: 1.000e+00
Coefficient: 7.09e−02
Value: C
Hymenobacter
2.0
1.5
1.0
0.5
0.0
A (n=15)
B (n=16)
C (n=14)
surface

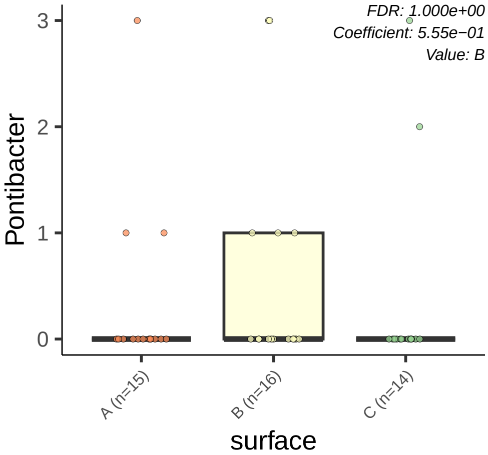

FDR: 1.000e+00
Coefficient: 5.55e−01
Value: B
Pontibacter
0
1
2
3
A (n=15)
B (n=16)
C (n=14)
surface

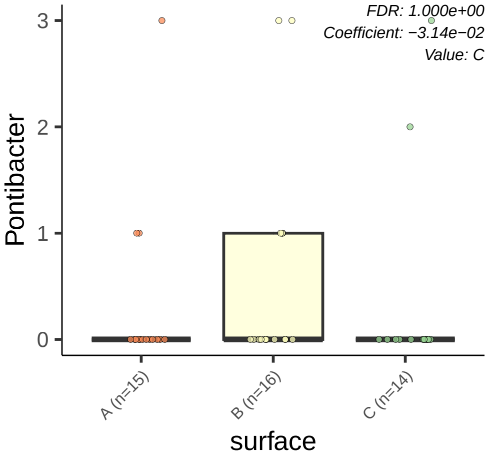
FDR: 1.000e+00
Coefficient: −3.14e−02
Value: C
Pontibacter
0
1
2
3
A (n=15)
B (n=16)
C (n=14)
surface

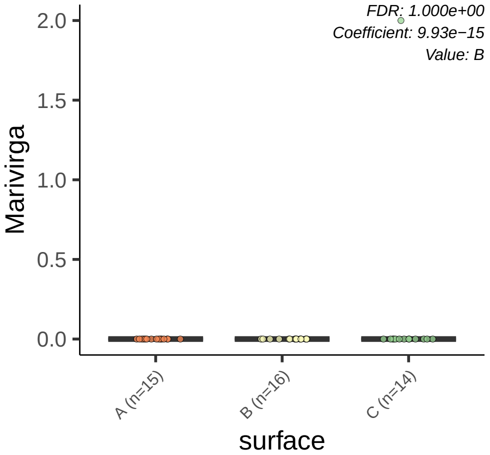

FDR: 1.000e+00
Coefficient: 9.93e−15
Value: B
Marivirga
2.0
1.5
1.0
0.5
0.0
A (n=15)
B (n=16)
C (n=14)
surface

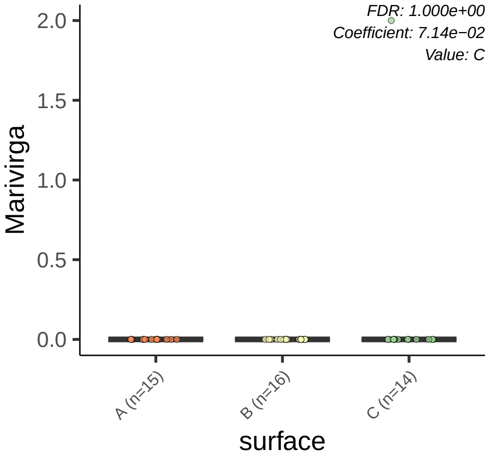

FDR: 1.000e+00
Coefficient: 7.14e−02
Value: C
2.0
1.5
1.0
0.5
0.0
Marivirga
A (n=15)
B (n=16)
C (n=14)
surface

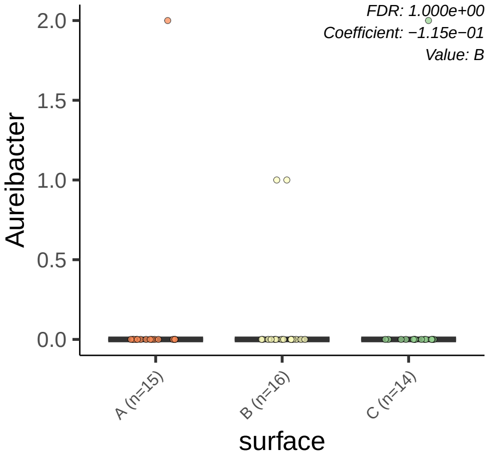
FDR: 1.000e+00
Coefficient: −1.15e−01
Value: B
2.0
1.5
1.0
0.5
0.0
Aureibacter
A (n=15)
B (n=16)
C (n=14)
surface

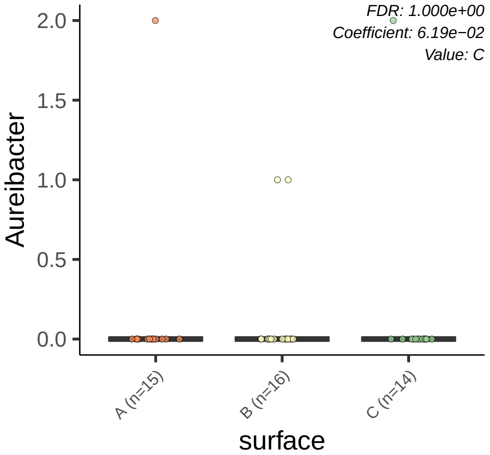

FDR: 1.000e+00
Coefficient: 6.19e−02
Value: C
Aureibacter
2.0
1.5
1.0
0.5
0.0
A (n=15)
B (n=16)
C (n=14)
surface

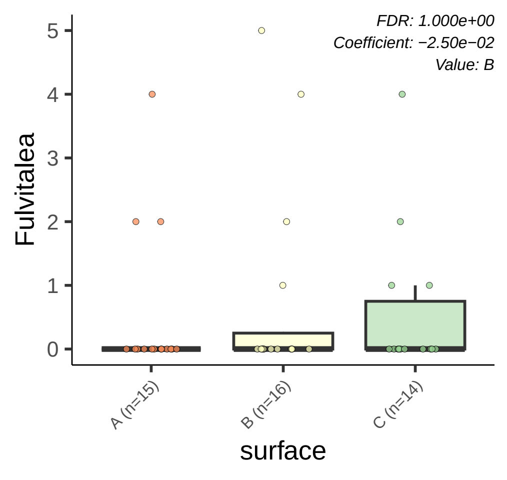

FDR: 1.000e+00
Coefficient: −2.50e−02
Value: B
Fulvitalea
0
1
2
3
4
5
A (n=15)
B (n=16)
C (n=14)
surface

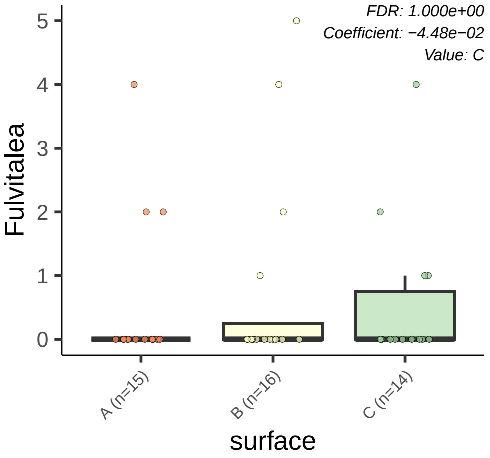

FDR: 1.000e+00
Coefficient: −4.48e−02
Value: C
Fulvitalea
0
1
2
3
4
5
A (n=15)
B (n=16)
C (n=14)
surface

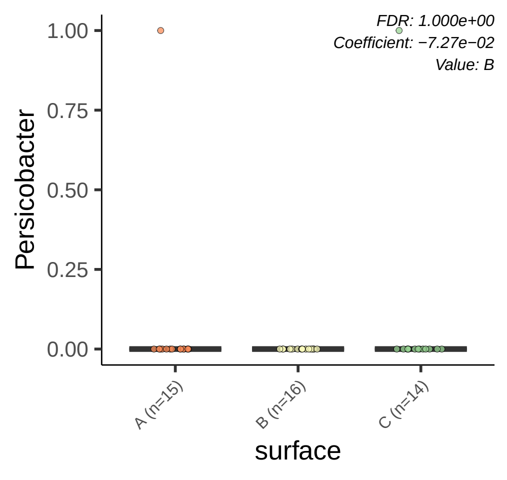
FDR: 1.000e+00
Coefficient: −7.27e−02
Value: B
Persicobacter
1.00
0.75
0.50
0.25
0.00
A (n=15)
B (n=16)
C (n=14)
surface

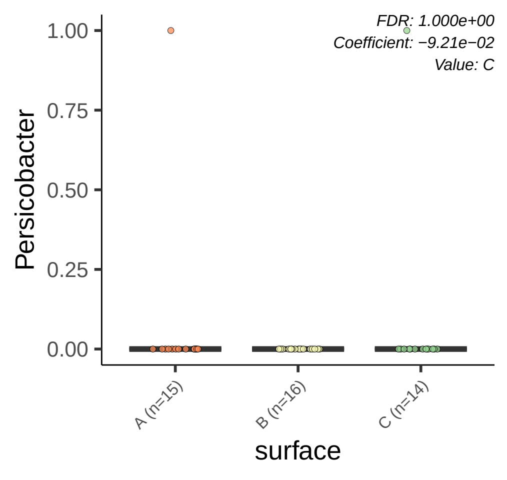
FDR: 1.000e+00
Coefficient: −9.21e−02
Value: C
Persicobacter
1.00
0.75
0.50
0.25
0.00
A (n=15)
B (n=16)
C (n=14)
surface

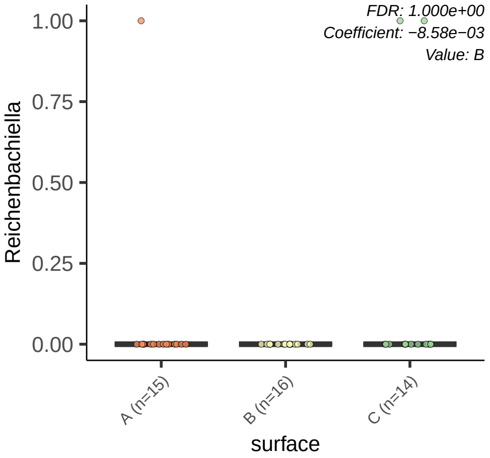

FDR: 1.000e+00
Coefficient: −8.58e−03
Value: B
Reichenbachiella
1.00
0.75
0.50
0.25
0.00
A (n=15)
B (n=16)
C (n=14)
surface

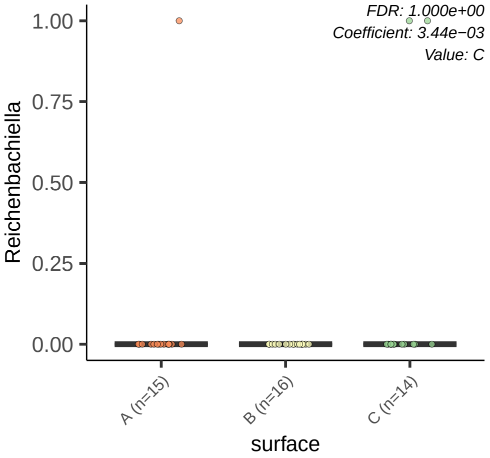
FDR: 1.000e+00
Coefficient: 3.44e−03
Value: C
Reichenbachiella
1.00
0.75
0.50
0.25
0.00
A (n=15)
B (n=16)
C (n=14)
surface

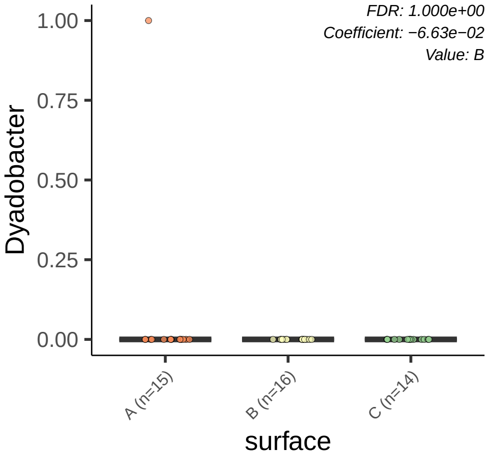
FDR: 1.000e+00
Coefficient: −6.63e−02
Value: B
Dyadobacter
1.00
0.75
0.50
0.25
0.00
A (n=15)
B (n=16)
C (n=14)
surface

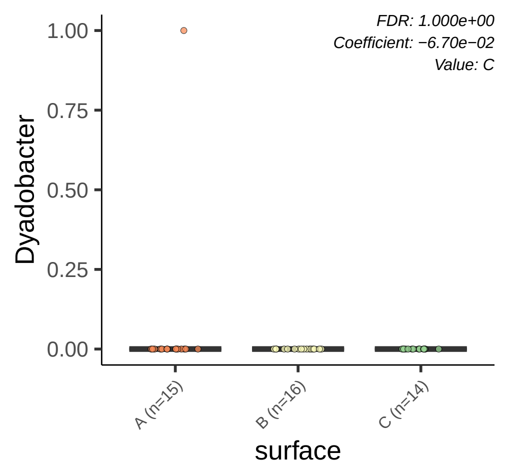
FDR: 1.000e+00
Coefficient: −6.70e−02
Value: C
1.00
0.75
0.50
0.25
0.00
Dyadobacter
A (n=15)
B (n=16)
C (n=14)
surface

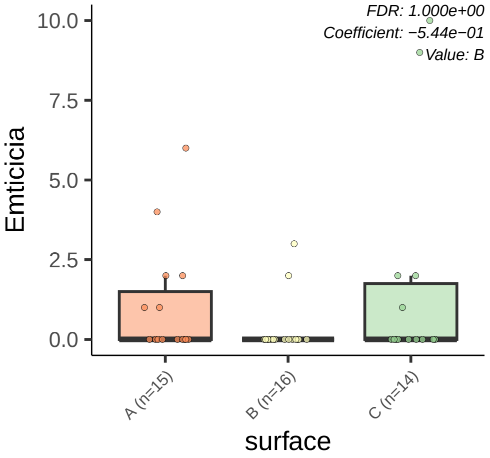

FDR: 1.000e+00
Coefficient: −5.44e−01
Value: B
Emticicia
10.0
7.5
5.0
2.5
0.0
A (n=15)
B (n=16)
C (n=14)
surface

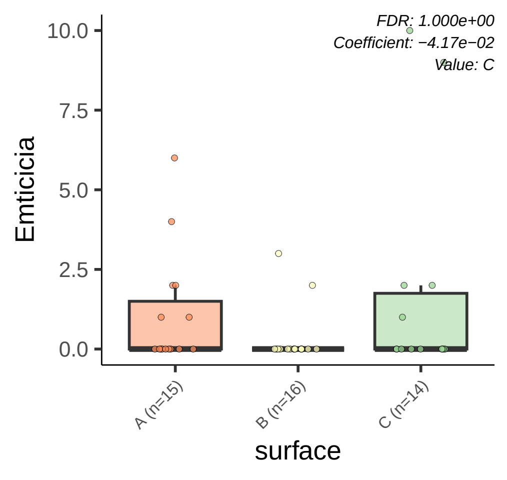

FDR: 1.000e+00
Coefficient: −4.17e−02
Value: C
10.0
7.5
5.0
2.5
0.0
Emticicia
A (n=15)
B (n=16)
C (n=14)
surface

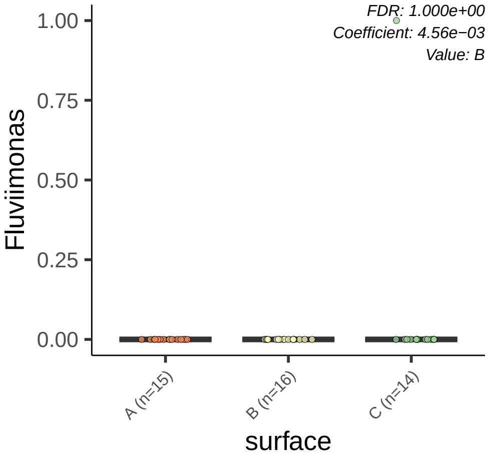

FDR: 1.000e+00
Coefficient: 4.56e−03
Value: B
1.00
0.75
0.50
0.25
0.00
Fluviimonas
A (n=15)
B (n=16)
C (n=14)
surface

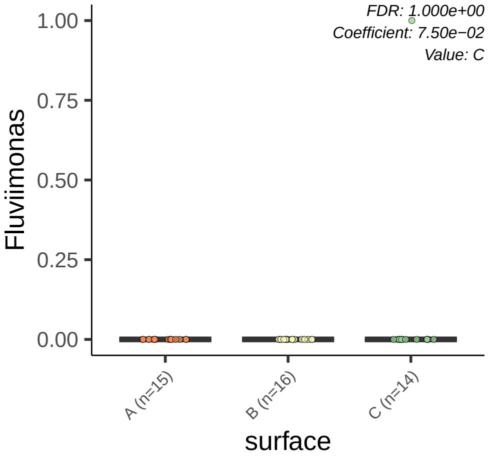

FDR: 1.000e+00
Coefficient: 7.50e−02
Value: C
Fluviimonas
1.00
0.75
0.50
0.25
0.00
A (n=15)
B (n=16)
C (n=14)
surface

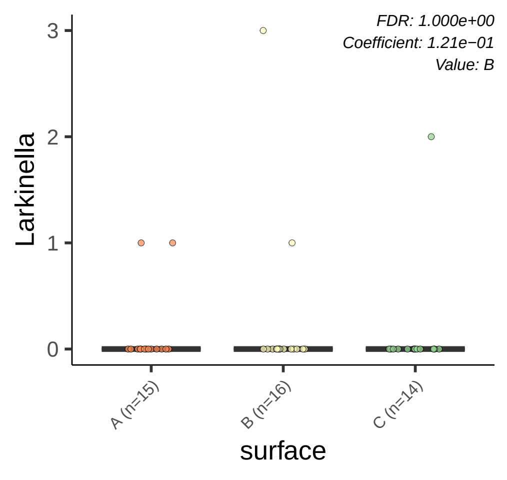

FDR: 1.000e+00
Coefficient: 1.21e−01
Value: B
Larkinella
0
1
2
3
A (n=15)
B (n=16)
C (n=14)
surface

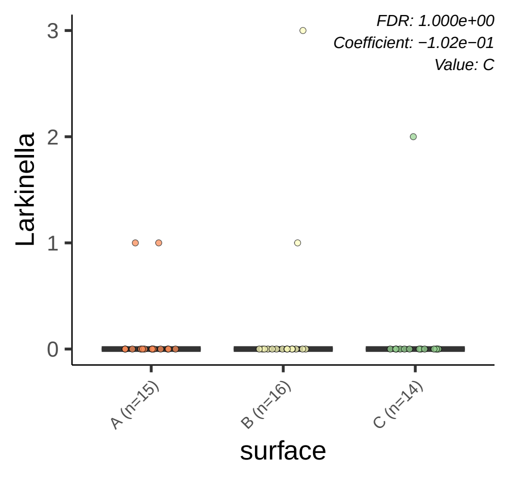
FDR: 1.000e+00
Coefficient: −1.02e−01
Value: C
Larkinella
0
1
2
3
A (n=15)
B (n=16)
C (n=14)
surface

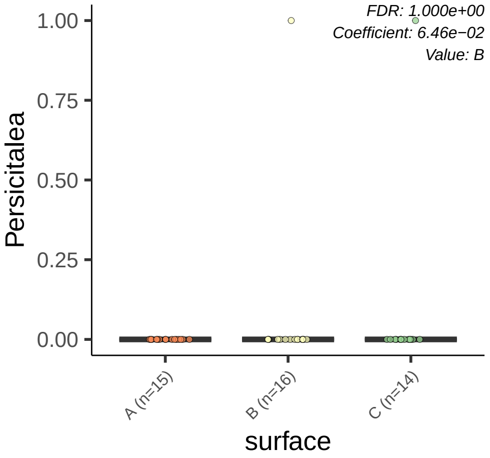
FDR: 1.000e+00
Coefficient: 6.46e−02
Value: B
Persicitalea
1.00
0.75
0.50
0.25
0.00
A (n=15)
B (n=16)
C (n=14)
surface

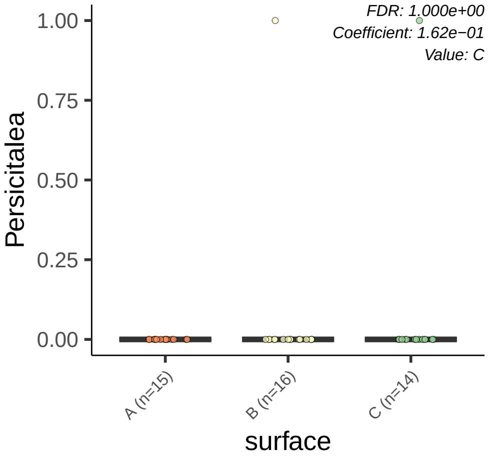

FDR: 1.000e+00
Coefficient: 1.62e−01
Value: C
1.00
0.75
0.50
0.25
0.00
Persicitalea
A (n=15)
B (n=16)
C (n=14)
surface

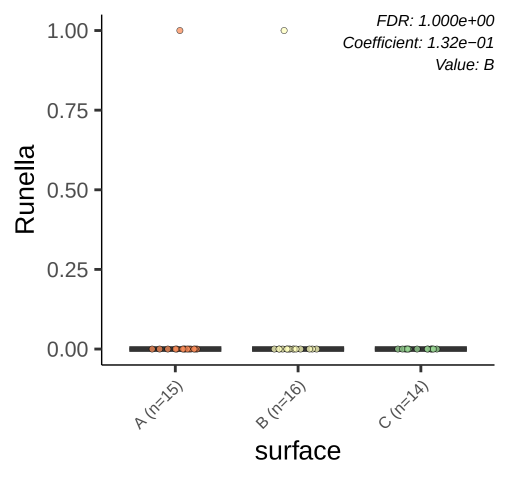

FDR: 1.000e+00
Coefficient: 1.32e−01
Value: B
Runella
1.00
0.75
0.50
0.25
0.00
A (n=15)
B (n=16)
C (n=14)
surface

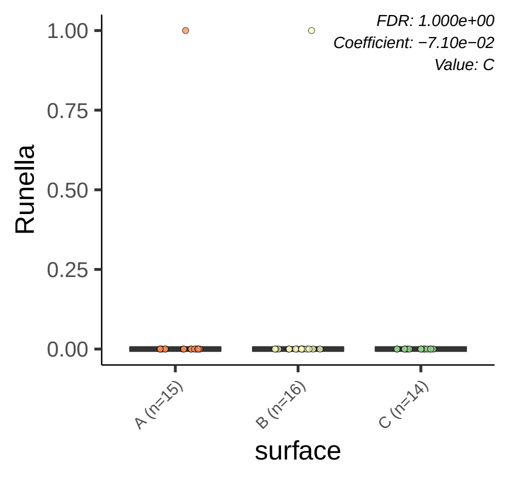

FDR: 1.000e+00
Coefficient: −7.10e−02
Value: C
Runella
1.00
0.75
0.50
0.25
0.00
A (n=15)
B (n=16)
C (n=14)
surface

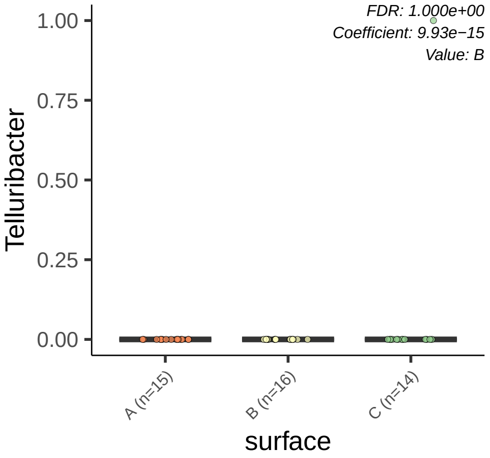
FDR: 1.000e+00
Coefficient: 9.93e−15
Value: B
Telluribacter
1.00
0.75
0.50
0.25
0.00
A (n=15)
B (n=16)
C (n=14)
surface

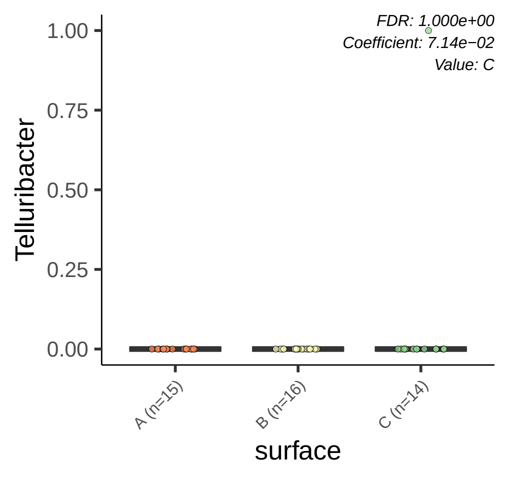
FDR: 1.000e+00
Coefficient: 7.14e−02
Value: C
Telluribacter
1.00
0.75
0.50
0.25
0.00
A (n=15)
B (n=16)
C (n=14)
surface

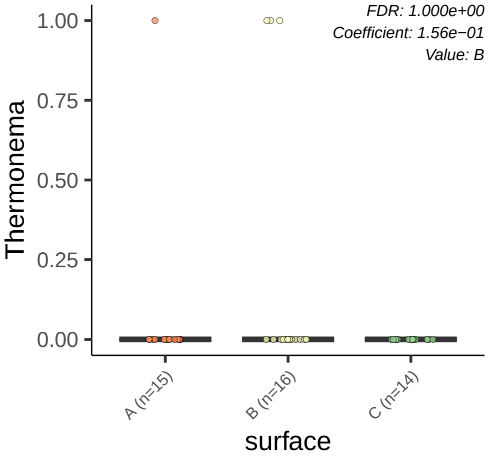

FDR: 1.000e+00
Coefficient: 1.56e−01
Value: B
Thermonema
1.00
0.75
0.50
0.25
0.00
A (n=15)
B (n=16)
C (n=14)
surface

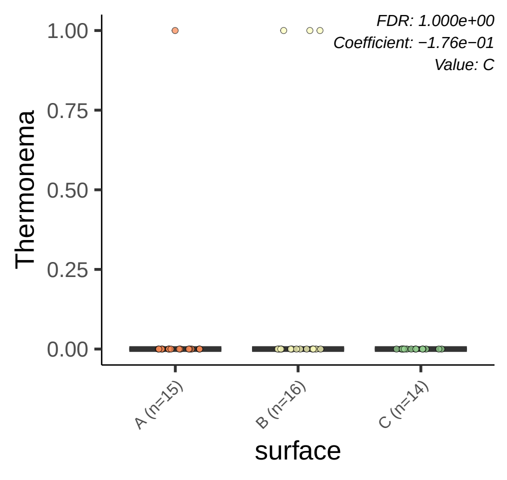

FDR: 1.000e+00
Coefficient: −1.76e−01
Value: C
Thermonema
1.00
0.75
0.50
0.25
0.00
A (n=15)
B (n=16)
C (n=14)
surface

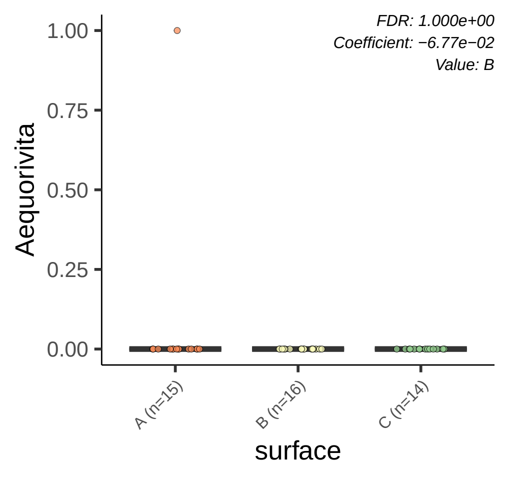

FDR: 1.000e+00
Coefficient: −6.77e−02
Value: B
Aequorivita
1.00
0.75
0.50
0.25
0.00
A (n=15)
B (n=16)
C (n=14)
surface

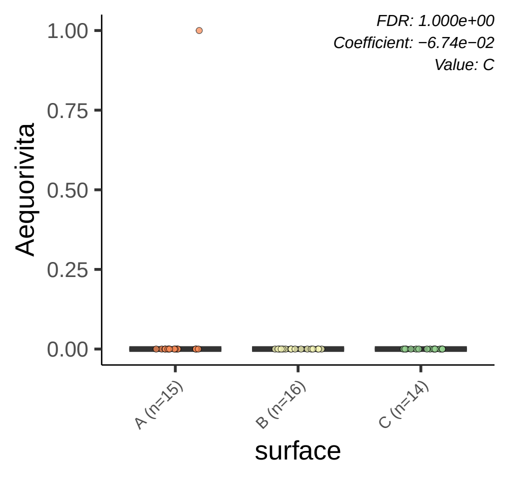

FDR: 1.000e+00
Coefficient: −6.74e−02
Value: C
Aequorivita
1.00
0.75
0.50
0.25
0.00
A (n=15)
B (n=16)
C (n=14)
surface

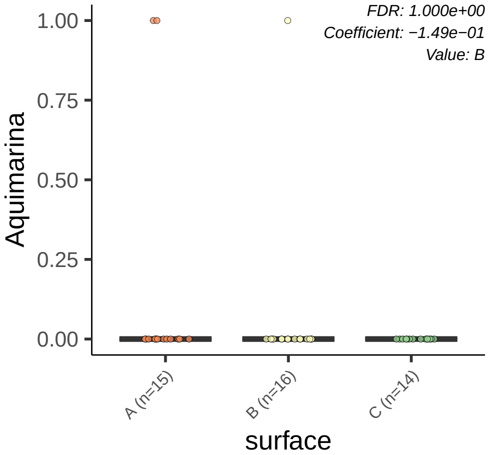
FDR: 1.000e+00
Coefficient: −1.49e−01
Value: B
Aquimarina
1.00
0.75
0.50
0.25
0.00
A (n=15)
B (n=16)
C (n=14)
surface

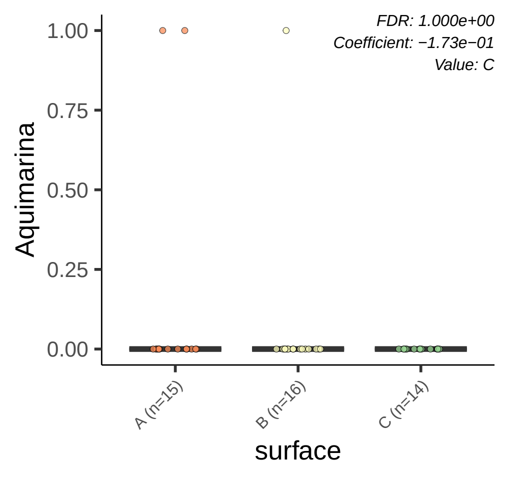
FDR: 1.000e+00
Coefficient: −1.73e−01
Value: C
Aquimarina
1.00
0.75
0.50
0.25
0.00
A (n=15)
B (n=16)
C (n=14)
surface

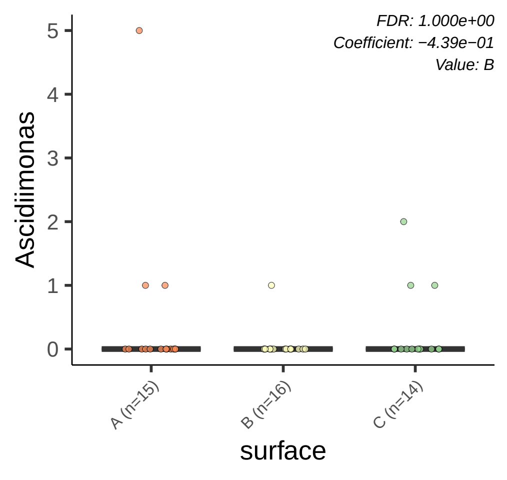
FDR: 1.000e+00
Coefficient: −4.39e−01
Value: B
Ascidimonas
5
4
3
2
1
0
A (n=15)
B (n=16)
C (n=14)
surface

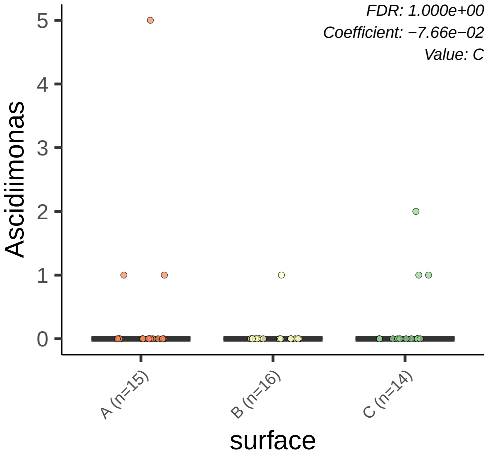

FDR: 1.000e+00
Coefficient: −7.66e−02
Value: C
Ascidimonas
0
1
2
3
4
5
A (n=15)
B (n=16)
C (n=14)
surface

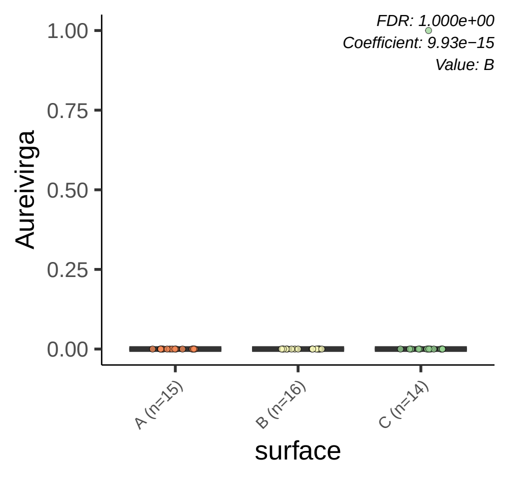

FDR: 1.000e+00
Coefficient: 9.93e−15
Value: B
Aureivirga
1.00
0.75
0.50
0.25
0.00
A (n=15)
B (n=16)
C (n=14)
surface

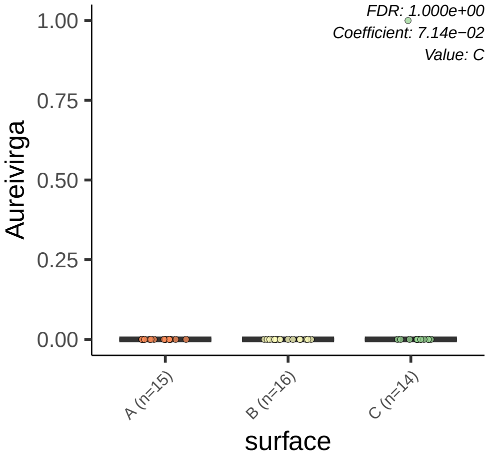

FDR: 1.000e+00
Coefficient: 7.14e−02
Value: C
1.00
0.75
0.50
0.25
0.00
Aureivirga
A (n=15)
B (n=16)
C (n=14)
surface

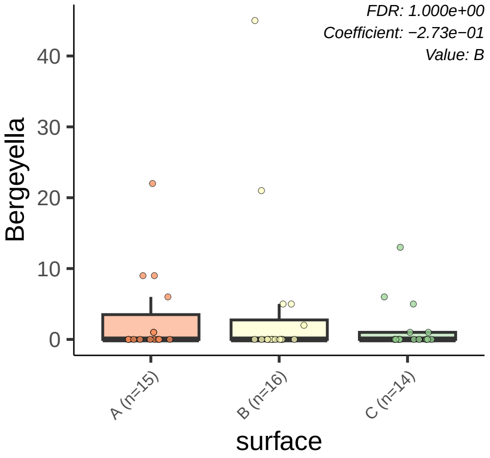
FDR: 1.000e+00
Coefficient: −2.73e−01
Value: B
Bergeyella
40
30
20
10
0
A (n=15)
B (n=16)
C (n=14)
surface

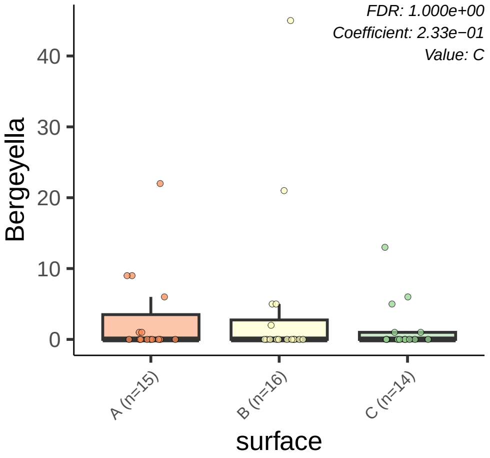
FDR: 1.000e+00
Coefficient: 2.33e−01
Value: C
Bergeyella
0
10
20
30
40
A (n=15)
B (n=16)
C (n=14)
surface

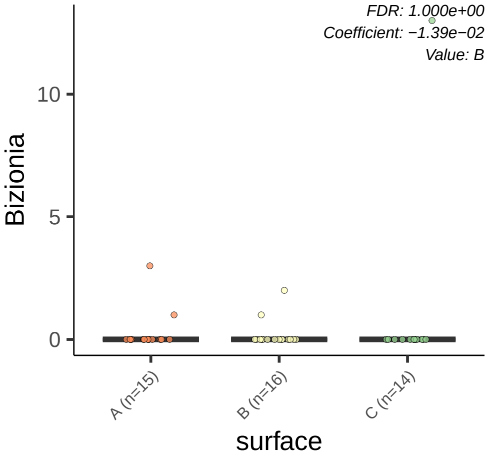
FDR: 1.000e+00
Coefficient: −1.39e−02
Value: B
Bizionia
10
5
0
A (n=15)
B (n=16)
C (n=14)
surface

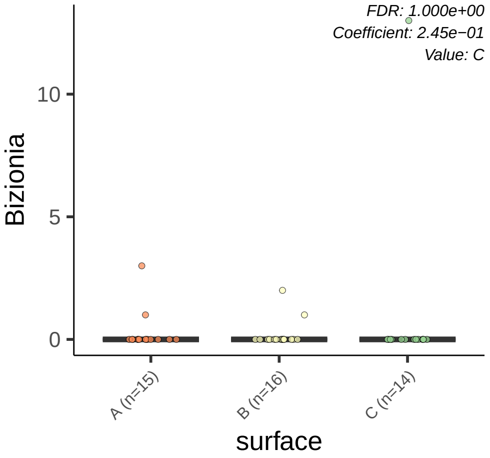
FDR: 1.000e+00
Coefficient: 2.45e−01
Value: C
Bizionia
10
5
0
A (n=15)
B (n=16)
C (n=14)
surface

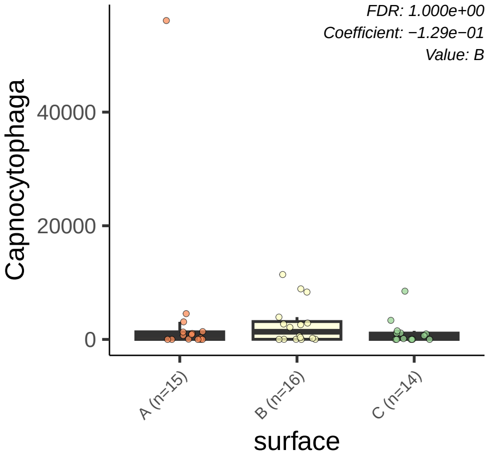

FDR: 1.000e+00
Coefficient: −1.29e−01
Value: B
Capnocytophaga
40000
20000
0
A (n=15)
B (n=16)
C (n=14)
surface

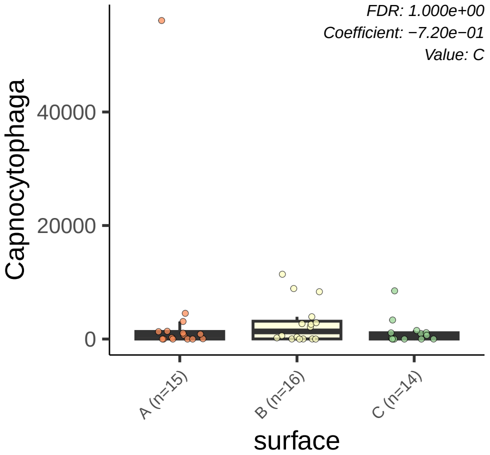
FDR: 1.000e+00
Coefficient: −7.20e−01
Value: C
Capnocytophaga
40000
20000
0
A (n=15)
B (n=16)
C (n=14)
surface

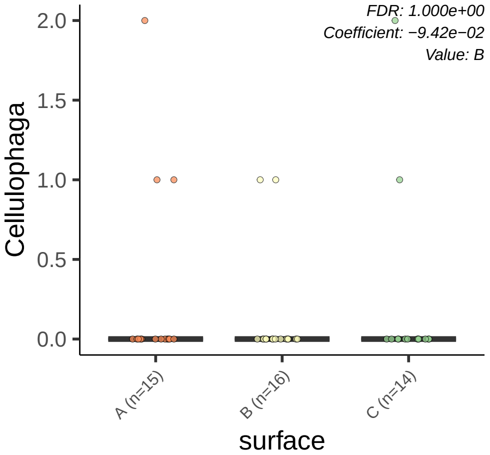
FDR: 1.000e+00
Coefficient: −9.42e−02
Value: B
Cellulophaga
2.0
1.5
1.0
0.5
0.0
A (n=15)
B (n=16)
C (n=14)
surface

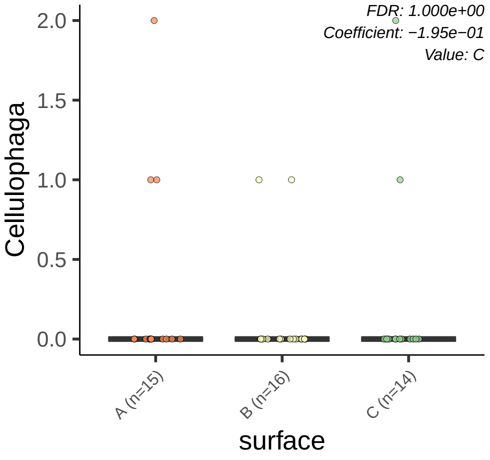
FDR: 1.000e+00
Coefficient: −1.95e−01
Value: C
Cellulophaga
2.0
1.5
1.0
0.5
0.0
A (n=15)
B (n=16)
C (n=14)
surface

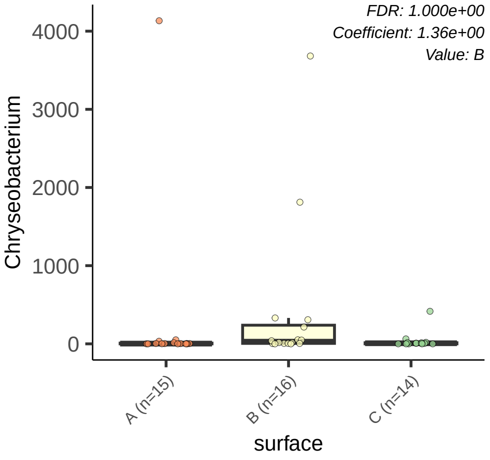

FDR: 1.000e+00
Coefficient: 1.36e+00
Value: B
Chryseobacterium
4000
3000
2000
1000
0
A (n=15)
B (n=16)
C (n=14)
surface

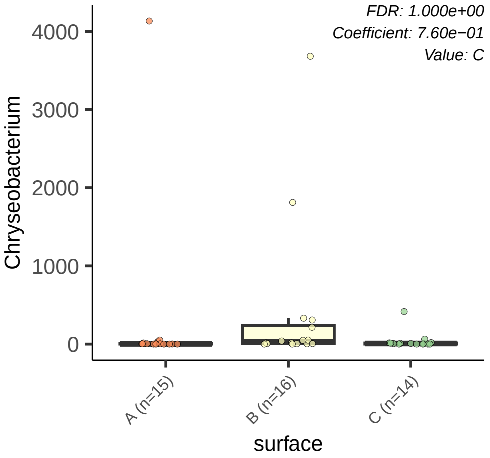

FDR: 1.000e+00
Coefficient: 7.60e−01
Value: C
Chryseobacterium
4000
3000
2000
1000
0
A (n=15)
B (n=16)
C (n=14)
surface

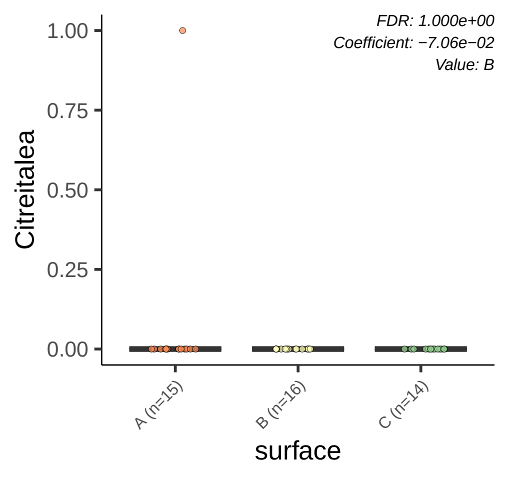
FDR: 1.000e+00
Coefficient: −7.06e−02
Value: B
Citreitalea
1.00
0.75
0.50
0.25
0.00
A (n=15)
B (n=16)
C (n=14)
surface

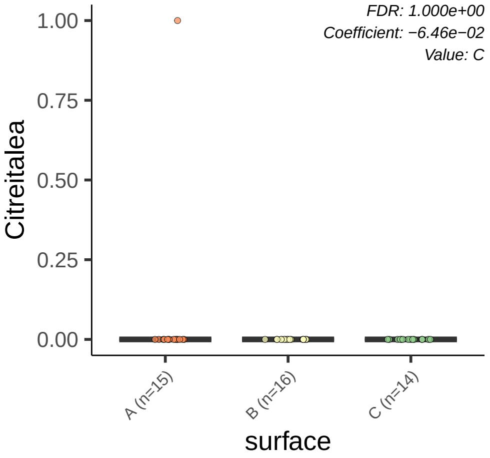
FDR: 1.000e+00
Coefficient: −6.46e−02
Value: C
Citreitalea
1.00
0.75
0.50
0.25
0.00
A (n=15)
B (n=16)
C (n=14)
surface

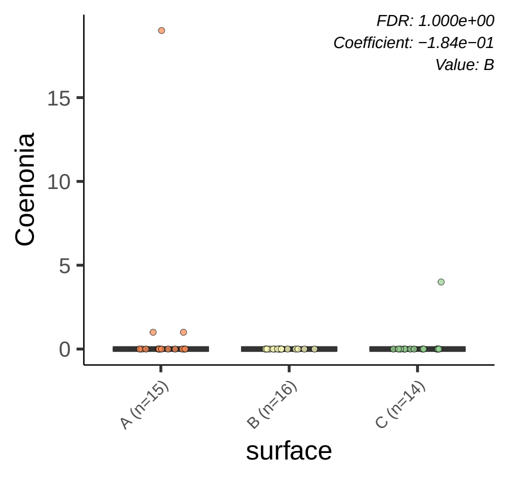
FDR: 1.000e+00
Coefficient: −1.84e−01
Value: B
Coenonia
15
10
5
0
A (n=15)
B (n=16)
C (n=14)
surface

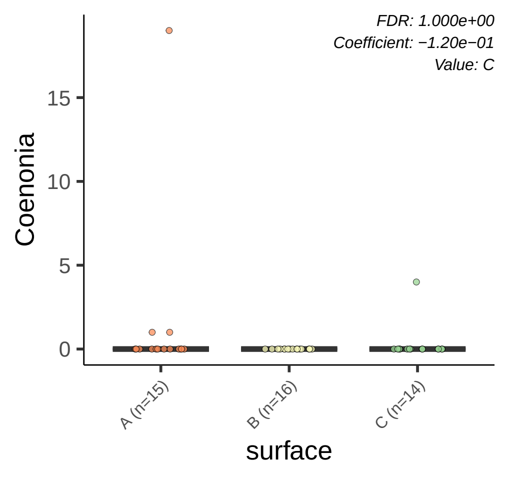

FDR: 1.000e+00
Coefficient: −1.20e−01
Value: C
Coenonia
0
5
10
15
A (n=15)
B (n=16)
C (n=14)
surface

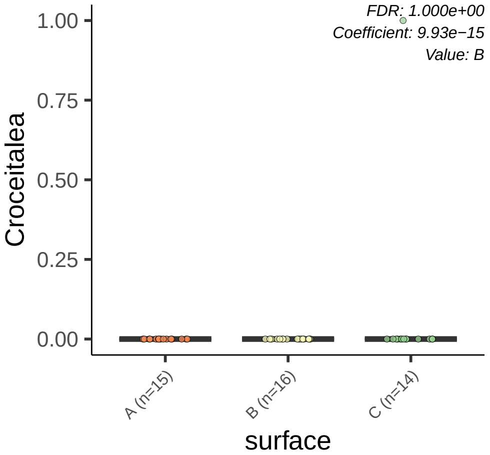

FDR: 1.000e+00
Coefficient: 9.93e−15
Value: B
1.00
0.75
0.50
0.25
0.00
Croceitalea
A (n=15)
B (n=16)
C (n=14)
surface

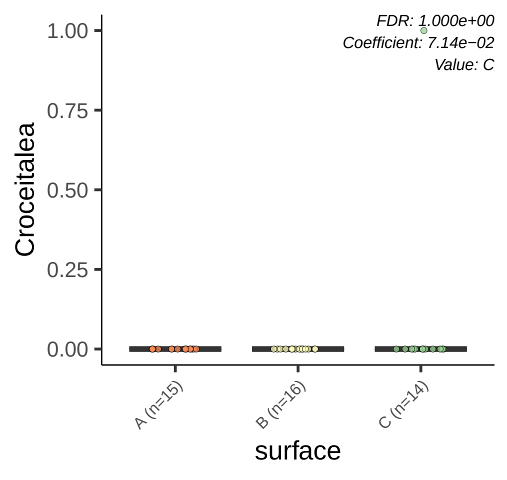
FDR: 1.000e+00
Coefficient: 7.14e−02
Value: C
Croceitalea
1.00
0.75
0.50
0.25
0.00
A (n=15)
B (n=16)
C (n=14)
surface

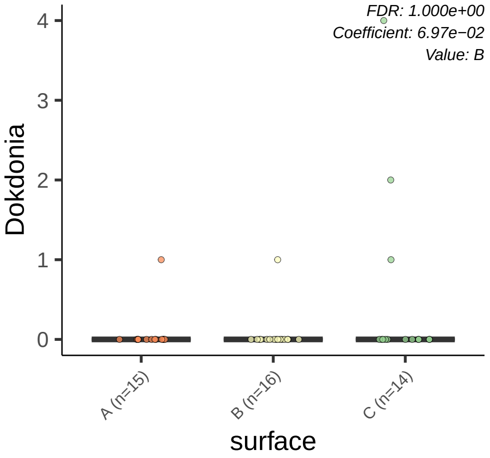
FDR: 1.000e+00
Coefficient: 6.97e−02
Value: B
Dokdonia
4
3
2
1
0
A (n=15)
B (n=16)
C (n=14)
surface

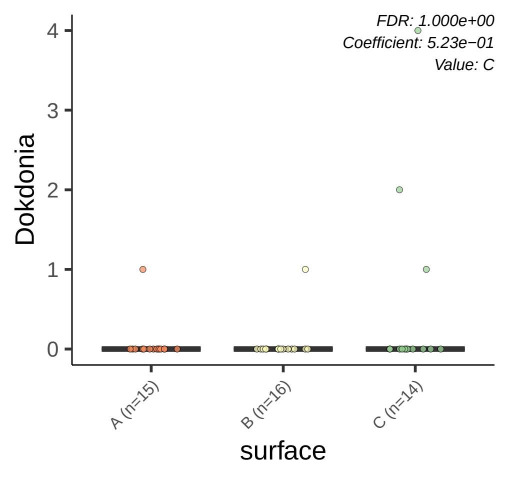
FDR: 1.000e+00
Coefficient: 5.23e−01
Value: C
Dokdonia
0
1
2
3
4
A (n=15)
B (n=16)
C (n=14)
surface

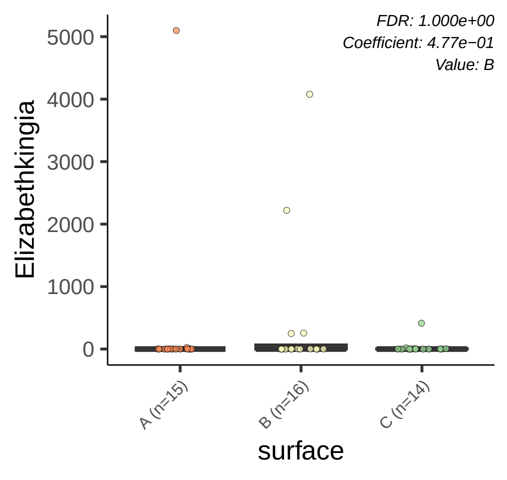
FDR: 1.000e+00
Coefficient: 4.77e−01
Value: B
Elizabethkingia
5000
4000
3000
2000
1000
0
A (n=15)
B (n=16)
C (n=14)
surface

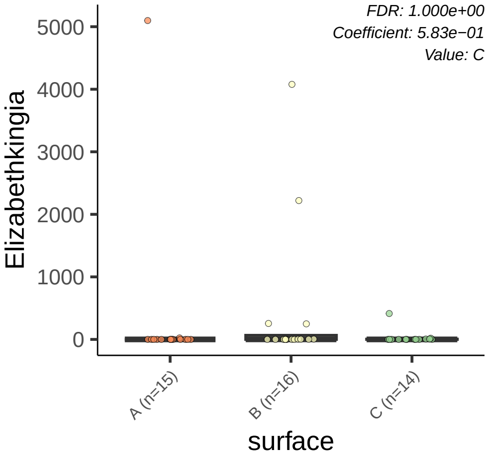

FDR: 1.000e+00
Coefficient: 5.83e−01
Value: C
Elizabethkingia
5000
4000
3000
2000
1000
0
A (n=15)
B (n=16)
C (n=14)
surface

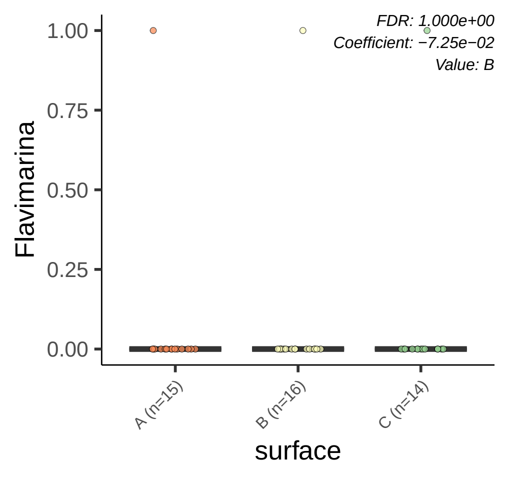

FDR: 1.000e+00
Coefficient: −7.25e−02
Value: B
Flavimarina
1.00
0.75
0.50
0.25
0.00
A (n=15)
B (n=16)
C (n=14)
surface

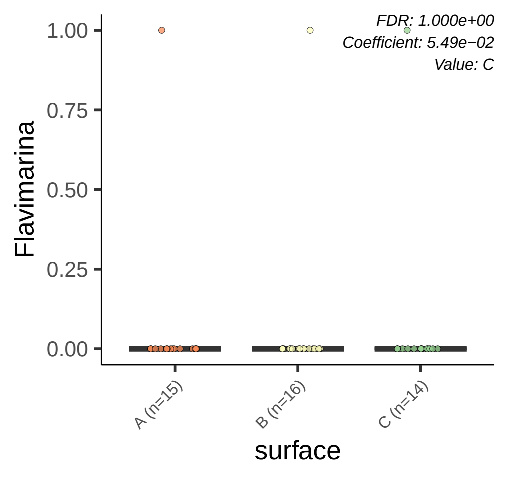
FDR: 1.000e+00
Coefficient: 5.49e−02
Value: C
Flavimarina
1.00
0.75
0.50
0.25
0.00
A (n=15)
B (n=16)
C (n=14)
surface

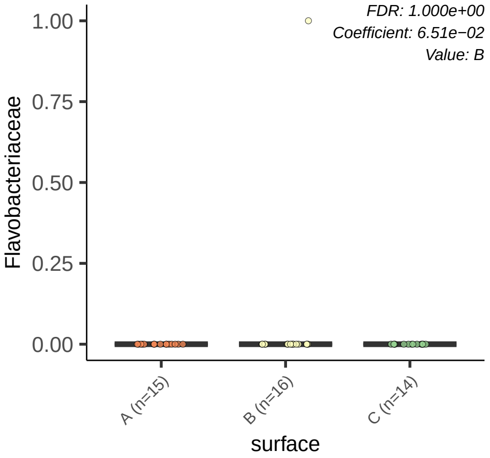

FDR: 1.000e+00
Coefficient: 6.51e−02
Value: B
Flavobacteriaceae
1.00
0.75
0.50
0.25
0.00
A (n=15)
B (n=16)
C (n=14)
surface

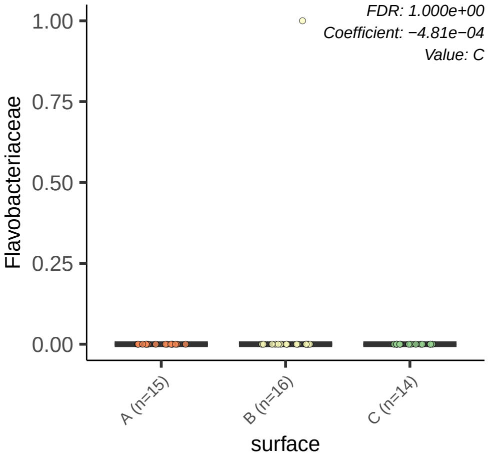
FDR: 1.000e+00
Coefficient: −4.81e−04
Value: C
Flavobacteriaceae
1.00
0.75
0.50
0.25
0.00
A (n=15)
B (n=16)
C (n=14)
surface

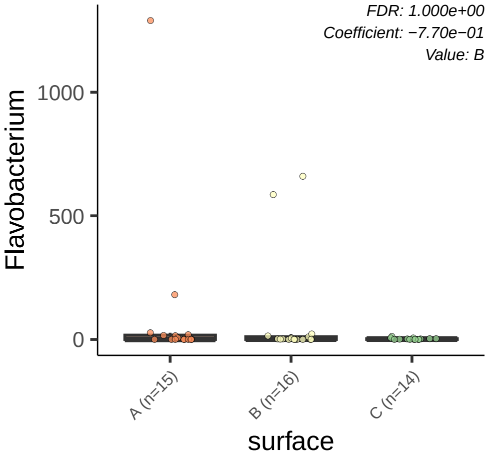
FDR: 1.000e+00
Coefficient: −7.70e−01
Value: B
Flavobacterium
1000
500
0
A (n=15)
B (n=16)
C (n=14)
surface

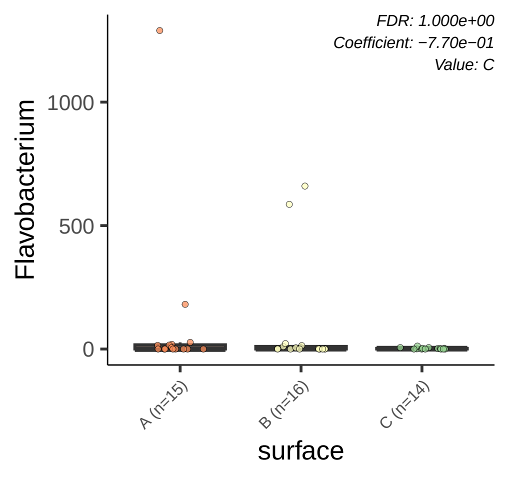
FDR: 1.000e+00
Coefficient: −7.70e−01
Value: C
Flavobacterium
1000
500
0
A (n=15)
B (n=16)
C (n=14)
surface

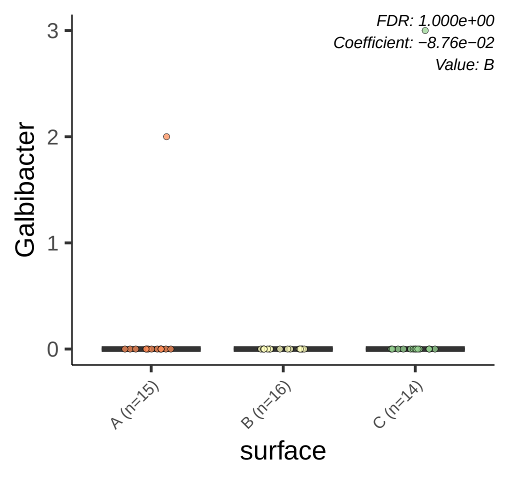
FDR: 1.000e+00
Coefficient: −8.76e−02
Value: B
Galbibacter
0
1
2
3
A (n=15)
B (n=16)
C (n=14)
surface

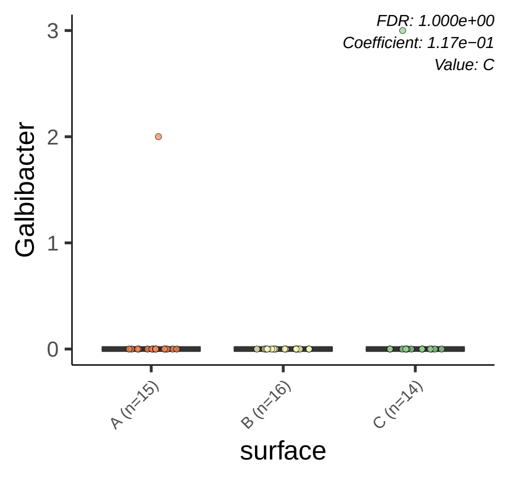
FDR: 1.000e+00
Coefficient: 1.17e−01
Value: C
Galbibacter
0
1
2
3
A (n=15)
B (n=16)
C (n=14)
surface

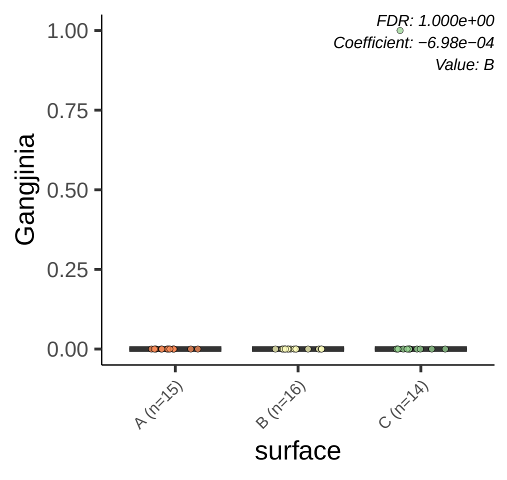

FDR: 1.000e+00
Coefficient: −6.98e−04
Value: B
1.00
0.75
0.50
0.25
0.00
Gangjinia
A (n=15)
B (n=16)
C (n=14)
surface

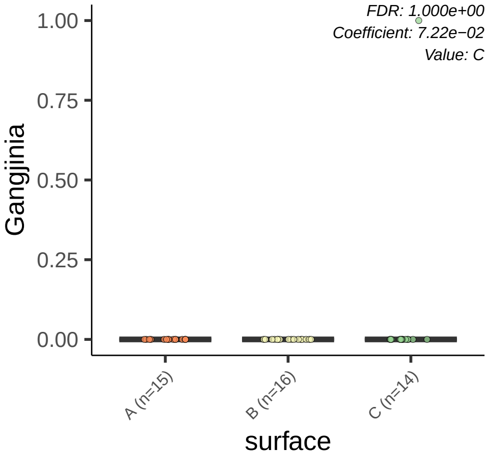
FDR: 1.000e+00
Coefficient: 7.22e−02
Value: C
1.00
0.75
0.50
0.25
0.00
Gangjinia
A (n=15)
B (n=16)
C (n=14)
surface

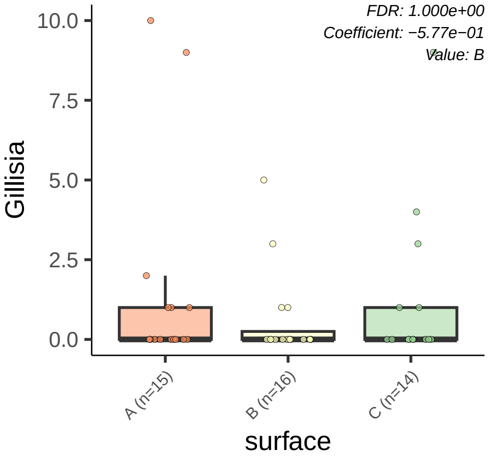

FDR: 1.000e+00
Coefficient: −5.77e−01
Value: B
10.0
7.5
5.0
2.5
0.0
Gillisia
A (n=15)
B (n=16)
C (n=14)
surface

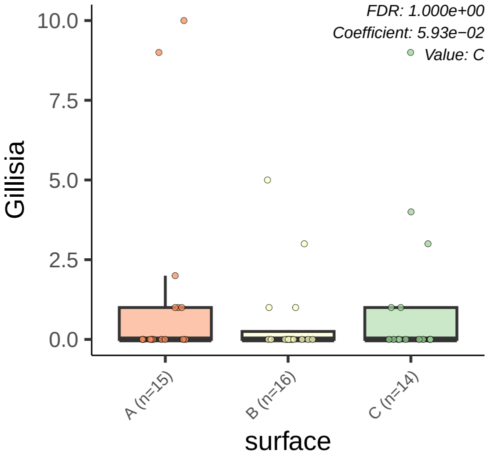
FDR: 1.000e+00
Coefficient: 5.93e−02
Value: C
Gillisia
10.0
7.5
5.0
2.5
0.0
A (n=15)
B (n=16)
C (n=14)
surface

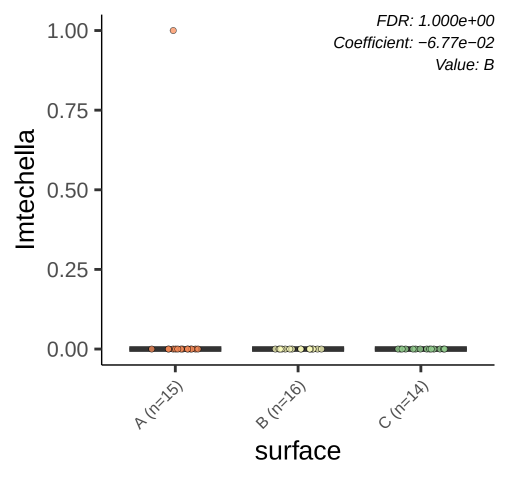

FDR: 1.000e+00
Coefficient: −6.77e−02
Value: B
Imtechella
1.00
0.75
0.50
0.25
0.00
A (n=15)
B (n=16)
C (n=14)
surface

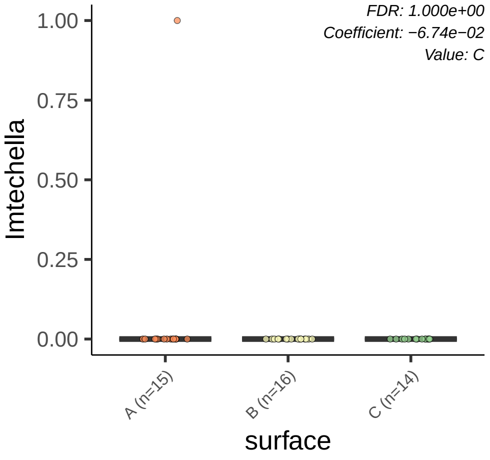

FDR: 1.000e+00
Coefficient: −6.74e−02
Value: C
Imtechella
1.00
0.75
0.50
0.25
0.00
A (n=15)
B (n=16)
C (n=14)
surface

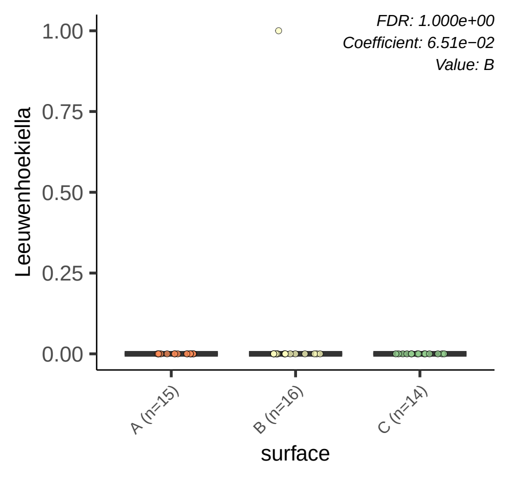
FDR: 1.000e+00
Coefficient: 6.51e−02
Value: B
Leeuwenhoekiella
1.00
0.75
0.50
0.25
0.00
A (n=15)
B (n=16)
C (n=14)
surface

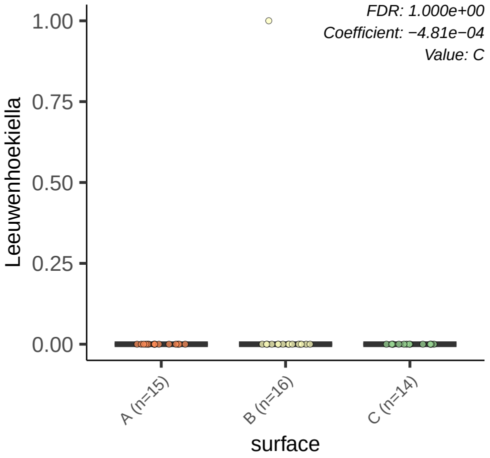
FDR: 1.000e+00
Coefficient: −4.81e−04
Value: C
Leeuwenhoekiella
1.00
0.75
0.50
0.25
0.00
A (n=15)
B (n=16)
C (n=14)
surface

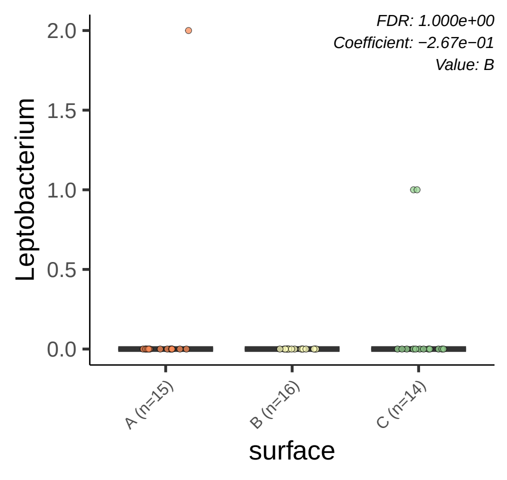

FDR: 1.000e+00
Coefficient: −2.67e−01
Value: B
Leptobacterium
2.0
1.5
1.0
0.5
0.0
A (n=15)
B (n=16)
C (n=14)
surface

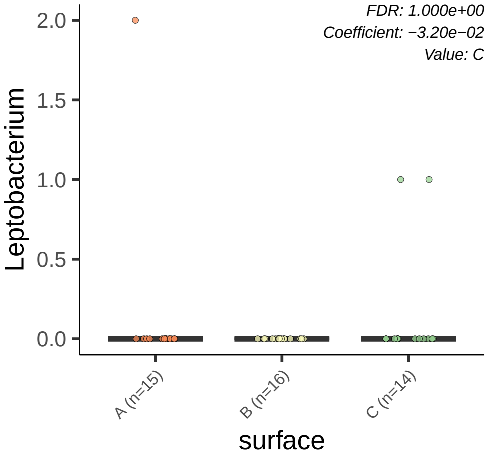
FDR: 1.000e+00
Coefficient: −3.20e−02
Value: C
Leptobacterium
2.0
1.5
1.0
0.5
0.0
A (n=15)
B (n=16)
C (n=14)
surface

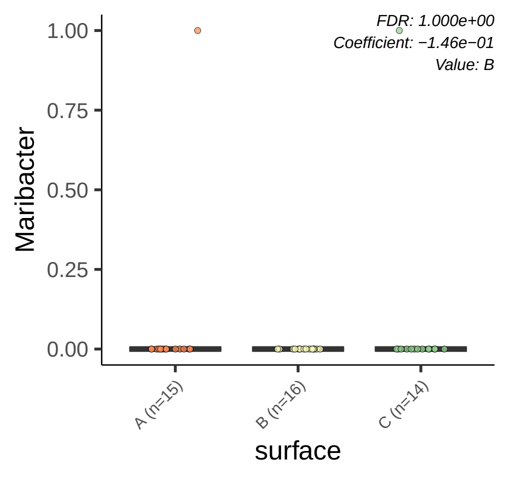
FDR: 1.000e+00
Coefficient: −1.46e−01
Value: B
Maribacter
1.00
0.75
0.50
0.25
0.00
A (n=15)
B (n=16)
C (n=14)
surface

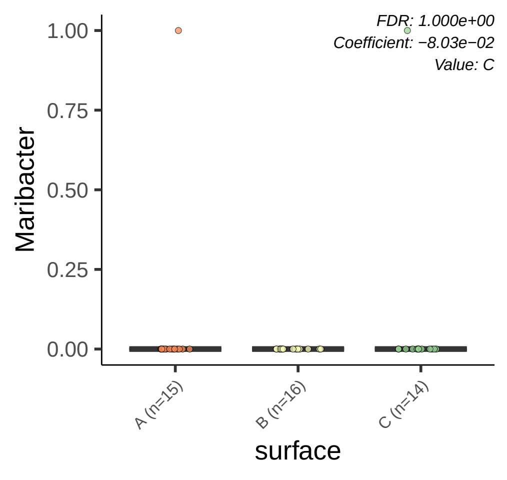
FDR: 1.000e+00
Coefficient: −8.03e−02
Value: C
Maribacter
1.00
0.75
0.50
0.25
0.00
A (n=15)
B (n=16)
C (n=14)
surface

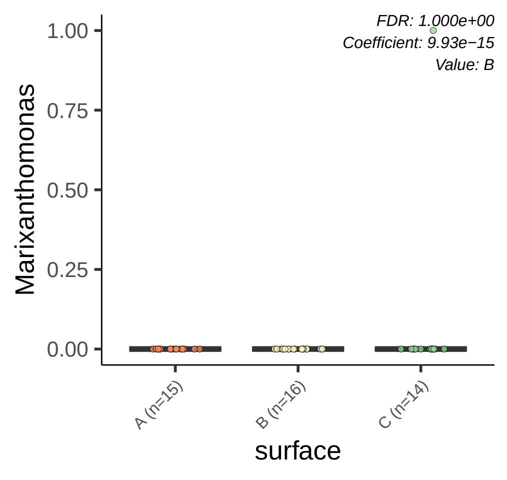
FDR: 1.000e+00
Coefficient: 9.93e−15
Value: B
Marixanthomonas
1.00
0.75
0.50
0.25
0.00
A (n=15)
B (n=16)
C (n=14)
surface

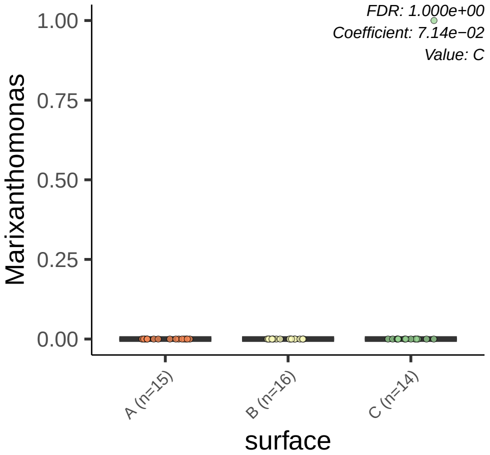

FDR: 1.000e+00
Coefficient: 7.14e−02
Value: C
Marixanthomonas
1.00
0.75
0.50
0.25
0.00
A (n=15)
B (n=16)
C (n=14)
surface

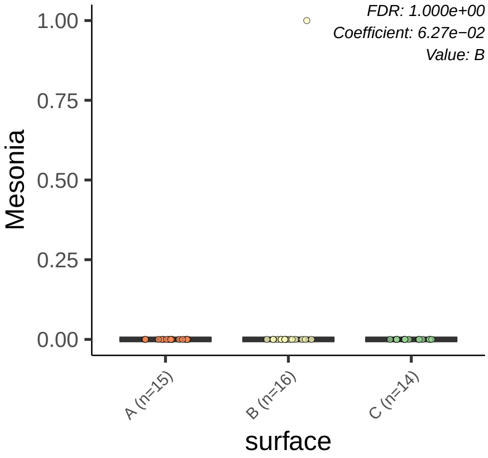
FDR: 1.000e+00
Coefficient: 6.27e−02
Value: B
Mesonia
1.00
0.75
0.50
0.25
0.00
A (n=15)
B (n=16)
C (n=14)
surface

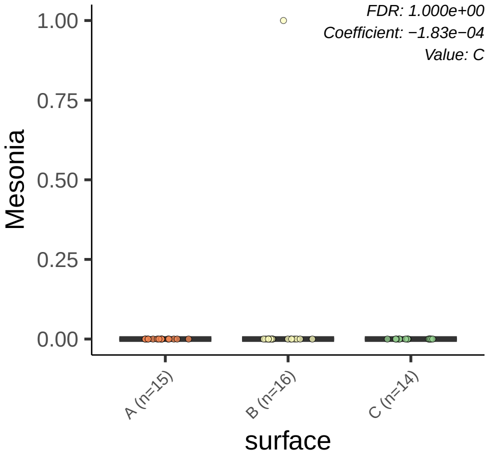
FDR: 1.000e+00
Coefficient: −1.83e−04
Value: C
Mesonia
1.00
0.75
0.50
0.25
0.00
A (n=15)
B (n=16)
C (n=14)
surface

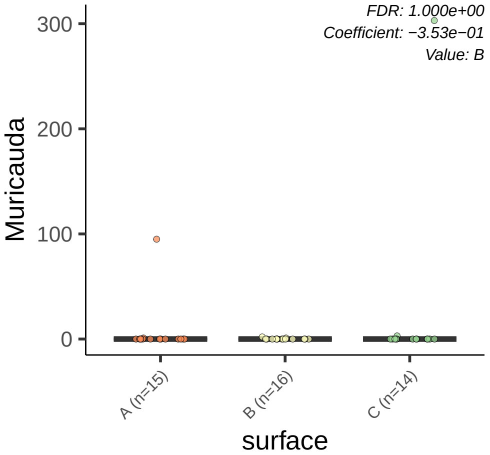
FDR: 1.000e+00
Coefficient: −3.53e−01
Value: B
Muricauda
300
200
100
0
A (n=15)
B (n=16)
C (n=14)
surface

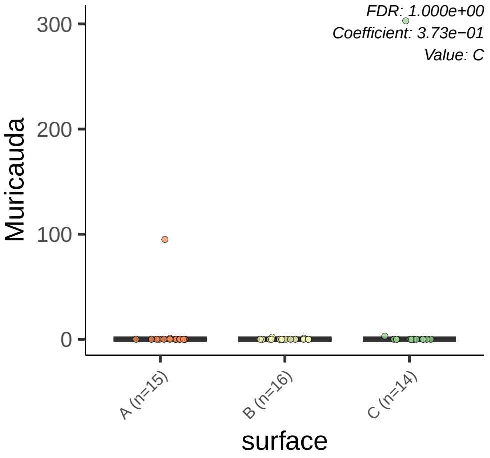
FDR: 1.000e+00
Coefficient: 3.73e−01
Value: C
Muricauda
300
200
100
0
A (n=15)
B (n=16)
C (n=14)
surface

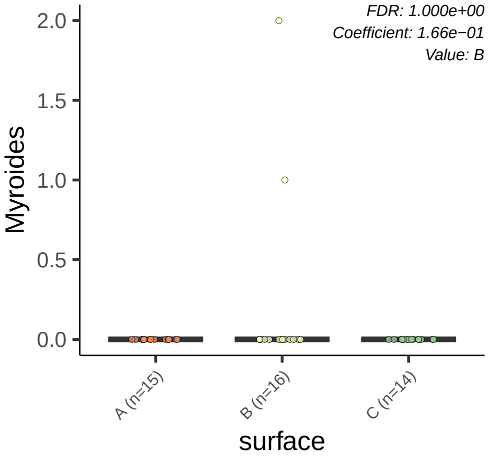

FDR: 1.000e+00
Coefficient: 1.66e−01
Value: B
Myroides
2.0
1.5
1.0
0.5
0.0
A (n=15)
B (n=16)
C (n=14)
surface

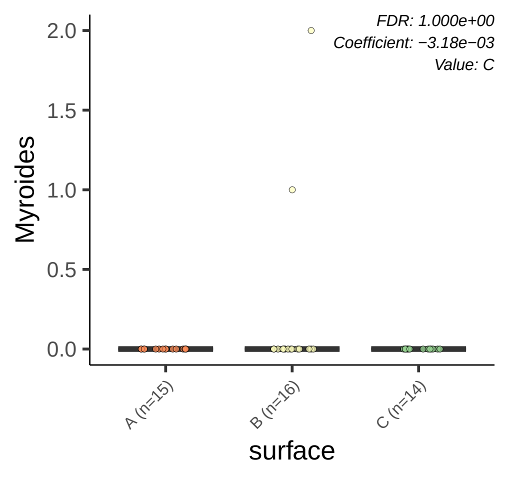

FDR: 1.000e+00
Coefficient: −3.18e−03
Value: C
Myroides
2.0
1.5
1.0
0.5
0.0
A (n=15)
B (n=16)
C (n=14)
surface

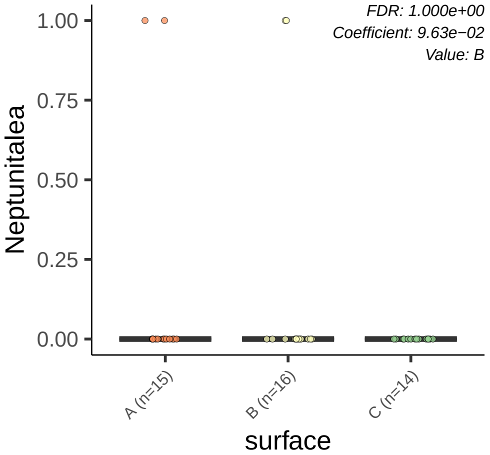

FDR: 1.000e+00
Coefficient: 9.63e−02
Value: B
Neptunitalea
1.00
0.75
0.50
0.25
0.00
A (n=15)
B (n=16)
C (n=14)
surface

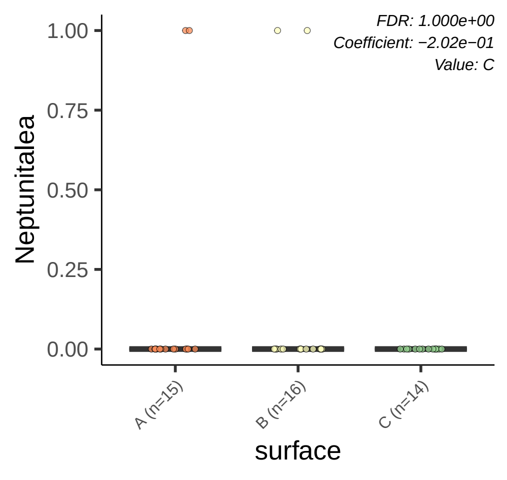

FDR: 1.000e+00
Coefficient: −2.02e−01
Value: C
1.00
0.75
0.50
0.25
0.00
Neptunitalea
A (n=15)
B (n=16)
C (n=14)
surface

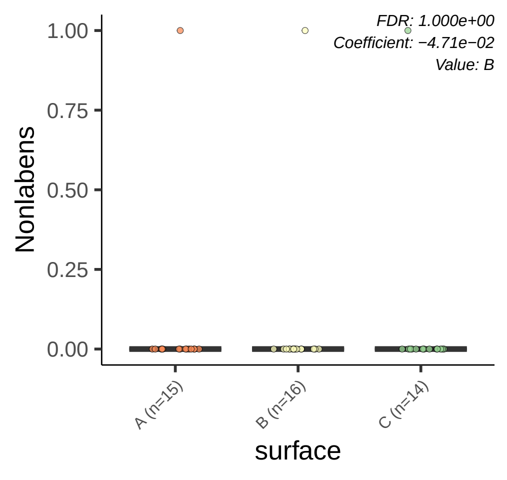

FDR: 1.000e+00
Coefficient: −4.71e−02
Value: B
Nonlabens
1.00
0.75
0.50
0.25
0.00
A (n=15)
B (n=16)
C (n=14)
surface

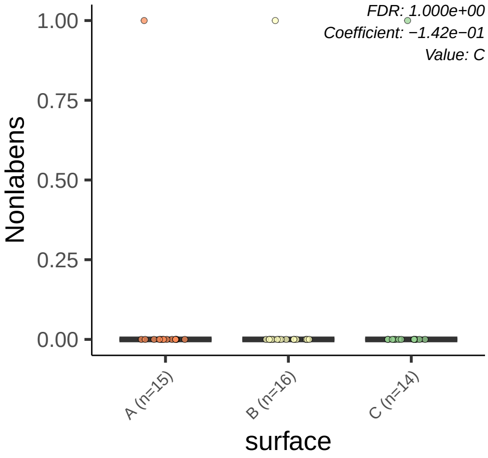

FDR: 1.000e+00
Coefficient: −1.42e−01
Value: C
Nonlabens
1.00
0.75
0.50
0.25
0.00
A (n=15)
B (n=16)
C (n=14)
surface

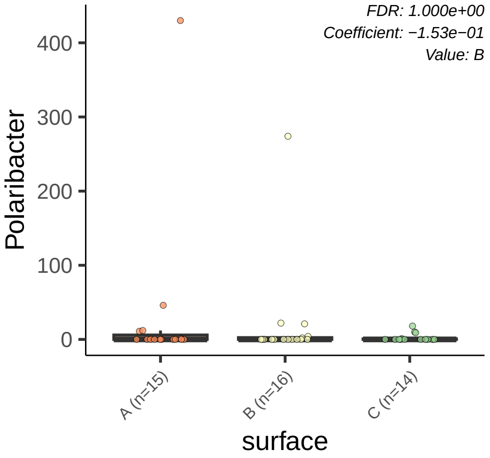
FDR: 1.000e+00
Coefficient: −1.53e−01
Value: B
Polaribacter
400
300
200
100
0
A (n=15)
B (n=16)
C (n=14)
surface

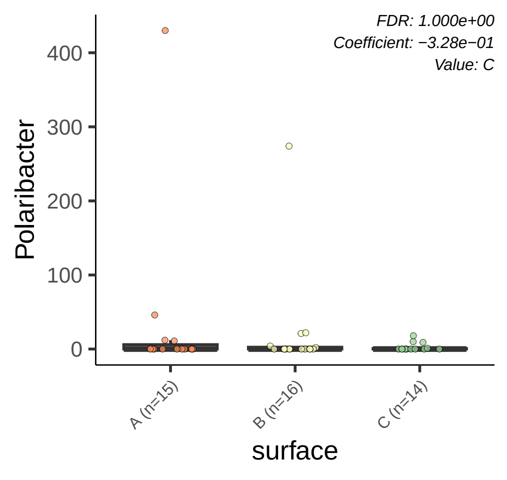

FDR: 1.000e+00
Coefficient: −3.28e−01
Value: C
Polaribacter
400
300
200
100
0
A (n=15)
B (n=16)
C (n=14)
surface

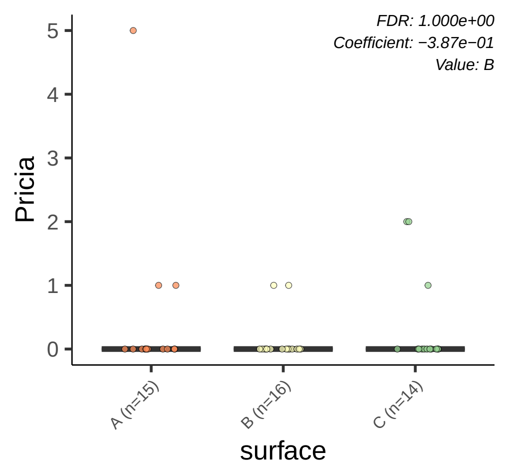
FDR: 1.000e+00
Coefficient: −3.87e−01
Value: B
Pricia
0
1
2
3
4
5
A (n=15)
B (n=16)
C (n=14)
surface

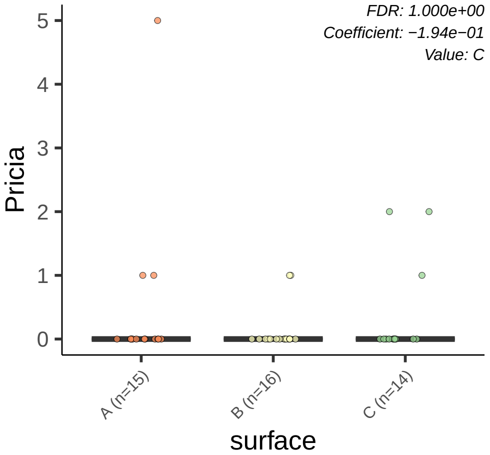
FDR: 1.000e+00
Coefficient: −1.94e−01
Value: C
Pricia
5
4
3
2
1
0
A (n=15)
B (n=16)
C (n=14)
surface

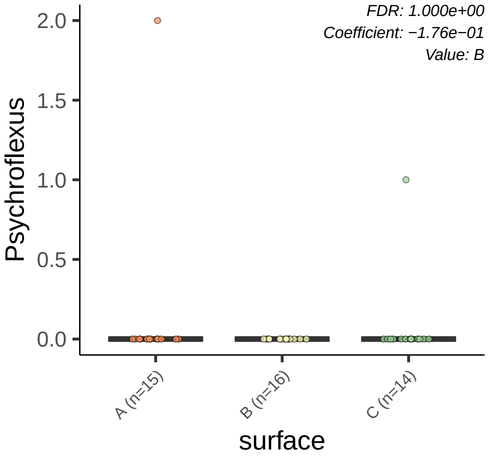

FDR: 1.000e+00
Coefficient: −1.76e−01
Value: B
Psychroflexus
2.0
1.5
1.0
0.5
0.0
A (n=15)
B (n=16)
C (n=14)
surface

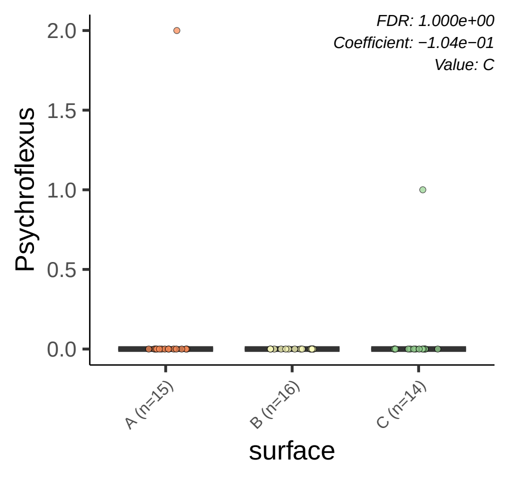
FDR: 1.000e+00
Coefficient: −1.04e−01
Value: C
Psychroflexus
2.0
1.5
1.0
0.5
0.0
A (n=15)
B (n=16)
C (n=14)
surface

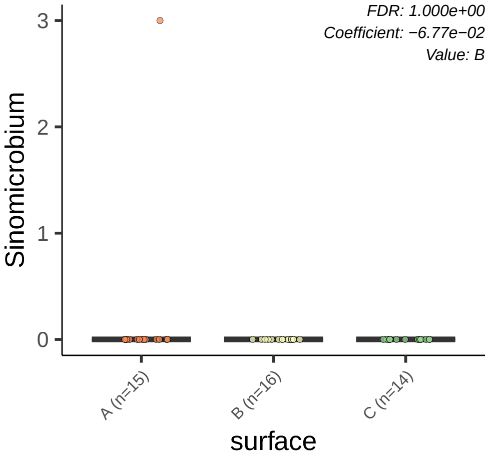

FDR: 1.000e+00
Coefficient: −6.77e−02
Value: B
Sinomicrobium
3
2
1
0
A (n=15)
B (n=16)
C (n=14)
surface

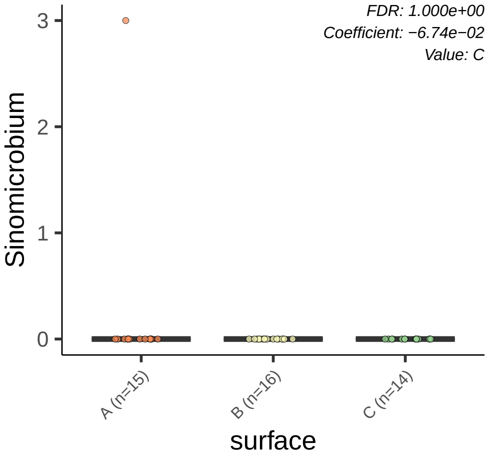
FDR: 1.000e+00
Coefficient: −6.74e−02
Value: C
Sinomicrobium
3
2
1
0
A (n=15)
B (n=16)
C (n=14)
surface

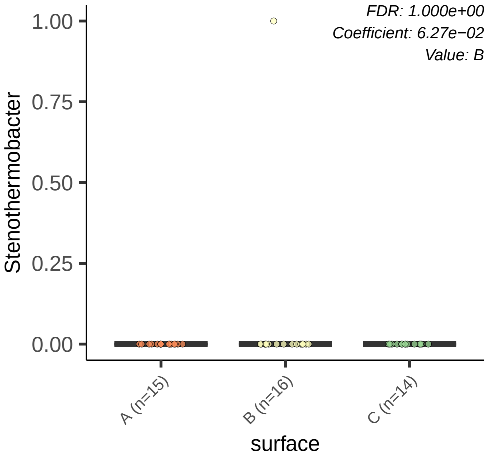
FDR: 1.000e+00
Coefficient: 6.27e−02
Value: B
Stenothermobacter
1.00
0.75
0.50
0.25
0.00
A (n=15)
B (n=16)
C (n=14)
surface

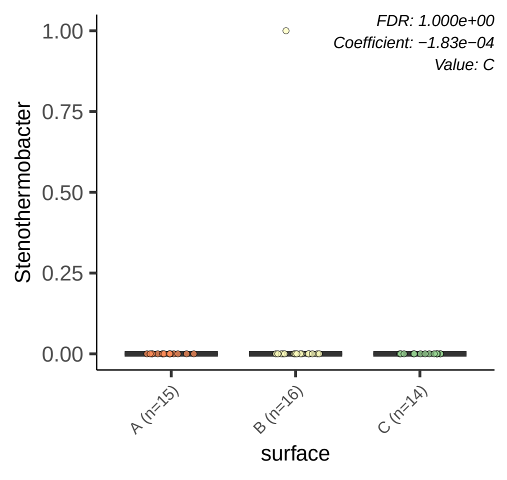
FDR: 1.000e+00
Coefficient: −1.83e−04
Value: C
Stenothermobacter
1.00
0.75
0.50
0.25
0.00
A (n=15)
B (n=16)
C (n=14)
surface

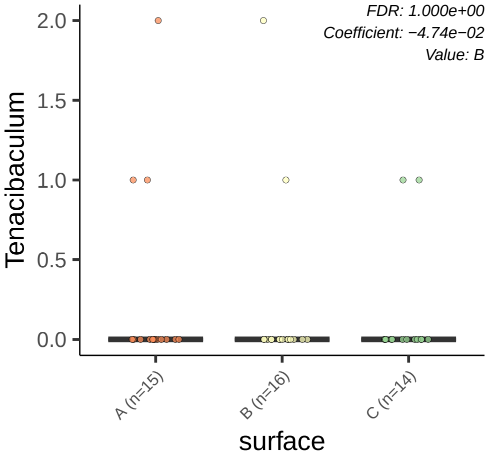
FDR: 1.000e+00
Coefficient: −4.74e−02
Value: B
Tenacibaculum
2.0
1.5
1.0
0.5
0.0
A (n=15)
B (n=16)
C (n=14)
surface

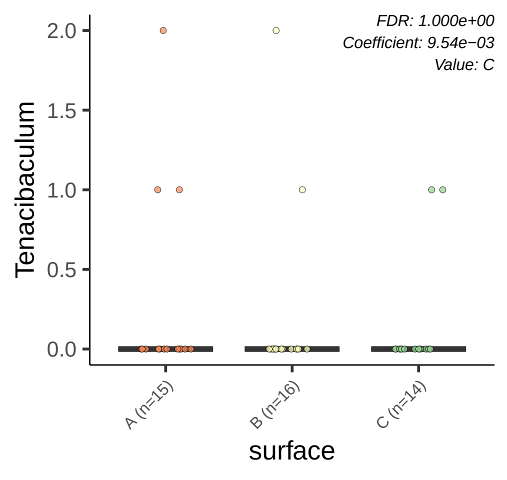
FDR: 1.000e+00
Coefficient: 9.54e−03
Value: C
Tenacibaculum
2.0
1.5
1.0
0.5
0.0
A (n=15)
B (n=16)
C (n=14)
surface

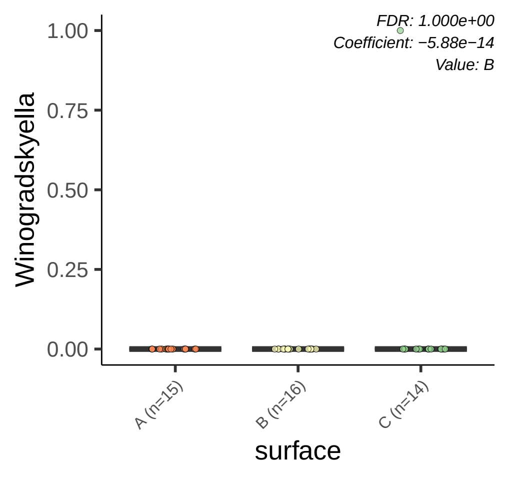
FDR: 1.000e+00
Coefficient: −5.88e−14
Value: B
Winogradskyella
1.00
0.75
0.50
0.25
0.00
A (n=15)
B (n=16)
C (n=14)
surface

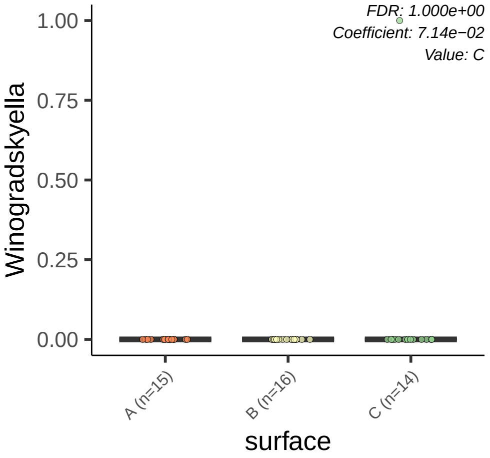

FDR: 1.000e+00
Coefficient: 7.14e−02
Value: C
Winogradskyella
1.00
0.75
0.50
0.25
0.00
A (n=15)
B (n=16)
C (n=14)
surface

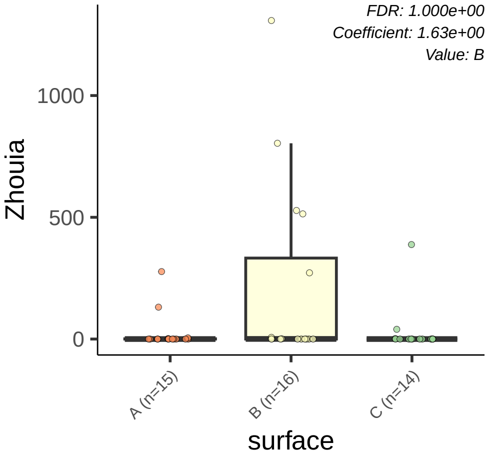
FDR: 1.000e+00
Coefficient: 1.63e+00
Value: B
Zhouia
1000
500
0
A (n=15)
B (n=16)
C (n=14)
surface

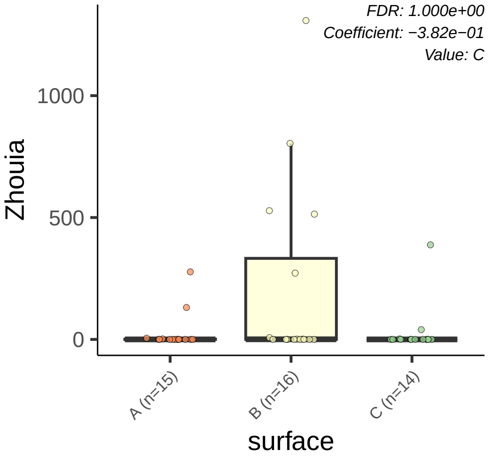

FDR: 1.000e+00
Coefficient: −3.82e−01
Value: C
Zhouia
1000
500
0
A (n=15)
B (n=16)
C (n=14)
surface

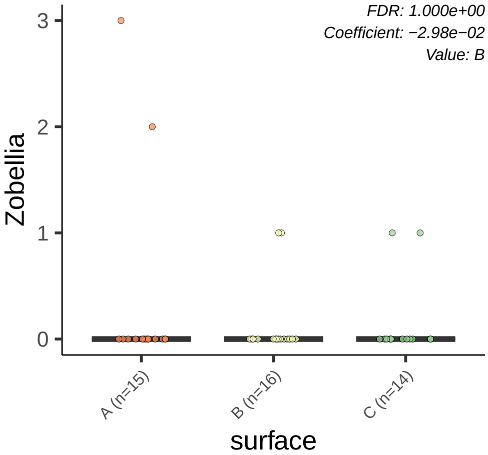

FDR: 1.000e+00
Coefficient: −2.98e−02
Value: B
Zobellia
3
2
1
0
A (n=15)
B (n=16)
C (n=14)
surface

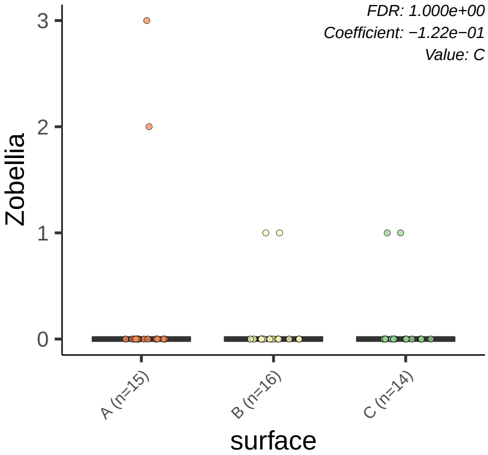
FDR: 1.000e+00
Coefficient: −1.22e−01
Value: C
Zobellia
3
2
1
0
A (n=15)
B (n=16)
C (n=14)
surface

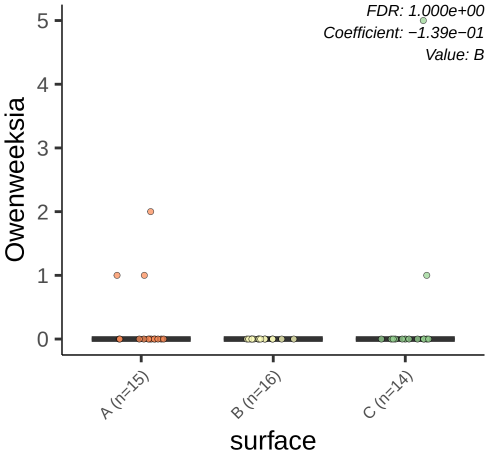

FDR: 1.000e+00
Coefficient: −1.39e−01
Value: B
Owenweeksia
0
1
2
3
4
5
A (n=15)
B (n=16)
C (n=14)
surface

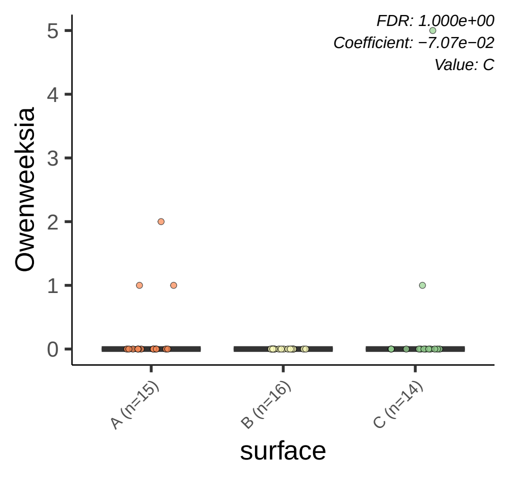
FDR: 1.000e+00
Coefficient: −7.07e−02
Value: C
Owenweeksia
5
4
3
2
1
0
A (n=15)
B (n=16)
C (n=14)
surface

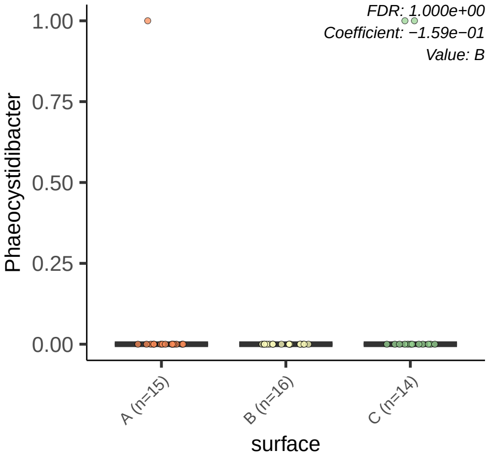

FDR: 1.000e+00
Coefficient: −1.59e−01
Value: B
Phaeocystidibacter
1.00
0.75
0.50
0.25
0.00
A (n=15)
B (n=16)
C (n=14)
surface

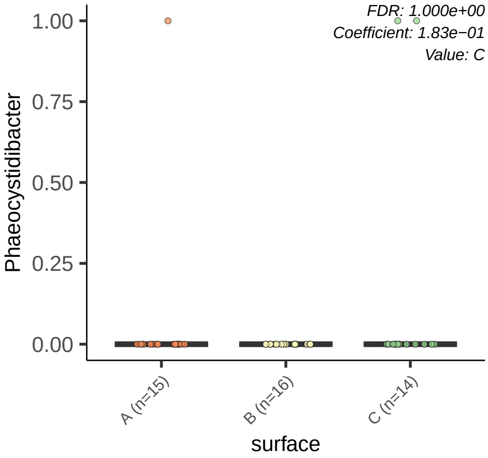

FDR: 1.000e+00
Coefficient: 1.83e−01
Value: C
Phaeocystidibacter
1.00
0.75
0.50
0.25
0.00
A (n=15)
B (n=16)
C (n=14)
surface

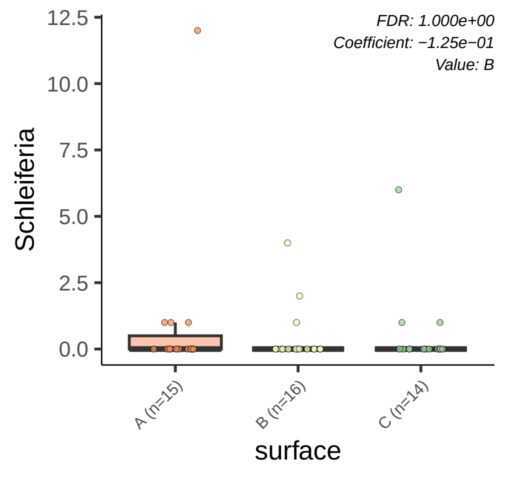
FDR: 1.000e+00
Coefficient: −1.25e−01
Value: B
Schleiferia
12.5
10.0
7.5
5.0
2.5
0.0
A (n=15)
B (n=16)
C (n=14)
surface

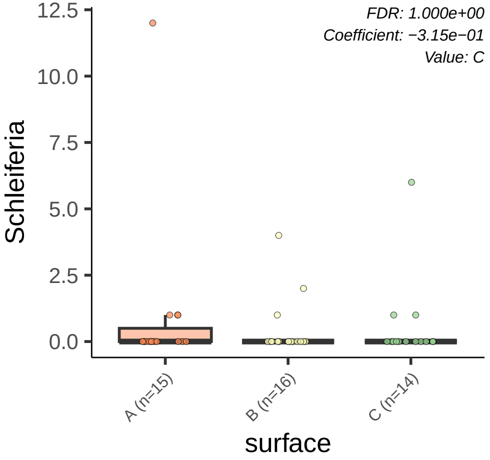

FDR: 1.000e+00
Coefficient: −3.15e−01
Value: C
Schleiferia
12.5
10.0
7.5
5.0
2.5
0.0
A (n=15)
B (n=16)
C (n=14)
surface

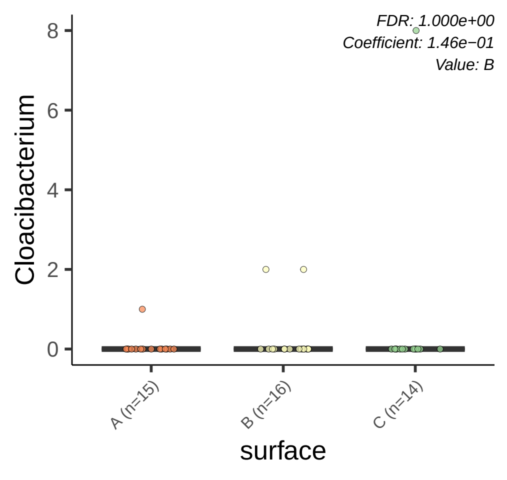

FDR: 1.000e+00
Coefficient: 1.46e−01
Value: B
Cloacibacterium
8
6
4
2
0
A (n=15)
B (n=16)
C (n=14)
surface

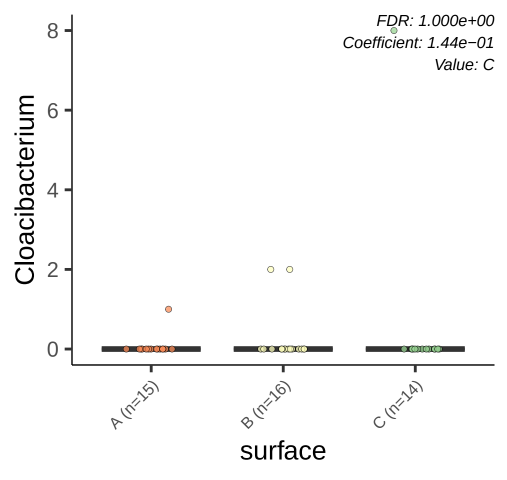
FDR: 1.000e+00
Coefficient: 1.44e−01
Value: C
Cloacibacterium
0
2
4
6
8
A (n=15)
B (n=16)
C (n=14)
surface

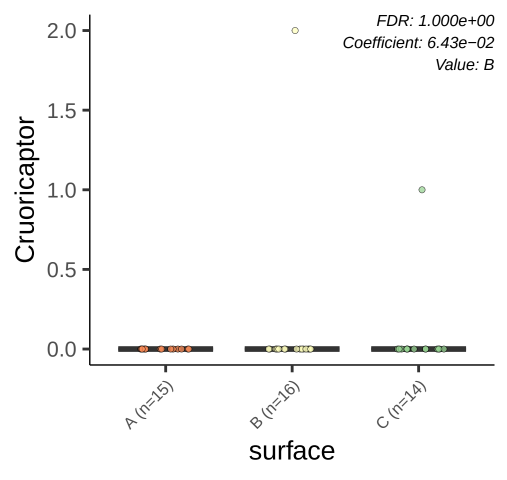
FDR: 1.000e+00
Coefficient: 6.43e−02
Value: B
Cruoricaptor
2.0
1.5
1.0
0.5
0.0
A (n=15)
B (n=16)
C (n=14)
surface

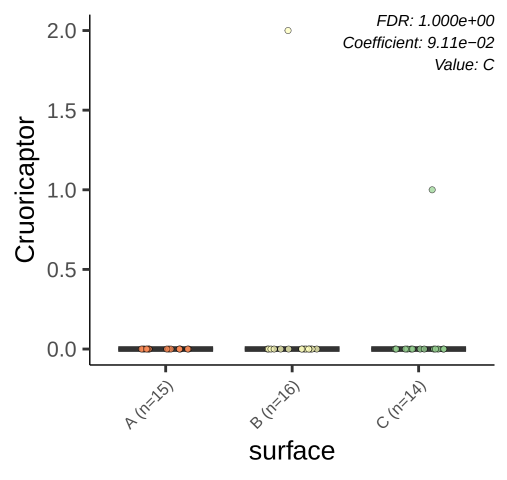
FDR: 1.000e+00
Coefficient: 9.11e−02
Value: C
Cruoricaptor
2.0
1.5
1.0
0.5
0.0
A (n=15)
B (n=16)
C (n=14)
surface

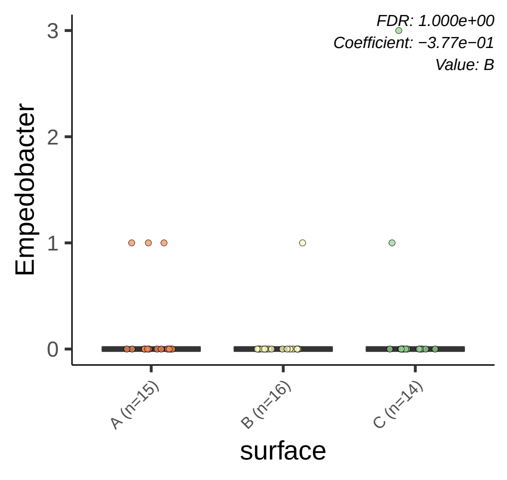

FDR: 1.000e+00
Coefficient: −3.77e−01
Value: B
Empedobacter
3
2
1
0
A (n=15)
B (n=16)
C (n=14)
surface

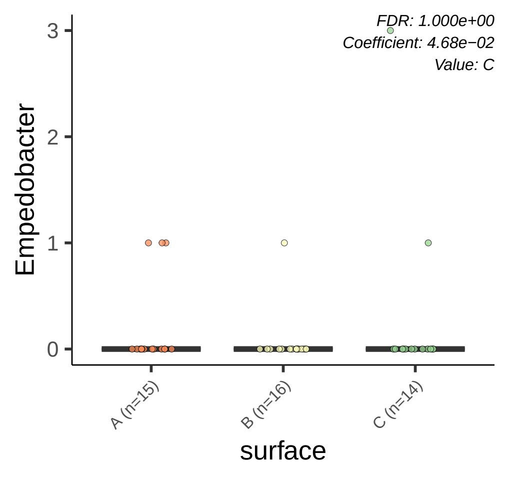
FDR: 1.000e+00
Coefficient: 4.68e−02
Value: C
Empedobacter
3
2
1
0
A (n=15)
B (n=16)
C (n=14)
surface

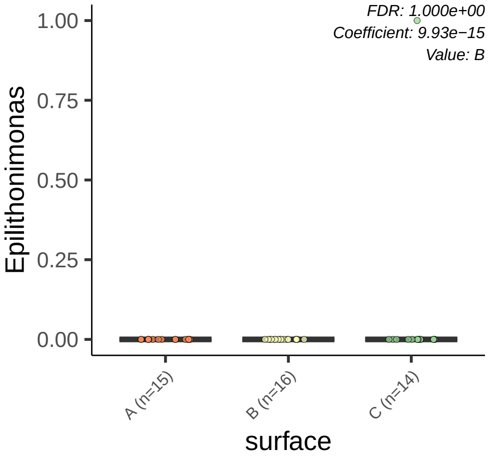
FDR: 1.000e+00
Coefficient: 9.93e−15
Value: B
Epilithonimonas
1.00
0.75
0.50
0.25
0.00
A (n=15)
B (n=16)
C (n=14)
surface

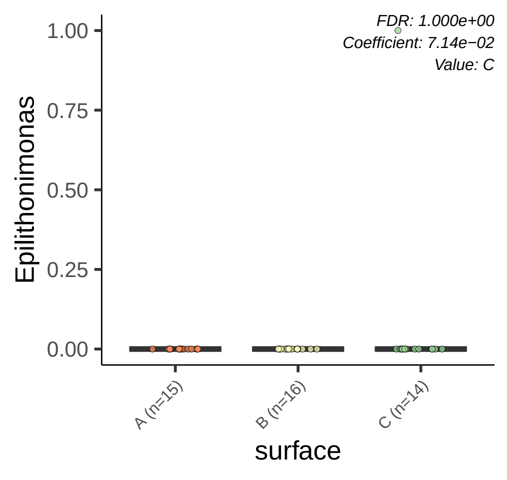
FDR: 1.000e+00
Coefficient: 7.14e−02
Value: C
1.00
0.75
0.50
0.25
0.00
Epilithonimonas
A (n=15)
B (n=16)
C (n=14)
surface

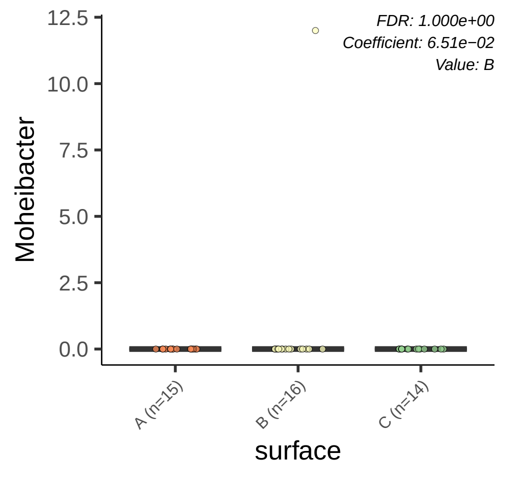

FDR: 1.000e+00
Coefficient: 6.51e−02
Value: B
Moheibacter
12.5
10.0
7.5
5.0
2.5
0.0
A (n=15)
B (n=16)
C (n=14)
surface

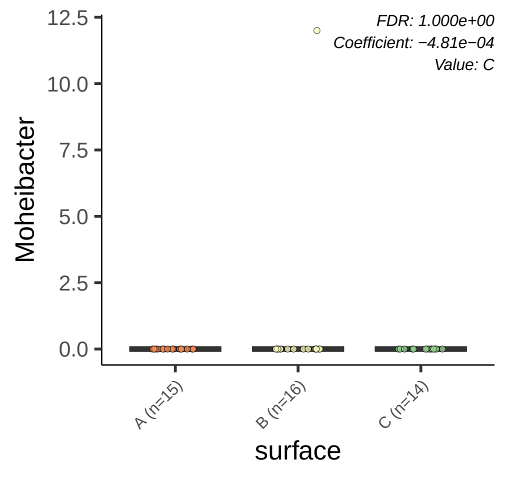
FDR: 1.000e+00
Coefficient: −4.81e−04
Value: C
Moheibacter
12.5
10.0
7.5
5.0
2.5
0.0
A (n=15)
B (n=16)
C (n=14)
surface

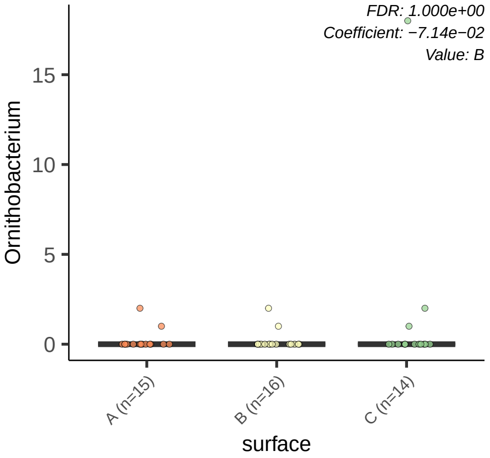

FDR: 1.000e+00
Coefficient: −7.14e−02
Value: B
Ornithobacterium
15
10
5
0
A (n=15)
B (n=16)
C (n=14)
surface

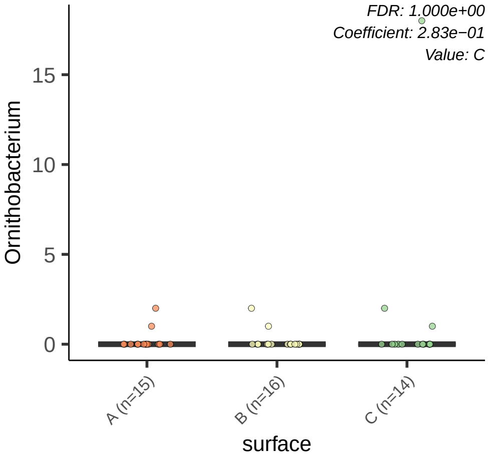

FDR: 1.000e+00
Coefficient: 2.83e−01
Value: C
Ornithobacterium
0
5
10
15
A (n=15)
B (n=16)
C (n=14)
surface

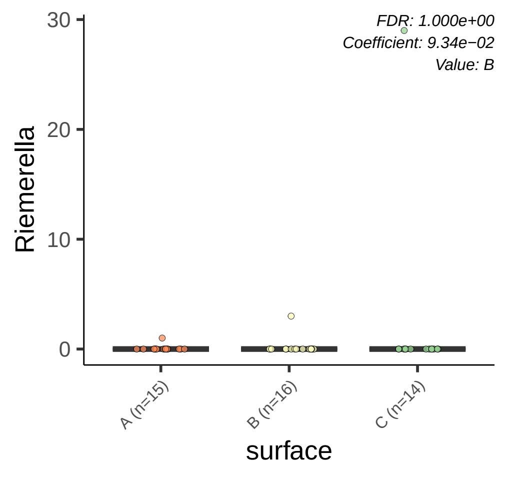

FDR: 1.000e+00
Coefficient: 9.34e−02
Value: B
Riemerella
30
20
10
0
A (n=15)
B (n=16)
C (n=14)
surface

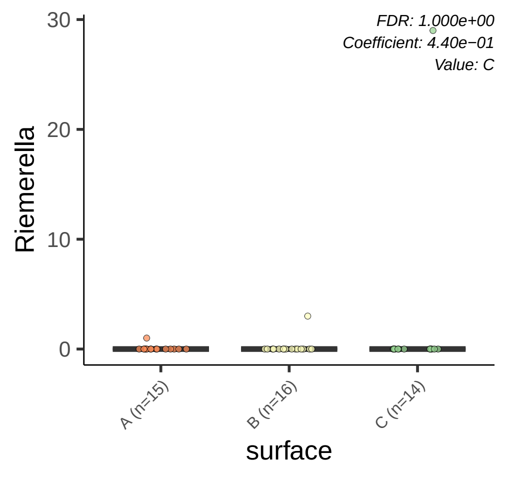
FDR: 1.000e+00
Coefficient: 4.40e−01
Value: C
Riemerella
30
20
10
0
A (n=15)
B (n=16)
C (n=14)
surface

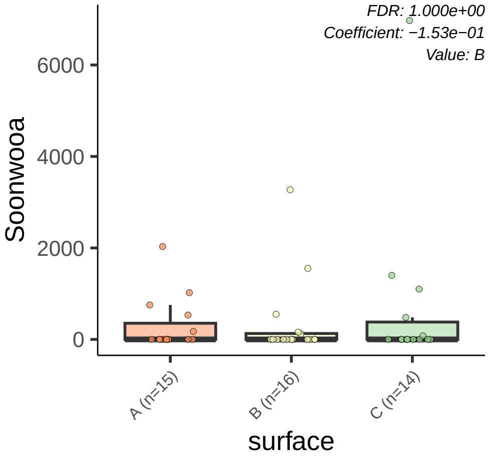

FDR: 1.000e+00
Coefficient: −1.53e−01
Value: B
6000
4000
2000
0
Soonwooa
A (n=15)
B (n=16)
C (n=14)
surface

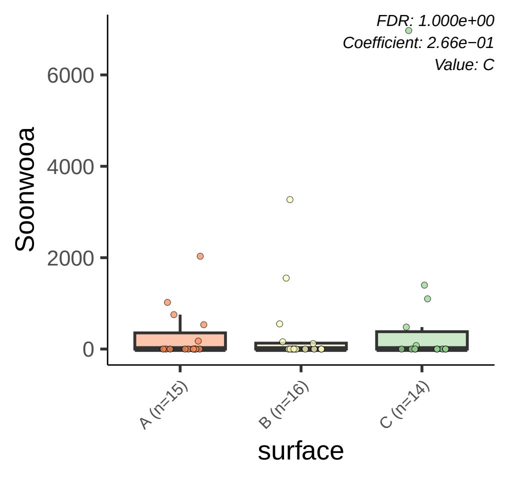
FDR: 1.000e+00
Coefficient: 2.66e−01
Value: C
Soonwooa
6000
4000
2000
0
A (n=15)
B (n=16)
C (n=14)
surface

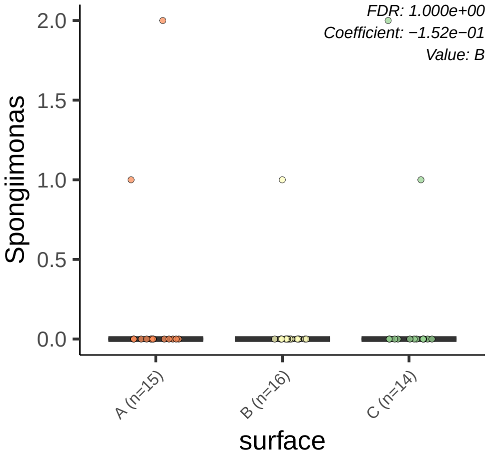
FDR: 1.000e+00
Coefficient: −1.52e−01
Value: B
2.0
1.5
1.0
0.5
0.0
Spongiimonas
A (n=15)
B (n=16)
C (n=14)
surface

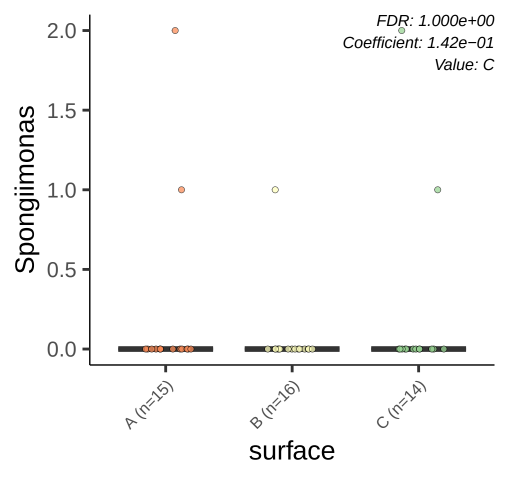
FDR: 1.000e+00
Coefficient: 1.42e−01
Value: C
Spongiimonas
2.0
1.5
1.0
0.5
0.0
A (n=15)
B (n=16)
C (n=14)
surface

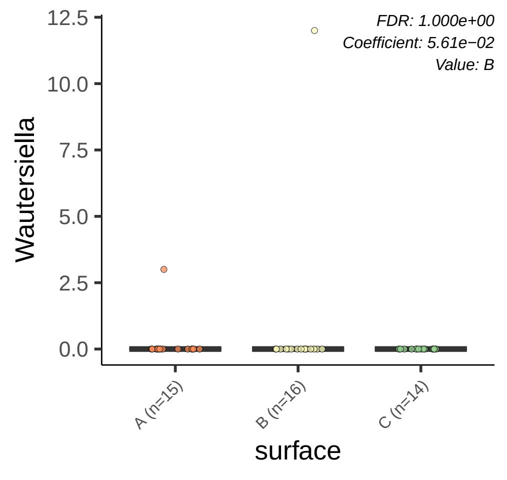
FDR: 1.000e+00
Coefficient: 5.61e−02
Value: B
12.5
10.0
7.5
5.0
2.5
0.0
Wautersiella
A (n=15)
B (n=16)
C (n=14)
surface

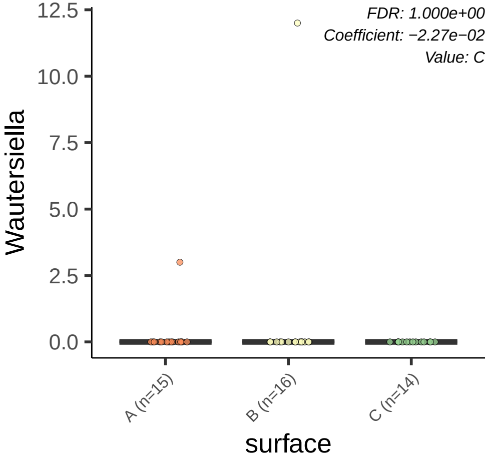

FDR: 1.000e+00
Coefficient: −2.27e−02
Value: C
Wautersiella
12.5
10.0
7.5
5.0
2.5
0.0
A (n=15)
B (n=16)
C (n=14)
surface

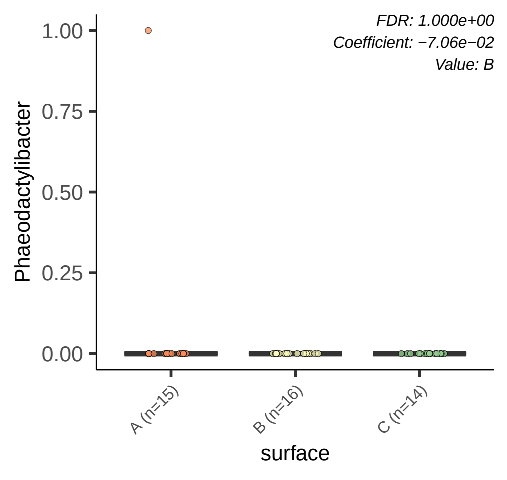
FDR: 1.000e+00
Coefficient: −7.06e−02
Value: B
Phaeodactylibacter
1.00
0.75
0.50
0.25
0.00
A (n=15)
B (n=16)
C (n=14)
surface

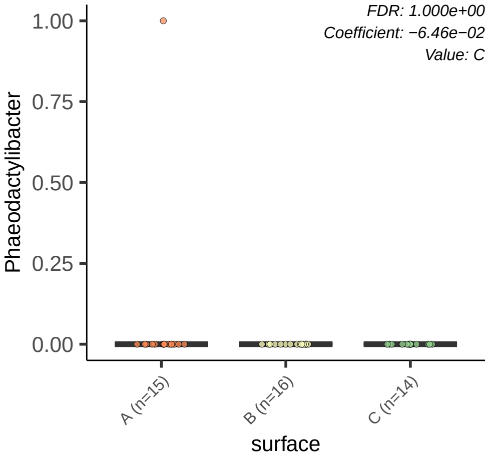
FDR: 1.000e+00
Coefficient: −6.46e−02
Value: C
Phaeodactylibacter
1.00
0.75
0.50
0.25
0.00
A (n=15)
B (n=16)
C (n=14)
surface

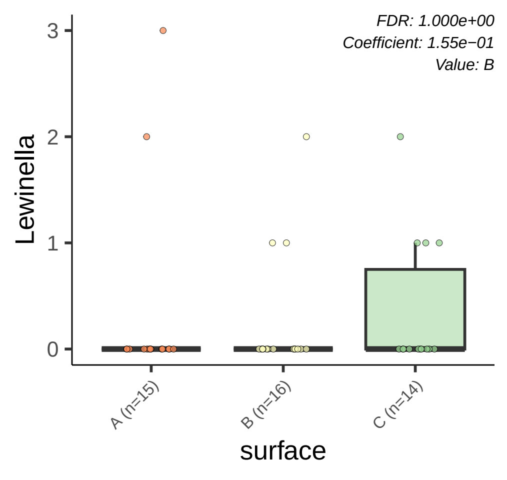
FDR: 1.000e+00
Coefficient: 1.55e−01
Value: B
Lewinella
0
1
2
3
A (n=15)
B (n=16)
C (n=14)
surface

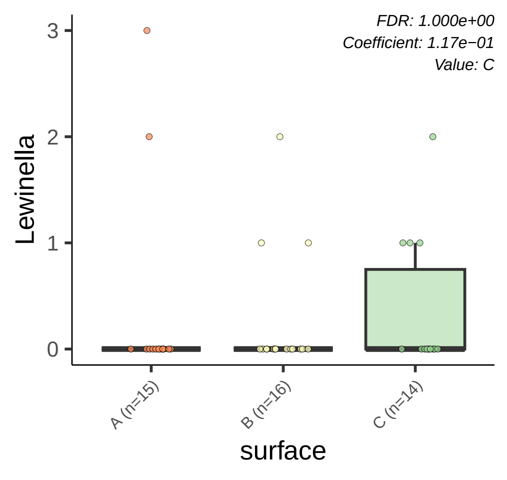

FDR: 1.000e+00
Coefficient: 1.17e−01
Value: C
Lewinella
3
2
1
0
A (n=15)
B (n=16)
C (n=14)
surface

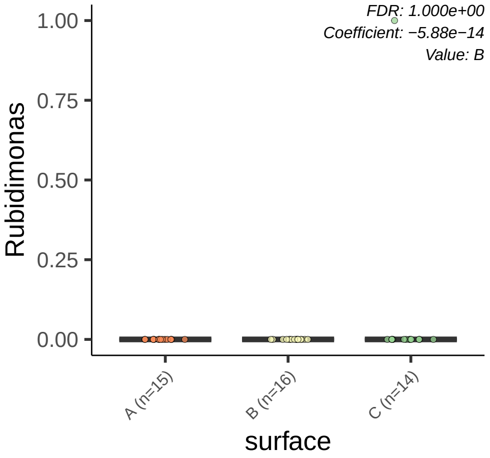

FDR: 1.000e+00
Coefficient: −5.88e−14
Value: B
Rubidimonas
1.00
0.75
0.50
0.25
0.00
A (n=15)
B (n=16)
C (n=14)
surface

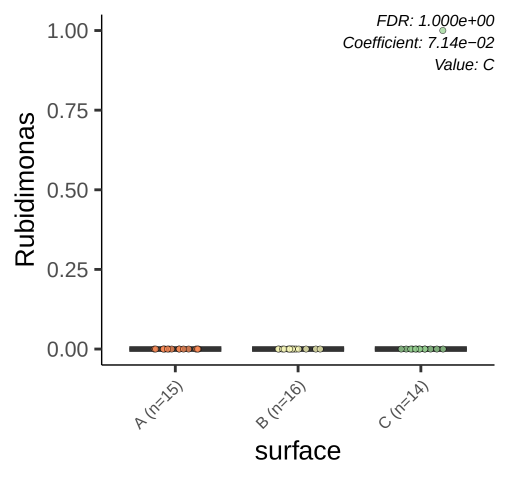
FDR: 1.000e+00
Coefficient: 7.14e−02
Value: C
1.00
0.75
0.50
0.25
0.00
Rubidimonas
A (n=15)
B (n=16)
C (n=14)
surface

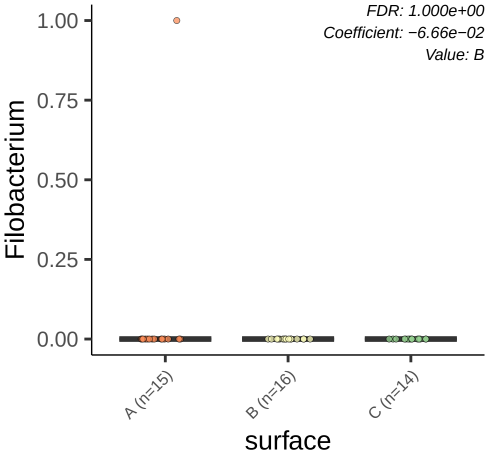
FDR: 1.000e+00
Coefficient: −6.66e−02
Value: B
Filobacterium
1.00
0.75
0.50
0.25
0.00
A (n=15)
B (n=16)
C (n=14)
surface

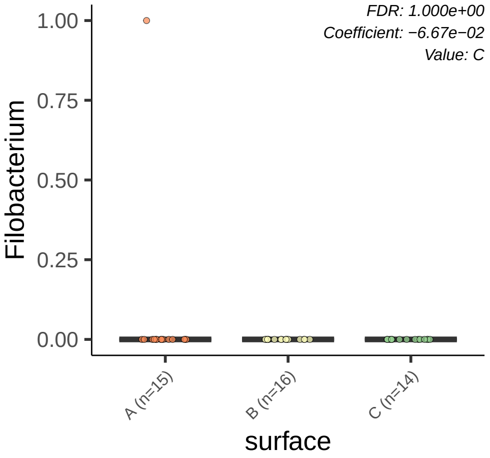
FDR: 1.000e+00
Coefficient: −6.67e−02
Value: C
Filobacterium
1.00
0.75
0.50
0.25
0.00
A (n=15)
B (n=16)
C (n=14)
surface

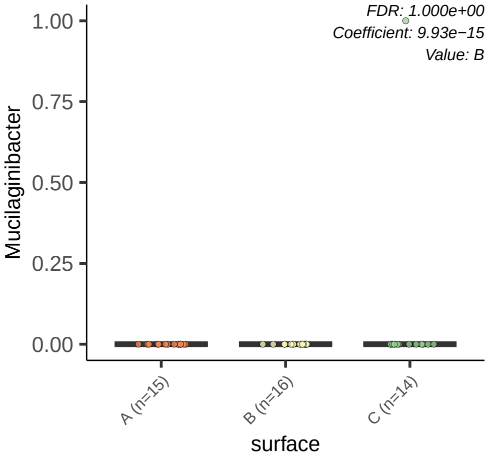

FDR: 1.000e+00
Coefficient: 9.93e−15
Value: B
Mucilaginibacter
1.00
0.75
0.50
0.25
0.00
A (n=15)
B (n=16)
C (n=14)
surface

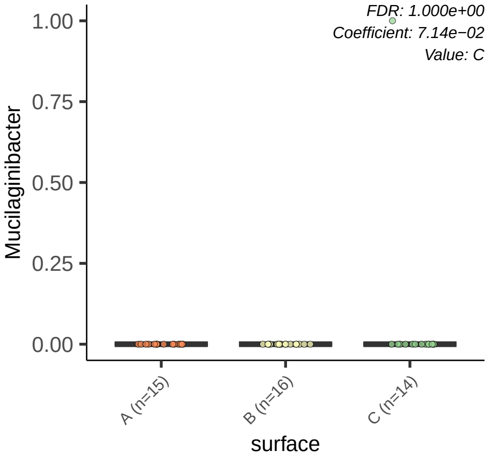
FDR: 1.000e+00
Coefficient: 7.14e−02
Value: C
Mucilaginibacter
1.00
0.75
0.50
0.25
0.00
A (n=15)
B (n=16)
C (n=14)
surface

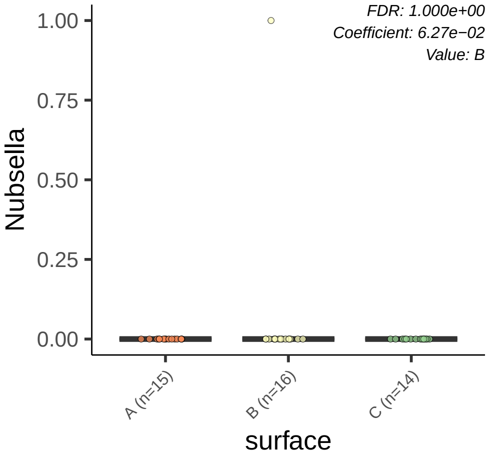
FDR: 1.000e+00
Coefficient: 6.27e−02
Value: B
Nubsella
1.00
0.75
0.50
0.25
0.00
A (n=15)
B (n=16)
C (n=14)
surface

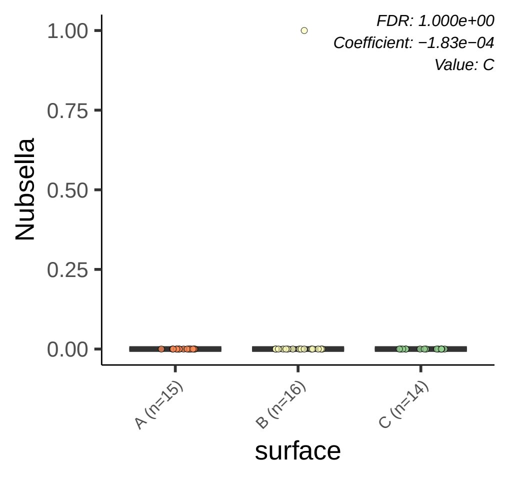
FDR: 1.000e+00
Coefficient: −1.83e−04
Value: C
Nubsella
1.00
0.75
0.50
0.25
0.00
A (n=15)
B (n=16)
C (n=14)
surface

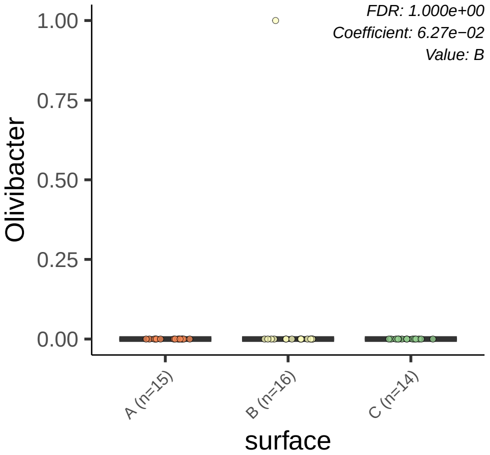
FDR: 1.000e+00
Coefficient: 6.27e−02
Value: B
Olivibacter
1.00
0.75
0.50
0.25
0.00
A (n=15)
B (n=16)
C (n=14)
surface

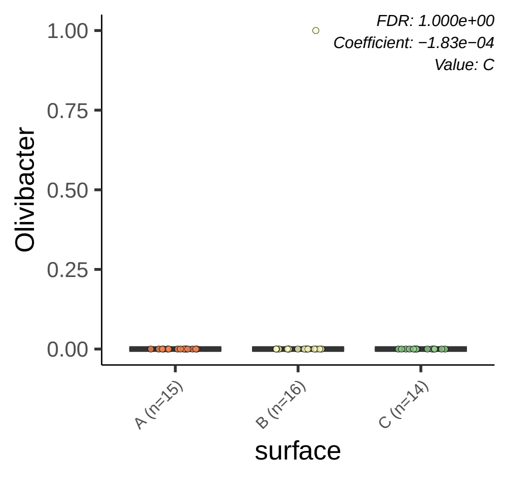
FDR: 1.000e+00
Coefficient: −1.83e−04
Value: C
Olivibacter
1.00
0.75
0.50
0.25
0.00
A (n=15)
B (n=16)
C (n=14)
surface

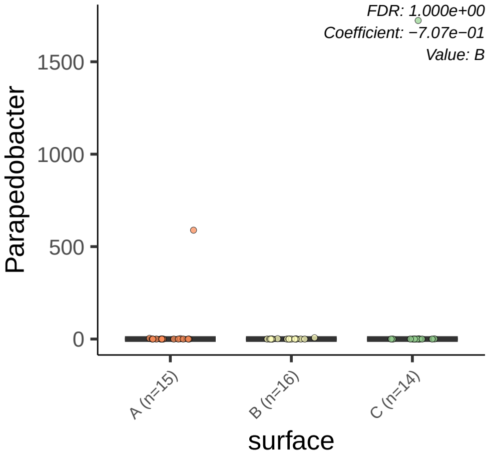
FDR: 1.000e+00
Coefficient: −7.07e−01
Value: B
Parapedobacter
1500
1000
500
0
A (n=15)
B (n=16)
C (n=14)
surface

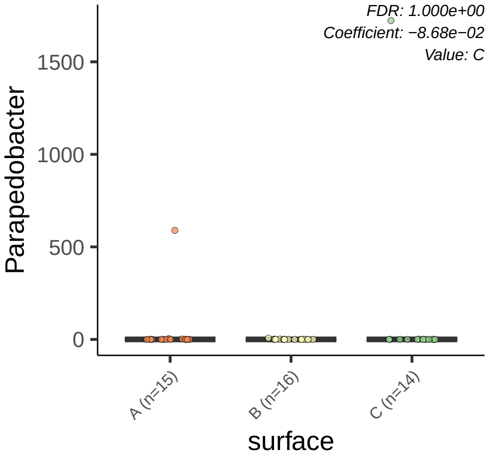

FDR: 1.000e+00
Coefficient: −8.68e−02
Value: C
Parapedobacter
1500
1000
500
0
A (n=15)
B (n=16)
C (n=14)
surface

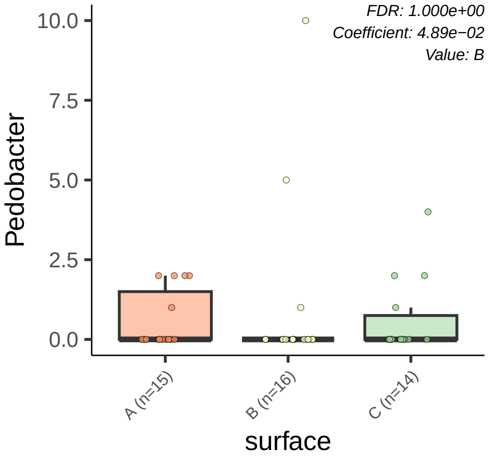

FDR: 1.000e+00
Coefficient: 4.89e−02
Value: B
Pedobacter
10.0
7.5
5.0
2.5
0.0
A (n=15)
B (n=16)
C (n=14)
surface

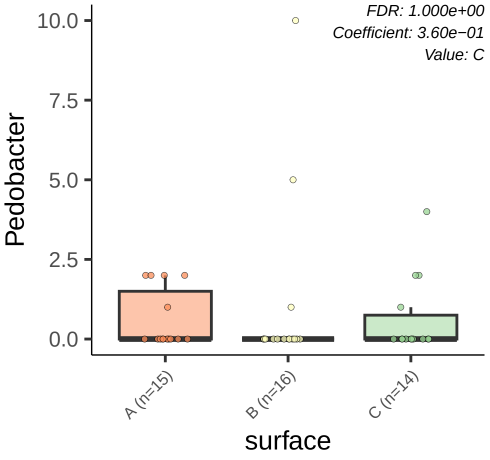

FDR: 1.000e+00
Coefficient: 3.60e−01
Value: C
Pedobacter
10.0
7.5
5.0
2.5
0.0
A (n=15)
B (n=16)
C (n=14)
surface

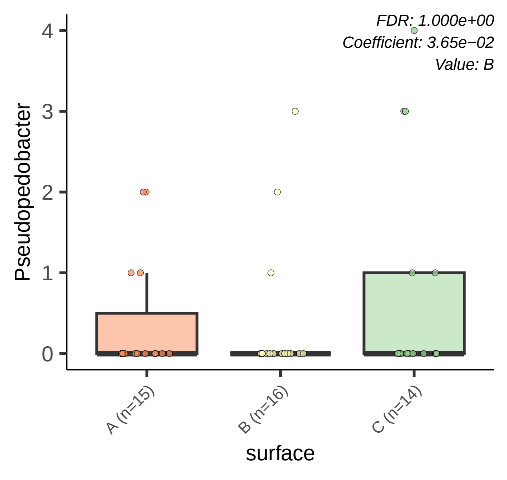

FDR: 1.000e+00
Coefficient: 3.65e−02
Value: B
Pseudopedobacter
4
3
2
1
0
A (n=15)
B (n=16)
C (n=14)
surface

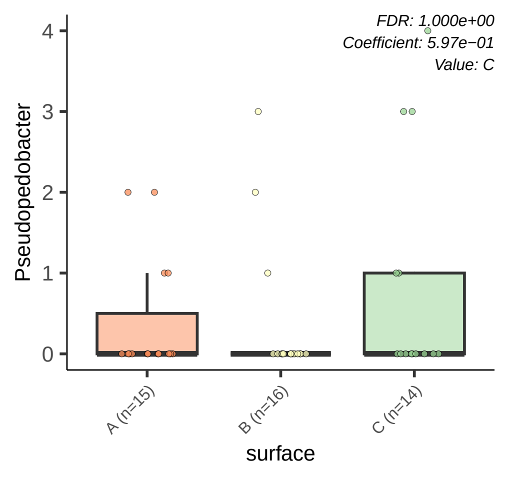

FDR: 1.000e+00
Coefficient: 5.97e−01
Value: C
Pseudopedobacter
0
1
2
3
4
A (n=15)
B (n=16)
C (n=14)
surface

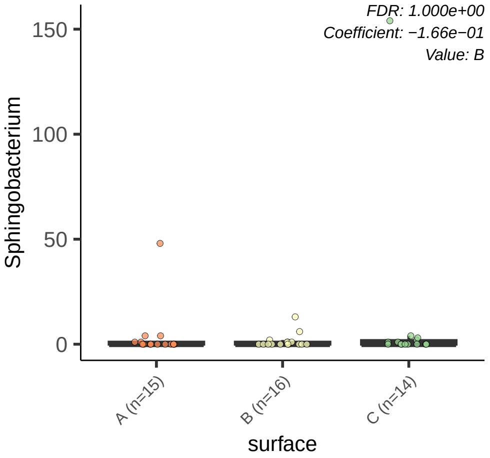
FDR: 1.000e+00
Coefficient: −1.66e−01
Value: B
Sphingobacterium
150
100
50
0
A (n=15)
B (n=16)
C (n=14)
surface

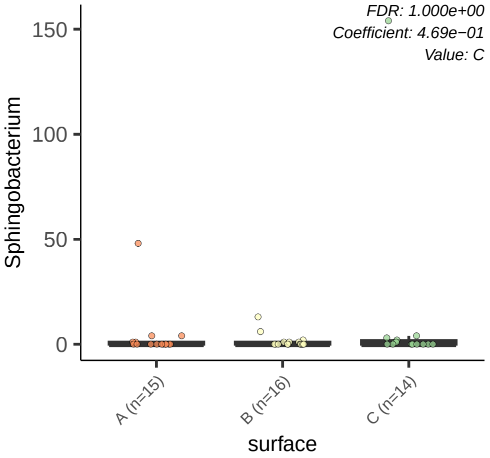

FDR: 1.000e+00
Coefficient: 4.69e−01
Value: C
Sphingobacterium
150
100
50
0
A (n=15)
B (n=16)
C (n=14)
surface

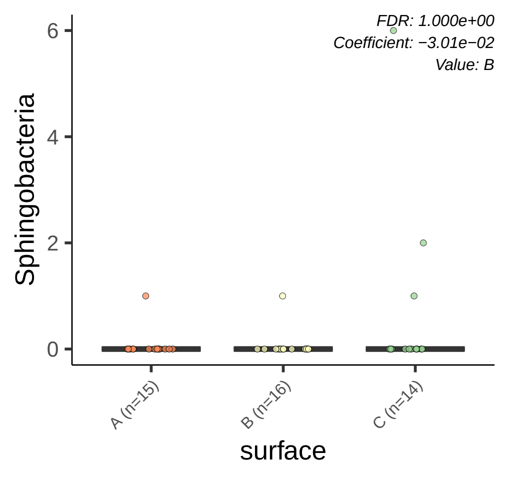
FDR: 1.000e+00
Coefficient: −3.01e−02
Value: B
Sphingobacteria
6
4
2
0
A (n=15)
B (n=16)
C (n=14)
surface

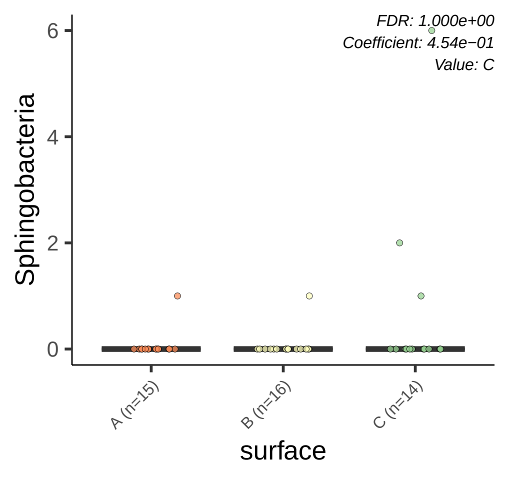
FDR: 1.000e+00
Coefficient: 4.54e−01
Value: C
Sphingobacteria
0
2
4
6
A (n=15)
B (n=16)
C (n=14)
surface

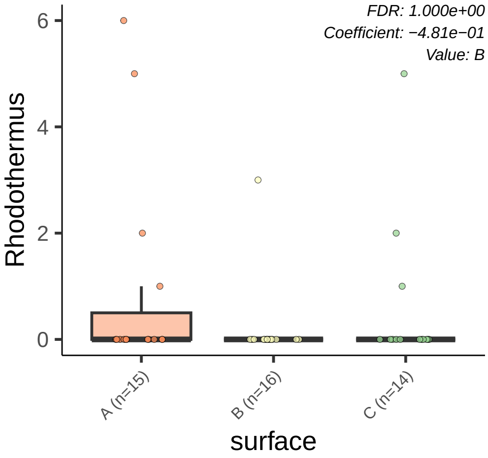
FDR: 1.000e+00
Coefficient: −4.81e−01
Value: B
Rhodothermus
0
2
4
6
A (n=15)
B (n=16)
C (n=14)
surface

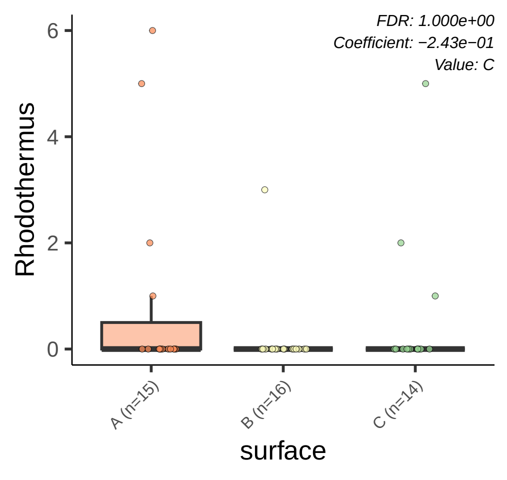
FDR: 1.000e+00
Coefficient: −2.43e−01
Value: C
Rhodothermus
0
2
4
6
A (n=15)
B (n=16)
C (n=14)
surface

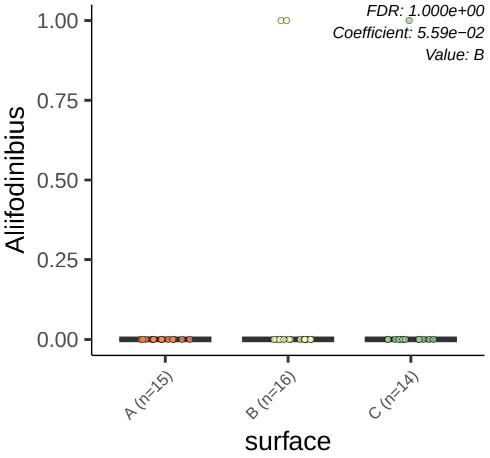
FDR: 1.000e+00
Coefficient: 5.59e−02
Value: B
Aliifodinibius
1.00
0.75
0.50
0.25
0.00
A (n=15)
B (n=16)
C (n=14)
surface

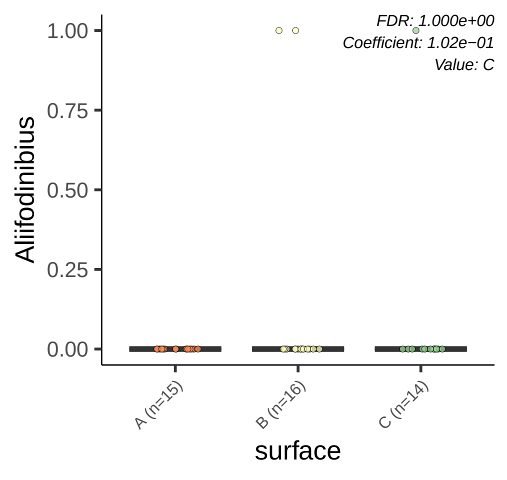

FDR: 1.000e+00
Coefficient: 1.02e−01
Value: C
1.00
0.75
0.50
0.25
0.00
Aliifodinibius
A (n=15)
B (n=16)
C (n=14)
surface

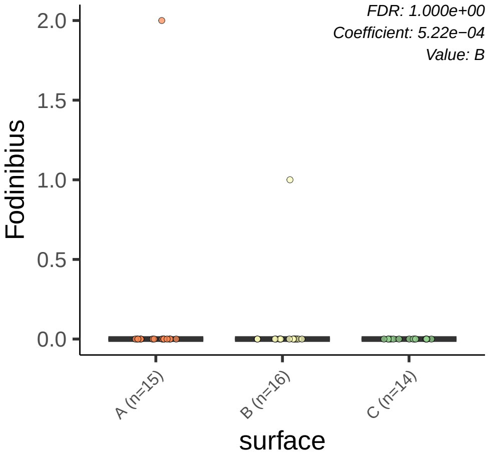

FDR: 1.000e+00
Coefficient: 5.22e−04
Value: B
Fodinibius
2.0
1.5
1.0
0.5
0.0
A (n=15)
B (n=16)
C (n=14)
surface

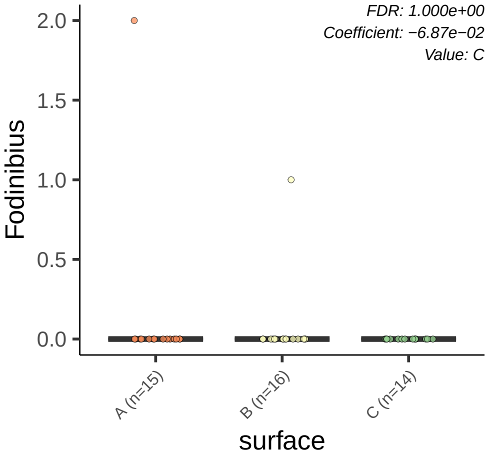

FDR: 1.000e+00
Coefficient: −6.87e−02
Value: C
2.0
1.5
1.0
0.5
0.0
Fodinibius
A (n=15)
B (n=16)
C (n=14)
surface

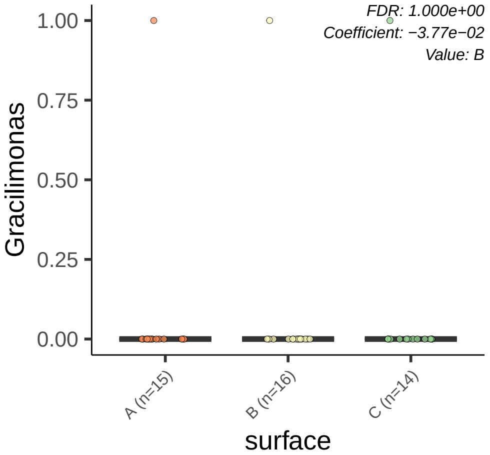

FDR: 1.000e+00
Coefficient: −3.77e−02
Value: B
Gracilimonas
1.00
0.75
0.50
0.25
0.00
A (n=15)
B (n=16)
C (n=14)
surface

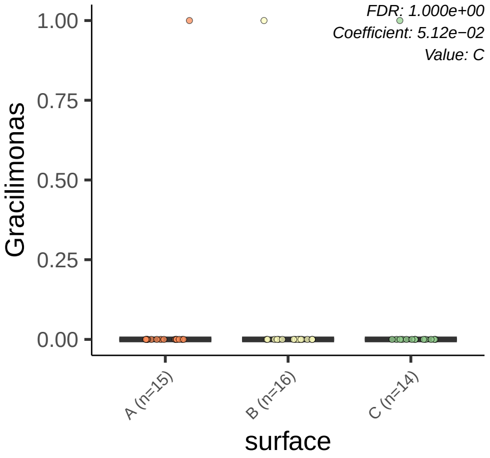
FDR: 1.000e+00
Coefficient: 5.12e−02
Value: C
Gracilimonas
1.00
0.75
0.50
0.25
0.00
A (n=15)
B (n=16)
C (n=14)
surface

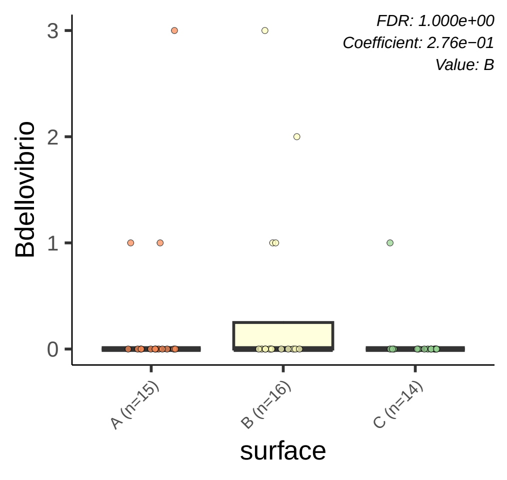
FDR: 1.000e+00
Coefficient: 2.76e−01
Value: B
Bdellovibrio
3
2
1
0
A (n=15)
B (n=16)
C (n=14)
surface

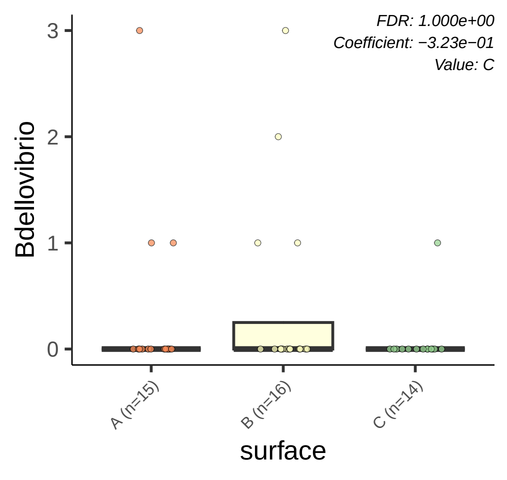
FDR: 1.000e+00
Coefficient: −3.23e−01
Value: C
Bdellovibrio
3
2
1
0
A (n=15)
B (n=16)
C (n=14)
surface

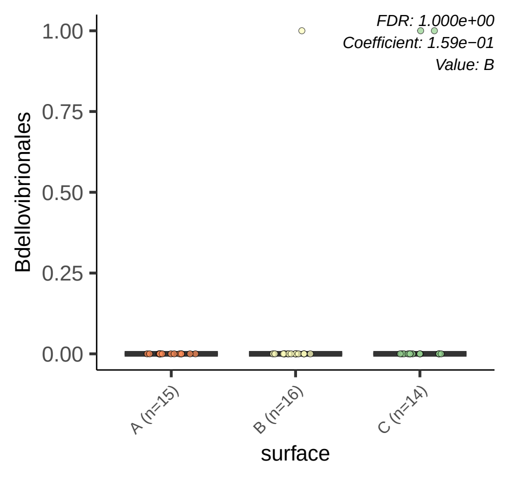
FDR: 1.000e+00
Coefficient: 1.59e−01
Value: B
Bdellovibrionales
1.00
0.75
0.50
0.25
0.00
A (n=15)
B (n=16)
C (n=14)
surface

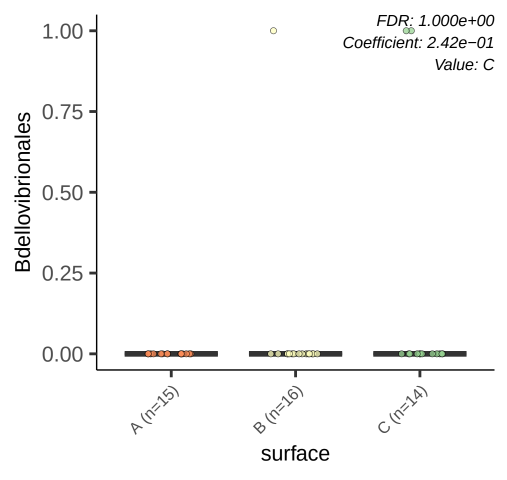
FDR: 1.000e+00
Coefficient: 2.42e−01
Value: C
Bdellovibrionales
1.00
0.75
0.50
0.25
0.00
A (n=15)
B (n=16)
C (n=14)
surface

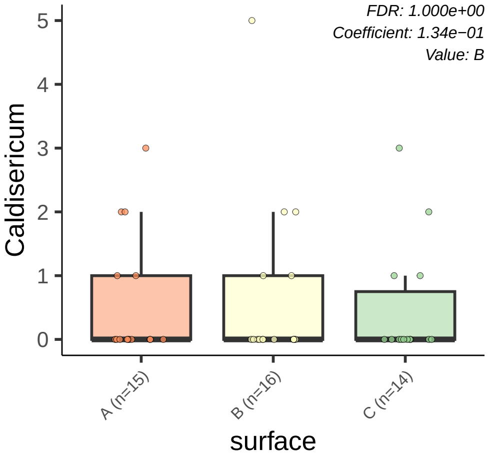
FDR: 1.000e+00
Coefficient: 1.34e−01
Value: B
Caldisericum
0
1
2
3
4
5
A (n=15)
B (n=16)
C (n=14)
surface

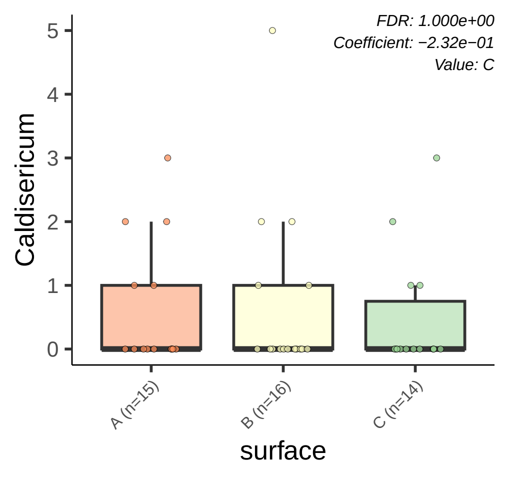
FDR: 1.000e+00
Coefficient: −2.32e−01
Value: C
Caldisericum
0
1
2
3
4
5
A (n=15)
B (n=16)
C (n=14)
surface

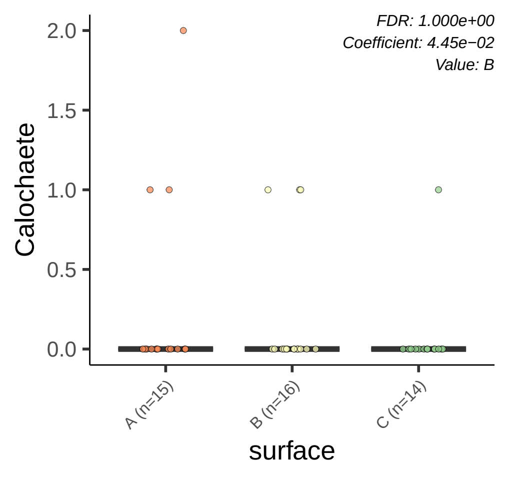

FDR: 1.000e+00
Coefficient: 4.45e−02
Value: B
Calochaete
2.0
1.5
1.0
0.5
0.0
A (n=15)
B (n=16)
C (n=14)
surface

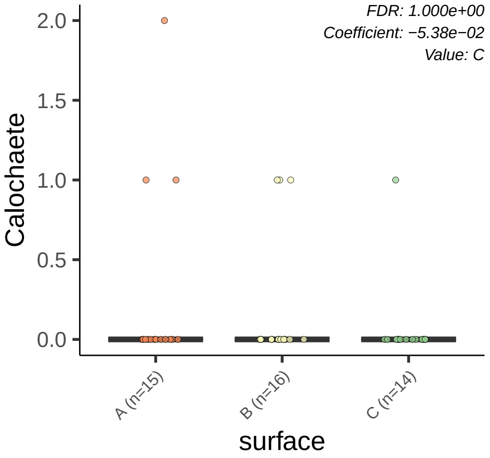
FDR: 1.000e+00
Coefficient: −5.38e−02
Value: C
Calochaete
2.0
1.5
1.0
0.5
0.0
A (n=15)
B (n=16)
C (n=14)
surface

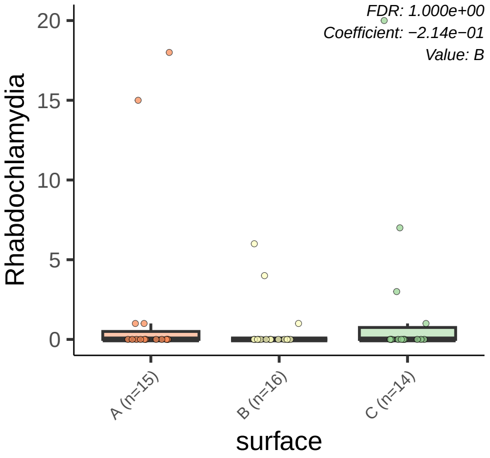
FDR: 1.000e+00
Coefficient: −2.14e−01
Value: B
Rhabdochlamydia
20
15
10
5
0
A (n=15)
B (n=16)
C (n=14)
surface

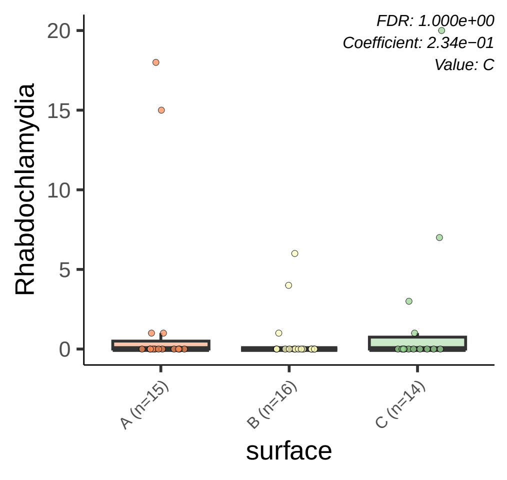

FDR: 1.000e+00
Coefficient: 2.34e−01
Value: C
Rhabdochlamydia
20
15
10
5
0
A (n=15)
B (n=16)
C (n=14)
surface

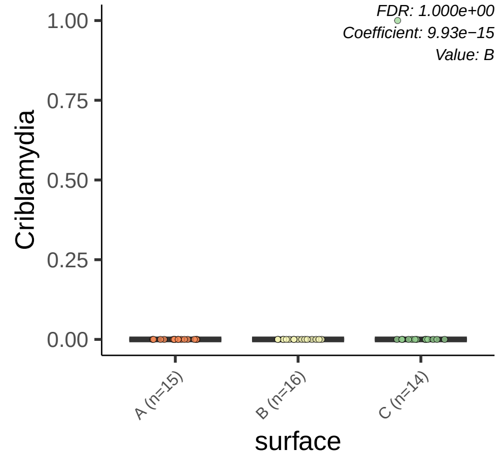

FDR: 1.000e+00
Coefficient: 9.93e−15
Value: B
1.00
0.75
0.50
0.25
0.00
Criblamydia
A (n=15)
B (n=16)
C (n=14)
surface

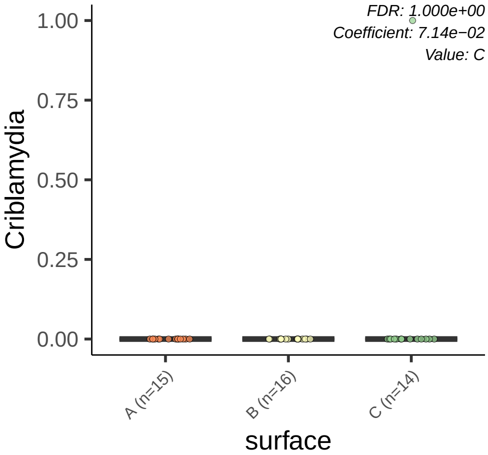
FDR: 1.000e+00
Coefficient: 7.14e−02
Value: C
Criblamydia
1.00
0.75
0.50
0.25
0.00
A (n=15)
B (n=16)
C (n=14)
surface

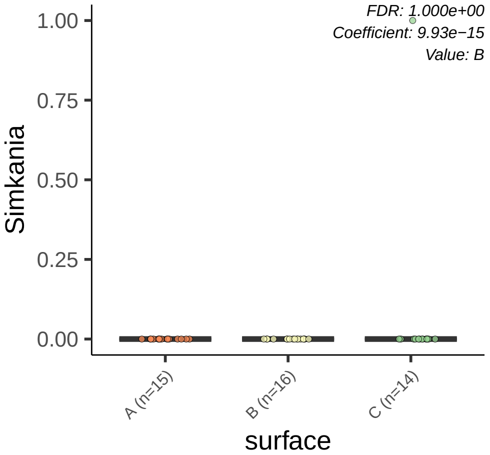
FDR: 1.000e+00
Coefficient: 9.93e−15
Value: B
1.00
0.75
0.50
0.25
0.00
Simkania
A (n=15)
B (n=16)
C (n=14)
surface

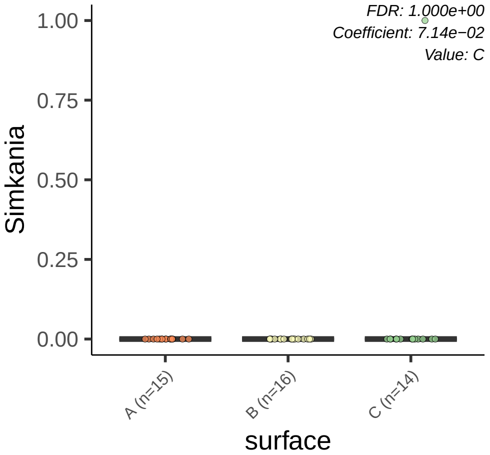
FDR: 1.000e+00
Coefficient: 7.14e−02
Value: C
Simkania
1.00
0.75
0.50
0.25
0.00
A (n=15)
B (n=16)
C (n=14)
surface

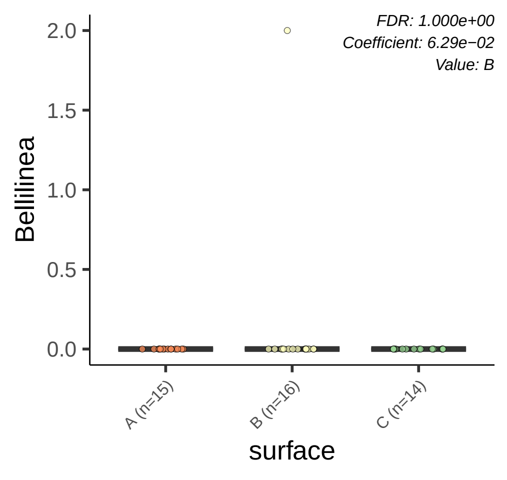
FDR: 1.000e+00
Coefficient: 6.29e−02
Value: B
2.0
1.5
1.0
0.5
0.0
Bellilinea
A (n=15)
B (n=16)
C (n=14)
surface

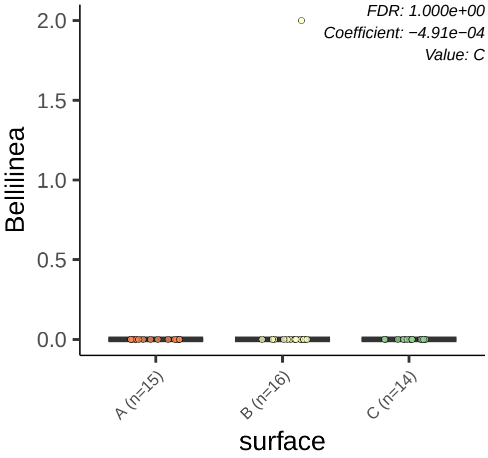
FDR: 1.000e+00
Coefficient: −4.91e−04
Value: C
Bellilinea
2.0
1.5
1.0
0.5
0.0
A (n=15)
B (n=16)
C (n=14)
surface

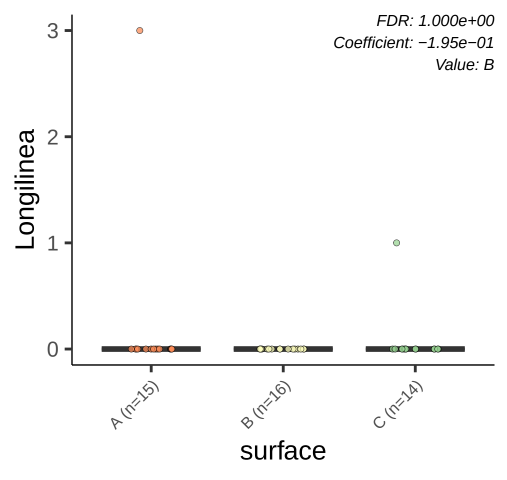

FDR: 1.000e+00
Coefficient: −1.95e−01
Value: B
Longilinea
3
2
1
0
A (n=15)
B (n=16)
C (n=14)
surface

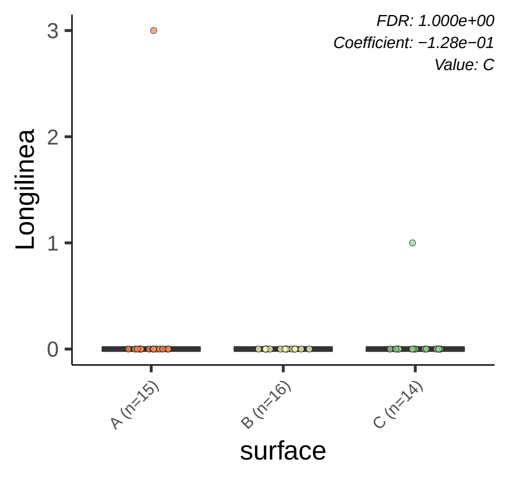
FDR: 1.000e+00
Coefficient: −1.28e−01
Value: C
Longilinea
3
2
1
0
A (n=15)
B (n=16)
C (n=14)
surface

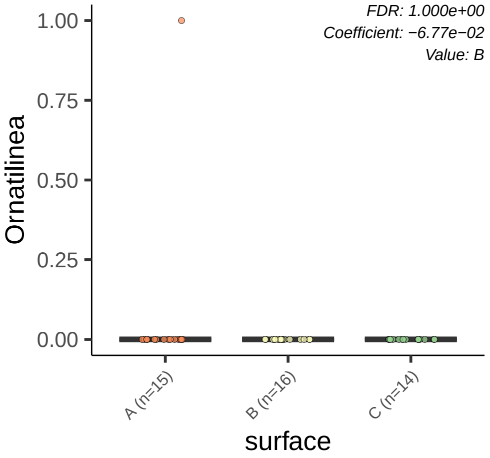
FDR: 1.000e+00
Coefficient: −6.77e−02
Value: B
Ornatilinea
1.00
0.75
0.50
0.25
0.00
A (n=15)
B (n=16)
C (n=14)
surface

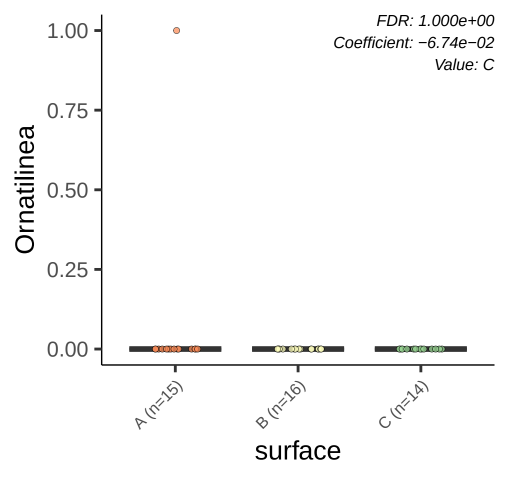

FDR: 1.000e+00
Coefficient: −6.74e−02
Value: C
Ornatilinea
1.00
0.75
0.50
0.25
0.00
A (n=15)
B (n=16)
C (n=14)
surface

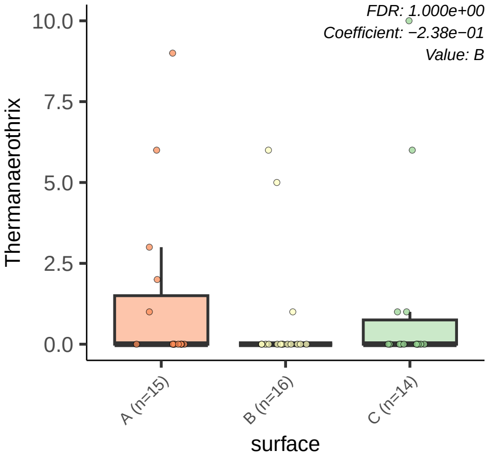
FDR: 1.000e+00
Coefficient: −2.38e−01
Value: B
Thermanaerothrix
10.0
7.5
5.0
2.5
0.0
A (n=15)
B (n=16)
C (n=14)
surface

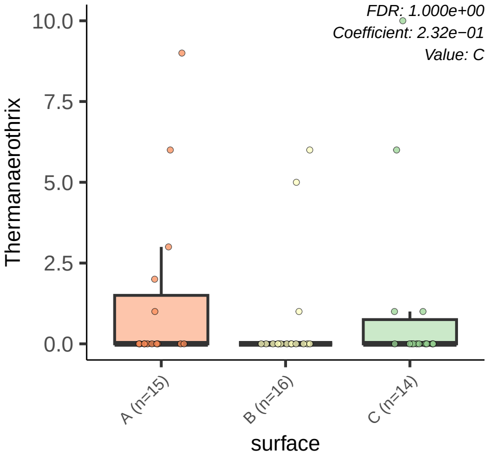

FDR: 1.000e+00
Coefficient: 2.32e−01
Value: C
Thermanaerothrix
10.0
7.5
5.0
2.5
0.0
A (n=15)
B (n=16)
C (n=14)
surface

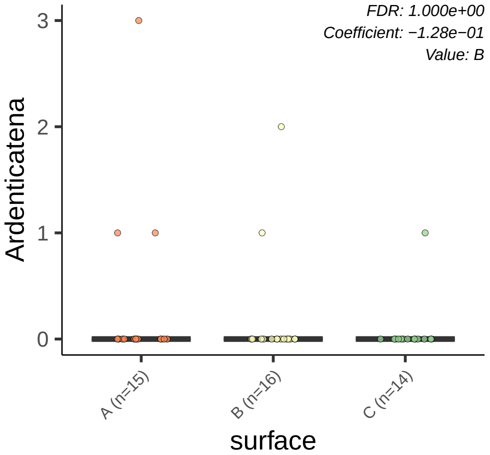
FDR: 1.000e+00
Coefficient: −1.28e−01
Value: B
Ardenticatena
3
2
1
0
A (n=15)
B (n=16)
C (n=14)
surface

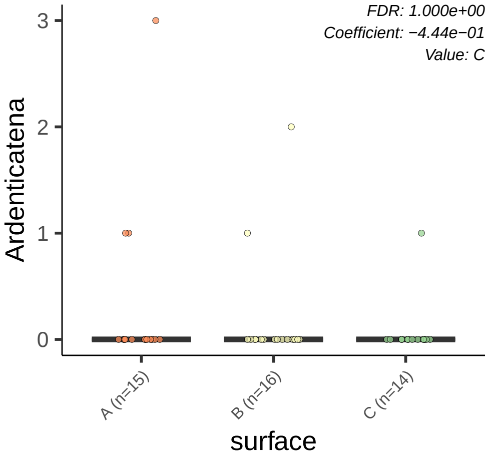
FDR: 1.000e+00
Coefficient: −4.44e−01
Value: C
Ardenticatena
3
2
1
0
A (n=15)
B (n=16)
C (n=14)
surface

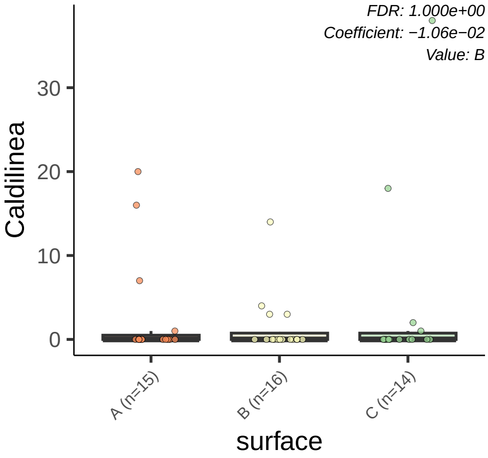

FDR: 1.000e+00
Coefficient: −1.06e−02
Value: B
Caldilinea
30
20
10
0
A (n=15)
B (n=16)
C (n=14)
surface

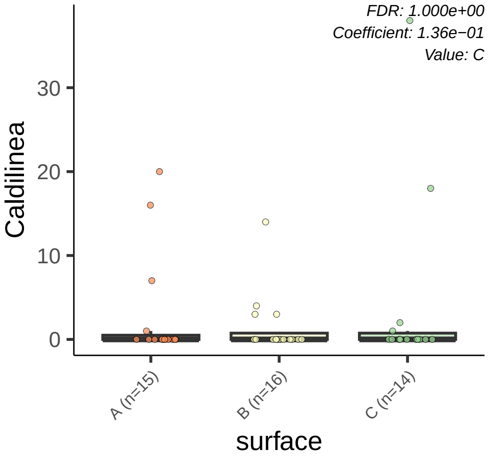

FDR: 1.000e+00
Coefficient: 1.36e−01
Value: C
Caldilinea
30
20
10
0
A (n=15)
B (n=16)
C (n=14)
surface

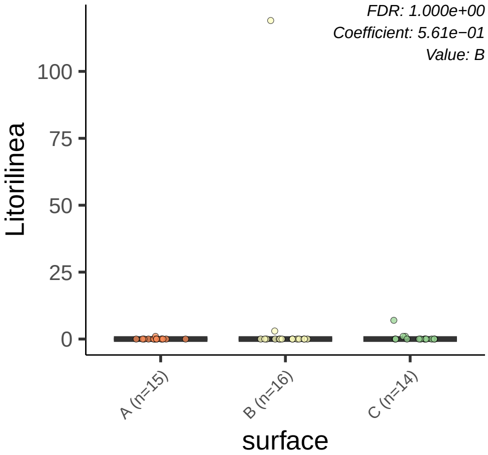
FDR: 1.000e+00
Coefficient: 5.61e−01
Value: B
Litorilinea
100
75
50
25
0
A (n=15)
B (n=16)
C (n=14)
surface

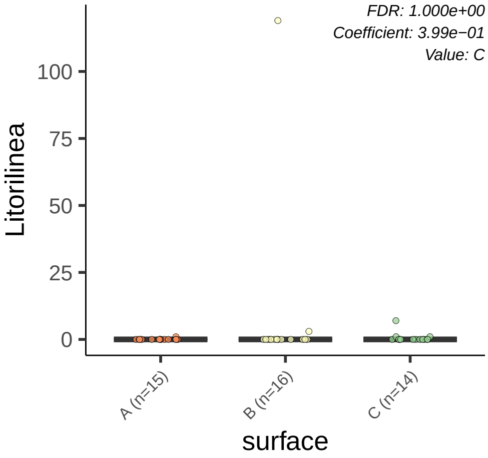

FDR: 1.000e+00
Coefficient: 3.99e−01
Value: C
Litorilinea
100
75
50
25
0
A (n=15)
B (n=16)
C (n=14)
surface

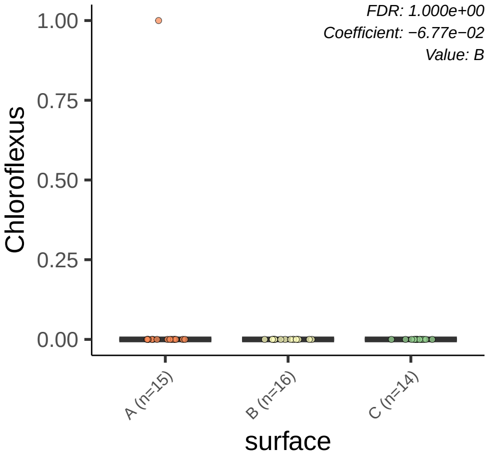
FDR: 1.000e+00
Coefficient: −6.77e−02
Value: B
Chloroflexus
1.00
0.75
0.50
0.25
0.00
A (n=15)
B (n=16)
C (n=14)
surface

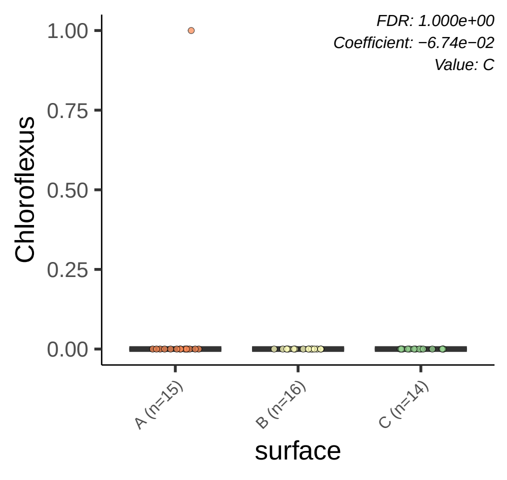

FDR: 1.000e+00
Coefficient: −6.74e−02
Value: C
Chloroflexus
1.00
0.75
0.50
0.25
0.00
A (n=15)
B (n=16)
C (n=14)
surface

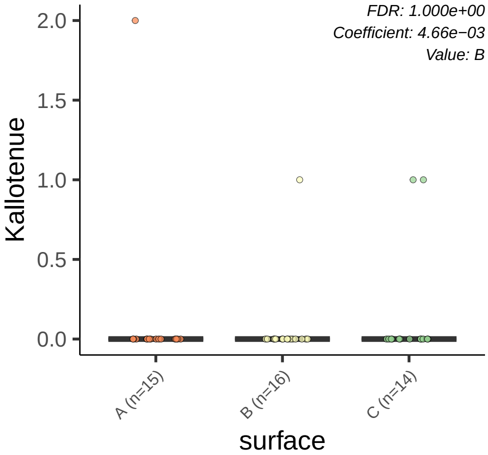
FDR: 1.000e+00
Coefficient: 4.66e−03
Value: B
Kallotenue
2.0
1.5
1.0
0.5
0.0
A (n=15)
B (n=16)
C (n=14)
surface

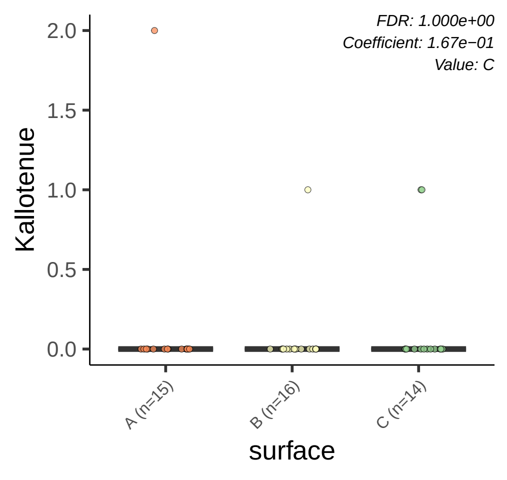
FDR: 1.000e+00
Coefficient: 1.67e−01
Value: C
Kallotenue
2.0
1.5
1.0
0.5
0.0
A (n=15)
B (n=16)
C (n=14)
surface

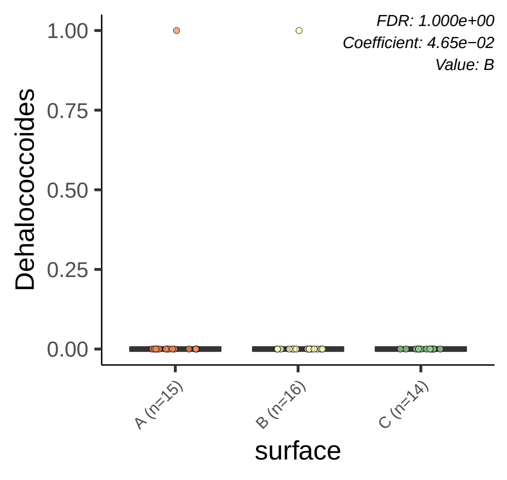

FDR: 1.000e+00
Coefficient: 4.65e−02
Value: B
Dehalococcoides
1.00
0.75
0.50
0.25
0.00
A (n=15)
B (n=16)
C (n=14)
surface

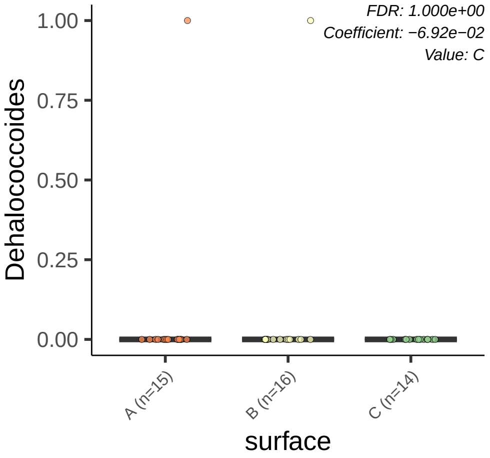
FDR: 1.000e+00
Coefficient: −6.92e−02
Value: C
Dehalococcoides
1.00
0.75
0.50
0.25
0.00
A (n=15)
B (n=16)
C (n=14)
surface

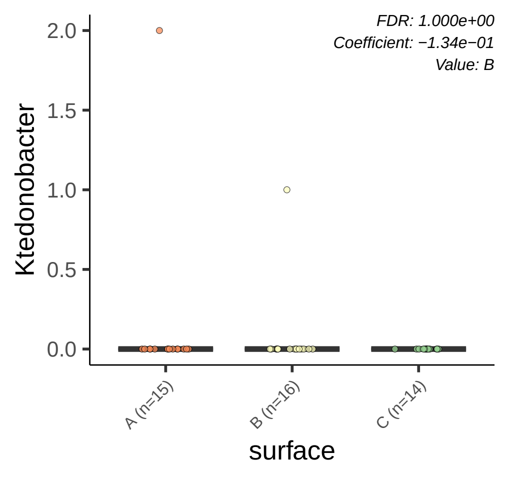

FDR: 1.000e+00
Coefficient: −1.34e−01
Value: B
Ktedonobacter
2.0
1.5
1.0
0.5
0.0
A (n=15)
B (n=16)
C (n=14)
surface

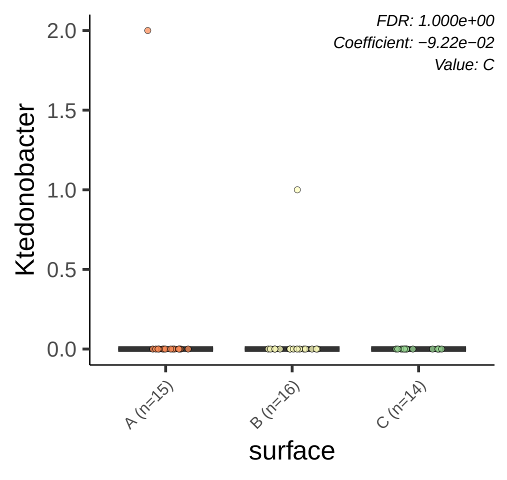
FDR: 1.000e+00
Coefficient: −9.22e−02
Value: C
Ktedonobacter
2.0
1.5
1.0
0.5
0.0
A (n=15)
B (n=16)
C (n=14)
surface

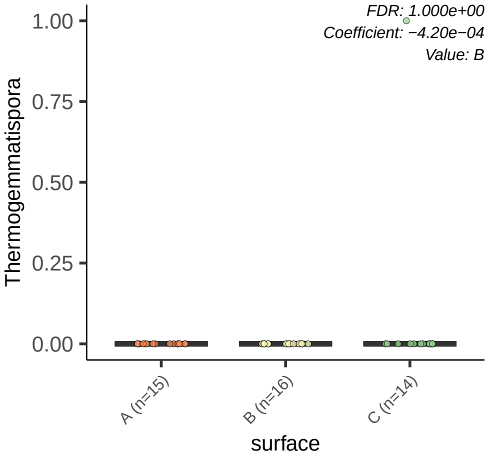
FDR: 1.000e+00
Coefficient: −4.20e−04
Value: B
Thermogemmatispora
1.00
0.75
0.50
0.25
0.00
A (n=15)
B (n=16)
C (n=14)
surface

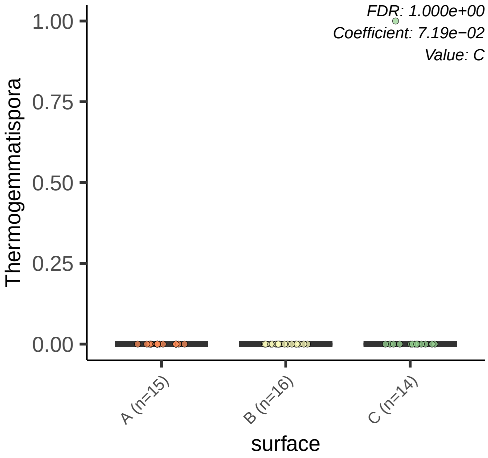

FDR: 1.000e+00
Coefficient: 7.19e−02
Value: C
Thermogemmatispora
1.00
0.75
0.50
0.25
0.00
A (n=15)
B (n=16)
C (n=14)
surface

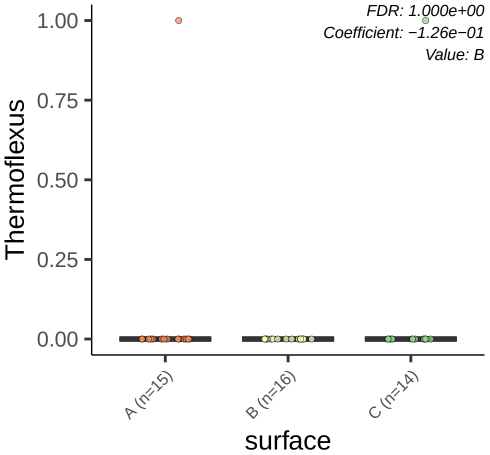

FDR: 1.000e+00
Coefficient: −1.26e−01
Value: B
Thermoflexus
1.00
0.75
0.50
0.25
0.00
A (n=15)
B (n=16)
C (n=14)
surface

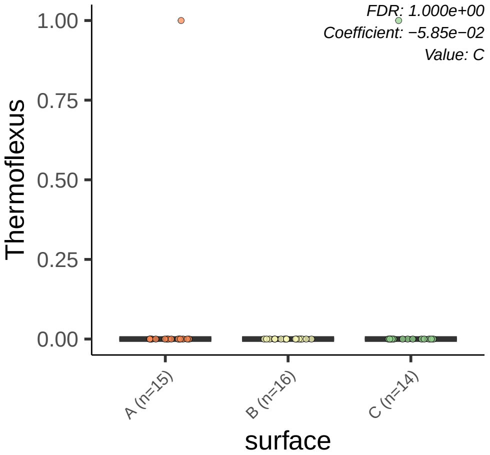

FDR: 1.000e+00
Coefficient: −5.85e−02
Value: C
Thermoflexus
1.00
0.75
0.50
0.25
0.00
A (n=15)
B (n=16)
C (n=14)
surface

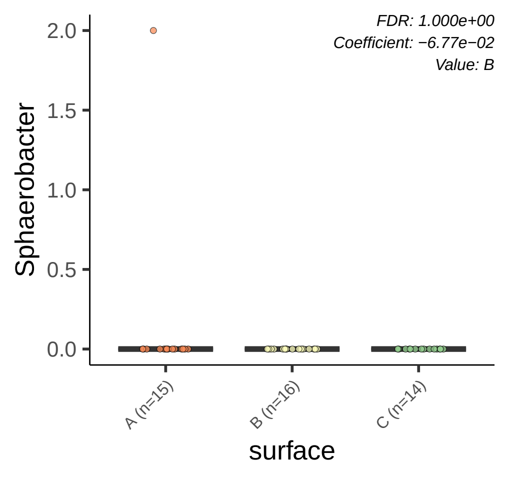
FDR: 1.000e+00
Coefficient: −6.77e−02
Value: B
Sphaerobacter
2.0
1.5
1.0
0.5
0.0
A (n=15)
B (n=16)
C (n=14)
surface

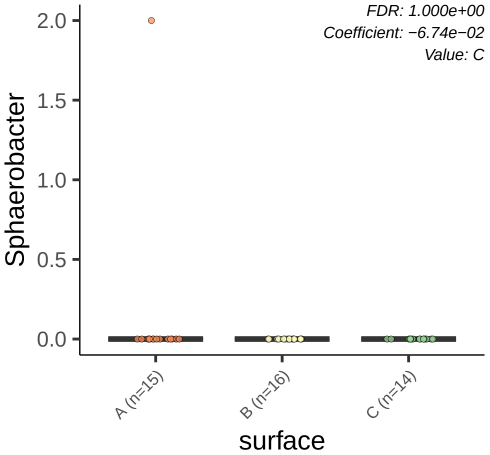

FDR: 1.000e+00
Coefficient: −6.74e−02
Value: C
Sphaerobacter
2.0
1.5
1.0
0.5
0.0
A (n=15)
B (n=16)
C (n=14)
surface

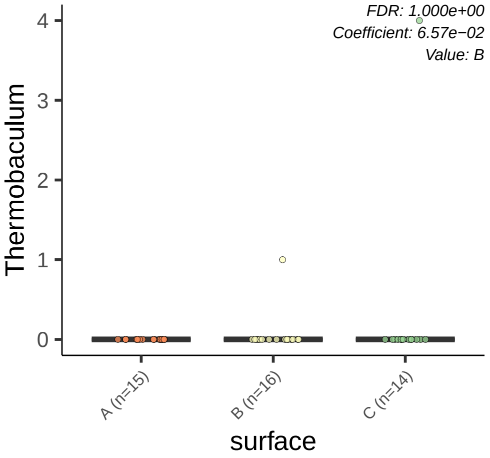
FDR: 1.000e+00
Coefficient: 6.57e−02
Value: B
Thermobaculum
4
3
2
1
0
A (n=15)
B (n=16)
C (n=14)
surface

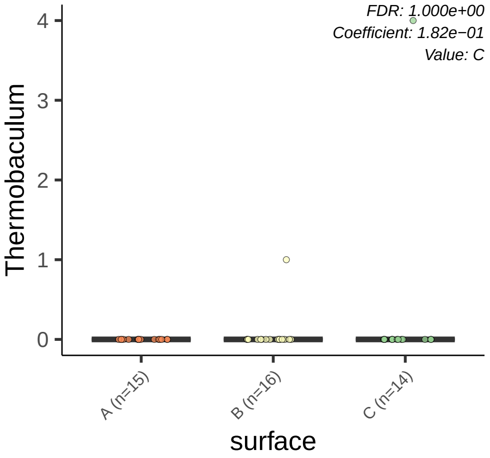

FDR: 1.000e+00
Coefficient: 1.82e−01
Value: C
Thermobaculum
0
1
2
3
4
A (n=15)
B (n=16)
C (n=14)
surface

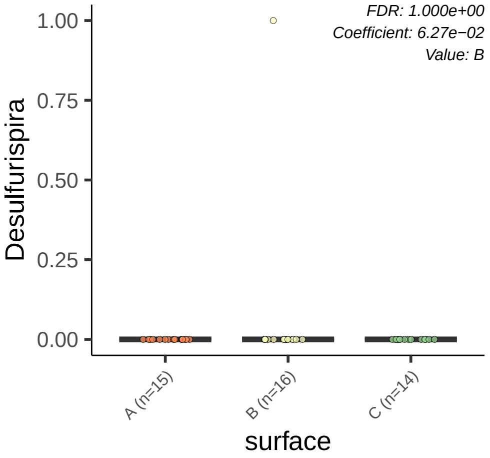
FDR: 1.000e+00
Coefficient: 6.27e−02
Value: B
Desulfurispira
1.00
0.75
0.50
0.25
0.00
A (n=15)
B (n=16)
C (n=14)
surface

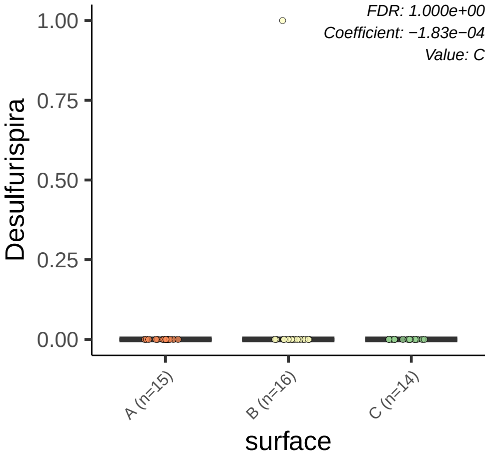

FDR: 1.000e+00
Coefficient: −1.83e−04
Value: C
Desulfurispira
1.00
0.75
0.50
0.25
0.00
A (n=15)
B (n=16)
C (n=14)
surface

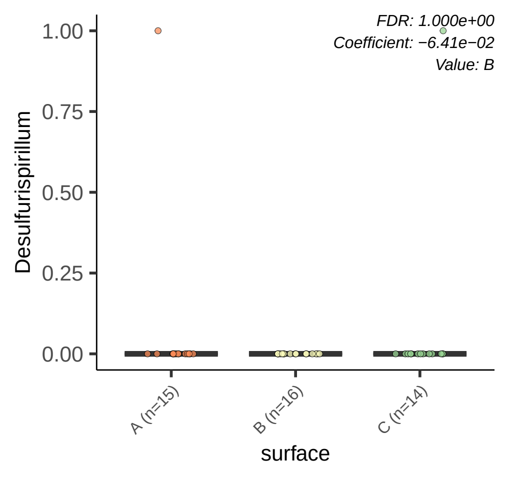

FDR: 1.000e+00
Coefficient: −6.41e−02
Value: B
1.00
0.75
0.50
0.25
0.00
Desulfurispirillum
A (n=15)
B (n=16)
C (n=14)
surface

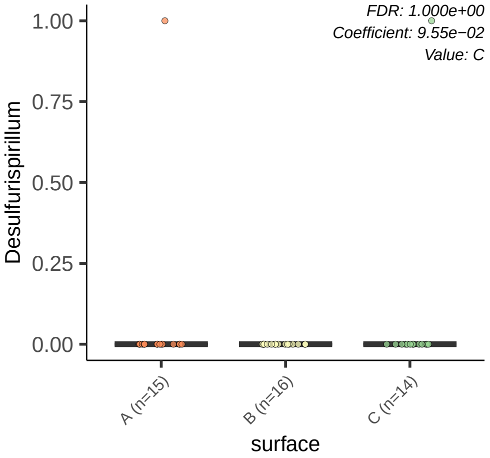
FDR: 1.000e+00
Coefficient: 9.55e−02
Value: C
Desulfurispirillum
1.00
0.75
0.50
0.25
0.00
A (n=15)
B (n=16)
C (n=14)
surface

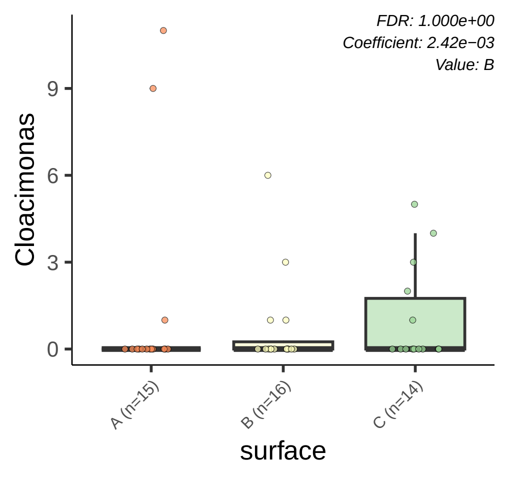
FDR: 1.000e+00
Coefficient: 2.42e−03
Value: B
Cloacimonas
0
3
6
9
A (n=15)
B (n=16)
C (n=14)
surface

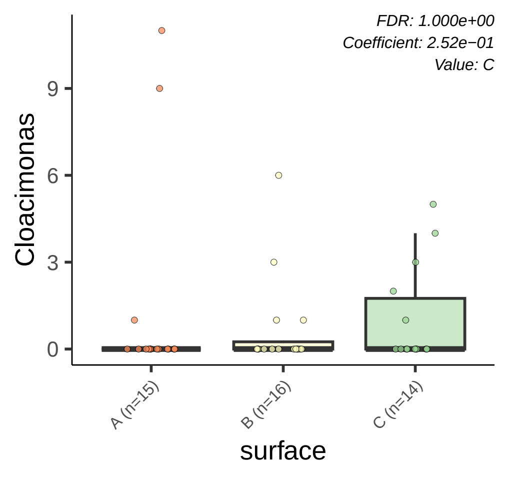
FDR: 1.000e+00
Coefficient: 2.52e−01
Value: C
Cloacimonas
0
3
6
9
A (n=15)
B (n=16)
C (n=14)
surface

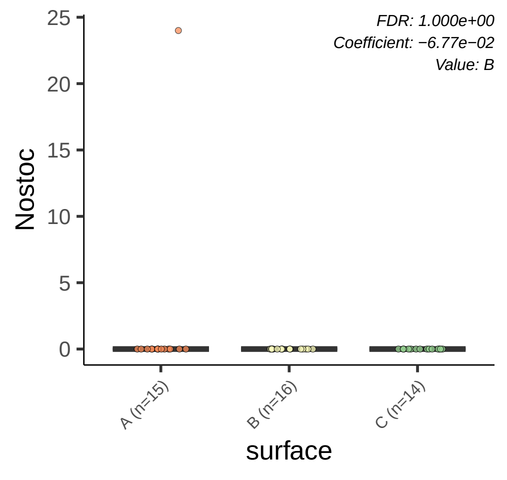

FDR: 1.000e+00
Coefficient: −6.77e−02
Value: B
Nostoc
25
20
15
10
5
0
A (n=15)
B (n=16)
C (n=14)
surface

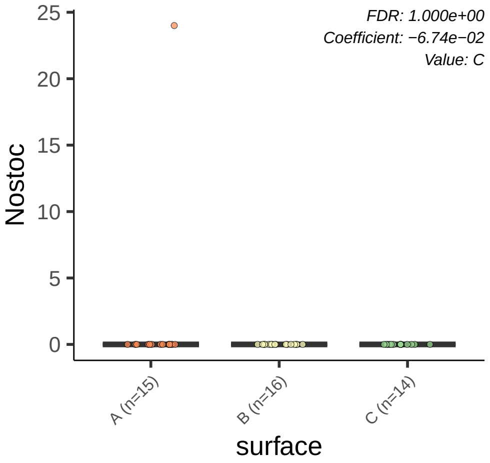
FDR: 1.000e+00
Coefficient: −6.74e−02
Value: C
Nostoc
25
20
15
10
5
0
A (n=15)
B (n=16)
C (n=14)
surface

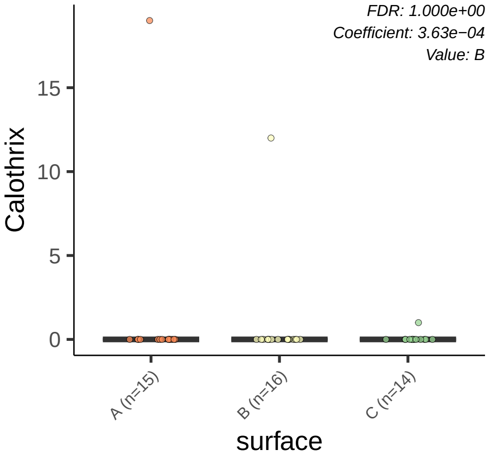

FDR: 1.000e+00
Coefficient: 3.63e−04
Value: B
Calothrix
15
10
5
0
A (n=15)
B (n=16)
C (n=14)
surface

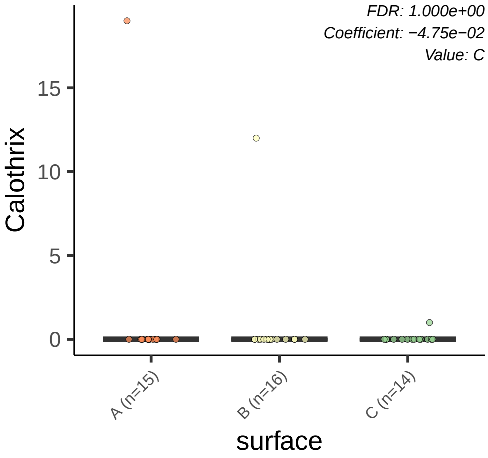
FDR: 1.000e+00
Coefficient: −4.75e−02
Value: C
Calothrix
15
10
5
0
A (n=15)
B (n=16)
C (n=14)
surface

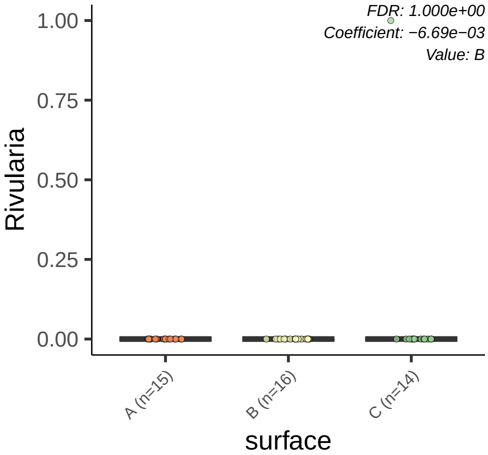
FDR: 1.000e+00
Coefficient: −6.69e−03
Value: B
1.00
0.75
0.50
0.25
0.00
Rivularia
A (n=15)
B (n=16)
C (n=14)
surface

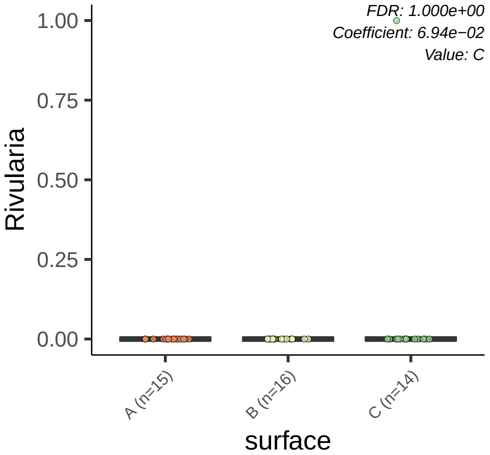

FDR: 1.000e+00
Coefficient: 6.94e−02
Value: C
1.00
0.75
0.50
0.25
0.00
Rivularia
A (n=15)
B (n=16)
C (n=14)
surface

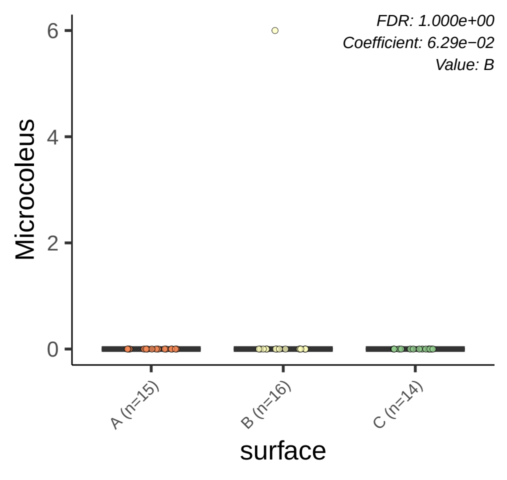

FDR: 1.000e+00
Coefficient: 6.29e−02
Value: B
Microcoleus
6
4
2
0
A (n=15)
B (n=16)
C (n=14)
surface

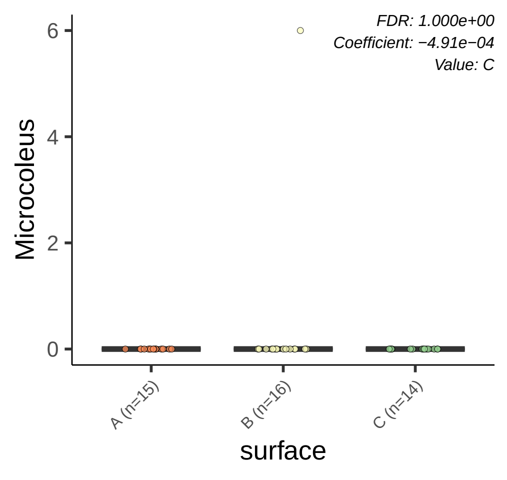
FDR: 1.000e+00
Coefficient: −4.91e−04
Value: C
Microcoleus
6
4
2
0
A (n=15)
B (n=16)
C (n=14)
surface

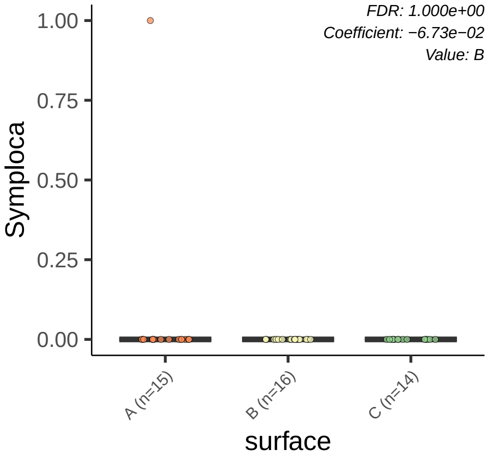

FDR: 1.000e+00
Coefficient: −6.73e−02
Value: B
Symploca
1.00
0.75
0.50
0.25
0.00
A (n=15)
B (n=16)
C (n=14)
surface

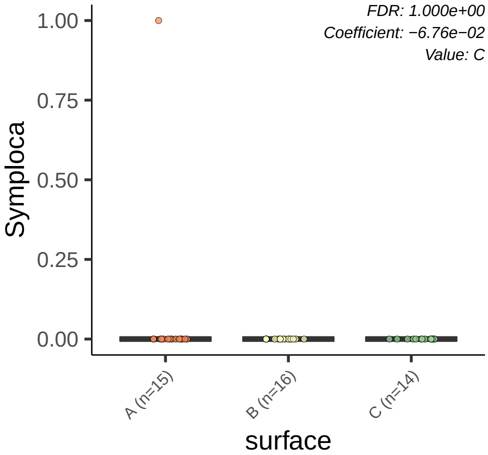

FDR: 1.000e+00
Coefficient: −6.76e−02
Value: C
Symploca
1.00
0.75
0.50
0.25
0.00
A (n=15)
B (n=16)
C (n=14)
surface

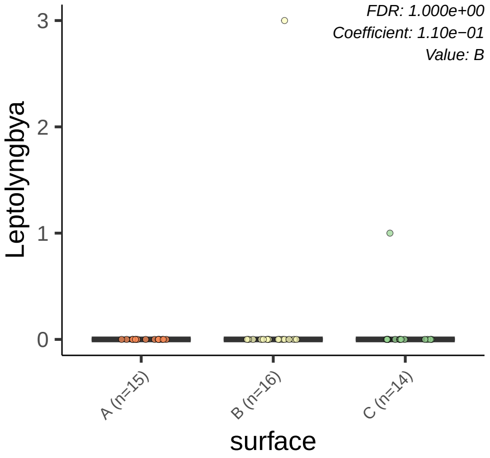
FDR: 1.000e+00
Coefficient: 1.10e−01
Value: B
Leptolyngbya
0
1
2
3
A (n=15)
B (n=16)
C (n=14)
surface

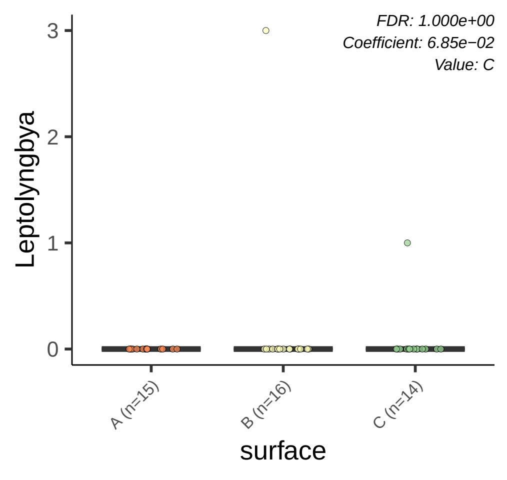
FDR: 1.000e+00
Coefficient: 6.85e−02
Value: C
Leptolyngbya
3
2
1
0
A (n=15)
B (n=16)
C (n=14)
surface

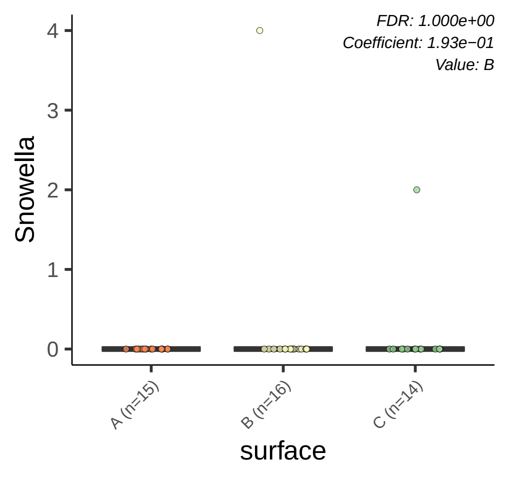

FDR: 1.000e+00
Coefficient: 1.93e−01
Value: B
Snowella
0
1
2
3
4
A (n=15)
B (n=16)
C (n=14)
surface

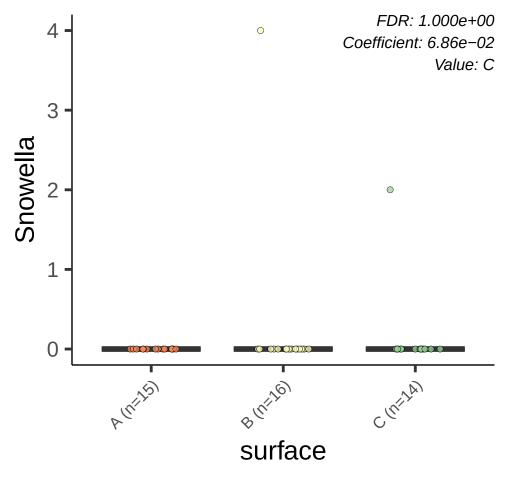

FDR: 1.000e+00
Coefficient: 6.86e−02
Value: C
Snowella
4
3
2
1
0
A (n=15)
B (n=16)
C (n=14)
surface

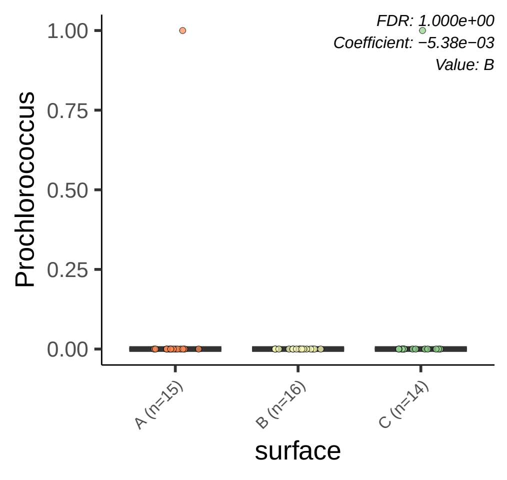

FDR: 1.000e+00
Coefficient: −5.38e−03
Value: B
Prochlorococcus
1.00
0.75
0.50
0.25
0.00
A (n=15)
B (n=16)
C (n=14)
surface

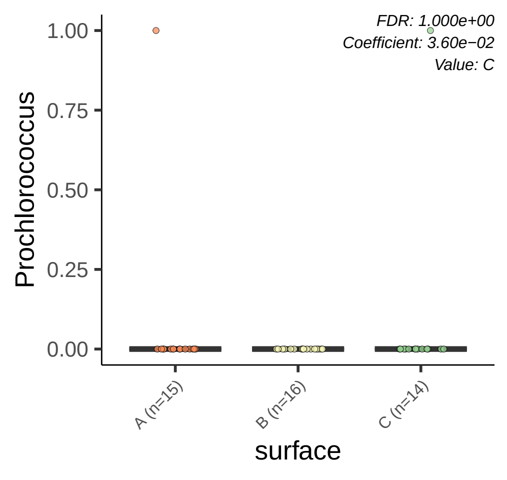
FDR: 1.000e+00
Coefficient: 3.60e−02
Value: C
Prochlorococcus
1.00
0.75
0.50
0.25
0.00
A (n=15)
B (n=16)
C (n=14)
surface

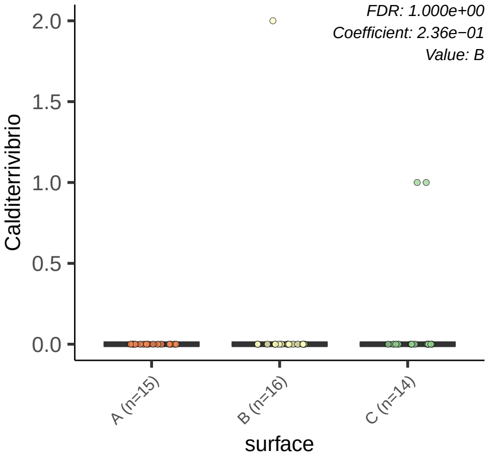
FDR: 1.000e+00
Coefficient: 2.36e−01
Value: B
2.0
1.5
1.0
0.5
0.0
Calditerrivibrio
A (n=15)
B (n=16)
C (n=14)
surface

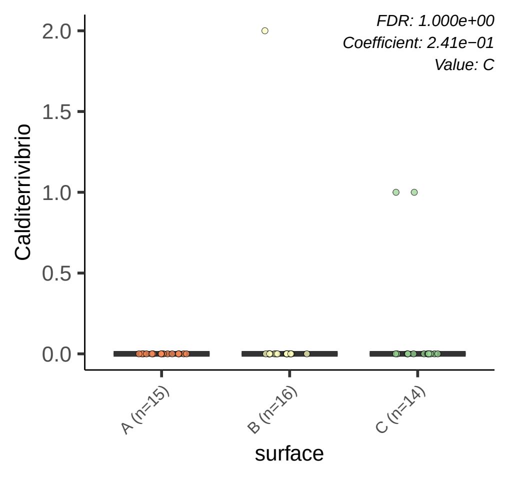
FDR: 1.000e+00
Coefficient: 2.41e−01
Value: C
Calditerrivibrio
2.0
1.5
1.0
0.5
0.0
A (n=15)
B (n=16)
C (n=14)
surface

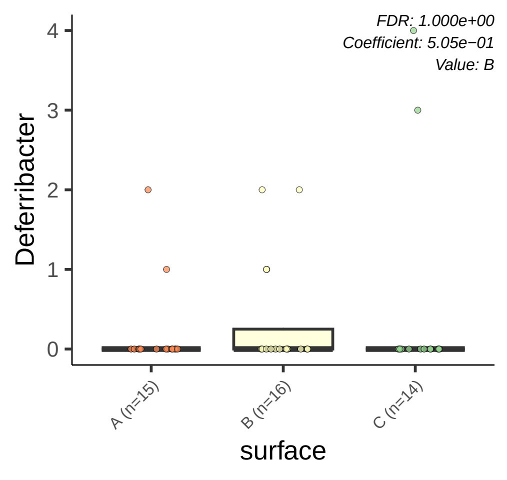
FDR: 1.000e+00
Coefficient: 5.05e−01
Value: B
Deferribacter
0
1
2
3
4
A (n=15)
B (n=16)
C (n=14)
surface

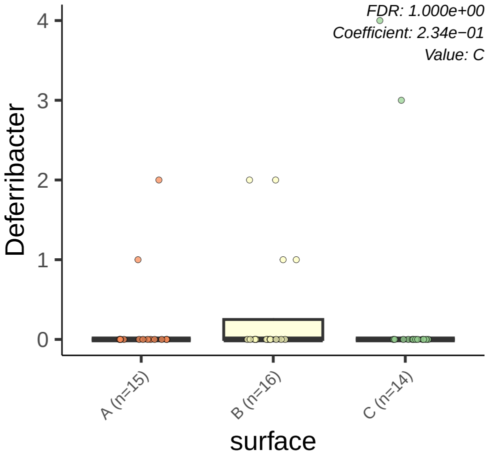

FDR: 1.000e+00
Coefficient: 2.34e−01
Value: C
Deferribacter
0
1
2
3
4
A (n=15)
B (n=16)
C (n=14)
surface

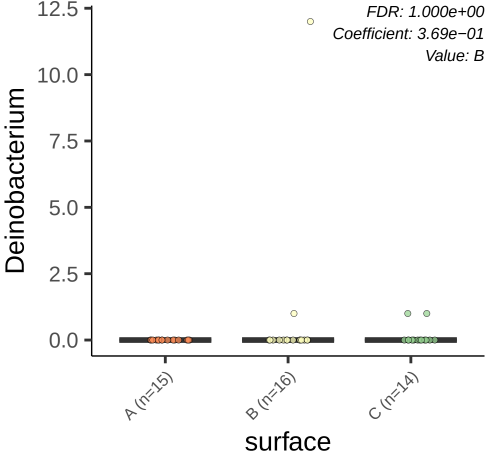
FDR: 1.000e+00
Coefficient: 3.69e−01
Value: B
Deinobacterium
12.5
10.0
7.5
5.0
2.5
0.0
A (n=15)
B (n=16)
C (n=14)
surface

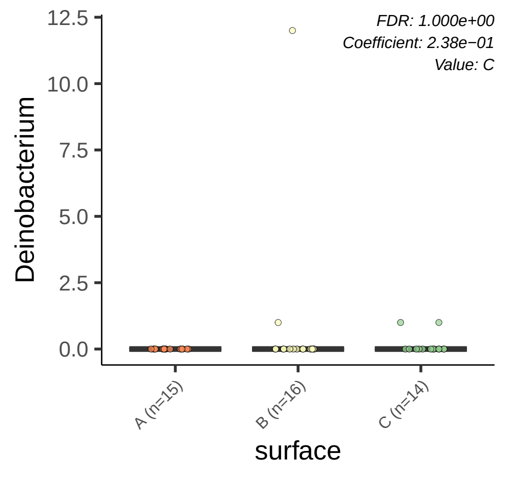
FDR: 1.000e+00
Coefficient: 2.38e−01
Value: C
Deinobacterium
12.5
10.0
7.5
5.0
2.5
0.0
A (n=15)
B (n=16)
C (n=14)
surface

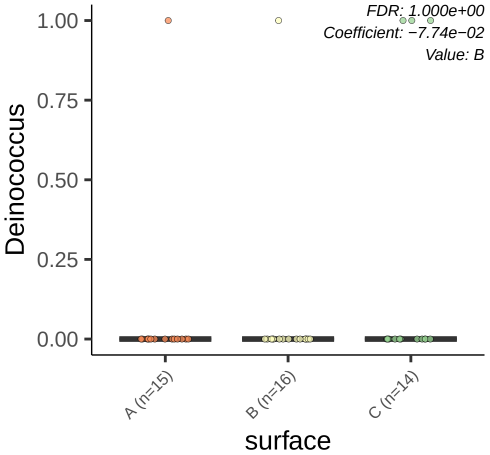

FDR: 1.000e+00
Coefficient: −7.74e−02
Value: B
Deinococcus
1.00
0.75
0.50
0.25
0.00
A (n=15)
B (n=16)
C (n=14)
surface

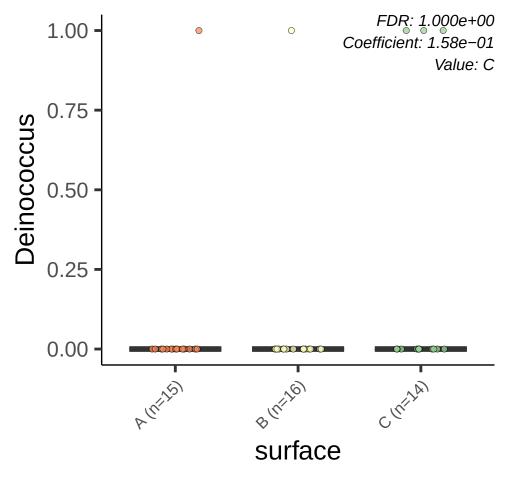
FDR: 1.000e+00
Coefficient: 1.58e−01
Value: C
Deinococcus
1.00
0.75
0.50
0.25
0.00
A (n=15)
B (n=16)
C (n=14)
surface

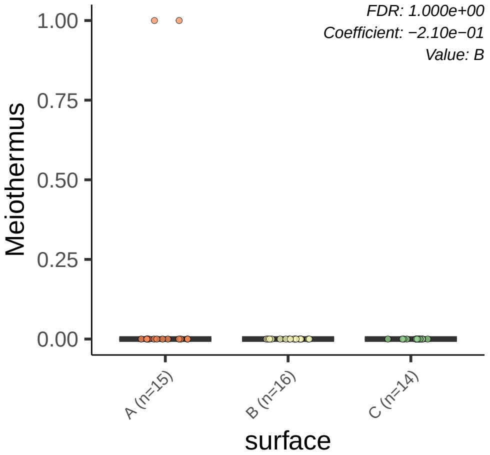
FDR: 1.000e+00
Coefficient: −2.10e−01
Value: B
Meiothermus
1.00
0.75
0.50
0.25
0.00
A (n=15)
B (n=16)
C (n=14)
surface

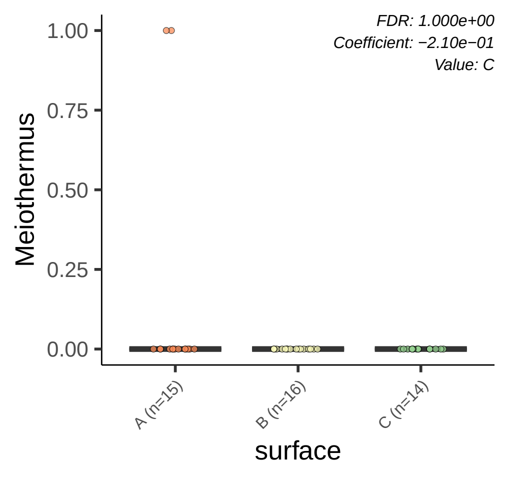

FDR: 1.000e+00
Coefficient: −2.10e−01
Value: C
Meiothermus
1.00
0.75
0.50
0.25
0.00
A (n=15)
B (n=16)
C (n=14)
surface

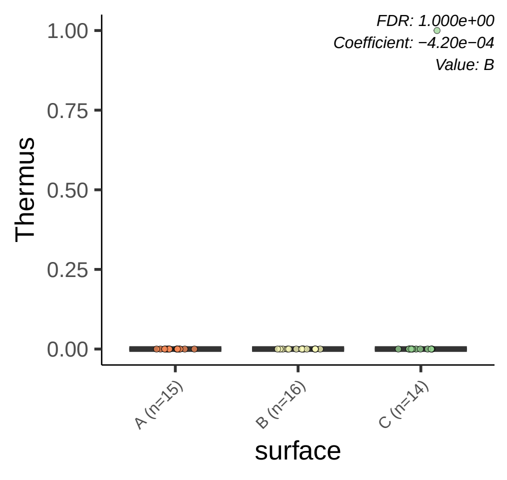
FDR: 1.000e+00
Coefficient: −4.20e−04
Value: B
Thermus
1.00
0.75
0.50
0.25
0.00
A (n=15)
B (n=16)
C (n=14)
surface

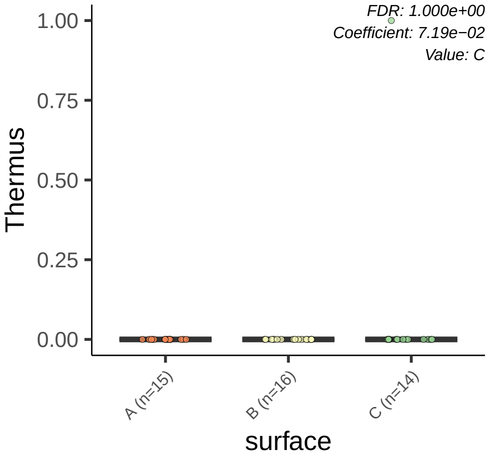
FDR: 1.000e+00
Coefficient: 7.19e−02
Value: C
1.00
0.75
0.50
0.25
0.00
Thermus
A (n=15)
B (n=16)
C (n=14)
surface

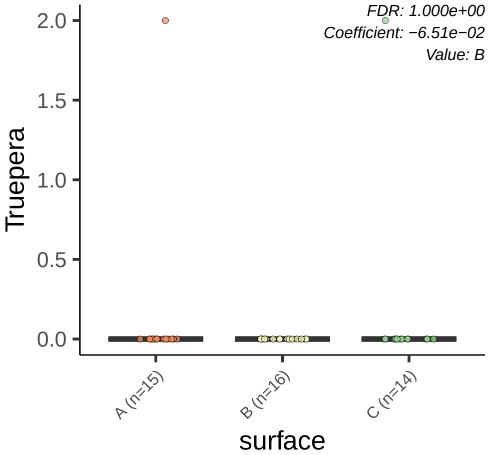
FDR: 1.000e+00
Coefficient: −6.51e−02
Value: B
Truepera
2.0
1.5
1.0
0.5
0.0
A (n=15)
B (n=16)
C (n=14)
surface

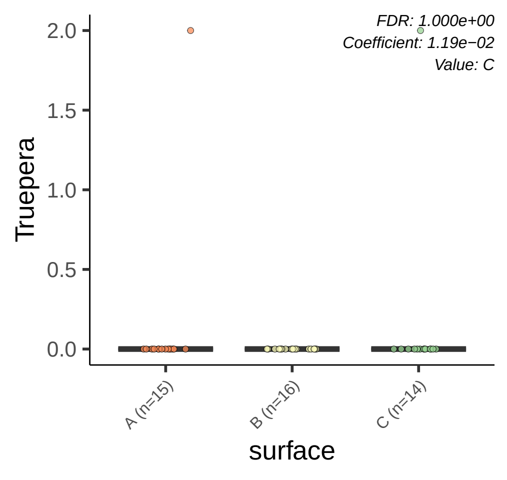
FDR: 1.000e+00
Coefficient: 1.19e−02
Value: C
Truepera
2.0
1.5
1.0
0.5
0.0
A (n=15)
B (n=16)
C (n=14)
surface

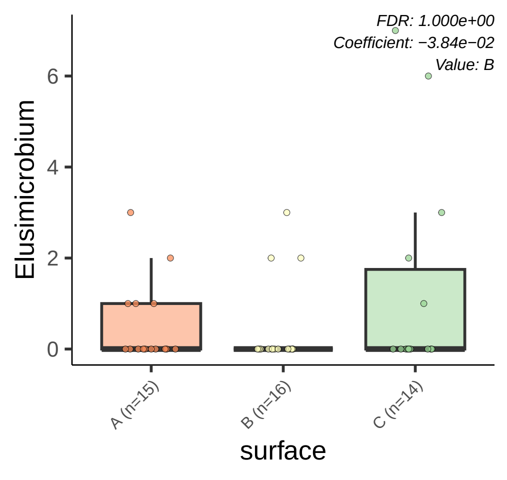
FDR: 1.000e+00
Coefficient: −3.84e−02
Value: B
Elusimicrobium
0
2
4
6
A (n=15)
B (n=16)
C (n=14)
surface

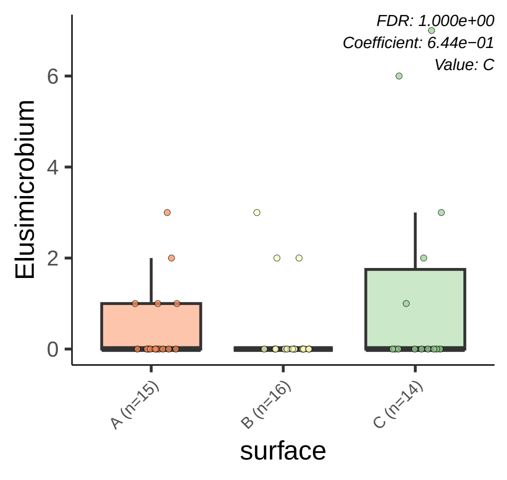
FDR: 1.000e+00
Coefficient: 6.44e−01
Value: C
Elusimicrobium
0
2
4
6
A (n=15)
B (n=16)
C (n=14)
surface

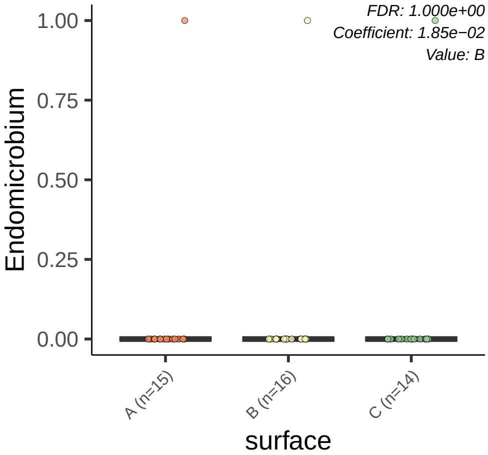
FDR: 1.000e+00
Coefficient: 1.85e−02
Value: B
1.00
0.75
0.50
0.25
0.00
Endomicrobium
A (n=15)
B (n=16)
C (n=14)
surface

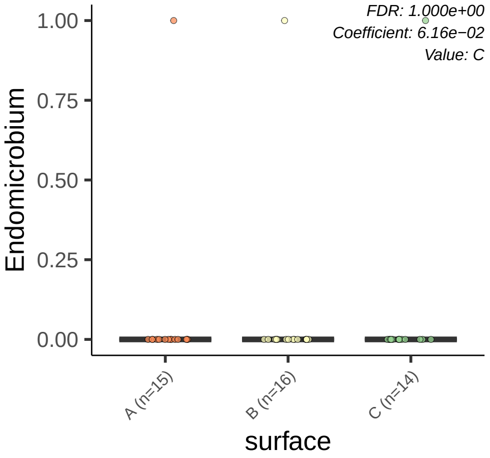
FDR: 1.000e+00
Coefficient: 6.16e−02
Value: C
Endomicrobium
1.00
0.75
0.50
0.25
0.00
A (n=15)
B (n=16)
C (n=14)
surface

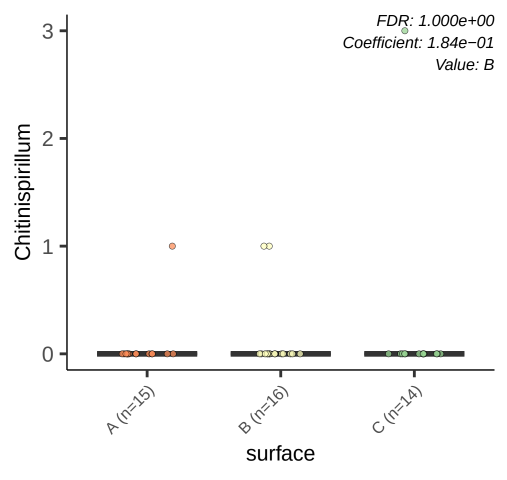

FDR: 1.000e+00
Coefficient: 1.84e−01
Value: B
Chitinispirillum
3
2
1
0
A (n=15)
B (n=16)
C (n=14)
surface

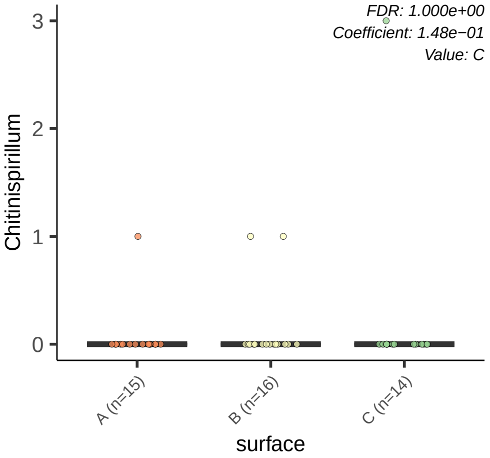
FDR: 1.000e+00
Coefficient: 1.48e−01
Value: C
Chitinispirillum
0
1
2
3
A (n=15)
B (n=16)
C (n=14)
surface

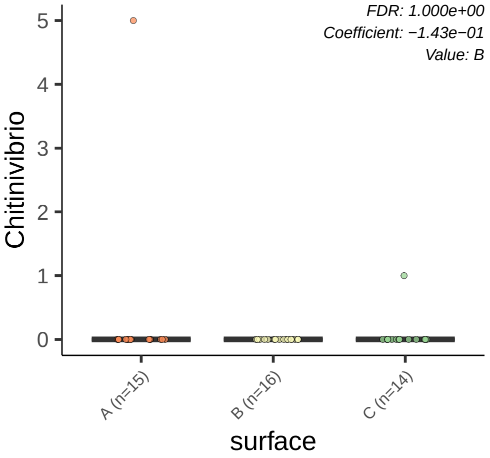
FDR: 1.000e+00
Coefficient: −1.43e−01
Value: B
Chitinivibrio
5
4
3
2
1
0
A (n=15)
B (n=16)
C (n=14)
surface

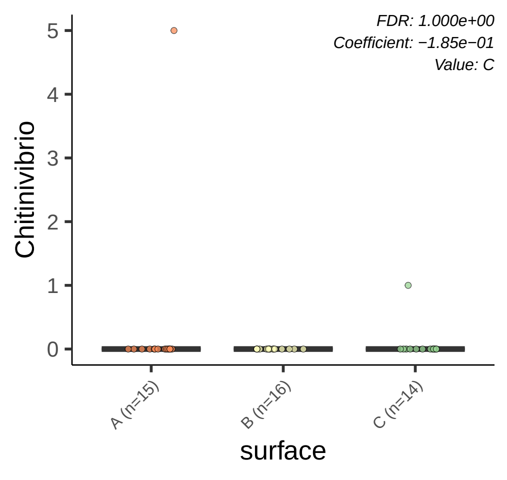

FDR: 1.000e+00
Coefficient: −1.85e−01
Value: C
Chitinivibrio
0
1
2
3
4
5
A (n=15)
B (n=16)
C (n=14)
surface

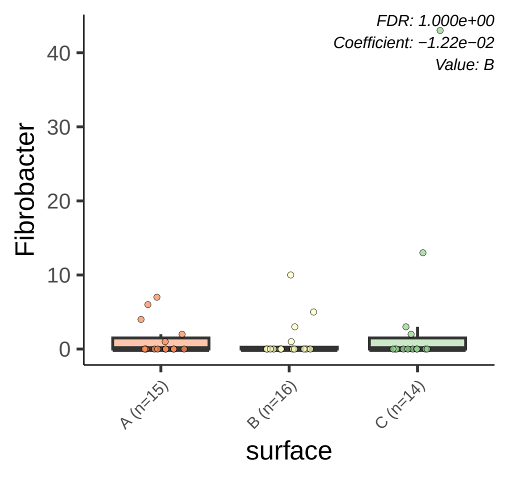

FDR: 1.000e+00
Coefficient: −1.22e−02
Value: B
Fibrobacter
40
30
20
10
0
A (n=15)
B (n=16)
C (n=14)
surface

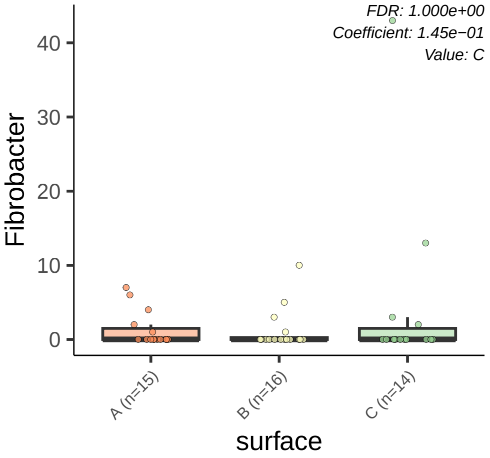

FDR: 1.000e+00
Coefficient: 1.45e−01
Value: C
Fibrobacter
40
30
20
10
0
A (n=15)
B (n=16)
C (n=14)
surface

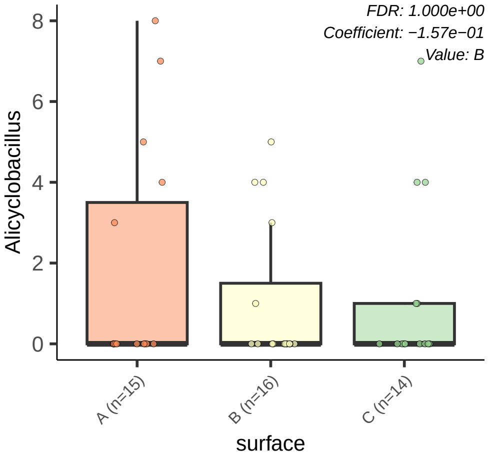

FDR: 1.000e+00
Coefficient: −1.57e−01
Value: B
Alicyclobacillus
8
6
4
2
0
A (n=15)
B (n=16)
C (n=14)
surface

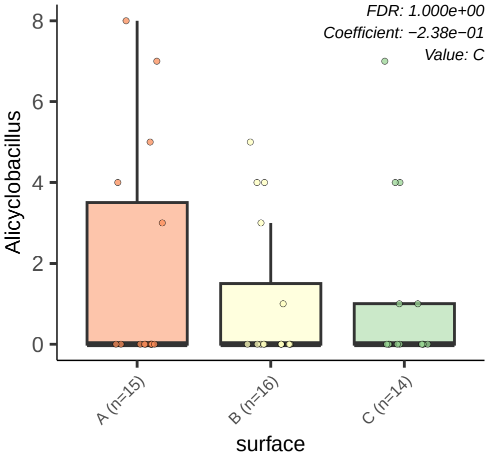
FDR: 1.000e+00
Coefficient: −2.38e−01
Value: C
Alicyclobacillus
8
6
4
2
0
A (n=15)
B (n=16)
C (n=14)
surface

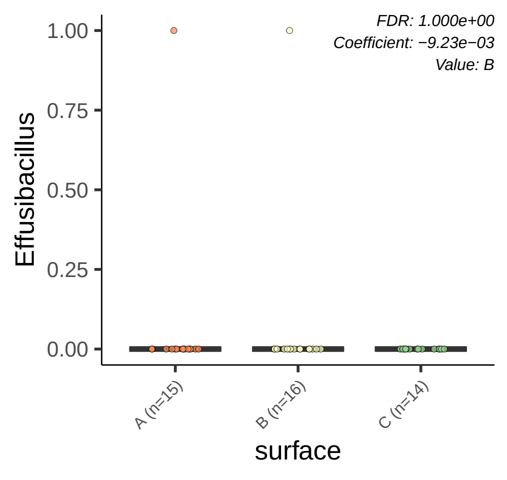

FDR: 1.000e+00
Coefficient: −9.23e−03
Value: B
Effusibacillus
1.00
0.75
0.50
0.25
0.00
A (n=15)
B (n=16)
C (n=14)
surface

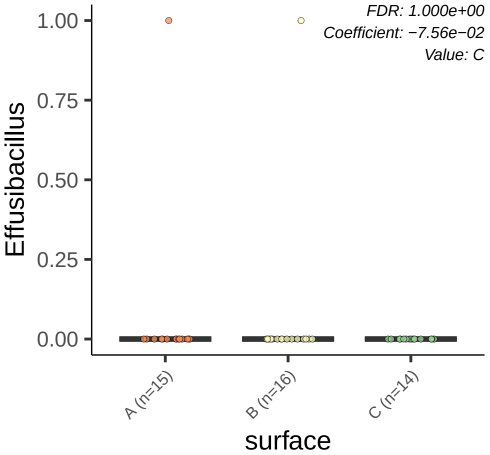

FDR: 1.000e+00
Coefficient: −7.56e−02
Value: C
Effusibacillus
1.00
0.75
0.50
0.25
0.00
A (n=15)
B (n=16)
C (n=14)
surface

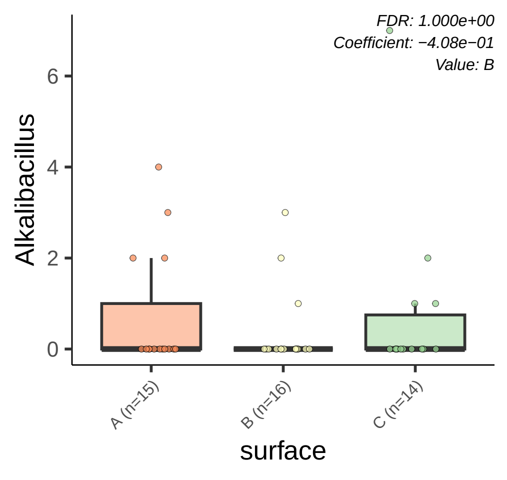
FDR: 1.000e+00
Coefficient: −4.08e−01
Value: B
Alkalibacillus
0
2
4
6
A (n=15)
B (n=16)
C (n=14)
surface

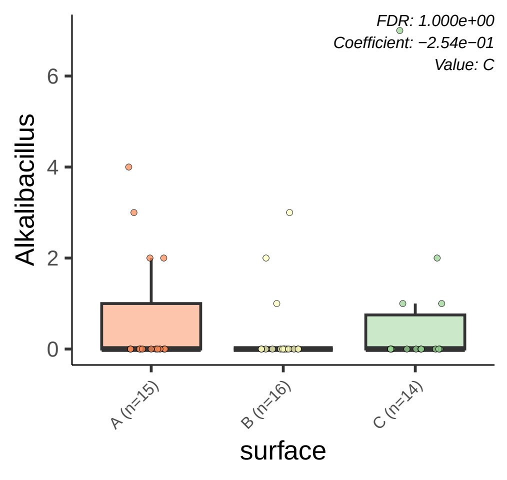
FDR: 1.000e+00
Coefficient: −2.54e−01
Value: C
Alkalibacillus
0
2
4
6
A (n=15)
B (n=16)
C (n=14)
surface

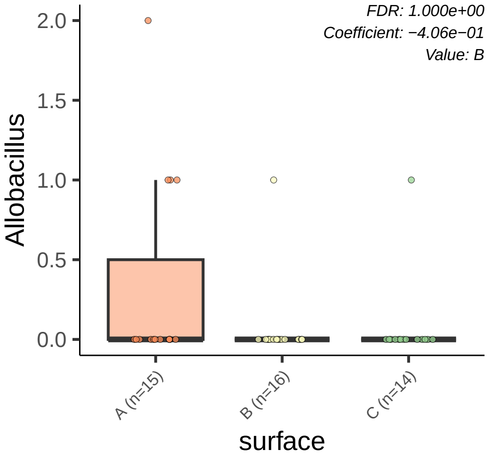

FDR: 1.000e+00
Coefficient: −4.06e−01
Value: B
Allobacillus
2.0
1.5
1.0
0.5
0.0
A (n=15)
B (n=16)
C (n=14)
surface

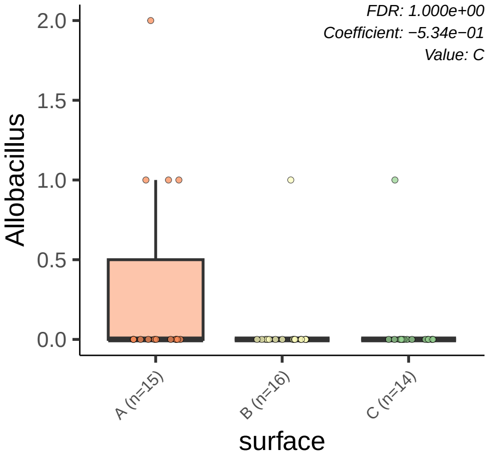
FDR: 1.000e+00
Coefficient: −5.34e−01
Value: C
Allobacillus
2.0
1.5
1.0
0.5
0.0
A (n=15)
B (n=16)
C (n=14)
surface

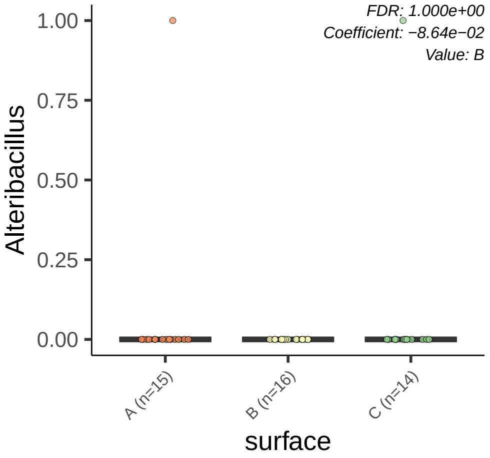
FDR: 1.000e+00
Coefficient: −8.64e−02
Value: B
1.00
0.75
0.50
0.25
0.00
Alteribacillus
A (n=15)
B (n=16)
C (n=14)
surface

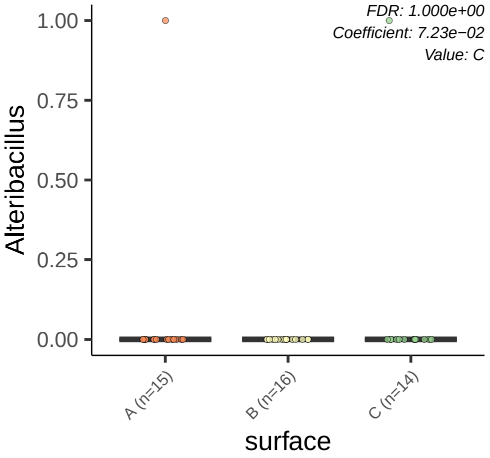
FDR: 1.000e+00
Coefficient: 7.23e−02
Value: C
Alteribacillus
1.00
0.75
0.50
0.25
0.00
A (n=15)
B (n=16)
C (n=14)
surface

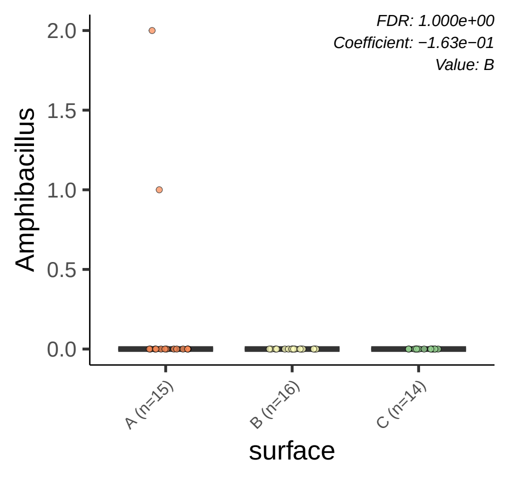

FDR: 1.000e+00
Coefficient: −1.63e−01
Value: B
Amphibacillus
2.0
1.5
1.0
0.5
0.0
A (n=15)
B (n=16)
C (n=14)
surface

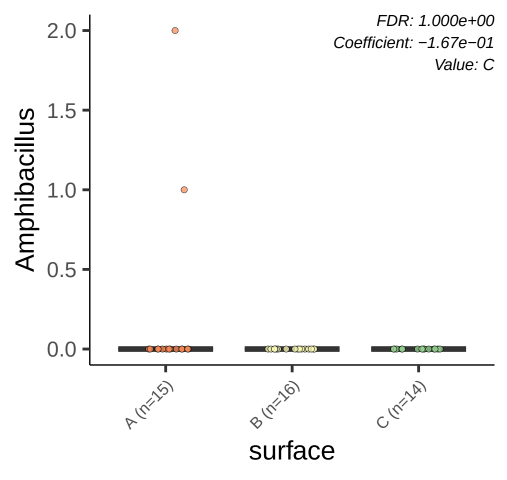

FDR: 1.000e+00
Coefficient: −1.67e−01
Value: C
Amphibacillus
2.0
1.5
1.0
0.5
0.0
A (n=15)
B (n=16)
C (n=14)
surface

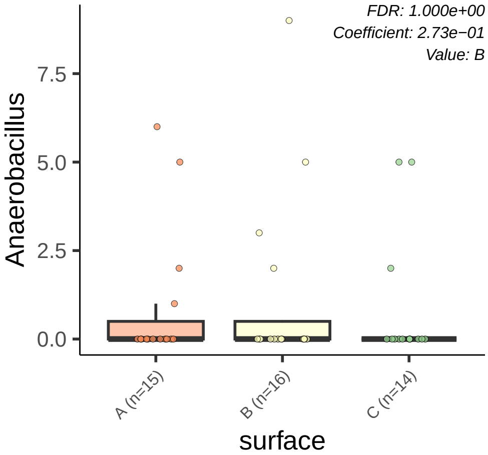

FDR: 1.000e+00
Coefficient: 2.73e−01
Value: B
Anaerobacillus
7.5
5.0
2.5
0.0
A (n=15)
B (n=16)
C (n=14)
surface

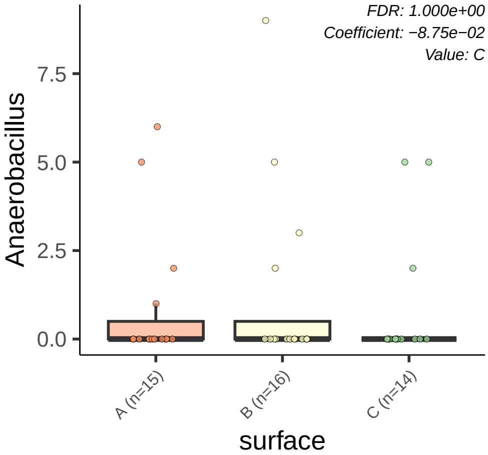

FDR: 1.000e+00
Coefficient: −8.75e−02
Value: C
Anaerobacillus
7.5
5.0
2.5
0.0
A (n=15)
B (n=16)
C (n=14)
surface

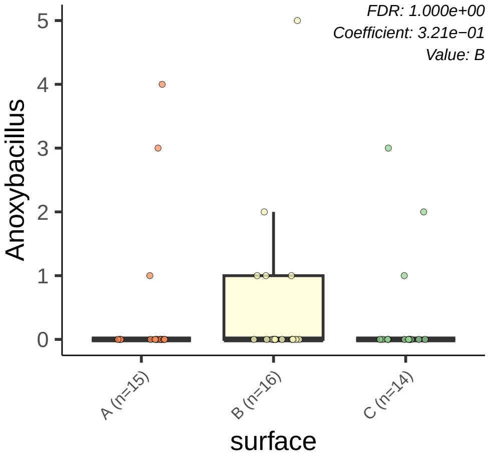
FDR: 1.000e+00
Coefficient: 3.21e−01
Value: B
Anoxybacillus
0
1
2
3
4
5
A (n=15)
B (n=16)
C (n=14)
surface

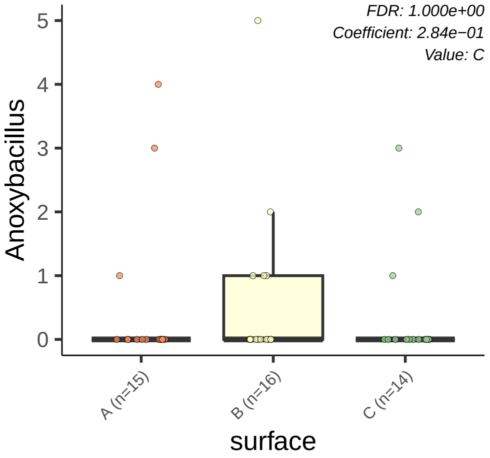
FDR: 1.000e+00
Coefficient: 2.84e−01
Value: C
Anoxybacillus
0
1
2
3
4
5
A (n=15)
B (n=16)
C (n=14)
surface

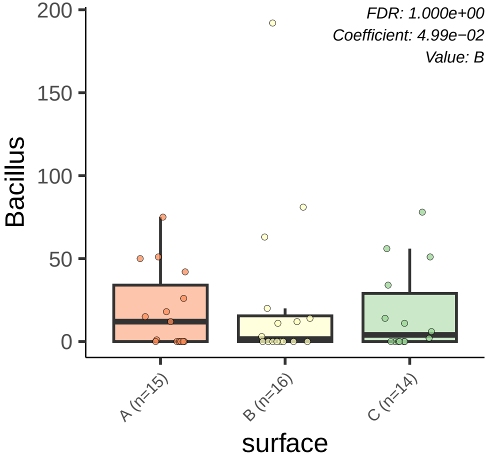

FDR: 1.000e+00
Coefficient: 4.99e−02
Value: B
200
150
100
50
0
Bacillus
A (n=15)
B (n=16)
C (n=14)
surface

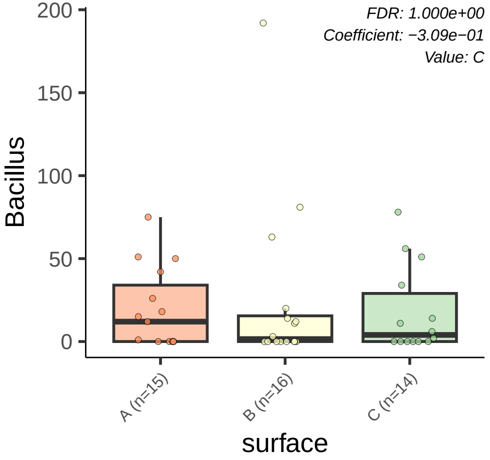

FDR: 1.000e+00
Coefficient: −3.09e−01
Value: C
Bacillus
200
150
100
50
0
A (n=15)
B (n=16)
C (n=14)
surface

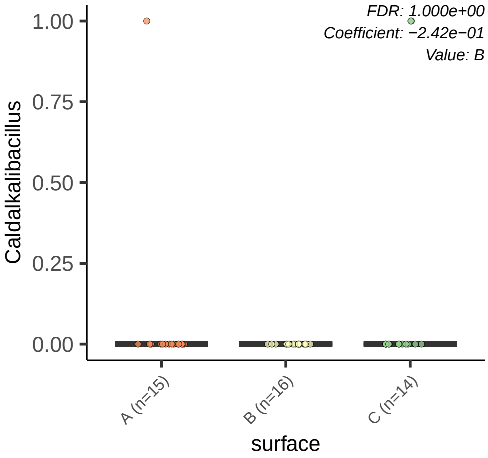

FDR: 1.000e+00
Coefficient: −2.42e−01
Value: B
Caldalkalibacillus
1.00
0.75
0.50
0.25
0.00
A (n=15)
B (n=16)
C (n=14)
surface

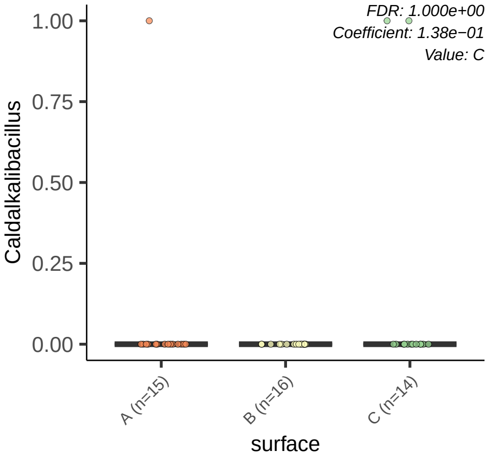
FDR: 1.000e+00
Coefficient: 1.38e−01
Value: C
Caldalkalibacillus
1.00
0.75
0.50
0.25
0.00
A (n=15)
B (n=16)
C (n=14)
surface

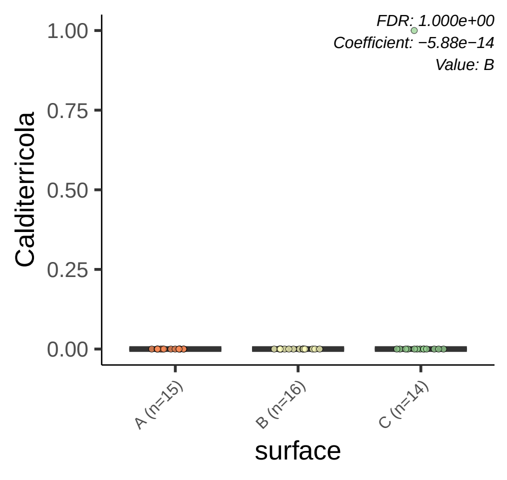
FDR: 1.000e+00
Coefficient: −5.88e−14
Value: B
1.00
0.75
0.50
0.25
0.00
Calditerricola
A (n=15)
B (n=16)
C (n=14)
surface

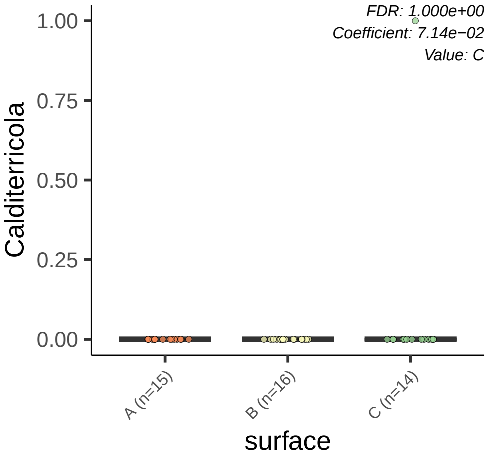
FDR: 1.000e+00
Coefficient: 7.14e−02
Value: C
1.00
0.75
0.50
0.25
0.00
Calditerricola
A (n=15)
B (n=16)
C (n=14)
surface

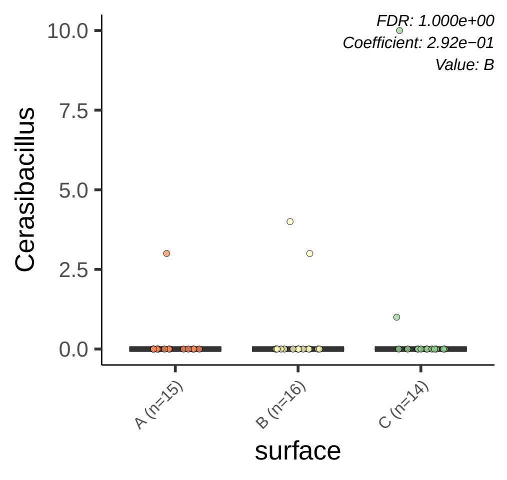
FDR: 1.000e+00
Coefficient: 2.92e−01
Value: B
Cerasibacillus
10.0
7.5
5.0
2.5
0.0
A (n=15)
B (n=16)
C (n=14)
surface

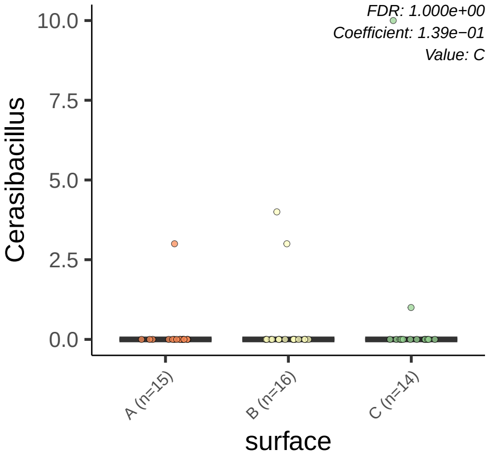

FDR: 1.000e+00
Coefficient: 1.39e−01
Value: C
Cerasibacillus
10.0
7.5
5.0
2.5
0.0
A (n=15)
B (n=16)
C (n=14)
surface

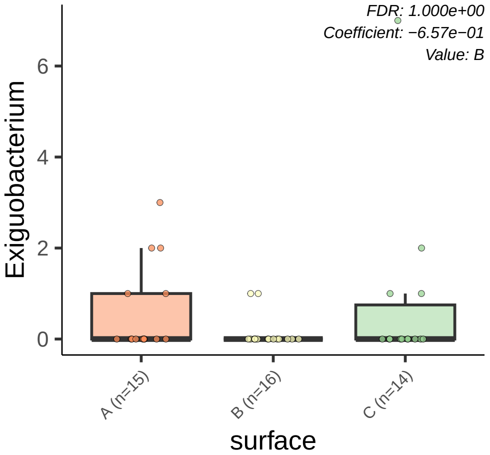

FDR: 1.000e+00
Coefficient: −6.57e−01
Value: B
Exiguobacterium
0
2
4
6
A (n=15)
B (n=16)
C (n=14)
surface

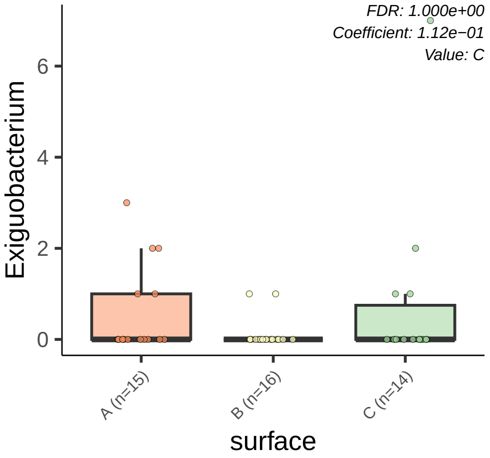

FDR: 1.000e+00
Coefficient: 1.12e−01
Value: C
Exiguobacterium
6
4
2
0
A (n=15)
B (n=16)
C (n=14)
surface

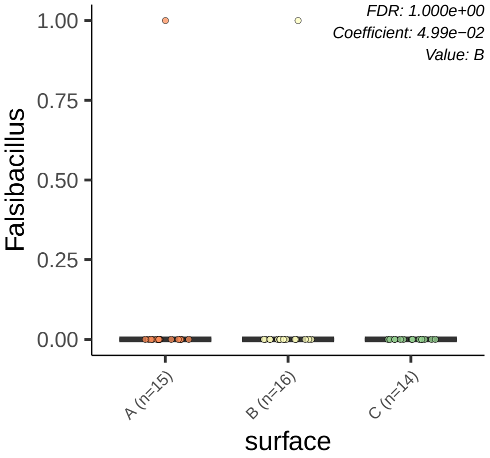

FDR: 1.000e+00
Coefficient: 4.99e−02
Value: B
1.00
0.75
0.50
0.25
0.00
Falsibacillus
A (n=15)
B (n=16)
C (n=14)
surface

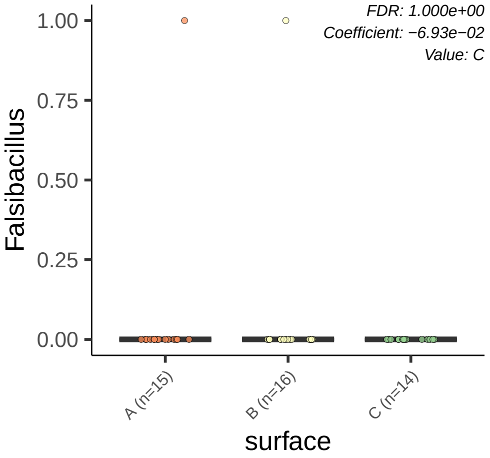
FDR: 1.000e+00
Coefficient: −6.93e−02
Value: C
1.00
0.75
0.50
0.25
0.00
Falsibacillus
A (n=15)
B (n=16)
C (n=14)
surface

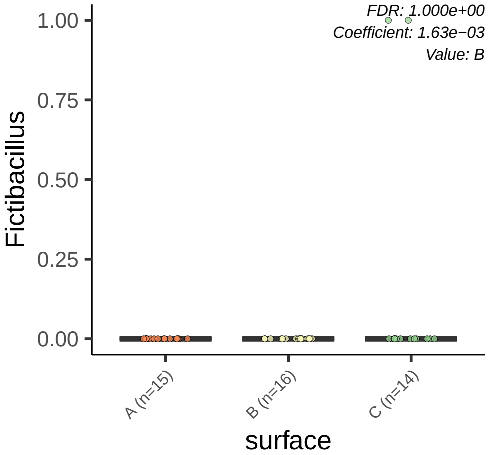

FDR: 1.000e+00
Coefficient: 1.63e−03
Value: B
1.00
0.75
0.50
0.25
0.00
Fictibacillus
A (n=15)
B (n=16)
C (n=14)
surface

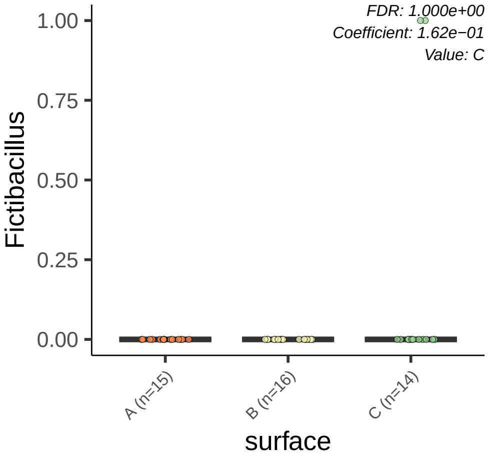
FDR: 1.000e+00
Coefficient: 1.62e−01
Value: C
Fictibacillus
1.00
0.75
0.50
0.25
0.00
A (n=15)
B (n=16)
C (n=14)
surface

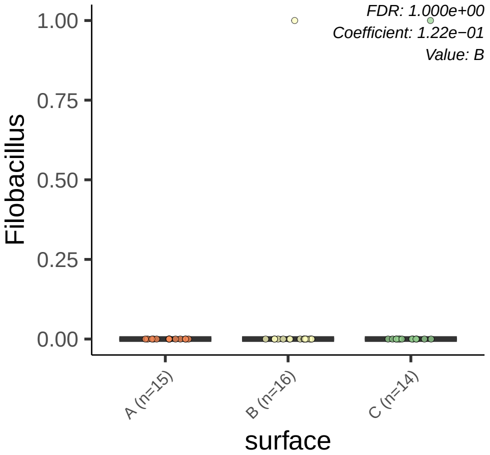
FDR: 1.000e+00
Coefficient: 1.22e−01
Value: B
Filobacillus
1.00
0.75
0.50
0.25
0.00
A (n=15)
B (n=16)
C (n=14)
surface

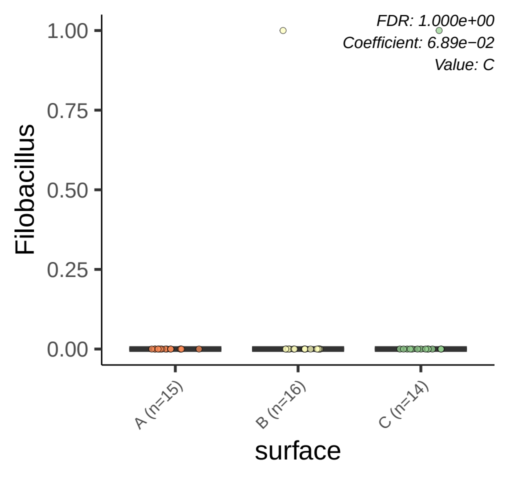
FDR: 1.000e+00
Coefficient: 6.89e−02
Value: C
Filobacillus
1.00
0.75
0.50
0.25
0.00
A (n=15)
B (n=16)
C (n=14)
surface

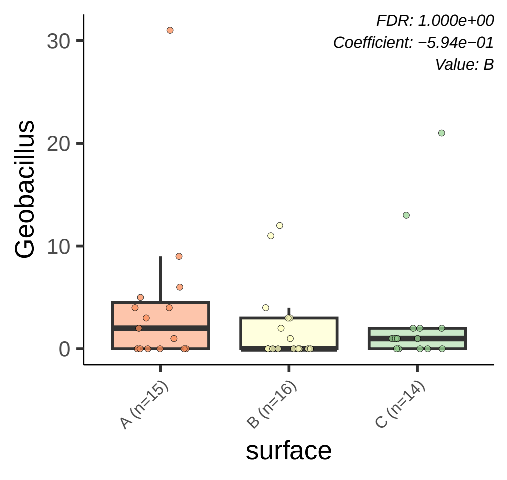

FDR: 1.000e+00
Coefficient: −5.94e−01
Value: B
Geobacillus
30
20
10
0
A (n=15)
B (n=16)
C (n=14)
surface

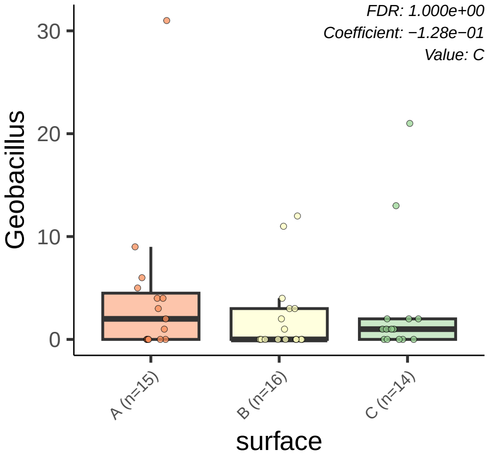

FDR: 1.000e+00
Coefficient: −1.28e−01
Value: C
Geobacillus
30
20
10
0
A (n=15)
B (n=16)
C (n=14)
surface

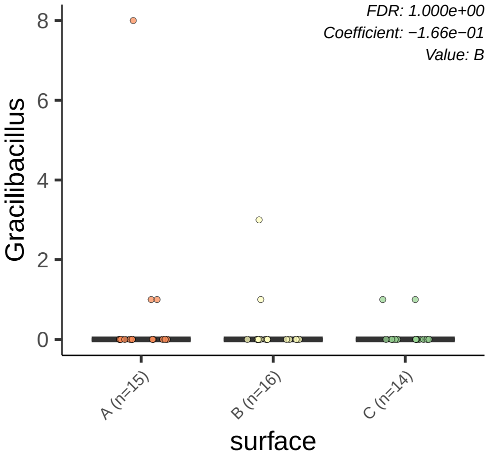
FDR: 1.000e+00
Coefficient: −1.66e−01
Value: B
Gracilibacillus
0
2
4
6
8
A (n=15)
B (n=16)
C (n=14)
surface

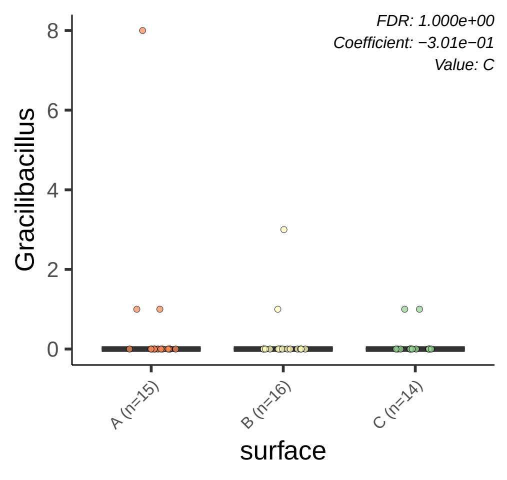

FDR: 1.000e+00
Coefficient: −3.01e−01
Value: C
Gracilibacillus
8
6
4
2
0
A (n=15)
B (n=16)
C (n=14)
surface

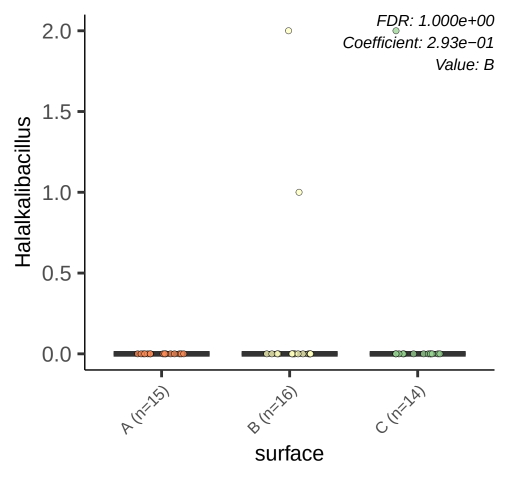

FDR: 1.000e+00
Coefficient: 2.93e−01
Value: B
2.0
1.5
1.0
0.5
0.0
Halalkalibacillus
A (n=15)
B (n=16)
C (n=14)
surface

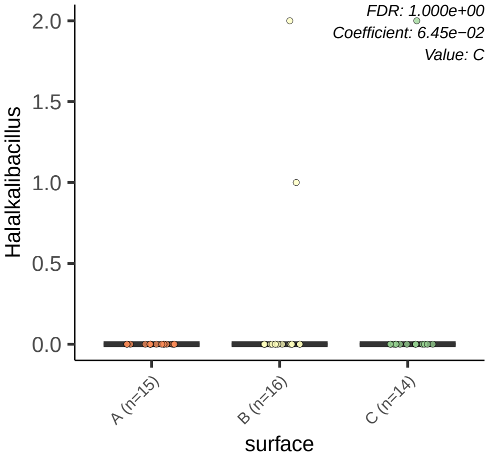
FDR: 1.000e+00
Coefficient: 6.45e−02
Value: C
Halalkalibacillus
2.0
1.5
1.0
0.5
0.0
A (n=15)
B (n=16)
C (n=14)
surface

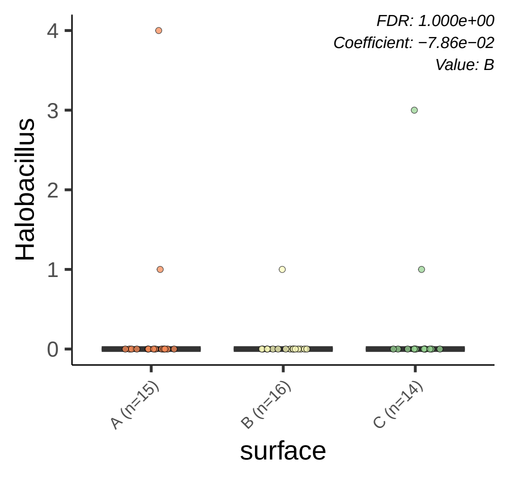
FDR: 1.000e+00
Coefficient: −7.86e−02
Value: B
Halobacillus
4
3
2
1
0
A (n=15)
B (n=16)
C (n=14)
surface

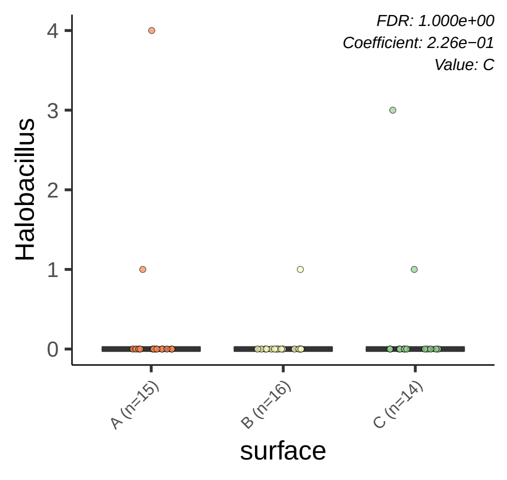
FDR: 1.000e+00
Coefficient: 2.26e−01
Value: C
Halobacillus
0
1
2
3
4
A (n=15)
B (n=16)
C (n=14)
surface

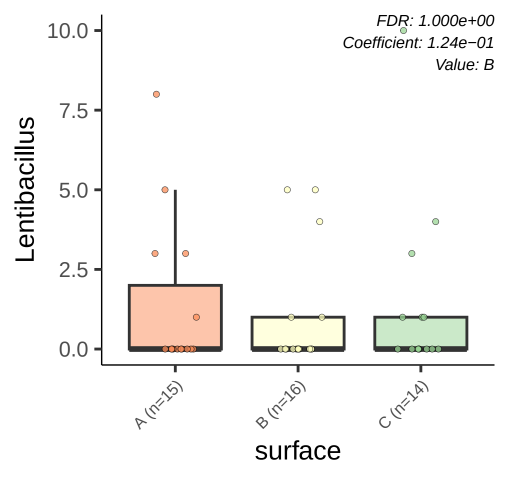
FDR: 1.000e+00
Coefficient: 1.24e−01
Value: B
Lentibacillus
10.0
7.5
5.0
2.5
0.0
A (n=15)
B (n=16)
C (n=14)
surface

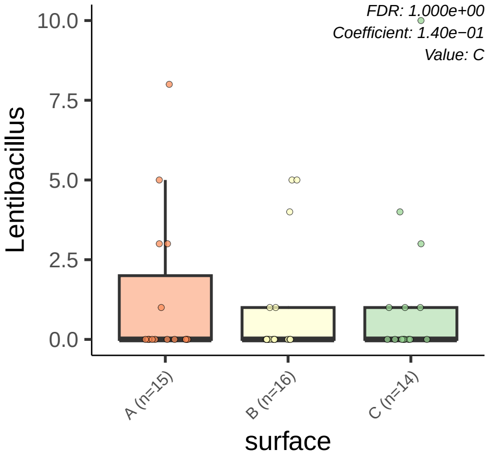
FDR: 1.000e+00
Coefficient: 1.40e−01
Value: C
Lentibacillus
10.0
7.5
5.0
2.5
0.0
A (n=15)
B (n=16)
C (n=14)
surface

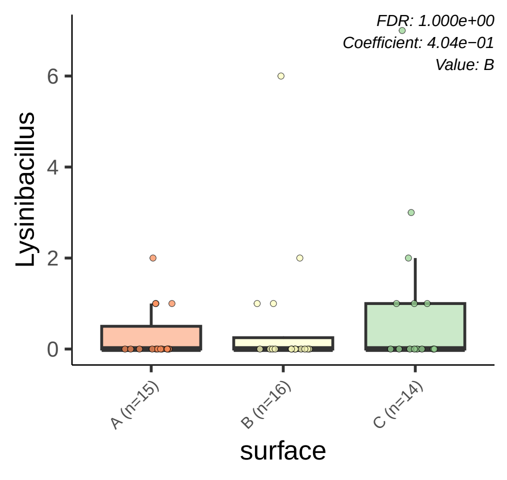

FDR: 1.000e+00
Coefficient: 4.04e−01
Value: B
Lysinibacillus
0
2
4
6
A (n=15)
B (n=16)
C (n=14)
surface

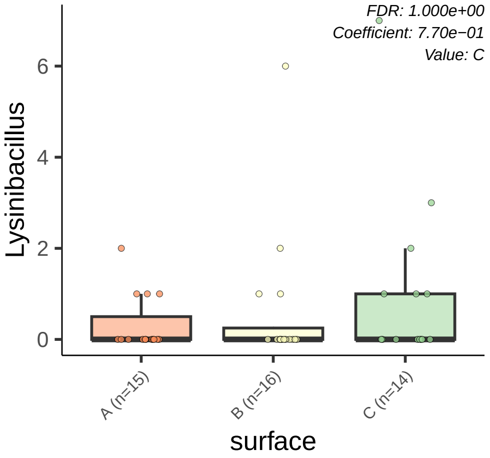
FDR: 1.000e+00
Coefficient: 7.70e−01
Value: C
Lysinibacillus
0
2
4
6
A (n=15)
B (n=16)
C (n=14)
surface

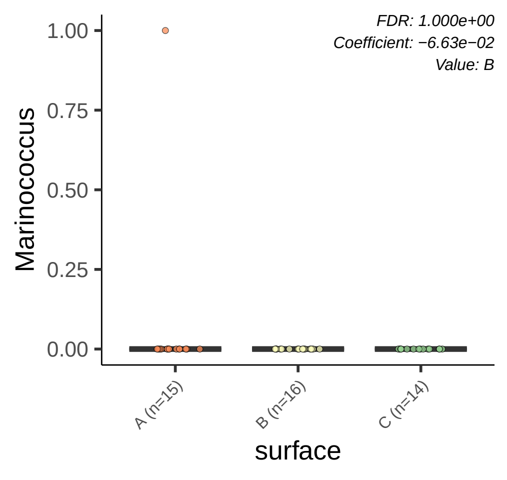
FDR: 1.000e+00
Coefficient: −6.63e−02
Value: B
Marinococcus
1.00
0.75
0.50
0.25
0.00
A (n=15)
B (n=16)
C (n=14)
surface

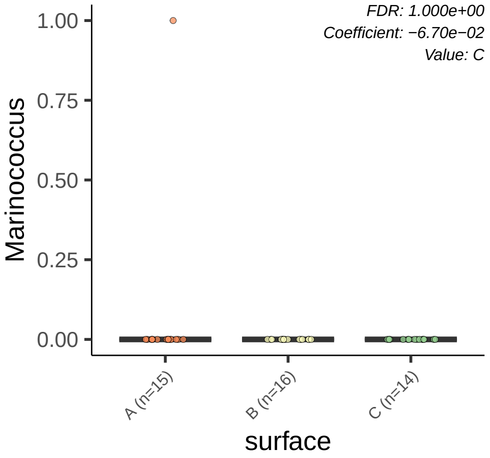
FDR: 1.000e+00
Coefficient: −6.70e−02
Value: C
Marinococcus
1.00
0.75
0.50
0.25
0.00
A (n=15)
B (n=16)
C (n=14)
surface

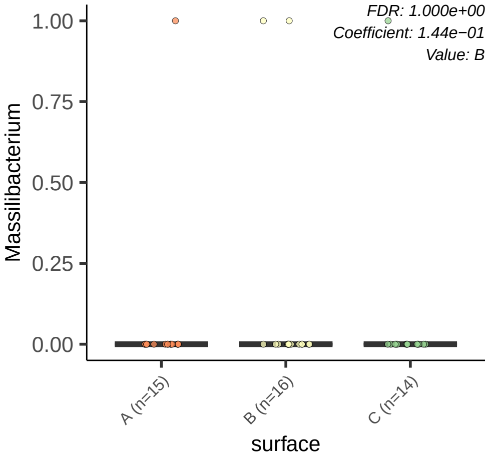

FDR: 1.000e+00
Coefficient: 1.44e−01
Value: B
Massilibacterium
1.00
0.75
0.50
0.25
0.00
A (n=15)
B (n=16)
C (n=14)
surface

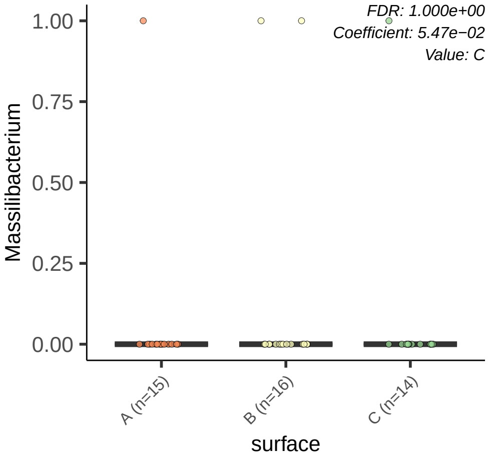
FDR: 1.000e+00
Coefficient: 5.47e−02
Value: C
Massilibacterium
1.00
0.75
0.50
0.25
0.00
A (n=15)
B (n=16)
C (n=14)
surface

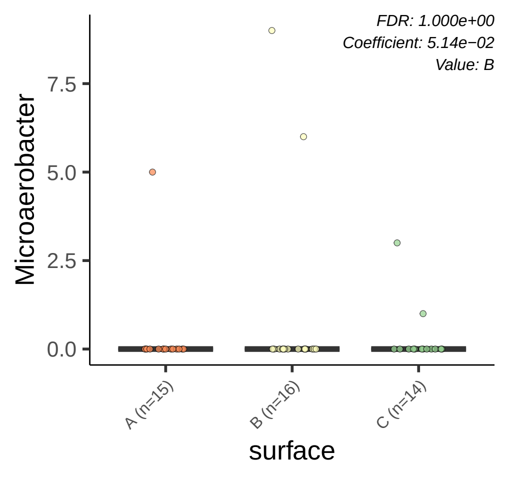

FDR: 1.000e+00
Coefficient: 5.14e−02
Value: B
Microaerobacter
7.5
5.0
2.5
0.0
A (n=15)
B (n=16)
C (n=14)
surface

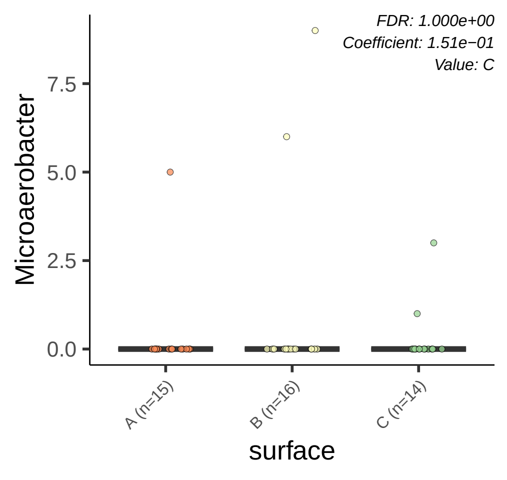
FDR: 1.000e+00
Coefficient: 1.51e−01
Value: C
Microaerobacter
7.5
5.0
2.5
0.0
A (n=15)
B (n=16)
C (n=14)
surface

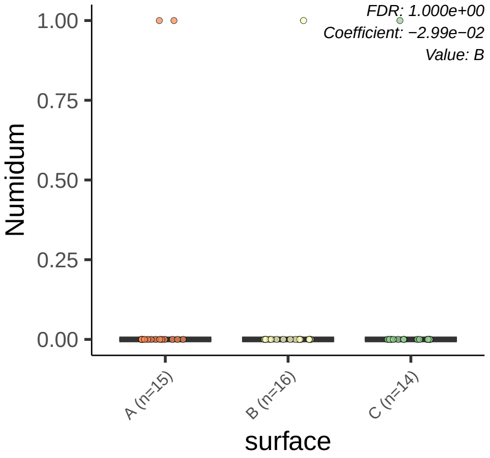

FDR: 1.000e+00
Coefficient: −2.99e−02
Value: B
Numidum
1.00
0.75
0.50
0.25
0.00
A (n=15)
B (n=16)
C (n=14)
surface

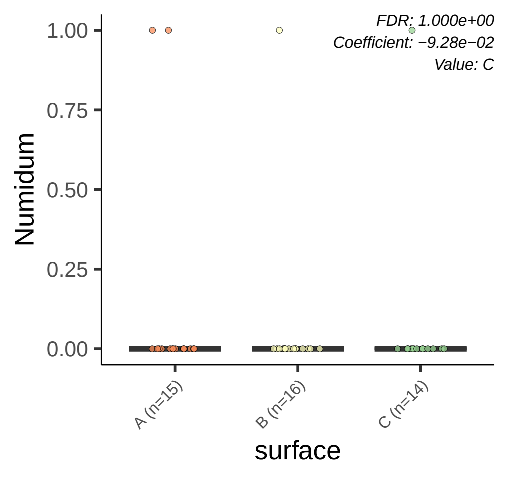
FDR: 1.000e+00
Coefficient: −9.28e−02
Value: C
Numidum
1.00
0.75
0.50
0.25
0.00
A (n=15)
B (n=16)
C (n=14)
surface

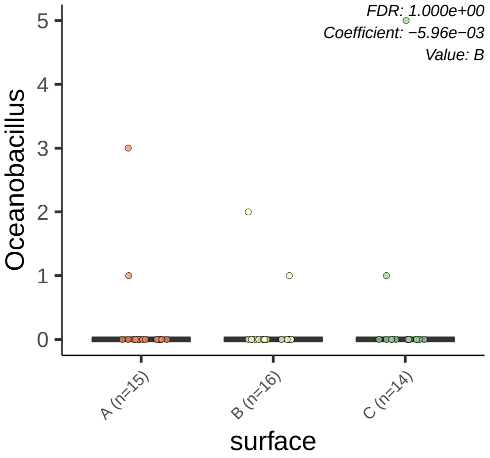

FDR: 1.000e+00
Coefficient: −5.96e−03
Value: B
Oceanobacillus
5
4
3
2
1
0
A (n=15)
B (n=16)
C (n=14)
surface

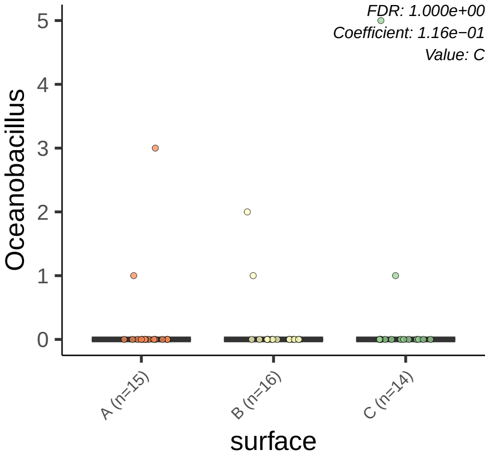

FDR: 1.000e+00
Coefficient: 1.16e−01
Value: C
Oceanobacillus
5
4
3
2
1
0
A (n=15)
B (n=16)
C (n=14)
surface

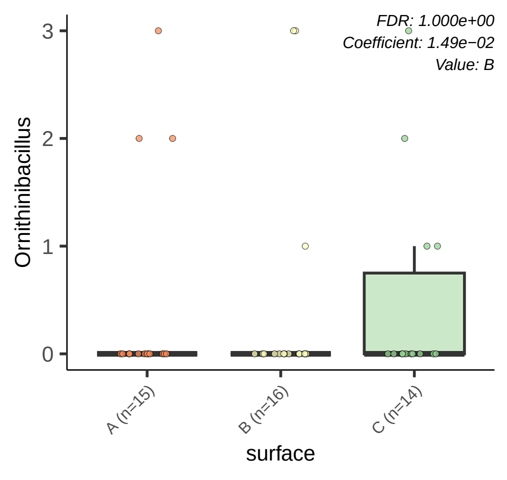
FDR: 1.000e+00
Coefficient: 1.49e−02
Value: B
Ornithinibacillus
3
2
1
0
A (n=15)
B (n=16)
C (n=14)
surface

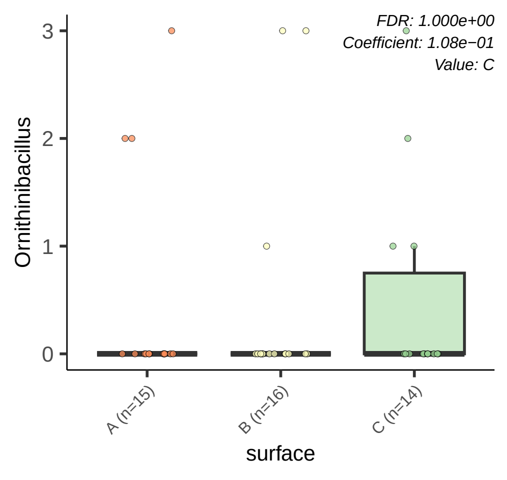
FDR: 1.000e+00
Coefficient: 1.08e−01
Value: C
Ornithinibacillus
0
1
2
3
A (n=15)
B (n=16)
C (n=14)
surface

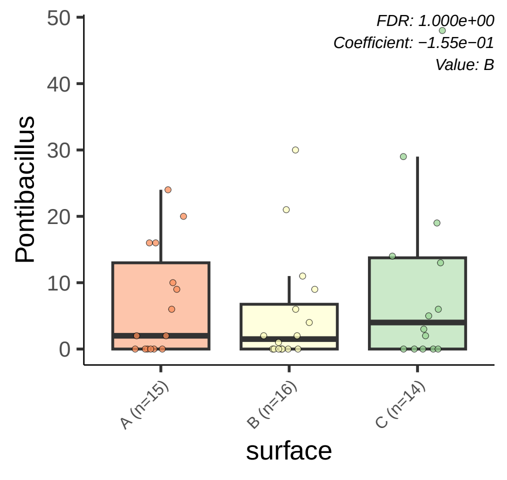

FDR: 1.000e+00
Coefficient: −1.55e−01
Value: B
Pontibacillus
0
10
20
30
40
50
A (n=15)
B (n=16)
C (n=14)
surface

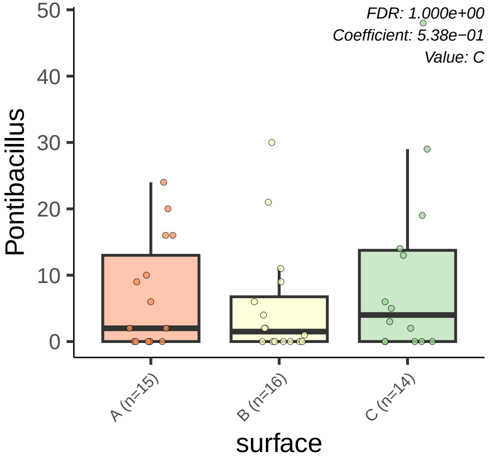

FDR: 1.000e+00
Coefficient: 5.38e−01
Value: C
Pontibacillus
0
10
20
30
40
50
A (n=15)
B (n=16)
C (n=14)
surface

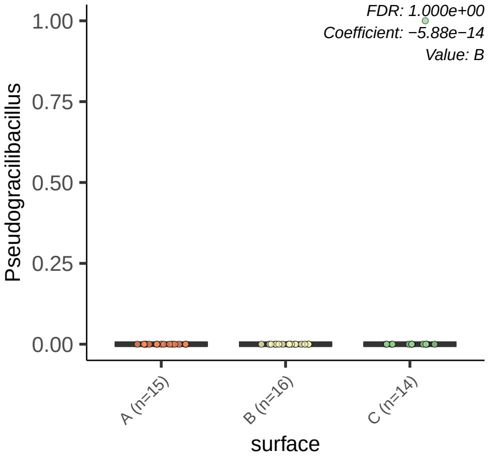

FDR: 1.000e+00
Coefficient: −5.88e−14
Value: B
1.00
0.75
0.50
0.25
0.00
Pseudogracilibacillus
A (n=15)
B (n=16)
C (n=14)
surface

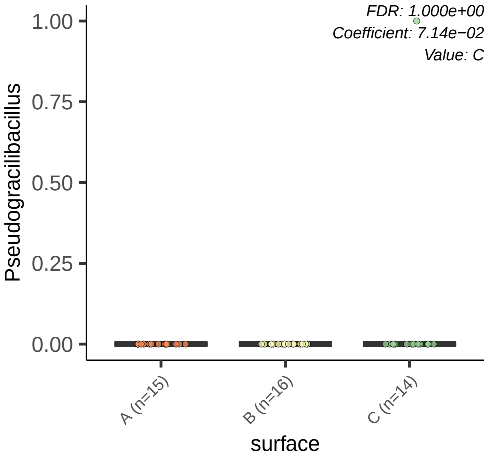

FDR: 1.000e+00
Coefficient: 7.14e−02
Value: C
Pseudogracilibacillus
1.00
0.75
0.50
0.25
0.00
A (n=15)
B (n=16)
C (n=14)
surface

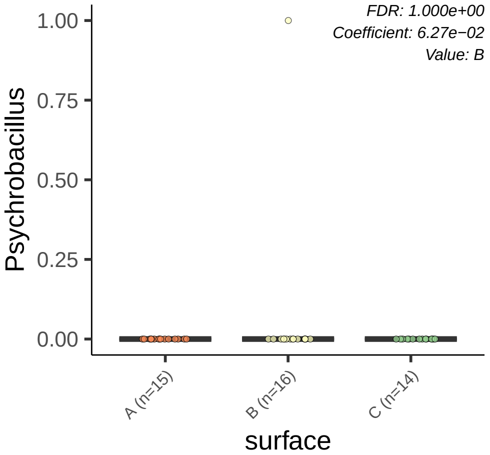
FDR: 1.000e+00
Coefficient: 6.27e−02
Value: B
Psychrobacillus
1.00
0.75
0.50
0.25
0.00
A (n=15)
B (n=16)
C (n=14)
surface

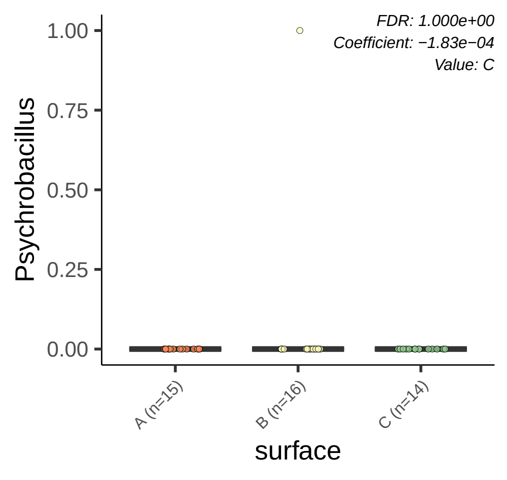

FDR: 1.000e+00
Coefficient: −1.83e−04
Value: C
Psychrobacillus
1.00
0.75
0.50
0.25
0.00
A (n=15)
B (n=16)
C (n=14)
surface

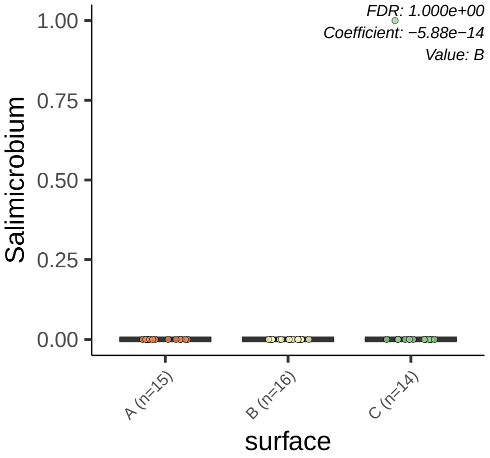

FDR: 1.000e+00
Coefficient: −5.88e−14
Value: B
Salimicrobium
1.00
0.75
0.50
0.25
0.00
A (n=15)
B (n=16)
C (n=14)
surface

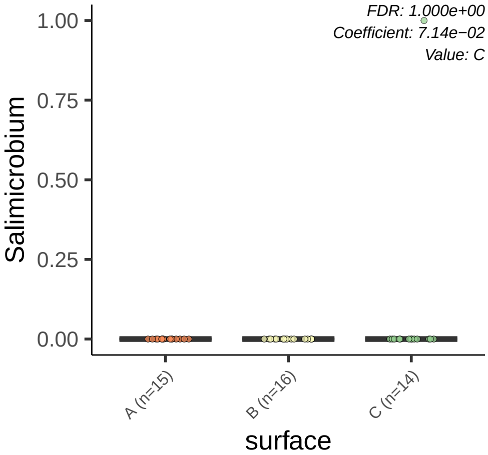

FDR: 1.000e+00
Coefficient: 7.14e−02
Value: C
Salimicrobium
1.00
0.75
0.50
0.25
0.00
A (n=15)
B (n=16)
C (n=14)
surface

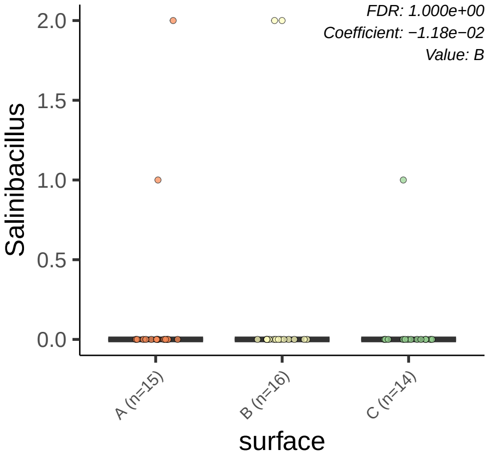
FDR: 1.000e+00
Coefficient: −1.18e−02
Value: B
Salinibacillus
2.0
1.5
1.0
0.5
0.0
A (n=15)
B (n=16)
C (n=14)
surface

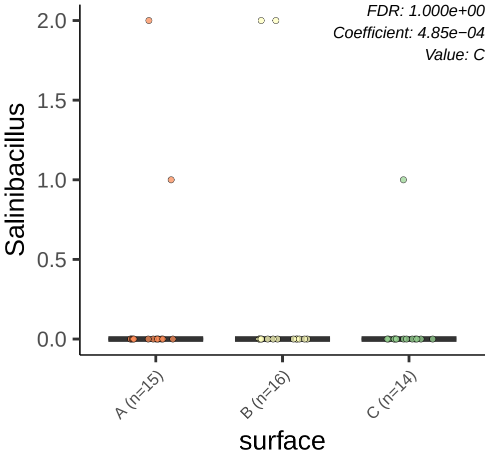
FDR: 1.000e+00
Coefficient: 4.85e−04
Value: C
Salinibacillus
2.0
1.5
1.0
0.5
0.0
A (n=15)
B (n=16)
C (n=14)
surface

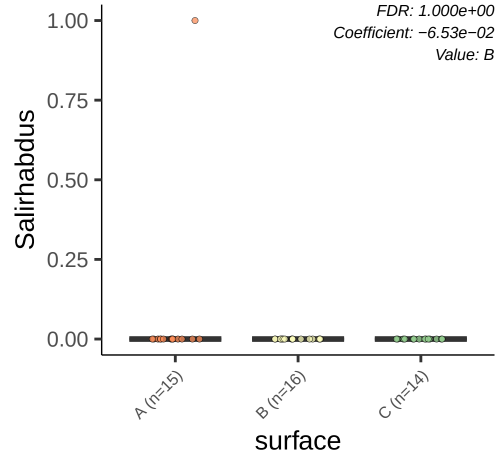
FDR: 1.000e+00
Coefficient: −6.53e−02
Value: B
1.00
0.75
0.50
0.25
0.00
Salirhabdus
A (n=15)
B (n=16)
C (n=14)
surface

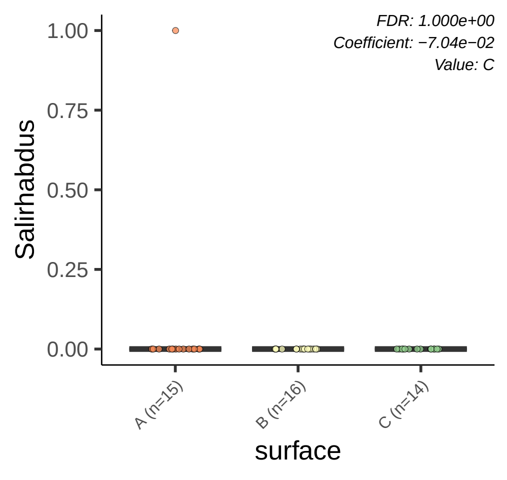
FDR: 1.000e+00
Coefficient: −7.04e−02
Value: C
Salirhabdus
1.00
0.75
0.50
0.25
0.00
A (n=15)
B (n=16)
C (n=14)
surface

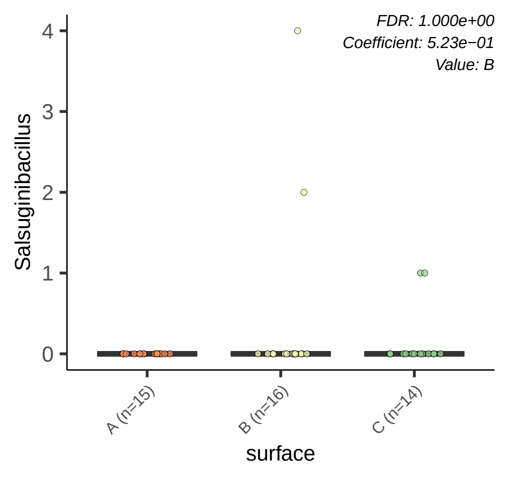
FDR: 1.000e+00
Coefficient: 5.23e−01
Value: B
Salsuginibacillus
0
1
2
3
4
A (n=15)
B (n=16)
C (n=14)
surface

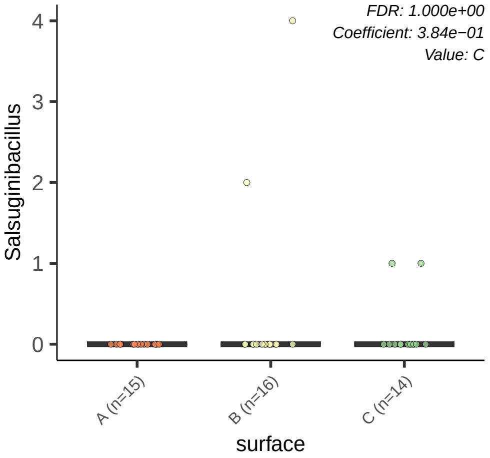

FDR: 1.000e+00
Coefficient: 3.84e−01
Value: C
Salsuginibacillus
0
1
2
3
4
A (n=15)
B (n=16)
C (n=14)
surface

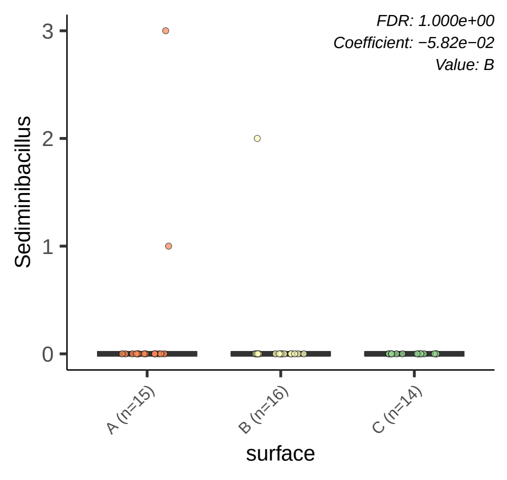

FDR: 1.000e+00
Coefficient: −5.82e−02
Value: B
Sediminibacillus
3
2
1
0
A (n=15)
B (n=16)
C (n=14)
surface

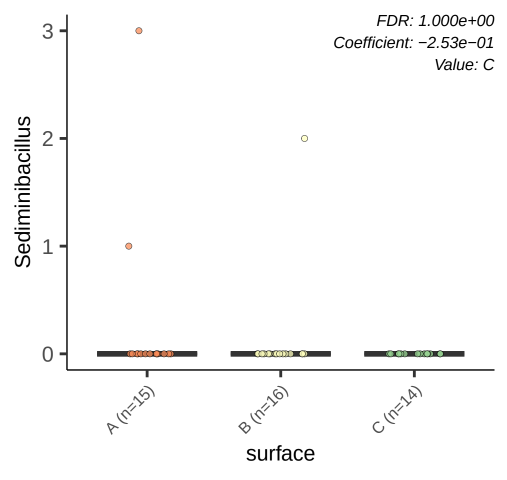
FDR: 1.000e+00
Coefficient: −2.53e−01
Value: C
Sediminibacillus
3
2
1
0
A (n=15)
B (n=16)
C (n=14)
surface

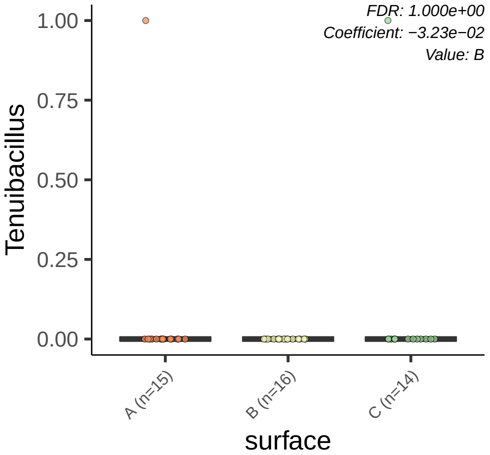
FDR: 1.000e+00
Coefficient: −3.23e−02
Value: B
Tenuibacillus
1.00
0.75
0.50
0.25
0.00
A (n=15)
B (n=16)
C (n=14)
surface

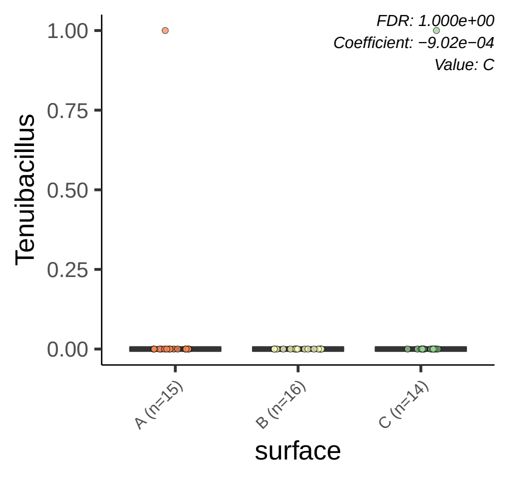

FDR: 1.000e+00
Coefficient: −9.02e−04
Value: C
Tenuibacillus
1.00
0.75
0.50
0.25
0.00
A (n=15)
B (n=16)
C (n=14)
surface

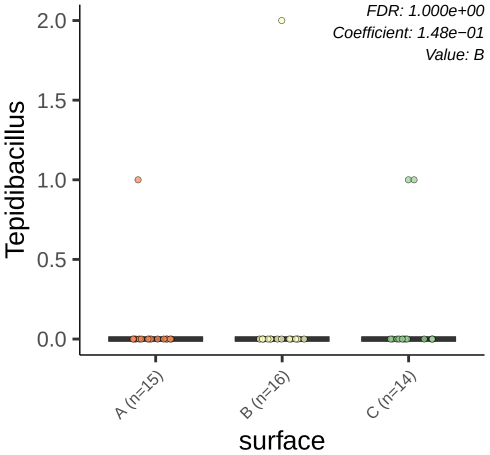
FDR: 1.000e+00
Coefficient: 1.48e−01
Value: B
Tepidibacillus
2.0
1.5
1.0
0.5
0.0
A (n=15)
B (n=16)
C (n=14)
surface

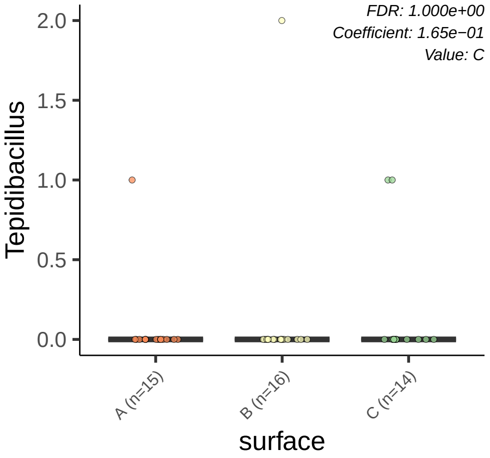

FDR: 1.000e+00
Coefficient: 1.65e−01
Value: C
Tepidibacillus
2.0
1.5
1.0
0.5
0.0
A (n=15)
B (n=16)
C (n=14)
surface

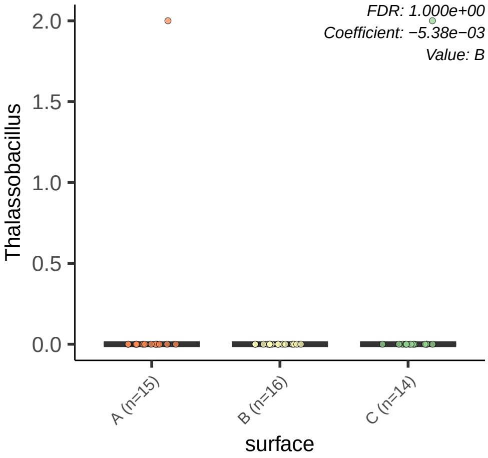
FDR: 1.000e+00
Coefficient: −5.38e−03
Value: B
Thalassobacillus
2.0
1.5
1.0
0.5
0.0
A (n=15)
B (n=16)
C (n=14)
surface

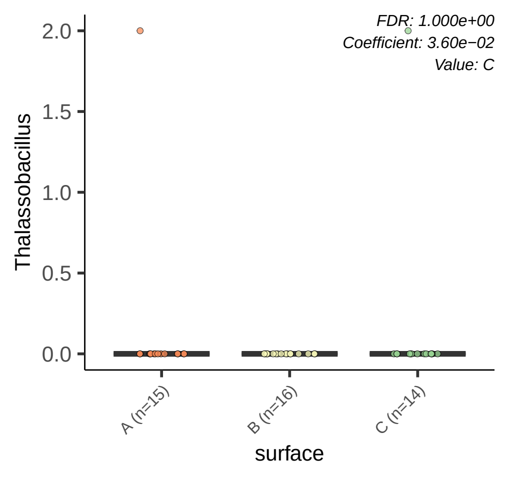

FDR: 1.000e+00
Coefficient: 3.60e−02
Value: C
Thalassobacillus
2.0
1.5
1.0
0.5
0.0
A (n=15)
B (n=16)
C (n=14)
surface

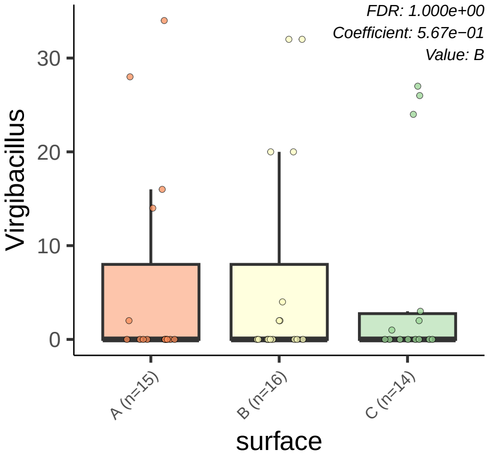

FDR: 1.000e+00
Coefficient: 5.67e−01
Value: B
Virgibacillus
0
10
20
30
A (n=15)
B (n=16)
C (n=14)
surface

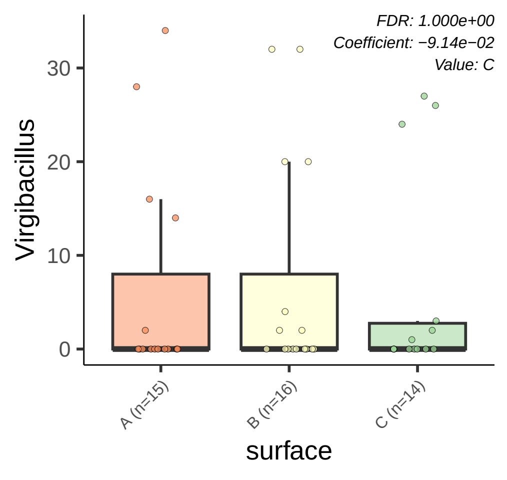
FDR: 1.000e+00
Coefficient: −9.14e−02
Value: C
Virgibacillus
0
10
20
30
A (n=15)
B (n=16)
C (n=14)
surface

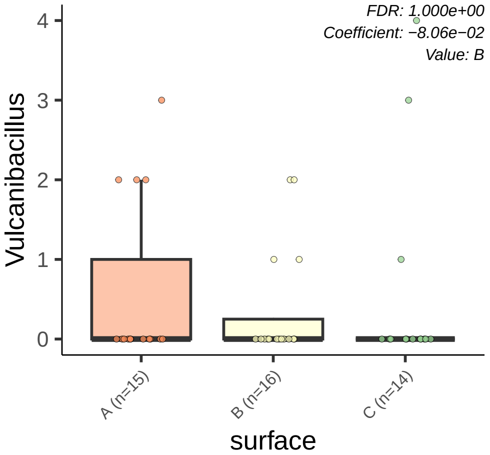
FDR: 1.000e+00
Coefficient: −8.06e−02
Value: B
Vulcanibacillus
0
1
2
3
4
A (n=15)
B (n=16)
C (n=14)
surface

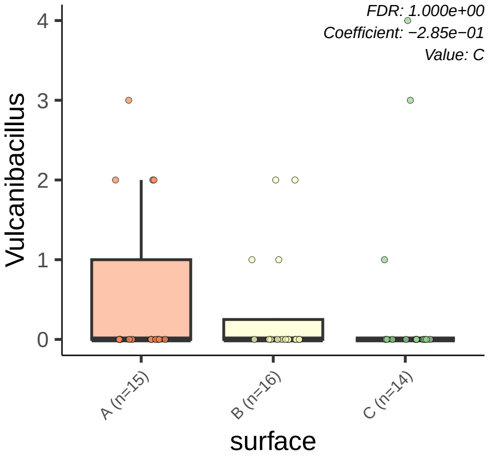
FDR: 1.000e+00
Coefficient: −2.85e−01
Value: C
Vulcanibacillus
0
1
2
3
4
A (n=15)
B (n=16)
C (n=14)
surface

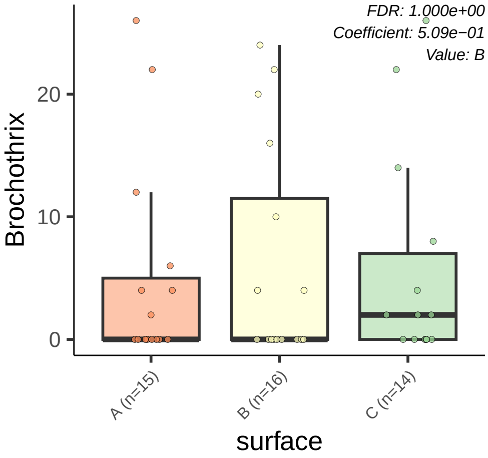

FDR: 1.000e+00
Coefficient: 5.09e−01
Value: B
Brochothrix
20
10
0
A (n=15)
B (n=16)
C (n=14)
surface

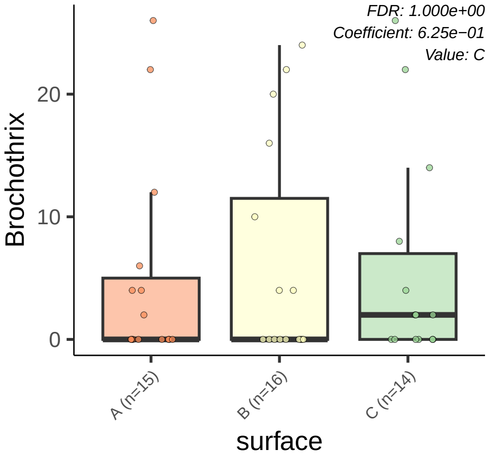

FDR: 1.000e+00
Coefficient: 6.25e−01
Value: C
Brochothrix
20
10
0
A (n=15)
B (n=16)
C (n=14)
surface

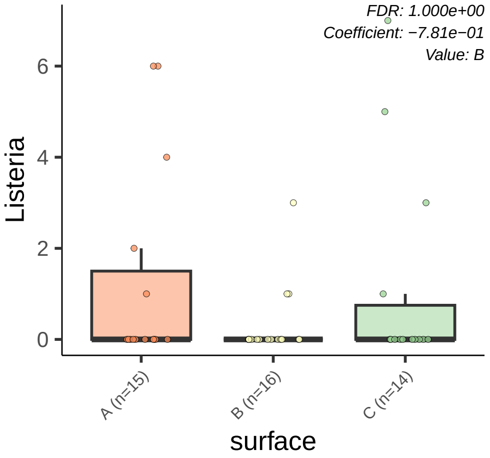

FDR: 1.000e+00
Coefficient: −7.81e−01
Value: B
Listeria
0
2
4
6
A (n=15)
B (n=16)
C (n=14)
surface

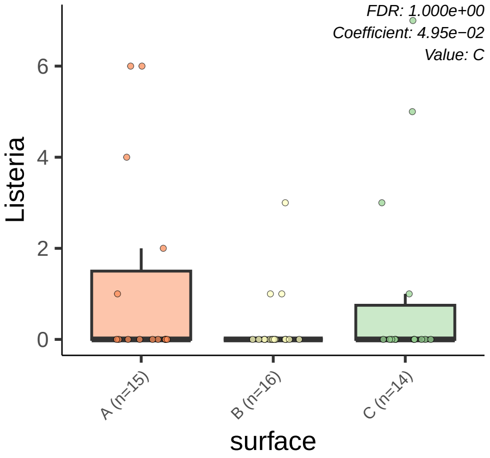
FDR: 1.000e+00
Coefficient: 4.95e−02
Value: C
Listeria
0
2
4
6
A (n=15)
B (n=16)
C (n=14)
surface

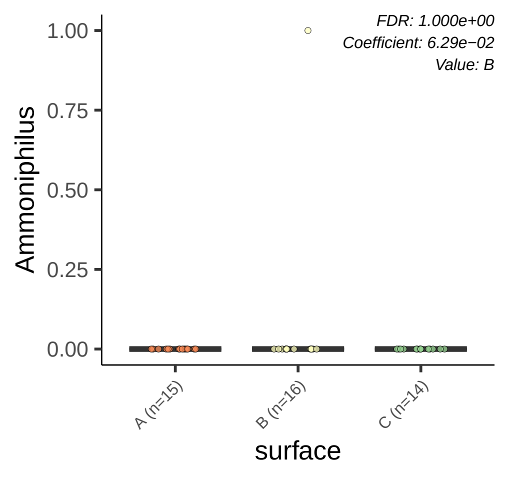

FDR: 1.000e+00
Coefficient: 6.29e−02
Value: B
Ammoniphilus
1.00
0.75
0.50
0.25
0.00
A (n=15)
B (n=16)
C (n=14)
surface

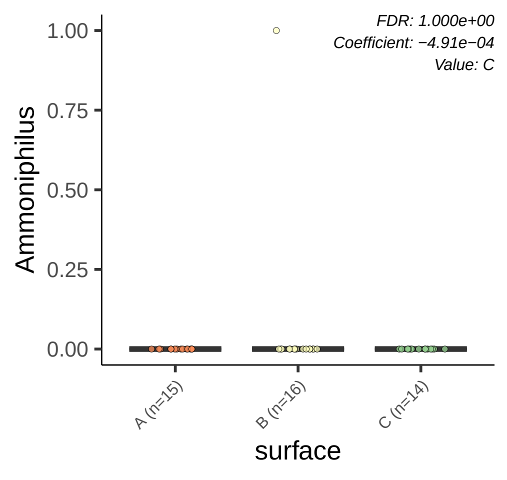
FDR: 1.000e+00
Coefficient: −4.91e−04
Value: C
Ammoniphilus
1.00
0.75
0.50
0.25
0.00
A (n=15)
B (n=16)
C (n=14)
surface

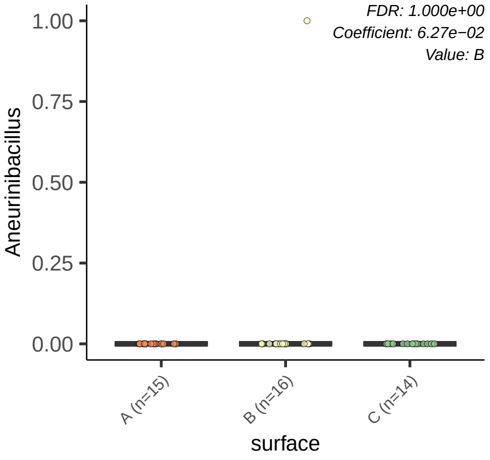
FDR: 1.000e+00
Coefficient: 6.27e−02
Value: B
Aneurinibacillus
1.00
0.75
0.50
0.25
0.00
A (n=15)
B (n=16)
C (n=14)
surface

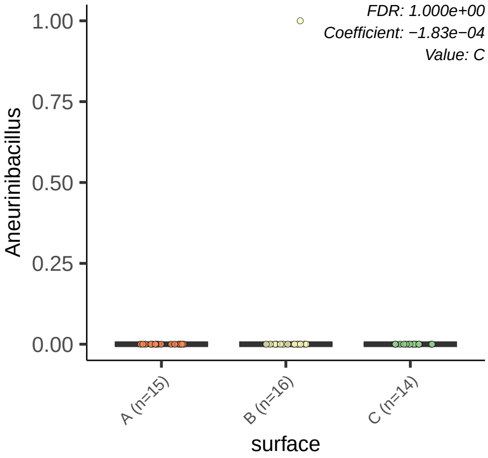

FDR: 1.000e+00
Coefficient: −1.83e−04
Value: C
1.00
0.75
0.50
0.25
0.00
Aneurinibacillus
A (n=15)
B (n=16)
C (n=14)
surface

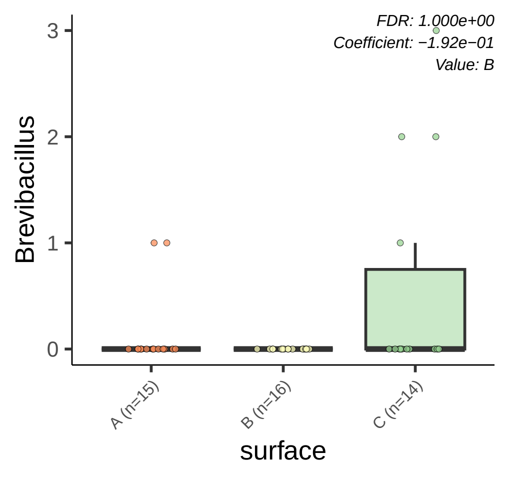
FDR: 1.000e+00
Coefficient: −1.92e−01
Value: B
Brevibacillus
3
2
1
0
A (n=15)
B (n=16)
C (n=14)
surface

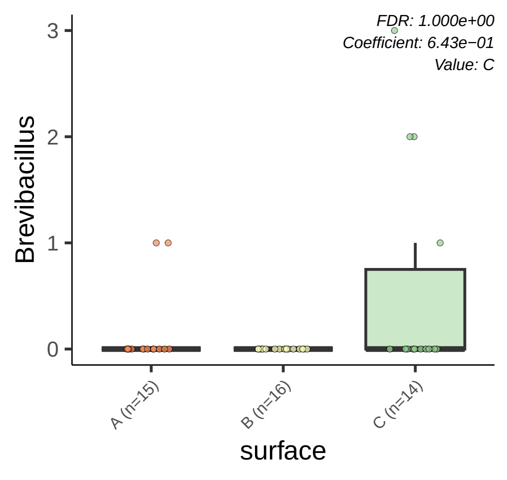

FDR: 1.000e+00
Coefficient: 6.43e−01
Value: C
Brevibacillus
3
2
1
0
A (n=15)
B (n=16)
C (n=14)
surface

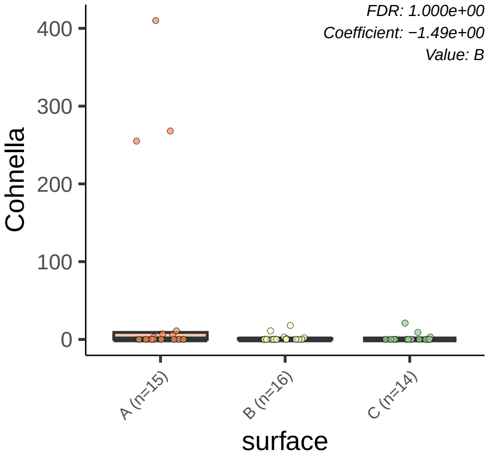
FDR: 1.000e+00
Coefficient: −1.49e+00
Value: B
Cohnella
400
300
200
100
0
A (n=15)
B (n=16)
C (n=14)
surface

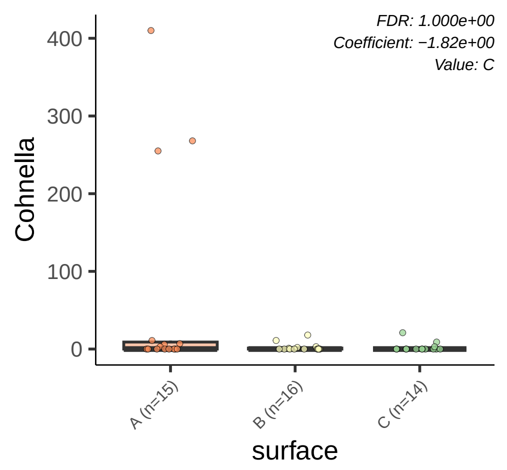

FDR: 1.000e+00
Coefficient: −1.82e+00
Value: C
Cohnella
400
300
200
100
0
A (n=15)
B (n=16)
C (n=14)
surface

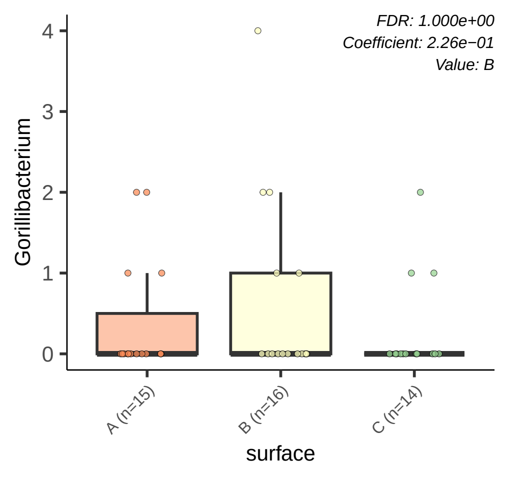
FDR: 1.000e+00
Coefficient: 2.26e−01
Value: B
Gorillibacterium
0
1
2
3
4
A (n=15)
B (n=16)
C (n=14)
surface

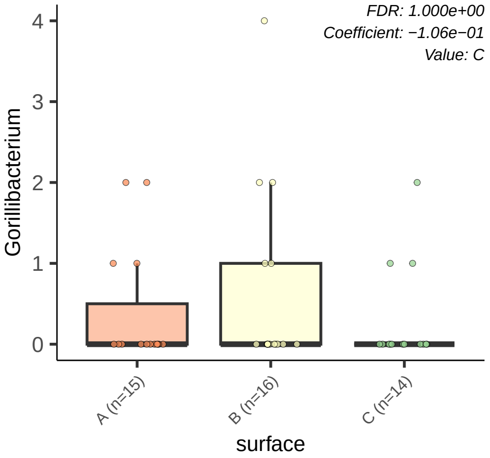

FDR: 1.000e+00
Coefficient: −1.06e−01
Value: C
Gorillibacterium
4
3
2
1
0
A (n=15)
B (n=16)
C (n=14)
surface

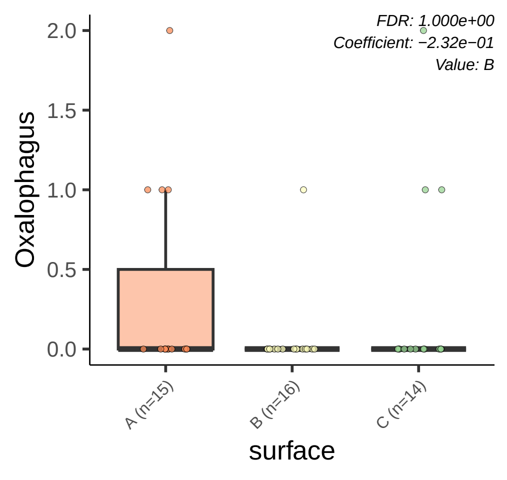
FDR: 1.000e+00
Coefficient: −2.32e−01
Value: B
Oxalophagus
2.0
1.5
1.0
0.5
0.0
A (n=15)
B (n=16)
C (n=14)
surface

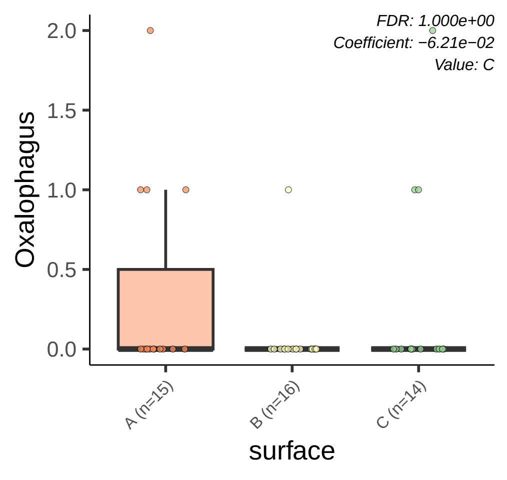

FDR: 1.000e+00
Coefficient: −6.21e−02
Value: C
Oxalophagus
2.0
1.5
1.0
0.5
0.0
A (n=15)
B (n=16)
C (n=14)
surface

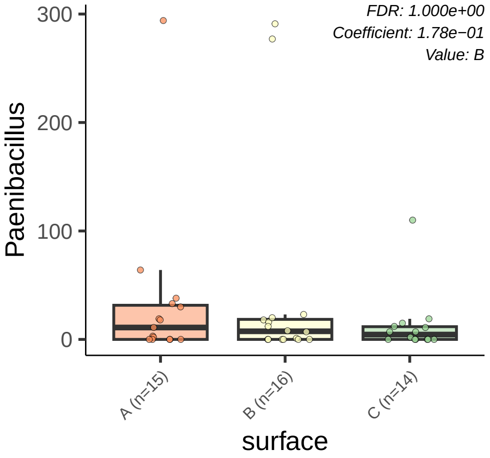
FDR: 1.000e+00
Coefficient: 1.78e−01
Value: B
Paenibacillus
300
200
100
0
A (n=15)
B (n=16)
C (n=14)
surface

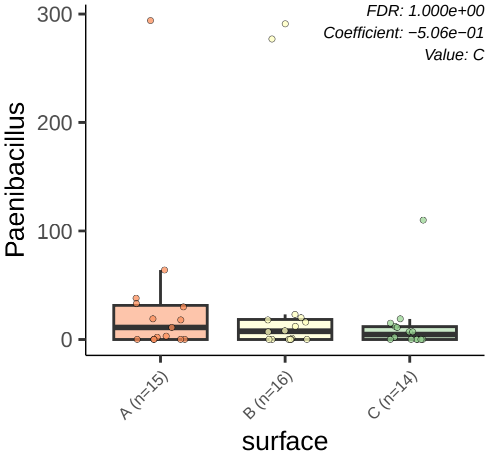
FDR: 1.000e+00
Coefficient: −5.06e−01
Value: C
Paenibacillus
300
200
100
0
A (n=15)
B (n=16)
C (n=14)
surface

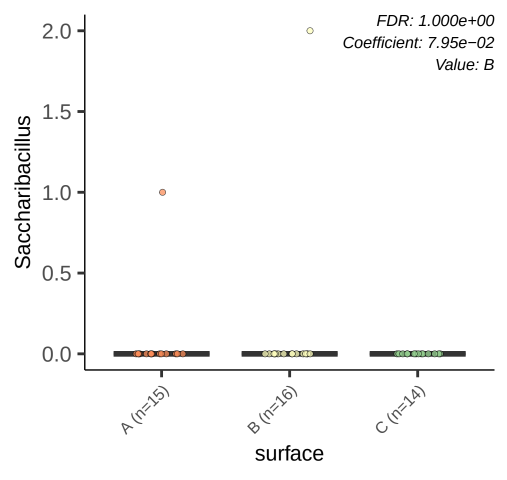
FDR: 1.000e+00
Coefficient: 7.95e−02
Value: B
Saccharibacillus
2.0
1.5
1.0
0.5
0.0
A (n=15)
B (n=16)
C (n=14)
surface

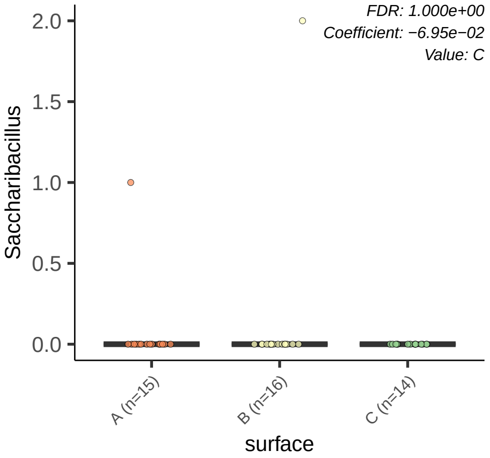

FDR: 1.000e+00
Coefficient: −6.95e−02
Value: C
Saccharibacillus
2.0
1.5
1.0
0.5
0.0
A (n=15)
B (n=16)
C (n=14)
surface

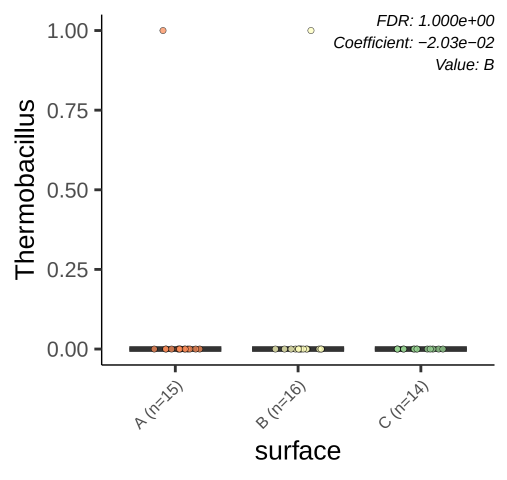
FDR: 1.000e+00
Coefficient: −2.03e−02
Value: B
Thermobacillus
1.00
0.75
0.50
0.25
0.00
A (n=15)
B (n=16)
C (n=14)
surface

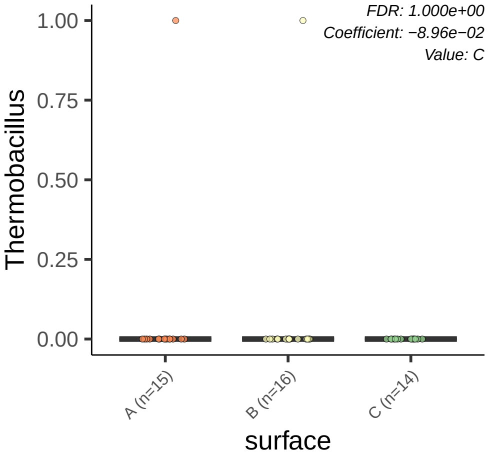

FDR: 1.000e+00
Coefficient: −8.96e−02
Value: C
1.00
0.75
0.50
0.25
0.00
Thermobacillus
A (n=15)
B (n=16)
C (n=14)
surface

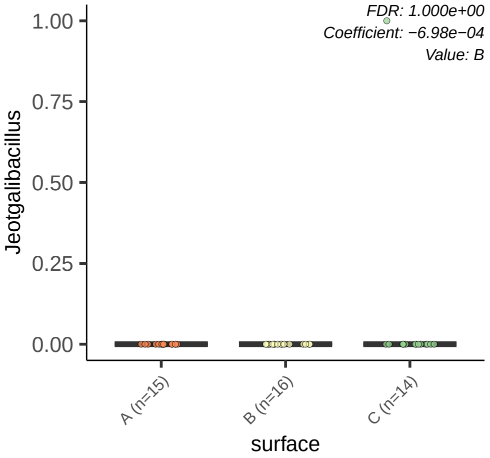
FDR: 1.000e+00
Coefficient: −6.98e−04
Value: B
Jeotgalibacillus
1.00
0.75
0.50
0.25
0.00
A (n=15)
B (n=16)
C (n=14)
surface

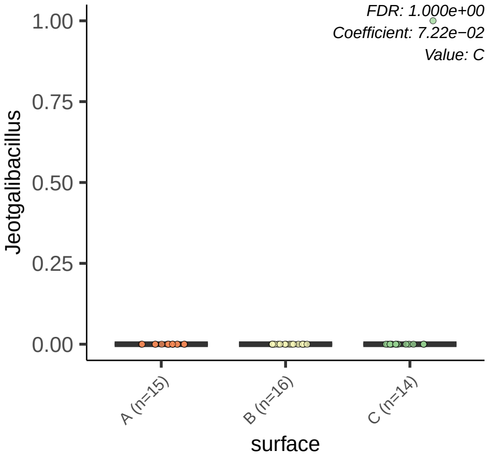
FDR: 1.000e+00
Coefficient: 7.22e−02
Value: C
Jeotgalibacillus
1.00
0.75
0.50
0.25
0.00
A (n=15)
B (n=16)
C (n=14)
surface

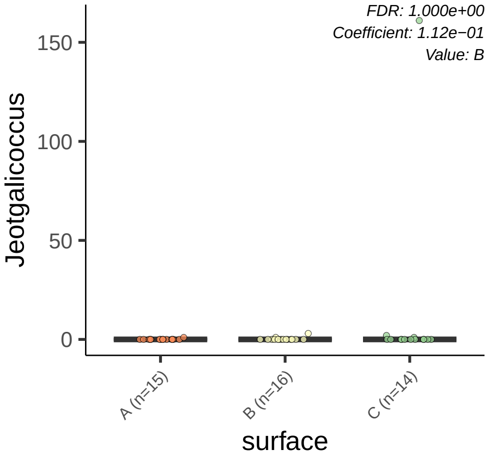

FDR: 1.000e+00
Coefficient: 1.12e−01
Value: B
Jeotgalicoccus
150
100
50
0
A (n=15)
B (n=16)
C (n=14)
surface

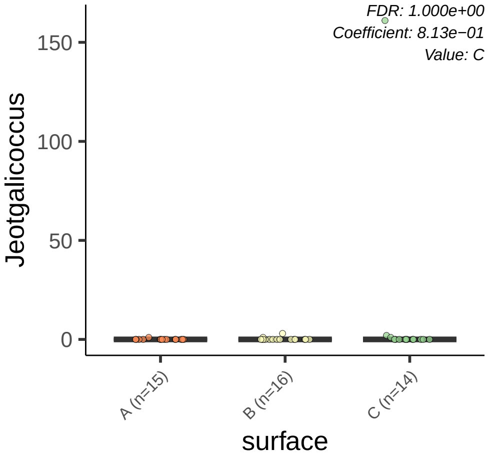
FDR: 1.000e+00
Coefficient: 8.13e−01
Value: C
Jeotgalicoccus
150
100
50
0
A (n=15)
B (n=16)
C (n=14)
surface

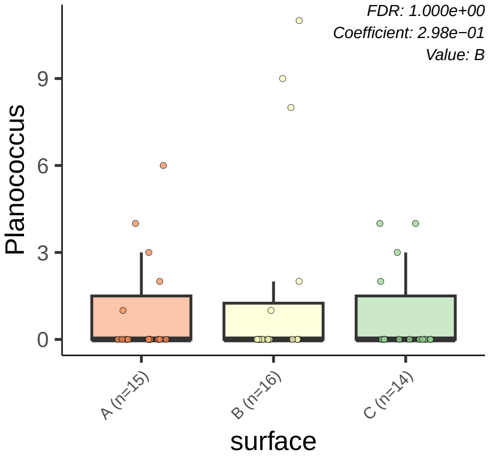

FDR: 1.000e+00
Coefficient: 2.98e−01
Value: B
Planococcus
0
3
6
9
A (n=15)
B (n=16)
C (n=14)
surface

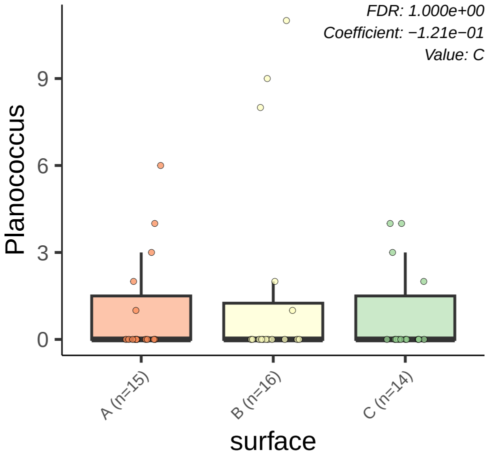
FDR: 1.000e+00
Coefficient: −1.21e−01
Value: C
Planococcus
0
3
6
9
A (n=15)
B (n=16)
C (n=14)
surface

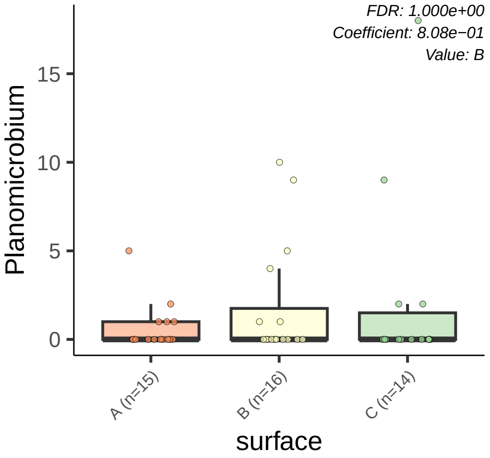

FDR: 1.000e+00
Coefficient: 8.08e−01
Value: B
Planomicrobium
15
10
5
0
A (n=15)
B (n=16)
C (n=14)
surface

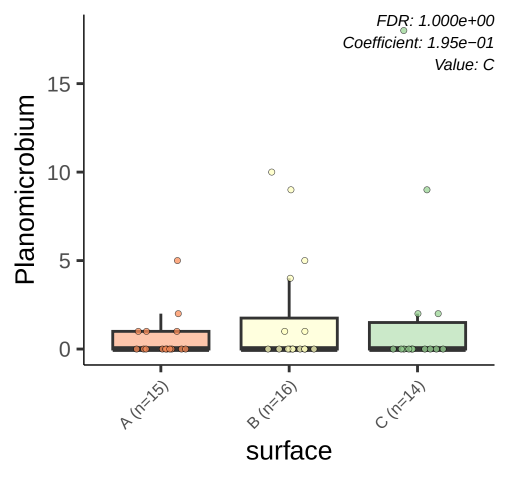
FDR: 1.000e+00
Coefficient: 1.95e−01
Value: C
Planomicrobium
15
10
5
0
A (n=15)
B (n=16)
C (n=14)
surface

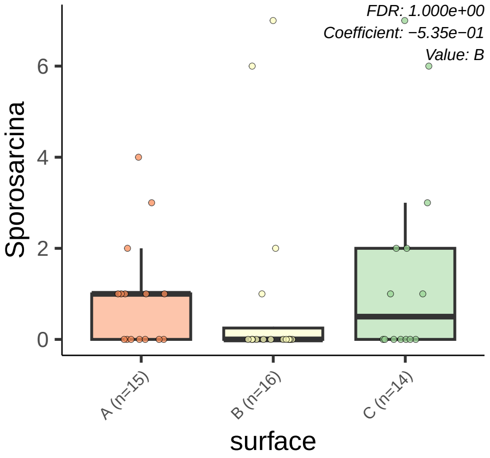

FDR: 1.000e+00
Coefficient: −5.35e−01
Value: B
Sporosarcina
0
2
4
6
A (n=15)
B (n=16)
C (n=14)
surface

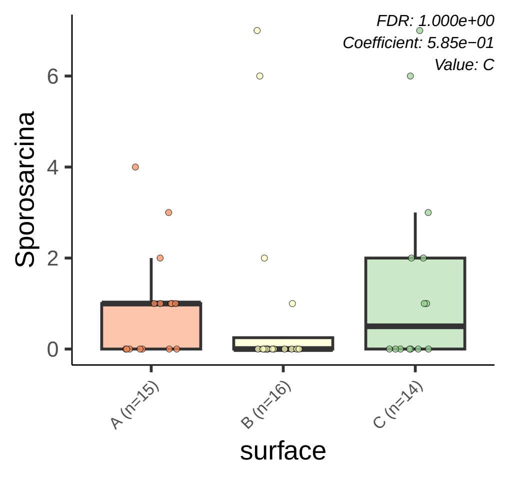

FDR: 1.000e+00
Coefficient: 5.85e−01
Value: C
Sporosarcina
0
2
4
6
A (n=15)
B (n=16)
C (n=14)
surface

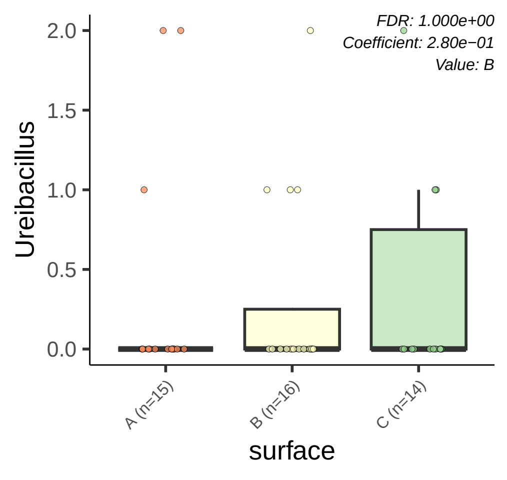

FDR: 1.000e+00
Coefficient: 2.80e−01
Value: B
Ureibacillus
2.0
1.5
1.0
0.5
0.0
A (n=15)
B (n=16)
C (n=14)
surface

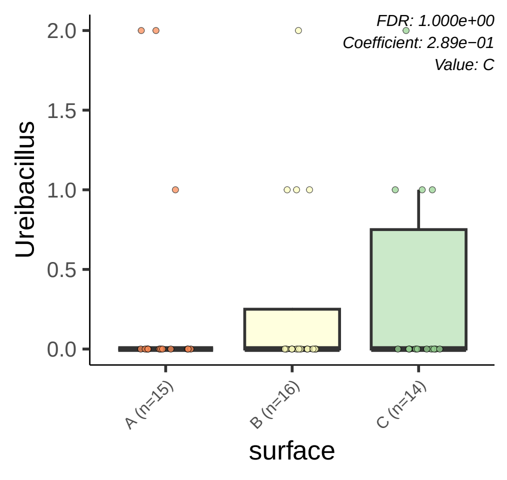

FDR: 1.000e+00
Coefficient: 2.89e−01
Value: C
Ureibacillus
2.0
1.5
1.0
0.5
0.0
A (n=15)
B (n=16)
C (n=14)
surface

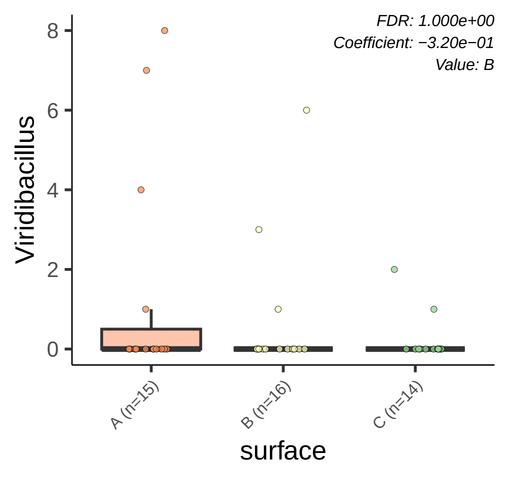
FDR: 1.000e+00
Coefficient: −3.20e−01
Value: B
Viridibacillus
0
2
4
6
8
A (n=15)
B (n=16)
C (n=14)
surface

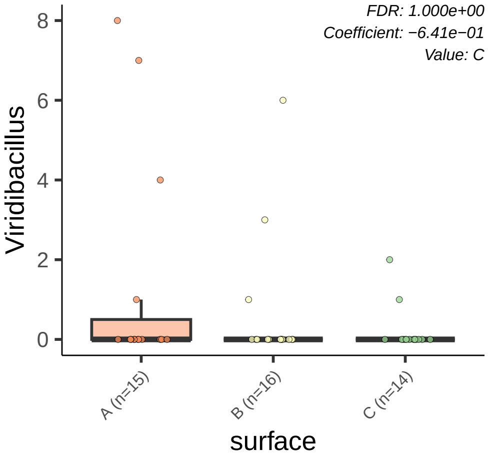

FDR: 1.000e+00
Coefficient: −6.41e−01
Value: C
Viridibacillus
8
6
4
2
0
A (n=15)
B (n=16)
C (n=14)
surface

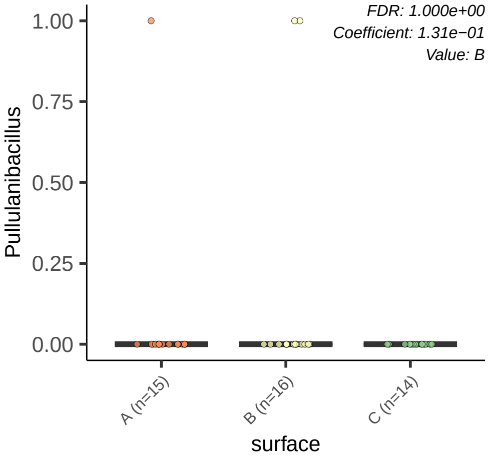

FDR: 1.000e+00
Coefficient: 1.31e−01
Value: B
Pullulanibacillus
1.00
0.75
0.50
0.25
0.00
A (n=15)
B (n=16)
C (n=14)
surface

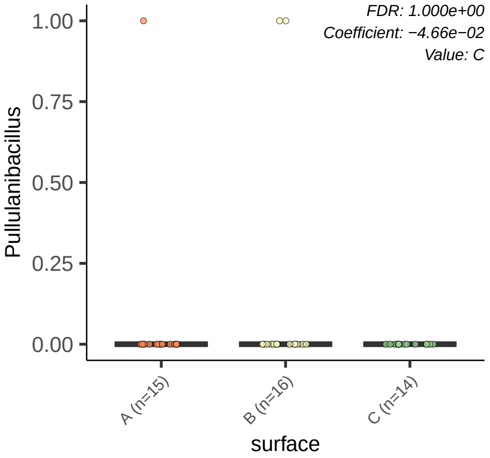

FDR: 1.000e+00
Coefficient: −4.66e−02
Value: C
Pullulanibacillus
1.00
0.75
0.50
0.25
0.00
A (n=15)
B (n=16)
C (n=14)
surface

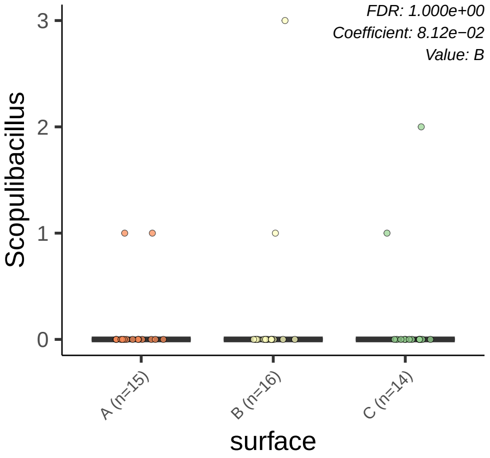

FDR: 1.000e+00
Coefficient: 8.12e−02
Value: B
Scopulibacillus
0
1
2
3
A (n=15)
B (n=16)
C (n=14)
surface

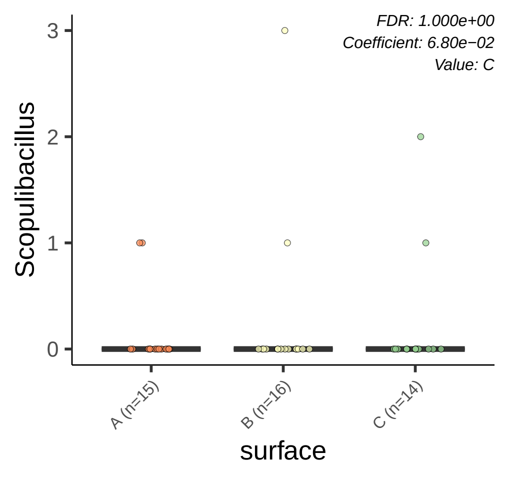

FDR: 1.000e+00
Coefficient: 6.80e−02
Value: C
Scopulibacillus
3
2
1
0
A (n=15)
B (n=16)
C (n=14)
surface

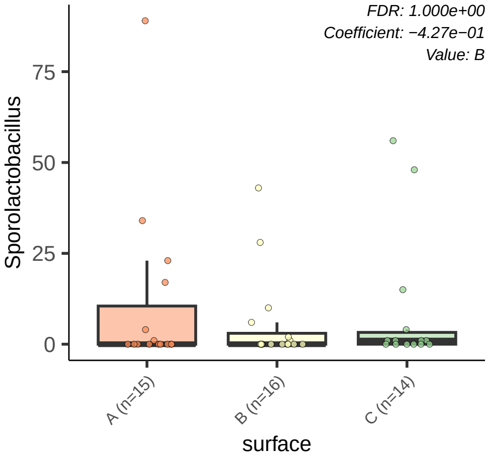
FDR: 1.000e+00
Coefficient: −4.27e−01
Value: B
Sporolactobacillus
75
50
25
0
A (n=15)
B (n=16)
C (n=14)
surface

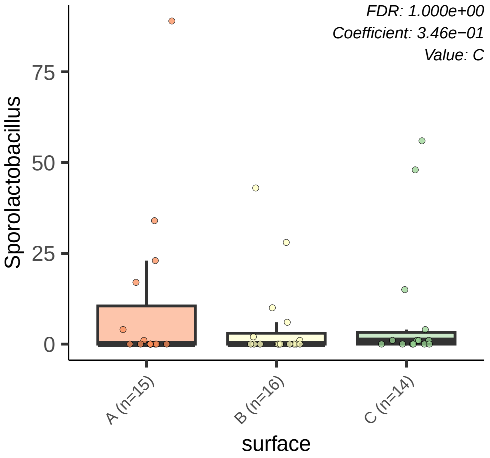
FDR: 1.000e+00
Coefficient: 3.46e−01
Value: C
Sporolactobacillus
0
25
50
75
A (n=15)
B (n=16)
C (n=14)
surface

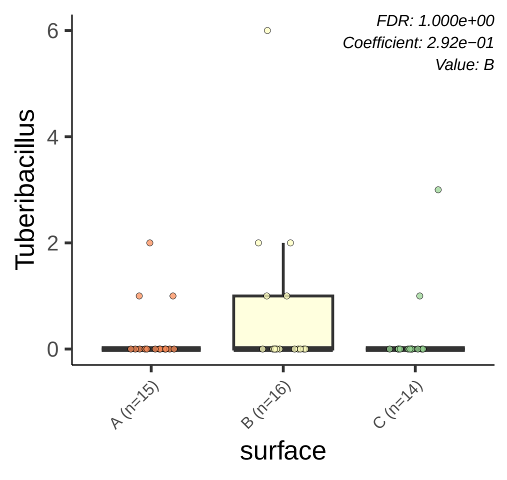

FDR: 1.000e+00
Coefficient: 2.92e−01
Value: B
Tuberibacillus
0
2
4
6
A (n=15)
B (n=16)
C (n=14)
surface

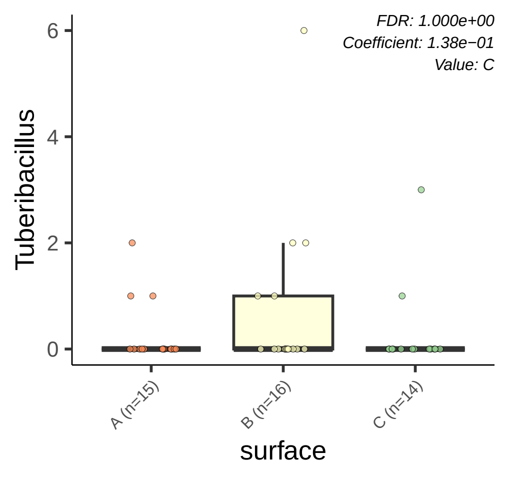
FDR: 1.000e+00
Coefficient: 1.38e−01
Value: C
Tuberibacillus
0
2
4
6
A (n=15)
B (n=16)
C (n=14)
surface

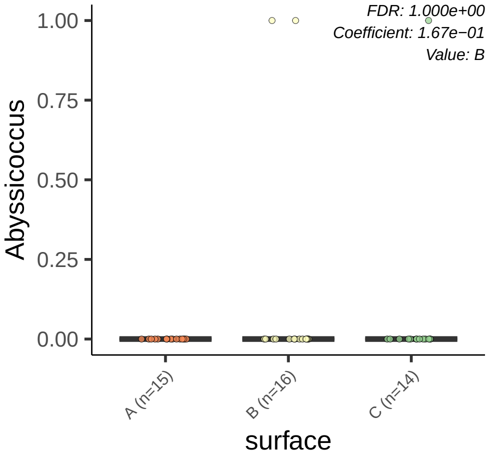

FDR: 1.000e+00
Coefficient: 1.67e−01
Value: B
Abyssicoccus
1.00
0.75
0.50
0.25
0.00
A (n=15)
B (n=16)
C (n=14)
surface

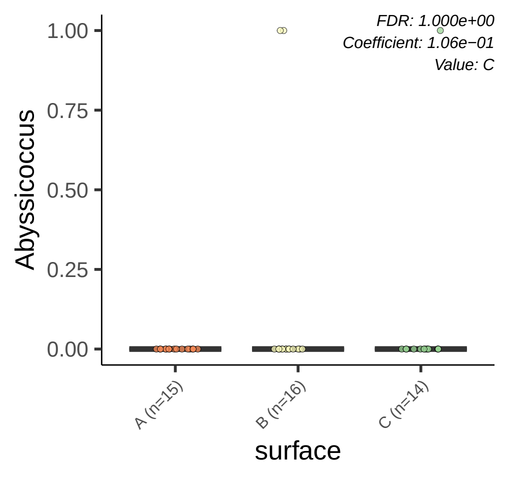
FDR: 1.000e+00
Coefficient: 1.06e−01
Value: C
Abyssicoccus
1.00
0.75
0.50
0.25
0.00
A (n=15)
B (n=16)
C (n=14)
surface

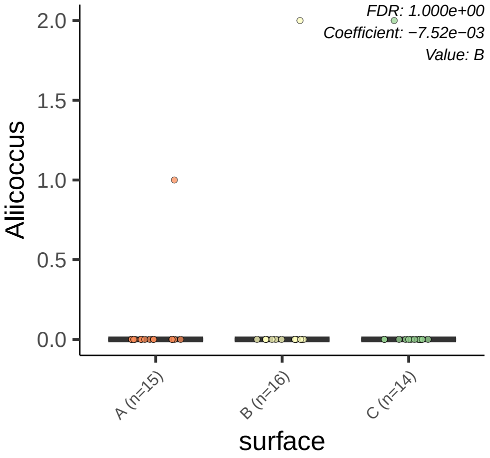

FDR: 1.000e+00
Coefficient: −7.52e−03
Value: B
2.0
1.5
1.0
0.5
0.0
Aliicoccus
A (n=15)
B (n=16)
C (n=14)
surface

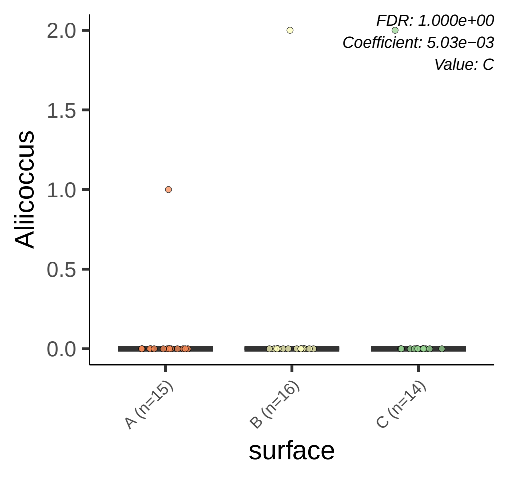
FDR: 1.000e+00
Coefficient: 5.03e−03
Value: C
Aliicoccus
2.0
1.5
1.0
0.5
0.0
A (n=15)
B (n=16)
C (n=14)
surface

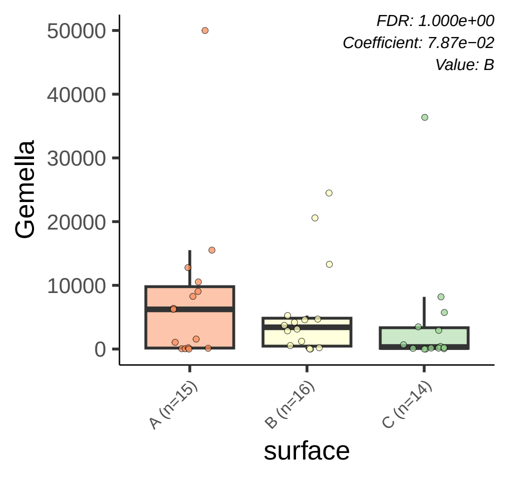
FDR: 1.000e+00
Coefficient: 7.87e−02
Value: B
Gemella
50000
40000
30000
20000
10000
0
A (n=15)
B (n=16)
C (n=14)
surface

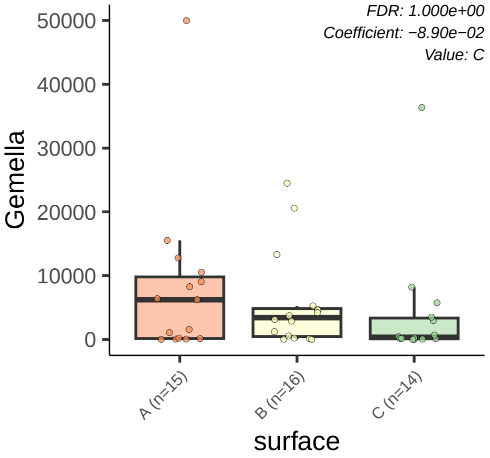
FDR: 1.000e+00
Coefficient: −8.90e−02
Value: C
50000
40000
30000
20000
10000
0
Gemella
A (n=15)
B (n=16)
C (n=14)
surface

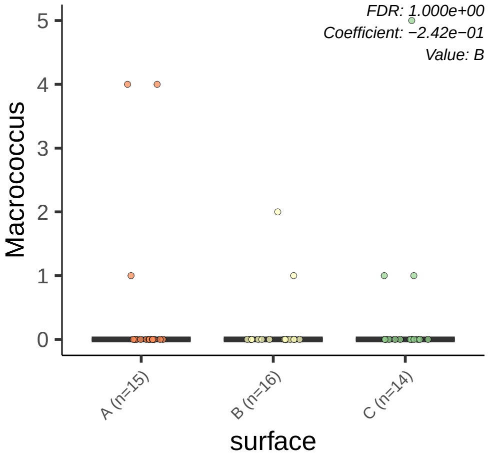
FDR: 1.000e+00
Coefficient: −2.42e−01
Value: B
Macrococcus
5
4
3
2
1
0
A (n=15)
B (n=16)
C (n=14)
surface

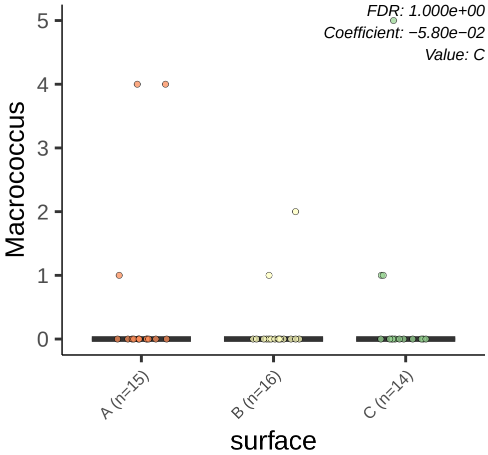

FDR: 1.000e+00
Coefficient: −5.80e−02
Value: C
Macrococcus
5
4
3
2
1
0
A (n=15)
B (n=16)
C (n=14)
surface

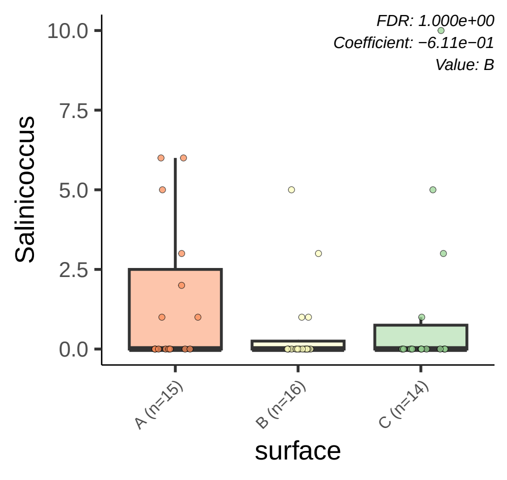
FDR: 1.000e+00
Coefficient: −6.11e−01
Value: B
10.0
7.5
5.0
2.5
0.0
Salinicoccus
A (n=15)
B (n=16)
C (n=14)
surface

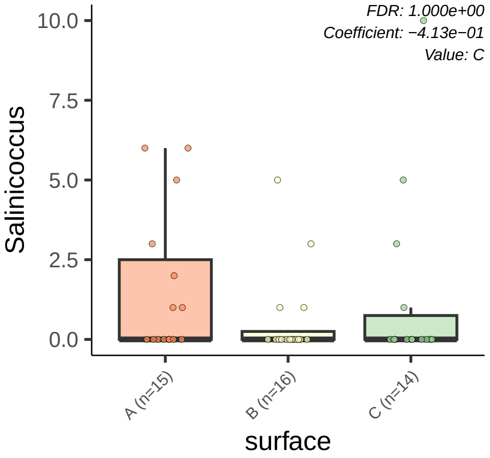
FDR: 1.000e+00
Coefficient: −4.13e−01
Value: C
Salinicoccus
10.0
7.5
5.0
2.5
0.0
A (n=15)
B (n=16)
C (n=14)
surface

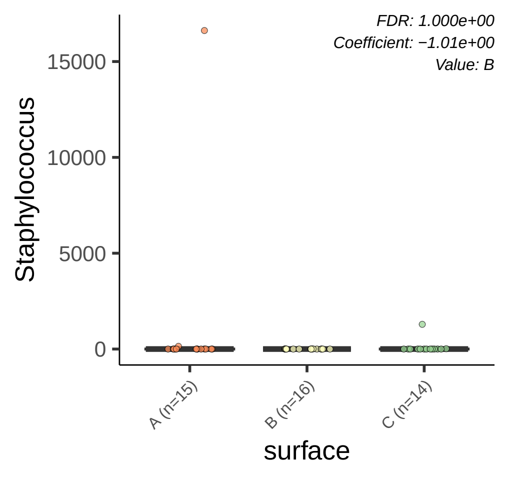
FDR: 1.000e+00
Coefficient: −1.01e+00
Value: B
Staphylococcus
15000
10000
5000
0
A (n=15)
B (n=16)
C (n=14)
surface

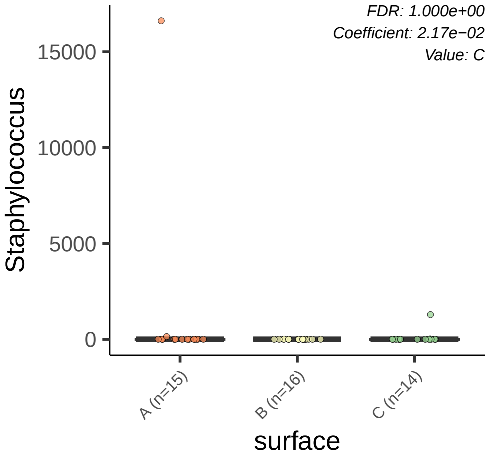
FDR: 1.000e+00
Coefficient: 2.17e−02
Value: C
Staphylococcus
15000
10000
5000
0
A (n=15)
B (n=16)
C (n=14)
surface

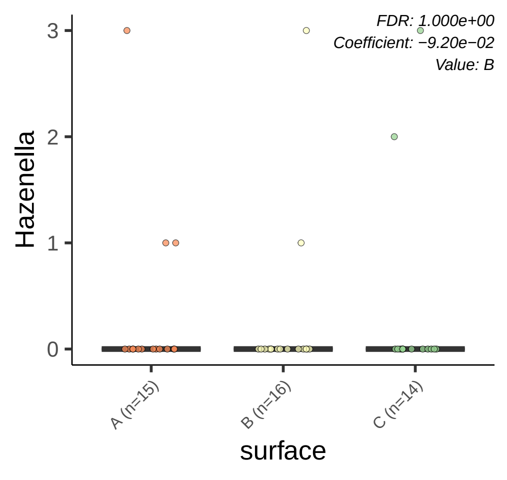

FDR: 1.000e+00
Coefficient: −9.20e−02
Value: B
Hazenella
0
1
2
3
A (n=15)
B (n=16)
C (n=14)
surface

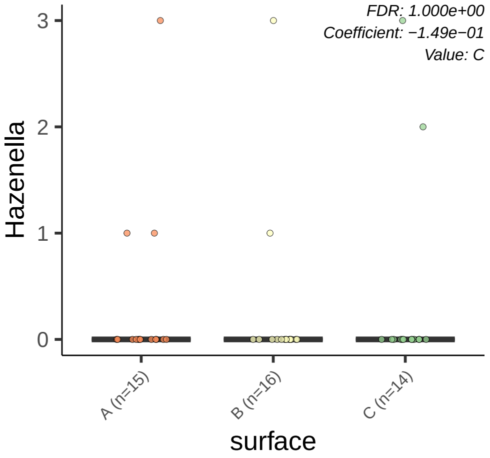

FDR: 1.000e+00
Coefficient: −1.49e−01
Value: C
Hazenella
0
1
2
3
A (n=15)
B (n=16)
C (n=14)
surface

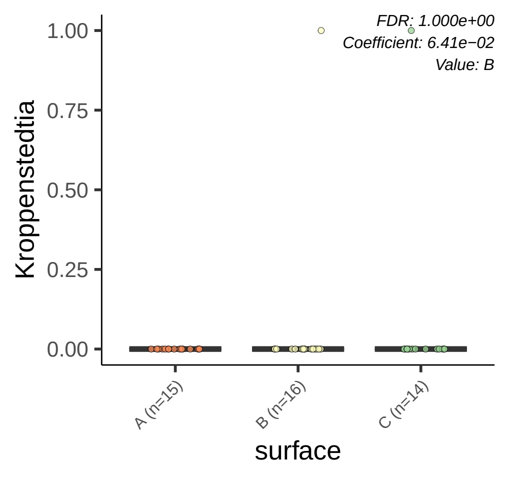
FDR: 1.000e+00
Coefficient: 6.41e−02
Value: B
Kroppenstedtia
1.00
0.75
0.50
0.25
0.00
A (n=15)
B (n=16)
C (n=14)
surface

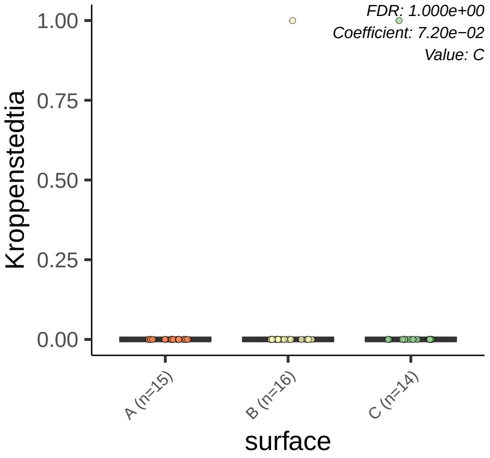

FDR: 1.000e+00
Coefficient: 7.20e−02
Value: C
Kroppenstedtia
1.00
0.75
0.50
0.25
0.00
A (n=15)
B (n=16)
C (n=14)
surface

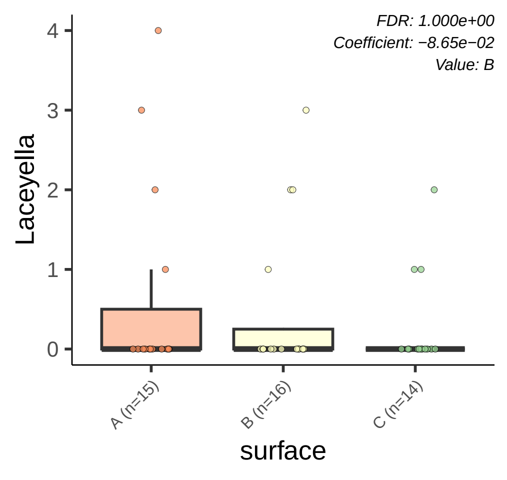
FDR: 1.000e+00
Coefficient: −8.65e−02
Value: B
Laceyella
0
1
2
3
4
A (n=15)
B (n=16)
C (n=14)
surface

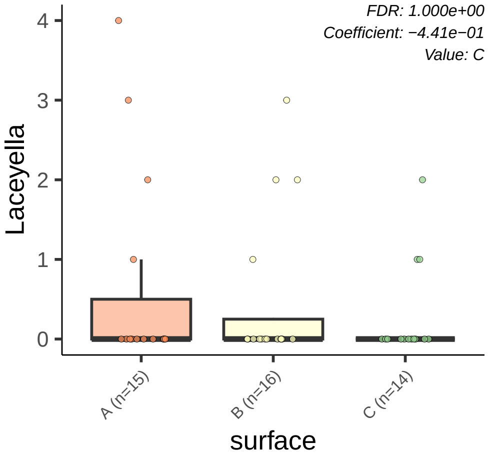

FDR: 1.000e+00
Coefficient: −4.41e−01
Value: C
Laceyella
0
1
2
3
4
A (n=15)
B (n=16)
C (n=14)
surface

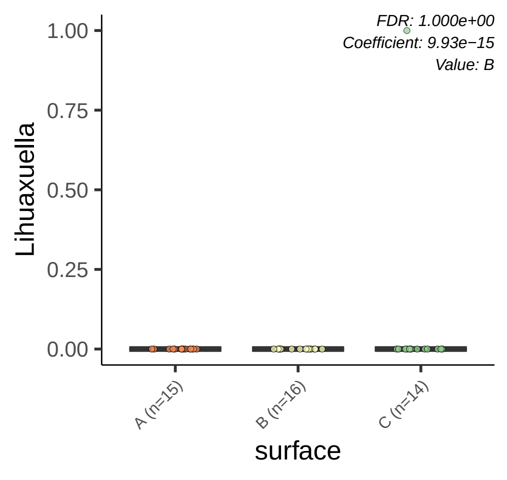
FDR: 1.000e+00
Coefficient: 9.93e−15
Value: B
1.00
0.75
0.50
0.25
0.00
Lihuaxuella
A (n=15)
B (n=16)
C (n=14)
surface

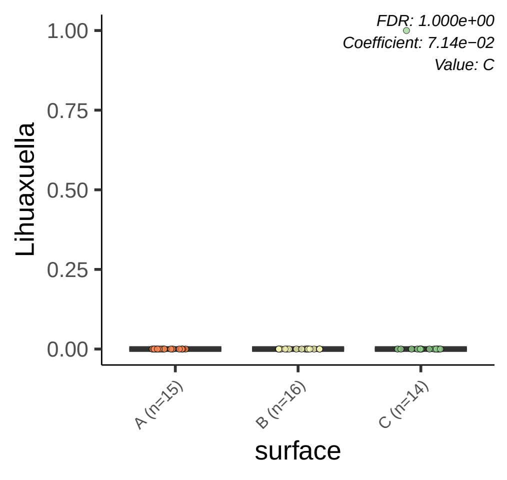

FDR: 1.000e+00
Coefficient: 7.14e−02
Value: C
Lihuaxuella
1.00
0.75
0.50
0.25
0.00
A (n=15)
B (n=16)
C (n=14)
surface

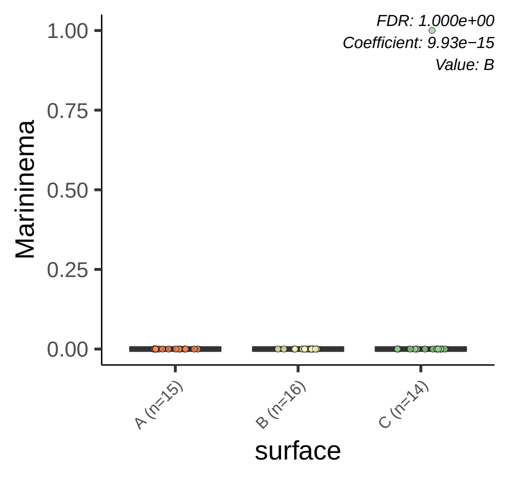
FDR: 1.000e+00
Coefficient: 9.93e−15
Value: B
Marininema
1.00
0.75
0.50
0.25
0.00
A (n=15)
B (n=16)
C (n=14)
surface

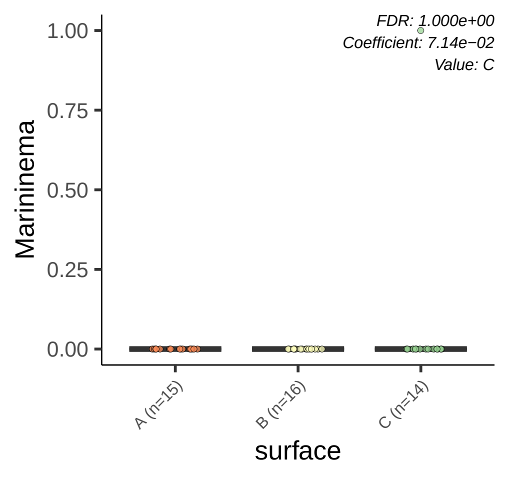
FDR: 1.000e+00
Coefficient: 7.14e−02
Value: C
Marininema
1.00
0.75
0.50
0.25
0.00
A (n=15)
B (n=16)
C (n=14)
surface

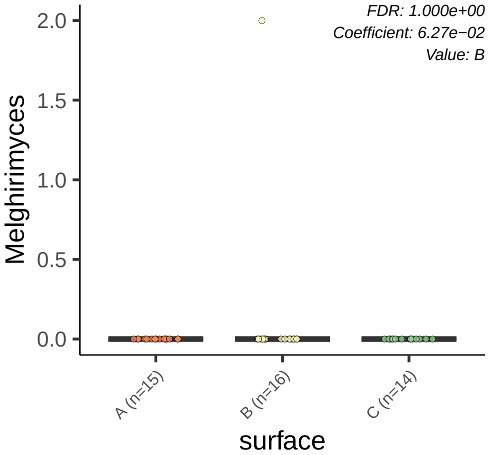

FDR: 1.000e+00
Coefficient: 6.27e−02
Value: B
Melghirimyces
2.0
1.5
1.0
0.5
0.0
A (n=15)
B (n=16)
C (n=14)
surface

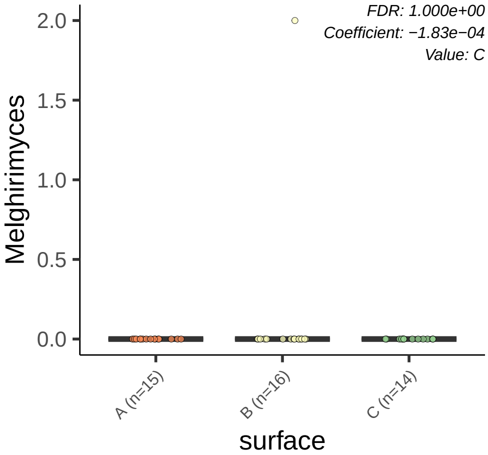
FDR: 1.000e+00
Coefficient: −1.83e−04
Value: C
Melghirimyces
2.0
1.5
1.0
0.5
0.0
A (n=15)
B (n=16)
C (n=14)
surface

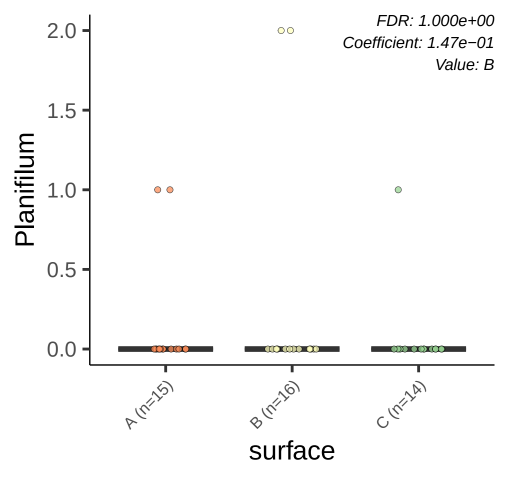

FDR: 1.000e+00
Coefficient: 1.47e−01
Value: B
Planifilum
2.0
1.5
1.0
0.5
0.0
A (n=15)
B (n=16)
C (n=14)
surface

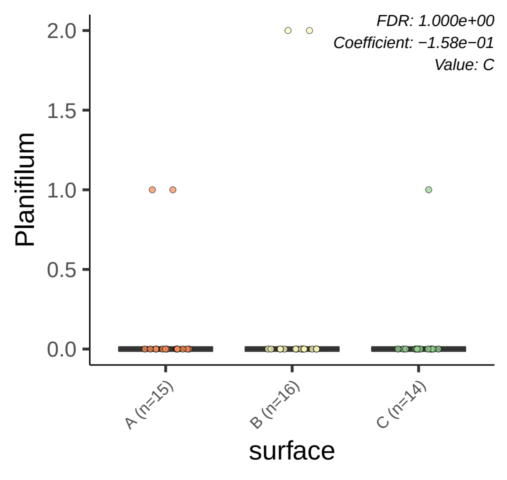

FDR: 1.000e+00
Coefficient: −1.58e−01
Value: C
Planifilum
2.0
1.5
1.0
0.5
0.0
A (n=15)
B (n=16)
C (n=14)
surface

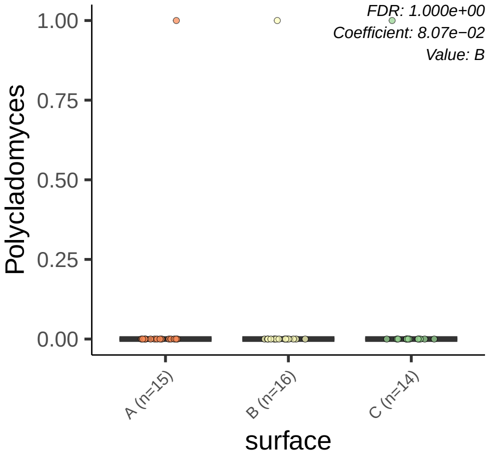
FDR: 1.000e+00
Coefficient: 8.07e−02
Value: B
Polycladomyces
1.00
0.75
0.50
0.25
0.00
A (n=15)
B (n=16)
C (n=14)
surface

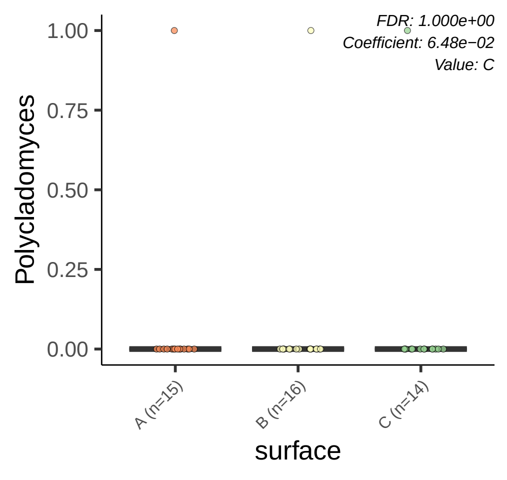
FDR: 1.000e+00
Coefficient: 6.48e−02
Value: C
Polycladomyces
1.00
0.75
0.50
0.25
0.00
A (n=15)
B (n=16)
C (n=14)
surface

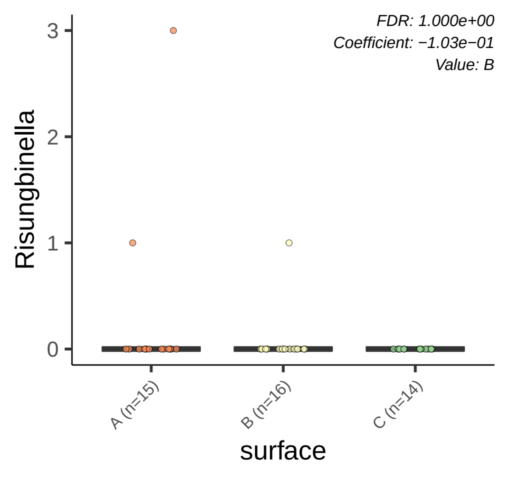

FDR: 1.000e+00
Coefficient: −1.03e−01
Value: B
Risungbinella
3
2
1
0
A (n=15)
B (n=16)
C (n=14)
surface

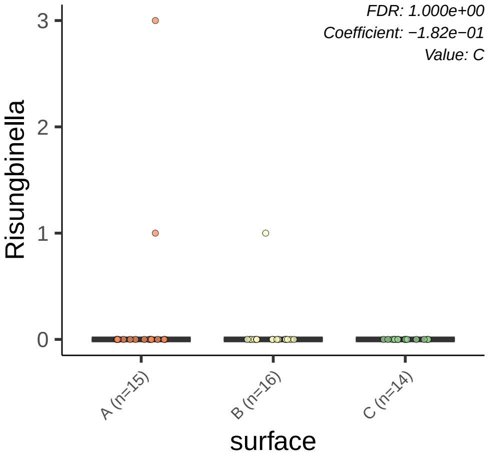
FDR: 1.000e+00
Coefficient: −1.82e−01
Value: C
Risungbinella
3
2
1
0
A (n=15)
B (n=16)
C (n=14)
surface

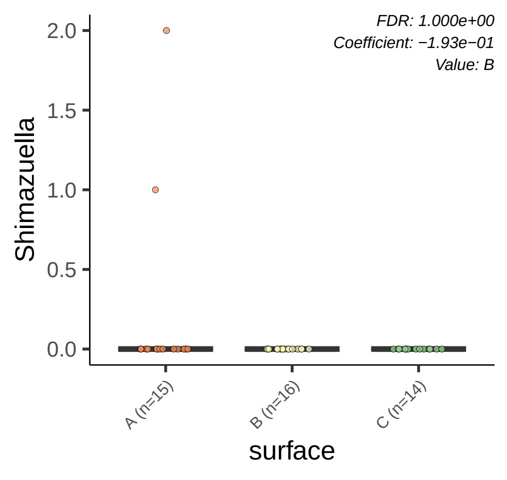
FDR: 1.000e+00
Coefficient: −1.93e−01
Value: B
Shimazuella
2.0
1.5
1.0
0.5
0.0
A (n=15)
B (n=16)
C (n=14)
surface

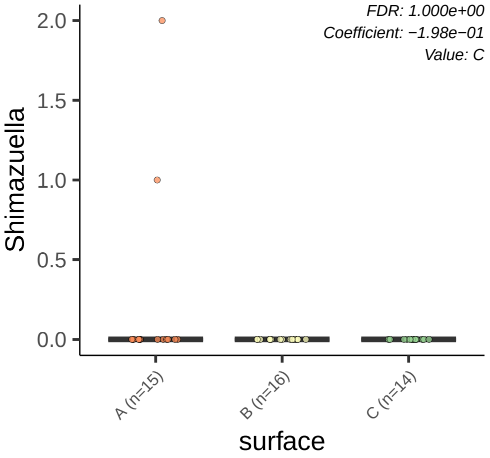

FDR: 1.000e+00
Coefficient: −1.98e−01
Value: C
Shimazuella
2.0
1.5
1.0
0.5
0.0
A (n=15)
B (n=16)
C (n=14)
surface

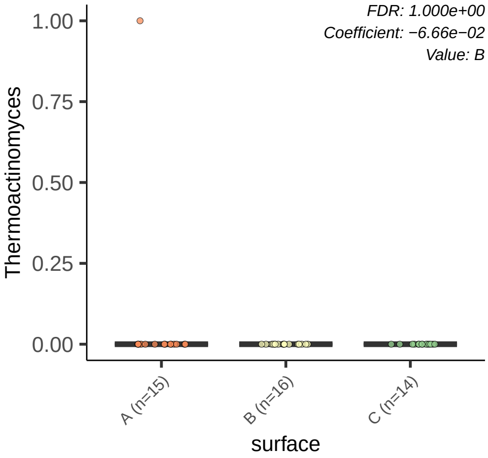

FDR: 1.000e+00
Coefficient: −6.66e−02
Value: B
Thermoactinomyces
1.00
0.75
0.50
0.25
0.00
A (n=15)
B (n=16)
C (n=14)
surface

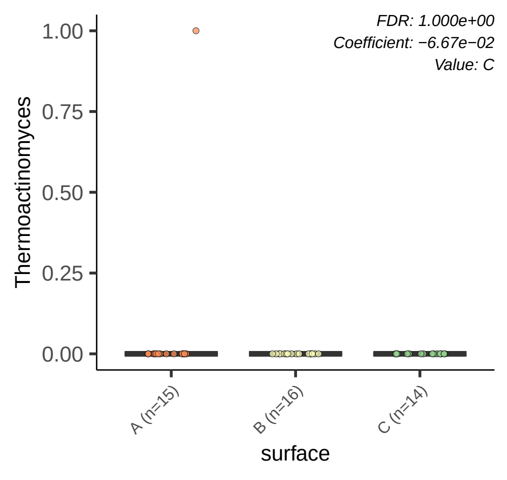
FDR: 1.000e+00
Coefficient: −6.67e−02
Value: C
Thermoactinomyces
1.00
0.75
0.50
0.25
0.00
A (n=15)
B (n=16)
C (n=14)
surface

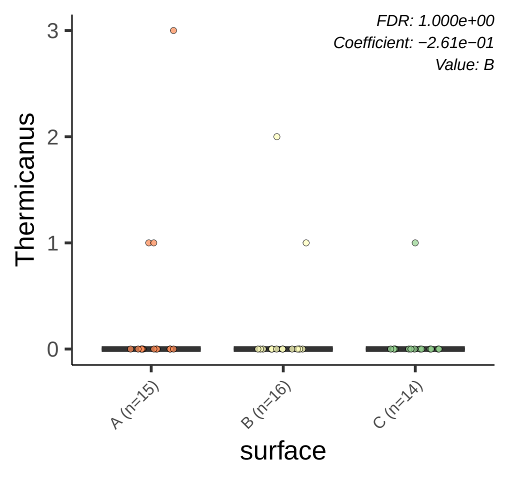

FDR: 1.000e+00
Coefficient: −2.61e−01
Value: B
Thermicanus
0
1
2
3
A (n=15)
B (n=16)
C (n=14)
surface

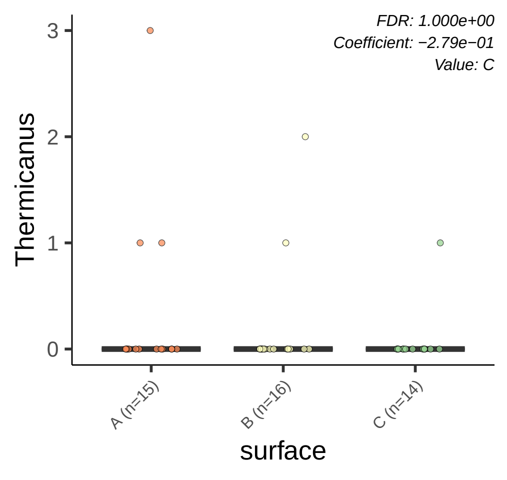

FDR: 1.000e+00
Coefficient: −2.79e−01
Value: C
Thermicanus
3
2
1
0
A (n=15)
B (n=16)
C (n=14)
surface

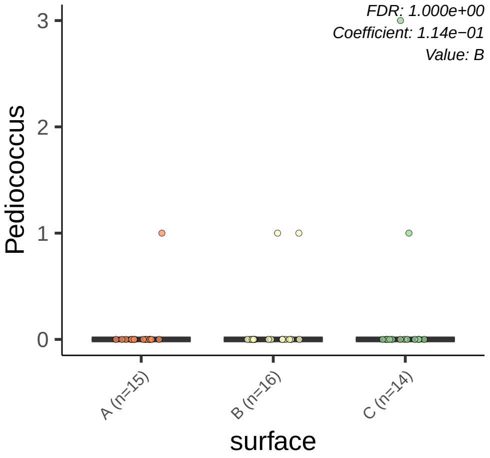

FDR: 1.000e+00
Coefficient: 1.14e−01
Value: B
Pediococcus
3
2
1
0
A (n=15)
B (n=16)
C (n=14)
surface

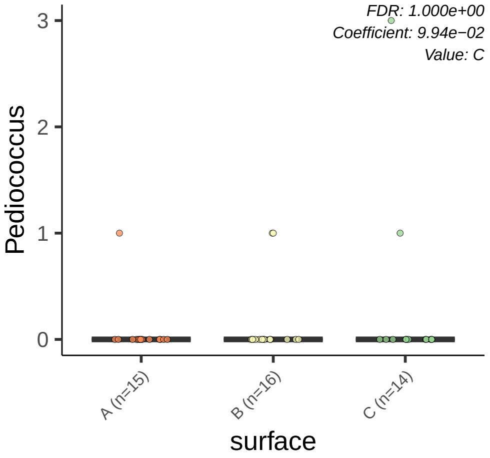
FDR: 1.000e+00
Coefficient: 9.94e−02
Value: C
Pediococcus
3
2
1
0
A (n=15)
B (n=16)
C (n=14)
surface

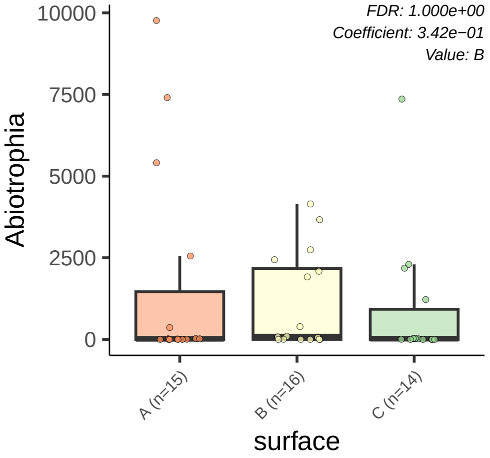
FDR: 1.000e+00
Coefficient: 3.42e−01
Value: B
Abiotrophia
10000
7500
5000
2500
0
A (n=15)
B (n=16)
C (n=14)
surface

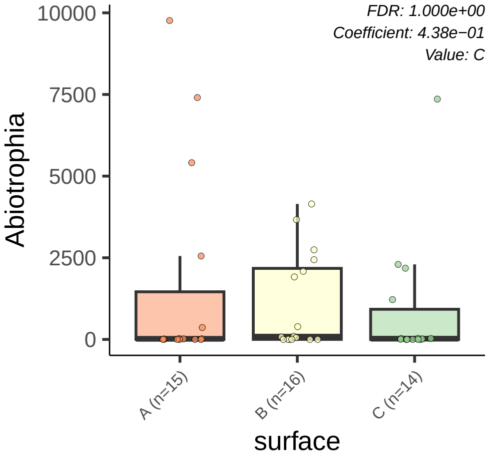

FDR: 1.000e+00
Coefficient: 4.38e−01
Value: C
10000
7500
5000
2500
0
Abiotrophia
A (n=15)
B (n=16)
C (n=14)
surface

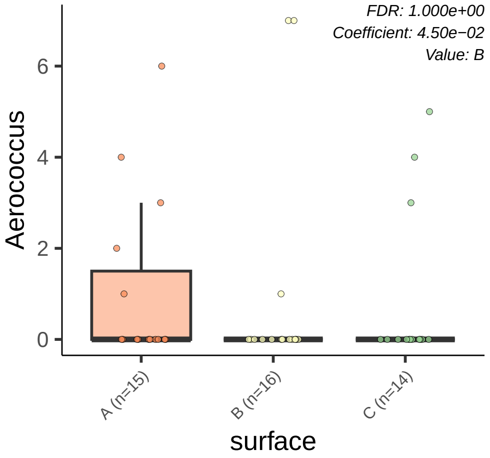

FDR: 1.000e+00
Coefficient: 4.50e−02
Value: B
Aerococcus
0
2
4
6
A (n=15)
B (n=16)
C (n=14)
surface

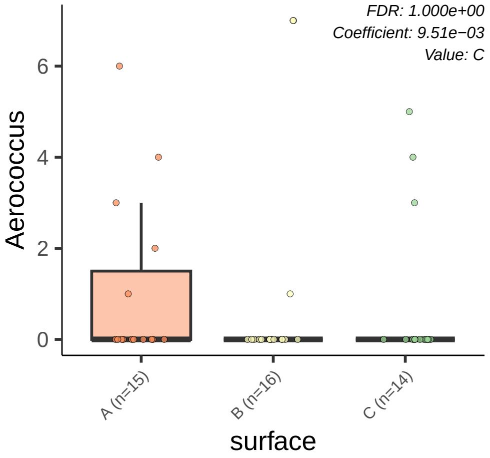

FDR: 1.000e+00
Coefficient: 9.51e−03
Value: C
Aerococcus
0
2
4
6
A (n=15)
B (n=16)
C (n=14)
surface

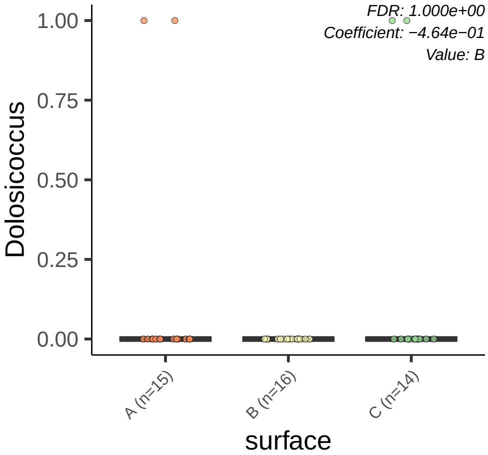
FDR: 1.000e+00
Coefficient: −4.64e−01
Value: B
Dolosicoccus
1.00
0.75
0.50
0.25
0.00
A (n=15)
B (n=16)
C (n=14)
surface

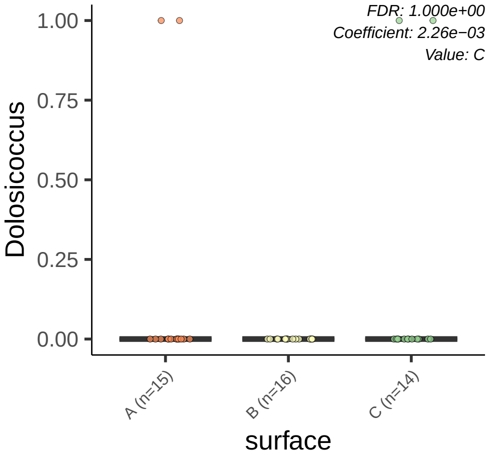
FDR: 1.000e+00
Coefficient: 2.26e−03
Value: C
Dolosicoccus
1.00
0.75
0.50
0.25
0.00
A (n=15)
B (n=16)
C (n=14)
surface

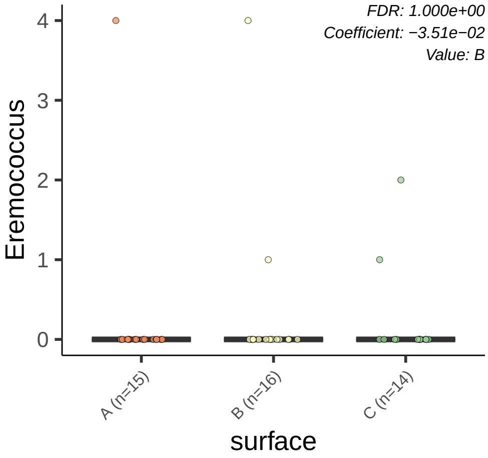

FDR: 1.000e+00
Coefficient: −3.51e−02
Value: B
Eremococcus
0
1
2
3
4
A (n=15)
B (n=16)
C (n=14)
surface

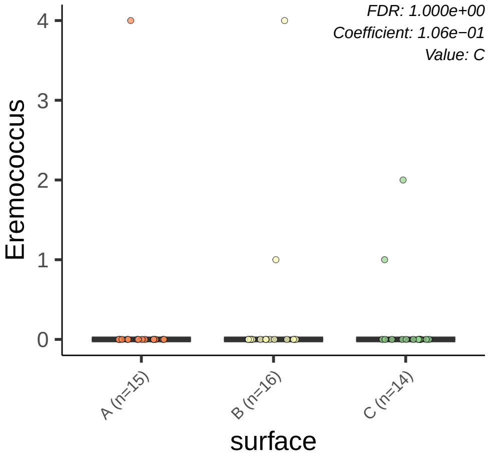

FDR: 1.000e+00
Coefficient: 1.06e−01
Value: C
Eremococcus
4
3
2
1
0
A (n=15)
B (n=16)
C (n=14)
surface

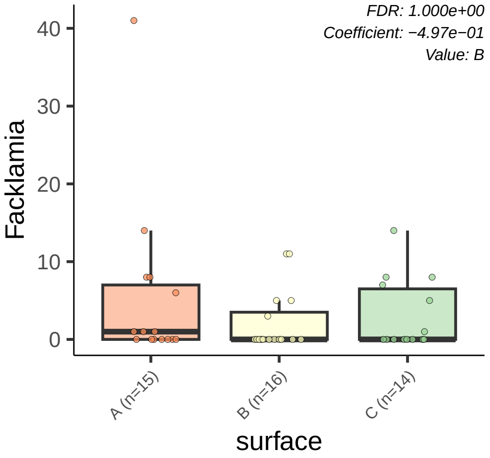
FDR: 1.000e+00
Coefficient: −4.97e−01
Value: B
Facklamia
40
30
20
10
0
A (n=15)
B (n=16)
C (n=14)
surface

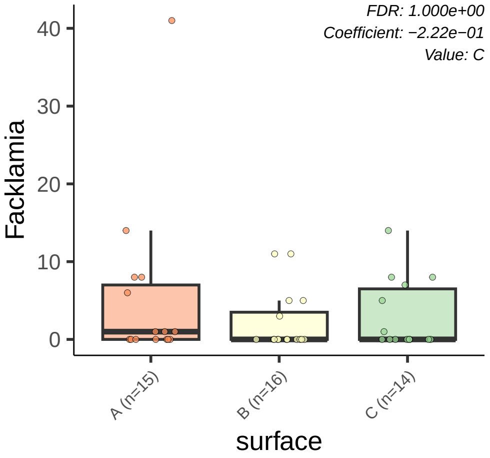

FDR: 1.000e+00
Coefficient: −2.22e−01
Value: C
Facklamia
40
30
20
10
0
A (n=15)
B (n=16)
C (n=14)
surface

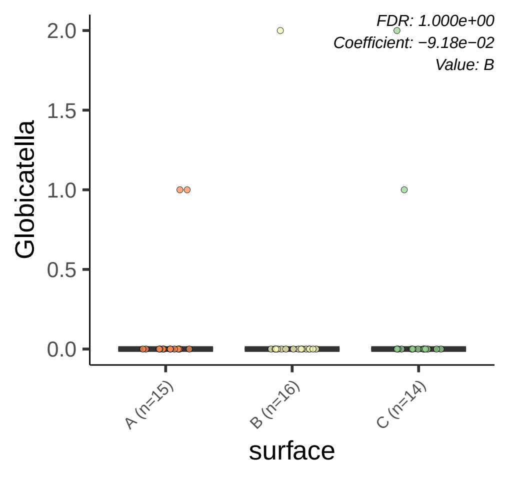
FDR: 1.000e+00
Coefficient: −9.18e−02
Value: B
Globicatella
2.0
1.5
1.0
0.5
0.0
A (n=15)
B (n=16)
C (n=14)
surface

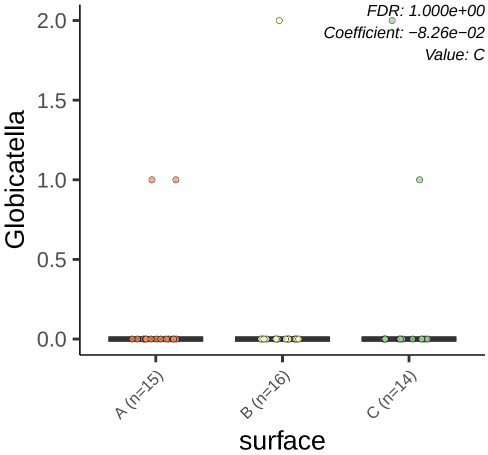
FDR: 1.000e+00
Coefficient: −8.26e−02
Value: C
Globicatella
2.0
1.5
1.0
0.5
0.0
A (n=15)
B (n=16)
C (n=14)
surface

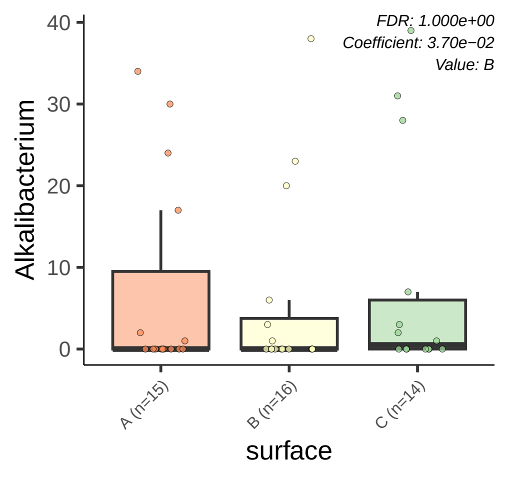
FDR: 1.000e+00
Coefficient: 3.70e−02
Value: B
Alkalibacterium
0
10
20
30
40
A (n=15)
B (n=16)
C (n=14)
surface

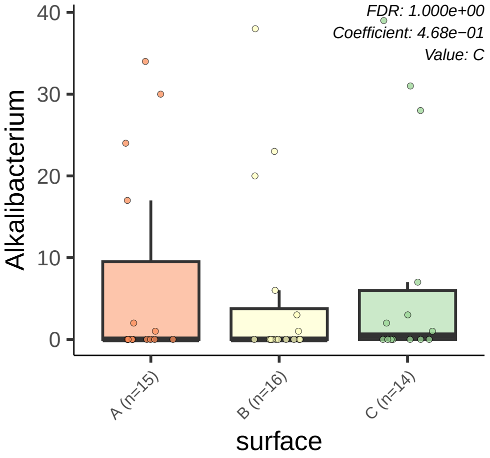

FDR: 1.000e+00
Coefficient: 4.68e−01
Value: C
Alkalibacterium
40
30
20
10
0
A (n=15)
B (n=16)
C (n=14)
surface

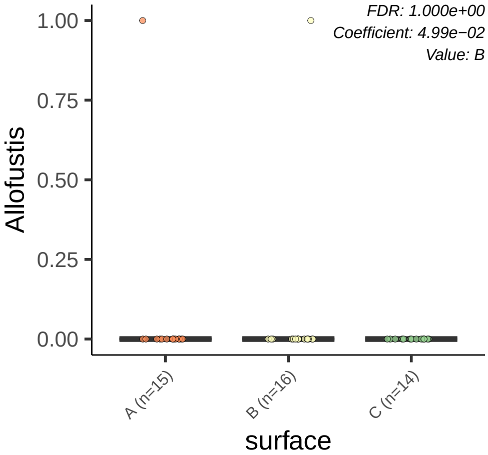

FDR: 1.000e+00
Coefficient: 4.99e−02
Value: B
1.00
0.75
0.50
0.25
0.00
Allofustis
A (n=15)
B (n=16)
C (n=14)
surface

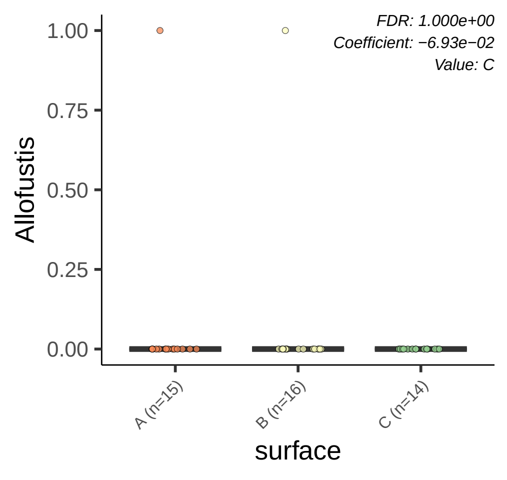
FDR: 1.000e+00
Coefficient: −6.93e−02
Value: C
Allofustis
1.00
0.75
0.50
0.25
0.00
A (n=15)
B (n=16)
C (n=14)
surface

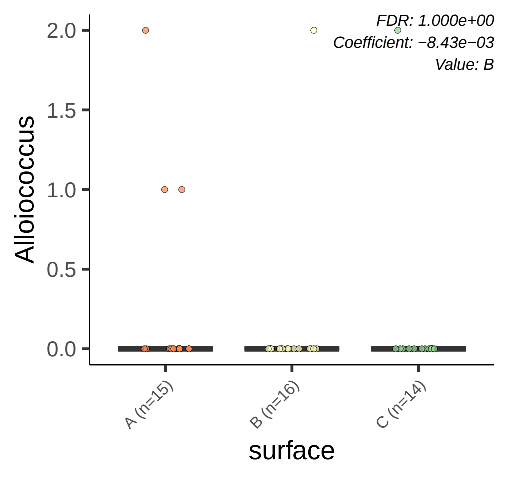
FDR: 1.000e+00
Coefficient: −8.43e−03
Value: B
Alloiococcus
2.0
1.5
1.0
0.5
0.0
A (n=15)
B (n=16)
C (n=14)
surface

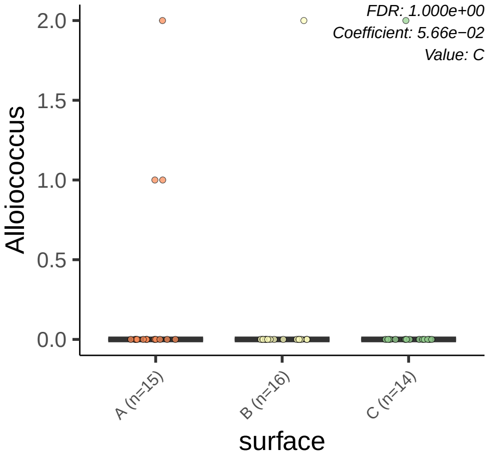
FDR: 1.000e+00
Coefficient: 5.66e−02
Value: C
Alloiococcus
2.0
1.5
1.0
0.5
0.0
A (n=15)
B (n=16)
C (n=14)
surface

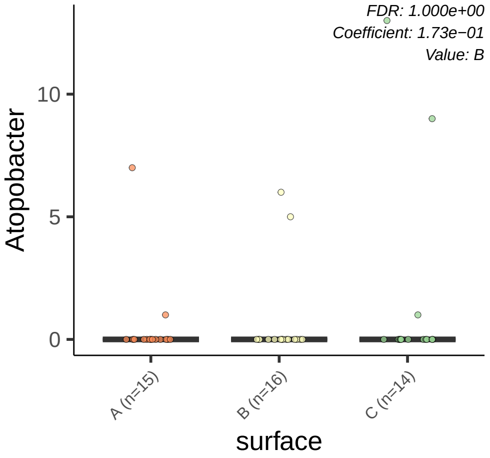
FDR: 1.000e+00
Coefficient: 1.73e−01
Value: B
Atopobacter
10
5
0
A (n=15)
B (n=16)
C (n=14)
surface

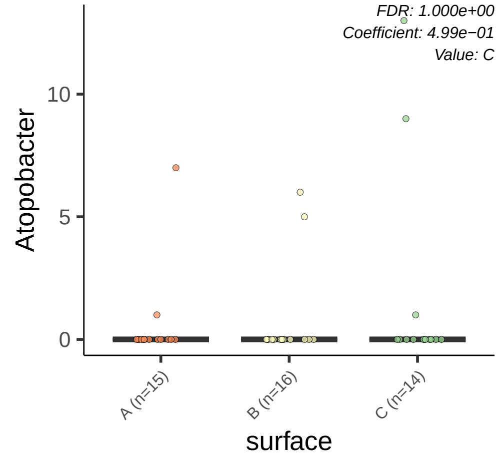
FDR: 1.000e+00
Coefficient: 4.99e−01
Value: C
Atopobacter
10
5
0
A (n=15)
B (n=16)
C (n=14)
surface

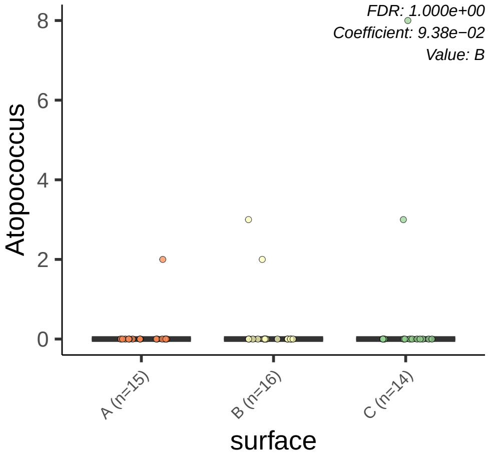

FDR: 1.000e+00
Coefficient: 9.38e−02
Value: B
Atopococcus
8
6
4
2
0
A (n=15)
B (n=16)
C (n=14)
surface

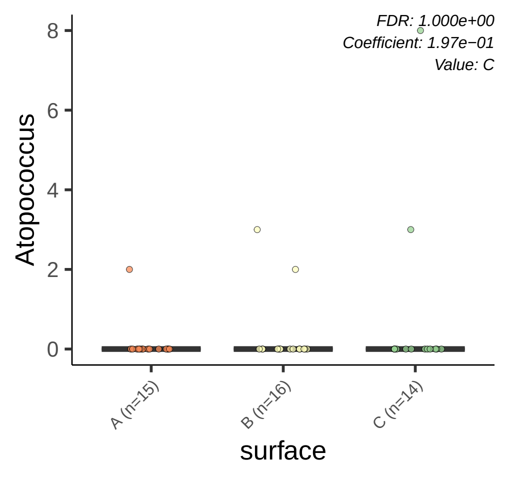
FDR: 1.000e+00
Coefficient: 1.97e−01
Value: C
Atopococcus
0
2
4
6
8
A (n=15)
B (n=16)
C (n=14)
surface

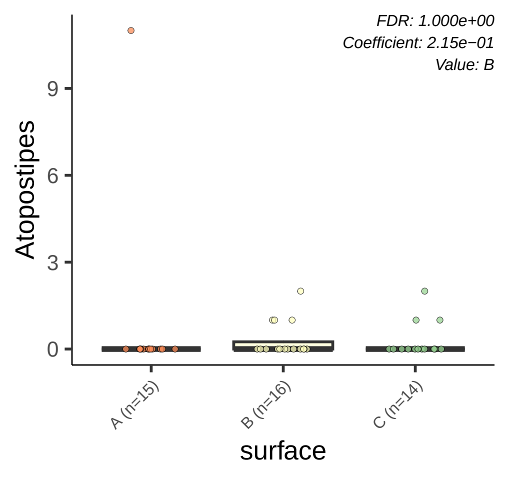

FDR: 1.000e+00
Coefficient: 2.15e−01
Value: B
Atopostipes
9
6
3
0
A (n=15)
B (n=16)
C (n=14)
surface

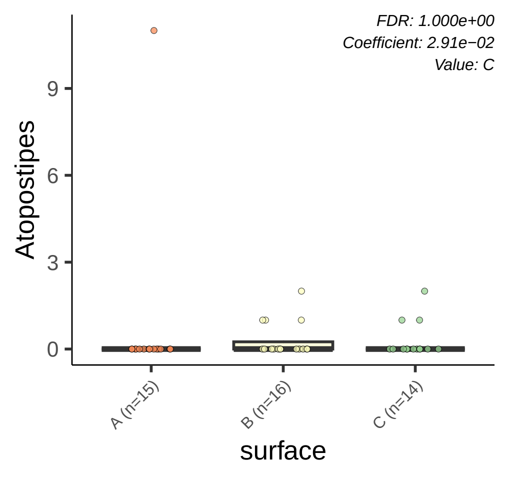

FDR: 1.000e+00
Coefficient: 2.91e−02
Value: C
Atopostipes
9
6
3
0
A (n=15)
B (n=16)
C (n=14)
surface

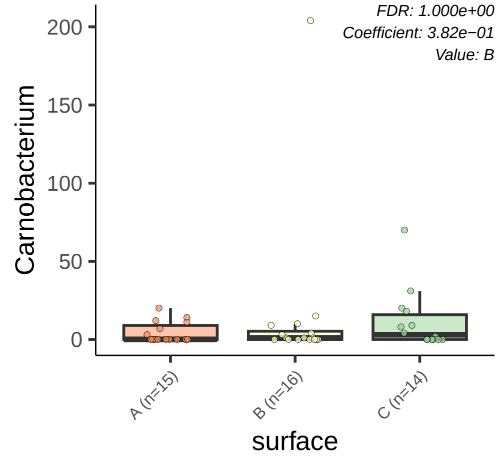

FDR: 1.000e+00
Coefficient: 3.82e−01
Value: B
Carnobacterium
200
150
100
50
0
A (n=15)
B (n=16)
C (n=14)
surface

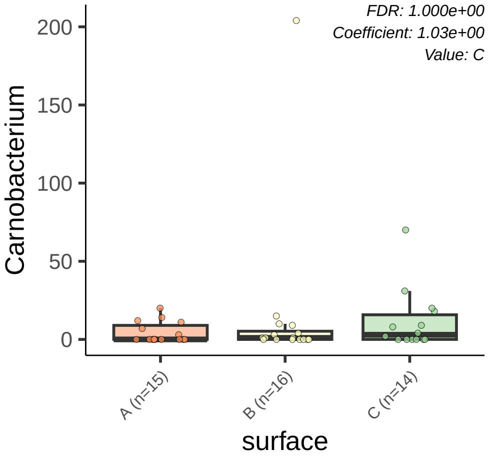
FDR: 1.000e+00
Coefficient: 1.03e+00
Value: C
Carnobacterium
200
150
100
50
0
A (n=15)
B (n=16)
C (n=14)
surface

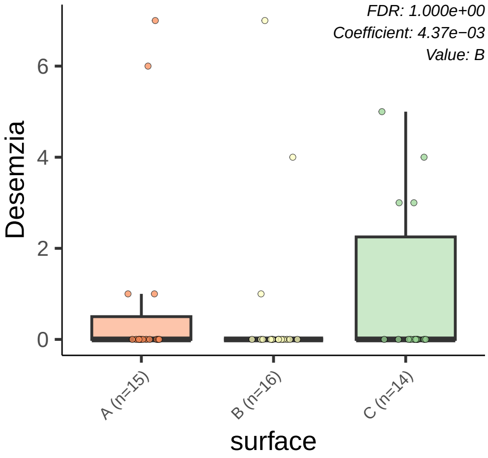
FDR: 1.000e+00
Coefficient: 4.37e−03
Value: B
Desemzia
0
2
4
6
A (n=15)
B (n=16)
C (n=14)
surface

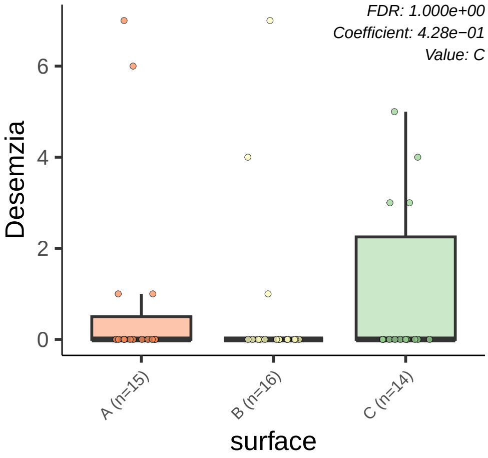

FDR: 1.000e+00
Coefficient: 4.28e−01
Value: C
Desemzia
0
2
4
6
A (n=15)
B (n=16)
C (n=14)
surface

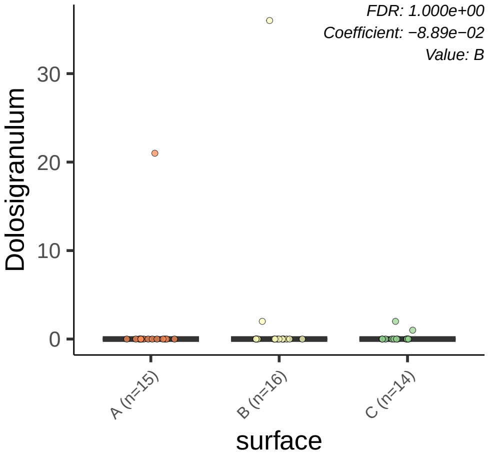

FDR: 1.000e+00
Coefficient: −8.89e−02
Value: B
Dolosigranulum
0
10
20
30
A (n=15)
B (n=16)
C (n=14)
surface

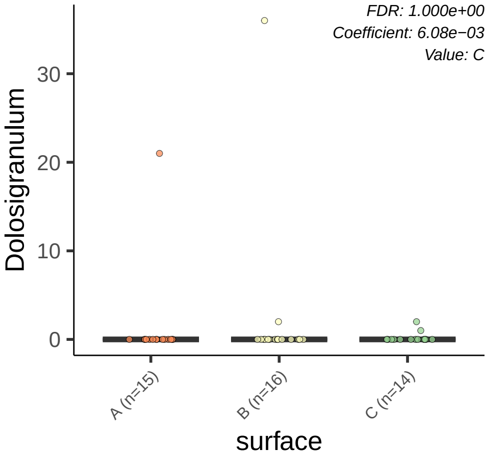
FDR: 1.000e+00
Coefficient: 6.08e−03
Value: C
Dolosigranulum
30
20
10
0
A (n=15)
B (n=16)
C (n=14)
surface

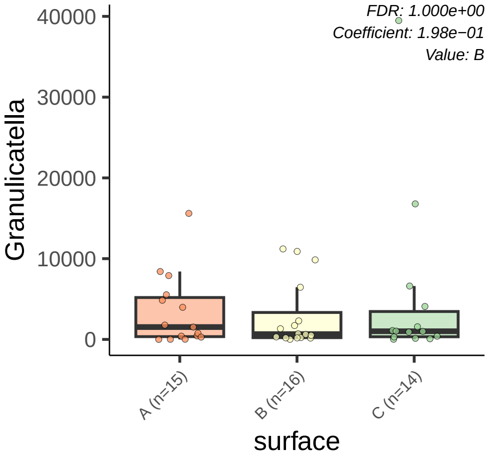

FDR: 1.000e+00
Coefficient: 1.98e−01
Value: B
40000
30000
20000
10000
0
Granulicatella
A (n=15)
B (n=16)
C (n=14)
surface

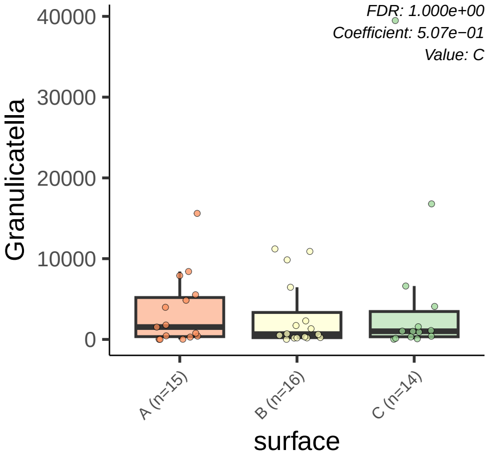

FDR: 1.000e+00
Coefficient: 5.07e−01
Value: C
Granulicatella
40000
30000
20000
10000
0
A (n=15)
B (n=16)
C (n=14)
surface

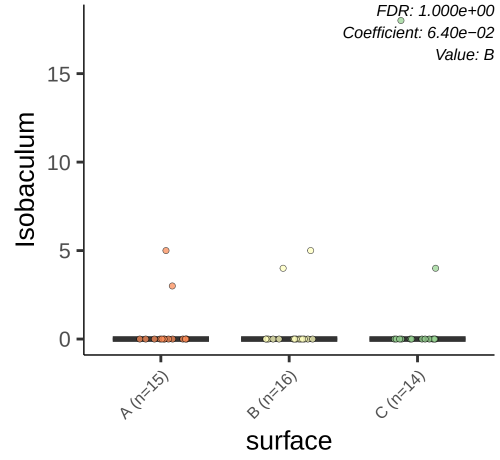
FDR: 1.000e+00
Coefficient: 6.40e−02
Value: B
Isobaculum
15
10
5
0
A (n=15)
B (n=16)
C (n=14)
surface

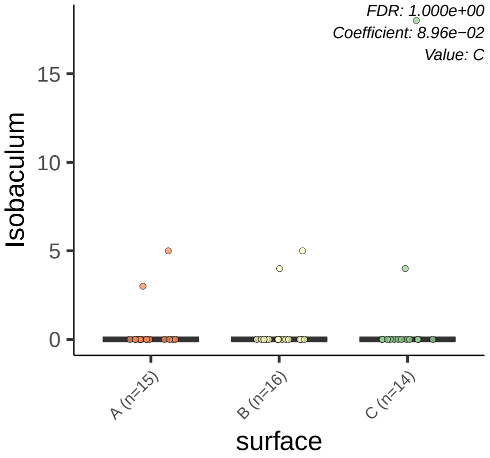
FDR: 1.000e+00
Coefficient: 8.96e−02
Value: C
Isobaculum
15
10
5
0
A (n=15)
B (n=16)
C (n=14)
surface

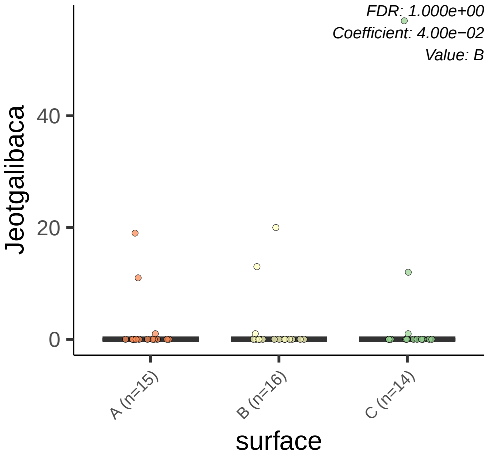
FDR: 1.000e+00
Coefficient: 4.00e−02
Value: B
Jeotgalibaca
0
20
40
A (n=15)
B (n=16)
C (n=14)
surface

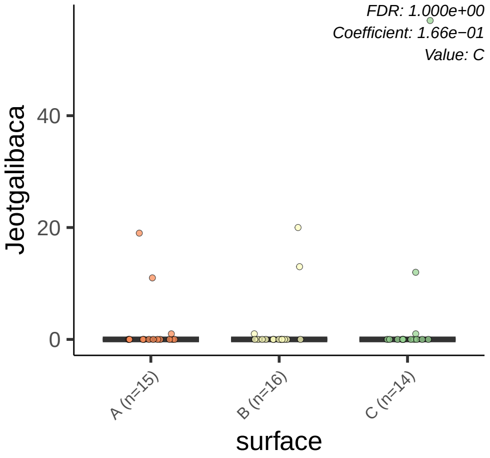

FDR: 1.000e+00
Coefficient: 1.66e−01
Value: C
Jeotgalibaca
0
20
40
A (n=15)
B (n=16)
C (n=14)
surface

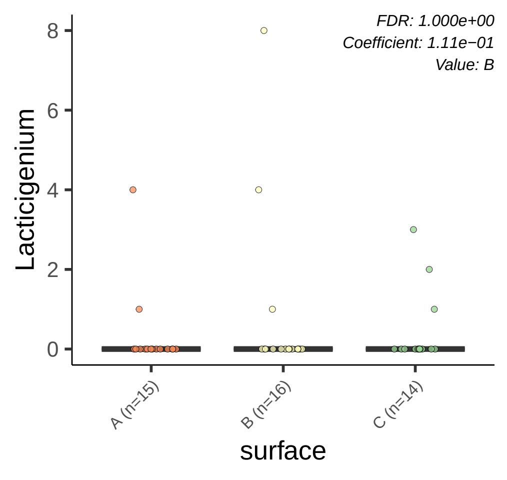

FDR: 1.000e+00
Coefficient: 1.11e−01
Value: B
Lacticigenium
8
6
4
2
0
A (n=15)
B (n=16)
C (n=14)
surface

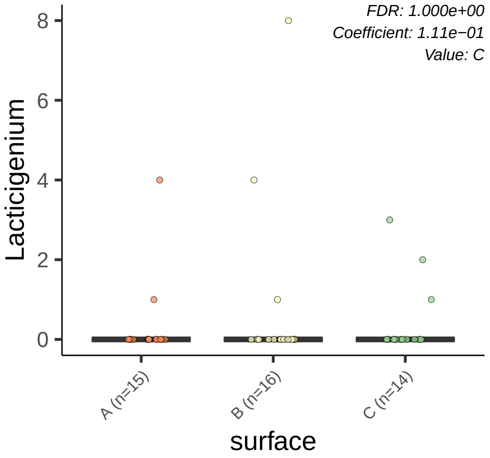
FDR: 1.000e+00
Coefficient: 1.11e−01
Value: C
Lacticigenium
0
2
4
6
8
A (n=15)
B (n=16)
C (n=14)
surface

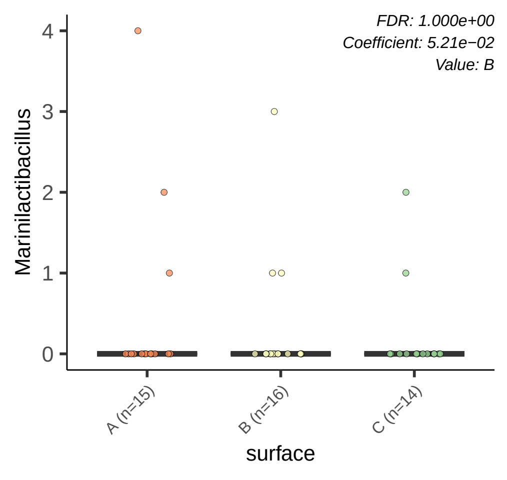
FDR: 1.000e+00
Coefficient: 5.21e−02
Value: B
Marinilactibacillus
4
3
2
1
0
A (n=15)
B (n=16)
C (n=14)
surface

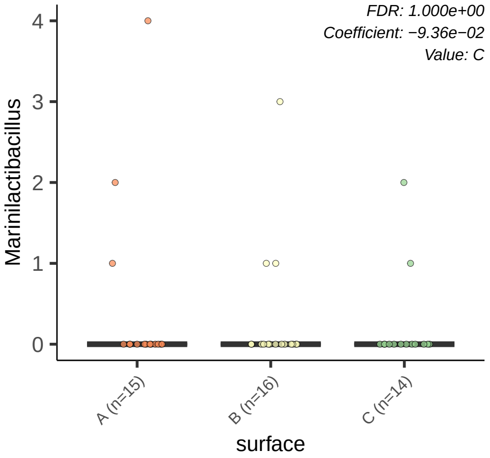
FDR: 1.000e+00
Coefficient: −9.36e−02
Value: C
Marinilactibacillus
4
3
2
1
0
A (n=15)
B (n=16)
C (n=14)
surface

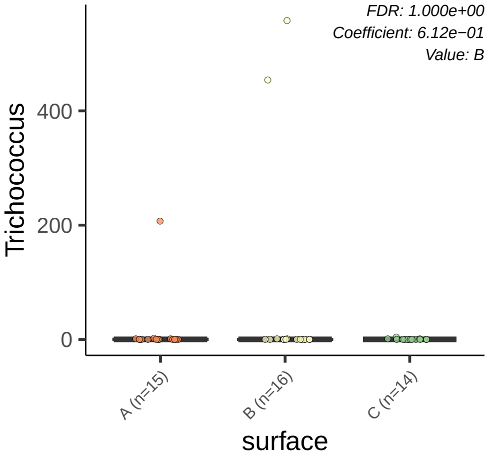

FDR: 1.000e+00
Coefficient: 6.12e−01
Value: B
Trichococcus
400
200
0
A (n=15)
B (n=16)
C (n=14)
surface

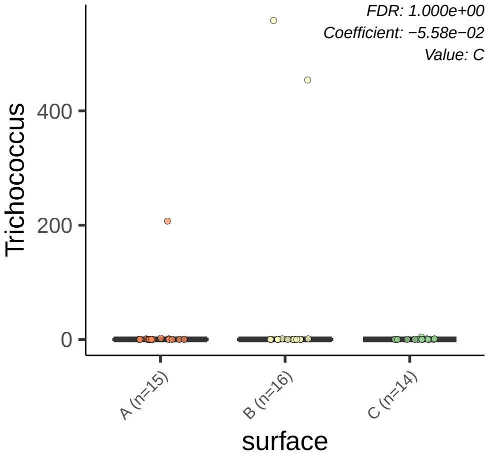
FDR: 1.000e+00
Coefficient: −5.58e−02
Value: C
Trichococcus
400
200
0
A (n=15)
B (n=16)
C (n=14)
surface

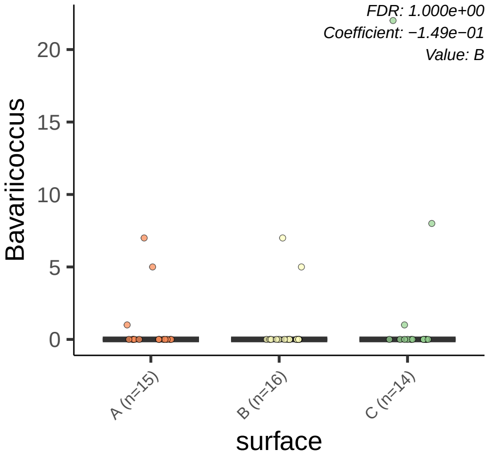

FDR: 1.000e+00
Coefficient: −1.49e−01
Value: B
Bavariicoccus
20
15
10
5
0
A (n=15)
B (n=16)
C (n=14)
surface

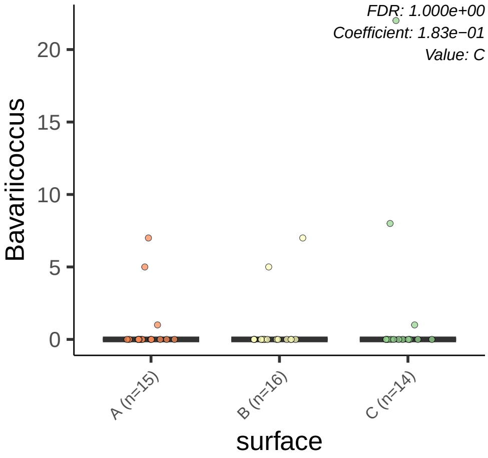

FDR: 1.000e+00
Coefficient: 1.83e−01
Value: C
Bavariicoccus
20
15
10
5
0
A (n=15)
B (n=16)
C (n=14)
surface

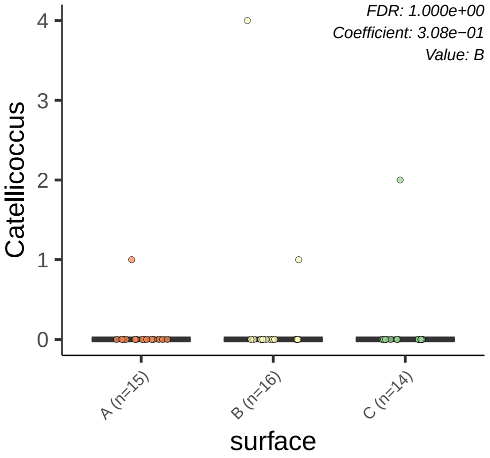
FDR: 1.000e+00
Coefficient: 3.08e−01
Value: B
Catellicoccus
0
1
2
3
4
A (n=15)
B (n=16)
C (n=14)
surface

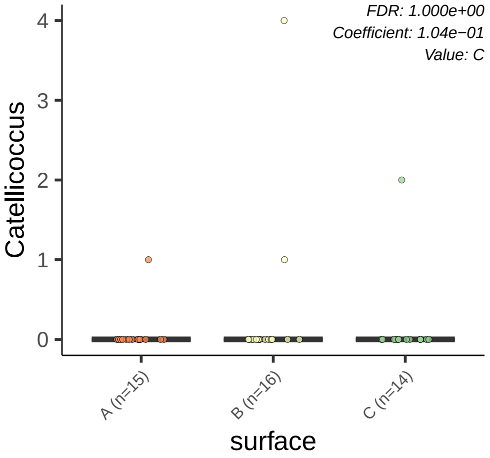
FDR: 1.000e+00
Coefficient: 1.04e−01
Value: C
Catellicoccus
4
3
2
1
0
A (n=15)
B (n=16)
C (n=14)
surface

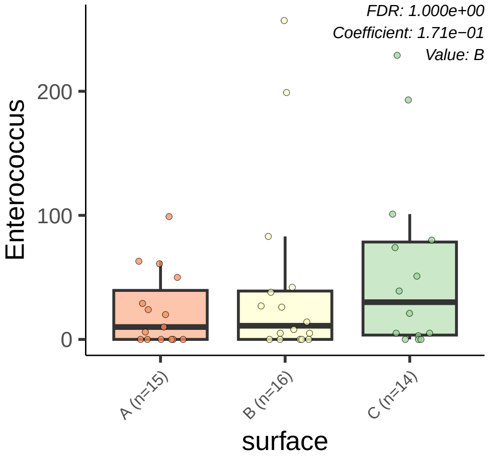

FDR: 1.000e+00
Coefficient: 1.71e−01
Value: B
Enterococcus
200
100
0
A (n=15)
B (n=16)
C (n=14)
surface

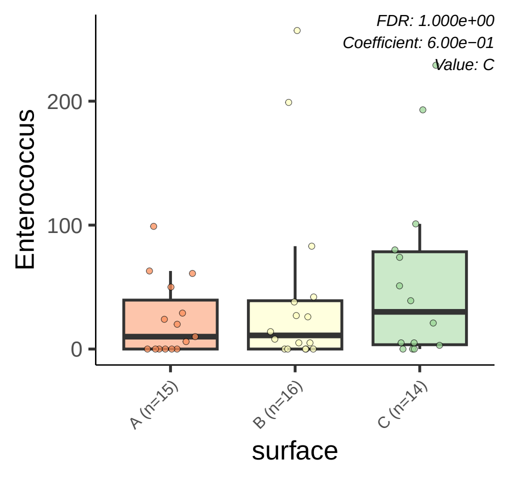
FDR: 1.000e+00
Coefficient: 6.00e−01
Value: C
Enterococcus
200
100
0
A (n=15)
B (n=16)
C (n=14)
surface

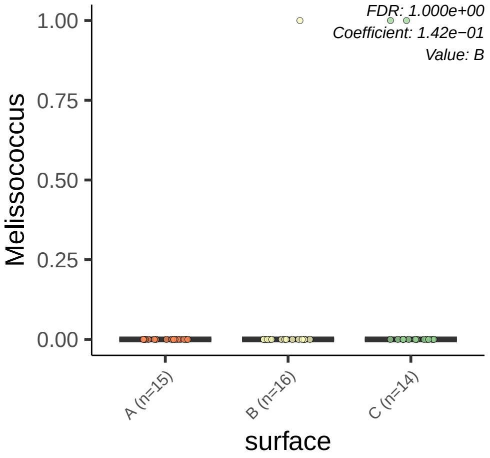

FDR: 1.000e+00
Coefficient: 1.42e−01
Value: B
Melissococcus
1.00
0.75
0.50
0.25
0.00
A (n=15)
B (n=16)
C (n=14)
surface

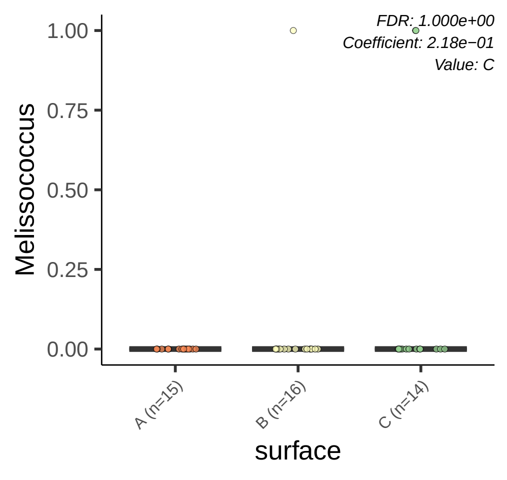
FDR: 1.000e+00
Coefficient: 2.18e−01
Value: C
1.00
0.75
0.50
0.25
0.00
Melissococcus
A (n=15)
B (n=16)
C (n=14)
surface

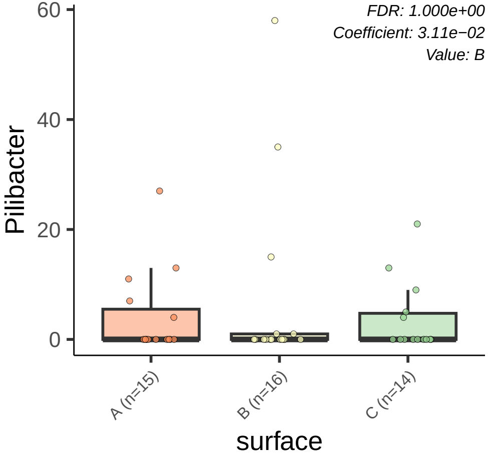
FDR: 1.000e+00
Coefficient: 3.11e−02
Value: B
Pilibacter
60
40
20
0
A (n=15)
B (n=16)
C (n=14)
surface

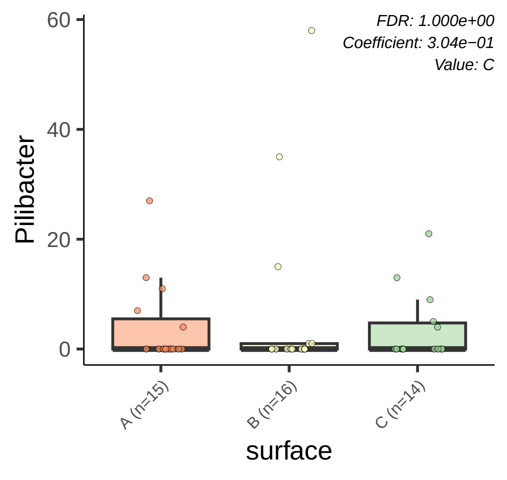

FDR: 1.000e+00
Coefficient: 3.04e−01
Value: C
Pilibacter
60
40
20
0
A (n=15)
B (n=16)
C (n=14)
surface

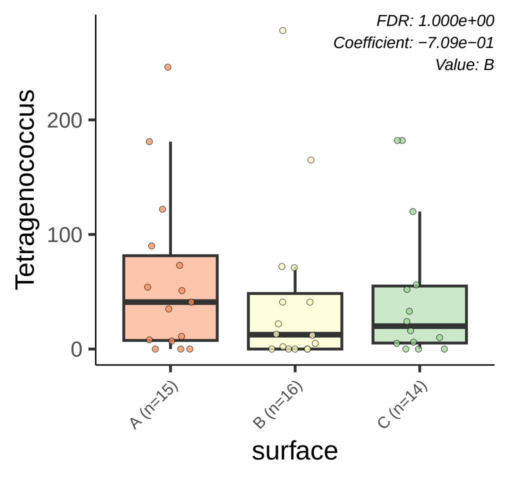
FDR: 1.000e+00
Coefficient: −7.09e−01
Value: B
Tetragenococcus
200
100
0
A (n=15)
B (n=16)
C (n=14)
surface

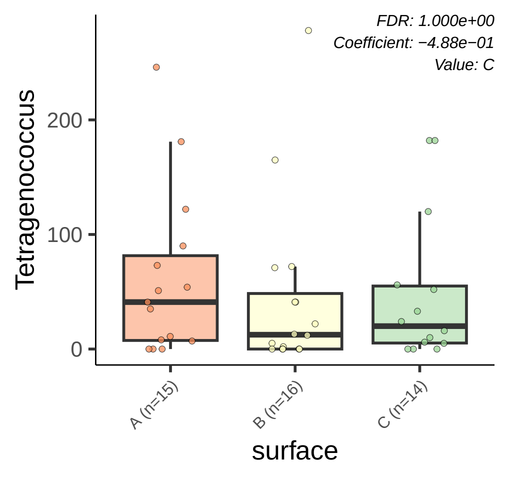
FDR: 1.000e+00
Coefficient: −4.88e−01
Value: C
Tetragenococcus
200
100
0
A (n=15)
B (n=16)
C (n=14)
surface

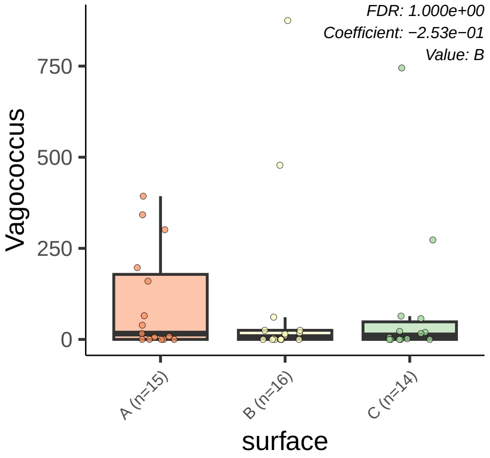

FDR: 1.000e+00
Coefficient: −2.53e−01
Value: B
Vagococcus
750
500
250
0
A (n=15)
B (n=16)
C (n=14)
surface

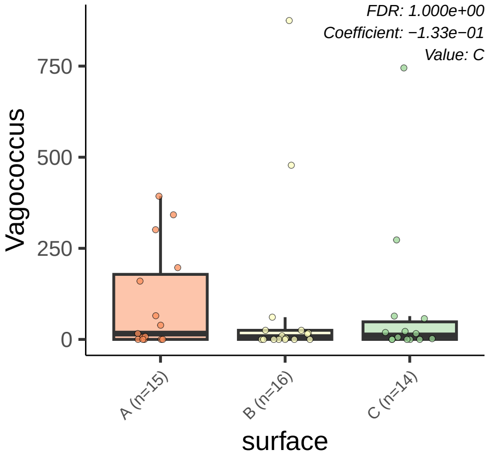

FDR: 1.000e+00
Coefficient: −1.33e−01
Value: C
Vagococcus
750
500
250
0
A (n=15)
B (n=16)
C (n=14)
surface

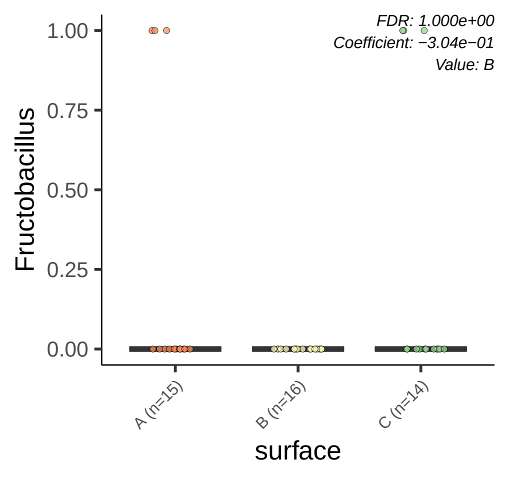
FDR: 1.000e+00
Coefficient: −3.04e−01
Value: B
Fructobacillus
1.00
0.75
0.50
0.25
0.00
A (n=15)
B (n=16)
C (n=14)
surface

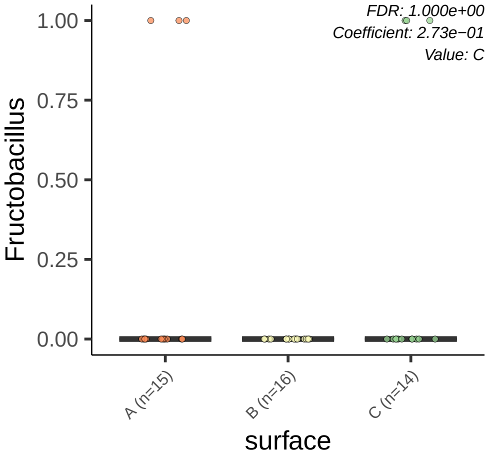

FDR: 1.000e+00
Coefficient: 2.73e−01
Value: C
Fructobacillus
1.00
0.75
0.50
0.25
0.00
A (n=15)
B (n=16)
C (n=14)
surface

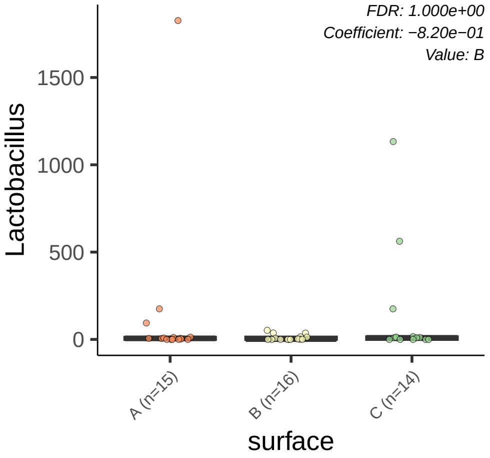
FDR: 1.000e+00
Coefficient: −8.20e−01
Value: B
Lactobacillus
1500
1000
500
0
A (n=15)
B (n=16)
C (n=14)
surface

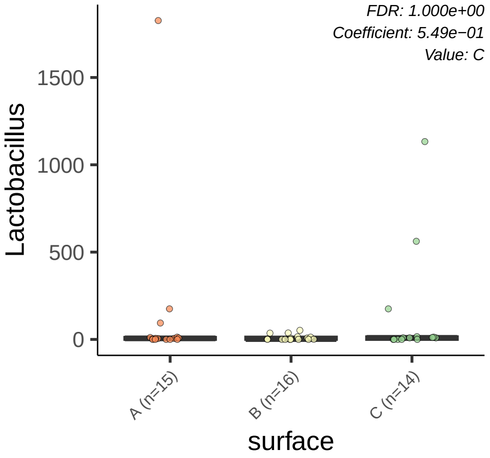
FDR: 1.000e+00
Coefficient: 5.49e−01
Value: C
Lactobacillus
1500
1000
500
0
A (n=15)
B (n=16)
C (n=14)
surface

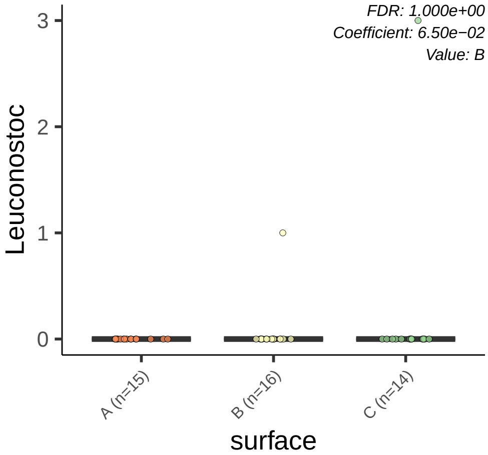
FDR: 1.000e+00
Coefficient: 6.50e−02
Value: B
Leuconostoc
3
2
1
0
A (n=15)
B (n=16)
C (n=14)
surface

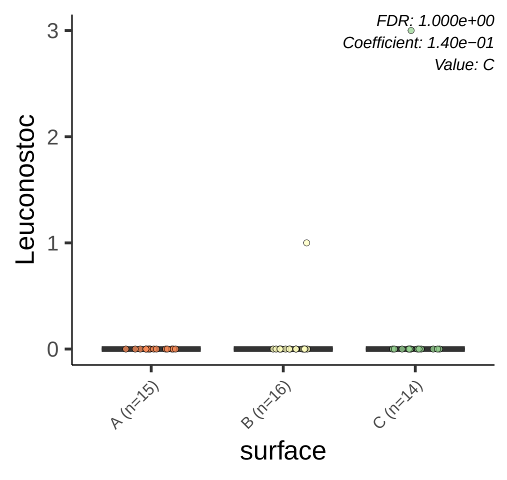
FDR: 1.000e+00
Coefficient: 1.40e−01
Value: C
Leuconostoc
3
2
1
0
A (n=15)
B (n=16)
C (n=14)
surface

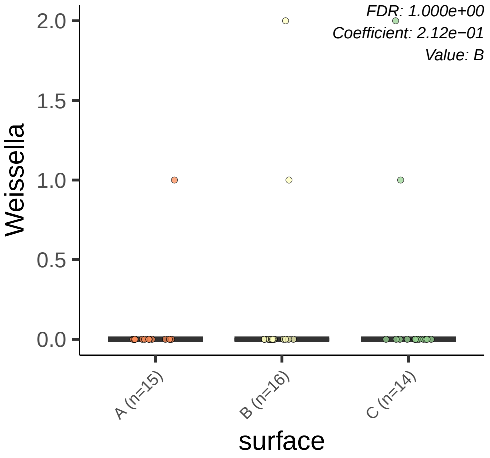
FDR: 1.000e+00
Coefficient: 2.12e−01
Value: B
Weissella
2.0
1.5
1.0
0.5
0.0
A (n=15)
B (n=16)
C (n=14)
surface

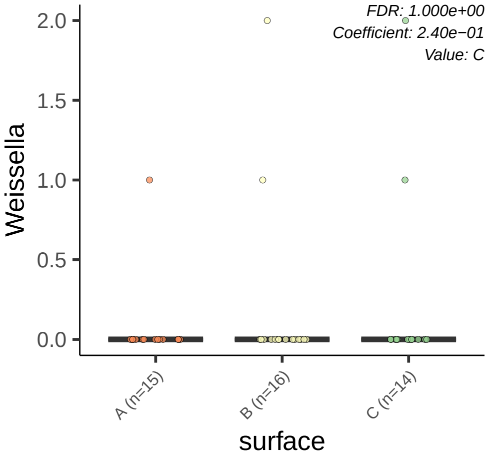
FDR: 1.000e+00
Coefficient: 2.40e−01
Value: C
Weissella
2.0
1.5
1.0
0.5
0.0
A (n=15)
B (n=16)
C (n=14)
surface

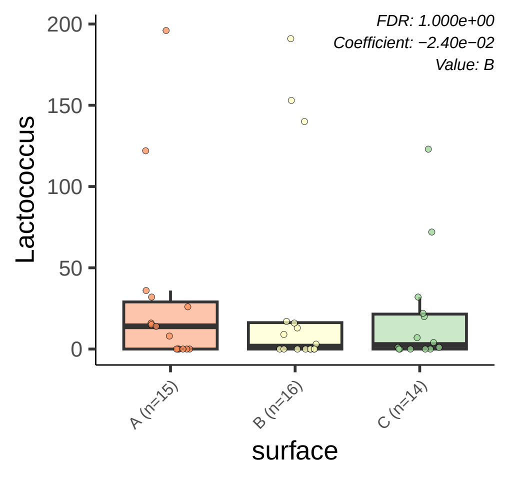

FDR: 1.000e+00
Coefficient: −2.40e−02
Value: B
Lactococcus
200
150
100
50
0
A (n=15)
B (n=16)
C (n=14)
surface

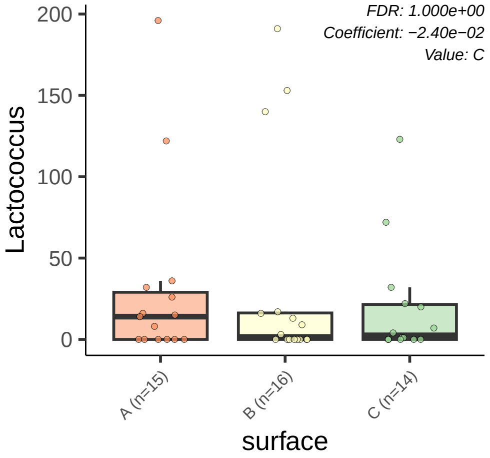

FDR: 1.000e+00
Coefficient: −2.40e−02
Value: C
Lactococcus
200
150
100
50
0
A (n=15)
B (n=16)
C (n=14)
surface

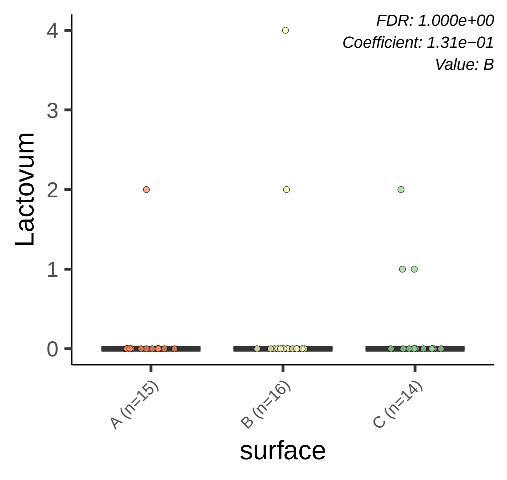
FDR: 1.000e+00
Coefficient: 1.31e−01
Value: B
Lactovum
0
1
2
3
4
A (n=15)
B (n=16)
C (n=14)
surface

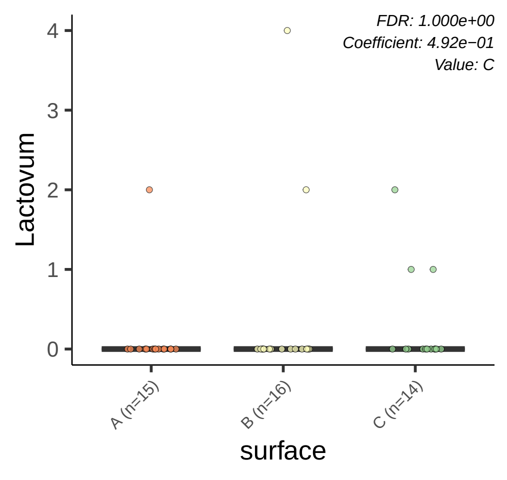
FDR: 1.000e+00
Coefficient: 4.92e−01
Value: C
Lactovum
4
3
2
1
0
A (n=15)
B (n=16)
C (n=14)
surface

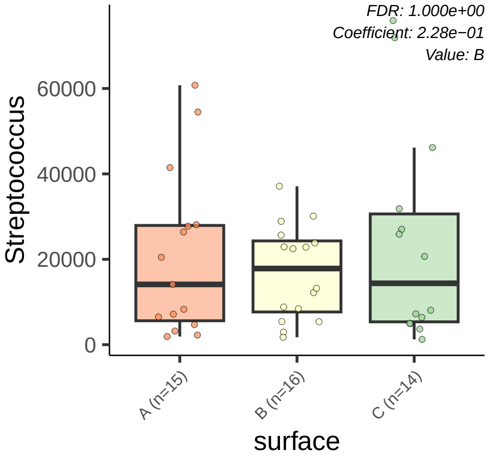

FDR: 1.000e+00
Coefficient: 2.28e−01
Value: B
Streptococcus
60000
40000
20000
0
A (n=15)
B (n=16)
C (n=14)
surface

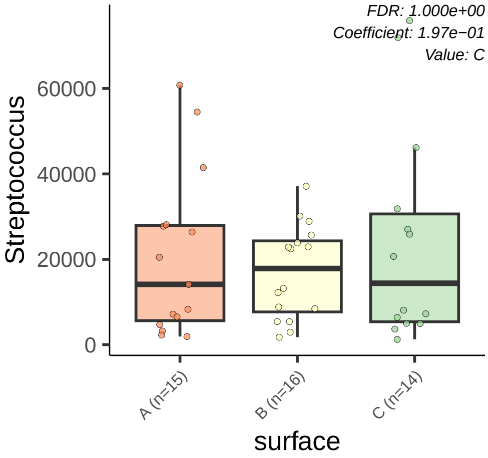

FDR: 1.000e+00
Coefficient: 1.97e−01
Value: C
Streptococcus
60000
40000
20000
0
A (n=15)
B (n=16)
C (n=14)
surface

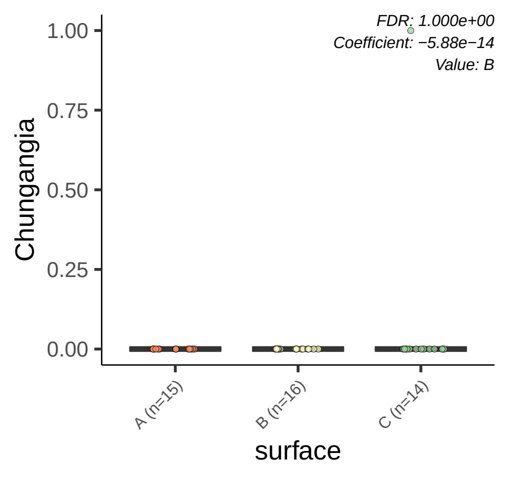
FDR: 1.000e+00
Coefficient: −5.88e−14
Value: B
Chungangia
1.00
0.75
0.50
0.25
0.00
A (n=15)
B (n=16)
C (n=14)
surface

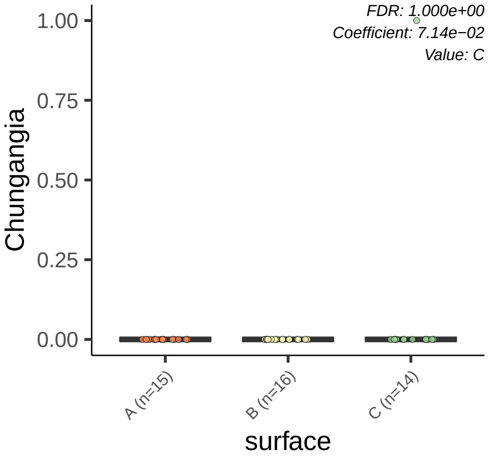
FDR: 1.000e+00
Coefficient: 7.14e−02
Value: C
Chungangia
1.00
0.75
0.50
0.25
0.00
A (n=15)
B (n=16)
C (n=14)
surface

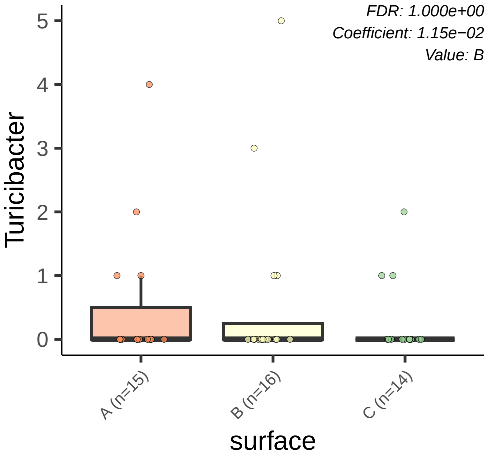
FDR: 1.000e+00
Coefficient: 1.15e−02
Value: B
Turicibacter
5
4
3
2
1
0
A (n=15)
B (n=16)
C (n=14)
surface

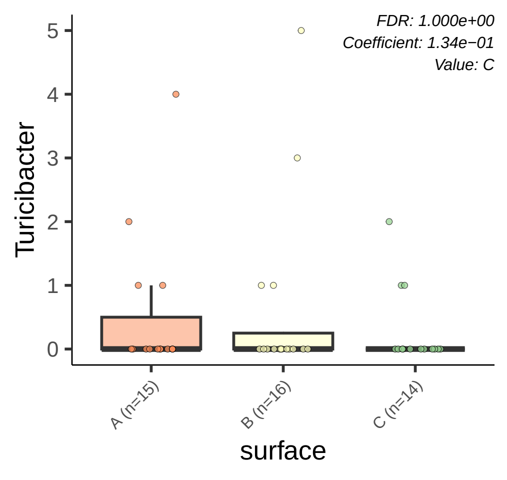

FDR: 1.000e+00
Coefficient: 1.34e−01
Value: C
Turicibacter
5
4
3
2
1
0
A (n=15)
B (n=16)
C (n=14)
surface

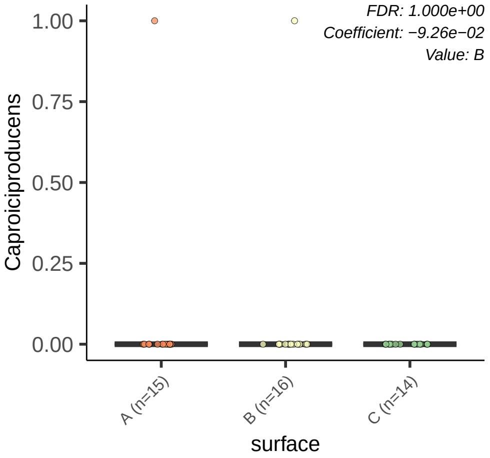
FDR: 1.000e+00
Coefficient: −9.26e−02
Value: B
1.00
0.75
0.50
0.25
0.00
Caproiciproducens
A (n=15)
B (n=16)
C (n=14)
surface

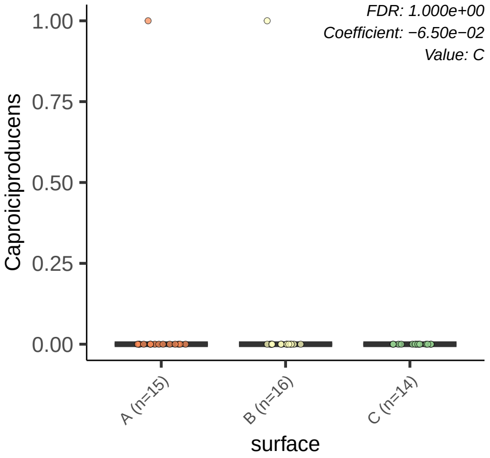

FDR: 1.000e+00
Coefficient: −6.50e−02
Value: C
Caproiciproducens
1.00
0.75
0.50
0.25
0.00
A (n=15)
B (n=16)
C (n=14)
surface

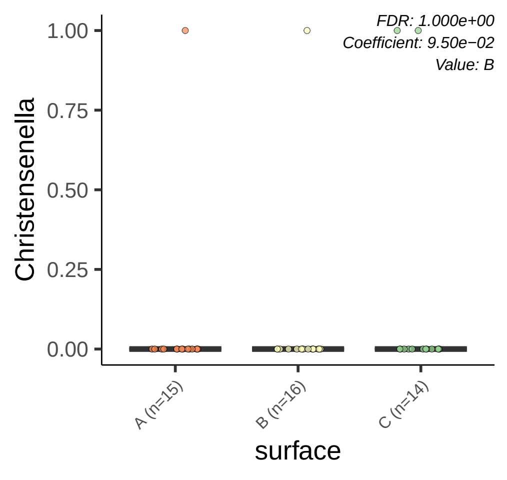
FDR: 1.000e+00
Coefficient: 9.50e−02
Value: B
Christensenella
1.00
0.75
0.50
0.25
0.00
A (n=15)
B (n=16)
C (n=14)
surface

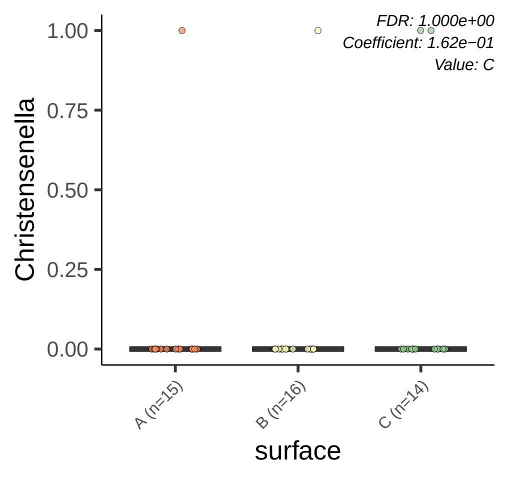

FDR: 1.000e+00
Coefficient: 1.62e−01
Value: C
Christensenella
1.00
0.75
0.50
0.25
0.00
A (n=15)
B (n=16)
C (n=14)
surface

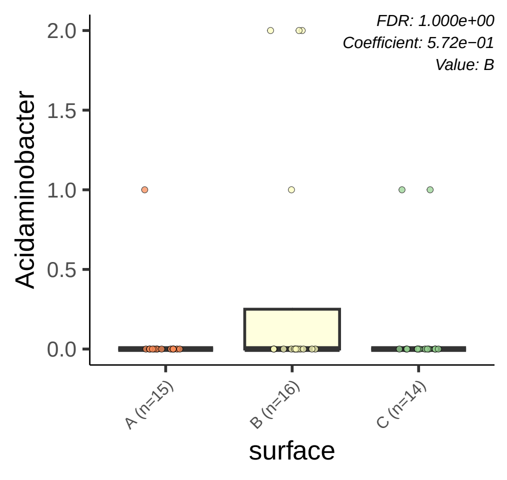
FDR: 1.000e+00
Coefficient: 5.72e−01
Value: B
Acidaminobacter
2.0
1.5
1.0
0.5
0.0
A (n=15)
B (n=16)
C (n=14)
surface

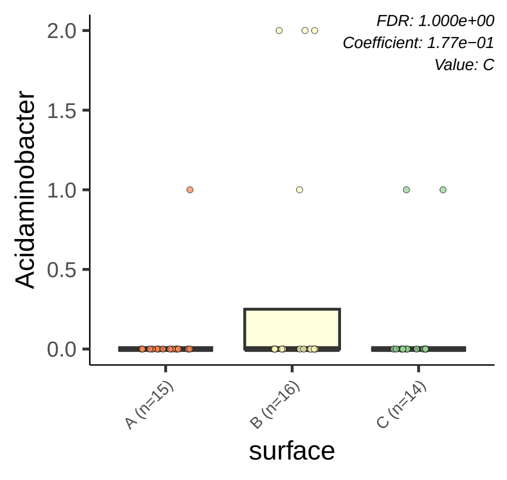
FDR: 1.000e+00
Coefficient: 1.77e−01
Value: C
Acidaminobacter
2.0
1.5
1.0
0.5
0.0
A (n=15)
B (n=16)
C (n=14)
surface

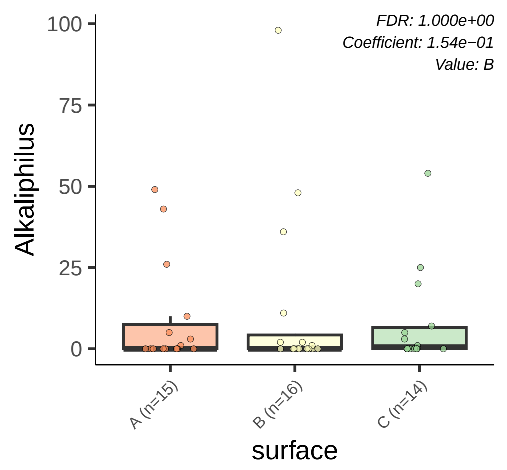

FDR: 1.000e+00
Coefficient: 1.54e−01
Value: B
Alkaliphilus
100
75
50
25
0
A (n=15)
B (n=16)
C (n=14)
surface

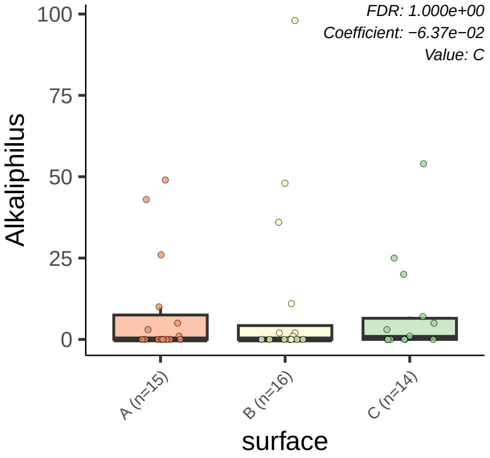

FDR: 1.000e+00
Coefficient: −6.37e−02
Value: C
Alkaliphilus
100
75
50
25
0
A (n=15)
B (n=16)
C (n=14)
surface

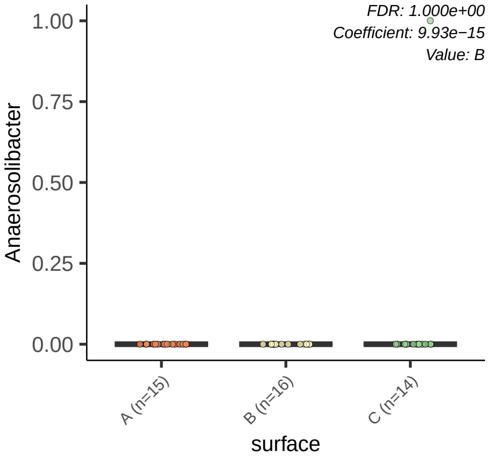
FDR: 1.000e+00
Coefficient: 9.93e−15
Value: B
Anaerosolibacter
1.00
0.75
0.50
0.25
0.00
A (n=15)
B (n=16)
C (n=14)
surface

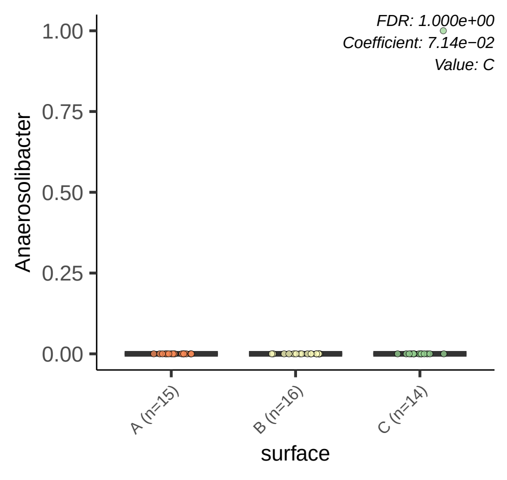
FDR: 1.000e+00
Coefficient: 7.14e−02
Value: C
1.00
0.75
0.50
0.25
0.00
Anaerosolibacter
A (n=15)
B (n=16)
C (n=14)
surface

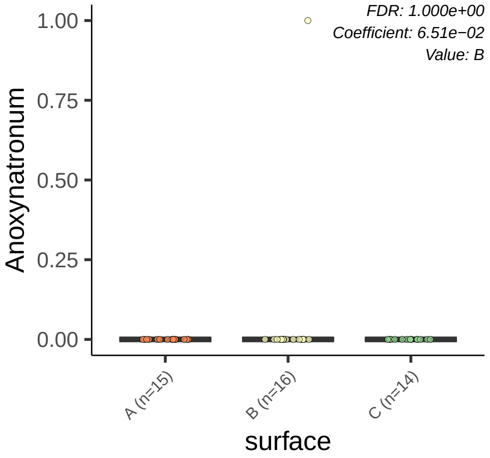

FDR: 1.000e+00
Coefficient: 6.51e−02
Value: B
Anoxynatronum
1.00
0.75
0.50
0.25
0.00
A (n=15)
B (n=16)
C (n=14)
surface

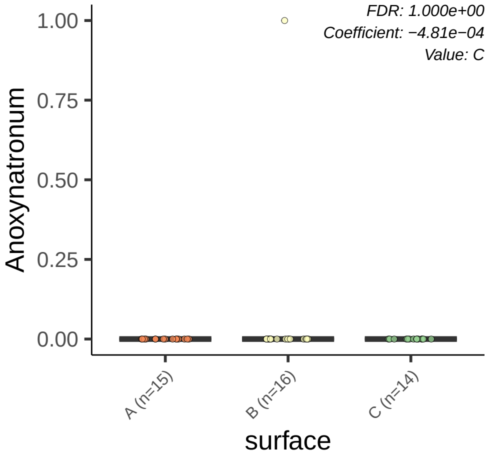
FDR: 1.000e+00
Coefficient: −4.81e−04
Value: C
Anoxynatronum
1.00
0.75
0.50
0.25
0.00
A (n=15)
B (n=16)
C (n=14)
surface

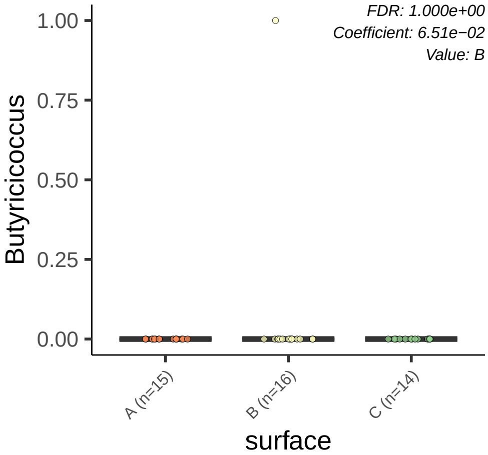

FDR: 1.000e+00
Coefficient: 6.51e−02
Value: B
Butyricicoccus
1.00
0.75
0.50
0.25
0.00
A (n=15)
B (n=16)
C (n=14)
surface

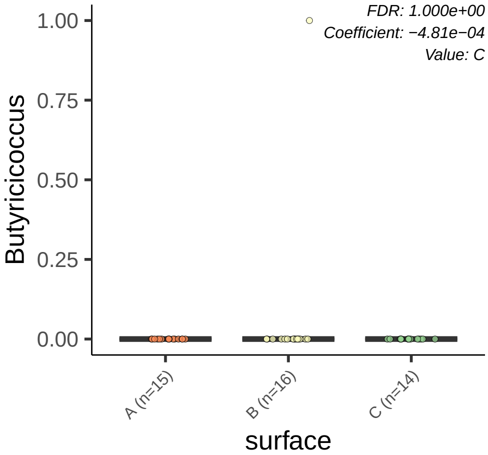
FDR: 1.000e+00
Coefficient: −4.81e−04
Value: C
Butyricicoccus
1.00
0.75
0.50
0.25
0.00
A (n=15)
B (n=16)
C (n=14)
surface

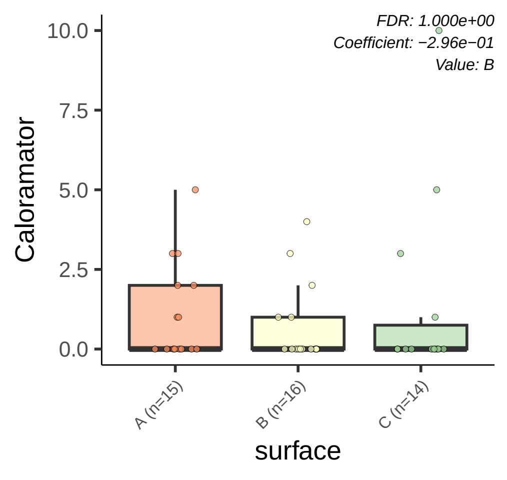
FDR: 1.000e+00
Coefficient: −2.96e−01
Value: B
Caloramator
10.0
7.5
5.0
2.5
0.0
A (n=15)
B (n=16)
C (n=14)
surface

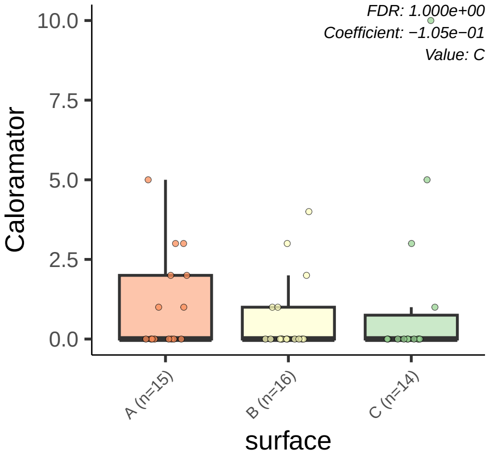

FDR: 1.000e+00
Coefficient: −1.05e−01
Value: C
Caloramator
10.0
7.5
5.0
2.5
0.0
A (n=15)
B (n=16)
C (n=14)
surface

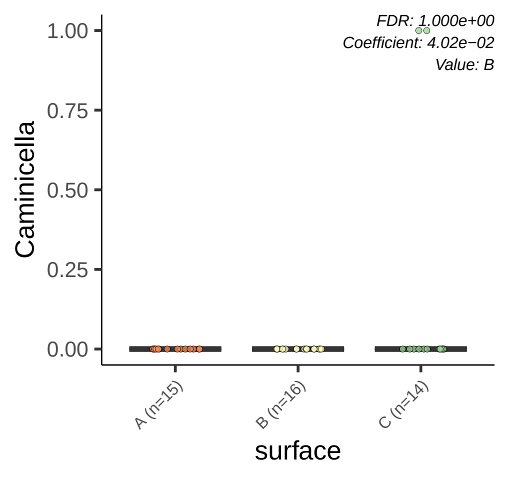

FDR: 1.000e+00
Coefficient: 4.02e−02
Value: B
Caminicella
1.00
0.75
0.50
0.25
0.00
A (n=15)
B (n=16)
C (n=14)
surface

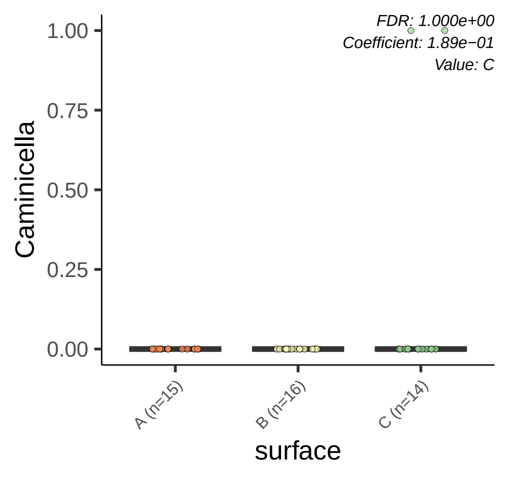

FDR: 1.000e+00
Coefficient: 1.89e−01
Value: C
Caminicella
1.00
0.75
0.50
0.25
0.00
A (n=15)
B (n=16)
C (n=14)
surface

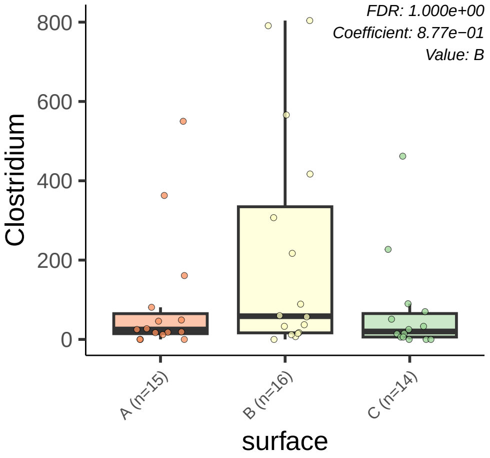
FDR: 1.000e+00
Coefficient: 8.77e−01
Value: B
Clostridium
0
200
400
600
800
A (n=15)
B (n=16)
C (n=14)
surface

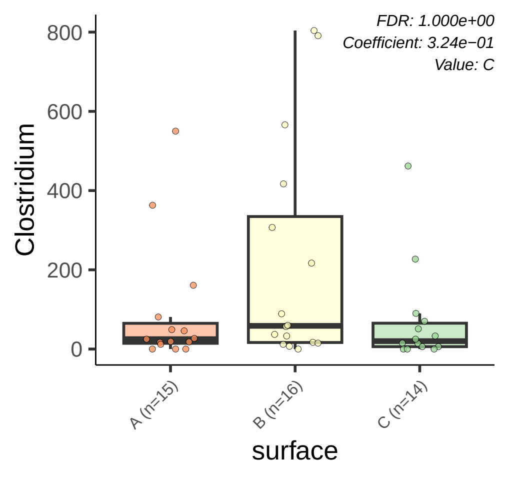

FDR: 1.000e+00
Coefficient: 3.24e−01
Value: C
Clostridium
800
600
400
200
0
A (n=15)
B (n=16)
C (n=14)
surface

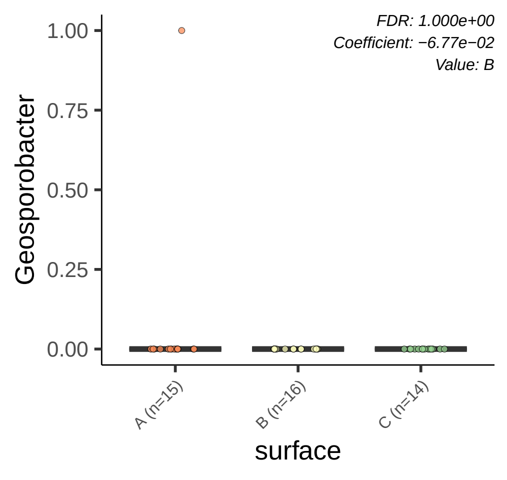

FDR: 1.000e+00
Coefficient: −6.77e−02
Value: B
Geosporobacter
1.00
0.75
0.50
0.25
0.00
A (n=15)
B (n=16)
C (n=14)
surface

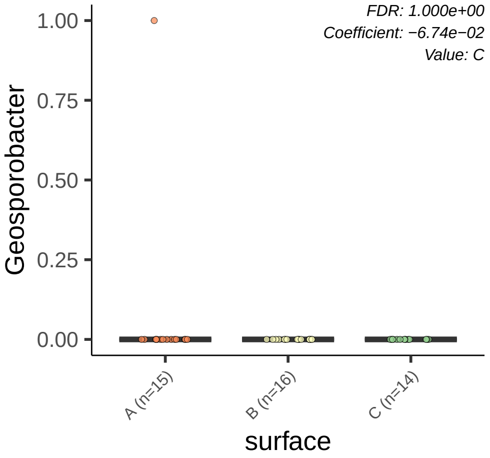
FDR: 1.000e+00
Coefficient: −6.74e−02
Value: C
Geosporobacter
1.00
0.75
0.50
0.25
0.00
A (n=15)
B (n=16)
C (n=14)
surface

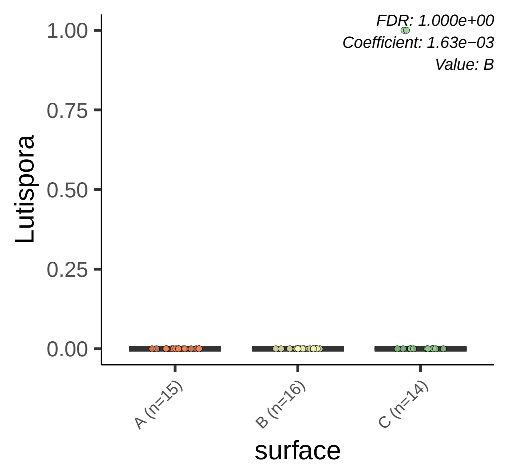

FDR: 1.000e+00
Coefficient: 1.63e−03
Value: B
1.00
0.75
0.50
0.25
0.00
Lutispora
A (n=15)
B (n=16)
C (n=14)
surface

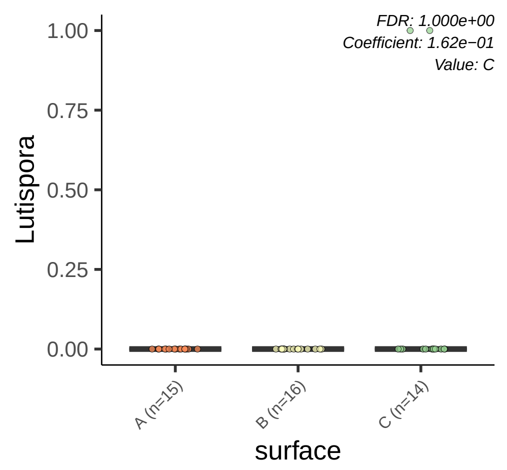
FDR: 1.000e+00
Coefficient: 1.62e−01
Value: C
Lutispora
1.00
0.75
0.50
0.25
0.00
A (n=15)
B (n=16)
C (n=14)
surface

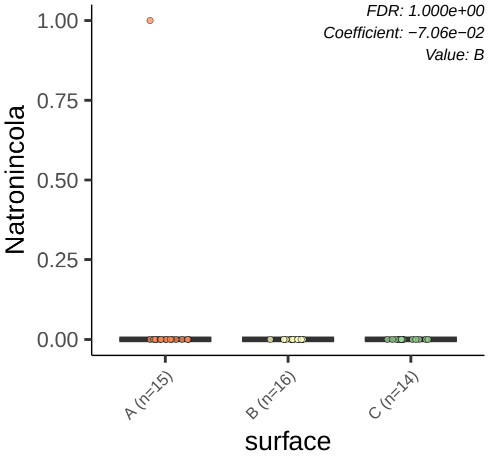
FDR: 1.000e+00
Coefficient: −7.06e−02
Value: B
Natronincola
1.00
0.75
0.50
0.25
0.00
A (n=15)
B (n=16)
C (n=14)
surface

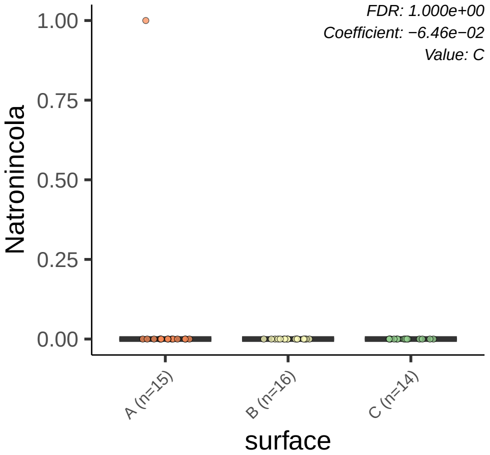
FDR: 1.000e+00
Coefficient: −6.46e−02
Value: C
Natronincola
1.00
0.75
0.50
0.25
0.00
A (n=15)
B (n=16)
C (n=14)
surface

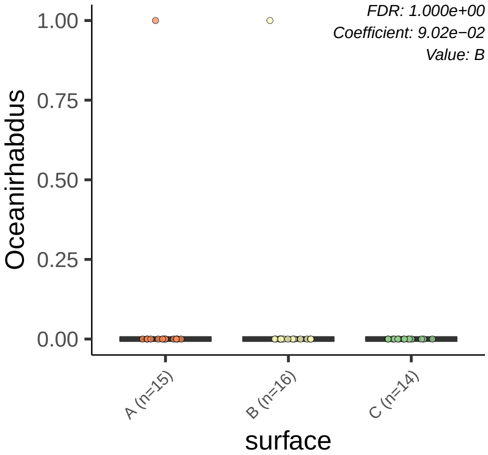

FDR: 1.000e+00
Coefficient: 9.02e−02
Value: B
1.00
0.75
0.50
0.25
0.00
Oceanirhabdus
A (n=15)
B (n=16)
C (n=14)
surface

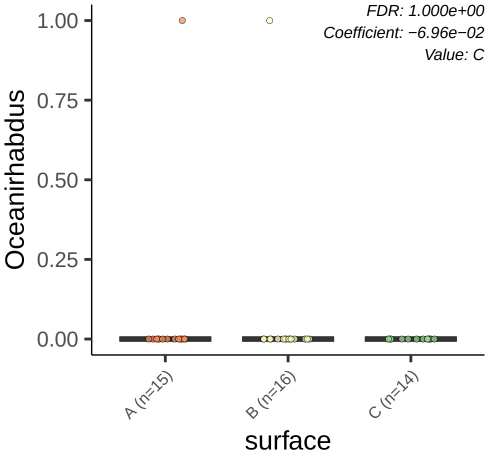
FDR: 1.000e+00
Coefficient: −6.96e−02
Value: C
Oceanirhabdus
1.00
0.75
0.50
0.25
0.00
A (n=15)
B (n=16)
C (n=14)
surface

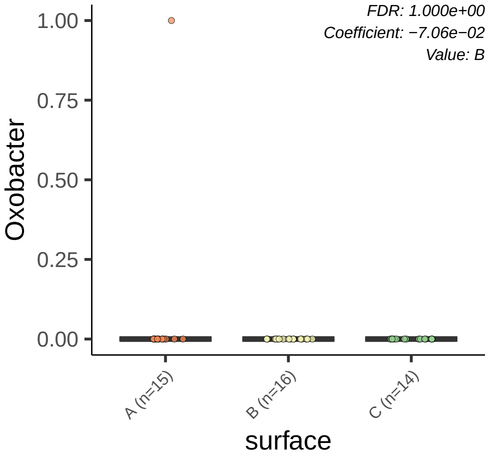
FDR: 1.000e+00
Coefficient: −7.06e−02
Value: B
Oxobacter
1.00
0.75
0.50
0.25
0.00
A (n=15)
B (n=16)
C (n=14)
surface

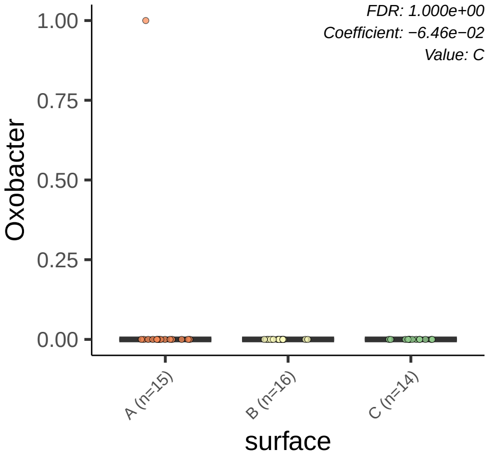

FDR: 1.000e+00
Coefficient: −6.46e−02
Value: C
Oxobacter
1.00
0.75
0.50
0.25
0.00
A (n=15)
B (n=16)
C (n=14)
surface

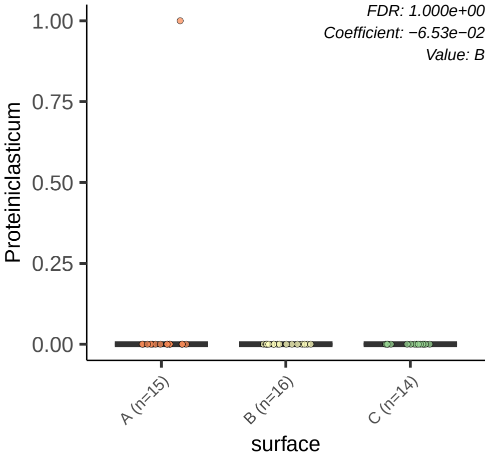

FDR: 1.000e+00
Coefficient: −6.53e−02
Value: B
Proteiniclasticum
1.00
0.75
0.50
0.25
0.00
A (n=15)
B (n=16)
C (n=14)
surface

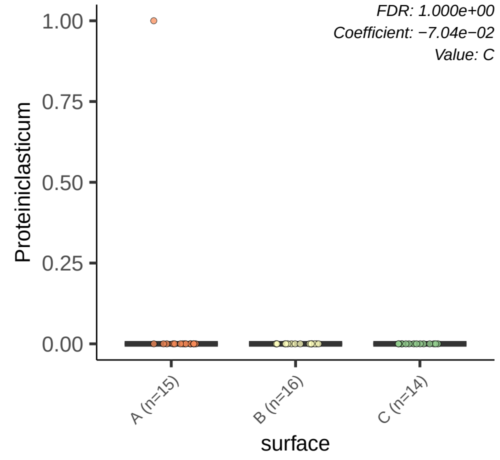

FDR: 1.000e+00
Coefficient: −7.04e−02
Value: C
Proteiniclasticum
1.00
0.75
0.50
0.25
0.00
A (n=15)
B (n=16)
C (n=14)
surface

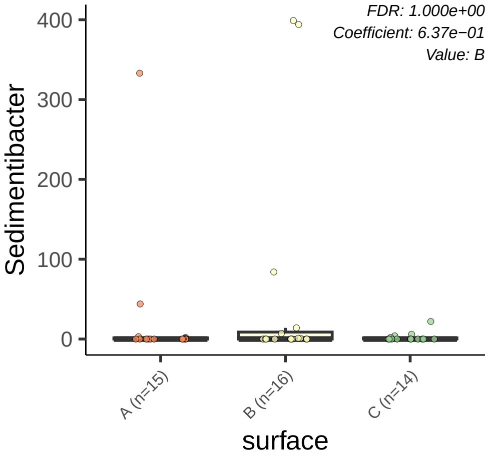
FDR: 1.000e+00
Coefficient: 6.37e−01
Value: B
Sedimentibacter
400
300
200
100
0
A (n=15)
B (n=16)
C (n=14)
surface

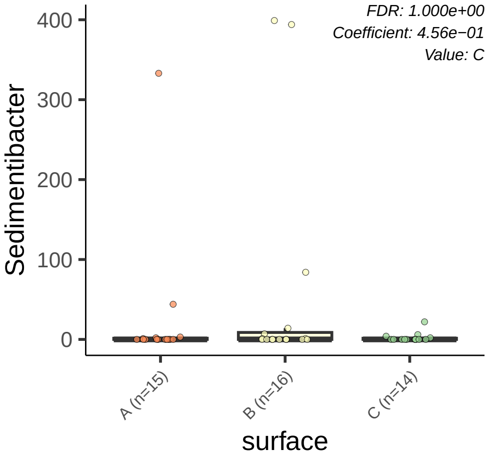

FDR: 1.000e+00
Coefficient: 4.56e−01
Value: C
Sedimentibacter
400
300
200
100
0
A (n=15)
B (n=16)
C (n=14)
surface

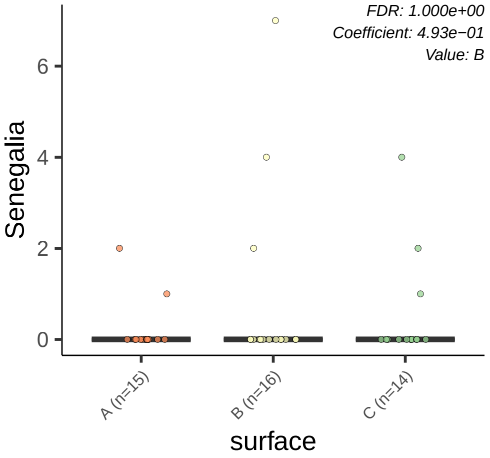
FDR: 1.000e+00
Coefficient: 4.93e−01
Value: B
Senegalia
0
2
4
6
A (n=15)
B (n=16)
C (n=14)
surface

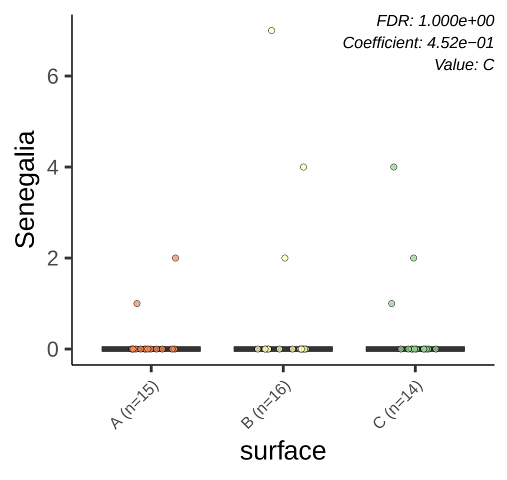

FDR: 1.000e+00
Coefficient: 4.52e−01
Value: C
Senegalia
0
2
4
6
A (n=15)
B (n=16)
C (n=14)
surface

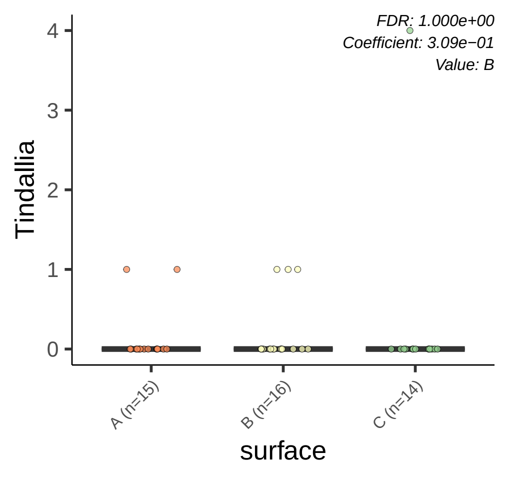
FDR: 1.000e+00
Coefficient: 3.09e−01
Value: B
Tindallia
4
3
2
1
0
A (n=15)
B (n=16)
C (n=14)
surface

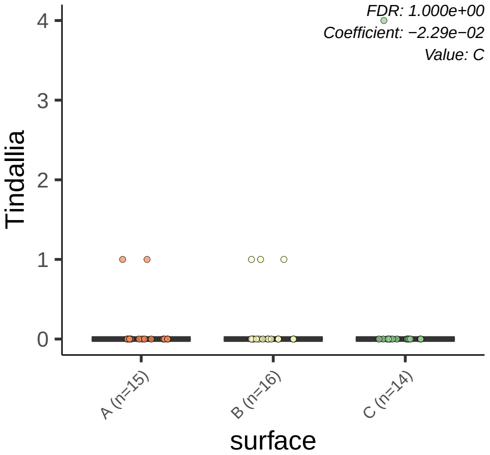

FDR: 1.000e+00
Coefficient: −2.29e−02
Value: C
Tindallia
4
3
2
1
0
A (n=15)
B (n=16)
C (n=14)
surface

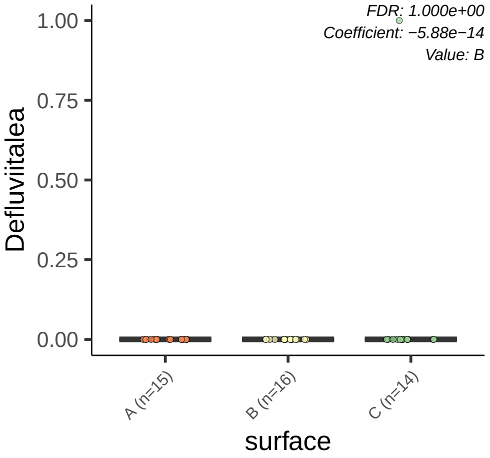

FDR: 1.000e+00
Coefficient: −5.88e−14
Value: B
1.00
0.75
0.50
0.25
0.00
Defluviitalea
A (n=15)
B (n=16)
C (n=14)
surface

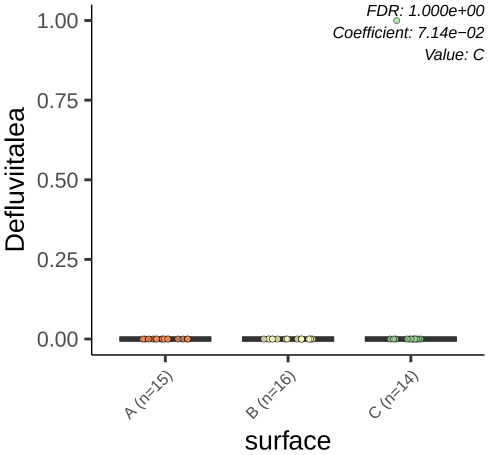
FDR: 1.000e+00
Coefficient: 7.14e−02
Value: C
1.00
0.75
0.50
0.25
0.00
Defluviitalea
A (n=15)
B (n=16)
C (n=14)
surface

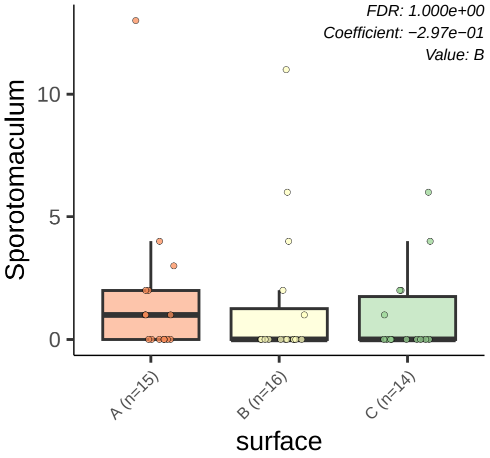

FDR: 1.000e+00
Coefficient: −2.97e−01
Value: B
Sporotomaculum
0
5
10
A (n=15)
B (n=16)
C (n=14)
surface

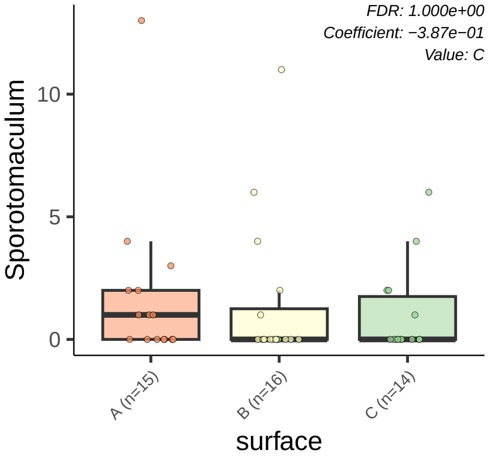

FDR: 1.000e+00
Coefficient: −3.87e−01
Value: C
Sporotomaculum
0
5
10
A (n=15)
B (n=16)
C (n=14)
surface

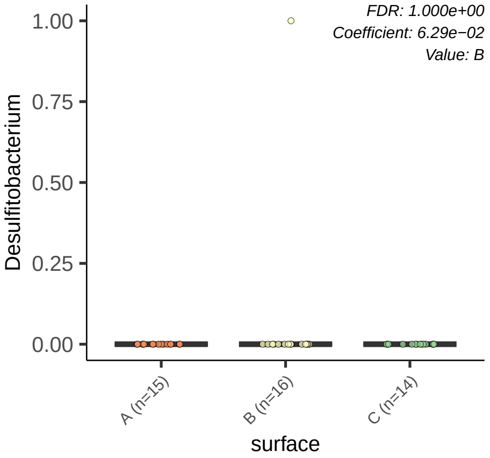
FDR: 1.000e+00
Coefficient: 6.29e−02
Value: B
Desulfitobacterium
1.00
0.75
0.50
0.25
0.00
A (n=15)
B (n=16)
C (n=14)
surface

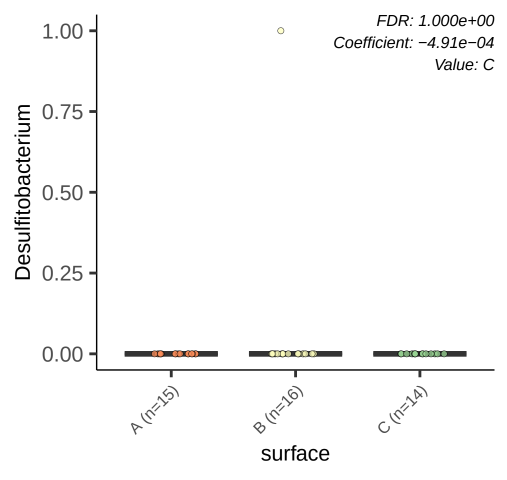
FDR: 1.000e+00
Coefficient: −4.91e−04
Value: C
Desulfitobacterium
1.00
0.75
0.50
0.25
0.00
A (n=15)
B (n=16)
C (n=14)
surface

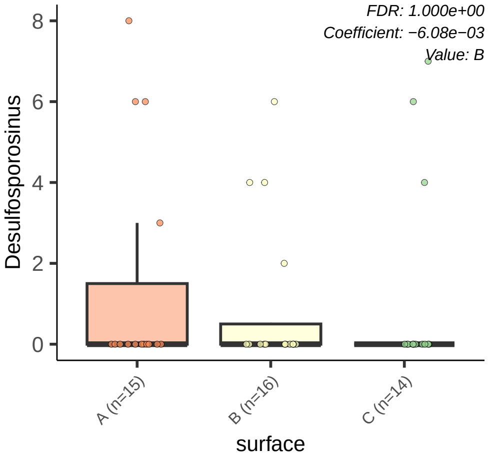

FDR: 1.000e+00
Coefficient: −6.08e−03
Value: B
Desulfosporosinus
8
6
4
2
0
A (n=15)
B (n=16)
C (n=14)
surface

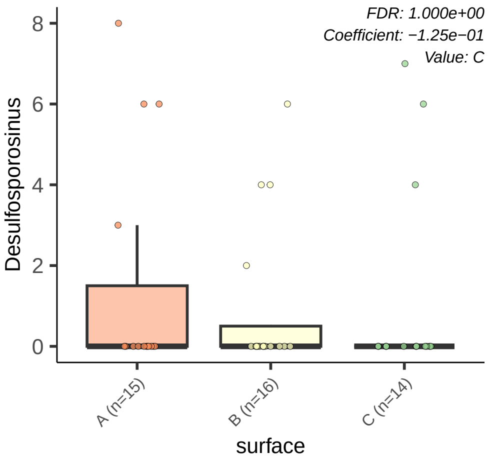
FDR: 1.000e+00
Coefficient: −1.25e−01
Value: C
Desulfosporosinus
0
2
4
6
8
A (n=15)
B (n=16)
C (n=14)
surface

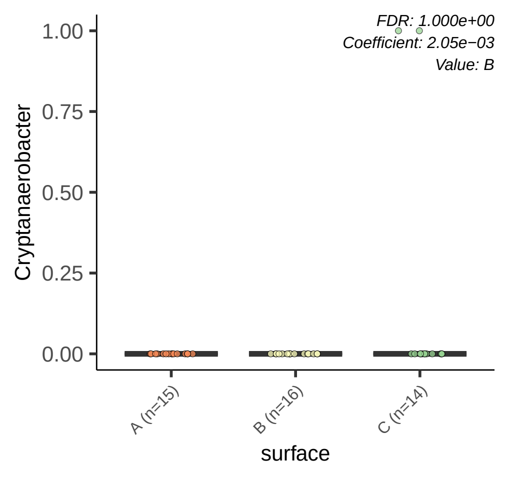
FDR: 1.000e+00
Coefficient: 2.05e−03
Value: B
1.00
0.75
0.50
0.25
0.00
Cryptanaerobacter
A (n=15)
B (n=16)
C (n=14)
surface

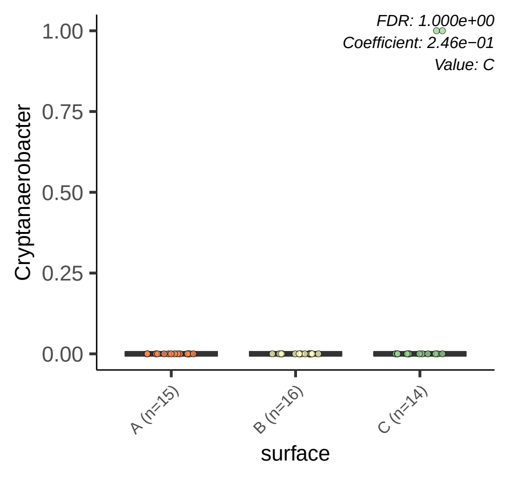

FDR: 1.000e+00
Coefficient: 2.46e−01
Value: C
Cryptanaerobacter
1.00
0.75
0.50
0.25
0.00
A (n=15)
B (n=16)
C (n=14)
surface

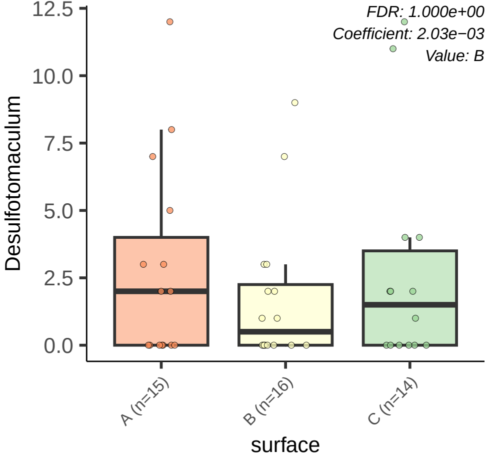

FDR: 1.000e+00
Coefficient: 2.03e−03
Value: B
Desulfotomaculum
12.5
10.0
7.5
5.0
2.5
0.0
A (n=15)
B (n=16)
C (n=14)
surface

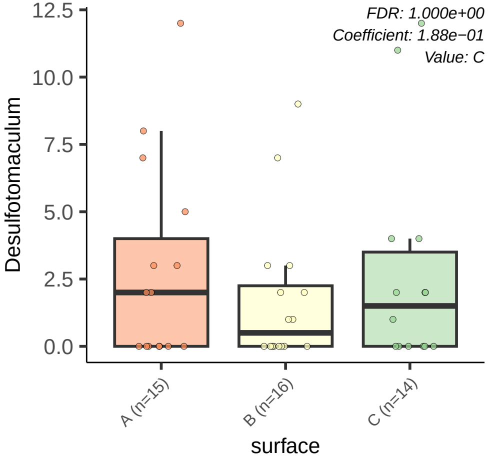
FDR: 1.000e+00
Coefficient: 1.88e−01
Value: C
Desulfotomaculum
12.5
10.0
7.5
5.0
2.5
0.0
A (n=15)
B (n=16)
C (n=14)
surface

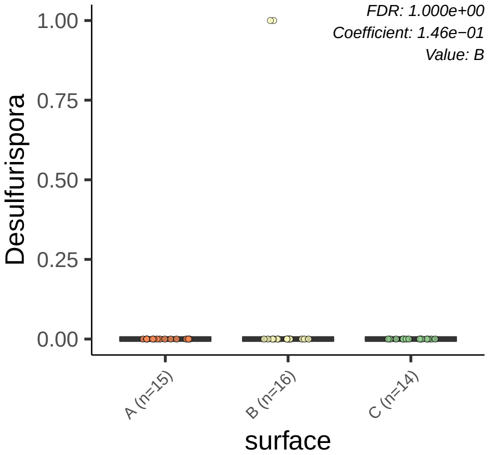
FDR: 1.000e+00
Coefficient: 1.46e−01
Value: B
Desulfurispora
1.00
0.75
0.50
0.25
0.00
A (n=15)
B (n=16)
C (n=14)
surface

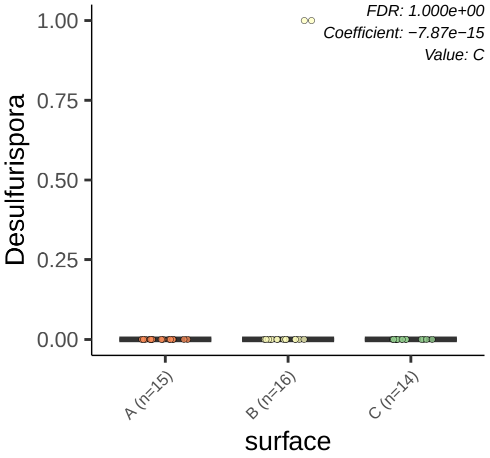

FDR: 1.000e+00
Coefficient: −7.87e−15
Value: C
Desulfurispora
1.00
0.75
0.50
0.25
0.00
A (n=15)
B (n=16)
C (n=14)
surface

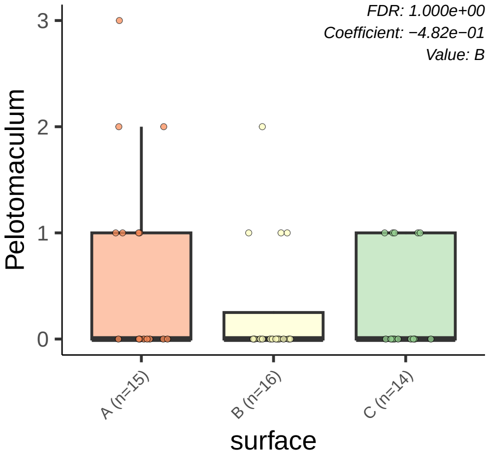

FDR: 1.000e+00
Coefficient: −4.82e−01
Value: B
Pelotomaculum
0
1
2
3
A (n=15)
B (n=16)
C (n=14)
surface

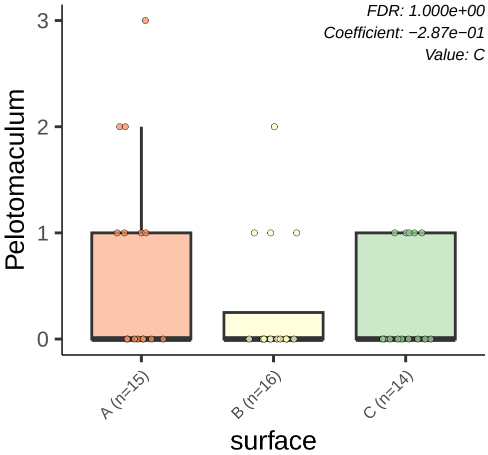

FDR: 1.000e+00
Coefficient: −2.87e−01
Value: C
Pelotomaculum
0
1
2
3
A (n=15)
B (n=16)
C (n=14)
surface

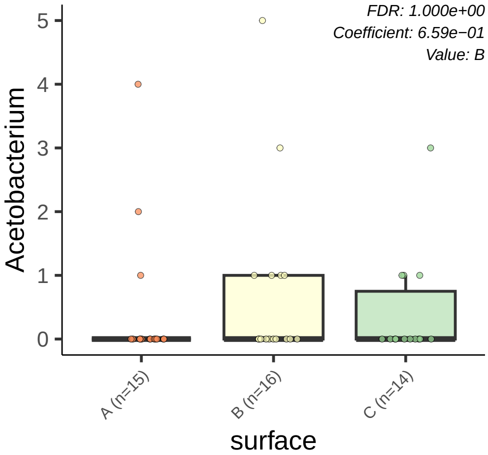

FDR: 1.000e+00
Coefficient: 6.59e−01
Value: B
Acetobacterium
0
1
2
3
4
5
A (n=15)
B (n=16)
C (n=14)
surface

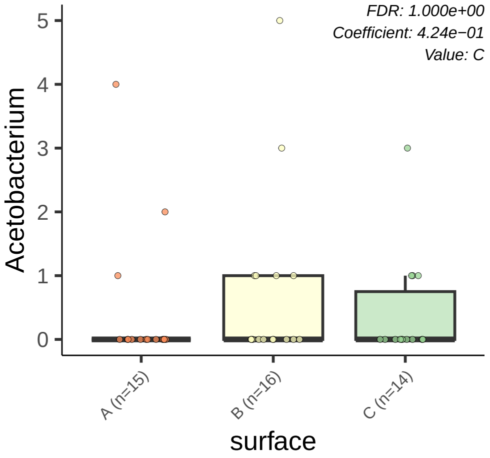

FDR: 1.000e+00
Coefficient: 4.24e−01
Value: C
Acetobacterium
0
1
2
3
4
5
A (n=15)
B (n=16)
C (n=14)
surface

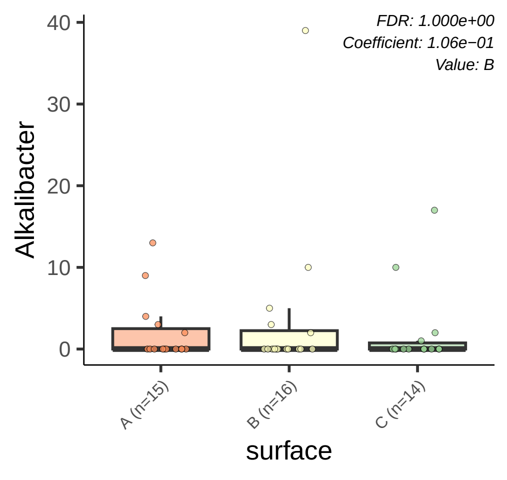
FDR: 1.000e+00
Coefficient: 1.06e−01
Value: B
Alkalibacter
40
30
20
10
0
A (n=15)
B (n=16)
C (n=14)
surface

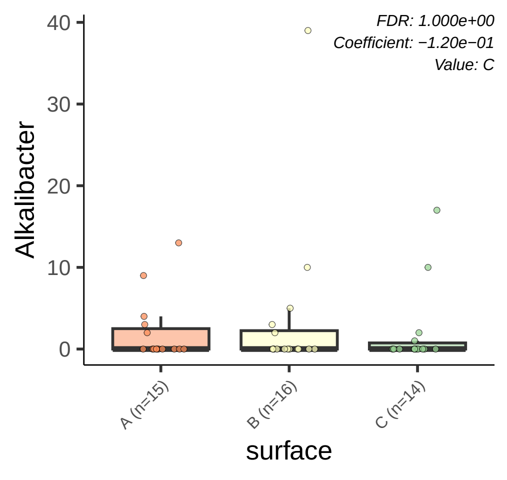

FDR: 1.000e+00
Coefficient: −1.20e−01
Value: C
Alkalibacter
40
30
20
10
0
A (n=15)
B (n=16)
C (n=14)
surface

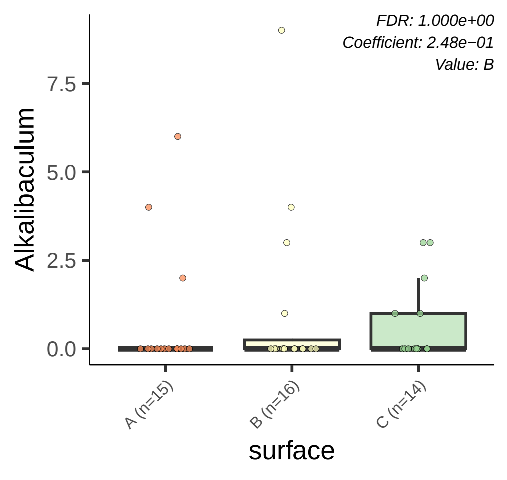

FDR: 1.000e+00
Coefficient: 2.48e−01
Value: B
Alkalibaculum
7.5
5.0
2.5
0.0
A (n=15)
B (n=16)
C (n=14)
surface

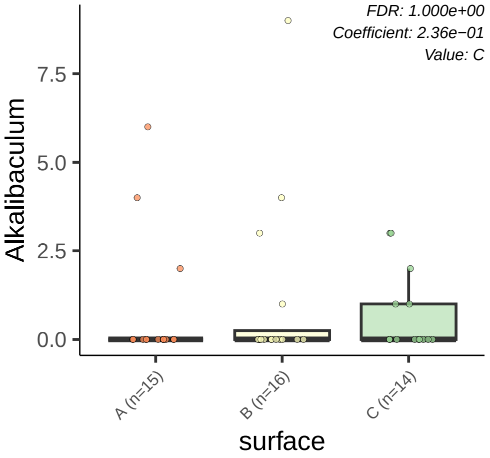

FDR: 1.000e+00
Coefficient: 2.36e−01
Value: C
Alkalibaculum
7.5
5.0
2.5
0.0
A (n=15)
B (n=16)
C (n=14)
surface

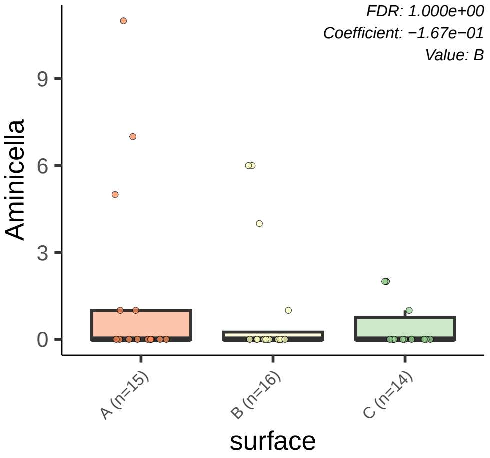
FDR: 1.000e+00
Coefficient: −1.67e−01
Value: B
Aminicella
9
6
3
0
A (n=15)
B (n=16)
C (n=14)
surface

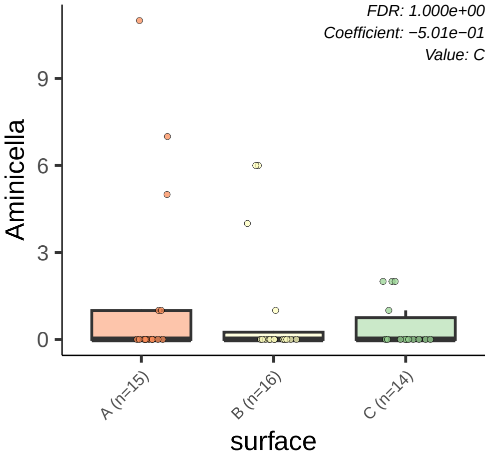
FDR: 1.000e+00
Coefficient: −5.01e−01
Value: C
Aminicella
9
6
3
0
A (n=15)
B (n=16)
C (n=14)
surface

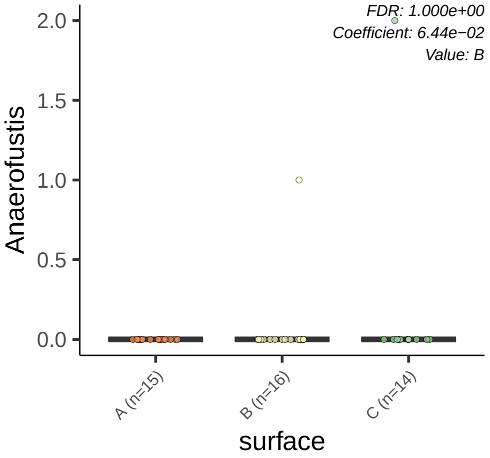

FDR: 1.000e+00
Coefficient: 6.44e−02
Value: B
Anaerofustis
2.0
1.5
1.0
0.5
0.0
A (n=15)
B (n=16)
C (n=14)
surface

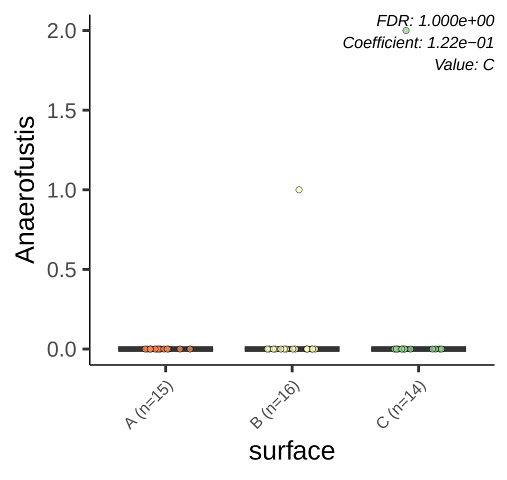
FDR: 1.000e+00
Coefficient: 1.22e−01
Value: C
Anaerofustis
2.0
1.5
1.0
0.5
0.0
A (n=15)
B (n=16)
C (n=14)
surface

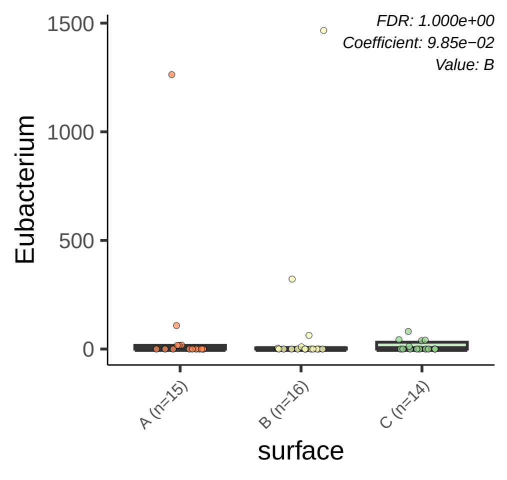
FDR: 1.000e+00
Coefficient: 9.85e−02
Value: B
Eubacterium
1500
1000
500
0
A (n=15)
B (n=16)
C (n=14)
surface

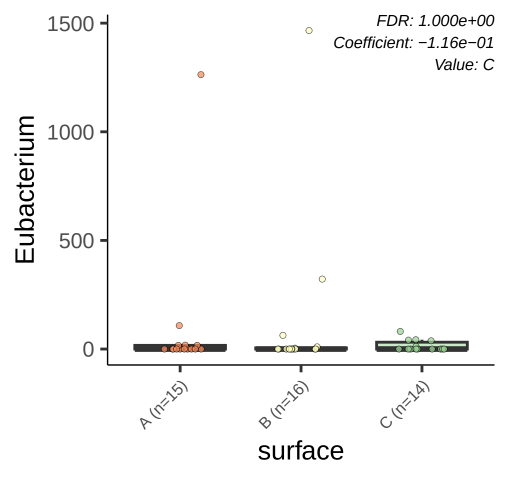

FDR: 1.000e+00
Coefficient: −1.16e−01
Value: C
1500
1000
500
0
Eubacterium
A (n=15)
B (n=16)
C (n=14)
surface

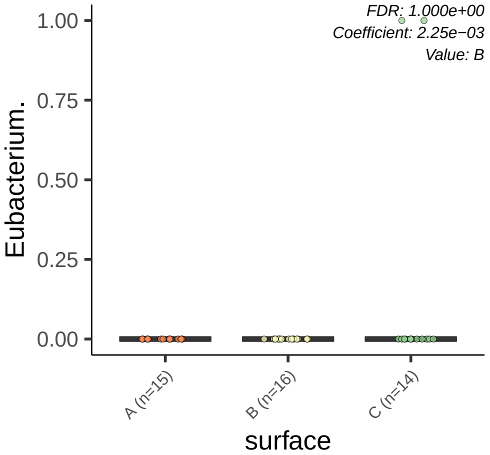

FDR: 1.000e+00
Coefficient: 2.25e−03
Value: B
Eubacterium.
1.00
0.75
0.50
0.25
0.00
A (n=15)
B (n=16)
C (n=14)
surface

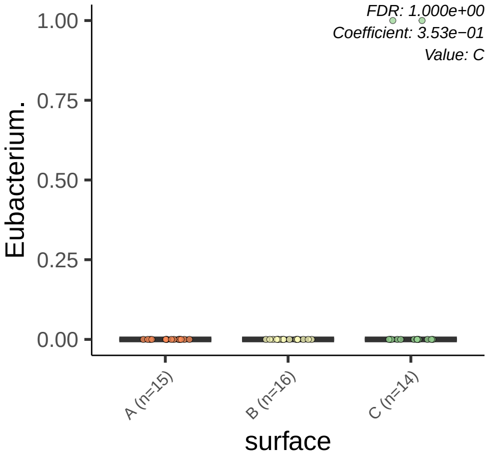
FDR: 1.000e+00
Coefficient: 3.53e−01
Value: C
1.00
0.75
0.50
0.25
0.00
Eubacterium.
A (n=15)
B (n=16)
C (n=14)
surface

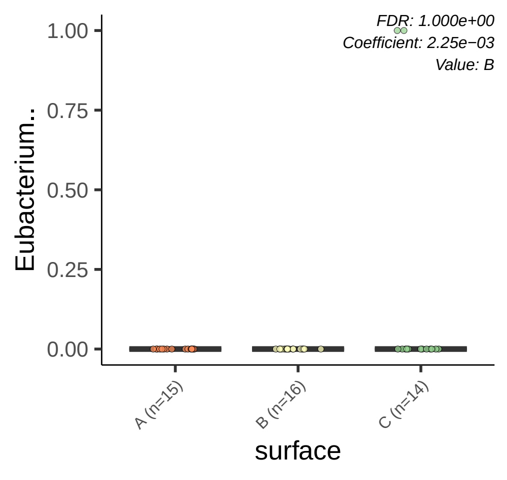
FDR: 1.000e+00
Coefficient: 2.25e−03
Value: B
1.00
0.75
0.50
0.25
0.00
Eubacterium..
A (n=15)
B (n=16)
C (n=14)
surface

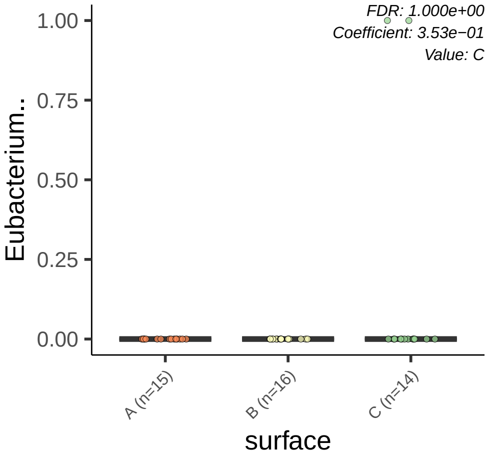

FDR: 1.000e+00
Coefficient: 3.53e−01
Value: C
Eubacterium..
1.00
0.75
0.50
0.25
0.00
A (n=15)
B (n=16)
C (n=14)
surface

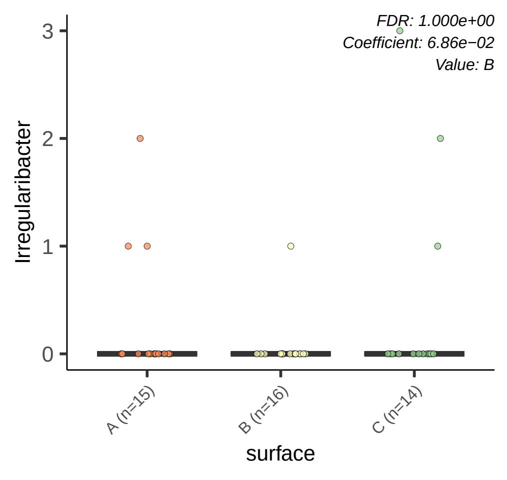
FDR: 1.000e+00
Coefficient: 6.86e−02
Value: B
Irregularibacter
3
2
1
0
A (n=15)
B (n=16)
C (n=14)
surface

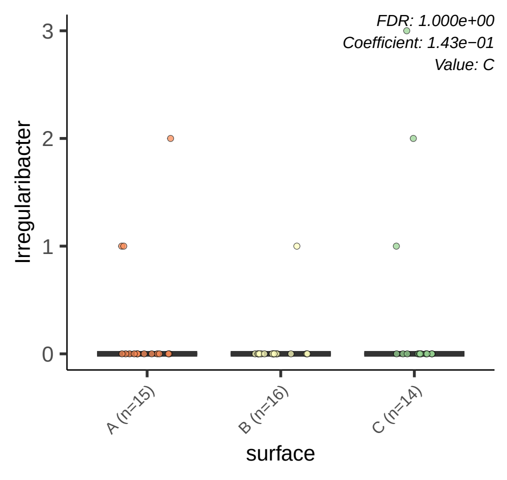
FDR: 1.000e+00
Coefficient: 1.43e−01
Value: C
Irregularibacter
3
2
1
0
A (n=15)
B (n=16)
C (n=14)
surface

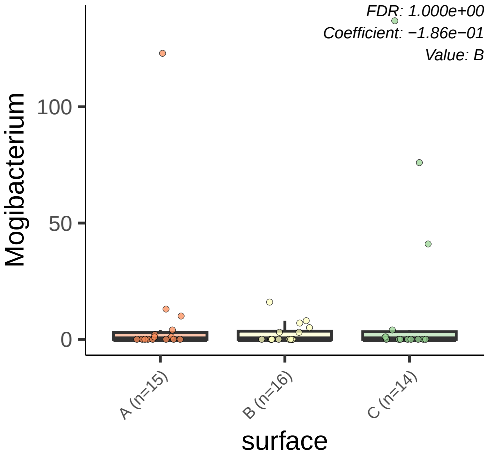

FDR: 1.000e+00
Coefficient: −1.86e−01
Value: B
Mogibacterium
100
50
0
A (n=15)
B (n=16)
C (n=14)
surface

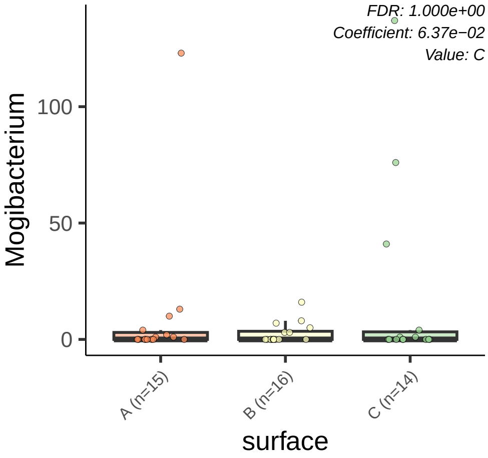
FDR: 1.000e+00
Coefficient: 6.37e−02
Value: C
Mogibacterium
100
50
0
A (n=15)
B (n=16)
C (n=14)
surface

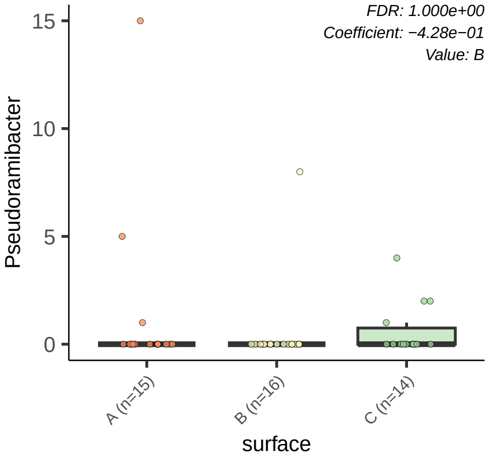
FDR: 1.000e+00
Coefficient: −4.28e−01
Value: B
Pseudoramibacter
15
10
5
0
A (n=15)
B (n=16)
C (n=14)
surface

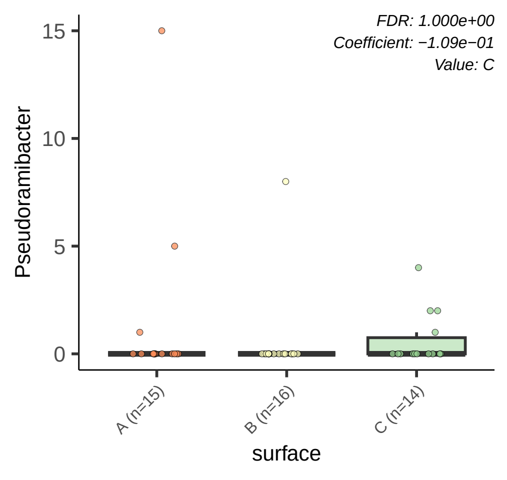
FDR: 1.000e+00
Coefficient: −1.09e−01
Value: C
Pseudoramibacter
15
10
5
0
A (n=15)
B (n=16)
C (n=14)
surface

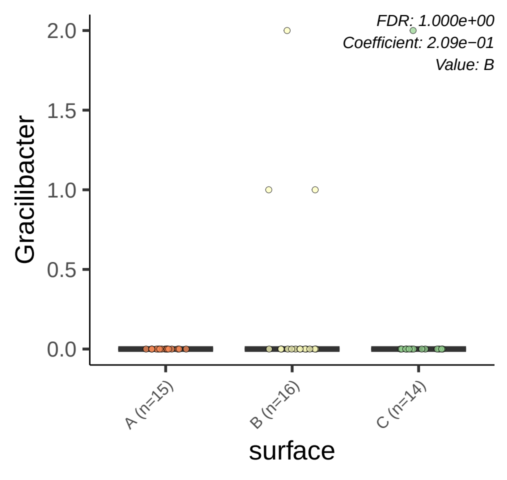

FDR: 1.000e+00
Coefficient: 2.09e−01
Value: B
Gracilibacter
2.0
1.5
1.0
0.5
0.0
A (n=15)
B (n=16)
C (n=14)
surface

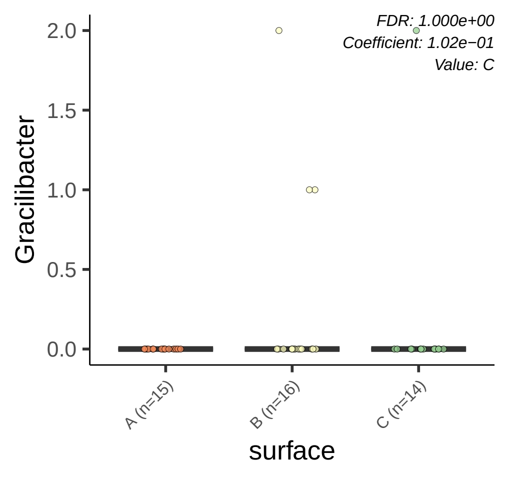
FDR: 1.000e+00
Coefficient: 1.02e−01
Value: C
Gracilibacter
2.0
1.5
1.0
0.5
0.0
A (n=15)
B (n=16)
C (n=14)
surface

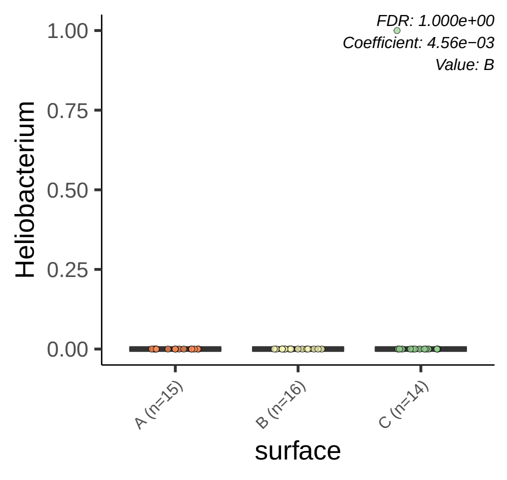
FDR: 1.000e+00
Coefficient: 4.56e−03
Value: B
Heliobacterium
1.00
0.75
0.50
0.25
0.00
A (n=15)
B (n=16)
C (n=14)
surface

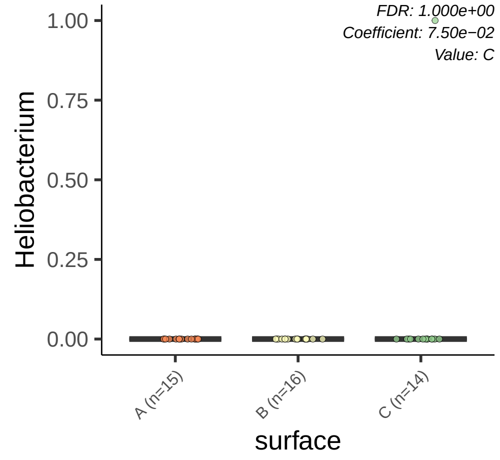

FDR: 1.000e+00
Coefficient: 7.50e−02
Value: C
Heliobacterium
1.00
0.75
0.50
0.25
0.00
A (n=15)
B (n=16)
C (n=14)
surface

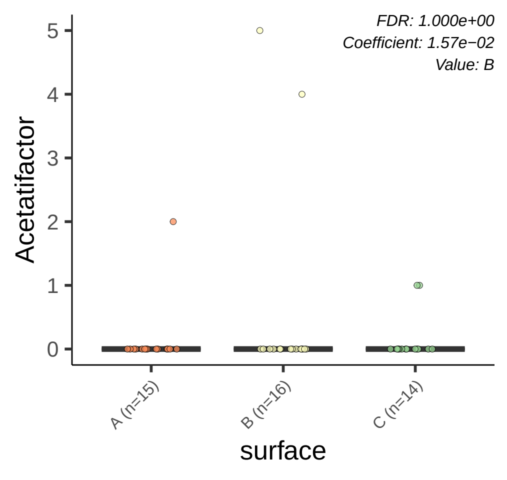
FDR: 1.000e+00
Coefficient: 1.57e−02
Value: B
Acetatifactor
5
4
3
2
1
0
A (n=15)
B (n=16)
C (n=14)
surface

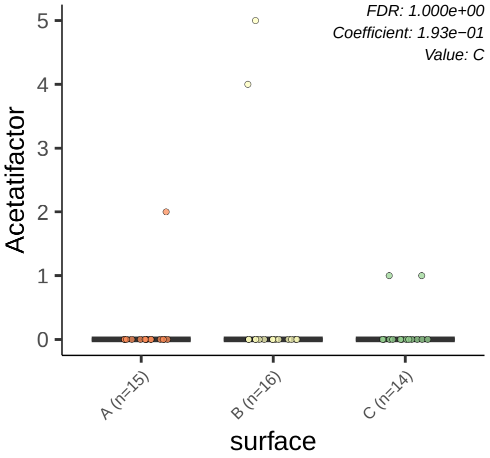
FDR: 1.000e+00
Coefficient: 1.93e−01
Value: C
Acetatifactor
5
4
3
2
1
0
A (n=15)
B (n=16)
C (n=14)
surface

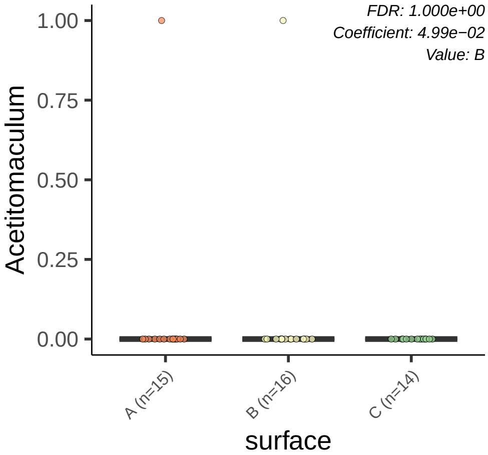
FDR: 1.000e+00
Coefficient: 4.99e−02
Value: B
Acetitomaculum
1.00
0.75
0.50
0.25
0.00
A (n=15)
B (n=16)
C (n=14)
surface

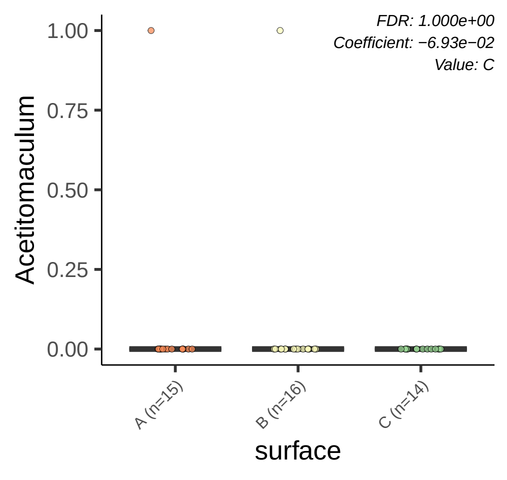
FDR: 1.000e+00
Coefficient: −6.93e−02
Value: C
Acetitomaculum
1.00
0.75
0.50
0.25
0.00
A (n=15)
B (n=16)
C (n=14)
surface

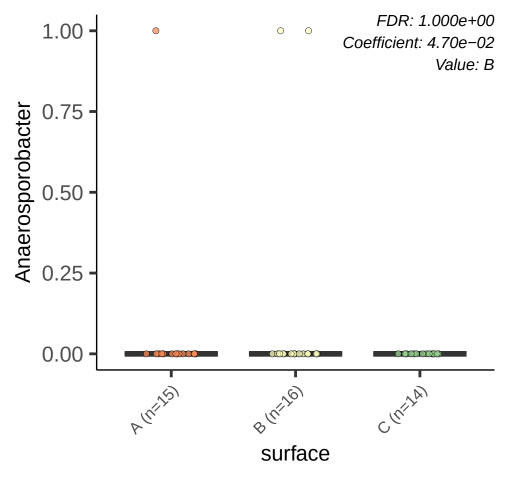
FDR: 1.000e+00
Coefficient: 4.70e−02
Value: B
Anaerosporobacter
1.00
0.75
0.50
0.25
0.00
A (n=15)
B (n=16)
C (n=14)
surface

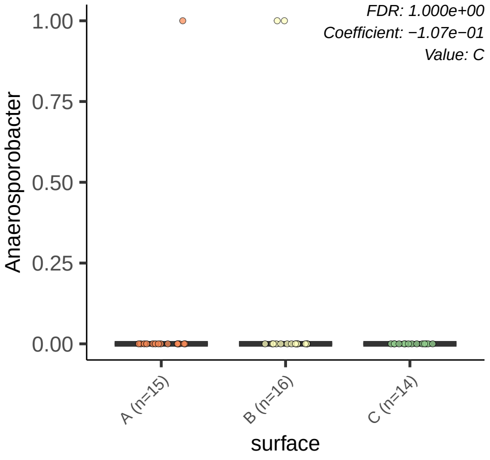
FDR: 1.000e+00
Coefficient: −1.07e−01
Value: C
Anaerosporobacter
1.00
0.75
0.50
0.25
0.00
A (n=15)
B (n=16)
C (n=14)
surface

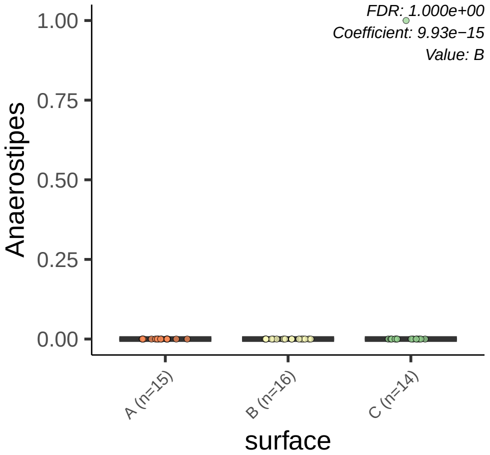
FDR: 1.000e+00
Coefficient: 9.93e−15
Value: B
Anaerostipes
1.00
0.75
0.50
0.25
0.00
A (n=15)
B (n=16)
C (n=14)
surface

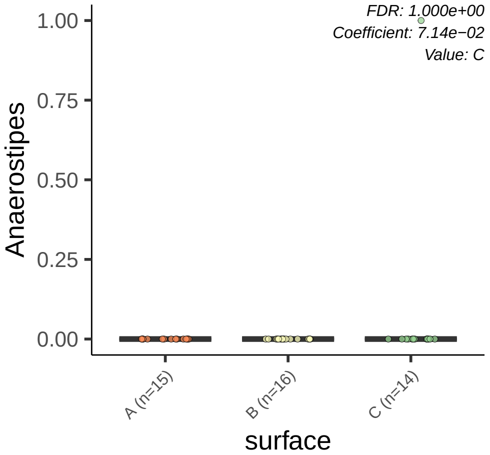
FDR: 1.000e+00
Coefficient: 7.14e−02
Value: C
Anaerostipes
1.00
0.75
0.50
0.25
0.00
A (n=15)
B (n=16)
C (n=14)
surface

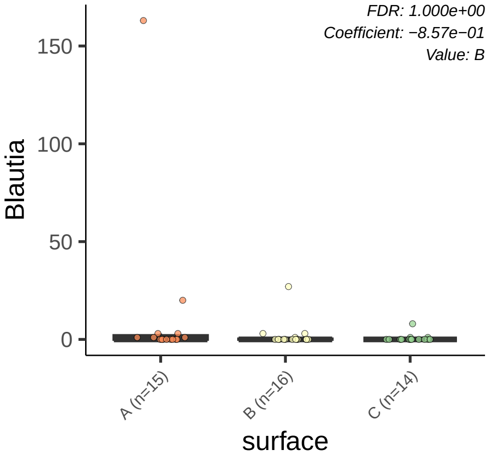

FDR: 1.000e+00
Coefficient: −8.57e−01
Value: B
Blautia
150
100
50
0
A (n=15)
B (n=16)
C (n=14)
surface

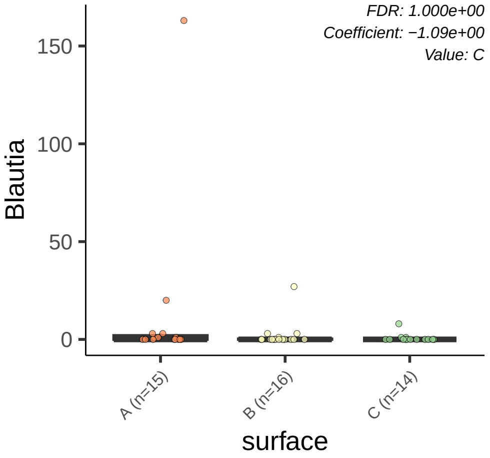

FDR: 1.000e+00
Coefficient: −1.09e+00
Value: C
Blautia
150
100
50
0
A (n=15)
B (n=16)
C (n=14)
surface

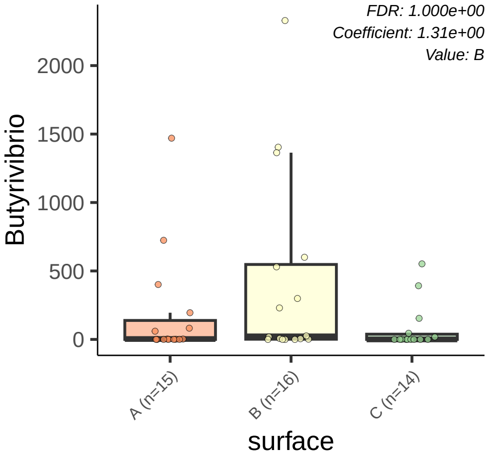

FDR: 1.000e+00
Coefficient: 1.31e+00
Value: B
Butyrivibrio
2000
1500
1000
500
0
A (n=15)
B (n=16)
C (n=14)
surface

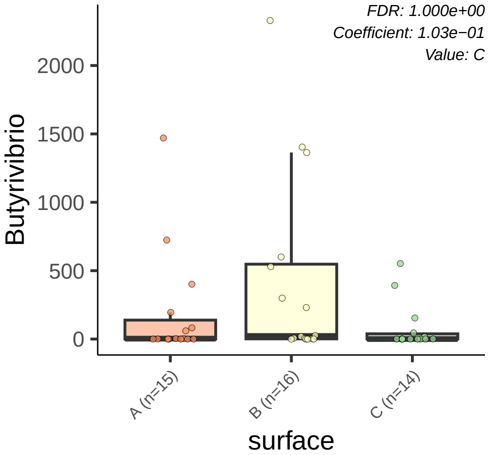
FDR: 1.000e+00
Coefficient: 1.03e−01
Value: C
Butyrivibrio
2000
1500
1000
500
0
A (n=15)
B (n=16)
C (n=14)
surface

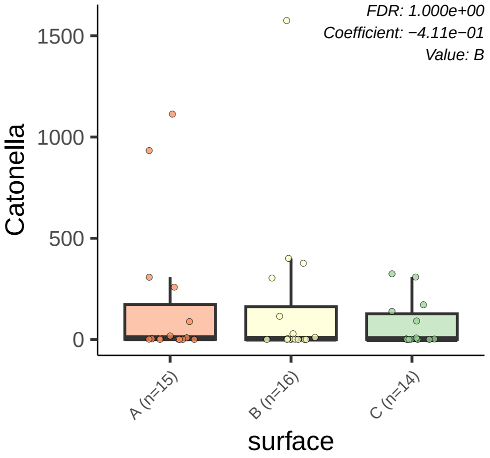

FDR: 1.000e+00
Coefficient: −4.11e−01
Value: B
Catonella
1500
1000
500
0
A (n=15)
B (n=16)
C (n=14)
surface

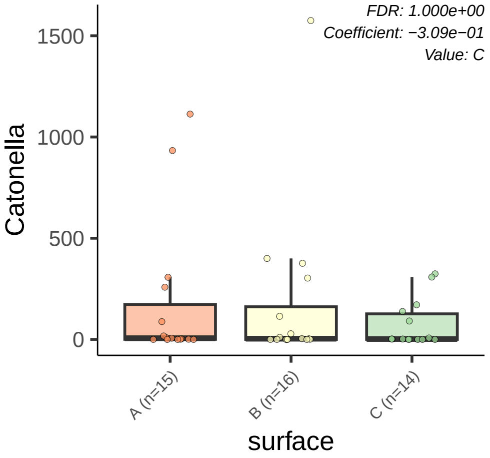

FDR: 1.000e+00
Coefficient: −3.09e−01
Value: C
1500
1000
500
0
Catonella
A (n=15)
B (n=16)
C (n=14)
surface

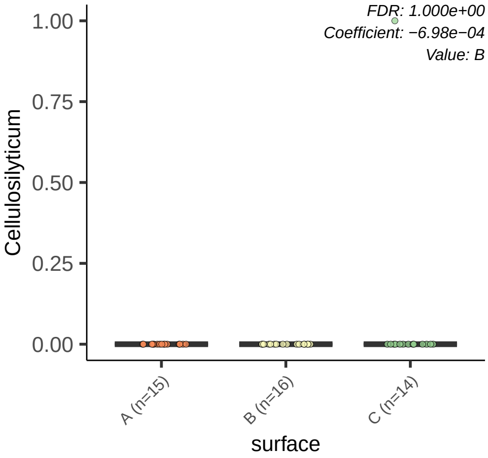

FDR: 1.000e+00
Coefficient: −6.98e−04
Value: B
Cellulosilyticum
1.00
0.75
0.50
0.25
0.00
A (n=15)
B (n=16)
C (n=14)
surface

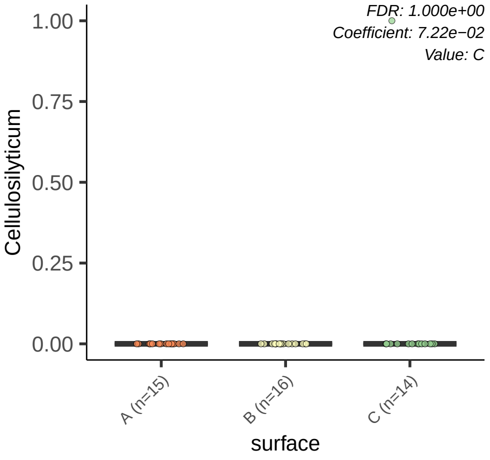

FDR: 1.000e+00
Coefficient: 7.22e−02
Value: C
1.00
0.75
0.50
0.25
0.00
Cellulosilyticum
A (n=15)
B (n=16)
C (n=14)
surface

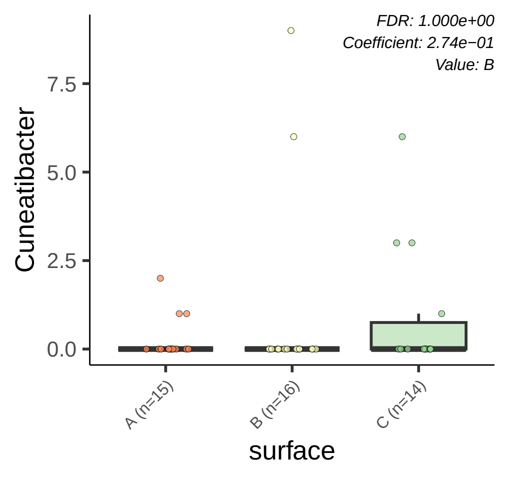

FDR: 1.000e+00
Coefficient: 2.74e−01
Value: B
Cuneatibacter
7.5
5.0
2.5
0.0
A (n=15)
B (n=16)
C (n=14)
surface

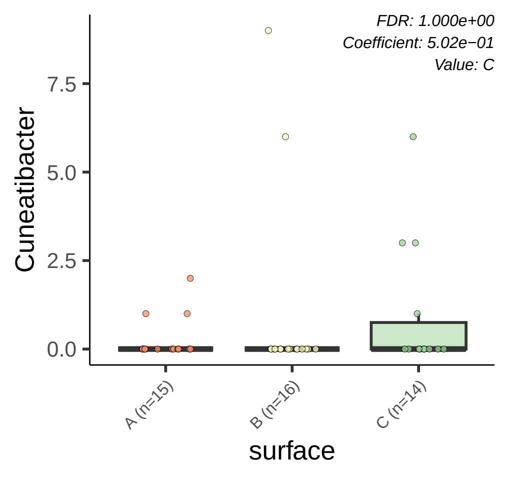

FDR: 1.000e+00
Coefficient: 5.02e−01
Value: C
Cuneatibacter
7.5
5.0
2.5
0.0
A (n=15)
B (n=16)
C (n=14)
surface

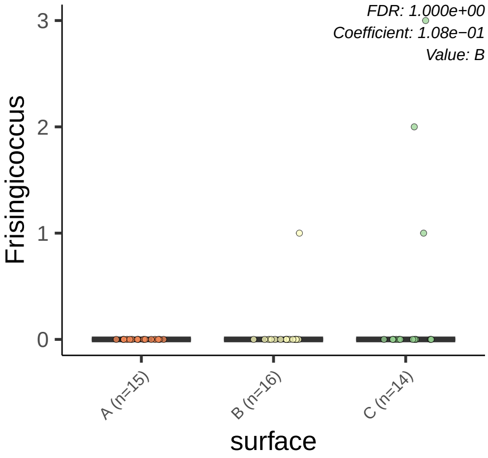

FDR: 1.000e+00
Coefficient: 1.08e−01
Value: B
Frisingicoccus
3
2
1
0
A (n=15)
B (n=16)
C (n=14)
surface

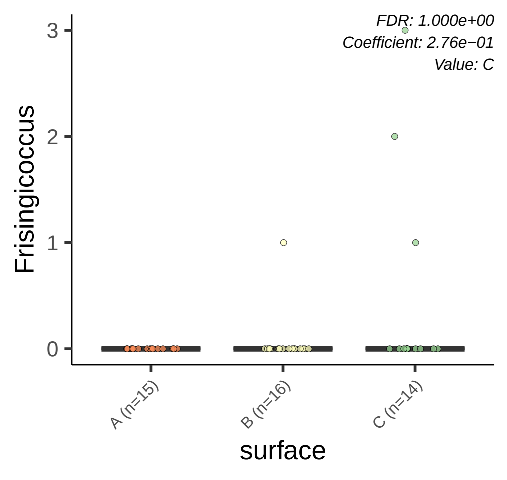
FDR: 1.000e+00
Coefficient: 2.76e−01
Value: C
Frisingicoccus
3
2
1
0
A (n=15)
B (n=16)
C (n=14)
surface

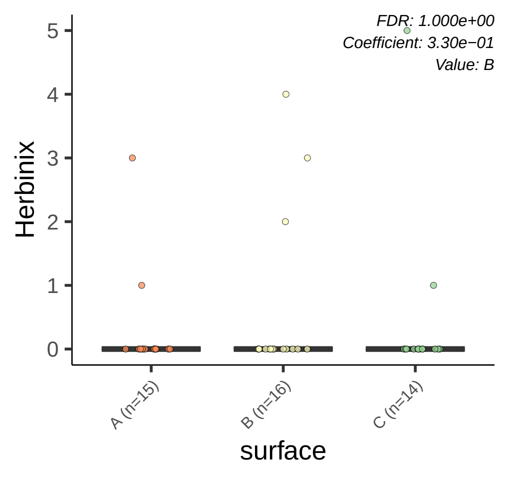
FDR: 1.000e+00
Coefficient: 3.30e−01
Value: B
Herbinix
0
1
2
3
4
5
A (n=15)
B (n=16)
C (n=14)
surface

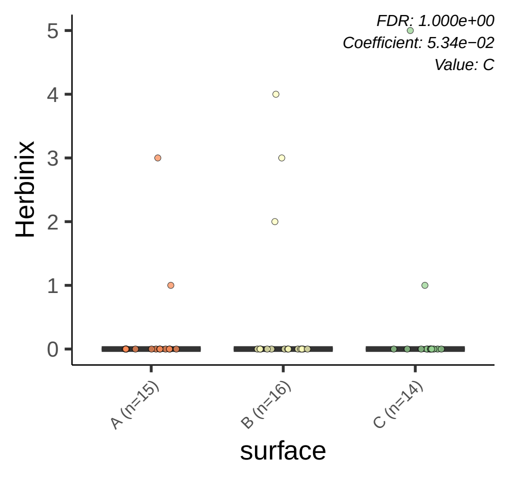
FDR: 1.000e+00
Coefficient: 5.34e−02
Value: C
Herbinix
0
1
2
3
4
5
A (n=15)
B (n=16)
C (n=14)
surface

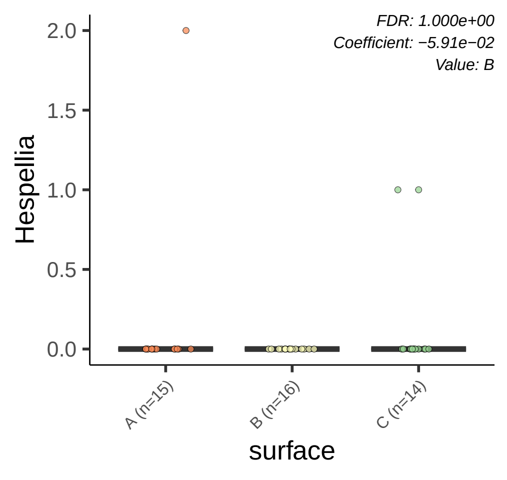
FDR: 1.000e+00
Coefficient: −5.91e−02
Value: B
Hespellia
2.0
1.5
1.0
0.5
0.0
A (n=15)
B (n=16)
C (n=14)
surface

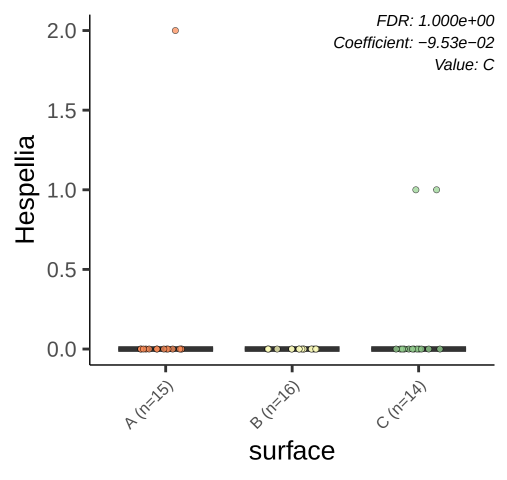

FDR: 1.000e+00
Coefficient: −9.53e−02
Value: C
Hespellia
2.0
1.5
1.0
0.5
0.0
A (n=15)
B (n=16)
C (n=14)
surface

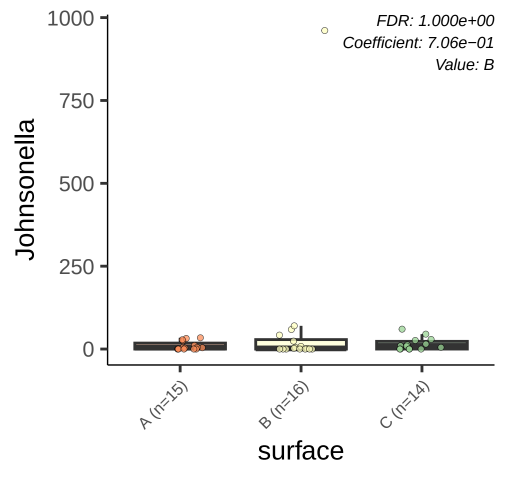

FDR: 1.000e+00
Coefficient: 7.06e−01
Value: B
Johnsonella
1000
750
500
250
0
A (n=15)
B (n=16)
C (n=14)
surface

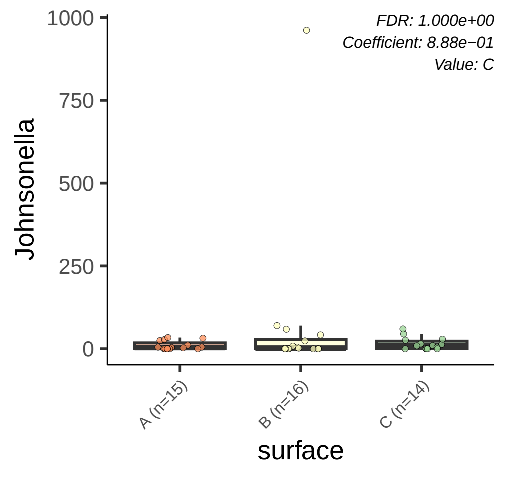
FDR: 1.000e+00
Coefficient: 8.88e−01
Value: C
Johnsonella
1000
750
500
250
0
A (n=15)
B (n=16)
C (n=14)
surface

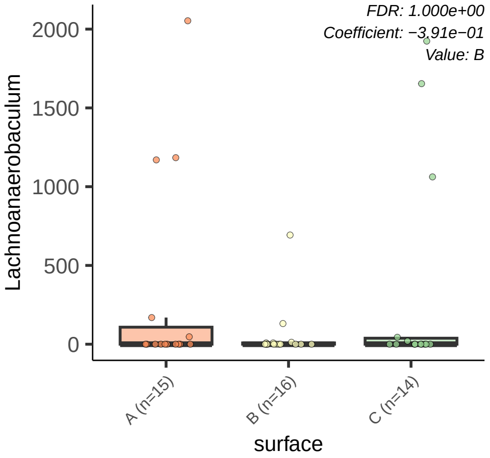
FDR: 1.000e+00
Coefficient: −3.91e−01
Value: B
Lachnoanaerobaculum
2000
1500
1000
500
0
A (n=15)
B (n=16)
C (n=14)
surface

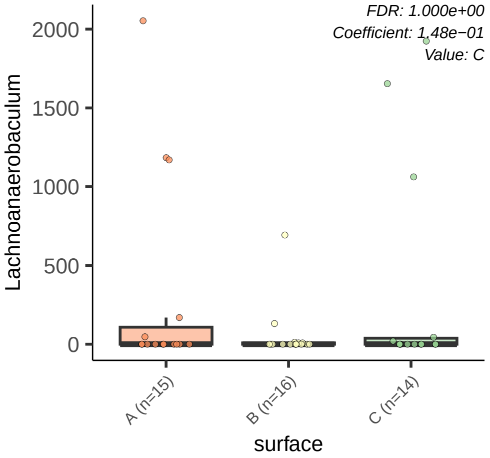

FDR: 1.000e+00
Coefficient: 1.48e−01
Value: C
Lachnoanaerobaculum
2000
1500
1000
500
0
A (n=15)
B (n=16)
C (n=14)
surface

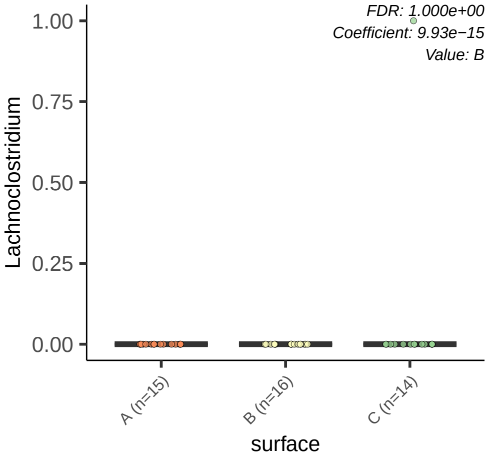
FDR: 1.000e+00
Coefficient: 9.93e−15
Value: B
Lachnoclostridium
1.00
0.75
0.50
0.25
0.00
A (n=15)
B (n=16)
C (n=14)
surface

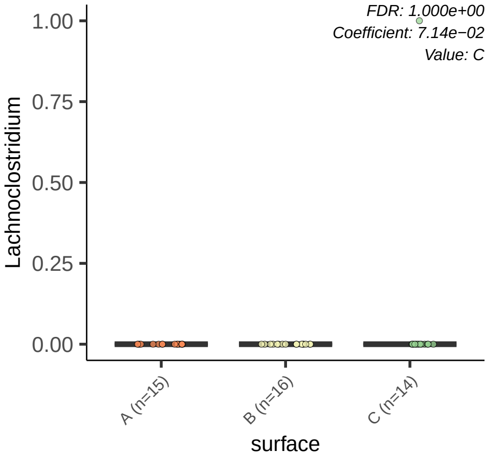
FDR: 1.000e+00
Coefficient: 7.14e−02
Value: C
Lachnoclostridium
1.00
0.75
0.50
0.25
0.00
A (n=15)
B (n=16)
C (n=14)
surface

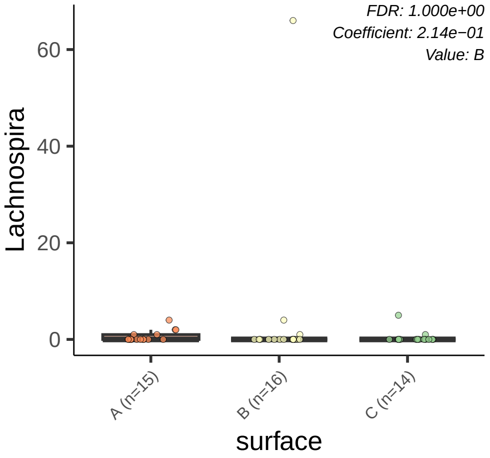
FDR: 1.000e+00
Coefficient: 2.14e−01
Value: B
Lachnospira
60
40
20
0
A (n=15)
B (n=16)
C (n=14)
surface

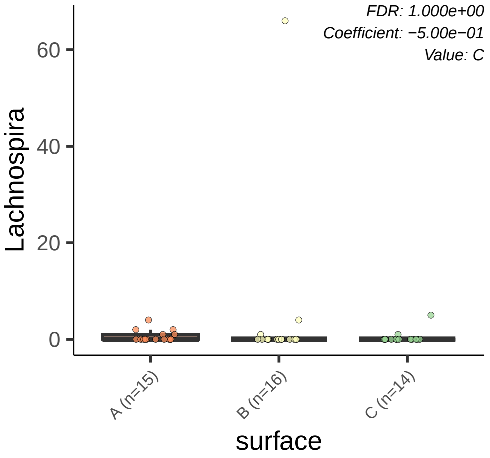

FDR: 1.000e+00
Coefficient: −5.00e−01
Value: C
Lachnospira
60
40
20
0
A (n=15)
B (n=16)
C (n=14)
surface

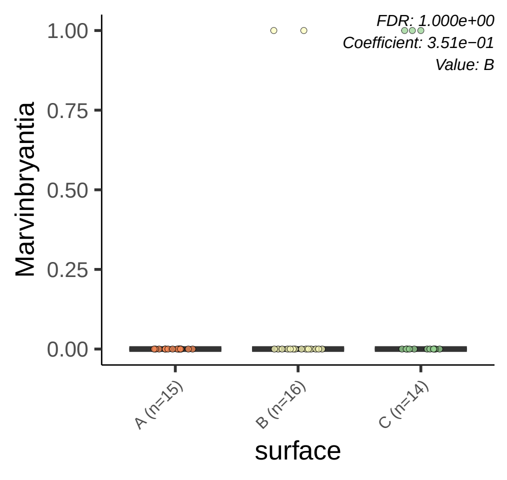
FDR: 1.000e+00
Coefficient: 3.51e−01
Value: B
Marvinbryantia
1.00
0.75
0.50
0.25
0.00
A (n=15)
B (n=16)
C (n=14)
surface

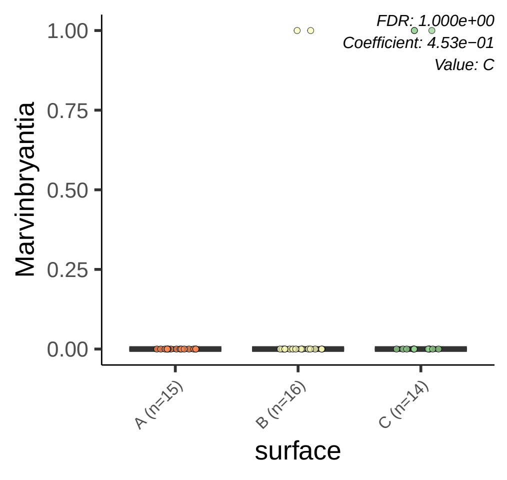

FDR: 1.000e+00
Coefficient: 4.53e−01
Value: C
Marvinbryantia
1.00
0.75
0.50
0.25
0.00
A (n=15)
B (n=16)
C (n=14)
surface

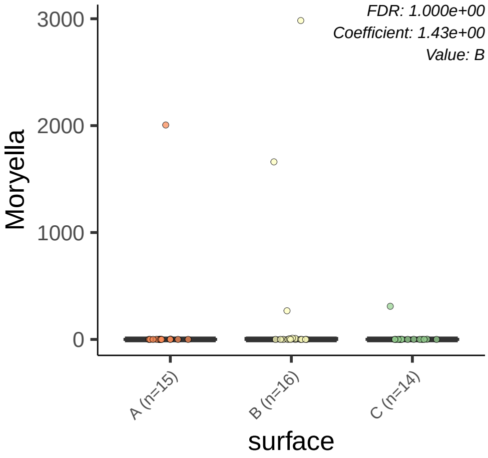
FDR: 1.000e+00
Coefficient: 1.43e+00
Value: B
3000
2000
1000
0
Moryella
A (n=15)
B (n=16)
C (n=14)
surface

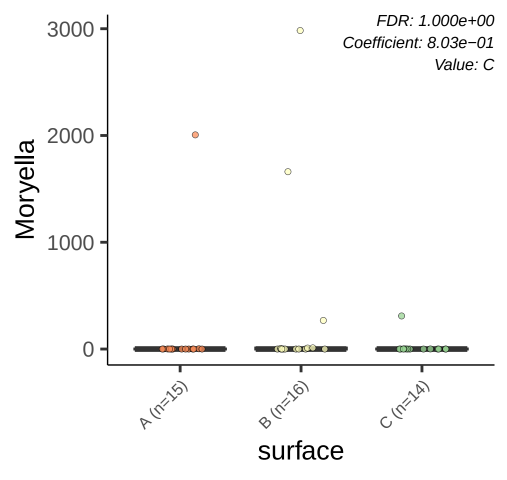
FDR: 1.000e+00
Coefficient: 8.03e−01
Value: C
Moryella
3000
2000
1000
0
A (n=15)
B (n=16)
C (n=14)
surface

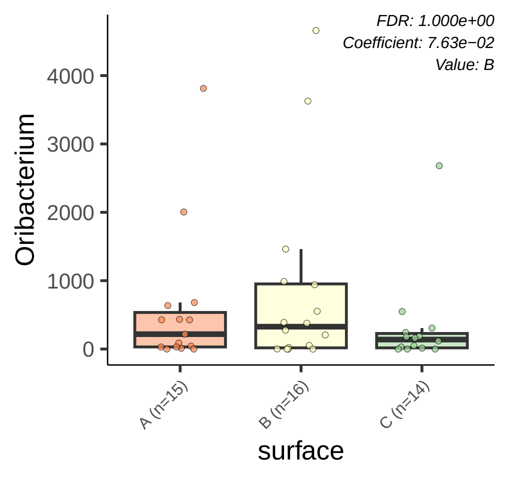
FDR: 1.000e+00
Coefficient: 7.63e−02
Value: B
Oribacterium
4000
3000
2000
1000
0
A (n=15)
B (n=16)
C (n=14)
surface

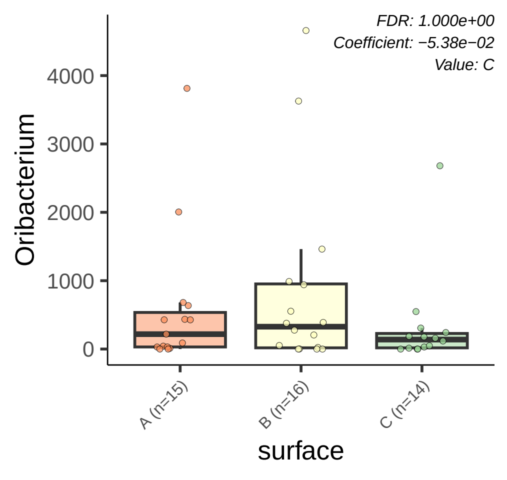
FDR: 1.000e+00
Coefficient: −5.38e−02
Value: C
Oribacterium
4000
3000
2000
1000
0
A (n=15)
B (n=16)
C (n=14)
surface

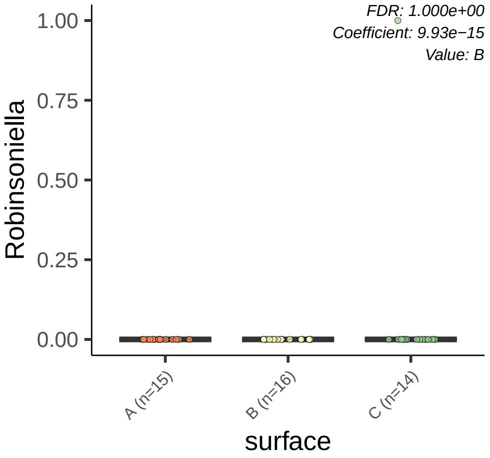
FDR: 1.000e+00
Coefficient: 9.93e−15
Value: B
Robinsoniella
1.00
0.75
0.50
0.25
0.00
A (n=15)
B (n=16)
C (n=14)
surface

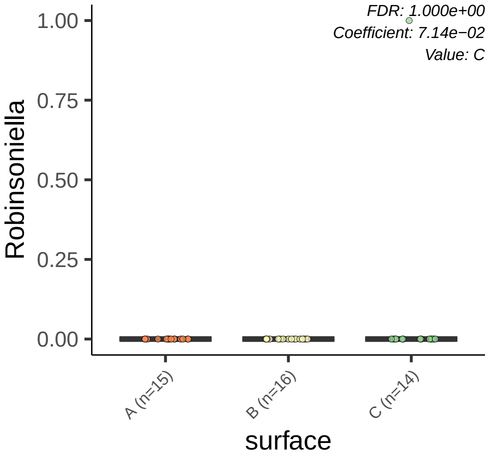

FDR: 1.000e+00
Coefficient: 7.14e−02
Value: C
Robinsoniella
1.00
0.75
0.50
0.25
0.00
A (n=15)
B (n=16)
C (n=14)
surface

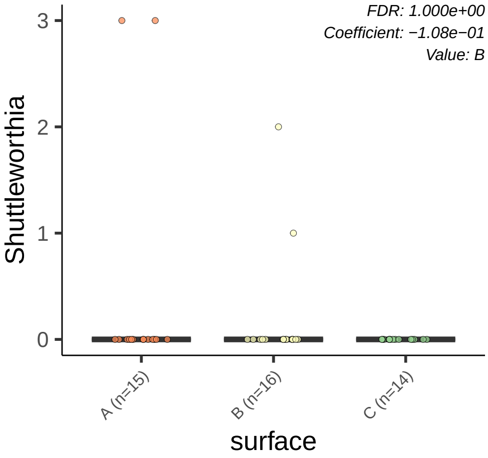
FDR: 1.000e+00
Coefficient: −1.08e−01
Value: B
Shuttleworthia
3
2
1
0
A (n=15)
B (n=16)
C (n=14)
surface

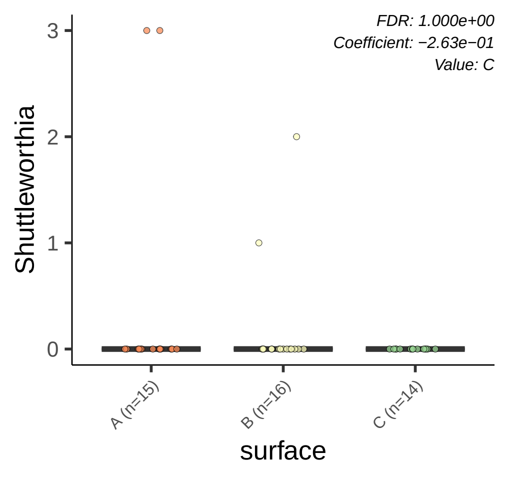

FDR: 1.000e+00
Coefficient: −2.63e−01
Value: C
Shuttleworthia
0
1
2
3
A (n=15)
B (n=16)
C (n=14)
surface

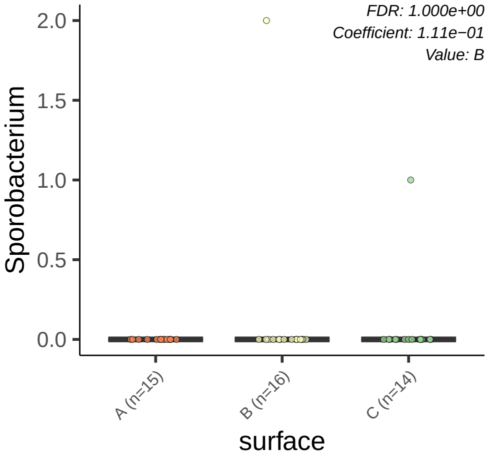

FDR: 1.000e+00
Coefficient: 1.11e−01
Value: B
Sporobacterium
2.0
1.5
1.0
0.5
0.0
A (n=15)
B (n=16)
C (n=14)
surface

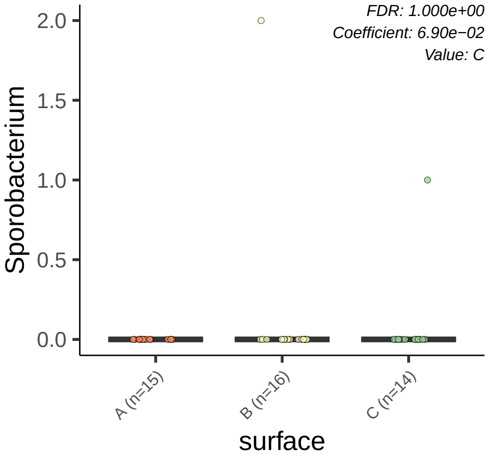

FDR: 1.000e+00
Coefficient: 6.90e−02
Value: C
Sporobacterium
2.0
1.5
1.0
0.5
0.0
A (n=15)
B (n=16)
C (n=14)
surface

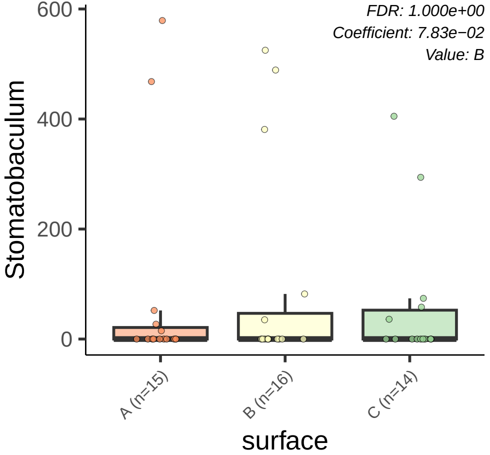
FDR: 1.000e+00
Coefficient: 7.83e−02
Value: B
Stomatobaculum
600
400
200
0
A (n=15)
B (n=16)
C (n=14)
surface

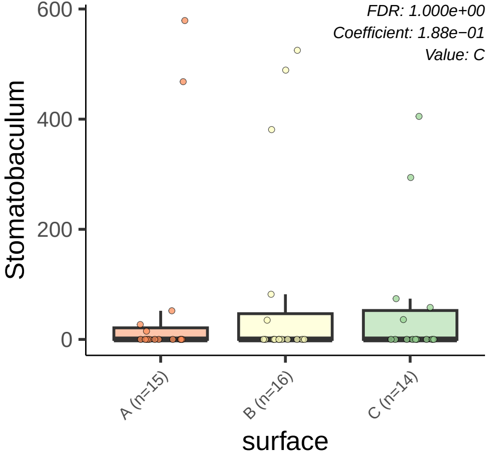

FDR: 1.000e+00
Coefficient: 1.88e−01
Value: C
Stomatobaculum
600
400
200
0
A (n=15)
B (n=16)
C (n=14)
surface

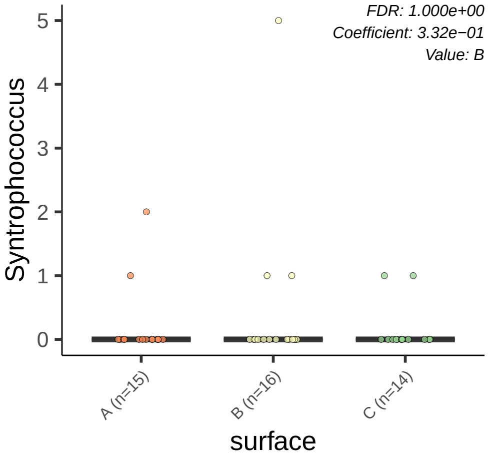
FDR: 1.000e+00
Coefficient: 3.32e−01
Value: B
Syntrophococcus
0
1
2
3
4
5
A (n=15)
B (n=16)
C (n=14)
surface

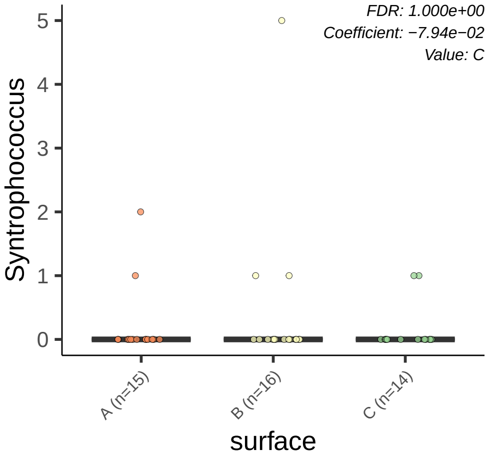
FDR: 1.000e+00
Coefficient: −7.94e−02
Value: C
Syntrophococcus
0
1
2
3
4
5
A (n=15)
B (n=16)
C (n=14)
surface

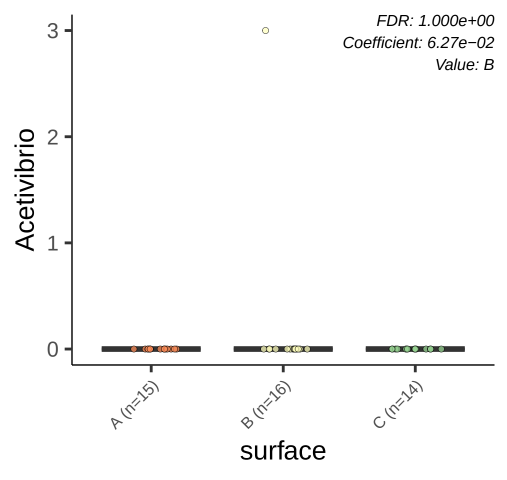

FDR: 1.000e+00
Coefficient: 6.27e−02
Value: B
Acetivibrio
3
2
1
0
A (n=15)
B (n=16)
C (n=14)
surface

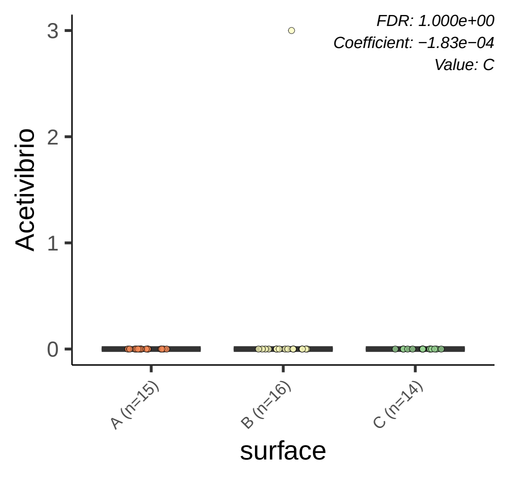

FDR: 1.000e+00
Coefficient: −1.83e−04
Value: C
Acetivibrio
0
1
2
3
A (n=15)
B (n=16)
C (n=14)
surface

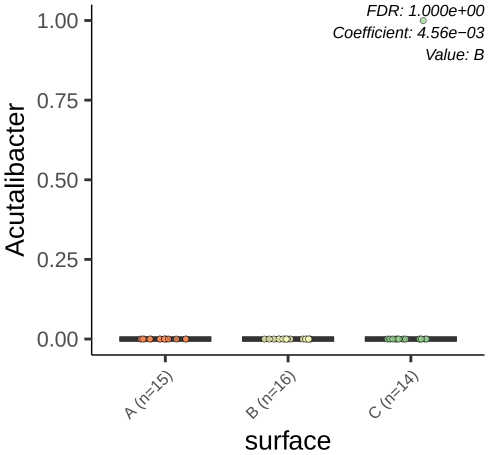
FDR: 1.000e+00
Coefficient: 4.56e−03
Value: B
Acutalibacter
1.00
0.75
0.50
0.25
0.00
A (n=15)
B (n=16)
C (n=14)
surface

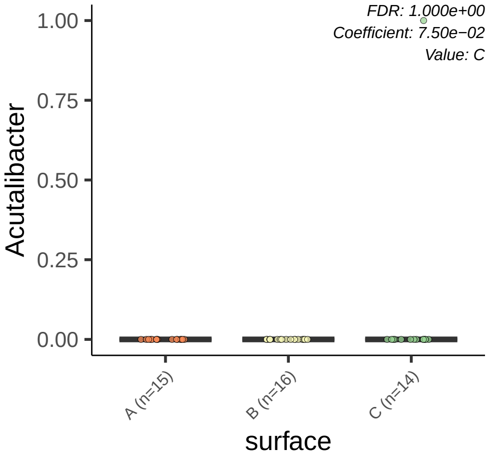

FDR: 1.000e+00
Coefficient: 7.50e−02
Value: C
Acutalibacter
1.00
0.75
0.50
0.25
0.00
A (n=15)
B (n=16)
C (n=14)
surface

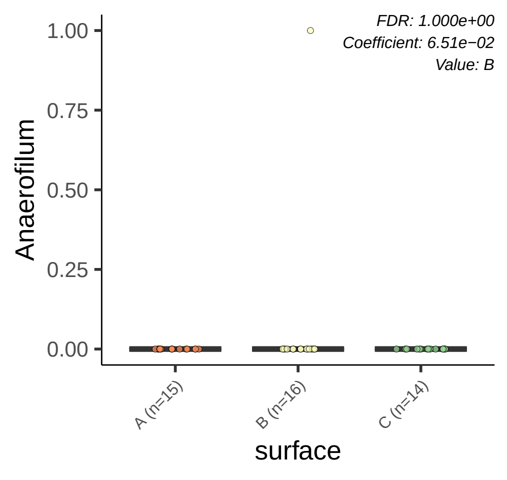

FDR: 1.000e+00
Coefficient: 6.51e−02
Value: B
Anaerofilum
1.00
0.75
0.50
0.25
0.00
A (n=15)
B (n=16)
C (n=14)
surface

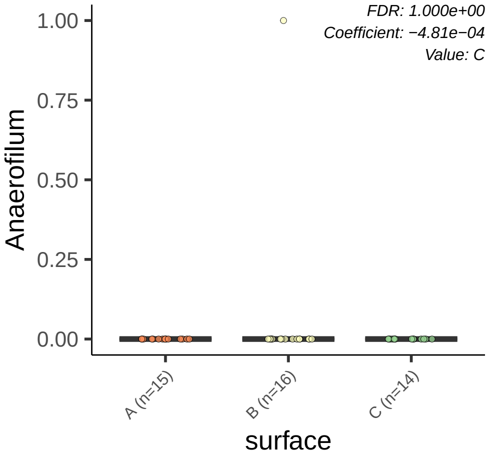

FDR: 1.000e+00
Coefficient: −4.81e−04
Value: C
Anaerofilum
1.00
0.75
0.50
0.25
0.00
A (n=15)
B (n=16)
C (n=14)
surface

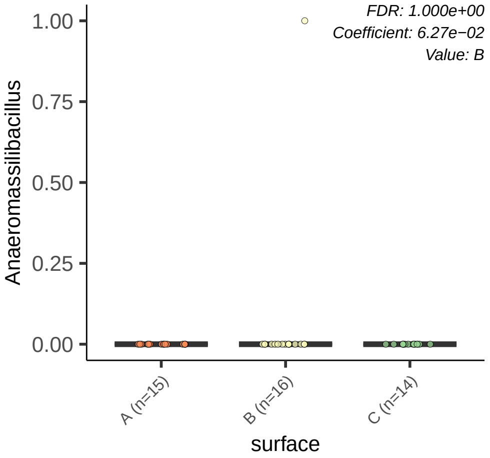
FDR: 1.000e+00
Coefficient: 6.27e−02
Value: B
Anaeromassilibacillus
1.00
0.75
0.50
0.25
0.00
A (n=15)
B (n=16)
C (n=14)
surface

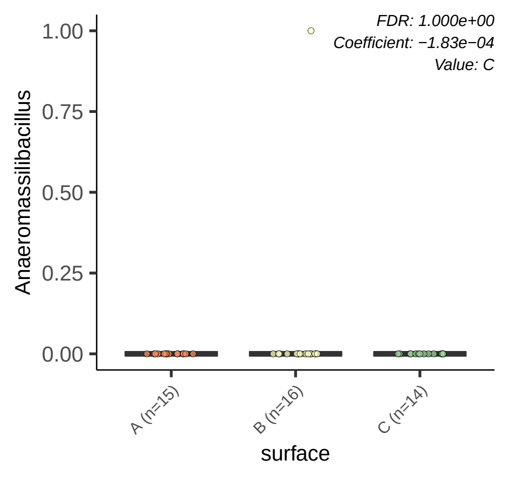
FDR: 1.000e+00
Coefficient: −1.83e−04
Value: C
Anaeromassilibacillus
1.00
0.75
0.50
0.25
0.00
A (n=15)
B (n=16)
C (n=14)
surface

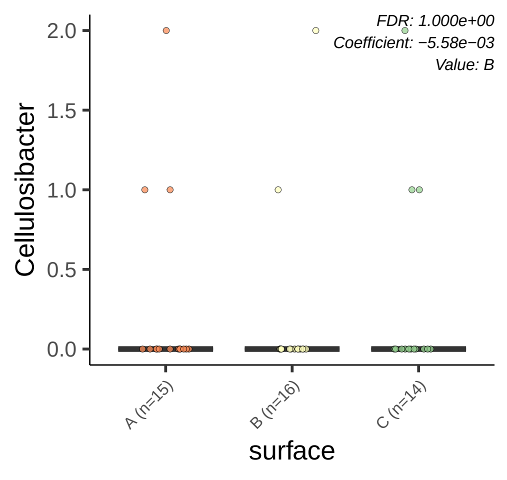
FDR: 1.000e+00
Coefficient: −5.58e−03
Value: B
Cellulosibacter
2.0
1.5
1.0
0.5
0.0
A (n=15)
B (n=16)
C (n=14)
surface

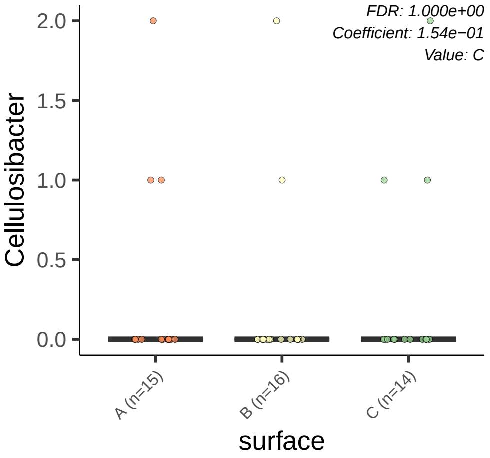

FDR: 1.000e+00
Coefficient: 1.54e−01
Value: C
Cellulosibacter
2.0
1.5
1.0
0.5
0.0
A (n=15)
B (n=16)
C (n=14)
surface

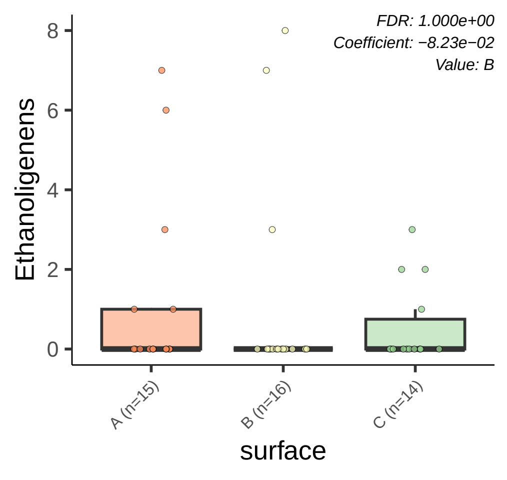
FDR: 1.000e+00
Coefficient: −8.23e−02
Value: B
Ethanoligenens
0
2
4
6
8
A (n=15)
B (n=16)
C (n=14)
surface

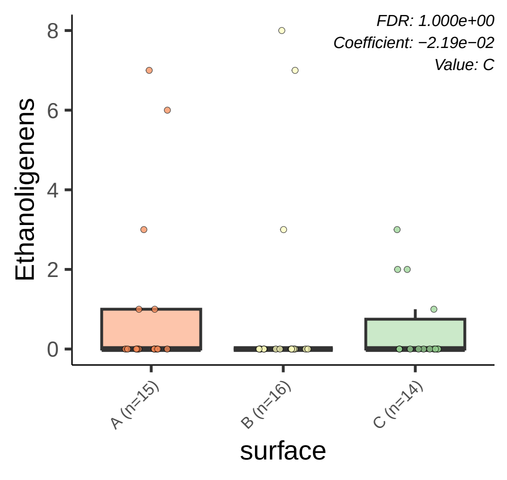

FDR: 1.000e+00
Coefficient: −2.19e−02
Value: C
Ethanoligenens
0
2
4
6
8
A (n=15)
B (n=16)
C (n=14)
surface

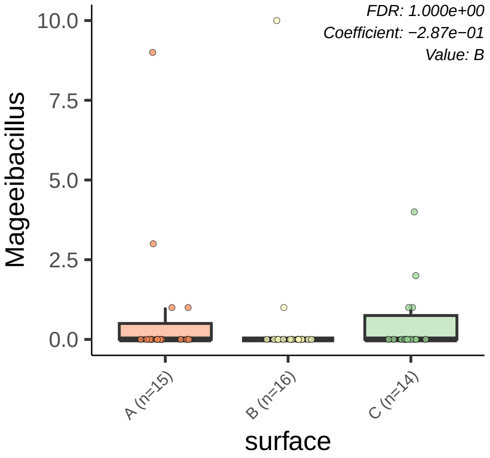

FDR: 1.000e+00
Coefficient: −2.87e−01
Value: B
Mageeibacillus
10.0
7.5
5.0
2.5
0.0
A (n=15)
B (n=16)
C (n=14)
surface

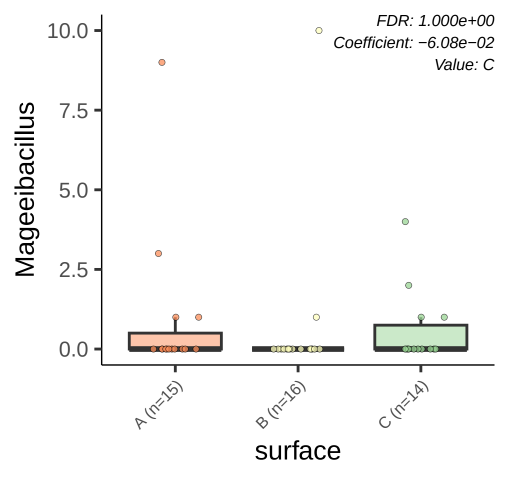

FDR: 1.000e+00
Coefficient: −6.08e−02
Value: C
Mageeibacillus
10.0
7.5
5.0
2.5
0.0
A (n=15)
B (n=16)
C (n=14)
surface

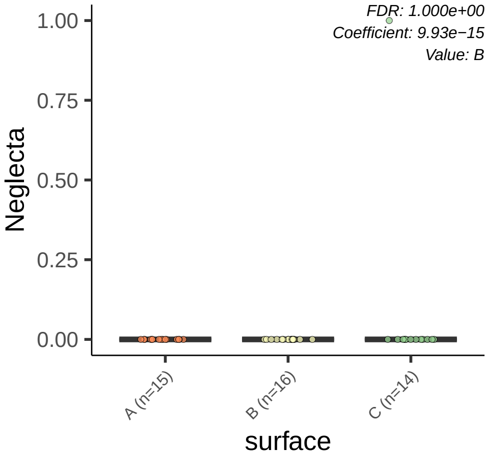
FDR: 1.000e+00
Coefficient: 9.93e−15
Value: B
1.00
0.75
0.50
0.25
0.00
Neglecta
A (n=15)
B (n=16)
C (n=14)
surface

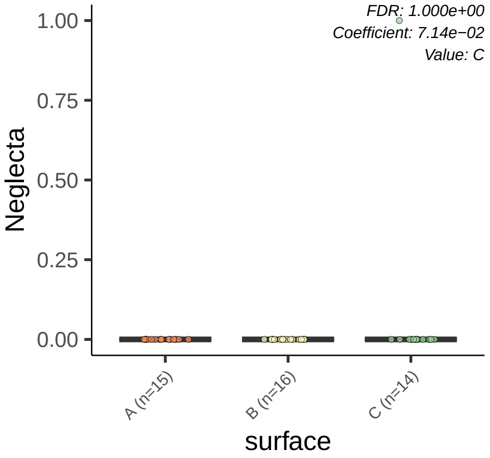
FDR: 1.000e+00
Coefficient: 7.14e−02
Value: C
Neglecta
1.00
0.75
0.50
0.25
0.00
A (n=15)
B (n=16)
C (n=14)
surface

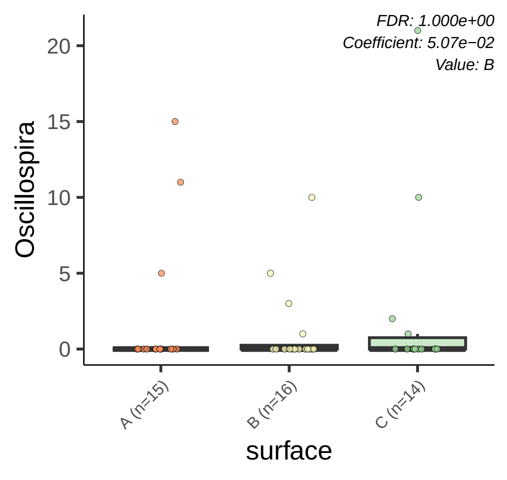

FDR: 1.000e+00
Coefficient: 5.07e−02
Value: B
Oscillospira
20
15
10
5
0
A (n=15)
B (n=16)
C (n=14)
surface

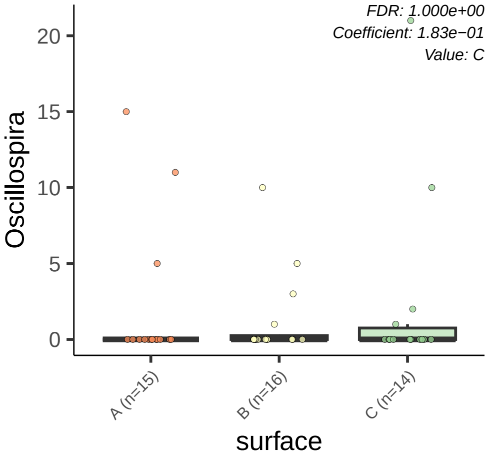
FDR: 1.000e+00
Coefficient: 1.83e−01
Value: C
Oscillospira
20
15
10
5
0
A (n=15)
B (n=16)
C (n=14)
surface

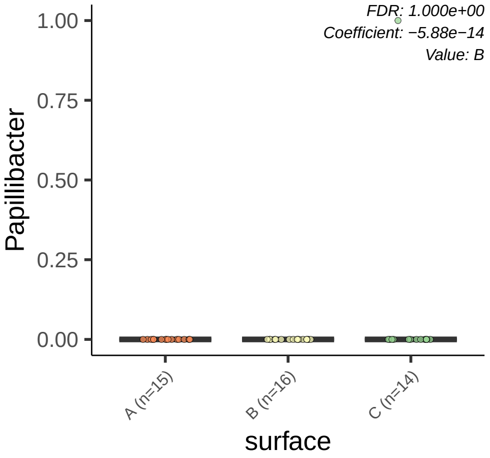
FDR: 1.000e+00
Coefficient: −5.88e−14
Value: B
Papillibacter
1.00
0.75
0.50
0.25
0.00
A (n=15)
B (n=16)
C (n=14)
surface

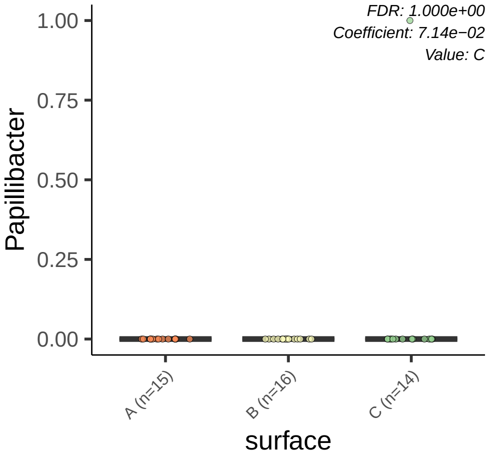

FDR: 1.000e+00
Coefficient: 7.14e−02
Value: C
1.00
0.75
0.50
0.25
0.00
Papillibacter
A (n=15)
B (n=16)
C (n=14)
surface

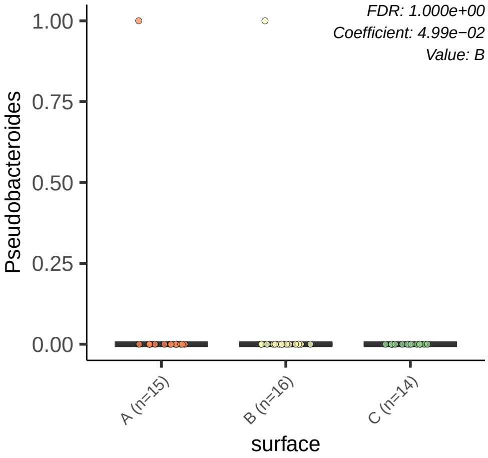
FDR: 1.000e+00
Coefficient: 4.99e−02
Value: B
Pseudobacteroides
1.00
0.75
0.50
0.25
0.00
A (n=15)
B (n=16)
C (n=14)
surface

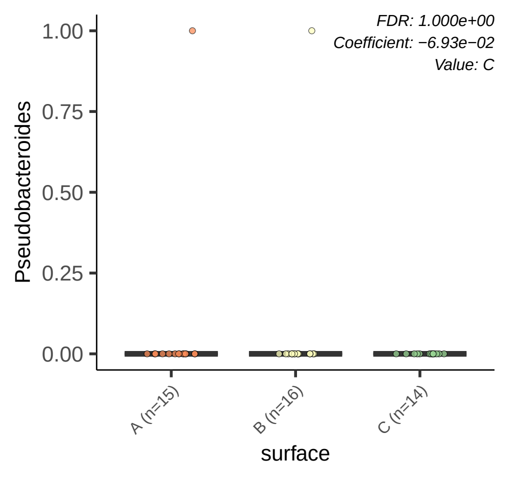

FDR: 1.000e+00
Coefficient: −6.93e−02
Value: C
Pseudobacteroides
1.00
0.75
0.50
0.25
0.00
A (n=15)
B (n=16)
C (n=14)
surface

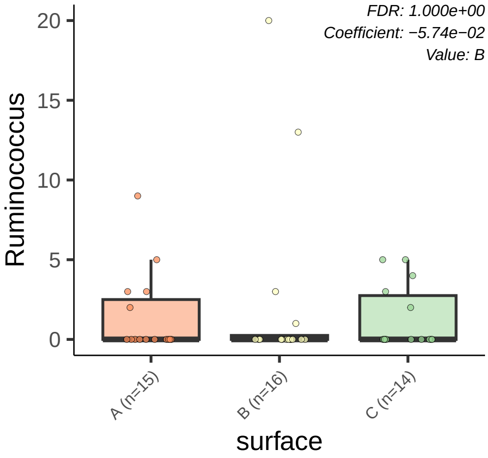
FDR: 1.000e+00
Coefficient: −5.74e−02
Value: B
Ruminococcus
20
15
10
5
0
A (n=15)
B (n=16)
C (n=14)
surface

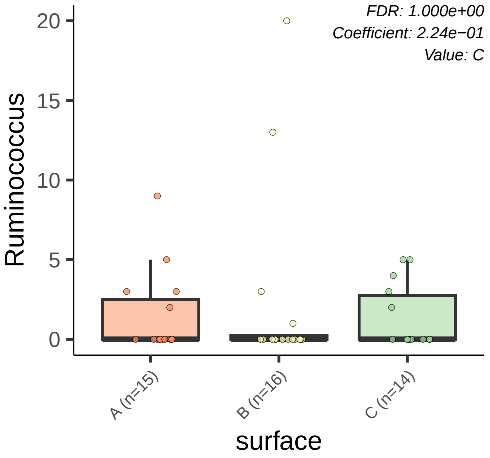

FDR: 1.000e+00
Coefficient: 2.24e−01
Value: C
20
15
10
5
0
Ruminococcus
A (n=15)
B (n=16)
C (n=14)
surface

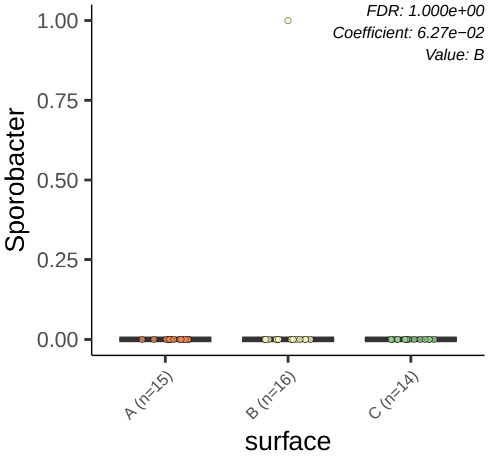
FDR: 1.000e+00
Coefficient: 6.27e−02
Value: B
Sporobacter
1.00
0.75
0.50
0.25
0.00
A (n=15)
B (n=16)
C (n=14)
surface

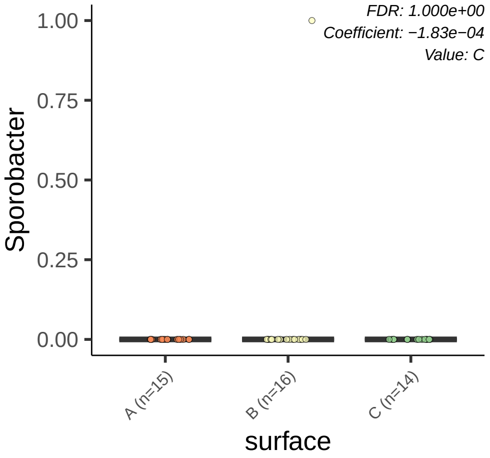

FDR: 1.000e+00
Coefficient: −1.83e−04
Value: C
Sporobacter
1.00
0.75
0.50
0.25
0.00
A (n=15)
B (n=16)
C (n=14)
surface

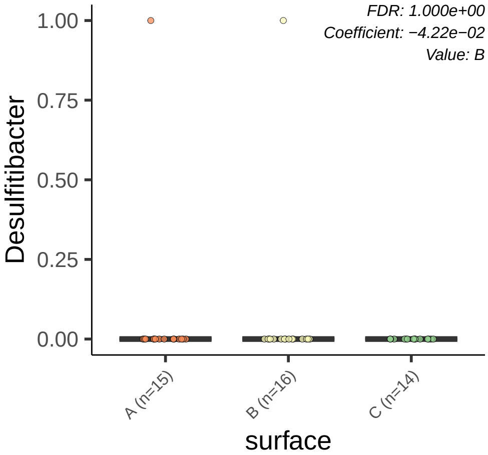
FDR: 1.000e+00
Coefficient: −4.22e−02
Value: B
Desulfitibacter
1.00
0.75
0.50
0.25
0.00
A (n=15)
B (n=16)
C (n=14)
surface

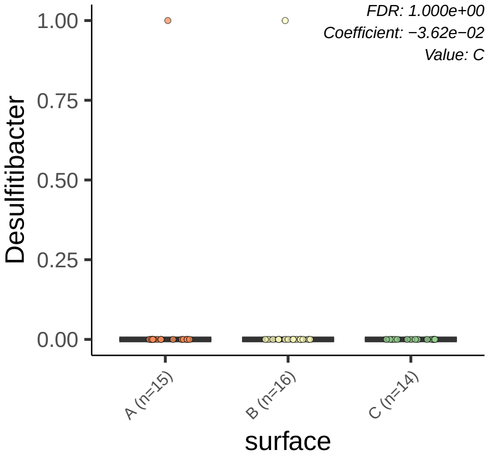
FDR: 1.000e+00
Coefficient: −3.62e−02
Value: C
Desulfitibacter
1.00
0.75
0.50
0.25
0.00
A (n=15)
B (n=16)
C (n=14)
surface

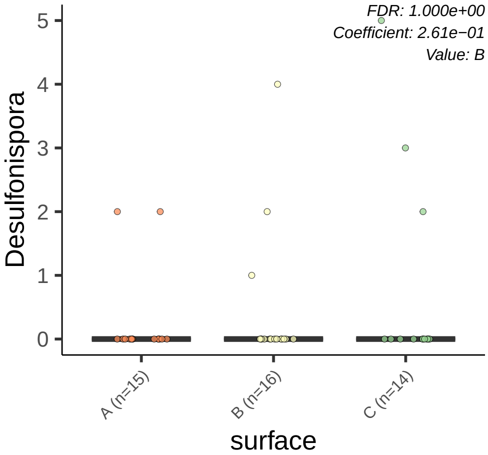

FDR: 1.000e+00
Coefficient: 2.61e−01
Value: B
Desulfonispora
0
1
2
3
4
5
A (n=15)
B (n=16)
C (n=14)
surface

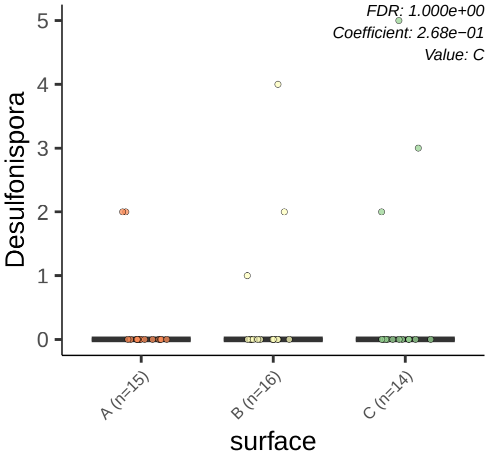
FDR: 1.000e+00
Coefficient: 2.68e−01
Value: C
Desulfonispora
0
1
2
3
4
5
A (n=15)
B (n=16)
C (n=14)
surface

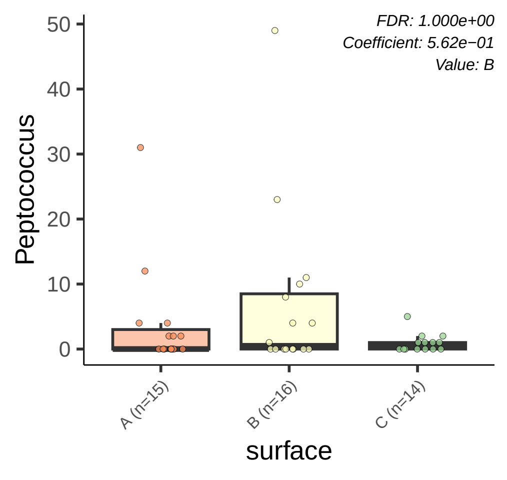
FDR: 1.000e+00
Coefficient: 5.62e−01
Value: B
Peptococcus
50
40
30
20
10
0
A (n=15)
B (n=16)
C (n=14)
surface

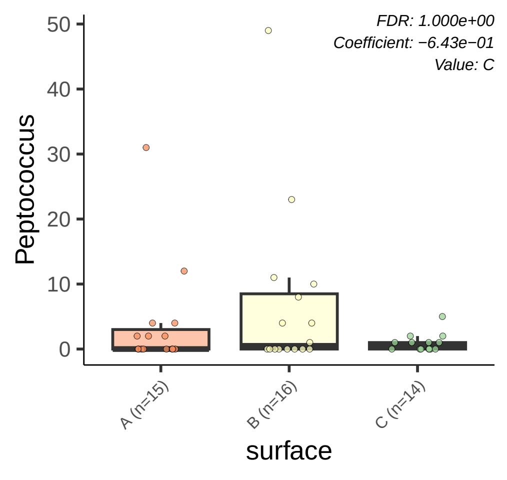
FDR: 1.000e+00
Coefficient: −6.43e−01
Value: C
Peptococcus
50
40
30
20
10
0
A (n=15)
B (n=16)
C (n=14)
surface

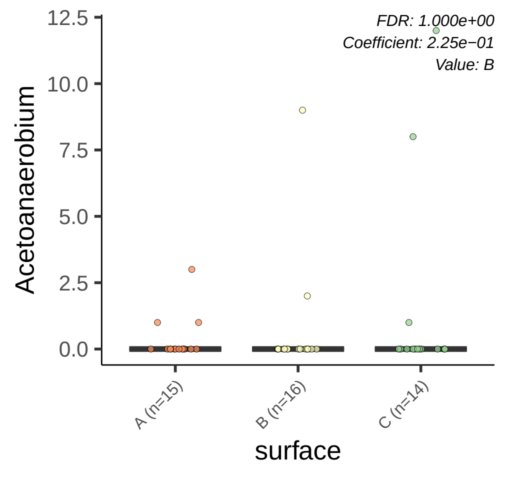
FDR: 1.000e+00
Coefficient: 2.25e−01
Value: B
Acetoanaerobium
12.5
10.0
7.5
5.0
2.5
0.0
A (n=15)
B (n=16)
C (n=14)
surface

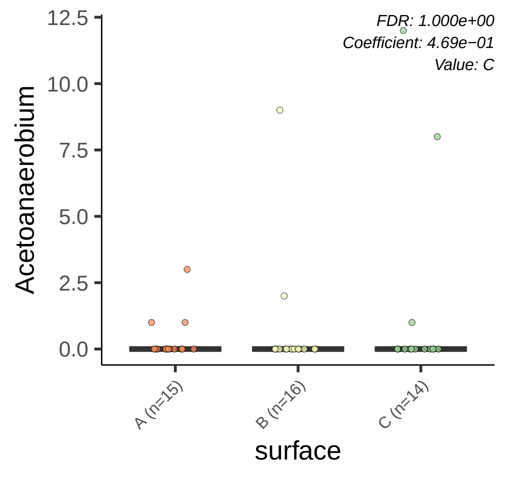

FDR: 1.000e+00
Coefficient: 4.69e−01
Value: C
Acetoanaerobium
12.5
10.0
7.5
5.0
2.5
0.0
A (n=15)
B (n=16)
C (n=14)
surface

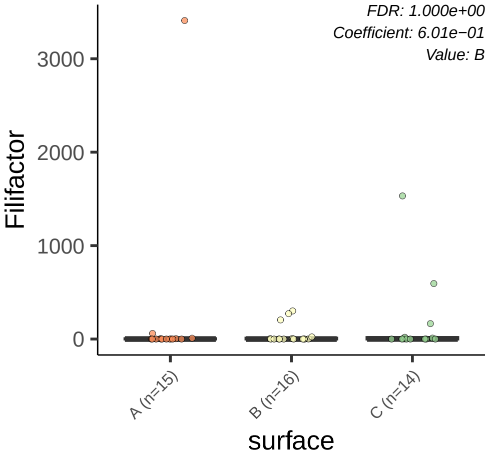
FDR: 1.000e+00
Coefficient: 6.01e−01
Value: B
Filifactor
3000
2000
1000
0
A (n=15)
B (n=16)
C (n=14)
surface

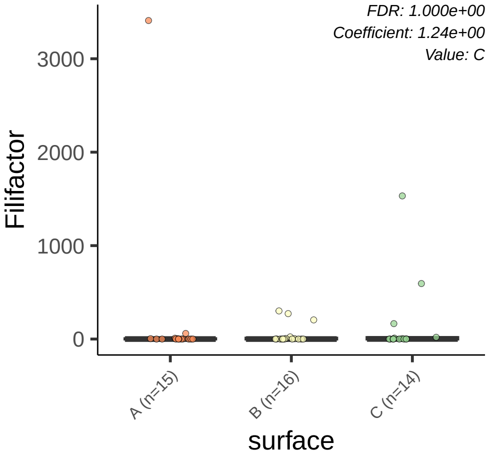
FDR: 1.000e+00
Coefficient: 1.24e+00
Value: C
Filifactor
3000
2000
1000
0
A (n=15)
B (n=16)
C (n=14)
surface

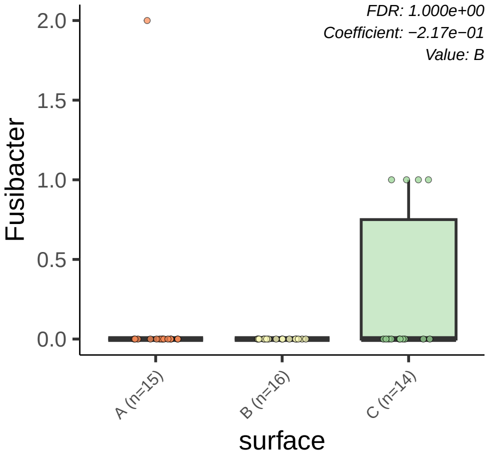
FDR: 1.000e+00
Coefficient: −2.17e−01
Value: B
Fusibacter
2.0
1.5
1.0
0.5
0.0
A (n=15)
B (n=16)
C (n=14)
surface

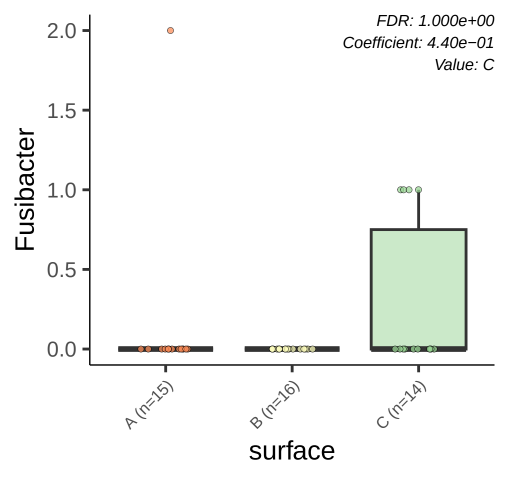

FDR: 1.000e+00
Coefficient: 4.40e−01
Value: C
Fusibacter
2.0
1.5
1.0
0.5
0.0
A (n=15)
B (n=16)
C (n=14)
surface

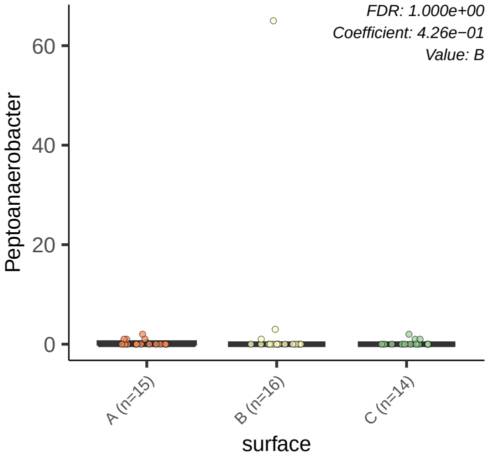
FDR: 1.000e+00
Coefficient: 4.26e−01
Value: B
Peptoanaerobacter
60
40
20
0
A (n=15)
B (n=16)
C (n=14)
surface

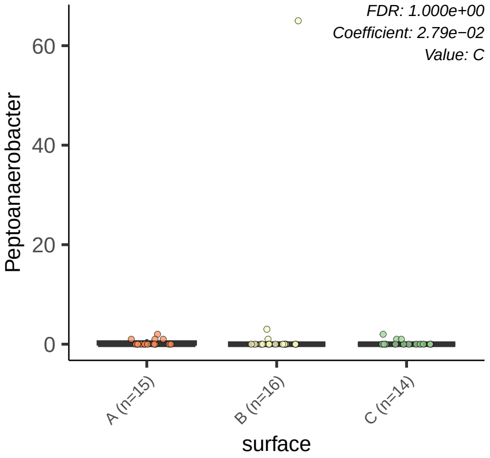

FDR: 1.000e+00
Coefficient: 2.79e−02
Value: C
Peptoanaerobacter
0
20
40
60
A (n=15)
B (n=16)
C (n=14)
surface

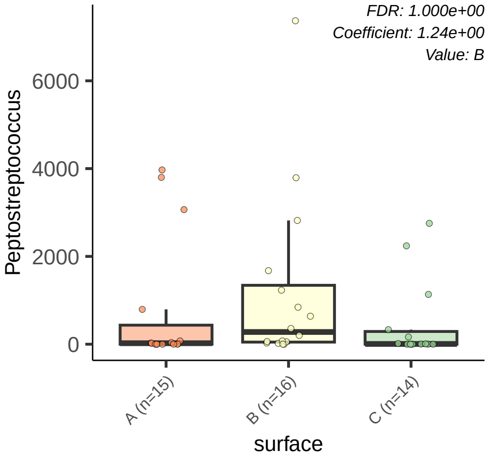

FDR: 1.000e+00
Coefficient: 1.24e+00
Value: B
Peptostreptococcus
6000
4000
2000
0
A (n=15)
B (n=16)
C (n=14)
surface

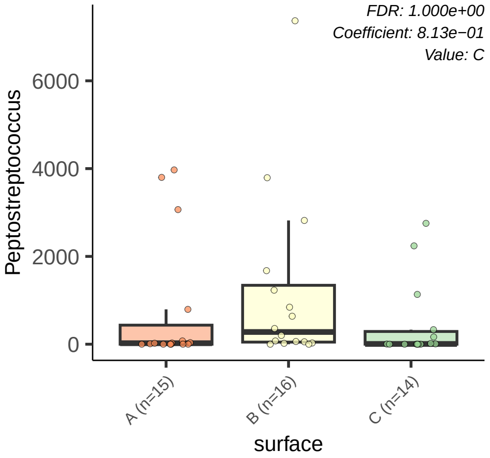
FDR: 1.000e+00
Coefficient: 8.13e−01
Value: C
Peptostreptococcus
6000
4000
2000
0
A (n=15)
B (n=16)
C (n=14)
surface

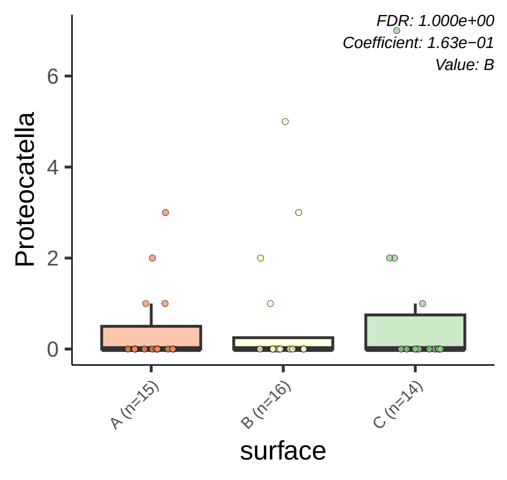
FDR: 1.000e+00
Coefficient: 1.63e−01
Value: B
Proteocatella
0
2
4
6
A (n=15)
B (n=16)
C (n=14)
surface

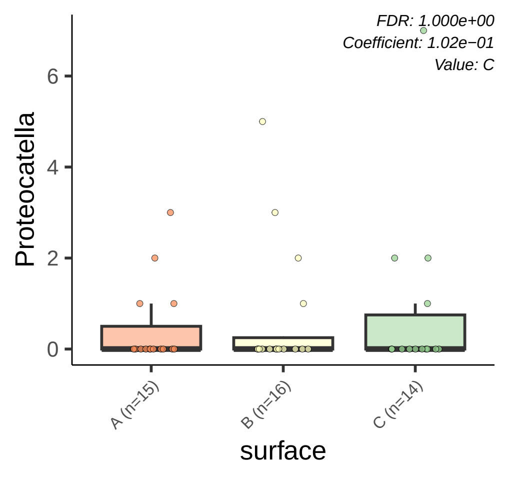

FDR: 1.000e+00
Coefficient: 1.02e−01
Value: C
Proteocatella
0
2
4
6
A (n=15)
B (n=16)
C (n=14)
surface

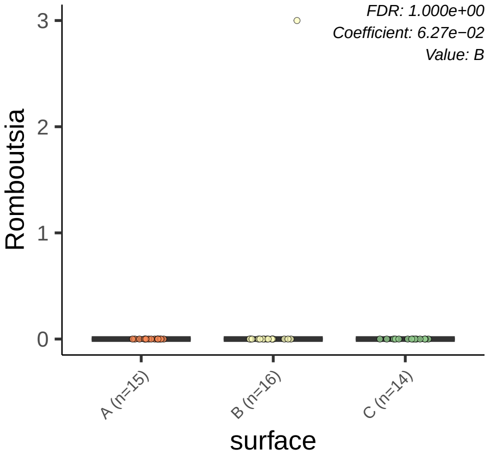

FDR: 1.000e+00
Coefficient: 6.27e−02
Value: B
Rombousia
3
2
1
0
A (n=15)
B (n=16)
C (n=14)
surface

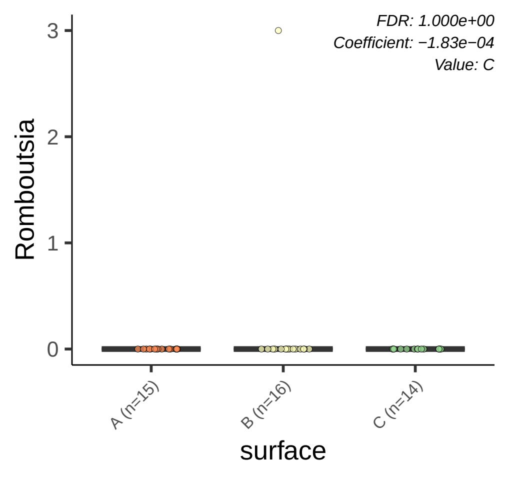

FDR: 1.000e+00
Coefficient: −1.83e−04
Value: C
Romboutsia
3
2
1
0
A (n=15)
B (n=16)
C (n=14)
surface

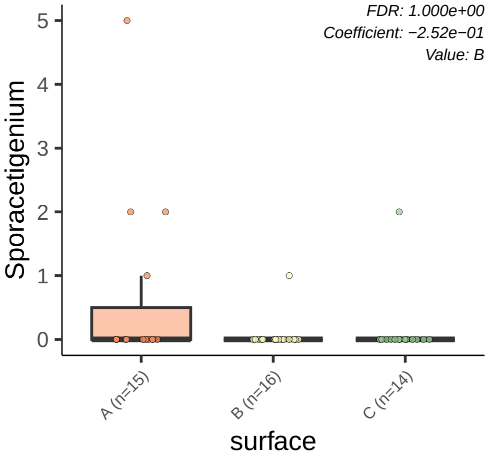
FDR: 1.000e+00
Coefficient: −2.52e−01
Value: B
Sporacetigenium
5
4
3
2
1
0
A (n=15)
B (n=16)
C (n=14)
surface

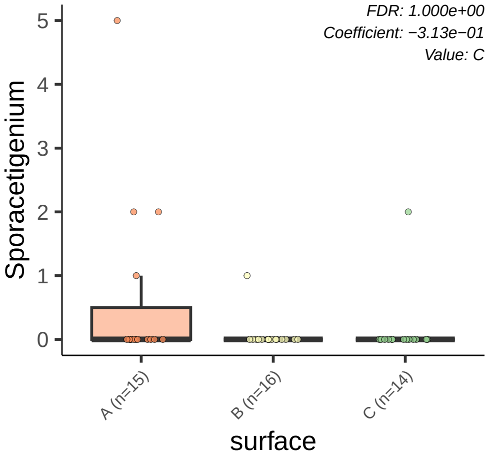
FDR: 1.000e+00
Coefficient: −3.13e−01
Value: C
Sporacetigenium
0
1
2
3
4
5
A (n=15)
B (n=16)
C (n=14)
surface

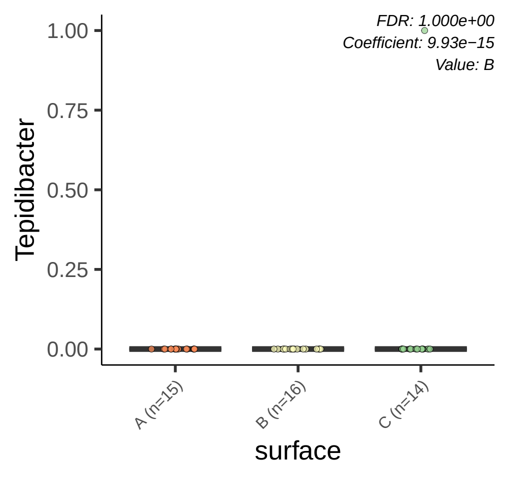
FDR: 1.000e+00
Coefficient: 9.93e−15
Value: B
Tepidibacter
1.00
0.75
0.50
0.25
0.00
A (n=15)
B (n=16)
C (n=14)
surface

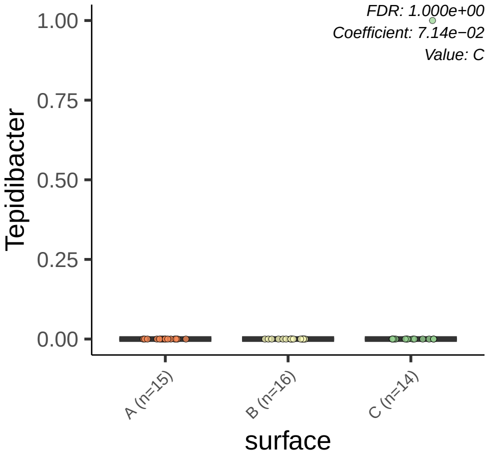
FDR: 1.000e+00
Coefficient: 7.14e−02
Value: C
1.00
0.75
0.50
0.25
0.00
Tepidibacter
A (n=15)
B (n=16)
C (n=14)
surface

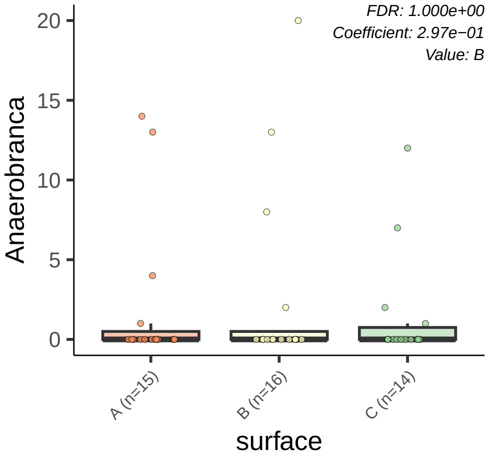
FDR: 1.000e+00
Coefficient: 2.97e−01
Value: B
Anaerobranca
20
15
10
5
0
A (n=15)
B (n=16)
C (n=14)
surface

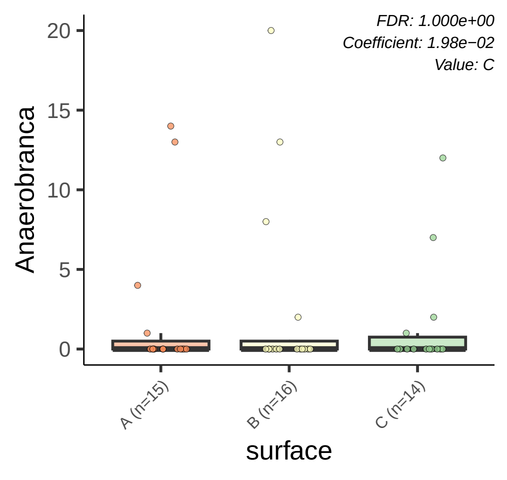

FDR: 1.000e+00
Coefficient: 1.98e−02
Value: C
Anaerobranca
20
15
10
5
0
A (n=15)
B (n=16)
C (n=14)
surface

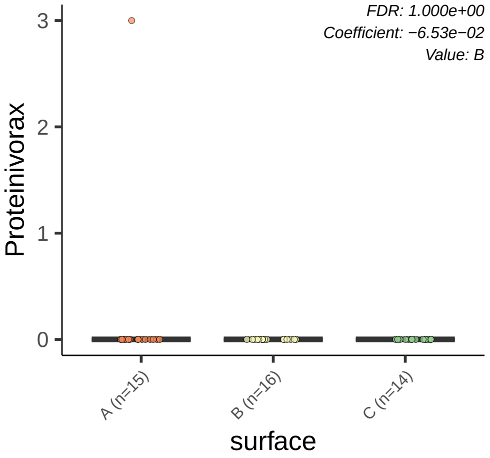

FDR: 1.000e+00
Coefficient: −6.53e−02
Value: B
Proteinivorax
3
2
1
0
A (n=15)
B (n=16)
C (n=14)
surface

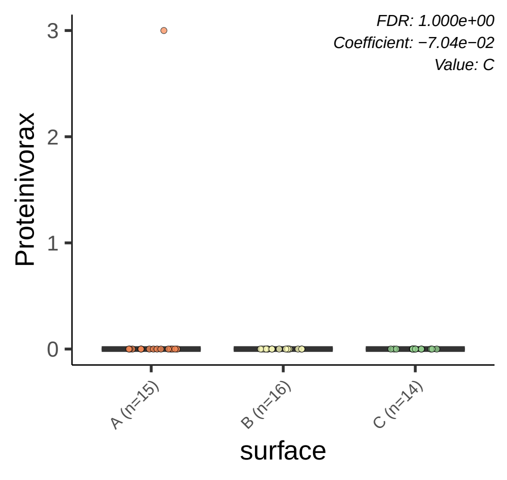

FDR: 1.000e+00
Coefficient: −7.04e−02
Value: C
Proteinivorax
3
2
1
0
A (n=15)
B (n=16)
C (n=14)
surface

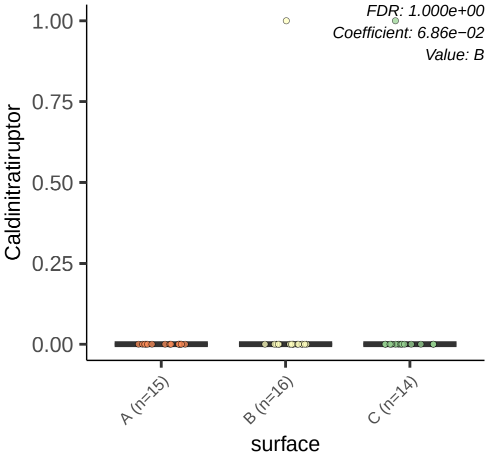
FDR: 1.000e+00
Coefficient: 6.86e−02
Value: B
Caldinitratiruptor
1.00
0.75
0.50
0.25
0.00
A (n=15)
B (n=16)
C (n=14)
surface

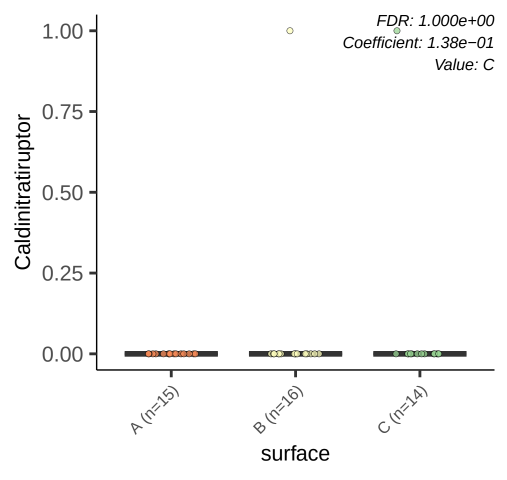

FDR: 1.000e+00
Coefficient: 1.38e−01
Value: C
Caldinitratiruptor
1.00
0.75
0.50
0.25
0.00
A (n=15)
B (n=16)
C (n=14)
surface

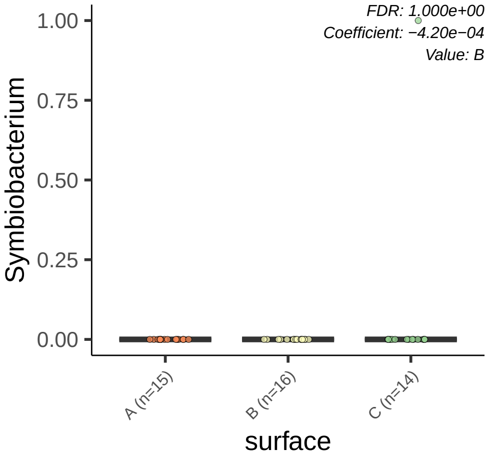
FDR: 1.000e+00
Coefficient: −4.20e−04
Value: B
Symbiobacterium
1.00
0.75
0.50
0.25
0.00
A (n=15)
B (n=16)
C (n=14)
surface

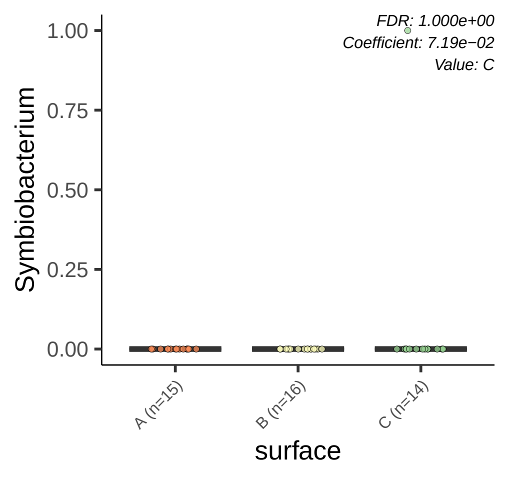

FDR: 1.000e+00
Coefficient: 7.19e−02
Value: C
Symbiobacterium
1.00
0.75
0.50
0.25
0.00
A (n=15)
B (n=16)
C (n=14)
surface

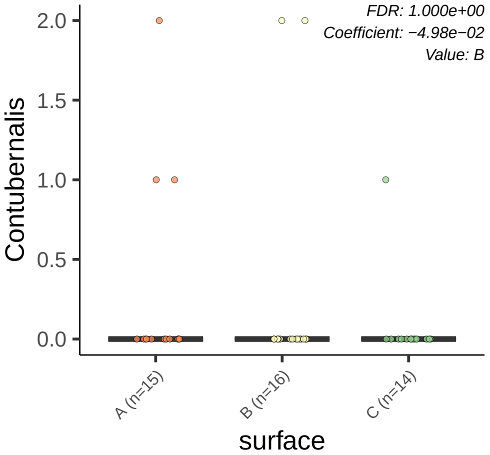
FDR: 1.000e+00
Coefficient: −4.98e−02
Value: B
Contubernalis
2.0
1.5
1.0
0.5
0.0
A (n=15)
B (n=16)
C (n=14)
surface

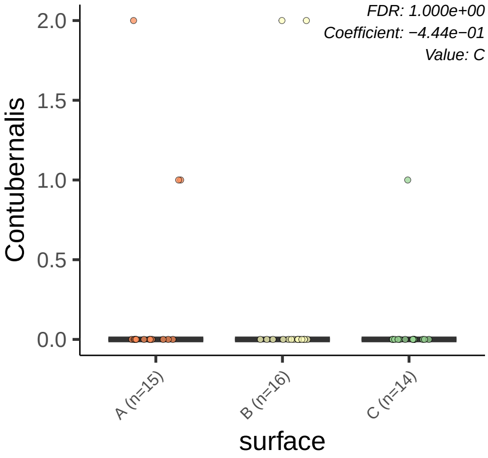
FDR: 1.000e+00
Coefficient: −4.44e−01
Value: C
Contubernalis
2.0
1.5
1.0
0.5
0.0
A (n=15)
B (n=16)
C (n=14)
surface

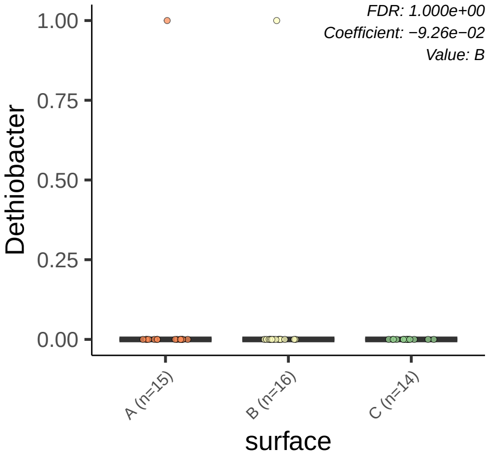
FDR: 1.000e+00
Coefficient: −9.26e−02
Value: B
Dethiobacter
1.00
0.75
0.50
0.25
0.00
A (n=15)
B (n=16)
C (n=14)
surface

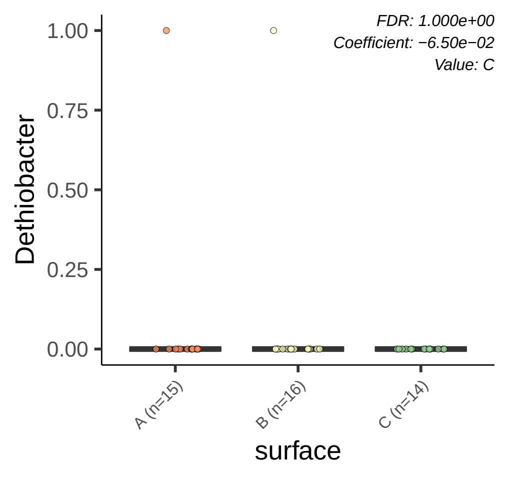
FDR: 1.000e+00
Coefficient: −6.50e−02
Value: C
1.00
0.75
0.50
0.25
0.00
Dethiobacter
A (n=15)
B (n=16)
C (n=14)
surface

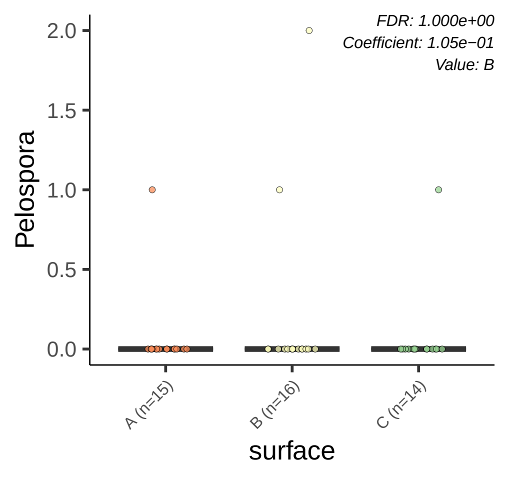
FDR: 1.000e+00
Coefficient: 1.05e−01
Value: B
Pelospora
2.0
1.5
1.0
0.5
0.0
A (n=15)
B (n=16)
C (n=14)
surface

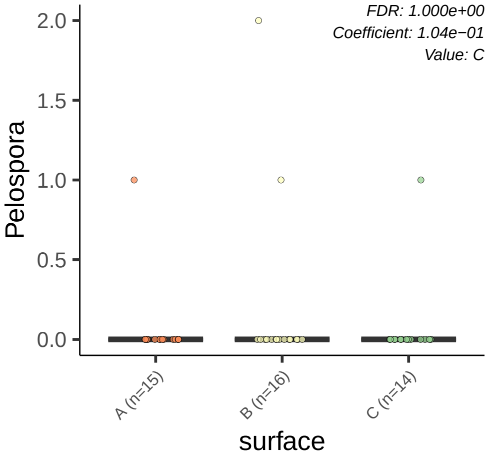
FDR: 1.000e+00
Coefficient: 1.04e−01
Value: C
Pelospora
2.0
1.5
1.0
0.5
0.0
A (n=15)
B (n=16)
C (n=14)
surface

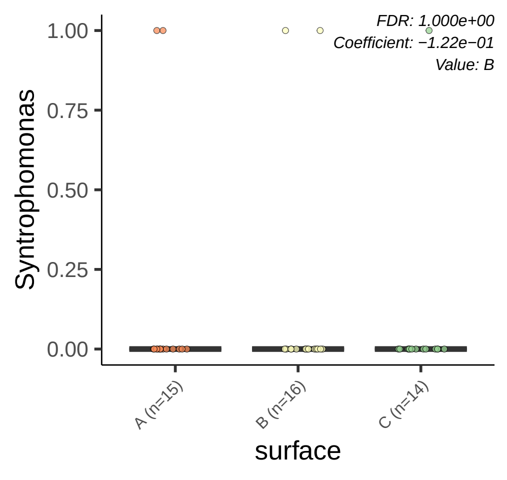
FDR: 1.000e+00
Coefficient: −1.22e−01
Value: B
Syntrophomonas
1.00
0.75
0.50
0.25
0.00
A (n=15)
B (n=16)
C (n=14)
surface

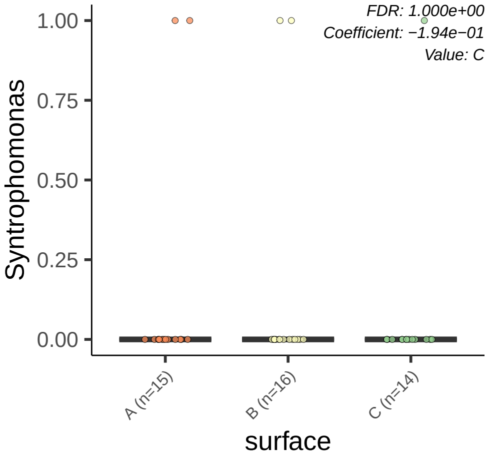

FDR: 1.000e+00
Coefficient: −1.94e−01
Value: C
Syntrophomonas
1.00
0.75
0.50
0.25
0.00
A (n=15)
B (n=16)
C (n=14)
surface

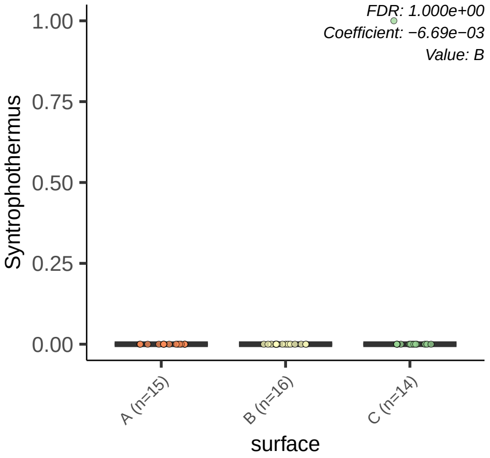
FDR: 1.000e+00
Coefficient: −6.69e−03
Value: B
1.00
0.75
0.50
0.25
0.00
Syntrophothermus
A (n=15)
B (n=16)
C (n=14)
surface

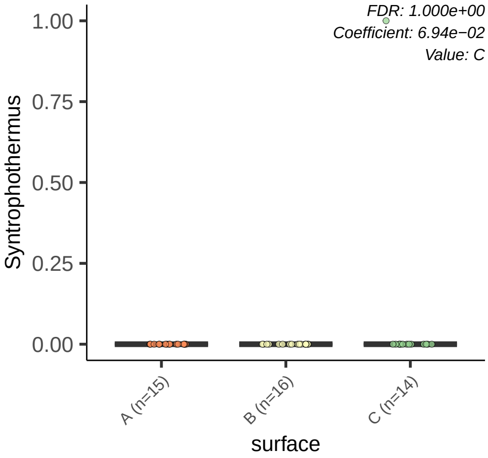

FDR: 1.000e+00
Coefficient: 6.94e−02
Value: C
Syntrophothermus
1.00
0.75
0.50
0.25
0.00
A (n=15)
B (n=16)
C (n=14)
surface

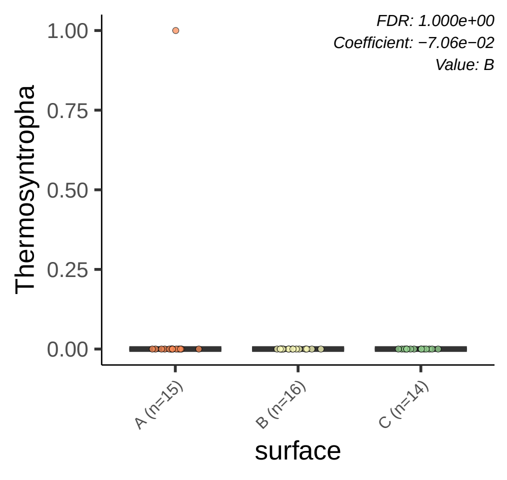
FDR: 1.000e+00
Coefficient: −7.06e−02
Value: B
Thermosyntropha
1.00
0.75
0.50
0.25
0.00
A (n=15)
B (n=16)
C (n=14)
surface

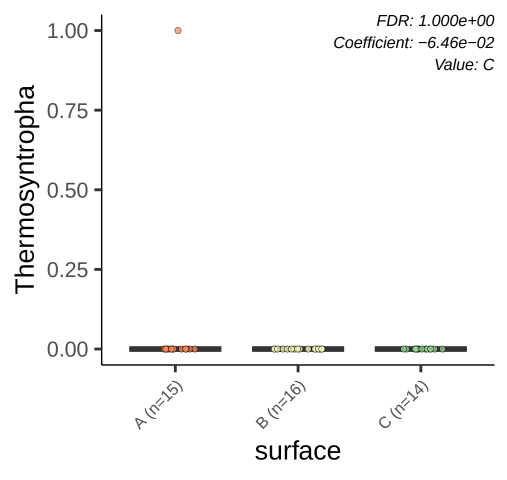

FDR: 1.000e+00
Coefficient: −6.46e−02
Value: C
Thermosyntropha
1.00
0.75
0.50
0.25
0.00
A (n=15)
B (n=16)
C (n=14)
surface

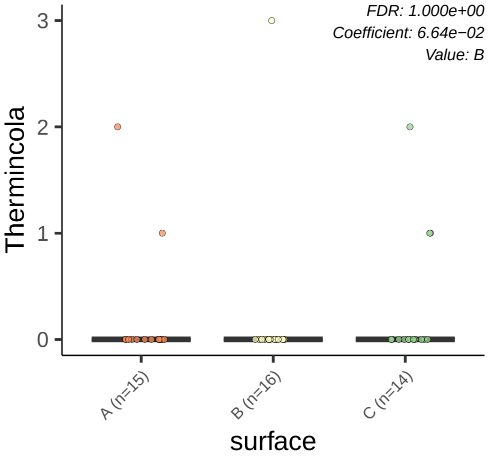

FDR: 1.000e+00
Coefficient: 6.64e−02
Value: B
Thermincola
3
2
1
0
A (n=15)
B (n=16)
C (n=14)
surface

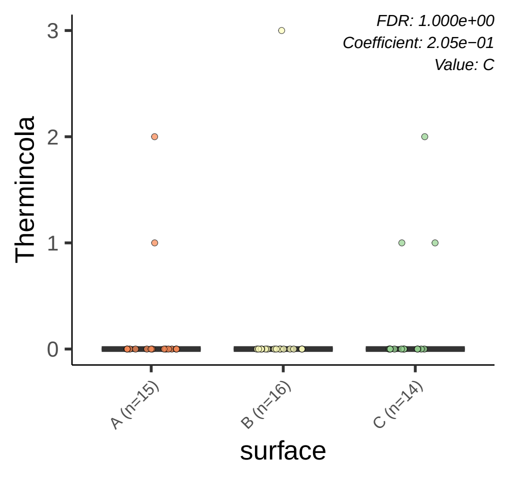

FDR: 1.000e+00
Coefficient: 2.05e−01
Value: C
Thermincola
3
2
1
0
A (n=15)
B (n=16)
C (n=14)
surface

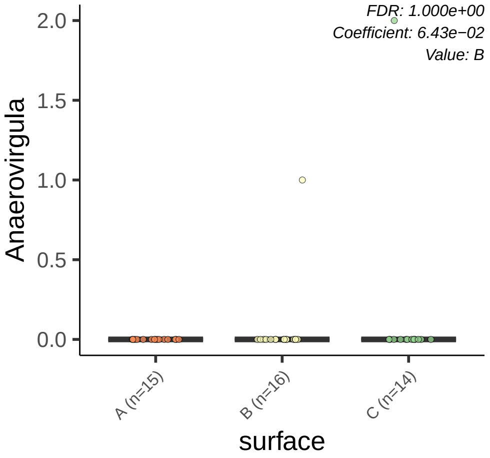
FDR: 1.000e+00
Coefficient: 6.43e−02
Value: B
Anaerovirgula
2.0
1.5
1.0
0.5
0.0
A (n=15)
B (n=16)
C (n=14)
surface

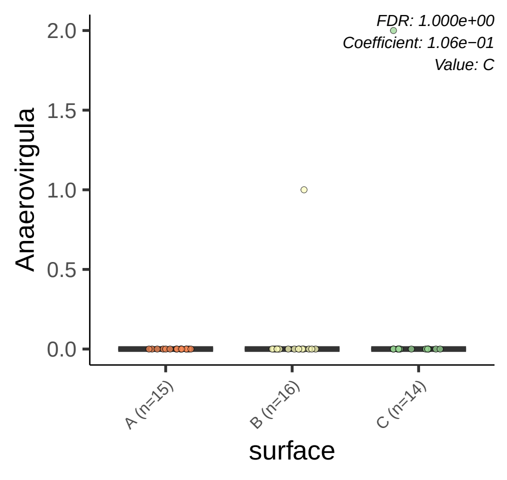
FDR: 1.000e+00
Coefficient: 1.06e−01
Value: C
Anaerovirgula
2.0
1.5
1.0
0.5
0.0
A (n=15)
B (n=16)
C (n=14)
surface

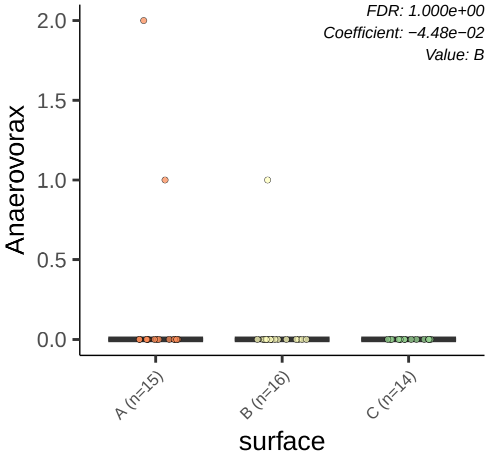

FDR: 1.000e+00
Coefficient: −4.48e−02
Value: B
Anaerovorax
2.0
1.5
1.0
0.5
0.0
A (n=15)
B (n=16)
C (n=14)
surface

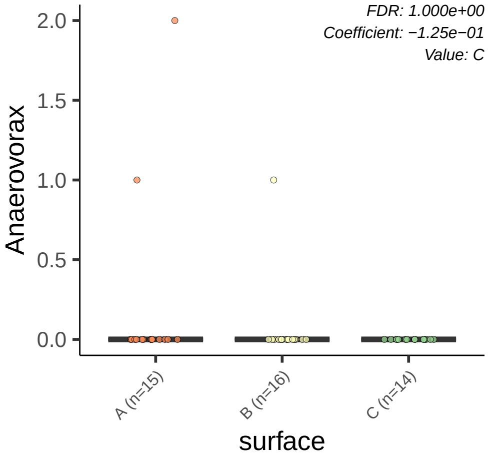
FDR: 1.000e+00
Coefficient: −1.25e−01
Value: C
Anaerovorax
2.0
1.5
1.0
0.5
0.0
A (n=15)
B (n=16)
C (n=14)
surface

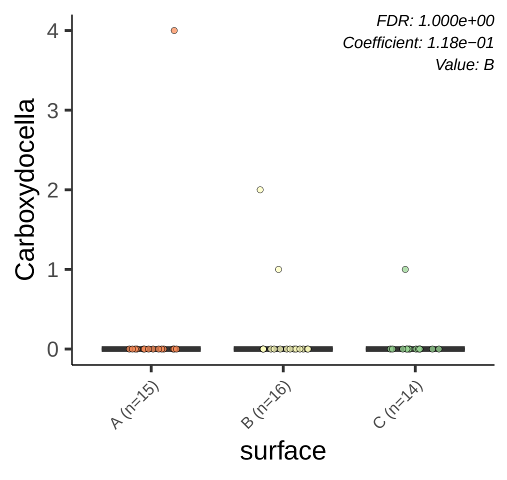
FDR: 1.000e+00
Coefficient: 1.18e−01
Value: B
Carboxydocella
0
1
2
3
4
A (n=15)
B (n=16)
C (n=14)
surface

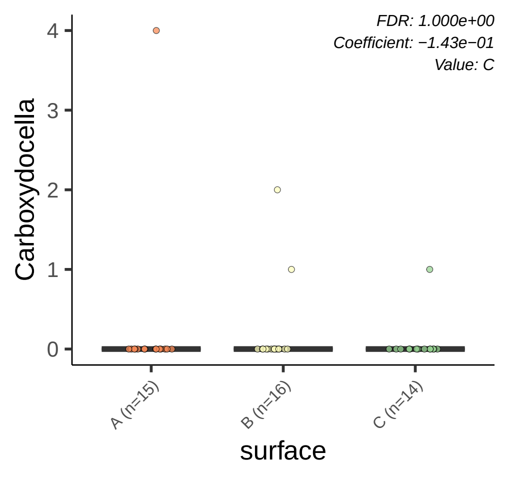

FDR: 1.000e+00
Coefficient: −1.43e−01
Value: C
Carboxydocella
4
3
2
1
0
A (n=15)
B (n=16)
C (n=14)
surface

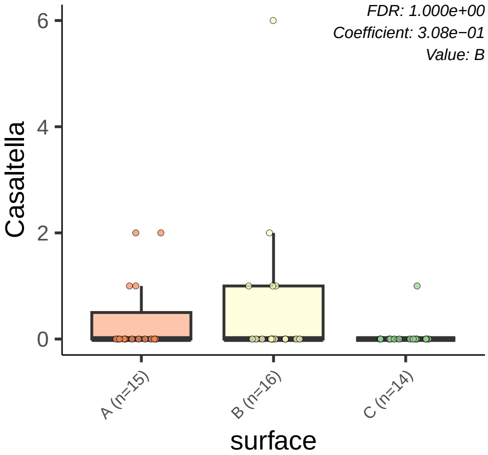
FDR: 1.000e+00
Coefficient: 3.08e−01
Value: B
Casaltella
6
4
2
0
A (n=15)
B (n=16)
C (n=14)
surface

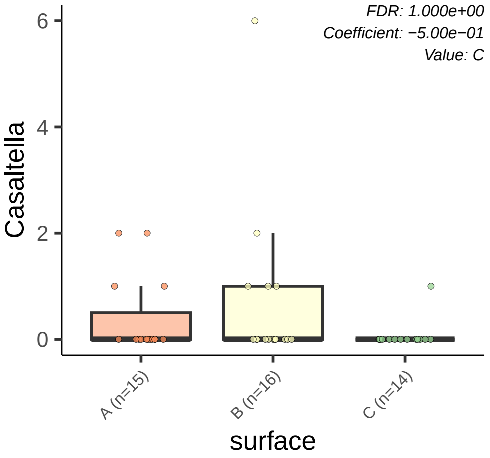
FDR: 1.000e+00
Coefficient: −5.00e−01
Value: C
Casaltella
0
2
4
6
A (n=15)
B (n=16)
C (n=14)
surface

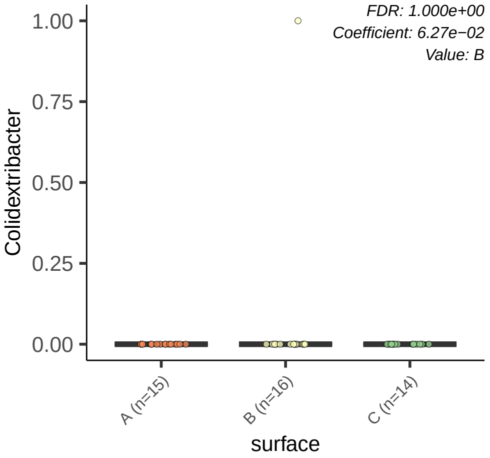
FDR: 1.000e+00
Coefficient: 6.27e−02
Value: B
1.00
0.75
0.50
0.25
0.00
Colidextribacter
A (n=15)
B (n=16)
C (n=14)
surface

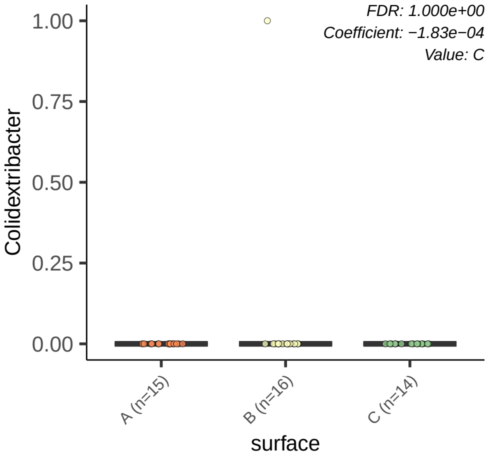

FDR: 1.000e+00
Coefficient: −1.83e−04
Value: C
Colidextribacter
1.00
0.75
0.50
0.25
0.00
A (n=15)
B (n=16)
C (n=14)
surface

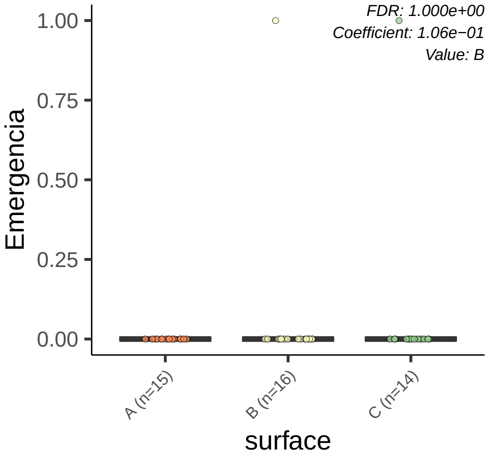

FDR: 1.000e+00
Coefficient: 1.06e−01
Value: B
Emergencia
1.00
0.75
0.50
0.25
0.00
A (n=15)
B (n=16)
C (n=14)
surface

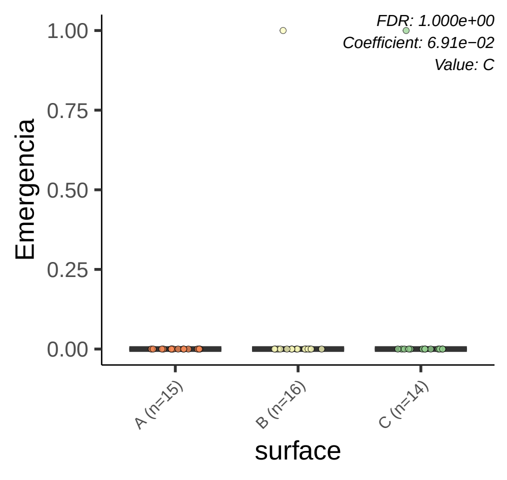
FDR: 1.000e+00
Coefficient: 6.91e−02
Value: C
Emergencia
1.00
0.75
0.50
0.25
0.00
A (n=15)
B (n=16)
C (n=14)
surface

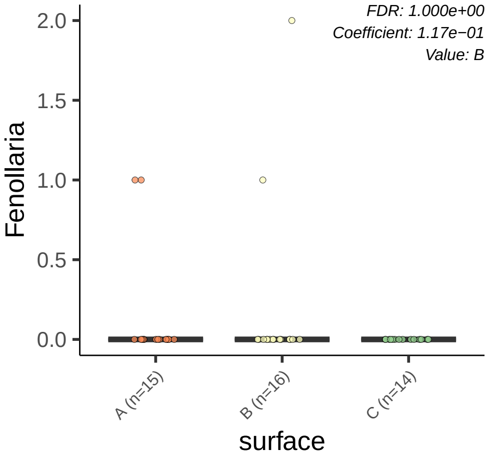

FDR: 1.000e+00
Coefficient: 1.17e−01
Value: B
Fenollaria
2.0
1.5
1.0
0.5
0.0
A (n=15)
B (n=16)
C (n=14)
surface

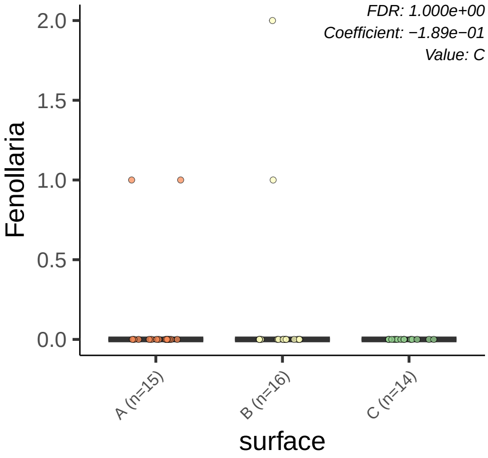
FDR: 1.000e+00
Coefficient: −1.89e−01
Value: C
Fenollaria
2.0
1.5
1.0
0.5
0.0
A (n=15)
B (n=16)
C (n=14)
surface

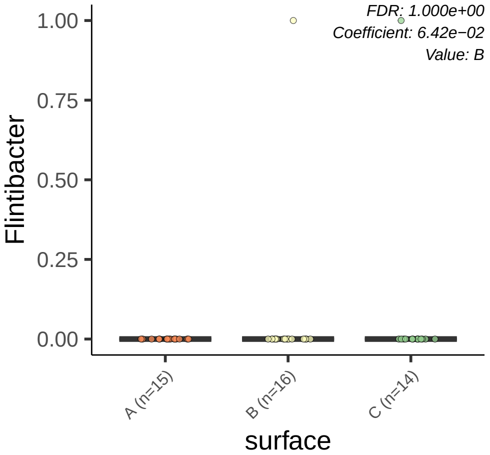
FDR: 1.000e+00
Coefficient: 6.42e−02
Value: B
Flintibacter
1.00
0.75
0.50
0.25
0.00
A (n=15)
B (n=16)
C (n=14)
surface

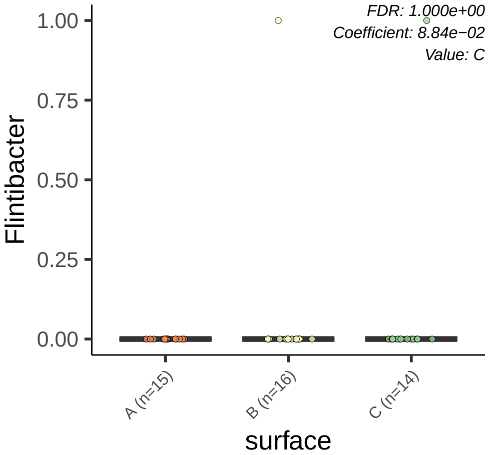

FDR: 1.000e+00
Coefficient: 8.84e−02
Value: C
Flintibacter
1.00
0.75
0.50
0.25
0.00
A (n=15)
B (n=16)
C (n=14)
surface

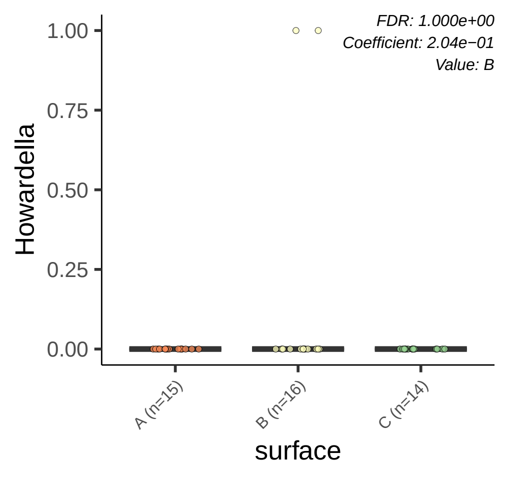
FDR: 1.000e+00
Coefficient: 2.04e−01
Value: B
Howardella
1.00
0.75
0.50
0.25
0.00
A (n=15)
B (n=16)
C (n=14)
surface

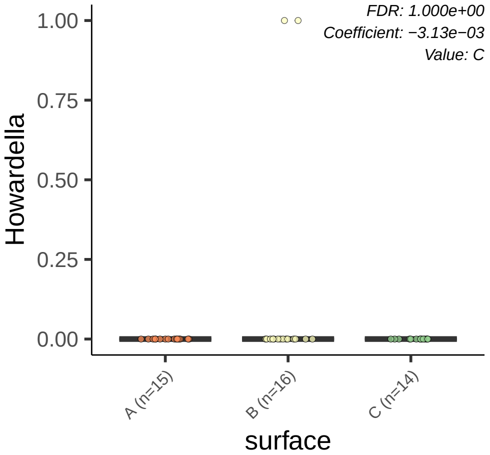

FDR: 1.000e+00
Coefficient: −3.13e−03
Value: C
Howardella
1.00
0.75
0.50
0.25
0.00
A (n=15)
B (n=16)
C (n=14)
surface

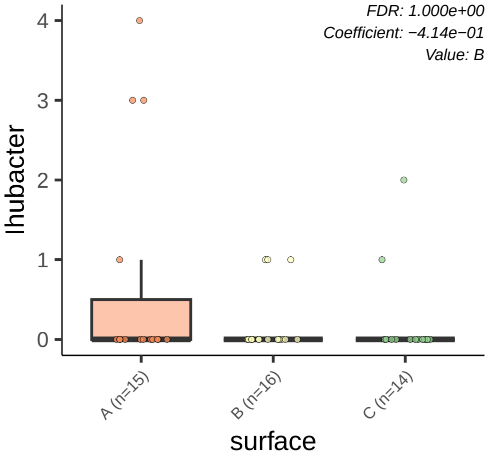

FDR: 1.000e+00
Coefficient: −4.14e−01
Value: B
Ihubacter
4
3
2
1
0
A (n=15)
B (n=16)
C (n=14)
surface

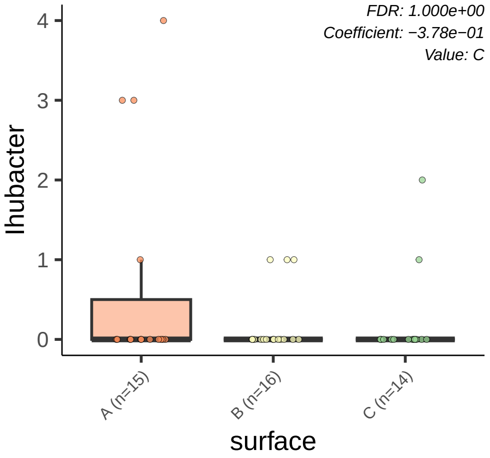

FDR: 1.000e+00
Coefficient: −3.78e−01
Value: C
Ihubacter
0
1
2
3
4
A (n=15)
B (n=16)
C (n=14)
surface

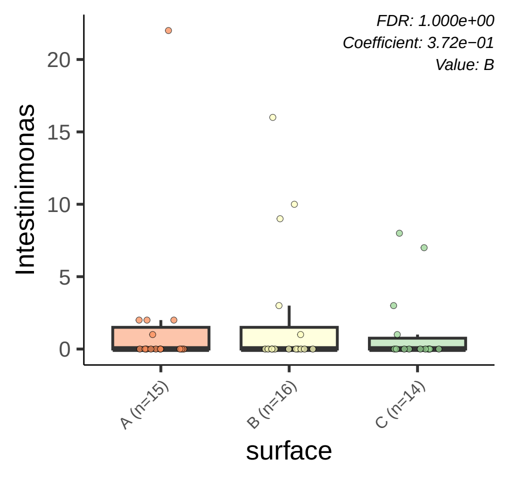

FDR: 1.000e+00
Coefficient: 3.72e−01
Value: B
Intestinimonas
20
15
10
5
0
A (n=15)
B (n=16)
C (n=14)
surface

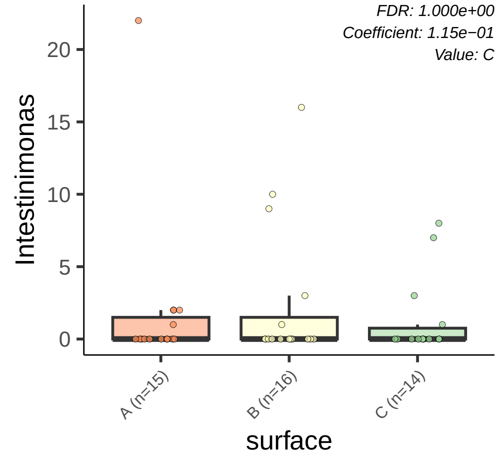

FDR: 1.000e+00
Coefficient: 1.15e−01
Value: C
Intestinimonas
0
5
10
15
20
A (n=15)
B (n=16)
C (n=14)
surface

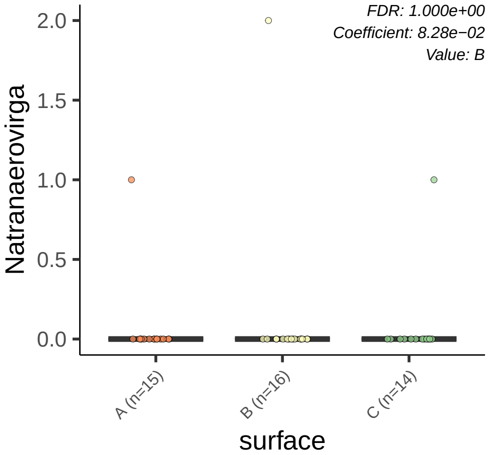
FDR: 1.000e+00
Coefficient: 8.28e−02
Value: B
Natranaerovirga
2.0
1.5
1.0
0.5
0.0
A (n=15)
B (n=16)
C (n=14)
surface

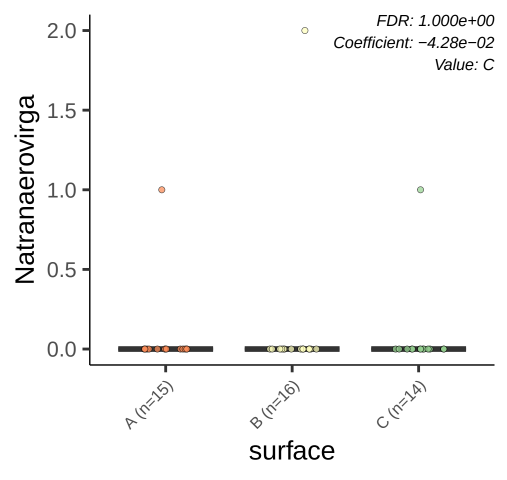
FDR: 1.000e+00
Coefficient: −4.28e−02
Value: C
Natranaerovirga
2.0
1.5
1.0
0.5
0.0
A (n=15)
B (n=16)
C (n=14)
surface

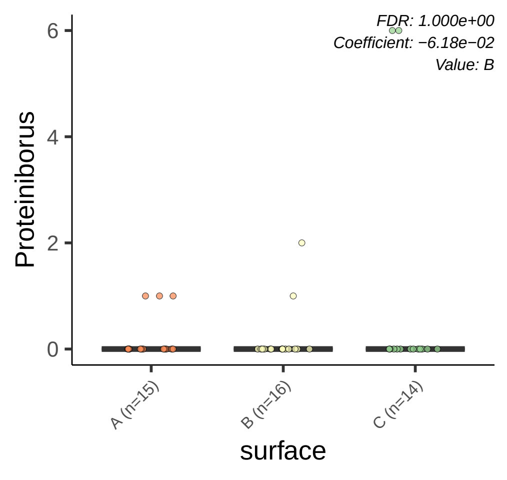

FDR: 1.000e+00
Coefficient: −6.18e−02
Value: B
Proteiniborus
6
4
2
0
A (n=15)
B (n=16)
C (n=14)
surface

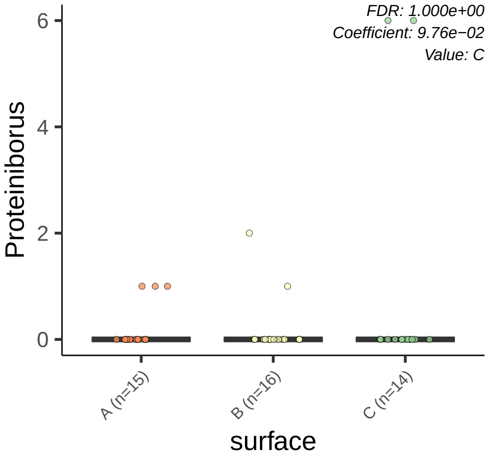
FDR: 1.000e+00
Coefficient: 9.76e−02
Value: C
Proteiniborus
6
4
2
0
A (n=15)
B (n=16)
C (n=14)
surface

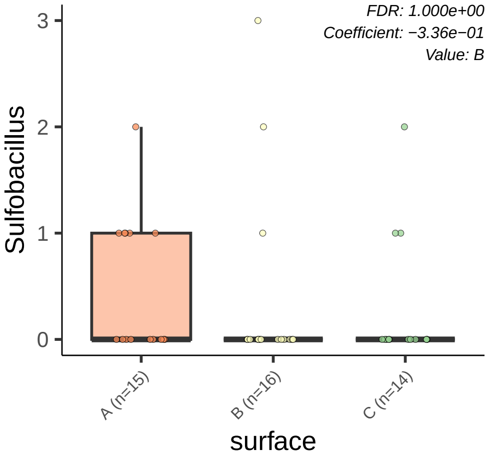
FDR: 1.000e+00
Coefficient: −3.36e−01
Value: B
Sulfobacillus
3
2
1
0
A (n=15)
B (n=16)
C (n=14)
surface

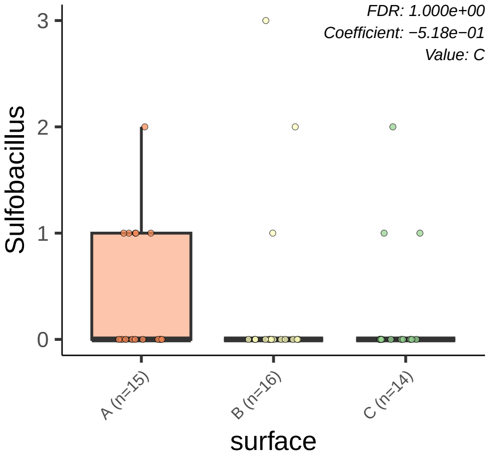
FDR: 1.000e+00
Coefficient: −5.18e−01
Value: C
Sulfobacillus
3
2
1
0
A (n=15)
B (n=16)
C (n=14)
surface

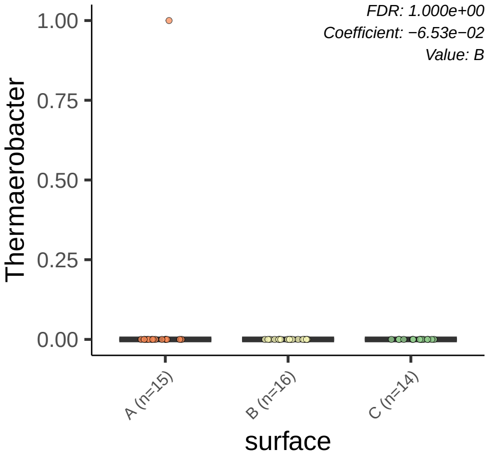

FDR: 1.000e+00
Coefficient: −6.53e−02
Value: B
Thermaerobacter
1.00
0.75
0.50
0.25
0.00
A (n=15)
B (n=16)
C (n=14)
surface

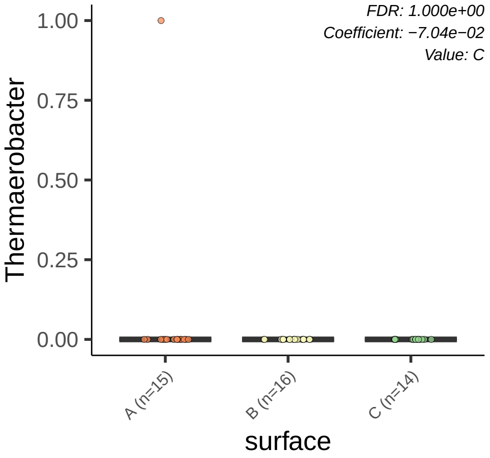

FDR: 1.000e+00
Coefficient: −7.04e−02
Value: C
Thermaerobacter
1.00
0.75
0.50
0.25
0.00
A (n=15)
B (n=16)
C (n=14)
surface

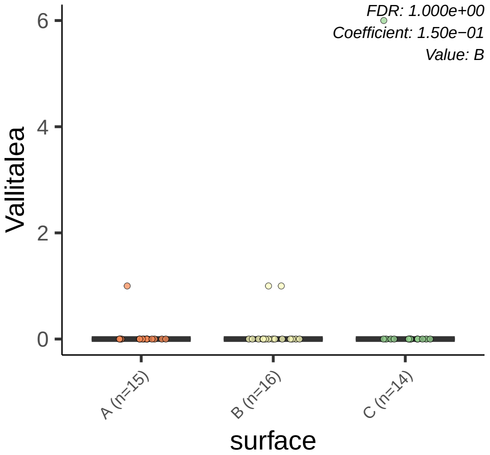
FDR: 1.000e+00
Coefficient: 1.50e−01
Value: B
Vallitalea
6
4
2
0
A (n=15)
B (n=16)
C (n=14)
surface

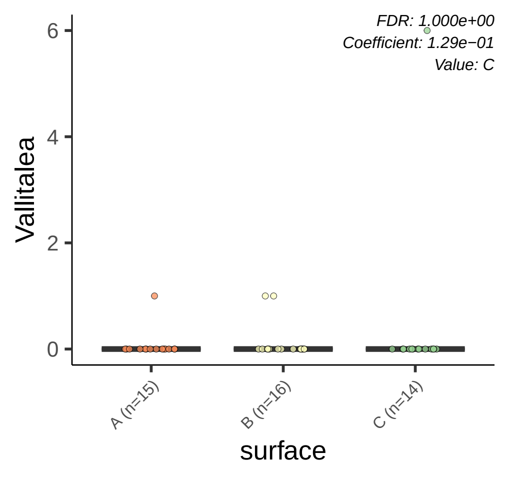

FDR: 1.000e+00
Coefficient: 1.29e−01
Value: C
Vallitalea
6
4
2
0
A (n=15)
B (n=16)
C (n=14)
surface

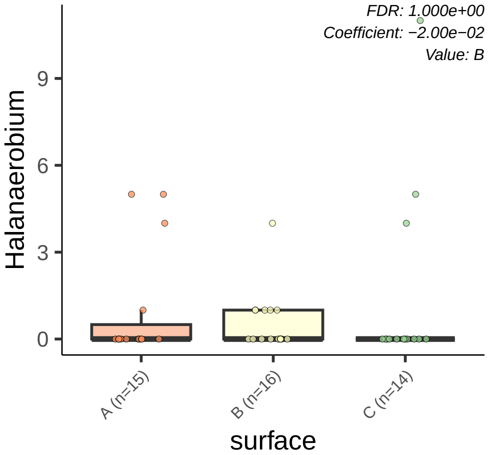
FDR: 1.000e+00
Coefficient: −2.00e−02
Value: B
Halanaerobium
0
3
6
9
A (n=15)
B (n=16)
C (n=14)
surface

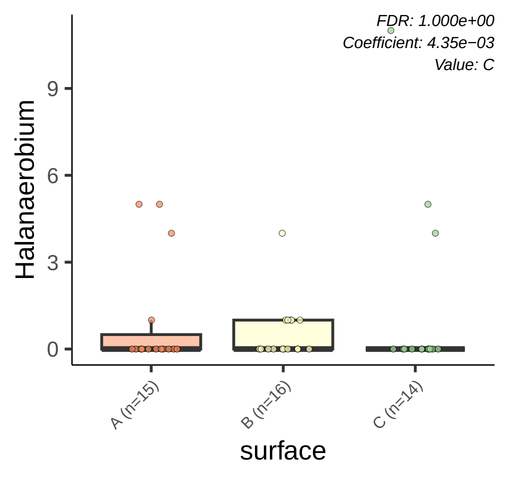

FDR: 1.000e+00
Coefficient: 4.35e−03
Value: C
Halanaerobium
9
6
3
0
A (n=15)
B (n=16)
C (n=14)
surface

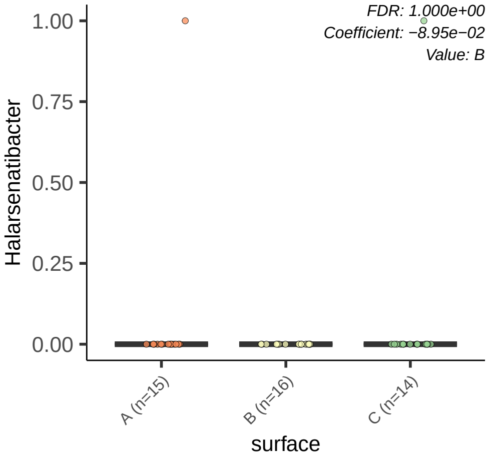

FDR: 1.000e+00
Coefficient: −8.95e−02
Value: B
Halarsenatibacter
1.00
0.75
0.50
0.25
0.00
A (n=15)
B (n=16)
C (n=14)
surface

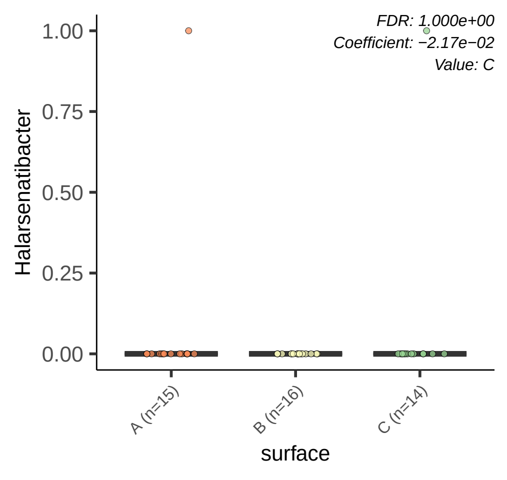
FDR: 1.000e+00
Coefficient: −2.17e−02
Value: C
Halarsenatibacter
1.00
0.75
0.50
0.25
0.00
A (n=15)
B (n=16)
C (n=14)
surface

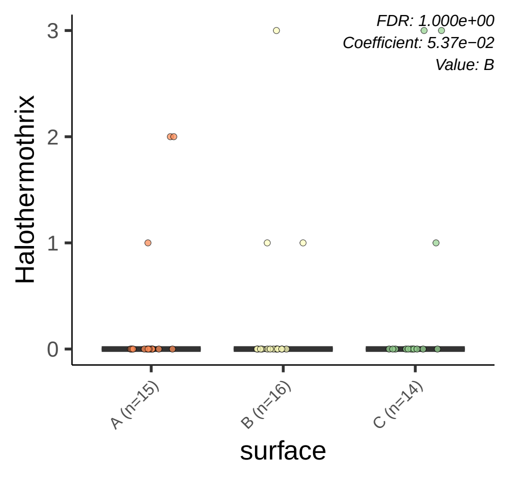
FDR: 1.000e+00
Coefficient: 5.37e−02
Value: B
Halothermothrix
3
2
1
0
A (n=15)
B (n=16)
C (n=14)
surface

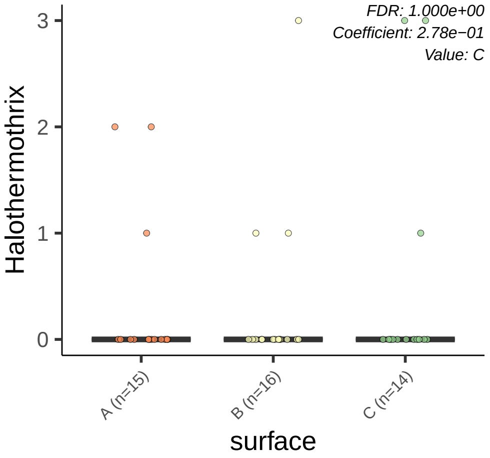
FDR: 1.000e+00
Coefficient: 2.78e−01
Value: C
Halothermothrix
3
2
1
0
A (n=15)
B (n=16)
C (n=14)
surface

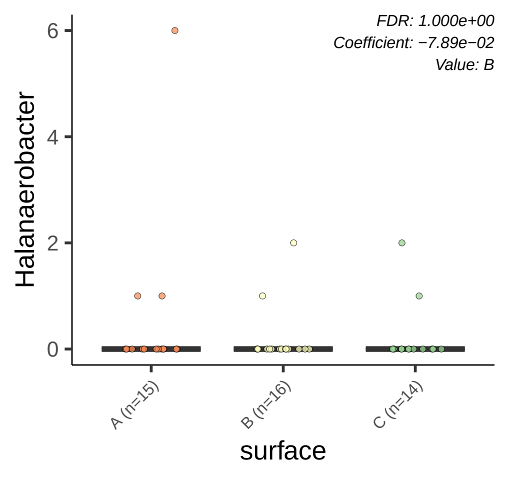

FDR: 1.000e+00
Coefficient: −7.89e−02
Value: B
Halanaerobacter
6
4
2
0
A (n=15)
B (n=16)
C (n=14)
surface

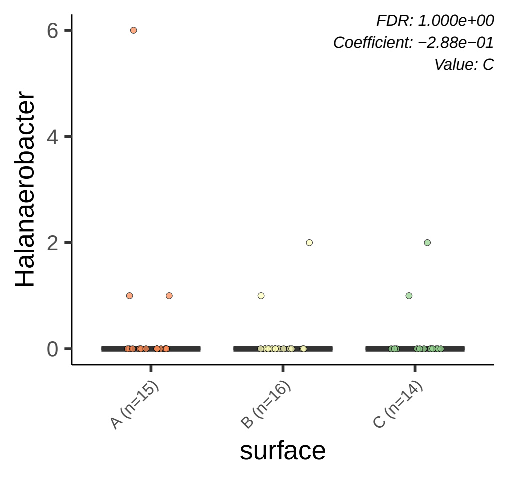
FDR: 1.000e+00
Coefficient: −2.88e−01
Value: C
Halanaerobacter
6
4
2
0
A (n=15)
B (n=16)
C (n=14)
surface

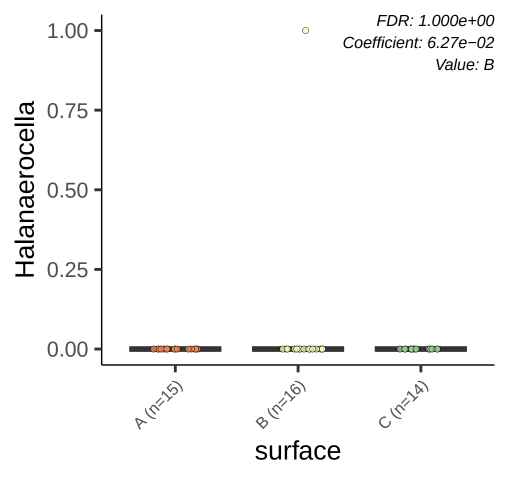
FDR: 1.000e+00
Coefficient: 6.27e−02
Value: B
Halanaerocella
1.00
0.75
0.50
0.25
0.00
A (n=15)
B (n=16)
C (n=14)
surface

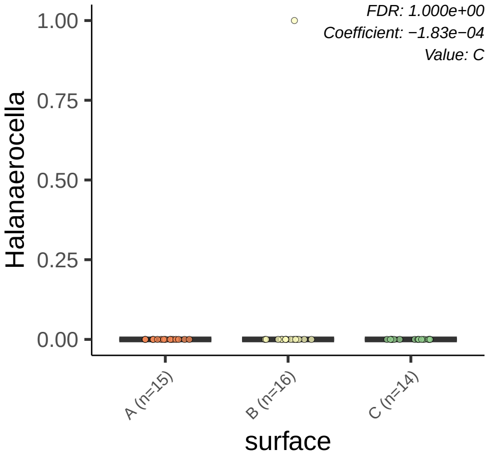
FDR: 1.000e+00
Coefficient: −1.83e−04
Value: C
Halanaerocella
1.00
0.75
0.50
0.25
0.00
A (n=15)
B (n=16)
C (n=14)
surface

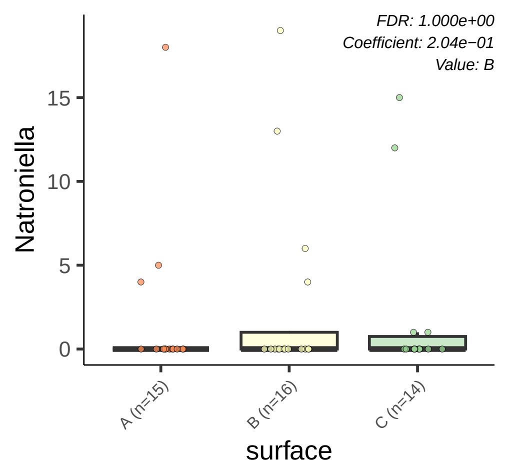

FDR: 1.000e+00
Coefficient: 2.04e−01
Value: B
Natroniella
15
10
5
0
A (n=15)
B (n=16)
C (n=14)
surface

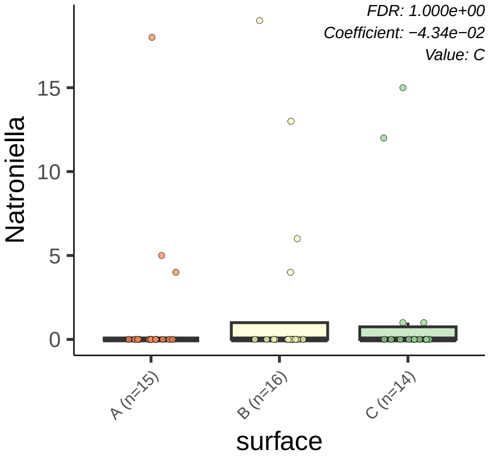

FDR: 1.000e+00
Coefficient: −4.34e−02
Value: C
Natroniella
0
5
10
15
A (n=15)
B (n=16)
C (n=14)
surface

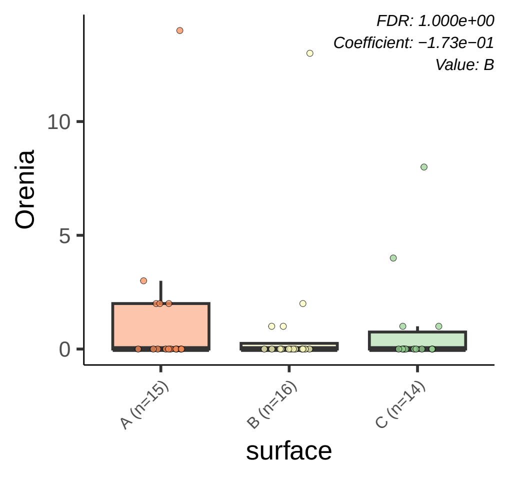

FDR: 1.000e+00
Coefficient: −1.73e−01
Value: B
Orenia
10
5
0
A (n=15)
B (n=16)
C (n=14)
surface

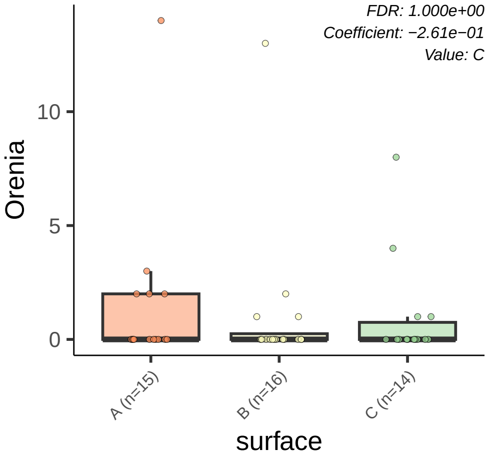

FDR: 1.000e+00
Coefficient: −2.61e−01
Value: C
Orenia
10
5
0
A (n=15)
B (n=16)
C (n=14)
surface

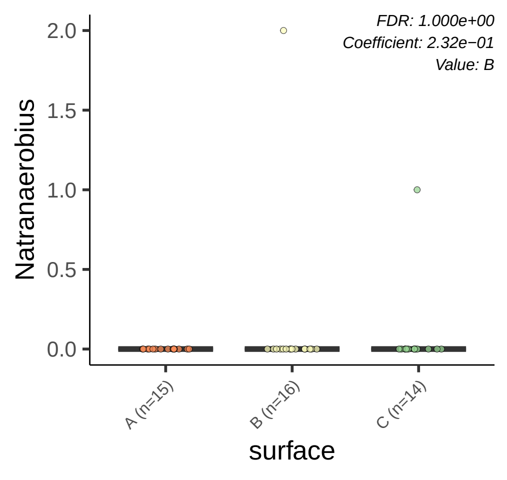

FDR: 1.000e+00
Coefficient: 2.32e−01
Value: B
Natranaerobius
2.0
1.5
1.0
0.5
0.0
A (n=15)
B (n=16)
C (n=14)
surface

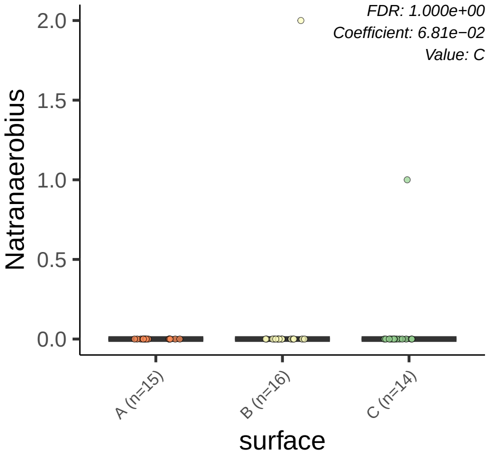

FDR: 1.000e+00
Coefficient: 6.81e−02
Value: C
Natranaerobius
2.0
1.5
1.0
0.5
0.0
A (n=15)
B (n=16)
C (n=14)
surface

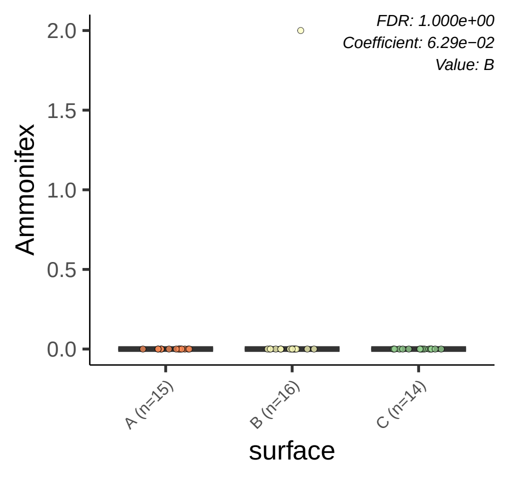

FDR: 1.000e+00
Coefficient: 6.29e−02
Value: B
Ammonifex
2.0
1.5
1.0
0.5
0.0
A (n=15)
B (n=16)
C (n=14)
surface

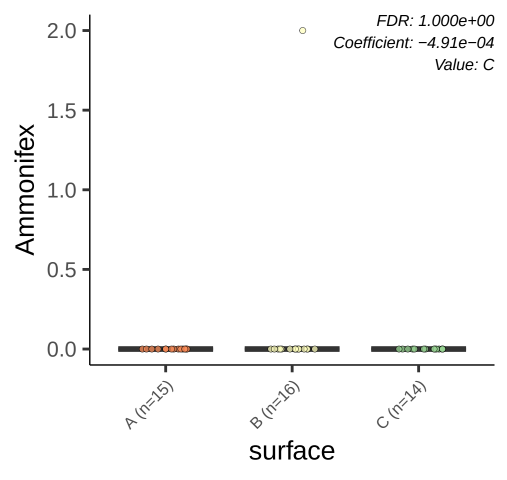

FDR: 1.000e+00
Coefficient: −4.91e−04
Value: C
2.0
1.5
1.0
0.5
0.0
Ammonifex
A (n=15)
B (n=16)
C (n=14)
surface

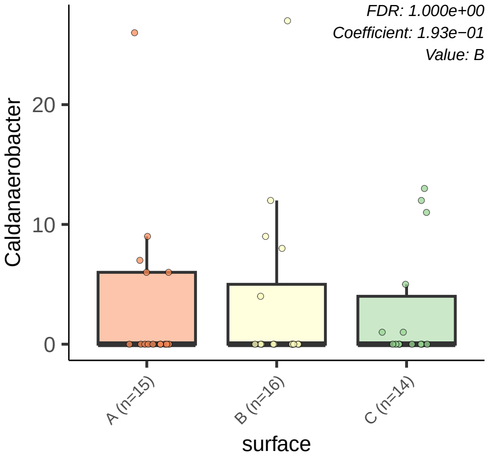
FDR: 1.000e+00
Coefficient: 1.93e−01
Value: B
Caldanaerobacter
0
10
20
A (n=15)
B (n=16)
C (n=14)
surface

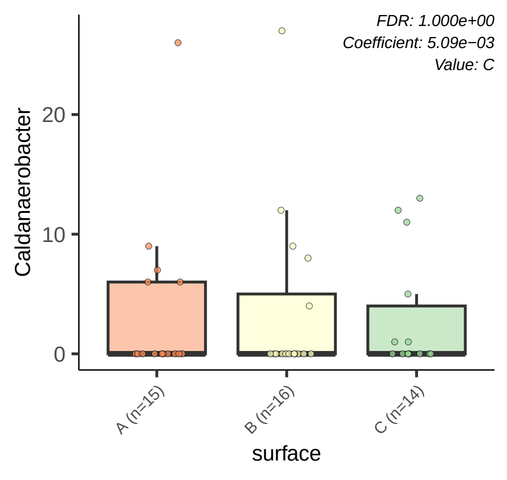
FDR: 1.000e+00
Coefficient: 5.09e−03
Value: C
Caldanaerobacter
0
10
20
A (n=15)
B (n=16)
C (n=14)
surface

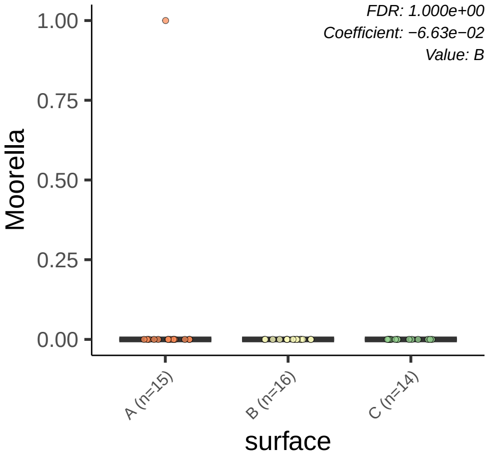

FDR: 1.000e+00
Coefficient: −6.63e−02
Value: B
Moorella
1.00
0.75
0.50
0.25
0.00
A (n=15)
B (n=16)
C (n=14)
surface

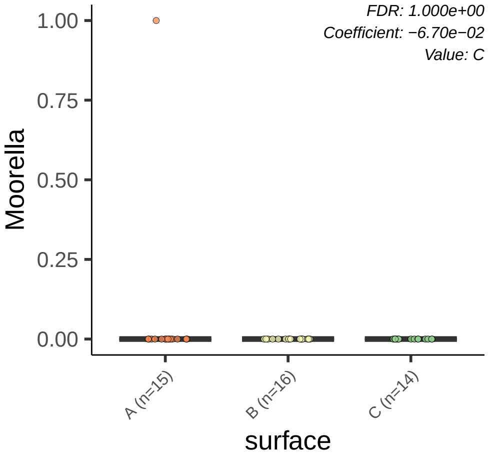
FDR: 1.000e+00
Coefficient: −6.70e−02
Value: C
Moorella
1.00
0.75
0.50
0.25
0.00
A (n=15)
B (n=16)
C (n=14)
surface

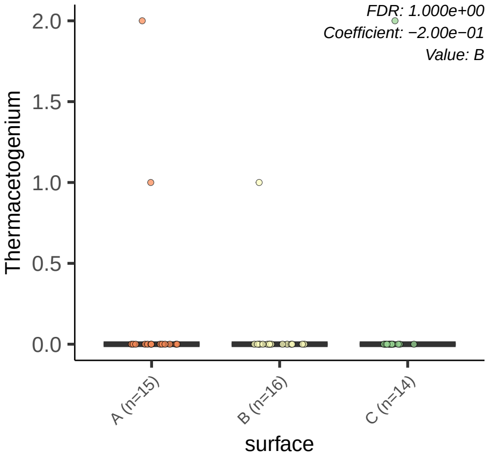

FDR: 1.000e+00
Coefficient: −2.00e−01
Value: B
Thermacetogenium
2.0
1.5
1.0
0.5
0.0
A (n=15)
B (n=16)
C (n=14)
surface

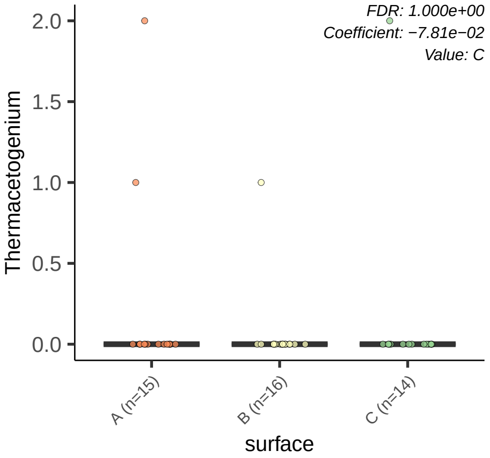
FDR: 1.000e+00
Coefficient: −7.81e−02
Value: C
Thermacetogenium
2.0
1.5
1.0
0.5
0.0
A (n=15)
B (n=16)
C (n=14)
surface

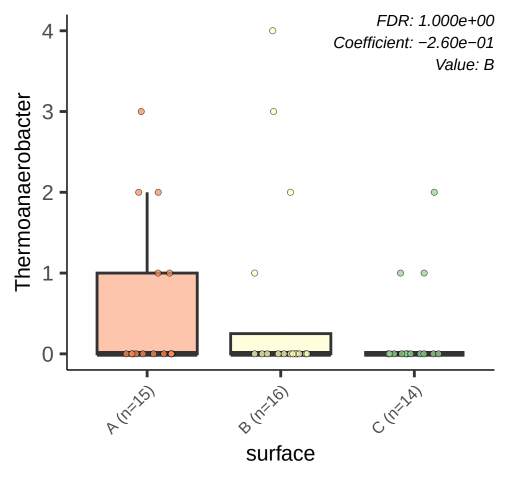

FDR: 1.000e+00
Coefficient: −2.60e−01
Value: B
Thermoanaerobacter
0
1
2
3
4
A (n=15)
B (n=16)
C (n=14)
surface

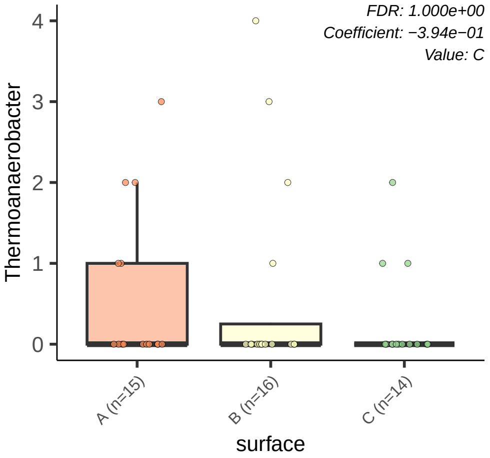
FDR: 1.000e+00
Coefficient: −3.94e−01
Value: C
Thermoanaerobacter
0
1
2
3
4
A (n=15)
B (n=16)
C (n=14)
surface

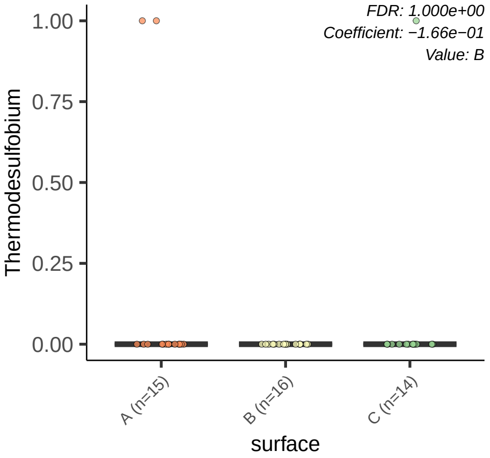

FDR: 1.000e+00
Coefficient: −1.66e−01
Value: B
Thermodesulfobium
1.00
0.75
0.50
0.25
0.00
A (n=15)
B (n=16)
C (n=14)
surface

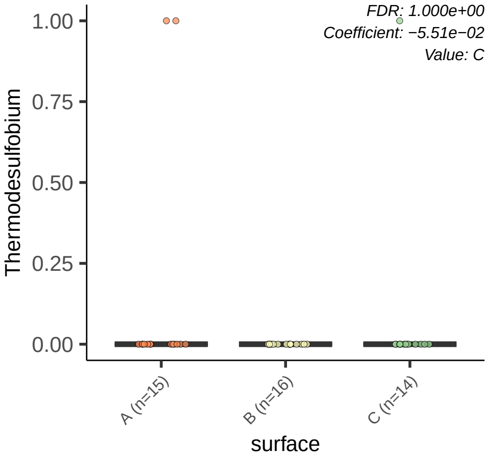
FDR: 1.000e+00
Coefficient: −5.51e−02
Value: C
Thermodesulfobium
1.00
0.75
0.50
0.25
0.00
A (n=15)
B (n=16)
C (n=14)
surface

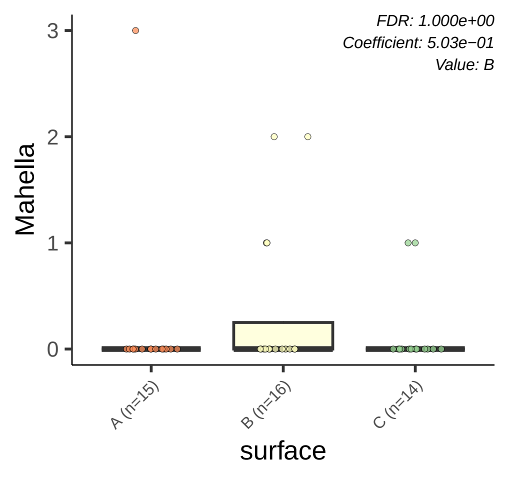
FDR: 1.000e+00
Coefficient: 5.03e−01
Value: B
Mahella
3
2
1
0
A (n=15)
B (n=16)
C (n=14)
surface

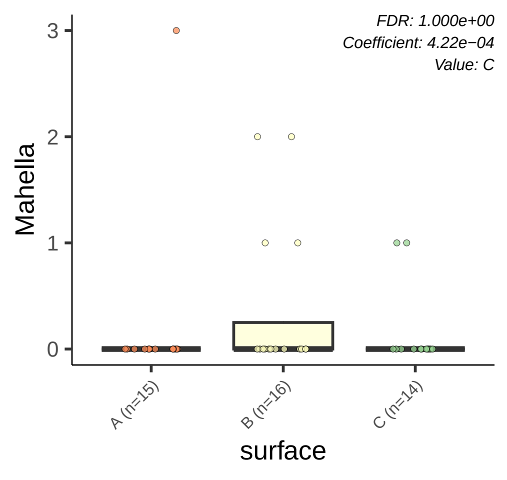
FDR: 1.000e+00
Coefficient: 4.22e−04
Value: C
Mahella
3
2
1
0
A (n=15)
B (n=16)
C (n=14)
surface

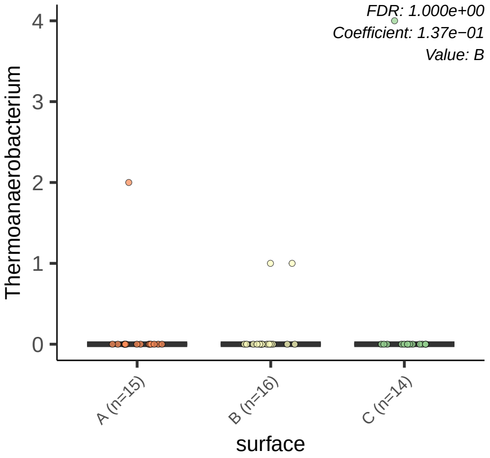

FDR: 1.000e+00
Coefficient: 1.37e−01
Value: B
Thermoanaerobacterium
0
1
2
3
4
A (n=15)
B (n=16)
C (n=14)
surface

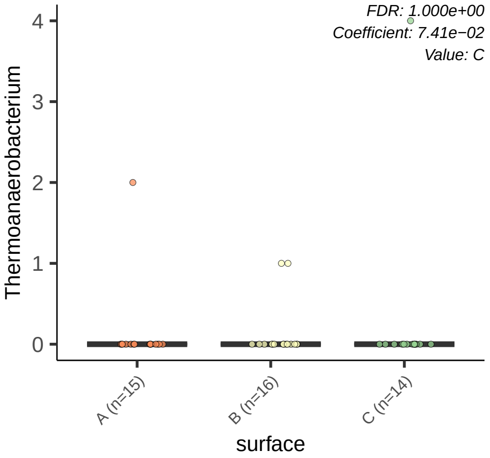

FDR: 1.000e+00
Coefficient: 7.41e−02
Value: C
Thermoanaerobacterium
0
1
2
3
4
A (n=15)
B (n=16)
C (n=14)
surface

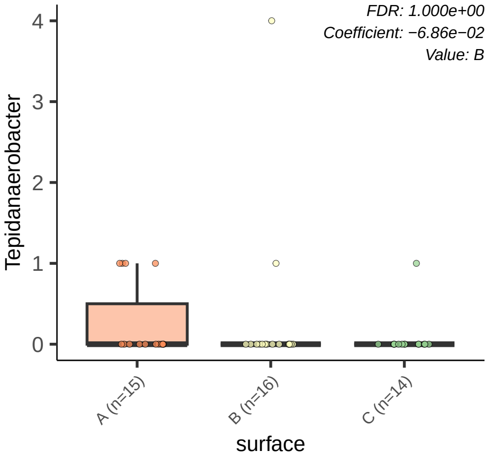
FDR: 1.000e+00
Coefficient: −6.86e−02
Value: B
Tepidanaerobacter
4
3
2
1
0
A (n=15)
B (n=16)
C (n=14)
surface

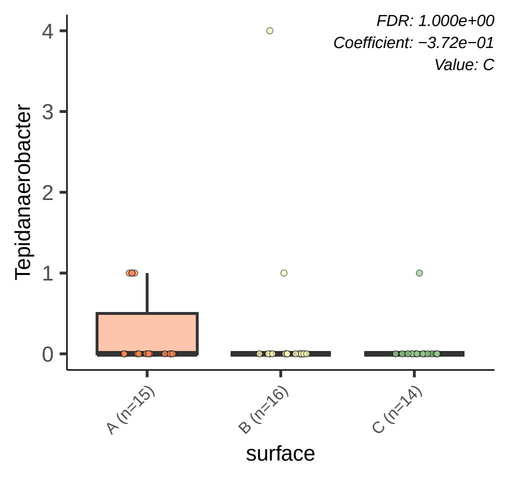
FDR: 1.000e+00
Coefficient: −3.72e−01
Value: C
Tepidanaerobacter
4
3
2
1
0
A (n=15)
B (n=16)
C (n=14)
surface

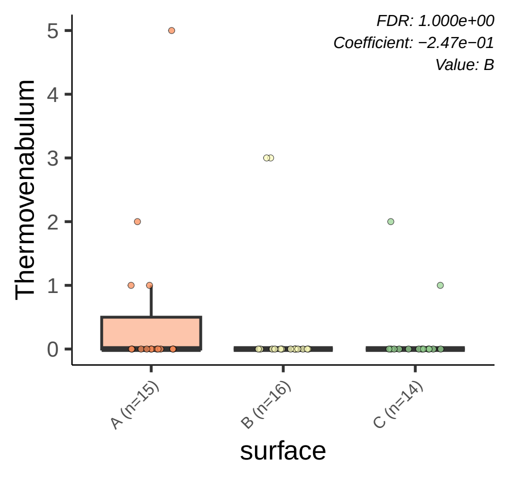
FDR: 1.000e+00
Coefficient: −2.47e−01
Value: B
Thermovenabulum
0
1
2
3
4
5
A (n=15)
B (n=16)
C (n=14)
surface

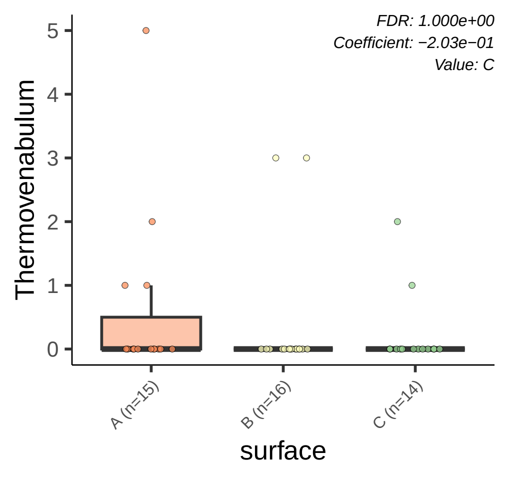
FDR: 1.000e+00
Coefficient: −2.03e−01
Value: C
Thermovenabulum
0
1
2
3
4
5
A (n=15)
B (n=16)
C (n=14)
surface

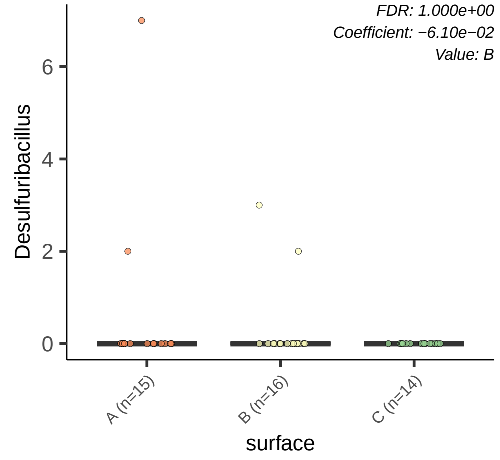

FDR: 1.000e+00
Coefficient: −6.10e−02
Value: B
Desulfuribacillus
0
2
4
6
A (n=15)
B (n=16)
C (n=14)
surface

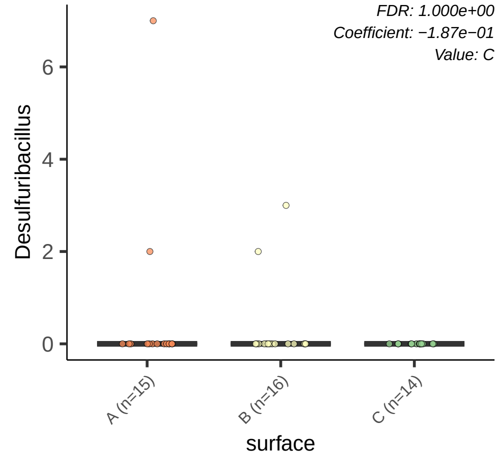
FDR: 1.000e+00
Coefficient: −1.87e−01
Value: C
Desulfuribacillus
0
2
4
6
A (n=15)
B (n=16)
C (n=14)
surface

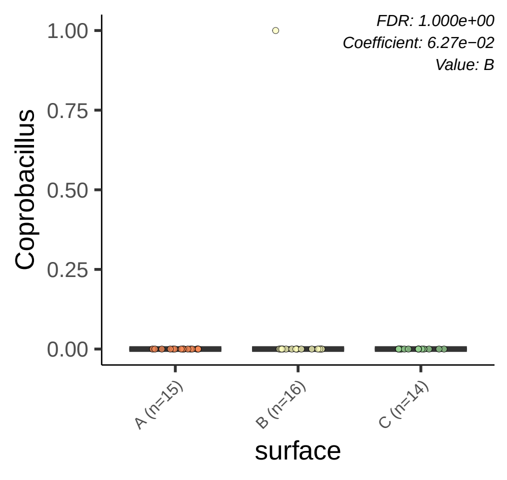
FDR: 1.000e+00
Coefficient: 6.27e−02
Value: B
Coprobacillus
1.00
0.75
0.50
0.25
0.00
A (n=15)
B (n=16)
C (n=14)
surface

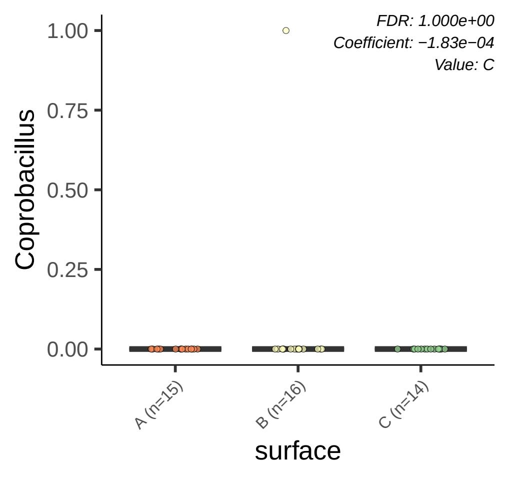

FDR: 1.000e+00
Coefficient: −1.83e−04
Value: C
Coprobacillus
1.00
0.75
0.50
0.25
0.00
A (n=15)
B (n=16)
C (n=14)
surface

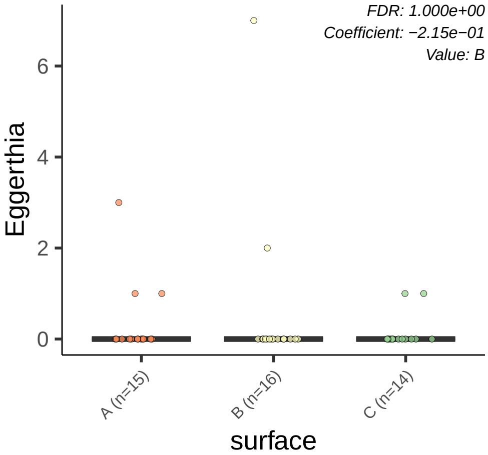

FDR: 1.000e+00
Coefficient: −2.15e−01
Value: B
Eggerthia
0
2
4
6
A (n=15)
B (n=16)
C (n=14)
surface

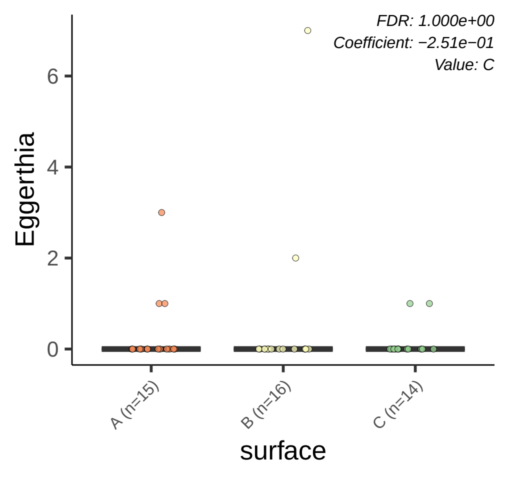

FDR: 1.000e+00
Coefficient: −2.51e−01
Value: C
Eggerthia
0
2
4
6
A (n=15)
B (n=16)
C (n=14)
surface

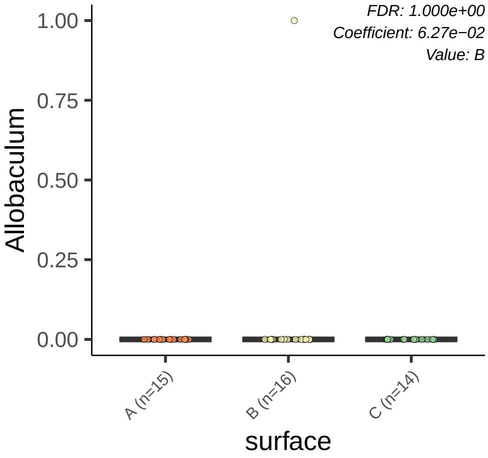
FDR: 1.000e+00
Coefficient: 6.27e−02
Value: B
Allobaculum
1.00
0.75
0.50
0.25
0.00
A (n=15)
B (n=16)
C (n=14)
surface

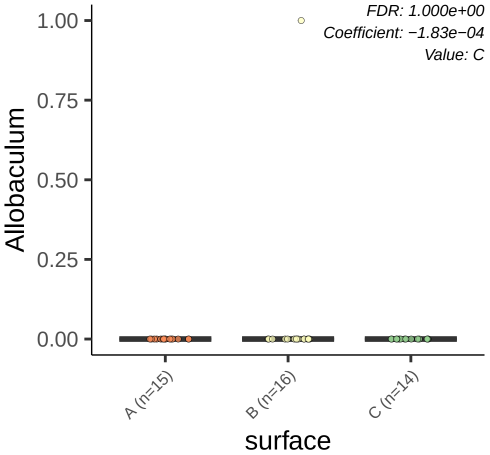
FDR: 1.000e+00
Coefficient: −1.83e−04
Value: C
1.00
0.75
0.50
0.25
0.00
Allobaculum
A (n=15)
B (n=16)
C (n=14)
surface

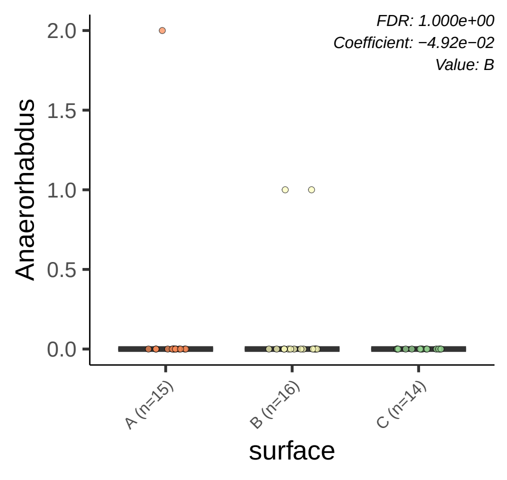
FDR: 1.000e+00
Coefficient: −4.92e−02
Value: B
Anaerorhabdus
2.0
1.5
1.0
0.5
0.0
A (n=15)
B (n=16)
C (n=14)
surface

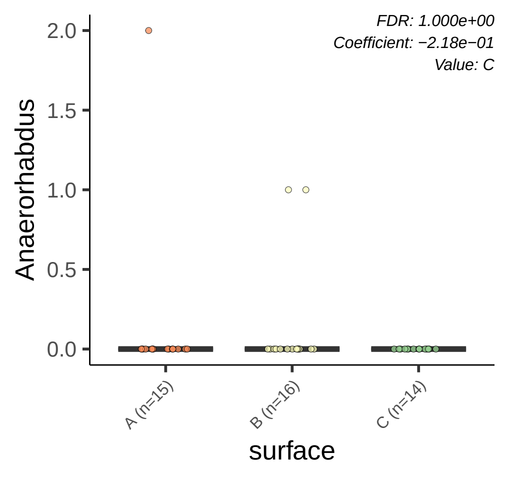

FDR: 1.000e+00
Coefficient: −2.18e−01
Value: C
Anaerorhabdus
2.0
1.5
1.0
0.5
0.0
A (n=15)
B (n=16)
C (n=14)
surface

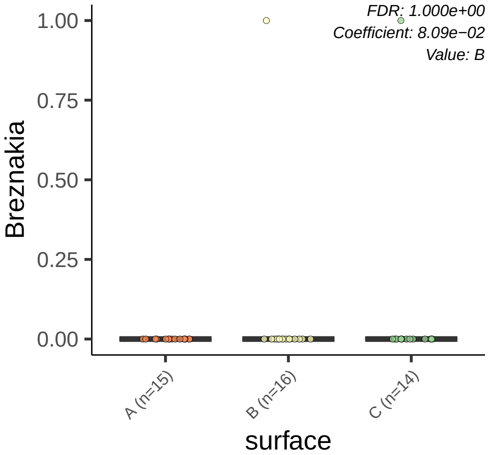
FDR: 1.000e+00
Coefficient: 8.09e−02
Value: B
Breznakia
1.00
0.75
0.50
0.25
0.00
A (n=15)
B (n=16)
C (n=14)
surface

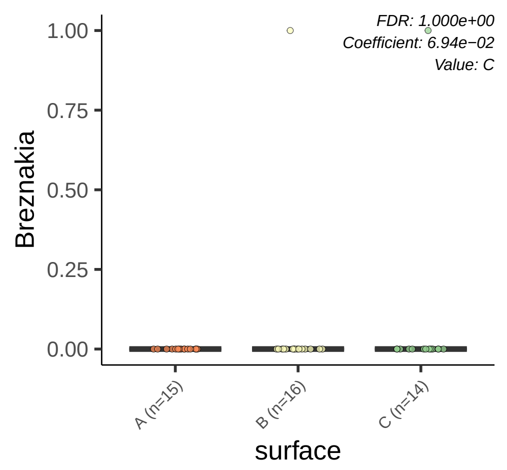
FDR: 1.000e+00
Coefficient: 6.94e−02
Value: C
Breznakia
1.00
0.75
0.50
0.25
0.00
A (n=15)
B (n=16)
C (n=14)
surface

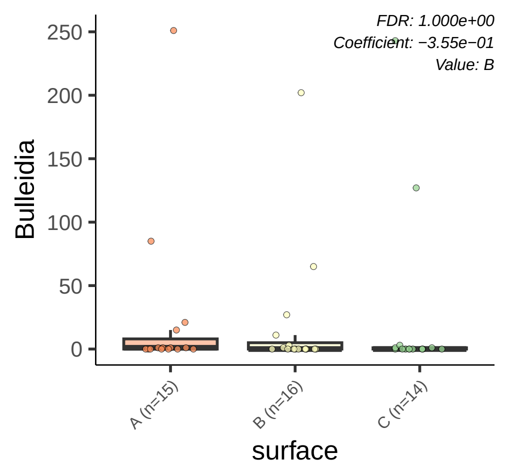
FDR: 1.000e+00
Coefficient: −3.55e−01
Value: B
Bulleidia
250
200
150
100
50
0
A (n=15)
B (n=16)
C (n=14)
surface

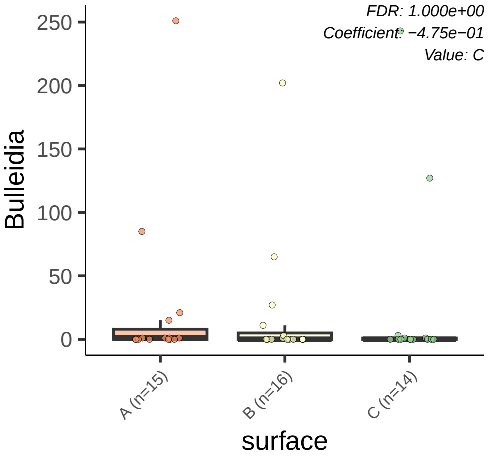

FDR: 1.000e+00
Coefficient: −4.75e−01
Value: C
Bulleidia
250
200
150
100
50
0
A (n=15)
B (n=16)
C (n=14)
surface

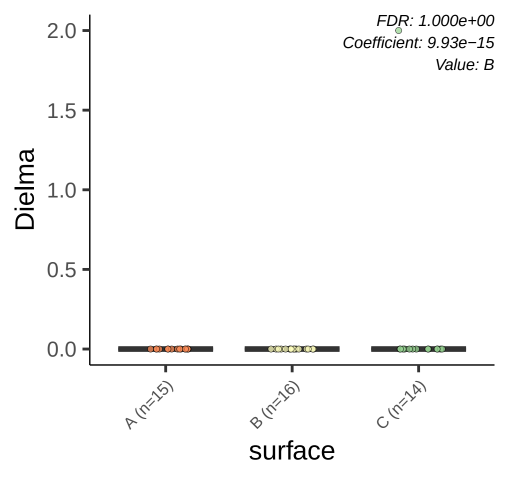
FDR: 1.000e+00
Coefficient: 9.93e−15
Value: B
Dielma
2.0
1.5
1.0
0.5
0.0
A (n=15)
B (n=16)
C (n=14)
surface

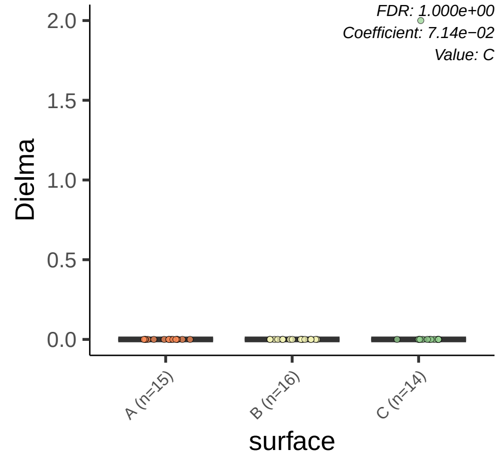

FDR: 1.000e+00
Coefficient: 7.14e−02
Value: C
2.0
1.5
1.0
0.5
0.0
Dielma
A (n=15)
B (n=16)
C (n=14)
surface

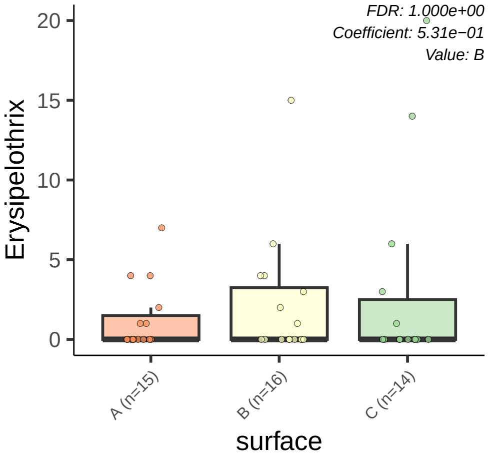

FDR: 1.000e+00
Coefficient: 5.31e−01
Value: B
Erysipelothrix
20
15
10
5
0
A (n=15)
B (n=16)
C (n=14)
surface

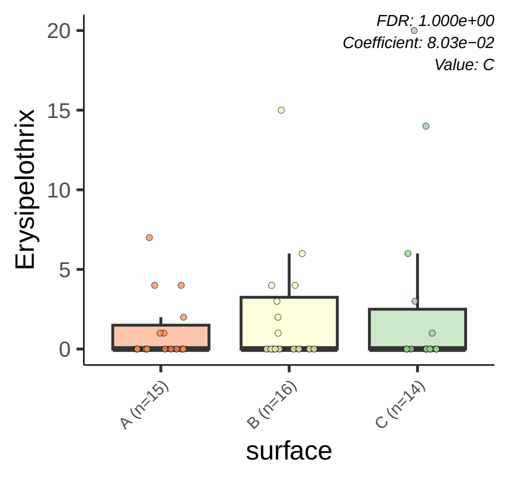
FDR: 1.000e+00
Coefficient: 8.03e−02
Value: C
Erysipelothrix
0
5
10
15
20
A (n=15)
B (n=16)
C (n=14)
surface

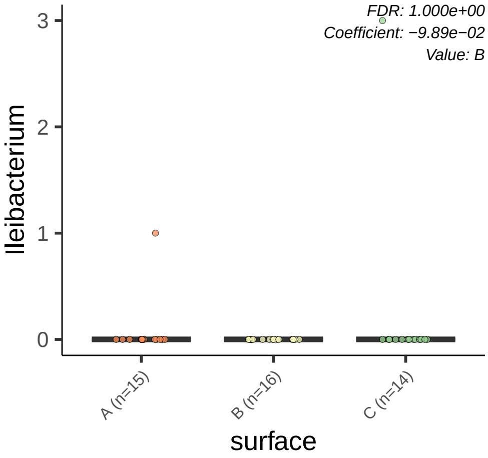

FDR: 1.000e+00
Coefficient: −9.89e−02
Value: B
Ileibacterium
3
2
1
0
A (n=15)
B (n=16)
C (n=14)
surface

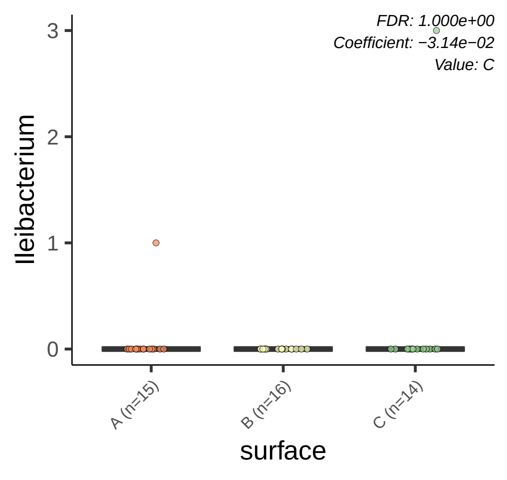
FDR: 1.000e+00
Coefficient: −3.14e−02
Value: C
Ileibacterium
3
2
1
0
A (n=15)
B (n=16)
C (n=14)
surface

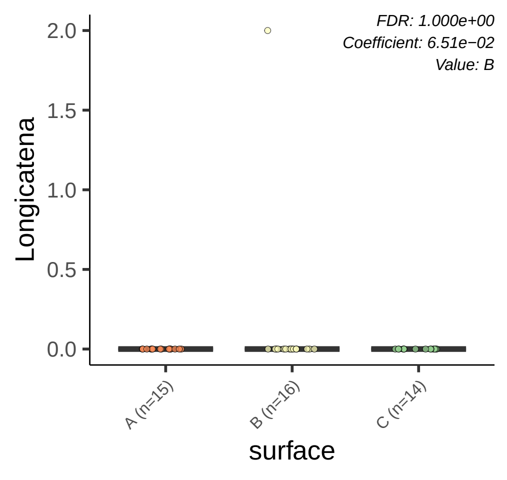
FDR: 1.000e+00
Coefficient: 6.51e−02
Value: B
Longicatena
2.0
1.5
1.0
0.5
0.0
A (n=15)
B (n=16)
C (n=14)
surface

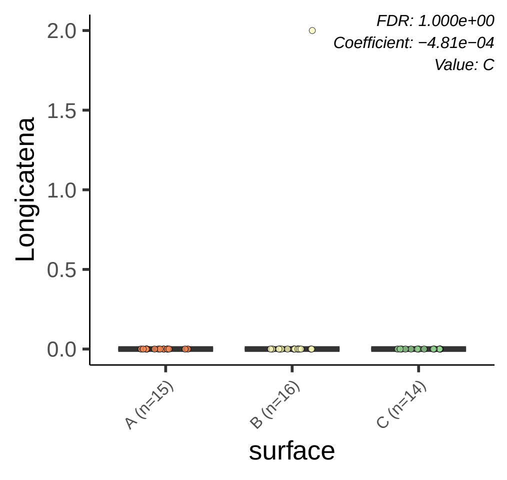

FDR: 1.000e+00
Coefficient: −4.81e−04
Value: C
Longicatena
2.0
1.5
1.0
0.5
0.0
A (n=15)
B (n=16)
C (n=14)
surface

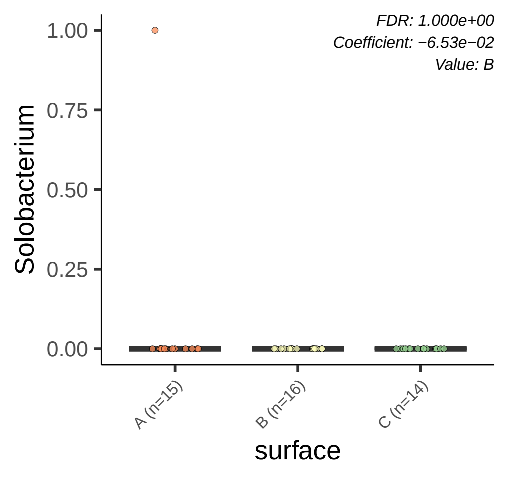

FDR: 1.000e+00
Coefficient: −6.53e−02
Value: B
Solobacterium
1.00
0.75
0.50
0.25
0.00
A (n=15)
B (n=16)
C (n=14)
surface

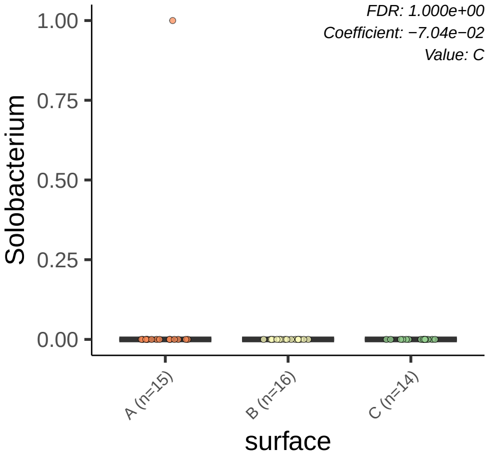

FDR: 1.000e+00
Coefficient: −7.04e−02
Value: C
Solobacterium
1.00
0.75
0.50
0.25
0.00
A (n=15)
B (n=16)
C (n=14)
surface

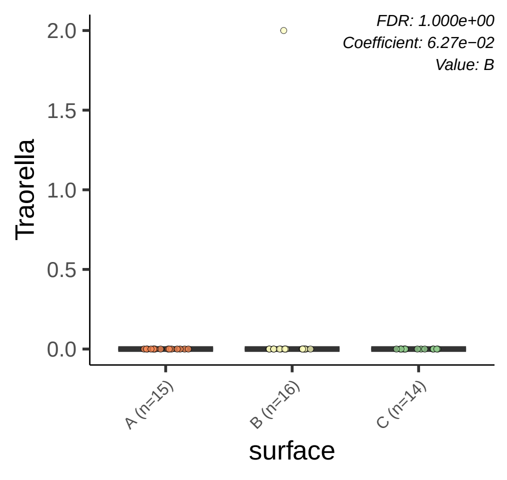
FDR: 1.000e+00
Coefficient: 6.27e−02
Value: B
Traorella
2.0
1.5
1.0
0.5
0.0
A (n=15)
B (n=16)
C (n=14)
surface

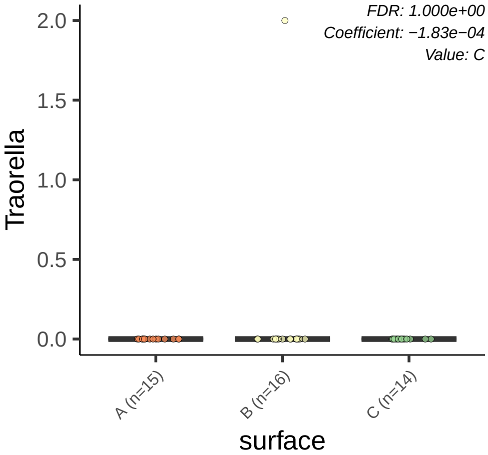
FDR: 1.000e+00
Coefficient: −1.83e−04
Value: C
Traorella
2.0
1.5
1.0
0.5
0.0
A (n=15)
B (n=16)
C (n=14)
surface

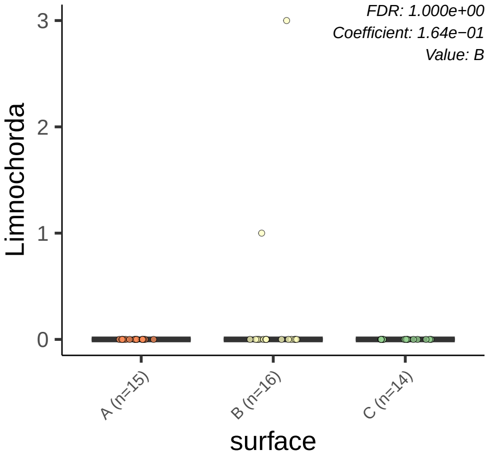
FDR: 1.000e+00
Coefficient: 1.64e−01
Value: B
Limnochorda
3
2
1
0
A (n=15)
B (n=16)
C (n=14)
surface

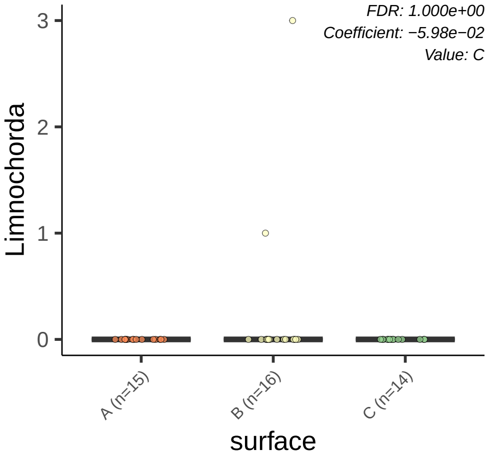
FDR: 1.000e+00
Coefficient: −5.98e−02
Value: C
Limnochorda
3
2
1
0
A (n=15)
B (n=16)
C (n=14)
surface

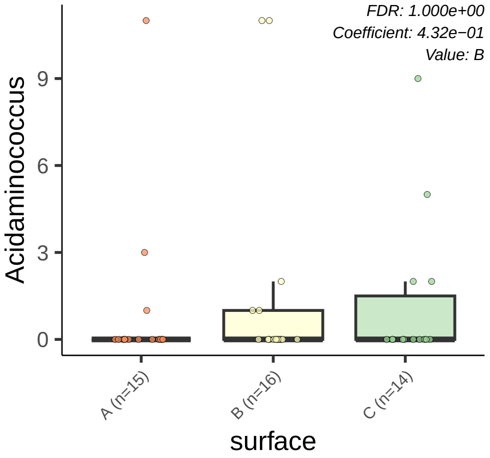

FDR: 1.000e+00
Coefficient: 4.32e−01
Value: B
Acidaminococcus
0
3
6
9
A (n=15)
B (n=16)
C (n=14)
surface

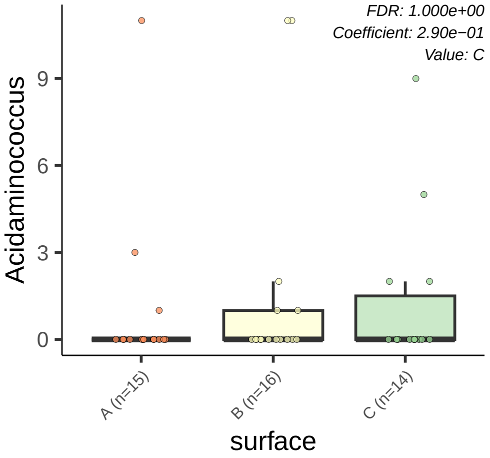

FDR: 1.000e+00
Coefficient: 2.90e−01
Value: C
Acidaminococcus
0
3
6
9
A (n=15)
B (n=16)
C (n=14)
surface

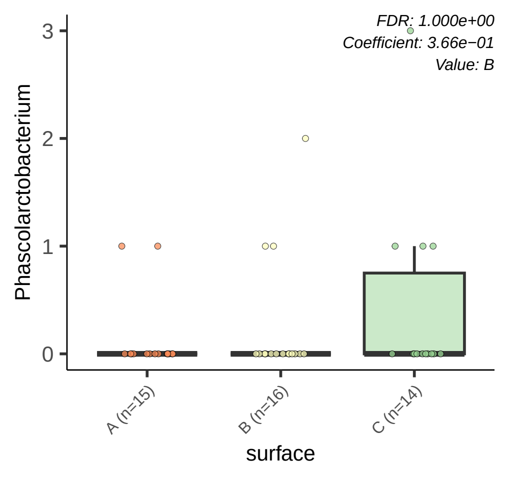

FDR: 1.000e+00
Coefficient: 3.66e−01
Value: B
Phascolarctobacterium
3
2
1
0
A (n=15)
B (n=16)
C (n=14)
surface

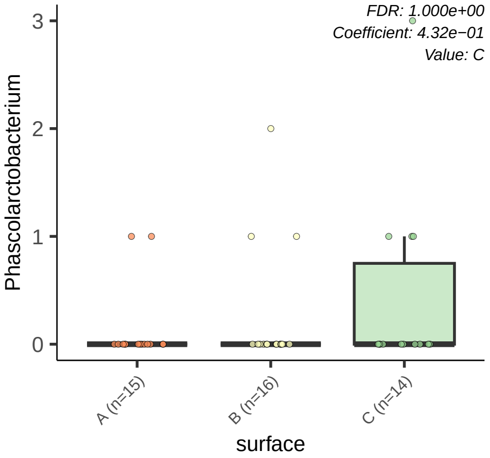
FDR: 1.000e+00
Coefficient: 4.32e−01
Value: C
Phascolarctobacterium
3
2
1
0
A (n=15)
B (n=16)
C (n=14)
surface

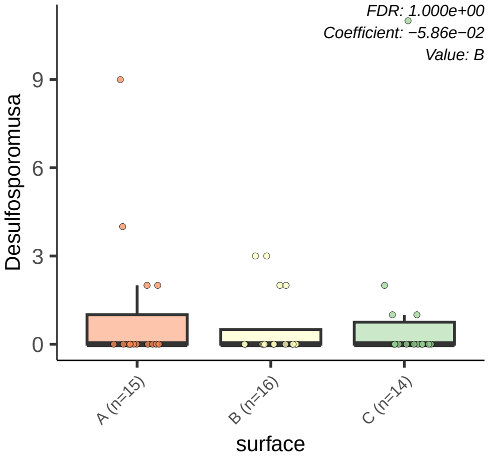
FDR: 1.000e+00
Coefficient: −5.86e−02
Value: B
Desulfosporomusa
9
6
3
0
A (n=15)
B (n=16)
C (n=14)
surface

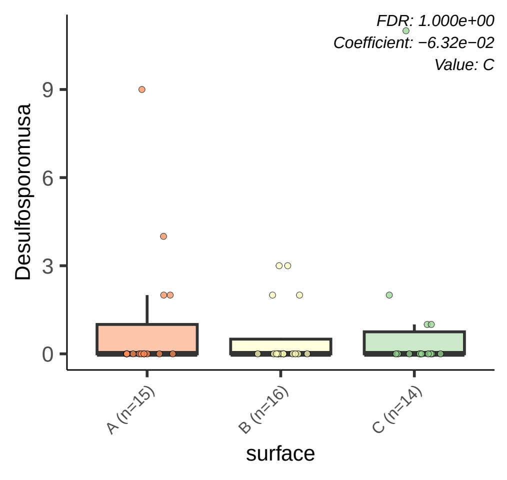
FDR: 1.000e+00
Coefficient: −6.32e−02
Value: C
Desulfosporomusa
9
6
3
0
A (n=15)
B (n=16)
C (n=14)
surface

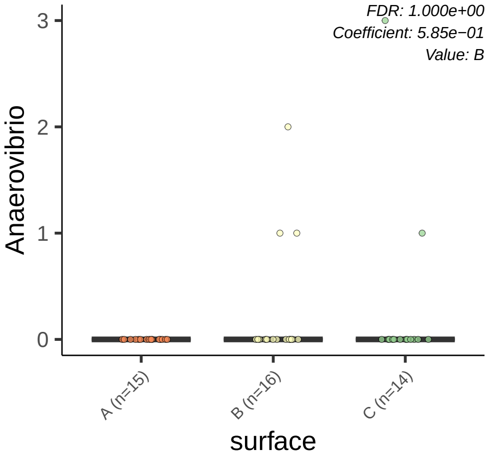

FDR: 1.000e+00
Coefficient: 5.85e−01
Value: B
Anaerovibrio
3
2
1
0
A (n=15)
B (n=16)
C (n=14)
surface

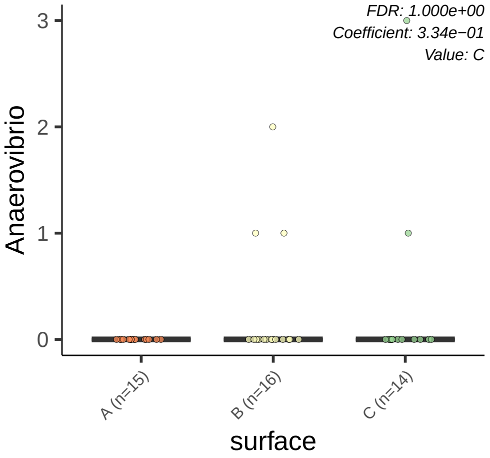
FDR: 1.000e+00
Coefficient: 3.34e−01
Value: C
Anaerovibrio
0
1
2
3
A (n=15)
B (n=16)
C (n=14)
surface

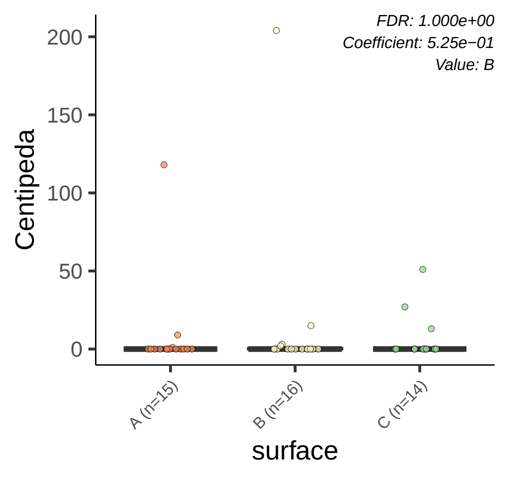

FDR: 1.000e+00
Coefficient: 5.25e−01
Value: B
Centipeda
200
150
100
50
0
A (n=15)
B (n=16)
C (n=14)
surface

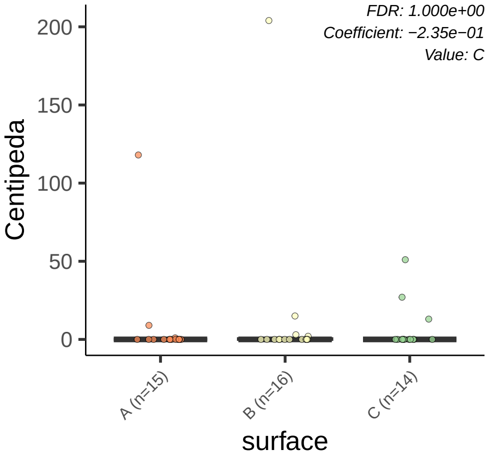
FDR: 1.000e+00
Coefficient: −2.35e−01
Value: C
Centipeda
200
150
100
50
0
A (n=15)
B (n=16)
C (n=14)
surface

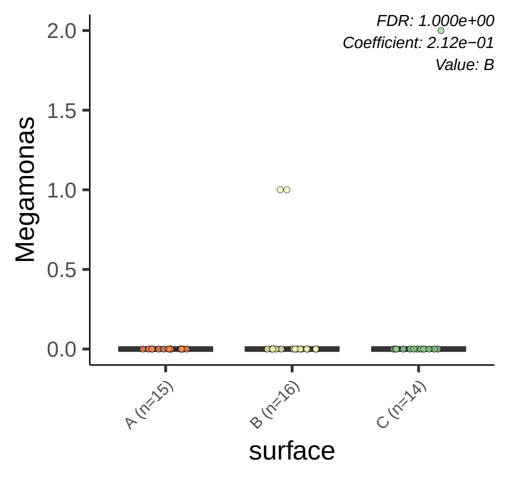

FDR: 1.000e+00
Coefficient: 2.12e−01
Value: B
Megamonas
2.0
1.5
1.0
0.5
0.0
A (n=15)
B (n=16)
C (n=14)
surface

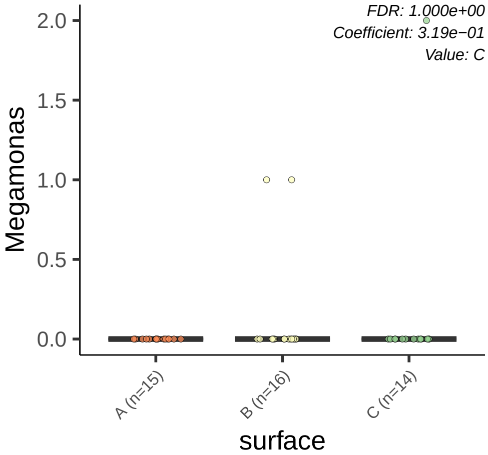
FDR: 1.000e+00
Coefficient: 3.19e−01
Value: C
Megamonas
2.0
1.5
1.0
0.5
0.0
A (n=15)
B (n=16)
C (n=14)
surface

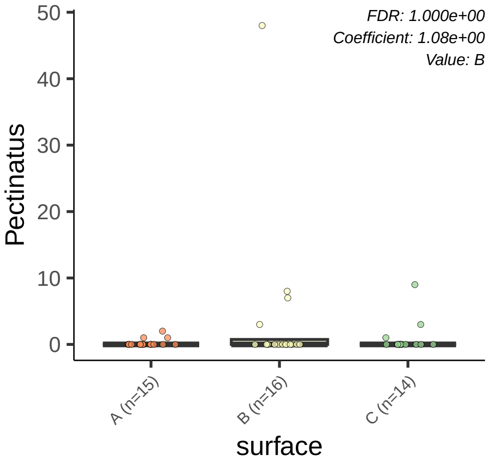

FDR: 1.000e+00
Coefficient: 1.08e+00
Value: B
Pectinatus
50
40
30
20
10
0
A (n=15)
B (n=16)
C (n=14)
surface

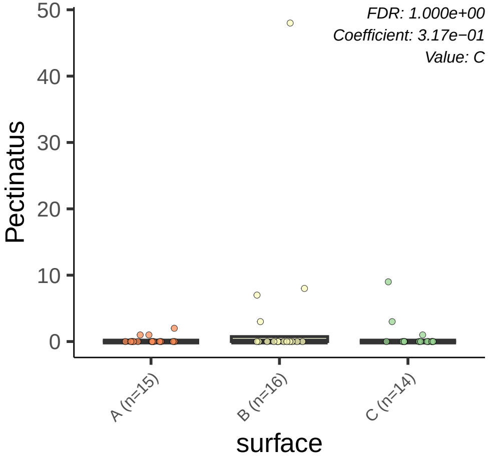

FDR: 1.000e+00
Coefficient: 3.17e−01
Value: C
Pectinatus
0
10
20
30
40
50
A (n=15)
B (n=16)
C (n=14)
surface

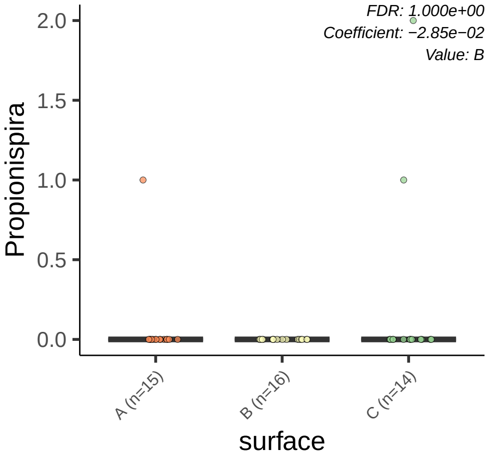

FDR: 1.000e+00
Coefficient: −2.85e−02
Value: B
Propionispira
2.0
1.5
1.0
0.5
0.0
A (n=15)
B (n=16)
C (n=14)
surface

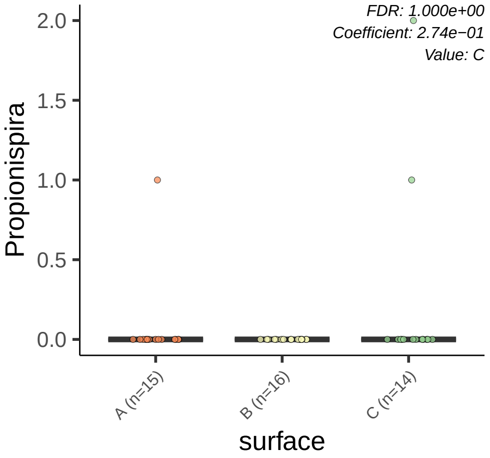

FDR: 1.000e+00
Coefficient: 2.74e−01
Value: C
Propionispira
2.0
1.5
1.0
0.5
0.0
A (n=15)
B (n=16)
C (n=14)
surface

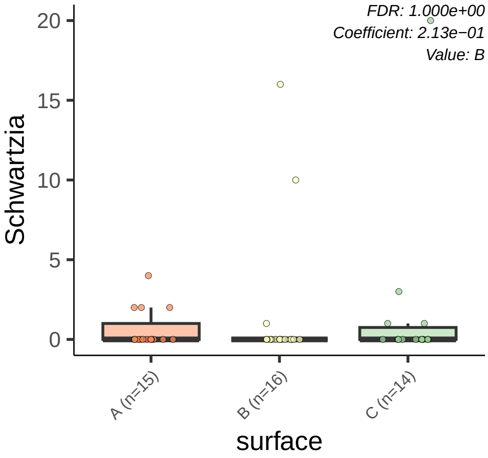

FDR: 1.000e+00
Coefficient: 2.13e−01
Value: B
Schwartzia
20
15
10
5
0
A (n=15)
B (n=16)
C (n=14)
surface

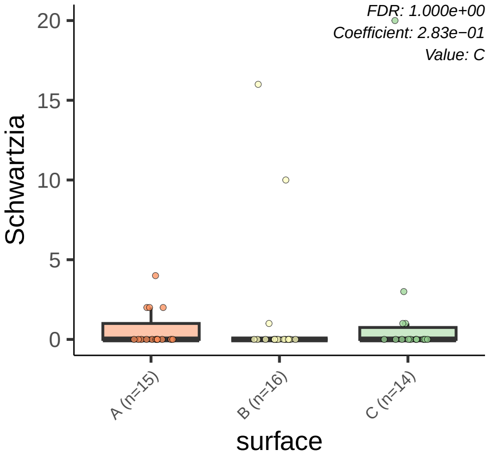
FDR: 1.000e+00
Coefficient: 2.83e−01
Value: C
Schwartzia
20
15
10
5
0
A (n=15)
B (n=16)
C (n=14)
surface

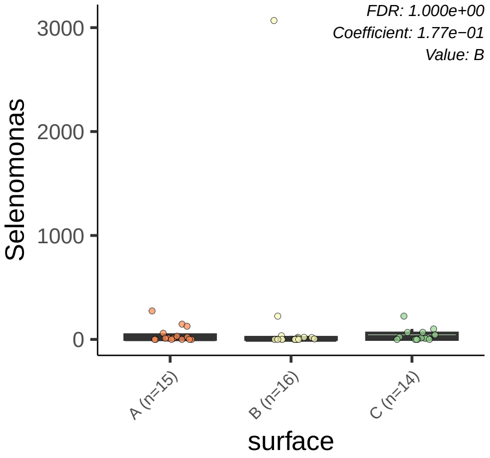

FDR: 1.000e+00
Coefficient: 1.77e−01
Value: B
3000
2000
1000
0
Selenomonas
A (n=15)
B (n=16)
C (n=14)
surface

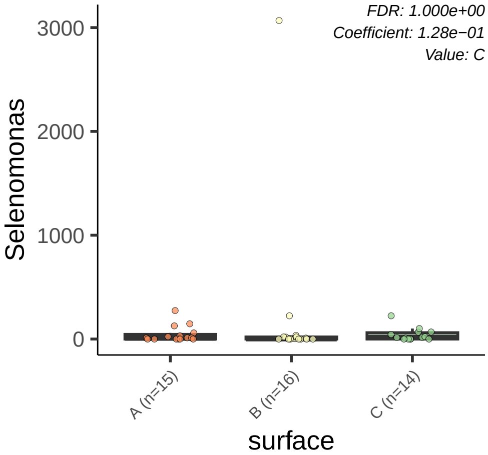
FDR: 1.000e+00
Coefficient: 1.28e−01
Value: C
3000
2000
1000
0
Selenomonas
A (n=15)
B (n=16)
C (n=14)
surface

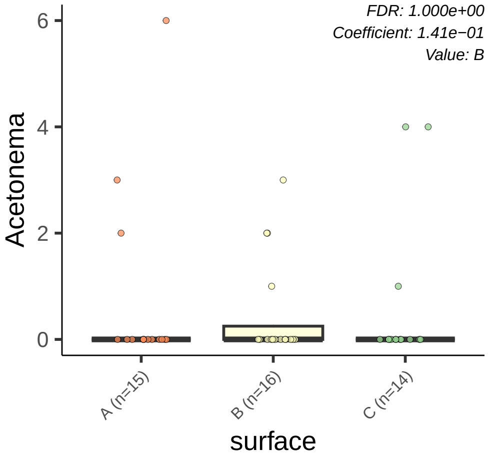
FDR: 1.000e+00
Coefficient: 1.41e−01
Value: B
Acetonema
6
4
2
0
A (n=15)
B (n=16)
C (n=14)
surface

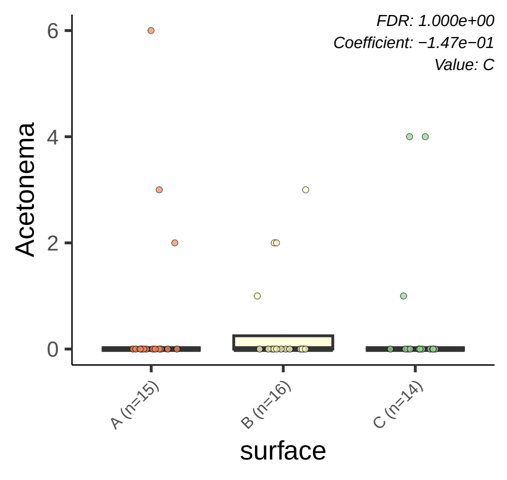

FDR: 1.000e+00
Coefficient: −1.47e−01
Value: C
Acetonema
6
4
2
0
A (n=15)
B (n=16)
C (n=14)
surface

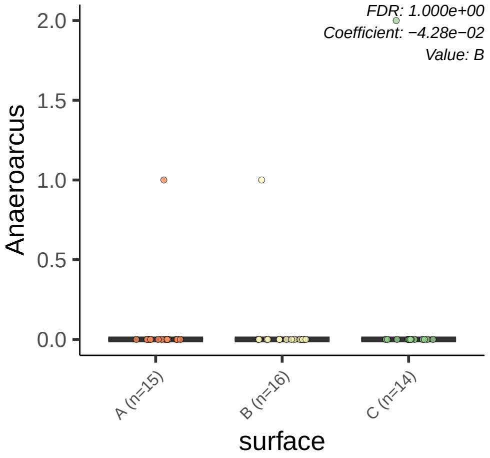
FDR: 1.000e+00
Coefficient: −4.28e−02
Value: B
Anaeroarcus
2.0
1.5
1.0
0.5
0.0
A (n=15)
B (n=16)
C (n=14)
surface

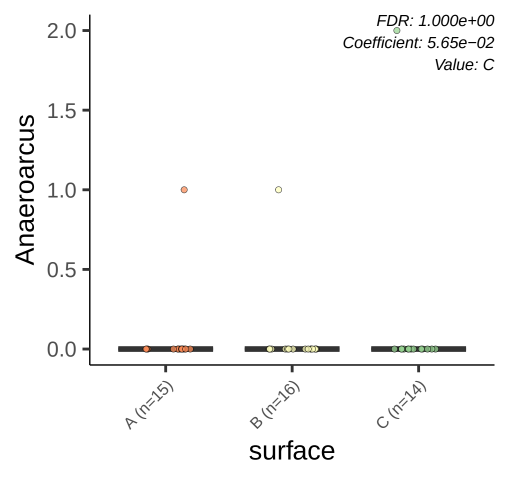

FDR: 1.000e+00
Coefficient: 5.65e−02
Value: C
Anaeroarcus
2.0
1.5
1.0
0.5
0.0
A (n=15)
B (n=16)
C (n=14)
surface

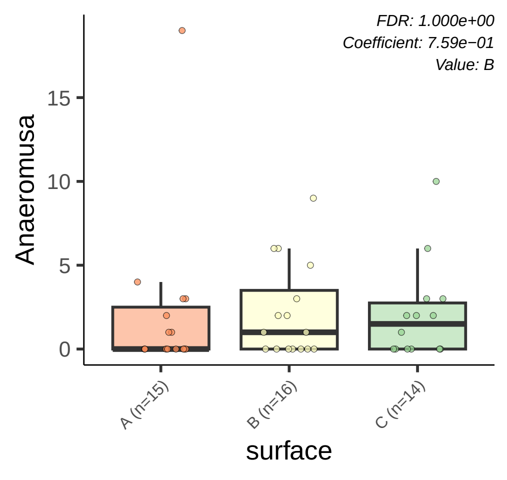

FDR: 1.000e+00
Coefficient: 7.59e−01
Value: B
Anaeromusa
0
5
10
15
A (n=15)
B (n=16)
C (n=14)
surface

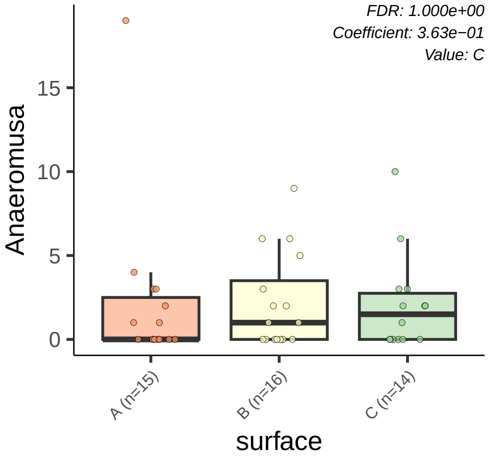
FDR: 1.000e+00
Coefficient: 3.63e−01
Value: C
Anaeromusa
0
5
10
15
A (n=15)
B (n=16)
C (n=14)
surface

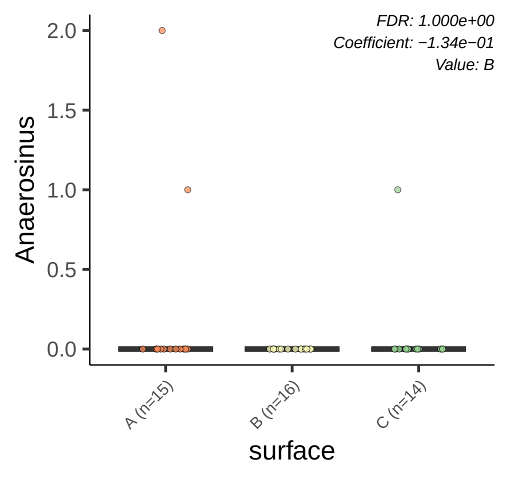
FDR: 1.000e+00
Coefficient: −1.34e−01
Value: B
Anaerosinus
2.0
1.5
1.0
0.5
0.0
A (n=15)
B (n=16)
C (n=14)
surface

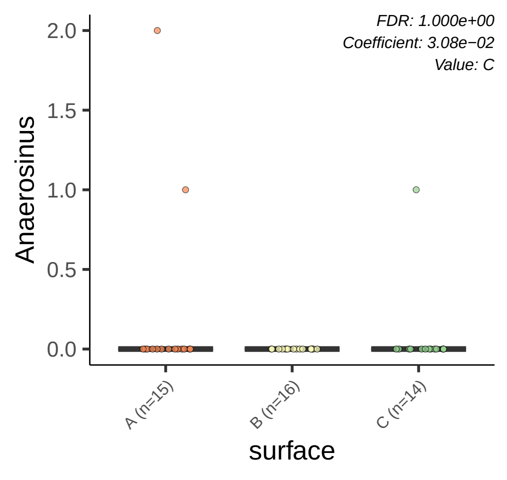

FDR: 1.000e+00
Coefficient: 3.08e−02
Value: C
Anaerosinus
2.0
1.5
1.0
0.5
0.0
A (n=15)
B (n=16)
C (n=14)
surface

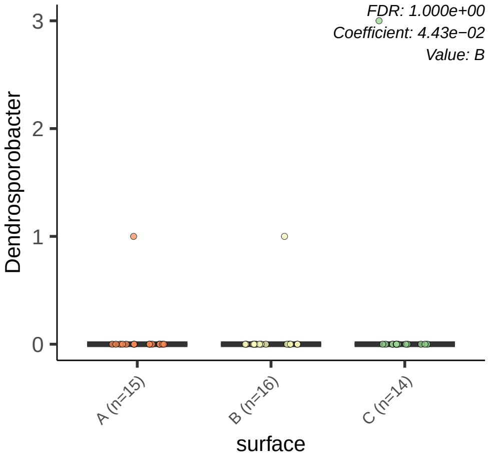

FDR: 1.000e+00
Coefficient: 4.43e−02
Value: B
Dendrosporobacter
0
1
2
3
A (n=15)
B (n=16)
C (n=14)
surface

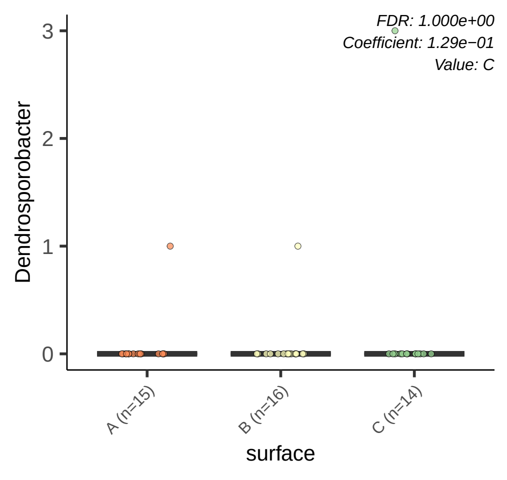

FDR: 1.000e+00
Coefficient: 1.29e−01
Value: C
Dendrosporobacter
3
2
1
0
A (n=15)
B (n=16)
C (n=14)
surface

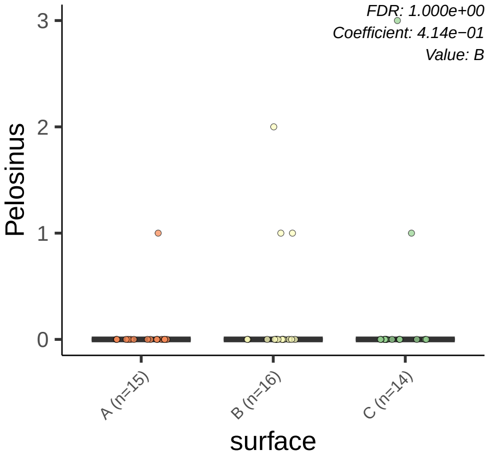

FDR: 1.000e+00
Coefficient: 4.14e−01
Value: B
Pelosinus
3
2
1
0
A (n=15)
B (n=16)
C (n=14)
surface

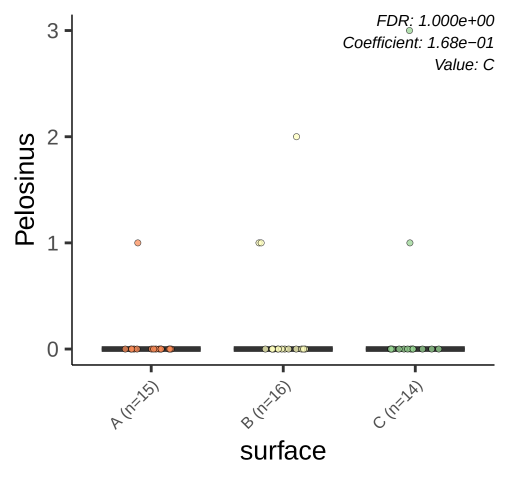
FDR: 1.000e+00
Coefficient: 1.68e−01
Value: C
Pelosinus
0
1
2
3
A (n=15)
B (n=16)
C (n=14)
surface

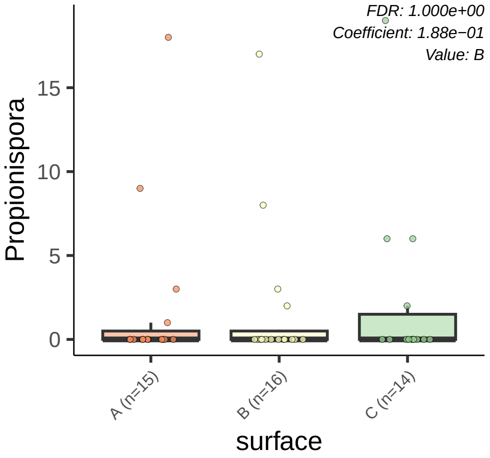
FDR: 1.000e+00
Coefficient: 1.88e−01
Value: B
Propionispora
0
5
10
15
A (n=15)
B (n=16)
C (n=14)
surface

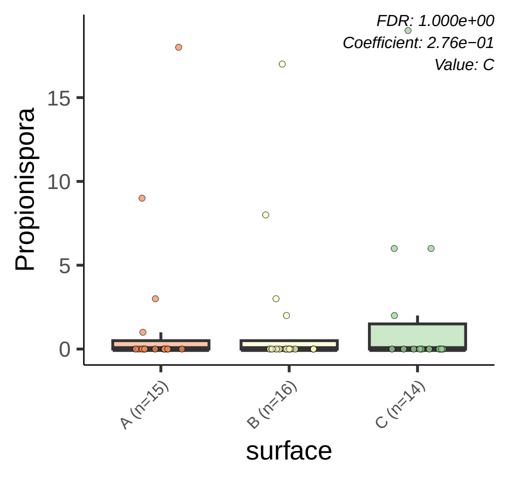

FDR: 1.000e+00
Coefficient: 2.76e−01
Value: C
Propionispora
0
5
10
15
A (n=15)
B (n=16)
C (n=14)
surface

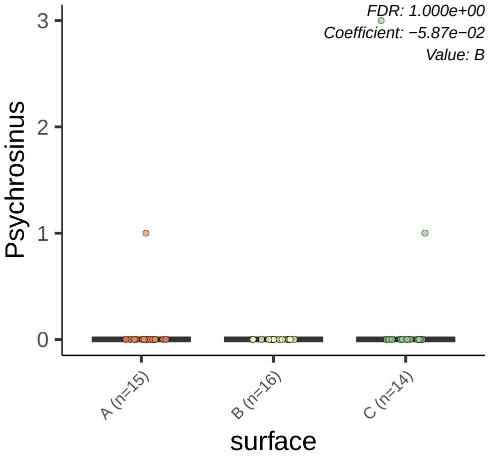

FDR: 1.000e+00
Coefficient: −5.87e−02
Value: B
Psychrosinus
3
2
1
0
A (n=15)
B (n=16)
C (n=14)
surface

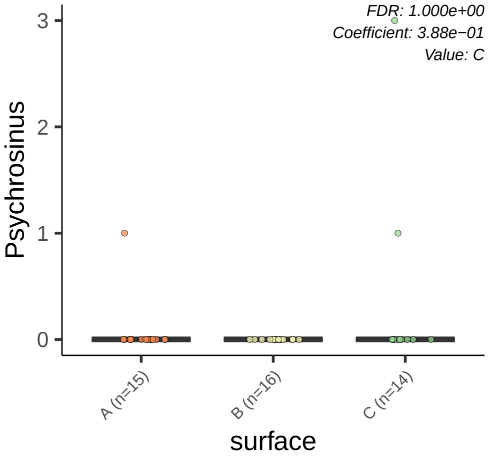
FDR: 1.000e+00
Coefficient: 3.88e−01
Value: C
Psychrosinus
3
2
1
0
A (n=15)
B (n=16)
C (n=14)
surface

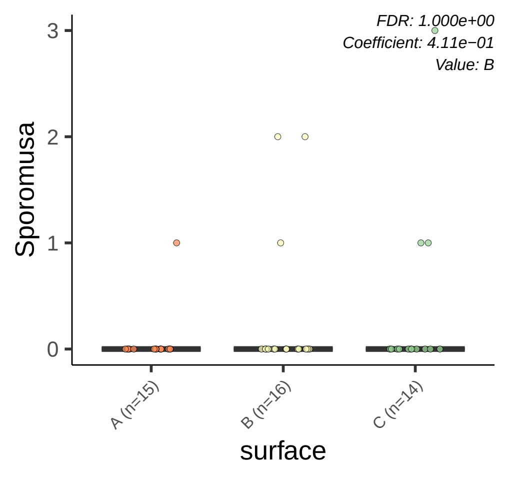

FDR: 1.000e+00
Coefficient: 4.11e−01
Value: B
Sporomusa
3
2
1
0
A (n=15)
B (n=16)
C (n=14)
surface

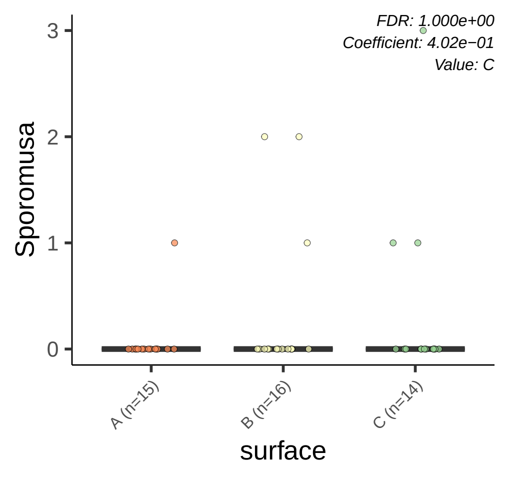
FDR: 1.000e+00
Coefficient: 4.02e−01
Value: C
Sporomusa
3
2
1
0
A (n=15)
B (n=16)
C (n=14)
surface

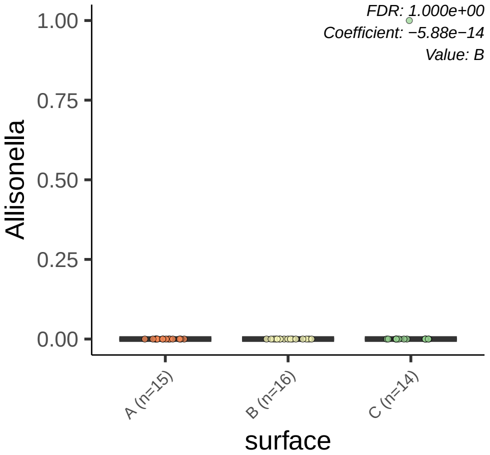

FDR: 1.000e+00
Coefficient: −5.88e−14
Value: B
1.00
0.75
0.50
0.25
0.00
Allisonella
A (n=15)
B (n=16)
C (n=14)
surface

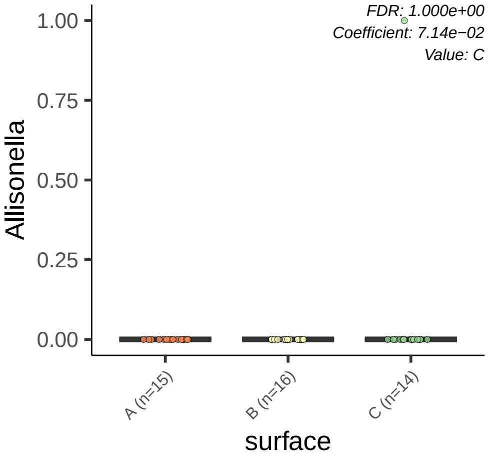

FDR: 1.000e+00
Coefficient: 7.14e−02
Value: C
Allisonella
1.00
0.75
0.50
0.25
0.00
A (n=15)
B (n=16)
C (n=14)
surface

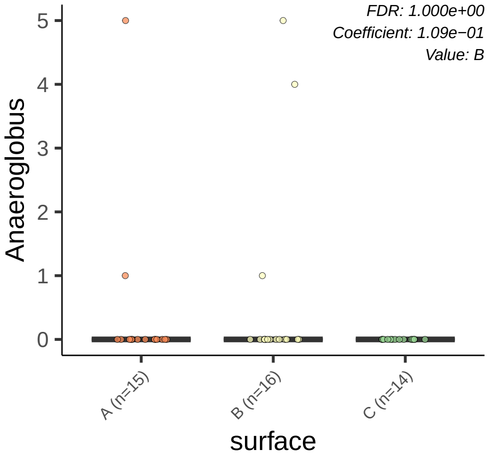
FDR: 1.000e+00
Coefficient: 1.09e−01
Value: B
Anaeroglobus
0
1
2
3
4
5
A (n=15)
B (n=16)
C (n=14)
surface

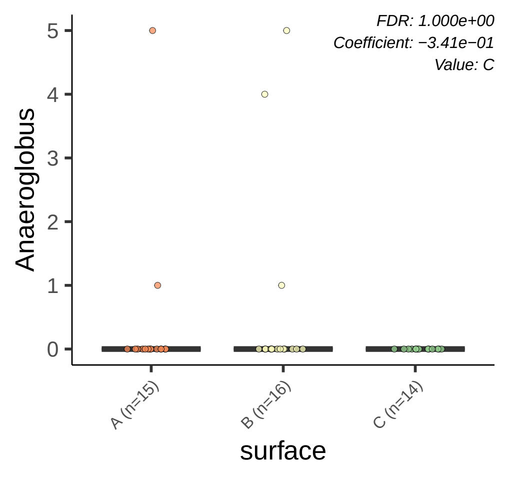
FDR: 1.000e+00
Coefficient: −3.41e−01
Value: C
Anaeroglobus
5
4
3
2
1
0
A (n=15)
B (n=16)
C (n=14)
surface

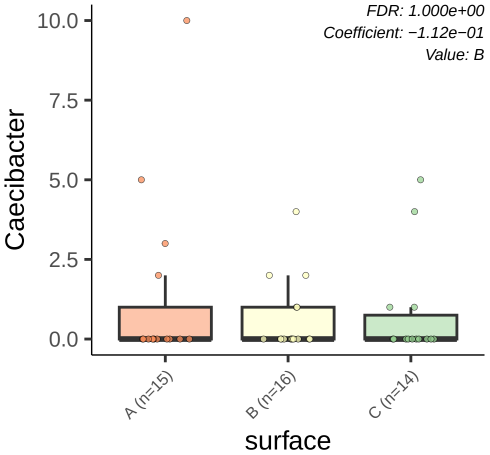
FDR: 1.000e+00
Coefficient: −1.12e−01
Value: B
Caecibacter
10.0
7.5
5.0
2.5
0.0
A (n=15)
B (n=16)
C (n=14)
surface

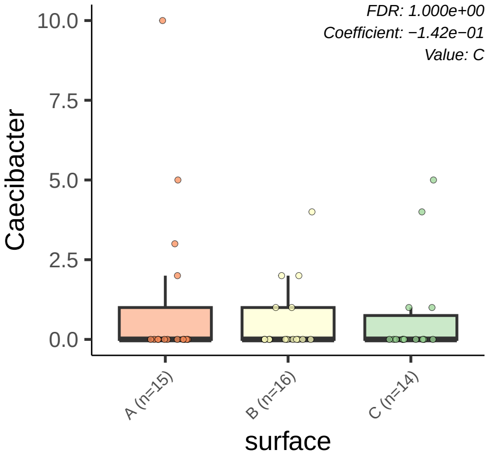
FDR: 1.000e+00
Coefficient: −1.42e−01
Value: C
Caecibacter
10.0
7.5
5.0
2.5
0.0
A (n=15)
B (n=16)
C (n=14)
surface

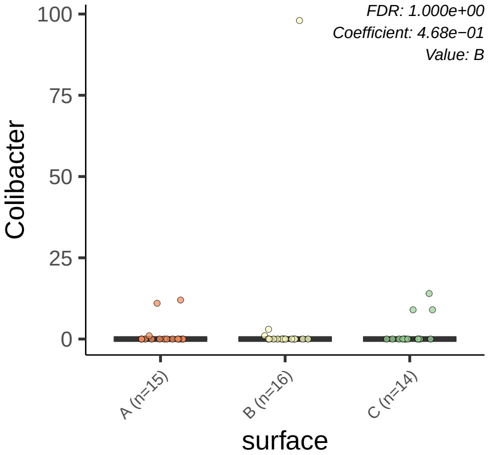
FDR: 1.000e+00
Coefficient: 4.68e−01
Value: B
Colibacter
100
75
50
25
0
A (n=15)
B (n=16)
C (n=14)
surface

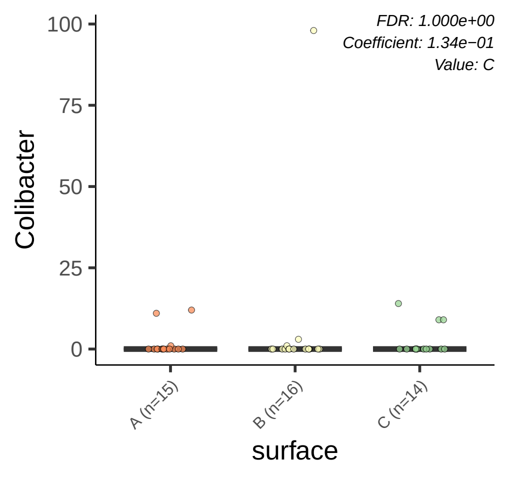
FDR: 1.000e+00
Coefficient: 1.34e−01
Value: C
Colibacter
100
75
50
25
0
A (n=15)
B (n=16)
C (n=14)
surface

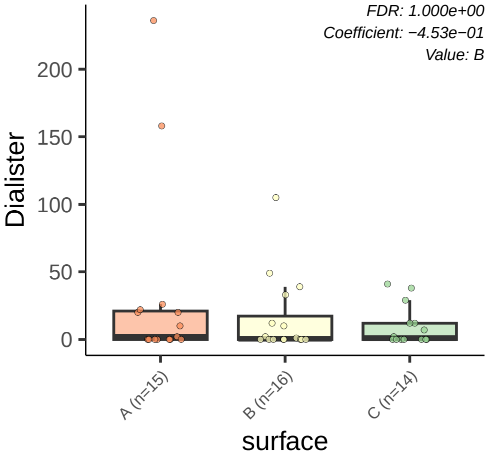
FDR: 1.000e+00
Coefficient: −4.53e−01
Value: B
Dialister
200
150
100
50
0
A (n=15)
B (n=16)
C (n=14)
surface

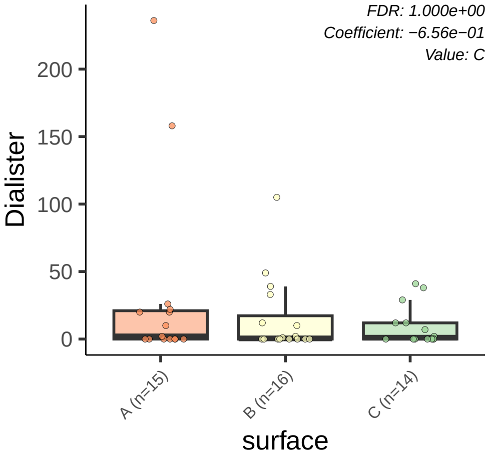

FDR: 1.000e+00
Coefficient: −6.56e−01
Value: C
Dialister
200
150
100
50
0
A (n=15)
B (n=16)
C (n=14)
surface

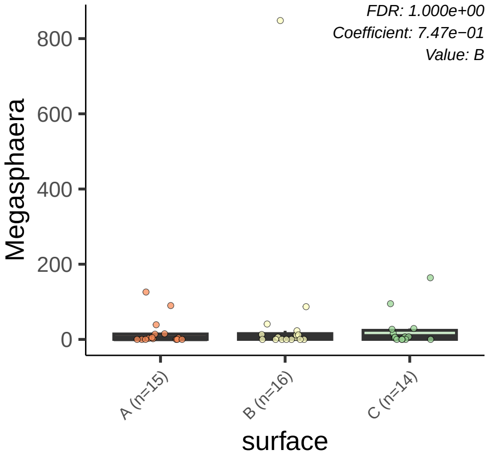
FDR: 1.000e+00
Coefficient: 7.47e−01
Value: B
Megasphaera
800
600
400
200
0
A (n=15)
B (n=16)
C (n=14)
surface

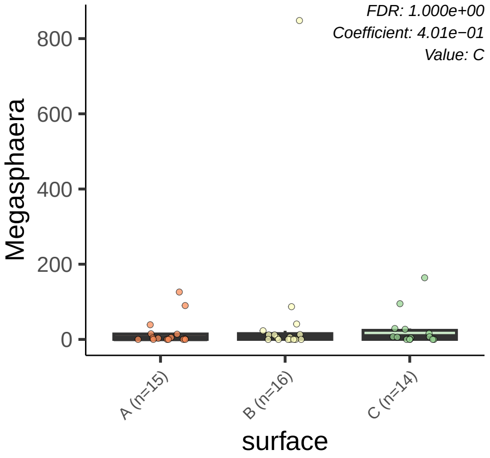

FDR: 1.000e+00
Coefficient: 4.01e−01
Value: C
Megasphaera
800
600
400
200
0
A (n=15)
B (n=16)
C (n=14)
surface

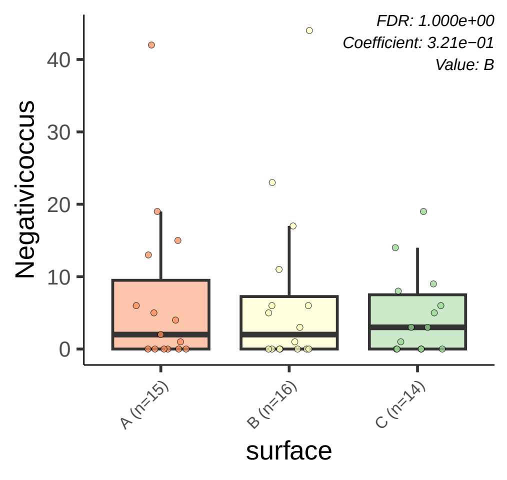

FDR: 1.000e+00
Coefficient: 3.21e−01
Value: B
Negativicoccus
0
10
20
30
40
A (n=15)
B (n=16)
C (n=14)
surface

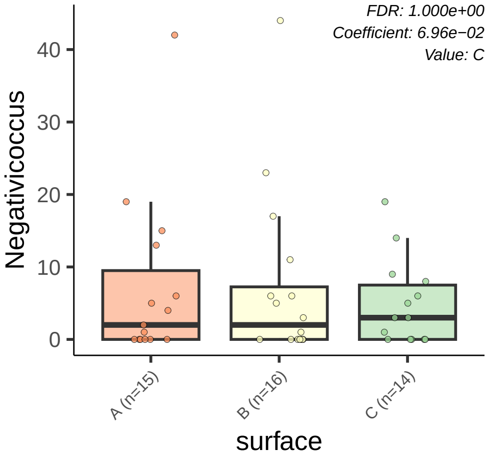
FDR: 1.000e+00
Coefficient: 6.96e−02
Value: C
Negativicoccus
0
10
20
30
40
A (n=15)
B (n=16)
C (n=14)
surface

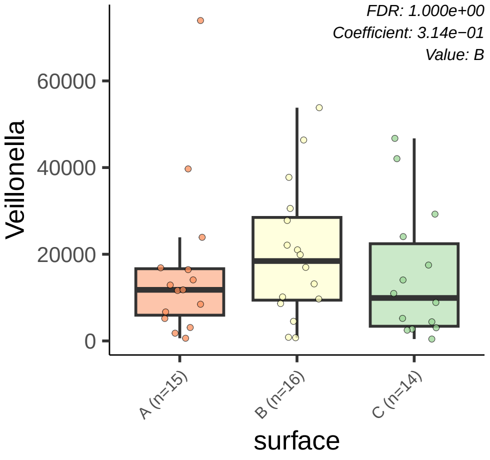

FDR: 1.000e+00
Coefficient: 3.14e−01
Value: B
Veillonella
60000
40000
20000
0
A (n=15)
B (n=16)
C (n=14)
surface

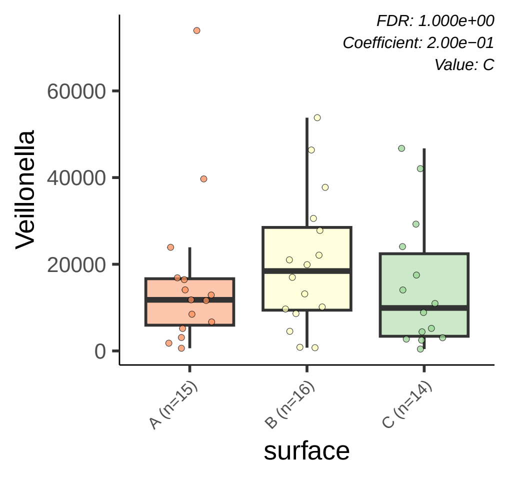
FDR: 1.000e+00
Coefficient: 2.00e−01
Value: C
Veillonella
60000
40000
20000
0
A (n=15)
B (n=16)
C (n=14)
surface

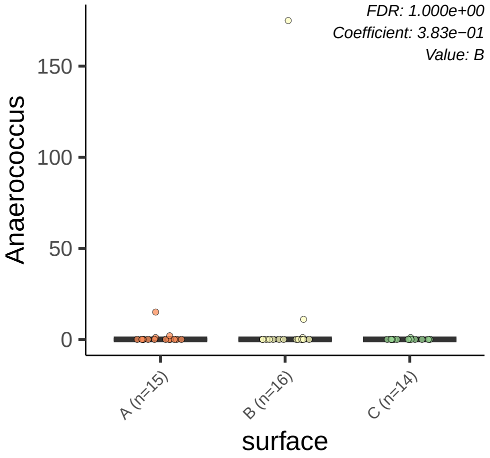
FDR: 1.000e+00
Coefficient: 3.83e−01
Value: B
Anaerococcus
150
100
50
0
A (n=15)
B (n=16)
C (n=14)
surface

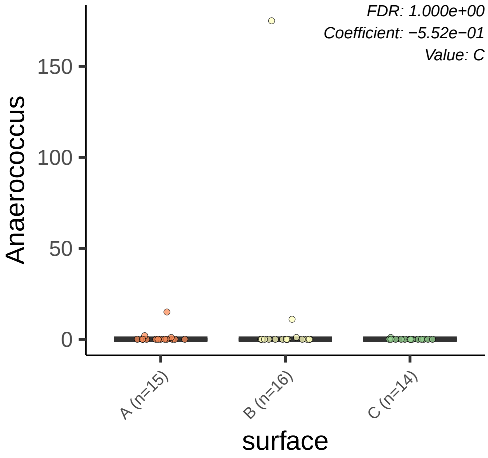
FDR: 1.000e+00
Coefficient: −5.52e−01
Value: C
Anaerococcus
150
100
50
0
A (n=15)
B (n=16)
C (n=14)
surface

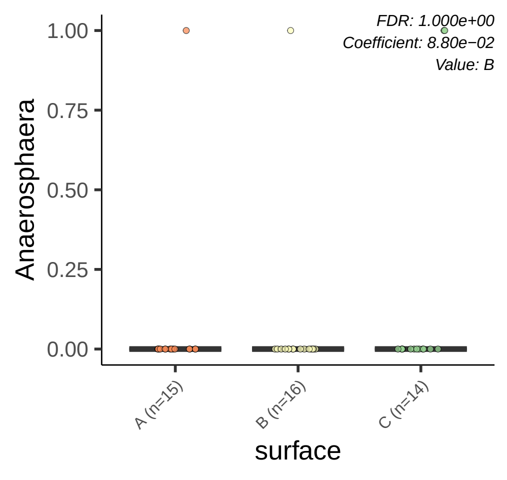
FDR: 1.000e+00
Coefficient: 8.80e−02
Value: B
Anaerosphaera
1.00
0.75
0.50
0.25
0.00
A (n=15)
B (n=16)
C (n=14)
surface

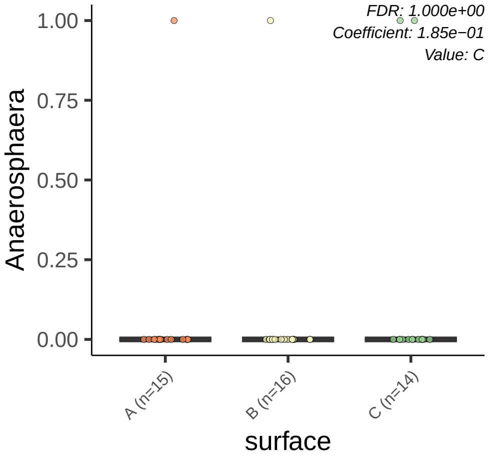
FDR: 1.000e+00
Coefficient: 1.85e−01
Value: C
Anaerosphaera
1.00
0.75
0.50
0.25
0.00
A (n=15)
B (n=16)
C (n=14)
surface

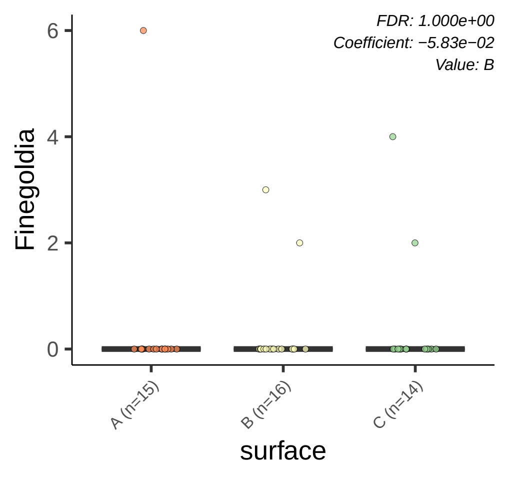
FDR: 1.000e+00
Coefficient: −5.83e−02
Value: B
Finegoldia
6
4
2
0
A (n=15)
B (n=16)
C (n=14)
surface

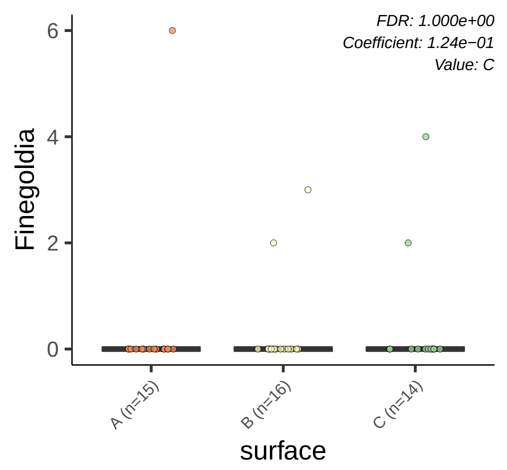
FDR: 1.000e+00
Coefficient: 1.24e−01
Value: C
Finegoldia
6
4
2
0
A (n=15)
B (n=16)
C (n=14)
surface

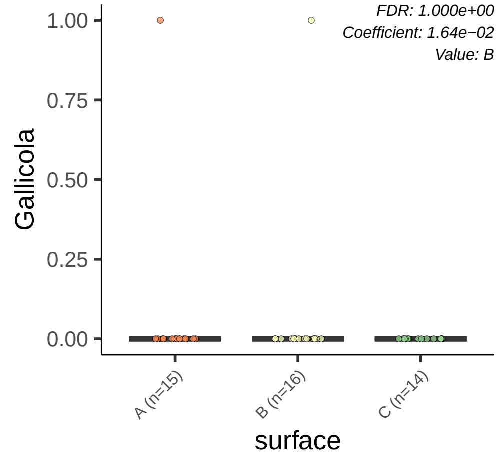

FDR: 1.000e+00
Coefficient: 1.64e−02
Value: B
Gallicola
1.00
0.75
0.50
0.25
0.00
A (n=15)
B (n=16)
C (n=14)
surface

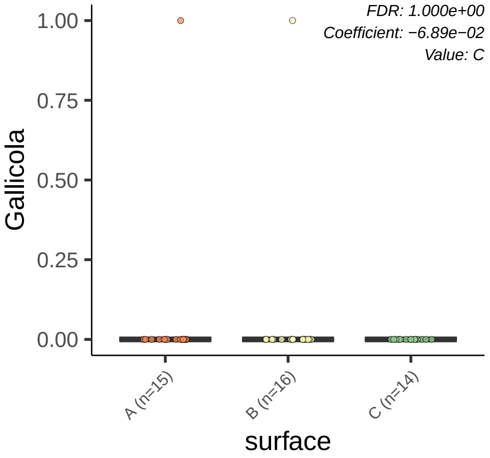
FDR: 1.000e+00
Coefficient: −6.89e−02
Value: C
Gallicola
1.00
0.75
0.50
0.25
0.00
A (n=15)
B (n=16)
C (n=14)
surface

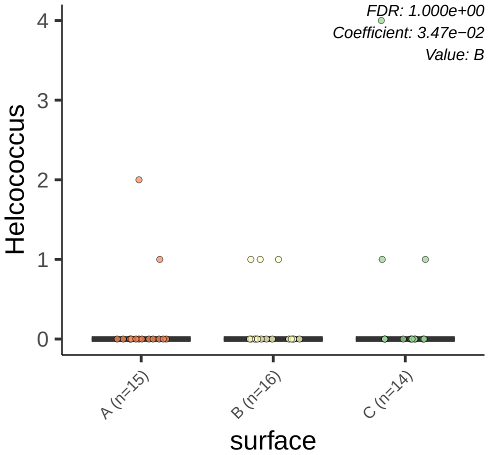
FDR: 1.000e+00
Coefficient: 3.47e−02
Value: B
Helcococcus
0
1
2
3
4
A (n=15)
B (n=16)
C (n=14)
surface

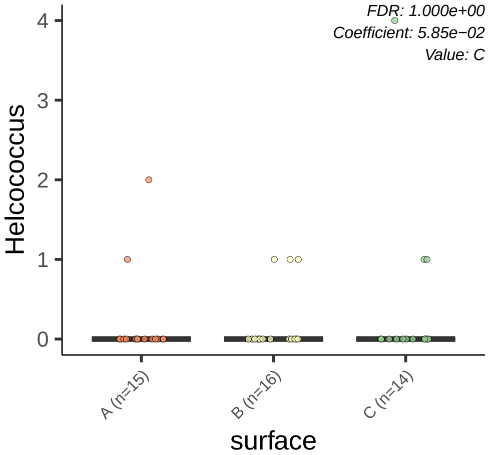

FDR: 1.000e+00
Coefficient: 5.85e−02
Value: C
Helcococcus
4
3
2
1
0
A (n=15)
B (n=16)
C (n=14)
surface

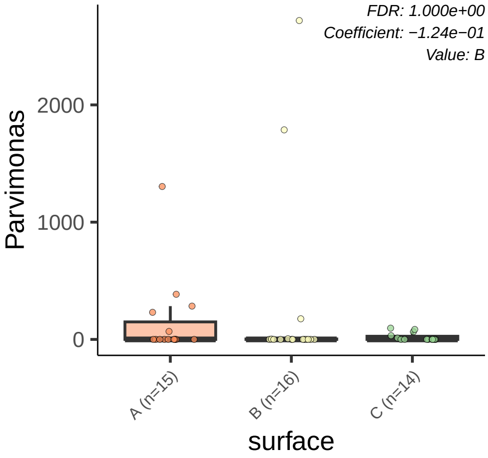
FDR: 1.000e+00
Coefficient: −1.24e−01
Value: B
Parvimonas
2000
1000
0
A (n=15)
B (n=16)
C (n=14)
surface

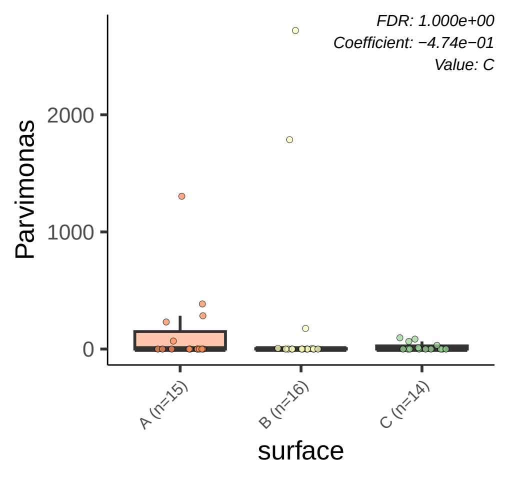
FDR: 1.000e+00
Coefficient: −4.74e−01
Value: C
Parvimonas
2000
1000
0
A (n=15)
B (n=16)
C (n=14)
surface

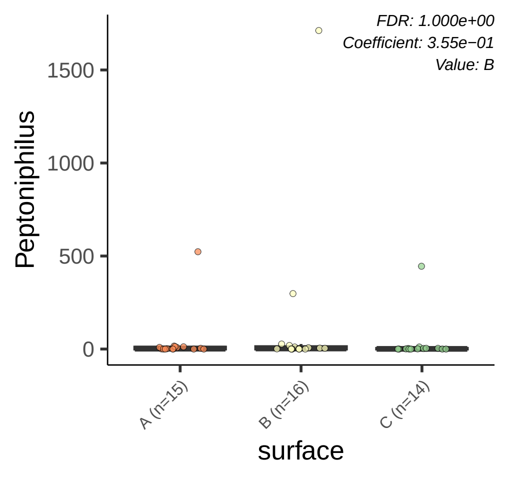

FDR: 1.000e+00
Coefficient: 3.55e−01
Value: B
Peptoniphilus
1500
1000
500
0
A (n=15)
B (n=16)
C (n=14)
surface

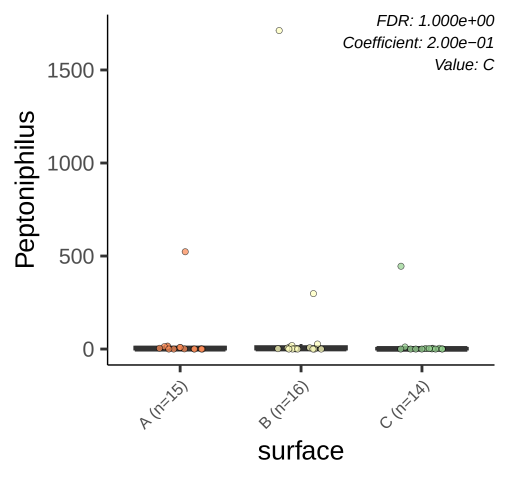
FDR: 1.000e+00
Coefficient: 2.00e−01
Value: C
Peptoniphilus
1500
1000
500
0
A (n=15)
B (n=16)
C (n=14)
surface

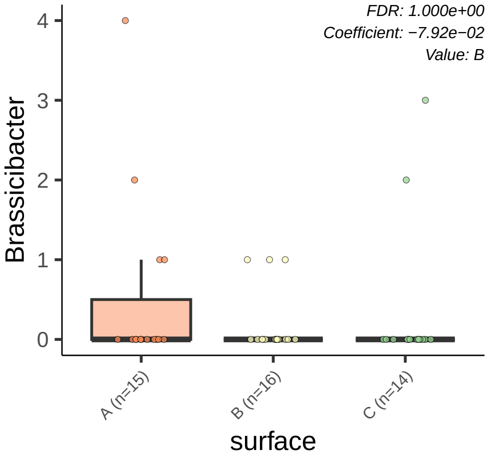

FDR: 1.000e+00
Coefficient: −7.92e−02
Value: B
Brassicibacter
0
1
2
3
4
A (n=15)
B (n=16)
C (n=14)
surface

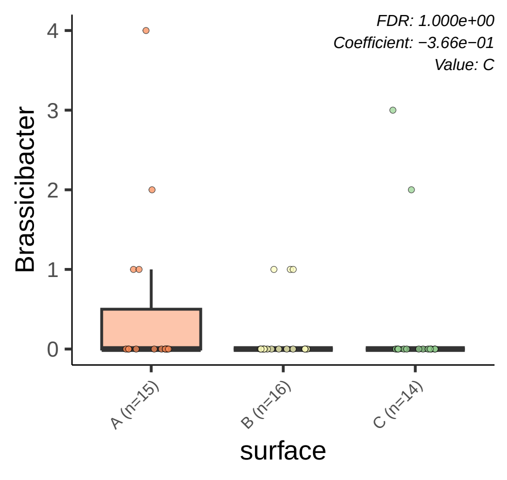

FDR: 1.000e+00
Coefficient: −3.66e−01
Value: C
Brassicibacter
4
3
2
1
0
A (n=15)
B (n=16)
C (n=14)
surface

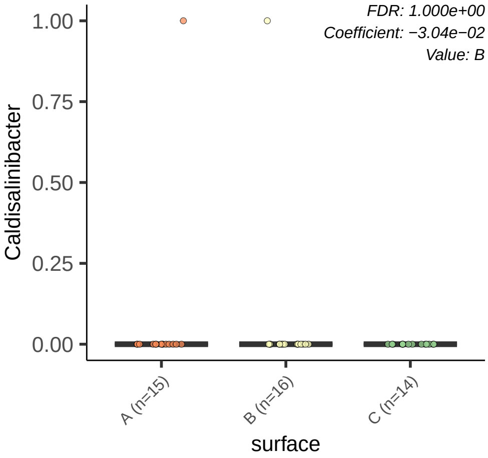

FDR: 1.000e+00
Coefficient: −3.04e−02
Value: B
Caldisalinibacter
1.00
0.75
0.50
0.25
0.00
A (n=15)
B (n=16)
C (n=14)
surface

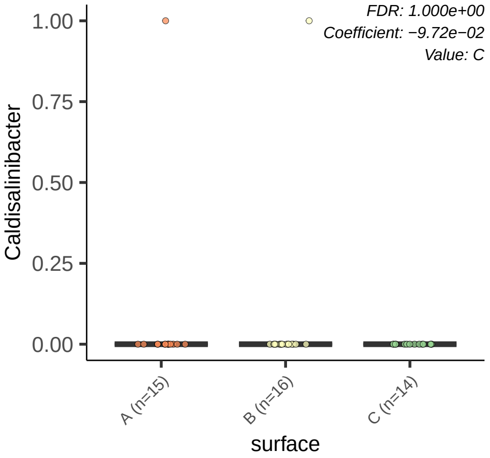

FDR: 1.000e+00
Coefficient: −9.72e−02
Value: C
Caldisalinibacter
1.00
0.75
0.50
0.25
0.00
A (n=15)
B (n=16)
C (n=14)
surface

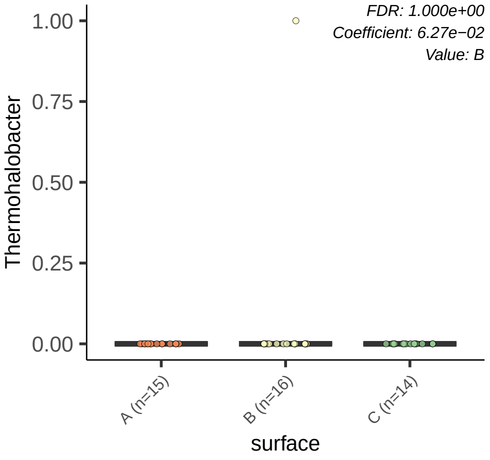

FDR: 1.000e+00
Coefficient: 6.27e−02
Value: B
Thermohalobacter
1.00
0.75
0.50
0.25
0.00
A (n=15)
B (n=16)
C (n=14)
surface

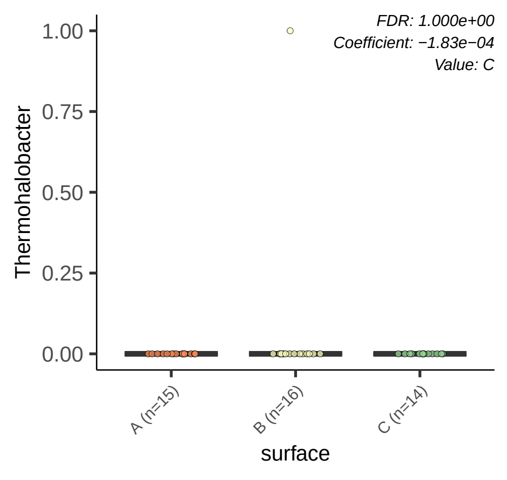
FDR: 1.000e+00
Coefficient: −1.83e−04
Value: C
Thermohalobacter
1.00
0.75
0.50
0.25
0.00
A (n=15)
B (n=16)
C (n=14)
surface

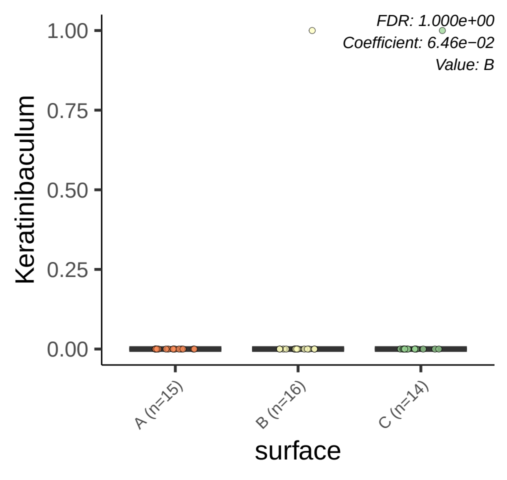
FDR: 1.000e+00
Coefficient: 6.46e−02
Value: B
Keratinibaculum
1.00
0.75
0.50
0.25
0.00
A (n=15)
B (n=16)
C (n=14)
surface

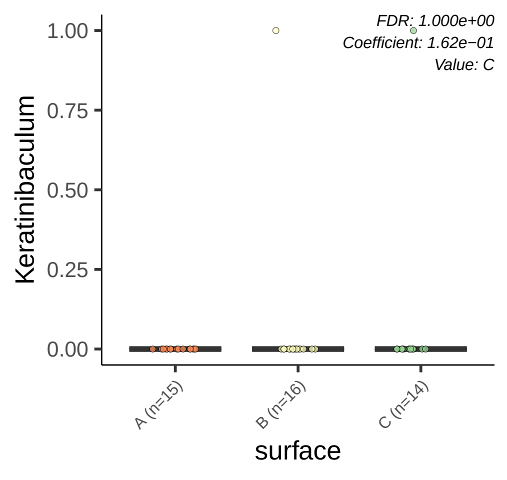

FDR: 1.000e+00
Coefficient: 1.62e−01
Value: C
Keratinibaculum
1.00
0.75
0.50
0.25
0.00
A (n=15)
B (n=16)
C (n=14)
surface

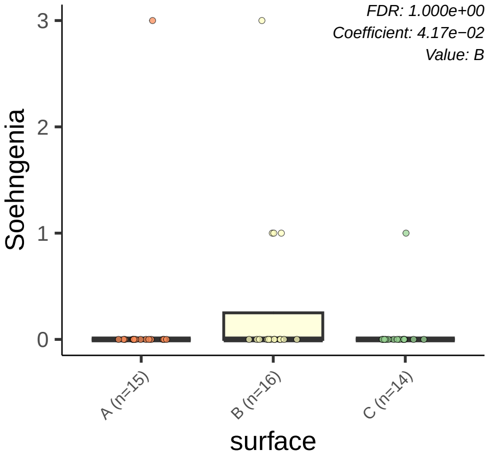

FDR: 1.000e+00
Coefficient: 4.17e−02
Value: B
Soehngenia
3
2
1
0
A (n=15)
B (n=16)
C (n=14)
surface

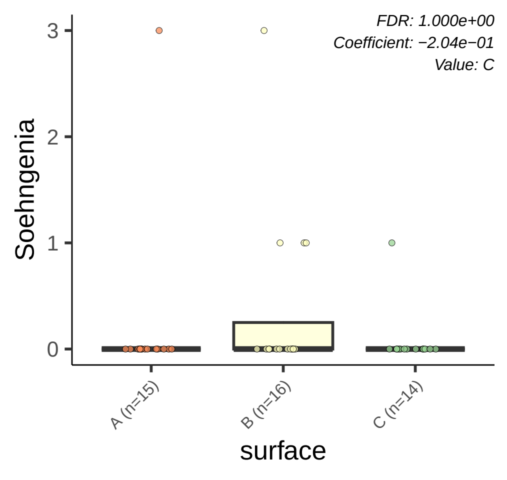

FDR: 1.000e+00
Coefficient: −2.04e−01
Value: C
Soehngenia
3
2
1
0
A (n=15)
B (n=16)
C (n=14)
surface

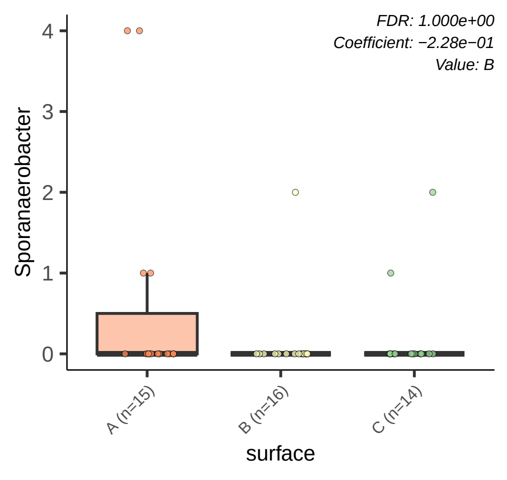
FDR: 1.000e+00
Coefficient: −2.28e−01
Value: B
Sporanaerobacter
0
1
2
3
4
A (n=15)
B (n=16)
C (n=14)
surface

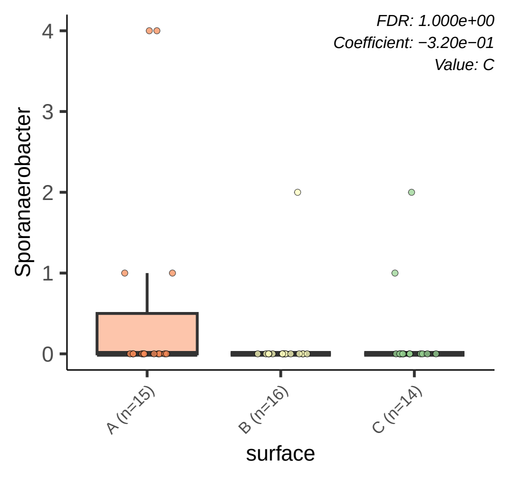

FDR: 1.000e+00
Coefficient: −3.20e−01
Value: C
Sporanaerobacter
4
3
2
1
0
A (n=15)
B (n=16)
C (n=14)
surface

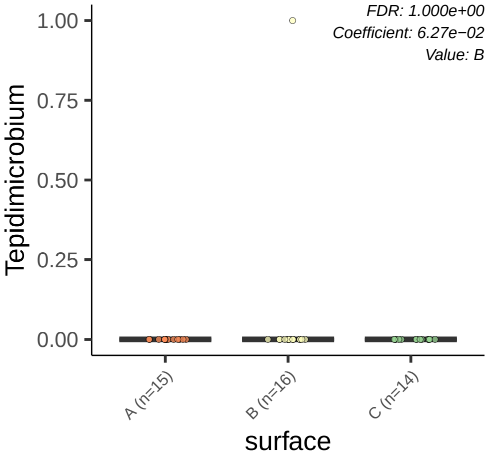
FDR: 1.000e+00
Coefficient: 6.27e−02
Value: B
Tepidimicrobium
1.00
0.75
0.50
0.25
0.00
A (n=15)
B (n=16)
C (n=14)
surface

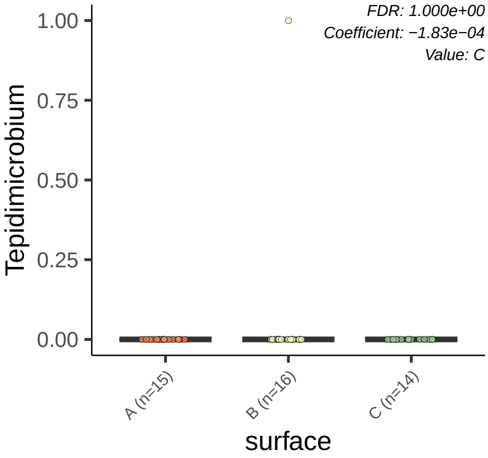
FDR: 1.000e+00
Coefficient: −1.83e−04
Value: C
Tepidimicrobium
1.00
0.75
0.50
0.25
0.00
A (n=15)
B (n=16)
C (n=14)
surface

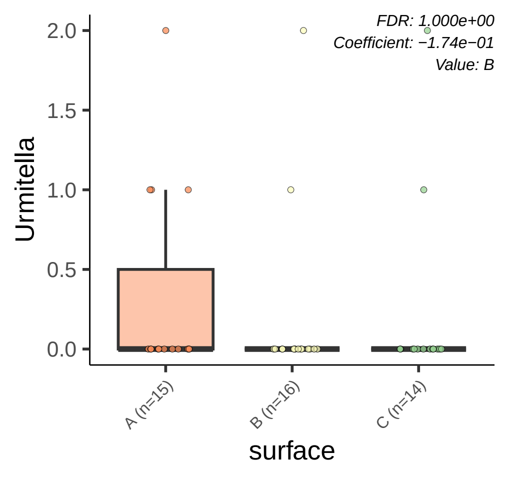
FDR: 1.000e+00
Coefficient: −1.74e−01
Value: B
Urmitella
2.0
1.5
1.0
0.5
0.0
A (n=15)
B (n=16)
C (n=14)
surface

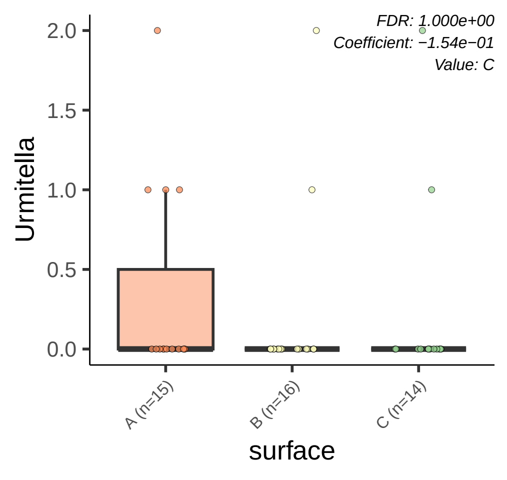
FDR: 1.000e+00
Coefficient: −1.54e−01
Value: C
Urmitella
2.0
1.5
1.0
0.5
0.0
A (n=15)
B (n=16)
C (n=14)
surface

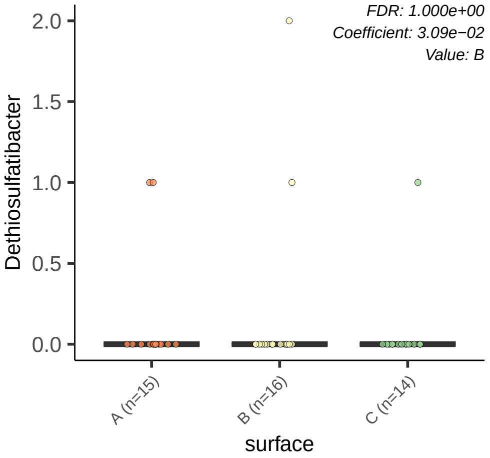

FDR: 1.000e+00
Coefficient: 3.09e−02
Value: B
Dethiosulfatibacter
2.0
1.5
1.0
0.5
0.0
A (n=15)
B (n=16)
C (n=14)
surface

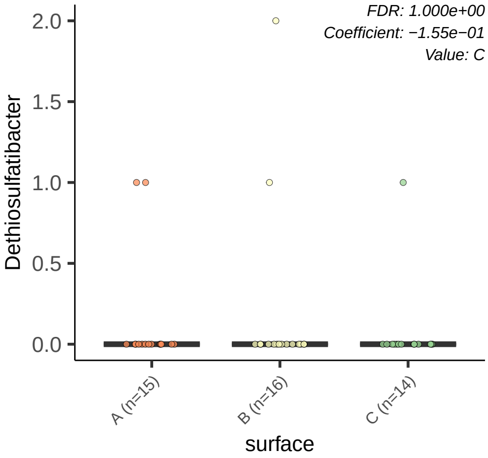

FDR: 1.000e+00
Coefficient: −1.55e−01
Value: C
Dethiosulfatibacter
2.0
1.5
1.0
0.5
0.0
A (n=15)
B (n=16)
C (n=14)
surface

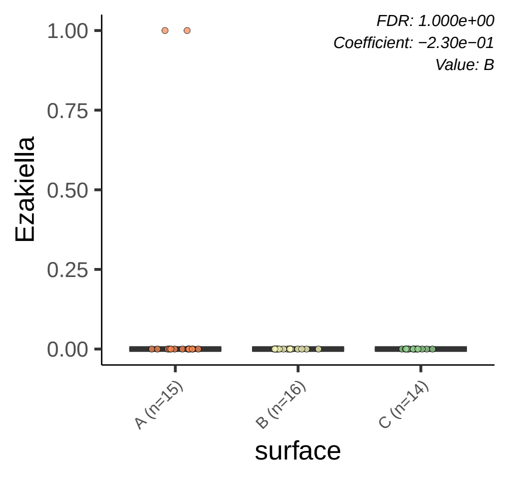

FDR: 1.000e+00
Coefficient: −2.30e−01
Value: B
Ezakiella
1.00
0.75
0.50
0.25
0.00
A (n=15)
B (n=16)
C (n=14)
surface

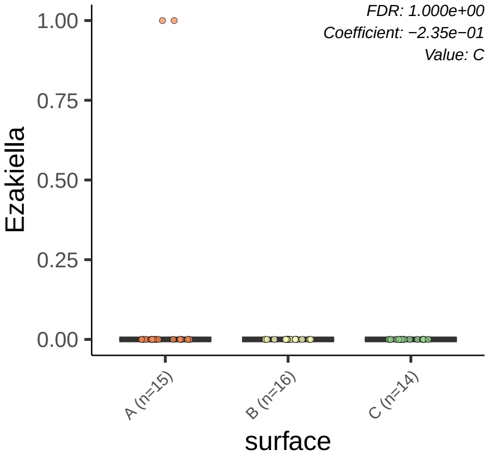

FDR: 1.000e+00
Coefficient: −2.35e−01
Value: C
Ezakiella
1.00
0.75
0.50
0.25
0.00
A (n=15)
B (n=16)
C (n=14)
surface

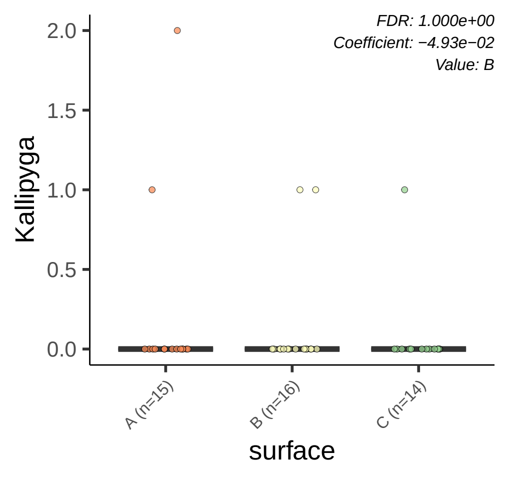

FDR: 1.000e+00
Coefficient: −4.93e−02
Value: B
Kallipyga
2.0
1.5
1.0
0.5
0.0
A (n=15)
B (n=16)
C (n=14)
surface

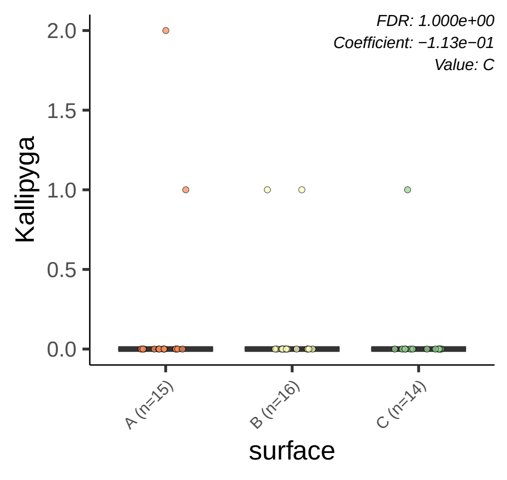

FDR: 1.000e+00
Coefficient: −1.13e−01
Value: C
Kallipyga
2.0
1.5
1.0
0.5
0.0
A (n=15)
B (n=16)
C (n=14)
surface

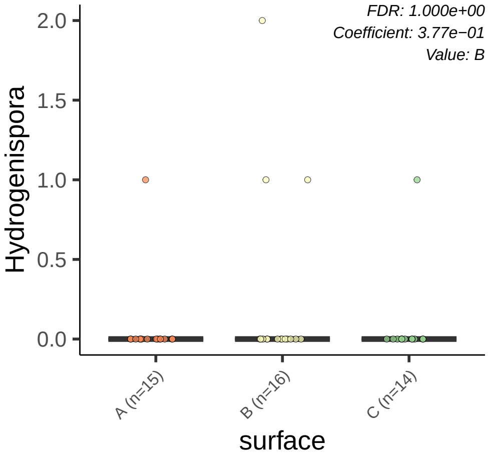
FDR: 1.000e+00
Coefficient: 3.77e−01
Value: B
Hydrogenispora
2.0
1.5
1.0
0.5
0.0
A (n=15)
B (n=16)
C (n=14)
surface

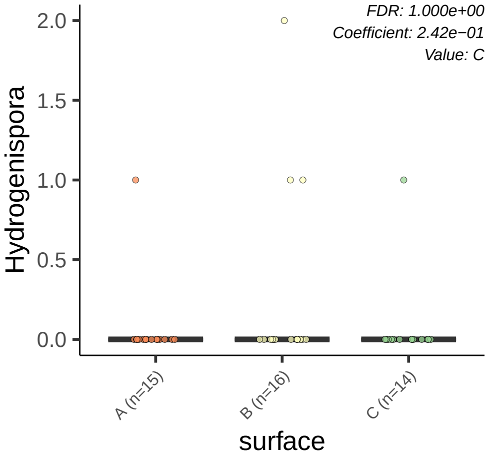

FDR: 1.000e+00
Coefficient: 2.42e−01
Value: C
Hydrogenispora
2.0
1.5
1.0
0.5
0.0
A (n=15)
B (n=16)
C (n=14)
surface

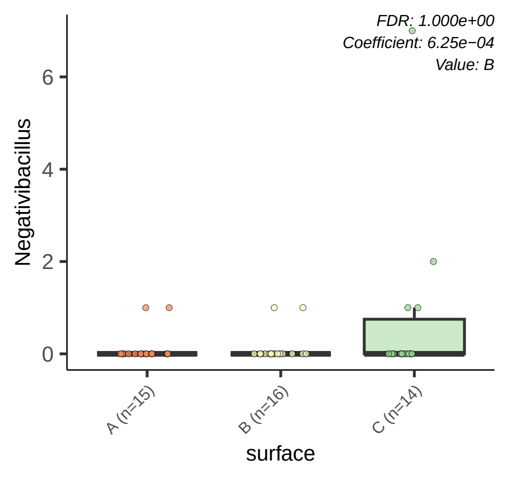
FDR: 1.000e+00
Coefficient: 6.25e−04
Value: B
Negativibacillus
0
2
4
6
A (n=15)
B (n=16)
C (n=14)
surface

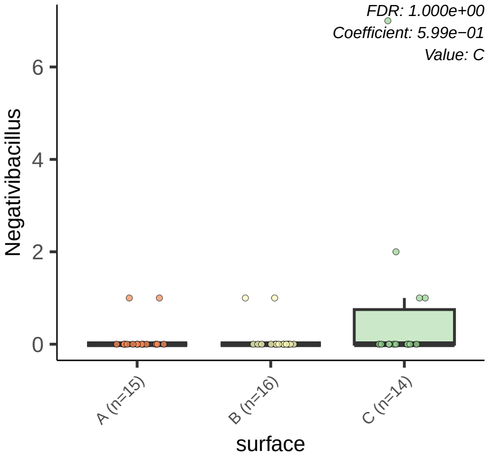
FDR: 1.000e+00
Coefficient: 5.99e−01
Value: C
Negativibacillus
0
2
4
6
A (n=15)
B (n=16)
C (n=14)
surface

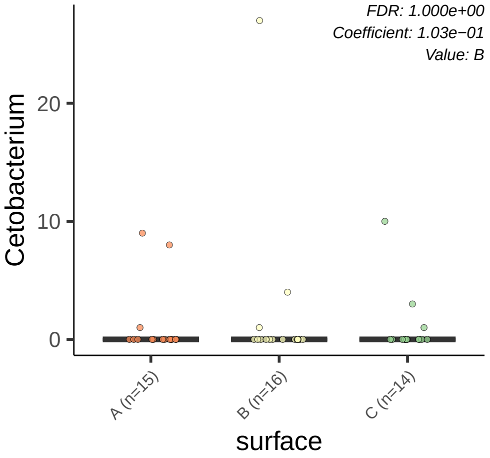

FDR: 1.000e+00
Coefficient: 1.03e−01
Value: B
Cetobacterium
20
10
0
A (n=15)
B (n=16)
C (n=14)
surface

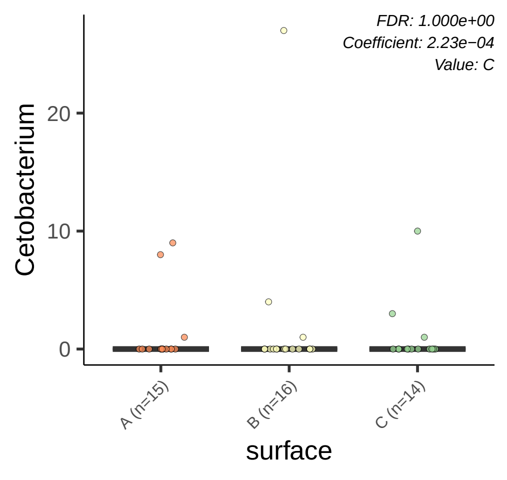

FDR: 1.000e+00
Coefficient: 2.23e−04
Value: C
Cetobacterium
20
10
0
A (n=15)
B (n=16)
C (n=14)
surface

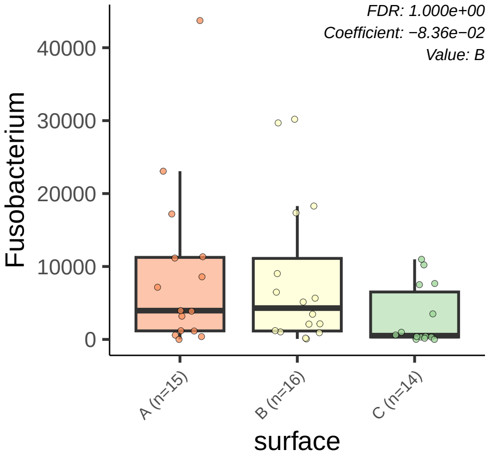

FDR: 1.000e+00
Coefficient: −8.36e−02
Value: B
Fusobacterium
40000
30000
20000
10000
0
A (n=15)
B (n=16)
C (n=14)
surface

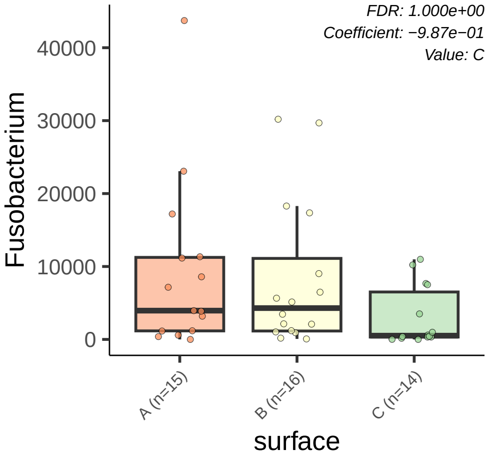
FDR: 1.000e+00
Coefficient: −9.87e−01
Value: C
Fusobacterium
40000
30000
20000
10000
0
A (n=15)
B (n=16)
C (n=14)
surface

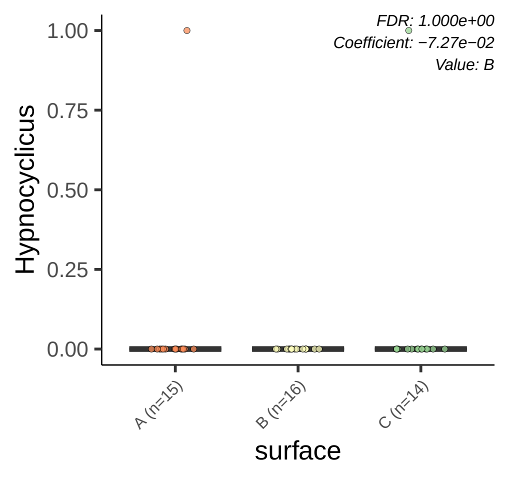
FDR: 1.000e+00
Coefficient: −7.27e−02
Value: B
Hypnocyclicus
1.00
0.75
0.50
0.25
0.00
A (n=15)
B (n=16)
C (n=14)
surface

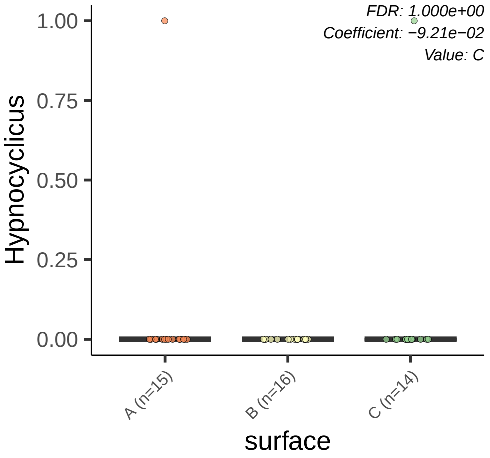

FDR: 1.000e+00
Coefficient: −9.21e−02
Value: C
Hypnocyclicus
1.00
0.75
0.50
0.25
0.00
A (n=15)
B (n=16)
C (n=14)
surface

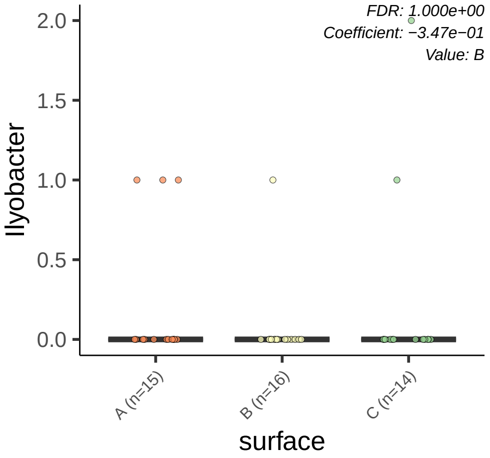

FDR: 1.000e+00
Coefficient: −3.47e−01
Value: B
Ilyobacter
2.0
1.5
1.0
0.5
0.0
A (n=15)
B (n=16)
C (n=14)
surface

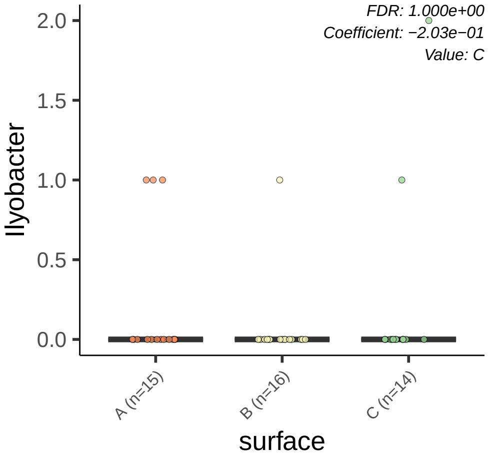

FDR: 1.000e+00
Coefficient: −2.03e−01
Value: C
Ilyobacter
2.0
1.5
1.0
0.5
0.0
A (n=15)
B (n=16)
C (n=14)
surface

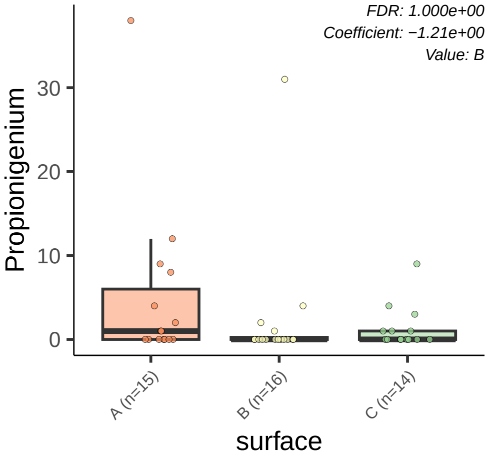

FDR: 1.000e+00
Coefficient: −1.21e+00
Value: B
Propionigenium
0
10
20
30
A (n=15)
B (n=16)
C (n=14)
surface

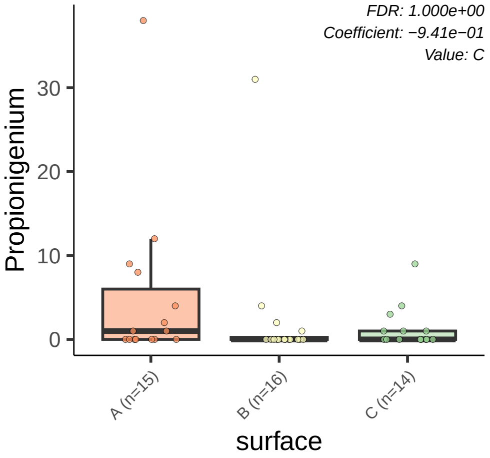

FDR: 1.000e+00
Coefficient: −9.41e−01
Value: C
Propionigenium
0
10
20
30
A (n=15)
B (n=16)
C (n=14)
surface

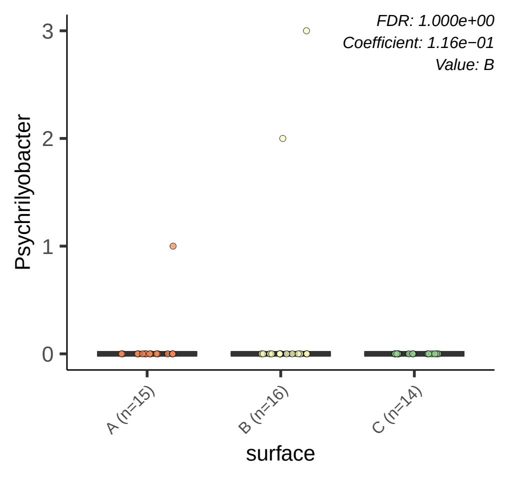
FDR: 1.000e+00
Coefficient: 1.16e−01
Value: B
Psychrilyobacter
0
1
2
3
A (n=15)
B (n=16)
C (n=14)
surface

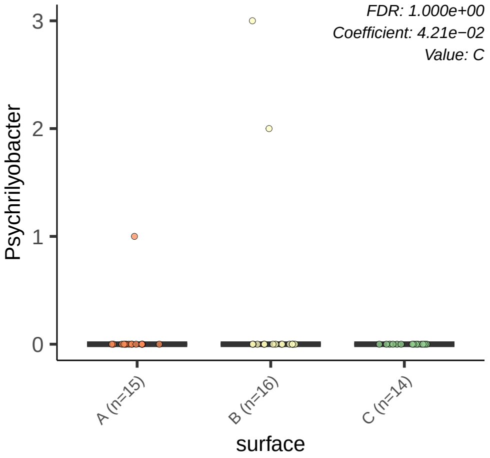

FDR: 1.000e+00
Coefficient: 4.21e−02
Value: C
Psychrilyobacter
0
1
2
3
A (n=15)
B (n=16)
C (n=14)
surface

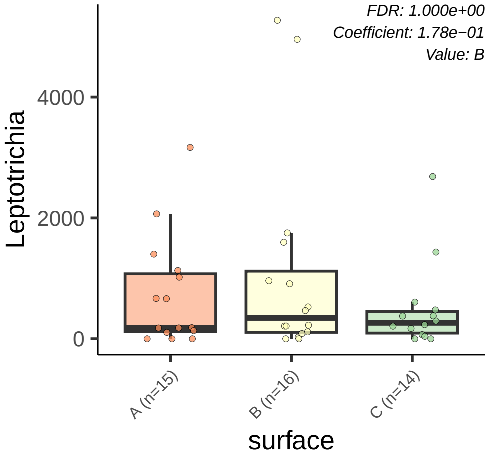

FDR: 1.000e+00
Coefficient: 1.78e−01
Value: B
Leptotrichia
4000
2000
0
A (n=15)
B (n=16)
C (n=14)
surface

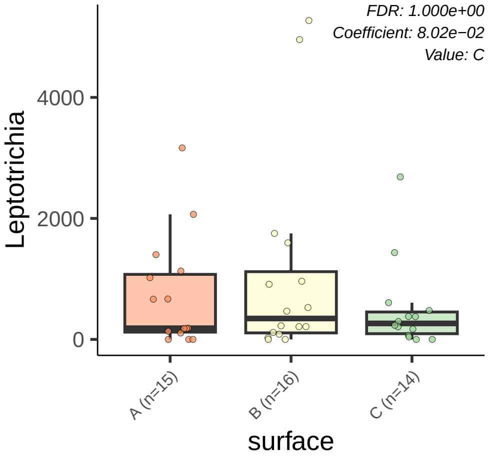

FDR: 1.000e+00
Coefficient: 8.02e−02
Value: C
4000
2000
0
Leptotrichia
A (n=15)
B (n=16)
C (n=14)
surface

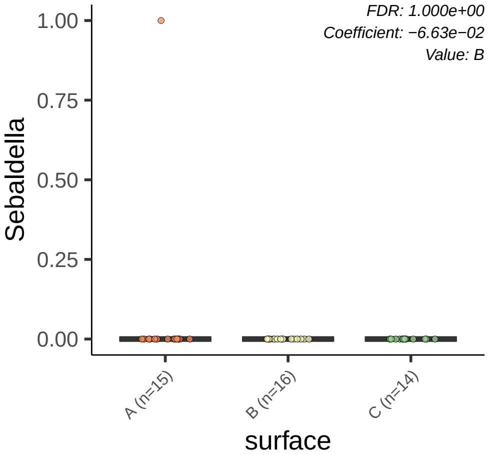

FDR: 1.000e+00
Coefficient: −6.63e−02
Value: B
1.00
0.75
0.50
0.25
0.00
Sebaldella
A (n=15)
B (n=16)
C (n=14)
surface

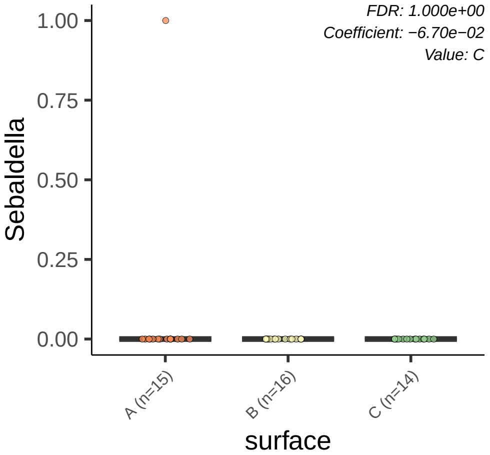

FDR: 1.000e+00
Coefficient: −6.70e−02
Value: C
Sebaldella
1.00
0.75
0.50
0.25
0.00
A (n=15)
B (n=16)
C (n=14)
surface

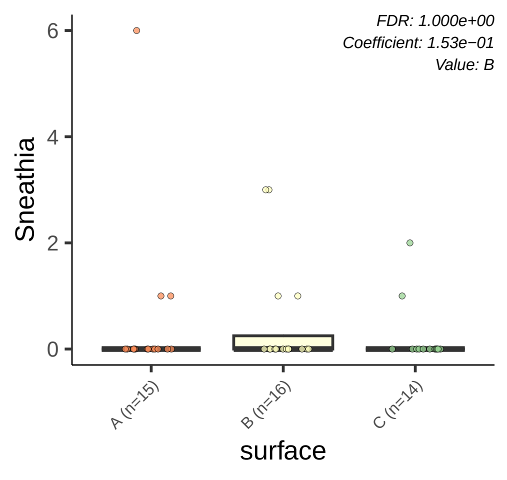
FDR: 1.000e+00
Coefficient: 1.53e−01
Value: B
Sneathia
0
2
4
6
A (n=15)
B (n=16)
C (n=14)
surface

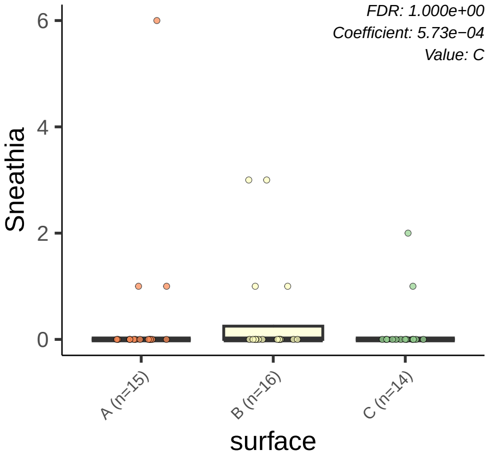

FDR: 1.000e+00
Coefficient: 5.73e−04
Value: C
Sneathia
6
4
2
0
A (n=15)
B (n=16)
C (n=14)
surface

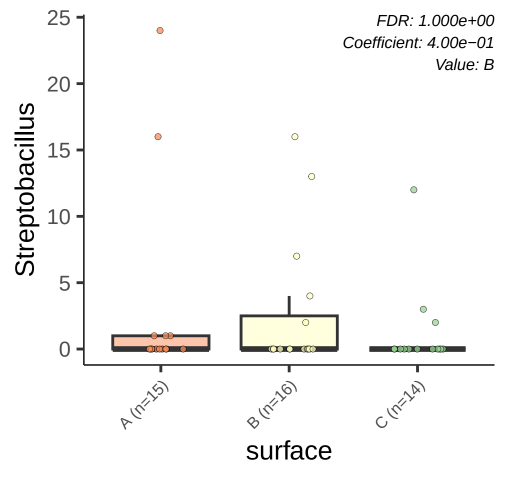

FDR: 1.000e+00
Coefficient: 4.00e−01
Value: B
Streptobacillus
25
20
15
10
5
0
A (n=15)
B (n=16)
C (n=14)
surface

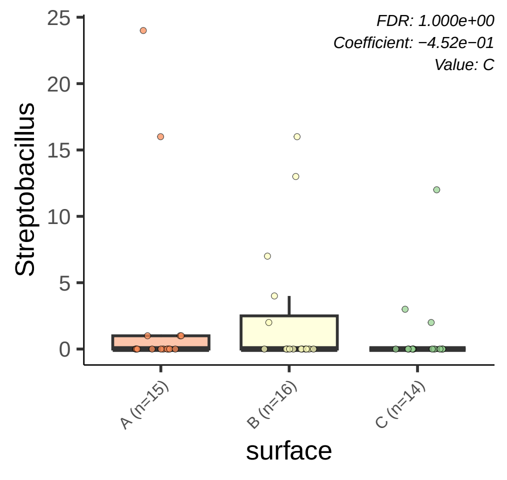
FDR: 1.000e+00
Coefficient: −4.52e−01
Value: C
Streptobacillus
0
5
10
15
20
25
A (n=15)
B (n=16)
C (n=14)
surface

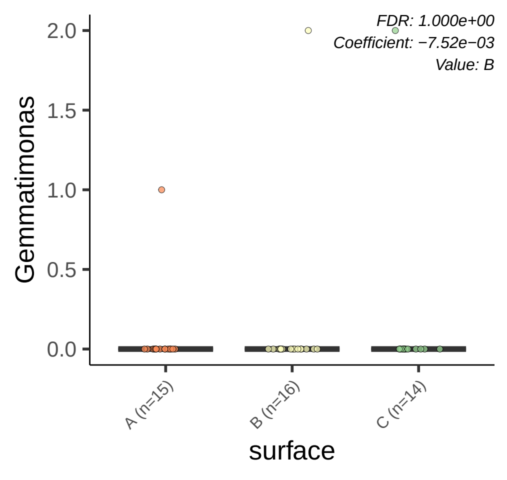

FDR: 1.000e+00
Coefficient: −7.52e−03
Value: B
Gemmatimonas
2.0
1.5
1.0
0.5
0.0
A (n=15)
B (n=16)
C (n=14)
surface

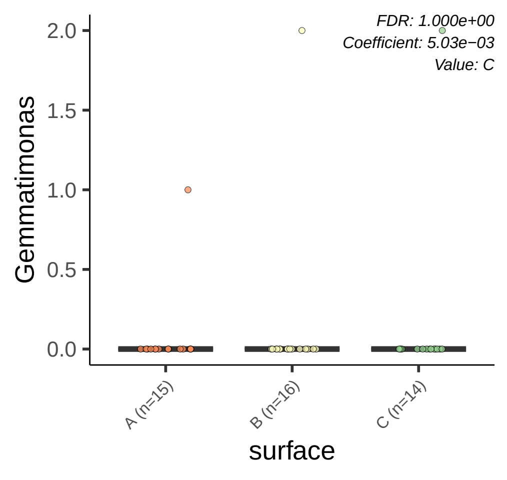
FDR: 1.000e+00
Coefficient: 5.03e−03
Value: C
Gemmatimonas
2.0
1.5
1.0
0.5
0.0
A (n=15)
B (n=16)
C (n=14)
surface

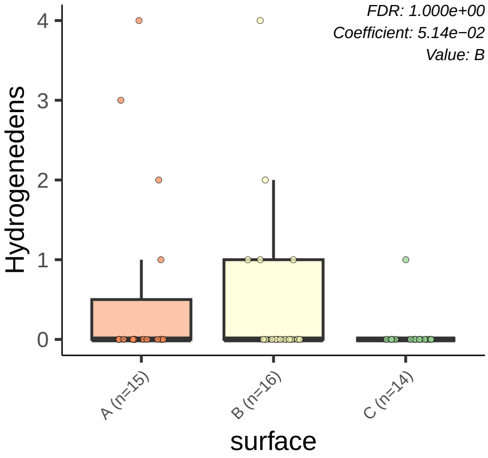

FDR: 1.000e+00
Coefficient: 5.14e−02
Value: B
Hydrogenedens
4
3
2
1
0
A (n=15)
B (n=16)
C (n=14)
surface

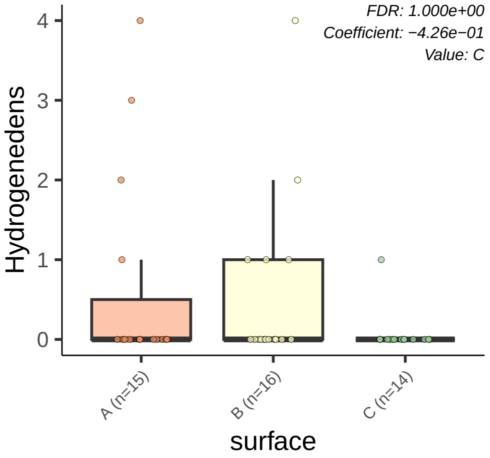

FDR: 1.000e+00
Coefficient: −4.26e−01
Value: C
Hydrogenedens
0
1
2
3
4
A (n=15)
B (n=16)
C (n=14)
surface

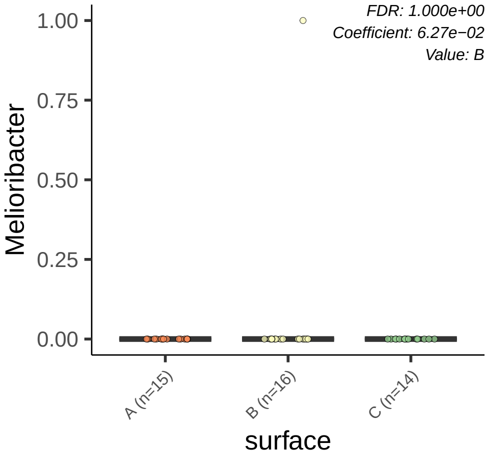
FDR: 1.000e+00
Coefficient: 6.27e−02
Value: B
Melioribacter
1.00
0.75
0.50
0.25
0.00
A (n=15)
B (n=16)
C (n=14)
surface

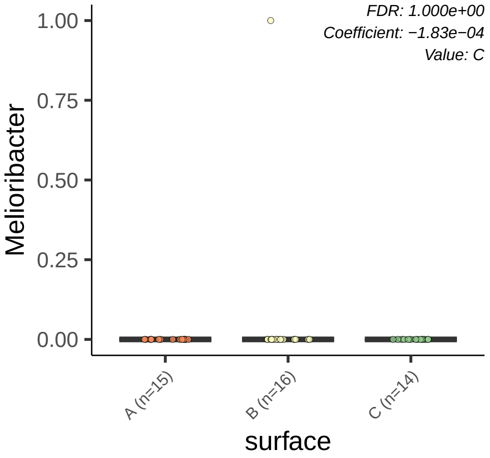
FDR: 1.000e+00
Coefficient: −1.83e−04
Value: C
Melioribacter
1.00
0.75
0.50
0.25
0.00
A (n=15)
B (n=16)
C (n=14)
surface

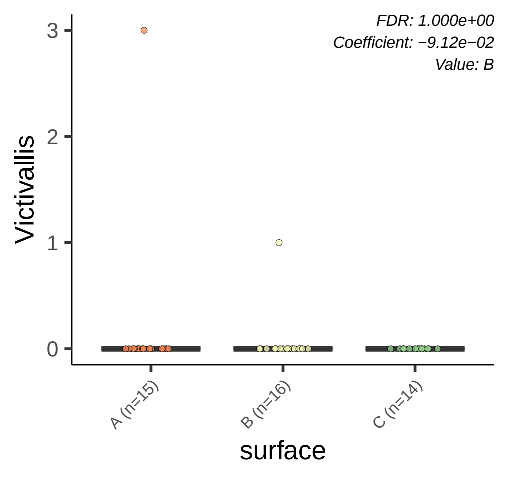

FDR: 1.000e+00
Coefficient: −9.12e−02
Value: B
Victivallis
3
2
1
0
A (n=15)
B (n=16)
C (n=14)
surface

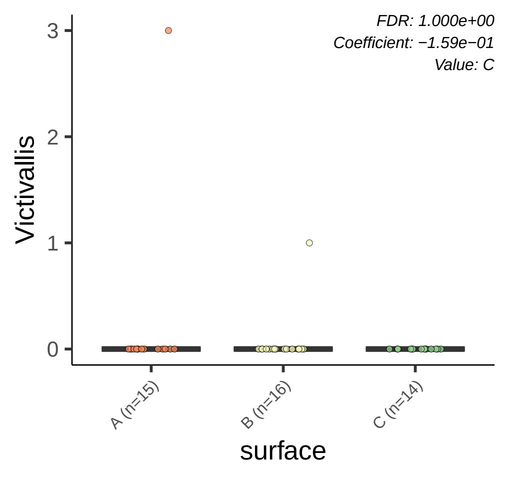
FDR: 1.000e+00
Coefficient: −1.59e−01
Value: C
Victivallis
0
1
2
3
A (n=15)
B (n=16)
C (n=14)
surface

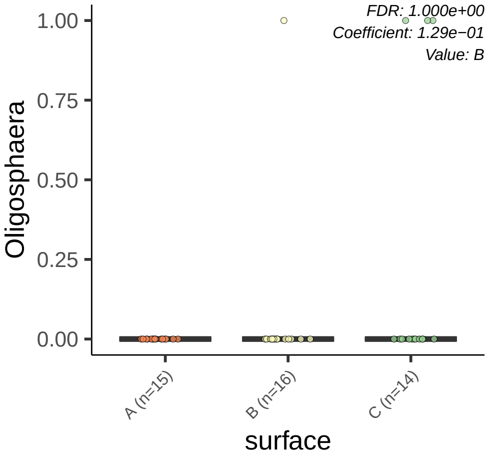
FDR: 1.000e+00
Coefficient: 1.29e−01
Value: B
1.00
0.75
0.50
0.25
0.00
Oligosphaera
A (n=15)
B (n=16)
C (n=14)
surface

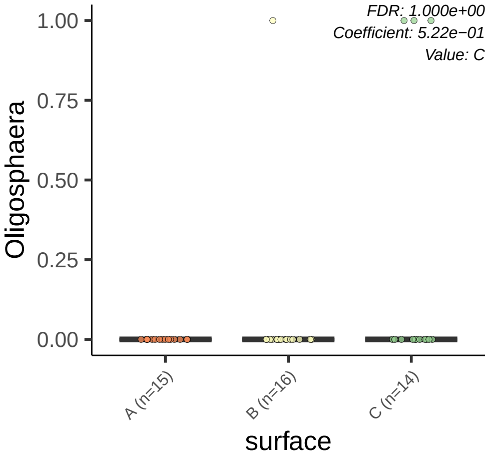

FDR: 1.000e+00
Coefficient: 5.22e−01
Value: C
Oligosphaera
1.00
0.75
0.50
0.25
0.00
A (n=15)
B (n=16)
C (n=14)
surface

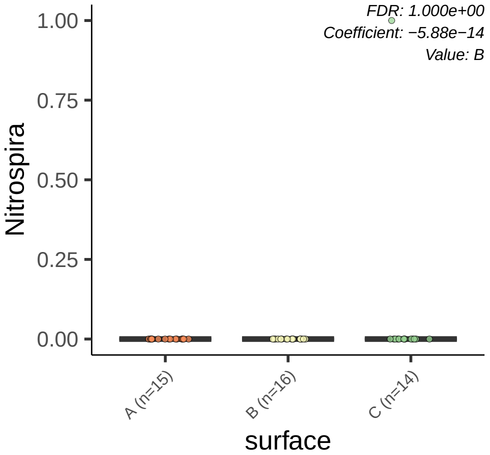

FDR: 1.000e+00
Coefficient: −5.88e−14
Value: B
1.00
0.75
0.50
0.25
0.00
Nitrospira
A (n=15)
B (n=16)
C (n=14)
surface

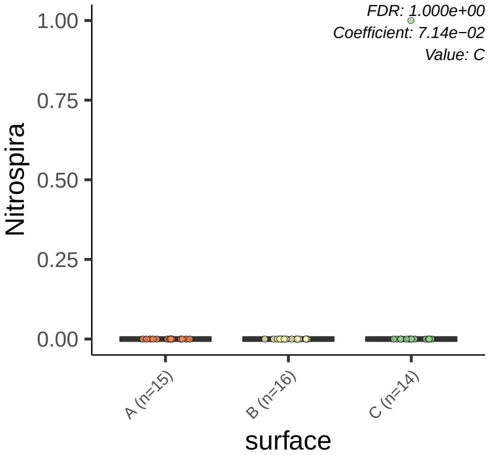

FDR: 1.000e+00
Coefficient: 7.14e−02
Value: C
Nitrospira
1.00
0.75
0.50
0.25
0.00
A (n=15)
B (n=16)
C (n=14)
surface

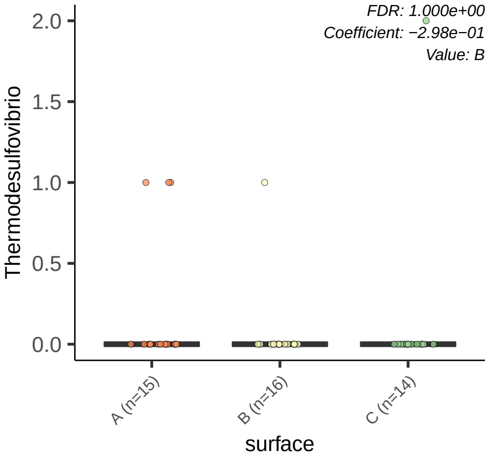
FDR: 1.000e+00
Coefficient: −2.98e−01
Value: B
Thermodesulfovibrio
2.0
1.5
1.0
0.5
0.0
A (n=15)
B (n=16)
C (n=14)
surface

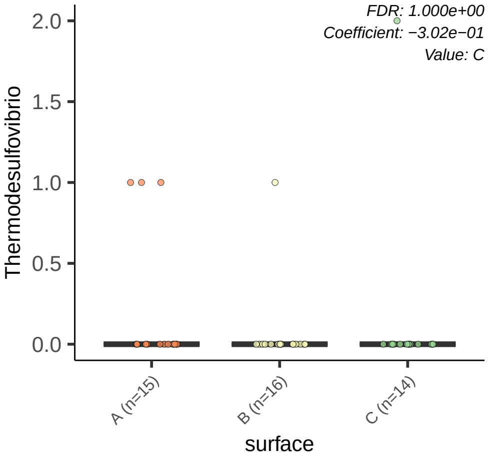
FDR: 1.000e+00
Coefficient: −3.02e−01
Value: C
Thermodesulfovibrio
2.0
1.5
1.0
0.5
0.0
A (n=15)
B (n=16)
C (n=14)
surface

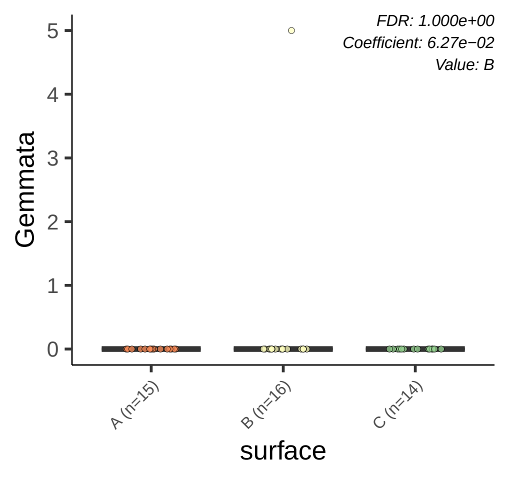
FDR: 1.000e+00
Coefficient: 6.27e−02
Value: B
Gemmata
5
4
3
2
1
0
A (n=15)
B (n=16)
C (n=14)
surface

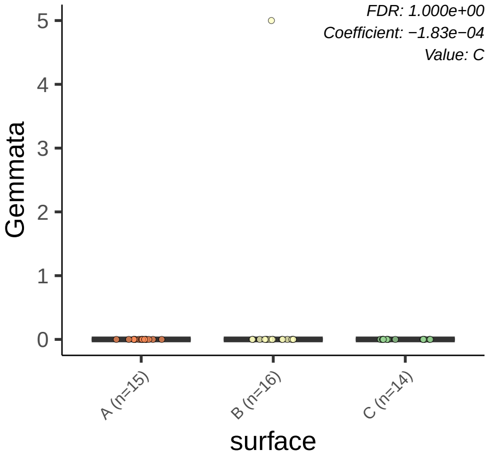
FDR: 1.000e+00
Coefficient: −1.83e−04
Value: C
Gemmata
5
4
3
2
1
0
A (n=15)
B (n=16)
C (n=14)
surface

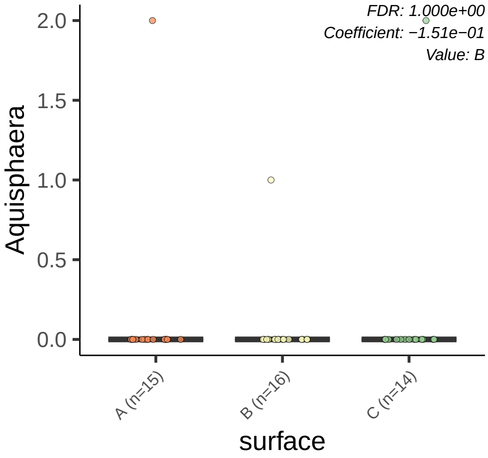
FDR: 1.000e+00
Coefficient: −1.51e−01
Value: B
Aquisphaera
2.0
1.5
1.0
0.5
0.0
A (n=15)
B (n=16)
C (n=14)
surface

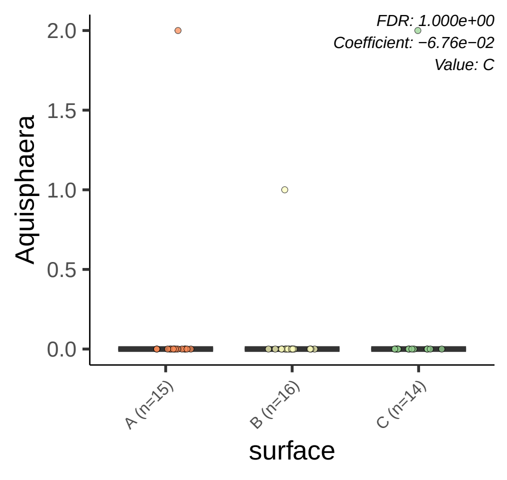

FDR: 1.000e+00
Coefficient: −6.76e−02
Value: C
Aquisphaera
2.0
1.5
1.0
0.5
0.0
A (n=15)
B (n=16)
C (n=14)
surface

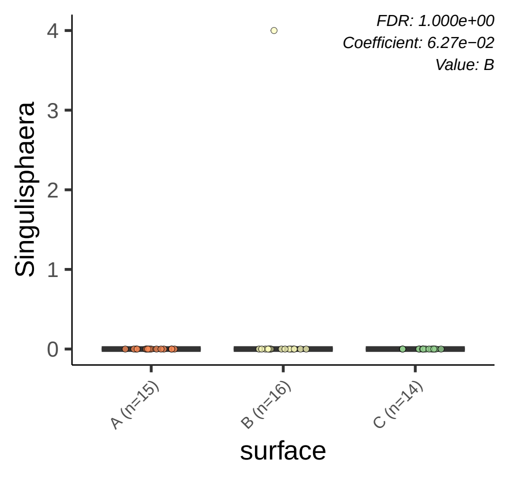
FDR: 1.000e+00
Coefficient: 6.27e−02
Value: B
Singulisphaera
4
3
2
1
0
A (n=15)
B (n=16)
C (n=14)
surface

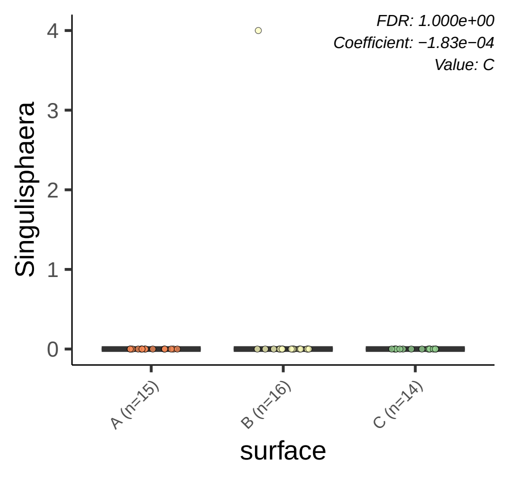
FDR: 1.000e+00
Coefficient: −1.83e−04
Value: C
Singulisphaera
4
3
2
1
0
A (n=15)
B (n=16)
C (n=14)
surface

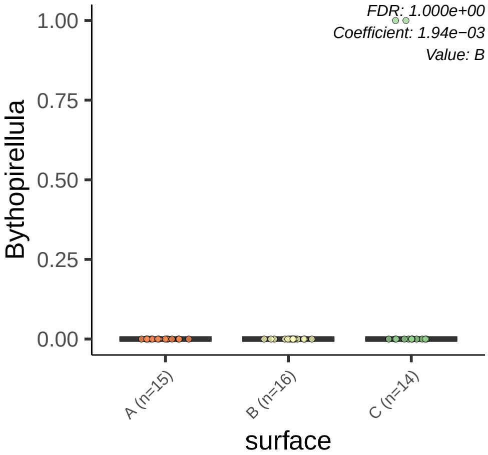

FDR: 1.000e+00
Coefficient: 1.94e−03
Value: B
Bythopirellula
1.00
0.75
0.50
0.25
0.00
A (n=15)
B (n=16)
C (n=14)
surface

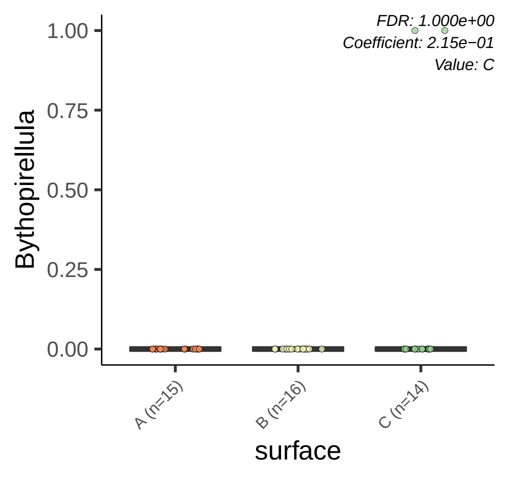
FDR: 1.000e+00
Coefficient: 2.15e−01
Value: C
Bythopirellula
1.00
0.75
0.50
0.25
0.00
A (n=15)
B (n=16)
C (n=14)
surface

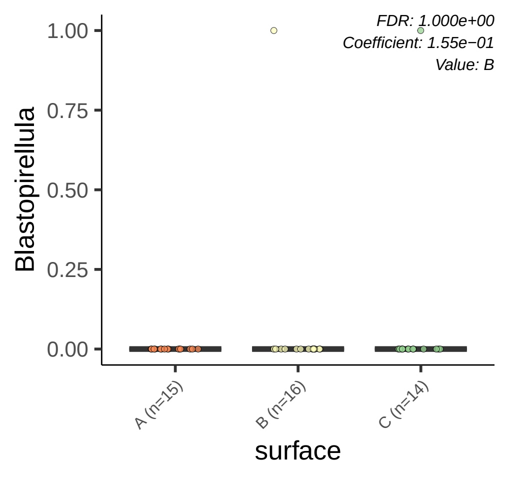
FDR: 1.000e+00
Coefficient: 1.55e−01
Value: B
Blastopirellula
1.00
0.75
0.50
0.25
0.00
A (n=15)
B (n=16)
C (n=14)
surface

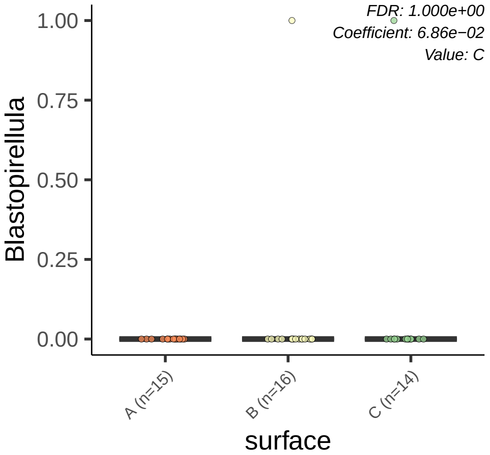
FDR: 1.000e+00
Coefficient: 6.86e−02
Value: C
Blastopirellula
1.00
0.75
0.50
0.25
0.00
A (n=15)
B (n=16)
C (n=14)
surface

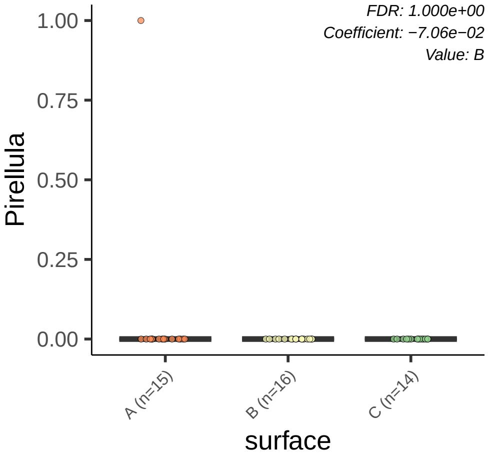
FDR: 1.000e+00
Coefficient: −7.06e−02
Value: B
Pirellula
1.00
0.75
0.50
0.25
0.00
A (n=15)
B (n=16)
C (n=14)
surface

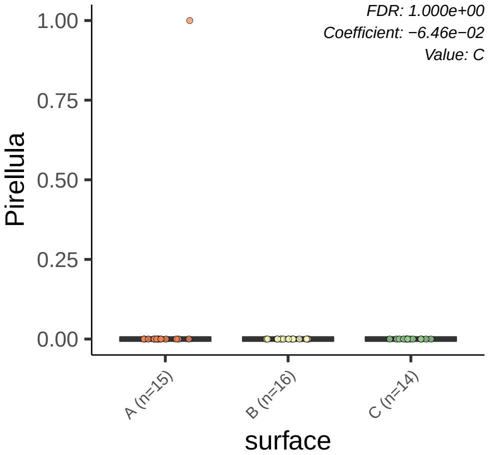

FDR: 1.000e+00
Coefficient: −6.46e−02
Value: C
Pirellula
1.00
0.75
0.50
0.25
0.00
A (n=15)
B (n=16)
C (n=14)
surface

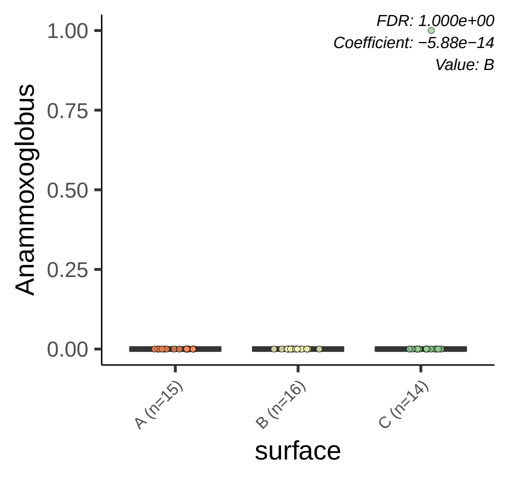
FDR: 1.000e+00
Coefficient: −5.88e−14
Value: B
Anammoxoglobus
1.00
0.75
0.50
0.25
0.00
A (n=15)
B (n=16)
C (n=14)
surface

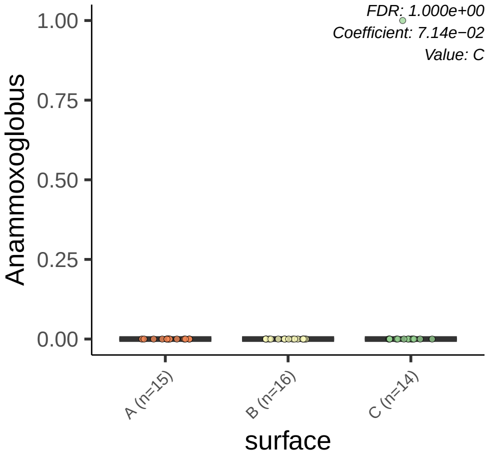
FDR: 1.000e+00
Coefficient: 7.14e−02
Value: C
Anammoxoglobus
1.00
0.75
0.50
0.25
0.00
A (n=15)
B (n=16)
C (n=14)
surface

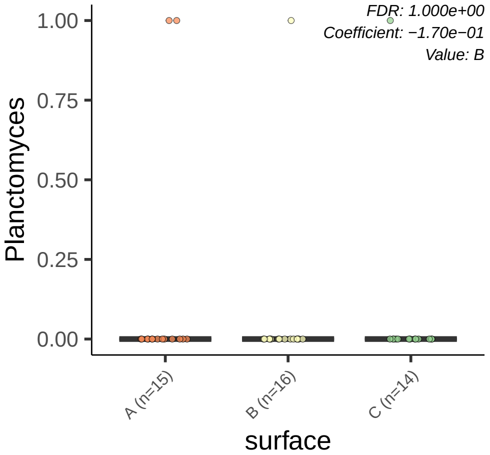

FDR: 1.000e+00
Coefficient: −1.70e−01
Value: B
Planctomyces
1.00
0.75
0.50
0.25
0.00
A (n=15)
B (n=16)
C (n=14)
surface

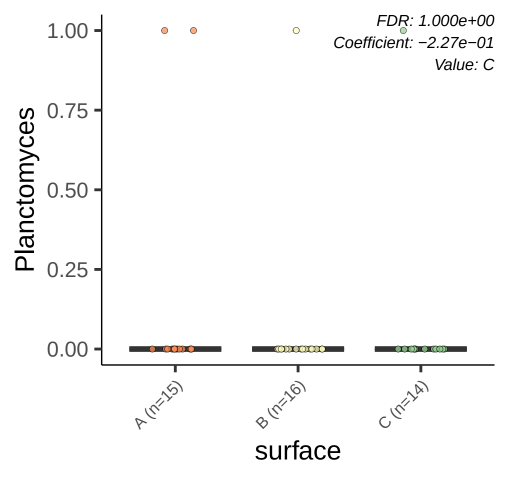

FDR: 1.000e+00
Coefficient: −2.27e−01
Value: C
Planctomyces
1.00
0.75
0.50
0.25
0.00
A (n=15)
B (n=16)
C (n=14)
surface

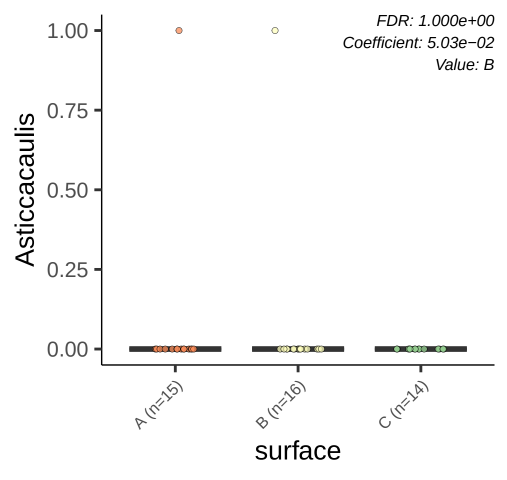
FDR: 1.000e+00
Coefficient: 5.03e−02
Value: B
Asticcacaulis
1.00
0.75
0.50
0.25
0.00
A (n=15)
B (n=16)
C (n=14)
surface

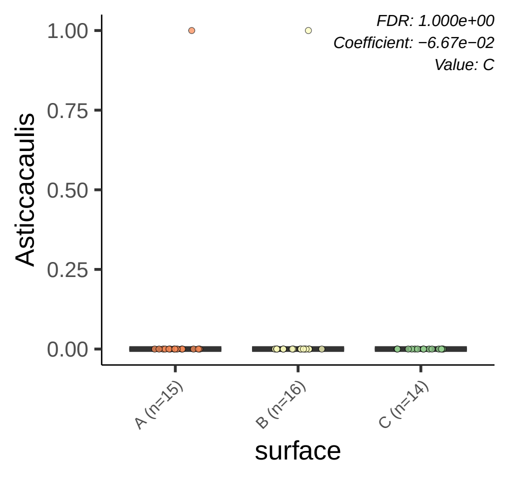
FDR: 1.000e+00
Coefficient: −6.67e−02
Value: C
Asticcacaulis
1.00
0.75
0.50
0.25
0.00
A (n=15)
B (n=16)
C (n=14)
surface

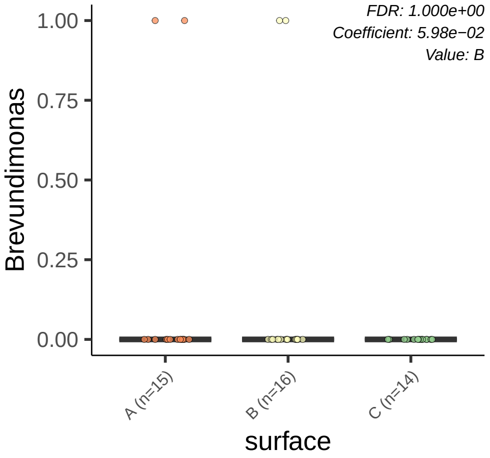
FDR: 1.000e+00
Coefficient: 5.98e−02
Value: B
Brevundimonas
1.00
0.75
0.50
0.25
0.00
A (n=15)
B (n=16)
C (n=14)
surface

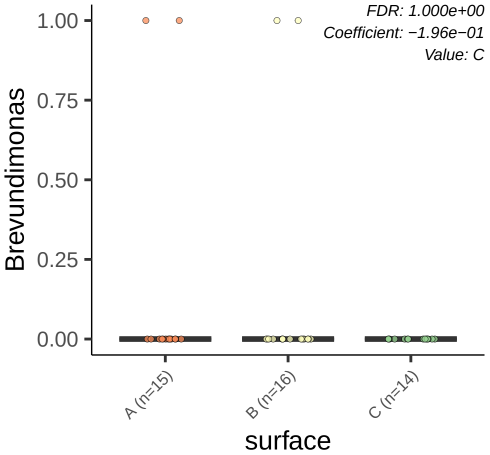

FDR: 1.000e+00
Coefficient: −1.96e−01
Value: C
Brevundimonas
1.00
0.75
0.50
0.25
0.00
A (n=15)
B (n=16)
C (n=14)
surface

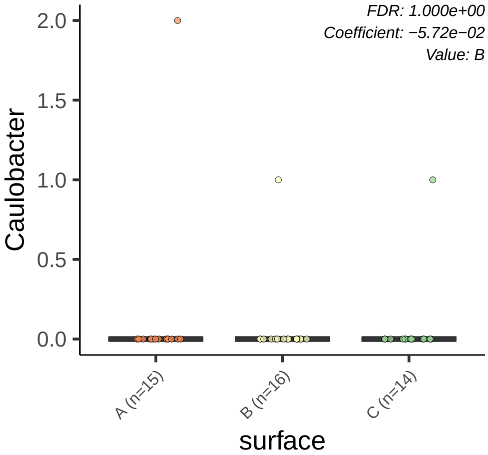

FDR: 1.000e+00
Coefficient: −5.72e−02
Value: B
Caulobacter
2.0
1.5
1.0
0.5
0.0
A (n=15)
B (n=16)
C (n=14)
surface

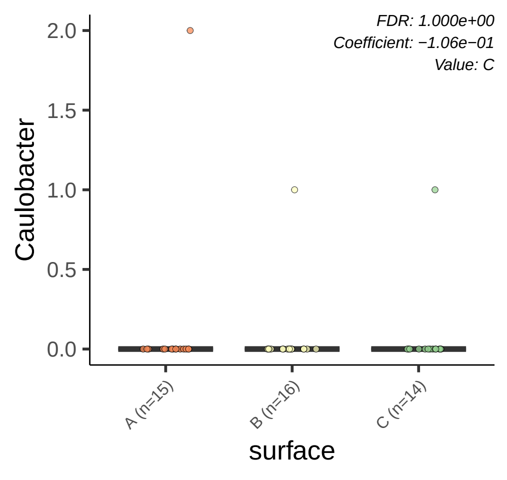
FDR: 1.000e+00
Coefficient: −1.06e−01
Value: C
Caulobacter
2.0
1.5
1.0
0.5
0.0
A (n=15)
B (n=16)
C (n=14)
surface

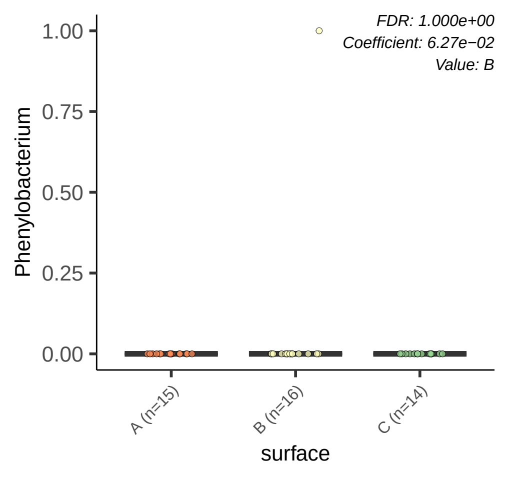
FDR: 1.000e+00
Coefficient: 6.27e−02
Value: B
Phenylobacterium
1.00
0.75
0.50
0.25
0.00
A (n=15)
B (n=16)
C (n=14)
surface

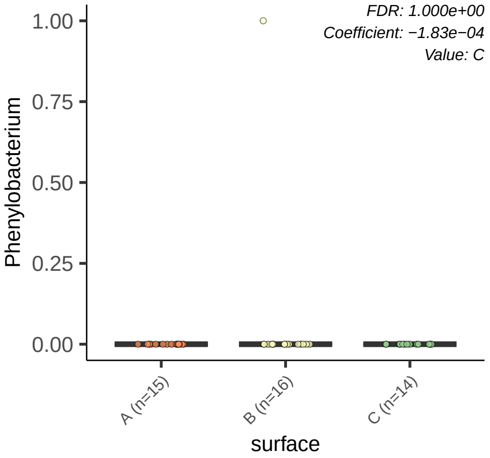

FDR: 1.000e+00
Coefficient: −1.83e−04
Value: C
Phenylobacterium
1.00
0.75
0.50
0.25
0.00
A (n=15)
B (n=16)
C (n=14)
surface

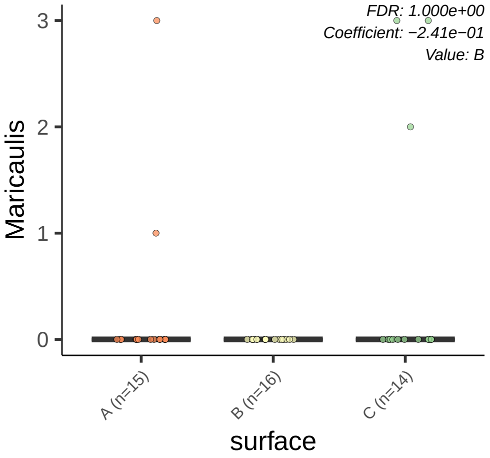

FDR: 1.000e+00
Coefficient: −2.41e−01
Value: B
Maricaulis
0
1
2
3
A (n=15)
B (n=16)
C (n=14)
surface

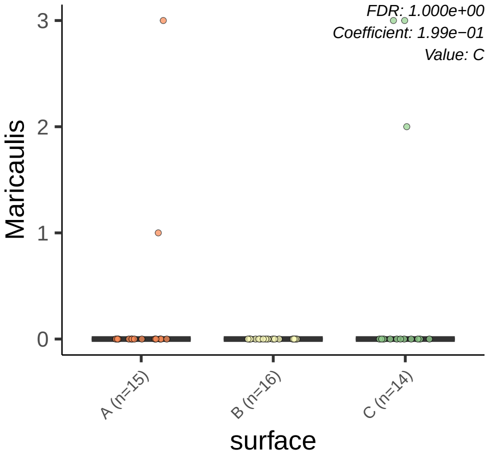
FDR: 1.000e+00
Coefficient: 1.99e−01
Value: C
Maricaulis
0
1
2
3
A (n=15)
B (n=16)
C (n=14)
surface

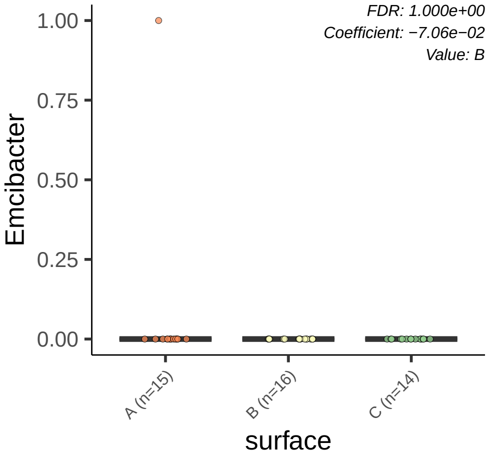
FDR: 1.000e+00
Coefficient: −7.06e−02
Value: B
Emcibacter
1.00
0.75
0.50
0.25
0.00
A (n=15)
B (n=16)
C (n=14)
surface

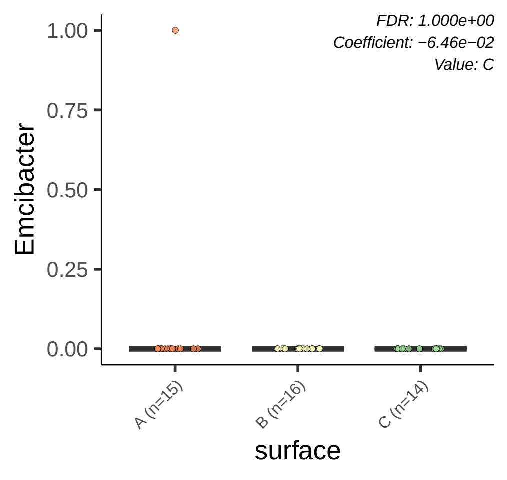

FDR: 1.000e+00
Coefficient: −6.46e−02
Value: C
1.00
0.75
0.50
0.25
0.00
Emcibacter
A (n=15)
B (n=16)
C (n=14)
surface

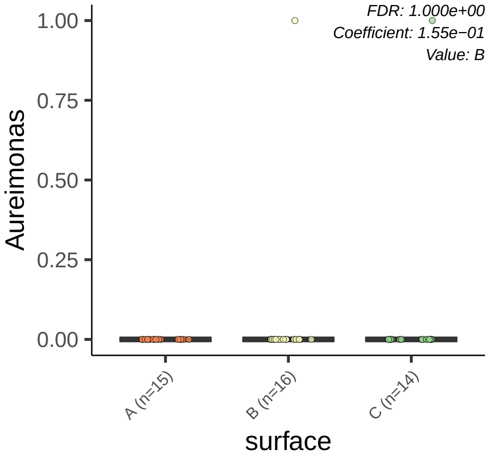

FDR: 1.000e+00
Coefficient: 1.55e−01
Value: B
Aureimonas
1.00
0.75
0.50
0.25
0.00
A (n=15)
B (n=16)
C (n=14)
surface

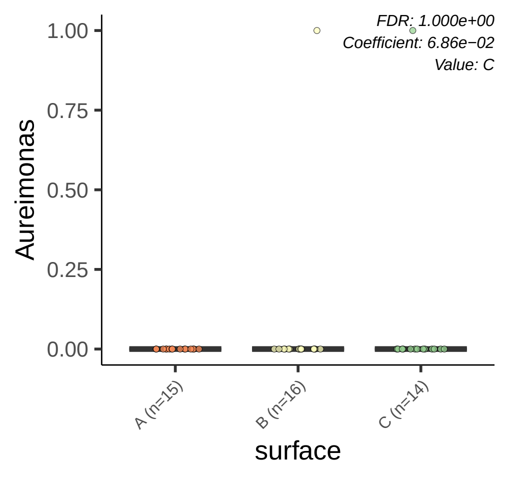
FDR: 1.000e+00
Coefficient: 6.86e−02
Value: C
1.00
0.75
0.50
0.25
0.00
Aureimonas
A (n=15)
B (n=16)
C (n=14)
surface

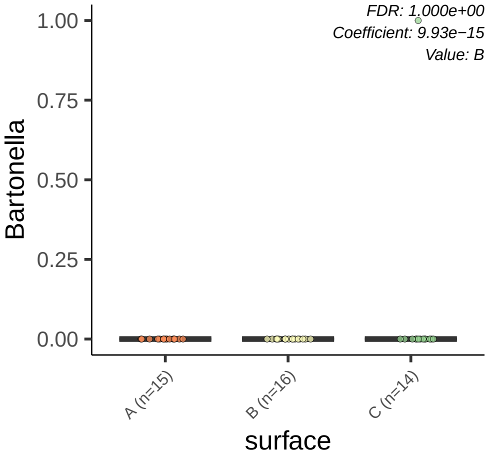

FDR: 1.000e+00
Coefficient: 9.93e−15
Value: B
Bartonella
1.00
0.75
0.50
0.25
0.00
A (n=15)
B (n=16)
C (n=14)
surface

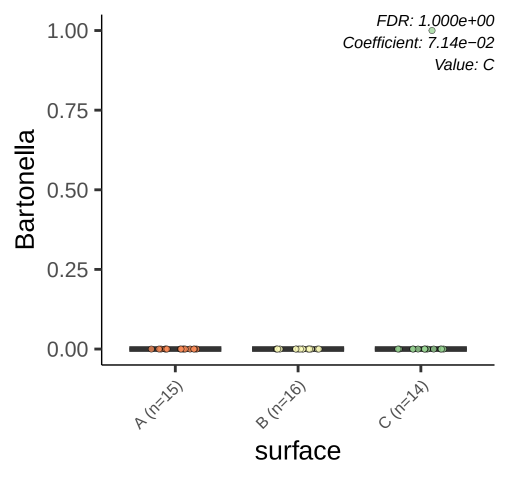
FDR: 1.000e+00
Coefficient: 7.14e−02
Value: C
Bartonella
1.00
0.75
0.50
0.25
0.00
A (n=15)
B (n=16)
C (n=14)
surface

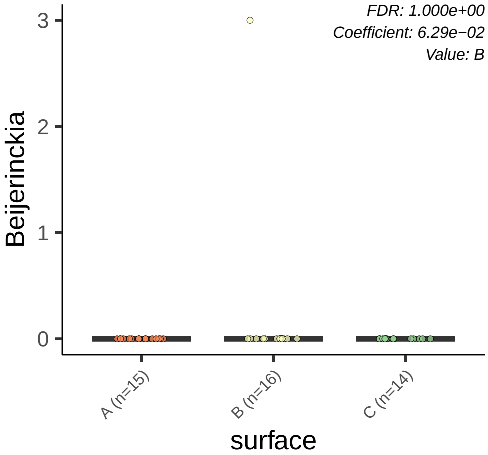

FDR: 1.000e+00
Coefficient: 6.29e−02
Value: B
Beijerinckia
3
2
1
0
A (n=15)
B (n=16)
C (n=14)
surface

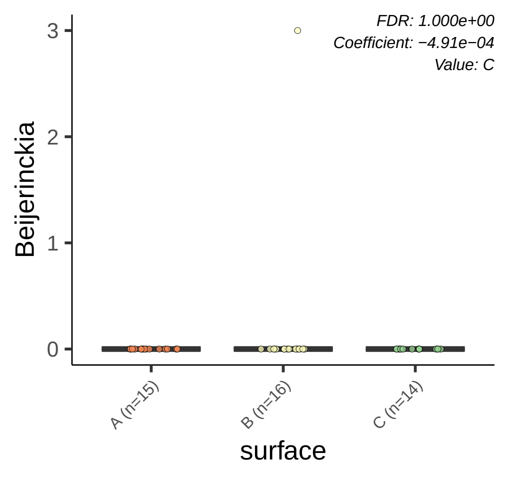

FDR: 1.000e+00
Coefficient: −4.91e−04
Value: C
Beijerinckia
0
1
2
3
A (n=15)
B (n=16)
C (n=14)
surface

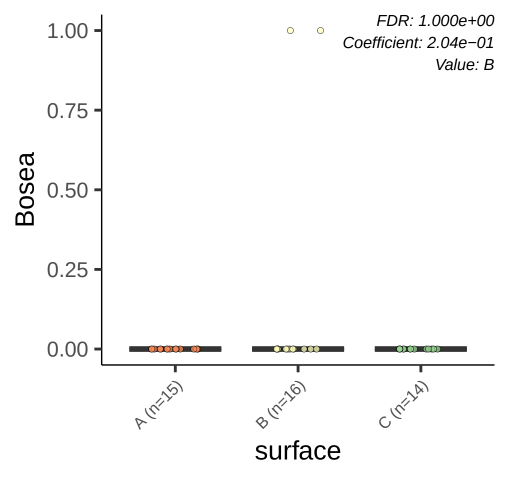
FDR: 1.000e+00
Coefficient: 2.04e−01
Value: B
Bosea
1.00
0.75
0.50
0.25
0.00
A (n=15)
B (n=16)
C (n=14)
surface

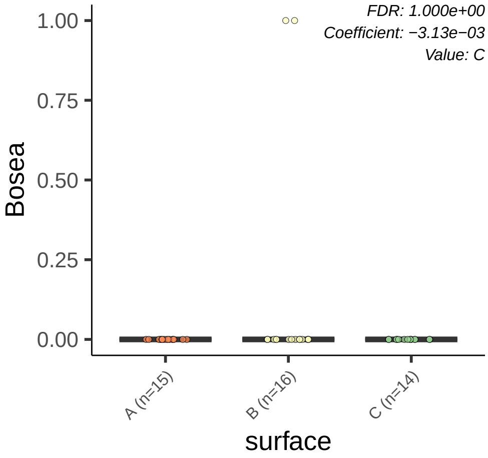
FDR: 1.000e+00
Coefficient: −3.13e−03
Value: C
1.00
0.75
0.50
0.25
0.00
Bosea
A (n=15)
B (n=16)
C (n=14)
surface

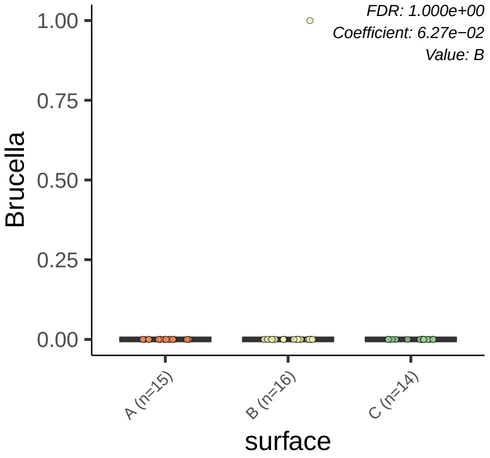
FDR: 1.000e+00
Coefficient: 6.27e−02
Value: B
Brucella
1.00
0.75
0.50
0.25
0.00
A (n=15)
B (n=16)
C (n=14)
surface

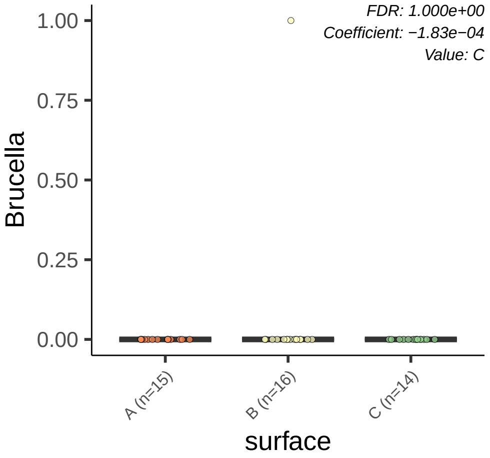
FDR: 1.000e+00
Coefficient: −1.83e−04
Value: C
Brucella
1.00
0.75
0.50
0.25
0.00
A (n=15)
B (n=16)
C (n=14)
surface

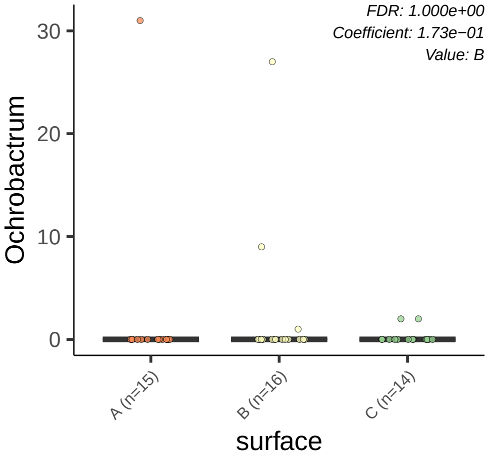
FDR: 1.000e+00
Coefficient: 1.73e−01
Value: B
Ochrobactrum
30
20
10
0
A (n=15)
B (n=16)
C (n=14)
surface

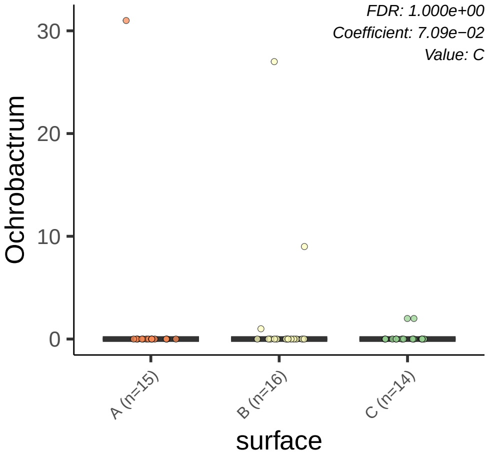

FDR: 1.000e+00
Coefficient: 7.09e−02
Value: C
Ochrobactrum
30
20
10
0
A (n=15)
B (n=16)
C (n=14)
surface

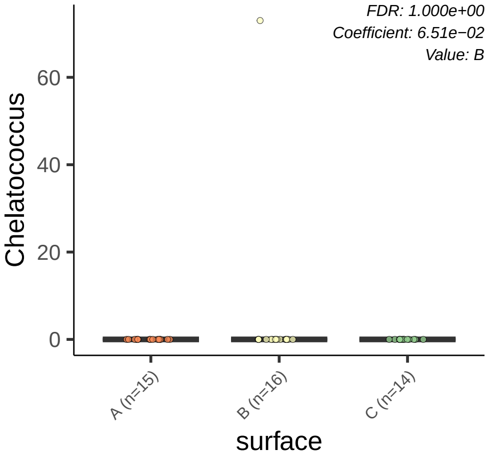
FDR: 1.000e+00
Coefficient: 6.51e−02
Value: B
Chelatococcus
0
20
40
60
A (n=15)
B (n=16)
C (n=14)
surface

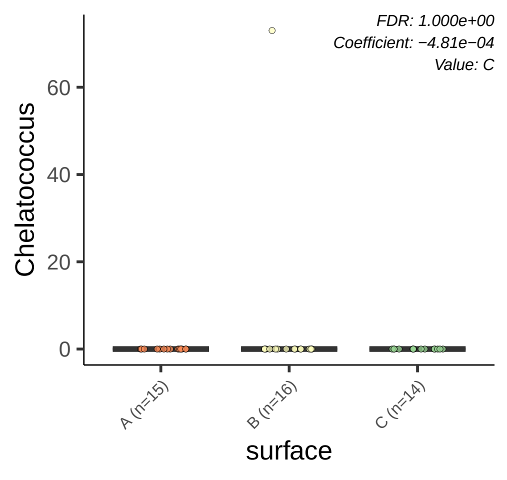
FDR: 1.000e+00
Coefficient: −4.81e−04
Value: C
Chelatococcus
0
20
40
60
A (n=15)
B (n=16)
C (n=14)
surface

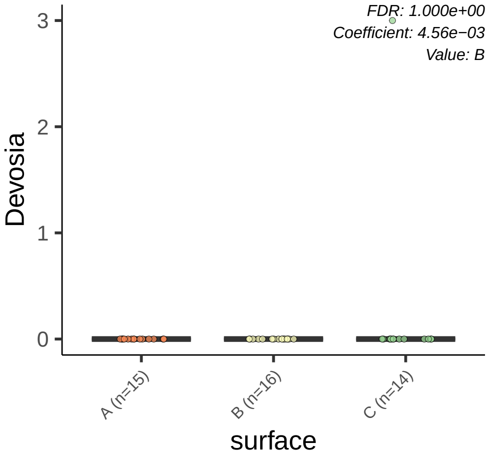
FDR: 1.000e+00
Coefficient: 4.56e−03
Value: B
Devosia
3
2
1
0
A (n=15)
B (n=16)
C (n=14)
surface

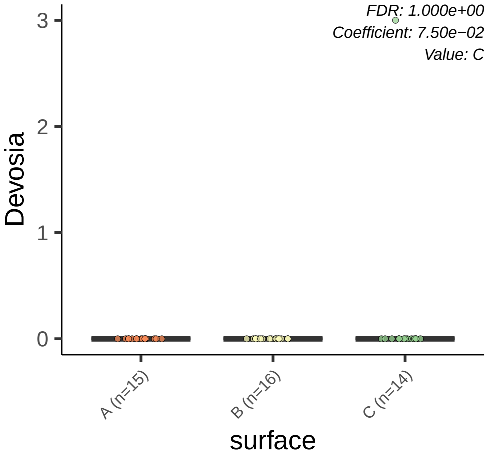

FDR: 1.000e+00
Coefficient: 7.50e−02
Value: C
3
2
1
0
Devosia
A (n=15)
B (n=16)
C (n=14)
surface

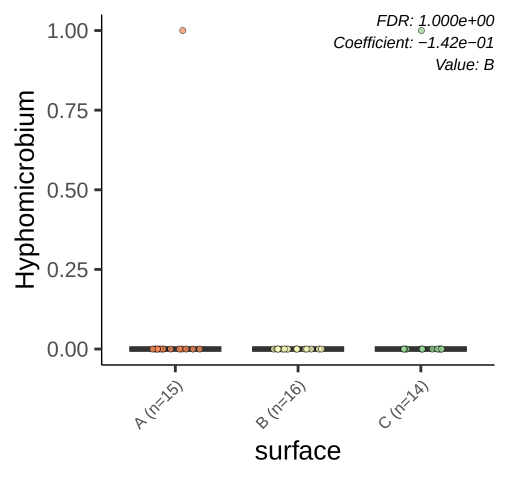

FDR: 1.000e+00
Coefficient: −1.42e−01
Value: B
Hyphomicrobium
1.00
0.75
0.50
0.25
0.00
A (n=15)
B (n=16)
C (n=14)
surface

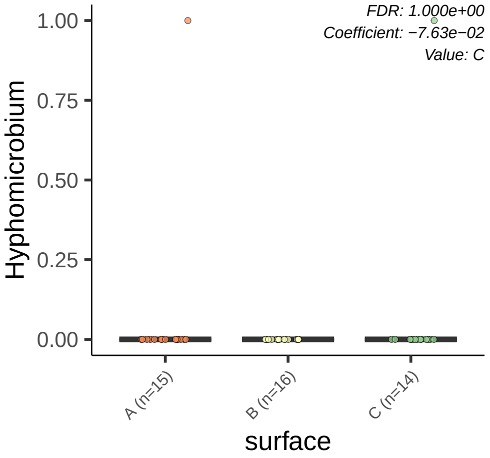
FDR: 1.000e+00
Coefficient: −7.63e−02
Value: C
Hyphomicrobium
1.00
0.75
0.50
0.25
0.00
A (n=15)
B (n=16)
C (n=14)
surface

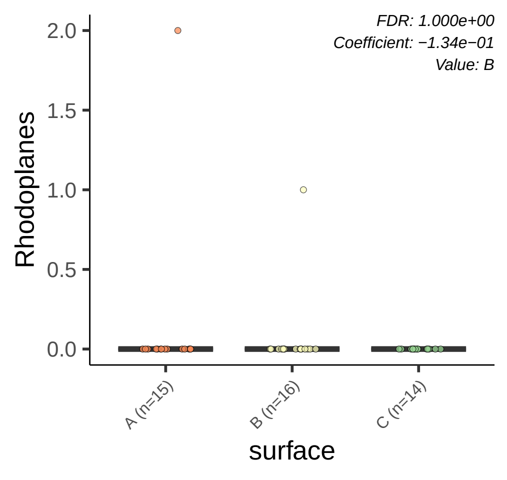
FDR: 1.000e+00
Coefficient: −1.34e−01
Value: B
Rhodoplanes
2.0
1.5
1.0
0.5
0.0
A (n=15)
B (n=16)
C (n=14)
surface

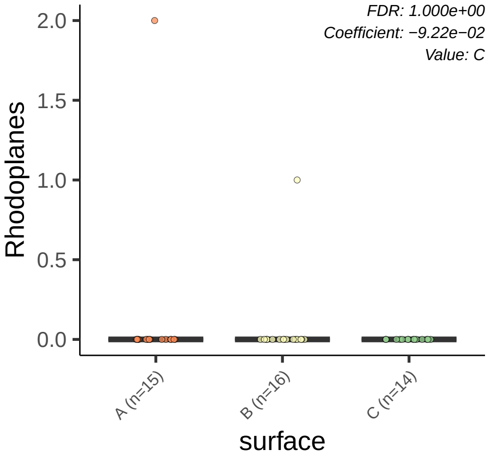
FDR: 1.000e+00
Coefficient: −9.22e−02
Value: C
Rhodoplanes
2.0
1.5
1.0
0.5
0.0
A (n=15)
B (n=16)
C (n=14)
surface

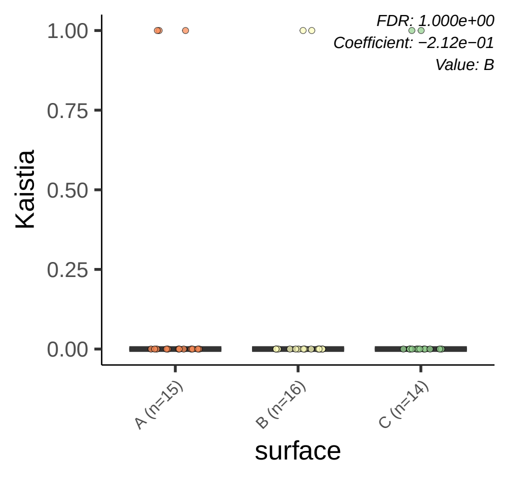
FDR: 1.000e+00
Coefficient: −2.12e−01
Value: B
Kaistia
1.00
0.75
0.50
0.25
0.00
A (n=15)
B (n=16)
C (n=14)
surface

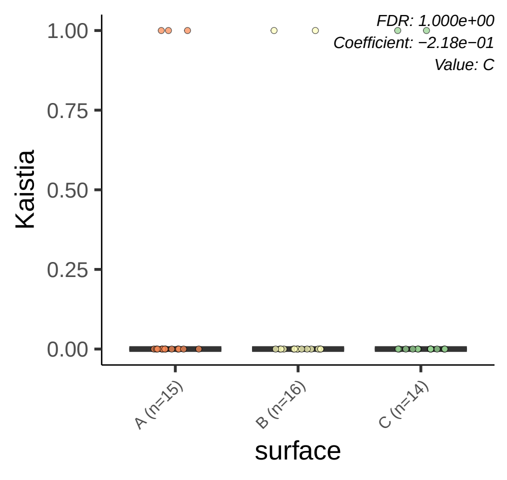

FDR: 1.000e+00
Coefficient: −2.18e−01
Value: C
Kaistia
1.00
0.75
0.50
0.25
0.00
A (n=15)
B (n=16)
C (n=14)
surface

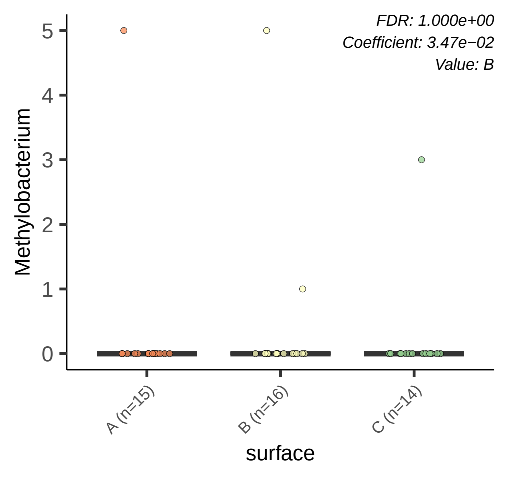
FDR: 1.000e+00
Coefficient: 3.47e−02
Value: B
Methylobacterium
0
1
2
3
4
5
A (n=15)
B (n=16)
C (n=14)
surface

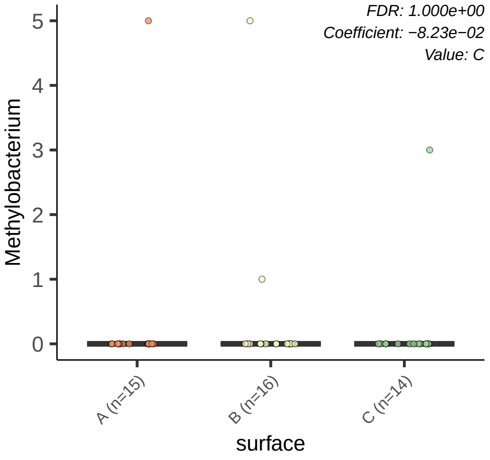

FDR: 1.000e+00
Coefficient: −8.23e−02
Value: C
Methylobacterium
0
1
2
3
4
5
A (n=15)
B (n=16)
C (n=14)
surface

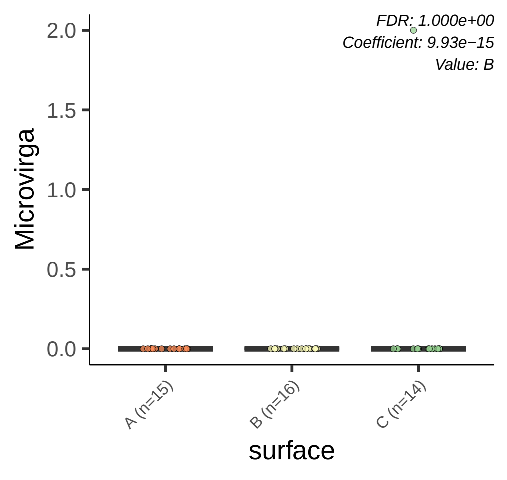
FDR: 1.000e+00
Coefficient: 9.93e−15
Value: B
Microvirga
2.0
1.5
1.0
0.5
0.0
A (n=15)
B (n=16)
C (n=14)
surface

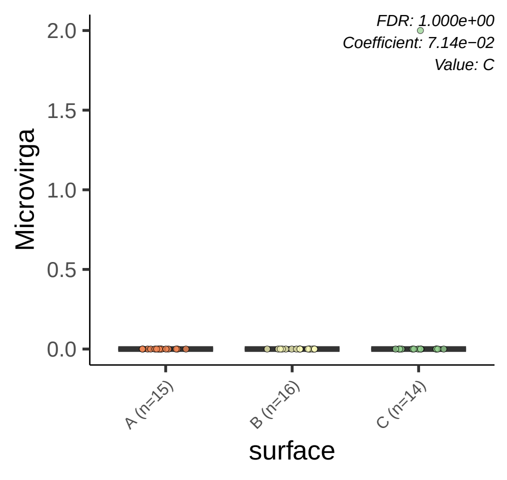
FDR: 1.000e+00
Coefficient: 7.14e−02
Value: C
Microvirga
2.0
1.5
1.0
0.5
0.0
A (n=15)
B (n=16)
C (n=14)
surface

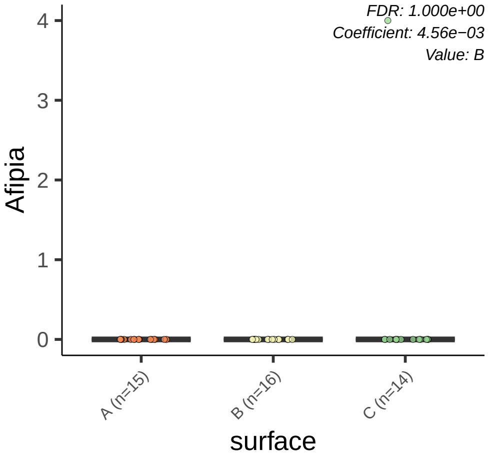
FDR: 1.000e+00
Coefficient: 4.56e−03
Value: B
Afipia
4
3
2
1
0
A (n=15)
B (n=16)
C (n=14)
surface

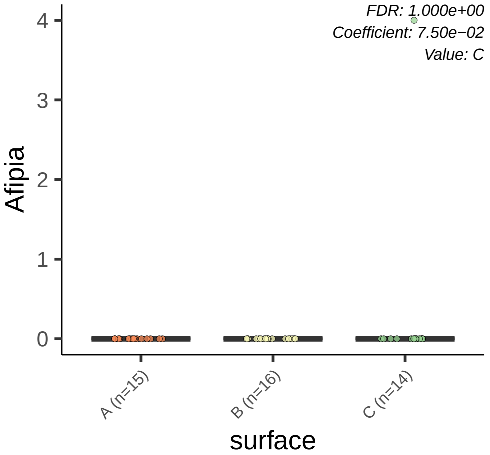
FDR: 1.000e+00
Coefficient: 7.50e−02
Value: C
Afipia
4
3
2
1
0
A (n=15)
B (n=16)
C (n=14)
surface

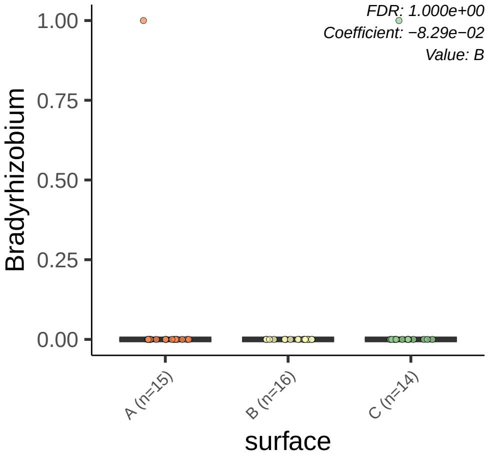

FDR: 1.000e+00
Coefficient: −8.29e−02
Value: B
Bradyrhizobium
1.00
0.75
0.50
0.25
0.00
A (n=15)
B (n=16)
C (n=14)
surface

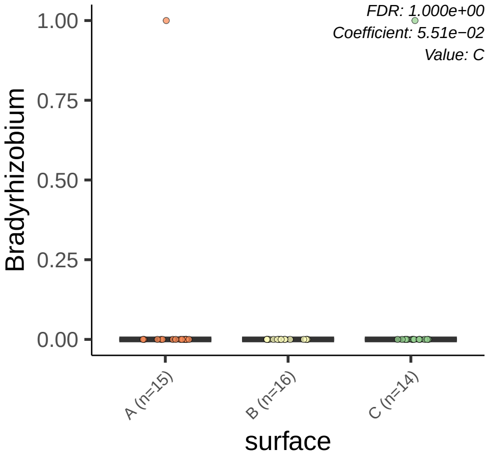

FDR: 1.000e+00
Coefficient: 5.51e−02
Value: C
Bradyrhizobium
1.00
0.75
0.50
0.25
0.00
A (n=15)
B (n=16)
C (n=14)
surface

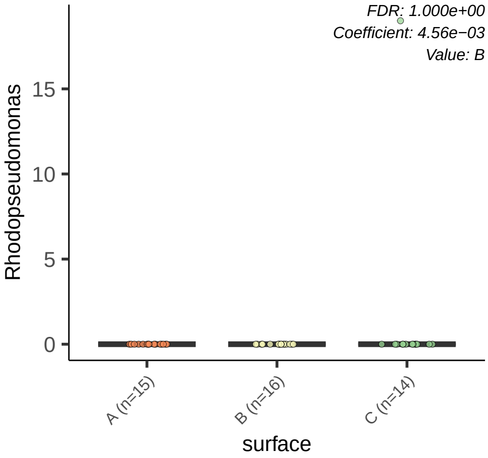

FDR: 1.000e+00
Coefficient: 4.56e−03
Value: B
Rhodopseudomonas
15
10
5
0
A (n=15)
B (n=16)
C (n=14)
surface

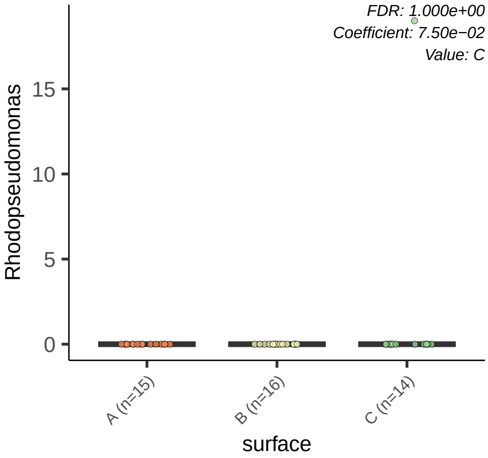

FDR: 1.000e+00
Coefficient: 7.50e−02
Value: C
Rhodopseudomonas
15
10
5
0
A (n=15)
B (n=16)
C (n=14)
surface

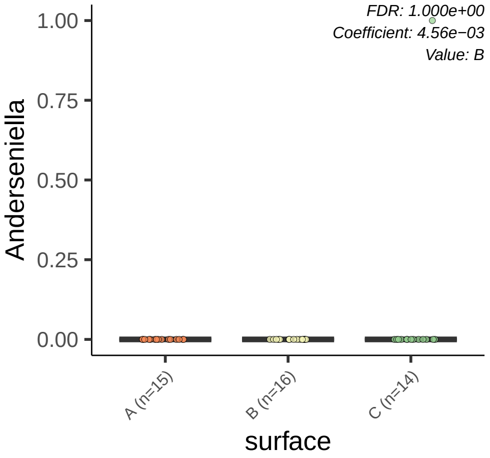

FDR: 1.000e+00
Coefficient: 4.56e−03
Value: B
Anderseniella
1.00
0.75
0.50
0.25
0.00
A (n=15)
B (n=16)
C (n=14)
surface

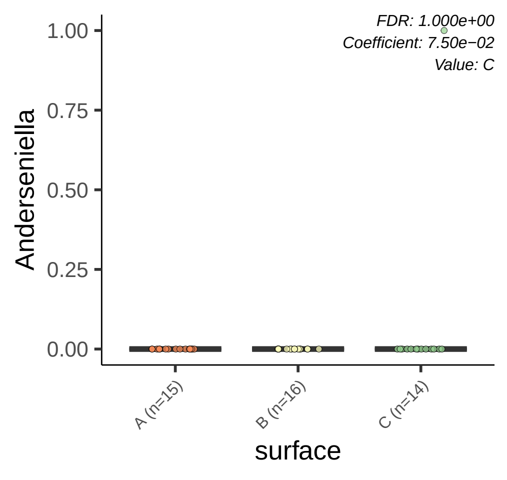

FDR: 1.000e+00
Coefficient: 7.50e−02
Value: C
1.00
0.75
0.50
0.25
0.00
Anderseniella
A (n=15)
B (n=16)
C (n=14)
surface

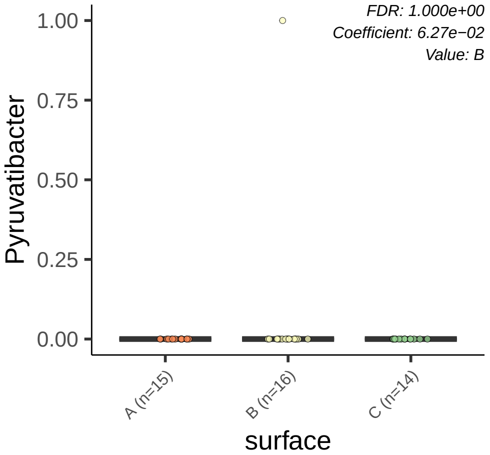
FDR: 1.000e+00
Coefficient: 6.27e−02
Value: B
Pyruvatibacter
1.00
0.75
0.50
0.25
0.00
A (n=15)
B (n=16)
C (n=14)
surface

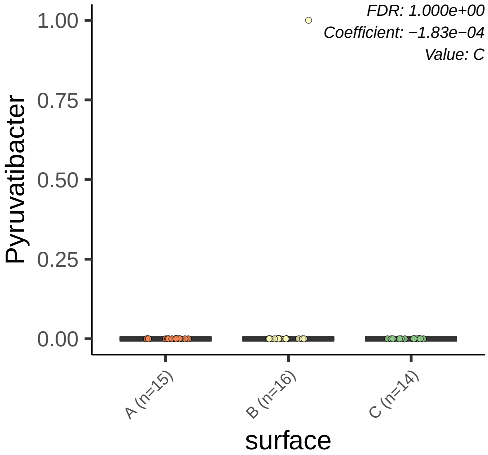

FDR: 1.000e+00
Coefficient: −1.83e−04
Value: C
Pyruvatibacter
1.00
0.75
0.50
0.25
0.00
A (n=15)
B (n=16)
C (n=14)
surface

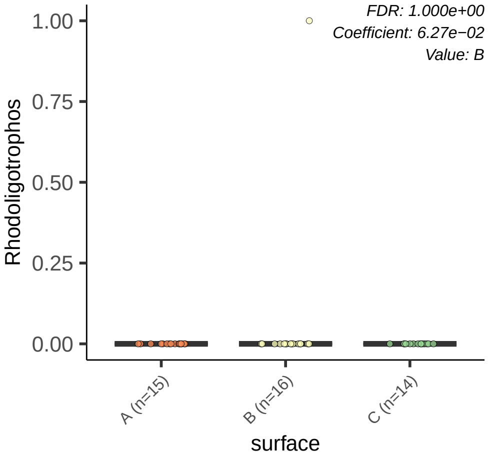
FDR: 1.000e+00
Coefficient: 6.27e−02
Value: B
Rhodoligotrophos
1.00
0.75
0.50
0.25
0.00
A (n=15)
B (n=16)
C (n=14)
surface

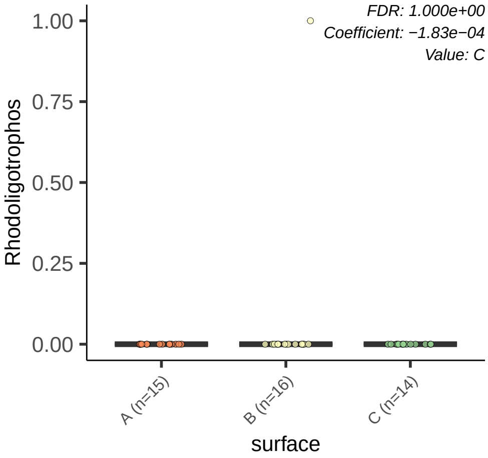

FDR: 1.000e+00
Coefficient: −1.83e−04
Value: C
Rhodoligotrophos
1.00
0.75
0.50
0.25
0.00
A (n=15)
B (n=16)
C (n=14)
surface

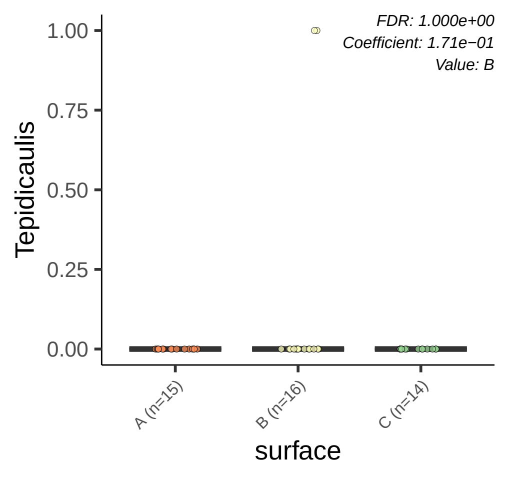

FDR: 1.000e+00
Coefficient: 1.71e−01
Value: B
1.00
0.75
0.50
0.25
0.00
Tepidicaulis
A (n=15)
B (n=16)
C (n=14)
surface

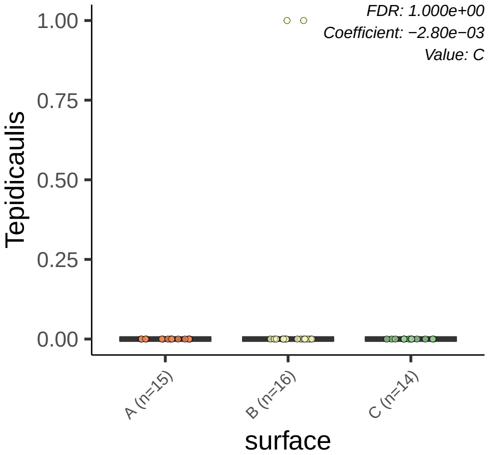
FDR: 1.000e+00
Coefficient: −2.80e−03
Value: C
Tepidicaulis
1.00
0.75
0.50
0.25
0.00
A (n=15)
B (n=16)
C (n=14)
surface

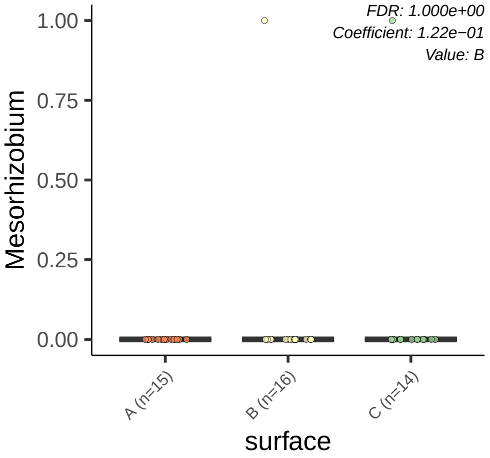
FDR: 1.000e+00
Coefficient: 1.22e−01
Value: B
Mesorhizobium
1.00
0.75
0.50
0.25
0.00
A (n=15)
B (n=16)
C (n=14)
surface

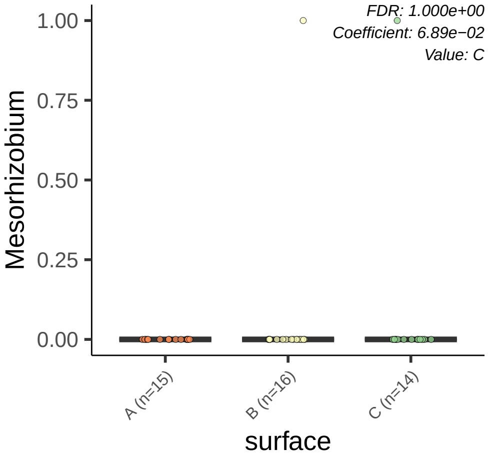
FDR: 1.000e+00
Coefficient: 6.89e−02
Value: C
Mesorhizobium
1.00
0.75
0.50
0.25
0.00
A (n=15)
B (n=16)
C (n=14)
surface

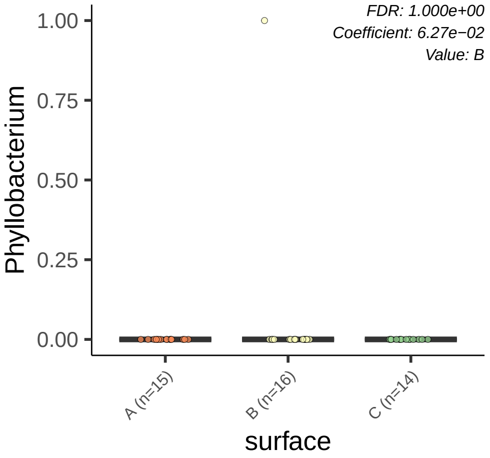

FDR: 1.000e+00
Coefficient: 6.27e−02
Value: B
Phyllobacterium
1.00
0.75
0.50
0.25
0.00
A (n=15)
B (n=16)
C (n=14)
surface

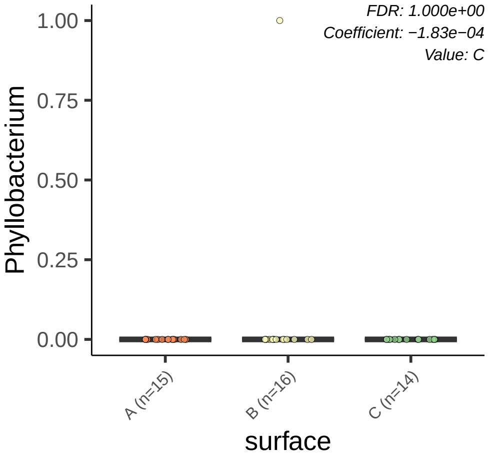
FDR: 1.000e+00
Coefficient: −1.83e−04
Value: C
Phyllobacterium
1.00
0.75
0.50
0.25
0.00
A (n=15)
B (n=16)
C (n=14)
surface

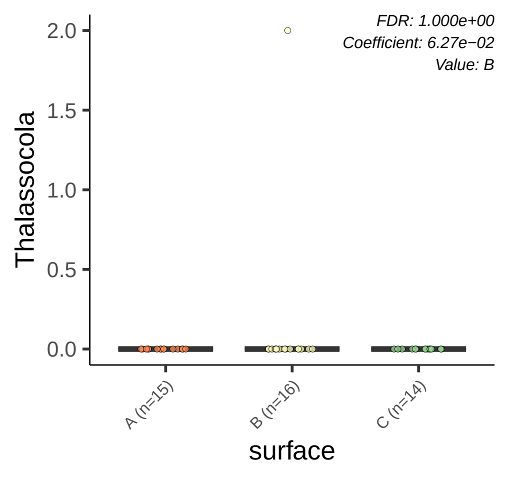

FDR: 1.000e+00
Coefficient: 6.27e−02
Value: B
Thalassocola
2.0
1.5
1.0
0.5
0.0
A (n=15)
B (n=16)
C (n=14)
surface

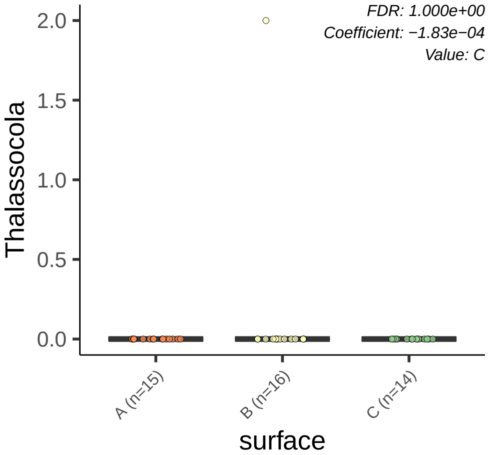

FDR: 1.000e+00
Coefficient: −1.83e−04
Value: C
Thalassocola
2.0
1.5
1.0
0.5
0.0
A (n=15)
B (n=16)
C (n=14)
surface

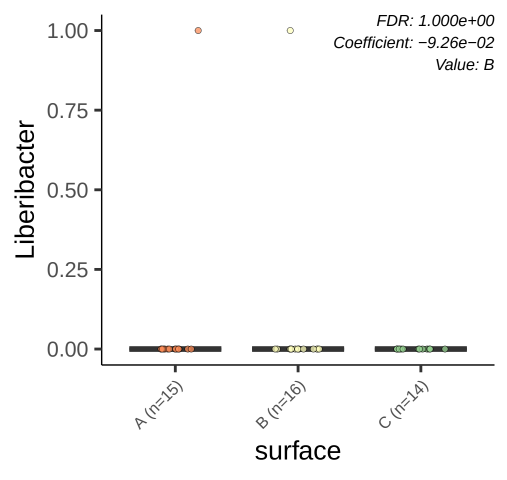
FDR: 1.000e+00
Coefficient: −9.26e−02
Value: B
Liberibacter
1.00
0.75
0.50
0.25
0.00
A (n=15)
B (n=16)
C (n=14)
surface

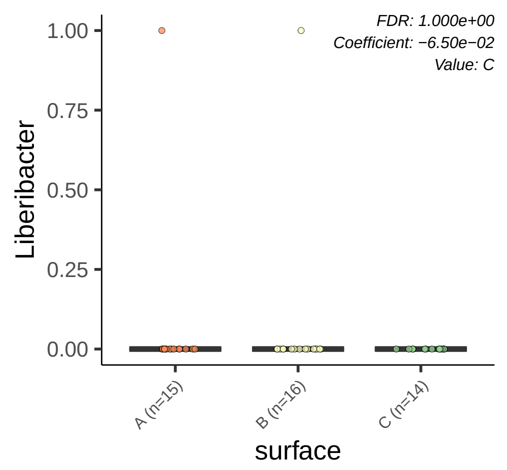

FDR: 1.000e+00
Coefficient: −6.50e−02
Value: C
1.00
0.75
0.50
0.25
0.00
Liberibacter
A (n=15)
B (n=16)
C (n=14)
surface

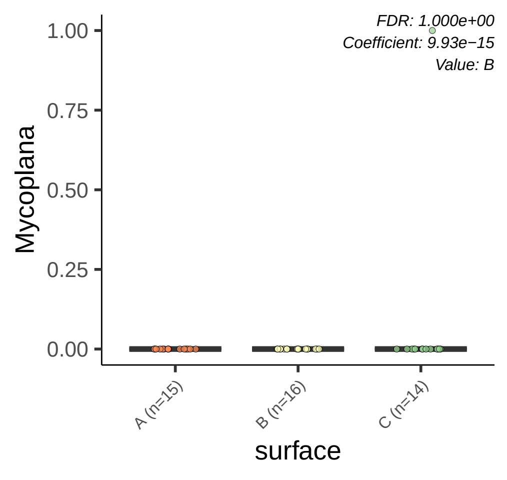
FDR: 1.000e+00
Coefficient: 9.93e−15
Value: B
Mycoplana
1.00
0.75
0.50
0.25
0.00
A (n=15)
B (n=16)
C (n=14)
surface

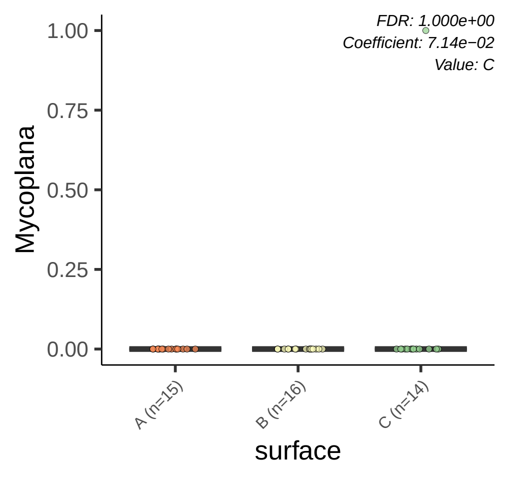
FDR: 1.000e+00
Coefficient: 7.14e−02
Value: C
Mycoplana
1.00
0.75
0.50
0.25
0.00
A (n=15)
B (n=16)
C (n=14)
surface

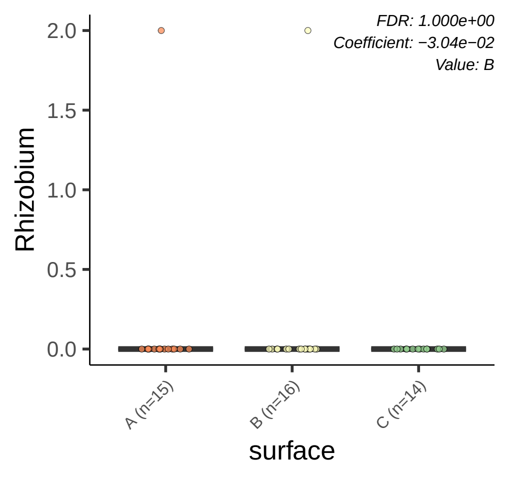

FDR: 1.000e+00
Coefficient: −3.04e−02
Value: B
Rhizobium
2.0
1.5
1.0
0.5
0.0
A (n=15)
B (n=16)
C (n=14)
surface

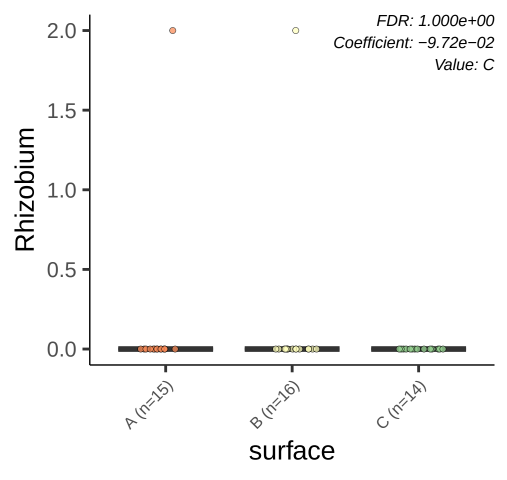

FDR: 1.000e+00
Coefficient: −9.72e−02
Value: C
Rhizobium
2.0
1.5
1.0
0.5
0.0
A (n=15)
B (n=16)
C (n=14)
surface

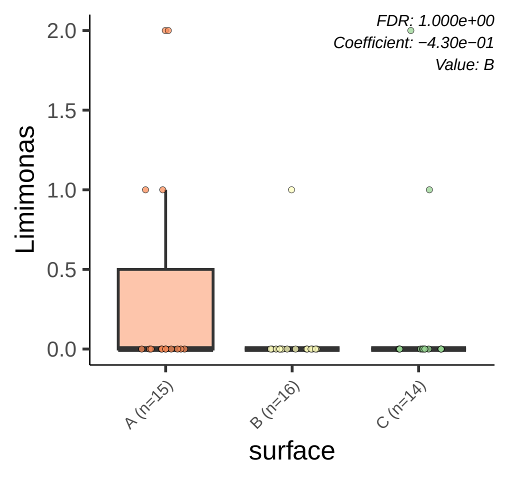

FDR: 1.000e+00
Coefficient: −4.30e−01
Value: B
Limimonas
2.0
1.5
1.0
0.5
0.0
A (n=15)
B (n=16)
C (n=14)
surface

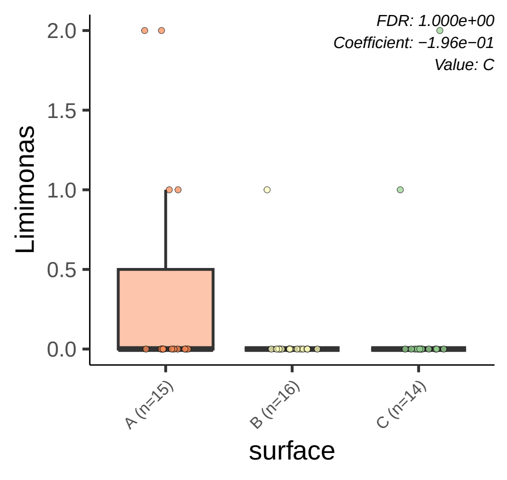
FDR: 1.000e+00
Coefficient: −1.96e−01
Value: C
Limimonas
2.0
1.5
1.0
0.5
0.0
A (n=15)
B (n=16)
C (n=14)
surface

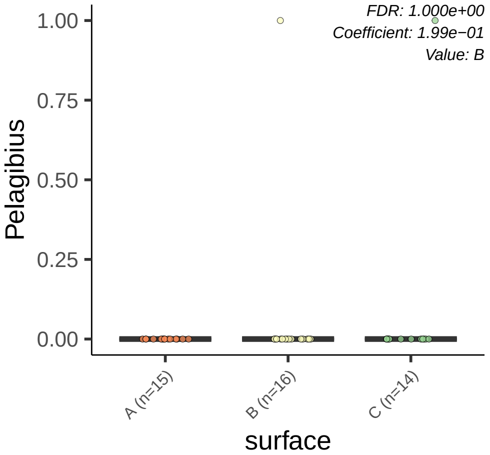

FDR: 1.000e+00
Coefficient: 1.99e−01
Value: B
Pelagibius
1.00
0.75
0.50
0.25
0.00
A (n=15)
B (n=16)
C (n=14)
surface

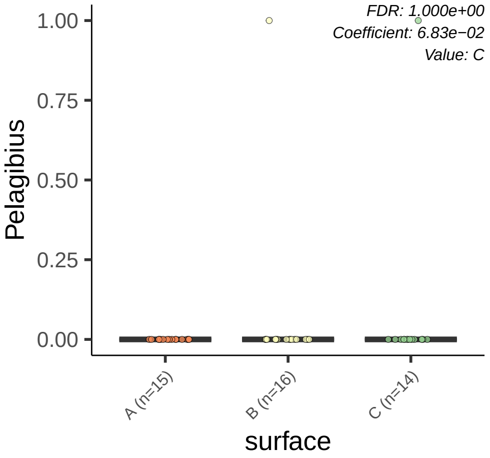
FDR: 1.000e+00
Coefficient: 6.83e−02
Value: C
Pelagibius
1.00
0.75
0.50
0.25
0.00
A (n=15)
B (n=16)
C (n=14)
surface

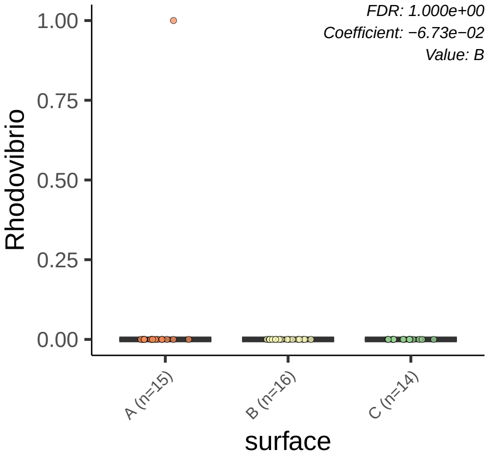

FDR: 1.000e+00
Coefficient: −6.73e−02
Value: B
Rhodovibrio
1.00
0.75
0.50
0.25
0.00
A (n=15)
B (n=16)
C (n=14)
surface

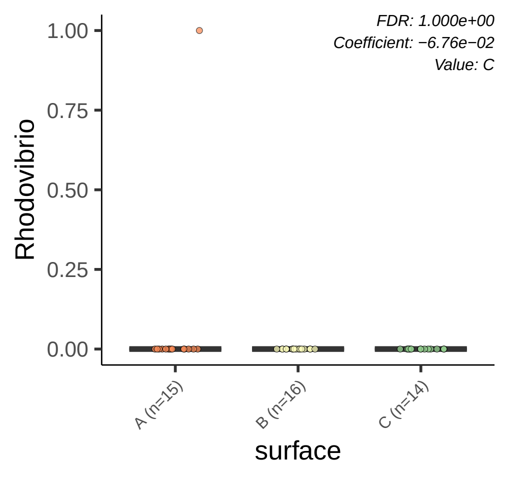

FDR: 1.000e+00
Coefficient: −6.76e−02
Value: C
Rhodovibrio
1.00
0.75
0.50
0.25
0.00
A (n=15)
B (n=16)
C (n=14)
surface

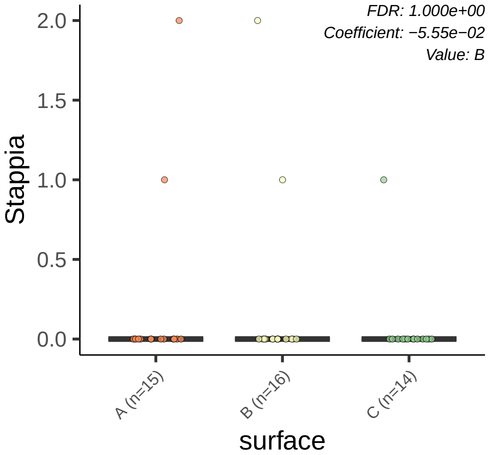
FDR: 1.000e+00
Coefficient: −5.55e−02
Value: B
Stappia
2.0
1.5
1.0
0.5
0.0
A (n=15)
B (n=16)
C (n=14)
surface

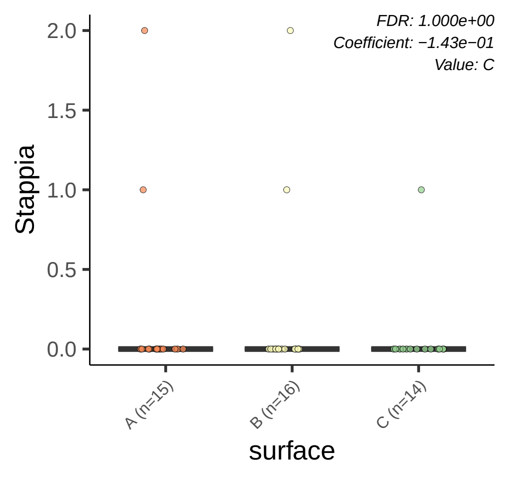
FDR: 1.000e+00
Coefficient: −1.43e−01
Value: C
Stappia
2.0
1.5
1.0
0.5
0.0
A (n=15)
B (n=16)
C (n=14)
surface

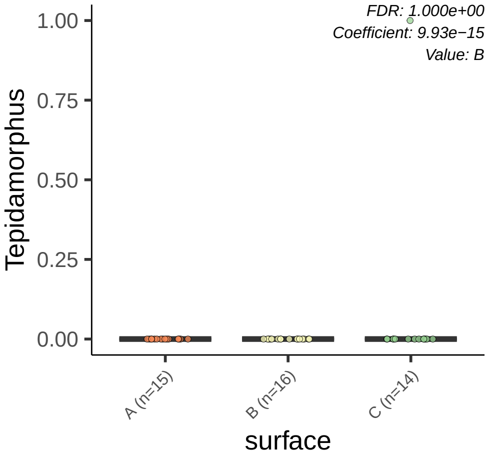

FDR: 1.000e+00
Coefficient: 9.93e−15
Value: B
Tepidamorphus
1.00
0.75
0.50
0.25
0.00
A (n=15)
B (n=16)
C (n=14)
surface

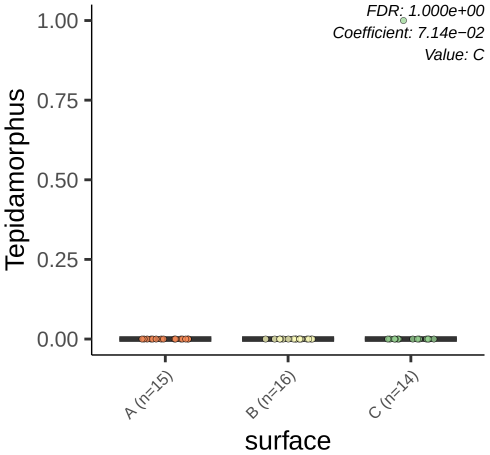

FDR: 1.000e+00
Coefficient: 7.14e−02
Value: C
Tepidamorphus
1.00
0.75
0.50
0.25
0.00
A (n=15)
B (n=16)
C (n=14)
surface

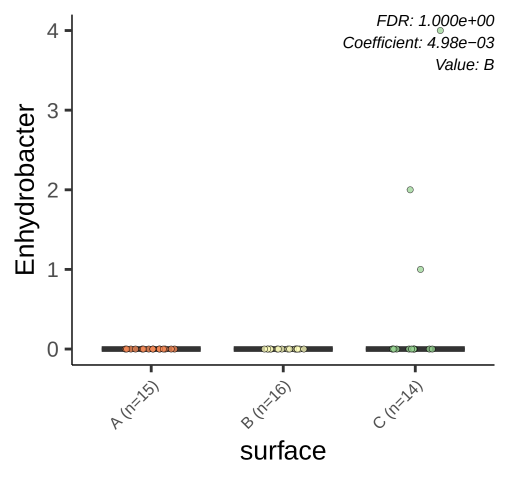

FDR: 1.000e+00
Coefficient: 4.98e−03
Value: B
Enhydrobacter
4
3
2
1
0
A (n=15)
B (n=16)
C (n=14)
surface

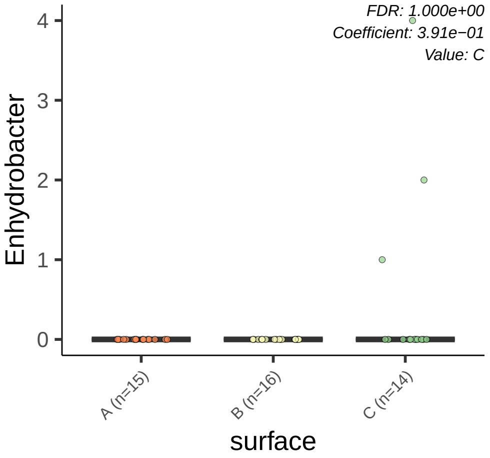
FDR: 1.000e+00
Coefficient: 3.91e−01
Value: C
Enhydrobacter
0
1
2
3
4
A (n=15)
B (n=16)
C (n=14)
surface

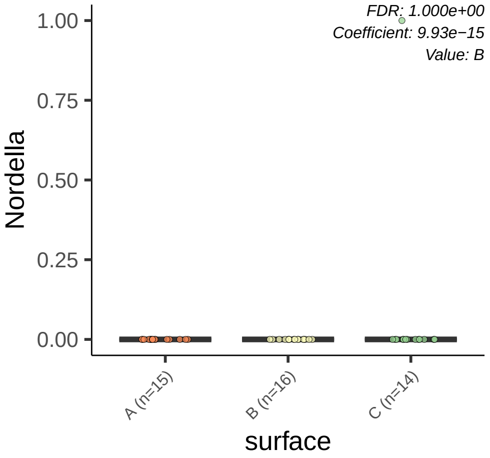
FDR: 1.000e+00
Coefficient: 9.93e−15
Value: B
Nordella
1.00
0.75
0.50
0.25
0.00
A (n=15)
B (n=16)
C (n=14)
surface

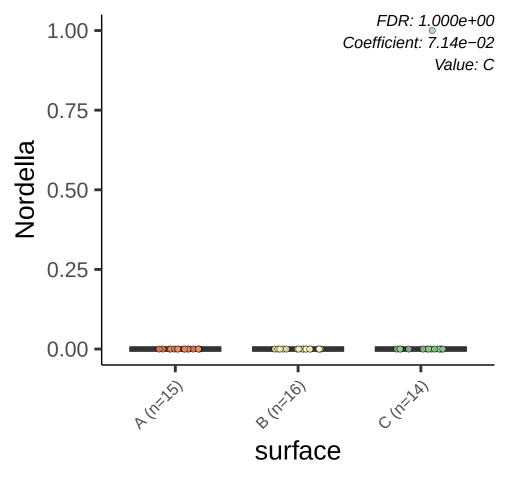

FDR: 1.000e+00
Coefficient: 7.14e−02
Value: C
1.00
0.75
0.50
0.25
0.00
Nordella
A (n=15)
B (n=16)
C (n=14)
surface

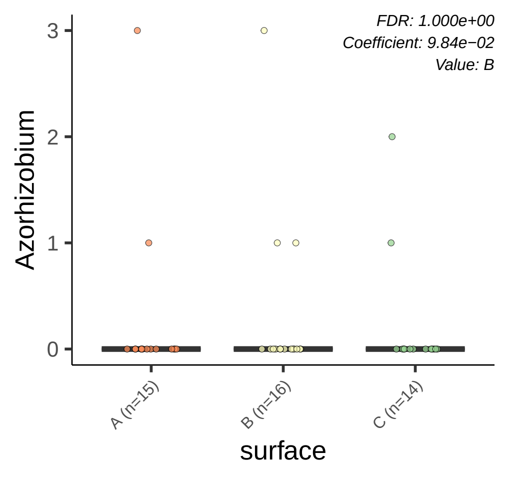

FDR: 1.000e+00
Coefficient: 9.84e−02
Value: B
Azorhizobium
3
2
1
0
A (n=15)
B (n=16)
C (n=14)
surface

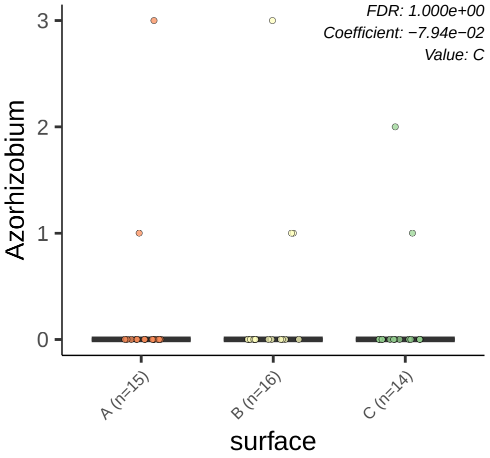
FDR: 1.000e+00
Coefficient: −7.94e−02
Value: C
Azorhizobium
3
2
1
0
A (n=15)
B (n=16)
C (n=14)
surface

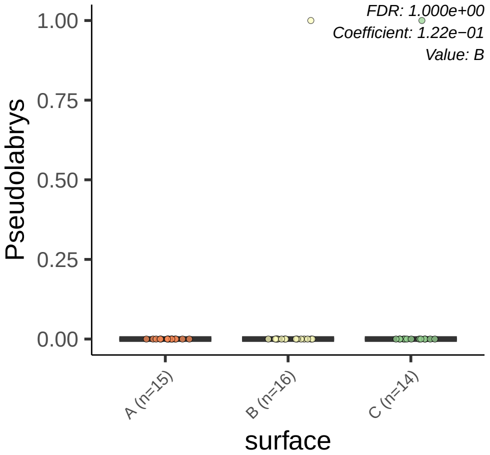

FDR: 1.000e+00
Coefficient: 1.22e−01
Value: B
Pseudolabrys
1.00
0.75
0.50
0.25
0.00
A (n=15)
B (n=16)
C (n=14)
surface

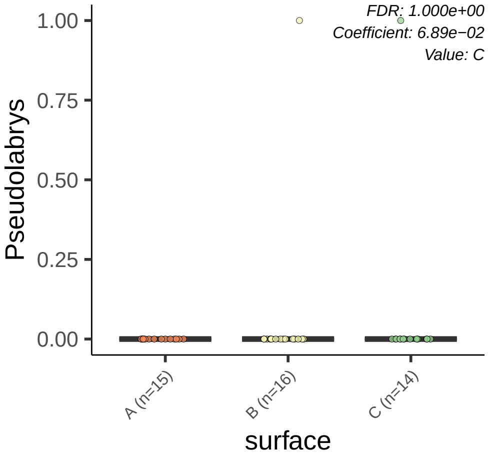

FDR: 1.000e+00
Coefficient: 6.89e−02
Value: C
Pseudolabrys
1.00
0.75
0.50
0.25
0.00
A (n=15)
B (n=16)
C (n=14)
surface

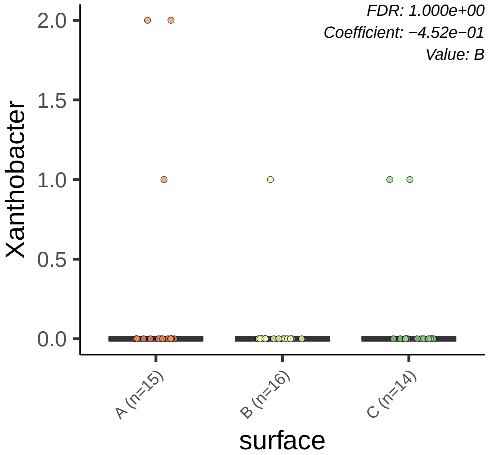
FDR: 1.000e+00
Coefficient: −4.52e−01
Value: B
Xanthobacter
2.0
1.5
1.0
0.5
0.0
A (n=15)
B (n=16)
C (n=14)
surface

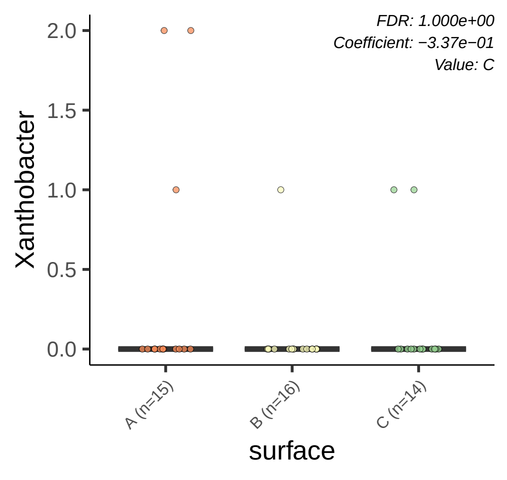

FDR: 1.000e+00
Coefficient: −3.37e−01
Value: C
Xanthobacter
2.0
1.5
1.0
0.5
0.0
A (n=15)
B (n=16)
C (n=14)
surface

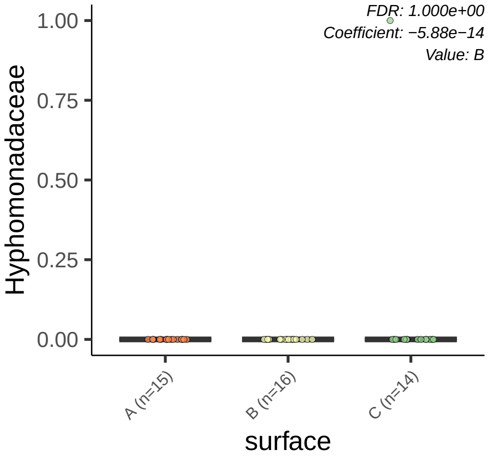

FDR: 1.000e+00
Coefficient: −5.88e−14
Value: B
1.00
0.75
0.50
0.25
0.00
Hyphomonadaceae
A (n=15)
B (n=16)
C (n=14)
surface

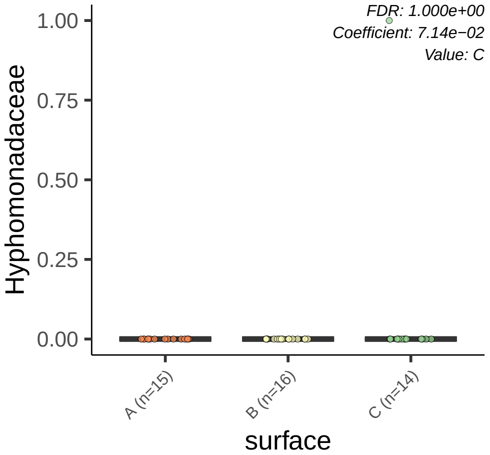
FDR: 1.000e+00
Coefficient: 7.14e−02
Value: C
Hyphomonadaceae
1.00
0.75
0.50
0.25
0.00
A (n=15)
B (n=16)
C (n=14)
surface

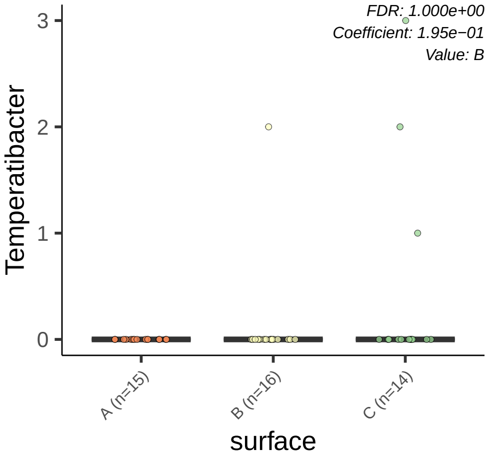FDR: 1.000e+00
Coefficient: 1.95e−01
Value: B
Temperatibacter
3
2
1
0
A (n=15)
B (n=16)
C (n=14)
surface

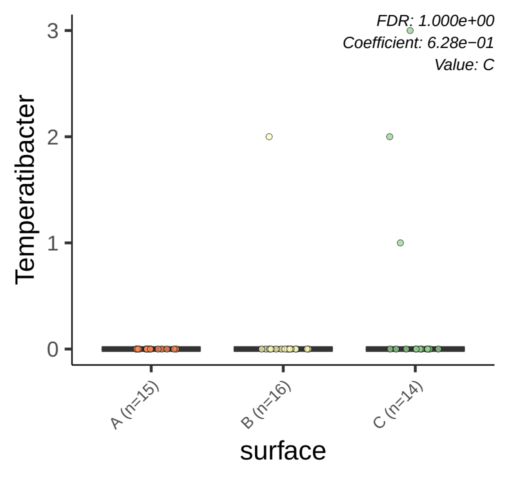
FDR: 1.000e+00
Coefficient: 6.28e−01
Value: C
Temperatibacter
3
2
1
0
A (n=15)
B (n=16)
C (n=14)
surface

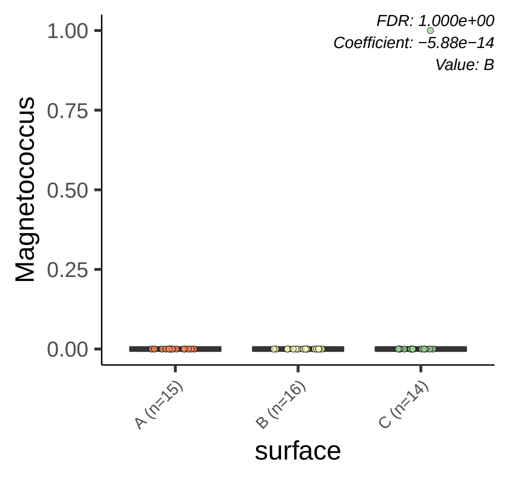

FDR: 1.000e+00
Coefficient: −5.88e−14
Value: B
Magnetococcus
1.00
0.75
0.50
0.25
0.00
A (n=15)
B (n=16)
C (n=14)
surface

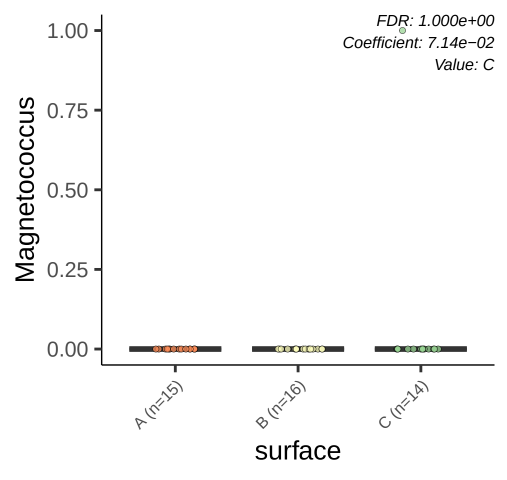
FDR: 1.000e+00
Coefficient: 7.14e−02
Value: C
Magnetococcus
1.00
0.75
0.50
0.25
0.00
A (n=15)
B (n=16)
C (n=14)
surface

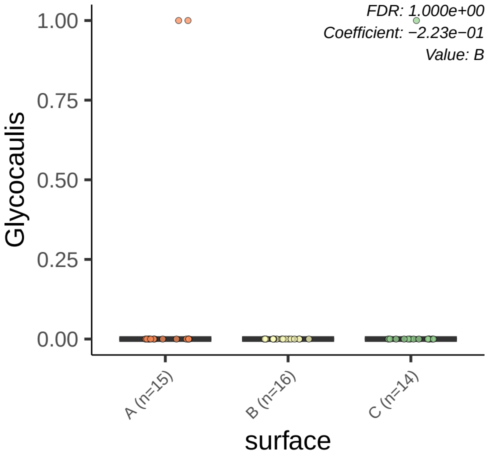
FDR: 1.000e+00
Coefficient: −2.23e−01
Value: B
Glycocaulis
1.00
0.75
0.50
0.25
0.00
A (n=15)
B (n=16)
C (n=14)
surface

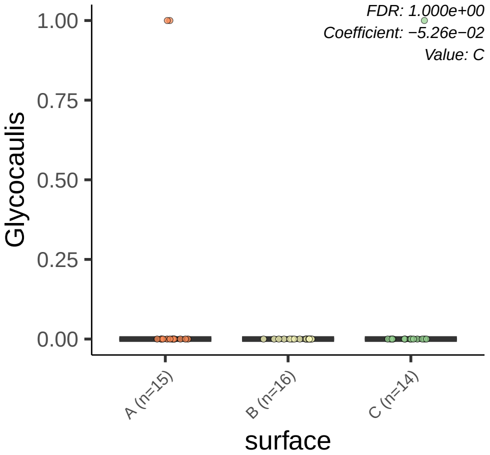

FDR: 1.000e+00
Coefficient: −5.26e−02
Value: C
Glycocaulis
1.00
0.75
0.50
0.25
0.00
A (n=15)
B (n=16)
C (n=14)
surface

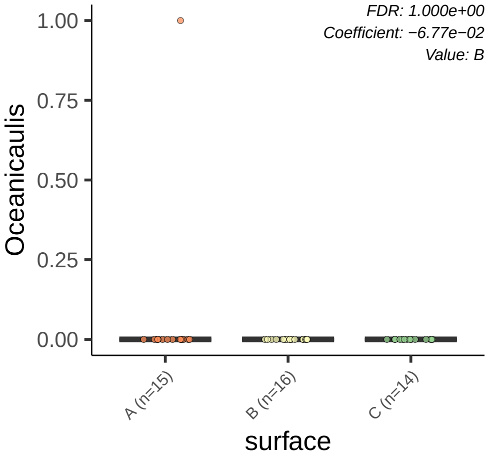

FDR: 1.000e+00
Coefficient: −6.77e−02
Value: B
Oceanicaulis
1.00
0.75
0.50
0.25
0.00
A (n=15)
B (n=16)
C (n=14)
surface

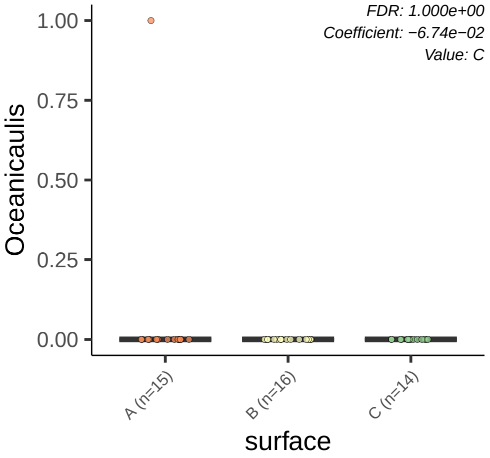

FDR: 1.000e+00
Coefficient: −6.74e−02
Value: C
Oceanicaulis
1.00
0.75
0.50
0.25
0.00
A (n=15)
B (n=16)
C (n=14)
surface

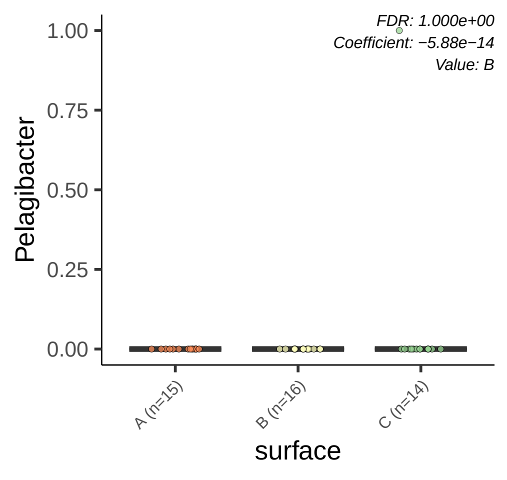
FDR: 1.000e+00
Coefficient: −5.88e−14
Value: B
Pelagibacter
1.00
0.75
0.50
0.25
0.00
A (n=15)
B (n=16)
C (n=14)
surface

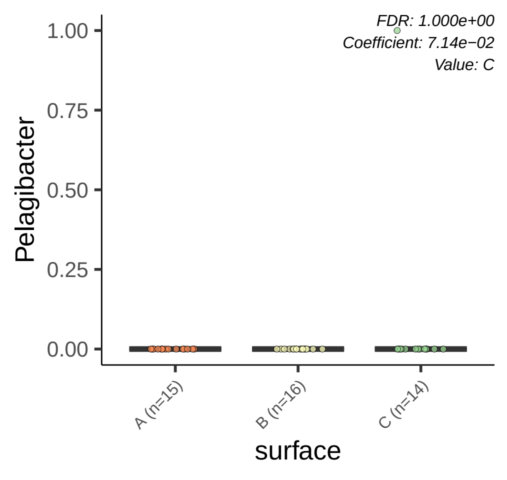
FDR: 1.000e+00
Coefficient: 7.14e−02
Value: C
1.00
0.75
0.50
0.25
0.00
Pelagibacter
A (n=15)
B (n=16)
C (n=14)
surface

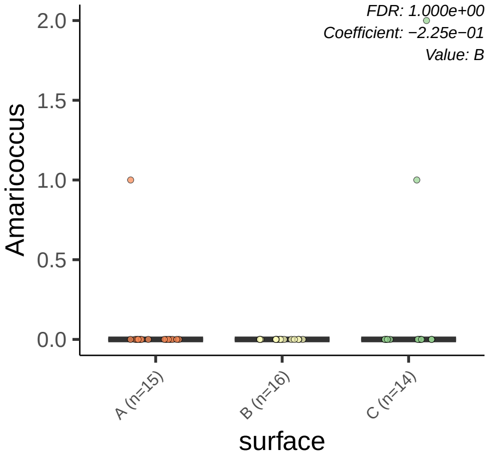
FDR: 1.000e+00
Coefficient: −2.25e−01
Value: B
Amaricoccus
2.0
1.5
1.0
0.5
0.0
A (n=15)
B (n=16)
C (n=14)
surface

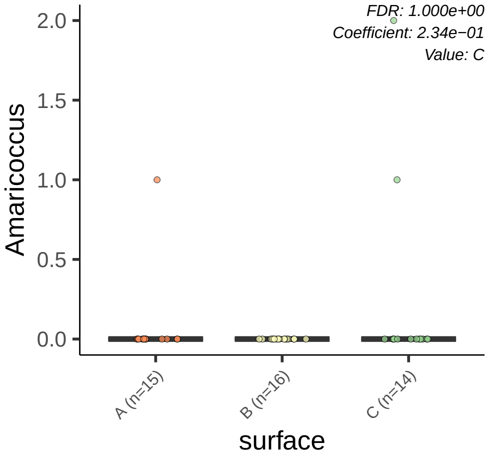
FDR: 1.000e+00
Coefficient: 2.34e−01
Value: C
Amaricoccus
2.0
1.5
1.0
0.5
0.0
A (n=15)
B (n=16)
C (n=14)
surface

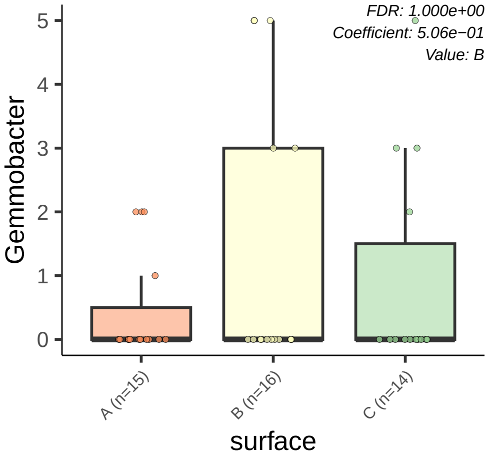
FDR: 1.000e+00
Coefficient: 5.06e−01
Value: B
Gemmobacter
0
1
2
3
4
5
A (n=15)
B (n=16)
C (n=14)
surface

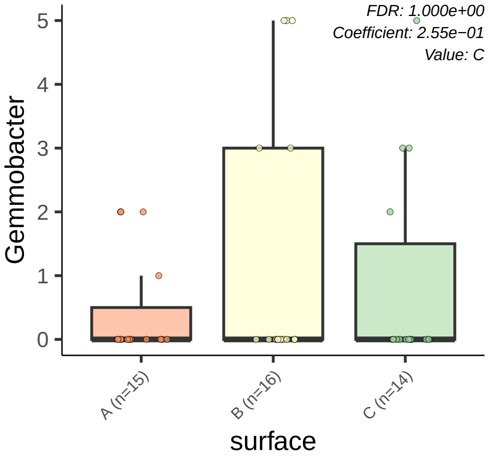

FDR: 1.000e+00
Coefficient: 2.55e−01
Value: C
Gemmobacter
0
1
2
3
4
5
A (n=15)
B (n=16)
C (n=14)
surface

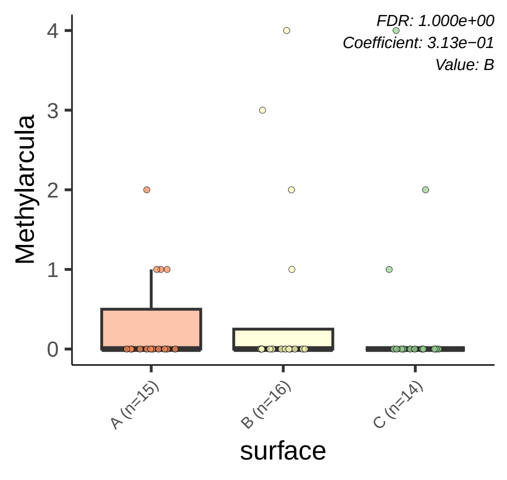
FDR: 1.000e+00
Coefficient: 3.13e−01
Value: B
Methylarcula
0
1
2
3
4
A (n=15)
B (n=16)
C (n=14)
surface

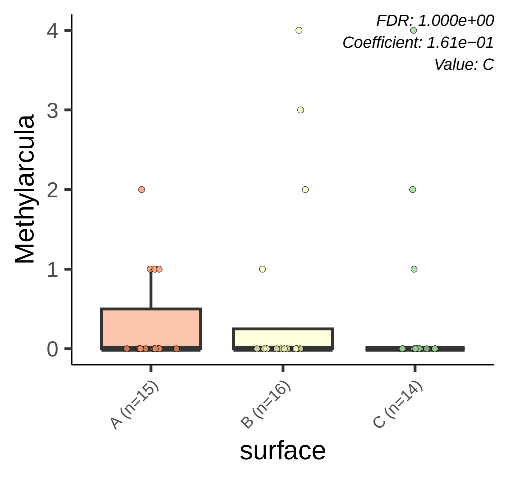

FDR: 1.000e+00
Coefficient: 1.61e−01
Value: C
Methylarcula
4
3
2
1
0
A (n=15)
B (n=16)
C (n=14)
surface

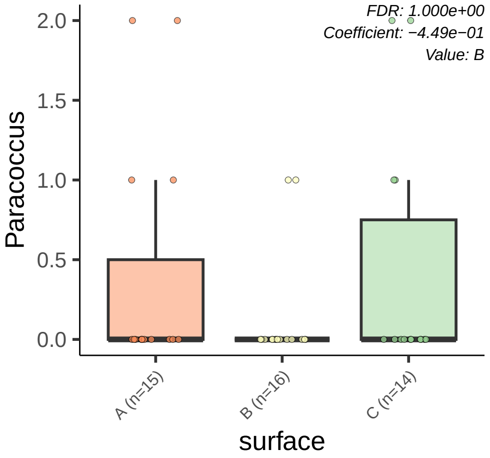

FDR: 1.000e+00
Coefficient: −4.49e−01
Value: B
Paracoccus
2.0
1.5
1.0
0.5
0.0
A (n=15)
B (n=16)
C (n=14)
surface

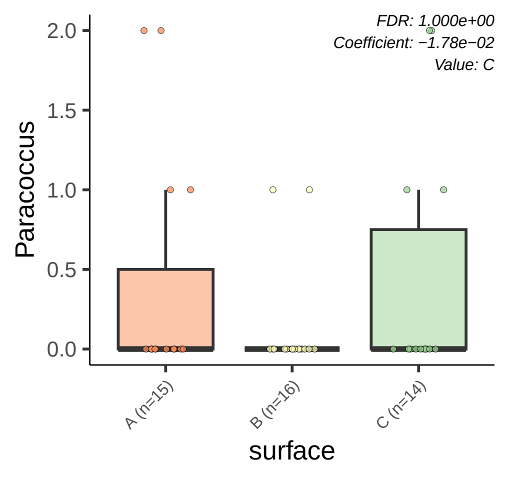
FDR: 1.000e+00
Coefficient: −1.78e−02
Value: C
Paracoccus
2.0
1.5
1.0
0.5
0.0
A (n=15)
B (n=16)
C (n=14)
surface

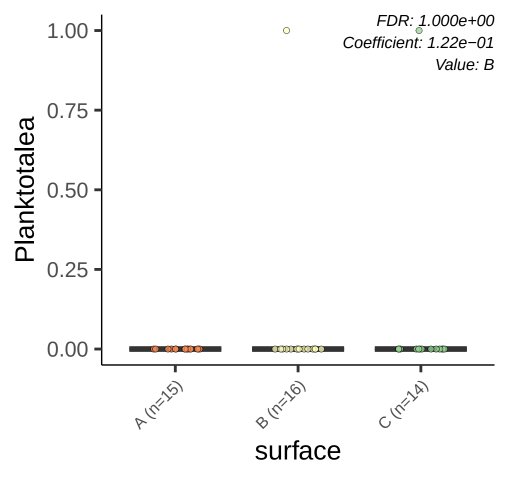
FDR: 1.000e+00
Coefficient: 1.22e−01
Value: B
Planktotalea
1.00
0.75
0.50
0.25
0.00
A (n=15)
B (n=16)
C (n=14)
surface

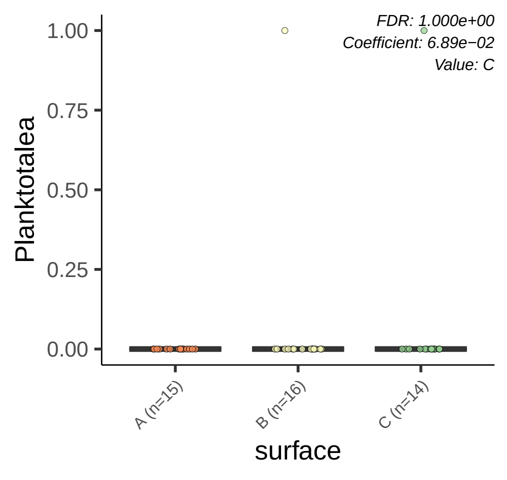
FDR: 1.000e+00
Coefficient: 6.89e−02
Value: C
Planktotalea
1.00
0.75
0.50
0.25
0.00
A (n=15)
B (n=16)
C (n=14)
surface

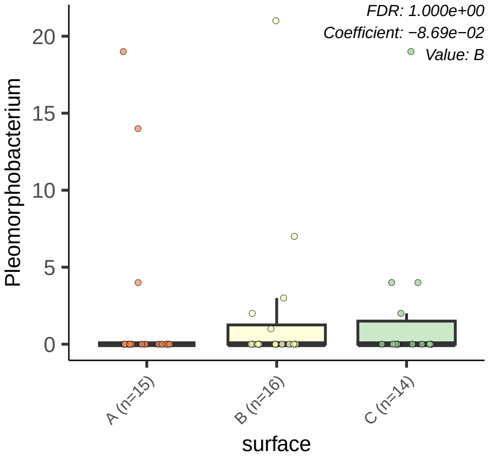

FDR: 1.000e+00
Coefficient: −8.69e−02
Value: B
Pleomorphobacterium
20
15
10
5
0
A (n=15)
B (n=16)
C (n=14)
surface

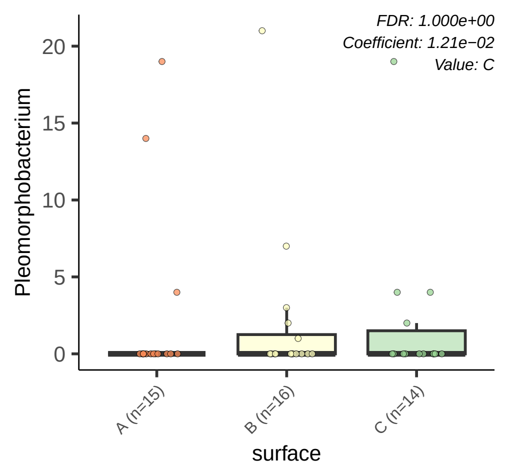

FDR: 1.000e+00
Coefficient: 1.21e−02
Value: C
Pleomorphobacterium
0
5
10
15
20
A (n=15)
B (n=16)
C (n=14)
surface

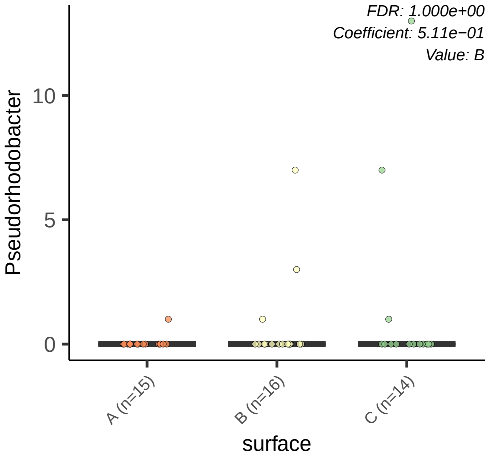
FDR: 1.000e+00
Coefficient: 5.11e−01
Value: B
Pseudorhodobacter
10
5
0
A (n=15)
B (n=16)
C (n=14)
surface

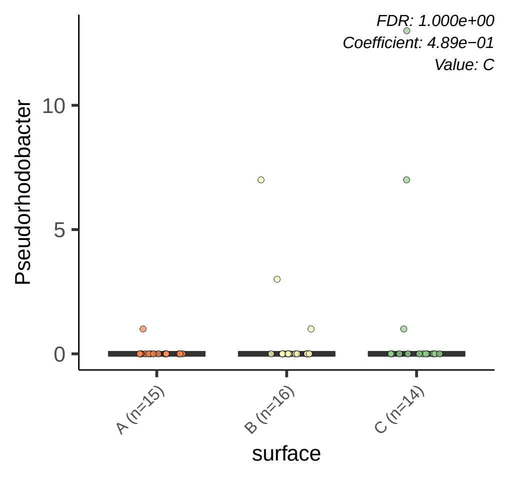

FDR: 1.000e+00
Coefficient: 4.89e−01
Value: C
Pseudorhodobacter
10
5
0
A (n=15)
B (n=16)
C (n=14)
surface

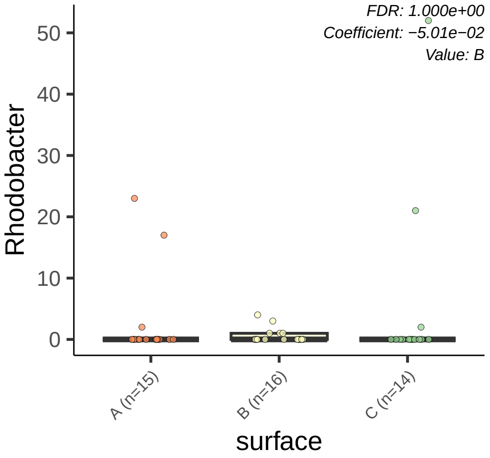
FDR: 1.000e+00
Coefficient: −5.01e−02
Value: B
Rhodobacter
50
40
30
20
10
0
A (n=15)
B (n=16)
C (n=14)
surface

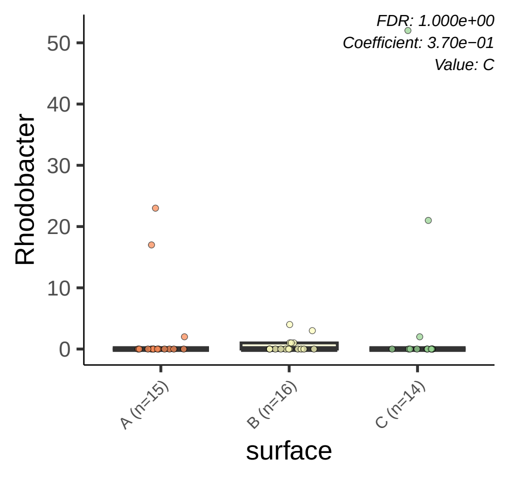

FDR: 1.000e+00
Coefficient: 3.70e−01
Value: C
Rhodobacter
50
40
30
20
10
0
A (n=15)
B (n=16)
C (n=14)
surface

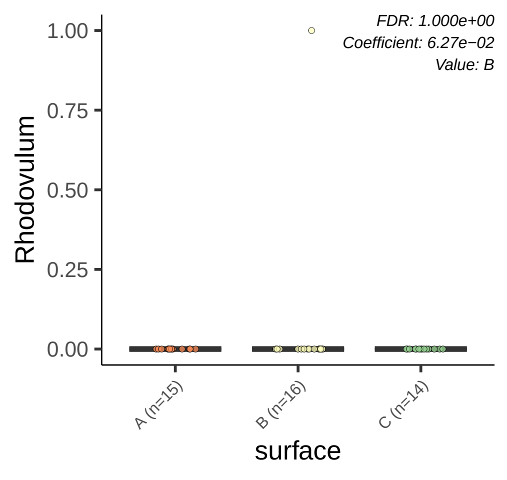
FDR: 1.000e+00
Coefficient: 6.27e−02
Value: B
Rhodovulum
1.00
0.75
0.50
0.25
0.00
A (n=15)
B (n=16)
C (n=14)
surface

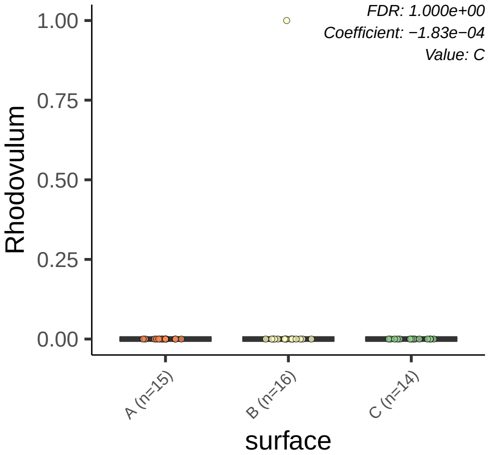
FDR: 1.000e+00
Coefficient: −1.83e−04
Value: C
Rhodovulum
1.00
0.75
0.50
0.25
0.00
A (n=15)
B (n=16)
C (n=14)
surface

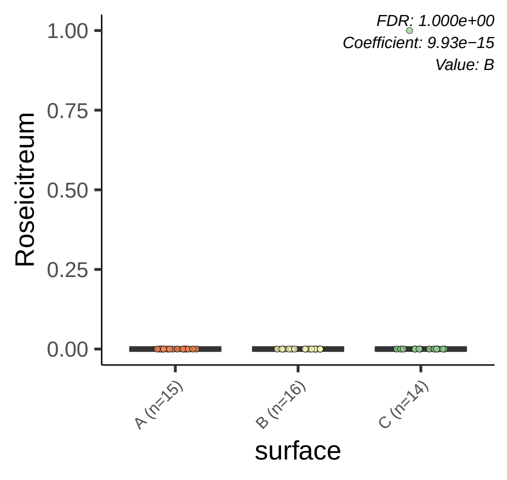
FDR: 1.000e+00
Coefficient: 9.93e−15
Value: B
Roseicitreum
1.00
0.75
0.50
0.25
0.00
A (n=15)
B (n=16)
C (n=14)
surface

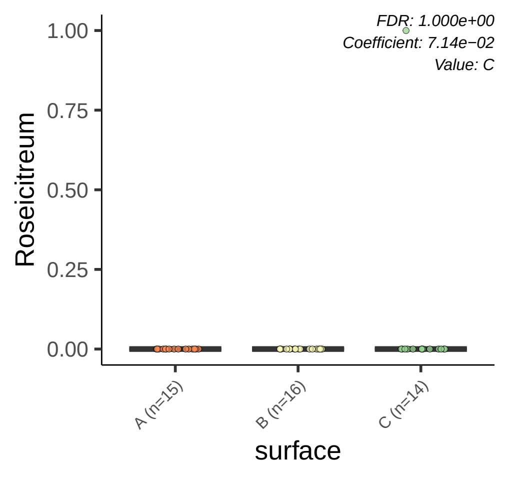
FDR: 1.000e+00
Coefficient: 7.14e−02
Value: C
Roseicitreum
1.00
0.75
0.50
0.25
0.00
A (n=15)
B (n=16)
C (n=14)
surface

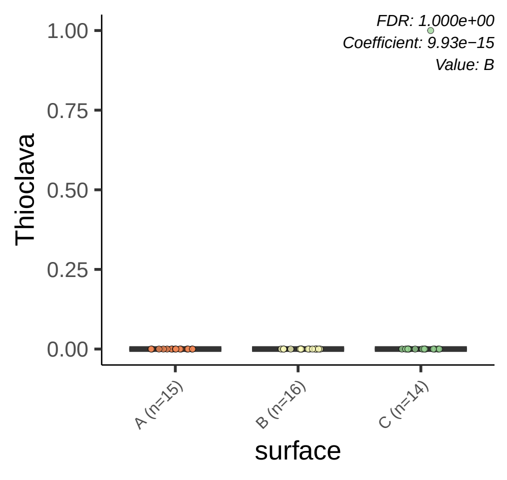
FDR: 1.000e+00
Coefficient: 9.93e−15
Value: B
Thioclava
1.00
0.75
0.50
0.25
0.00
A (n=15)
B (n=16)
C (n=14)
surface

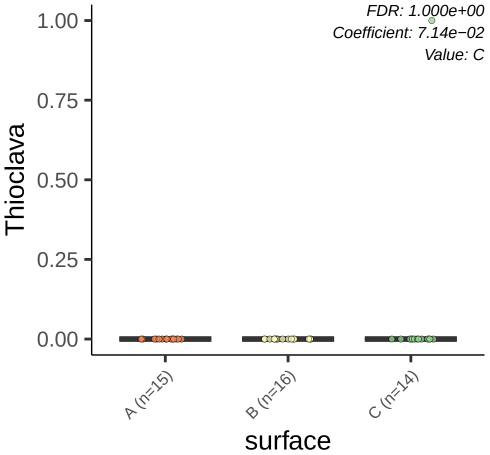
FDR: 1.000e+00
Coefficient: 7.14e−02
Value: C
1.00
0.75
0.50
0.25
0.00
Thioclava
A (n=15)
B (n=16)
C (n=14)
surface

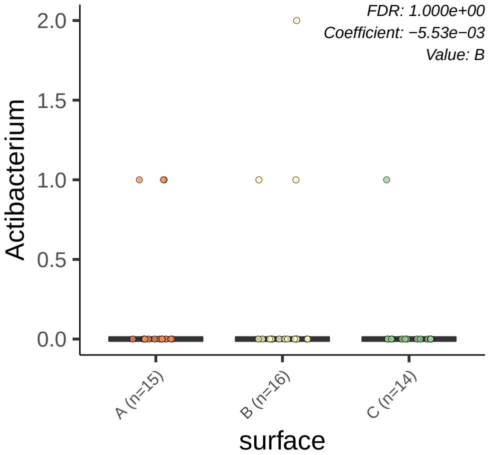
FDR: 1.000e+00
Coefficient: −5.53e−03
Value: B
Actibacterium
2.0
1.5
1.0
0.5
0.0
A (n=15)
B (n=16)
C (n=14)
surface

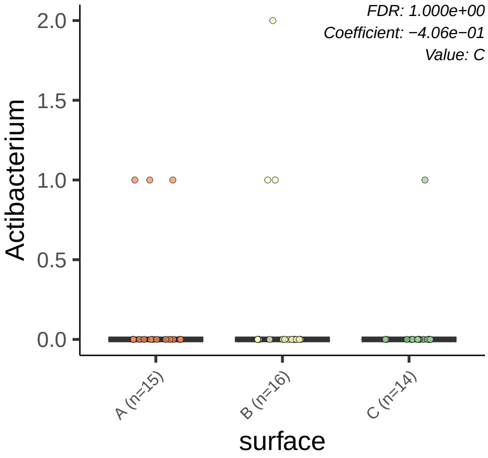
FDR: 1.000e+00
Coefficient: −4.06e−01
Value: C
Actibacterium
2.0
1.5
1.0
0.5
0.0
A (n=15)
B (n=16)
C (n=14)
surface

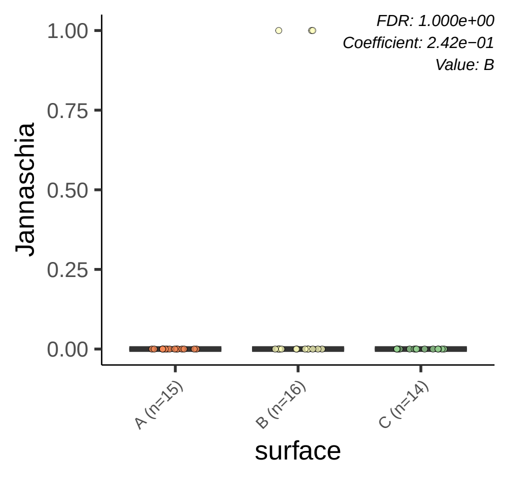
FDR: 1.000e+00
Coefficient: 2.42e−01
Value: B
1.00
0.75
0.50
0.25
0.00
Jannaschia
A (n=15)
B (n=16)
C (n=14)
surface

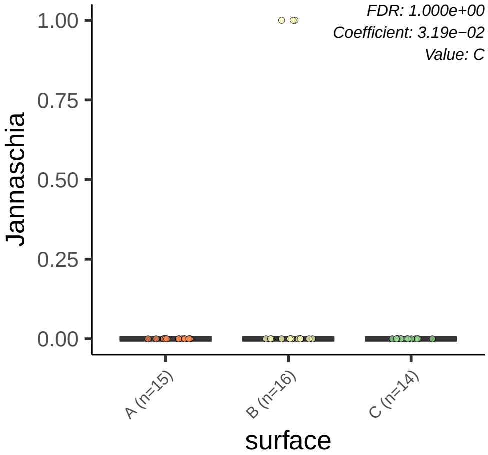

FDR: 1.000e+00
Coefficient: 3.19e−02
Value: C
Jannaschia
1.00
0.75
0.50
0.25
0.00
A (n=15)
B (n=16)
C (n=14)
surface

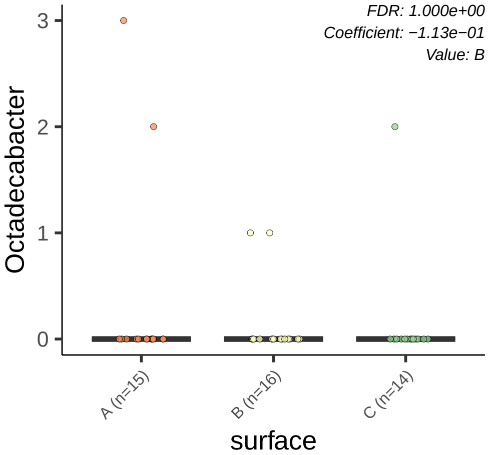

FDR: 1.000e+00
Coefficient: −1.13e−01
Value: B
Octadecabacter
3
2
1
0
A (n=15)
B (n=16)
C (n=14)
surface

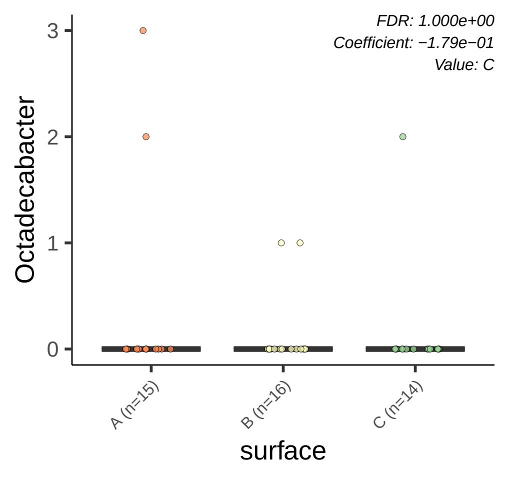
FDR: 1.000e+00
Coefficient: −1.79e−01
Value: C
Octadecabacter
0
1
2
3
A (n=15)
B (n=16)
C (n=14)
surface

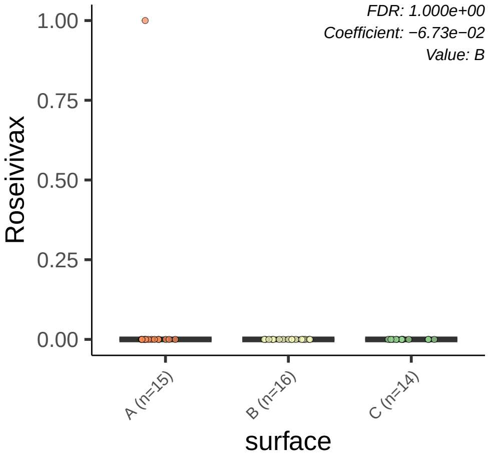
FDR: 1.000e+00
Coefficient: −6.73e−02
Value: B
Roseivivax
1.00
0.75
0.50
0.25
0.00
A (n=15)
B (n=16)
C (n=14)
surface

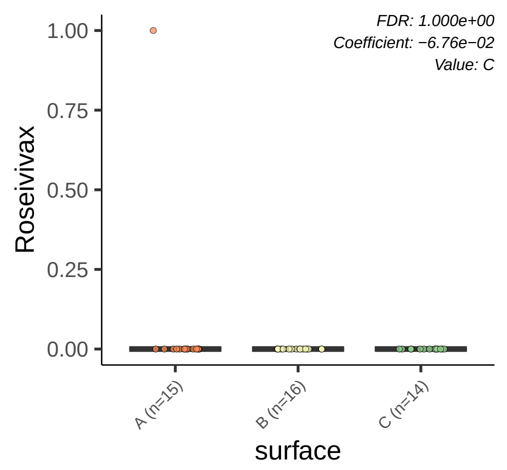
FDR: 1.000e+00
Coefficient: −6.76e−02
Value: C
Roseivivax
1.00
0.75
0.50
0.25
0.00
A (n=15)
B (n=16)
C (n=14)
surface

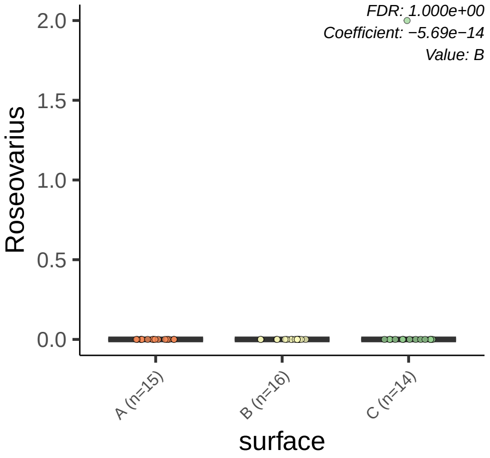
FDR: 1.000e+00
Coefficient: −5.69e−14
Value: B
Roseovarius
2.0
1.5
1.0
0.5
0.0
A (n=15)
B (n=16)
C (n=14)
surface

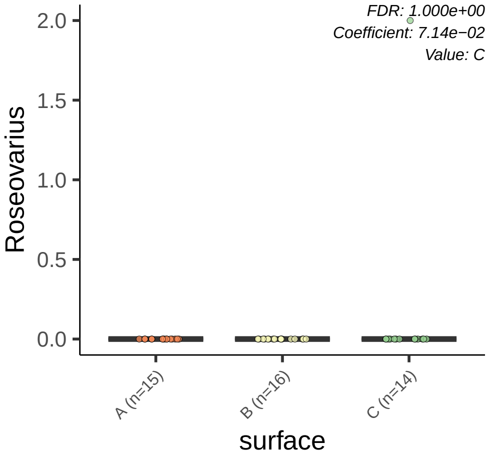

FDR: 1.000e+00
Coefficient: 7.14e−02
Value: C
Roseovarius
2.0
1.5
1.0
0.5
0.0
A (n=15)
B (n=16)
C (n=14)
surface

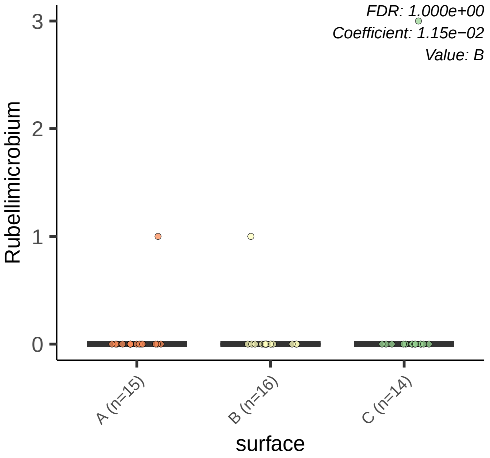

FDR: 1.000e+00
Coefficient: 1.15e−02
Value: B
Rubellimicrobium
0
1
2
3
A (n=15)
B (n=16)
C (n=14)
surface

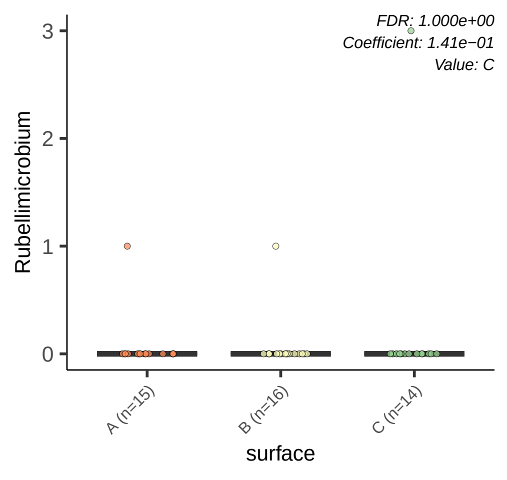

FDR: 1.000e+00
Coefficient: 1.41e−01
Value: C
Rubellimicrobium
3
2
1
0
A (n=15)
B (n=16)
C (n=14)
surface

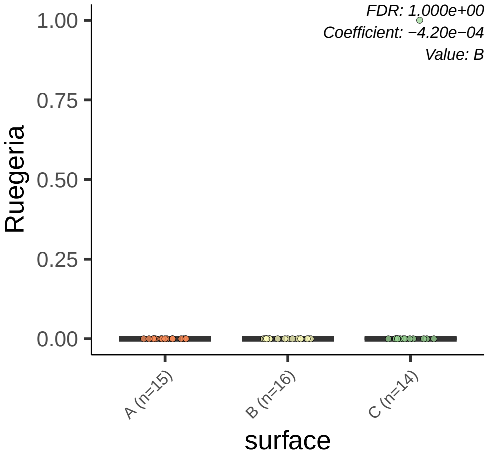

FDR: 1.000e+00
Coefficient: −4.20e−04
Value: B
1.00
0.75
0.50
0.25
0.00
Ruegeria
A (n=15)
B (n=16)
C (n=14)
surface

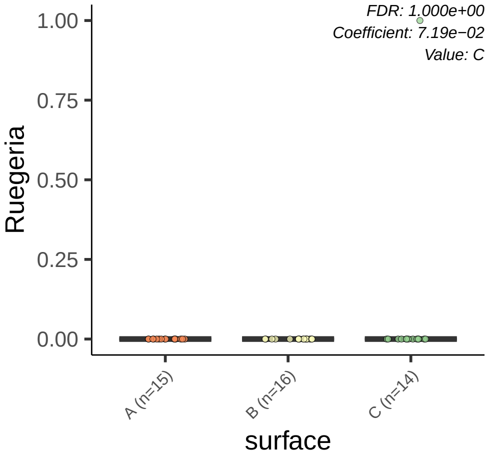
FDR: 1.000e+00
Coefficient: 7.19e−02
Value: C
Ruegeria
1.00
0.75
0.50
0.25
0.00
A (n=15)
B (n=16)
C (n=14)
surface

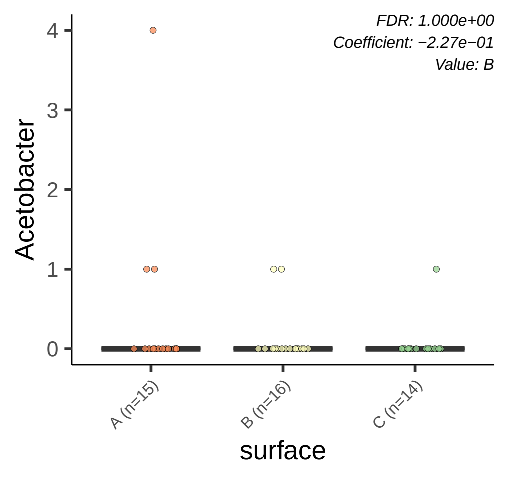

FDR: 1.000e+00
Coefficient: −2.27e−01
Value: B
Acetobacter
4
3
2
1
0
A (n=15)
B (n=16)
C (n=14)
surface

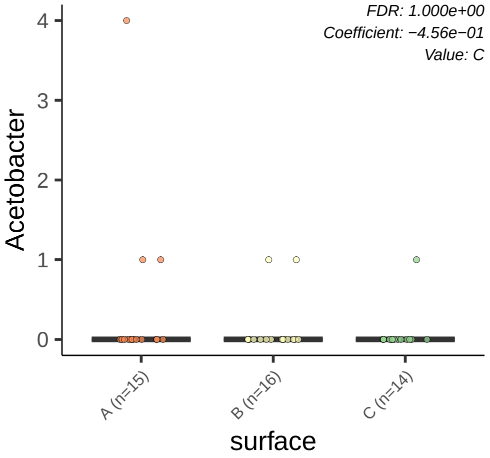

FDR: 1.000e+00
Coefficient: −4.56e−01
Value: C
Acetobacter
4
3
2
1
0
A (n=15)
B (n=16)
C (n=14)
surface

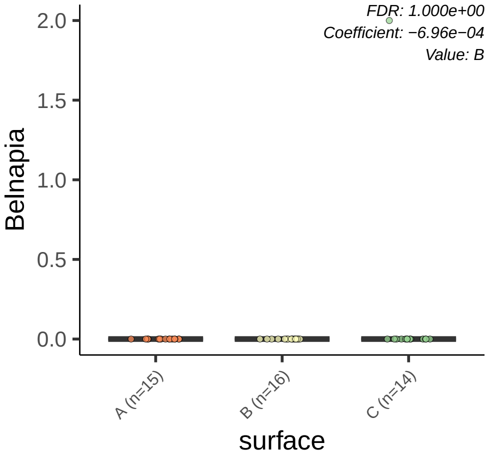

FDR: 1.000e+00
Coefficient: −6.96e−04
Value: B
2.0
1.5
1.0
0.5
0.0
Belnapia
A (n=15)
B (n=16)
C (n=14)
surface

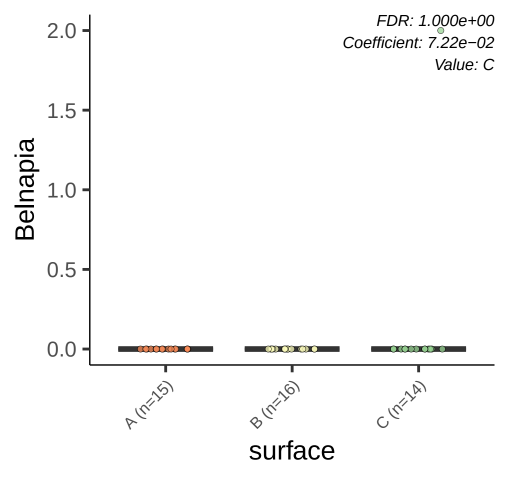

FDR: 1.000e+00
Coefficient: 7.22e−02
Value: C
2.0
1.5
1.0
0.5
0.0
Belnapia
A (n=15)
B (n=16)
C (n=14)
surface

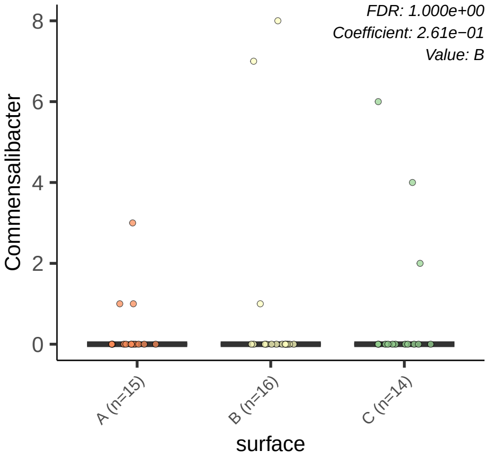

FDR: 1.000e+00
Coefficient: 2.61e−01
Value: B
Commensalibacter
0
2
4
6
8
A (n=15)
B (n=16)
C (n=14)
surface

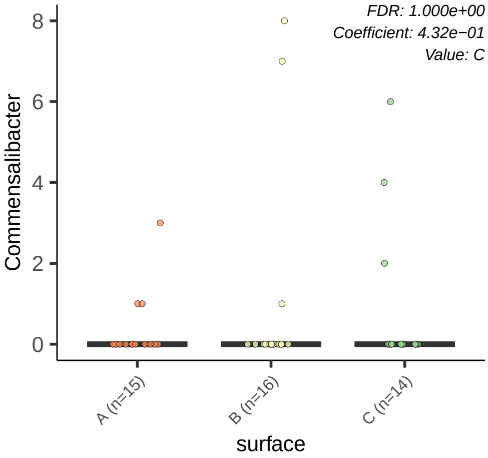
FDR: 1.000e+00
Coefficient: 4.32e−01
Value: C
Commensalibacter
0
2
4
6
8
A (n=15)
B (n=16)
C (n=14)
surface

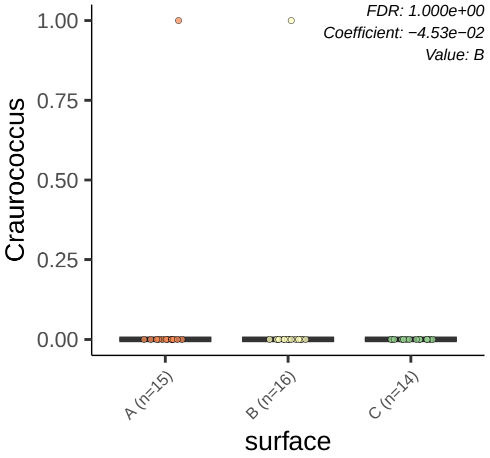

FDR: 1.000e+00
Coefficient: −4.53e−02
Value: B
Craurococcus
1.00
0.75
0.50
0.25
0.00
A (n=15)
B (n=16)
C (n=14)
surface

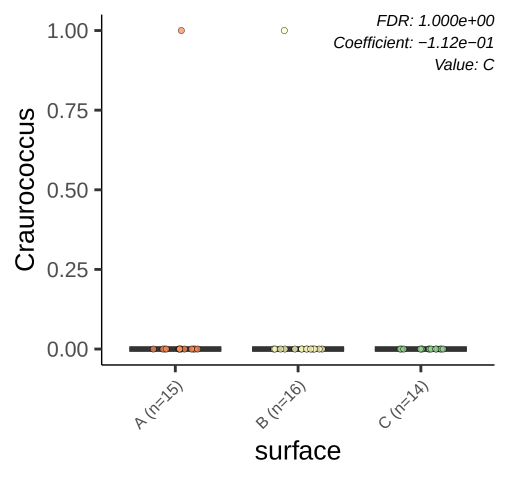
FDR: 1.000e+00
Coefficient: −1.12e−01
Value: C
Craurococcus
1.00
0.75
0.50
0.25
0.00
A (n=15)
B (n=16)
C (n=14)
surface

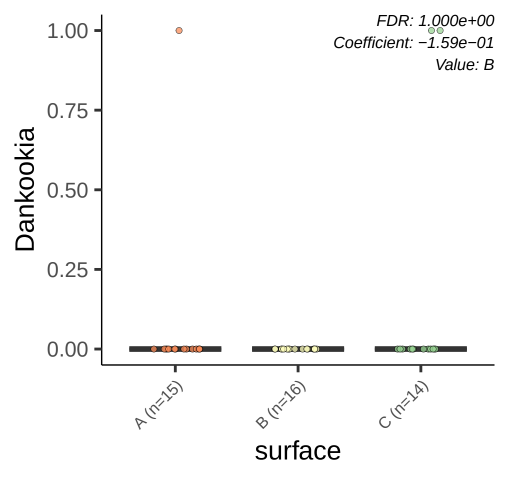
FDR: 1.000e+00
Coefficient: −1.59e−01
Value: B
Dankookia
1.00
0.75
0.50
0.25
0.00
A (n=15)
B (n=16)
C (n=14)
surface

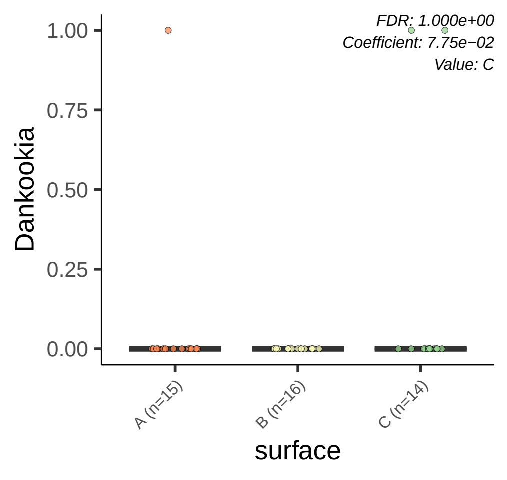
FDR: 1.000e+00
Coefficient: 7.75e−02
Value: C
Dankookia
1.00
0.75
0.50
0.25
0.00
A (n=15)
B (n=16)
C (n=14)
surface

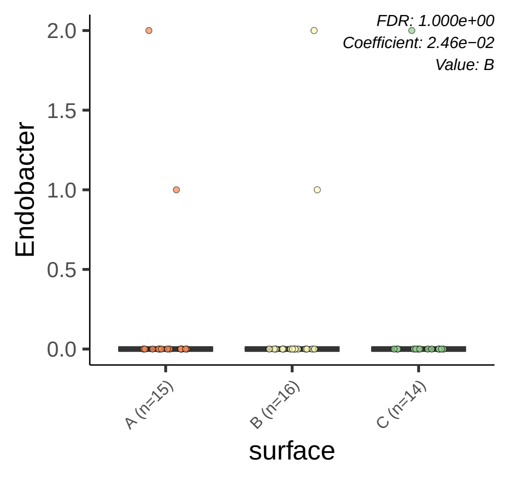

FDR: 1.000e+00
Coefficient: 2.46e−02
Value: B
Endobacter
2.0
1.5
1.0
0.5
0.0
A (n=15)
B (n=16)
C (n=14)
surface

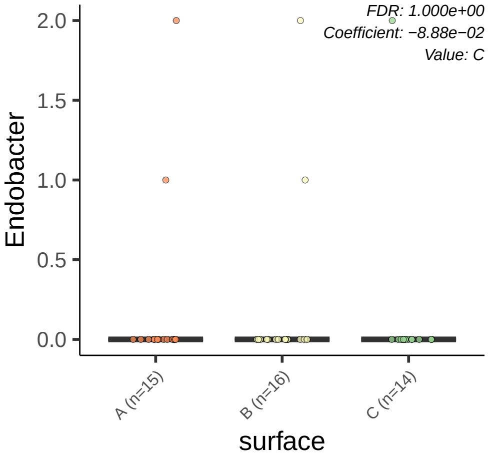
FDR: 1.000e+00
Coefficient: −8.88e−02
Value: C
Endobacter
2.0
1.5
1.0
0.5
0.0
A (n=15)
B (n=16)
C (n=14)
surface

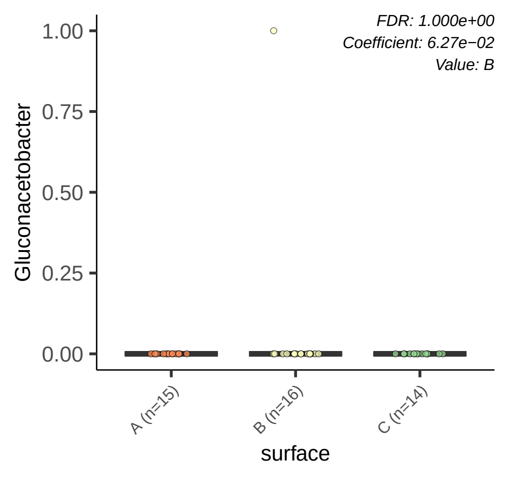

FDR: 1.000e+00
Coefficient: 6.27e−02
Value: B
Gluconacetobacter
1.00
0.75
0.50
0.25
0.00
A (n=15)
B (n=16)
C (n=14)
surface

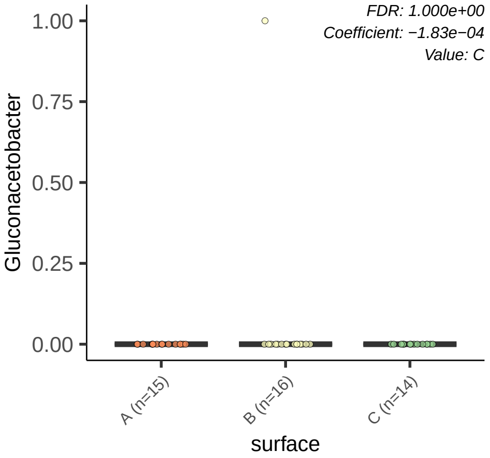

FDR: 1.000e+00
Coefficient: −1.83e−04
Value: C
Gluconacetobacter
1.00
0.75
0.50
0.25
0.00
A (n=15)
B (n=16)
C (n=14)
surface

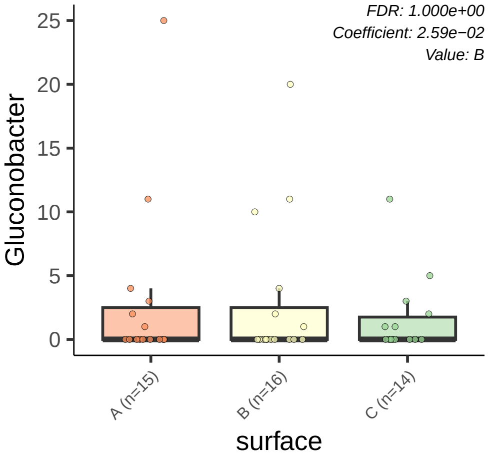

FDR: 1.000e+00
Coefficient: 2.59e−02
Value: B
Gluconobacter
25
20
15
10
5
0
A (n=15)
B (n=16)
C (n=14)
surface

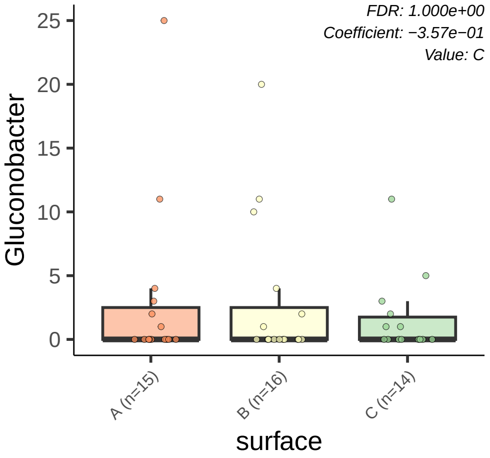
FDR: 1.000e+00
Coefficient: −3.57e−01
Value: C
Gluconobacter
25
20
15
10
5
0
A (n=15)
B (n=16)
C (n=14)
surface

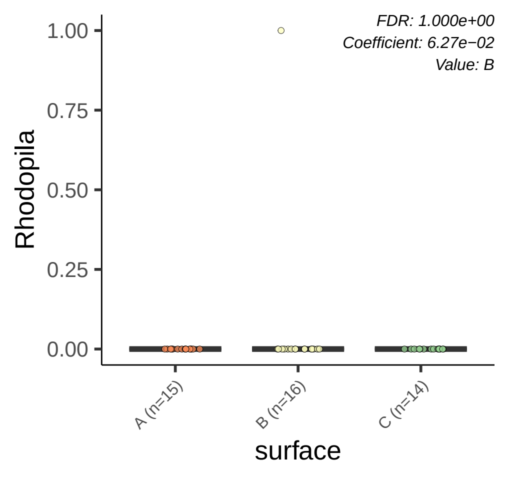

FDR: 1.000e+00
Coefficient: 6.27e−02
Value: B
Rhodopila
1.00
0.75
0.50
0.25
0.00
A (n=15)
B (n=16)
C (n=14)
surface

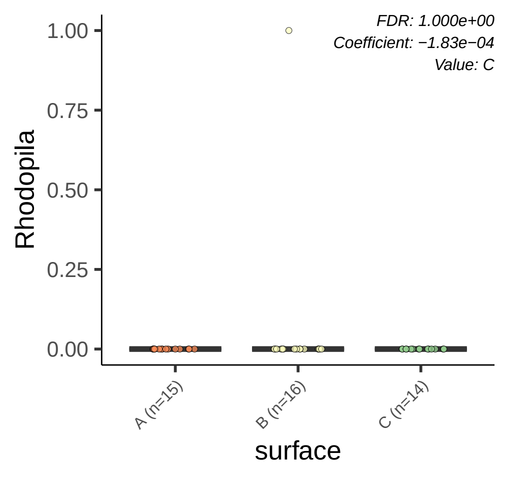

FDR: 1.000e+00
Coefficient: −1.83e−04
Value: C
Rhodopila
1.00
0.75
0.50
0.25
0.00
A (n=15)
B (n=16)
C (n=14)
surface

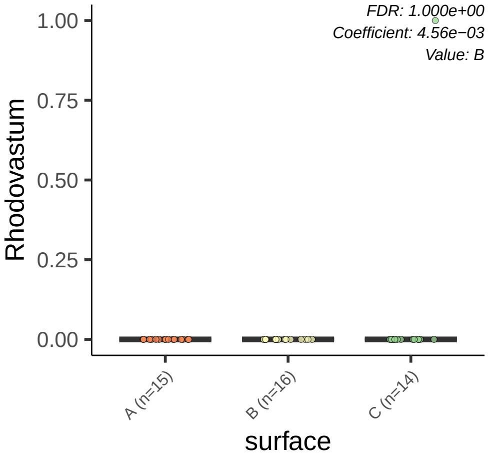

FDR: 1.000e+00
Coefficient: 4.56e−03
Value: B
Rhodovastum
1.00
0.75
0.50
0.25
0.00
A (n=15)
B (n=16)
C (n=14)
surface

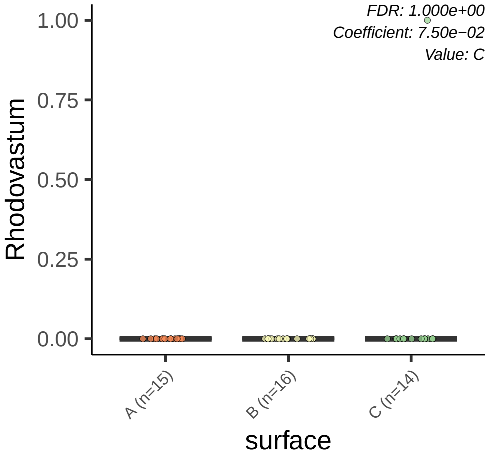
FDR: 1.000e+00
Coefficient: 7.50e−02
Value: C
Rhodovastum
1.00
0.75
0.50
0.25
0.00
A (n=15)
B (n=16)
C (n=14)
surface

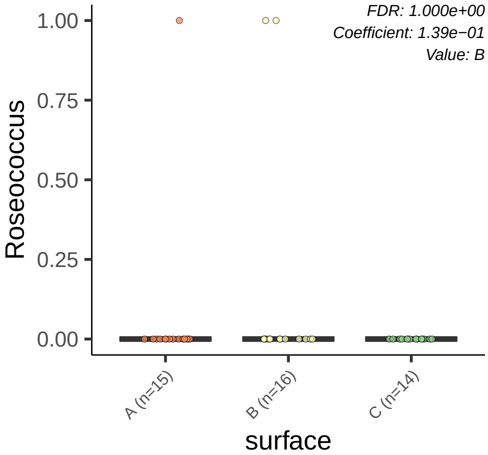

FDR: 1.000e+00
Coefficient: 1.39e−01
Value: B
Roseococcus
1.00
0.75
0.50
0.25
0.00
A (n=15)
B (n=16)
C (n=14)
surface

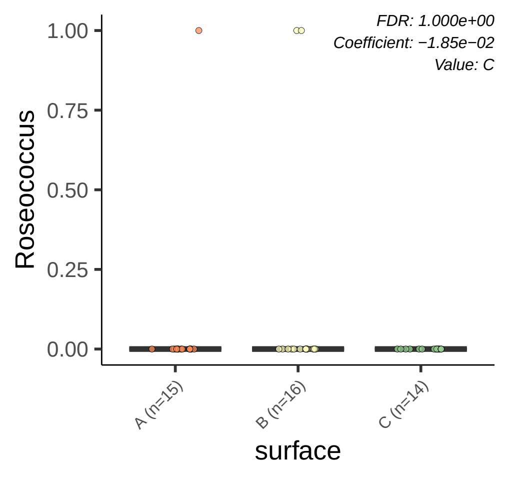
FDR: 1.000e+00
Coefficient: −1.85e−02
Value: C
Roseococcus
1.00
0.75
0.50
0.25
0.00
A (n=15)
B (n=16)
C (n=14)
surface

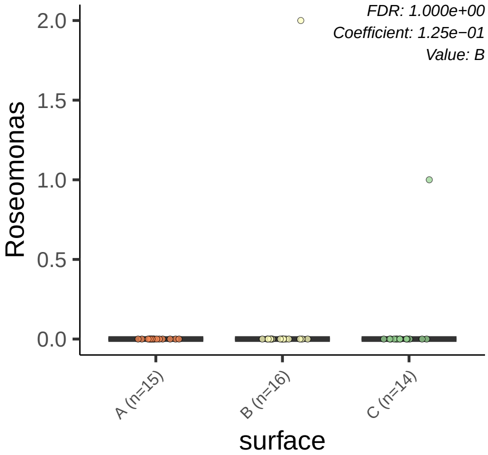
FDR: 1.000e+00
Coefficient: 1.25e−01
Value: B
Roseomonas
2.0
1.5
1.0
0.5
0.0
A (n=15)
B (n=16)
C (n=14)
surface

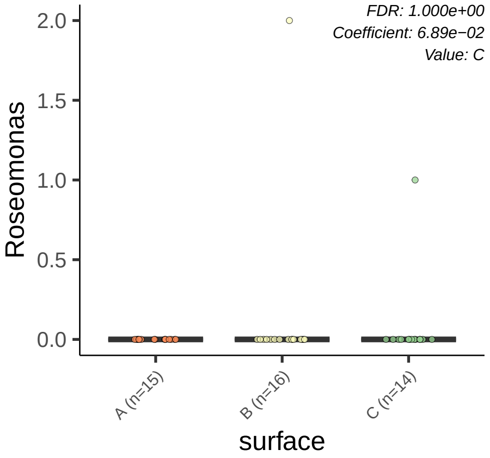

FDR: 1.000e+00
Coefficient: 6.89e−02
Value: C
Roseomonas
2.0
1.5
1.0
0.5
0.0
A (n=15)
B (n=16)
C (n=14)
surface

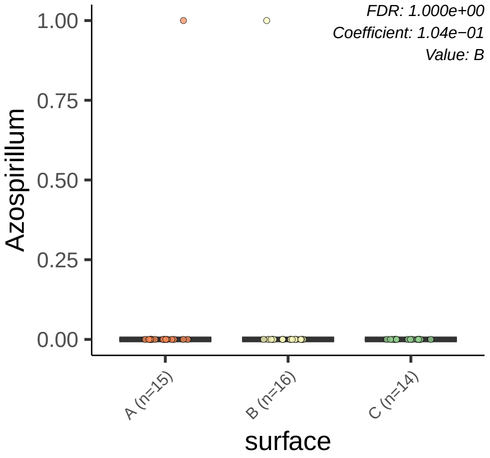
FDR: 1.000e+00
Coefficient: 1.04e−01
Value: B
Azospirillum
1.00
0.75
0.50
0.25
0.00
A (n=15)
B (n=16)
C (n=14)
surface

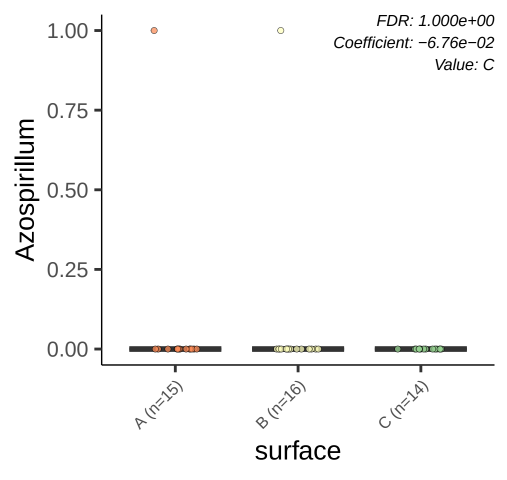
FDR: 1.000e+00
Coefficient: −6.76e−02
Value: C
Azospirillum
1.00
0.75
0.50
0.25
0.00
A (n=15)
B (n=16)
C (n=14)
surface

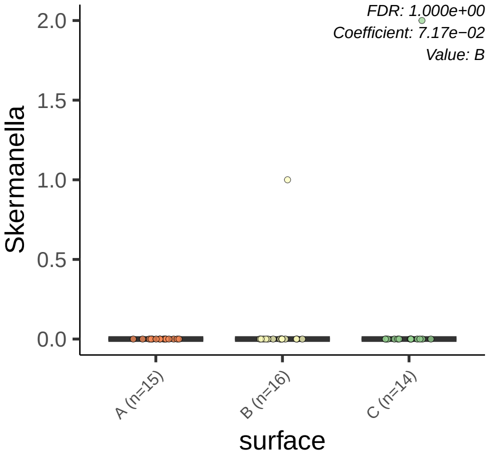
FDR: 1.000e+00
Coefficient: 7.17e−02
Value: B
Skermanella
2.0
1.5
1.0
0.5
0.0
A (n=15)
B (n=16)
C (n=14)
surface

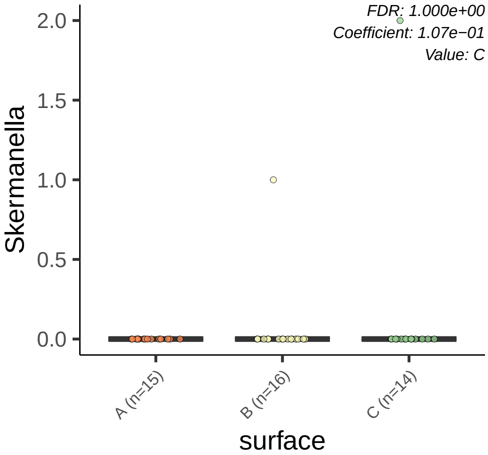
FDR: 1.000e+00
Coefficient: 1.07e−01
Value: C
Skermanella
2.0
1.5
1.0
0.5
0.0
A (n=15)
B (n=16)
C (n=14)
surface

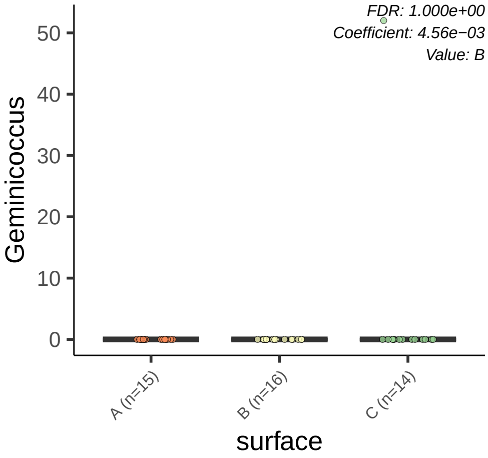

FDR: 1.000e+00
Coefficient: 4.56e−03
Value: B
Geminicoccus
0
10
20
30
40
50
A (n=15)
B (n=16)
C (n=14)
surface

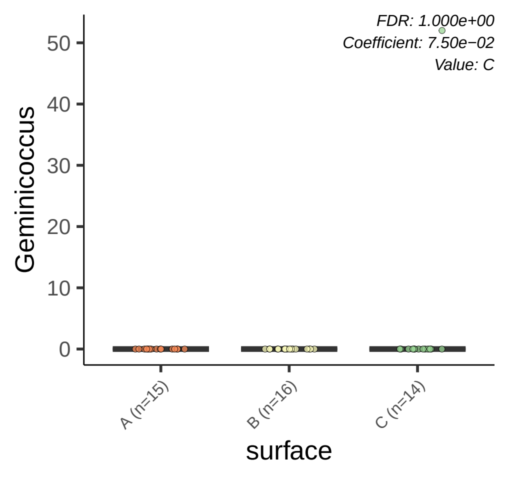

FDR: 1.000e+00
Coefficient: 7.50e−02
Value: C
Geminicoccus
50
40
30
20
10
0
A (n=15)
B (n=16)
C (n=14)
surface

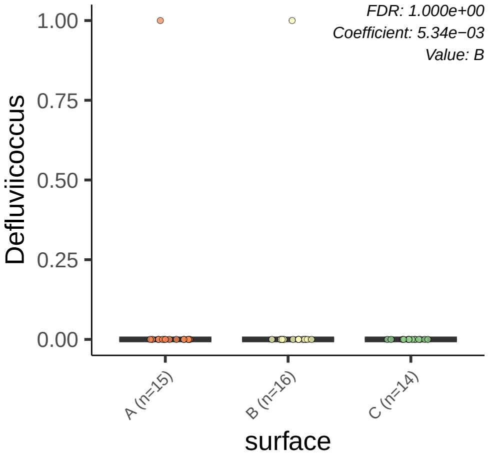
FDR: 1.000e+00
Coefficient: 5.34e−03
Value: B
Defluviicoccus
1.00
0.75
0.50
0.25
0.00
A (n=15)
B (n=16)
C (n=14)
surface

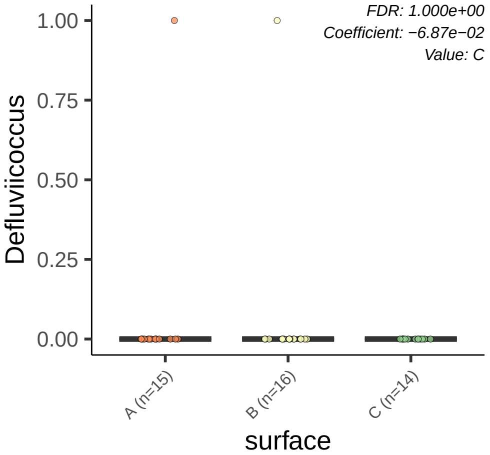

FDR: 1.000e+00
Coefficient: −6.87e−02
Value: C
Defluviicoccus
1.00
0.75
0.50
0.25
0.00
A (n=15)
B (n=16)
C (n=14)
surface

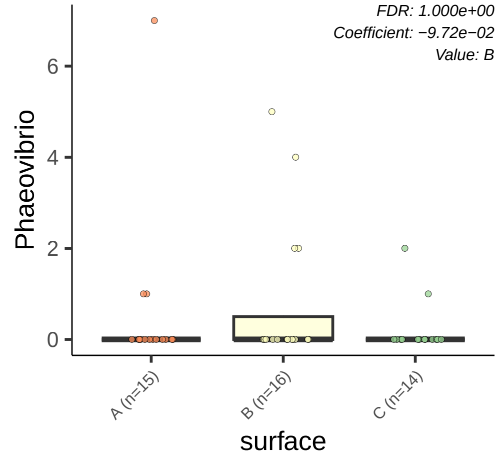

FDR: 1.000e+00
Coefficient: −9.72e−02
Value: B
Phaeovibrio
0
2
4
6
A (n=15)
B (n=16)
C (n=14)
surface

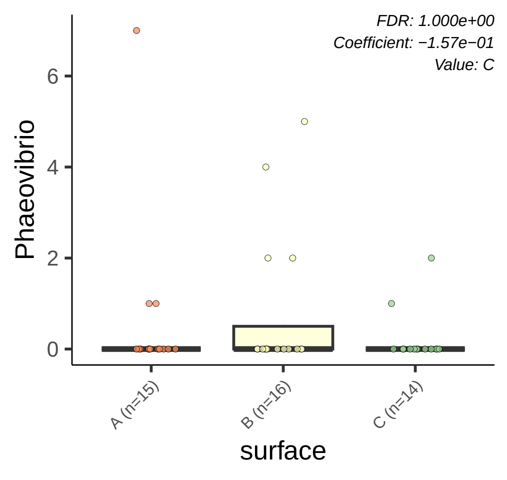

FDR: 1.000e+00
Coefficient: −1.57e−01
Value: C
Phaeovibrio
6
4
2
0
A (n=15)
B (n=16)
C (n=14)
surface

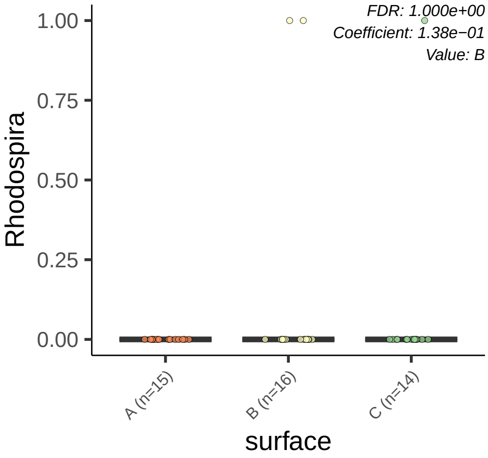

FDR: 1.000e+00
Coefficient: 1.38e−01
Value: B
Rhodospira
1.00
0.75
0.50
0.25
0.00
A (n=15)
B (n=16)
C (n=14)
surface

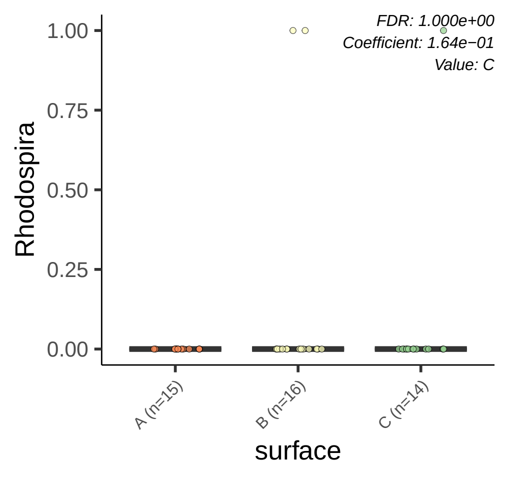

FDR: 1.000e+00
Coefficient: 1.64e−01
Value: C
Rhodospira
1.00
0.75
0.50
0.25
0.00
A (n=15)
B (n=16)
C (n=14)
surface

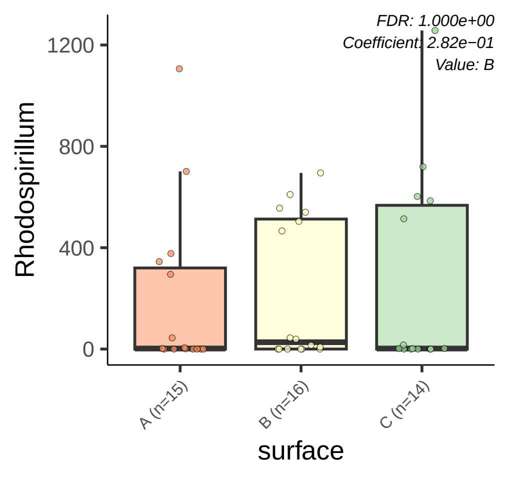

FDR: 1.000e+00
Coefficient: 2.82e−01
Value: B
Rhodospirillum
0
400
800
1200
A (n=15)
B (n=16)
C (n=14)
surface

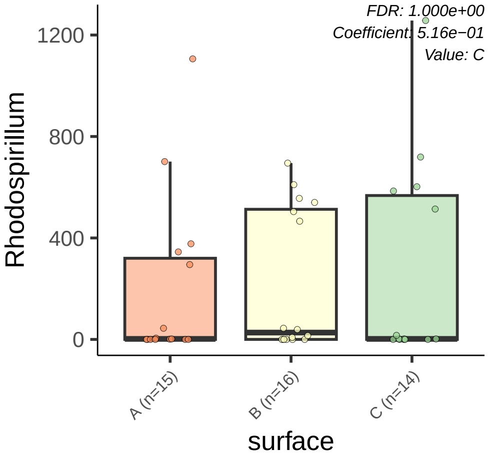

FDR: 1.000e+00
Coefficient: 5.16e−01
Value: C
Rhodospirillum
1200
800
400
0
A (n=15)
B (n=16)
C (n=14)
surface

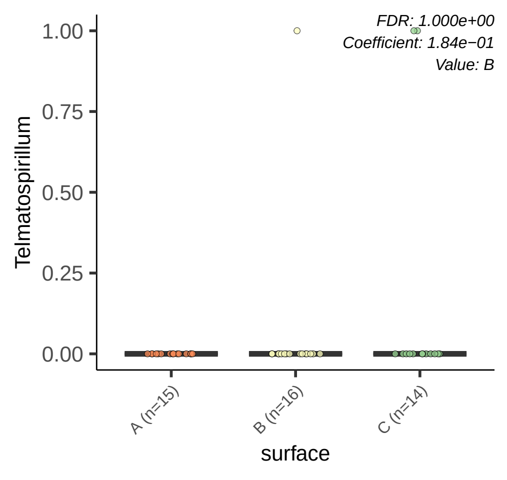

FDR: 1.000e+00
Coefficient: 1.84e−01
Value: B
1.00
0.75
0.50
0.25
0.00
Telmatospirillum
A (n=15)
B (n=16)
C (n=14)
surface

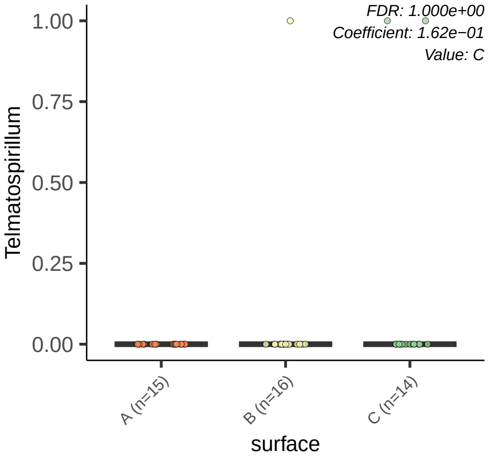

FDR: 1.000e+00
Coefficient: 1.62e−01
Value: C
1.00
0.75
0.50
0.25
0.00
Telmatospirillum
A (n=15)
B (n=16)
C (n=14)
surface

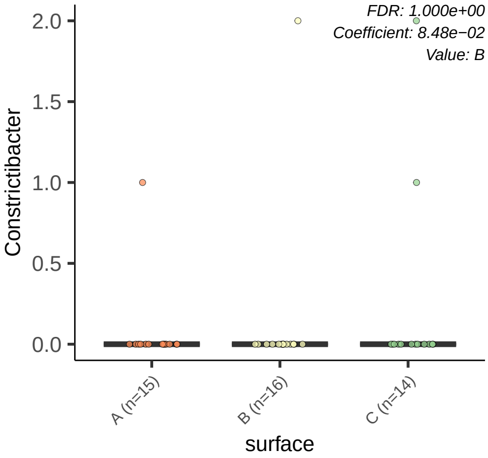
FDR: 1.000e+00
Coefficient: 8.48e−02
Value: B
Constrictibacter
2.0
1.5
1.0
0.5
0.0
A (n=15)
B (n=16)
C (n=14)
surface

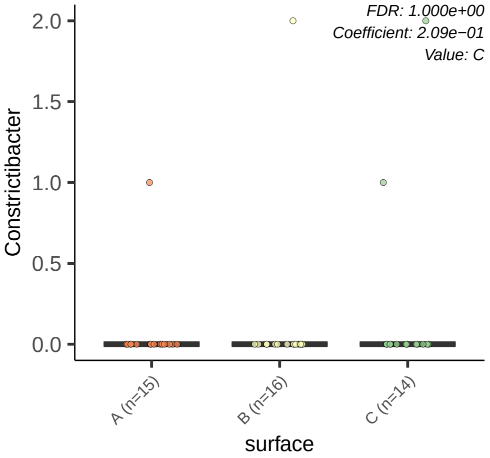
FDR: 1.000e+00
Coefficient: 2.09e−01
Value: C
Constrictibacter
2.0
1.5
1.0
0.5
0.0
A (n=15)
B (n=16)
C (n=14)
surface

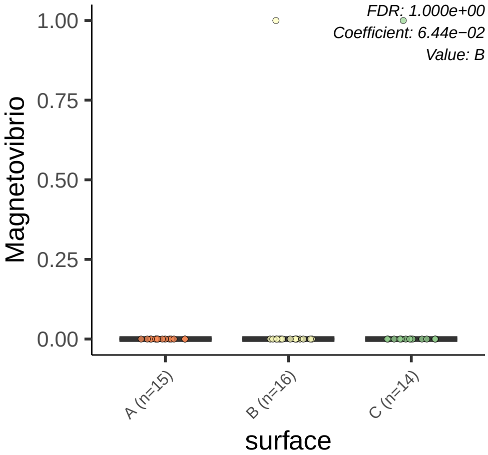
FDR: 1.000e+00
Coefficient: 6.44e−02
Value: B
Magnetovibrio
1.00
0.75
0.50
0.25
0.00
A (n=15)
B (n=16)
C (n=14)
surface

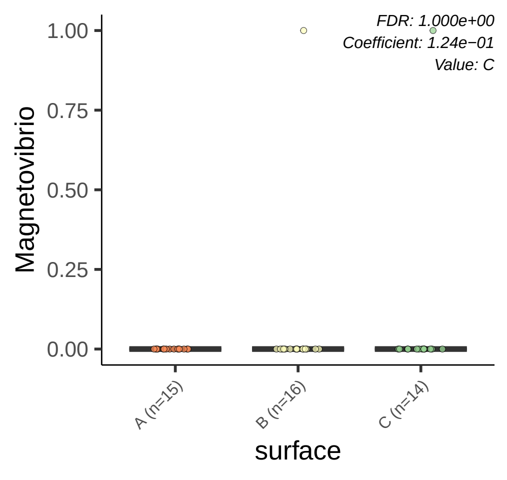
FDR: 1.000e+00
Coefficient: 1.24e−01
Value: C
Magnetovibrio
1.00
0.75
0.50
0.25
0.00
A (n=15)
B (n=16)
C (n=14)
surface

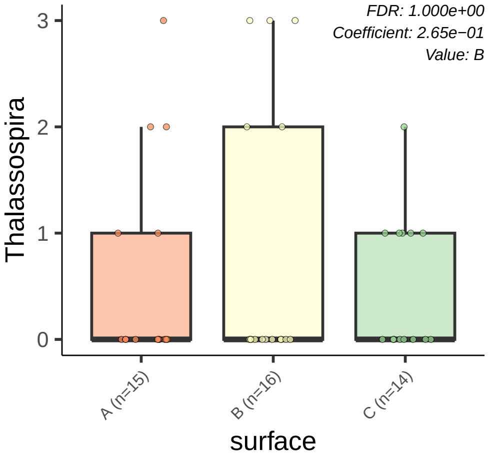

FDR: 1.000e+00
Coefficient: 2.65e−01
Value: B
Thalassospira
3
2
1
0
A (n=15)
B (n=16)
C (n=14)
surface

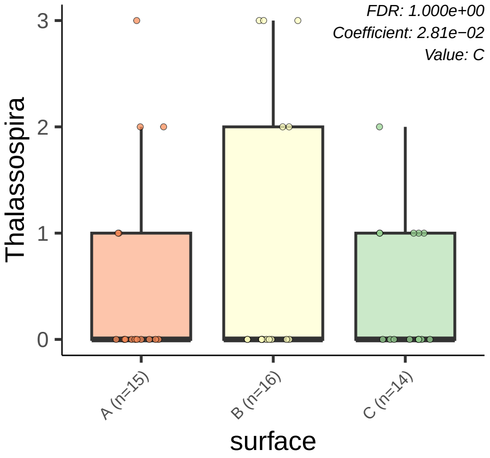

FDR: 1.000e+00
Coefficient: 2.81e−02
Value: C
Thalassospira
0
1
2
3
A (n=15)
B (n=16)
C (n=14)
surface

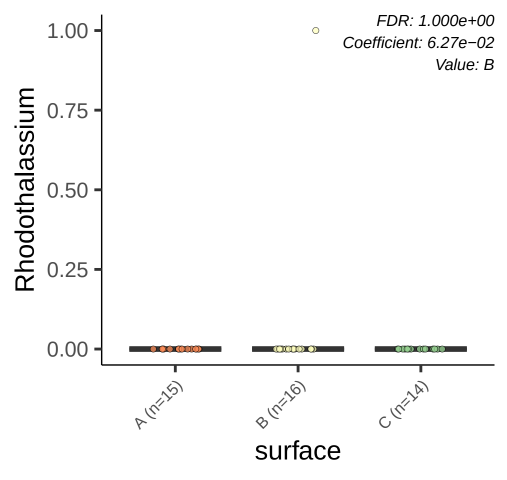
FDR: 1.000e+00
Coefficient: 6.27e−02
Value: B
Rhodothalassium
1.00
0.75
0.50
0.25
0.00
A (n=15)
B (n=16)
C (n=14)
surface

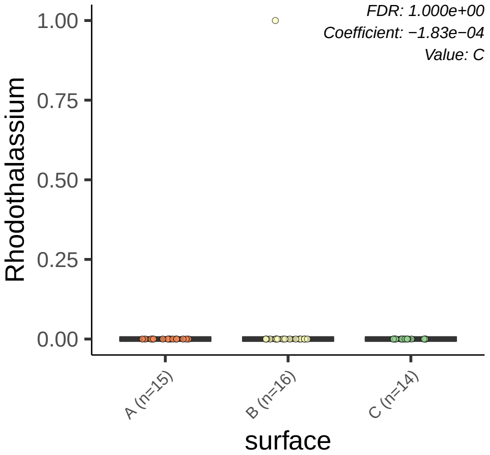

FDR: 1.000e+00
Coefficient: −1.83e−04
Value: C
Rhodothalassium
1.00
0.75
0.50
0.25
0.00
A (n=15)
B (n=16)
C (n=14)
surface

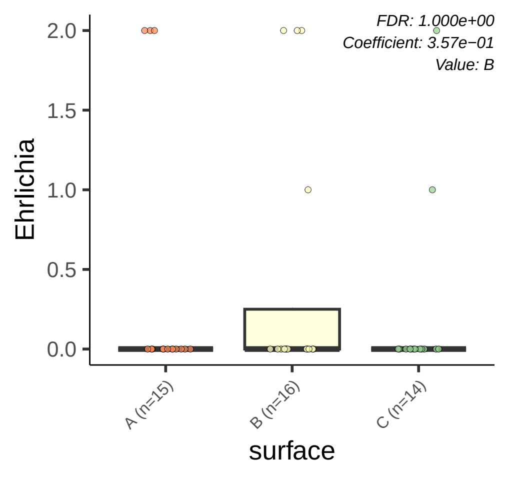

FDR: 1.000e+00
Coefficient: 3.57e−01
Value: B
Ehrlichia
2.0
1.5
1.0
0.5
0.0
A (n=15)
B (n=16)
C (n=14)
surface

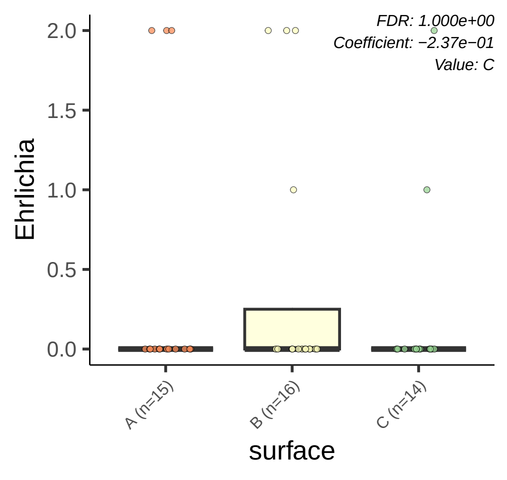
FDR: 1.000e+00
Coefficient: −2.37e−01
Value: C
Ehrlichia
2.0
1.5
1.0
0.5
0.0
A (n=15)
B (n=16)
C (n=14)
surface

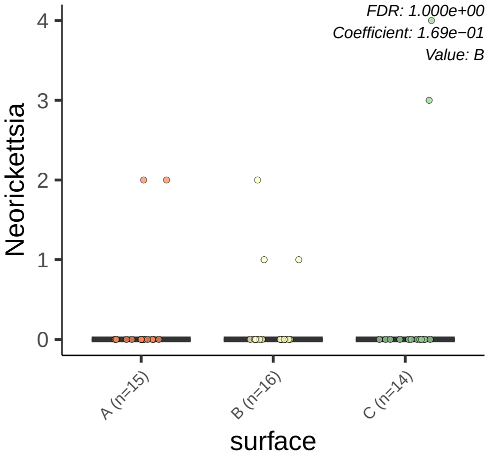

FDR: 1.000e+00
Coefficient: 1.69e−01
Value: B
Neorickettsia
0
1
2
3
4
A (n=15)
B (n=16)
C (n=14)
surface

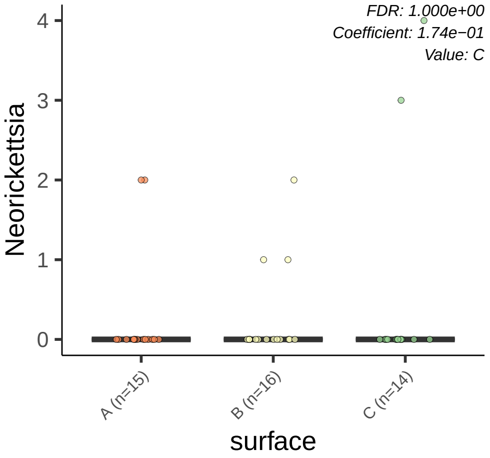
FDR: 1.000e+00
Coefficient: 1.74e−01
Value: C
Neorickettsia
0
1
2
3
4
A (n=15)
B (n=16)
C (n=14)
surface

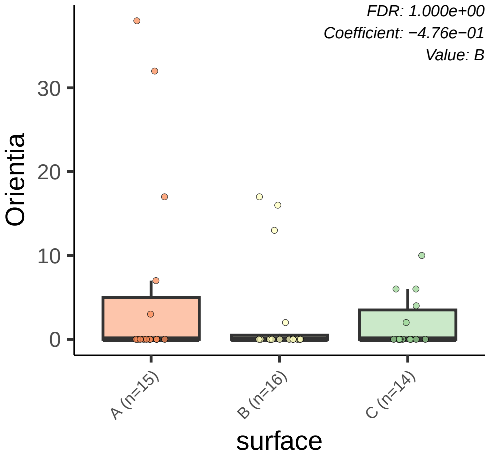

FDR: 1.000e+00
Coefficient: −4.76e−01
Value: B
Orientia
30
20
10
0
A (n=15)
B (n=16)
C (n=14)
surface

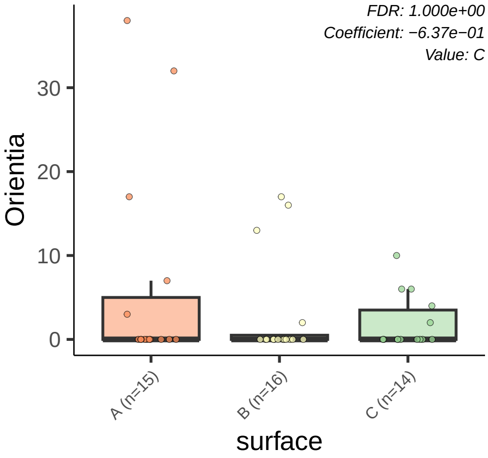
FDR: 1.000e+00
Coefficient: −6.37e−01
Value: C
Orientia
30
20
10
0
A (n=15)
B (n=16)
C (n=14)
surface

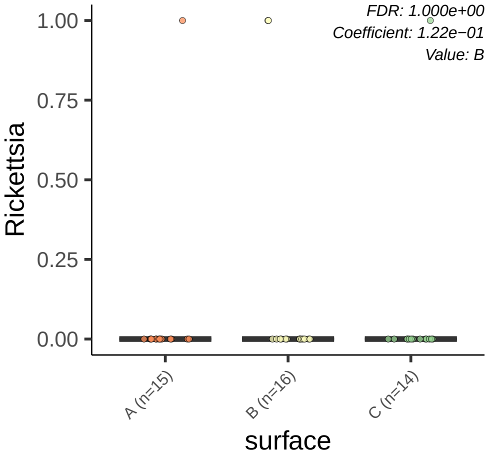
FDR: 1.000e+00
Coefficient: 1.22e−01
Value: B
1.00
0.75
0.50
0.25
0.00
Rickettsia
A (n=15)
B (n=16)
C (n=14)
surface

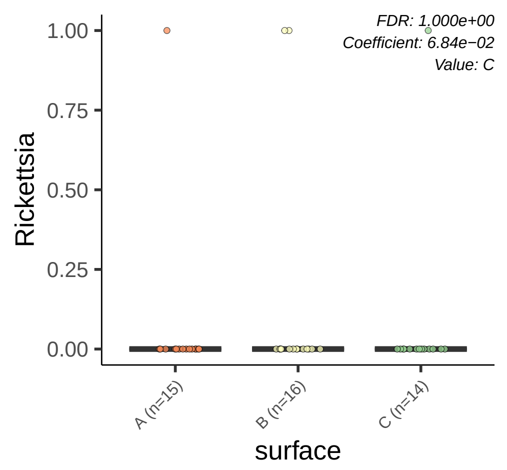
FDR: 1.000e+00
Coefficient: 6.84e−02
Value: C
Rickettsia
1.00
0.75
0.50
0.25
0.00
A (n=15)
B (n=16)
C (n=14)
surface

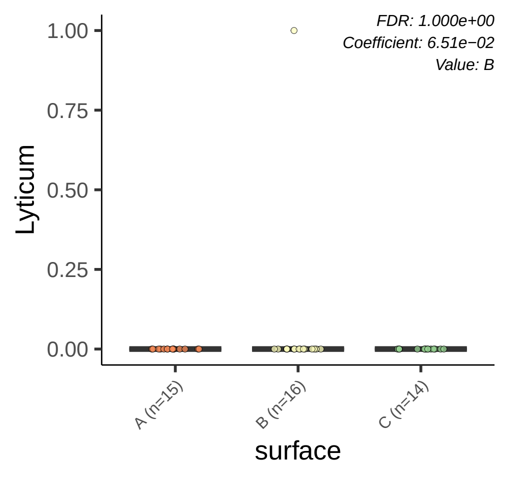

FDR: 1.000e+00
Coefficient: 6.51e−02
Value: B
Lyticum
1.00
0.75
0.50
0.25
0.00
A (n=15)
B (n=16)
C (n=14)
surface

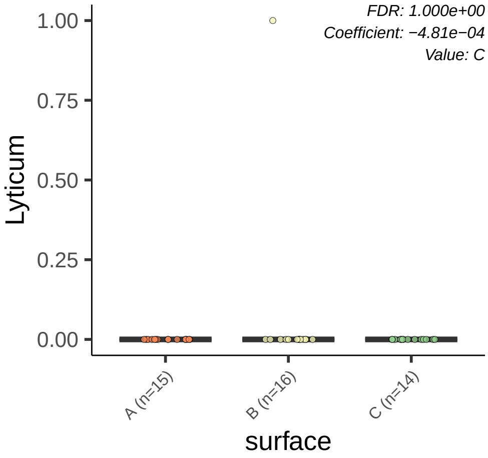

FDR: 1.000e+00
Coefficient: −4.81e−04
Value: C
Lyticum
1.00
0.75
0.50
0.25
0.00
A (n=15)
B (n=16)
C (n=14)
surface

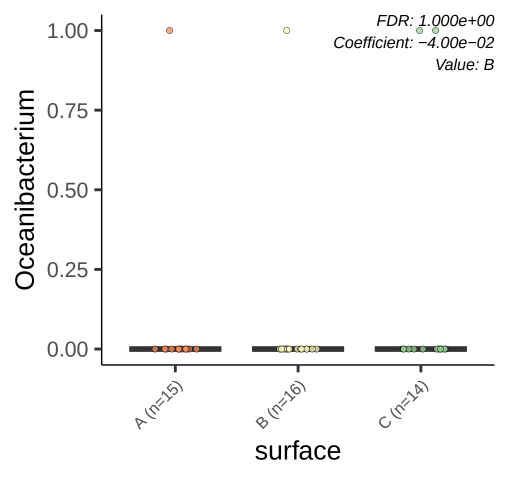

FDR: 1.000e+00
Coefficient: −4.00e−02
Value: B
Oceanibacterium
1.00
0.75
0.50
0.25
0.00
A (n=15)
B (n=16)
C (n=14)
surface

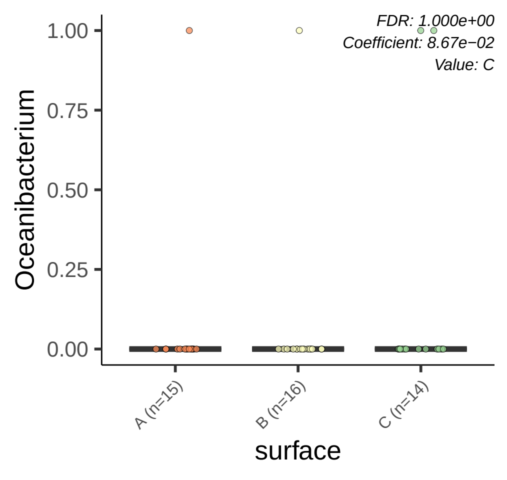

FDR: 1.000e+00
Coefficient: 8.67e−02
Value: C
1.00
0.75
0.50
0.25
0.00
Oceanibacterium
A (n=15)
B (n=16)
C (n=14)
surface

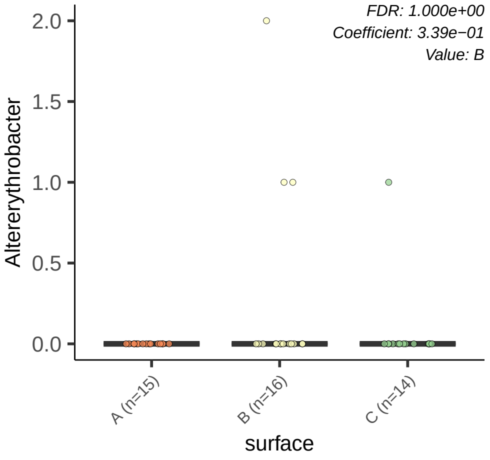
FDR: 1.000e+00
Coefficient: 3.39e−01
Value: B
Altererythrobacter
2.0
1.5
1.0
0.5
0.0
A (n=15)
B (n=16)
C (n=14)
surface

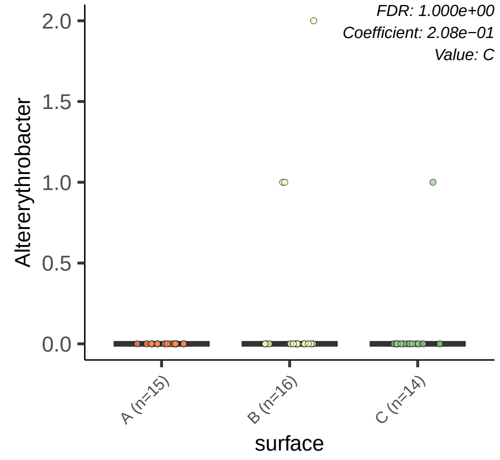
FDR: 1.000e+00
Coefficient: 2.08e−01
Value: C
Altererythrobacter
2.0
1.5
1.0
0.5
0.0
A (n=15)
B (n=16)
C (n=14)
surface

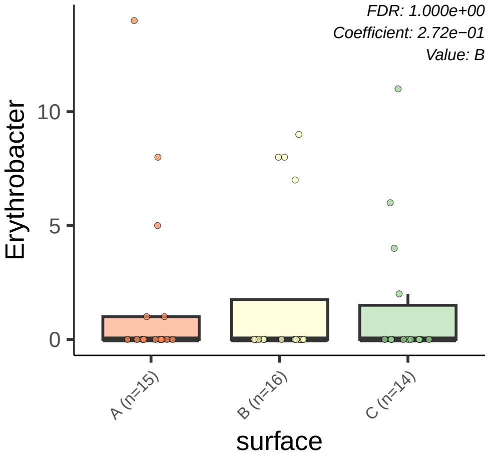

FDR: 1.000e+00
Coefficient: 2.72e−01
Value: B
Erythrobacter
10
5
0
A (n=15)
B (n=16)
C (n=14)
surface

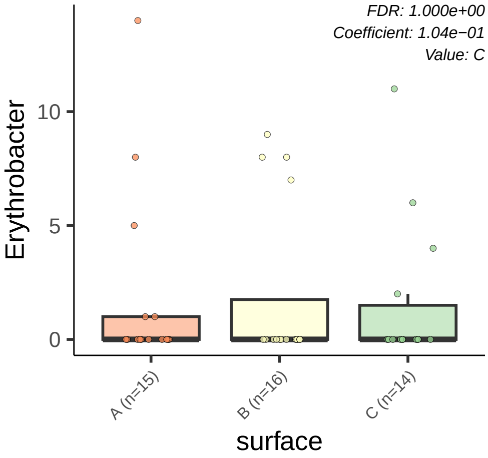

FDR: 1.000e+00
Coefficient: 1.04e−01
Value: C
Erythrobacter
0
5
10
A (n=15)
B (n=16)
C (n=14)
surface

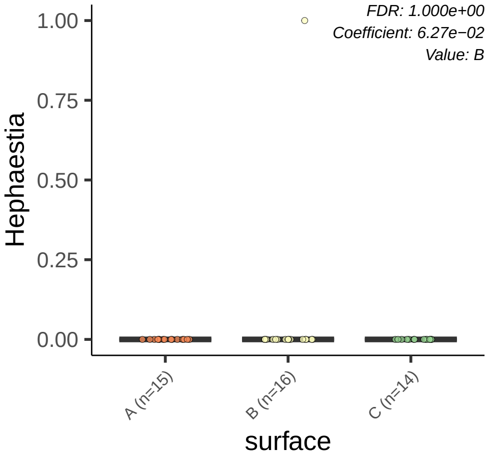

FDR: 1.000e+00
Coefficient: 6.27e−02
Value: B
Hephaestia
1.00
0.75
0.50
0.25
0.00
A (n=15)
B (n=16)
C (n=14)
surface

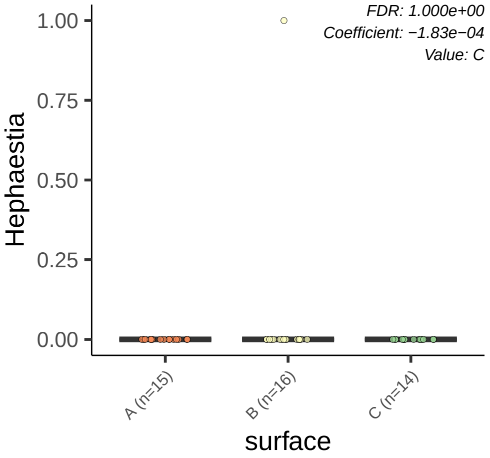

FDR: 1.000e+00
Coefficient: −1.83e−04
Value: C
1.00
0.75
0.50
0.25
0.00
Hephaestia
A (n=15)
B (n=16)
C (n=14)
surface

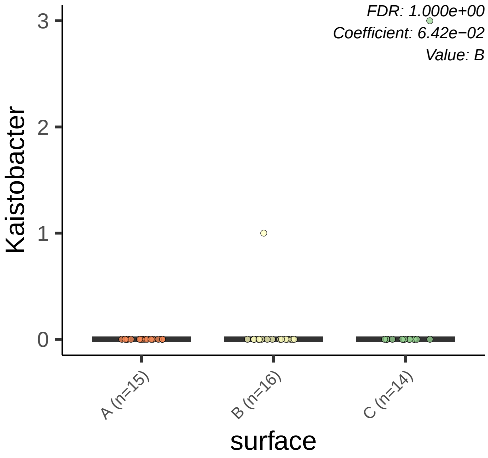

FDR: 1.000e+00
Coefficient: 6.42e−02
Value: B
Kaistobacter
3
2
1
0
A (n=15)
B (n=16)
C (n=14)
surface

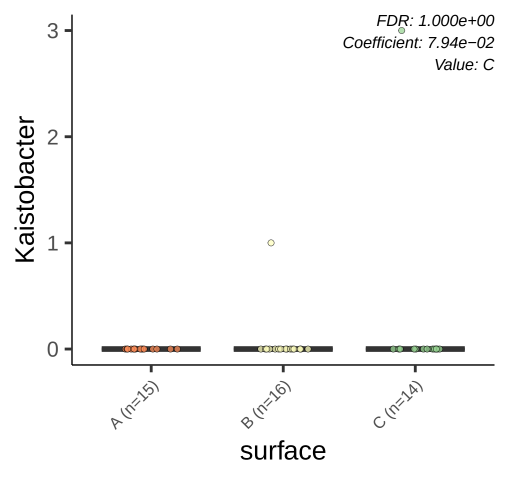

FDR: 1.000e+00
Coefficient: 7.94e−02
Value: C
Kaistobacter
3
2
1
0
A (n=15)
B (n=16)
C (n=14)
surface

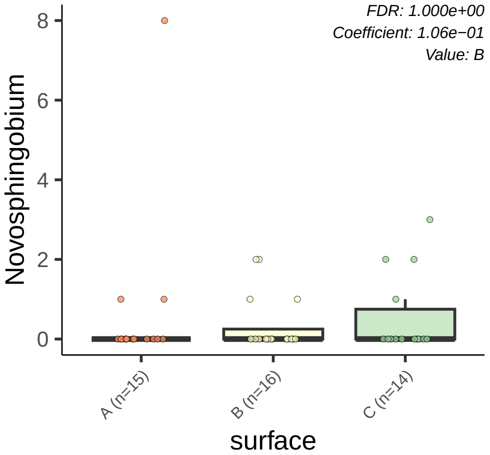

FDR: 1.000e+00
Coefficient: 1.06e−01
Value: B
Novosphingobium
8
6
4
2
0
A (n=15)
B (n=16)
C (n=14)
surface

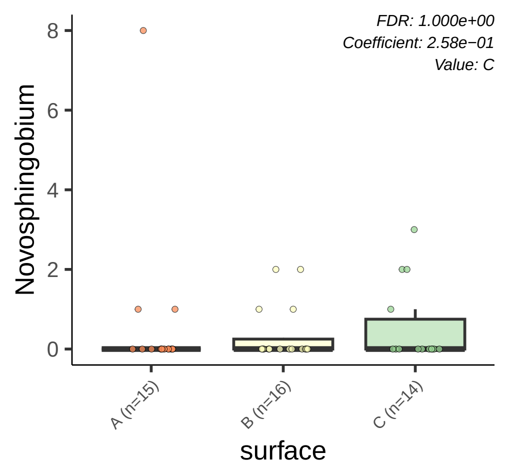

FDR: 1.000e+00
Coefficient: 2.58e−01
Value: C
Novosphingobium
0
2
4
6
8
A (n=15)
B (n=16)
C (n=14)
surface

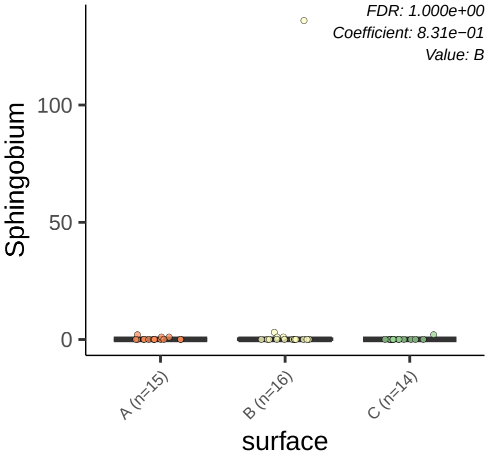
FDR: 1.000e+00
Coefficient: 8.31e−01
Value: B
Sphingobium
100
50
0
A (n=15)
B (n=16)
C (n=14)
surface

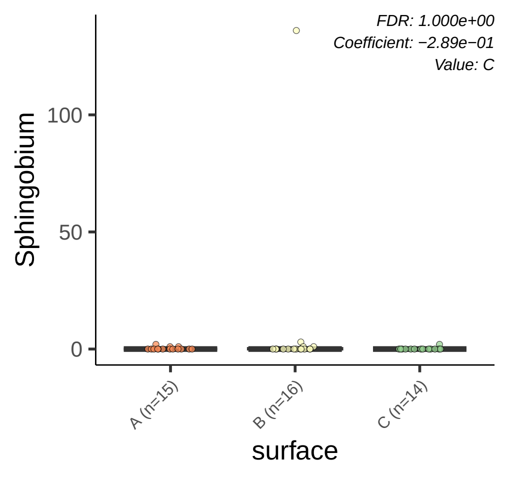

FDR: 1.000e+00
Coefficient: −2.89e−01
Value: C
Sphingobium
100
50
0
A (n=15)
B (n=16)
C (n=14)
surface

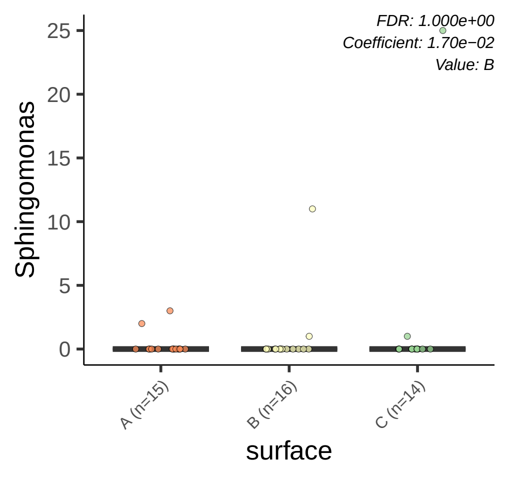
FDR: 1.000e+00
Coefficient: 1.70e−02
Value: B
Sphingomonas
25
20
15
10
5
0
A (n=15)
B (n=16)
C (n=14)
surface

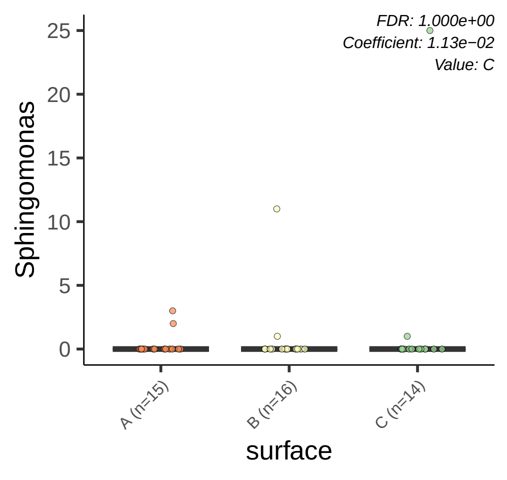
FDR: 1.000e+00
Coefficient: 1.13e−02
Value: C
Sphingomonas
25
20
15
10
5
0
A (n=15)
B (n=16)
C (n=14)
surface

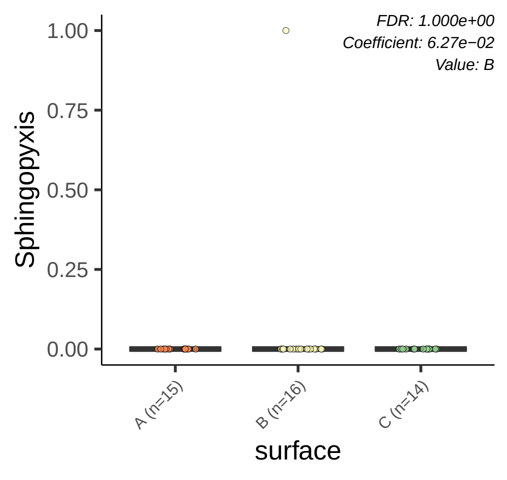
FDR: 1.000e+00
Coefficient: 6.27e−02
Value: B
Sphingopyxis
1.00
0.75
0.50
0.25
0.00
A (n=15)
B (n=16)
C (n=14)
surface

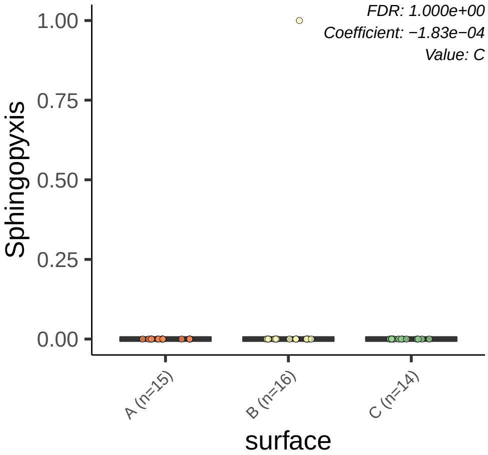
FDR: 1.000e+00
Coefficient: −1.83e−04
Value: C
Sphingopyxis
1.00
0.75
0.50
0.25
0.00
A (n=15)
B (n=16)
C (n=14)
surface

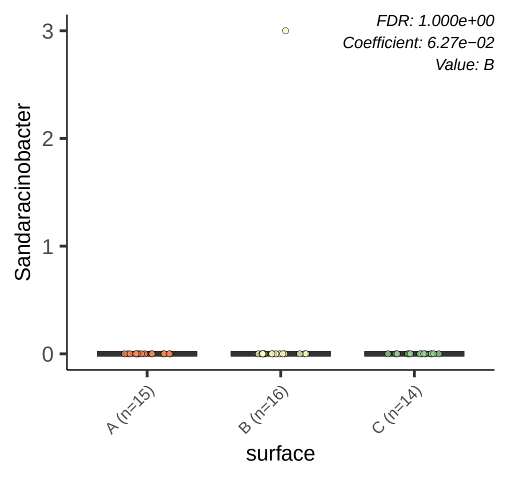

FDR: 1.000e+00
Coefficient: 6.27e−02
Value: B
Sandaracinobacter
3
2
1
0
A (n=15)
B (n=16)
C (n=14)
surface

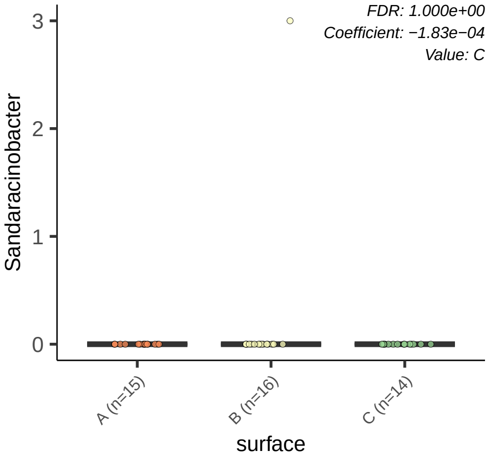

FDR: 1.000e+00
Coefficient: −1.83e−04
Value: C
Sandaracinobacter
3
2
1
0
A (n=15)
B (n=16)
C (n=14)
surface

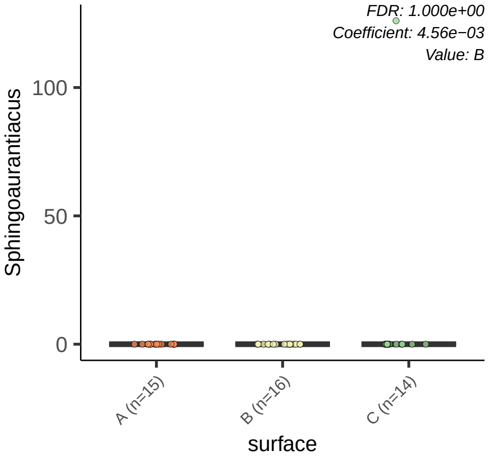
FDR: 1.000e+00
Coefficient: 4.56e−03
Value: B
Sphingoaurantiacus
100
50
0
A (n=15)
B (n=16)
C (n=14)
surface

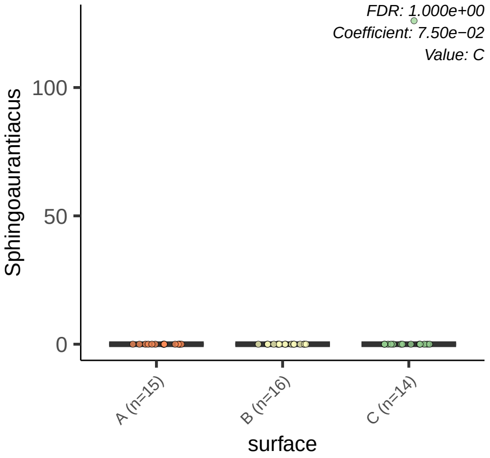
FDR: 1.000e+00
Coefficient: 7.50e−02
Value: C
Sphingoaurantiacus
100
50
0
A (n=15)
B (n=16)
C (n=14)
surface

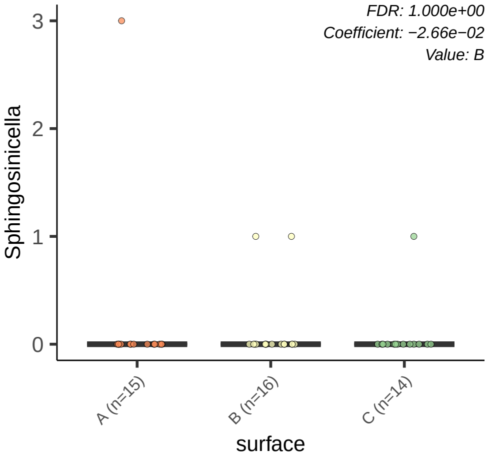
FDR: 1.000e+00
Coefficient: −2.66e−02
Value: B
Sphingosinicella
3
2
1
0
A (n=15)
B (n=16)
C (n=14)
surface

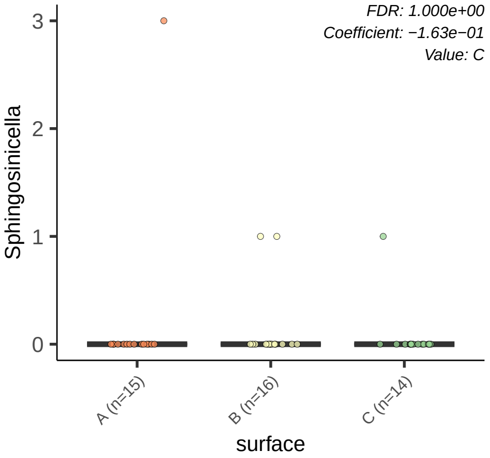

FDR: 1.000e+00
Coefficient: −1.63e−01
Value: C
Sphingosinicella
3
2
1
0
A (n=15)
B (n=16)
C (n=14)
surface

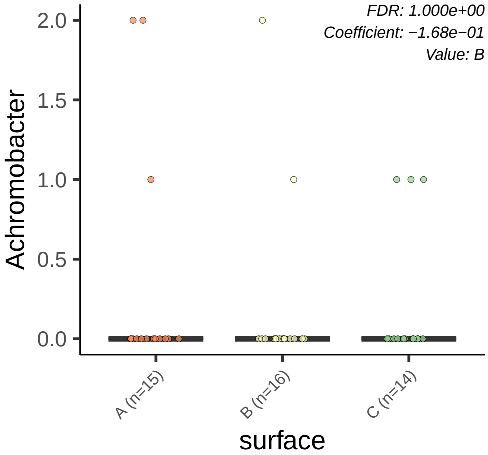
FDR: 1.000e+00
Coefficient: −1.68e−01
Value: B
Achromobacter
2.0
1.5
1.0
0.5
0.0
A (n=15)
B (n=16)
C (n=14)
surface

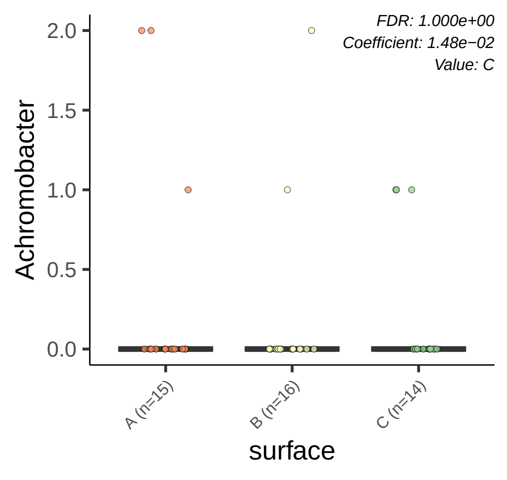

FDR: 1.000e+00
Coefficient: 1.48e−02
Value: C
Achromobacter
2.0
1.5
1.0
0.5
0.0
A (n=15)
B (n=16)
C (n=14)
surface

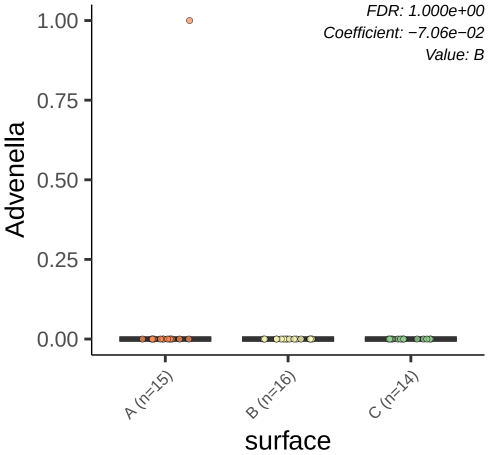
FDR: 1.000e+00
Coefficient: −7.06e−02
Value: B
1.00
0.75
0.50
0.25
0.00
Advenella
A (n=15)
B (n=16)
C (n=14)
surface

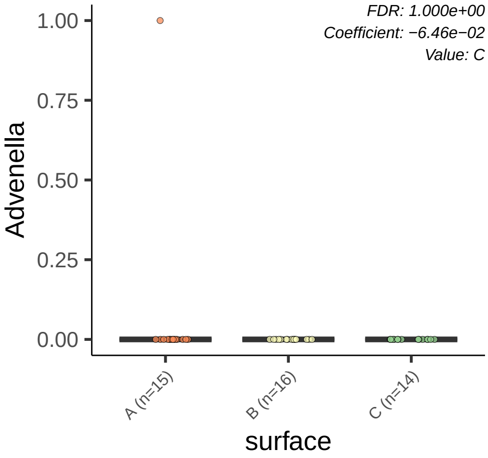

FDR: 1.000e+00
Coefficient: −6.46e−02
Value: C
Advenella
1.00
0.75
0.50
0.25
0.00
A (n=15)
B (n=16)
C (n=14)
surface

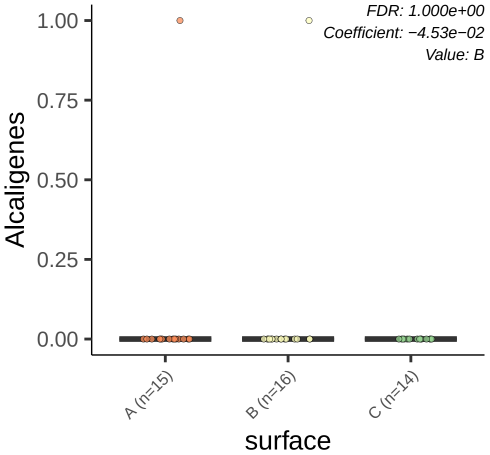
FDR: 1.000e+00
Coefficient: −4.53e−02
Value: B
Alcaligenes
1.00
0.75
0.50
0.25
0.00
A (n=15)
B (n=16)
C (n=14)
surface

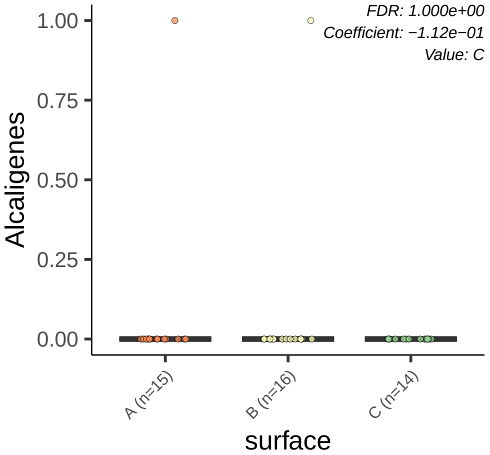
FDR: 1.000e+00
Coefficient: −1.12e−01
Value: C
Alcaligenes
1.00
0.75
0.50
0.25
0.00
A (n=15)
B (n=16)
C (n=14)
surface

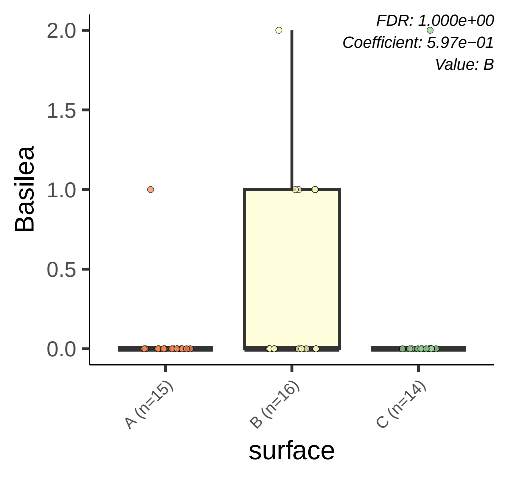

FDR: 1.000e+00
Coefficient: 5.97e−01
Value: B
Basilea
2.0
1.5
1.0
0.5
0.0
A (n=15)
B (n=16)
C (n=14)
surface

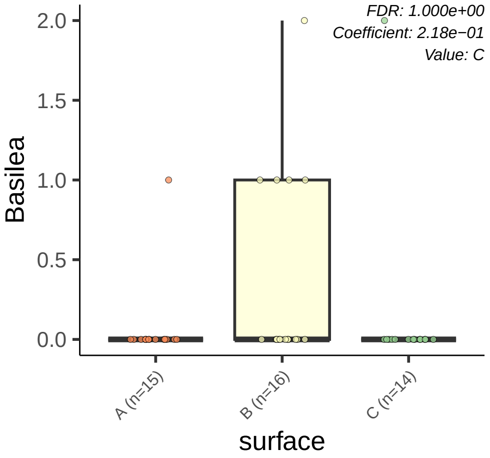
FDR: 1.000e+00
Coefficient: 2.18e−01
Value: C
Basilea
2.0
1.5
1.0
0.5
0.0
A (n=15)
B (n=16)
C (n=14)
surface

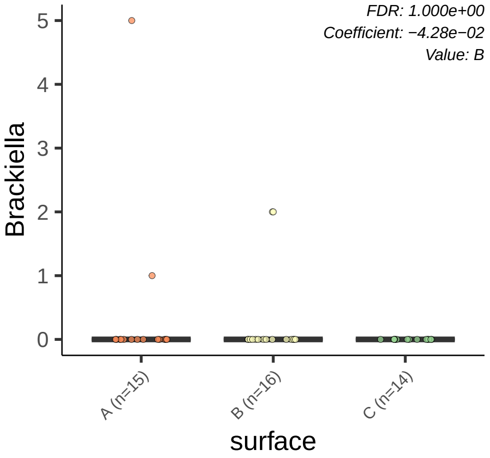
FDR: 1.000e+00
Coefficient: −4.28e−02
Value: B
Brackiella
5
4
3
2
1
0
A (n=15)
B (n=16)
C (n=14)
surface

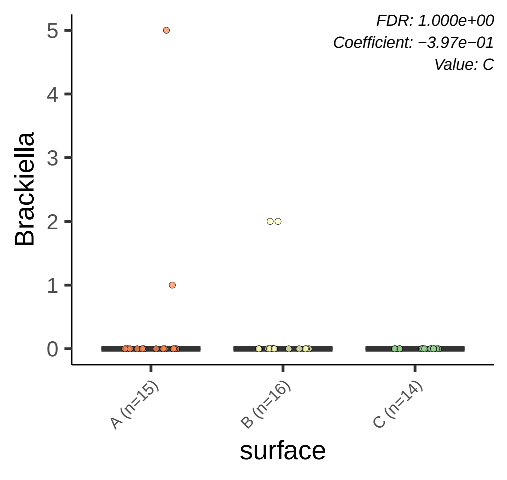
FDR: 1.000e+00
Coefficient: −3.97e−01
Value: C
Brackiella
0
1
2
3
4
5
A (n=15)
B (n=16)
C (n=14)
surface

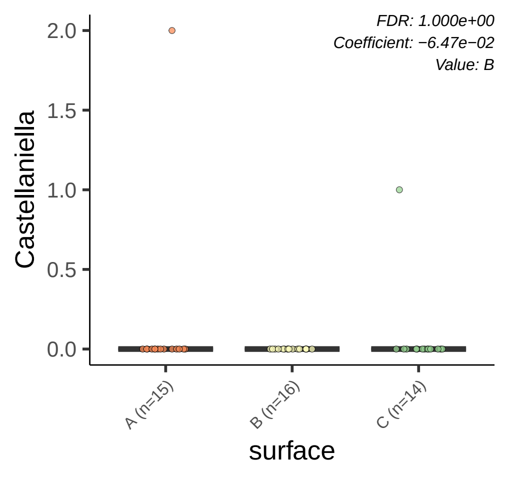
FDR: 1.000e+00
Coefficient: −6.47e−02
Value: B
Castellaniella
2.0
1.5
1.0
0.5
0.0
A (n=15)
B (n=16)
C (n=14)
surface

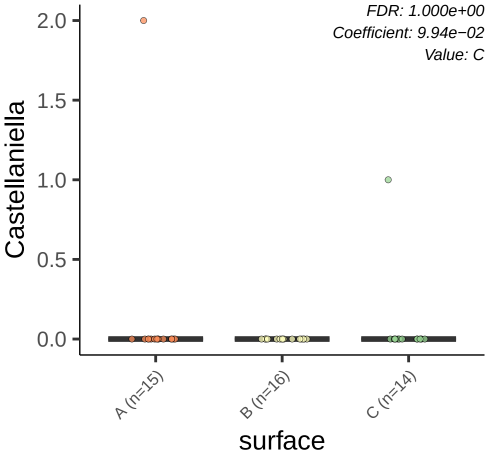

FDR: 1.000e+00
Coefficient: 9.94e−02
Value: C
Castellaniella
2.0
1.5
1.0
0.5
0.0
A (n=15)
B (n=16)
C (n=14)
surface

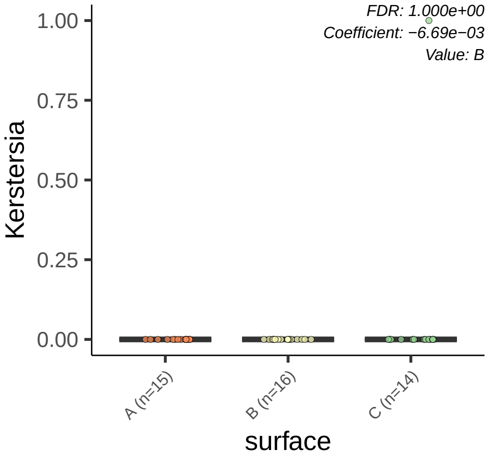

FDR: 1.000e+00
Coefficient: −6.69e−03
Value: B
Kerstersia
1.00
0.75
0.50
0.25
0.00
A (n=15)
B (n=16)
C (n=14)
surface

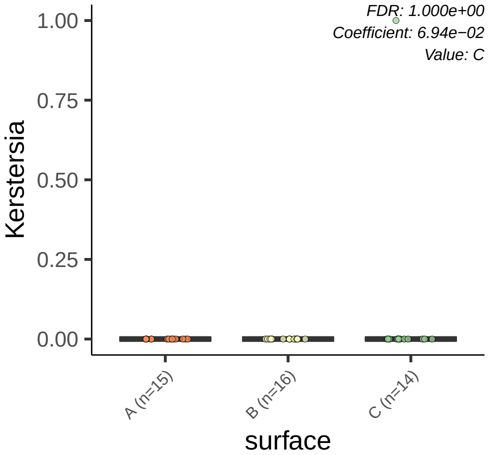
FDR: 1.000e+00
Coefficient: 6.94e−02
Value: C
1.00
0.75
0.50
0.25
0.00
Kerstersia
A (n=15)
B (n=16)
C (n=14)
surface

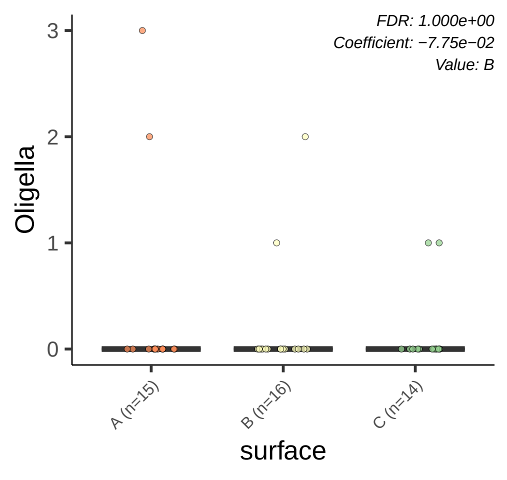
FDR: 1.000e+00
Coefficient: −7.75e−02
Value: B
Oligella
3
2
1
0
A (n=15)
B (n=16)
C (n=14)
surface

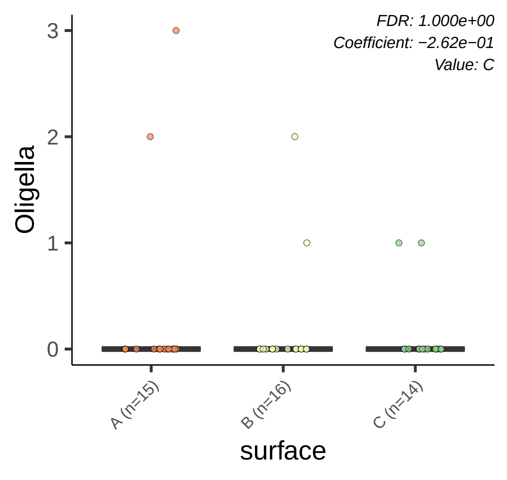
FDR: 1.000e+00
Coefficient: −2.62e−01
Value: C
Oligella
0
1
2
3
A (n=15)
B (n=16)
C (n=14)
surface

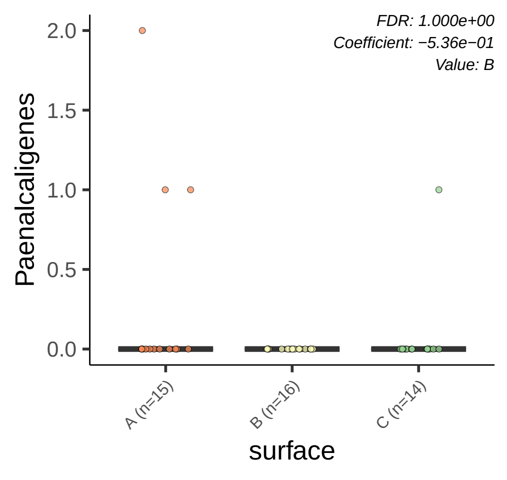
FDR: 1.000e+00
Coefficient: −5.36e−01
Value: B
Paenalcaligenes
2.0
1.5
1.0
0.5
0.0
A (n=15)
B (n=16)
C (n=14)
surface

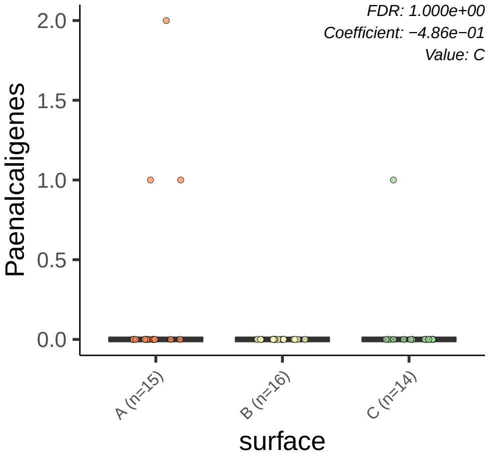

FDR: 1.000e+00
Coefficient: −4.86e−01
Value: C
Paenalcaligenes
2.0
1.5
1.0
0.5
0.0
A (n=15)
B (n=16)
C (n=14)
surface

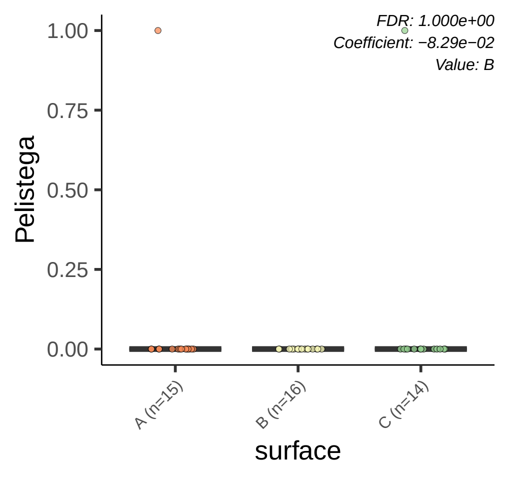

FDR: 1.000e+00
Coefficient: −8.29e−02
Value: B
Pelistega
1.00
0.75
0.50
0.25
0.00
A (n=15)
B (n=16)
C (n=14)
surface

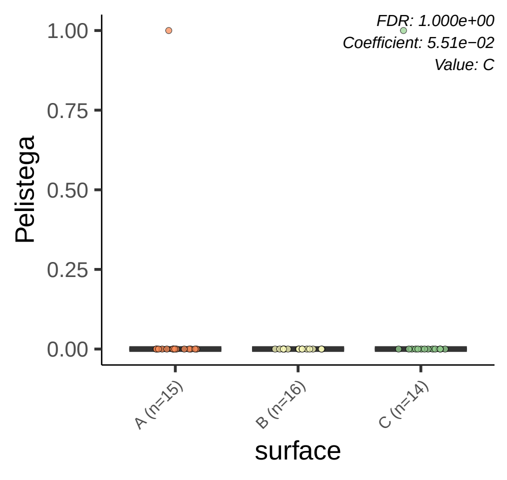
FDR: 1.000e+00
Coefficient: 5.51e−02
Value: C
Pelistega
1.00
0.75
0.50
0.25
0.00
A (n=15)
B (n=16)
C (n=14)
surface

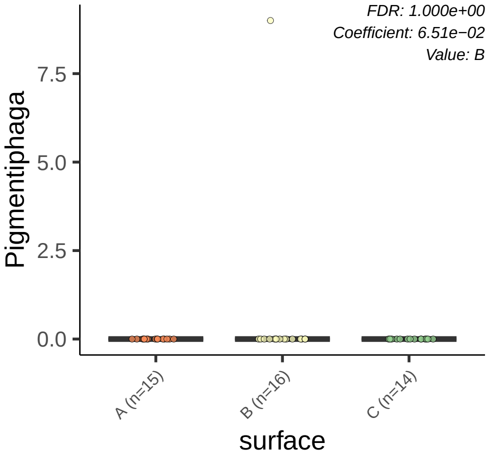
FDR: 1.000e+00
Coefficient: 6.51e−02
Value: B
Pigmentiphaga
7.5
5.0
2.5
0.0
A (n=15)
B (n=16)
C (n=14)
surface

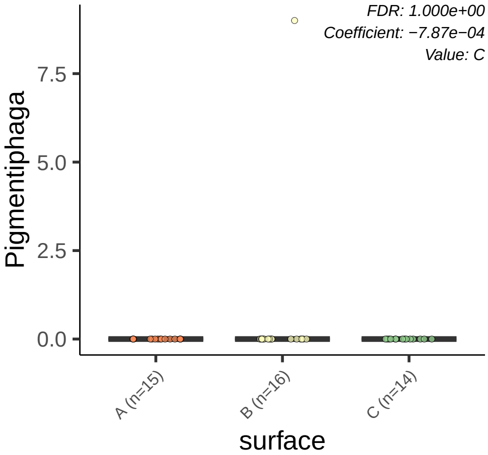

FDR: 1.000e+00
Coefficient: −7.87e−04
Value: C
Pigmentiphaga
7.5
5.0
2.5
0.0
A (n=15)
B (n=16)
C (n=14)
surface

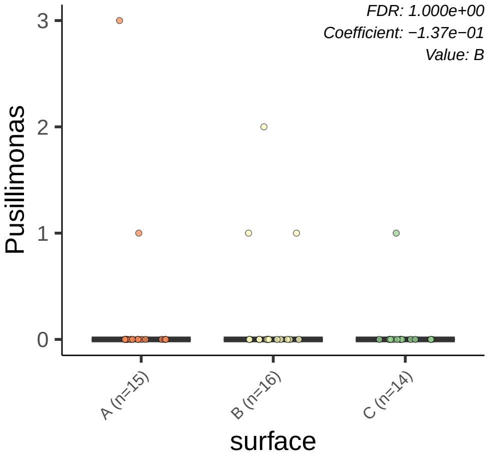

FDR: 1.000e+00
Coefficient: −1.37e−01
Value: B
Pusillimonas
3
2
1
0
A (n=15)
B (n=16)
C (n=14)
surface

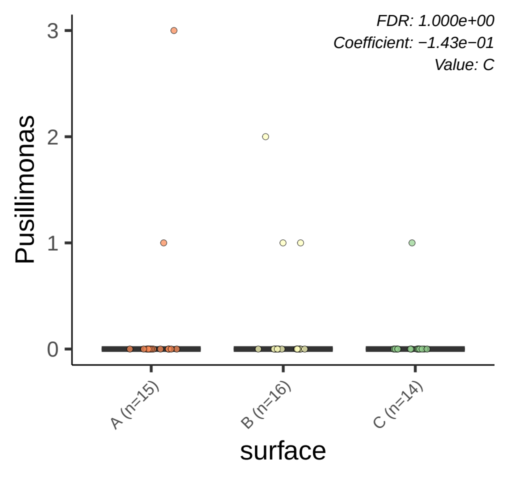
FDR: 1.000e+00
Coefficient: −1.43e−01
Value: C
Pusillimonas
3
2
1
0
A (n=15)
B (n=16)
C (n=14)
surface

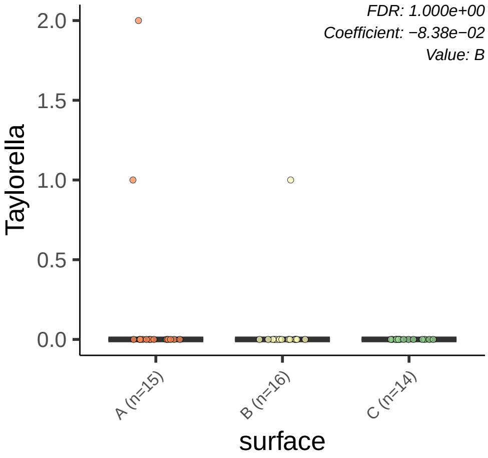

FDR: 1.000e+00
Coefficient: −8.38e−02
Value: B
Taylorella
2.0
1.5
1.0
0.5
0.0
A (n=15)
B (n=16)
C (n=14)
surface

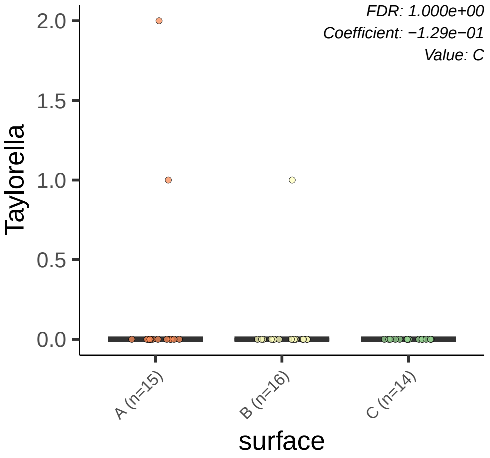
FDR: 1.000e+00
Coefficient: −1.29e−01
Value: C
Taylorella
2.0
1.5
1.0
0.5
0.0
A (n=15)
B (n=16)
C (n=14)
surface

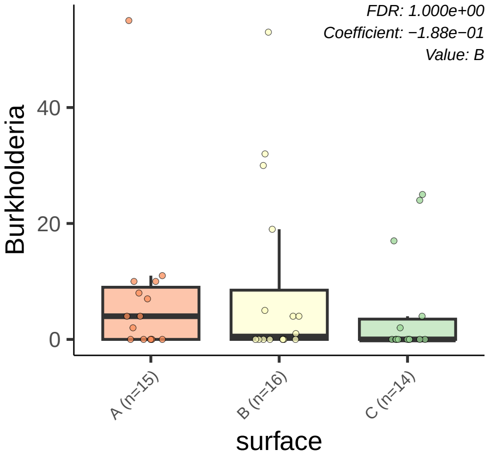
FDR: 1.000e+00
Coefficient: −1.88e−01
Value: B
Burkholderia
0
20
40
A (n=15)
B (n=16)
C (n=14)
surface

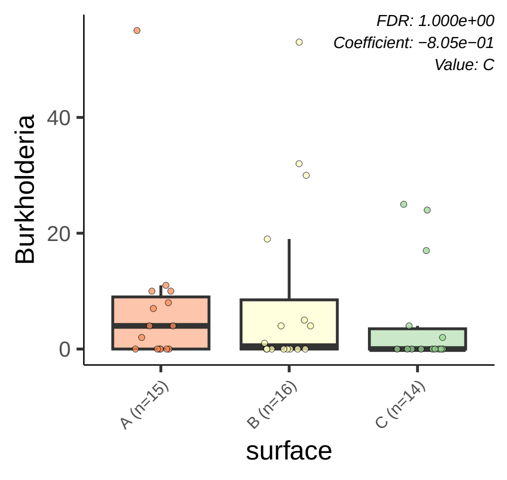
FDR: 1.000e+00
Coefficient: −8.05e−01
Value: C
Burkholderia
0
20
40
A (n=15)
B (n=16)
C (n=14)
surface

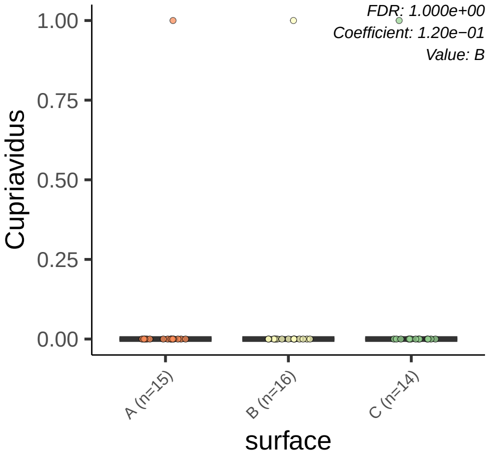
FDR: 1.000e+00
Coefficient: 1.20e−01
Value: B
1.00
0.75
0.50
0.25
0.00
Cupriavidus
A (n=15)
B (n=16)
C (n=14)
surface

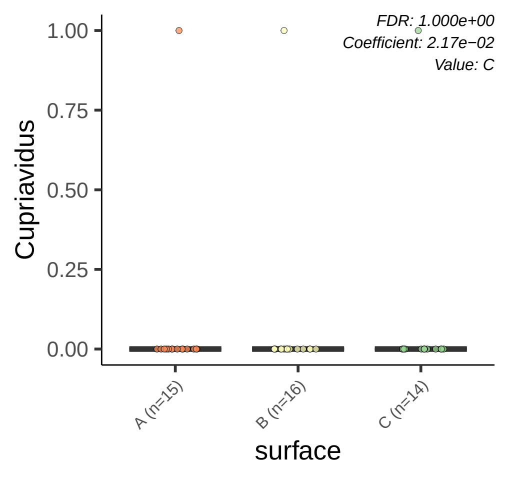
FDR: 1.000e+00
Coefficient: 2.17e−02
Value: C
Cupriavidus
1.00
0.75
0.50
0.25
0.00
A (n=15)
B (n=16)
C (n=14)
surface

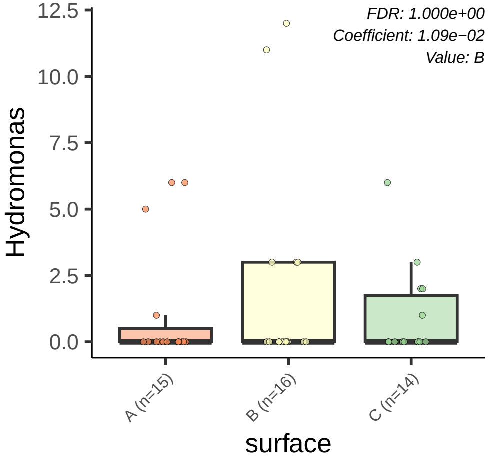

FDR: 1.000e+00
Coefficient: 1.09e−02
Value: B
Hydromonas
12.5
10.0
7.5
5.0
2.5
0.0
A (n=15)
B (n=16)
C (n=14)
surface

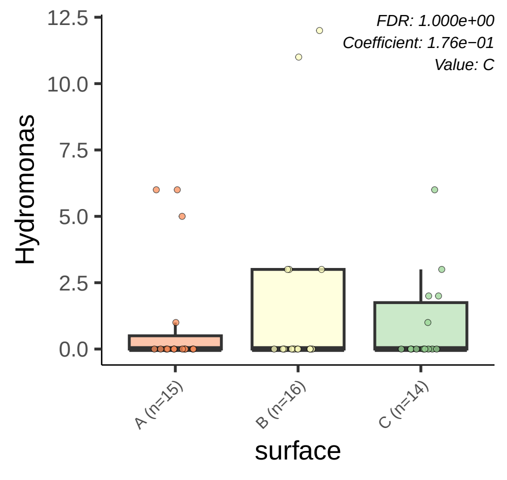
FDR: 1.000e+00
Coefficient: 1.76e−01
Value: C
Hydromonas
12.5
10.0
7.5
5.0
2.5
0.0
A (n=15)
B (n=16)
C (n=14)
surface

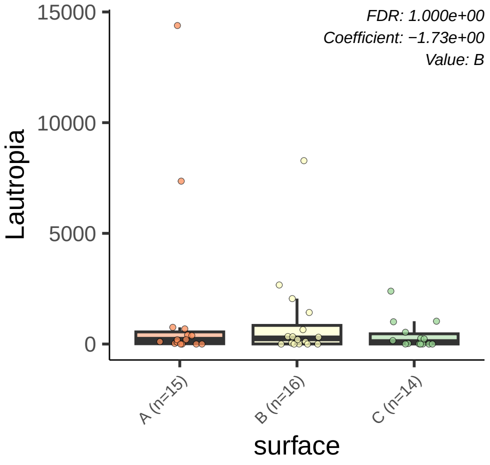

FDR: 1.000e+00
Coefficient: −1.73e+00
Value: B
15000
10000
5000
0
Lautropia
A (n=15)
B (n=16)
C (n=14)
surface

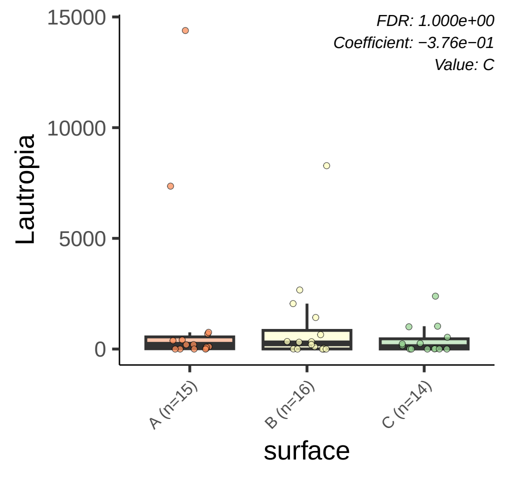

FDR: 1.000e+00
Coefficient: −3.76e−01
Value: C
15000
10000
5000
0
Lautropia
A (n=15)
B (n=16)
C (n=14)
surface

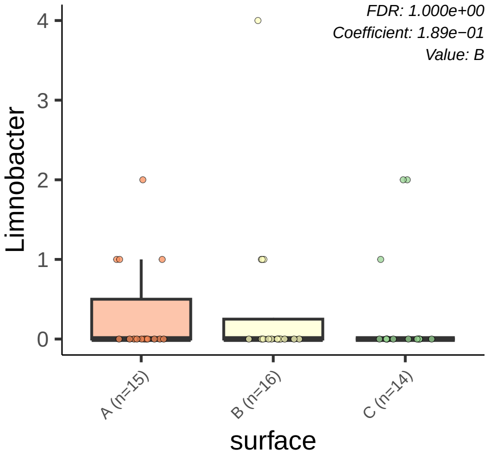
FDR: 1.000e+00
Coefficient: 1.89e−01
Value: B
Limnobacter
0
1
2
3
4
A (n=15)
B (n=16)
C (n=14)
surface

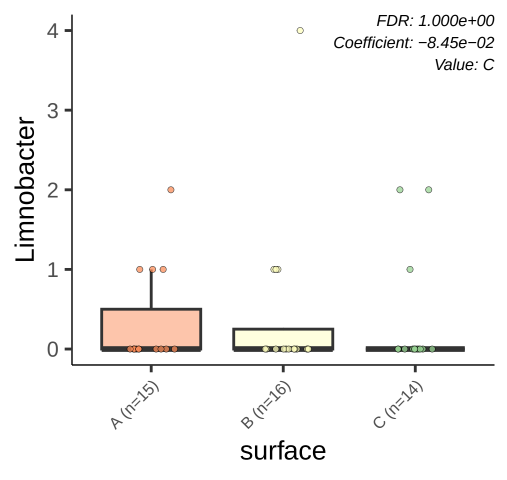

FDR: 1.000e+00
Coefficient: −8.45e−02
Value: C
Limnobacter
0
1
2
3
4
A (n=15)
B (n=16)
C (n=14)
surface

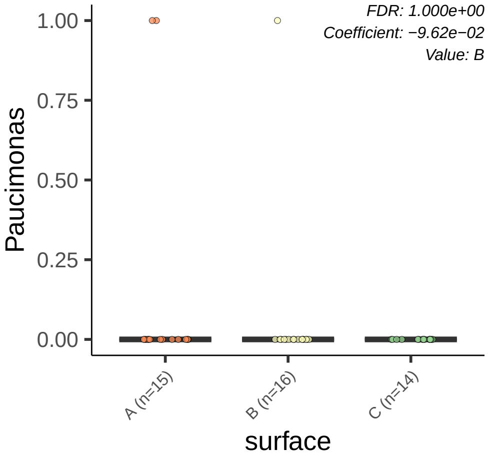
FDR: 1.000e+00
Coefficient: −9.62e−02
Value: B
Paucimonas
1.00
0.75
0.50
0.25
0.00
A (n=15)
B (n=16)
C (n=14)
surface

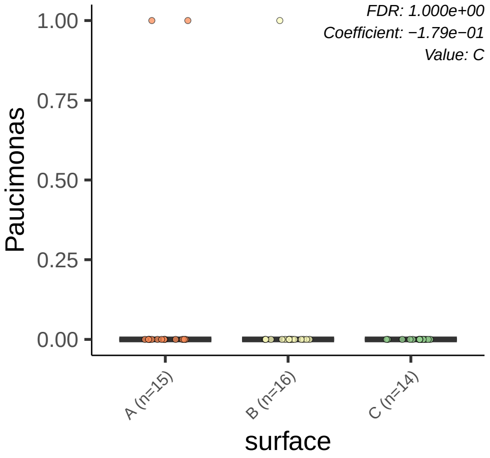

FDR: 1.000e+00
Coefficient: −1.79e−01
Value: C
Paucimonas
1.00
0.75
0.50
0.25
0.00
A (n=15)
B (n=16)
C (n=14)
surface

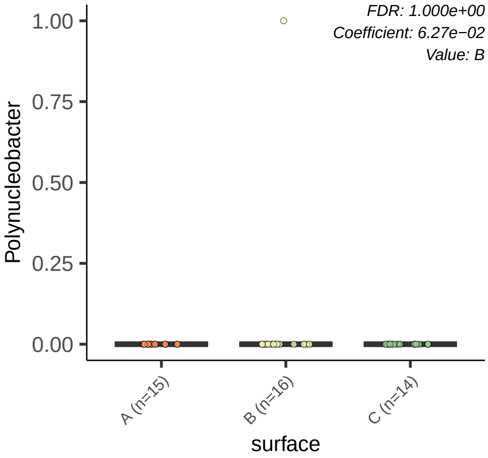

FDR: 1.000e+00
Coefficient: 6.27e−02
Value: B
Polynucleobacter
1.00
0.75
0.50
0.25
0.00
A (n=15)
B (n=16)
C (n=14)
surface

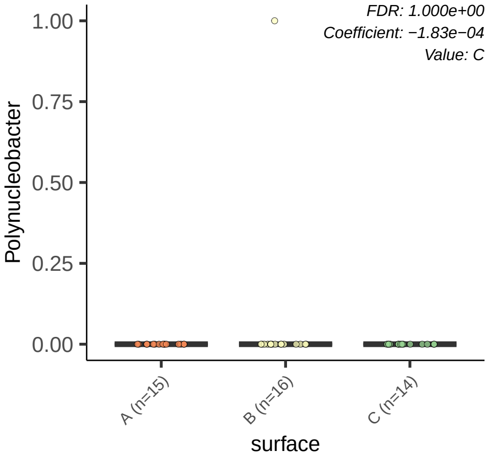

FDR: 1.000e+00
Coefficient: −1.83e−04
Value: C
Polynucleobacter
1.00
0.75
0.50
0.25
0.00
A (n=15)
B (n=16)
C (n=14)
surface

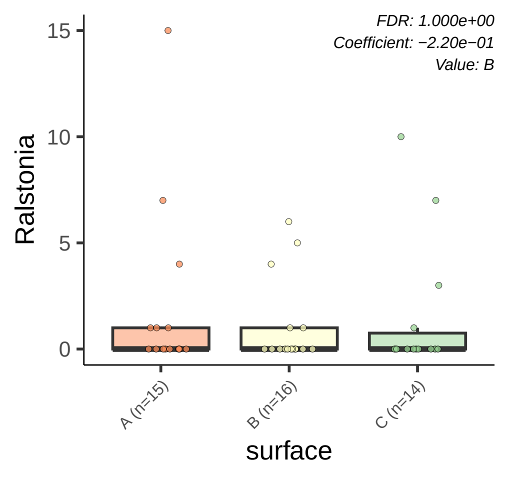

FDR: 1.000e+00
Coefficient: −2.20e−01
Value: B
15
10
5
0
Ralstonia
A (n=15)
B (n=16)
C (n=14)
surface

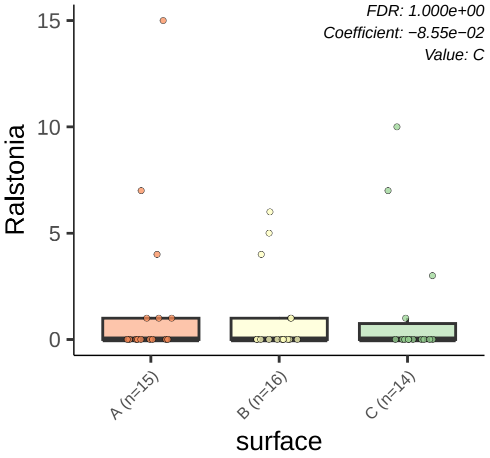
FDR: 1.000e+00
Coefficient: −8.55e−02
Value: C
Ralstonia
15
10
5
0
A (n=15)
B (n=16)
C (n=14)
surface

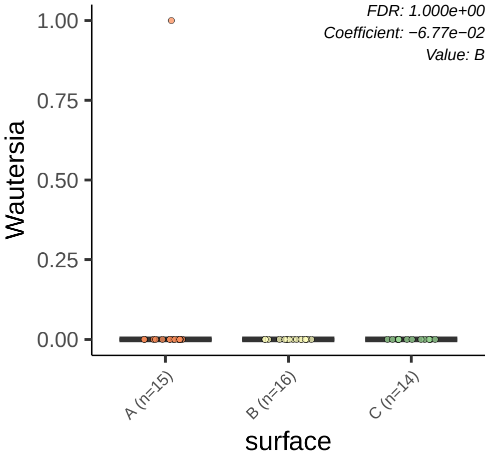
FDR: 1.000e+00
Coefficient: −6.77e−02
Value: B
Wautersia
1.00
0.75
0.50
0.25
0.00
A (n=15)
B (n=16)
C (n=14)
surface

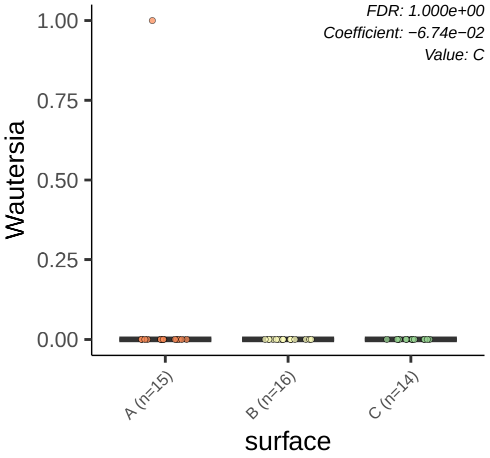
FDR: 1.000e+00
Coefficient: −6.74e−02
Value: C
1.00
0.75
0.50
0.25
0.00
Wautersia
A (n=15)
B (n=16)
C (n=14)
surface

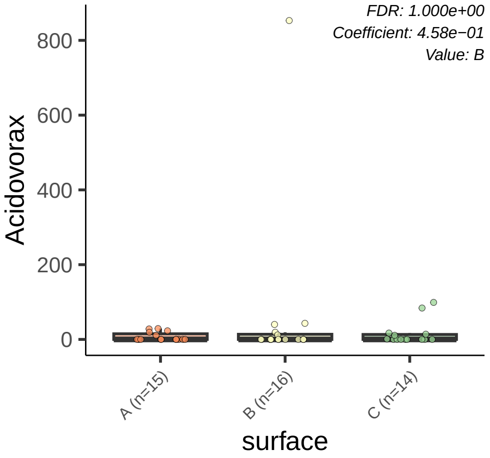
FDR: 1.000e+00
Coefficient: 4.58e−01
Value: B
Acidovorax
800
600
400
200
0
A (n=15)
B (n=16)
C (n=14)
surface

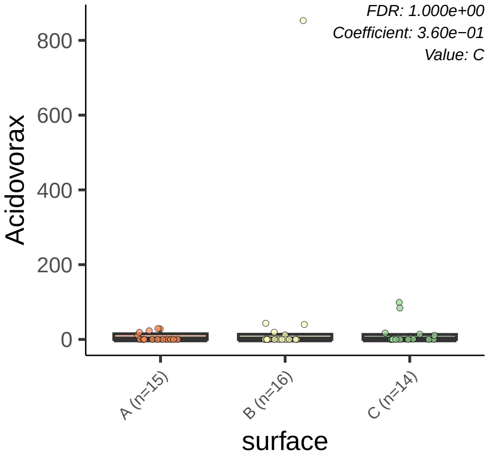

FDR: 1.000e+00
Coefficient: 3.60e−01
Value: C
Acidovorax
800
600
400
200
0
A (n=15)
B (n=16)
C (n=14)
surface

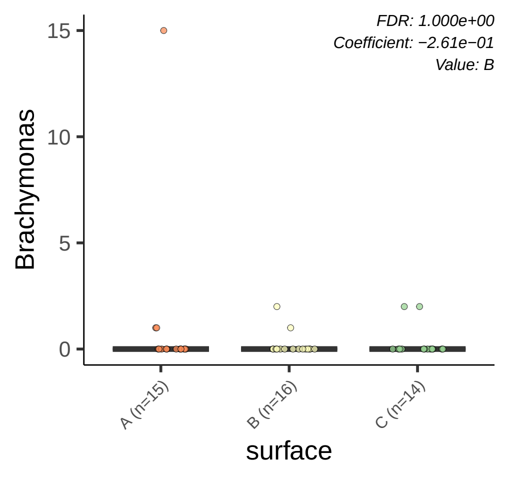

FDR: 1.000e+00
Coefficient: −2.61e−01
Value: B
Brachymonas
15
10
5
0
A (n=15)
B (n=16)
C (n=14)
surface

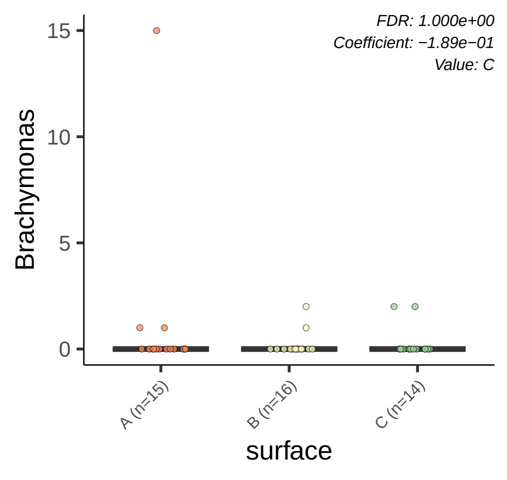
FDR: 1.000e+00
Coefficient: −1.89e−01
Value: C
Brachymonas
15
10
5
0
A (n=15)
B (n=16)
C (n=14)
surface

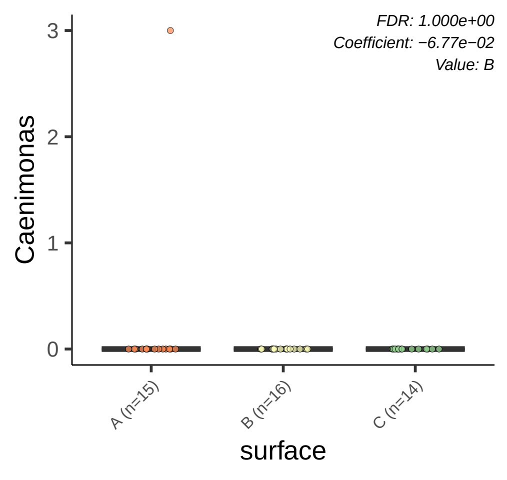
FDR: 1.000e+00
Coefficient: −6.77e−02
Value: B
Caenimonas
3
2
1
0
A (n=15)
B (n=16)
C (n=14)
surface

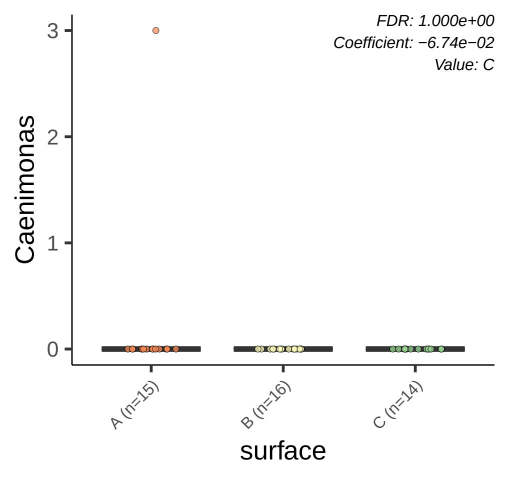

FDR: 1.000e+00
Coefficient: −6.74e−02
Value: C
Caenimonas
3
2
1
0
A (n=15)
B (n=16)
C (n=14)
surface

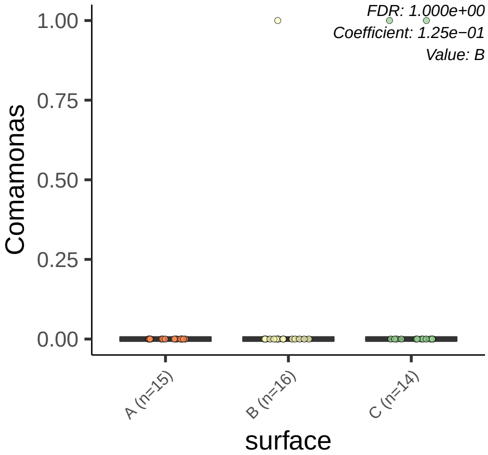

FDR: 1.000e+00
Coefficient: 1.25e−01
Value: B
1.00
0.75
0.50
0.25
0.00
Comamonas
A (n=15)
B (n=16)
C (n=14)
surface

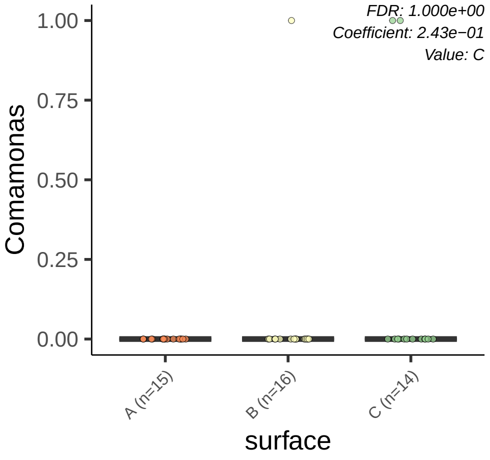
FDR: 1.000e+00
Coefficient: 2.43e−01
Value: C
Comamonas
1.00
0.75
0.50
0.25
0.00
A (n=15)
B (n=16)
C (n=14)
surface

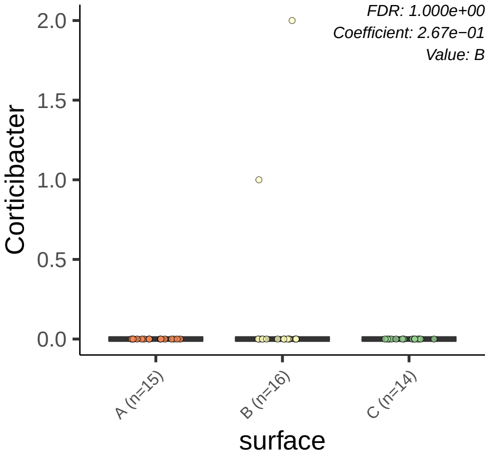
FDR: 1.000e+00
Coefficient: 2.67e−01
Value: B
Corticibacter
2.0
1.5
1.0
0.5
0.0
A (n=15)
B (n=16)
C (n=14)
surface

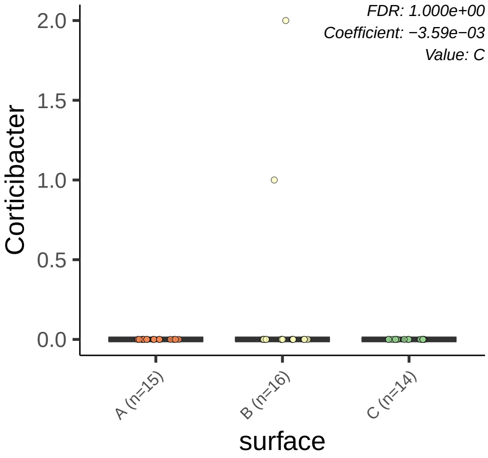
FDR: 1.000e+00
Coefficient: −3.59e−03
Value: C
Corticibacter
2.0
1.5
1.0
0.5
0.0
A (n=15)
B (n=16)
C (n=14)
surface

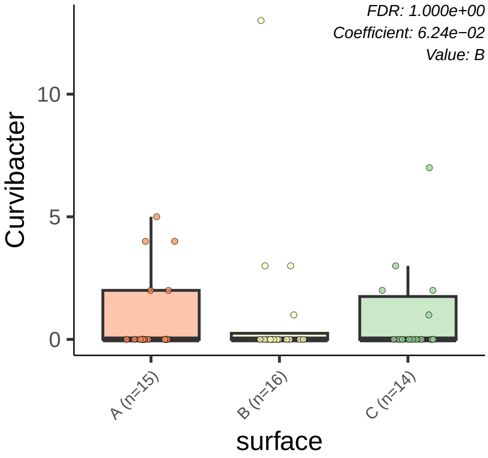

FDR: 1.000e+00
Coefficient: 6.24e−02
Value: B
Curvibacter
0
5
10
A (n=15)
B (n=16)
C (n=14)
surface

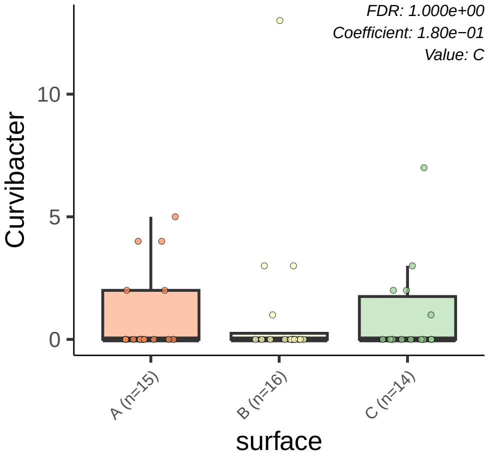
FDR: 1.000e+00
Coefficient: 1.80e−01
Value: C
Curvibacter
10
5
0
A (n=15)
B (n=16)
C (n=14)
surface

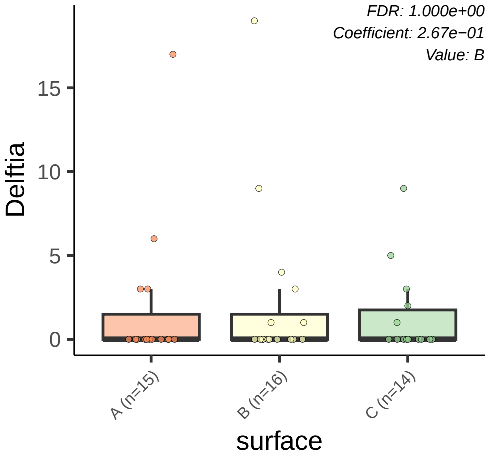

FDR: 1.000e+00
Coefficient: 2.67e−01
Value: B
Delftia
15
10
5
0
A (n=15)
B (n=16)
C (n=14)
surface

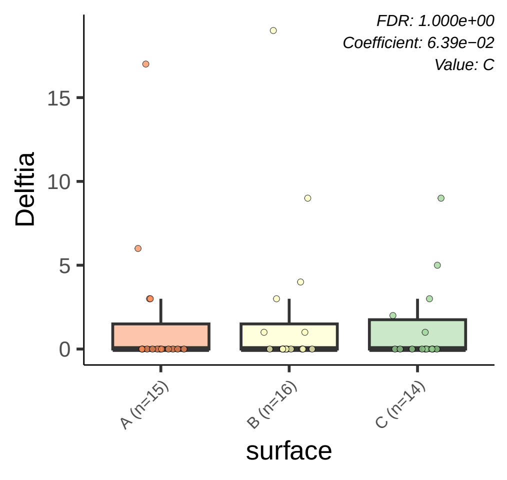

FDR: 1.000e+00
Coefficient: 6.39e−02
Value: C
Delftia
15
10
5
0
A (n=15)
B (n=16)
C (n=14)
surface

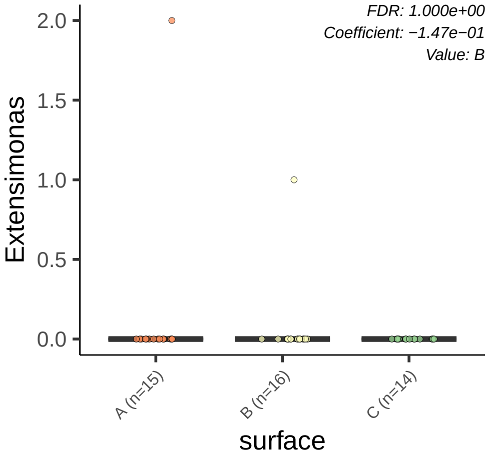
FDR: 1.000e+00
Coefficient: −1.47e−01
Value: B
Extensimonas
2.0
1.5
1.0
0.5
0.0
A (n=15)
B (n=16)
C (n=14)
surface

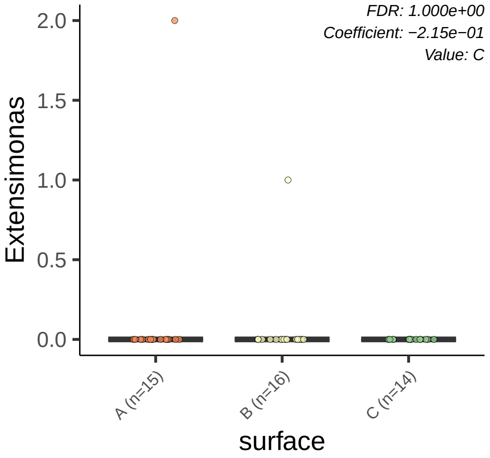
FDR: 1.000e+00
Coefficient: −2.15e−01
Value: C
Extensimonas
2.0
1.5
1.0
0.5
0.0
A (n=15)
B (n=16)
C (n=14)
surface

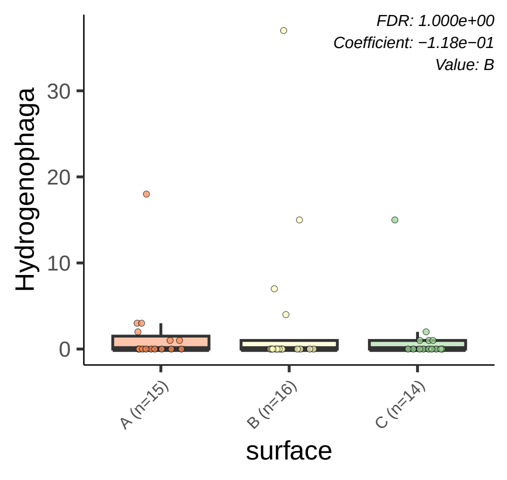

FDR: 1.000e+00
Coefficient: −1.18e−01
Value: B
Hydrogenophaga
0
10
20
30
A (n=15)
B (n=16)
C (n=14)
surface

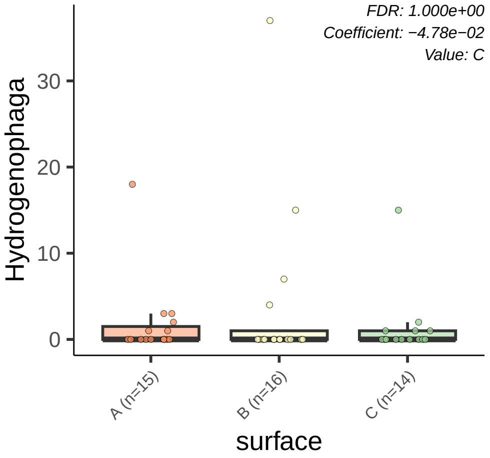
FDR: 1.000e+00
Coefficient: −4.78e−02
Value: C
Hydrogenophaga
30
20
10
0
A (n=15)
B (n=16)
C (n=14)
surface

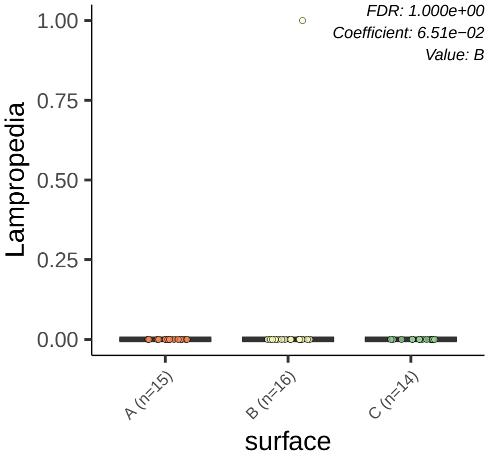
FDR: 1.000e+00
Coefficient: 6.51e−02
Value: B
Lampropedia
1.00
0.75
0.50
0.25
0.00
A (n=15)
B (n=16)
C (n=14)
surface

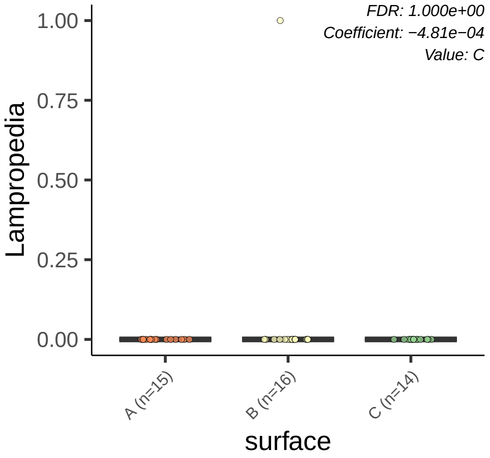
FDR: 1.000e+00
Coefficient: −4.81e−04
Value: C
Lampropedia
1.00
0.75
0.50
0.25
0.00
A (n=15)
B (n=16)
C (n=14)
surface

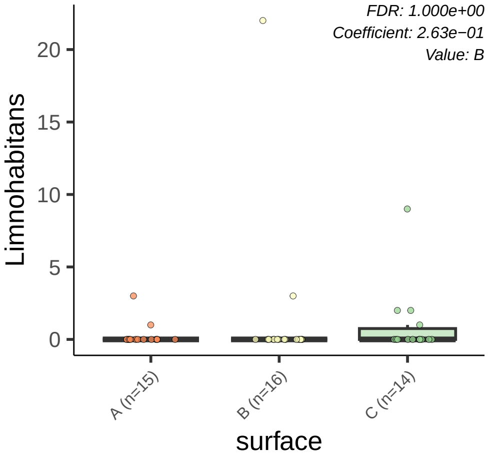
FDR: 1.000e+00
Coefficient: 2.63e−01
Value: B
Limnohabitans
20
15
10
5
0
A (n=15)
B (n=16)
C (n=14)
surface

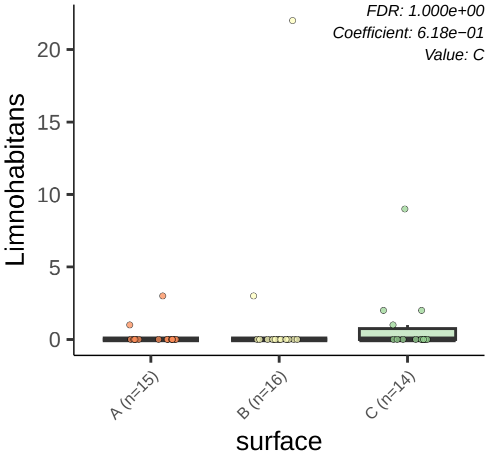
FDR: 1.000e+00
Coefficient: 6.18e−01
Value: C
Limnohabitans
0
5
10
15
20
A (n=15)
B (n=16)
C (n=14)
surface

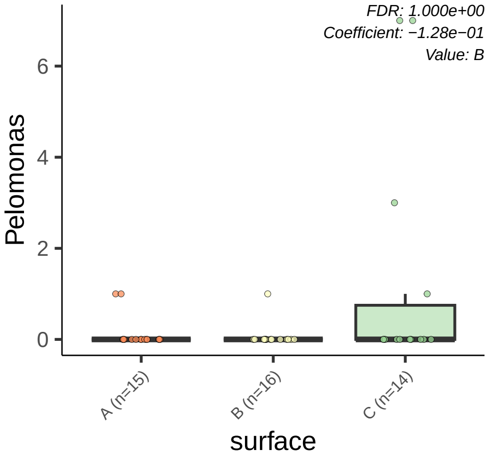
FDR: 1.000e+00
Coefficient: −1.28e−01
Value: B
Pelomonas
0
2
4
6
A (n=15)
B (n=16)
C (n=14)
surface

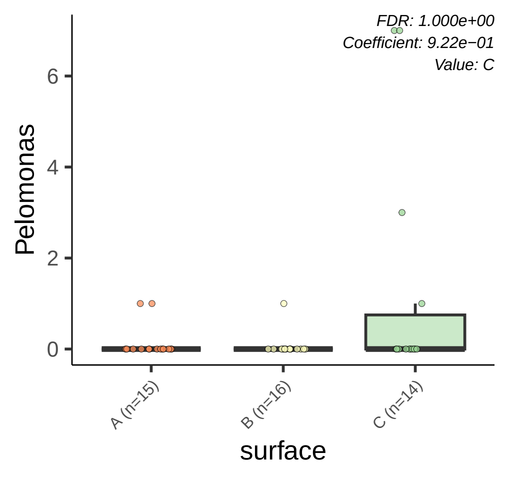

FDR: 1.000e+00
Coefficient: 9.22e−01
Value: C
Pelomonas
0
2
4
6
A (n=15)
B (n=16)
C (n=14)
surface

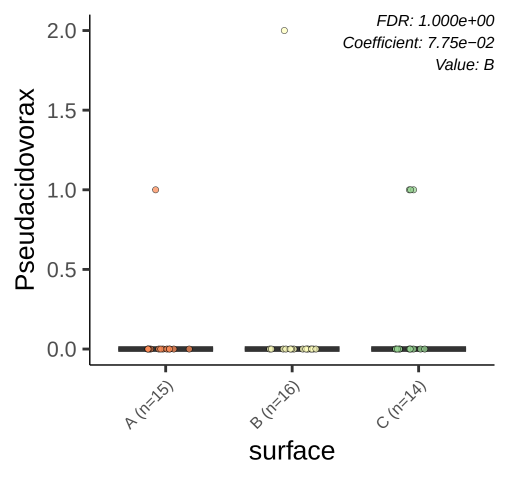
FDR: 1.000e+00
Coefficient: 7.75e−02
Value: B
Pseudacidovorax
2.0
1.5
1.0
0.5
0.0
A (n=15)
B (n=16)
C (n=14)
surface

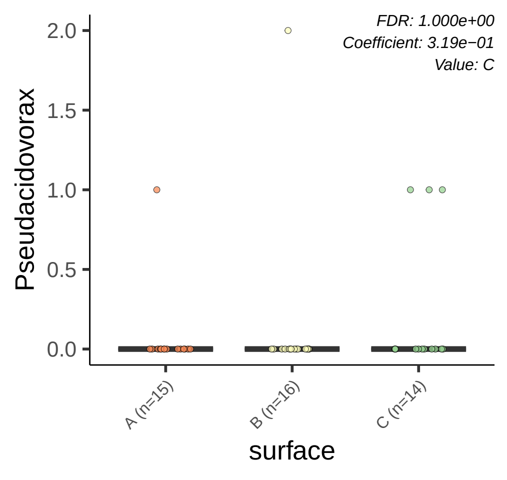
FDR: 1.000e+00
Coefficient: 3.19e−01
Value: C
Pseudacidovorax
2.0
1.5
1.0
0.5
0.0
A (n=15)
B (n=16)
C (n=14)
surface

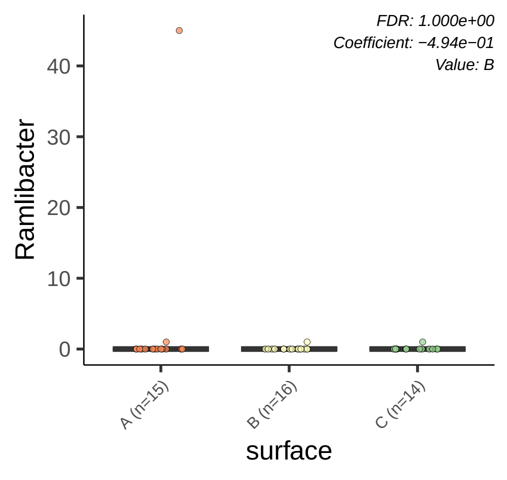

FDR: 1.000e+00
Coefficient: −4.94e−01
Value: B
Ramlibacter
40
30
20
10
0
A (n=15)
B (n=16)
C (n=14)
surface

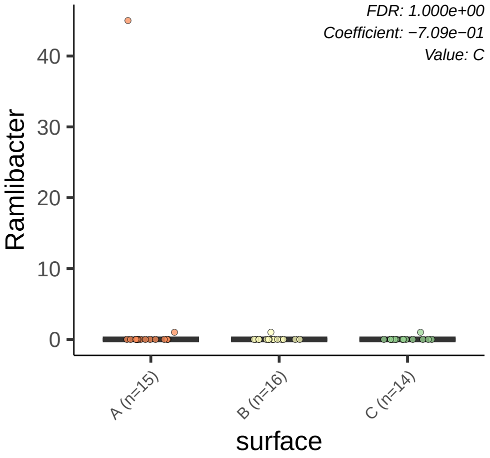

FDR: 1.000e+00
Coefficient: −7.09e−01
Value: C
Ramlibacter
0
10
20
30
40
A (n=15)
B (n=16)
C (n=14)
surface

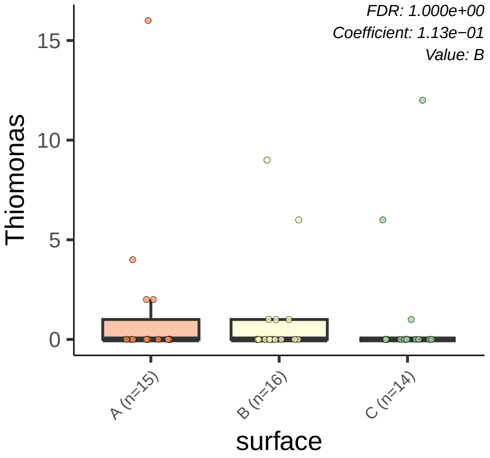

FDR: 1.000e+00
Coefficient: 1.13e−01
Value: B
Thiomonas
15
10
5
0
A (n=15)
B (n=16)
C (n=14)
surface

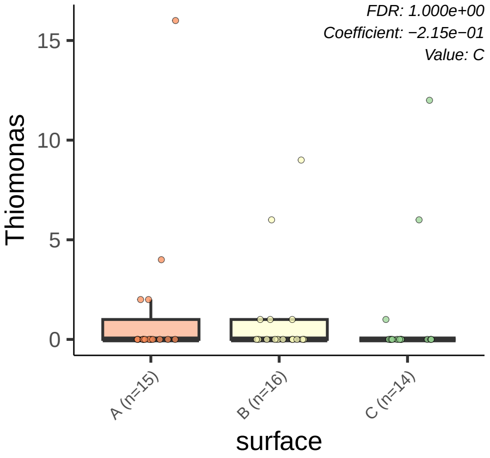
FDR: 1.000e+00
Coefficient: −2.15e−01
Value: C
Thiomonas
15
10
5
0
A (n=15)
B (n=16)
C (n=14)
surface

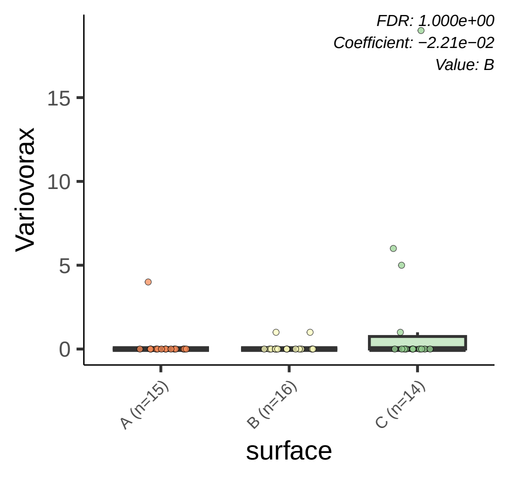

FDR: 1.000e+00
Coefficient: −2.21e−02
Value: B
Variovorax
15
10
5
0
A (n=15)
B (n=16)
C (n=14)
surface

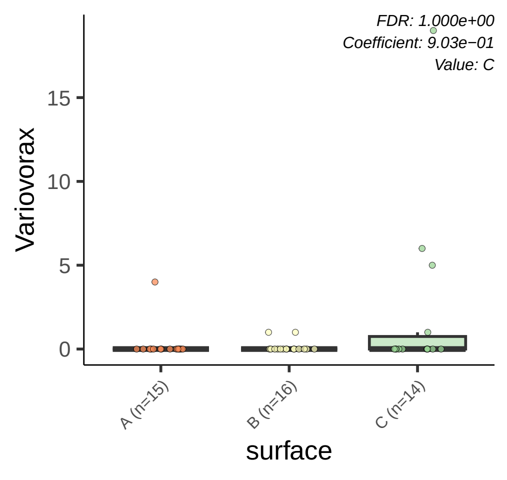

FDR: 1.000e+00
Coefficient: 9.03e−01
Value: C
Variovorax
15
10
5
0
A (n=15)
B (n=16)
C (n=14)
surface

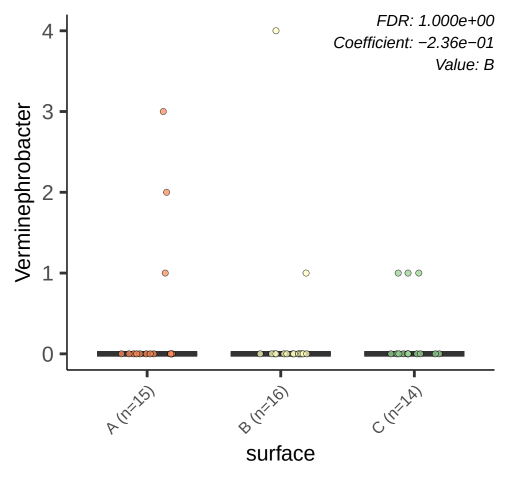
FDR: 1.000e+00
Coefficient: −2.36e−01
Value: B
Verminephrobacter
0
1
2
3
4
A (n=15)
B (n=16)
C (n=14)
surface

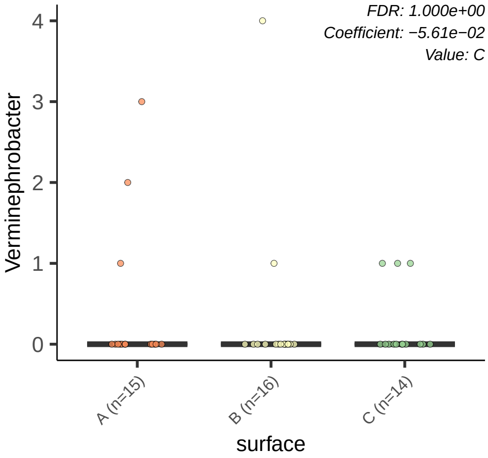

FDR: 1.000e+00
Coefficient: −5.61e−02
Value: C
Verminephrobacter
4
3
2
1
0
A (n=15)
B (n=16)
C (n=14)
surface

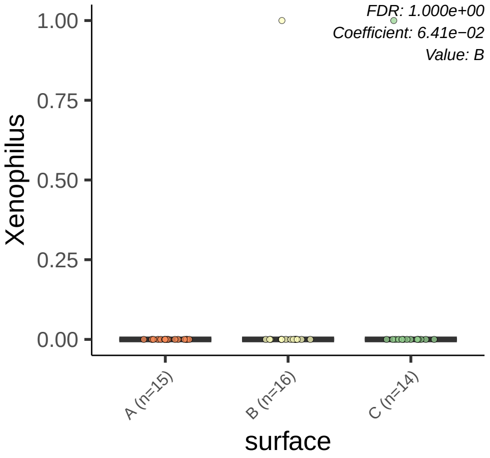
FDR: 1.000e+00
Coefficient: 6.41e−02
Value: B
Xenophilus
1.00
0.75
0.50
0.25
0.00
A (n=15)
B (n=16)
C (n=14)
surface

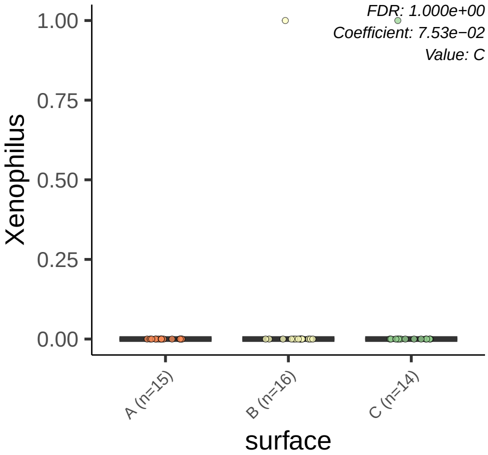
FDR: 1.000e+00
Coefficient: 7.53e−02
Value: C
Xenophilus
1.00
0.75
0.50
0.25
0.00
A (n=15)
B (n=16)
C (n=14)
surface

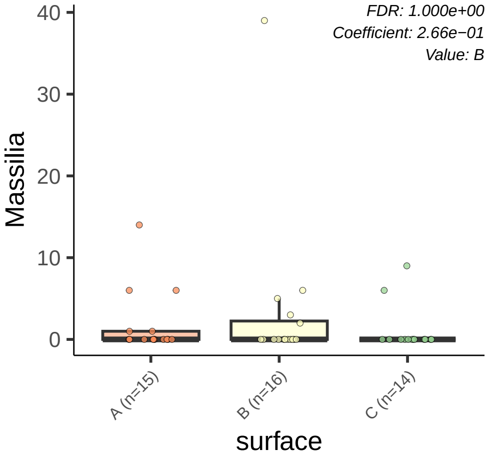
FDR: 1.000e+00
Coefficient: 2.66e−01
Value: B
Massilia
40
30
20
10
0
A (n=15)
B (n=16)
C (n=14)
surface

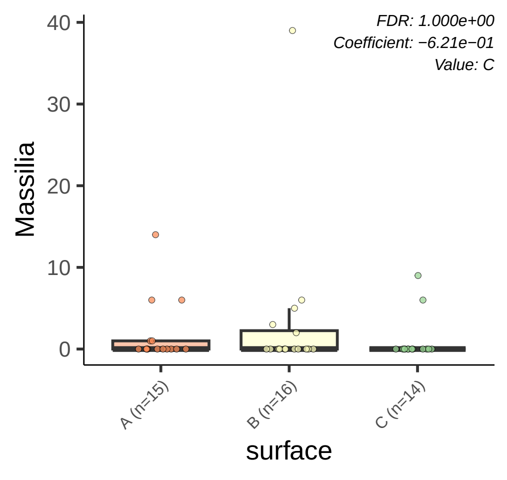
FDR: 1.000e+00
Coefficient: −6.21e−01
Value: C
40
30
20
10
0
Massilia
A (n=15)
B (n=16)
C (n=14)
surface

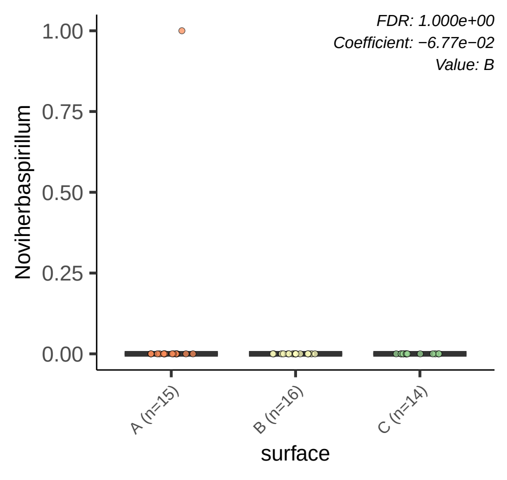

FDR: 1.000e+00
Coefficient: −6.77e−02
Value: B
Noviherbaspirillum
1.00
0.75
0.50
0.25
0.00
A (n=15)
B (n=16)
C (n=14)
surface

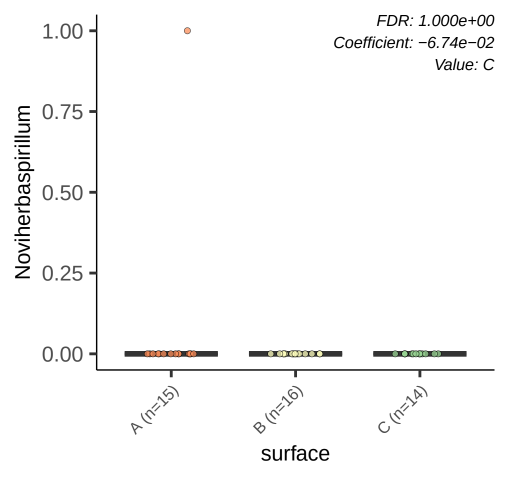

FDR: 1.000e+00
Coefficient: −6.74e−02
Value: C
Noviherbaspirillum
1.00
0.75
0.50
0.25
0.00
A (n=15)
B (n=16)
C (n=14)
surface

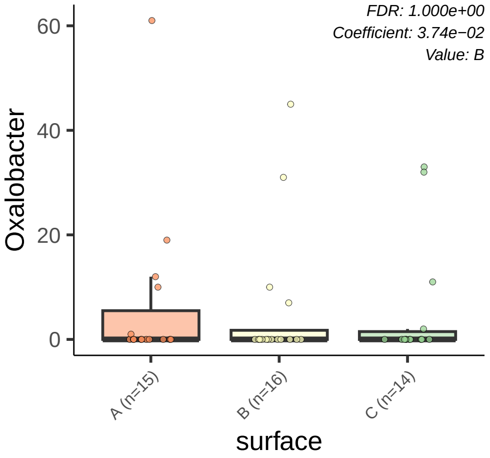

FDR: 1.000e+00
Coefficient: 3.74e−02
Value: B
Oxalobacter
60
40
20
0
A (n=15)
B (n=16)
C (n=14)
surface

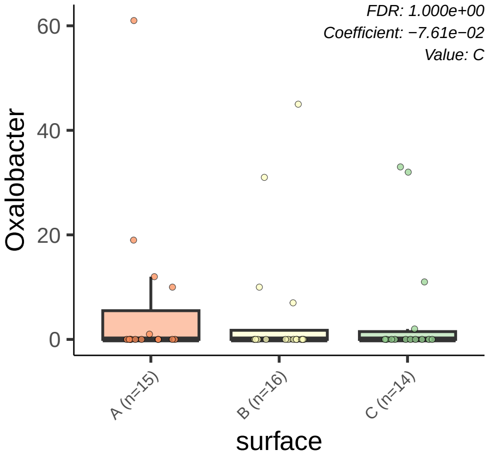

FDR: 1.000e+00
Coefficient: −7.61e−02
Value: C
Oxalobacter
60
40
20
0
A (n=15)
B (n=16)
C (n=14)
surface

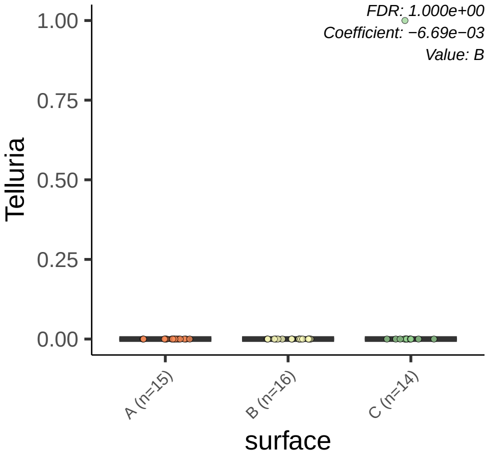

FDR: 1.000e+00
Coefficient: −6.69e−03
Value: B
Telluria
1.00
0.75
0.50
0.25
0.00
A (n=15)
B (n=16)
C (n=14)
surface

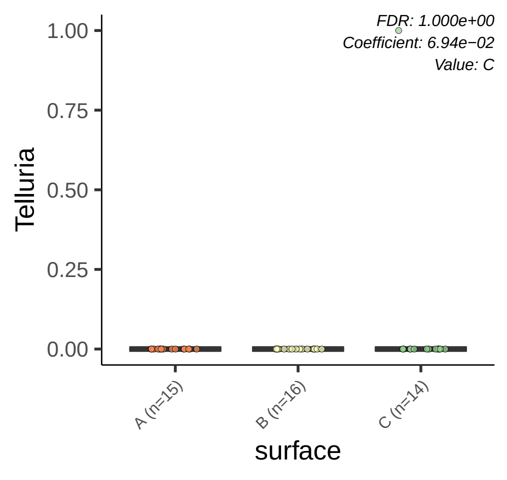

FDR: 1.000e+00
Coefficient: 6.94e−02
Value: C
1.00
0.75
0.50
0.25
0.00
Telluria
A (n=15)
B (n=16)
C (n=14)
surface

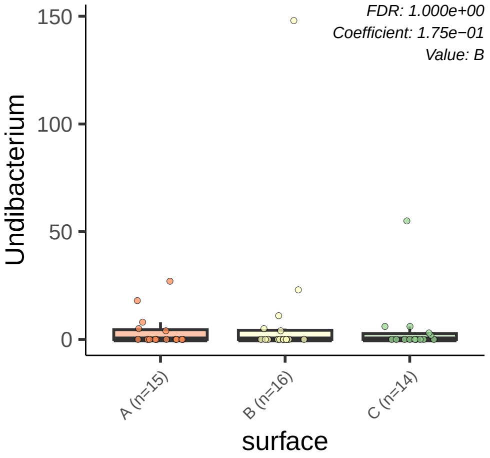

FDR: 1.000e+00
Coefficient: 1.75e−01
Value: B
Undibacterium
150
100
50
0
A (n=15)
B (n=16)
C (n=14)
surface

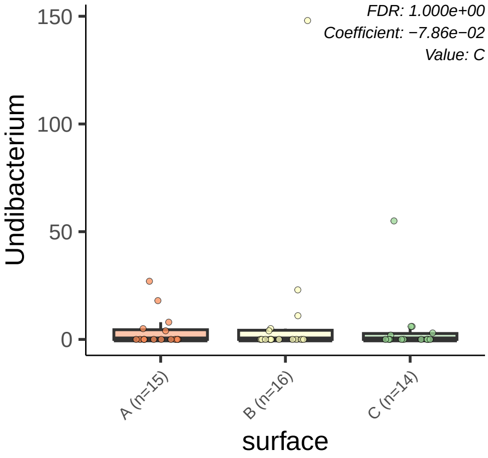
FDR: 1.000e+00
Coefficient: −7.86e−02
Value: C
Undibacterium
150
100
50
0
A (n=15)
B (n=16)
C (n=14)
surface

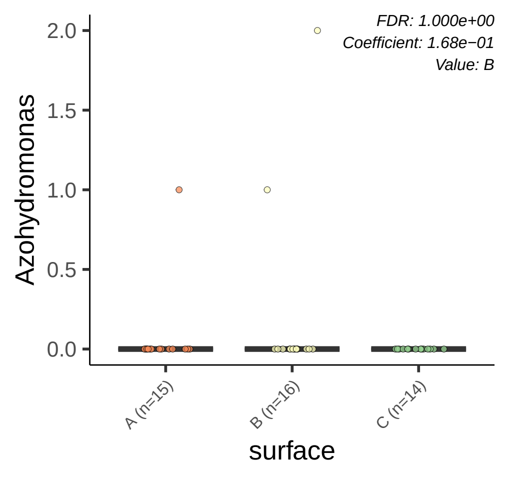

FDR: 1.000e+00
Coefficient: 1.68e−01
Value: B
Azohydromonas
2.0
1.5
1.0
0.5
0.0
A (n=15)
B (n=16)
C (n=14)
surface

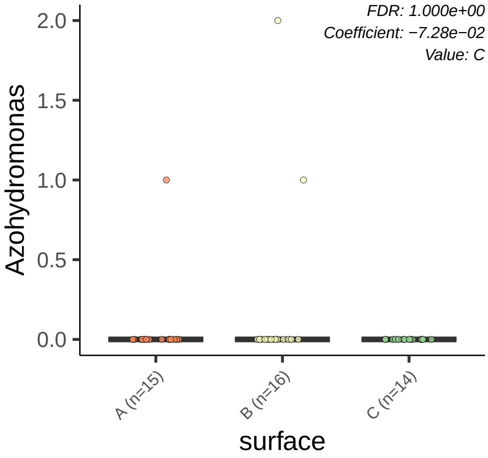

FDR: 1.000e+00
Coefficient: −7.28e−02
Value: C
Azohydromonas
2.0
1.5
1.0
0.5
0.0
A (n=15)
B (n=16)
C (n=14)
surface

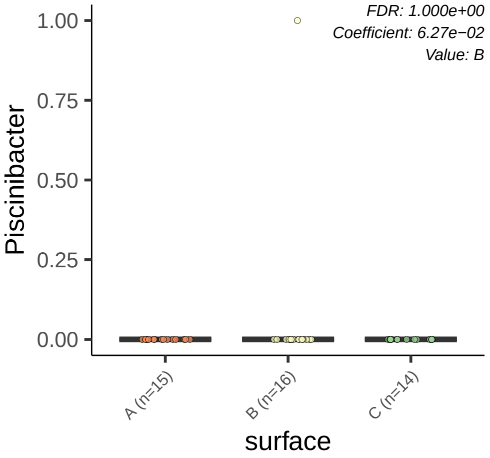

FDR: 1.000e+00
Coefficient: 6.27e−02
Value: B
Piscinibacter
1.00
0.75
0.50
0.25
0.00
A (n=15)
B (n=16)
C (n=14)
surface

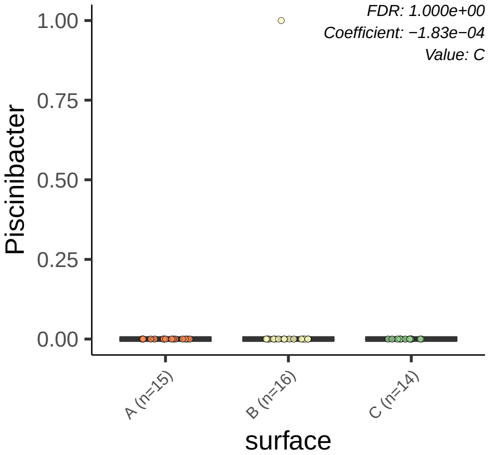

FDR: 1.000e+00
Coefficient: −1.83e−04
Value: C
Piscinibacter
1.00
0.75
0.50
0.25
0.00
A (n=15)
B (n=16)
C (n=14)
surface

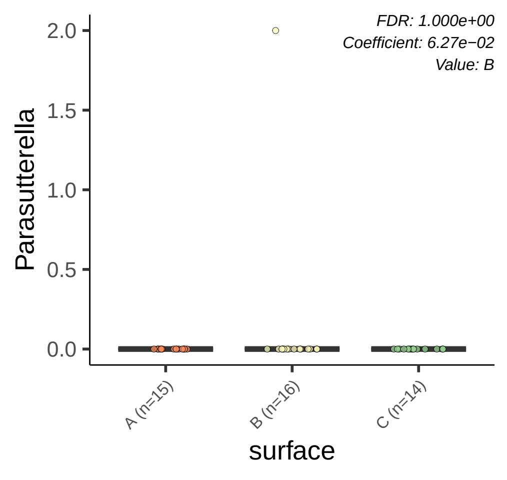
FDR: 1.000e+00
Coefficient: 6.27e−02
Value: B
Parasutterella
2.0
1.5
1.0
0.5
0.0
A (n=15)
B (n=16)
C (n=14)
surface

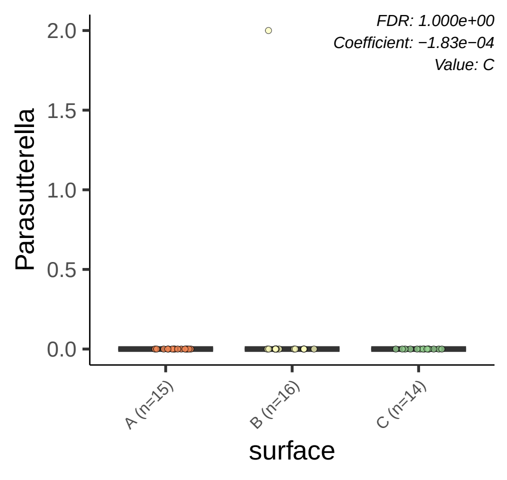

FDR: 1.000e+00
Coefficient: −1.83e−04
Value: C
Parasutterella
2.0
1.5
1.0
0.5
0.0
A (n=15)
B (n=16)
C (n=14)
surface

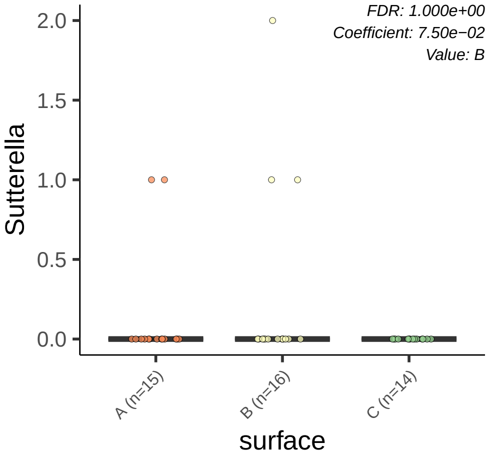

FDR: 1.000e+00
Coefficient: 7.50e−02
Value: B
Sutterella
2.0
1.5
1.0
0.5
0.0
A (n=15)
B (n=16)
C (n=14)
surface

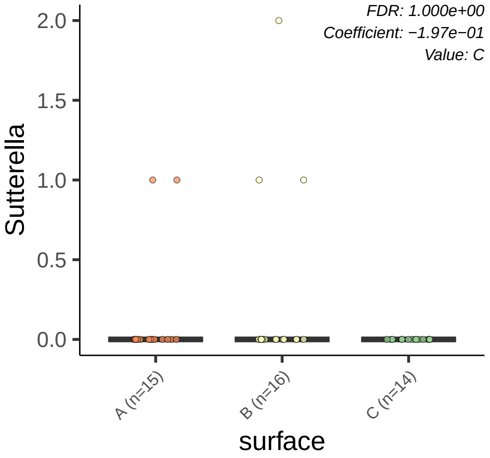
FDR: 1.000e+00
Coefficient: −1.97e−01
Value: C
Sutterella
2.0
1.5
1.0
0.5
0.0
A (n=15)
B (n=16)
C (n=14)
surface

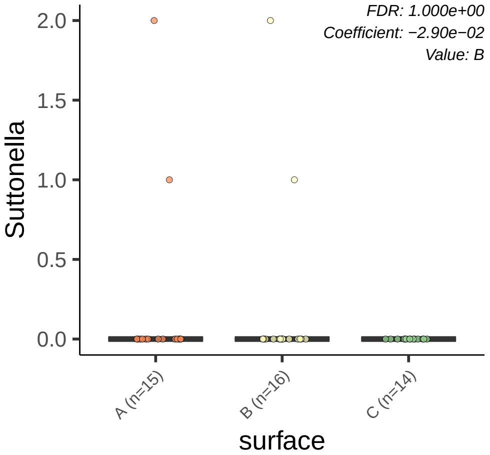
FDR: 1.000e+00
Coefficient: −2.90e−02
Value: B
Suttonella
2.0
1.5
1.0
0.5
0.0
A (n=15)
B (n=16)
C (n=14)
surface

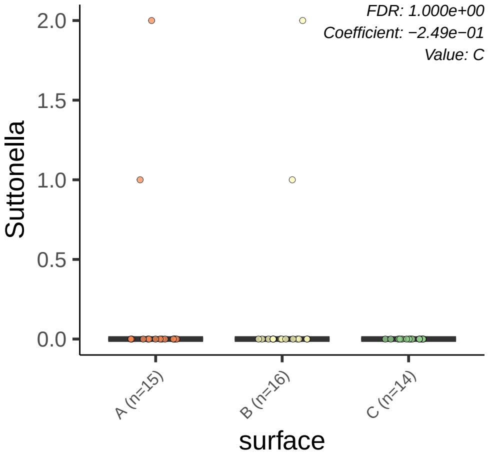

FDR: 1.000e+00
Coefficient: −2.49e−01
Value: C
Suttonella
2.0
1.5
1.0
0.5
0.0
A (n=15)
B (n=16)
C (n=14)
surface

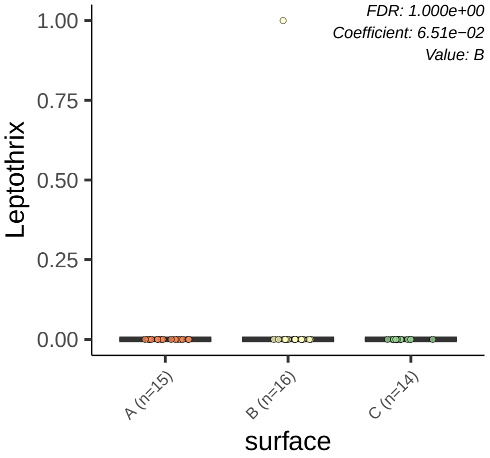
FDR: 1.000e+00
Coefficient: 6.51e−02
Value: B
Leptothrix
1.00
0.75
0.50
0.25
0.00
A (n=15)
B (n=16)
C (n=14)
surface

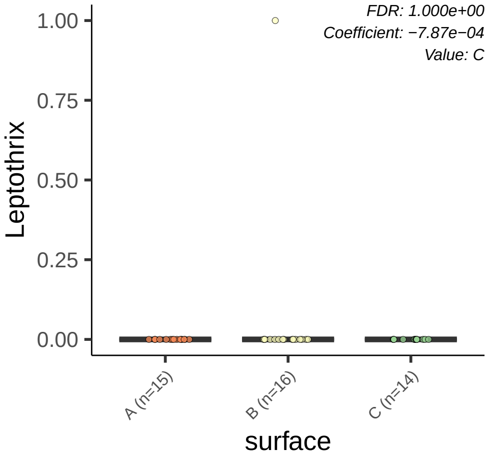
FDR: 1.000e+00
Coefficient: −7.87e−04
Value: C
Leptothrix
1.00
0.75
0.50
0.25
0.00
A (n=15)
B (n=16)
C (n=14)
surface

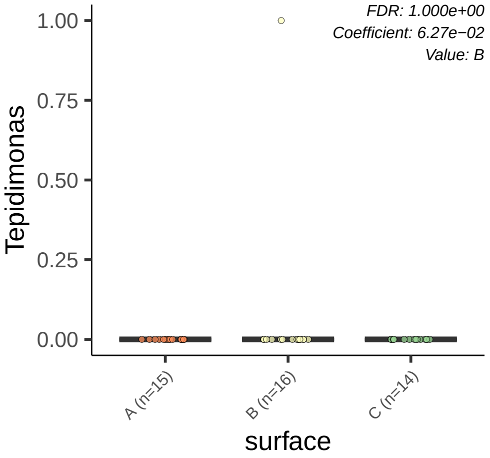
FDR: 1.000e+00
Coefficient: 6.27e−02
Value: B
Tepidimonas
1.00
0.75
0.50
0.25
0.00
A (n=15)
B (n=16)
C (n=14)
surface

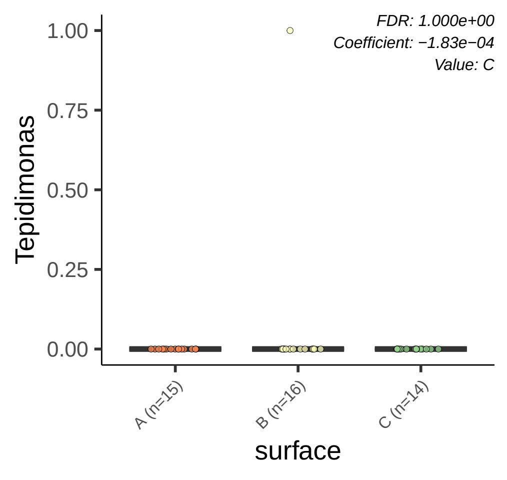
FDR: 1.000e+00
Coefficient: −1.83e−04
Value: C
Tepidimonas
1.00
0.75
0.50
0.25
0.00
A (n=15)
B (n=16)
C (n=14)
surface

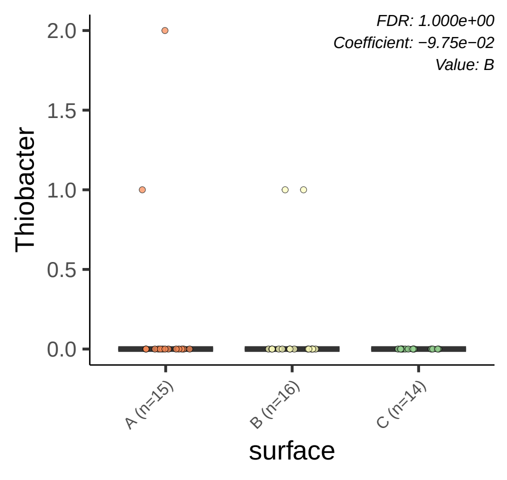

FDR: 1.000e+00
Coefficient: −9.75e−02
Value: B
Thiobacter
2.0
1.5
1.0
0.5
0.0
A (n=15)
B (n=16)
C (n=14)
surface

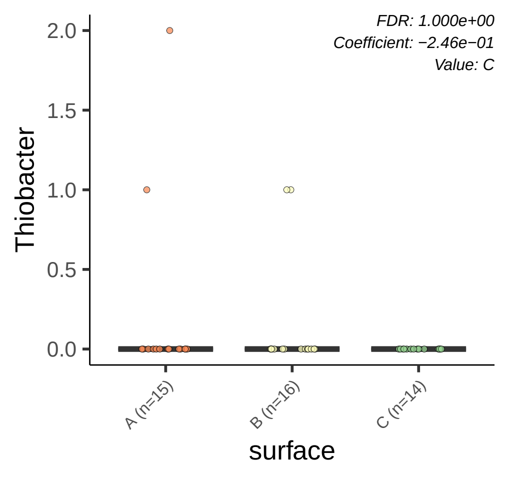

FDR: 1.000e+00
Coefficient: −2.46e−01
Value: C
Thiobacter
2.0
1.5
1.0
0.5
0.0
A (n=15)
B (n=16)
C (n=14)
surface

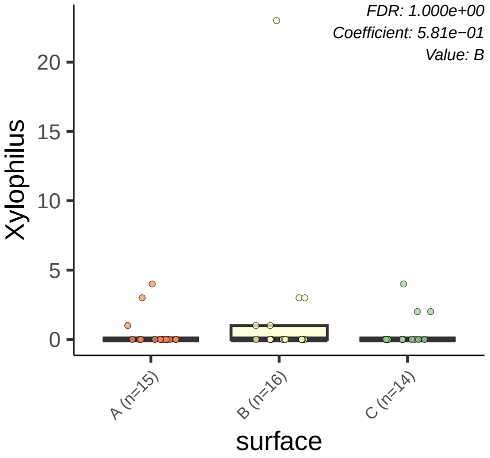
FDR: 1.000e+00
Coefficient: 5.81e−01
Value: B
Xylophilus
20
15
10
5
0
A (n=15)
B (n=16)
C (n=14)
surface

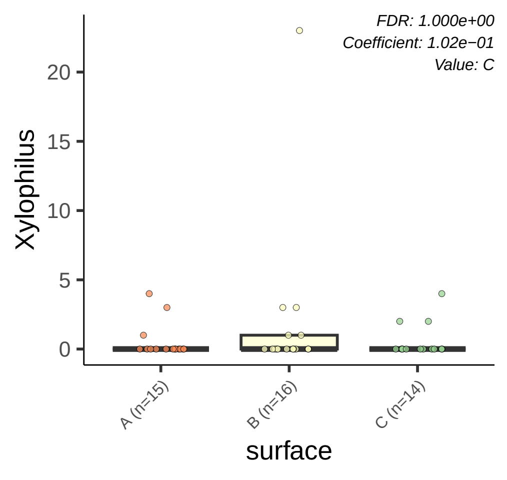

FDR: 1.000e+00
Coefficient: 1.02e−01
Value: C
Xylophilus
0
5
10
15
20
A (n=15)
B (n=16)
C (n=14)
surface

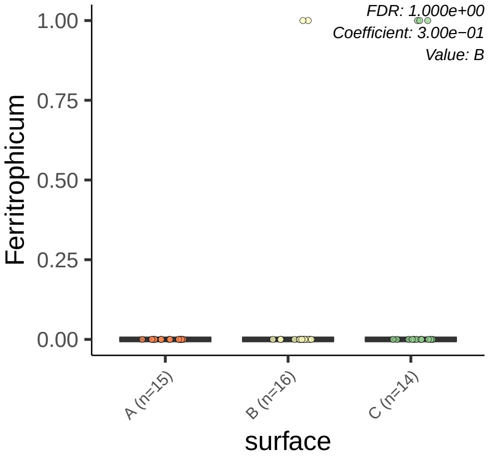
FDR: 1.000e+00
Coefficient: 3.00e−01
Value: B
Ferritrophicum
1.00
0.75
0.50
0.25
0.00
A (n=15)
B (n=16)
C (n=14)
surface

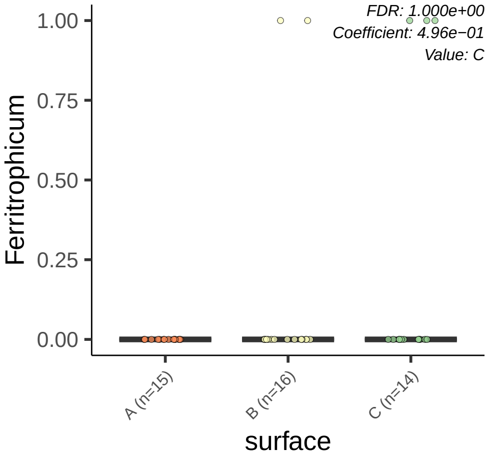
FDR: 1.000e+00
Coefficient: 4.96e−01
Value: C
Ferritrophicum
1.00
0.75
0.50
0.25
0.00
A (n=15)
B (n=16)
C (n=14)
surface

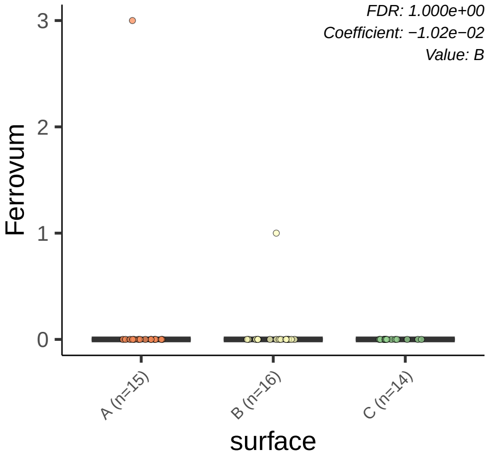
FDR: 1.000e+00
Coefficient: −1.02e−02
Value: B
Ferrovum
3
2
1
0
A (n=15)
B (n=16)
C (n=14)
surface

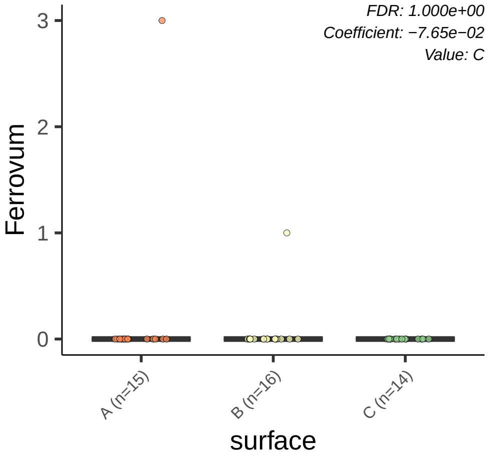

FDR: 1.000e+00
Coefficient: −7.65e−02
Value: C
Ferrovum
3
2
1
0
A (n=15)
B (n=16)
C (n=14)
surface

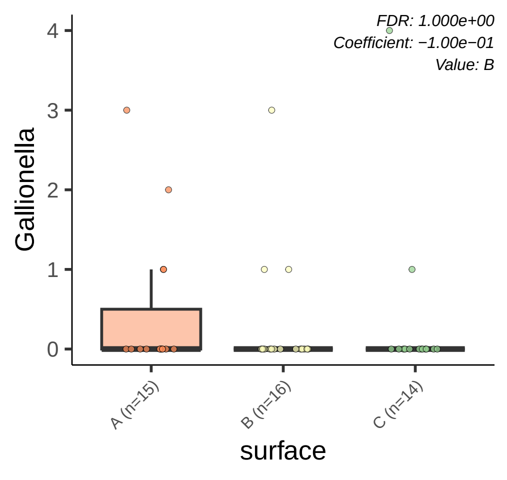
FDR: 1.000e+00
Coefficient: −1.00e−01
Value: B
Gallionella
0
1
2
3
4
A (n=15)
B (n=16)
C (n=14)
surface

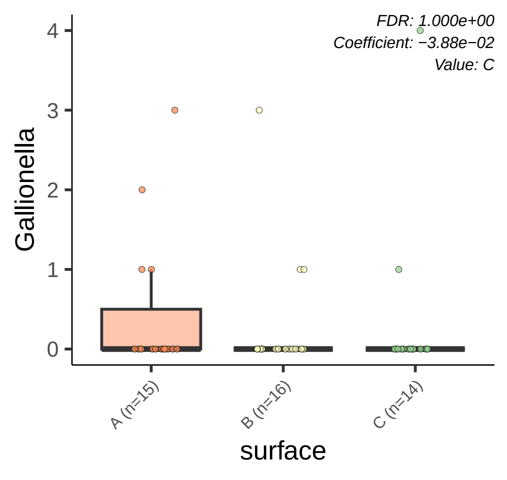
FDR: 1.000e+00
Coefficient: −3.88e−02
Value: C
Gallionella
0
1
2
3
4
A (n=15)
B (n=16)
C (n=14)
surface

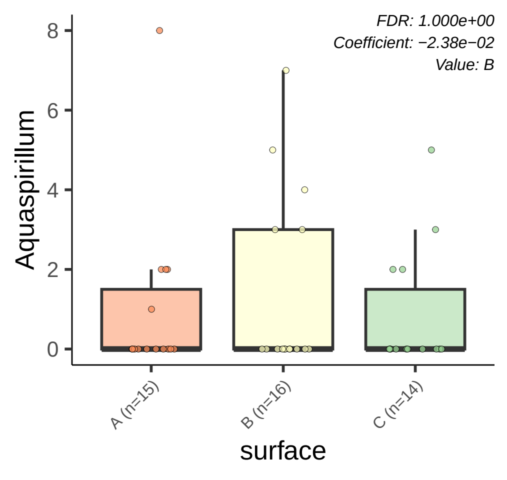

FDR: 1.000e+00
Coefficient: −2.38e−02
Value: B
Aquaspirillum
0
2
4
6
8
A (n=15)
B (n=16)
C (n=14)
surface

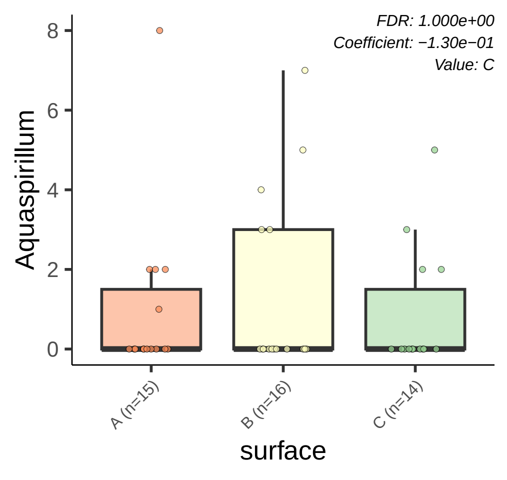
FDR: 1.000e+00
Coefficient: −1.30e−01
Value: C
Aquaspirillum
0
2
4
6
8
A (n=15)
B (n=16)
C (n=14)
surface

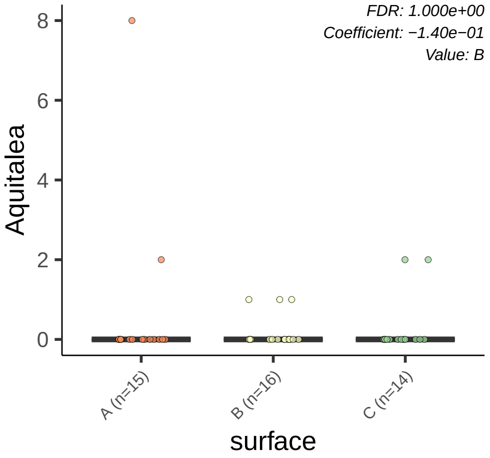
FDR: 1.000e+00
Coefficient: −1.40e−01
Value: B
Aquitalea
8
6
4
2
0
A (n=15)
B (n=16)
C (n=14)
surface

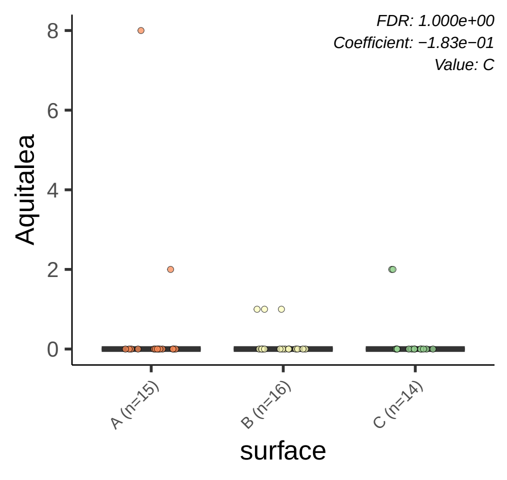

FDR: 1.000e+00
Coefficient: −1.83e−01
Value: C
Aquitalea
8
6
4
2
0
A (n=15)
B (n=16)
C (n=14)
surface

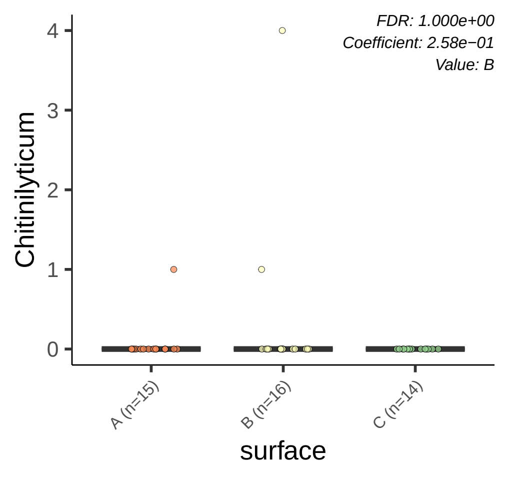
FDR: 1.000e+00
Coefficient: 2.58e−01
Value: B
Chitinilyticum
4
3
2
1
0
A (n=15)
B (n=16)
C (n=14)
surface

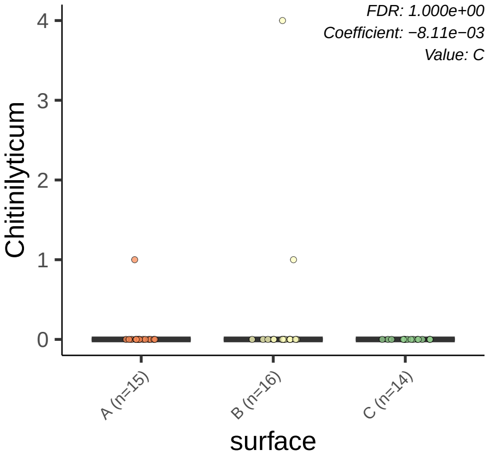
FDR: 1.000e+00
Coefficient: −8.11e−03
Value: C
Chitinilyticum
4
3
2
1
0
A (n=15)
B (n=16)
C (n=14)
surface

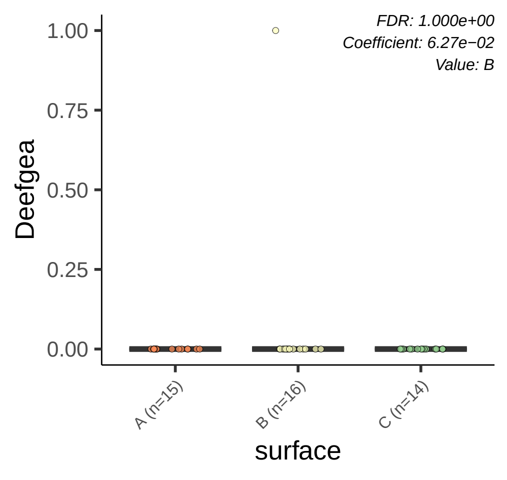

FDR: 1.000e+00
Coefficient: 6.27e−02
Value: B
1.00
0.75
0.50
0.25
0.00
Deefgea
A (n=15)
B (n=16)
C (n=14)
surface

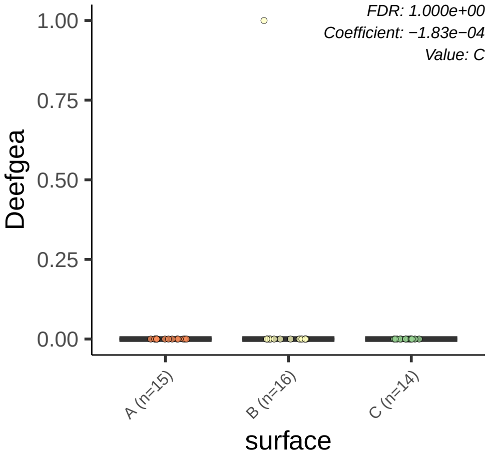
FDR: 1.000e+00
Coefficient: −1.83e−04
Value: C
Deefgea
1.00
0.75
0.50
0.25
0.00
A (n=15)
B (n=16)
C (n=14)
surface

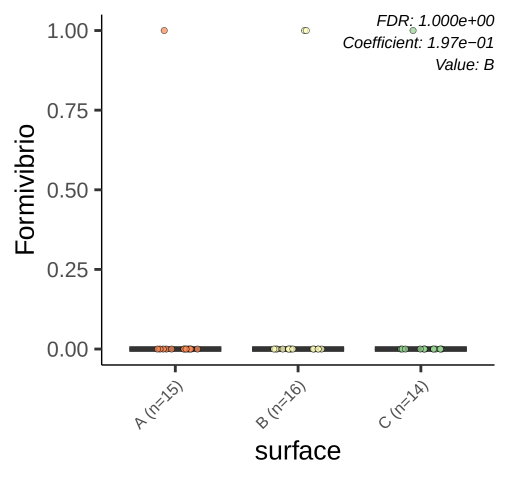
FDR: 1.000e+00
Coefficient: 1.97e−01
Value: B
Formivibrio
1.00
0.75
0.50
0.25
0.00
A (n=15)
B (n=16)
C (n=14)
surface

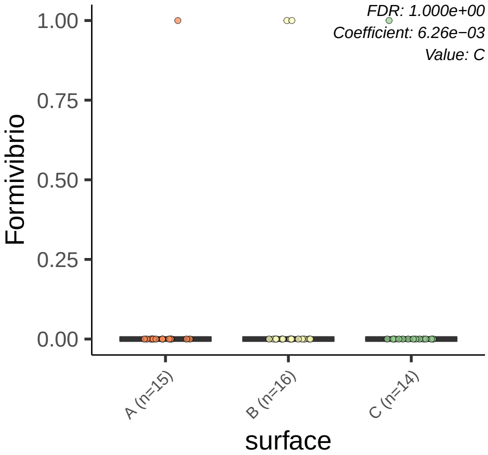
FDR: 1.000e+00
Coefficient: 6.26e−03
Value: C
Formivibrio
1.00
0.75
0.50
0.25
0.00
A (n=15)
B (n=16)
C (n=14)
surface

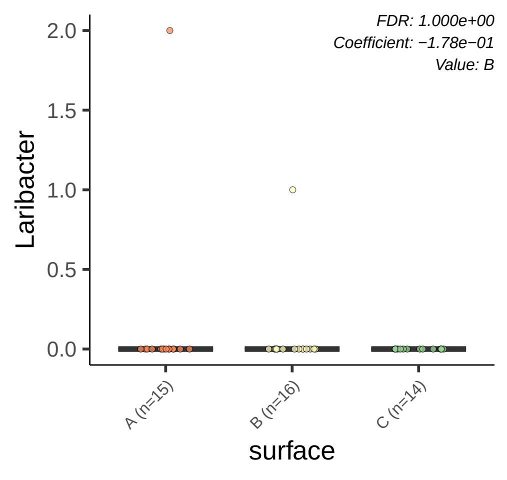
FDR: 1.000e+00
Coefficient: −1.78e−01
Value: B
Laribacter
2.0
1.5
1.0
0.5
0.0
A (n=15)
B (n=16)
C (n=14)
surface

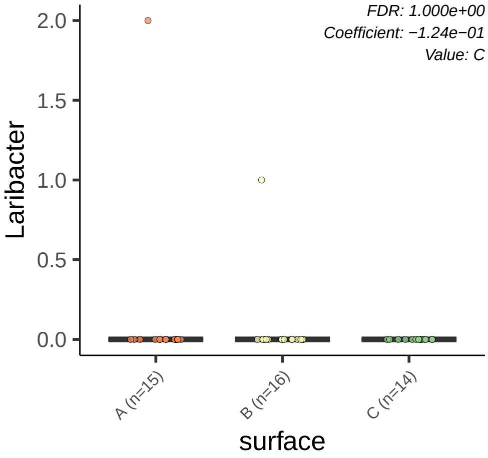

FDR: 1.000e+00
Coefficient: −1.24e−01
Value: C
Laribacter
2.0
1.5
1.0
0.5
0.0
A (n=15)
B (n=16)
C (n=14)
surface

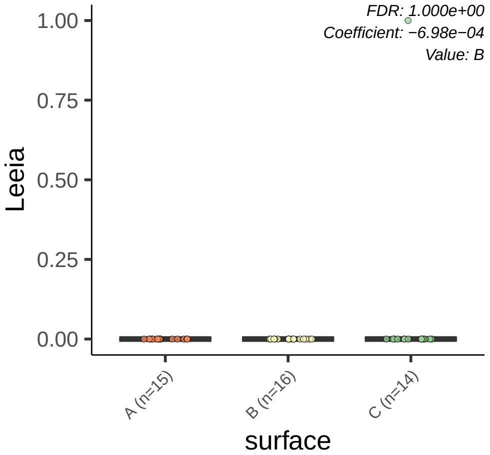

FDR: 1.000e+00
Coefficient: −6.98e−04
Value: B
1.00
0.75
0.50
0.25
0.00
Leeia
A (n=15)
B (n=16)
C (n=14)
surface

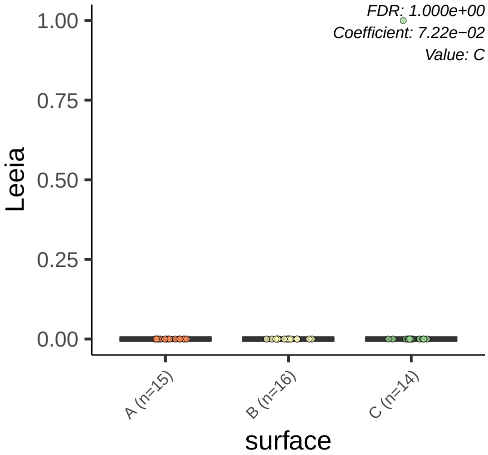

FDR: 1.000e+00
Coefficient: 7.22e−02
Value: C
1.00
0.75
0.50
0.25
0.00
Leeia
A (n=15)
B (n=16)
C (n=14)
surface

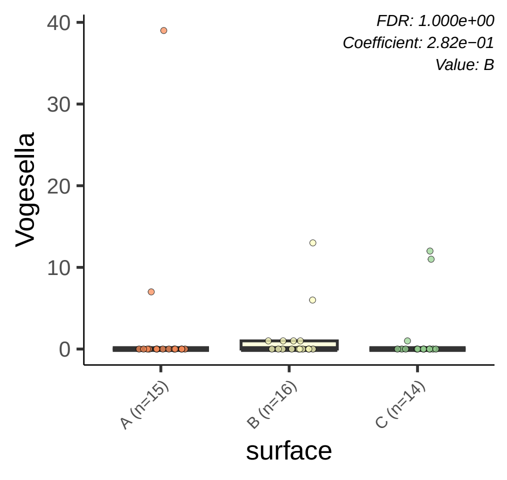

FDR: 1.000e+00
Coefficient: 2.82e−01
Value: B
Vogesella
40
30
20
10
0
A (n=15)
B (n=16)
C (n=14)
surface

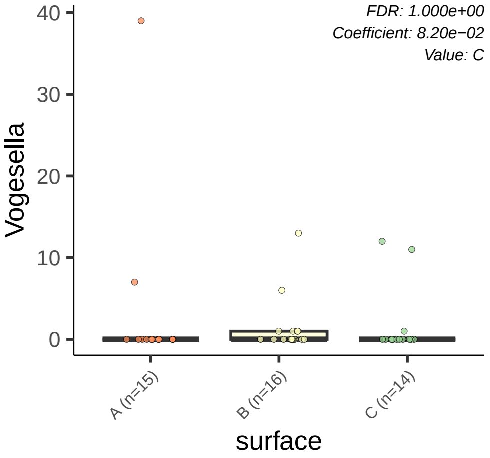
FDR: 1.000e+00
Coefficient: 8.20e−02
Value: C
Vogesella
40
30
20
10
0
A (n=15)
B (n=16)
C (n=14)
surface

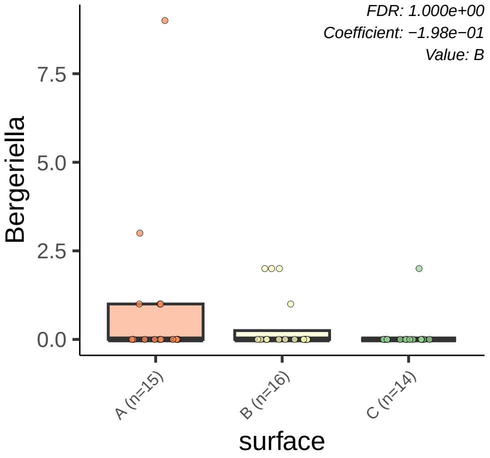

FDR: 1.000e+00
Coefficient: −1.98e−01
Value: B
Bergeriella
7.5
5.0
2.5
0.0
A (n=15)
B (n=16)
C (n=14)
surface

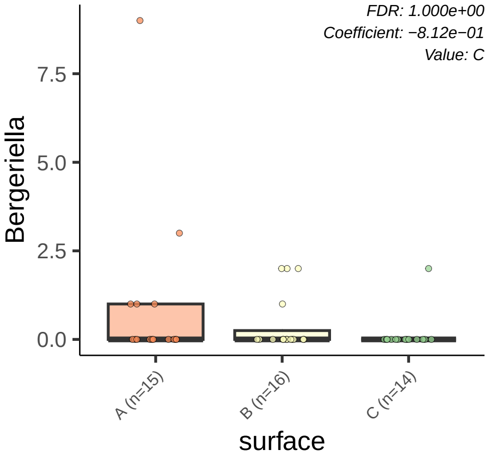

FDR: 1.000e+00
Coefficient: −8.12e−01
Value: C
Bergeriella
7.5
5.0
2.5
0.0
A (n=15)
B (n=16)
C (n=14)
surface

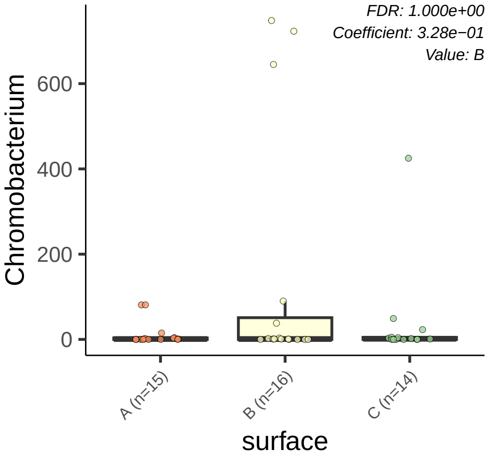

FDR: 1.000e+00
Coefficient: 3.28e−01
Value: B
Chromobacterium
600
400
200
0
A (n=15)
B (n=16)
C (n=14)
surface

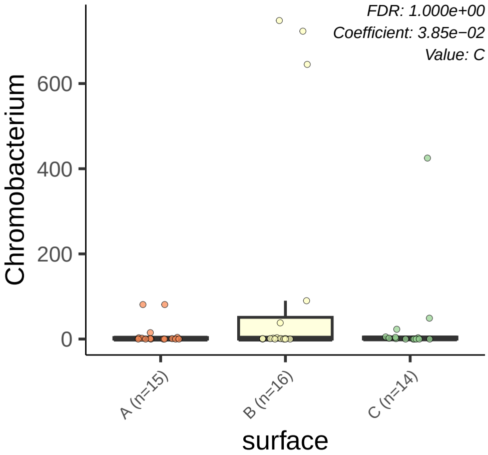
FDR: 1.000e+00
Coefficient: 3.85e−02
Value: C
Chromobacterium
0
200
400
600
A (n=15)
B (n=16)
C (n=14)
surface

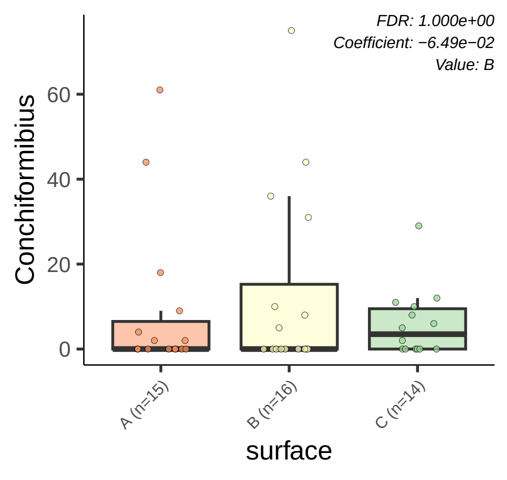
FDR: 1.000e+00
Coefficient: −6.49e−02
Value: B
Conchiformibius
0
20
40
60
A (n=15)
B (n=16)
C (n=14)
surface

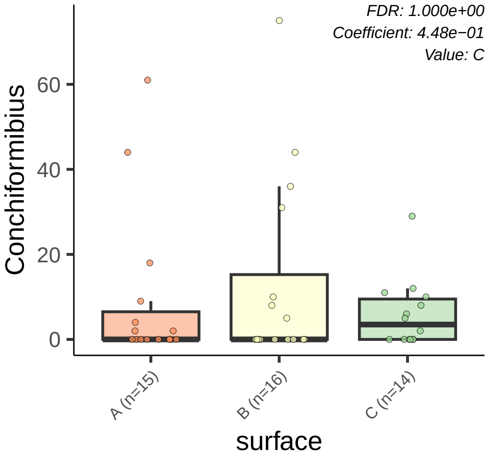

FDR: 1.000e+00
Coefficient: 4.48e−01
Value: C
Conchiformibius
0
20
40
60
A (n=15)
B (n=16)
C (n=14)
surface

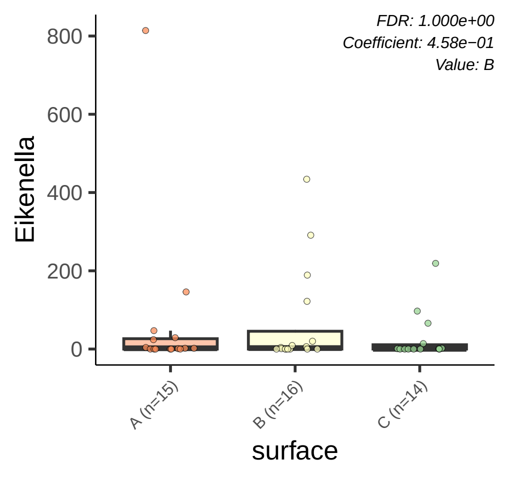
FDR: 1.000e+00
Coefficient: 4.58e−01
Value: B
Eikenella
800
600
400
200
0
A (n=15)
B (n=16)
C (n=14)
surface

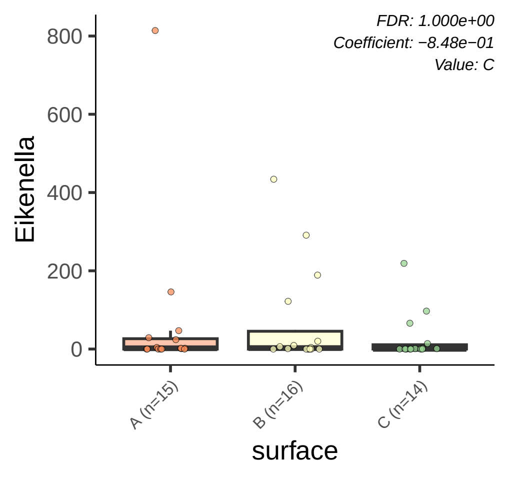
FDR: 1.000e+00
Coefficient: −8.48e−01
Value: C
800
600
400
200
0
Eikenella
A (n=15)
B (n=16)
C (n=14)
surface

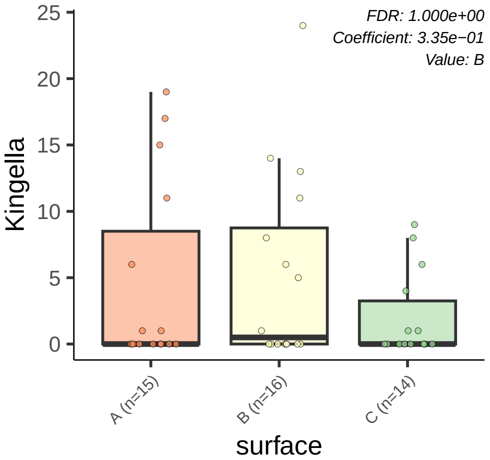

FDR: 1.000e+00
Coefficient: 3.35e−01
Value: B
Kingella
25
20
15
10
5
0
A (n=15)
B (n=16)
C (n=14)
surface

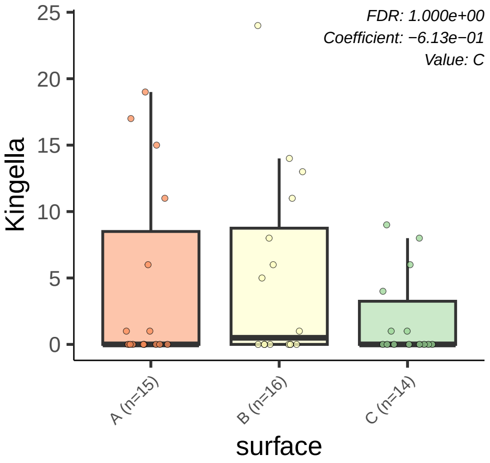

FDR: 1.000e+00
Coefficient: −6.13e−01
Value: C
Kingella
25
20
15
10
5
0
A (n=15)
B (n=16)
C (n=14)
surface

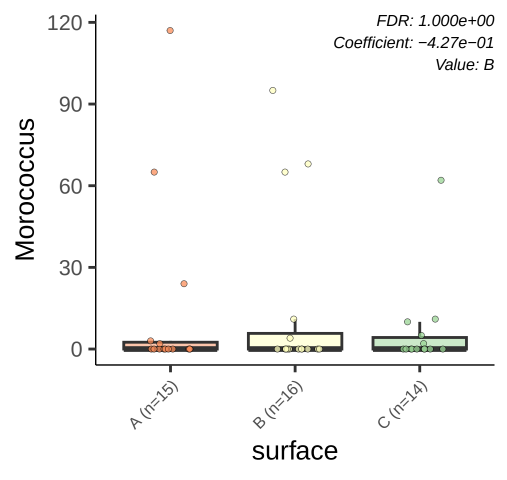

FDR: 1.000e+00
Coefficient: −4.27e−01
Value: B
Morococcus
120
90
60
30
0
A (n=15)
B (n=16)
C (n=14)
surface

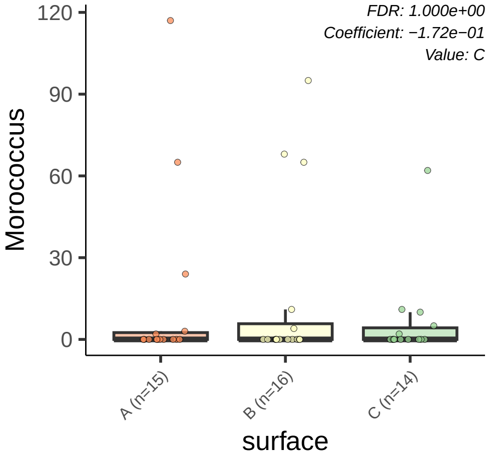

FDR: 1.000e+00
Coefficient: −1.72e−01
Value: C
Morococcus
120
90
60
30
0
A (n=15)
B (n=16)
C (n=14)
surface

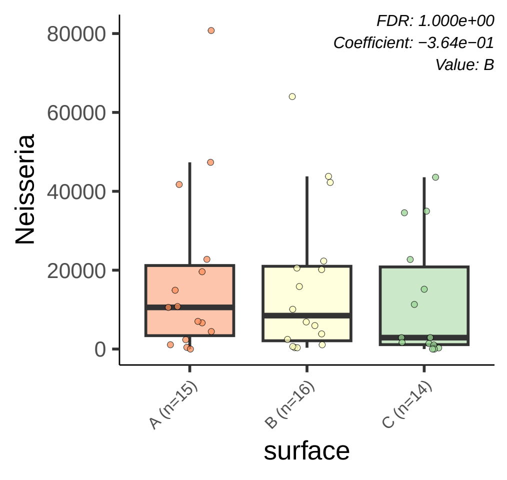
FDR: 1.000e+00
Coefficient: −3.64e−01
Value: B
Neisseria
80000
60000
40000
20000
0
A (n=15)
B (n=16)
C (n=14)
surface

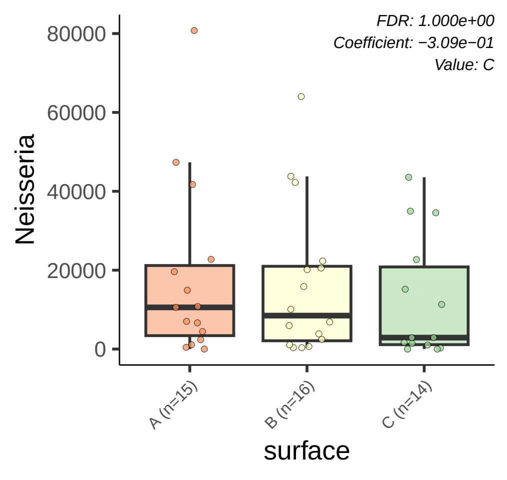
FDR: 1.000e+00
Coefficient: −3.09e−01
Value: C
80000
60000
40000
20000
0
Neisseria
A (n=15)
B (n=16)
C (n=14)
surface

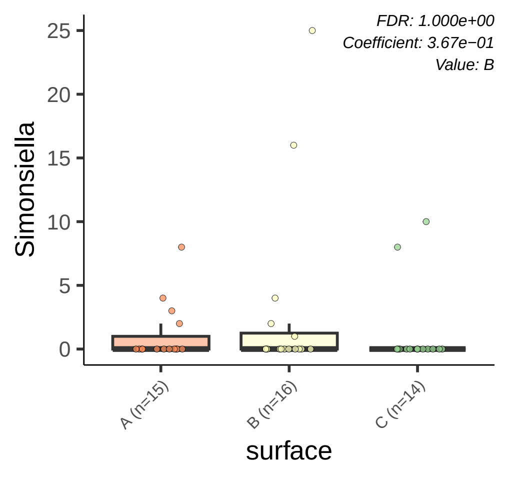

FDR: 1.000e+00
Coefficient: 3.67e−01
Value: B
Simonsiella
0
5
10
15
20
25
A (n=15)
B (n=16)
C (n=14)
surface

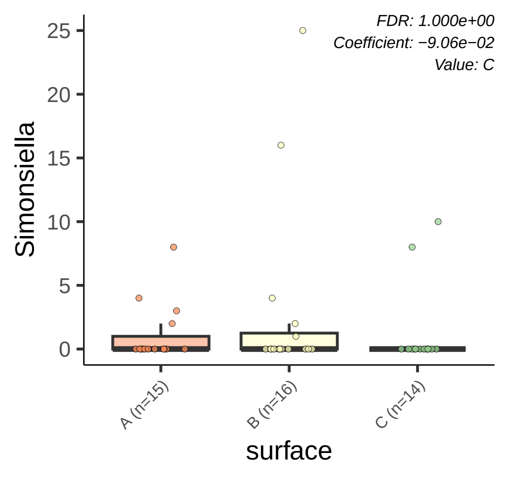
FDR: 1.000e+00
Coefficient: −9.06e−02
Value: C
Simonsiella
0
5
10
15
20
25
A (n=15)
B (n=16)
C (n=14)
surface

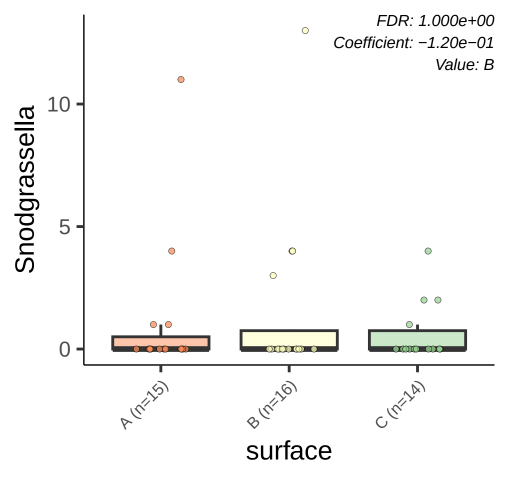

FDR: 1.000e+00
Coefficient: −1.20e−01
Value: B
Snodgrassella
10
5
0
A (n=15)
B (n=16)
C (n=14)
surface

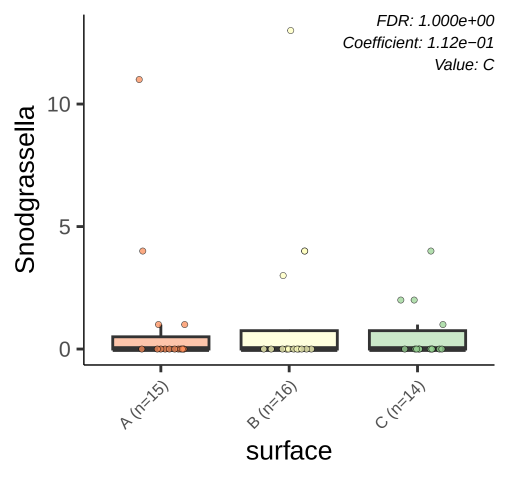
FDR: 1.000e+00
Coefficient: 1.12e−01
Value: C
Snodgrassella
0
5
10
A (n=15)
B (n=16)
C (n=14)
surface

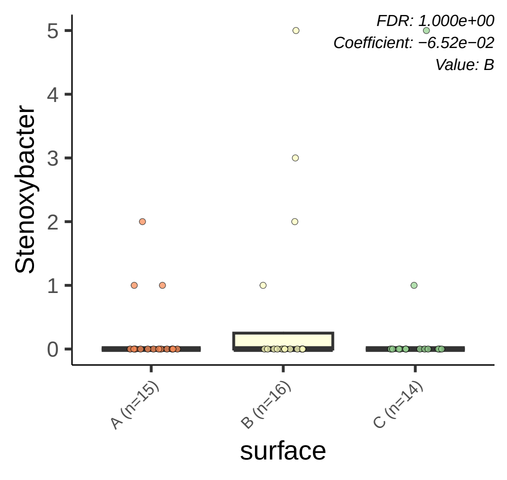
FDR: 1.000e+00
Coefficient: −6.52e−02
Value: B
Stenoxybacter
0
1
2
3
4
5
A (n=15)
B (n=16)
C (n=14)
surface

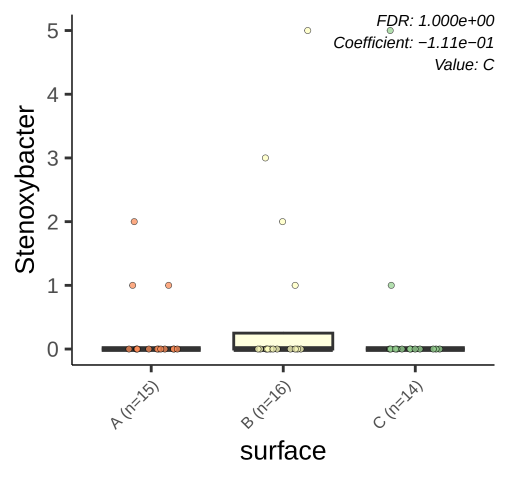
FDR: 1.000e+00
Coefficient: −1.11e−01
Value: C
Stenoxybacter
0
1
2
3
4
5
A (n=15)
B (n=16)
C (n=14)
surface

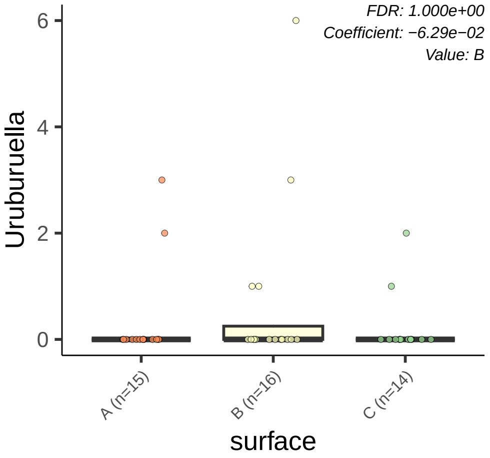
FDR: 1.000e+00
Coefficient: −6.29e−02
Value: B
Uruburuella
0
2
4
6
A (n=15)
B (n=16)
C (n=14)
surface

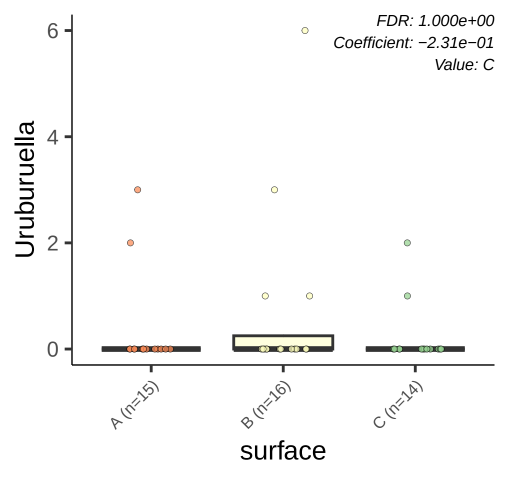
FDR: 1.000e+00
Coefficient: −2.31e−01
Value: C
Uruburuella
0
2
4
6
A (n=15)
B (n=16)
C (n=14)
surface

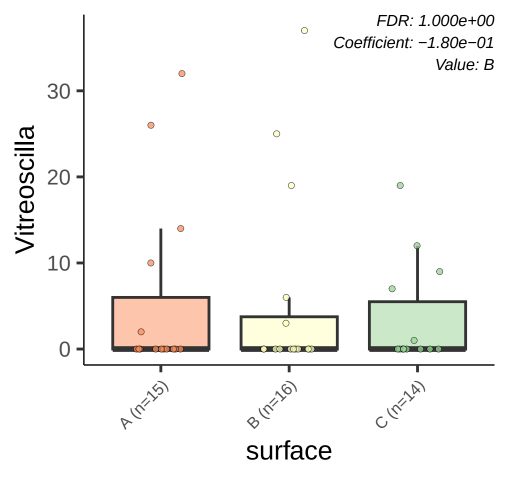

FDR: 1.000e+00
Coefficient: −1.80e−01
Value: B
Vitreoscilla
0
10
20
30
A (n=15)
B (n=16)
C (n=14)
surface

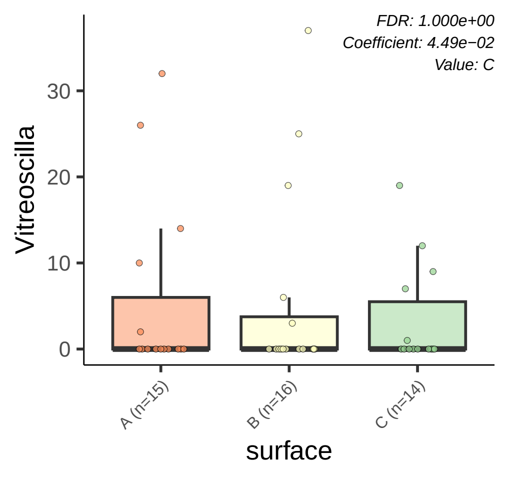
FDR: 1.000e+00
Coefficient: 4.49e−02
Value: C
Vitreoscilla
30
20
10
0
A (n=15)
B (n=16)
C (n=14)
surface

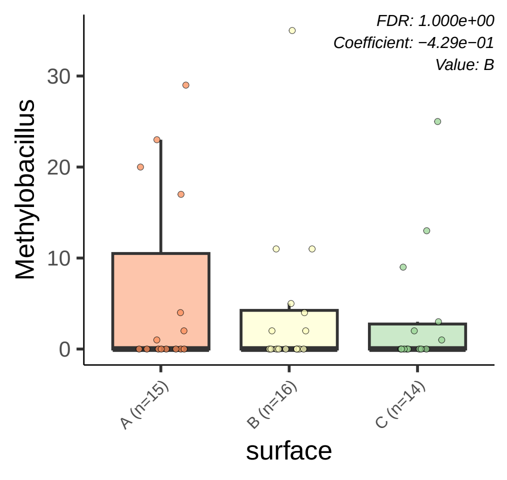
FDR: 1.000e+00
Coefficient: −4.29e−01
Value: B
Methylobacillus
0
10
20
30
A (n=15)
B (n=16)
C (n=14)
surface

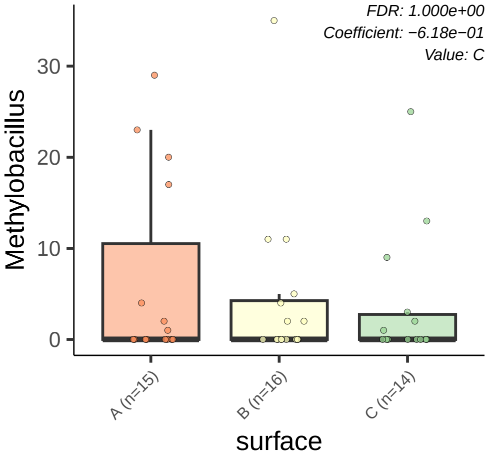
FDR: 1.000e+00
Coefficient: −6.18e−01
Value: C
Methylobacillus
0
10
20
30
A (n=15)
B (n=16)
C (n=14)
surface

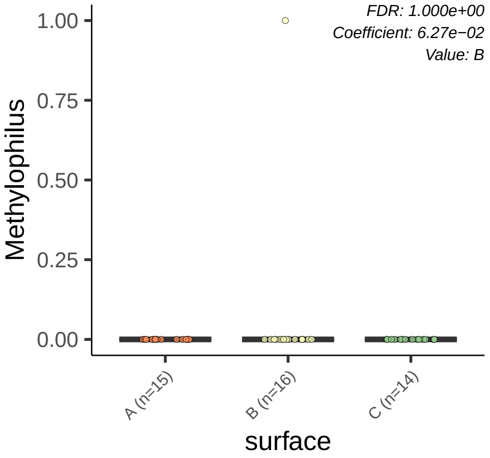
FDR: 1.000e+00
Coefficient: 6.27e−02
Value: B
Methylophilus
1.00
0.75
0.50
0.25
0.00
A (n=15)
B (n=16)
C (n=14)
surface

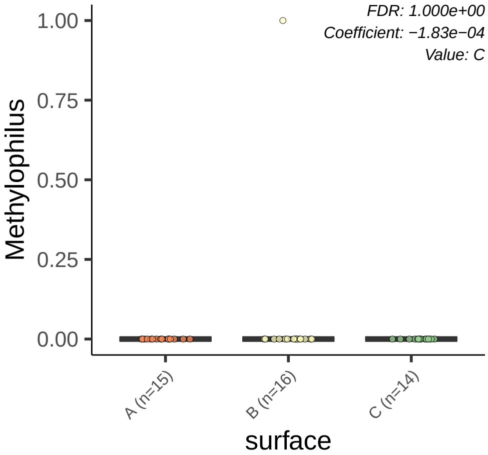

FDR: 1.000e+00
Coefficient: −1.83e−04
Value: C
Methylophilus
1.00
0.75
0.50
0.25
0.00
A (n=15)
B (n=16)
C (n=14)
surface

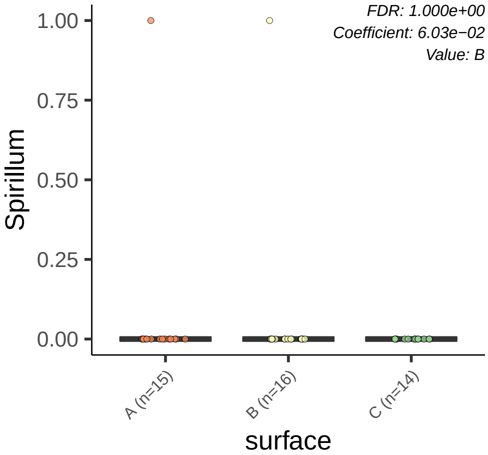

FDR: 1.000e+00
Coefficient: 6.03e−02
Value: B
1.00
0.75
0.50
0.25
0.00
Spirillum
A (n=15)
B (n=16)
C (n=14)
surface

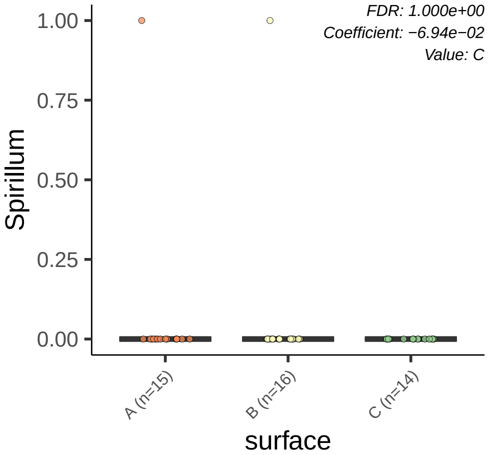

FDR: 1.000e+00
Coefficient: −6.94e−02
Value: C
1.00
0.75
0.50
0.25
0.00
Spirillum
A (n=15)
B (n=16)
C (n=14)
surface

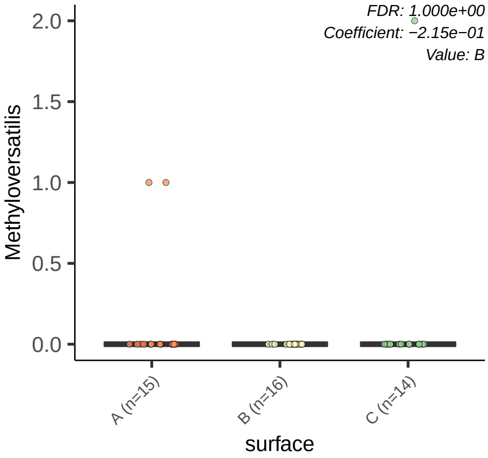

FDR: 1.000e+00
Coefficient: −2.15e−01
Value: B
Methyloversatilis
2.0
1.5
1.0
0.5
0.0
A (n=15)
B (n=16)
C (n=14)
surface

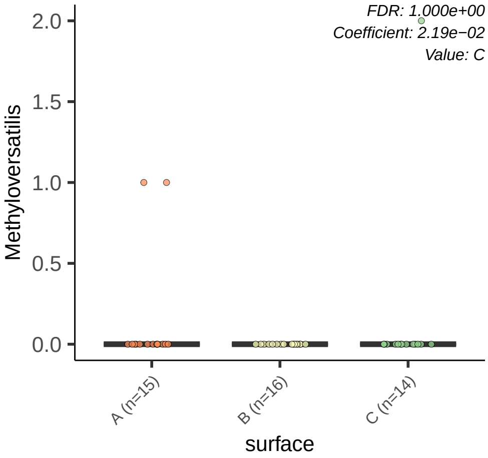

FDR: 1.000e+00
Coefficient: 2.19e−02
Value: C
Methyloversatilis
2.0
1.5
1.0
0.5
0.0
A (n=15)
B (n=16)
C (n=14)
surface

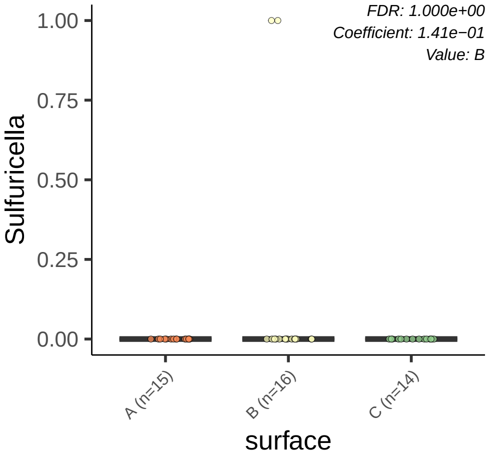
FDR: 1.000e+00
Coefficient: 1.41e−01
Value: B
1.00
0.75
0.50
0.25
0.00
Sulfuricella
A (n=15)
B (n=16)
C (n=14)
surface

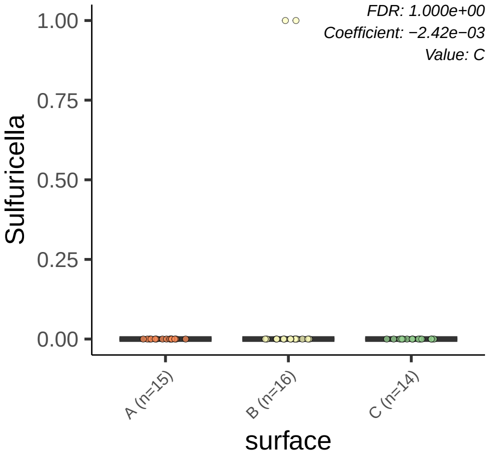
FDR: 1.000e+00
Coefficient: −2.42e−03
Value: C
Sulfuricella
1.00
0.75
0.50
0.25
0.00
A (n=15)
B (n=16)
C (n=14)
surface

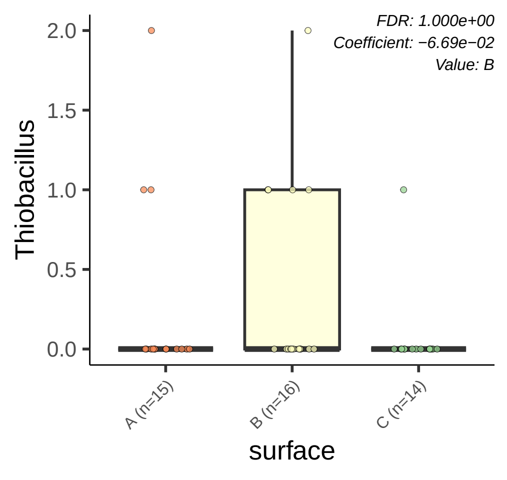
FDR: 1.000e+00
Coefficient: −6.69e−02
Value: B
Thiobacillus
2.0
1.5
1.0
0.5
0.0
A (n=15)
B (n=16)
C (n=14)
surface

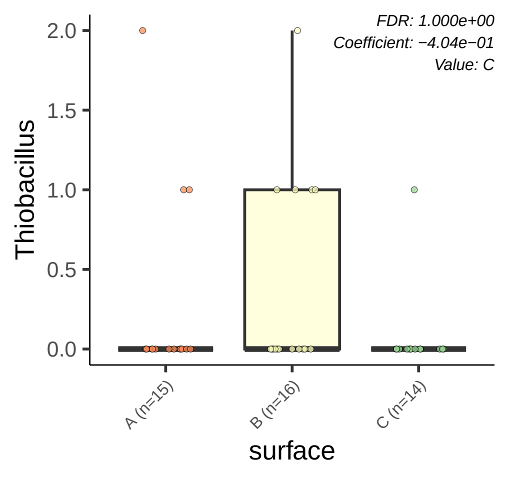

FDR: 1.000e+00
Coefficient: −4.04e−01
Value: C
Thiobacillus
2.0
1.5
1.0
0.5
0.0
A (n=15)
B (n=16)
C (n=14)
surface

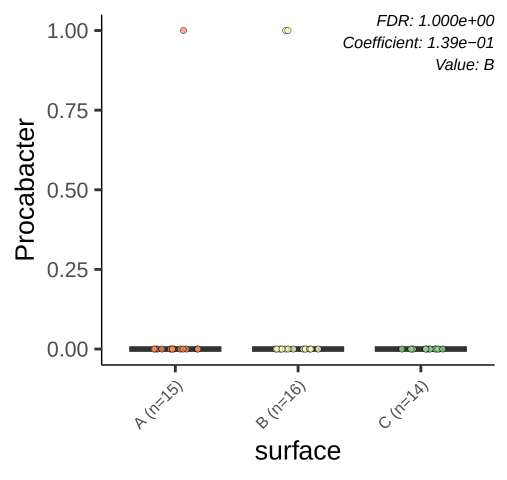

FDR: 1.000e+00
Coefficient: 1.39e−01
Value: B
Procabacter
1.00
0.75
0.50
0.25
0.00
A (n=15)
B (n=16)
C (n=14)
surface

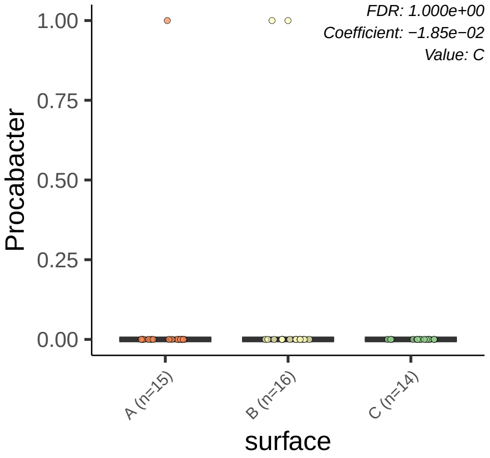
FDR: 1.000e+00
Coefficient: −1.85e−02
Value: C
Procabacter
1.00
0.75
0.50
0.25
0.00
A (n=15)
B (n=16)
C (n=14)
surface

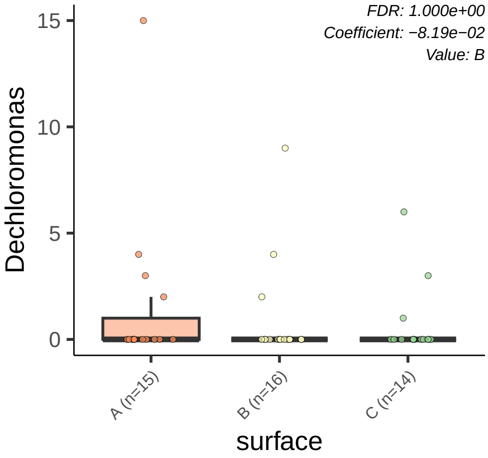
FDR: 1.000e+00
Coefficient: −8.19e−02
Value: B
Dechloromonas
15
10
5
0
A (n=15)
B (n=16)
C (n=14)
surface

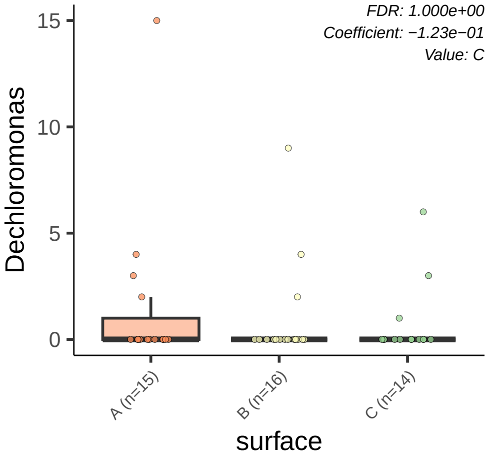

FDR: 1.000e+00
Coefficient: −1.23e−01
Value: C
Dechloromonas
15
10
5
0
A (n=15)
B (n=16)
C (n=14)
surface

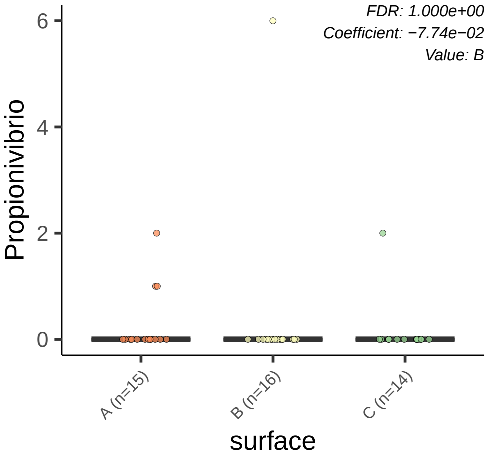

FDR: 1.000e+00
Coefficient: −7.74e−02
Value: B
Propionivibrio
6
4
2
0
A (n=15)
B (n=16)
C (n=14)
surface

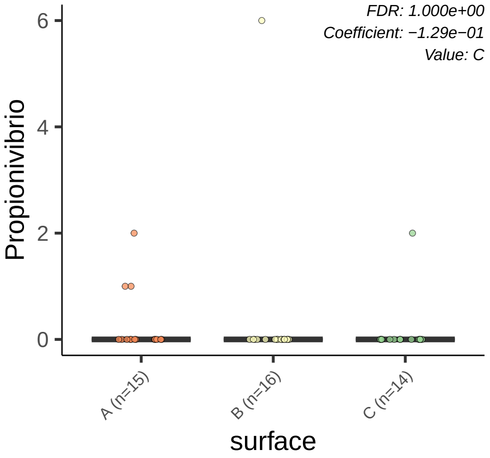

FDR: 1.000e+00
Coefficient: −1.29e−01
Value: C
Propionivibrio
0
2
4
6
A (n=15)
B (n=16)
C (n=14)
surface

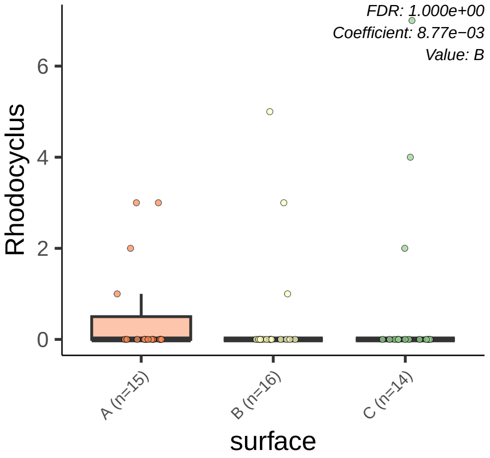

FDR: 1.000e+00
Coefficient: 8.77e−03
Value: B
Rhodocyclus
0
2
4
6
A (n=15)
B (n=16)
C (n=14)
surface

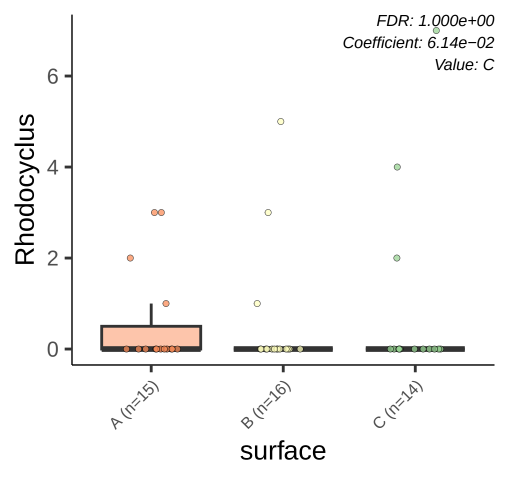
FDR: 1.000e+00
Coefficient: 6.14e−02
Value: C
Rhodocyclus
0
2
4
6
A (n=15)
B (n=16)
C (n=14)
surface

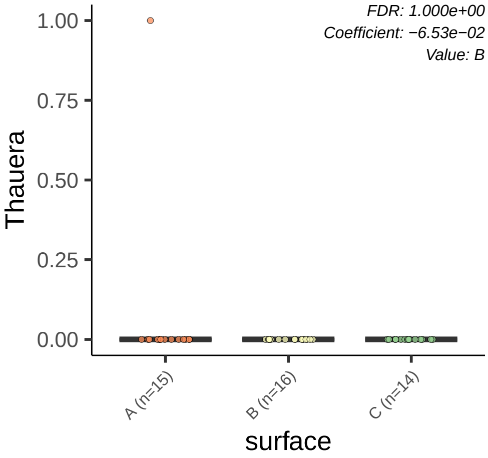

FDR: 1.000e+00
Coefficient: −6.53e−02
Value: B
Thauera
1.00
0.75
0.50
0.25
0.00
A (n=15)
B (n=16)
C (n=14)
surface

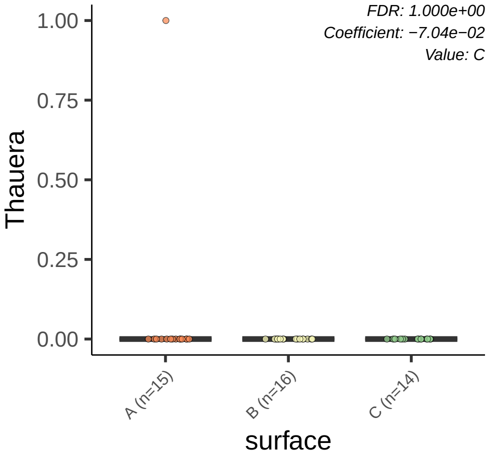

FDR: 1.000e+00
Coefficient: −7.04e−02
Value: C
1.00
0.75
0.50
0.25
0.00
Thauera
A (n=15)
B (n=16)
C (n=14)
surface

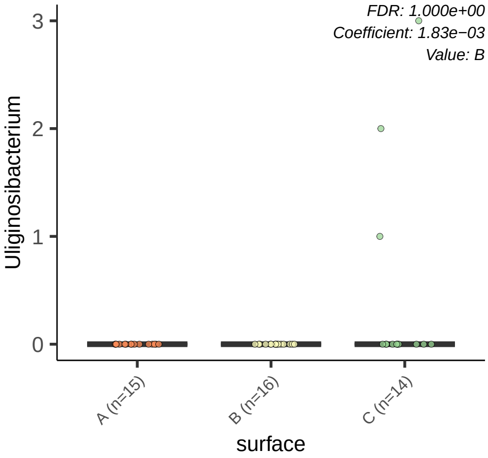

FDR: 1.000e+00
Coefficient: 1.83e−03
Value: B
Uliginosibacterium
3
2
1
0
A (n=15)
B (n=16)
C (n=14)
surface

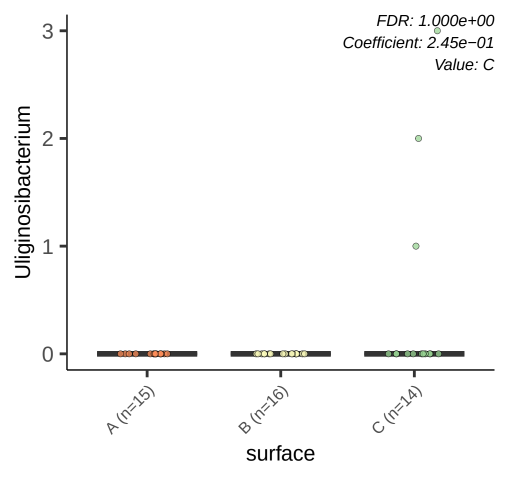
FDR: 1.000e+00
Coefficient: 2.45e−01
Value: C
Uliginosibacterium
3
2
1
0
A (n=15)
B (n=16)
C (n=14)
surface

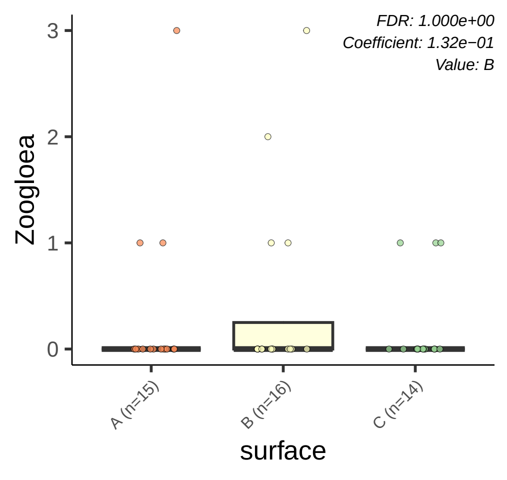

FDR: 1.000e+00
Coefficient: 1.32e−01
Value: B
Zoogloea
0
1
2
3
A (n=15)
B (n=16)
C (n=14)
surface

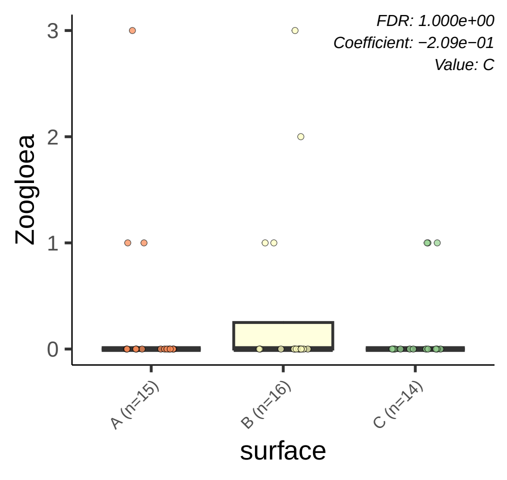

FDR: 1.000e+00
Coefficient: −2.09e−01
Value: C
Zoogloea
0
1
2
3
A (n=15)
B (n=16)
C (n=14)
surface

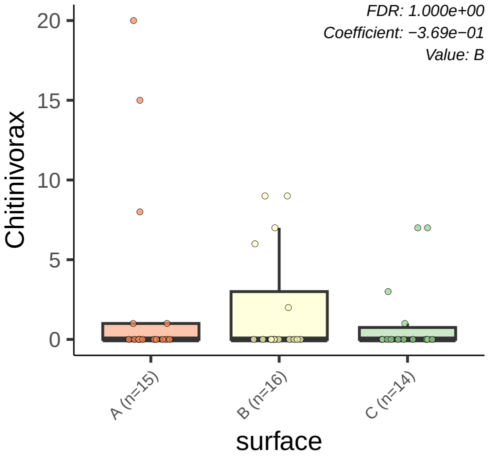
FDR: 1.000e+00
Coefficient: −3.69e−01
Value: B
Chitinivorax
20
15
10
5
0
A (n=15)
B (n=16)
C (n=14)
surface

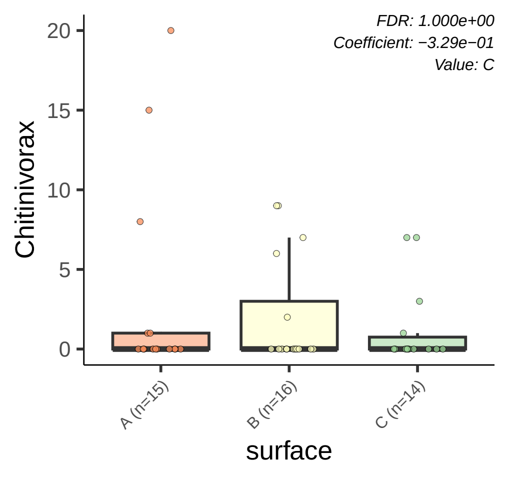
FDR: 1.000e+00
Coefficient: −3.29e−01
Value: C
Chitinivorax
20
15
10
5
0
A (n=15)
B (n=16)
C (n=14)
surface

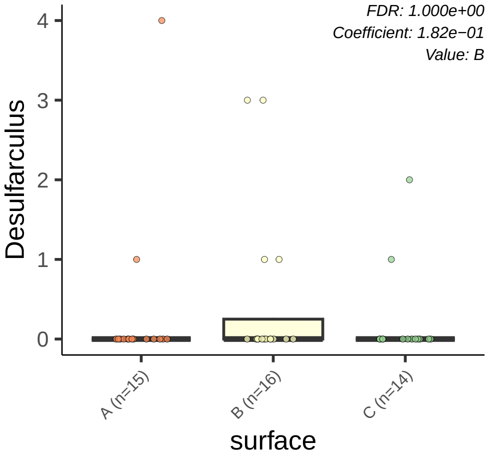

FDR: 1.000e+00
Coefficient: 1.82e−01
Value: B
Desulfarculus
0
1
2
3
4
A (n=15)
B (n=16)
C (n=14)
surface

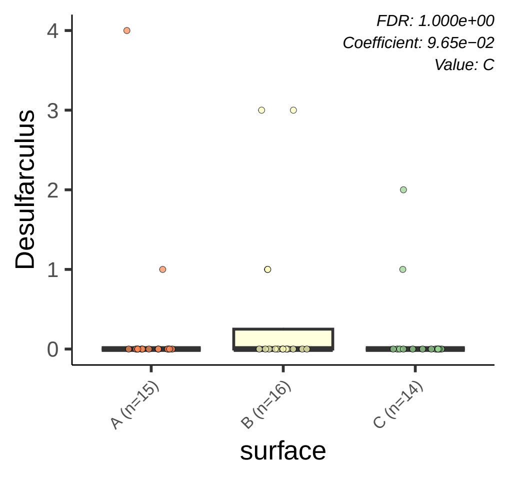

FDR: 1.000e+00
Coefficient: 9.65e−02
Value: C
Desulfarculus
0
1
2
3
4
A (n=15)
B (n=16)
C (n=14)
surface

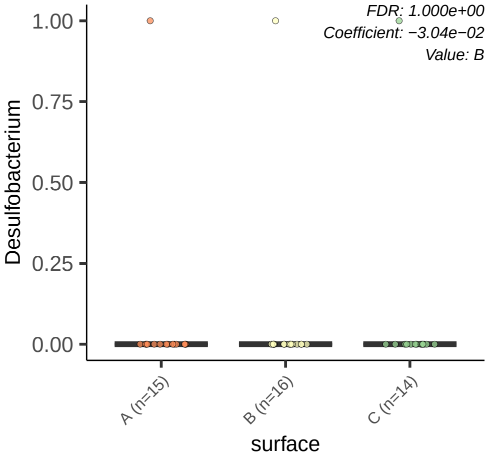
FDR: 1.000e+00
Coefficient: −3.04e−02
Value: B
Desulfobacterium
1.00
0.75
0.50
0.25
0.00
A (n=15)
B (n=16)
C (n=14)
surface

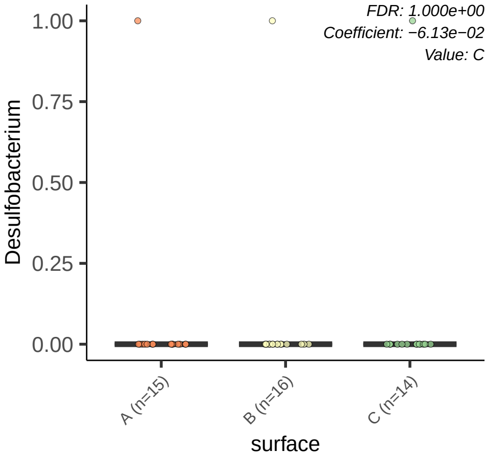
FDR: 1.000e+00
Coefficient: −6.13e−02
Value: C
Desulfobacterium
1.00
0.75
0.50
0.25
0.00
A (n=15)
B (n=16)
C (n=14)
surface

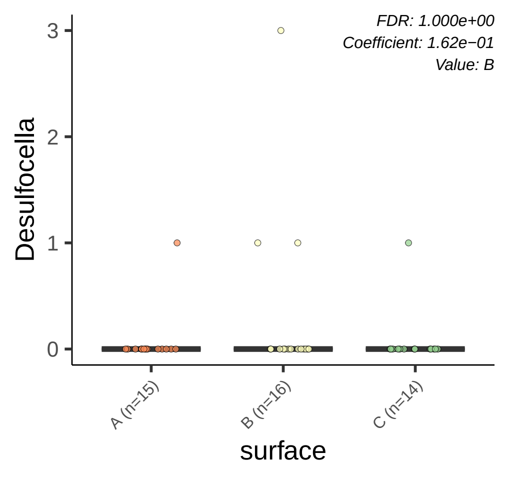
FDR: 1.000e+00
Coefficient: 1.62e−01
Value: B
Desulfocella
3
2
1
0
A (n=15)
B (n=16)
C (n=14)
surface

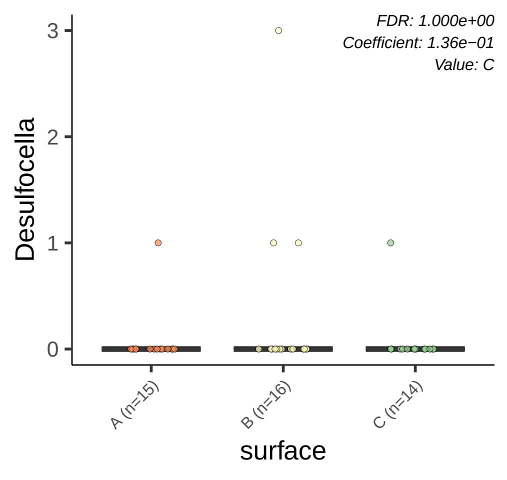

FDR: 1.000e+00
Coefficient: 1.36e−01
Value: C
Desulfocella
3
2
1
0
A (n=15)
B (n=16)
C (n=14)
surface

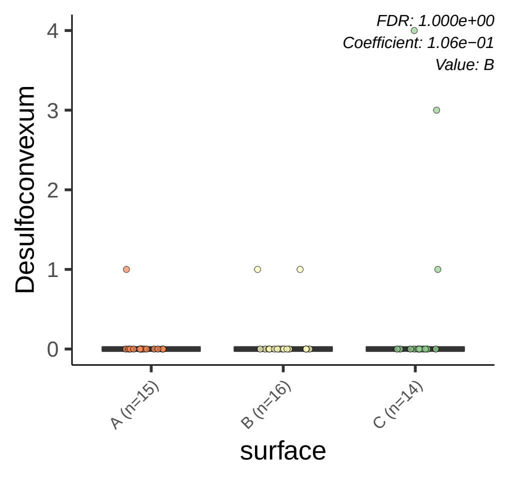
FDR: 1.000e+00
Coefficient: 1.06e−01
Value: B
Desulfoconvexum
0
1
2
3
4
A (n=15)
B (n=16)
C (n=14)
surface

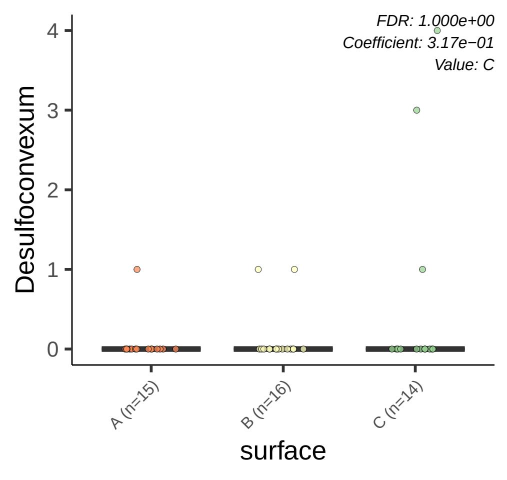

FDR: 1.000e+00
Coefficient: 3.17e−01
Value: C
Desulfoconvexum
4
3
2
1
0
A (n=15)
B (n=16)
C (n=14)
surface

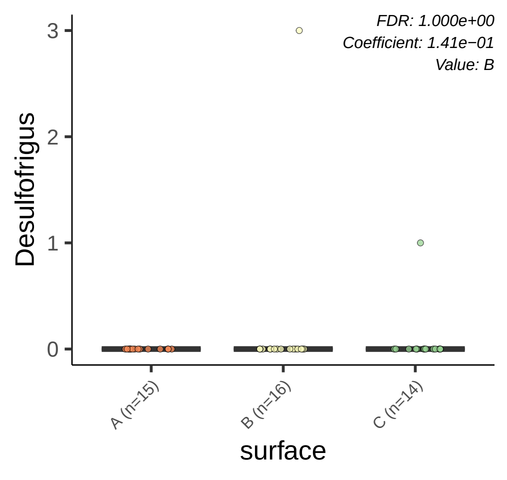
FDR: 1.000e+00
Coefficient: 1.41e−01
Value: B
Desulfofrigus
3
2
1
0
A (n=15)
B (n=16)
C (n=14)
surface

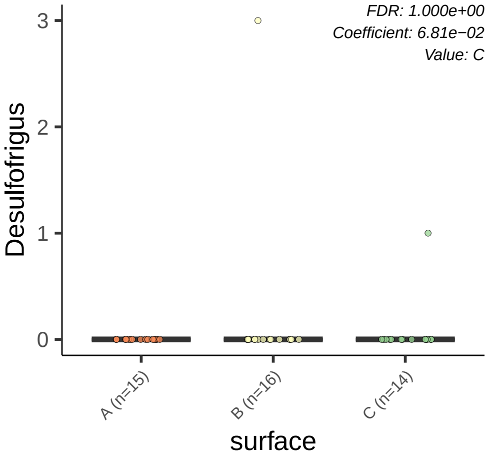
FDR: 1.000e+00
Coefficient: 6.81e−02
Value: C
Desulfofrigus
3
2
1
0
A (n=15)
B (n=16)
C (n=14)
surface

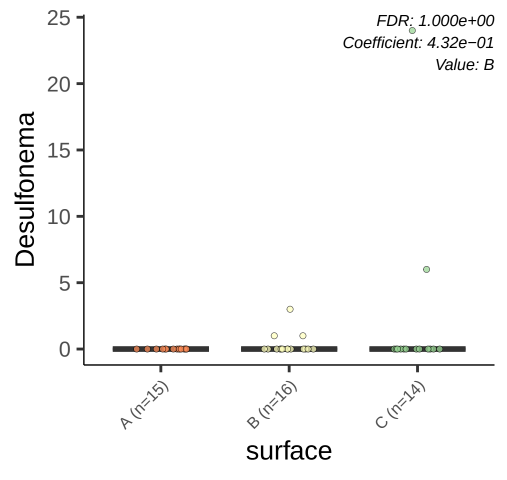

FDR: 1.000e+00
Coefficient: 4.32e−01
Value: B
Desulfonema
25
20
15
10
5
0
A (n=15)
B (n=16)
C (n=14)
surface

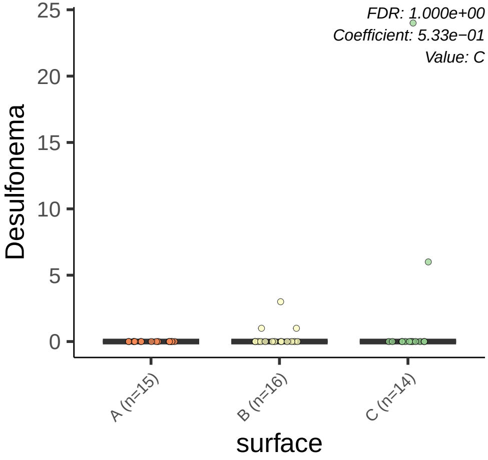

FDR: 1.000e+00
Coefficient: 5.33e−01
Value: C
Desulfonema
25
20
15
10
5
0
A (n=15)
B (n=16)
C (n=14)
surface

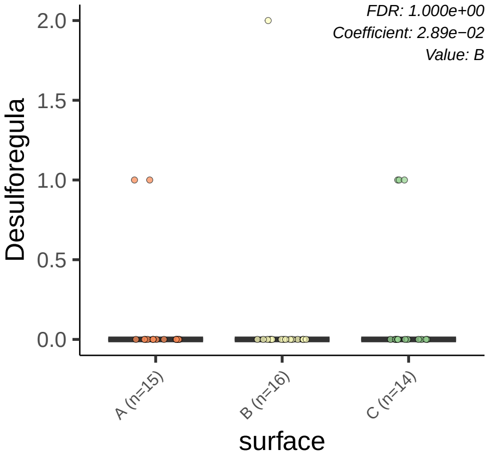
FDR: 1.000e+00
Coefficient: 2.89e−02
Value: B
Desulforegula
2.0
1.5
1.0
0.5
0.0
A (n=15)
B (n=16)
C (n=14)
surface

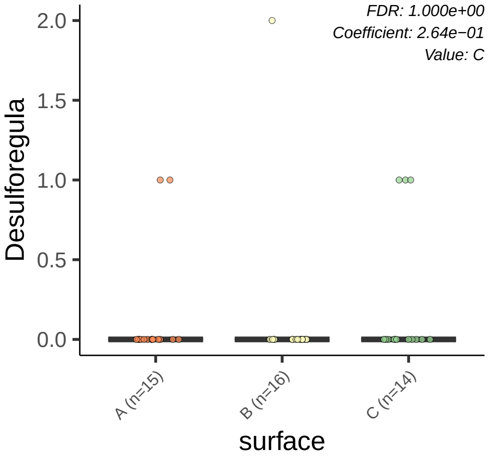

FDR: 1.000e+00
Coefficient: 2.64e−01
Value: C
Desulforegula
2.0
1.5
1.0
0.5
0.0
A (n=15)
B (n=16)
C (n=14)
surface

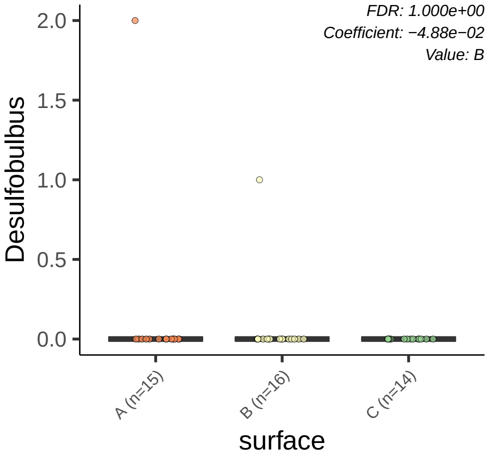
FDR: 1.000e+00
Coefficient: −4.88e−02
Value: B
Desulfobulbus
2.0
1.5
1.0
0.5
0.0
A (n=15)
B (n=16)
C (n=14)
surface

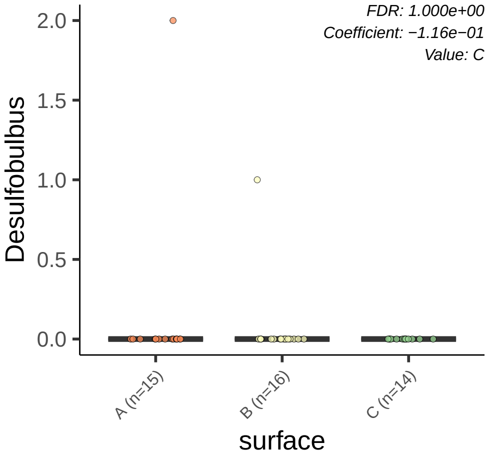

FDR: 1.000e+00
Coefficient: −1.16e−01
Value: C
Desulfobulbus
2.0
1.5
1.0
0.5
0.0
A (n=15)
B (n=16)
C (n=14)
surface

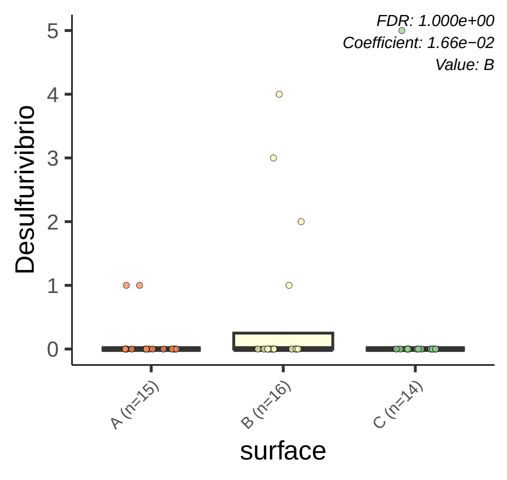

FDR: 1.000e+00
Coefficient: 1.66e−02
Value: B
Desulfurivibrio
0
1
2
3
4
5
A (n=15)
B (n=16)
C (n=14)
surface

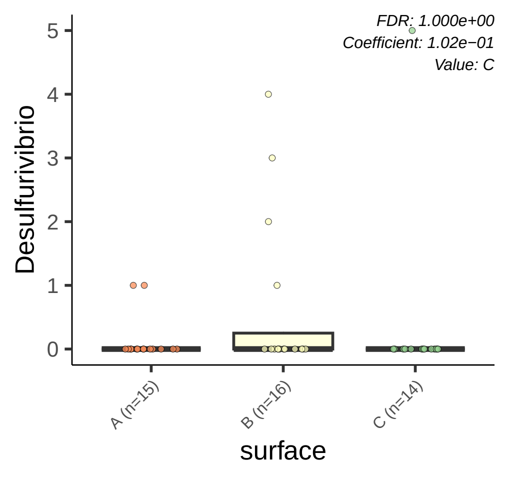

FDR: 1.000e+00
Coefficient: 1.02e−01
Value: C
Desulfurivibrio
5
4
3
2
1
0
A (n=15)
B (n=16)
C (n=14)
surface

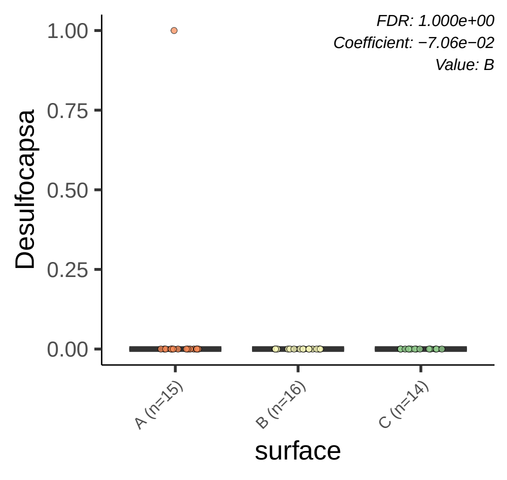

FDR: 1.000e+00
Coefficient: −7.06e−02
Value: B
Desulfocapsa
1.00
0.75
0.50
0.25
0.00
A (n=15)
B (n=16)
C (n=14)
surface

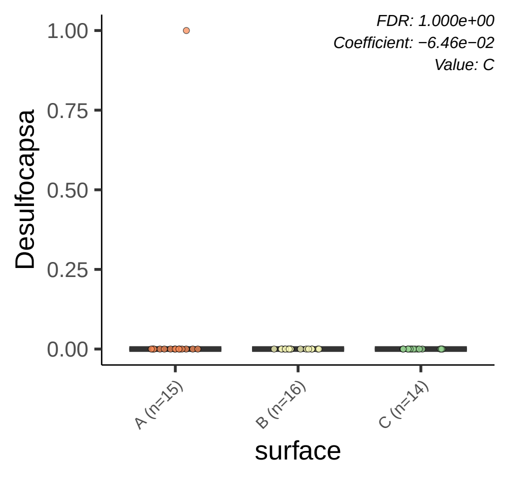
FDR: 1.000e+00
Coefficient: −6.46e−02
Value: C
Desulfocapsa
1.00
0.75
0.50
0.25
0.00
A (n=15)
B (n=16)
C (n=14)
surface

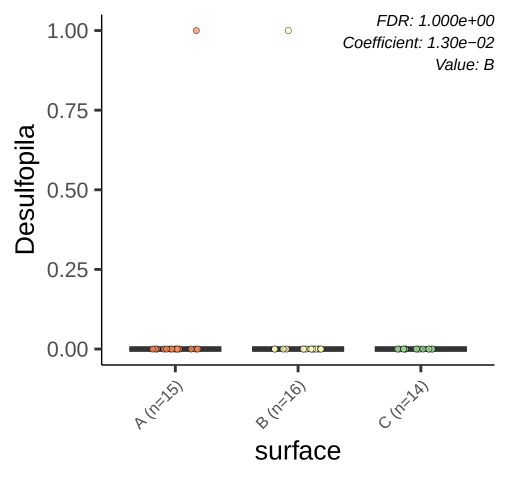
FDR: 1.000e+00
Coefficient: 1.30e−02
Value: B
Desulfopila
1.00
0.75
0.50
0.25
0.00
A (n=15)
B (n=16)
C (n=14)
surface

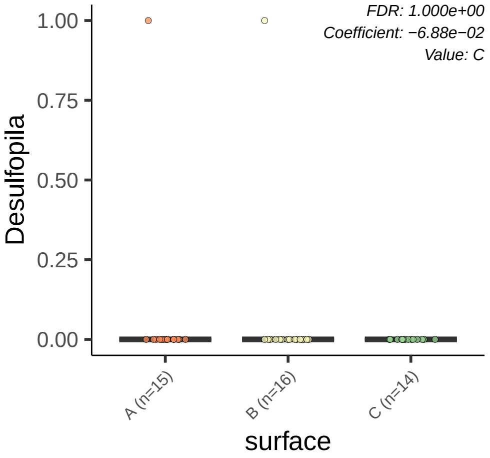
FDR: 1.000e+00
Coefficient: −6.88e−02
Value: C
Desulfopila
1.00
0.75
0.50
0.25
0.00
A (n=15)
B (n=16)
C (n=14)
surface

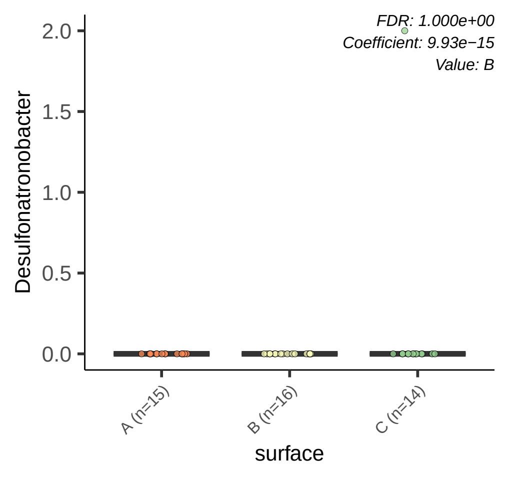
FDR: 1.000e+00
Coefficient: 9.93e−15
Value: B
Desulfonatronobacter
2.0
1.5
1.0
0.5
0.0
A (n=15)
B (n=16)
C (n=14)
surface

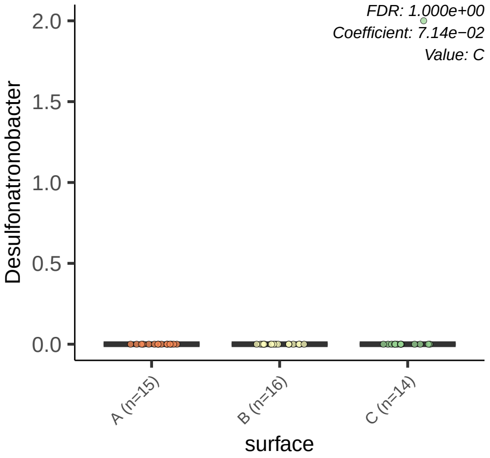
FDR: 1.000e+00
Coefficient: 7.14e−02
Value: C
Desulfonatronobacter
2.0
1.5
1.0
0.5
0.0
A (n=15)
B (n=16)
C (n=14)
surface

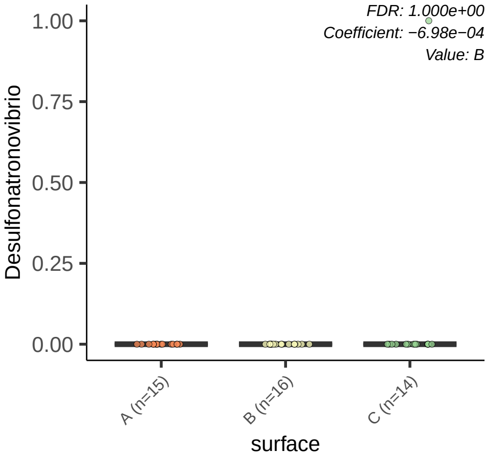
FDR: 1.000e+00
Coefficient: −6.98e−04
Value: B
Desulfonatronovibrio
1.00
0.75
0.50
0.25
0.00
A (n=15)
B (n=16)
C (n=14)
surface

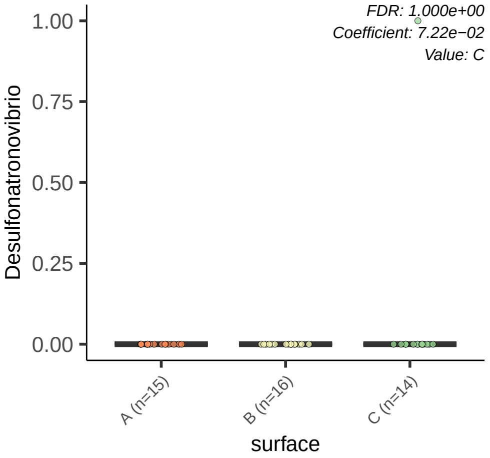
FDR: 1.000e+00
Coefficient: 7.22e−02
Value: C
Desulfonatronovibrio
1.00
0.75
0.50
0.25
0.00
A (n=15)
B (n=16)
C (n=14)
surface

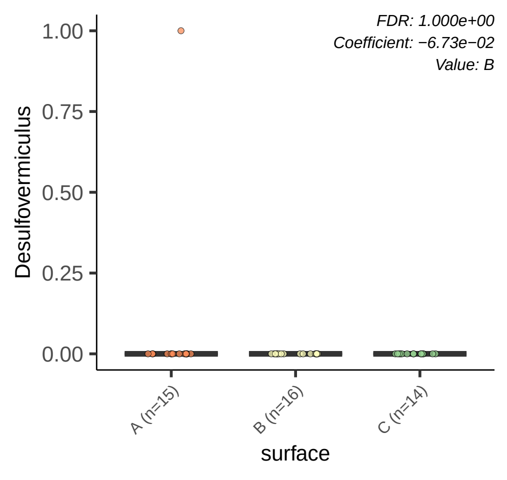
FDR: 1.000e+00
Coefficient: −6.73e−02
Value: B
Desulfovermiculus
1.00
0.75
0.50
0.25
0.00
A (n=15)
B (n=16)
C (n=14)
surface

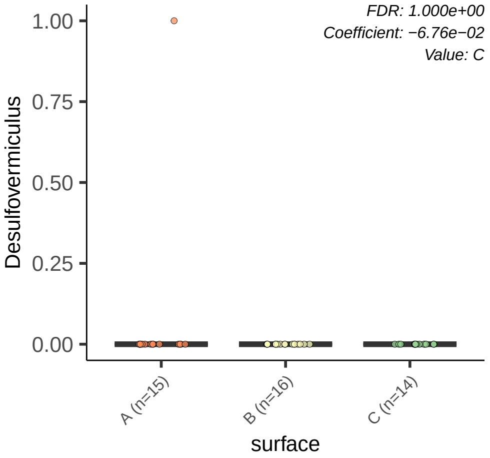

FDR: 1.000e+00
Coefficient: −6.76e−02
Value: C
Desulfovermiculus
1.00
0.75
0.50
0.25
0.00
A (n=15)
B (n=16)
C (n=14)
surface

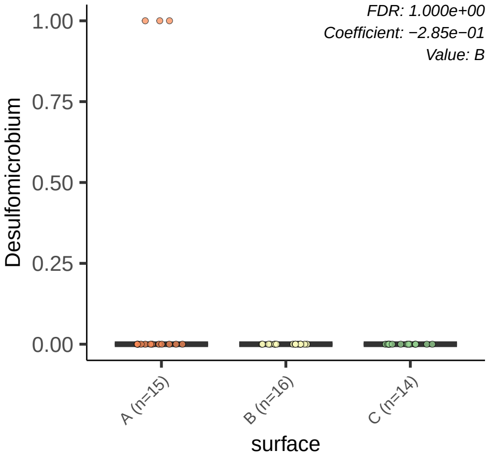
FDR: 1.000e+00
Coefficient: −2.85e−01
Value: B
Desulfomicrobium
1.00
0.75
0.50
0.25
0.00
A (n=15)
B (n=16)
C (n=14)
surface

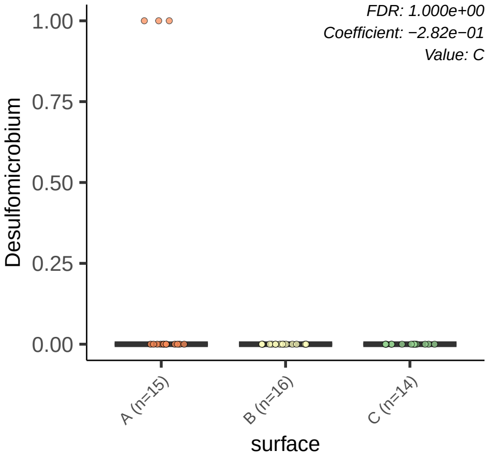
FDR: 1.000e+00
Coefficient: −2.82e−01
Value: C
Desulfomicrobium
1.00
0.75
0.50
0.25
0.00
A (n=15)
B (n=16)
C (n=14)
surface

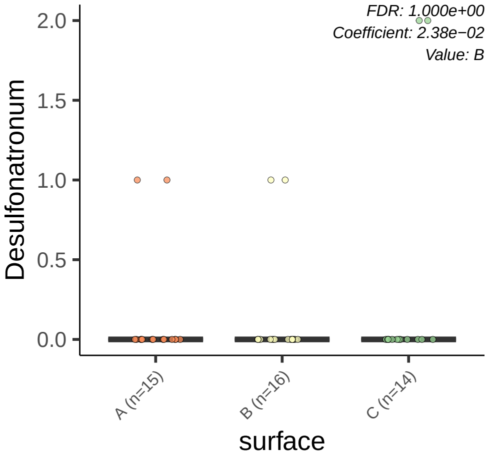
FDR: 1.000e+00
Coefficient: 2.38e−02
Value: B
Desulfonatronum
2.0
1.5
1.0
0.5
0.0
A (n=15)
B (n=16)
C (n=14)
surface

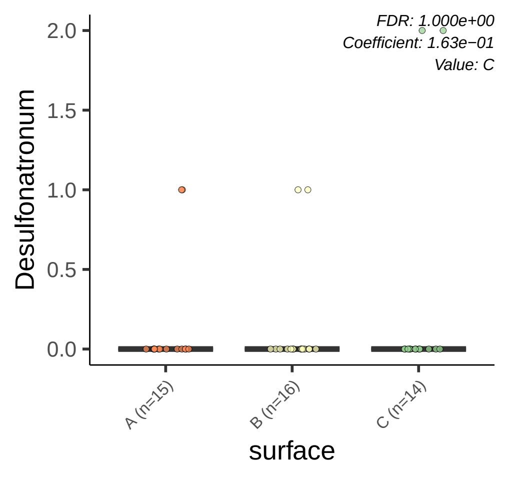
FDR: 1.000e+00
Coefficient: 1.63e−01
Value: C
2.0
1.5
1.0
0.5
0.0
Desulfonatronum
A (n=15)
B (n=16)
C (n=14)
surface

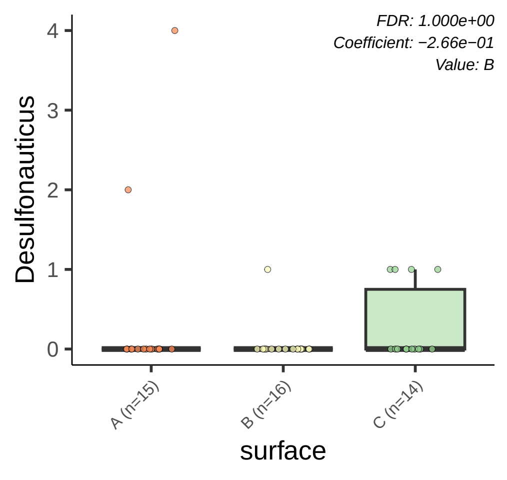
FDR: 1.000e+00
Coefficient: −2.66e−01
Value: B
Desulfonauticus
0
1
2
3
4
A (n=15)
B (n=16)
C (n=14)
surface

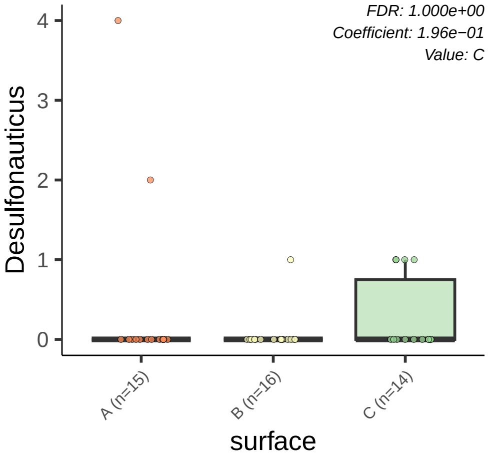
FDR: 1.000e+00
Coefficient: 1.96e−01
Value: C
Desulfonauticus
0
1
2
3
4
A (n=15)
B (n=16)
C (n=14)
surface

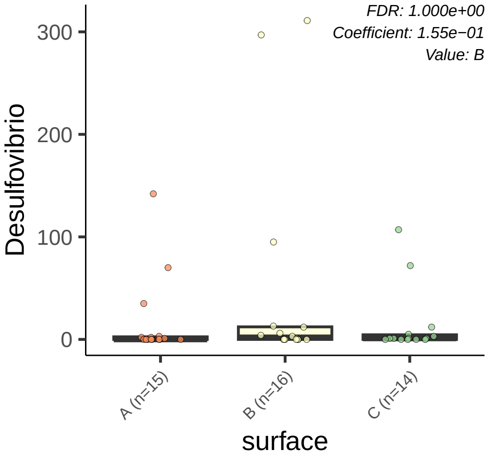
FDR: 1.000e+00
Coefficient: 1.55e−01
Value: B
Desulfovibrio
300
200
100
0
A (n=15)
B (n=16)
C (n=14)
surface

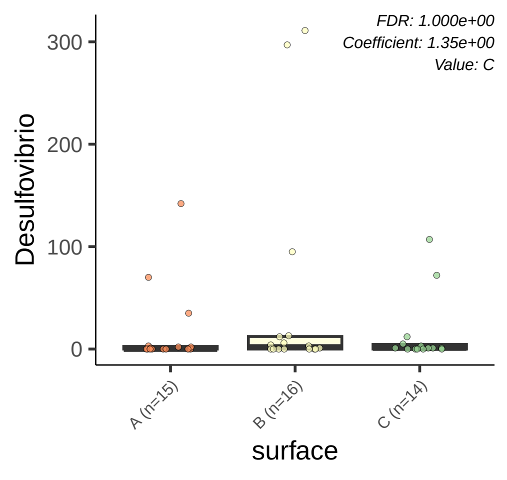

FDR: 1.000e+00
Coefficient: 1.35e+00
Value: C
Desulfovibrio
300
200
100
0
A (n=15)
B (n=16)
C (n=14)
surface

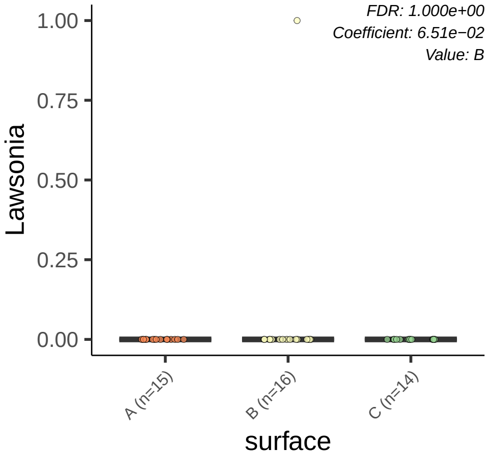
FDR: 1.000e+00
Coefficient: 6.51e−02
Value: B
Lawsonia
1.00
0.75
0.50
0.25
0.00
A (n=15)
B (n=16)
C (n=14)
surface

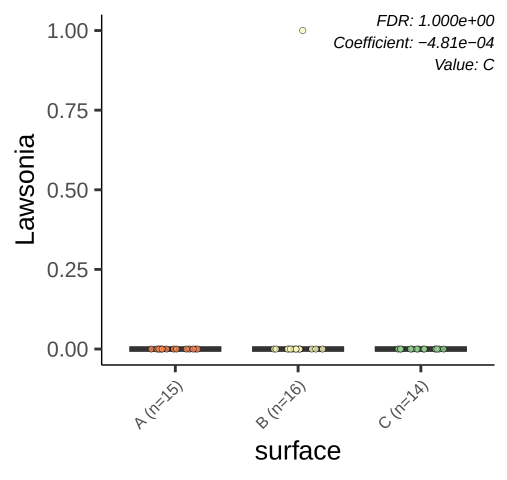
FDR: 1.000e+00
Coefficient: −4.81e−04
Value: C
Lawsonia
1.00
0.75
0.50
0.25
0.00
A (n=15)
B (n=16)
C (n=14)
surface

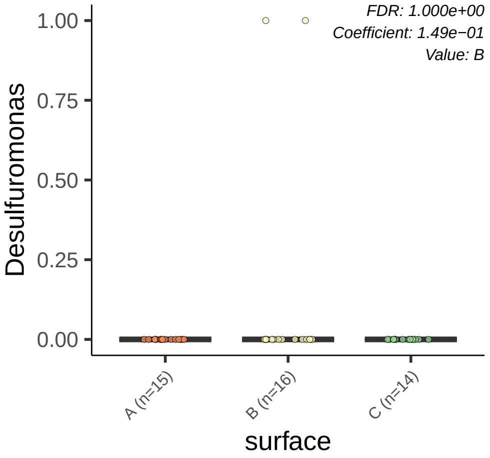
FDR: 1.000e+00
Coefficient: 1.49e−01
Value: B
Desulfuromonas
1.00
0.75
0.50
0.25
0.00
A (n=15)
B (n=16)
C (n=14)
surface

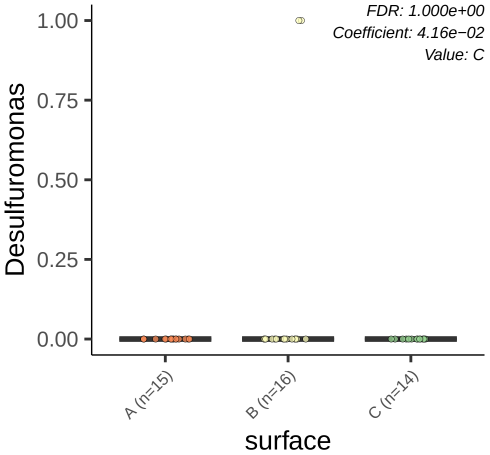
FDR: 1.000e+00
Coefficient: 4.16e−02
Value: C
Desulfuromonas
1.00
0.75
0.50
0.25
0.00
A (n=15)
B (n=16)
C (n=14)
surface

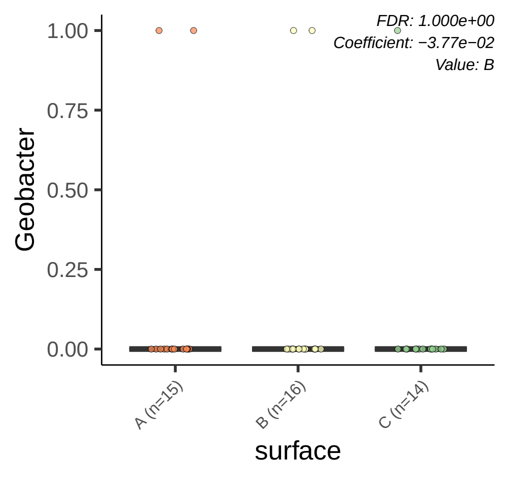
FDR: 1.000e+00
Coefficient: −3.77e−02
Value: B
Geobacter
1.00
0.75
0.50
0.25
0.00
A (n=15)
B (n=16)
C (n=14)
surface

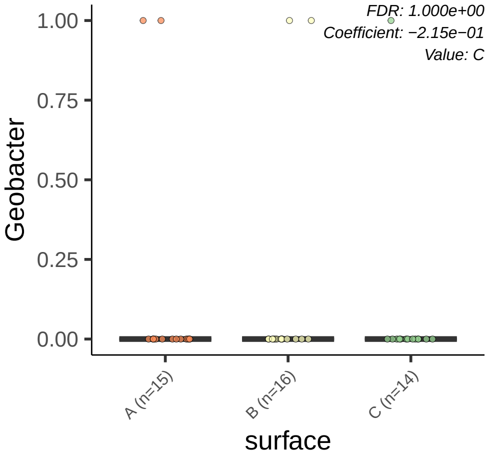
FDR: 1.000e+00
Coefficient: −2.15e−01
Value: C
1.00
0.75
0.50
0.25
0.00
Geobacter
A (n=15)
B (n=16)
C (n=14)
surface

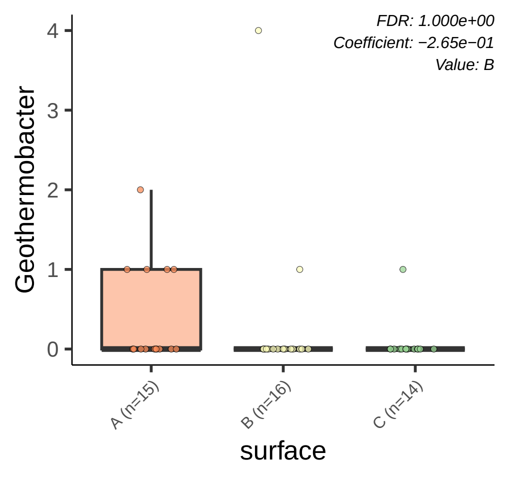

FDR: 1.000e+00
Coefficient: −2.65e−01
Value: B
Geothermobacter
0
1
2
3
4
A (n=15)
B (n=16)
C (n=14)
surface

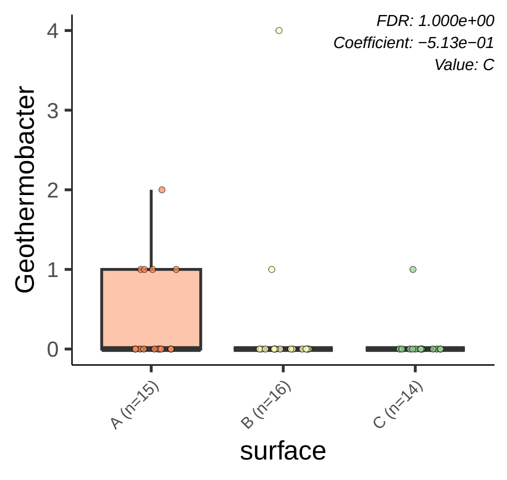

FDR: 1.000e+00
Coefficient: −5.13e−01
Value: C
Geothermobacter
0
1
2
3
4
A (n=15)
B (n=16)
C (n=14)
surface

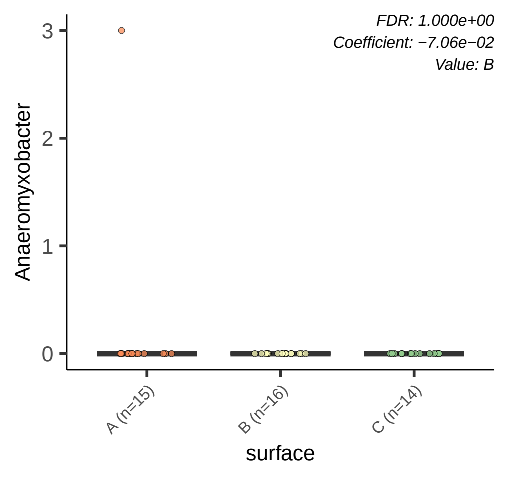

FDR: 1.000e+00
Coefficient: −7.06e−02
Value: B
Anaeromyxobacter
3
2
1
0
A (n=15)
B (n=16)
C (n=14)
surface

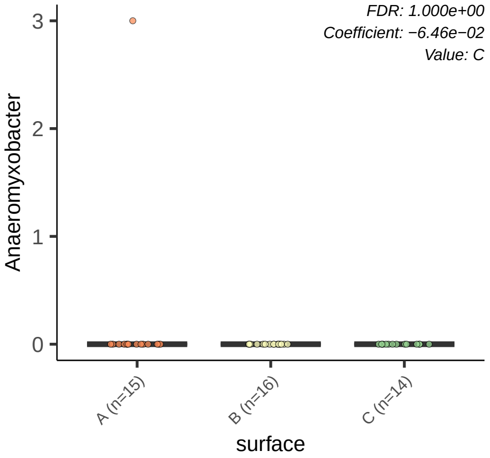

FDR: 1.000e+00
Coefficient: −6.46e−02
Value: C
Anaeromyxobacter
3
2
1
0
A (n=15)
B (n=16)
C (n=14)
surface

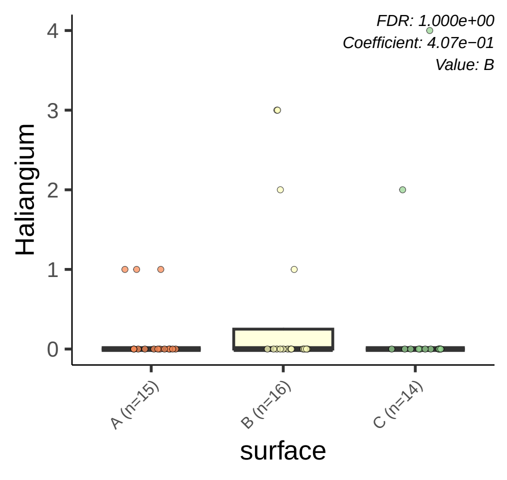
FDR: 1.000e+00
Coefficient: 4.07e−01
Value: B
Haliangium
0
1
2
3
4
A (n=15)
B (n=16)
C (n=14)
surface

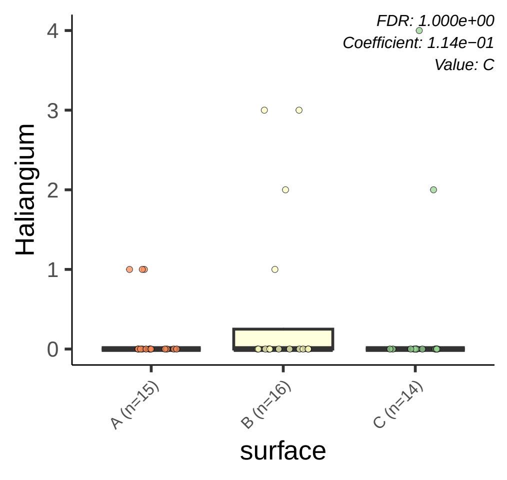
FDR: 1.000e+00
Coefficient: 1.14e−01
Value: C
Haliangium
4
3
2
1
0
A (n=15)
B (n=16)
C (n=14)
surface

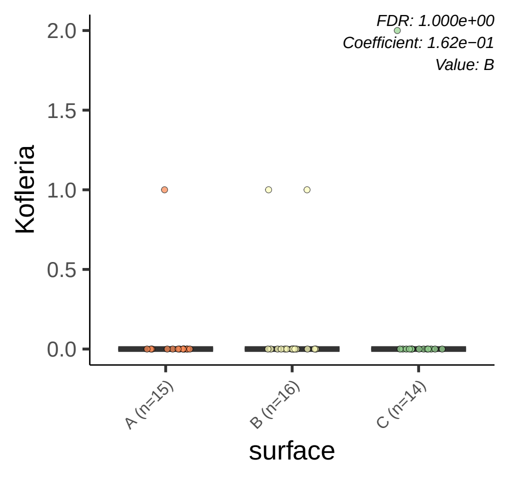

FDR: 1.000e+00
Coefficient: 1.62e−01
Value: B
Kofleria
2.0
1.5
1.0
0.5
0.0
A (n=15)
B (n=16)
C (n=14)
surface

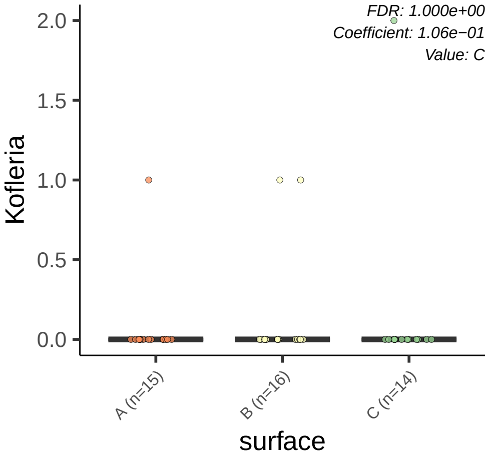
FDR: 1.000e+00
Coefficient: 1.06e−01
Value: C
Kofleria
2.0
1.5
1.0
0.5
0.0
A (n=15)
B (n=16)
C (n=14)
surface

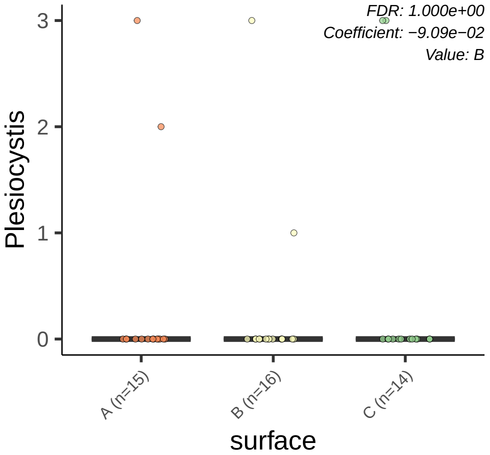
FDR: 1.000e+00
Coefficient: −9.09e−02
Value: B
Plesiocystis
0
1
2
3
A (n=15)
B (n=16)
C (n=14)
surface

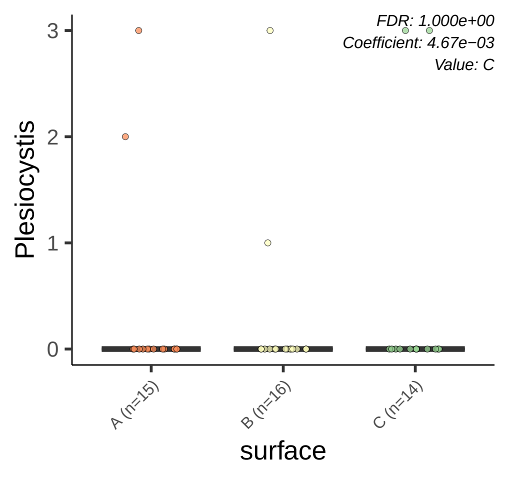

FDR: 1.000e+00
Coefficient: 4.67e−03
Value: C
Plesiocystis
3
2
1
0
A (n=15)
B (n=16)
C (n=14)
surface

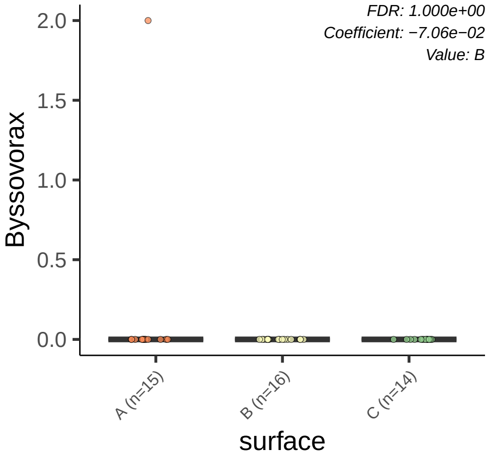

FDR: 1.000e+00
Coefficient: −7.06e−02
Value: B
Byssovorax
2.0
1.5
1.0
0.5
0.0
A (n=15)
B (n=16)
C (n=14)
surface

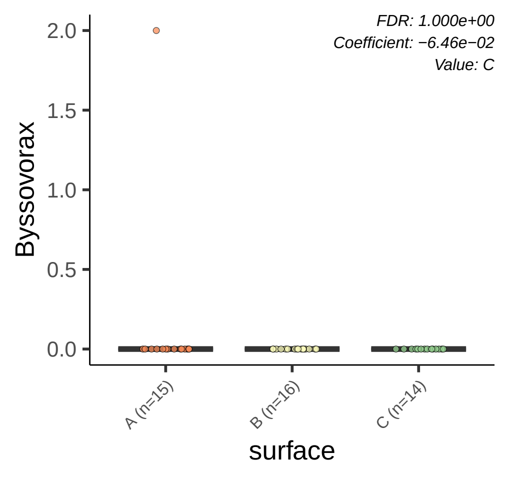
FDR: 1.000e+00
Coefficient: −6.46e−02
Value: C
Byssovorax
2.0
1.5
1.0
0.5
0.0
A (n=15)
B (n=16)
C (n=14)
surface

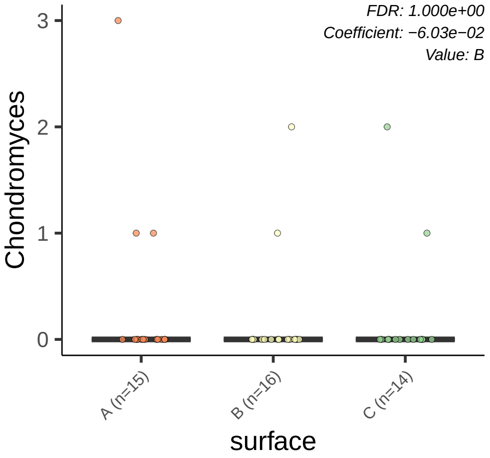

FDR: 1.000e+00
Coefficient: −6.03e−02
Value: B
Chondromyces
3
2
1
0
A (n=15)
B (n=16)
C (n=14)
surface

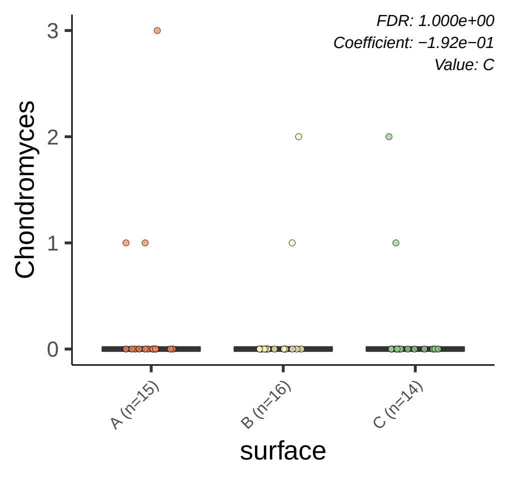

FDR: 1.000e+00
Coefficient: −1.92e−01
Value: C
Chondromyces
3
2
1
0
A (n=15)
B (n=16)
C (n=14)
surface

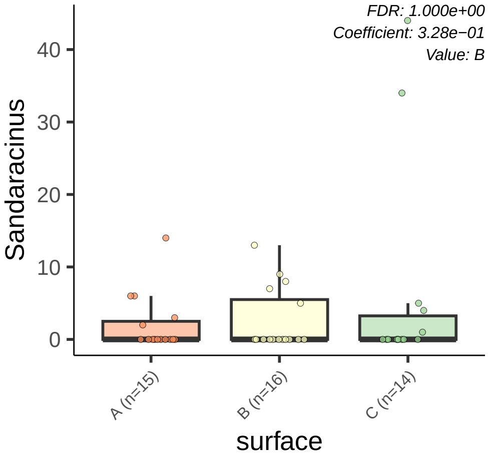

FDR: 1.000e+00
Coefficient: 3.28e−01
Value: B
Sandaracinus
0
10
20
30
40
A (n=15)
B (n=16)
C (n=14)
surface

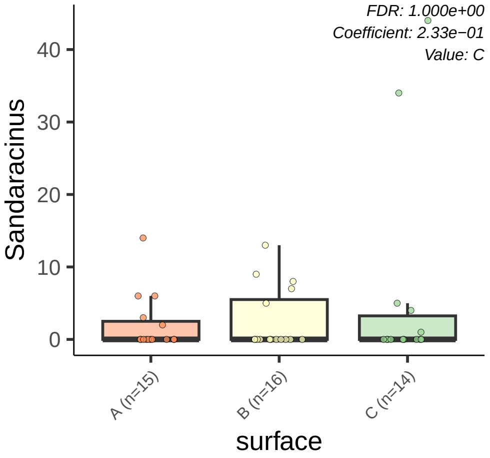

FDR: 1.000e+00
Coefficient: 2.33e−01
Value: C
Sandaracinus
0
10
20
30
40
A (n=15)
B (n=16)
C (n=14)
surface

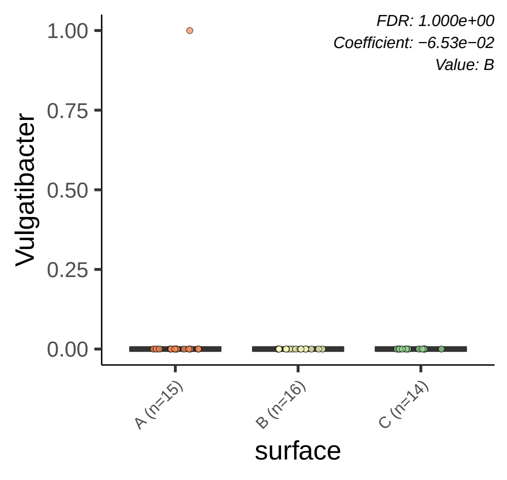

FDR: 1.000e+00
Coefficient: −6.53e−02
Value: B
Vulgatibacter
1.00
0.75
0.50
0.25
0.00
A (n=15)
B (n=16)
C (n=14)
surface

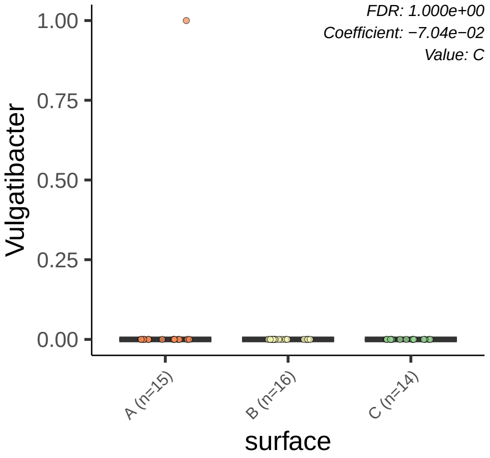

FDR: 1.000e+00
Coefficient: −7.04e−02
Value: C
Vulgatibacter
1.00
0.75
0.50
0.25
0.00
A (n=15)
B (n=16)
C (n=14)
surface

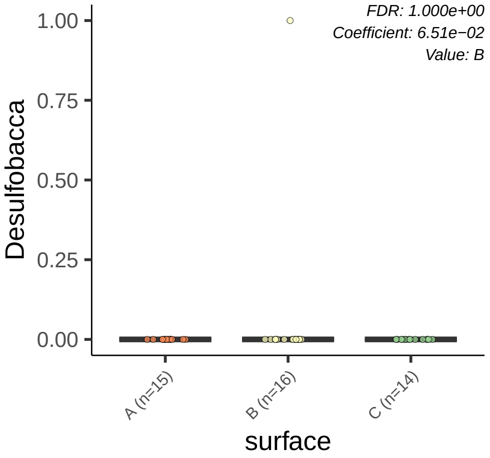
FDR: 1.000e+00
Coefficient: 6.51e−02
Value: B
Desulfobacca
1.00
0.75
0.50
0.25
0.00
A (n=15)
B (n=16)
C (n=14)
surface

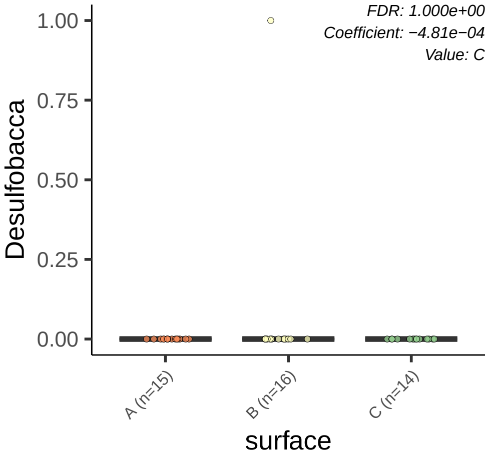

FDR: 1.000e+00
Coefficient: −4.81e−04
Value: C
Desulfobacca
1.00
0.75
0.50
0.25
0.00
A (n=15)
B (n=16)
C (n=14)
surface

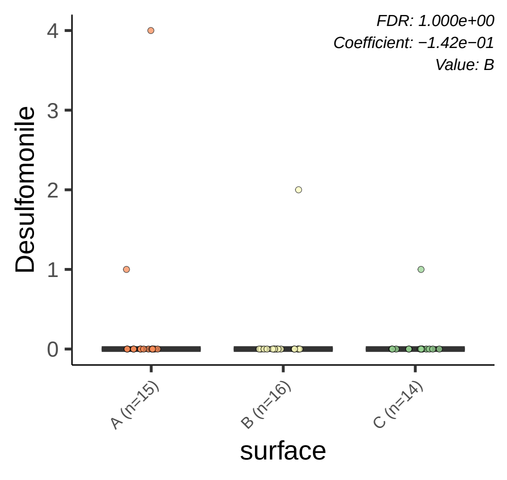
FDR: 1.000e+00
Coefficient: −1.42e−01
Value: B
Desulfomonile
4
3
2
1
0
A (n=15)
B (n=16)
C (n=14)
surface

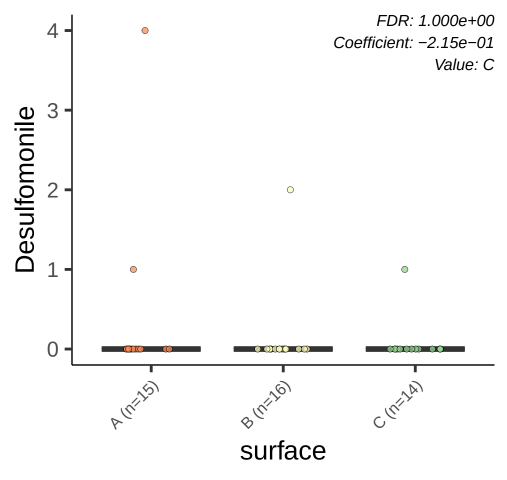
FDR: 1.000e+00
Coefficient: −2.15e−01
Value: C
Desulfomonile
4
3
2
1
0
A (n=15)
B (n=16)
C (n=14)
surface

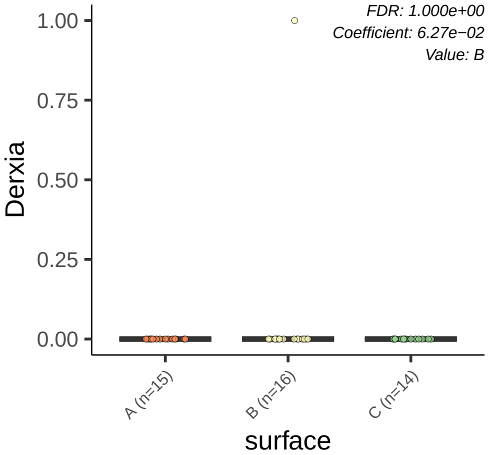
FDR: 1.000e+00
Coefficient: 6.27e−02
Value: B
Derxia
1.00
0.75
0.50
0.25
0.00
A (n=15)
B (n=16)
C (n=14)
surface

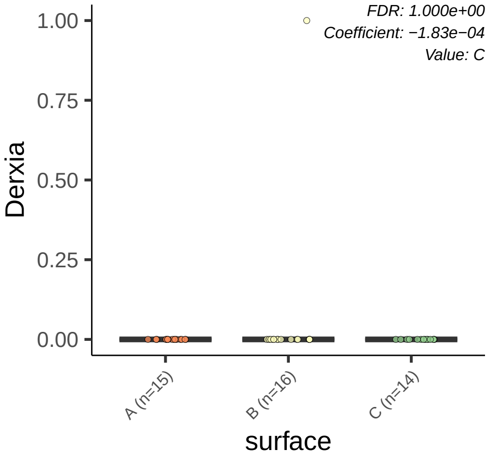

FDR: 1.000e+00
Coefficient: −1.83e−04
Value: C
Derxia
1.00
0.75
0.50
0.25
0.00
A (n=15)
B (n=16)
C (n=14)
surface

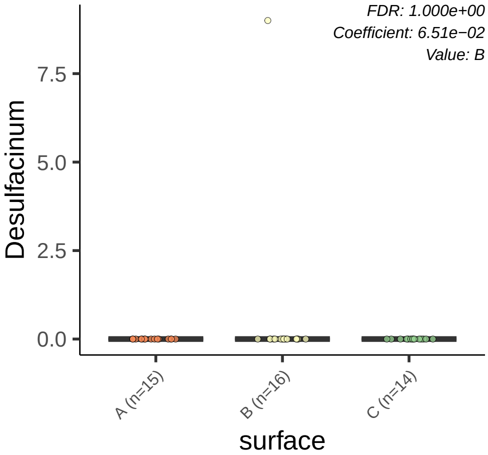

FDR: 1.000e+00
Coefficient: 6.51e−02
Value: B
Desulfacinum
7.5
5.0
2.5
0.0
A (n=15)
B (n=16)
C (n=14)
surface

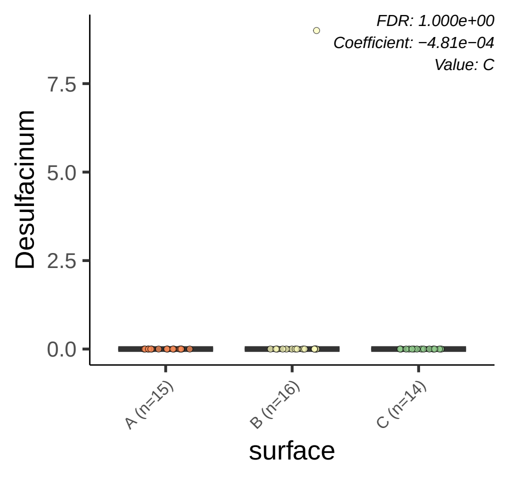

FDR: 1.000e+00
Coefficient: −4.81e−04
Value: C
Desulfacinum
7.5
5.0
2.5
0.0
A (n=15)
B (n=16)
C (n=14)
surface

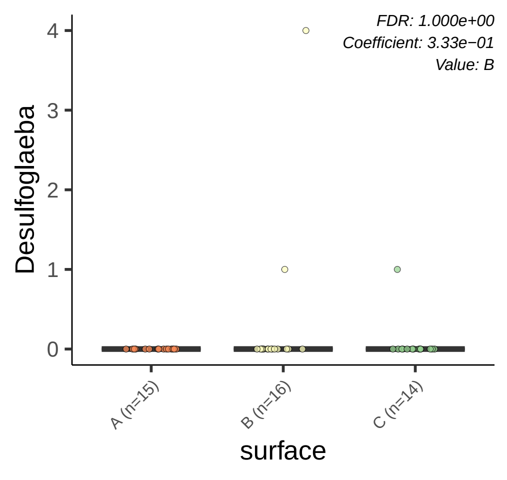

FDR: 1.000e+00
Coefficient: 3.33e−01
Value: B
Desulfoglaeba
0
1
2
3
4
A (n=15)
B (n=16)
C (n=14)
surface

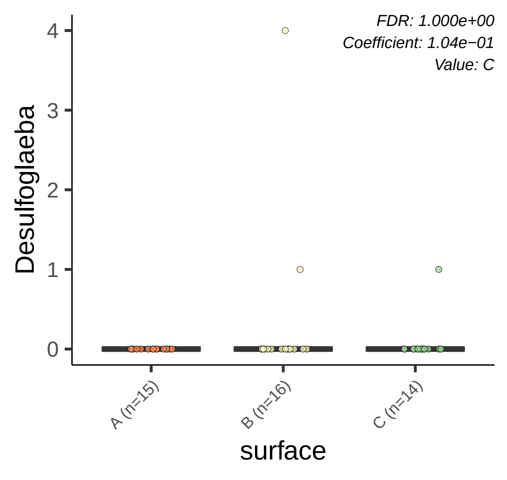
FDR: 1.000e+00
Coefficient: 1.04e−01
Value: C
Desulfoglaeba
4
3
2
1
0
A (n=15)
B (n=16)
C (n=14)
surface

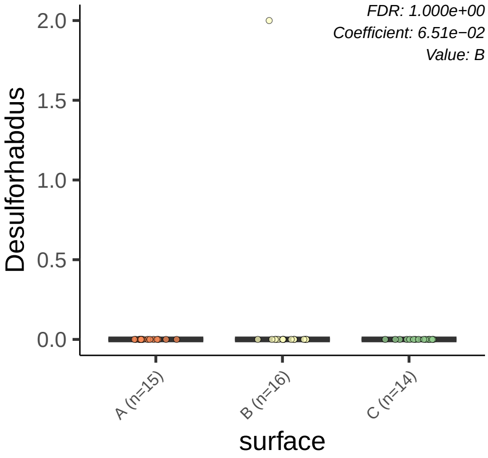
FDR: 1.000e+00
Coefficient: 6.51e−02
Value: B
Desulforhabdus
2.0
1.5
1.0
0.5
0.0
A (n=15)
B (n=16)
C (n=14)
surface

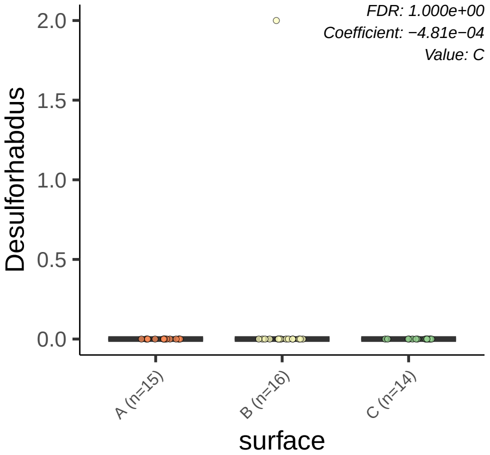
FDR: 1.000e+00
Coefficient: −4.81e−04
Value: C
Desulforhabdus
2.0
1.5
1.0
0.5
0.0
A (n=15)
B (n=16)
C (n=14)
surface

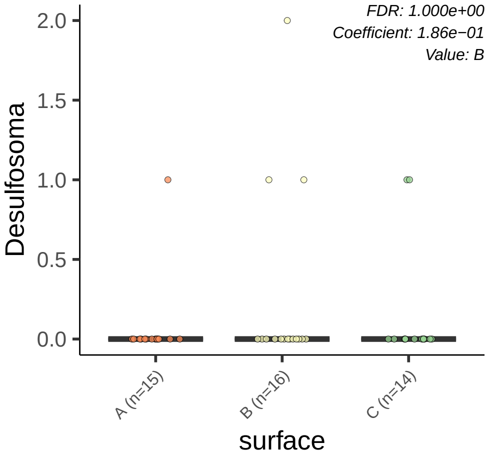
FDR: 1.000e+00
Coefficient: 1.86e−01
Value: B
Desulfosoma
2.0
1.5
1.0
0.5
0.0
A (n=15)
B (n=16)
C (n=14)
surface

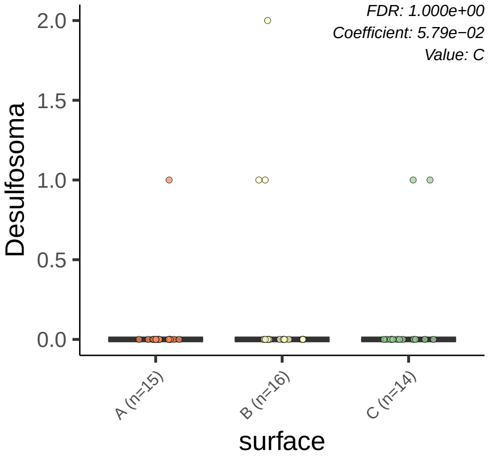
FDR: 1.000e+00
Coefficient: 5.79e−02
Value: C
Desulfosoma
2.0
1.5
1.0
0.5
0.0
A (n=15)
B (n=16)
C (n=14)
surface

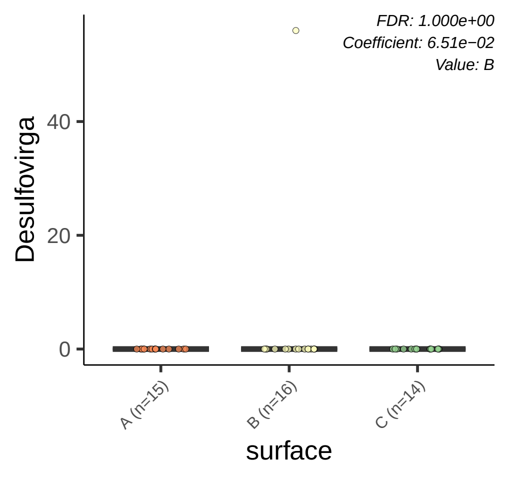
FDR: 1.000e+00
Coefficient: 6.51e−02
Value: B
Desulfovirga
40
20
0
A (n=15)
B (n=16)
C (n=14)
surface

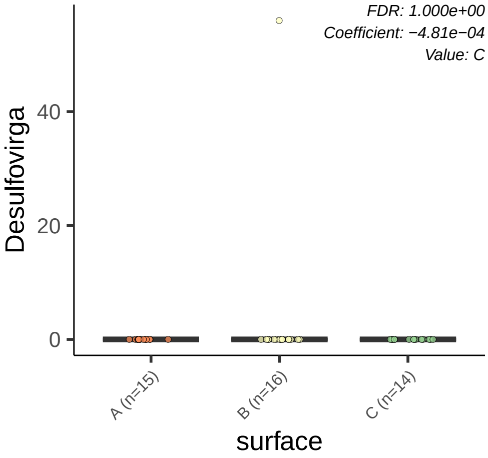
FDR: 1.000e+00
Coefficient: −4.81e−04
Value: C
Desulfovirga
40
20
0
A (n=15)
B (n=16)
C (n=14)
surface

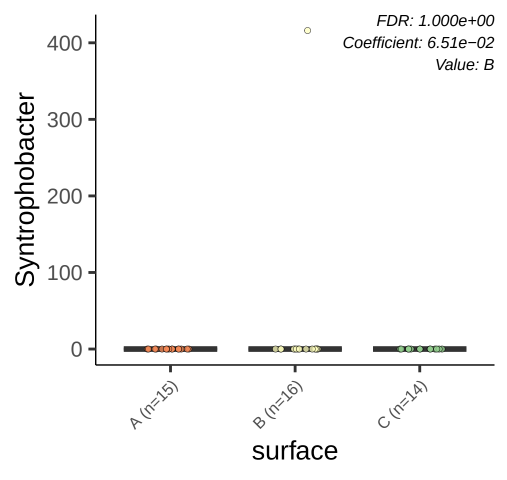
FDR: 1.000e+00
Coefficient: 6.51e−02
Value: B
Syntrophobacter
400
300
200
100
0
A (n=15)
B (n=16)
C (n=14)
surface

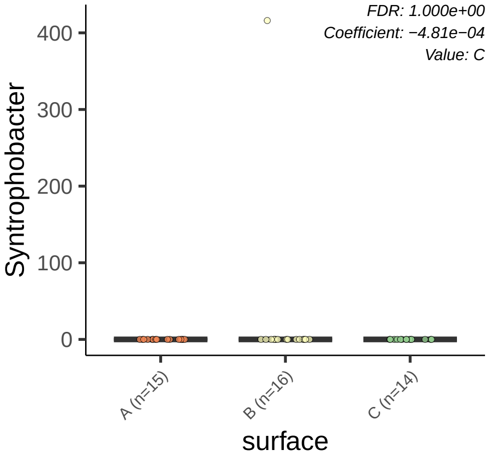
FDR: 1.000e+00
Coefficient: −4.81e−04
Value: C
Syntrophobacter
400
300
200
100
0
A (n=15)
B (n=16)
C (n=14)
surface

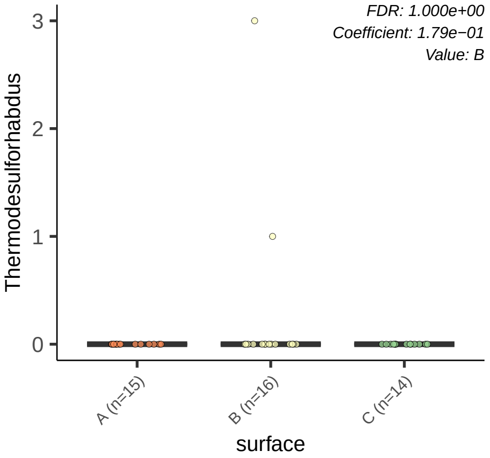
FDR: 1.000e+00
Coefficient: 1.79e−01
Value: B
Thermodesulforhabdus
3
2
1
0
A (n=15)
B (n=16)
C (n=14)
surface

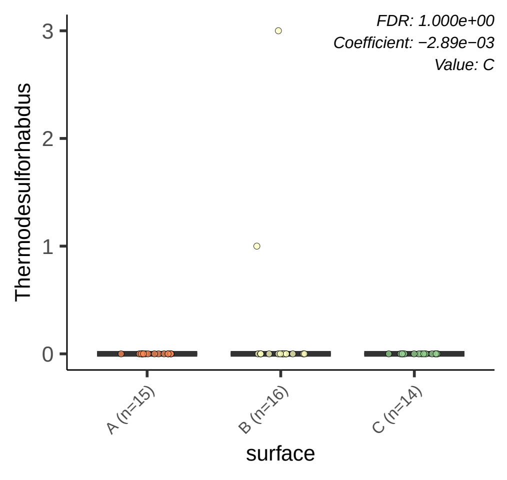
FDR: 1.000e+00
Coefficient: −2.89e−03
Value: C
Thermodesulforhabdus
3
2
1
0
A (n=15)
B (n=16)
C (n=14)
surface

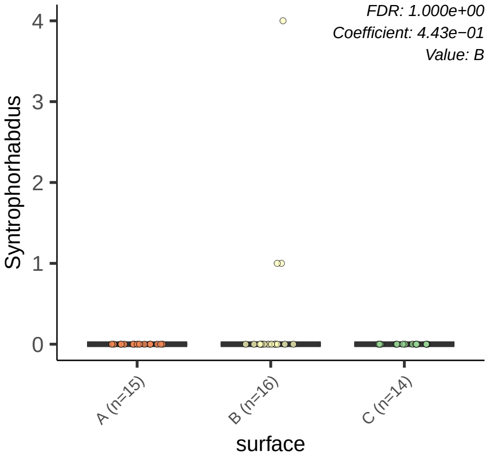
FDR: 1.000e+00
Coefficient: 4.43e−01
Value: B
Syntrophorhabdus
0
1
2
3
4
A (n=15)
B (n=16)
C (n=14)
surface

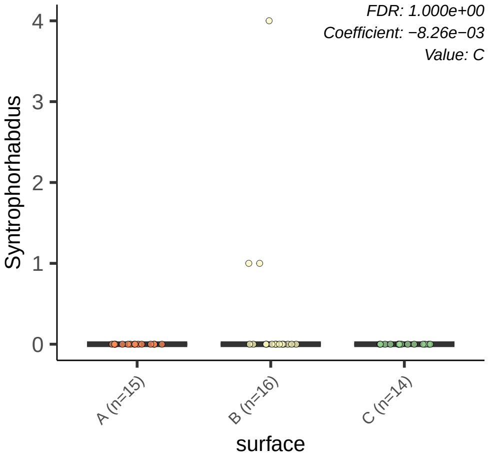
FDR: 1.000e+00
Coefficient: −8.26e−03
Value: C
Syntrophorhabdus
4
3
2
1
0
A (n=15)
B (n=16)
C (n=14)
surface

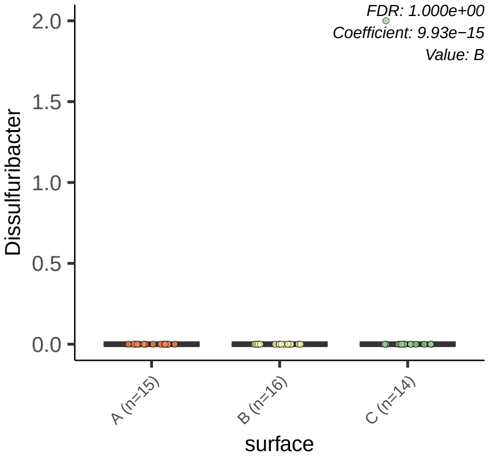
FDR: 1.000e+00
Coefficient: 9.93e−15
Value: B
Dissulfuribacter
2.0
1.5
1.0
0.5
0.0
A (n=15)
B (n=16)
C (n=14)
surface

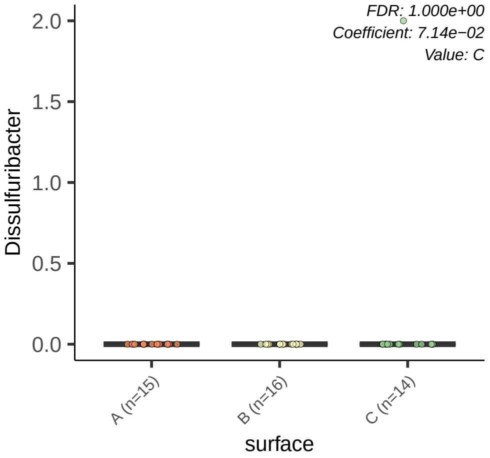

FDR: 1.000e+00
Coefficient: 7.14e−02
Value: C
Dissulfuribacter
2.0
1.5
1.0
0.5
0.0
A (n=15)
B (n=16)
C (n=14)
surface

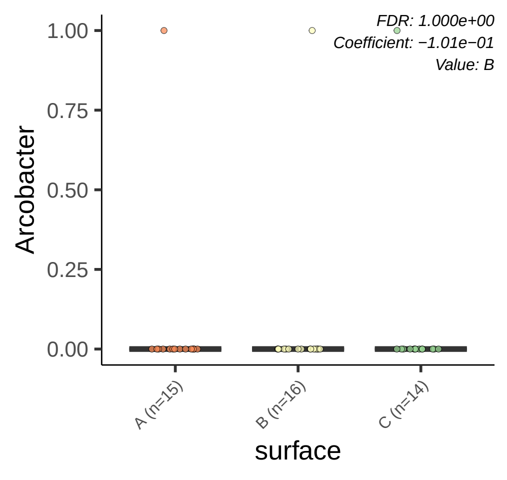
FDR: 1.000e+00
Coefficient: −1.01e−01
Value: B
1.00
0.75
0.50
0.25
0.00
Arcobacter
A (n=15)
B (n=16)
C (n=14)
surface

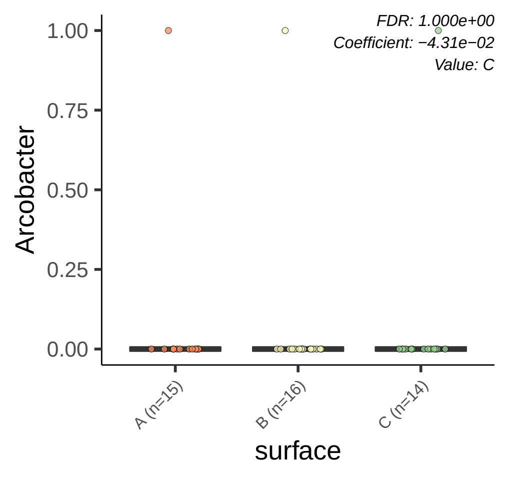
FDR: 1.000e+00
Coefficient: −4.31e−02
Value: C
Arcobacter
1.00
0.75
0.50
0.25
0.00
A (n=15)
B (n=16)
C (n=14)
surface

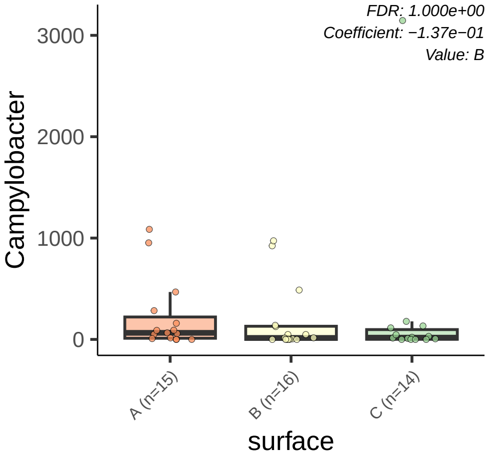
FDR: 1.000e+00
Coefficient: −1.37e−01
Value: B
3000
2000
1000
0
Campylobacter
A (n=15)
B (n=16)
C (n=14)
surface

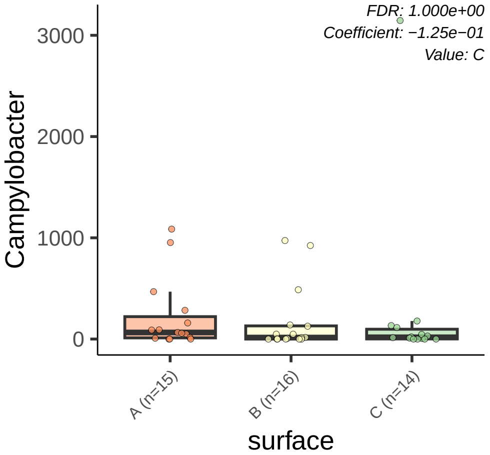
FDR: 1.000e+00
Coefficient: −1.25e−01
Value: C
Campylobacter
3000
2000
1000
0
A (n=15)
B (n=16)
C (n=14)
surface

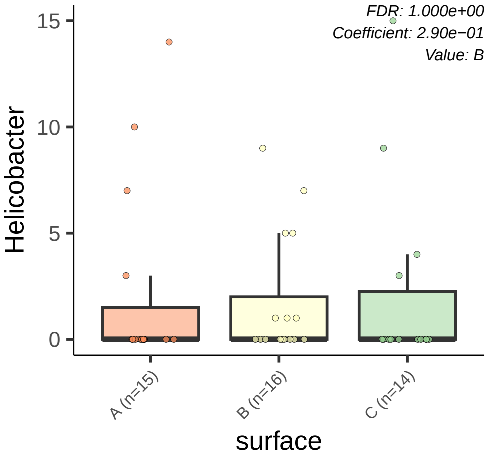
FDR: 1.000e+00
Coefficient: 2.90e−01
Value: B
Helicobacter
15
10
5
0
A (n=15)
B (n=16)
C (n=14)
surface

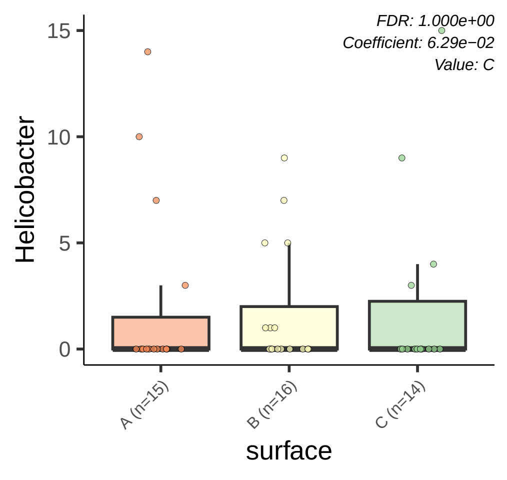

FDR: 1.000e+00
Coefficient: 6.29e−02
Value: C
Helicobacter
15
10
5
0
A (n=15)
B (n=16)
C (n=14)
surface

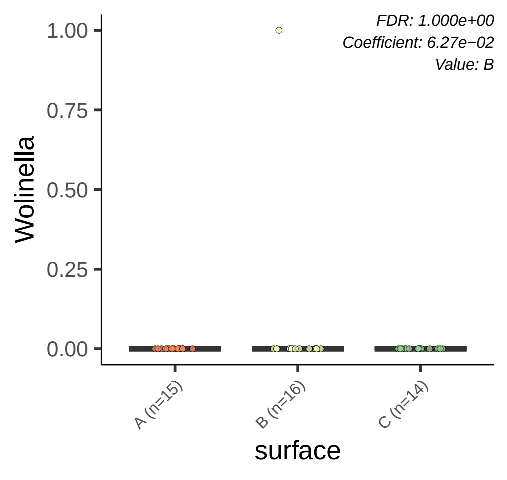
FDR: 1.000e+00
Coefficient: 6.27e−02
Value: B
1.00
0.75
0.50
0.25
0.00
Wolinella
A (n=15)
B (n=16)
C (n=14)
surface

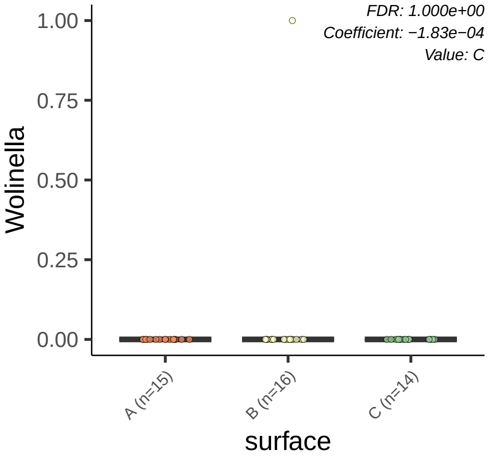

FDR: 1.000e+00
Coefficient: −1.83e−04
Value: C
1.00
0.75
0.50
0.25
0.00
Wolinella
A (n=15)
B (n=16)
C (n=14)
surface

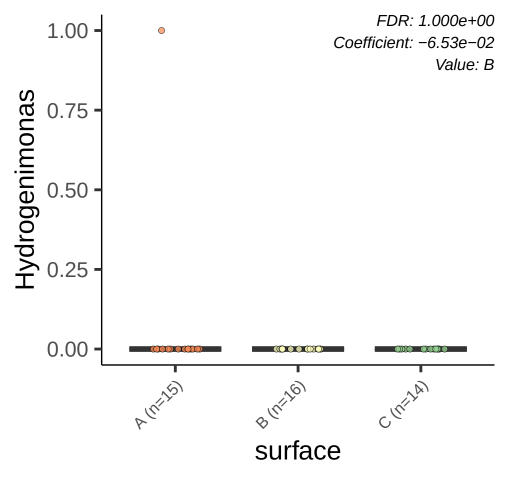

FDR: 1.000e+00
Coefficient: −6.53e−02
Value: B
Hydrogenimonas
1.00
0.75
0.50
0.25
0.00
A (n=15)
B (n=16)
C (n=14)
surface

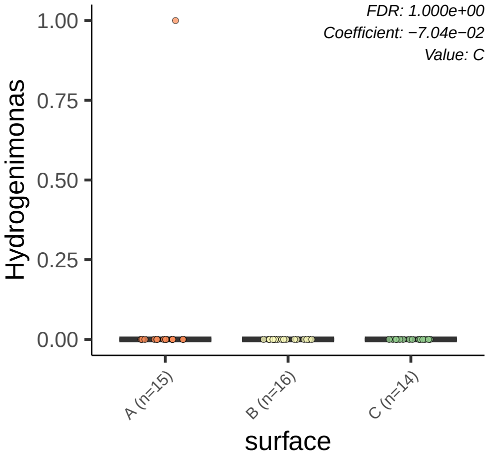

FDR: 1.000e+00
Coefficient: −7.04e−02
Value: C
Hydrogenimonas
1.00
0.75
0.50
0.25
0.00
A (n=15)
B (n=16)
C (n=14)
surface

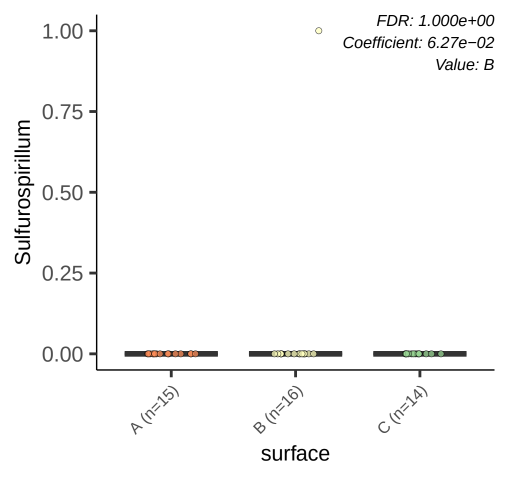
FDR: 1.000e+00
Coefficient: 6.27e−02
Value: B
Sulfurospirillum
1.00
0.75
0.50
0.25
0.00
A (n=15)
B (n=16)
C (n=14)
surface

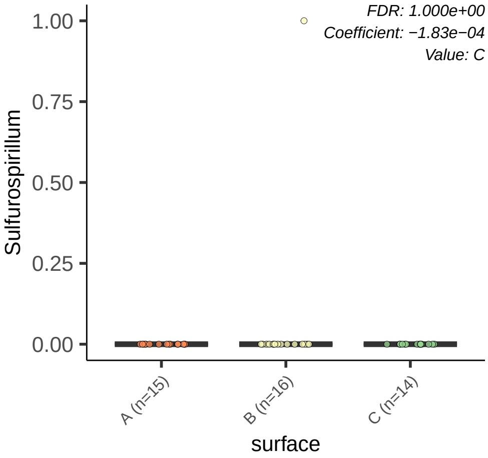

FDR: 1.000e+00
Coefficient: −1.83e−04
Value: C
Sulfurospirillum
1.00
0.75
0.50
0.25
0.00
A (n=15)
B (n=16)
C (n=14)
surface

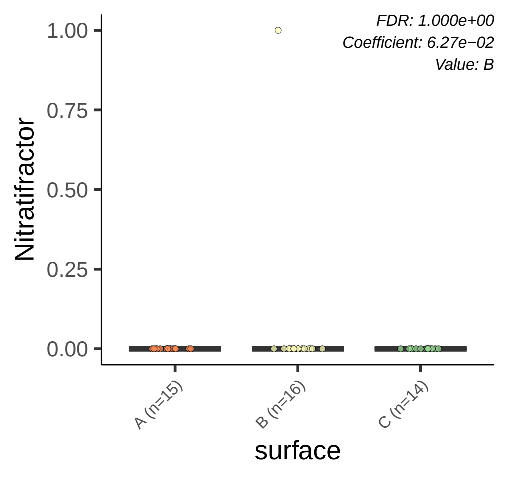
FDR: 1.000e+00
Coefficient: 6.27e−02
Value: B
Nitratifractor
1.00
0.75
0.50
0.25
0.00
A (n=15)
B (n=16)
C (n=14)
surface

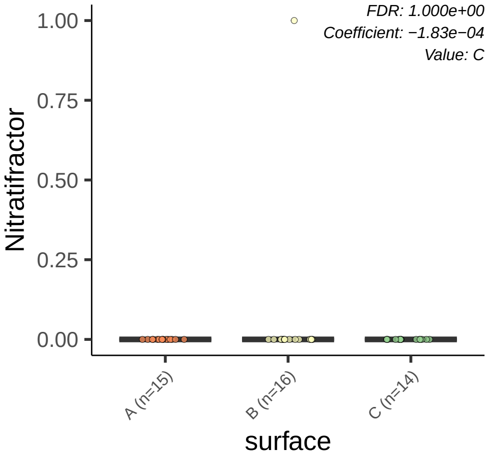

FDR: 1.000e+00
Coefficient: −1.83e−04
Value: C
Nitratifractor
1.00
0.75
0.50
0.25
0.00
A (n=15)
B (n=16)
C (n=14)
surface

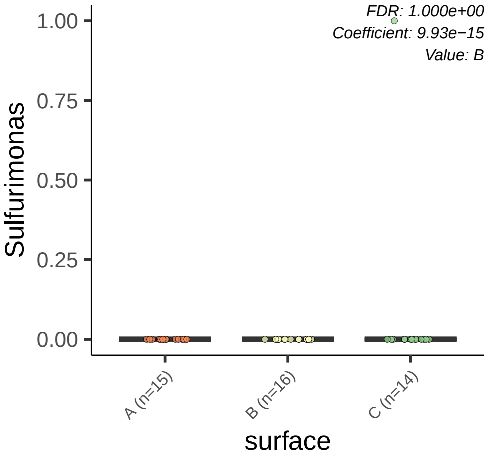

FDR: 1.000e+00
Coefficient: 9.93e−15
Value: B
Sulfurimonas
1.00
0.75
0.50
0.25
0.00
A (n=15)
B (n=16)
C (n=14)
surface

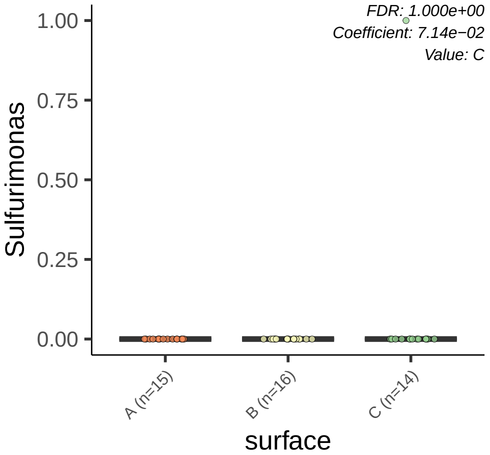

FDR: 1.000e+00
Coefficient: 7.14e−02
Value: C
Sulfurimonas
1.00
0.75
0.50
0.25
0.00
A (n=15)
B (n=16)
C (n=14)
surface

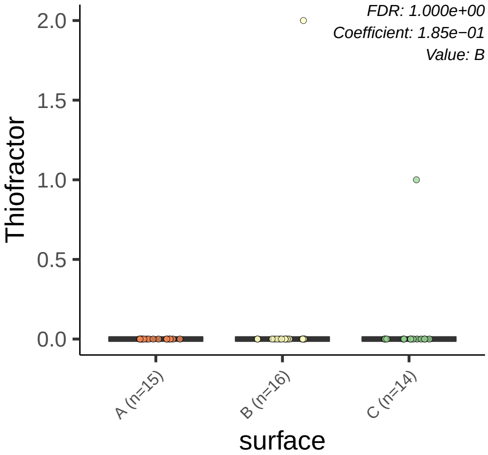

FDR: 1.000e+00
Coefficient: 1.85e−01
Value: B
Thiofractor
2.0
1.5
1.0
0.5
0.0
A (n=15)
B (n=16)
C (n=14)
surface

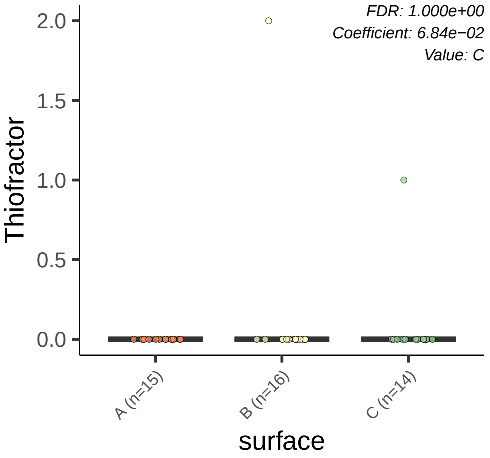

FDR: 1.000e+00
Coefficient: 6.84e−02
Value: C
Thiofractor
2.0
1.5
1.0
0.5
0.0
A (n=15)
B (n=16)
C (n=14)
surface

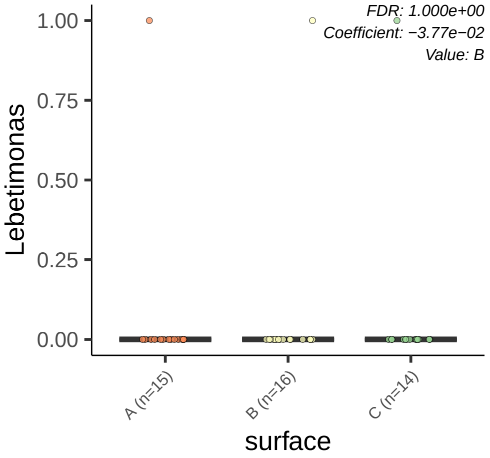
FDR: 1.000e+00
Coefficient: −3.77e−02
Value: B
Lebetimonas
1.00
0.75
0.50
0.25
0.00
A (n=15)
B (n=16)
C (n=14)
surface

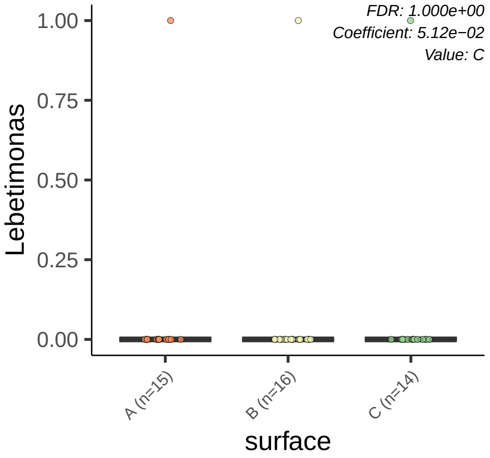

FDR: 1.000e+00
Coefficient: 5.12e−02
Value: C
Lebetimonas
1.00
0.75
0.50
0.25
0.00
A (n=15)
B (n=16)
C (n=14)
surface

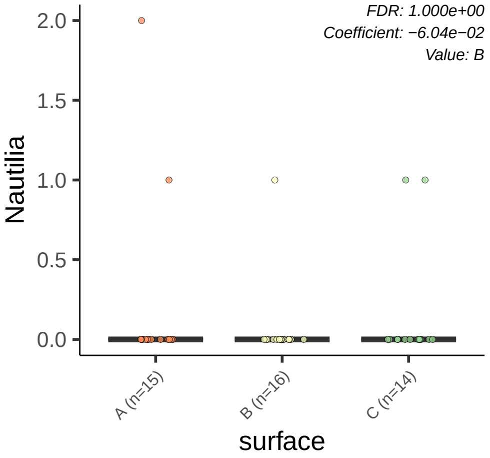
FDR: 1.000e+00
Coefficient: −6.04e−02
Value: B
Nautilia
2.0
1.5
1.0
0.5
0.0
A (n=15)
B (n=16)
C (n=14)
surface

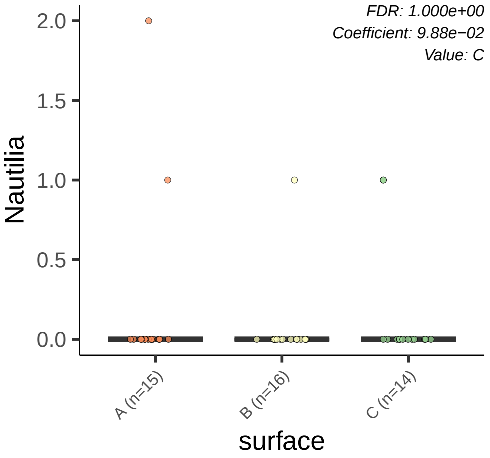

FDR: 1.000e+00
Coefficient: 9.88e−02
Value: C
Nautilia
2.0
1.5
1.0
0.5
0.0
A (n=15)
B (n=16)
C (n=14)
surface

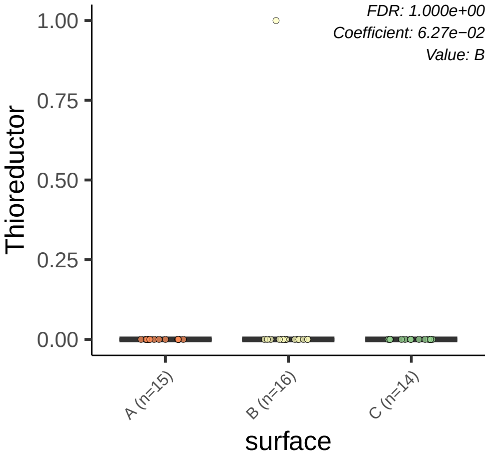
FDR: 1.000e+00
Coefficient: 6.27e−02
Value: B
1.00
0.75
0.50
0.25
0.00
Thioreductor
A (n=15)
B (n=16)
C (n=14)
surface

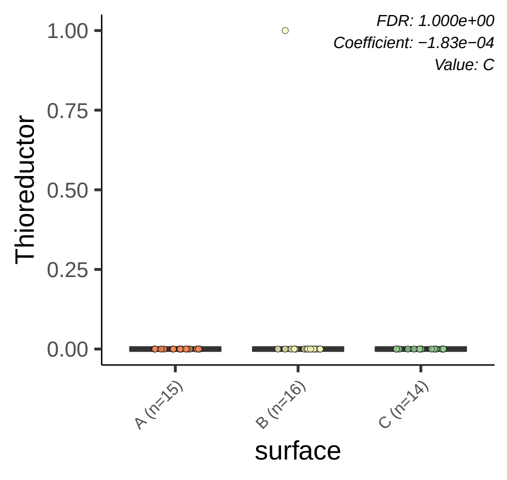
FDR: 1.000e+00
Coefficient: −1.83e−04
Value: C
Thioreductor
1.00
0.75
0.50
0.25
0.00
A (n=15)
B (n=16)
C (n=14)
surface

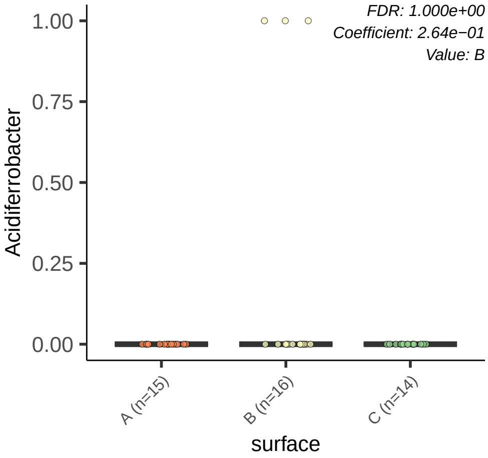
FDR: 1.000e+00
Coefficient: 2.64e−01
Value: B
Acidiferrobacter
1.00
0.75
0.50
0.25
0.00
A (n=15)
B (n=16)
C (n=14)
surface

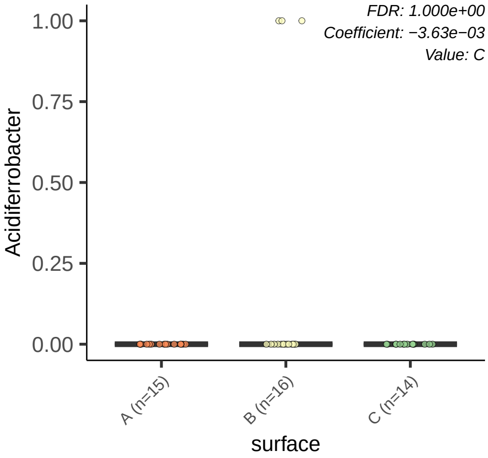

FDR: 1.000e+00
Coefficient: −3.63e−03
Value: C
Acidiferrobacter
1.00
0.75
0.50
0.25
0.00
A (n=15)
B (n=16)
C (n=14)
surface

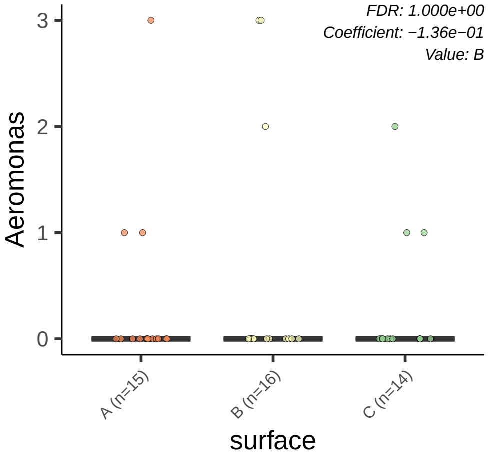
FDR: 1.000e+00
Coefficient: −1.36e−01
Value: B
Aeromonas
0
1
2
3
A (n=15)
B (n=16)
C (n=14)
surface

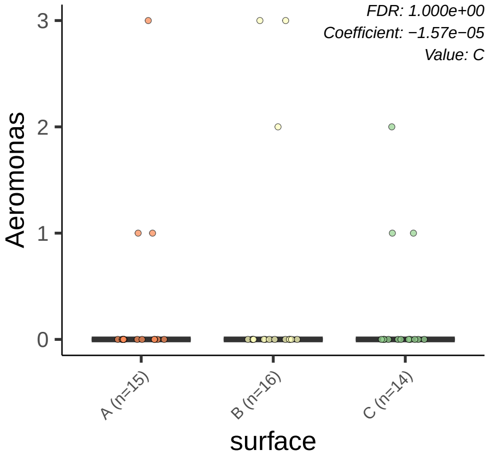
FDR: 1.000e+00
Coefficient: −1.57e−05
Value: C
Aeromonas
0
1
2
3
A (n=15)
B (n=16)
C (n=14)
surface

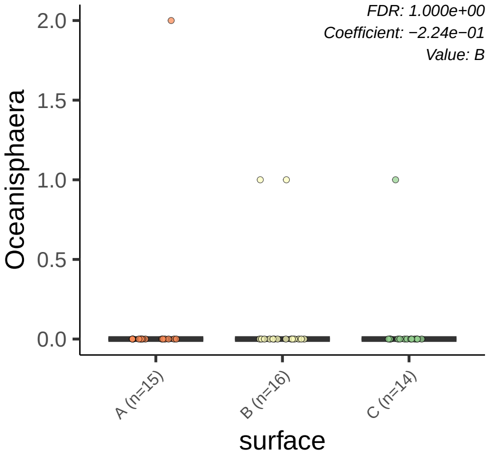
FDR: 1.000e+00
Coefficient: −2.24e−01
Value: B
Oceanisphaera
2.0
1.5
1.0
0.5
0.0
A (n=15)
B (n=16)
C (n=14)
surface

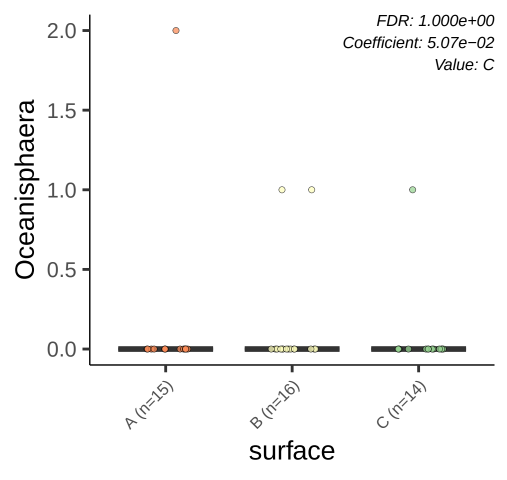

FDR: 1.000e+00
Coefficient: 5.07e−02
Value: C
Oceanisphaera
2.0
1.5
1.0
0.5
0.0
A (n=15)
B (n=16)
C (n=14)
surface

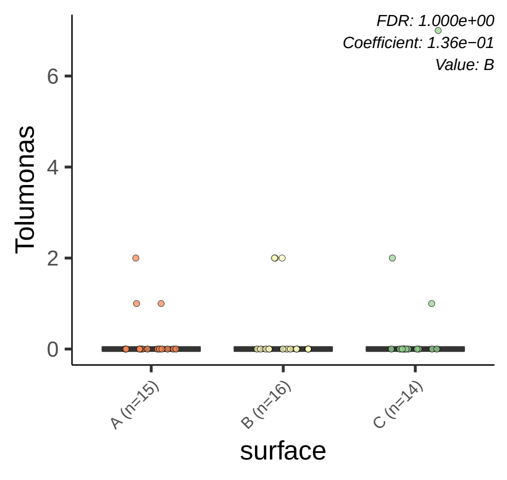

FDR: 1.000e+00
Coefficient: 1.36e−01
Value: B
Tolumonas
6
4
2
0
A (n=15)
B (n=16)
C (n=14)
surface

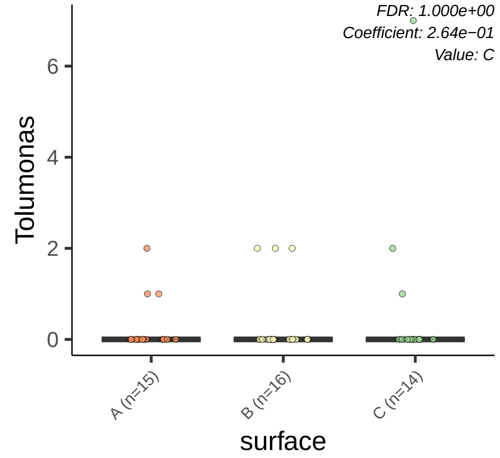
FDR: 1.000e+00
Coefficient: 2.64e−01
Value: C
Tolumonas
0
2
4
6
A (n=15)
B (n=16)
C (n=14)
surface

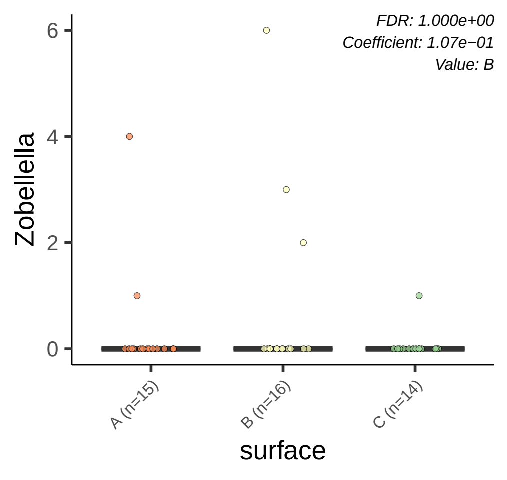
FDR: 1.000e+00
Coefficient: 1.07e−01
Value: B
Zobellella
0
2
4
6
A (n=15)
B (n=16)
C (n=14)
surface

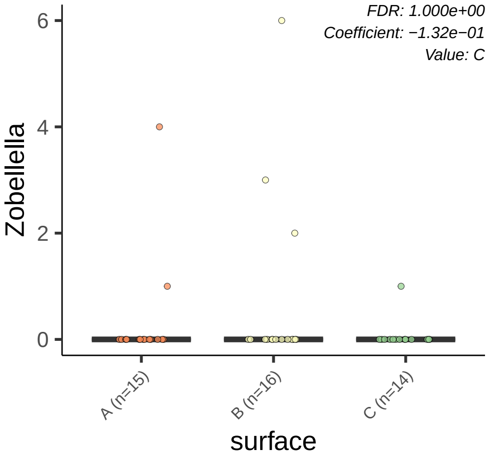
FDR: 1.000e+00
Coefficient: −1.32e−01
Value: C
Zobellella
0
2
4
6
A (n=15)
B (n=16)
C (n=14)
surface

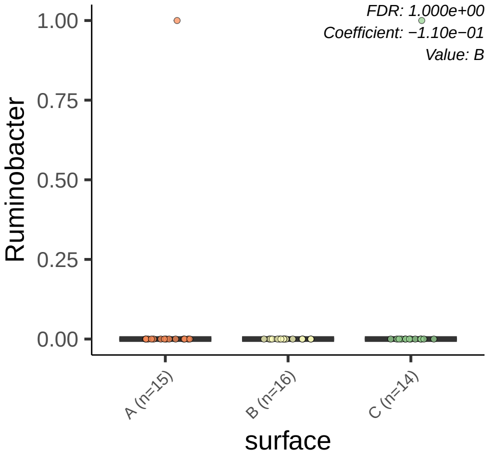

FDR: 1.000e+00
Coefficient: −1.10e−01
Value: B
Ruminobacter
1.00
0.75
0.50
0.25
0.00
A (n=15)
B (n=16)
C (n=14)
surface

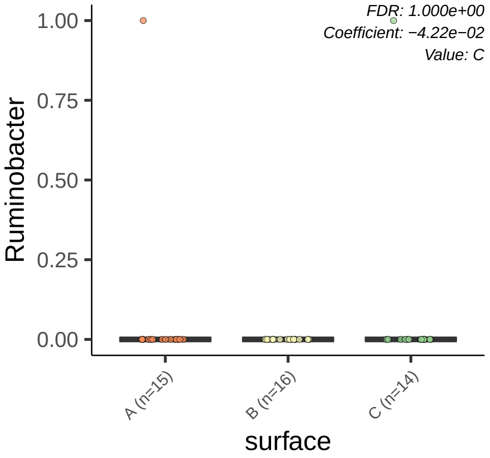
FDR: 1.000e+00
Coefficient: −4.22e−02
Value: C
Ruminobacter
1.00
0.75
0.50
0.25
0.00
A (n=15)
B (n=16)
C (n=14)
surface

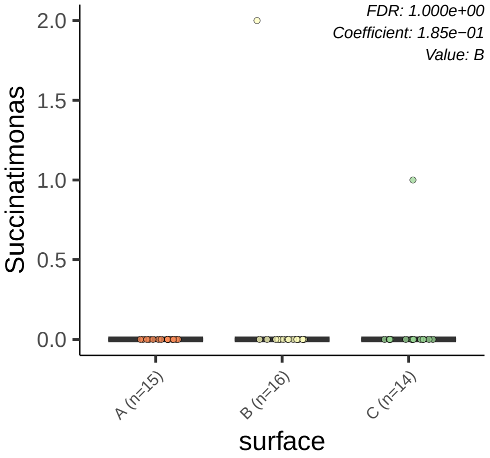
FDR: 1.000e+00
Coefficient: 1.85e−01
Value: B
Succinatimonas
2.0
1.5
1.0
0.5
0.0
A (n=15)
B (n=16)
C (n=14)
surface

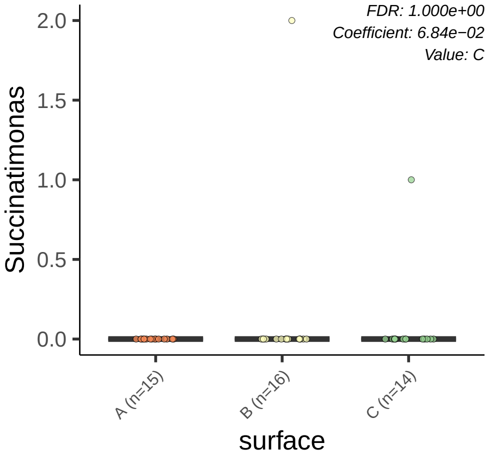
FDR: 1.000e+00
Coefficient: 6.84e−02
Value: C
Succinatimonas
2.0
1.5
1.0
0.5
0.0
A (n=15)
B (n=16)
C (n=14)
surface

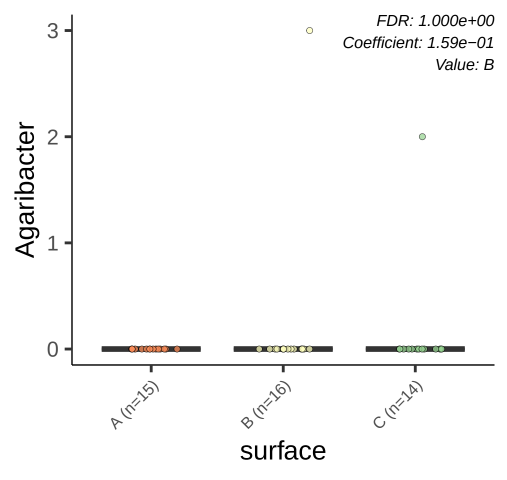
FDR: 1.000e+00
Coefficient: 1.59e−01
Value: B
Agaribacter
3
2
1
0
A (n=15)
B (n=16)
C (n=14)
surface

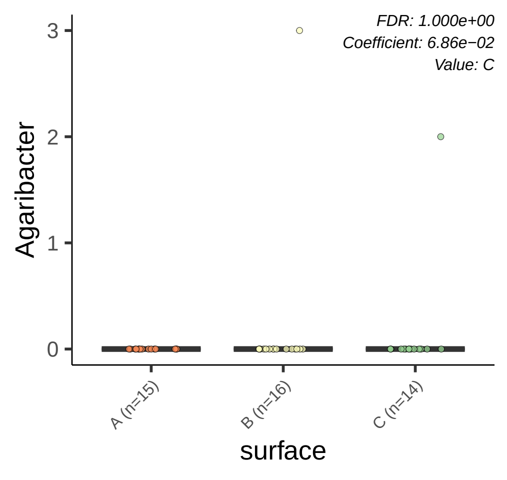
FDR: 1.000e+00
Coefficient: 6.86e−02
Value: C
Agaribacter
0
1
2
3
A (n=15)
B (n=16)
C (n=14)
surface

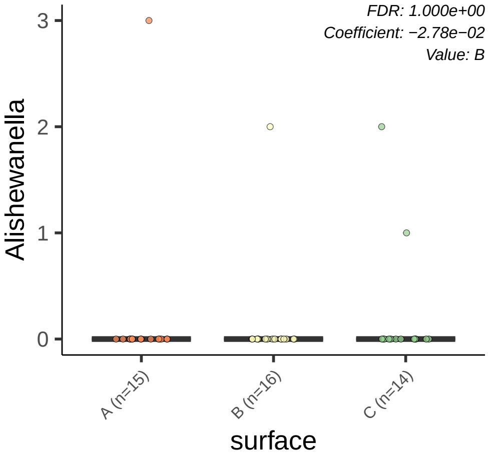
FDR: 1.000e+00
Coefficient: −2.78e−02
Value: B
Alishewanella
3
2
1
0
A (n=15)
B (n=16)
C (n=14)
surface

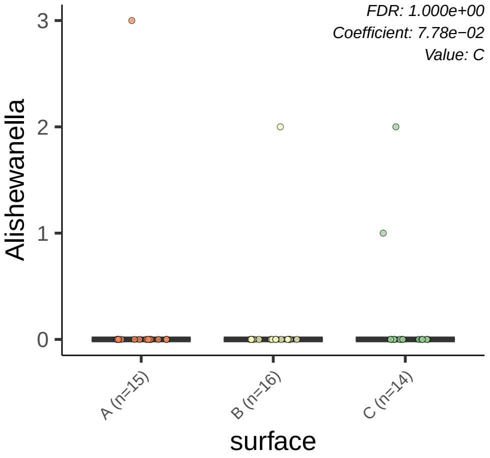
FDR: 1.000e+00
Coefficient: 7.78e−02
Value: C
Alishewanella
3
2
1
0
A (n=15)
B (n=16)
C (n=14)
surface

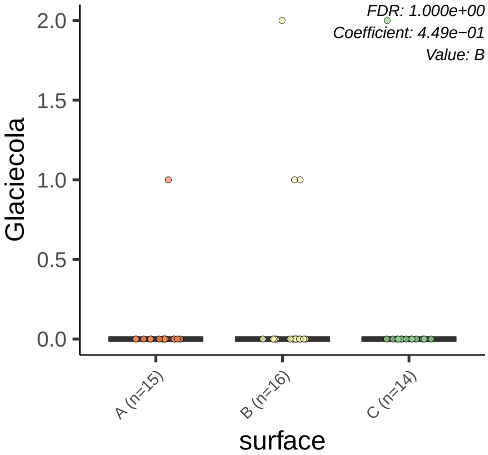
FDR: 1.000e+00
Coefficient: 4.49e−01
Value: B
Glaciecola
2.0
1.5
1.0
0.5
0.0
A (n=15)
B (n=16)
C (n=14)
surface

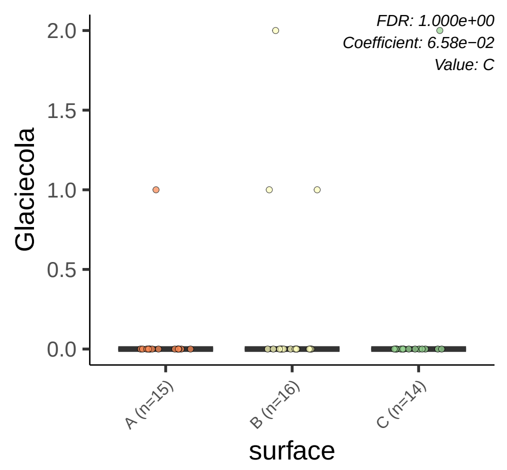
FDR: 1.000e+00
Coefficient: 6.58e−02
Value: C
Glaciecola
2.0
1.5
1.0
0.5
0.0
A (n=15)
B (n=16)
C (n=14)
surface

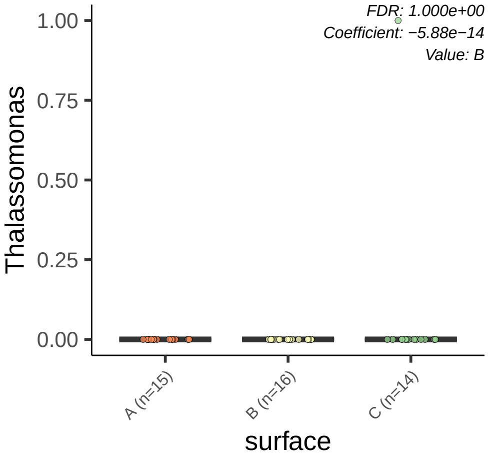

FDR: 1.000e+00
Coefficient: −5.88e−14
Value: B
Thalassomonas
1.00
0.75
0.50
0.25
0.00
A (n=15)
B (n=16)
C (n=14)
surface

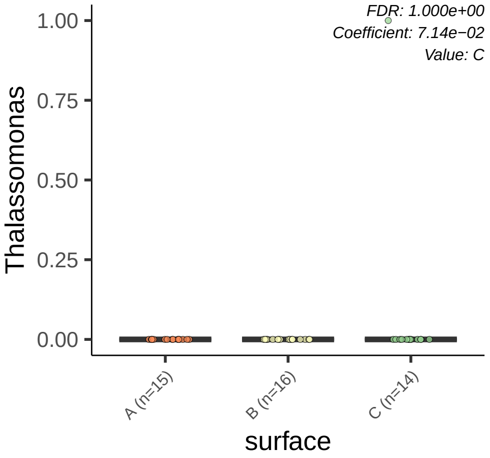
FDR: 1.000e+00
Coefficient: 7.14e−02
Value: C
Thalassomonas
1.00
0.75
0.50
0.25
0.00
A (n=15)
B (n=16)
C (n=14)
surface

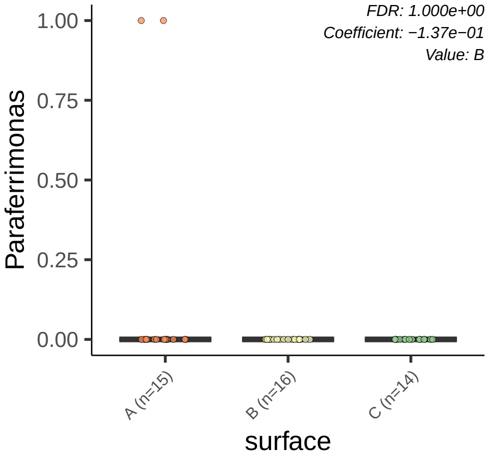

FDR: 1.000e+00
Coefficient: −1.37e−01
Value: B
Paraferrimonas
1.00
0.75
0.50
0.25
0.00
A (n=15)
B (n=16)
C (n=14)
surface

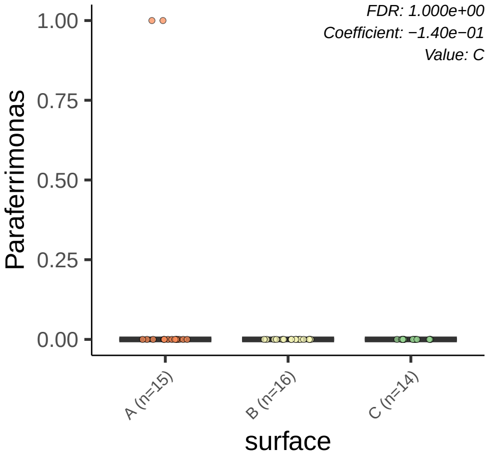
FDR: 1.000e+00
Coefficient: −1.40e−01
Value: C
Paraferrimonas
1.00
0.75
0.50
0.25
0.00
A (n=15)
B (n=16)
C (n=14)
surface

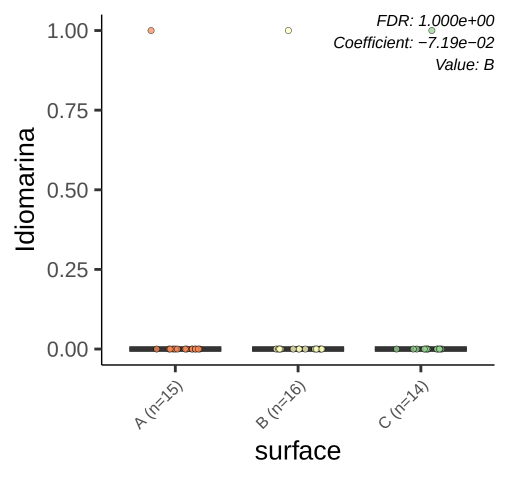
FDR: 1.000e+00
Coefficient: −7.19e−02
Value: B
Idiomarina
1.00
0.75
0.50
0.25
0.00
A (n=15)
B (n=16)
C (n=14)
surface

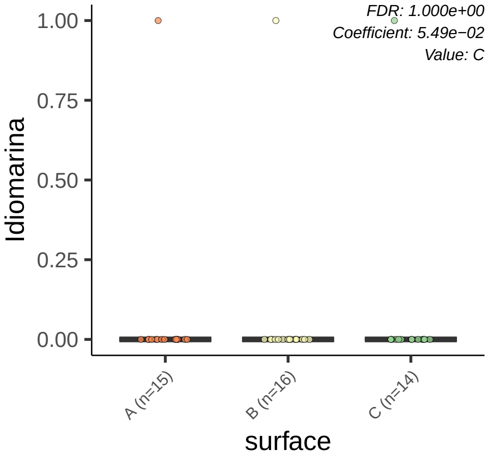
FDR: 1.000e+00
Coefficient: 5.49e−02
Value: C
Idiomarina
1.00
0.75
0.50
0.25
0.00
A (n=15)
B (n=16)
C (n=14)
surface

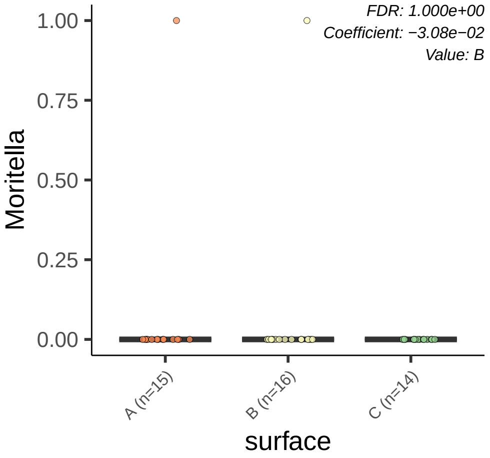

FDR: 1.000e+00
Coefficient: −3.08e−02
Value: B
Moritella
1.00
0.75
0.50
0.25
0.00
A (n=15)
B (n=16)
C (n=14)
surface

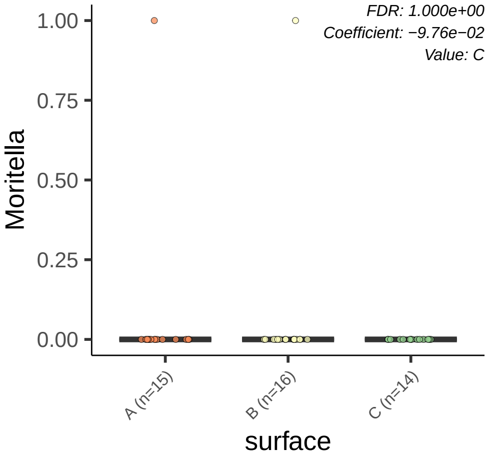
FDR: 1.000e+00
Coefficient: −9.76e−02
Value: C
Moritella
1.00
0.75
0.50
0.25
0.00
A (n=15)
B (n=16)
C (n=14)
surface

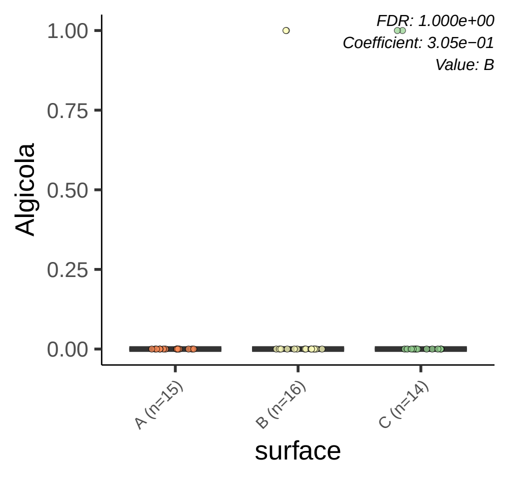

FDR: 1.000e+00
Coefficient: 3.05e−01
Value: B
Algicola
1.00
0.75
0.50
0.25
0.00
A (n=15)
B (n=16)
C (n=14)
surface

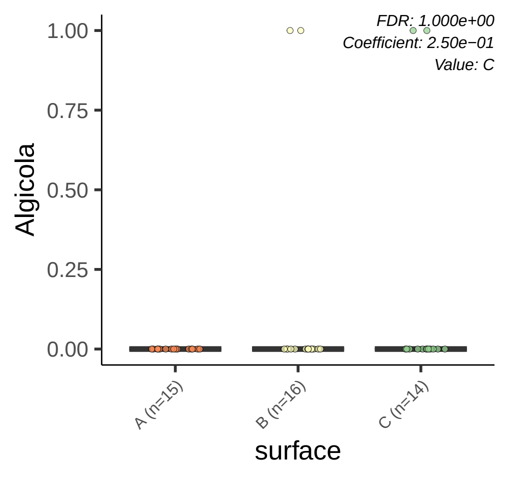
FDR: 1.000e+00
Coefficient: 2.50e−01
Value: C
Algicola
1.00
0.75
0.50
0.25
0.00
A (n=15)
B (n=16)
C (n=14)
surface

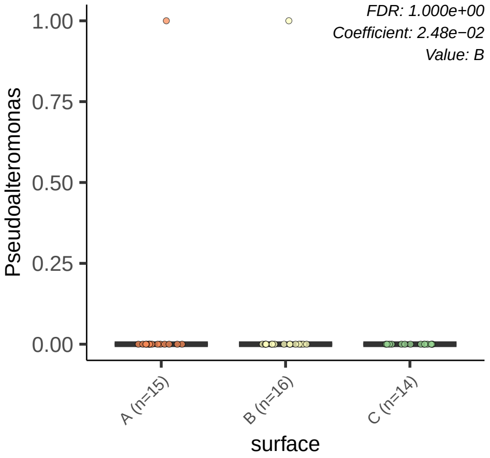
FDR: 1.000e+00
Coefficient: 2.48e−02
Value: B
Pseudoalteromonas
1.00
0.75
0.50
0.25
0.00
A (n=15)
B (n=16)
C (n=14)
surface

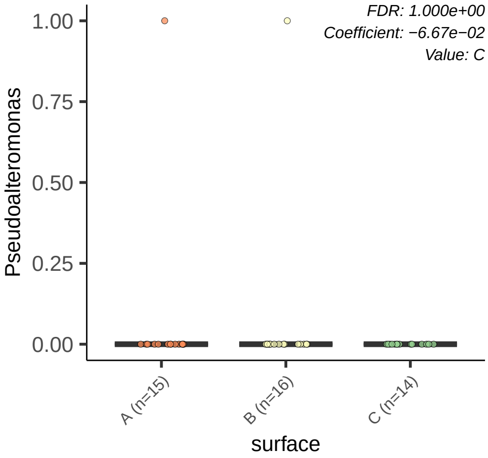

FDR: 1.000e+00
Coefficient: −6.67e−02
Value: C
Pseudoalteromonas
1.00
0.75
0.50
0.25
0.00
A (n=15)
B (n=16)
C (n=14)
surface

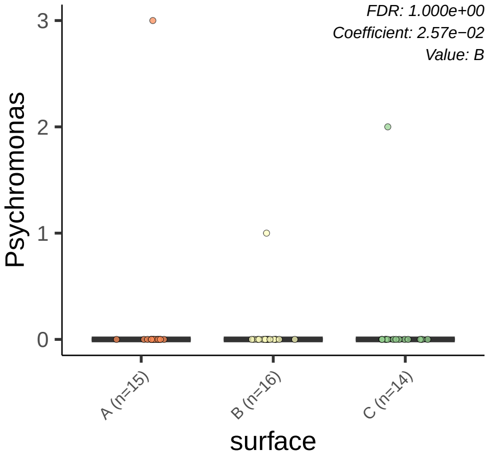

FDR: 1.000e+00
Coefficient: 2.57e−02
Value: B
Psychromonas
3
2
1
0
A (n=15)
B (n=16)
C (n=14)
surface

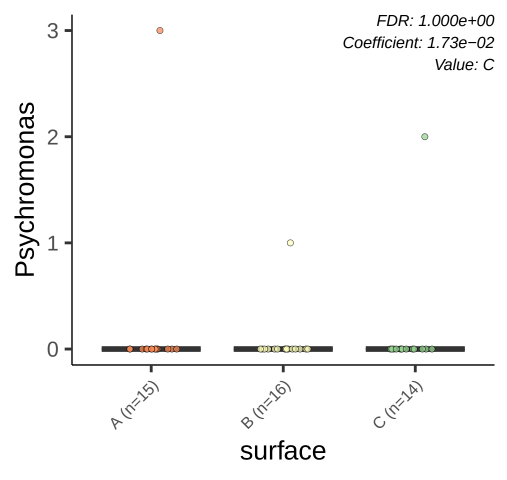

FDR: 1.000e+00
Coefficient: 1.73e−02
Value: C
Psychromonas
3
2
1
0
A (n=15)
B (n=16)
C (n=14)
surface

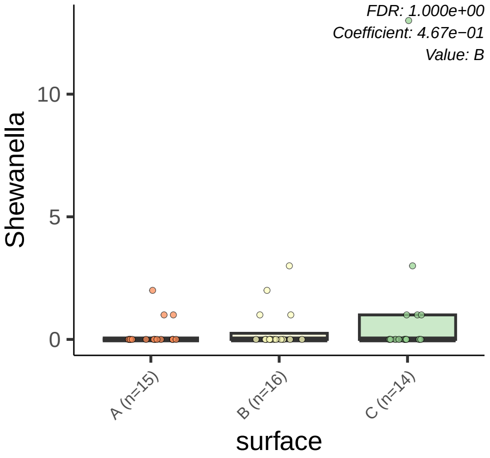
FDR: 1.000e+00
Coefficient: 4.67e−01
Value: B
Shewanella
10
5
0
A (n=15)
B (n=16)
C (n=14)
surface

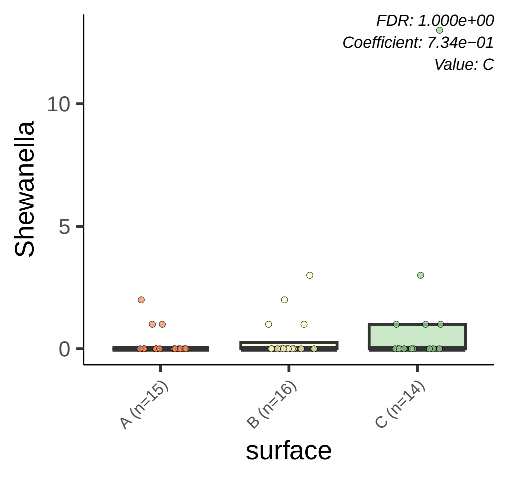

FDR: 1.000e+00
Coefficient: 7.34e−01
Value: C
Shewanella
10
5
0
A (n=15)
B (n=16)
C (n=14)
surface

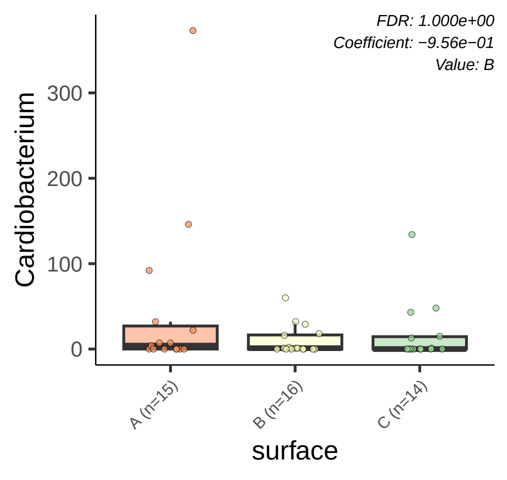

FDR: 1.000e+00
Coefficient: −9.56e−01
Value: B
Cardiobacterium
300
200
100
0
A (n=15)
B (n=16)
C (n=14)
surface

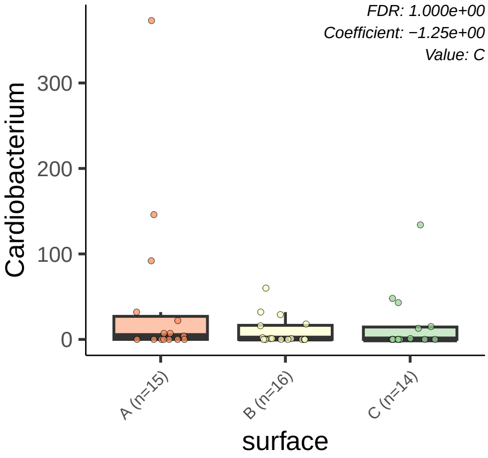
FDR: 1.000e+00
Coefficient: −1.25e+00
Value: C
Cardiobacterium
300
200
100
0
A (n=15)
B (n=16)
C (n=14)
surface

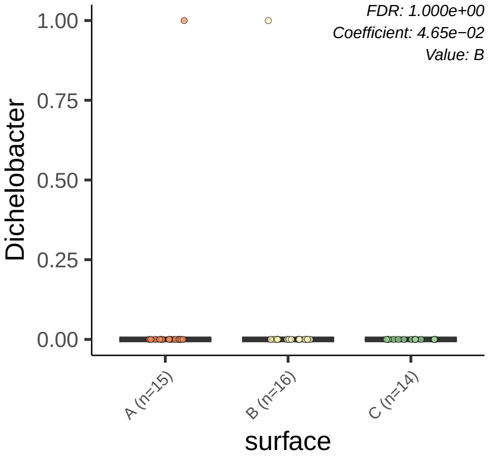

FDR: 1.000e+00
Coefficient: 4.65e−02
Value: B
1.00
0.75
0.50
0.25
0.00
Dichelobacter
A (n=15)
B (n=16)
C (n=14)
surface

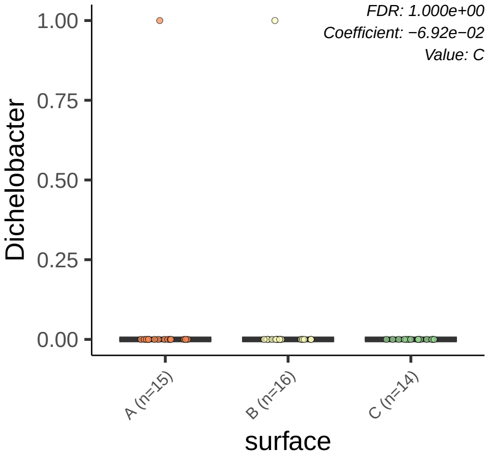

FDR: 1.000e+00
Coefficient: −6.92e−02
Value: C
Dichelobacter
1.00
0.75
0.50
0.25
0.00
A (n=15)
B (n=16)
C (n=14)
surface

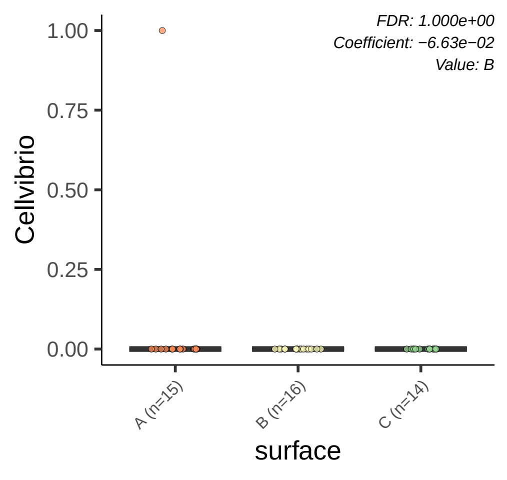

FDR: 1.000e+00
Coefficient: −6.63e−02
Value: B
Cellvibrio
1.00
0.75
0.50
0.25
0.00
A (n=15)
B (n=16)
C (n=14)
surface

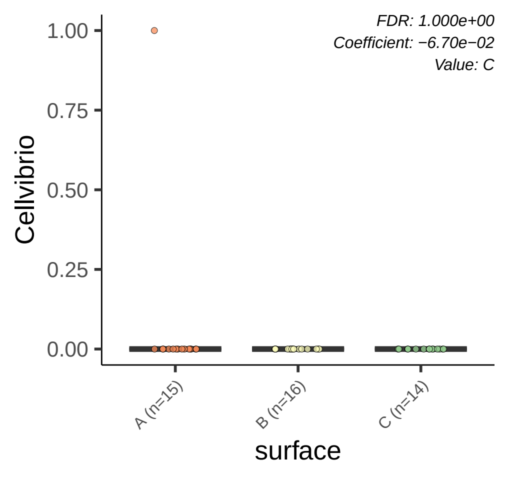
FDR: 1.000e+00
Coefficient: −6.70e−02
Value: C
Cellvibrio
1.00
0.75
0.50
0.25
0.00
A (n=15)
B (n=16)
C (n=14)
surface

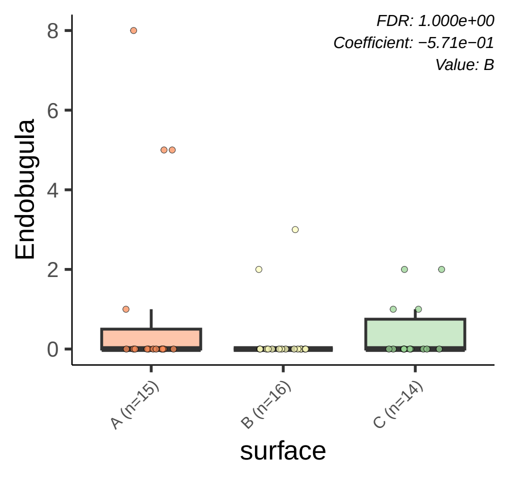

FDR: 1.000e+00
Coefficient: −5.71e−01
Value: B
Endobugula
8
6
4
2
0
A (n=15)
B (n=16)
C (n=14)
surface

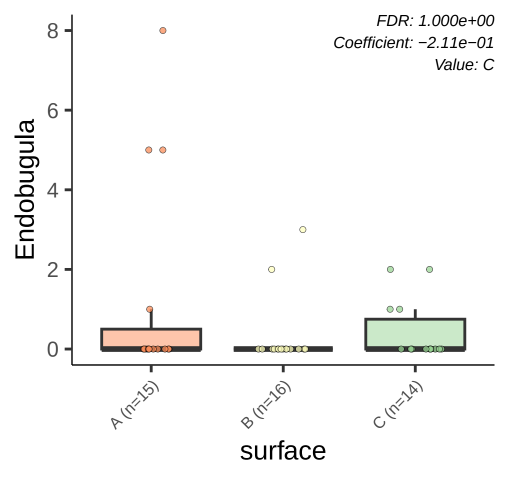
FDR: 1.000e+00
Coefficient: −2.11e−01
Value: C
Endobugula
0
2
4
6
8
A (n=15)
B (n=16)
C (n=14)
surface

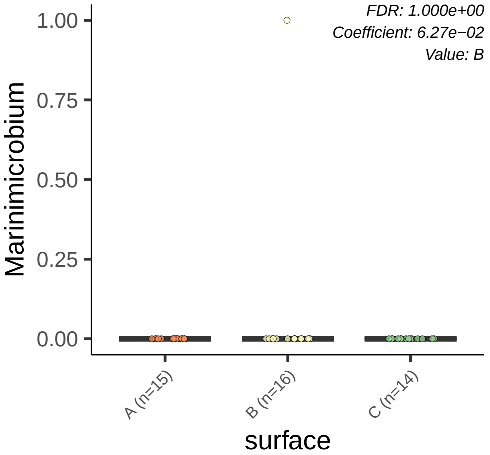
FDR: 1.000e+00
Coefficient: 6.27e−02
Value: B
Marinimicrobium
1.00
0.75
0.50
0.25
0.00
A (n=15)
B (n=16)
C (n=14)
surface

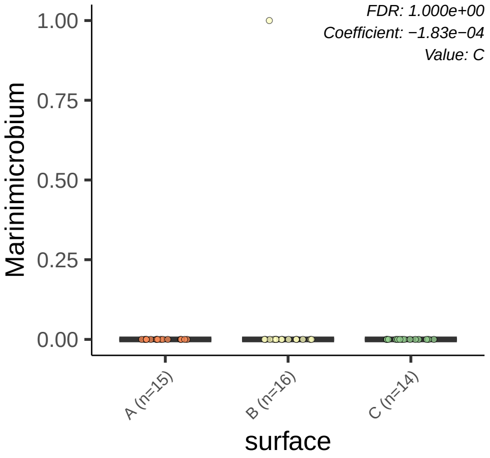
FDR: 1.000e+00
Coefficient: −1.83e−04
Value: C
Marinimicrobium
1.00
0.75
0.50
0.25
0.00
A (n=15)
B (n=16)
C (n=14)
surface

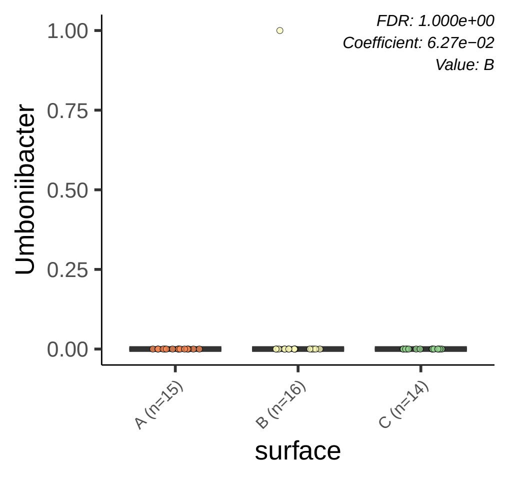
FDR: 1.000e+00
Coefficient: 6.27e−02
Value: B
Umboniibacter
1.00
0.75
0.50
0.25
0.00
A (n=15)
B (n=16)
C (n=14)
surface

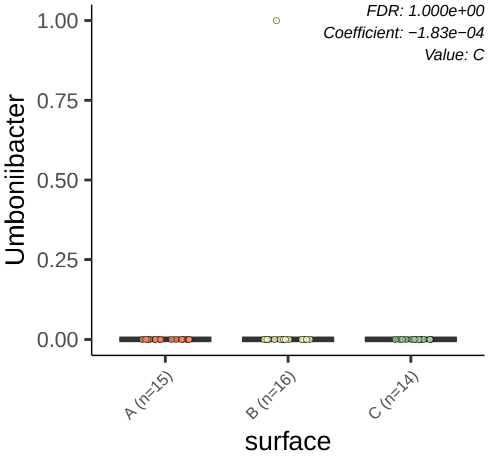
FDR: 1.000e+00
Coefficient: −1.83e−04
Value: C
Umboniibacter
1.00
0.75
0.50
0.25
0.00
A (n=15)
B (n=16)
C (n=14)
surface

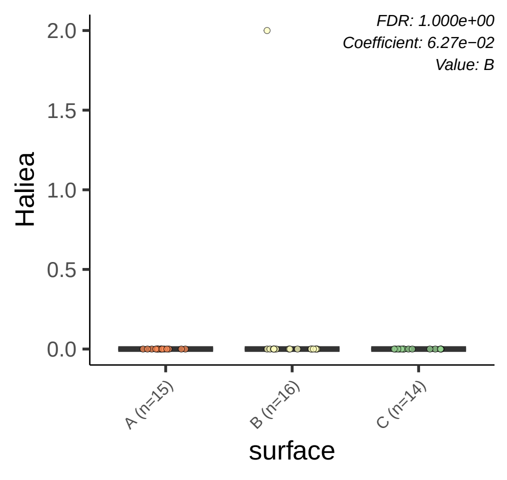
FDR: 1.000e+00
Coefficient: 6.27e−02
Value: B
Haliea
2.0
1.5
1.0
0.5
0.0
A (n=15)
B (n=16)
C (n=14)
surface

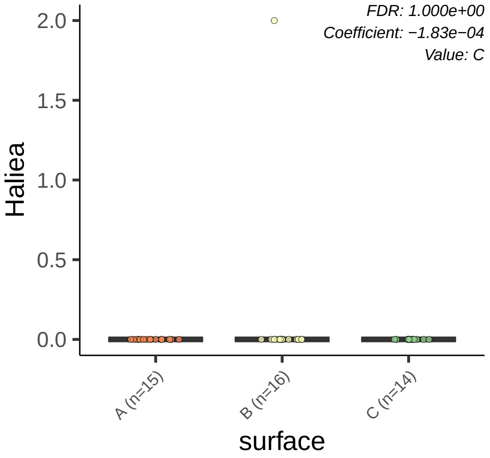

FDR: 1.000e+00
Coefficient: −1.83e−04
Value: C
Haliea
2.0
1.5
1.0
0.5
0.0
A (n=15)
B (n=16)
C (n=14)
surface

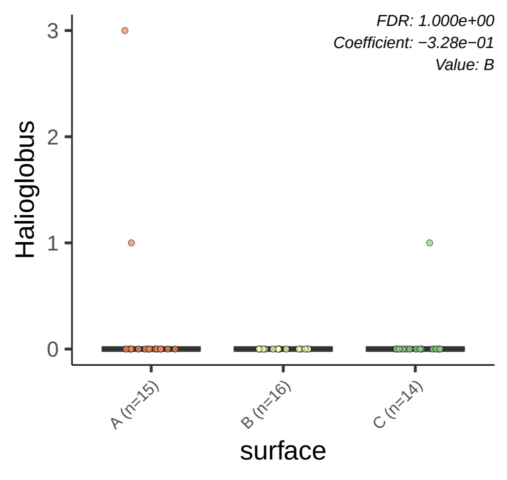
FDR: 1.000e+00
Coefficient: −3.28e−01
Value: B
Halioglobus
3
2
1
0
A (n=15)
B (n=16)
C (n=14)
surface

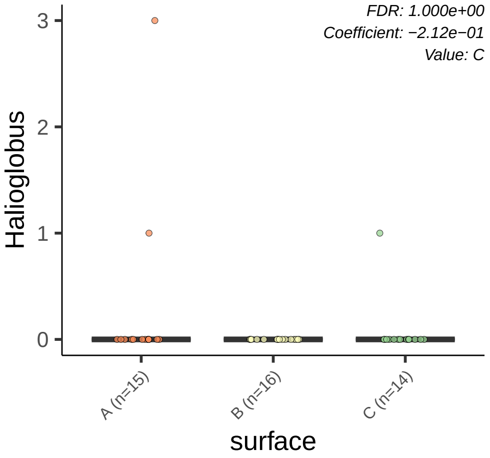
FDR: 1.000e+00
Coefficient: −2.12e−01
Value: C
Halioglobus
3
2
1
0
A (n=15)
B (n=16)
C (n=14)
surface

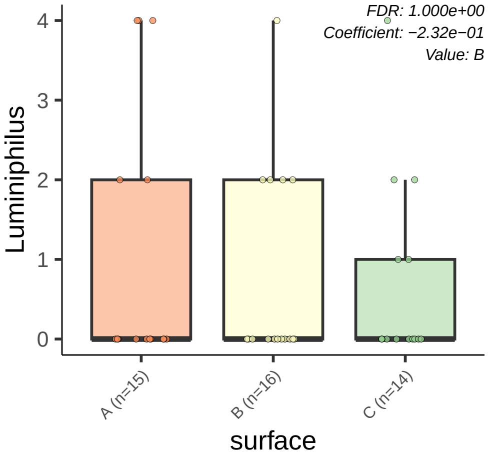

FDR: 1.000e+00
Coefficient: −2.32e−01
Value: B
Luminiphilus
0
1
2
3
4
A (n=15)
B (n=16)
C (n=14)
surface

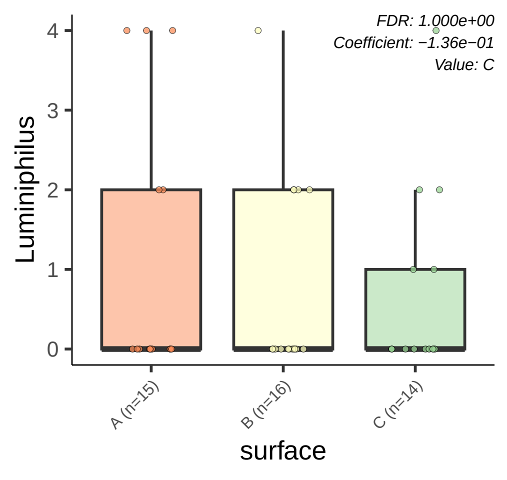
FDR: 1.000e+00
Coefficient: −1.36e−01
Value: C
Luminiphilus
4
3
2
1
0
A (n=15)
B (n=16)
C (n=14)
surface

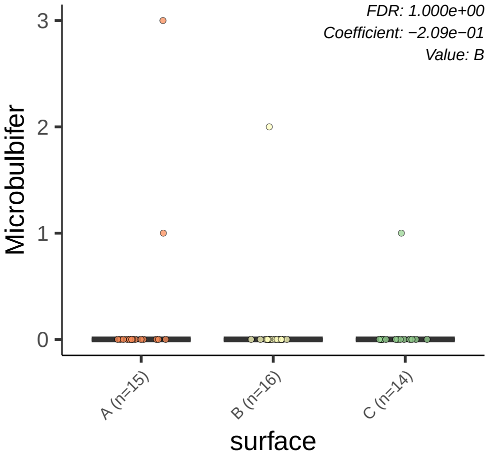

FDR: 1.000e+00
Coefficient: −2.09e−01
Value: B
Microbulbifer
3
2
1
0
A (n=15)
B (n=16)
C (n=14)
surface

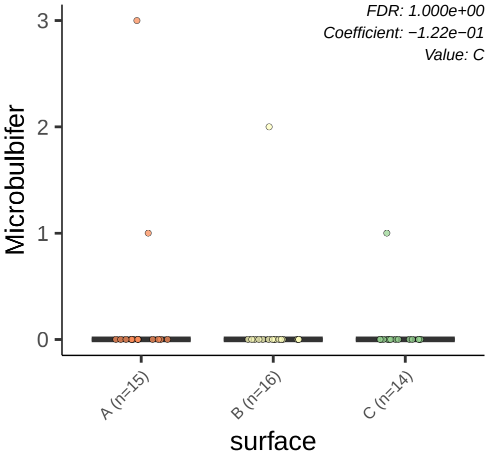
FDR: 1.000e+00
Coefficient: −1.22e−01
Value: C
Microbulbifer
3
2
1
0
A (n=15)
B (n=16)
C (n=14)
surface

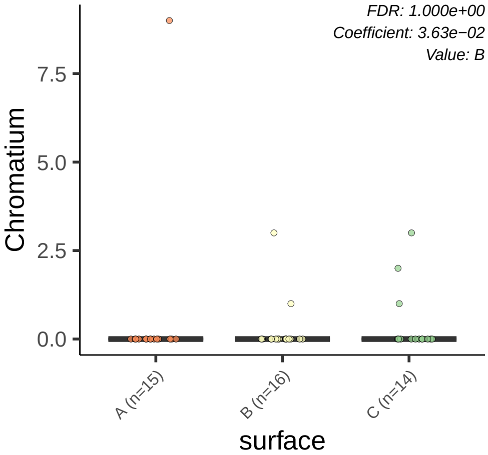

FDR: 1.000e+00
Coefficient: 3.63e−02
Value: B
Chromatium
7.5
5.0
2.5
0.0
A (n=15)
B (n=16)
C (n=14)
surface

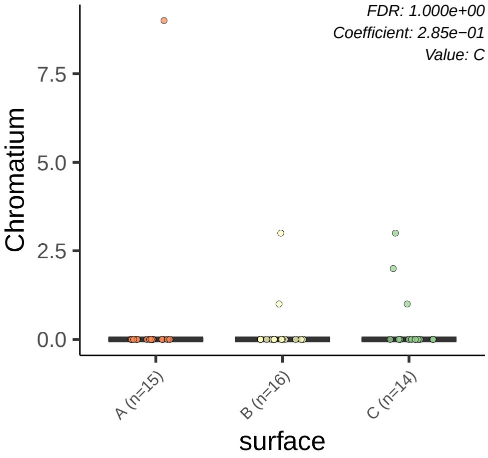

FDR: 1.000e+00
Coefficient: 2.85e−01
Value: C
Chromatium
7.5
5.0
2.5
0.0
A (n=15)
B (n=16)
C (n=14)
surface

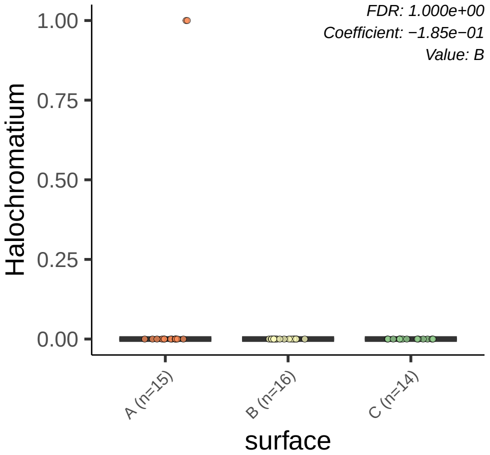

FDR: 1.000e+00
Coefficient: −1.85e−01
Value: B
Halochromatium
1.00
0.75
0.50
0.25
0.00
A (n=15)
B (n=16)
C (n=14)
surface

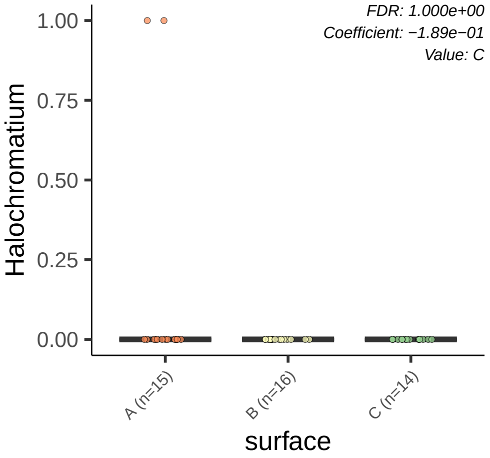
FDR: 1.000e+00
Coefficient: −1.89e−01
Value: C
Halochromatium
1.00
0.75
0.50
0.25
0.00
A (n=15)
B (n=16)
C (n=14)
surface

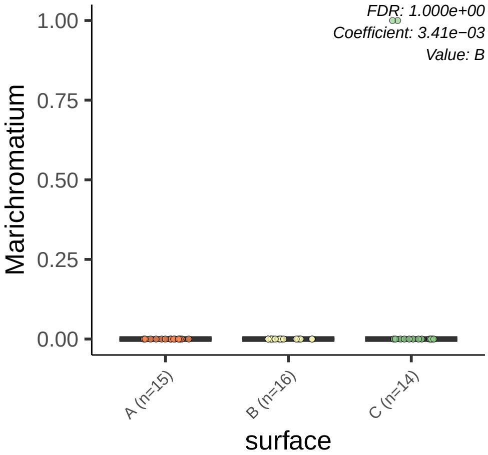
FDR: 1.000e+00
Coefficient: 3.41e−03
Value: B
Marichromatium
1.00
0.75
0.50
0.25
0.00
A (n=15)
B (n=16)
C (n=14)
surface

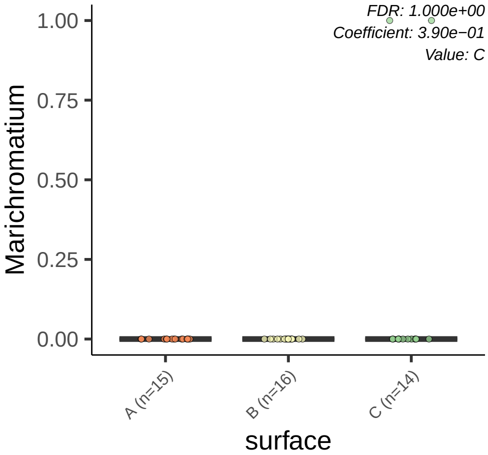
FDR: 1.000e+00
Coefficient: 3.90e−01
Value: C
Marichromatium
1.00
0.75
0.50
0.25
0.00
A (n=15)
B (n=16)
C (n=14)
surface

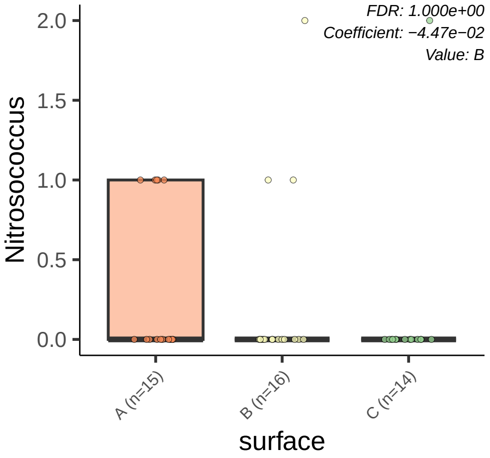
FDR: 1.000e+00
Coefficient: −4.47e−02
Value: B
Nitrosococcus
2.0
1.5
1.0
0.5
0.0
A (n=15)
B (n=16)
C (n=14)
surface

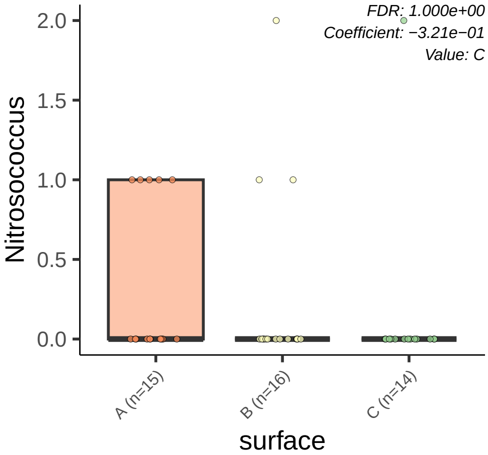

FDR: 1.000e+00
Coefficient: −3.21e−01
Value: C
Nitrosococcus
2.0
1.5
1.0
0.5
0.0
A (n=15)
B (n=16)
C (n=14)
surface

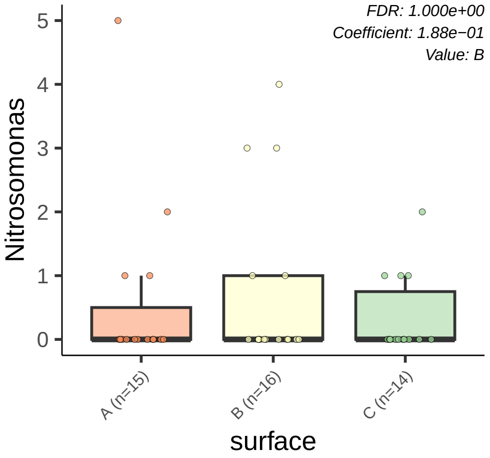

FDR: 1.000e+00
Coefficient: 1.88e−01
Value: B
Nitrosomonas
0
1
2
3
4
5
A (n=15)
B (n=16)
C (n=14)
surface

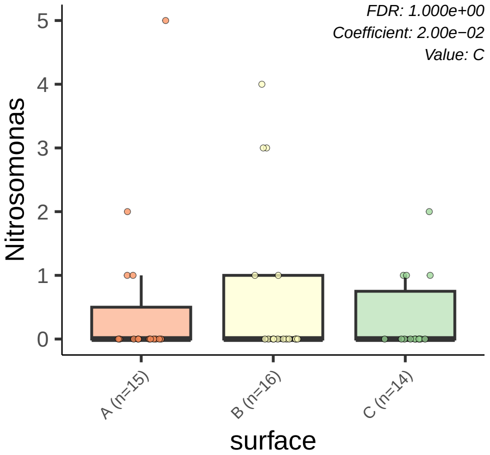

FDR: 1.000e+00
Coefficient: 2.00e−02
Value: C
Nitrosomonas
0
1
2
3
4
5
A (n=15)
B (n=16)
C (n=14)
surface

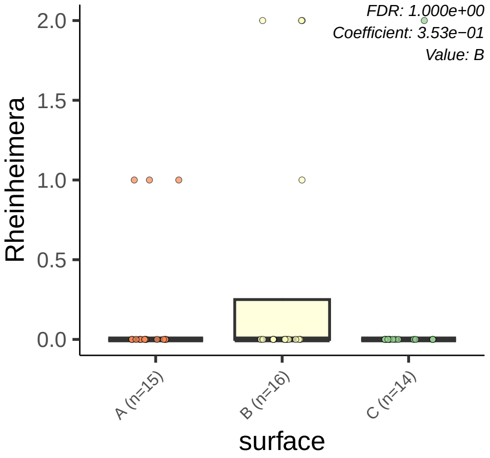

FDR: 1.000e+00
Coefficient: 3.53e−01
Value: B
Rheinheimera
2.0
1.5
1.0
0.5
0.0
A (n=15)
B (n=16)
C (n=14)
surface

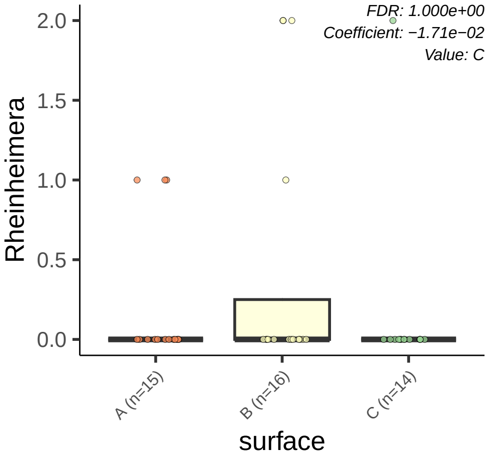

FDR: 1.000e+00
Coefficient: −1.71e−02
Value: C
Rheinheimera
2.0
1.5
1.0
0.5
0.0
A (n=15)
B (n=16)
C (n=14)
surface

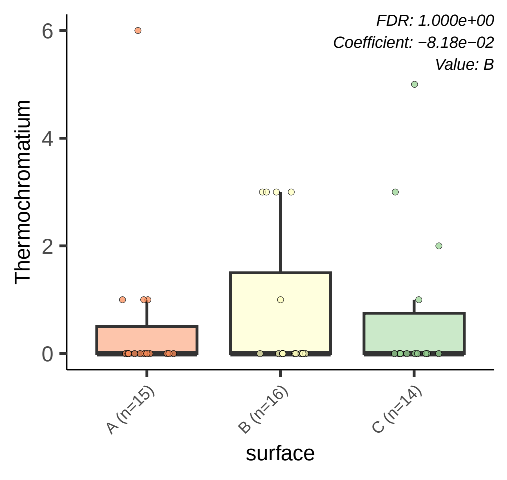

FDR: 1.000e+00
Coefficient: −8.18e−02
Value: B
Thermochromatium
6
4
2
0
A (n=15)
B (n=16)
C (n=14)
surface

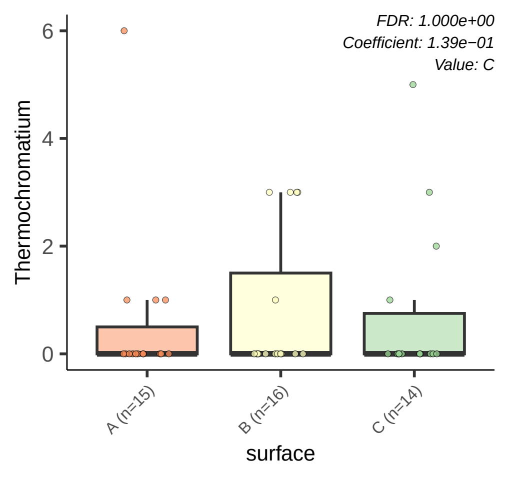

FDR: 1.000e+00
Coefficient: 1.39e−01
Value: C
Thermochromatium
0
2
4
6
A (n=15)
B (n=16)
C (n=14)
surface

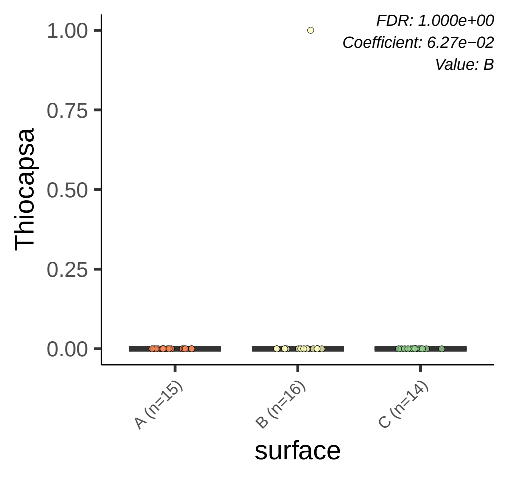
FDR: 1.000e+00
Coefficient: 6.27e−02
Value: B
1.00
0.75
0.50
0.25
0.00
Thiocapsa
A (n=15)
B (n=16)
C (n=14)
surface

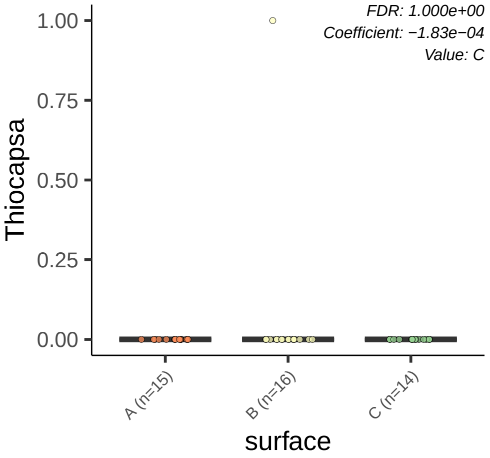

FDR: 1.000e+00
Coefficient: −1.83e−04
Value: C
Thiocapsa
1.00
0.75
0.50
0.25
0.00
A (n=15)
B (n=16)
C (n=14)
surface

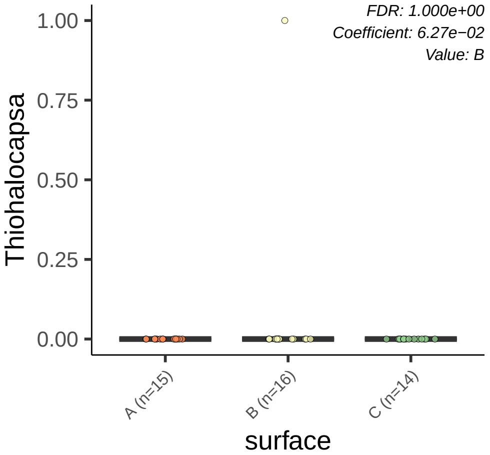
FDR: 1.000e+00
Coefficient: 6.27e−02
Value: B
Thiohalocapsa
1.00
0.75
0.50
0.25
0.00
A (n=15)
B (n=16)
C (n=14)
surface

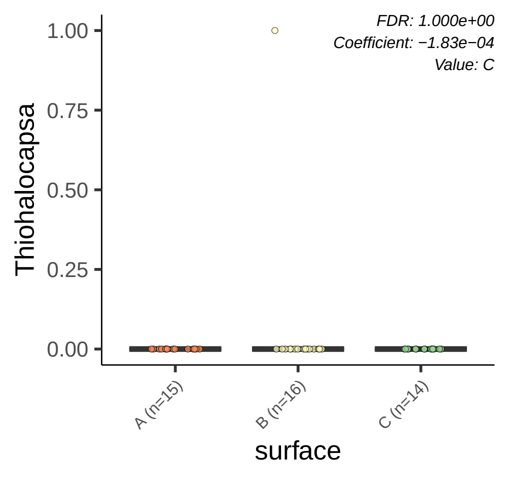
FDR: 1.000e+00
Coefficient: −1.83e−04
Value: C
Thiohalocapsa
1.00
0.75
0.50
0.25
0.00
A (n=15)
B (n=16)
C (n=14)
surface

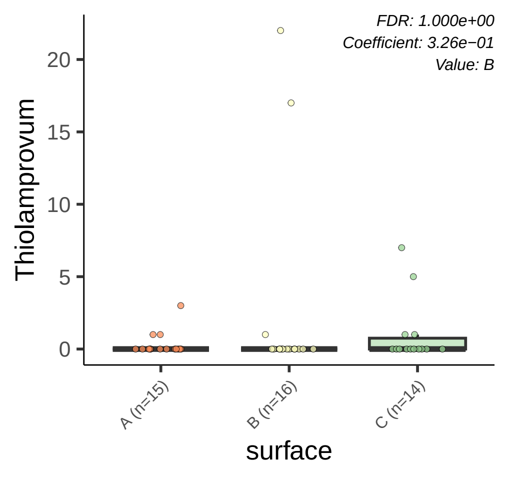
FDR: 1.000e+00
Coefficient: 3.26e−01
Value: B
Thiolamprovum
0
5
10
15
20
A (n=15)
B (n=16)
C (n=14)
surface

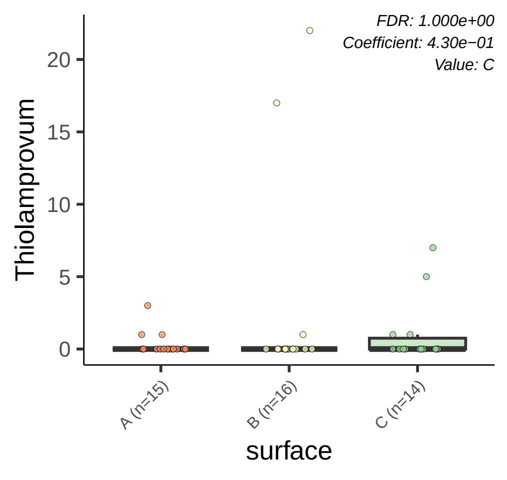
FDR: 1.000e+00
Coefficient: 4.30e−01
Value: C
Thiolamprovum
0
5
10
15
20
A (n=15)
B (n=16)
C (n=14)
surface

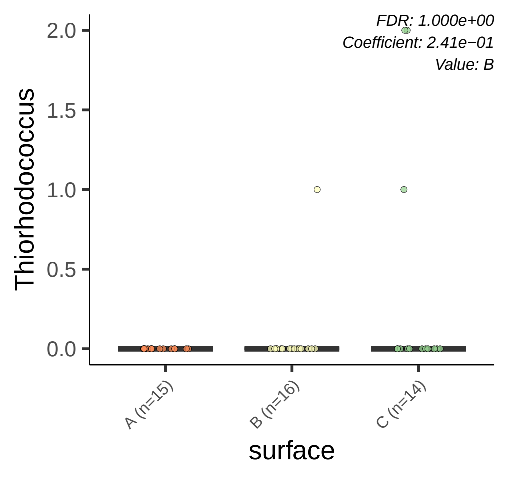

FDR: 1.000e+00
Coefficient: 2.41e−01
Value: B
Thiorhodococcus
2.0
1.5
1.0
0.5
0.0
A (n=15)
B (n=16)
C (n=14)
surface

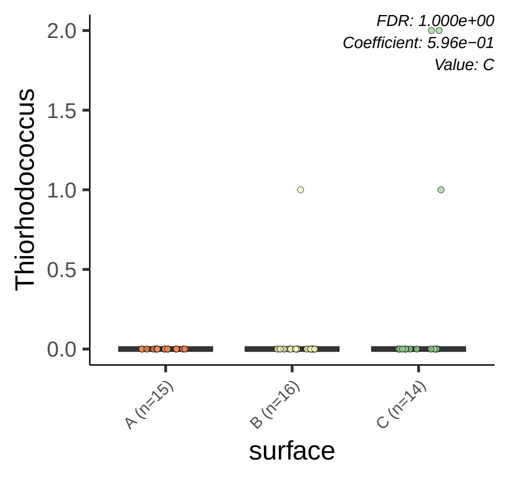
FDR: 1.000e+00
Coefficient: 5.96e−01
Value: C
Thiorhodococcus
2.0
1.5
1.0
0.5
0.0
A (n=15)
B (n=16)
C (n=14)
surface

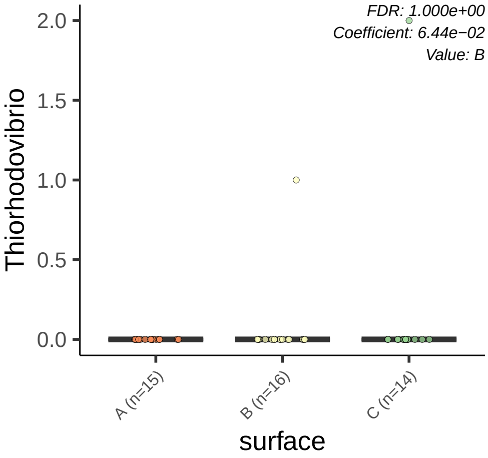
FDR: 1.000e+00
Coefficient: 6.44e−02
Value: B
Thiorhodovibrio
2.0
1.5
1.0
0.5
0.0
A (n=15)
B (n=16)
C (n=14)
surface

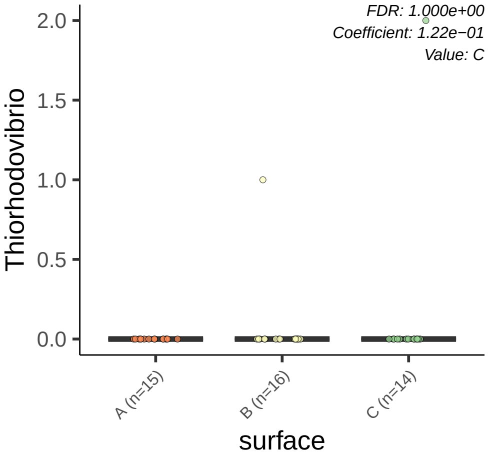
FDR: 1.000e+00
Coefficient: 1.22e−01
Value: C
Thiorhodovibrio
2.0
1.5
1.0
0.5
0.0
A (n=15)
B (n=16)
C (n=14)
surface

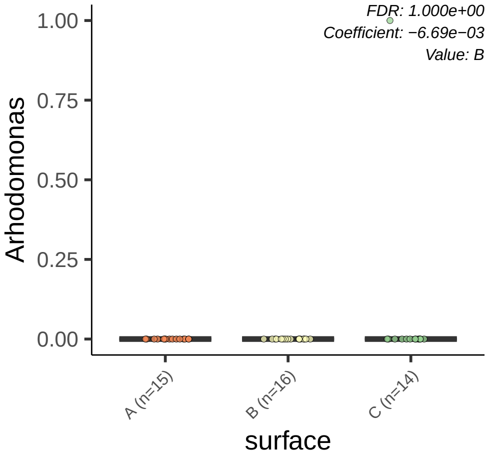
FDR: 1.000e+00
Coefficient: −6.69e−03
Value: B
1.00
0.75
0.50
0.25
0.00
Arhodomonas
A (n=15)
B (n=16)
C (n=14)
surface

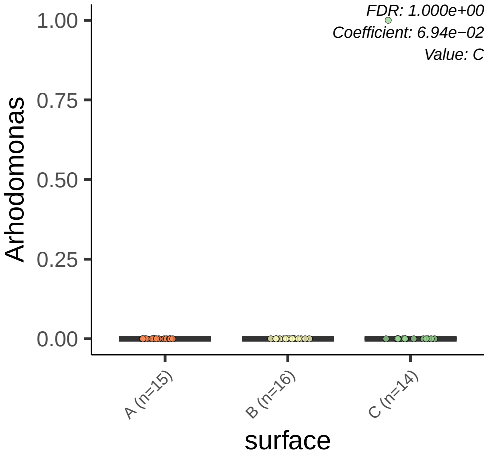

FDR: 1.000e+00
Coefficient: 6.94e−02
Value: C
Arhodomonas
1.00
0.75
0.50
0.25
0.00
A (n=15)
B (n=16)
C (n=14)
surface

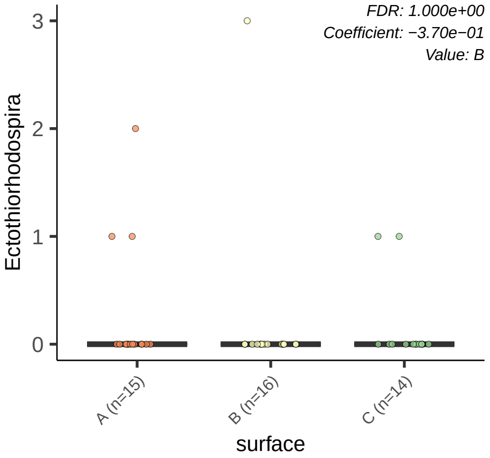
FDR: 1.000e+00
Coefficient: −3.70e−01
Value: B
Ectothiorhodospira
3
2
1
0
A (n=15)
B (n=16)
C (n=14)
surface

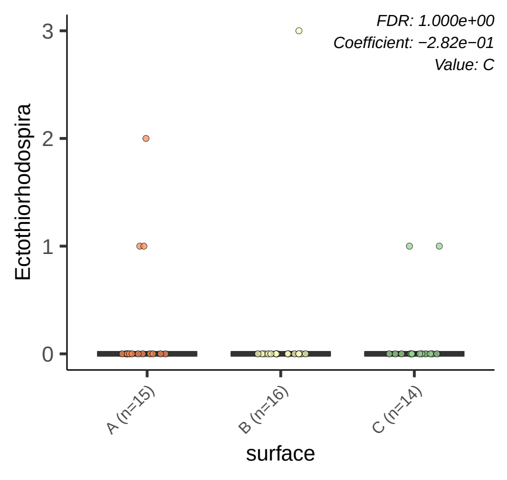

FDR: 1.000e+00
Coefficient: −2.82e−01
Value: C
Ectothiorhodospira
3
2
1
0
A (n=15)
B (n=16)
C (n=14)
surface

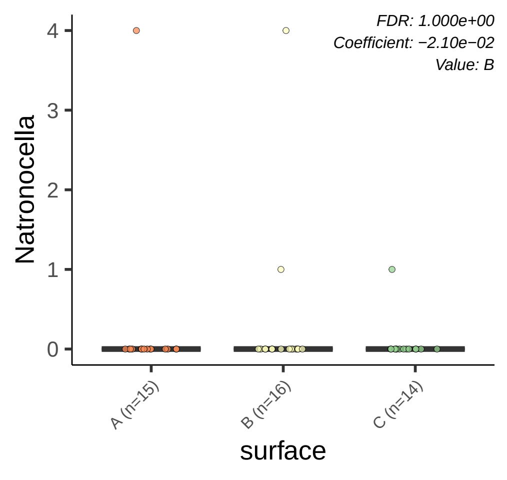
FDR: 1.000e+00
Coefficient: −2.10e−02
Value: B
Natronocella
0
1
2
3
4
A (n=15)
B (n=16)
C (n=14)
surface

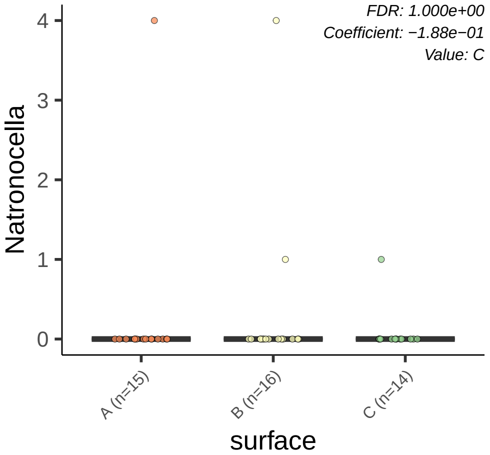
FDR: 1.000e+00
Coefficient: −1.88e−01
Value: C
Natronocella
4
3
2
1
0
A (n=15)
B (n=16)
C (n=14)
surface

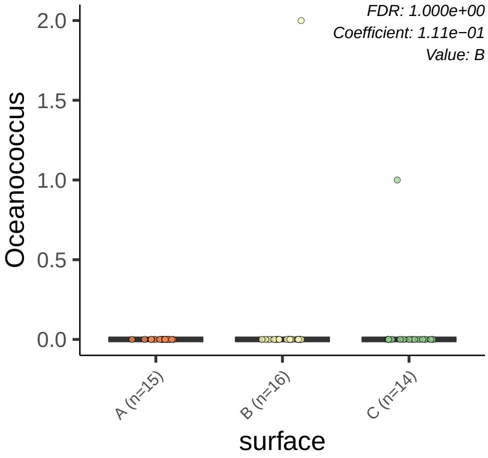
FDR: 1.000e+00
Coefficient: 1.11e−01
Value: B
Oceanococcus
2.0
1.5
1.0
0.5
0.0
A (n=15)
B (n=16)
C (n=14)
surface

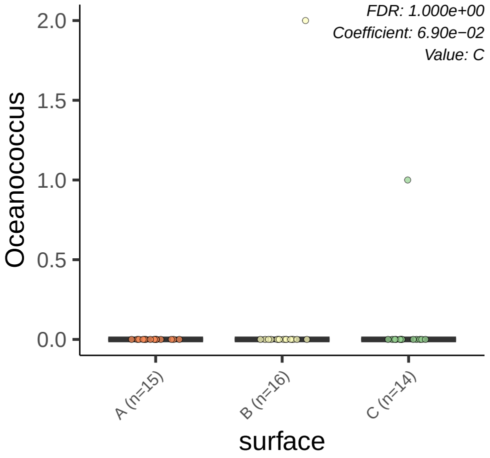

FDR: 1.000e+00
Coefficient: 6.90e−02
Value: C
Oceanococcus
2.0
1.5
1.0
0.5
0.0
A (n=15)
B (n=16)
C (n=14)
surface

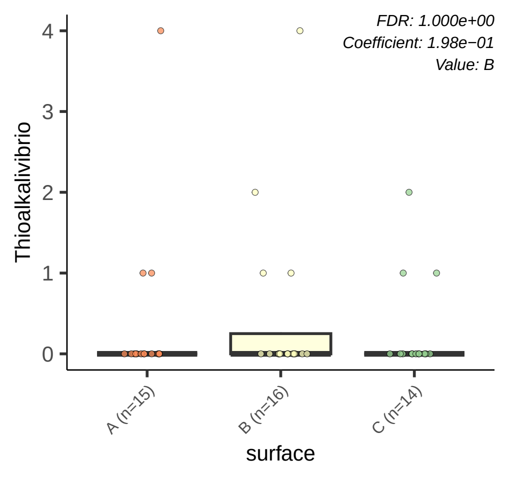

FDR: 1.000e+00
Coefficient: 1.98e−01
Value: B
Thioalkalivibrio
0
1
2
3
4
A (n=15)
B (n=16)
C (n=14)
surface

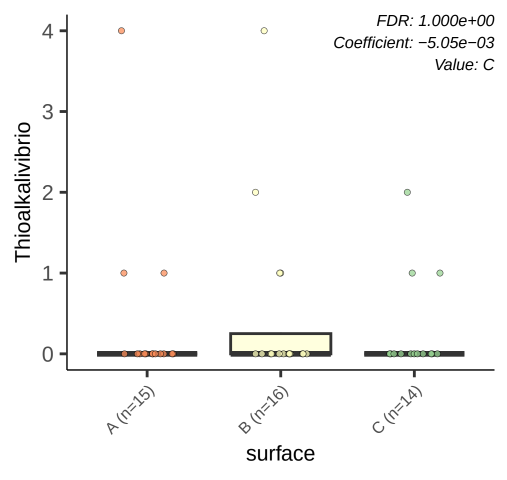

FDR: 1.000e+00
Coefficient: −5.05e−03
Value: C
Thioalkalivibrio
0
1
2
3
4
A (n=15)
B (n=16)
C (n=14)
surface

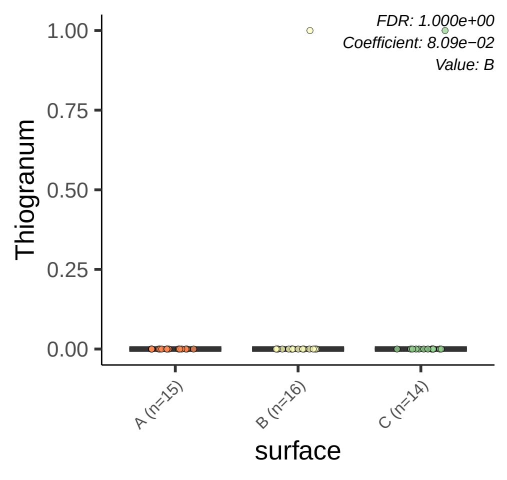

FDR: 1.000e+00
Coefficient: 8.09e−02
Value: B
Thiogranum
1.00
0.75
0.50
0.25
0.00
A (n=15)
B (n=16)
C (n=14)
surface

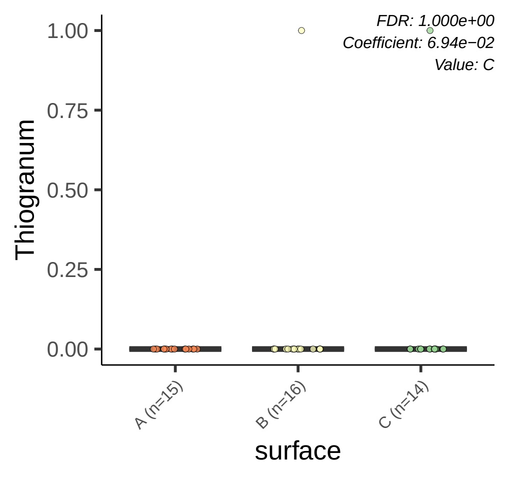
FDR: 1.000e+00
Coefficient: 6.94e−02
Value: C
Thiogranum
1.00
0.75
0.50
0.25
0.00
A (n=15)
B (n=16)
C (n=14)
surface

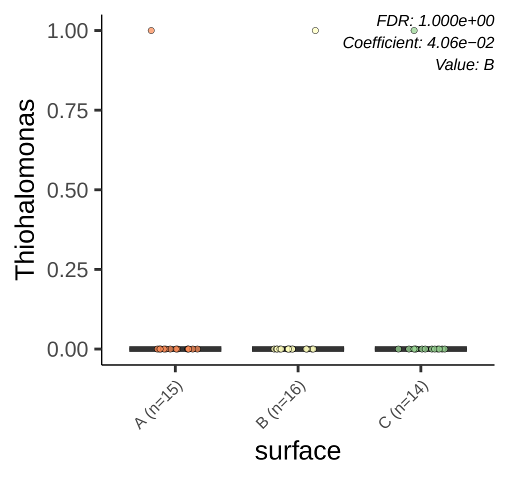
FDR: 1.000e+00
Coefficient: 4.06e−02
Value: B
Thiohalomonas
1.00
0.75
0.50
0.25
0.00
A (n=15)
B (n=16)
C (n=14)
surface

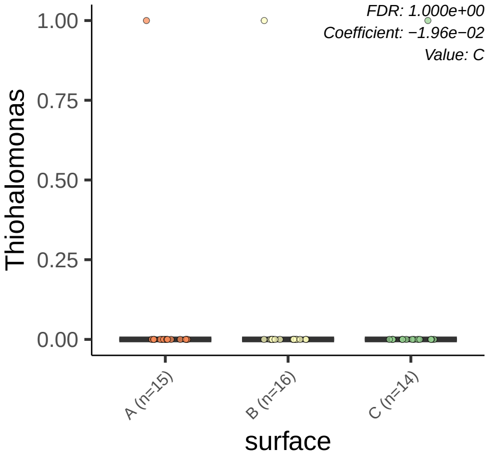
FDR: 1.000e+00
Coefficient: −1.96e−02
Value: C
Thiohalomonas
1.00
0.75
0.50
0.25
0.00
A (n=15)
B (n=16)
C (n=14)
surface

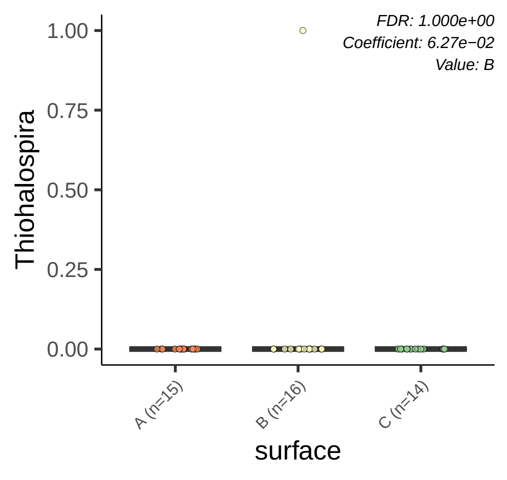

FDR: 1.000e+00
Coefficient: 6.27e−02
Value: B
Thiohalospira
1.00
0.75
0.50
0.25
0.00
A (n=15)
B (n=16)
C (n=14)
surface

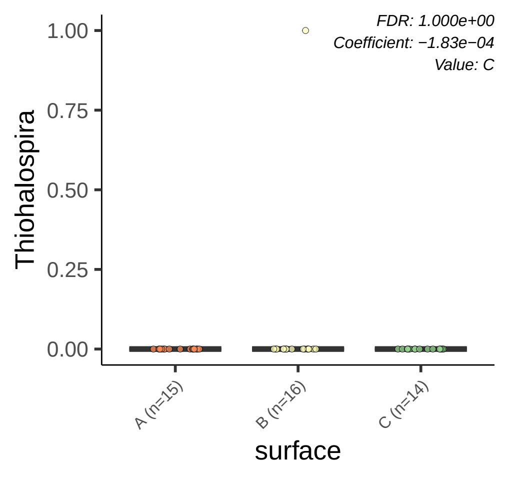
FDR: 1.000e+00
Coefficient: −1.83e−04
Value: C
Thiohalospira
1.00
0.75
0.50
0.25
0.00
A (n=15)
B (n=16)
C (n=14)
surface

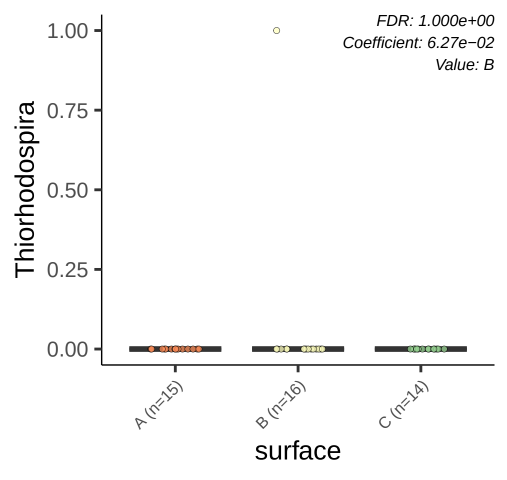
FDR: 1.000e+00
Coefficient: 6.27e−02
Value: B
Thiorhodospira
1.00
0.75
0.50
0.25
0.00
A (n=15)
B (n=16)
C (n=14)
surface

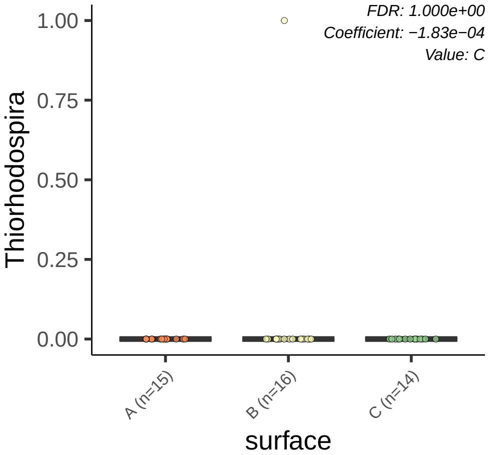

FDR: 1.000e+00
Coefficient: −1.83e−04
Value: C
Thiorhodospira
1.00
0.75
0.50
0.25
0.00
A (n=15)
B (n=16)
C (n=14)
surface

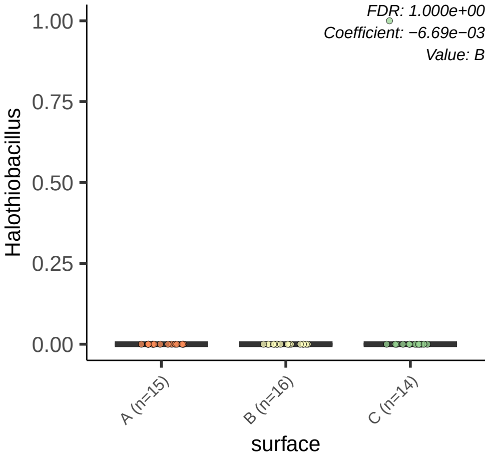

FDR: 1.000e+00
Coefficient: −6.69e−03
Value: B
Halothiobacillus
1.00
0.75
0.50
0.25
0.00
A (n=15)
B (n=16)
C (n=14)
surface

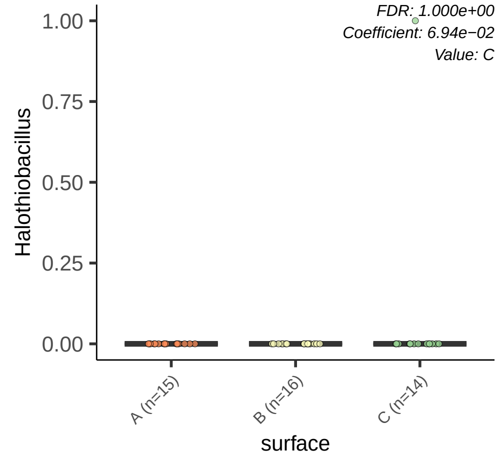
FDR: 1.000e+00
Coefficient: 6.94e−02
Value: C
Halothiobacillus
1.00
0.75
0.50
0.25
0.00
A (n=15)
B (n=16)
C (n=14)
surface

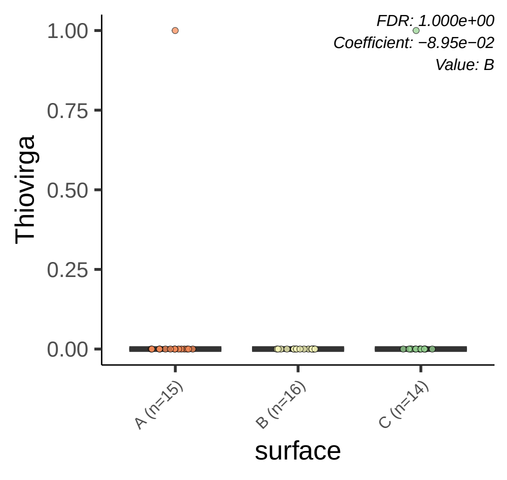

FDR: 1.000e+00
Coefficient: −8.95e−02
Value: B
Thiovirga
1.00
0.75
0.50
0.25
0.00
A (n=15)
B (n=16)
C (n=14)
surface

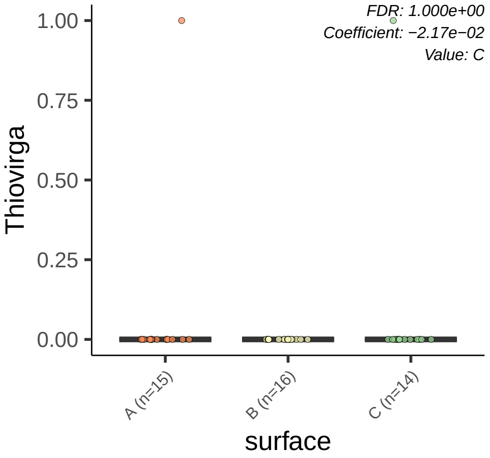
FDR: 1.000e+00
Coefficient: −2.17e−02
Value: C
1.00
0.75
0.50
0.25
0.00
Thiovirga
A (n=15)
B (n=16)
C (n=14)
surface

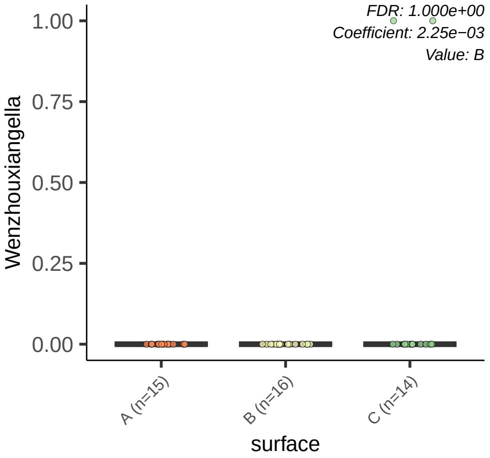
FDR: 1.000e+00
Coefficient: 2.25e−03
Value: B
Wenzhouxiangella
1.00
0.75
0.50
0.25
0.00
A (n=15)
B (n=16)
C (n=14)
surface

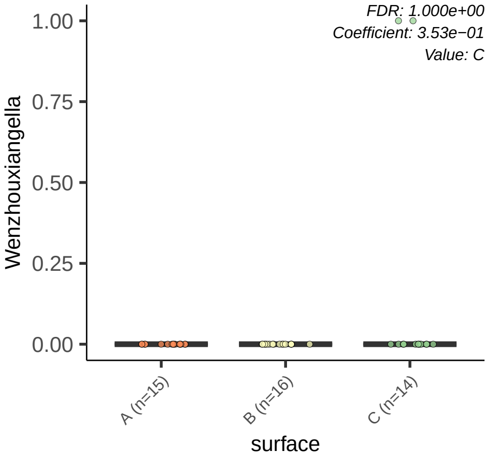
FDR: 1.000e+00
Coefficient: 3.53e−01
Value: C
Wenzhouxiangella
1.00
0.75
0.50
0.25
0.00
A (n=15)
B (n=16)
C (n=14)
surface

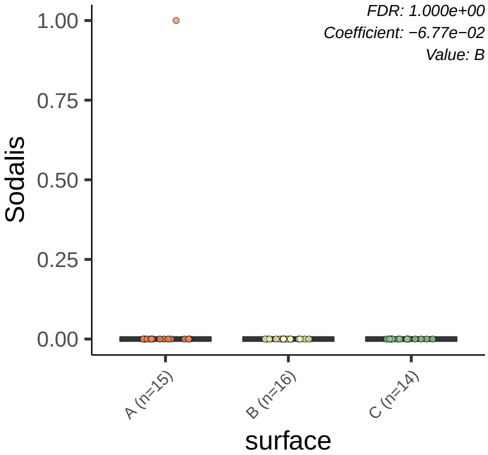
FDR: 1.000e+00
Coefficient: −6.77e−02
Value: B
Sodalis
1.00
0.75
0.50
0.25
0.00
A (n=15)
B (n=16)
C (n=14)
surface

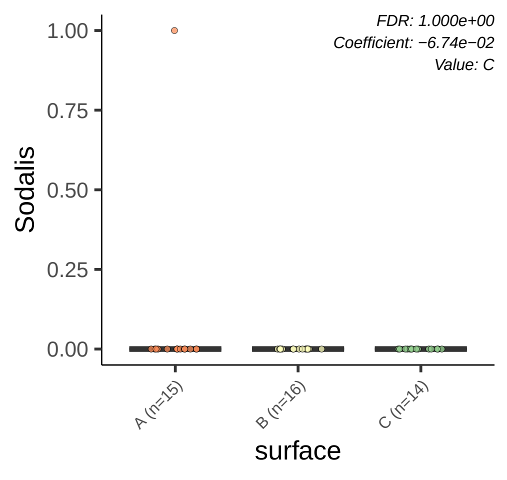
FDR: 1.000e+00
Coefficient: −6.74e−02
Value: C
Sodalis
1.00
0.75
0.50
0.25
0.00
A (n=15)
B (n=16)
C (n=14)
surface

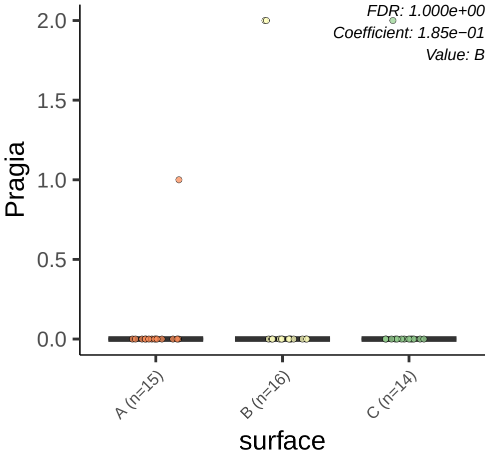
FDR: 1.000e+00
Coefficient: 1.85e−01
Value: B
Pragia
2.0
1.5
1.0
0.5
0.0
A (n=15)
B (n=16)
C (n=14)
surface

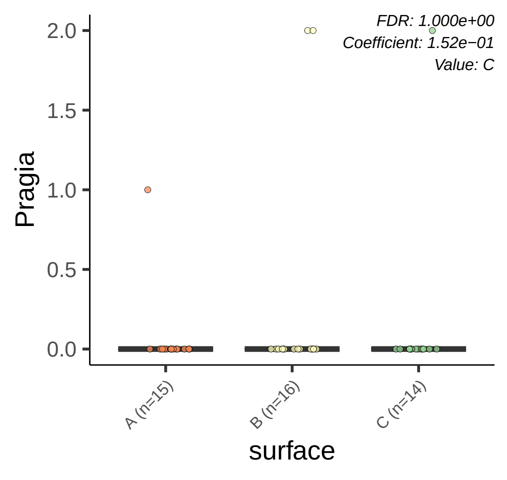
FDR: 1.000e+00
Coefficient: 1.52e−01
Value: C
Pragia
2.0
1.5
1.0
0.5
0.0
A (n=15)
B (n=16)
C (n=14)
surface

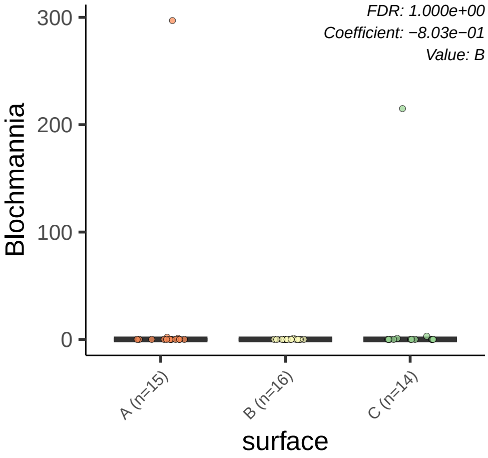

FDR: 1.000e+00
Coefficient: −8.03e−01
Value: B
Blochmannia
300
200
100
0
A (n=15)
B (n=16)
C (n=14)
surface

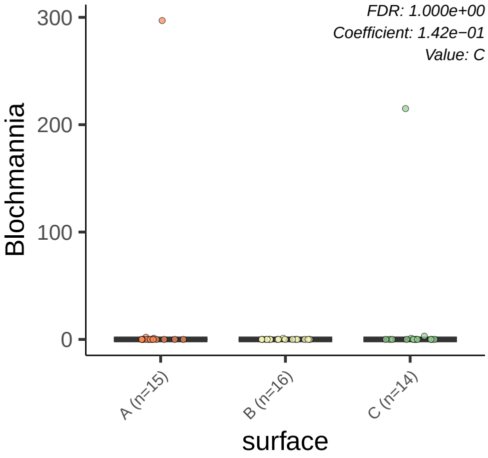

FDR: 1.000e+00
Coefficient: 1.42e−01
Value: C
300
200
100
0
Blochmannia
A (n=15)
B (n=16)
C (n=14)
surface

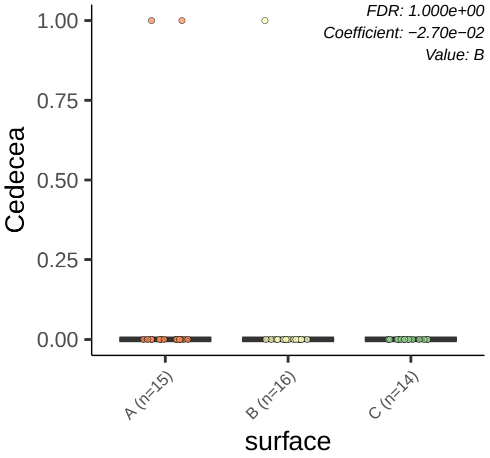
FDR: 1.000e+00
Coefficient: −2.70e−02
Value: B
Cedecea
1.00
0.75
0.50
0.25
0.00
A (n=15)
B (n=16)
C (n=14)
surface

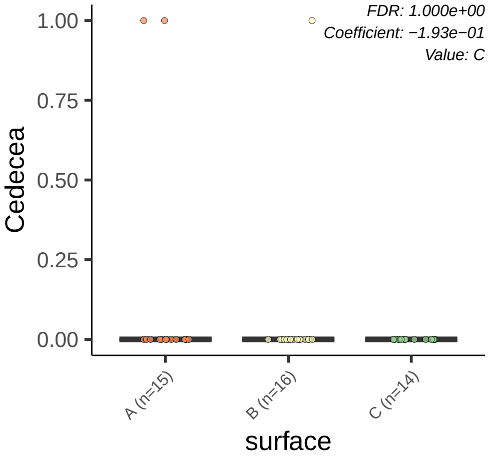

FDR: 1.000e+00
Coefficient: −1.93e−01
Value: C
Cedecea
1.00
0.75
0.50
0.25
0.00
A (n=15)
B (n=16)
C (n=14)
surface

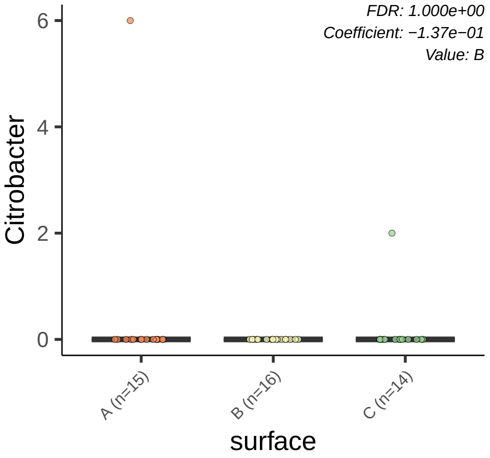
FDR: 1.000e+00
Coefficient: −1.37e−01
Value: B
Citrobacter
6
4
2
0
A (n=15)
B (n=16)
C (n=14)
surface

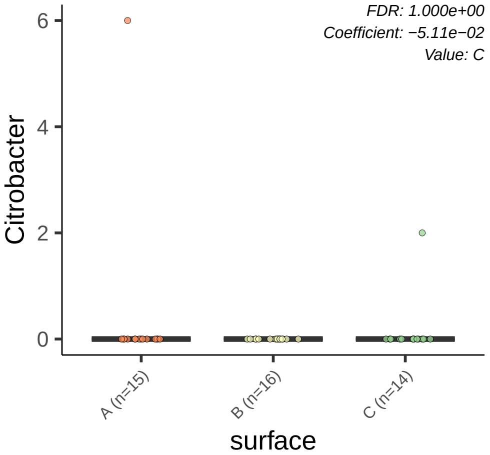
FDR: 1.000e+00
Coefficient: −5.11e−02
Value: C
Citrobacter
0
2
4
6
A (n=15)
B (n=16)
C (n=14)
surface

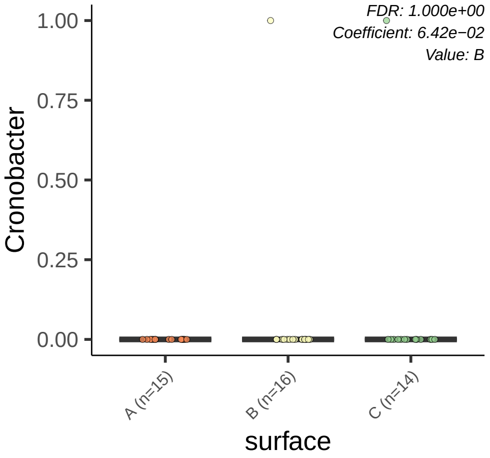

FDR: 1.000e+00
Coefficient: 6.42e−02
Value: B
1.00
0.75
0.50
0.25
0.00
Cronobacter
A (n=15)
B (n=16)
C (n=14)
surface

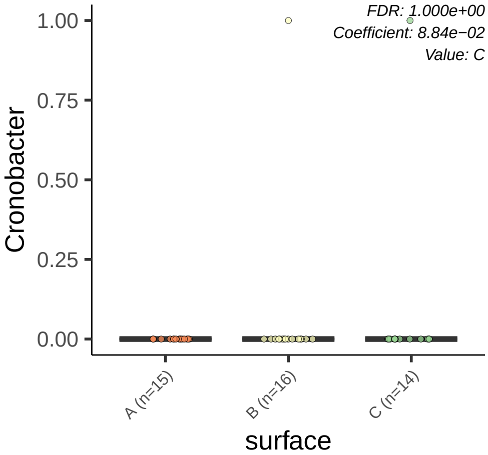
FDR: 1.000e+00
Coefficient: 8.84e−02
Value: C
Cronobacter
1.00
0.75
0.50
0.25
0.00
A (n=15)
B (n=16)
C (n=14)
surface

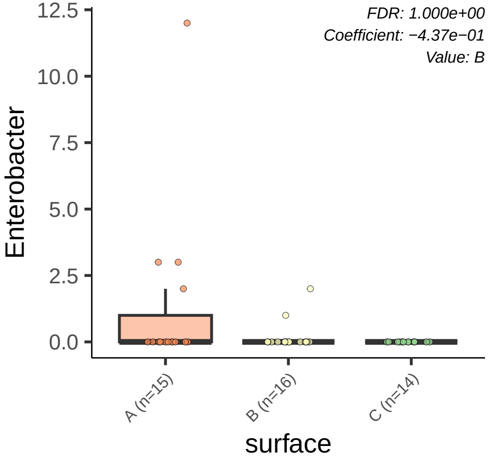

FDR: 1.000e+00
Coefficient: −4.37e−01
Value: B
Enterobacter
12.5
10.0
7.5
5.0
2.5
0.0
A (n=15)
B (n=16)
C (n=14)
surface

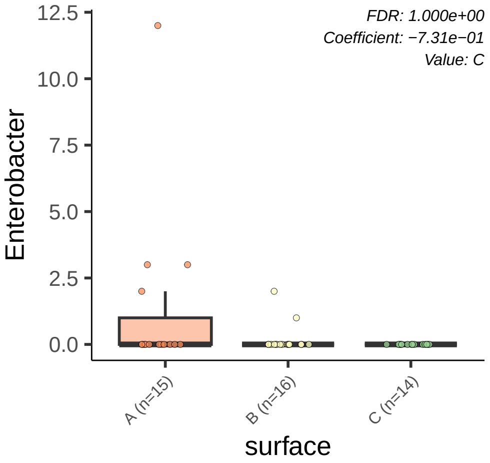

FDR: 1.000e+00
Coefficient: −7.31e−01
Value: C
Enterobacter
12.5
10.0
7.5
5.0
2.5
0.0
A (n=15)
B (n=16)
C (n=14)
surface

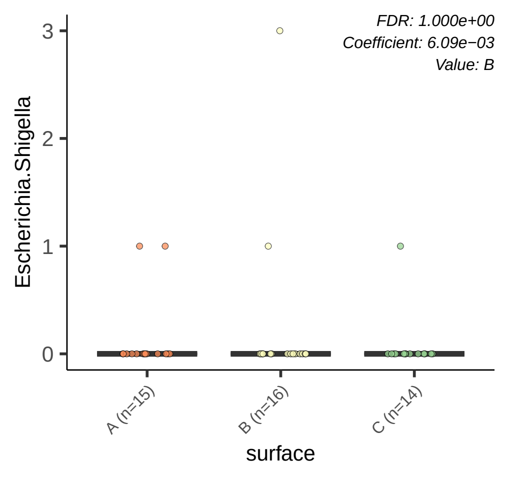

FDR: 1.000e+00
Coefficient: 6.09e−03
Value: B
Escherichia.Shigella
3
2
1
0
A (n=15)
B (n=16)
C (n=14)
surface

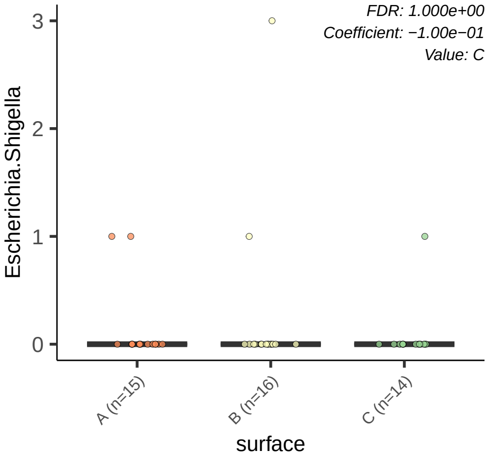

FDR: 1.000e+00
Coefficient: −1.00e−01
Value: C
Escherichia.Shigella
3
2
1
0
A (n=15)
B (n=16)
C (n=14)
surface

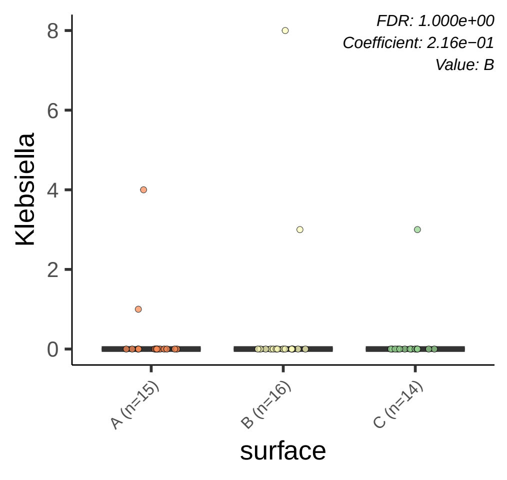
FDR: 1.000e+00
Coefficient: 2.16e−01
Value: B
Klebsiella
0
2
4
6
8
A (n=15)
B (n=16)
C (n=14)
surface

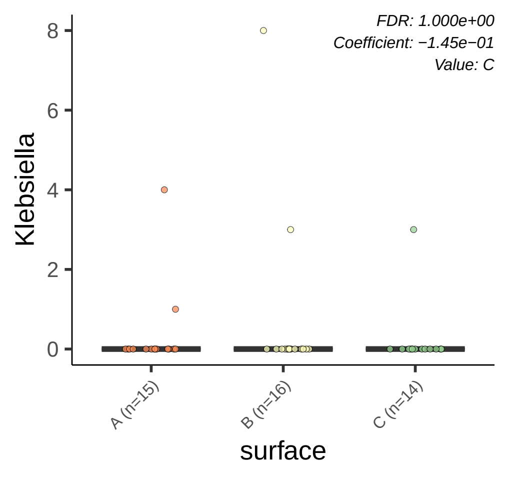
FDR: 1.000e+00
Coefficient: −1.45e−01
Value: C
Klebsiella
8
6
4
2
0
A (n=15)
B (n=16)
C (n=14)
surface

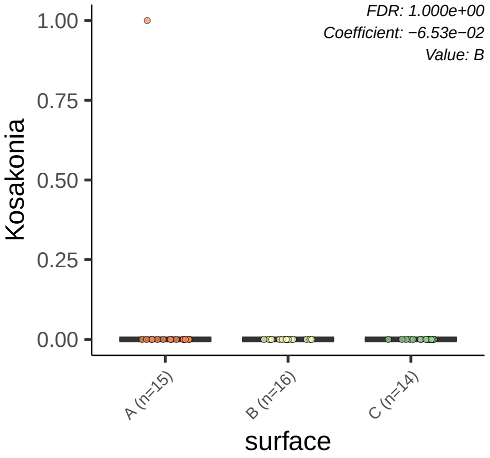

FDR: 1.000e+00
Coefficient: −6.53e−02
Value: B
Kosakonia
1.00
0.75
0.50
0.25
0.00
A (n=15)
B (n=16)
C (n=14)
surface

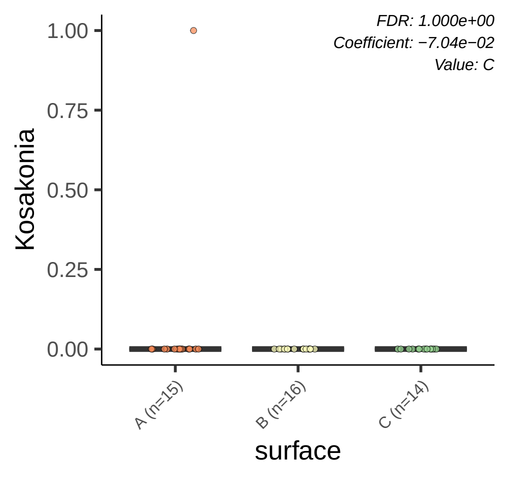
FDR: 1.000e+00
Coefficient: −7.04e−02
Value: C
Kosakonia
1.00
0.75
0.50
0.25
0.00
A (n=15)
B (n=16)
C (n=14)
surface

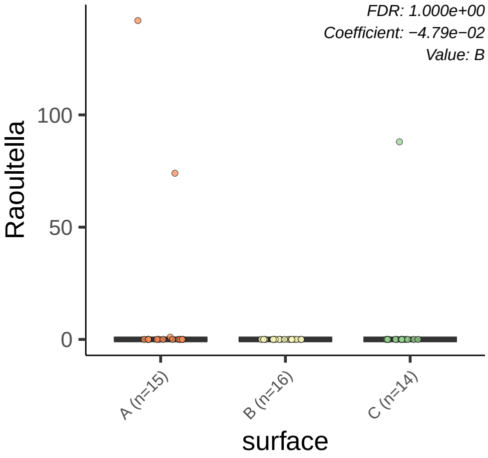

FDR: 1.000e+00
Coefficient: −4.79e−02
Value: B
Raoultella
100
50
0
A (n=15)
B (n=16)
C (n=14)
surface

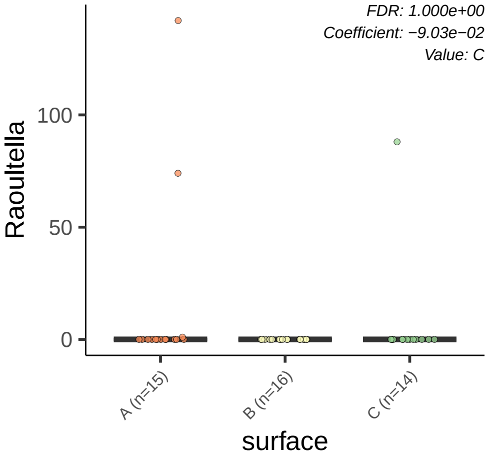

FDR: 1.000e+00
Coefficient: −9.03e−02
Value: C
Raoultella
100
50
0
A (n=15)
B (n=16)
C (n=14)
surface

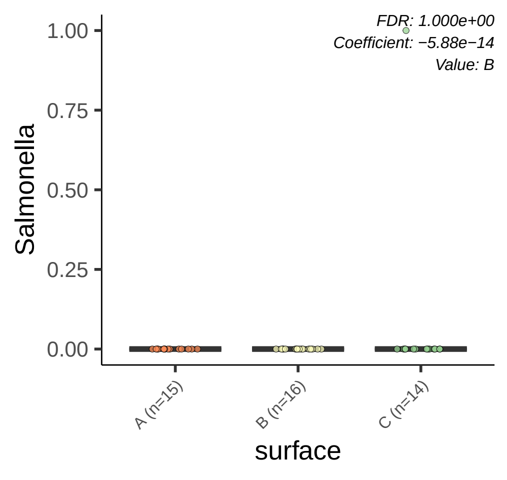
FDR: 1.000e+00
Coefficient: −5.88e−14
Value: B
Salmonella
1.00
0.75
0.50
0.25
0.00
A (n=15)
B (n=16)
C (n=14)
surface

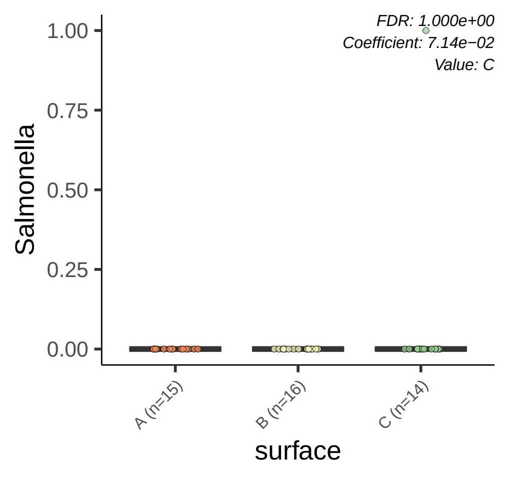
FDR: 1.000e+00
Coefficient: 7.14e−02
Value: C
Salmonella
1.00
0.75
0.50
0.25
0.00
A (n=15)
B (n=16)
C (n=14)
surface

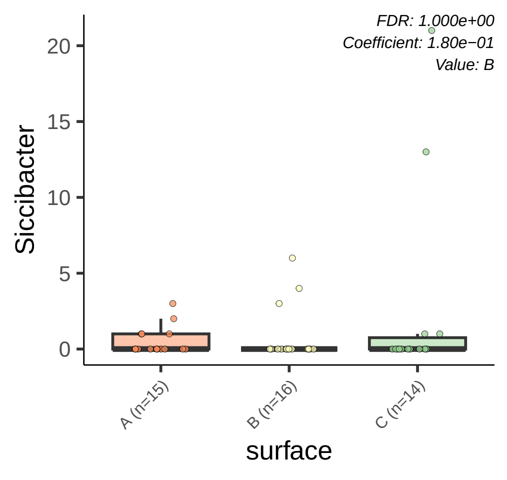
FDR: 1.000e+00
Coefficient: 1.80e−01
Value: B
Siccibacter
20
15
10
5
0
A (n=15)
B (n=16)
C (n=14)
surface

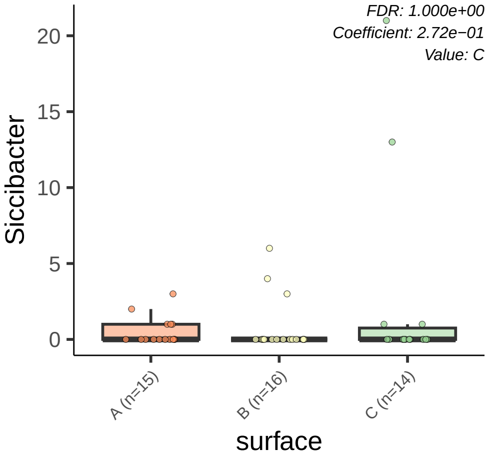
FDR: 1.000e+00
Coefficient: 2.72e−01
Value: C
Siccibacter
20
15
10
5
0
A (n=15)
B (n=16)
C (n=14)
surface

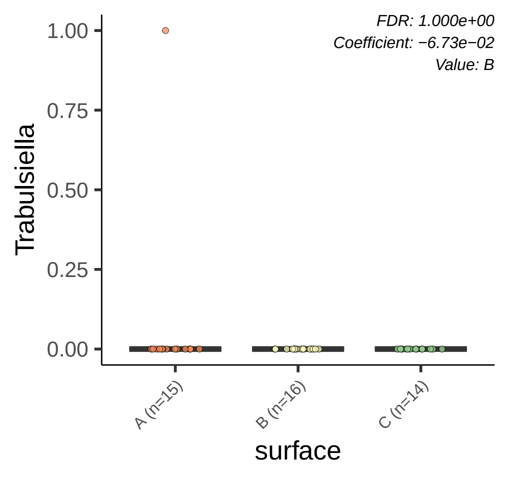

FDR: 1.000e+00
Coefficient: −6.73e−02
Value: B
Trabulsiella
1.00
0.75
0.50
0.25
0.00
A (n=15)
B (n=16)
C (n=14)
surface

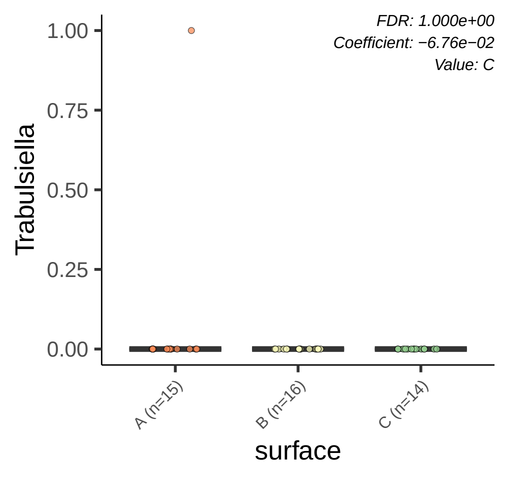
FDR: 1.000e+00
Coefficient: −6.76e−02
Value: C
Trabulsiella
1.00
0.75
0.50
0.25
0.00
A (n=15)
B (n=16)
C (n=14)
surface

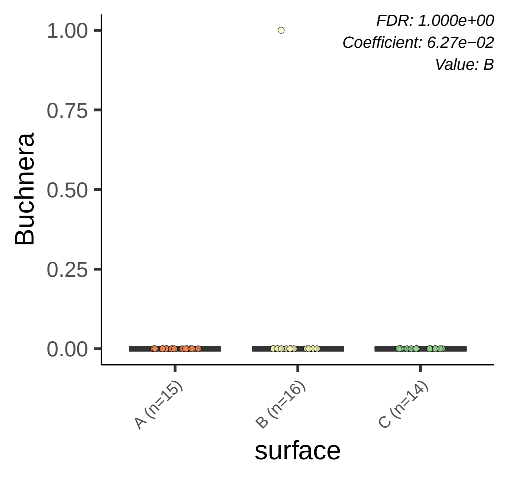

FDR: 1.000e+00
Coefficient: 6.27e−02
Value: B
Buchnera
1.00
0.75
0.50
0.25
0.00
A (n=15)
B (n=16)
C (n=14)
surface

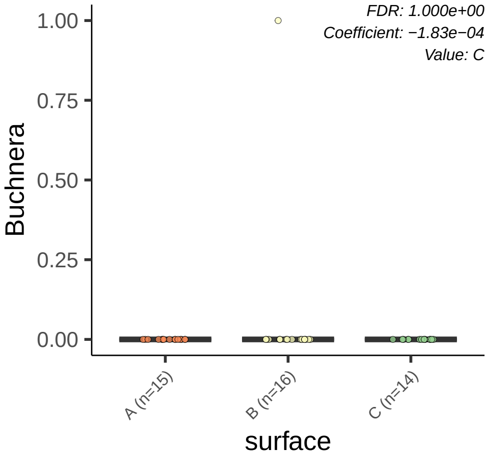
FDR: 1.000e+00
Coefficient: −1.83e−04
Value: C
Buchnera
1.00
0.75
0.50
0.25
0.00
A (n=15)
B (n=16)
C (n=14)
surface

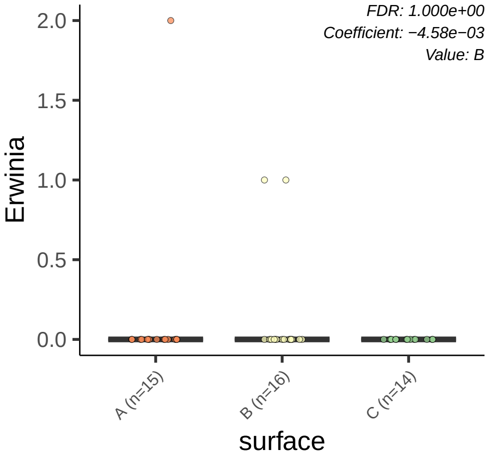

FDR: 1.000e+00
Coefficient: −4.58e−03
Value: B
Erwinia
2.0
1.5
1.0
0.5
0.0
A (n=15)
B (n=16)
C (n=14)
surface

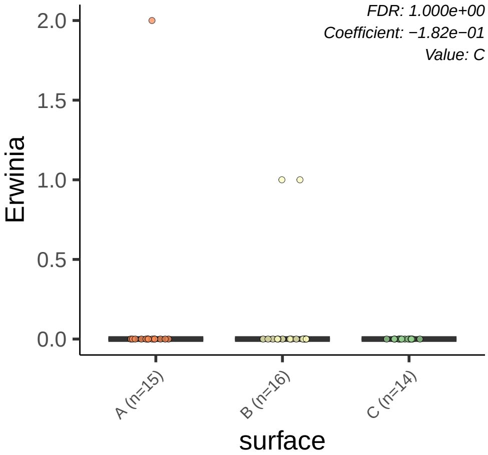
FDR: 1.000e+00
Coefficient: −1.82e−01
Value: C
Erwinia
2.0
1.5
1.0
0.5
0.0
A (n=15)
B (n=16)
C (n=14)
surface

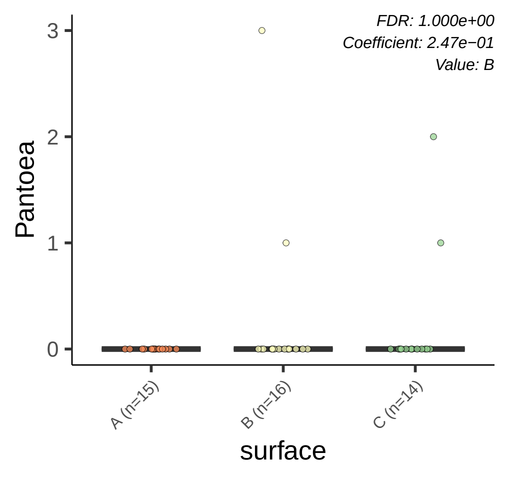
FDR: 1.000e+00
Coefficient: 2.47e−01
Value: B
Pantoea
3
2
1
0
A (n=15)
B (n=16)
C (n=14)
surface

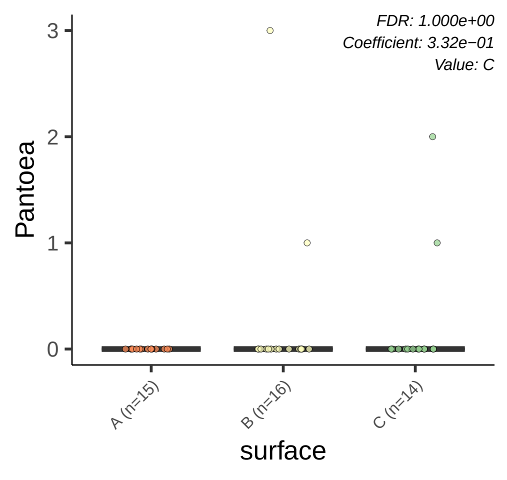

FDR: 1.000e+00
Coefficient: 3.32e−01
Value: C
Pantoea
3
2
1
0
A (n=15)
B (n=16)
C (n=14)
surface

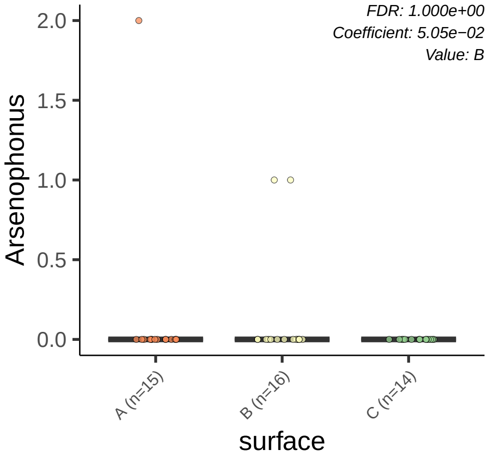
FDR: 1.000e+00
Coefficient: 5.05e−02
Value: B
Arsenophonus
2.0
1.5
1.0
0.5
0.0
A (n=15)
B (n=16)
C (n=14)
surface

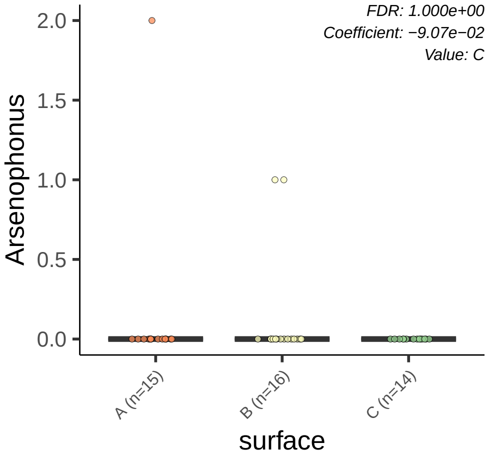
FDR: 1.000e+00
Coefficient: −9.07e−02
Value: C
Arsenophonus
2.0
1.5
1.0
0.5
0.0
A (n=15)
B (n=16)
C (n=14)
surface

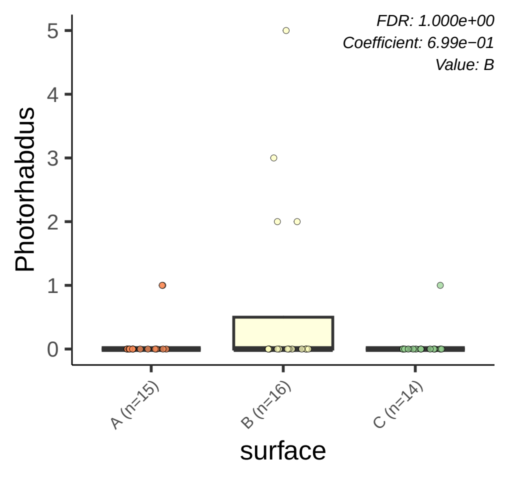

FDR: 1.000e+00
Coefficient: 6.99e−01
Value: B
Photorhabdus
5
4
3
2
1
0
A (n=15)
B (n=16)
C (n=14)
surface

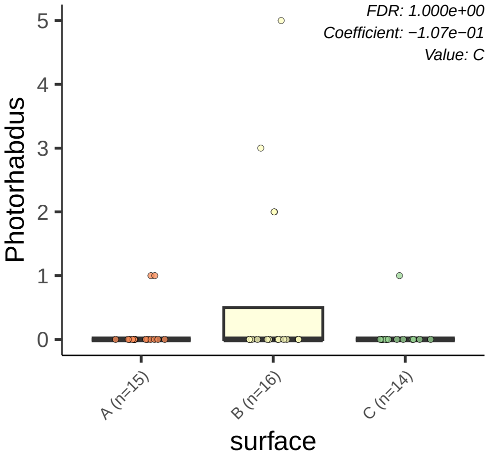

FDR: 1.000e+00
Coefficient: −1.07e−01
Value: C
Photorhabdus
0
1
2
3
4
5
A (n=15)
B (n=16)
C (n=14)
surface

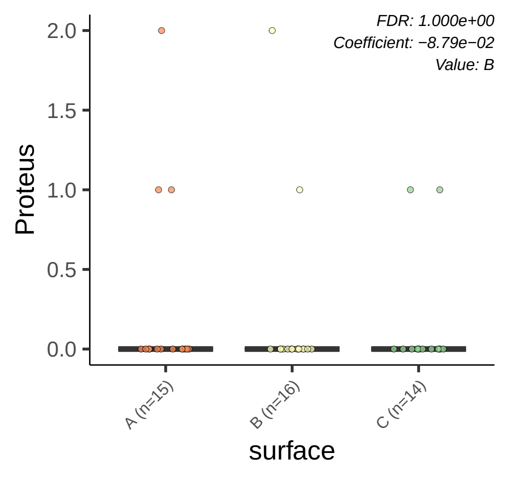

FDR: 1.000e+00
Coefficient: −8.79e−02
Value: B
Proteus
2.0
1.5
1.0
0.5
0.0
A (n=15)
B (n=16)
C (n=14)
surface

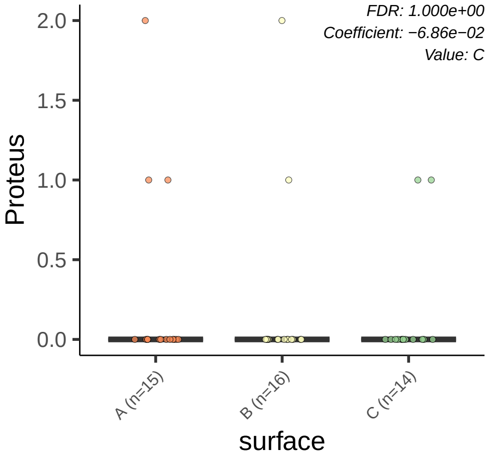

FDR: 1.000e+00
Coefficient: −6.86e−02
Value: C
Proteus
2.0
1.5
1.0
0.5
0.0
A (n=15)
B (n=16)
C (n=14)
surface

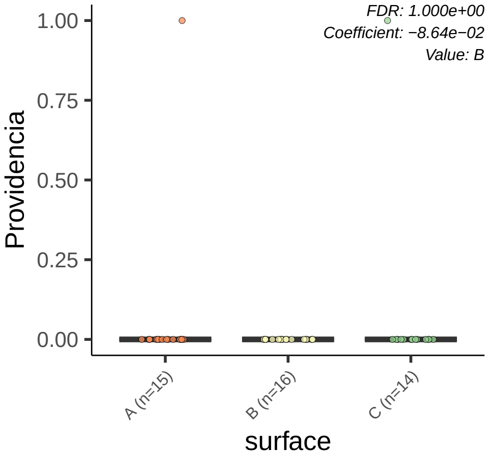
FDR: 1.000e+00
Coefficient: −8.64e−02
Value: B
Providencia
1.00
0.75
0.50
0.25
0.00
A (n=15)
B (n=16)
C (n=14)
surface

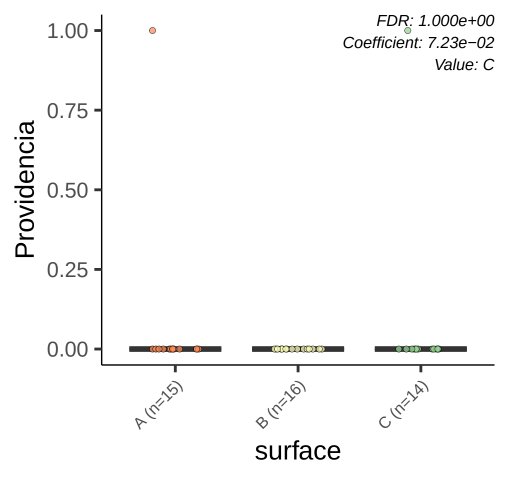

FDR: 1.000e+00
Coefficient: 7.23e−02
Value: C
Providencia
1.00
0.75
0.50
0.25
0.00
A (n=15)
B (n=16)
C (n=14)
surface

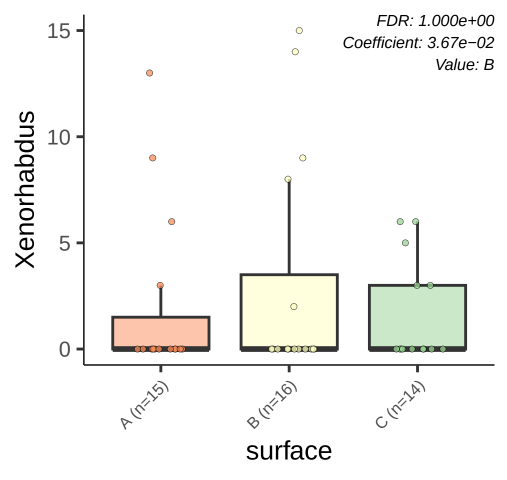

FDR: 1.000e+00
Coefficient: 3.67e−02
Value: B
Xenorhabdus
15
10
5
0
A (n=15)
B (n=16)
C (n=14)
surface

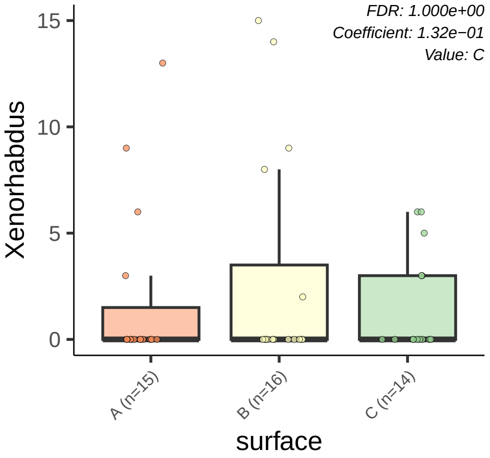

FDR: 1.000e+00
Coefficient: 1.32e−01
Value: C
Xenorhabdus
0
5
10
15
A (n=15)
B (n=16)
C (n=14)
surface

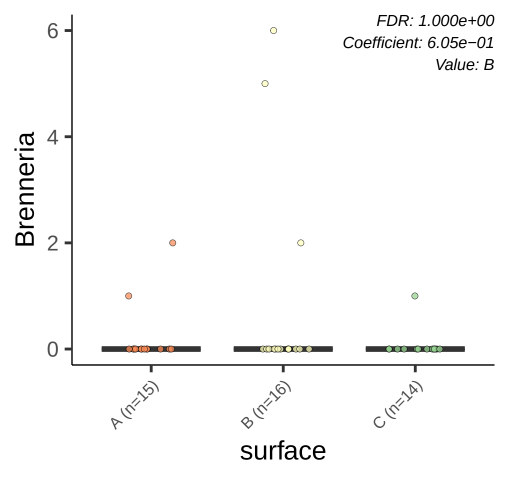
FDR: 1.000e+00
Coefficient: 6.05e−01
Value: B
Brenneria
0
2
4
6
A (n=15)
B (n=16)
C (n=14)
surface

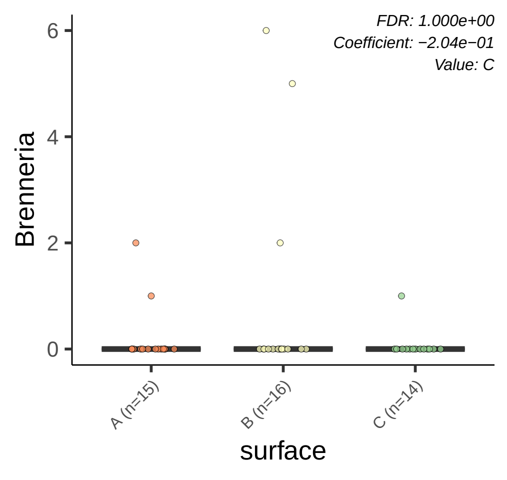

FDR: 1.000e+00
Coefficient: −2.04e−01
Value: C
Brenneria
0
2
4
6
A (n=15)
B (n=16)
C (n=14)
surface

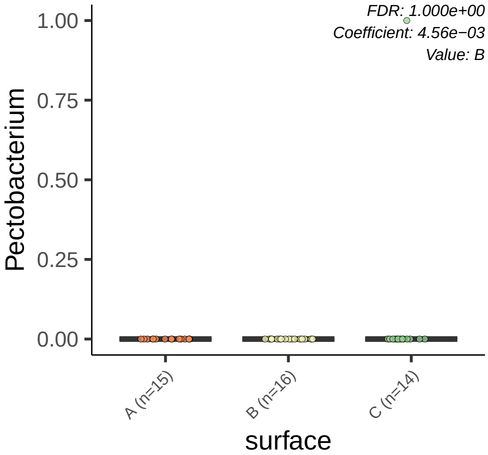
FDR: 1.000e+00
Coefficient: 4.56e−03
Value: B
Pectobacterium
1.00
0.75
0.50
0.25
0.00
A (n=15)
B (n=16)
C (n=14)
surface

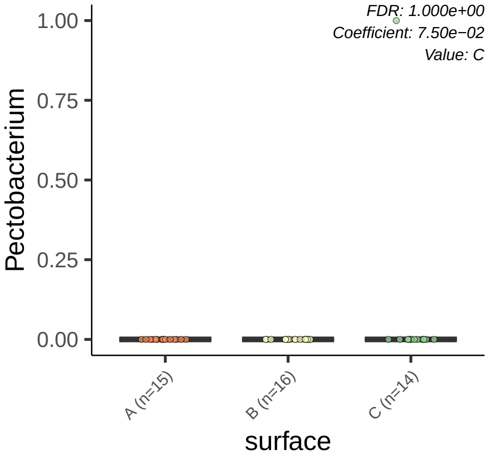

FDR: 1.000e+00
Coefficient: 7.50e−02
Value: C
Pectobacterium
1.00
0.75
0.50
0.25
0.00
A (n=15)
B (n=16)
C (n=14)
surface

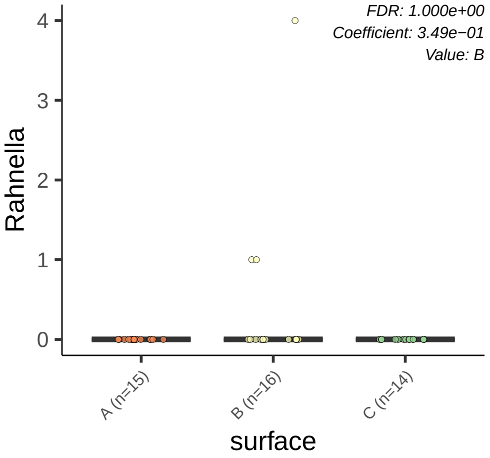
FDR: 1.000e+00
Coefficient: 3.49e−01
Value: B
Rahnella
0
1
2
3
4
A (n=15)
B (n=16)
C (n=14)
surface

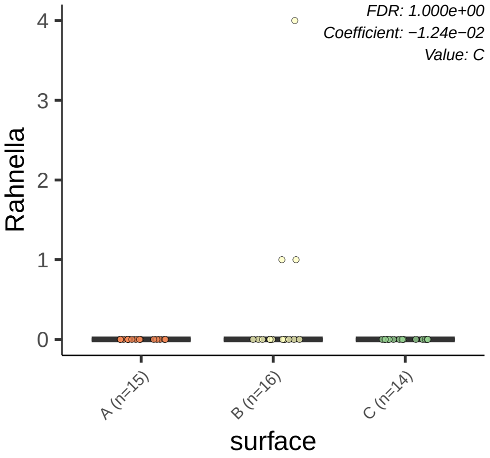

FDR: 1.000e+00
Coefficient: −1.24e−02
Value: C
Rahnella
0
1
2
3
4
A (n=15)
B (n=16)
C (n=14)
surface

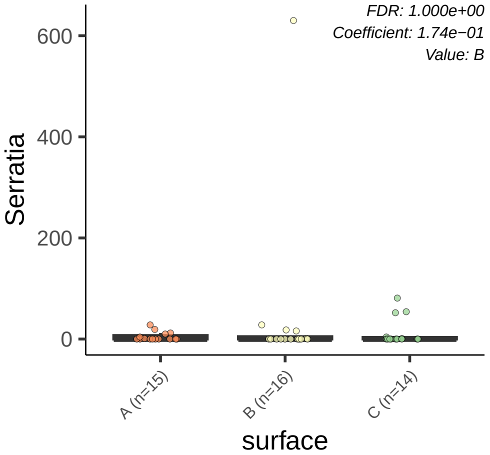
FDR: 1.000e+00
Coefficient: 1.74e−01
Value: B
Serratia
600
400
200
0
A (n=15)
B (n=16)
C (n=14)
surface

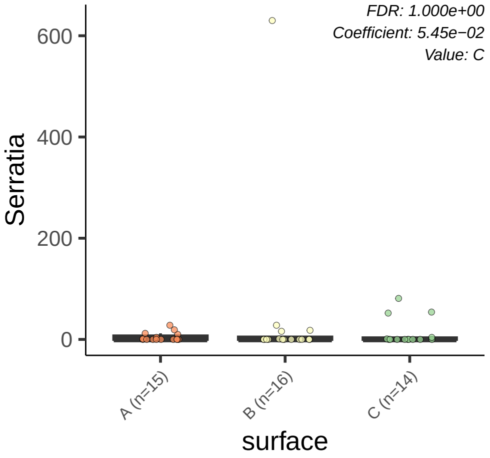

FDR: 1.000e+00
Coefficient: 5.45e−02
Value: C
600
400
200
0
Serratia
A (n=15)
B (n=16)
C (n=14)
surface

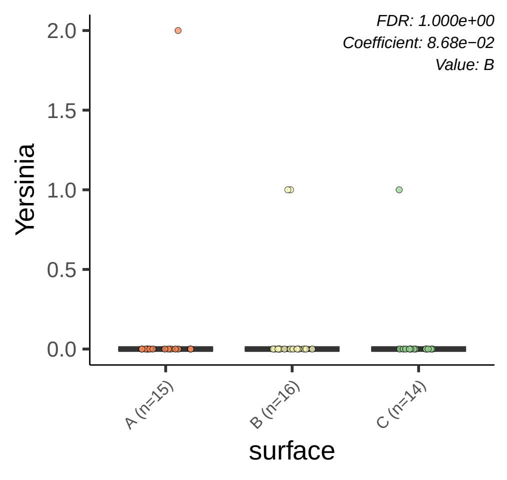
FDR: 1.000e+00
Coefficient: 8.68e−02
Value: B
Yersinia
2.0
1.5
1.0
0.5
0.0
A (n=15)
B (n=16)
C (n=14)
surface

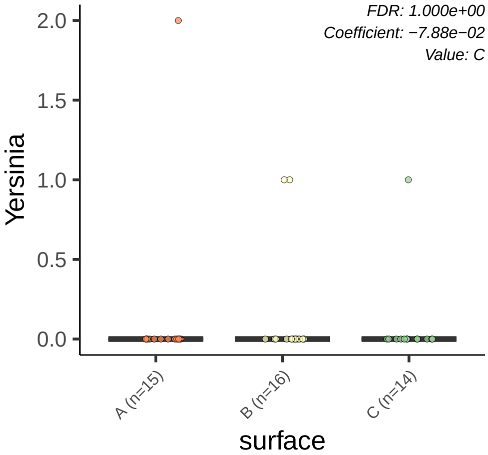
FDR: 1.000e+00
Coefficient: −7.88e−02
Value: C
Yersinia
2.0
1.5
1.0
0.5
0.0
A (n=15)
B (n=16)
C (n=14)
surface

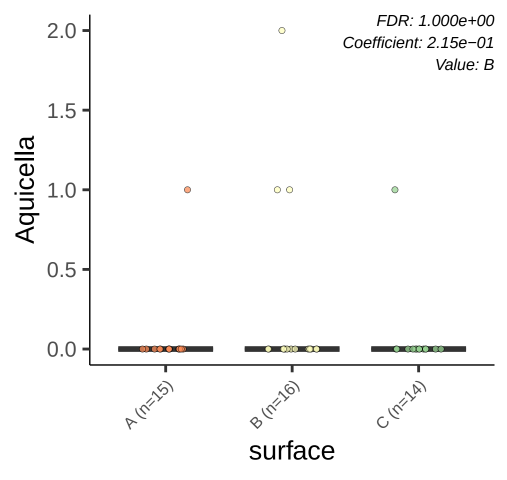
FDR: 1.000e+00
Coefficient: 2.15e−01
Value: B
Aquicella
2.0
1.5
1.0
0.5
0.0
A (n=15)
B (n=16)
C (n=14)
surface

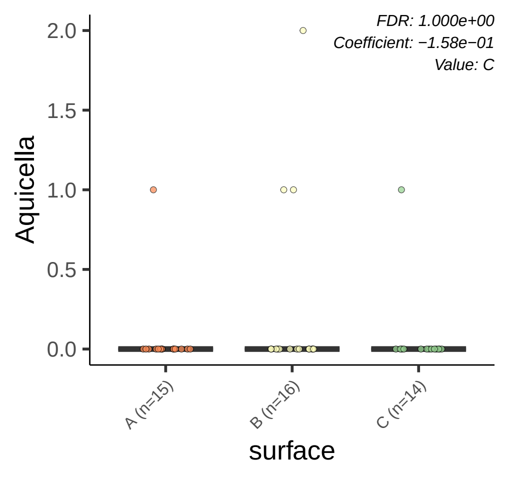
FDR: 1.000e+00
Coefficient: −1.58e−01
Value: C
Aquicella
2.0
1.5
1.0
0.5
0.0
A (n=15)
B (n=16)
C (n=14)
surface

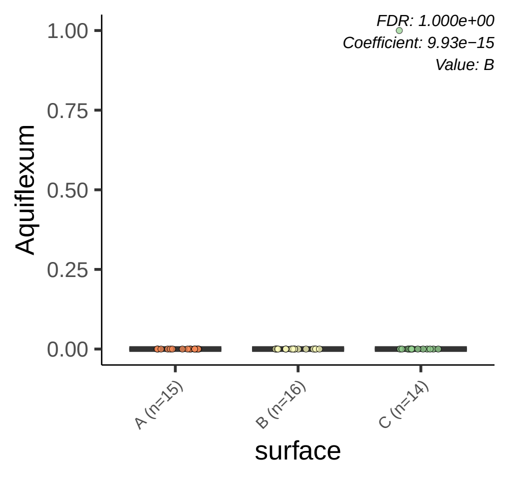

FDR: 1.000e+00
Coefficient: 9.93e−15
Value: B
1.00
0.75
0.50
0.25
0.00
Aquiflexum
A (n=15)
B (n=16)
C (n=14)
surface

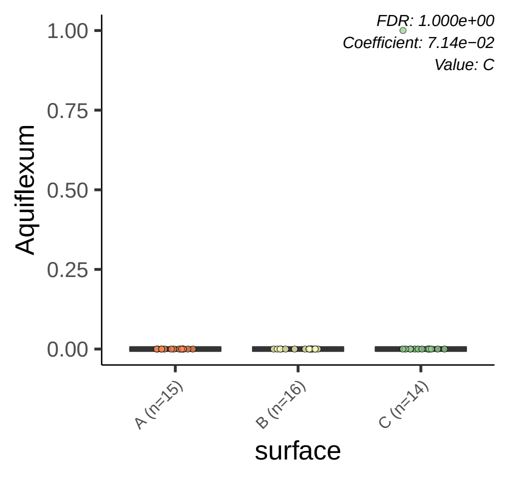

FDR: 1.000e+00
Coefficient: 7.14e−02
Value: C
1.00
0.75
0.50
0.25
0.00
Aquiflexum
A (n=15)
B (n=16)
C (n=14)
surface

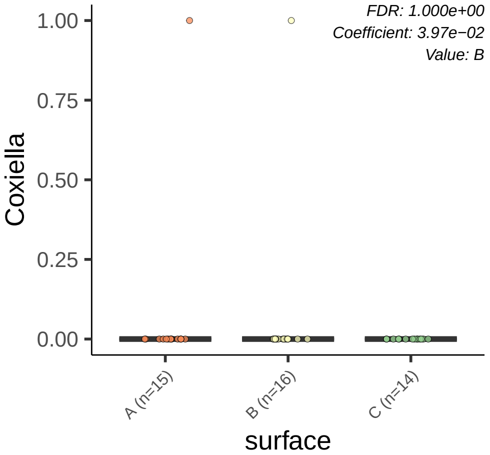
FDR: 1.000e+00
Coefficient: 3.97e−02
Value: B
Coxiella
1.00
0.75
0.50
0.25
0.00
A (n=15)
B (n=16)
C (n=14)
surface

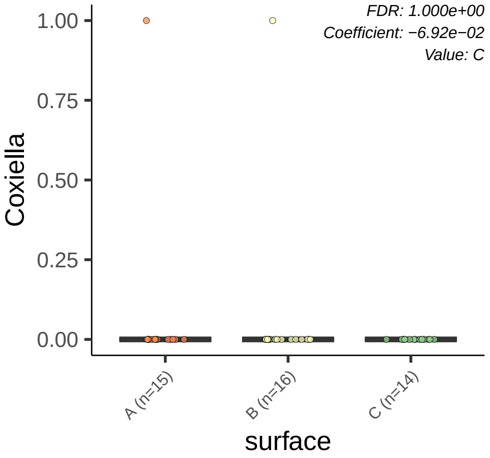

FDR: 1.000e+00
Coefficient: −6.92e−02
Value: C
Coxiella
1.00
0.75
0.50
0.25
0.00
A (n=15)
B (n=16)
C (n=14)
surface

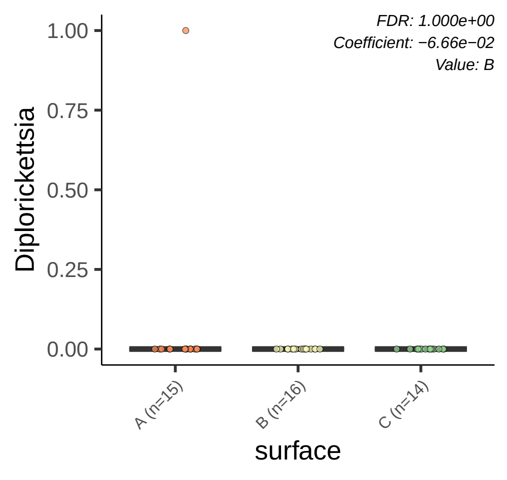

FDR: 1.000e+00
Coefficient: −6.66e−02
Value: B
Diploricketsia
1.00
0.75
0.50
0.25
0.00
A (n=15)
B (n=16)
C (n=14)
surface

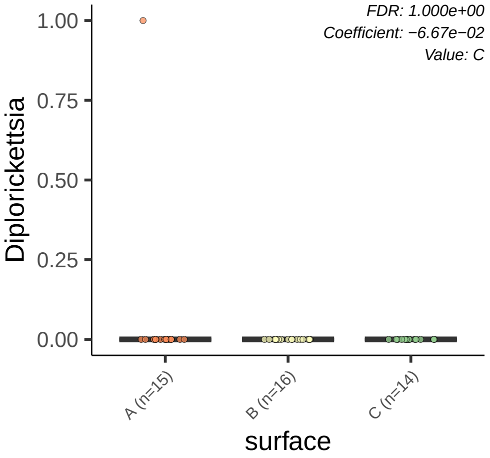
FDR: 1.000e+00
Coefficient: −6.67e−02
Value: C
Diploricketttsia
1.00
0.75
0.50
0.25
0.00
A (n=15)
B (n=16)
C (n=14)
surface

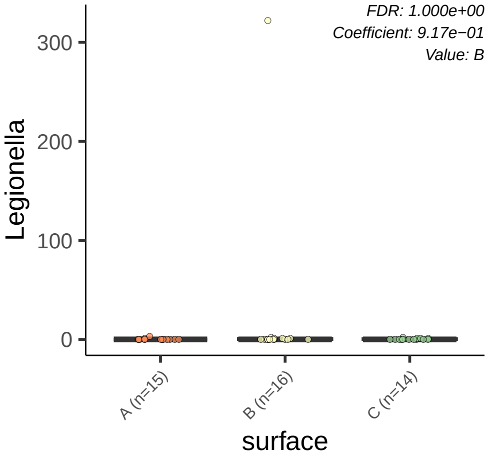

FDR: 1.000e+00
Coefficient: 9.17e−01
Value: B
Legionella
300
200
100
0
A (n=15)
B (n=16)
C (n=14)
surface

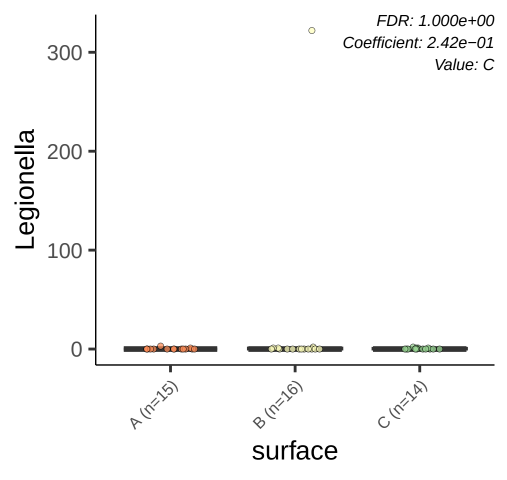

FDR: 1.000e+00
Coefficient: 2.42e−01
Value: C
300
200
100
0
Legionella
A (n=15)
B (n=16)
C (n=14)
surface

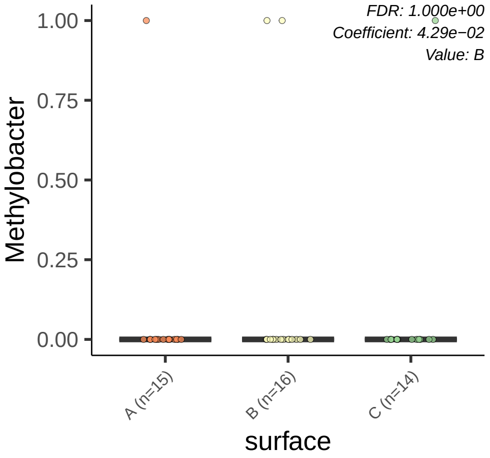

FDR: 1.000e+00
Coefficient: 4.29e−02
Value: B
Methylobacter
1.00
0.75
0.50
0.25
0.00
A (n=15)
B (n=16)
C (n=14)
surface

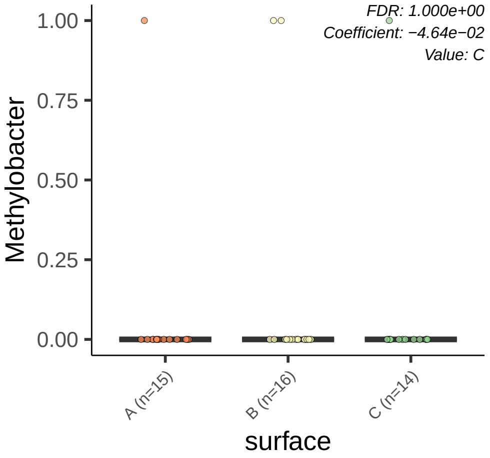

FDR: 1.000e+00
Coefficient: −4.64e−02
Value: C
Methylobacter
1.00
0.75
0.50
0.25
0.00
A (n=15)
B (n=16)
C (n=14)
surface

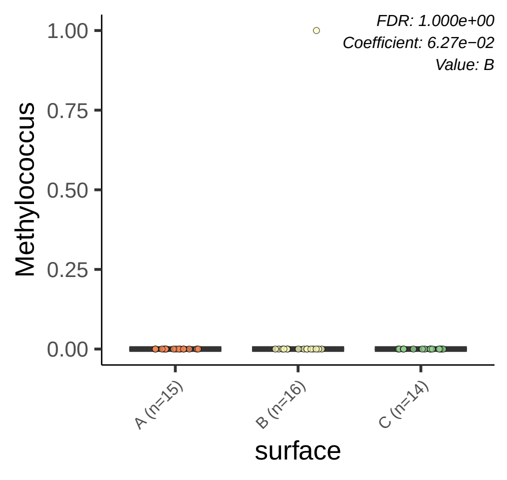

FDR: 1.000e+00
Coefficient: 6.27e−02
Value: B
Methylococcus
1.00
0.75
0.50
0.25
0.00
A (n=15)
B (n=16)
C (n=14)
surface

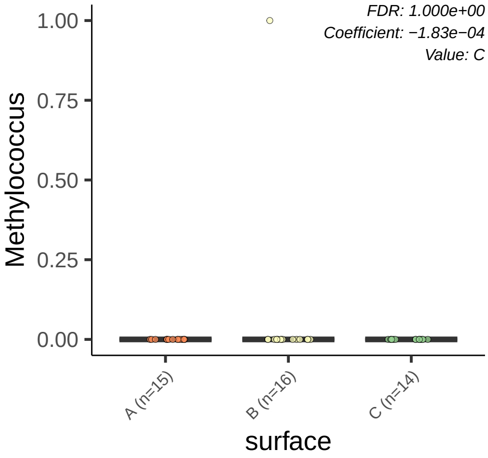

FDR: 1.000e+00
Coefficient: −1.83e−04
Value: C
Methylococcus
1.00
0.75
0.50
0.25
0.00
A (n=15)
B (n=16)
C (n=14)
surface

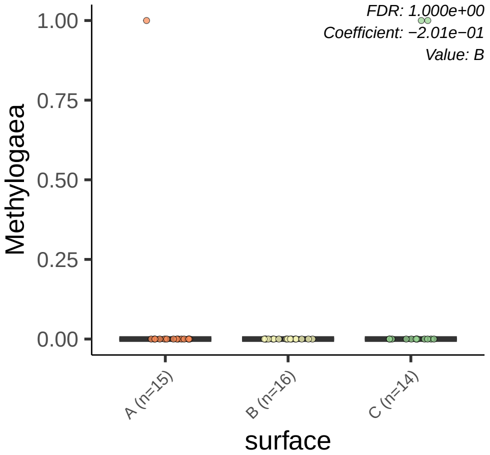

FDR: 1.000e+00
Coefficient: −2.01e−01
Value: B
Methylogaea
1.00
0.75
0.50
0.25
0.00
A (n=15)
B (n=16)
C (n=14)
surface

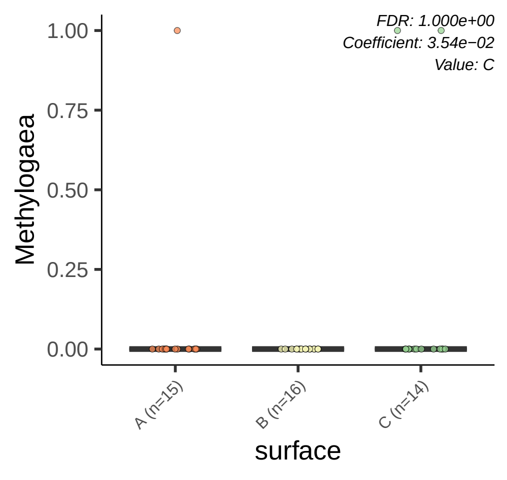

FDR: 1.000e+00
Coefficient: 3.54e−02
Value: C
Methylogaea
1.00
0.75
0.50
0.25
0.00
A (n=15)
B (n=16)
C (n=14)
surface

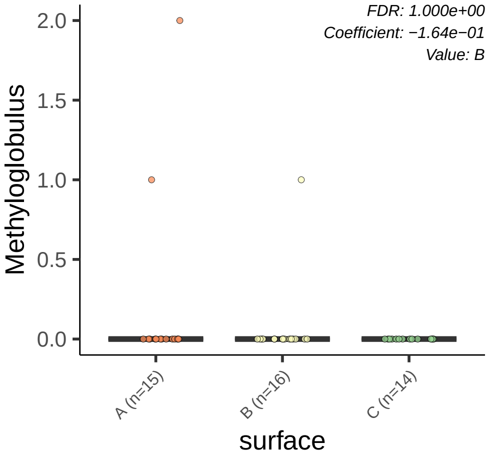
FDR: 1.000e+00
Coefficient: −1.64e−01
Value: B
Methyloglobulus
2.0
1.5
1.0
0.5
0.0
A (n=15)
B (n=16)
C (n=14)
surface

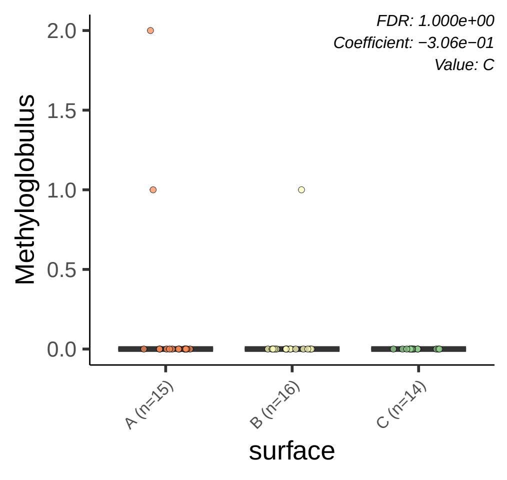
FDR: 1.000e+00
Coefficient: −3.06e−01
Value: C
Methyloglobulus
2.0
1.5
1.0
0.5
0.0
A (n=15)
B (n=16)
C (n=14)
surface

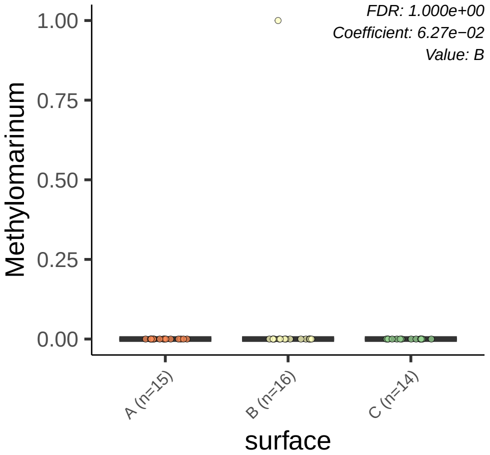
FDR: 1.000e+00
Coefficient: 6.27e−02
Value: B
Methylomarinum
1.00
0.75
0.50
0.25
0.00
A (n=15)
B (n=16)
C (n=14)
surface

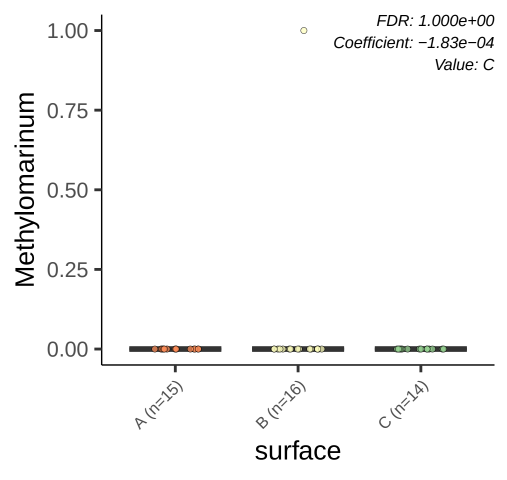
FDR: 1.000e+00
Coefficient: −1.83e−04
Value: C
Methylomarinum
1.00
0.75
0.50
0.25
0.00
A (n=15)
B (n=16)
C (n=14)
surface

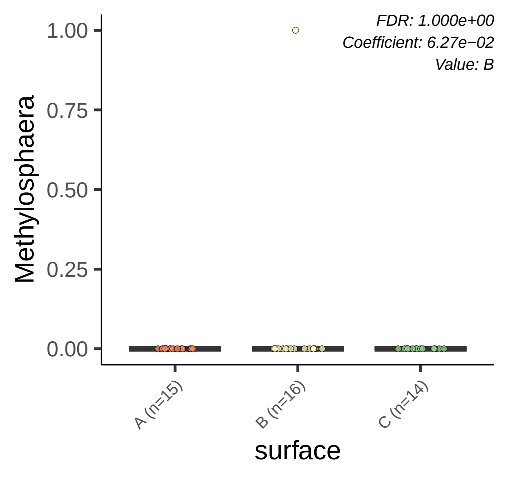

FDR: 1.000e+00
Coefficient: 6.27e−02
Value: B
Methylosphaera
1.00
0.75
0.50
0.25
0.00
A (n=15)
B (n=16)
C (n=14)
surface

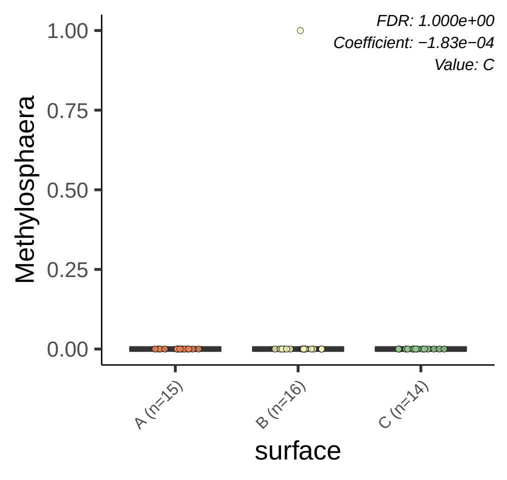
FDR: 1.000e+00
Coefficient: −1.83e−04
Value: C
Methylosphaera
1.00
0.75
0.50
0.25
0.00
A (n=15)
B (n=16)
C (n=14)
surface

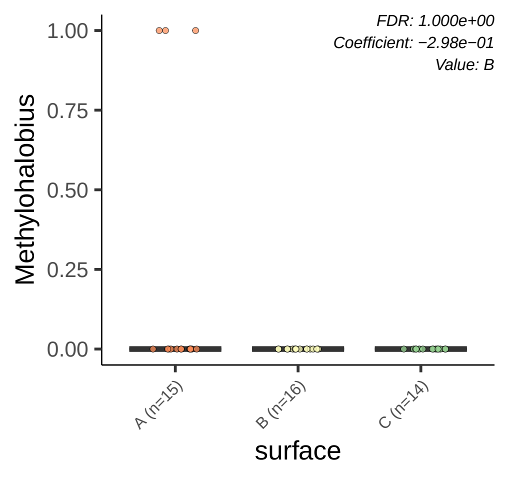

FDR: 1.000e+00
Coefficient: −2.98e−01
Value: B
Methylohalobius
1.00
0.75
0.50
0.25
0.00
A (n=15)
B (n=16)
C (n=14)
surface

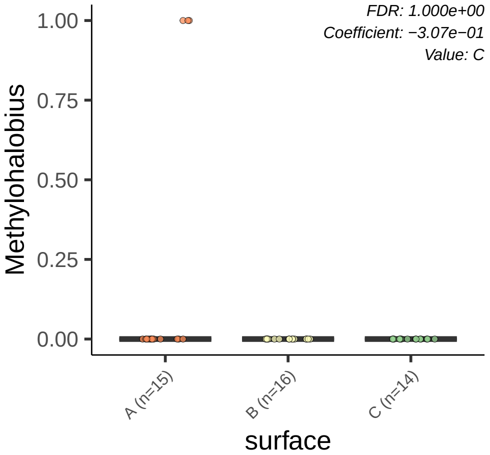
FDR: 1.000e+00
Coefficient: −3.07e−01
Value: C
Methylohalobius
1.00
0.75
0.50
0.25
0.00
A (n=15)
B (n=16)
C (n=14)
surface

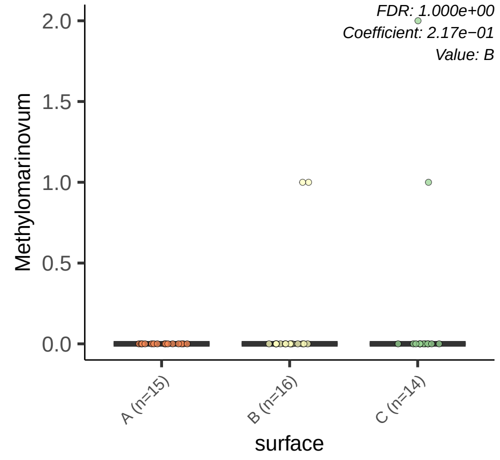
FDR: 1.000e+00
Coefficient: 2.17e−01
Value: B
Methylomarinovum
2.0
1.5
1.0
0.5
0.0
A (n=15)
B (n=16)
C (n=14)
surface

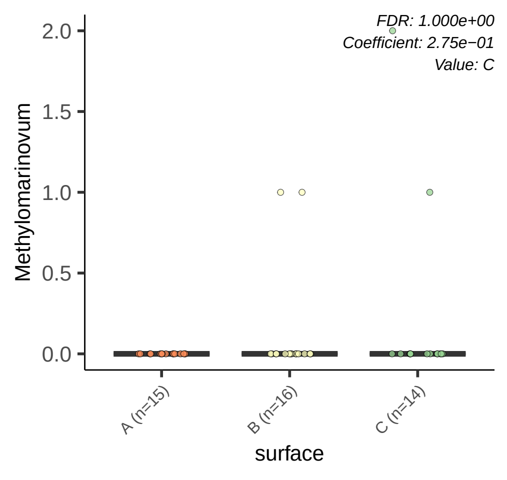

FDR: 1.000e+00
Coefficient: 2.75e−01
Value: C
Methylomarinovum
2.0
1.5
1.0
0.5
0.0
A (n=15)
B (n=16)
C (n=14)
surface

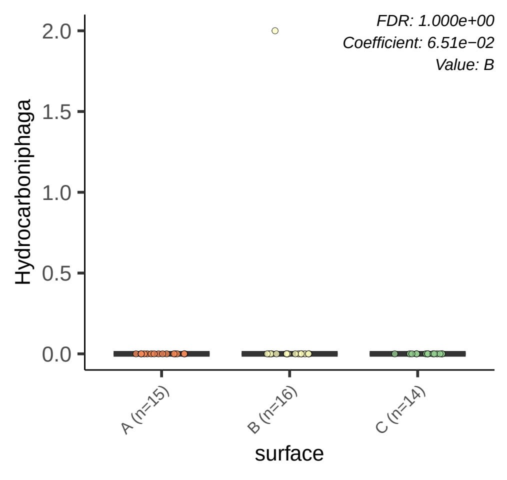

FDR: 1.000e+00
Coefficient: 6.51e−02
Value: B
Hydrocarboniphaga
2.0
1.5
1.0
0.5
0.0
A (n=15)
B (n=16)
C (n=14)
surface

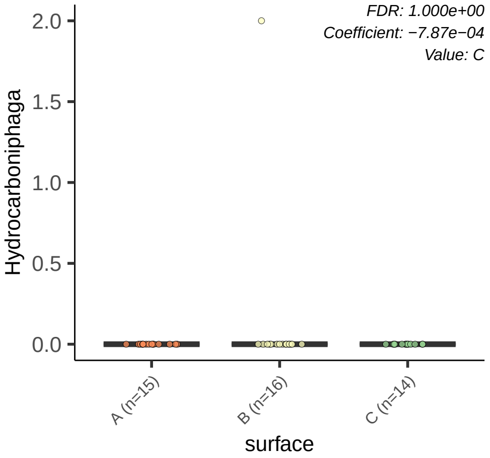

FDR: 1.000e+00
Coefficient: −7.87e−04
Value: C
Hydrocarboniphaga
2.0
1.5
1.0
0.5
0.0
A (n=15)
B (n=16)
C (n=14)
surface

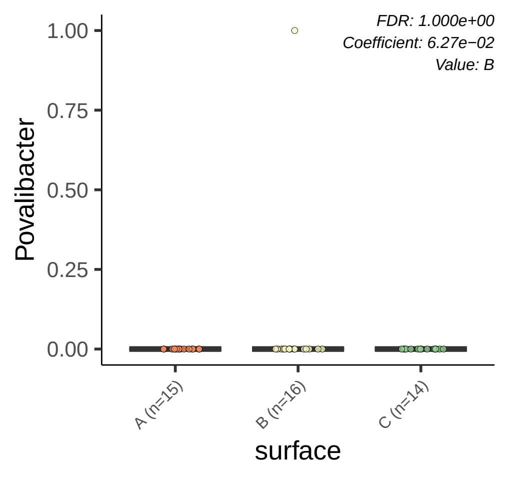

FDR: 1.000e+00
Coefficient: 6.27e−02
Value: B
Povalibacter
1.00
0.75
0.50
0.25
0.00
A (n=15)
B (n=16)
C (n=14)
surface

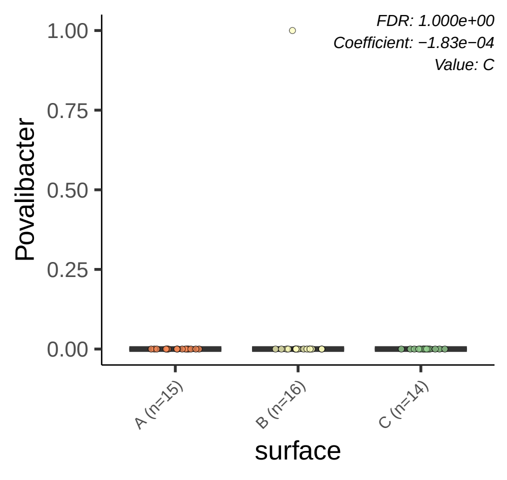

FDR: 1.000e+00
Coefficient: −1.83e−04
Value: C
1.00
0.75
0.50
0.25
0.00
Povalibacter
A (n=15)
B (n=16)
C (n=14)
surface

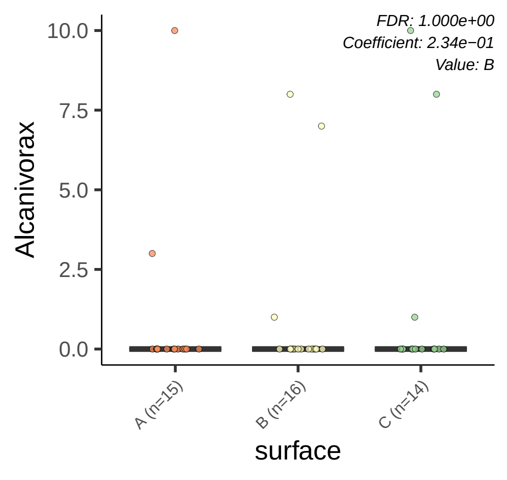
FDR: 1.000e+00
Coefficient: 2.34e−01
Value: B
Alcanivorax
10.0
7.5
5.0
2.5
0.0
A (n=15)
B (n=16)
C (n=14)
surface

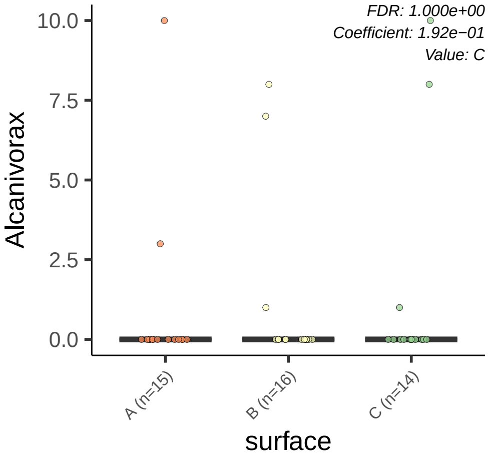

FDR: 1.000e+00
Coefficient: 1.92e−01
Value: C
Alcanivorax
10.0
7.5
5.0
2.5
0.0
A (n=15)
B (n=16)
C (n=14)
surface

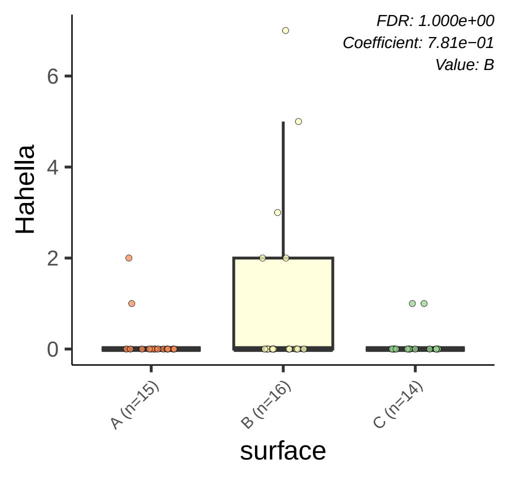

FDR: 1.000e+00
Coefficient: 7.81e−01
Value: B
Hahella
0
2
4
6
A (n=15)
B (n=16)
C (n=14)
surface

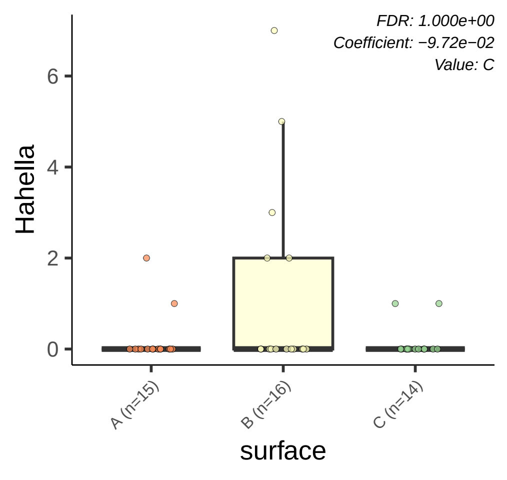
FDR: 1.000e+00
Coefficient: −9.72e−02
Value: C
Hahella
0
2
4
6
A (n=15)
B (n=16)
C (n=14)
surface

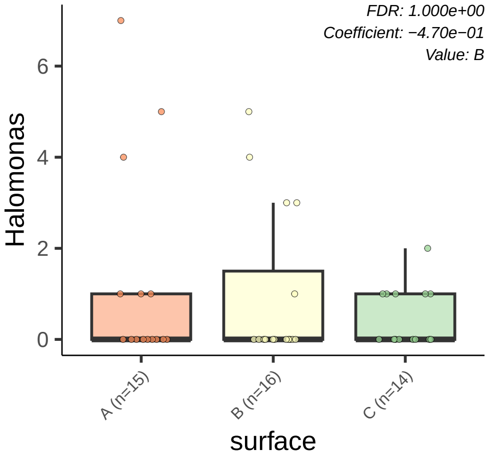

FDR: 1.000e+00
Coefficient: −4.70e−01
Value: B
Halomonas
0
2
4
6
A (n=15)
B (n=16)
C (n=14)
surface

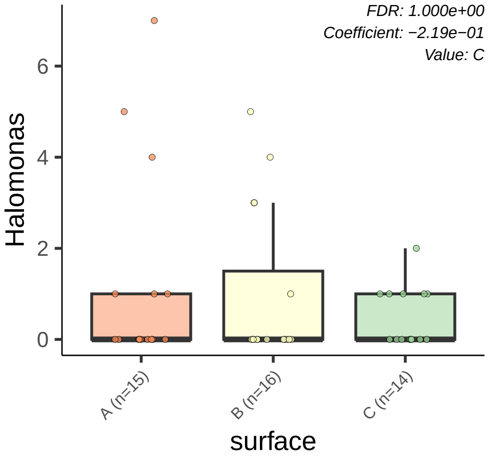
FDR: 1.000e+00
Coefficient: −2.19e−01
Value: C
Halomonas
0
2
4
6
A (n=15)
B (n=16)
C (n=14)
surface

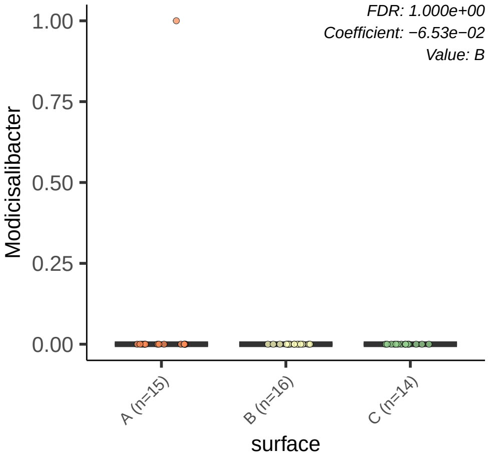
FDR: 1.000e+00
Coefficient: −6.53e−02
Value: B
Modicisalibacter
1.00
0.75
0.50
0.25
0.00
A (n=15)
B (n=16)
C (n=14)
surface

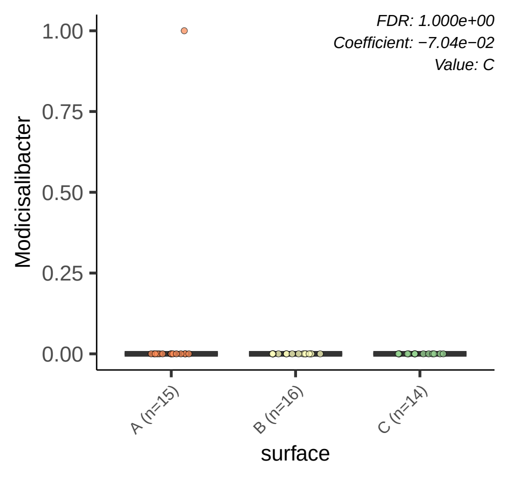
FDR: 1.000e+00
Coefficient: −7.04e−02
Value: C
Modicisalibacter
1.00
0.75
0.50
0.25
0.00
A (n=15)
B (n=16)
C (n=14)
surface

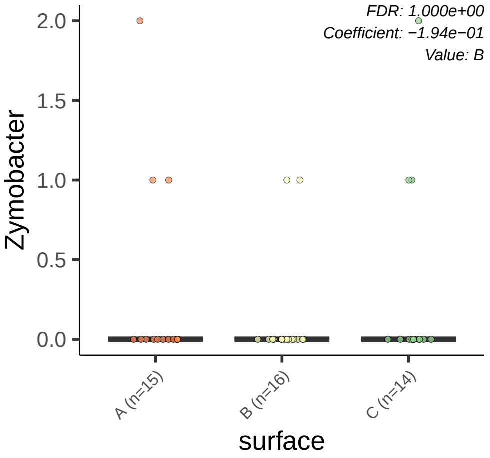
FDR: 1.000e+00
Coefficient: −1.94e−01
Value: B
Zymobacter
2.0
1.5
1.0
0.5
0.0
A (n=15)
B (n=16)
C (n=14)
surface

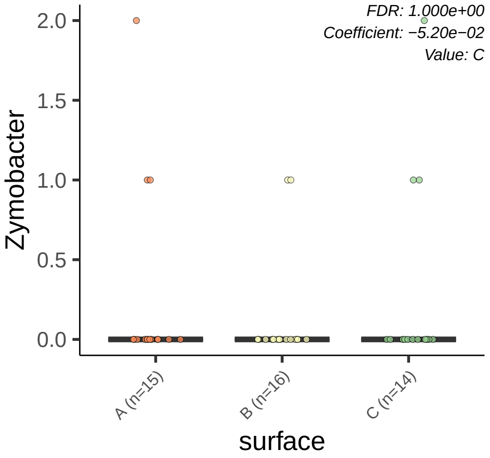
FDR: 1.000e+00
Coefficient: −5.20e−02
Value: C
Zymobacter
2.0
1.5
1.0
0.5
0.0
A (n=15)
B (n=16)
C (n=14)
surface

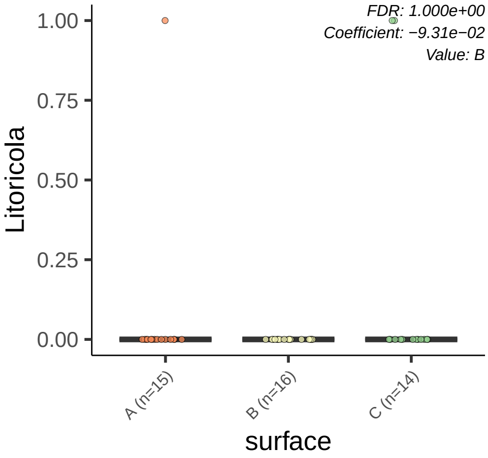

FDR: 1.000e+00
Coefficient: −9.31e−02
Value: B
Litoricola
1.00
0.75
0.50
0.25
0.00
A (n=15)
B (n=16)
C (n=14)
surface

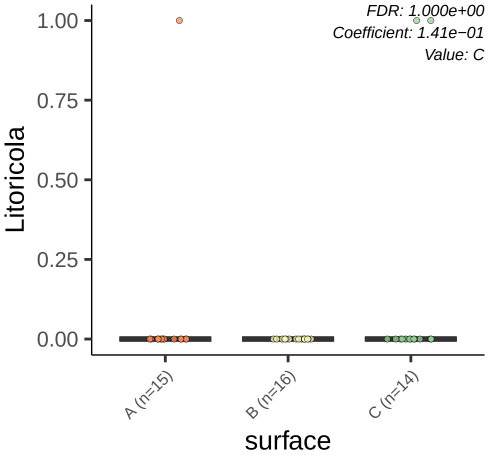
FDR: 1.000e+00
Coefficient: 1.41e−01
Value: C
Litoricola
1.00
0.75
0.50
0.25
0.00
A (n=15)
B (n=16)
C (n=14)
surface

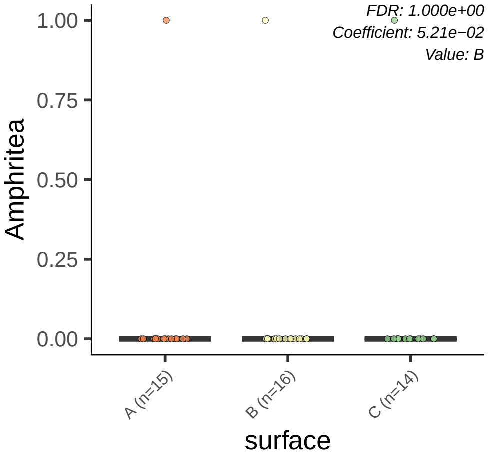

FDR: 1.000e+00
Coefficient: 5.21e−02
Value: B
Amphritea
1.00
0.75
0.50
0.25
0.00
A (n=15)
B (n=16)
C (n=14)
surface

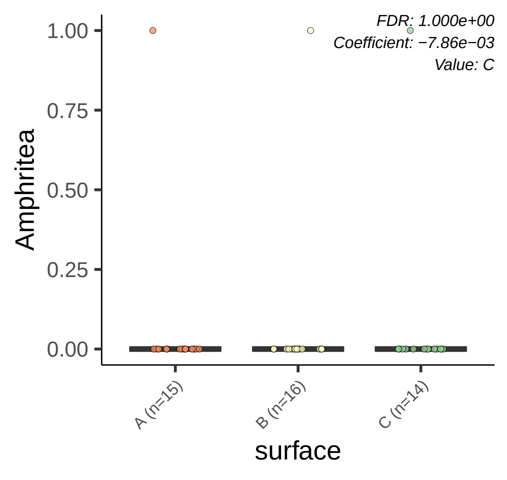

FDR: 1.000e+00
Coefficient: −7.86e−03
Value: C
Amphritea
1.00
0.75
0.50
0.25
0.00
A (n=15)
B (n=16)
C (n=14)
surface

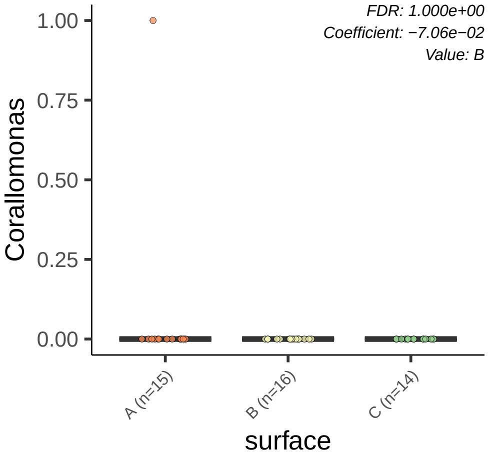
FDR: 1.000e+00
Coefficient: −7.06e−02
Value: B
Corallomonas
1.00
0.75
0.50
0.25
0.00
A (n=15)
B (n=16)
C (n=14)
surface

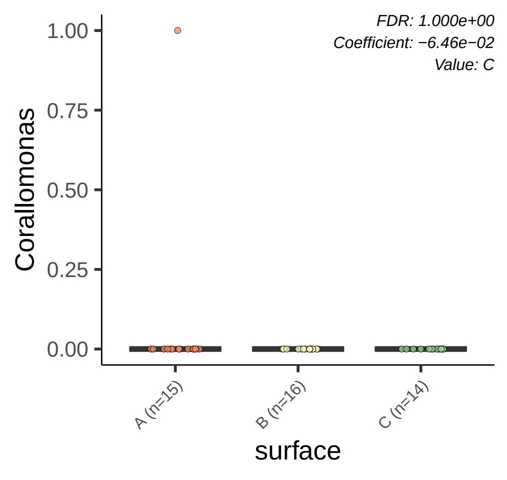
FDR: 1.000e+00
Coefficient: −6.46e−02
Value: C
Corallomonas
1.00
0.75
0.50
0.25
0.00
A (n=15)
B (n=16)
C (n=14)
surface

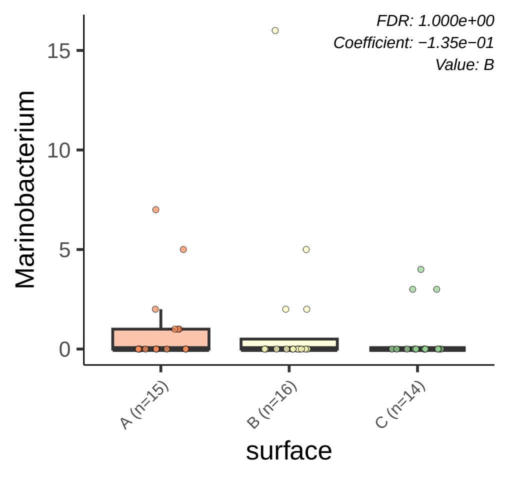

FDR: 1.000e+00
Coefficient: −1.35e−01
Value: B
Marinobacterium
15
10
5
0
A (n=15)
B (n=16)
C (n=14)
surface

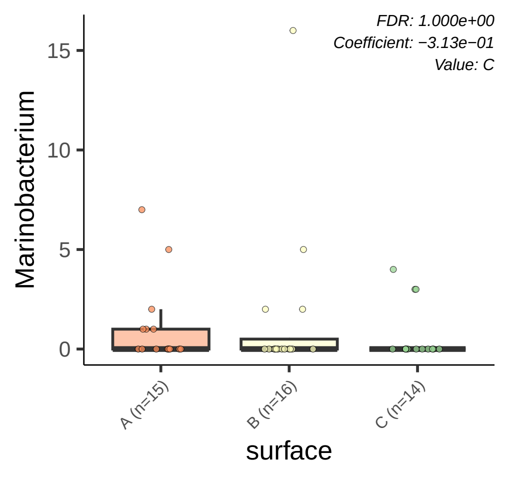
FDR: 1.000e+00
Coefficient: −3.13e−01
Value: C
Marinobacterium
0
5
10
15
A (n=15)
B (n=16)
C (n=14)
surface

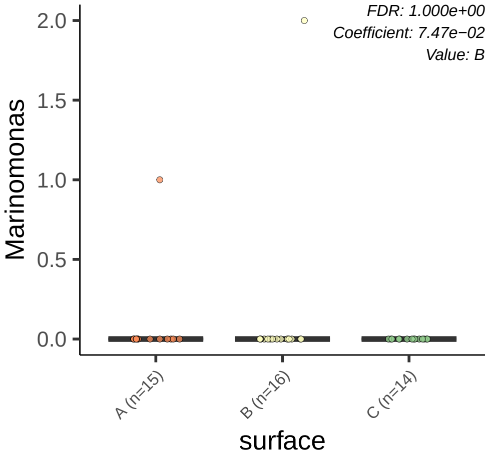

FDR: 1.000e+00
Coefficient: 7.47e−02
Value: B
Marinomonas
2.0
1.5
1.0
0.5
0.0
A (n=15)
B (n=16)
C (n=14)
surface

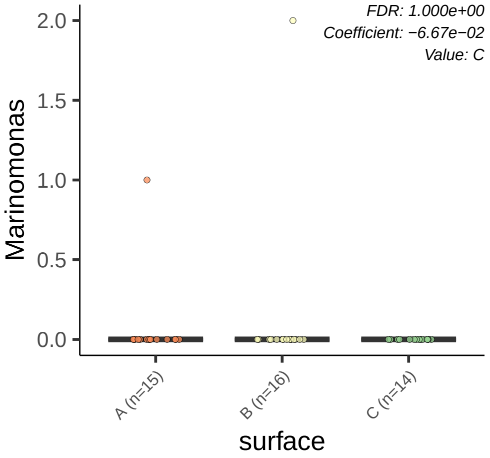
FDR: 1.000e+00
Coefficient: −6.67e−02
Value: C
Marinomonas
2.0
1.5
1.0
0.5
0.0
A (n=15)
B (n=16)
C (n=14)
surface

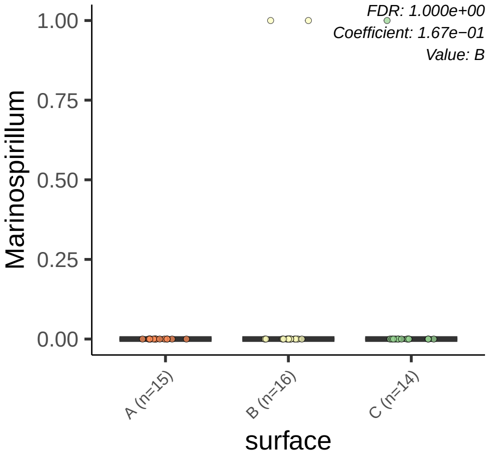

FDR: 1.000e+00
Coefficient: 1.67e−01
Value: B
Marinospirillum
1.00
0.75
0.50
0.25
0.00
A (n=15)
B (n=16)
C (n=14)
surface

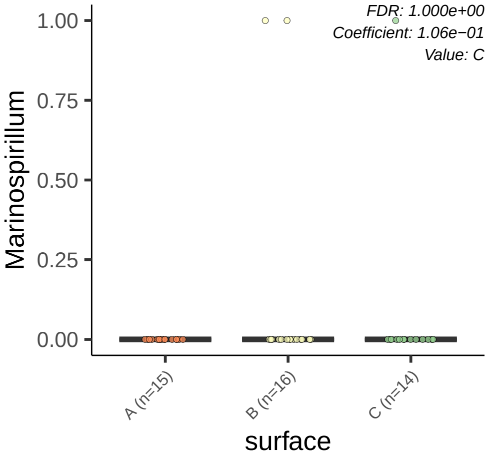
FDR: 1.000e+00
Coefficient: 1.06e−01
Value: C
Marinospirillum
1.00
0.75
0.50
0.25
0.00
A (n=15)
B (n=16)
C (n=14)
surface

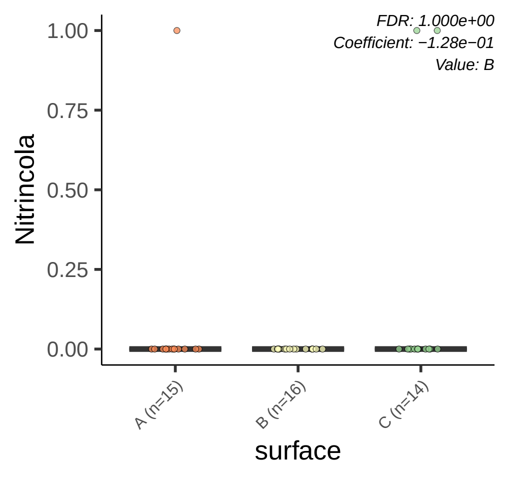
FDR: 1.000e+00
Coefficient: −1.28e−01
Value: B
Nitrincola
1.00
0.75
0.50
0.25
0.00
A (n=15)
B (n=16)
C (n=14)
surface

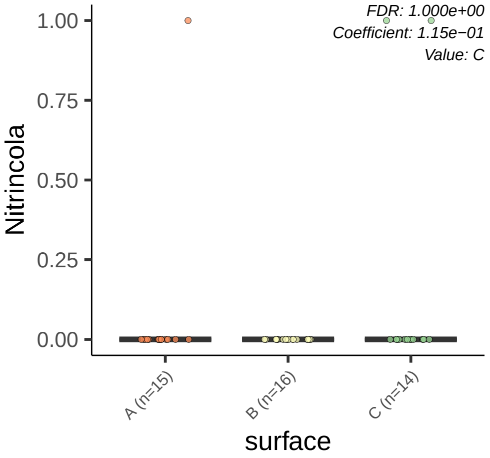
FDR: 1.000e+00
Coefficient: 1.15e−01
Value: C
Nitrincola
1.00
0.75
0.50
0.25
0.00
A (n=15)
B (n=16)
C (n=14)
surface

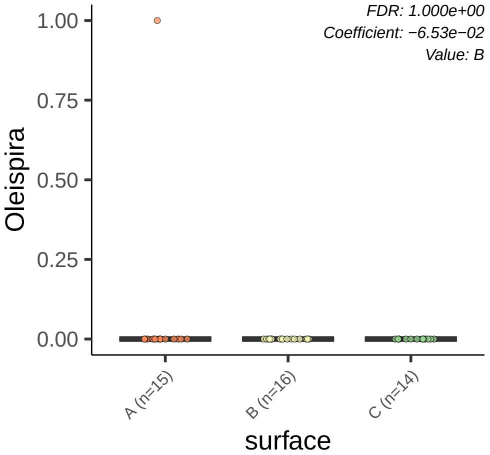
FDR: 1.000e+00
Coefficient: −6.53e−02
Value: B
Oleispira
1.00
0.75
0.50
0.25
0.00
A (n=15)
B (n=16)
C (n=14)
surface

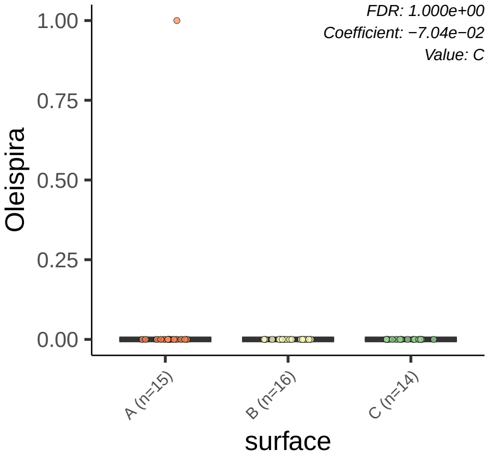
FDR: 1.000e+00
Coefficient: −7.04e−02
Value: C
Oleispira
1.00
0.75
0.50
0.25
0.00
A (n=15)
B (n=16)
C (n=14)
surface

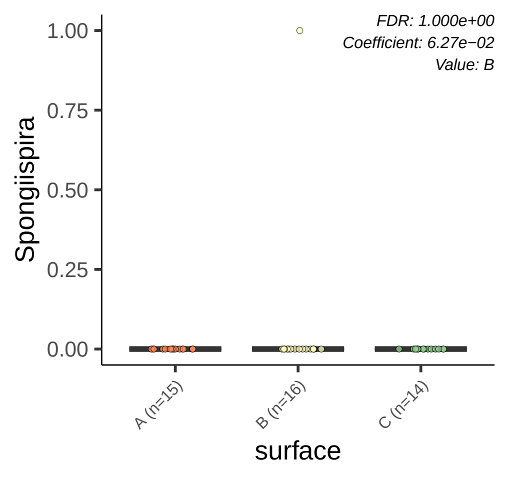

FDR: 1.000e+00
Coefficient: 6.27e−02
Value: B
Spongiispira
1.00
0.75
0.50
0.25
0.00
A (n=15)
B (n=16)
C (n=14)
surface

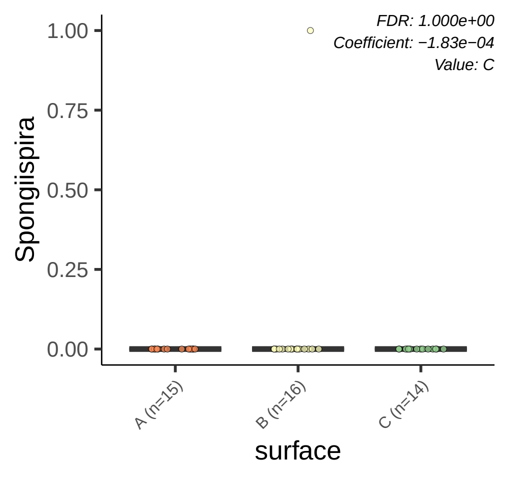
FDR: 1.000e+00
Coefficient: −1.83e−04
Value: C
Spongiispira
1.00
0.75
0.50
0.25
0.00
A (n=15)
B (n=16)
C (n=14)
surface

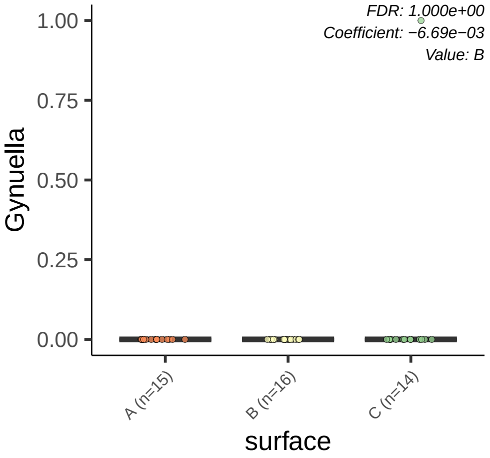
FDR: 1.000e+00
Coefficient: −6.69e−03
Value: B
Gynuella
1.00
0.75
0.50
0.25
0.00
A (n=15)
B (n=16)
C (n=14)
surface

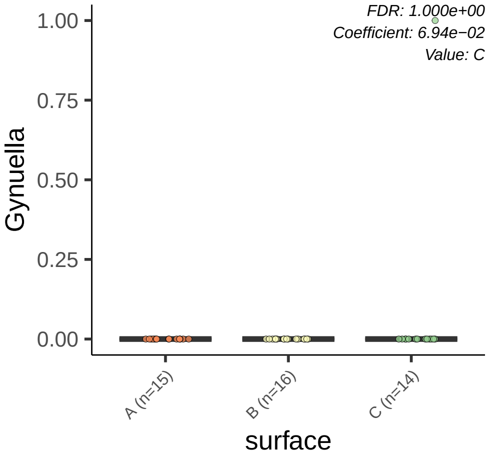

FDR: 1.000e+00
Coefficient: 6.94e−02
Value: C
Gynuella
1.00
0.75
0.50
0.25
0.00
A (n=15)
B (n=16)
C (n=14)
surface

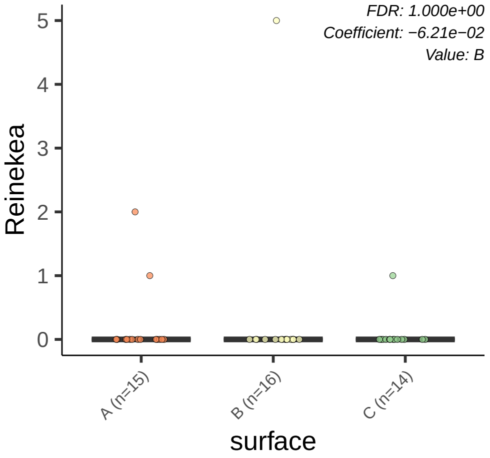
FDR: 1.000e+00
Coefficient: −6.21e−02
Value: B
Reinekea
5
4
3
2
1
0
A (n=15)
B (n=16)
C (n=14)
surface

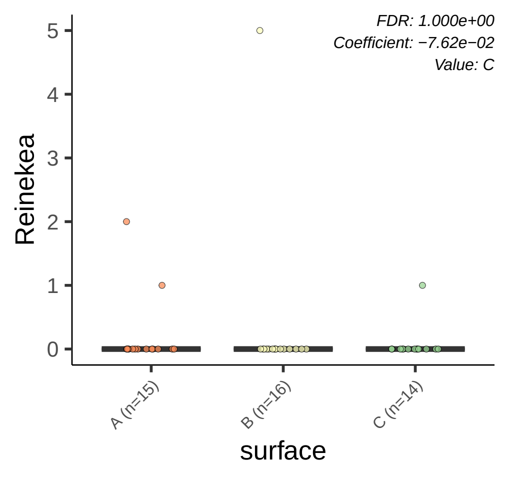

FDR: 1.000e+00
Coefficient: −7.62e−02
Value: C
Reinekea
5
4
3
2
1
0
A (n=15)
B (n=16)
C (n=14)
surface

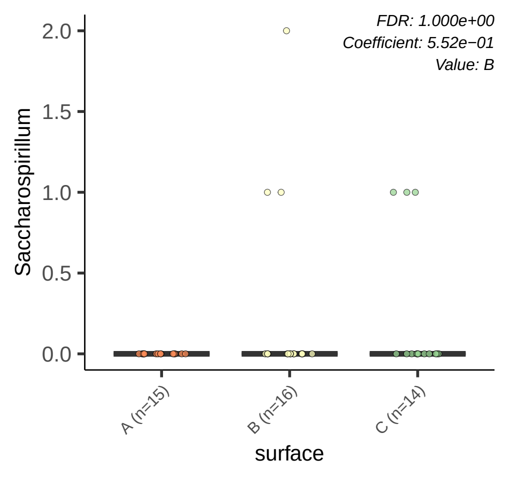
FDR: 1.000e+00
Coefficient: 5.52e−01
Value: B
Saccharospirillum
2.0
1.5
1.0
0.5
0.0
A (n=15)
B (n=16)
C (n=14)
surface

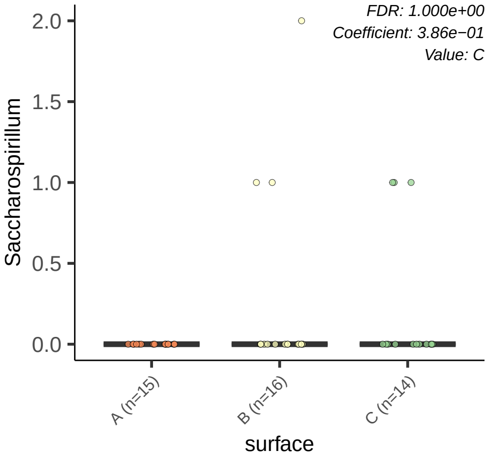

FDR: 1.000e+00
Coefficient: 3.86e−01
Value: C
Saccharospirillum
2.0
1.5
1.0
0.5
0.0
A (n=15)
B (n=16)
C (n=14)
surface

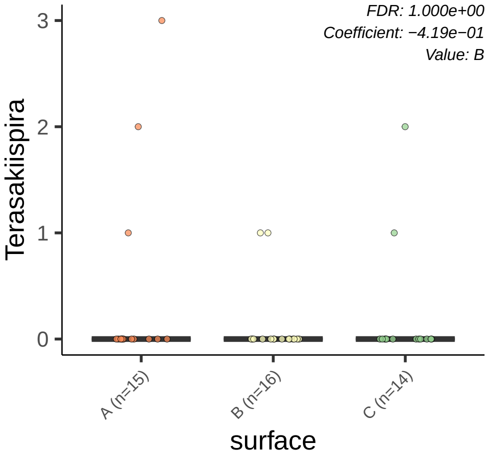
FDR: 1.000e+00
Coefficient: −4.19e−01
Value: B
Terasakiispira
3
2
1
0
A (n=15)
B (n=16)
C (n=14)
surface

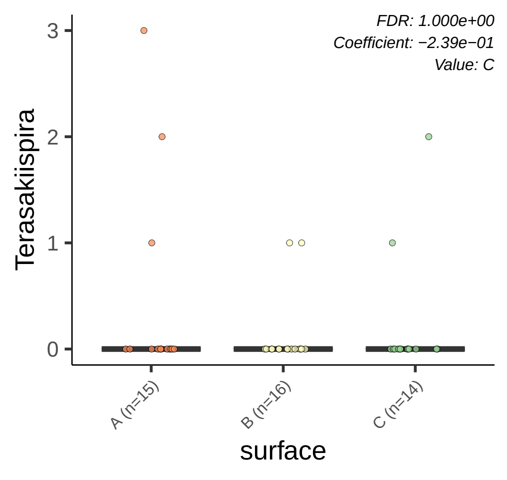
FDR: 1.000e+00
Coefficient: −2.39e−01
Value: C
Terasakiispira
3
2
1
0
A (n=15)
B (n=16)
C (n=14)
surface

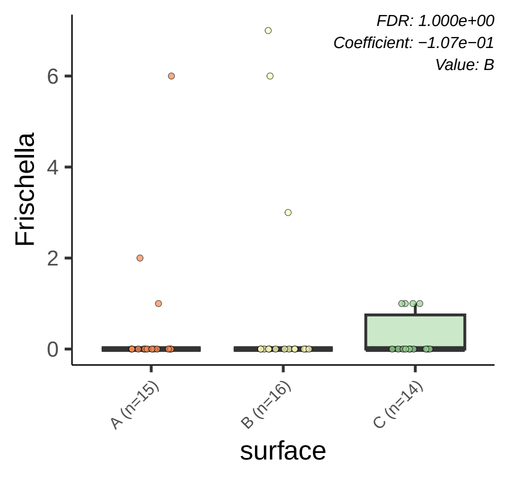
FDR: 1.000e+00
Coefficient: −1.07e−01
Value: B
Frischella
0
2
4
6
A (n=15)
B (n=16)
C (n=14)
surface

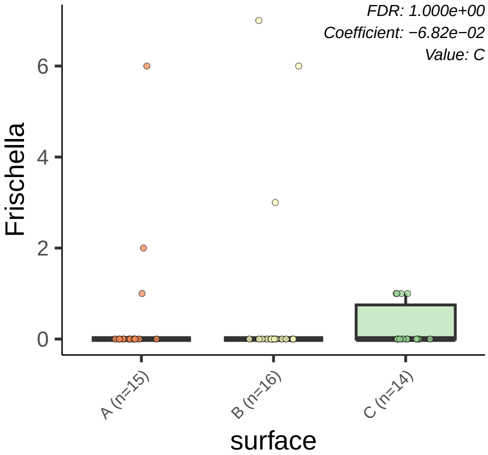

FDR: 1.000e+00
Coefficient: −6.82e−02
Value: C
Frischella
0
2
4
6
A (n=15)
B (n=16)
C (n=14)
surface

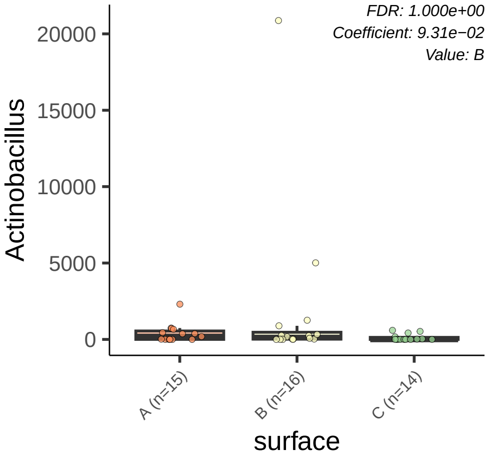

FDR: 1.000e+00
Coefficient: 9.31e−02
Value: B
Actinobacillus
20000
15000
10000
5000
0
A (n=15)
B (n=16)
C (n=14)
surface

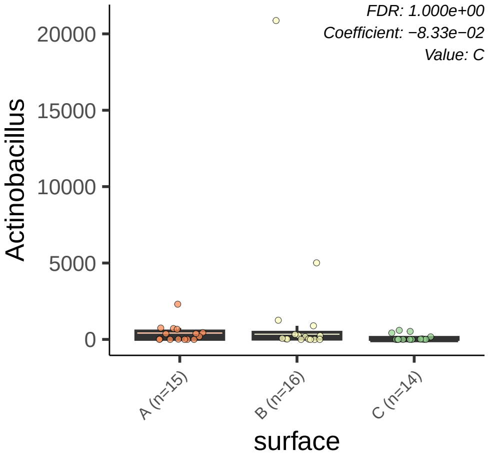
FDR: 1.000e+00
Coefficient: −8.33e−02
Value: C
Actinobacillus
20000
15000
10000
5000
0
A (n=15)
B (n=16)
C (n=14)
surface

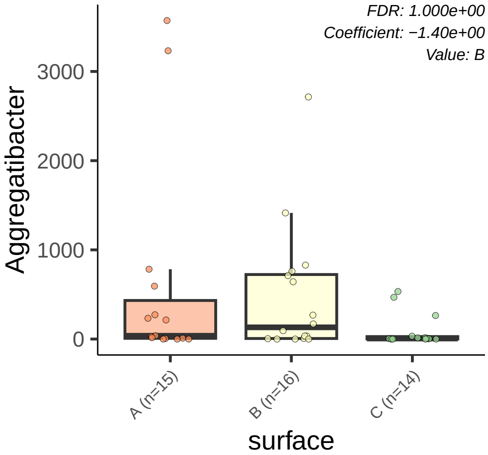
FDR: 1.000e+00
Coefficient: −1.40e+00
Value: B
Aggregatibacter
3000
2000
1000
0
A (n=15)
B (n=16)
C (n=14)
surface

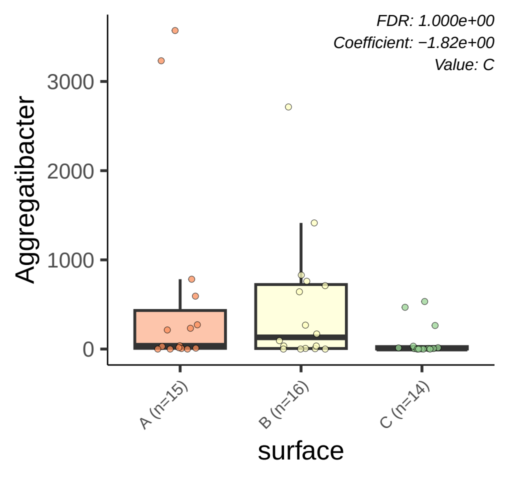

FDR: 1.000e+00
Coefficient: −1.82e+00
Value: C
Aggregatibacter
3000
2000
1000
0
A (n=15)
B (n=16)
C (n=14)
surface

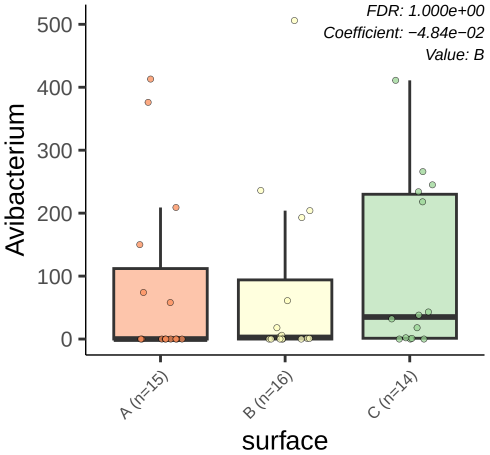
FDR: 1.000e+00
Coefficient: −4.84e−02
Value: B
Avibacterium
500
400
300
200
100
0
A (n=15)
B (n=16)
C (n=14)
surface

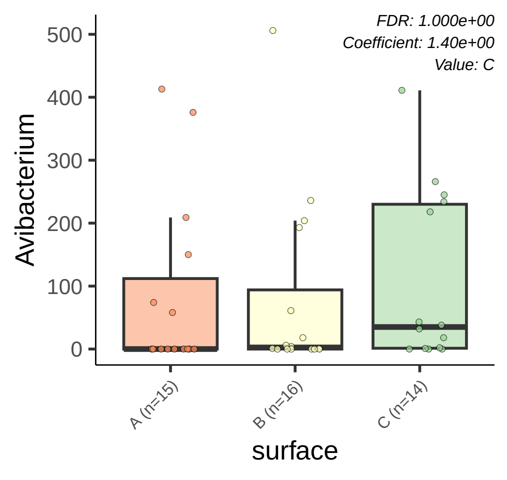

FDR: 1.000e+00
Coefficient: 1.40e+00
Value: C
Avibacterium
0
100
200
300
400
500
A (n=15)
B (n=16)
C (n=14)
surface

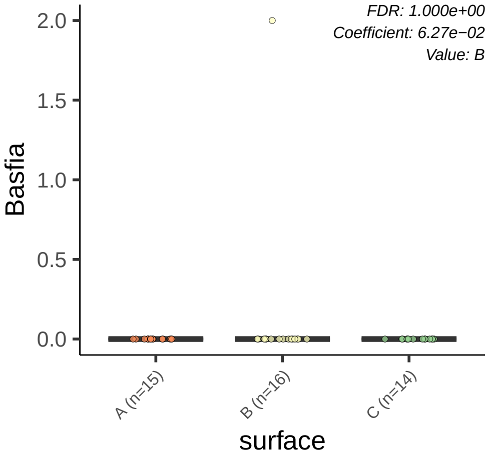
FDR: 1.000e+00
Coefficient: 6.27e−02
Value: B
Basfia
2.0
1.5
1.0
0.5
0.0
A (n=15)
B (n=16)
C (n=14)
surface

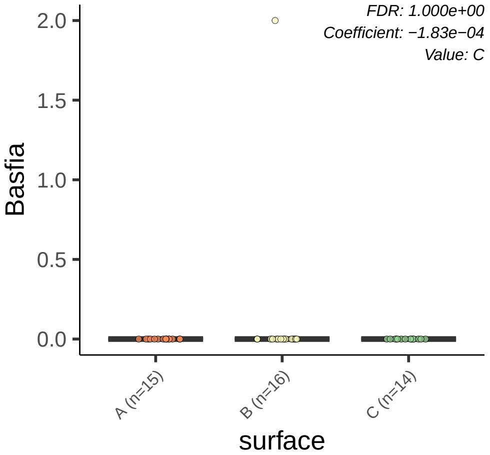
FDR: 1.000e+00
Coefficient: −1.83e−04
Value: C
Basfia
2.0
1.5
1.0
0.5
0.0
A (n=15)
B (n=16)
C (n=14)
surface

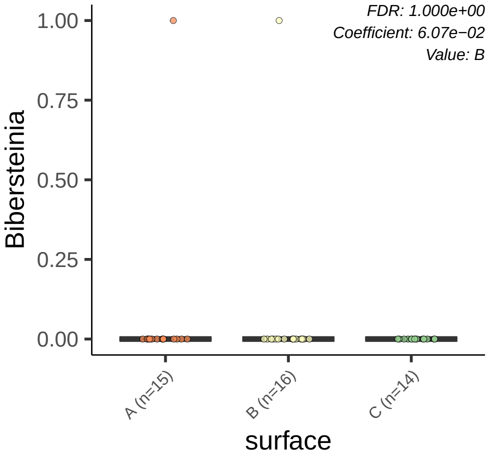
FDR: 1.000e+00
Coefficient: 6.07e−02
Value: B
Bibersteinia
1.00
0.75
0.50
0.25
0.00
A (n=15)
B (n=16)
C (n=14)
surface

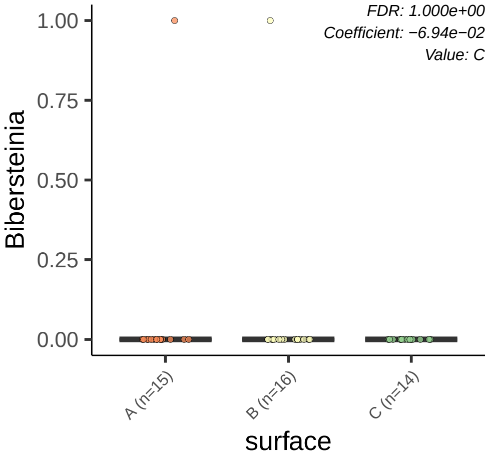
FDR: 1.000e+00
Coefficient: −6.94e−02
Value: C
Bibersteinia
1.00
0.75
0.50
0.25
0.00
A (n=15)
B (n=16)
C (n=14)
surface

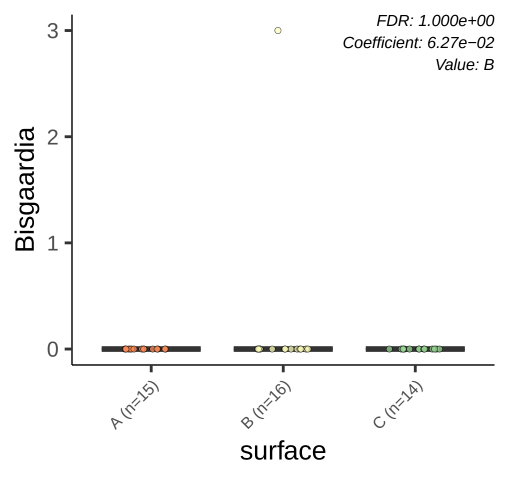

FDR: 1.000e+00
Coefficient: 6.27e−02
Value: B
Bisgaardia
3
2
1
0
A (n=15)
B (n=16)
C (n=14)
surface

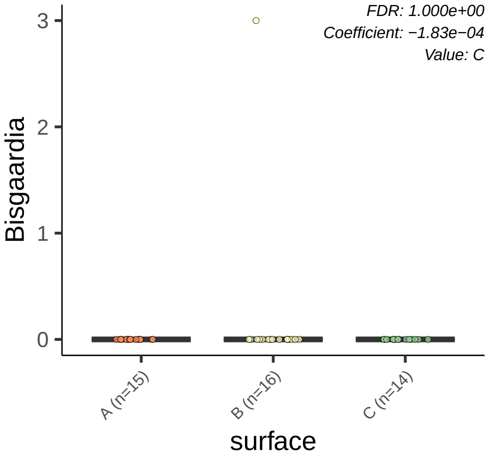
FDR: 1.000e+00
Coefficient: −1.83e−04
Value: C
Bisgaardia
3
2
1
0
A (n=15)
B (n=16)
C (n=14)
surface

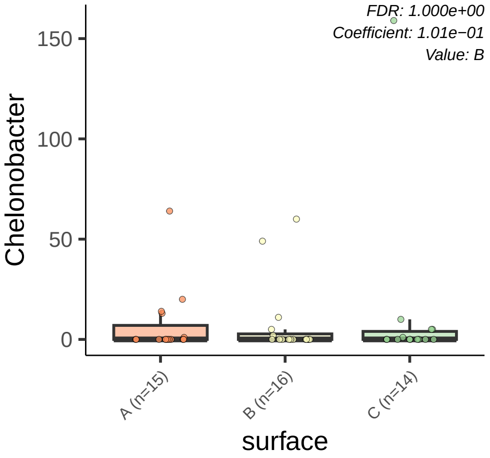
FDR: 1.000e+00
Coefficient: 1.01e−01
Value: B
Chelonobacter
150
100
50
0
A (n=15)
B (n=16)
C (n=14)
surface

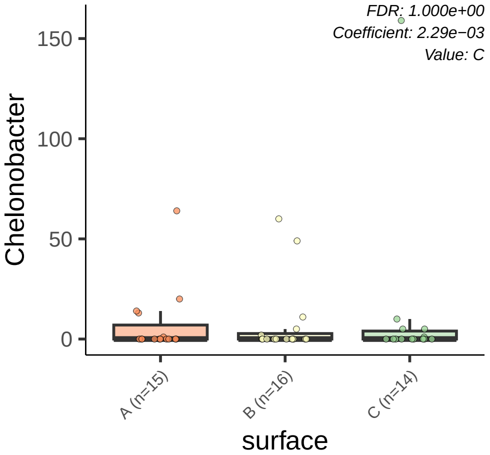
FDR: 1.000e+00
Coefficient: 2.29e−03
Value: C
Chelonobacter
150
100
50
0
A (n=15)
B (n=16)
C (n=14)
surface

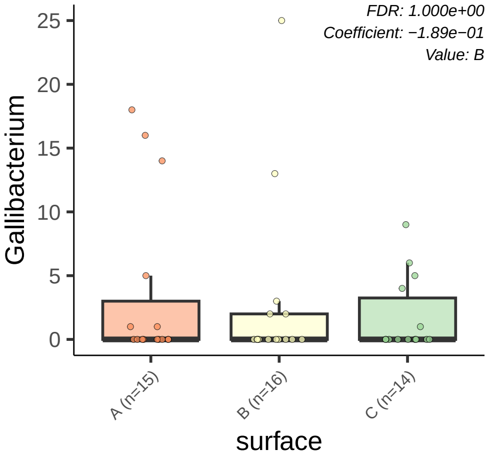

FDR: 1.000e+00
Coefficient: −1.89e−01
Value: B
Gallibacterium
25
20
15
10
5
0
A (n=15)
B (n=16)
C (n=14)
surface

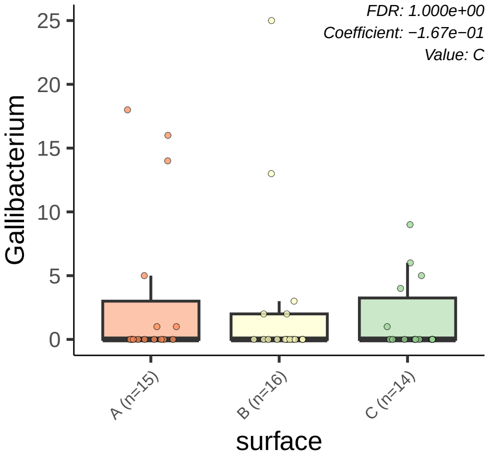
FDR: 1.000e+00
Coefficient: −1.67e−01
Value: C
Gallibacterium
0
5
10
15
20
25
A (n=15)
B (n=16)
C (n=14)
surface

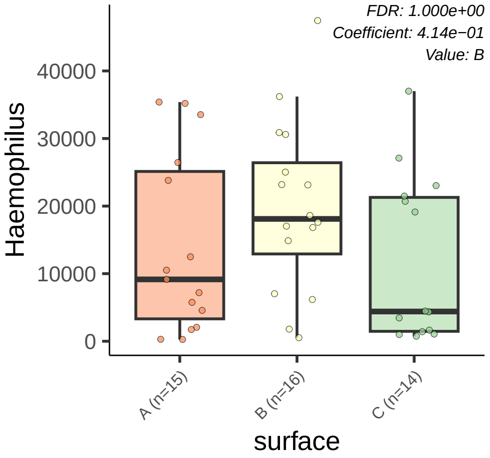
FDR: 1.000e+00
Coefficient: 4.14e−01
Value: B
Haemophilus
40000
30000
20000
10000
0
A (n=15)
B (n=16)
C (n=14)
surface

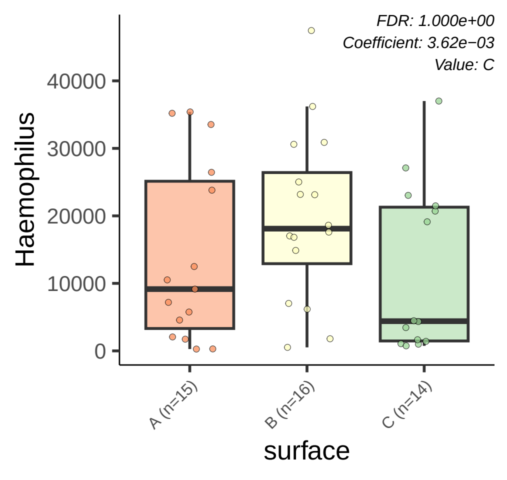

FDR: 1.000e+00
Coefficient: 3.62e−03
Value: C
Haemophilus
0
10000
20000
30000
40000
A (n=15)
B (n=16)
C (n=14)
surface

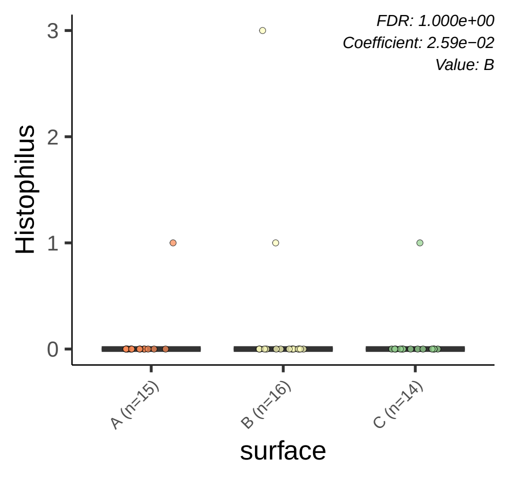
FDR: 1.000e+00
Coefficient: 2.59e−02
Value: B
Histophilus
0
1
2
3
A (n=15)
B (n=16)
C (n=14)
surface

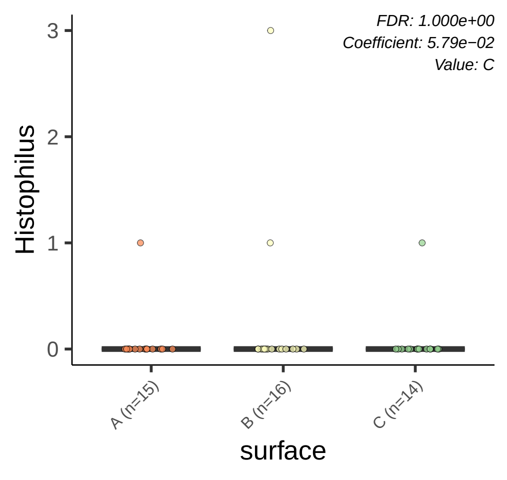

FDR: 1.000e+00
Coefficient: 5.79e−02
Value: C
Histophilus
3
2
1
0
A (n=15)
B (n=16)
C (n=14)
surface

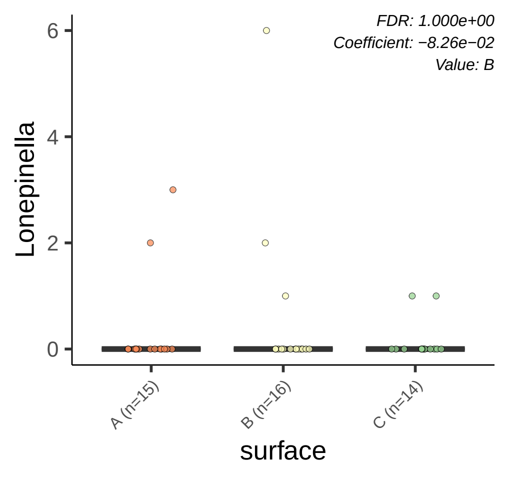

FDR: 1.000e+00
Coefficient: −8.26e−02
Value: B
Lonepinella
6
4
2
0
A (n=15)
B (n=16)
C (n=14)
surface

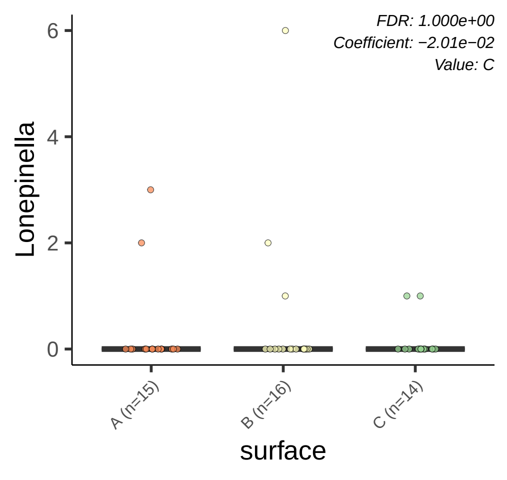

FDR: 1.000e+00
Coefficient: −2.01e−02
Value: C
Lonepinella
6
4
2
0
A (n=15)
B (n=16)
C (n=14)
surface

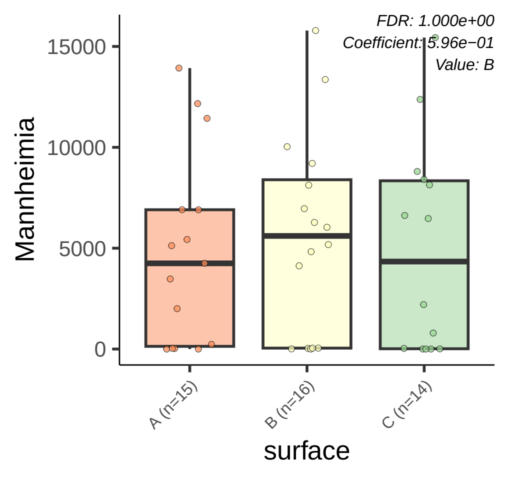
FDR: 1.000e+00
Coefficient: 5.96e−01
Value: B
Mannheimia
15000
10000
5000
0
A (n=15)
B (n=16)
C (n=14)
surface

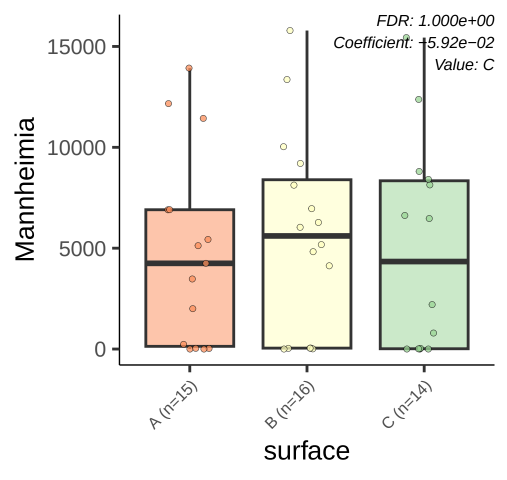

FDR: 1.000e+00
Coefficient: −5.92e−02
Value: C
Mannheimia
15000
10000
5000
0
A (n=15)
B (n=16)
C (n=14)
surface

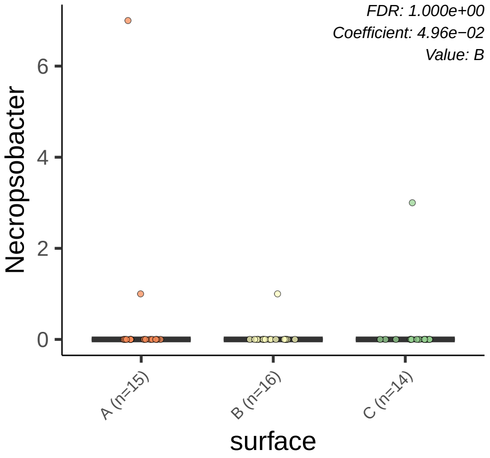
FDR: 1.000e+00
Coefficient: 4.96e−02
Value: B
Necropsobacter
0
2
4
6
A (n=15)
B (n=16)
C (n=14)
surface

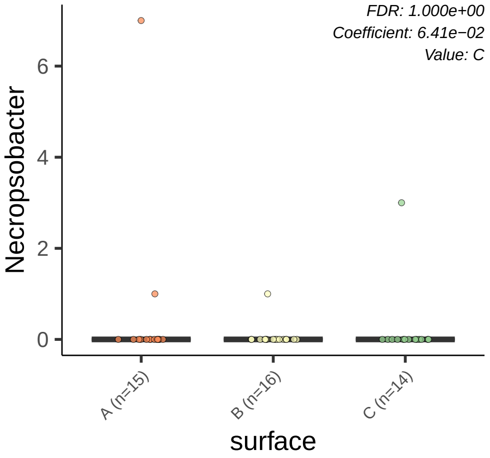
FDR: 1.000e+00
Coefficient: 6.41e−02
Value: C
Necropsobacter
0
2
4
6
A (n=15)
B (n=16)
C (n=14)
surface

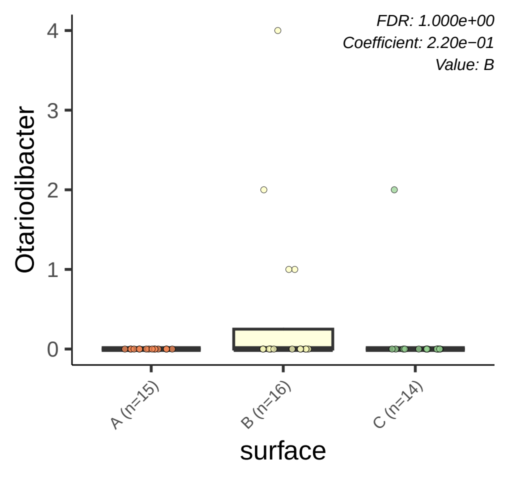

FDR: 1.000e+00
Coefficient: 2.20e−01
Value: B
Otariodibacter
0
1
2
3
4
A (n=15)
B (n=16)
C (n=14)
surface

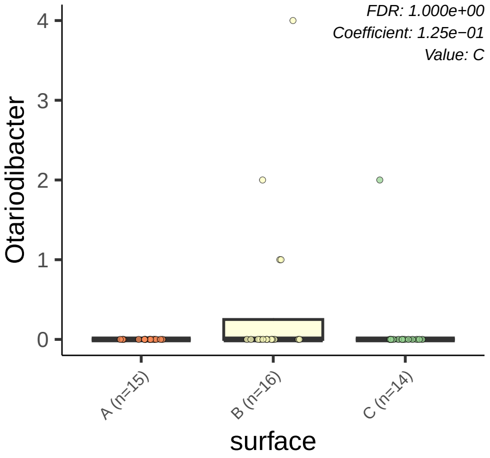

FDR: 1.000e+00
Coefficient: 1.25e−01
Value: C
Otariodibacter
0
1
2
3
4
A (n=15)
B (n=16)
C (n=14)
surface

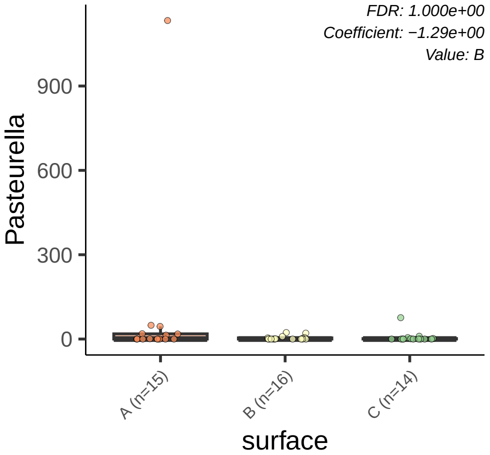

FDR: 1.000e+00
Coefficient: −1.29e+00
Value: B
Pasteurella
900
600
300
0
A (n=15)
B (n=16)
C (n=14)
surface

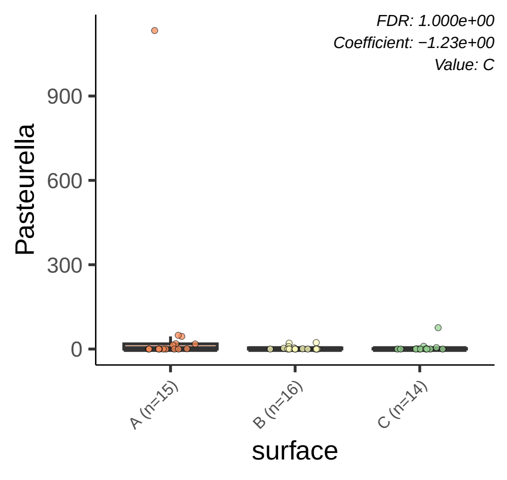
FDR: 1.000e+00
Coefficient: −1.23e+00
Value: C
Pasteurella
900
600
300
0
A (n=15)
B (n=16)
C (n=14)
surface

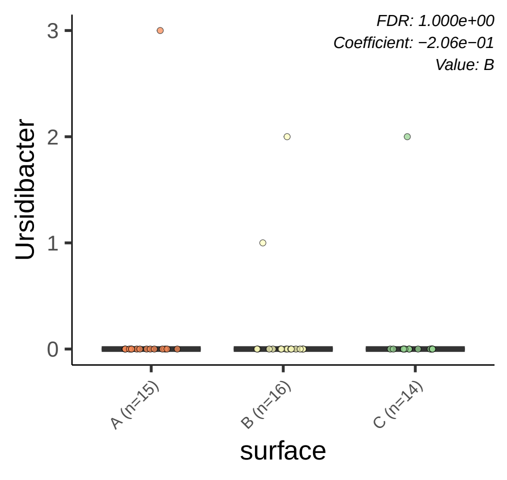

FDR: 1.000e+00
Coefficient: −2.06e−01
Value: B
Ursidibacter
0
1
2
3
A (n=15)
B (n=16)
C (n=14)
surface

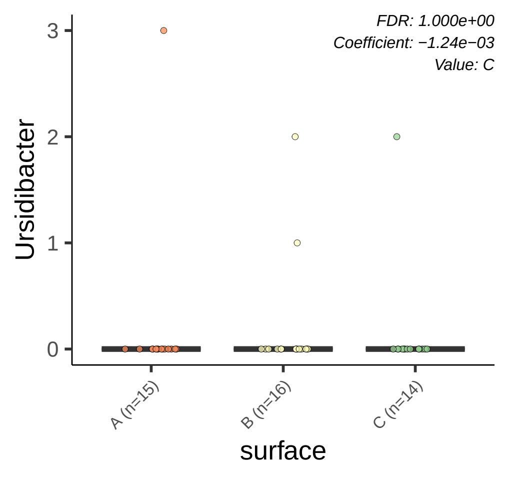
FDR: 1.000e+00
Coefficient: −1.24e−03
Value: C
Ursidibacter
3
2
1
0
A (n=15)
B (n=16)
C (n=14)
surface

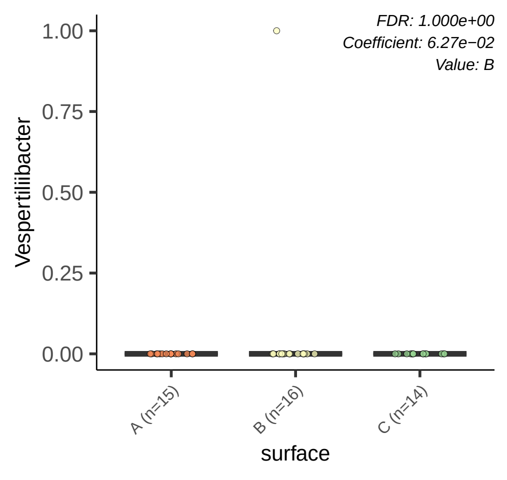

FDR: 1.000e+00
Coefficient: 6.27e−02
Value: B
Vespertiliibacter
1.00
0.75
0.50
0.25
0.00
A (n=15)
B (n=16)
C (n=14)
surface

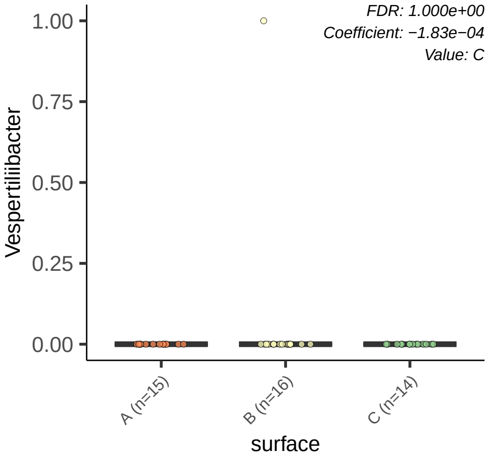

FDR: 1.000e+00
Coefficient: −1.83e−04
Value: C
Vespertiliibacter
1.00
0.75
0.50
0.25
0.00
A (n=15)
B (n=16)
C (n=14)
surface

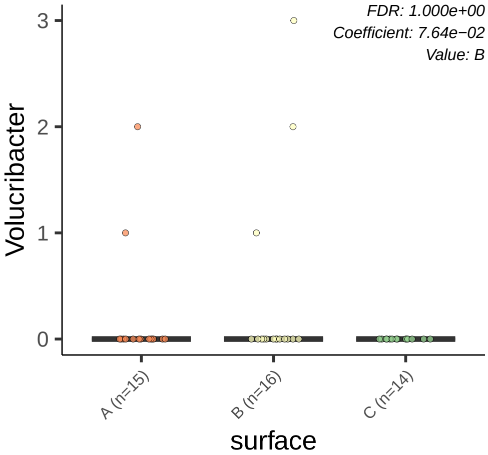

FDR: 1.000e+00
Coefficient: 7.64e−02
Value: B
Volucribacter
3
2
1
0
A (n=15)
B (n=16)
C (n=14)
surface

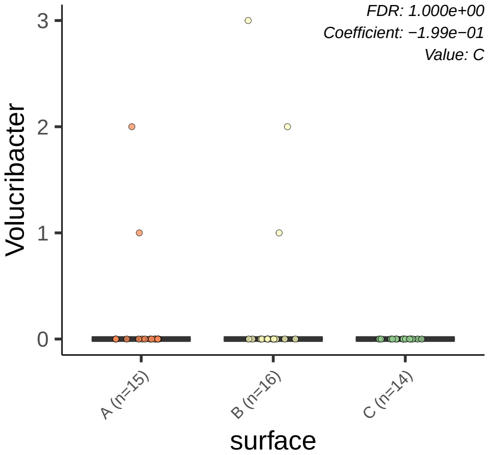
FDR: 1.000e+00
Coefficient: −1.99e−01
Value: C
Volucribacter
3
2
1
0
A (n=15)
B (n=16)
C (n=14)
surface

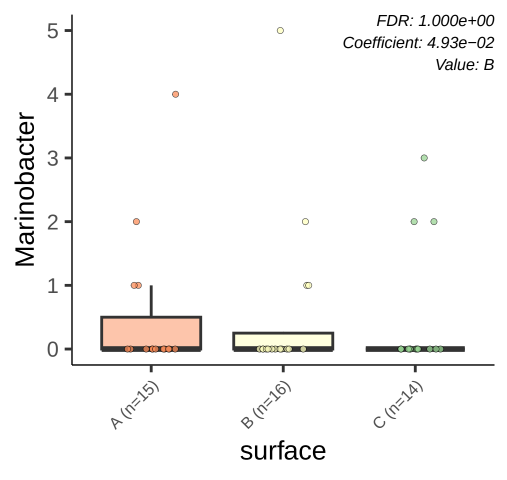
FDR: 1.000e+00
Coefficient: 4.93e−02
Value: B
Marinobacter
0
1
2
3
4
5
A (n=15)
B (n=16)
C (n=14)
surface

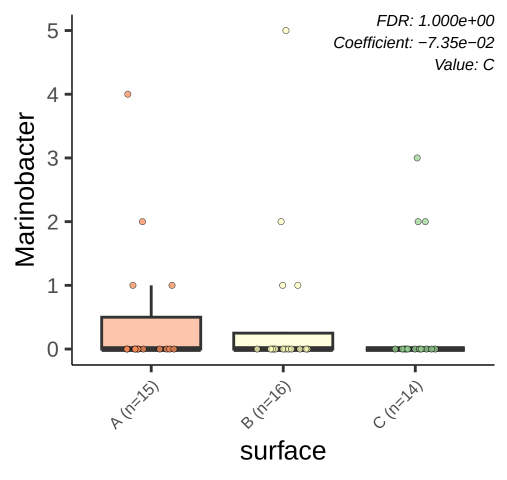

FDR: 1.000e+00
Coefficient: −7.35e−02
Value: C
Marinobacter
0
1
2
3
4
5
A (n=15)
B (n=16)
C (n=14)
surface

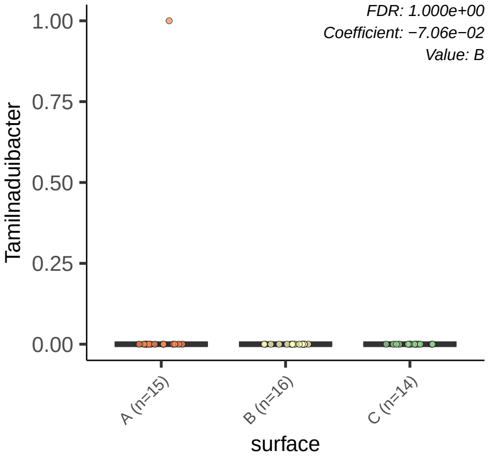

FDR: 1.000e+00
Coefficient: −7.06e−02
Value: B
Tamilnaduibacter
1.00
0.75
0.50
0.25
0.00
A (n=15)
B (n=16)
C (n=14)
surface

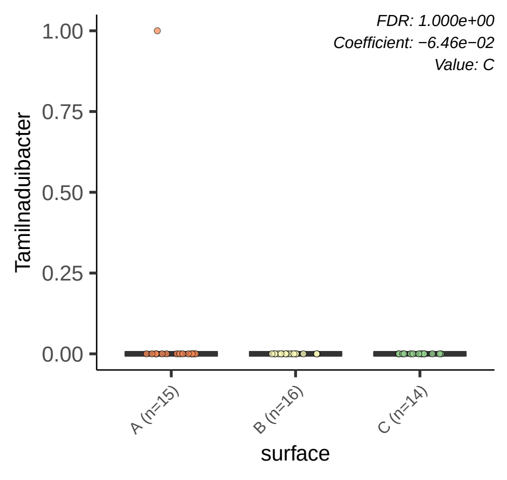
FDR: 1.000e+00
Coefficient: −6.46e−02
Value: C
Tamilnaduibacter
1.00
0.75
0.50
0.25
0.00
A (n=15)
B (n=16)
C (n=14)
surface

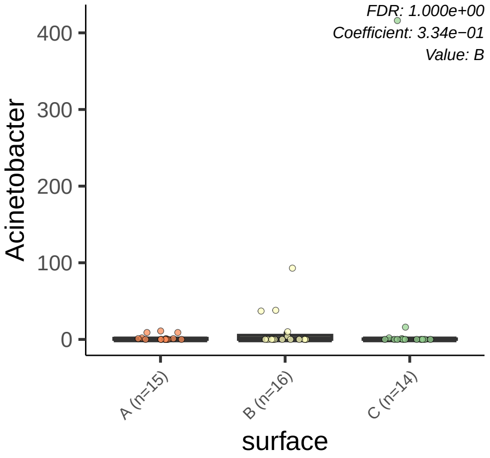

FDR: 1.000e+00
Coefficient: 3.34e−01
Value: B
Acinetobacter
400
300
200
100
0
A (n=15)
B (n=16)
C (n=14)
surface

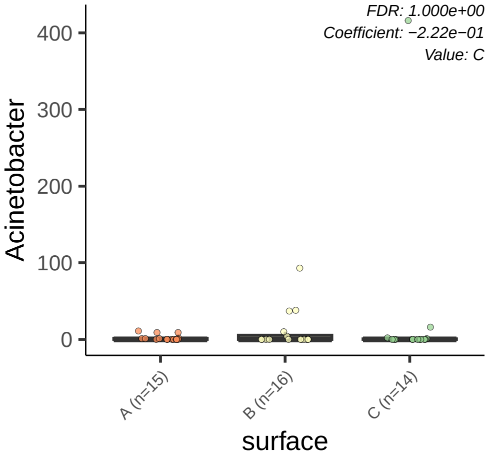
FDR: 1.000e+00
Coefficient: −2.22e−01
Value: C
Acinetobacter
400
300
200
100
0
A (n=15)
B (n=16)
C (n=14)
surface

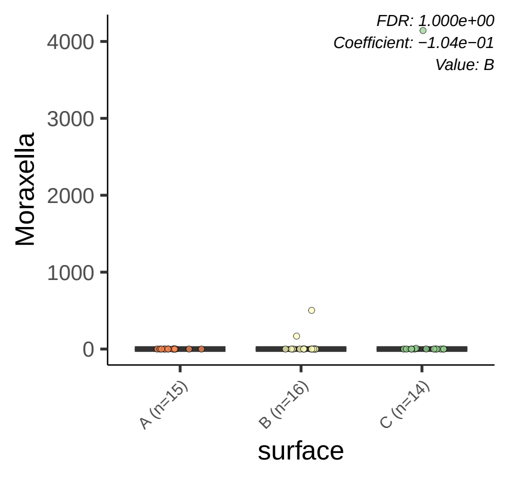
FDR: 1.000e+00
Coefficient: −1.04e−01
Value: B
4000
3000
2000
1000
0
Moraxella
A (n=15)
B (n=16)
C (n=14)
surface

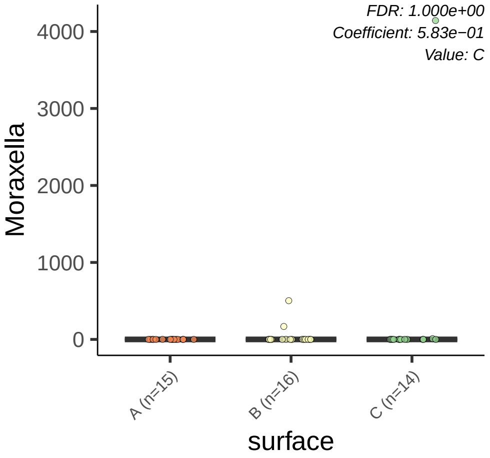

FDR: 1.000e+00
Coefficient: 5.83e−01
Value: C
4000
3000
2000
1000
0
Moraxella
A (n=15)
B (n=16)
C (n=14)
surface

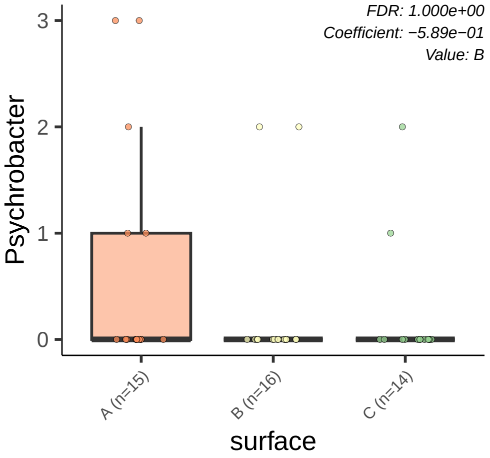
FDR: 1.000e+00
Coefficient: −5.89e−01
Value: B
Psychrobacter
3
2
1
0
A (n=15)
B (n=16)
C (n=14)
surface

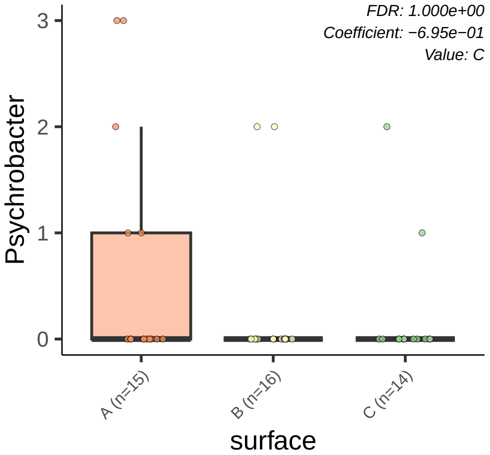

FDR: 1.000e+00
Coefficient: −6.95e−01
Value: C
Psychrobacter
3
2
1
0
A (n=15)
B (n=16)
C (n=14)
surface

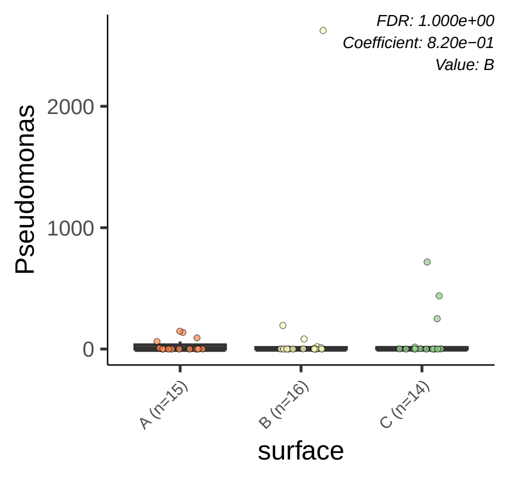
FDR: 1.000e+00
Coefficient: 8.20e−01
Value: B
Pseudomonas
2000
1000
0
A (n=15)
B (n=16)
C (n=14)
surface

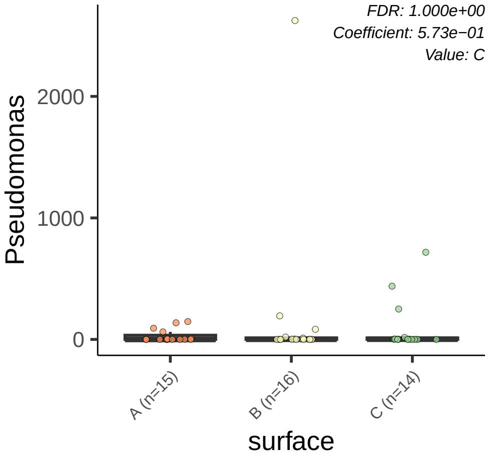

FDR: 1.000e+00
Coefficient: 5.73e−01
Value: C
Pseudomonas
2000
1000
0
A (n=15)
B (n=16)
C (n=14)
surface

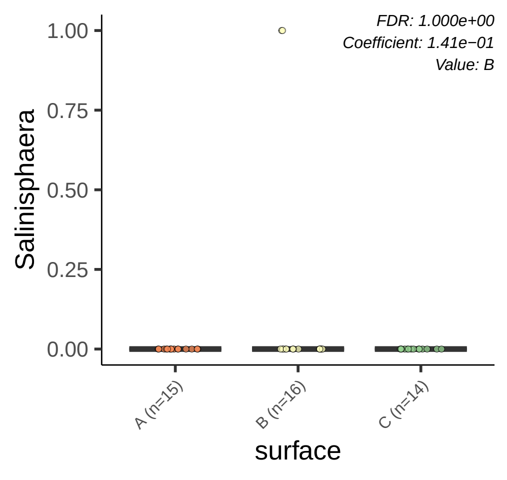
FDR: 1.000e+00
Coefficient: 1.41e−01
Value: B
Salinisphaera
1.00
0.75
0.50
0.25
0.00
A (n=15)
B (n=16)
C (n=14)
surface

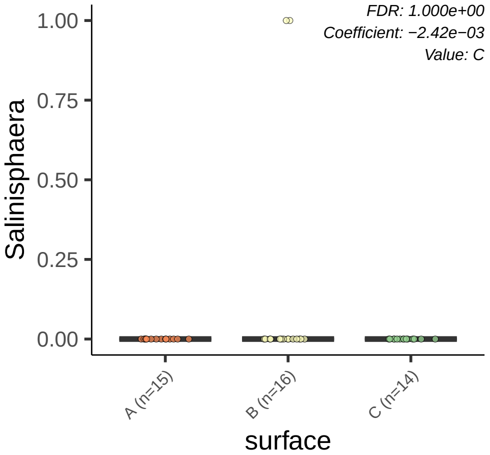
FDR: 1.000e+00
Coefficient: −2.42e−03
Value: C
Salinisphaera
1.00
0.75
0.50
0.25
0.00
A (n=15)
B (n=16)
C (n=14)
surface

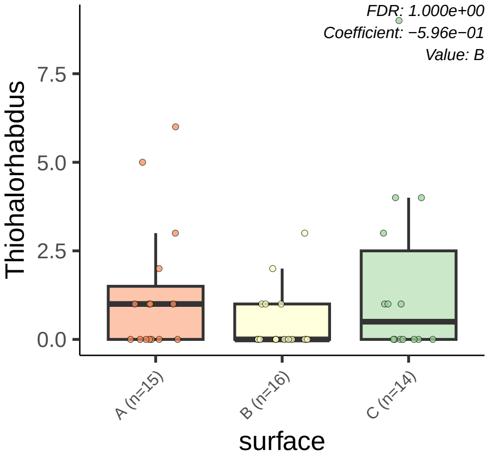
FDR: 1.000e+00
Coefficient: −5.96e−01
Value: B
Thiohalorhabdus
7.5
5.0
2.5
0.0
A (n=15)
B (n=16)
C (n=14)
surface

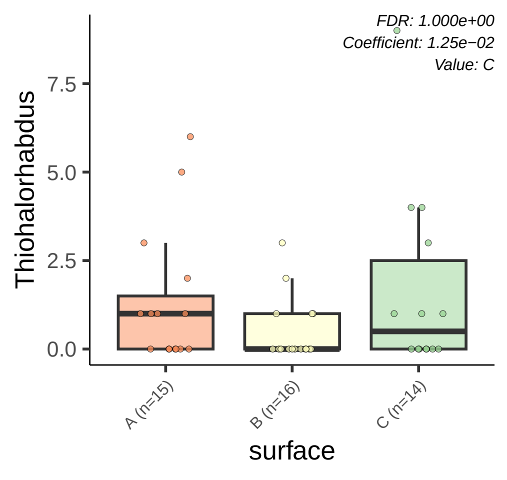
FDR: 1.000e+00
Coefficient: 1.25e−02
Value: C
Thiohalorhabdus
7.5
5.0
2.5
0.0
A (n=15)
B (n=16)
C (n=14)
surface

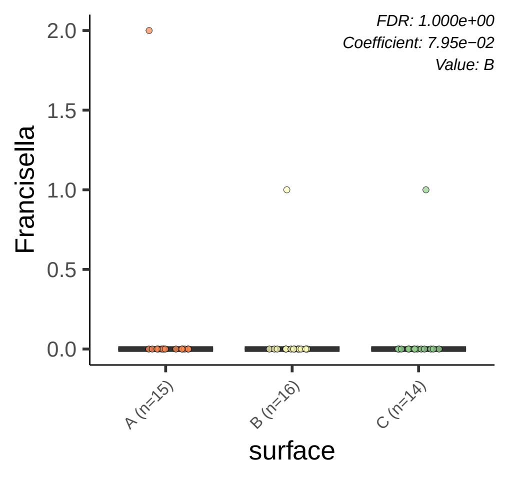

FDR: 1.000e+00
Coefficient: 7.95e−02
Value: B
Francisella
2.0
1.5
1.0
0.5
0.0
A (n=15)
B (n=16)
C (n=14)
surface

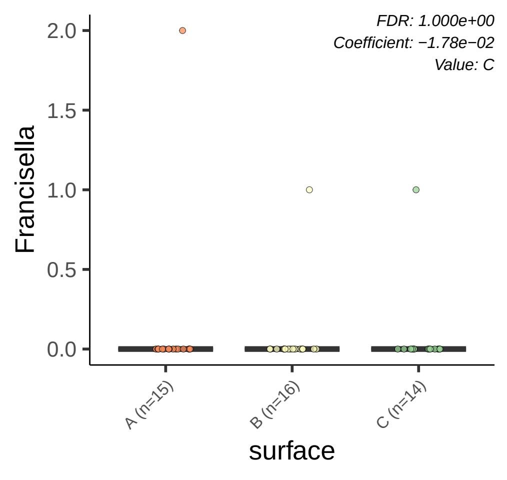
FDR: 1.000e+00
Coefficient: −1.78e−02
Value: C
Francisella
2.0
1.5
1.0
0.5
0.0
A (n=15)
B (n=16)
C (n=14)
surface

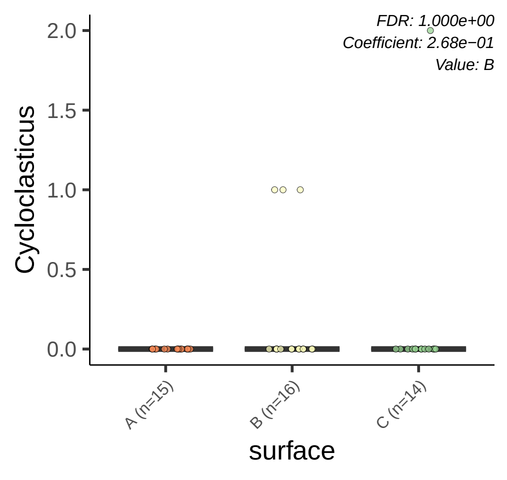
FDR: 1.000e+00
Coefficient: 2.68e−01
Value: B
Cycloclasticus
2.0
1.5
1.0
0.5
0.0
A (n=15)
B (n=16)
C (n=14)
surface

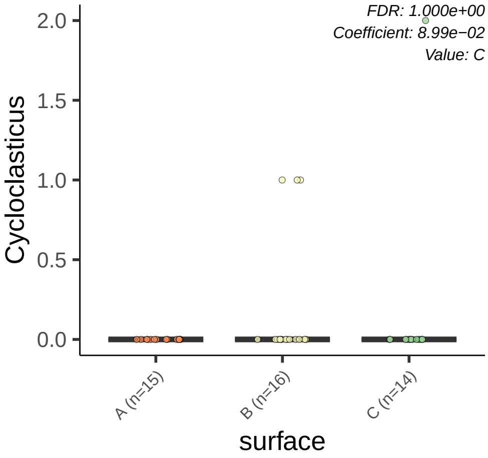
FDR: 1.000e+00
Coefficient: 8.99e−02
Value: C
Cycloclasticus
2.0
1.5
1.0
0.5
0.0
A (n=15)
B (n=16)
C (n=14)
surface

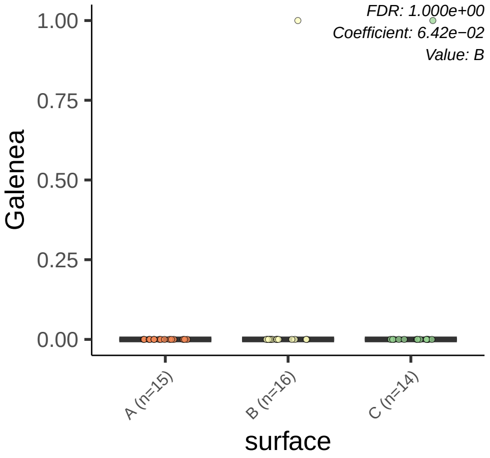
FDR: 1.000e+00
Coefficient: 6.42e−02
Value: B
Galenea
1.00
0.75
0.50
0.25
0.00
A (n=15)
B (n=16)
C (n=14)
surface

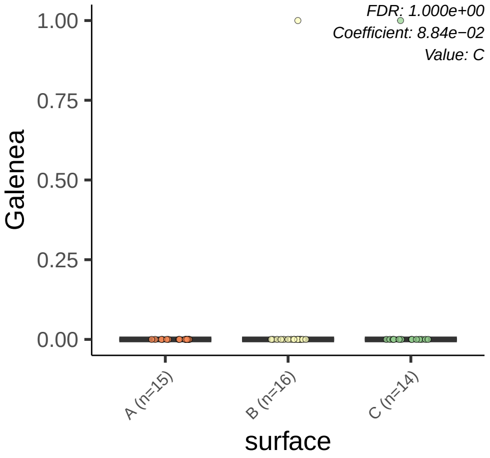
FDR: 1.000e+00
Coefficient: 8.84e−02
Value: C
1.00
0.75
0.50
0.25
0.00
Galenea
A (n=15)
B (n=16)
C (n=14)
surface

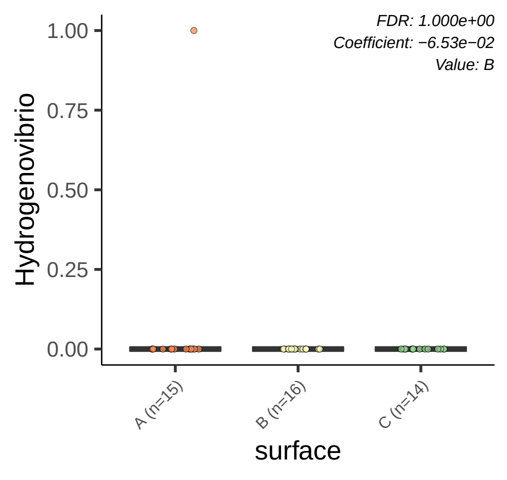
FDR: 1.000e+00
Coefficient: −6.53e−02
Value: B
Hydrogenovibrio
1.00
0.75
0.50
0.25
0.00
A (n=15)
B (n=16)
C (n=14)
surface

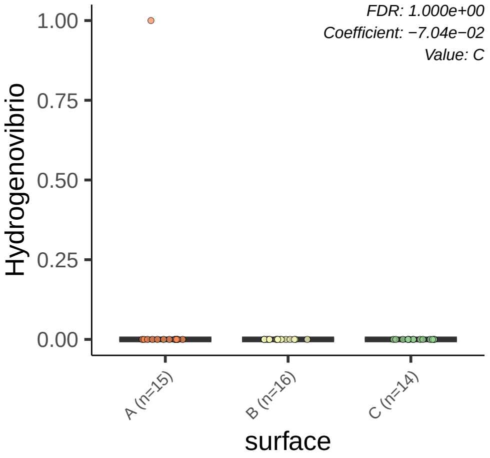
FDR: 1.000e+00
Coefficient: −7.04e−02
Value: C
Hydrogenovibrio
1.00
0.75
0.50
0.25
0.00
A (n=15)
B (n=16)
C (n=14)
surface

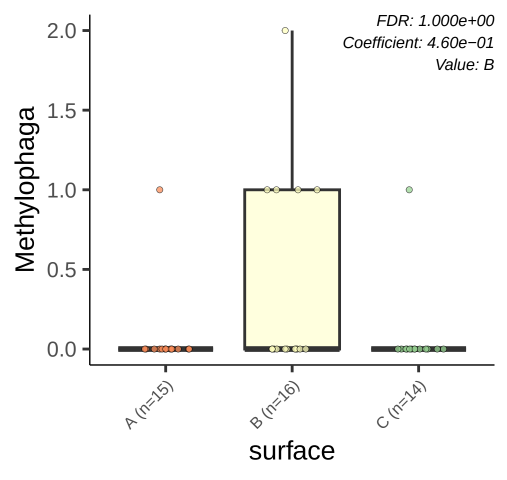
FDR: 1.000e+00
Coefficient: 4.60e−01
Value: B
Methylophaga
2.0
1.5
1.0
0.5
0.0
A (n=15)
B (n=16)
C (n=14)
surface

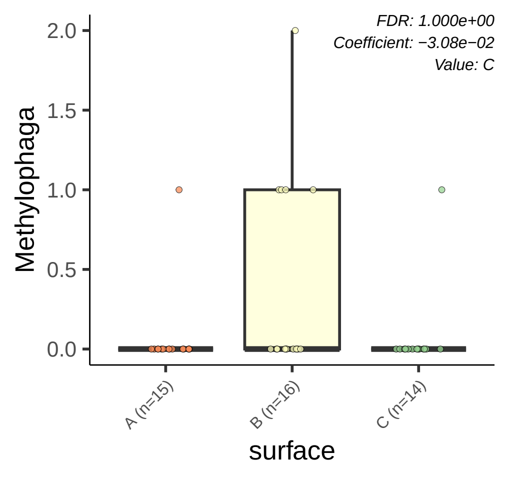
FDR: 1.000e+00
Coefficient: −3.08e−02
Value: C
Methylophaga
2.0
1.5
1.0
0.5
0.0
A (n=15)
B (n=16)
C (n=14)
surface

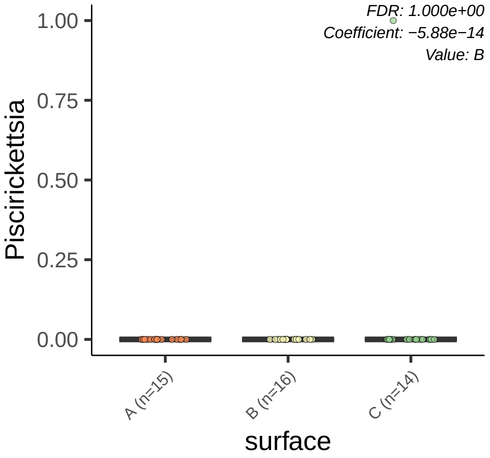
FDR: 1.000e+00
Coefficient: −5.88e−14
Value: B
1.00
0.75
0.50
0.25
0.00
Piscirickettsia
A (n=15)
B (n=16)
C (n=14)
surface

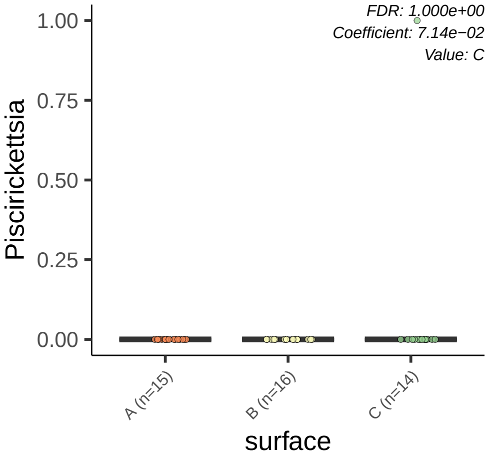
FDR: 1.000e+00
Coefficient: 7.14e−02
Value: C
Piscirickettsia
1.00
0.75
0.50
0.25
0.00
A (n=15)
B (n=16)
C (n=14)
surface

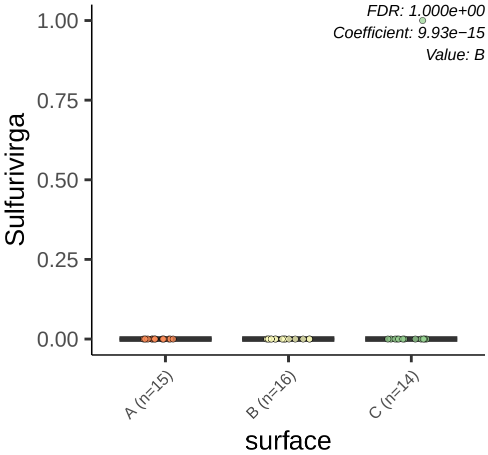

FDR: 1.000e+00
Coefficient: 9.93e−15
Value: B
Sulfurivirga
1.00
0.75
0.50
0.25
0.00
A (n=15)
B (n=16)
C (n=14)
surface

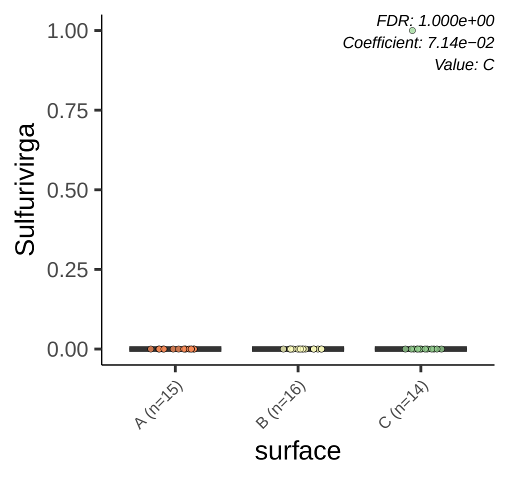
FDR: 1.000e+00
Coefficient: 7.14e−02
Value: C
Sulfurivirga
1.00
0.75
0.50
0.25
0.00
A (n=15)
B (n=16)
C (n=14)
surface

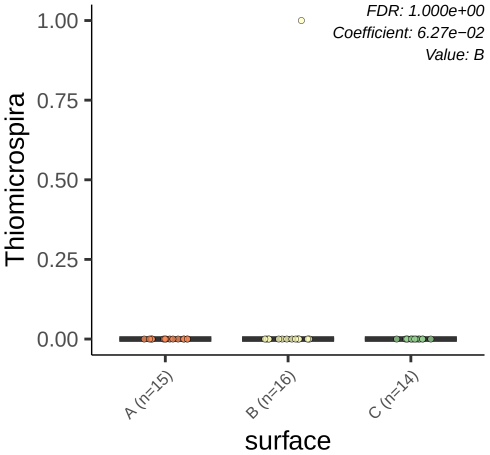
FDR: 1.000e+00
Coefficient: 6.27e−02
Value: B
Thiomicrospira
1.00
0.75
0.50
0.25
0.00
A (n=15)
B (n=16)
C (n=14)
surface

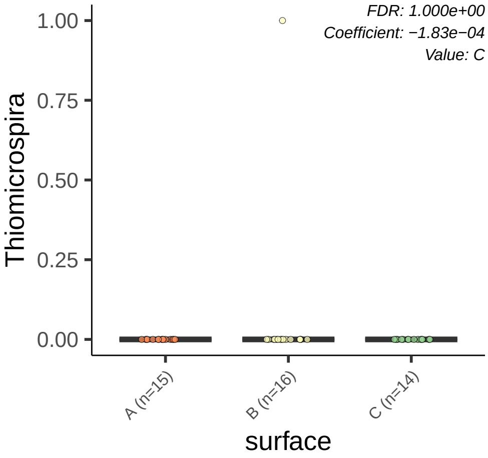

FDR: 1.000e+00
Coefficient: −1.83e−04
Value: C
Thiomicrospira
1.00
0.75
0.50
0.25
0.00
A (n=15)
B (n=16)
C (n=14)
surface

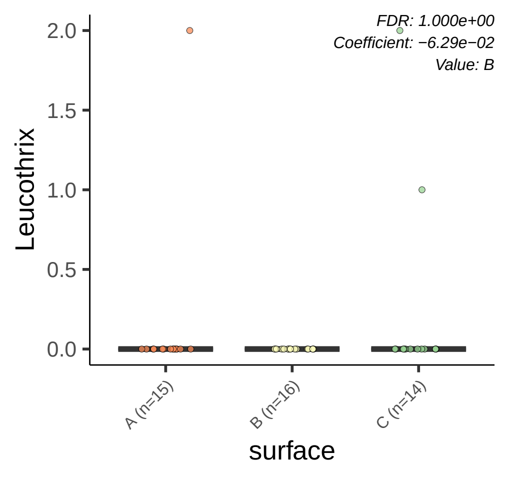
FDR: 1.000e+00
Coefficient: −6.29e−02
Value: B
Leucothrix
2.0
1.5
1.0
0.5
0.0
A (n=15)
B (n=16)
C (n=14)
surface

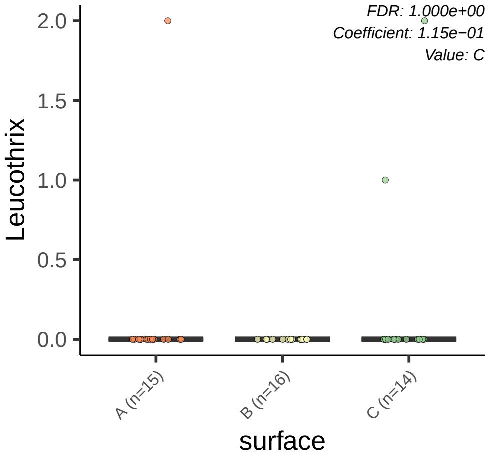
FDR: 1.000e+00
Coefficient: 1.15e−01
Value: C
Leucothrix
2.0
1.5
1.0
0.5
0.0
A (n=15)
B (n=16)
C (n=14)
surface

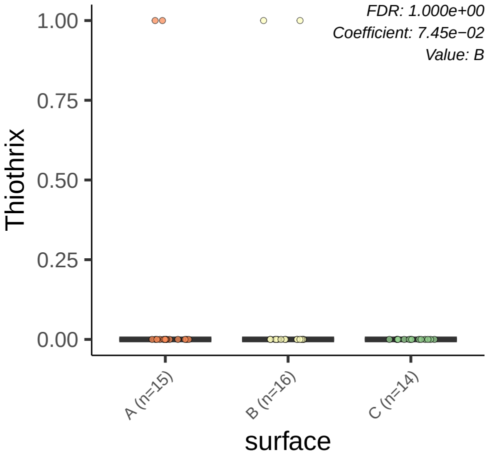

FDR: 1.000e+00
Coefficient: 7.45e−02
Value: B
1.00
0.75
0.50
0.25
0.00
Thiothrix
A (n=15)
B (n=16)
C (n=14)
surface

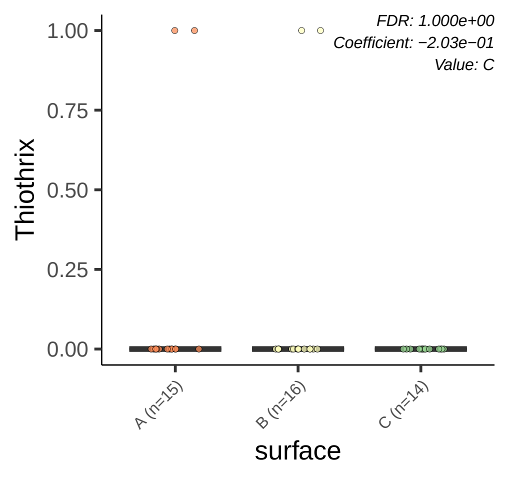

FDR: 1.000e+00
Coefficient: −2.03e−01
Value: C
1.00
0.75
0.50
0.25
0.00
Thiothrix
A (n=15)
B (n=16)
C (n=14)
surface

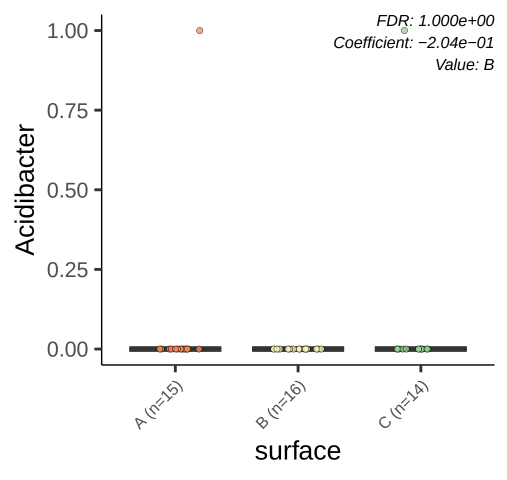

FDR: 1.000e+00
Coefficient: −2.04e−01
Value: B
Acidibacter
1.00
0.75
0.50
0.25
0.00
A (n=15)
B (n=16)
C (n=14)
surface

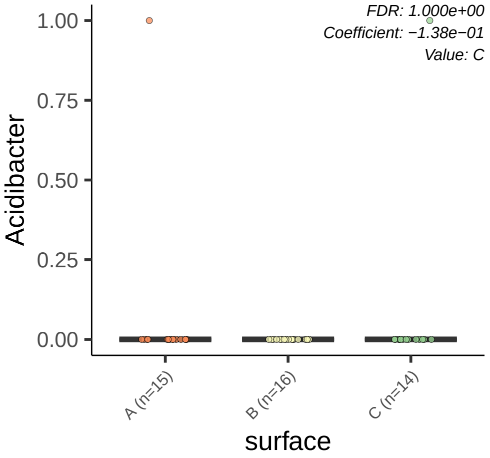

FDR: 1.000e+00
Coefficient: −1.38e−01
Value: C
Acidibacter
1.00
0.75
0.50
0.25
0.00
A (n=15)
B (n=16)
C (n=14)
surface

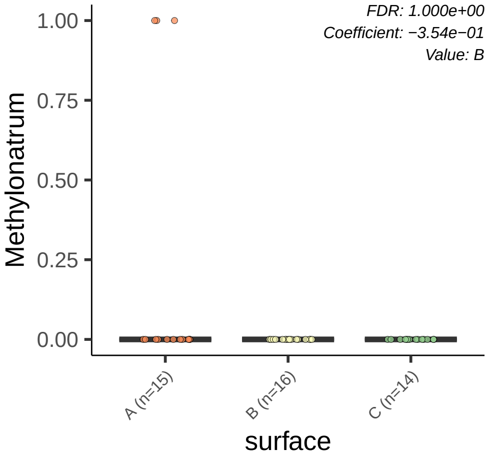
FDR: 1.000e+00
Coefficient: −3.54e−01
Value: B
Methylonatrum
1.00
0.75
0.50
0.25
0.00
A (n=15)
B (n=16)
C (n=14)
surface

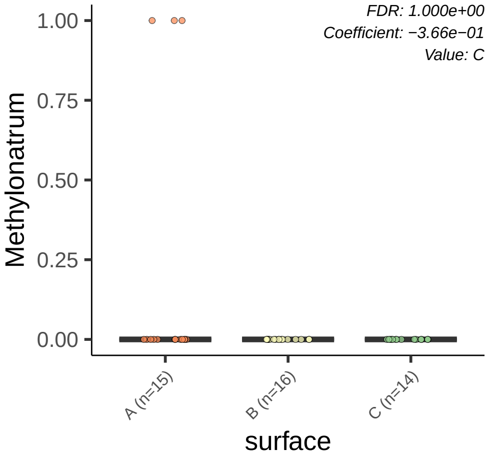
FDR: 1.000e+00
Coefficient: −3.66e−01
Value: C
Methylonatrum
1.00
0.75
0.50
0.25
0.00
A (n=15)
B (n=16)
C (n=14)
surface

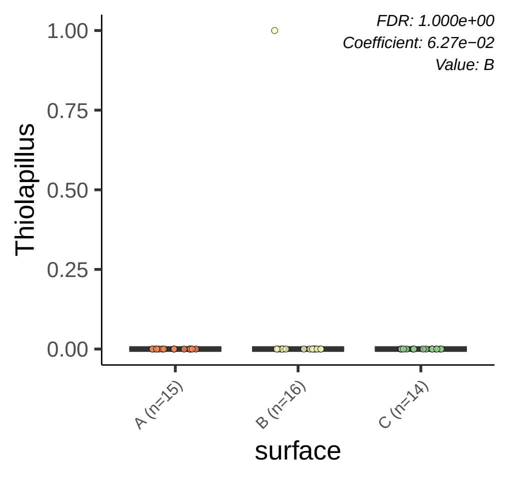
FDR: 1.000e+00
Coefficient: 6.27e−02
Value: B
Thiolapillus
1.00
0.75
0.50
0.25
0.00
A (n=15)
B (n=16)
C (n=14)
surface

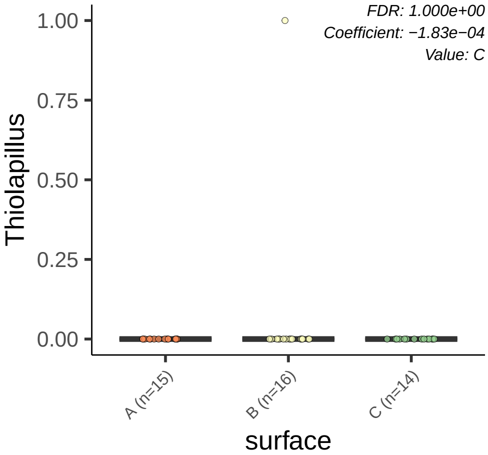

FDR: 1.000e+00
Coefficient: −1.83e−04
Value: C
Thiolapillus
1.00
0.75
0.50
0.25
0.00
A (n=15)
B (n=16)
C (n=14)
surface

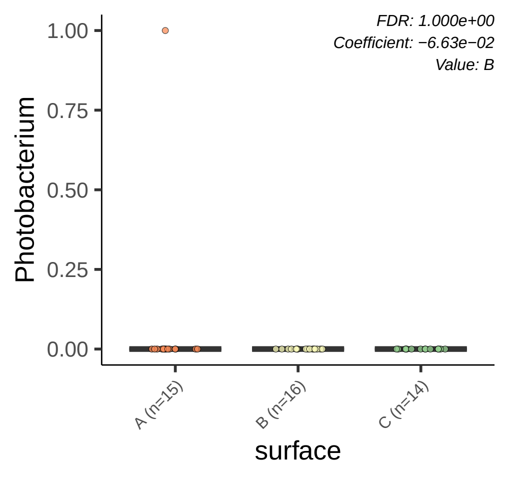

FDR: 1.000e+00
Coefficient: −6.63e−02
Value: B
Photobacterium
1.00
0.75
0.50
0.25
0.00
A (n=15)
B (n=16)
C (n=14)
surface

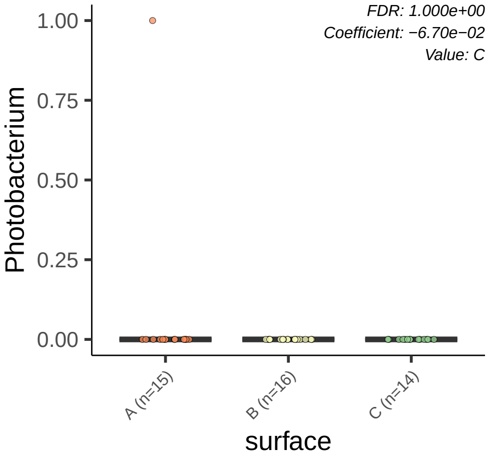
FDR: 1.000e+00
Coefficient: −6.70e−02
Value: C
Photobacterium
1.00
0.75
0.50
0.25
0.00
A (n=15)
B (n=16)
C (n=14)
surface

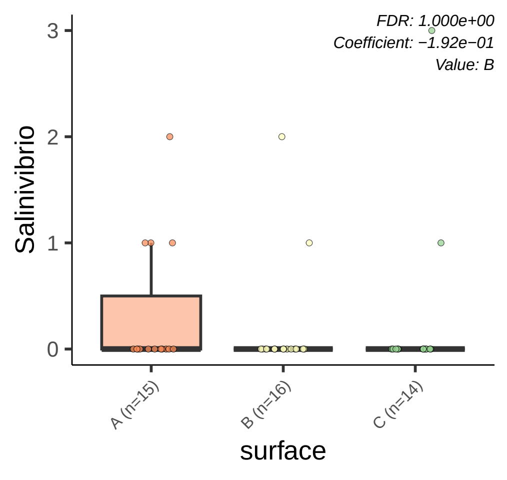

FDR: 1.000e+00
Coefficient: −1.92e−01
Value: B
Salinivibrio
0
1
2
3
A (n=15)
B (n=16)
C (n=14)
surface

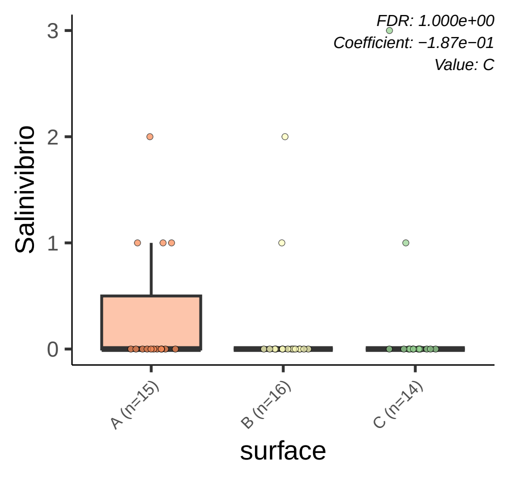

FDR: 1.000e+00
Coefficient: −1.87e−01
Value: C
Salinivibrio
3
2
1
0
A (n=15)
B (n=16)
C (n=14)
surface

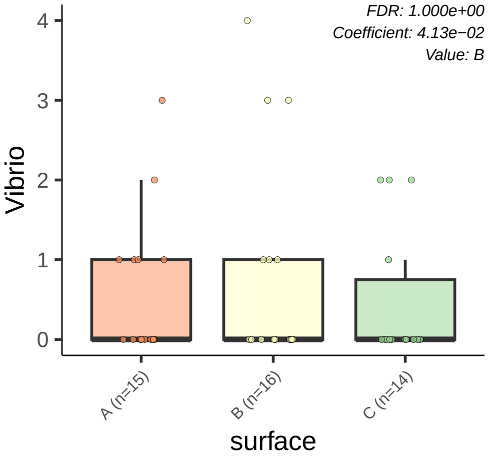
FDR: 1.000e+00
Coefficient: 4.13e−02
Value: B
Vibrio
4
3
2
1
0
A (n=15)
B (n=16)
C (n=14)
surface

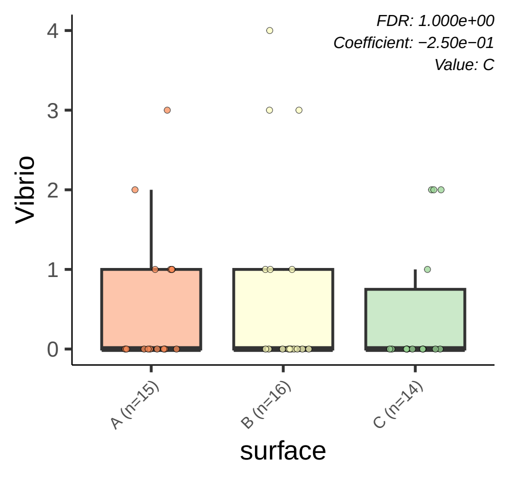
FDR: 1.000e+00
Coefficient: −2.50e−01
Value: C
Vibrio
4
3
2
1
0
A (n=15)
B (n=16)
C (n=14)
surface

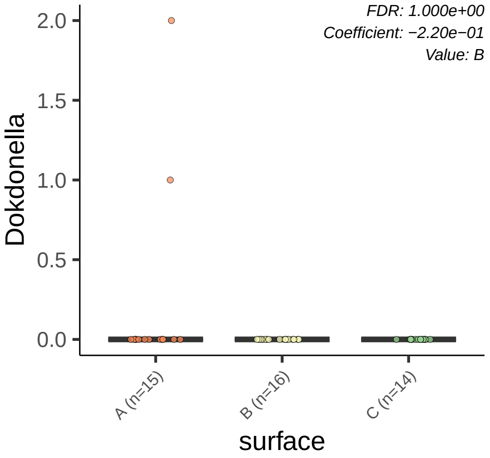

FDR: 1.000e+00
Coefficient: −2.20e−01
Value: B
Dokdonella
2.0
1.5
1.0
0.5
0.0
A (n=15)
B (n=16)
C (n=14)
surface

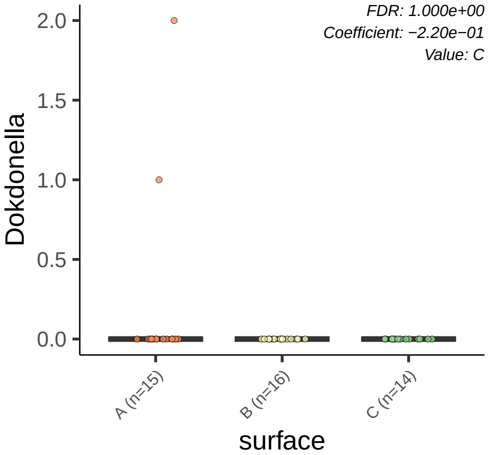

FDR: 1.000e+00
Coefficient: −2.20e−01
Value: C
Dokdonella
2.0
1.5
1.0
0.5
0.0
A (n=15)
B (n=16)
C (n=14)
surface

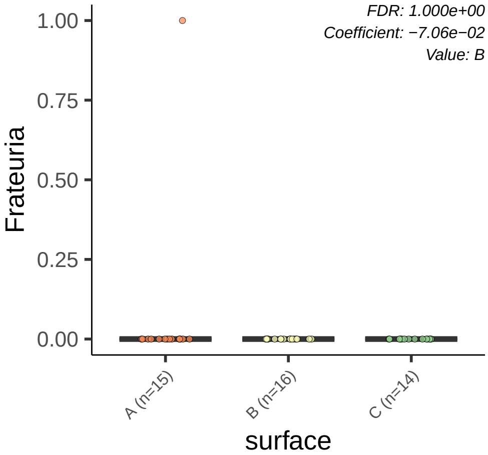

FDR: 1.000e+00
Coefficient: −7.06e−02
Value: B
Frateuria
1.00
0.75
0.50
0.25
0.00
A (n=15)
B (n=16)
C (n=14)
surface

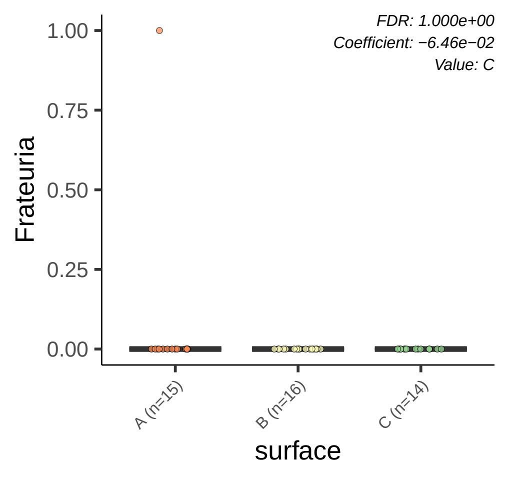
FDR: 1.000e+00
Coefficient: −6.46e−02
Value: C
1.00
0.75
0.50
0.25
0.00
Frateuria
A (n=15)
B (n=16)
C (n=14)
surface

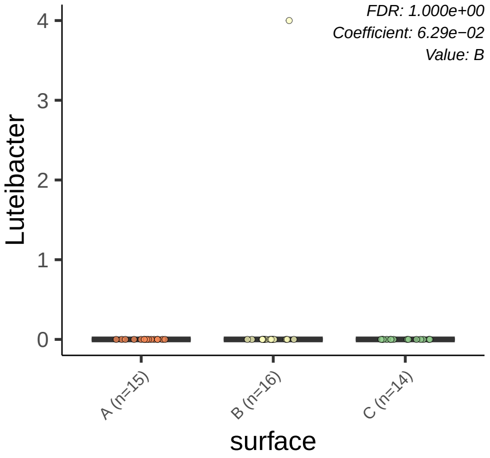
FDR: 1.000e+00
Coefficient: 6.29e−02
Value: B
Luteibacter
0
1
2
3
4
A (n=15)
B (n=16)
C (n=14)
surface

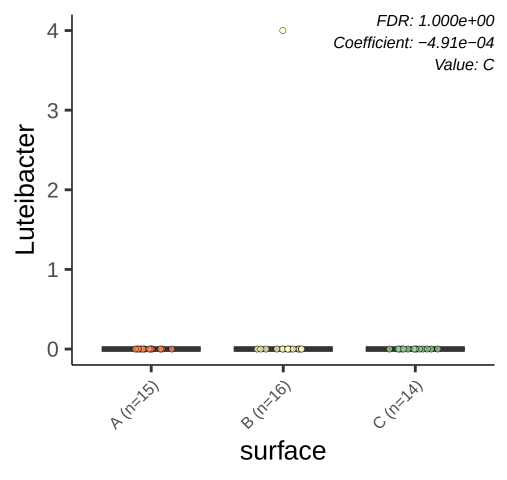
FDR: 1.000e+00
Coefficient: −4.91e−04
Value: C
Luteibacter
0
1
2
3
4
A (n=15)
B (n=16)
C (n=14)
surface

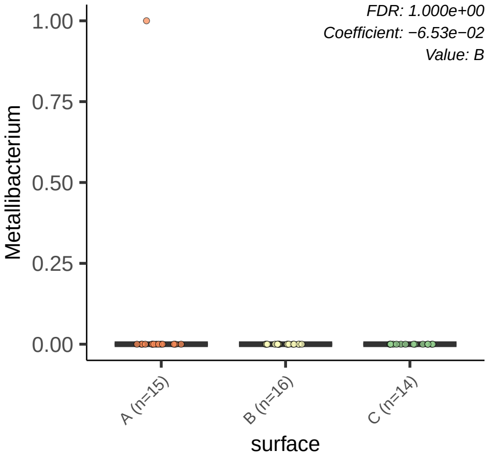
FDR: 1.000e+00
Coefficient: −6.53e−02
Value: B
Metallibacterium
1.00
0.75
0.50
0.25
0.00
A (n=15)
B (n=16)
C (n=14)
surface

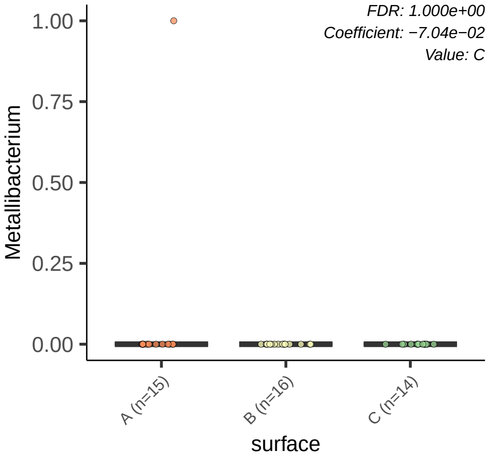
FDR: 1.000e+00
Coefficient: −7.04e−02
Value: C
Metallibacterium
1.00
0.75
0.50
0.25
0.00
A (n=15)
B (n=16)
C (n=14)
surface

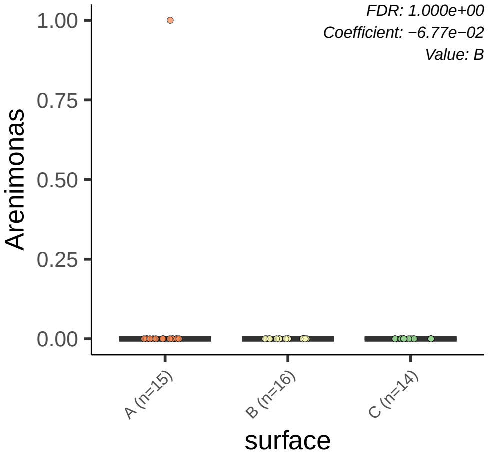

FDR: 1.000e+00
Coefficient: −6.77e−02
Value: B
Arenimonas
1.00
0.75
0.50
0.25
0.00
A (n=15)
B (n=16)
C (n=14)
surface

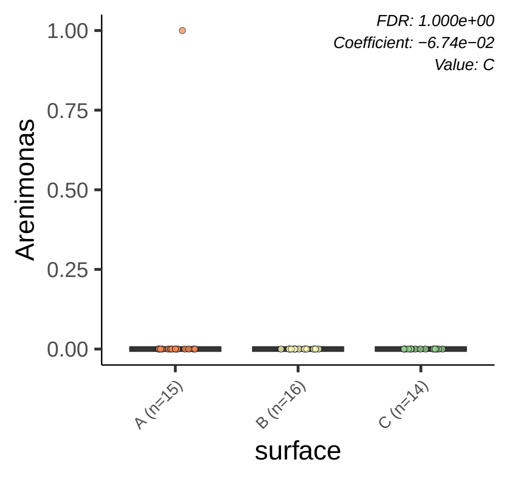

FDR: 1.000e+00
Coefficient: −6.74e−02
Value: C
Arenimonas
1.00
0.75
0.50
0.25
0.00
A (n=15)
B (n=16)
C (n=14)
surface

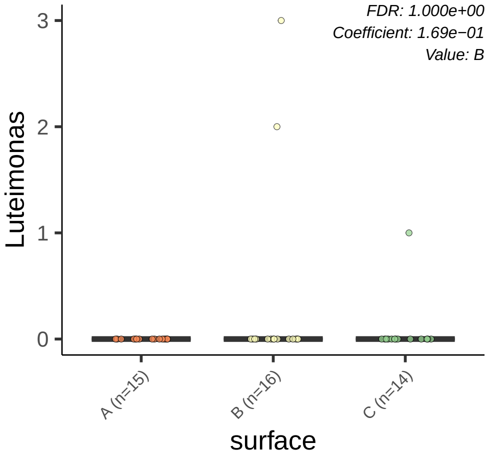

FDR: 1.000e+00
Coefficient: 1.69e−01
Value: B
Luteimonas
3
2
1
0
A (n=15)
B (n=16)
C (n=14)
surface

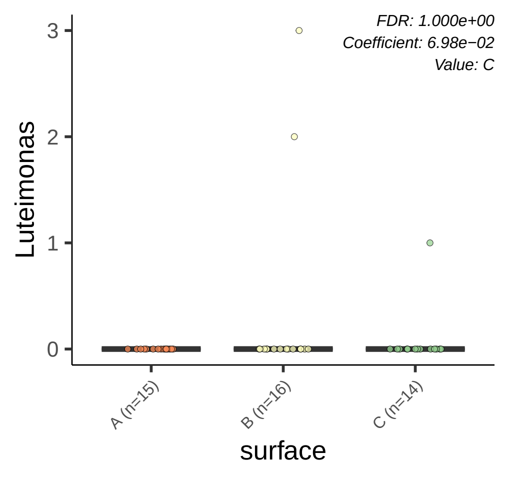
FDR: 1.000e+00
Coefficient: 6.98e−02
Value: C
Luteimonas
0
1
2
3
A (n=15)
B (n=16)
C (n=14)
surface

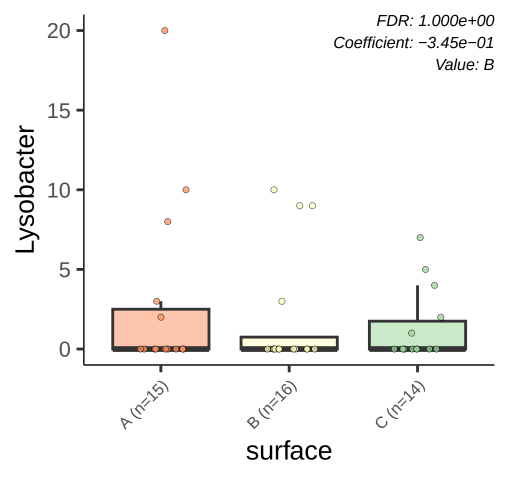
FDR: 1.000e+00
Coefficient: −3.45e−01
Value: B
Lysobacter
20
15
10
5
0
A (n=15)
B (n=16)
C (n=14)
surface

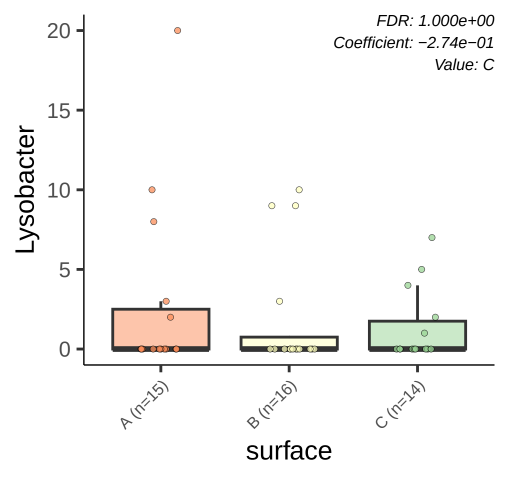
FDR: 1.000e+00
Coefficient: −2.74e−01
Value: C
Lysobacter
20
15
10
5
0
A (n=15)
B (n=16)
C (n=14)
surface

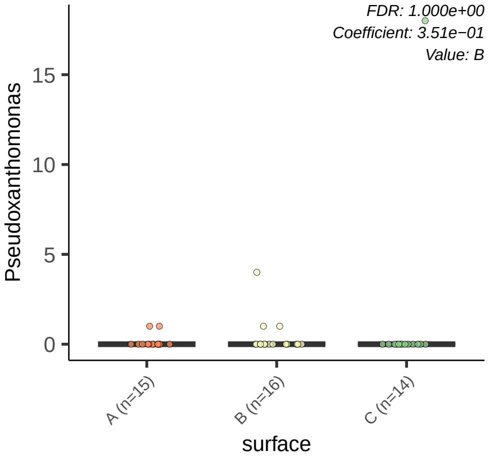

FDR: 1.000e+00
Coefficient: 3.51e−01
Value: B
Pseudoxanthomonas
0
5
10
15
A (n=15)
B (n=16)
C (n=14)
surface

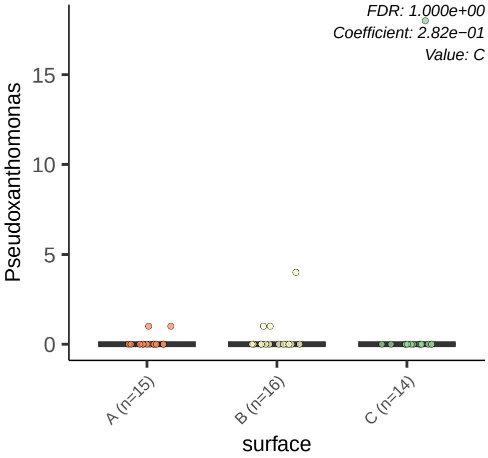
FDR: 1.000e+00
Coefficient: 2.82e−01
Value: C
Pseudoxanthomonas
15
10
5
0
A (n=15)
B (n=16)
C (n=14)
surface

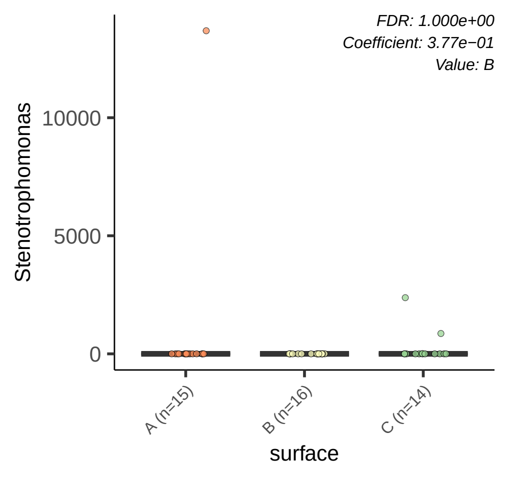
FDR: 1.000e+00
Coefficient: 3.77e−01
Value: B
Stenotrophomonas
10000
5000
0
A (n=15)
B (n=16)
C (n=14)
surface

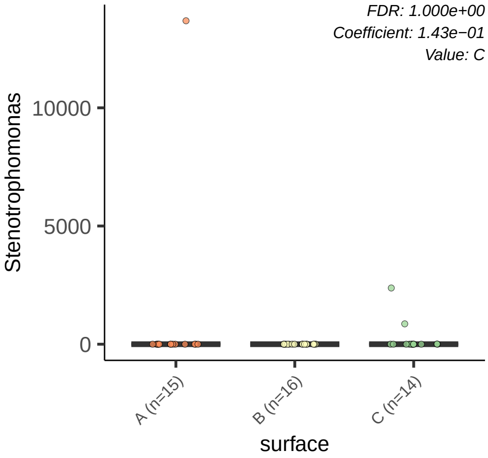

FDR: 1.000e+00
Coefficient: 1.43e−01
Value: C
Stenotrophomonas
10000
5000
0
A (n=15)
B (n=16)
C (n=14)
surface

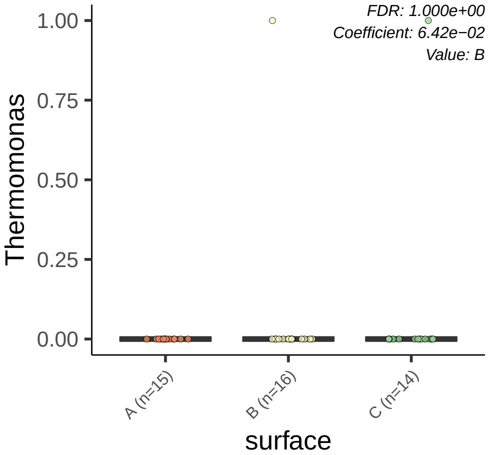

FDR: 1.000e+00
Coefficient: 6.42e−02
Value: B
Thermomonas
1.00
0.75
0.50
0.25
0.00
A (n=15)
B (n=16)
C (n=14)
surface

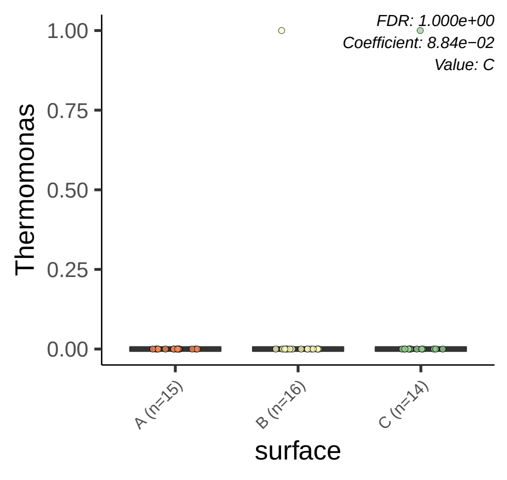
FDR: 1.000e+00
Coefficient: 8.84e−02
Value: C
Thermomonas
1.00
0.75
0.50
0.25
0.00
A (n=15)
B (n=16)
C (n=14)
surface

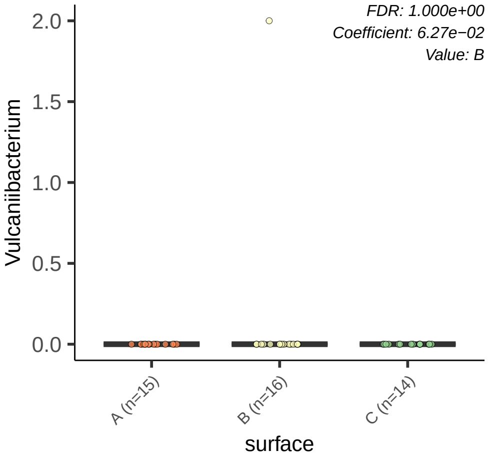
FDR: 1.000e+00
Coefficient: 6.27e−02
Value: B
Vulcaniibacterium
2.0
1.5
1.0
0.5
0.0
A (n=15)
B (n=16)
C (n=14)
surface

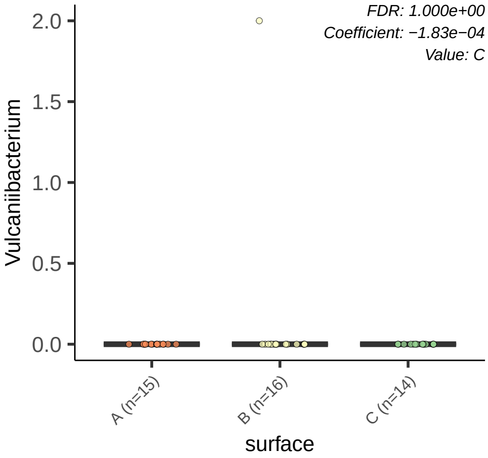

FDR: 1.000e+00
Coefficient: −1.83e−04
Value: C
Vulcaniibacterium
2.0
1.5
1.0
0.5
0.0
A (n=15)
B (n=16)
C (n=14)
surface

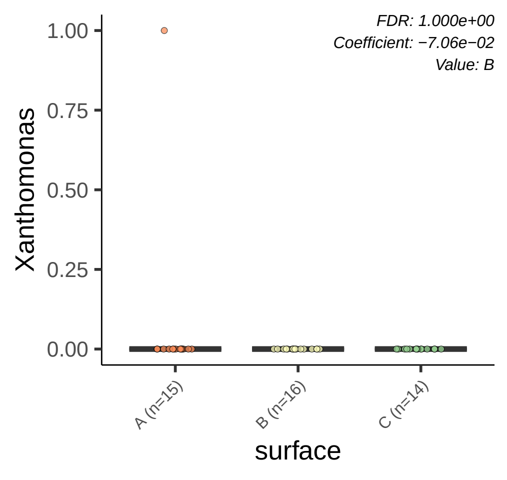
FDR: 1.000e+00
Coefficient: −7.06e−02
Value: B
1.00
0.75
0.50
0.25
0.00
Xanthomonas
A (n=15)
B (n=16)
C (n=14)
surface

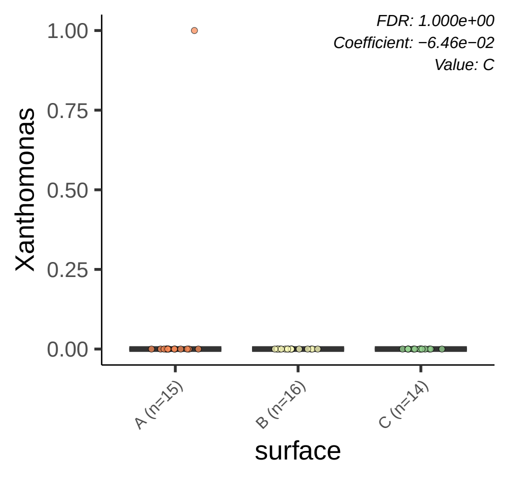

FDR: 1.000e+00
Coefficient: −6.46e−02
Value: C
1.00
0.75
0.50
0.25
0.00
Xanthomonas
A (n=15)
B (n=16)
C (n=14)
surface

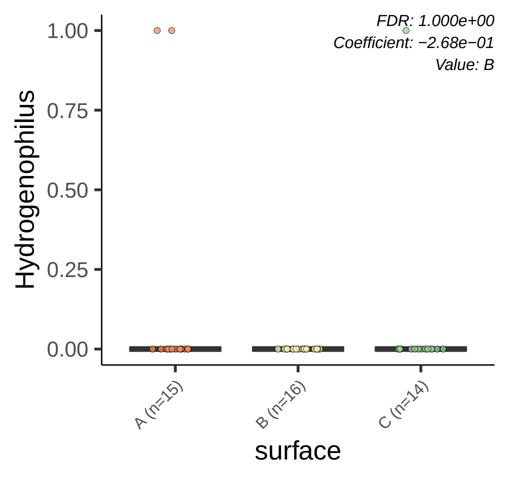

FDR: 1.000e+00
Coefficient: −2.68e−01
Value: B
Hydrogenophilus
1.00
0.75
0.50
0.25
0.00
A (n=15)
B (n=16)
C (n=14)
surface

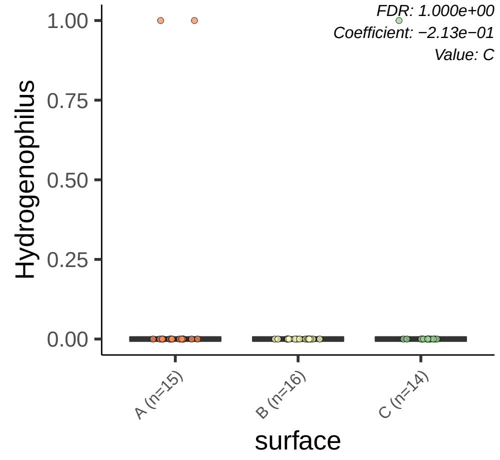

FDR: 1.000e+00
Coefficient: −2.13e−01
Value: C
Hydrogenophilus
1.00
0.75
0.50
0.25
0.00
A (n=15)
B (n=16)
C (n=14)
surface

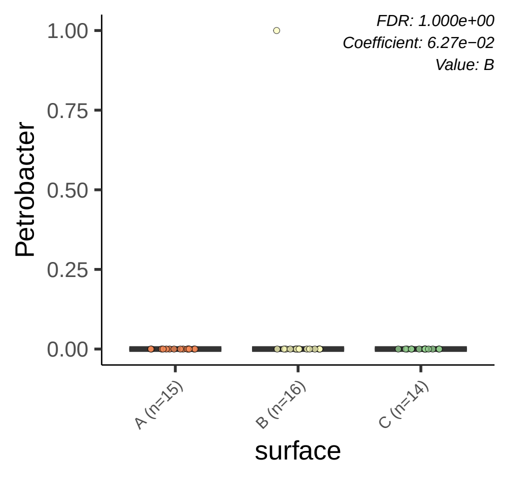
FDR: 1.000e+00
Coefficient: 6.27e−02
Value: B
Petrobacter
1.00
0.75
0.50
0.25
0.00
A (n=15)
B (n=16)
C (n=14)
surface

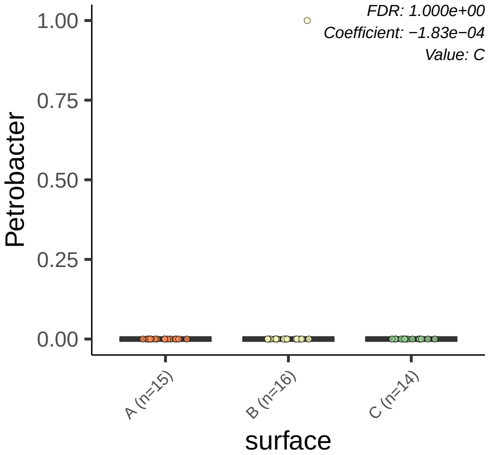
FDR: 1.000e+00
Coefficient: −1.83e−04
Value: C
1.00
0.75
0.50
0.25
0.00
Petrobacter
A (n=15)
B (n=16)
C (n=14)
surface

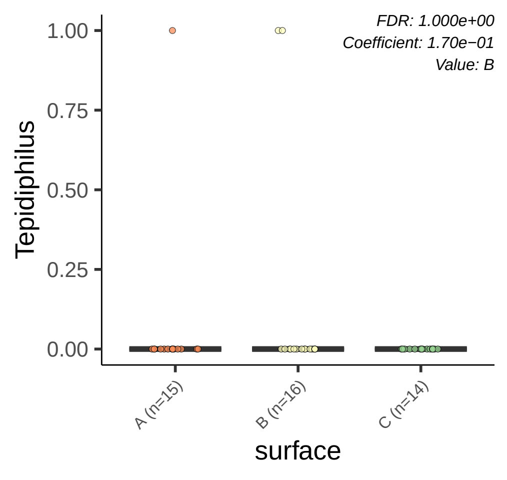
FDR: 1.000e+00
Coefficient: 1.70e−01
Value: B
Tepidiphilus
1.00
0.75
0.50
0.25
0.00
A (n=15)
B (n=16)
C (n=14)
surface

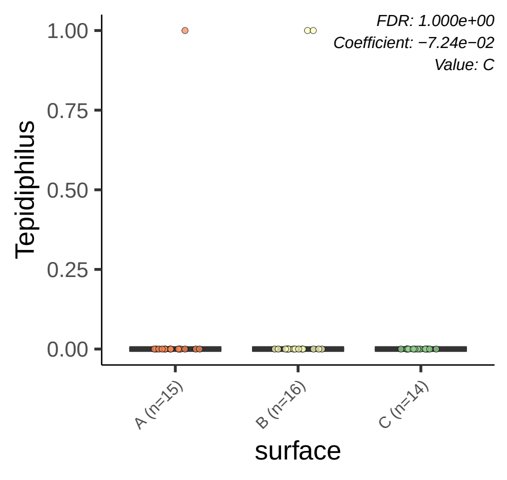

FDR: 1.000e+00
Coefficient: −7.24e−02
Value: C
Tepidiphilus
1.00
0.75
0.50
0.25
0.00
A (n=15)
B (n=16)
C (n=14)
surface

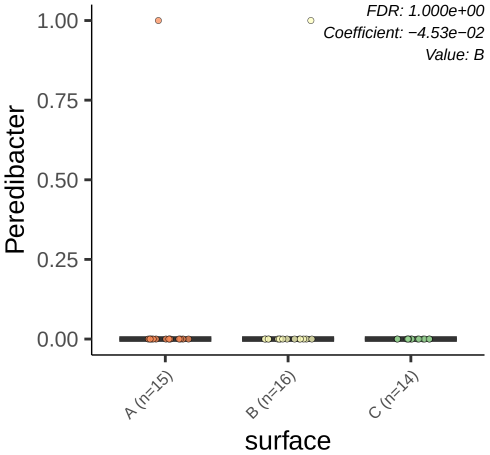

FDR: 1.000e+00
Coefficient: −4.53e−02
Value: B
Peredibacter
1.00
0.75
0.50
0.25
0.00
A (n=15)
B (n=16)
C (n=14)
surface

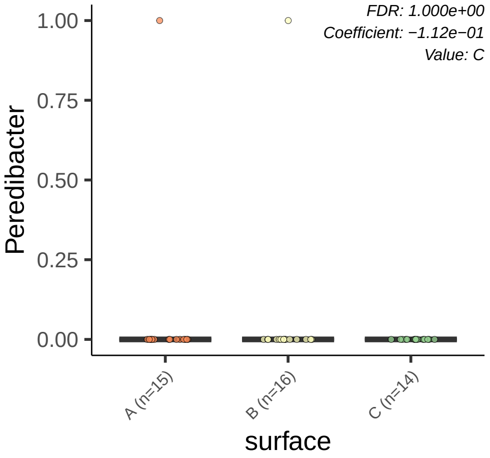
FDR: 1.000e+00
Coefficient: −1.12e−01
Value: C
Peredibacter
1.00
0.75
0.50
0.25
0.00
A (n=15)
B (n=16)
C (n=14)
surface

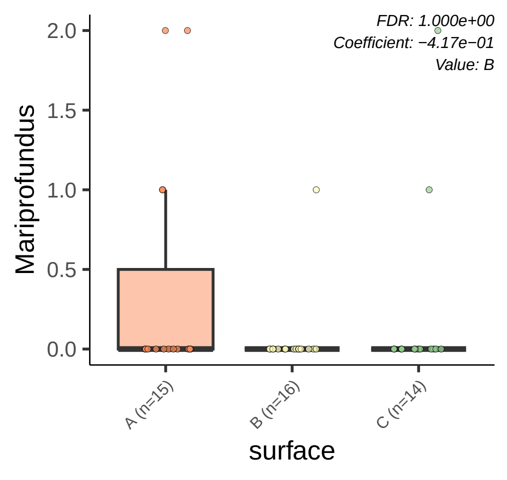

FDR: 1.000e+00
Coefficient: −4.17e−01
Value: B
Mariprofundus
2.0
1.5
1.0
0.5
0.0
A (n=15)
B (n=16)
C (n=14)
surface

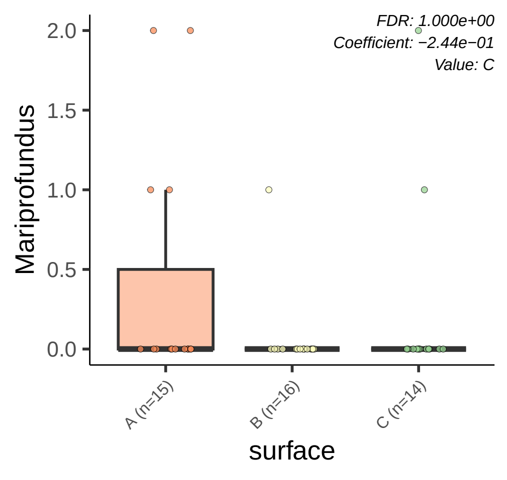

FDR: 1.000e+00
Coefficient: −2.44e−01
Value: C
Mariprofundus
2.0
1.5
1.0
0.5
0.0
A (n=15)
B (n=16)
C (n=14)
surface

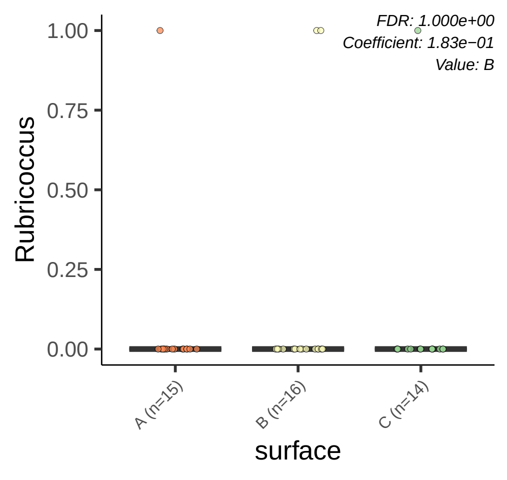
FDR: 1.000e+00
Coefficient: 1.83e−01
Value: B
Rubricoccus
1.00
0.75
0.50
0.25
0.00
A (n=15)
B (n=16)
C (n=14)
surface

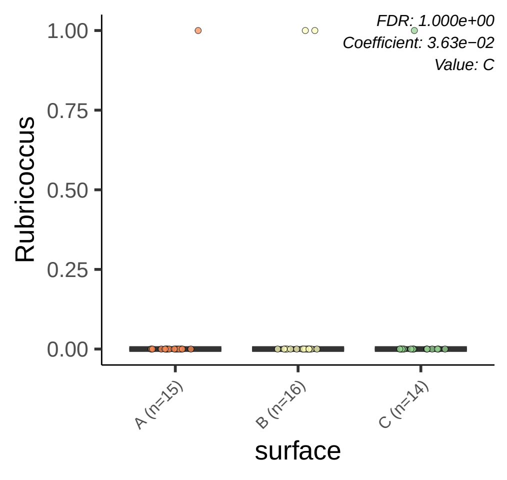

FDR: 1.000e+00
Coefficient: 3.63e−02
Value: C
1.00
0.75
0.50
0.25
0.00
Rubricoccus
A (n=15)
B (n=16)
C (n=14)
surface

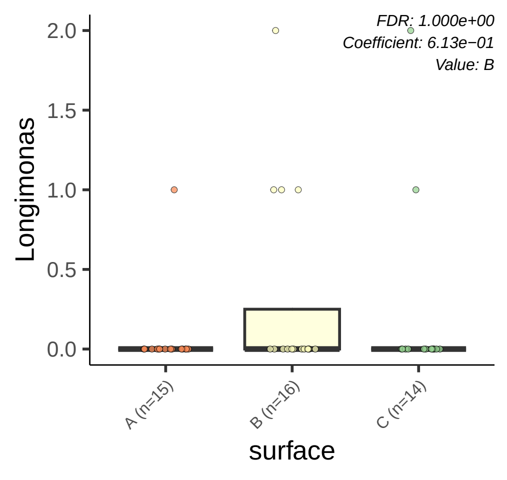
FDR: 1.000e+00
Coefficient: 6.13e−01
Value: B
Longimonas
2.0
1.5
1.0
0.5
0.0
A (n=15)
B (n=16)
C (n=14)
surface

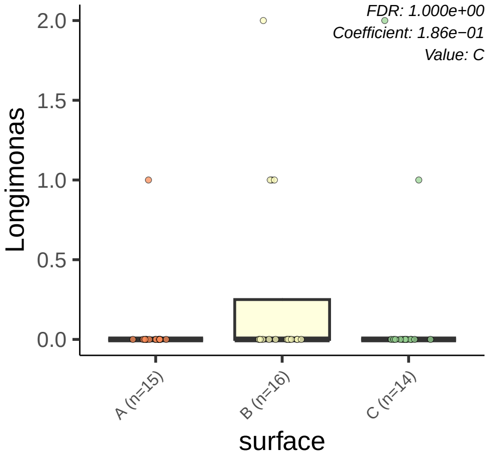

FDR: 1.000e+00
Coefficient: 1.86e−01
Value: C
Longimonas
2.0
1.5
1.0
0.5
0.0
A (n=15)
B (n=16)
C (n=14)
surface

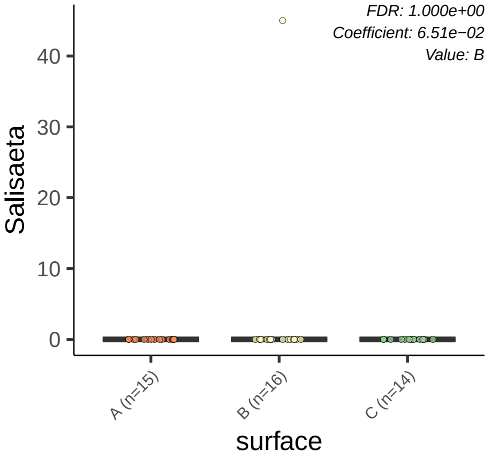

FDR: 1.000e+00
Coefficient: 6.51e−02
Value: B
Salisaeta
40
30
20
10
0
A (n=15)
B (n=16)
C (n=14)
surface

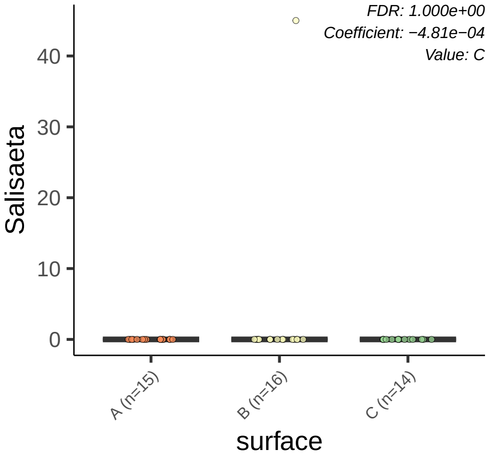
FDR: 1.000e+00
Coefficient: −4.81e−04
Value: C
Salisaeta
40
30
20
10
0
A (n=15)
B (n=16)
C (n=14)
surface

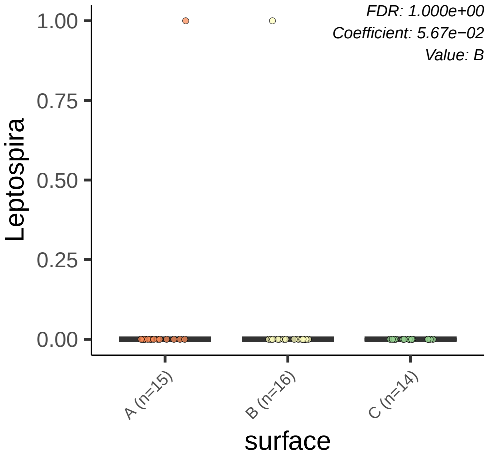
FDR: 1.000e+00
Coefficient: 5.67e−02
Value: B
Leptospira
1.00
0.75
0.50
0.25
0.00
A (n=15)
B (n=16)
C (n=14)
surface

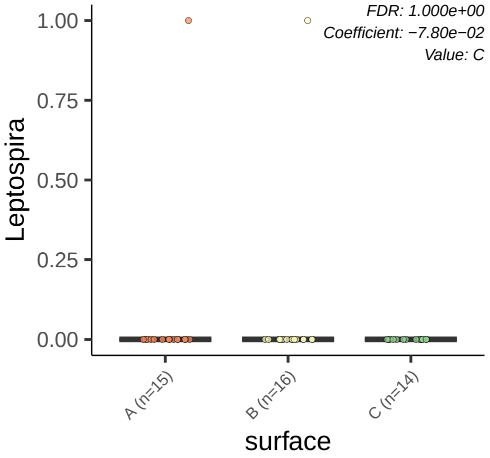
FDR: 1.000e+00
Coefficient: −7.80e−02
Value: C
Leptospira
1.00
0.75
0.50
0.25
0.00
A (n=15)
B (n=16)
C (n=14)
surface

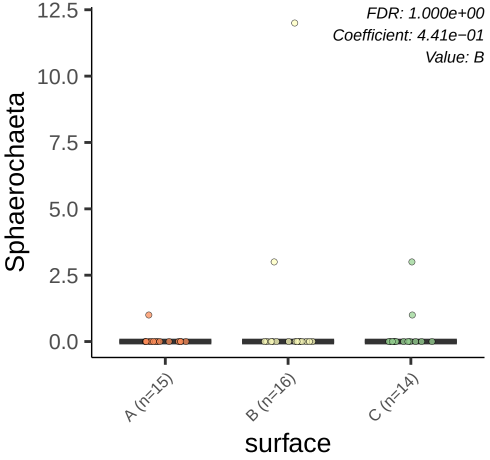

FDR: 1.000e+00
Coefficient: 4.41e−01
Value: B
Sphaerochaeta
12.5
10.0
7.5
5.0
2.5
0.0
A (n=15)
B (n=16)
C (n=14)
surface

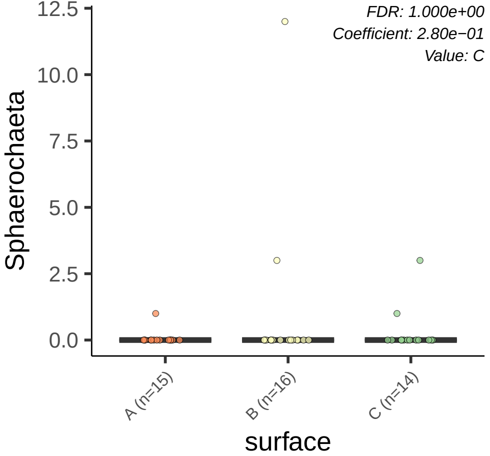

FDR: 1.000e+00
Coefficient: 2.80e−01
Value: C
Sphaerochaeta
12.5
10.0
7.5
5.0
2.5
0.0
A (n=15)
B (n=16)
C (n=14)
surface

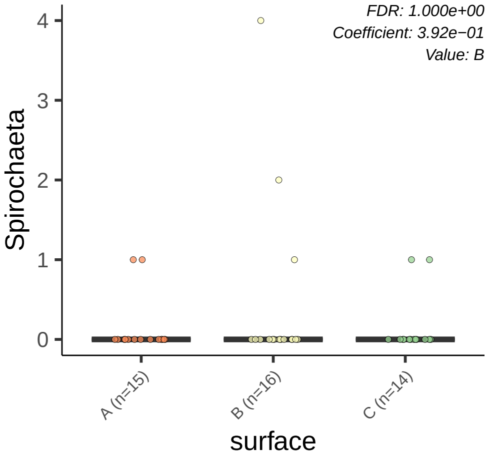
FDR: 1.000e+00
Coefficient: 3.92e−01
Value: B
Spirochaeta
4
3
2
1
0
A (n=15)
B (n=16)
C (n=14)
surface

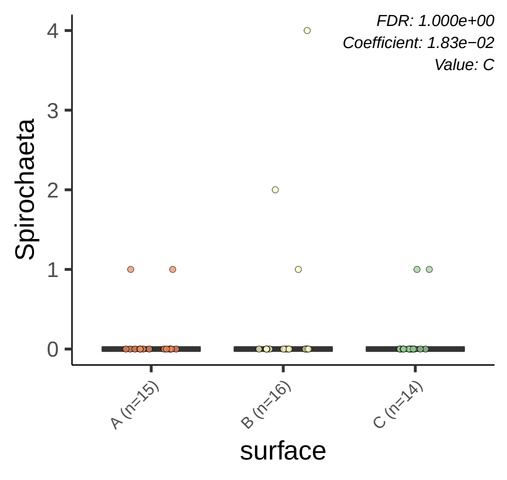
FDR: 1.000e+00
Coefficient: 1.83e−02
Value: C
Spirochaeta
4
3
2
1
0
A (n=15)
B (n=16)
C (n=14)
surface

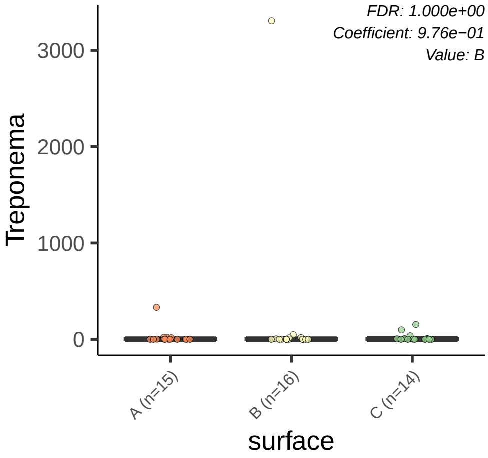
FDR: 1.000e+00
Coefficient: 9.76e−01
Value: B
Treponema
3000
2000
1000
0
A (n=15)
B (n=16)
C (n=14)
surface

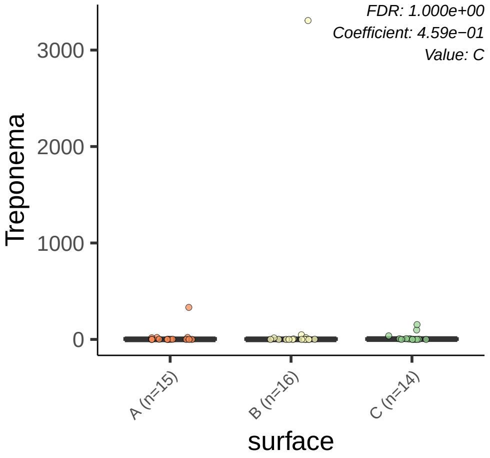

FDR: 1.000e+00
Coefficient: 4.59e−01
Value: C
Treponema
3000
2000
1000
0
A (n=15)
B (n=16)
C (n=14)
surface

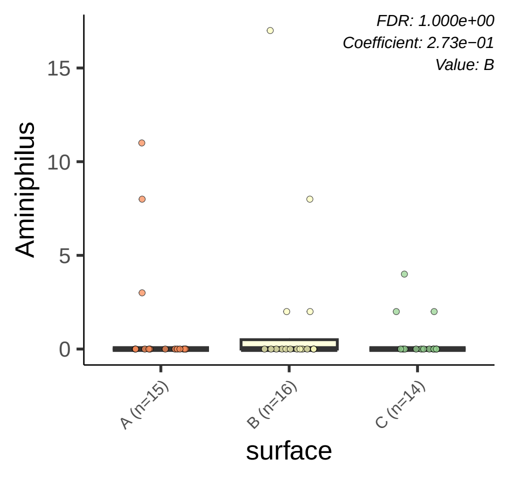

FDR: 1.000e+00
Coefficient: 2.73e−01
Value: B
Aminiphilus
15
10
5
0
A (n=15)
B (n=16)
C (n=14)
surface

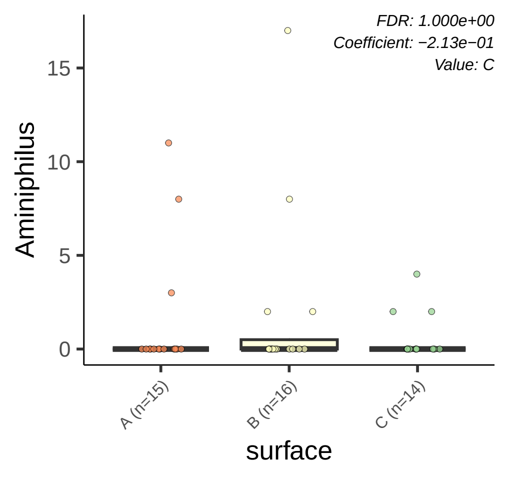

FDR: 1.000e+00
Coefficient: −2.13e−01
Value: C
Aminiphilus
0
5
10
15
A (n=15)
B (n=16)
C (n=14)
surface

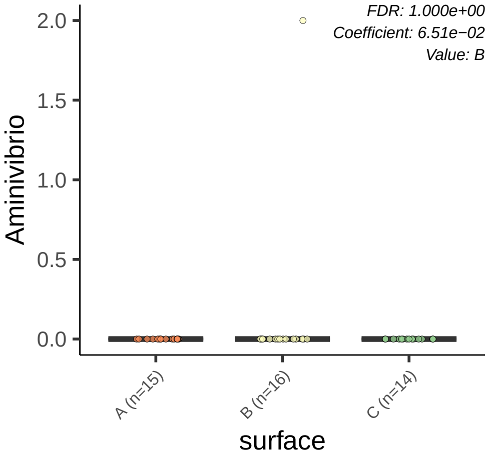
FDR: 1.000e+00
Coefficient: 6.51e−02
Value: B
Aminivibrio
2.0
1.5
1.0
0.5
0.0
A (n=15)
B (n=16)
C (n=14)
surface

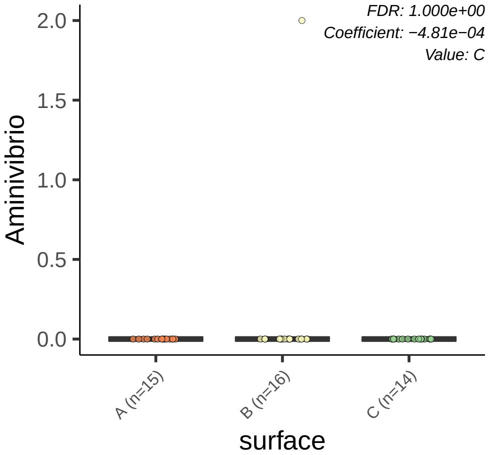
FDR: 1.000e+00
Coefficient: −4.81e−04
Value: C
2.0
1.5
1.0
0.5
0.0
Aminivibrio
A (n=15)
B (n=16)
C (n=14)
surface

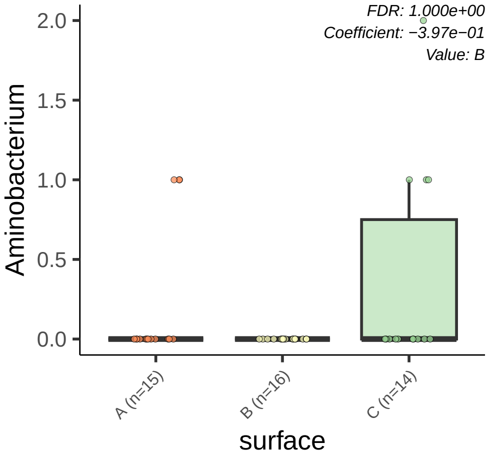
FDR: 1.000e+00
Coefficient: −3.97e−01
Value: B
Aminobacterium
2.0
1.5
1.0
0.5
0.0
A (n=15)
B (n=16)
C (n=14)
surface

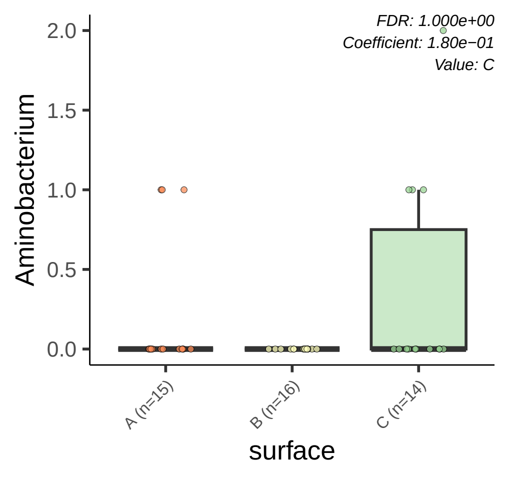
FDR: 1.000e+00
Coefficient: 1.80e−01
Value: C
Aminobacterium
2.0
1.5
1.0
0.5
0.0
A (n=15)
B (n=16)
C (n=14)
surface

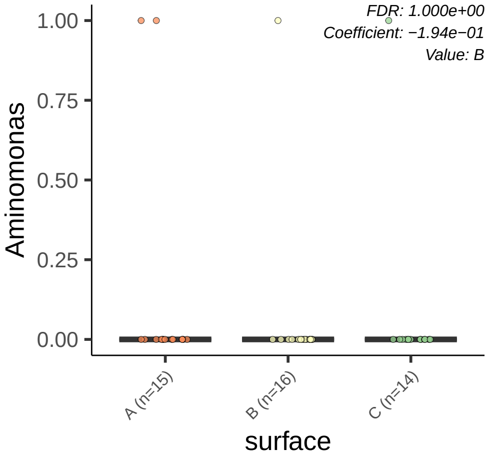
FDR: 1.000e+00
Coefficient: −1.94e−01
Value: B
Aminomonas
1.00
0.75
0.50
0.25
0.00
A (n=15)
B (n=16)
C (n=14)
surface

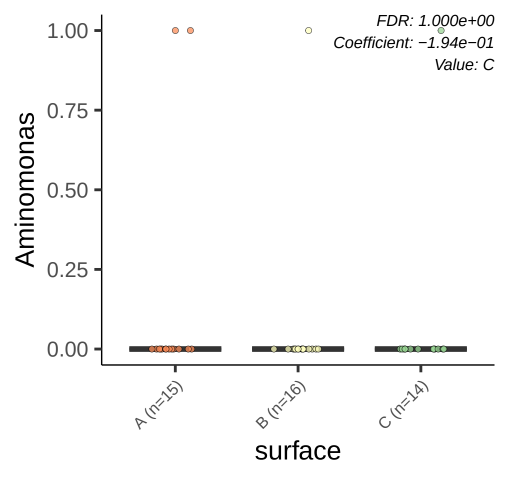

FDR: 1.000e+00
Coefficient: −1.94e−01
Value: C
Aminomonas
1.00
0.75
0.50
0.25
0.00
A (n=15)
B (n=16)
C (n=14)
surface

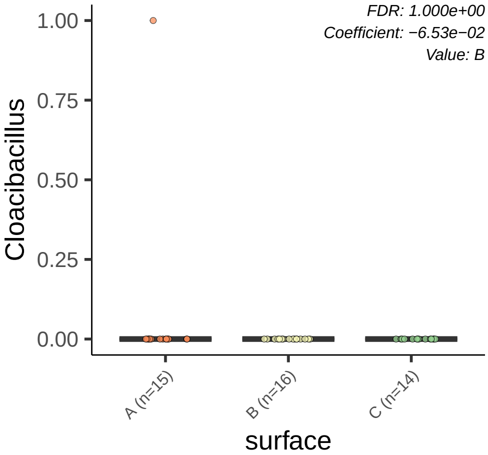
FDR: 1.000e+00
Coefficient: −6.53e−02
Value: B
Cloacibacillus
1.00
0.75
0.50
0.25
0.00
A (n=15)
B (n=16)
C (n=14)
surface

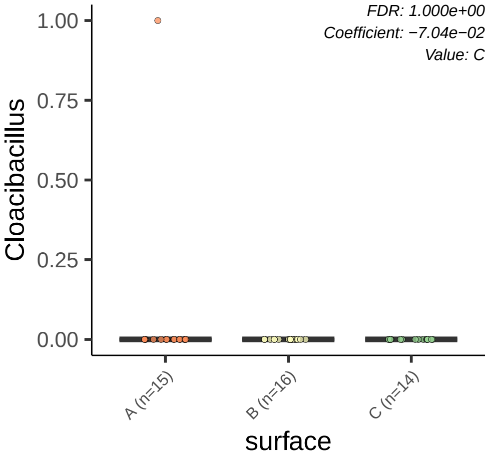

FDR: 1.000e+00
Coefficient: −7.04e−02
Value: C
Cloacibacillus
1.00
0.75
0.50
0.25
0.00
A (n=15)
B (n=16)
C (n=14)
surface

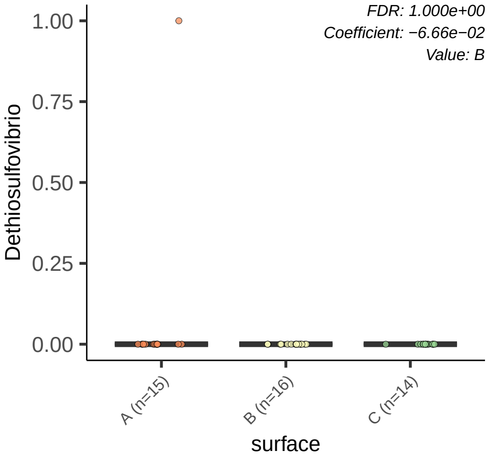
FDR: 1.000e+00
Coefficient: −6.66e−02
Value: B
Dethiosulfovibrio
1.00
0.75
0.50
0.25
0.00
A (n=15)
B (n=16)
C (n=14)
surface

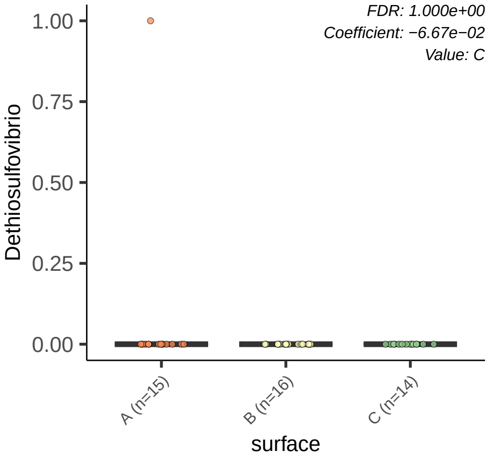
FDR: 1.000e+00
Coefficient: −6.67e−02
Value: C
Dethiosulfovibrio
1.00
0.75
0.50
0.25
0.00
A (n=15)
B (n=16)
C (n=14)
surface

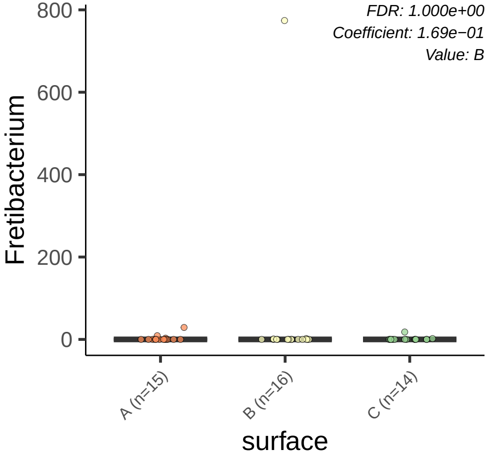

FDR: 1.000e+00
Coefficient: 1.69e−01
Value: B
Fretibacterium
800
600
400
200
0
A (n=15)
B (n=16)
C (n=14)
surface

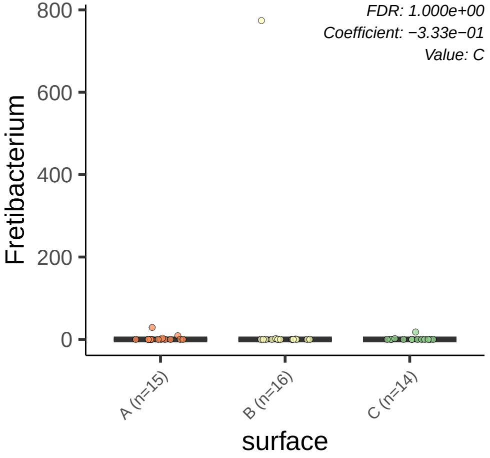

FDR: 1.000e+00
Coefficient: −3.33e−01
Value: C
Fretibacterium
800
600
400
200
0
A (n=15)
B (n=16)
C (n=14)
surface

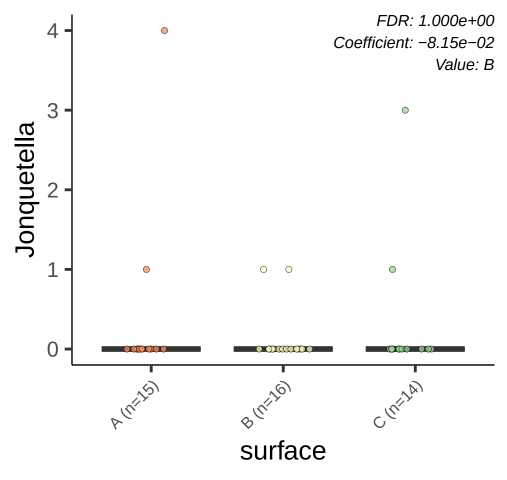

FDR: 1.000e+00
Coefficient: −8.15e−02
Value: B
Jonquetella
0
1
2
3
4
A (n=15)
B (n=16)
C (n=14)
surface

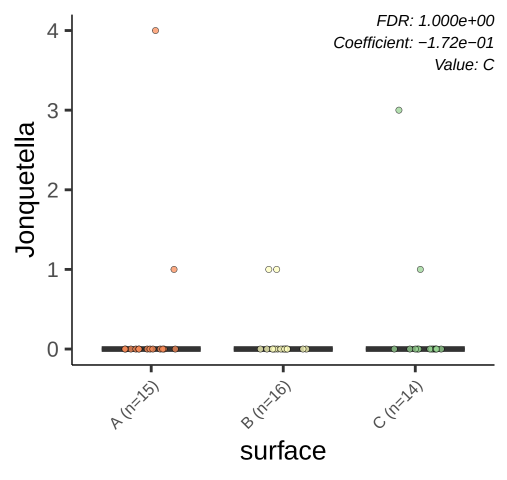
FDR: 1.000e+00
Coefficient: −1.72e−01
Value: C
Jonquetella
4
3
2
1
0
A (n=15)
B (n=16)
C (n=14)
surface

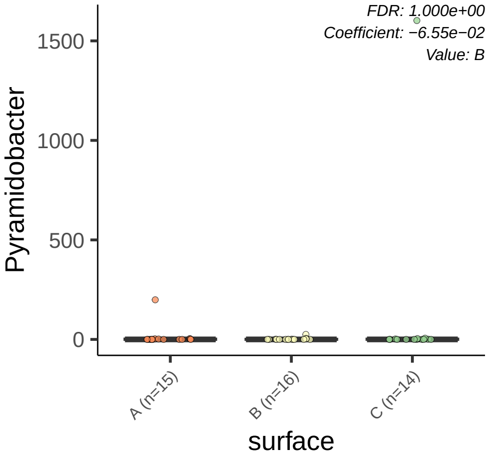

FDR: 1.000e+00
Coefficient: −6.55e−02
Value: B
Pyramidobacter
1500
1000
500
0
A (n=15)
B (n=16)
C (n=14)
surface

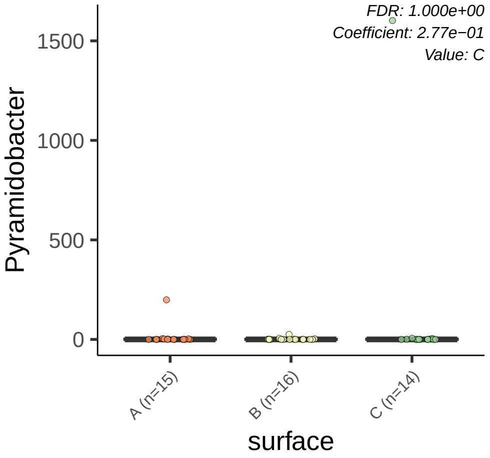

FDR: 1.000e+00
Coefficient: 2.77e−01
Value: C
Pyramidobacter
1500
1000
500
0
A (n=15)
B (n=16)
C (n=14)
surface

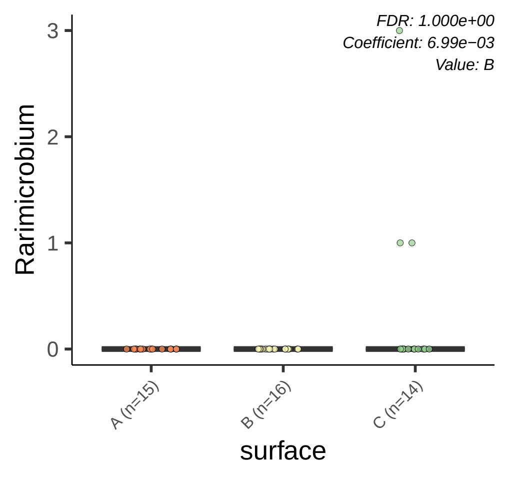

FDR: 1.000e+00
Coefficient: 6.99e−03
Value: B
Rarimicrobium
3
2
1
0
A (n=15)
B (n=16)
C (n=14)
surface

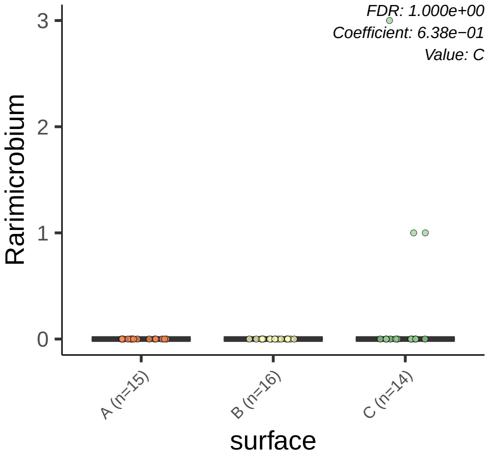
FDR: 1.000e+00
Coefficient: 6.38e−01
Value: C
Rarimicrobium
3
2
1
0
A (n=15)
B (n=16)
C (n=14)
surface

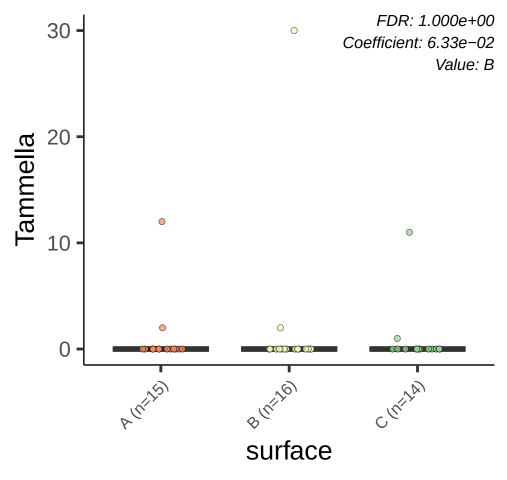

FDR: 1.000e+00
Coefficient: 6.33e−02
Value: B
Tammella
30
20
10
0
A (n=15)
B (n=16)
C (n=14)
surface

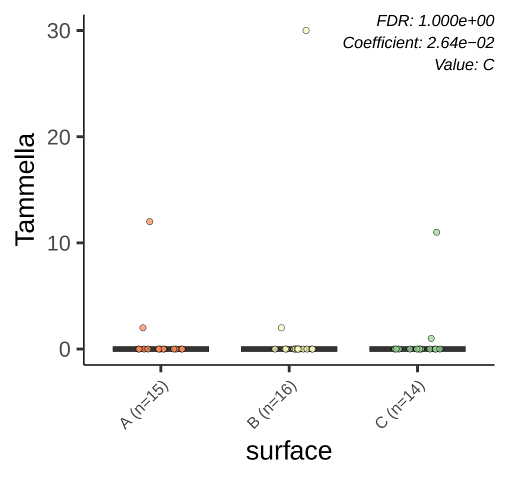

FDR: 1.000e+00
Coefficient: 2.64e−02
Value: C
30
20
10
0
Tammella
A (n=15)
B (n=16)
C (n=14)
surface

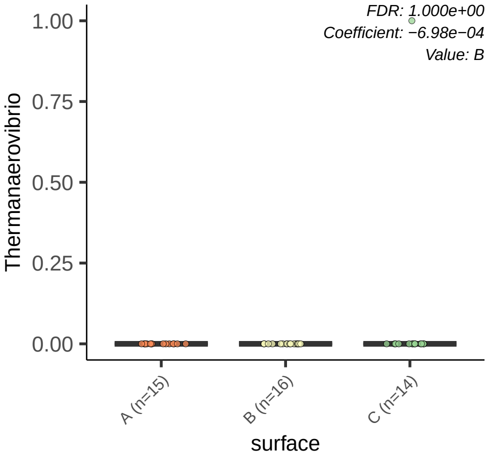

FDR: 1.000e+00
Coefficient: −6.98e−04
Value: B
Thermanaerovibrio
1.00
0.75
0.50
0.25
0.00
A (n=15)
B (n=16)
C (n=14)
surface

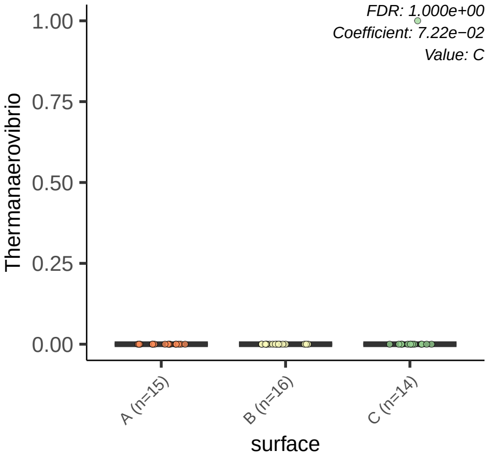

FDR: 1.000e+00
Coefficient: 7.22e−02
Value: C
1.00
0.75
0.50
0.25
0.00
Thermanaerovibrio
A (n=15)
B (n=16)
C (n=14)
surface

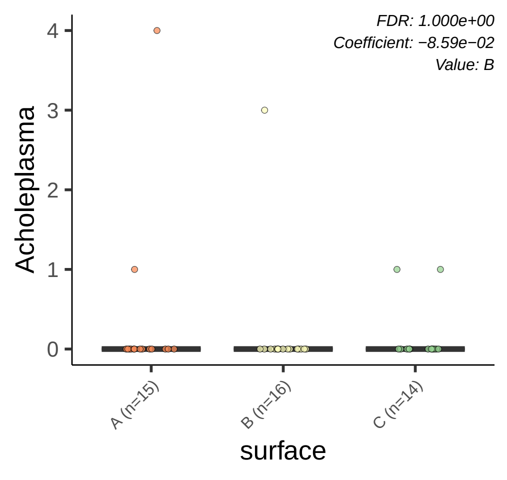
FDR: 1.000e+00
Coefficient: −8.59e−02
Value: B
Acholeplasma
4
3
2
1
0
A (n=15)
B (n=16)
C (n=14)
surface

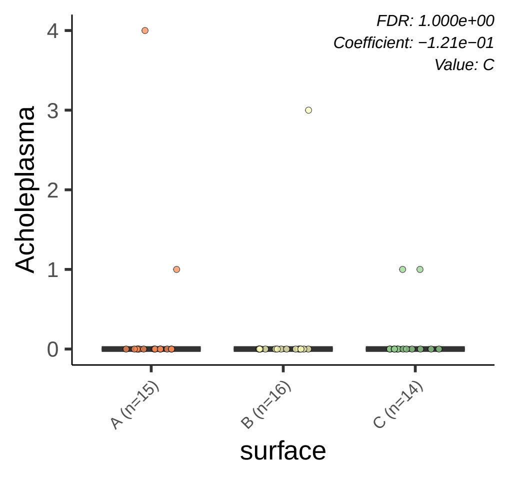
FDR: 1.000e+00
Coefficient: −1.21e−01
Value: C
Acholeplasma
0
1
2
3
4
A (n=15)
B (n=16)
C (n=14)
surface

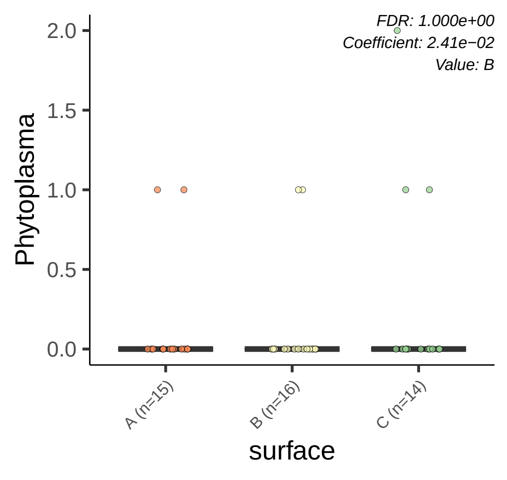
FDR: 1.000e+00
Coefficient: 2.41e−02
Value: B
Phytoplasma
2.0
1.5
1.0
0.5
0.0
A (n=15)
B (n=16)
C (n=14)
surface

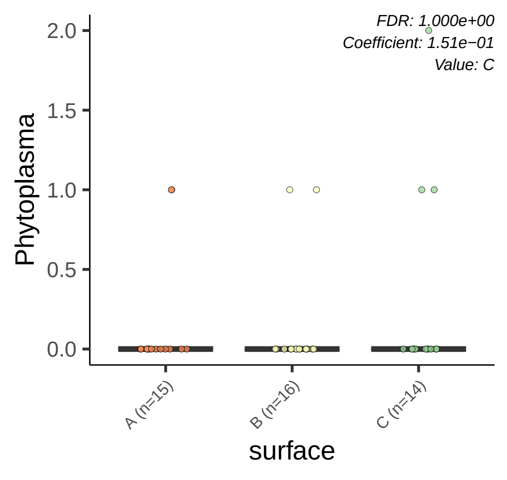
FDR: 1.000e+00
Coefficient: 1.51e−01
Value: C
Phytoplasma
2.0
1.5
1.0
0.5
0.0
A (n=15)
B (n=16)
C (n=14)
surface

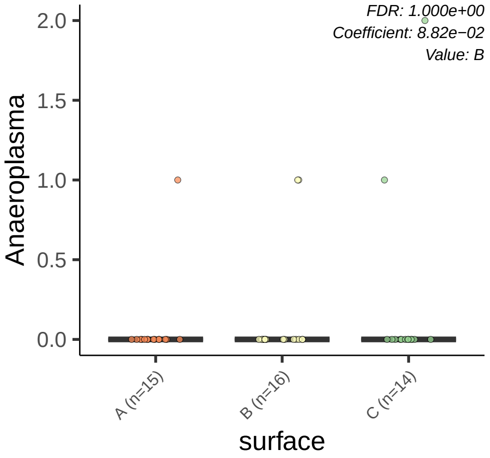

FDR: 1.000e+00
Coefficient: 8.82e−02
Value: B
Anaeroplasma
2.0
1.5
1.0
0.5
0.0
A (n=15)
B (n=16)
C (n=14)
surface

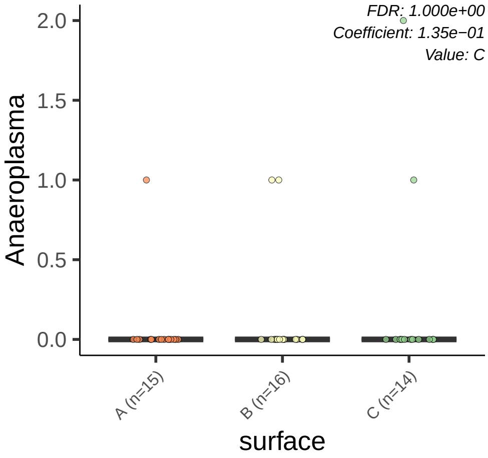
FDR: 1.000e+00
Coefficient: 1.35e−01
Value: C
Anaeroplasma
2.0
1.5
1.0
0.5
0.0
A (n=15)
B (n=16)
C (n=14)
surface

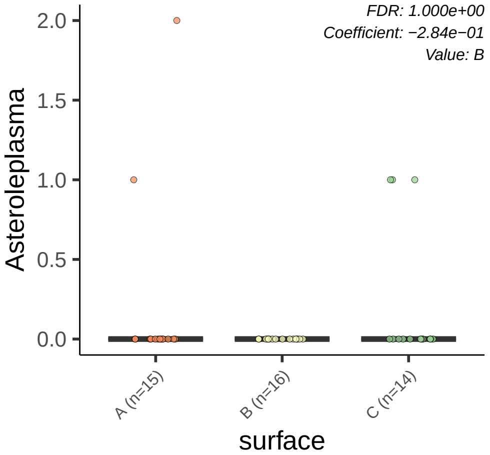

FDR: 1.000e+00
Coefficient: −2.84e−01
Value: B
Asteroleplasma
2.0
1.5
1.0
0.5
0.0
A (n=15)
B (n=16)
C (n=14)
surface

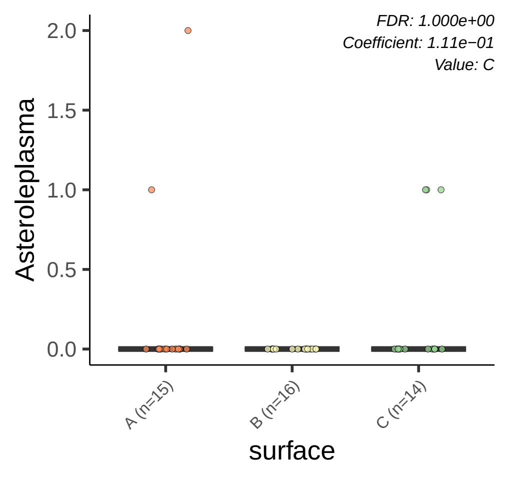

FDR: 1.000e+00
Coefficient: 1.11e−01
Value: C
Asteroleplasma
2.0
1.5
1.0
0.5
0.0
A (n=15)
B (n=16)
C (n=14)
surface

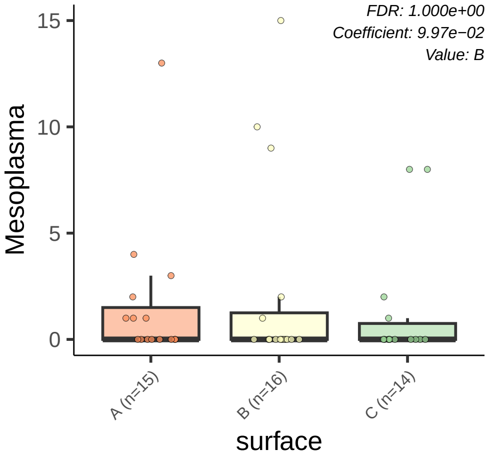

FDR: 1.000e+00
Coefficient: 9.97e−02
Value: B
Mesoplasma
15
10
5
0
A (n=15)
B (n=16)
C (n=14)
surface

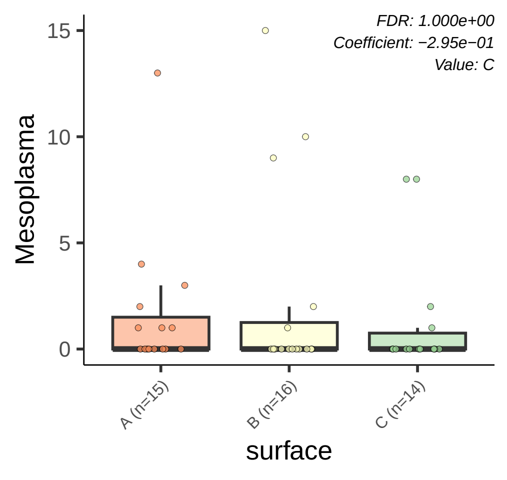

FDR: 1.000e+00
Coefficient: −2.95e−01
Value: C
Mesoplasma
15
10
5
0
A (n=15)
B (n=16)
C (n=14)
surface

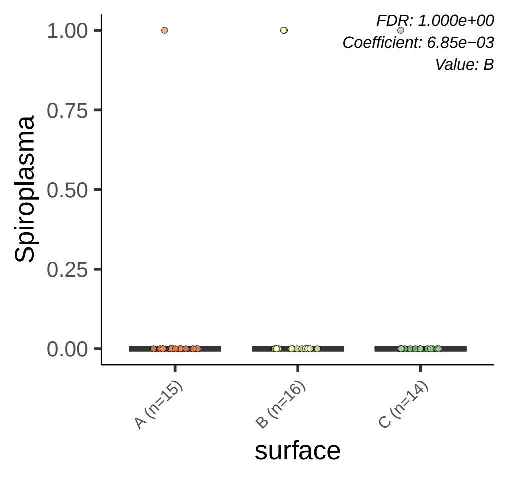

FDR: 1.000e+00
Coefficient: 6.85e−03
Value: B
1.00
0.75
0.50
0.25
0.00
Spiroplasma
A (n=15)
B (n=16)
C (n=14)
surface

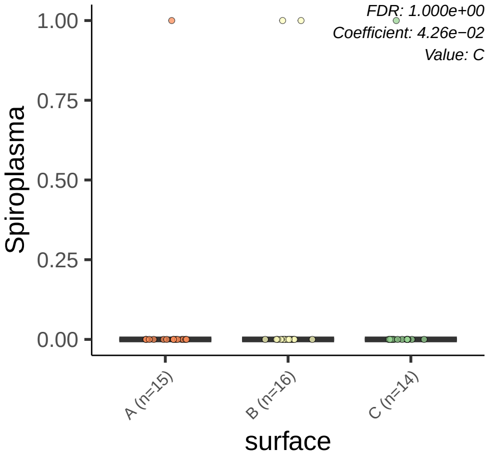
FDR: 1.000e+00
Coefficient: 4.26e−02
Value: C
Spiroplasma
1.00
0.75
0.50
0.25
0.00
A (n=15)
B (n=16)
C (n=14)
surface

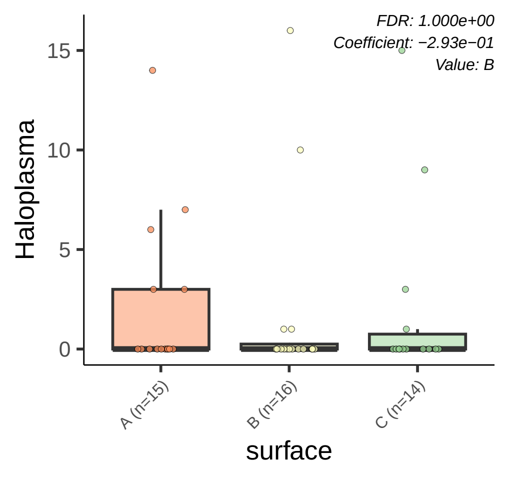
FDR: 1.000e+00
Coefficient: −2.93e−01
Value: B
Haloplasma
15
10
5
0
A (n=15)
B (n=16)
C (n=14)
surface

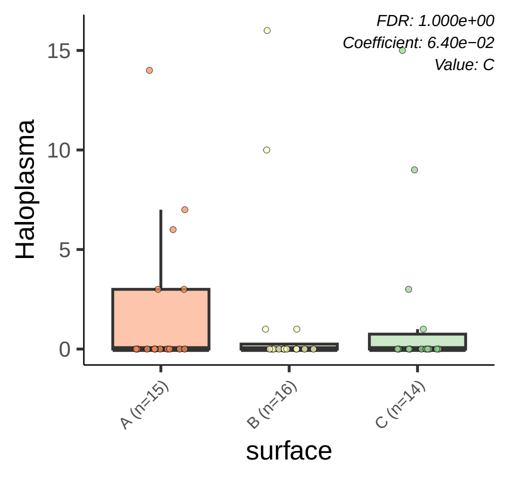

FDR: 1.000e+00
Coefficient: 6.40e−02
Value: C
Haloplasma
15
10
5
0
A (n=15)
B (n=16)
C (n=14)
surface

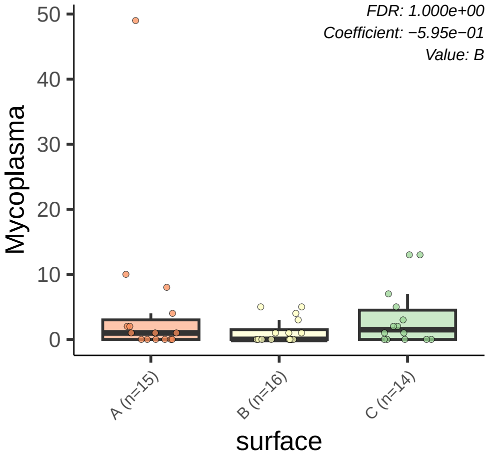

FDR: 1.000e+00
Coefficient: −5.95e−01
Value: B
Mycoplasma
50
40
30
20
10
0
A (n=15)
B (n=16)
C (n=14)
surface

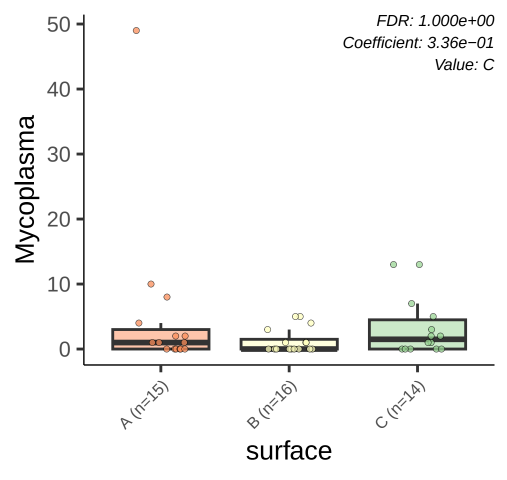

FDR: 1.000e+00
Coefficient: 3.36e−01
Value: C
Mycoplasma
50
40
30
20
10
0
A (n=15)
B (n=16)
C (n=14)
surface

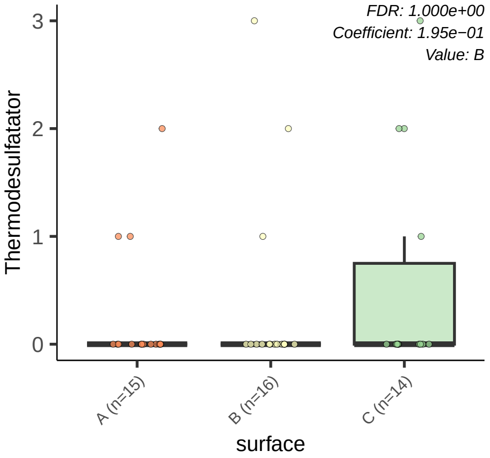
FDR: 1.000e+00
Coefficient: 1.95e−01
Value: B
Thermodesulfatator
3
2
1
0
A (n=15)
B (n=16)
C (n=14)
surface

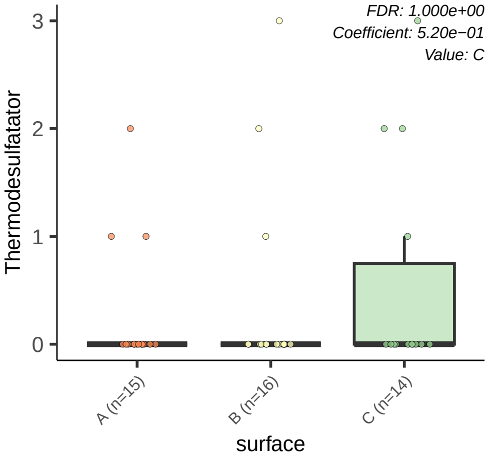
FDR: 1.000e+00
Coefficient: 5.20e−01
Value: C
Thermodesulfatator
3
2
1
0
A (n=15)
B (n=16)
C (n=14)
surface

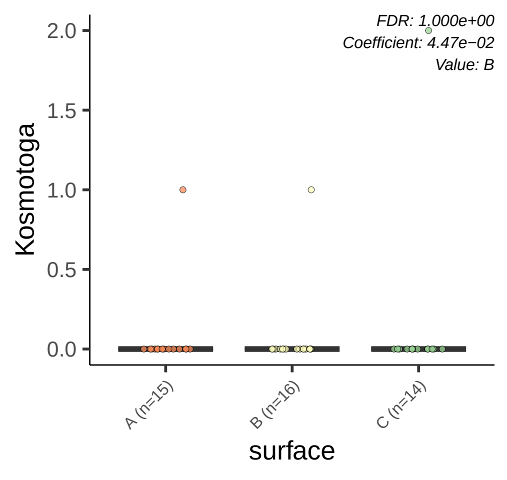

FDR: 1.000e+00
Coefficient: 4.47e−02
Value: B
2.0
1.5
1.0
0.5
0.0
Kosmotoga
A (n=15)
B (n=16)
C (n=14)
surface

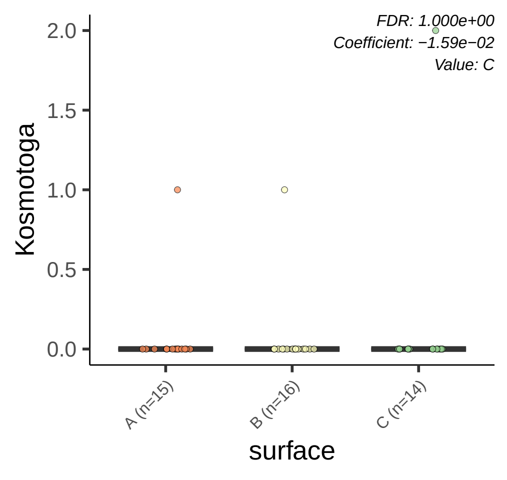
FDR: 1.000e+00
Coefficient: −1.59e−02
Value: C
Kosmotoga
2.0
1.5
1.0
0.5
0.0
A (n=15)
B (n=16)
C (n=14)
surface

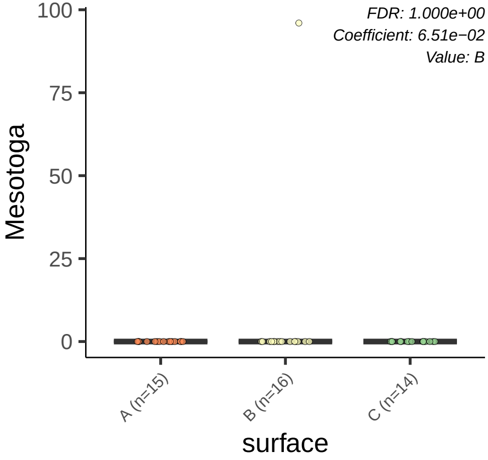
FDR: 1.000e+00
Coefficient: 6.51e−02
Value: B
Mesotoga
100
75
50
25
0
A (n=15)
B (n=16)
C (n=14)
surface

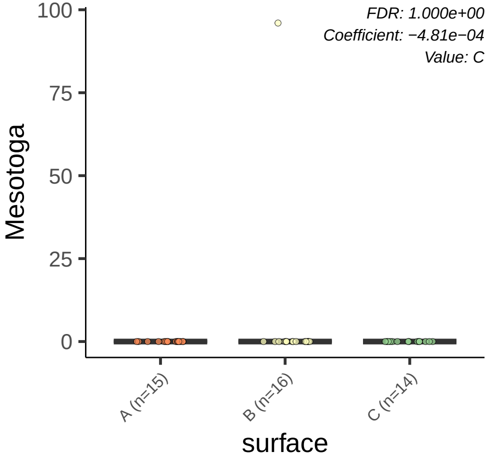
FDR: 1.000e+00
Coefficient: −4.81e−04
Value: C
Mesotoga
100
75
50
25
0
A (n=15)
B (n=16)
C (n=14)
surface

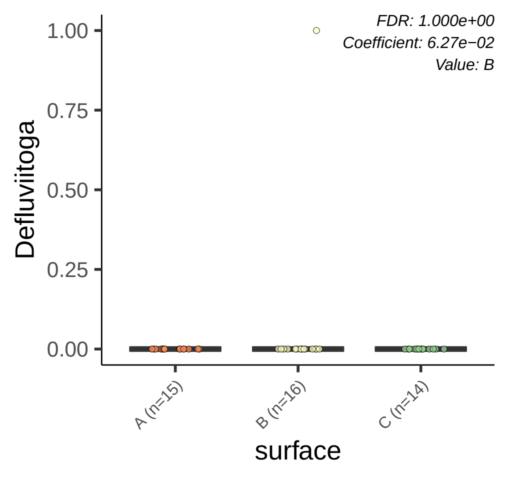
FDR: 1.000e+00
Coefficient: 6.27e−02
Value: B
1.00
0.75
0.50
0.25
0.00
Defluviitoga
A (n=15)
B (n=16)
C (n=14)
surface

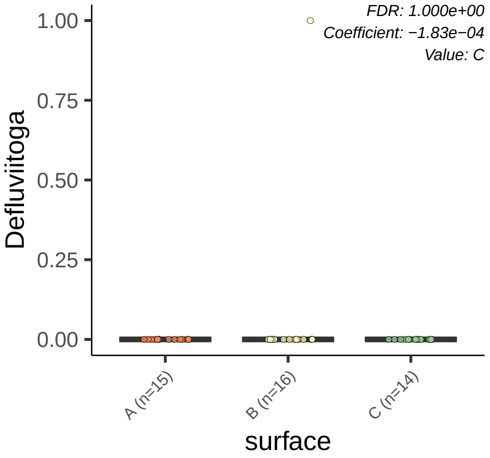
FDR: 1.000e+00
Coefficient: −1.83e−04
Value: C
Defluviitoga
1.00
0.75
0.50
0.25
0.00
A (n=15)
B (n=16)
C (n=14)
surface

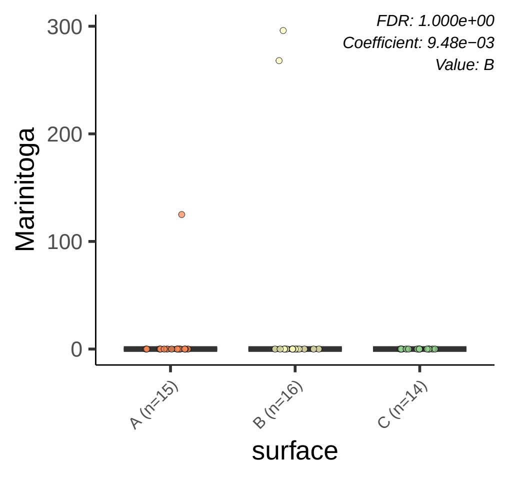
FDR: 1.000e+00
Coefficient: 9.48e−03
Value: B
Marinitoga
300
200
100
0
A (n=15)
B (n=16)
C (n=14)
surface

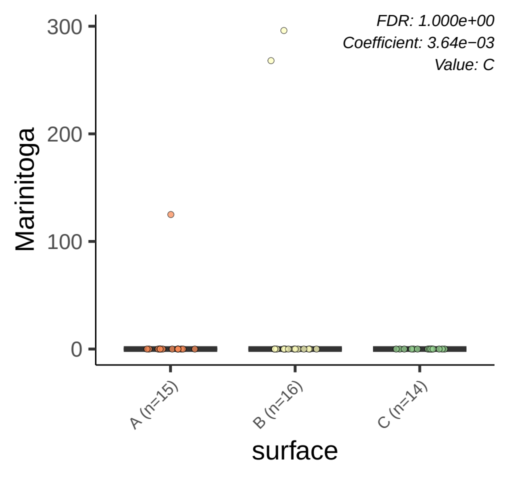
FDR: 1.000e+00
Coefficient: 3.64e−03
Value: C
Marinitoga
300
200
100
0
A (n=15)
B (n=16)
C (n=14)
surface

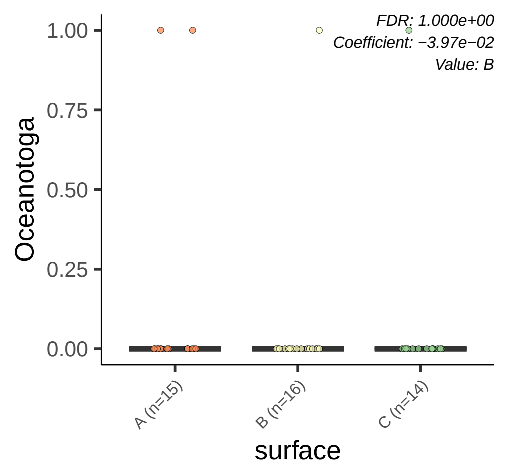

FDR: 1.000e+00
Coefficient: −3.97e−02
Value: B
Oceanotoga
1.00
0.75
0.50
0.25
0.00
A (n=15)
B (n=16)
C (n=14)
surface

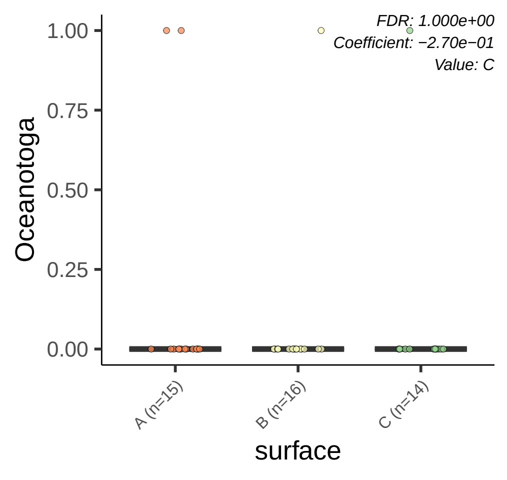

FDR: 1.000e+00
Coefficient: −2.70e−01
Value: C
Oceanotoga
1.00
0.75
0.50
0.25
0.00
A (n=15)
B (n=16)
C (n=14)
surface

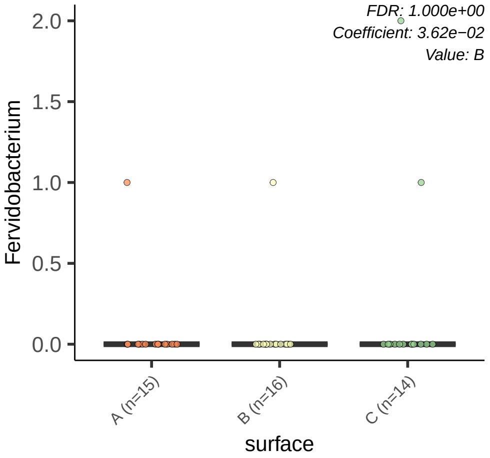

FDR: 1.000e+00
Coefficient: 3.62e−02
Value: B
Fervidobacterium
2.0
1.5
1.0
0.5
0.0
A (n=15)
B (n=16)
C (n=14)
surface

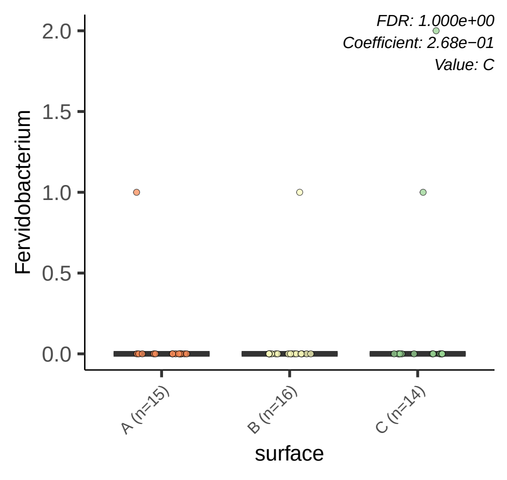
FDR: 1.000e+00
Coefficient: 2.68e−01
Value: C
Fervidobacterium
2.0
1.5
1.0
0.5
0.0
A (n=15)
B (n=16)
C (n=14)
surface

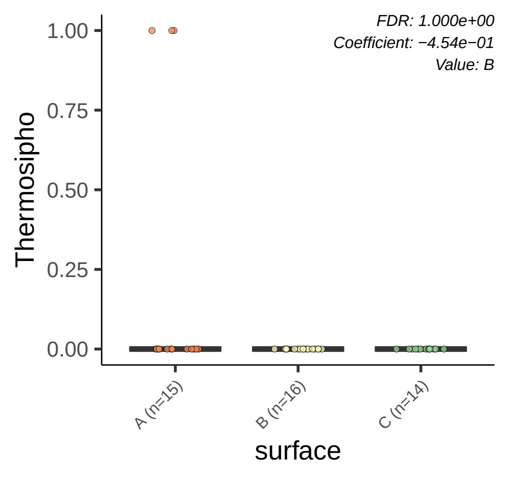
FDR: 1.000e+00
Coefficient: −4.54e−01
Value: B
Thermosipho
1.00
0.75
0.50
0.25
0.00
A (n=15)
B (n=16)
C (n=14)
surface

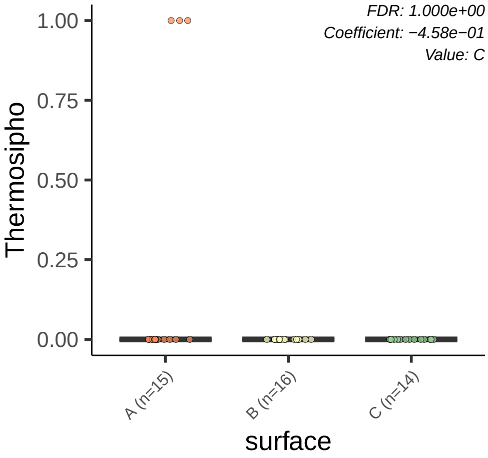
FDR: 1.000e+00
Coefficient: −4.58e−01
Value: C
Thermosipho
1.00
0.75
0.50
0.25
0.00
A (n=15)
B (n=16)
C (n=14)
surface

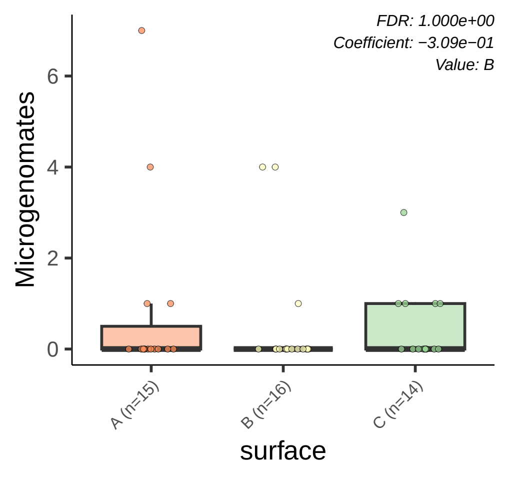

FDR: 1.000e+00
Coefficient: −3.09e−01
Value: B
Microgenomates
0
2
4
6
A (n=15)
B (n=16)
C (n=14)
surface

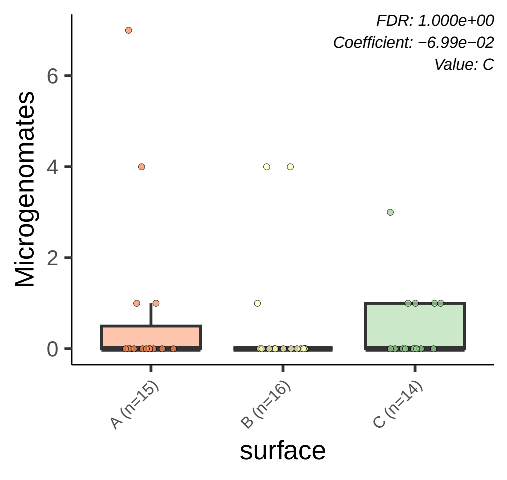

FDR: 1.000e+00
Coefficient: −6.99e−02
Value: C
Microgenomates
0
2
4
6
A (n=15)
B (n=16)
C (n=14)
surface

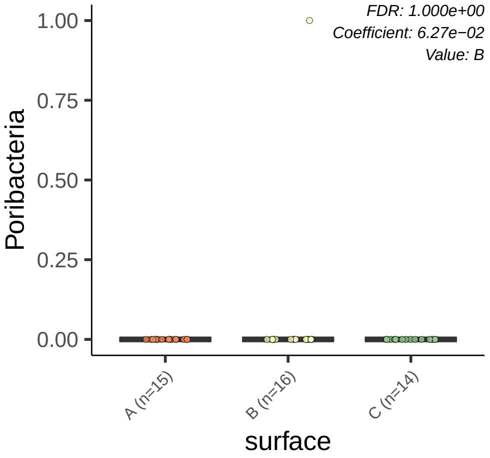
FDR: 1.000e+00
Coefficient: 6.27e−02
Value: B
Poribacteria
1.00
0.75
0.50
0.25
0.00
A (n=15)
B (n=16)
C (n=14)
surface

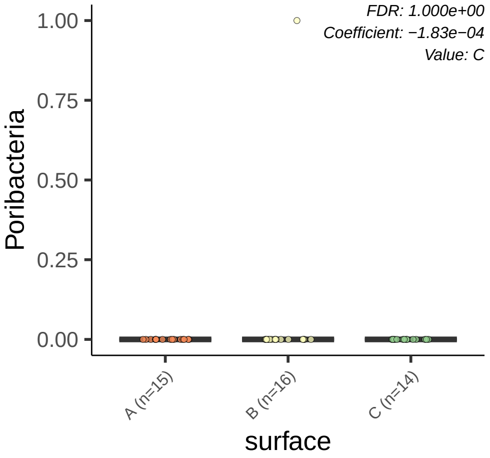
FDR: 1.000e+00
Coefficient: −1.83e−04
Value: C
Poribacteria
1.00
0.75
0.50
0.25
0.00
A (n=15)
B (n=16)
C (n=14)
surface

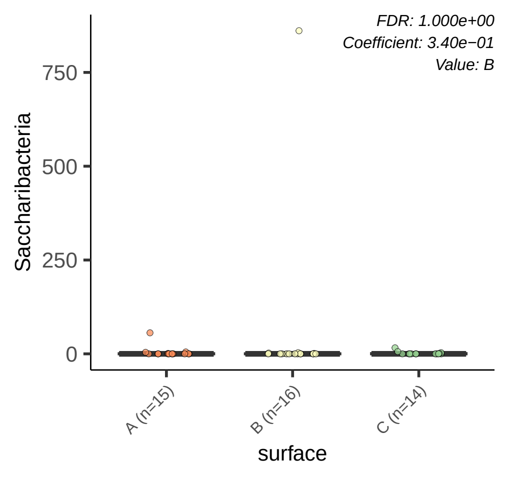
FDR: 1.000e+00
Coefficient: 3.40e−01
Value: B
Saccharibacteria
750
500
250
0
A (n=15)
B (n=16)
C (n=14)
surface

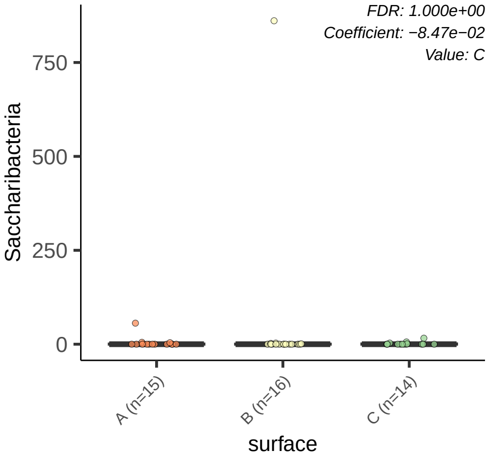
FDR: 1.000e+00
Coefficient: −8.47e−02
Value: C
Saccharibacteria
750
500
250
0
A (n=15)
B (n=16)
C (n=14)
surface

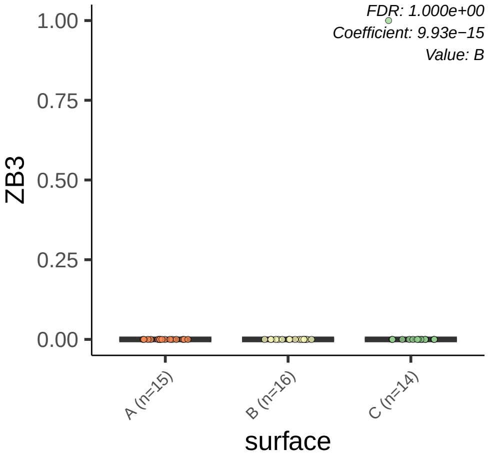
FDR: 1.000e+00
Coefficient: 9.93e−15
Value: B
ZB3
1.00
0.75
0.50
0.25
0.00
A (n=15)
B (n=16)
C (n=14)
surface

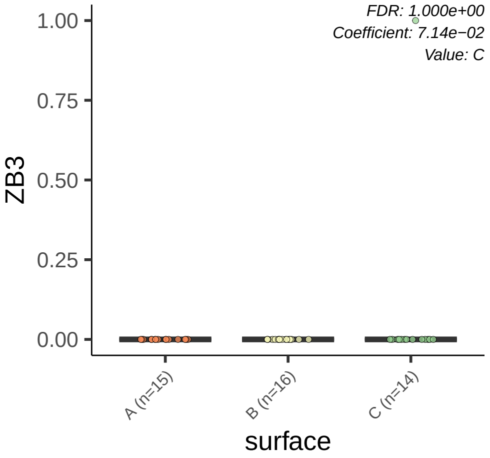
FDR: 1.000e+00
Coefficient: 7.14e−02
Value: C
ZB3
1.00
0.75
0.50
0.25
0.00
A (n=15)
B (n=16)
C (n=14)
surface

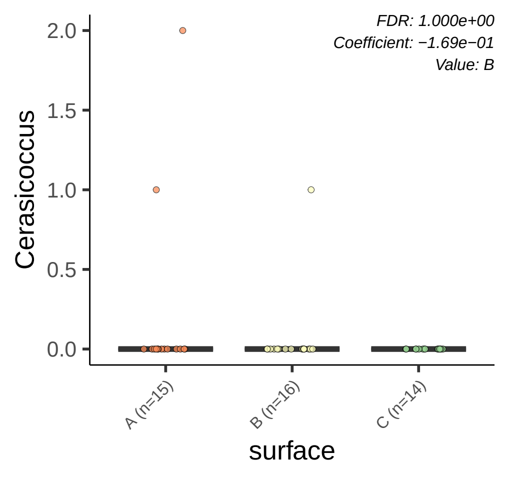
FDR: 1.000e+00
Coefficient: −1.69e−01
Value: B
Cerasicoccus
2.0
1.5
1.0
0.5
0.0
A (n=15)
B (n=16)
C (n=14)
surface

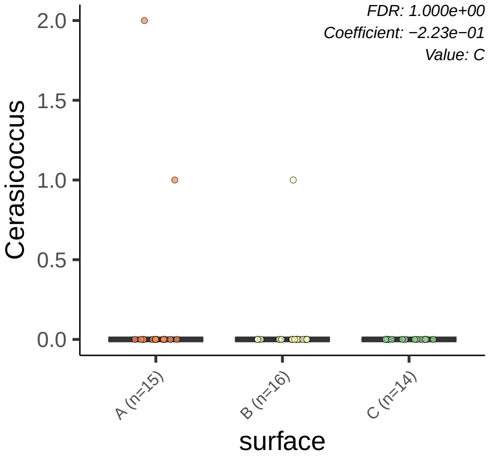
FDR: 1.000e+00
Coefficient: −2.23e−01
Value: C
Cerasicoccus
2.0
1.5
1.0
0.5
0.0
A (n=15)
B (n=16)
C (n=14)
surface

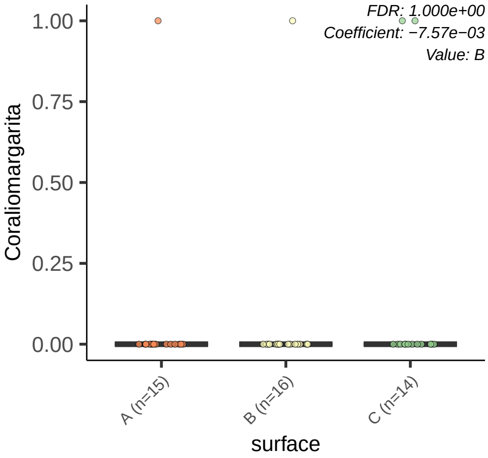
FDR: 1.000e+00
Coefficient: −7.57e−03
Value: B
Coraliomargarita
1.00
0.75
0.50
0.25
0.00
A (n=15)
B (n=16)
C (n=14)
surface

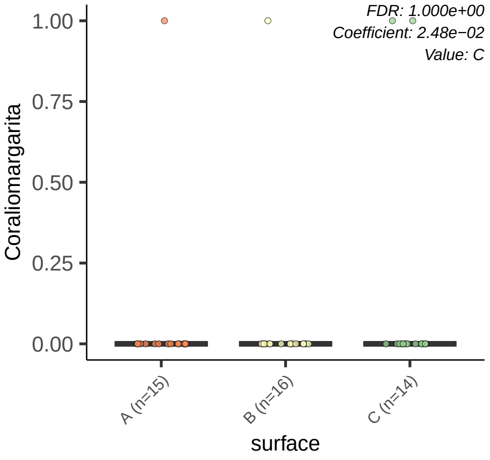

FDR: 1.000e+00
Coefficient: 2.48e−02
Value: C
1.00
0.75
0.50
0.25
0.00
Coraliomargarita
A (n=15)
B (n=16)
C (n=14)
surface

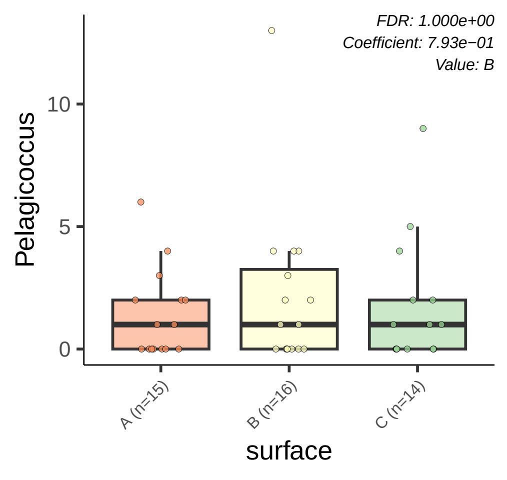
FDR: 1.000e+00
Coefficient: 7.93e−01
Value: B
Pelagicoccus
10
5
0
A (n=15)
B (n=16)
C (n=14)
surface

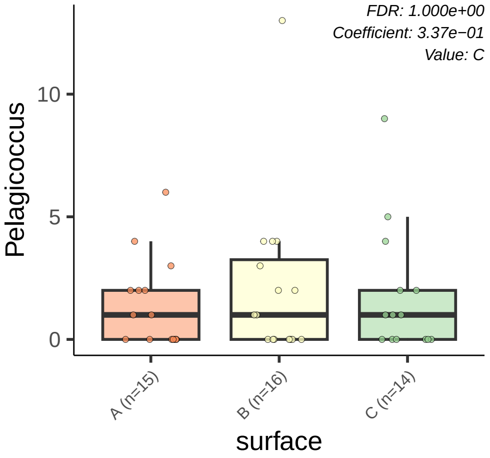
FDR: 1.000e+00
Coefficient: 3.37e−01
Value: C
Pelagicoccus
10
5
0
A (n=15)
B (n=16)
C (n=14)
surface

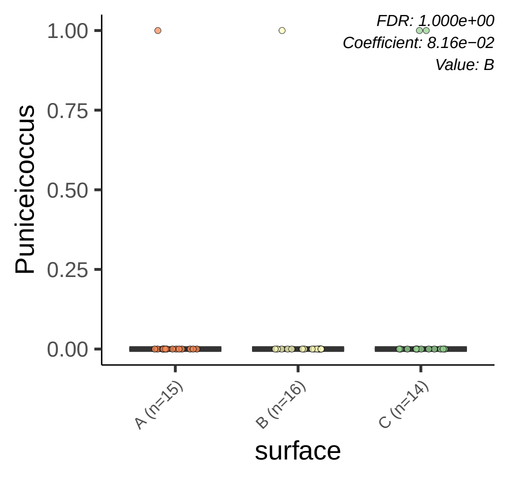
FDR: 1.000e+00
Coefficient: 8.16e−02
Value: B
Puniceicoccus
1.00
0.75
0.50
0.25
0.00
A (n=15)
B (n=16)
C (n=14)
surface

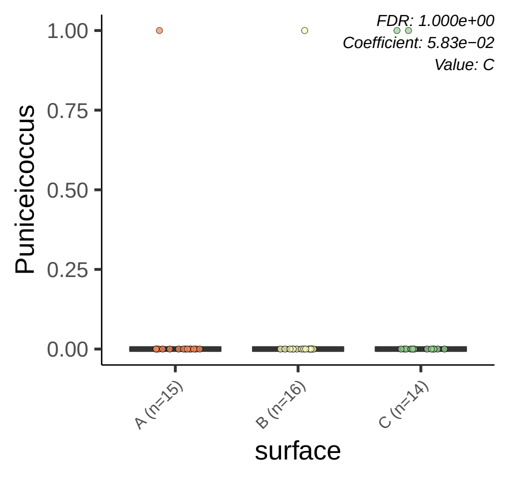

FDR: 1.000e+00
Coefficient: 5.83e−02
Value: C
Puniceicoccus
1.00
0.75
0.50
0.25
0.00
A (n=15)
B (n=16)
C (n=14)
surface

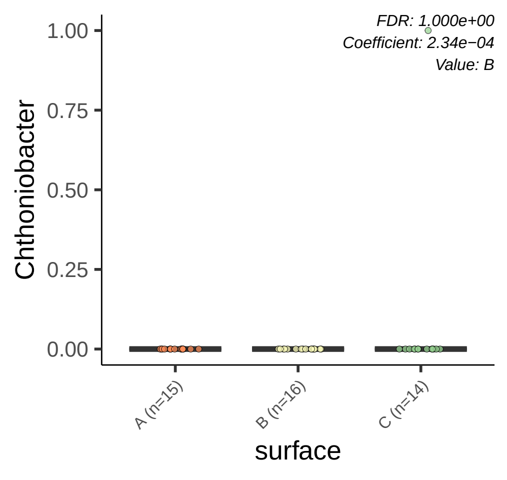
FDR: 1.000e+00
Coefficient: 2.34e−04
Value: B
Chthoniobacter
1.00
0.75
0.50
0.25
0.00
A (n=15)
B (n=16)
C (n=14)
surface

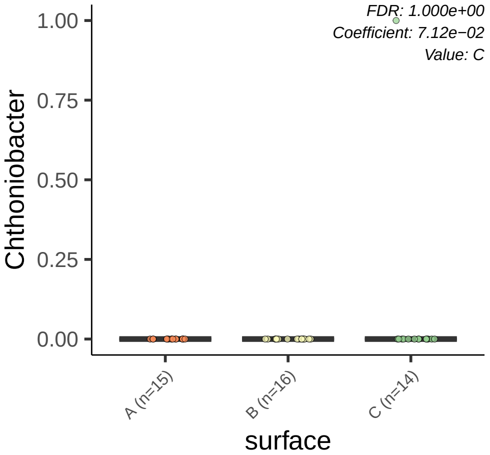

FDR: 1.000e+00
Coefficient: 7.12e−02
Value: C
Chthoniobacter
1.00
0.75
0.50
0.25
0.00
A (n=15)
B (n=16)
C (n=14)
surface

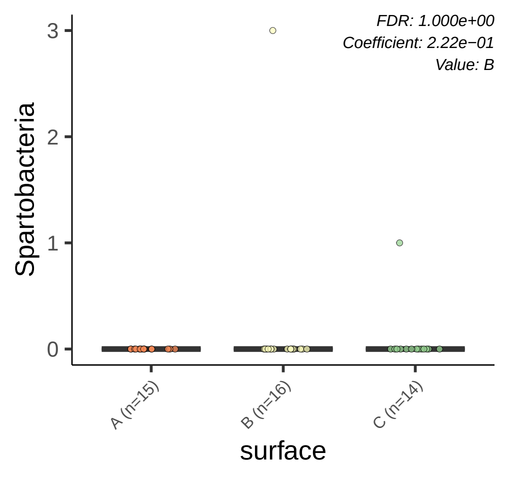
FDR: 1.000e+00
Coefficient: 2.22e−01
Value: B
Spartobacteria
3
2
1
0
A (n=15)
B (n=16)
C (n=14)
surface

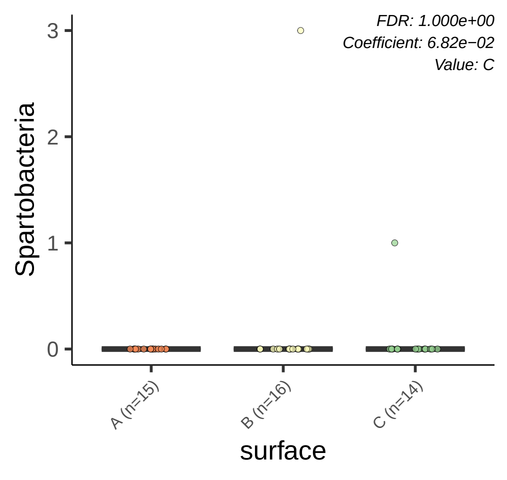
FDR: 1.000e+00
Coefficient: 6.82e−02
Value: C
Spartobacteria
3
2
1
0
A (n=15)
B (n=16)
C (n=14)
surface

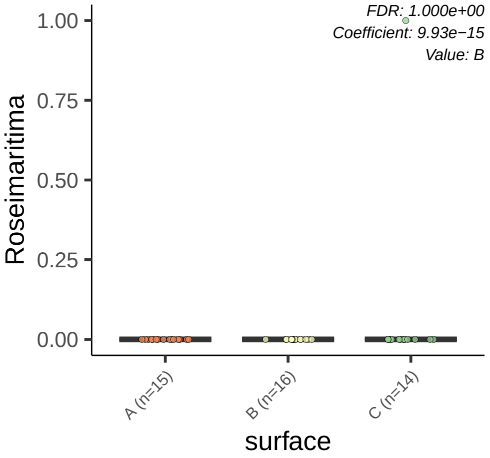

FDR: 1.000e+00
Coefficient: 9.93e−15
Value: B
Roseimaritima
1.00
0.75
0.50
0.25
0.00
A (n=15)
B (n=16)
C (n=14)
surface

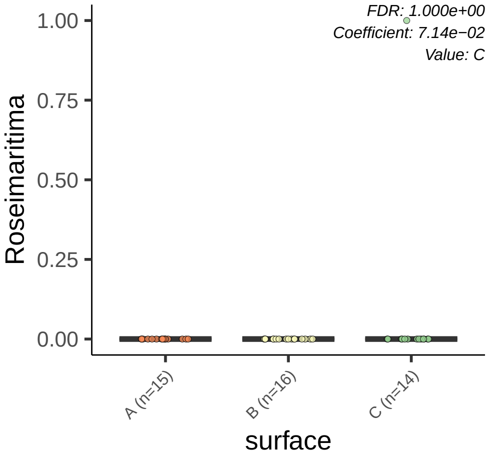

FDR: 1.000e+00
Coefficient: 7.14e−02
Value: C
Roseimaritima
1.00
0.75
0.50
0.25
0.00
A (n=15)
B (n=16)
C (n=14)
surface

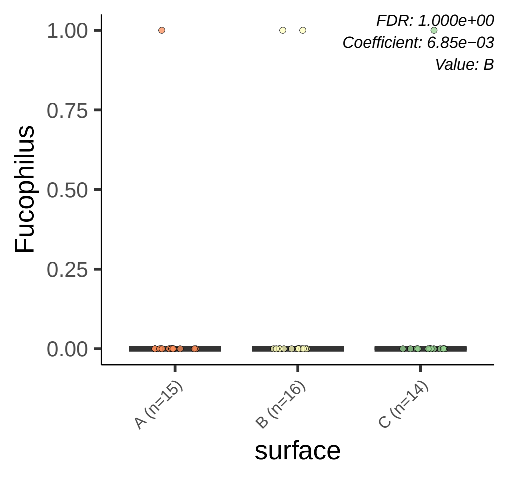
FDR: 1.000e+00
Coefficient: 6.85e−03
Value: B
Fucophilus
1.00
0.75
0.50
0.25
0.00
A (n=15)
B (n=16)
C (n=14)
surface

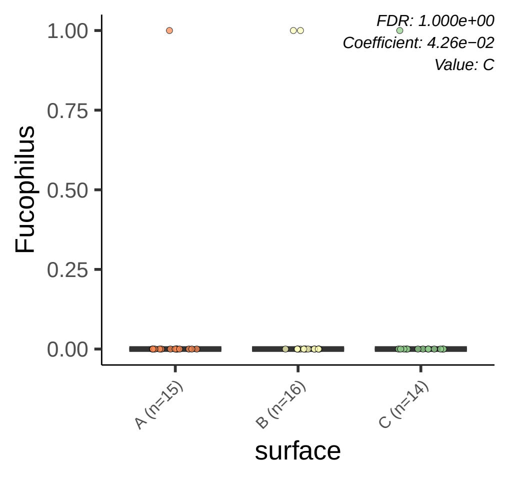

FDR: 1.000e+00
Coefficient: 4.26e−02
Value: C
1.00
0.75
0.50
0.25
0.00
Fucophilus
A (n=15)
B (n=16)
C (n=14)
surface

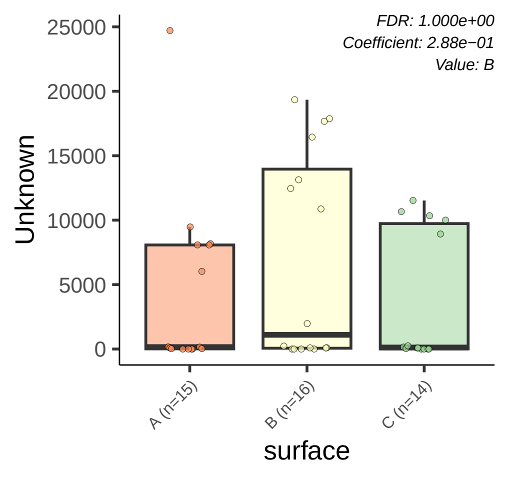

FDR: 1.000e+00
Coefficient: 2.88e−01
Value: B
Unknown
25000
20000
15000
10000
5000
0
A (n=15)
B (n=16)
C (n=14)
surface

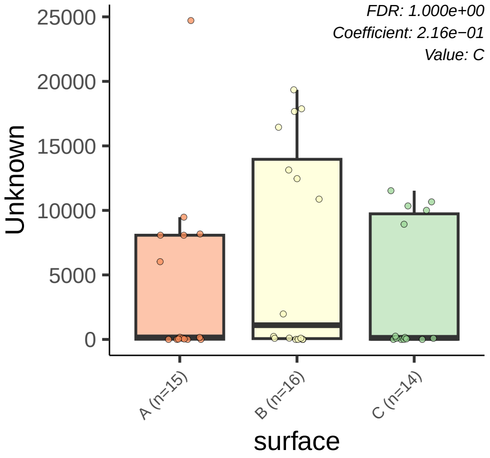
FDR: 1.000e+00
Coefficient: 2.16e−01
Value: C
Unknown
25000
20000
15000
10000
5000
0
A (n=15)
B (n=16)
C (n=14)
surface

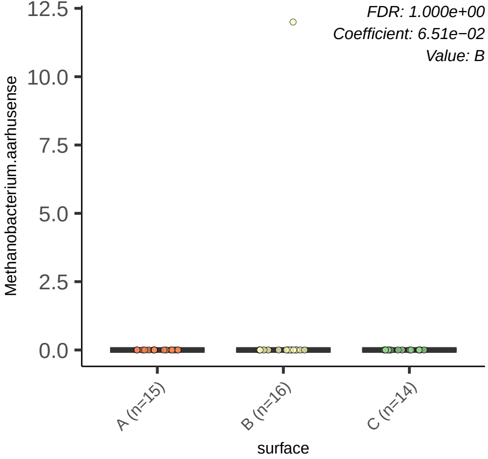
FDR: 1.000e+00
Coefficient: 6.51e−02
Value: B
Methanobacterium.aarhusense
12.5
10.0
7.5
5.0
2.5
0.0
A (n=15)
B (n=16)
C (n=14)
surface

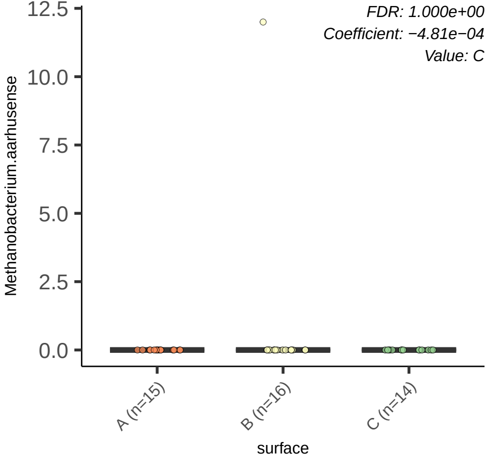

FDR: 1.000e+00
Coefficient: −4.81e−04
Value: C
Methanobacterium.aarhusense
12.5
10.0
7.5
5.0
2.5
0.0
A (n=15)
B (n=16)
C (n=14)
surface

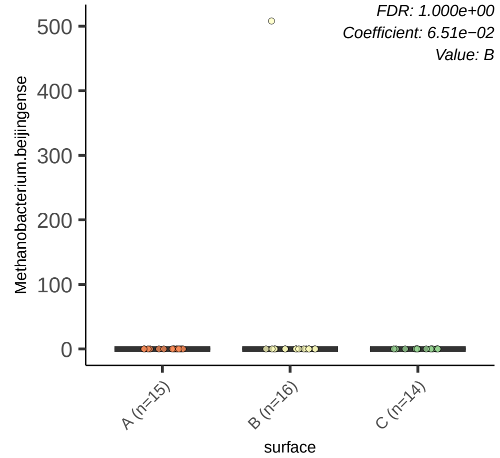

FDR: 1.000e+00
Coefficient: 6.51e−02
Value: B
Methanobacterium.beijingense
500
400
300
200
100
0
A (n=15)
B (n=16)
C (n=14)
surface

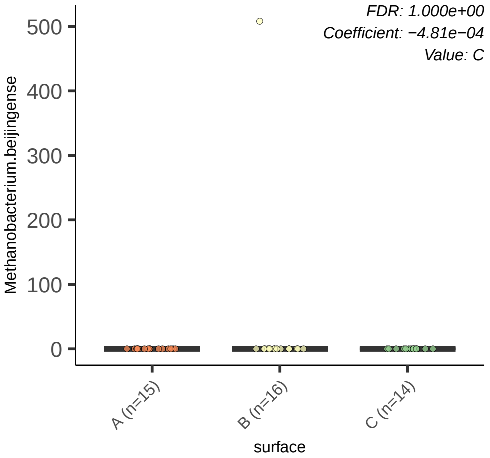
FDR: 1.000e+00
Coefficient: −4.81e−04
Value: C
Methanobacterium.beijingense
500
400
300
200
100
0
A (n=15)
B (n=16)
C (n=14)
surface

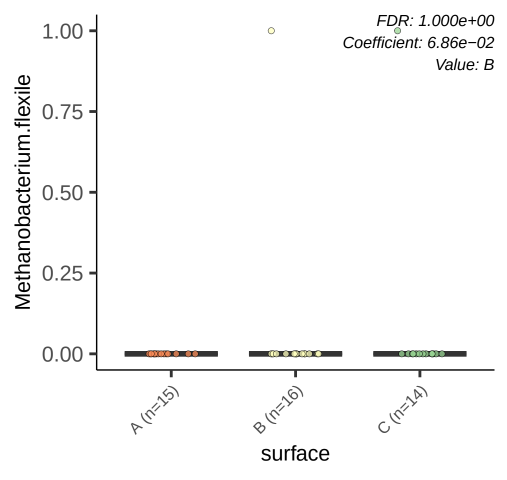
FDR: 1.000e+00
Coefficient: 6.86e−02
Value: B
Methanobacterium.flexile
1.00
0.75
0.50
0.25
0.00
A (n=15)
B (n=16)
C (n=14)
surface

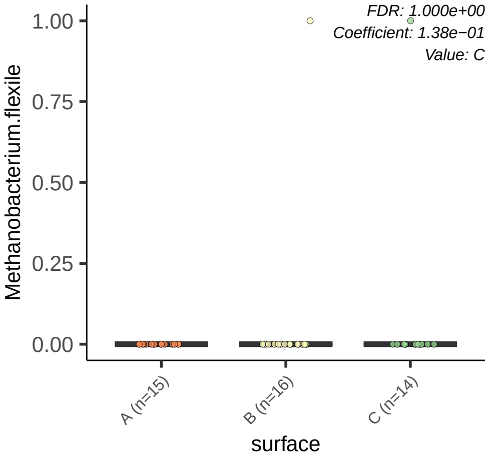
FDR: 1.000e+00
Coefficient: 1.38e−01
Value: C
Methanobacterium.flexile
1.00
0.75
0.50
0.25
0.00
A (n=15)
B (n=16)
C (n=14)
surface

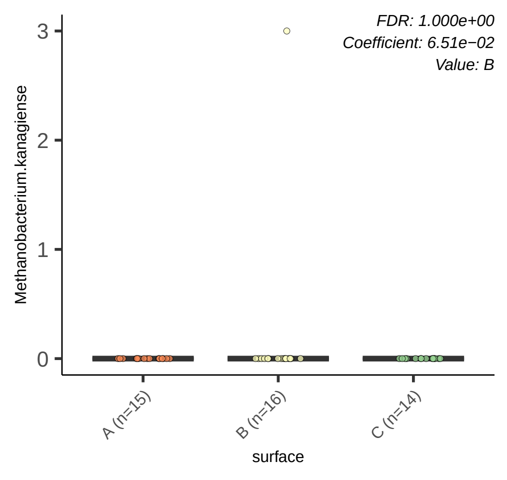
FDR: 1.000e+00
Coefficient: 6.51e−02
Value: B
Methanobacterium.kanagiense
3
2
1
0
A (n=15)
B (n=16)
C (n=14)
surface

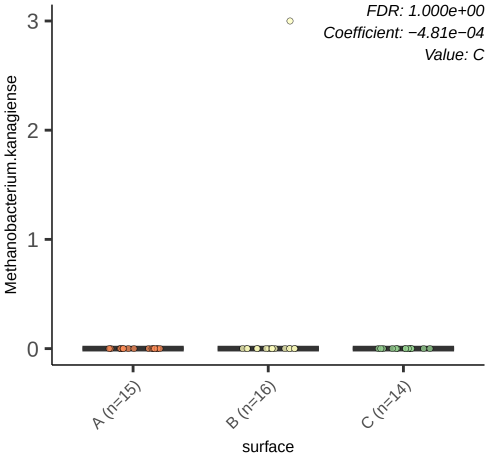

FDR: 1.000e+00
Coefficient: −4.81e−04
Value: C
Methanobacterium.kanagiense
3
2
1
0
A (n=15)
B (n=16)
C (n=14)
surface

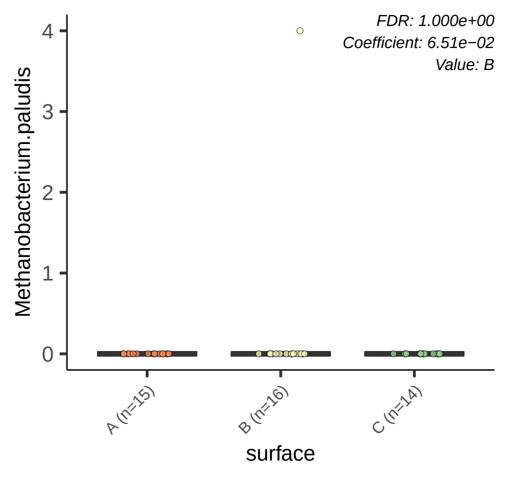

FDR: 1.000e+00
Coefficient: 6.51e−02
Value: B
Methanobacterium.paludis
0
1
2
3
4
A (n=15)
B (n=16)
C (n=14)
surface

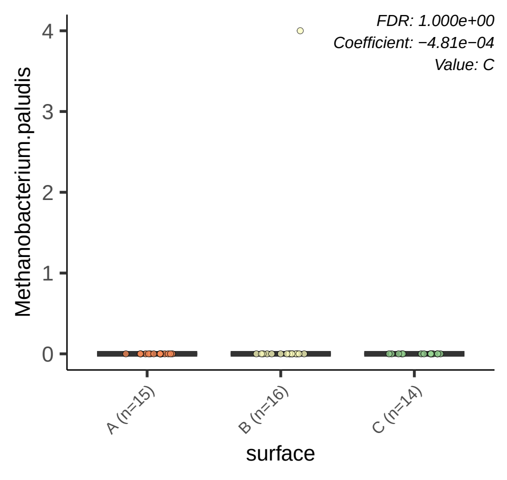
FDR: 1.000e+00
Coefficient: −4.81e−04
Value: C
Methanobacterium.paludis
4
3
2
1
0
A (n=15)
B (n=16)
C (n=14)
surface

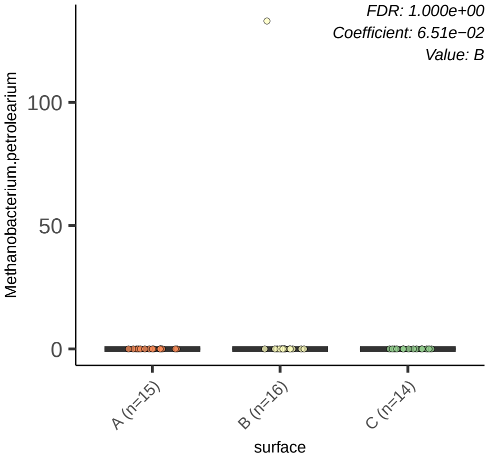
FDR: 1.000e+00
Coefficient: 6.51e−02
Value: B
Methanobacterium.petrolearium
100
50
0
A (n=15)
B (n=16)
C (n=14)
surface

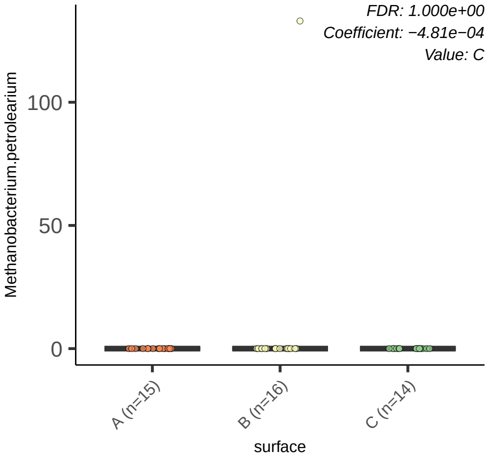

FDR: 1.000e+00
Coefficient: −4.81e−04
Value: C
Methanobacterium.petrolearium
100
50
0
A (n=15)
B (n=16)
C (n=14)
surface

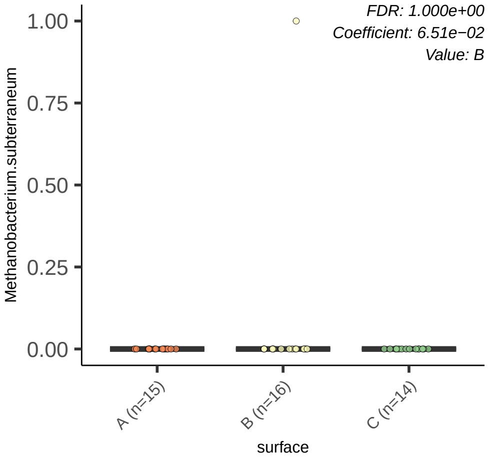

FDR: 1.000e+00
Coefficient: 6.51e−02
Value: B
1.00
0.75
0.50
0.25
0.00
Methanobacterium.subterraneum
A (n=15)
B (n=16)
C (n=14)
surface

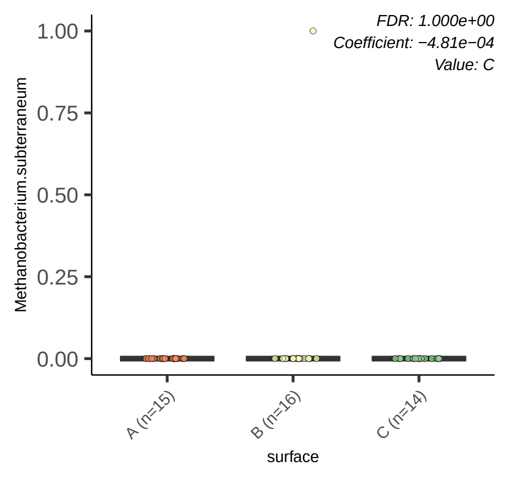
FDR: 1.000e+00
Coefficient: −4.81e−04
Value: C
Methanobacterium.subterraneum
1.00
0.75
0.50
0.25
0.00
A (n=15)
B (n=16)
C (n=14)
surface

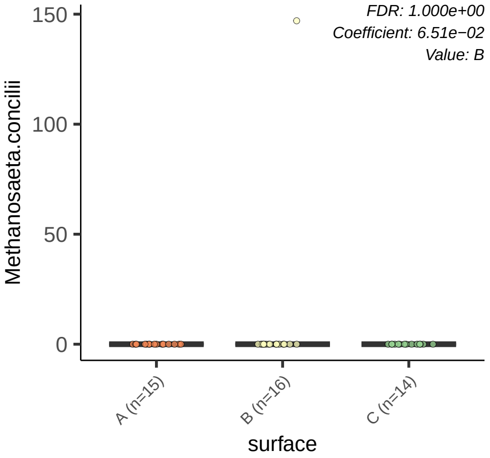

FDR: 1.000e+00
Coefficient: 6.51e−02
Value: B
Methanosaeta.concilii
150
100
50
0
A (n=15)
B (n=16)
C (n=14)
surface

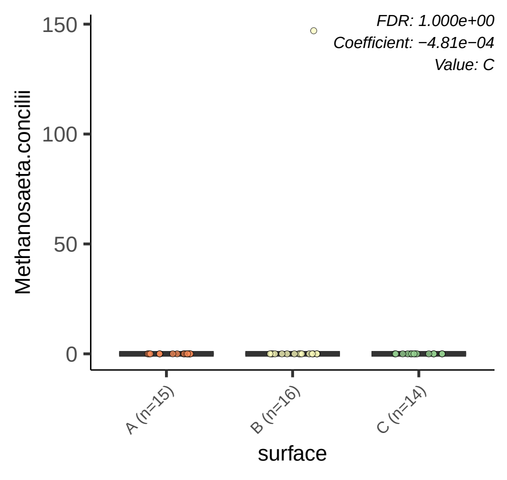

FDR: 1.000e+00
Coefficient: −4.81e−04
Value: C
Methanosaeta.concilii
150
100
50
0
A (n=15)
B (n=16)
C (n=14)
surface

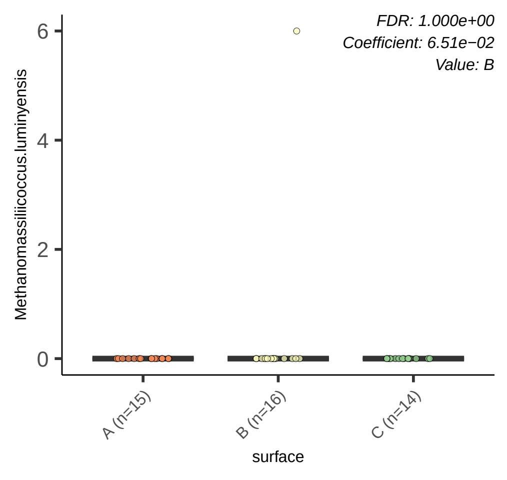
FDR: 1.000e+00
Coefficient: 6.51e−02
Value: B
Methanomassiliicoccus.luminyensis
0
2
4
6
A (n=15)
B (n=16)
C (n=14)
surface

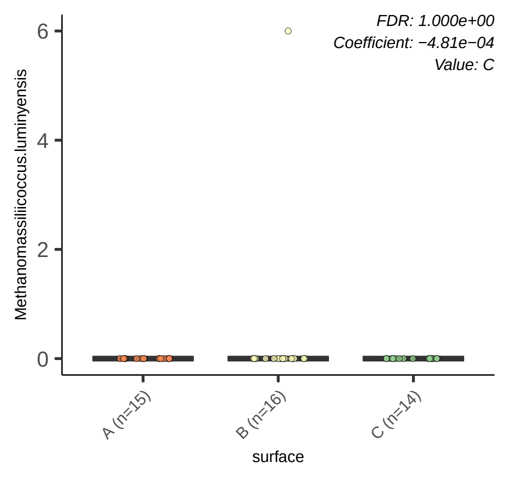
FDR: 1.000e+00
Coefficient: −4.81e−04
Value: C
Methanomassiliicoccus.luminyensis
0
2
4
6
A (n=15)
B (n=16)
C (n=14)
surface

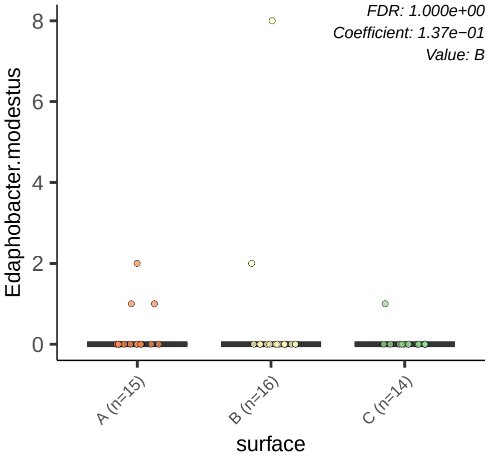

FDR: 1.000e+00
Coefficient: 1.37e−01
Value: B
Edaphobacter.modestus
0
2
4
6
8
A (n=15)
B (n=16)
C (n=14)
surface

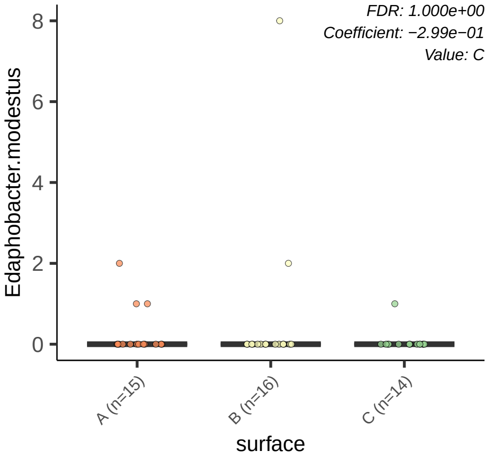

FDR: 1.000e+00
Coefficient: −2.99e−01
Value: C
Edaphobacter.modestus
0
2
4
6
8
A (n=15)
B (n=16)
C (n=14)
surface

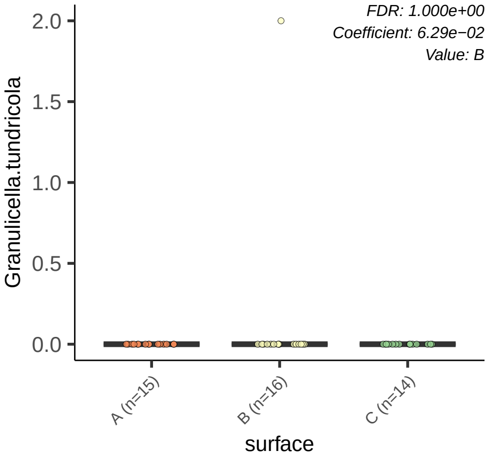

FDR: 1.000e+00
Coefficient: 6.29e−02
Value: B
Granulicella.tundricola
2.0
1.5
1.0
0.5
0.0
A (n=15)
B (n=16)
C (n=14)
surface

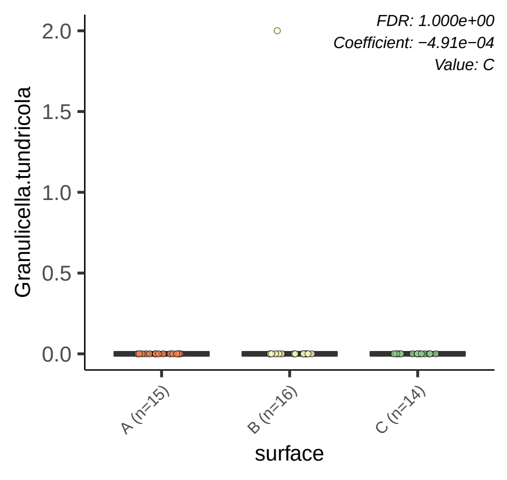
FDR: 1.000e+00
Coefficient: −4.91e−04
Value: C
Granulicella.tundricola
2.0
1.5
1.0
0.5
0.0
A (n=15)
B (n=16)
C (n=14)
surface

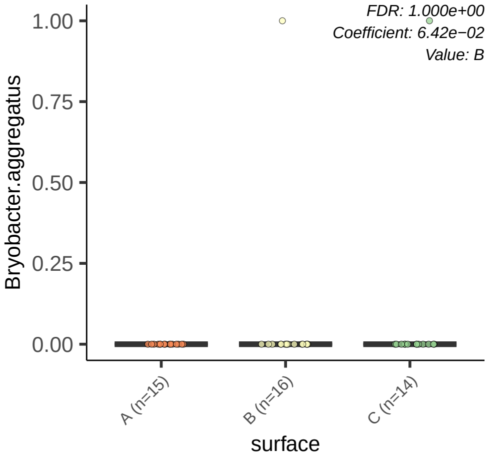

FDR: 1.000e+00
Coefficient: 6.42e−02
Value: B
Bryobacter.aggregatus
1.00
0.75
0.50
0.25
0.00
A (n=15)
B (n=16)
C (n=14)
surface

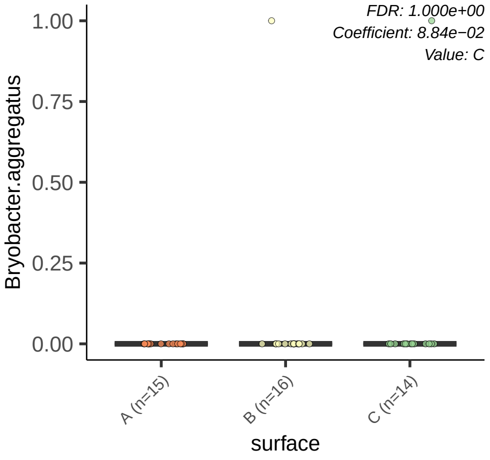
FDR: 1.000e+00
Coefficient: 8.84e−02
Value: C
Bryobacter.aggregatus
1.00
0.75
0.50
0.25
0.00
A (n=15)
B (n=16)
C (n=14)
surface

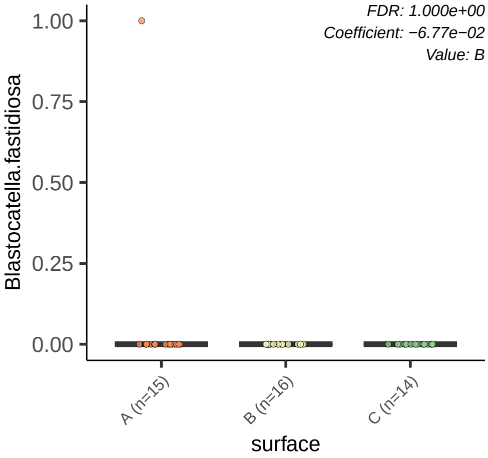
FDR: 1.000e+00
Coefficient: −6.77e−02
Value: B
Blastocatella.fastidiosa
1.00
0.75
0.50
0.25
0.00
A (n=15)
B (n=16)
C (n=14)
surface

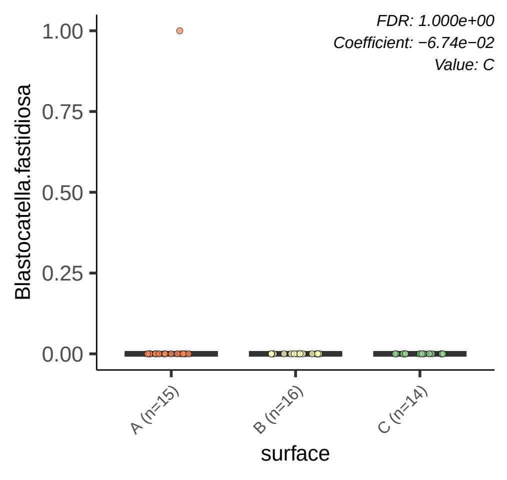
FDR: 1.000e+00
Coefficient: −6.74e−02
Value: C
Blastocatella.fastidiosa
1.00
0.75
0.50
0.25
0.00
A (n=15)
B (n=16)
C (n=14)
surface

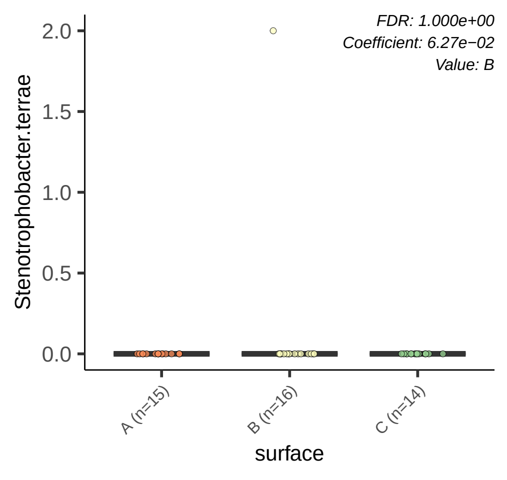
FDR: 1.000e+00
Coefficient: 6.27e−02
Value: B
Stenotrophobacter.terrae
2.0
1.5
1.0
0.5
0.0
A (n=15)
B (n=16)
C (n=14)
surface

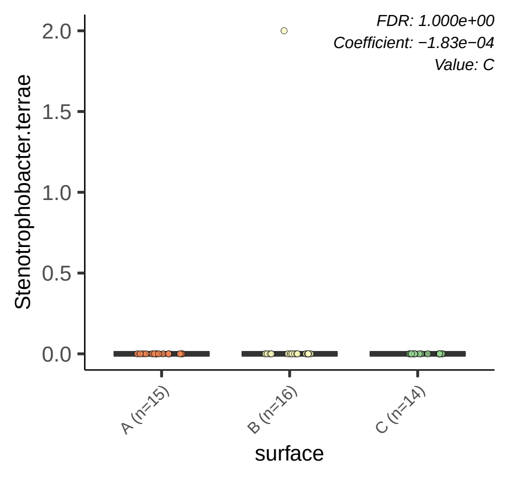
FDR: 1.000e+00
Coefficient: −1.83e−04
Value: C
Stenotrophobacter.terrae
2.0
1.5
1.0
0.5
0.0
A (n=15)
B (n=16)
C (n=14)
surface

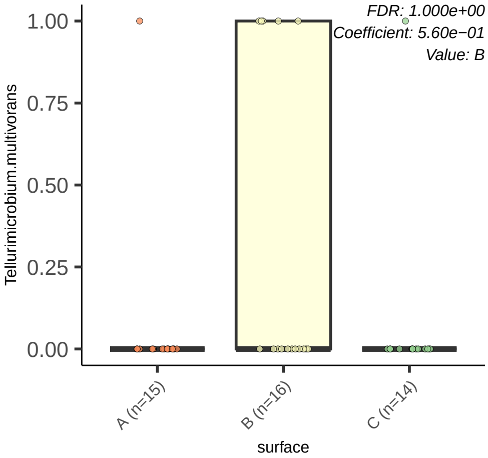
FDR: 1.000e+00
Coefficient: 5.60e−01
Value: B
Tellurimicrobium.multivorans
1.00
0.75
0.50
0.25
0.00
A (n=15)
B (n=16)
C (n=14)
surface

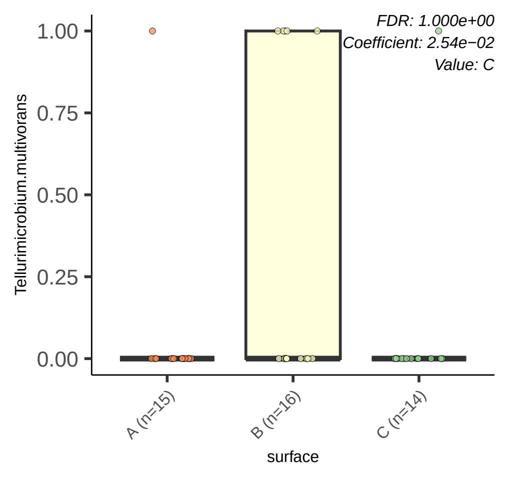

FDR: 1.000e+00
Coefficient: 2.54e−02
Value: C
1.00
0.75
0.50
0.25
0.00
Tellurimicrobium.multivorans
A (n=15)
B (n=16)
C (n=14)
surface

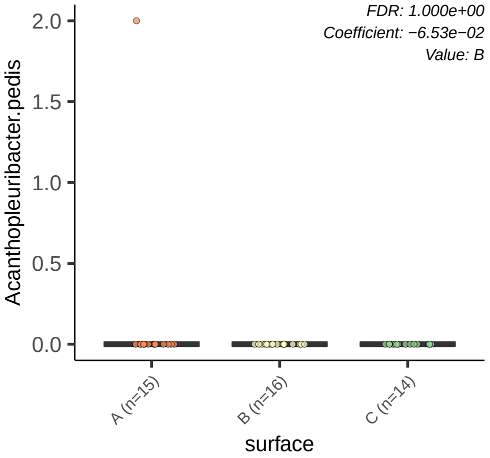

FDR: 1.000e+00
Coefficient: −6.53e−02
Value: B
Acanthopleuribacter.pedis
2.0
1.5
1.0
0.5
0.0
A (n=15)
B (n=16)
C (n=14)
surface

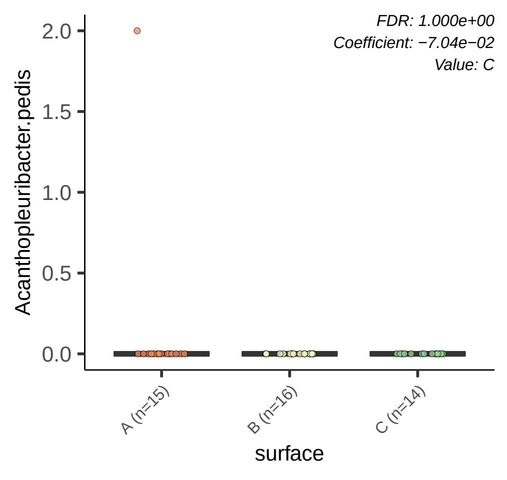

FDR: 1.000e+00
Coefficient: −7.04e−02
Value: C
Acanthopleuribacter.pedis
2.0
1.5
1.0
0.5
0.0
A (n=15)
B (n=16)
C (n=14)
surface

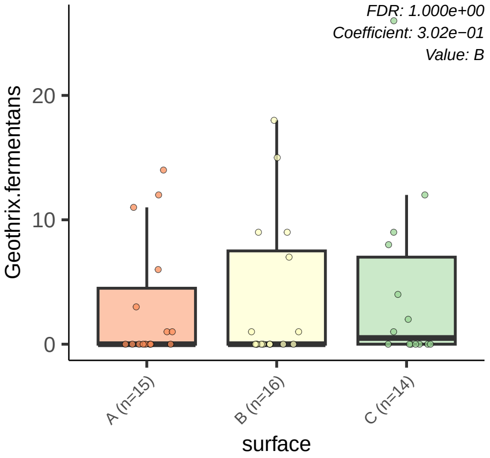
FDR: 1.000e+00
Coefficient: 3.02e−01
Value: B
Geothrix.fermentans
0
10
20
A (n=15)
B (n=16)
C (n=14)
surface

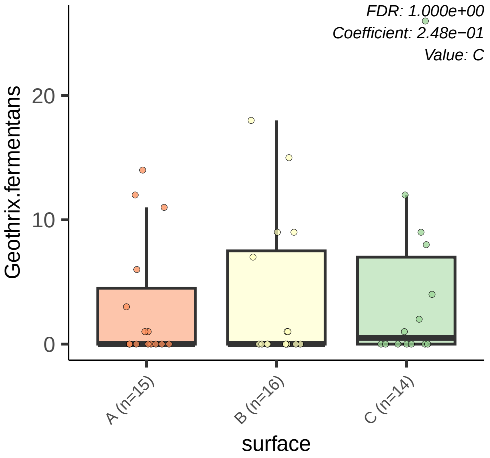

FDR: 1.000e+00
Coefficient: 2.48e−01
Value: C
Geothrix.fermentans
20
10
0
A (n=15)
B (n=16)
C (n=14)
surface

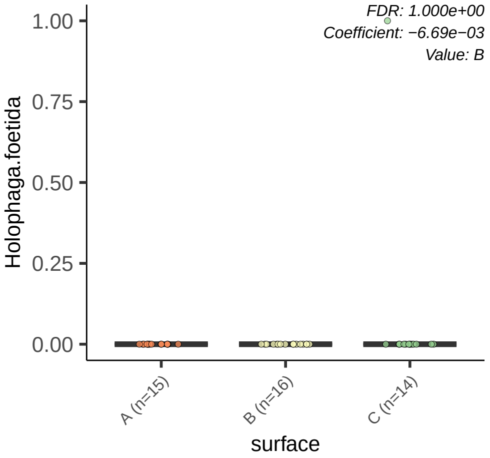

FDR: 1.000e+00
Coefficient: −6.69e−03
Value: B
Holophaga.foetida
1.00
0.75
0.50
0.25
0.00
A (n=15)
B (n=16)
C (n=14)
surface

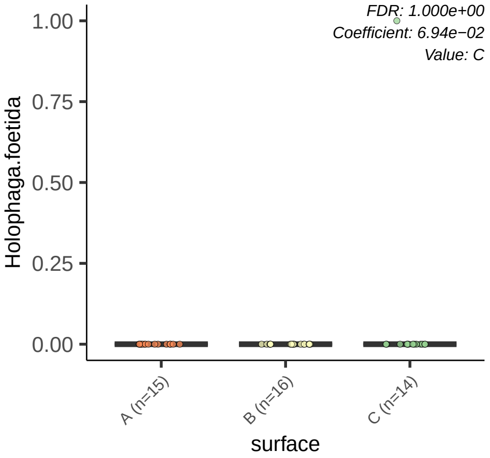
FDR: 1.000e+00
Coefficient: 6.94e−02
Value: C
Holophaga.foetida
1.00
0.75
0.50
0.25
0.00
A (n=15)
B (n=16)
C (n=14)
surface

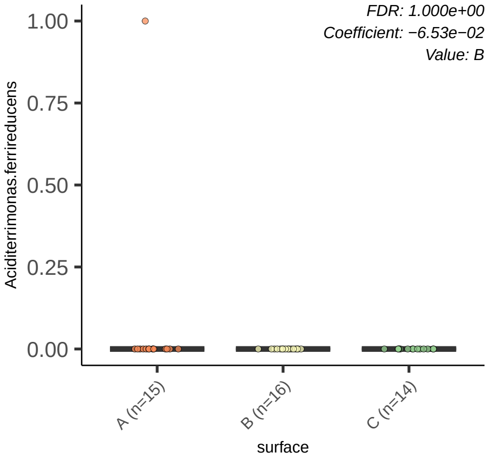

FDR: 1.000e+00
Coefficient: −6.53e−02
Value: B
1.00
0.75
0.50
0.25
0.00
Aciditerrimonas.ferrireducens
A (n=15)
B (n=16)
C (n=14)
surface

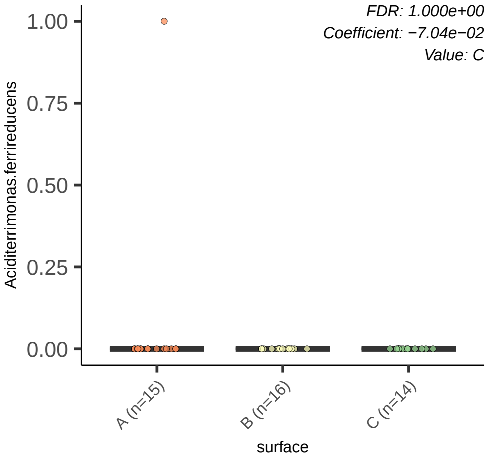

FDR: 1.000e+00
Coefficient: −7.04e−02
Value: C
Aciditerrimonas.ferrireducens
1.00
0.75
0.50
0.25
0.00
A (n=15)
B (n=16)
C (n=14)
surface

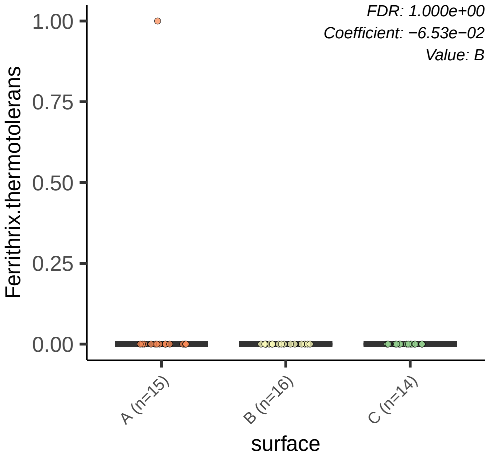
FDR: 1.000e+00
Coefficient: −6.53e−02
Value: B
Ferrithrix.thermotolerans
1.00
0.75
0.50
0.25
0.00
A (n=15)
B (n=16)
C (n=14)
surface

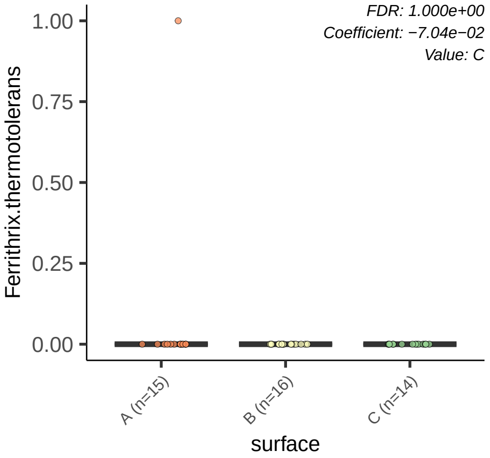
FDR: 1.000e+00
Coefficient: −7.04e−02
Value: C
1.00
0.75
0.50
0.25
0.00
Ferrithrix.thermotolerans
A (n=15)
B (n=16)
C (n=14)
surface

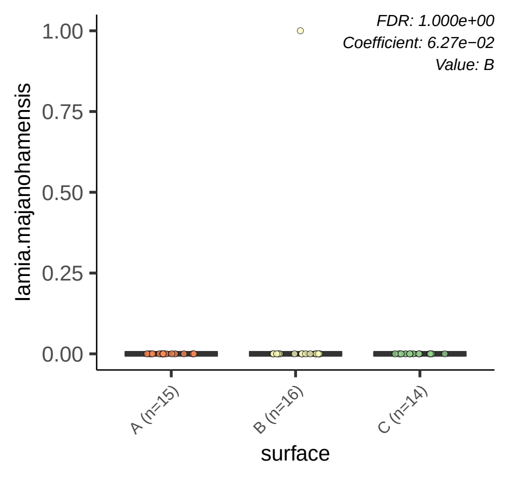
FDR: 1.000e+00
Coefficient: 6.27e−02
Value: B
lamia.majanohamensis
1.00
0.75
0.50
0.25
0.00
A (n=15)
B (n=16)
C (n=14)
surface

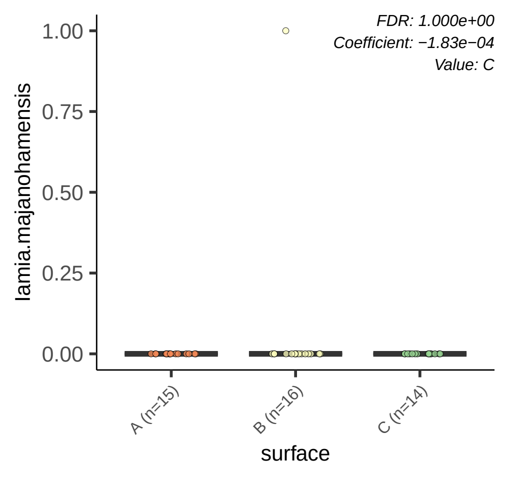
FDR: 1.000e+00
Coefficient: −1.83e−04
Value: C
lamia.majanohamensis
1.00
0.75
0.50
0.25
0.00
A (n=15)
B (n=16)
C (n=14)
surface

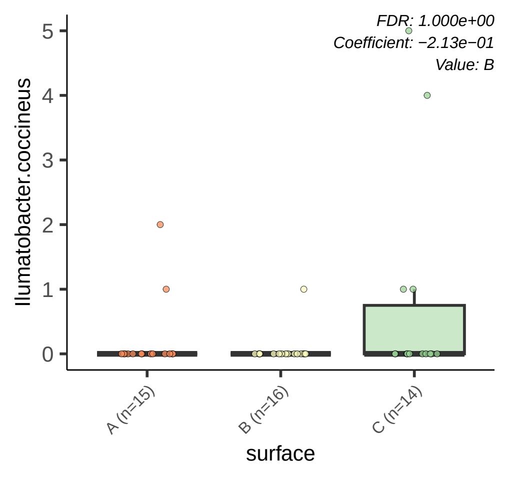
FDR: 1.000e+00
Coefficient: −2.13e−01
Value: B
Ilumatobacter.coccineus
0
1
2
3
4
5
A (n=15)
B (n=16)
C (n=14)
surface

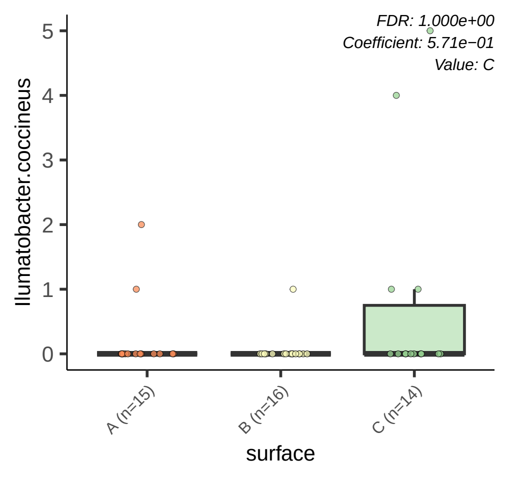
FDR: 1.000e+00
Coefficient: 5.71e−01
Value: C
Ilumatobacter.coccineus
0
1
2
3
4
5
A (n=15)
B (n=16)
C (n=14)
surface

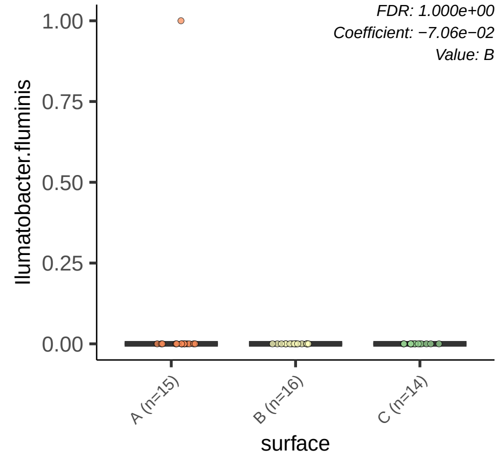
FDR: 1.000e+00
Coefficient: −7.06e−02
Value: B
Ilumatobacter.fluminis
1.00
0.75
0.50
0.25
0.00
A (n=15)
B (n=16)
C (n=14)
surface

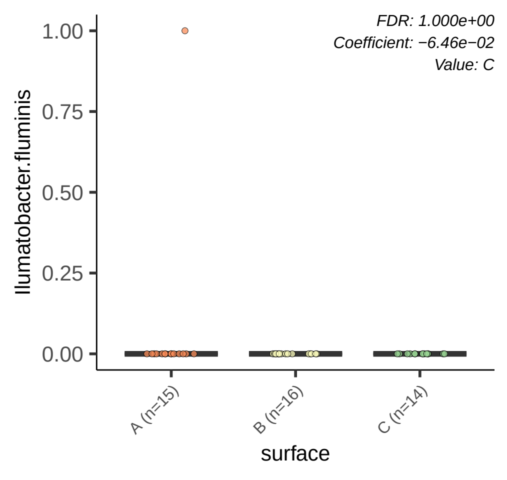
FDR: 1.000e+00
Coefficient: −6.46e−02
Value: C
Ilumatobacter.fluminis
1.00
0.75
0.50
0.25
0.00
A (n=15)
B (n=16)
C (n=14)
surface

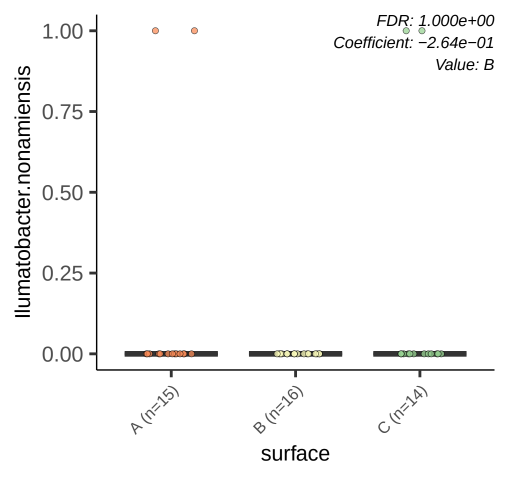
FDR: 1.000e+00
Coefficient: −2.64e−01
Value: B
Ilumatobacter.nonamiensis
1.00
0.75
0.50
0.25
0.00
A (n=15)
B (n=16)
C (n=14)
surface

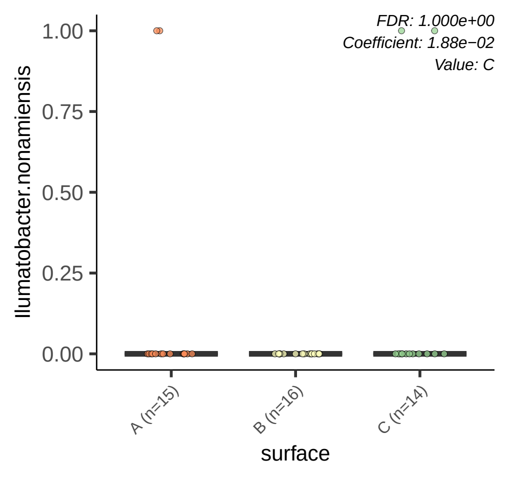
FDR: 1.000e+00
Coefficient: 1.88e−02
Value: C
Ilumatobacter.nonamiensis
1.00
0.75
0.50
0.25
0.00
A (n=15)
B (n=16)
C (n=14)
surface

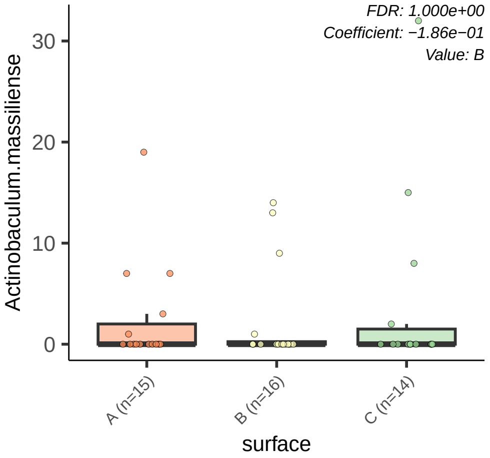
FDR: 1.000e+00
Coefficient: −1.86e−01
Value: B
Actinobaculum.massiliense
30
20
10
0
A (n=15)
B (n=16)
C (n=14)
surface

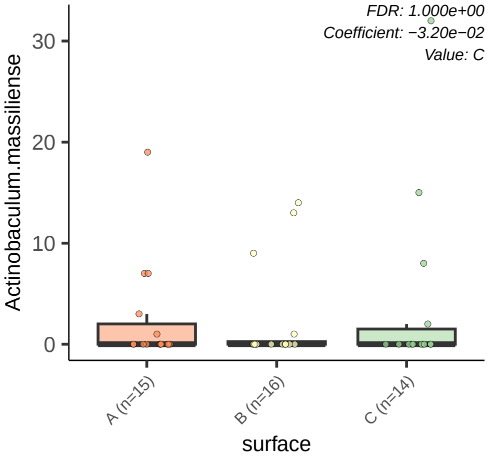
FDR: 1.000e+00
Coefficient: −3.20e−02
Value: C
Actinobaculum.massiliense
30
20
10
0
A (n=15)
B (n=16)
C (n=14)
surface

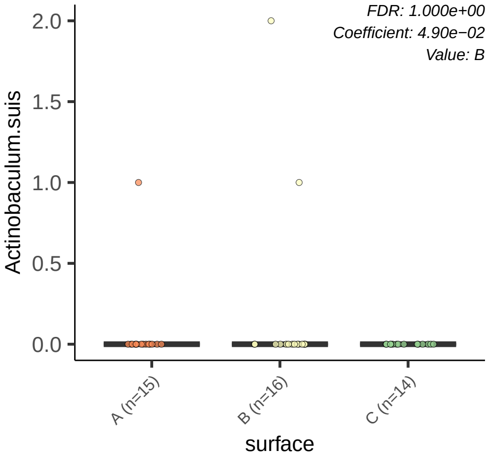
FDR: 1.000e+00
Coefficient: 4.90e−02
Value: B
Actinobaculum.suis
2.0
1.5
1.0
0.5
0.0
A (n=15)
B (n=16)
C (n=14)
surface

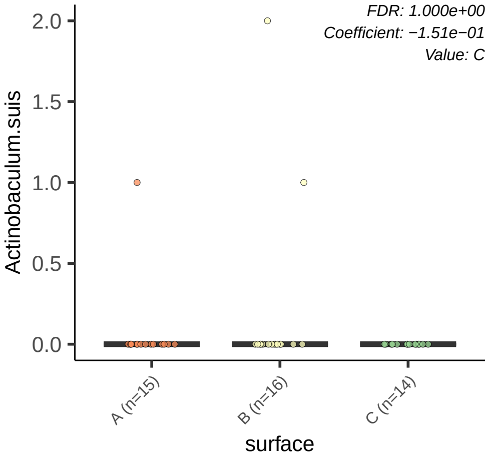

FDR: 1.000e+00
Coefficient: −1.51e−01
Value: C
Actinobaculum.suis
2.0
1.5
1.0
0.5
0.0
A (n=15)
B (n=16)
C (n=14)
surface

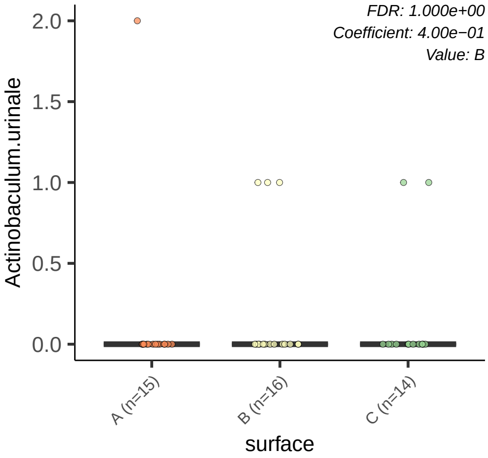

FDR: 1.000e+00
Coefficient: 4.00e−01
Value: B
Actinobaculum.urinale
2.0
1.5
1.0
0.5
0.0
A (n=15)
B (n=16)
C (n=14)
surface

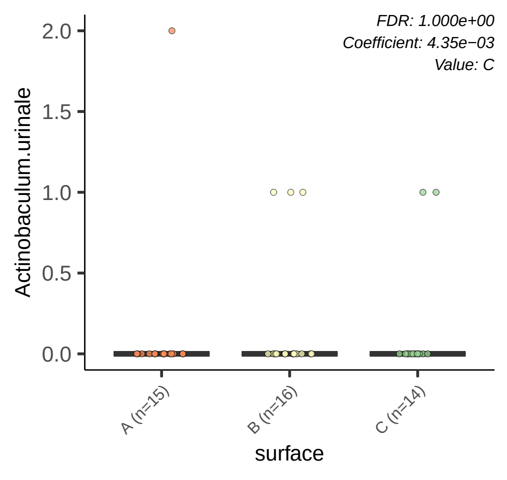
FDR: 1.000e+00
Coefficient: 4.35e−03
Value: C
Actinobaculum.urinale
2.0
1.5
1.0
0.5
0.0
A (n=15)
B (n=16)
C (n=14)
surface

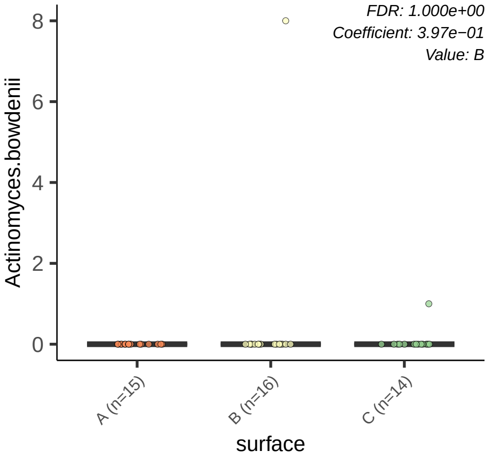
FDR: 1.000e+00
Coefficient: 3.97e−01
Value: B
Actinomyces.bowdenii
8
6
4
2
0
A (n=15)
B (n=16)
C (n=14)
surface

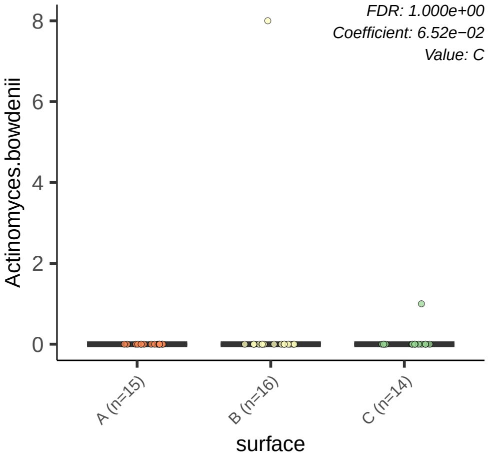
FDR: 1.000e+00
Coefficient: 6.52e−02
Value: C
Actinomyces.bowdenii
8
6
4
2
0
A (n=15)
B (n=16)
C (n=14)
surface

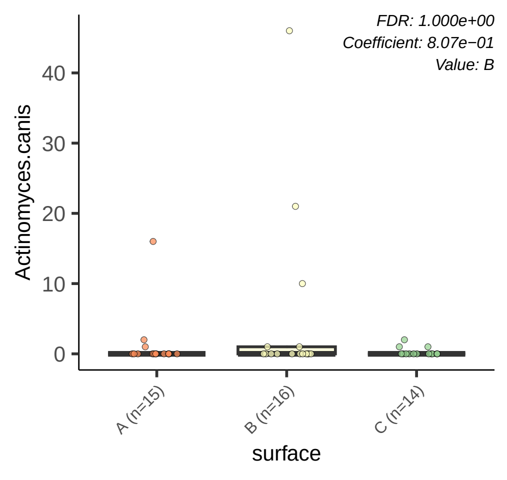

FDR: 1.000e+00
Coefficient: 8.07e−01
Value: B
Actinomyces.canis
0
10
20
30
40
A (n=15)
B (n=16)
C (n=14)
surface

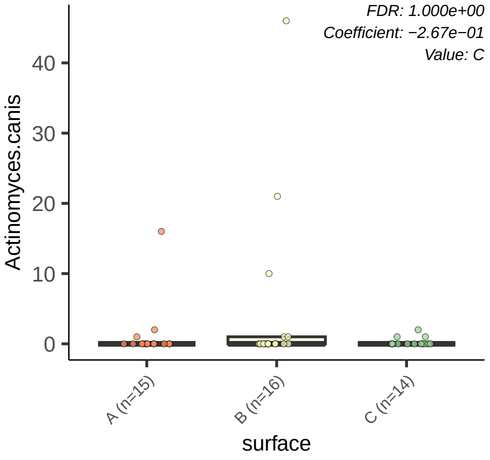

FDR: 1.000e+00
Coefficient: −2.67e−01
Value: C
Actinomyces.canis
0
10
20
30
40
A (n=15)
B (n=16)
C (n=14)
surface

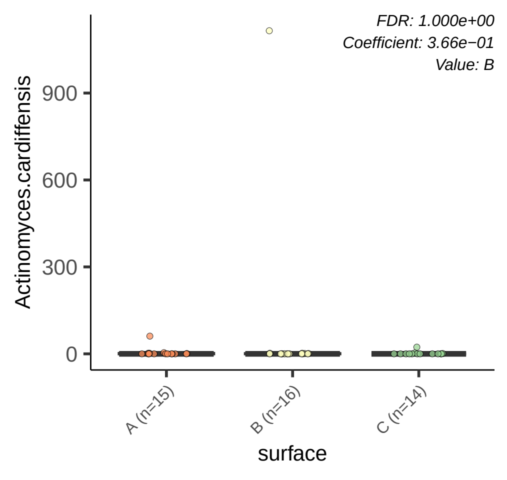

FDR: 1.000e+00
Coefficient: 3.66e−01
Value: B
Actinomyces.cardiffensis
900
600
300
0
A (n=15)
B (n=16)
C (n=14)
surface

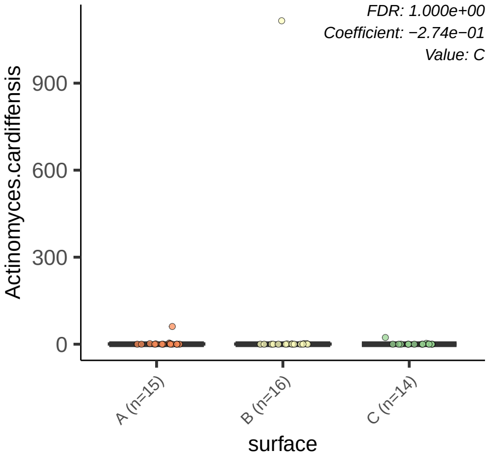

FDR: 1.000e+00
Coefficient: −2.74e−01
Value: C
Actinomyces.cardiffensis
900
600
300
0
A (n=15)
B (n=16)
C (n=14)
surface

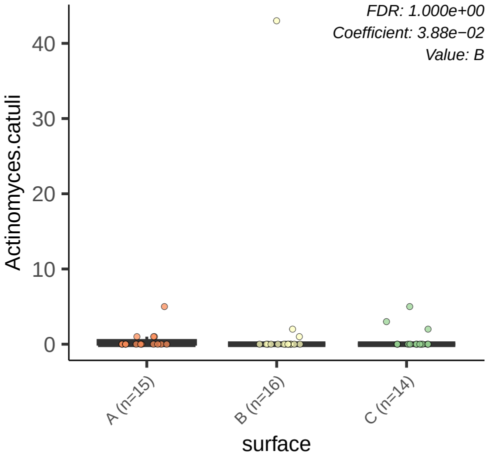

FDR: 1.000e+00
Coefficient: 3.88e−02
Value: B
Actinomyces.catuli
0
10
20
30
40
A (n=15)
B (n=16)
C (n=14)
surface

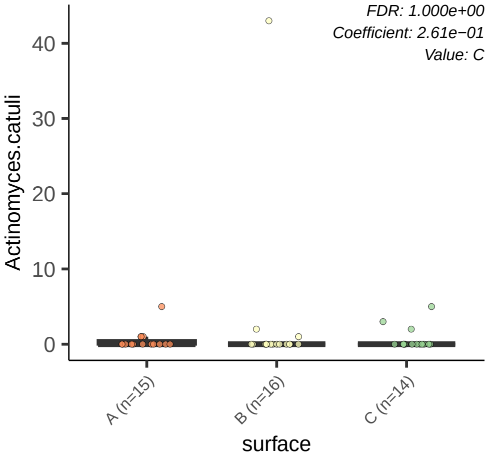

FDR: 1.000e+00
Coefficient: 2.61e−01
Value: C
Actinomyces.catuli
0
10
20
30
40
A (n=15)
B (n=16)
C (n=14)
surface

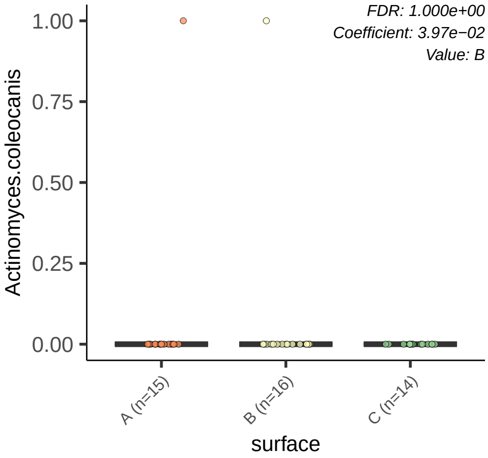

FDR: 1.000e+00
Coefficient: 3.97e−02
Value: B
Actinomyces.coleocanis
1.00
0.75
0.50
0.25
0.00
A (n=15)
B (n=16)
C (n=14)
surface

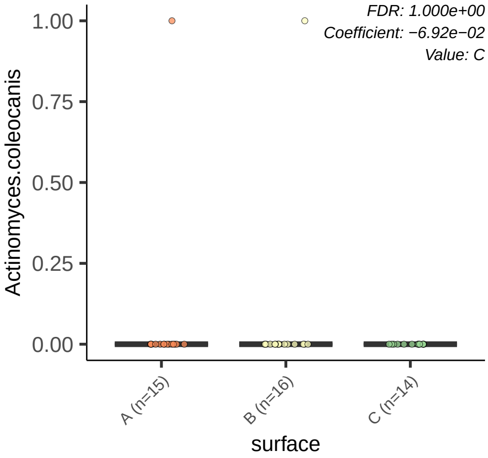
FDR: 1.000e+00
Coefficient: −6.92e−02
Value: C
Actinomyces.coleocanis
1.00
0.75
0.50
0.25
0.00
A (n=15)
B (n=16)
C (n=14)
surface

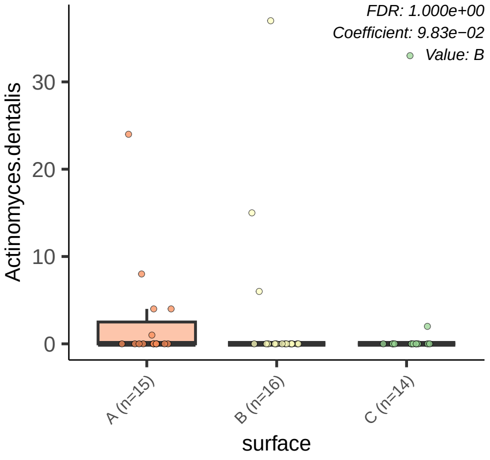

FDR: 1.000e+00
Coefficient: 9.83e−02
Value: B
Actinomyces.dentalis
0
10
20
30
A (n=15)
B (n=16)
C (n=14)
surface

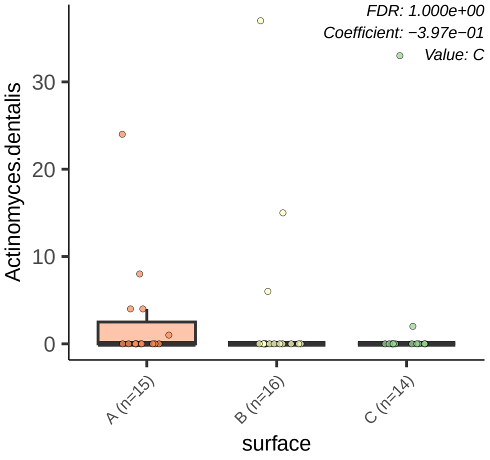
FDR: 1.000e+00
Coefficient: −3.97e−01
Value: C
Actinomyces.dentalis
0
10
20
30
A (n=15)
B (n=16)
C (n=14)
surface

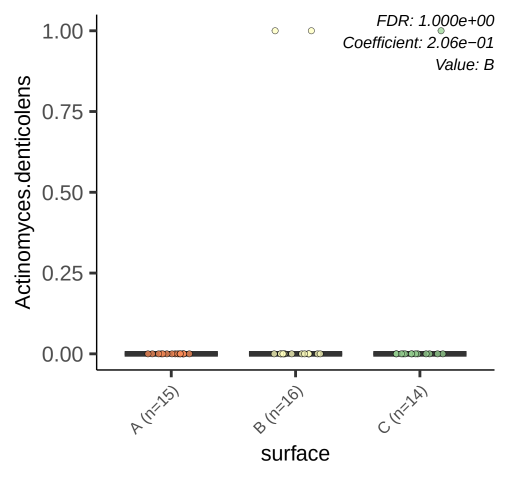
FDR: 1.000e+00
Coefficient: 2.06e−01
Value: B
Actinomyces.denticolens
1.00
0.75
0.50
0.25
0.00
A (n=15)
B (n=16)
C (n=14)
surface

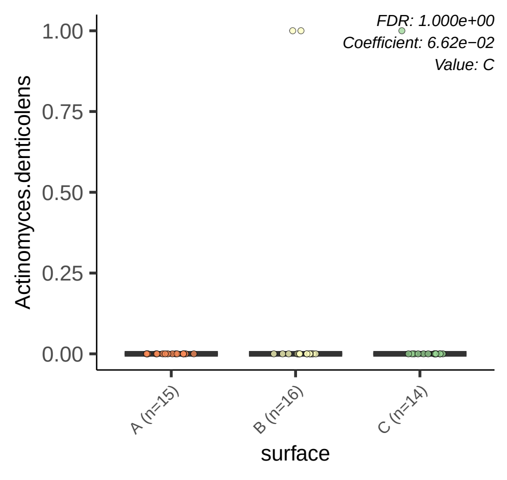

FDR: 1.000e+00
Coefficient: 6.62e−02
Value: C
Actinomyces.denticolens
1.00
0.75
0.50
0.25
0.00
A (n=15)
B (n=16)
C (n=14)
surface

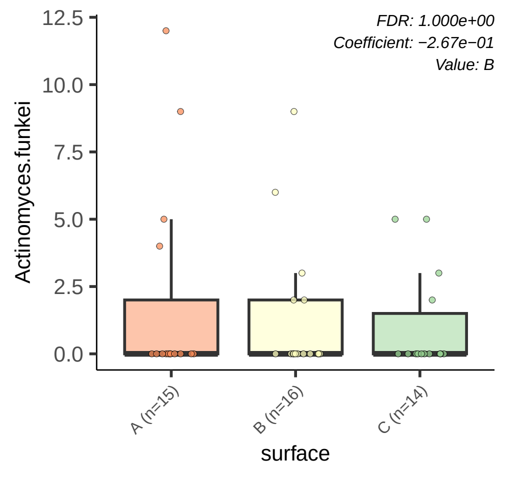
FDR: 1.000e+00
Coefficient: −2.67e−01
Value: B
Actinomyces.funkei
12.5
10.0
7.5
5.0
2.5
0.0
A (n=15)
B (n=16)
C (n=14)
surface

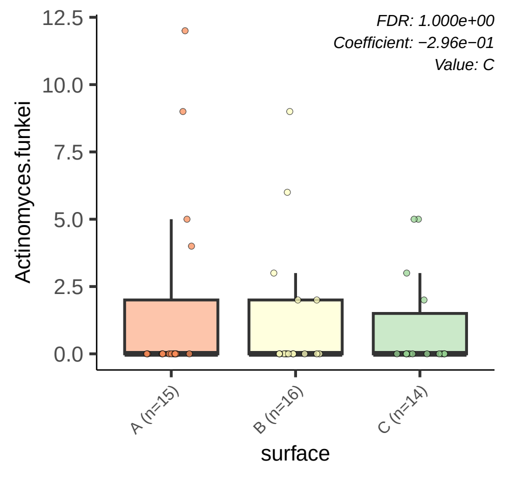
FDR: 1.000e+00
Coefficient: −2.96e−01
Value: C
Actinomyces.funkei
12.5
10.0
7.5
5.0
2.5
0.0
A (n=15)
B (n=16)
C (n=14)
surface

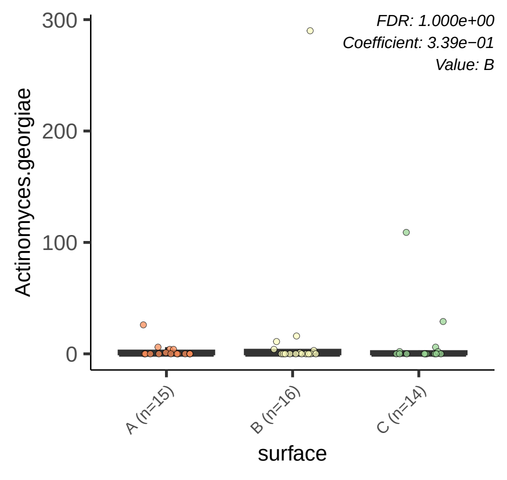
FDR: 1.000e+00
Coefficient: 3.39e−01
Value: B
Actinomyces.georgiae
300
200
100
0
A (n=15)
B (n=16)
C (n=14)
surface

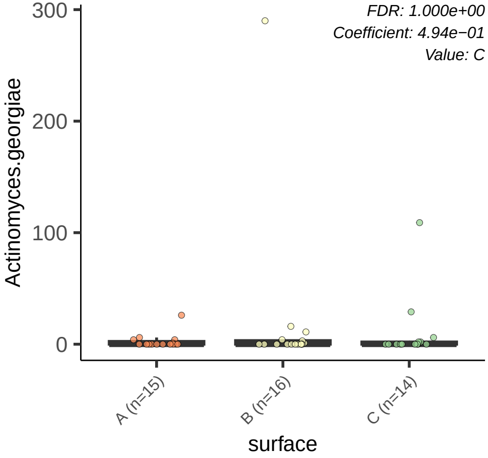

FDR: 1.000e+00
Coefficient: 4.94e−01
Value: C
300
200
100
0
Actinomyces.georgiae
A (n=15)
B (n=16)
C (n=14)
surface

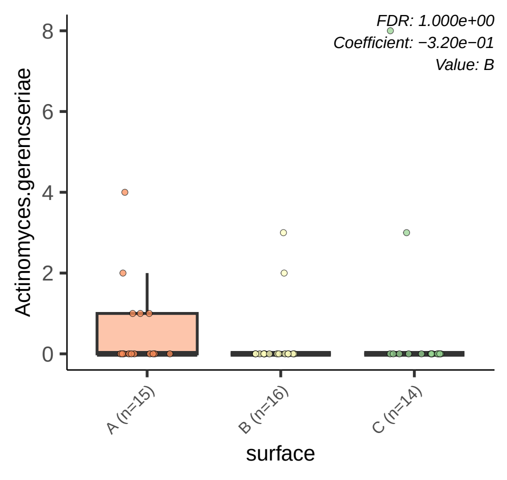
FDR: 1.000e+00
Coefficient: −3.20e−01
Value: B
Actinomyces.gerencseriae
8
6
4
2
0
A (n=15)
B (n=16)
C (n=14)
surface

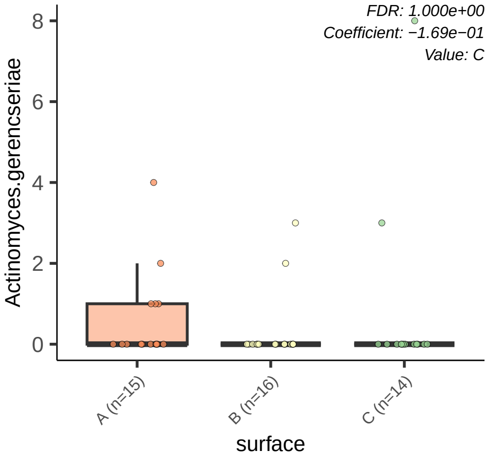
FDR: 1.000e+00
Coefficient: −1.69e−01
Value: C
Actinomyces.gerencseriae
0
2
4
6
8
A (n=15)
B (n=16)
C (n=14)
surface

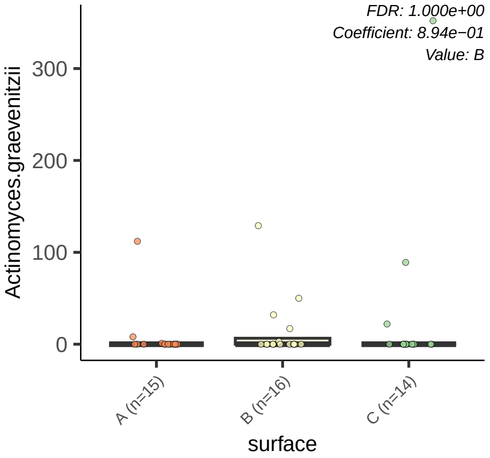

FDR: 1.000e+00
Coefficient: 8.94e−01
Value: B
Actinomyces.graevenitzii
300
200
100
0
A (n=15)
B (n=16)
C (n=14)
surface

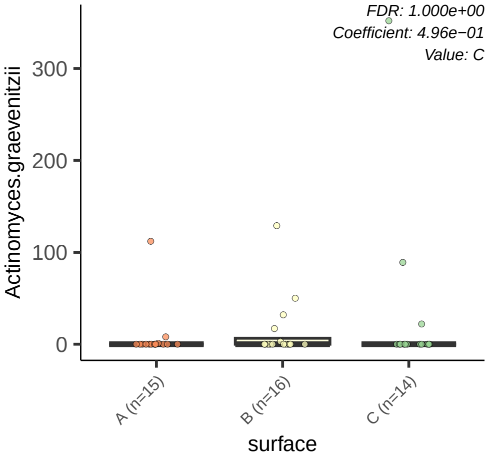
FDR: 1.000e+00
Coefficient: 4.96e−01
Value: C
Actinomyces.graevenitzii
300
200
100
0
A (n=15)
B (n=16)
C (n=14)
surface

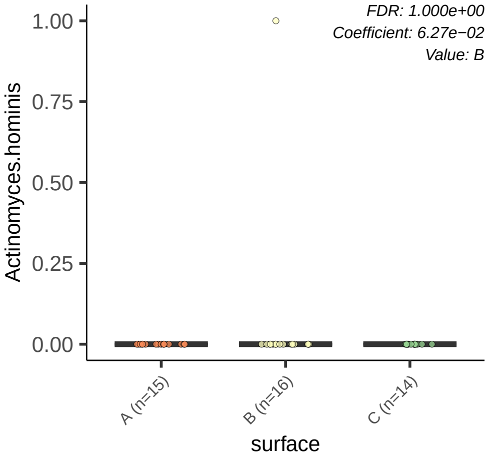

FDR: 1.000e+00
Coefficient: 6.27e−02
Value: B
Actinomyces.hominis
1.00
0.75
0.50
0.25
0.00
A (n=15)
B (n=16)
C (n=14)
surface

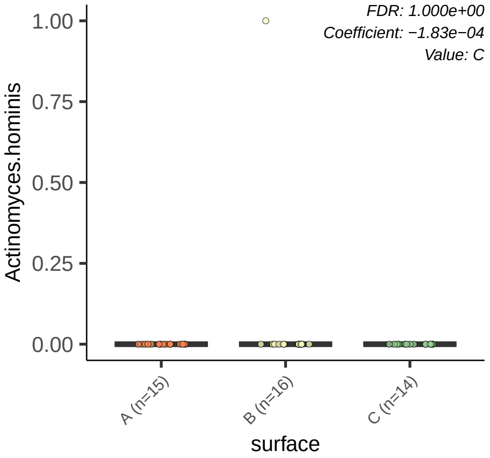
FDR: 1.000e+00
Coefficient: −1.83e−04
Value: C
Actinomyces.hominis
1.00
0.75
0.50
0.25
0.00
A (n=15)
B (n=16)
C (n=14)
surface

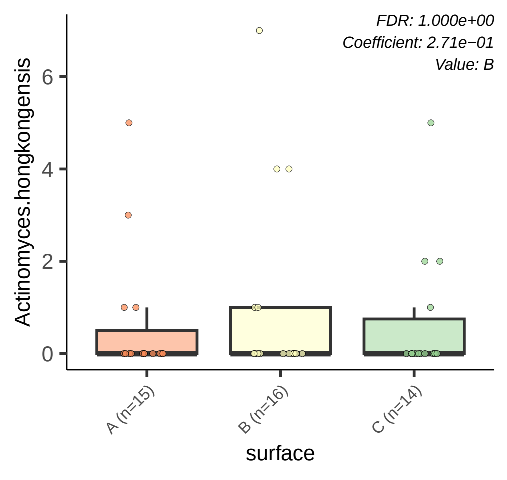
FDR: 1.000e+00
Coefficient: 2.71e−01
Value: B
Actinomyces.hongkongensis
0
2
4
6
A (n=15)
B (n=16)
C (n=14)
surface

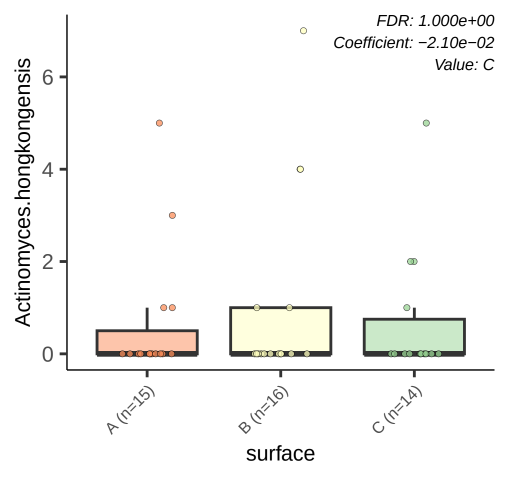

FDR: 1.000e+00
Coefficient: −2.10e−02
Value: C
Actinomyces.hongkongensis
0
2
4
6
A (n=15)
B (n=16)
C (n=14)
surface

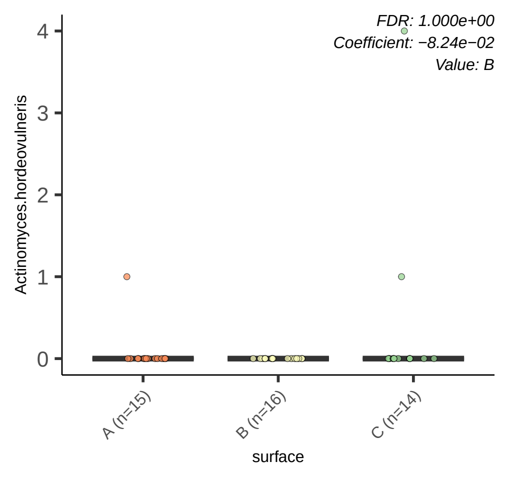
FDR: 1.000e+00
Coefficient: −8.24e−02
Value: B
Actinomyces.hordeovulneris
4
3
2
1
0
A (n=15)
B (n=16)
C (n=14)
surface

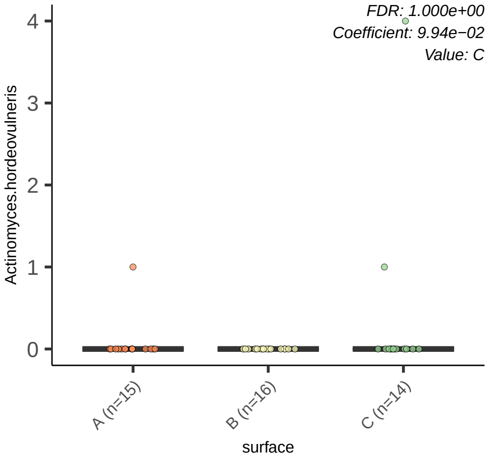
FDR: 1.000e+00
Coefficient: 9.94e−02
Value: C
Actinomyces.hordeovulneris
0
1
2
3
4
A (n=15)
B (n=16)
C (n=14)
surface

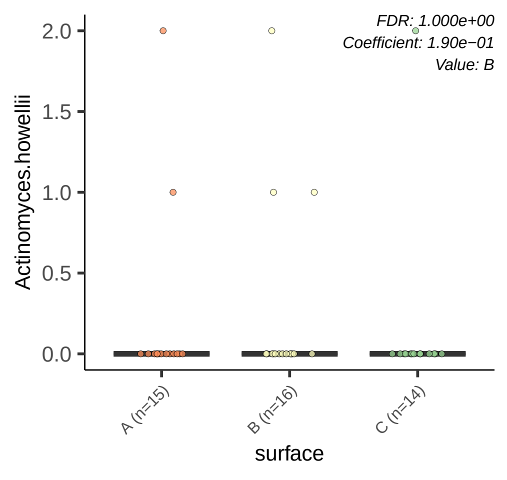

FDR: 1.000e+00
Coefficient: 1.90e−01
Value: B
2.0
1.5
1.0
0.5
0.0
Actinomyces.howellii
A (n=15)
B (n=16)
C (n=14)
surface

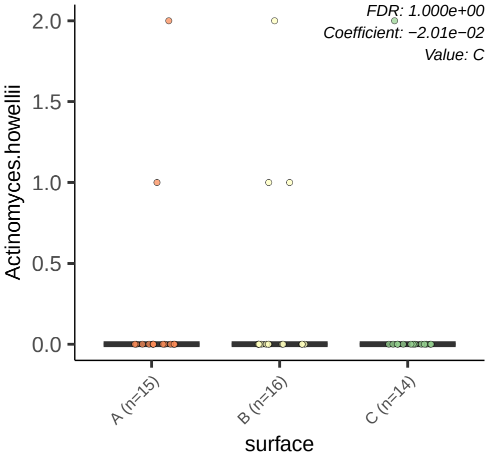

FDR: 1.000e+00
Coefficient: −2.01e−02
Value: C
Actinomyces.howellii
2.0
1.5
1.0
0.5
0.0
A (n=15)
B (n=16)
C (n=14)
surface

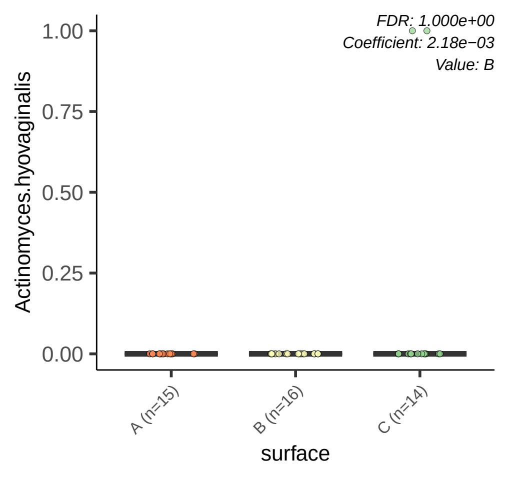
FDR: 1.000e+00
Coefficient: 2.18e−03
Value: B
Actinomyces.hyovaginalis
1.00
0.75
0.50
0.25
0.00
A (n=15)
B (n=16)
C (n=14)
surface

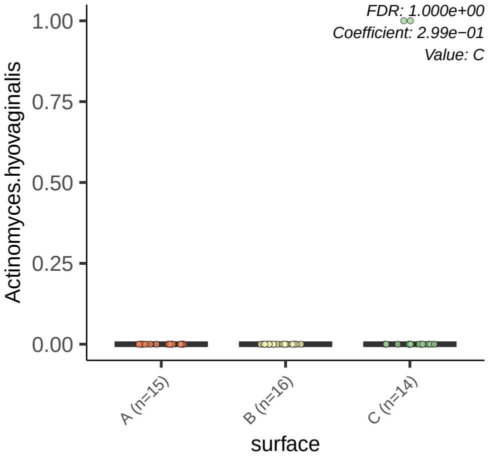

FDR: 1.000e+00
Coefficient: 2.99e−01
Value: C
Actinomyces.hyovaginalis
1.00
0.75
0.50
0.25
0.00
A (n=15)
B (n=16)
C (n=14)
surface

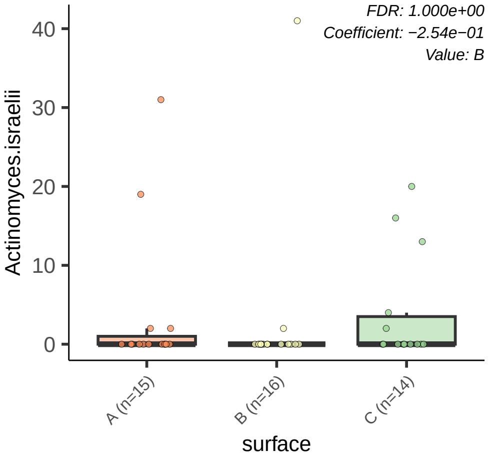

FDR: 1.000e+00
Coefficient: −2.54e−01
Value: B
Actinomyces.israelii
0
10
20
30
40
A (n=15)
B (n=16)
C (n=14)
surface

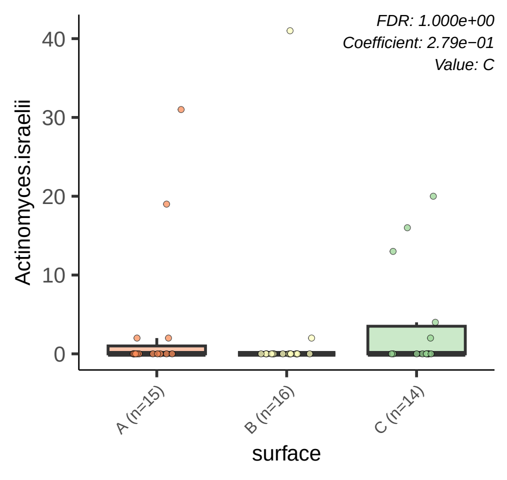
FDR: 1.000e+00
Coefficient: 2.79e−01
Value: C
Actinomyces.israelii
0
10
20
30
40
A (n=15)
B (n=16)
C (n=14)
surface

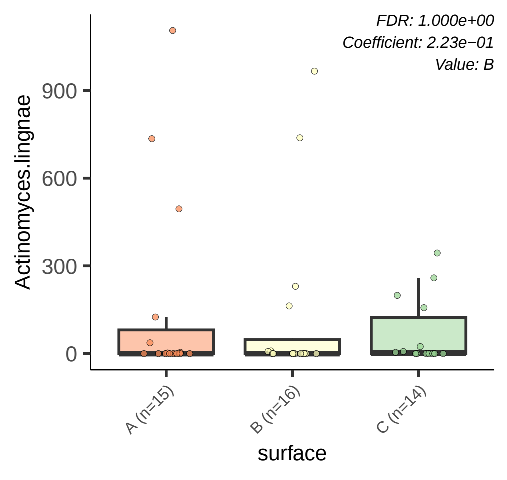
FDR: 1.000e+00
Coefficient: 2.23e−01
Value: B
Actinomyces.lingnae
900
600
300
0
A (n=15)
B (n=16)
C (n=14)
surface

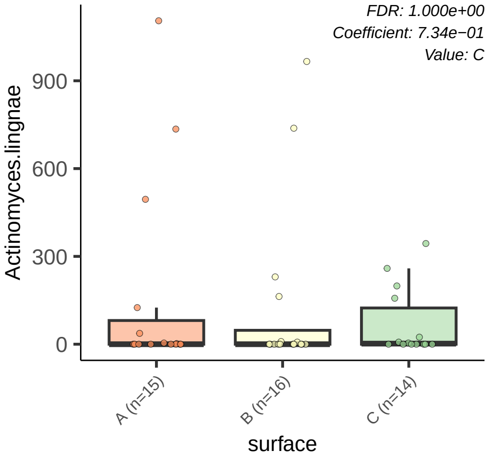
FDR: 1.000e+00
Coefficient: 7.34e−01
Value: C
Actinomyces.lingnae
900
600
300
0
A (n=15)
B (n=16)
C (n=14)
surface

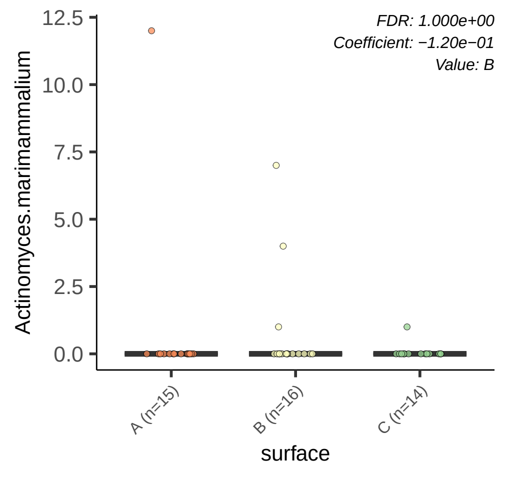
FDR: 1.000e+00
Coefficient: −1.20e−01
Value: B
Actinomyces.marimammalium
12.5
10.0
7.5
5.0
2.5
0.0
A (n=15)
B (n=16)
C (n=14)
surface

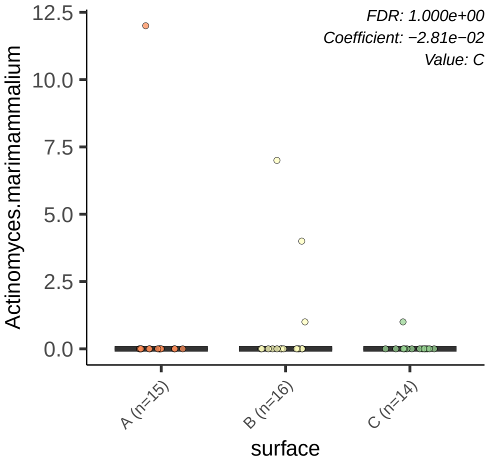

FDR: 1.000e+00
Coefficient: −2.81e−02
Value: C
Actinomyces.marimammalium
12.5
10.0
7.5
5.0
2.5
0.0
A (n=15)
B (n=16)
C (n=14)
surface

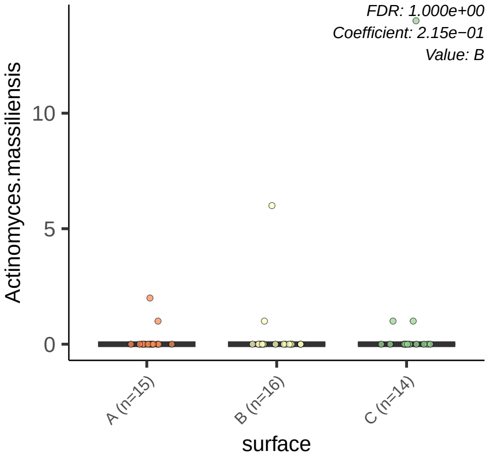
FDR: 1.000e+00
Coefficient: 2.15e−01
Value: B
Actinomyces.massiliensis
10
5
0
A (n=15)
B (n=16)
C (n=14)
surface

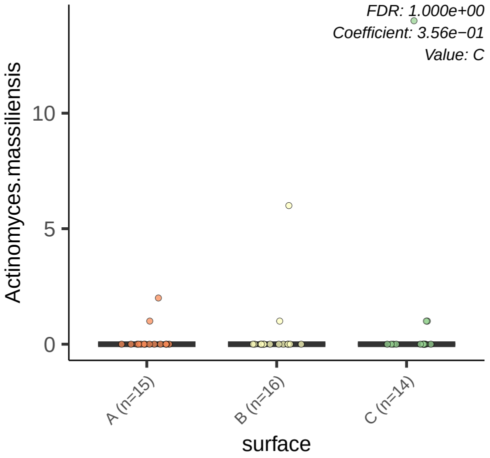

FDR: 1.000e+00
Coefficient: 3.56e−01
Value: C
Actinomyces.massiliensis
10
5
0
A (n=15)
B (n=16)
C (n=14)
surface

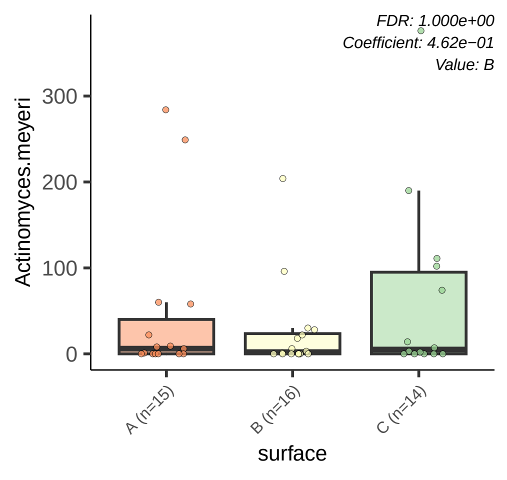
FDR: 1.000e+00
Coefficient: 4.62e−01
Value: B
Actinomyces.meyeri
300
200
100
0
A (n=15)
B (n=16)
C (n=14)
surface

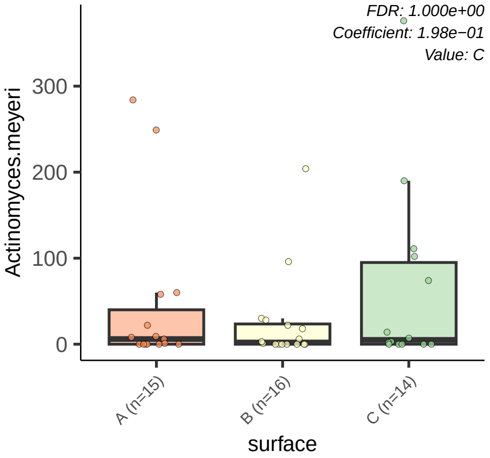
FDR: 1.000e+00
Coefficient: 1.98e−01
Value: C
Actinomyces.meyeri
300
200
100
0
A (n=15)
B (n=16)
C (n=14)
surface

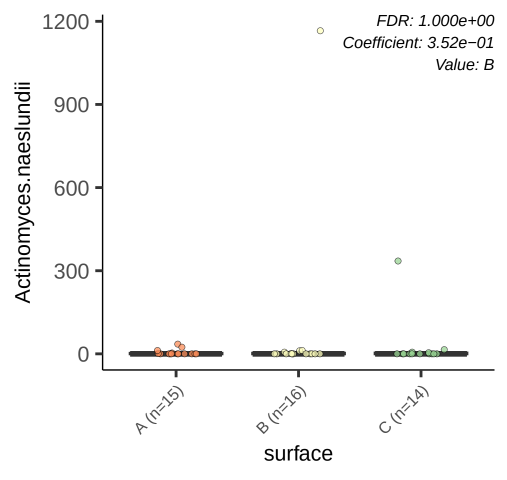
FDR: 1.000e+00
Coefficient: 3.52e−01
Value: B
Actinomyces.naeslundii
1200
900
600
300
0
A (n=15)
B (n=16)
C (n=14)
surface

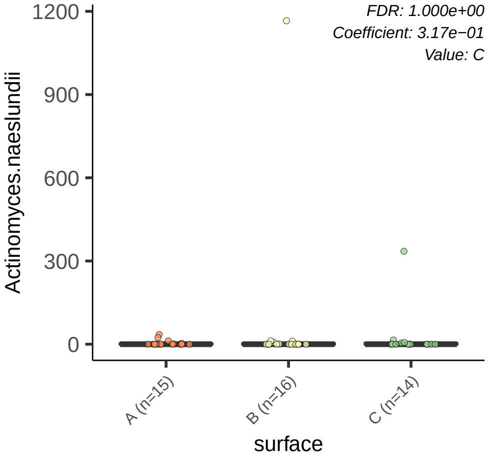
FDR: 1.000e+00
Coefficient: 3.17e−01
Value: C
Actinomyces.naeslundii
1200
900
600
300
0
A (n=15)
B (n=16)
C (n=14)
surface

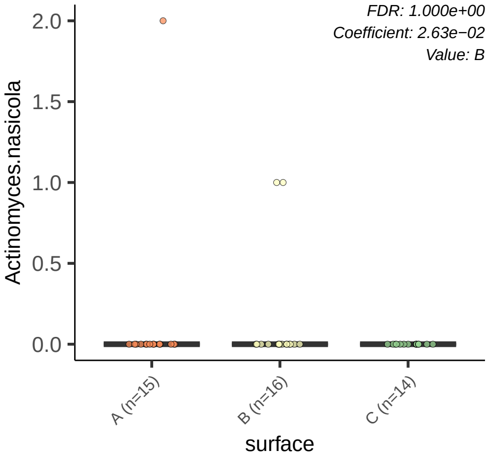
FDR: 1.000e+00
Coefficient: 2.63e−02
Value: B
Actinomyces.nasicola
2.0
1.5
1.0
0.5
0.0
A (n=15)
B (n=16)
C (n=14)
surface

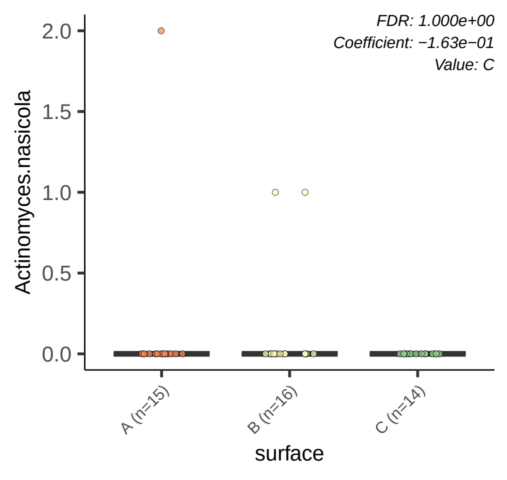

FDR: 1.000e+00
Coefficient: −1.63e−01
Value: C
Actinomyces.nasicola
2.0
1.5
1.0
0.5
0.0
A (n=15)
B (n=16)
C (n=14)
surface

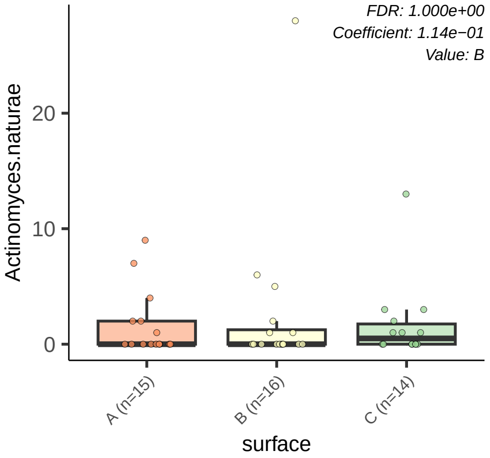

FDR: 1.000e+00
Coefficient: 1.14e−01
Value: B
Actinomyces.naturae
0
10
20
A (n=15)
B (n=16)
C (n=14)
surface

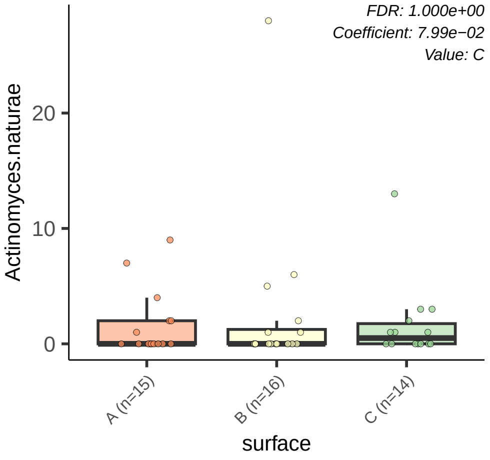
FDR: 1.000e+00
Coefficient: 7.99e−02
Value: C
Actinomyces.naturae
0
10
20
A (n=15)
B (n=16)
C (n=14)
surface

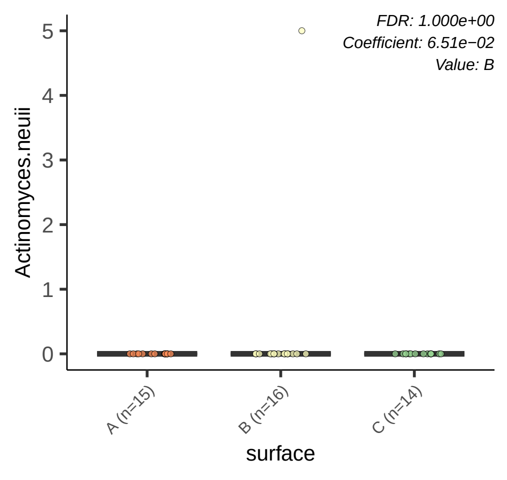
FDR: 1.000e+00
Coefficient: 6.51e−02
Value: B
Actinomyces.neuii
0
1
2
3
4
5
A (n=15)
B (n=16)
C (n=14)
surface

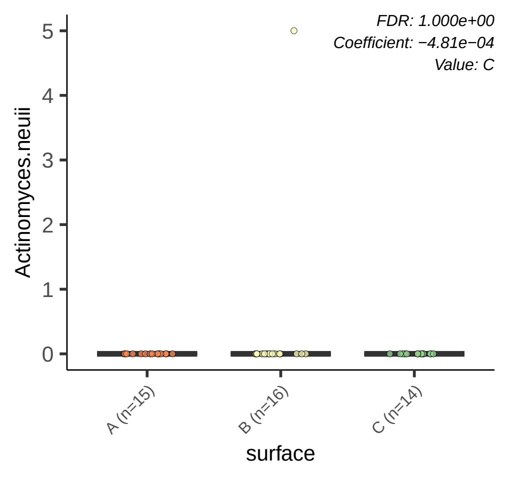

FDR: 1.000e+00
Coefficient: −4.81e−04
Value: C
Actinomyces.neuii
0
1
2
3
4
5
A (n=15)
B (n=16)
C (n=14)
surface

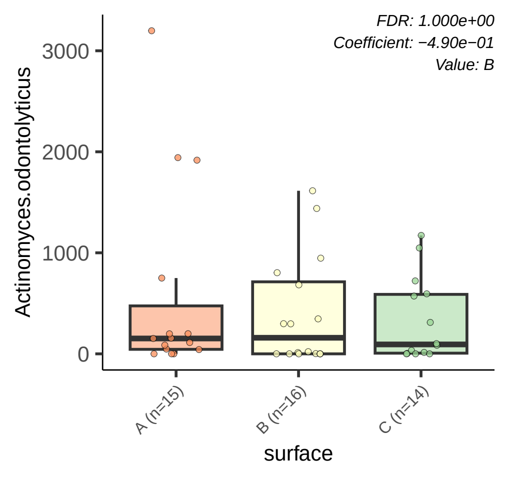

FDR: 1.000e+00
Coefficient: −4.90e−01
Value: B
Actinomyces.odontolyticus
3000
2000
1000
0
A (n=15)
B (n=16)
C (n=14)
surface

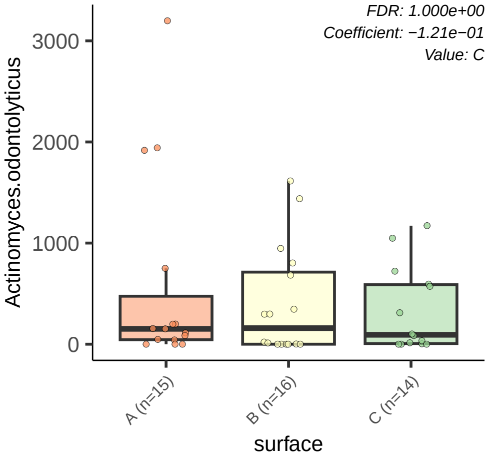

FDR: 1.000e+00
Coefficient: −1.21e−01
Value: C
Actinomyces.odontolyticus
0
1000
2000
3000
A (n=15)
B (n=16)
C (n=14)
surface

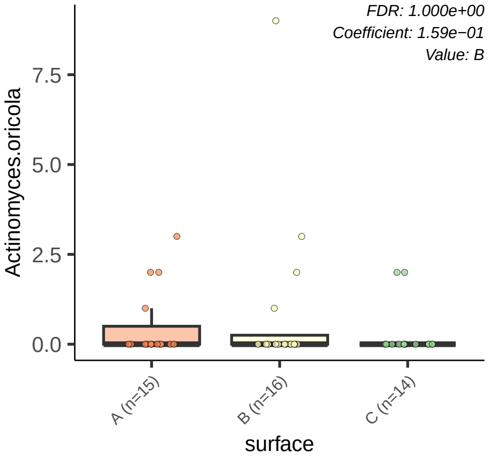

FDR: 1.000e+00
Coefficient: 1.59e−01
Value: B
Actinomyces.oricola
7.5
5.0
2.5
0.0
A (n=15)
B (n=16)
C (n=14)
surface

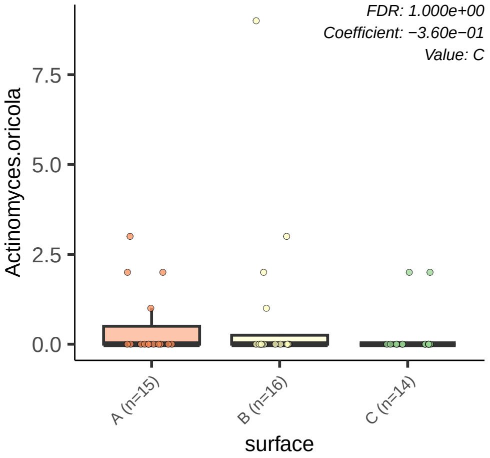

FDR: 1.000e+00
Coefficient: −3.60e−01
Value: C
7.5
5.0
2.5
0.0
Actinomyces.oricola
A (n=15)
B (n=16)
C (n=14)
surface

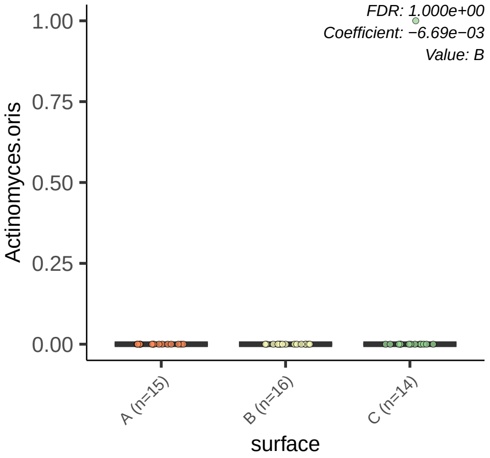
FDR: 1.000e+00
Coefficient: −6.69e−03
Value: B
Actinomyces.oris
1.00
0.75
0.50
0.25
0.00
A (n=15)
B (n=16)
C (n=14)
surface

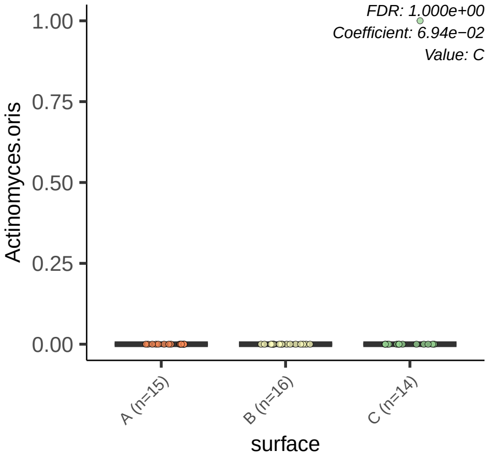

FDR: 1.000e+00
Coefficient: 6.94e−02
Value: C
Actinomyces.oris
1.00
0.75
0.50
0.25
0.00
A (n=15)
B (n=16)
C (n=14)
surface

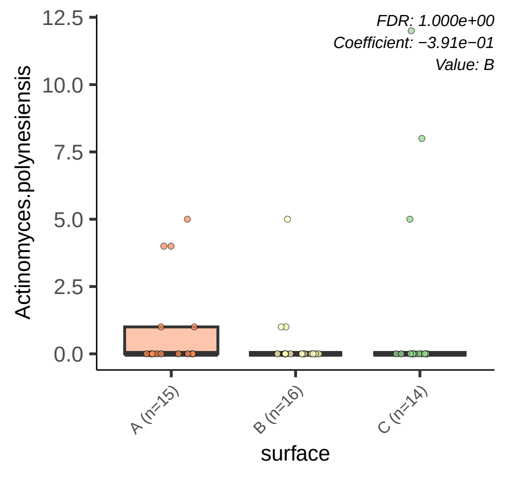

FDR: 1.000e+00
Coefficient: −3.91e−01
Value: B
Actinomyces.polynesiensis
12.5
10.0
7.5
5.0
2.5
0.0
A (n=15)
B (n=16)
C (n=14)
surface

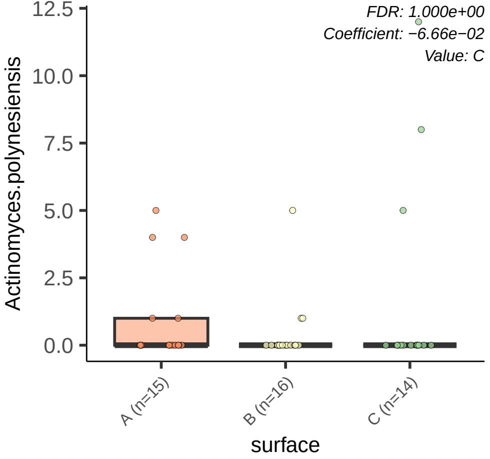
FDR: 1.000e+00
Coefficient: −6.66e−02
Value: C
Actinomyces.polynesiensis
12.5
10.0
7.5
5.0
2.5
0.0
A (n=15)
B (n=16)
C (n=14)
surface

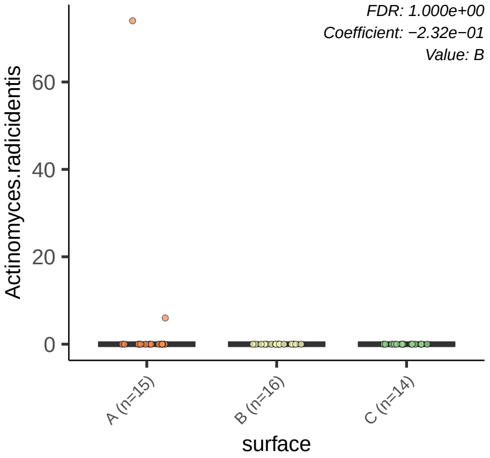
FDR: 1.000e+00
Coefficient: −2.32e−01
Value: B
Actinomyces.radicidentis
60
40
20
0
A (n=15)
B (n=16)
C (n=14)
surface

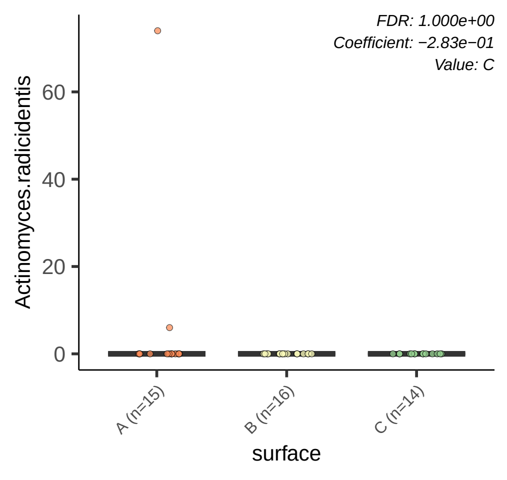
FDR: 1.000e+00
Coefficient: −2.83e−01
Value: C
Actinomyces.radicidentis
0
20
40
60
A (n=15)
B (n=16)
C (n=14)
surface

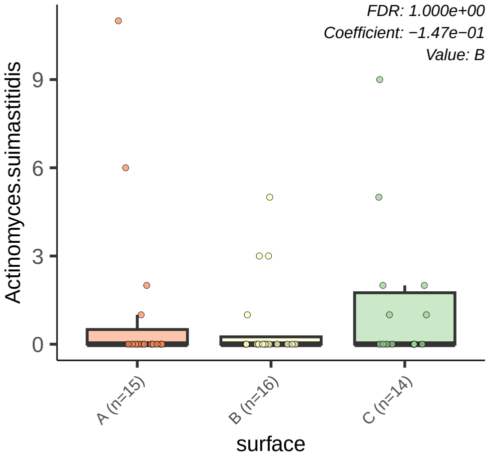

FDR: 1.000e+00
Coefficient: −1.47e−01
Value: B
Actinomyces.suimastitidis
0
3
6
9
A (n=15)
B (n=16)
C (n=14)
surface

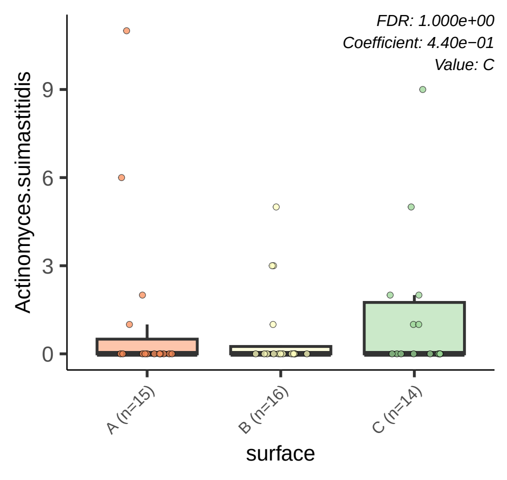

FDR: 1.000e+00
Coefficient: 4.40e−01
Value: C
Actinomyces.suimastitidis
0
3
6
9
A (n=15)
B (n=16)
C (n=14)
surface

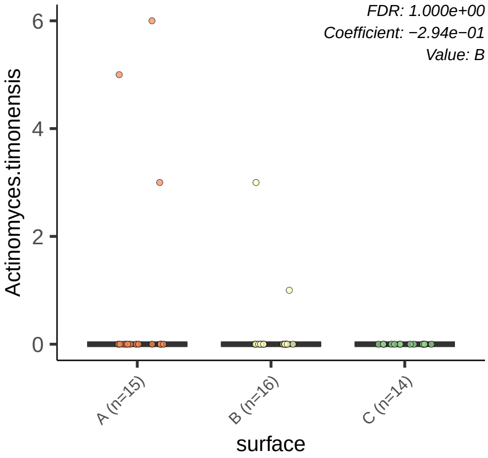

FDR: 1.000e+00
Coefficient: −2.94e−01
Value: B
Actinomyces.timonensis
6
4
2
0
A (n=15)
B (n=16)
C (n=14)
surface

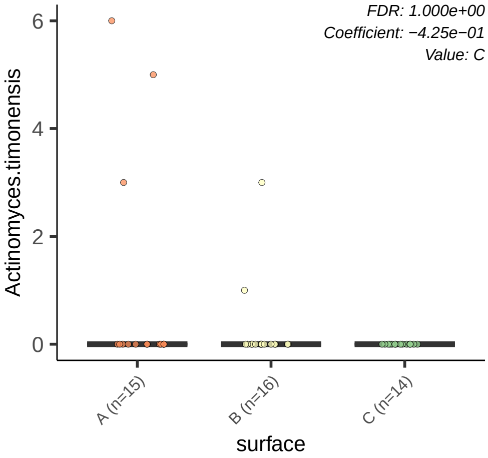
FDR: 1.000e+00
Coefficient: −4.25e−01
Value: C
Actinomyces.timonensis
6
4
2
0
A (n=15)
B (n=16)
C (n=14)
surface

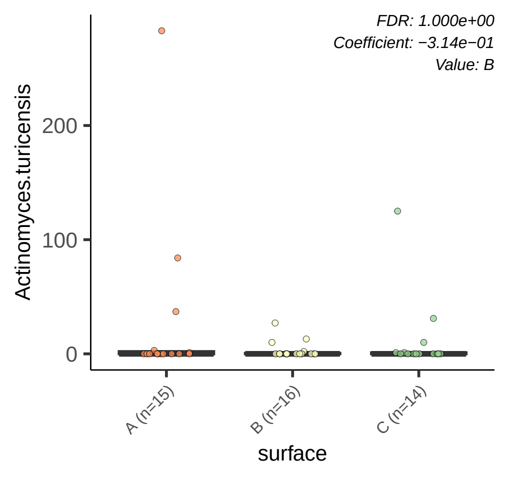
FDR: 1.000e+00
Coefficient: −3.14e−01
Value: B
Actinomyces.turicensis
200
100
0
A (n=15)
B (n=16)
C (n=14)
surface

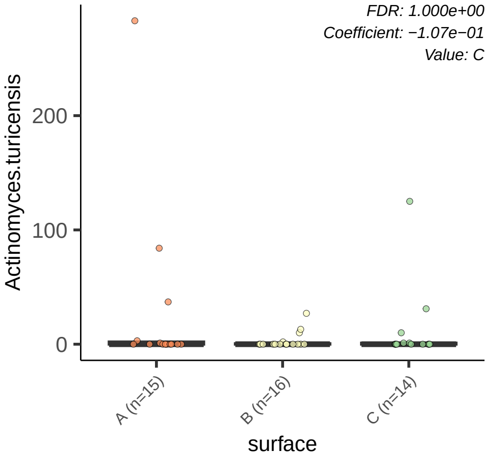

FDR: 1.000e+00
Coefficient: −1.07e−01
Value: C
Actinomyces.turicensis
200
100
0
A (n=15)
B (n=16)
C (n=14)
surface

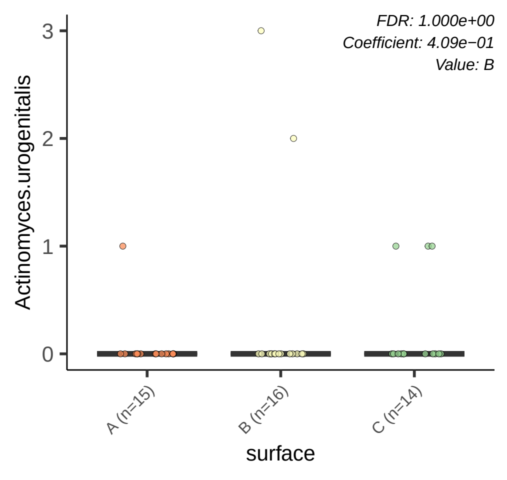

FDR: 1.000e+00
Coefficient: 4.09e−01
Value: B
Actinomyces.urogenitalis
0
1
2
3
A (n=15)
B (n=16)
C (n=14)
surface

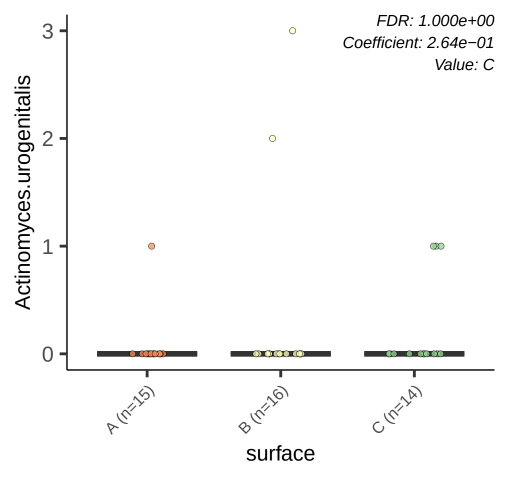

FDR: 1.000e+00
Coefficient: 2.64e−01
Value: C
Actinomyces.urogenitalis
3
2
1
0
A (n=15)
B (n=16)
C (n=14)
surface

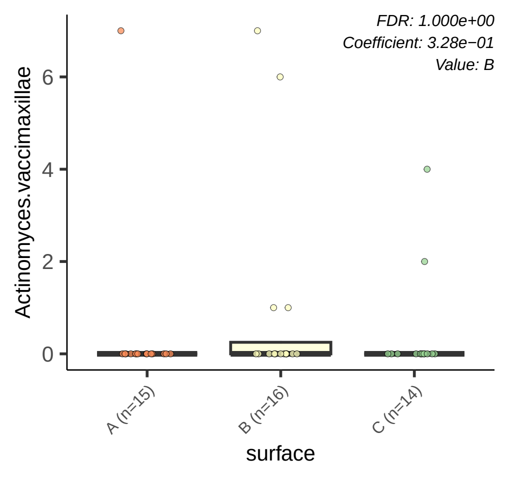
FDR: 1.000e+00
Coefficient: 3.28e−01
Value: B
Actinomyces.vaccimaxillae
0
2
4
6
A (n=15)
B (n=16)
C (n=14)
surface

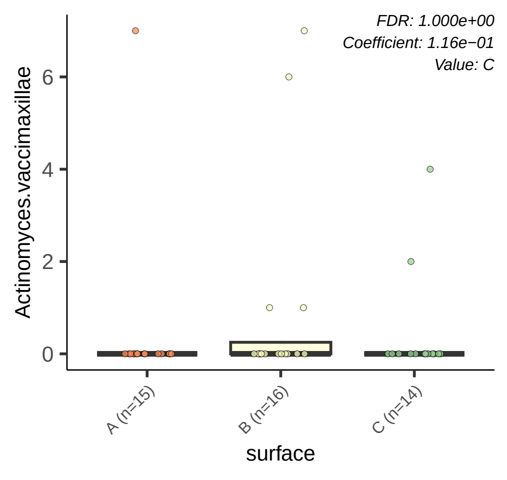

FDR: 1.000e+00
Coefficient: 1.16e−01
Value: C
Actinomyces.vaccimaxillae
0
2
4
6
A (n=15)
B (n=16)
C (n=14)
surface

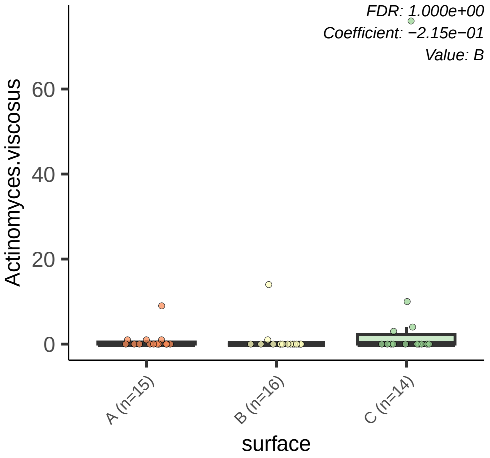

FDR: 1.000e+00
Coefficient: −2.15e−01
Value: B
Actinomyces.viscosus
0
20
40
60
A (n=15)
B (n=16)
C (n=14)
surface

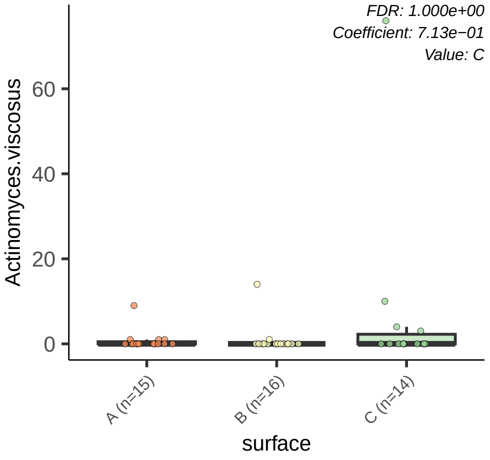
FDR: 1.000e+00
Coefficient: 7.13e−01
Value: C
Actinomyces.viscosus
60
40
20
0
A (n=15)
B (n=16)
C (n=14)
surface

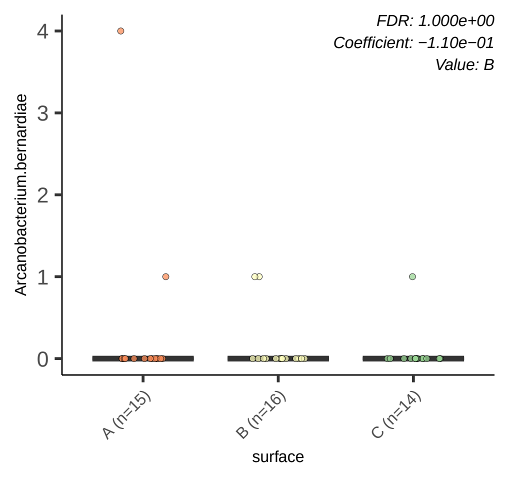
FDR: 1.000e+00
Coefficient: −1.10e−01
Value: B
Arcanobacterium.bernardiae
0
1
2
3
4
A (n=15)
B (n=16)
C (n=14)
surface

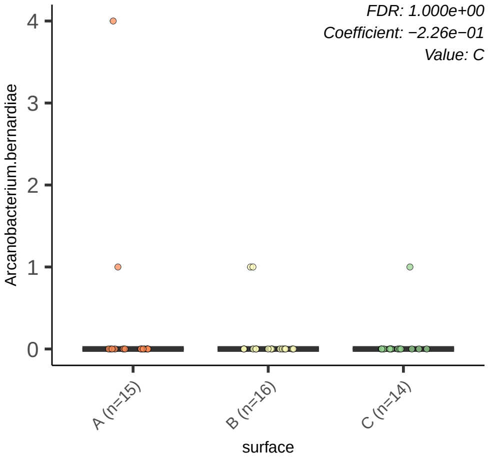
FDR: 1.000e+00
Coefficient: −2.26e−01
Value: C
Arcanobacterium.bernardiae
4
3
2
1
0
A (n=15)
B (n=16)
C (n=14)
surface

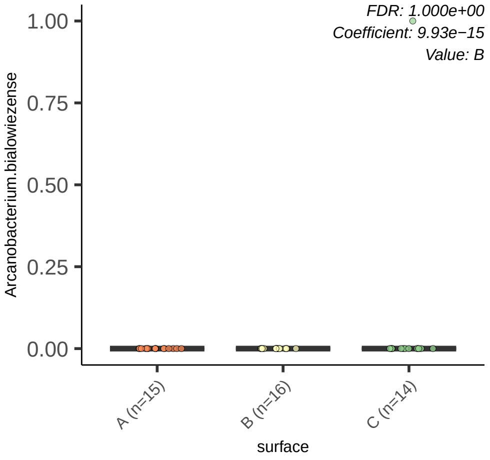
FDR: 1.000e+00
Coefficient: 9.93e−15
Value: B
Arcanobacterium.bialowiezense
1.00
0.75
0.50
0.25
0.00
A (n=15)
B (n=16)
C (n=14)
surface

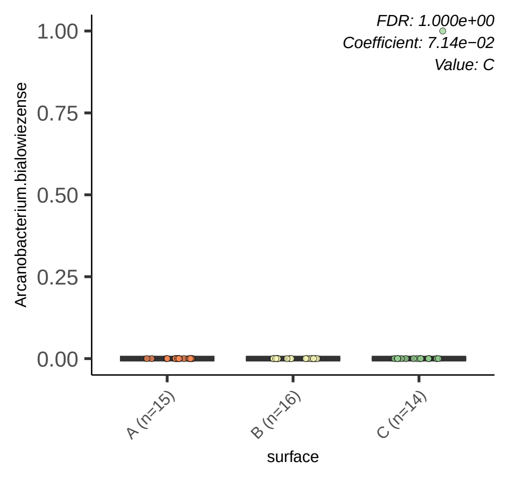
FDR: 1.000e+00
Coefficient: 7.14e−02
Value: C
Arcanobacterium.bialowiezense
1.00
0.75
0.50
0.25
0.00
A (n=15)
B (n=16)
C (n=14)
surface

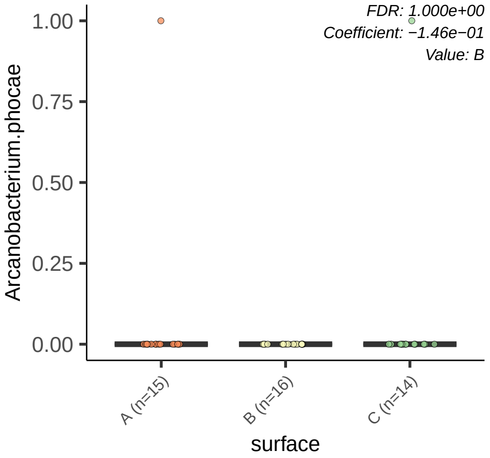

FDR: 1.000e+00
Coefficient: −1.46e−01
Value: B
Arcanobacterium.phocae
1.00
0.75
0.50
0.25
0.00
A (n=15)
B (n=16)
C (n=14)
surface

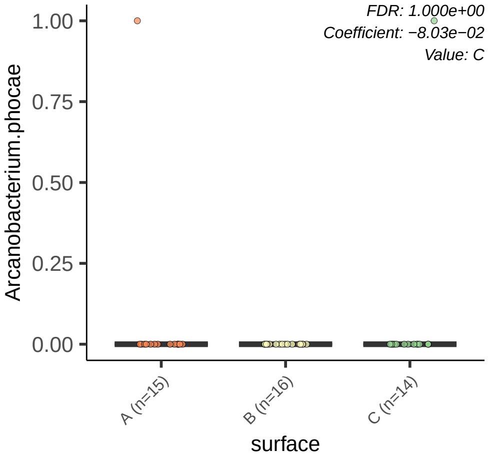
FDR: 1.000e+00
Coefficient: −8.03e−02
Value: C
Arcanobacterium.phocae
1.00
0.75
0.50
0.25
0.00
A (n=15)
B (n=16)
C (n=14)
surface

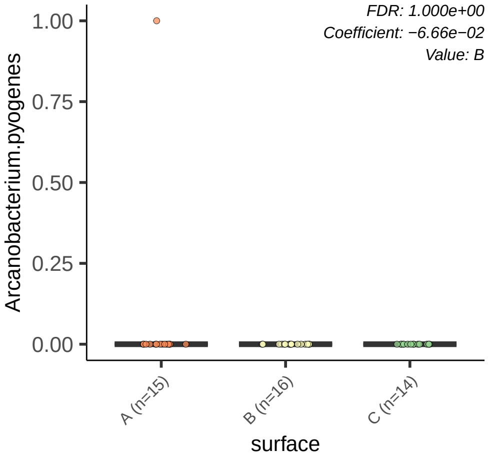

FDR: 1.000e+00
Coefficient: −6.66e−02
Value: B
Arcanobacterium.pyogenes
1.00
0.75
0.50
0.25
0.00
A (n=15)
B (n=16)
C (n=14)
surface

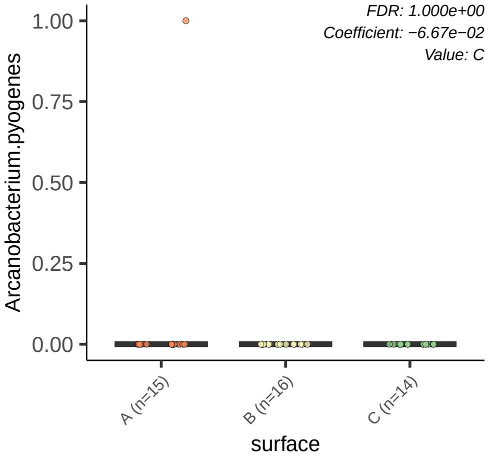

FDR: 1.000e+00
Coefficient: −6.67e−02
Value: C
Arcanobacterium.pyogenes
1.00
0.75
0.50
0.25
0.00
A (n=15)
B (n=16)
C (n=14)
surface

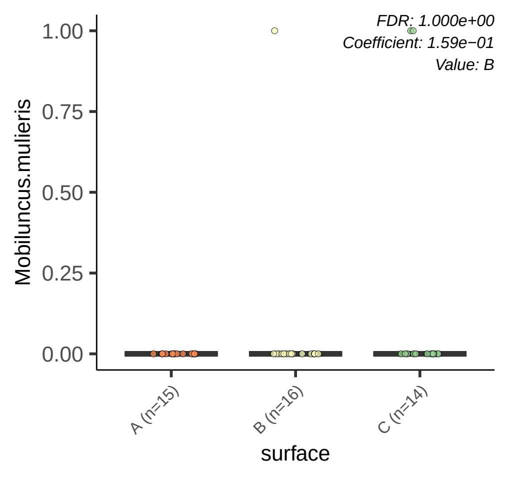

FDR: 1.000e+00
Coefficient: 1.59e−01
Value: B
Mobiluncus.mulieris
1.00
0.75
0.50
0.25
0.00
A (n=15)
B (n=16)
C (n=14)
surface

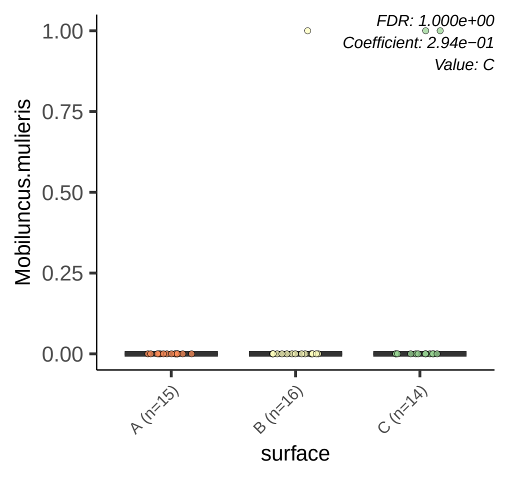
FDR: 1.000e+00
Coefficient: 2.94e−01
Value: C
Mobiluncus.mulieris
1.00
0.75
0.50
0.25
0.00
A (n=15)
B (n=16)
C (n=14)
surface

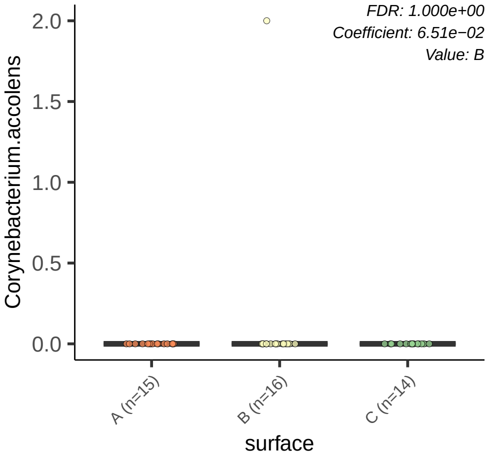
FDR: 1.000e+00
Coefficient: 6.51e−02
Value: B
2.0
1.5
1.0
0.5
0.0
Corynebacterium.accolens
A (n=15)
B (n=16)
C (n=14)
surface

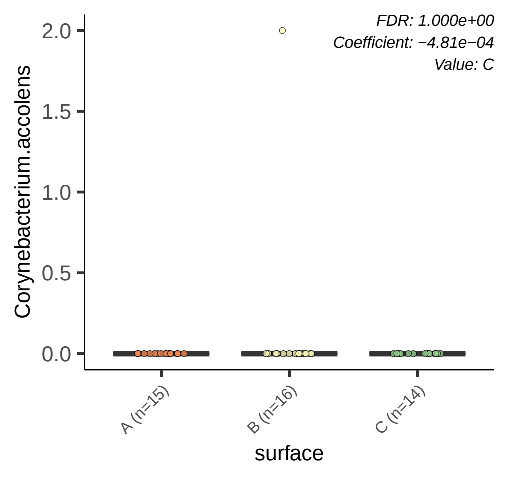
FDR: 1.000e+00
Coefficient: −4.81e−04
Value: C
Corynebacterium.accolens
2.0
1.5
1.0
0.5
0.0
A (n=15)
B (n=16)
C (n=14)
surface

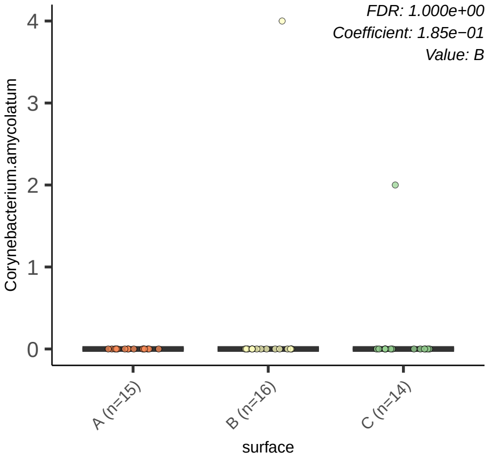
FDR: 1.000e+00
Coefficient: 1.85e−01
Value: B
Corynebacterium.amycolatum
0
1
2
3
4
A (n=15)
B (n=16)
C (n=14)
surface

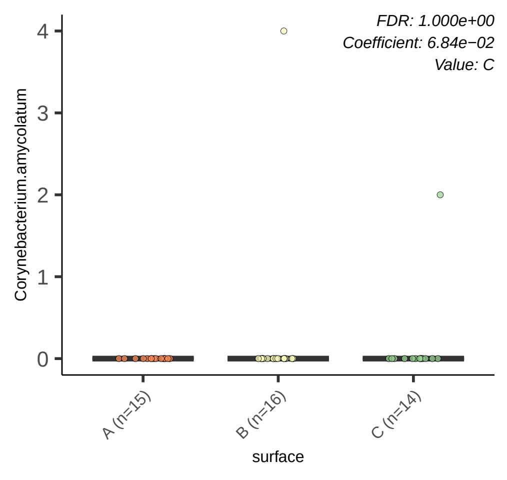
FDR: 1.000e+00
Coefficient: 6.84e−02
Value: C
Corynebacterium.amycolatum
4
3
2
1
0
A (n=15)
B (n=16)
C (n=14)
surface

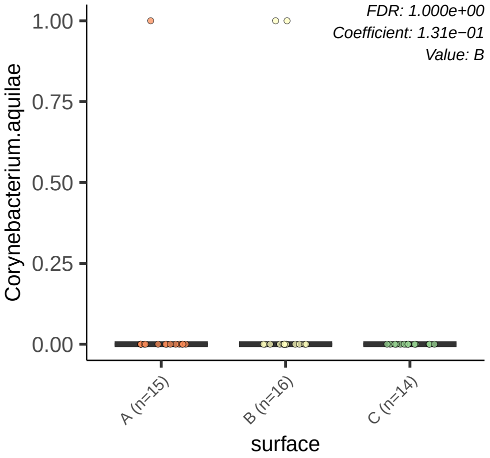
FDR: 1.000e+00
Coefficient: 1.31e−01
Value: B
Corynebacterium.aquilae
1.00
0.75
0.50
0.25
0.00
A (n=15)
B (n=16)
C (n=14)
surface

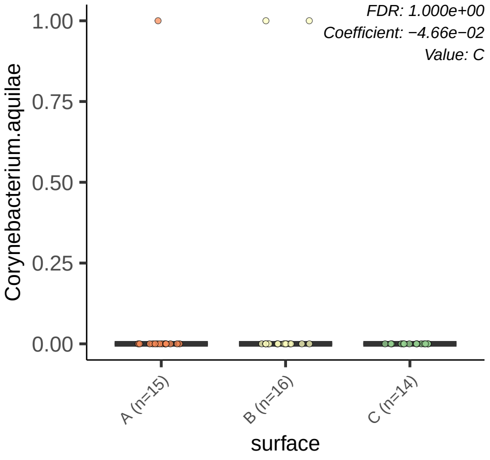

FDR: 1.000e+00
Coefficient: −4.66e−02
Value: C
Corynebacterium.aquilae
1.00
0.75
0.50
0.25
0.00
A (n=15)
B (n=16)
C (n=14)
surface

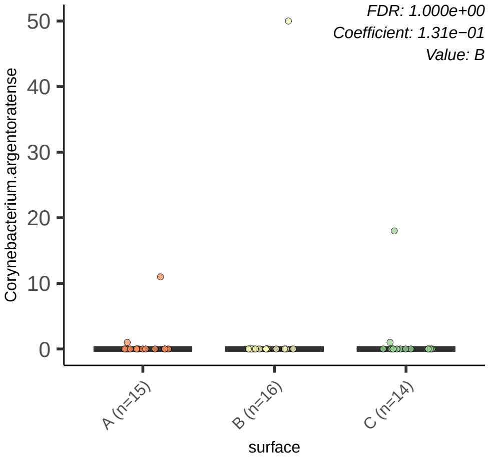
FDR: 1.000e+00
Coefficient: 1.31e−01
Value: B
Corynebacterium.argentoratense
0
10
20
30
40
50
A (n=15)
B (n=16)
C (n=14)
surface

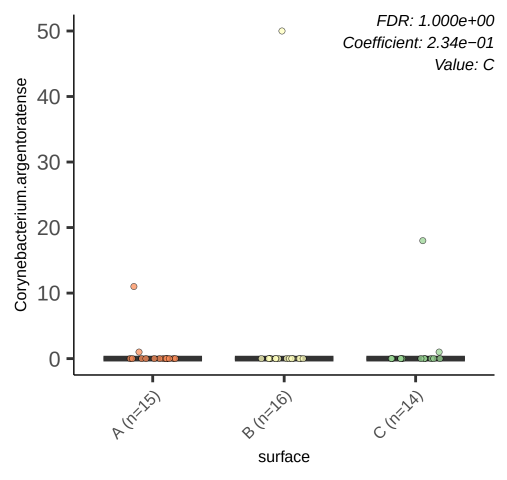

FDR: 1.000e+00
Coefficient: 2.34e−01
Value: C
Corynebacterium.argentoratense
50
40
30
20
10
0
A (n=15)
B (n=16)
C (n=14)
surface

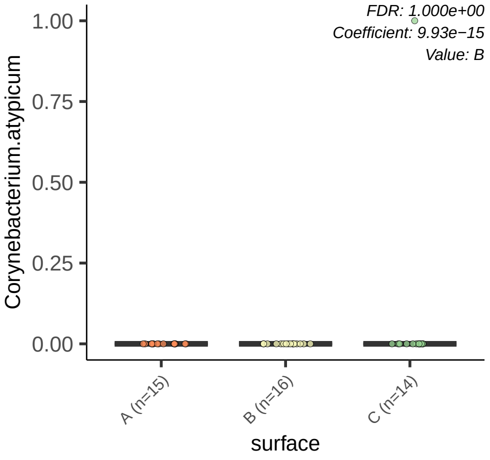

FDR: 1.000e+00
Coefficient: 9.93e−15
Value: B
1.00
0.75
0.50
0.25
0.00
Corynebacterium.atypicum
A (n=15)
B (n=16)
C (n=14)
surface

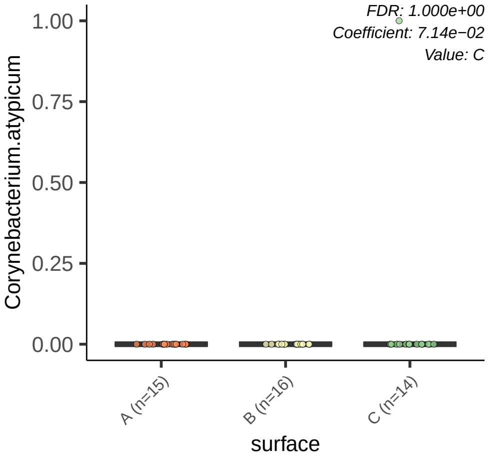
FDR: 1.000e+00
Coefficient: 7.14e−02
Value: C
Corynebacterium.atypicum
1.00
0.75
0.50
0.25
0.00
A (n=15)
B (n=16)
C (n=14)
surface

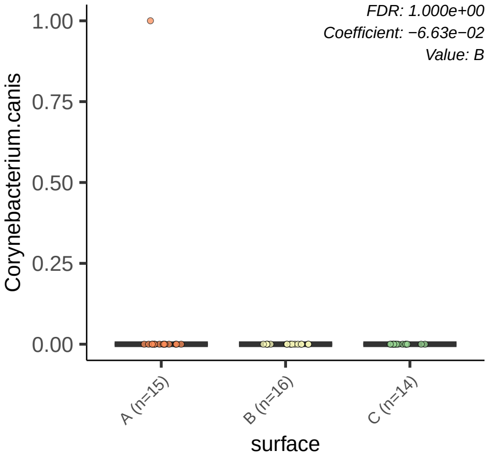
FDR: 1.000e+00
Coefficient: −6.63e−02
Value: B
Corynebacterium.canis
1.00
0.75
0.50
0.25
0.00
A (n=15)
B (n=16)
C (n=14)
surface

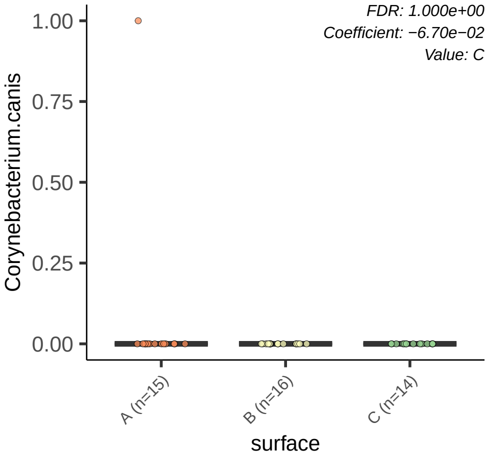

FDR: 1.000e+00
Coefficient: −6.70e−02
Value: C
Corynebacterium.canis
1.00
0.75
0.50
0.25
0.00
A (n=15)
B (n=16)
C (n=14)
surface

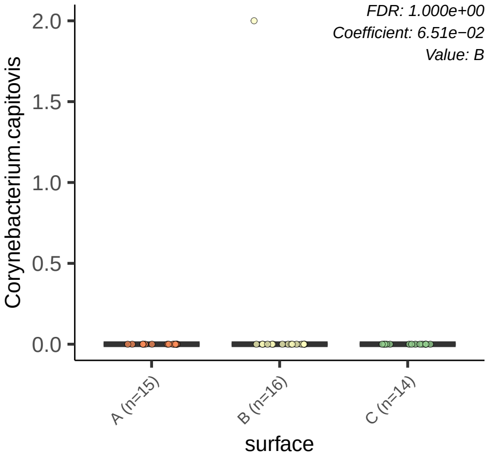

FDR: 1.000e+00
Coefficient: 6.51e−02
Value: B
Corynebacterium.capitovis
2.0
1.5
1.0
0.5
0.0
A (n=15)
B (n=16)
C (n=14)
surface

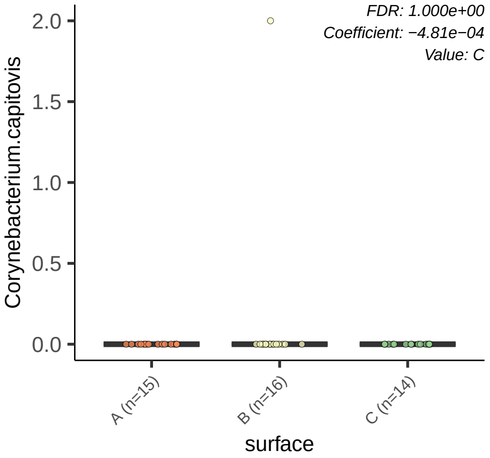
FDR: 1.000e+00
Coefficient: −4.81e−04
Value: C
Corynebacterium.capitovis
2.0
1.5
1.0
0.5
0.0
A (n=15)
B (n=16)
C (n=14)
surface

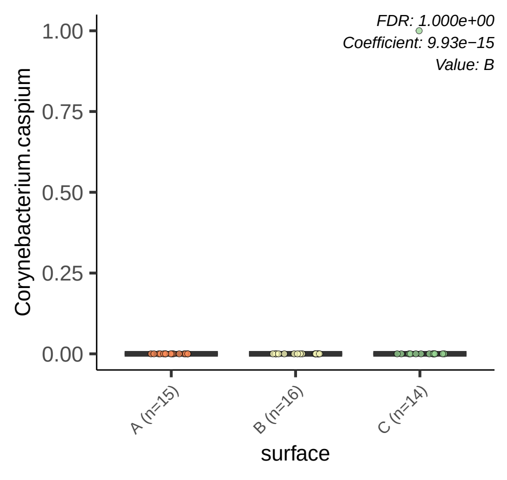
FDR: 1.000e+00
Coefficient: 9.93e−15
Value: B
Corynebacterium.caspium
1.00
0.75
0.50
0.25
0.00
A (n=15)
B (n=16)
C (n=14)
surface

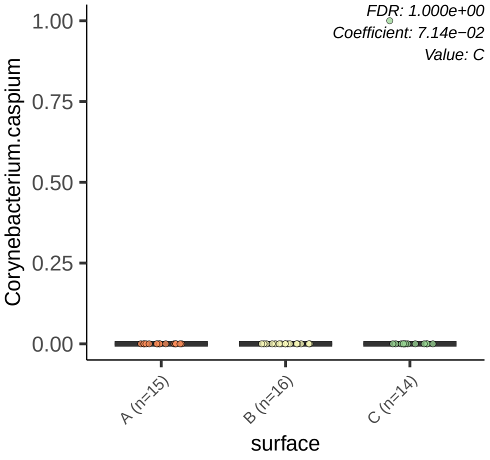
FDR: 1.000e+00
Coefficient: 7.14e−02
Value: C
Corynebacterium.caspium
1.00
0.75
0.50
0.25
0.00
A (n=15)
B (n=16)
C (n=14)
surface

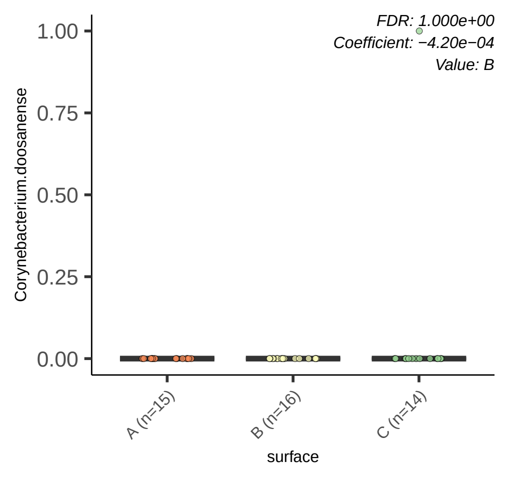
FDR: 1.000e+00
Coefficient: −4.20e−04
Value: B
1.00
0.75
0.50
0.25
0.00
Corynebacterium.doosanense
A (n=15)
B (n=16)
C (n=14)
surface

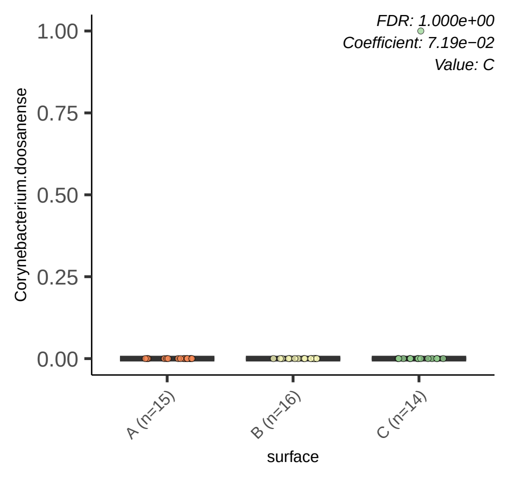

FDR: 1.000e+00
Coefficient: 7.19e−02
Value: C
1.00
0.75
0.50
0.25
0.00
Corynebacterium.doosanense
A (n=15)
B (n=16)
C (n=14)
surface

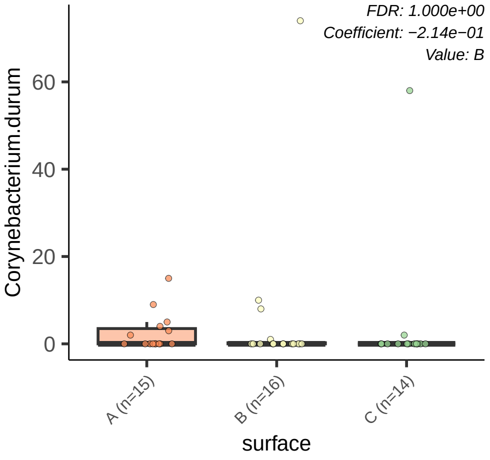

FDR: 1.000e+00
Coefficient: −2.14e−01
Value: B
Corynebacterium.durum
0
20
40
60
A (n=15)
B (n=16)
C (n=14)
surface

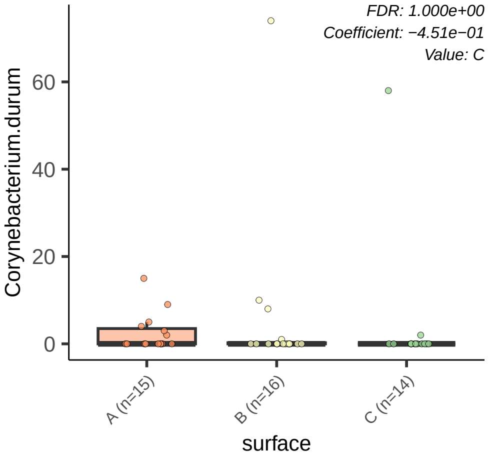

FDR: 1.000e+00
Coefficient: −4.51e−01
Value: C
Corynebacterium.durum
0
20
40
60
A (n=15)
B (n=16)
C (n=14)
surface

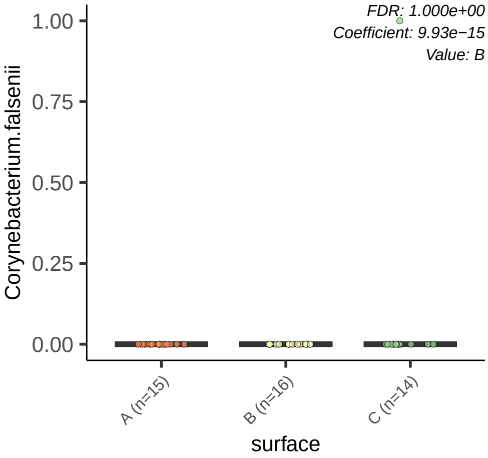

FDR: 1.000e+00
Coefficient: 9.93e−15
Value: B
Corynebacterium.falsenii
1.00
0.75
0.50
0.25
0.00
A (n=15)
B (n=16)
C (n=14)
surface

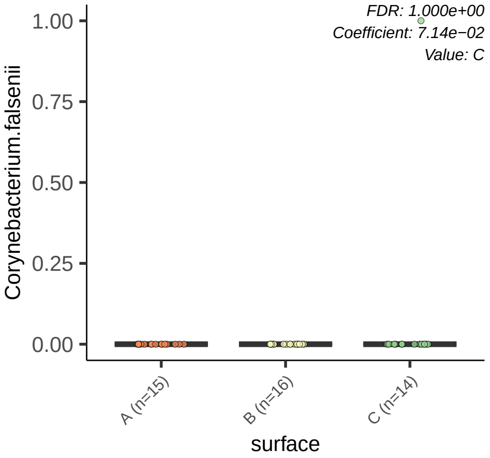
FDR: 1.000e+00
Coefficient: 7.14e−02
Value: C
Corynebacterium.falsenii
1.00
0.75
0.50
0.25
0.00
A (n=15)
B (n=16)
C (n=14)
surface

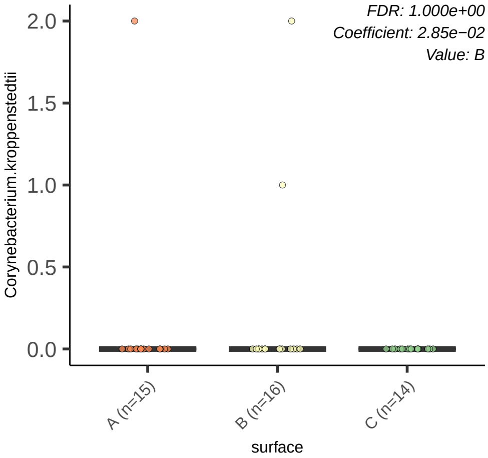

FDR: 1.000e+00
Coefficient: 2.85e−02
Value: B
Corynebacterium.kroppenstedtii
2.0
1.5
1.0
0.5
0.0
A (n=15)
B (n=16)
C (n=14)
surface

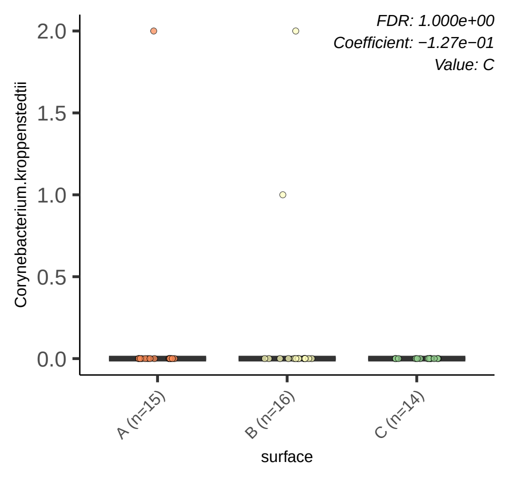
FDR: 1.000e+00
Coefficient: −1.27e−01
Value: C
Corynebacterium.kroppenstedtii
2.0
1.5
1.0
0.5
0.0
A (n=15)
B (n=16)
C (n=14)
surface

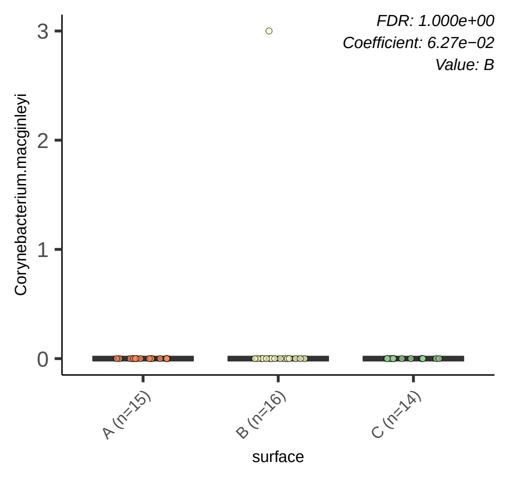

FDR: 1.000e+00
Coefficient: 6.27e−02
Value: B
Corynebacterium.macginleyi
3
2
1
0
A (n=15)
B (n=16)
C (n=14)
surface

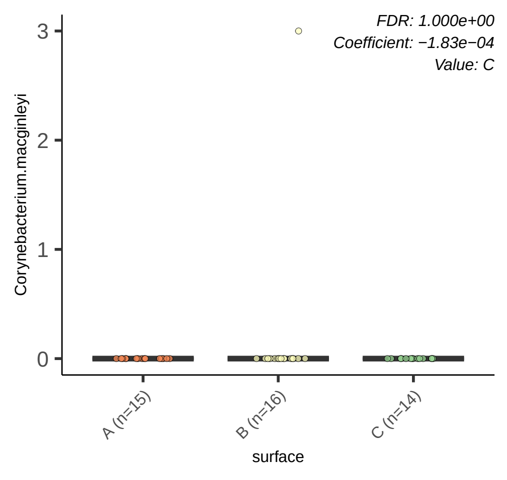
FDR: 1.000e+00
Coefficient: −1.83e−04
Value: C
Corynebacterium.macginleyi
3
2
1
0
A (n=15)
B (n=16)
C (n=14)
surface

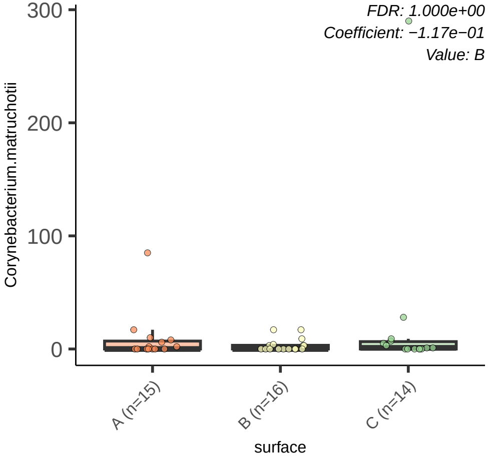

FDR: 1.000e+00
Coefficient: −1.17e−01
Value: B
300
200
100
0
Corynebacterium.matruchotii
A (n=15)
B (n=16)
C (n=14)
surface

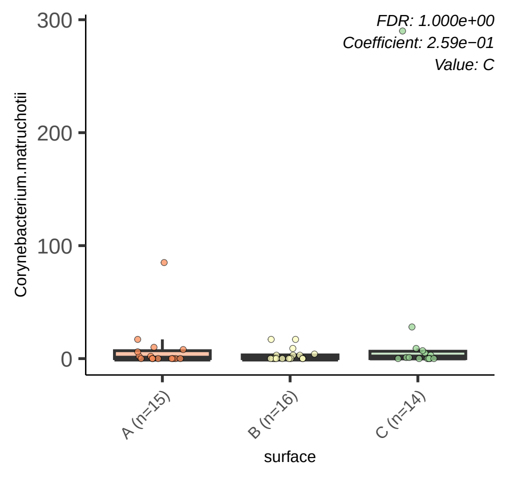
FDR: 1.000e+00
Coefficient: 2.59e−01
Value: C
Corynebacterium.matruchotii
300
200
100
0
A (n=15)
B (n=16)
C (n=14)
surface

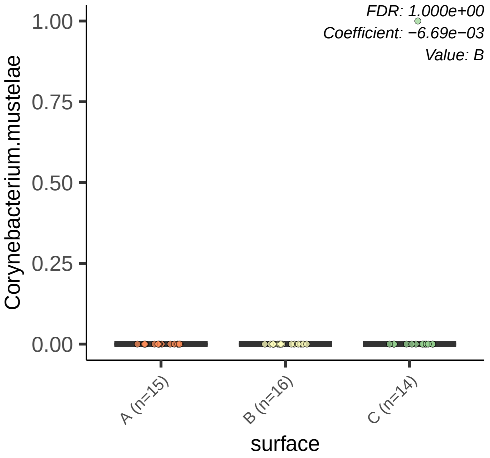
FDR: 1.000e+00
Coefficient: −6.69e−03
Value: B
Corynebacterium.mustelae
1.00
0.75
0.50
0.25
0.00
A (n=15)
B (n=16)
C (n=14)
surface

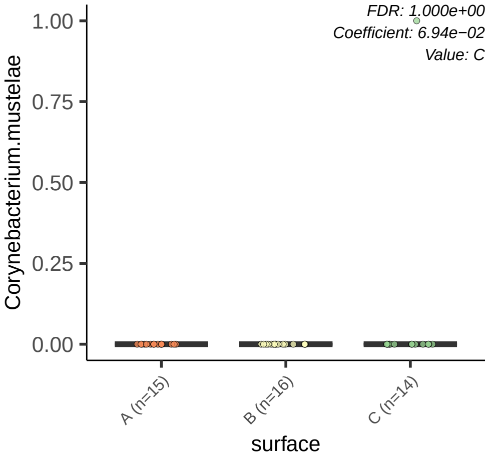

FDR: 1.000e+00
Coefficient: 6.94e−02
Value: C
Corynebacterium.mustelae
1.00
0.75
0.50
0.25
0.00
A (n=15)
B (n=16)
C (n=14)
surface

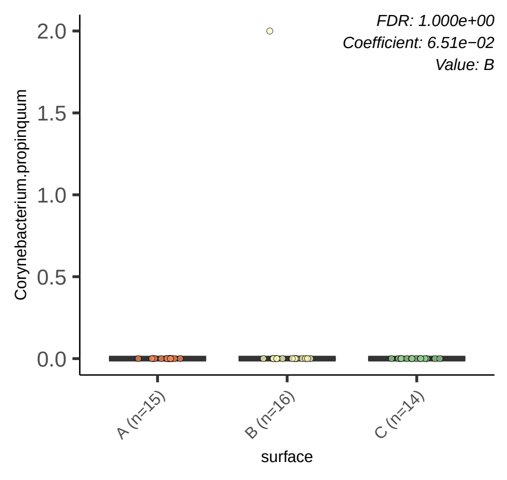

FDR: 1.000e+00
Coefficient: 6.51e−02
Value: B
Corynebacterium.propinquum
2.0
1.5
1.0
0.5
0.0
A (n=15)
B (n=16)
C (n=14)
surface

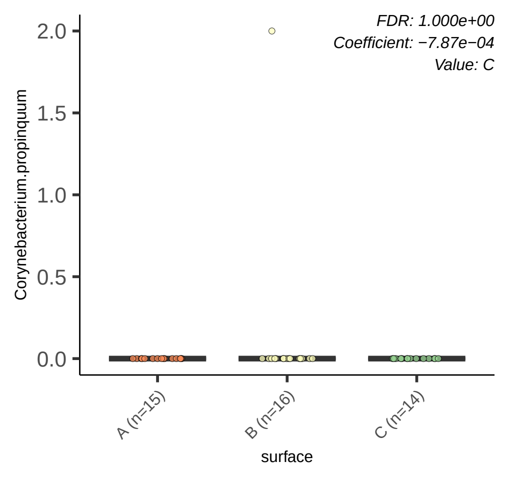
FDR: 1.000e+00
Coefficient: −7.87e−04
Value: C
Corynebacterium.propinquum
2.0
1.5
1.0
0.5
0.0
A (n=15)
B (n=16)
C (n=14)
surface

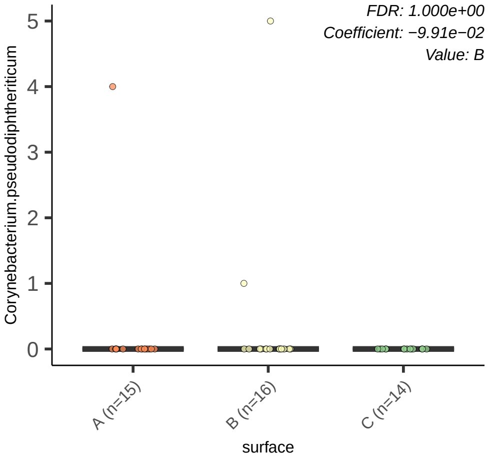

FDR: 1.000e+00
Coefficient: −9.91e−02
Value: B
Corynebacterium.pseudodiphtheriticum
0
1
2
3
4
5
A (n=15)
B (n=16)
C (n=14)
surface

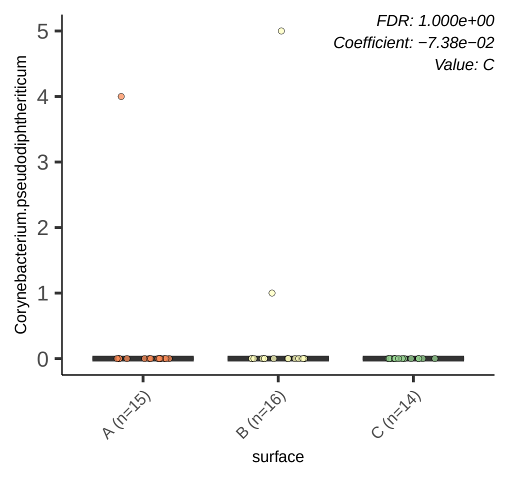

FDR: 1.000e+00
Coefficient: −7.38e−02
Value: C
Corynebacterium.pseudodiphtheriticum
0
1
2
3
4
5
A (n=15)
B (n=16)
C (n=14)
surface

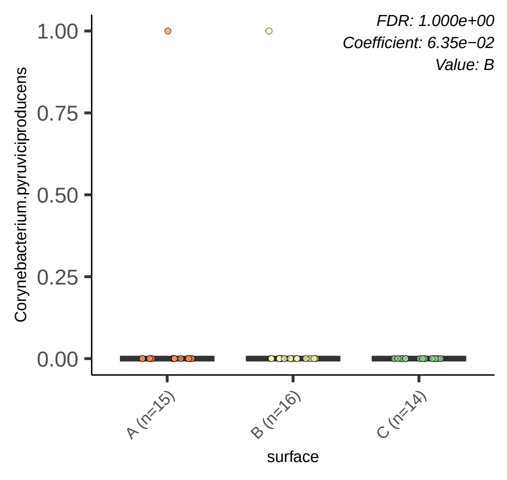

FDR: 1.000e+00
Coefficient: 6.35e−02
Value: B
1.00
0.75
0.50
0.25
0.00
Corynebacterium.pyruviciproducens
A (n=15)
B (n=16)
C (n=14)
surface

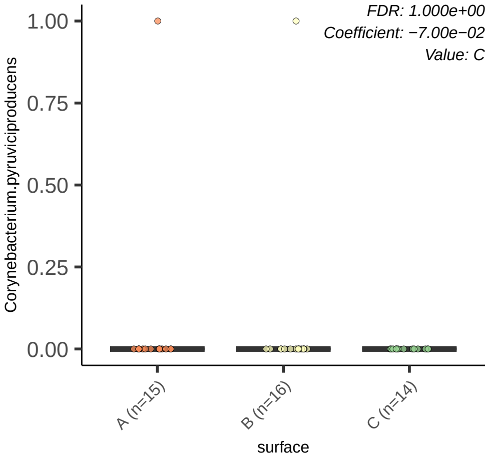
FDR: 1.000e+00
Coefficient: −7.00e−02
Value: C
Corynebacterium.pyruviciproducens
1.00
0.75
0.50
0.25
0.00
A (n=15)
B (n=16)
C (n=14)
surface

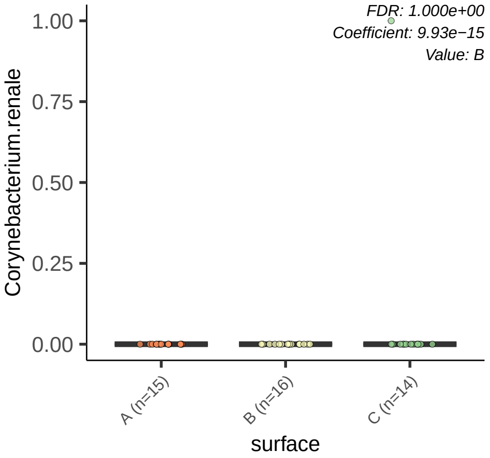
FDR: 1.000e+00
Coefficient: 9.93e−15
Value: B
Corynebacterium.renale
1.00
0.75
0.50
0.25
0.00
A (n=15)
B (n=16)
C (n=14)
surface

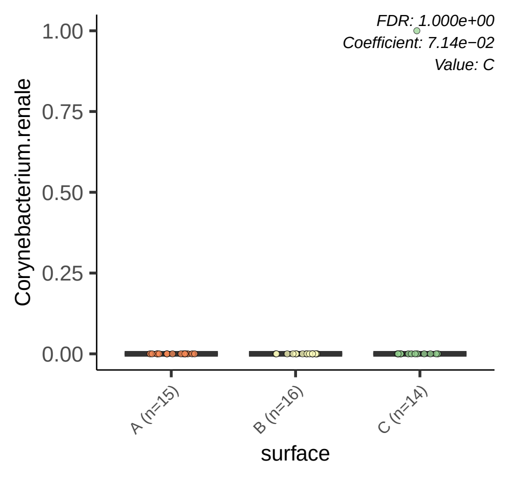
FDR: 1.000e+00
Coefficient: 7.14e−02
Value: C
1.00
0.75
0.50
0.25
0.00
Corynebacterium.renale
A (n=15)
B (n=16)
C (n=14)
surface

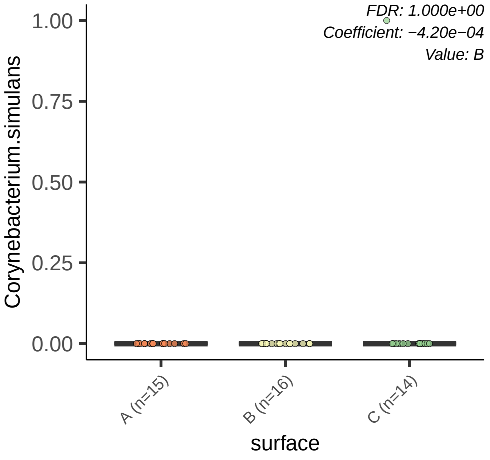
FDR: 1.000e+00
Coefficient: −4.20e−04
Value: B
Corynebacterium.simulans
1.00
0.75
0.50
0.25
0.00
A (n=15)
B (n=16)
C (n=14)
surface

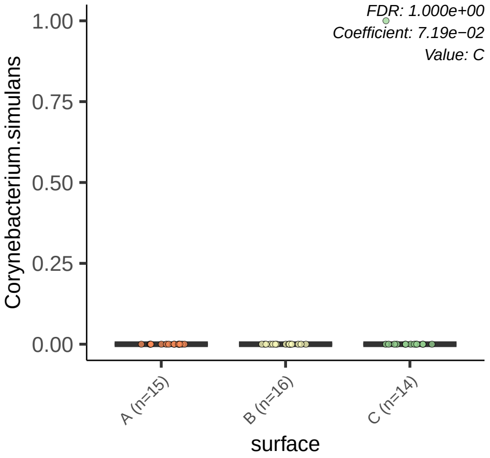

FDR: 1.000e+00
Coefficient: 7.19e−02
Value: C
Corynebacterium.simulans
1.00
0.75
0.50
0.25
0.00
A (n=15)
B (n=16)
C (n=14)
surface

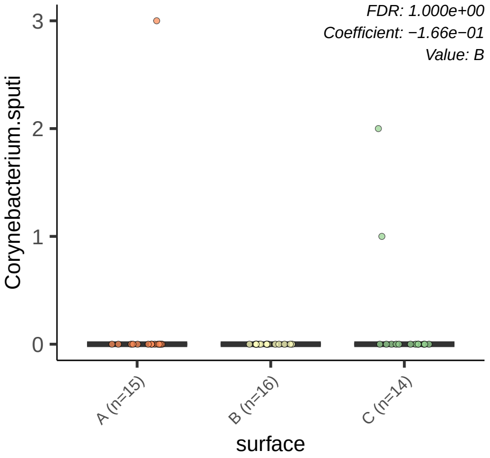
FDR: 1.000e+00
Coefficient: −1.66e−01
Value: B
Corynebacterium.sputi
3
2
1
0
A (n=15)
B (n=16)
C (n=14)
surface

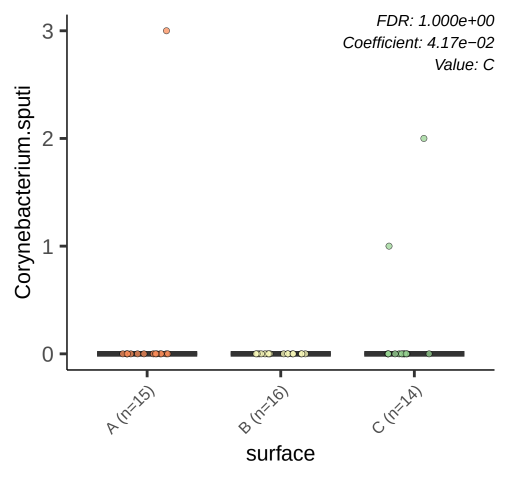
FDR: 1.000e+00
Coefficient: 4.17e−02
Value: C
Corynebacterium.sputi
0
1
2
3
A (n=15)
B (n=16)
C (n=14)
surface

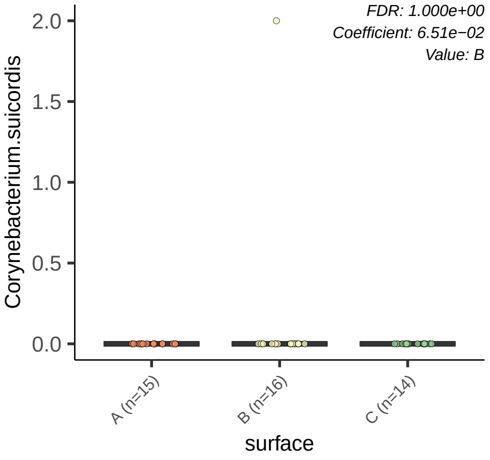

FDR: 1.000e+00
Coefficient: 6.51e−02
Value: B
Corynebacterium.suicordis
2.0
1.5
1.0
0.5
0.0
A (n=15)
B (n=16)
C (n=14)
surface

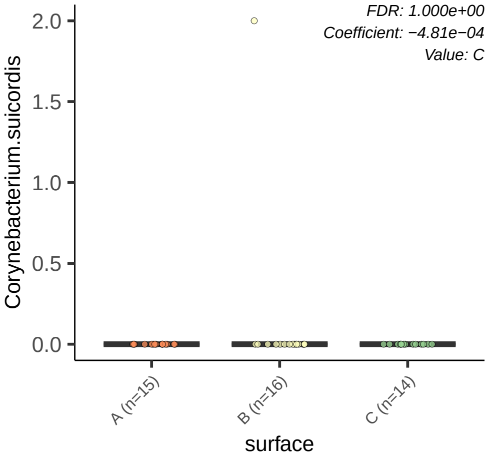
FDR: 1.000e+00
Coefficient: −4.81e−04
Value: C
Corynebacterium.suicordis
2.0
1.5
1.0
0.5
0.0
A (n=15)
B (n=16)
C (n=14)
surface

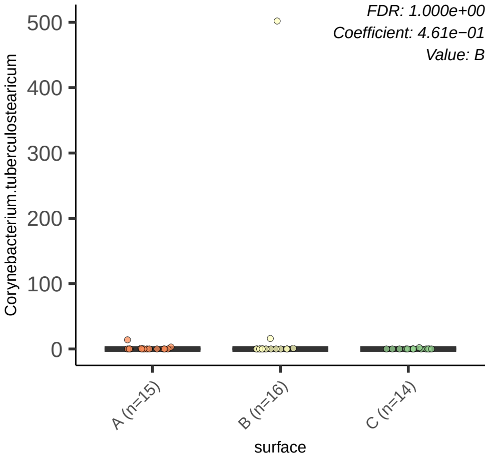

FDR: 1.000e+00
Coefficient: 4.61e−01
Value: B
Corynebacterium.tuberculostearicum
500
400
300
200
100
0
A (n=15)
B (n=16)
C (n=14)
surface

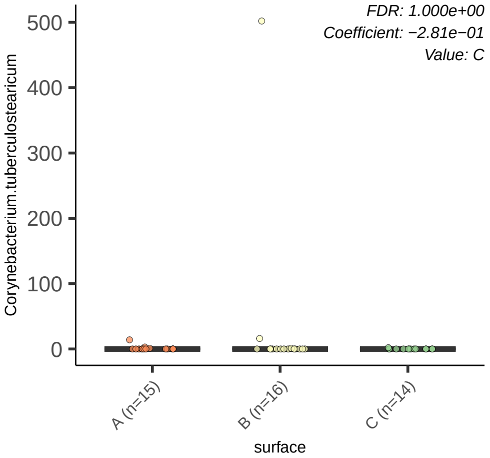
FDR: 1.000e+00
Coefficient: −2.81e−01
Value: C
Corynebacterium.tuberculostearicum
500
400
300
200
100
0
A (n=15)
B (n=16)
C (n=14)
surface

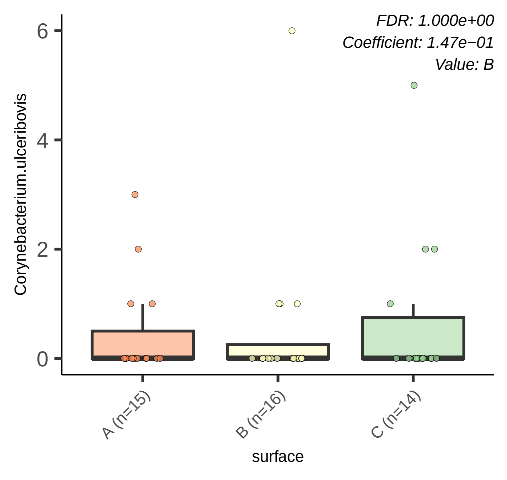

FDR: 1.000e+00
Coefficient: 1.47e−01
Value: B
Corynebacterium.ulceribovis
0
2
4
6
A (n=15)
B (n=16)
C (n=14)
surface

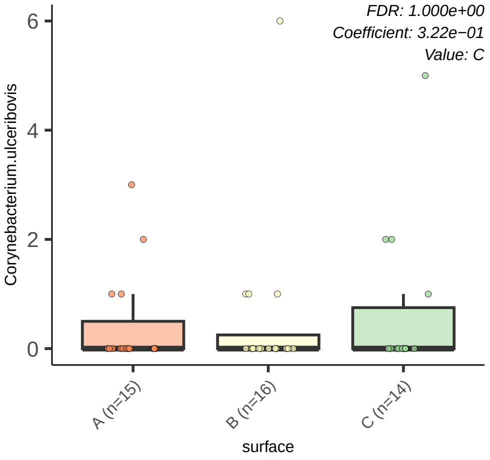
FDR: 1.000e+00
Coefficient: 3.22e−01
Value: C
Corynebacterium.ulceribovis
0
2
4
6
A (n=15)
B (n=16)
C (n=14)
surface

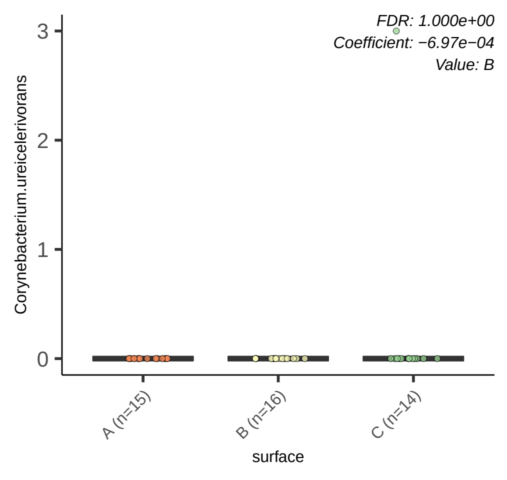
FDR: 1.000e+00
Coefficient: −6.97e−04
Value: B
Corynebacterium.ureicelerivorans
3
2
1
0
A (n=15)
B (n=16)
C (n=14)
surface

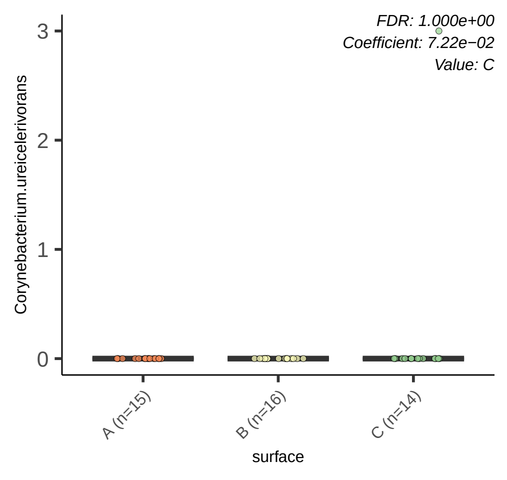
FDR: 1.000e+00
Coefficient: 7.22e−02
Value: C
Corynebacterium.ureicelerivorans
3
2
1
0
A (n=15)
B (n=16)
C (n=14)
surface

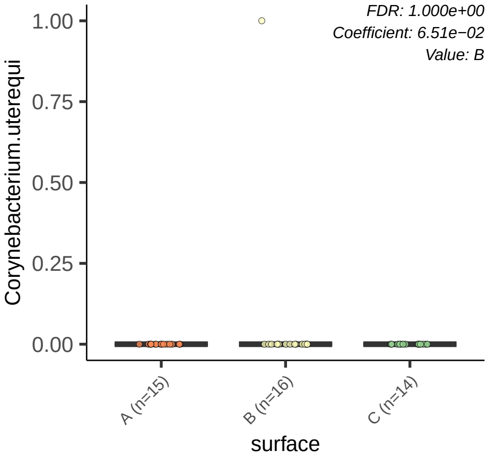
FDR: 1.000e+00
Coefficient: 6.51e−02
Value: B
Corynebacterium.uterequi
1.00
0.75
0.50
0.25
0.00
A (n=15)
B (n=16)
C (n=14)
surface

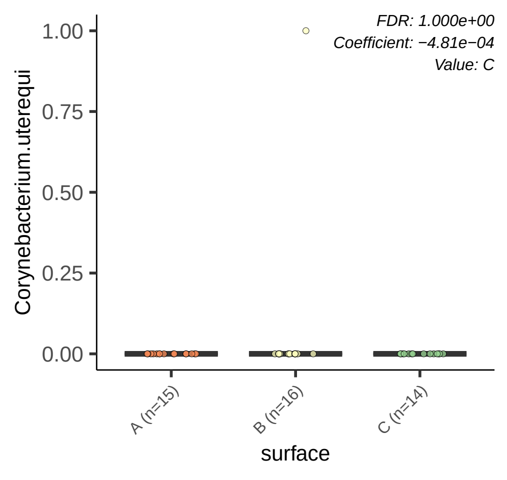
FDR: 1.000e+00
Coefficient: −4.81e−04
Value: C
Corynebacterium.uterequi
1.00
0.75
0.50
0.25
0.00
A (n=15)
B (n=16)
C (n=14)
surface

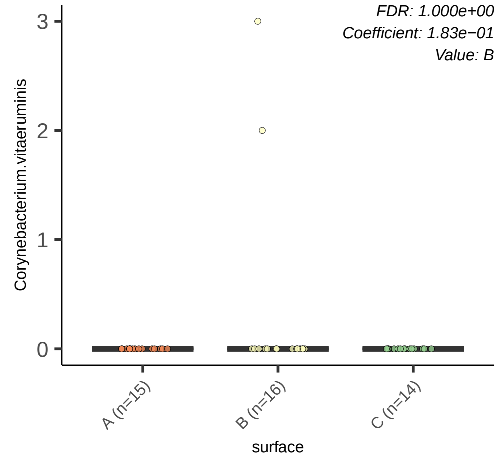

FDR: 1.000e+00
Coefficient: 1.83e−01
Value: B
Corynebacterium.vitaeruminis
3
2
1
0
A (n=15)
B (n=16)
C (n=14)
surface

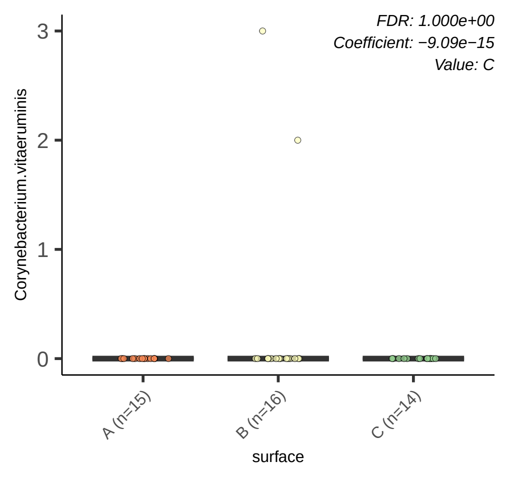
FDR: 1.000e+00
Coefficient: −9.09e−15
Value: C
Corynebacterium.vitaeruminis
3
2
1
0
A (n=15)
B (n=16)
C (n=14)
surface

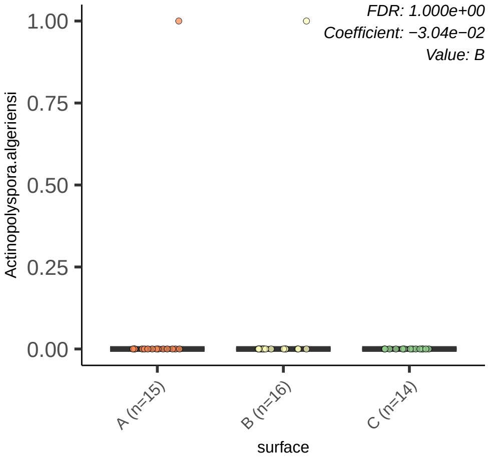

FDR: 1.000e+00
Coefficient: −3.04e−02
Value: B
Actinopolyspora.algeriensi
1.00
0.75
0.50
0.25
0.00
A (n=15)
B (n=16)
C (n=14)
surface

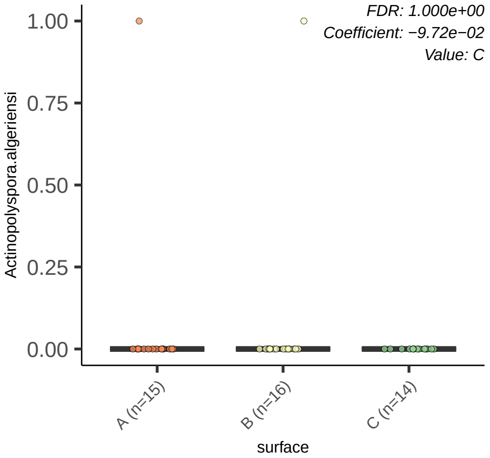

FDR: 1.000e+00
Coefficient: −9.72e−02
Value: C
Actinopolyspora.algeriensi
1.00
0.75
0.50
0.25
0.00
A (n=15)
B (n=16)
C (n=14)
surface

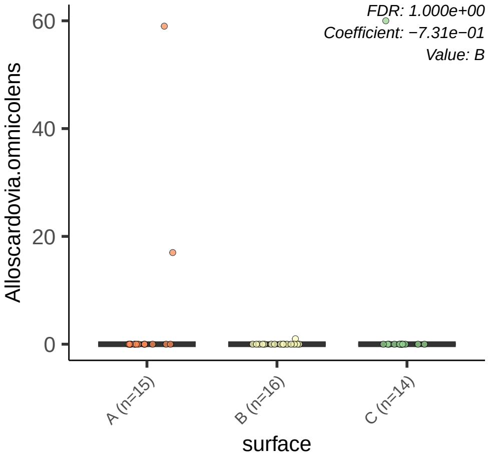

FDR: 1.000e+00
Coefficient: −7.31e−01
Value: B
Alloscardovia.omnicolens
60
40
20
0
A (n=15)
B (n=16)
C (n=14)
surface

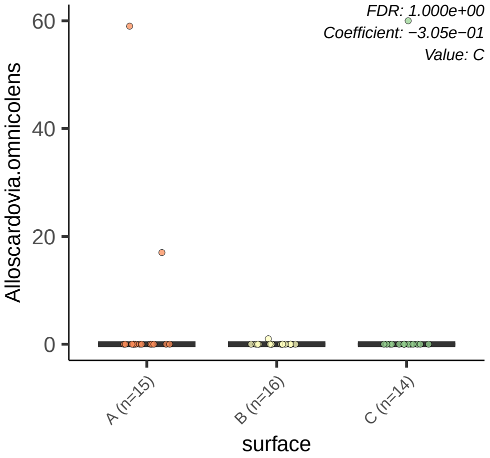
FDR: 1.000e+00
Coefficient: −3.05e−01
Value: C
Alloscardovia.omnicolens
60
40
20
0
A (n=15)
B (n=16)
C (n=14)
surface

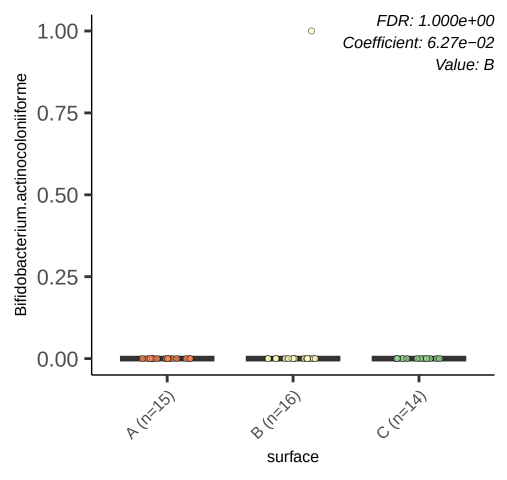

FDR: 1.000e+00
Coefficient: 6.27e−02
Value: B
Bifidobacterium.actinocoloniiforme
1.00
0.75
0.50
0.25
0.00
A (n=15)
B (n=16)
C (n=14)
surface

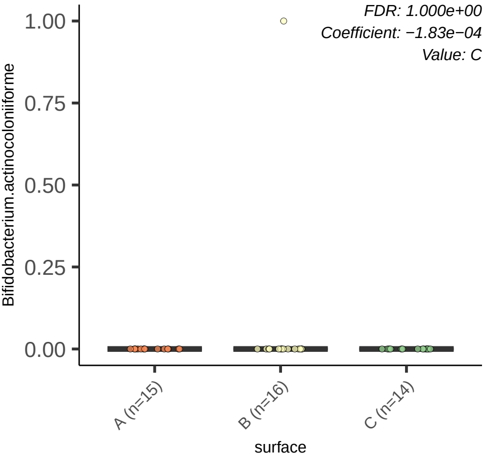
FDR: 1.000e+00
Coefficient: −1.83e−04
Value: C
1.00
0.75
0.50
0.25
0.00
Bifidobacterium.actinocoloniiforme
A (n=15)
B (n=16)
C (n=14)
surface

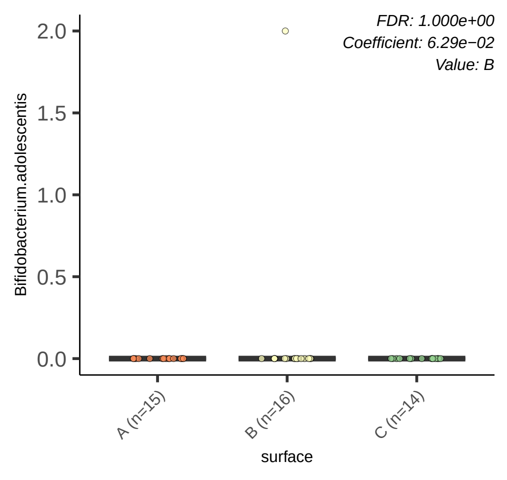

FDR: 1.000e+00
Coefficient: 6.29e−02
Value: B
Bifidobacterium.adolescentis
2.0
1.5
1.0
0.5
0.0
A (n=15)
B (n=16)
C (n=14)
surface

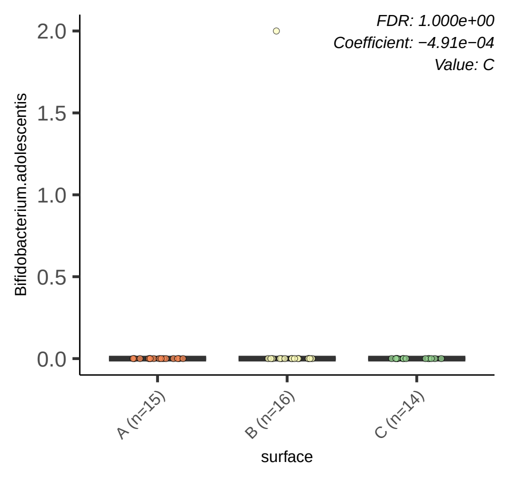
FDR: 1.000e+00
Coefficient: −4.91e−04
Value: C
Bifidobacterium.adolescentis
2.0
1.5
1.0
0.5
0.0
A (n=15)
B (n=16)
C (n=14)
surface

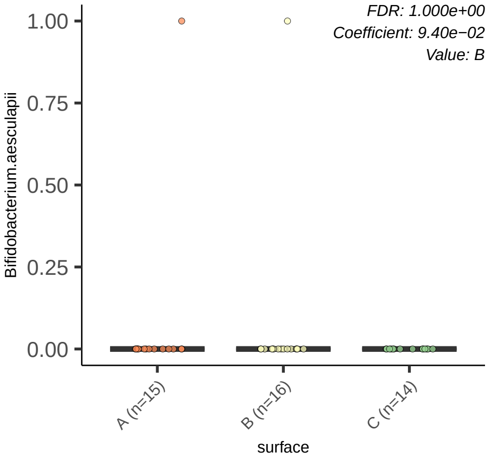

FDR: 1.000e+00
Coefficient: 9.40e−02
Value: B
Bifidobacterium.aesculapii
1.00
0.75
0.50
0.25
0.00
A (n=15)
B (n=16)
C (n=14)
surface

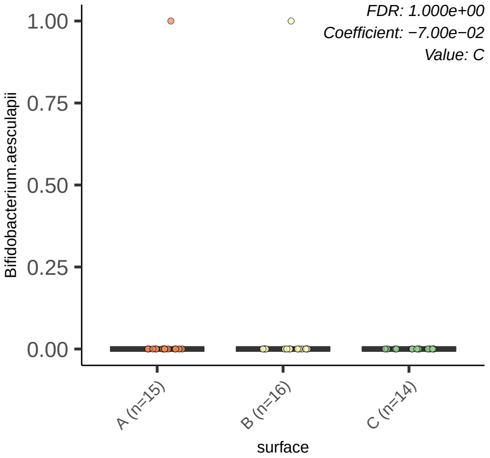
FDR: 1.000e+00
Coefficient: −7.00e−02
Value: C
1.00
0.75
0.50
0.25
0.00
Bifidobacterium.aesculapii
A (n=15)
B (n=16)
C (n=14)
surface

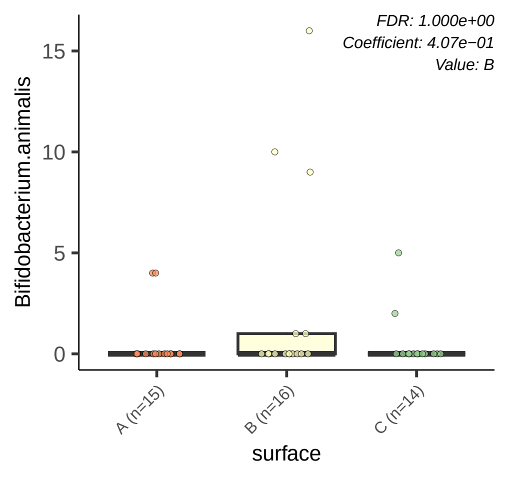

FDR: 1.000e+00
Coefficient: 4.07e−01
Value: B
Bifidobacterium.animalis
15
10
5
0
A (n=15)
B (n=16)
C (n=14)
surface

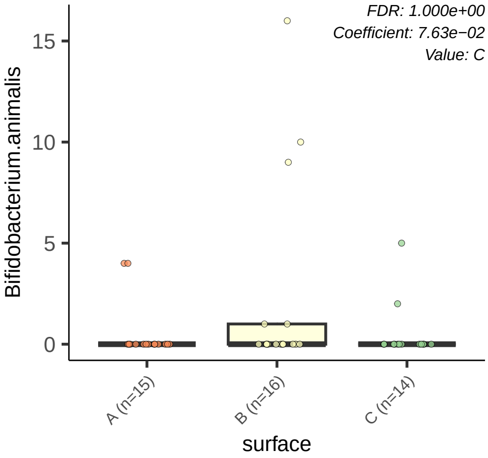

FDR: 1.000e+00
Coefficient: 7.63e−02
Value: C
Bifidobacterium.animalis
0
5
10
15
A (n=15)
B (n=16)
C (n=14)
surface

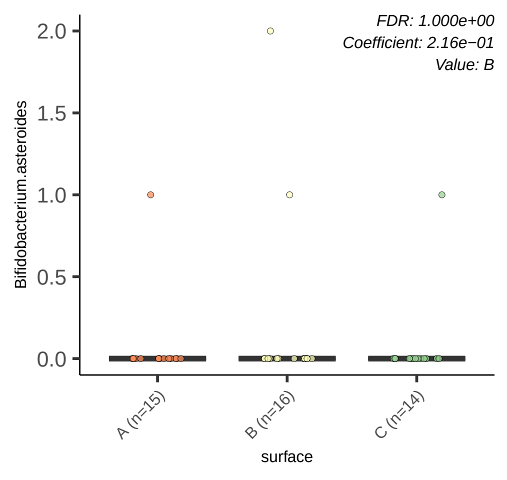
FDR: 1.000e+00
Coefficient: 2.16e−01
Value: B
Bifidobacterium.asteroides
2.0
1.5
1.0
0.5
0.0
A (n=15)
B (n=16)
C (n=14)
surface

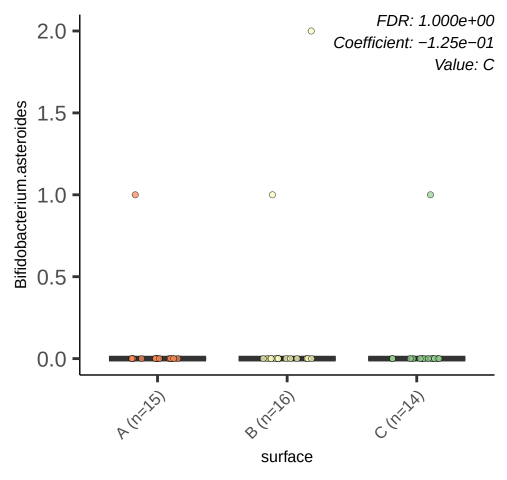
FDR: 1.000e+00
Coefficient: −1.25e−01
Value: C
Bifidobacterium.asteroides
2.0
1.5
1.0
0.5
0.0
A (n=15)
B (n=16)
C (n=14)
surface

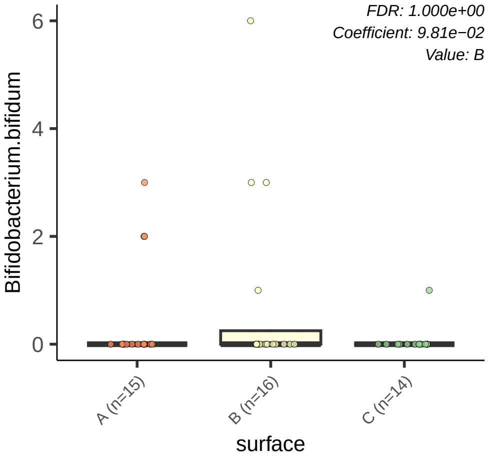
FDR: 1.000e+00
Coefficient: 9.81e−02
Value: B
Bifidobacterium.bifidum
0
2
4
6
A (n=15)
B (n=16)
C (n=14)
surface

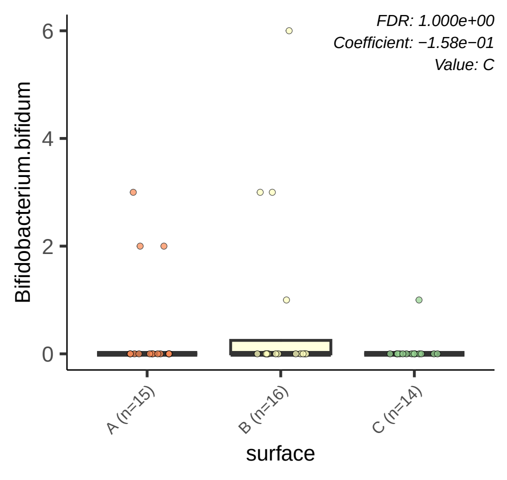

FDR: 1.000e+00
Coefficient: −1.58e−01
Value: C
Bifidobacterium.bifidum
0
2
4
6
A (n=15)
B (n=16)
C (n=14)
surface

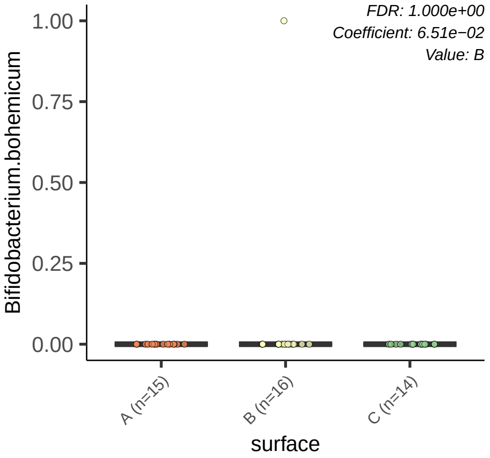

FDR: 1.000e+00
Coefficient: 6.51e−02
Value: B
Bifidobacterium.bohemicum
1.00
0.75
0.50
0.25
0.00
A (n=15)
B (n=16)
C (n=14)
surface

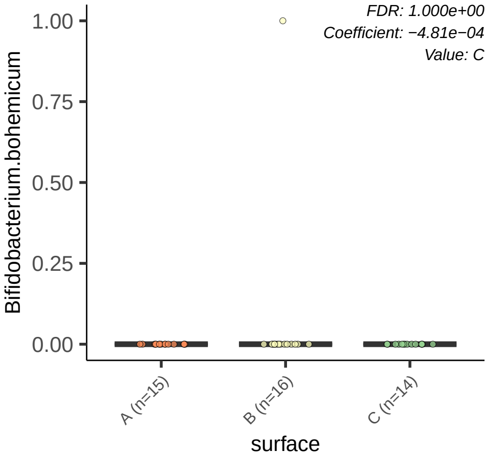

FDR: 1.000e+00
Coefficient: −4.81e−04
Value: C
Bifidobacterium.bohemicum
1.00
0.75
0.50
0.25
0.00
A (n=15)
B (n=16)
C (n=14)
surface

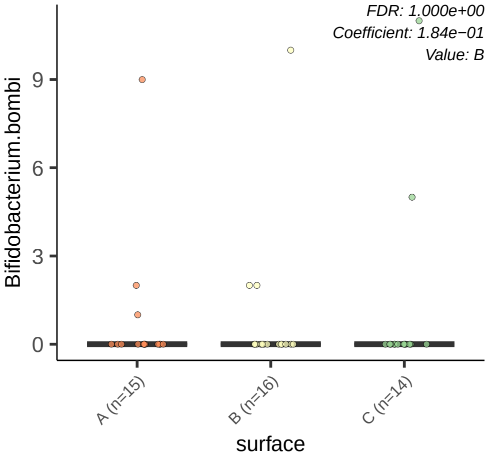

FDR: 1.000e+00
Coefficient: 1.84e−01
Value: B
Bifidobacterium.bombi
0
3
6
9
A (n=15)
B (n=16)
C (n=14)
surface

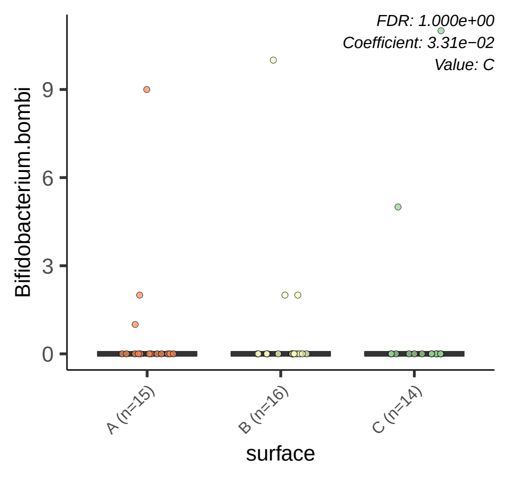

FDR: 1.000e+00
Coefficient: 3.31e−02
Value: C
Bifidobacterium.bombi
0
3
6
9
A (n=15)
B (n=16)
C (n=14)
surface

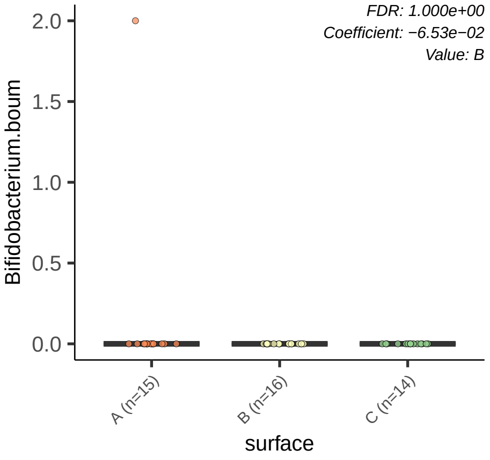
FDR: 1.000e+00
Coefficient: −6.53e−02
Value: B
2.0
1.5
1.0
0.5
0.0
Bifidobacterium.boum
A (n=15)
B (n=16)
C (n=14)
surface

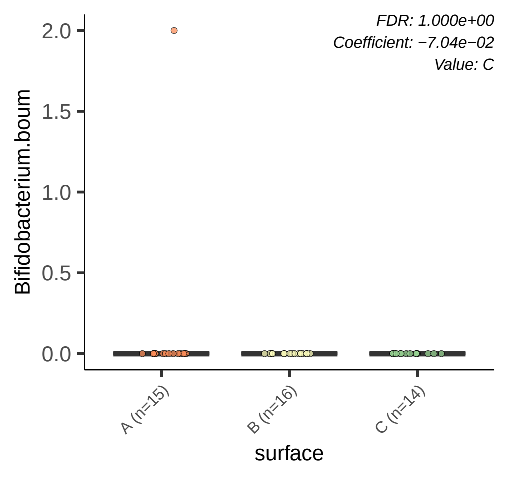
FDR: 1.000e+00
Coefficient: −7.04e−02
Value: C
Bifidobacterium.boum
2.0
1.5
1.0
0.5
0.0
A (n=15)
B (n=16)
C (n=14)
surface

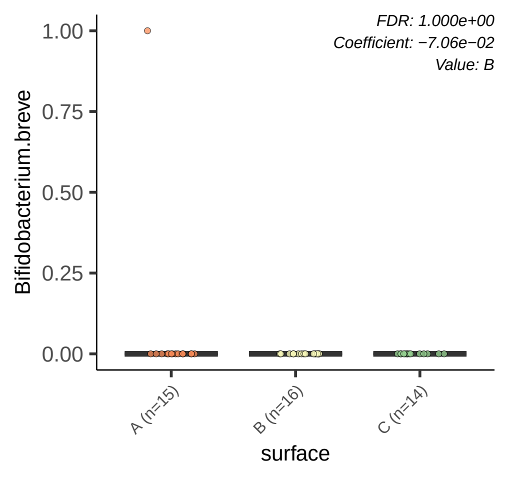

FDR: 1.000e+00
Coefficient: −7.06e−02
Value: B
Bifidobacterium.breve
1.00
0.75
0.50
0.25
0.00
A (n=15)
B (n=16)
C (n=14)
surface

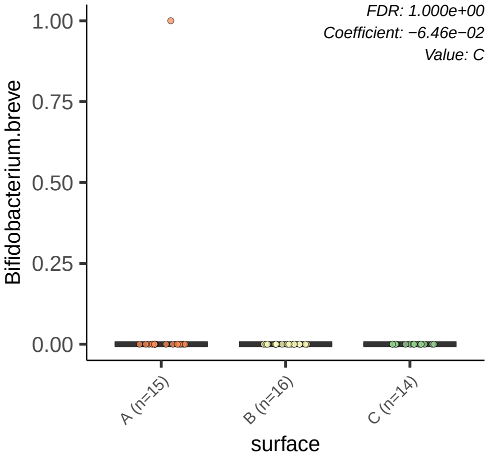

FDR: 1.000e+00
Coefficient: −6.46e−02
Value: C
1.00
0.75
0.50
0.25
0.00
Bifidobacterium.breve
A (n=15)
B (n=16)
C (n=14)
surface

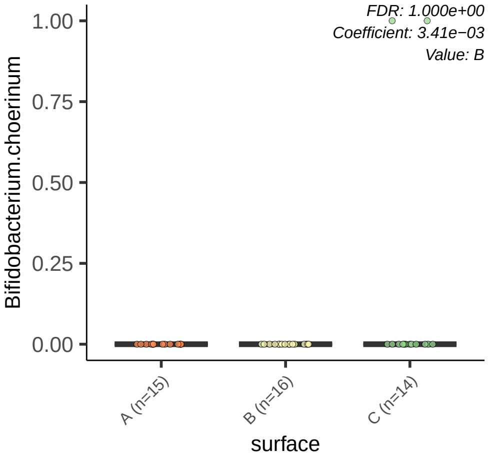

FDR: 1.000e+00
Coefficient: 3.41e−03
Value: B
1.00
0.75
0.50
0.25
0.00
Bifidobacterium.choerinum
A (n=15)
B (n=16)
C (n=14)
surface

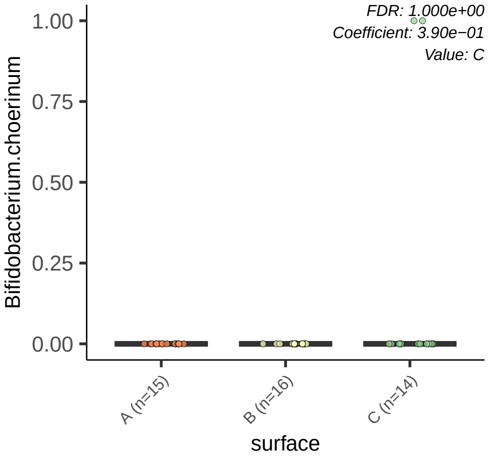

FDR: 1.000e+00
Coefficient: 3.90e−01
Value: C
Bifidobacterium.choerinum
1.00
0.75
0.50
0.25
0.00
A (n=15)
B (n=16)
C (n=14)
surface

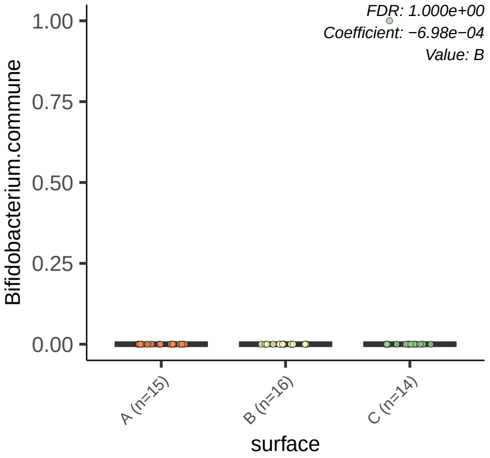
FDR: 1.000e+00
Coefficient: −6.98e−04
Value: B
Bifidobacterium.commune
1.00
0.75
0.50
0.25
0.00
A (n=15)
B (n=16)
C (n=14)
surface

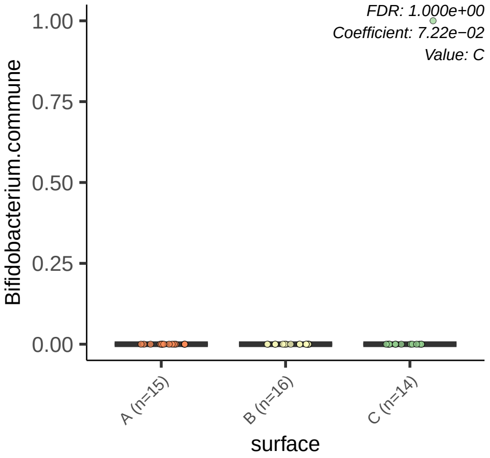
FDR: 1.000e+00
Coefficient: 7.22e−02
Value: C
Bifidobacterium.commune
1.00
0.75
0.50
0.25
0.00
A (n=15)
B (n=16)
C (n=14)
surface

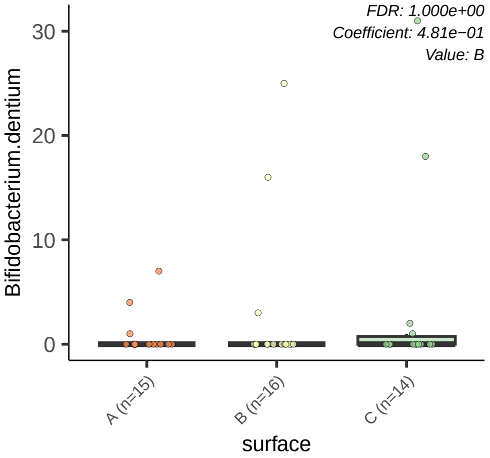

FDR: 1.000e+00
Coefficient: 4.81e−01
Value: B
Bifidobacterium.dentium
0
10
20
30
A (n=15)
B (n=16)
C (n=14)
surface

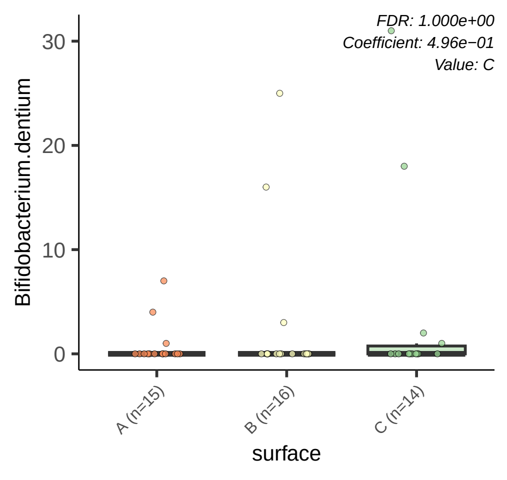

FDR: 1.000e+00
Coefficient: 4.96e−01
Value: C
Bifidobacterium.dentium
30
20
10
0
A (n=15)
B (n=16)
C (n=14)
surface

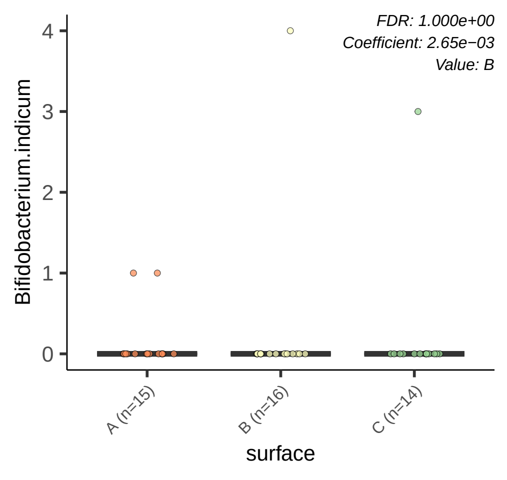

FDR: 1.000e+00
Coefficient: 2.65e−03
Value: B
Bifidobacterium.indicum
4
3
2
1
0
A (n=15)
B (n=16)
C (n=14)
surface

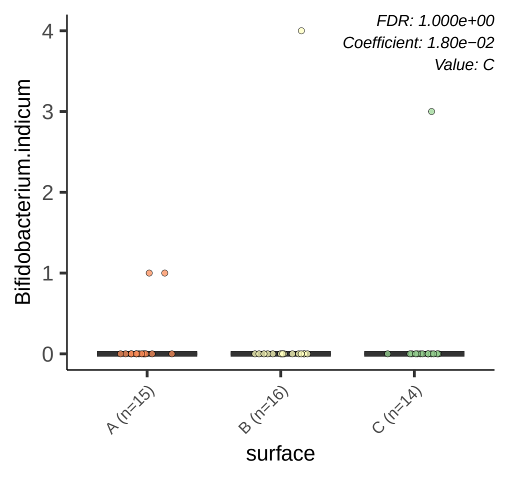

FDR: 1.000e+00
Coefficient: 1.80e−02
Value: C
Bifidobacterium.indicum
4
3
2
1
0
A (n=15)
B (n=16)
C (n=14)
surface

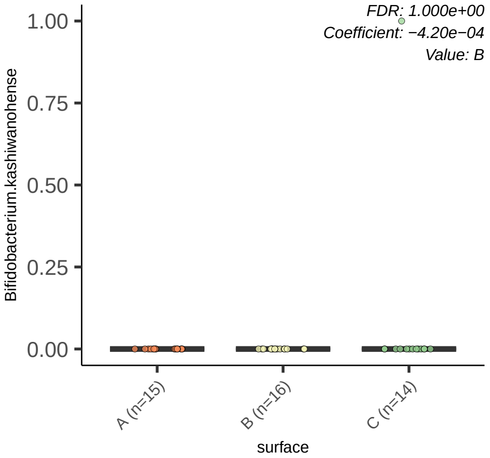
FDR: 1.000e+00
Coefficient: −4.20e−04
Value: B
Bifidobacterium.kashiwanohense
1.00
0.75
0.50
0.25
0.00
A (n=15)
B (n=16)
C (n=14)
surface

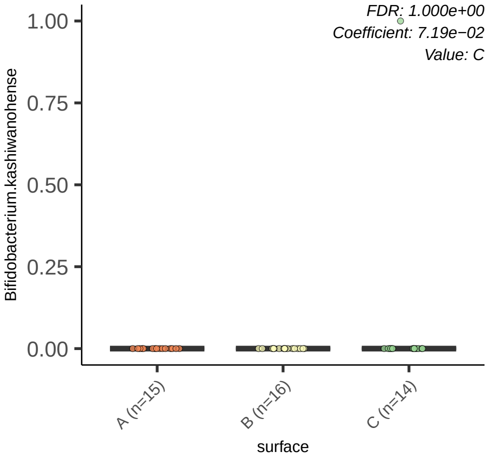

FDR: 1.000e+00
Coefficient: 7.19e−02
Value: C
1.00
0.75
0.50
0.25
0.00
Bifidobacterium.kashiwanohense
A (n=15)
B (n=16)
C (n=14)
surface

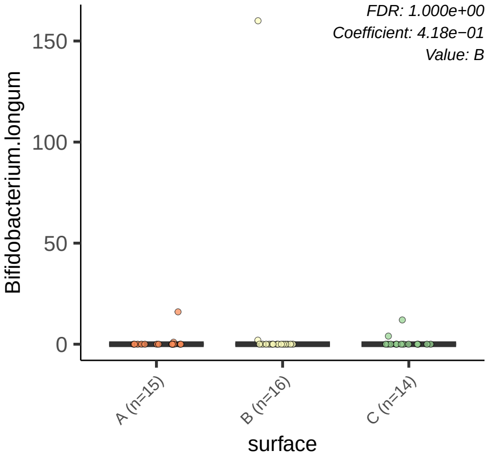

FDR: 1.000e+00
Coefficient: 4.18e−01
Value: B
Bifidobacterium.longum
150
100
50
0
A (n=15)
B (n=16)
C (n=14)
surface

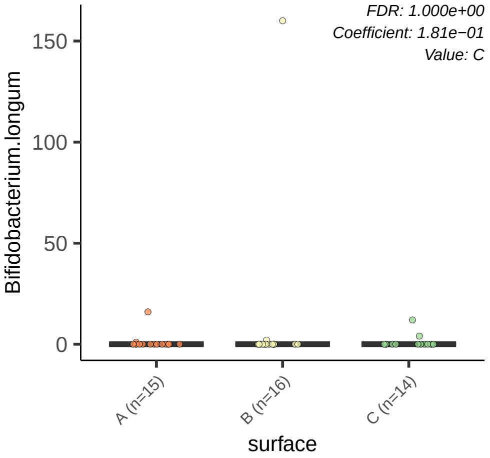

FDR: 1.000e+00
Coefficient: 1.81e−01
Value: C
Bifidobacterium.longum
150
100
50
0
A (n=15)
B (n=16)
C (n=14)
surface

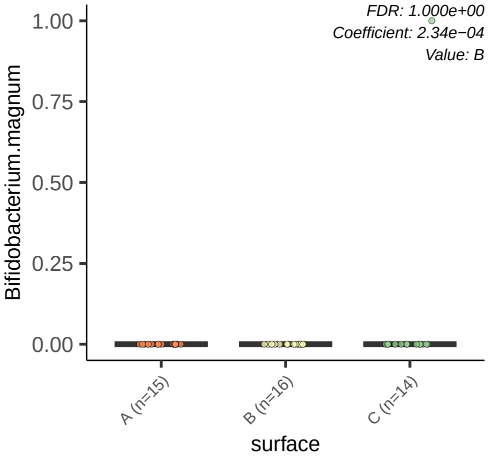

FDR: 1.000e+00
Coefficient: 2.34e−04
Value: B
Bifidobacterium.magnum
1.00
0.75
0.50
0.25
0.00
A (n=15)
B (n=16)
C (n=14)
surface

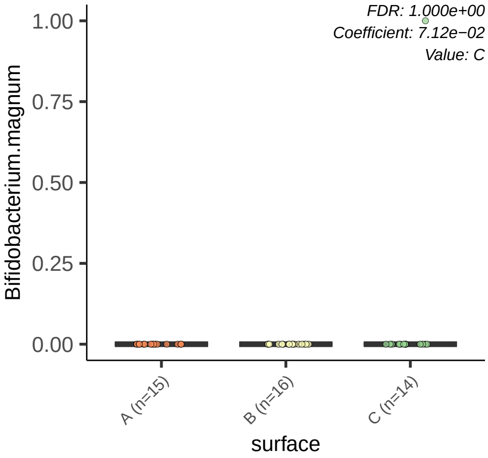

FDR: 1.000e+00
Coefficient: 7.12e−02
Value: C
Bifidobacterium.magnum
1.00
0.75
0.50
0.25
0.00
A (n=15)
B (n=16)
C (n=14)
surface

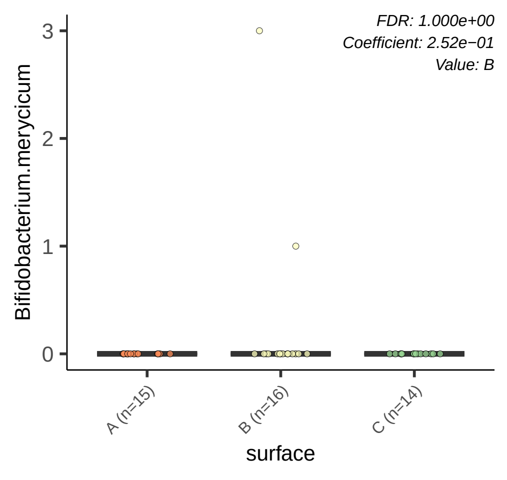
FDR: 1.000e+00
Coefficient: 2.52e−01
Value: B
Bifidobacterium.merycicum
3
2
1
0
A (n=15)
B (n=16)
C (n=14)
surface

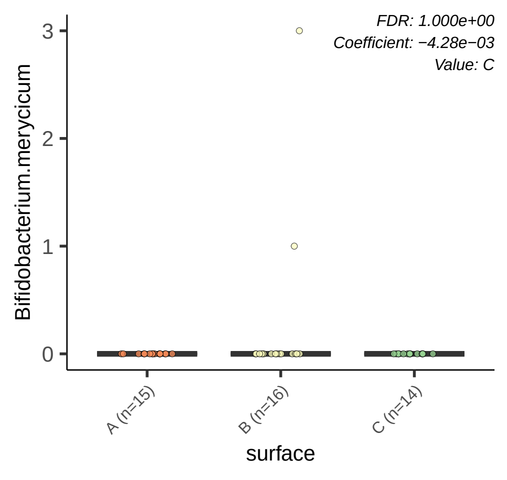

FDR: 1.000e+00
Coefficient: −4.28e−03
Value: C
Bifidobacterium.merycicum
3
2
1
0
A (n=15)
B (n=16)
C (n=14)
surface

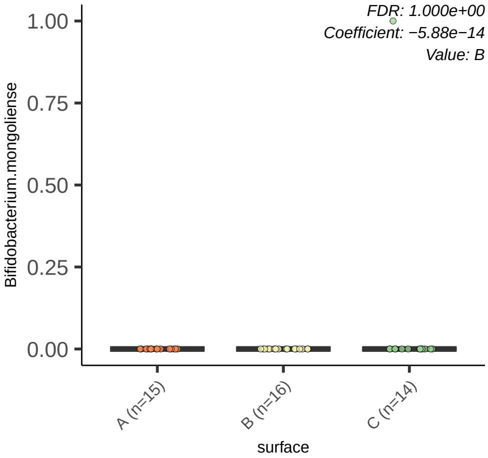
FDR: 1.000e+00
Coefficient: −5.88e−14
Value: B
1.00
0.75
0.50
0.25
0.00
Bifidobacterium.mongoliense
A (n=15)
B (n=16)
C (n=14)
surface

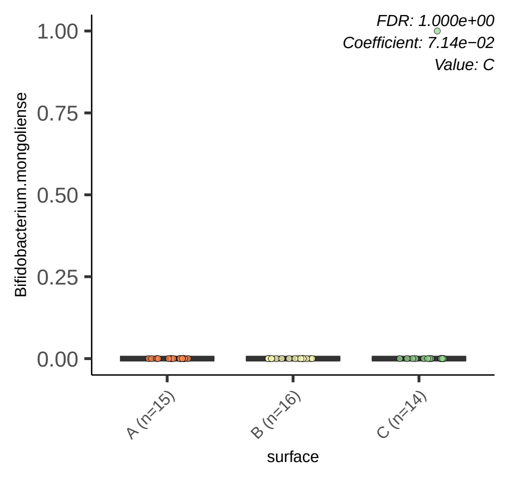
FDR: 1.000e+00
Coefficient: 7.14e−02
Value: C
1.00
0.75
0.50
0.25
0.00
Bifidobacterium.mongoliense
A (n=15)
B (n=16)
C (n=14)
surface

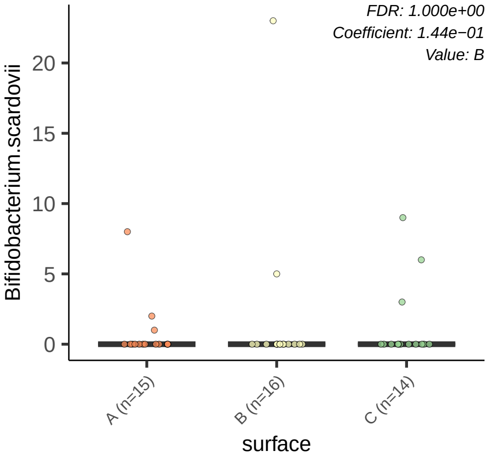

FDR: 1.000e+00
Coefficient: 1.44e−01
Value: B
Bifidobacterium.scardovii
20
15
10
5
0
A (n=15)
B (n=16)
C (n=14)
surface

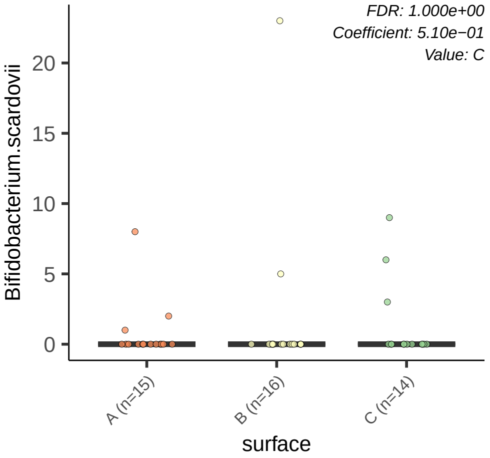

FDR: 1.000e+00
Coefficient: 5.10e−01
Value: C
Bifidobacterium.scardovii
20
15
10
5
0
A (n=15)
B (n=16)
C (n=14)
surface

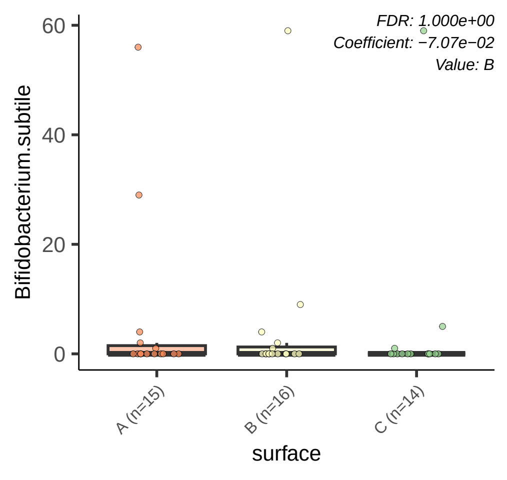

FDR: 1.000e+00
Coefficient: −7.07e−02
Value: B
Bifidobacterium.subtile
0
20
40
60
A (n=15)
B (n=16)
C (n=14)
surface

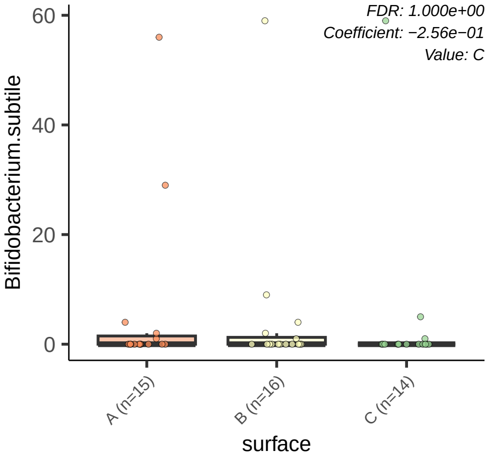

FDR: 1.000e+00
Coefficient: −2.56e−01
Value: C
Bifidobacterium.subtile
60
40
20
0
A (n=15)
B (n=16)
C (n=14)
surface

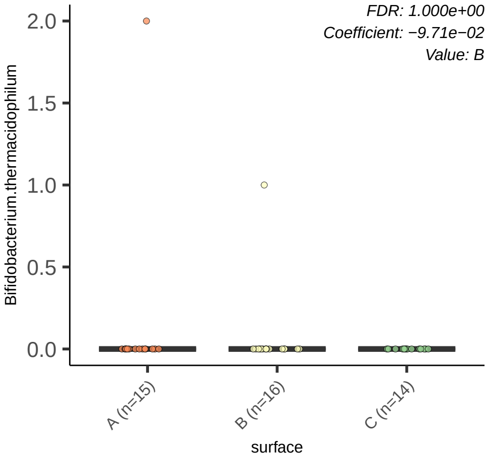

FDR: 1.000e+00
Coefficient: −9.71e−02
Value: B
Bifidobacterium.thermacidophilum
2.0
1.5
1.0
0.5
0.0
A (n=15)
B (n=16)
C (n=14)
surface

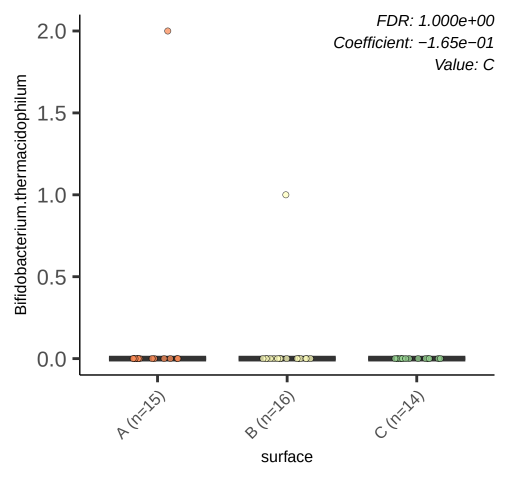
FDR: 1.000e+00
Coefficient: −1.65e−01
Value: C
Bifidobacterium.thermacidophilum
2.0
1.5
1.0
0.5
0.0
A (n=15)
B (n=16)
C (n=14)
surface

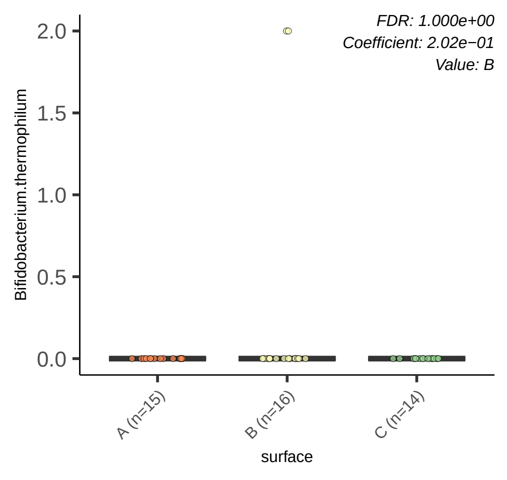

FDR: 1.000e+00
Coefficient: 2.02e−01
Value: B
Bifidobacterium.thermophilum
2.0
1.5
1.0
0.5
0.0
A (n=15)
B (n=16)
C (n=14)
surface

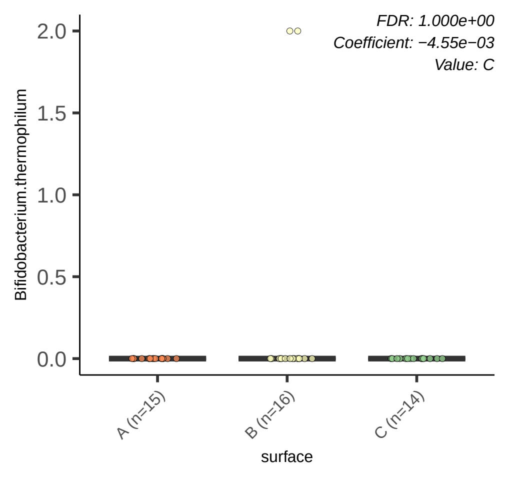

FDR: 1.000e+00
Coefficient: −4.55e−03
Value: C
Bifidobacterium.thermophilum
2.0
1.5
1.0
0.5
0.0
A (n=15)
B (n=16)
C (n=14)
surface

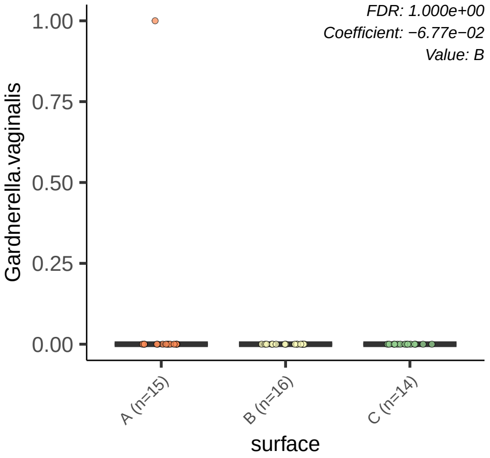

FDR: 1.000e+00
Coefficient: −6.77e−02
Value: B
1.00
0.75
0.50
0.25
0.00
Gardnerella.vaginalis
A (n=15)
B (n=16)
C (n=14)
surface

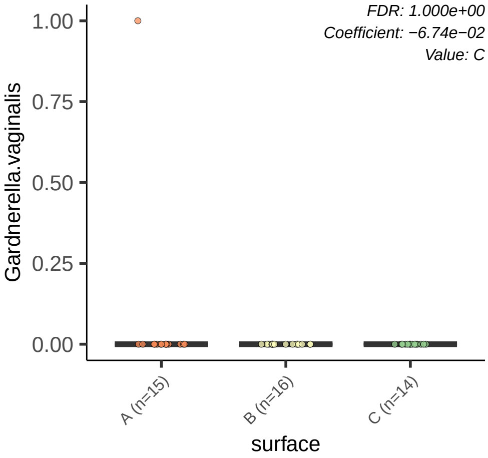
FDR: 1.000e+00
Coefficient: −6.74e−02
Value: C
1.00
0.75
0.50
0.25
0.00
Gardnerella.vaginalis
A (n=15)
B (n=16)
C (n=14)
surface

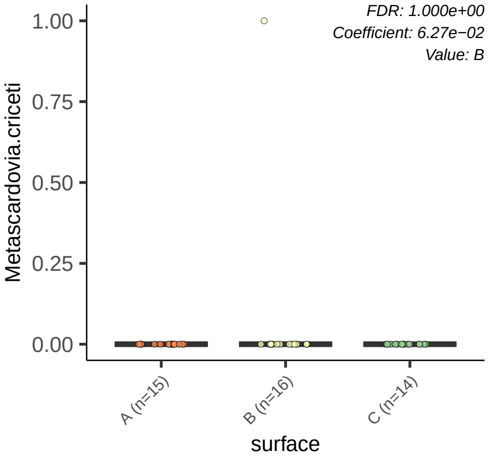
FDR: 1.000e+00
Coefficient: 6.27e−02
Value: B
Metascardovia.criceti
1.00
0.75
0.50
0.25
0.00
A (n=15)
B (n=16)
C (n=14)
surface

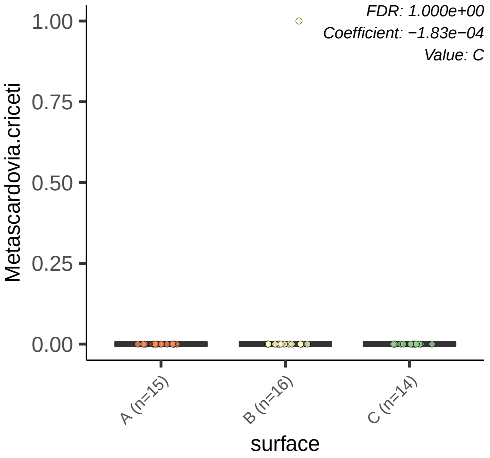

FDR: 1.000e+00
Coefficient: −1.83e−04
Value: C
Metascardovia.criceti
1.00
0.75
0.50
0.25
0.00
A (n=15)
B (n=16)
C (n=14)
surface

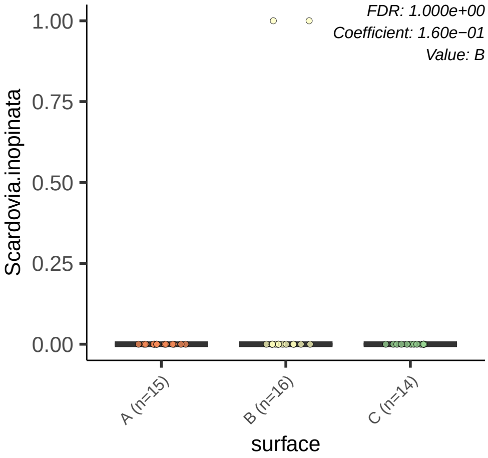
FDR: 1.000e+00
Coefficient: 1.60e−01
Value: B
Scardovia.inopinata
1.00
0.75
0.50
0.25
0.00
A (n=15)
B (n=16)
C (n=14)
surface

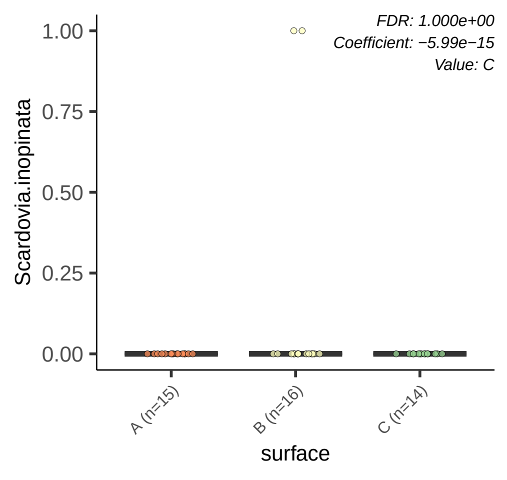

FDR: 1.000e+00
Coefficient: −5.99e−15
Value: C
Scardovia.inopinata
1.00
0.75
0.50
0.25
0.00
A (n=15)
B (n=16)
C (n=14)
surface

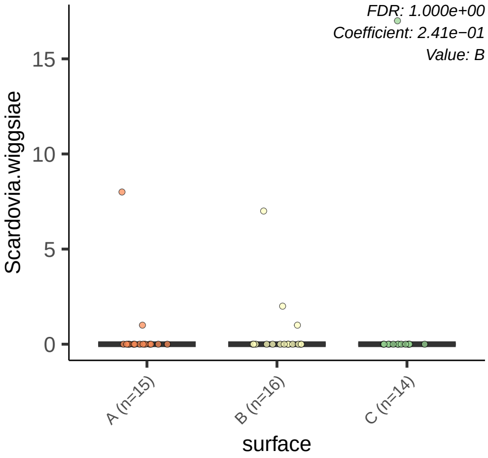
FDR: 1.000e+00
Coefficient: 2.41e−01
Value: B
Scardovia.wiggsiae
0
5
10
15
A (n=15)
B (n=16)
C (n=14)
surface

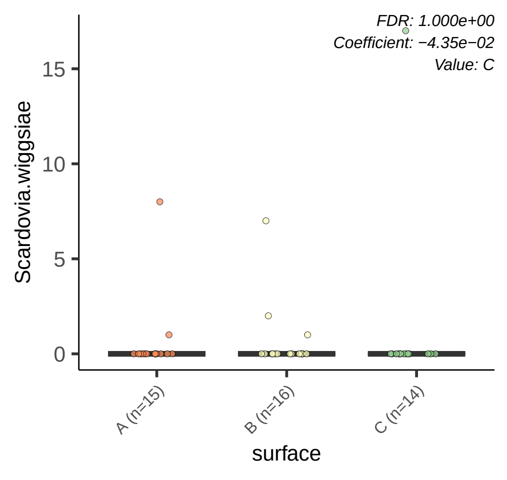

FDR: 1.000e+00
Coefficient: −4.35e−02
Value: C
Scardovia.wiggsiae
0
5
10
15
A (n=15)
B (n=16)
C (n=14)
surface

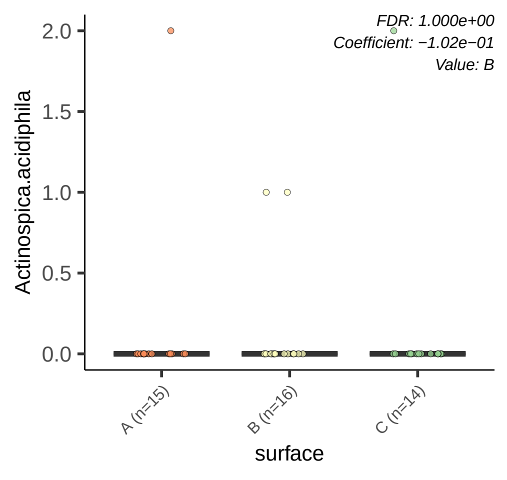
FDR: 1.000e+00
Coefficient: −1.02e−01
Value: B
Actinospica.acidiphila
2.0
1.5
1.0
0.5
0.0
A (n=15)
B (n=16)
C (n=14)
surface

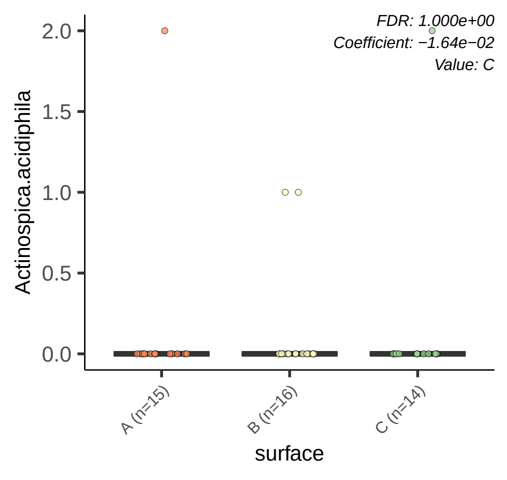
FDR: 1.000e+00
Coefficient: −1.64e−02
Value: C
Actinospica.acidiphila
2.0
1.5
1.0
0.5
0.0
A (n=15)
B (n=16)
C (n=14)
surface

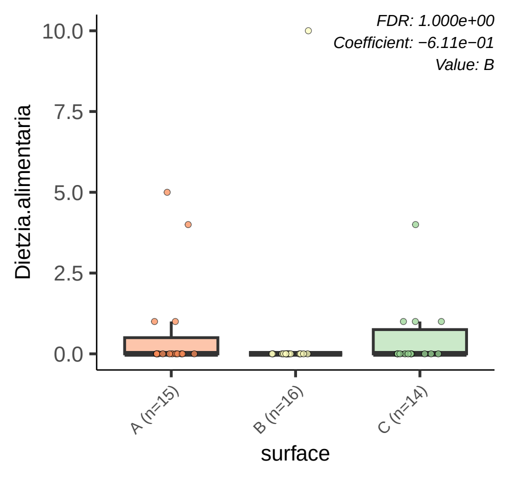
FDR: 1.000e+00
Coefficient: −6.11e−01
Value: B
Dietzia.alimentaria
10.0
7.5
5.0
2.5
0.0
A (n=15)
B (n=16)
C (n=14)
surface

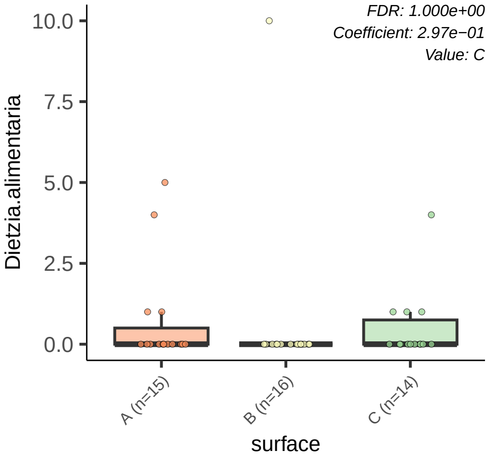

FDR: 1.000e+00
Coefficient: 2.97e−01
Value: C
Dietzia.alimentaria
10.0
7.5
5.0
2.5
0.0
A (n=15)
B (n=16)
C (n=14)
surface

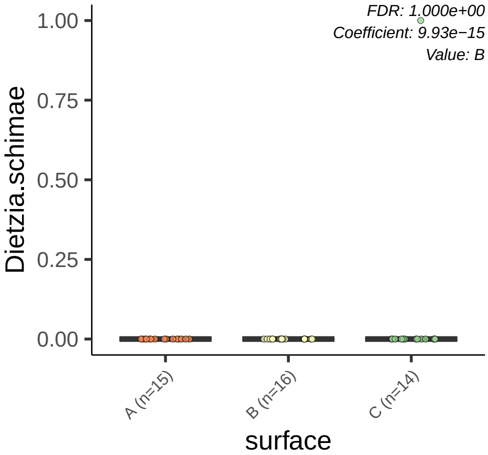

FDR: 1.000e+00
Coefficient: 9.93e−15
Value: B
Dietzia.schimae
1.00
0.75
0.50
0.25
0.00
A (n=15)
B (n=16)
C (n=14)
surface

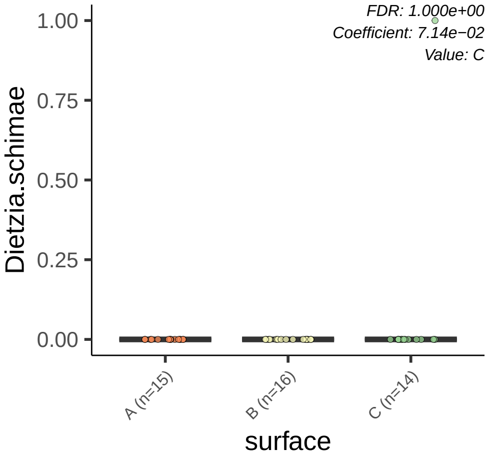
FDR: 1.000e+00
Coefficient: 7.14e−02
Value: C
Dietzia.schimae
1.00
0.75
0.50
0.25
0.00
A (n=15)
B (n=16)
C (n=14)
surface

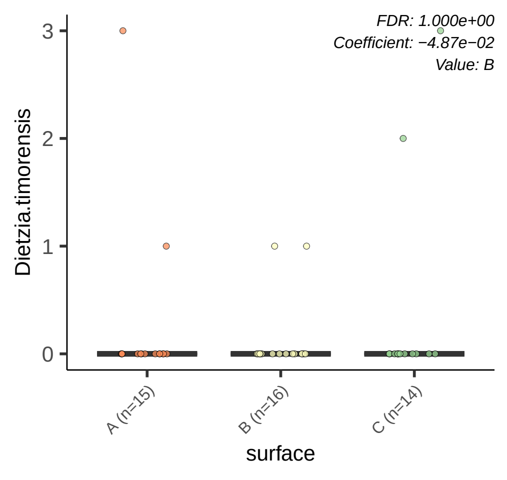

FDR: 1.000e+00
Coefficient: −4.87e−02
Value: B
Dietzia.timorensis
3
2
1
0
A (n=15)
B (n=16)
C (n=14)
surface

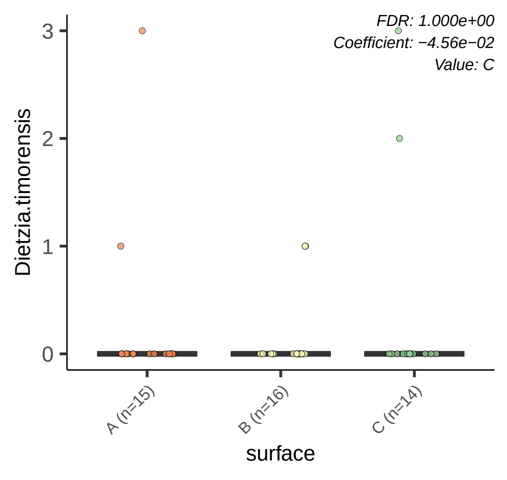

FDR: 1.000e+00
Coefficient: −4.56e−02
Value: C
Dietzia.timorensis
3
2
1
0
A (n=15)
B (n=16)
C (n=14)
surface

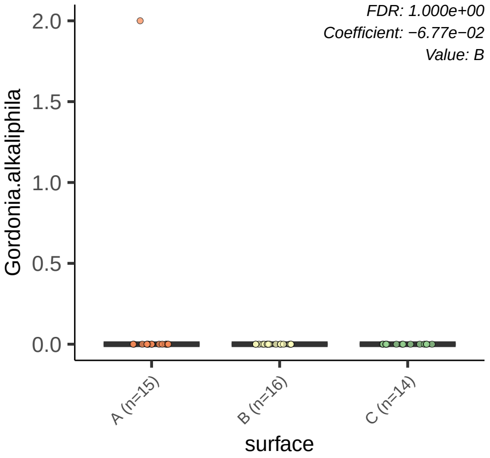
FDR: 1.000e+00
Coefficient: −6.77e−02
Value: B
Gordonia.alkaliphila
2.0
1.5
1.0
0.5
0.0
A (n=15)
B (n=16)
C (n=14)
surface

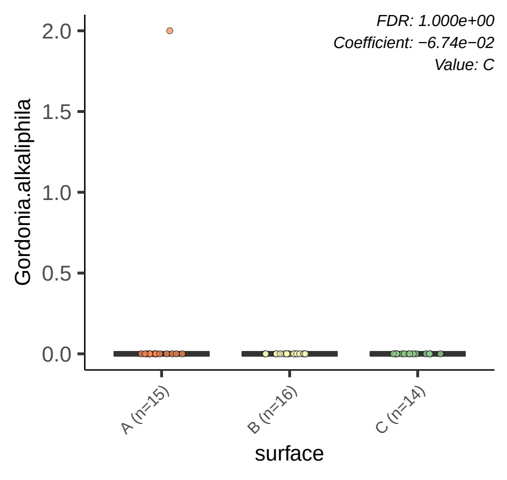
FDR: 1.000e+00
Coefficient: −6.74e−02
Value: C
Gordonia.alkaliphila
2.0
1.5
1.0
0.5
0.0
A (n=15)
B (n=16)
C (n=14)
surface

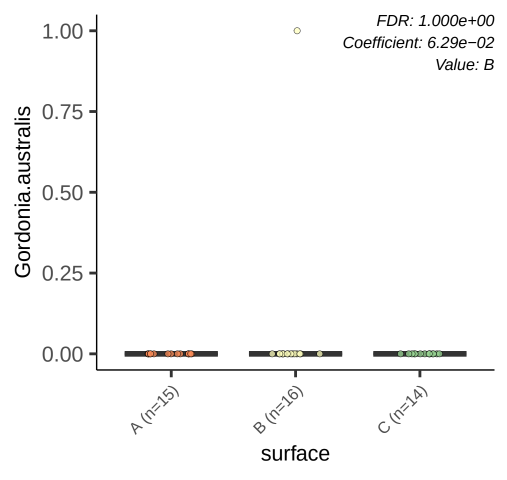
FDR: 1.000e+00
Coefficient: 6.29e−02
Value: B
1.00
0.75
0.50
0.25
0.00
Gordonia.australis
A (n=15)
B (n=16)
C (n=14)
surface

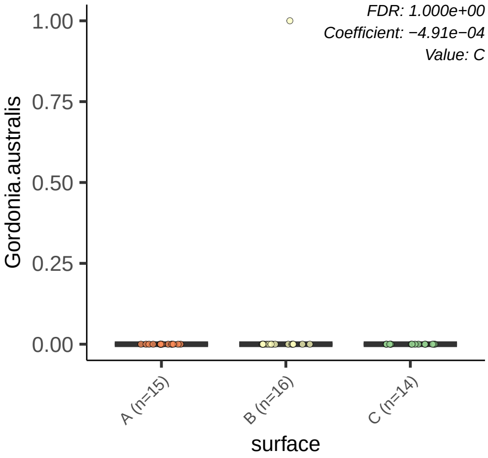
FDR: 1.000e+00
Coefficient: −4.91e−04
Value: C
Gordonia.australis
1.00
0.75
0.50
0.25
0.00
A (n=15)
B (n=16)
C (n=14)
surface

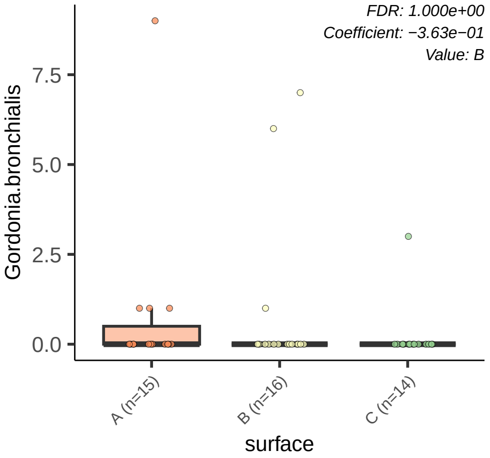

FDR: 1.000e+00
Coefficient: −3.63e−01
Value: B
Gordonia.bronchialis
7.5
5.0
2.5
0.0
A (n=15)
B (n=16)
C (n=14)
surface

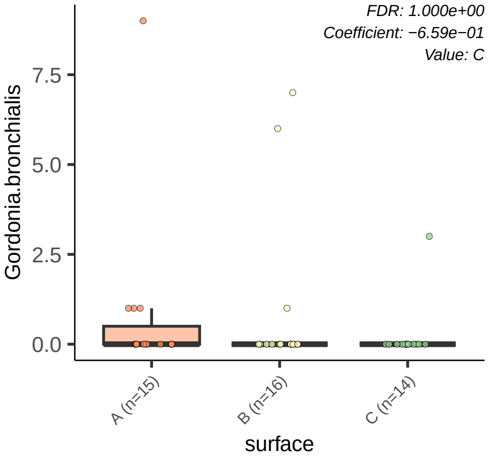

FDR: 1.000e+00
Coefficient: −6.59e−01
Value: C
Gordonia.bronchialis
7.5
5.0
2.5
0.0
A (n=15)
B (n=16)
C (n=14)
surface

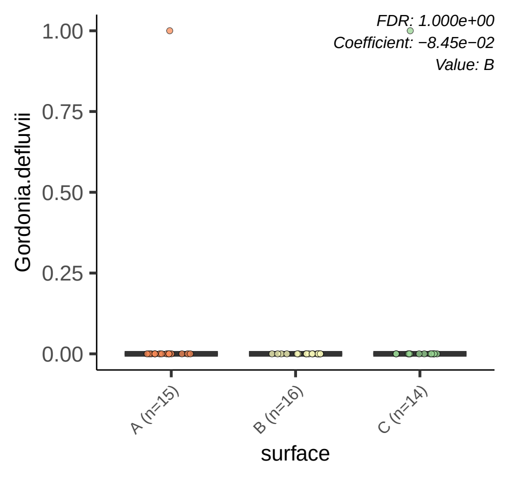
FDR: 1.000e+00
Coefficient: −8.45e−02
Value: B
Gordonia.defluvii
1.00
0.75
0.50
0.25
0.00
A (n=15)
B (n=16)
C (n=14)
surface

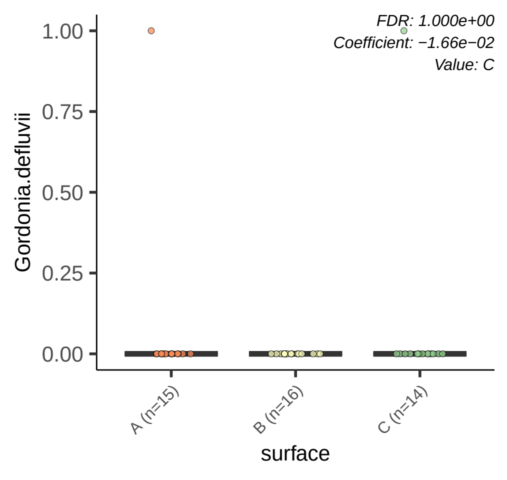

FDR: 1.000e+00
Coefficient: −1.66e−02
Value: C
Gordonia.defluvii
1.00
0.75
0.50
0.25
0.00
A (n=15)
B (n=16)
C (n=14)
surface

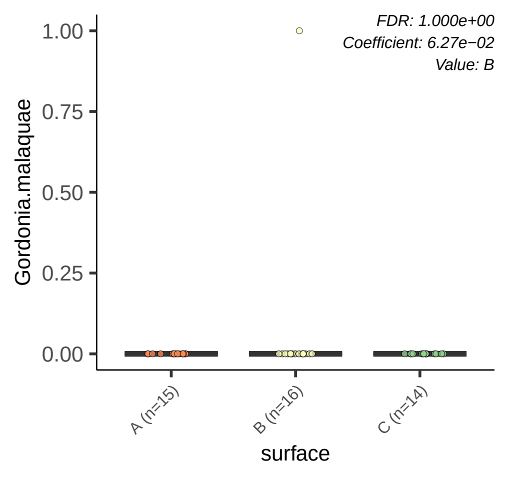

FDR: 1.000e+00
Coefficient: 6.27e−02
Value: B
Gordonia.malaquae
1.00
0.75
0.50
0.25
0.00
A (n=15)
B (n=16)
C (n=14)
surface

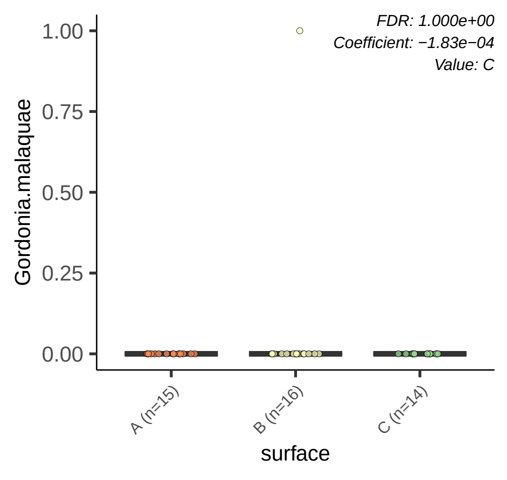
FDR: 1.000e+00
Coefficient: −1.83e−04
Value: C
Gordonia.malaquae
1.00
0.75
0.50
0.25
0.00
A (n=15)
B (n=16)
C (n=14)
surface

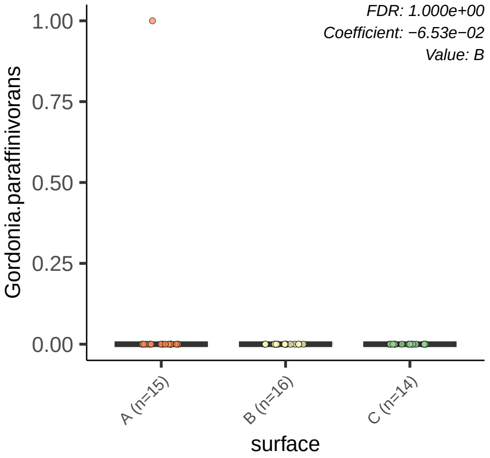
FDR: 1.000e+00
Coefficient: −6.53e−02
Value: B
Gordonia.paraffinivorans
1.00
0.75
0.50
0.25
0.00
A (n=15)
B (n=16)
C (n=14)
surface

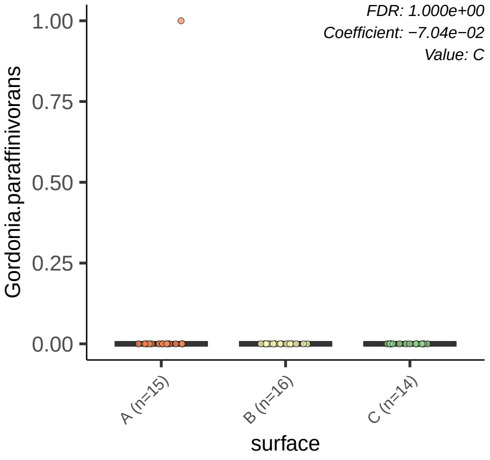
FDR: 1.000e+00
Coefficient: −7.04e−02
Value: C
Gordonia.paraffinivorans
1.00
0.75
0.50
0.25
0.00
A (n=15)
B (n=16)
C (n=14)
surface

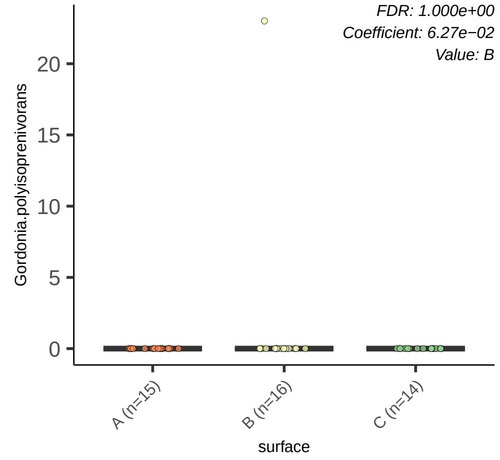
FDR: 1.000e+00
Coefficient: 6.27e−02
Value: B
Gordonia.polyisoprenivorans
20
15
10
5
0
A (n=15)
B (n=16)
C (n=14)
surface

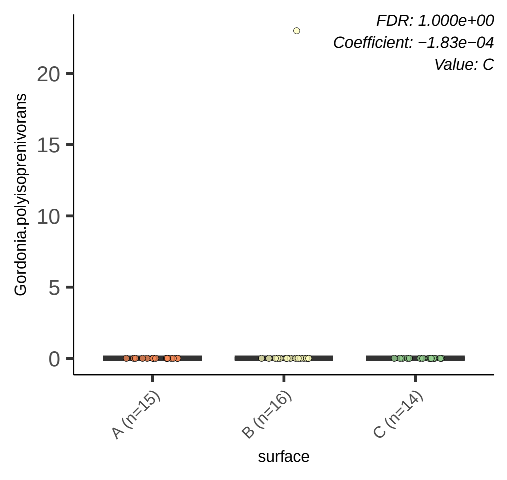
FDR: 1.000e+00
Coefficient: −1.83e−04
Value: C
Gordonia.polyisoprenivorans
0
5
10
15
20
A (n=15)
B (n=16)
C (n=14)
surface

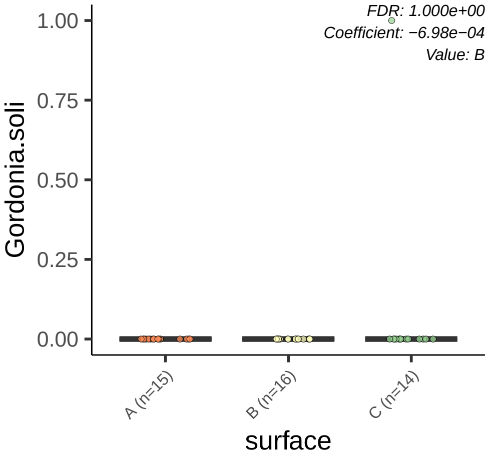
FDR: 1.000e+00
Coefficient: −6.98e−04
Value: B
Gordonia.soli
1.00
0.75
0.50
0.25
0.00
A (n=15)
B (n=16)
C (n=14)
surface

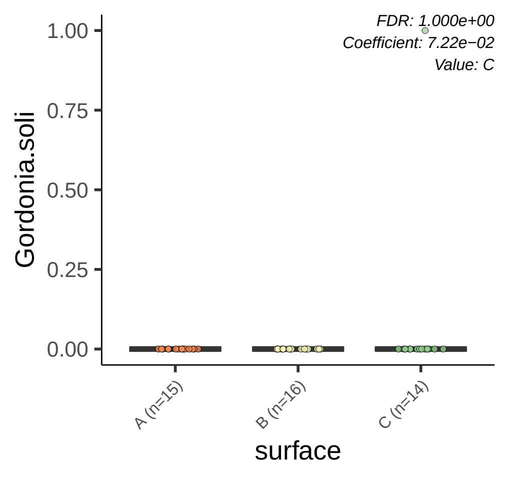
FDR: 1.000e+00
Coefficient: 7.22e−02
Value: C
Gordonia.soli
1.00
0.75
0.50
0.25
0.00
A (n=15)
B (n=16)
C (n=14)
surface

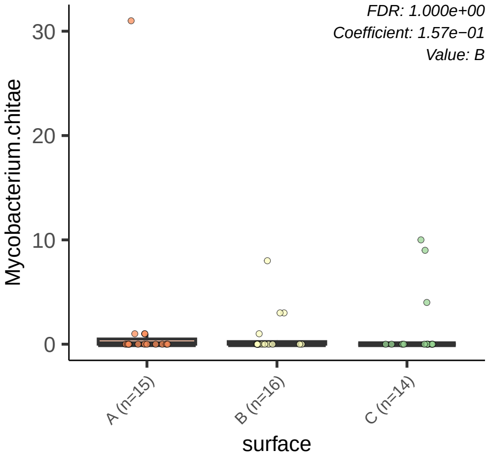
FDR: 1.000e+00
Coefficient: 1.57e−01
Value: B
Mycobacterium.chitae
30
20
10
0
A (n=15)
B (n=16)
C (n=14)
surface

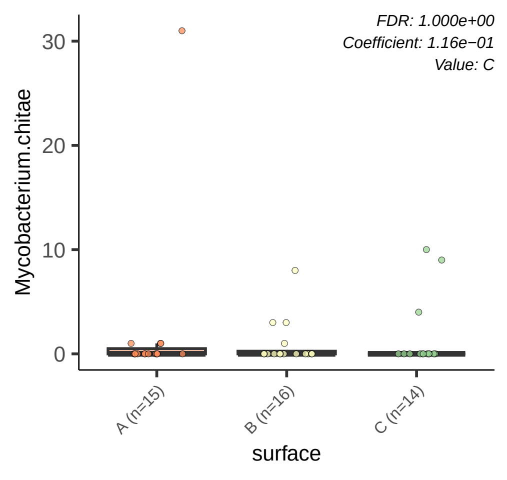
FDR: 1.000e+00
Coefficient: 1.16e−01
Value: C
Mycobacterium.chitae
30
20
10
0
A (n=15)
B (n=16)
C (n=14)
surface

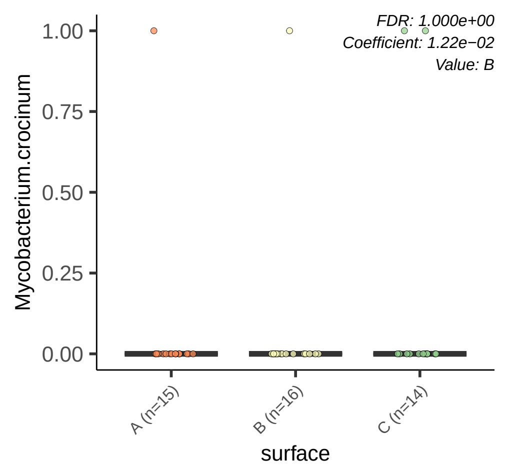

FDR: 1.000e+00
Coefficient: 1.22e−02
Value: B
Mycobacterium.crocinum
1.00
0.75
0.50
0.25
0.00
A (n=15)
B (n=16)
C (n=14)
surface

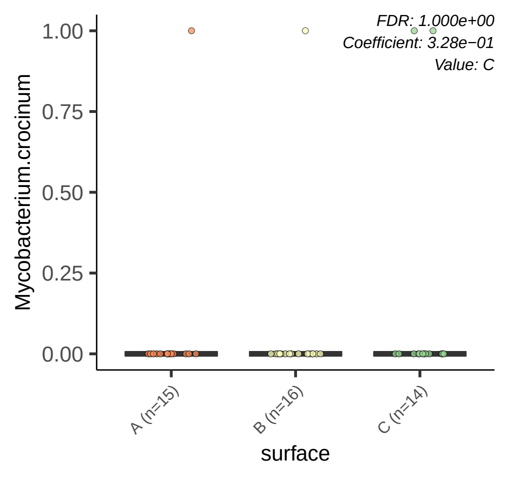
FDR: 1.000e+00
Coefficient: 3.28e−01
Value: C
Mycobacterium.crocinum
1.00
0.75
0.50
0.25
0.00
A (n=15)
B (n=16)
C (n=14)
surface

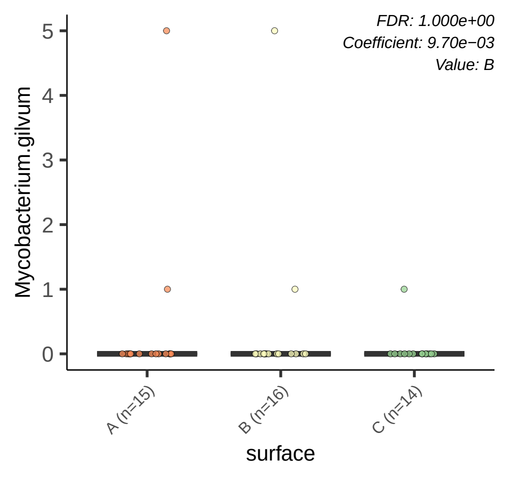
FDR: 1.000e+00
Coefficient: 9.70e−03
Value: B
Mycobacterium.gilvum
0
1
2
3
4
5
A (n=15)
B (n=16)
C (n=14)
surface

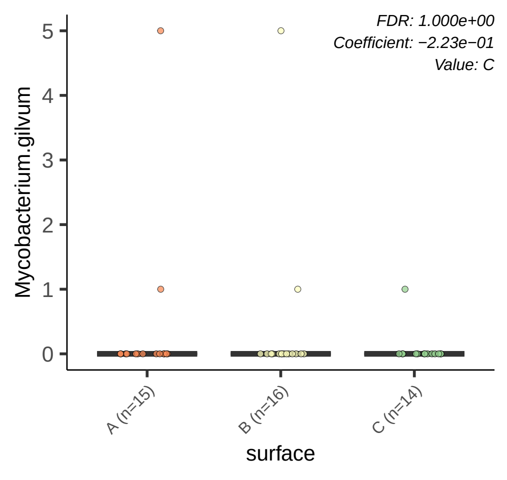
FDR: 1.000e+00
Coefficient: −2.23e−01
Value: C
Mycobacterium.gilvum
0
1
2
3
4
5
A (n=15)
B (n=16)
C (n=14)
surface

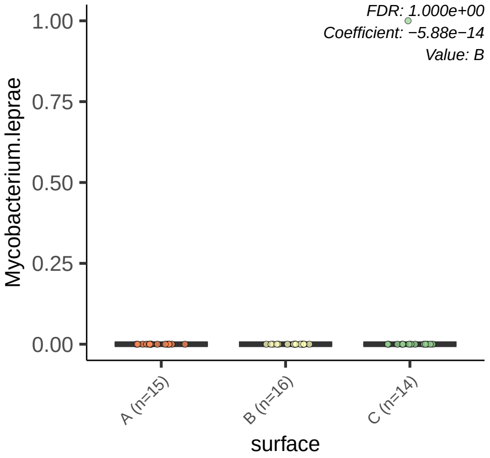

FDR: 1.000e+00
Coefficient: −5.88e−14
Value: B
Mycobacterium.leprae
1.00
0.75
0.50
0.25
0.00
A (n=15)
B (n=16)
C (n=14)
surface

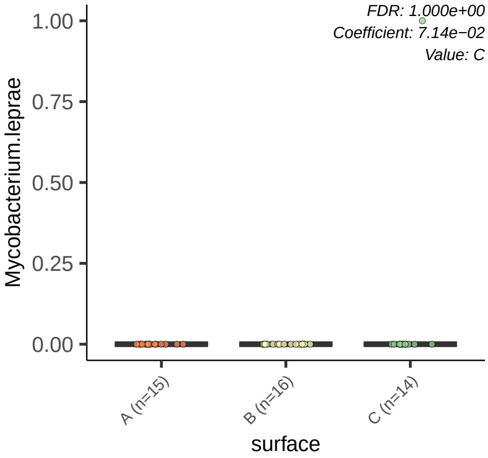
FDR: 1.000e+00
Coefficient: 7.14e−02
Value: C
Mycobacterium.leprae
1.00
0.75
0.50
0.25
0.00
A (n=15)
B (n=16)
C (n=14)
surface

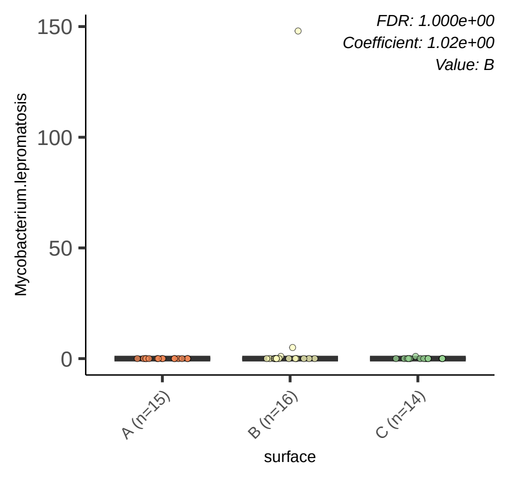

FDR: 1.000e+00
Coefficient: 1.02e+00
Value: B
150
100
50
0
Mycobacterium.lepromatosis
A (n=15)
B (n=16)
C (n=14)
surface

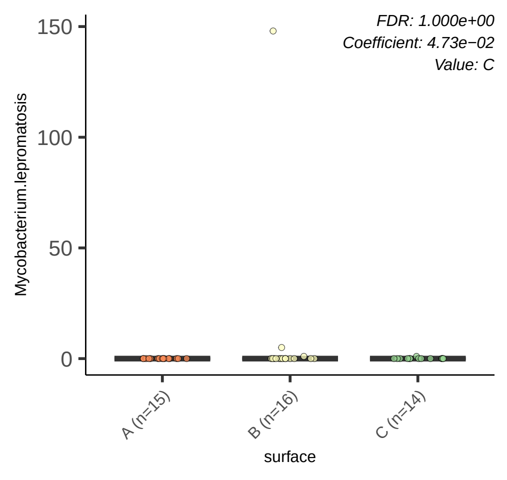

FDR: 1.000e+00
Coefficient: 4.73e−02
Value: C
150
100
50
0
Mycobacterium.lepromatosis
A (n=15)
B (n=16)
C (n=14)
surface

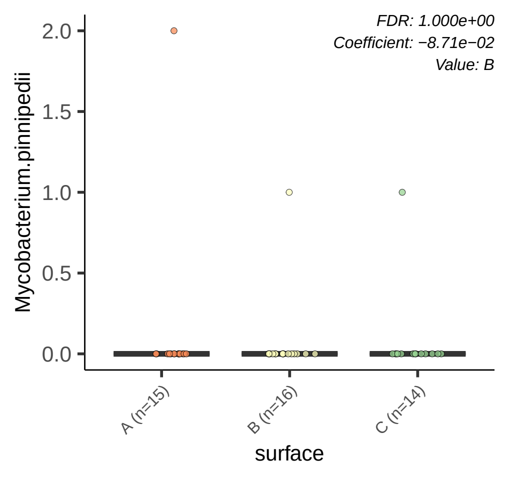

FDR: 1.000e+00
Coefficient: −8.71e−02
Value: B
Mycobacterium.pinnipedii
2.0
1.5
1.0
0.5
0.0
A (n=15)
B (n=16)
C (n=14)
surface

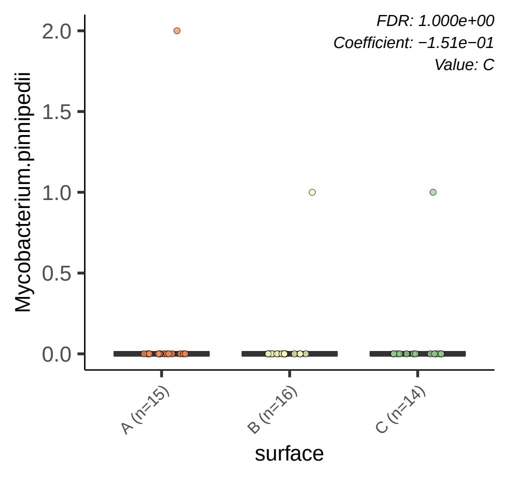
FDR: 1.000e+00
Coefficient: −1.51e−01
Value: C
Mycobacterium.pinnipedii
2.0
1.5
1.0
0.5
0.0
A (n=15)
B (n=16)
C (n=14)
surface

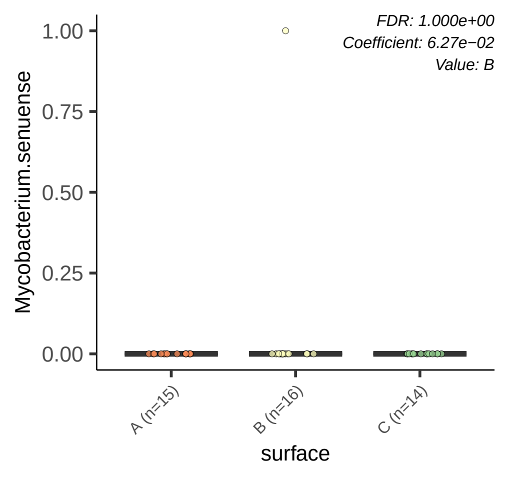

FDR: 1.000e+00
Coefficient: 6.27e−02
Value: B
Mycobacterium.senuense
1.00
0.75
0.50
0.25
0.00
A (n=15)
B (n=16)
C (n=14)
surface

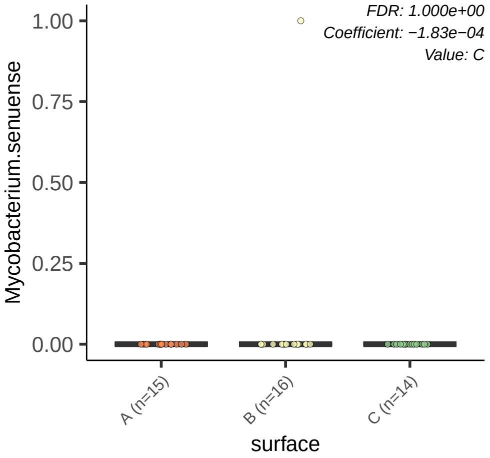
FDR: 1.000e+00
Coefficient: −1.83e−04
Value: C
Mycobacterium.senuense
1.00
0.75
0.50
0.25
0.00
A (n=15)
B (n=16)
C (n=14)
surface

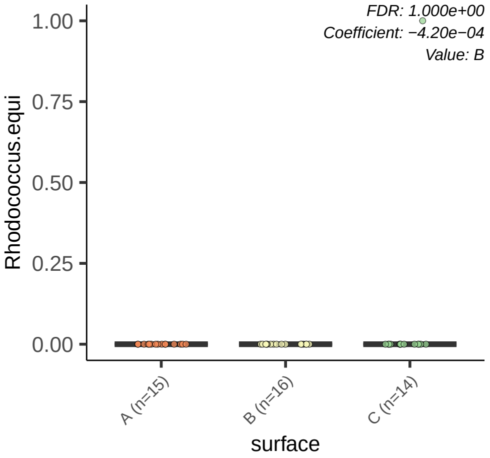

FDR: 1.000e+00
Coefficient: −4.20e−04
Value: B
Rhodococcus.equi
1.00
0.75
0.50
0.25
0.00
A (n=15)
B (n=16)
C (n=14)
surface

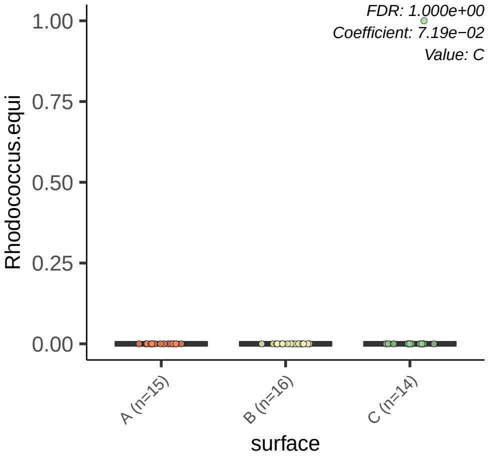

FDR: 1.000e+00
Coefficient: 7.19e−02
Value: C
Rhodococcus.equi
1.00
0.75
0.50
0.25
0.00
A (n=15)
B (n=16)
C (n=14)
surface

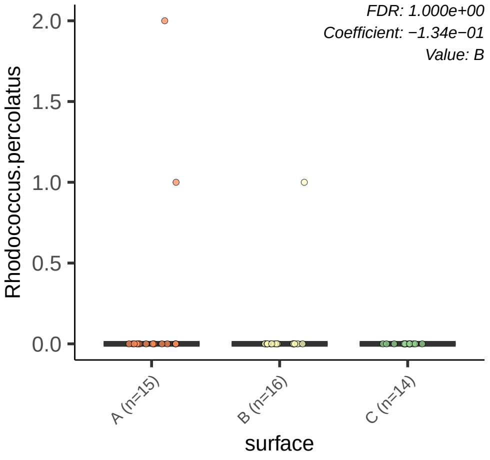

FDR: 1.000e+00
Coefficient: −1.34e−01
Value: B
Rhodococcus.percolatus
2.0
1.5
1.0
0.5
0.0
A (n=15)
B (n=16)
C (n=14)
surface

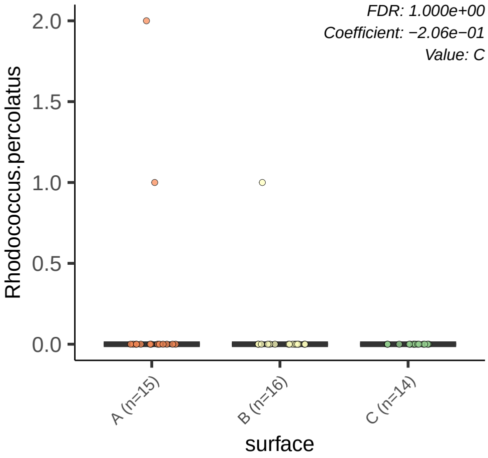

FDR: 1.000e+00
Coefficient: −2.06e−01
Value: C
Rhodococcus.percolatus
2.0
1.5
1.0
0.5
0.0
A (n=15)
B (n=16)
C (n=14)
surface

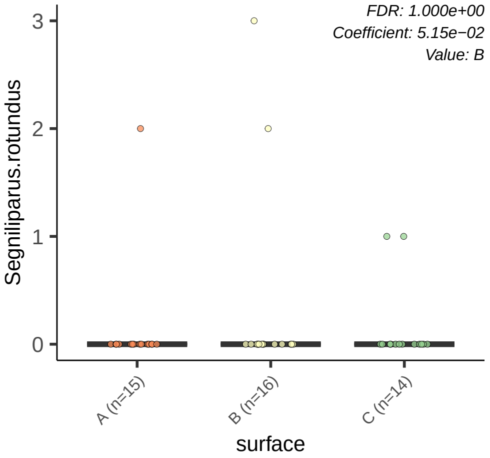
FDR: 1.000e+00
Coefficient: 5.15e−02
Value: B
Segniliparus.rotundus
0
1
2
3
A (n=15)
B (n=16)
C (n=14)
surface

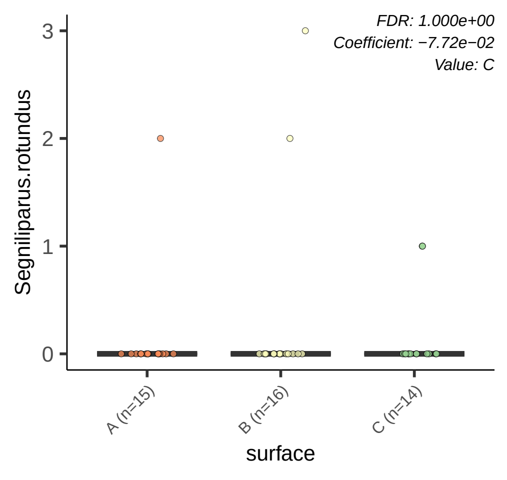
FDR: 1.000e+00
Coefficient: −7.72e−02
Value: C
Segniliparus.rotundus
3
2
1
0
A (n=15)
B (n=16)
C (n=14)
surface

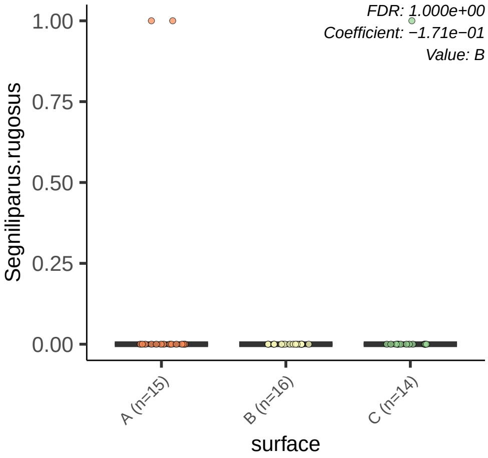

FDR: 1.000e+00
Coefficient: −1.71e−01
Value: B
Segniliparus.rugosus
1.00
0.75
0.50
0.25
0.00
A (n=15)
B (n=16)
C (n=14)
surface

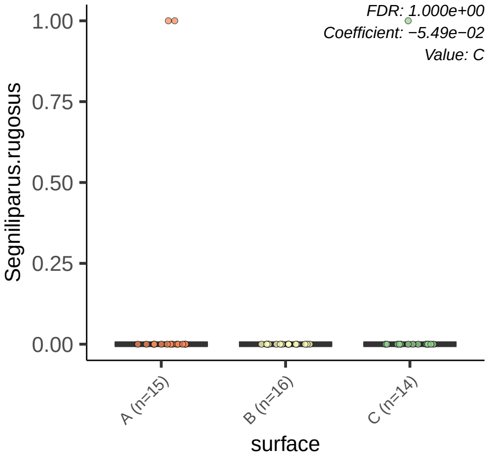

FDR: 1.000e+00
Coefficient: −5.49e−02
Value: C
Segniliparus.rugosus
1.00
0.75
0.50
0.25
0.00
A (n=15)
B (n=16)
C (n=14)
surface

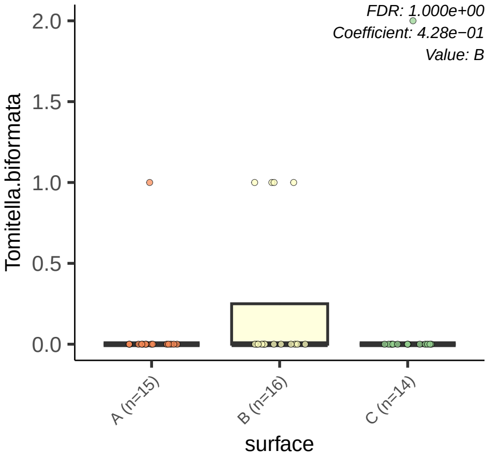
FDR: 1.000e+00
Coefficient: 4.28e−01
Value: B
Tomitella.biformata
2.0
1.5
1.0
0.5
0.0
A (n=15)
B (n=16)
C (n=14)
surface

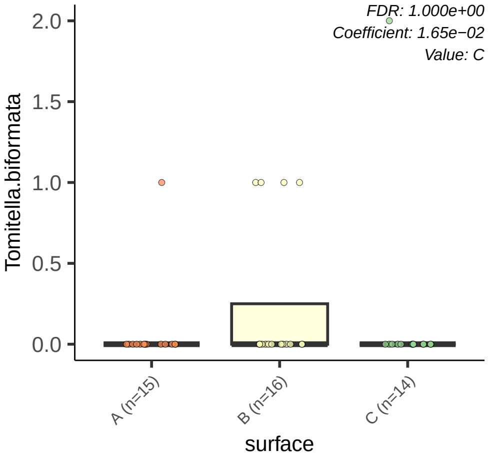
FDR: 1.000e+00
Coefficient: 1.65e−02
Value: C
Tomitella.biformata
2.0
1.5
1.0
0.5
0.0
A (n=15)
B (n=16)
C (n=14)
surface

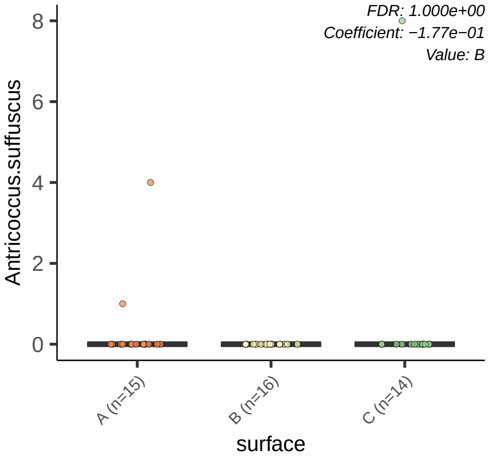
FDR: 1.000e+00
Coefficient: −1.77e−01
Value: B
Antricoccus.suffuscus
8
6
4
2
0
A (n=15)
B (n=16)
C (n=14)
surface

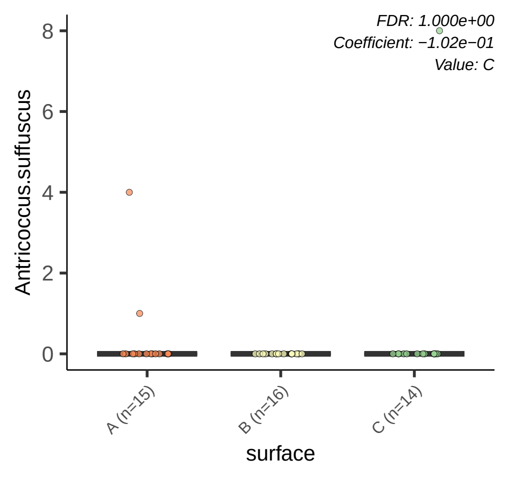
FDR: 1.000e+00
Coefficient: −1.02e−01
Value: C
Antricoccus.suffuscus
0
2
4
6
8
A (n=15)
B (n=16)
C (n=14)
surface

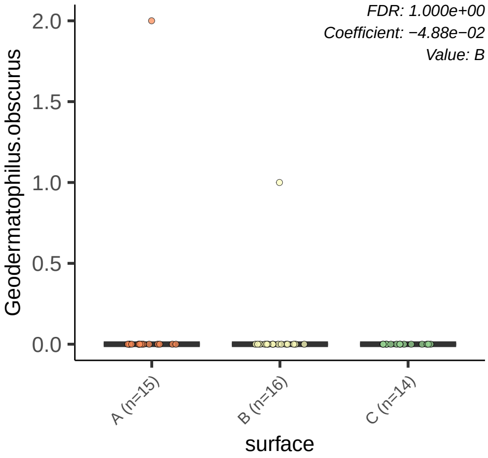
FDR: 1.000e+00
Coefficient: −4.88e−02
Value: B
Geodermatophilus.obscurus
2.0
1.5
1.0
0.5
0.0
A (n=15)
B (n=16)
C (n=14)
surface

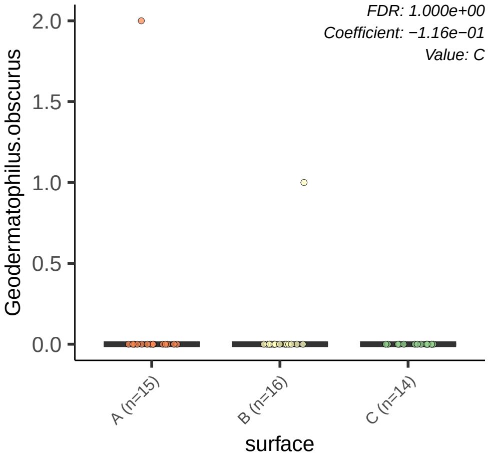

FDR: 1.000e+00
Coefficient: −1.16e−01
Value: C
Geodermatophilus.obscurus
2.0
1.5
1.0
0.5
0.0
A (n=15)
B (n=16)
C (n=14)
surface

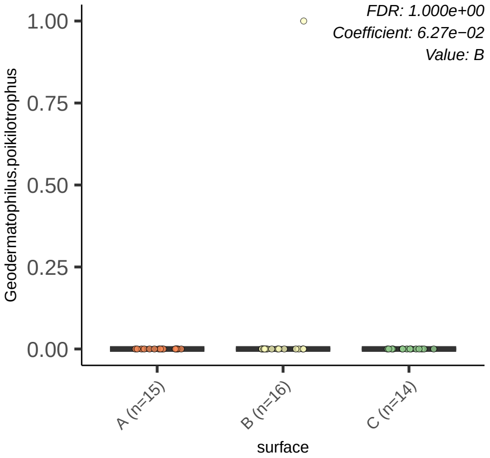

FDR: 1.000e+00
Coefficient: 6.27e−02
Value: B
1.00
0.75
0.50
0.25
0.00
Geodermatophilus.poikilotrophus
A (n=15)
B (n=16)
C (n=14)
surface

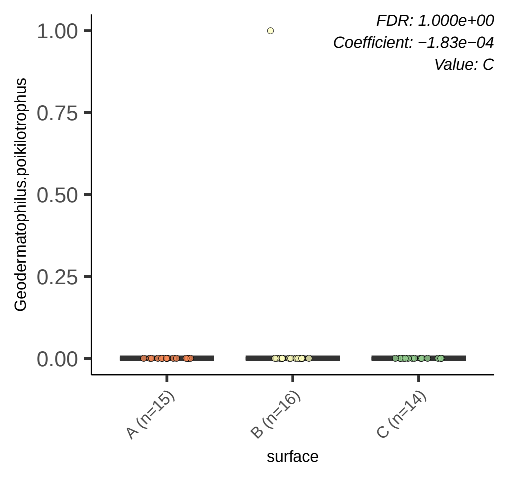
FDR: 1.000e+00
Coefficient: −1.83e−04
Value: C
Geodermatophilus.poikilotrophus
1.00
0.75
0.50
0.25
0.00
A (n=15)
B (n=16)
C (n=14)
surface

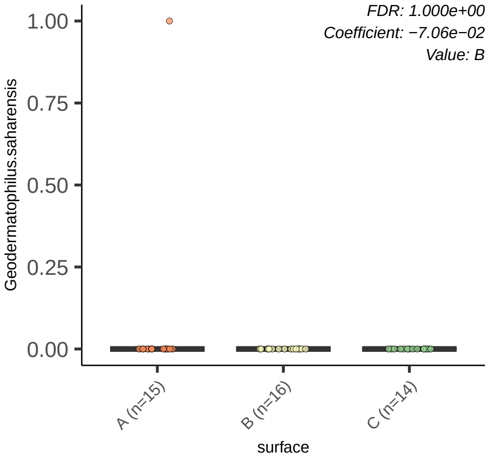
FDR: 1.000e+00
Coefficient: −7.06e−02
Value: B
1.00
0.75
0.50
0.25
0.00
Geodermatophilus.saharensis
A (n=15)
B (n=16)
C (n=14)
surface

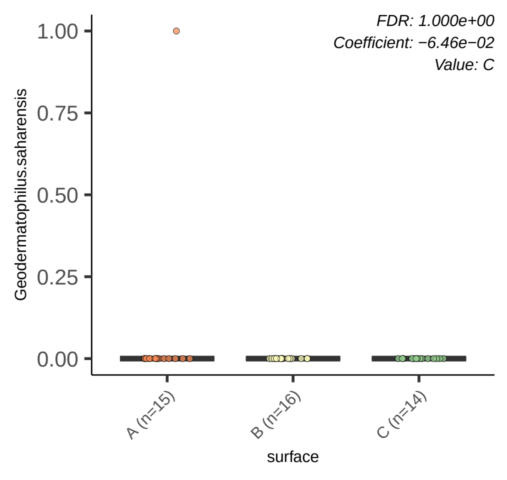
FDR: 1.000e+00
Coefficient: −6.46e−02
Value: C
1.00
0.75
0.50
0.25
0.00
Geodermatophilus.saharensis
A (n=15)
B (n=16)
C (n=14)
surface

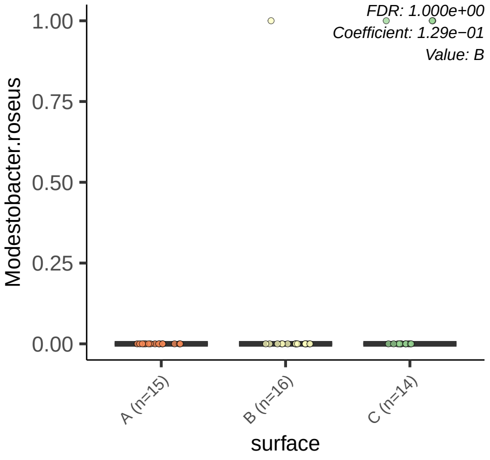
FDR: 1.000e+00
Coefficient: 1.29e−01
Value: B
Modestobacter.roseus
1.00
0.75
0.50
0.25
0.00
A (n=15)
B (n=16)
C (n=14)
surface

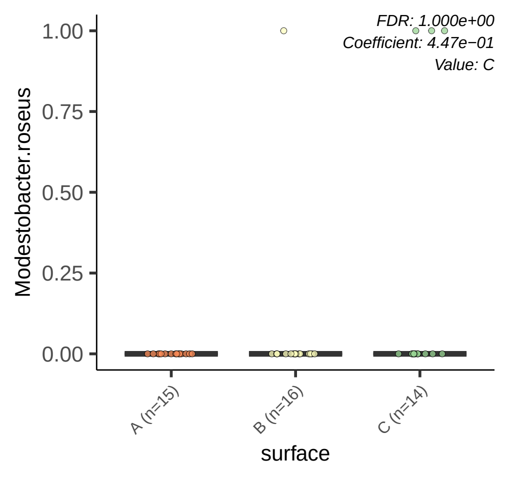
FDR: 1.000e+00
Coefficient: 4.47e−01
Value: C
Modestobacter.roseus
1.00
0.75
0.50
0.25
0.00
A (n=15)
B (n=16)
C (n=14)
surface

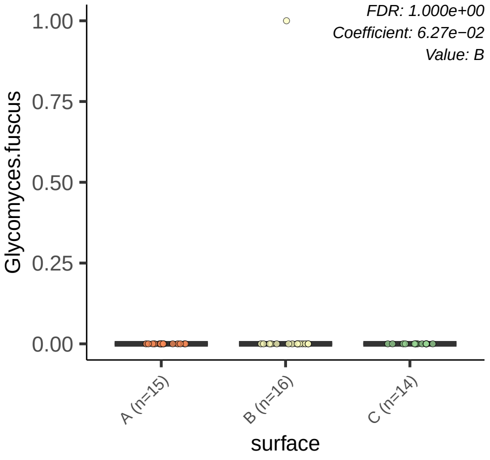

FDR: 1.000e+00
Coefficient: 6.27e−02
Value: B
Glycomyces.fuscus
1.00
0.75
0.50
0.25
0.00
A (n=15)
B (n=16)
C (n=14)
surface

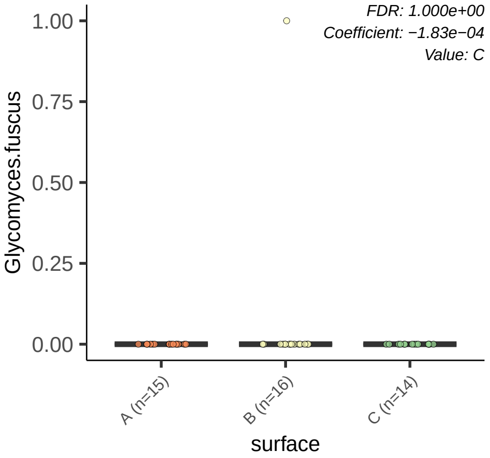

FDR: 1.000e+00
Coefficient: −1.83e−04
Value: C
Glycomyces.fuscus
1.00
0.75
0.50
0.25
0.00
A (n=15)
B (n=16)
C (n=14)
surface

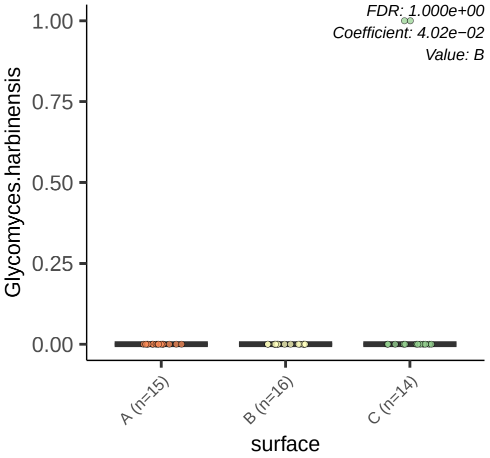

FDR: 1.000e+00
Coefficient: 4.02e−02
Value: B
Glycomyces.harbinensis
1.00
0.75
0.50
0.25
0.00
A (n=15)
B (n=16)
C (n=14)
surface

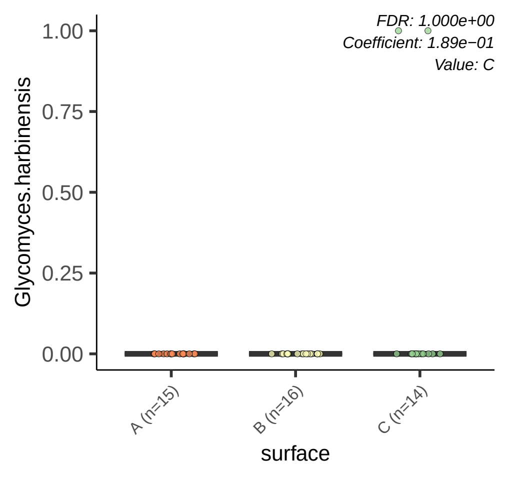

FDR: 1.000e+00
Coefficient: 1.89e−01
Value: C
1.00
0.75
0.50
0.25
0.00
Glycomyces.harbinensis
A (n=15)
B (n=16)
C (n=14)
surface

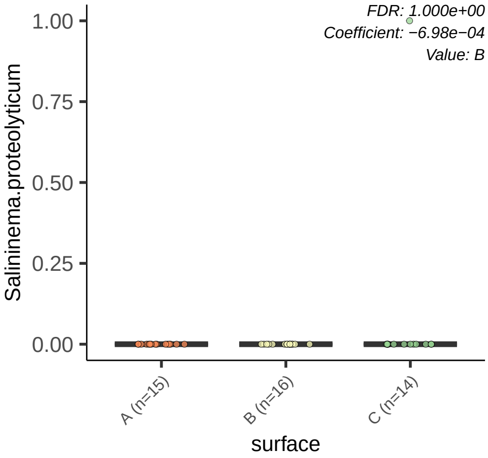
FDR: 1.000e+00
Coefficient: −6.98e−04
Value: B
Salininema.proteolyticum
1.00
0.75
0.50
0.25
0.00
A (n=15)
B (n=16)
C (n=14)
surface

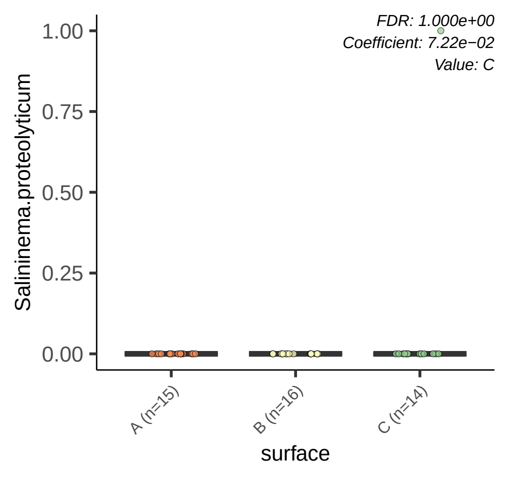
FDR: 1.000e+00
Coefficient: 7.22e−02
Value: C
1.00
0.75
0.50
0.25
0.00
Salininema.proteolyticum
A (n=15)
B (n=16)
C (n=14)
surface

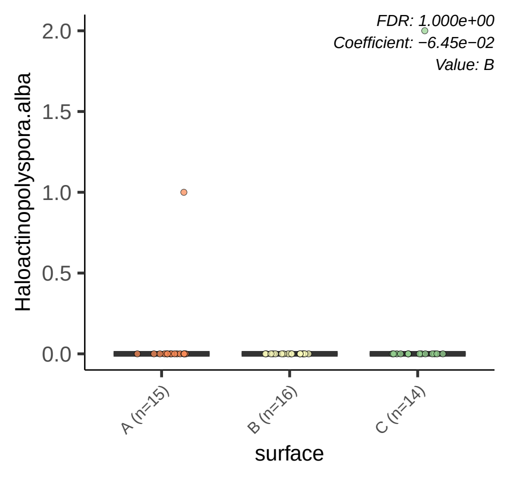
FDR: 1.000e+00
Coefficient: −6.45e−02
Value: B
Haloactinopolyspora.alba
2.0
1.5
1.0
0.5
0.0
A (n=15)
B (n=16)
C (n=14)
surface

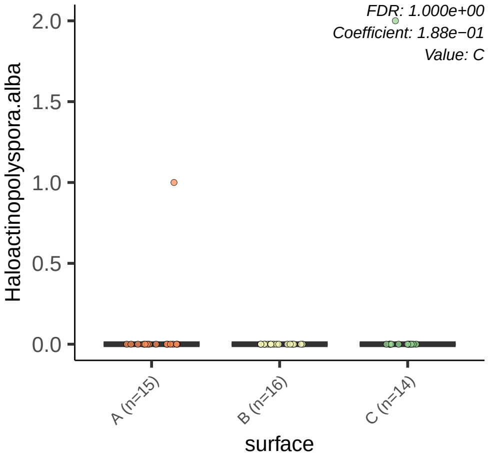
FDR: 1.000e+00
Coefficient: 1.88e−01
Value: C
Haloactinopolyspora.alba
2.0
1.5
1.0
0.5
0.0
A (n=15)
B (n=16)
C (n=14)
surface

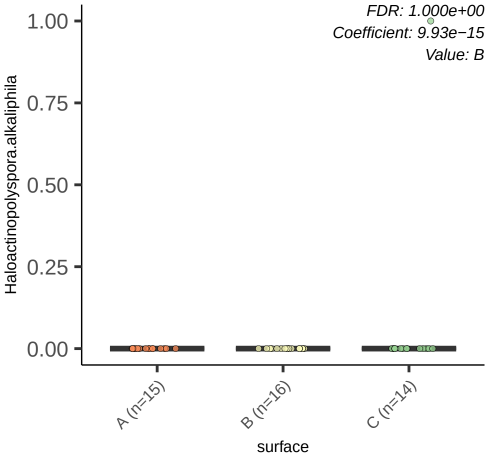

FDR: 1.000e+00
Coefficient: 9.93e−15
Value: B
1.00
0.75
0.50
0.25
0.00
Haloactinopolyspora.alkaliphila
A (n=15)
B (n=16)
C (n=14)
surface

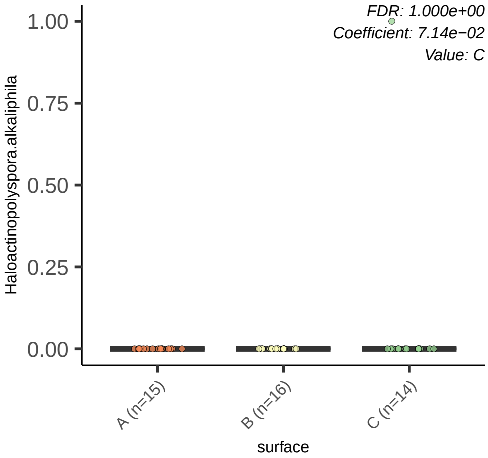
FDR: 1.000e+00
Coefficient: 7.14e−02
Value: C
Haloactinopolyspora.alkaliphila
1.00
0.75
0.50
0.25
0.00
A (n=15)
B (n=16)
C (n=14)
surface

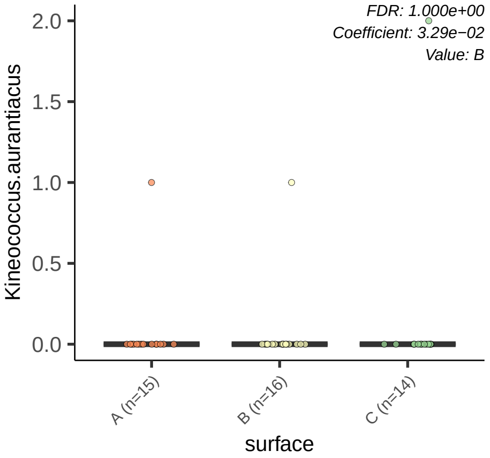

FDR: 1.000e+00
Coefficient: 3.29e−02
Value: B
Kineococcus.aurantiacus
2.0
1.5
1.0
0.5
0.0
A (n=15)
B (n=16)
C (n=14)
surface

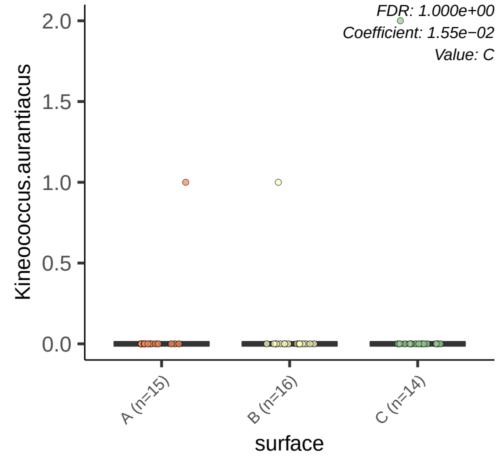

FDR: 1.000e+00
Coefficient: 1.55e−02
Value: C
Kineococcus.aurantiacus
2.0
1.5
1.0
0.5
0.0
A (n=15)
B (n=16)
C (n=14)
surface

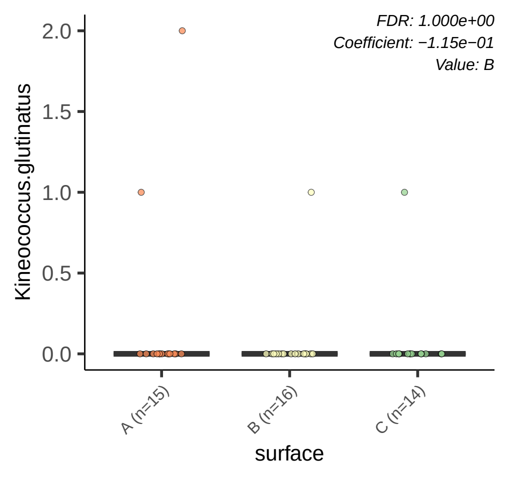
FDR: 1.000e+00
Coefficient: −1.15e−01
Value: B
Kineococcus.glutinatus
2.0
1.5
1.0
0.5
0.0
A (n=15)
B (n=16)
C (n=14)
surface

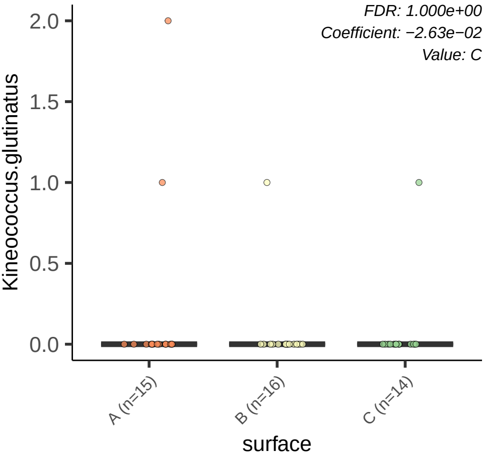

FDR: 1.000e+00
Coefficient: −2.63e−02
Value: C
Kineococcus.glutinatus
2.0
1.5
1.0
0.5
0.0
A (n=15)
B (n=16)
C (n=14)
surface

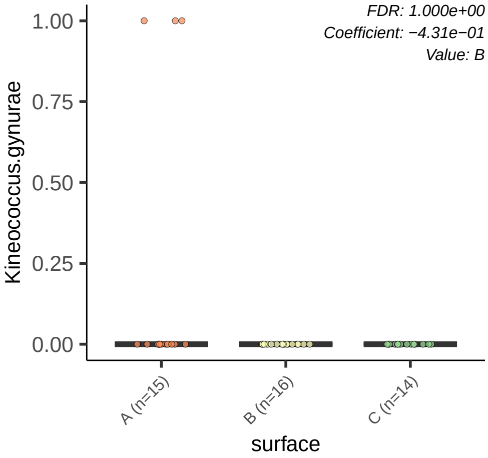
FDR: 1.000e+00
Coefficient: −4.31e−01
Value: B
Kineococcus.gynurae
1.00
0.75
0.50
0.25
0.00
A (n=15)
B (n=16)
C (n=14)
surface

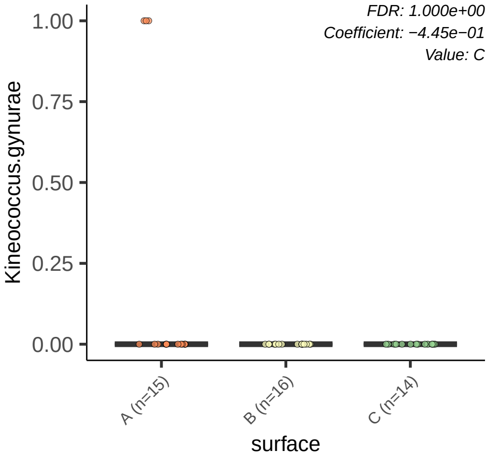
FDR: 1.000e+00
Coefficient: −4.45e−01
Value: C
Kineococcus.gynurae
1.00
0.75
0.50
0.25
0.00
A (n=15)
B (n=16)
C (n=14)
surface

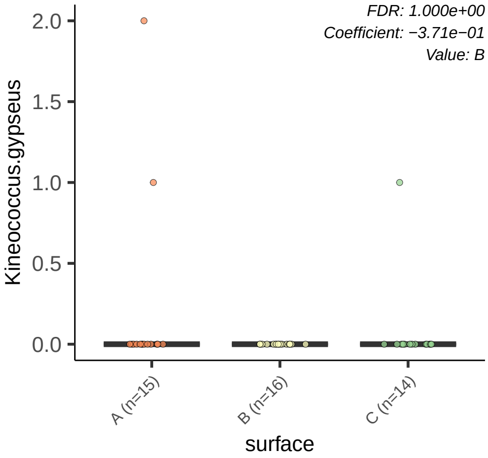
FDR: 1.000e+00
Coefficient: −3.71e−01
Value: B
Kineococcus.gypseus
2.0
1.5
1.0
0.5
0.0
A (n=15)
B (n=16)
C (n=14)
surface

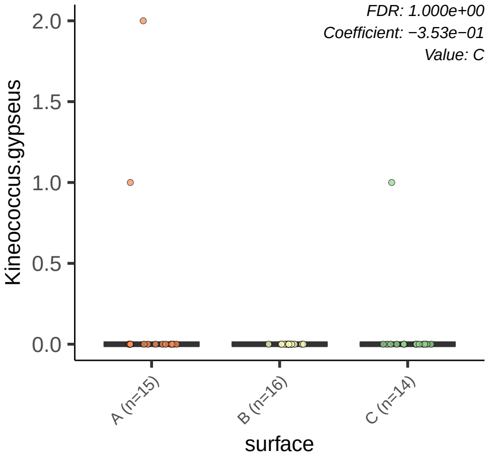

FDR: 1.000e+00
Coefficient: −3.53e−01
Value: C
Kineococcus.gypseus
2.0
1.5
1.0
0.5
0.0
A (n=15)
B (n=16)
C (n=14)
surface

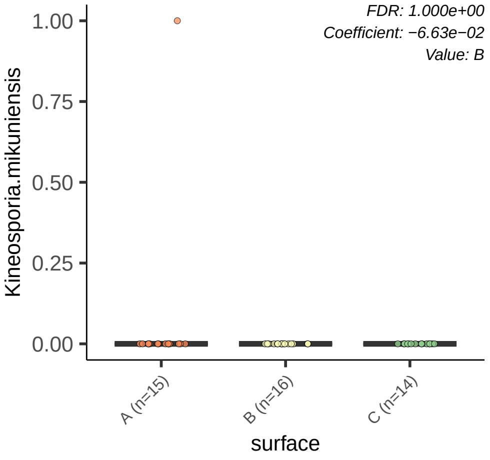

FDR: 1.000e+00
Coefficient: −6.63e−02
Value: B
Kineosporia.mikuniensis
1.00
0.75
0.50
0.25
0.00
A (n=15)
B (n=16)
C (n=14)
surface

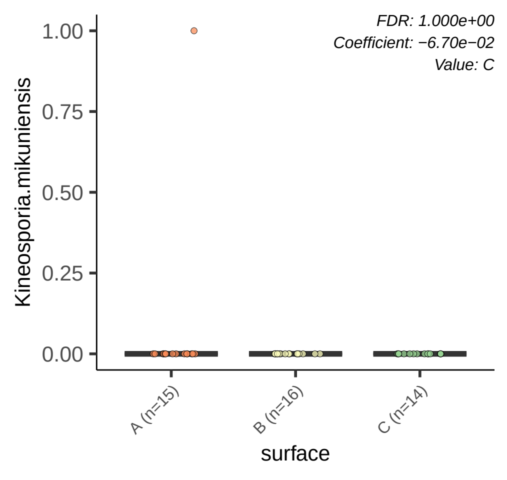

FDR: 1.000e+00
Coefficient: −6.70e−02
Value: C
Kineosporia.mikuniensis
1.00
0.75
0.50
0.25
0.00
A (n=15)
B (n=16)
C (n=14)
surface

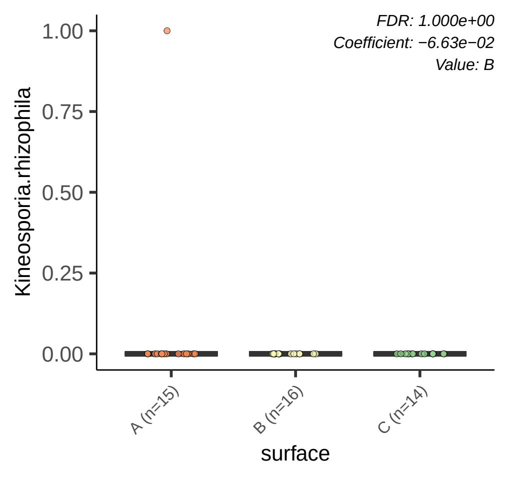

FDR: 1.000e+00
Coefficient: −6.63e−02
Value: B
Kineosporia.rhizophila
1.00
0.75
0.50
0.25
0.00
A (n=15)
B (n=16)
C (n=14)
surface

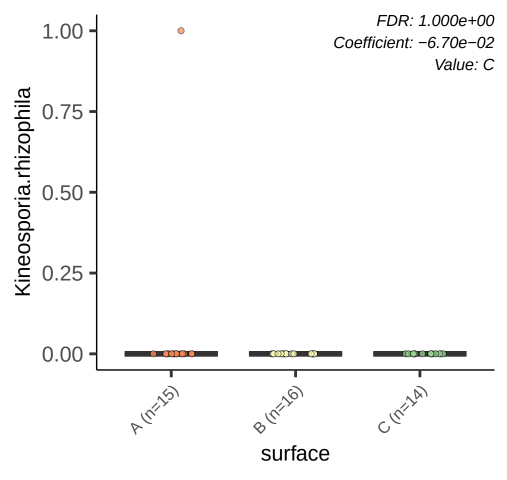
FDR: 1.000e+00
Coefficient: −6.70e−02
Value: C
Kineosporia.rhizophila
1.00
0.75
0.50
0.25
0.00
A (n=15)
B (n=16)
C (n=14)
surface

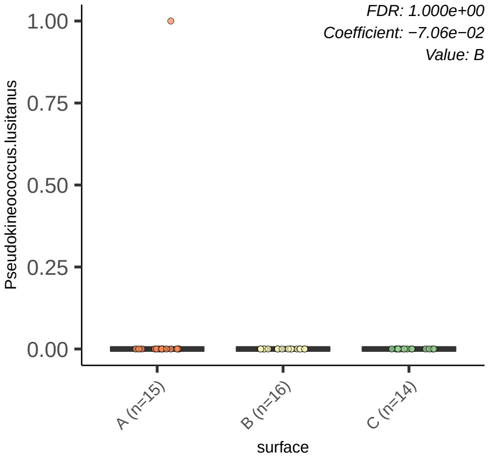

FDR: 1.000e+00
Coefficient: −7.06e−02
Value: B
1.00
0.75
0.50
0.25
0.00
Pseudokineococcus.lusitanus
A (n=15)
B (n=16)
C (n=14)
surface

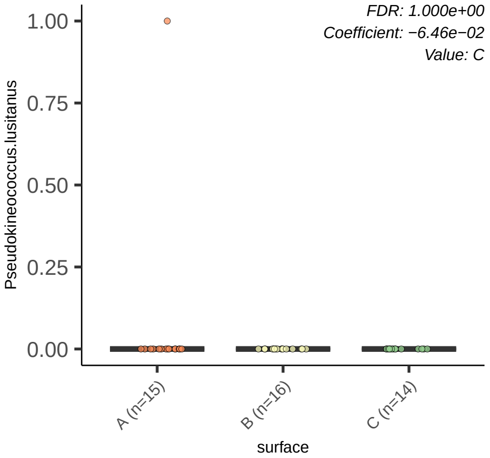
FDR: 1.000e+00
Coefficient: −6.46e−02
Value: C
Pseudokineococcus.lusitanus
1.00
0.75
0.50
0.25
0.00
A (n=15)
B (n=16)
C (n=14)
surface

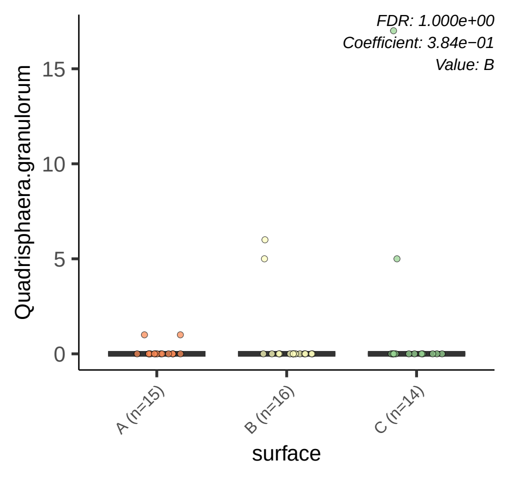

FDR: 1.000e+00
Coefficient: 3.84e−01
Value: B
Quadrisphaera.granulorum
15
10
5
0
A (n=15)
B (n=16)
C (n=14)
surface

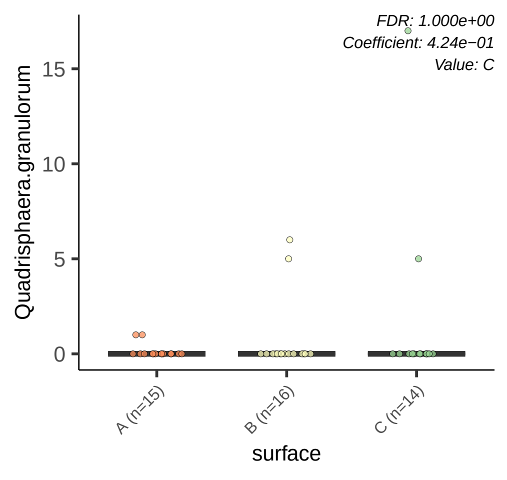
FDR: 1.000e+00
Coefficient: 4.24e−01
Value: C
Quadrisphaera.granulorum
15
10
5
0
A (n=15)
B (n=16)
C (n=14)
surface

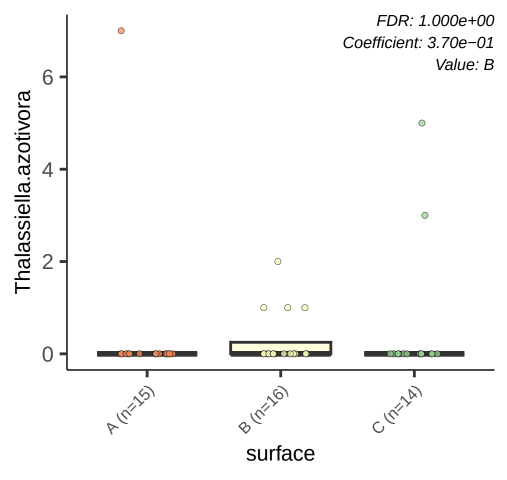

FDR: 1.000e+00
Coefficient: 3.70e−01
Value: B
Thalassiella.azotivora
0
2
4
6
A (n=15)
B (n=16)
C (n=14)
surface

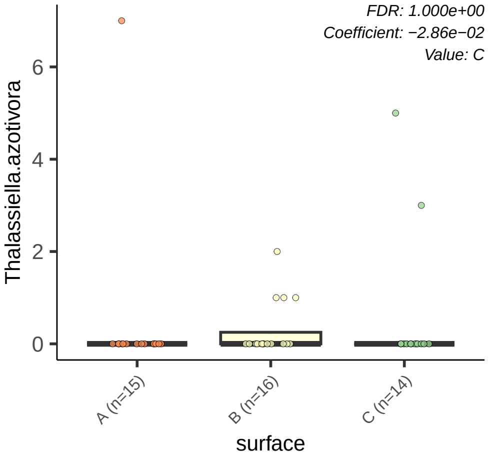
FDR: 1.000e+00
Coefficient: −2.86e−02
Value: C
Thalassiella.azotivora
0
2
4
6
A (n=15)
B (n=16)
C (n=14)
surface

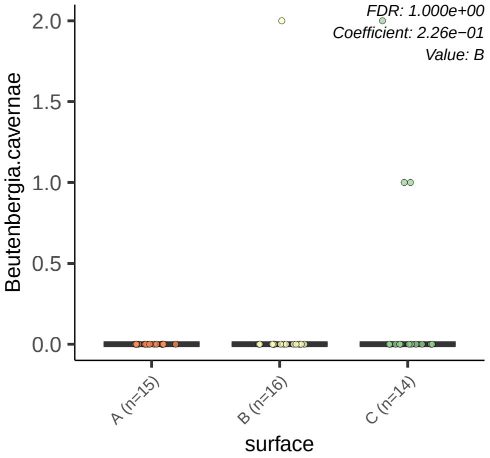
FDR: 1.000e+00
Coefficient: 2.26e−01
Value: B
Beutenbergia.cavernae
2.0
1.5
1.0
0.5
0.0
A (n=15)
B (n=16)
C (n=14)
surface

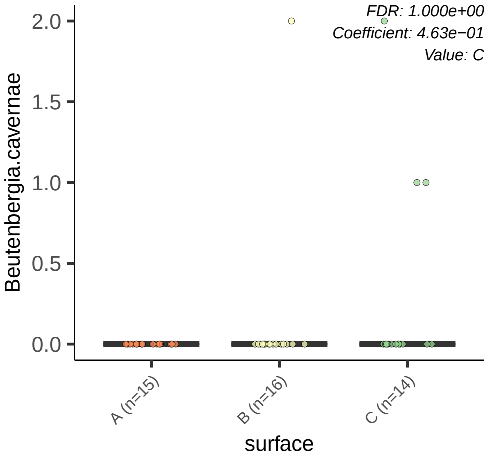
FDR: 1.000e+00
Coefficient: 4.63e−01
Value: C
Beutenbergia.cavernae
2.0
1.5
1.0
0.5
0.0
A (n=15)
B (n=16)
C (n=14)
surface

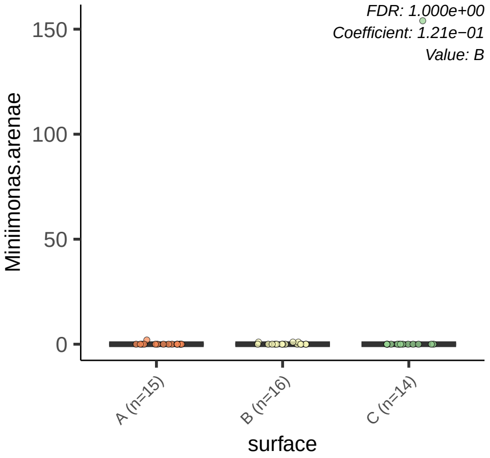
FDR: 1.000e+00
Coefficient: 1.21e−01
Value: B
Miniimonas.arenae
150
100
50
0
A (n=15)
B (n=16)
C (n=14)
surface

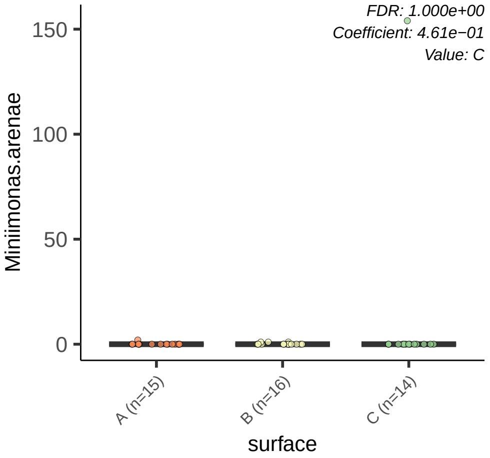

FDR: 1.000e+00
Coefficient: 4.61e−01
Value: C
Miniimonas.arenae
150
100
50
0
A (n=15)
B (n=16)
C (n=14)
surface

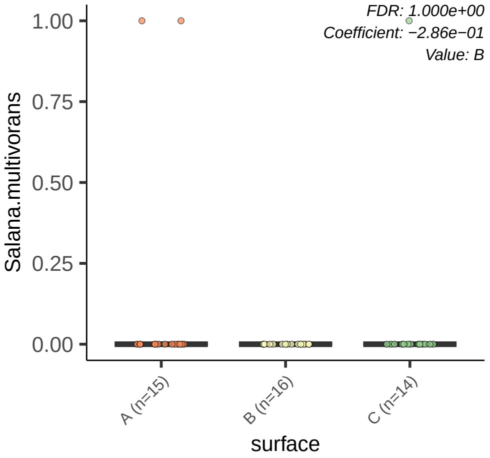
FDR: 1.000e+00
Coefficient: −2.86e−01
Value: B
Salana.multivorans
1.00
0.75
0.50
0.25
0.00
A (n=15)
B (n=16)
C (n=14)
surface

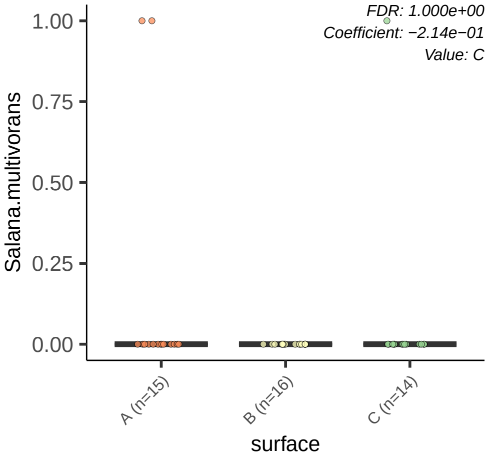

FDR: 1.000e+00
Coefficient: −2.14e−01
Value: C
Salana.multivorans
1.00
0.75
0.50
0.25
0.00
A (n=15)
B (n=16)
C (n=14)
surface

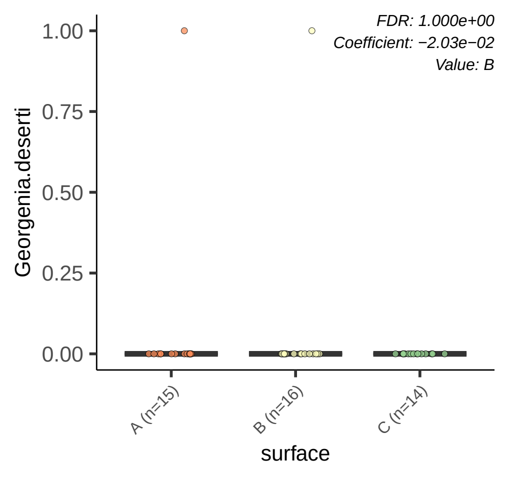
FDR: 1.000e+00
Coefficient: −2.03e−02
Value: B
Georgenia.deserti
1.00
0.75
0.50
0.25
0.00
A (n=15)
B (n=16)
C (n=14)
surface

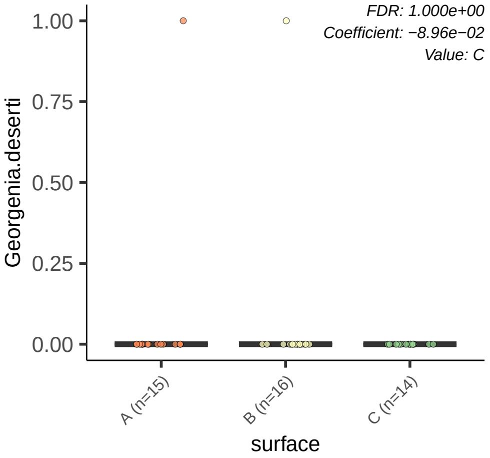

FDR: 1.000e+00
Coefficient: −8.96e−02
Value: C
1.00
0.75
0.50
0.25
0.00
Georgenia.deserti
A (n=15)
B (n=16)
C (n=14)
surface

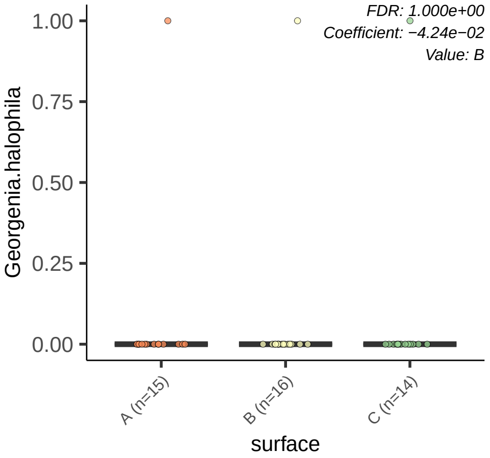

FDR: 1.000e+00
Coefficient: −4.24e−02
Value: B
Georgenia.halophila
1.00
0.75
0.50
0.25
0.00
A (n=15)
B (n=16)
C (n=14)
surface

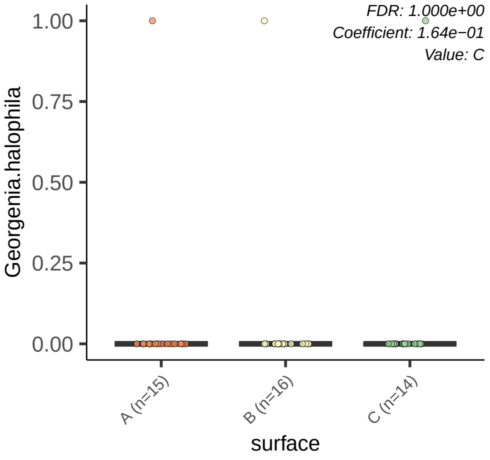
FDR: 1.000e+00
Coefficient: 1.64e−01
Value: C
Georgenia.halophila
1.00
0.75
0.50
0.25
0.00
A (n=15)
B (n=16)
C (n=14)
surface

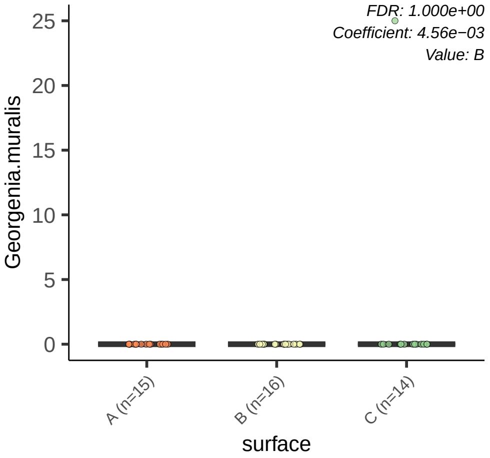
FDR: 1.000e+00
Coefficient: 4.56e−03
Value: B
Georgenia.muralis
25
20
15
10
5
0
A (n=15)
B (n=16)
C (n=14)
surface

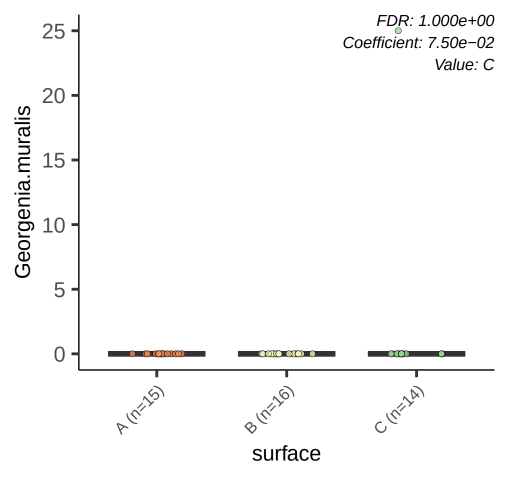

FDR: 1.000e+00
Coefficient: 7.50e−02
Value: C
Georgenia.muralis
25
20
15
10
5
0
A (n=15)
B (n=16)
C (n=14)
surface

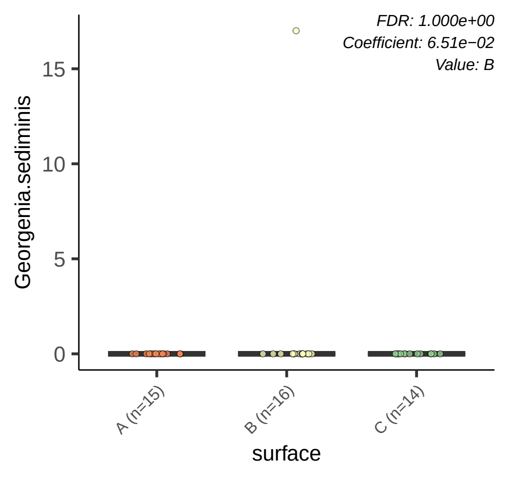
FDR: 1.000e+00
Coefficient: 6.51e−02
Value: B
Georgenia.sediminis
0
5
10
15
A (n=15)
B (n=16)
C (n=14)
surface

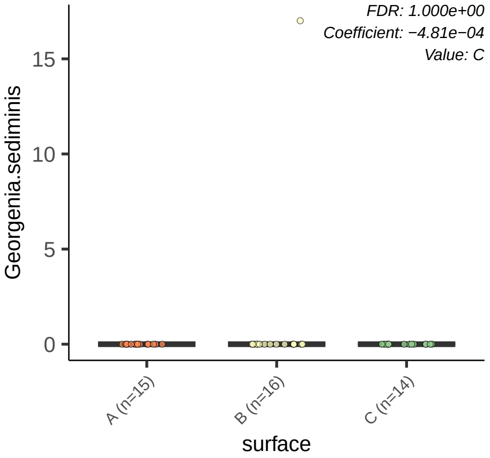
FDR: 1.000e+00
Coefficient: −4.81e−04
Value: C
Georgenia.sediminis
0
5
10
15
A (n=15)
B (n=16)
C (n=14)
surface

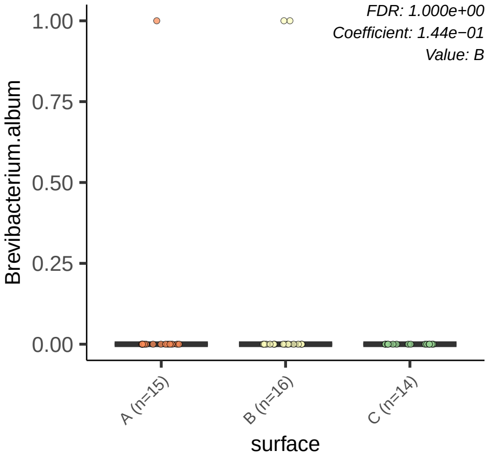

FDR: 1.000e+00
Coefficient: 1.44e−01
Value: B
Brevibacterium.album
1.00
0.75
0.50
0.25
0.00
A (n=15)
B (n=16)
C (n=14)
surface

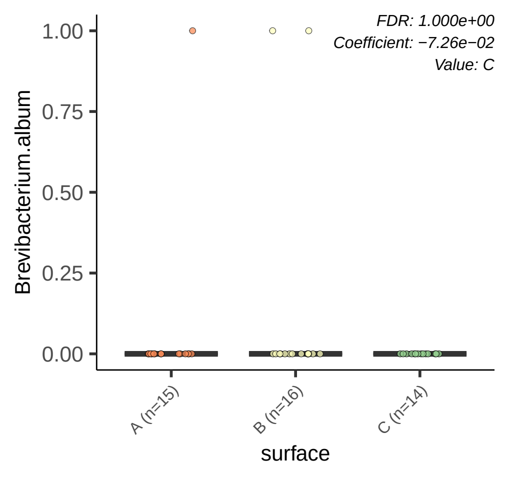
FDR: 1.000e+00
Coefficient: −7.26e−02
Value: C
Brevibacterium.album
1.00
0.75
0.50
0.25
0.00
A (n=15)
B (n=16)
C (n=14)
surface

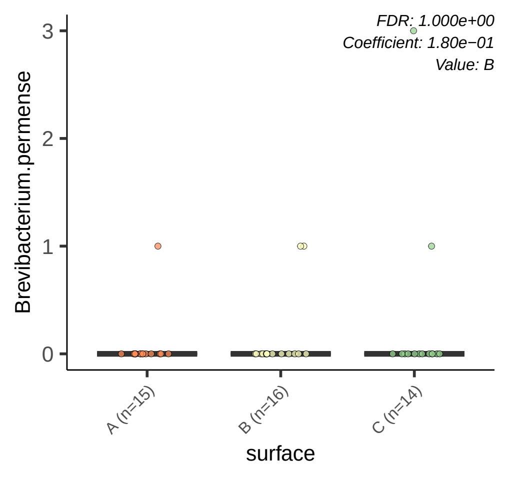

FDR: 1.000e+00
Coefficient: 1.80e−01
Value: B
Brevibacterium.permense
3
2
1
0
A (n=15)
B (n=16)
C (n=14)
surface

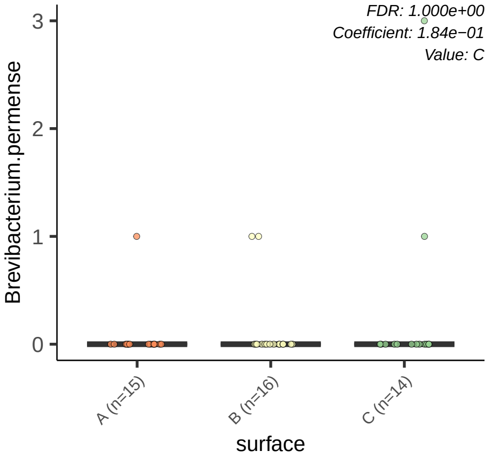

FDR: 1.000e+00
Coefficient: 1.84e−01
Value: C
Brevibacterium.permense
3
2
1
0
A (n=15)
B (n=16)
C (n=14)
surface

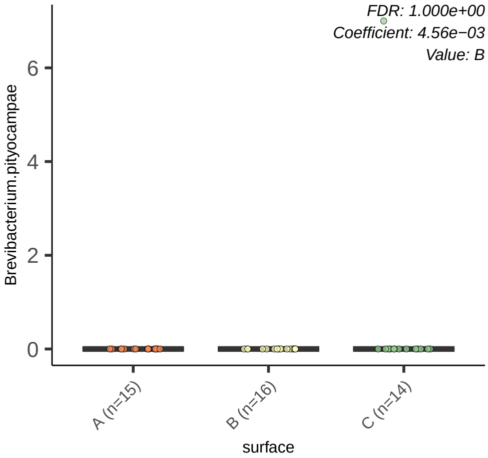

FDR: 1.000e+00
Coefficient: 4.56e−03
Value: B
Brevibacterium.pityocampae
6
4
2
0
A (n=15)
B (n=16)
C (n=14)
surface

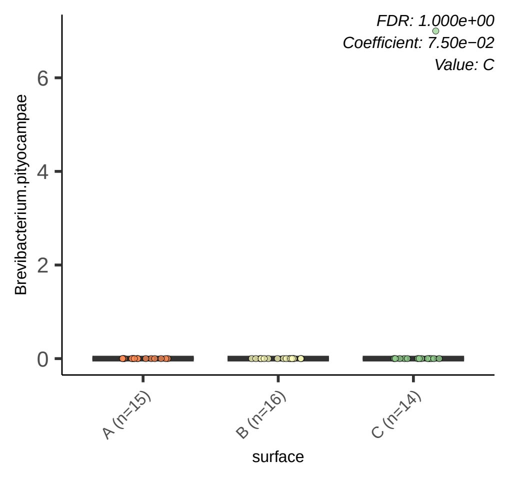

FDR: 1.000e+00
Coefficient: 7.50e−02
Value: C
Brevibacterium.pityocampae
6
4
2
0
A (n=15)
B (n=16)
C (n=14)
surface

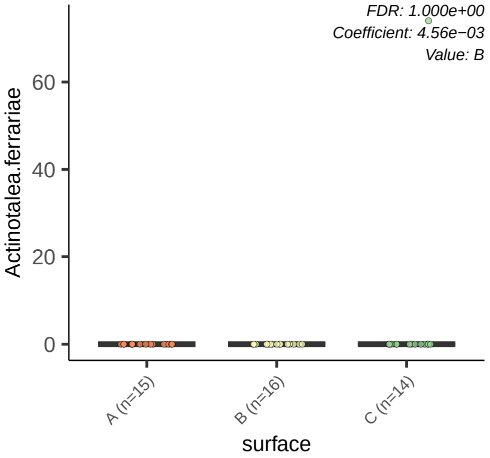

FDR: 1.000e+00
Coefficient: 4.56e−03
Value: B
Actinotalea.ferrariae
60
40
20
0
A (n=15)
B (n=16)
C (n=14)
surface

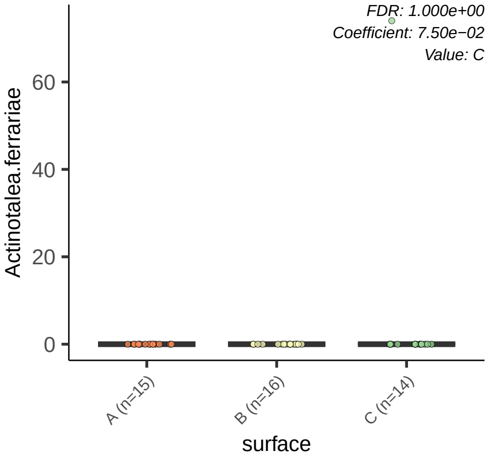

FDR: 1.000e+00
Coefficient: 7.50e−02
Value: C
Actinotalea.ferrariae
60
40
20
0
A (n=15)
B (n=16)
C (n=14)
surface

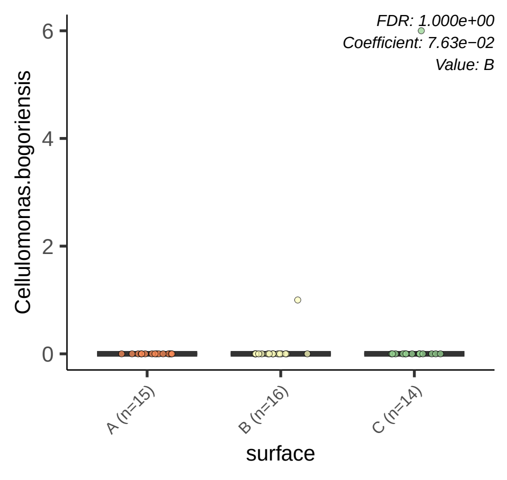

FDR: 1.000e+00
Coefficient: 7.63e−02
Value: B
Cellulomonas.bogoriensis
0
2
4
6
A (n=15)
B (n=16)
C (n=14)
surface

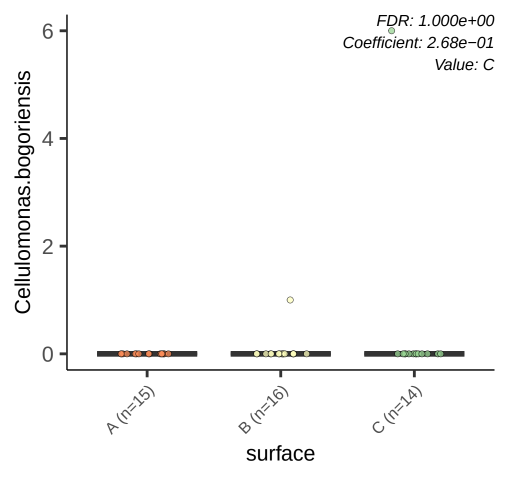

FDR: 1.000e+00
Coefficient: 2.68e−01
Value: C
Cellulomonas.bogoriensis
6
4
2
0
A (n=15)
B (n=16)
C (n=14)
surface

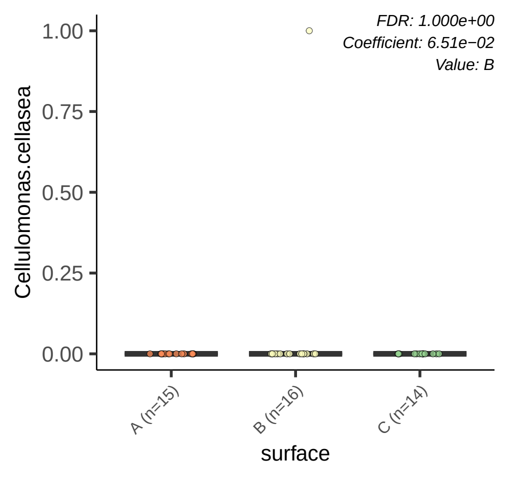
FDR: 1.000e+00
Coefficient: 6.51e−02
Value: B
Cellulomonas.cellasea
1.00
0.75
0.50
0.25
0.00
A (n=15)
B (n=16)
C (n=14)
surface

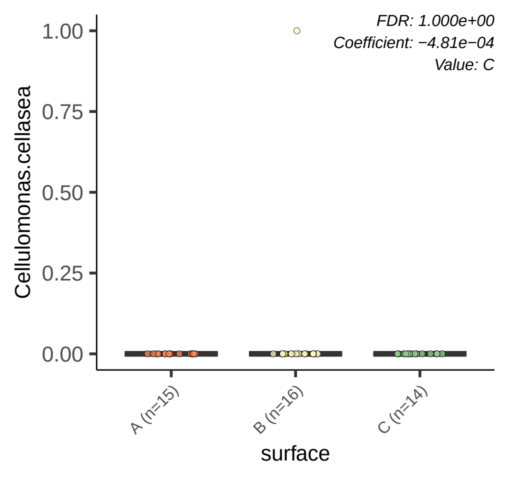
FDR: 1.000e+00
Coefficient: −4.81e−04
Value: C
Cellulomonas.cellasea
1.00
0.75
0.50
0.25
0.00
A (n=15)
B (n=16)
C (n=14)
surface

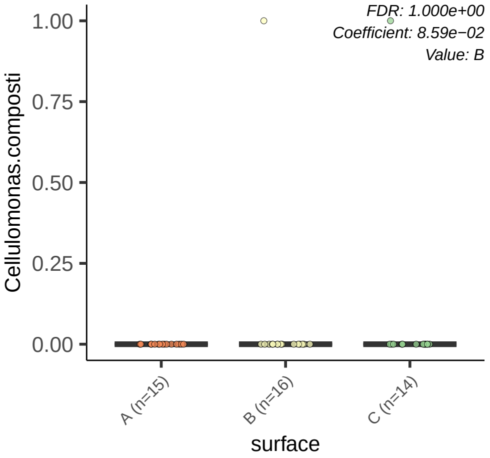
FDR: 1.000e+00
Coefficient: 8.59e−02
Value: B
Cellulomonas.composti
1.00
0.75
0.50
0.25
0.00
A (n=15)
B (n=16)
C (n=14)
surface

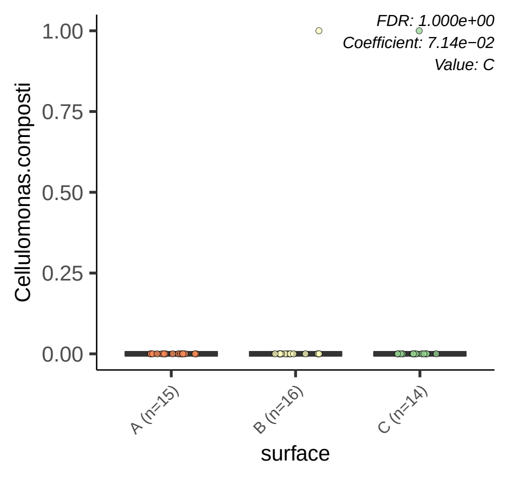

FDR: 1.000e+00
Coefficient: 7.14e−02
Value: C
Cellulomonas.composti
1.00
0.75
0.50
0.25
0.00
A (n=15)
B (n=16)
C (n=14)
surface

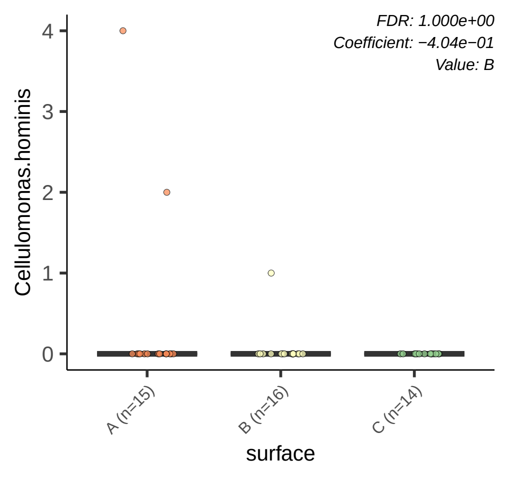
FDR: 1.000e+00
Coefficient: −4.04e−01
Value: B
Cellulomonas.hominis
0
1
2
3
4
A (n=15)
B (n=16)
C (n=14)
surface

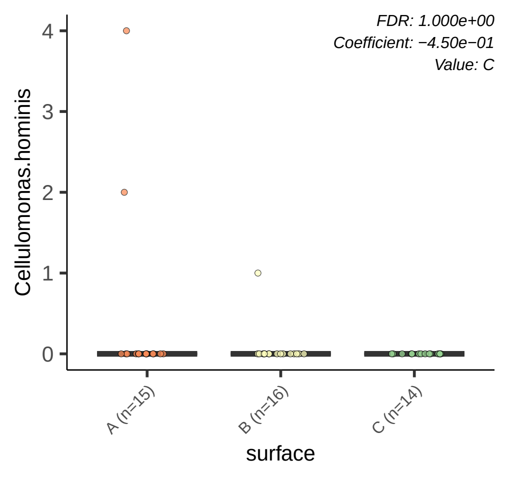
FDR: 1.000e+00
Coefficient: −4.50e−01
Value: C
Cellulomonas.hominis
0
1
2
3
4
A (n=15)
B (n=16)
C (n=14)
surface

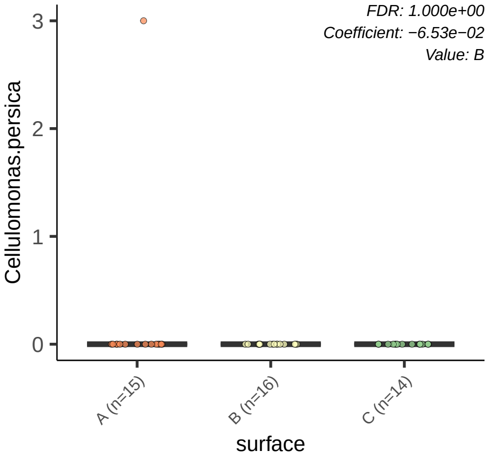
FDR: 1.000e+00
Coefficient: −6.53e−02
Value: B
Cellulomonas.persica
3
2
1
0
A (n=15)
B (n=16)
C (n=14)
surface

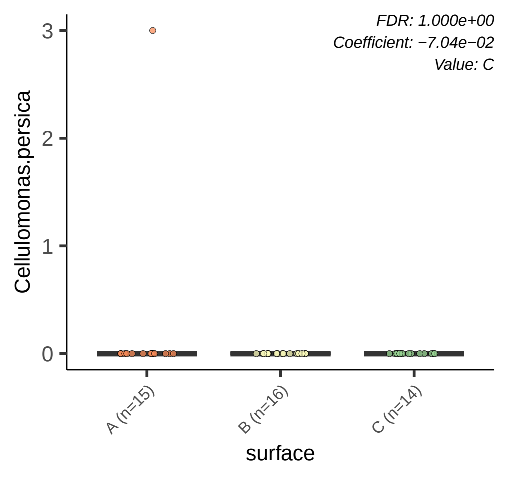

FDR: 1.000e+00
Coefficient: −7.04e−02
Value: C
Cellulomonas.persica
3
2
1
0
A (n=15)
B (n=16)
C (n=14)
surface

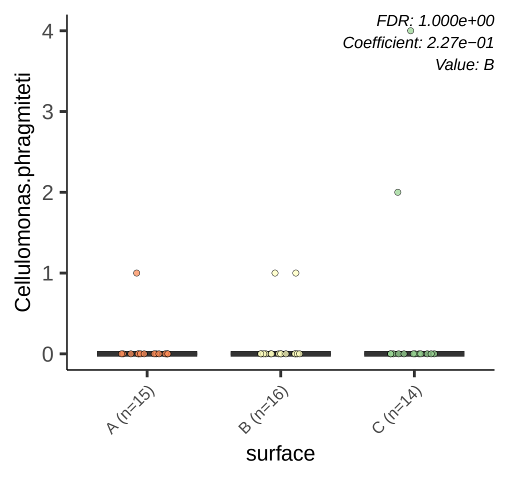
FDR: 1.000e+00
Coefficient: 2.27e−01
Value: B
Cellulomonas.phragmiteti
4
3
2
1
0
A (n=15)
B (n=16)
C (n=14)
surface

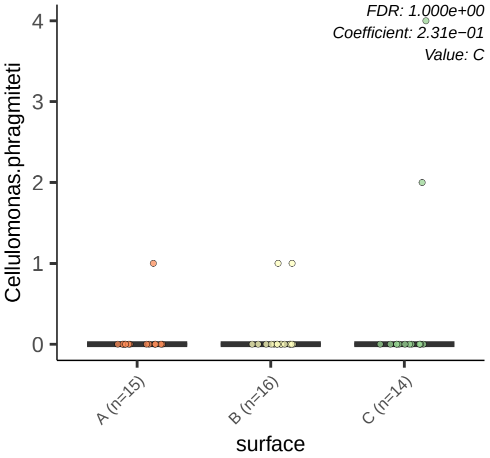
FDR: 1.000e+00
Coefficient: 2.31e−01
Value: C
Cellulomonas.phragmiteti
0
1
2
3
4
A (n=15)
B (n=16)
C (n=14)
surface

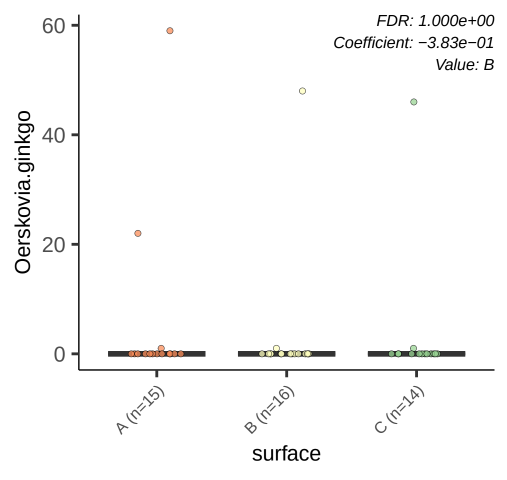
FDR: 1.000e+00
Coefficient: −3.83e−01
Value: B
Oerskovia.ginkgo
0
20
40
60
A (n=15)
B (n=16)
C (n=14)
surface

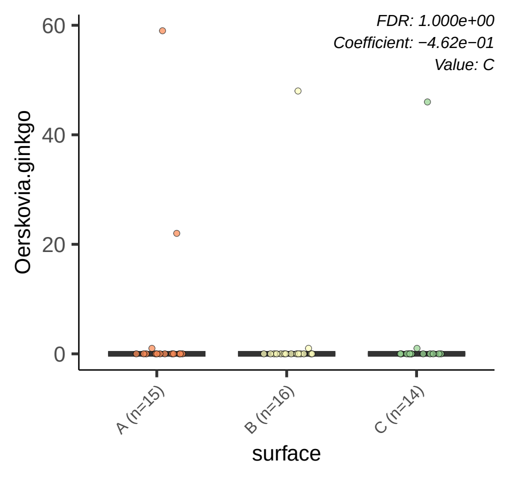
FDR: 1.000e+00
Coefficient: −4.62e−01
Value: C
Oerskovia.ginkgo
0
20
40
60
A (n=15)
B (n=16)
C (n=14)
surface

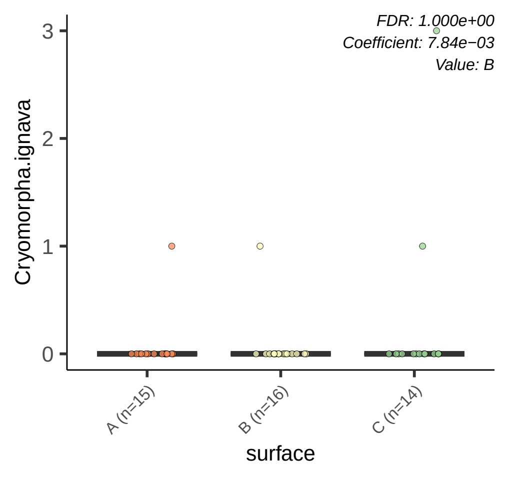
FDR: 1.000e+00
Coefficient: 7.84e−03
Value: B
Cryomorpha.ignava
3
2
1
0
A (n=15)
B (n=16)
C (n=14)
surface

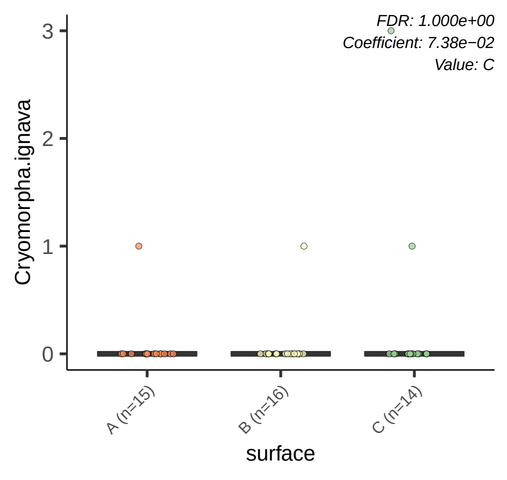
FDR: 1.000e+00
Coefficient: 7.38e−02
Value: C
Cryomorpha.ignava
3
2
1
0
A (n=15)
B (n=16)
C (n=14)
surface

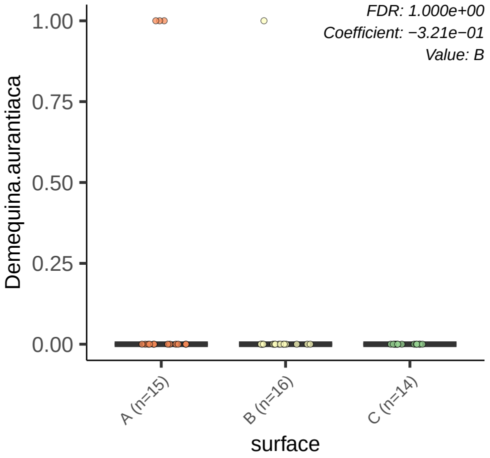

FDR: 1.000e+00
Coefficient: −3.21e−01
Value: B
Demequina.aurantiaca
1.00
0.75
0.50
0.25
0.00
A (n=15)
B (n=16)
C (n=14)
surface

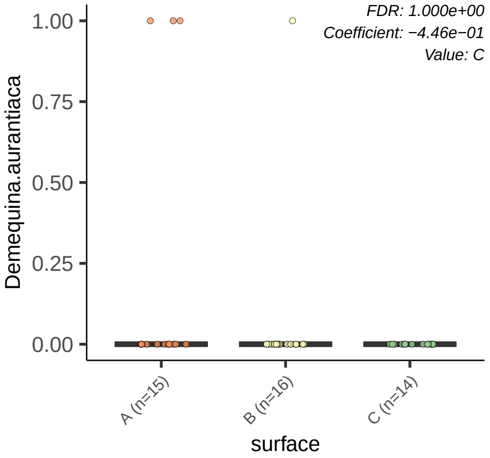
FDR: 1.000e+00
Coefficient: −4.46e−01
Value: C
Demequina.aurantiaca
1.00
0.75
0.50
0.25
0.00
A (n=15)
B (n=16)
C (n=14)
surface

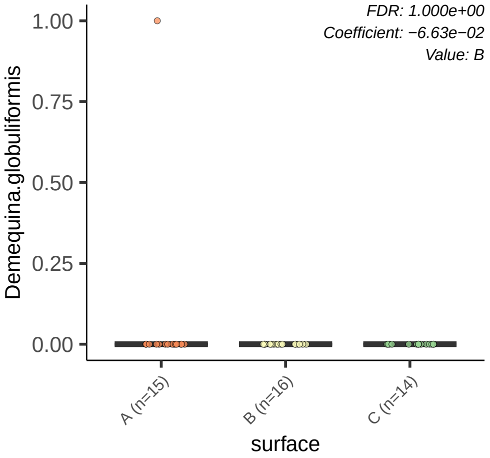
FDR: 1.000e+00
Coefficient: −6.63e−02
Value: B
Demequina.globuliformis
1.00
0.75
0.50
0.25
0.00
A (n=15)
B (n=16)
C (n=14)
surface

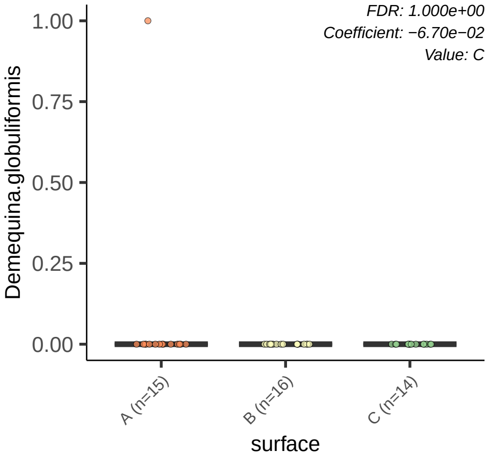
FDR: 1.000e+00
Coefficient: −6.70e−02
Value: C
1.00
0.75
0.50
0.25
0.00
Demequina.globuliformis
A (n=15)
B (n=16)
C (n=14)
surface

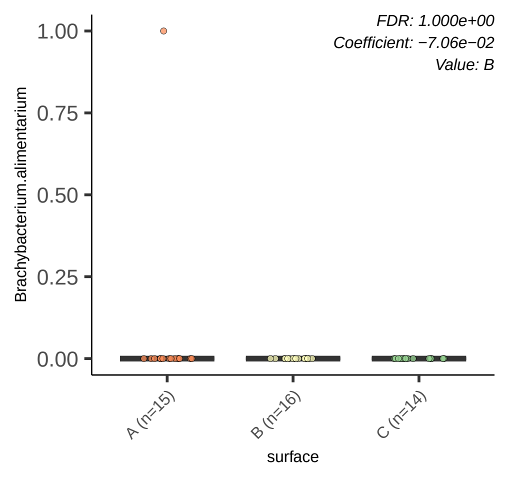

FDR: 1.000e+00
Coefficient: −7.06e−02
Value: B
1.00
0.75
0.50
0.25
0.00
Brachybacterium.alimentarium
A (n=15)
B (n=16)
C (n=14)
surface

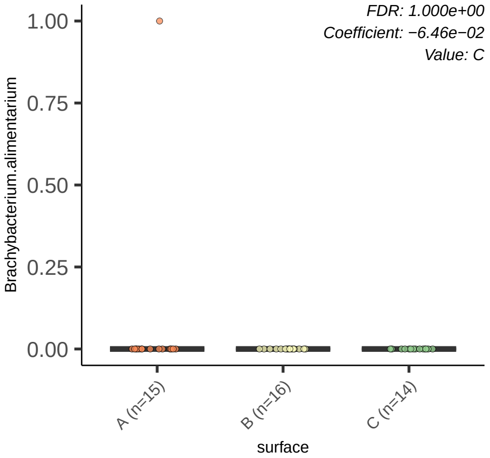

FDR: 1.000e+00
Coefficient: −6.46e−02
Value: C
1.00
0.75
0.50
0.25
0.00
Brachybacterium.alimentarium
A (n=15)
B (n=16)
C (n=14)
surface

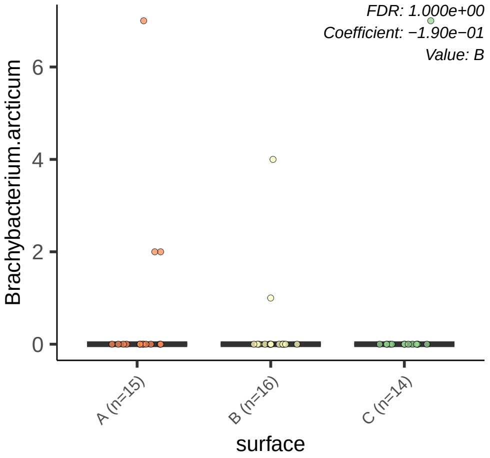
FDR: 1.000e+00
Coefficient: −1.90e−01
Value: B
Brachybacterium.arcticum
0
2
4
6
A (n=15)
B (n=16)
C (n=14)
surface

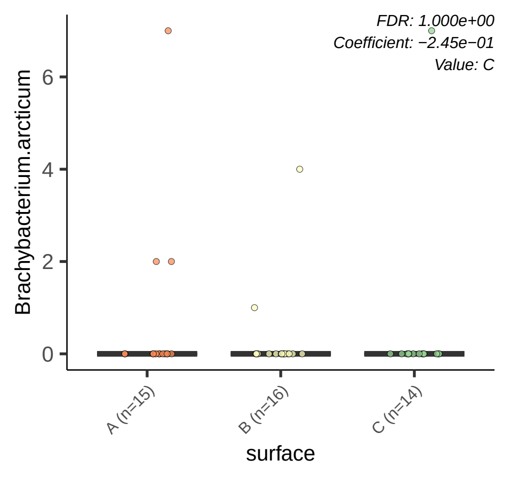

FDR: 1.000e+00
Coefficient: −2.45e−01
Value: C
Brachybacterium.arcticum
0
2
4
6
A (n=15)
B (n=16)
C (n=14)
surface

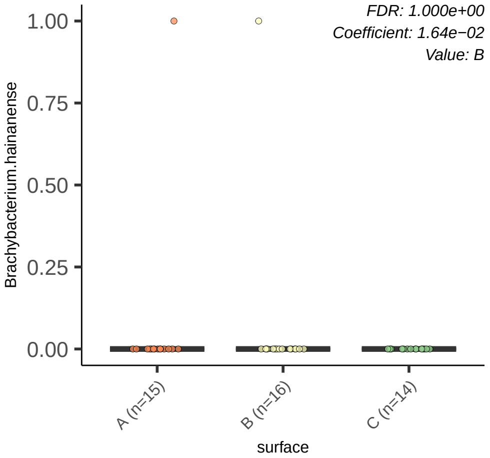

FDR: 1.000e+00
Coefficient: 1.64e−02
Value: B
Brachybacterium.hainanense
1.00
0.75
0.50
0.25
0.00
A (n=15)
B (n=16)
C (n=14)
surface

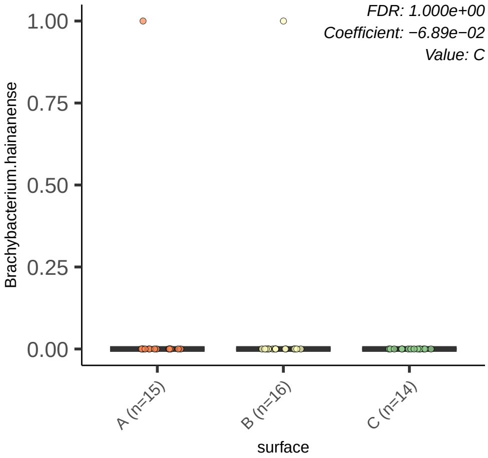
FDR: 1.000e+00
Coefficient: −6.89e−02
Value: C
Brachybacterium.hainanense
1.00
0.75
0.50
0.25
0.00
A (n=15)
B (n=16)
C (n=14)
surface

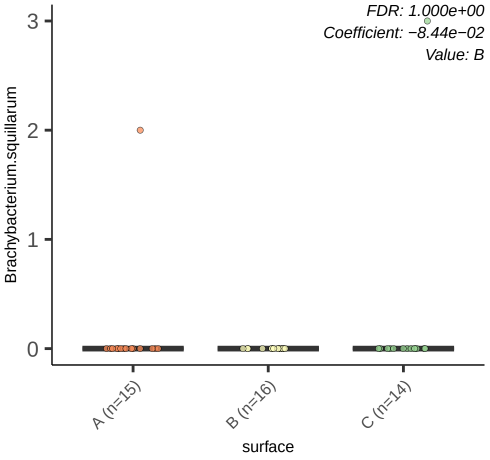

FDR: 1.000e+00
Coefficient: −8.44e−02
Value: B
Brachybacterium.squillarum
0
1
2
3
A (n=15)
B (n=16)
C (n=14)
surface

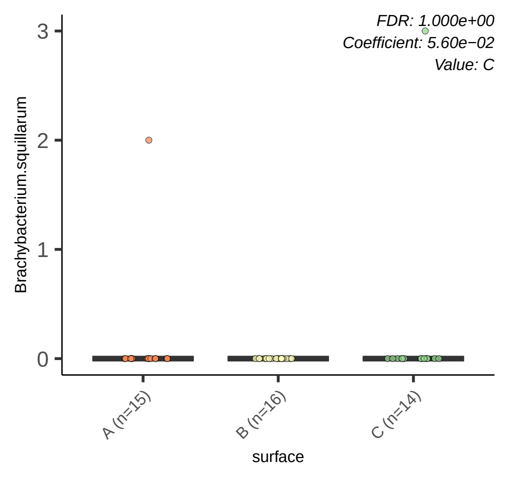

FDR: 1.000e+00
Coefficient: 5.60e−02
Value: C
Brachybacterium.squillarum
0
1
2
3
A (n=15)
B (n=16)
C (n=14)
surface

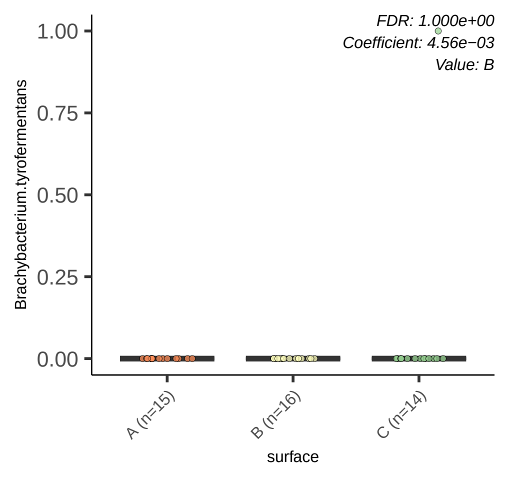
FDR: 1.000e+00
Coefficient: 4.56e−03
Value: B
1.00
0.75
0.50
0.25
0.00
Brachybacterium.tyrofermentans
A (n=15)
B (n=16)
C (n=14)
surface

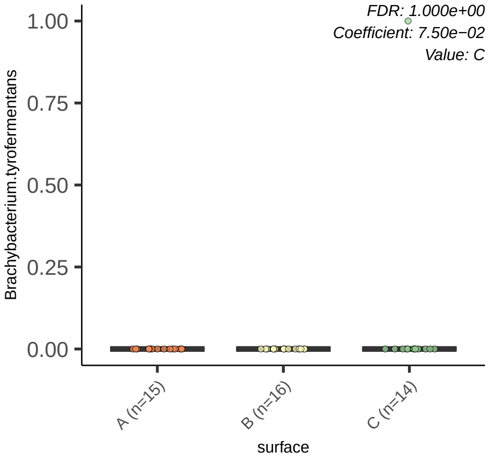
FDR: 1.000e+00
Coefficient: 7.50e−02
Value: C
1.00
0.75
0.50
0.25
0.00
Brachybacterium.tyrofermentans
A (n=15)
B (n=16)
C (n=14)
surface

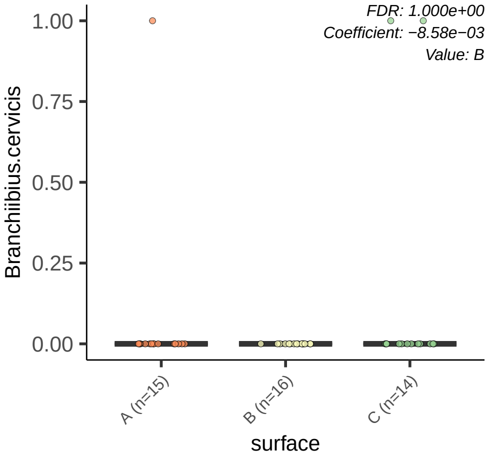

FDR: 1.000e+00
Coefficient: −8.58e−03
Value: B
Branchiibius.cervicis
1.00
0.75
0.50
0.25
0.00
A (n=15)
B (n=16)
C (n=14)
surface

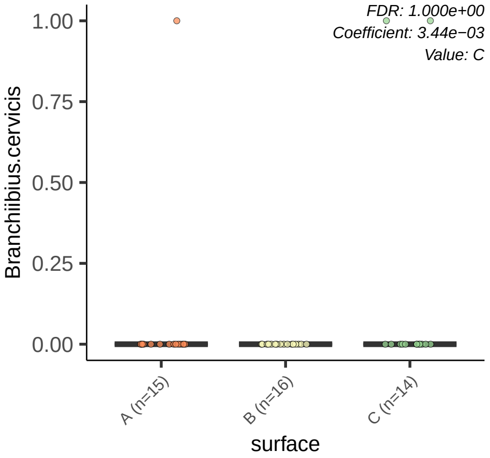

FDR: 1.000e+00
Coefficient: 3.44e−03
Value: C
1.00
0.75
0.50
0.25
0.00
Branchiibius.cervicis
A (n=15)
B (n=16)
C (n=14)
surface

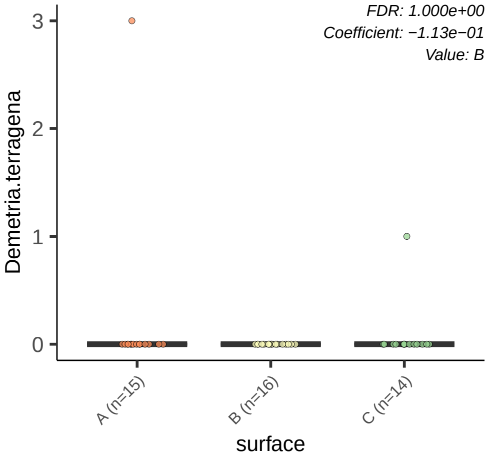
FDR: 1.000e+00
Coefficient: −1.13e−01
Value: B
Demetria.terragena
3
2
1
0
A (n=15)
B (n=16)
C (n=14)
surface

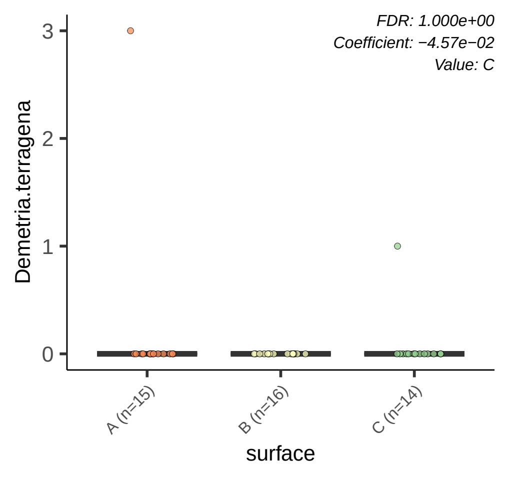
FDR: 1.000e+00
Coefficient: −4.57e−02
Value: C
Demetria.terragena
3
2
1
0
A (n=15)
B (n=16)
C (n=14)
surface

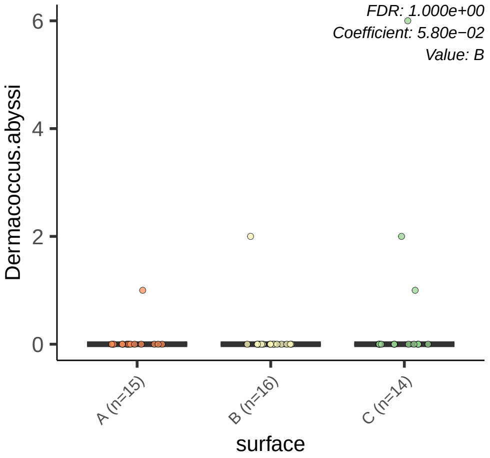
FDR: 1.000e+00
Coefficient: 5.80e−02
Value: B
Dermacoccus.abyssi
0
2
4
6
A (n=15)
B (n=16)
C (n=14)
surface

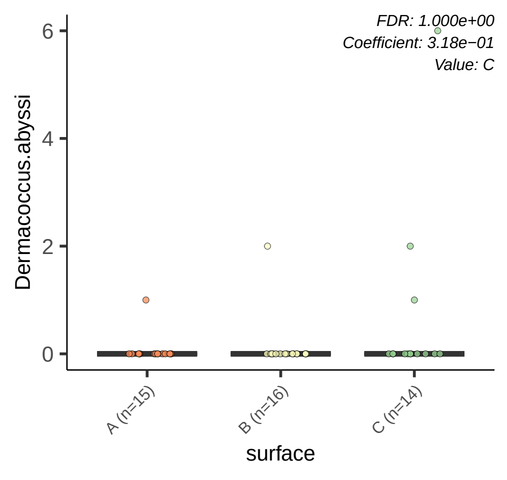
FDR: 1.000e+00
Coefficient: 3.18e−01
Value: C
Dermacoccus.abyssi
6
4
2
0
A (n=15)
B (n=16)
C (n=14)
surface

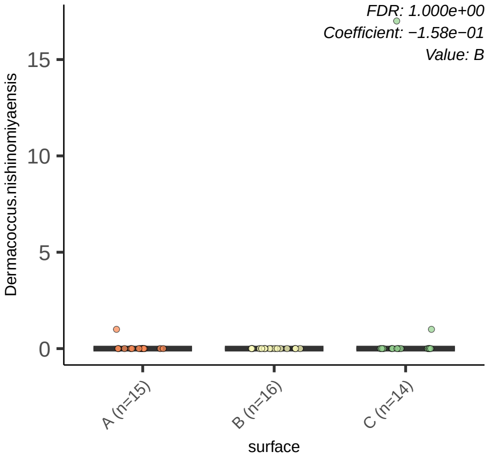

FDR: 1.000e+00
Coefficient: −1.58e−01
Value: B
Dermacoccus.nishinomiyaensis
15
10
5
0
A (n=15)
B (n=16)
C (n=14)
surface

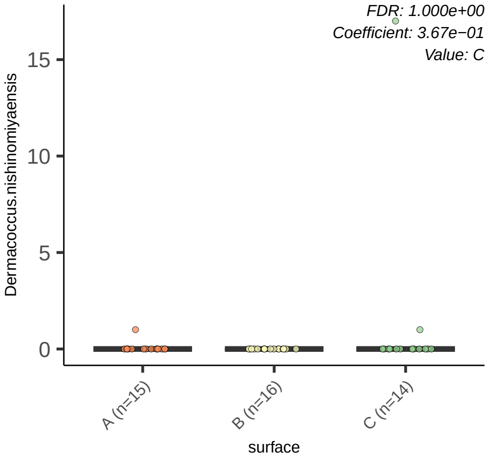
FDR: 1.000e+00
Coefficient: 3.67e−01
Value: C
Dermacoccus.nishinomiyaensis
0
5
10
15
A (n=15)
B (n=16)
C (n=14)
surface

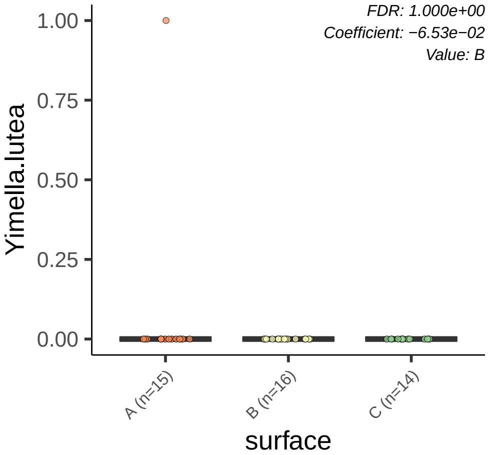

FDR: 1.000e+00
Coefficient: −6.53e−02
Value: B
Yimella.lutea
1.00
0.75
0.50
0.25
0.00
A (n=15)
B (n=16)
C (n=14)
surface

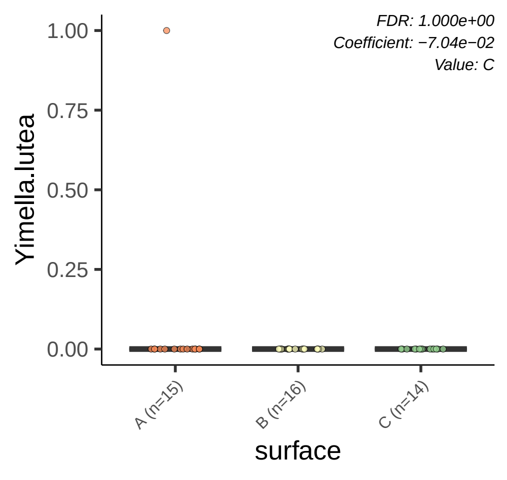
FDR: 1.000e+00
Coefficient: −7.04e−02
Value: C
Yimella.lutea
1.00
0.75
0.50
0.25
0.00
A (n=15)
B (n=16)
C (n=14)
surface

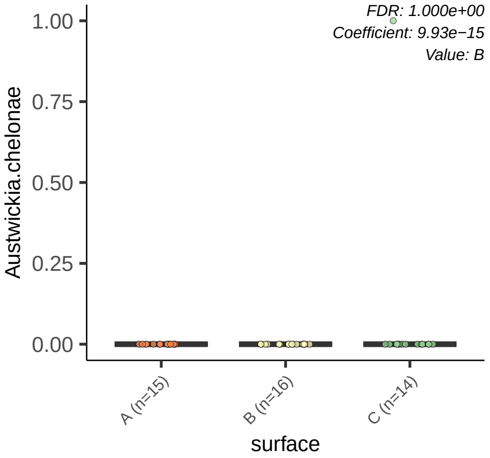

FDR: 1.000e+00
Coefficient: 9.93e−15
Value: B
Austwickia.chelonae
1.00
0.75
0.50
0.25
0.00
A (n=15)
B (n=16)
C (n=14)
surface

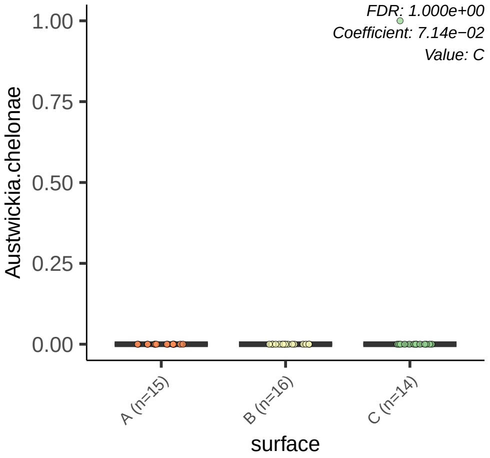
FDR: 1.000e+00
Coefficient: 7.14e−02
Value: C
Austwickia.chelonae
1.00
0.75
0.50
0.25
0.00
A (n=15)
B (n=16)
C (n=14)
surface

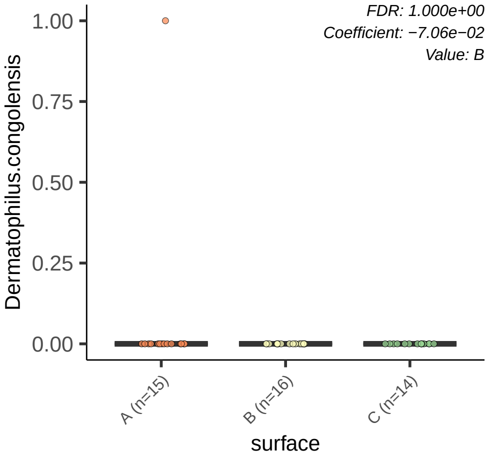

FDR: 1.000e+00
Coefficient: −7.06e−02
Value: B
Dermatophilus.congolensis
1.00
0.75
0.50
0.25
0.00
A (n=15)
B (n=16)
C (n=14)
surface

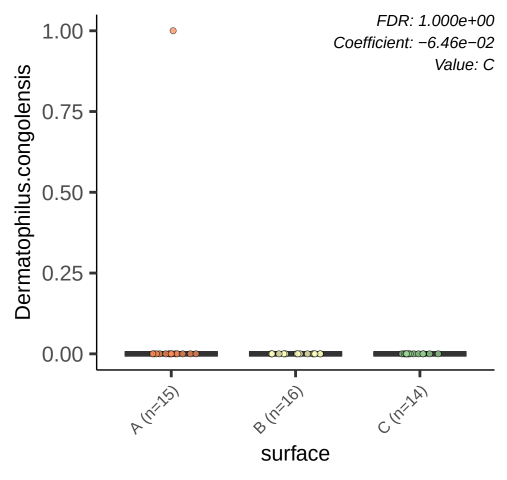
FDR: 1.000e+00
Coefficient: −6.46e−02
Value: C
Dermatophilus.congolensis
1.00
0.75
0.50
0.25
0.00
A (n=15)
B (n=16)
C (n=14)
surface

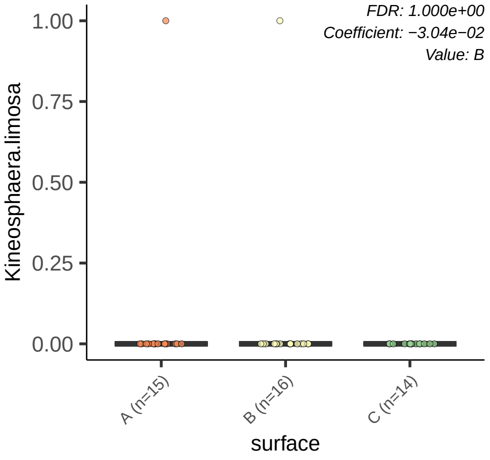

FDR: 1.000e+00
Coefficient: −3.04e−02
Value: B
Kineosphaera.limosa
1.00
0.75
0.50
0.25
0.00
A (n=15)
B (n=16)
C (n=14)
surface

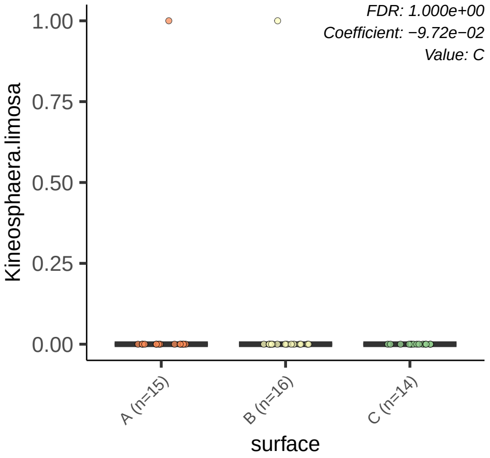

FDR: 1.000e+00
Coefficient: −9.72e−02
Value: C
Kineosphaera.limosa
1.00
0.75
0.50
0.25
0.00
A (n=15)
B (n=16)
C (n=14)
surface

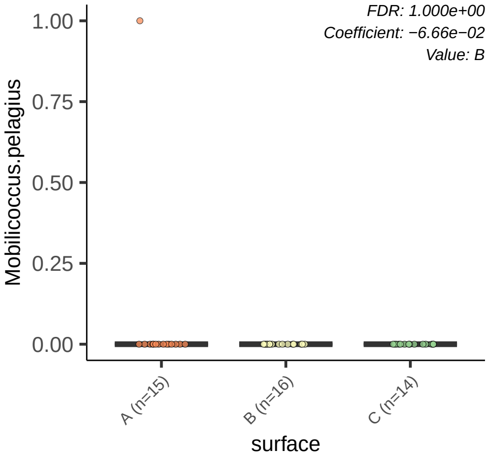

FDR: 1.000e+00
Coefficient: −6.66e−02
Value: B
Mobilicoccus.pelagius
1.00
0.75
0.50
0.25
0.00
A (n=15)
B (n=16)
C (n=14)
surface

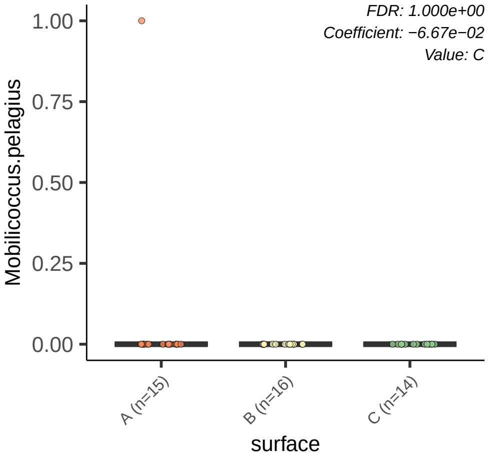
FDR: 1.000e+00
Coefficient: −6.67e−02
Value: C
Mobilicoccus.pelagius
1.00
0.75
0.50
0.25
0.00
A (n=15)
B (n=16)
C (n=14)
surface

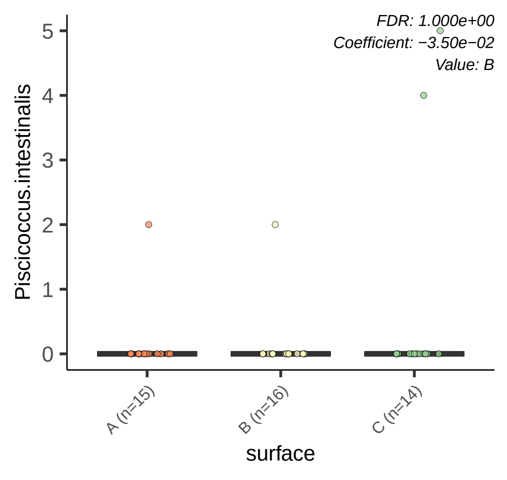
FDR: 1.000e+00
Coefficient: −3.50e−02
Value: B
Piscicoccus.intestinalis
0
1
2
3
4
5
A (n=15)
B (n=16)
C (n=14)
surface

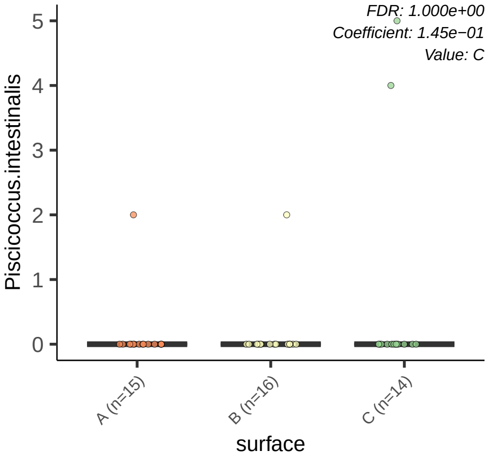
FDR: 1.000e+00
Coefficient: 1.45e−01
Value: C
Piscicoccus.intestinalis
0
1
2
3
4
5
A (n=15)
B (n=16)
C (n=14)
surface

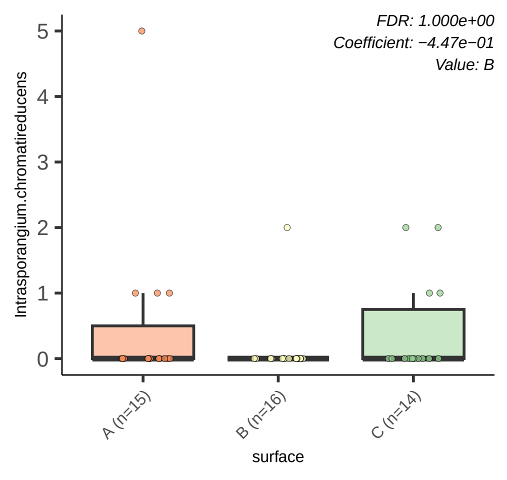
FDR: 1.000e+00
Coefficient: −4.47e−01
Value: B
Intrasporangium.chromatireducens
0
1
2
3
4
5
A (n=15)
B (n=16)
C (n=14)
surface

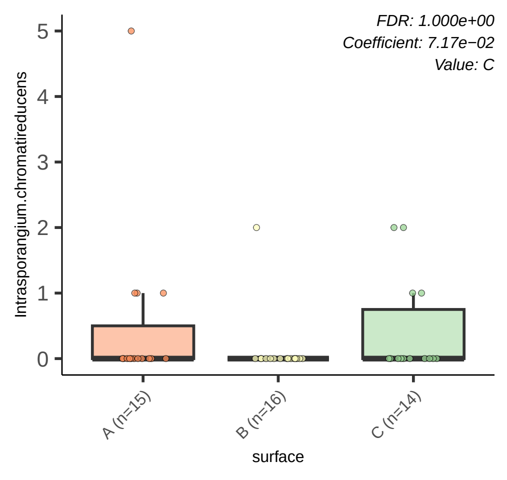

FDR: 1.000e+00
Coefficient: 7.17e−02
Value: C
Intrasporangium.chromatireducens
0
1
2
3
4
5
A (n=15)
B (n=16)
C (n=14)
surface

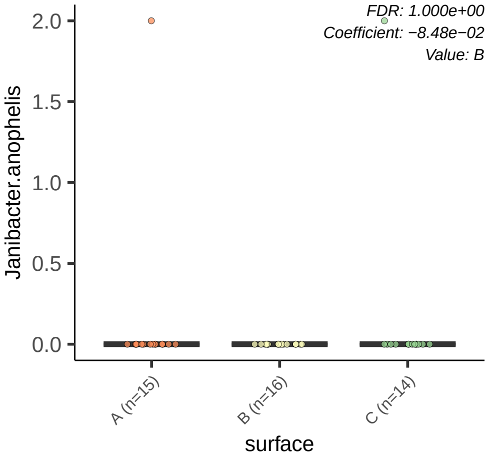

FDR: 1.000e+00
Coefficient: −8.48e−02
Value: B
Janibacter.anophelis
2.0
1.5
1.0
0.5
0.0
A (n=15)
B (n=16)
C (n=14)
surface

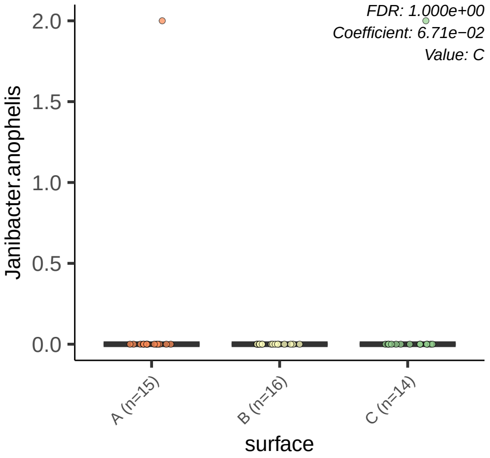
FDR: 1.000e+00
Coefficient: 6.71e−02
Value: C
Janibacter.anophelis
2.0
1.5
1.0
0.5
0.0
A (n=15)
B (n=16)
C (n=14)
surface

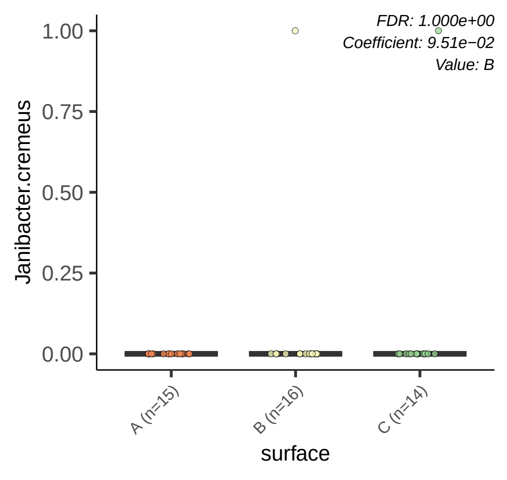
FDR: 1.000e+00
Coefficient: 9.51e−02
Value: B
Janibacter.cremeus
1.00
0.75
0.50
0.25
0.00
A (n=15)
B (n=16)
C (n=14)
surface

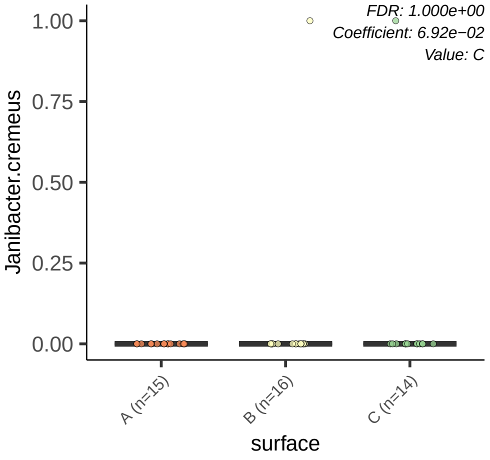
FDR: 1.000e+00
Coefficient: 6.92e−02
Value: C
Janibacter.cremeus
1.00
0.75
0.50
0.25
0.00
A (n=15)
B (n=16)
C (n=14)
surface

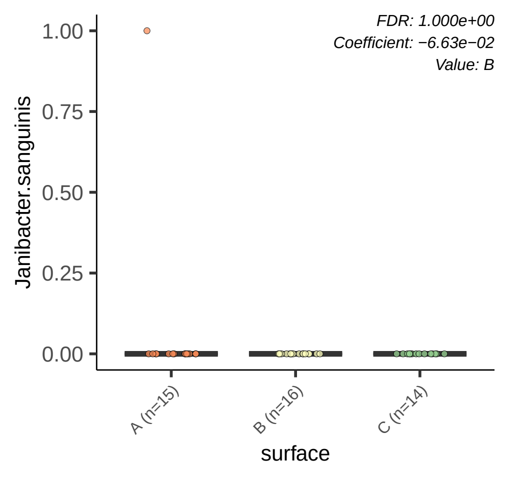

FDR: 1.000e+00
Coefficient: −6.63e−02
Value: B
Janibacter.sanguinis
1.00
0.75
0.50
0.25
0.00
A (n=15)
B (n=16)
C (n=14)
surface

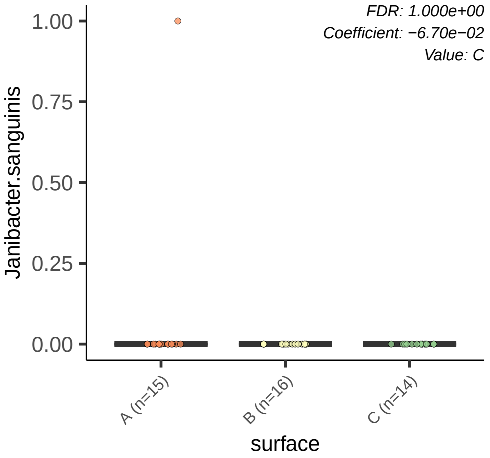

FDR: 1.000e+00
Coefficient: −6.70e−02
Value: C
Janibacter.sanguinis
1.00
0.75
0.50
0.25
0.00
A (n=15)
B (n=16)
C (n=14)
surface

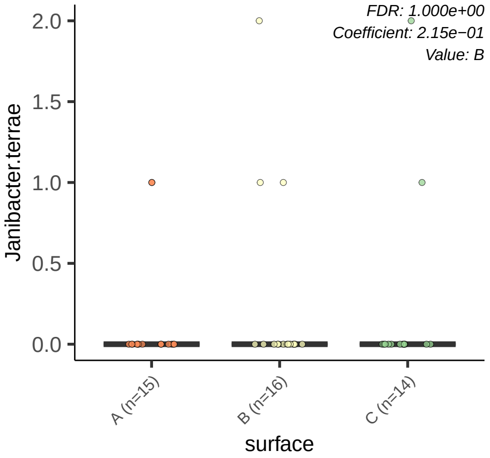
FDR: 1.000e+00
Coefficient: 2.15e−01
Value: B
Janibacter.terrae
2.0
1.5
1.0
0.5
0.0
A (n=15)
B (n=16)
C (n=14)
surface

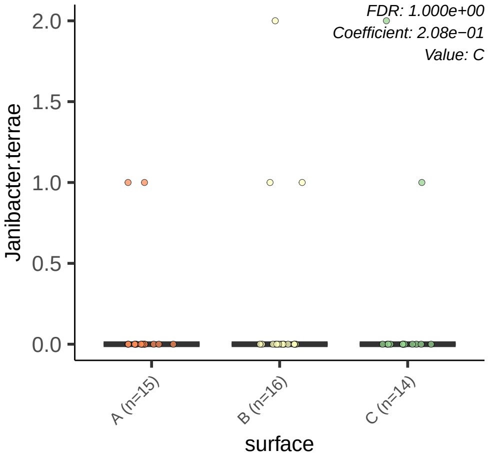
FDR: 1.000e+00
Coefficient: 2.08e−01
Value: C
Janibacter.terrae
2.0
1.5
1.0
0.5
0.0
A (n=15)
B (n=16)
C (n=14)
surface

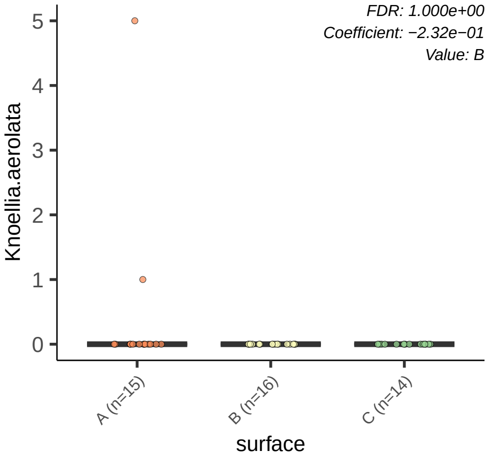

FDR: 1.000e+00
Coefficient: −2.32e−01
Value: B
Knoellia.aerolata
0
1
2
3
4
5
A (n=15)
B (n=16)
C (n=14)
surface

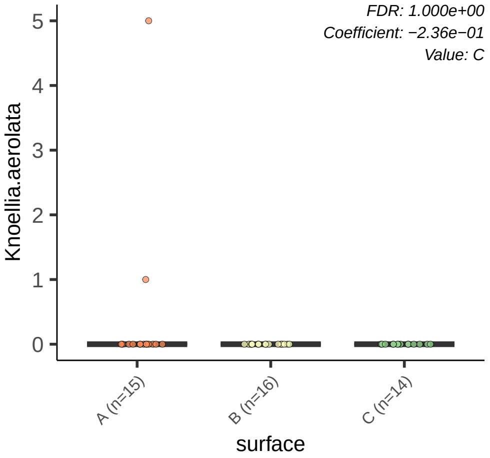
FDR: 1.000e+00
Coefficient: −2.36e−01
Value: C
Knoellia.aerolata
0
1
2
3
4
5
A (n=15)
B (n=16)
C (n=14)
surface

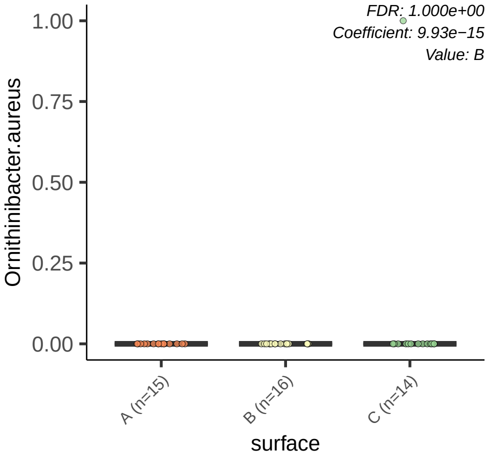
FDR: 1.000e+00
Coefficient: 9.93e−15
Value: B
Ornithinibacter.aureus
1.00
0.75
0.50
0.25
0.00
A (n=15)
B (n=16)
C (n=14)
surface

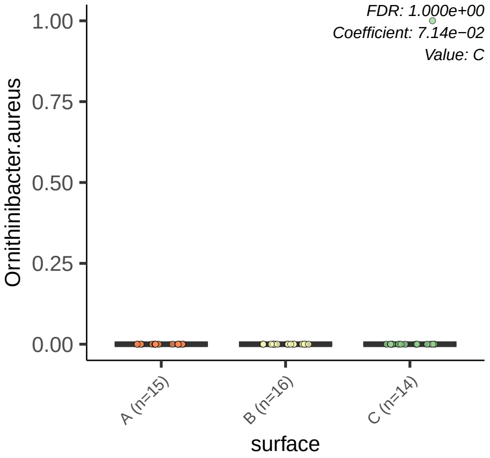

FDR: 1.000e+00
Coefficient: 7.14e−02
Value: C
Ornithinibacter.aureus
1.00
0.75
0.50
0.25
0.00
A (n=15)
B (n=16)
C (n=14)
surface

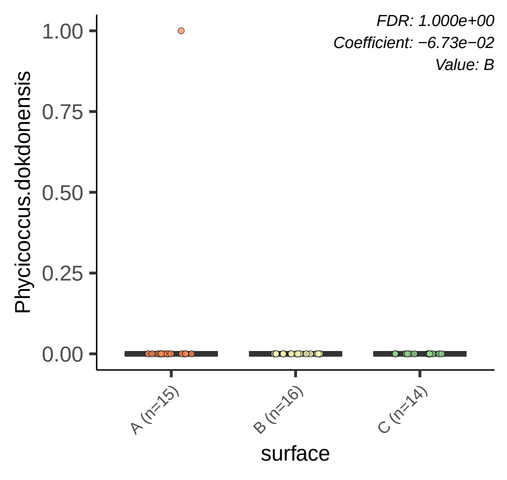

FDR: 1.000e+00
Coefficient: −6.73e−02
Value: B
Phycicoccus.dokdonensis
1.00
0.75
0.50
0.25
0.00
A (n=15)
B (n=16)
C (n=14)
surface

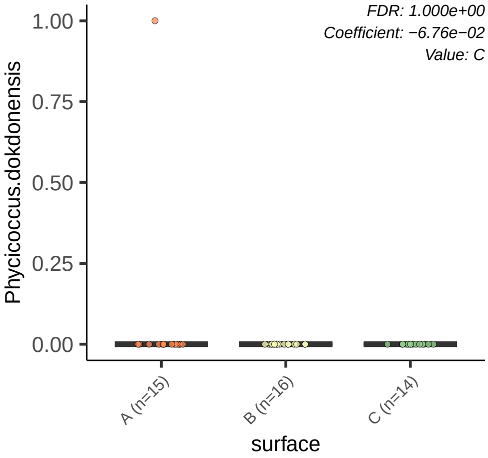
FDR: 1.000e+00
Coefficient: −6.76e−02
Value: C
Phycicoccus.dokdonensis
1.00
0.75
0.50
0.25
0.00
A (n=15)
B (n=16)
C (n=14)
surface

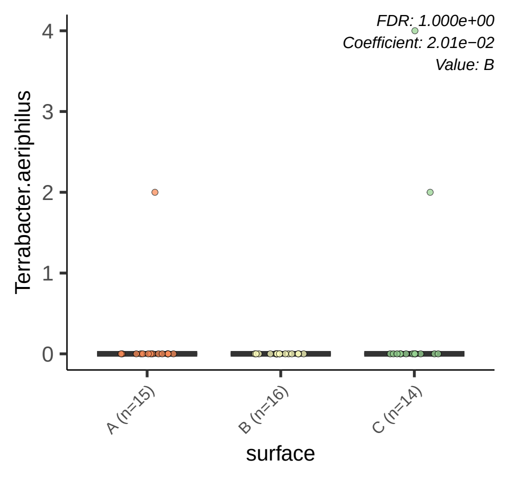

FDR: 1.000e+00
Coefficient: 2.01e−02
Value: B
Terrabacter.aeriphilus
4
3
2
1
0
A (n=15)
B (n=16)
C (n=14)
surface

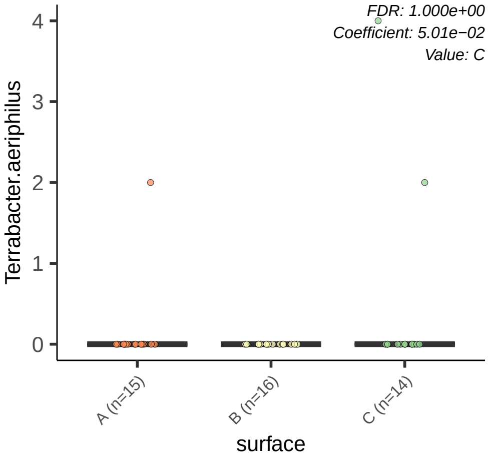
FDR: 1.000e+00
Coefficient: 5.01e−02
Value: C
Terrabacter.aeriphilus
0
1
2
3
4
A (n=15)
B (n=16)
C (n=14)
surface

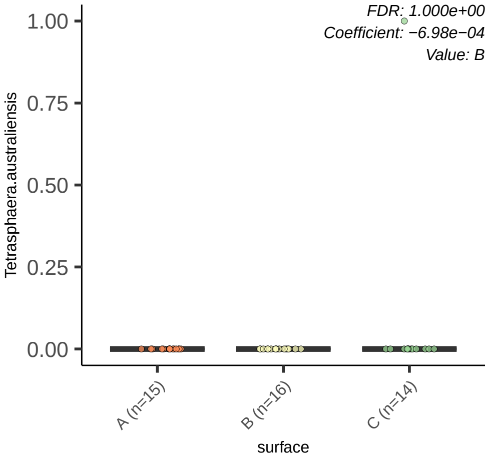
FDR: 1.000e+00
Coefficient: −6.98e−04
Value: B
Tetrasphaera.australiensis
1.00
0.75
0.50
0.25
0.00
A (n=15)
B (n=16)
C (n=14)
surface

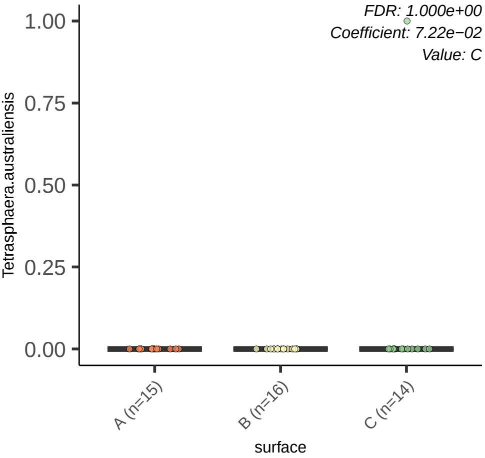

FDR: 1.000e+00
Coefficient: 7.22e−02
Value: C
Tetrasphaera.australiensis
1.00
0.75
0.50
0.25
0.00
A (n=15)
B (n=16)
C (n=14)
surface

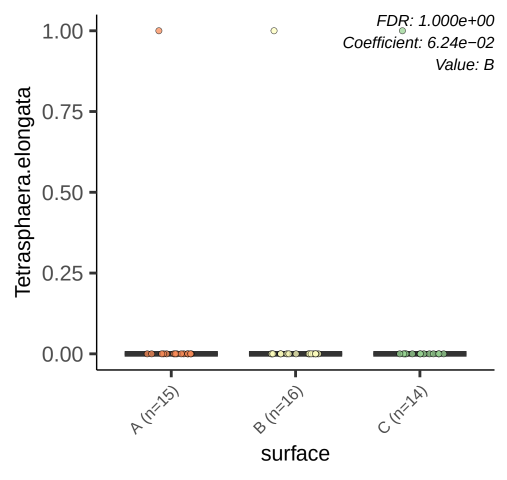

FDR: 1.000e+00
Coefficient: 6.24e−02
Value: B
Tetrasphaera.elongata
1.00
0.75
0.50
0.25
0.00
A (n=15)
B (n=16)
C (n=14)
surface

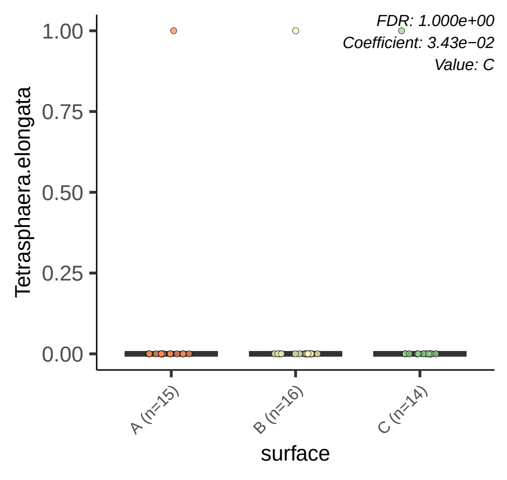
FDR: 1.000e+00
Coefficient: 3.43e−02
Value: C
1.00
0.75
0.50
0.25
0.00
Tetrasphaera.elongata
A (n=15)
B (n=16)
C (n=14)
surface

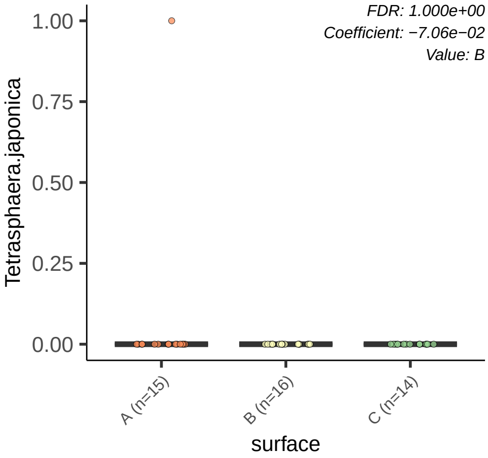
FDR: 1.000e+00
Coefficient: −7.06e−02
Value: B
Tetrasphaera.japonica
1.00
0.75
0.50
0.25
0.00
A (n=15)
B (n=16)
C (n=14)
surface

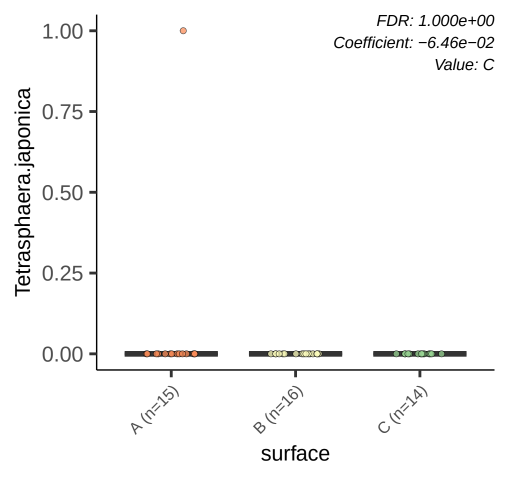
FDR: 1.000e+00
Coefficient: −6.46e−02
Value: C
Tetrasphaera.japonica
1.00
0.75
0.50
0.25
0.00
A (n=15)
B (n=16)
C (n=14)
surface

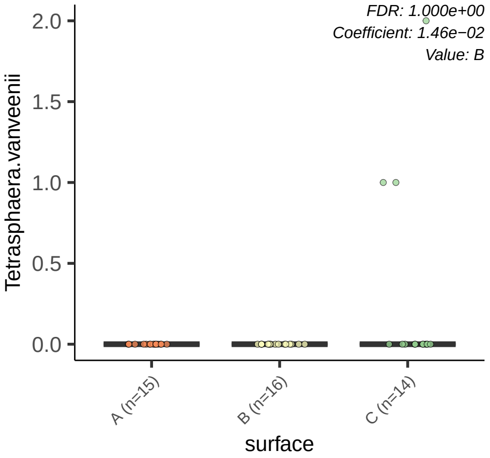
FDR: 1.000e+00
Coefficient: 1.46e−02
Value: B
Tetrasphaera.vanveenii
2.0
1.5
1.0
0.5
0.0
A (n=15)
B (n=16)
C (n=14)
surface

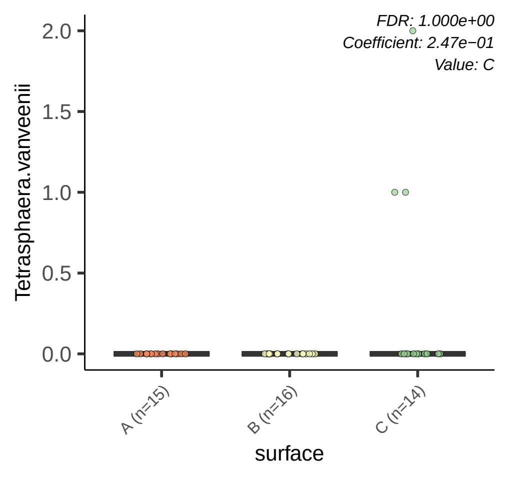
FDR: 1.000e+00
Coefficient: 2.47e−01
Value: C
Tetrasphaera.vanveenii
2.0
1.5
1.0
0.5
0.0
A (n=15)
B (n=16)
C (n=14)
surface

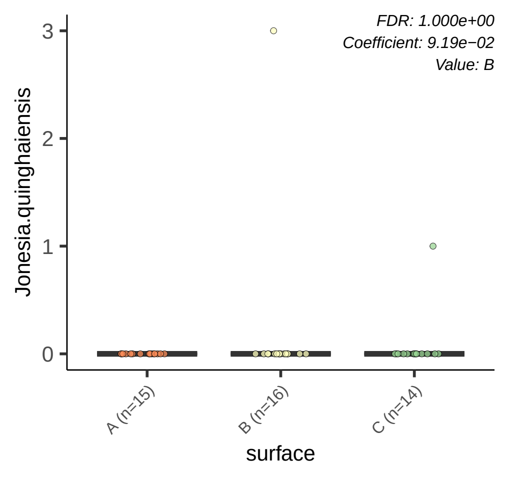

FDR: 1.000e+00
Coefficient: 9.19e−02
Value: B
Jonesia.quinghaiensis
0
1
2
3
A (n=15)
B (n=16)
C (n=14)
surface

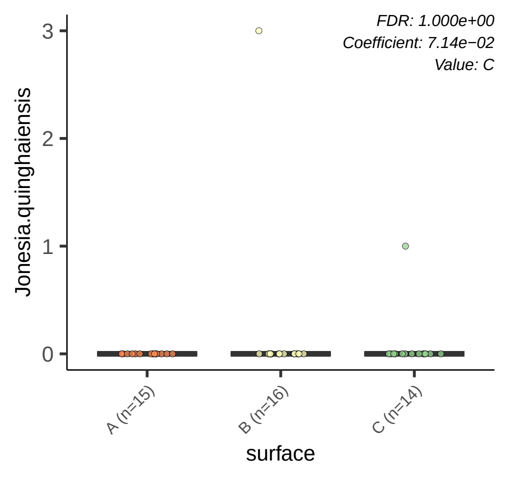
FDR: 1.000e+00
Coefficient: 7.14e−02
Value: C
Jonesia.quinghaiensis
3
2
1
0
A (n=15)
B (n=16)
C (n=14)
surface

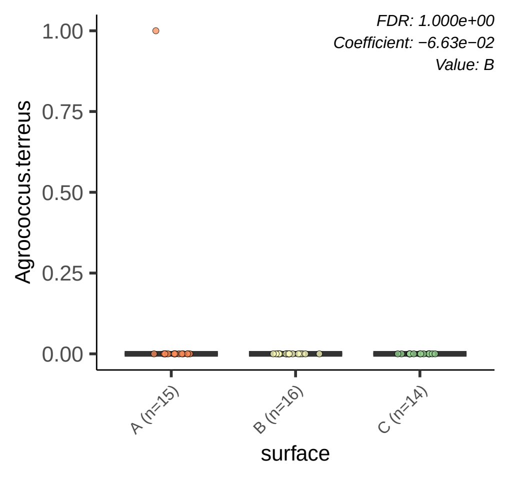

FDR: 1.000e+00
Coefficient: −6.63e−02
Value: B
Agrococcus.terreus
1.00
0.75
0.50
0.25
0.00
A (n=15)
B (n=16)
C (n=14)
surface

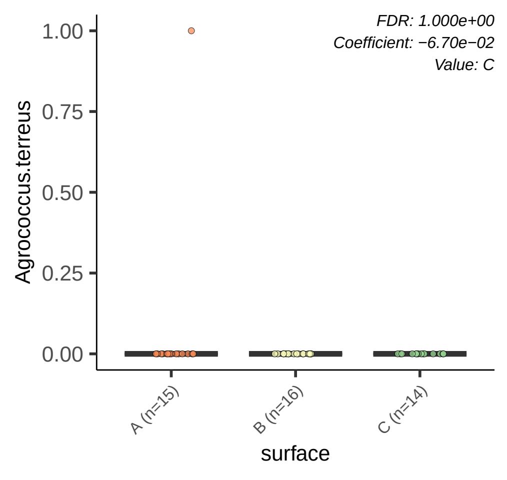
FDR: 1.000e+00
Coefficient: −6.70e−02
Value: C
Agrococcus.terreus
1.00
0.75
0.50
0.25
0.00
A (n=15)
B (n=16)
C (n=14)
surface

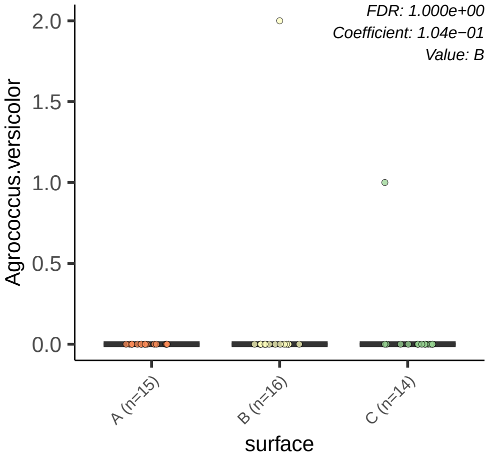
FDR: 1.000e+00
Coefficient: 1.04e−01
Value: B
Agrococcus.versicolor
2.0
1.5
1.0
0.5
0.0
A (n=15)
B (n=16)
C (n=14)
surface

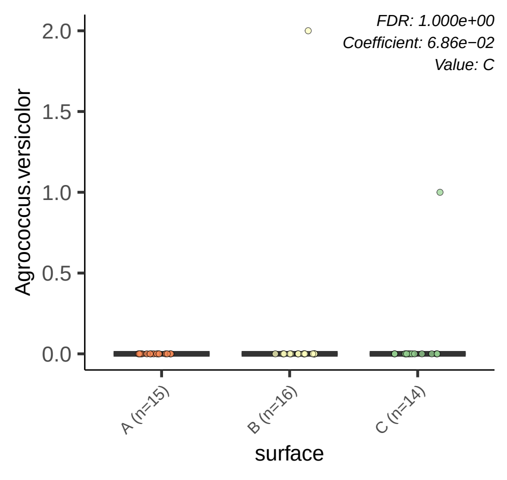

FDR: 1.000e+00
Coefficient: 6.86e−02
Value: C
Agrococcus.versicolor
2.0
1.5
1.0
0.5
0.0
A (n=15)
B (n=16)
C (n=14)
surface

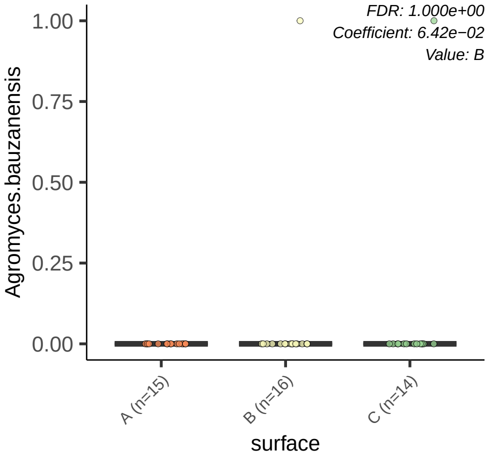
FDR: 1.000e+00
Coefficient: 6.42e−02
Value: B
Agromyces.bauzanensis
1.00
0.75
0.50
0.25
0.00
A (n=15)
B (n=16)
C (n=14)
surface

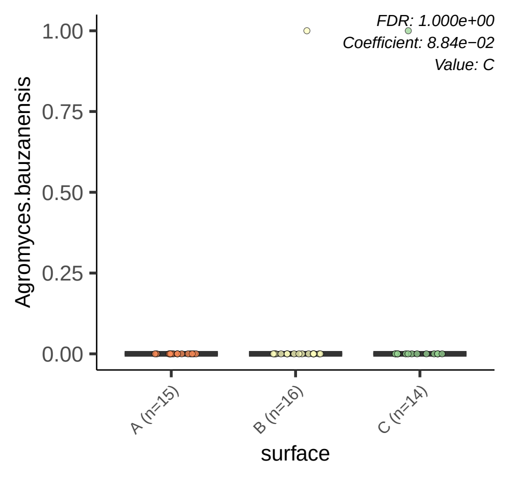
FDR: 1.000e+00
Coefficient: 8.84e−02
Value: C
Agromyces.bauzanensis
1.00
0.75
0.50
0.25
0.00
A (n=15)
B (n=16)
C (n=14)
surface

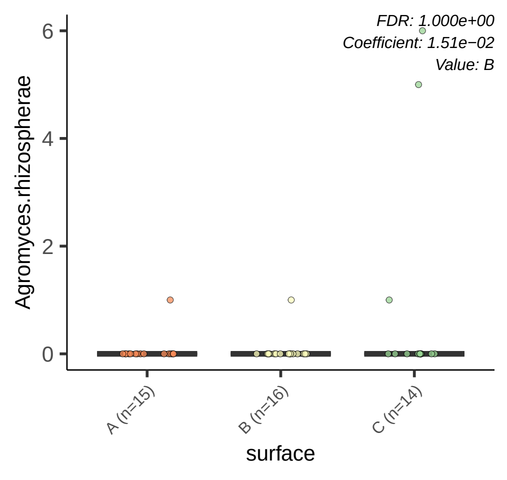

FDR: 1.000e+00
Coefficient: 1.51e−02
Value: B
Agromyces.rhizospherae
6
4
2
0
A (n=15)
B (n=16)
C (n=14)
surface

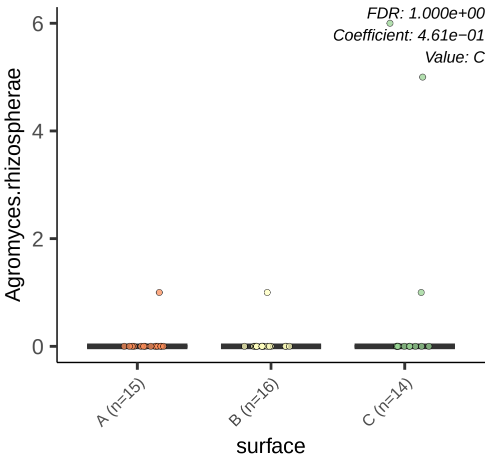

FDR: 1.000e+00
Coefficient: 4.61e−01
Value: C
Agromyces.rhizospherae
6
4
2
0
A (n=15)
B (n=16)
C (n=14)
surface

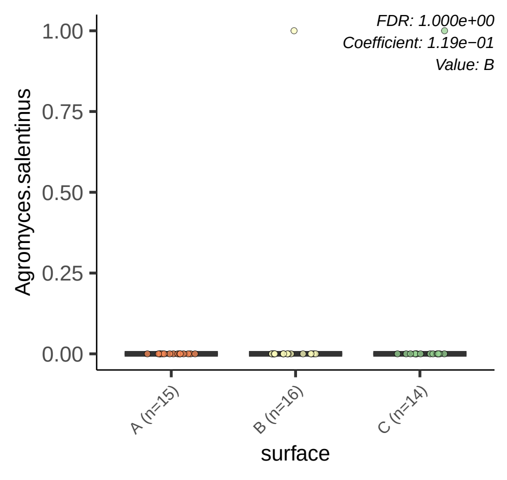

FDR: 1.000e+00
Coefficient: 1.19e−01
Value: B
Agromyces.salentinus
1.00
0.75
0.50
0.25
0.00
A (n=15)
B (n=16)
C (n=14)
surface

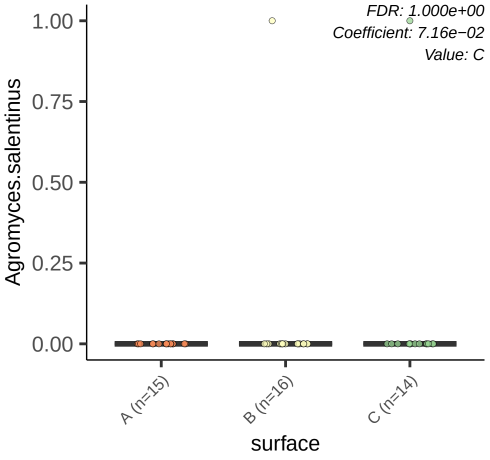
FDR: 1.000e+00
Coefficient: 7.16e−02
Value: C
Agromyces.salentinus
1.00
0.75
0.50
0.25
0.00
A (n=15)
B (n=16)
C (n=14)
surface

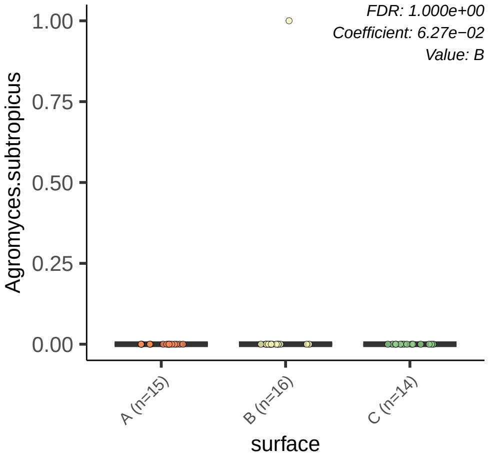

FDR: 1.000e+00
Coefficient: 6.27e−02
Value: B
Agromyces.subtropicus
1.00
0.75
0.50
0.25
0.00
A (n=15)
B (n=16)
C (n=14)
surface

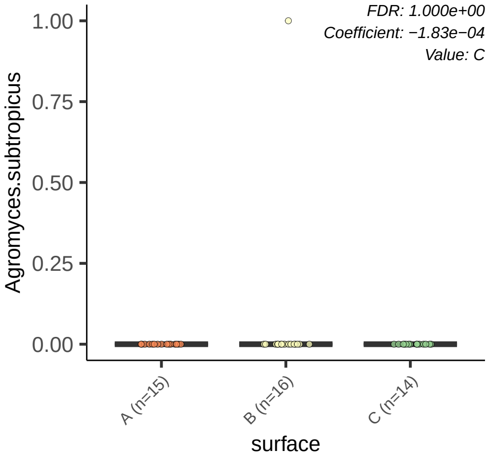

FDR: 1.000e+00
Coefficient: −1.83e−04
Value: C
Agromyces.subtropicus
1.00
0.75
0.50
0.25
0.00
A (n=15)
B (n=16)
C (n=14)
surface

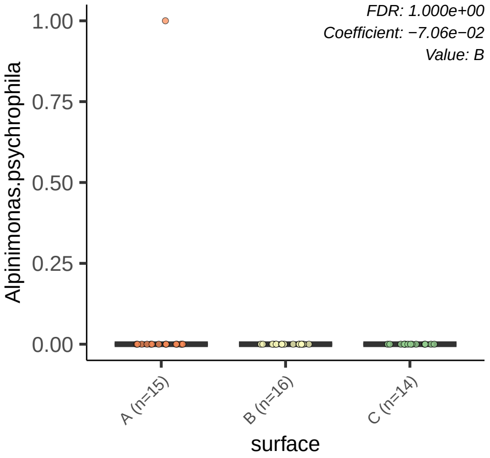

FDR: 1.000e+00
Coefficient: −7.06e−02
Value: B
Alpinimonas.psychrophila
1.00
0.75
0.50
0.25
0.00
A (n=15)
B (n=16)
C (n=14)
surface

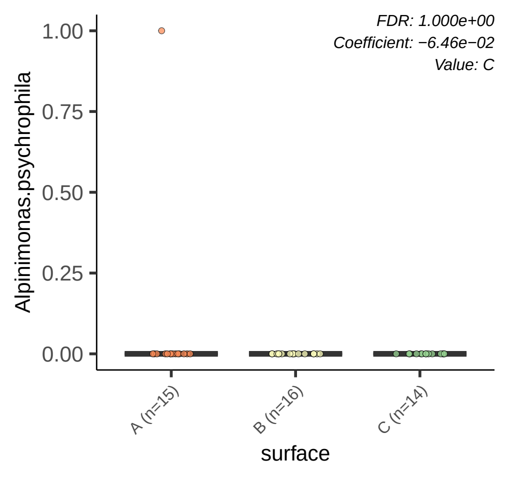
FDR: 1.000e+00
Coefficient: −6.46e−02
Value: C
Alpinimonas.psychrophila
1.00
0.75
0.50
0.25
0.00
A (n=15)
B (n=16)
C (n=14)
surface

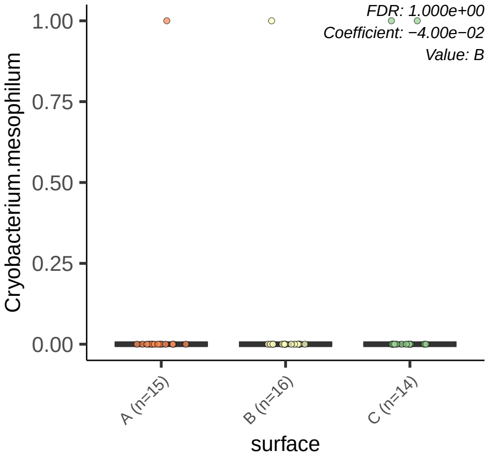
FDR: 1.000e+00
Coefficient: −4.00e−02
Value: B
Cryobacterium.mesophilum
1.00
0.75
0.50
0.25
0.00
A (n=15)
B (n=16)
C (n=14)
surface

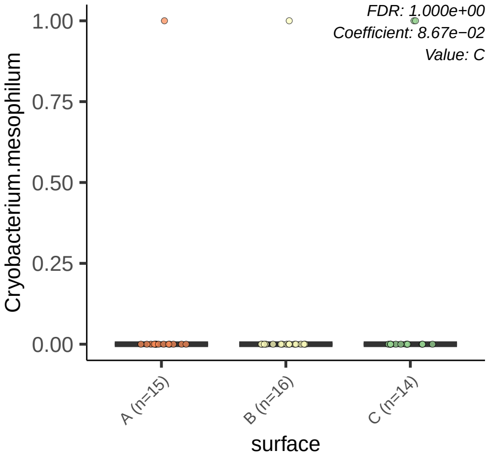

FDR: 1.000e+00
Coefficient: 8.67e−02
Value: C
Cryobacterium.mesophilum
1.00
0.75
0.50
0.25
0.00
A (n=15)
B (n=16)
C (n=14)
surface

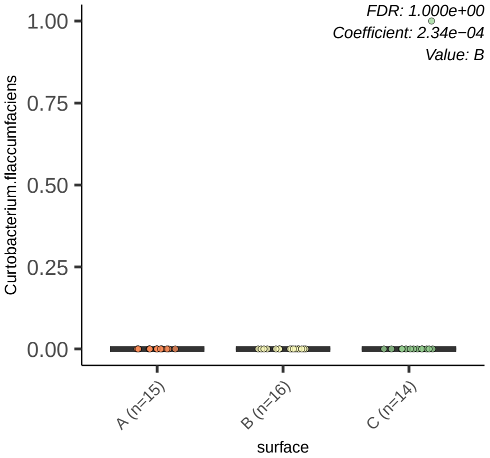
FDR: 1.000e+00
Coefficient: 2.34e−04
Value: B
1.00
0.75
0.50
0.25
0.00
Curtobacterium.flaccumfaciens
A (n=15)
B (n=16)
C (n=14)
surface

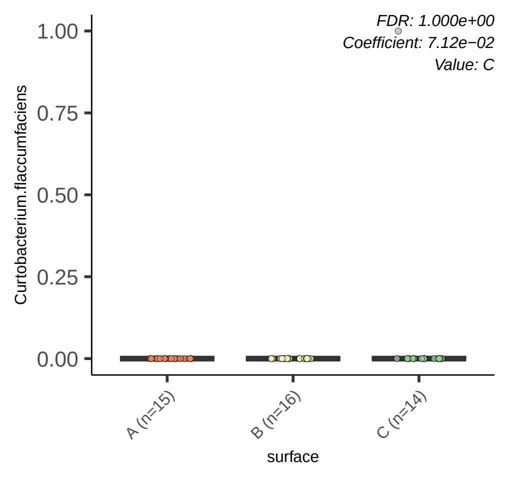
FDR: 1.000e+00
Coefficient: 7.12e−02
Value: C
1.00
0.75
0.50
0.25
0.00
Curtobacterium.flaccumfaciens
A (n=15)
B (n=16)
C (n=14)
surface

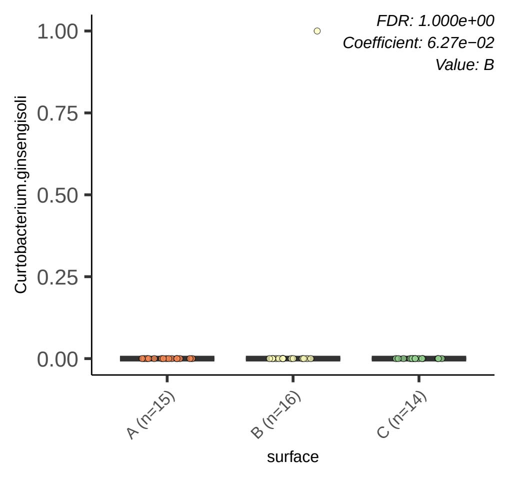
FDR: 1.000e+00
Coefficient: 6.27e−02
Value: B
1.00
0.75
0.50
0.25
0.00
Curtobacterium.ginsengisoli
A (n=15)
B (n=16)
C (n=14)
surface

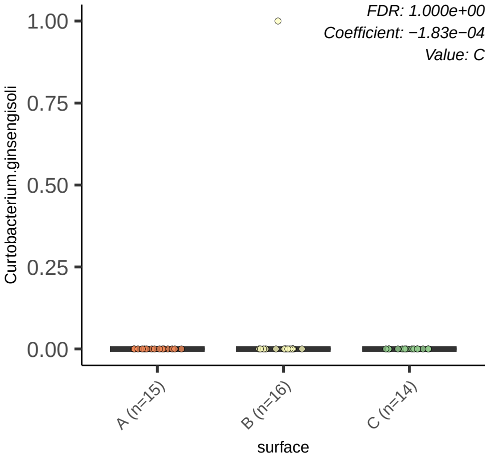

FDR: 1.000e+00
Coefficient: −1.83e−04
Value: C
1.00
0.75
0.50
0.25
0.00
Curtobacterium.ginsengisoli
A (n=15)
B (n=16)
C (n=14)
surface

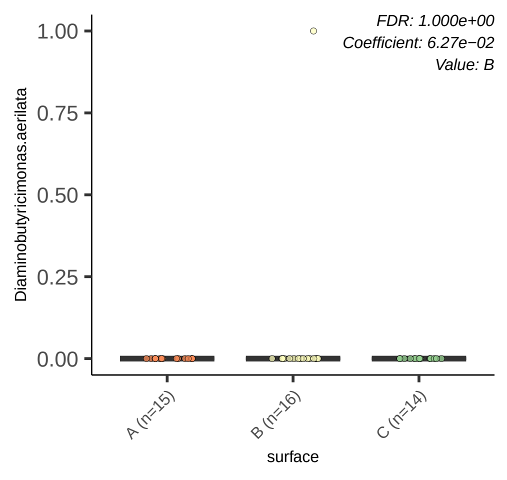
FDR: 1.000e+00
Coefficient: 6.27e−02
Value: B
Diaminobutyricimonas.aerilata
1.00
0.75
0.50
0.25
0.00
A (n=15)
B (n=16)
C (n=14)
surface

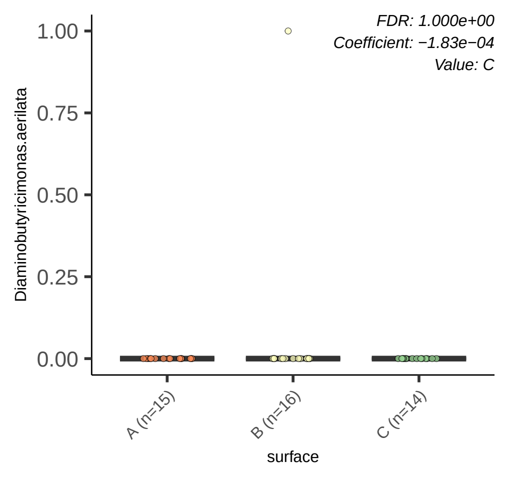
FDR: 1.000e+00
Coefficient: −1.83e−04
Value: C
1.00
0.75
0.50
0.25
0.00
Diaminobutyricimonas.aerilata
A (n=15)
B (n=16)
C (n=14)
surface

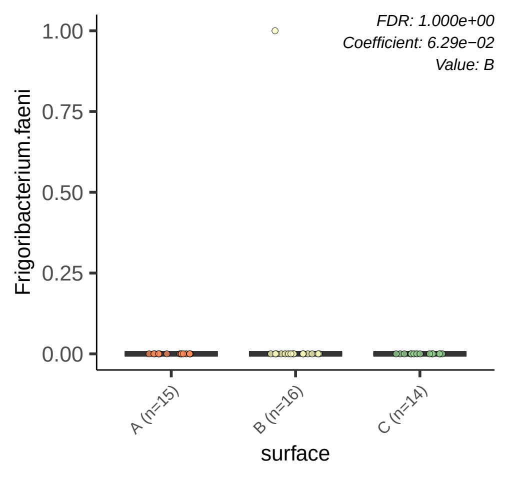
FDR: 1.000e+00
Coefficient: 6.29e−02
Value: B
Frigoribacterium.faeni
1.00
0.75
0.50
0.25
0.00
A (n=15)
B (n=16)
C (n=14)
surface

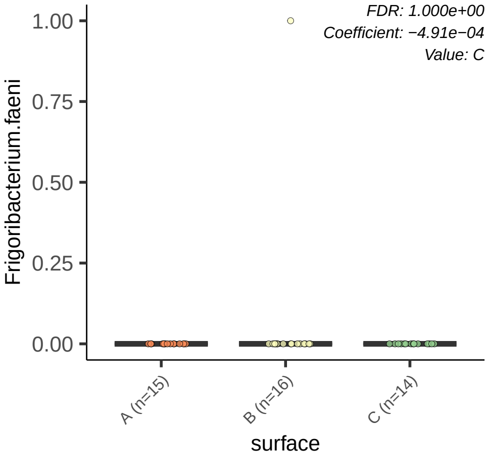
FDR: 1.000e+00
Coefficient: −4.91e−04
Value: C
Frigoribacterium.faeni
1.00
0.75
0.50
0.25
0.00
A (n=15)
B (n=16)
C (n=14)
surface

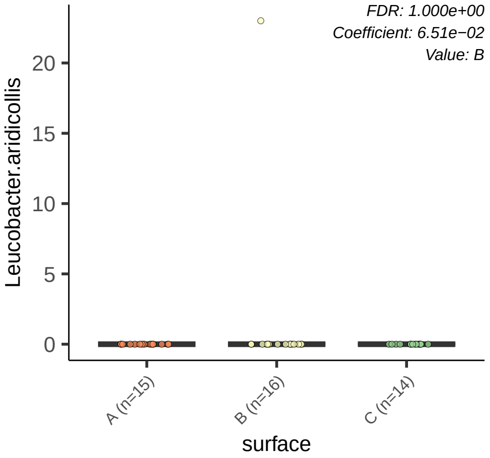

FDR: 1.000e+00
Coefficient: 6.51e−02
Value: B
Leucobacter.aridicollis
0
5
10
15
20
A (n=15)
B (n=16)
C (n=14)
surface

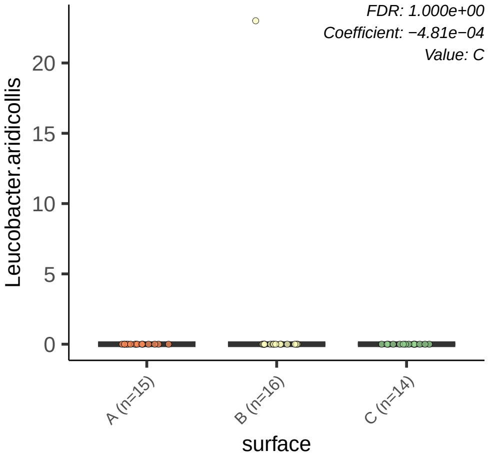

FDR: 1.000e+00
Coefficient: −4.81e−04
Value: C
Leucobacter.aridicollis
20
15
10
5
0
A (n=15)
B (n=16)
C (n=14)
surface

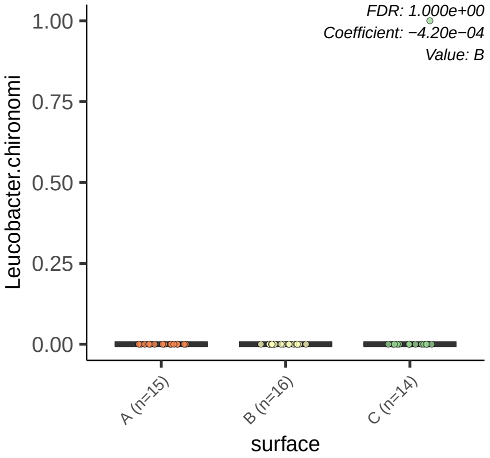

FDR: 1.000e+00
Coefficient: −4.20e−04
Value: B
Leucobacter.chironomi
1.00
0.75
0.50
0.25
0.00
A (n=15)
B (n=16)
C (n=14)
surface

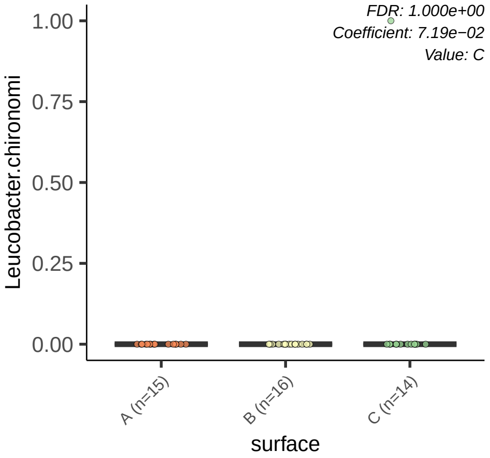
FDR: 1.000e+00
Coefficient: 7.19e−02
Value: C
Leucobacter.chironomi
1.00
0.75
0.50
0.25
0.00
A (n=15)
B (n=16)
C (n=14)
surface

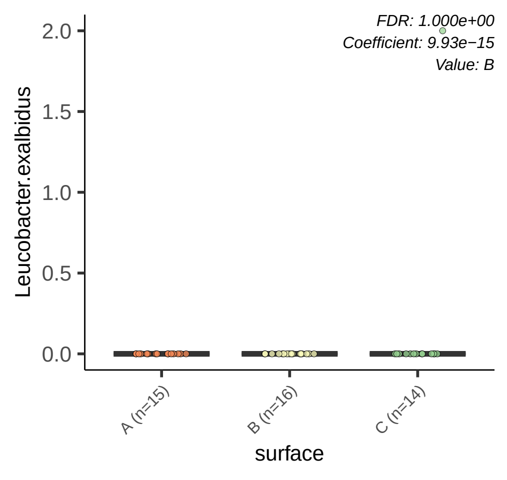
FDR: 1.000e+00
Coefficient: 9.93e−15
Value: B
Leucobacter.exalbidus
2.0
1.5
1.0
0.5
0.0
A (n=15)
B (n=16)
C (n=14)
surface

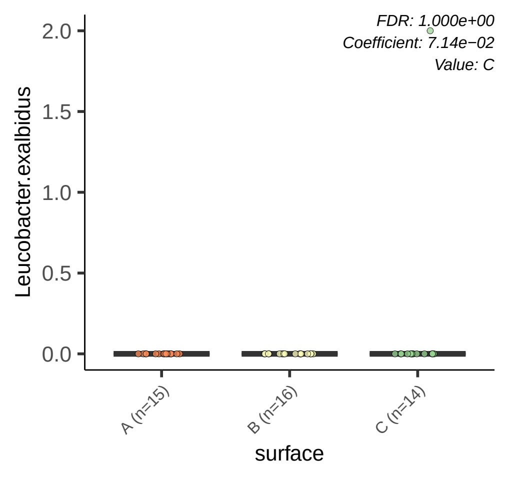
FDR: 1.000e+00
Coefficient: 7.14e−02
Value: C
Leucobacter.exalbidus
2.0
1.5
1.0
0.5
0.0
A (n=15)
B (n=16)
C (n=14)
surface

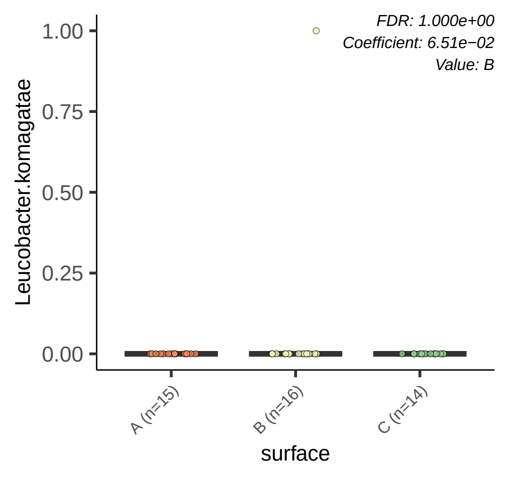

FDR: 1.000e+00
Coefficient: 6.51e−02
Value: B
Leucobacter.komagatae
1.00
0.75
0.50
0.25
0.00
A (n=15)
B (n=16)
C (n=14)
surface

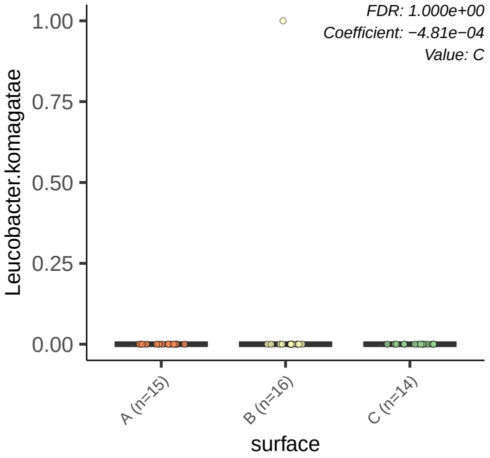
FDR: 1.000e+00
Coefficient: −4.81e−04
Value: C
Leucobacter.komagatae
1.00
0.75
0.50
0.25
0.00
A (n=15)
B (n=16)
C (n=14)
surface

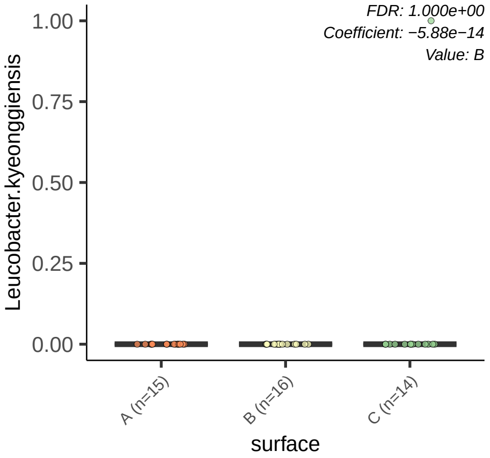

FDR: 1.000e+00
Coefficient: −5.88e−14
Value: B
Leucobacter.kyeonggiensis
1.00
0.75
0.50
0.25
0.00
A (n=15)
B (n=16)
C (n=14)
surface

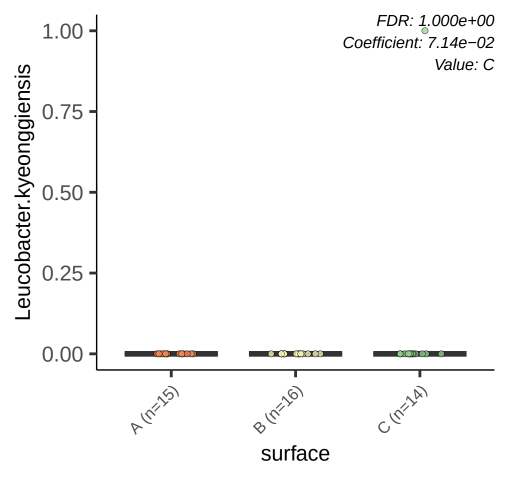

FDR: 1.000e+00
Coefficient: 7.14e−02
Value: C
Leucobacter.kyeonggiensis
1.00
0.75
0.50
0.25
0.00
A (n=15)
B (n=16)
C (n=14)
surface

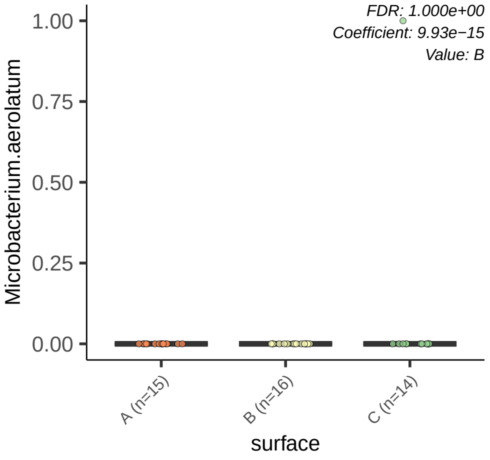

FDR: 1.000e+00
Coefficient: 9.93e−15
Value: B
1.00
0.75
0.50
0.25
0.00
Microbacterium.aerolatum
A (n=15)
B (n=16)
C (n=14)
surface

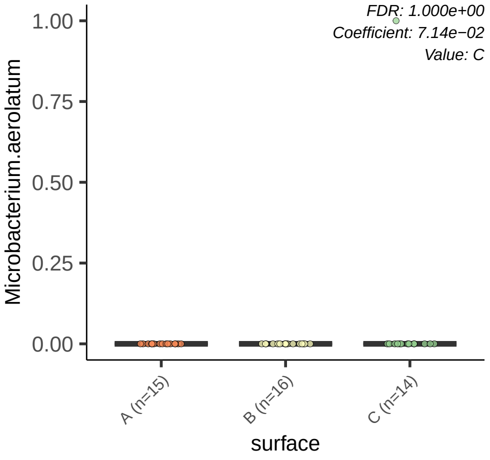

FDR: 1.000e+00
Coefficient: 7.14e−02
Value: C
Microbacterium.aerolatum
1.00
0.75
0.50
0.25
0.00
A (n=15)
B (n=16)
C (n=14)
surface

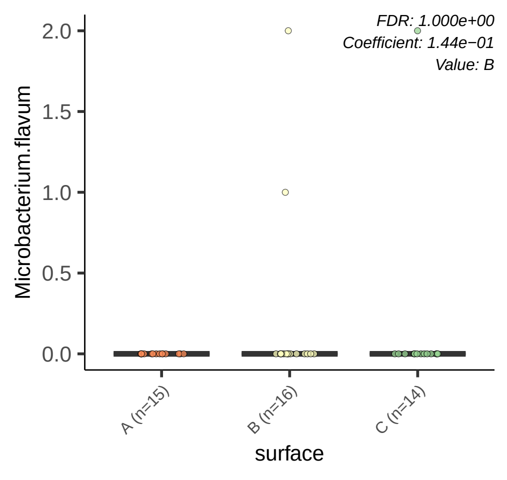
FDR: 1.000e+00
Coefficient: 1.44e−01
Value: B
Microbacterium.flavum
2.0
1.5
1.0
0.5
0.0
A (n=15)
B (n=16)
C (n=14)
surface

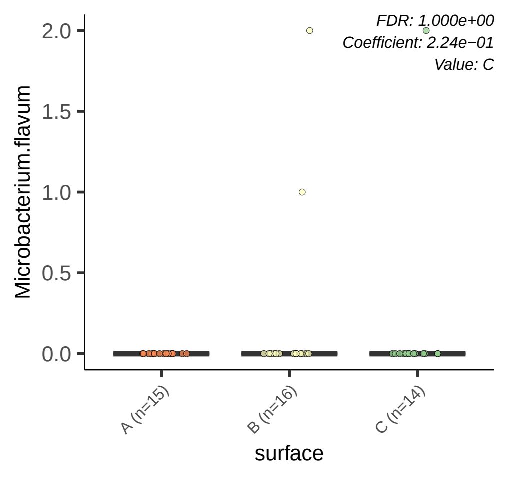
FDR: 1.000e+00
Coefficient: 2.24e−01
Value: C
Microbacterium.flavum
2.0
1.5
1.0
0.5
0.0
A (n=15)
B (n=16)
C (n=14)
surface

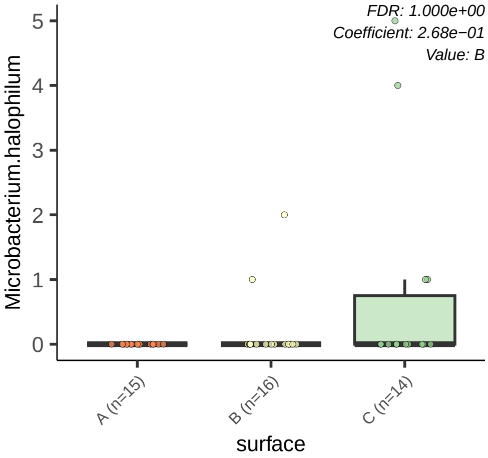

FDR: 1.000e+00
Coefficient: 2.68e−01
Value: B
Microbacterium.halophilum
0
1
2
3
4
5
A (n=15)
B (n=16)
C (n=14)
surface

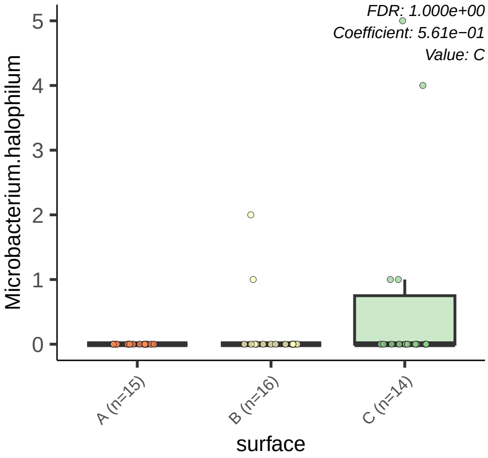

FDR: 1.000e+00
Coefficient: 5.61e−01
Value: C
Microbacterium.halophilum
0
1
2
3
4
5
A (n=15)
B (n=16)
C (n=14)
surface

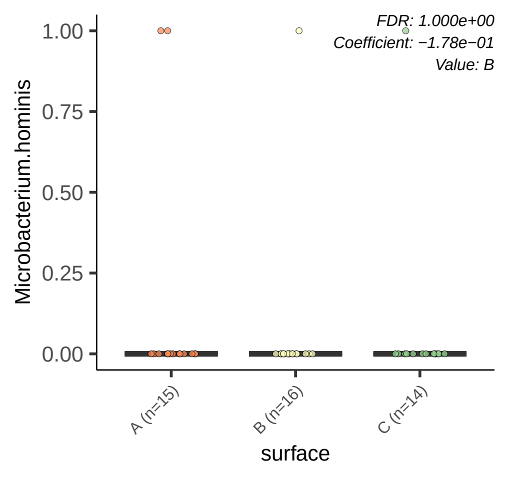

FDR: 1.000e+00
Coefficient: −1.78e−01
Value: B
Microbacterium.hominis
1.00
0.75
0.50
0.25
0.00
A (n=15)
B (n=16)
C (n=14)
surface

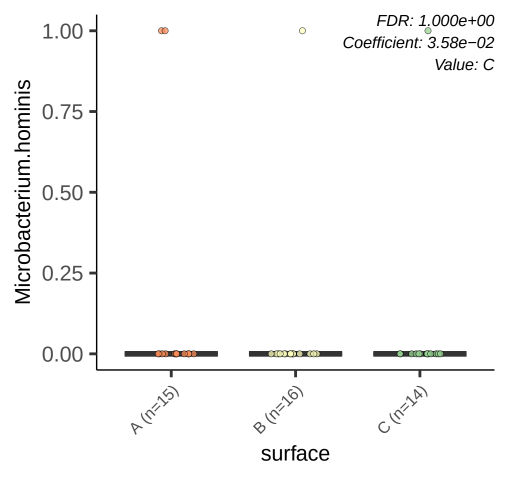
FDR: 1.000e+00
Coefficient: 3.58e−02
Value: C
Microbacterium.hominis
1.00
0.75
0.50
0.25
0.00
A (n=15)
B (n=16)
C (n=14)
surface

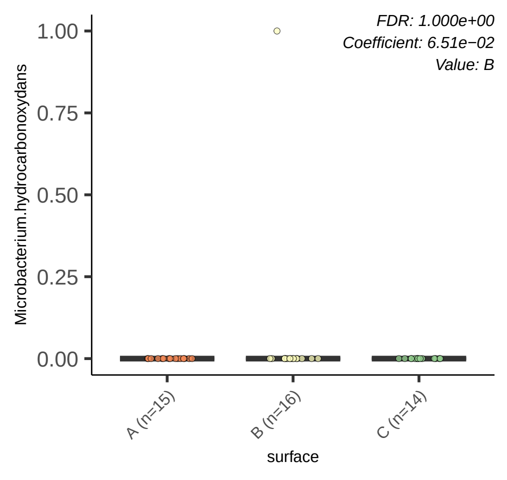
FDR: 1.000e+00
Coefficient: 6.51e−02
Value: B
Microbacterium.hydrocarbonoxydans
1.00
0.75
0.50
0.25
0.00
A (n=15)
B (n=16)
C (n=14)
surface

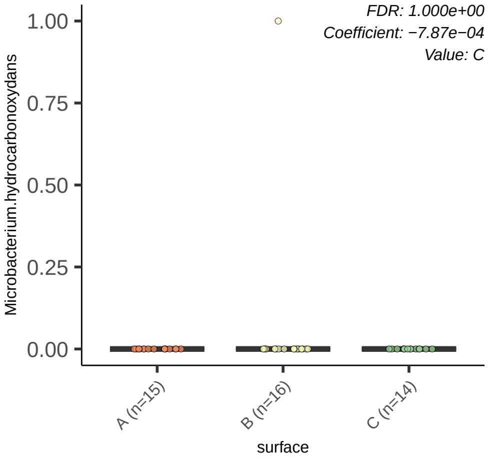
FDR: 1.000e+00
Coefficient: −7.87e−04
Value: C
Microbacterium.hydrocarbonoxydans
1.00
0.75
0.50
0.25
0.00
A (n=15)
B (n=16)
C (n=14)
surface

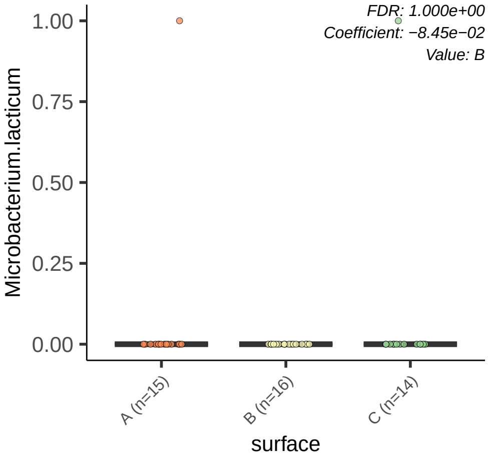

FDR: 1.000e+00
Coefficient: −8.45e−02
Value: B
Microbacterium.lacticum
1.00
0.75
0.50
0.25
0.00
A (n=15)
B (n=16)
C (n=14)
surface

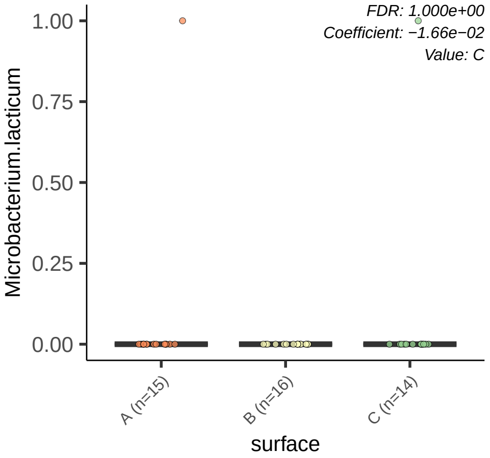
FDR: 1.000e+00
Coefficient: −1.66e−02
Value: C
Microbacterium.lacticum
1.00
0.75
0.50
0.25
0.00
A (n=15)
B (n=16)
C (n=14)
surface

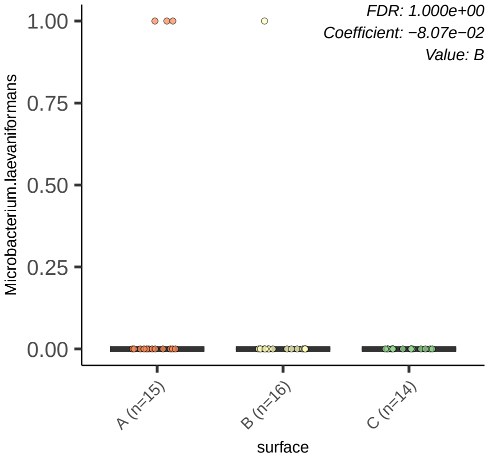
FDR: 1.000e+00
Coefficient: −8.07e−02
Value: B
Microbacterium.laevaniformans
1.00
0.75
0.50
0.25
0.00
A (n=15)
B (n=16)
C (n=14)
surface

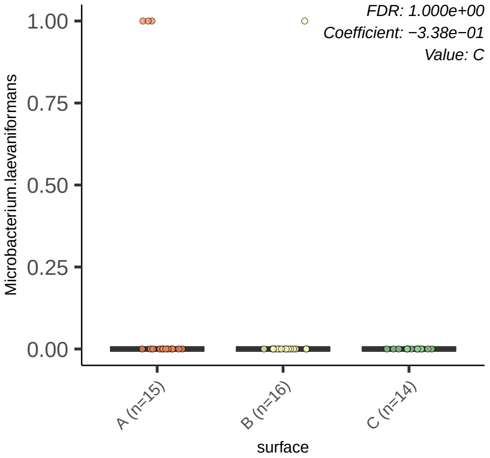

FDR: 1.000e+00
Coefficient: −3.38e−01
Value: C
Microbacterium.laevaniformans
1.00
0.75
0.50
0.25
0.00
A (n=15)
B (n=16)
C (n=14)
surface

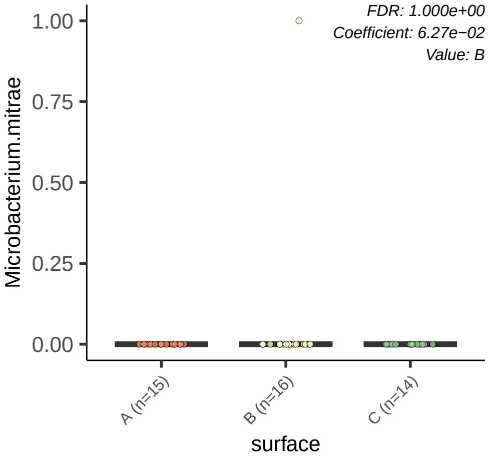

FDR: 1.000e+00
Coefficient: 6.27e−02
Value: B
Microbacterium.mitrae
1.00
0.75
0.50
0.25
0.00
A (n=15)
B (n=16)
C (n=14)
surface

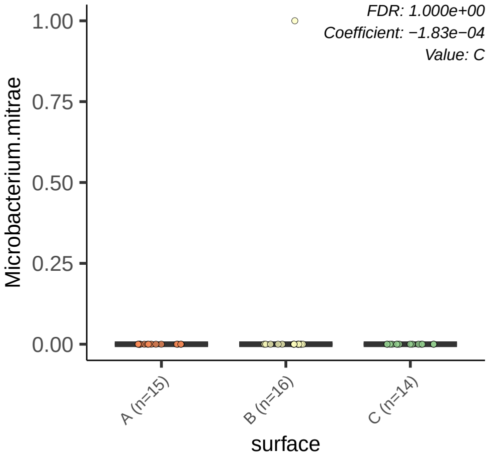

FDR: 1.000e+00
Coefficient: −1.83e−04
Value: C
Microbacterium.mitrae
1.00
0.75
0.50
0.25
0.00
A (n=15)
B (n=16)
C (n=14)
surface

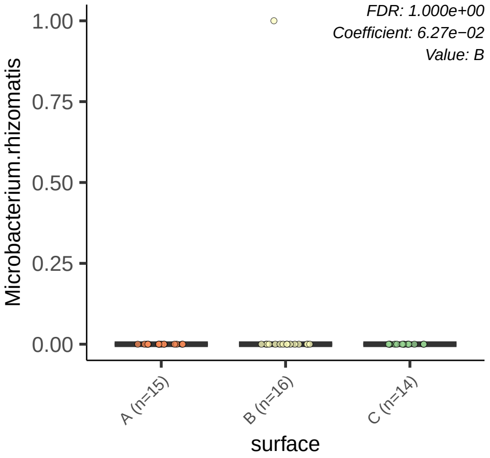

FDR: 1.000e+00
Coefficient: 6.27e−02
Value: B
Microbacterium.rhizomatis
1.00
0.75
0.50
0.25
0.00
A (n=15)
B (n=16)
C (n=14)
surface

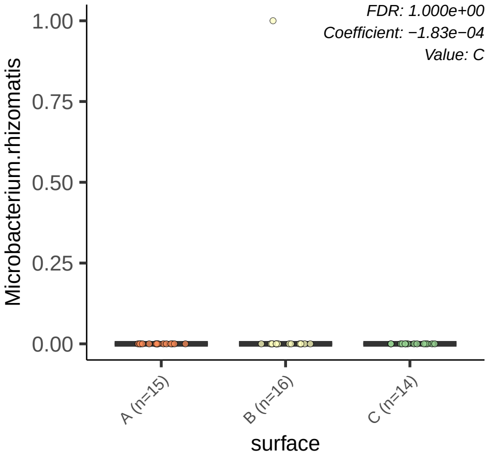

FDR: 1.000e+00
Coefficient: −1.83e−04
Value: C
Microbacterium.rhizomatis
1.00
0.75
0.50
0.25
0.00
A (n=15)
B (n=16)
C (n=14)
surface

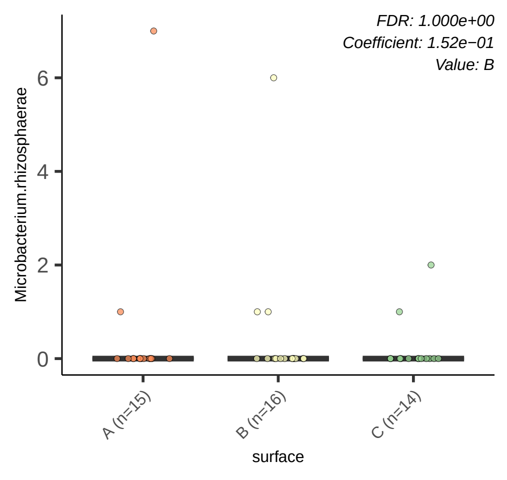
FDR: 1.000e+00
Coefficient: 1.52e−01
Value: B
Microbacterium.rhizosphaerae
0
2
4
6
A (n=15)
B (n=16)
C (n=14)
surface

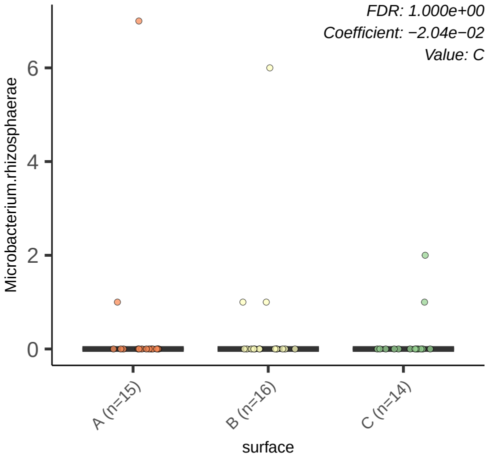
FDR: 1.000e+00
Coefficient: −2.04e−02
Value: C
Microbacterium.rhizosphaerae
0
2
4
6
A (n=15)
B (n=16)
C (n=14)
surface

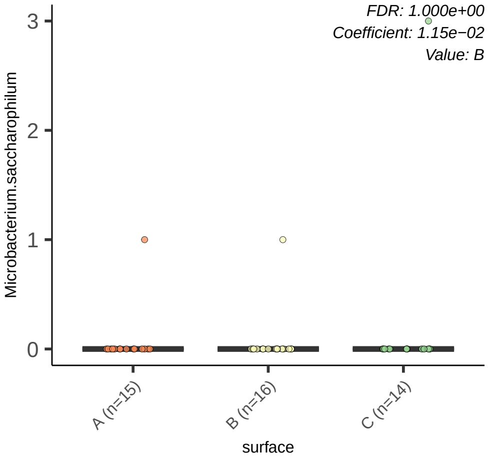
FDR: 1.000e+00
Coefficient: 1.15e−02
Value: B
Microbacterium.saccharophilum
0
1
2
3
A (n=15)
B (n=16)
C (n=14)
surface

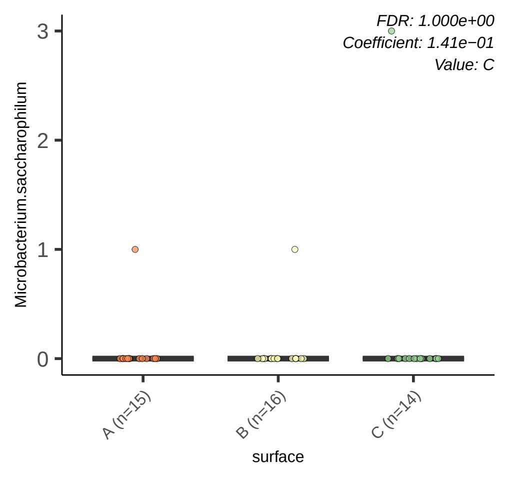

FDR: 1.000e+00
Coefficient: 1.41e−01
Value: C
Microbacterium.saccharophilum
3
2
1
0
A (n=15)
B (n=16)
C (n=14)
surface

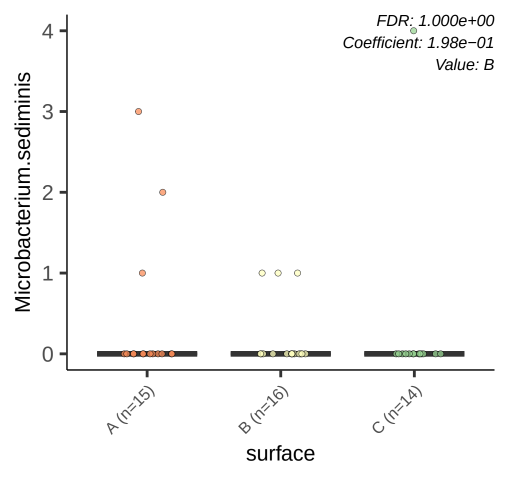
FDR: 1.000e+00
Coefficient: 1.98e−01
Value: B
Microbacterium.sedimininis
0
1
2
3
4
A (n=15)
B (n=16)
C (n=14)
surface

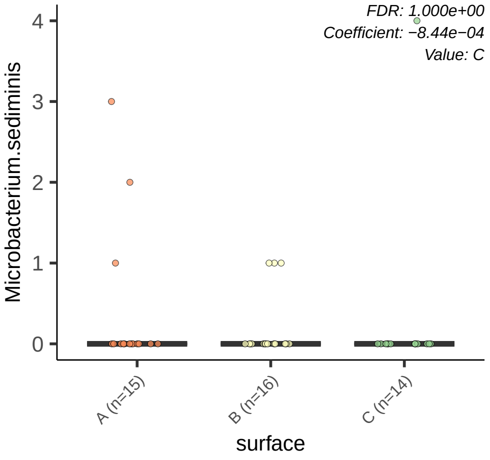

FDR: 1.000e+00
Coefficient: −8.44e−04
Value: C
Microbacterium.sedimini
0
1
2
3
4
A (n=15)
B (n=16)
C (n=14)
surface

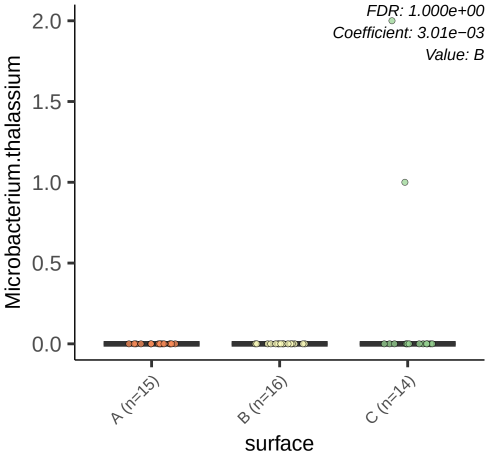

FDR: 1.000e+00
Coefficient: 3.01e−03
Value: B
2.0
1.5
1.0
0.5
0.0
Microbacterium.thalassium
A (n=15)
B (n=16)
C (n=14)
surface

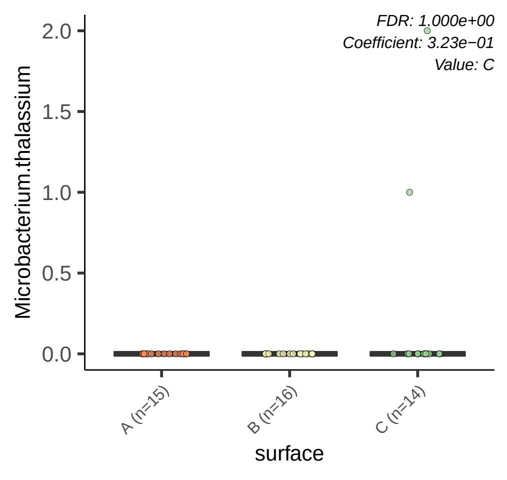
FDR: 1.000e+00
Coefficient: 3.23e−01
Value: C
Microbacterium.thalassium
2.0
1.5
1.0
0.5
0.0
A (n=15)
B (n=16)
C (n=14)
surface

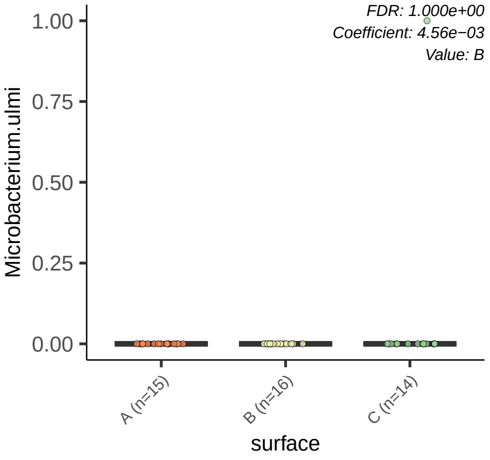

FDR: 1.000e+00
Coefficient: 4.56e−03
Value: B
Microbacterium.ulmi
1.00
0.75
0.50
0.25
0.00
A (n=15)
B (n=16)
C (n=14)
surface

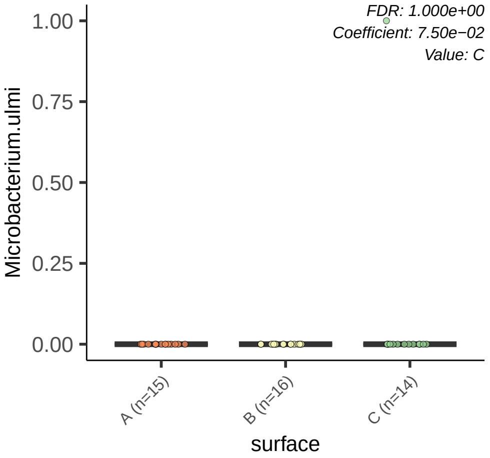

FDR: 1.000e+00
Coefficient: 7.50e−02
Value: C
Microbacterium.ulmi
1.00
0.75
0.50
0.25
0.00
A (n=15)
B (n=16)
C (n=14)
surface

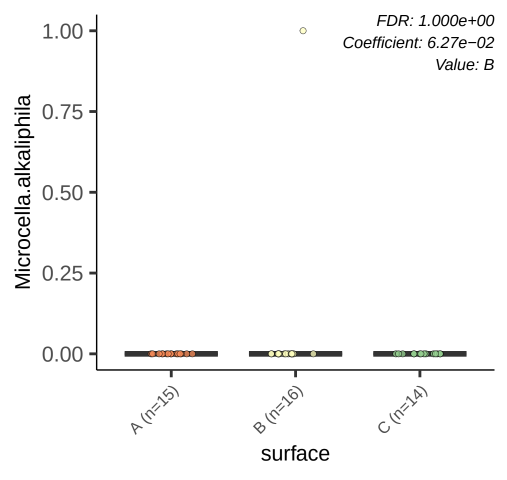

FDR: 1.000e+00
Coefficient: 6.27e−02
Value: B
Microcella.alkaliphila
1.00
0.75
0.50
0.25
0.00
A (n=15)
B (n=16)
C (n=14)
surface

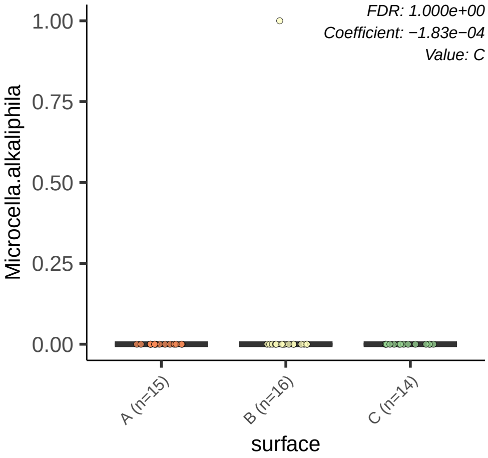
FDR: 1.000e+00
Coefficient: −1.83e−04
Value: C
Microcella.alkaliphila
1.00
0.75
0.50
0.25
0.00
A (n=15)
B (n=16)
C (n=14)
surface

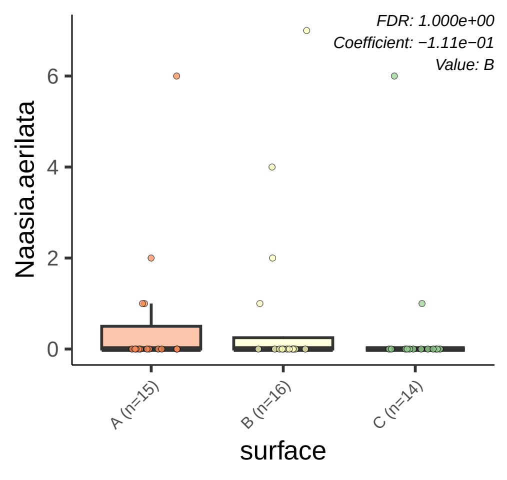
FDR: 1.000e+00
Coefficient: −1.11e−01
Value: B
Naasia.aerilata
0
2
4
6
A (n=15)
B (n=16)
C (n=14)
surface

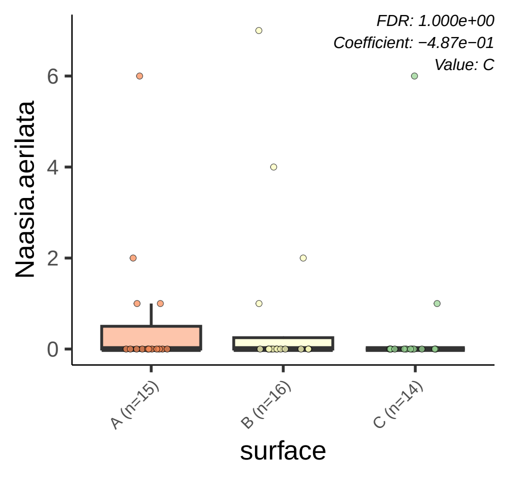
FDR: 1.000e+00
Coefficient: −4.87e−01
Value: C
Naasia.aerilata
0
2
4
6
A (n=15)
B (n=16)
C (n=14)
surface

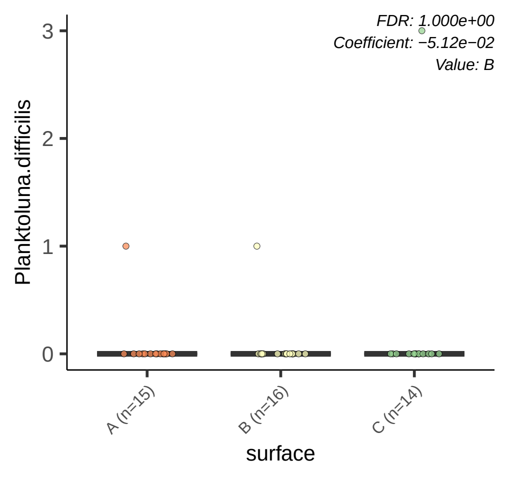
FDR: 1.000e+00
Coefficient: −5.12e−02
Value: B
Planktoluna.difficilis
0
1
2
3
A (n=15)
B (n=16)
C (n=14)
surface

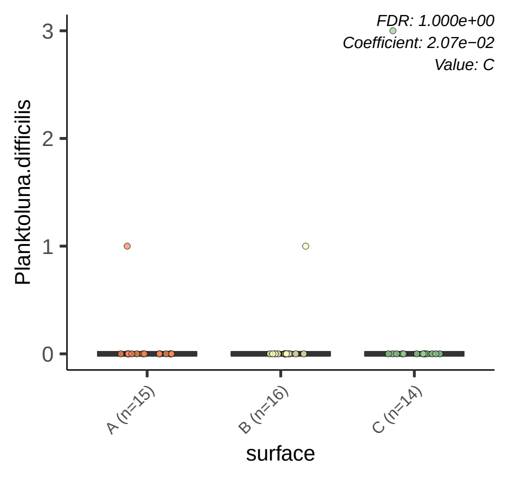

FDR: 1.000e+00
Coefficient: 2.07e−02
Value: C
Planktoluna.difficilis
0
1
2
3
A (n=15)
B (n=16)
C (n=14)
surface

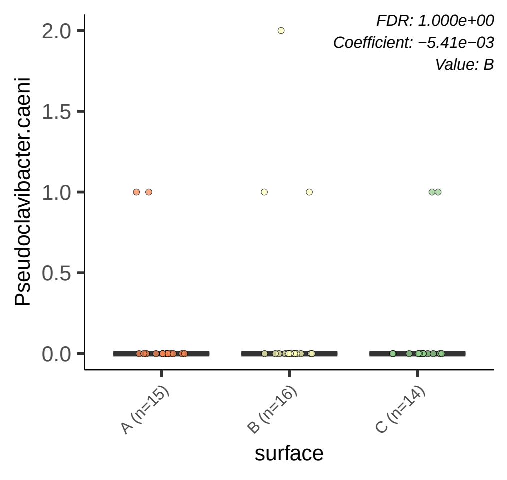

FDR: 1.000e+00
Coefficient: −5.41e−03
Value: B
2.0
1.5
1.0
0.5
0.0
Pseudoclavibacter.caeni
A (n=15)
B (n=16)
C (n=14)
surface

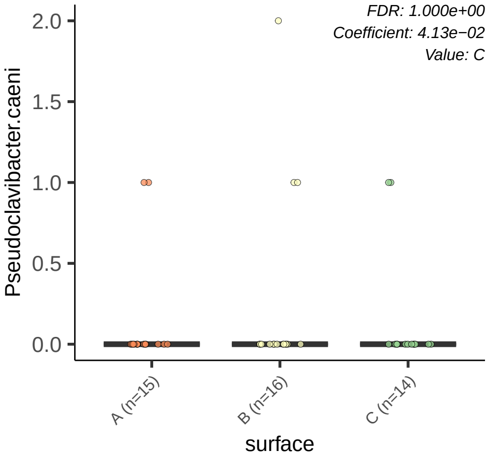

FDR: 1.000e+00
Coefficient: 4.13e−02
Value: C
Pseudoclavibacter.caeni
2.0
1.5
1.0
0.5
0.0
A (n=15)
B (n=16)
C (n=14)
surface

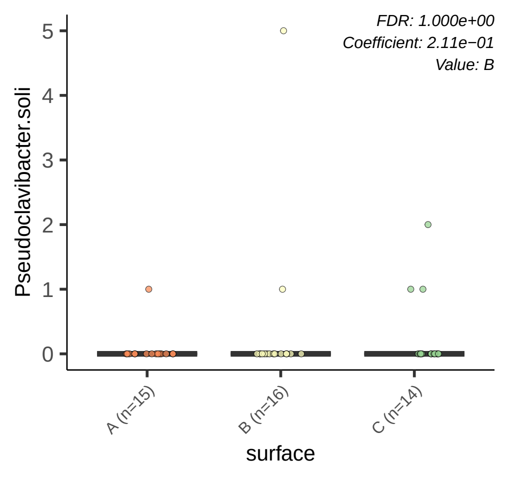

FDR: 1.000e+00
Coefficient: 2.11e−01
Value: B
Pseudoclavibacter.soli
0
1
2
3
4
5
A (n=15)
B (n=16)
C (n=14)
surface

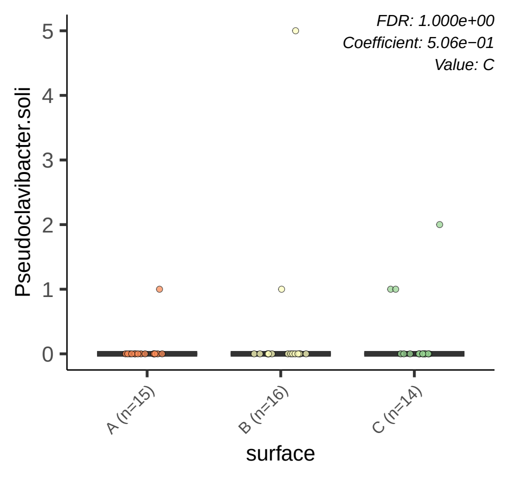
FDR: 1.000e+00
Coefficient: 5.06e−01
Value: C
Pseudoclavibacter.soli
0
1
2
3
4
5
A (n=15)
B (n=16)
C (n=14)
surface

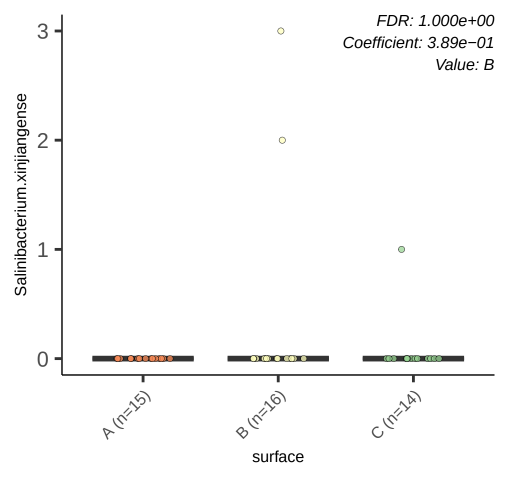
FDR: 1.000e+00
Coefficient: 3.89e−01
Value: B
Salinibacterium.xinjiangense
0
1
2
3
A (n=15)
B (n=16)
C (n=14)
surface

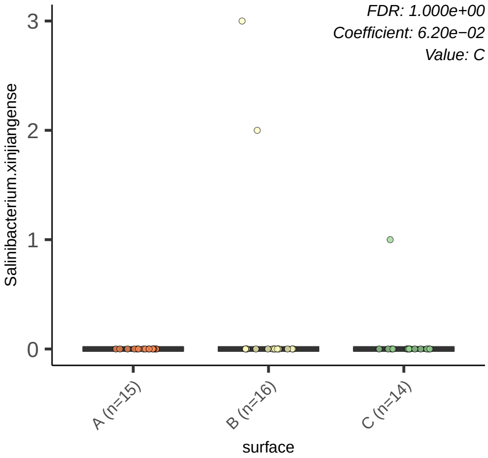

FDR: 1.000e+00
Coefficient: 6.20e−02
Value: C
Salinibacterium.xinjiangense
3
2
1
0
A (n=15)
B (n=16)
C (n=14)
surface

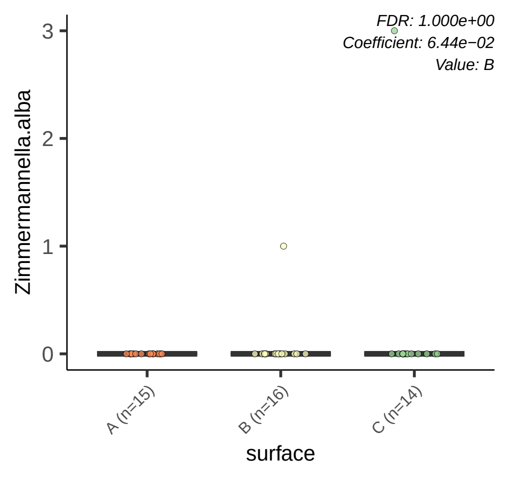

FDR: 1.000e+00
Coefficient: 6.44e−02
Value: B
Zimmermannella.alba
3
2
1
0
A (n=15)
B (n=16)
C (n=14)
surface

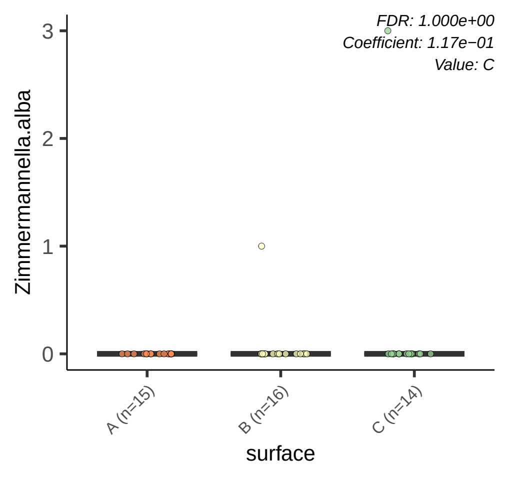
FDR: 1.000e+00
Coefficient: 1.17e−01
Value: C
Zimmermannella.alba
0
1
2
3
A (n=15)
B (n=16)
C (n=14)
surface

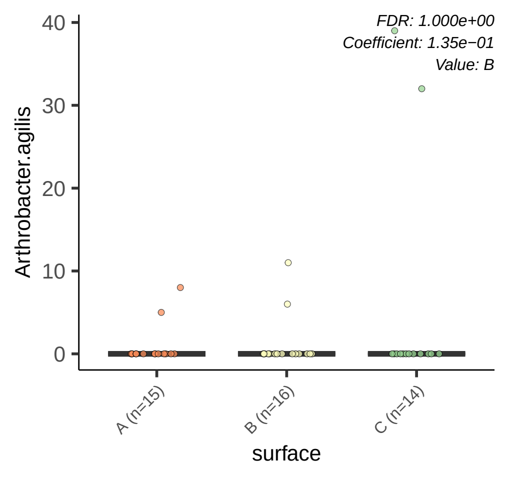
FDR: 1.000e+00
Coefficient: 1.35e−01
Value: B
Arthrobacter.agilis
40
30
20
10
0
A (n=15)
B (n=16)
C (n=14)
surface

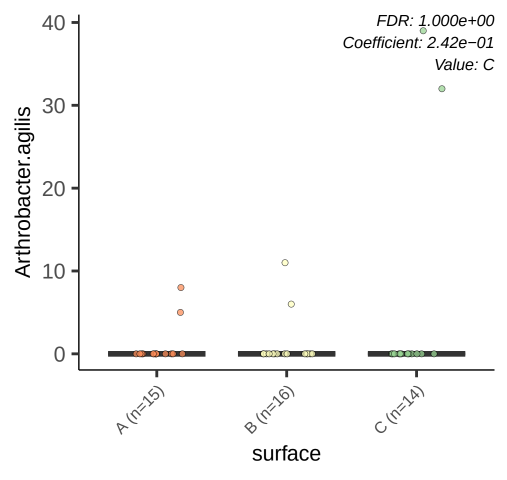

FDR: 1.000e+00
Coefficient: 2.42e−01
Value: C
Arthrobacter.agilis
0
10
20
30
40
A (n=15)
B (n=16)
C (n=14)
surface

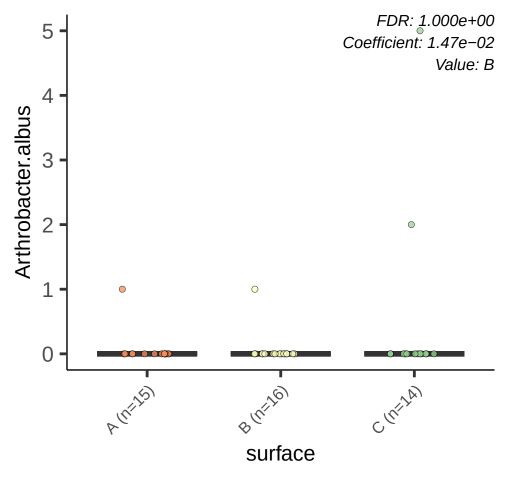

FDR: 1.000e+00
Coefficient: 1.47e−02
Value: B
Arthrobacter.albus
0
1
2
3
4
5
A (n=15)
B (n=16)
C (n=14)
surface

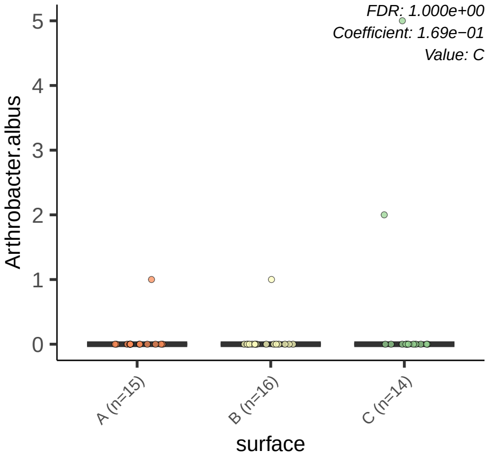
FDR: 1.000e+00
Coefficient: 1.69e−01
Value: C
Arthrobacter.albus
0
1
2
3
4
5
A (n=15)
B (n=16)
C (n=14)
surface

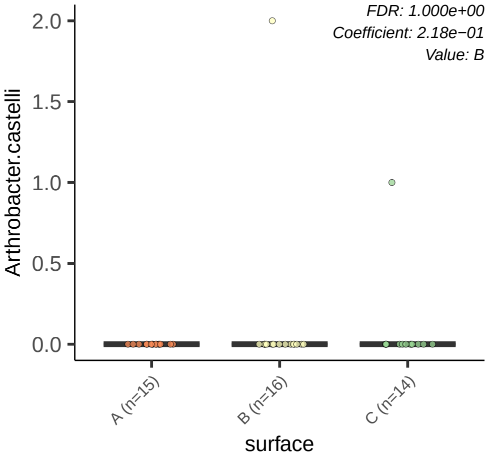
FDR: 1.000e+00
Coefficient: 2.18e−01
Value: B
Arthrobacter.castelli
2.0
1.5
1.0
0.5
0.0
A (n=15)
B (n=16)
C (n=14)
surface

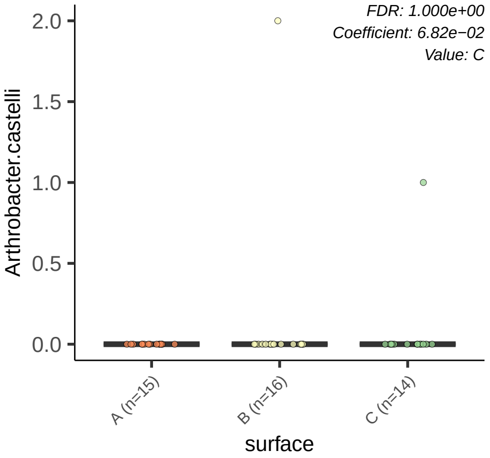

FDR: 1.000e+00
Coefficient: 6.82e−02
Value: C
Arthrobacter.castelli
2.0
1.5
1.0
0.5
0.0
A (n=15)
B (n=16)
C (n=14)
surface

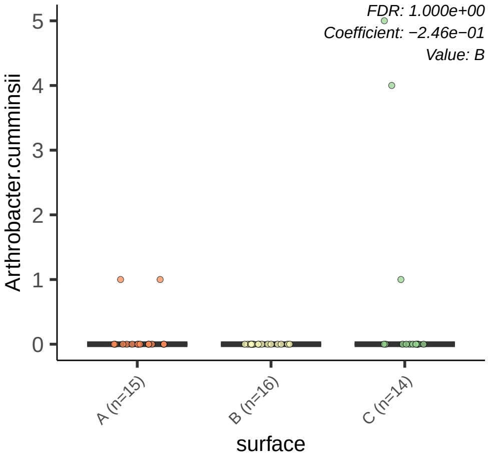
FDR: 1.000e+00
Coefficient: −2.46e−01
Value: B
Arthrobacter.cumminsii
0
1
2
3
4
5
A (n=15)
B (n=16)
C (n=14)
surface

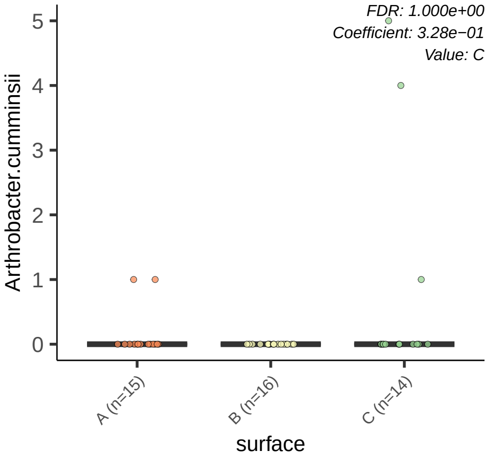
FDR: 1.000e+00
Coefficient: 3.28e−01
Value: C
Arthrobacter.cumminsii
0
1
2
3
4
5
A (n=15)
B (n=16)
C (n=14)
surface

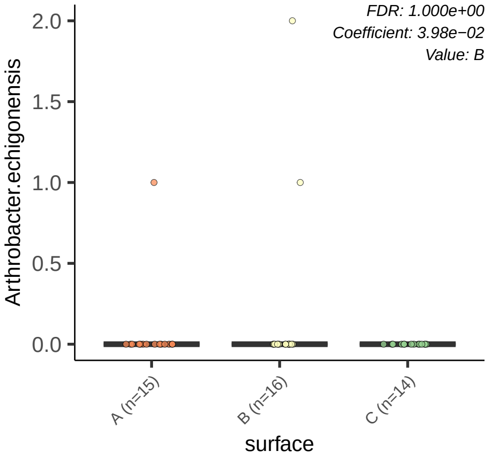

FDR: 1.000e+00
Coefficient: 3.98e−02
Value: B
Arthrobacter.echigonensis
2.0
1.5
1.0
0.5
0.0
A (n=15)
B (n=16)
C (n=14)
surface

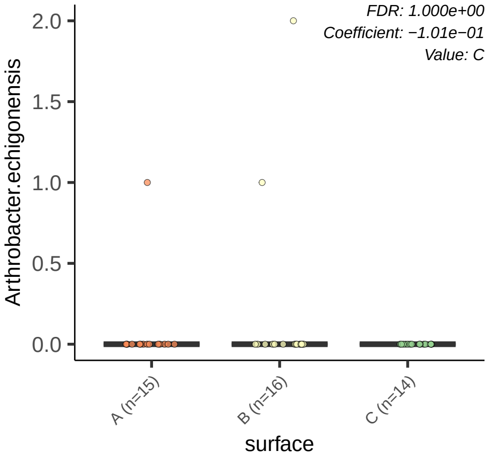

FDR: 1.000e+00
Coefficient: −1.01e−01
Value: C
Arthrobacter.echigonensis
2.0
1.5
1.0
0.5
0.0
A (n=15)
B (n=16)
C (n=14)
surface

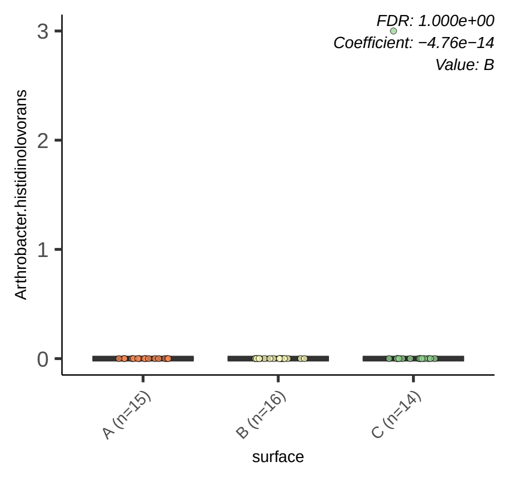

FDR: 1.000e+00
Coefficient: −4.76e−14
Value: B
Arthrobacter.histidinolovorans
3
2
1
0
A (n=15)
B (n=16)
C (n=14)
surface

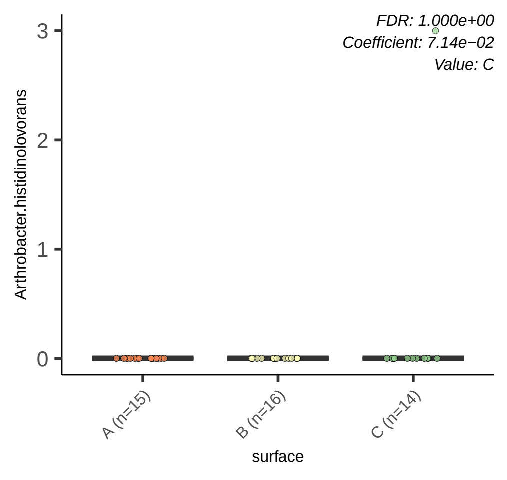
FDR: 1.000e+00
Coefficient: 7.14e−02
Value: C
Arthrobacter.histidinolovorans
0
1
2
3
A (n=15)
B (n=16)
C (n=14)
surface

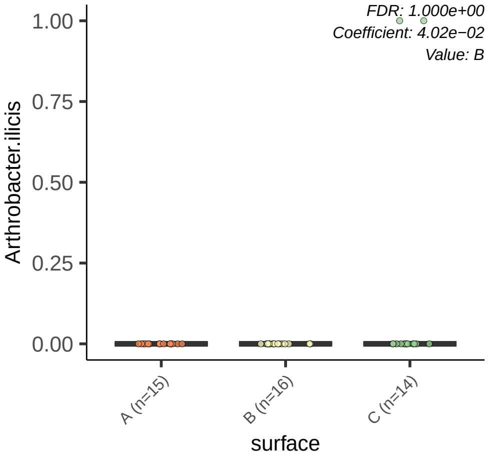
FDR: 1.000e+00
Coefficient: 4.02e−02
Value: B
Arthrobacter.ilicis
1.00
0.75
0.50
0.25
0.00
A (n=15)
B (n=16)
C (n=14)
surface

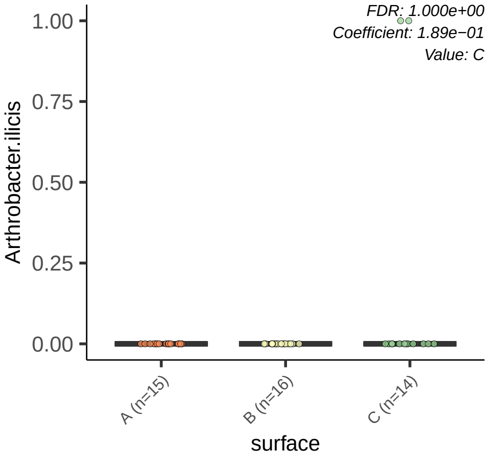
FDR: 1.000e+00
Coefficient: 1.89e−01
Value: C
1.00
0.75
0.50
0.25
0.00
Arthrobacter.ilicis
A (n=15)
B (n=16)
C (n=14)
surface

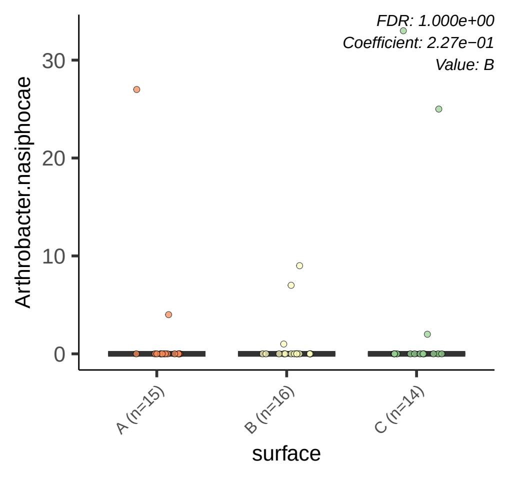
FDR: 1.000e+00
Coefficient: 2.27e−01
Value: B
Arthrobacter.nasiphocae
0
10
20
30
A (n=15)
B (n=16)
C (n=14)
surface

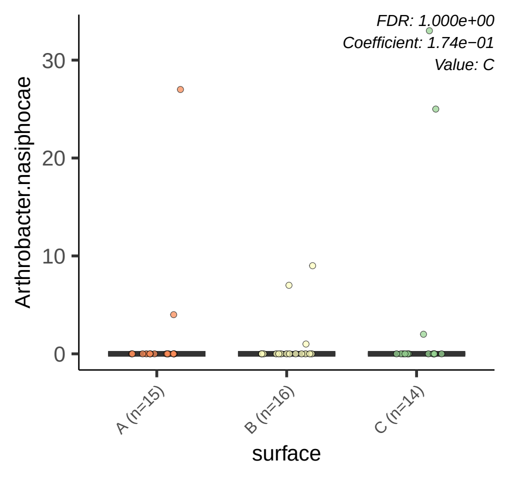

FDR: 1.000e+00
Coefficient: 1.74e−01
Value: C
Arthrobacter.nasiphocae
0
10
20
30
A (n=15)
B (n=16)
C (n=14)
surface

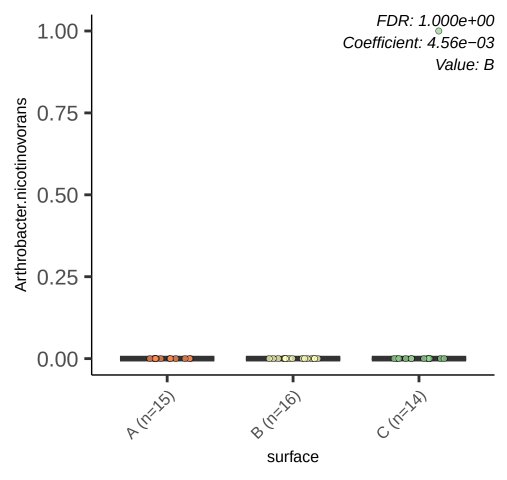
FDR: 1.000e+00
Coefficient: 4.56e−03
Value: B
1.00
0.75
0.50
0.25
0.00
Arthrobacter.nicotinovorans
A (n=15)
B (n=16)
C (n=14)
surface

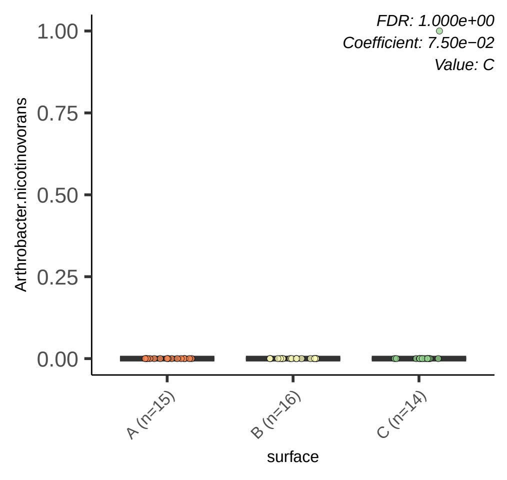
FDR: 1.000e+00
Coefficient: 7.50e−02
Value: C
Arthrobacter.nicotinovorans
1.00
0.75
0.50
0.25
0.00
A (n=15)
B (n=16)
C (n=14)
surface

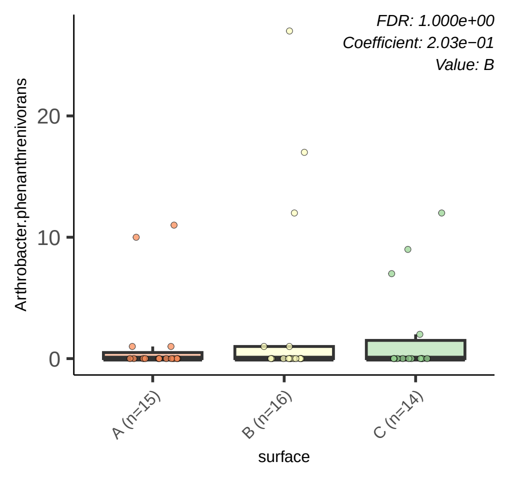
FDR: 1.000e+00
Coefficient: 2.03e−01
Value: B
Arthrobacter.phenanthrenivorans
20
10
0
A (n=15)
B (n=16)
C (n=14)
surface

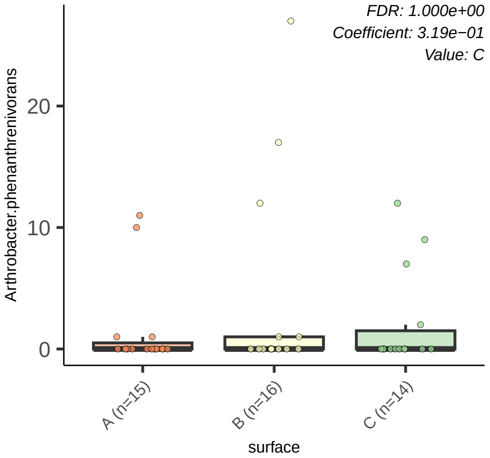

FDR: 1.000e+00
Coefficient: 3.19e−01
Value: C
Arthrobacter.phenanthrenivorans
0
10
20
A (n=15)
B (n=16)
C (n=14)
surface

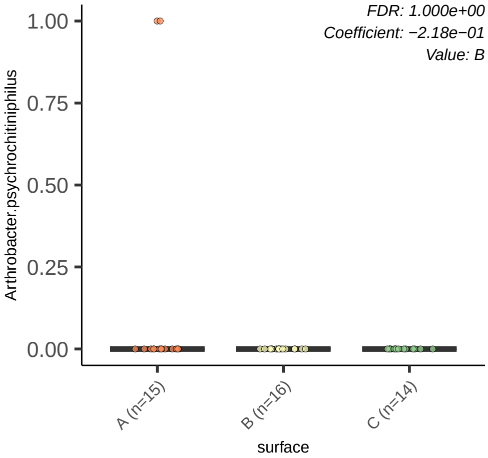

FDR: 1.000e+00
Coefficient: −2.18e−01
Value: B
1.00
0.75
0.50
0.25
0.00
Arthrobacter.psychrochitiniphilus
A (n=15)
B (n=16)
C (n=14)
surface

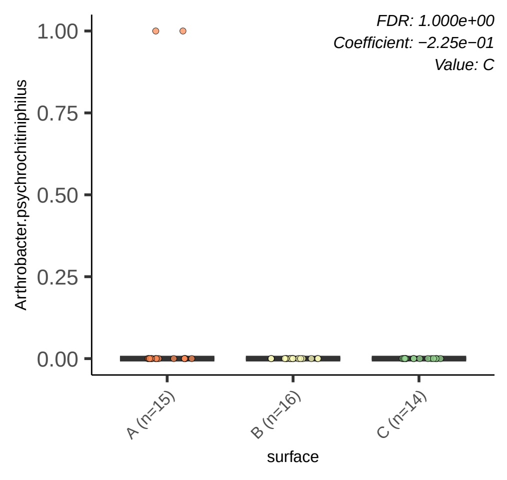
FDR: 1.000e+00
Coefficient: −2.25e−01
Value: C
Arthrobacter.psychrochitiniphilus
1.00
0.75
0.50
0.25
0.00
A (n=15)
B (n=16)
C (n=14)
surface

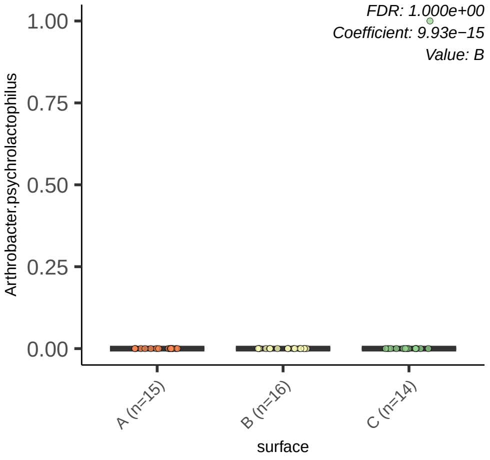

FDR: 1.000e+00
Coefficient: 9.93e−15
Value: B
Arthrobacter.psychrolactophilus
1.00
0.75
0.50
0.25
0.00
A (n=15)
B (n=16)
C (n=14)
surface

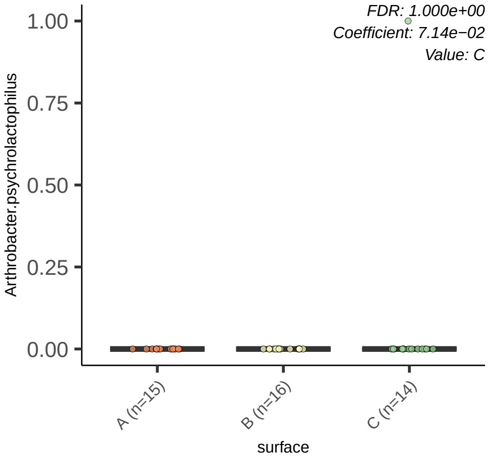

FDR: 1.000e+00
Coefficient: 7.14e−02
Value: C
Arthrobacter.psychrolactophilus
1.00
0.75
0.50
0.25
0.00
A (n=15)
B (n=16)
C (n=14)
surface

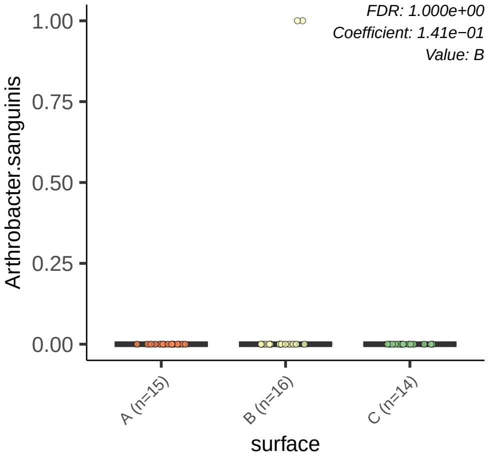
FDR: 1.000e+00
Coefficient: 1.41e−01
Value: B
Arthrobacter.sanguinis
1.00
0.75
0.50
0.25
0.00
A (n=15)
B (n=16)
C (n=14)
surface

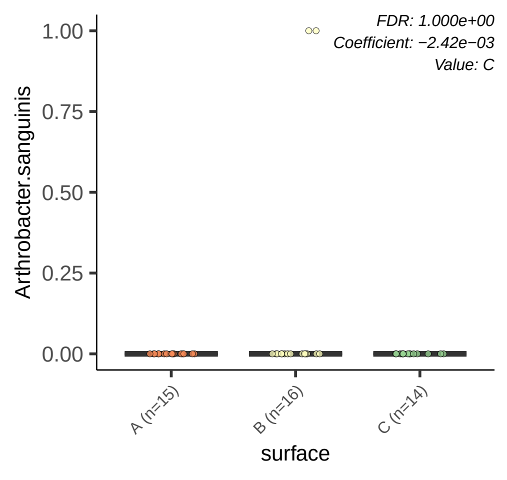

FDR: 1.000e+00
Coefficient: −2.42e−03
Value: C
Arthrobacter.sanguinis
1.00
0.75
0.50
0.25
0.00
A (n=15)
B (n=16)
C (n=14)
surface

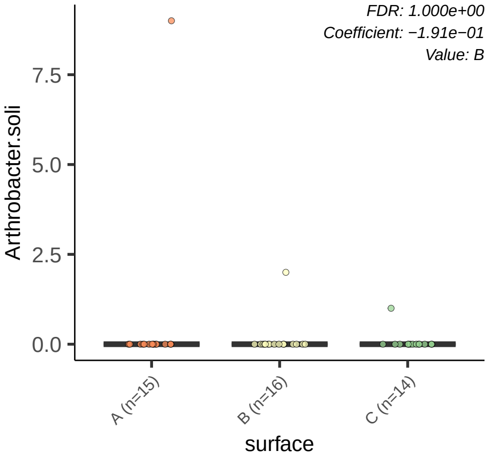
FDR: 1.000e+00
Coefficient: −1.91e−01
Value: B
Arthrobacter.soli
7.5
5.0
2.5
0.0
A (n=15)
B (n=16)
C (n=14)
surface

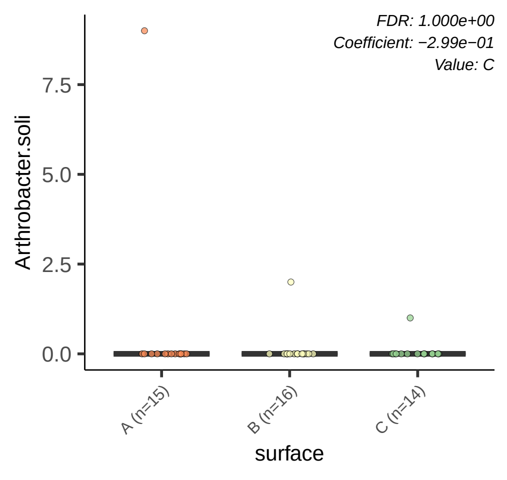
FDR: 1.000e+00
Coefficient: −2.99e−01
Value: C
Arthrobacter.soli
0.0
2.5
5.0
7.5
A (n=15)
B (n=16)
C (n=14)
surface

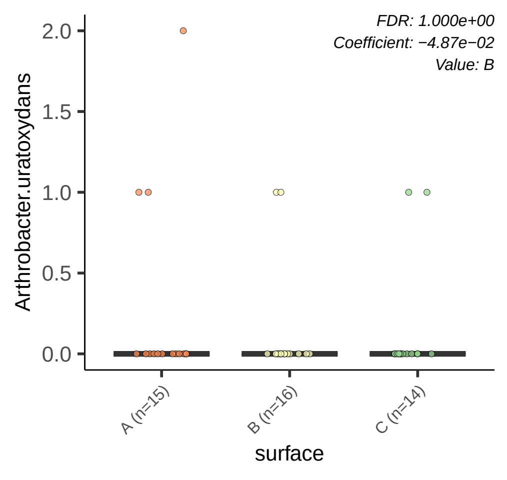

FDR: 1.000e+00
Coefficient: −4.87e−02
Value: B
Arthrobacter.uratoxydans
2.0
1.5
1.0
0.5
0.0
A (n=15)
B (n=16)
C (n=14)
surface

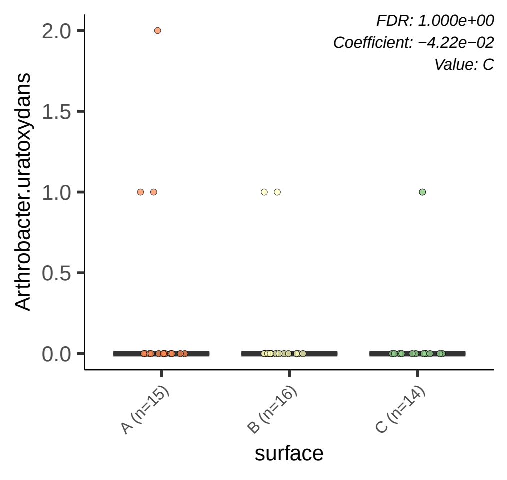
FDR: 1.000e+00
Coefficient: −4.22e−02
Value: C
Arthrobacter.uratoxydans
2.0
1.5
1.0
0.5
0.0
A (n=15)
B (n=16)
C (n=14)
surface

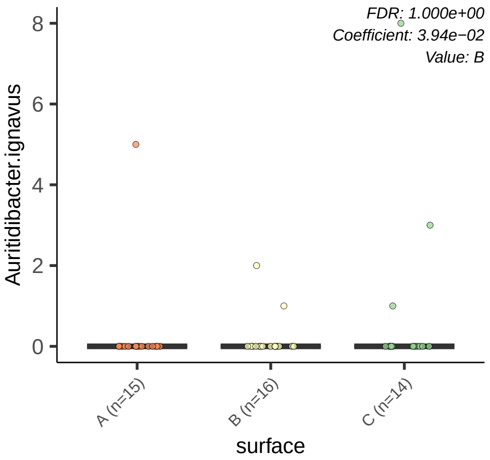
FDR: 1.000e+00
Coefficient: 3.94e−02
Value: B
Auritidibacter.ignavus
8
6
4
2
0
A (n=15)
B (n=16)
C (n=14)
surface

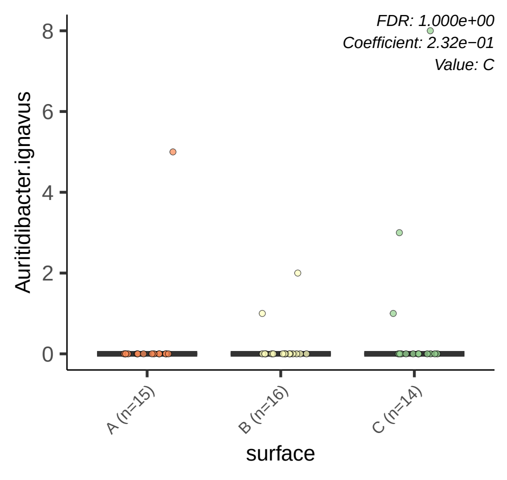

FDR: 1.000e+00
Coefficient: 2.32e−01
Value: C
Auritidibacter.ignavus
8
6
4
2
0
A (n=15)
B (n=16)
C (n=14)
surface

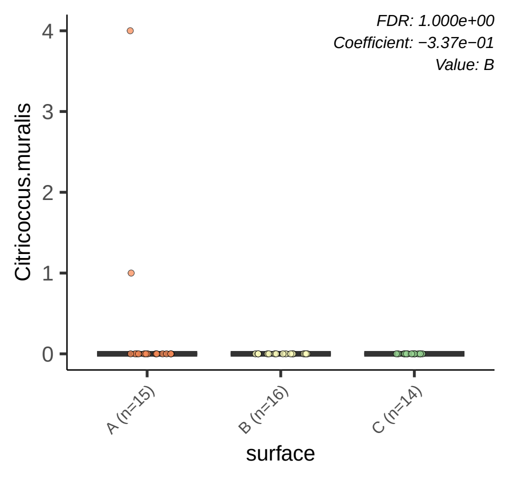

FDR: 1.000e+00
Coefficient: −3.37e−01
Value: B
Citricoccus.muralis
4
3
2
1
0
A (n=15)
B (n=16)
C (n=14)
surface

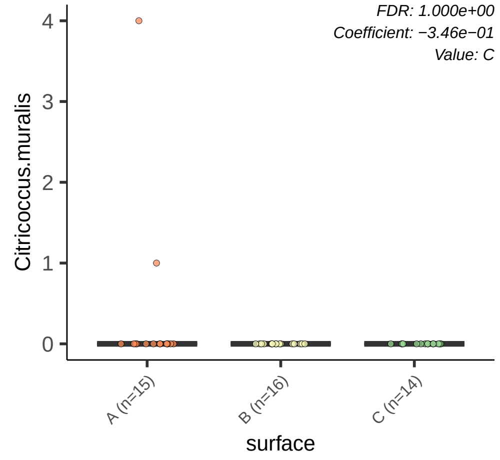
FDR: 1.000e+00
Coefficient: −3.46e−01
Value: C
Citricoccus.muralis
4
3
2
1
0
A (n=15)
B (n=16)
C (n=14)
surface

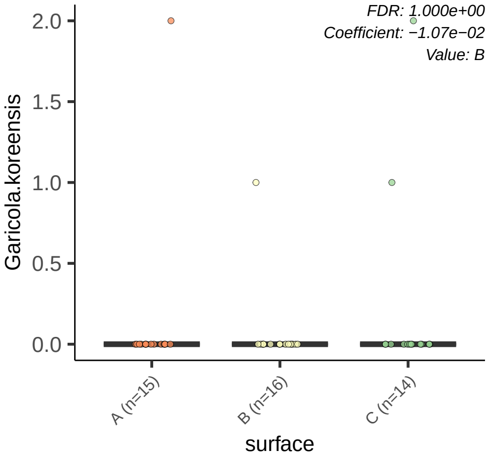

FDR: 1.000e+00
Coefficient: −1.07e−02
Value: B
Garicola.koreensis
2.0
1.5
1.0
0.5
0.0
A (n=15)
B (n=16)
C (n=14)
surface

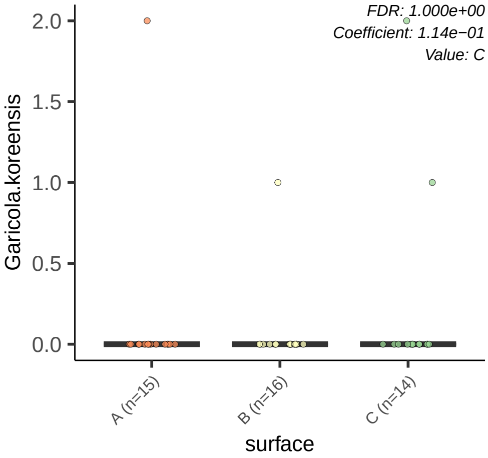
FDR: 1.000e+00
Coefficient: 1.14e−01
Value: C
Garicola.koreensis
2.0
1.5
1.0
0.5
0.0
A (n=15)
B (n=16)
C (n=14)
surface

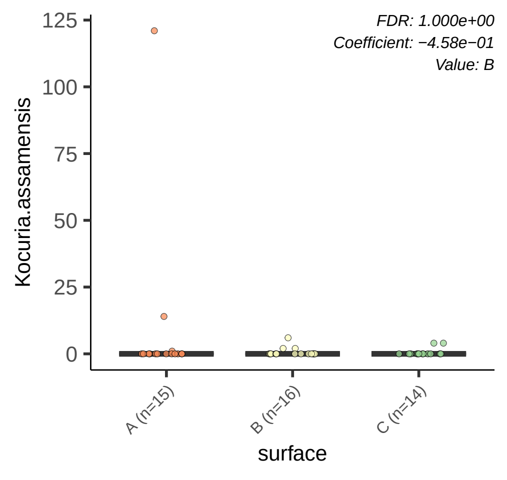
FDR: 1.000e+00
Coefficient: −4.58e−01
Value: B
Kocuria.assamensis
125
100
75
50
25
0
A (n=15)
B (n=16)
C (n=14)
surface

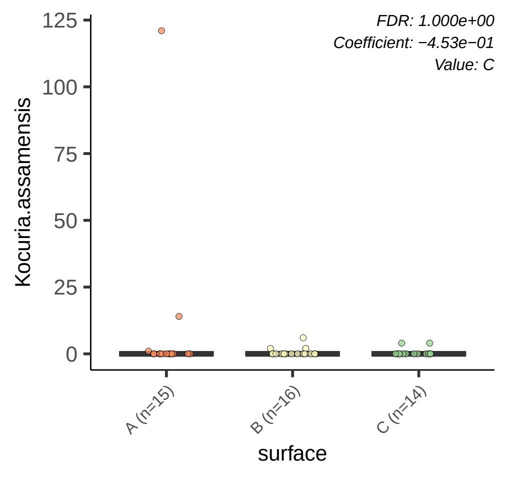
FDR: 1.000e+00
Coefficient: −4.53e−01
Value: C
Kocuria.assamensis
125
100
75
50
25
0
A (n=15)
B (n=16)
C (n=14)
surface

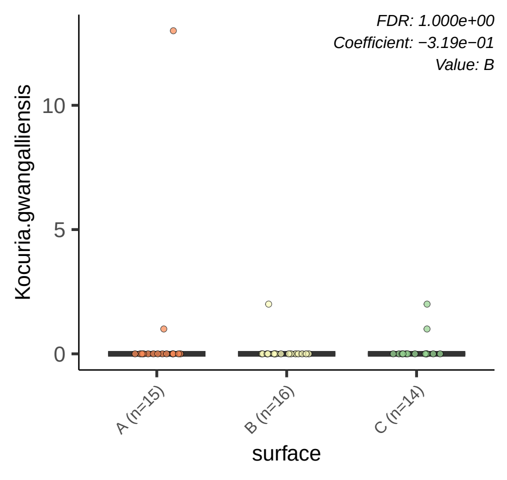
FDR: 1.000e+00
Coefficient: −3.19e−01
Value: B
Kocuria.gwangalliensis
0
5
10
A (n=15)
B (n=16)
C (n=14)
surface

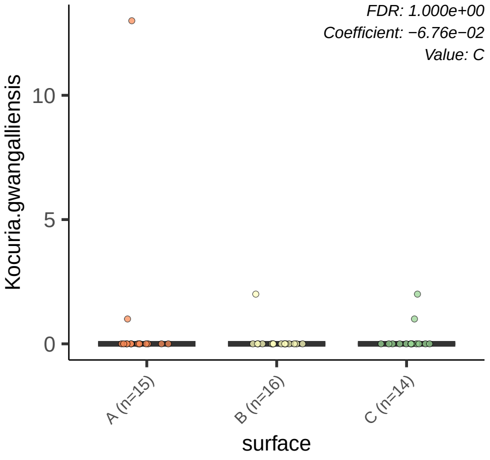

FDR: 1.000e+00
Coefficient: −6.76e−02
Value: C
Kocuria.gwangalliensis
10
5
0
A (n=15)
B (n=16)
C (n=14)
surface

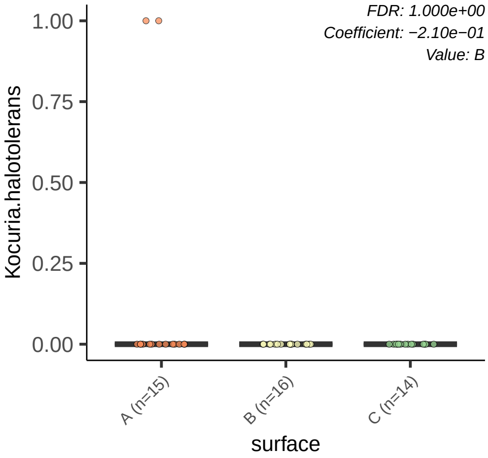
FDR: 1.000e+00
Coefficient: −2.10e−01
Value: B
Kocuria.halotolerans
1.00
0.75
0.50
0.25
0.00
A (n=15)
B (n=16)
C (n=14)
surface

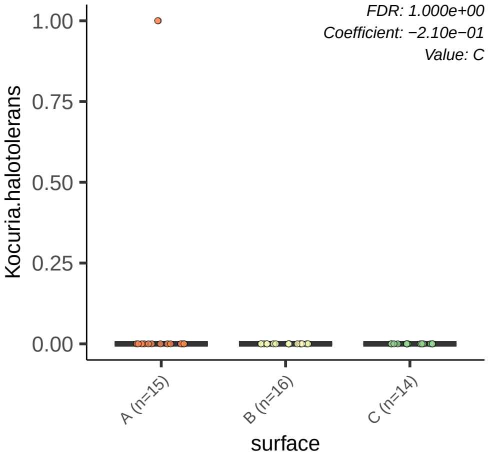
FDR: 1.000e+00
Coefficient: −2.10e−01
Value: C
Kocuria.halotolerans
1.00
0.75
0.50
0.25
0.00
A (n=15)
B (n=16)
C (n=14)
surface

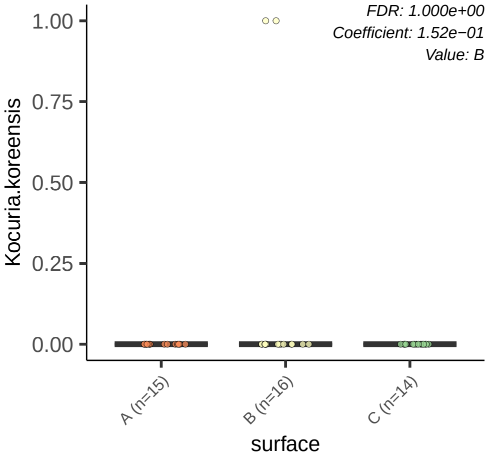

FDR: 1.000e+00
Coefficient: 1.52e−01
Value: B
Kocuria.koreensis
1.00
0.75
0.50
0.25
0.00
A (n=15)
B (n=16)
C (n=14)
surface

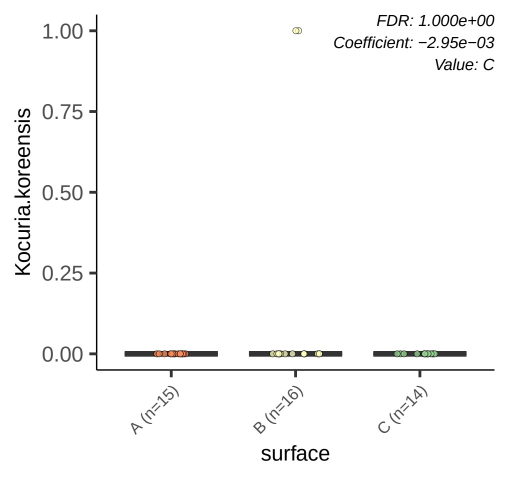
FDR: 1.000e+00
Coefficient: −2.95e−03
Value: C
1.00
0.75
0.50
0.25
0.00
Kocuria.koreensis
A (n=15)
B (n=16)
C (n=14)
surface

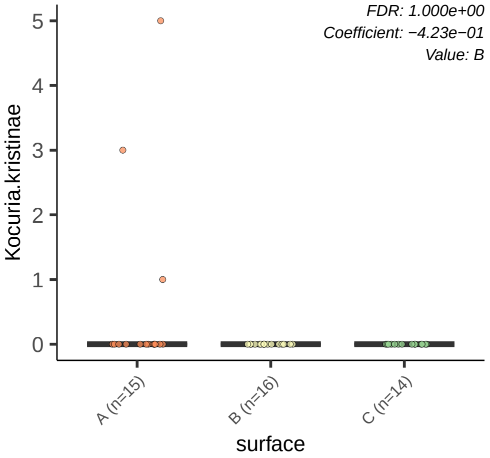

FDR: 1.000e+00
Coefficient: −4.23e−01
Value: B
Kocuria.kristinae
0
1
2
3
4
5
A (n=15)
B (n=16)
C (n=14)
surface

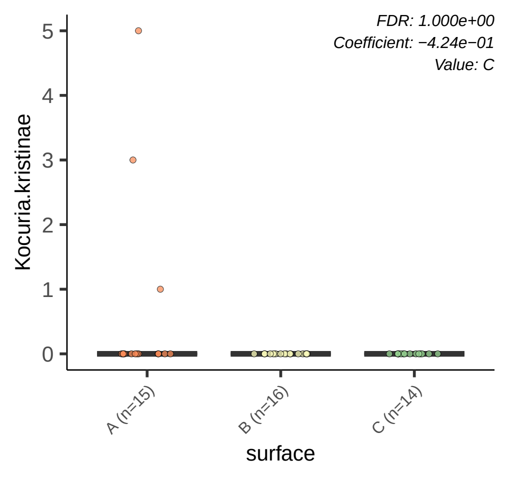

FDR: 1.000e+00
Coefficient: −4.24e−01
Value: C
Kocuria.kristinae
0
1
2
3
4
5
A (n=15)
B (n=16)
C (n=14)
surface

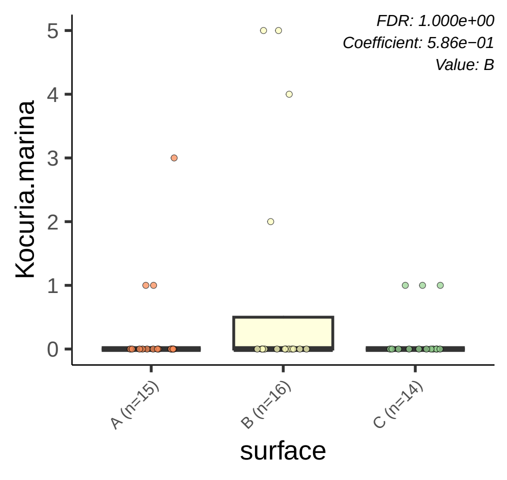
FDR: 1.000e+00
Coefficient: 5.86e−01
Value: B
Kocuria.marina
0
1
2
3
4
5
A (n=15)
B (n=16)
C (n=14)
surface

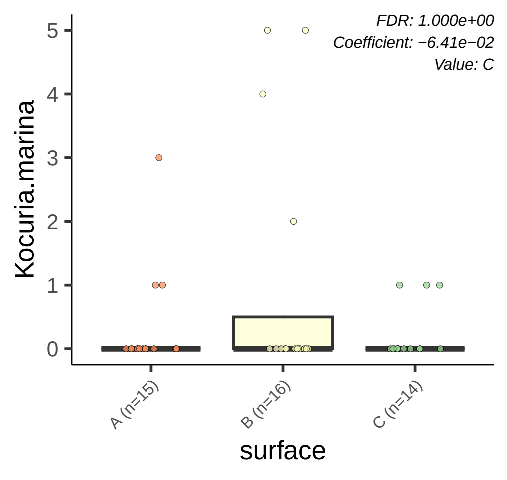
FDR: 1.000e+00
Coefficient: −6.41e−02
Value: C
Kocuria.marina
0
1
2
3
4
5
A (n=15)
B (n=16)
C (n=14)
surface

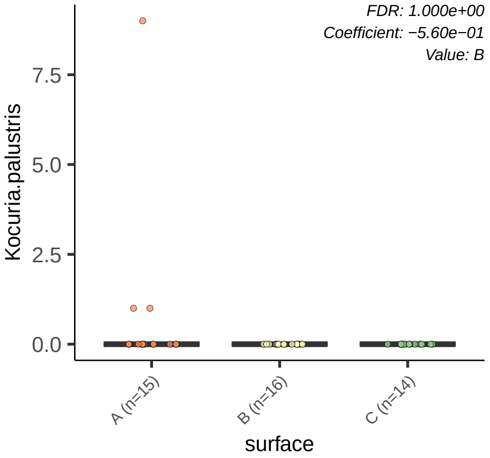
FDR: 1.000e+00
Coefficient: −5.60e−01
Value: B
Kocuria.palustris
7.5
5.0
2.5
0.0
A (n=15)
B (n=16)
C (n=14)
surface

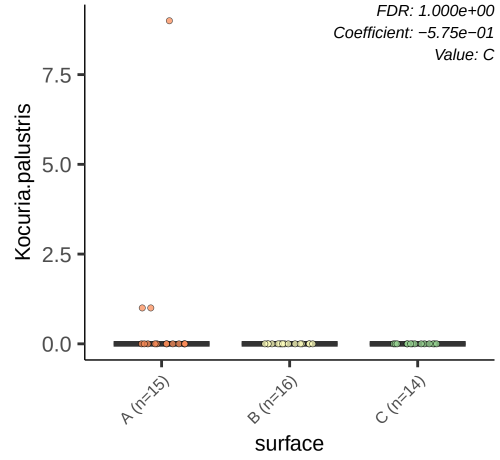
FDR: 1.000e+00
Coefficient: −5.75e−01
Value: C
Kocuria.palustris
7.5
5.0
2.5
0.0
A (n=15)
B (n=16)
C (n=14)
surface

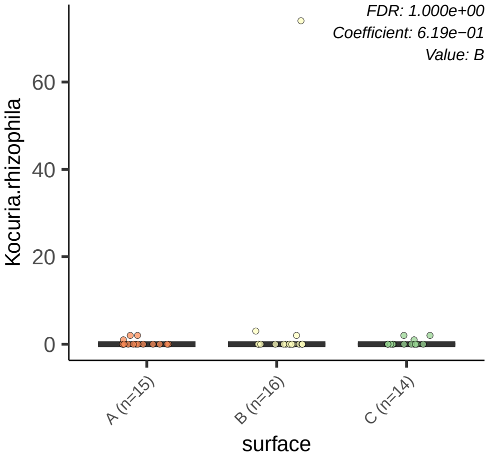

FDR: 1.000e+00
Coefficient: 6.19e−01
Value: B
Kocuria.rhizophila
0
20
40
60
A (n=15)
B (n=16)
C (n=14)
surface

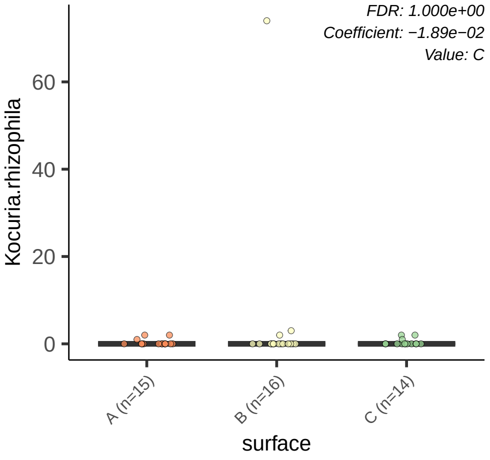
FDR: 1.000e+00
Coefficient: −1.89e−02
Value: C
Kocuria.rhizophila
60
40
20
0
A (n=15)
B (n=16)
C (n=14)
surface

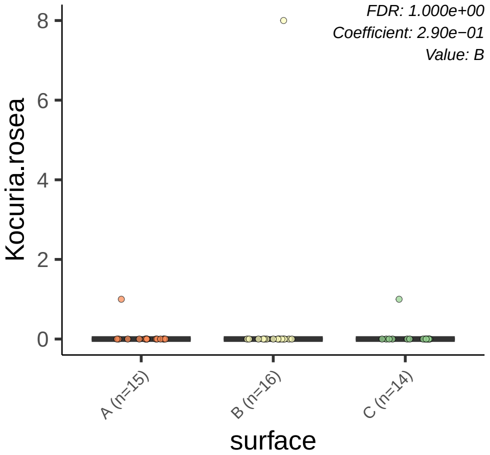
FDR: 1.000e+00
Coefficient: 2.90e−01
Value: B
Kocuria.rosea
0
2
4
6
8
A (n=15)
B (n=16)
C (n=14)
surface

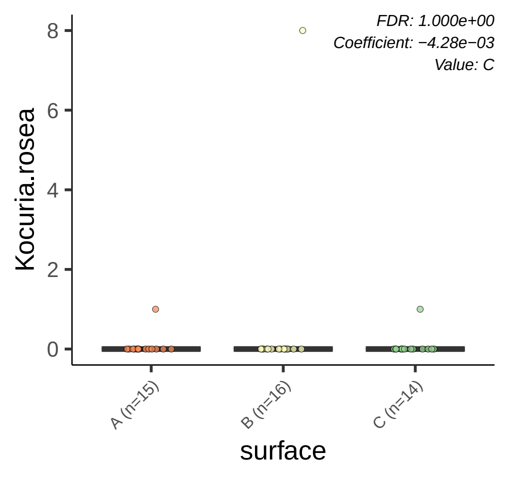
FDR: 1.000e+00
Coefficient: −4.28e−03
Value: C
Kocuria.rosea
8
6
4
2
0
A (n=15)
B (n=16)
C (n=14)
surface

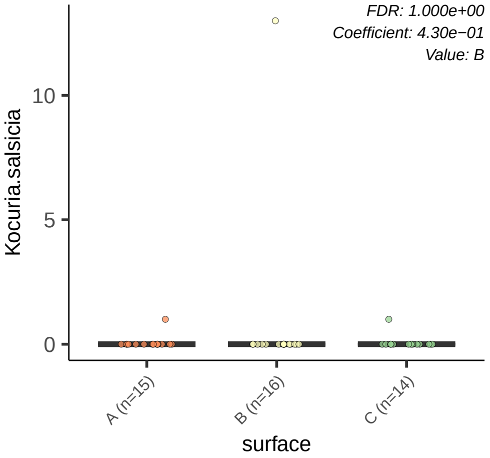
FDR: 1.000e+00
Coefficient: 4.30e−01
Value: B
Kocuria.salsicia
0
5
10
A (n=15)
B (n=16)
C (n=14)
surface

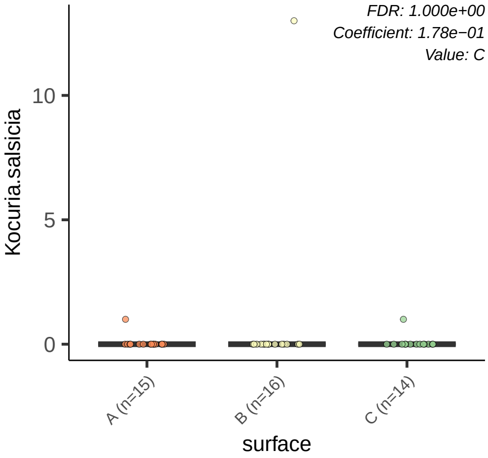

FDR: 1.000e+00
Coefficient: 1.78e−01
Value: C
10
5
0
Kocuria.salsicia
A (n=15)
B (n=16)
C (n=14)
surface

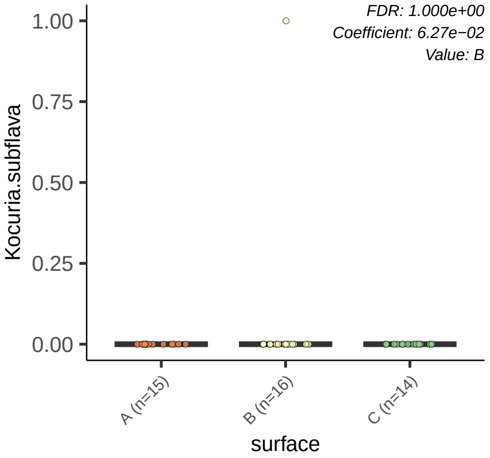
FDR: 1.000e+00
Coefficient: 6.27e−02
Value: B
1.00
0.75
0.50
0.25
0.00
Kocuria.subflava
A (n=15)
B (n=16)
C (n=14)
surface

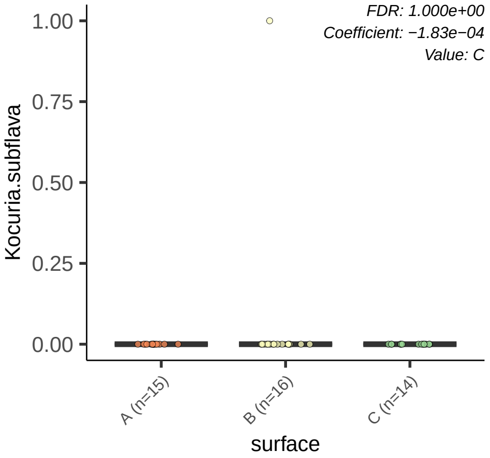
FDR: 1.000e+00
Coefficient: −1.83e−04
Value: C
Kocuria.subflava
1.00
0.75
0.50
0.25
0.00
A (n=15)
B (n=16)
C (n=14)
surface

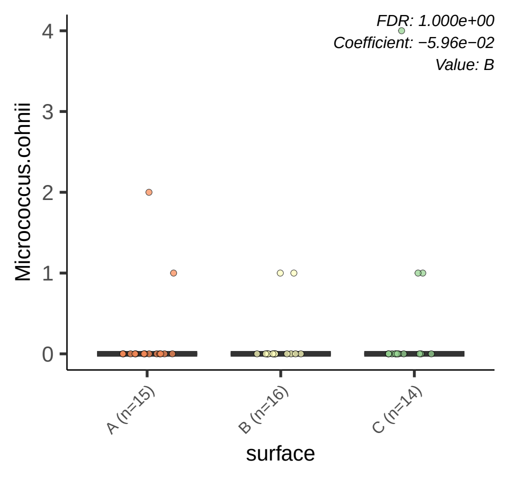
FDR: 1.000e+00
Coefficient: −5.96e−02
Value: B
Micrococcus.cohnii
0
1
2
3
4
A (n=15)
B (n=16)
C (n=14)
surface

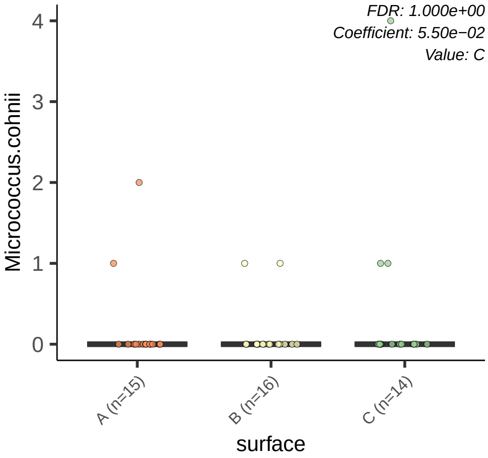
FDR: 1.000e+00
Coefficient: 5.50e−02
Value: C
Micrococcus.cohnii
4
3
2
1
0
A (n=15)
B (n=16)
C (n=14)
surface

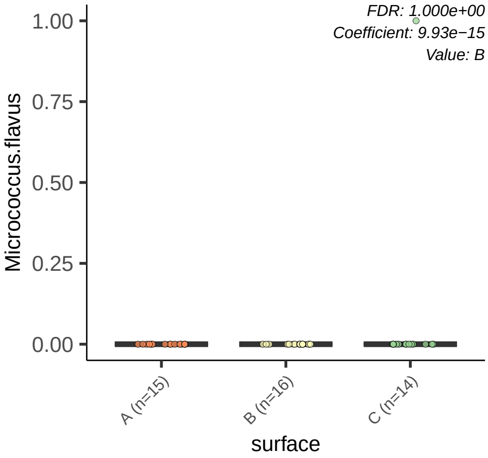

FDR: 1.000e+00
Coefficient: 9.93e−15
Value: B
Micrococcus.flavus
1.00
0.75
0.50
0.25
0.00
A (n=15)
B (n=16)
C (n=14)
surface

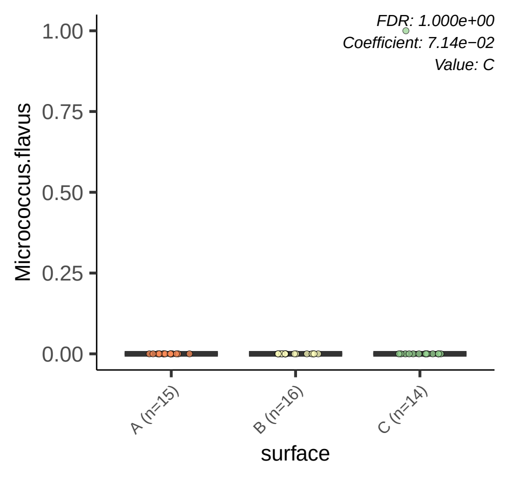
FDR: 1.000e+00
Coefficient: 7.14e−02
Value: C
Micrococcus.flavus
1.00
0.75
0.50
0.25
0.00
A (n=15)
B (n=16)
C (n=14)
surface

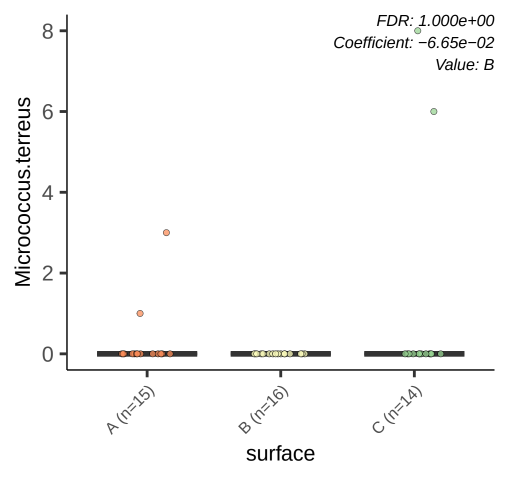

FDR: 1.000e+00
Coefficient: −6.65e−02
Value: B
Micrococcus.terreus
8
6
4
2
0
A (n=15)
B (n=16)
C (n=14)
surface

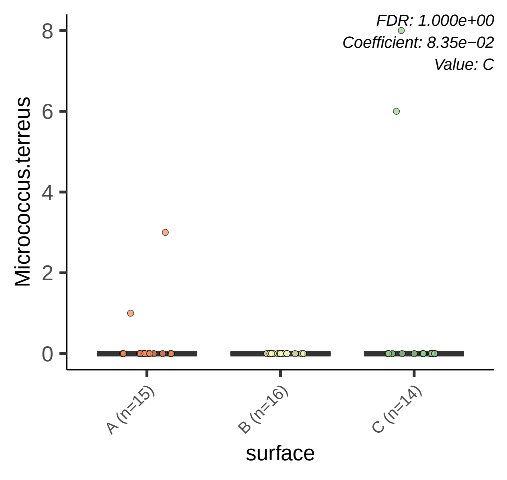

FDR: 1.000e+00
Coefficient: 8.35e−02
Value: C
Micrococcus.terreus
0
2
4
6
8
A (n=15)
B (n=16)
C (n=14)
surface

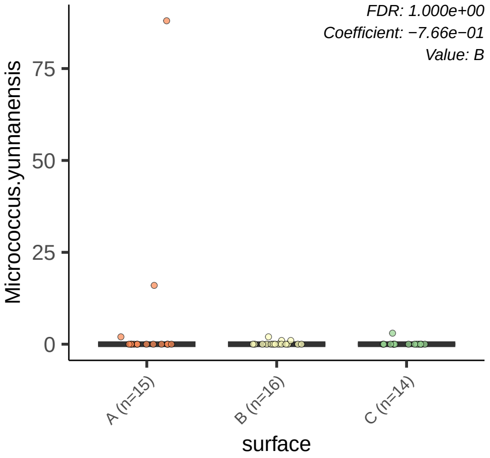

FDR: 1.000e+00
Coefficient: −7.66e−01
Value: B
Micrococcus.yunnanensis
75
50
25
0
A (n=15)
B (n=16)
C (n=14)
surface

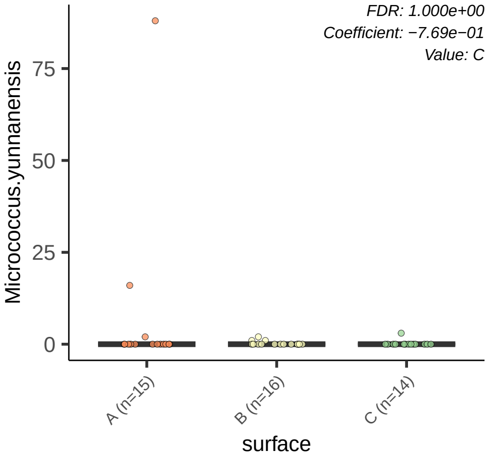
FDR: 1.000e+00
Coefficient: −7.69e−01
Value: C
Micrococcus.yunnanensis
75
50
25
0
A (n=15)
B (n=16)
C (n=14)
surface

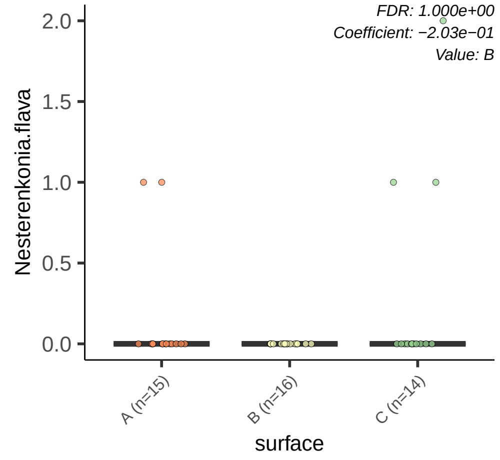

FDR: 1.000e+00
Coefficient: −2.03e−01
Value: B
Nesterenkonia.flava
2.0
1.5
1.0
0.5
0.0
A (n=15)
B (n=16)
C (n=14)
surface

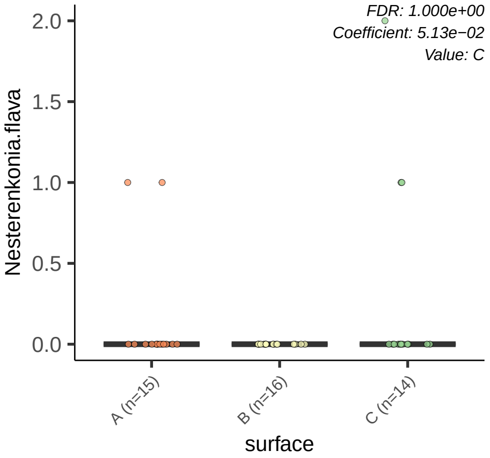

FDR: 1.000e+00
Coefficient: 5.13e−02
Value: C
Nesterenkonia.flava
2.0
1.5
1.0
0.5
0.0
A (n=15)
B (n=16)
C (n=14)
surface

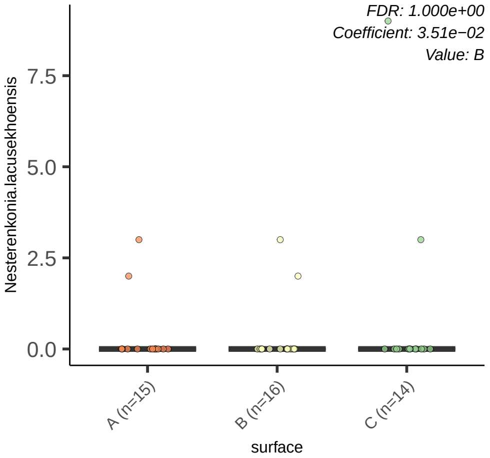
FDR: 1.000e+00
Coefficient: 3.51e−02
Value: B
Nesterenkonia.lacusekhoensis
7.5
5.0
2.5
0.0
A (n=15)
B (n=16)
C (n=14)
surface

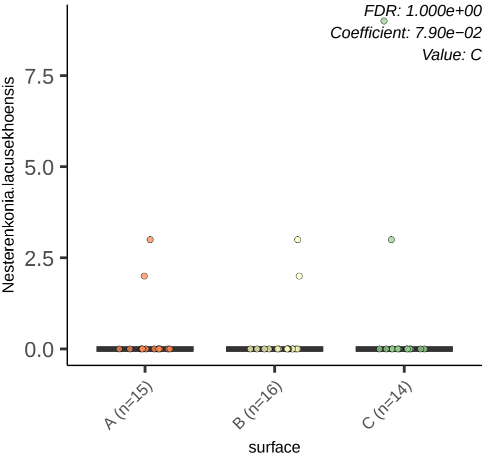
FDR: 1.000e+00
Coefficient: 7.90e−02
Value: C
Nesterenkonia.lacusekhoensis
7.5
5.0
2.5
0.0
A (n=15)
B (n=16)
C (n=14)
surface

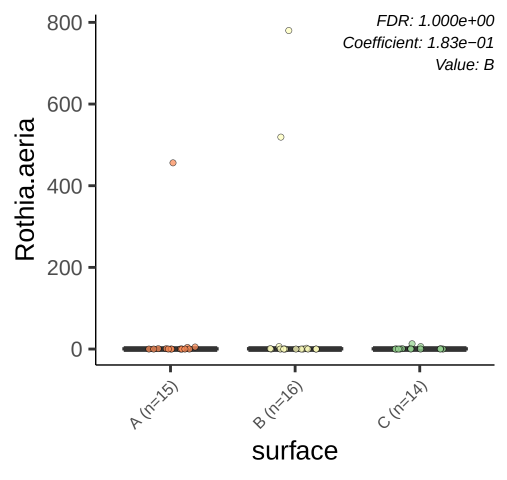

FDR: 1.000e+00
Coefficient: 1.83e−01
Value: B
Rothia.aeria
800
600
400
200
0
A (n=15)
B (n=16)
C (n=14)
surface

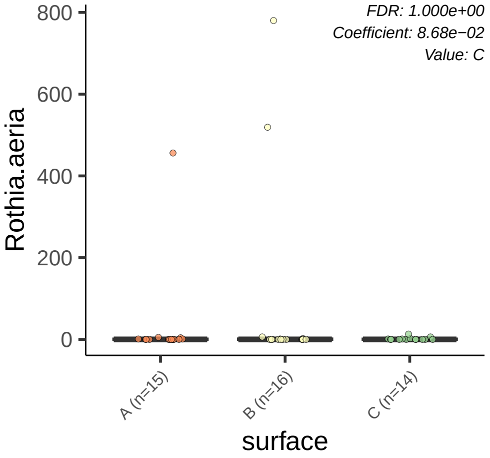
FDR: 1.000e+00
Coefficient: 8.68e−02
Value: C
800
600
400
200
0
Rothia.aeria
A (n=15)
B (n=16)
C (n=14)
surface

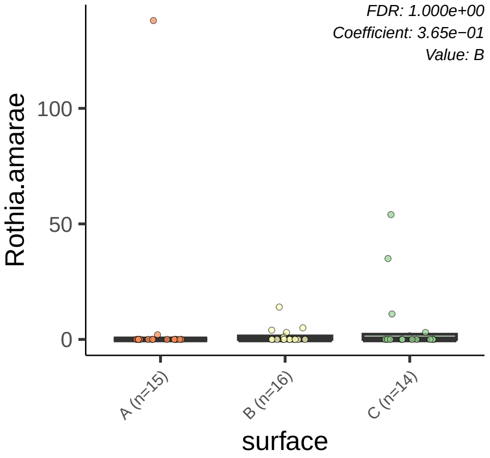

FDR: 1.000e+00
Coefficient: 3.65e−01
Value: B
Rothia.amarae
100
50
0
A (n=15)
B (n=16)
C (n=14)
surface

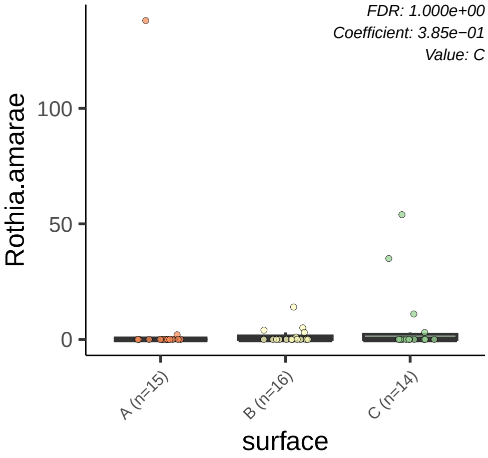
FDR: 1.000e+00
Coefficient: 3.85e−01
Value: C
Rothia.amarae
100
50
0
A (n=15)
B (n=16)
C (n=14)
surface

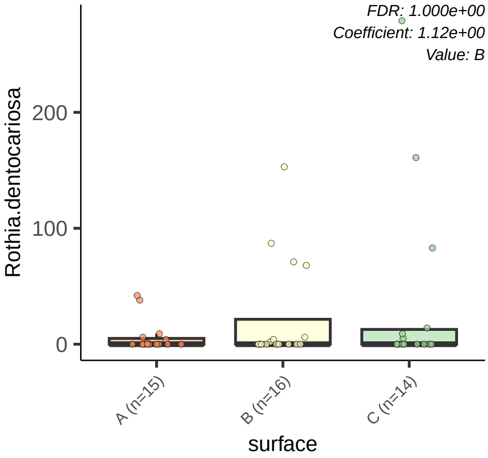
FDR: 1.000e+00
Coefficient: 1.12e+00
Value: B
Rothia.dentocariosa
200
100
0
A (n=15)
B (n=16)
C (n=14)
surface

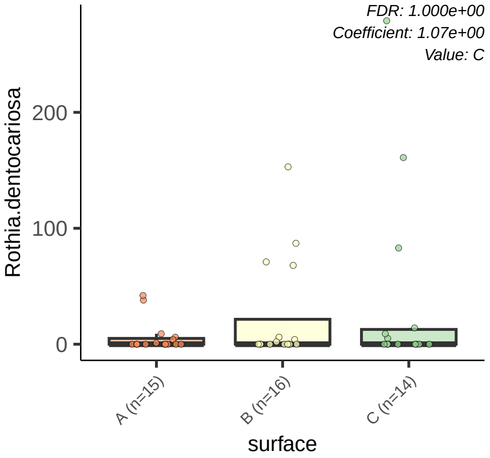

FDR: 1.000e+00
Coefficient: 1.07e+00
Value: C
Rothia.dentocariosa
200
100
0
A (n=15)
B (n=16)
C (n=14)
surface

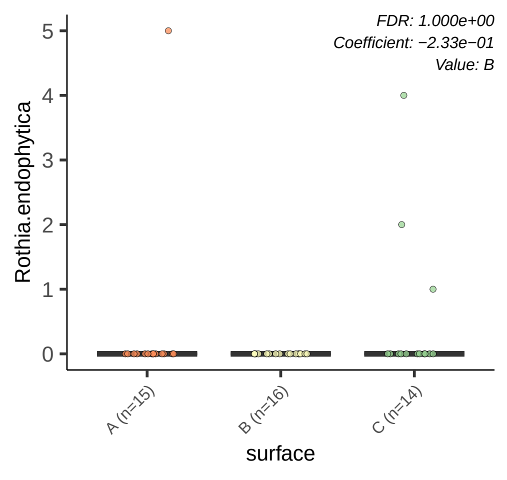
FDR: 1.000e+00
Coefficient: −2.33e−01
Value: B
Rothia.endophytica
0
1
2
3
4
5
A (n=15)
B (n=16)
C (n=14)
surface

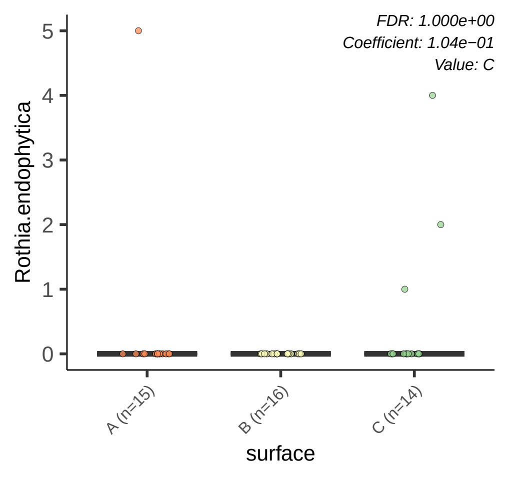
FDR: 1.000e+00
Coefficient: 1.04e−01
Value: C
Rothia.endophytica
0
1
2
3
4
5
A (n=15)
B (n=16)
C (n=14)
surface

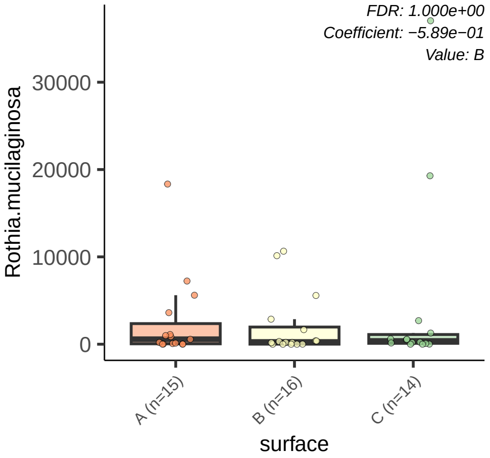

FDR: 1.000e+00
Coefficient: −5.89e−01
Value: B
Rothia.mucilaginosa
30000
20000
10000
0
A (n=15)
B (n=16)
C (n=14)
surface

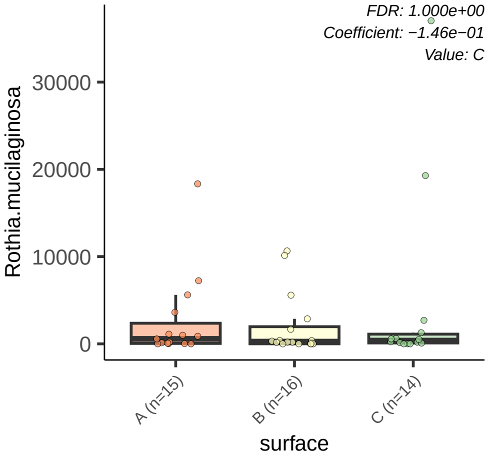

FDR: 1.000e+00
Coefficient: −1.46e−01
Value: C
Rothia.mucilaginosa
30000
20000
10000
0
A (n=15)
B (n=16)
C (n=14)
surface

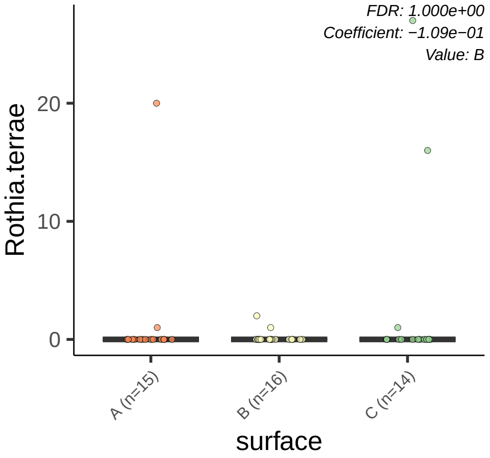
FDR: 1.000e+00
Coefficient: −1.09e−01
Value: B
Rothia.terrae
20
10
0
A (n=15)
B (n=16)
C (n=14)
surface

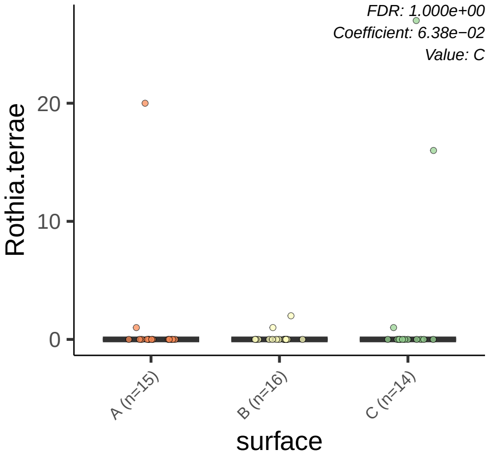
FDR: 1.000e+00
Coefficient: 6.38e−02
Value: C
Rothia.terrae
0
10
20
A (n=15)
B (n=16)
C (n=14)
surface

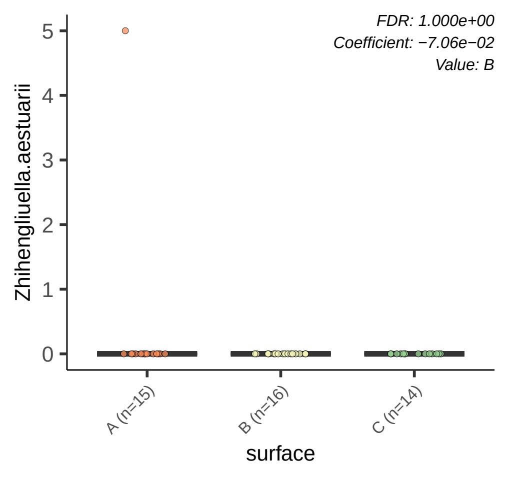

FDR: 1.000e+00
Coefficient: −7.06e−02
Value: B
Zhihengliuella.aestuarii
5
4
3
2
1
0
A (n=15)
B (n=16)
C (n=14)
surface

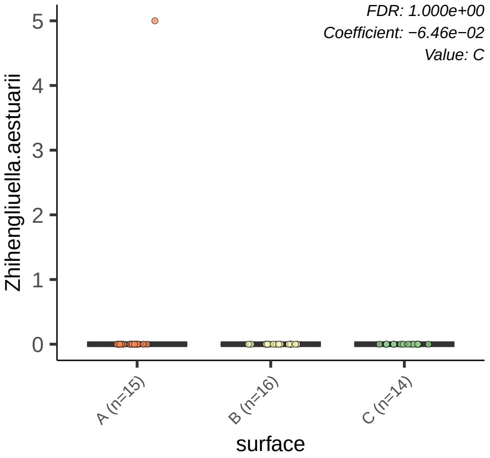

FDR: 1.000e+00
Coefficient: −6.46e−02
Value: C
Zhihengliuella.aestuarii
0
1
2
3
4
5
A (n=15)
B (n=16)
C (n=14)
surface

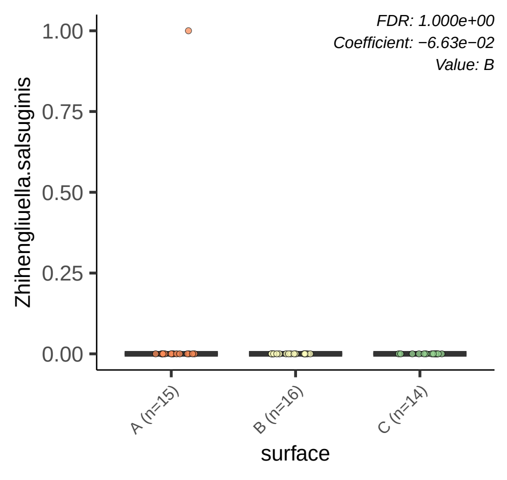
FDR: 1.000e+00
Coefficient: −6.63e−02
Value: B
Zhihengliuella.salsuginis
1.00
0.75
0.50
0.25
0.00
A (n=15)
B (n=16)
C (n=14)
surface

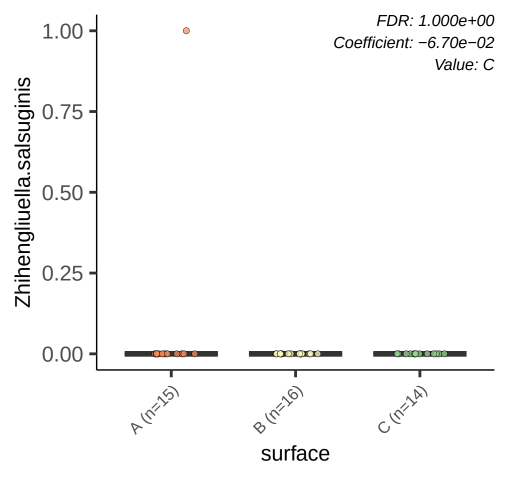

FDR: 1.000e+00
Coefficient: −6.70e−02
Value: C
Zhihengliuella.salsuginis
1.00
0.75
0.50
0.25
0.00
A (n=15)
B (n=16)
C (n=14)
surface

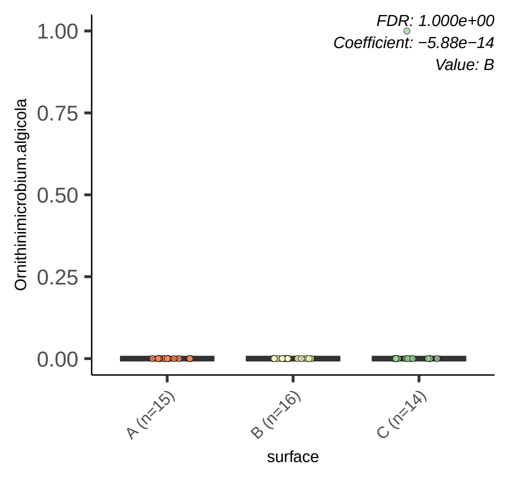
FDR: 1.000e+00
Coefficient: −5.88e−14
Value: B
Ornithinimicrobium.algicola
1.00
0.75
0.50
0.25
0.00
A (n=15)
B (n=16)
C (n=14)
surface

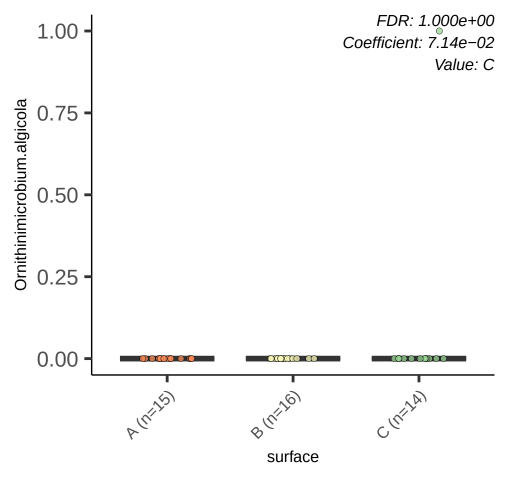
FDR: 1.000e+00
Coefficient: 7.14e−02
Value: C
Ornithinimicrobium.algicola
1.00
0.75
0.50
0.25
0.00
A (n=15)
B (n=16)
C (n=14)
surface

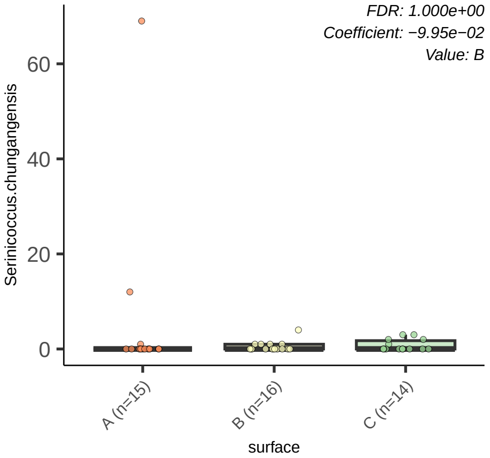

FDR: 1.000e+00
Coefficient: −9.95e−02
Value: B
Serinicoccus.chungangensis
0
20
40
60
A (n=15)
B (n=16)
C (n=14)
surface

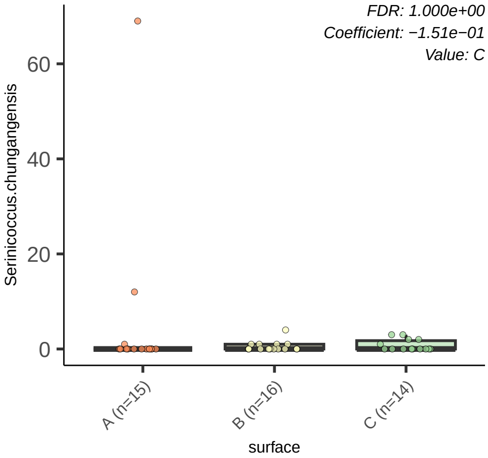

FDR: 1.000e+00
Coefficient: −1.51e−01
Value: C
Serinicoccus.chungangensis
60
40
20
0
A (n=15)
B (n=16)
C (n=14)
surface

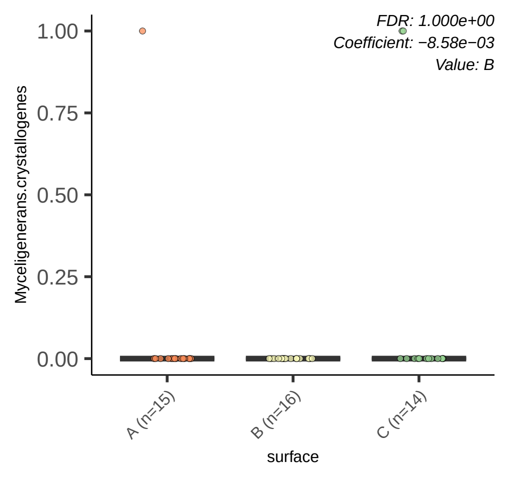

FDR: 1.000e+00
Coefficient: −8.58e−03
Value: B
Myceligenerans.crystallogenes
1.00
0.75
0.50
0.25
0.00
A (n=15)
B (n=16)
C (n=14)
surface

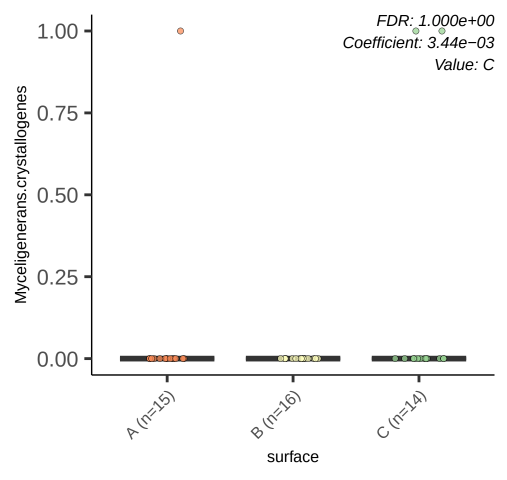
FDR: 1.000e+00
Coefficient: 3.44e−03
Value: C
Myceligenerans.crystallogenes
1.00
0.75
0.50
0.25
0.00
A (n=15)
B (n=16)
C (n=14)
surface

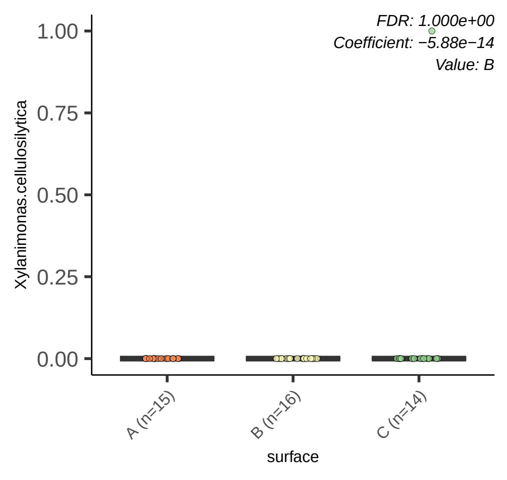

FDR: 1.000e+00
Coefficient: −5.88e−14
Value: B
Xylanimonas.cellulosilytica
1.00
0.75
0.50
0.25
0.00
A (n=15)
B (n=16)
C (n=14)
surface

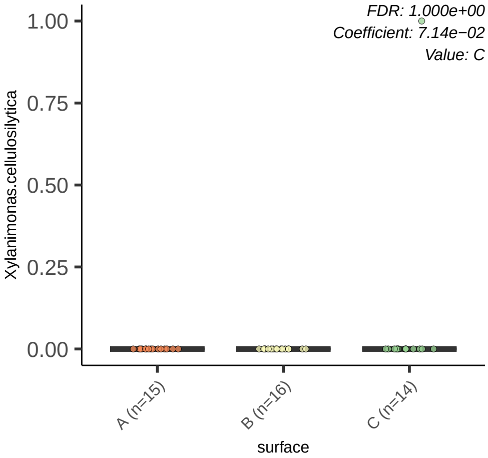
FDR: 1.000e+00
Coefficient: 7.14e−02
Value: C
1.00
0.75
0.50
0.25
0.00
Xylanimonas.cellulosilytica
A (n=15)
B (n=16)
C (n=14)
surface

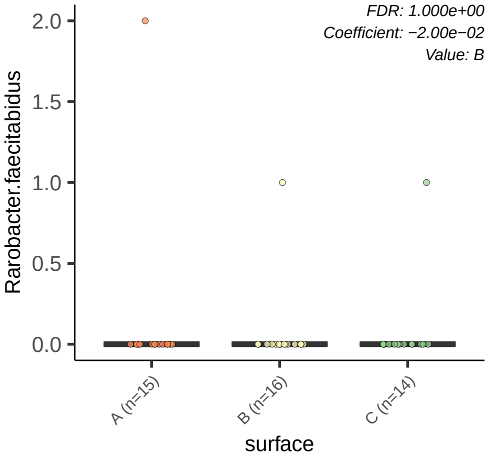

FDR: 1.000e+00
Coefficient: −2.00e−02
Value: B
Rarobacter.faecitabidus
2.0
1.5
1.0
0.5
0.0
A (n=15)
B (n=16)
C (n=14)
surface

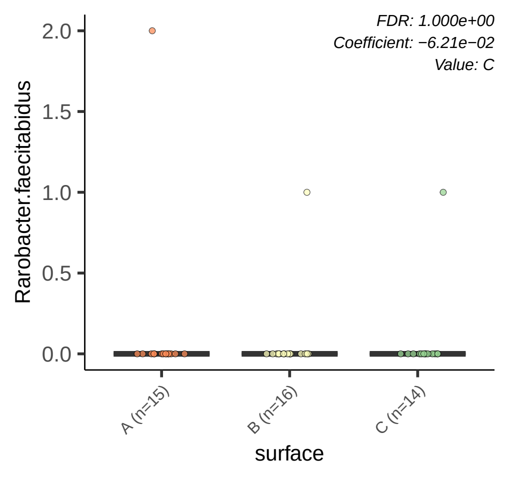
FDR: 1.000e+00
Coefficient: −6.21e−02
Value: C
Rarobacter.faecitabidus
2.0
1.5
1.0
0.5
0.0
A (n=15)
B (n=16)
C (n=14)
surface

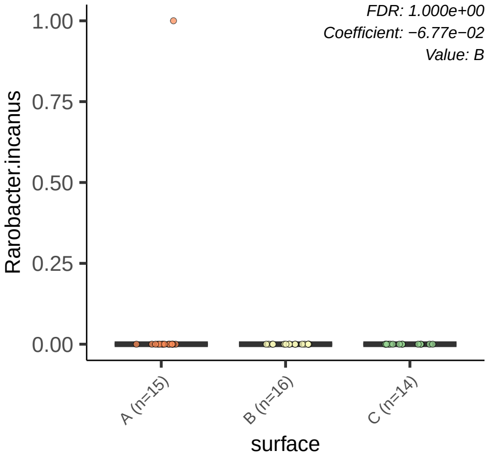
FDR: 1.000e+00
Coefficient: −6.77e−02
Value: B
Rarobacter.incanus
1.00
0.75
0.50
0.25
0.00
A (n=15)
B (n=16)
C (n=14)
surface

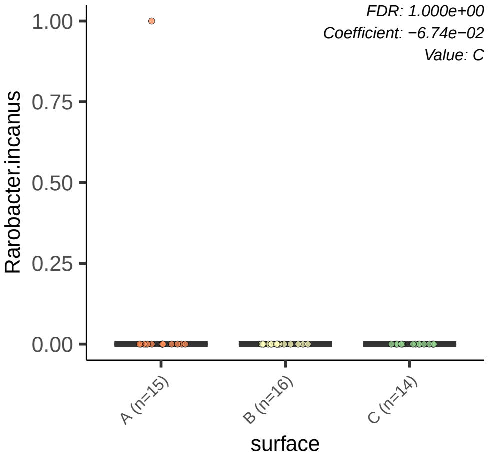

FDR: 1.000e+00
Coefficient: −6.74e−02
Value: C
Rarobacter.incanus
1.00
0.75
0.50
0.25
0.00
A (n=15)
B (n=16)
C (n=14)
surface

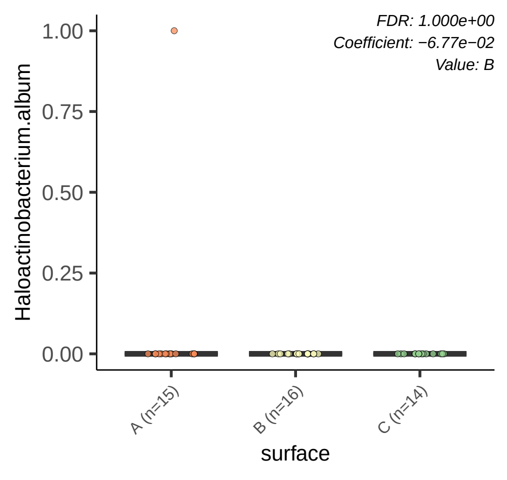
FDR: 1.000e+00
Coefficient: −6.77e−02
Value: B
Haloactinobacterium.album
1.00
0.75
0.50
0.25
0.00
A (n=15)
B (n=16)
C (n=14)
surface

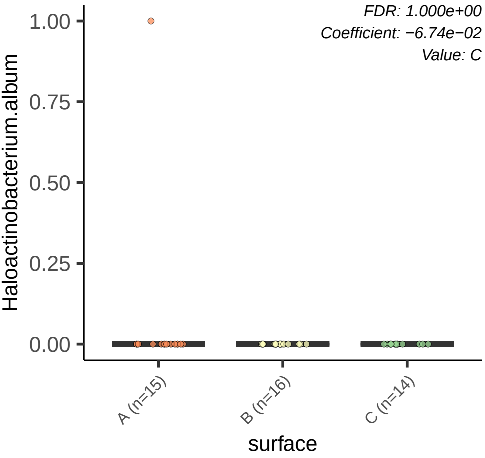

FDR: 1.000e+00
Coefficient: −6.74e−02
Value: C
Haloactinobacterium.album
1.00
0.75
0.50
0.25
0.00
A (n=15)
B (n=16)
C (n=14)
surface

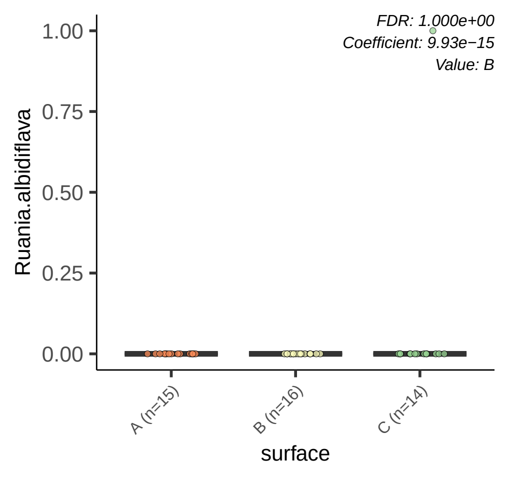
FDR: 1.000e+00
Coefficient: 9.93e−15
Value: B
Ruania.albidiflava
1.00
0.75
0.50
0.25
0.00
A (n=15)
B (n=16)
C (n=14)
surface

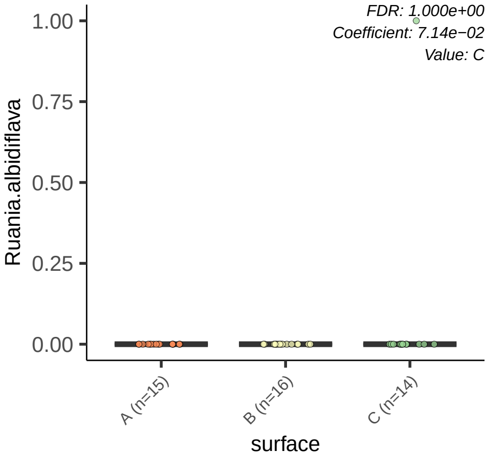
FDR: 1.000e+00
Coefficient: 7.14e−02
Value: C
Ruania.albidiflava
1.00
0.75
0.50
0.25
0.00
A (n=15)
B (n=16)
C (n=14)
surface

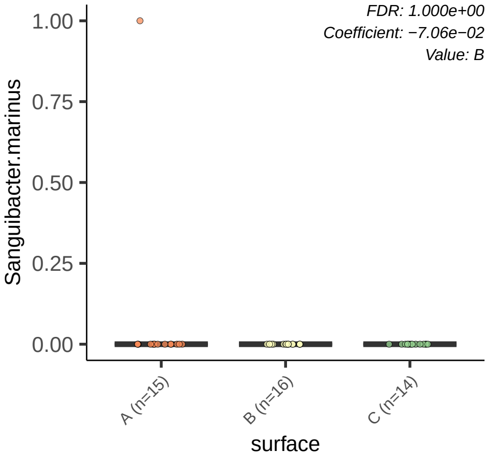

FDR: 1.000e+00
Coefficient: −7.06e−02
Value: B
Sanguibacter.marinus
1.00
0.75
0.50
0.25
0.00
A (n=15)
B (n=16)
C (n=14)
surface

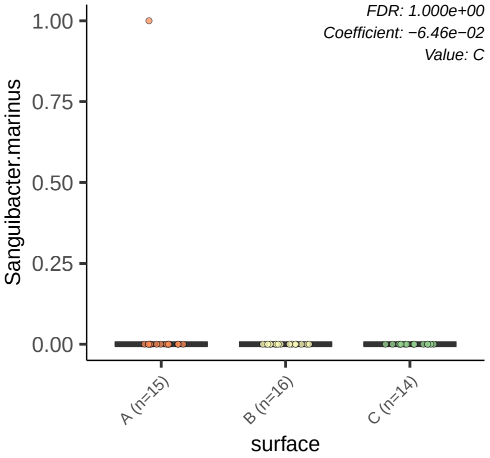

FDR: 1.000e+00
Coefficient: −6.46e−02
Value: C
Sanguibacter.marinus
1.00
0.75
0.50
0.25
0.00
A (n=15)
B (n=16)
C (n=14)
surface

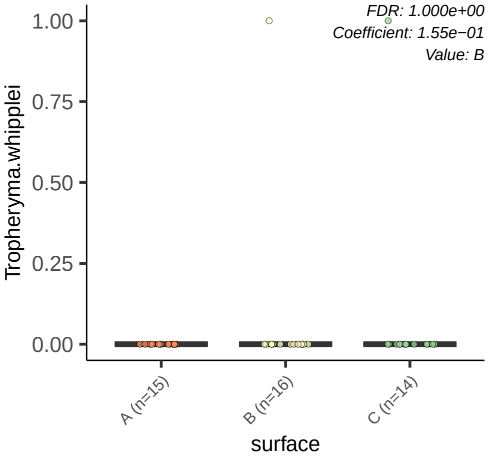
FDR: 1.000e+00
Coefficient: 1.55e−01
Value: B
Tropheryma.whipplei
1.00
0.75
0.50
0.25
0.00
A (n=15)
B (n=16)
C (n=14)
surface

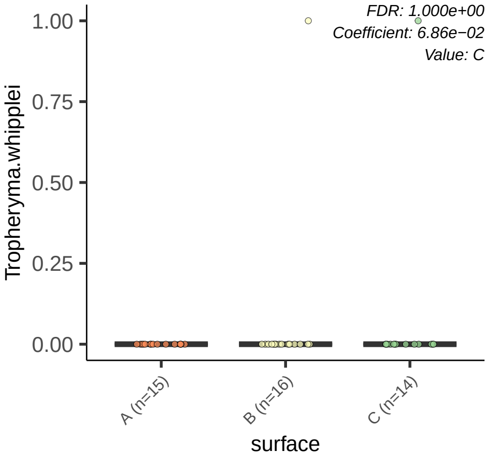

FDR: 1.000e+00
Coefficient: 6.86e−02
Value: C
Tropheryma.whipplei
1.00
0.75
0.50
0.25
0.00
A (n=15)
B (n=16)
C (n=14)
surface

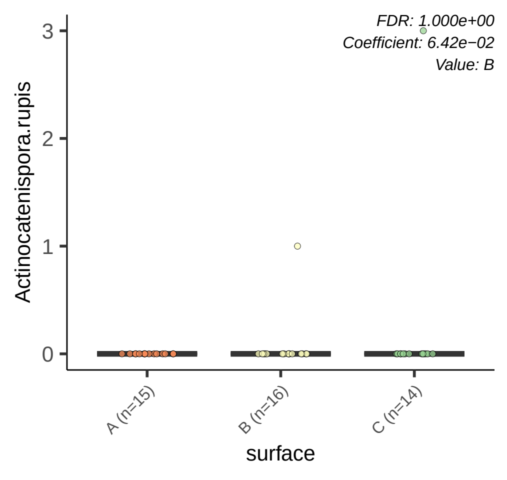
FDR: 1.000e+00
Coefficient: 6.42e−02
Value: B
Actinocatenispora.rupis
0
1
2
3
A (n=15)
B (n=16)
C (n=14)
surface

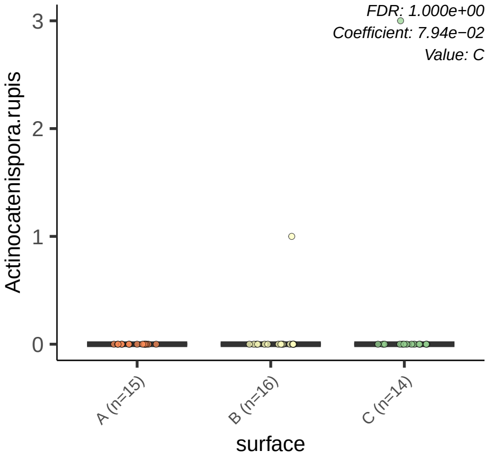

FDR: 1.000e+00
Coefficient: 7.94e−02
Value: C
Actinocatenispora.rupis
3
2
1
0
A (n=15)
B (n=16)
C (n=14)
surface

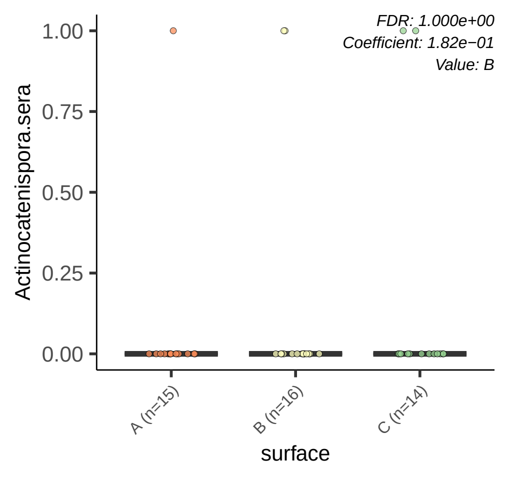
FDR: 1.000e+00
Coefficient: 1.82e−01
Value: B
Actinocatenispora.sera
1.00
0.75
0.50
0.25
0.00
A (n=15)
B (n=16)
C (n=14)
surface

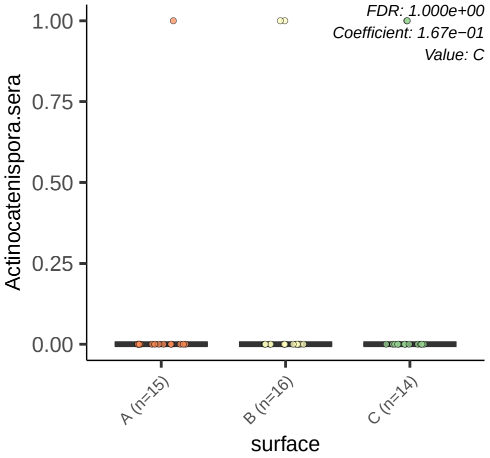

FDR: 1.000e+00
Coefficient: 1.67e−01
Value: C
Actinocatenispora.sera
1.00
0.75
0.50
0.25
0.00
A (n=15)
B (n=16)
C (n=14)
surface

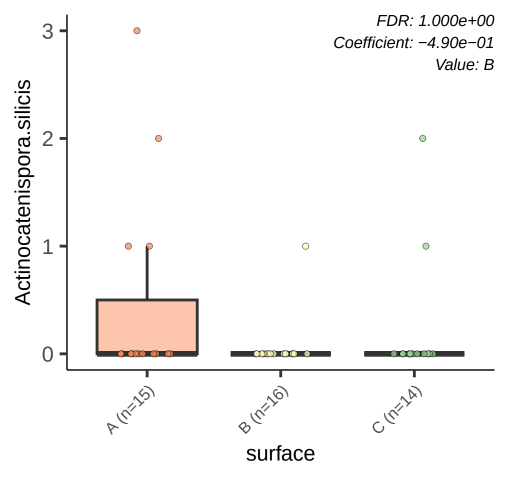
FDR: 1.000e+00
Coefficient: −4.90e−01
Value: B
Actinocatenispora.silicis
0
1
2
3
A (n=15)
B (n=16)
C (n=14)
surface

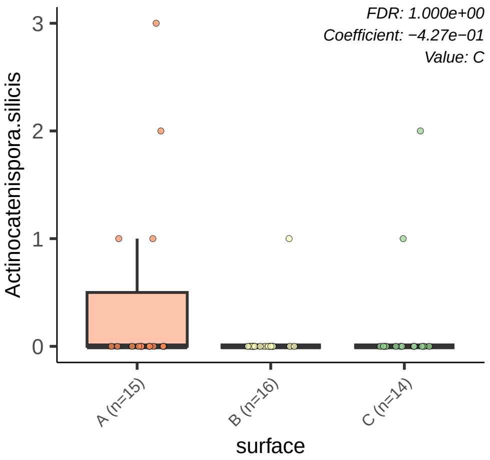
FDR: 1.000e+00
Coefficient: −4.27e−01
Value: C
Actinocatenispora.silicis
0
1
2
3
A (n=15)
B (n=16)
C (n=14)
surface

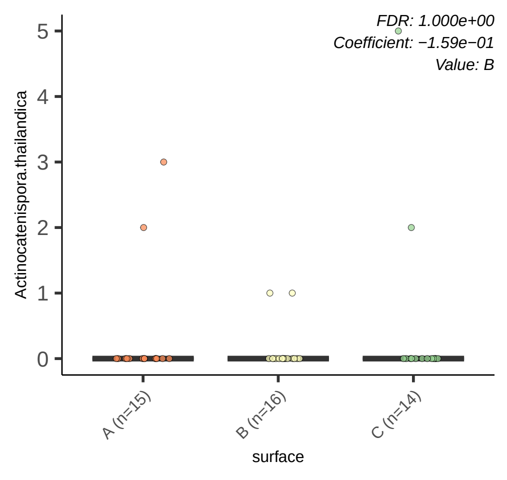

FDR: 1.000e+00
Coefficient: −1.59e−01
Value: B
Actinocatenispora.thailandica
0
1
2
3
4
5
A (n=15)
B (n=16)
C (n=14)
surface

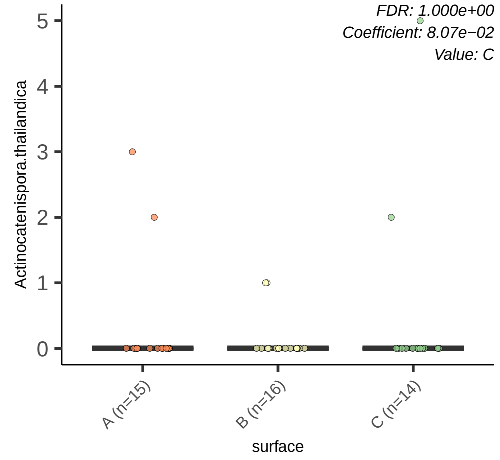

FDR: 1.000e+00
Coefficient: 8.07e−02
Value: C
Actinocatenispora.thailandica
0
1
2
3
4
5
A (n=15)
B (n=16)
C (n=14)
surface

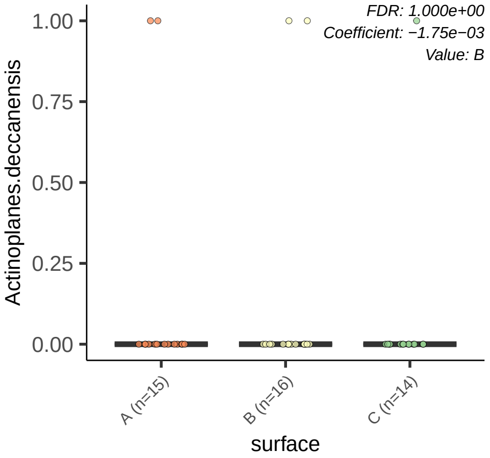
FDR: 1.000e+00
Coefficient: −1.75e−03
Value: B
Actinoplanes.deccanensis
1.00
0.75
0.50
0.25
0.00
A (n=15)
B (n=16)
C (n=14)
surface

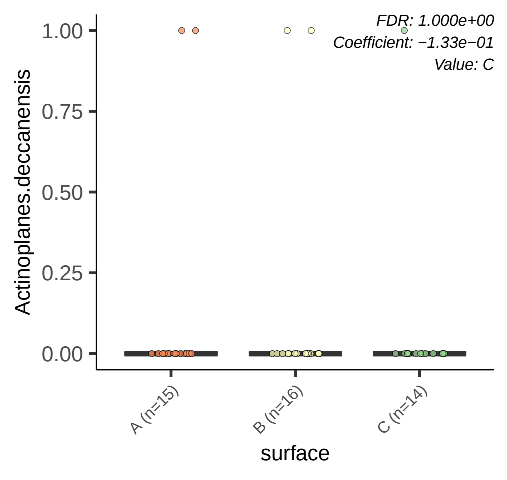

FDR: 1.000e+00
Coefficient: −1.33e−01
Value: C
Actinoplanes.deccanensis
1.00
0.75
0.50
0.25
0.00
A (n=15)
B (n=16)
C (n=14)
surface

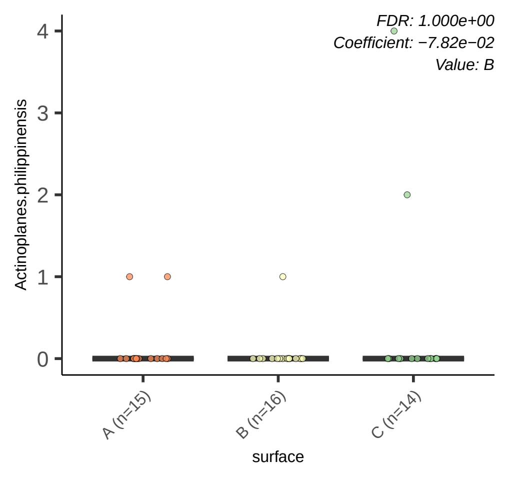
FDR: 1.000e+00
Coefficient: −7.82e−02
Value: B
Actinoplanes.philippinensis
4
3
2
1
0
A (n=15)
B (n=16)
C (n=14)
surface

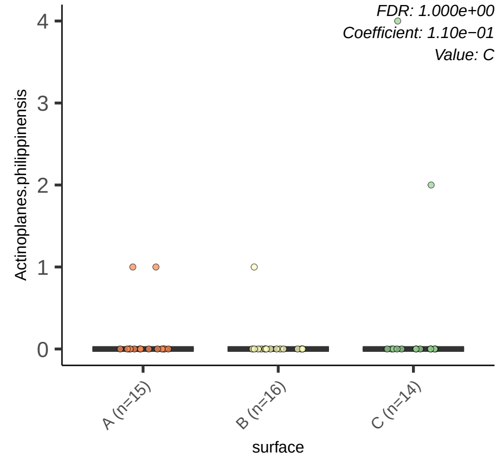

FDR: 1.000e+00
Coefficient: 1.10e−01
Value: C
Actinoplanes.philippinensis
0
1
2
3
4
A (n=15)
B (n=16)
C (n=14)
surface

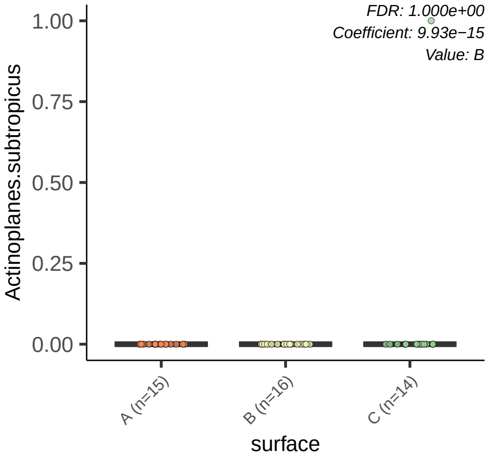
FDR: 1.000e+00
Coefficient: 9.93e−15
Value: B
Actinoplanes.subtropicus
1.00
0.75
0.50
0.25
0.00
A (n=15)
B (n=16)
C (n=14)
surface

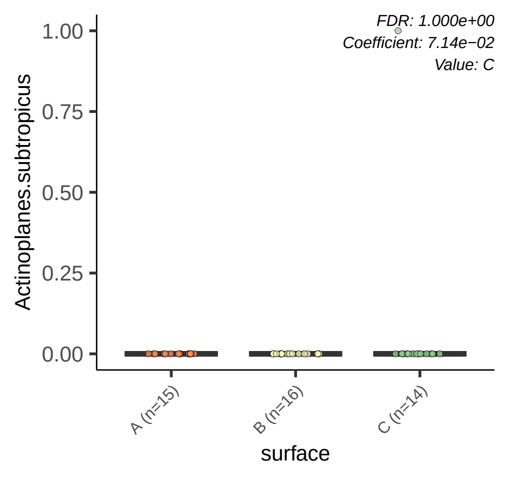

FDR: 1.000e+00
Coefficient: 7.14e−02
Value: C
Actinoplanes.subtropicus
1.00
0.75
0.50
0.25
0.00
A (n=15)
B (n=16)
C (n=14)
surface

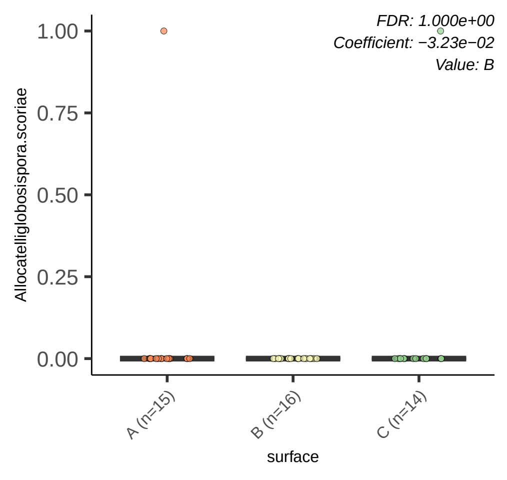
FDR: 1.000e+00
Coefficient: −3.23e−02
Value: B
Allocatelliglobosispora.scoriae
1.00
0.75
0.50
0.25
0.00
A (n=15)
B (n=16)
C (n=14)
surface

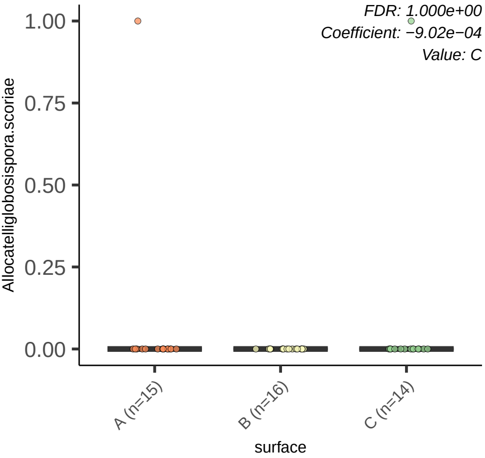

FDR: 1.000e+00
Coefficient: −9.02e−04
Value: C
1.00
0.75
0.50
0.25
0.00
Allocatelliglobosispora.scoriae
A (n=15)
B (n=16)
C (n=14)
surface

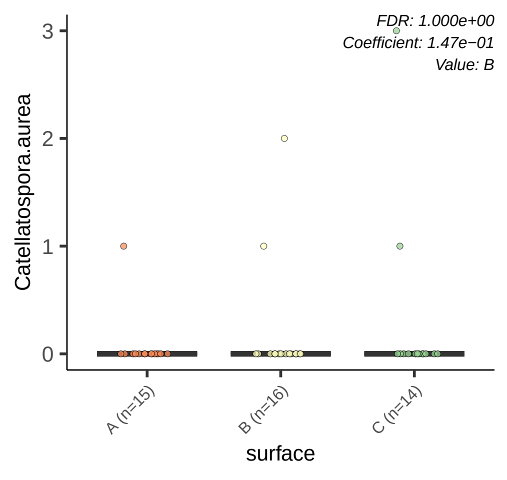
FDR: 1.000e+00
Coefficient: 1.47e−01
Value: B
Catellatospora.aurea
3
2
1
0
A (n=15)
B (n=16)
C (n=14)
surface

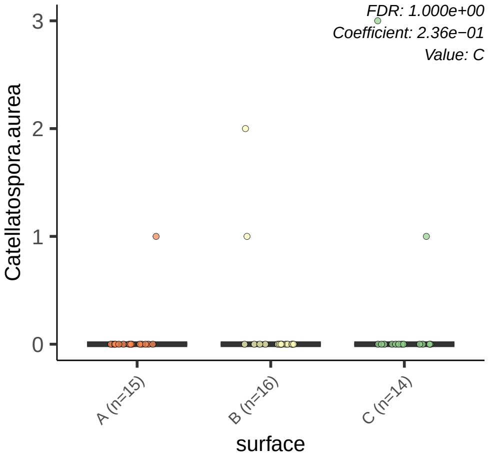

FDR: 1.000e+00
Coefficient: 2.36e−01
Value: C
Catellatospora.aurea
3
2
1
0
A (n=15)
B (n=16)
C (n=14)
surface

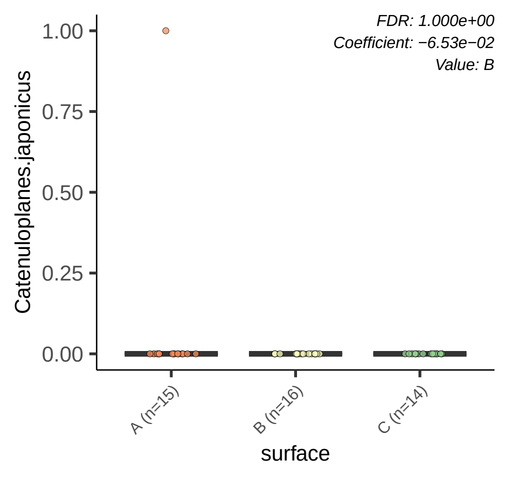

FDR: 1.000e+00
Coefficient: −6.53e−02
Value: B
Catenuloplanes.japonicus
1.00
0.75
0.50
0.25
0.00
A (n=15)
B (n=16)
C (n=14)
surface

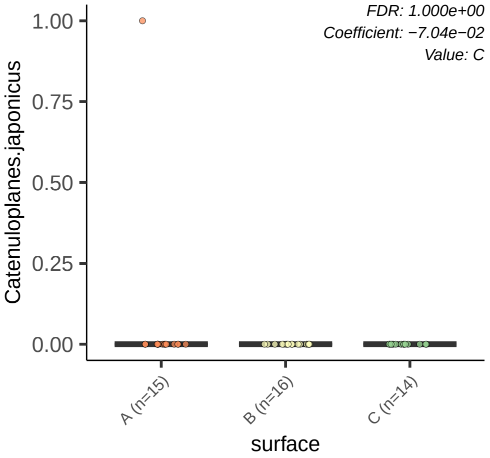
FDR: 1.000e+00
Coefficient: −7.04e−02
Value: C
Catenuloplanes.japonicus
1.00
0.75
0.50
0.25
0.00
A (n=15)
B (n=16)
C (n=14)
surface

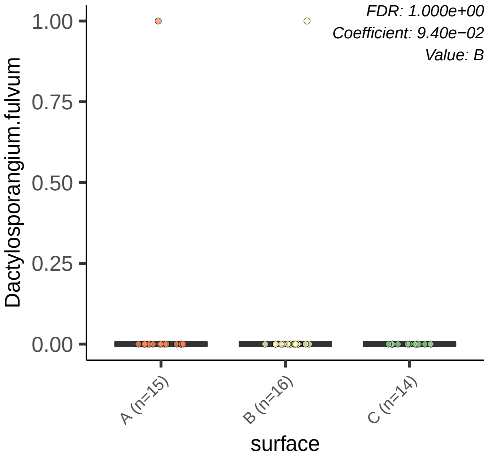
FDR: 1.000e+00
Coefficient: 9.40e−02
Value: B
Dactylosporangium.fulvum
1.00
0.75
0.50
0.25
0.00
A (n=15)
B (n=16)
C (n=14)
surface

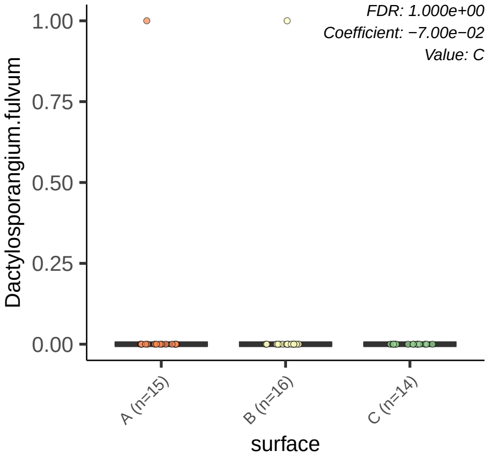

FDR: 1.000e+00
Coefficient: −7.00e−02
Value: C
1.00
0.75
0.50
0.25
0.00
Dactylosporangium.fulvum
A (n=15)
B (n=16)
C (n=14)
surface

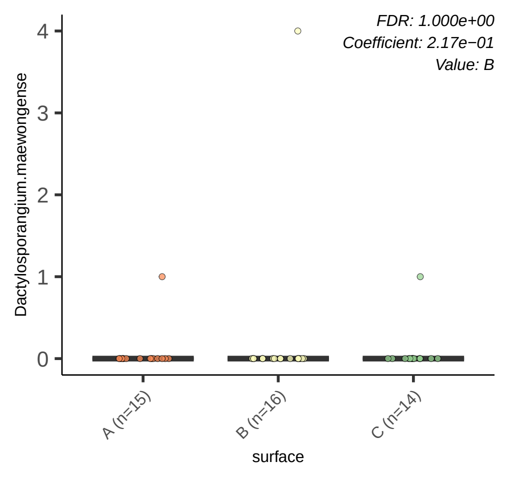

FDR: 1.000e+00
Coefficient: 2.17e−01
Value: B
Dactylosporangium.maewongense
0
1
2
3
4
A (n=15)
B (n=16)
C (n=14)
surface

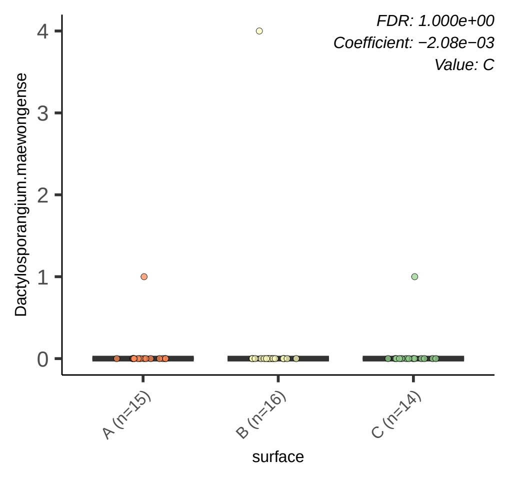

FDR: 1.000e+00
Coefficient: −2.08e−03
Value: C
Dactylosporangium.maewongense
4
3
2
1
0
A (n=15)
B (n=16)
C (n=14)
surface

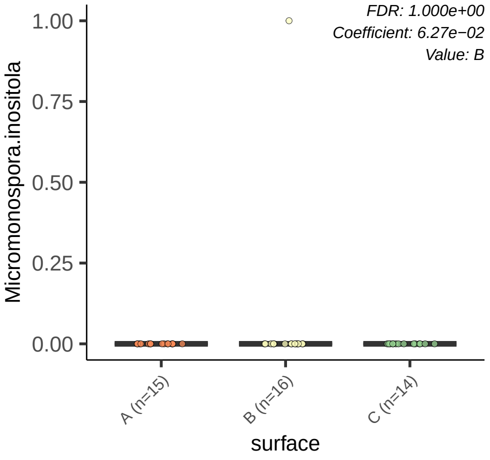

FDR: 1.000e+00
Coefficient: 6.27e−02
Value: B
Micromonospora.inositola
1.00
0.75
0.50
0.25
0.00
A (n=15)
B (n=16)
C (n=14)
surface

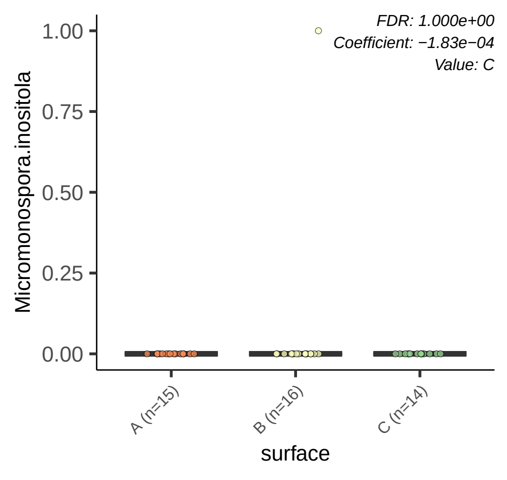
FDR: 1.000e+00
Coefficient: −1.83e−04
Value: C
Micromonospora.inositola
1.00
0.75
0.50
0.25
0.00
A (n=15)
B (n=16)
C (n=14)
surface

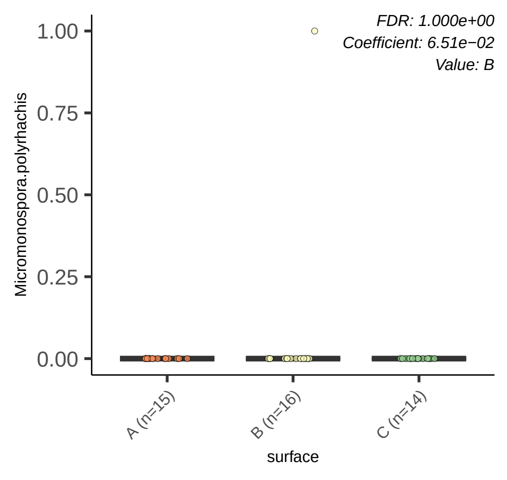
FDR: 1.000e+00
Coefficient: 6.51e−02
Value: B
Micromonospora.polyrhachis
1.00
0.75
0.50
0.25
0.00
A (n=15)
B (n=16)
C (n=14)
surface

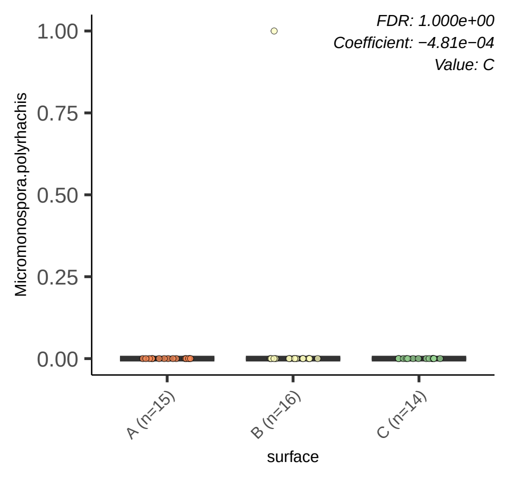
FDR: 1.000e+00
Coefficient: −4.81e−04
Value: C
Micromonospora.polyrhachis
1.00
0.75
0.50
0.25
0.00
A (n=15)
B (n=16)
C (n=14)
surface

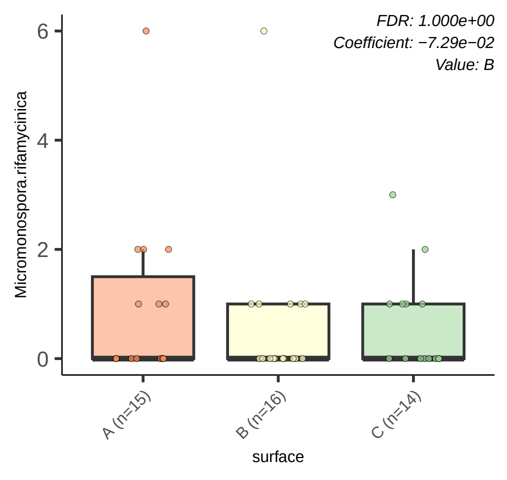
FDR: 1.000e+00
Coefficient: −7.29e−02
Value: B
Micromonospora.rifamycinica
0
2
4
6
A (n=15)
B (n=16)
C (n=14)
surface

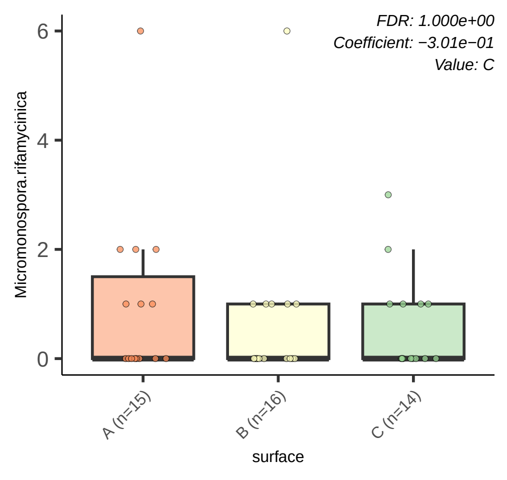
FDR: 1.000e+00
Coefficient: −3.01e−01
Value: C
Micromonospora.rifamycinica
0
2
4
6
A (n=15)
B (n=16)
C (n=14)
surface

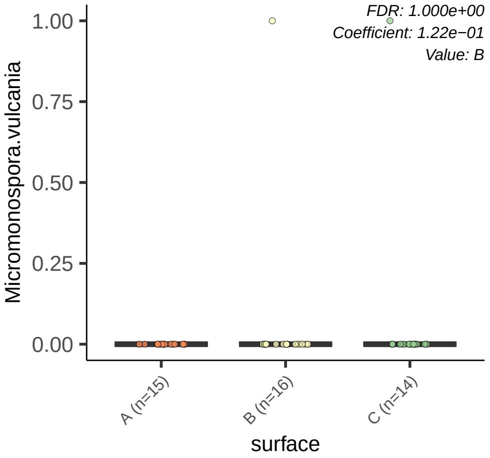
FDR: 1.000e+00
Coefficient: 1.22e−01
Value: B
Micromonospora.vulcania
1.00
0.75
0.50
0.25
0.00
A (n=15)
B (n=16)
C (n=14)
surface

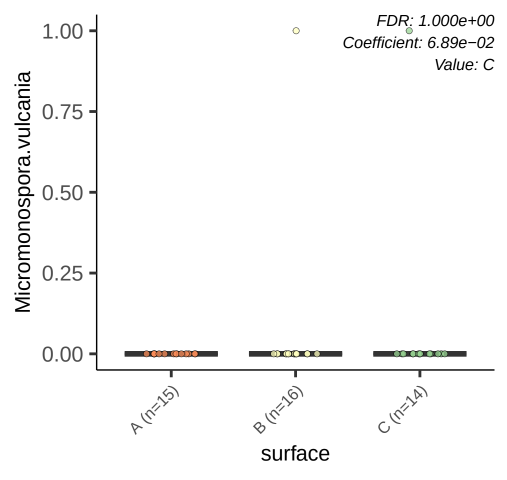
FDR: 1.000e+00
Coefficient: 6.89e−02
Value: C
Micromonospora.vulcania
1.00
0.75
0.50
0.25
0.00
A (n=15)
B (n=16)
C (n=14)
surface

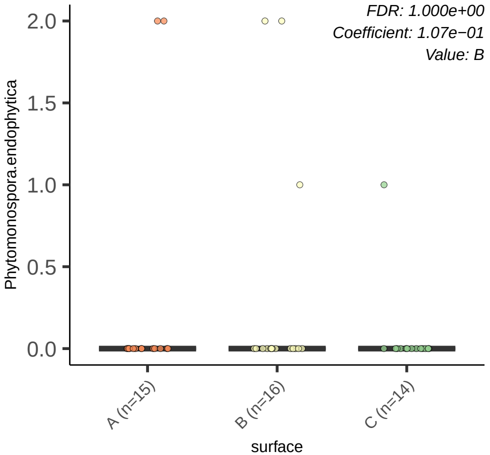

FDR: 1.000e+00
Coefficient: 1.07e−01
Value: B
2.0
1.5
1.0
0.5
0.0
Phytomonospora.endophytica
A (n=15)
B (n=16)
C (n=14)
surface

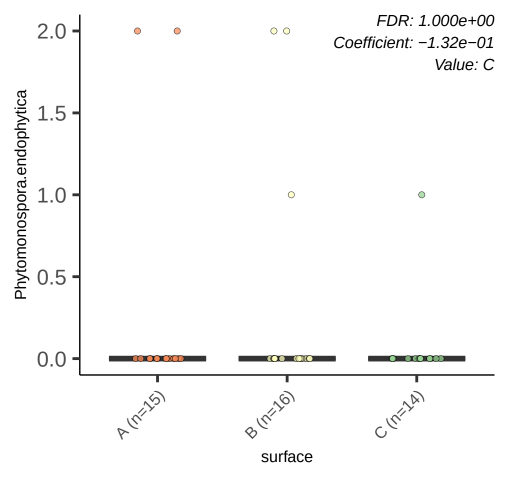
FDR: 1.000e+00
Coefficient: −1.32e−01
Value: C
Phytomonospora.endophytica
2.0
1.5
1.0
0.5
0.0
A (n=15)
B (n=16)
C (n=14)
surface

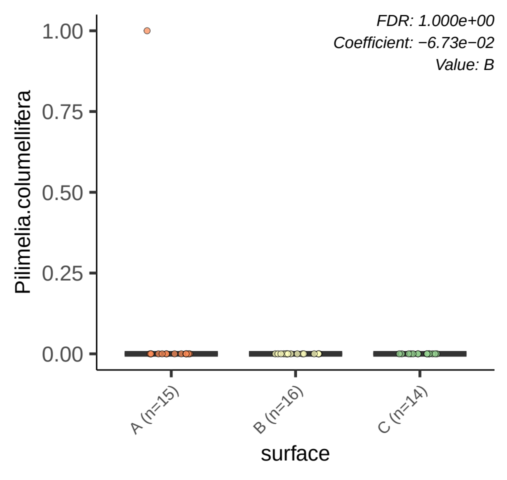
FDR: 1.000e+00
Coefficient: −6.73e−02
Value: B
Pilimelia.columellifera
1.00
0.75
0.50
0.25
0.00
A (n=15)
B (n=16)
C (n=14)
surface

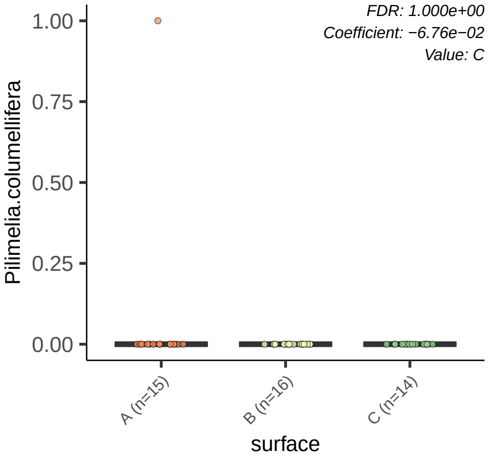
FDR: 1.000e+00
Coefficient: −6.76e−02
Value: C
Pilimelia.columellifera
1.00
0.75
0.50
0.25
0.00
A (n=15)
B (n=16)
C (n=14)
surface

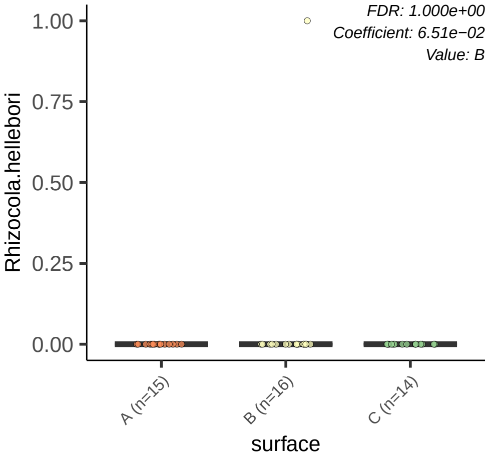

FDR: 1.000e+00
Coefficient: 6.51e−02
Value: B
1.00
0.75
0.50
0.25
0.00
Rhizocola.hellebori
A (n=15)
B (n=16)
C (n=14)
surface

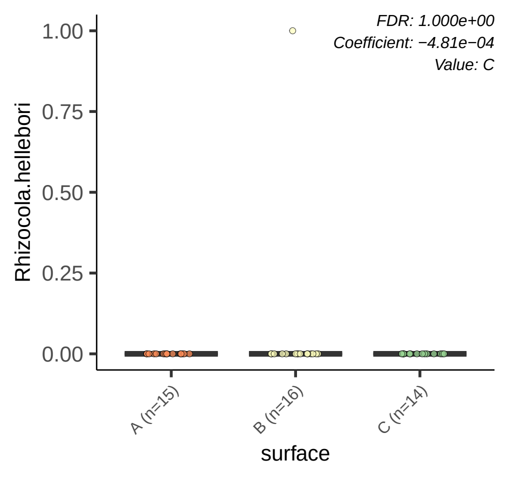
FDR: 1.000e+00
Coefficient: −4.81e−04
Value: C
Rhizocola.hellebori
1.00
0.75
0.50
0.25
0.00
A (n=15)
B (n=16)
C (n=14)
surface

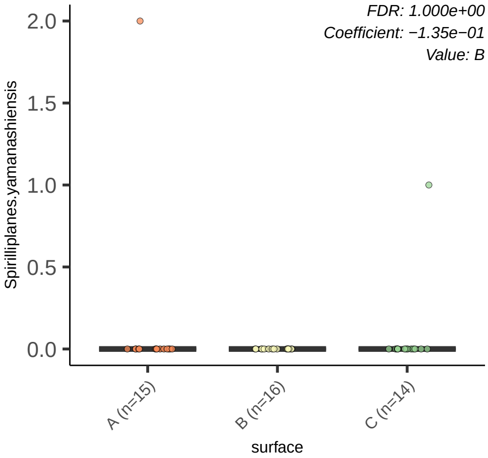
FDR: 1.000e+00
Coefficient: −1.35e−01
Value: B
Spirilliplanes.yamanashiensis
2.0
1.5
1.0
0.5
0.0
A (n=15)
B (n=16)
C (n=14)
surface

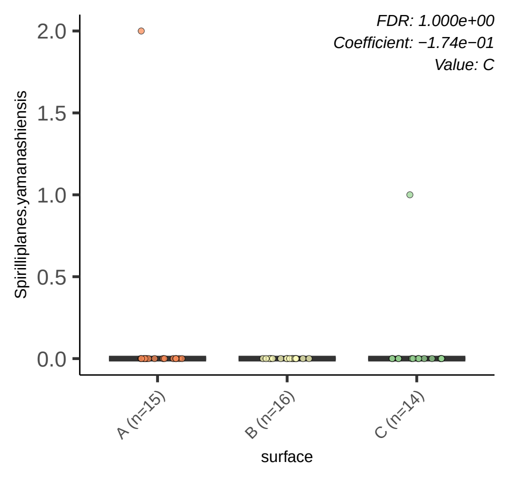

FDR: 1.000e+00
Coefficient: −1.74e−01
Value: C
Spirilliplanes.yamanashiensis
2.0
1.5
1.0
0.5
0.0
A (n=15)
B (n=16)
C (n=14)
surface

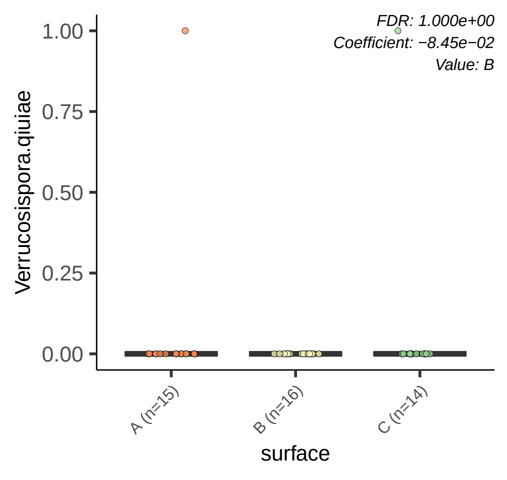

FDR: 1.000e+00
Coefficient: −8.45e−02
Value: B
Verrucosispora.qiuiae
1.00
0.75
0.50
0.25
0.00
A (n=15)
B (n=16)
C (n=14)
surface

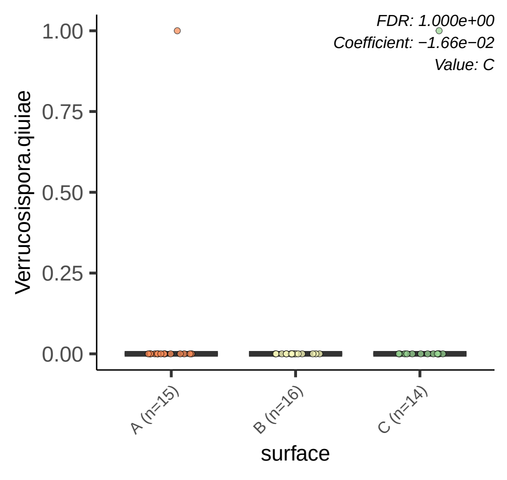
FDR: 1.000e+00
Coefficient: −1.66e−02
Value: C
Verrucosispora.qiuiae
1.00
0.75
0.50
0.25
0.00
A (n=15)
B (n=16)
C (n=14)
surface

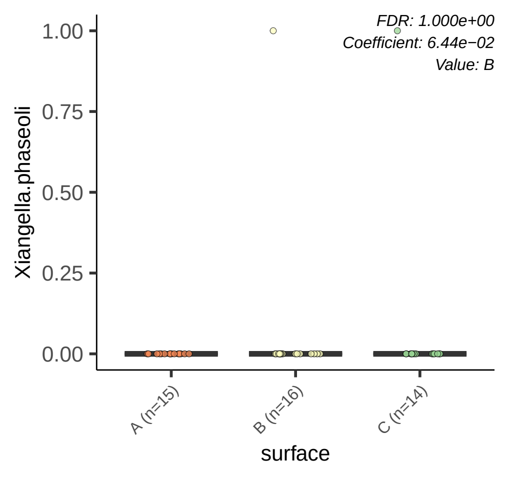
FDR: 1.000e+00
Coefficient: 6.44e−02
Value: B
Xiangella.phaseoli
1.00
0.75
0.50
0.25
0.00
A (n=15)
B (n=16)
C (n=14)
surface

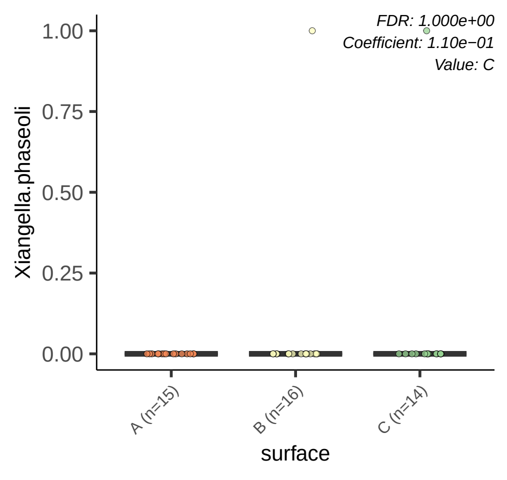

FDR: 1.000e+00
Coefficient: 1.10e−01
Value: C
Xiangella.phaseoli
1.00
0.75
0.50
0.25
0.00
A (n=15)
B (n=16)
C (n=14)
surface

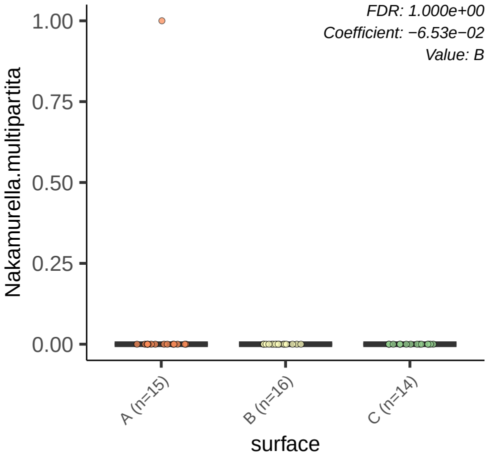
FDR: 1.000e+00
Coefficient: −6.53e−02
Value: B
Nakamurella.multipartita
1.00
0.75
0.50
0.25
0.00
A (n=15)
B (n=16)
C (n=14)
surface

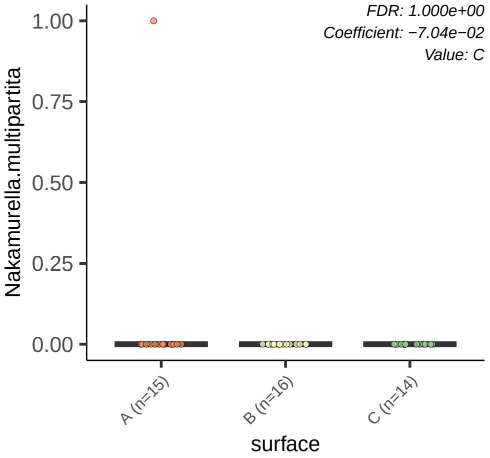
FDR: 1.000e+00
Coefficient: −7.04e−02
Value: C
Nakamurella.multipartita
1.00
0.75
0.50
0.25
0.00
A (n=15)
B (n=16)
C (n=14)
surface

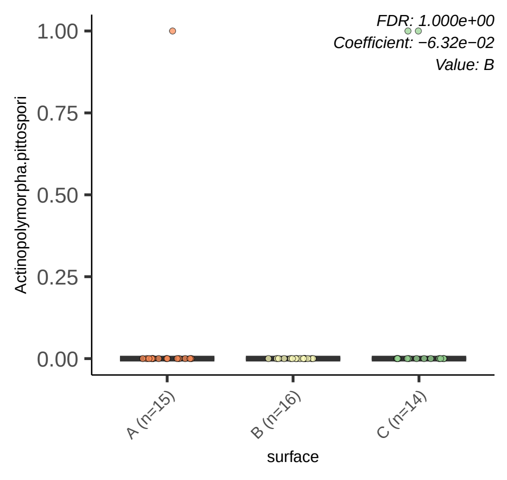
FDR: 1.000e+00
Coefficient: −6.32e−02
Value: B
1.00
0.75
0.50
0.25
0.00
Actinopolymorpha.pittospori
A (n=15)
B (n=16)
C (n=14)
surface

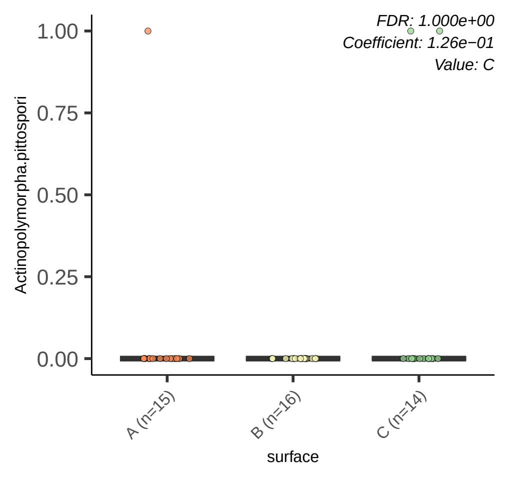

FDR: 1.000e+00
Coefficient: 1.26e−01
Value: C
Actinopolymorpha.pittospori
1.00
0.75
0.50
0.25
0.00
A (n=15)
B (n=16)
C (n=14)
surface

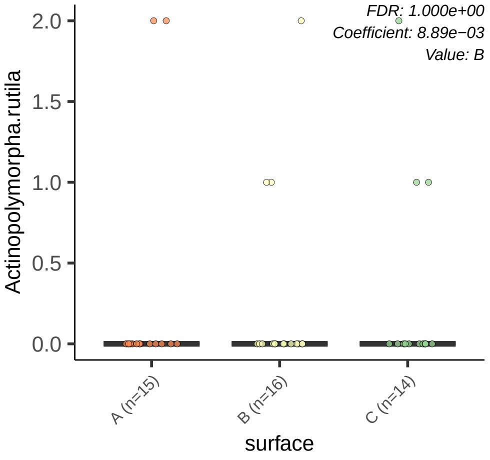
FDR: 1.000e+00
Coefficient: 8.89e−03
Value: B
Actinopolymorpha.rutila
2.0
1.5
1.0
0.5
0.0
A (n=15)
B (n=16)
C (n=14)
surface

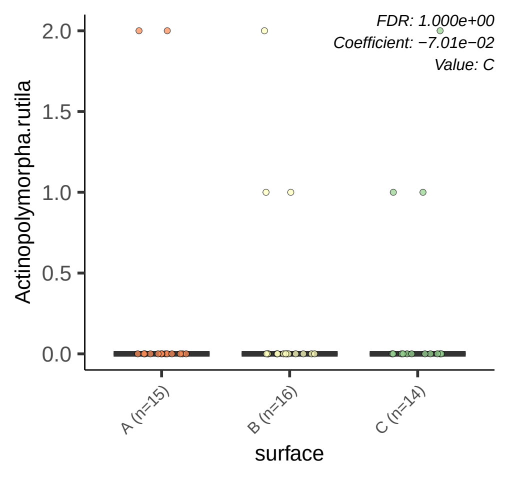
FDR: 1.000e+00
Coefficient: −7.01e−02
Value: C
Actinopolymorpha.rutila
2.0
1.5
1.0
0.5
0.0
A (n=15)
B (n=16)
C (n=14)
surface

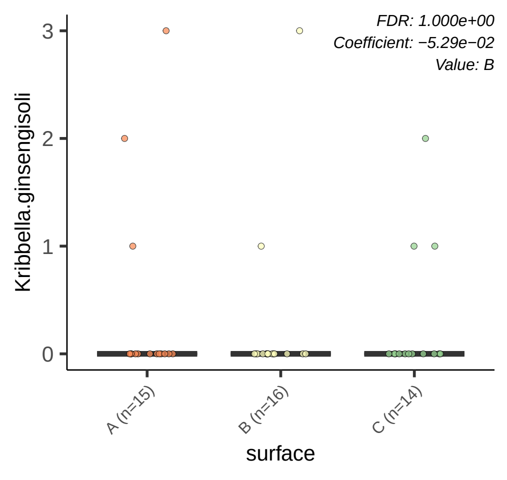

FDR: 1.000e+00
Coefficient: −5.29e−02
Value: B
Kribbella.ginsengisoli
3
2
1
0
A (n=15)
B (n=16)
C (n=14)
surface

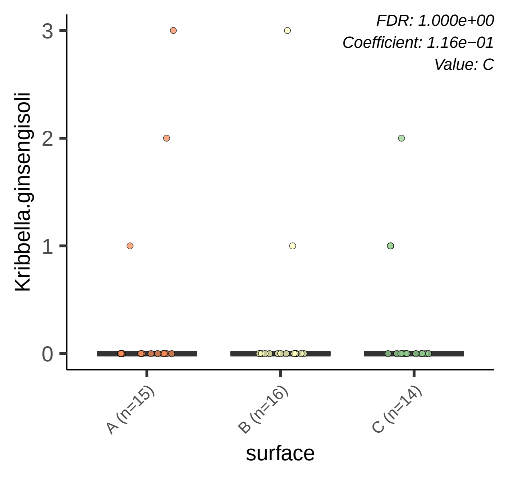

FDR: 1.000e+00
Coefficient: 1.16e−01
Value: C
Kribbella.ginsengisoli
3
2
1
0
A (n=15)
B (n=16)
C (n=14)
surface

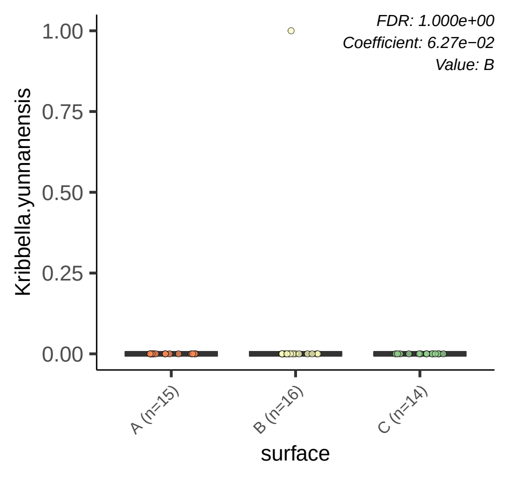

FDR: 1.000e+00
Coefficient: 6.27e−02
Value: B
Kribbella.yunnanensis
1.00
0.75
0.50
0.25
0.00
A (n=15)
B (n=16)
C (n=14)
surface

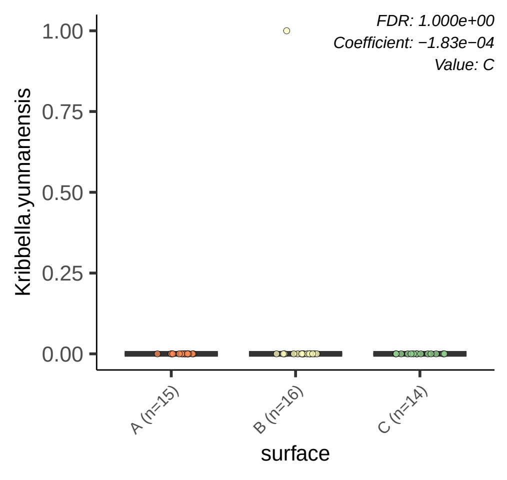

FDR: 1.000e+00
Coefficient: −1.83e−04
Value: C
Kribbella.yunnanensis
1.00
0.75
0.50
0.25
0.00
A (n=15)
B (n=16)
C (n=14)
surface

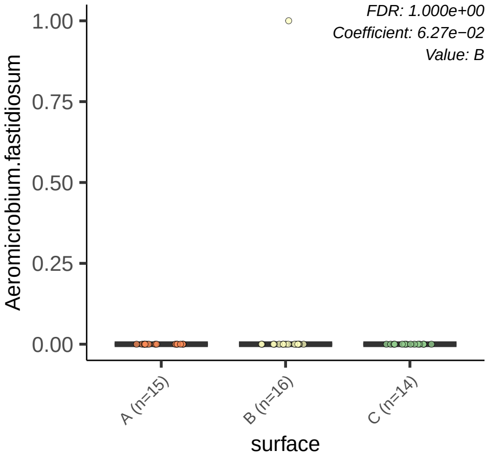

FDR: 1.000e+00
Coefficient: 6.27e−02
Value: B
Aeromicrobium.fastidiosum
1.00
0.75
0.50
0.25
0.00
A (n=15)
B (n=16)
C (n=14)
surface

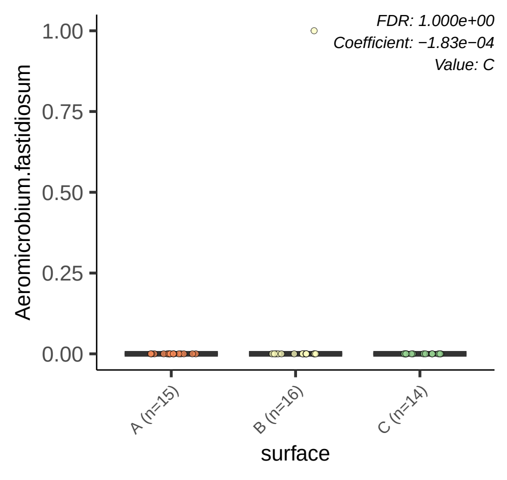
FDR: 1.000e+00
Coefficient: −1.83e−04
Value: C
1.00
0.75
0.50
0.25
0.00
Aeromicrobium.fastidiosum
A (n=15)
B (n=16)
C (n=14)
surface

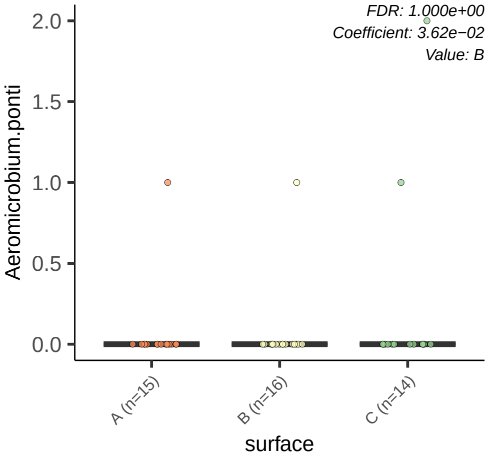
FDR: 1.000e+00
Coefficient: 3.62e−02
Value: B
Aeromicrobium.ponti
2.0
1.5
1.0
0.5
0.0
A (n=15)
B (n=16)
C (n=14)
surface

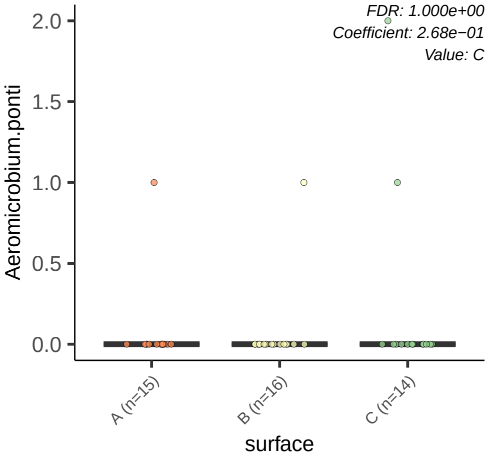
FDR: 1.000e+00
Coefficient: 2.68e−01
Value: C
Aeromicrobium.ponti
2.0
1.5
1.0
0.5
0.0
A (n=15)
B (n=16)
C (n=14)
surface

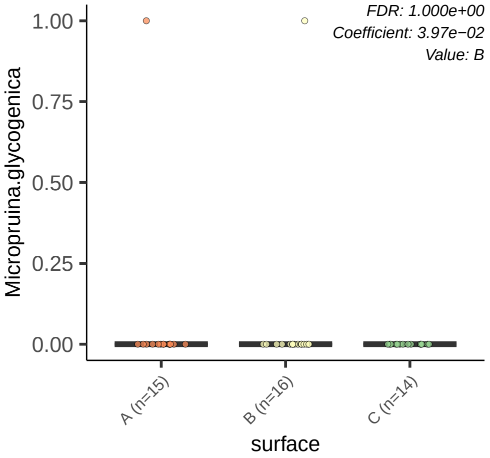

FDR: 1.000e+00
Coefficient: 3.97e−02
Value: B
Micropruina.glycogenica
1.00
0.75
0.50
0.25
0.00
A (n=15)
B (n=16)
C (n=14)
surface

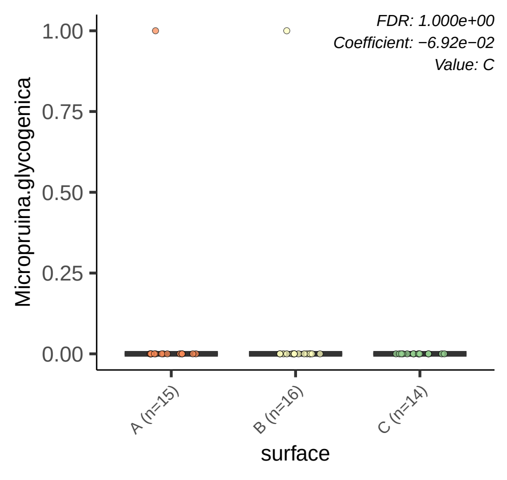

FDR: 1.000e+00
Coefficient: −6.92e−02
Value: C
Micropruina.glycogenica
1.00
0.75
0.50
0.25
0.00
A (n=15)
B (n=16)
C (n=14)
surface

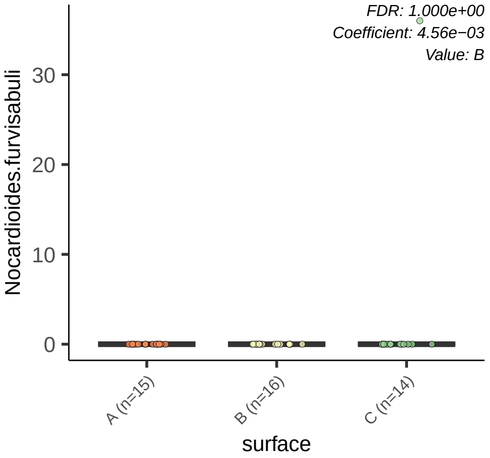

FDR: 1.000e+00
Coefficient: 4.56e−03
Value: B
Nocardioides.furvisabuli
0
10
20
30
A (n=15)
B (n=16)
C (n=14)
surface

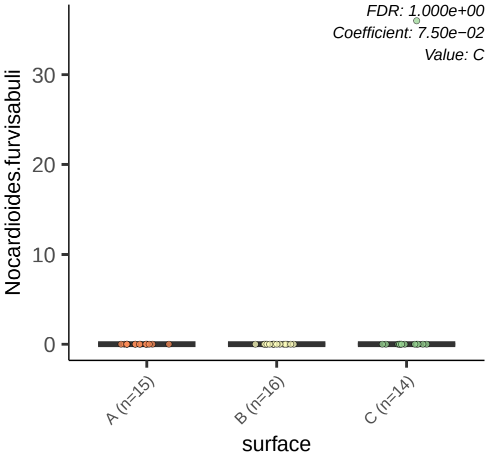

FDR: 1.000e+00
Coefficient: 7.50e−02
Value: C
Nocardioides.furvisabuli
0
10
20
30
A (n=15)
B (n=16)
C (n=14)
surface

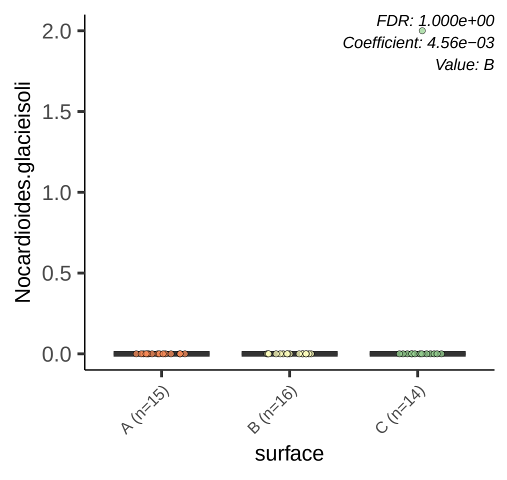

FDR: 1.000e+00
Coefficient: 4.56e−03
Value: B
Nocardioides.glacieisoli
2.0
1.5
1.0
0.5
0.0
A (n=15)
B (n=16)
C (n=14)
surface

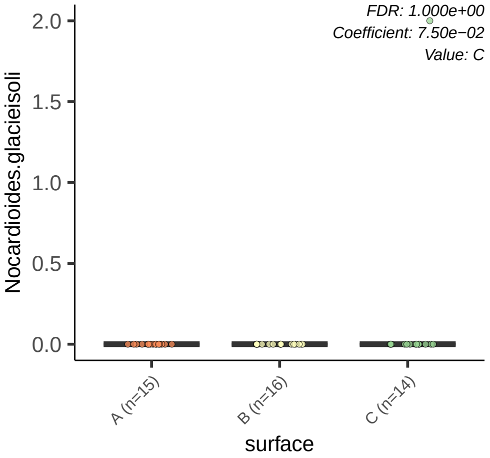

FDR: 1.000e+00
Coefficient: 7.50e−02
Value: C
Nocardioides.glacieisoli
2.0
1.5
1.0
0.5
0.0
A (n=15)
B (n=16)
C (n=14)
surface

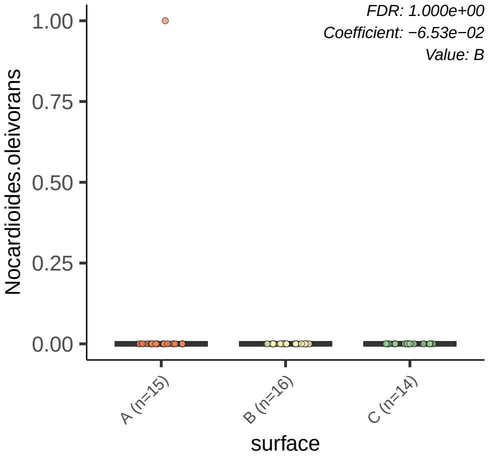
FDR: 1.000e+00
Coefficient: −6.53e−02
Value: B
Nocardioides.oleivorans
1.00
0.75
0.50
0.25
0.00
A (n=15)
B (n=16)
C (n=14)
surface

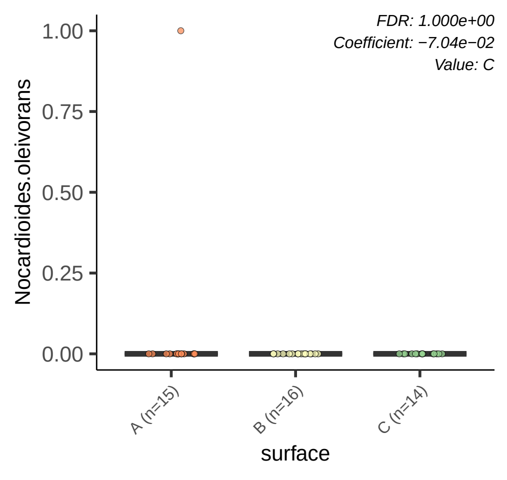
FDR: 1.000e+00
Coefficient: −7.04e−02
Value: C
Nocardioides.oleivorans
1.00
0.75
0.50
0.25
0.00
A (n=15)
B (n=16)
C (n=14)
surface

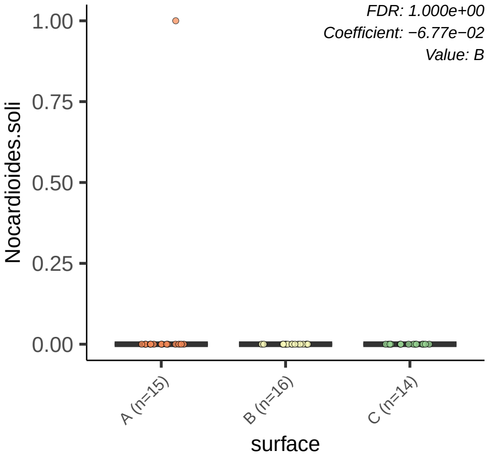

FDR: 1.000e+00
Coefficient: −6.77e−02
Value: B
Nocardioides.soli
1.00
0.75
0.50
0.25
0.00
A (n=15)
B (n=16)
C (n=14)
surface

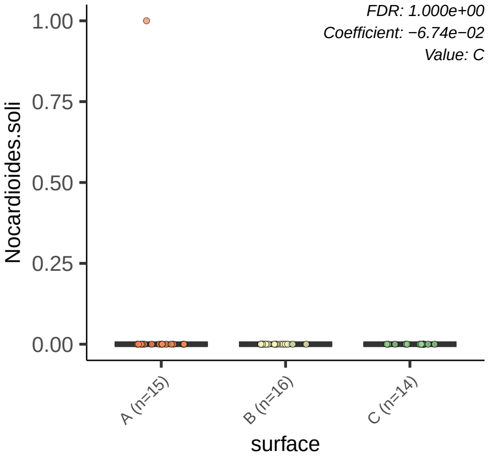
FDR: 1.000e+00
Coefficient: −6.74e−02
Value: C
1.00
0.75
0.50
0.25
0.00
Nocardioides.soli
A (n=15)
B (n=16)
C (n=14)
surface

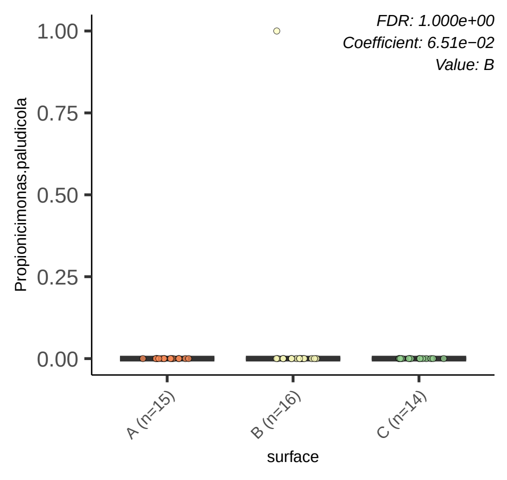
FDR: 1.000e+00
Coefficient: 6.51e−02
Value: B
1.00
0.75
0.50
0.25
0.00
Propionicimonas.paludicola
A (n=15)
B (n=16)
C (n=14)
surface

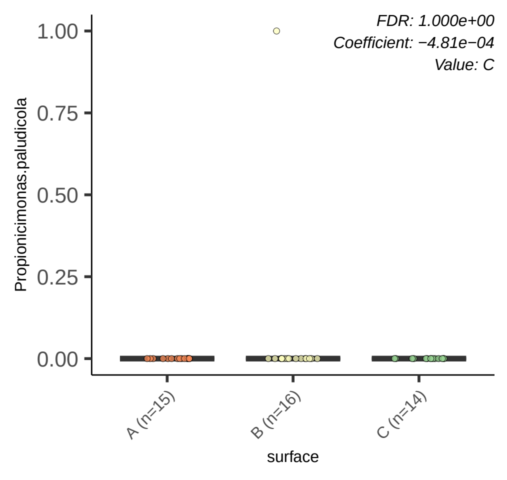
FDR: 1.000e+00
Coefficient: −4.81e−04
Value: C
Propionicimonas.paludicola
1.00
0.75
0.50
0.25
0.00
A (n=15)
B (n=16)
C (n=14)
surface

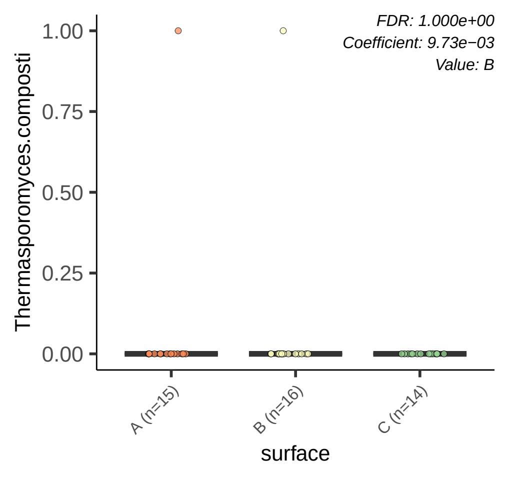

FDR: 1.000e+00
Coefficient: 9.73e−03
Value: B
Thermasporomyces.composti
1.00
0.75
0.50
0.25
0.00
A (n=15)
B (n=16)
C (n=14)
surface

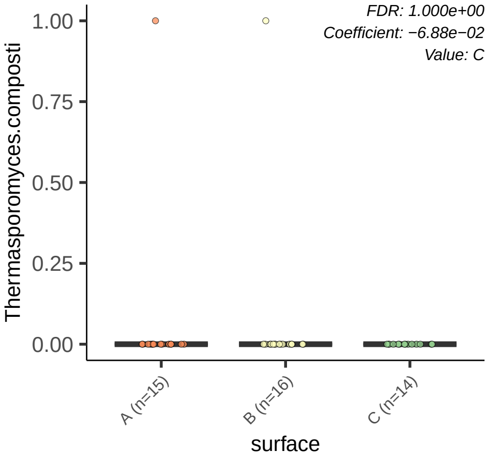

FDR: 1.000e+00
Coefficient: −6.88e−02
Value: C
Thermasporomyces.composti
1.00
0.75
0.50
0.25
0.00
A (n=15)
B (n=16)
C (n=14)
surface

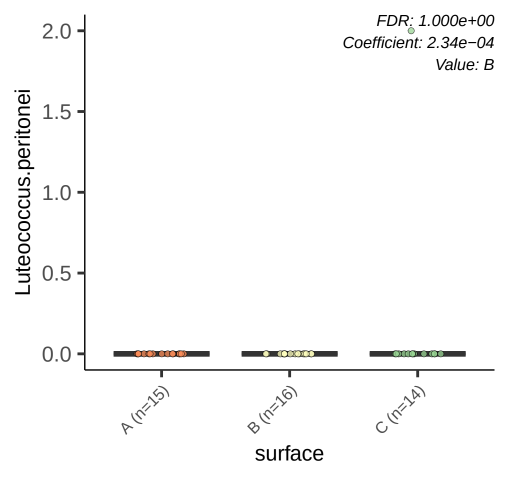
FDR: 1.000e+00
Coefficient: 2.34e−04
Value: B
Luteococcus.peritonei
2.0
1.5
1.0
0.5
0.0
A (n=15)
B (n=16)
C (n=14)
surface

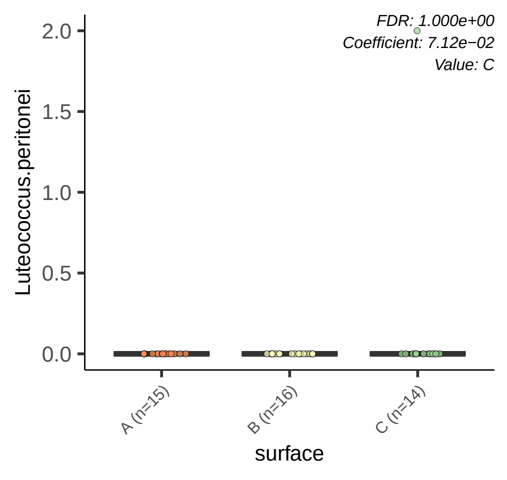

FDR: 1.000e+00
Coefficient: 7.12e−02
Value: C
Luteococcus.peritonei
2.0
1.5
1.0
0.5
0.0
A (n=15)
B (n=16)
C (n=14)
surface

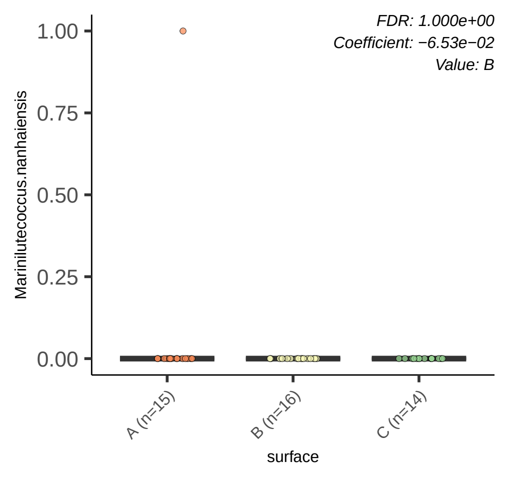
FDR: 1.000e+00
Coefficient: −6.53e−02
Value: B
1.00
0.75
0.50
0.25
0.00
Marinilutecoccus.nanhaiensis
A (n=15)
B (n=16)
C (n=14)
surface

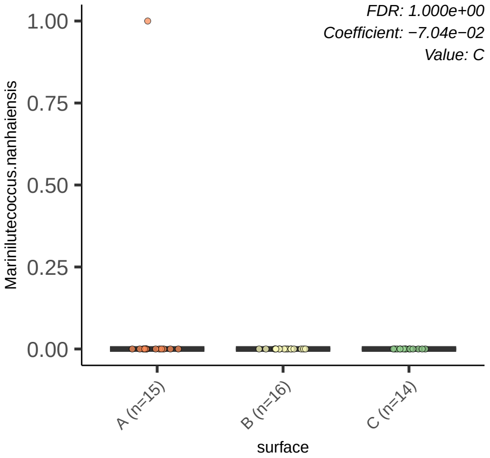
FDR: 1.000e+00
Coefficient: −7.04e−02
Value: C
Marinilutecoccus.nanhaiensis
1.00
0.75
0.50
0.25
0.00
A (n=15)
B (n=16)
C (n=14)
surface

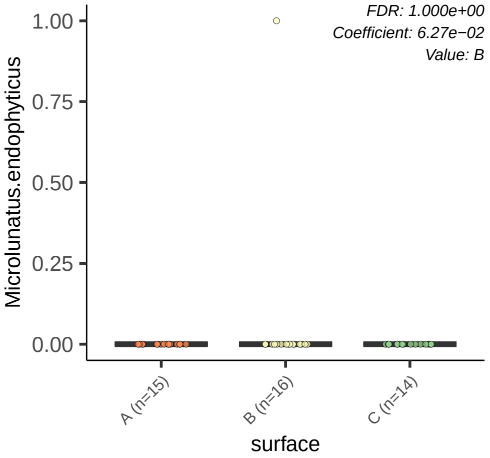

FDR: 1.000e+00
Coefficient: 6.27e−02
Value: B
Microlunatus.endophyticus
1.00
0.75
0.50
0.25
0.00
A (n=15)
B (n=16)
C (n=14)
surface

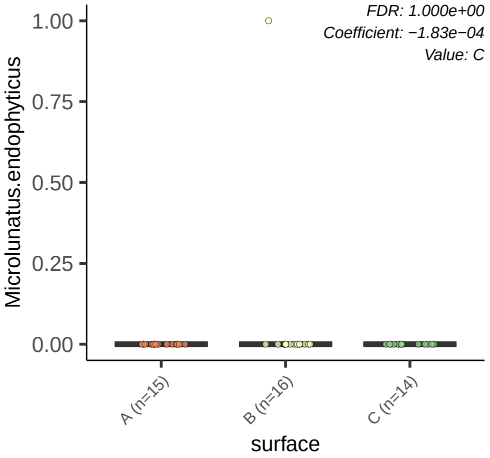
FDR: 1.000e+00
Coefficient: −1.83e−04
Value: C
Microlunatus.endophyticus
1.00
0.75
0.50
0.25
0.00
A (n=15)
B (n=16)
C (n=14)
surface

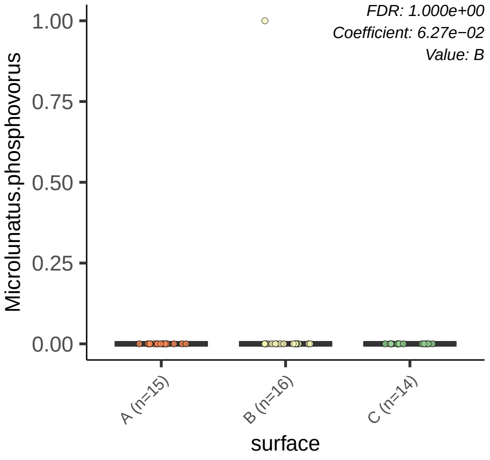

FDR: 1.000e+00
Coefficient: 6.27e−02
Value: B
Microlunatus.phosphovorus
1.00
0.75
0.50
0.25
0.00
A (n=15)
B (n=16)
C (n=14)
surface

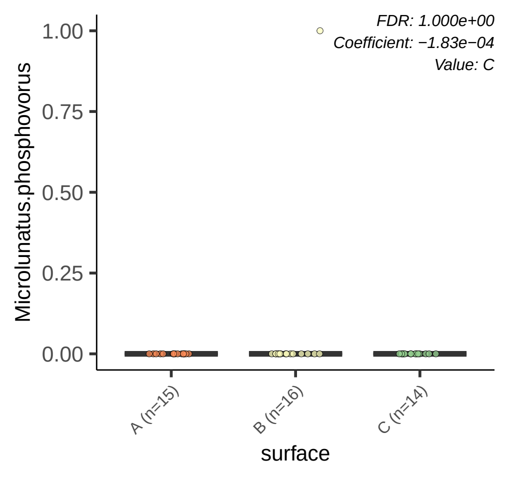
FDR: 1.000e+00
Coefficient: −1.83e−04
Value: C
Microlunatus.phosphovorus
1.00
0.75
0.50
0.25
0.00
A (n=15)
B (n=16)
C (n=14)
surface

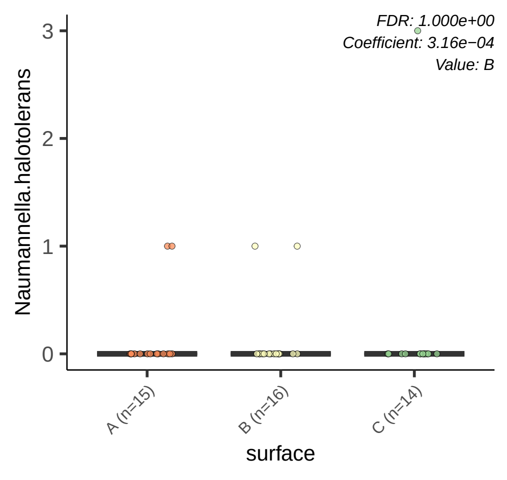

FDR: 1.000e+00
Coefficient: 3.16e−04
Value: B
Naumannella.halotolerans
3
2
1
0
A (n=15)
B (n=16)
C (n=14)
surface

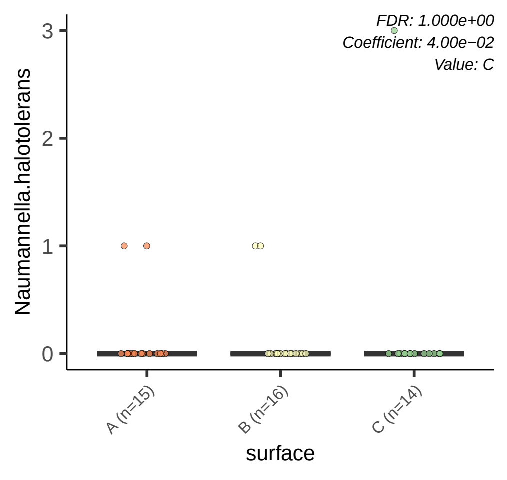

FDR: 1.000e+00
Coefficient: 4.00e−02
Value: C
Naumannella.halotolerans
3
2
1
0
A (n=15)
B (n=16)
C (n=14)
surface

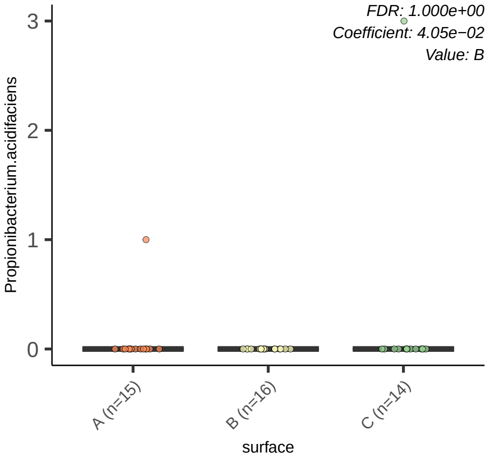

FDR: 1.000e+00
Coefficient: 4.05e−02
Value: B
Propionibacterium.acidifaciens
0
1
2
3
A (n=15)
B (n=16)
C (n=14)
surface

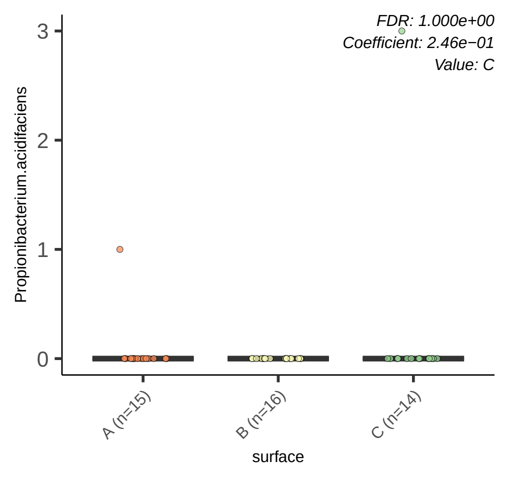

FDR: 1.000e+00
Coefficient: 2.46e−01
Value: C
Propionibacterium.acidifaciens
3
2
1
0
A (n=15)
B (n=16)
C (n=14)
surface

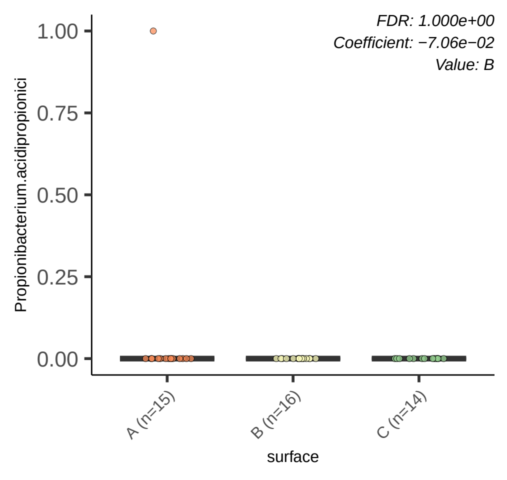
FDR: 1.000e+00
Coefficient: −7.06e−02
Value: B
Propionibacterium.acidipropionici
1.00
0.75
0.50
0.25
0.00
A (n=15)
B (n=16)
C (n=14)
surface

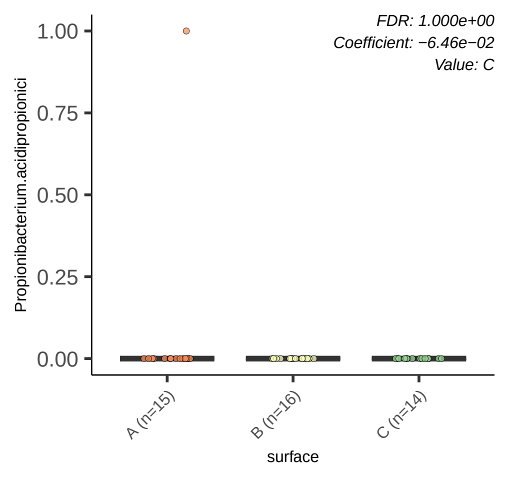
FDR: 1.000e+00
Coefficient: −6.46e−02
Value: C
Propionibacterium.acidipropionici
1.00
0.75
0.50
0.25
0.00
A (n=15)
B (n=16)
C (n=14)
surface

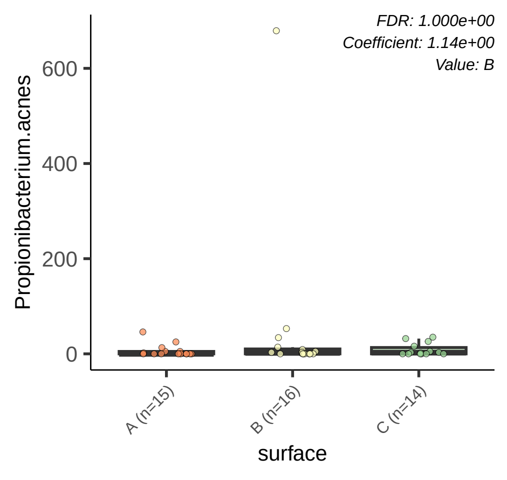
FDR: 1.000e+00
Coefficient: 1.14e+00
Value: B
Propionibacterium.acnes
600
400
200
0
A (n=15)
B (n=16)
C (n=14)
surface

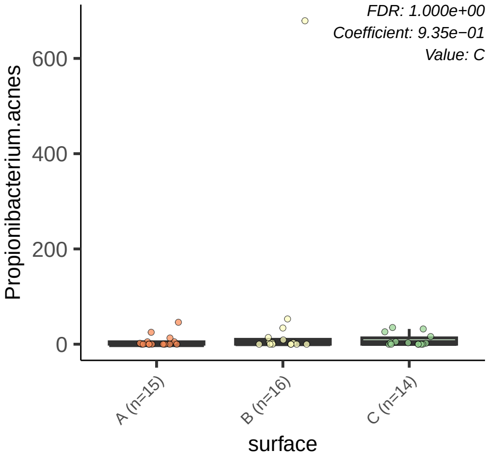
FDR: 1.000e+00
Coefficient: 9.35e−01
Value: C
Propionibacterium.acnes
0
200
400
600
A (n=15)
B (n=16)
C (n=14)
surface

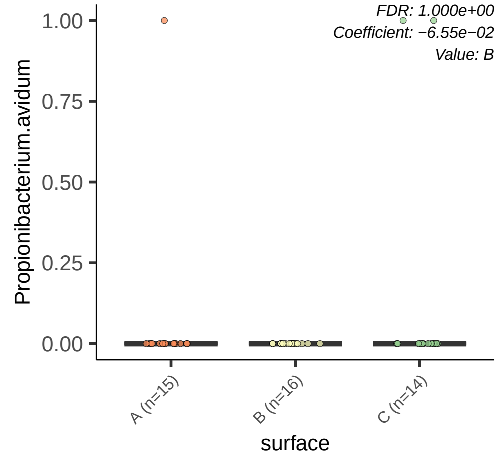
FDR: 1.000e+00
Coefficient: −6.55e−02
Value: B
Propionibacterium.avidum
1.00
0.75
0.50
0.25
0.00
A (n=15)
B (n=16)
C (n=14)
surface

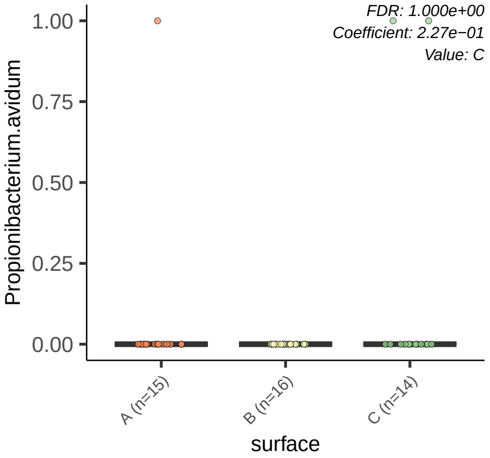

FDR: 1.000e+00
Coefficient: 2.27e−01
Value: C
Propionibacterium.avidum
1.00
0.75
0.50
0.25
0.00
A (n=15)
B (n=16)
C (n=14)
surface

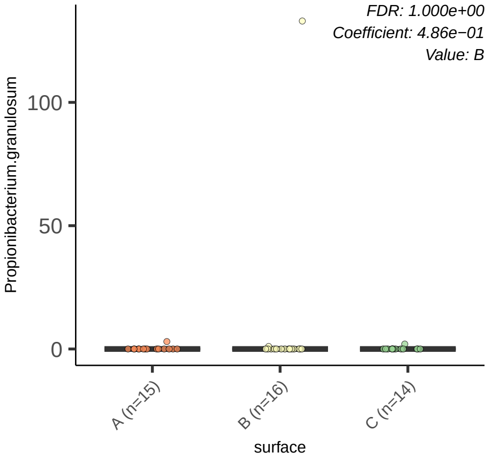
FDR: 1.000e+00
Coefficient: 4.86e−01
Value: B
Propionibacterium.granulosum
100
50
0
A (n=15)
B (n=16)
C (n=14)
surface

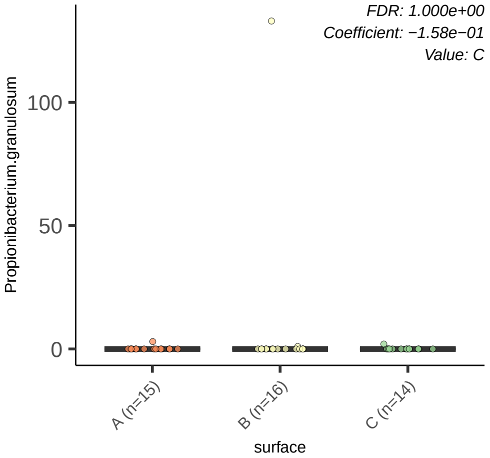

FDR: 1.000e+00
Coefficient: −1.58e−01
Value: C
Propionibacterium.granulosum
100
50
0
A (n=15)
B (n=16)
C (n=14)
surface

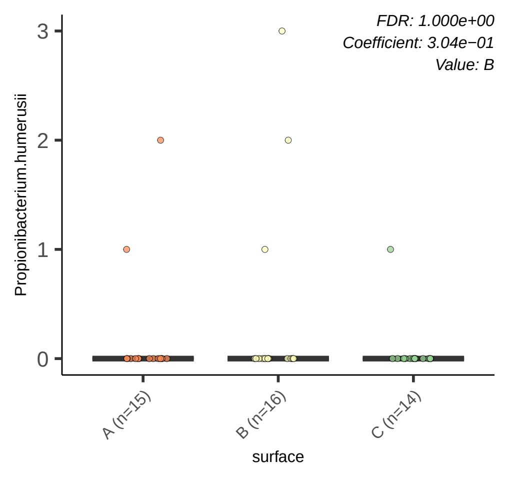
FDR: 1.000e+00
Coefficient: 3.04e−01
Value: B
Propionibacterium.humerusii
0
1
2
3
A (n=15)
B (n=16)
C (n=14)
surface

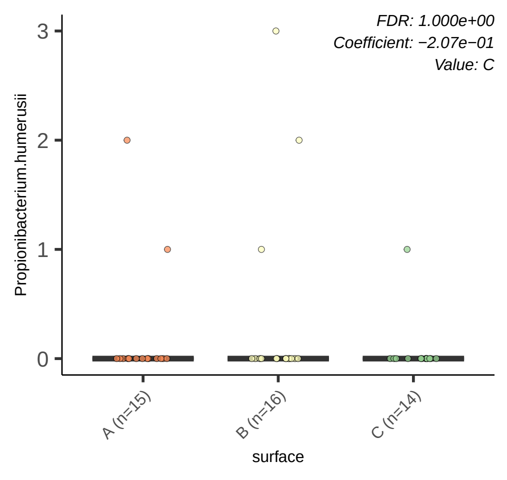

FDR: 1.000e+00
Coefficient: −2.07e−01
Value: C
Propionibacterium.humerusii
3
2
1
0
A (n=15)
B (n=16)
C (n=14)
surface

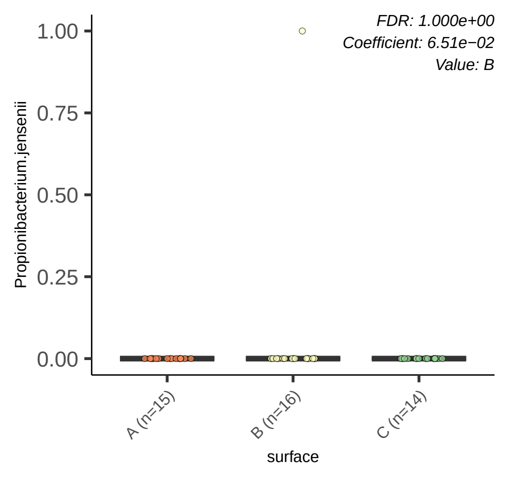
FDR: 1.000e+00
Coefficient: 6.51e−02
Value: B
Propionibacterium.jensenii
1.00
0.75
0.50
0.25
0.00
A (n=15)
B (n=16)
C (n=14)
surface

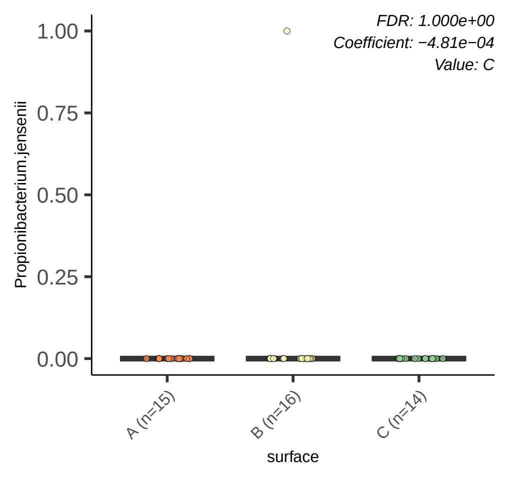

FDR: 1.000e+00
Coefficient: −4.81e−04
Value: C
Propionibacterium.jensenii
1.00
0.75
0.50
0.25
0.00
A (n=15)
B (n=16)
C (n=14)
surface

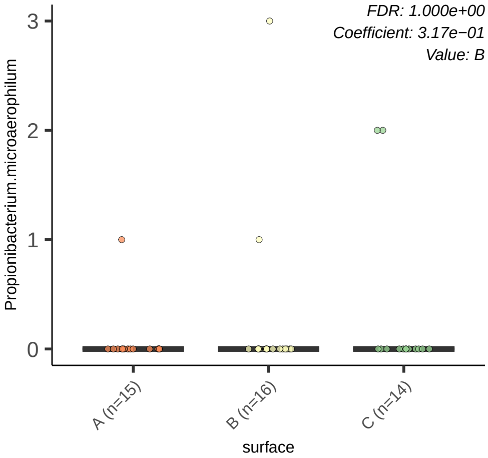
FDR: 1.000e+00
Coefficient: 3.17e−01
Value: B
Propionibacterium.microaerophilum
0
1
2
3
A (n=15)
B (n=16)
C (n=14)
surface

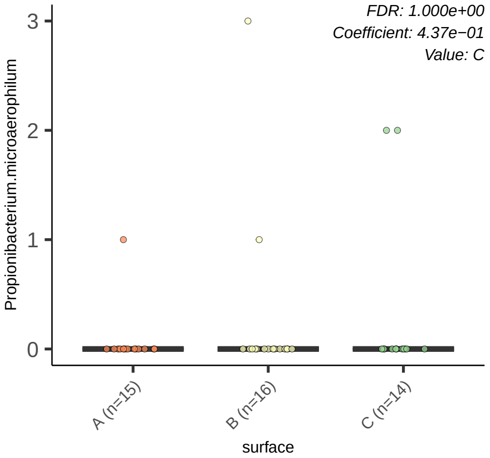
FDR: 1.000e+00
Coefficient: 4.37e−01
Value: C
Propionibacterium.microaerophilum
3
2
1
0
A (n=15)
B (n=16)
C (n=14)
surface

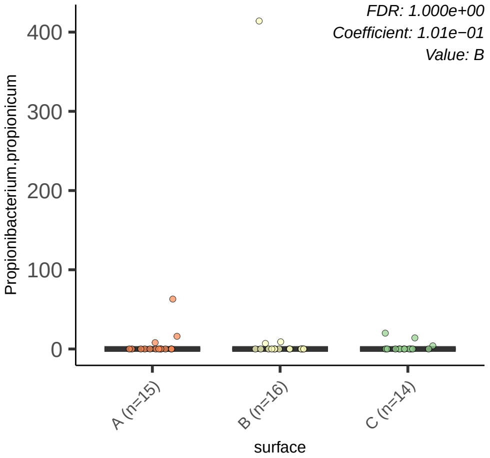
FDR: 1.000e+00
Coefficient: 1.01e−01
Value: B
Propionibacterium.propionicum
400
300
200
100
0
A (n=15)
B (n=16)
C (n=14)
surface

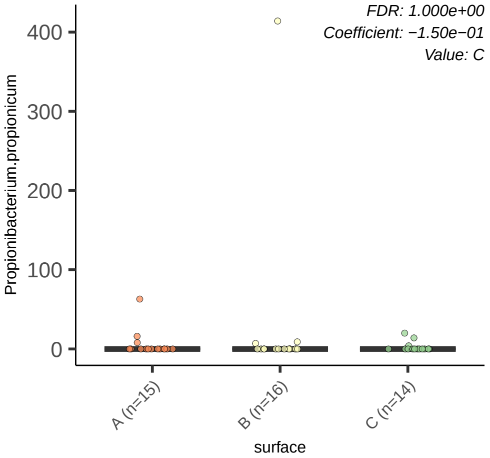

FDR: 1.000e+00
Coefficient: −1.50e−01
Value: C
Propionibacterium.propionicum
400
300
200
100
0
A (n=15)
B (n=16)
C (n=14)
surface

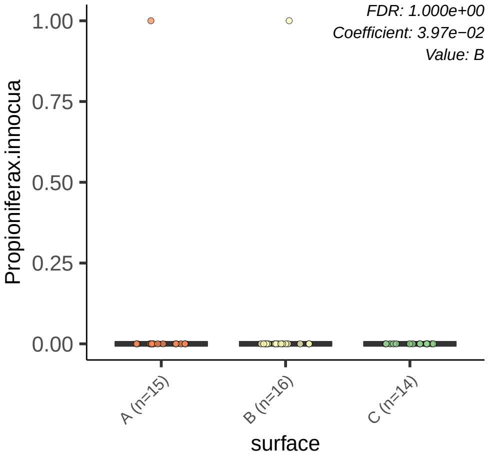
FDR: 1.000e+00
Coefficient: 3.97e−02
Value: B
Propioniferax.innocua
1.00
0.75
0.50
0.25
0.00
A (n=15)
B (n=16)
C (n=14)
surface

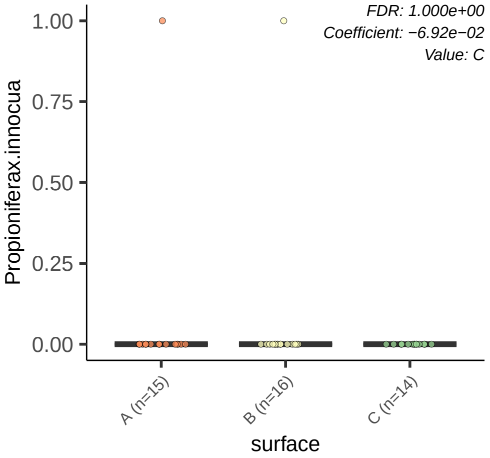
FDR: 1.000e+00
Coefficient: −6.92e−02
Value: C
Propioniferax.innocua
1.00
0.75
0.50
0.25
0.00
A (n=15)
B (n=16)
C (n=14)
surface

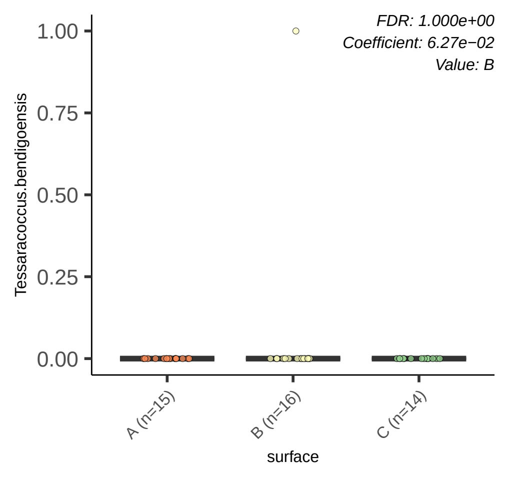
FDR: 1.000e+00
Coefficient: 6.27e−02
Value: B
1.00
0.75
0.50
0.25
0.00
Tessaracoccus.bendigoensis
A (n=15)
B (n=16)
C (n=14)
surface

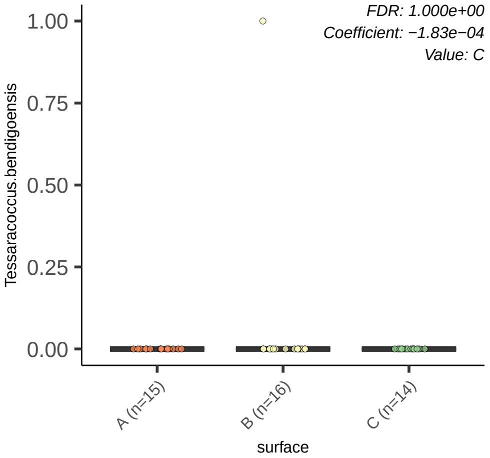
FDR: 1.000e+00
Coefficient: −1.83e−04
Value: C
1.00
0.75
0.50
0.25
0.00
Tessaracoccus.bendigoensis
A (n=15)
B (n=16)
C (n=14)
surface

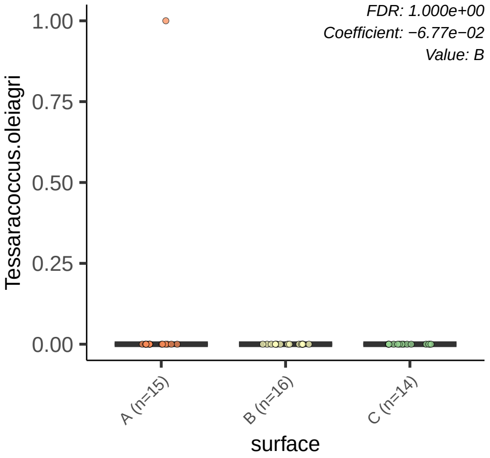

FDR: 1.000e+00
Coefficient: −6.77e−02
Value: B
Tessaracoccus.oleiagri
1.00
0.75
0.50
0.25
0.00
A (n=15)
B (n=16)
C (n=14)
surface

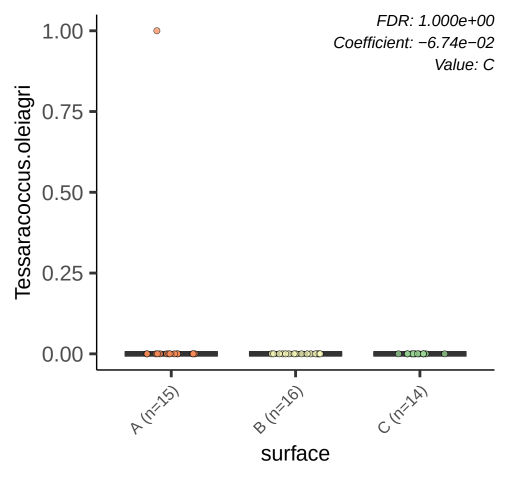
FDR: 1.000e+00
Coefficient: −6.74e−02
Value: C
Tessaracoccus.oleiagri
1.00
0.75
0.50
0.25
0.00
A (n=15)
B (n=16)
C (n=14)
surface

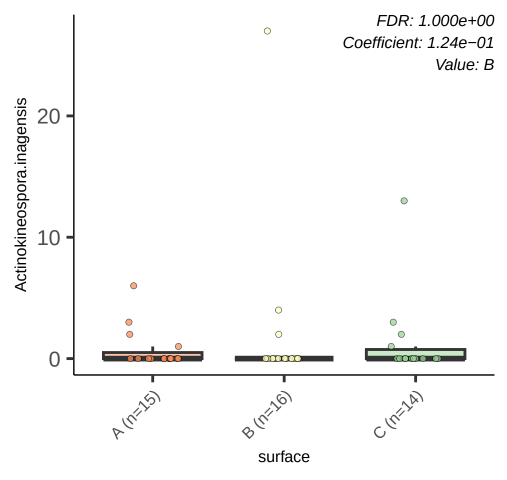
FDR: 1.000e+00
Coefficient: 1.24e−01
Value: B
Actinokineospora.inagensis
20
10
0
A (n=15)
B (n=16)
C (n=14)
surface

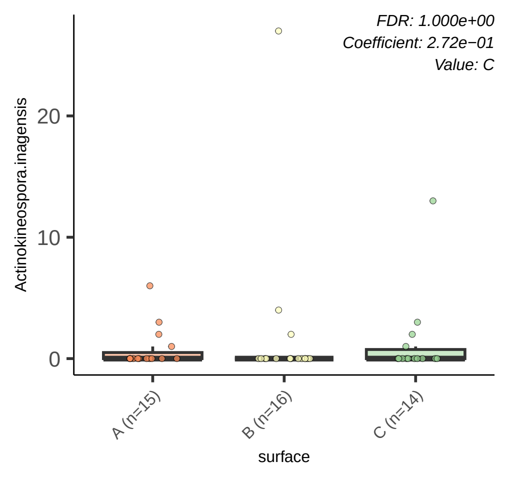
FDR: 1.000e+00
Coefficient: 2.72e−01
Value: C
Actinokineospora.inagensis
0
10
20
A (n=15)
B (n=16)
C (n=14)
surface

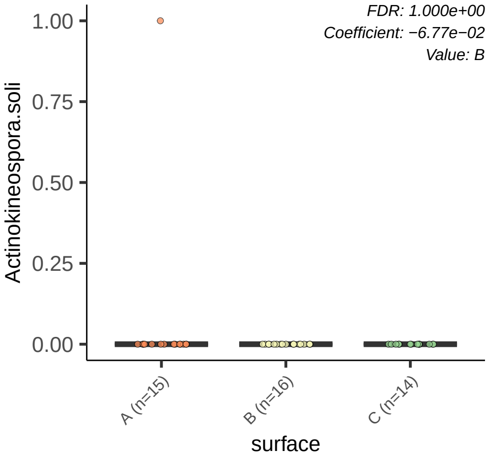

FDR: 1.000e+00
Coefficient: −6.77e−02
Value: B
1.00
0.75
0.50
0.25
0.00
Actinokineospora.soli
A (n=15)
B (n=16)
C (n=14)
surface

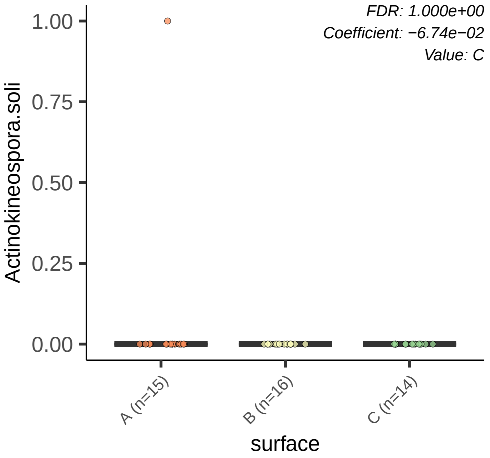
FDR: 1.000e+00
Coefficient: −6.74e−02
Value: C
Actinokineospora.soli
1.00
0.75
0.50
0.25
0.00
A (n=15)
B (n=16)
C (n=14)
surface

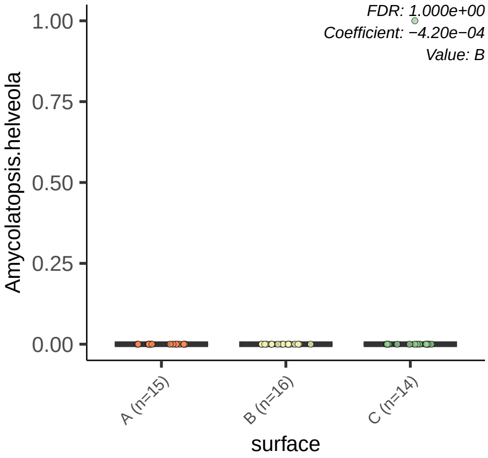
FDR: 1.000e+00
Coefficient: −4.20e−04
Value: B
Amycolatopsis.helveola
1.00
0.75
0.50
0.25
0.00
A (n=15)
B (n=16)
C (n=14)
surface

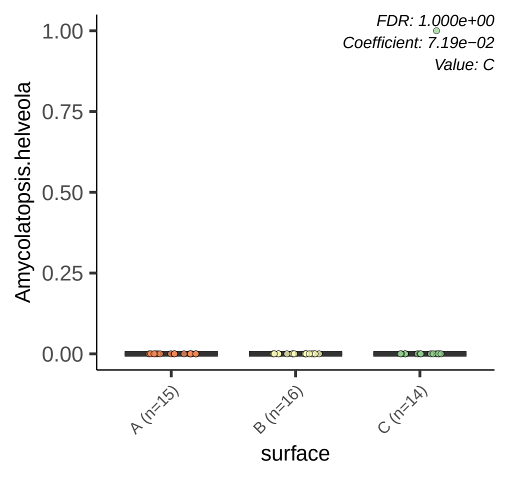
FDR: 1.000e+00
Coefficient: 7.19e−02
Value: C
Amycolatopsis.helveola
1.00
0.75
0.50
0.25
0.00
A (n=15)
B (n=16)
C (n=14)
surface

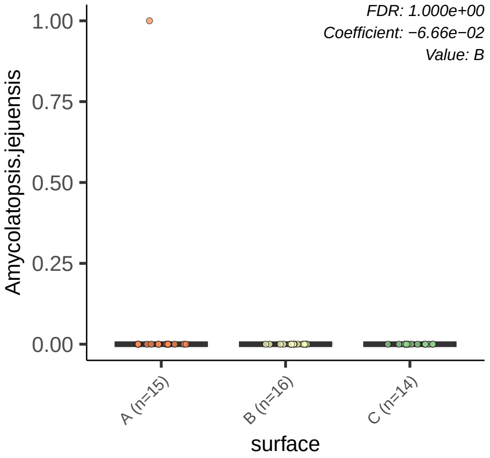
FDR: 1.000e+00
Coefficient: −6.66e−02
Value: B
1.00
0.75
0.50
0.25
0.00
Amycolatopsis.jejuensis
A (n=15)
B (n=16)
C (n=14)
surface

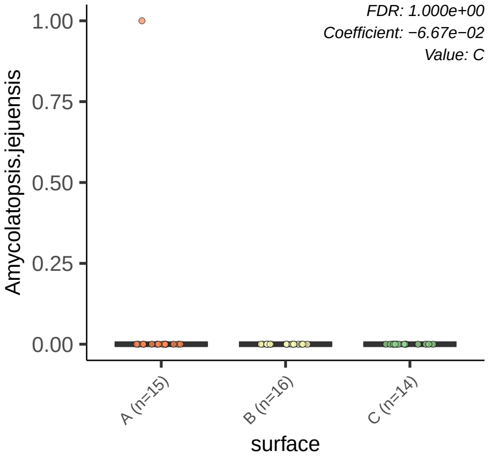

FDR: 1.000e+00
Coefficient: −6.67e−02
Value: C
Amycolatopsis.jejuensis
1.00
0.75
0.50
0.25
0.00
A (n=15)
B (n=16)
C (n=14)
surface

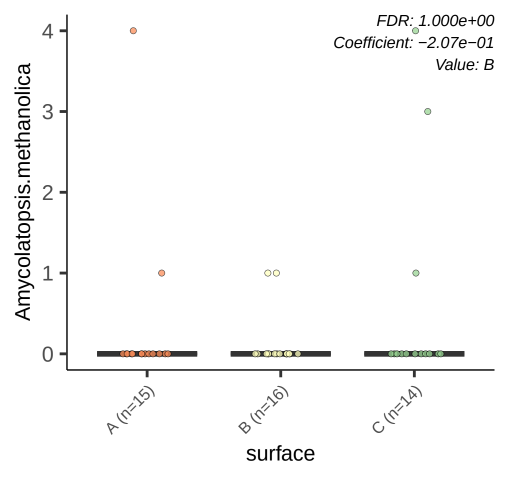
FDR: 1.000e+00
Coefficient: −2.07e−01
Value: B
Amycolatopsis.methanolica
0
1
2
3
4
A (n=15)
B (n=16)
C (n=14)
surface

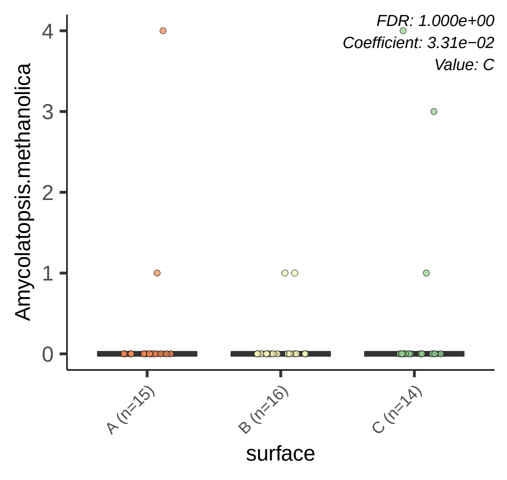
FDR: 1.000e+00
Coefficient: 3.31e−02
Value: C
Amycolatopsis.methanolica
4
3
2
1
0
A (n=15)
B (n=16)
C (n=14)
surface

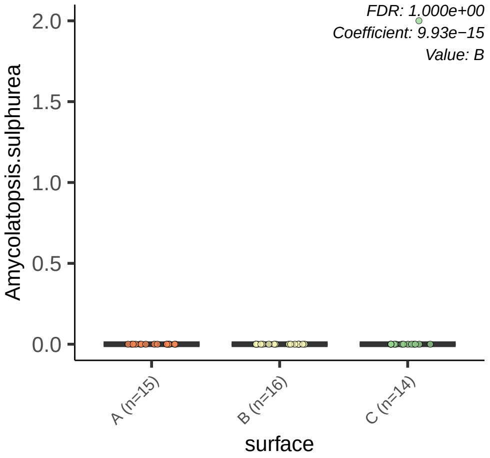

FDR: 1.000e+00
Coefficient: 9.93e−15
Value: B
Amycolatopsis.sulphurea
2.0
1.5
1.0
0.5
0.0
A (n=15)
B (n=16)
C (n=14)
surface

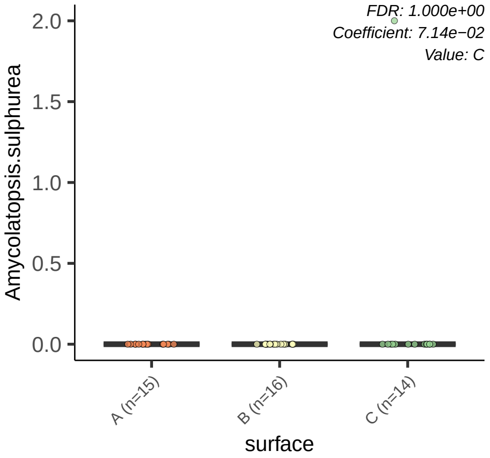
FDR: 1.000e+00
Coefficient: 7.14e−02
Value: C
Amycolatopsis.sulphurea
2.0
1.5
1.0
0.5
0.0
A (n=15)
B (n=16)
C (n=14)
surface

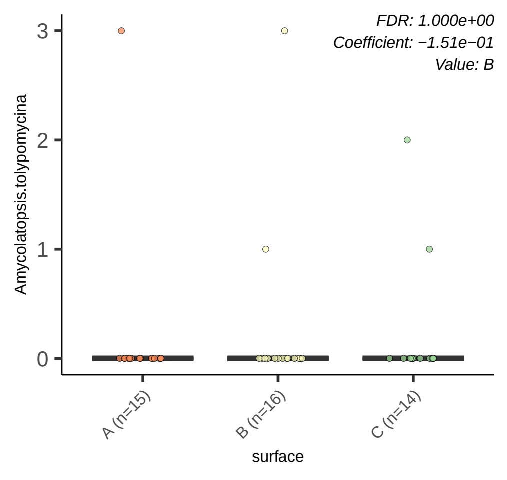
FDR: 1.000e+00
Coefficient: −1.51e−01
Value: B
Amycolatopsis.tolypomycina
3
2
1
0
A (n=15)
B (n=16)
C (n=14)
surface

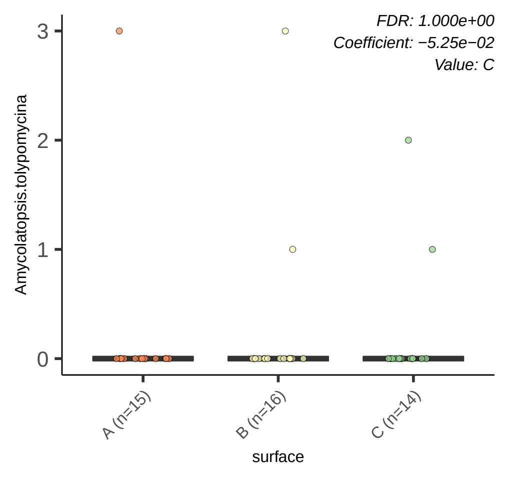

FDR: 1.000e+00
Coefficient: −5.25e−02
Value: C
Amycolatopsis.tolypomycina
3
2
1
0
A (n=15)
B (n=16)
C (n=14)
surface

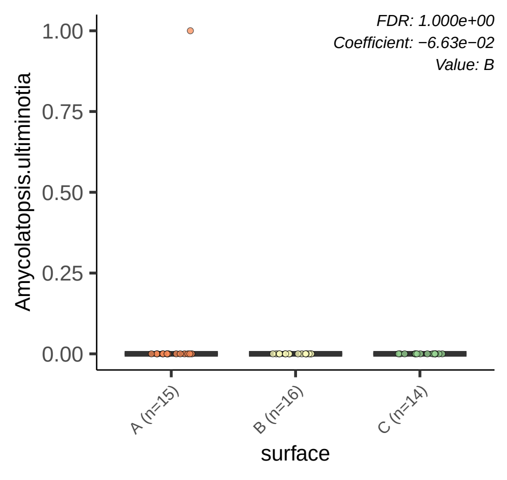
FDR: 1.000e+00
Coefficient: −6.63e−02
Value: B
Amycolatopsis.ultiminotia
1.00
0.75
0.50
0.25
0.00
A (n=15)
B (n=16)
C (n=14)
surface

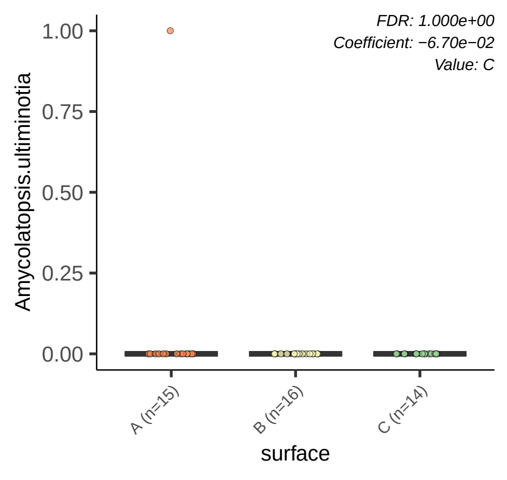
FDR: 1.000e+00
Coefficient: −6.70e−02
Value: C
Amycolatopsis.ultiminotia
1.00
0.75
0.50
0.25
0.00
A (n=15)
B (n=16)
C (n=14)
surface

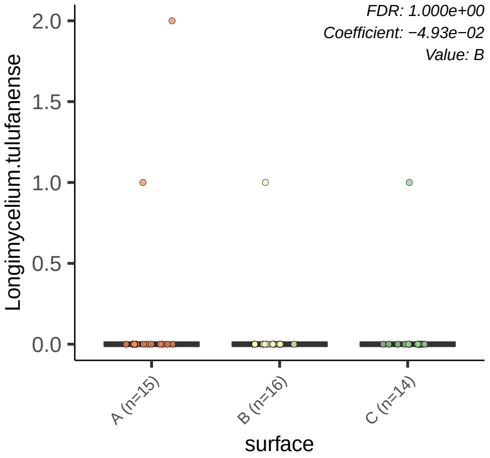

FDR: 1.000e+00
Coefficient: −4.93e−02
Value: B
Longimycelium.tulufanense
2.0
1.5
1.0
0.5
0.0
A (n=15)
B (n=16)
C (n=14)
surface

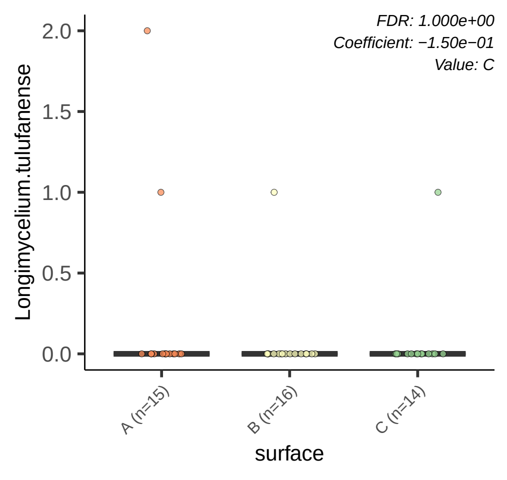

FDR: 1.000e+00
Coefficient: −1.50e−01
Value: C
Longimycelium.tulufanense
2.0
1.5
1.0
0.5
0.0
A (n=15)
B (n=16)
C (n=14)
surface

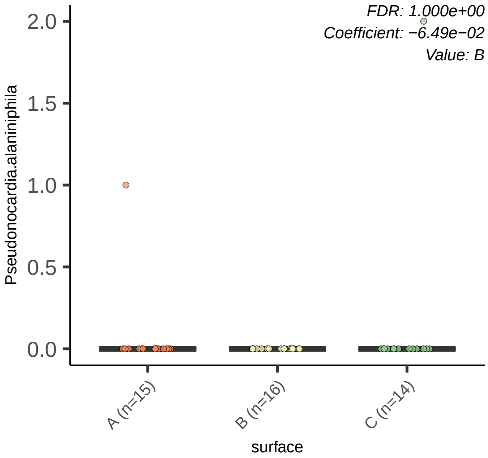
FDR: 1.000e+00
Coefficient: −6.49e−02
Value: B
Pseudonocardia.alaniniphila
2.0
1.5
1.0
0.5
0.0
A (n=15)
B (n=16)
C (n=14)
surface

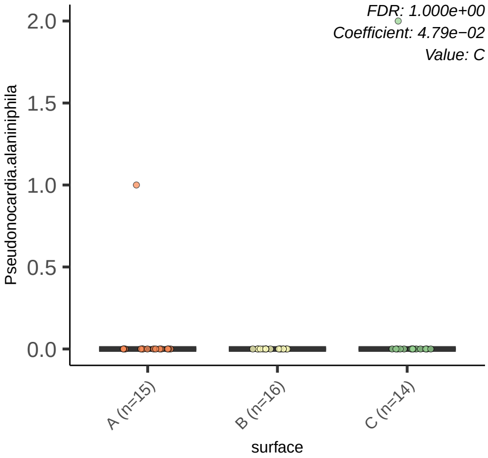

FDR: 1.000e+00
Coefficient: 4.79e−02
Value: C
2.0
1.5
1.0
0.5
0.0
Pseudonocardia.alaniniphila
A (n=15)
B (n=16)
C (n=14)
surface

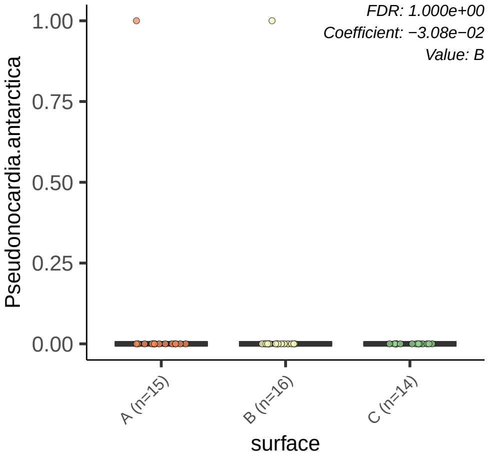
FDR: 1.000e+00
Coefficient: −3.08e−02
Value: B
Pseudonocardia.antarctica
1.00
0.75
0.50
0.25
0.00
A (n=15)
B (n=16)
C (n=14)
surface

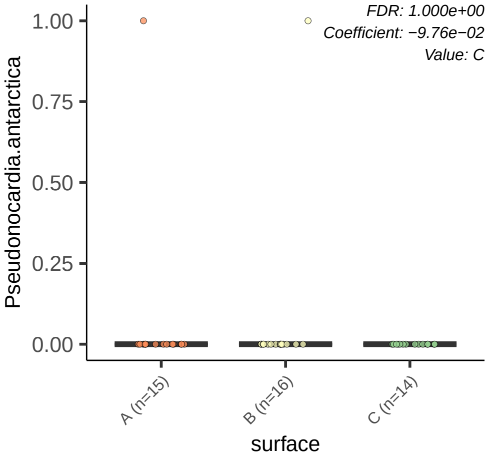

FDR: 1.000e+00
Coefficient: −9.76e−02
Value: C
Pseudonocardia.antarctica
1.00
0.75
0.50
0.25
0.00
A (n=15)
B (n=16)
C (n=14)
surface

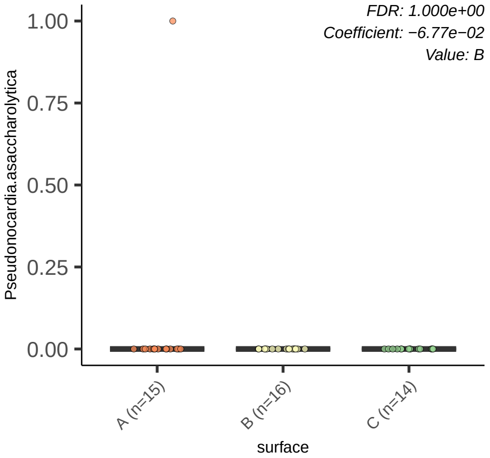
FDR: 1.000e+00
Coefficient: −6.77e−02
Value: B
1.00
0.75
0.50
0.25
0.00
Pseudonocardia.asaccharolytica
A (n=15)
B (n=16)
C (n=14)
surface

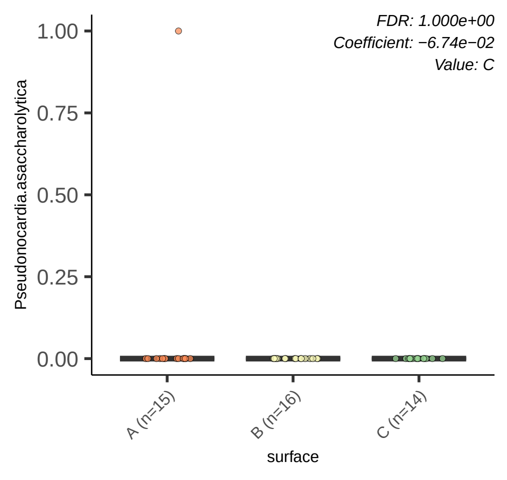

FDR: 1.000e+00
Coefficient: −6.74e−02
Value: C
1.00
0.75
0.50
0.25
0.00
Pseudonocardia.asaccharolytica
A (n=15)
B (n=16)
C (n=14)
surface

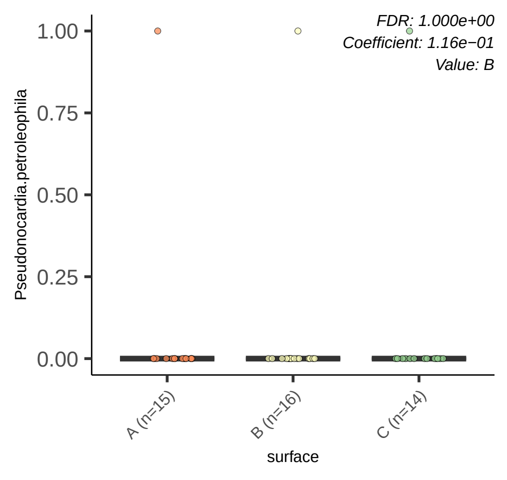
FDR: 1.000e+00
Coefficient: 1.16e−01
Value: B
1.00
0.75
0.50
0.25
0.00
Pseudonocardia.petroleophila
A (n=15)
B (n=16)
C (n=14)
surface

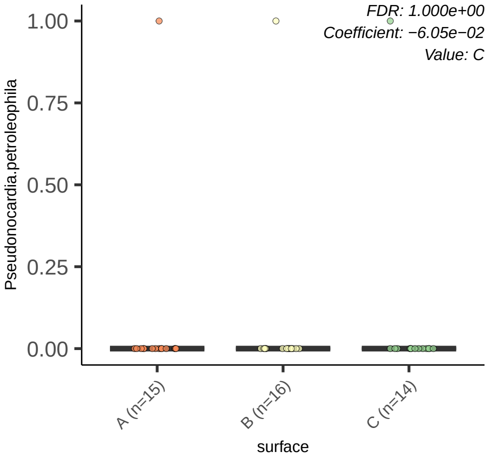
FDR: 1.000e+00
Coefficient: −6.05e−02
Value: C
Pseudonocardia.petroleophila
1.00
0.75
0.50
0.25
0.00
A (n=15)
B (n=16)
C (n=14)
surface

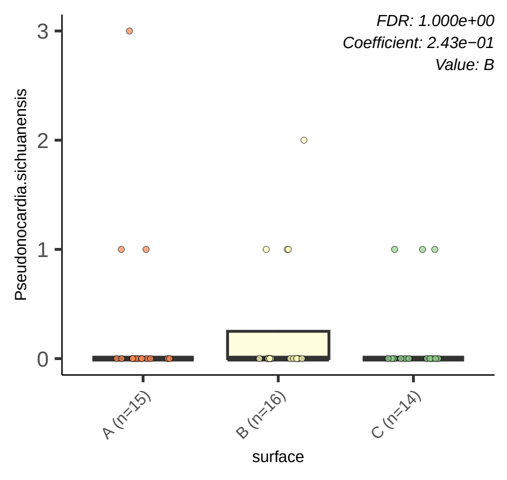
FDR: 1.000e+00
Coefficient: 2.43e−01
Value: B
Pseudonocardia.sichuanensis
0
1
2
3
A (n=15)
B (n=16)
C (n=14)
surface

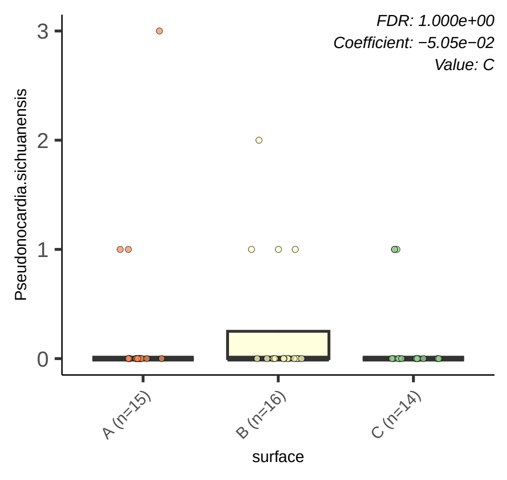
FDR: 1.000e+00
Coefficient: −5.05e−02
Value: C
Pseudonocardia.sichuanensis
0
1
2
3
A (n=15)
B (n=16)
C (n=14)
surface

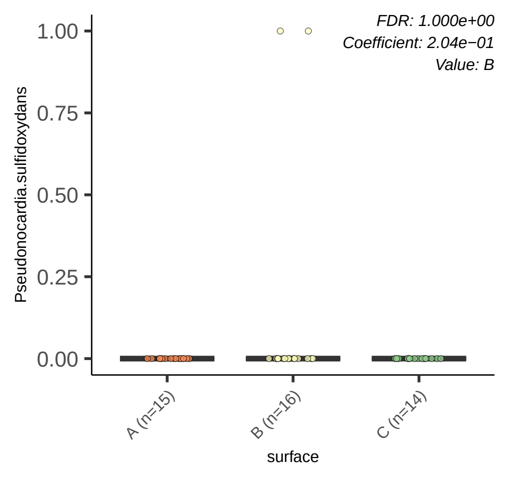

FDR: 1.000e+00
Coefficient: 2.04e−01
Value: B
Pseudonocardia.sulfidoxydans
1.00
0.75
0.50
0.25
0.00
A (n=15)
B (n=16)
C (n=14)
surface

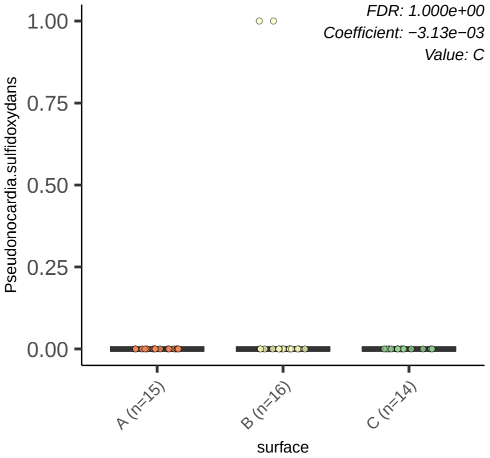
FDR: 1.000e+00
Coefficient: −3.13e−03
Value: C
Pseudonocardia.sulfidoxydans
1.00
0.75
0.50
0.25
0.00
A (n=15)
B (n=16)
C (n=14)
surface

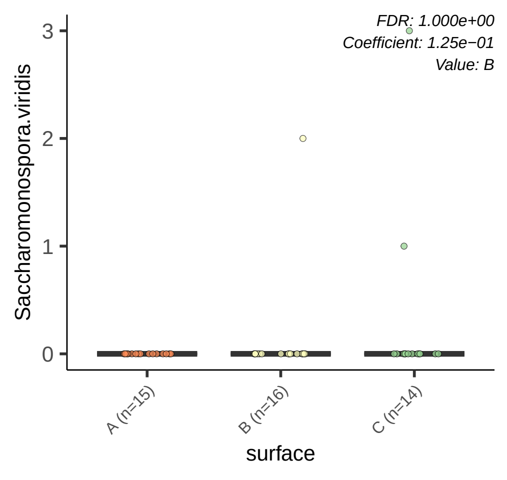

FDR: 1.000e+00
Coefficient: 1.25e−01
Value: B
Saccharomonospora.viridis
3
2
1
0
A (n=15)
B (n=16)
C (n=14)
surface

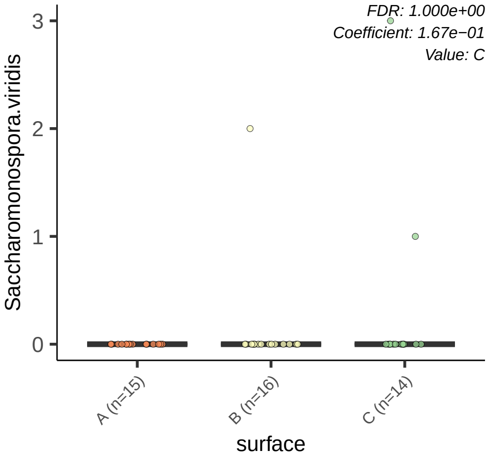

FDR: 1.000e+00
Coefficient: 1.67e−01
Value: C
Saccharomonospora.viridis
3
2
1
0
A (n=15)
B (n=16)
C (n=14)
surface

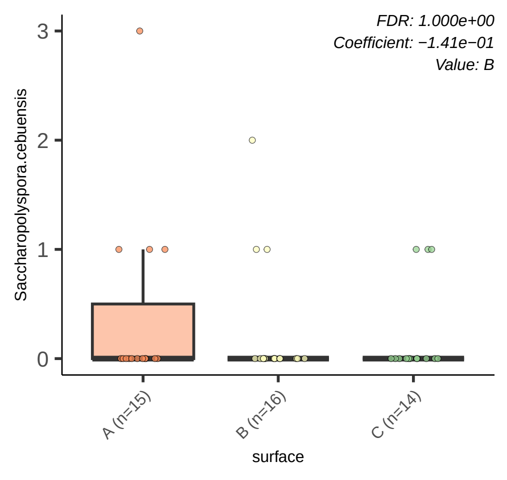
FDR: 1.000e+00
Coefficient: −1.41e−01
Value: B
Saccharopolyspora.cebuensis
0
1
2
3
A (n=15)
B (n=16)
C (n=14)
surface

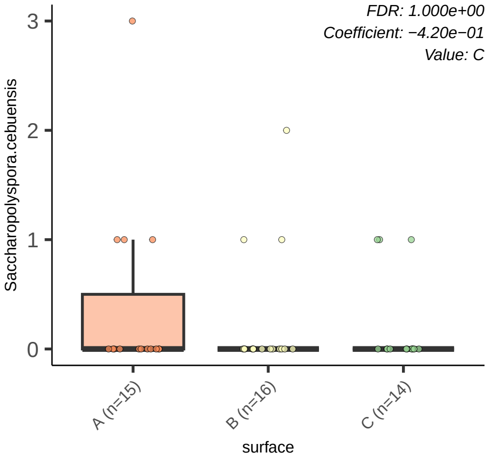
FDR: 1.000e+00
Coefficient: −4.20e−01
Value: C
Saccharopolyspora.cebuensis
3
2
1
0
A (n=15)
B (n=16)
C (n=14)
surface

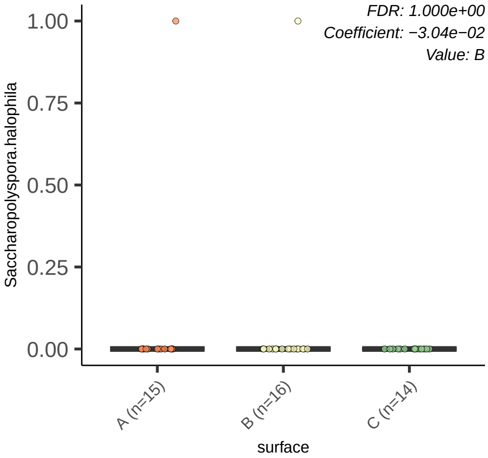
FDR: 1.000e+00
Coefficient: −3.04e−02
Value: B
Saccharopolyspora.halophila
1.00
0.75
0.50
0.25
0.00
A (n=15)
B (n=16)
C (n=14)
surface

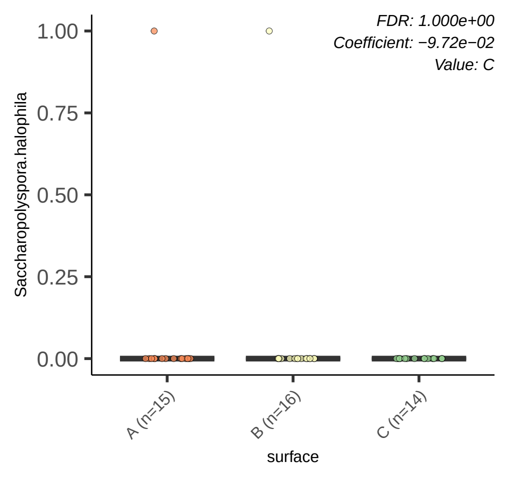

FDR: 1.000e+00
Coefficient: −9.72e−02
Value: C
1.00
0.75
0.50
0.25
0.00
Saccharopolyspora.halophila
A (n=15)
B (n=16)
C (n=14)
surface

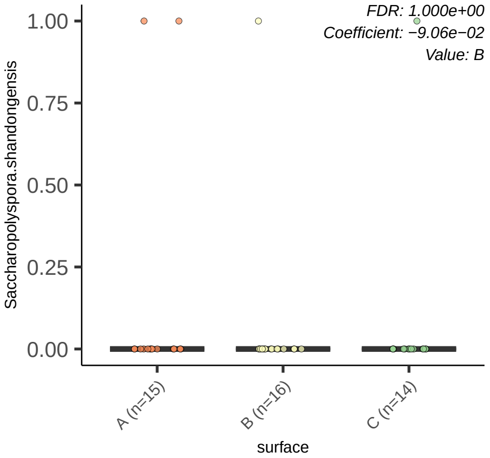

FDR: 1.000e+00
Coefficient: −9.06e−02
Value: B
Saccharopolyspora.shandongensis
1.00
0.75
0.50
0.25
0.00
A (n=15)
B (n=16)
C (n=14)
surface

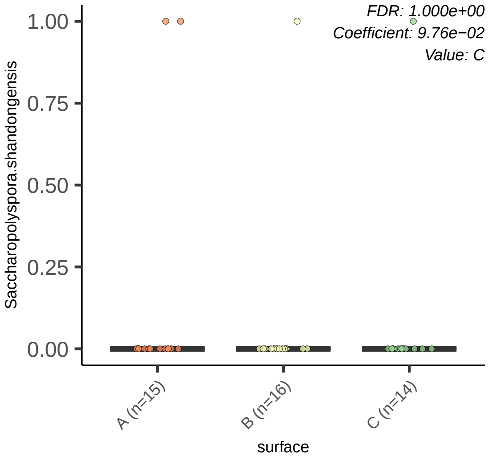

FDR: 1.000e+00
Coefficient: 9.76e−02
Value: C
Saccharopolyspora.shandongensis
1.00
0.75
0.50
0.25
0.00
A (n=15)
B (n=16)
C (n=14)
surface

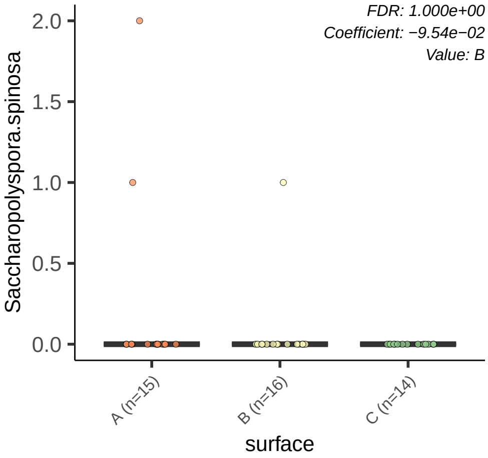
FDR: 1.000e+00
Coefficient: −9.54e−02
Value: B
Saccharopolyspora.spinosa
2.0
1.5
1.0
0.5
0.0
A (n=15)
B (n=16)
C (n=14)
surface

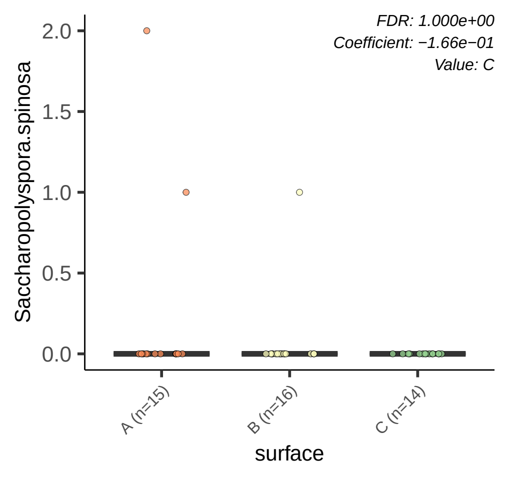
FDR: 1.000e+00
Coefficient: −1.66e−01
Value: C
Saccharopolyspora.spinosa
2.0
1.5
1.0
0.5
0.0
A (n=15)
B (n=16)
C (n=14)
surface

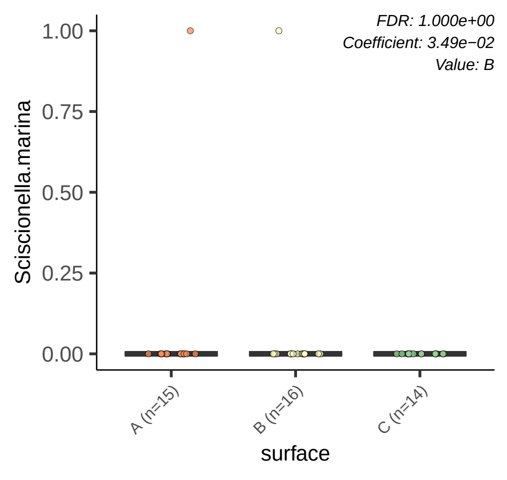
FDR: 1.000e+00
Coefficient: 3.49e−02
Value: B
Sciscionella.marina
1.00
0.75
0.50
0.25
0.00
A (n=15)
B (n=16)
C (n=14)
surface

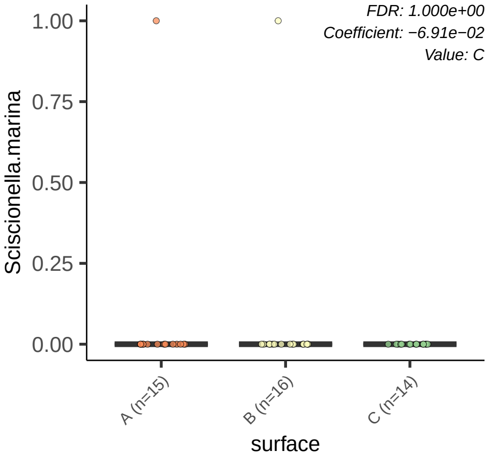

FDR: 1.000e+00
Coefficient: −6.91e−02
Value: C
Sciscionella.marina
1.00
0.75
0.50
0.25
0.00
A (n=15)
B (n=16)
C (n=14)
surface

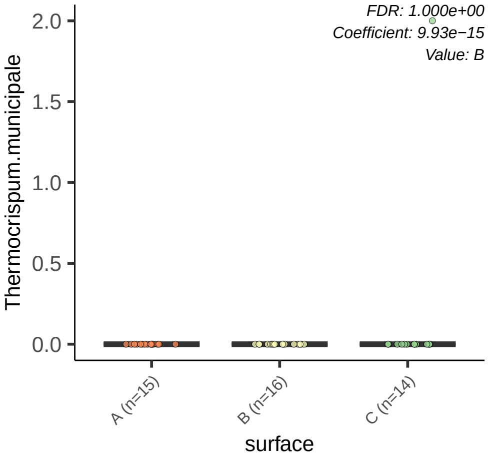
FDR: 1.000e+00
Coefficient: 9.93e−15
Value: B
Thermocrispum.municipale
2.0
1.5
1.0
0.5
0.0
A (n=15)
B (n=16)
C (n=14)
surface

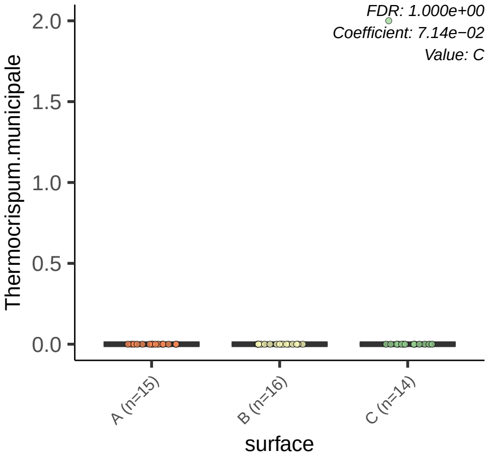

FDR: 1.000e+00
Coefficient: 7.14e−02
Value: C
Thermocrispum.municipale
2.0
1.5
1.0
0.5
0.0
A (n=15)
B (n=16)
C (n=14)
surface

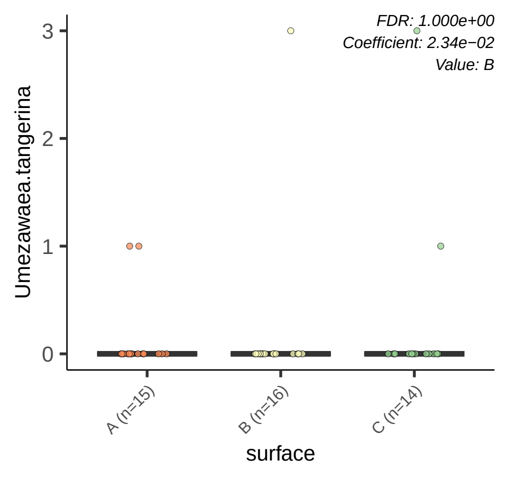

FDR: 1.000e+00
Coefficient: 2.34e−02
Value: B
Umezawaea.tangerina
0
1
2
3
A (n=15)
B (n=16)
C (n=14)
surface

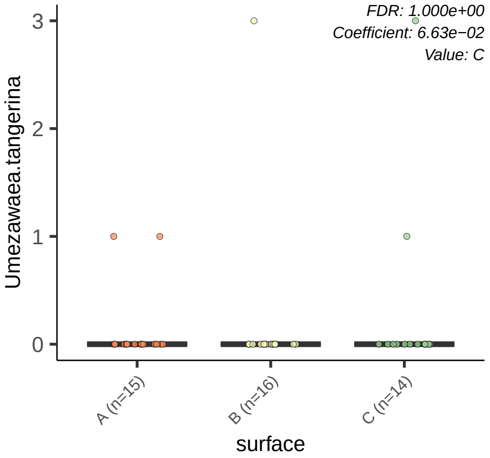
FDR: 1.000e+00
Coefficient: 6.63e−02
Value: C
Umezawaea.tangerina
3
2
1
0
A (n=15)
B (n=16)
C (n=14)
surface

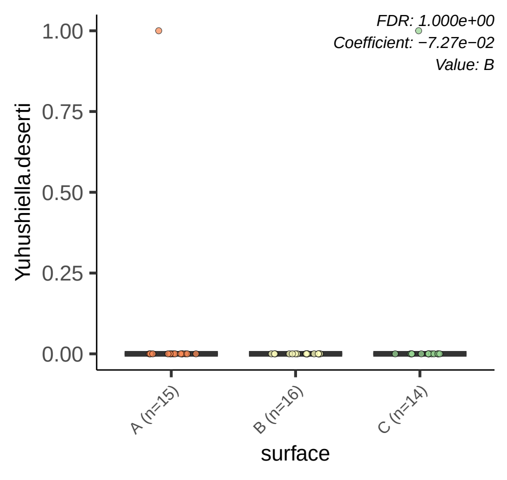
FDR: 1.000e+00
Coefficient: −7.27e−02
Value: B
Yuhushiella.deserti
1.00
0.75
0.50
0.25
0.00
A (n=15)
B (n=16)
C (n=14)
surface

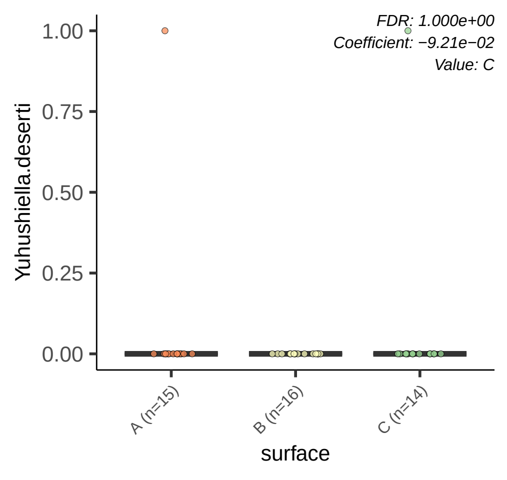

FDR: 1.000e+00
Coefficient: −9.21e−02
Value: C
Yuhushiella.deserti
1.00
0.75
0.50
0.25
0.00
A (n=15)
B (n=16)
C (n=14)
surface

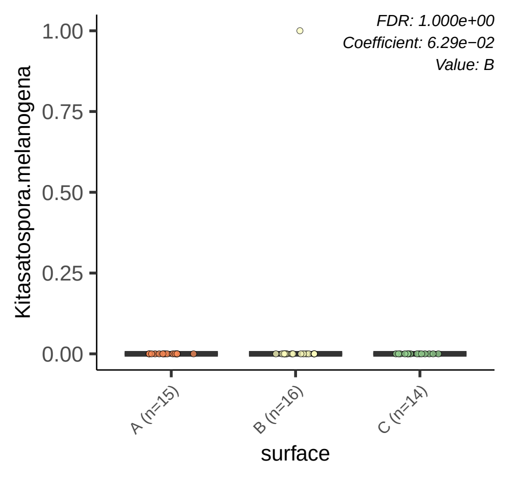

FDR: 1.000e+00
Coefficient: 6.29e−02
Value: B
1.00
0.75
0.50
0.25
0.00
Kitasatospora.melanogena
A (n=15)
B (n=16)
C (n=14)
surface

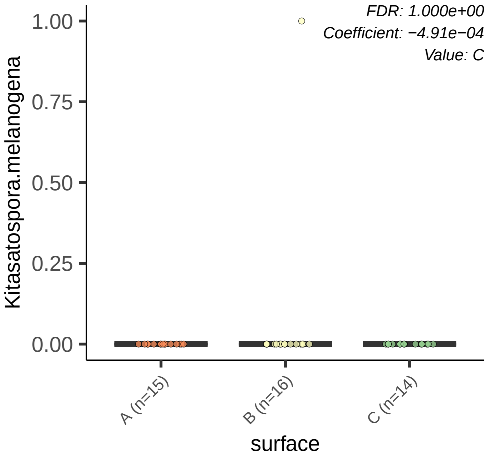

FDR: 1.000e+00
Coefficient: −4.91e−04
Value: C
Kitasatospora.melanogena
1.00
0.75
0.50
0.25
0.00
A (n=15)
B (n=16)
C (n=14)
surface

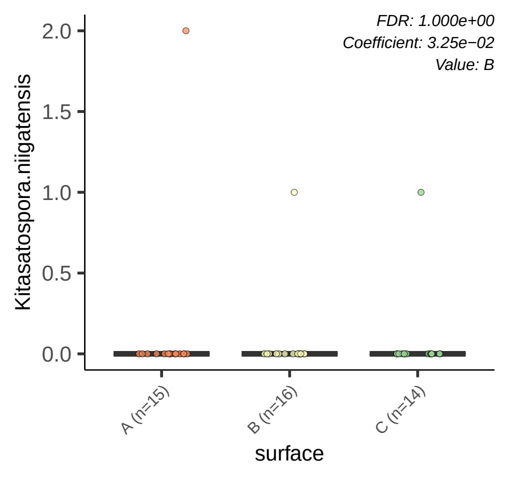
FDR: 1.000e+00
Coefficient: 3.25e−02
Value: B
Kitasatospora.niigatensis
2.0
1.5
1.0
0.5
0.0
A (n=15)
B (n=16)
C (n=14)
surface

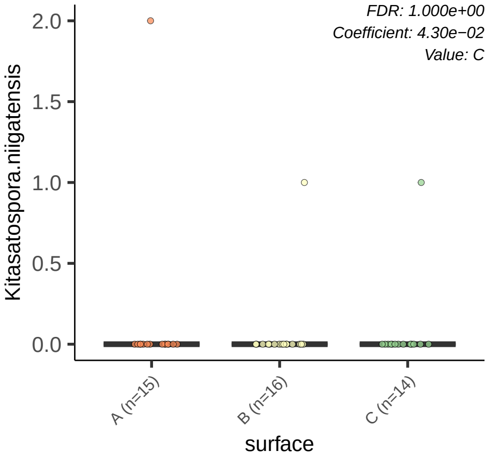
FDR: 1.000e+00
Coefficient: 4.30e−02
Value: C
Kitasatospora.niigatensis
2.0
1.5
1.0
0.5
0.0
A (n=15)
B (n=16)
C (n=14)
surface

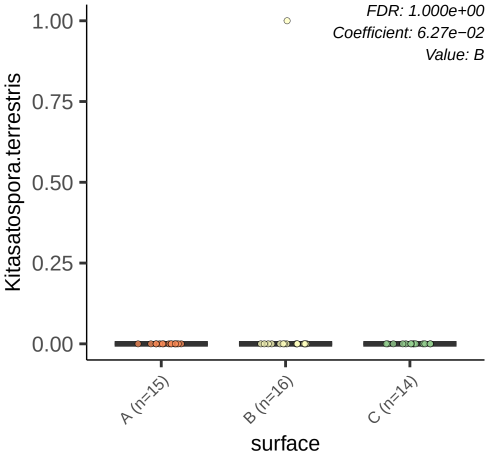

FDR: 1.000e+00
Coefficient: 6.27e−02
Value: B
Kitasatospora.terrestris
1.00
0.75
0.50
0.25
0.00
A (n=15)
B (n=16)
C (n=14)
surface

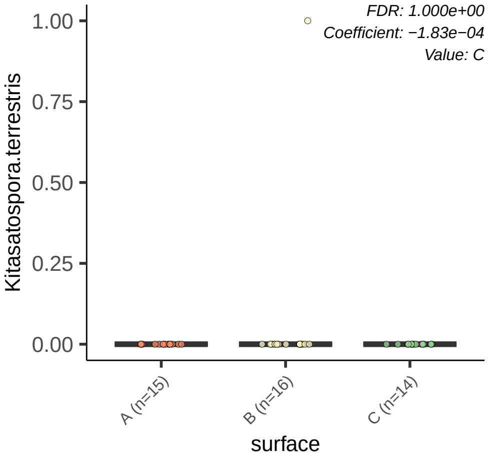
FDR: 1.000e+00
Coefficient: −1.83e−04
Value: C
Kitasatospora.terrestris
1.00
0.75
0.50
0.25
0.00
A (n=15)
B (n=16)
C (n=14)
surface

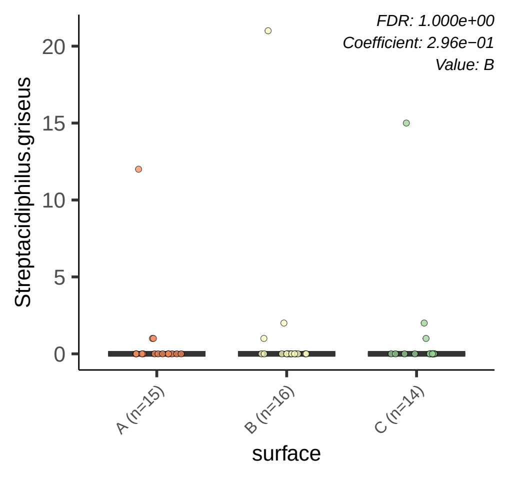
FDR: 1.000e+00
Coefficient: 2.96e−01
Value: B
Streptacidiphilus.griseus
0
5
10
15
20
A (n=15)
B (n=16)
C (n=14)
surface

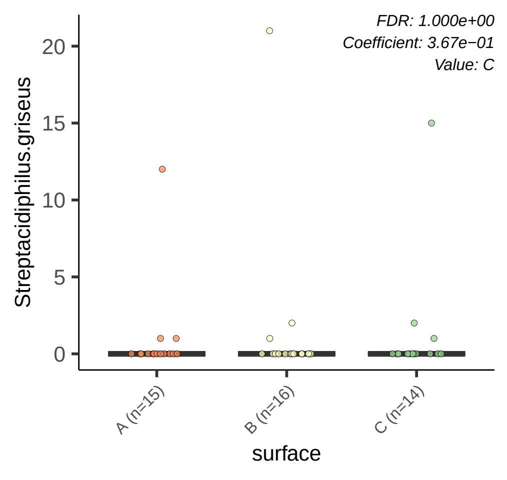

FDR: 1.000e+00
Coefficient: 3.67e−01
Value: C
Streptacidiphilus.griseus
0
5
10
15
20
A (n=15)
B (n=16)
C (n=14)
surface

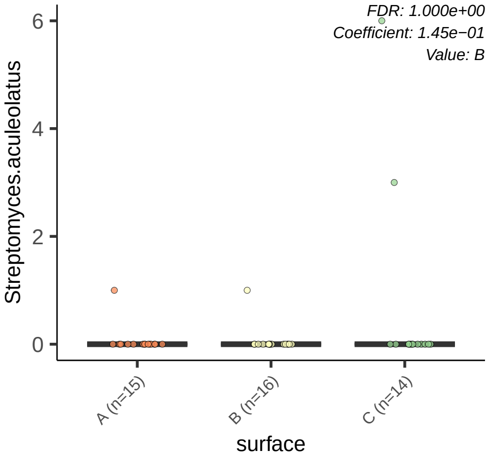

FDR: 1.000e+00
Coefficient: 1.45e−01
Value: B
Streptomyces.aculeolatus
0
2
4
6
A (n=15)
B (n=16)
C (n=14)
surface

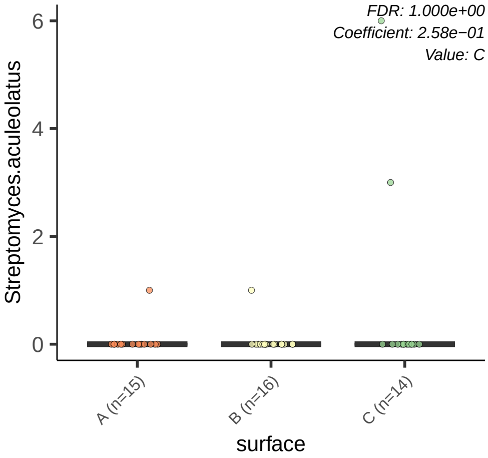

FDR: 1.000e+00
Coefficient: 2.58e−01
Value: C
Streptomyces.aculeolatus
0
2
4
6
A (n=15)
B (n=16)
C (n=14)
surface

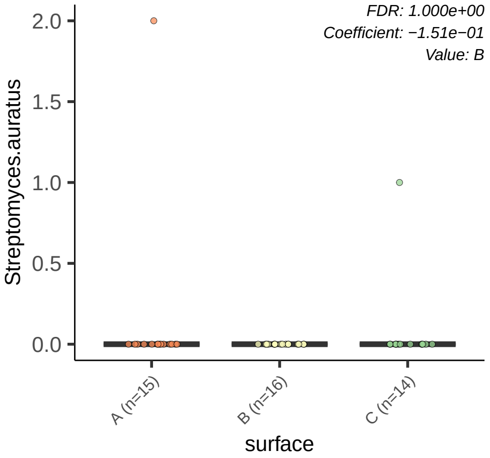

FDR: 1.000e+00
Coefficient: −1.51e−01
Value: B
Streptomyces.auratus
2.0
1.5
1.0
0.5
0.0
A (n=15)
B (n=16)
C (n=14)
surface

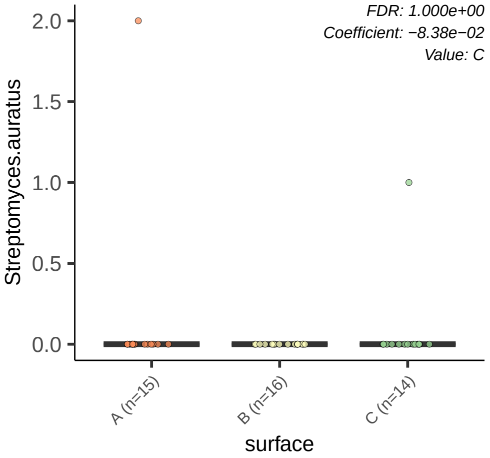
FDR: 1.000e+00
Coefficient: −8.38e−02
Value: C
Streptomyces.auratus
2.0
1.5
1.0
0.5
0.0
A (n=15)
B (n=16)
C (n=14)
surface

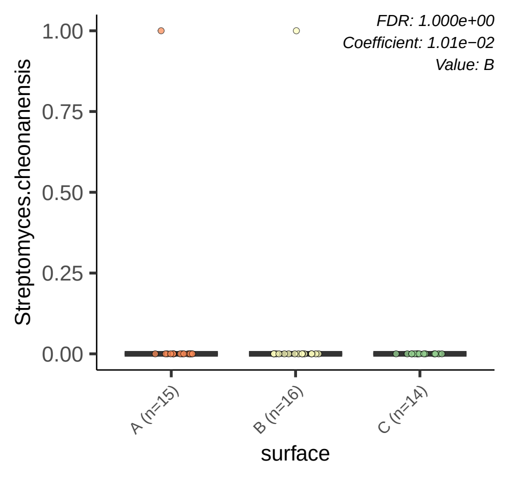

FDR: 1.000e+00
Coefficient: 1.01e−02
Value: B
Streptomyces.cheonanensis
1.00
0.75
0.50
0.25
0.00
A (n=15)
B (n=16)
C (n=14)
surface

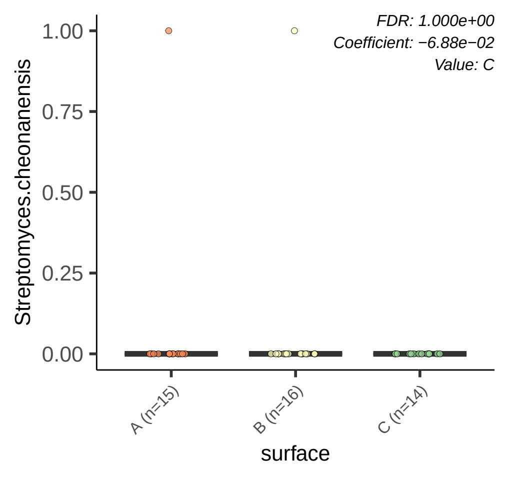
FDR: 1.000e+00
Coefficient: −6.88e−02
Value: C
Streptomyces.cheonanensis
1.00
0.75
0.50
0.25
0.00
A (n=15)
B (n=16)
C (n=14)
surface

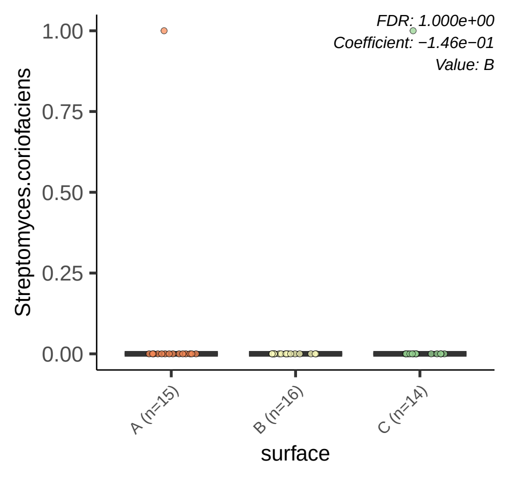
FDR: 1.000e+00
Coefficient: −1.46e−01
Value: B
Streptomyces.coriofaciens
1.00
0.75
0.50
0.25
0.00
A (n=15)
B (n=16)
C (n=14)
surface

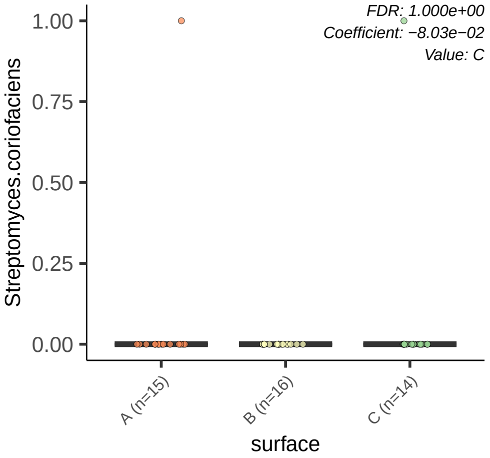
FDR: 1.000e+00
Coefficient: −8.03e−02
Value: C
Streptomyces.coriofaciens
1.00
0.75
0.50
0.25
0.00
A (n=15)
B (n=16)
C (n=14)
surface

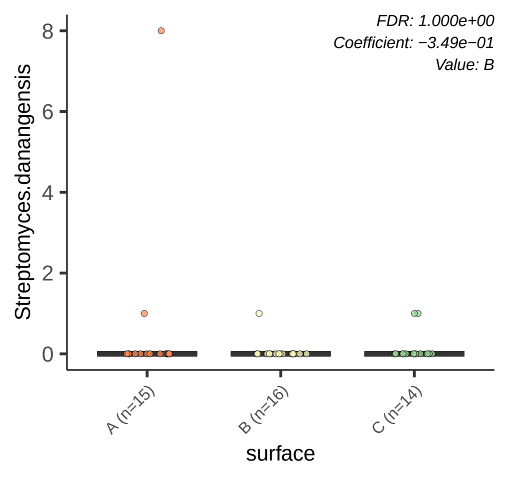
FDR: 1.000e+00
Coefficient: −3.49e−01
Value: B
Streptomyces.danangensis
8
6
4
2
0
A (n=15)
B (n=16)
C (n=14)
surface

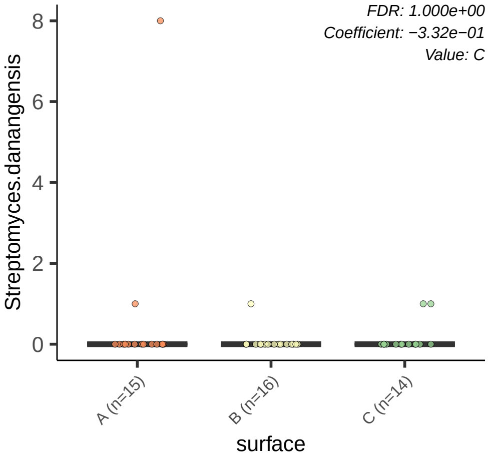

FDR: 1.000e+00
Coefficient: −3.32e−01
Value: C
Streptomyces.danangensis
0
2
4
6
8
A (n=15)
B (n=16)
C (n=14)
surface

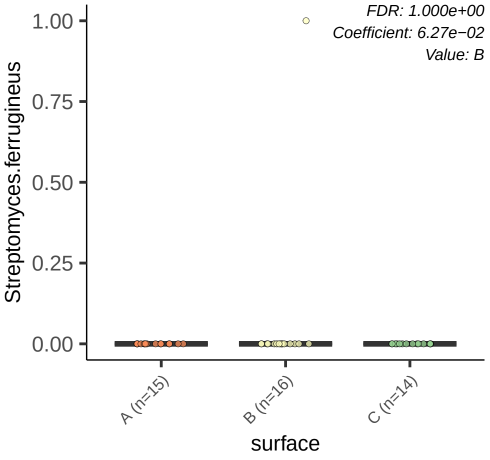

FDR: 1.000e+00
Coefficient: 6.27e−02
Value: B
Streptomyces.ferrugineus
1.00
0.75
0.50
0.25
0.00
A (n=15)
B (n=16)
C (n=14)
surface

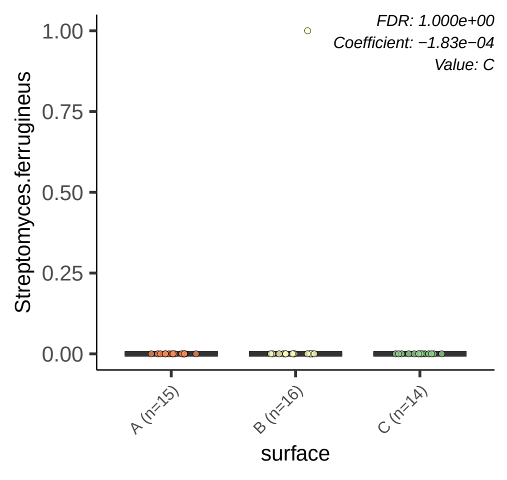
FDR: 1.000e+00
Coefficient: −1.83e−04
Value: C
Streptomyces.ferrugineus
1.00
0.75
0.50
0.25
0.00
A (n=15)
B (n=16)
C (n=14)
surface

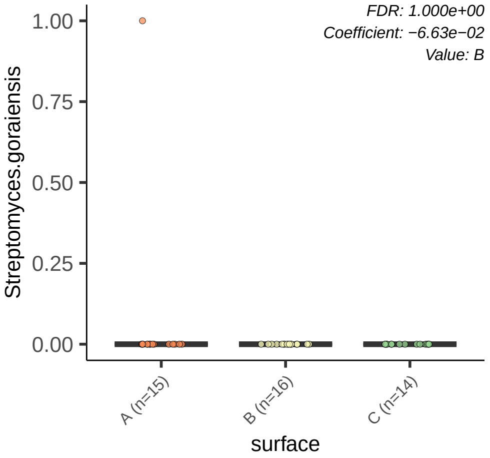
FDR: 1.000e+00
Coefficient: −6.63e−02
Value: B
1.00
0.75
0.50
0.25
0.00
Streptomyces.goraiensis
A (n=15)
B (n=16)
C (n=14)
surface

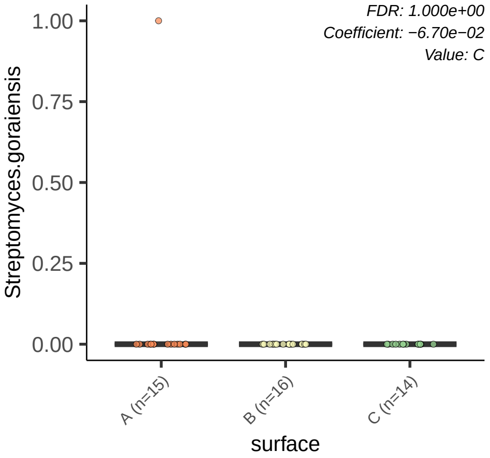
FDR: 1.000e+00
Coefficient: −6.70e−02
Value: C
Streptomyces.goraiensis
1.00
0.75
0.50
0.25
0.00
A (n=15)
B (n=16)
C (n=14)
surface

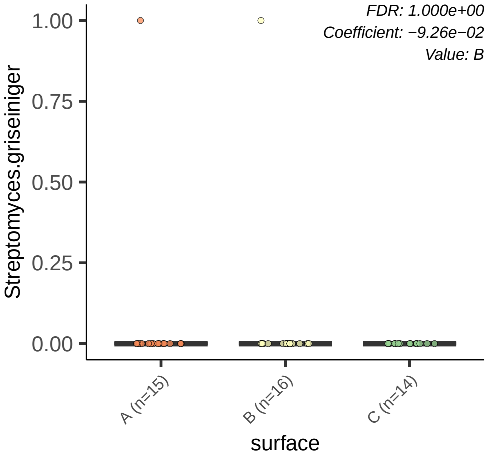
FDR: 1.000e+00
Coefficient: −9.26e−02
Value: B
Streptomyces.griseiniger
1.00
0.75
0.50
0.25
0.00
A (n=15)
B (n=16)
C (n=14)
surface

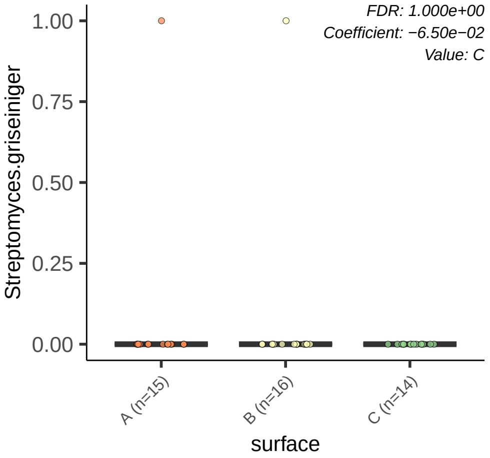

FDR: 1.000e+00
Coefficient: −6.50e−02
Value: C
Streptomyces.griseiniger
1.00
0.75
0.50
0.25
0.00
A (n=15)
B (n=16)
C (n=14)
surface

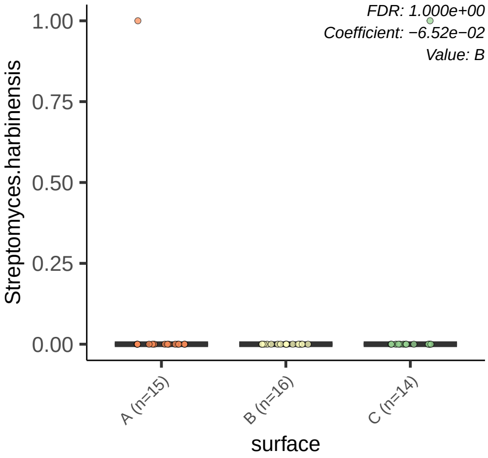
FDR: 1.000e+00
Coefficient: −6.52e−02
Value: B
Streptomyces.harbinensis
1.00
0.75
0.50
0.25
0.00
A (n=15)
B (n=16)
C (n=14)
surface

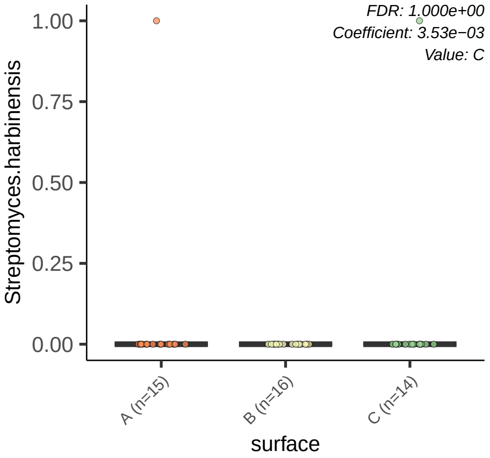

FDR: 1.000e+00
Coefficient: 3.53e−03
Value: C
Streptomyces.harbinensis
1.00
0.75
0.50
0.25
0.00
A (n=15)
B (n=16)
C (n=14)
surface

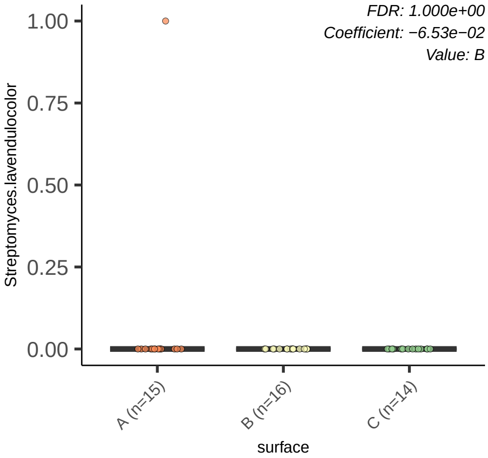
FDR: 1.000e+00
Coefficient: −6.53e−02
Value: B
Streptomyces.lavendulocolor
1.00
0.75
0.50
0.25
0.00
A (n=15)
B (n=16)
C (n=14)
surface

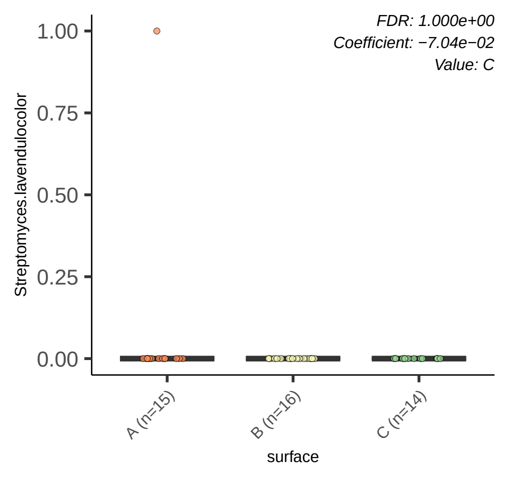
FDR: 1.000e+00
Coefficient: −7.04e−02
Value: C
1.00
0.75
0.50
0.25
0.00
Streptomyces.lavendulocolor
A (n=15)
B (n=16)
C (n=14)
surface

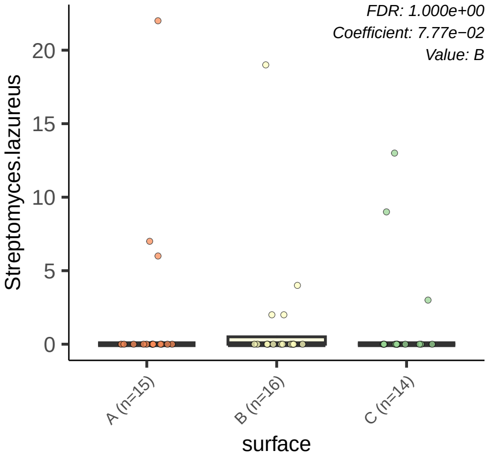

FDR: 1.000e+00
Coefficient: 7.77e−02
Value: B
Streptomyces.lazureus
0
5
10
15
20
A (n=15)
B (n=16)
C (n=14)
surface

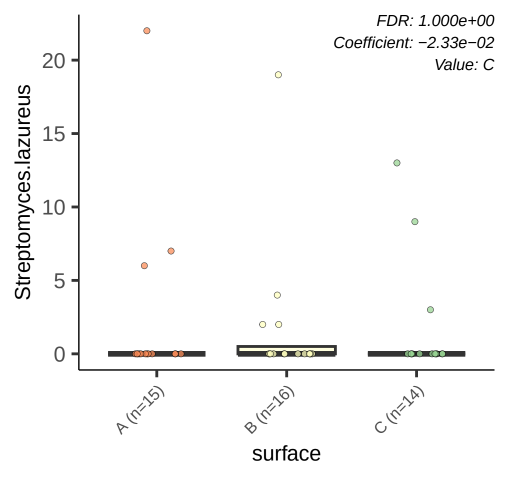

FDR: 1.000e+00
Coefficient: −2.33e−02
Value: C
Streptomyces.lazureus
0
5
10
15
20
A (n=15)
B (n=16)
C (n=14)
surface

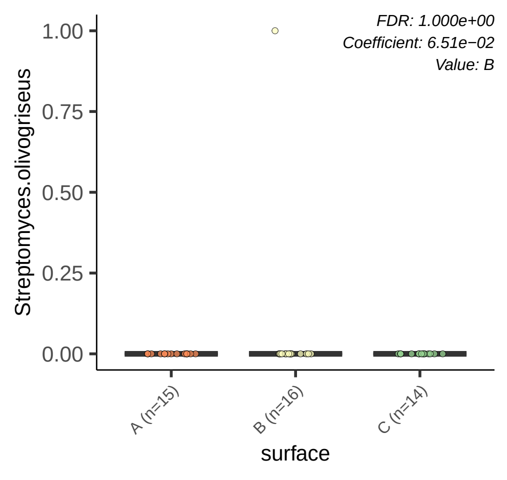

FDR: 1.000e+00
Coefficient: 6.51e−02
Value: B
Streptomyces.olivogriseus
1.00
0.75
0.50
0.25
0.00
A (n=15)
B (n=16)
C (n=14)
surface

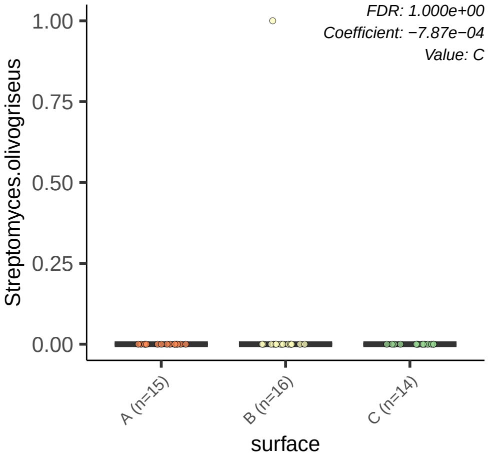
FDR: 1.000e+00
Coefficient: −7.87e−04
Value: C
Streptomyces.olivogriseus
1.00
0.75
0.50
0.25
0.00
A (n=15)
B (n=16)
C (n=14)
surface

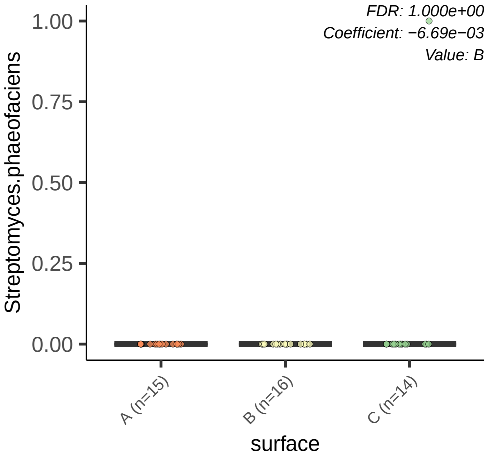

FDR: 1.000e+00
Coefficient: −6.69e−03
Value: B
Streptomyces.phaeofaciens
1.00
0.75
0.50
0.25
0.00
A (n=15)
B (n=16)
C (n=14)
surface

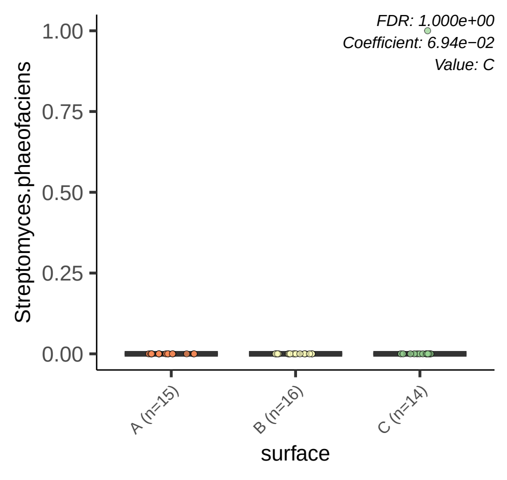
FDR: 1.000e+00
Coefficient: 6.94e−02
Value: C
Streptomyces.phaeofaciens
1.00
0.75
0.50
0.25
0.00
A (n=15)
B (n=16)
C (n=14)
surface

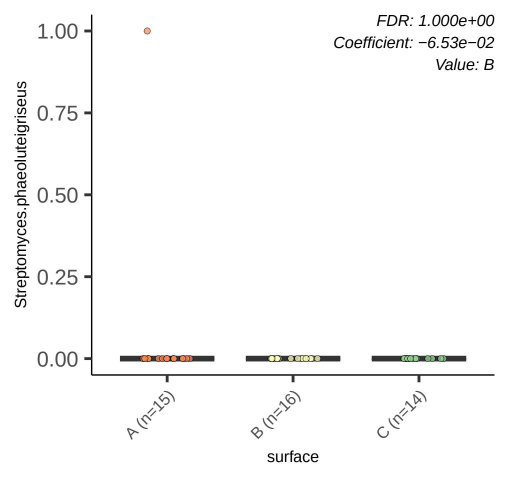
FDR: 1.000e+00
Coefficient: −6.53e−02
Value: B
Streptomyces.phaeoluteigriseus
1.00
0.75
0.50
0.25
0.00
A (n=15)
B (n=16)
C (n=14)
surface

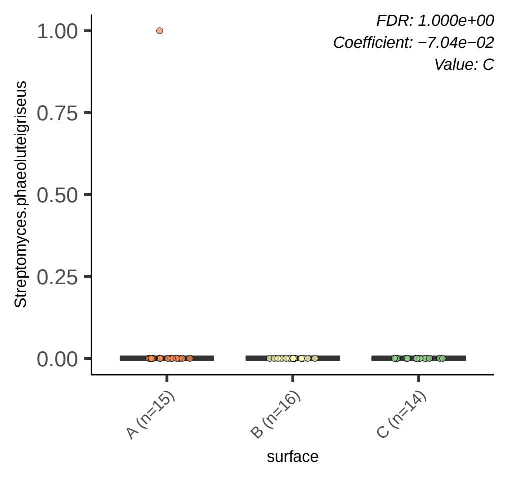
FDR: 1.000e+00
Coefficient: −7.04e−02
Value: C
1.00
0.75
0.50
0.25
0.00
Streptomyces.phaeoluteigriseus
A (n=15)
B (n=16)
C (n=14)
surface

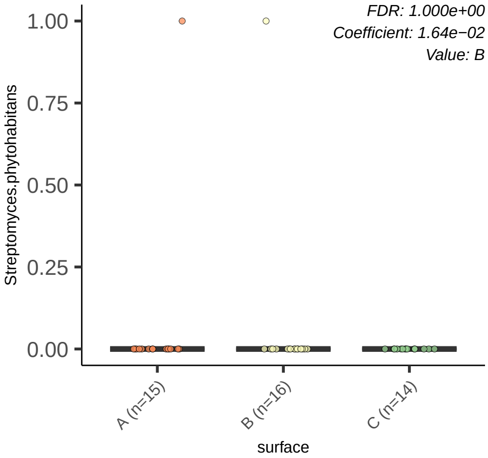
FDR: 1.000e+00
Coefficient: 1.64e−02
Value: B
Streptomyces.phytohabitans
1.00
0.75
0.50
0.25
0.00
A (n=15)
B (n=16)
C (n=14)
surface

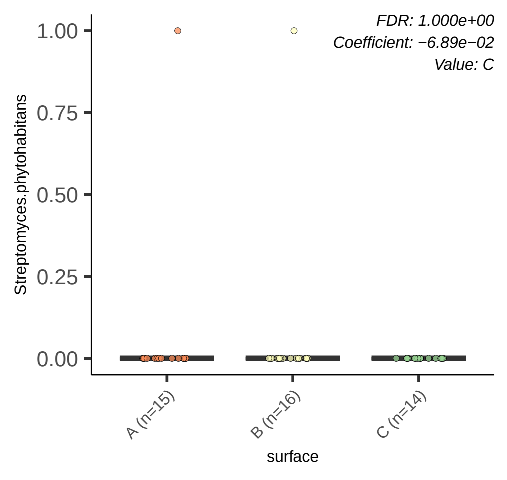
FDR: 1.000e+00
Coefficient: −6.89e−02
Value: C
Streptomyces.phytohabitans
1.00
0.75
0.50
0.25
0.00
A (n=15)
B (n=16)
C (n=14)
surface

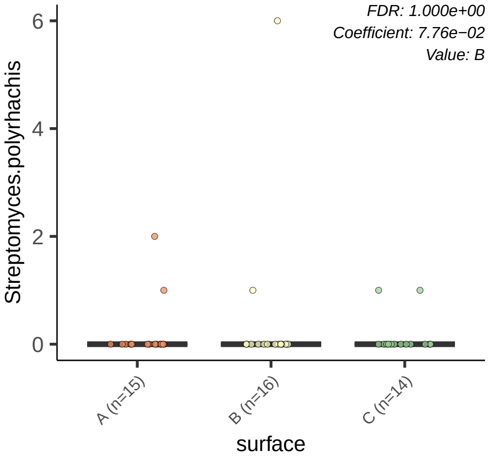

FDR: 1.000e+00
Coefficient: 7.76e−02
Value: B
Streptomyces.polyrhachis
6
4
2
0
A (n=15)
B (n=16)
C (n=14)
surface

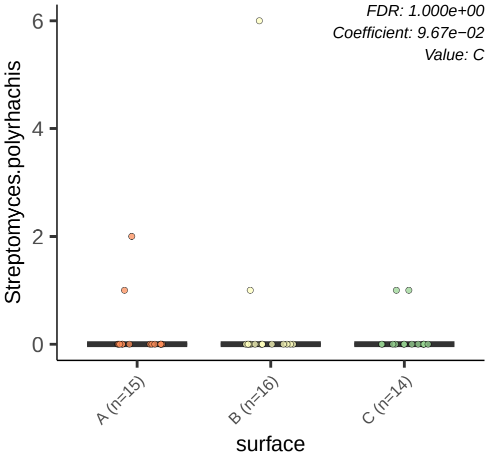
FDR: 1.000e+00
Coefficient: 9.67e−02
Value: C
Streptomyces.polyrhachis
0
2
4
6
A (n=15)
B (n=16)
C (n=14)
surface

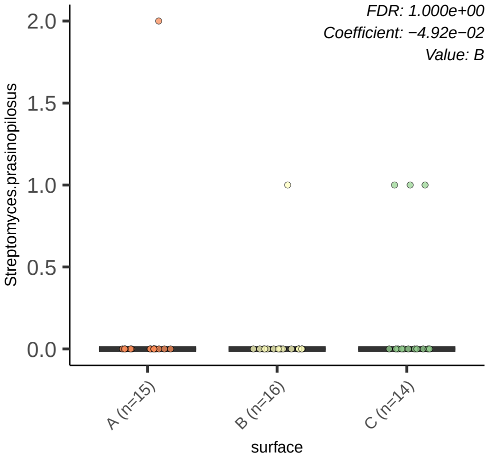

FDR: 1.000e+00
Coefficient: −4.92e−02
Value: B
Streptomyces.prasinopilosus
2.0
1.5
1.0
0.5
0.0
A (n=15)
B (n=16)
C (n=14)
surface

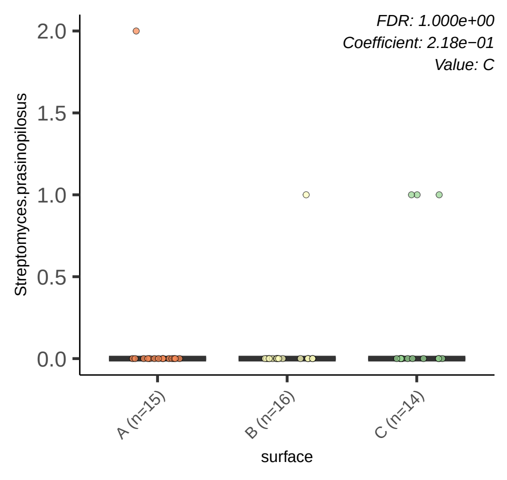
FDR: 1.000e+00
Coefficient: 2.18e−01
Value: C
Streptomyces.prasinopilosus
2.0
1.5
1.0
0.5
0.0
A (n=15)
B (n=16)
C (n=14)
surface

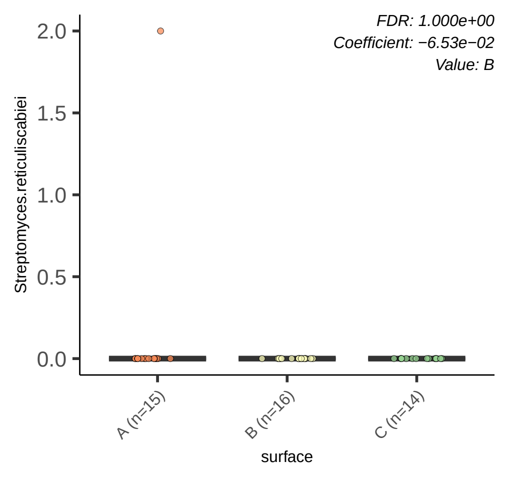

FDR: 1.000e+00
Coefficient: −6.53e−02
Value: B
Streptomyces.reticuliscabiei
2.0
1.5
1.0
0.5
0.0
A (n=15)
B (n=16)
C (n=14)
surface

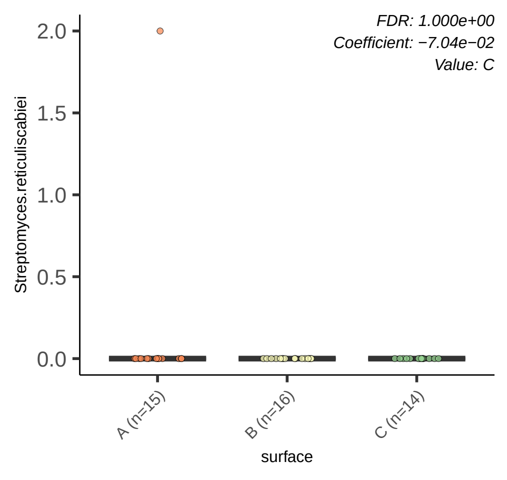
FDR: 1.000e+00
Coefficient: −7.04e−02
Value: C
Streptomyces.reticuliscabiei
2.0
1.5
1.0
0.5
0.0
A (n=15)
B (n=16)
C (n=14)
surface

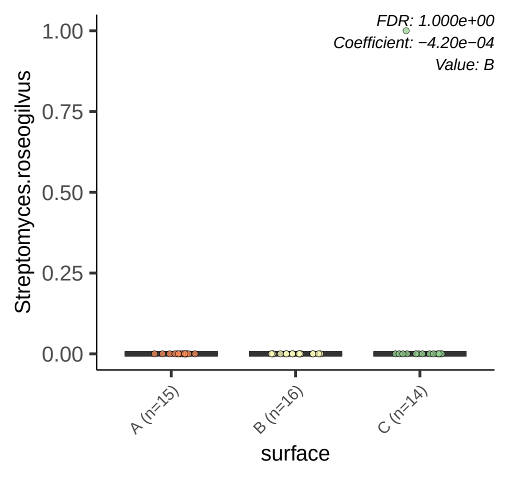
FDR: 1.000e+00
Coefficient: −4.20e−04
Value: B
Streptomyces.roseogilvus
1.00
0.75
0.50
0.25
0.00
A (n=15)
B (n=16)
C (n=14)
surface

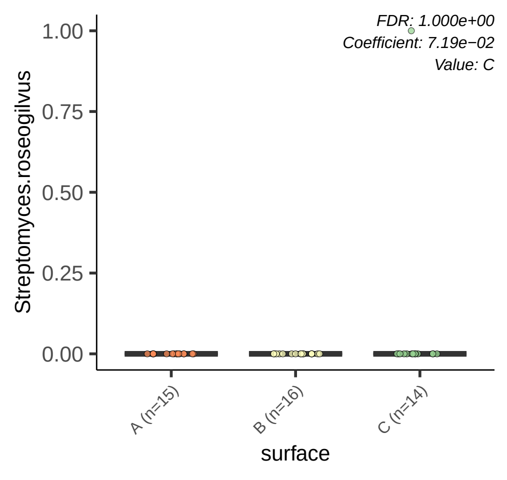

FDR: 1.000e+00
Coefficient: 7.19e−02
Value: C
Streptomyces.roseogilvus
1.00
0.75
0.50
0.25
0.00
A (n=15)
B (n=16)
C (n=14)
surface

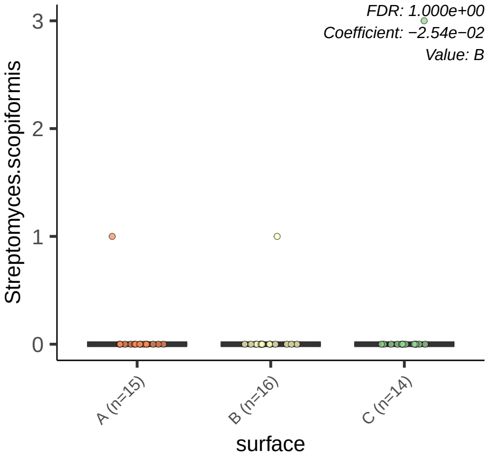
FDR: 1.000e+00
Coefficient: −2.54e−02
Value: B
Streptomyces.scopiformis
0
1
2
3
A (n=15)
B (n=16)
C (n=14)
surface

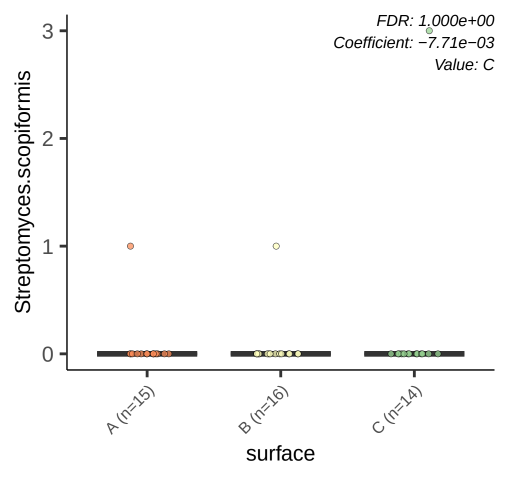
FDR: 1.000e+00
Coefficient: −7.71e−03
Value: C
Streptomyces.scopiformis
0
1
2
3
A (n=15)
B (n=16)
C (n=14)
surface

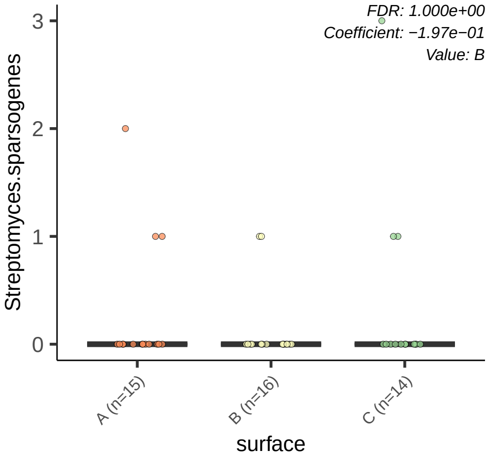

FDR: 1.000e+00
Coefficient: −1.97e−01
Value: B
Streptomyces.sparsogenes
0
1
2
3
A (n=15)
B (n=16)
C (n=14)
surface

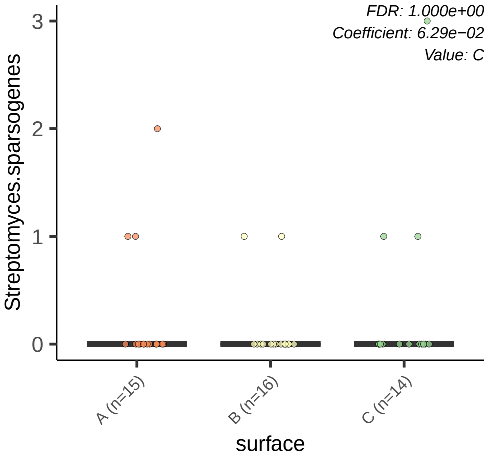
FDR: 1.000e+00
Coefficient: 6.29e−02
Value: C
Streptomyces.sparsogenes
3
2
1
0
A (n=15)
B (n=16)
C (n=14)
surface

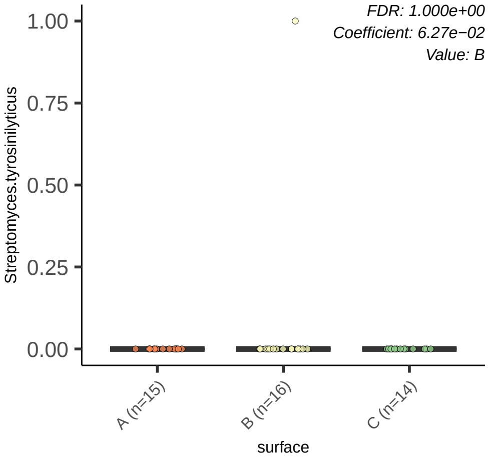
FDR: 1.000e+00
Coefficient: 6.27e−02
Value: B
Streptomyces.tyrosinilyticus
1.00
0.75
0.50
0.25
0.00
A (n=15)
B (n=16)
C (n=14)
surface

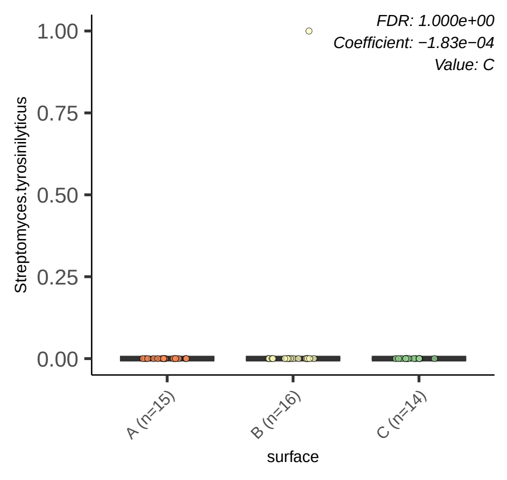
FDR: 1.000e+00
Coefficient: −1.83e−04
Value: C
1.00
0.75
0.50
0.25
0.00
Streptomyces.tyrosinilyticus
A (n=15)
B (n=16)
C (n=14)
surface

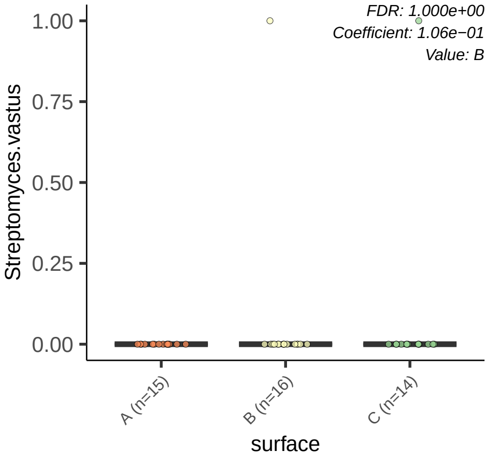

FDR: 1.000e+00
Coefficient: 1.06e−01
Value: B
Streptomyces.vastus
1.00
0.75
0.50
0.25
0.00
A (n=15)
B (n=16)
C (n=14)
surface

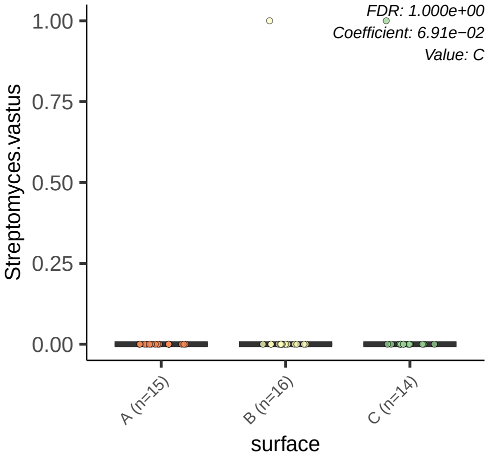
FDR: 1.000e+00
Coefficient: 6.91e−02
Value: C
Streptomyces.vastus
1.00
0.75
0.50
0.25
0.00
A (n=15)
B (n=16)
C (n=14)
surface

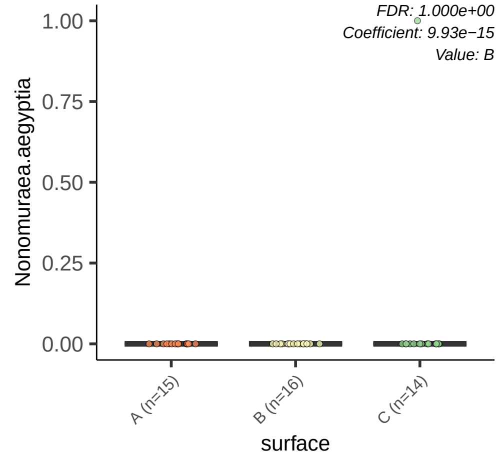
FDR: 1.000e+00
Coefficient: 9.93e−15
Value: B
1.00
0.75
0.50
0.25
0.00
Nonomuraea.aegyptia
A (n=15)
B (n=16)
C (n=14)
surface

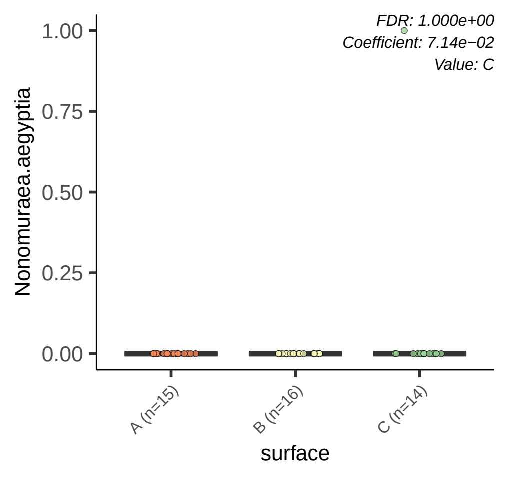
FDR: 1.000e+00
Coefficient: 7.14e−02
Value: C
Nonomuraea.aegyptia
1.00
0.75
0.50
0.25
0.00
A (n=15)
B (n=16)
C (n=14)
surface

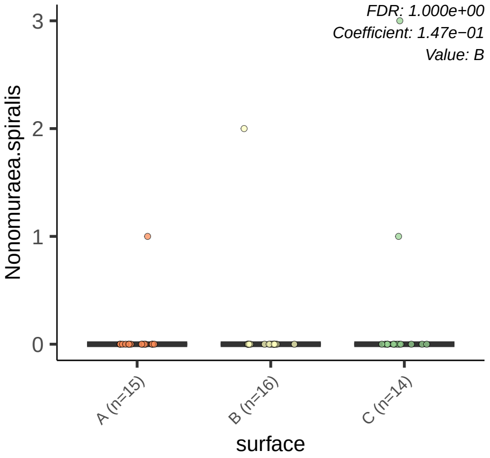

FDR: 1.000e+00
Coefficient: 1.47e−01
Value: B
Nonomuraea.spiralis
3
2
1
0
A (n=15)
B (n=16)
C (n=14)
surface

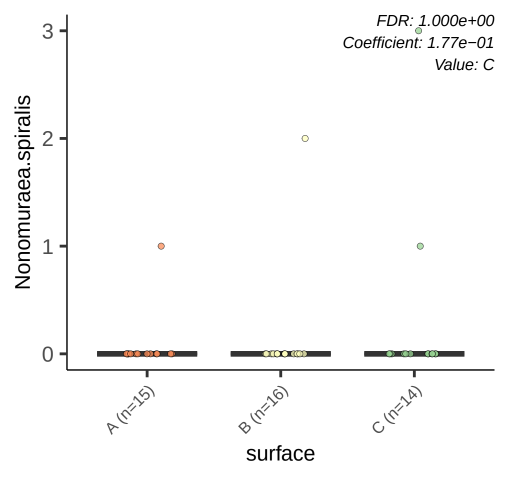
FDR: 1.000e+00
Coefficient: 1.77e−01
Value: C
Nonomuraea.spiralis
0
1
2
3
A (n=15)
B (n=16)
C (n=14)
surface

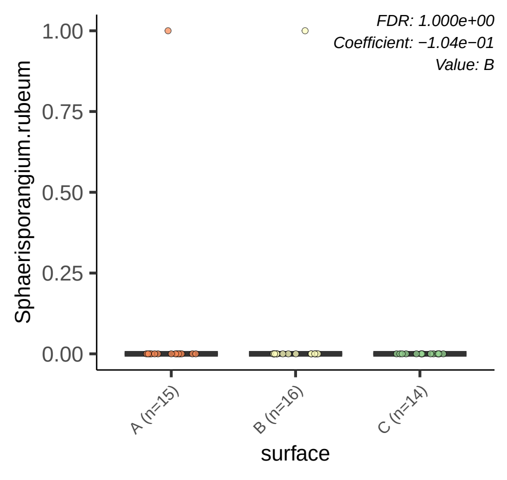

FDR: 1.000e+00
Coefficient: −1.04e−01
Value: B
Sphaerisporangium.rubeum
1.00
0.75
0.50
0.25
0.00
A (n=15)
B (n=16)
C (n=14)
surface

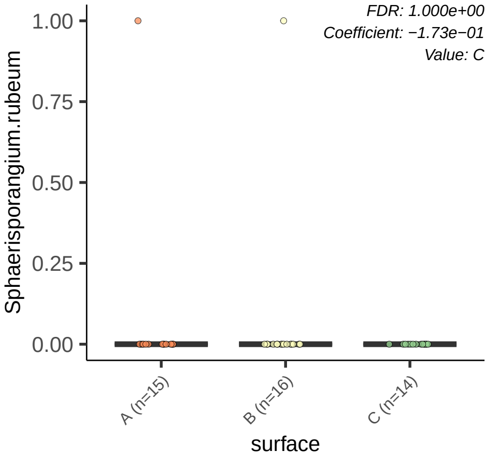
FDR: 1.000e+00
Coefficient: −1.73e−01
Value: C
Sphaerisporangium.rubeum
1.00
0.75
0.50
0.25
0.00
A (n=15)
B (n=16)
C (n=14)
surface

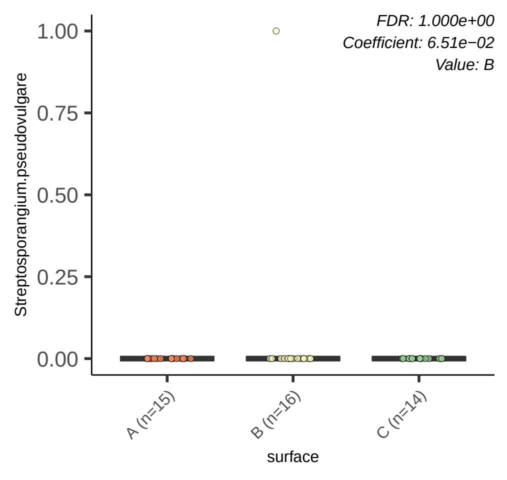
FDR: 1.000e+00
Coefficient: 6.51e−02
Value: B
Streptosporangium.pseudovulgare
1.00
0.75
0.50
0.25
0.00
A (n=15)
B (n=16)
C (n=14)
surface

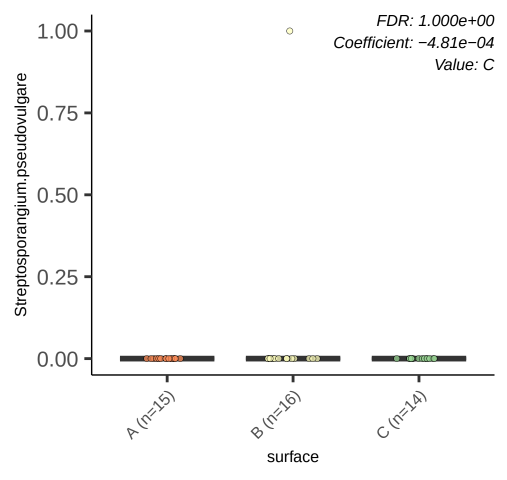
FDR: 1.000e+00
Coefficient: −4.81e−04
Value: C
Streptosporangium.pseudovulgare
1.00
0.75
0.50
0.25
0.00
A (n=15)
B (n=16)
C (n=14)
surface

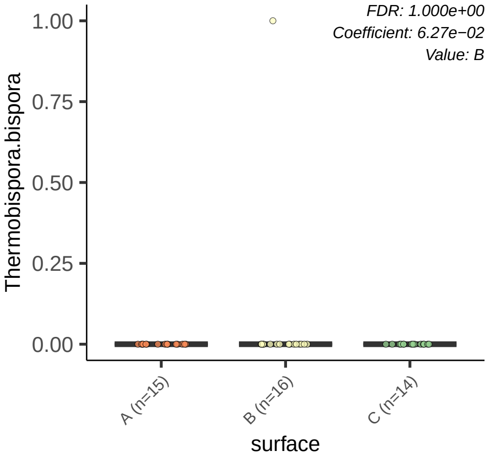
FDR: 1.000e+00
Coefficient: 6.27e−02
Value: B
Thermobispora.bispora
1.00
0.75
0.50
0.25
0.00
A (n=15)
B (n=16)
C (n=14)
surface

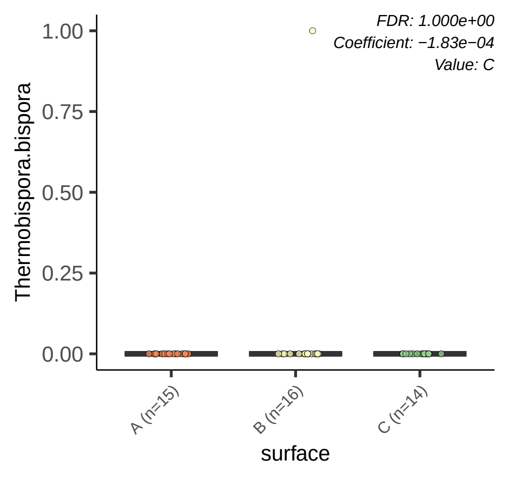
FDR: 1.000e+00
Coefficient: −1.83e−04
Value: C
Thermobispora.bispora
1.00
0.75
0.50
0.25
0.00
A (n=15)
B (n=16)
C (n=14)
surface

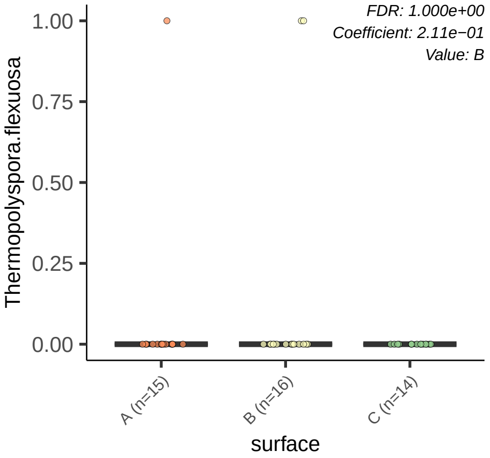

FDR: 1.000e+00
Coefficient: 2.11e−01
Value: B
Thermopolyspora.flexuosa
1.00
0.75
0.50
0.25
0.00
A (n=15)
B (n=16)
C (n=14)
surface

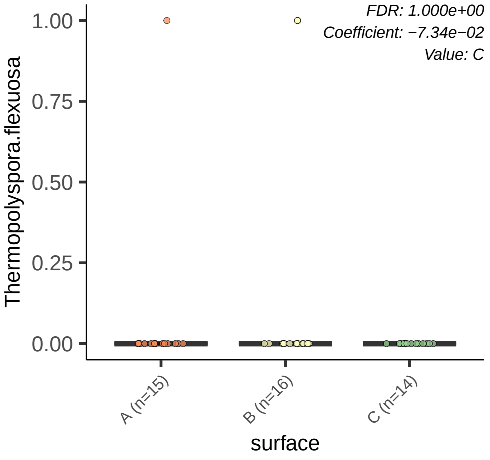
FDR: 1.000e+00
Coefficient: −7.34e−02
Value: C
Thermopolyspora.flexuosa
1.00
0.75
0.50
0.25
0.00
A (n=15)
B (n=16)
C (n=14)
surface

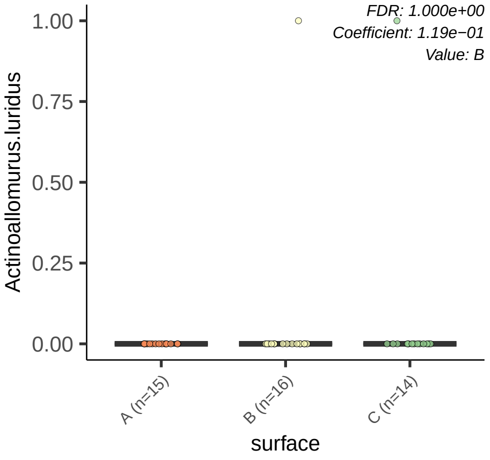
FDR: 1.000e+00
Coefficient: 1.19e−01
Value: B
Actinoallomurus.luridus
1.00
0.75
0.50
0.25
0.00
A (n=15)
B (n=16)
C (n=14)
surface

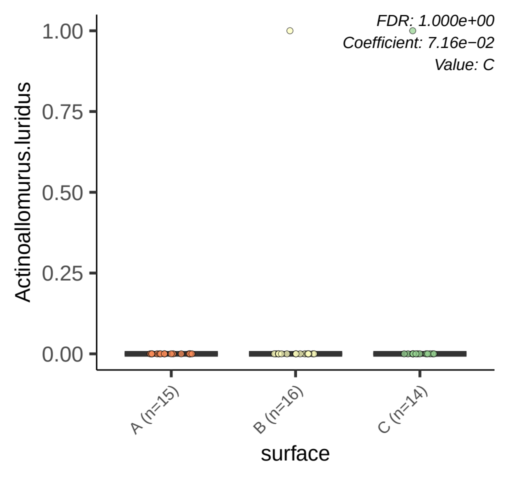

FDR: 1.000e+00
Coefficient: 7.16e−02
Value: C
Actinoallomurus.luridus
1.00
0.75
0.50
0.25
0.00
A (n=15)
B (n=16)
C (n=14)
surface

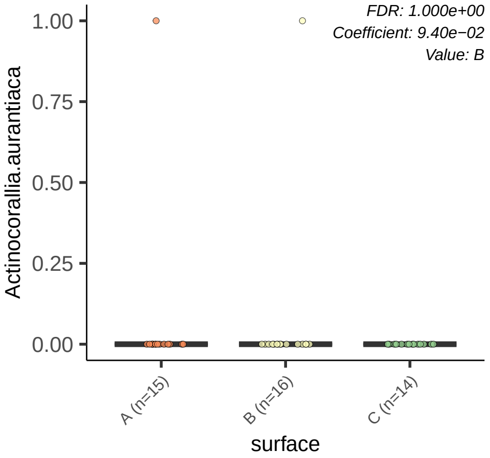
FDR: 1.000e+00
Coefficient: 9.40e−02
Value: B
Actinocorallia.aurantiaca
1.00
0.75
0.50
0.25
0.00
A (n=15)
B (n=16)
C (n=14)
surface

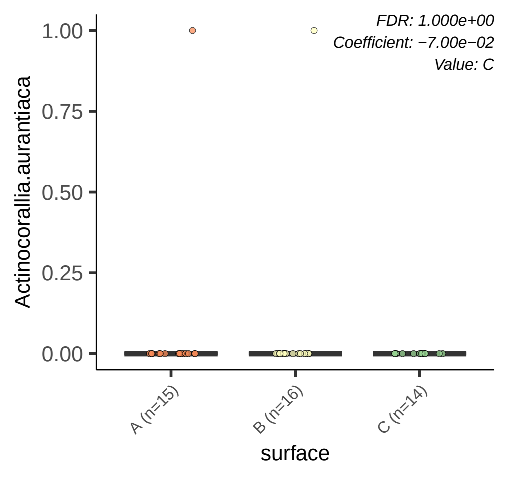
FDR: 1.000e+00
Coefficient: −7.00e−02
Value: C
Actinocorallia.aurantiaca
1.00
0.75
0.50
0.25
0.00
A (n=15)
B (n=16)
C (n=14)
surface

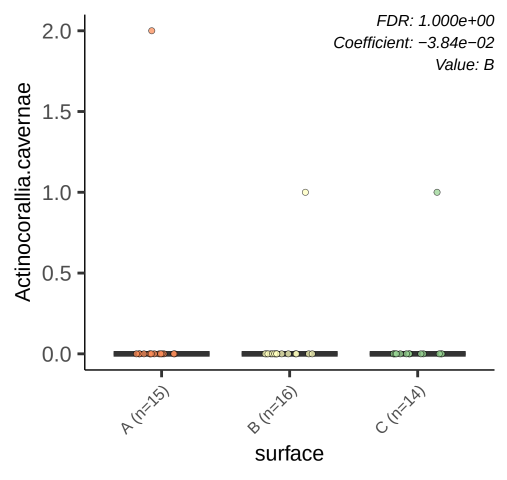
FDR: 1.000e+00
Coefficient: −3.84e−02
Value: B
Actinocorallia.cavernae
2.0
1.5
1.0
0.5
0.0
A (n=15)
B (n=16)
C (n=14)
surface

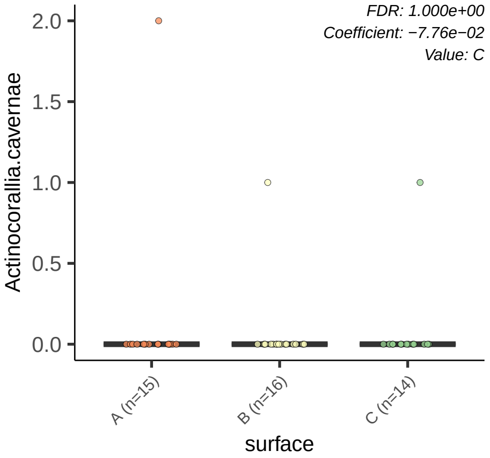
FDR: 1.000e+00
Coefficient: −7.76e−02
Value: C
Actinocorallia.cavernae
2.0
1.5
1.0
0.5
0.0
A (n=15)
B (n=16)
C (n=14)
surface

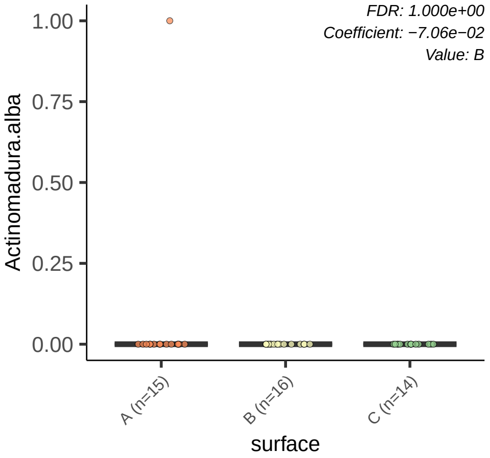
FDR: 1.000e+00
Coefficient: −7.06e−02
Value: B
Actinomadura.alba
1.00
0.75
0.50
0.25
0.00
A (n=15)
B (n=16)
C (n=14)
surface

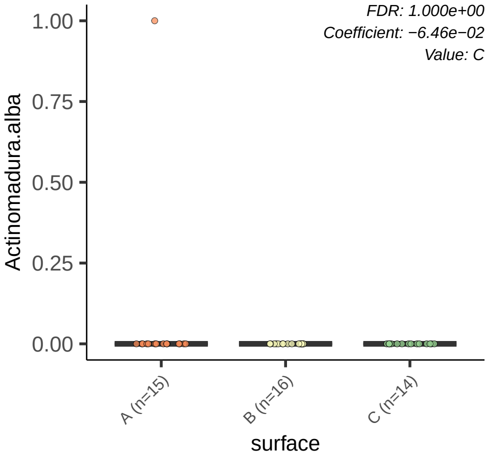

FDR: 1.000e+00
Coefficient: −6.46e−02
Value: C
Actinomadura.alba
1.00
0.75
0.50
0.25
0.00
A (n=15)
B (n=16)
C (n=14)
surface

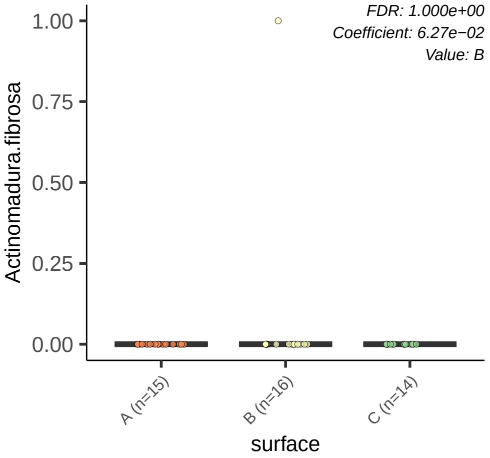

FDR: 1.000e+00
Coefficient: 6.27e−02
Value: B
Actinomadura.fibrosa
1.00
0.75
0.50
0.25
0.00
A (n=15)
B (n=16)
C (n=14)
surface

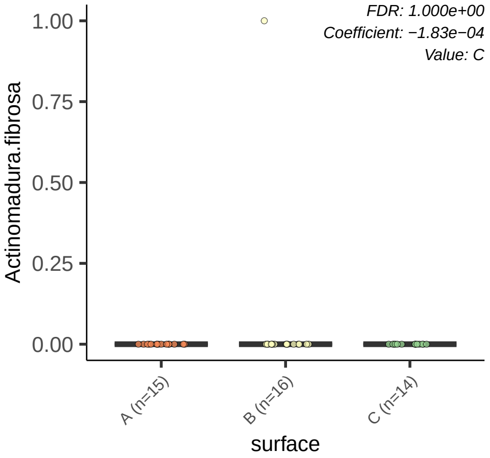

FDR: 1.000e+00
Coefficient: −1.83e−04
Value: C
Actinomadura.fibrosa
1.00
0.75
0.50
0.25
0.00
A (n=15)
B (n=16)
C (n=14)
surface

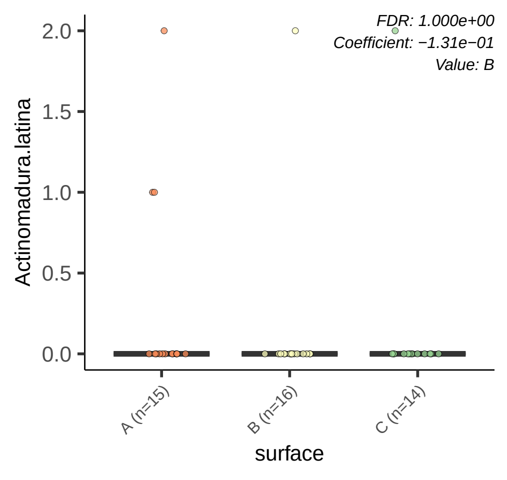

FDR: 1.000e+00
Coefficient: −1.31e−01
Value: B
Actinomadura.latina
2.0
1.5
1.0
0.5
0.0
A (n=15)
B (n=16)
C (n=14)
surface

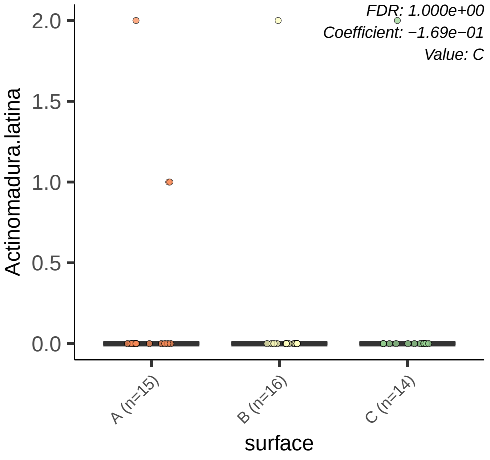
FDR: 1.000e+00
Coefficient: −1.69e−01
Value: C
Actinomadura.latina
2.0
1.5
1.0
0.5
0.0
A (n=15)
B (n=16)
C (n=14)
surface

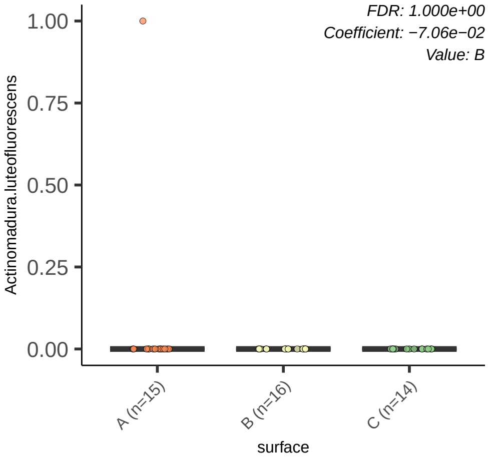

FDR: 1.000e+00
Coefficient: −7.06e−02
Value: B
Actinomadura.luteofluorescens
1.00
0.75
0.50
0.25
0.00
A (n=15)
B (n=16)
C (n=14)
surface

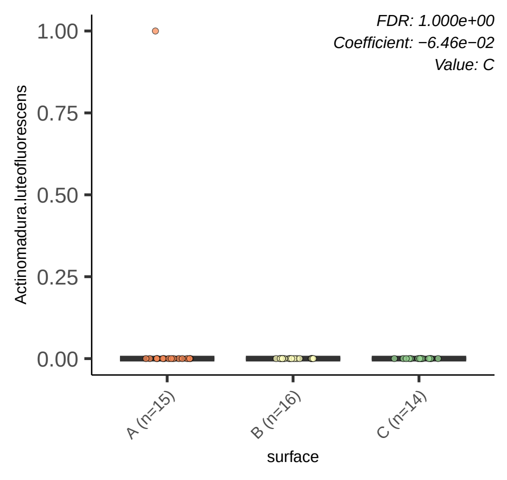

FDR: 1.000e+00
Coefficient: −6.46e−02
Value: C
Actinomadura.luteofluorescens
1.00
0.75
0.50
0.25
0.00
A (n=15)
B (n=16)
C (n=14)
surface

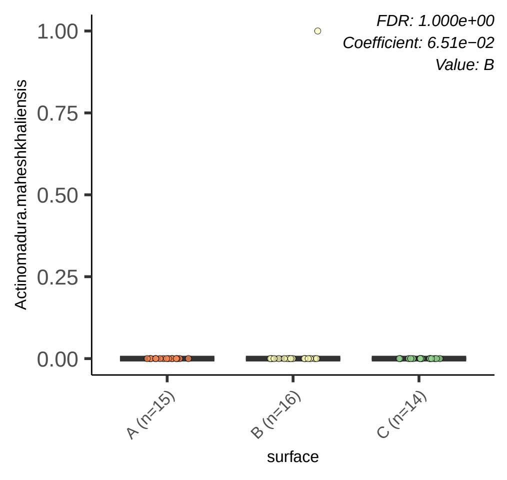

FDR: 1.000e+00
Coefficient: 6.51e−02
Value: B
1.00
0.75
0.50
0.25
0.00
Actinomadura.maheshkhaliensis
A (n=15)
B (n=16)
C (n=14)
surface

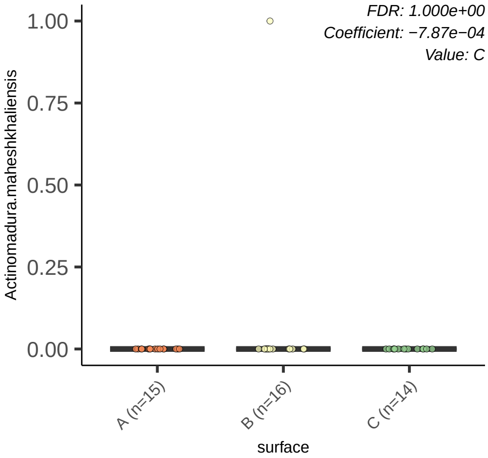
FDR: 1.000e+00
Coefficient: −7.87e−04
Value: C
Actinomadura.maheshkhaliensis
1.00
0.75
0.50
0.25
0.00
A (n=15)
B (n=16)
C (n=14)
surface

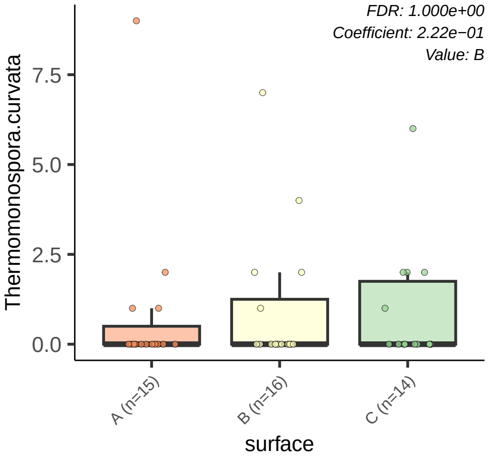

FDR: 1.000e+00
Coefficient: 2.22e−01
Value: B
Thermomonospora.curvata
7.5
5.0
2.5
0.0
A (n=15)
B (n=16)
C (n=14)
surface

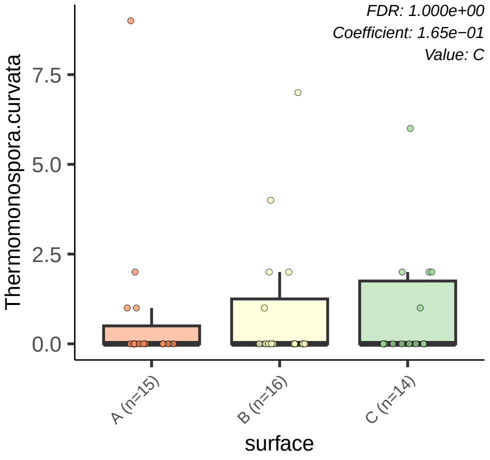

FDR: 1.000e+00
Coefficient: 1.65e−01
Value: C
Thermomonospora.curvata
7.5
5.0
2.5
0.0
A (n=15)
B (n=16)
C (n=14)
surface

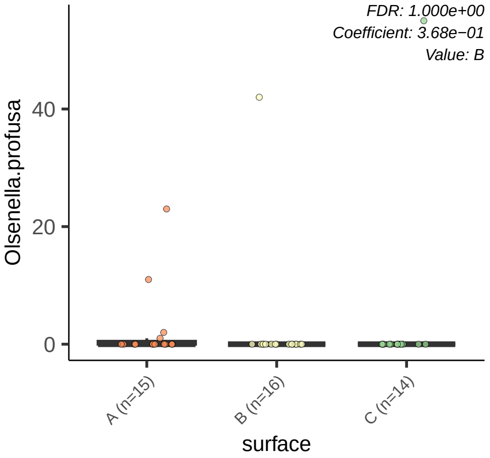
FDR: 1.000e+00
Coefficient: 3.68e−01
Value: B
Olsenella.profusa
0
20
40
A (n=15)
B (n=16)
C (n=14)
surface

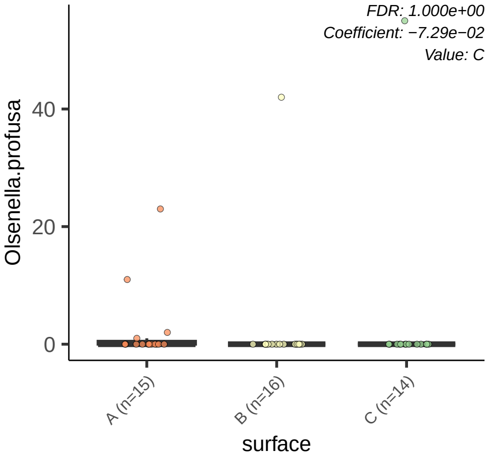

FDR: 1.000e+00
Coefficient: −7.29e−02
Value: C
Olsenella.profusa
0
20
40
A (n=15)
B (n=16)
C (n=14)
surface

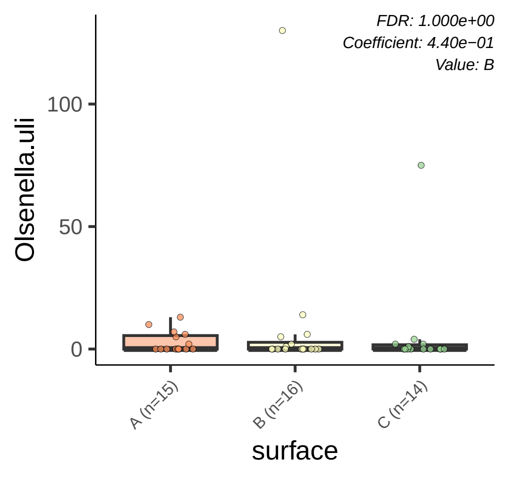
FDR: 1.000e+00
Coefficient: 4.40e−01
Value: B
Olsenella.uli
100
50
0
A (n=15)
B (n=16)
C (n=14)
surface

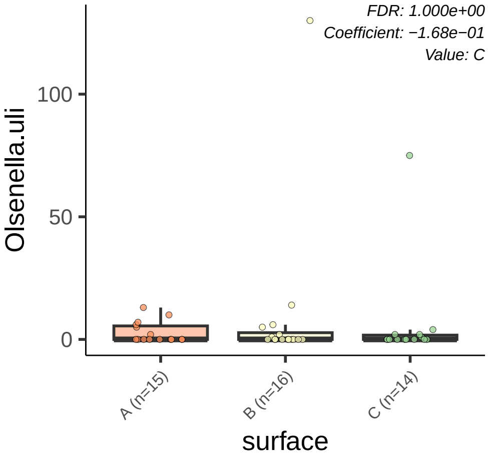
FDR: 1.000e+00
Coefficient: −1.68e−01
Value: C
Olsenella.uli
100
50
0
A (n=15)
B (n=16)
C (n=14)
surface

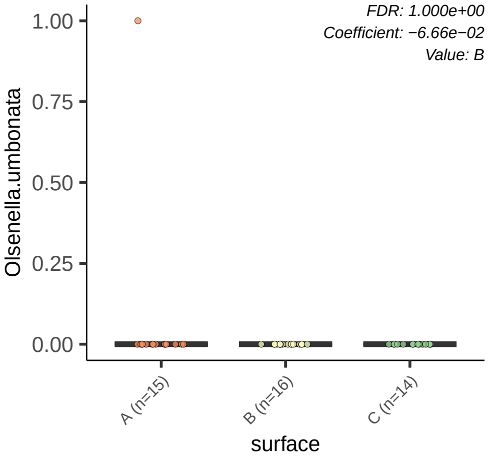
FDR: 1.000e+00
Coefficient: −6.66e−02
Value: B
Olsenella.umbonata
1.00
0.75
0.50
0.25
0.00
A (n=15)
B (n=16)
C (n=14)
surface

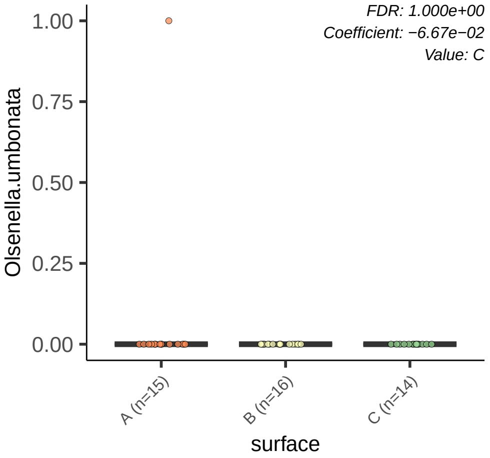
FDR: 1.000e+00
Coefficient: −6.67e−02
Value: C
Olsenella.umbonata
1.00
0.75
0.50
0.25
0.00
A (n=15)
B (n=16)
C (n=14)
surface

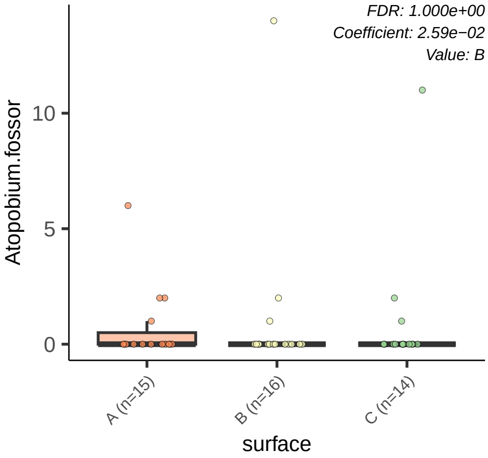

FDR: 1.000e+00
Coefficient: 2.59e−02
Value: B
Atopobium.fossor
10
5
0
A (n=15)
B (n=16)
C (n=14)
surface

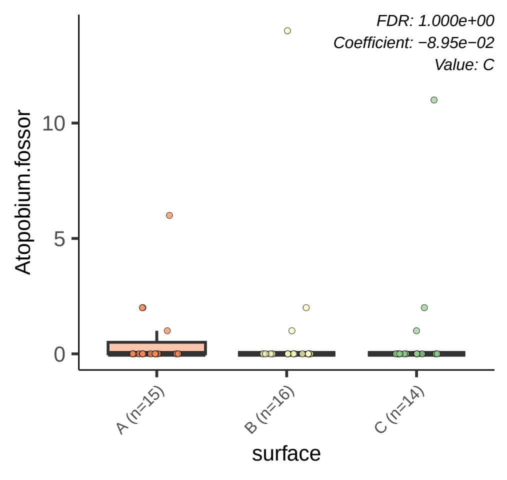
FDR: 1.000e+00
Coefficient: −8.95e−02
Value: C
Atopobium.fossor
0
5
10
A (n=15)
B (n=16)
C (n=14)
surface

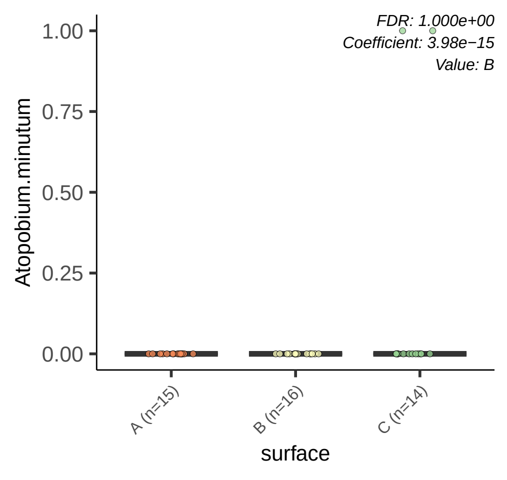
FDR: 1.000e+00
Coefficient: 3.98e−15
Value: B
1.00
0.75
0.50
0.25
0.00
Atopobium.minutum
A (n=15)
B (n=16)
C (n=14)
surface

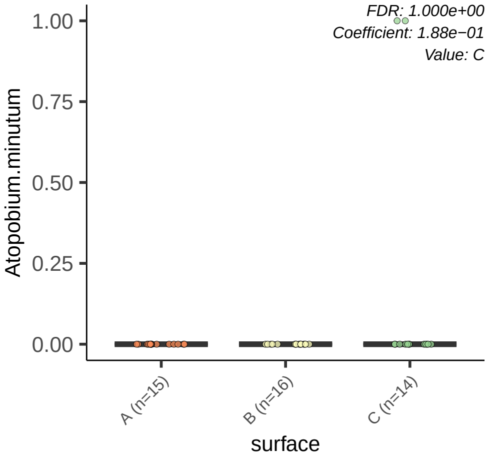

FDR: 1.000e+00
Coefficient: 1.88e−01
Value: C
Atopobium.minutum
1.00
0.75
0.50
0.25
0.00
A (n=15)
B (n=16)
C (n=14)
surface

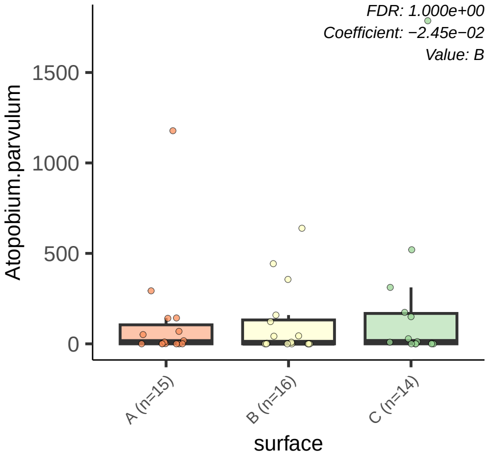
FDR: 1.000e+00
Coefficient: −2.45e−02
Value: B
Atopobium.parvulum
1500
1000
500
0
A (n=15)
B (n=16)
C (n=14)
surface

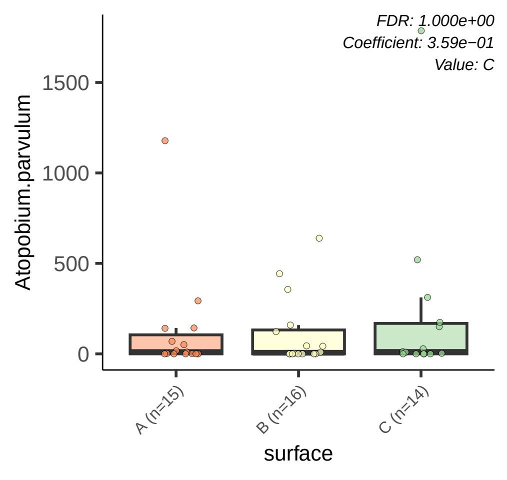

FDR: 1.000e+00
Coefficient: 3.59e−01
Value: C
Atopobium.parvulum
1500
1000
500
0
A (n=15)
B (n=16)
C (n=14)
surface

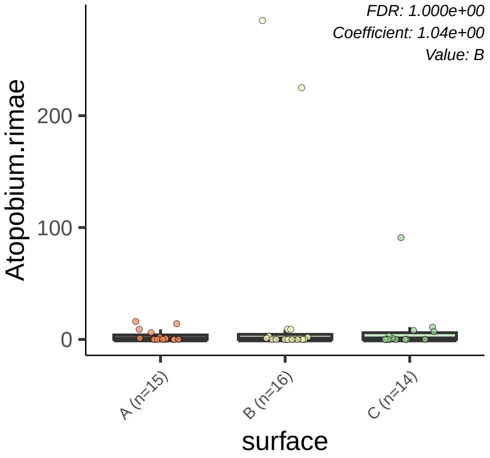

FDR: 1.000e+00
Coefficient: 1.04e+00
Value: B
Atopobium.rimae
200
100
0
A (n=15)
B (n=16)
C (n=14)
surface

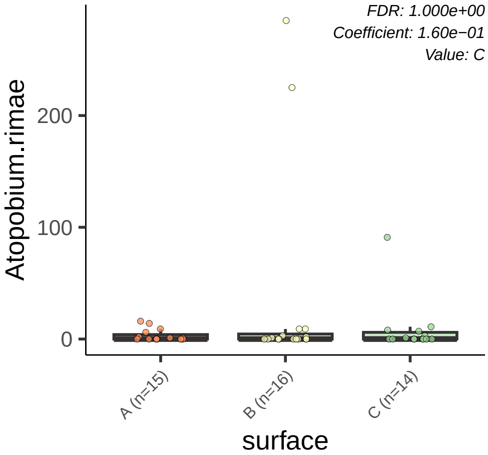
FDR: 1.000e+00
Coefficient: 1.60e−01
Value: C
Atopobium.rimae
200
100
0
A (n=15)
B (n=16)
C (n=14)
surface

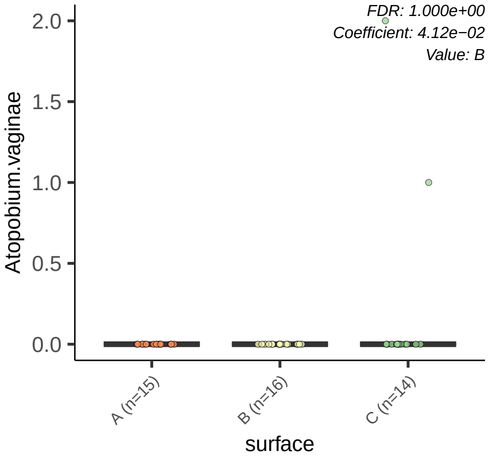

FDR: 1.000e+00
Coefficient: 4.12e−02
Value: B
Atopobium.vaginae
2.0
1.5
1.0
0.5
0.0
A (n=15)
B (n=16)
C (n=14)
surface

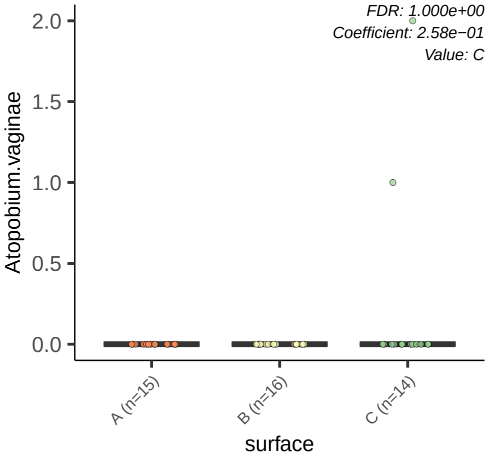

FDR: 1.000e+00
Coefficient: 2.58e−01
Value: C
Atopobium.vaginae
2.0
1.5
1.0
0.5
0.0
A (n=15)
B (n=16)
C (n=14)
surface

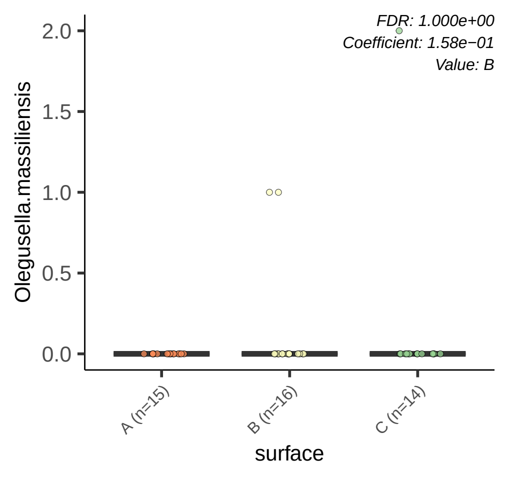
FDR: 1.000e+00
Coefficient: 1.58e−01
Value: B
Oleguscella.massiliensis
2.0
1.5
1.0
0.5
0.0
A (n=15)
B (n=16)
C (n=14)
surface

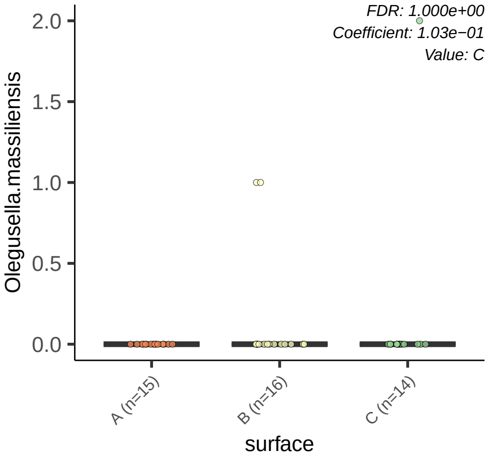

FDR: 1.000e+00
Coefficient: 1.03e−01
Value: C
Olegusella.massiliensis
2.0
1.5
1.0
0.5
0.0
A (n=15)
B (n=16)
C (n=14)
surface

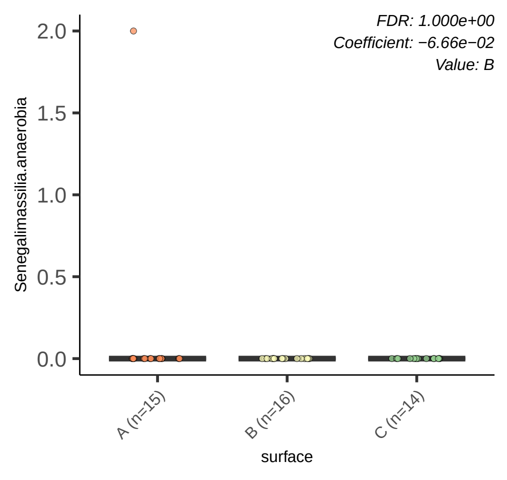

FDR: 1.000e+00
Coefficient: −6.66e−02
Value: B
Senegalimassilia.anaerobia
2.0
1.5
1.0
0.5
0.0
A (n=15)
B (n=16)
C (n=14)
surface

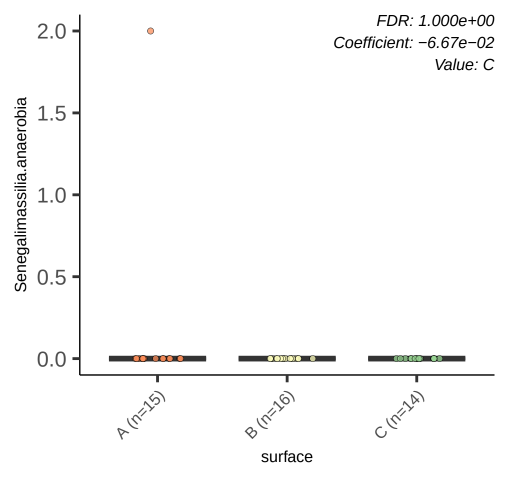

FDR: 1.000e+00
Coefficient: −6.67e−02
Value: C
Senegalimassilia.anaerobia
2.0
1.5
1.0
0.5
0.0
A (n=15)
B (n=16)
C (n=14)
surface

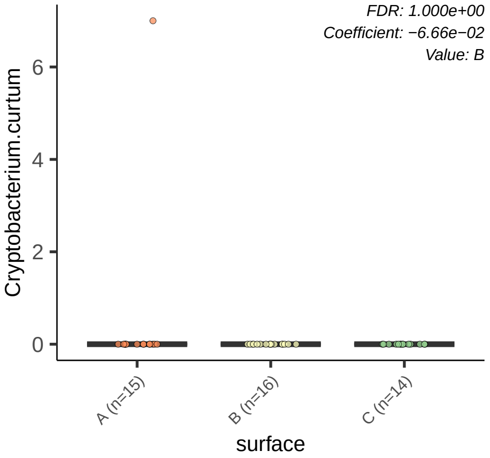

FDR: 1.000e+00
Coefficient: −6.66e−02
Value: B
Cryptobacterium.curtum
0
2
4
6
A (n=15)
B (n=16)
C (n=14)
surface

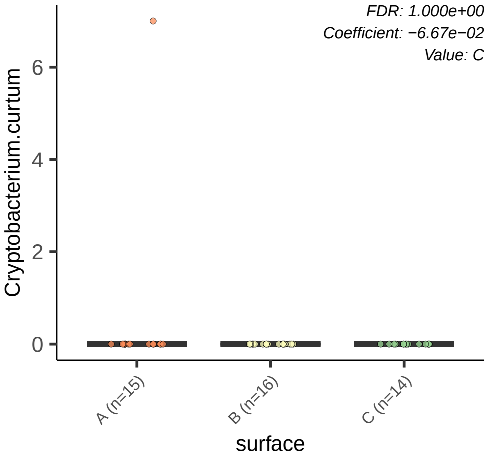
FDR: 1.000e+00
Coefficient: −6.67e−02
Value: C
Cryptobacterium.curtum
0
2
4
6
A (n=15)
B (n=16)
C (n=14)
surface

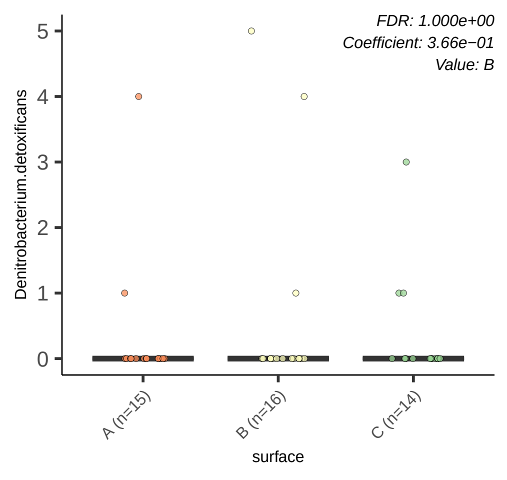

FDR: 1.000e+00
Coefficient: 3.66e−01
Value: B
Denitrobacterium.detoxificans
0
1
2
3
4
5
A (n=15)
B (n=16)
C (n=14)
surface

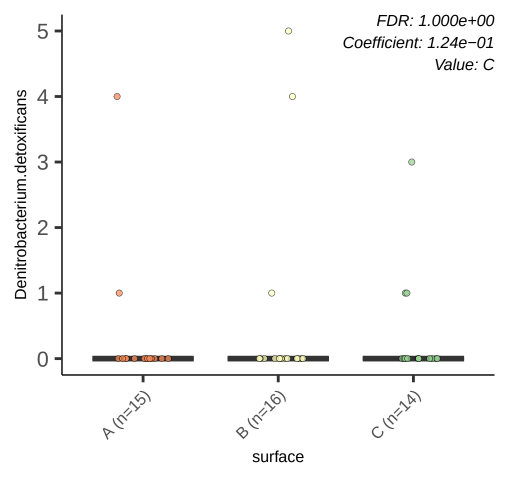
FDR: 1.000e+00
Coefficient: 1.24e−01
Value: C
Denitrobacterium.detoxificans
0
1
2
3
4
5
A (n=15)
B (n=16)
C (n=14)
surface

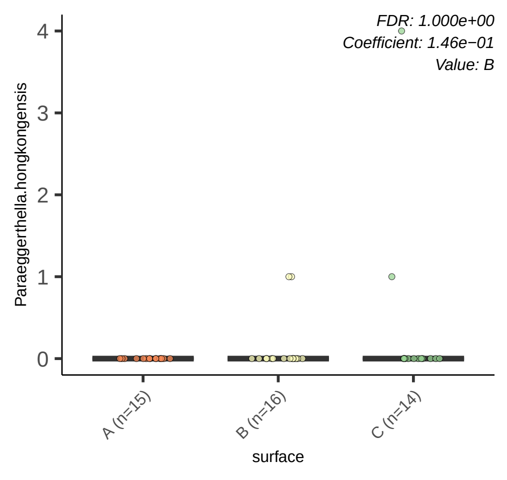

FDR: 1.000e+00
Coefficient: 1.46e−01
Value: B
Paraeggerthella.hongkongensis
4
3
2
1
0
A (n=15)
B (n=16)
C (n=14)
surface

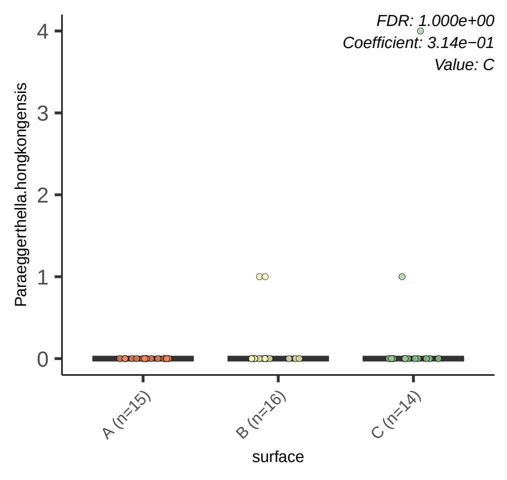

FDR: 1.000e+00
Coefficient: 3.14e−01
Value: C
Paraeggerthella.hongkongensis
0
1
2
3
4
A (n=15)
B (n=16)
C (n=14)
surface

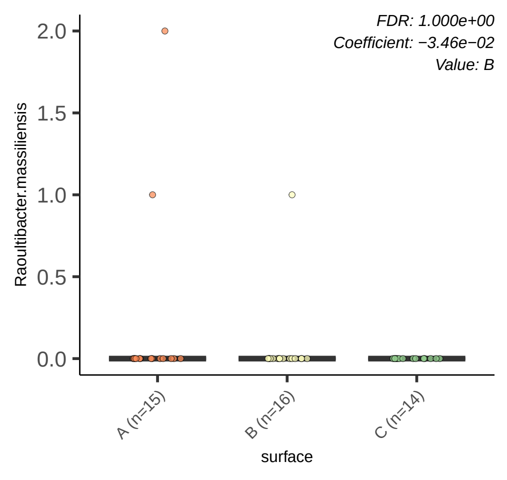

FDR: 1.000e+00
Coefficient: −3.46e−02
Value: B
Raoultibacter.massiliensis
2.0
1.5
1.0
0.5
0.0
A (n=15)
B (n=16)
C (n=14)
surface

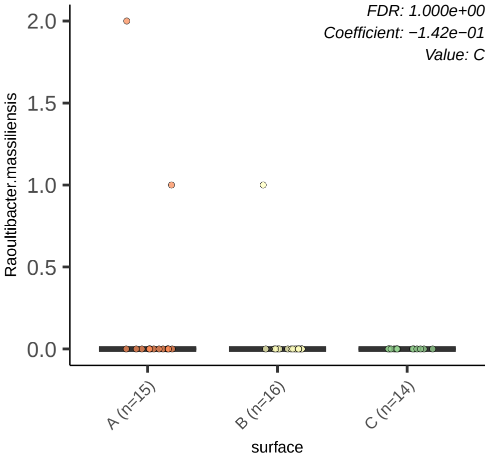
FDR: 1.000e+00
Coefficient: −1.42e−01
Value: C
Raoultibacter.massiliensis
2.0
1.5
1.0
0.5
0.0
A (n=15)
B (n=16)
C (n=14)
surface

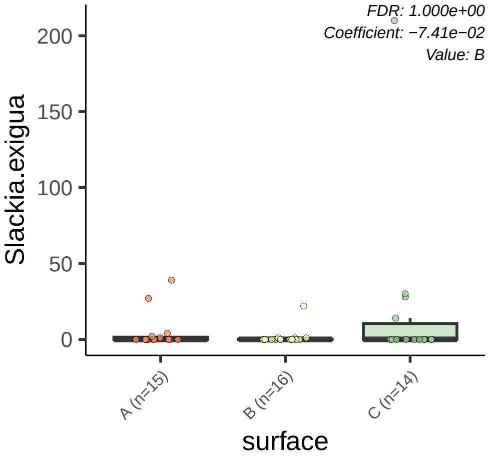
FDR: 1.000e+00
Coefficient: −7.41e−02
Value: B
Slackia.exigua
200
150
100
50
0
A (n=15)
B (n=16)
C (n=14)
surface

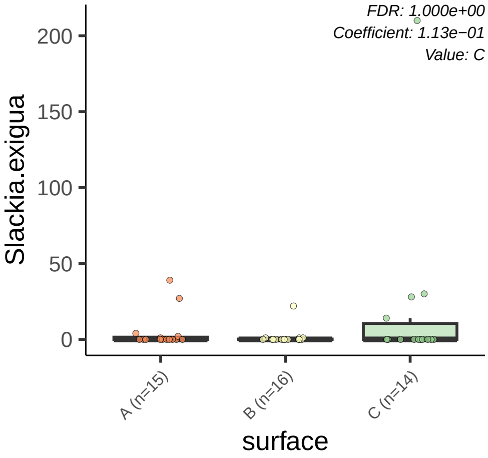

FDR: 1.000e+00
Coefficient: 1.13e−01
Value: C
Slackia.exigua
200
150
100
50
0
A (n=15)
B (n=16)
C (n=14)
surface

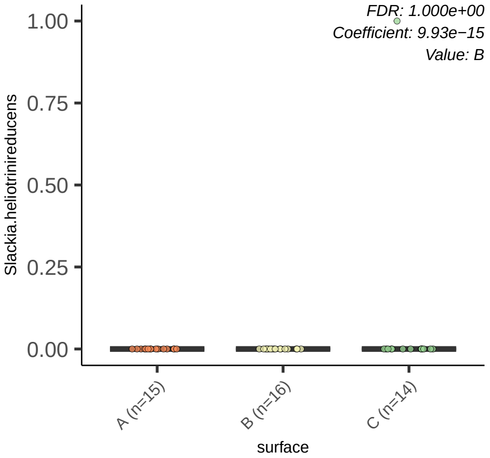

FDR: 1.000e+00
Coefficient: 9.93e−15
Value: B
1.00
0.75
0.50
0.25
0.00
Slackia.heliotrinireducens
A (n=15)
B (n=16)
C (n=14)
surface

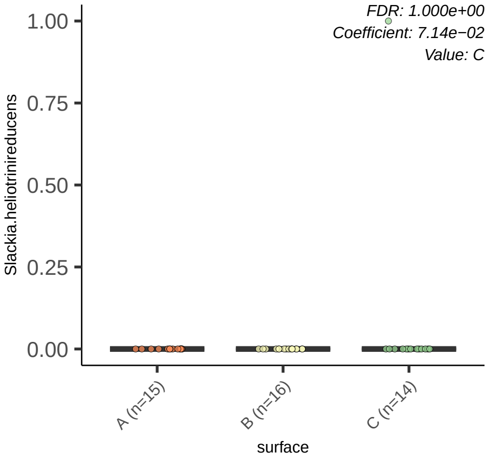

FDR: 1.000e+00
Coefficient: 7.14e−02
Value: C
1.00
0.75
0.50
0.25
0.00
Slackia.heliotrinireducens
A (n=15)
B (n=16)
C (n=14)
surface

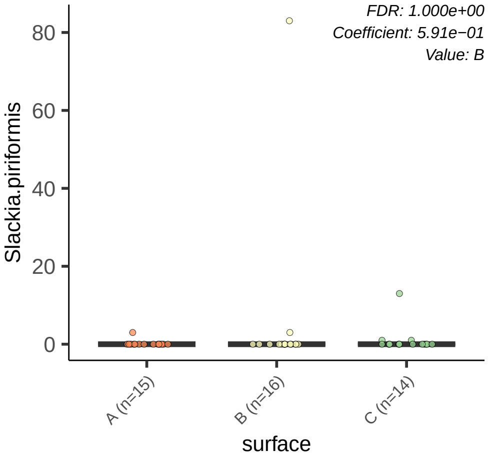
FDR: 1.000e+00
Coefficient: 5.91e−01
Value: B
Slackia.piriformis
0
20
40
60
80
A (n=15)
B (n=16)
C (n=14)
surface

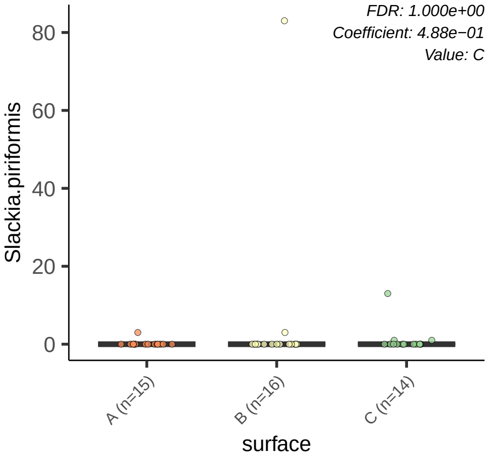

FDR: 1.000e+00
Coefficient: 4.88e−01
Value: C
Slackia.piriformis
0
20
40
60
80
A (n=15)
B (n=16)
C (n=14)
surface

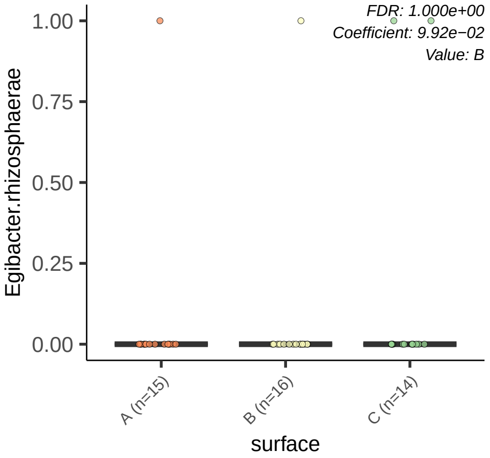

FDR: 1.000e+00
Coefficient: 9.92e−02
Value: B
Egibacter.rhizosphaerae
1.00
0.75
0.50
0.25
0.00
A (n=15)
B (n=16)
C (n=14)
surface

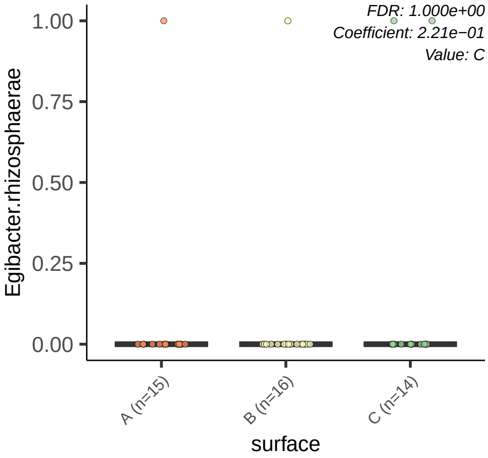
FDR: 1.000e+00
Coefficient: 2.21e−01
Value: C
Egibacter.rhizosphaerae
1.00
0.75
0.50
0.25
0.00
A (n=15)
B (n=16)
C (n=14)
surface

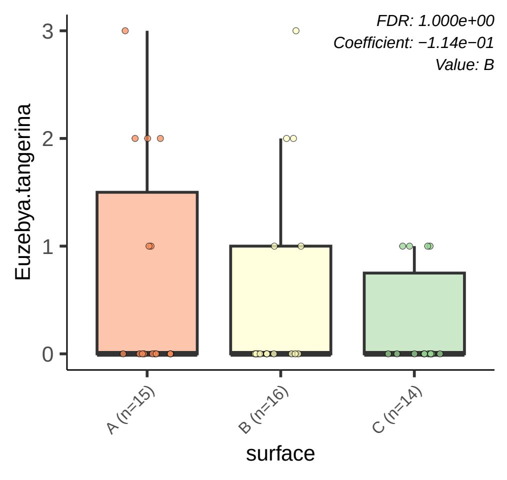
FDR: 1.000e+00
Coefficient: −1.14e−01
Value: B
Euzebya.tangerina
3
2
1
0
A (n=15)
B (n=16)
C (n=14)
surface

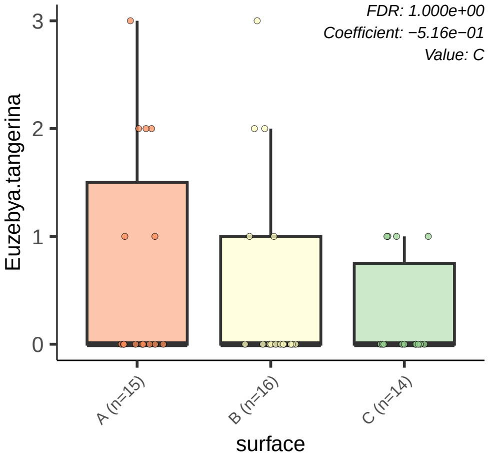

FDR: 1.000e+00
Coefficient: −5.16e−01
Value: C
Euzebya.tangerina
0
1
2
3
A (n=15)
B (n=16)
C (n=14)
surface

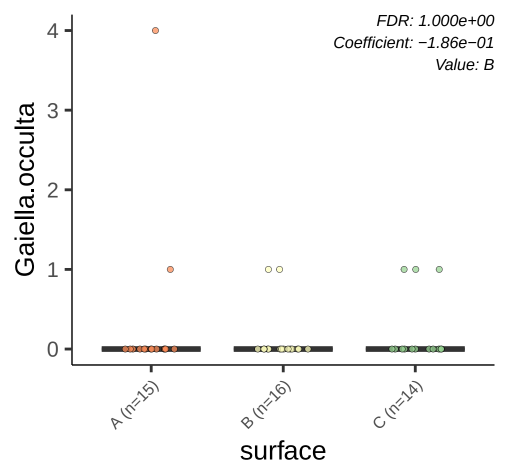

FDR: 1.000e+00
Coefficient: −1.86e−01
Value: B
Gaiella.occulta
0
1
2
3
4
A (n=15)
B (n=16)
C (n=14)
surface

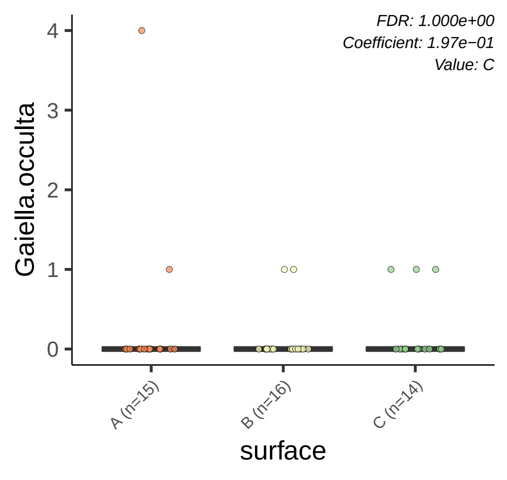
FDR: 1.000e+00
Coefficient: 1.97e−01
Value: C
Gaiella.occulta
4
3
2
1
0
A (n=15)
B (n=16)
C (n=14)
surface

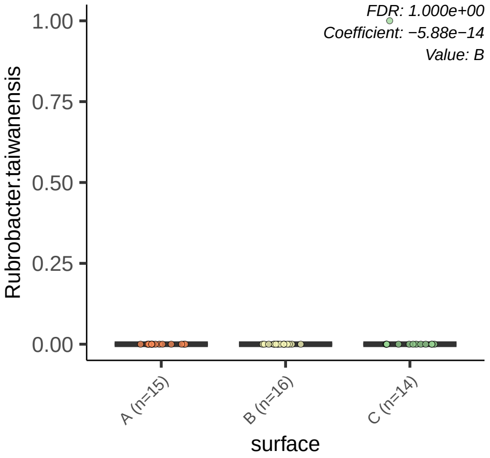
FDR: 1.000e+00
Coefficient: −5.88e−14
Value: B
Rubrobacter.taiwanensis
1.00
0.75
0.50
0.25
0.00
A (n=15)
B (n=16)
C (n=14)
surface

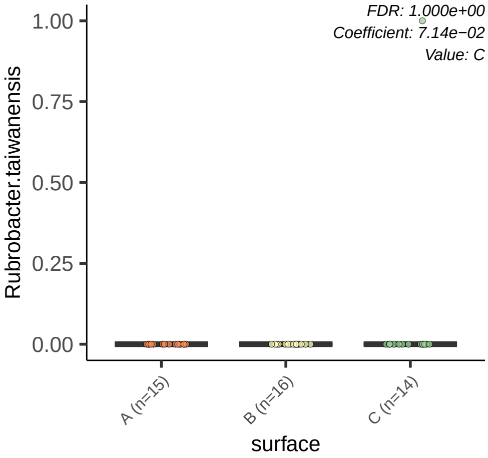
FDR: 1.000e+00
Coefficient: 7.14e−02
Value: C
Rubrobacter.taiwanensis
1.00
0.75
0.50
0.25
0.00
A (n=15)
B (n=16)
C (n=14)
surface

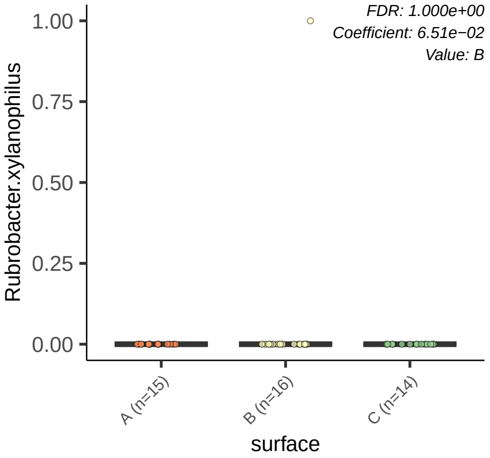
FDR: 1.000e+00
Coefficient: 6.51e−02
Value: B
Rubrobacter.xylanophilus
1.00
0.75
0.50
0.25
0.00
A (n=15)
B (n=16)
C (n=14)
surface

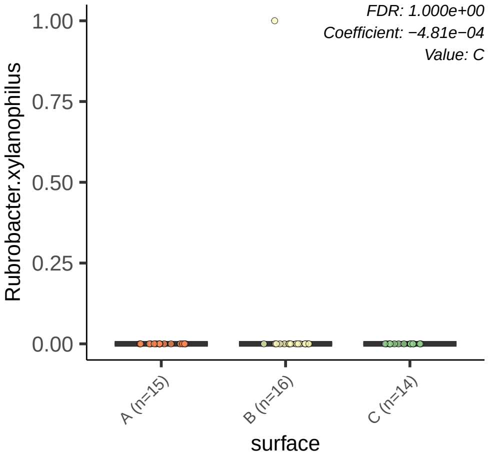

FDR: 1.000e+00
Coefficient: −4.81e−04
Value: C
Rubrobacter.xylanophilus
1.00
0.75
0.50
0.25
0.00
A (n=15)
B (n=16)
C (n=14)
surface

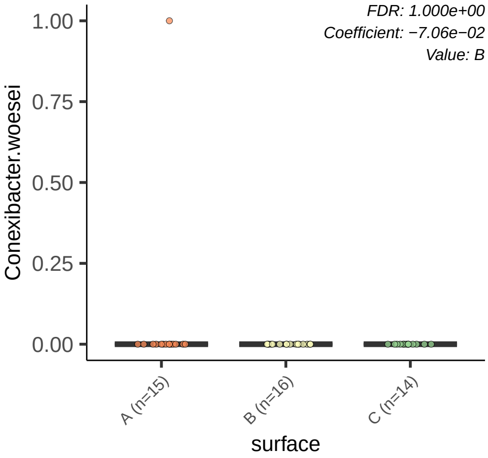
FDR: 1.000e+00
Coefficient: −7.06e−02
Value: B
Conexibacter.woesei
1.00
0.75
0.50
0.25
0.00
A (n=15)
B (n=16)
C (n=14)
surface

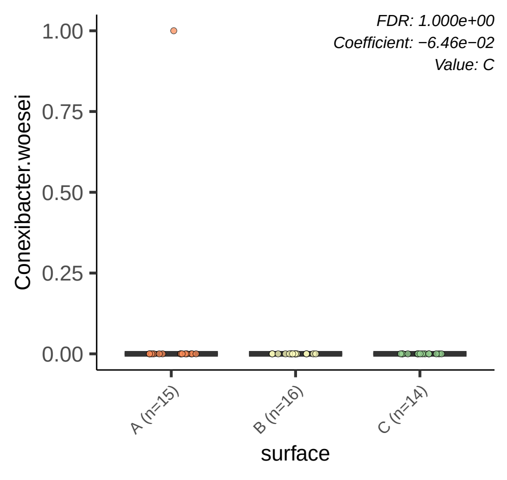

FDR: 1.000e+00
Coefficient: −6.46e−02
Value: C
Conexibacter.woesei
1.00
0.75
0.50
0.25
0.00
A (n=15)
B (n=16)
C (n=14)
surface

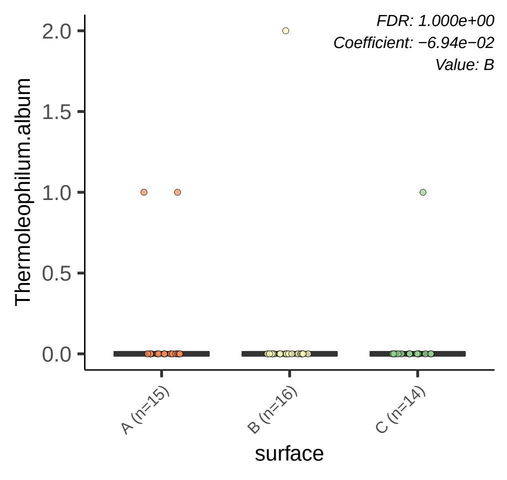
FDR: 1.000e+00
Coefficient: −6.94e−02
Value: B
Thermoleophilum.album
2.0
1.5
1.0
0.5
0.0
A (n=15)
B (n=16)
C (n=14)
surface

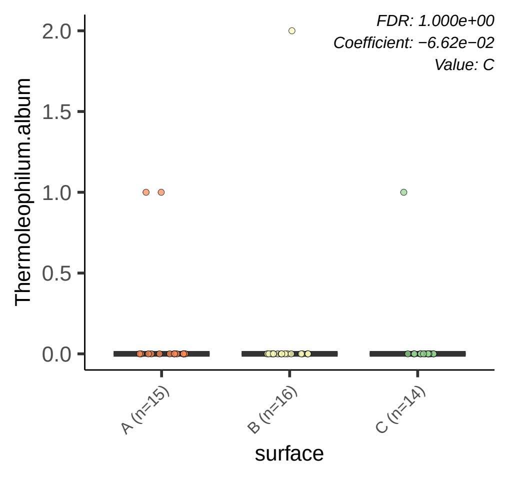
FDR: 1.000e+00
Coefficient: −6.62e−02
Value: C
Thermoleophilum.album
2.0
1.5
1.0
0.5
0.0
A (n=15)
B (n=16)
C (n=14)
surface

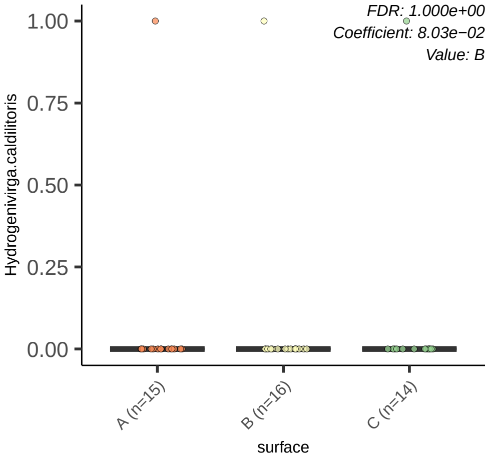

FDR: 1.000e+00
Coefficient: 8.03e−02
Value: B
Hydrogenivirga.caldilitoris
1.00
0.75
0.50
0.25
0.00
A (n=15)
B (n=16)
C (n=14)
surface

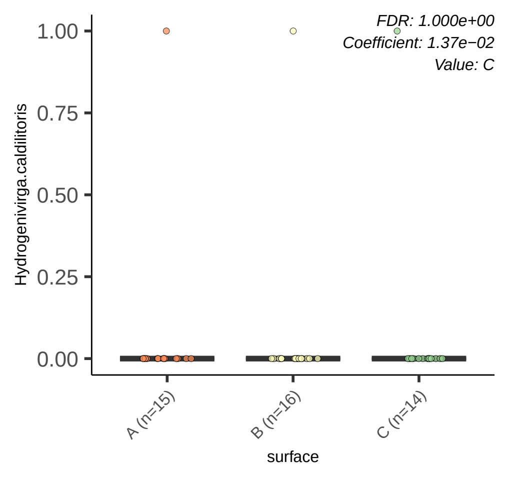
FDR: 1.000e+00
Coefficient: 1.37e−02
Value: C
1.00
0.75
0.50
0.25
0.00
Hydrogenivirga.caldilitoris
A (n=15)
B (n=16)
C (n=14)
surface

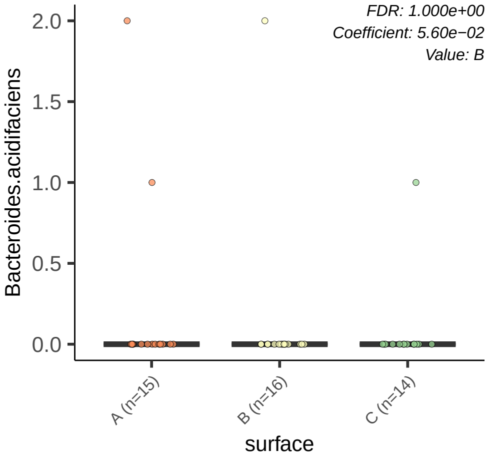
FDR: 1.000e+00
Coefficient: 5.60e−02
Value: B
Bacteroides.acidifaciens
2.0
1.5
1.0
0.5
0.0
A (n=15)
B (n=16)
C (n=14)
surface

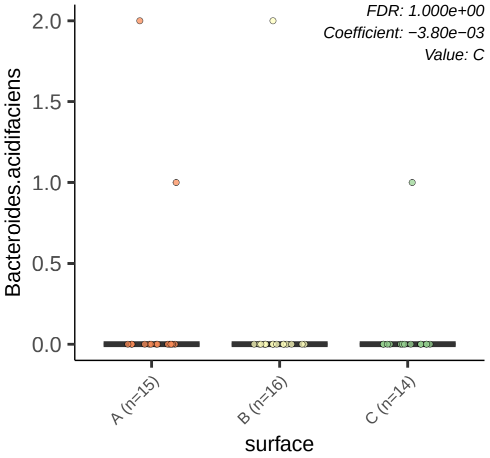
FDR: 1.000e+00
Coefficient: −3.80e−03
Value: C
Bacteroides.acidifaciens
2.0
1.5
1.0
0.5
0.0
A (n=15)
B (n=16)
C (n=14)
surface

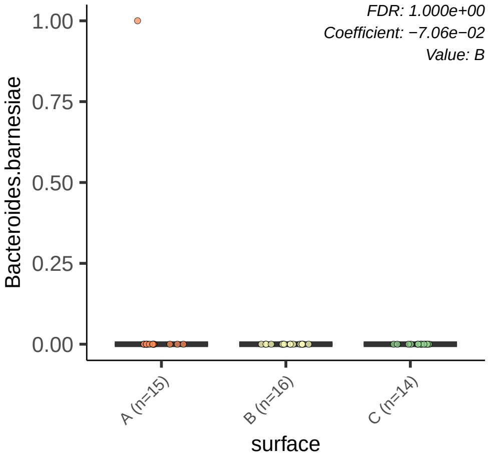
FDR: 1.000e+00
Coefficient: −7.06e−02
Value: B
1.00
0.75
0.50
0.25
0.00
Bacteroides.barnesiae
A (n=15)
B (n=16)
C (n=14)
surface

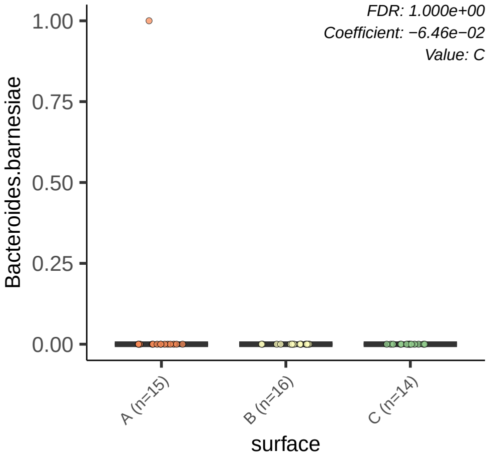

FDR: 1.000e+00
Coefficient: −6.46e−02
Value: C
Bacteroides.barnesiae
1.00
0.75
0.50
0.25
0.00
A (n=15)
B (n=16)
C (n=14)
surface

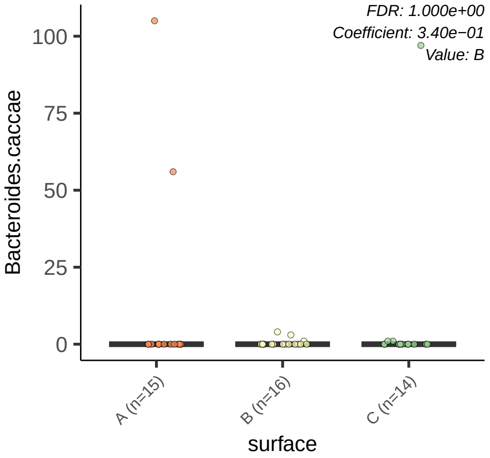

FDR: 1.000e+00
Coefficient: 3.40e−01
Value: B
Bacteroides.caccae
100
75
50
25
0
A (n=15)
B (n=16)
C (n=14)
surface

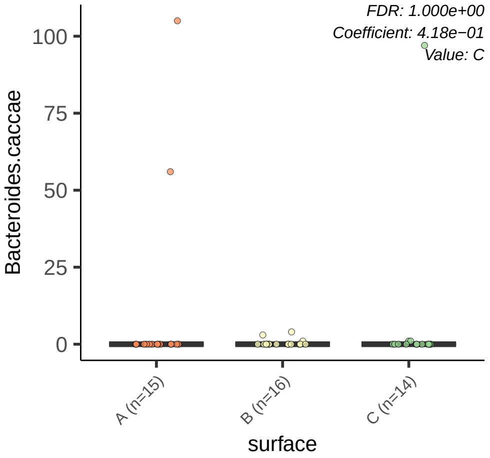
FDR: 1.000e+00
Coefficient: 4.18e−01
Value: C
Bacteroides.caccae
100
75
50
25
0
A (n=15)
B (n=16)
C (n=14)
surface

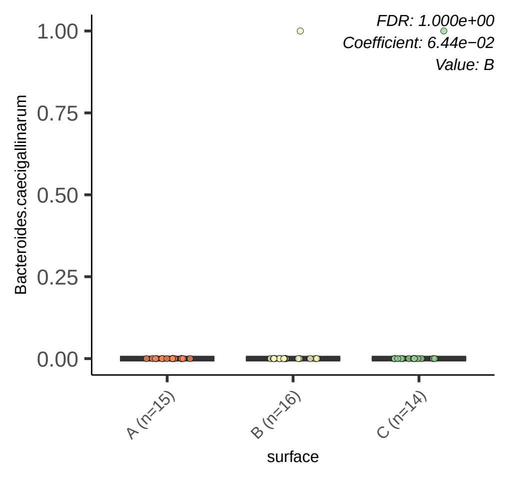
FDR: 1.000e+00
Coefficient: 6.44e−02
Value: B
Bacteroides.caecigallinarum
1.00
0.75
0.50
0.25
0.00
A (n=15)
B (n=16)
C (n=14)
surface

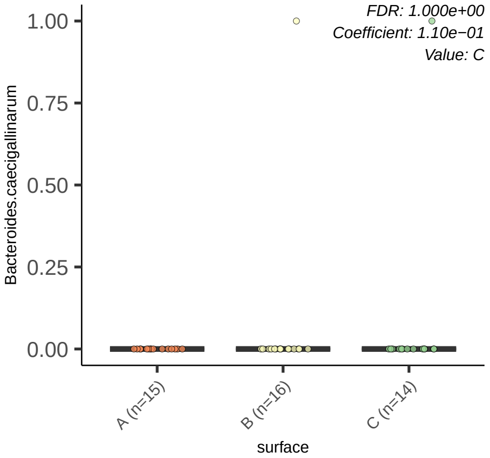
FDR: 1.000e+00
Coefficient: 1.10e−01
Value: C
Bacteroides.caecigallinarum
1.00
0.75
0.50
0.25
0.00
A (n=15)
B (n=16)
C (n=14)
surface

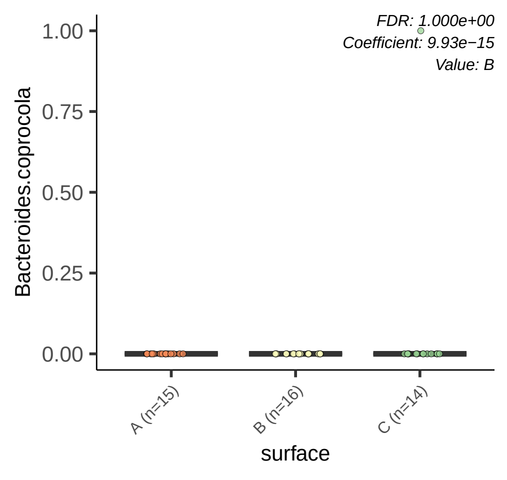
FDR: 1.000e+00
Coefficient: 9.93e−15
Value: B
Bacteroides.coprocola
1.00
0.75
0.50
0.25
0.00
A (n=15)
B (n=16)
C (n=14)
surface

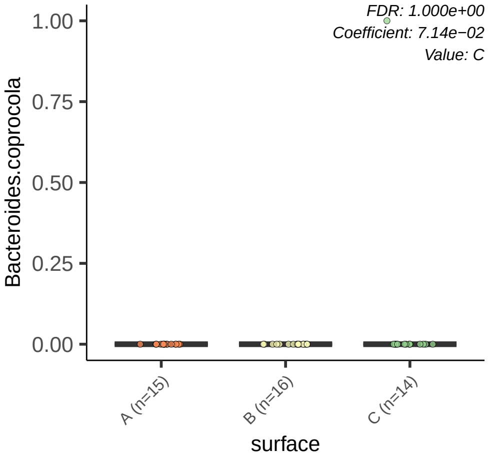
FDR: 1.000e+00
Coefficient: 7.14e−02
Value: C
Bacteroides.coprocola
1.00
0.75
0.50
0.25
0.00
A (n=15)
B (n=16)
C (n=14)
surface

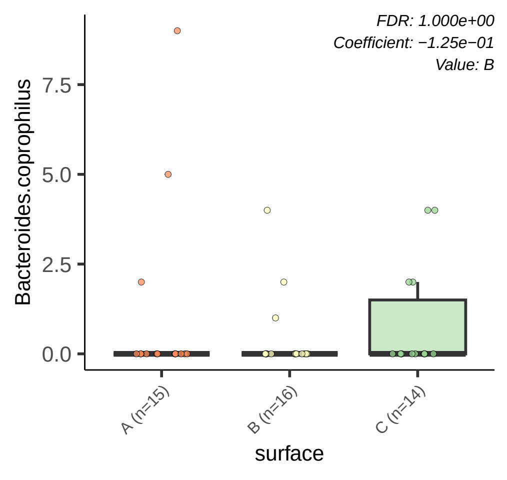

FDR: 1.000e+00
Coefficient: −1.25e−01
Value: B
Bacteroides.coprophilus
7.5
5.0
2.5
0.0
A (n=15)
B (n=16)
C (n=14)
surface

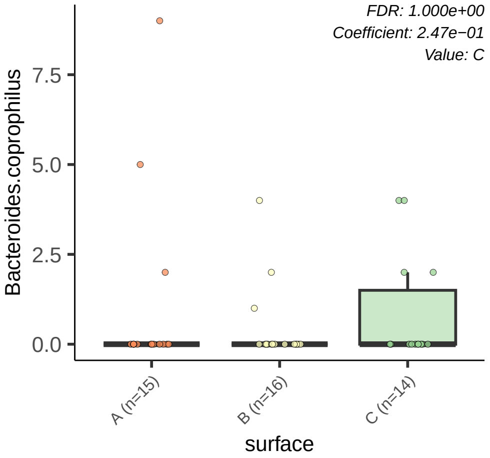

FDR: 1.000e+00
Coefficient: 2.47e−01
Value: C
Bacteroides.coprophilus
7.5
5.0
2.5
0.0
A (n=15)
B (n=16)
C (n=14)
surface

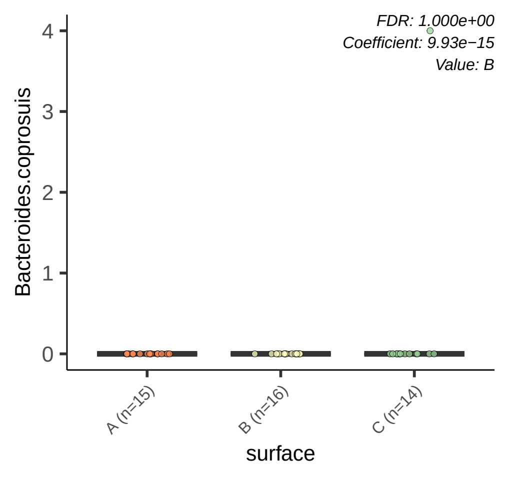
FDR: 1.000e+00
Coefficient: 9.93e−15
Value: B
Bacteroides.coprosuis
0
1
2
3
4
A (n=15)
B (n=16)
C (n=14)
surface

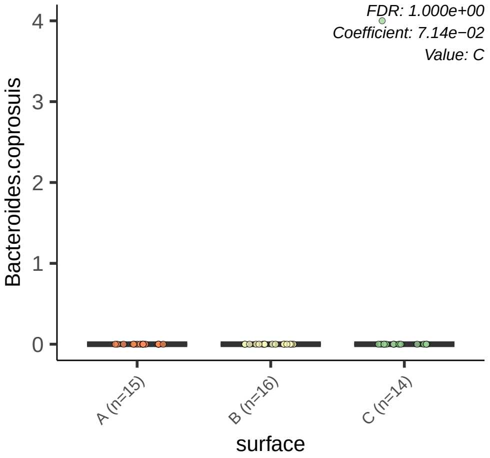

FDR: 1.000e+00
Coefficient: 7.14e−02
Value: C
Bacteroides.coprosuis
4
3
2
1
0
A (n=15)
B (n=16)
C (n=14)
surface

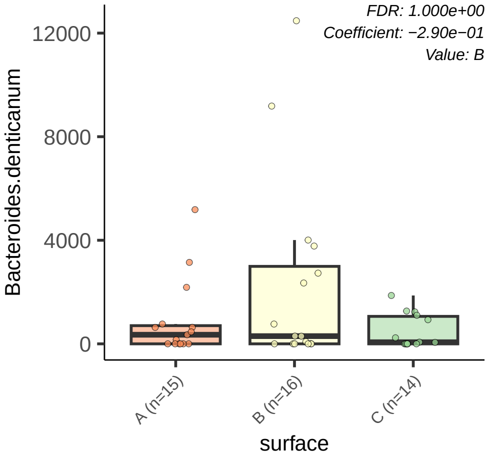

FDR: 1.000e+00
Coefficient: −2.90e−01
Value: B
Bacteroides.denticanum
12000
8000
4000
0
A (n=15)
B (n=16)
C (n=14)
surface

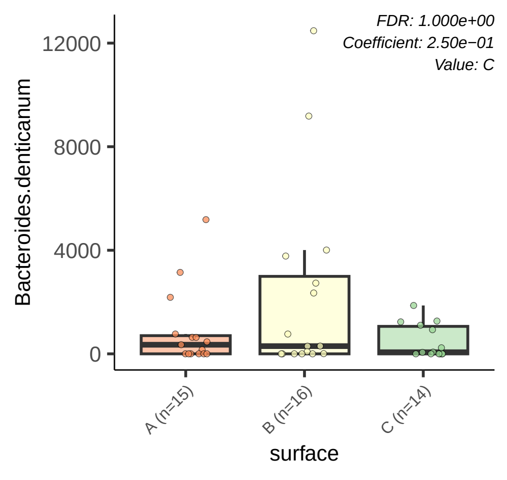
FDR: 1.000e+00
Coefficient: 2.50e−01
Value: C
Bacteroides.denticanum
12000
8000
4000
0
A (n=15)
B (n=16)
C (n=14)
surface

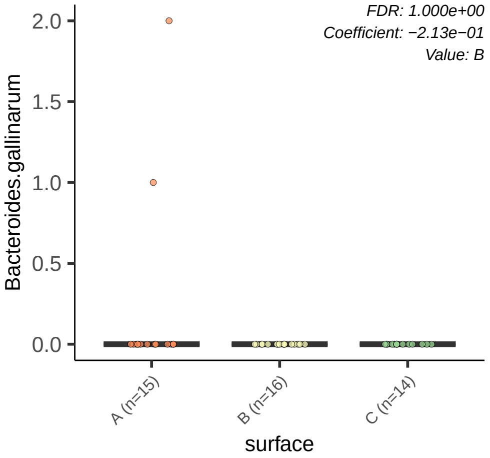
FDR: 1.000e+00
Coefficient: −2.13e−01
Value: B
Bacteroides.gallinarum
2.0
1.5
1.0
0.5
0.0
A (n=15)
B (n=16)
C (n=14)
surface

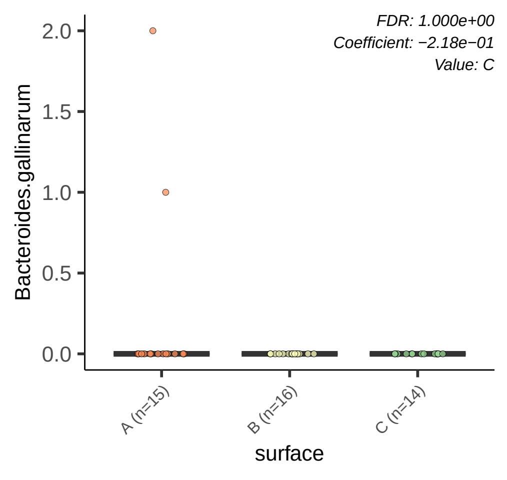
FDR: 1.000e+00
Coefficient: −2.18e−01
Value: C
Bacteroides.gallinarum
2.0
1.5
1.0
0.5
0.0
A (n=15)
B (n=16)
C (n=14)
surface

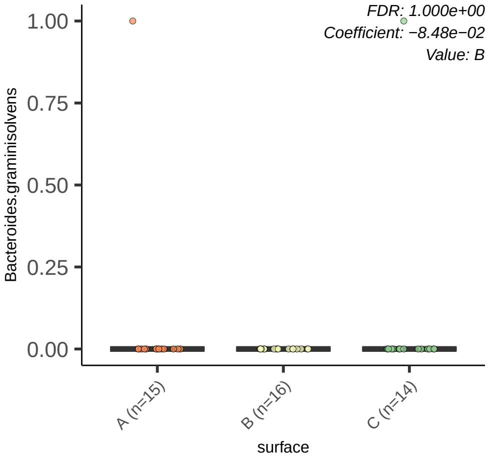
FDR: 1.000e+00
Coefficient: −8.48e−02
Value: B
1.00
0.75
0.50
0.25
0.00
Bacteroides.graminisolvens
A (n=15)
B (n=16)
C (n=14)
surface

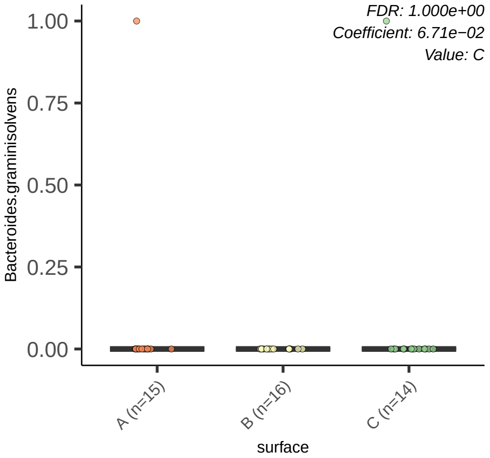

FDR: 1.000e+00
Coefficient: 6.71e−02
Value: C
1.00
0.75
0.50
0.25
0.00
Bacteroides.graminisolvens
A (n=15)
B (n=16)
C (n=14)
surface

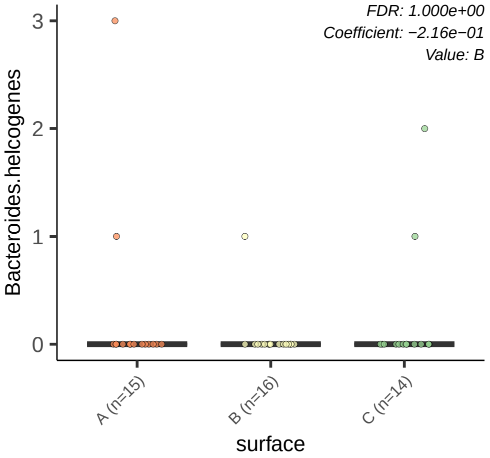
FDR: 1.000e+00
Coefficient: −2.16e−01
Value: B
Bacteroides.helcogenes
0
1
2
3
A (n=15)
B (n=16)
C (n=14)
surface

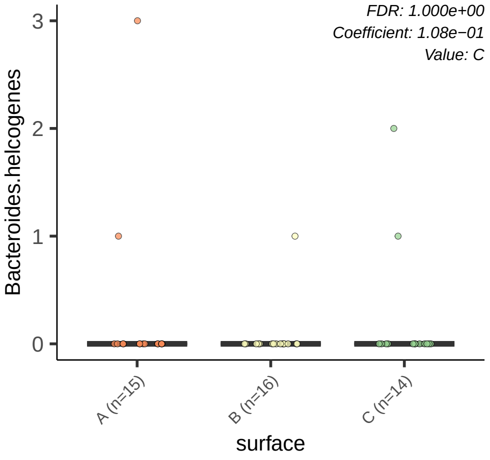
FDR: 1.000e+00
Coefficient: 1.08e−01
Value: C
Bacteroides.helcogenes
0
1
2
3
A (n=15)
B (n=16)
C (n=14)
surface

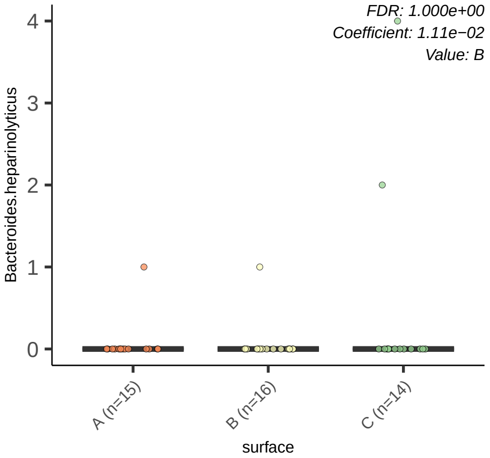

FDR: 1.000e+00
Coefficient: 1.11e−02
Value: B
Bacteroides.heparinolyticus
0
1
2
3
4
A (n=15)
B (n=16)
C (n=14)
surface

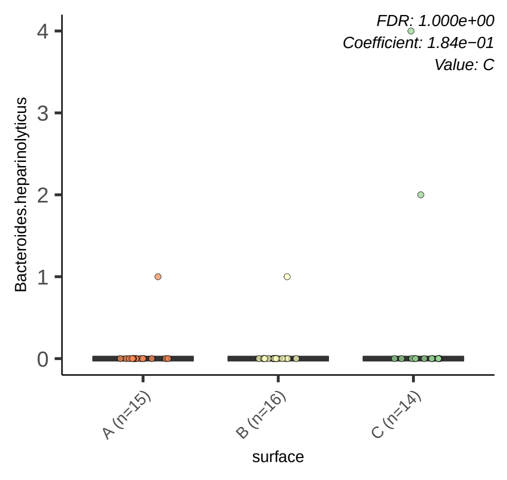
FDR: 1.000e+00
Coefficient: 1.84e−01
Value: C
Bacteroides.heparinolyticus
0
1
2
3
4
A (n=15)
B (n=16)
C (n=14)
surface

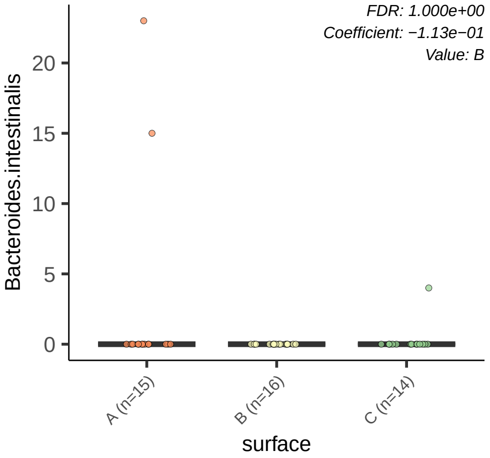

FDR: 1.000e+00
Coefficient: −1.13e−01
Value: B
Bacteroides.intestinalis
0
5
10
15
20
A (n=15)
B (n=16)
C (n=14)
surface

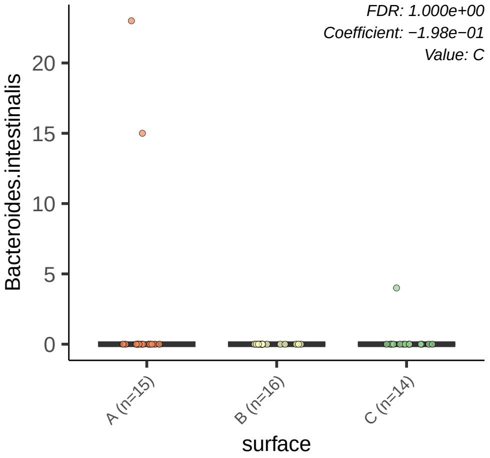

FDR: 1.000e+00
Coefficient: −1.98e−01
Value: C
Bacteroides.intestinalis
0
5
10
15
20
A (n=15)
B (n=16)
C (n=14)
surface

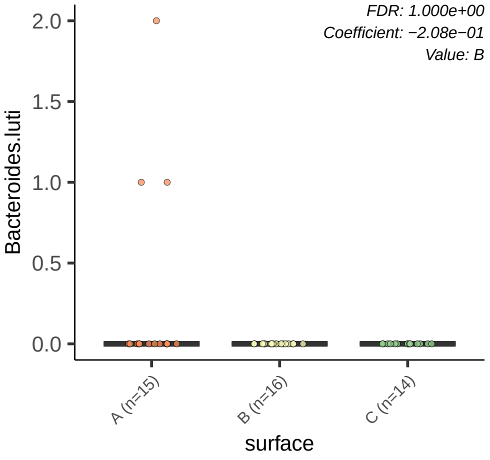

FDR: 1.000e+00
Coefficient: −2.08e−01
Value: B
Bacteroides.luti
2.0
1.5
1.0
0.5
0.0
A (n=15)
B (n=16)
C (n=14)
surface

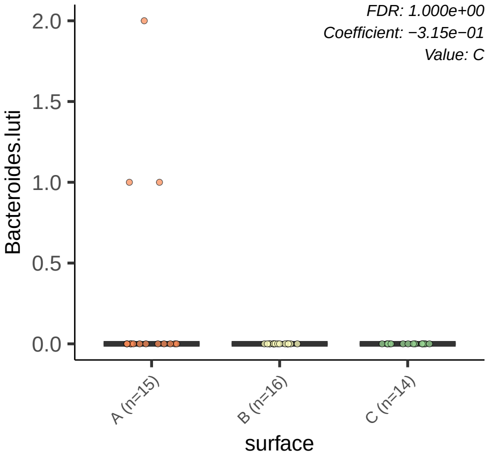
FDR: 1.000e+00
Coefficient: −3.15e−01
Value: C
Bacteroides.luti
2.0
1.5
1.0
0.5
0.0
A (n=15)
B (n=16)
C (n=14)
surface

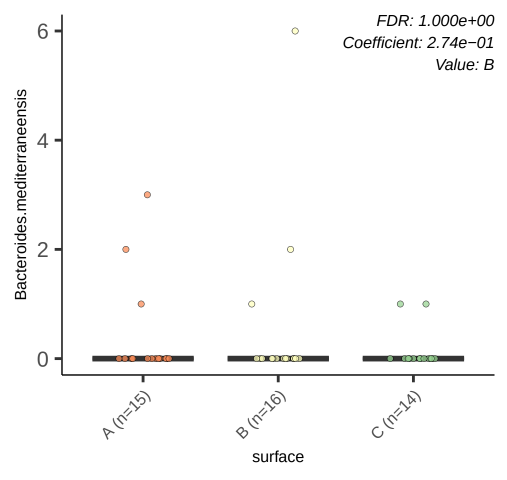
FDR: 1.000e+00
Coefficient: 2.74e−01
Value: B
Bacteroides.mediterraneensis
0
2
4
6
A (n=15)
B (n=16)
C (n=14)
surface

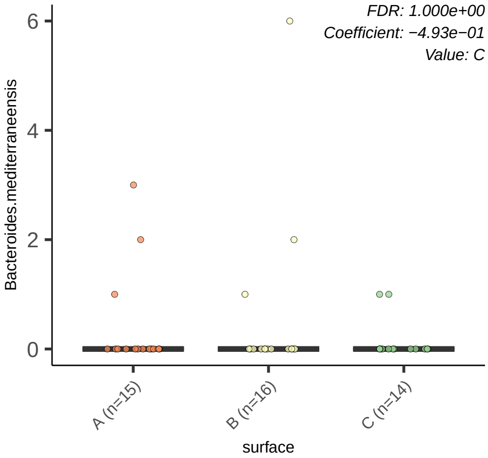

FDR: 1.000e+00
Coefficient: −4.93e−01
Value: C
Bacteroides.mediterraneensis
0
2
4
6
A (n=15)
B (n=16)
C (n=14)
surface

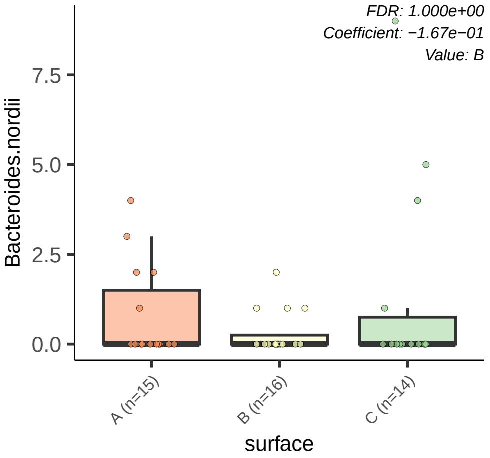

FDR: 1.000e+00
Coefficient: −1.67e−01
Value: B
Bacteroides.nordii
7.5
5.0
2.5
0.0
A (n=15)
B (n=16)
C (n=14)
surface

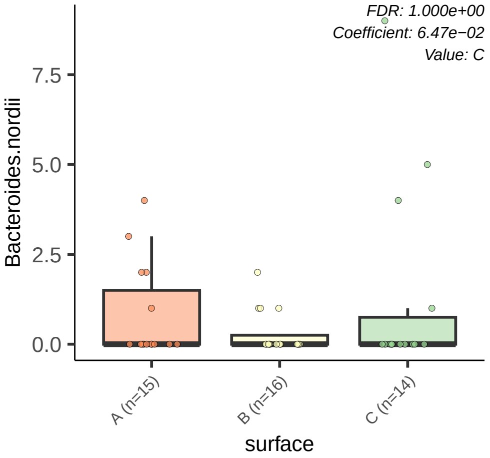
FDR: 1.000e+00
Coefficient: 6.47e−02
Value: C
Bacteroides.nordii
7.5
5.0
2.5
0.0
A (n=15)
B (n=16)
C (n=14)
surface

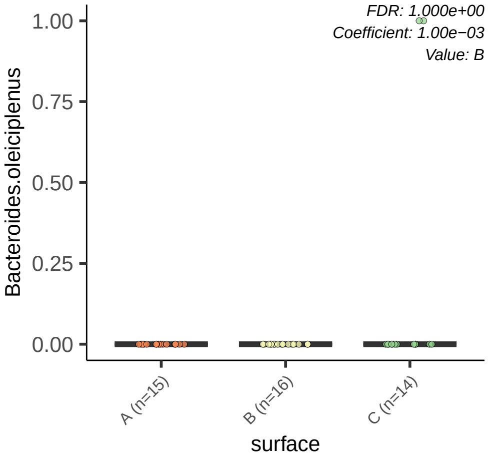

FDR: 1.000e+00
Coefficient: 1.00e−03
Value: B
1.00
0.75
0.50
0.25
0.00
Bacteroides.oleiciplenus
A (n=15)
B (n=16)
C (n=14)
surface

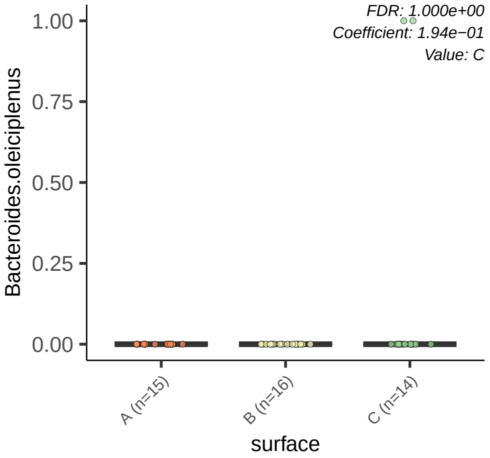

FDR: 1.000e+00
Coefficient: 1.94e−01
Value: C
Bacteroides.oleiciplenus
1.00
0.75
0.50
0.25
0.00
A (n=15)
B (n=16)
C (n=14)
surface

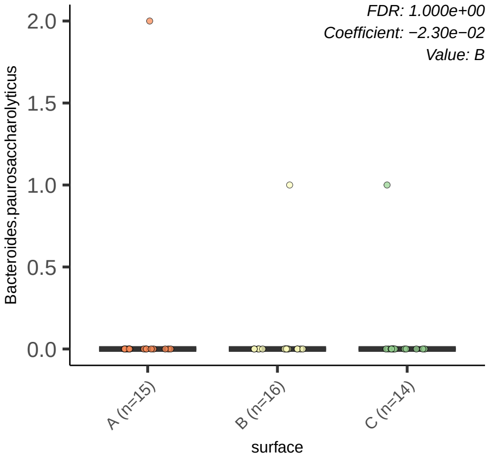
FDR: 1.000e+00
Coefficient: −2.30e−02
Value: B
Bacteroides.paurosaccharolyticus
2.0
1.5
1.0
0.5
0.0
A (n=15)
B (n=16)
C (n=14)
surface

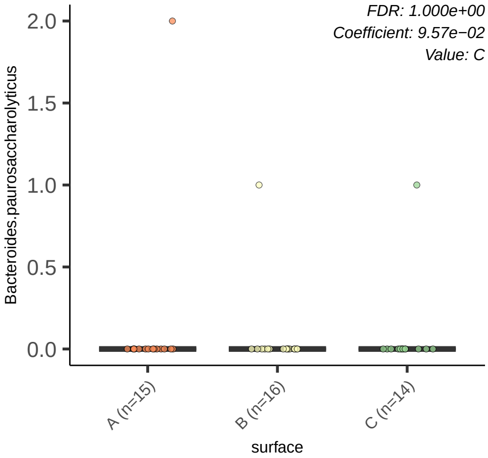

FDR: 1.000e+00
Coefficient: 9.57e−02
Value: C
Bacteroides.paurosaccharolyticus
2.0
1.5
1.0
0.5
0.0
A (n=15)
B (n=16)
C (n=14)
surface

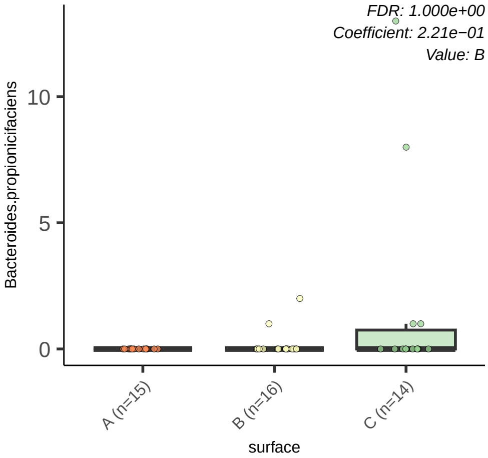
FDR: 1.000e+00
Coefficient: 2.21e−01
Value: B
Bacteroides.propionicifaciens
10
5
0
A (n=15)
B (n=16)
C (n=14)
surface

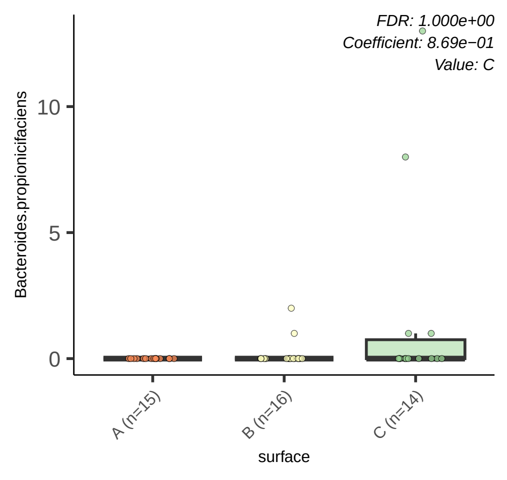

FDR: 1.000e+00
Coefficient: 8.69e−01
Value: C
Bacteroides.propionicifaciens
10
5
0
A (n=15)
B (n=16)
C (n=14)
surface

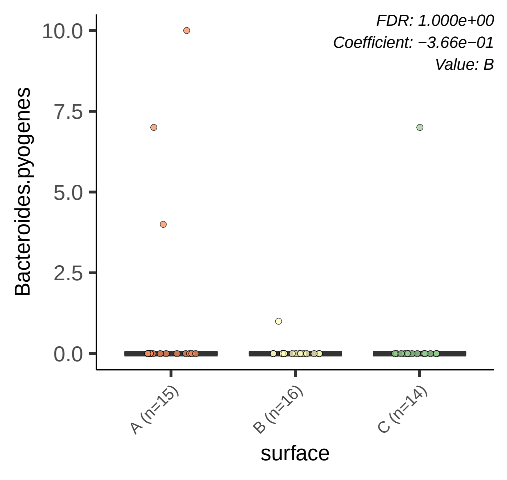
FDR: 1.000e+00
Coefficient: −3.66e−01
Value: B
Bacteroides.pyogenes
10.0
7.5
5.0
2.5
0.0
A (n=15)
B (n=16)
C (n=14)
surface

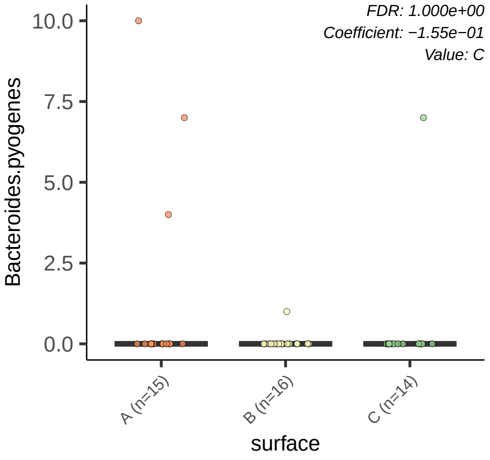
FDR: 1.000e+00
Coefficient: −1.55e−01
Value: C
Bacteroides.pyogenes
10.0
7.5
5.0
2.5
0.0
A (n=15)
B (n=16)
C (n=14)
surface

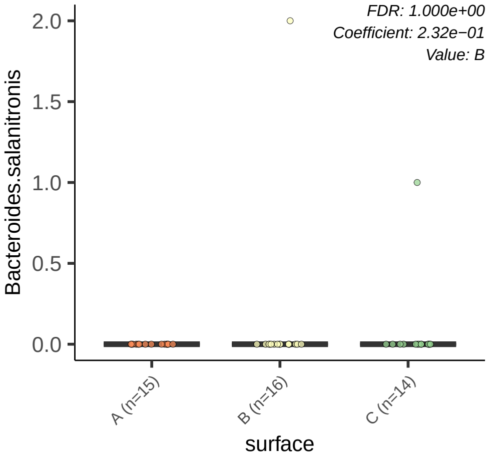

FDR: 1.000e+00
Coefficient: 2.32e−01
Value: B
Bacteroides.salanitronis
2.0
1.5
1.0
0.5
0.0
A (n=15)
B (n=16)
C (n=14)
surface

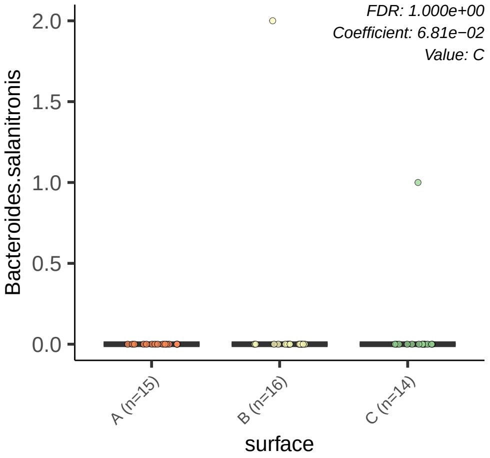

FDR: 1.000e+00
Coefficient: 6.81e−02
Value: C
Bacteroides.salanitronis
2.0
1.5
1.0
0.5
0.0
A (n=15)
B (n=16)
C (n=14)
surface

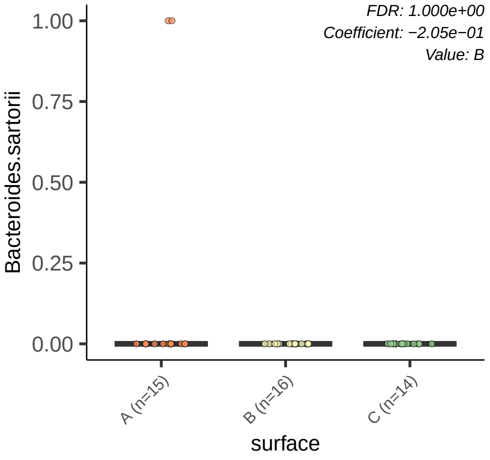
FDR: 1.000e+00
Coefficient: −2.05e−01
Value: B
Bacteroides.sartorii
1.00
0.75
0.50
0.25
0.00
A (n=15)
B (n=16)
C (n=14)
surface

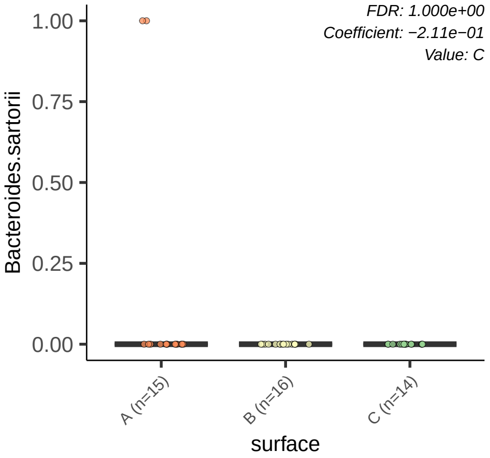
FDR: 1.000e+00
Coefficient: −2.11e−01
Value: C
1.00
0.75
0.50
0.25
0.00
Bacteroides.sartorii
A (n=15)
B (n=16)
C (n=14)
surface

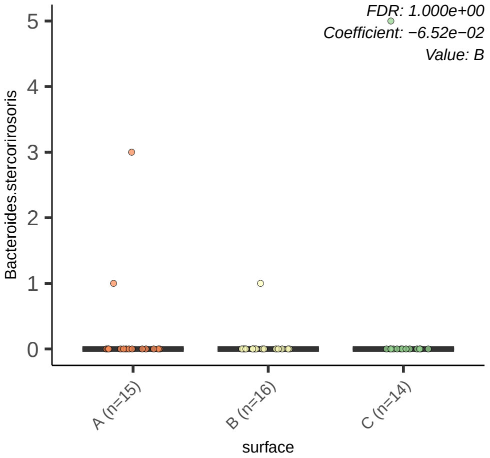

FDR: 1.000e+00
Coefficient: −6.52e−02
Value: B
Bacteroides.stercorirosoris
0
1
2
3
4
5
A (n=15)
B (n=16)
C (n=14)
surface

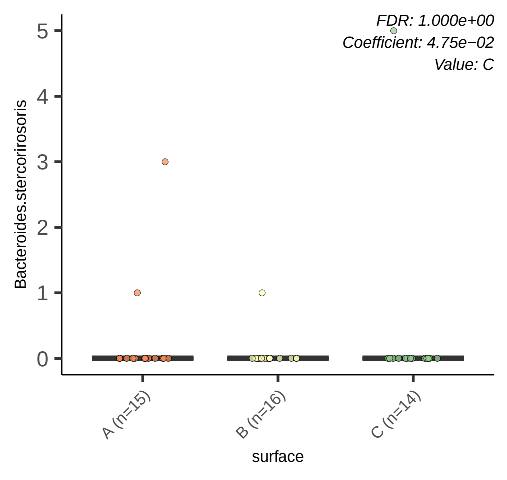
FDR: 1.000e+00
Coefficient: 4.75e−02
Value: C
Bacteroides.stercorirosoris
0
1
2
3
4
5
A (n=15)
B (n=16)
C (n=14)
surface

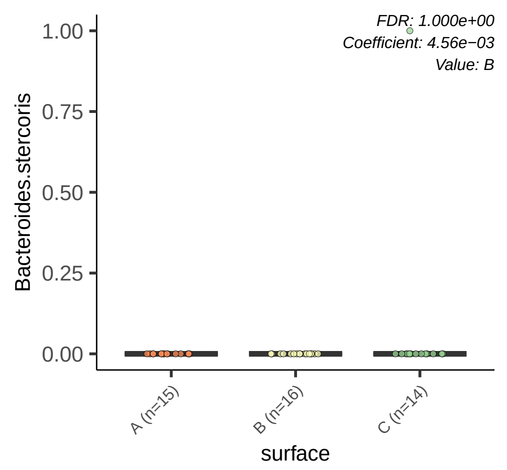

FDR: 1.000e+00
Coefficient: 4.56e−03
Value: B
Bacteroides.stercoris
1.00
0.75
0.50
0.25
0.00
A (n=15)
B (n=16)
C (n=14)
surface

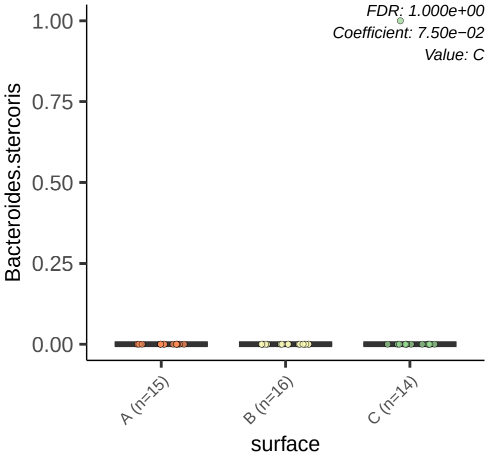
FDR: 1.000e+00
Coefficient: 7.50e−02
Value: C
Bacteroides.stercoris
1.00
0.75
0.50
0.25
0.00
A (n=15)
B (n=16)
C (n=14)
surface

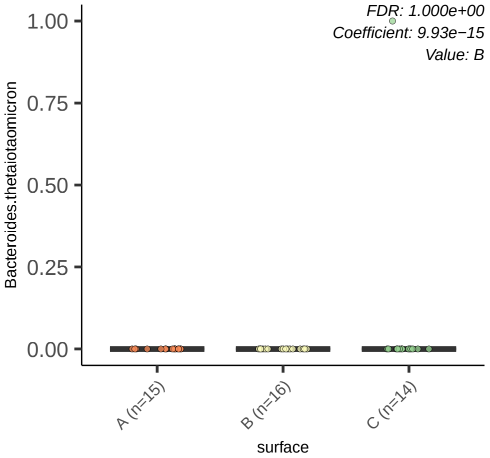
FDR: 1.000e+00
Coefficient: 9.93e−15
Value: B
Bacteroides.thetaiotaomicron
1.00
0.75
0.50
0.25
0.00
A (n=15)
B (n=16)
C (n=14)
surface

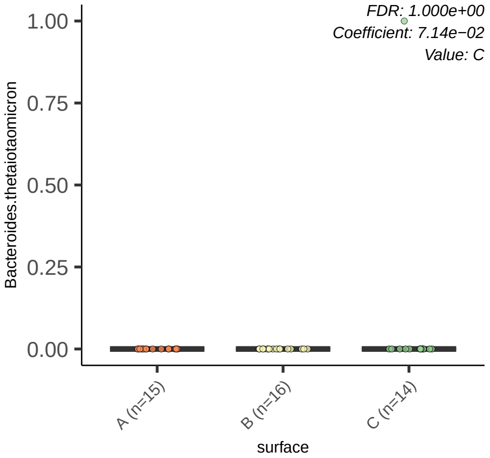
FDR: 1.000e+00
Coefficient: 7.14e−02
Value: C
1.00
0.75
0.50
0.25
0.00
Bacteroides.thetaiotaomicron
A (n=15)
B (n=16)
C (n=14)
surface

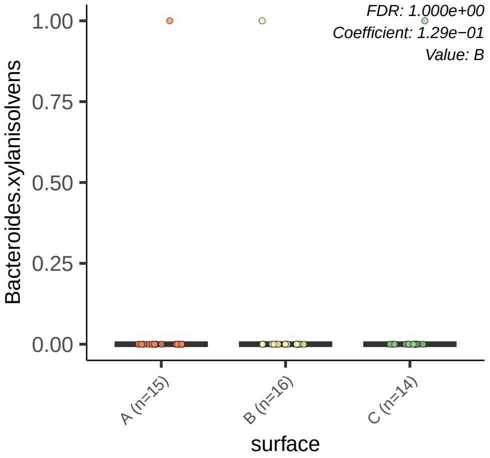
FDR: 1.000e+00
Coefficient: 1.29e−01
Value: B
Bacteroides.xylanisolvens
1.00
0.75
0.50
0.25
0.00
A (n=15)
B (n=16)
C (n=14)
surface

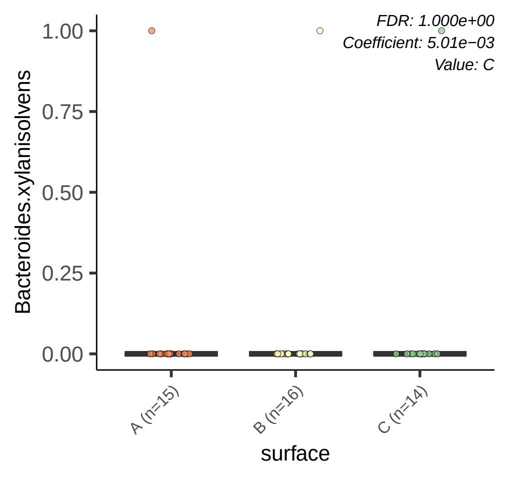
FDR: 1.000e+00
Coefficient: 5.01e−03
Value: C
Bacteroides.xylanisolvens
1.00
0.75
0.50
0.25
0.00
A (n=15)
B (n=16)
C (n=14)
surface

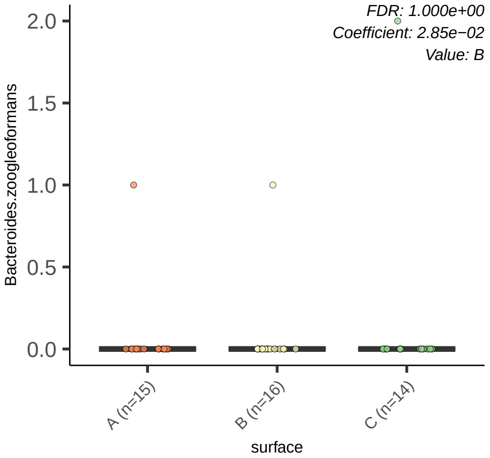
FDR: 1.000e+00
Coefficient: 2.85e−02
Value: B
2.0
1.5
1.0
0.5
0.0
Bacteroides.zoogleoformans
A (n=15)
B (n=16)
C (n=14)
surface

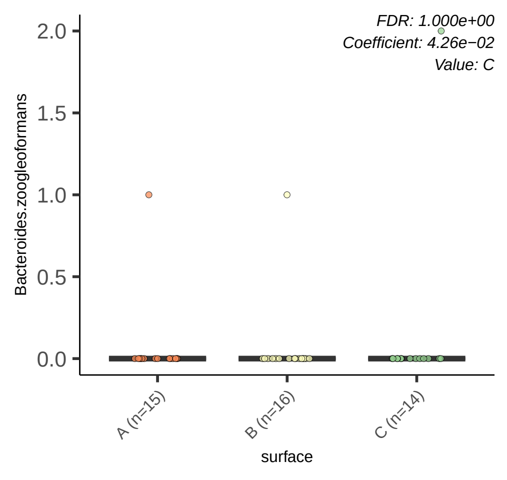

FDR: 1.000e+00
Coefficient: 4.26e−02
Value: C
Bacteroides.zoogleoformans
2.0
1.5
1.0
0.5
0.0
A (n=15)
B (n=16)
C (n=14)
surface

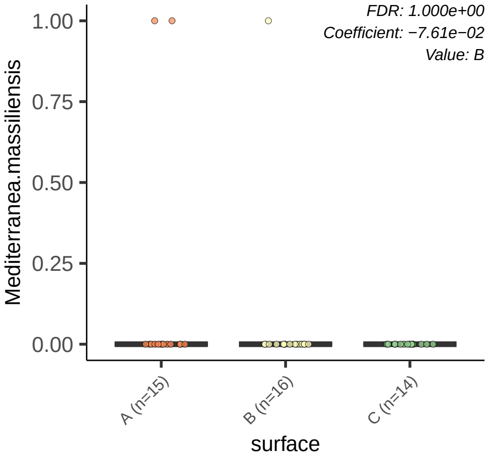
FDR: 1.000e+00
Coefficient: −7.61e−02
Value: B
Mediterranea.massiliensis
1.00
0.75
0.50
0.25
0.00
A (n=15)
B (n=16)
C (n=14)
surface

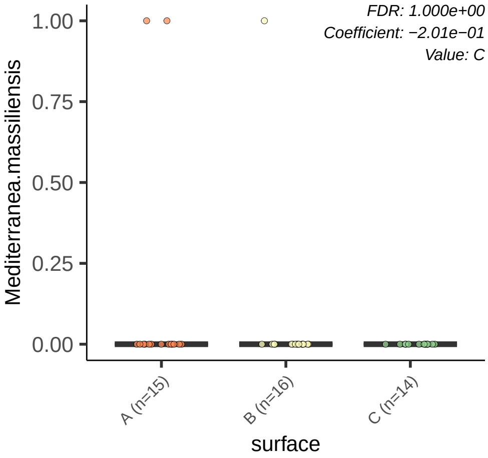
FDR: 1.000e+00
Coefficient: −2.01e−01
Value: C
Mediterranea.massiliensis
1.00
0.75
0.50
0.25
0.00
A (n=15)
B (n=16)
C (n=14)
surface

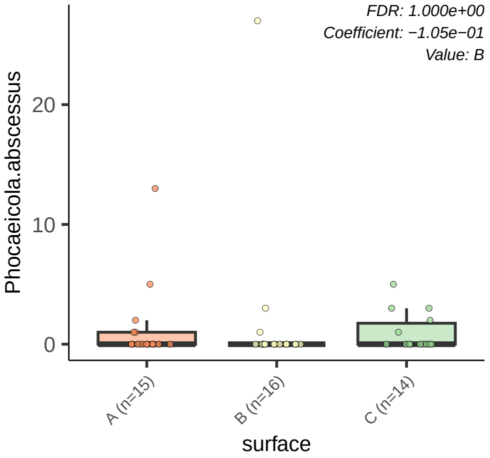
FDR: 1.000e+00
Coefficient: −1.05e−01
Value: B
Phocaeicola.abscessus
0
10
20
A (n=15)
B (n=16)
C (n=14)
surface

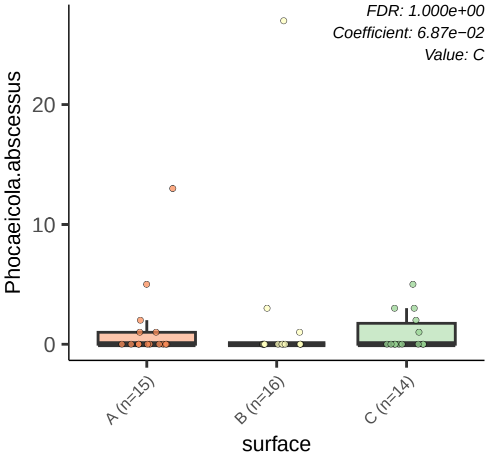
FDR: 1.000e+00
Coefficient: 6.87e−02
Value: C
Phocaeicola.abscessus
0
10
20
A (n=15)
B (n=16)
C (n=14)
surface

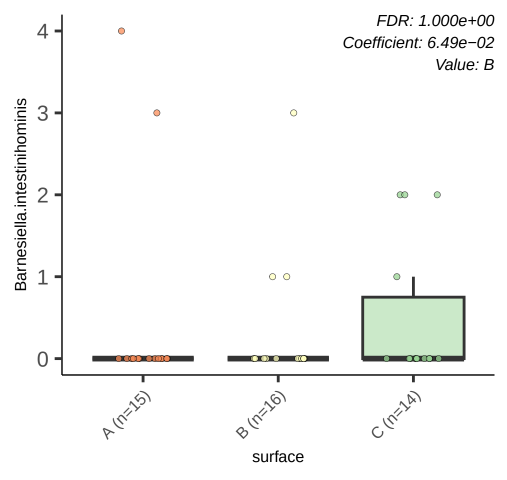
FDR: 1.000e+00
Coefficient: 6.49e−02
Value: B
Barnesiella.intestinihominis
4
3
2
1
0
A (n=15)
B (n=16)
C (n=14)
surface

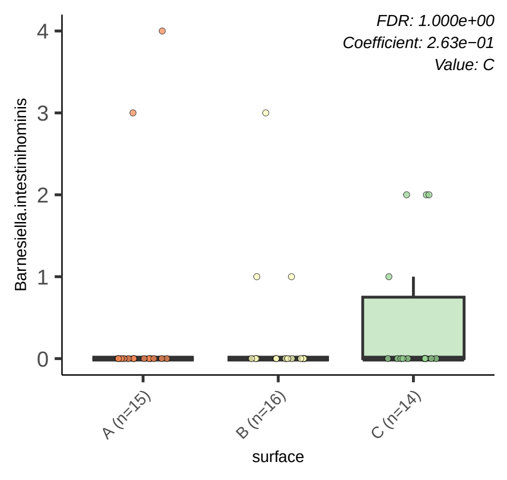
FDR: 1.000e+00
Coefficient: 2.63e−01
Value: C
Barnesiella.intestinihominis
0
1
2
3
4
A (n=15)
B (n=16)
C (n=14)
surface

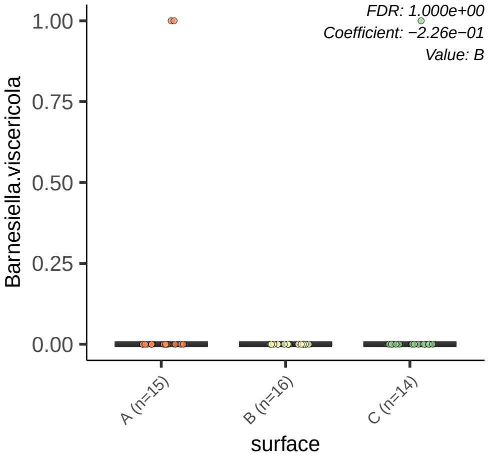
FDR: 1.000e+00
Coefficient: −2.26e−01
Value: B
Barnesiella.viscericola
1.00
0.75
0.50
0.25
0.00
A (n=15)
B (n=16)
C (n=14)
surface

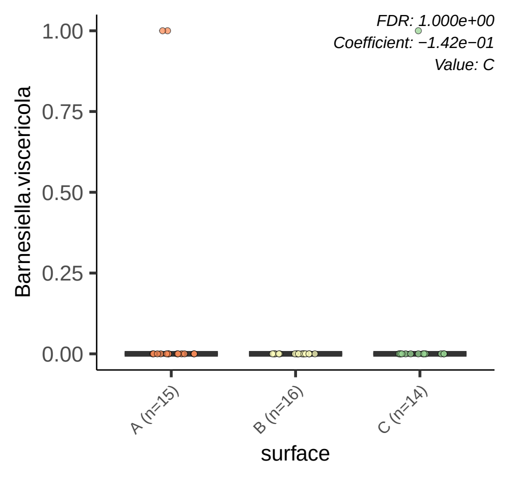
FDR: 1.000e+00
Coefficient: −1.42e−01
Value: C
Barnesiella.viscericola
1.00
0.75
0.50
0.25
0.00
A (n=15)
B (n=16)
C (n=14)
surface

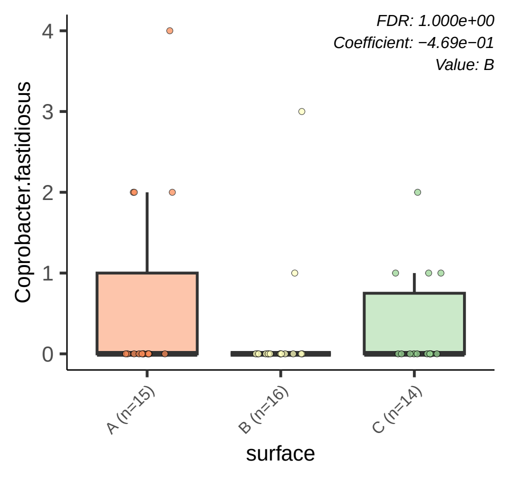
FDR: 1.000e+00
Coefficient: −4.69e−01
Value: B
Coprobacter.fastidiosus
0
1
2
3
4
A (n=15)
B (n=16)
C (n=14)
surface

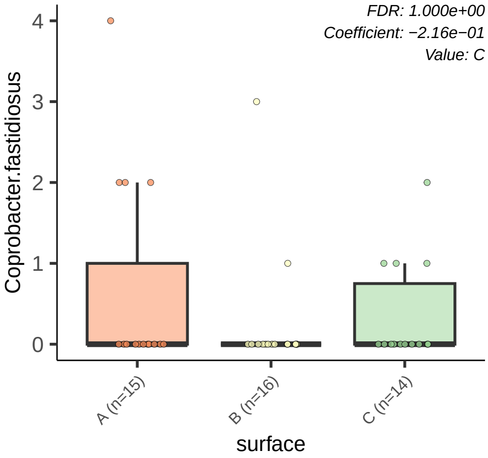

FDR: 1.000e+00
Coefficient: −2.16e−01
Value: C
Coprobacter.fastidiosus
0
1
2
3
4
A (n=15)
B (n=16)
C (n=14)
surface

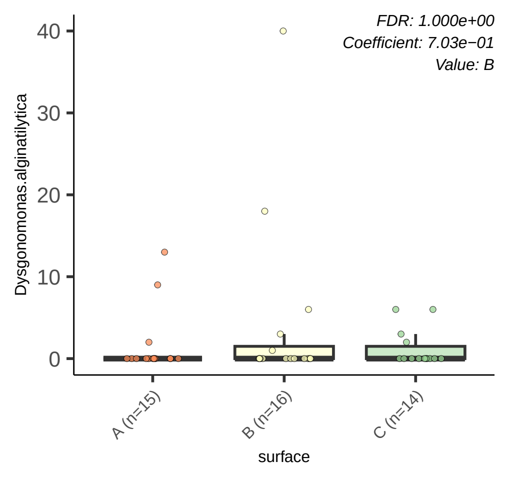
FDR: 1.000e+00
Coefficient: 7.03e−01
Value: B
Dysgonomonas.alginatilytica
0
10
20
30
40
A (n=15)
B (n=16)
C (n=14)
surface

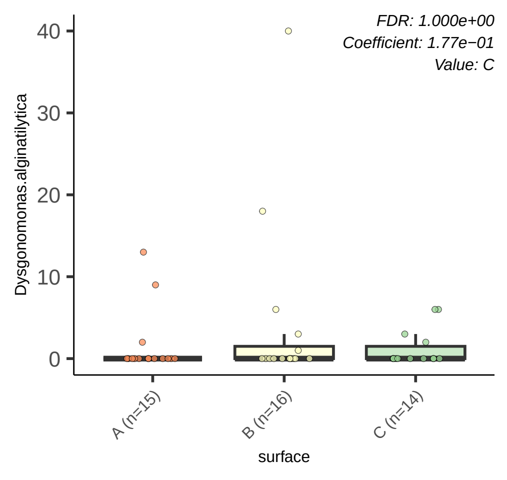
FDR: 1.000e+00
Coefficient: 1.77e−01
Value: C
Dysgonomonas.alginatilytica
40
30
20
10
0
A (n=15)
B (n=16)
C (n=14)
surface

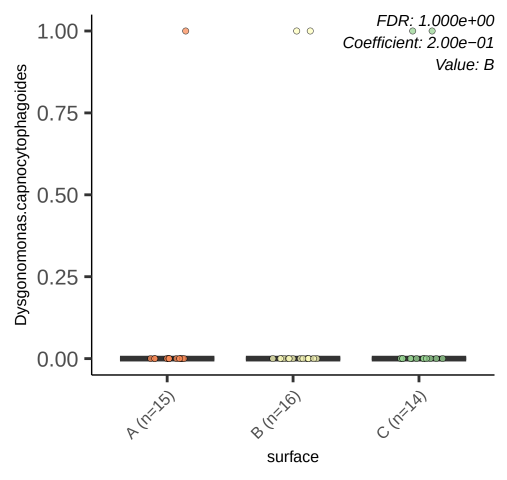

FDR: 1.000e+00
Coefficient: 2.00e−01
Value: B
Dysgonomonas.capnocytophagoides
1.00
0.75
0.50
0.25
0.00
A (n=15)
B (n=16)
C (n=14)
surface

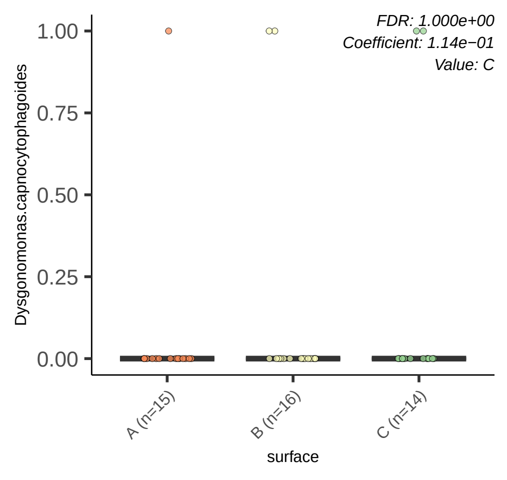
FDR: 1.000e+00
Coefficient: 1.14e−01
Value: C
Dysgonomonas.capnocytophagoides
1.00
0.75
0.50
0.25
0.00
A (n=15)
B (n=16)
C (n=14)
surface

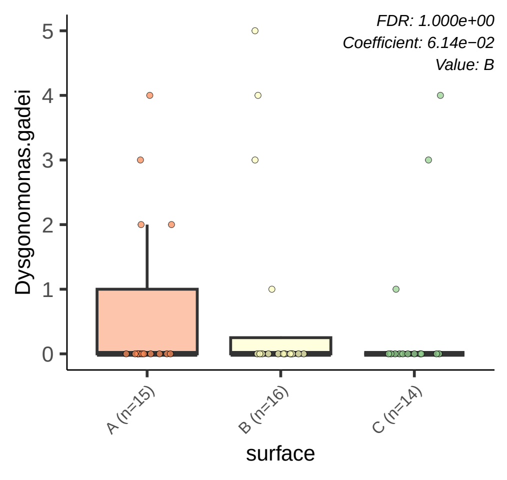
FDR: 1.000e+00
Coefficient: 6.14e−02
Value: B
Dysgonomonas.gadei
0
1
2
3
4
5
A (n=15)
B (n=16)
C (n=14)
surface

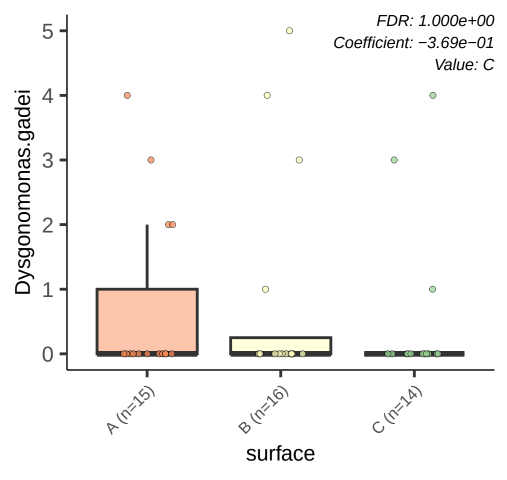
FDR: 1.000e+00
Coefficient: −3.69e−01
Value: C
Dysgonomonas.gadei
0
1
2
3
4
5
A (n=15)
B (n=16)
C (n=14)
surface

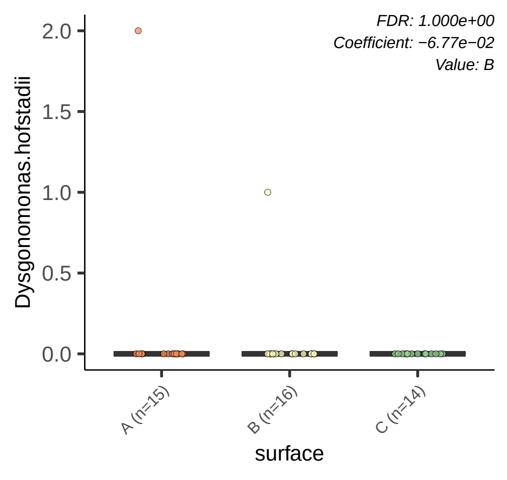
FDR: 1.000e+00
Coefficient: −6.77e−02
Value: B
Dysgonomonas.hofstadii
2.0
1.5
1.0
0.5
0.0
A (n=15)
B (n=16)
C (n=14)
surface

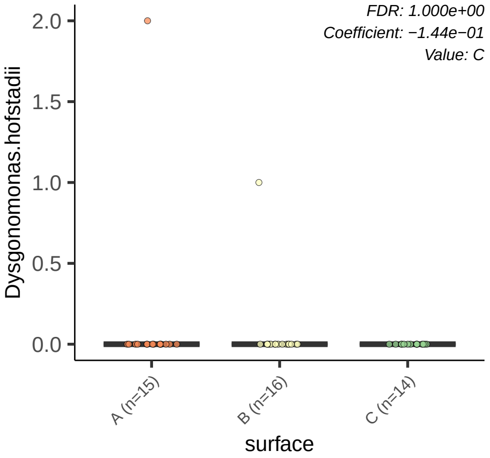
FDR: 1.000e+00
Coefficient: −1.44e−01
Value: C
Dysgonomonas.hofstadii
2.0
1.5
1.0
0.5
0.0
A (n=15)
B (n=16)
C (n=14)
surface

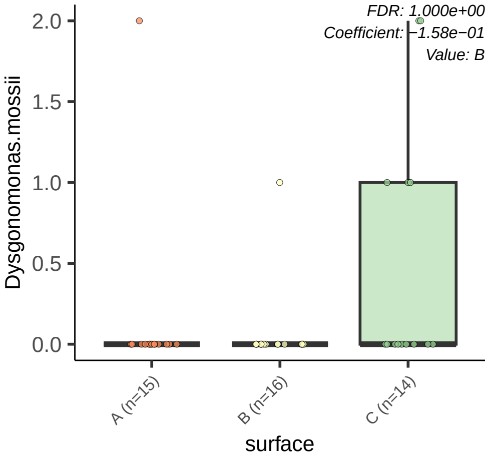
FDR: 1.000e+00
Coefficient: −1.58e−01
Value: B
Dysgonomonas.mossii
2.0
1.5
1.0
0.5
0.0
A (n=15)
B (n=16)
C (n=14)
surface

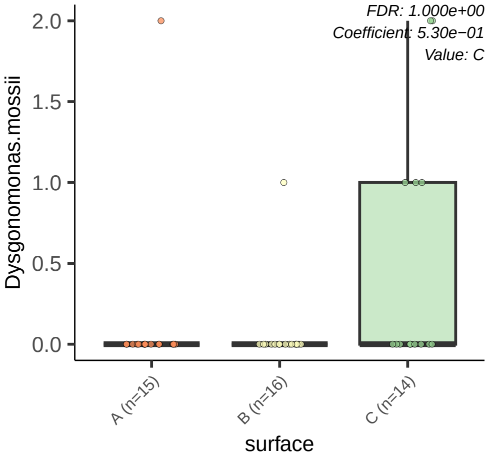

FDR: 1.000e+00
Coefficient: 5.30e−01
Value: C
Dysgonomonas.mossii
2.0
1.5
1.0
0.5
0.0
A (n=15)
B (n=16)
C (n=14)
surface

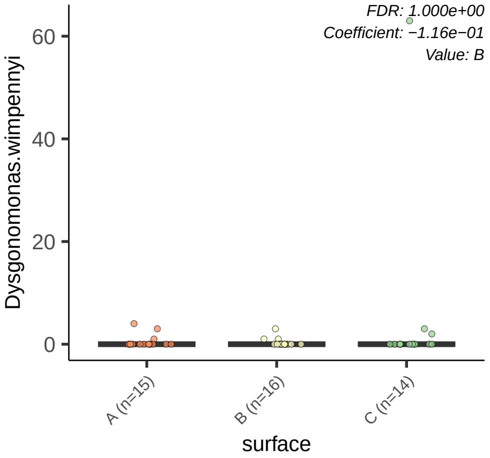

FDR: 1.000e+00
Coefficient: −1.16e−01
Value: B
Dysgonomonas.wimpennyi
60
40
20
0
A (n=15)
B (n=16)
C (n=14)
surface

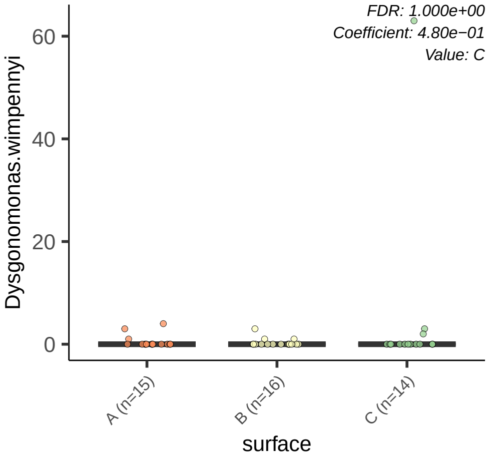
FDR: 1.000e+00
Coefficient: 4.80e−01
Value: C
Dysgonomonas.wimpennyi
0
20
40
60
A (n=15)
B (n=16)
C (n=14)
surface

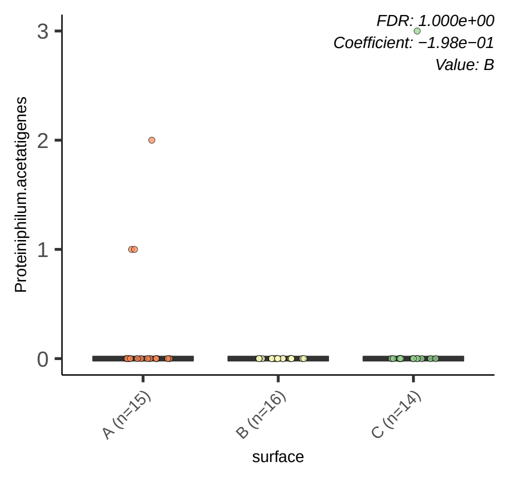
FDR: 1.000e+00
Coefficient: −1.98e−01
Value: B
Proteiniphilum.acetatigenes
0
1
2
3
A (n=15)
B (n=16)
C (n=14)
surface

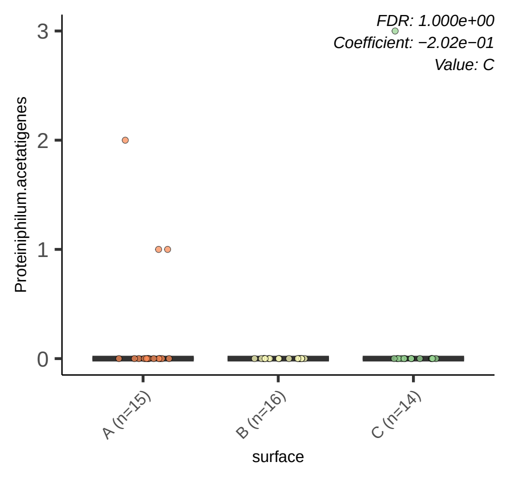
FDR: 1.000e+00
Coefficient: −2.02e−01
Value: C
Proteiniphilum.acetatigenes
3
2
1
0
A (n=15)
B (n=16)
C (n=14)
surface

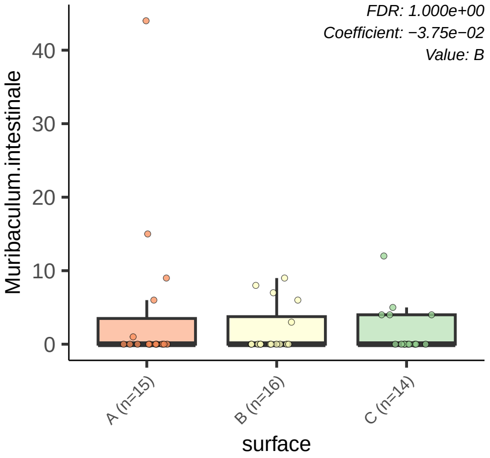
FDR: 1.000e+00
Coefficient: −3.75e−02
Value: B
Muribaculum.intestinale
0
10
20
30
40
A (n=15)
B (n=16)
C (n=14)
surface

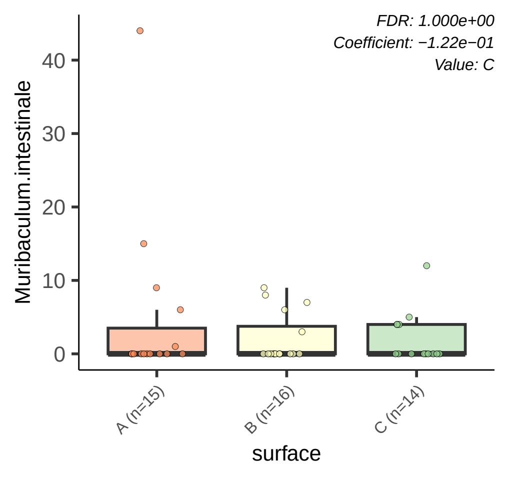
FDR: 1.000e+00
Coefficient: −1.22e−01
Value: C
Muribaculum.intestinale
0
10
20
30
40
A (n=15)
B (n=16)
C (n=14)
surface

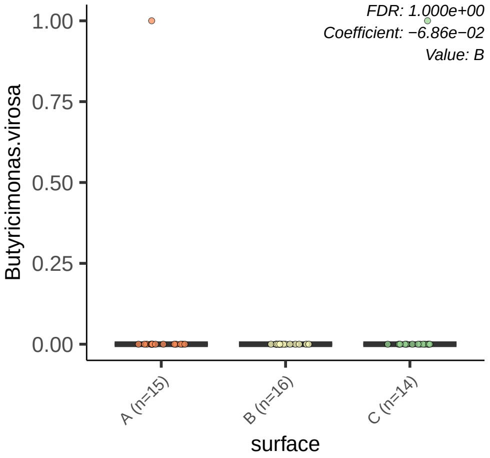
FDR: 1.000e+00
Coefficient: −6.86e−02
Value: B
Butyricimonas.virosa
1.00
0.75
0.50
0.25
0.00
A (n=15)
B (n=16)
C (n=14)
surface

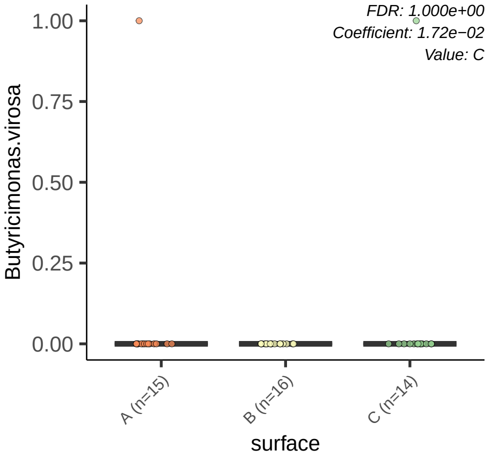
FDR: 1.000e+00
Coefficient: 1.72e−02
Value: C
Butyricimonas.virosa
1.00
0.75
0.50
0.25
0.00
A (n=15)
B (n=16)
C (n=14)
surface

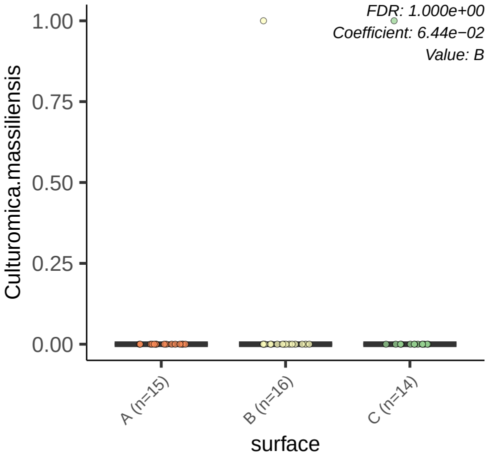

FDR: 1.000e+00
Coefficient: 6.44e−02
Value: B
Culturomica.massiliensis
1.00
0.75
0.50
0.25
0.00
A (n=15)
B (n=16)
C (n=14)
surface

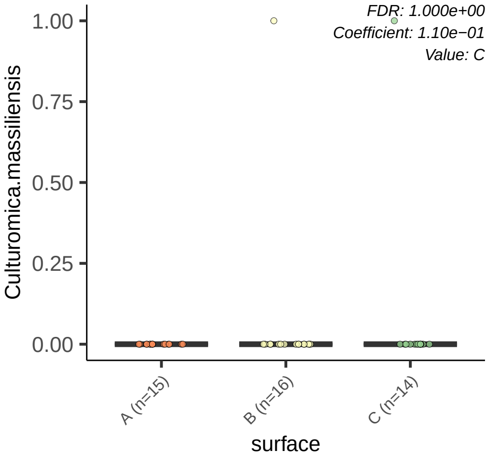
FDR: 1.000e+00
Coefficient: 1.10e−01
Value: C
Culturomica.massiliensis
1.00
0.75
0.50
0.25
0.00
A (n=15)
B (n=16)
C (n=14)
surface

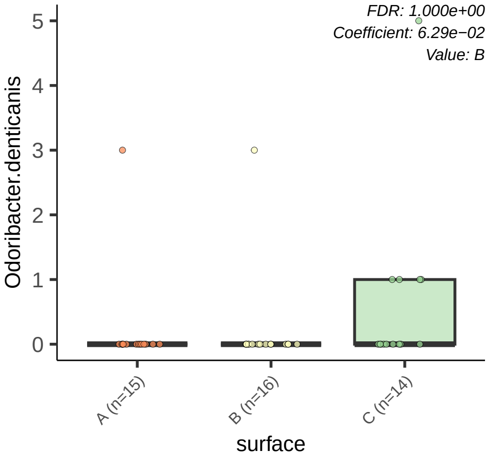

FDR: 1.000e+00
Coefficient: 6.29e−02
Value: B
Odoribacter.denticanis
5
4
3
2
1
0
A (n=15)
B (n=16)
C (n=14)
surface

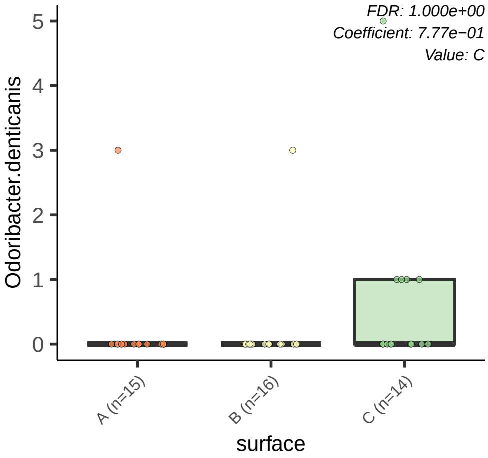
FDR: 1.000e+00
Coefficient: 7.77e−01
Value: C
Odoribacter.denticanis
0
1
2
3
4
5
A (n=15)
B (n=16)
C (n=14)
surface

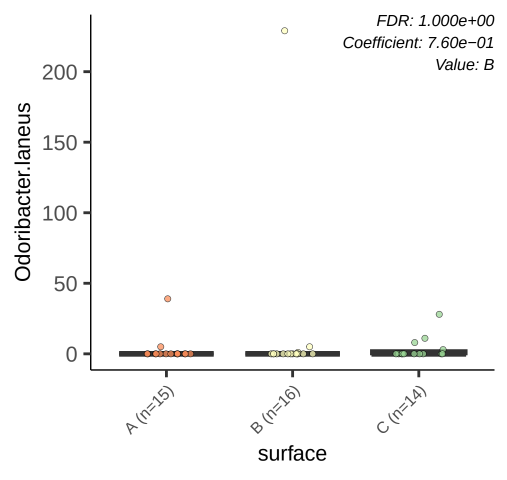
FDR: 1.000e+00
Coefficient: 7.60e−01
Value: B
Odoribacter.laneus
0
50
100
150
200
A (n=15)
B (n=16)
C (n=14)
surface

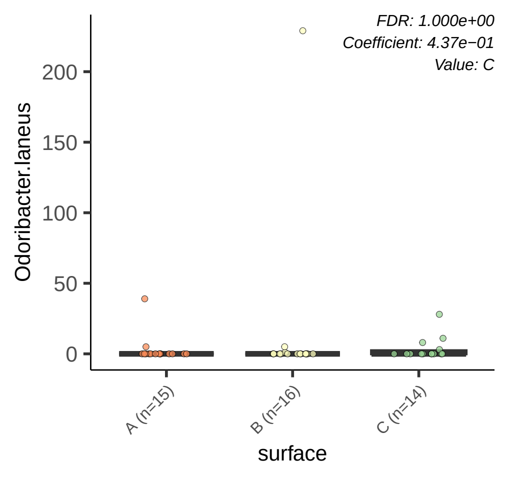
FDR: 1.000e+00
Coefficient: 4.37e−01
Value: C
Odoribacter.laneus
200
150
100
50
0
A (n=15)
B (n=16)
C (n=14)
surface

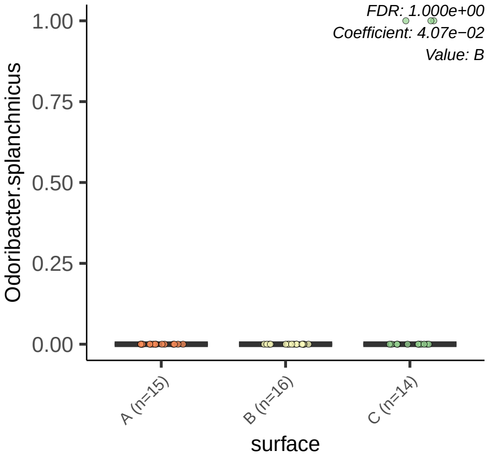
FDR: 1.000e+00
Coefficient: 4.07e−02
Value: B
Odoribacter.splanchnicus
1.00
0.75
0.50
0.25
0.00
A (n=15)
B (n=16)
C (n=14)
surface

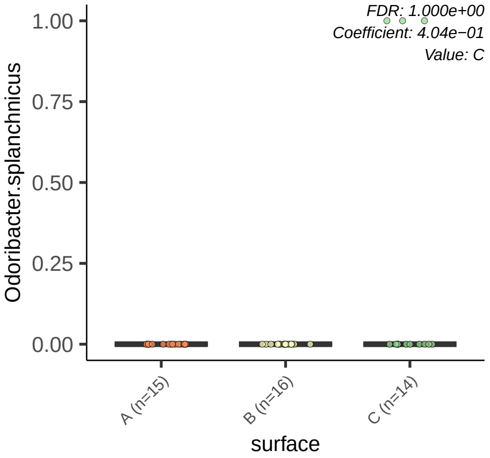

FDR: 1.000e+00
Coefficient: 4.04e−01
Value: C
Odoribacter.splanchnicus
1.00
0.75
0.50
0.25
0.00
A (n=15)
B (n=16)
C (n=14)
surface

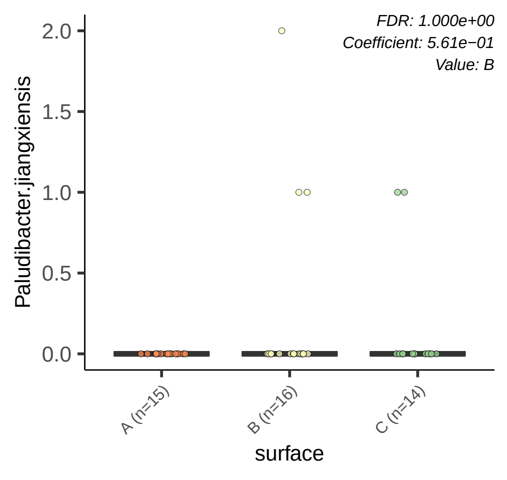

FDR: 1.000e+00
Coefficient: 5.61e−01
Value: B
2.0
1.5
1.0
0.5
0.0
Paludibacter.jiangxiensis
A (n=15)
B (n=16)
C (n=14)
surface

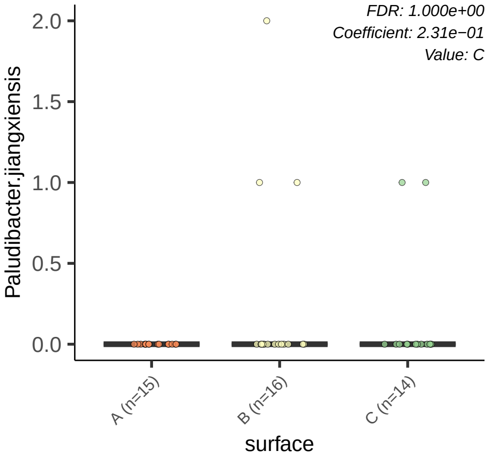
FDR: 1.000e+00
Coefficient: 2.31e−01
Value: C
Paludibacter.jiangxiensis
2.0
1.5
1.0
0.5
0.0
A (n=15)
B (n=16)
C (n=14)
surface

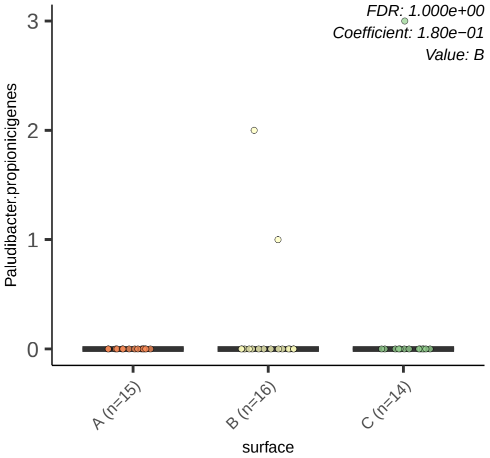

FDR: 1.000e+00
Coefficient: 1.80e−01
Value: B
Paludibacter.propionicigenes
0
1
2
3
A (n=15)
B (n=16)
C (n=14)
surface

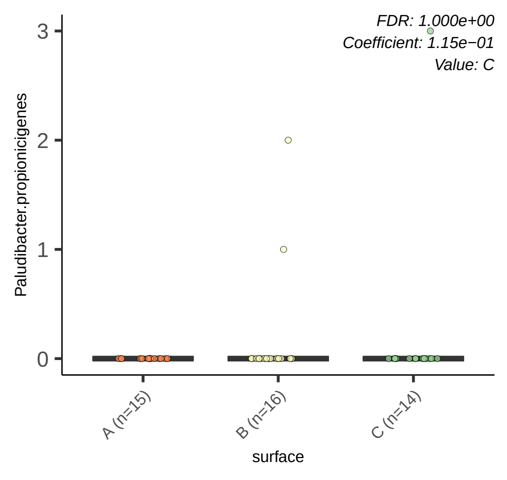
FDR: 1.000e+00
Coefficient: 1.15e−01
Value: C
Paludibacter.propionicigenes
3
2
1
0
A (n=15)
B (n=16)
C (n=14)
surface

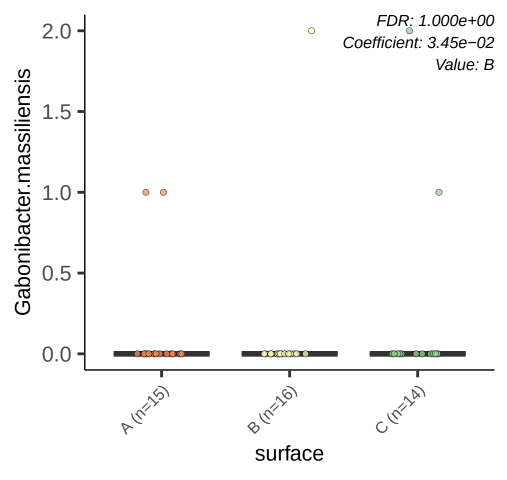

FDR: 1.000e+00
Coefficient: 3.45e−02
Value: B
Gabonibacter.massiliensis
2.0
1.5
1.0
0.5
0.0
A (n=15)
B (n=16)
C (n=14)
surface

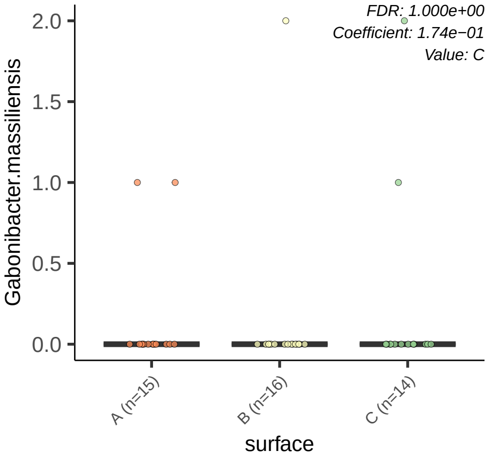

FDR: 1.000e+00
Coefficient: 1.74e−01
Value: C
Gabonibacter.massiliensis
2.0
1.5
1.0
0.5
0.0
A (n=15)
B (n=16)
C (n=14)
surface

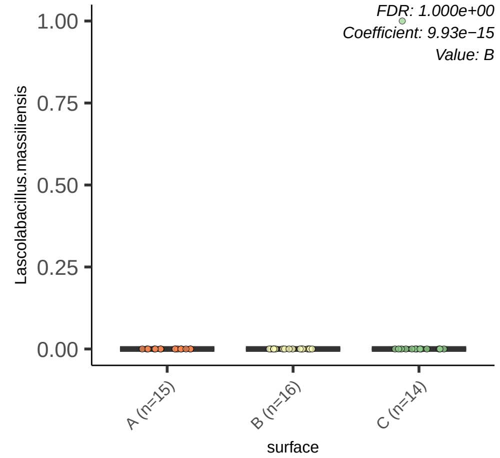

FDR: 1.000e+00
Coefficient: 9.93e−15
Value: B
Lascolabacillus.massiliensis
1.00
0.75
0.50
0.25
0.00
A (n=15)
B (n=16)
C (n=14)
surface

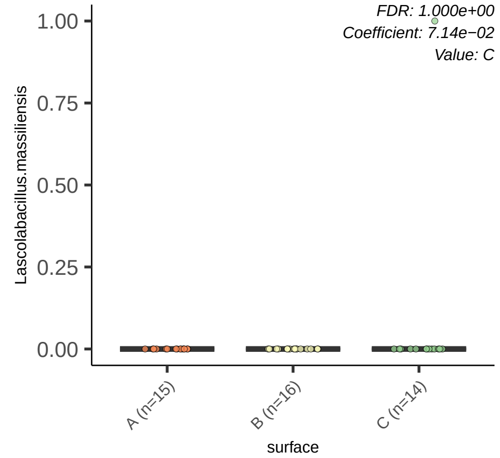
FDR: 1.000e+00
Coefficient: 7.14e−02
Value: C
Lascolabacillus.massiliensis
1.00
0.75
0.50
0.25
0.00
A (n=15)
B (n=16)
C (n=14)
surface

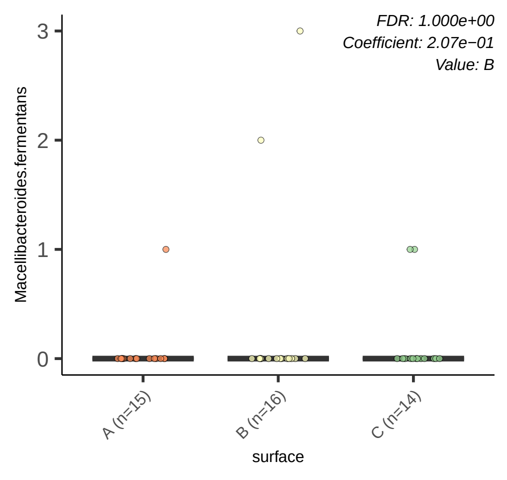
FDR: 1.000e+00
Coefficient: 2.07e−01
Value: B
Macellibacteroides.fermentans
0
1
2
3
A (n=15)
B (n=16)
C (n=14)
surface

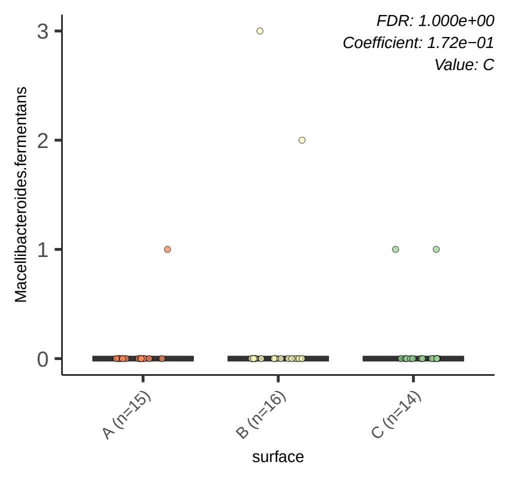

FDR: 1.000e+00
Coefficient: 1.72e−01
Value: C
Macellibacteroides.fermentans
0
1
2
3
A (n=15)
B (n=16)
C (n=14)
surface

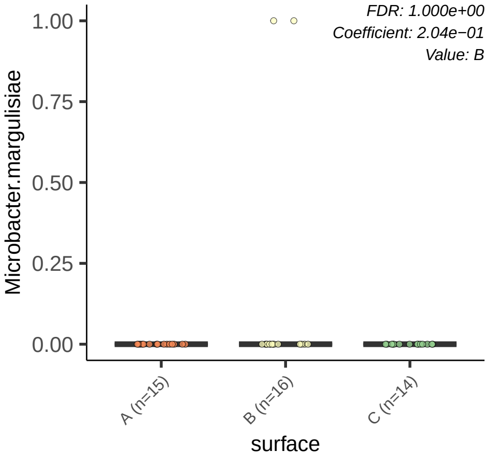

FDR: 1.000e+00
Coefficient: 2.04e−01
Value: B
Microbacter.margulisiae
1.00
0.75
0.50
0.25
0.00
A (n=15)
B (n=16)
C (n=14)
surface

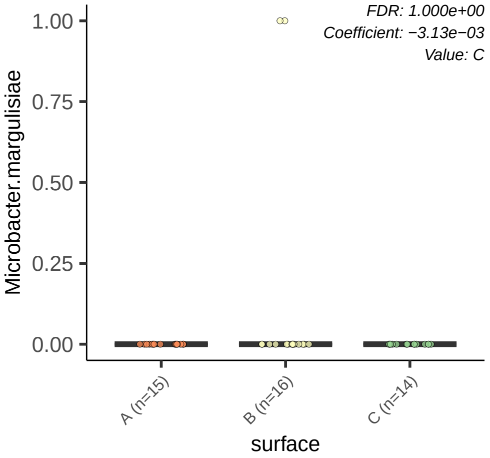
FDR: 1.000e+00
Coefficient: −3.13e−03
Value: C
Microbacter.margulisiae
1.00
0.75
0.50
0.25
0.00
A (n=15)
B (n=16)
C (n=14)
surface

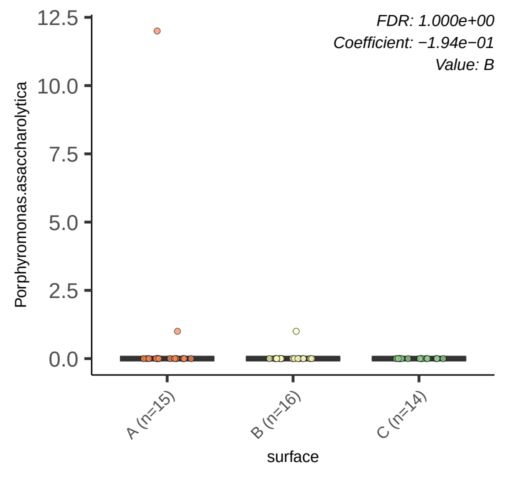
FDR: 1.000e+00
Coefficient: −1.94e−01
Value: B
Porphyromonas.asaccharolytica
12.5
10.0
7.5
5.0
2.5
0.0
A (n=15)
B (n=16)
C (n=14)
surface

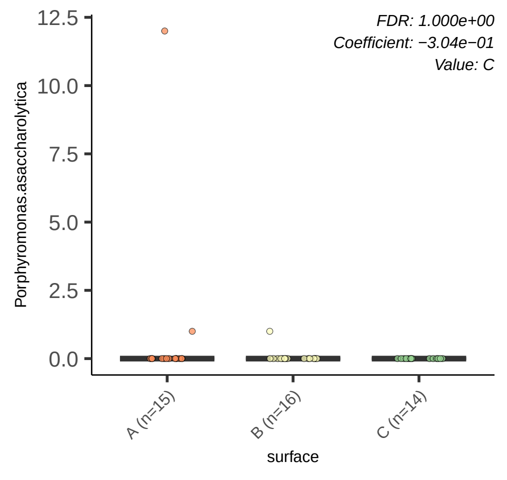
FDR: 1.000e+00
Coefficient: −3.04e−01
Value: C
Porphyromonas.asaccharolytica
12.5
10.0
7.5
5.0
2.5
0.0
A (n=15)
B (n=16)
C (n=14)
surface

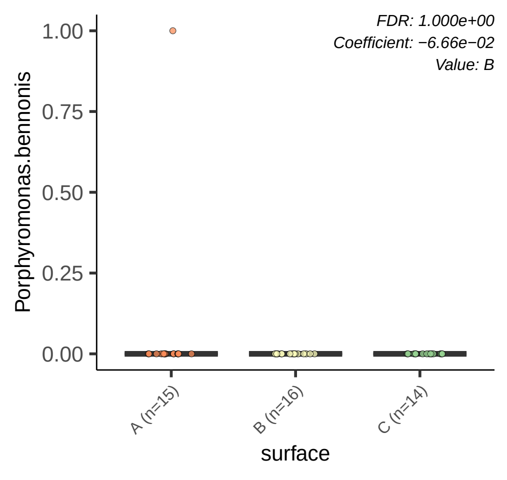
FDR: 1.000e+00
Coefficient: −6.66e−02
Value: B
Porphyromonas.bennonis
1.00
0.75
0.50
0.25
0.00
A (n=15)
B (n=16)
C (n=14)
surface

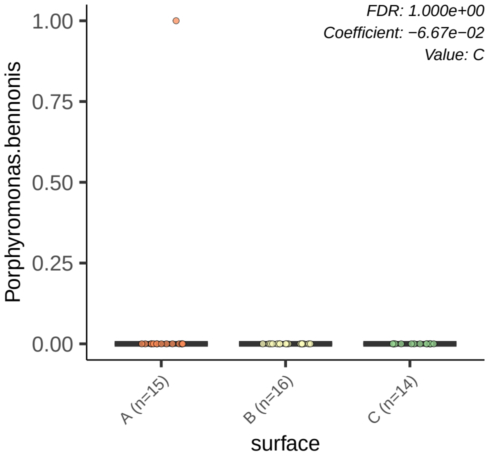

FDR: 1.000e+00
Coefficient: −6.67e−02
Value: C
Porphyromonas.bennonis
1.00
0.75
0.50
0.25
0.00
A (n=15)
B (n=16)
C (n=14)
surface

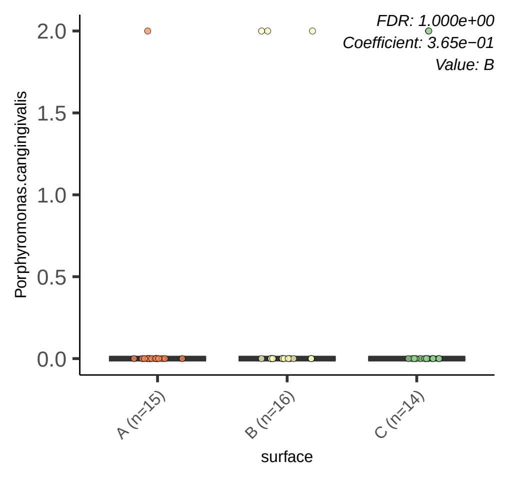
FDR: 1.000e+00
Coefficient: 3.65e−01
Value: B
Porphyromonas.cangingivalis
2.0
1.5
1.0
0.5
0.0
A (n=15)
B (n=16)
C (n=14)
surface

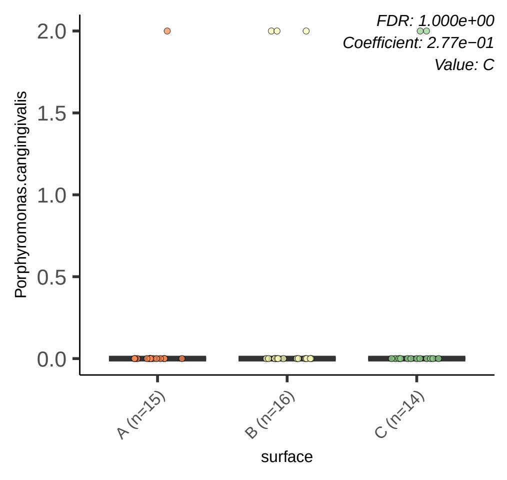

FDR: 1.000e+00
Coefficient: 2.77e−01
Value: C
Porphyromonas.cangingivalis
2.0
1.5
1.0
0.5
0.0
A (n=15)
B (n=16)
C (n=14)
surface

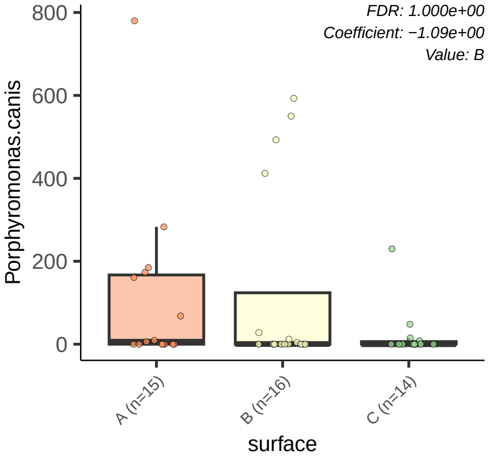

FDR: 1.000e+00
Coefficient: −1.09e+00
Value: B
Porphyromonas.canis
800
600
400
200
0
A (n=15)
B (n=16)
C (n=14)
surface

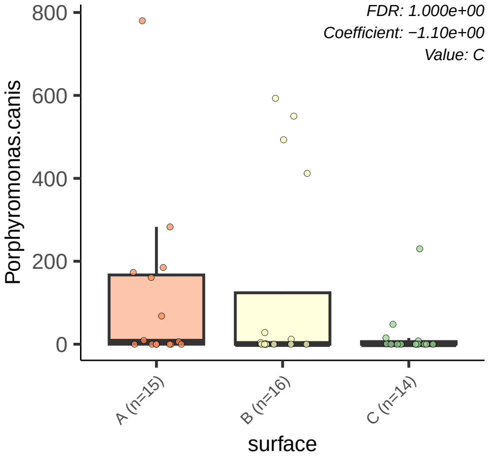

FDR: 1.000e+00
Coefficient: −1.10e+00
Value: C
Porphyromonas.canis
800
600
400
200
0
A (n=15)
B (n=16)
C (n=14)
surface

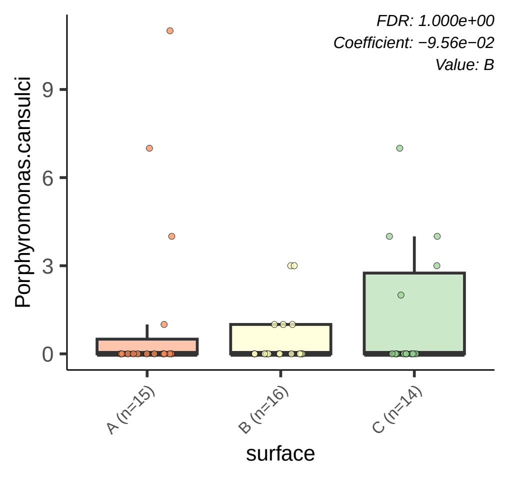

FDR: 1.000e+00
Coefficient: −9.56e−02
Value: B
Porphyromonas.cansulci
0
3
6
9
A (n=15)
B (n=16)
C (n=14)
surface

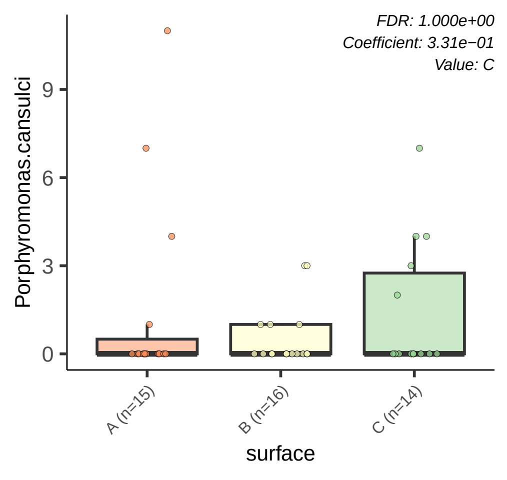

FDR: 1.000e+00
Coefficient: 3.31e−01
Value: C
Porphyromonas.cansulci
0
3
6
9
A (n=15)
B (n=16)
C (n=14)
surface

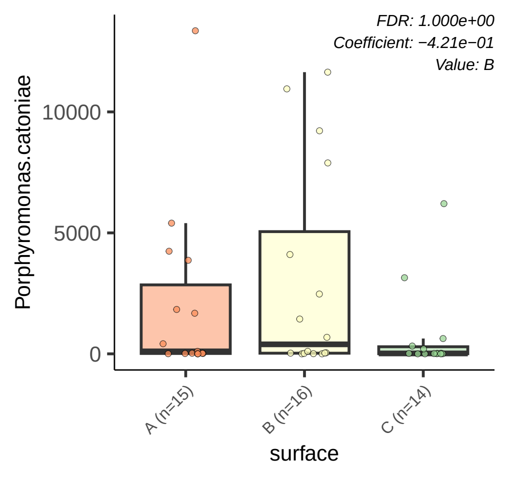
FDR: 1.000e+00
Coefficient: −4.21e−01
Value: B
Porphyromonas.catoniae
10000
5000
0
A (n=15)
B (n=16)
C (n=14)
surface

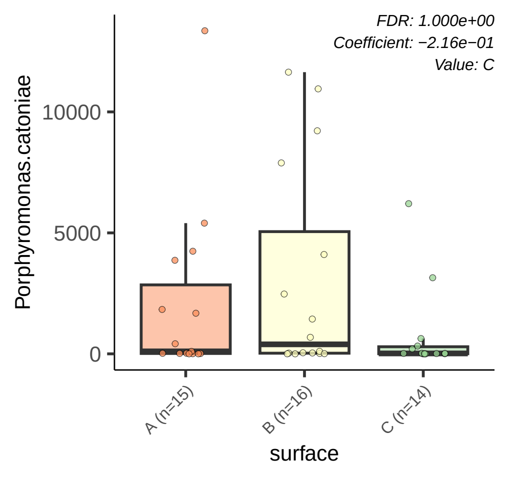
FDR: 1.000e+00
Coefficient: −2.16e−01
Value: C
Porphyromonas.catoniae
10000
5000
0
A (n=15)
B (n=16)
C (n=14)
surface

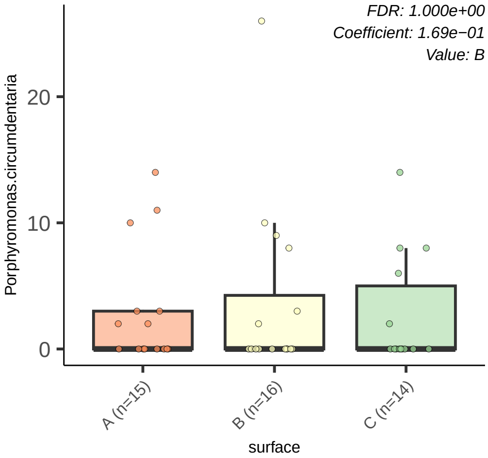
FDR: 1.000e+00
Coefficient: 1.69e−01
Value: B
Porphyromonas.circumdentaria
0
10
20
A (n=15)
B (n=16)
C (n=14)
surface

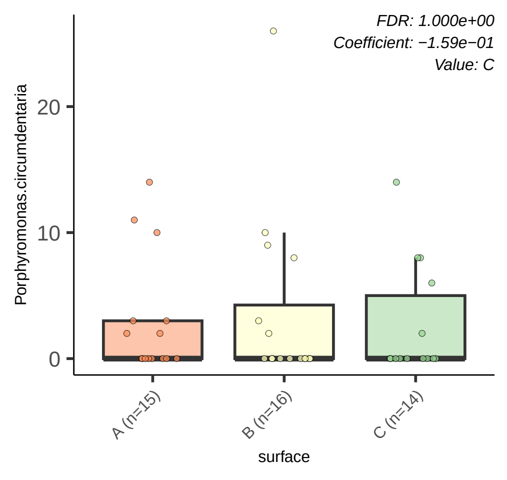
FDR: 1.000e+00
Coefficient: −1.59e−01
Value: C
Porphyromonas.circumdentaria
0
10
20
A (n=15)
B (n=16)
C (n=14)
surface

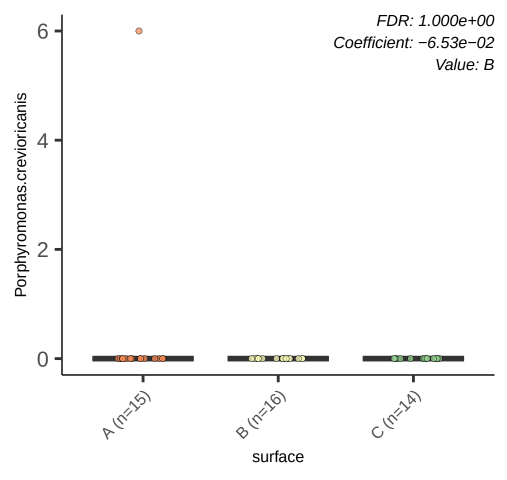
FDR: 1.000e+00
Coefficient: −6.53e−02
Value: B
Porphyromonas.creviorican is
6
4
2
0
A (n=15)
B (n=16)
C (n=14)
surface

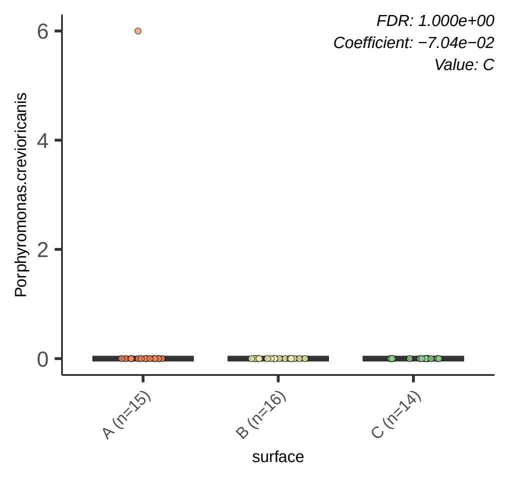
FDR: 1.000e+00
Coefficient: −7.04e−02
Value: C
Porphyromonas.crevioricanis
6
4
2
0
A (n=15)
B (n=16)
C (n=14)
surface

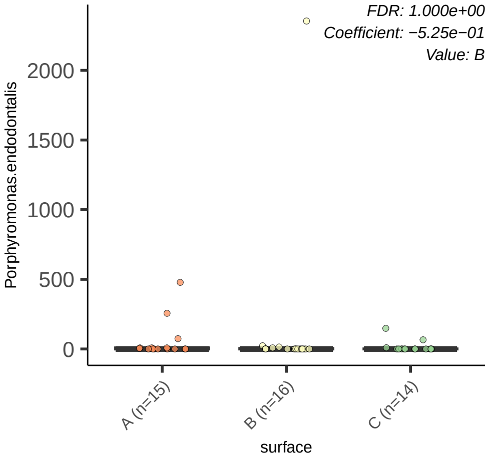
FDR: 1.000e+00
Coefficient: −5.25e−01
Value: B
2000
1500
1000
500
0
Porphyromonas.endodontalis
A (n=15)
B (n=16)
C (n=14)
surface

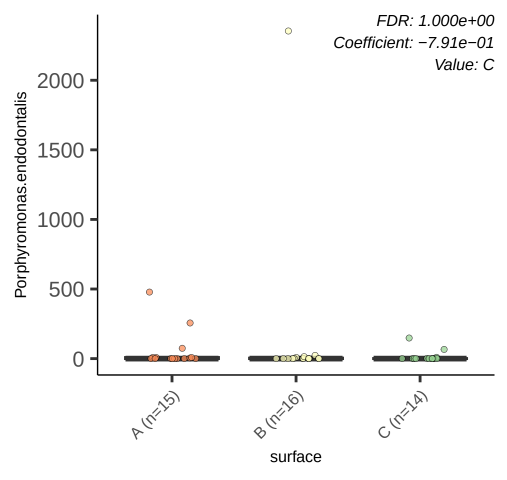
FDR: 1.000e+00
Coefficient: −7.91e−01
Value: C
Porphyromonas.endodontalis
2000
1500
1000
500
0
A (n=15)
B (n=16)
C (n=14)
surface

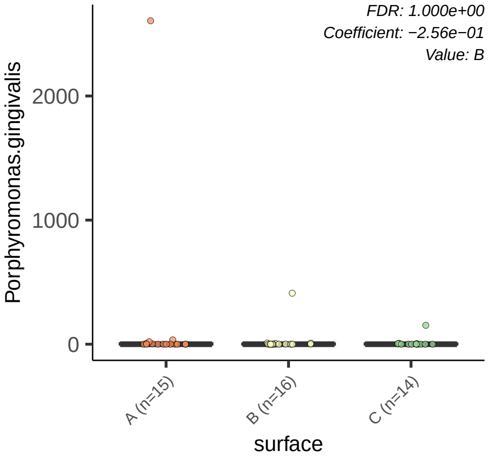

FDR: 1.000e+00
Coefficient: −2.56e−01
Value: B
Porphyromonas.gingivalis
2000
1000
0
A (n=15)
B (n=16)
C (n=14)
surface

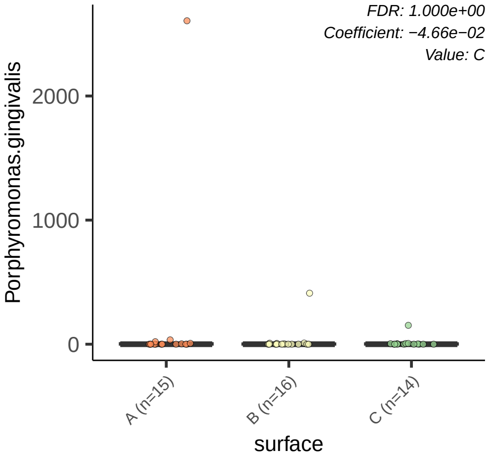
FDR: 1.000e+00
Coefficient: −4.66e−02
Value: C
Porphyromonas.gingivalis
2000
1000
0
A (n=15)
B (n=16)
C (n=14)
surface

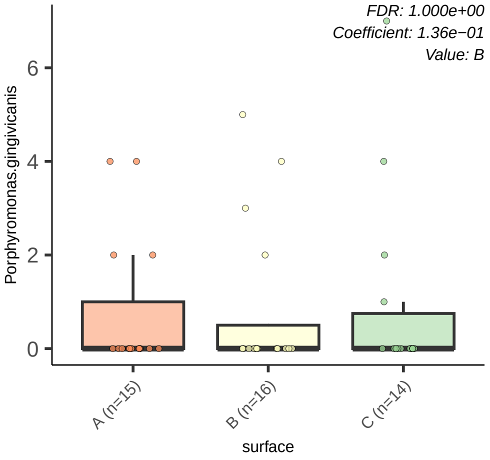

FDR: 1.000e+00
Coefficient: 1.36e−01
Value: B
Porphyromonas.gingivicanis
0
2
4
6
A (n=15)
B (n=16)
C (n=14)
surface

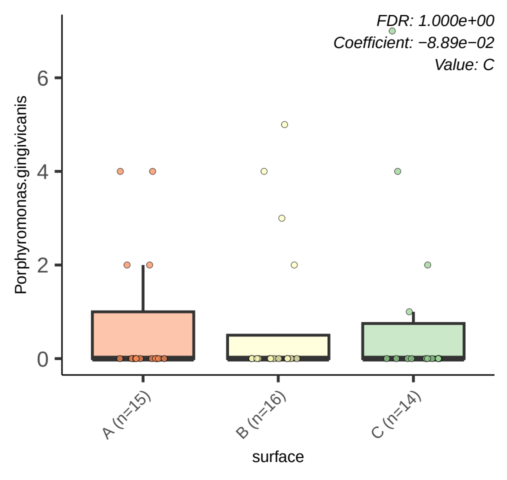

FDR: 1.000e+00
Coefficient: −8.89e−02
Value: C
Porphyromonas.gingivicanis
0
2
4
6
A (n=15)
B (n=16)
C (n=14)
surface

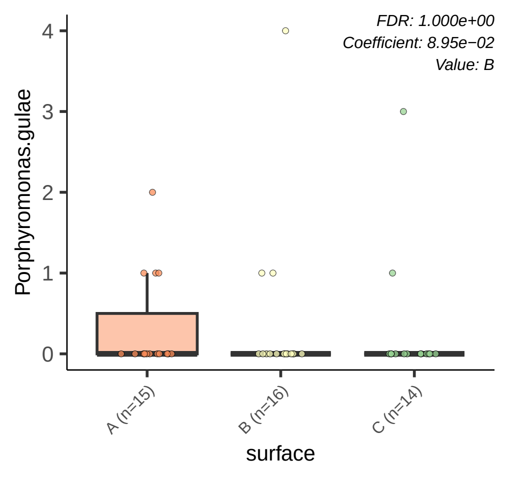
FDR: 1.000e+00
Coefficient: 8.95e−02
Value: B
Porphyromonas.gulae
0
1
2
3
4
A (n=15)
B (n=16)
C (n=14)
surface

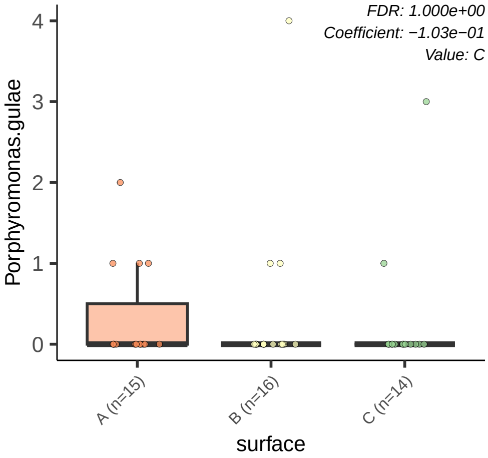
FDR: 1.000e+00
Coefficient: −1.03e−01
Value: C
Porphyromonas.gulae
4
3
2
1
0
A (n=15)
B (n=16)
C (n=14)
surface

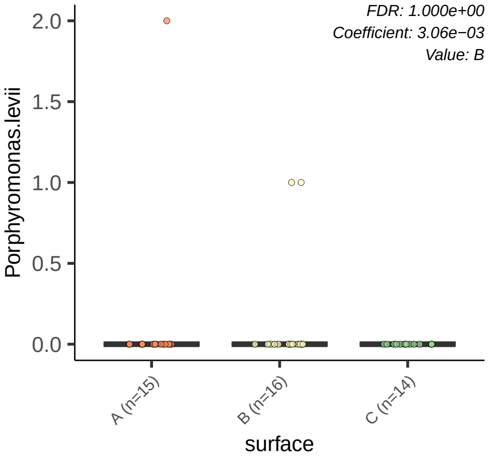
FDR: 1.000e+00
Coefficient: 3.06e−03
Value: B
2.0
1.5
1.0
0.5
0.0
Porphyromonas.levii
A (n=15)
B (n=16)
C (n=14)
surface

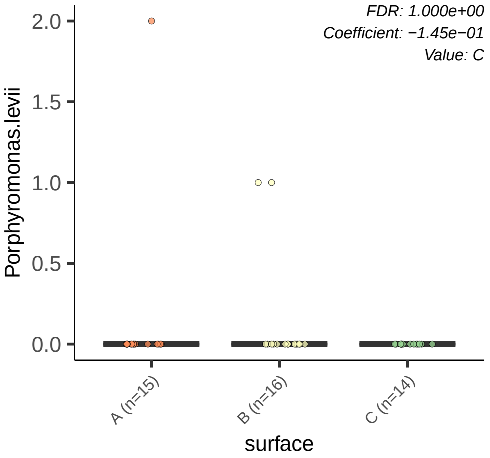

FDR: 1.000e+00
Coefficient: −1.45e−01
Value: C
Porphyromonas.levii
2.0
1.5
1.0
0.5
0.0
A (n=15)
B (n=16)
C (n=14)
surface

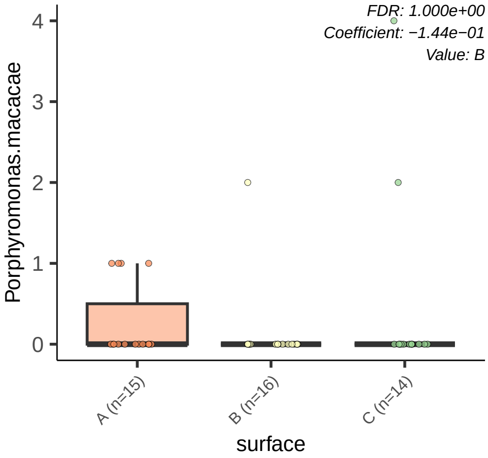
FDR: 1.000e+00
Coefficient: −1.44e−01
Value: B
Porphyromonas.macacae
0
1
2
3
4
A (n=15)
B (n=16)
C (n=14)
surface

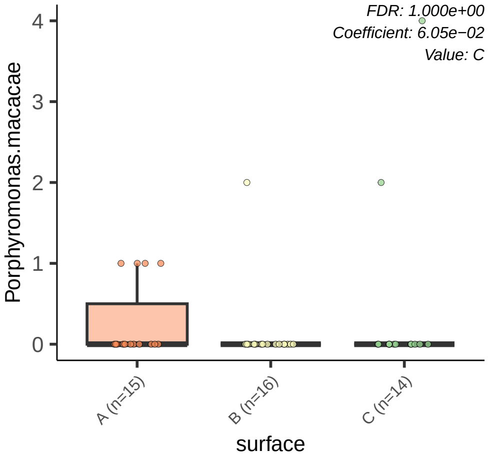

FDR: 1.000e+00
Coefficient: 6.05e−02
Value: C
Porphyromonas.macacae
0
1
2
3
4
A (n=15)
B (n=16)
C (n=14)
surface

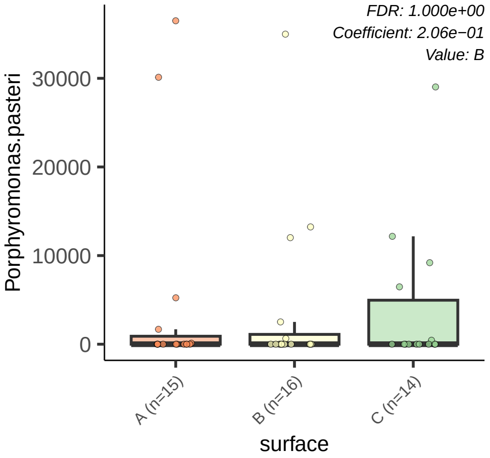

FDR: 1.000e+00
Coefficient: 2.06e−01
Value: B
30000
20000
10000
0
Porphyromonas.pasteri
A (n=15)
B (n=16)
C (n=14)
surface

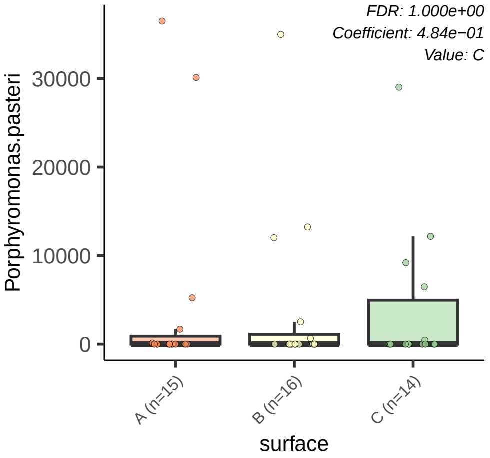
FDR: 1.000e+00
Coefficient: 4.84e−01
Value: C
Porphyromonas.pasteri
30000
20000
10000
0
A (n=15)
B (n=16)
C (n=14)
surface

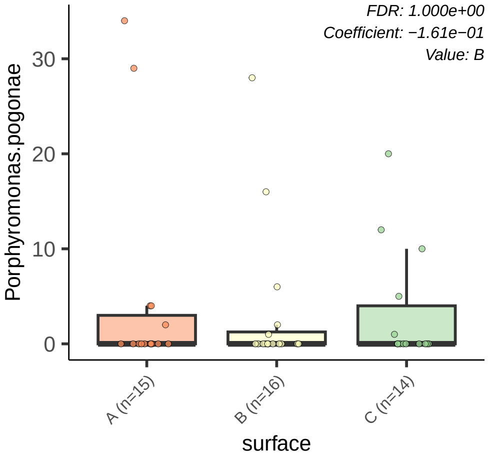
FDR: 1.000e+00
Coefficient: −1.61e−01
Value: B
Porphyromonas.pogonae
30
20
10
0
A (n=15)
B (n=16)
C (n=14)
surface

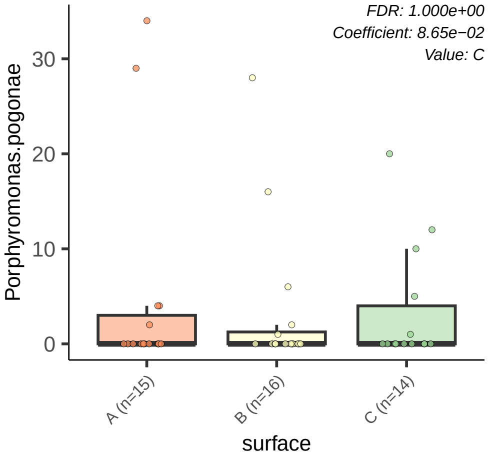

FDR: 1.000e+00
Coefficient: 8.65e−02
Value: C
Porphyromonas.pogonae
30
20
10
0
A (n=15)
B (n=16)
C (n=14)
surface

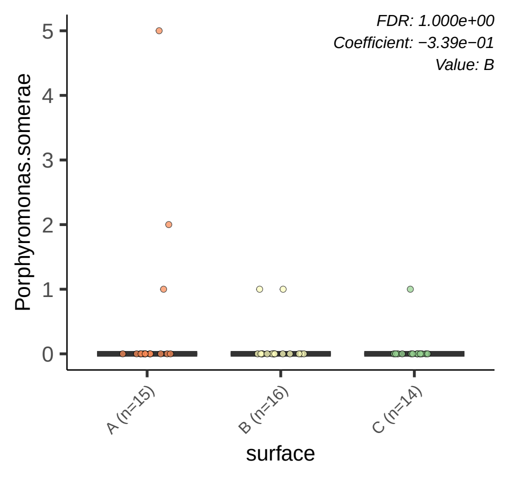
FDR: 1.000e+00
Coefficient: −3.39e−01
Value: B
Porphyromonas.somerae
0
1
2
3
4
5
A (n=15)
B (n=16)
C (n=14)
surface

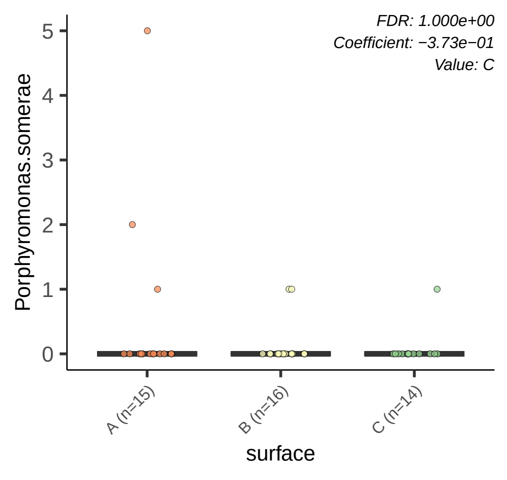
FDR: 1.000e+00
Coefficient: −3.73e−01
Value: C
Porphyromonas.somerae
0
1
2
3
4
5
A (n=15)
B (n=16)
C (n=14)
surface

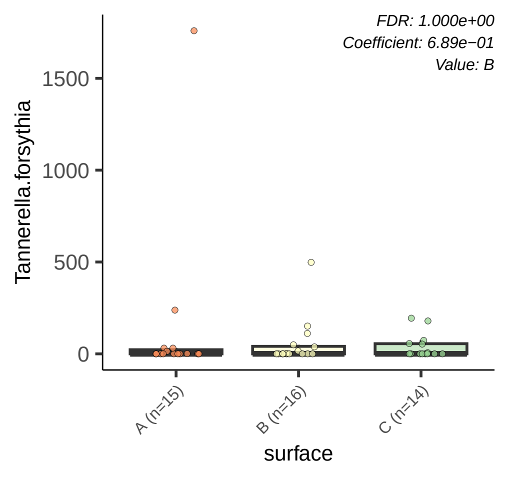
FDR: 1.000e+00
Coefficient: 6.89e−01
Value: B
Tannerella.forsythia
1500
1000
500
0
A (n=15)
B (n=16)
C (n=14)
surface

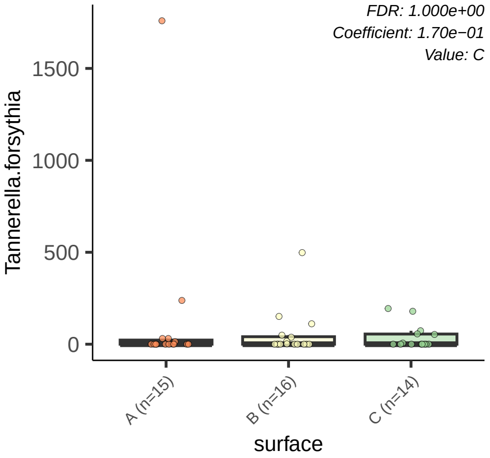
FDR: 1.000e+00
Coefficient: 1.70e−01
Value: C
Tannerella.forsythia
1500
1000
500
0
A (n=15)
B (n=16)
C (n=14)
surface

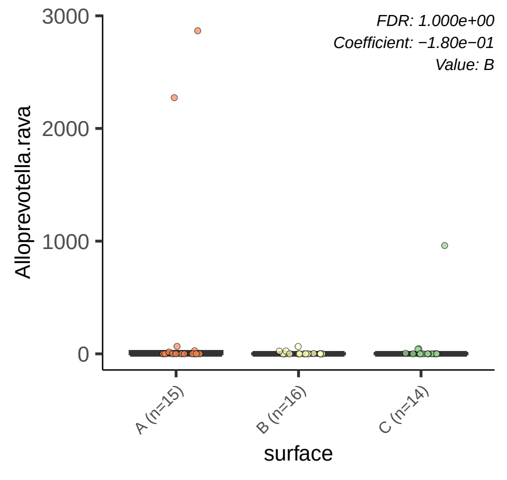
FDR: 1.000e+00
Coefficient: −1.80e−01
Value: B
Alloprevotella.rava
3000
2000
1000
0
A (n=15)
B (n=16)
C (n=14)
surface

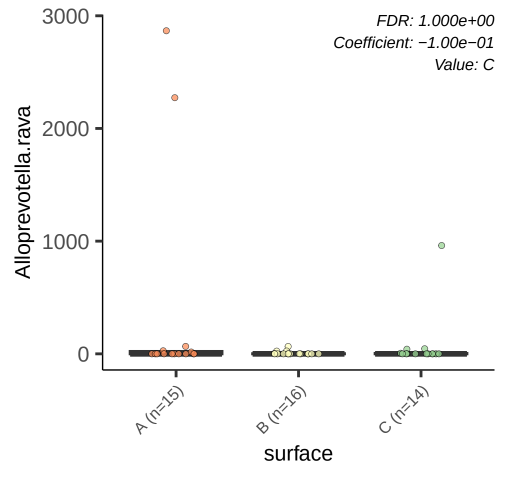

FDR: 1.000e+00
Coefficient: −1.00e−01
Value: C
Alloprevotella.rava
3000
2000
1000
0
A (n=15)
B (n=16)
C (n=14)
surface

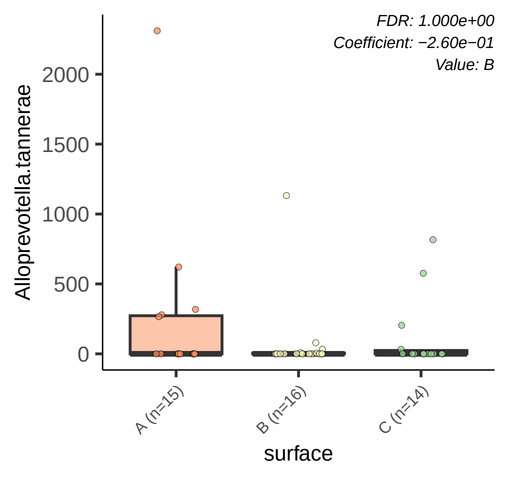
FDR: 1.000e+00
Coefficient: −2.60e−01
Value: B
Alloprevotella.tannerae
2000
1500
1000
500
0
A (n=15)
B (n=16)
C (n=14)
surface

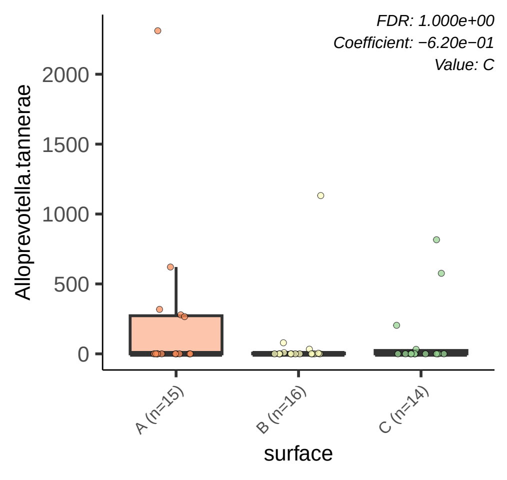
FDR: 1.000e+00
Coefficient: −6.20e−01
Value: C
Alloprevotella.tannerae
2000
1500
1000
500
0
A (n=15)
B (n=16)
C (n=14)
surface

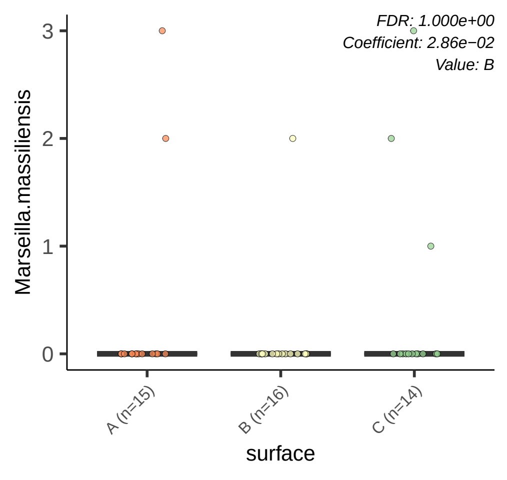

FDR: 1.000e+00
Coefficient: 2.86e−02
Value: B
Marseilla.massiliensis
0
1
2
3
A (n=15)
B (n=16)
C (n=14)
surface

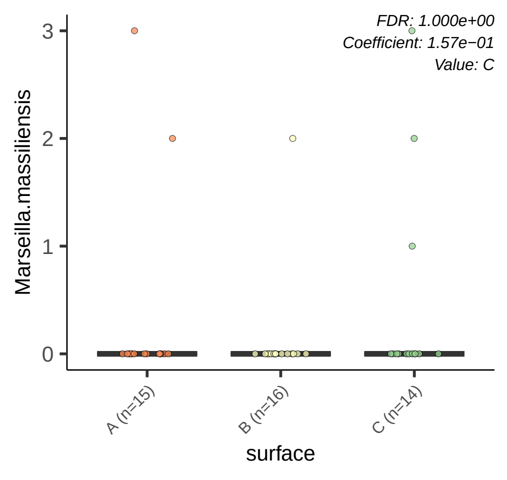
FDR: 1.000e+00
Coefficient: 1.57e−01
Value: C
Marseilla.massiliensis
3
2
1
0
A (n=15)
B (n=16)
C (n=14)
surface

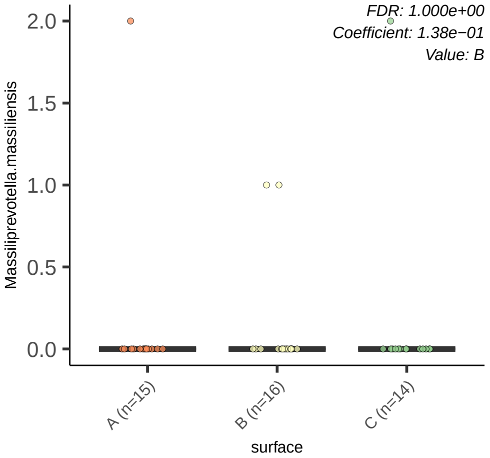

FDR: 1.000e+00
Coefficient: 1.38e−01
Value: B
2.0
1.5
1.0
0.5
0.0
Massiliprevotella.massiliensis
A (n=15)
B (n=16)
C (n=14)
surface

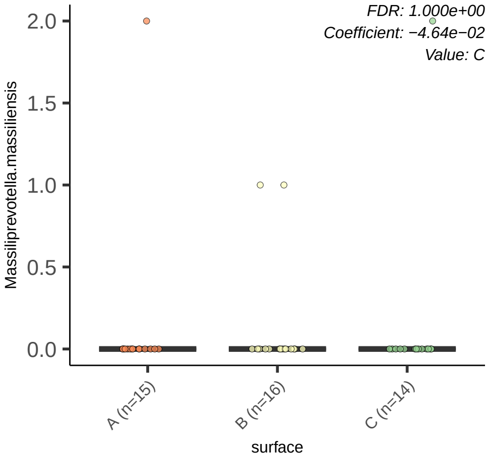

FDR: 1.000e+00
Coefficient: −4.64e−02
Value: C
Massiliprevotella.massiliensis
2.0
1.5
1.0
0.5
0.0
A (n=15)
B (n=16)
C (n=14)
surface

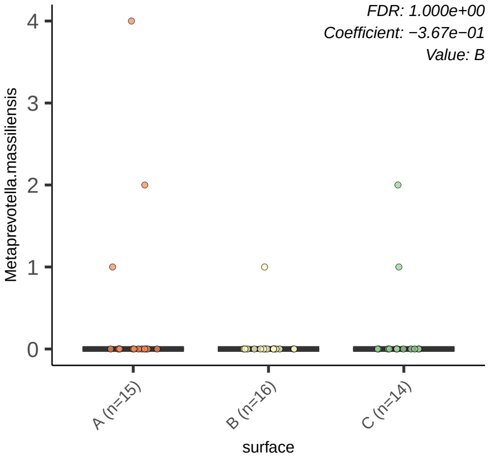

FDR: 1.000e+00
Coefficient: −3.67e−01
Value: B
Metaprevotella.massiliensis
4
3
2
1
0
A (n=15)
B (n=16)
C (n=14)
surface

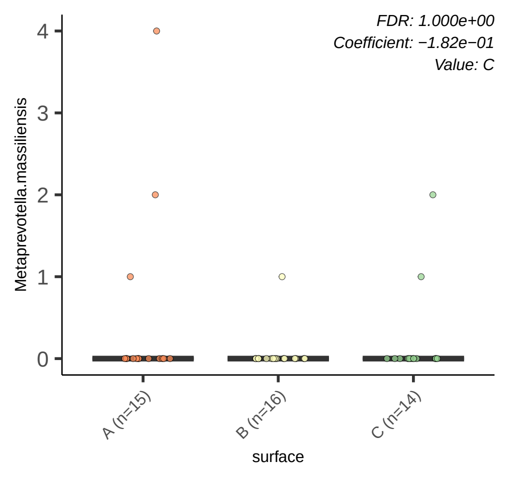

FDR: 1.000e+00
Coefficient: −1.82e−01
Value: C
Metaprevotella.massiliensis
4
3
2
1
0
A (n=15)
B (n=16)
C (n=14)
surface

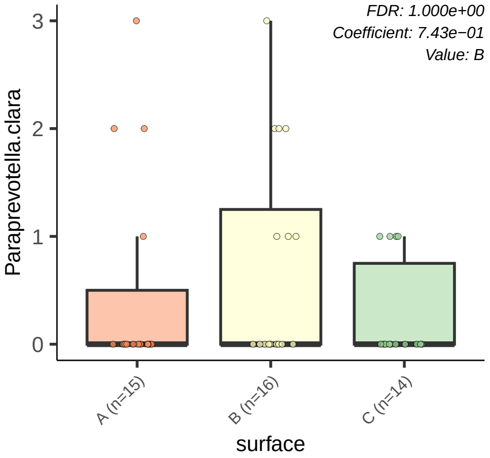

FDR: 1.000e+00
Coefficient: 7.43e−01
Value: B
Paraprevotella.clara
0
1
2
3
A (n=15)
B (n=16)
C (n=14)
surface

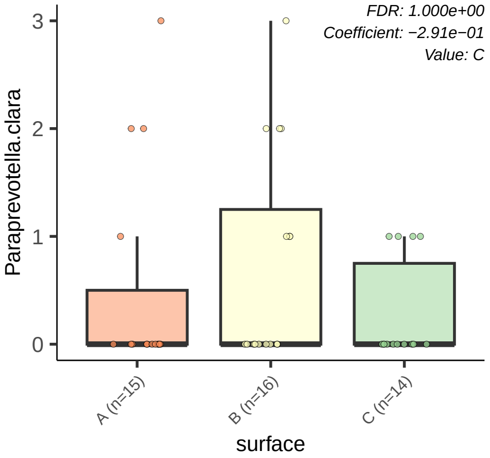

FDR: 1.000e+00
Coefficient: −2.91e−01
Value: C
Paraprevotella.clara
0
1
2
3
A (n=15)
B (n=16)
C (n=14)
surface

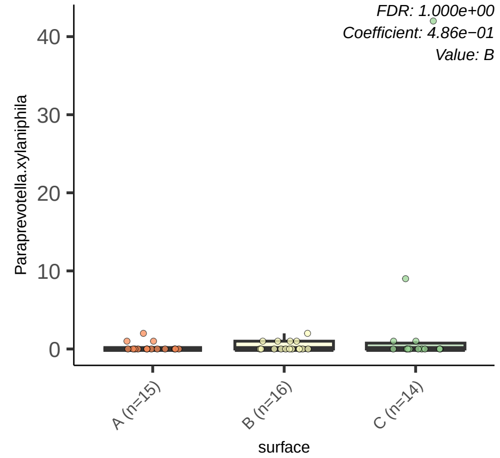
FDR: 1.000e+00
Coefficient: 4.86e−01
Value: B
Paraprevotella.xylaniphila
40
30
20
10
0
A (n=15)
B (n=16)
C (n=14)
surface

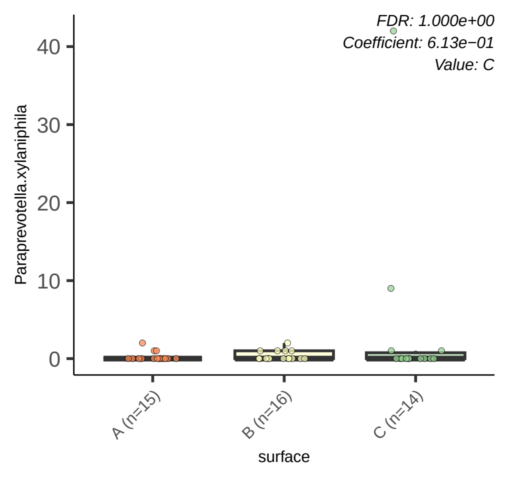

FDR: 1.000e+00
Coefficient: 6.13e−01
Value: C
Paraprevotella.xylaniphila
40
30
20
10
0
A (n=15)
B (n=16)
C (n=14)
surface

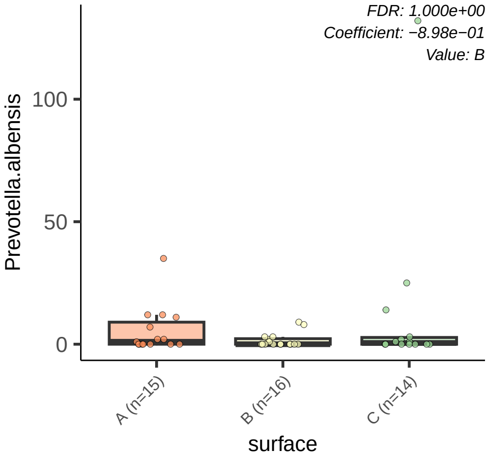

FDR: 1.000e+00
Coefficient: −8.98e−01
Value: B
Prevotella.albensis
100
50
0
A (n=15)
B (n=16)
C (n=14)
surface

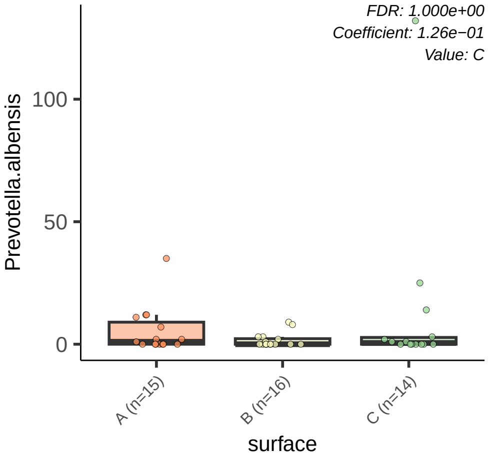
FDR: 1.000e+00
Coefficient: 1.26e−01
Value: C
Prevotella.albensis
100
50
0
A (n=15)
B (n=16)
C (n=14)
surface

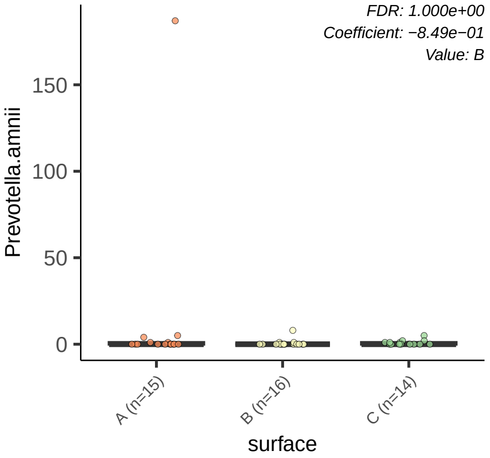

FDR: 1.000e+00
Coefficient: −8.49e−01
Value: B
Prevotella.amnii
150
100
50
0
A (n=15)
B (n=16)
C (n=14)
surface

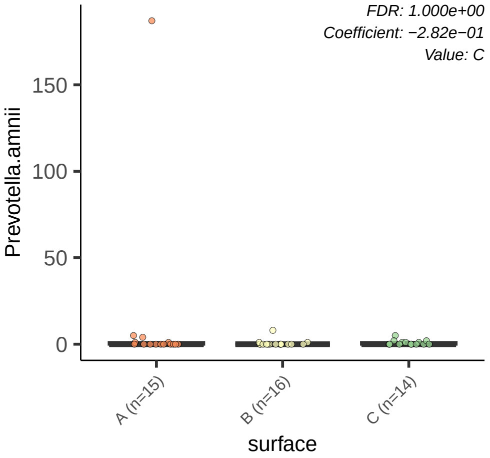
FDR: 1.000e+00
Coefficient: −2.82e−01
Value: C
Prevotella.amnii
150
100
50
0
A (n=15)
B (n=16)
C (n=14)
surface

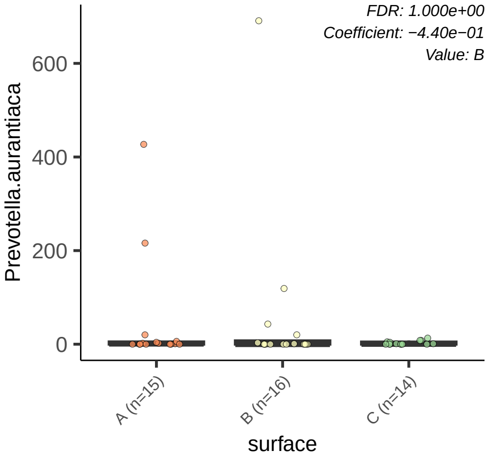
FDR: 1.000e+00
Coefficient: −4.40e−01
Value: B
Prevotella.aurantiaca
600
400
200
0
A (n=15)
B (n=16)
C (n=14)
surface

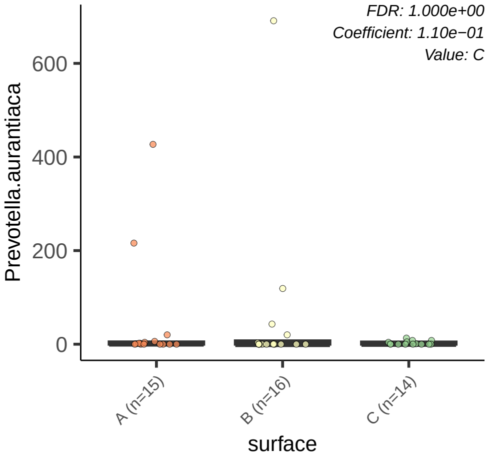
FDR: 1.000e+00
Coefficient: 1.10e−01
Value: C
600
400
200
0
Prevotella.aurantiaca
A (n=15)
B (n=16)
C (n=14)
surface

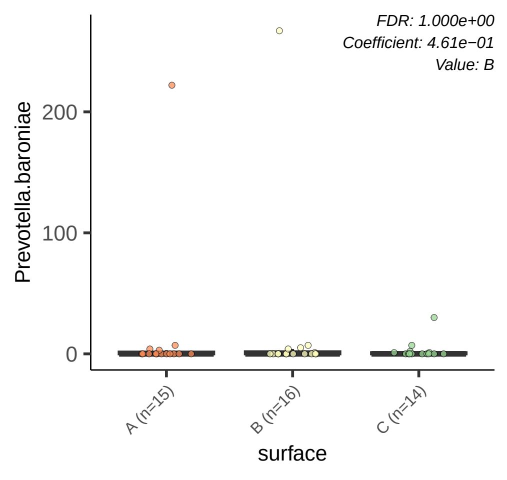

FDR: 1.000e+00
Coefficient: 4.61e−01
Value: B
Prevotella.baroniae
200
100
0
A (n=15)
B (n=16)
C (n=14)
surface

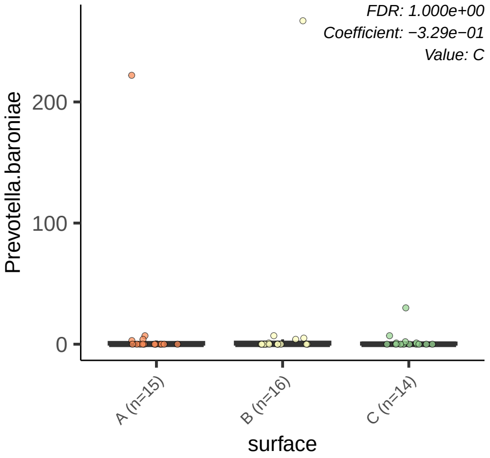

FDR: 1.000e+00
Coefficient: −3.29e−01
Value: C
Prevotella.baroniae
200
100
0
A (n=15)
B (n=16)
C (n=14)
surface

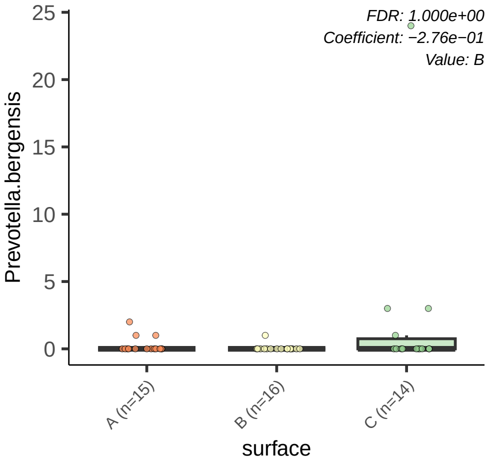
FDR: 1.000e+00
Coefficient: −2.76e−01
Value: B
Prevotella.bergensis
25
20
15
10
5
0
A (n=15)
B (n=16)
C (n=14)
surface

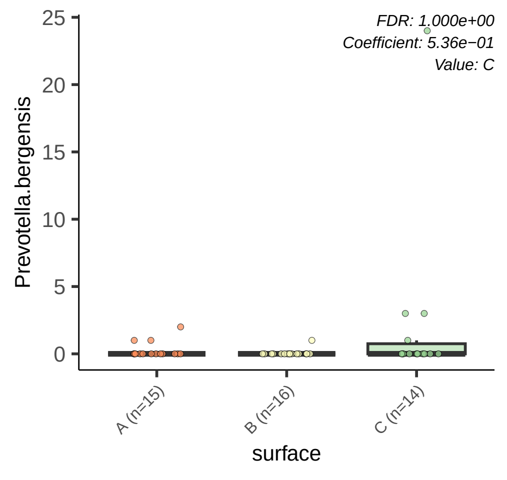
FDR: 1.000e+00
Coefficient: 5.36e−01
Value: C
Prevotella.bergensis
25
20
15
10
5
0
A (n=15)
B (n=16)
C (n=14)
surface

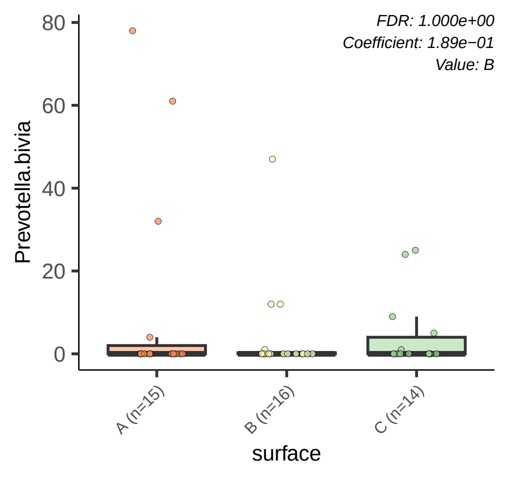
FDR: 1.000e+00
Coefficient: 1.89e−01
Value: B
Prevotella.bivia
80
60
40
20
0
A (n=15)
B (n=16)
C (n=14)
surface

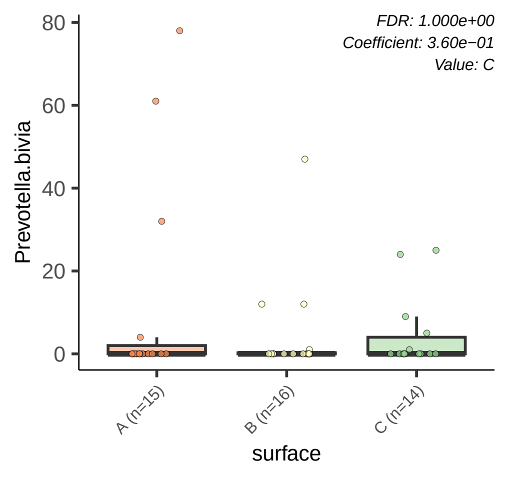
FDR: 1.000e+00
Coefficient: 3.60e−01
Value: C
Prevotella.bivia
80
60
40
20
0
A (n=15)
B (n=16)
C (n=14)
surface

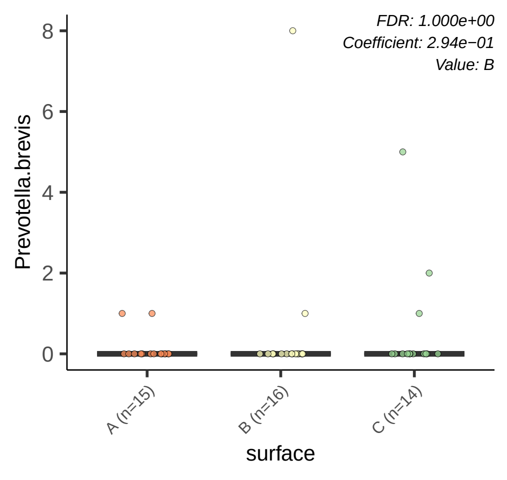
FDR: 1.000e+00
Coefficient: 2.94e−01
Value: B
Prevotella.brevis
0
2
4
6
8
A (n=15)
B (n=16)
C (n=14)
surface

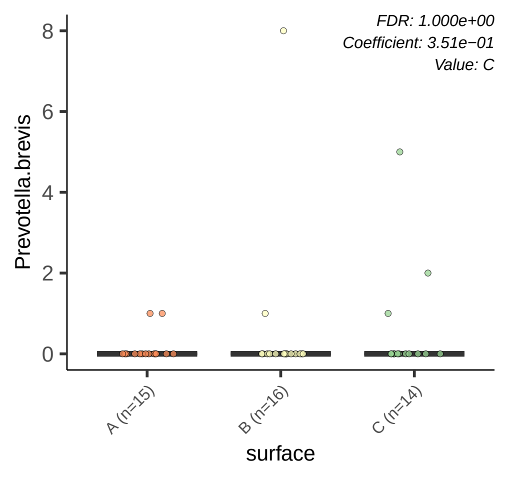
FDR: 1.000e+00
Coefficient: 3.51e−01
Value: C
Prevotella.brevis
0
2
4
6
8
A (n=15)
B (n=16)
C (n=14)
surface

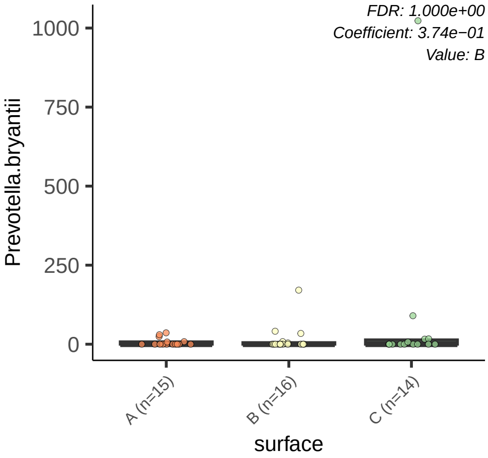
FDR: 1.000e+00
Coefficient: 3.74e−01
Value: B
Prevotella.bryantii
1000
750
500
250
0
A (n=15)
B (n=16)
C (n=14)
surface

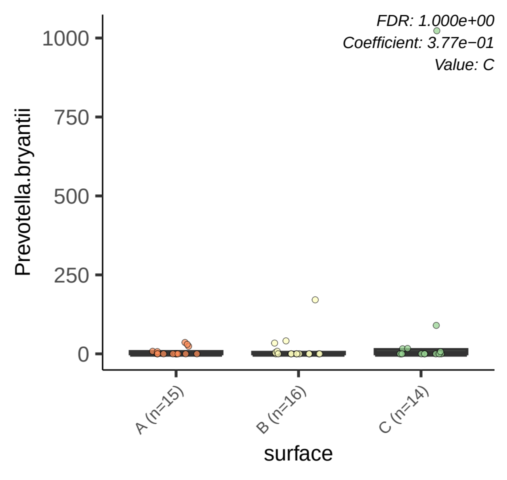
FDR: 1.000e+00
Coefficient: 3.77e−01
Value: C
Prevotella.bryantii
1000
750
500
250
0
A (n=15)
B (n=16)
C (n=14)
surface

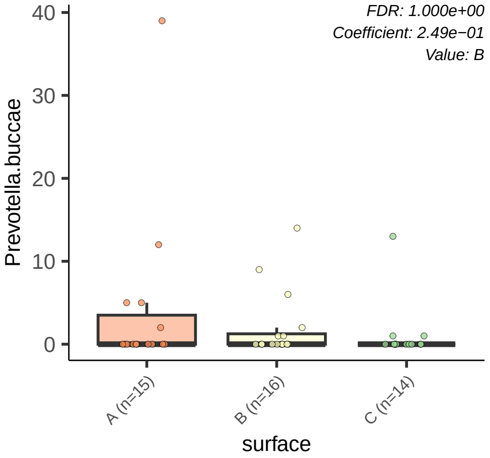
FDR: 1.000e+00
Coefficient: 2.49e−01
Value: B
Prevotella.buccae
0
10
20
30
40
A (n=15)
B (n=16)
C (n=14)
surface

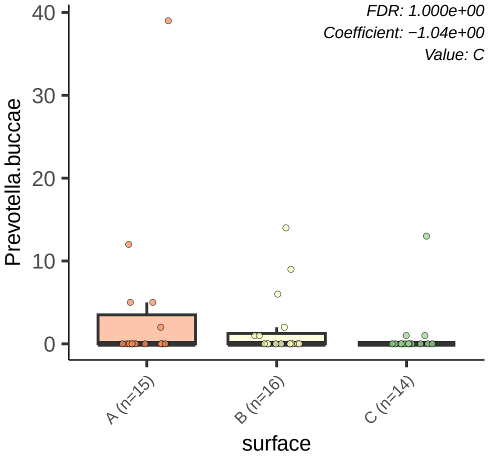
FDR: 1.000e+00
Coefficient: −1.04e+00
Value: C
Prevotella.buccae
40
30
20
10
0
A (n=15)
B (n=16)
C (n=14)
surface

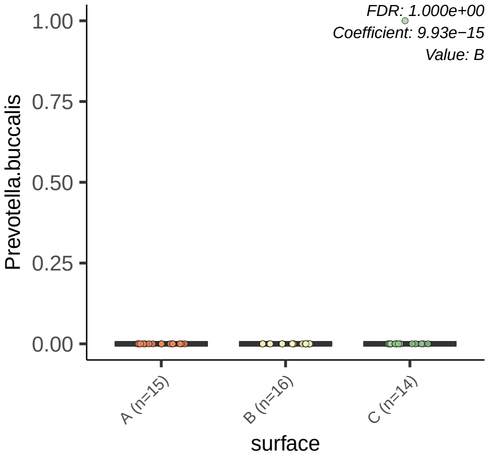

FDR: 1.000e+00
Coefficient: 9.93e−15
Value: B
Prevotella.buccalis
1.00
0.75
0.50
0.25
0.00
A (n=15)
B (n=16)
C (n=14)
surface

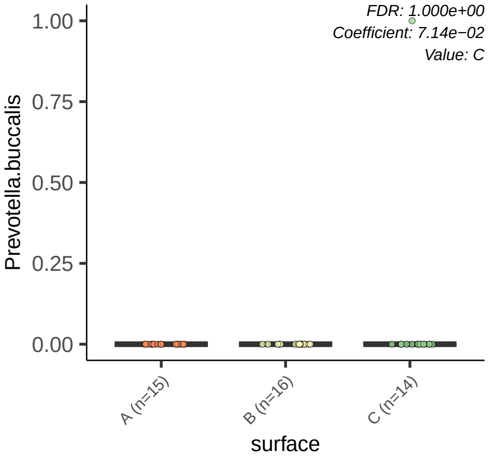

FDR: 1.000e+00
Coefficient: 7.14e−02
Value: C
Prevotella.buccalis
1.00
0.75
0.50
0.25
0.00
A (n=15)
B (n=16)
C (n=14)
surface

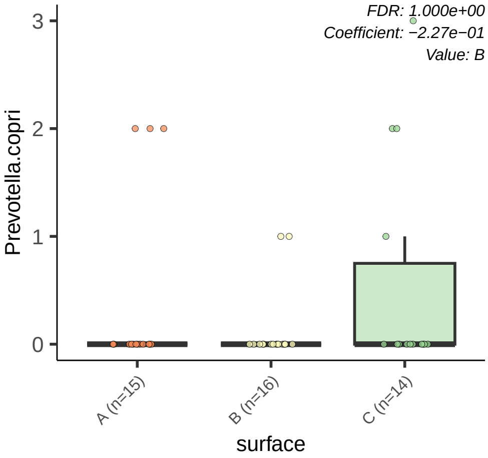

FDR: 1.000e+00
Coefficient: −2.27e−01
Value: B
Prevotella.copri
3
2
1
0
A (n=15)
B (n=16)
C (n=14)
surface

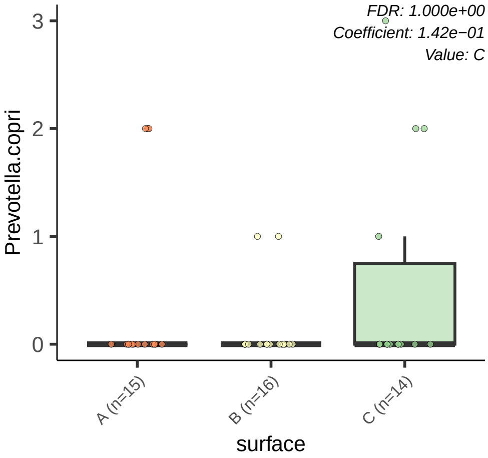
FDR: 1.000e+00
Coefficient: 1.42e−01
Value: C
Prevotella.copri
3
2
1
0
A (n=15)
B (n=16)
C (n=14)
surface

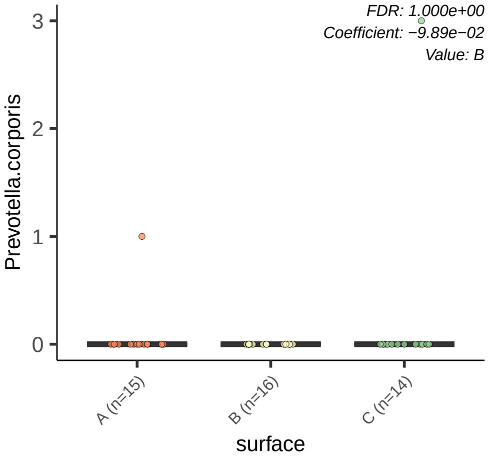

FDR: 1.000e+00
Coefficient: −9.89e−02
Value: B
Prevotella.corporis
0
1
2
3
A (n=15)
B (n=16)
C (n=14)
surface

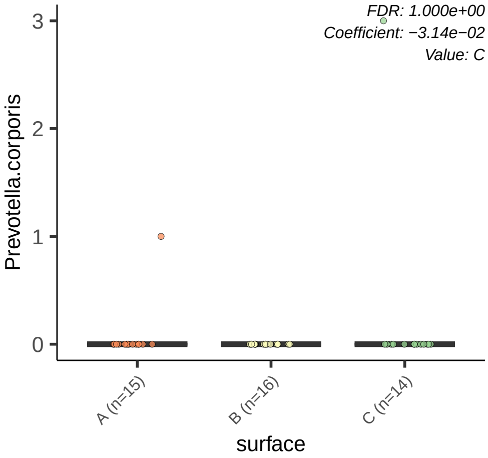
FDR: 1.000e+00
Coefficient: −3.14e−02
Value: C
Prevotella.corporis
3
2
1
0
A (n=15)
B (n=16)
C (n=14)
surface

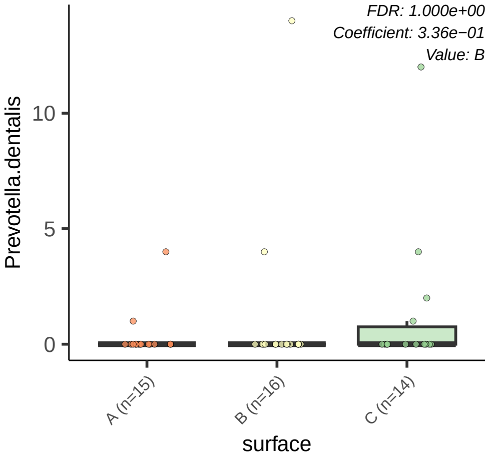
FDR: 1.000e+00
Coefficient: 3.36e−01
Value: B
Prevotella.dentalis
0
5
10
A (n=15)
B (n=16)
C (n=14)
surface

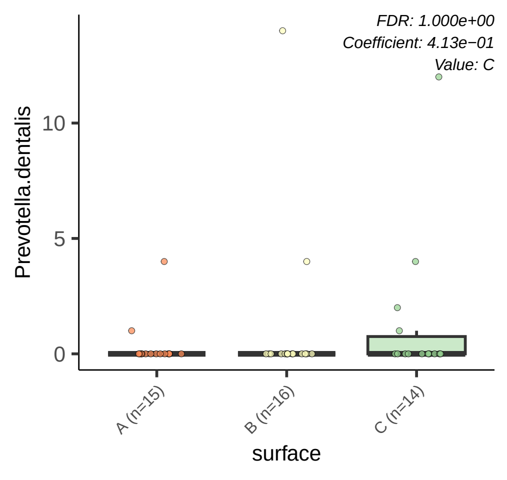
FDR: 1.000e+00
Coefficient: 4.13e−01
Value: C
Prevotella.dentalis
10
5
0
A (n=15)
B (n=16)
C (n=14)
surface

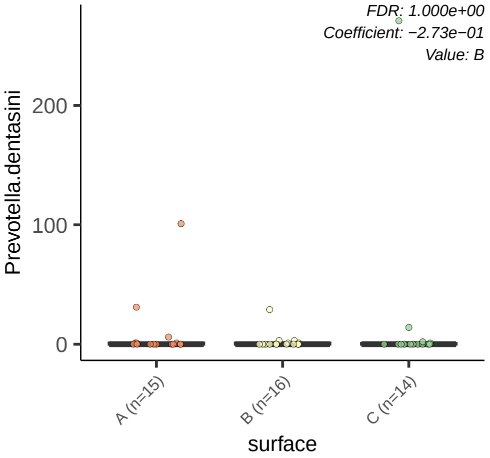

FDR: 1.000e+00
Coefficient: −2.73e−01
Value: B
Prevotella.dentasini
200
100
0
A (n=15)
B (n=16)
C (n=14)
surface

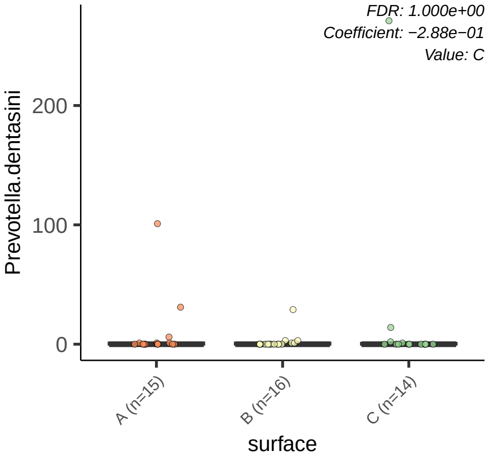
FDR: 1.000e+00
Coefficient: −2.88e−01
Value: C
Prevotella.dentasini
200
100
0
A (n=15)
B (n=16)
C (n=14)
surface

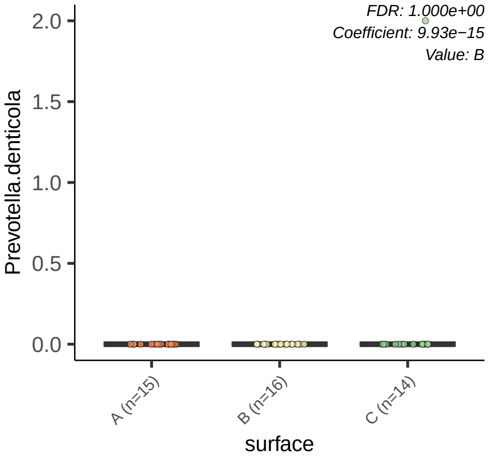

FDR: 1.000e+00
Coefficient: 9.93e−15
Value: B
Prevotella.denticola
2.0
1.5
1.0
0.5
0.0
A (n=15)
B (n=16)
C (n=14)
surface

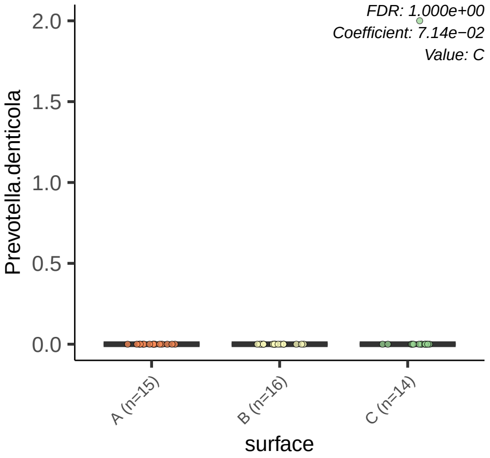
FDR: 1.000e+00
Coefficient: 7.14e−02
Value: C
Prevotella.denticola
2.0
1.5
1.0
0.5
0.0
A (n=15)
B (n=16)
C (n=14)
surface

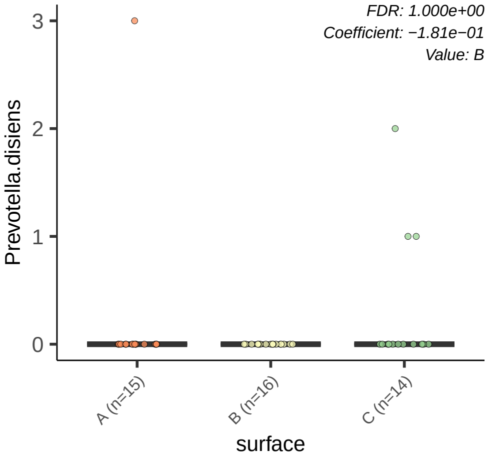

FDR: 1.000e+00
Coefficient: −1.81e−01
Value: B
Prevotella.disiens
3
2
1
0
A (n=15)
B (n=16)
C (n=14)
surface

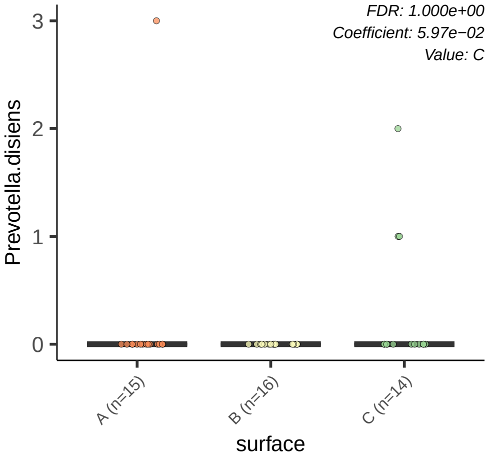

FDR: 1.000e+00
Coefficient: 5.97e−02
Value: C
Prevotella.disiens
0
1
2
3
A (n=15)
B (n=16)
C (n=14)
surface

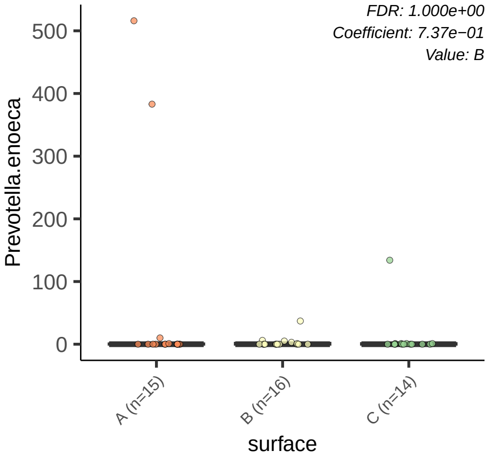

FDR: 1.000e+00
Coefficient: 7.37e−01
Value: B
Prevotella.enoeca
500
400
300
200
100
0
A (n=15)
B (n=16)
C (n=14)
surface

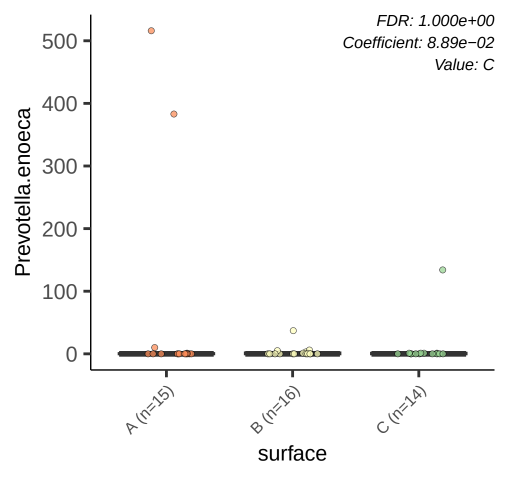
FDR: 1.000e+00
Coefficient: 8.89e−02
Value: C
Prevotella.enoeca
500
400
300
200
100
0
A (n=15)
B (n=16)
C (n=14)
surface

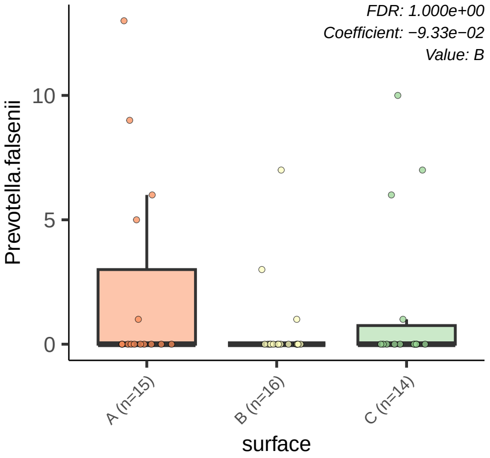

FDR: 1.000e+00
Coefficient: −9.33e−02
Value: B
Prevotella.falsenii
0
5
10
A (n=15)
B (n=16)
C (n=14)
surface

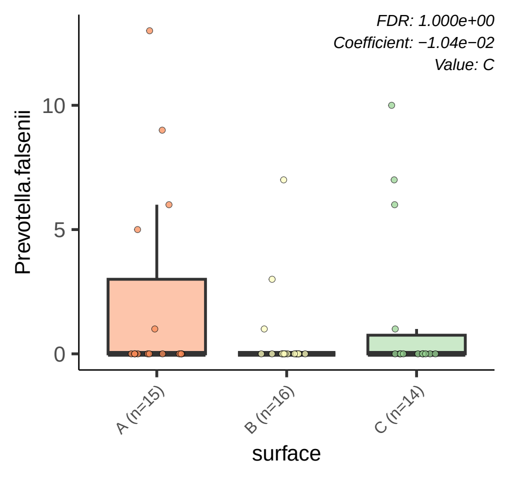
FDR: 1.000e+00
Coefficient: −1.04e−02
Value: C
Prevotella.falsenii
10
5
0
A (n=15)
B (n=16)
C (n=14)
surface

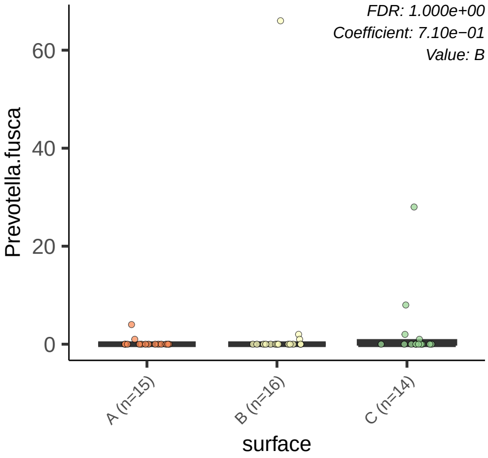

FDR: 1.000e+00
Coefficient: 7.10e−01
Value: B
Prevotella.fusca
60
40
20
0
A (n=15)
B (n=16)
C (n=14)
surface

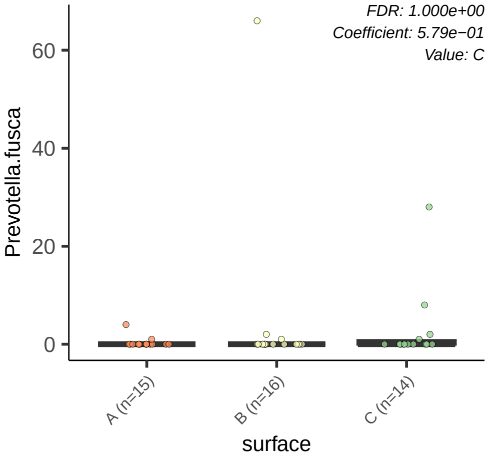

FDR: 1.000e+00
Coefficient: 5.79e−01
Value: C
Prevotella.fusca
60
40
20
0
A (n=15)
B (n=16)
C (n=14)
surface

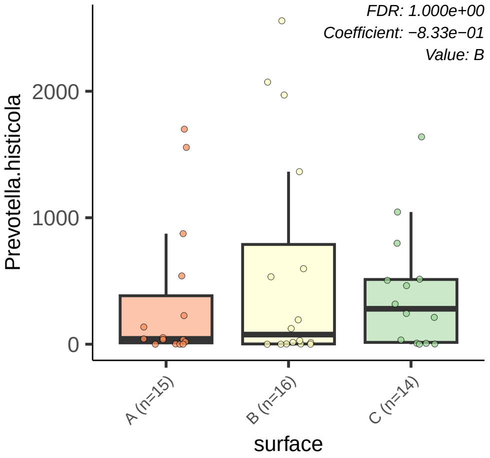
FDR: 1.000e+00
Coefficient: −8.33e−01
Value: B
Prevotella.histicola
2000
1000
0
A (n=15)
B (n=16)
C (n=14)
surface

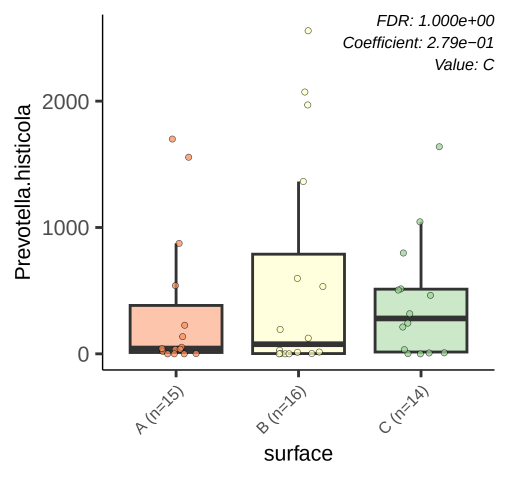

FDR: 1.000e+00
Coefficient: 2.79e−01
Value: C
Prevotella.histicola
2000
1000
0
A (n=15)
B (n=16)
C (n=14)
surface

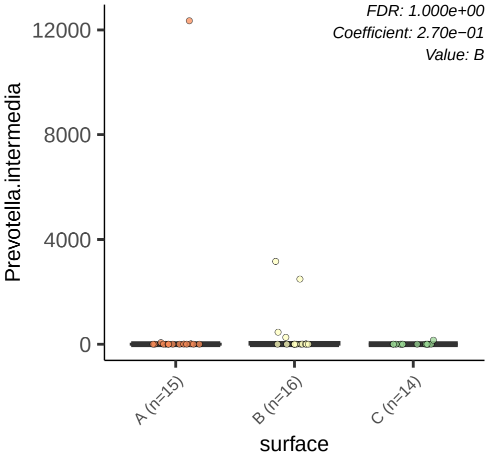
FDR: 1.000e+00
Coefficient: 2.70e−01
Value: B
Prevotella.intermedia
12000
8000
4000
0
A (n=15)
B (n=16)
C (n=14)
surface

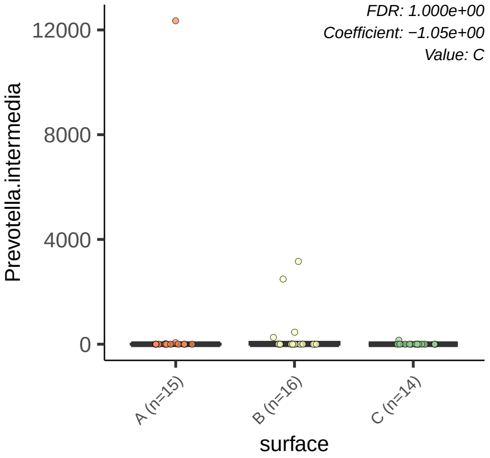

FDR: 1.000e+00
Coefficient: −1.05e+00
Value: C
12000
8000
4000
0
Prevotella.intermedia
A (n=15)
B (n=16)
C (n=14)
surface

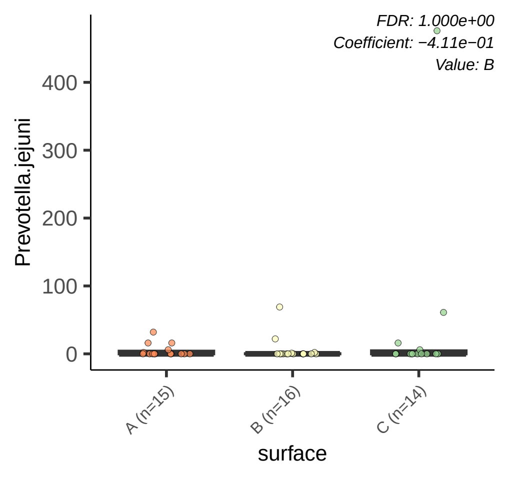
FDR: 1.000e+00
Coefficient: −4.11e−01
Value: B
Prevotella.jejuni
400
300
200
100
0
A (n=15)
B (n=16)
C (n=14)
surface

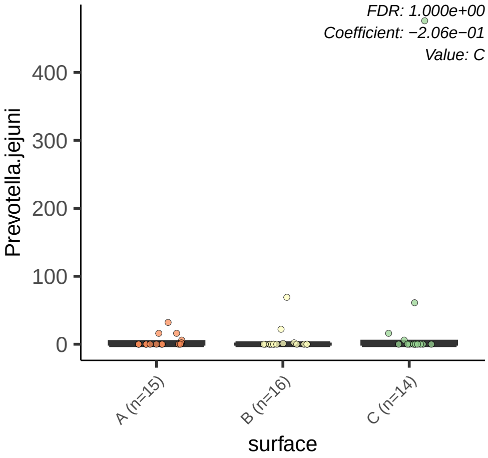
FDR: 1.000e+00
Coefficient: −2.06e−01
Value: C
Prevotella.jejuni
400
300
200
100
0
A (n=15)
B (n=16)
C (n=14)
surface

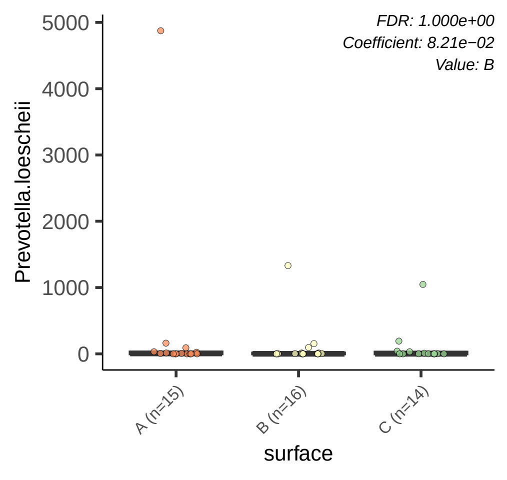
FDR: 1.000e+00
Coefficient: 8.21e−02
Value: B
Prevotella.loescheii
5000
4000
3000
2000
1000
0
A (n=15)
B (n=16)
C (n=14)
surface

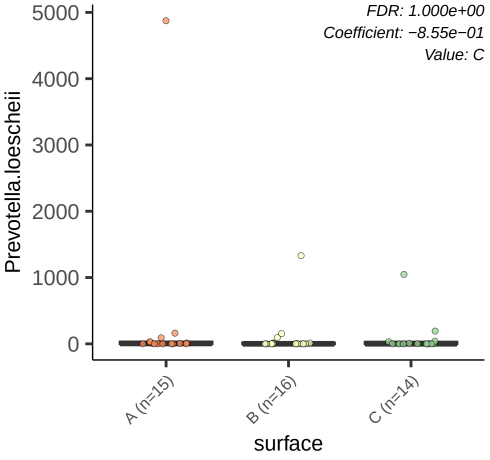
FDR: 1.000e+00
Coefficient: −8.55e−01
Value: C
Prevotella.loescheii
5000
4000
3000
2000
1000
0
A (n=15)
B (n=16)
C (n=14)
surface

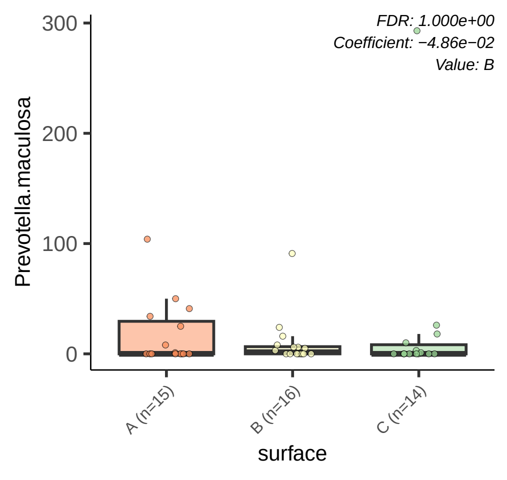
FDR: 1.000e+00
Coefficient: −4.86e−02
Value: B
Prevotella.maculosa
300
200
100
0
A (n=15)
B (n=16)
C (n=14)
surface

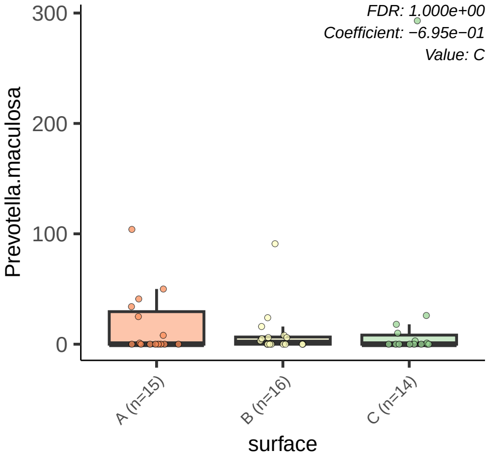

FDR: 1.000e+00
Coefficient: −6.95e−01
Value: C
Prevotella.maculosa
300
200
100
0
A (n=15)
B (n=16)
C (n=14)
surface

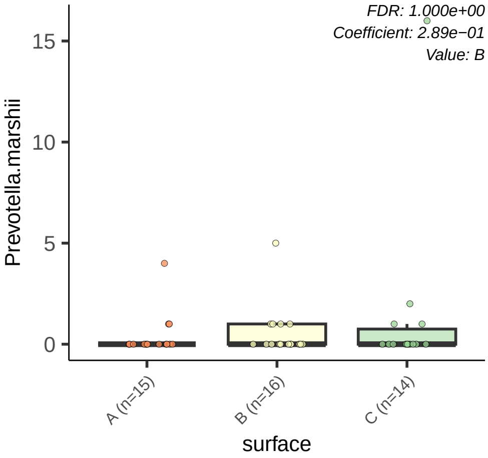

FDR: 1.000e+00
Coefficient: 2.89e−01
Value: B
Prevotella.marshii
0
5
10
15
A (n=15)
B (n=16)
C (n=14)
surface

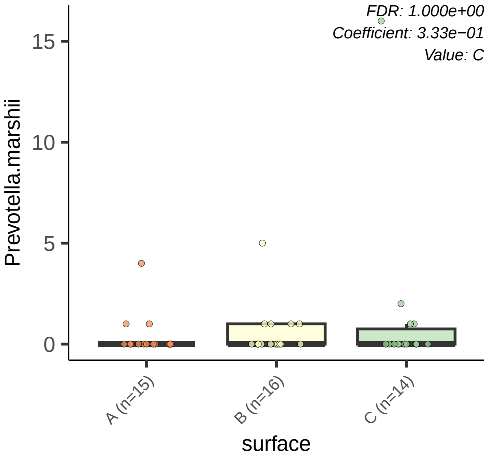

FDR: 1.000e+00
Coefficient: 3.33e−01
Value: C
Prevotella.marshii
15
10
5
0
A (n=15)
B (n=16)
C (n=14)
surface

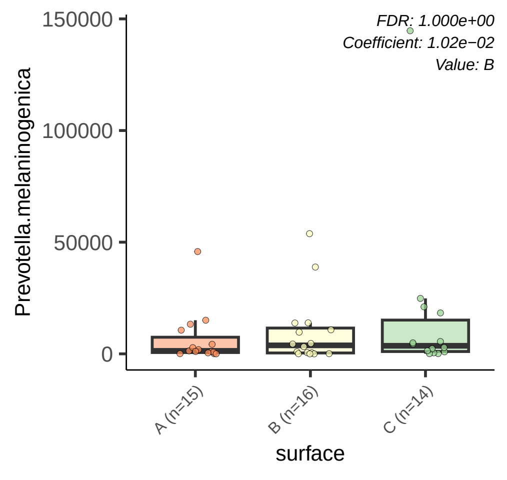
FDR: 1.000e+00
Coefficient: 1.02e−02
Value: B
150000
100000
50000
0
Prevotella.melaninogenica
A (n=15)
B (n=16)
C (n=14)
surface

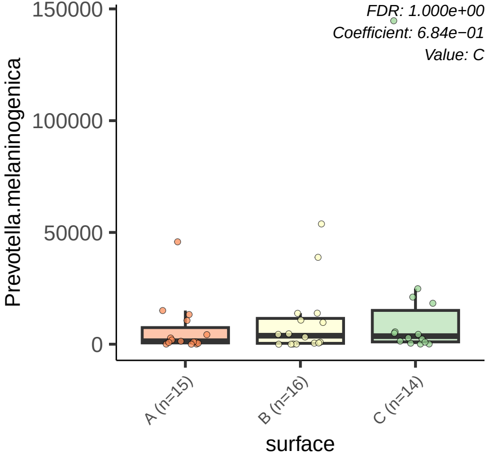
FDR: 1.000e+00
Coefficient: 6.84e−01
Value: C
150000
100000
50000
0
Prevotella.melaninogenica
A (n=15)
B (n=16)
C (n=14)
surface

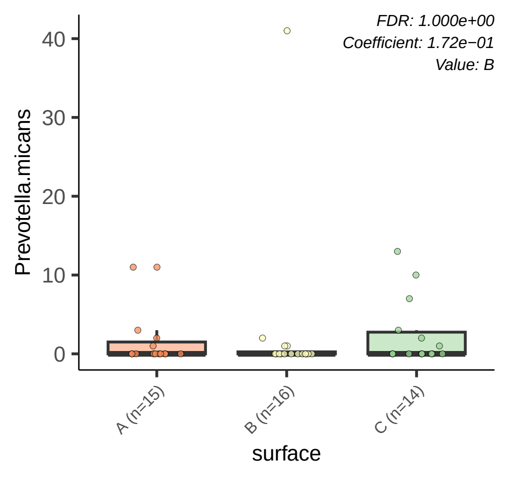
FDR: 1.000e+00
Coefficient: 1.72e−01
Value: B
Prevotella.micans
0
10
20
30
40
A (n=15)
B (n=16)
C (n=14)
surface

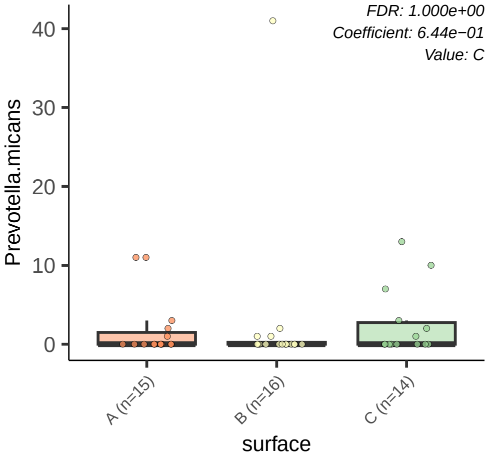
FDR: 1.000e+00
Coefficient: 6.44e−01
Value: C
Prevotella.micans
0
10
20
30
40
A (n=15)
B (n=16)
C (n=14)
surface

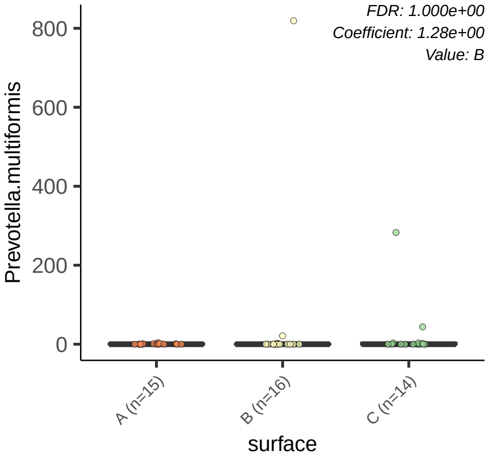

FDR: 1.000e+00
Coefficient: 1.28e+00
Value: B
800
600
400
200
0
Prevotella.multiformis
A (n=15)
B (n=16)
C (n=14)
surface

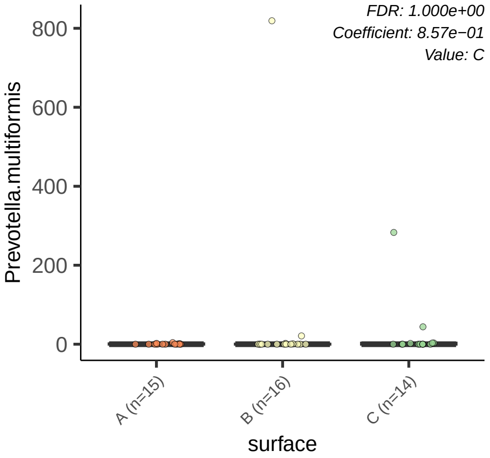
FDR: 1.000e+00
Coefficient: 8.57e−01
Value: C
Prevotella.multiformis
800
600
400
200
0
A (n=15)
B (n=16)
C (n=14)
surface

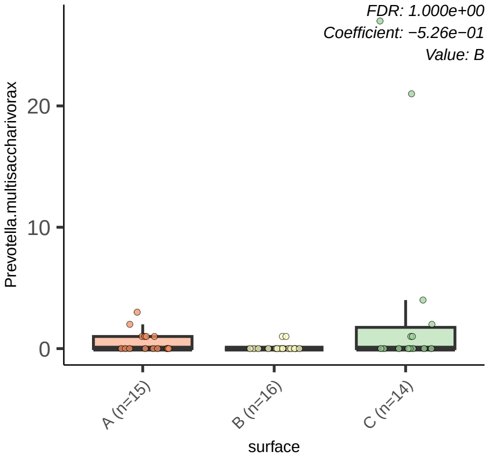
FDR: 1.000e+00
Coefficient: −5.26e−01
Value: B
Prevotella.multisaccharivorax
20
10
0
A (n=15)
B (n=16)
C (n=14)
surface

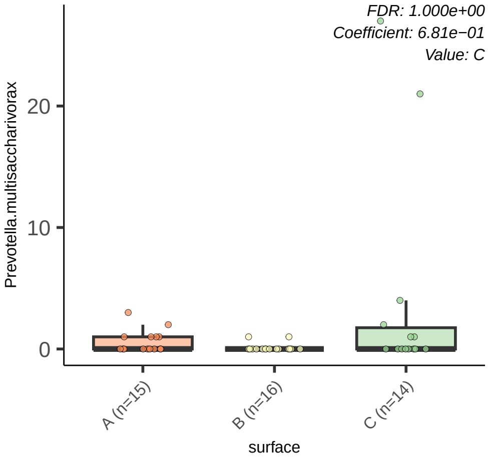
FDR: 1.000e+00
Coefficient: 6.81e−01
Value: C
Prevotella.multisaccharivorax
20
10
0
A (n=15)
B (n=16)
C (n=14)
surface

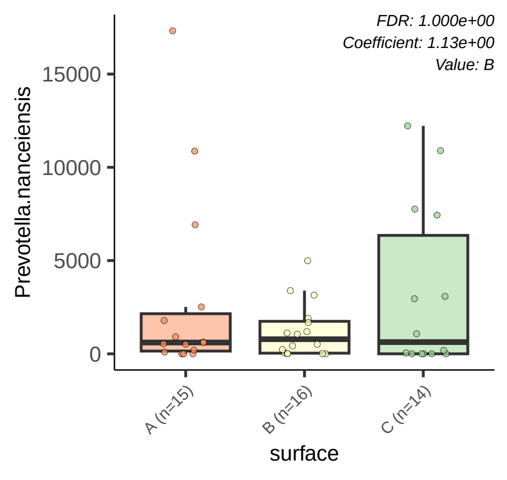

FDR: 1.000e+00
Coefficient: 1.13e+00
Value: B
15000
10000
5000
0
Prevotella.nanceiensis
A (n=15)
B (n=16)
C (n=14)
surface

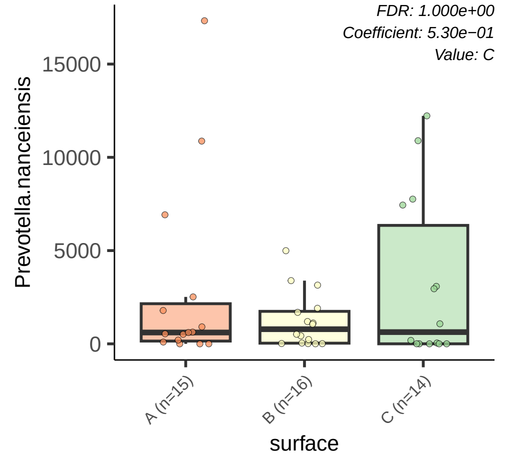
FDR: 1.000e+00
Coefficient: 5.30e−01
Value: C
Prevotella.nanceiensis
15000
10000
5000
0
A (n=15)
B (n=16)
C (n=14)
surface

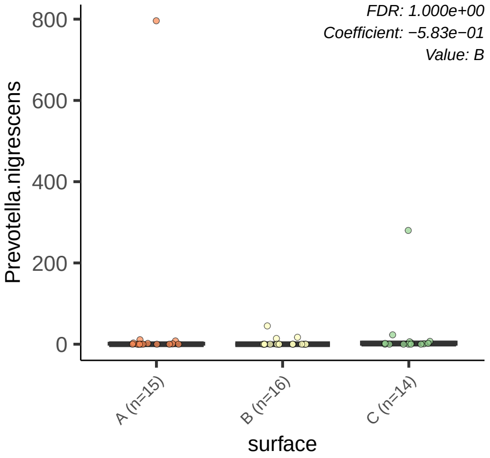

FDR: 1.000e+00
Coefficient: −5.83e−01
Value: B
Prevotella.nigrescens
800
600
400
200
0
A (n=15)
B (n=16)
C (n=14)
surface

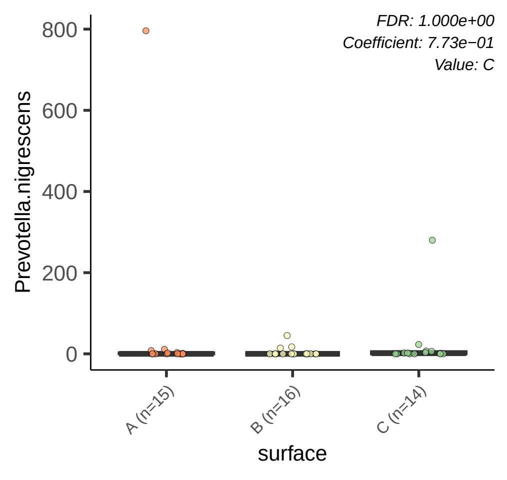

FDR: 1.000e+00
Coefficient: 7.73e−01
Value: C
Prevotella.nigrescens
800
600
400
200
0
A (n=15)
B (n=16)
C (n=14)
surface

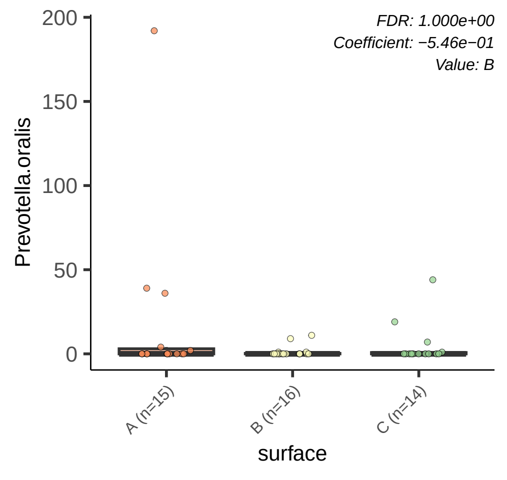

FDR: 1.000e+00
Coefficient: −5.46e−01
Value: B
Prevotella.oralis
200
150
100
50
0
A (n=15)
B (n=16)
C (n=14)
surface

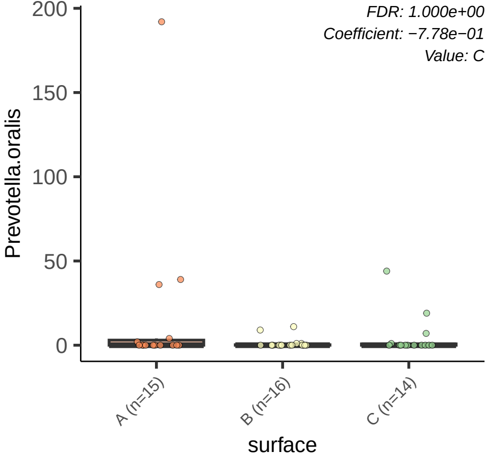

FDR: 1.000e+00
Coefficient: −7.78e−01
Value: C
200
150
100
50
0
Prevotella.oralis
A (n=15)
B (n=16)
C (n=14)
surface

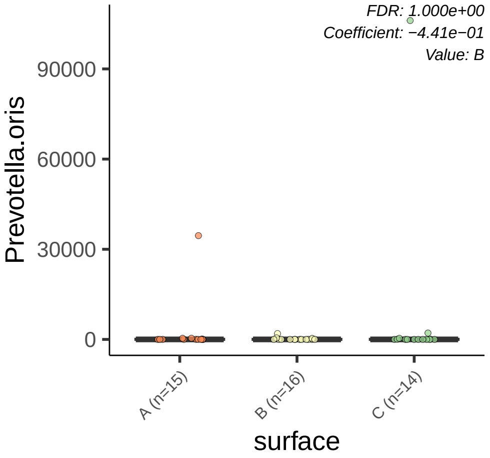

FDR: 1.000e+00
Coefficient: −4.41e−01
Value: B
Prevotella.oris
90000
60000
30000
0
A (n=15)
B (n=16)
C (n=14)
surface

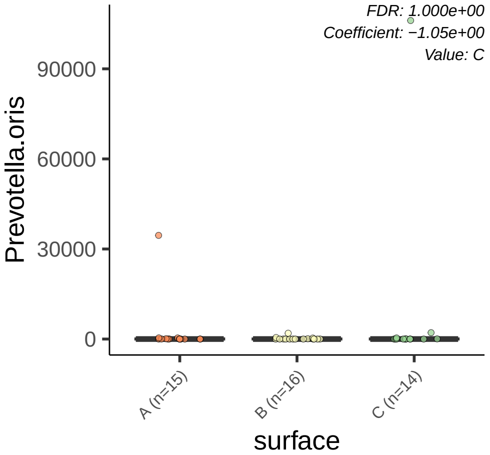

FDR: 1.000e+00
Coefficient: −1.05e+00
Value: C
Prevotella.oris
90000
60000
30000
0
A (n=15)
B (n=16)
C (n=14)
surface

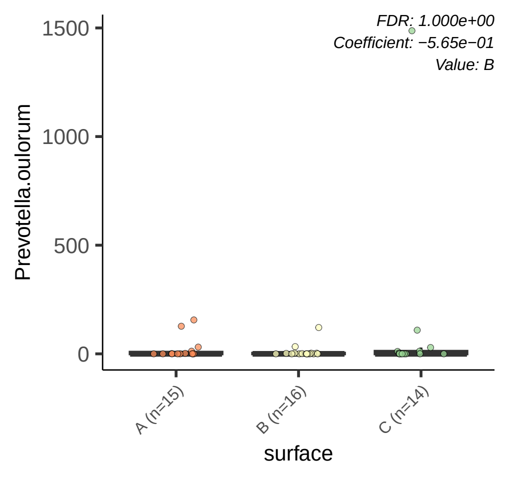
FDR: 1.000e+00
Coefficient: −5.65e−01
Value: B
1500
1000
500
0
Prevotella.oulorum
A (n=15)
B (n=16)
C (n=14)
surface

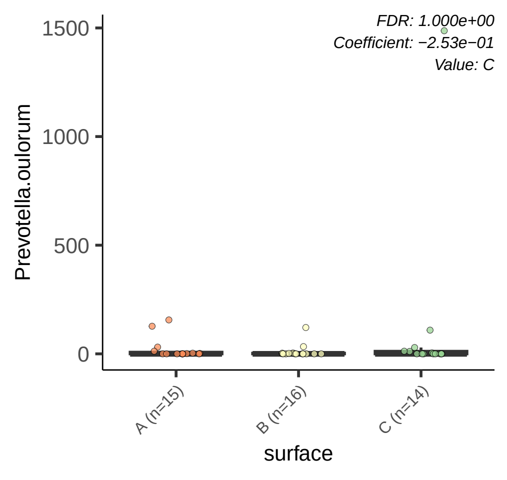

FDR: 1.000e+00
Coefficient: −2.53e−01
Value: C
Prevotella.oulorum
1500
1000
500
0
A (n=15)
B (n=16)
C (n=14)
surface

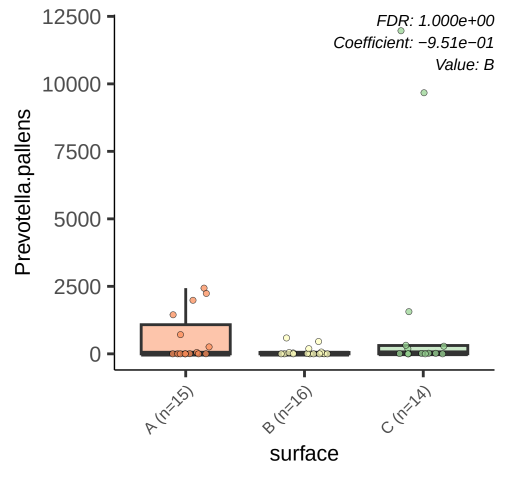

FDR: 1.000e+00
Coefficient: −9.51e−01
Value: B
Prevotella.pallens
12500
10000
7500
5000
2500
0
A (n=15)
B (n=16)
C (n=14)
surface

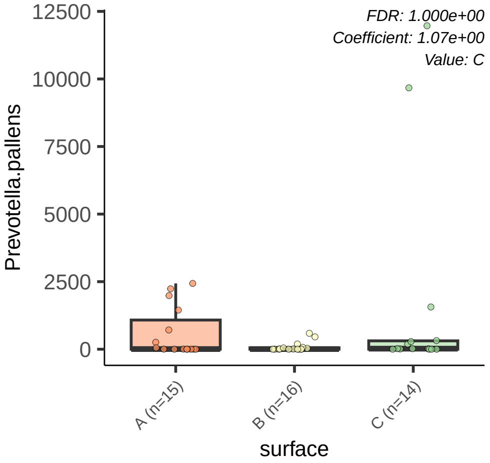

FDR: 1.000e+00
Coefficient: 1.07e+00
Value: C
Prevotella.pallens
12500
10000
7500
5000
2500
0
A (n=15)
B (n=16)
C (n=14)
surface

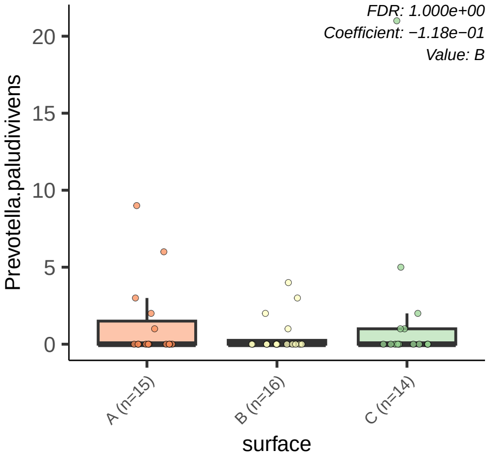

FDR: 1.000e+00
Coefficient: −1.18e−01
Value: B
Prevotella.paludivivens
0
5
10
15
20
A (n=15)
B (n=16)
C (n=14)
surface

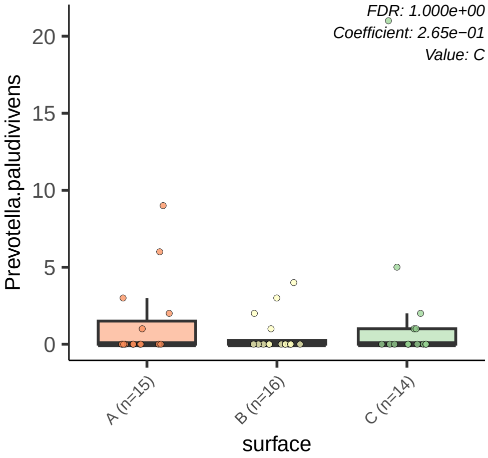
FDR: 1.000e+00
Coefficient: 2.65e−01
Value: C
Prevotella.paludivivens
0
5
10
15
20
A (n=15)
B (n=16)
C (n=14)
surface

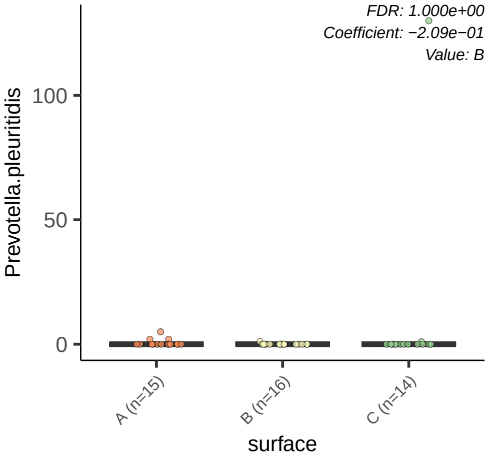

FDR: 1.000e+00
Coefficient: −2.09e−01
Value: B
Prevotella.pleuritidis
100
50
0
A (n=15)
B (n=16)
C (n=14)
surface

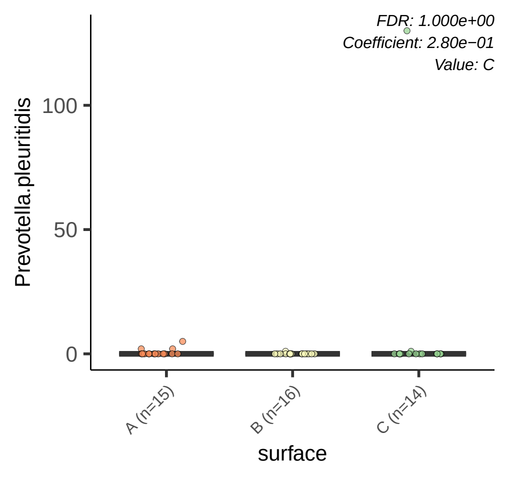
FDR: 1.000e+00
Coefficient: 2.80e−01
Value: C
Prevotella.pleuritidis
100
50
0
A (n=15)
B (n=16)
C (n=14)
surface

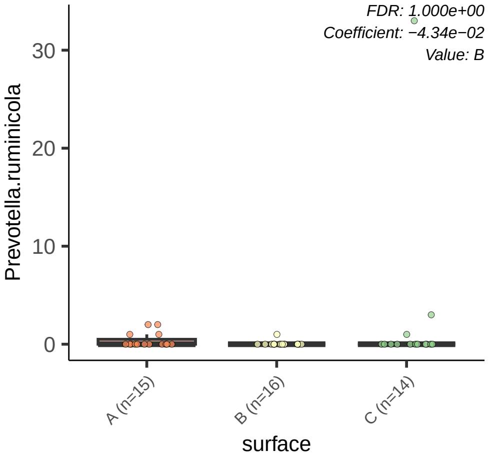
FDR: 1.000e+00
Coefficient: −4.34e−02
Value: B
Prevotella.ruminicola
0
10
20
30
A (n=15)
B (n=16)
C (n=14)
surface

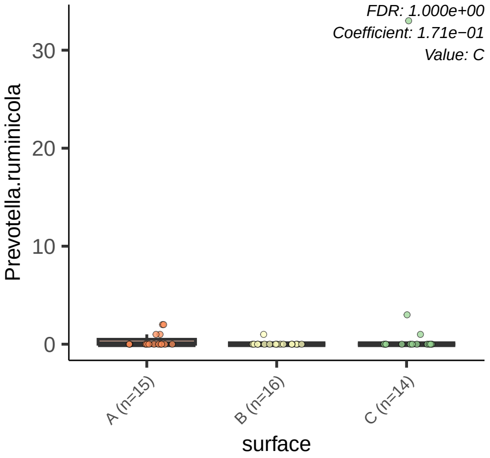
FDR: 1.000e+00
Coefficient: 1.71e−01
Value: C
Prevotella.ruminicola
30
20
10
0
A (n=15)
B (n=16)
C (n=14)
surface

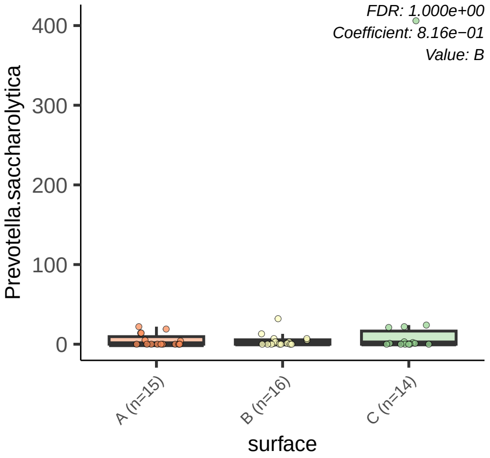

FDR: 1.000e+00
Coefficient: 8.16e−01
Value: B
Prevotella.saccharolytica
400
300
200
100
0
A (n=15)
B (n=16)
C (n=14)
surface

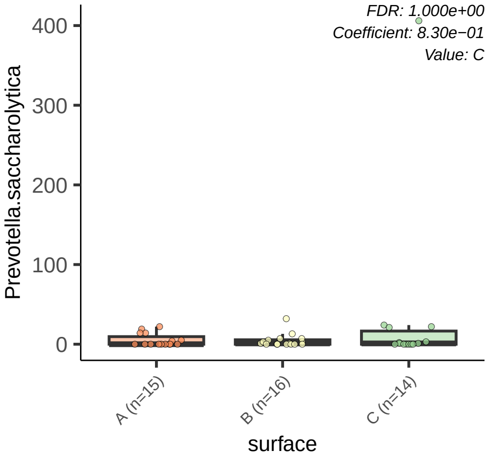
FDR: 1.000e+00
Coefficient: 8.30e−01
Value: C
Prevotella.saccharolytica
400
300
200
100
0
A (n=15)
B (n=16)
C (n=14)
surface

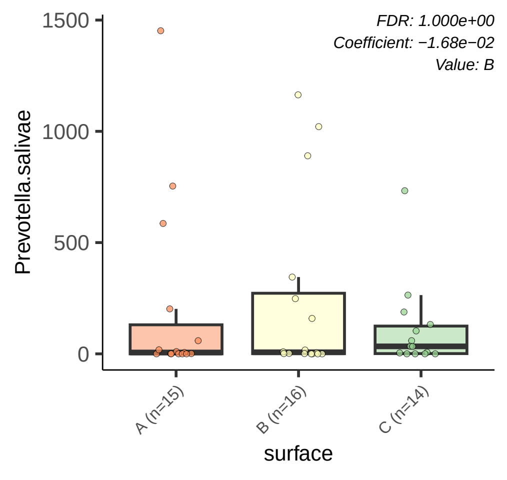
FDR: 1.000e+00
Coefficient: −1.68e−02
Value: B
Prevotella.salivae
1500
1000
500
0
A (n=15)
B (n=16)
C (n=14)
surface

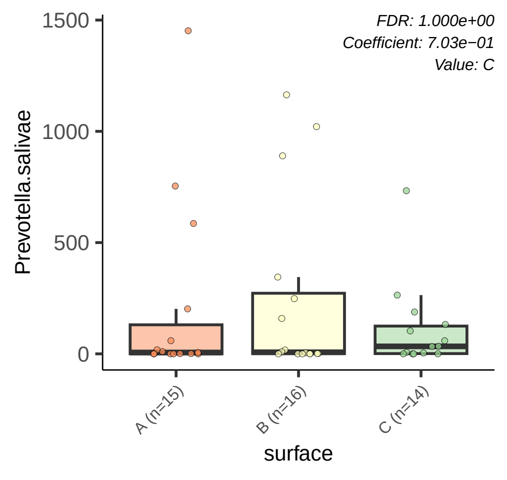

FDR: 1.000e+00
Coefficient: 7.03e−01
Value: C
Prevotella.salivae
1500
1000
500
0
A (n=15)
B (n=16)
C (n=14)
surface

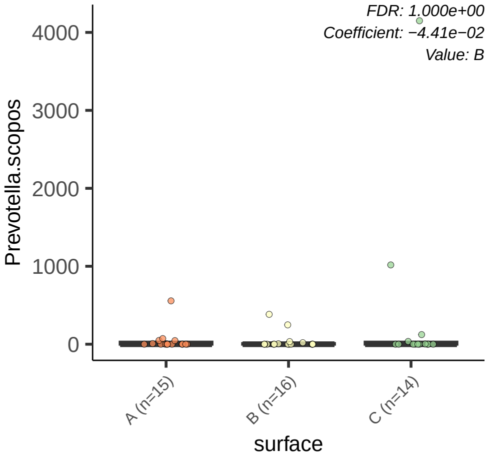

FDR: 1.000e+00
Coefficient: −4.41e−02
Value: B
Prevotella.scopos
4000
3000
2000
1000
0
A (n=15)
B (n=16)
C (n=14)
surface

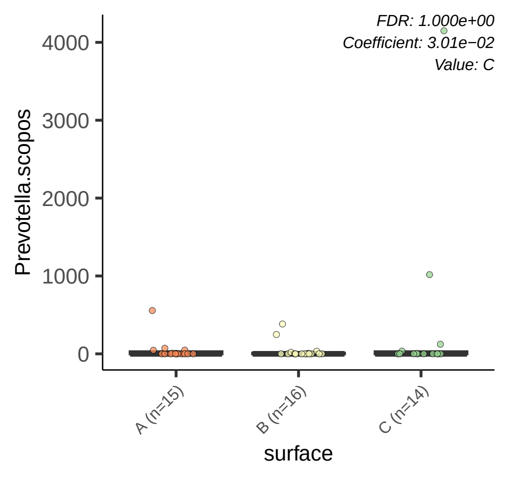

FDR: 1.000e+00
Coefficient: 3.01e−02
Value: C
Prevotella.scopos
4000
3000
2000
1000
0
A (n=15)
B (n=16)
C (n=14)
surface

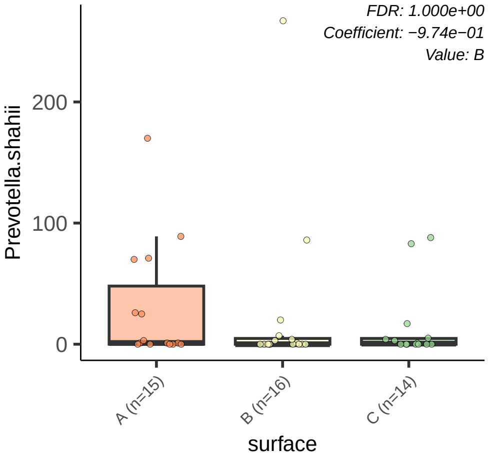

FDR: 1.000e+00
Coefficient: −9.74e−01
Value: B
Prevotella.shahii
200
100
0
A (n=15)
B (n=16)
C (n=14)
surface

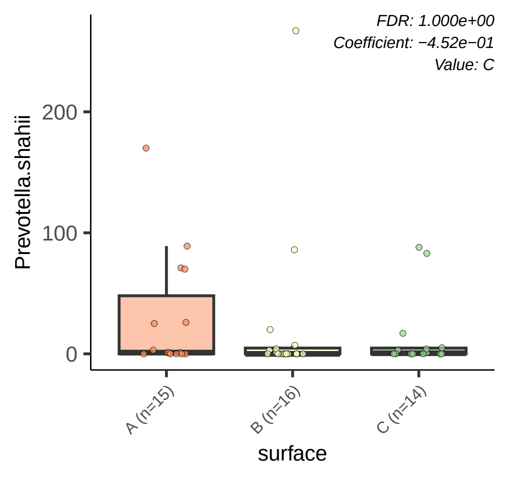
FDR: 1.000e+00
Coefficient: −4.52e−01
Value: C
Prevotella.shahii
200
100
0
A (n=15)
B (n=16)
C (n=14)
surface

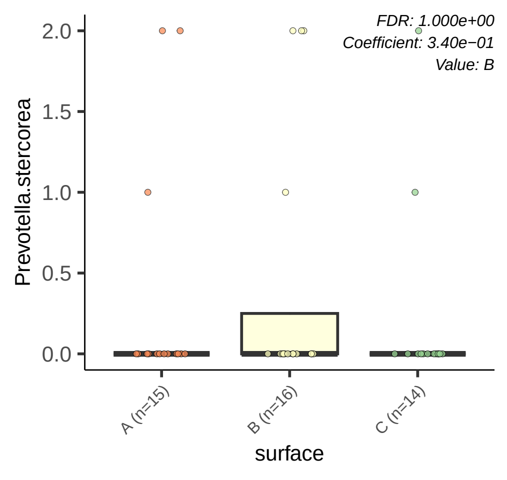
FDR: 1.000e+00
Coefficient: 3.40e−01
Value: B
Prevotella.stercorea
2.0
1.5
1.0
0.5
0.0
A (n=15)
B (n=16)
C (n=14)
surface

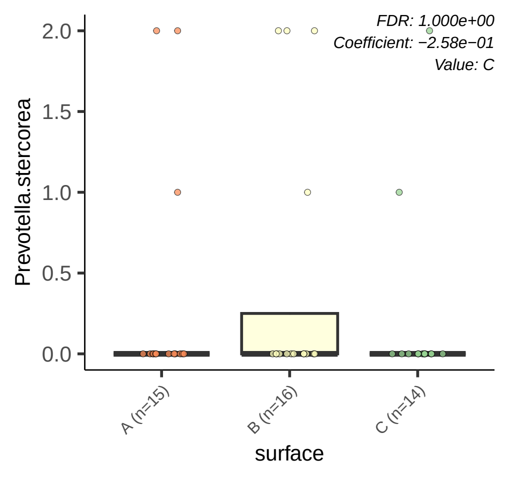

FDR: 1.000e+00
Coefficient: −2.58e−01
Value: C
Prevotella.stercorea
2.0
1.5
1.0
0.5
0.0
A (n=15)
B (n=16)
C (n=14)
surface

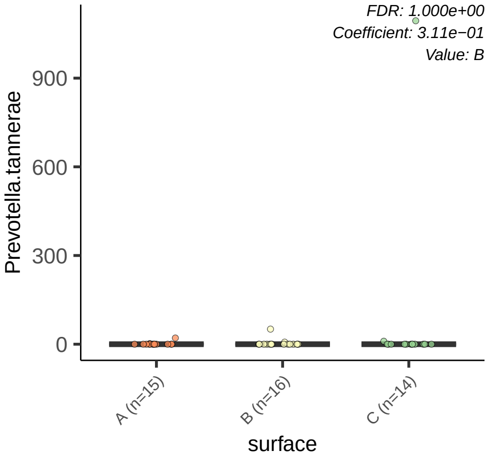
FDR: 1.000e+00
Coefficient: 3.11e−01
Value: B
900
600
300
0
Prevotella.tannerae
A (n=15)
B (n=16)
C (n=14)
surface

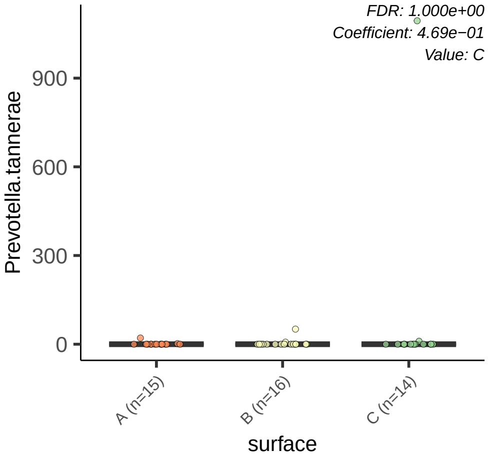
FDR: 1.000e+00
Coefficient: 4.69e−01
Value: C
900
600
300
0
Prevotella.tannerae
A (n=15)
B (n=16)
C (n=14)
surface

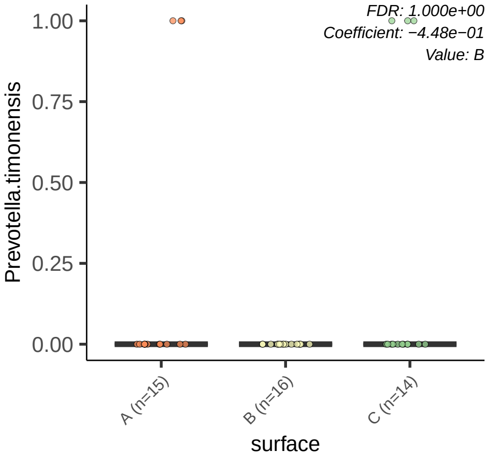
FDR: 1.000e+00
Coefficient: −4.48e−01
Value: B
Prevotella.timonensis
1.00
0.75
0.50
0.25
0.00
A (n=15)
B (n=16)
C (n=14)
surface

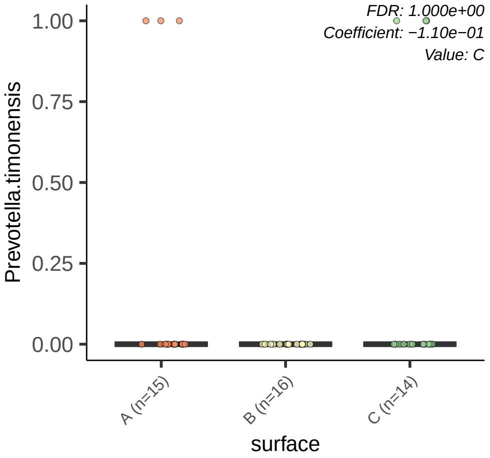

FDR: 1.000e+00
Coefficient: −1.10e−01
Value: C
Prevotella.timonensis
1.00
0.75
0.50
0.25
0.00
A (n=15)
B (n=16)
C (n=14)
surface

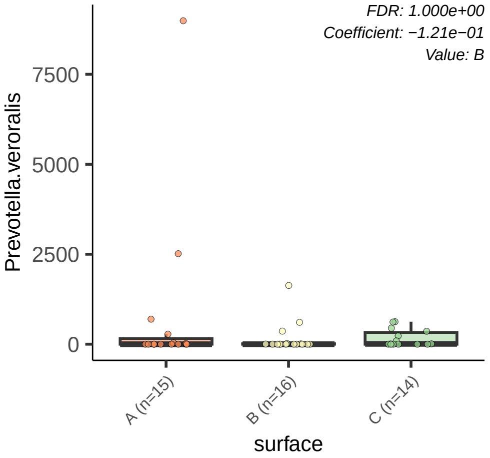

FDR: 1.000e+00
Coefficient: −1.21e−01
Value: B
Prevotella.veroralis
7500
5000
2500
0
A (n=15)
B (n=16)
C (n=14)
surface

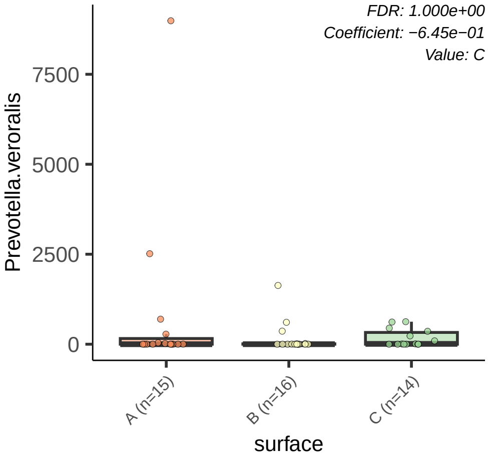
FDR: 1.000e+00
Coefficient: −6.45e−01
Value: C
Prevotella.veroralis
7500
5000
2500
0
A (n=15)
B (n=16)
C (n=14)
surface

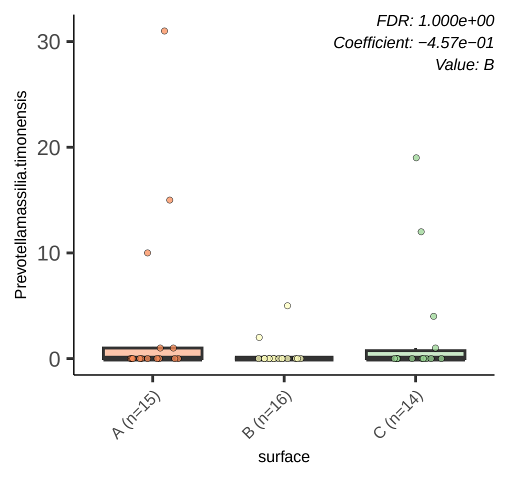

FDR: 1.000e+00
Coefficient: −4.57e−01
Value: B
Prevotellamassilia.timonensis
30
20
10
0
A (n=15)
B (n=16)
C (n=14)
surface

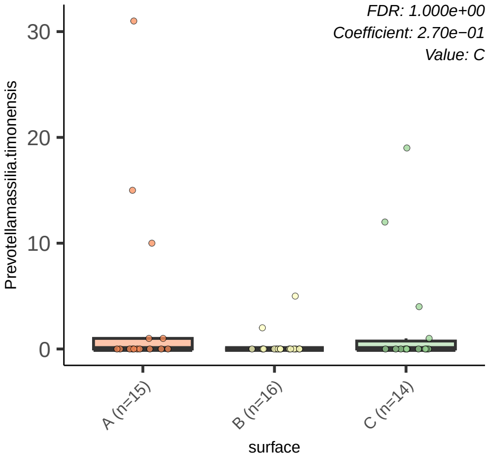

FDR: 1.000e+00
Coefficient: 2.70e−01
Value: C
Prevotellamassilia.timonensis
30
20
10
0
A (n=15)
B (n=16)
C (n=14)
surface

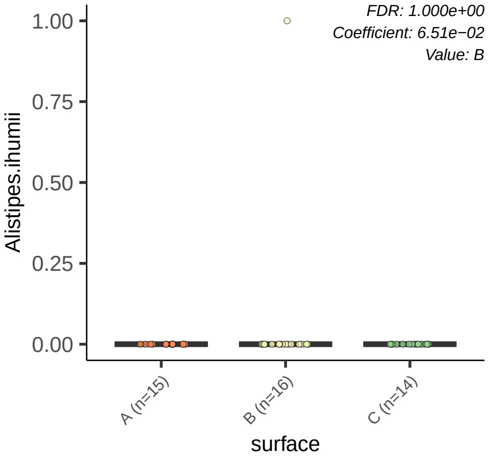

FDR: 1.000e+00
Coefficient: 6.51e−02
Value: B
1.00
0.75
0.50
0.25
0.00
Alistipes.ihumii
A (n=15)
B (n=16)
C (n=14)
surface

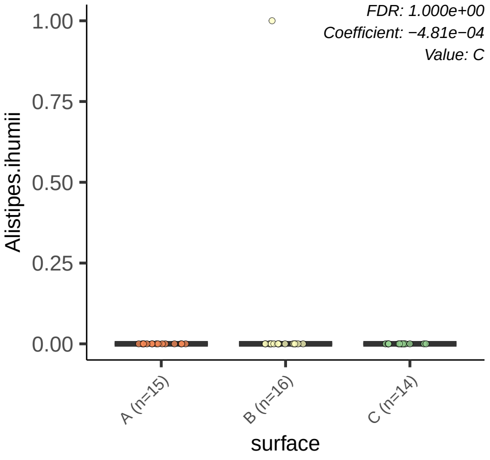
FDR: 1.000e+00
Coefficient: −4.81e−04
Value: C
Alistipes.ihumii
1.00
0.75
0.50
0.25
0.00
A (n=15)
B (n=16)
C (n=14)
surface

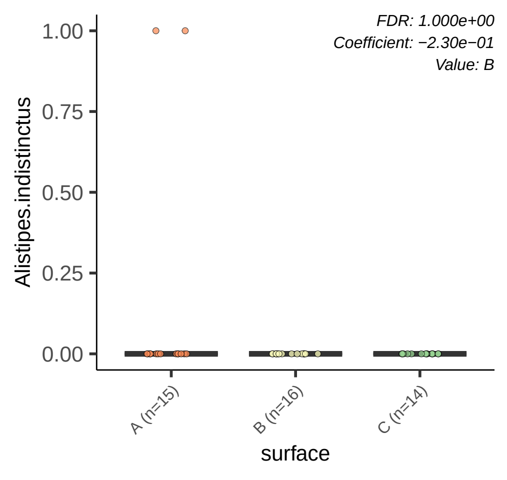
FDR: 1.000e+00
Coefficient: −2.30e−01
Value: B
Alistipes.indistinctus
1.00
0.75
0.50
0.25
0.00
A (n=15)
B (n=16)
C (n=14)
surface

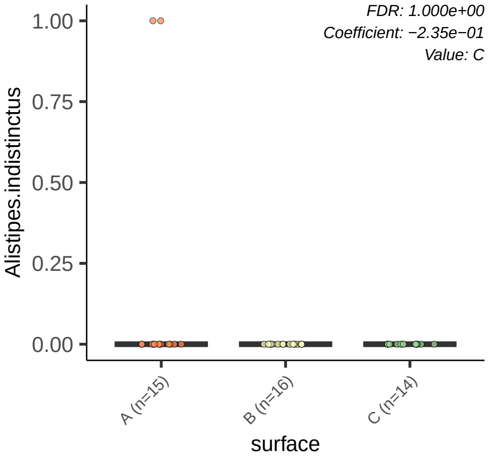
FDR: 1.000e+00
Coefficient: −2.35e−01
Value: C
Alistipes.indistinctus
1.00
0.75
0.50
0.25
0.00
A (n=15)
B (n=16)
C (n=14)
surface

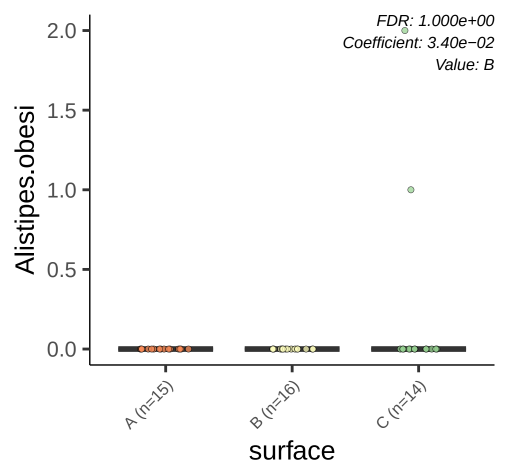
FDR: 1.000e+00
Coefficient: 3.40e−02
Value: B
Alistipes.obesi
2.0
1.5
1.0
0.5
0.0
A (n=15)
B (n=16)
C (n=14)
surface

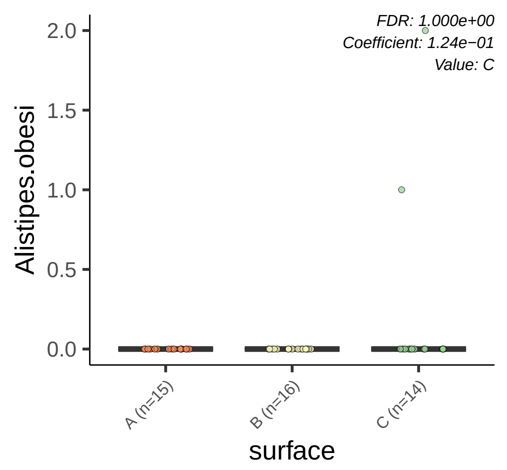
FDR: 1.000e+00
Coefficient: 1.24e−01
Value: C
Alistipes.obesi
2.0
1.5
1.0
0.5
0.0
A (n=15)
B (n=16)
C (n=14)
surface

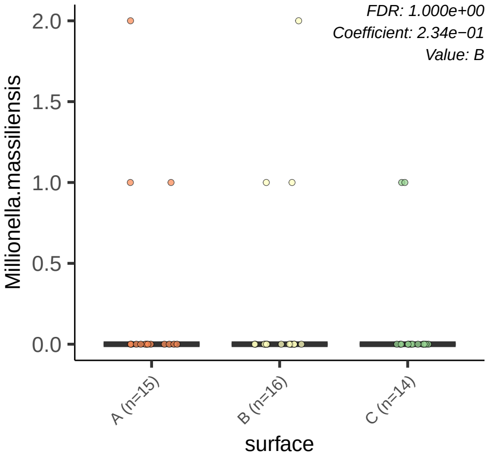

FDR: 1.000e+00
Coefficient: 2.34e−01
Value: B
Millionella.massiliensis
2.0
1.5
1.0
0.5
0.0
A (n=15)
B (n=16)
C (n=14)
surface

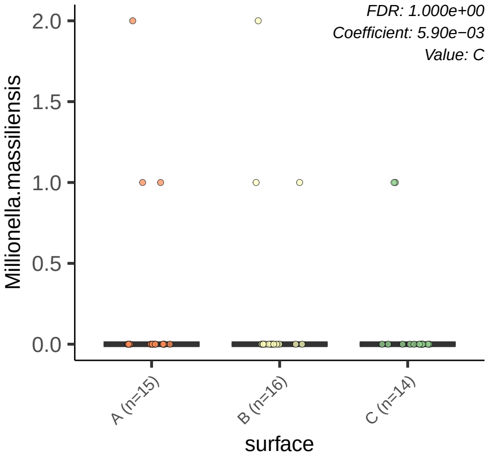
FDR: 1.000e+00
Coefficient: 5.90e−03
Value: C
Millionella.massiliensis
2.0
1.5
1.0
0.5
0.0
A (n=15)
B (n=16)
C (n=14)
surface

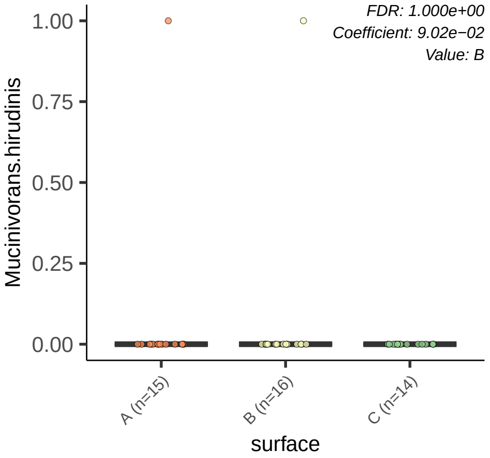

FDR: 1.000e+00
Coefficient: 9.02e−02
Value: B
Mucinivorans.hirudinis
1.00
0.75
0.50
0.25
0.00
A (n=15)
B (n=16)
C (n=14)
surface

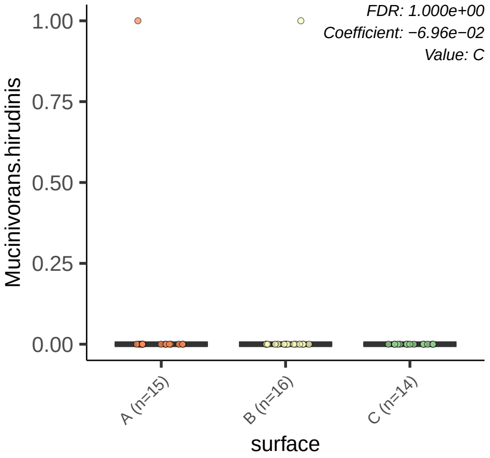
FDR: 1.000e+00
Coefficient: −6.96e−02
Value: C
Mucinivorans.hirudinis
1.00
0.75
0.50
0.25
0.00
A (n=15)
B (n=16)
C (n=14)
surface

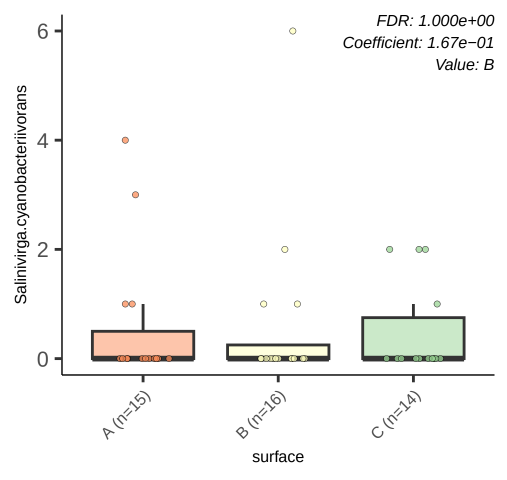
FDR: 1.000e+00
Coefficient: 1.67e−01
Value: B
Saliniviirga.cyanobacteriivorans
0
2
4
6
A (n=15)
B (n=16)
C (n=14)
surface

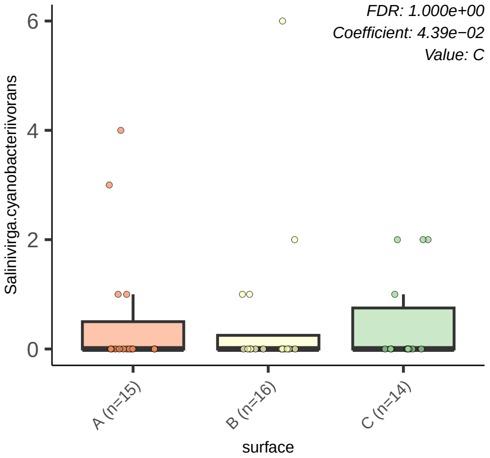

FDR: 1.000e+00
Coefficient: 4.39e−02
Value: C
Saliniviirga.cyanobacteriivorans
6
4
2
0
A (n=15)
B (n=16)
C (n=14)
surface

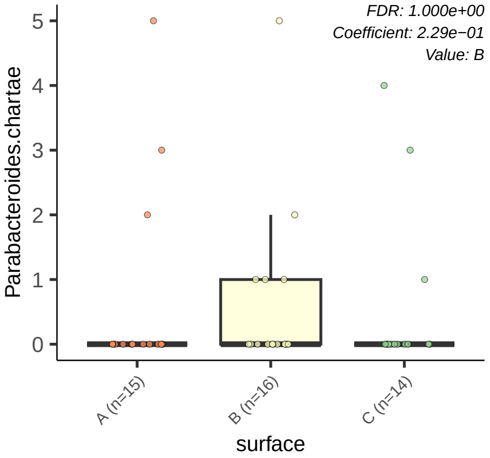

FDR: 1.000e+00
Coefficient: 2.29e−01
Value: B
Parabacteroides.chartae
0
1
2
3
4
5
A (n=15)
B (n=16)
C (n=14)
surface

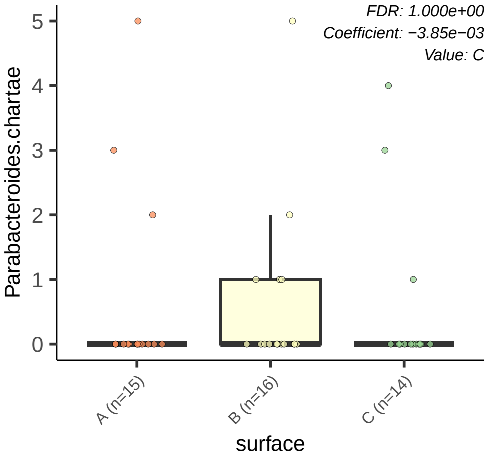
FDR: 1.000e+00
Coefficient: −3.85e−03
Value: C
Parabacteroides.chartae
0
1
2
3
4
5
A (n=15)
B (n=16)
C (n=14)
surface

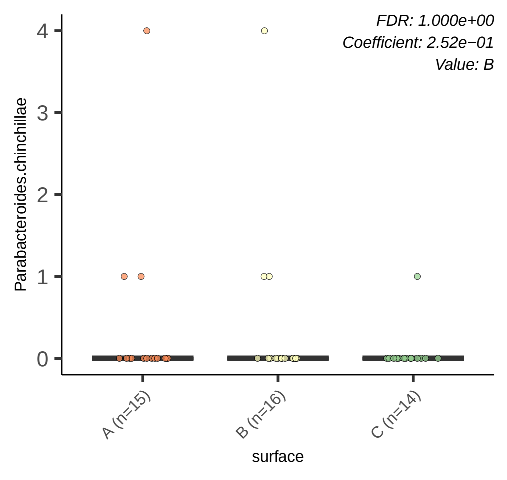
FDR: 1.000e+00
Coefficient: 2.52e−01
Value: B
Parabacteroides.chinchillae
0
1
2
3
4
A (n=15)
B (n=16)
C (n=14)
surface

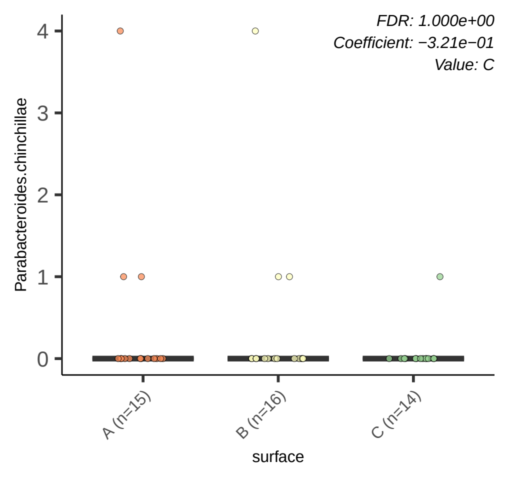

FDR: 1.000e+00
Coefficient: −3.21e−01
Value: C
Parabacteroides.chinchillae
4
3
2
1
0
A (n=15)
B (n=16)
C (n=14)
surface

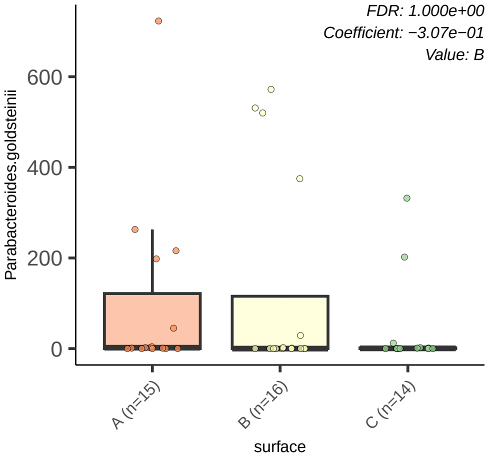
FDR: 1.000e+00
Coefficient: −3.07e−01
Value: B
Parabacteroides.goldsteinii
600
400
200
0
A (n=15)
B (n=16)
C (n=14)
surface

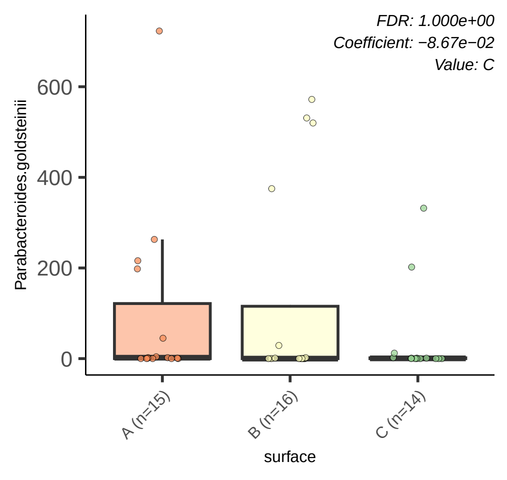
FDR: 1.000e+00
Coefficient: −8.67e−02
Value: C
Parabacteroides.goldsteinii
0
200
400
600
A (n=15)
B (n=16)
C (n=14)
surface

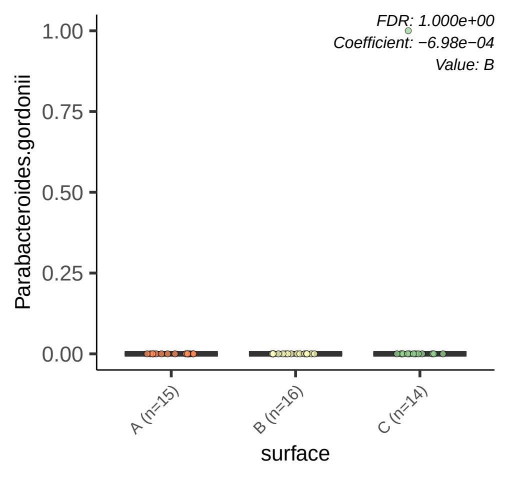

FDR: 1.000e+00
Coefficient: −6.98e−04
Value: B
Parabacteroides.gordonii
1.00
0.75
0.50
0.25
0.00
A (n=15)
B (n=16)
C (n=14)
surface

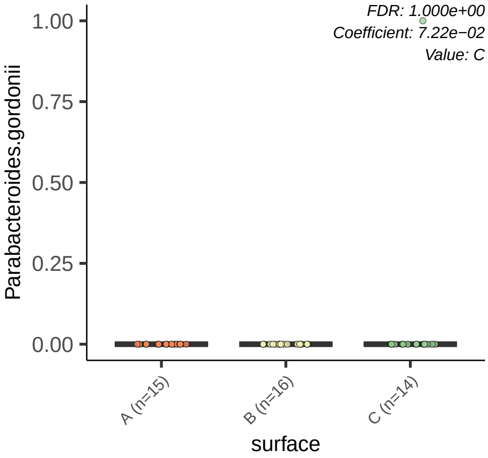
FDR: 1.000e+00
Coefficient: 7.22e−02
Value: C
Parabacteroides.gordonii
1.00
0.75
0.50
0.25
0.00
A (n=15)
B (n=16)
C (n=14)
surface

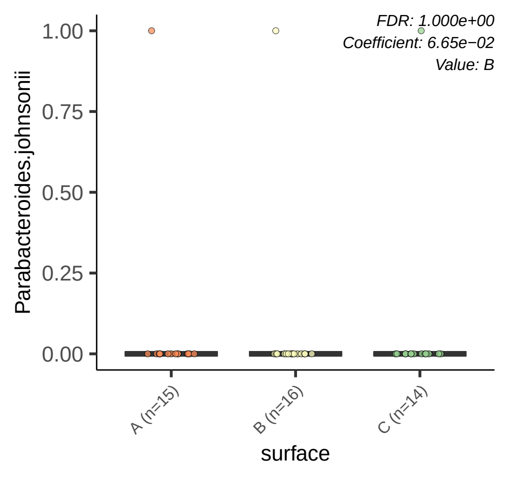

FDR: 1.000e+00
Coefficient: 6.65e−02
Value: B
Parabacteroides.johnsonii
1.00
0.75
0.50
0.25
0.00
A (n=15)
B (n=16)
C (n=14)
surface

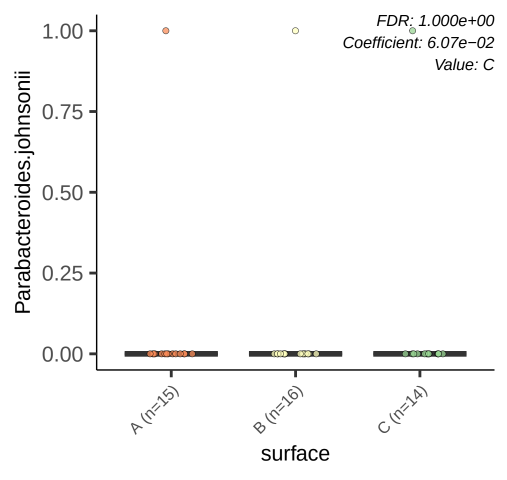
FDR: 1.000e+00
Coefficient: 6.07e−02
Value: C
Parabacteroides.johnsonii
1.00
0.75
0.50
0.25
0.00
A (n=15)
B (n=16)
C (n=14)
surface

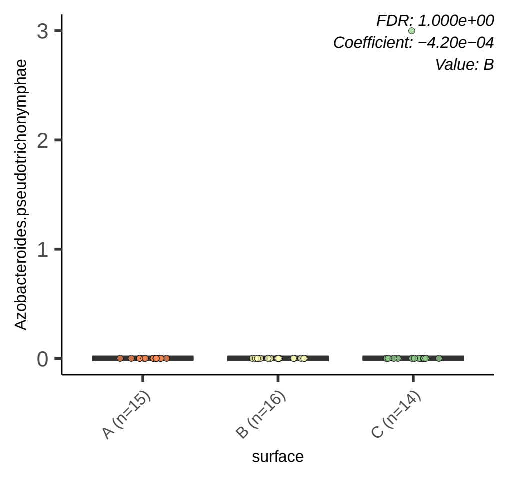
FDR: 1.000e+00
Coefficient: −4.20e−04
Value: B
Azobacteroides.pseudotrichonymphae
3
2
1
0
A (n=15)
B (n=16)
C (n=14)
surface

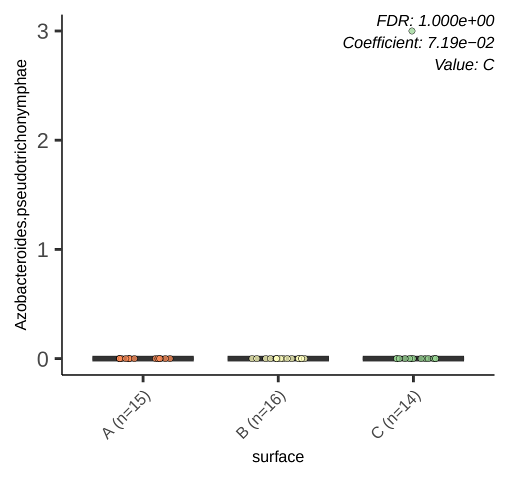
FDR: 1.000e+00
Coefficient: 7.19e−02
Value: C
Azobacteroides.pseudotrichonymphae
3
2
1
0
A (n=15)
B (n=16)
C (n=14)
surface

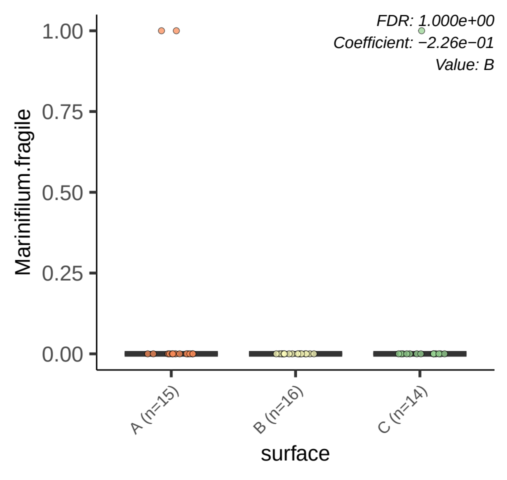
FDR: 1.000e+00
Coefficient: −2.26e−01
Value: B
Marinifilum.fragile
1.00
0.75
0.50
0.25
0.00
A (n=15)
B (n=16)
C (n=14)
surface

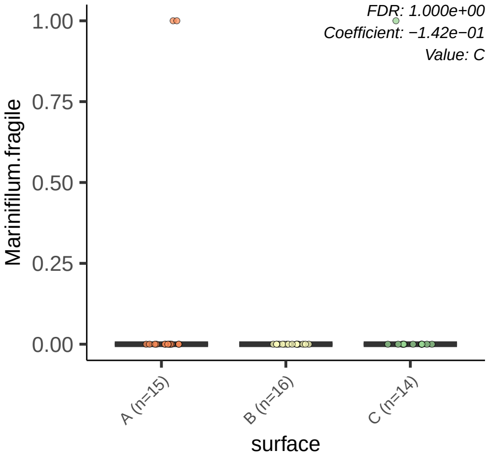

FDR: 1.000e+00
Coefficient: −1.42e−01
Value: C
Marinifilum.fragile
1.00
0.75
0.50
0.25
0.00
A (n=15)
B (n=16)
C (n=14)
surface

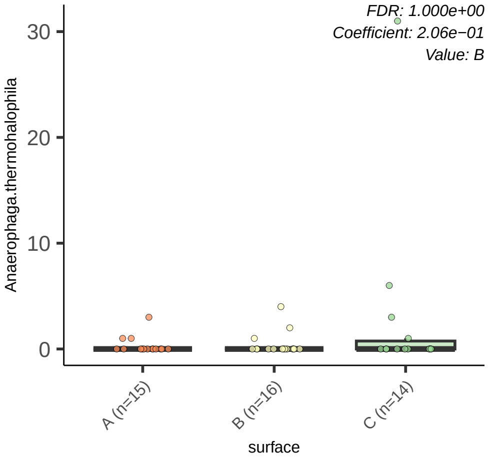
FDR: 1.000e+00
Coefficient: 2.06e−01
Value: B
Anaerophaga.thermohalophila
30
20
10
0
A (n=15)
B (n=16)
C (n=14)
surface

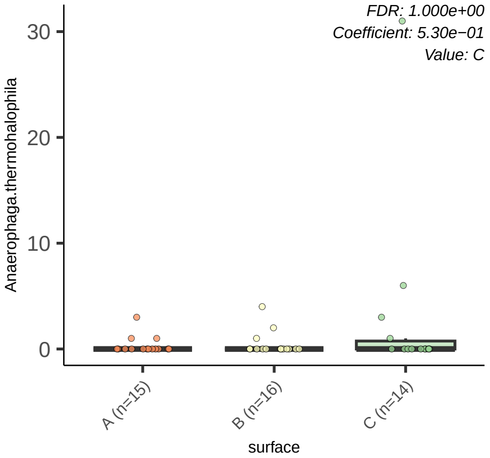

FDR: 1.000e+00
Coefficient: 5.30e−01
Value: C
Anaerophaga.thermohalophila
30
20
10
0
A (n=15)
B (n=16)
C (n=14)
surface

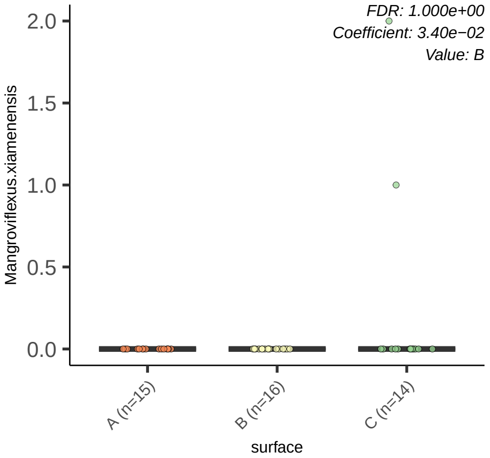

FDR: 1.000e+00
Coefficient: 3.40e−02
Value: B
Mangroviflexus.xiamenensis
2.0
1.5
1.0
0.5
0.0
A (n=15)
B (n=16)
C (n=14)
surface

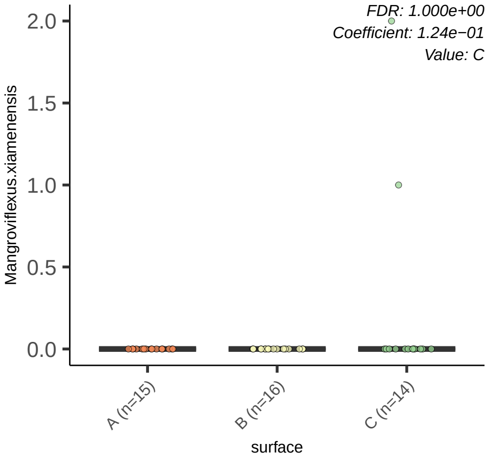
FDR: 1.000e+00
Coefficient: 1.24e−01
Value: C
Mangroviflexus.xiamenensis
2.0
1.5
1.0
0.5
0.0
A (n=15)
B (n=16)
C (n=14)
surface

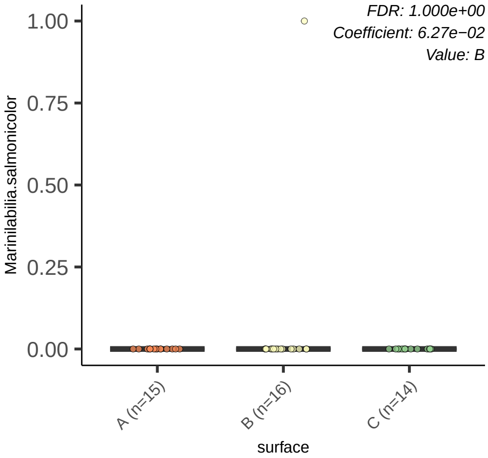

FDR: 1.000e+00
Coefficient: 6.27e−02
Value: B
Marinilabilia.salmonicolor
1.00
0.75
0.50
0.25
0.00
A (n=15)
B (n=16)
C (n=14)
surface

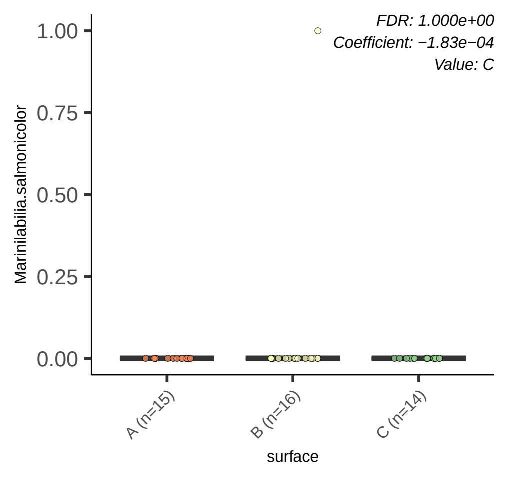
FDR: 1.000e+00
Coefficient: −1.83e−04
Value: C
1.00
0.75
0.50
0.25
0.00
Marinilabilia.salmonicolor
A (n=15)
B (n=16)
C (n=14)
surface

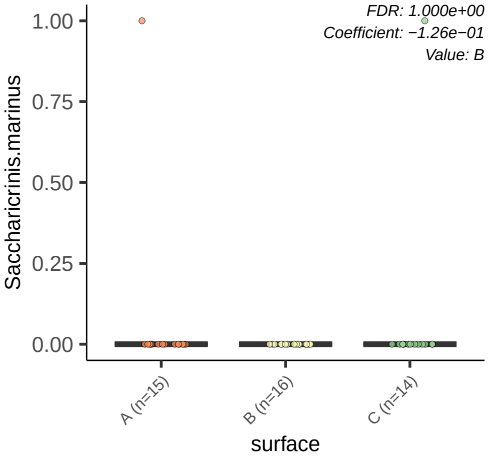
FDR: 1.000e+00
Coefficient: −1.26e−01
Value: B
Saccharicrinis.marinus
1.00
0.75
0.50
0.25
0.00
A (n=15)
B (n=16)
C (n=14)
surface

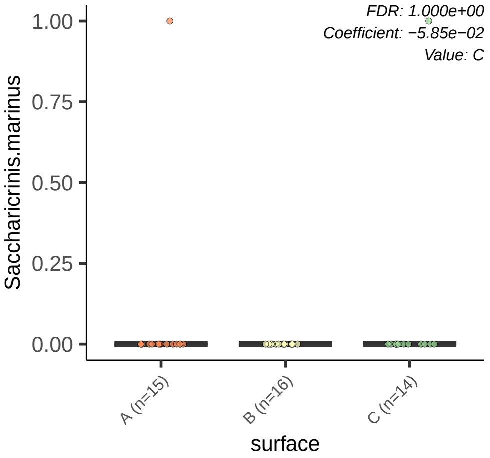
FDR: 1.000e+00
Coefficient: −5.85e−02
Value: C
Saccharicrinis.marinus
1.00
0.75
0.50
0.25
0.00
A (n=15)
B (n=16)
C (n=14)
surface

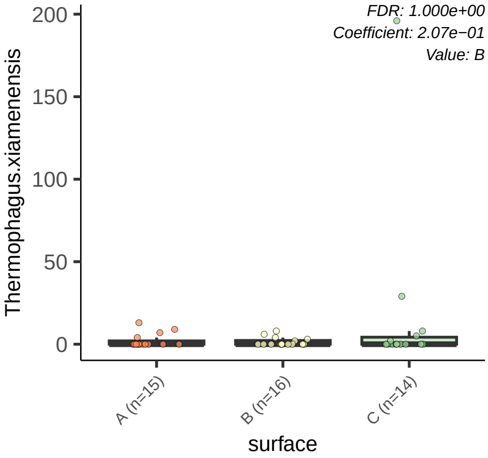

FDR: 1.000e+00
Coefficient: 2.07e−01
Value: B
Thermophagus.xiamenensis
200
150
100
50
0
A (n=15)
B (n=16)
C (n=14)
surface

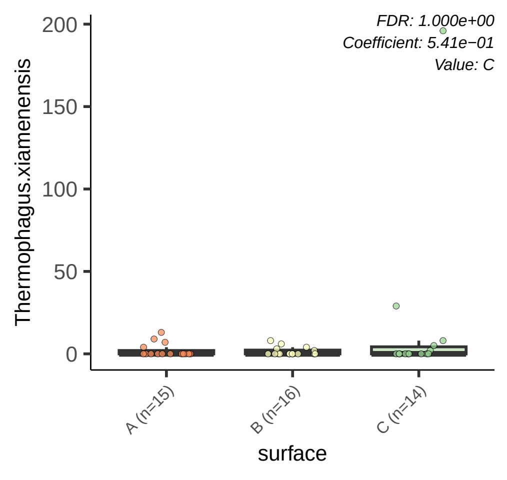
FDR: 1.000e+00
Coefficient: 5.41e−01
Value: C
Thermophagus.xiamenensis
200
150
100
50
0
A (n=15)
B (n=16)
C (n=14)
surface

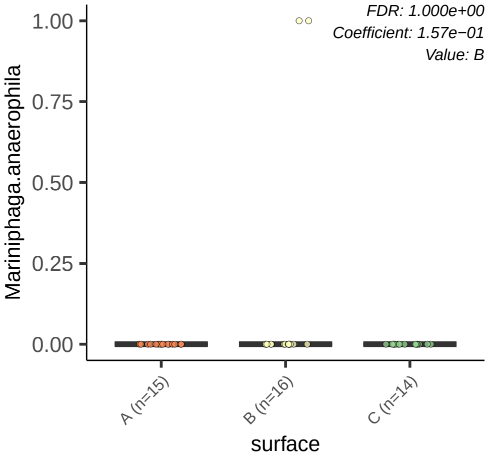
FDR: 1.000e+00
Coefficient: 1.57e−01
Value: B
Mariniphaga.anaerophila
1.00
0.75
0.50
0.25
0.00
A (n=15)
B (n=16)
C (n=14)
surface

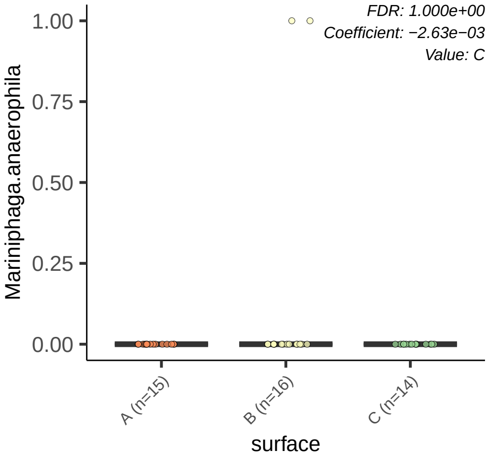
FDR: 1.000e+00
Coefficient: −2.63e−03
Value: C
Mariniphaga.anaerophila
1.00
0.75
0.50
0.25
0.00
A (n=15)
B (n=16)
C (n=14)
surface

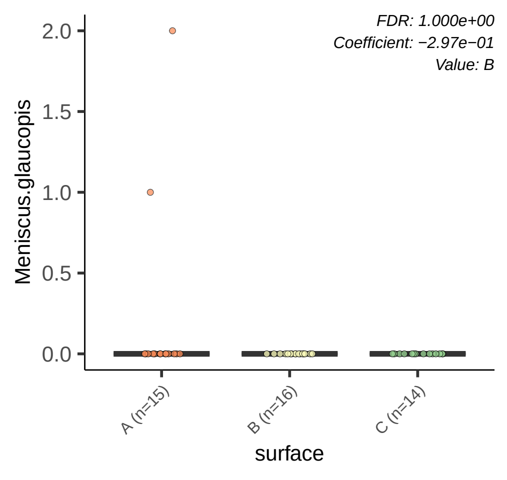

FDR: 1.000e+00
Coefficient: −2.97e−01
Value: B
Meniscus.glaucopis
2.0
1.5
1.0
0.5
0.0
A (n=15)
B (n=16)
C (n=14)
surface

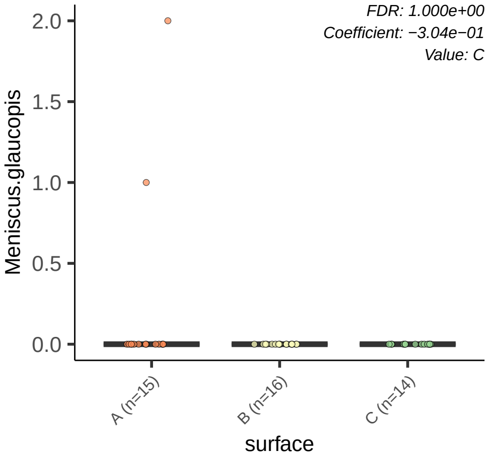

FDR: 1.000e+00
Coefficient: −3.04e−01
Value: C
Meniscus.glaucopis
2.0
1.5
1.0
0.5
0.0
A (n=15)
B (n=16)
C (n=14)
surface

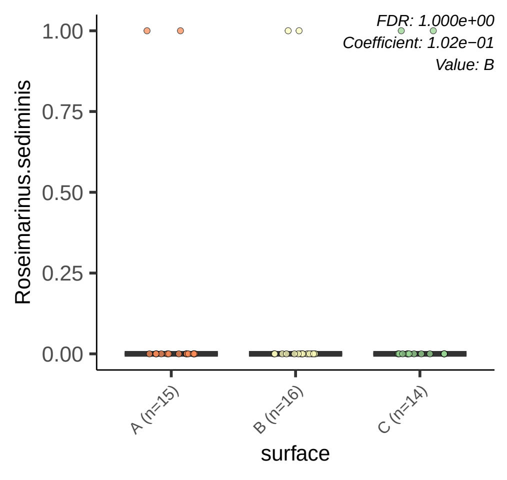
FDR: 1.000e+00
Coefficient: 1.02e−01
Value: B
Roseimarinus.sedimini s
1.00
0.75
0.50
0.25
0.00
A (n=15)
B (n=16)
C (n=14)
surface

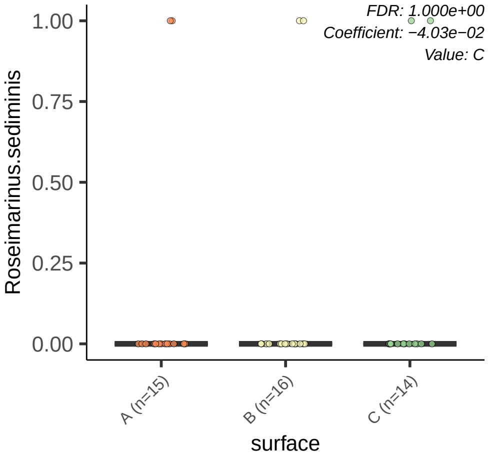

FDR: 1.000e+00
Coefficient: −4.03e−02
Value: C
1.00
0.75
0.50
0.25
0.00
Roseimarinus.sedíminis
A (n=15)
B (n=16)
C (n=14)
surface

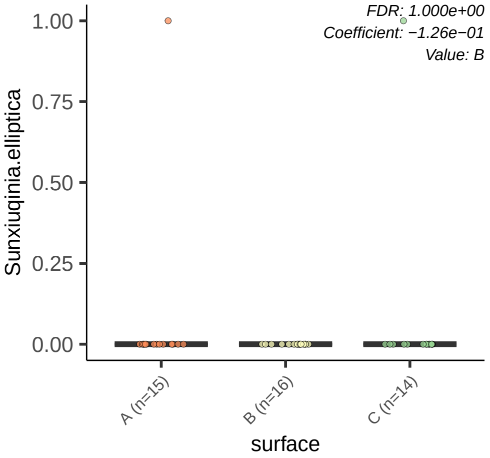
FDR: 1.000e+00
Coefficient: −1.26e−01
Value: B
Sunxiuqinia.elliptica
1.00
0.75
0.50
0.25
0.00
A (n=15)
B (n=16)
C (n=14)
surface

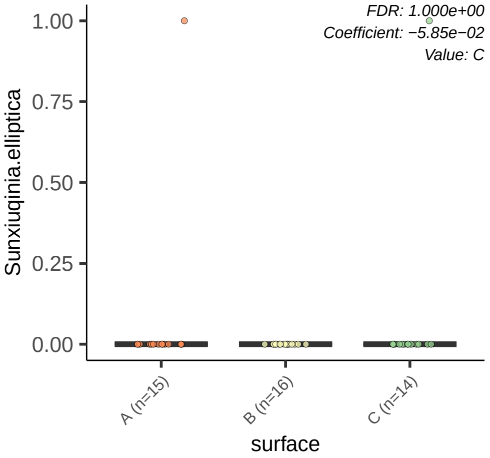
FDR: 1.000e+00
Coefficient: −5.85e−02
Value: C
Sunxiuqinia.elliptica
1.00
0.75
0.50
0.25
0.00
A (n=15)
B (n=16)
C (n=14)
surface

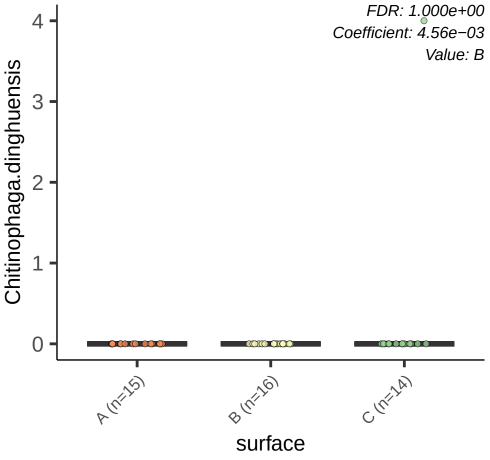
FDR: 1.000e+00
Coefficient: 4.56e−03
Value: B
Chitinophaga.dinghuensis
0
1
2
3
4
A (n=15)
B (n=16)
C (n=14)
surface

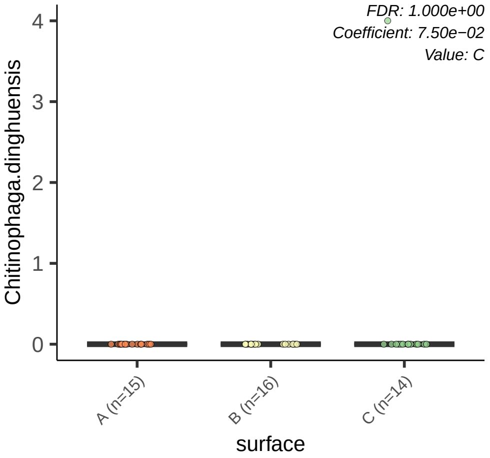

FDR: 1.000e+00
Coefficient: 7.50e−02
Value: C
Chitinophaga.dinghuensis
0
1
2
3
4
A (n=15)
B (n=16)
C (n=14)
surface

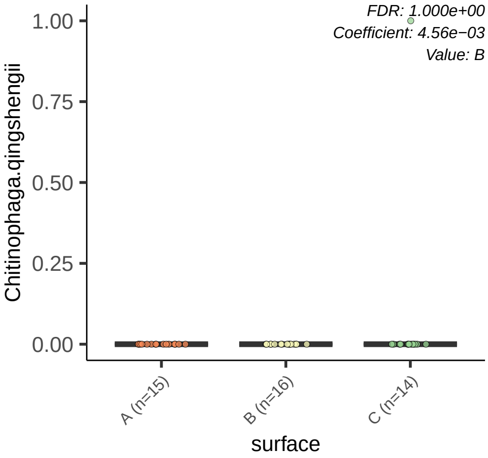
FDR: 1.000e+00
Coefficient: 4.56e−03
Value: B
Chitinophaga.qingshengii
1.00
0.75
0.50
0.25
0.00
A (n=15)
B (n=16)
C (n=14)
surface

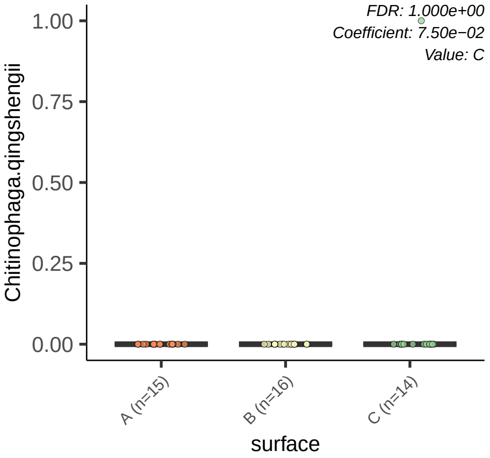
FDR: 1.000e+00
Coefficient: 7.50e−02
Value: C
Chitinophaga.qingshengii
1.00
0.75
0.50
0.25
0.00
A (n=15)
B (n=16)
C (n=14)
surface

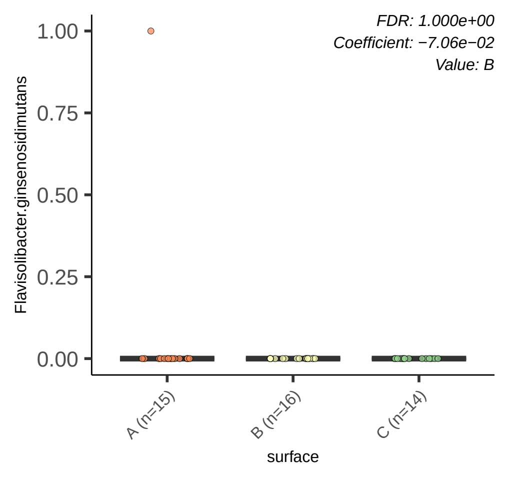
FDR: 1.000e+00
Coefficient: −7.06e−02
Value: B
1.00
0.75
0.50
0.25
0.00
Flavisolibacter.ginsenosidimutans
A (n=15)
B (n=16)
C (n=14)
surface

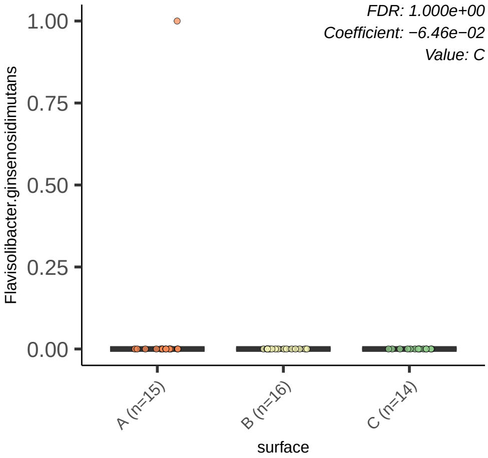

FDR: 1.000e+00
Coefficient: −6.46e−02
Value: C
1.00
0.75
0.50
0.25
0.00
Flavisolibacter.ginsenosidimutans
A (n=15)
B (n=16)
C (n=14)
surface

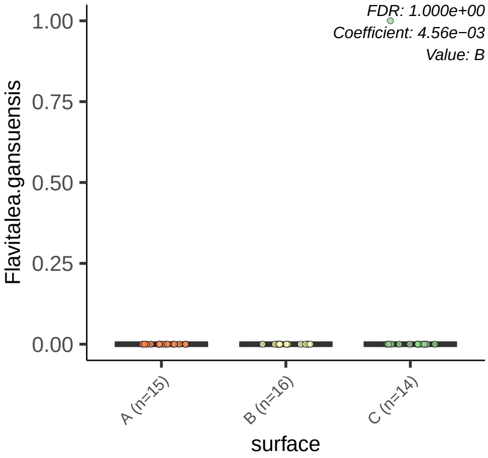
FDR: 1.000e+00
Coefficient: 4.56e−03
Value: B
Flavitalea.gansuensis
1.00
0.75
0.50
0.25
0.00
A (n=15)
B (n=16)
C (n=14)
surface

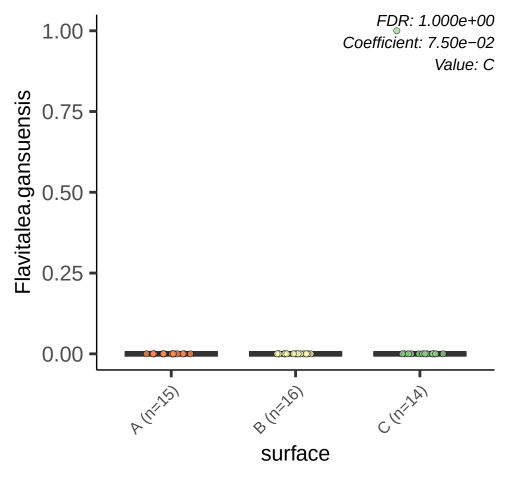
FDR: 1.000e+00
Coefficient: 7.50e−02
Value: C
1.00
0.75
0.50
0.25
0.00
Flavitalea.gansuensis
A (n=15)
B (n=16)
C (n=14)
surface

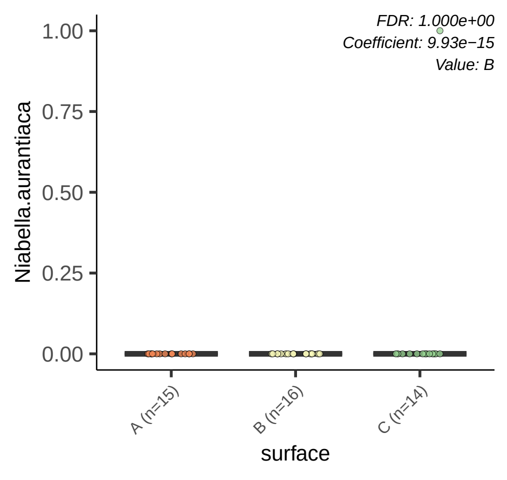
FDR: 1.000e+00
Coefficient: 9.93e−15
Value: B
Niabella.aurantiaca
1.00
0.75
0.50
0.25
0.00
A (n=15)
B (n=16)
C (n=14)
surface

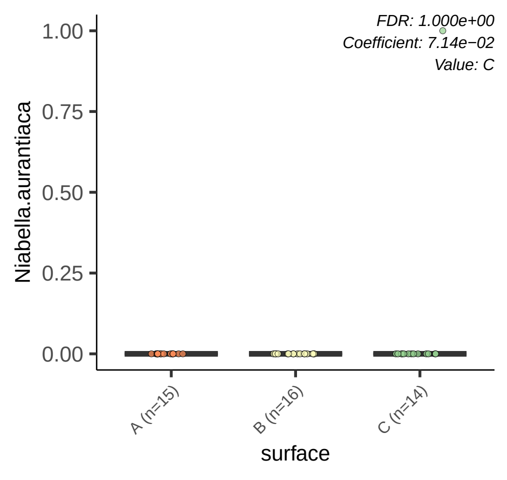
FDR: 1.000e+00
Coefficient: 7.14e−02
Value: C
Niabella.aurantiaca
1.00
0.75
0.50
0.25
0.00
A (n=15)
B (n=16)
C (n=14)
surface

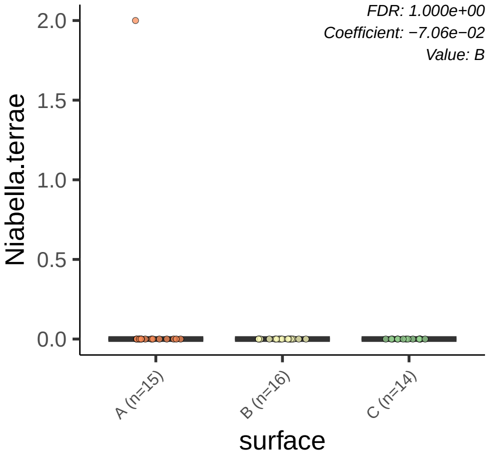

FDR: 1.000e+00
Coefficient: −7.06e−02
Value: B
Niabella.terrae
2.0
1.5
1.0
0.5
0.0
A (n=15)
B (n=16)
C (n=14)
surface

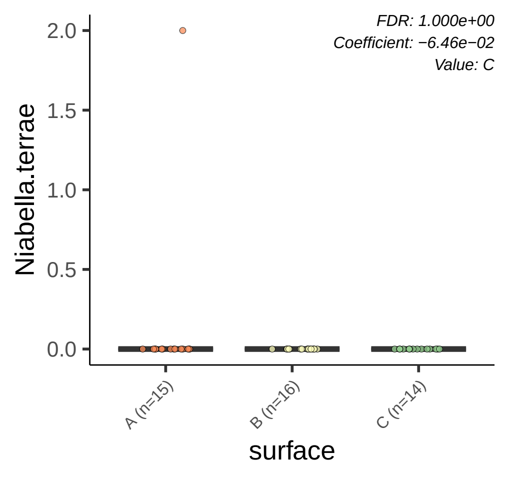
FDR: 1.000e+00
Coefficient: −6.46e−02
Value: C
Niabella.terrae
2.0
1.5
1.0
0.5
0.0
A (n=15)
B (n=16)
C (n=14)
surface

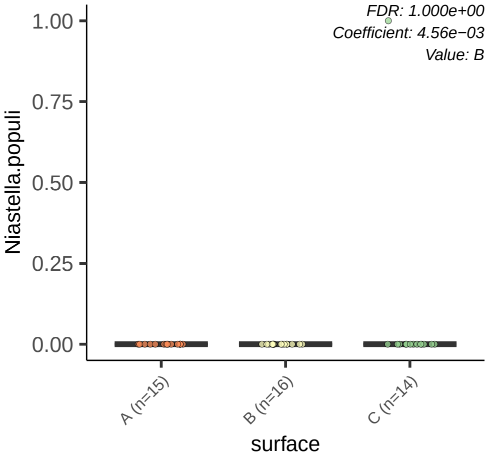

FDR: 1.000e+00
Coefficient: 4.56e−03
Value: B
Niastella.populi
1.00
0.75
0.50
0.25
0.00
A (n=15)
B (n=16)
C (n=14)
surface

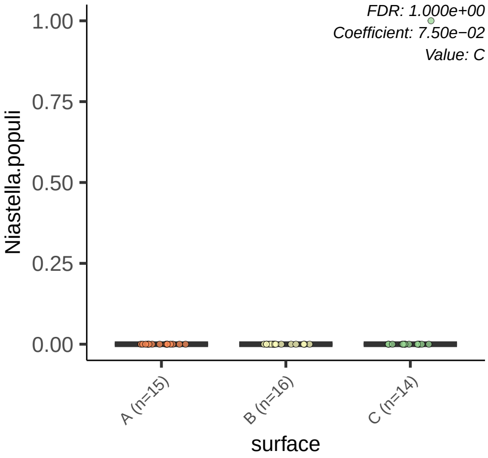
FDR: 1.000e+00
Coefficient: 7.50e−02
Value: C
1.00
0.75
0.50
0.25
0.00
Niastella.populi
A (n=15)
B (n=16)
C (n=14)
surface

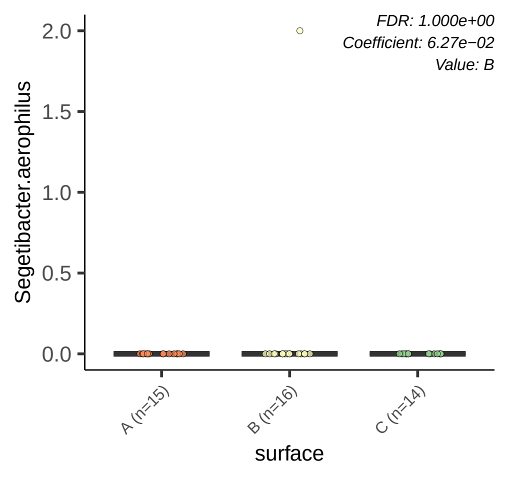
FDR: 1.000e+00
Coefficient: 6.27e−02
Value: B
Segetibacter.aerophilus
2.0
1.5
1.0
0.5
0.0
A (n=15)
B (n=16)
C (n=14)
surface

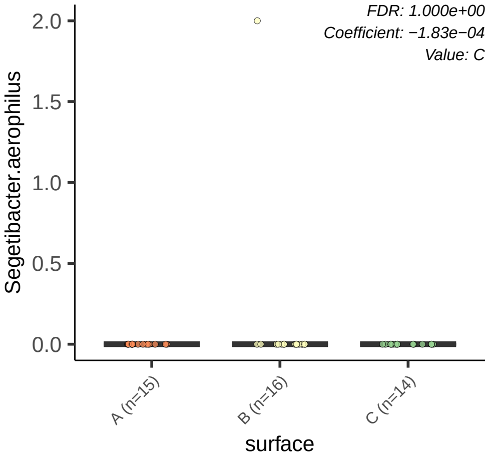
FDR: 1.000e+00
Coefficient: −1.83e−04
Value: C
Segetibacter.aerophilus
2.0
1.5
1.0
0.5
0.0
A (n=15)
B (n=16)
C (n=14)
surface

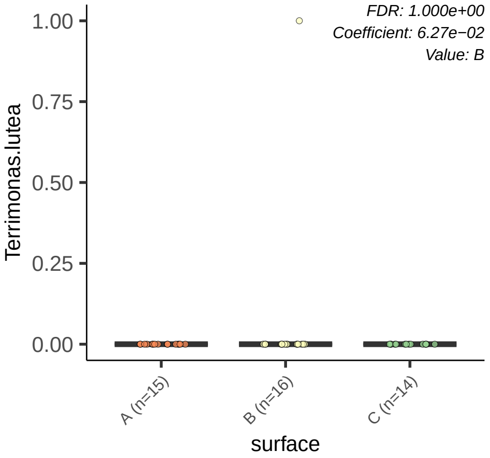
FDR: 1.000e+00
Coefficient: 6.27e−02
Value: B
1.00
0.75
0.50
0.25
0.00
Terrimonas.lutea
A (n=15)
B (n=16)
C (n=14)
surface

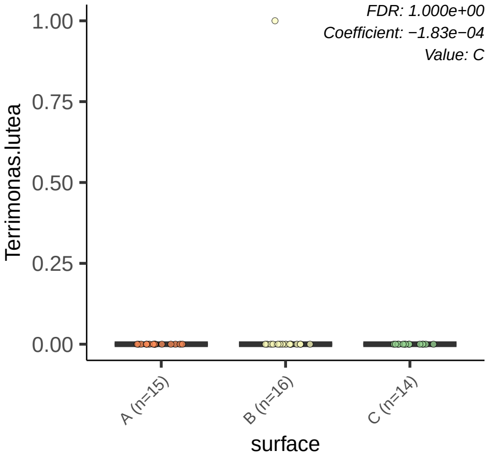

FDR: 1.000e+00
Coefficient: −1.83e−04
Value: C
Terrimonas.lutea
1.00
0.75
0.50
0.25
0.00
A (n=15)
B (n=16)
C (n=14)
surface

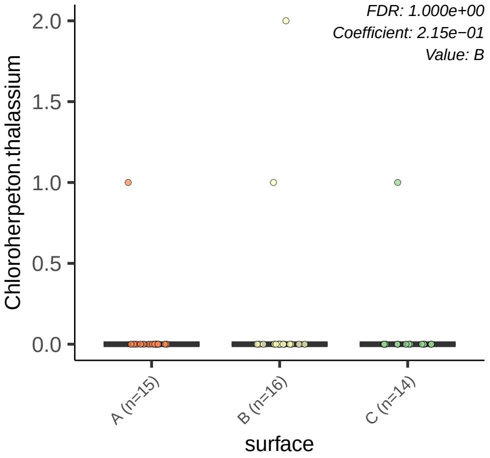

FDR: 1.000e+00
Coefficient: 2.15e−01
Value: B
Chloroherpeton.thalassium
2.0
1.5
1.0
0.5
0.0
A (n=15)
B (n=16)
C (n=14)
surface

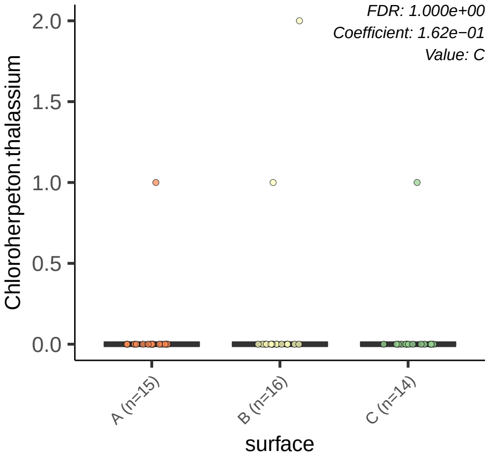

FDR: 1.000e+00
Coefficient: 1.62e−01
Value: C
Chloroherpeton.thalassium
2.0
1.5
1.0
0.5
0.0
A (n=15)
B (n=16)
C (n=14)
surface

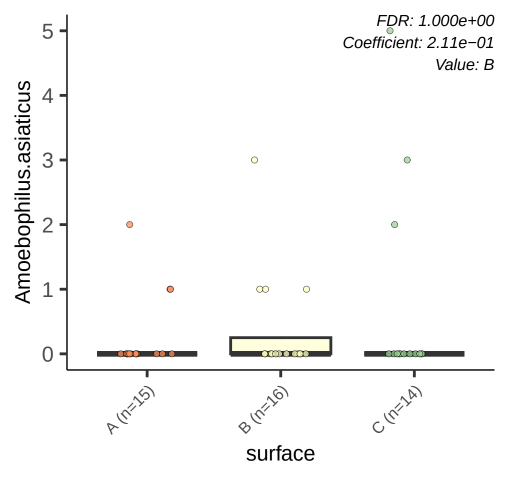
FDR: 1.000e+00
Coefficient: 2.11e−01
Value: B
Amoebophilus.asiaticus
5
4
3
2
1
0
A (n=15)
B (n=16)
C (n=14)
surface

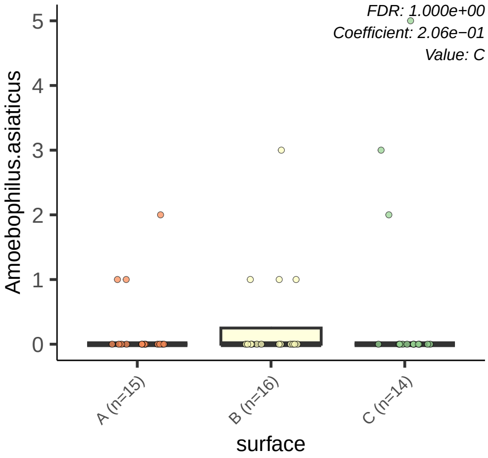

FDR: 1.000e+00
Coefficient: 2.06e−01
Value: C
Amoebophilus.asiaticus
0
1
2
3
4
5
A (n=15)
B (n=16)
C (n=14)
surface

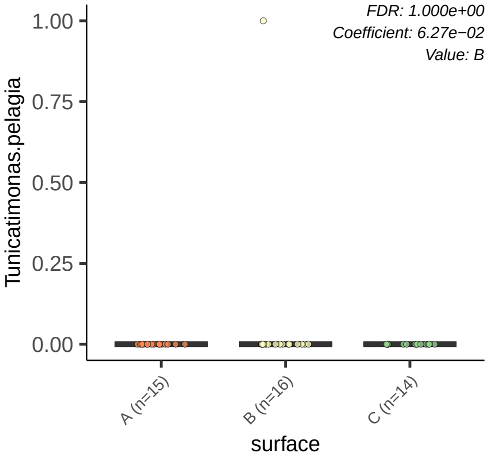

FDR: 1.000e+00
Coefficient: 6.27e−02
Value: B
Tunicatimonas.pelagia
1.00
0.75
0.50
0.25
0.00
A (n=15)
B (n=16)
C (n=14)
surface

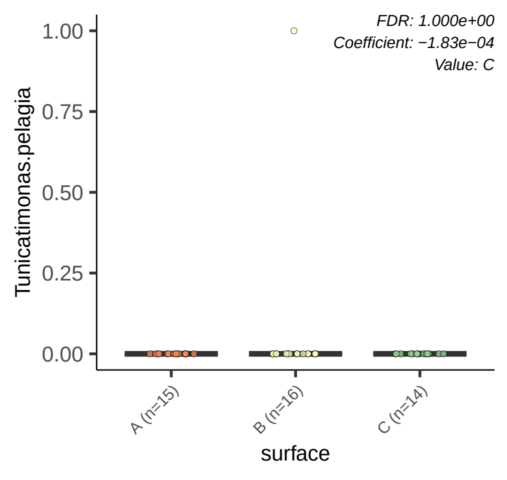
FDR: 1.000e+00
Coefficient: −1.83e−04
Value: C
Tunicatimonas.pelagia
1.00
0.75
0.50
0.25
0.00
A (n=15)
B (n=16)
C (n=14)
surface

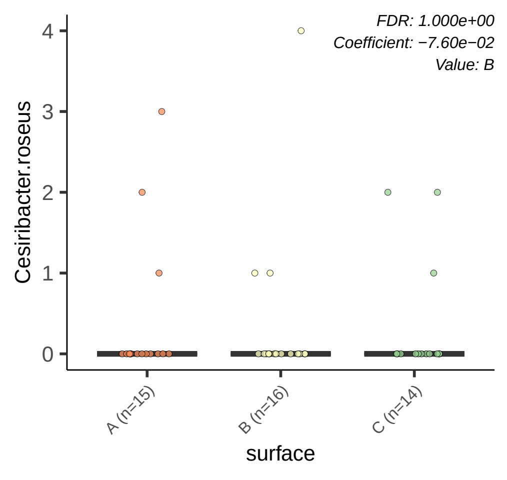
FDR: 1.000e+00
Coefficient: −7.60e−02
Value: B
Cesiribacter.roseus
4
3
2
1
0
A (n=15)
B (n=16)
C (n=14)
surface

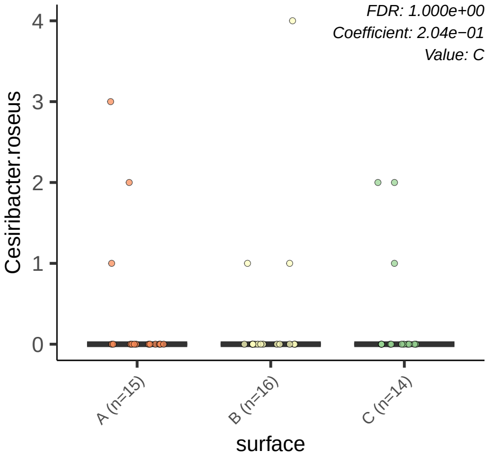

FDR: 1.000e+00
Coefficient: 2.04e−01
Value: C
Cesiribacter.roseus
0
1
2
3
4
A (n=15)
B (n=16)
C (n=14)
surface

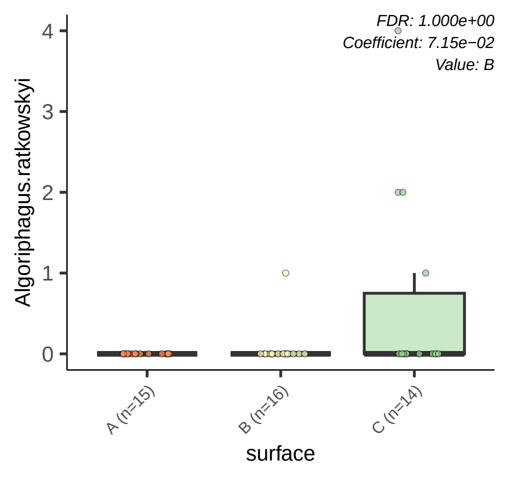
FDR: 1.000e+00
Coefficient: 7.15e−02
Value: B
Algoriphagus.ratkowskyi
4
3
2
1
0
A (n=15)
B (n=16)
C (n=14)
surface

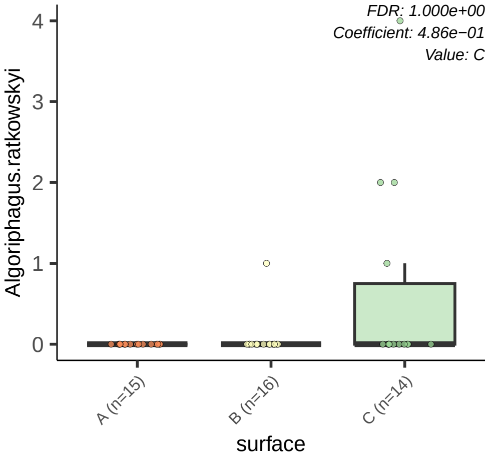

FDR: 1.000e+00
Coefficient: 4.86e−01
Value: C
Algoriphagus.ratkowskyi
0
1
2
3
4
A (n=15)
B (n=16)
C (n=14)
surface

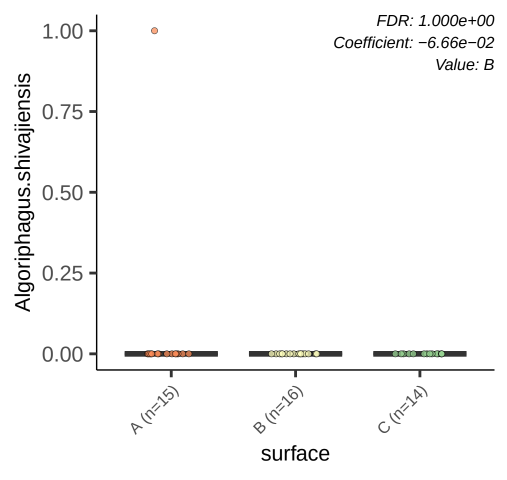
FDR: 1.000e+00
Coefficient: −6.66e−02
Value: B
Algoriphagus.shivajiensis
1.00
0.75
0.50
0.25
0.00
A (n=15)
B (n=16)
C (n=14)
surface

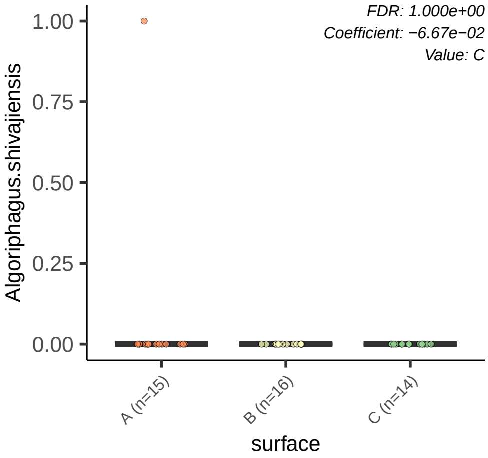
FDR: 1.000e+00
Coefficient: −6.67e−02
Value: C
Algoriphagus.shivajiensis
1.00
0.75
0.50
0.25
0.00
A (n=15)
B (n=16)
C (n=14)
surface

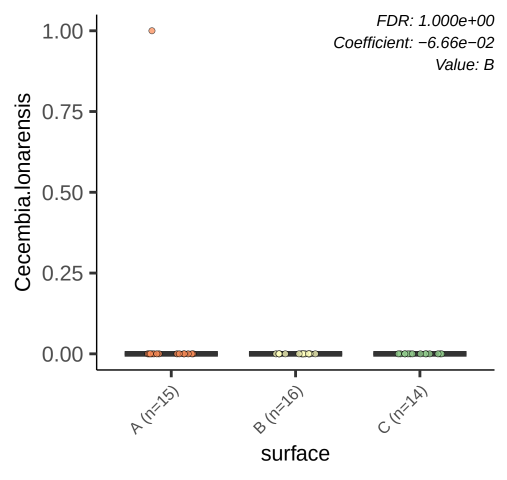
FDR: 1.000e+00
Coefficient: −6.66e−02
Value: B
1.00
0.75
0.50
0.25
0.00
Cecembia.lonarensis
A (n=15)
B (n=16)
C (n=14)
surface

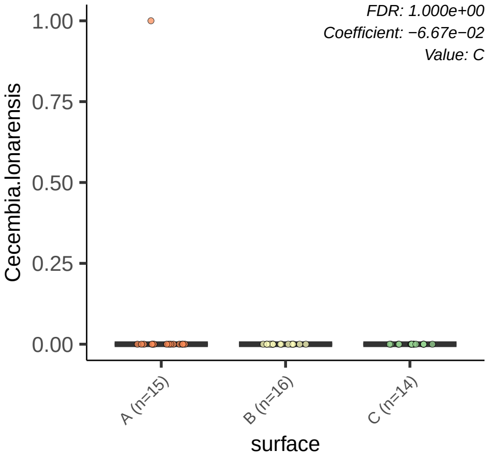
FDR: 1.000e+00
Coefficient: −6.67e−02
Value: C
Cecembia.lonarensis
1.00
0.75
0.50
0.25
0.00
A (n=15)
B (n=16)
C (n=14)
surface

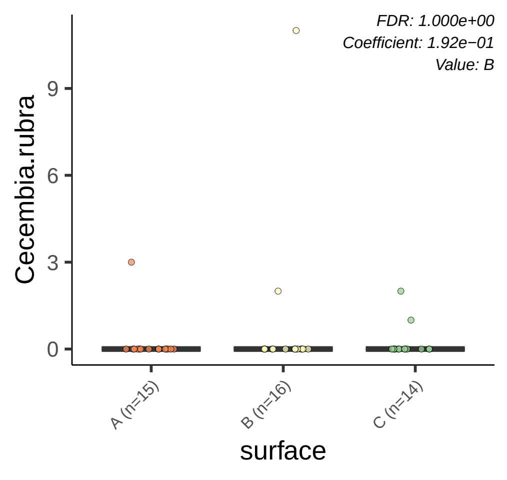
FDR: 1.000e+00
Coefficient: 1.92e−01
Value: B
Cecembia.rubra
0
3
6
9
A (n=15)
B (n=16)
C (n=14)
surface

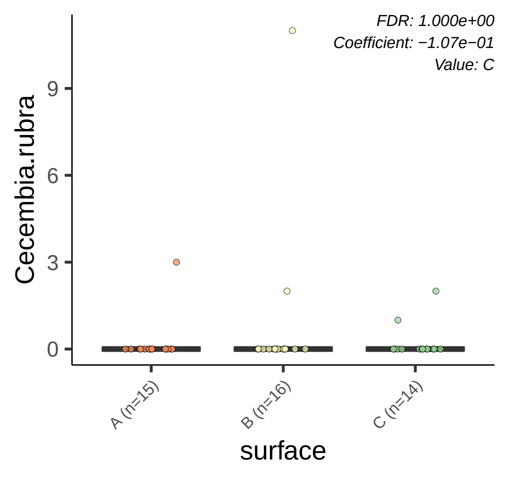

FDR: 1.000e+00
Coefficient: −1.07e−01
Value: C
Cecembia.rubra
9
6
3
0
A (n=15)
B (n=16)
C (n=14)
surface

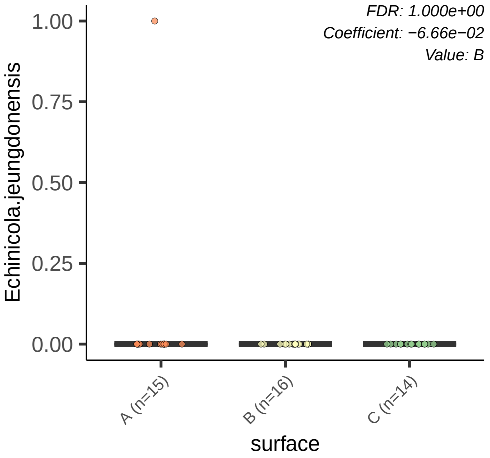
FDR: 1.000e+00
Coefficient: −6.66e−02
Value: B
Echinicola.jeungdonensis
1.00
0.75
0.50
0.25
0.00
A (n=15)
B (n=16)
C (n=14)
surface

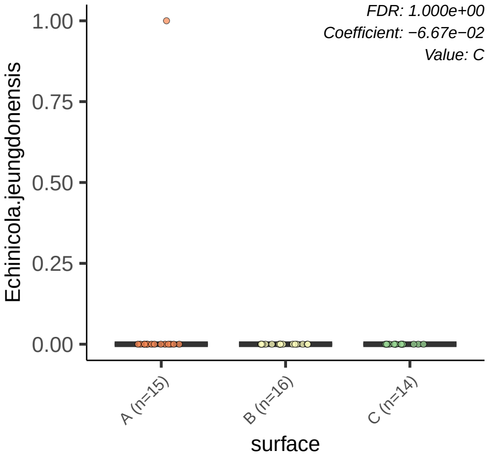
FDR: 1.000e+00
Coefficient: −6.67e−02
Value: C
Echinicola.jeungdonensis
1.00
0.75
0.50
0.25
0.00
A (n=15)
B (n=16)
C (n=14)
surface

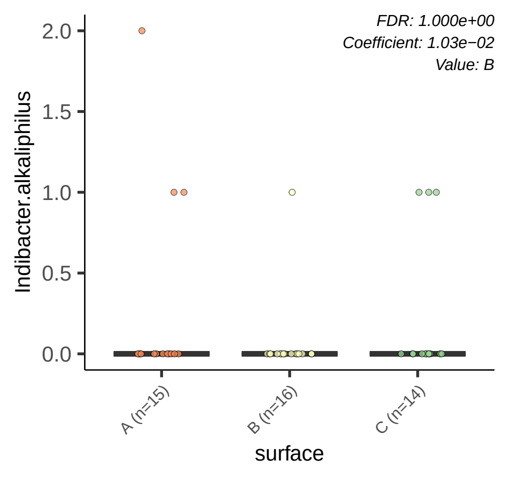
FDR: 1.000e+00
Coefficient: 1.03e−02
Value: B
Indibacter.alkaliphilus
2.0
1.5
1.0
0.5
0.0
A (n=15)
B (n=16)
C (n=14)
surface

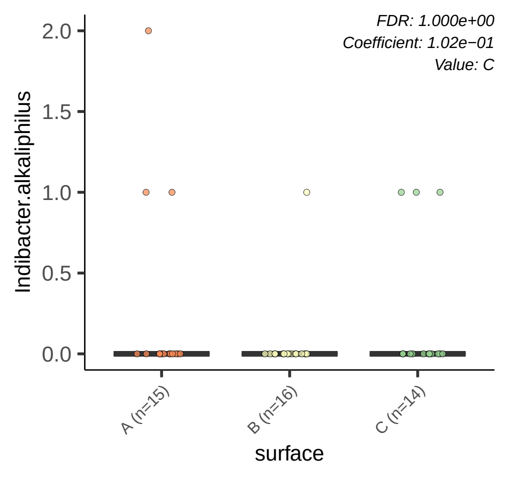

FDR: 1.000e+00
Coefficient: 1.02e−01
Value: C
Indibacter.alkaliphilus
2.0
1.5
1.0
0.5
0.0
A (n=15)
B (n=16)
C (n=14)
surface

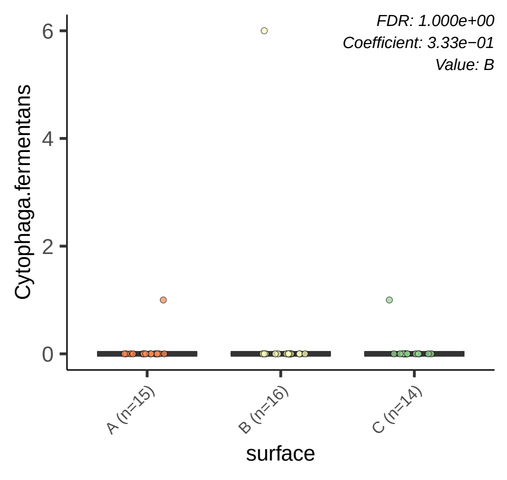
FDR: 1.000e+00
Coefficient: 3.33e−01
Value: B
6
4
2
0
Cytophaga.fermentans
A (n=15)
B (n=16)
C (n=14)
surface

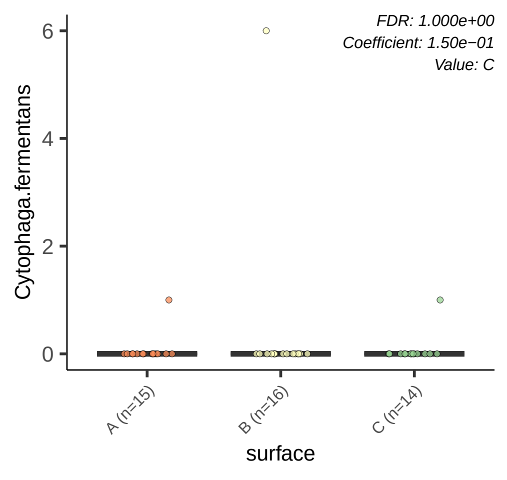
FDR: 1.000e+00
Coefficient: 1.50e−01
Value: C
Cytophaga.fermentans
6
4
2
0
A (n=15)
B (n=16)
C (n=14)
surface

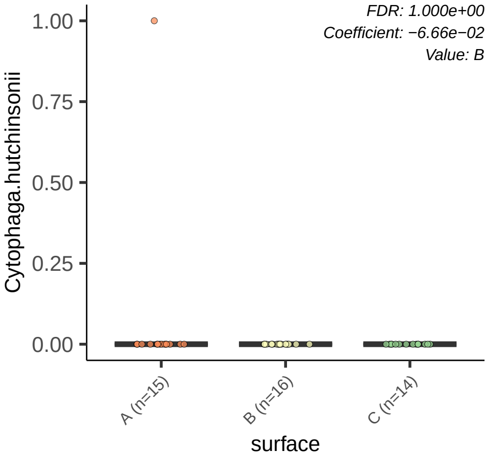

FDR: 1.000e+00
Coefficient: −6.66e−02
Value: B
Cytophaga.hutchinsonii
1.00
0.75
0.50
0.25
0.00
A (n=15)
B (n=16)
C (n=14)
surface

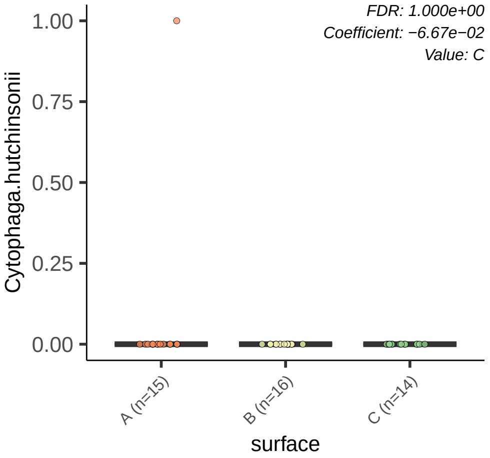

FDR: 1.000e+00
Coefficient: −6.67e−02
Value: C
Cytophaga.hutchinsonii
1.00
0.75
0.50
0.25
0.00
A (n=15)
B (n=16)
C (n=14)
surface

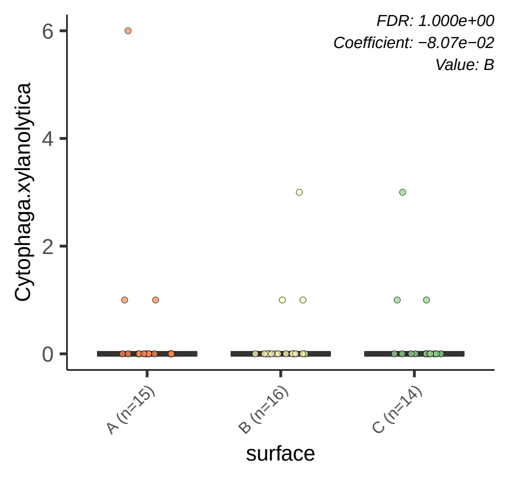
FDR: 1.000e+00
Coefficient: −8.07e−02
Value: B
Cytophaga.xylanolytica
6
4
2
0
A (n=15)
B (n=16)
C (n=14)
surface

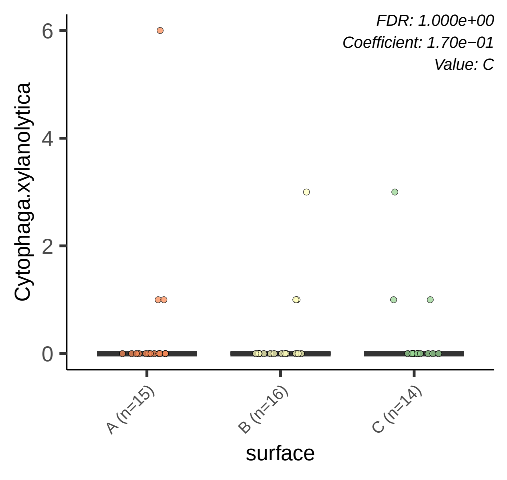

FDR: 1.000e+00
Coefficient: 1.70e−01
Value: C
Cytophaga.xylanolytica
6
4
2
0
A (n=15)
B (n=16)
C (n=14)
surface

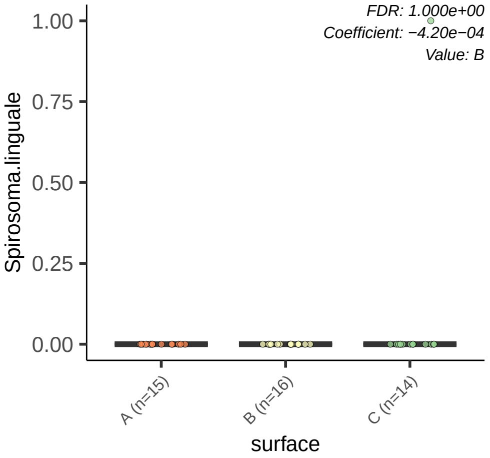
FDR: 1.000e+00
Coefficient: −4.20e−04
Value: B
1.00
0.75
0.50
0.25
0.00
Spirosoma.linguale
A (n=15)
B (n=16)
C (n=14)
surface

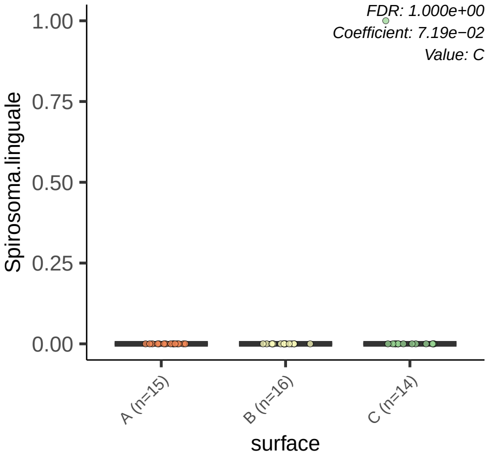
FDR: 1.000e+00
Coefficient: 7.19e−02
Value: C
1.00
0.75
0.50
0.25
0.00
Spirosoma.linguale
A (n=15)
B (n=16)
C (n=14)
surface

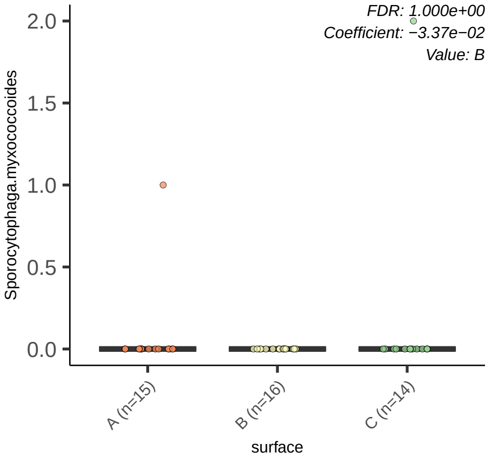

FDR: 1.000e+00
Coefficient: −3.37e−02
Value: B
2.0
1.5
1.0
0.5
0.0
Sporocytophaga.myxococcoides
A (n=15)
B (n=16)
C (n=14)
surface

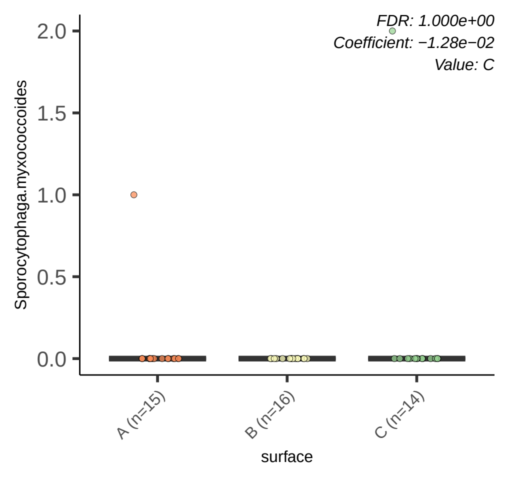
FDR: 1.000e+00
Coefficient: −1.28e−02
Value: C
2.0
1.5
1.0
0.5
0.0
Sporocytophaga.myxococcoides
A (n=15)
B (n=16)
C (n=14)
surface

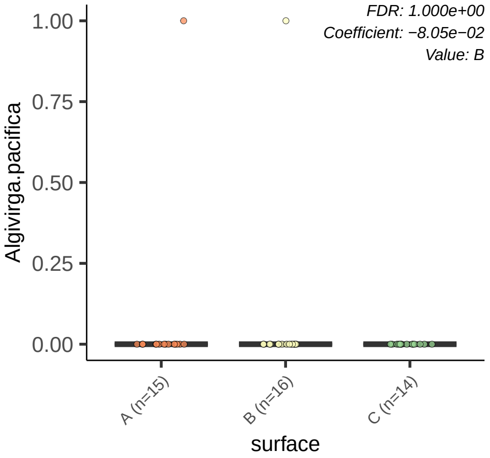

FDR: 1.000e+00
Coefficient: −8.05e−02
Value: B
Algivirga.pacifica
1.00
0.75
0.50
0.25
0.00
A (n=15)
B (n=16)
C (n=14)
surface

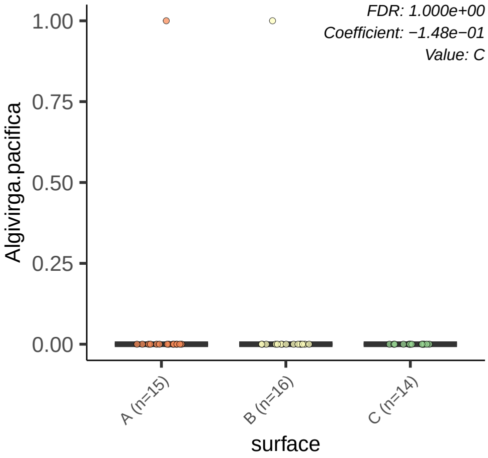
FDR: 1.000e+00
Coefficient: −1.48e−01
Value: C
Algivirga.pacifica
1.00
0.75
0.50
0.25
0.00
A (n=15)
B (n=16)
C (n=14)
surface

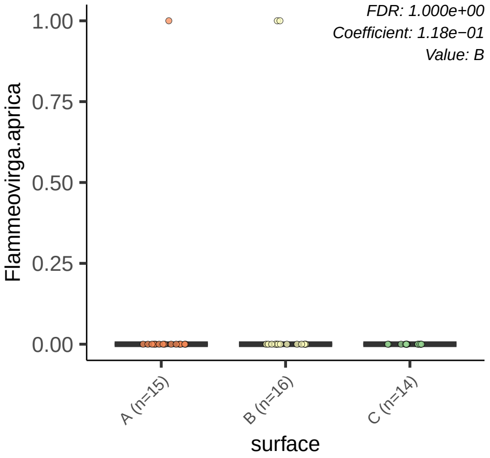
FDR: 1.000e+00
Coefficient: 1.18e−01
Value: B
Flammeovirga.aprica
1.00
0.75
0.50
0.25
0.00
A (n=15)
B (n=16)
C (n=14)
surface

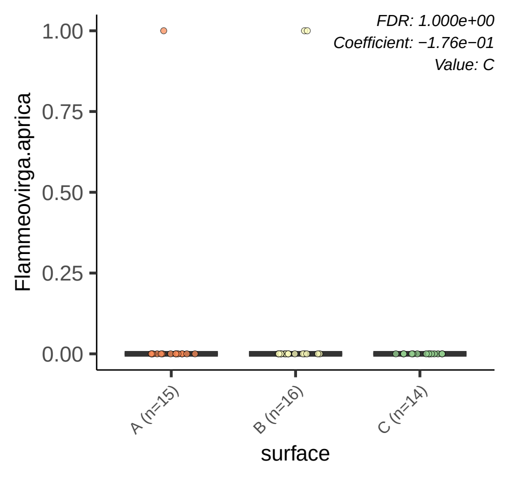

FDR: 1.000e+00
Coefficient: −1.76e−01
Value: C
Flammeovirga.aprica
1.00
0.75
0.50
0.25
0.00
A (n=15)
B (n=16)
C (n=14)
surface

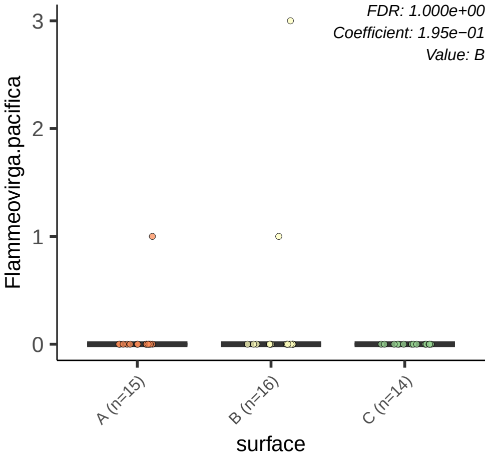

FDR: 1.000e+00
Coefficient: 1.95e−01
Value: B
Flammeovirga.pacifica
3
2
1
0
A (n=15)
B (n=16)
C (n=14)
surface

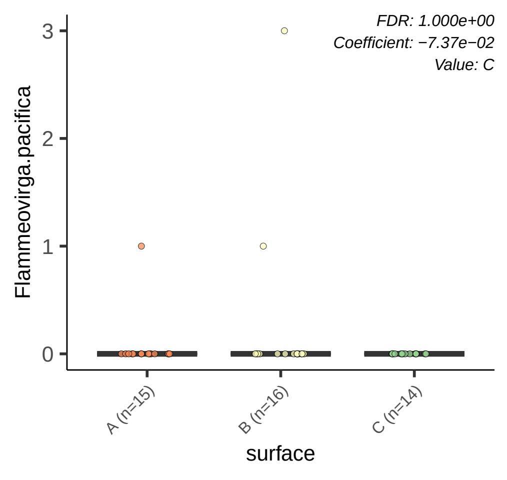

FDR: 1.000e+00
Coefficient: −7.37e−02
Value: C
Flammeovirga.pacifica
3
2
1
0
A (n=15)
B (n=16)
C (n=14)
surface

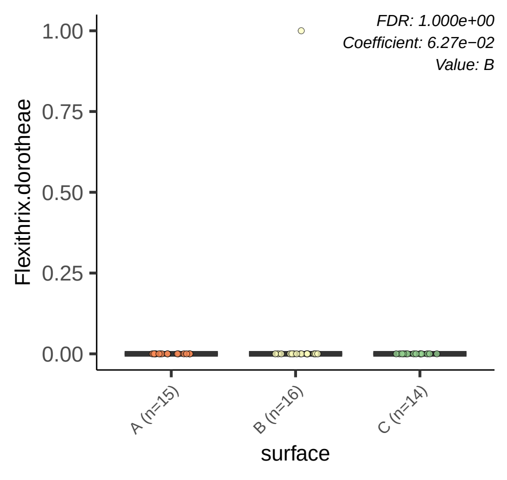
FDR: 1.000e+00
Coefficient: 6.27e−02
Value: B
Flexithrix.dorotheae
1.00
0.75
0.50
0.25
0.00
A (n=15)
B (n=16)
C (n=14)
surface

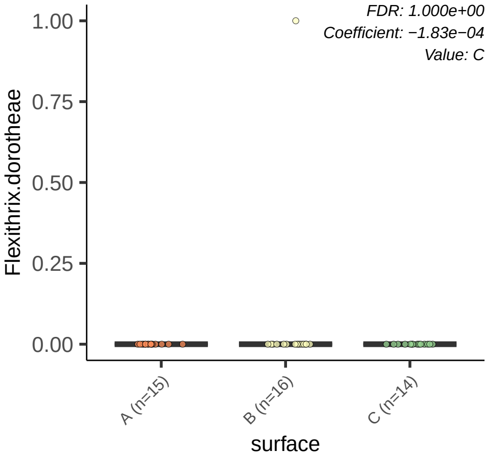
FDR: 1.000e+00
Coefficient: −1.83e−04
Value: C
Flexithrix.dorotheae
1.00
0.75
0.50
0.25
0.00
A (n=15)
B (n=16)
C (n=14)
surface

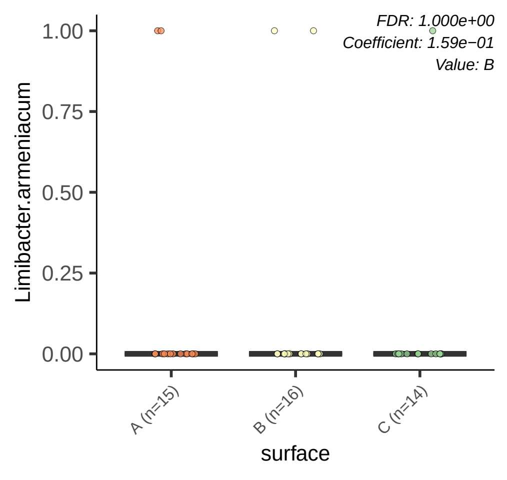
FDR: 1.000e+00
Coefficient: 1.59e−01
Value: B
Limibacter.armeniacum
1.00
0.75
0.50
0.25
0.00
A (n=15)
B (n=16)
C (n=14)
surface

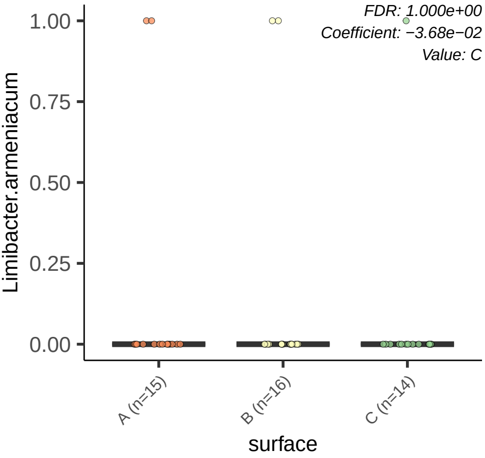

FDR: 1.000e+00
Coefficient: −3.68e−02
Value: C
Limibacter.armeniacum
1.00
0.75
0.50
0.25
0.00
A (n=15)
B (n=16)
C (n=14)
surface

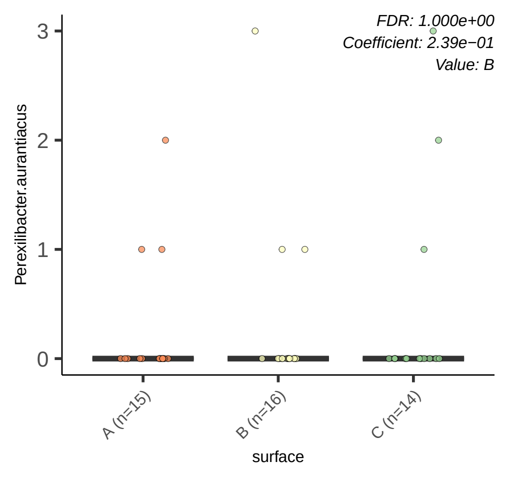
FDR: 1.000e+00
Coefficient: 2.39e−01
Value: B
Perexilibacter.aurantiacus
0
1
2
3
A (n=15)
B (n=16)
C (n=14)
surface

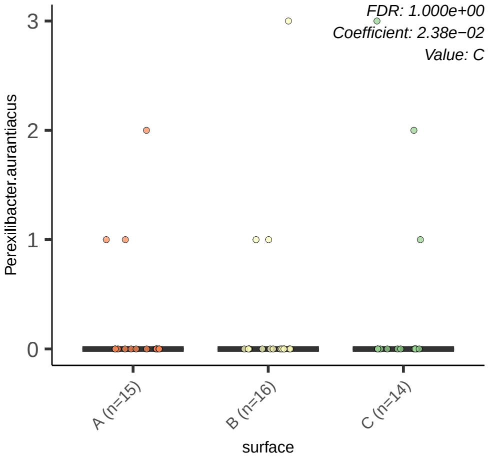
FDR: 1.000e+00
Coefficient: 2.38e−02
Value: C
Perexilibacter.aurantiacus
3
2
1
0
A (n=15)
B (n=16)
C (n=14)
surface

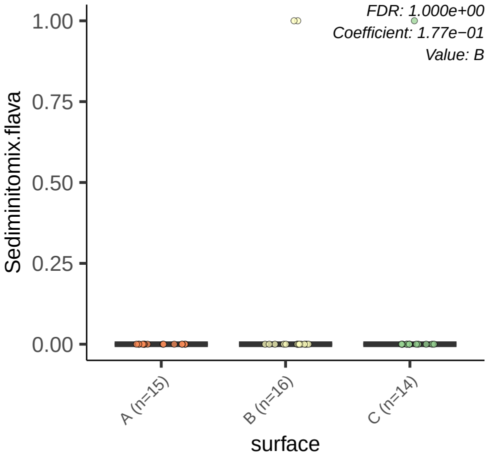

FDR: 1.000e+00
Coefficient: 1.77e−01
Value: B
Sediminitomix.flava
1.00
0.75
0.50
0.25
0.00
A (n=15)
B (n=16)
C (n=14)
surface

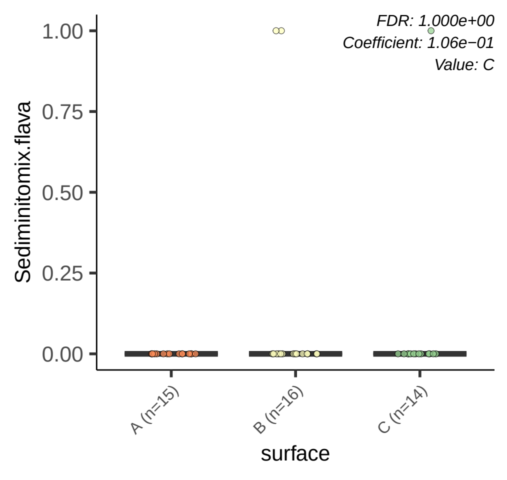
FDR: 1.000e+00
Coefficient: 1.06e−01
Value: C
Sediminitomix.flava
1.00
0.75
0.50
0.25
0.00
A (n=15)
B (n=16)
C (n=14)
surface

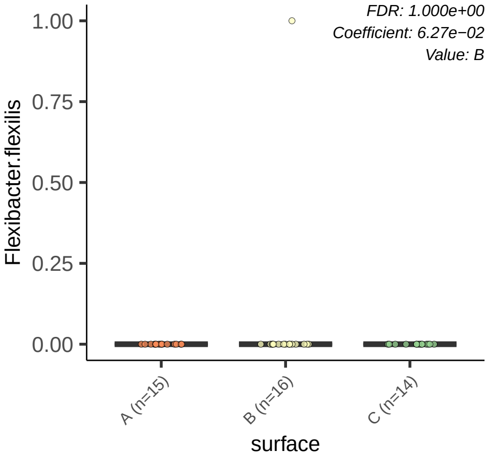
FDR: 1.000e+00
Coefficient: 6.27e−02
Value: B
Flexibacter.flexilis
1.00
0.75
0.50
0.25
0.00
A (n=15)
B (n=16)
C (n=14)
surface

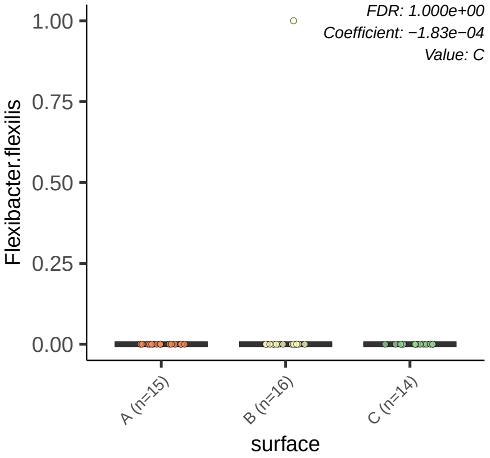

FDR: 1.000e+00
Coefficient: −1.83e−04
Value: C
Flexibacter.flexilis
1.00
0.75
0.50
0.25
0.00
A (n=15)
B (n=16)
C (n=14)
surface

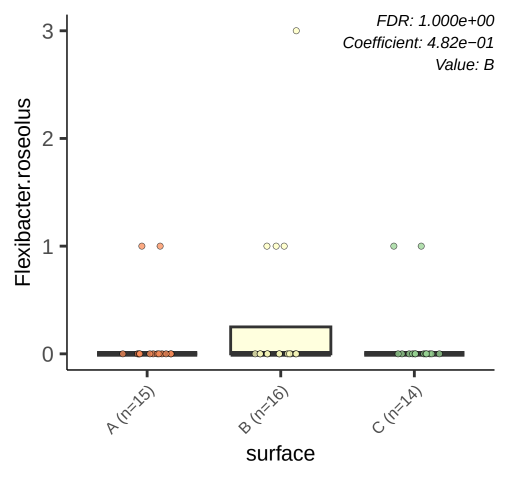
FDR: 1.000e+00
Coefficient: 4.82e−01
Value: B
Flexibacter.roseolus
3
2
1
0
A (n=15)
B (n=16)
C (n=14)
surface

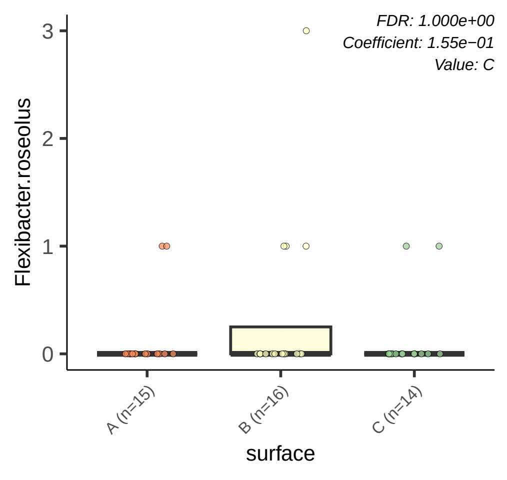

FDR: 1.000e+00
Coefficient: 1.55e−01
Value: C
Flexibacter.roseolus
3
2
1
0
A (n=15)
B (n=16)
C (n=14)
surface

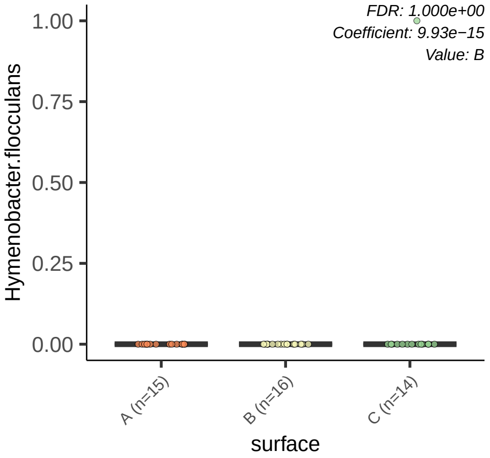

FDR: 1.000e+00
Coefficient: 9.93e−15
Value: B
Hymenobacter.flocculans
1.00
0.75
0.50
0.25
0.00
A (n=15)
B (n=16)
C (n=14)
surface

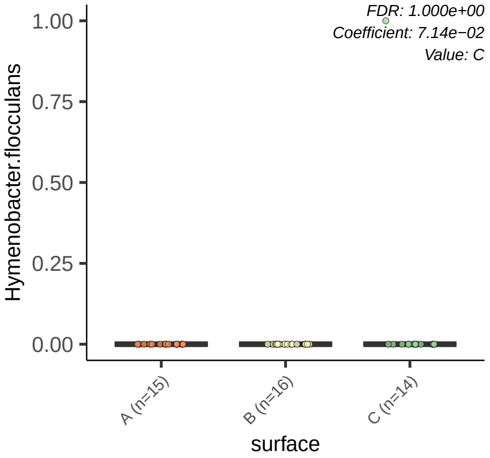

FDR: 1.000e+00
Coefficient: 7.14e−02
Value: C
1.00
0.75
0.50
0.25
0.00
Hymenobacter.flocculans
A (n=15)
B (n=16)
C (n=14)
surface

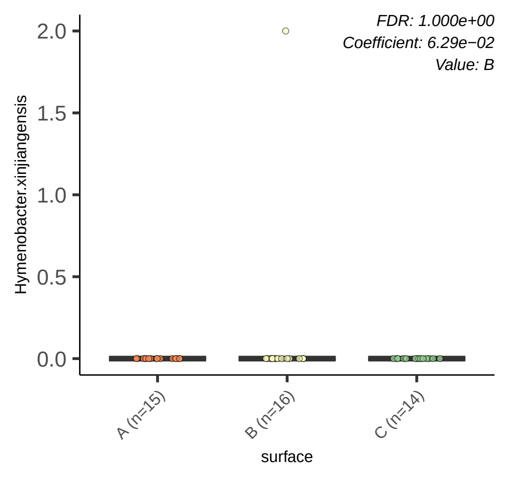
FDR: 1.000e+00
Coefficient: 6.29e−02
Value: B
Hymenobacter.xinjiangensis
2.0
1.5
1.0
0.5
0.0
A (n=15)
B (n=16)
C (n=14)
surface

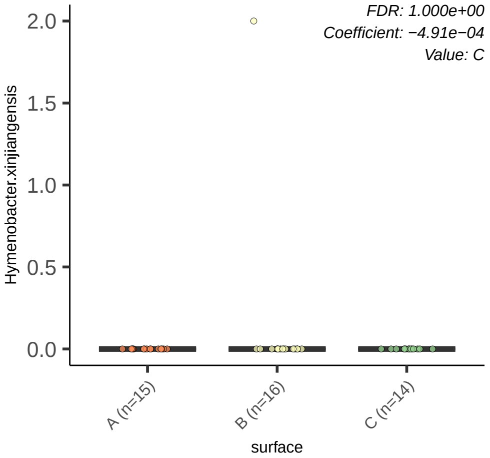

FDR: 1.000e+00
Coefficient: −4.91e−04
Value: C
Hymenobacter.xinjiangensis
2.0
1.5
1.0
0.5
0.0
A (n=15)
B (n=16)
C (n=14)
surface

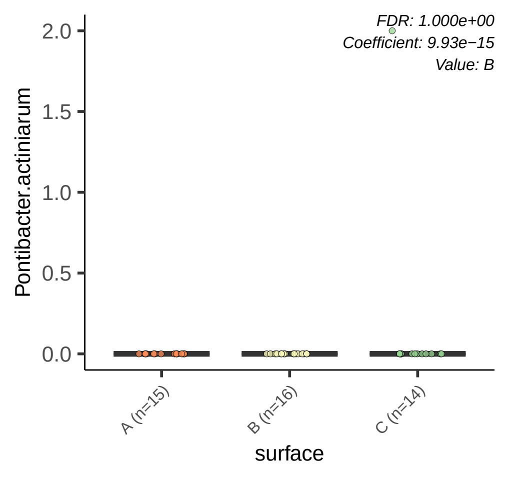

FDR: 1.000e+00
Coefficient: 9.93e−15
Value: B
Pontibacter.actiniarum
2.0
1.5
1.0
0.5
0.0
A (n=15)
B (n=16)
C (n=14)
surface

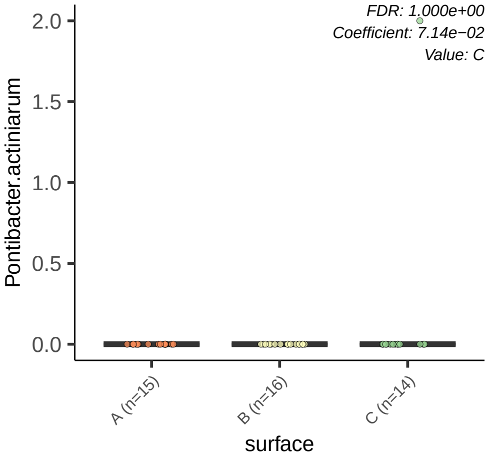

FDR: 1.000e+00
Coefficient: 7.14e−02
Value: C
Pontibacter.actiniarum
2.0
1.5
1.0
0.5
0.0
A (n=15)
B (n=16)
C (n=14)
surface

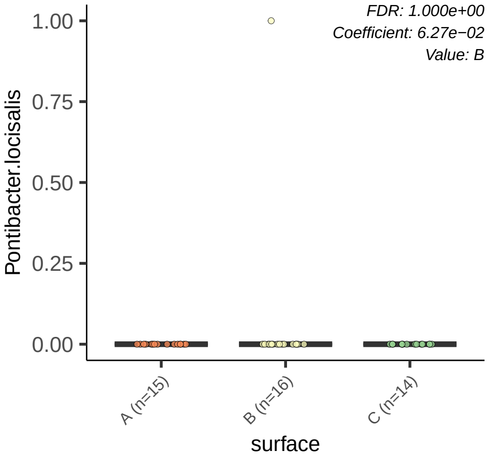
FDR: 1.000e+00
Coefficient: 6.27e−02
Value: B
Pontibacter.locisalis
1.00
0.75
0.50
0.25
0.00
A (n=15)
B (n=16)
C (n=14)
surface

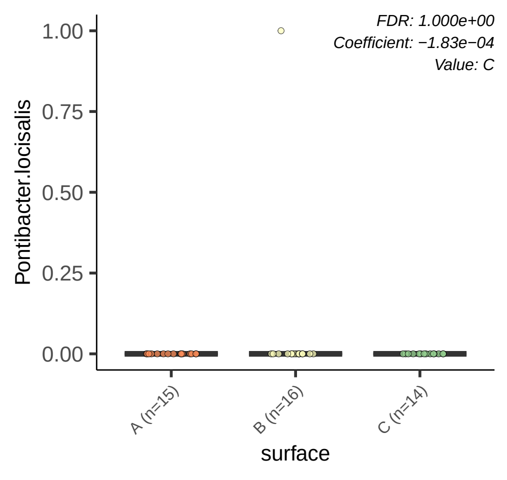

FDR: 1.000e+00
Coefficient: −1.83e−04
Value: C
Pontibacter.locisalis
1.00
0.75
0.50
0.25
0.00
A (n=15)
B (n=16)
C (n=14)
surface

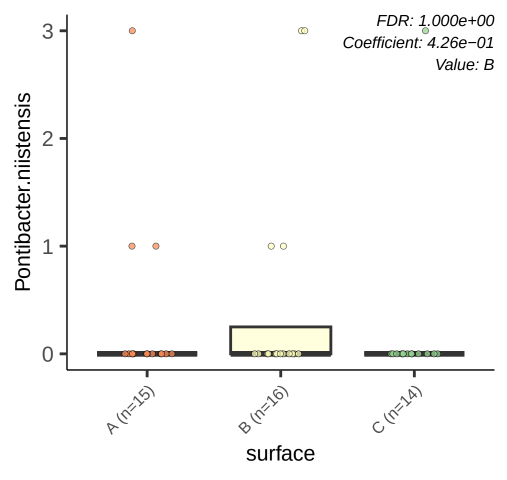

FDR: 1.000e+00
Coefficient: 4.26e−01
Value: B
Pontibacter.niistensis
3
2
1
0
A (n=15)
B (n=16)
C (n=14)
surface

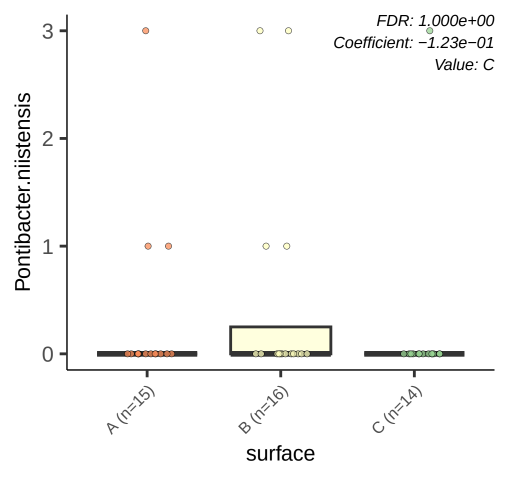
FDR: 1.000e+00
Coefficient: −1.23e−01
Value: C
Pontibacter.niistensis
3
2
1
0
A (n=15)
B (n=16)
C (n=14)
surface

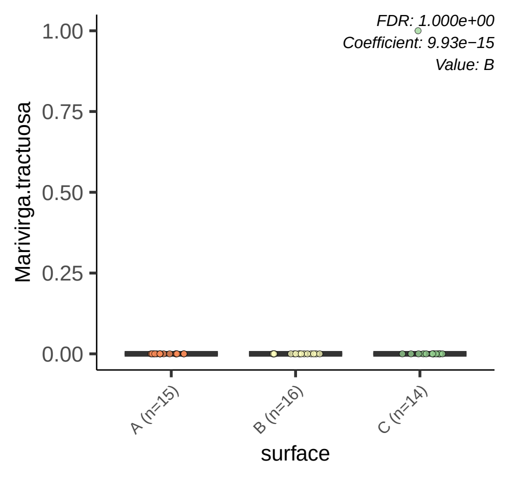
FDR: 1.000e+00
Coefficient: 9.93e−15
Value: B
Marivirga.tractuosa
1.00
0.75
0.50
0.25
0.00
A (n=15)
B (n=16)
C (n=14)
surface

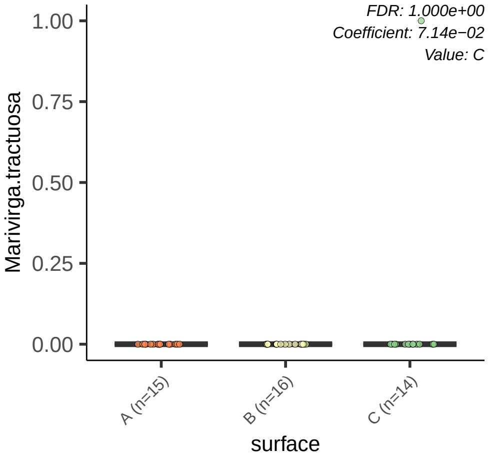
FDR: 1.000e+00
Coefficient: 7.14e−02
Value: C
Marivirga.tractuosa
1.00
0.75
0.50
0.25
0.00
A (n=15)
B (n=16)
C (n=14)
surface

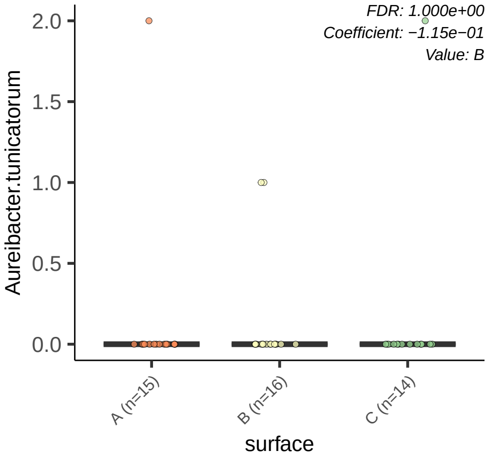

FDR: 1.000e+00
Coefficient: −1.15e−01
Value: B
Aureibacter.tunicatorum
2.0
1.5
1.0
0.5
0.0
A (n=15)
B (n=16)
C (n=14)
surface

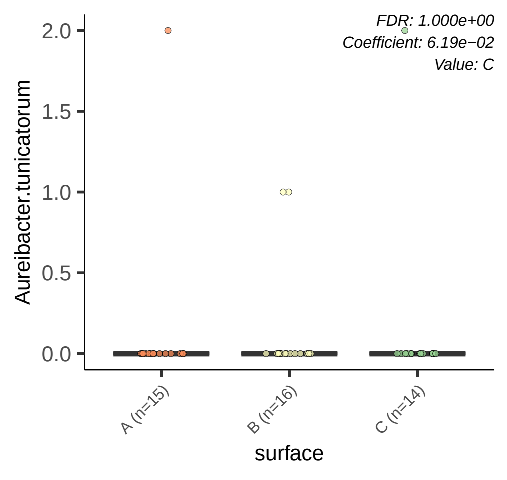

FDR: 1.000e+00
Coefficient: 6.19e−02
Value: C
Aureibacter.tunicatorum
2.0
1.5
1.0
0.5
0.0
A (n=15)
B (n=16)
C (n=14)
surface

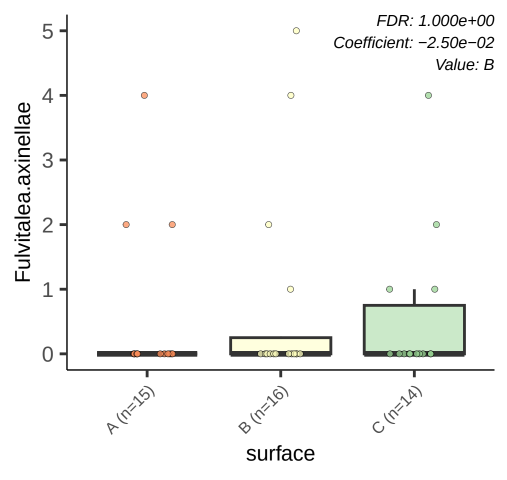
FDR: 1.000e+00
Coefficient: −2.50e−02
Value: B
Fulvitalea.axinellae
0
1
2
3
4
5
A (n=15)
B (n=16)
C (n=14)
surface

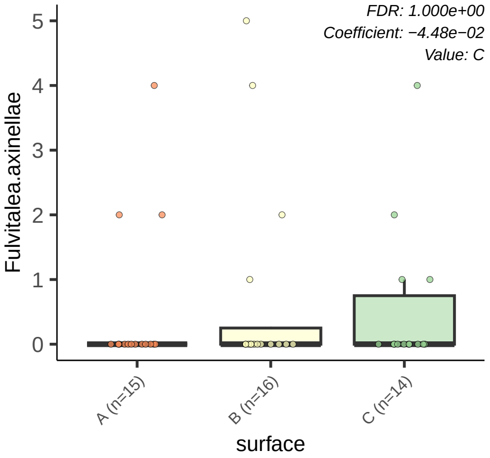

FDR: 1.000e+00
Coefficient: −4.48e−02
Value: C
Fulvitalea.axinellae
0
1
2
3
4
5
A (n=15)
B (n=16)
C (n=14)
surface

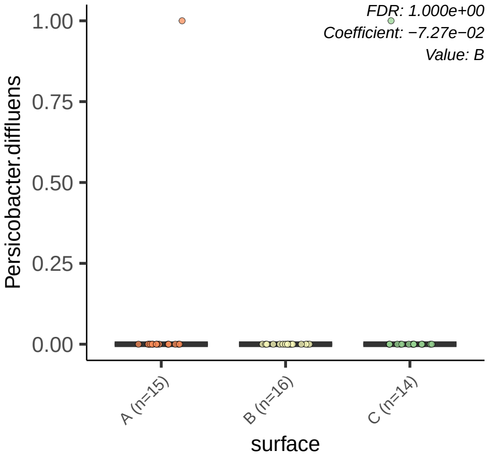
FDR: 1.000e+00
Coefficient: −7.27e−02
Value: B
Persicobacter.diffluens
1.00
0.75
0.50
0.25
0.00
A (n=15)
B (n=16)
C (n=14)
surface

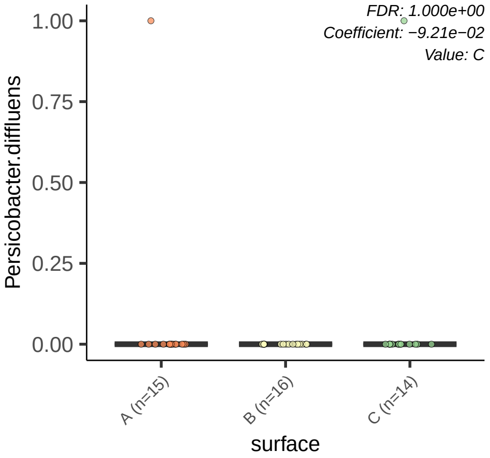
FDR: 1.000e+00
Coefficient: −9.21e−02
Value: C
Persicobacter.diffluens
1.00
0.75
0.50
0.25
0.00
A (n=15)
B (n=16)
C (n=14)
surface

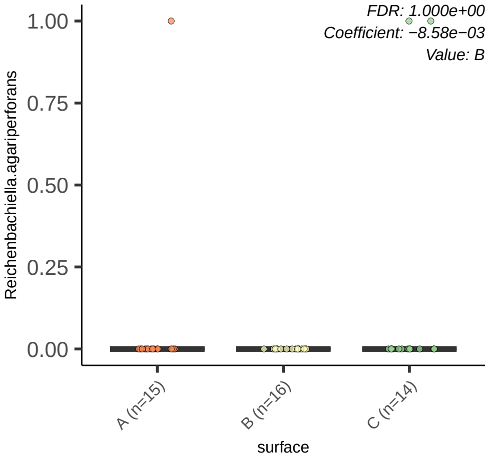
FDR: 1.000e+00
Coefficient: −8.58e−03
Value: B
1.00
0.75
0.50
0.25
0.00
Reichenbachiella.agariperforans
A (n=15)
B (n=16)
C (n=14)
surface

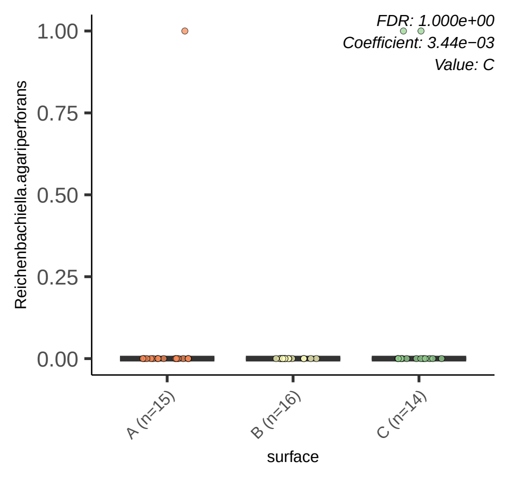
FDR: 1.000e+00
Coefficient: 3.44e−03
Value: C
Reichenbachiella.agariperforans
1.00
0.75
0.50
0.25
0.00
A (n=15)
B (n=16)
C (n=14)
surface

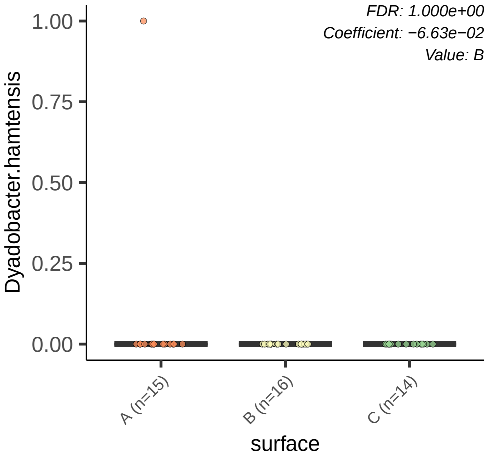
FDR: 1.000e+00
Coefficient: −6.63e−02
Value: B
Dyadobacter.hamtensis
1.00
0.75
0.50
0.25
0.00
A (n=15)
B (n=16)
C (n=14)
surface

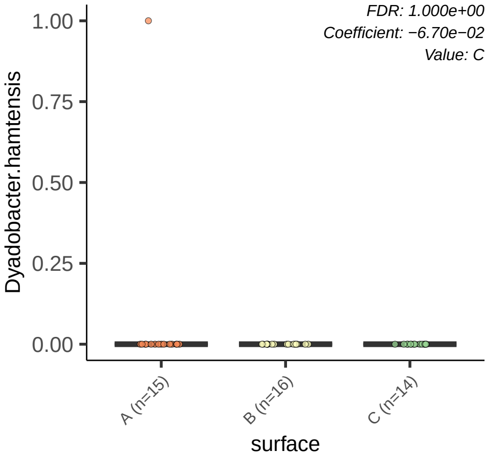
FDR: 1.000e+00
Coefficient: −6.70e−02
Value: C
Dyadobacter.hamtensis
1.00
0.75
0.50
0.25
0.00
A (n=15)
B (n=16)
C (n=14)
surface

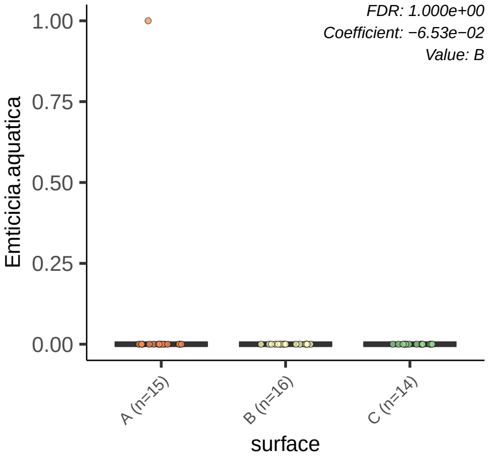
FDR: 1.000e+00
Coefficient: −6.53e−02
Value: B
Emticicia.aquatica
1.00
0.75
0.50
0.25
0.00
A (n=15)
B (n=16)
C (n=14)
surface

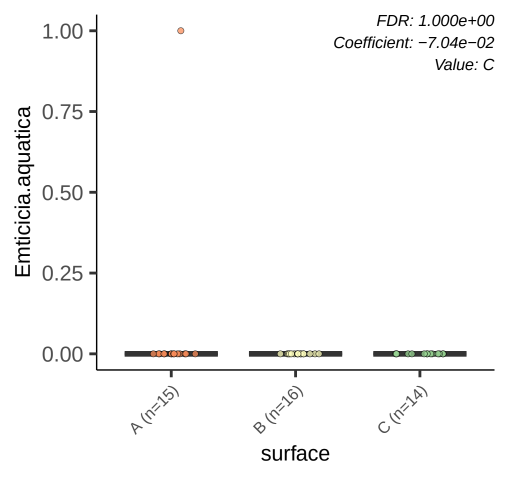
FDR: 1.000e+00
Coefficient: −7.04e−02
Value: C
Emticicia.aquatica
1.00
0.75
0.50
0.25
0.00
A (n=15)
B (n=16)
C (n=14)
surface

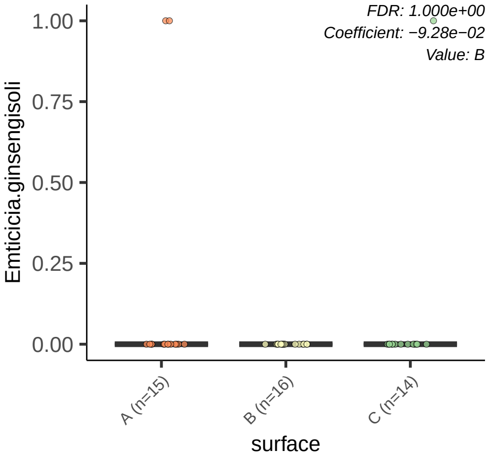

FDR: 1.000e+00
Coefficient: −9.28e−02
Value: B
Emticicia.ginsengisoli
1.00
0.75
0.50
0.25
0.00
A (n=15)
B (n=16)
C (n=14)
surface

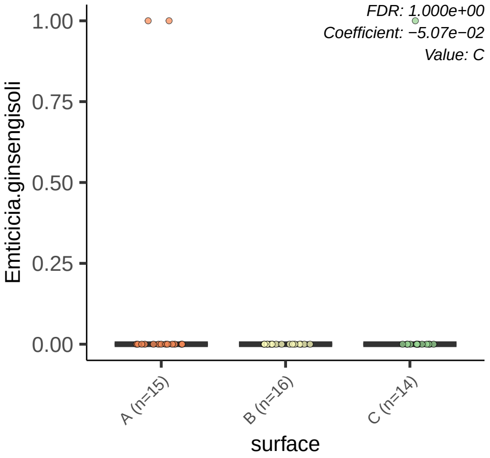

FDR: 1.000e+00
Coefficient: −5.07e−02
Value: C
Emticicia.ginsengisoli
1.00
0.75
0.50
0.25
0.00
A (n=15)
B (n=16)
C (n=14)
surface

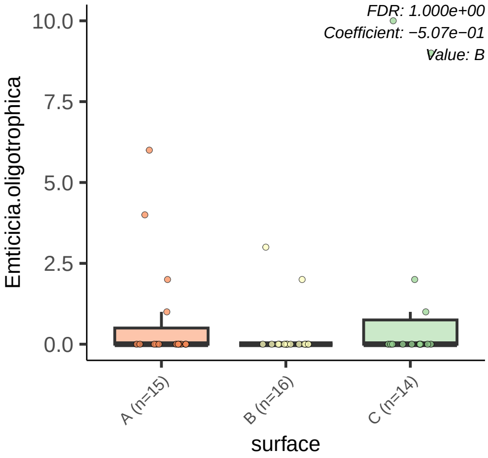

FDR: 1.000e+00
Coefficient: −5.07e−01
Value: B
Emticicia.oligotrophica
10.0
7.5
5.0
2.5
0.0
A (n=15)
B (n=16)
C (n=14)
surface

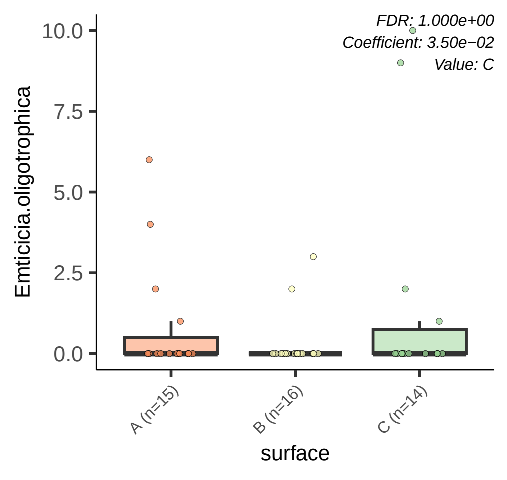
FDR: 1.000e+00
Coefficient: 3.50e−02
Value: C
Emticicia.oligotrophica
10.0
7.5
5.0
2.5
0.0
A (n=15)
B (n=16)
C (n=14)
surface

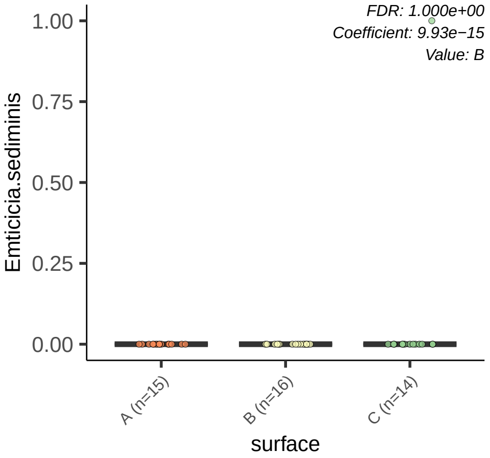

FDR: 1.000e+00
Coefficient: 9.93e−15
Value: B
Emticicia.sedіminis
1.00
0.75
0.50
0.25
0.00
A (n=15)
B (n=16)
C (n=14)
surface

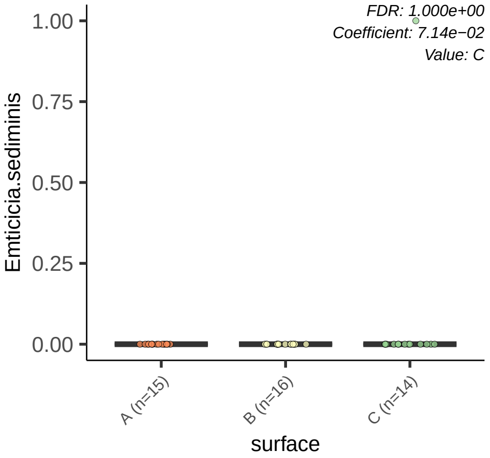
FDR: 1.000e+00
Coefficient: 7.14e−02
Value: C
Emticicia.sedimini s
1.00
0.75
0.50
0.25
0.00
A (n=15)
B (n=16)
C (n=14)
surface

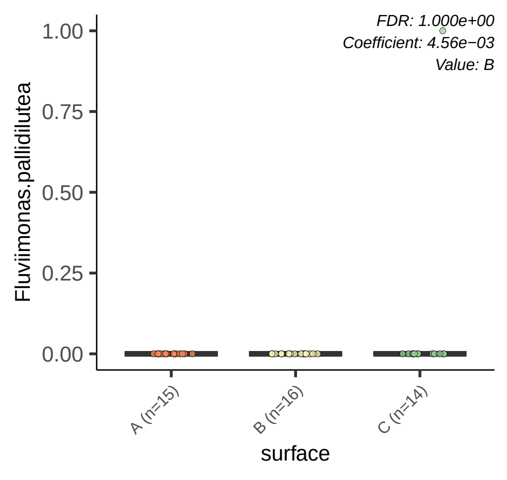
FDR: 1.000e+00
Coefficient: 4.56e−03
Value: B
Fluviimonas.pallidilutea
1.00
0.75
0.50
0.25
0.00
A (n=15)
B (n=16)
C (n=14)
surface

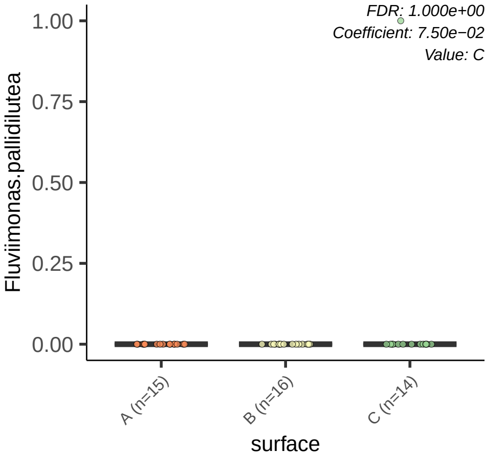

FDR: 1.000e+00
Coefficient: 7.50e−02
Value: C
1.00
0.75
0.50
0.25
0.00
Fluviimonas.pallidilutea
A (n=15)
B (n=16)
C (n=14)
surface

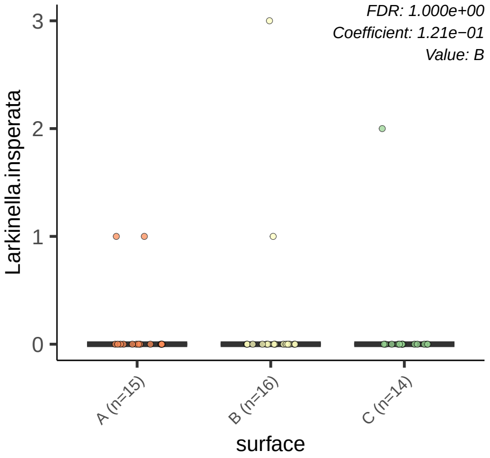
FDR: 1.000e+00
Coefficient: 1.21e−01
Value: B
Larkinella.insperata
0
1
2
3
A (n=15)
B (n=16)
C (n=14)
surface

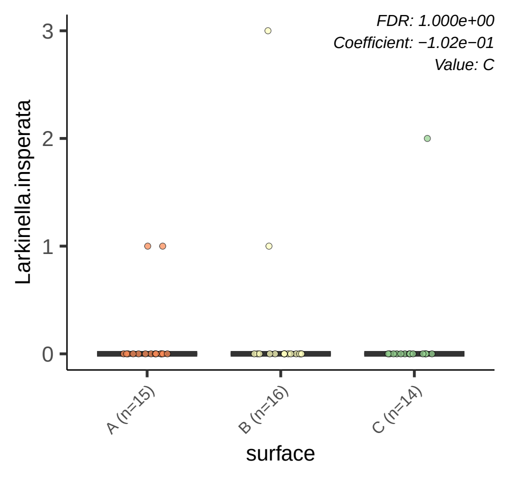

FDR: 1.000e+00
Coefficient: −1.02e−01
Value: C
Larkinella.insperata
3
2
1
0
A (n=15)
B (n=16)
C (n=14)
surface

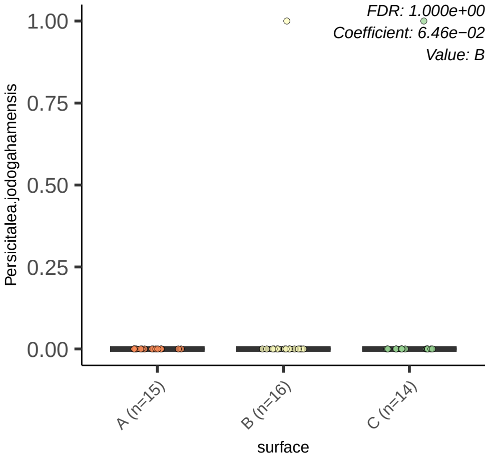

FDR: 1.000e+00
Coefficient: 6.46e−02
Value: B
1.00
0.75
0.50
0.25
0.00
Persicitalea.jodogahamensis
A (n=15)
B (n=16)
C (n=14)
surface

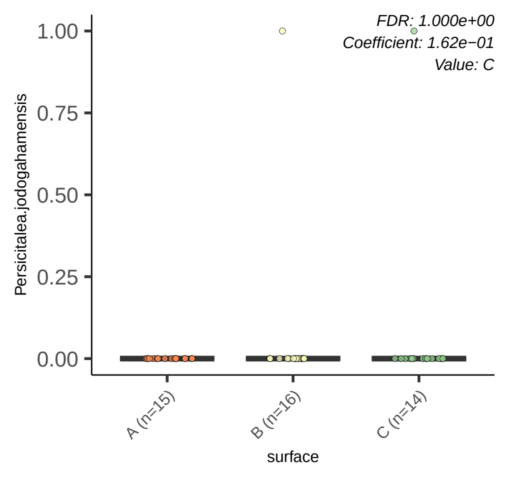
FDR: 1.000e+00
Coefficient: 1.62e−01
Value: C
Persicitalea.jodogahamensis
1.00
0.75
0.50
0.25
0.00
A (n=15)
B (n=16)
C (n=14)
surface

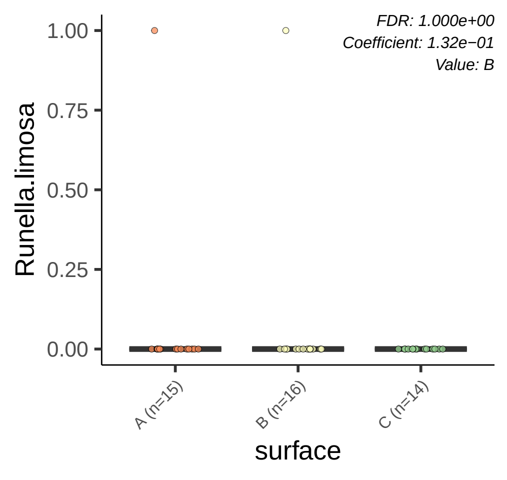
FDR: 1.000e+00
Coefficient: 1.32e−01
Value: B
Runella.limosa
1.00
0.75
0.50
0.25
0.00
A (n=15)
B (n=16)
C (n=14)
surface

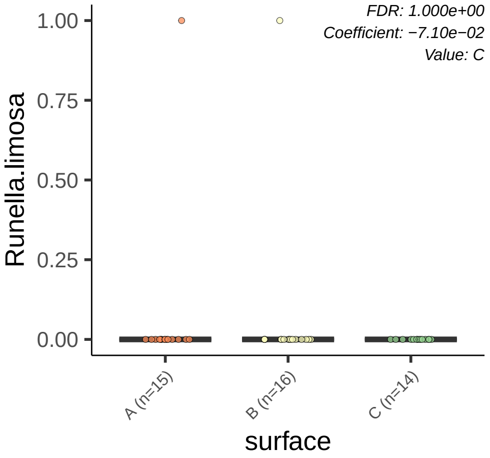
FDR: 1.000e+00
Coefficient: −7.10e−02
Value: C
Runella.limosa
1.00
0.75
0.50
0.25
0.00
A (n=15)
B (n=16)
C (n=14)
surface

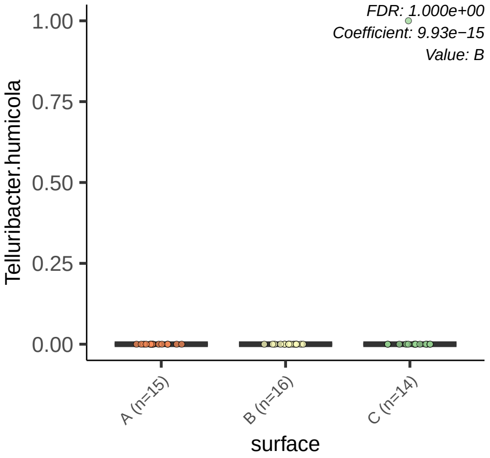
FDR: 1.000e+00
Coefficient: 9.93e−15
Value: B
Telluribacter.humicola
1.00
0.75
0.50
0.25
0.00
A (n=15)
B (n=16)
C (n=14)
surface

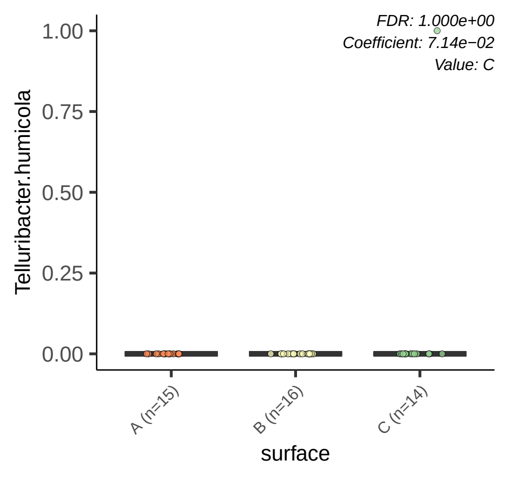
FDR: 1.000e+00
Coefficient: 7.14e−02
Value: C
Telluribacter.humicola
1.00
0.75
0.50
0.25
0.00
A (n=15)
B (n=16)
C (n=14)
surface

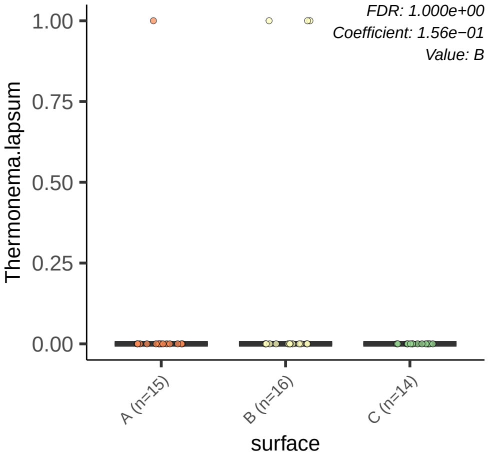
FDR: 1.000e+00
Coefficient: 1.56e−01
Value: B
1.00
0.75
0.50
0.25
0.00
Thermonema.lapsum
A (n=15)
B (n=16)
C (n=14)
surface

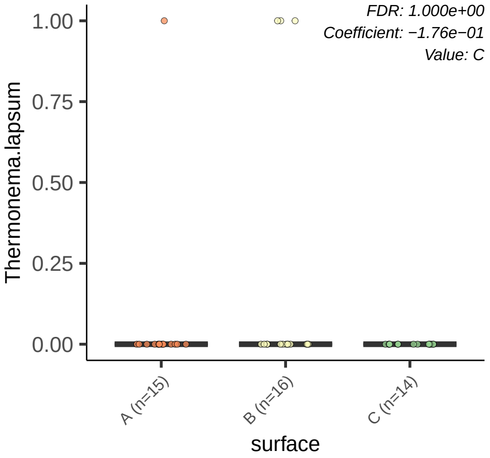
FDR: 1.000e+00
Coefficient: −1.76e−01
Value: C
Thermonema.lapsum
1.00
0.75
0.50
0.25
0.00
A (n=15)
B (n=16)
C (n=14)
surface

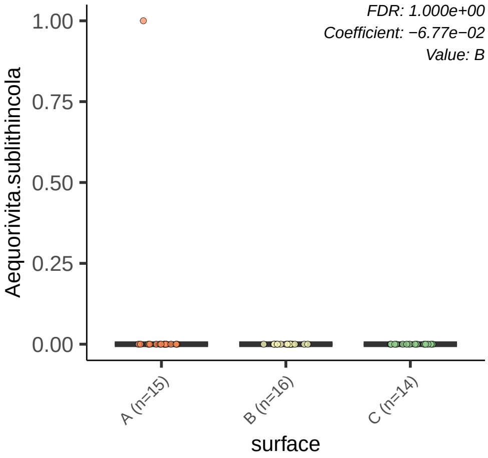

FDR: 1.000e+00
Coefficient: −6.77e−02
Value: B
Aequorivita.sublithincola
1.00
0.75
0.50
0.25
0.00
A (n=15)
B (n=16)
C (n=14)
surface

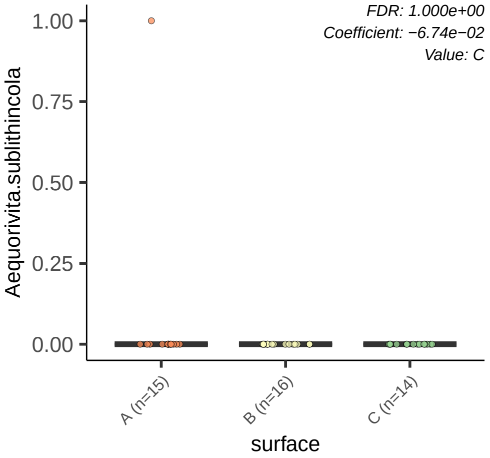
FDR: 1.000e+00
Coefficient: −6.74e−02
Value: C
Aequorivita.sublithincola
1.00
0.75
0.50
0.25
0.00
A (n=15)
B (n=16)
C (n=14)
surface

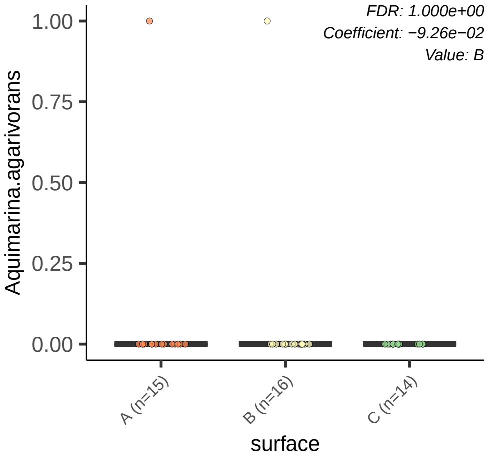

FDR: 1.000e+00
Coefficient: −9.26e−02
Value: B
Aquimarina.agarivorans
1.00
0.75
0.50
0.25
0.00
A (n=15)
B (n=16)
C (n=14)
surface

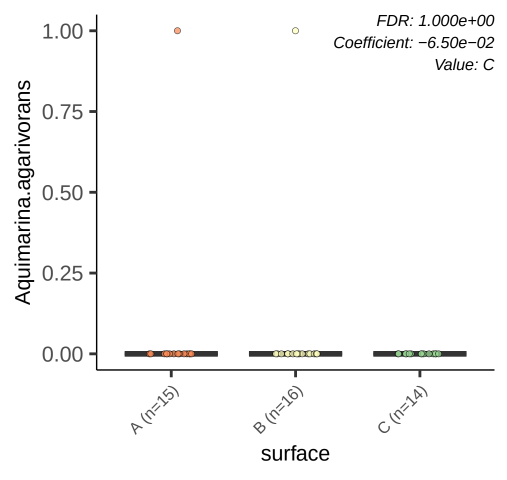

FDR: 1.000e+00
Coefficient: −6.50e−02
Value: C
Aquimarina.agarivorans
1.00
0.75
0.50
0.25
0.00
A (n=15)
B (n=16)
C (n=14)
surface

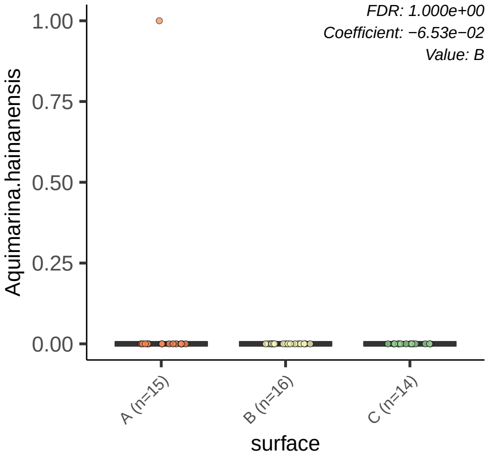
FDR: 1.000e+00
Coefficient: −6.53e−02
Value: B
Aquimarina.hainanensis
1.00
0.75
0.50
0.25
0.00
A (n=15)
B (n=16)
C (n=14)
surface

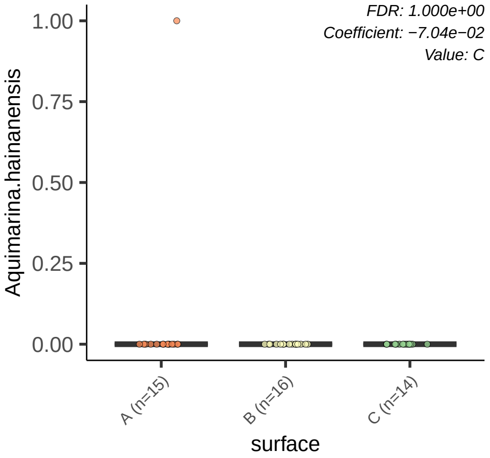

FDR: 1.000e+00
Coefficient: −7.04e−02
Value: C
Aquimarina.hainanensis
1.00
0.75
0.50
0.25
0.00
A (n=15)
B (n=16)
C (n=14)
surface

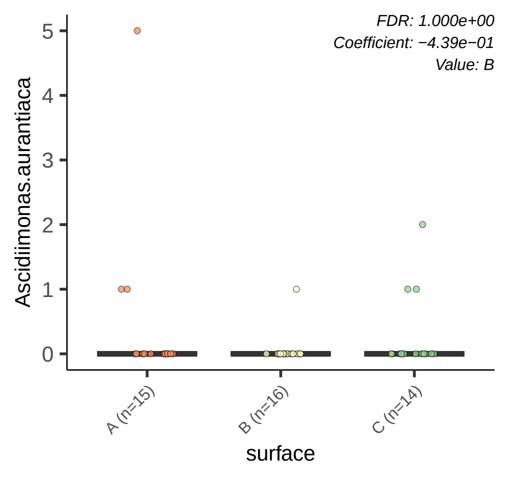

FDR: 1.000e+00
Coefficient: −4.39e−01
Value: B
Ascidiimonas.aurantiaca
0
1
2
3
4
5
A (n=15)
B (n=16)
C (n=14)
surface

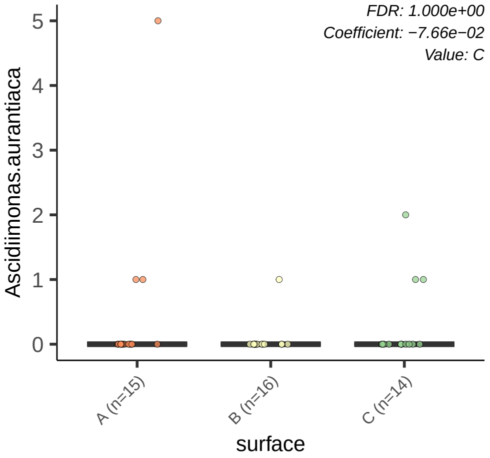
FDR: 1.000e+00
Coefficient: −7.66e−02
Value: C
Ascidiimonas.aurantiaca
0
1
2
3
4
5
A (n=15)
B (n=16)
C (n=14)
surface

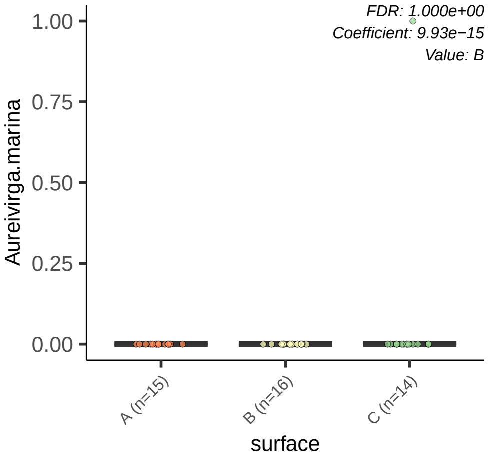
FDR: 1.000e+00
Coefficient: 9.93e−15
Value: B
Aureivirga.marina
1.00
0.75
0.50
0.25
0.00
A (n=15)
B (n=16)
C (n=14)
surface

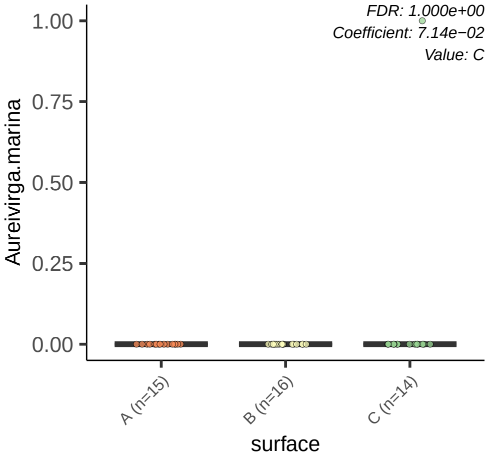
FDR: 1.000e+00
Coefficient: 7.14e−02
Value: C
Aureivirga.marina
1.00
0.75
0.50
0.25
0.00
A (n=15)
B (n=16)
C (n=14)
surface

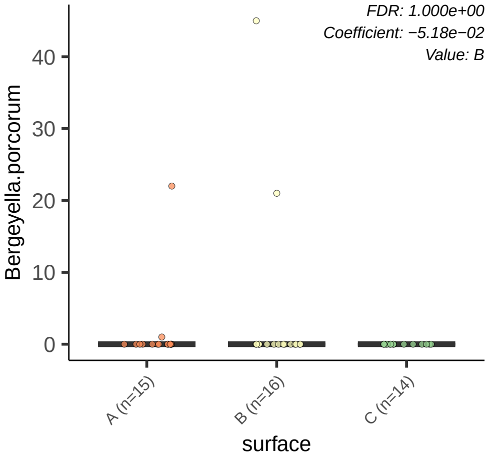
FDR: 1.000e+00
Coefficient: −5.18e−02
Value: B
Bergeyella.porcorum
0
10
20
30
40
A (n=15)
B (n=16)
C (n=14)
surface

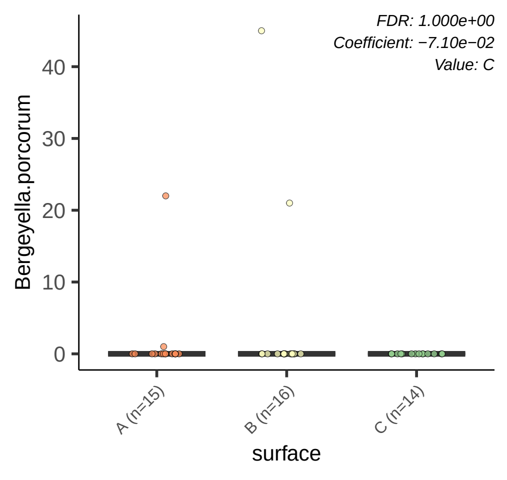

FDR: 1.000e+00
Coefficient: −7.10e−02
Value: C
Bergeyella.porcorum
0
10
20
30
40
A (n=15)
B (n=16)
C (n=14)
surface

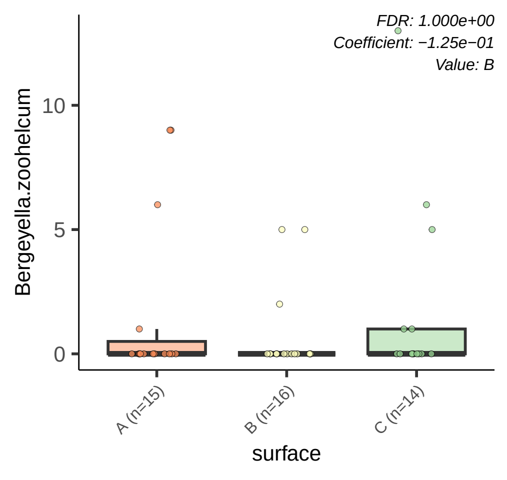

FDR: 1.000e+00
Coefficient: −1.25e−01
Value: B
Bergeyella.zoohelcum
10
5
0
A (n=15)
B (n=16)
C (n=14)
surface

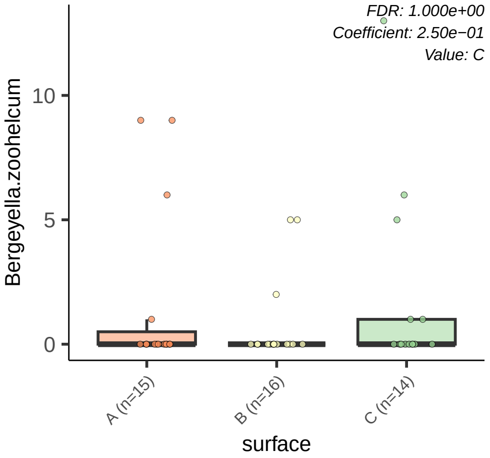

FDR: 1.000e+00
Coefficient: 2.50e−01
Value: C
Bergeyella.zoohelcum
10
5
0
A (n=15)
B (n=16)
C (n=14)
surface

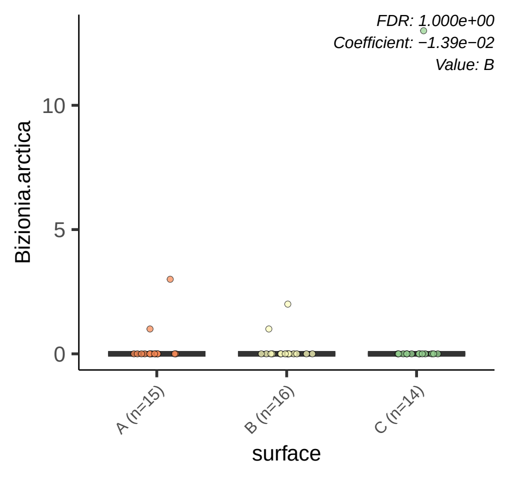
FDR: 1.000e+00
Coefficient: −1.39e−02
Value: B
Bizionia.arctica
10
5
0
A (n=15)
B (n=16)
C (n=14)
surface

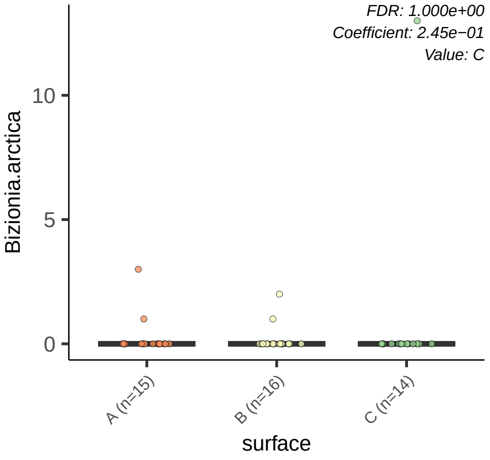
FDR: 1.000e+00
Coefficient: 2.45e−01
Value: C
Bizionia.arctica
10
5
0
A (n=15)
B (n=16)
C (n=14)
surface

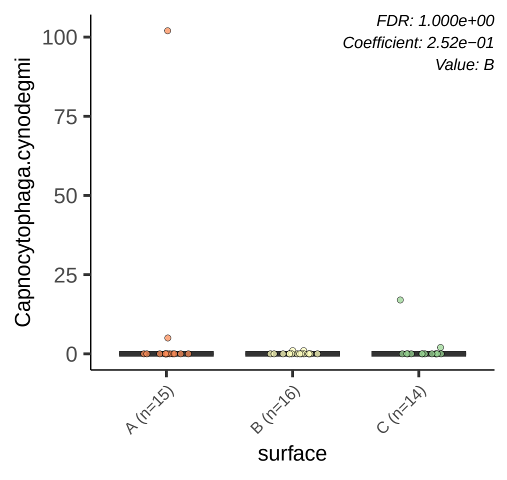

FDR: 1.000e+00
Coefficient: 2.52e−01
Value: B
Capnocytophaga.cynodegmi
100
75
50
25
0
A (n=15)
B (n=16)
C (n=14)
surface

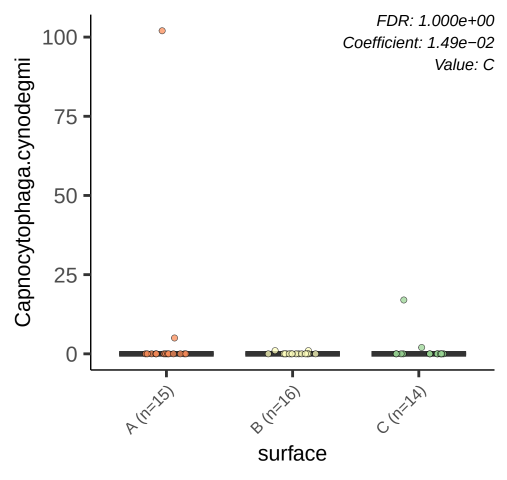
FDR: 1.000e+00
Coefficient: 1.49e−02
Value: C
Capnocytophaga.cynodegmi
100
75
50
25
0
A (n=15)
B (n=16)
C (n=14)
surface

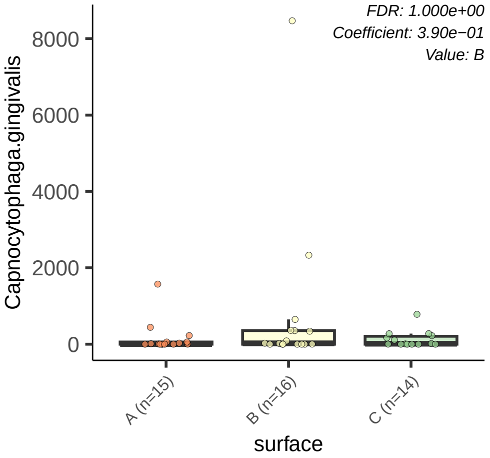
FDR: 1.000e+00
Coefficient: 3.90e−01
Value: B
Capnocytophaga.gingivalis
8000
6000
4000
2000
0
A (n=15)
B (n=16)
C (n=14)
surface

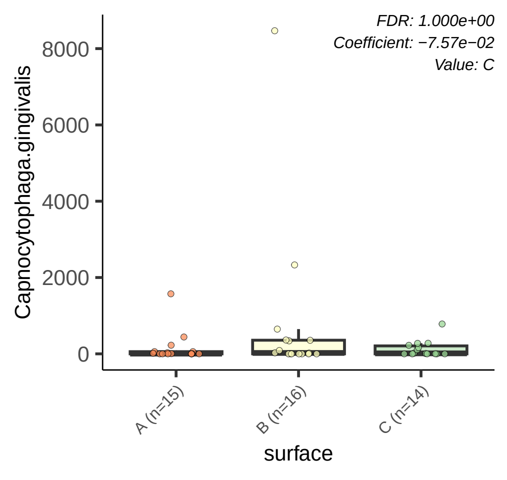
FDR: 1.000e+00
Coefficient: −7.57e−02
Value: C
Capnocytophaga.gingivalis
8000
6000
4000
2000
0
A (n=15)
B (n=16)
C (n=14)
surface

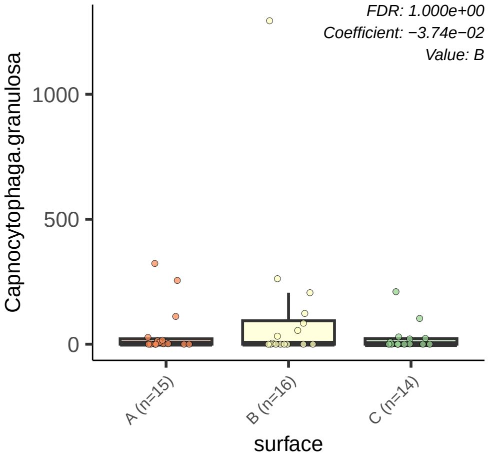

FDR: 1.000e+00
Coefficient: −3.74e−02
Value: B
Capnocytophaga.granulosa
1000
500
0
A (n=15)
B (n=16)
C (n=14)
surface

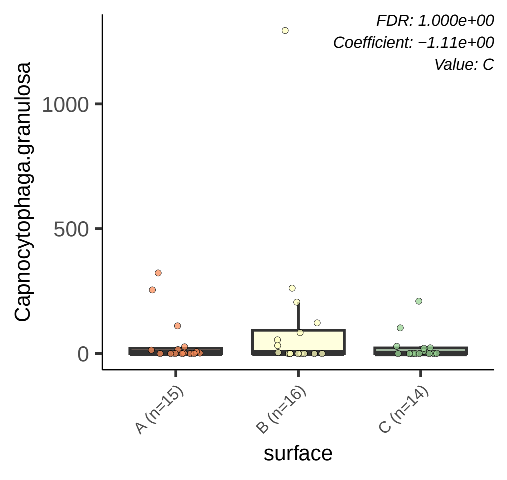
FDR: 1.000e+00
Coefficient: −1.11e+00
Value: C
Capnocytophaga.granulosa
1000
500
0
A (n=15)
B (n=16)
C (n=14)
surface

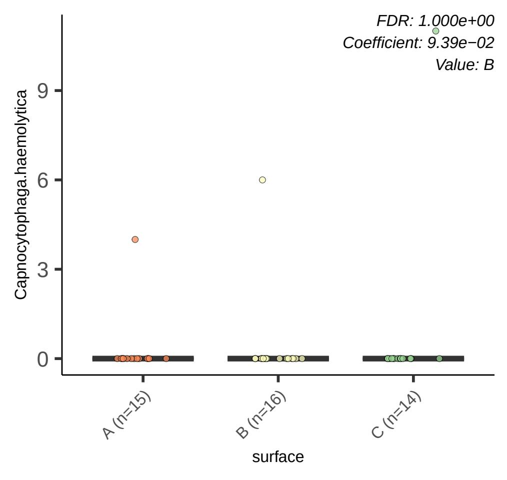
FDR: 1.000e+00
Coefficient: 9.39e−02
Value: B
Capnocytophaga.haemolytica
0
3
6
9
A (n=15)
B (n=16)
C (n=14)
surface

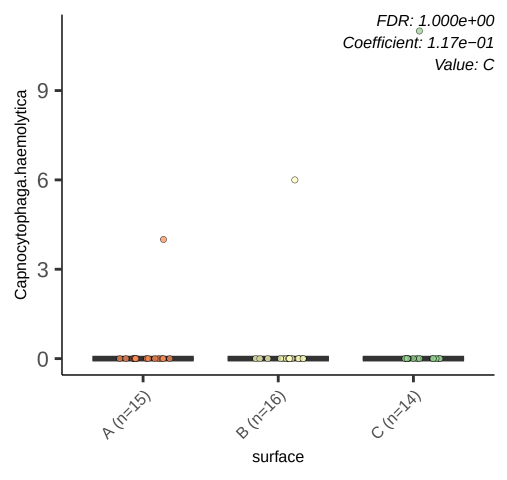

FDR: 1.000e+00
Coefficient: 1.17e−01
Value: C
Capnocytophaga.haemolytica
0
3
6
9
A (n=15)
B (n=16)
C (n=14)
surface

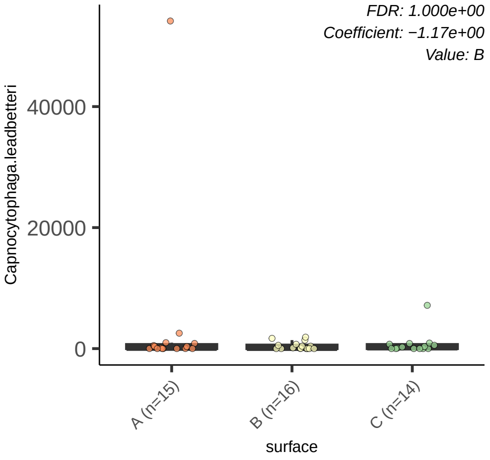
FDR: 1.000e+00
Coefficient: −1.17e+00
Value: B
Capnocytophaga.leadbetteri
40000
20000
0
A (n=15)
B (n=16)
C (n=14)
surface

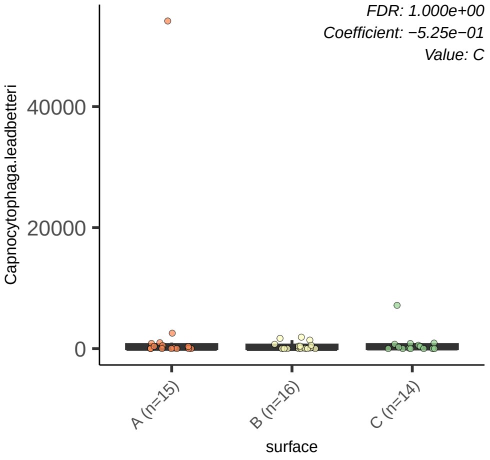
FDR: 1.000e+00
Coefficient: −5.25e−01
Value: C
Capnocytophaga.leadbetteri
40000
20000
0
A (n=15)
B (n=16)
C (n=14)
surface

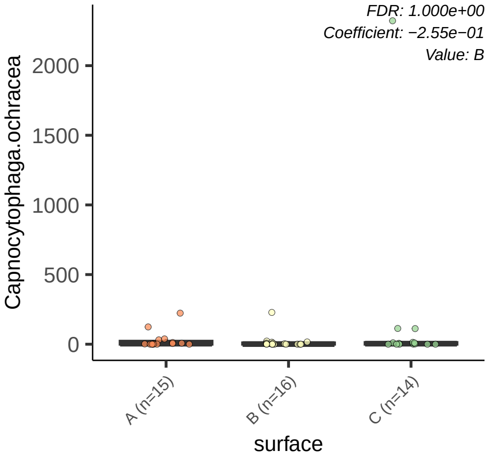
FDR: 1.000e+00
Coefficient: −2.55e−01
Value: B
Capnocytophaga.ochracea
2000
1500
1000
500
0
A (n=15)
B (n=16)
C (n=14)
surface

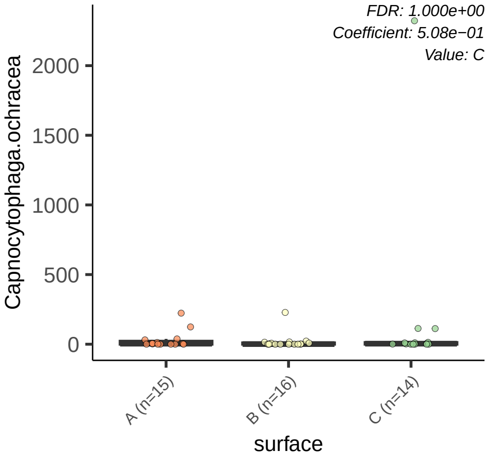

FDR: 1.000e+00
Coefficient: 5.08e−01
Value: C
Capnocytophaga.ochracea
2000
1500
1000
500
0
A (n=15)
B (n=16)
C (n=14)
surface

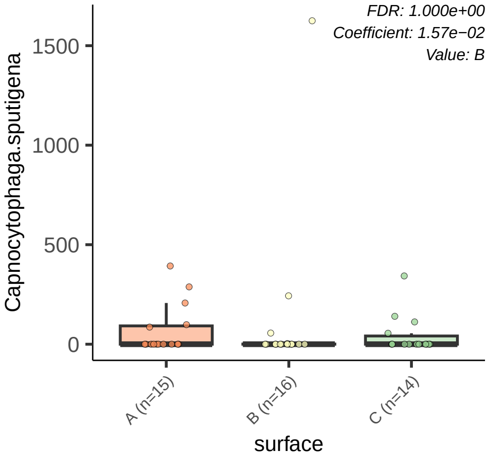
FDR: 1.000e+00
Coefficient: 1.57e−02
Value: B
Capnocytophaga.sputigena
1500
1000
500
0
A (n=15)
B (n=16)
C (n=14)
surface

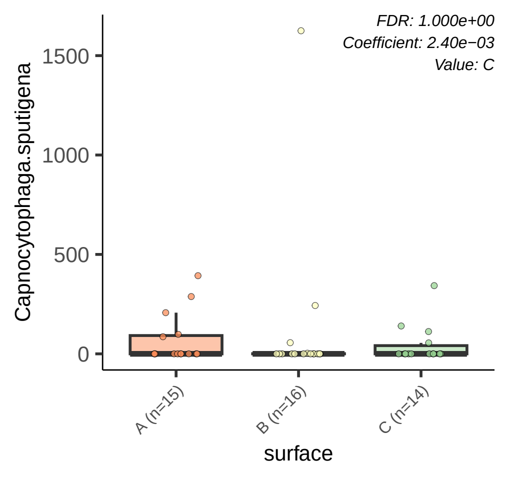
FDR: 1.000e+00
Coefficient: 2.40e−03
Value: C
Capnocytophaga.sputigena
1500
1000
500
0
A (n=15)
B (n=16)
C (n=14)
surface

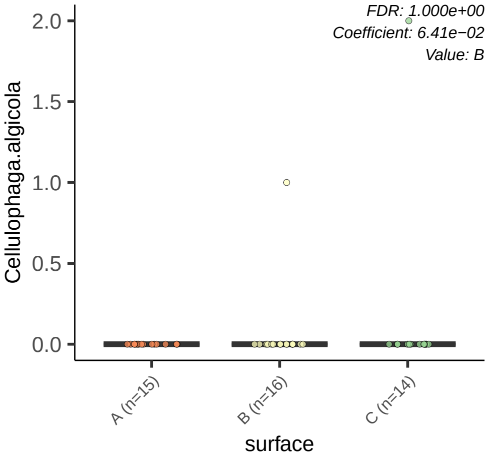

FDR: 1.000e+00
Coefficient: 6.41e−02
Value: B
Cellulophaga.algicola
2.0
1.5
1.0
0.5
0.0
A (n=15)
B (n=16)
C (n=14)
surface

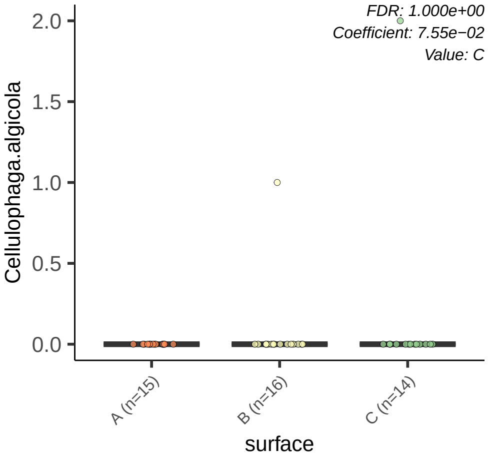
FDR: 1.000e+00
Coefficient: 7.55e−02
Value: C
Cellulophaga.algicola
2.0
1.5
1.0
0.5
0.0
A (n=15)
B (n=16)
C (n=14)
surface

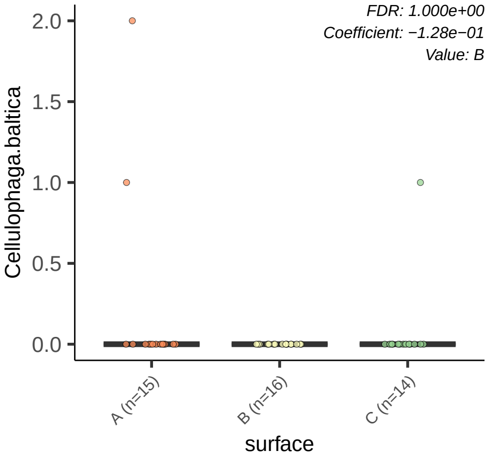
FDR: 1.000e+00
Coefficient: −1.28e−01
Value: B
Cellulophaga.baltica
2.0
1.5
1.0
0.5
0.0
A (n=15)
B (n=16)
C (n=14)
surface

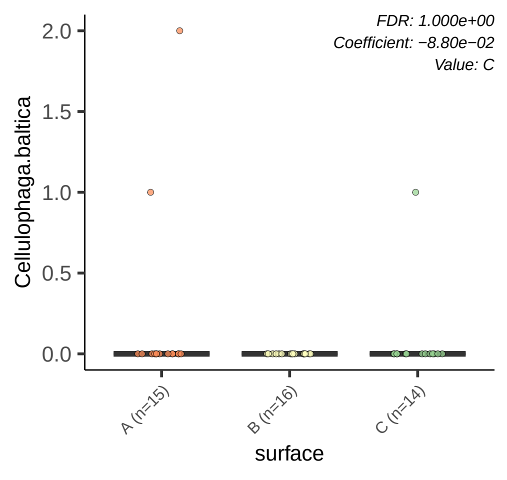
FDR: 1.000e+00
Coefficient: −8.80e−02
Value: C
Cellulophaga.baltica
2.0
1.5
1.0
0.5
0.0
A (n=15)
B (n=16)
C (n=14)
surface

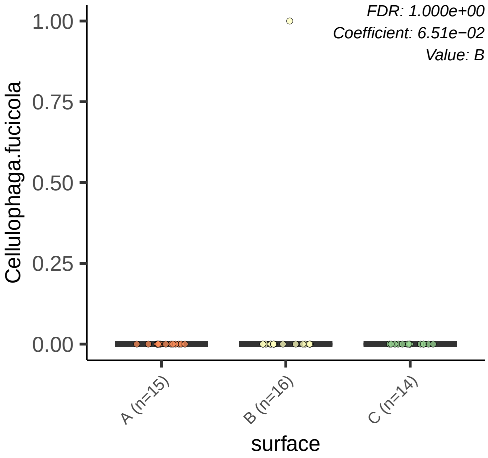

FDR: 1.000e+00
Coefficient: 6.51e−02
Value: B
Cellulophaga.fucicola
1.00
0.75
0.50
0.25
0.00
A (n=15)
B (n=16)
C (n=14)
surface

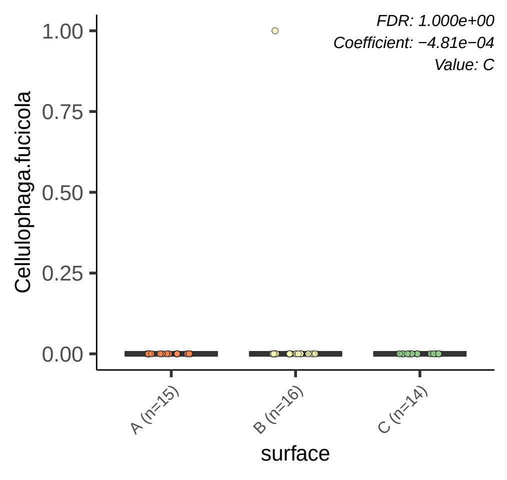
FDR: 1.000e+00
Coefficient: −4.81e−04
Value: C
Cellulophaga.fucicola
1.00
0.75
0.50
0.25
0.00
A (n=15)
B (n=16)
C (n=14)
surface

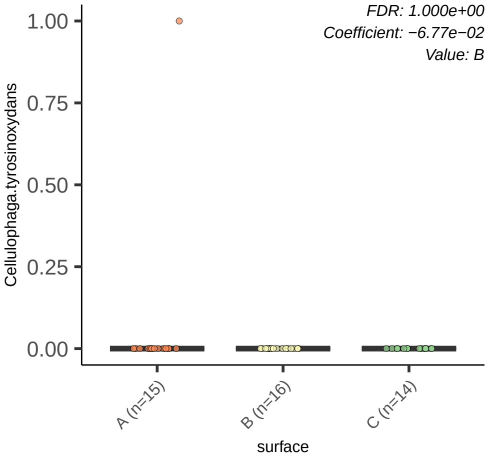
FDR: 1.000e+00
Coefficient: −6.77e−02
Value: B
Cellulophaga.tyrosinoxydans
1.00
0.75
0.50
0.25
0.00
A (n=15)
B (n=16)
C (n=14)
surface

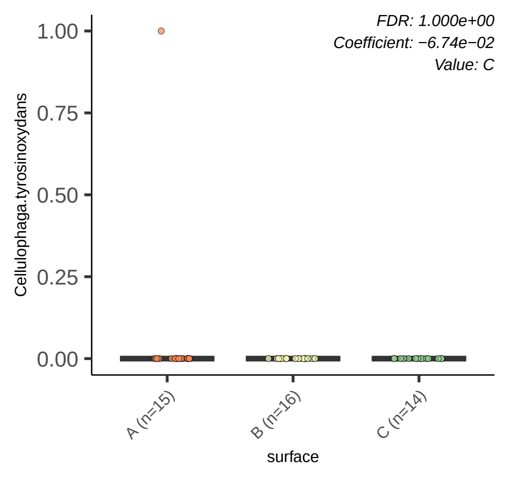
FDR: 1.000e+00
Coefficient: −6.74e−02
Value: C
1.00
0.75
0.50
0.25
0.00
Cellulophaga.tyrosinoxydans
A (n=15)
B (n=16)
C (n=14)
surface

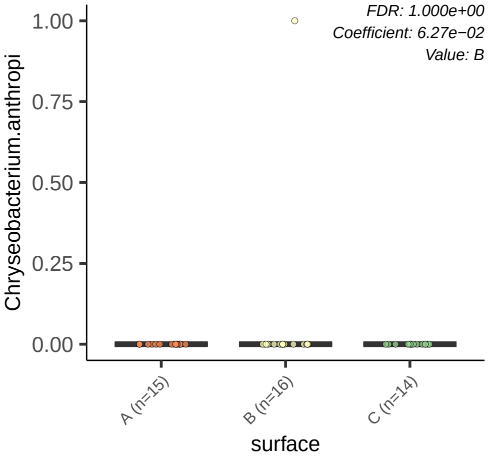

FDR: 1.000e+00
Coefficient: 6.27e−02
Value: B
Chryseobacterium.anthropi
1.00
0.75
0.50
0.25
0.00
A (n=15)
B (n=16)
C (n=14)
surface

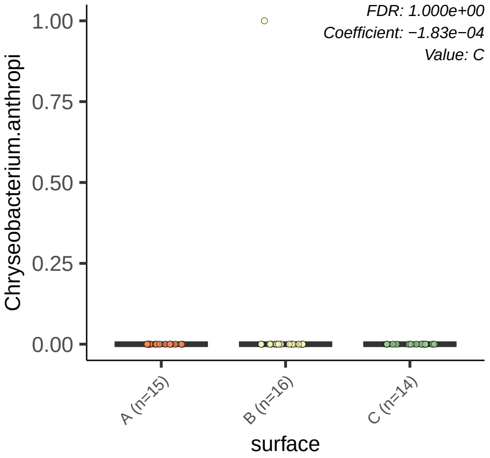
FDR: 1.000e+00
Coefficient: −1.83e−04
Value: C
Chryseobacterium.anthropi
1.00
0.75
0.50
0.25
0.00
A (n=15)
B (n=16)
C (n=14)
surface

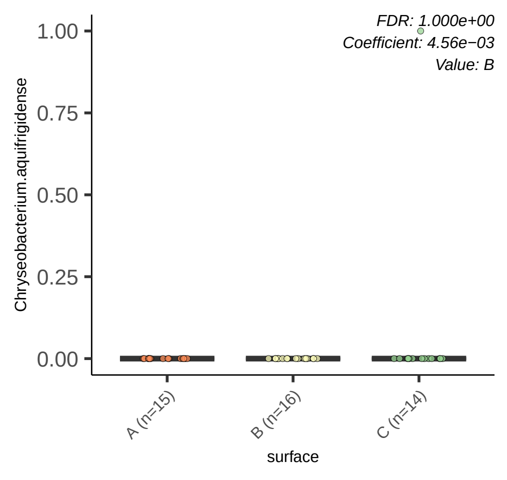
FDR: 1.000e+00
Coefficient: 4.56e−03
Value: B
1.00
0.75
0.50
0.25
0.00
Chryseobacterium.aquifrigidense
A (n=15)
B (n=16)
C (n=14)
surface

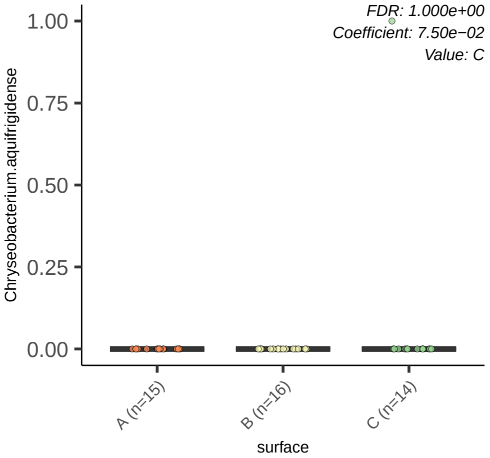

FDR: 1.000e+00
Coefficient: 7.50e−02
Value: C
1.00
0.75
0.50
0.25
0.00
Chryseobacterium.aquifrigidense
A (n=15)
B (n=16)
C (n=14)
surface

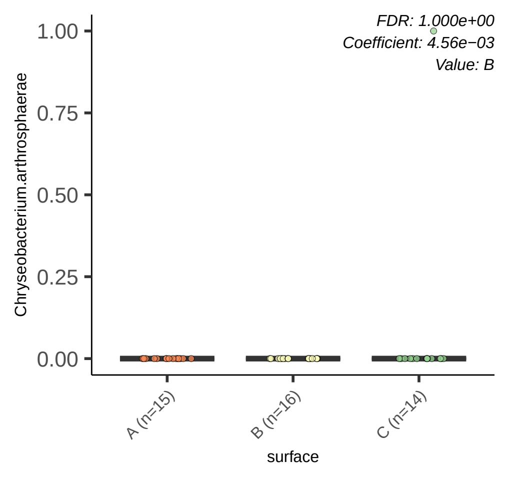
FDR: 1.000e+00
Coefficient: 4.56e−03
Value: B
1.00
0.75
0.50
0.25
0.00
Chryseobacterium.arthrosphaerae
A (n=15)
B (n=16)
C (n=14)
surface

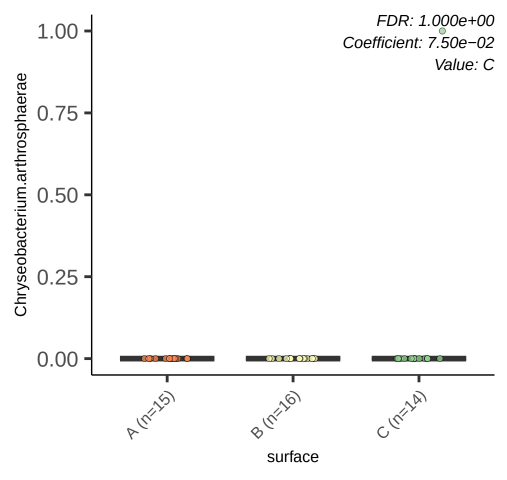
FDR: 1.000e+00
Coefficient: 7.50e−02
Value: C
1.00
0.75
0.50
0.25
0.00
Chryseobacterium.arthrosphaerae
A (n=15)
B (n=16)
C (n=14)
surface

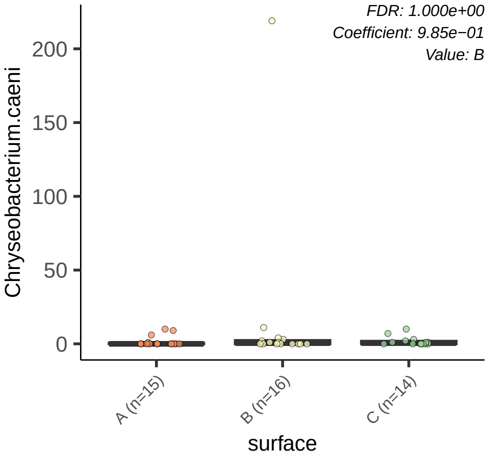

FDR: 1.000e+00
Coefficient: 9.85e−01
Value: B
Chryseobacterium.caeni
200
150
100
50
0
A (n=15)
B (n=16)
C (n=14)
surface

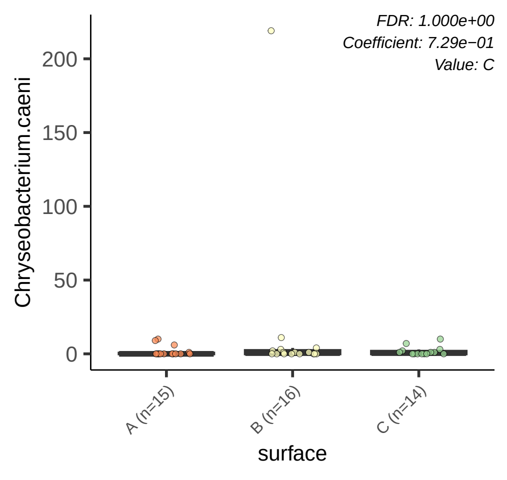
FDR: 1.000e+00
Coefficient: 7.29e−01
Value: C
Chryseobacterium.caeni
0
50
100
150
200
A (n=15)
B (n=16)
C (n=14)
surface

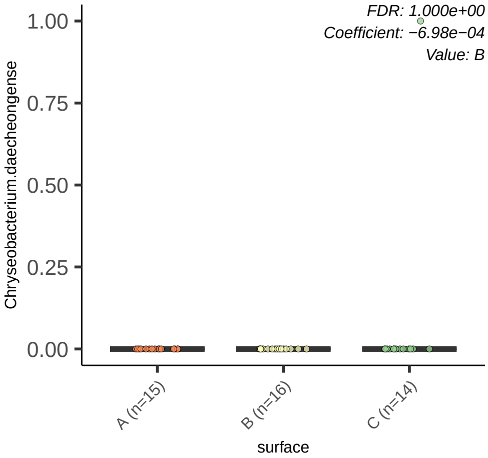

FDR: 1.000e+00
Coefficient: −6.98e−04
Value: B
1.00
0.75
0.50
0.25
0.00
Chryseobacterium.daecheongense
A (n=15)
B (n=16)
C (n=14)
surface

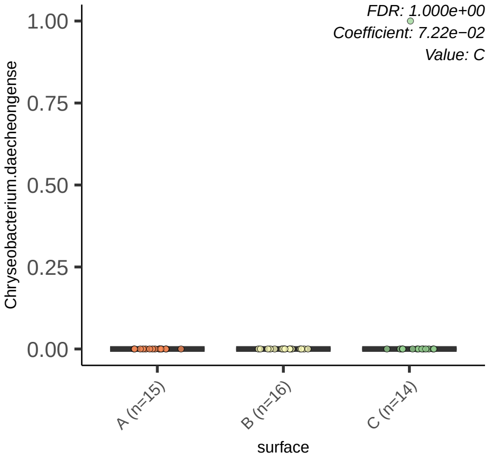

FDR: 1.000e+00
Coefficient: 7.22e−02
Value: C
1.00
0.75
0.50
0.25
0.00
Chryseobacterium.daecheongense
A (n=15)
B (n=16)
C (n=14)
surface

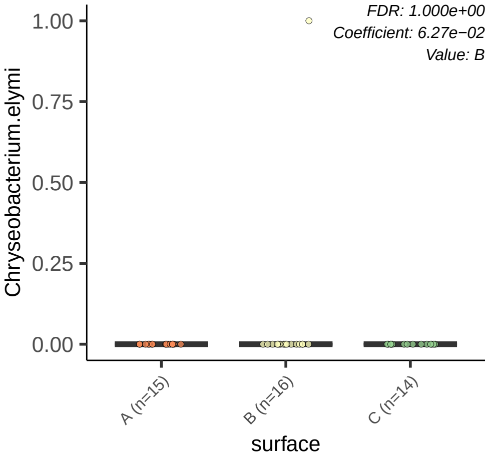
FDR: 1.000e+00
Coefficient: 6.27e−02
Value: B
Chryseobacterium.elymi
1.00
0.75
0.50
0.25
0.00
A (n=15)
B (n=16)
C (n=14)
surface

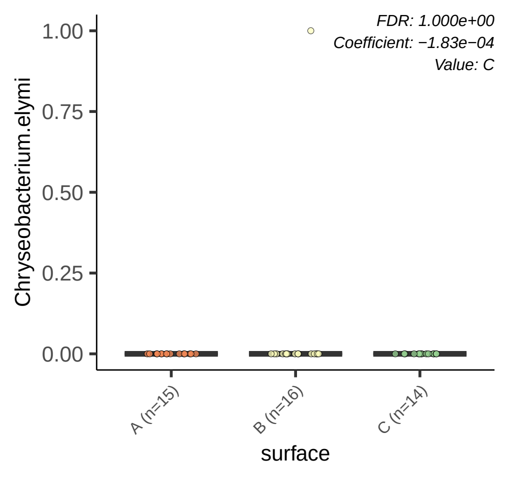

FDR: 1.000e+00
Coefficient: −1.83e−04
Value: C
Chryseobacterium.elymi
1.00
0.75
0.50
0.25
0.00
A (n=15)
B (n=16)
C (n=14)
surface

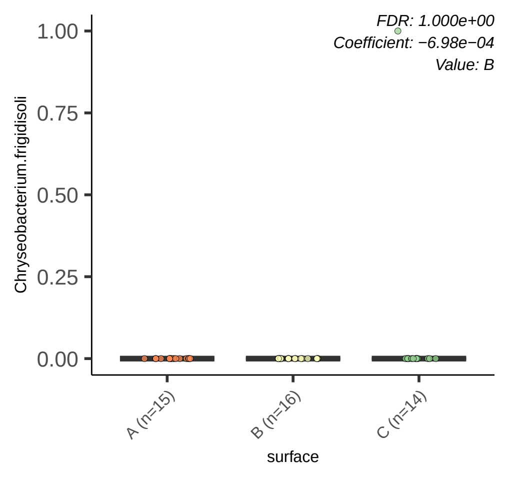
FDR: 1.000e+00
Coefficient: −6.98e−04
Value: B
Chryseobacterium.frigidisoli
1.00
0.75
0.50
0.25
0.00
A (n=15)
B (n=16)
C (n=14)
surface

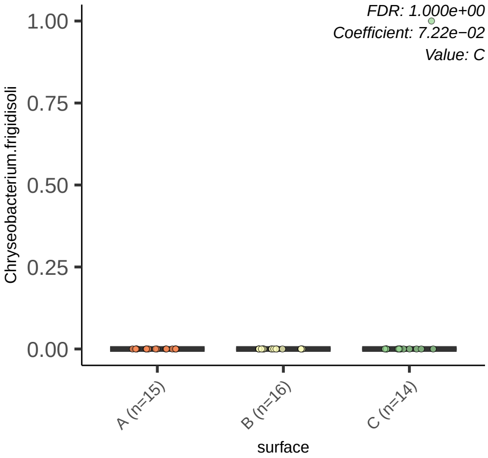

FDR: 1.000e+00
Coefficient: 7.22e−02
Value: C
1.00
0.75
0.50
0.25
0.00
Chryseobacterium.frigidisoli
A (n=15)
B (n=16)
C (n=14)
surface

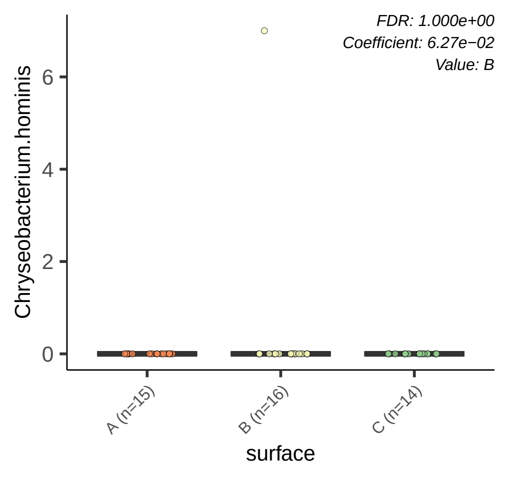
FDR: 1.000e+00
Coefficient: 6.27e−02
Value: B
Chryseobacterium.hominis
0
2
4
6
A (n=15)
B (n=16)
C (n=14)
surface

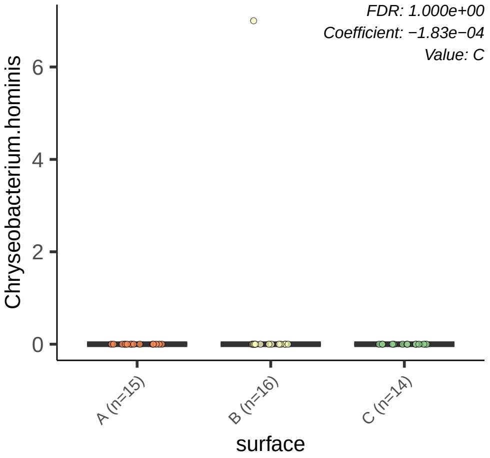
FDR: 1.000e+00
Coefficient: −1.83e−04
Value: C
Chryseobacterium.hominis
0
2
4
6
A (n=15)
B (n=16)
C (n=14)
surface

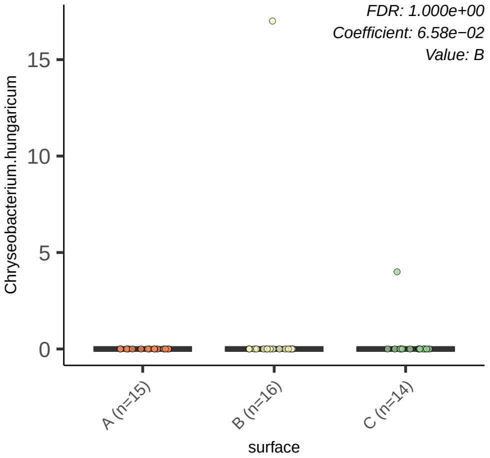
FDR: 1.000e+00
Coefficient: 6.58e−02
Value: B
Chryseobacterium.hungaricum
15
10
5
0
A (n=15)
B (n=16)
C (n=14)
surface

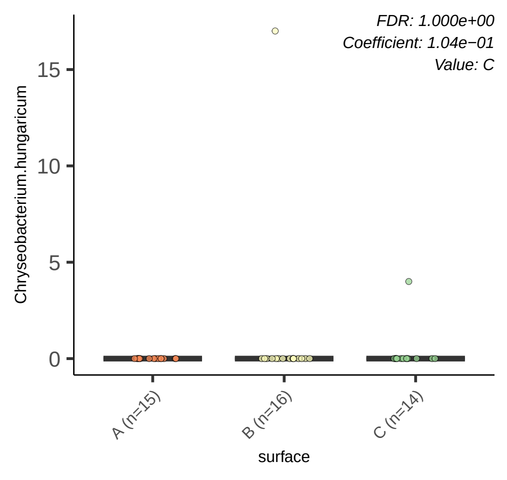
FDR: 1.000e+00
Coefficient: 1.04e−01
Value: C
Chryseobacterium.hungaricum
15
10
5
0
A (n=15)
B (n=16)
C (n=14)
surface

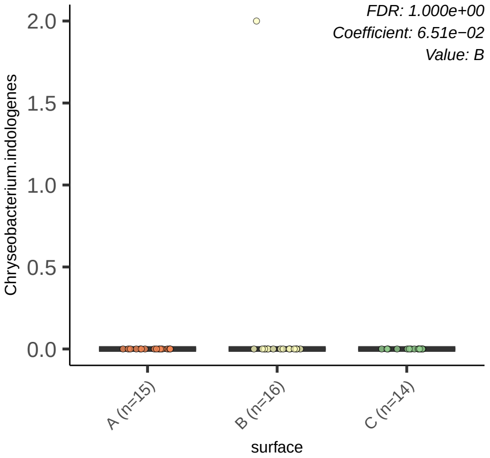

FDR: 1.000e+00
Coefficient: 6.51e−02
Value: B
Chryseobacterium.indologenes
2.0
1.5
1.0
0.5
0.0
A (n=15)
B (n=16)
C (n=14)
surface

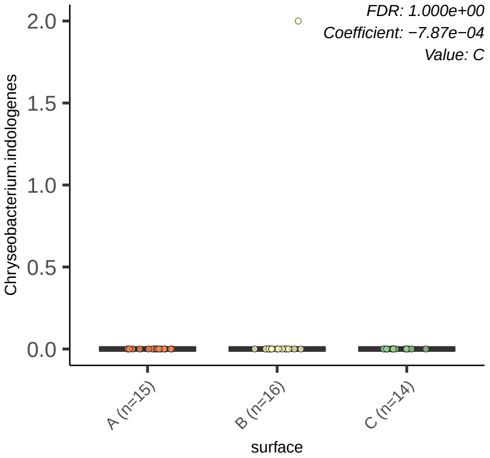
FDR: 1.000e+00
Coefficient: −7.87e−04
Value: C
Chryseobacterium.indologenes
2.0
1.5
1.0
0.5
0.0
A (n=15)
B (n=16)
C (n=14)
surface

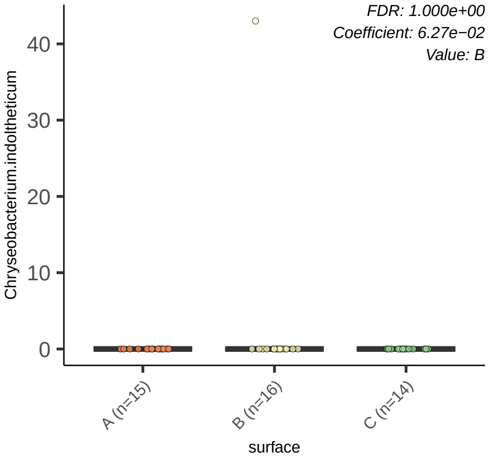

FDR: 1.000e+00
Coefficient: 6.27e−02
Value: B
Chryseobacterium.indoltheticum
40
30
20
10
0
A (n=15)
B (n=16)
C (n=14)
surface

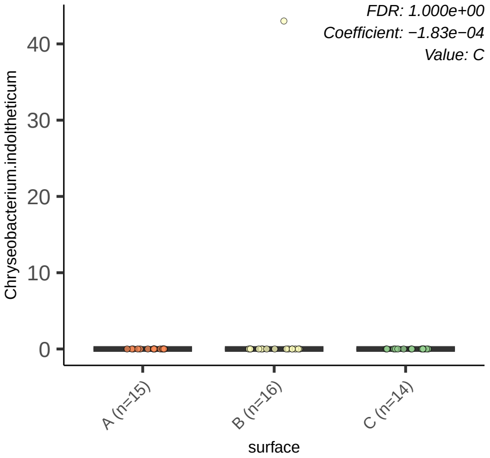
FDR: 1.000e+00
Coefficient: −1.83e−04
Value: C
Chryseobacterium.indoltheticum
40
30
20
10
0
A (n=15)
B (n=16)
C (n=14)
surface

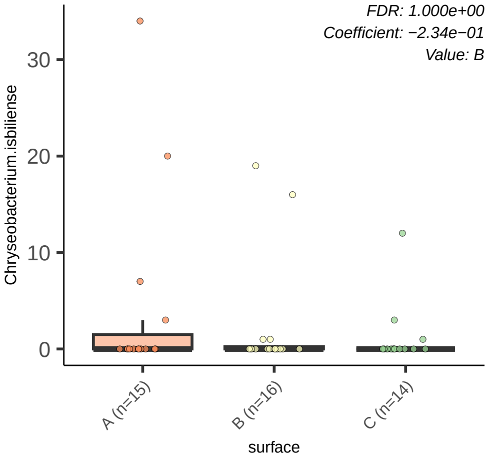
FDR: 1.000e+00
Coefficient: −2.34e−01
Value: B
Chryseobacterium.isbiliense
30
20
10
0
A (n=15)
B (n=16)
C (n=14)
surface

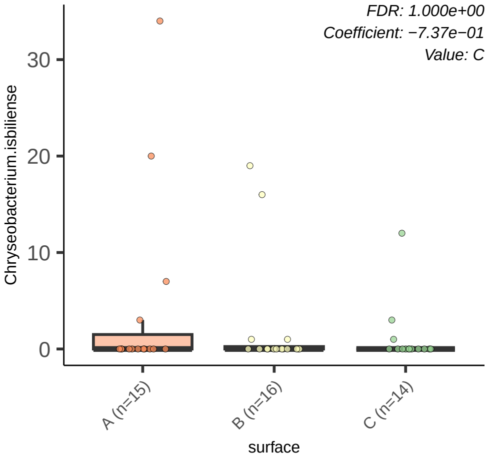

FDR: 1.000e+00
Coefficient: −7.37e−01
Value: C
Chryseobacterium.isbiliense
30
20
10
0
A (n=15)
B (n=16)
C (n=14)
surface

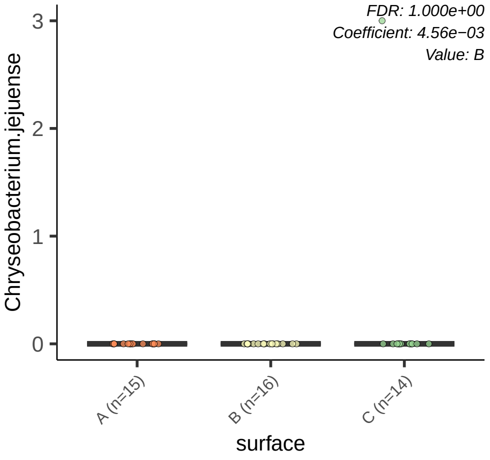

FDR: 1.000e+00
Coefficient: 4.56e−03
Value: B
Chryseobacterium.jejuense
3
2
1
0
A (n=15)
B (n=16)
C (n=14)
surface

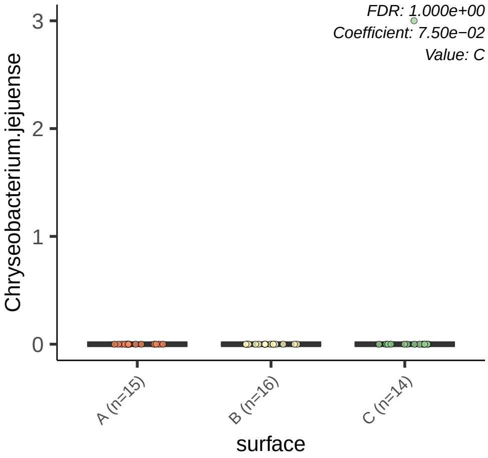

FDR: 1.000e+00
Coefficient: 7.50e−02
Value: C
Chryseobacterium.jejuense
3
2
1
0
A (n=15)
B (n=16)
C (n=14)
surface

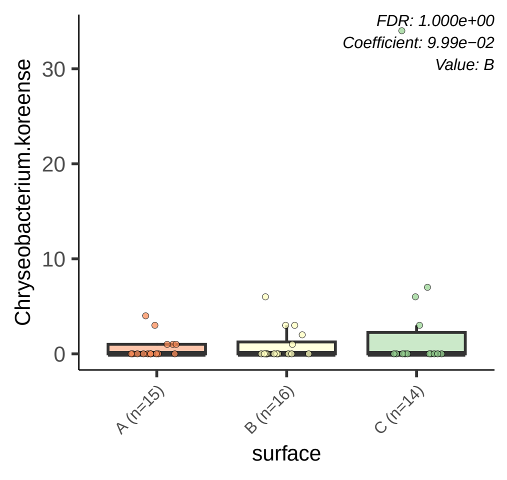
FDR: 1.000e+00
Coefficient: 9.99e−02
Value: B
Chryseobacterium.koreense
0
10
20
30
A (n=15)
B (n=16)
C (n=14)
surface

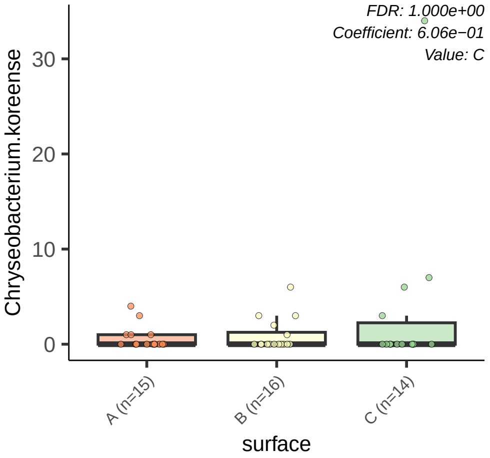
FDR: 1.000e+00
Coefficient: 6.06e−01
Value: C
Chryseobacterium.koreense
30
20
10
0
A (n=15)
B (n=16)
C (n=14)
surface

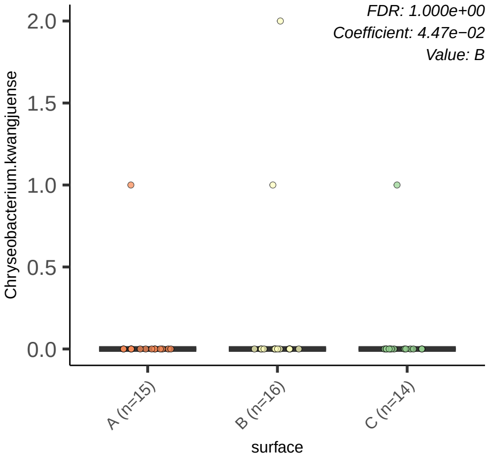

FDR: 1.000e+00
Coefficient: 4.47e−02
Value: B
Chryseobacterium.kwangjuense
2.0
1.5
1.0
0.5
0.0
A (n=15)
B (n=16)
C (n=14)
surface

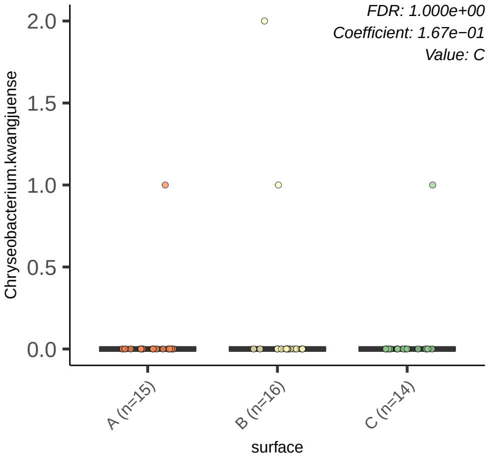
FDR: 1.000e+00
Coefficient: 1.67e−01
Value: C
Chryseobacterium.kwangjuense
2.0
1.5
1.0
0.5
0.0
A (n=15)
B (n=16)
C (n=14)
surface

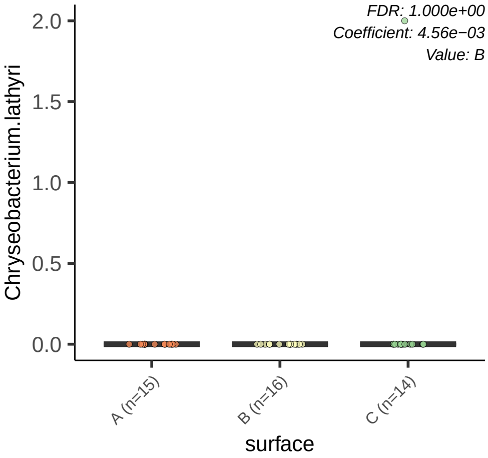
FDR: 1.000e+00
Coefficient: 4.56e−03
Value: B
Chryseobacterium.lathyri
2.0
1.5
1.0
0.5
0.0
A (n=15)
B (n=16)
C (n=14)
surface

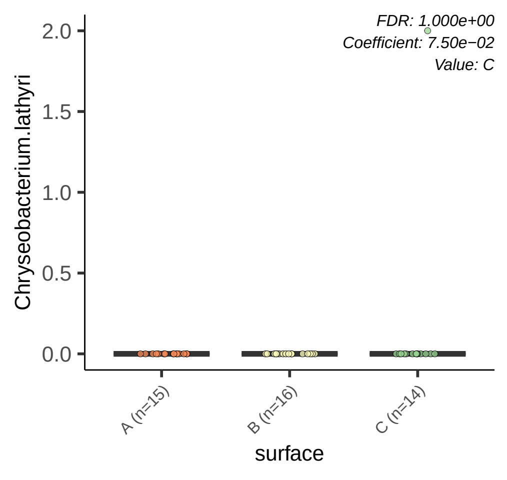
FDR: 1.000e+00
Coefficient: 7.50e−02
Value: C
Chryseobacterium.lathyri
2.0
1.5
1.0
0.5
0.0
A (n=15)
B (n=16)
C (n=14)
surface

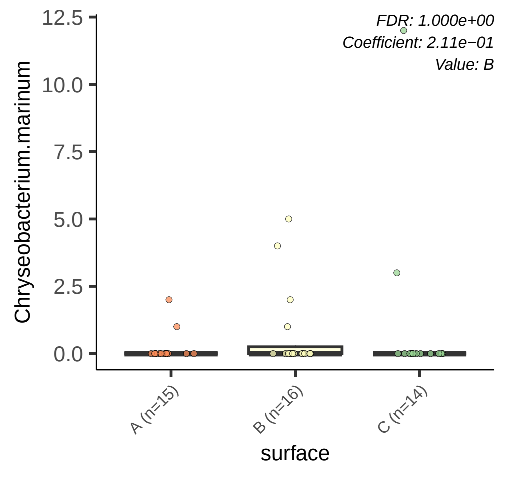
FDR: 1.000e+00
Coefficient: 2.11e−01
Value: B
Chryseobacterium.marinum
12.5
10.0
7.5
5.0
2.5
0.0
A (n=15)
B (n=16)
C (n=14)
surface

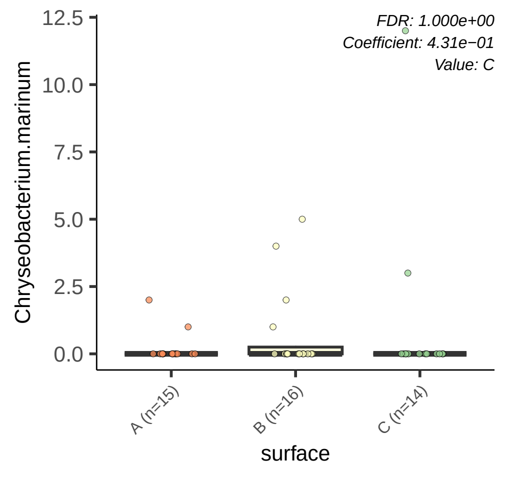

FDR: 1.000e+00
Coefficient: 4.31e−01
Value: C
Chryseobacterium.marinum
12.5
10.0
7.5
5.0
2.5
0.0
A (n=15)
B (n=16)
C (n=14)
surface

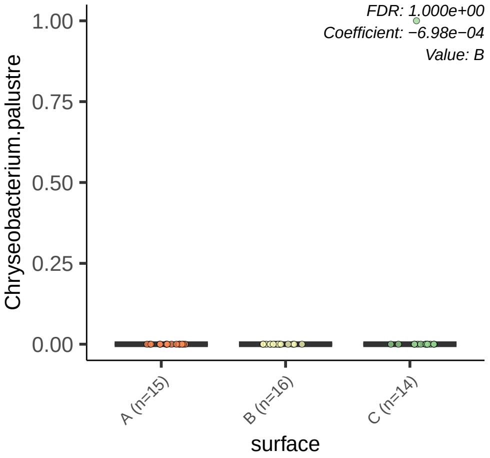
FDR: 1.000e+00
Coefficient: −6.98e−04
Value: B
Chryseobacterium.palustre
1.00
0.75
0.50
0.25
0.00
A (n=15)
B (n=16)
C (n=14)
surface

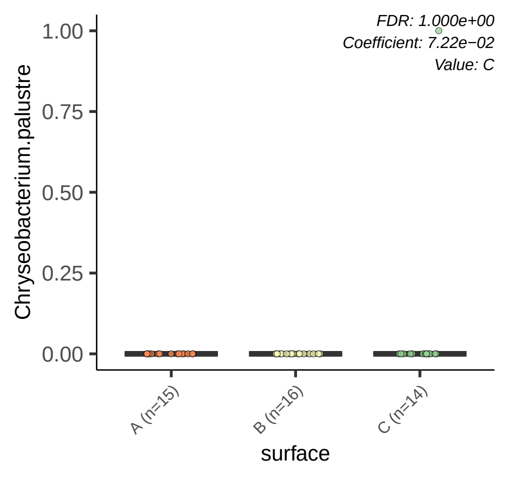
FDR: 1.000e+00
Coefficient: 7.22e−02
Value: C
Chryseobacterium.palustre
1.00
0.75
0.50
0.25
0.00
A (n=15)
B (n=16)
C (n=14)
surface

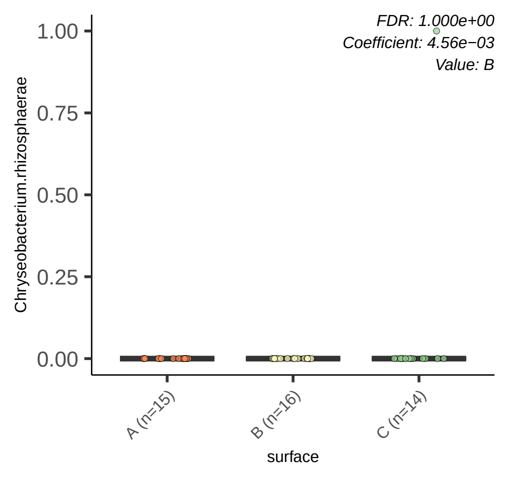

FDR: 1.000e+00
Coefficient: 4.56e−03
Value: B
1.00
0.75
0.50
0.25
0.00
Chryseobacterium.rhizosphaerae
A (n=15)
B (n=16)
C (n=14)
surface

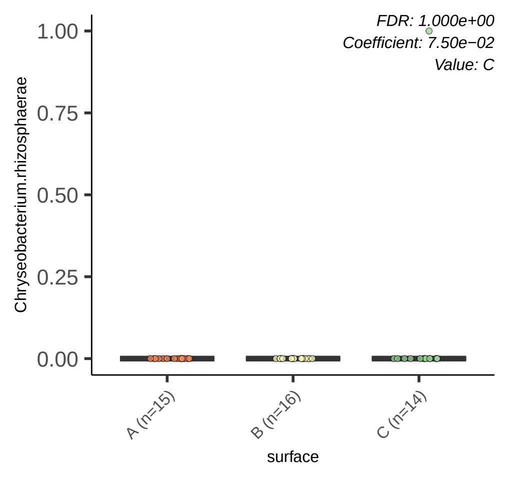
FDR: 1.000e+00
Coefficient: 7.50e−02
Value: C
1.00
0.75
0.50
0.25
0.00
Chryseobacterium.rhizosphaerae
A (n=15)
B (n=16)
C (n=14)
surface

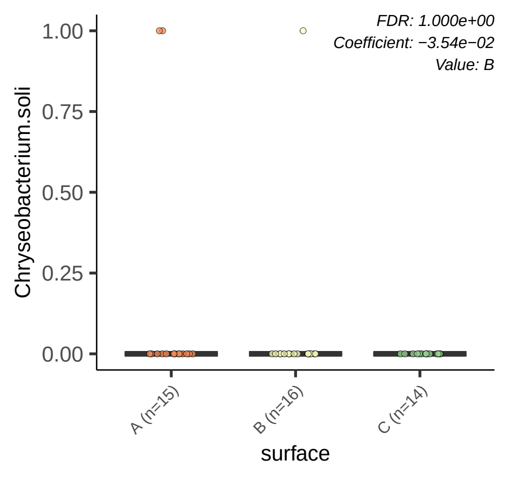
FDR: 1.000e+00
Coefficient: −3.54e−02
Value: B
Chryseobacterium.soli
1.00
0.75
0.50
0.25
0.00
A (n=15)
B (n=16)
C (n=14)
surface

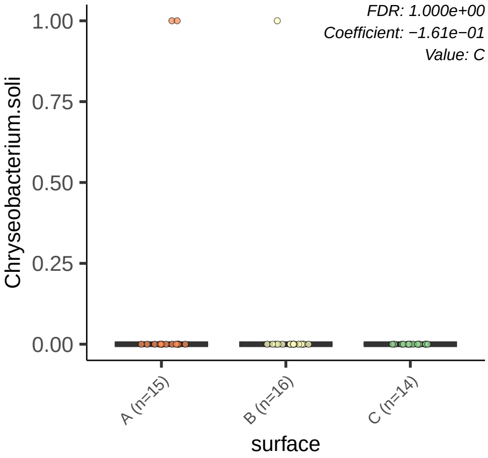

FDR: 1.000e+00
Coefficient: −1.61e−01
Value: C
Chryseobacterium.soli
1.00
0.75
0.50
0.25
0.00
A (n=15)
B (n=16)
C (n=14)
surface

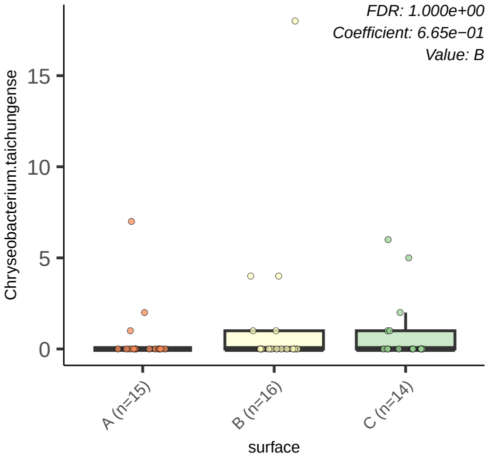

FDR: 1.000e+00
Coefficient: 6.65e−01
Value: B
Chryseobacterium.taichungense
0
5
10
15
A (n=15)
B (n=16)
C (n=14)
surface

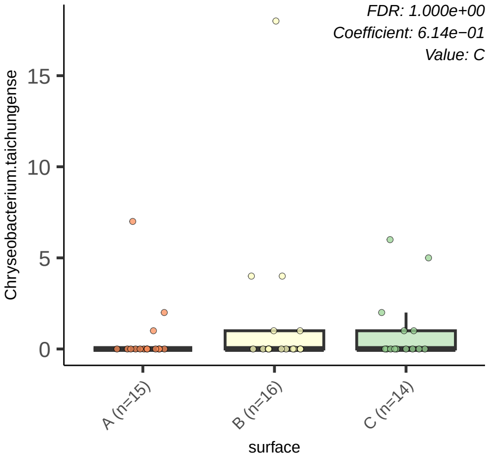

FDR: 1.000e+00
Coefficient: 6.14e−01
Value: C
Chryseobacterium.taichungense
0
5
10
15
A (n=15)
B (n=16)
C (n=14)
surface

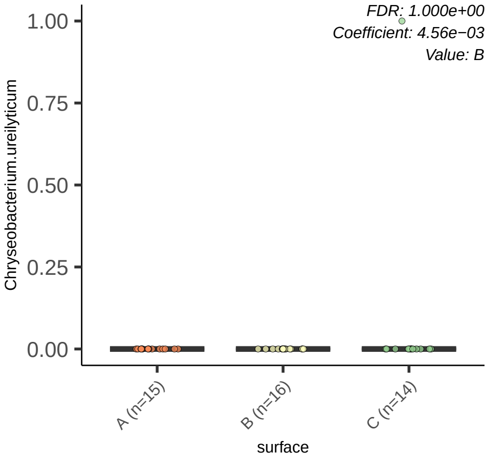

FDR: 1.000e+00
Coefficient: 4.56e−03
Value: B
1.00
0.75
0.50
0.25
0.00
Chryseobacterium.ureilyticum
A (n=15)
B (n=16)
C (n=14)
surface

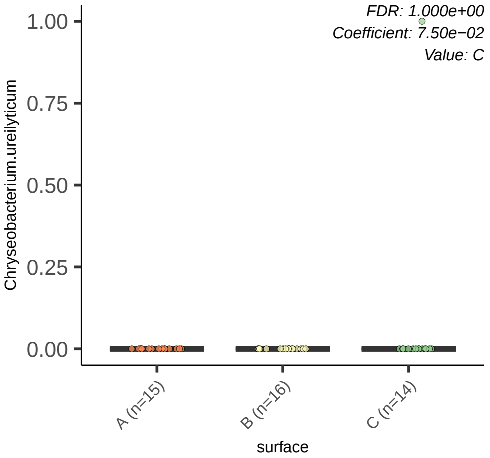

FDR: 1.000e+00
Coefficient: 7.50e−02
Value: C
1.00
0.75
0.50
0.25
0.00
Chryseobacterium.ureilyticum
A (n=15)
B (n=16)
C (n=14)
surface

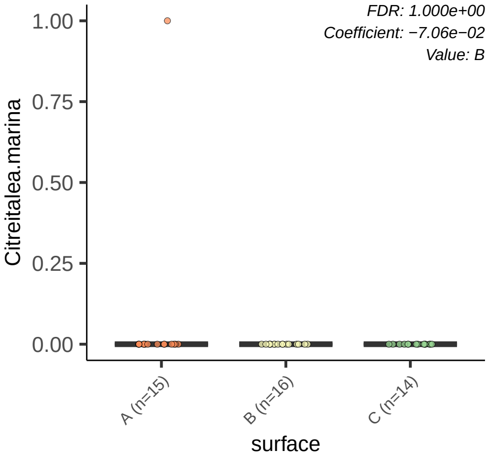
FDR: 1.000e+00
Coefficient: −7.06e−02
Value: B
Citreitalea.marina
1.00
0.75
0.50
0.25
0.00
A (n=15)
B (n=16)
C (n=14)
surface

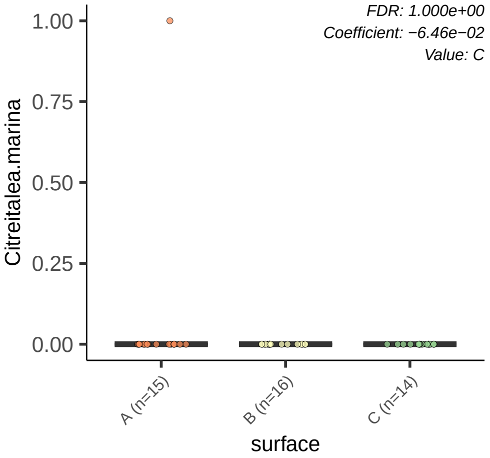

FDR: 1.000e+00
Coefficient: −6.46e−02
Value: C
Citreitalea.marina
1.00
0.75
0.50
0.25
0.00
A (n=15)
B (n=16)
C (n=14)
surface

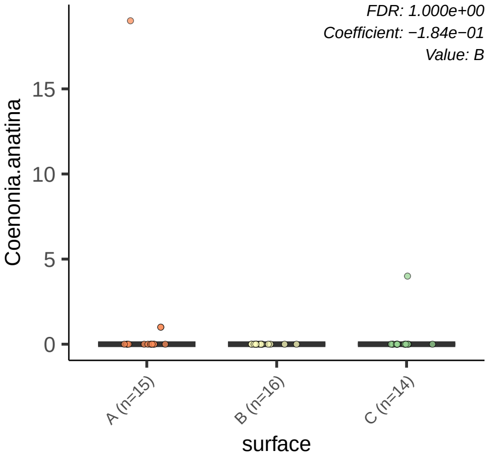
FDR: 1.000e+00
Coefficient: −1.84e−01
Value: B
Coenonia.anatina
0
5
10
15
A (n=15)
B (n=16)
C (n=14)
surface

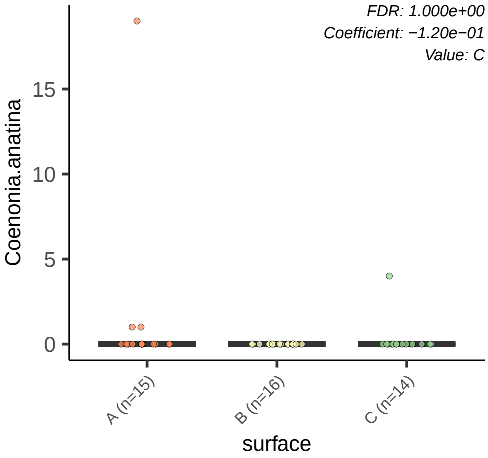
FDR: 1.000e+00
Coefficient: −1.20e−01
Value: C
Coenonia.anatina
0
5
10
15
A (n=15)
B (n=16)
C (n=14)
surface

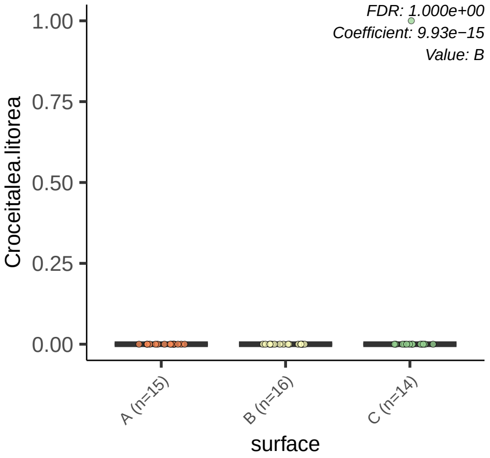
FDR: 1.000e+00
Coefficient: 9.93e−15
Value: B
Croceitalea.litorea
1.00
0.75
0.50
0.25
0.00
A (n=15)
B (n=16)
C (n=14)
surface

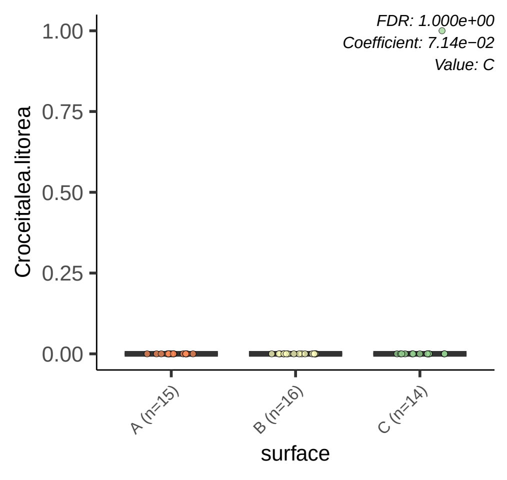

FDR: 1.000e+00
Coefficient: 7.14e−02
Value: C
Croceitalea.litorea
1.00
0.75
0.50
0.25
0.00
A (n=15)
B (n=16)
C (n=14)
surface

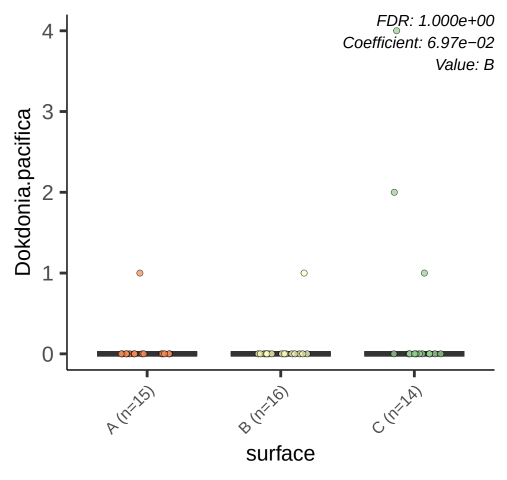
FDR: 1.000e+00
Coefficient: 6.97e−02
Value: B
Dokdonia.pacifica
0
1
2
3
4
A (n=15)
B (n=16)
C (n=14)
surface

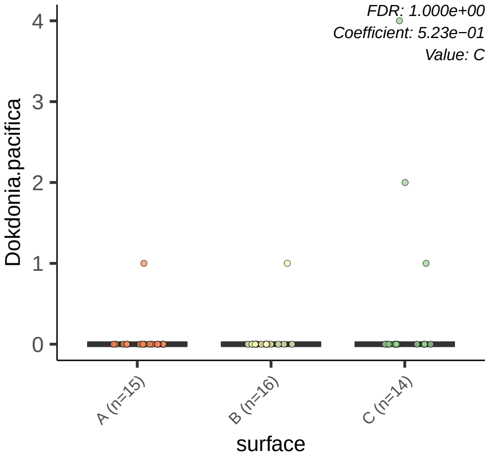

FDR: 1.000e+00
Coefficient: 5.23e−01
Value: C
Dokdonia.pacifica
0
1
2
3
4
A (n=15)
B (n=16)
C (n=14)
surface

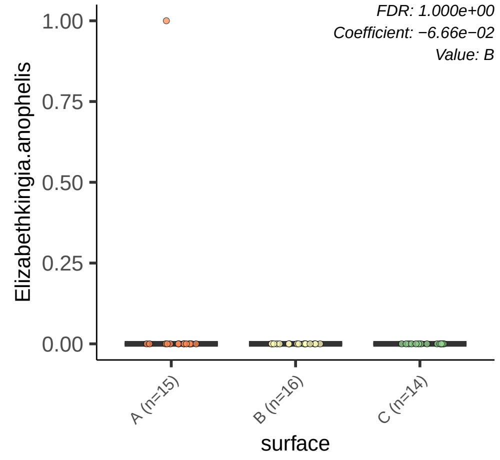
FDR: 1.000e+00
Coefficient: −6.66e−02
Value: B
Elizabethkingia.anophelis
1.00
0.75
0.50
0.25
0.00
A (n=15)
B (n=16)
C (n=14)
surface

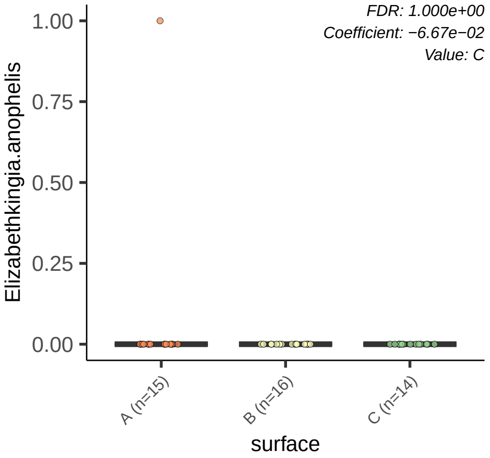

FDR: 1.000e+00
Coefficient: −6.67e−02
Value: C
Elizabethkingia.anophelis
1.00
0.75
0.50
0.25
0.00
A (n=15)
B (n=16)
C (n=14)
surface

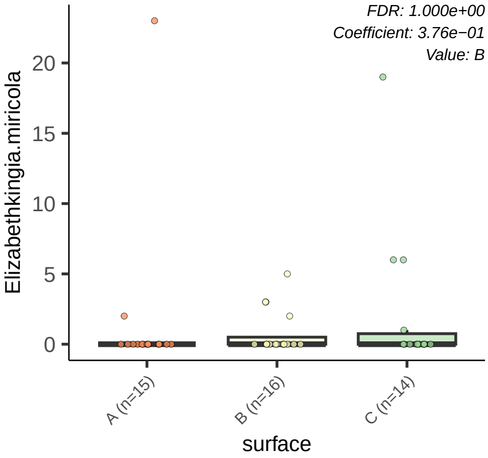

FDR: 1.000e+00
Coefficient: 3.76e−01
Value: B
Elizabethkingia.miricola
0
5
10
15
20
A (n=15)
B (n=16)
C (n=14)
surface

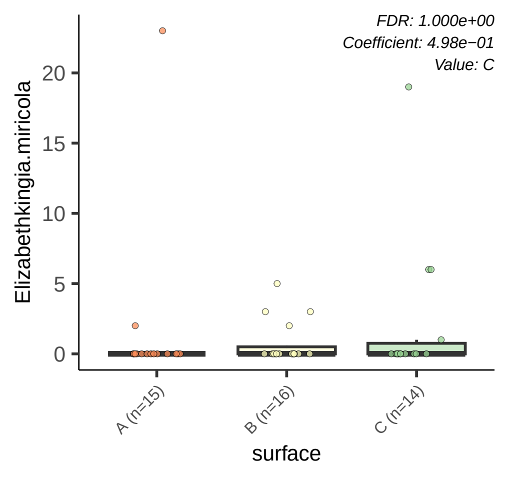
FDR: 1.000e+00
Coefficient: 4.98e−01
Value: C
Elizabethkingia.miricola
0
5
10
15
20
A (n=15)
B (n=16)
C (n=14)
surface

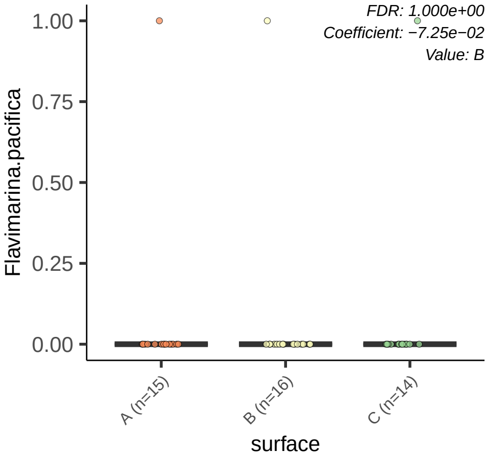

FDR: 1.000e+00
Coefficient: −7.25e−02
Value: B
1.00
0.75
0.50
0.25
0.00
Flavimarina.pacifica
A (n=15)
B (n=16)
C (n=14)
surface

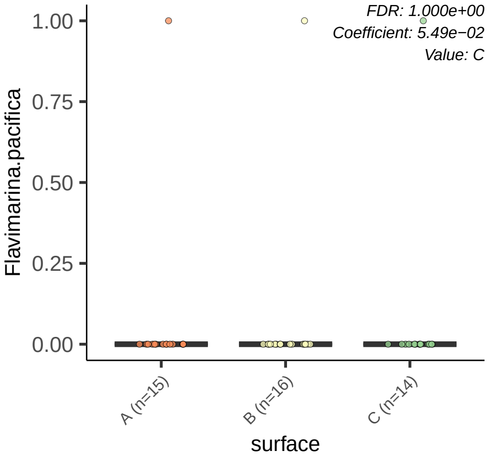
FDR: 1.000e+00
Coefficient: 5.49e−02
Value: C
Flavimarina.pacifica
1.00
0.75
0.50
0.25
0.00
A (n=15)
B (n=16)
C (n=14)
surface

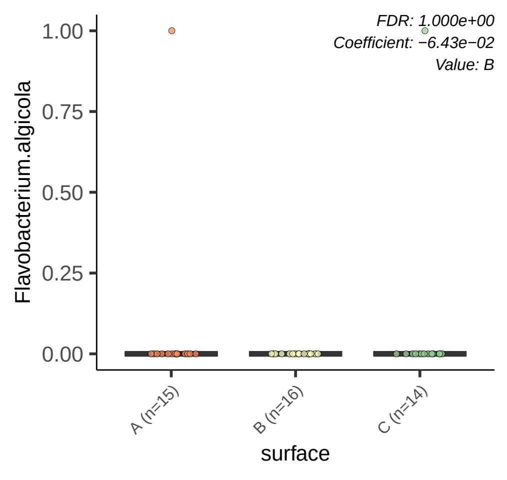
FDR: 1.000e+00
Coefficient: −6.43e−02
Value: B
Flavobacterium.algicola
1.00
0.75
0.50
0.25
0.00
A (n=15)
B (n=16)
C (n=14)
surface

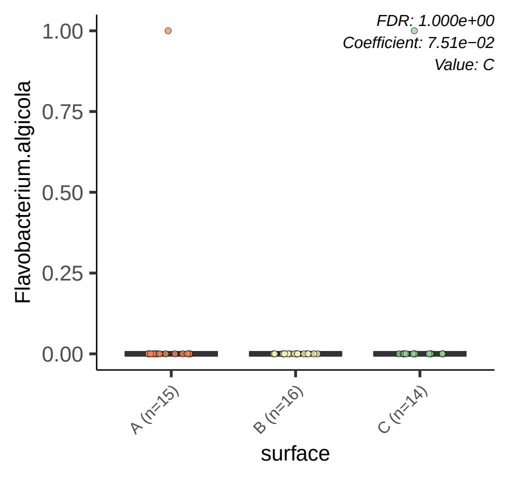
FDR: 1.000e+00
Coefficient: 7.51e−02
Value: C
Flavobacterium.algicola
1.00
0.75
0.50
0.25
0.00
A (n=15)
B (n=16)
C (n=14)
surface

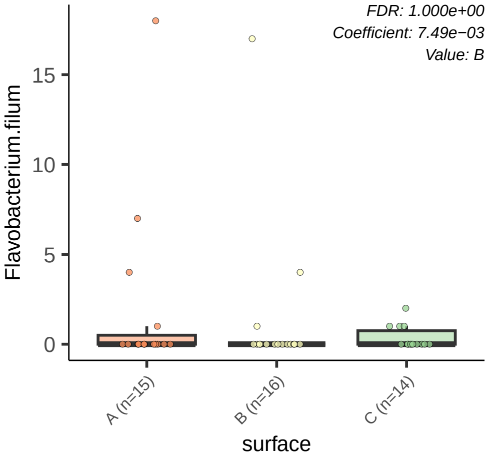

FDR: 1.000e+00
Coefficient: 7.49e−03
Value: B
Flavobacterium.filum
0
5
10
15
A (n=15)
B (n=16)
C (n=14)
surface

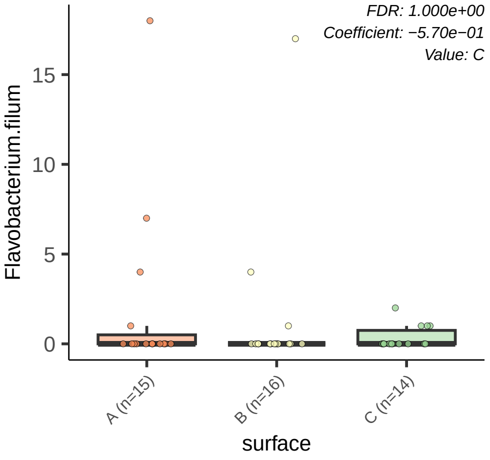
FDR: 1.000e+00
Coefficient: −5.70e−01
Value: C
Flavobacterium.filum
0
5
10
15
A (n=15)
B (n=16)
C (n=14)
surface

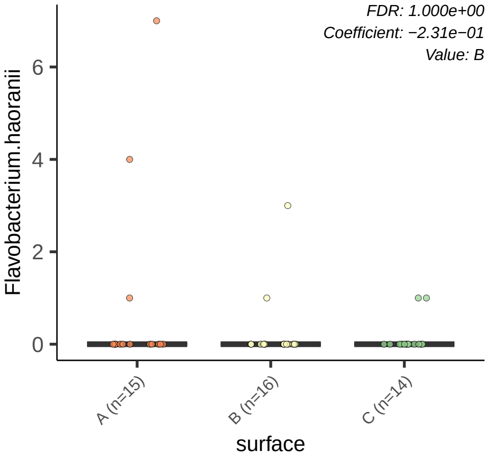

FDR: 1.000e+00
Coefficient: −2.31e−01
Value: B
Flavobacterium.haoranii
0
2
4
6
A (n=15)
B (n=16)
C (n=14)
surface

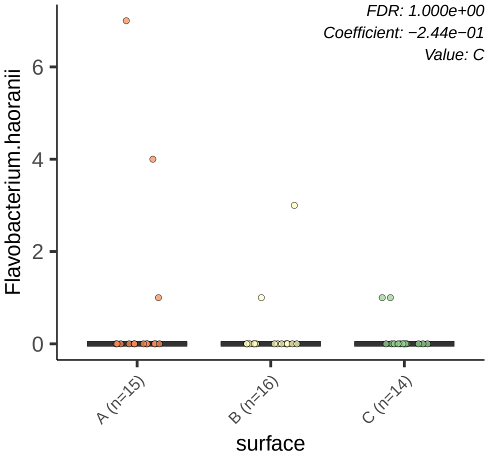

FDR: 1.000e+00
Coefficient: −2.44e−01
Value: C
Flavobacterium.haoranii
0
2
4
6
A (n=15)
B (n=16)
C (n=14)
surface

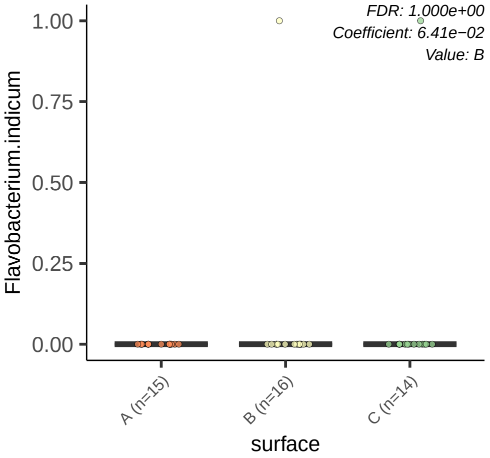

FDR: 1.000e+00
Coefficient: 6.41e−02
Value: B
Flavobacterium.indicum
1.00
0.75
0.50
0.25
0.00
A (n=15)
B (n=16)
C (n=14)
surface

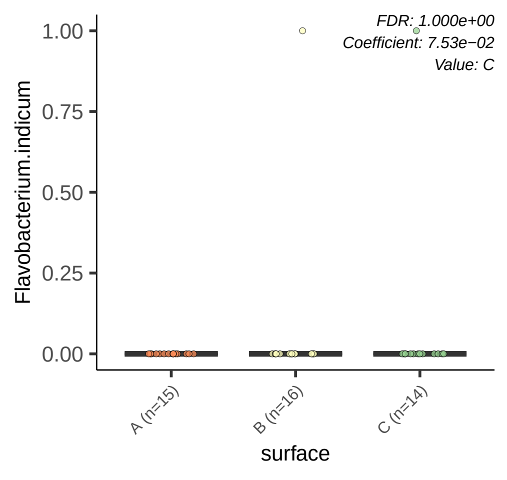
FDR: 1.000e+00
Coefficient: 7.53e−02
Value: C
Flavobacterium.indicum
1.00
0.75
0.50
0.25
0.00
A (n=15)
B (n=16)
C (n=14)
surface

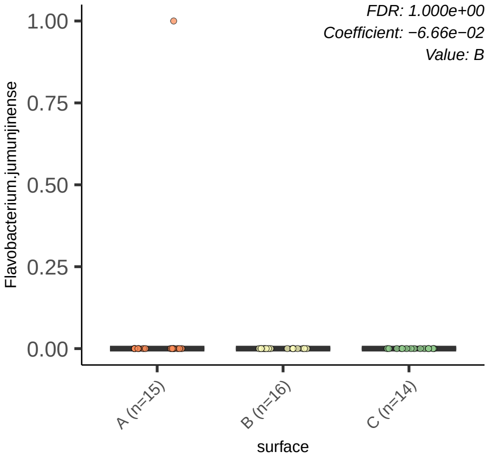
FDR: 1.000e+00
Coefficient: −6.66e−02
Value: B
Flavobacterium.jumunjinense
1.00
0.75
0.50
0.25
0.00
A (n=15)
B (n=16)
C (n=14)
surface

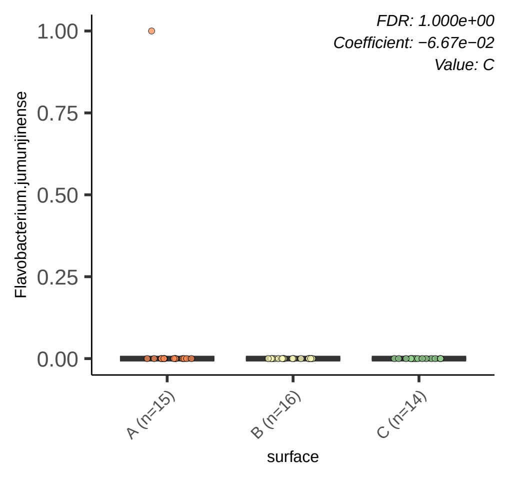

FDR: 1.000e+00
Coefficient: −6.67e−02
Value: C
Flavobacterium.jumunjinense
1.00
0.75
0.50
0.25
0.00
A (n=15)
B (n=16)
C (n=14)
surface

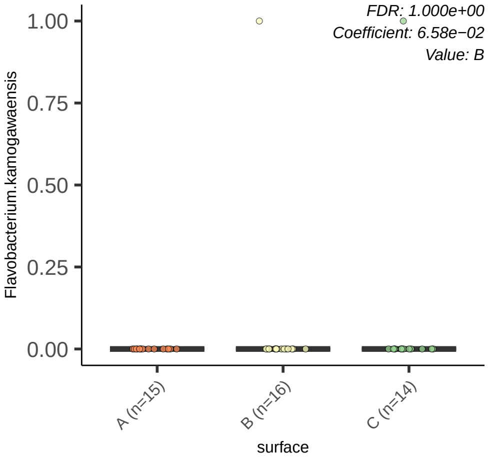

FDR: 1.000e+00
Coefficient: 6.58e−02
Value: B
Flavobacterium.kamogawaensis
1.00
0.75
0.50
0.25
0.00
A (n=15)
B (n=16)
C (n=14)
surface

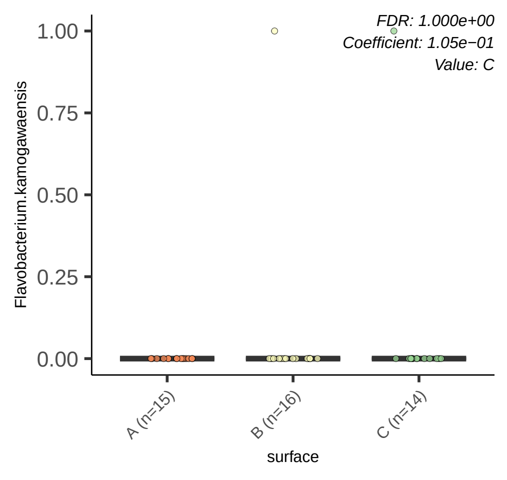

FDR: 1.000e+00
Coefficient: 1.05e−01
Value: C
1.00
0.75
0.50
0.25
0.00
Flavobacterium.kamogawaensis
A (n=15)
B (n=16)
C (n=14)
surface

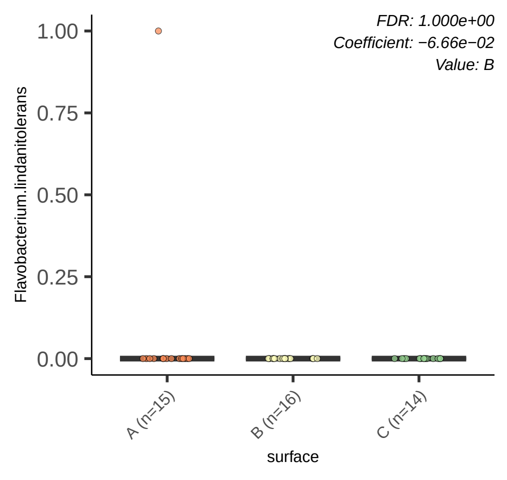

FDR: 1.000e+00
Coefficient: −6.66e−02
Value: B
Flavobacterium.lindanitolerans
1.00
0.75
0.50
0.25
0.00
A (n=15)
B (n=16)
C (n=14)
surface

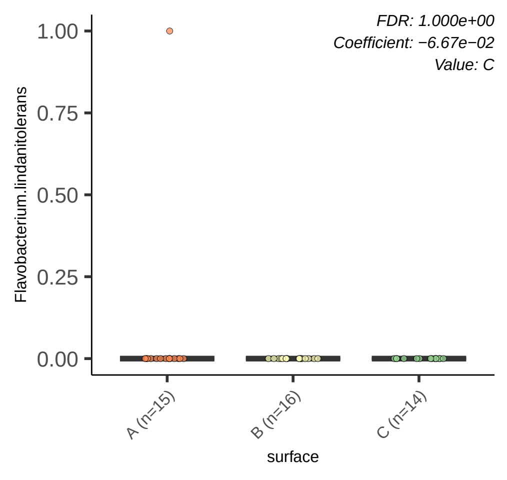
FDR: 1.000e+00
Coefficient: −6.67e−02
Value: C
1.00
0.75
0.50
0.25
0.00
Flavobacterium.lindanitolerans
A (n=15)
B (n=16)
C (n=14)
surface

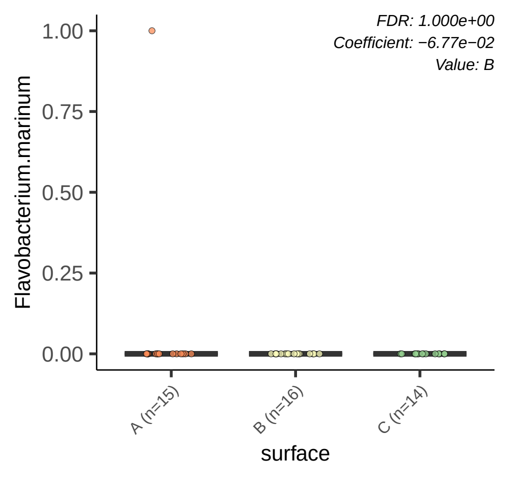
FDR: 1.000e+00
Coefficient: −6.77e−02
Value: B
Flavobacterium.marinum
1.00
0.75
0.50
0.25
0.00
A (n=15)
B (n=16)
C (n=14)
surface

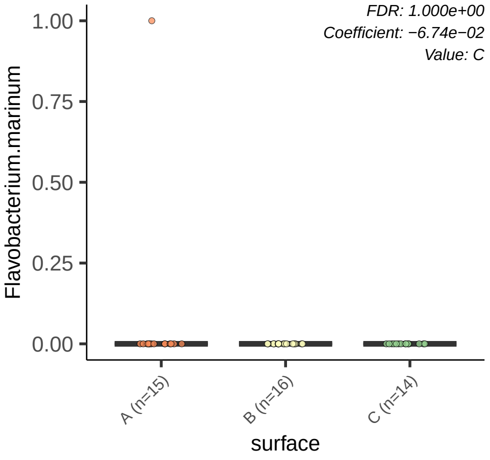

FDR: 1.000e+00
Coefficient: −6.74e−02
Value: C
Flavobacterium.marinum
1.00
0.75
0.50
0.25
0.00
A (n=15)
B (n=16)
C (n=14)
surface

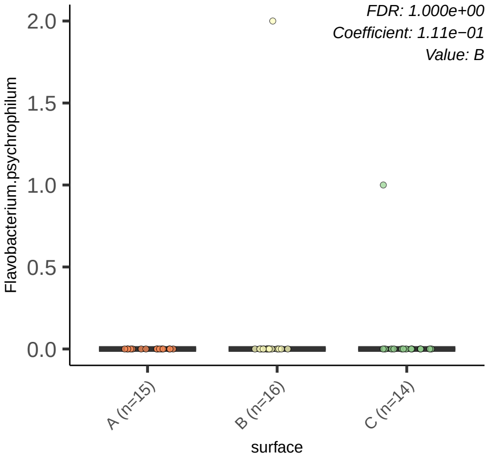
FDR: 1.000e+00
Coefficient: 1.11e−01
Value: B
Flavobacterium.psychrophilum
2.0
1.5
1.0
0.5
0.0
A (n=15)
B (n=16)
C (n=14)
surface

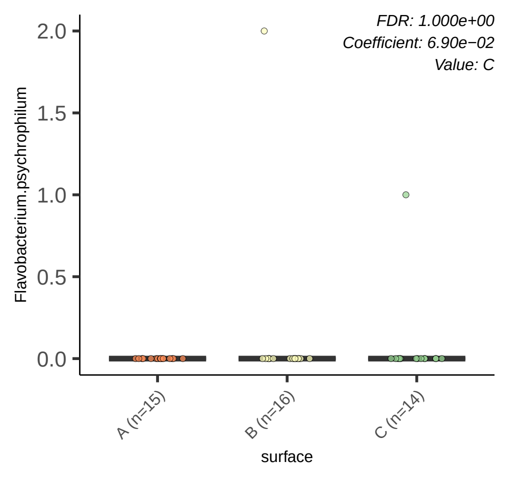

FDR: 1.000e+00
Coefficient: 6.90e−02
Value: C
Flavobacterium.psychrophilum
2.0
1.5
1.0
0.5
0.0
A (n=15)
B (n=16)
C (n=14)
surface

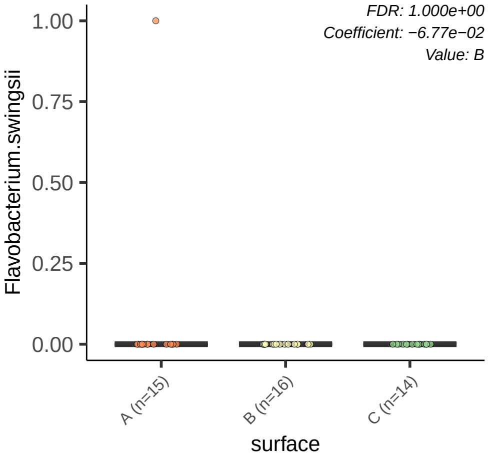

FDR: 1.000e+00
Coefficient: −6.77e−02
Value: B
1.00
0.75
0.50
0.25
0.00
Flavobacterium.swingsii
A (n=15)
B (n=16)
C (n=14)
surface

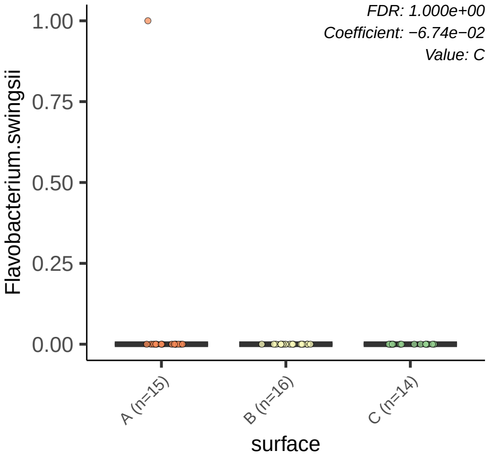

FDR: 1.000e+00
Coefficient: −6.74e−02
Value: C
Flavobacterium.swingsii
1.00
0.75
0.50
0.25
0.00
A (n=15)
B (n=16)
C (n=14)
surface

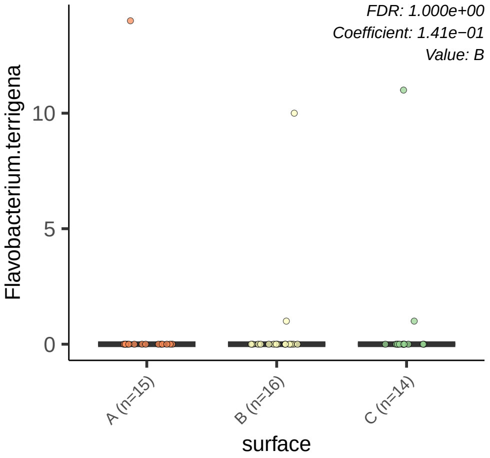

FDR: 1.000e+00
Coefficient: 1.41e−01
Value: B
Flavobacterium.terrigena
10
5
0
A (n=15)
B (n=16)
C (n=14)
surface

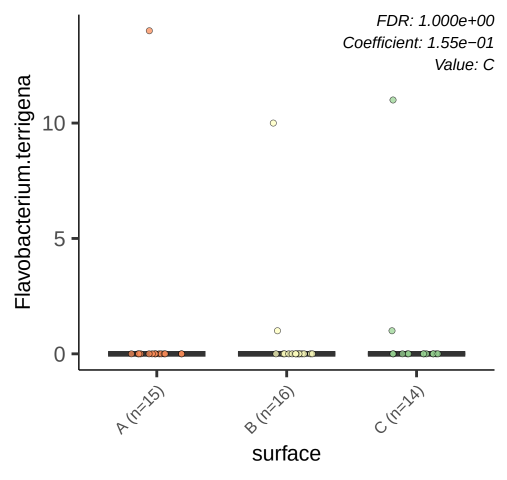

FDR: 1.000e+00
Coefficient: 1.55e−01
Value: C
Flavobacterium.terrigena
0
5
10
A (n=15)
B (n=16)
C (n=14)
surface

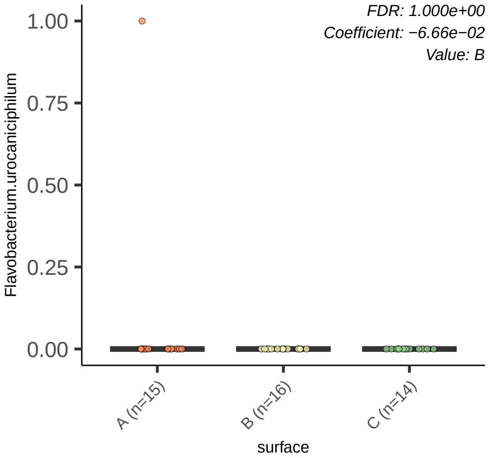
FDR: 1.000e+00
Coefficient: −6.66e−02
Value: B
Flavobacterium.urocaniciphilum
1.00
0.75
0.50
0.25
0.00
A (n=15)
B (n=16)
C (n=14)
surface

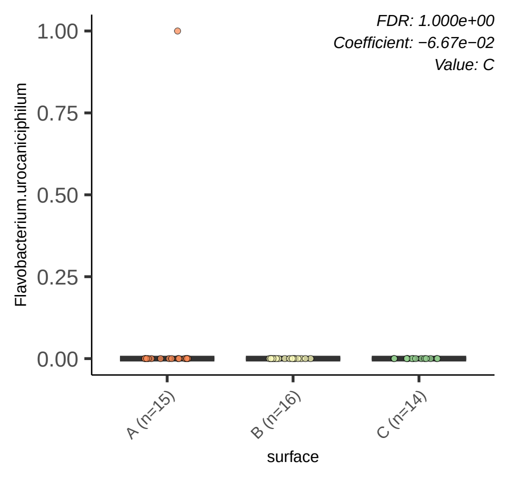
FDR: 1.000e+00
Coefficient: −6.67e−02
Value: C
1.00
0.75
0.50
0.25
0.00
Flavobacterium.urocaniciphilum
A (n=15)
B (n=16)
C (n=14)
surface

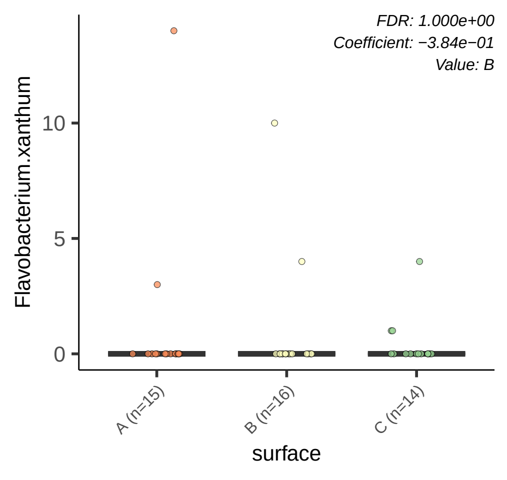
FDR: 1.000e+00
Coefficient: −3.84e−01
Value: B
Flavobacterium.xanthum
0
5
10
A (n=15)
B (n=16)
C (n=14)
surface

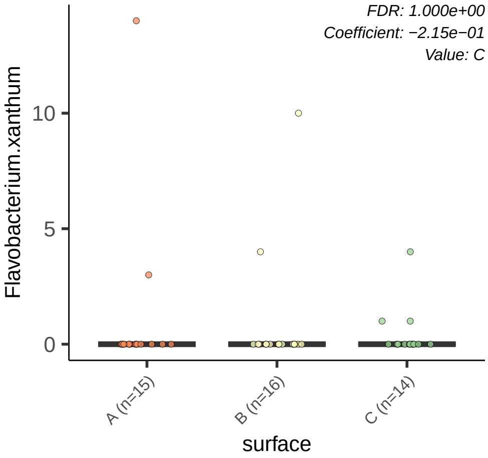

FDR: 1.000e+00
Coefficient: −2.15e−01
Value: C
Flavobacterium.xanthum
0
5
10
A (n=15)
B (n=16)
C (n=14)
surface

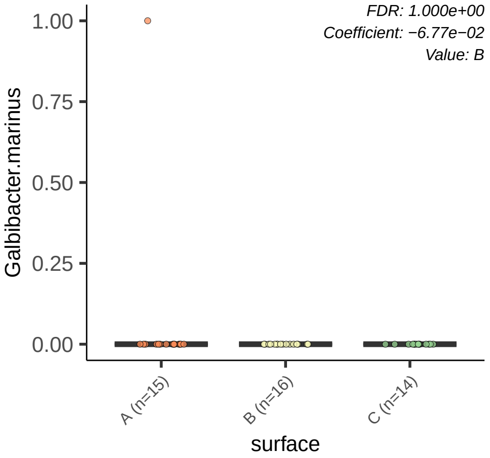

FDR: 1.000e+00
Coefficient: −6.77e−02
Value: B
Galbibacter.marinus
1.00
0.75
0.50
0.25
0.00
A (n=15)
B (n=16)
C (n=14)
surface

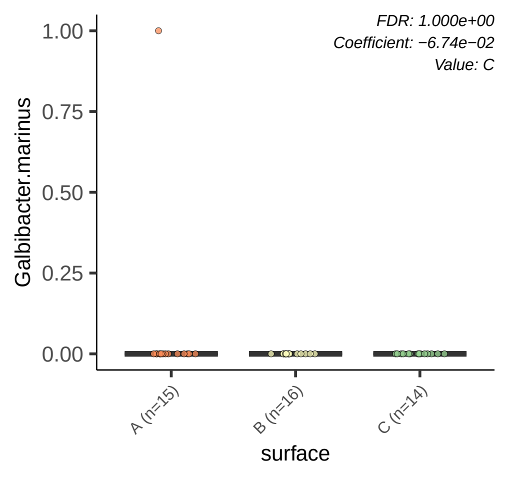

FDR: 1.000e+00
Coefficient: −6.74e−02
Value: C
Galbibacter.marinus
1.00
0.75
0.50
0.25
0.00
A (n=15)
B (n=16)
C (n=14)
surface

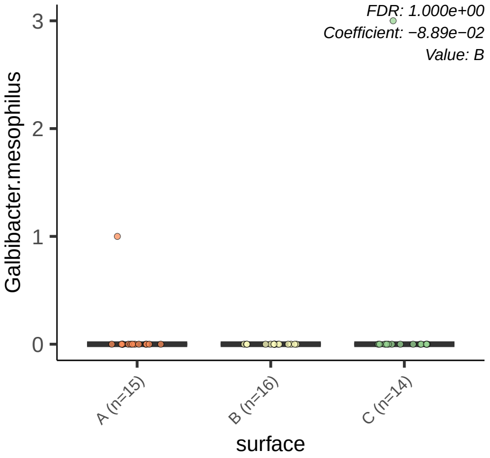

FDR: 1.000e+00
Coefficient: −8.89e−02
Value: B
Galbibacter.mesophilus
3
2
1
0
A (n=15)
B (n=16)
C (n=14)
surface

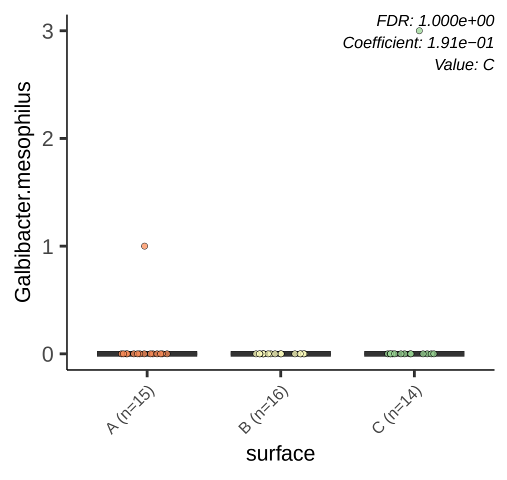
FDR: 1.000e+00
Coefficient: 1.91e−01
Value: C
Galbibacter.mesophilus
0
1
2
3
A (n=15)
B (n=16)
C (n=14)
surface

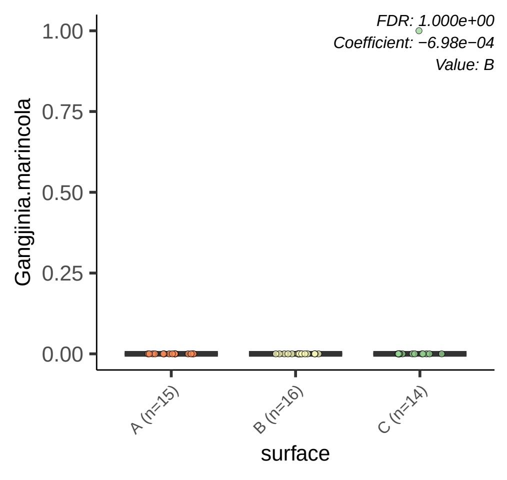
FDR: 1.000e+00
Coefficient: −6.98e−04
Value: B
1.00
0.75
0.50
0.25
0.00
Gangjinia.marincola
A (n=15)
B (n=16)
C (n=14)
surface

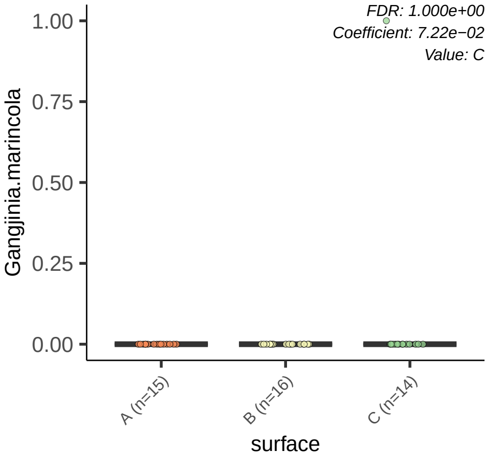

FDR: 1.000e+00
Coefficient: 7.22e−02
Value: C
1.00
0.75
0.50
0.25
0.00
Gangjinia.marincola
A (n=15)
B (n=16)
C (n=14)
surface

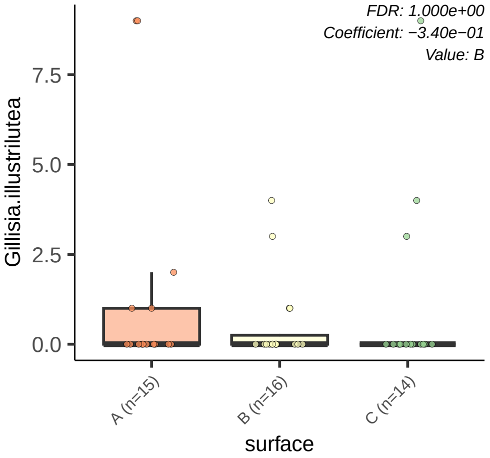
FDR: 1.000e+00
Coefficient: −3.40e−01
Value: B
Gillisia.illustrilutea
7.5
5.0
2.5
0.0
A (n=15)
B (n=16)
C (n=14)
surface

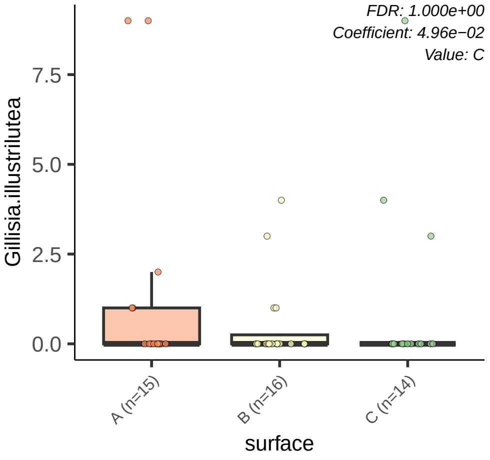
FDR: 1.000e+00
Coefficient: 4.96e−02
Value: C
Gillisia.illustrilutea
0.0
2.5
5.0
7.5
A (n=15)
B (n=16)
C (n=14)
surface

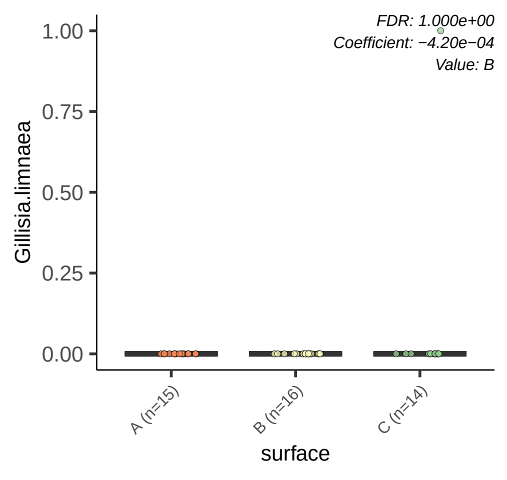

FDR: 1.000e+00
Coefficient: −4.20e−04
Value: B
Gillisia.limnaea
1.00
0.75
0.50
0.25
0.00
A (n=15)
B (n=16)
C (n=14)
surface

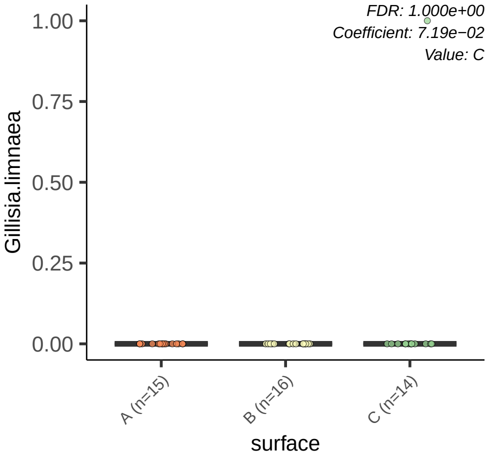
FDR: 1.000e+00
Coefficient: 7.19e−02
Value: C
Gillisia.limnaea
1.00
0.75
0.50
0.25
0.00
A (n=15)
B (n=16)
C (n=14)
surface

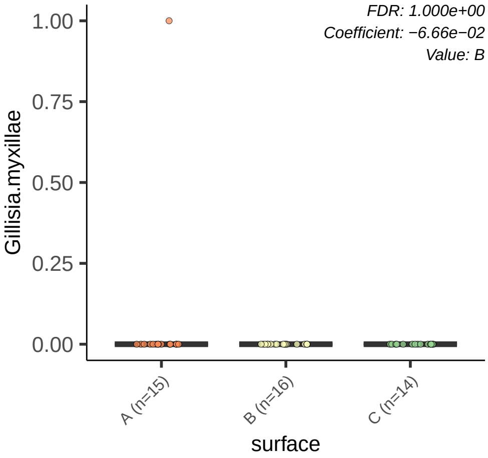

FDR: 1.000e+00
Coefficient: −6.66e−02
Value: B
Gillisia.myxillae
1.00
0.75
0.50
0.25
0.00
A (n=15)
B (n=16)
C (n=14)
surface

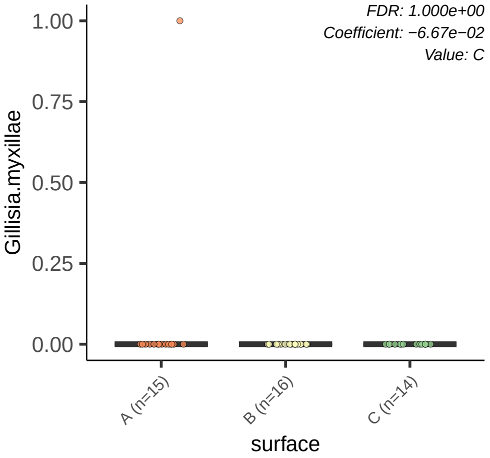
FDR: 1.000e+00
Coefficient: −6.67e−02
Value: C
Gillisia.myxillae
1.00
0.75
0.50
0.25
0.00
A (n=15)
B (n=16)
C (n=14)
surface

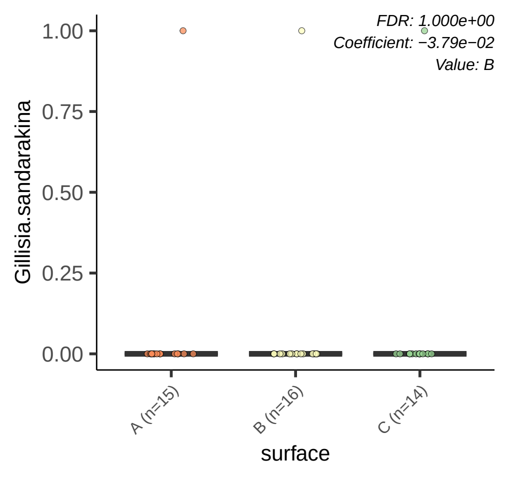
FDR: 1.000e+00
Coefficient: −3.79e−02
Value: B
Gillisia.sandarakina
1.00
0.75
0.50
0.25
0.00
A (n=15)
B (n=16)
C (n=14)
surface

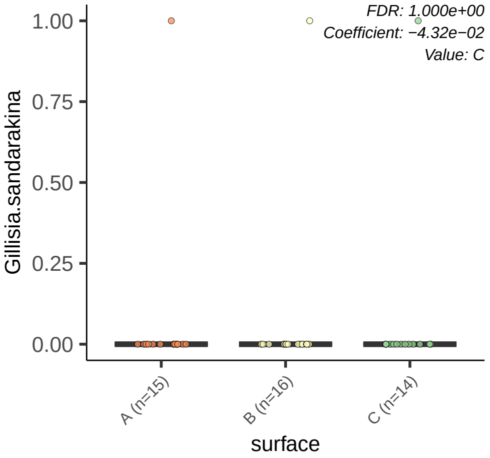
FDR: 1.000e+00
Coefficient: −4.32e−02
Value: C
Gillisia.sandarakina
1.00
0.75
0.50
0.25
0.00
A (n=15)
B (n=16)
C (n=14)
surface

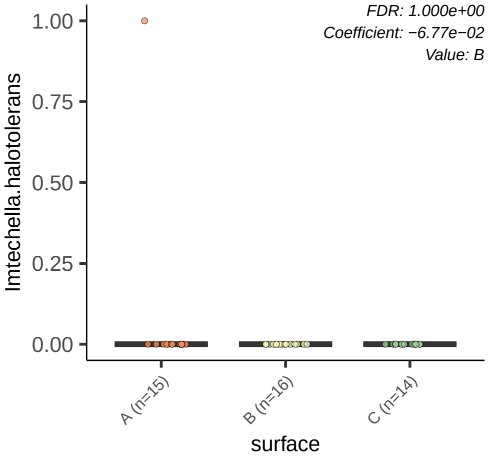
FDR: 1.000e+00
Coefficient: −6.77e−02
Value: B
Imtechella.halotolerans
1.00
0.75
0.50
0.25
0.00
A (n=15)
B (n=16)
C (n=14)
surface

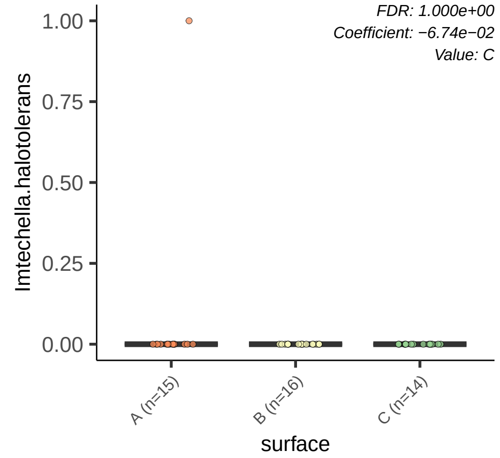

FDR: 1.000e+00
Coefficient: −6.74e−02
Value: C
Imtechella.halotolerans
1.00
0.75
0.50
0.25
0.00
A (n=15)
B (n=16)
C (n=14)
surface

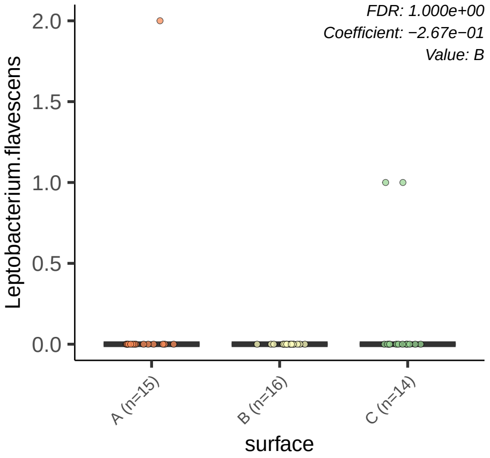
FDR: 1.000e+00
Coefficient: −2.67e−01
Value: B
Leptobacterium.flavescens
2.0
1.5
1.0
0.5
0.0
A (n=15)
B (n=16)
C (n=14)
surface

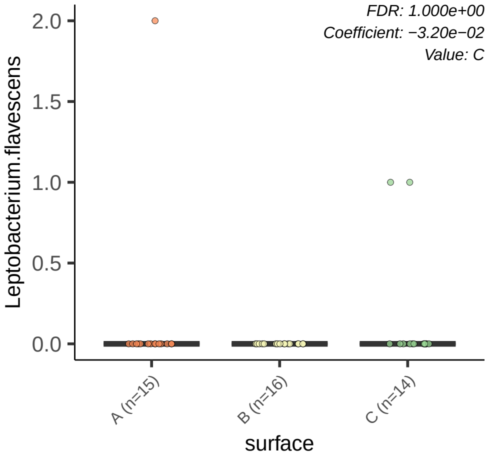
FDR: 1.000e+00
Coefficient: −3.20e−02
Value: C
Leptobacterium.flavescens
2.0
1.5
1.0
0.5
0.0
A (n=15)
B (n=16)
C (n=14)
surface

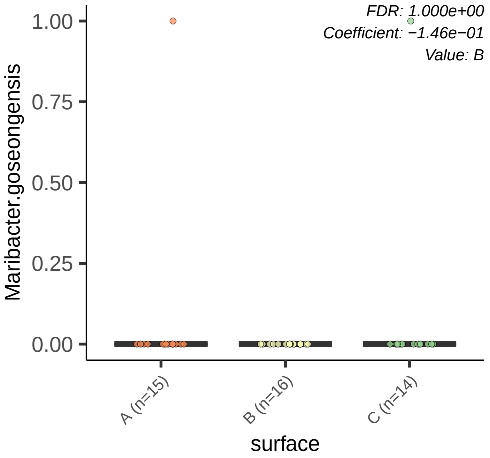

FDR: 1.000e+00
Coefficient: −1.46e−01
Value: B
Maribacter.goseongensis
1.00
0.75
0.50
0.25
0.00
A (n=15)
B (n=16)
C (n=14)
surface

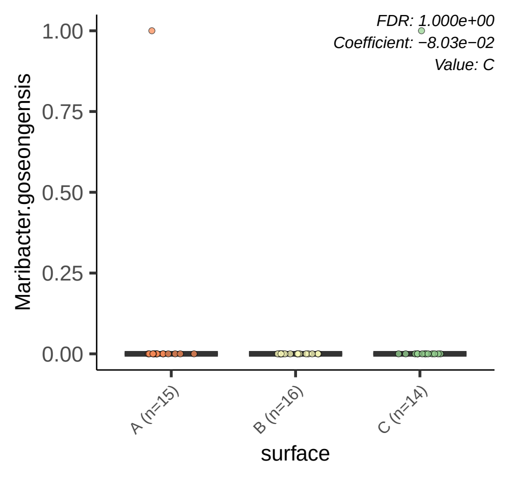
FDR: 1.000e+00
Coefficient: −8.03e−02
Value: C
Maribacter.goseongensis
1.00
0.75
0.50
0.25
0.00
A (n=15)
B (n=16)
C (n=14)
surface

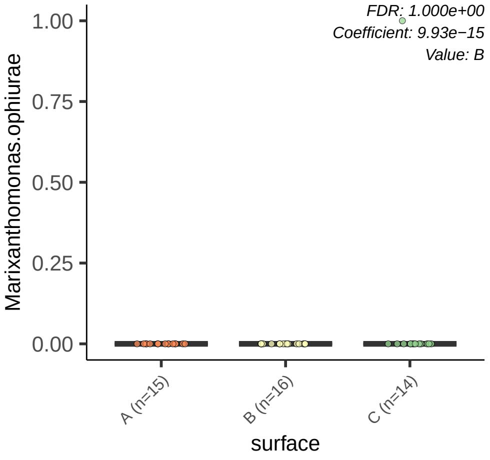

FDR: 1.000e+00
Coefficient: 9.93e−15
Value: B
Marixanthomonas.ophiurae
1.00
0.75
0.50
0.25
0.00
A (n=15)
B (n=16)
C (n=14)
surface

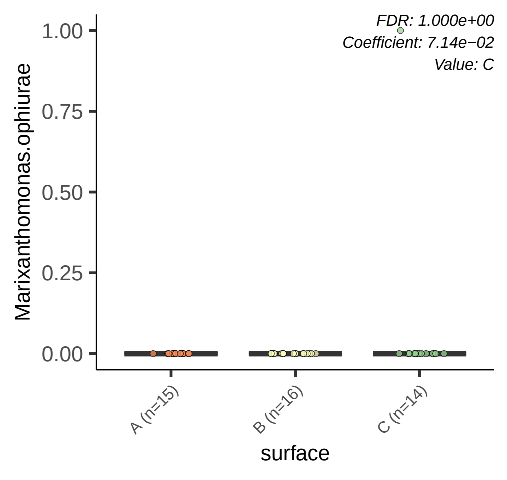

FDR: 1.000e+00
Coefficient: 7.14e−02
Value: C
Marixanthomonas.ophiurae
1.00
0.75
0.50
0.25
0.00
A (n=15)
B (n=16)
C (n=14)
surface

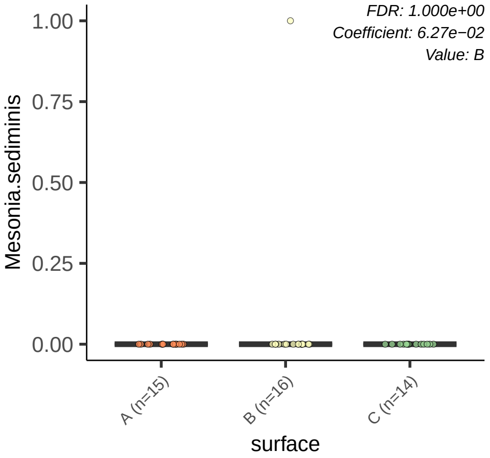
FDR: 1.000e+00
Coefficient: 6.27e−02
Value: B
1.00
0.75
0.50
0.25
0.00
Mesonia.sedimінis
A (n=15)
B (n=16)
C (n=14)
surface

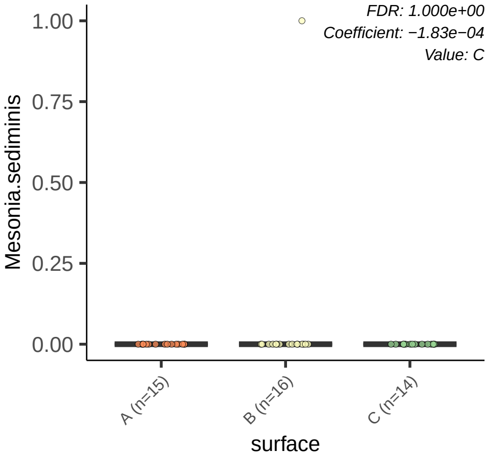
FDR: 1.000e+00
Coefficient: −1.83e−04
Value: C
Mesonia.sedimінis
1.00
0.75
0.50
0.25
0.00
A (n=15)
B (n=16)
C (n=14)
surface

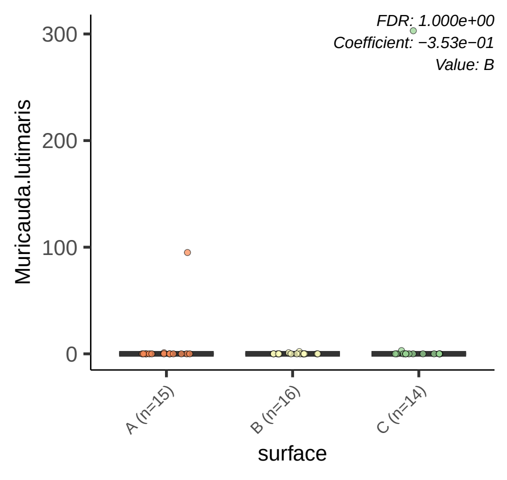
FDR: 1.000e+00
Coefficient: −3.53e−01
Value: B
Muricauda.lutimaris
300
200
100
0
A (n=15)
B (n=16)
C (n=14)
surface

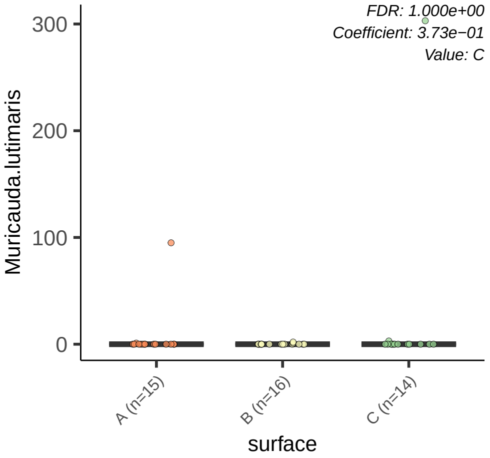

FDR: 1.000e+00
Coefficient: 3.73e−01
Value: C
Muricauda.lutimaris
300
200
100
0
A (n=15)
B (n=16)
C (n=14)
surface

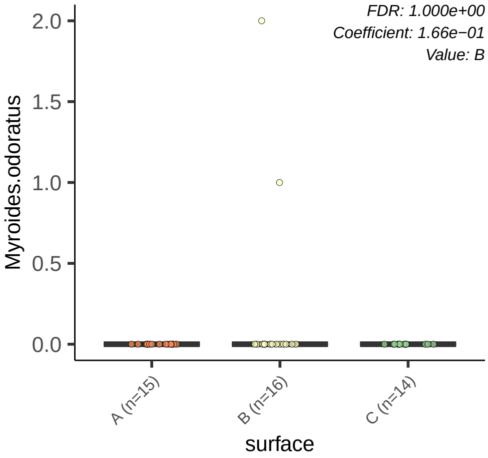

FDR: 1.000e+00
Coefficient: 1.66e−01
Value: B
Myroides.odoratus
2.0
1.5
1.0
0.5
0.0
A (n=15)
B (n=16)
C (n=14)
surface

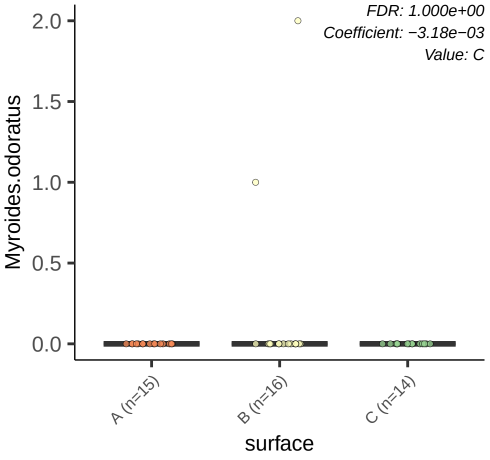

FDR: 1.000e+00
Coefficient: −3.18e−03
Value: C
Myroides.odoratus
2.0
1.5
1.0
0.5
0.0
A (n=15)
B (n=16)
C (n=14)
surface

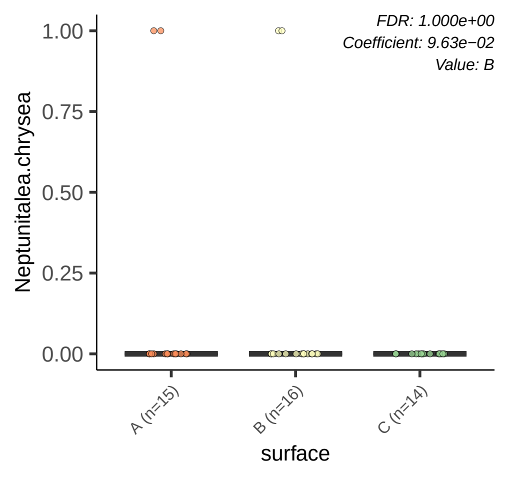

FDR: 1.000e+00
Coefficient: 9.63e−02
Value: B
Neptunitalea.chrysea
1.00
0.75
0.50
0.25
0.00
A (n=15)
B (n=16)
C (n=14)
surface

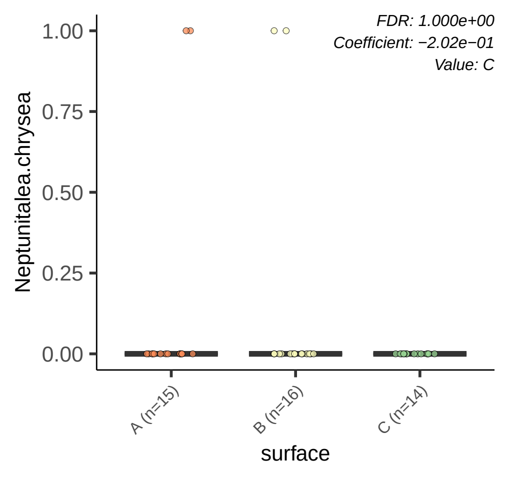

FDR: 1.000e+00
Coefficient: −2.02e−01
Value: C
Neptunitalea.chrysea
1.00
0.75
0.50
0.25
0.00
A (n=15)
B (n=16)
C (n=14)
surface

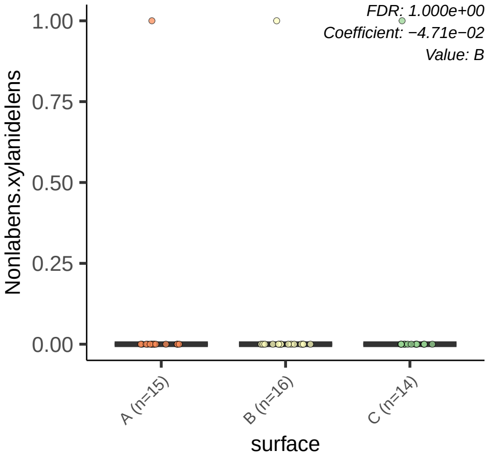
FDR: 1.000e+00
Coefficient: −4.71e−02
Value: B
Nonlabens.xylanidelens
1.00
0.75
0.50
0.25
0.00
A (n=15)
B (n=16)
C (n=14)
surface

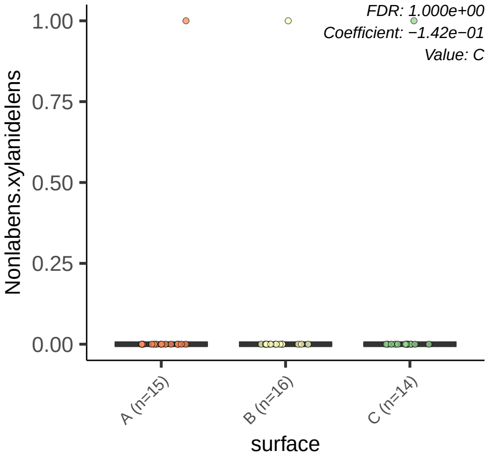
FDR: 1.000e+00
Coefficient: −1.42e−01
Value: C
Nonlabens.xylanidelens
1.00
0.75
0.50
0.25
0.00
A (n=15)
B (n=16)
C (n=14)
surface

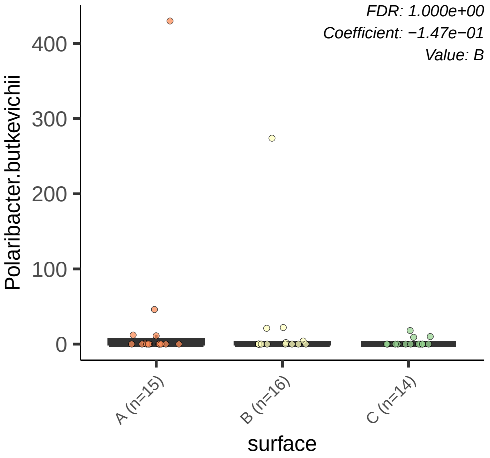
FDR: 1.000e+00
Coefficient: −1.47e−01
Value: B
Polaribacter.butkevichii
0
100
200
300
400
A (n=15)
B (n=16)
C (n=14)
surface

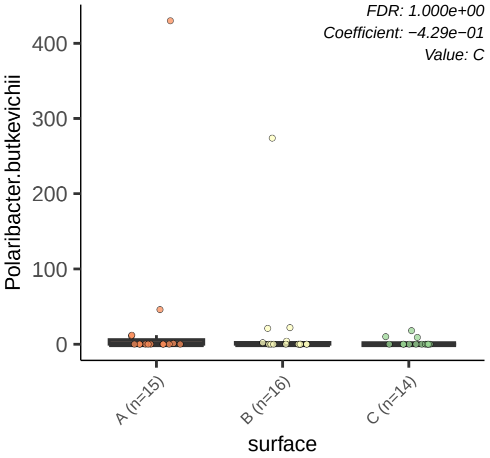

FDR: 1.000e+00
Coefficient: −4.29e−01
Value: C
Polaribacter.butkevichii
400
300
200
100
0
A (n=15)
B (n=16)
C (n=14)
surface

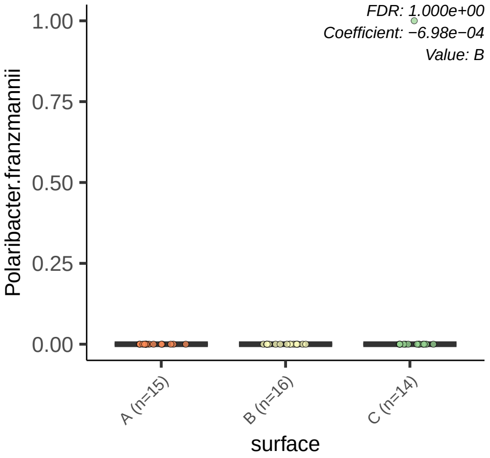

FDR: 1.000e+00
Coefficient: −6.98e−04
Value: B
Polaribacter.franzmannii
1.00
0.75
0.50
0.25
0.00
A (n=15)
B (n=16)
C (n=14)
surface

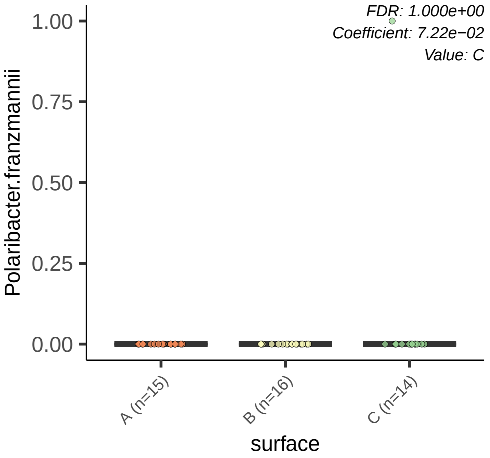
FDR: 1.000e+00
Coefficient: 7.22e−02
Value: C
Polaribacter.franzmannii
1.00
0.75
0.50
0.25
0.00
A (n=15)
B (n=16)
C (n=14)
surface

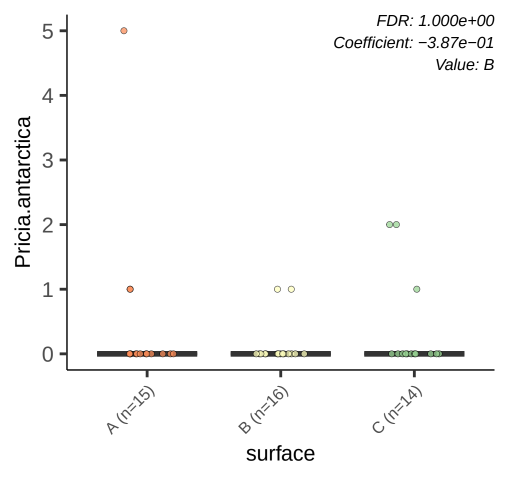
FDR: 1.000e+00
Coefficient: −3.87e−01
Value: B
Pricia.antarctica
0
1
2
3
4
5
A (n=15)
B (n=16)
C (n=14)
surface

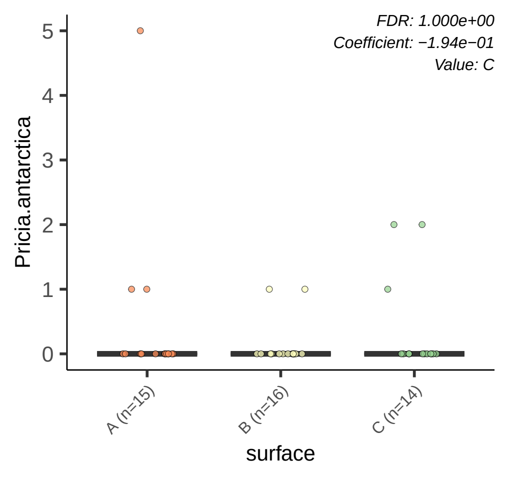
FDR: 1.000e+00
Coefficient: −1.94e−01
Value: C
Pricia.antarctica
0
1
2
3
4
5
A (n=15)
B (n=16)
C (n=14)
surface

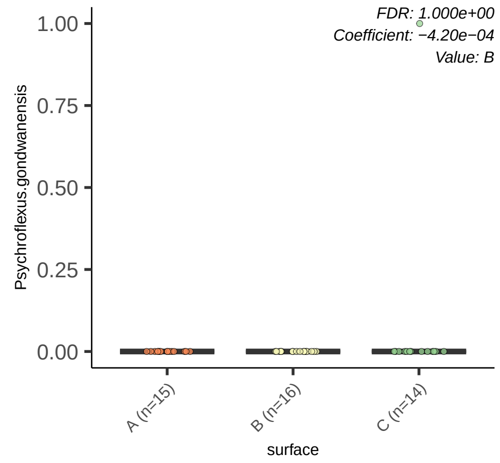
FDR: 1.000e+00
Coefficient: −4.20e−04
Value: B
1.00
0.75
0.50
0.25
0.00
Psychroflexus.gondwanensis
A (n=15)
B (n=16)
C (n=14)
surface

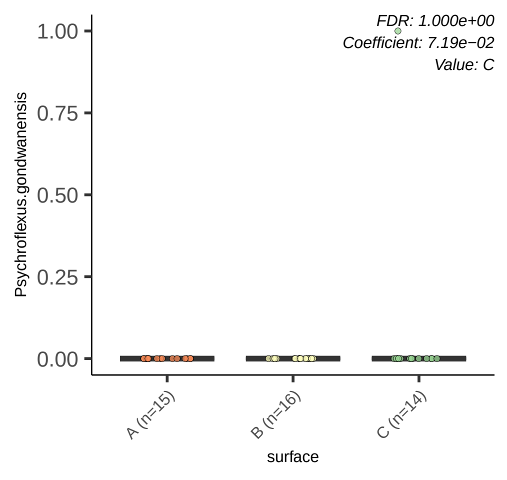
FDR: 1.000e+00
Coefficient: 7.19e−02
Value: C
Psychroflexus.gondwanensis
1.00
0.75
0.50
0.25
0.00
A (n=15)
B (n=16)
C (n=14)
surface

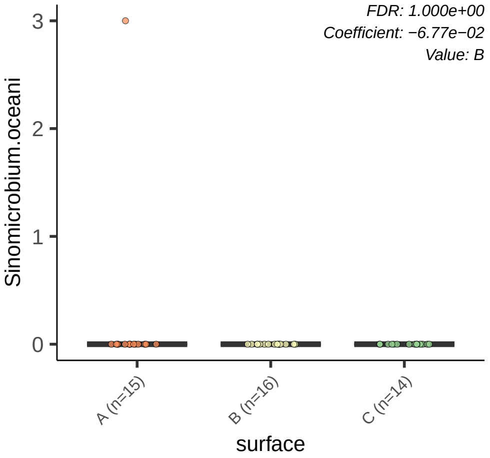
FDR: 1.000e+00
Coefficient: −6.77e−02
Value: B
Sinomicrobium.oceani
0
1
2
3
A (n=15)
B (n=16)
C (n=14)
surface

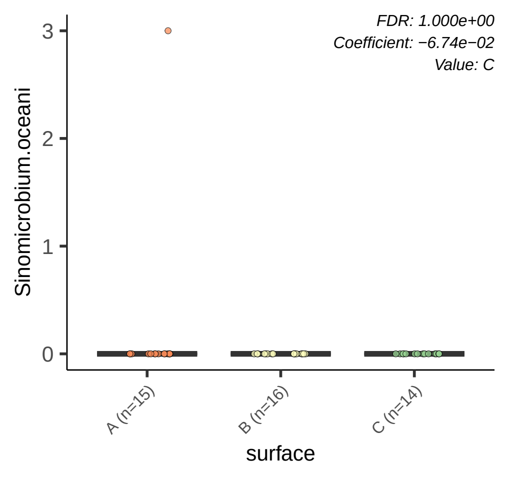
FDR: 1.000e+00
Coefficient: −6.74e−02
Value: C
Sinomicrobium.oceani
3
2
1
0
A (n=15)
B (n=16)
C (n=14)
surface

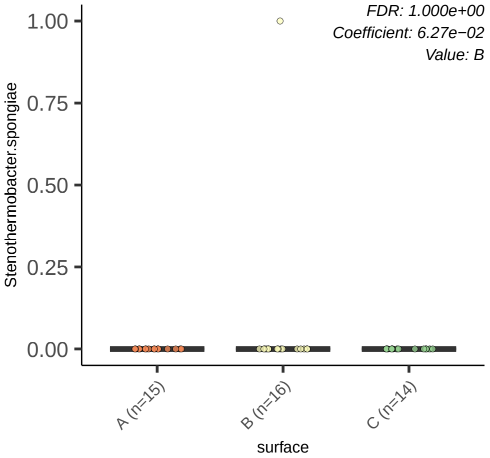

FDR: 1.000e+00
Coefficient: 6.27e−02
Value: B
1.00
0.75
0.50
0.25
0.00
Stenothermobacter.spongiae
A (n=15)
B (n=16)
C (n=14)
surface

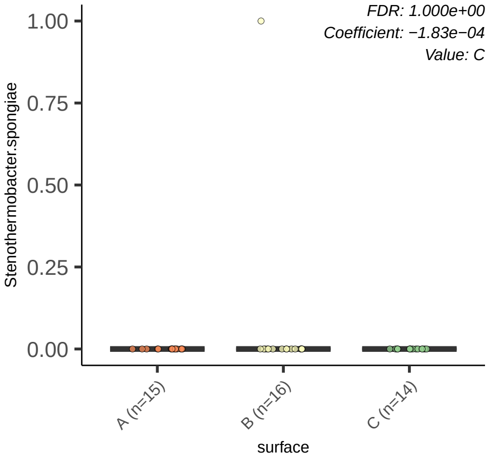
FDR: 1.000e+00
Coefficient: −1.83e−04
Value: C
1.00
0.75
0.50
0.25
0.00
Stenothermobacter.spongiae
A (n=15)
B (n=16)
C (n=14)
surface

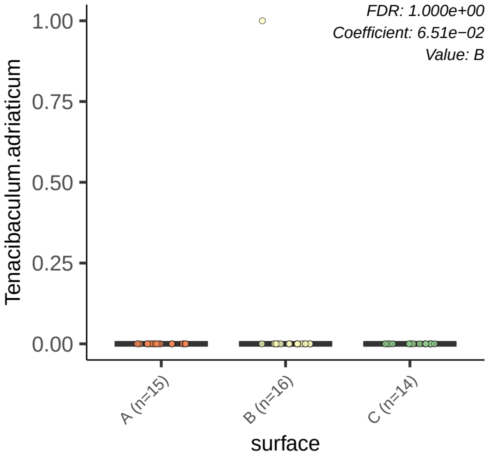
FDR: 1.000e+00
Coefficient: 6.51e−02
Value: B
Tenacibaculum.adriaticum
1.00
0.75
0.50
0.25
0.00
A (n=15)
B (n=16)
C (n=14)
surface

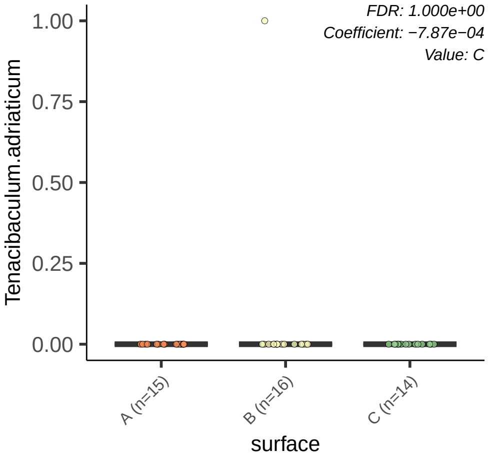

FDR: 1.000e+00
Coefficient: −7.87e−04
Value: C
Tenacibaculum.adriaticum
1.00
0.75
0.50
0.25
0.00
A (n=15)
B (n=16)
C (n=14)
surface

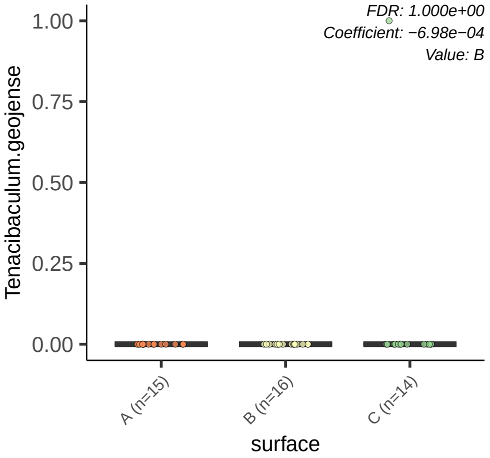
FDR: 1.000e+00
Coefficient: −6.98e−04
Value: B
Tenacibaculum.geojense
1.00
0.75
0.50
0.25
0.00
A (n=15)
B (n=16)
C (n=14)
surface

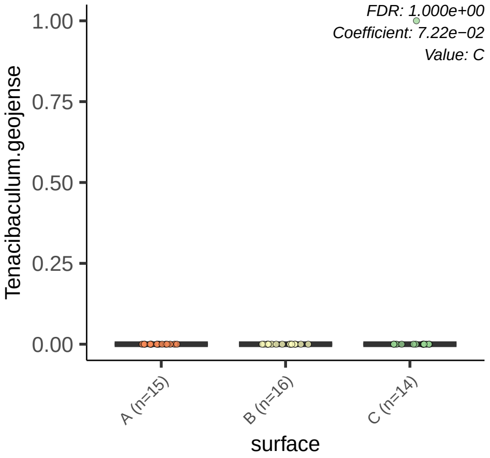
FDR: 1.000e+00
Coefficient: 7.22e−02
Value: C
1.00
0.75
0.50
0.25
0.00
Tenacibaculum.geojense
A (n=15)
B (n=16)
C (n=14)
surface

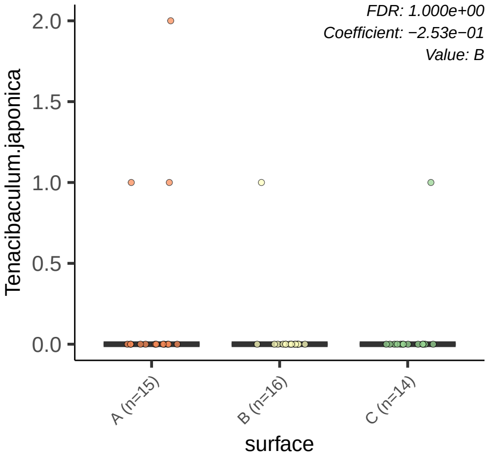

FDR: 1.000e+00
Coefficient: −2.53e−01
Value: B
Tenacibaculum.japonica
2.0
1.5
1.0
0.5
0.0
A (n=15)
B (n=16)
C (n=14)
surface

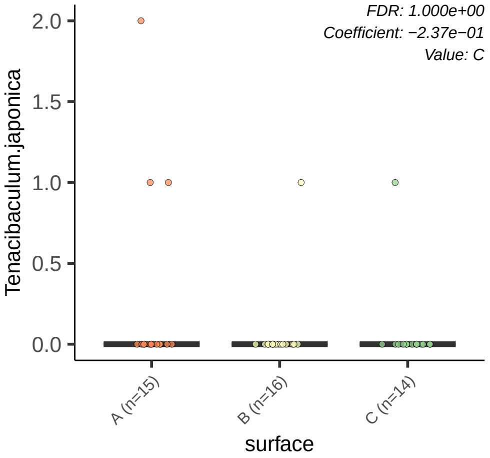
FDR: 1.000e+00
Coefficient: −2.37e−01
Value: C
Tenacibaculum.japonica
2.0
1.5
1.0
0.5
0.0
A (n=15)
B (n=16)
C (n=14)
surface

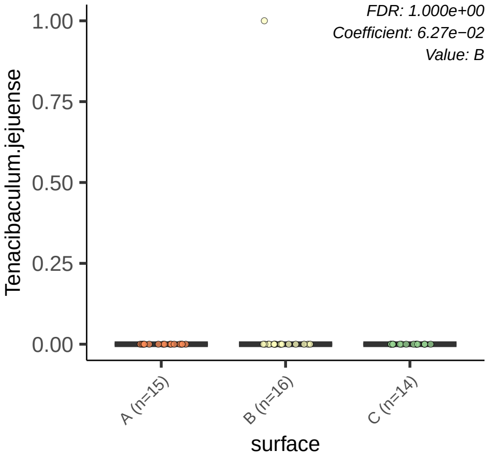

FDR: 1.000e+00
Coefficient: 6.27e−02
Value: B
Tenacibaculum.jejuense
1.00
0.75
0.50
0.25
0.00
A (n=15)
B (n=16)
C (n=14)
surface

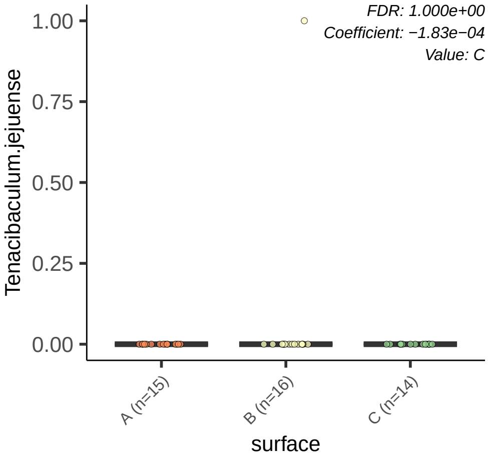

FDR: 1.000e+00
Coefficient: −1.83e−04
Value: C
Tenacibaculum.jejuense
1.00
0.75
0.50
0.25
0.00
A (n=15)
B (n=16)
C (n=14)
surface

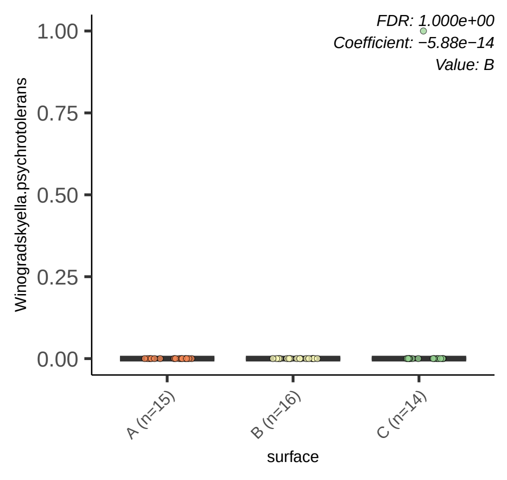
FDR: 1.000e+00
Coefficient: −5.88e−14
Value: B
Winogradskyella.psychrotolerans
1.00
0.75
0.50
0.25
0.00
A (n=15)
B (n=16)
C (n=14)
surface

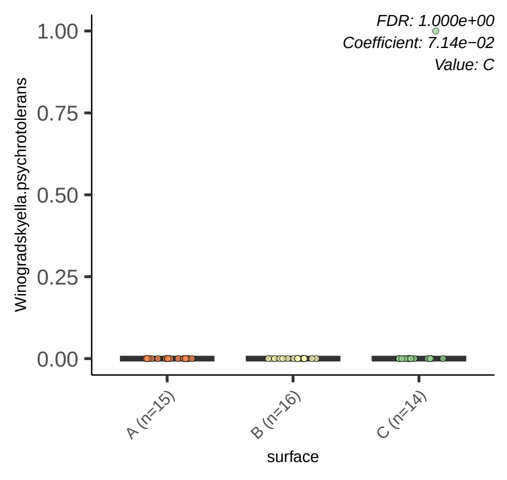

FDR: 1.000e+00
Coefficient: 7.14e−02
Value: C
1.00
0.75
0.50
0.25
0.00
Winogradskyella.psychrotolerans
A (n=15)
B (n=16)
C (n=14)
surface

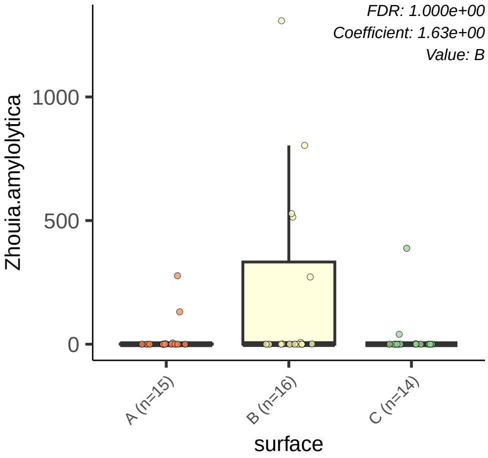

FDR: 1.000e+00
Coefficient: 1.63e+00
Value: B
Zhouia.amylolytica
1000
500
0
A (n=15)
B (n=16)
C (n=14)
surface

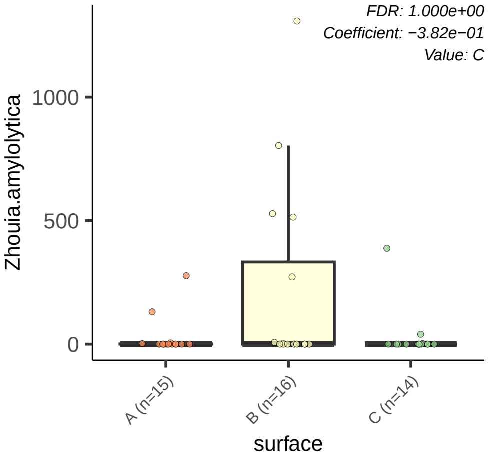
FDR: 1.000e+00
Coefficient: −3.82e−01
Value: C
Zhouia.amylolytica
1000
500
0
A (n=15)
B (n=16)
C (n=14)
surface

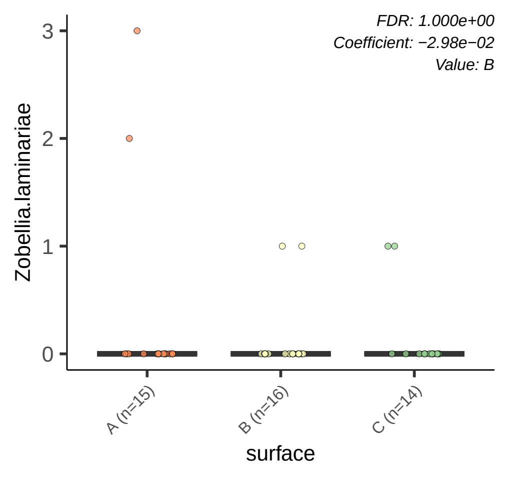
FDR: 1.000e+00
Coefficient: −2.98e−02
Value: B
Zobellia.laminariae
3
2
1
0
A (n=15)
B (n=16)
C (n=14)
surface

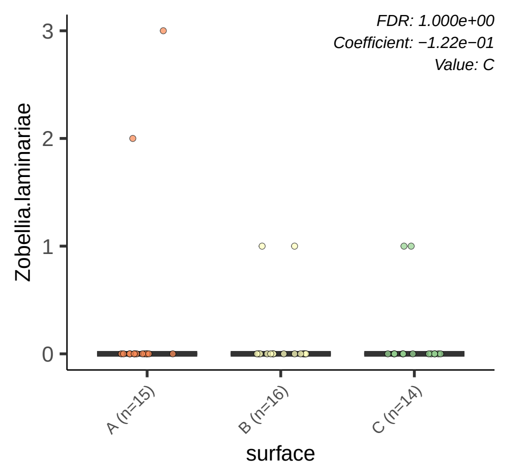

FDR: 1.000e+00
Coefficient: −1.22e−01
Value: C
Zobellia.laminariae
3
2
1
0
A (n=15)
B (n=16)
C (n=14)
surface

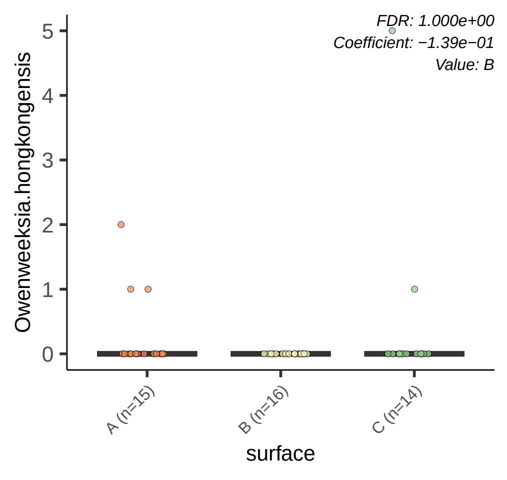
FDR: 1.000e+00
Coefficient: −1.39e−01
Value: B
Owenweeksia.hongkongensis
0
1
2
3
4
5
A (n=15)
B (n=16)
C (n=14)
surface

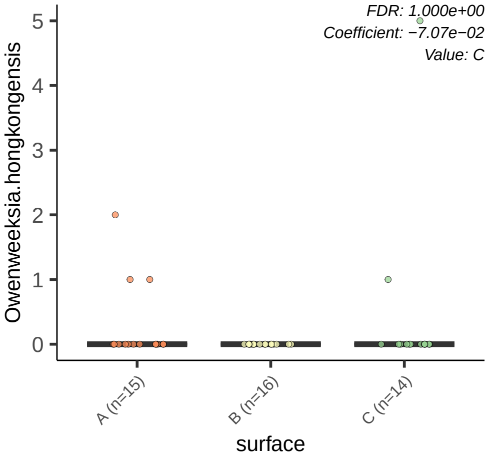
FDR: 1.000e+00
Coefficient: −7.07e−02
Value: C
Owenweeksia.hongkongensis
0
1
2
3
4
5
A (n=15)
B (n=16)
C (n=14)
surface

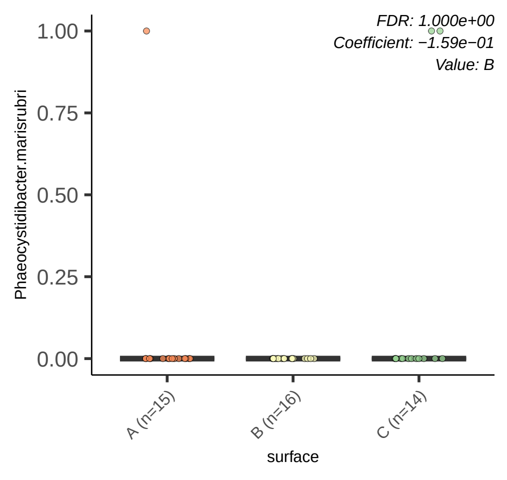
FDR: 1.000e+00
Coefficient: −1.59e−01
Value: B
Phaeocystidibacter.marisrubri
1.00
0.75
0.50
0.25
0.00
A (n=15)
B (n=16)
C (n=14)
surface

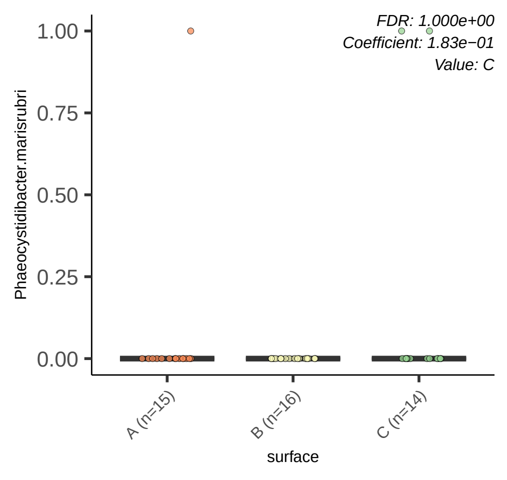
FDR: 1.000e+00
Coefficient: 1.83e−01
Value: C
1.00
0.75
0.50
0.25
0.00
Phaeocystidibacter.marisrubri
A (n=15)
B (n=16)
C (n=14)
surface

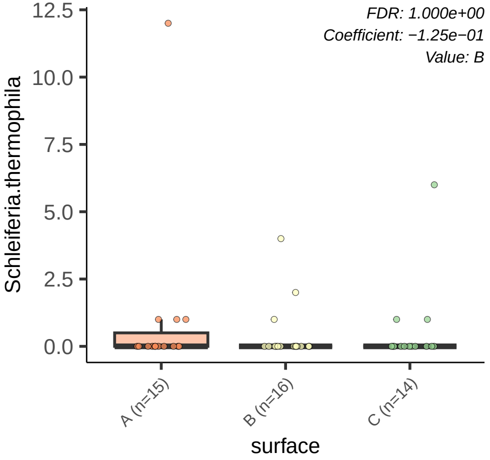

FDR: 1.000e+00
Coefficient: −1.25e−01
Value: B
Schleiferia.thermophila
12.5
10.0
7.5
5.0
2.5
0.0
A (n=15)
B (n=16)
C (n=14)
surface

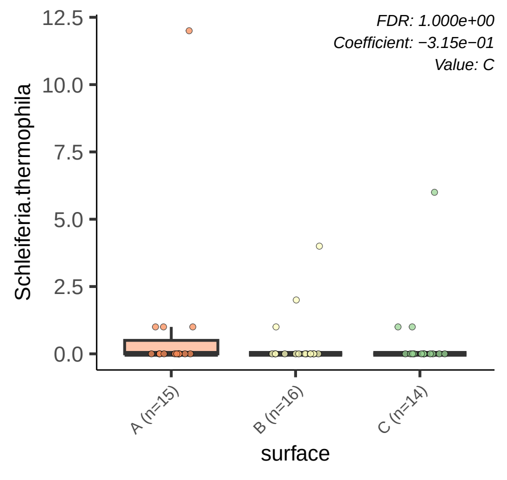

FDR: 1.000e+00
Coefficient: −3.15e−01
Value: C
12.5
10.0
7.5
5.0
2.5
0.0
Schleiferia.thermophila
A (n=15)
B (n=16)
C (n=14)
surface

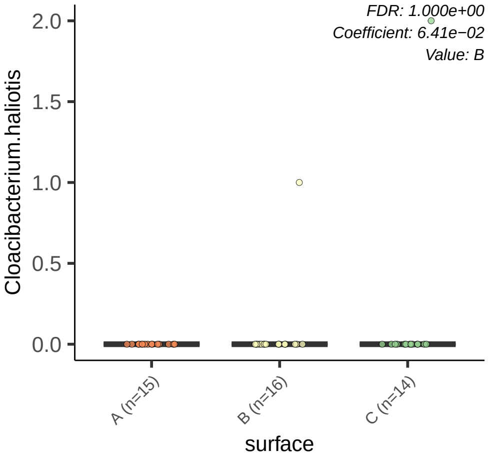

FDR: 1.000e+00
Coefficient: 6.41e−02
Value: B
Cloacibacterium.haliotis
2.0
1.5
1.0
0.5
0.0
A (n=15)
B (n=16)
C (n=14)
surface

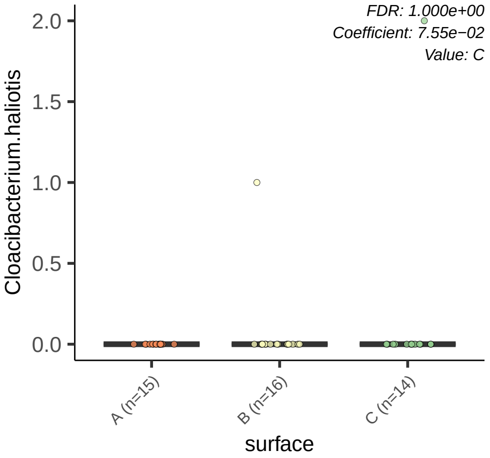
FDR: 1.000e+00
Coefficient: 7.55e−02
Value: C
Cloacibacterium.haliotis
2.0
1.5
1.0
0.5
0.0
A (n=15)
B (n=16)
C (n=14)
surface

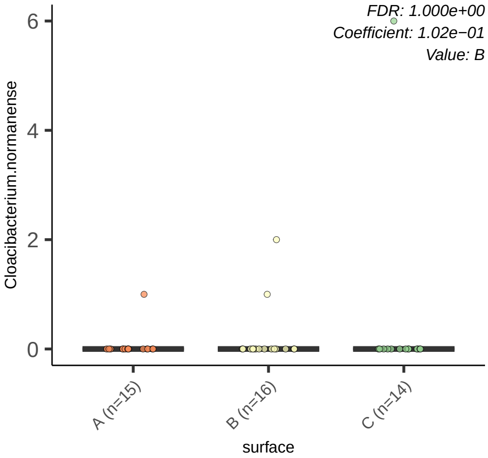
FDR: 1.000e+00
Coefficient: 1.02e−01
Value: B
Cloacibacterium.normanense
0
2
4
6
A (n=15)
B (n=16)
C (n=14)
surface

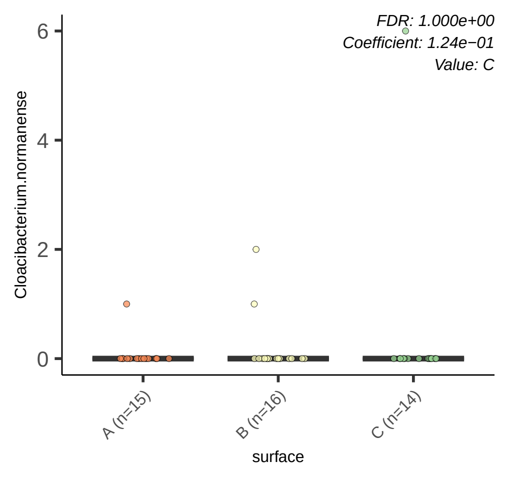

FDR: 1.000e+00
Coefficient: 1.24e−01
Value: C
Cloacibacterium.normanense
6
4
2
0
A (n=15)
B (n=16)
C (n=14)
surface

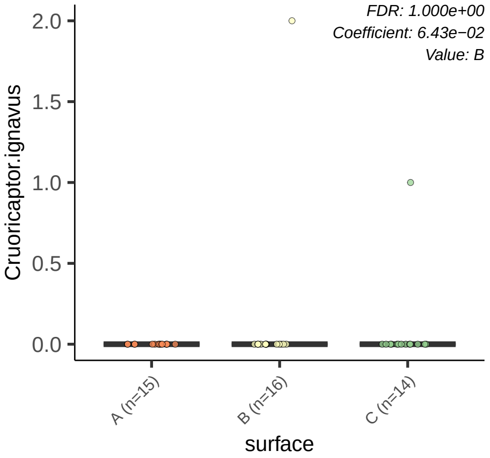
FDR: 1.000e+00
Coefficient: 6.43e−02
Value: B
Cruoricaptor.ignavus
2.0
1.5
1.0
0.5
0.0
A (n=15)
B (n=16)
C (n=14)
surface

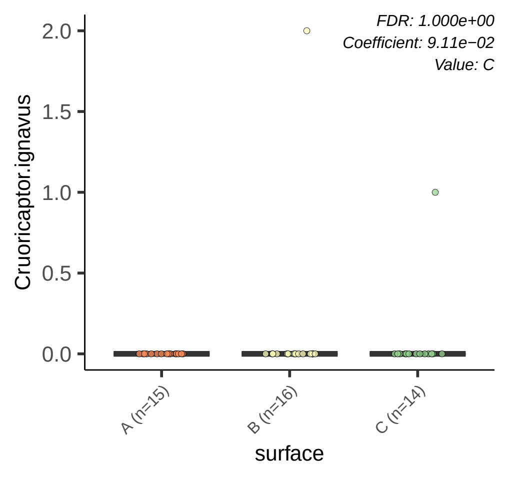
FDR: 1.000e+00
Coefficient: 9.11e−02
Value: C
Cruoricaptor.ignavus
2.0
1.5
1.0
0.5
0.0
A (n=15)
B (n=16)
C (n=14)
surface

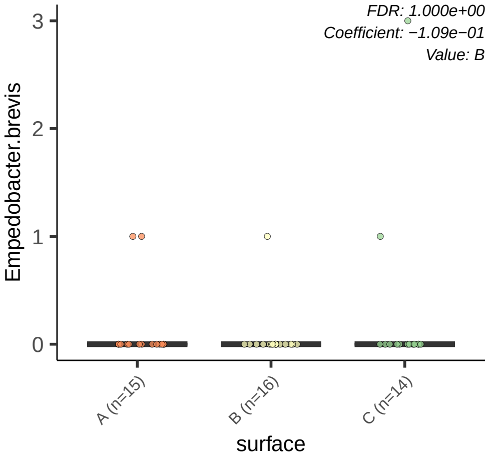

FDR: 1.000e+00
Coefficient: −1.09e−01
Value: B
Empedobacter.brevis
3
2
1
0
A (n=15)
B (n=16)
C (n=14)
surface

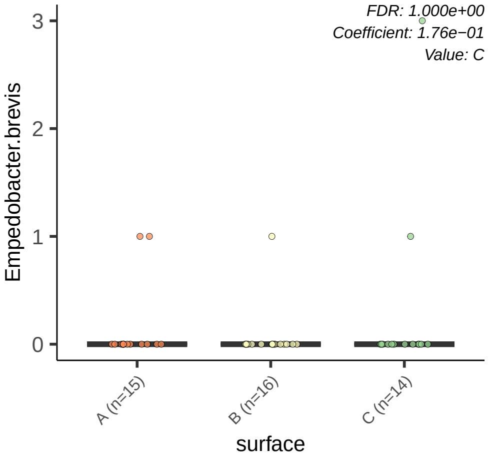
FDR: 1.000e+00
Coefficient: 1.76e−01
Value: C
Empedobacter.brevis
3
2
1
0
A (n=15)
B (n=16)
C (n=14)
surface

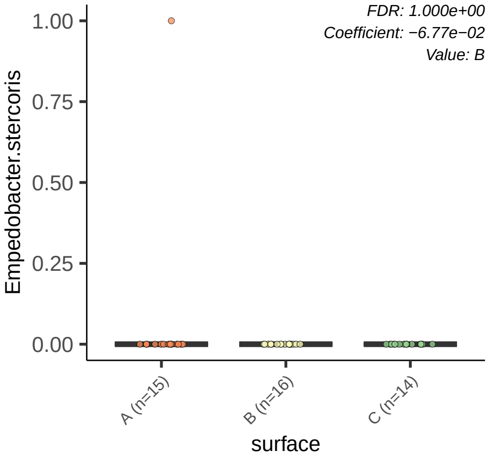
FDR: 1.000e+00
Coefficient: −6.77e−02
Value: B
Empedobacter.stercoris
1.00
0.75
0.50
0.25
0.00
A (n=15)
B (n=16)
C (n=14)
surface

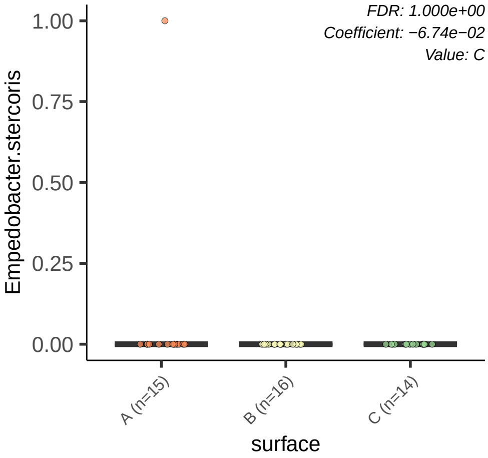
FDR: 1.000e+00
Coefficient: −6.74e−02
Value: C
Empedobacter.stercoris
1.00
0.75
0.50
0.25
0.00
A (n=15)
B (n=16)
C (n=14)
surface

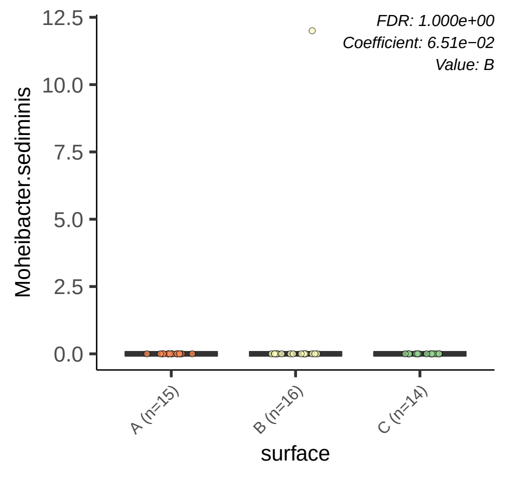
FDR: 1.000e+00
Coefficient: 6.51e−02
Value: B
Moheibacter.sedimminis
12.5
10.0
7.5
5.0
2.5
0.0
A (n=15)
B (n=16)
C (n=14)
surface

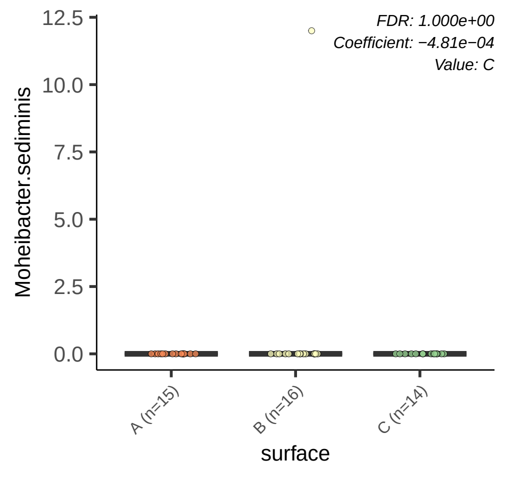
FDR: 1.000e+00
Coefficient: −4.81e−04
Value: C
Moheibacter.sedim inis
12.5
10.0
7.5
5.0
2.5
0.0
A (n=15)
B (n=16)
C (n=14)
surface

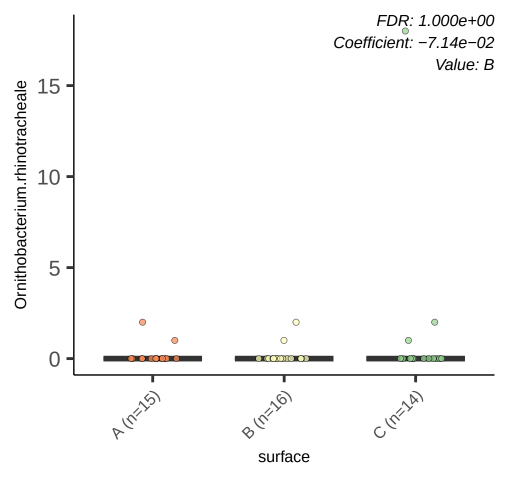

FDR: 1.000e+00
Coefficient: −7.14e−02
Value: B
Ornithobacterium.rhinotracheale
0
5
10
15
A (n=15)
B (n=16)
C (n=14)
surface

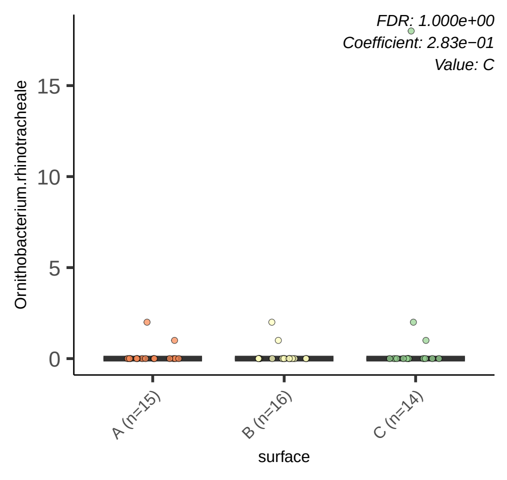

FDR: 1.000e+00
Coefficient: 2.83e−01
Value: C
Ornithobacterium.rhinotracheale
0
5
10
15
A (n=15)
B (n=16)
C (n=14)
surface

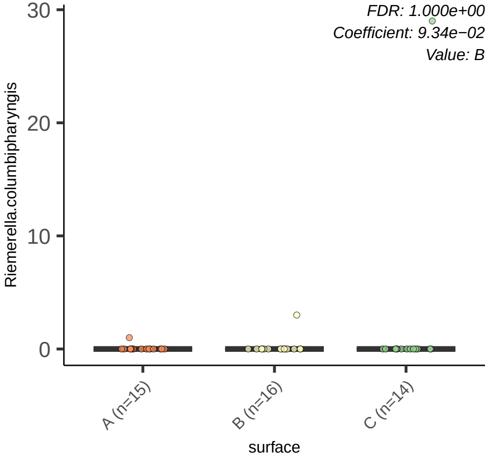

FDR: 1.000e+00
Coefficient: 9.34e−02
Value: B
Riemerella.columbipharyngis
30
20
10
0
A (n=15)
B (n=16)
C (n=14)
surface

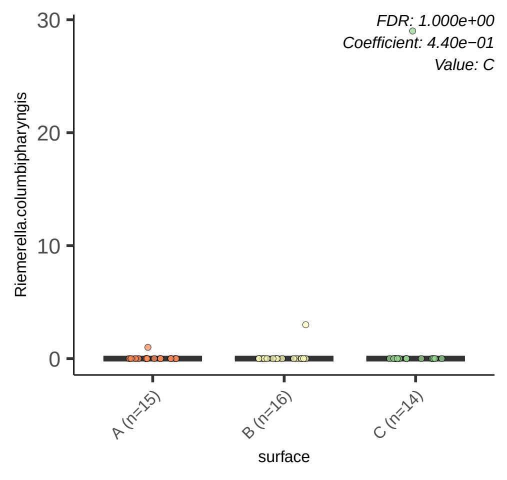
FDR: 1.000e+00
Coefficient: 4.40e−01
Value: C
30
20
10
0
Riemerella.columbipharyngis
A (n=15)
B (n=16)
C (n=14)
surface

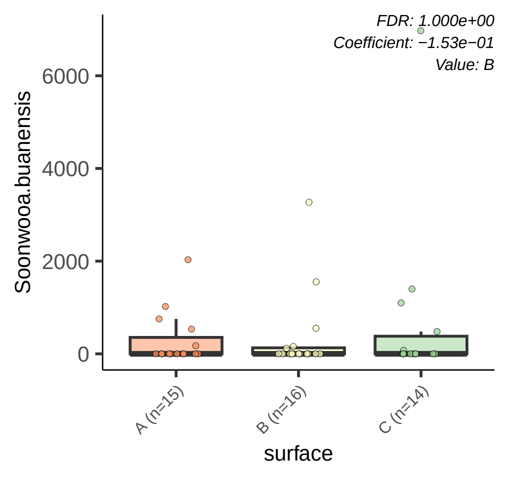
FDR: 1.000e+00
Coefficient: −1.53e−01
Value: B
6000
4000
2000
0
Soonwooa.buanensis
A (n=15)
B (n=16)
C (n=14)
surface

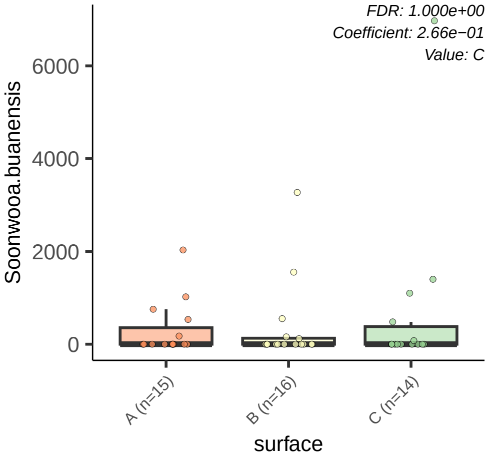
FDR: 1.000e+00
Coefficient: 2.66e−01
Value: C
Soonwooa.buanensis
0
2000
4000
6000
A (n=15)
B (n=16)
C (n=14)
surface

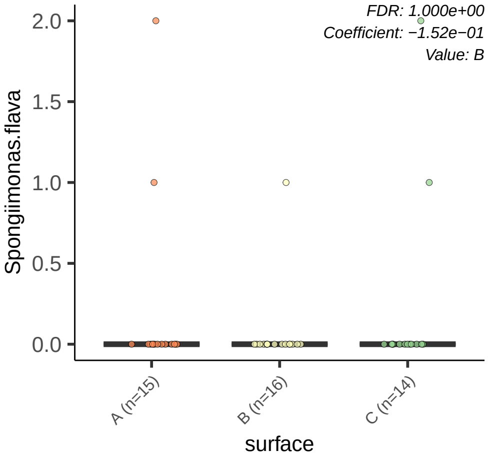

FDR: 1.000e+00
Coefficient: −1.52e−01
Value: B
Spongiimonas.flava
2.0
1.5
1.0
0.5
0.0
A (n=15)
B (n=16)
C (n=14)
surface

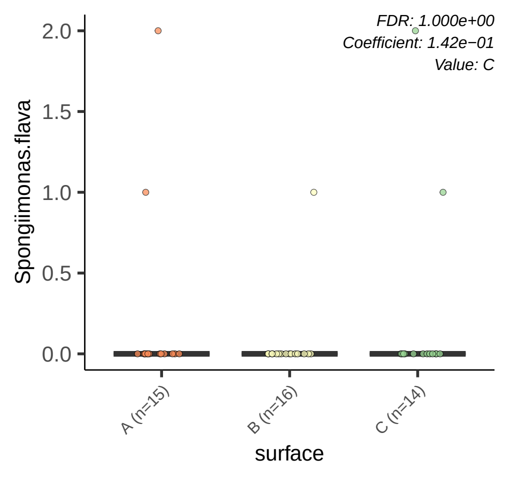
FDR: 1.000e+00
Coefficient: 1.42e−01
Value: C
Spongiimonas.flava
2.0
1.5
1.0
0.5
0.0
A (n=15)
B (n=16)
C (n=14)
surface

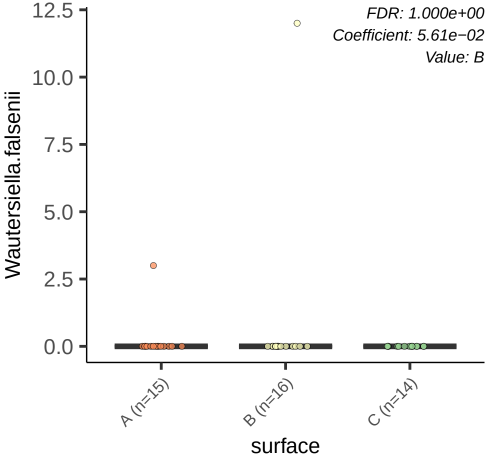

FDR: 1.000e+00
Coefficient: 5.61e−02
Value: B
Wautersiella.falsenii
12.5
10.0
7.5
5.0
2.5
0.0
A (n=15)
B (n=16)
C (n=14)
surface

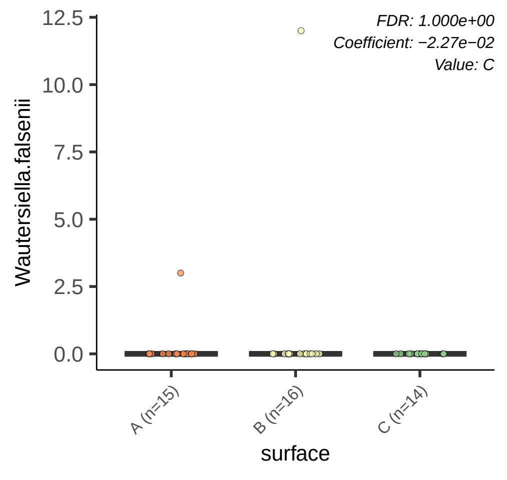
FDR: 1.000e+00
Coefficient: −2.27e−02
Value: C
Wautersiella.falsenii
12.5
10.0
7.5
5.0
2.5
0.0
A (n=15)
B (n=16)
C (n=14)
surface

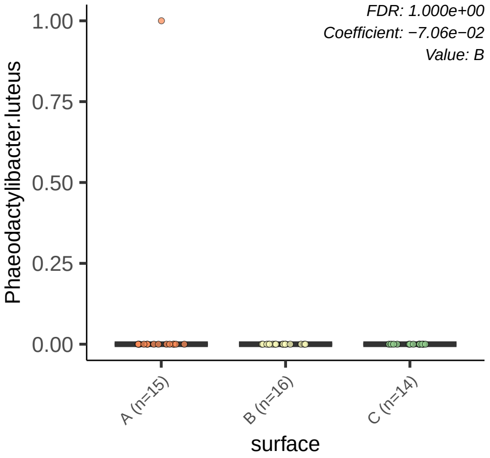
FDR: 1.000e+00
Coefficient: −7.06e−02
Value: B
Phaeodactylibacter.luteus
1.00
0.75
0.50
0.25
0.00
A (n=15)
B (n=16)
C (n=14)
surface

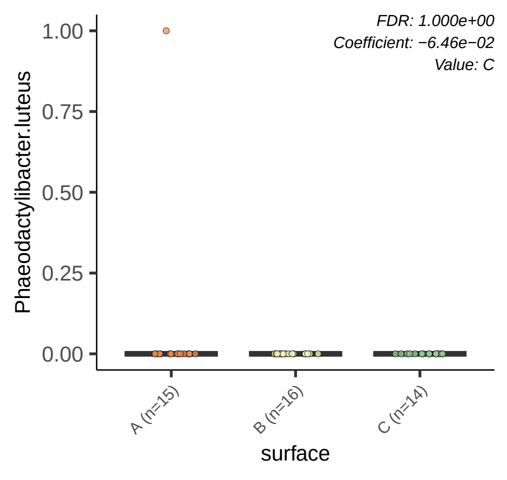
FDR: 1.000e+00
Coefficient: −6.46e−02
Value: C
Phaeodactylibacter.luteus
1.00
0.75
0.50
0.25
0.00
A (n=15)
B (n=16)
C (n=14)
surface

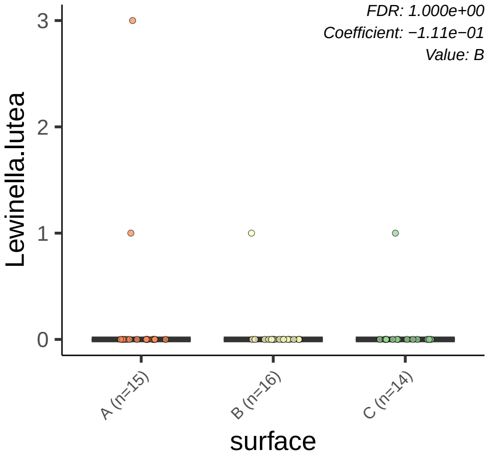
FDR: 1.000e+00
Coefficient: −1.11e−01
Value: B
Lewinella.lutea
3
2
1
0
A (n=15)
B (n=16)
C (n=14)
surface

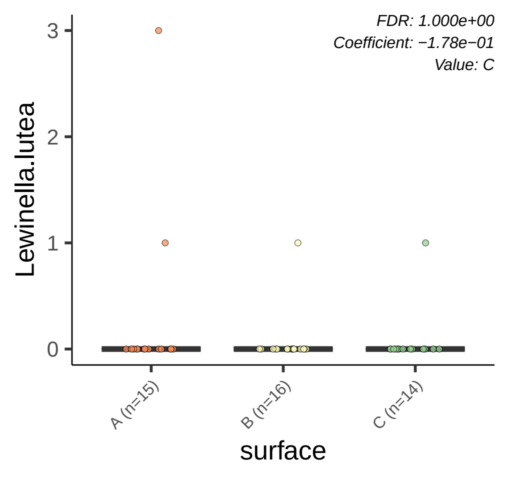
FDR: 1.000e+00
Coefficient: −1.78e−01
Value: C
Lewinella.lutea
3
2
1
0
A (n=15)
B (n=16)
C (n=14)
surface

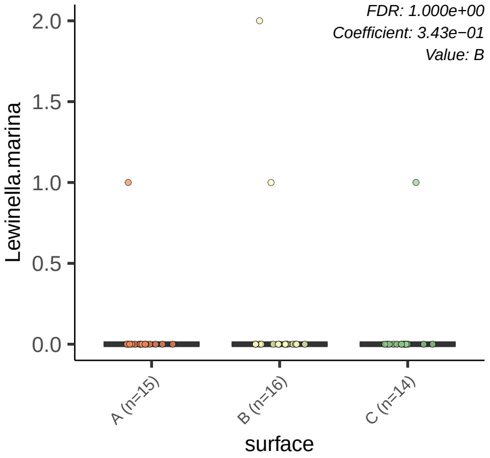

FDR: 1.000e+00
Coefficient: 3.43e−01
Value: B
Lewinella.marina
2.0
1.5
1.0
0.5
0.0
A (n=15)
B (n=16)
C (n=14)
surface

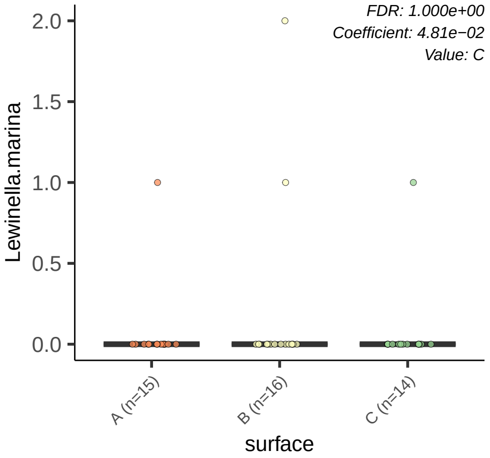
FDR: 1.000e+00
Coefficient: 4.81e−02
Value: C
Lewinella.marina
2.0
1.5
1.0
0.5
0.0
A (n=15)
B (n=16)
C (n=14)
surface

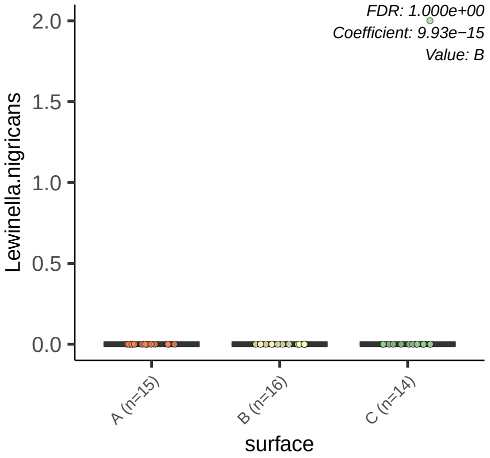
FDR: 1.000e+00
Coefficient: 9.93e−15
Value: B
Lewinella.nigricans
2.0
1.5
1.0
0.5
0.0
A (n=15)
B (n=16)
C (n=14)
surface

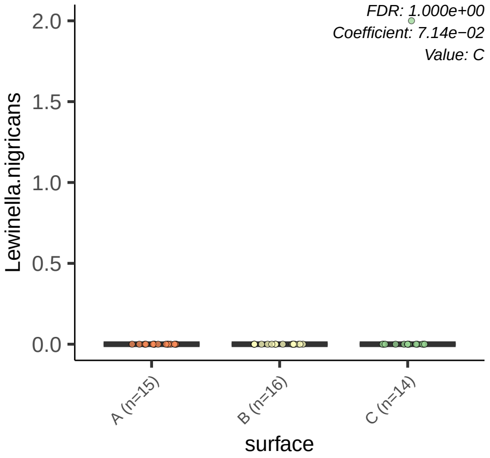
FDR: 1.000e+00
Coefficient: 7.14e−02
Value: C
Lewinella.nigricans
2.0
1.5
1.0
0.5
0.0
A (n=15)
B (n=16)
C (n=14)
surface

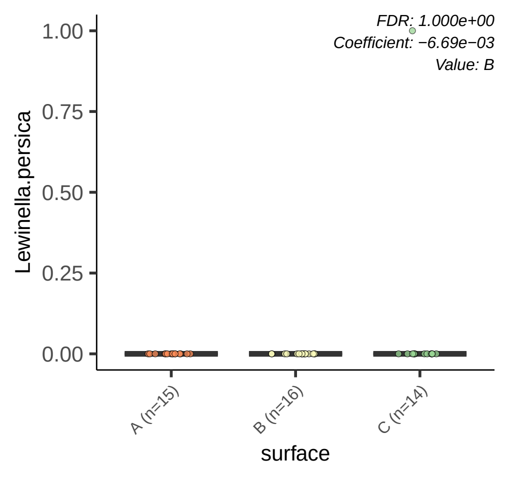

FDR: 1.000e+00
Coefficient: −6.69e−03
Value: B
Lewinella.persica
1.00
0.75
0.50
0.25
0.00
A (n=15)
B (n=16)
C (n=14)
surface

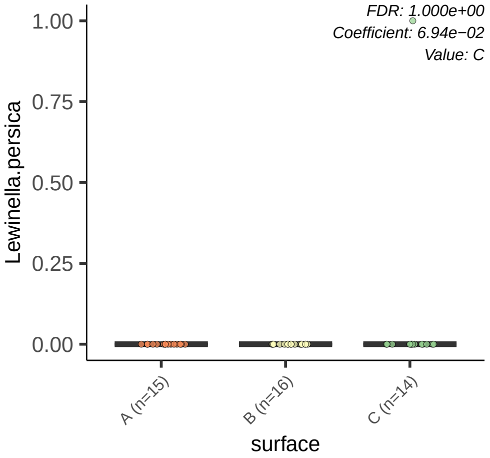

FDR: 1.000e+00
Coefficient: 6.94e−02
Value: C
Lewinella.persica
1.00
0.75
0.50
0.25
0.00
A (n=15)
B (n=16)
C (n=14)
surface

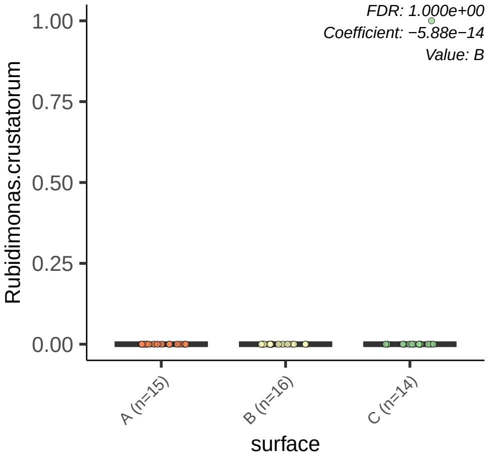

FDR: 1.000e+00
Coefficient: −5.88e−14
Value: B
Rubidimonas.crustatorum
1.00
0.75
0.50
0.25
0.00
A (n=15)
B (n=16)
C (n=14)
surface

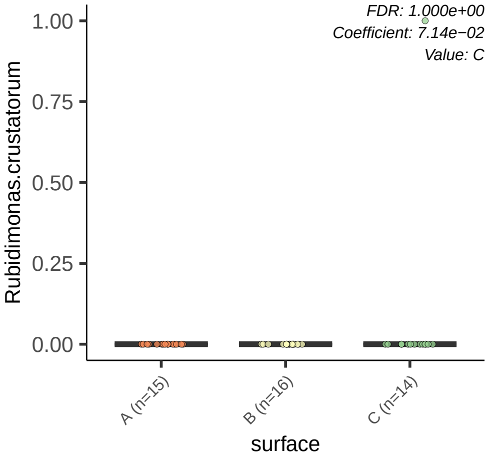
FDR: 1.000e+00
Coefficient: 7.14e−02
Value: C
Rubidimonas.crustatorum
1.00
0.75
0.50
0.25
0.00
A (n=15)
B (n=16)
C (n=14)
surface

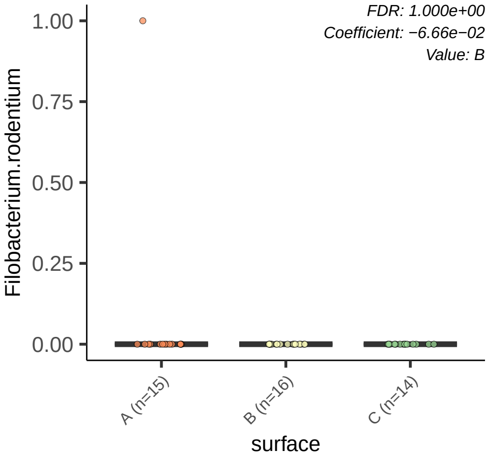

FDR: 1.000e+00
Coefficient: −6.66e−02
Value: B
Filobacterium.rodentium
1.00
0.75
0.50
0.25
0.00
A (n=15)
B (n=16)
C (n=14)
surface

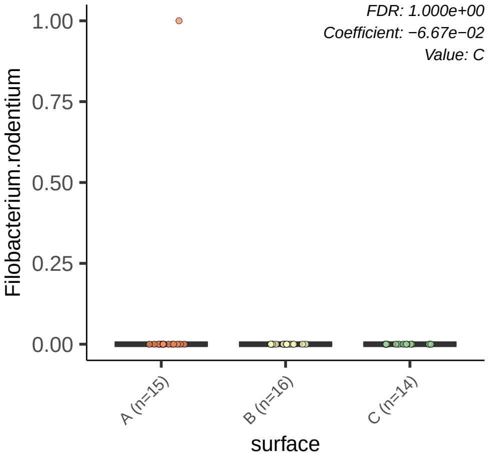

FDR: 1.000e+00
Coefficient: −6.67e−02
Value: C
Filobacterium.rodentium
1.00
0.75
0.50
0.25
0.00
A (n=15)
B (n=16)
C (n=14)
surface

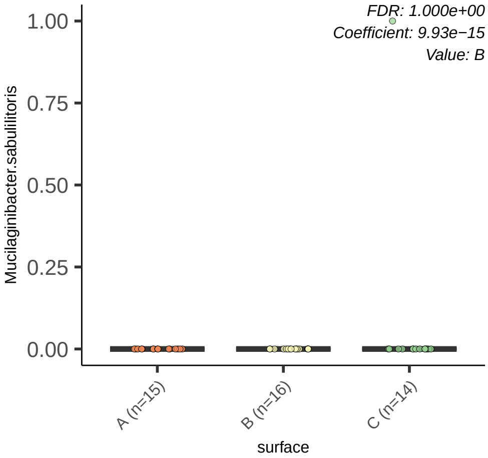

FDR: 1.000e+00
Coefficient: 9.93e−15
Value: B
Mucilaginibacter.sabulilitoris
1.00
0.75
0.50
0.25
0.00
A (n=15)
B (n=16)
C (n=14)
surface

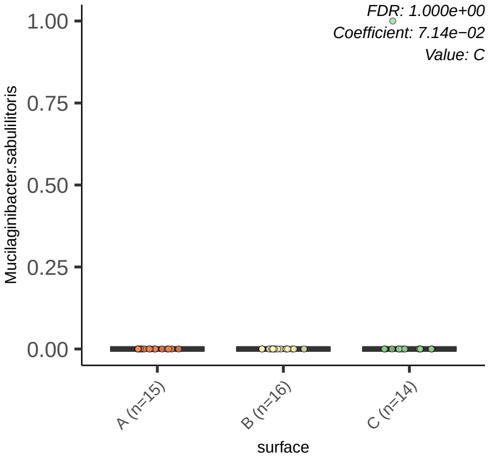

FDR: 1.000e+00
Coefficient: 7.14e−02
Value: C
1.00
0.75
0.50
0.25
0.00
Mucilaginibacter.sabulilitoris
A (n=15)
B (n=16)
C (n=14)
surface

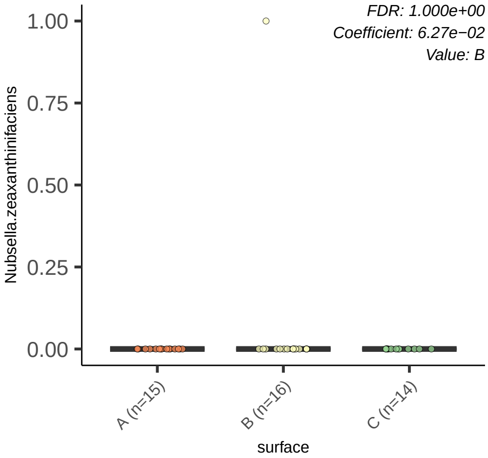
FDR: 1.000e+00
Coefficient: 6.27e−02
Value: B
Nubsella.zeaxanthinifaciens
1.00
0.75
0.50
0.25
0.00
A (n=15)
B (n=16)
C (n=14)
surface

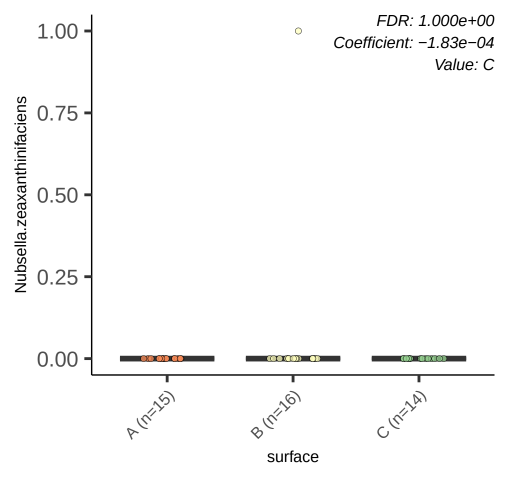
FDR: 1.000e+00
Coefficient: −1.83e−04
Value: C
Nubsella.zeaxanthinifaciens
1.00
0.75
0.50
0.25
0.00
A (n=15)
B (n=16)
C (n=14)
surface

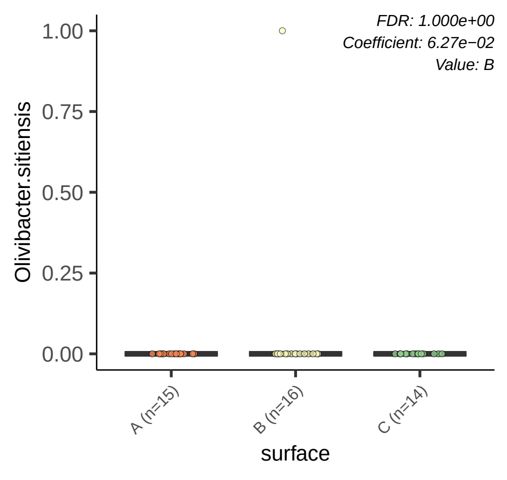
FDR: 1.000e+00
Coefficient: 6.27e−02
Value: B
Olivibacter.sitiensis
1.00
0.75
0.50
0.25
0.00
A (n=15)
B (n=16)
C (n=14)
surface

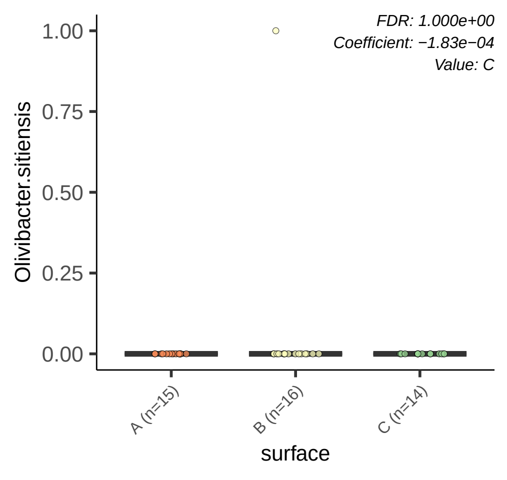

FDR: 1.000e+00
Coefficient: −1.83e−04
Value: C
Olivibacter.sitiensis
1.00
0.75
0.50
0.25
0.00
A (n=15)
B (n=16)
C (n=14)
surface

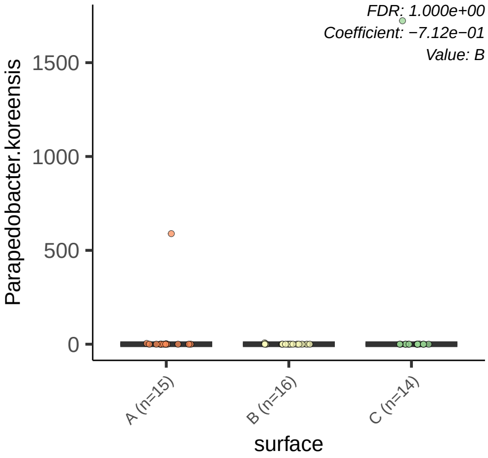
FDR: 1.000e+00
Coefficient: −7.12e−01
Value: B
Parapedobacter.koreensis
1500
1000
500
0
A (n=15)
B (n=16)
C (n=14)
surface

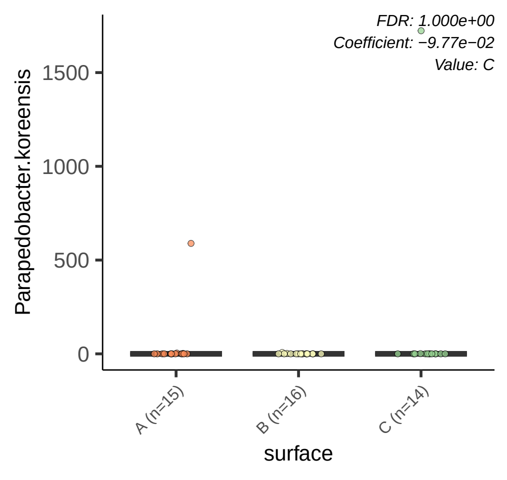

FDR: 1.000e+00
Coefficient: −9.77e−02
Value: C
Parapedobacter.koreensis
1500
1000
500
0
A (n=15)
B (n=16)
C (n=14)
surface

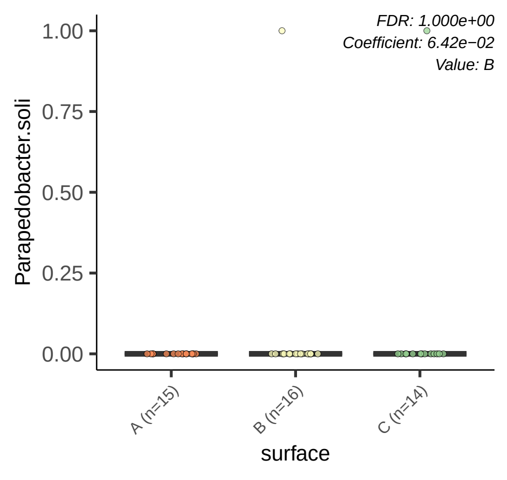

FDR: 1.000e+00
Coefficient: 6.42e−02
Value: B
Parapedobacter.soli
1.00
0.75
0.50
0.25
0.00
A (n=15)
B (n=16)
C (n=14)
surface

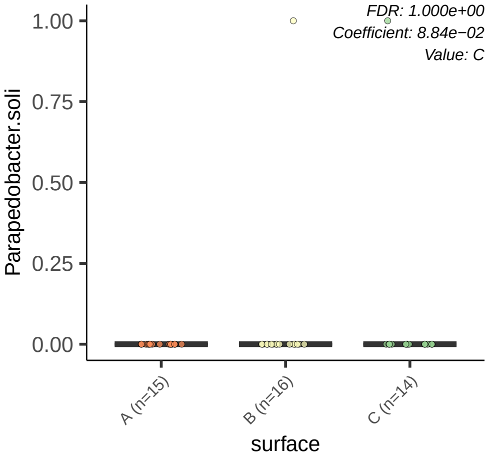
FDR: 1.000e+00
Coefficient: 8.84e−02
Value: C
Parapedobacter.soli
1.00
0.75
0.50
0.25
0.00
A (n=15)
B (n=16)
C (n=14)
surface

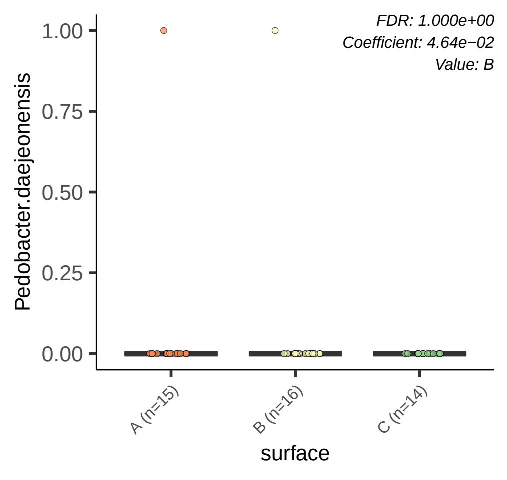
FDR: 1.000e+00
Coefficient: 4.64e−02
Value: B
Pedobacter.daejeonensis
1.00
0.75
0.50
0.25
0.00
A (n=15)
B (n=16)
C (n=14)
surface

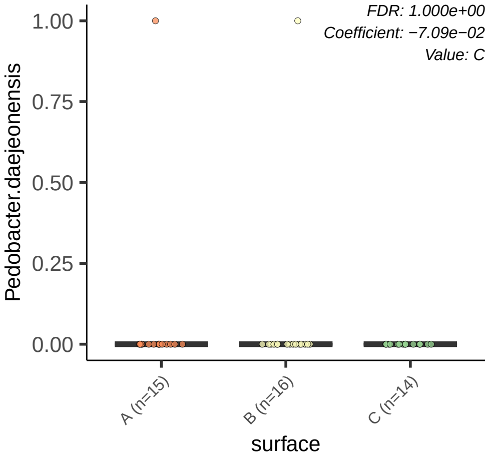
FDR: 1.000e+00
Coefficient: −7.09e−02
Value: C
Pedobacter.daejeonensis
1.00
0.75
0.50
0.25
0.00
A (n=15)
B (n=16)
C (n=14)
surface

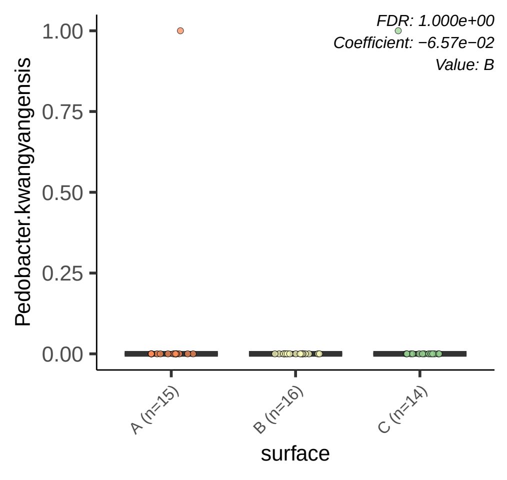

FDR: 1.000e+00
Coefficient: −6.57e−02
Value: B
Pedobacter.kwangyangensis
1.00
0.75
0.50
0.25
0.00
A (n=15)
B (n=16)
C (n=14)
surface

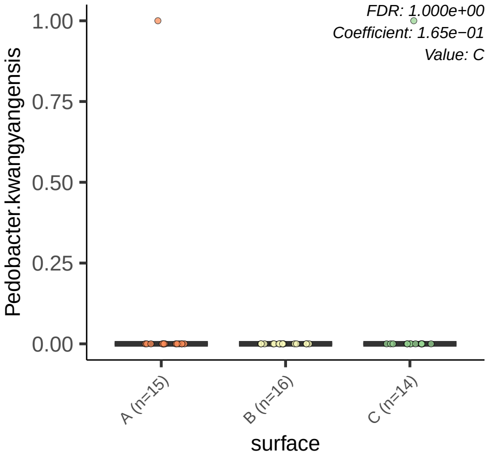
FDR: 1.000e+00
Coefficient: 1.65e−01
Value: C
Pedobacter.kwangyangensis
1.00
0.75
0.50
0.25
0.00
A (n=15)
B (n=16)
C (n=14)
surface

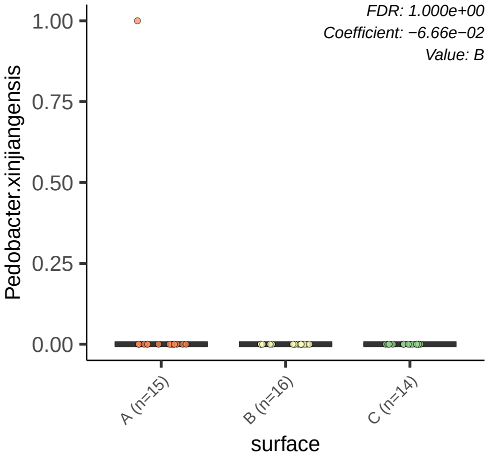
FDR: 1.000e+00
Coefficient: −6.66e−02
Value: B
Pedobacter.xinjiangensis
1.00
0.75
0.50
0.25
0.00
A (n=15)
B (n=16)
C (n=14)
surface

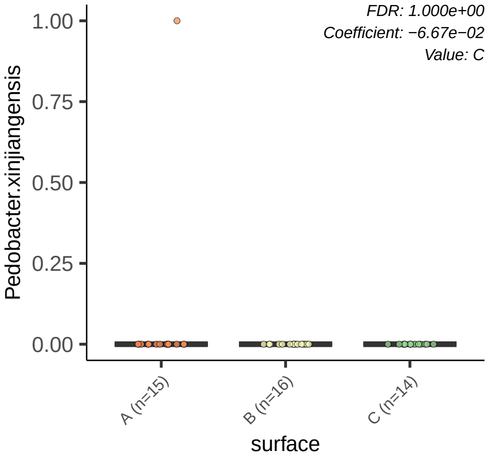
FDR: 1.000e+00
Coefficient: −6.67e−02
Value: C
Pedobacter.xinjiangensis
1.00
0.75
0.50
0.25
0.00
A (n=15)
B (n=16)
C (n=14)
surface

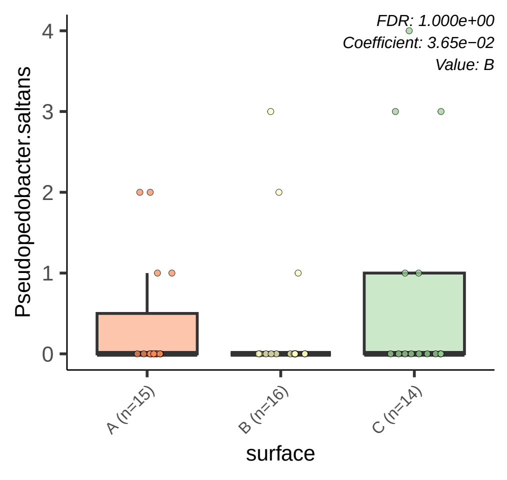
FDR: 1.000e+00
Coefficient: 3.65e−02
Value: B
Pseudopedobacter.saltans
0
1
2
3
4
A (n=15)
B (n=16)
C (n=14)
surface

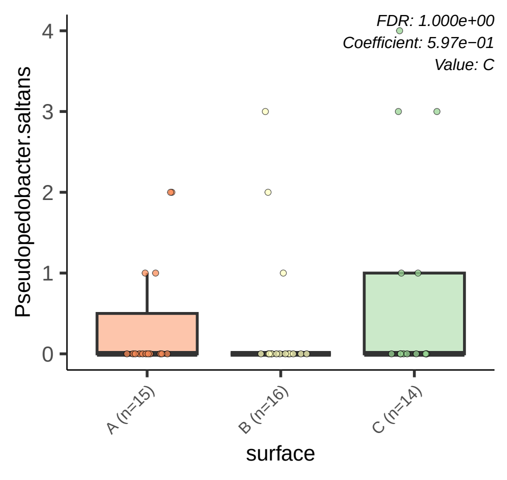

FDR: 1.000e+00
Coefficient: 5.97e−01
Value: C
Pseudopedobacter.saltans
0
1
2
3
4
A (n=15)
B (n=16)
C (n=14)
surface

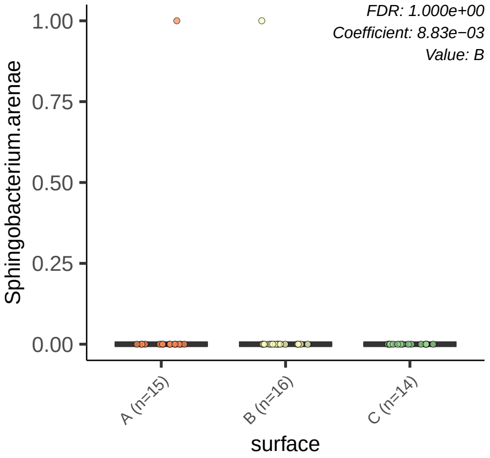

FDR: 1.000e+00
Coefficient: 8.83e−03
Value: B
Sphingobacterium.arenae
1.00
0.75
0.50
0.25
0.00
A (n=15)
B (n=16)
C (n=14)
surface

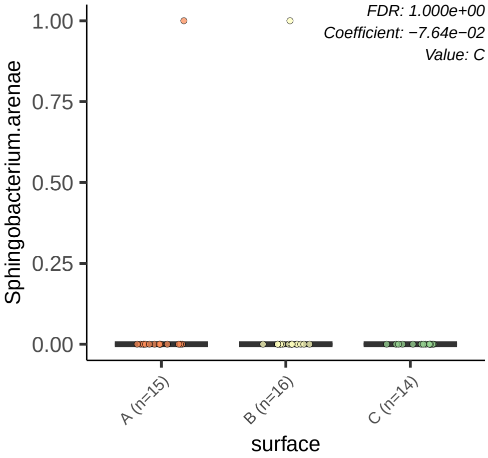

FDR: 1.000e+00
Coefficient: −7.64e−02
Value: C
1.00
0.75
0.50
0.25
0.00
Sphingobacterium.arenae
A (n=15)
B (n=16)
C (n=14)
surface

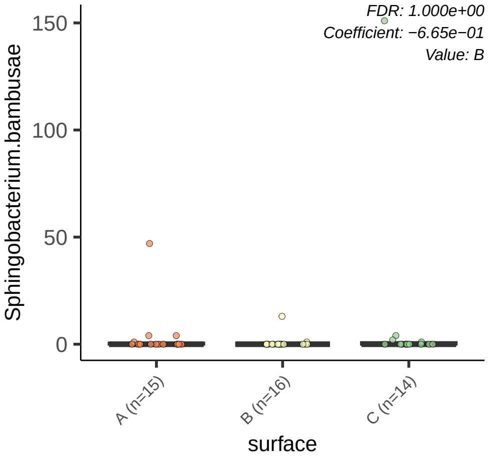
FDR: 1.000e+00
Coefficient: −6.65e−01
Value: B
Sphingobacterium.bambusae
150
100
50
0
A (n=15)
B (n=16)
C (n=14)
surface

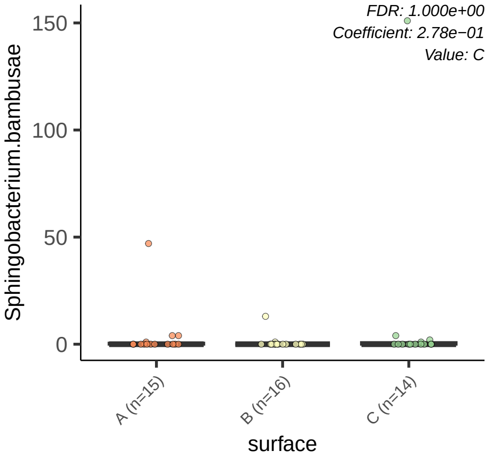

FDR: 1.000e+00
Coefficient: 2.78e−01
Value: C
Sphingobacterium.bambusae
150
100
50
0
A (n=15)
B (n=16)
C (n=14)
surface

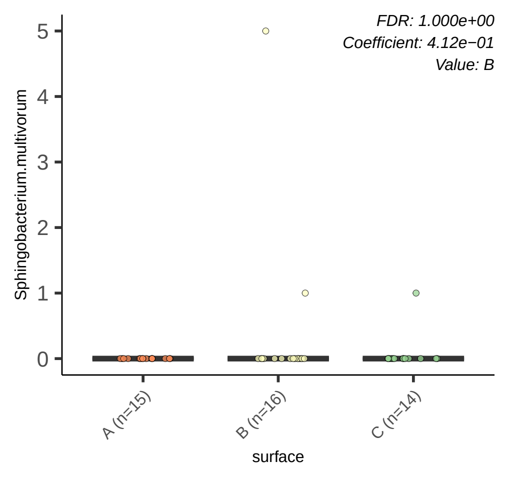
FDR: 1.000e+00
Coefficient: 4.12e−01
Value: B
Sphingobacterium.multivorum
0
1
2
3
4
5
A (n=15)
B (n=16)
C (n=14)
surface

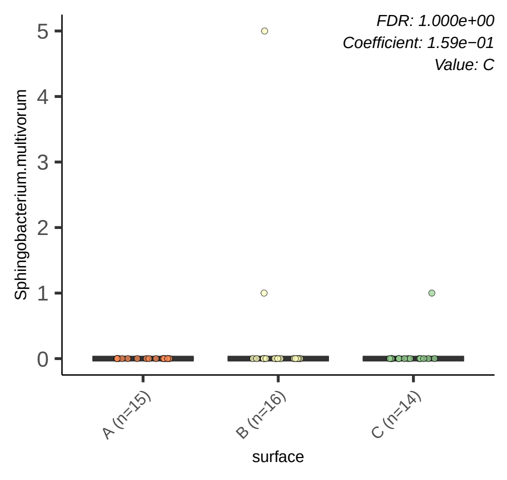
FDR: 1.000e+00
Coefficient: 1.59e−01
Value: C
Sphingobacterium.multivorum
0
1
2
3
4
5
A (n=15)
B (n=16)
C (n=14)
surface

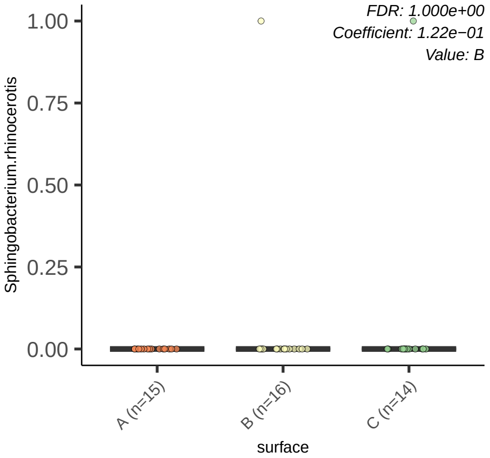
FDR: 1.000e+00
Coefficient: 1.22e−01
Value: B
Sphingobacterium.rhinocerotis
1.00
0.75
0.50
0.25
0.00
A (n=15)
B (n=16)
C (n=14)
surface

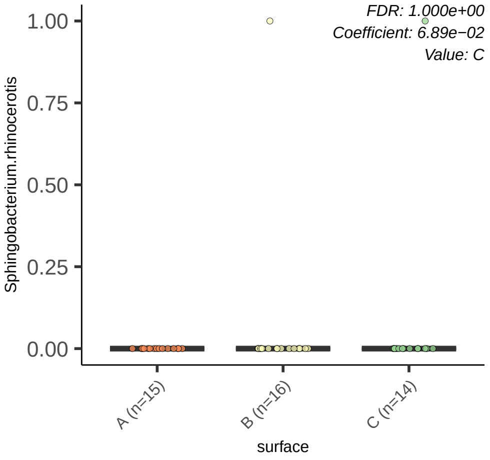

FDR: 1.000e+00
Coefficient: 6.89e−02
Value: C
Sphingobacterium.rhinocerotis
1.00
0.75
0.50
0.25
0.00
A (n=15)
B (n=16)
C (n=14)
surface

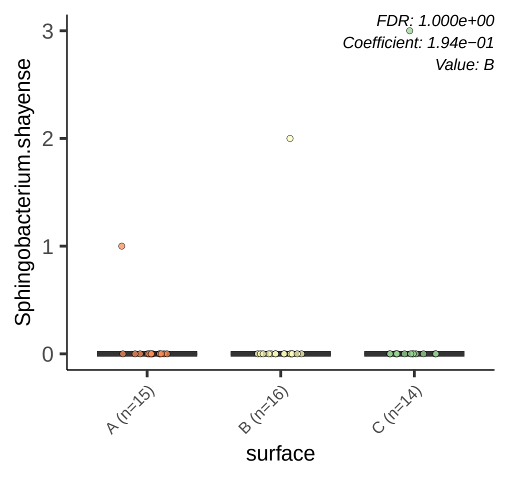
FDR: 1.000e+00
Coefficient: 1.94e−01
Value: B
Sphingobacterium.shayense
3
2
1
0
A (n=15)
B (n=16)
C (n=14)
surface

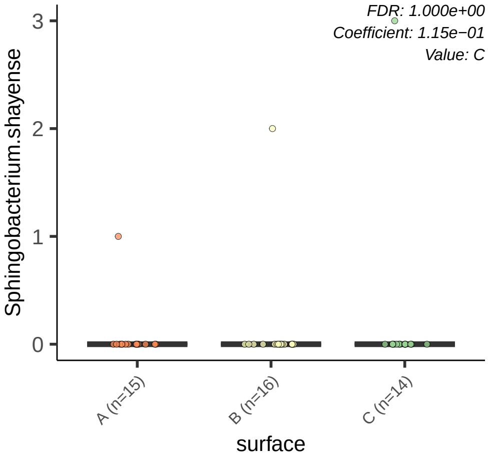
FDR: 1.000e+00
Coefficient: 1.15e−01
Value: C
Sphingobacterium.shayense
3
2
1
0
A (n=15)
B (n=16)
C (n=14)
surface

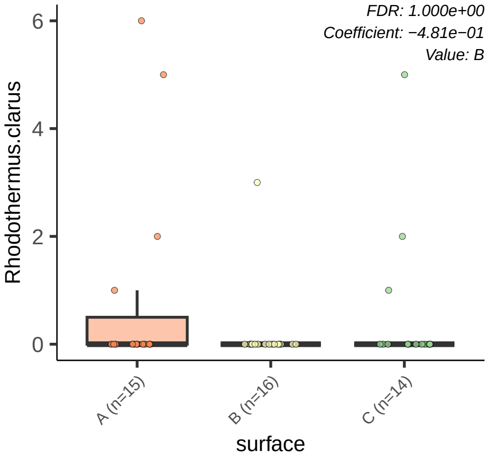

FDR: 1.000e+00
Coefficient: −4.81e−01
Value: B
Rhodothermus.clarus
0
2
4
6
A (n=15)
B (n=16)
C (n=14)
surface

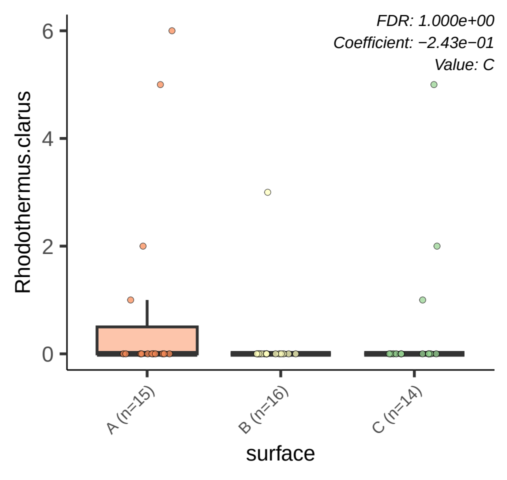

FDR: 1.000e+00
Coefficient: −2.43e−01
Value: C
Rhodothermus.clarus
0
2
4
6
A (n=15)
B (n=16)
C (n=14)
surface

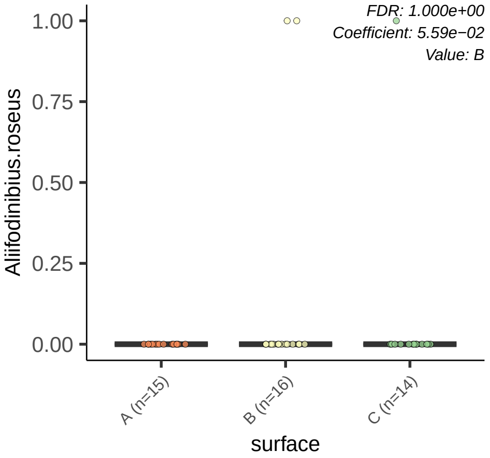
FDR: 1.000e+00
Coefficient: 5.59e−02
Value: B
Aliifodinibius.roseus
1.00
0.75
0.50
0.25
0.00
A (n=15)
B (n=16)
C (n=14)
surface

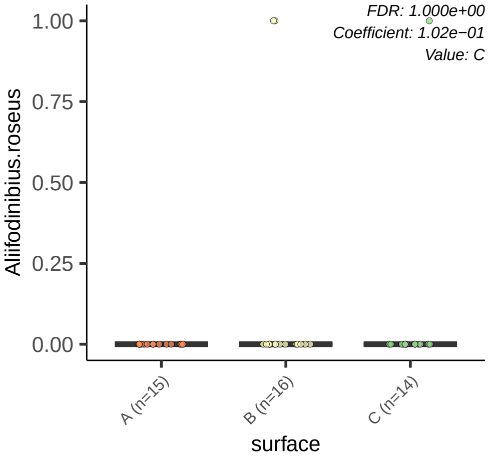

FDR: 1.000e+00
Coefficient: 1.02e−01
Value: C
Aliifodinibius.roseus
1.00
0.75
0.50
0.25
0.00
A (n=15)
B (n=16)
C (n=14)
surface

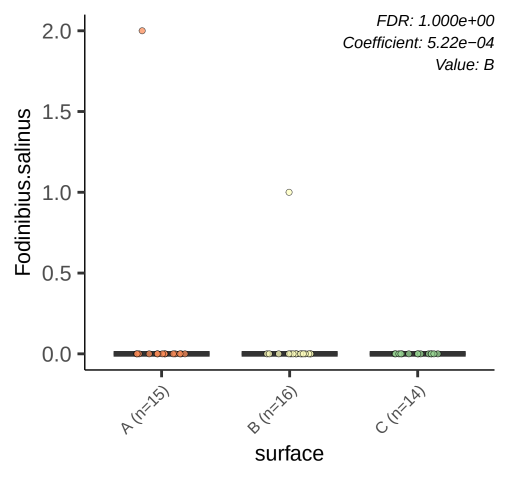
FDR: 1.000e+00
Coefficient: 5.22e−04
Value: B
Fodinibius.salinus
2.0
1.5
1.0
0.5
0.0
A (n=15)
B (n=16)
C (n=14)
surface

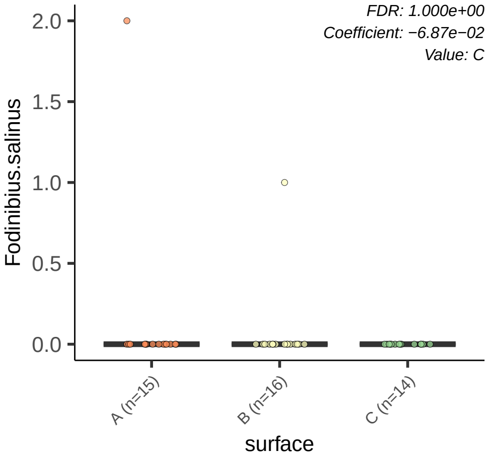
FDR: 1.000e+00
Coefficient: −6.87e−02
Value: C
Fodinibius.salinus
2.0
1.5
1.0
0.5
0.0
A (n=15)
B (n=16)
C (n=14)
surface

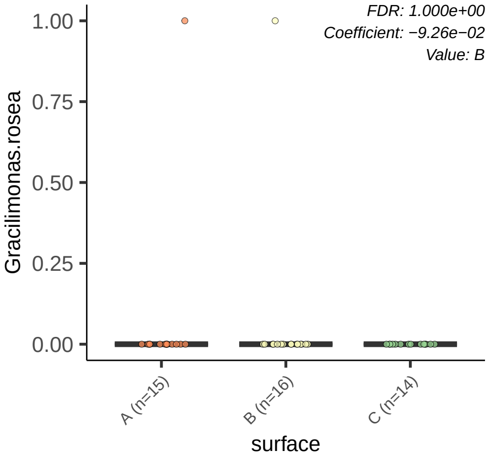
FDR: 1.000e+00
Coefficient: −9.26e−02
Value: B
Gracilimonas.rosea
1.00
0.75
0.50
0.25
0.00
A (n=15)
B (n=16)
C (n=14)
surface

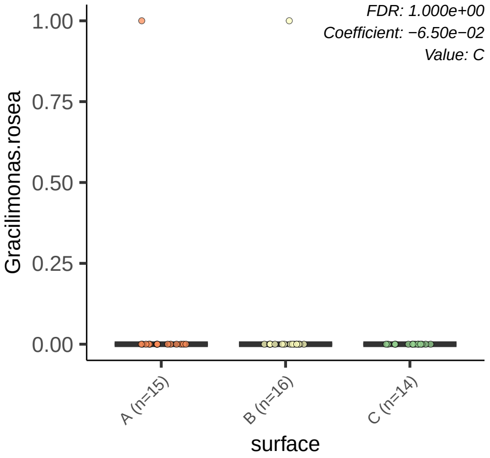

FDR: 1.000e+00
Coefficient: −6.50e−02
Value: C
Gracilimonas.rosea
1.00
0.75
0.50
0.25
0.00
A (n=15)
B (n=16)
C (n=14)
surface

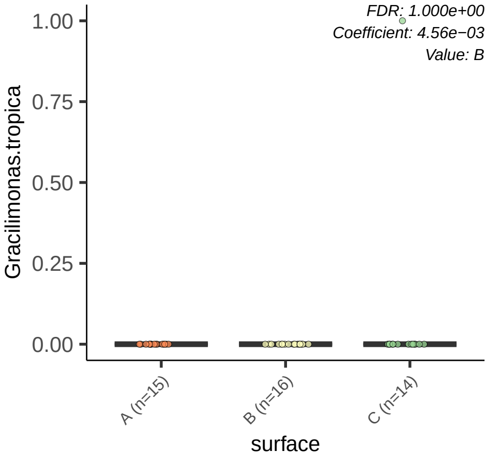
FDR: 1.000e+00
Coefficient: 4.56e−03
Value: B
Gracilimonas.tropica
1.00
0.75
0.50
0.25
0.00
A (n=15)
B (n=16)
C (n=14)
surface

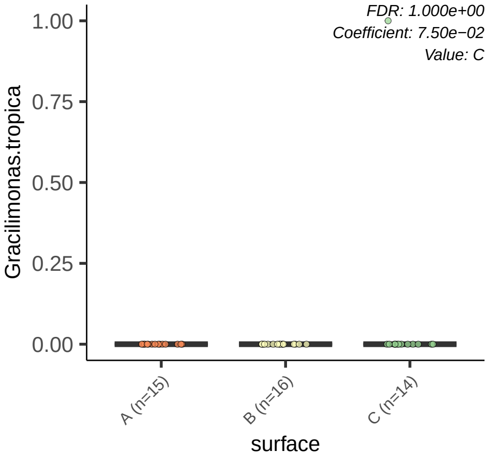
FDR: 1.000e+00
Coefficient: 7.50e−02
Value: C
Gracilimonas.tropica
1.00
0.75
0.50
0.25
0.00
A (n=15)
B (n=16)
C (n=14)
surface

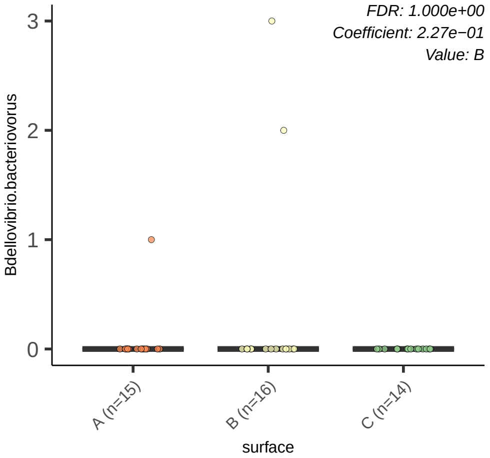
FDR: 1.000e+00
Coefficient: 2.27e−01
Value: B
Bdellovibrio.bacteriovorus
3
2
1
0
A (n=15)
B (n=16)
C (n=14)
surface

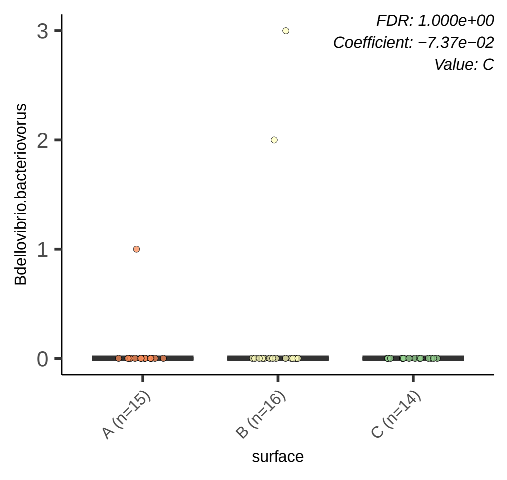
FDR: 1.000e+00
Coefficient: −7.37e−02
Value: C
Bdellovibrio.bacteriovorus
0
1
2
3
A (n=15)
B (n=16)
C (n=14)
surface

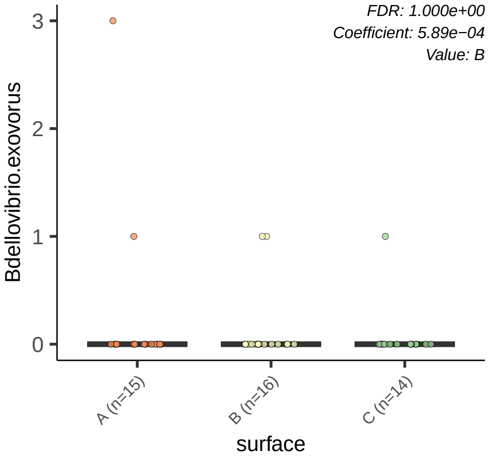
FDR: 1.000e+00
Coefficient: 5.89e−04
Value: B
Bdellovibrio.exovorus
3
2
1
0
A (n=15)
B (n=16)
C (n=14)
surface

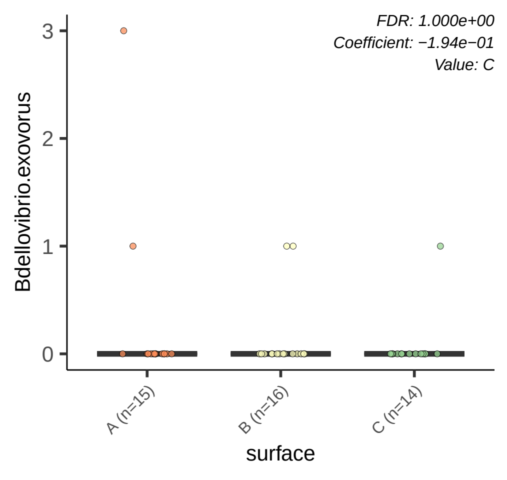
FDR: 1.000e+00
Coefficient: −1.94e−01
Value: C
Bdellovibrio.exovorus
3
2
1
0
A (n=15)
B (n=16)
C (n=14)
surface

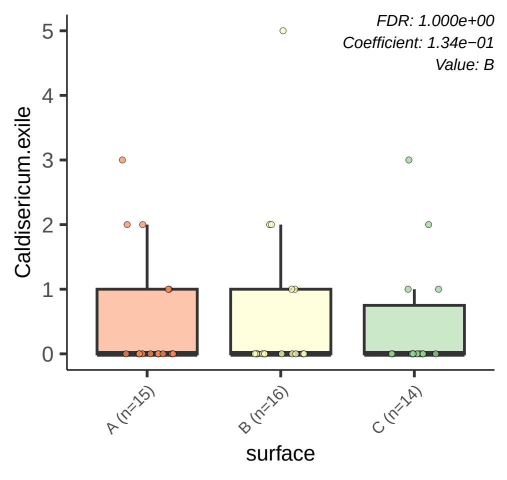
FDR: 1.000e+00
Coefficient: 1.34e−01
Value: B
Caldisericum.exile
0
1
2
3
4
5
A (n=15)
B (n=16)
C (n=14)
surface

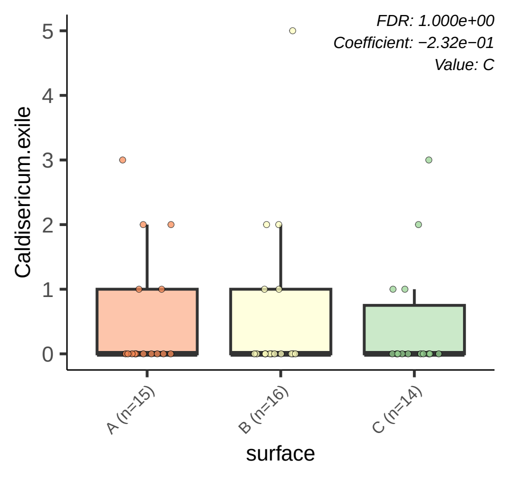

FDR: 1.000e+00
Coefficient: −2.32e−01
Value: C
Caldisericum.exile
0
1
2
3
4
5
A (n=15)
B (n=16)
C (n=14)
surface

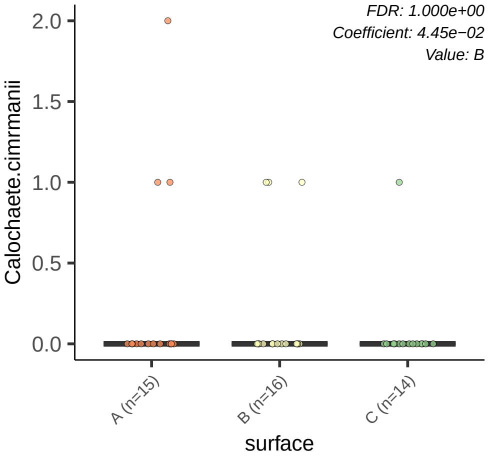

FDR: 1.000e+00
Coefficient: 4.45e−02
Value: B
Calochaete.cimrmanii
2.0
1.5
1.0
0.5
0.0
A (n=15)
B (n=16)
C (n=14)
surface

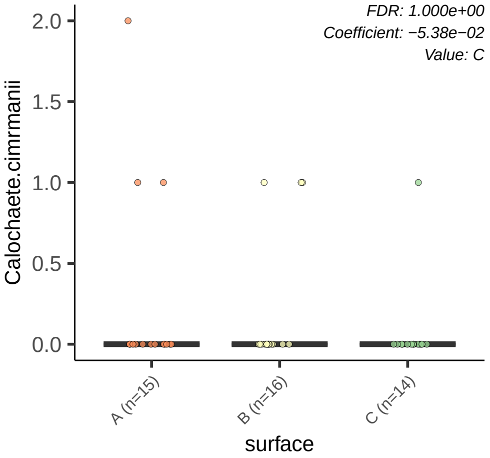

FDR: 1.000e+00
Coefficient: −5.38e−02
Value: C
Calochaete.cimrmanii
2.0
1.5
1.0
0.5
0.0
A (n=15)
B (n=16)
C (n=14)
surface

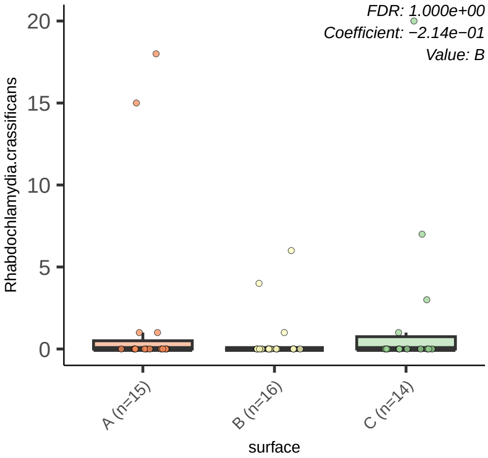
FDR: 1.000e+00
Coefficient: −2.14e−01
Value: B
Rhabdochlamydia.crassificans
20
15
10
5
0
A (n=15)
B (n=16)
C (n=14)
surface

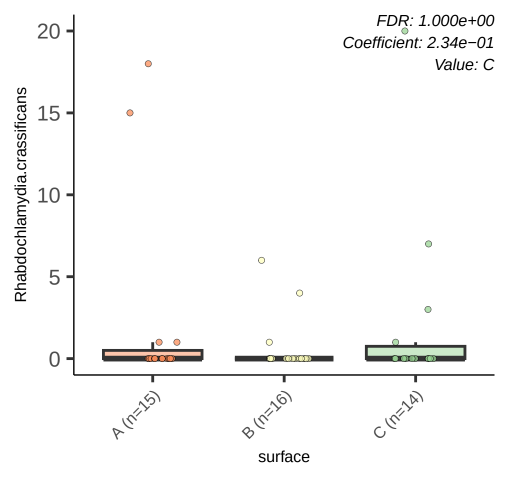
FDR: 1.000e+00
Coefficient: 2.34e−01
Value: C
Rhabdochlamydia.crassificans
20
15
10
5
0
A (n=15)
B (n=16)
C (n=14)
surface

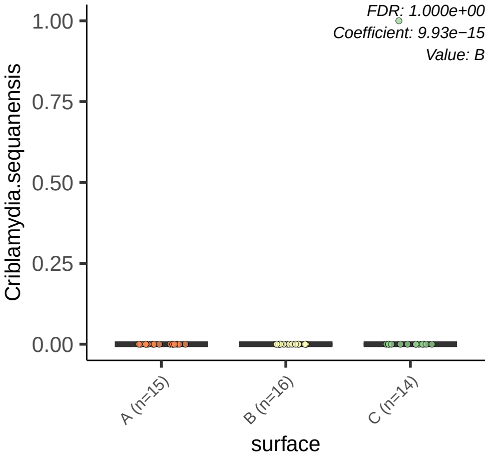

FDR: 1.000e+00
Coefficient: 9.93e−15
Value: B
Criblamydia.sequanensis
1.00
0.75
0.50
0.25
0.00
A (n=15)
B (n=16)
C (n=14)
surface

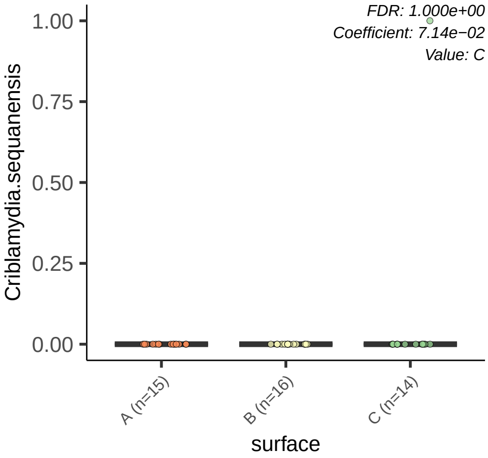
FDR: 1.000e+00
Coefficient: 7.14e−02
Value: C
Criblamydia.sequanensis
1.00
0.75
0.50
0.25
0.00
A (n=15)
B (n=16)
C (n=14)
surface

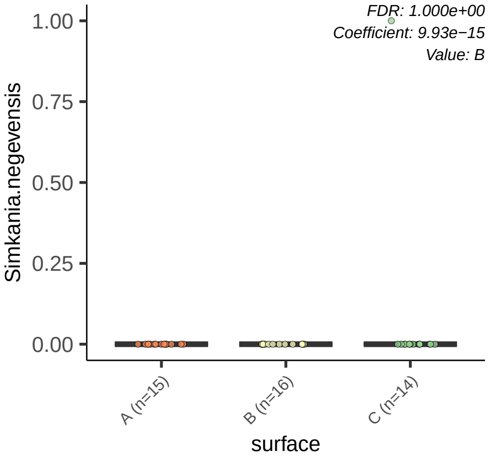

FDR: 1.000e+00
Coefficient: 9.93e−15
Value: B
Simkania.negevensis
1.00
0.75
0.50
0.25
0.00
A (n=15)
B (n=16)
C (n=14)
surface

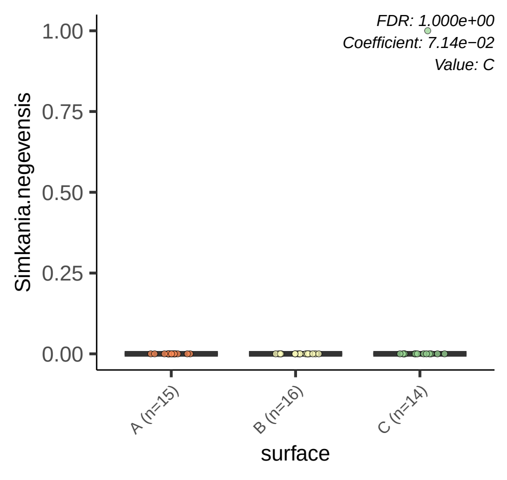
FDR: 1.000e+00
Coefficient: 7.14e−02
Value: C
Simkania.negevensis
1.00
0.75
0.50
0.25
0.00
A (n=15)
B (n=16)
C (n=14)
surface

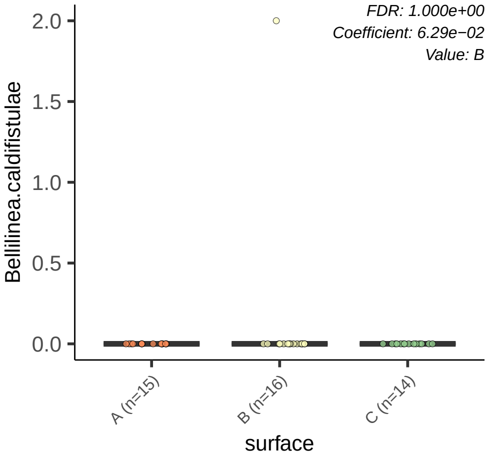

FDR: 1.000e+00
Coefficient: 6.29e−02
Value: B
Bellilinea.caldifistulae
2.0
1.5
1.0
0.5
0.0
A (n=15)
B (n=16)
C (n=14)
surface

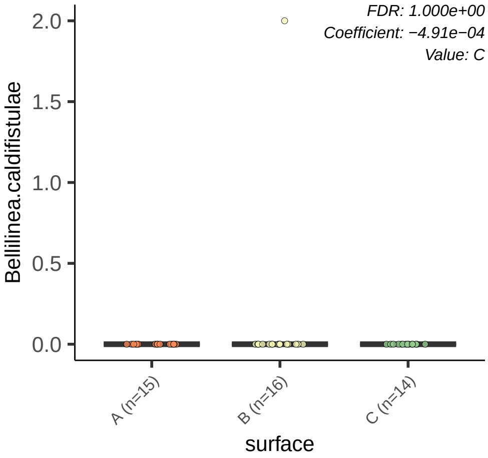
FDR: 1.000e+00
Coefficient: −4.91e−04
Value: C
2.0
1.5
1.0
0.5
0.0
Bellilinea.caldifistulae
A (n=15)
B (n=16)
C (n=14)
surface

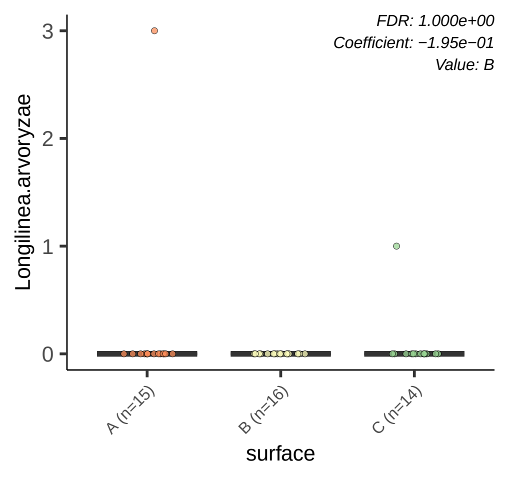

FDR: 1.000e+00
Coefficient: −1.95e−01
Value: B
Longilinea.arvoryzae
3
2
1
0
A (n=15)
B (n=16)
C (n=14)
surface

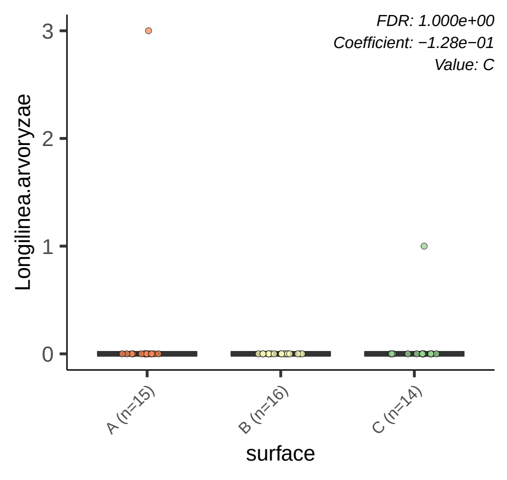
FDR: 1.000e+00
Coefficient: −1.28e−01
Value: C
Longilinea.arvoryzae
3
2
1
0
A (n=15)
B (n=16)
C (n=14)
surface

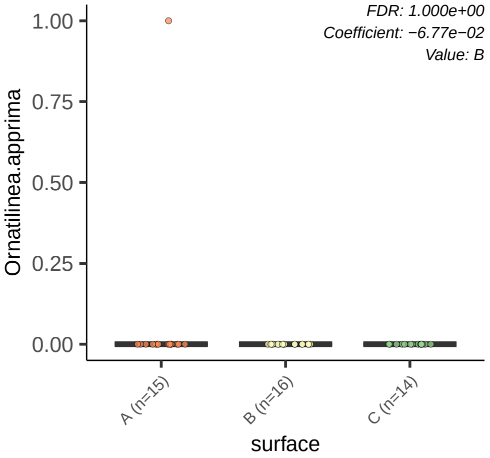
FDR: 1.000e+00
Coefficient: −6.77e−02
Value: B
Ornatilinea.apprima
1.00
0.75
0.50
0.25
0.00
A (n=15)
B (n=16)
C (n=14)
surface

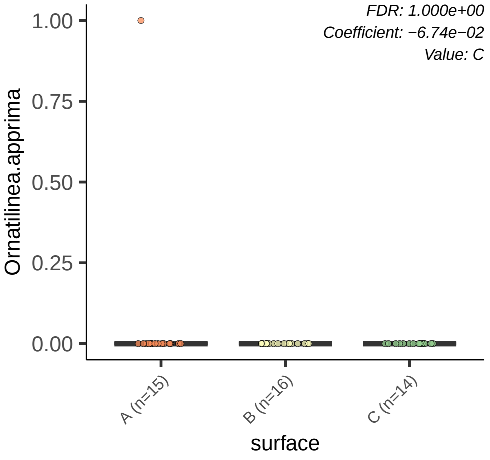
FDR: 1.000e+00
Coefficient: −6.74e−02
Value: C
1.00
0.75
0.50
0.25
0.00
Ornatilinea.apprima
A (n=15)
B (n=16)
C (n=14)
surface

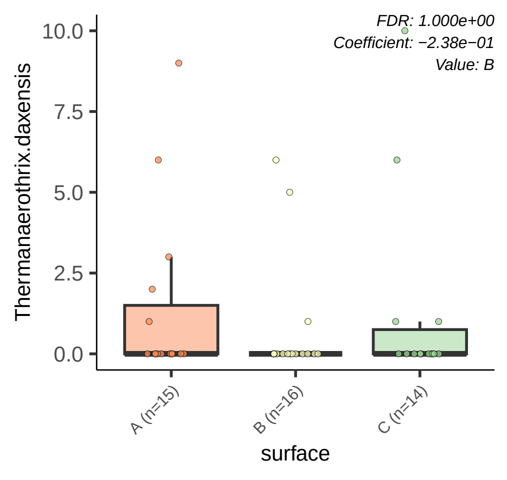

FDR: 1.000e+00
Coefficient: −2.38e−01
Value: B
Thermanaerothrix.daxensis
10.0
7.5
5.0
2.5
0.0
A (n=15)
B (n=16)
C (n=14)
surface

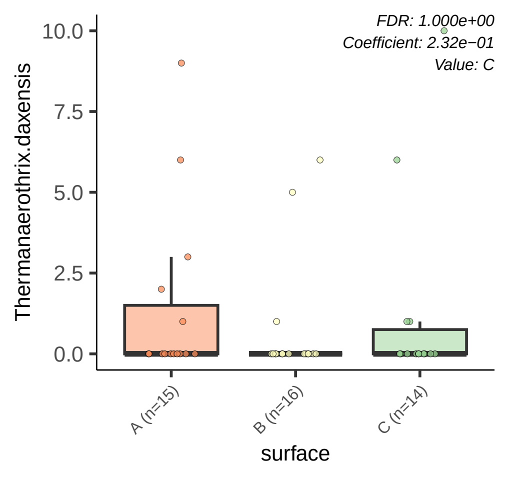
FDR: 1.000e+00
Coefficient: 2.32e−01
Value: C
10.0
7.5
5.0
2.5
0.0
Thermanaerothrix.daxensis
A (n=15)
B (n=16)
C (n=14)
surface

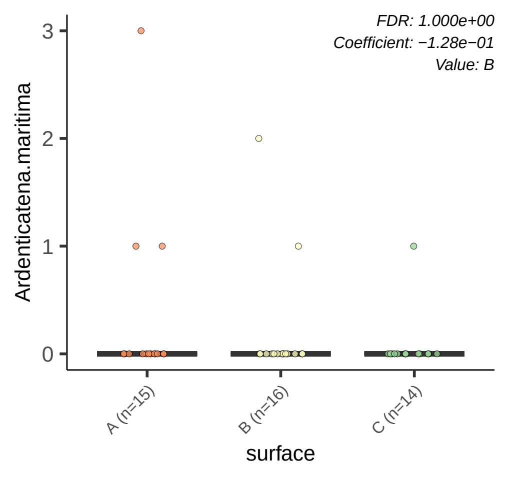

FDR: 1.000e+00
Coefficient: −1.28e−01
Value: B
Ardenticatena.maritima
3
2
1
0
A (n=15)
B (n=16)
C (n=14)
surface

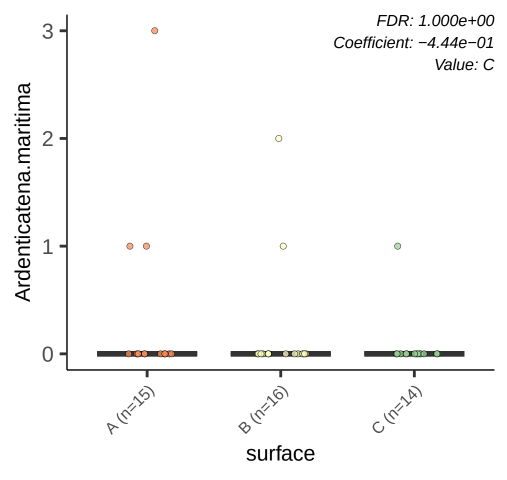

FDR: 1.000e+00
Coefficient: −4.44e−01
Value: C
Ardenticatena.maritima
0
1
2
3
A (n=15)
B (n=16)
C (n=14)
surface

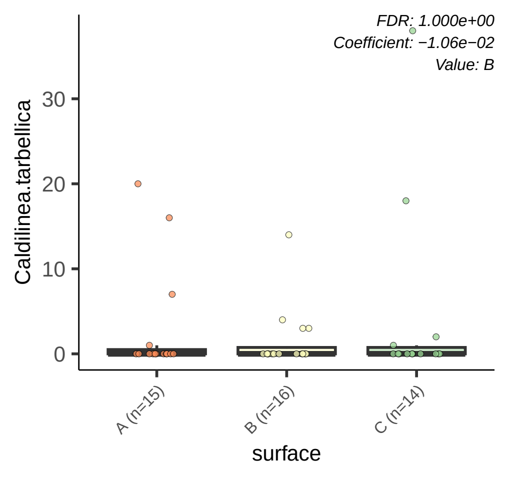
FDR: 1.000e+00
Coefficient: −1.06e−02
Value: B
Caldilinea.tarbellica
0
10
20
30
A (n=15)
B (n=16)
C (n=14)
surface

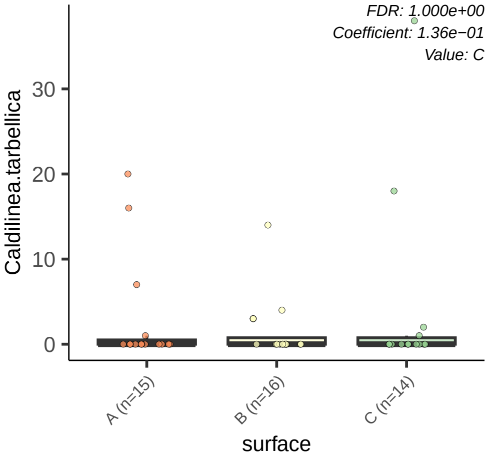

FDR: 1.000e+00
Coefficient: 1.36e−01
Value: C
Caldilinea.tarbellica
0
10
20
30
A (n=15)
B (n=16)
C (n=14)
surface

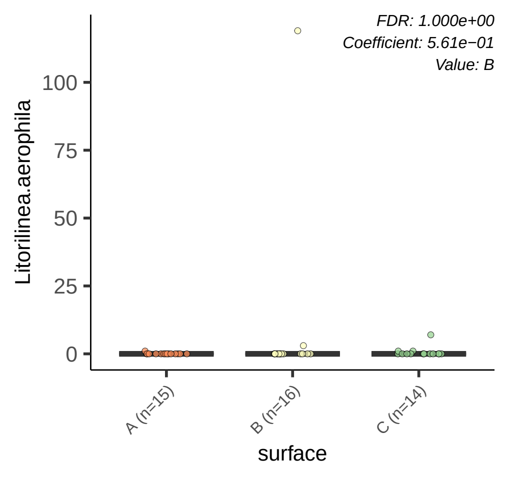
FDR: 1.000e+00
Coefficient: 5.61e−01
Value: B
Litorilinea.aerophila
100
75
50
25
0
A (n=15)
B (n=16)
C (n=14)
surface

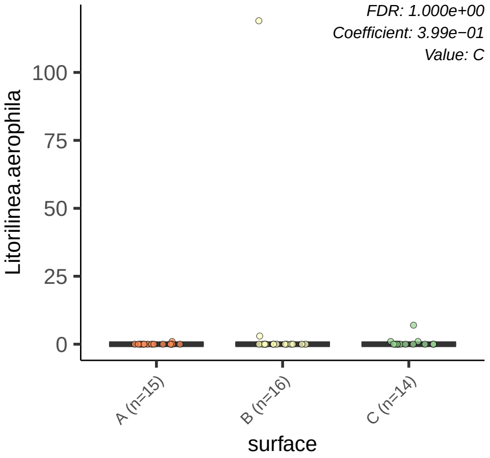
FDR: 1.000e+00
Coefficient: 3.99e−01
Value: C
Litorilinea.aerophila
100
75
50
25
0
A (n=15)
B (n=16)
C (n=14)
surface

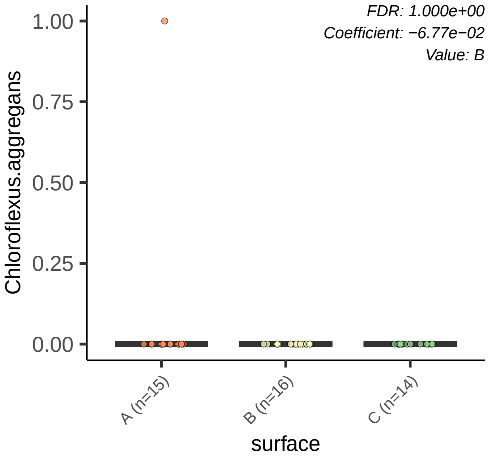
FDR: 1.000e+00
Coefficient: −6.77e−02
Value: B
Chloroflexus.aggregans
1.00
0.75
0.50
0.25
0.00
A (n=15)
B (n=16)
C (n=14)
surface

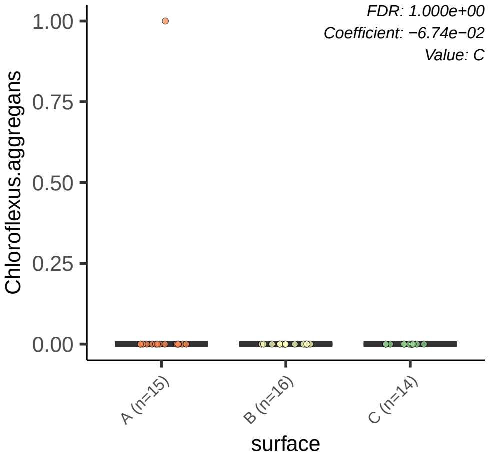

FDR: 1.000e+00
Coefficient: −6.74e−02
Value: C
Chloroflexus.aggregans
1.00
0.75
0.50
0.25
0.00
A (n=15)
B (n=16)
C (n=14)
surface

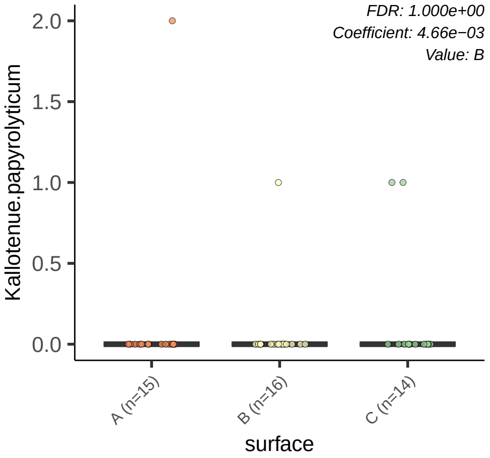
FDR: 1.000e+00
Coefficient: 4.66e−03
Value: B
Kallotenue.papyrolyticum
2.0
1.5
1.0
0.5
0.0
A (n=15)
B (n=16)
C (n=14)
surface

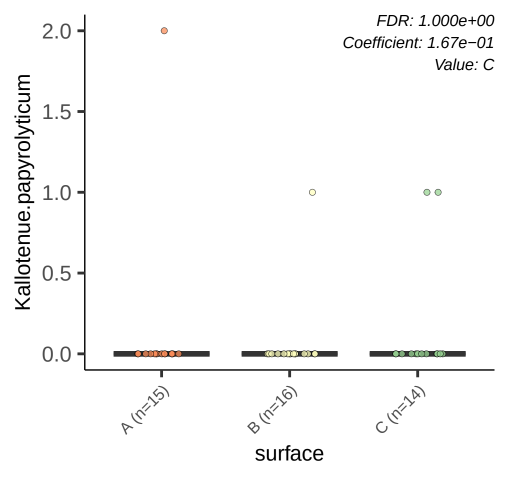
FDR: 1.000e+00
Coefficient: 1.67e−01
Value: C
Kallotenue.papyrolyticum
2.0
1.5
1.0
0.5
0.0
A (n=15)
B (n=16)
C (n=14)
surface

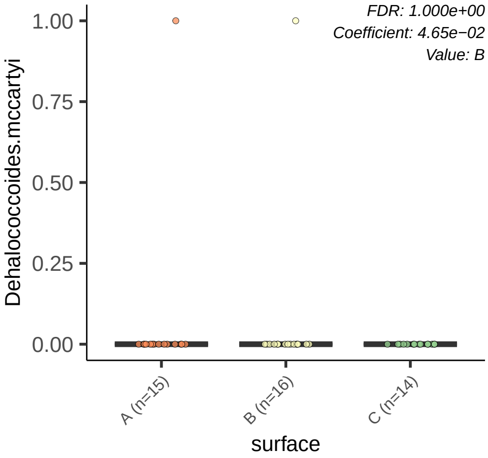
FDR: 1.000e+00
Coefficient: 4.65e−02
Value: B
Dehalococcoides.mccartyi
1.00
0.75
0.50
0.25
0.00
A (n=15)
B (n=16)
C (n=14)
surface

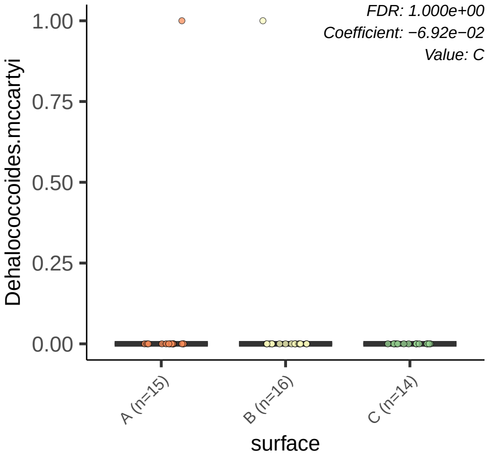

FDR: 1.000e+00
Coefficient: −6.92e−02
Value: C
Dehalococcoides.mccartyi
1.00
0.75
0.50
0.25
0.00
A (n=15)
B (n=16)
C (n=14)
surface

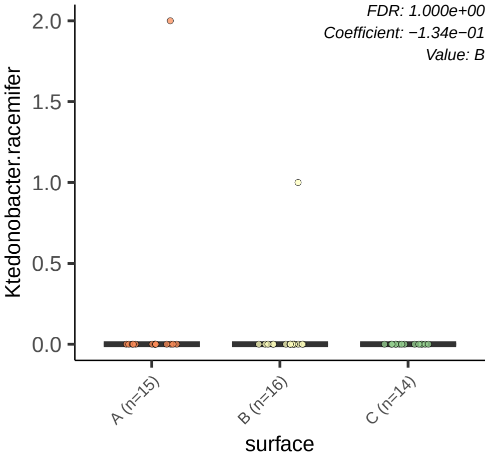

FDR: 1.000e+00
Coefficient: −1.34e−01
Value: B
Ktedonobacter.racemifer
2.0
1.5
1.0
0.5
0.0
A (n=15)
B (n=16)
C (n=14)
surface

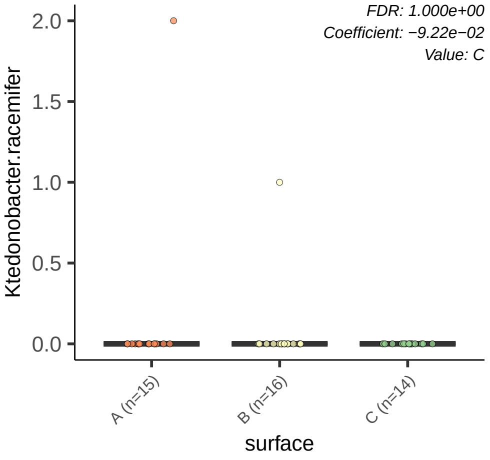

FDR: 1.000e+00
Coefficient: −9.22e−02
Value: C
Ktedonobacter.racemifer
2.0
1.5
1.0
0.5
0.0
A (n=15)
B (n=16)
C (n=14)
surface

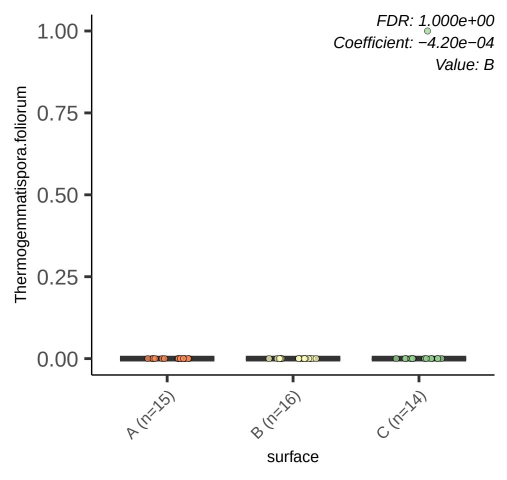
FDR: 1.000e+00
Coefficient: −4.20e−04
Value: B
1.00
0.75
0.50
0.25
0.00
Thermogemmatispora.foliorum
A (n=15)
B (n=16)
C (n=14)
surface

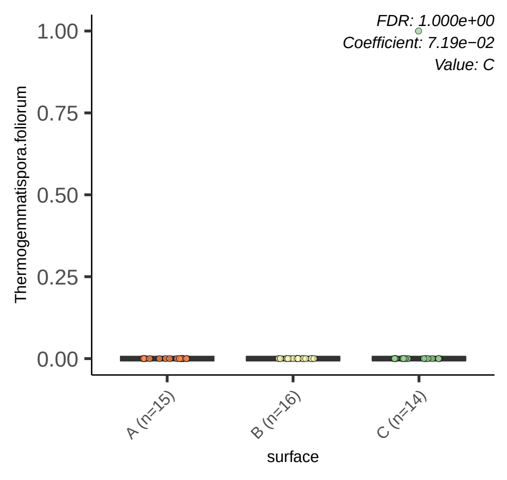
FDR: 1.000e+00
Coefficient: 7.19e−02
Value: C
1.00
0.75
0.50
0.25
0.00
Thermogemmatispora.foliorum
A (n=15)
B (n=16)
C (n=14)
surface

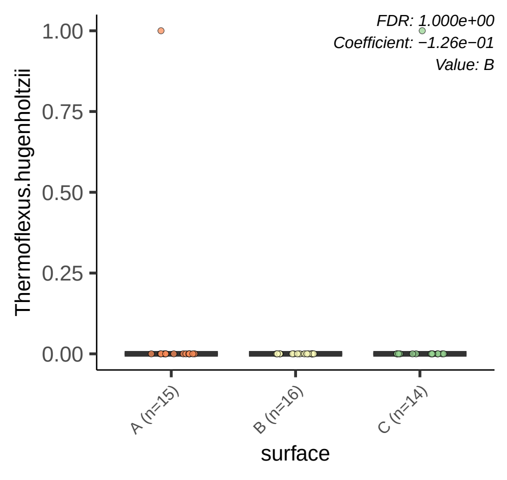
FDR: 1.000e+00
Coefficient: −1.26e−01
Value: B
Thermoflexus.hugenholtzii
1.00
0.75
0.50
0.25
0.00
A (n=15)
B (n=16)
C (n=14)
surface

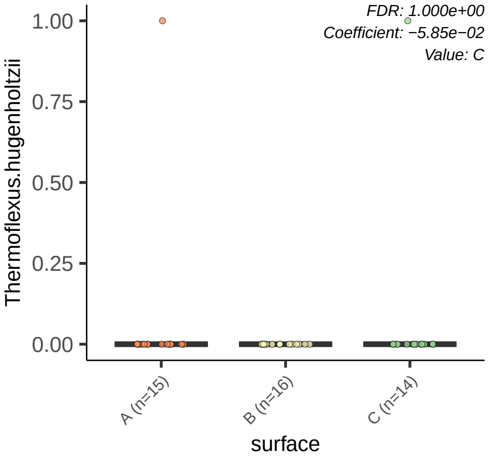
FDR: 1.000e+00
Coefficient: −5.85e−02
Value: C
Thermoflexus.hugenholtzii
1.00
0.75
0.50
0.25
0.00
A (n=15)
B (n=16)
C (n=14)
surface

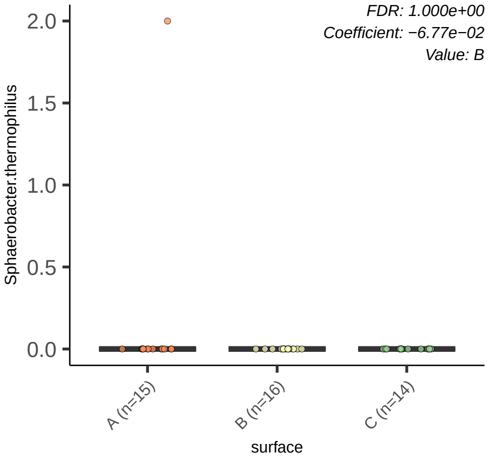

FDR: 1.000e+00
Coefficient: −6.77e−02
Value: B
Sphaerobacter.thermophilus
2.0
1.5
1.0
0.5
0.0
A (n=15)
B (n=16)
C (n=14)
surface

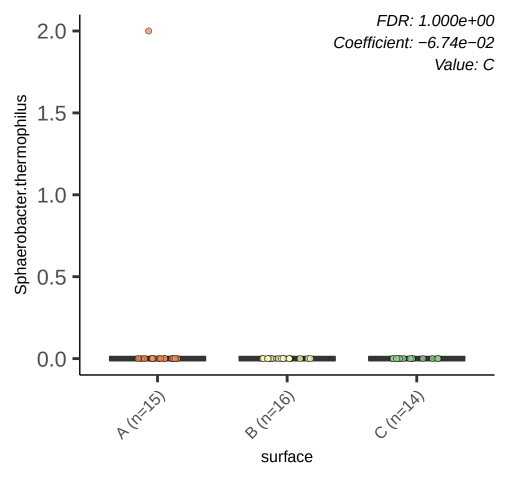
FDR: 1.000e+00
Coefficient: −6.74e−02
Value: C
Sphaerobacter.thermophilus
2.0
1.5
1.0
0.5
0.0
A (n=15)
B (n=16)
C (n=14)
surface

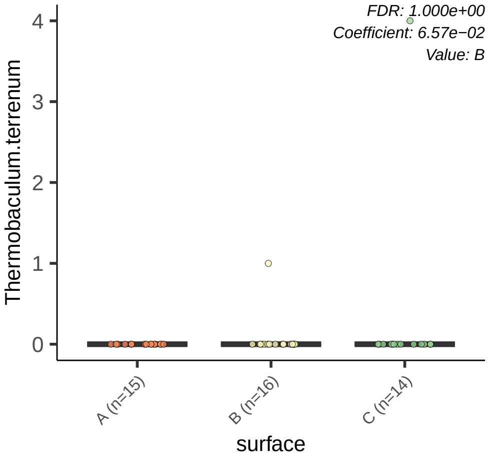

FDR: 1.000e+00
Coefficient: 6.57e−02
Value: B
Thermobaculum.terrenum
4
3
2
1
0
A (n=15)
B (n=16)
C (n=14)
surface

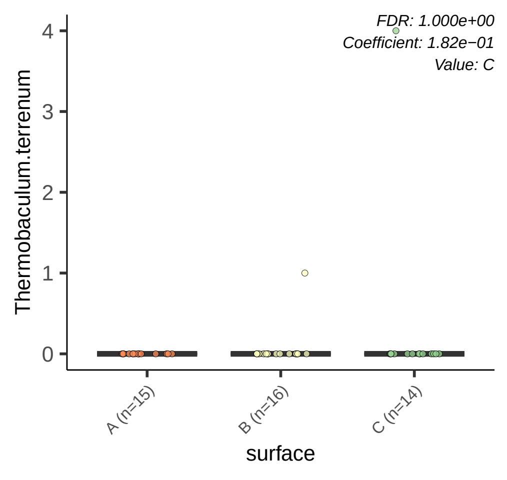

FDR: 1.000e+00
Coefficient: 1.82e−01
Value: C
Thermobaculum.terrenum
0
1
2
3
4
A (n=15)
B (n=16)
C (n=14)
surface

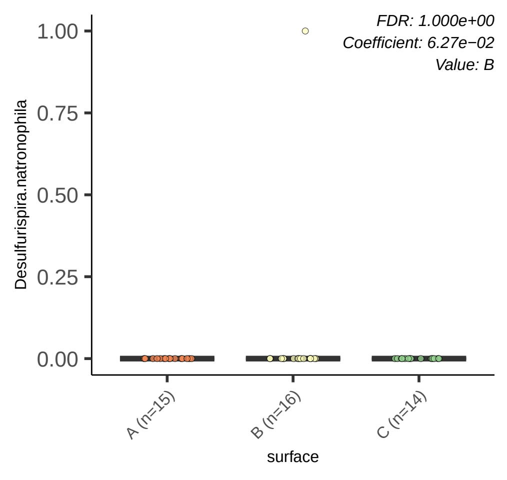
FDR: 1.000e+00
Coefficient: 6.27e−02
Value: B
Desulfurispira.natronophila
1.00
0.75
0.50
0.25
0.00
A (n=15)
B (n=16)
C (n=14)
surface

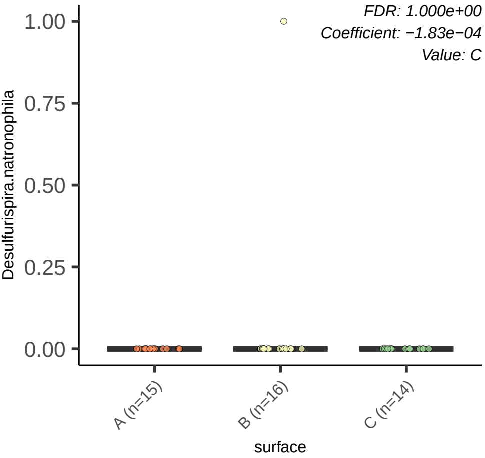

FDR: 1.000e+00
Coefficient: −1.83e−04
Value: C
Desulfurispira.natronophila
1.00
0.75
0.50
0.25
0.00
A (n=15)
B (n=16)
C (n=14)
surface

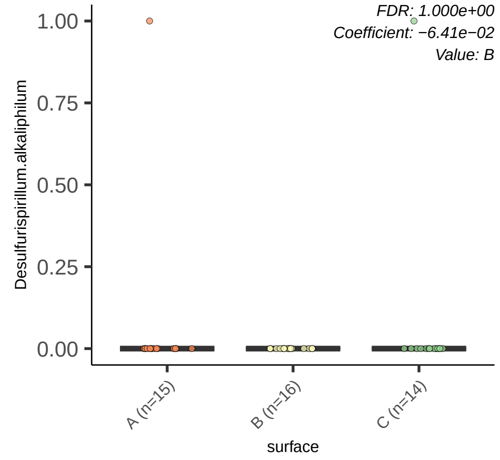

FDR: 1.000e+00
Coefficient: −6.41e−02
Value: B
1.00
0.75
0.50
0.25
0.00
Desulfurispirillum.alkaliphilum
A (n=15)
B (n=16)
C (n=14)
surface

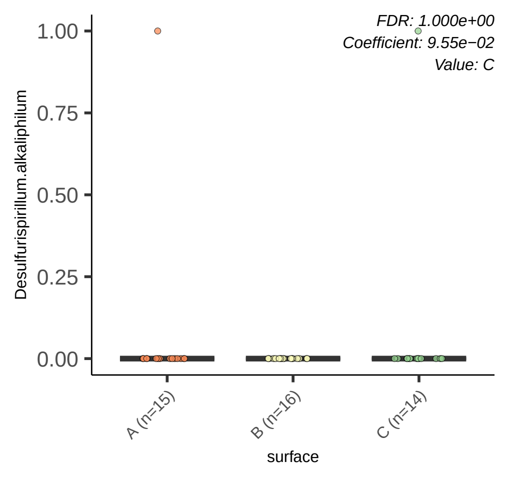
FDR: 1.000e+00
Coefficient: 9.55e−02
Value: C
Desulfurispirillum.alkaliphilum
1.00
0.75
0.50
0.25
0.00
A (n=15)
B (n=16)
C (n=14)
surface

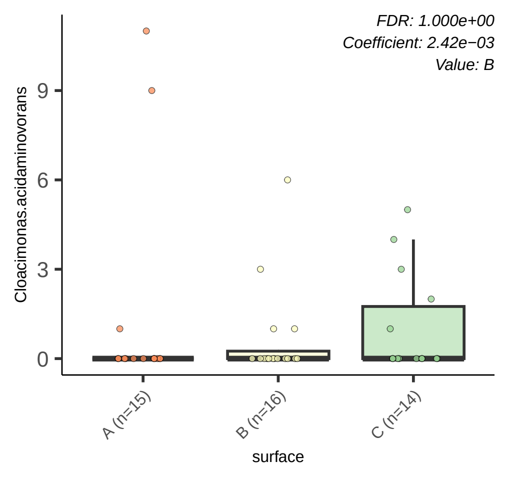
FDR: 1.000e+00
Coefficient: 2.42e−03
Value: B
Cloacimonas.acidaminovorans
0
3
6
9
A (n=15)
B (n=16)
C (n=14)
surface

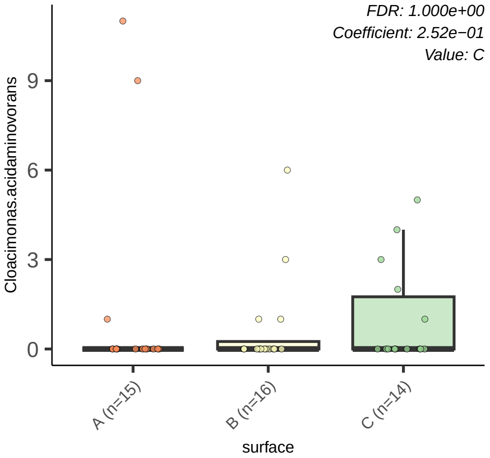
FDR: 1.000e+00
Coefficient: 2.52e−01
Value: C
Cloacimonas.acidaminovorans
0
3
6
9
A (n=15)
B (n=16)
C (n=14)
surface

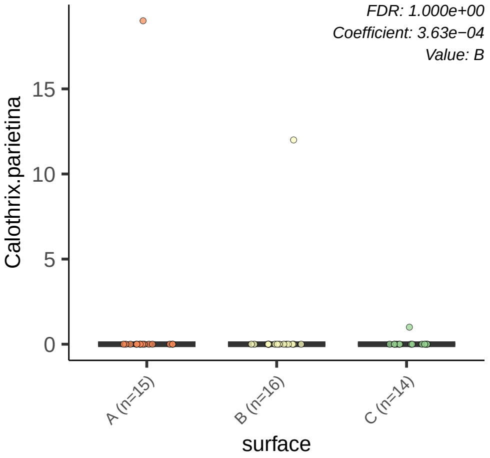
FDR: 1.000e+00
Coefficient: 3.63e−04
Value: B
Calothrix.parietina
15
10
5
0
A (n=15)
B (n=16)
C (n=14)
surface

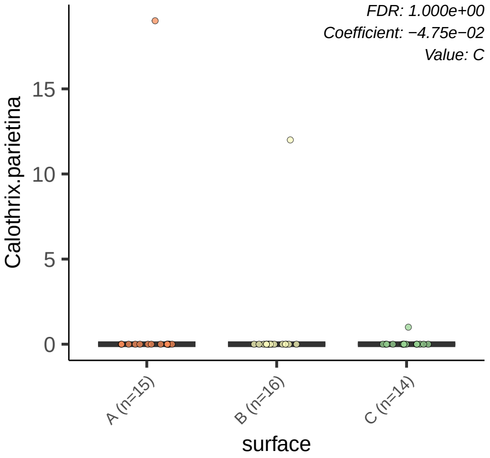
FDR: 1.000e+00
Coefficient: −4.75e−02
Value: C
Calothrix.parietina
0
5
10
15
A (n=15)
B (n=16)
C (n=14)
surface

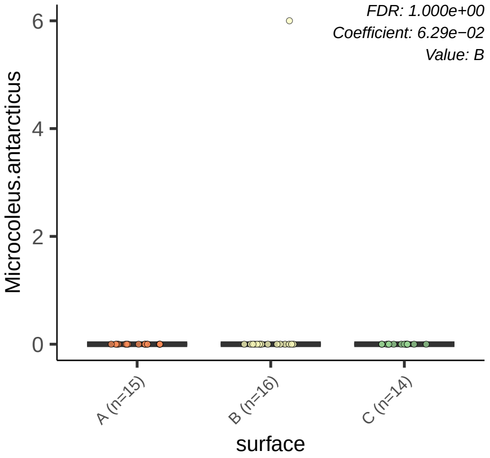
FDR: 1.000e+00
Coefficient: 6.29e−02
Value: B
Microcoleus.antarctius
0
2
4
6
A (n=15)
B (n=16)
C (n=14)
surface

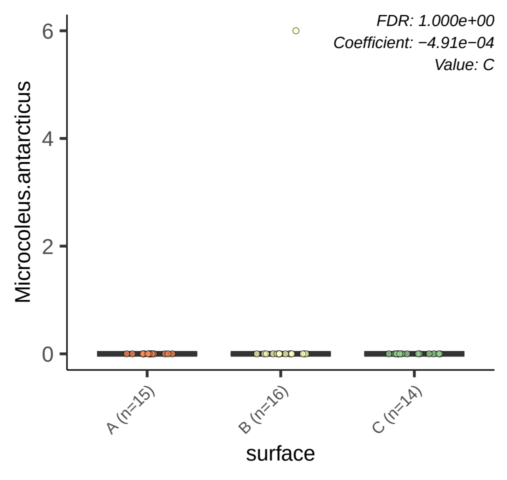
FDR: 1.000e+00
Coefficient: −4.91e−04
Value: C
Microcoleus.antarcticus
6
4
2
0
A (n=15)
B (n=16)
C (n=14)
surface

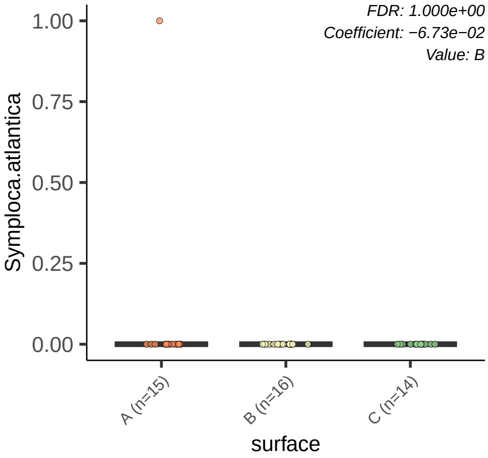
FDR: 1.000e+00
Coefficient: −6.73e−02
Value: B
Symploca.atlantica
1.00
0.75
0.50
0.25
0.00
A (n=15)
B (n=16)
C (n=14)
surface

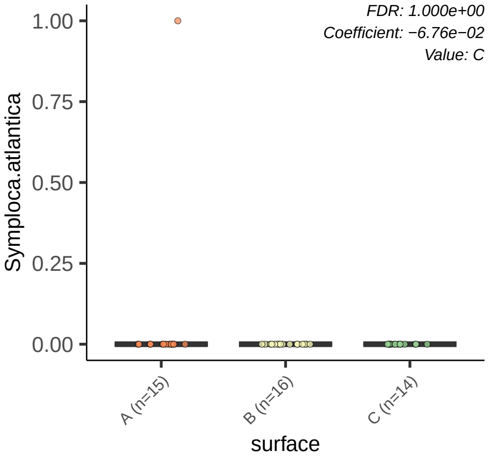
FDR: 1.000e+00
Coefficient: −6.76e−02
Value: C
Symploca.atlantica
1.00
0.75
0.50
0.25
0.00
A (n=15)
B (n=16)
C (n=14)
surface

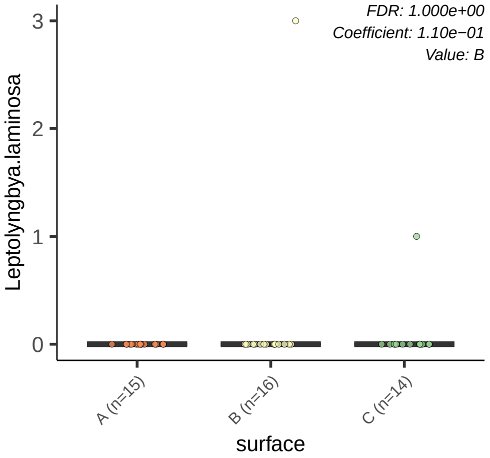

FDR: 1.000e+00
Coefficient: 1.10e−01
Value: B
Leptolyngbya.laminosa
3
2
1
0
A (n=15)
B (n=16)
C (n=14)
surface

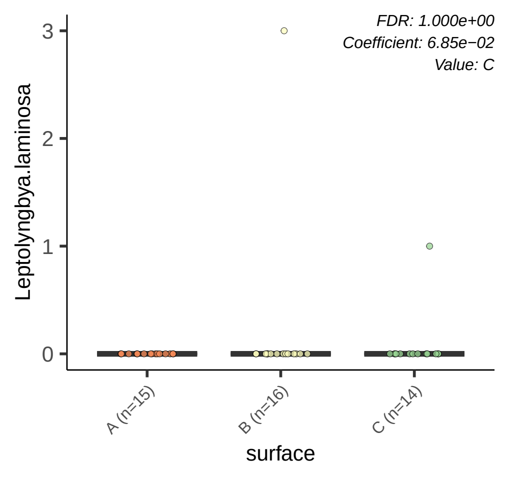

FDR: 1.000e+00
Coefficient: 6.85e−02
Value: C
Leptolyngbya.laminosa
3
2
1
0
A (n=15)
B (n=16)
C (n=14)
surface

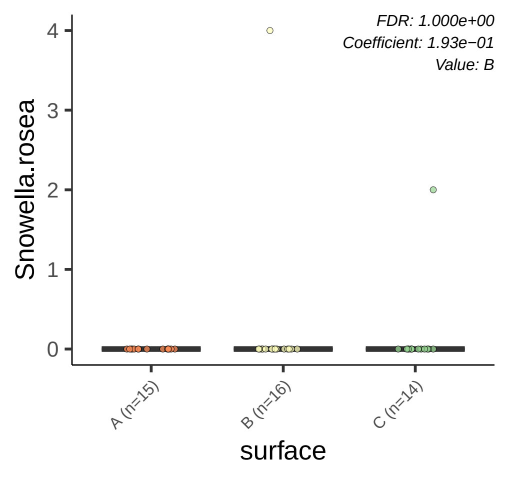
FDR: 1.000e+00
Coefficient: 1.93e−01
Value: B
Snowella.rosea
0
1
2
3
4
A (n=15)
B (n=16)
C (n=14)
surface

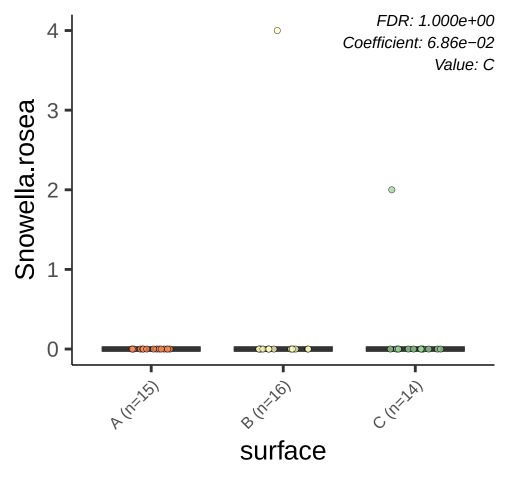

FDR: 1.000e+00
Coefficient: 6.86e−02
Value: C
Snowella.rosea
0
1
2
3
4
A (n=15)
B (n=16)
C (n=14)
surface

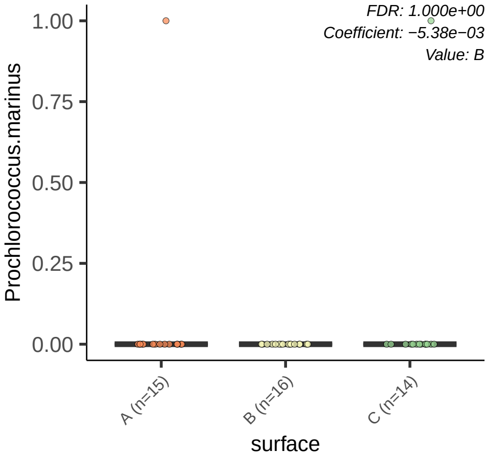
FDR: 1.000e+00
Coefficient: −5.38e−03
Value: B
Prochlorococcus.marinus
1.00
0.75
0.50
0.25
0.00
A (n=15)
B (n=16)
C (n=14)
surface

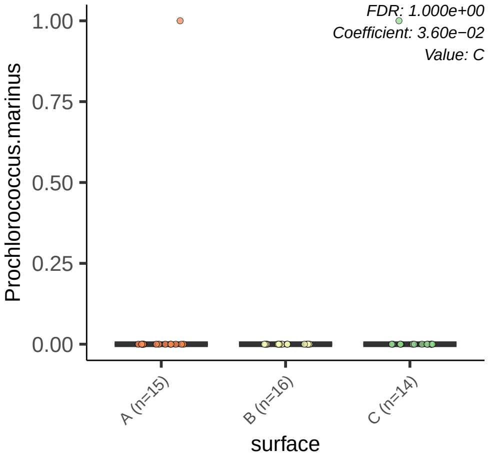
FDR: 1.000e+00
Coefficient: 3.60e−02
Value: C
Prochlorococcus.marinus
1.00
0.75
0.50
0.25
0.00
A (n=15)
B (n=16)
C (n=14)
surface

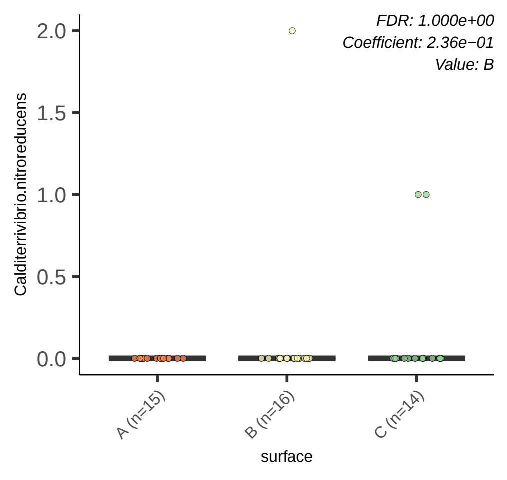

FDR: 1.000e+00
Coefficient: 2.36e−01
Value: B
2.0
1.5
1.0
0.5
0.0
Calditerrivibrio.nitroreducens
A (n=15)
B (n=16)
C (n=14)
surface

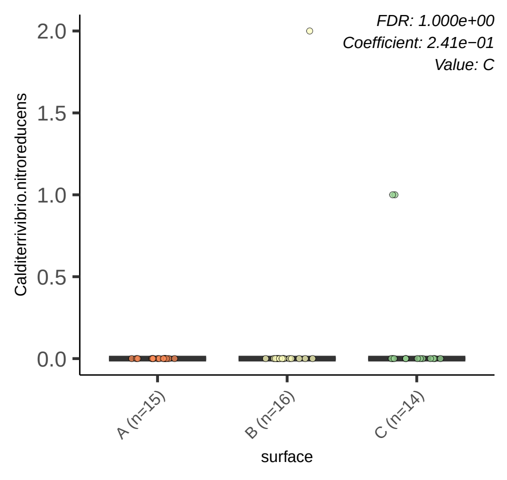

FDR: 1.000e+00
Coefficient: 2.41e−01
Value: C
Calditerrivibrio.nitroreducens
2.0
1.5
1.0
0.5
0.0
A (n=15)
B (n=16)
C (n=14)
surface

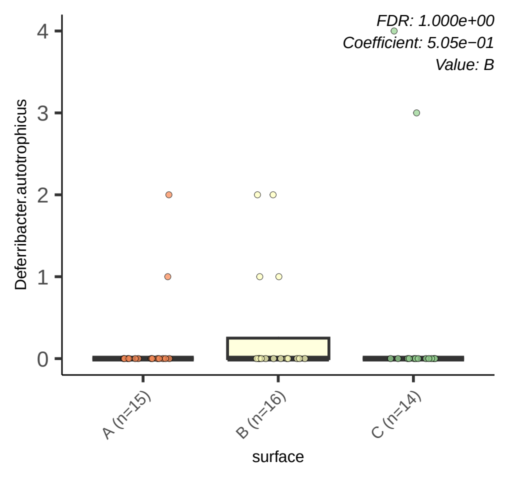
FDR: 1.000e+00
Coefficient: 5.05e−01
Value: B
Deferribacter.autotrophicus
0
1
2
3
4
A (n=15)
B (n=16)
C (n=14)
surface

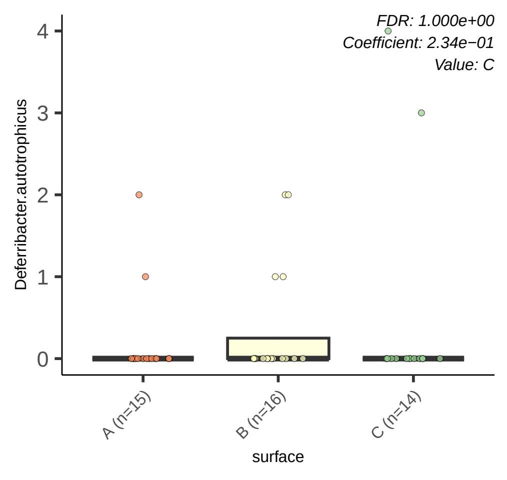
FDR: 1.000e+00
Coefficient: 2.34e−01
Value: C
Deferribacter.autotrophicus
0
1
2
3
4
A (n=15)
B (n=16)
C (n=14)
surface

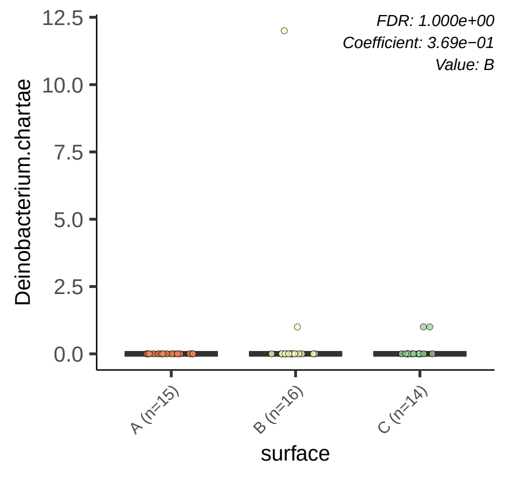
FDR: 1.000e+00
Coefficient: 3.69e−01
Value: B
Deinobacterium.chartae
12.5
10.0
7.5
5.0
2.5
0.0
A (n=15)
B (n=16)
C (n=14)
surface

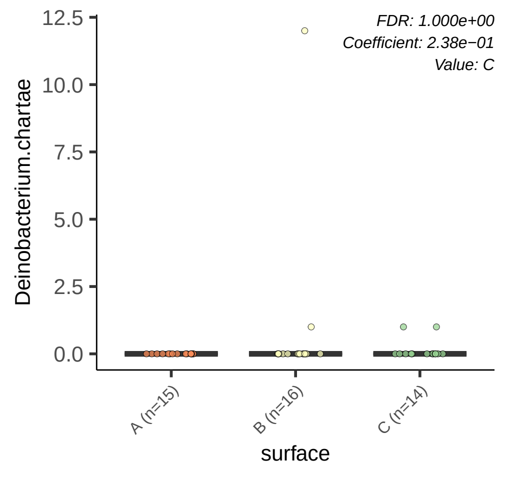

FDR: 1.000e+00
Coefficient: 2.38e−01
Value: C
Deinobacterium.chartae
12.5
10.0
7.5
5.0
2.5
0.0
A (n=15)
B (n=16)
C (n=14)
surface

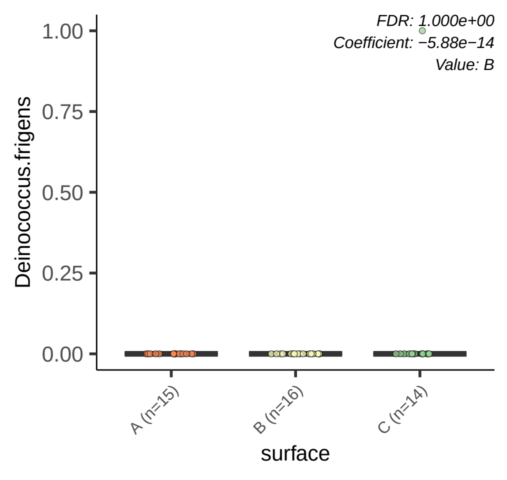

FDR: 1.000e+00
Coefficient: −5.88e−14
Value: B
1.00
0.75
0.50
0.25
0.00
Deinococcus.frigens
A (n=15)
B (n=16)
C (n=14)
surface

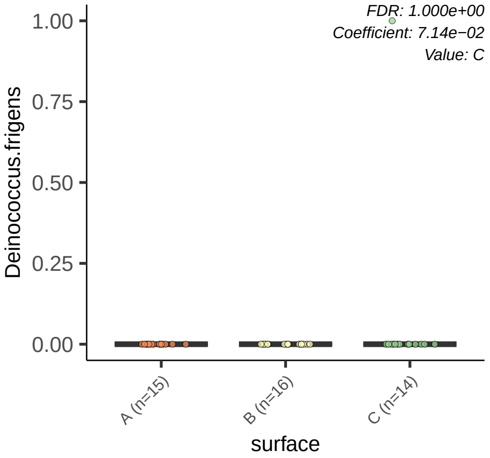

FDR: 1.000e+00
Coefficient: 7.14e−02
Value: C
Deinococcus.frigens
1.00
0.75
0.50
0.25
0.00
A (n=15)
B (n=16)
C (n=14)
surface

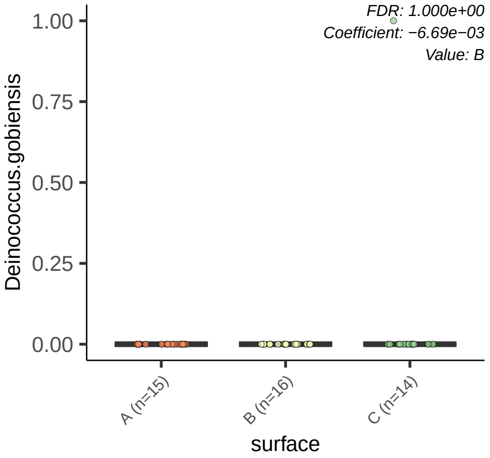

FDR: 1.000e+00
Coefficient: −6.69e−03
Value: B
1.00
0.75
0.50
0.25
0.00
Deinococcus.gobiensis
A (n=15)
B (n=16)
C (n=14)
surface

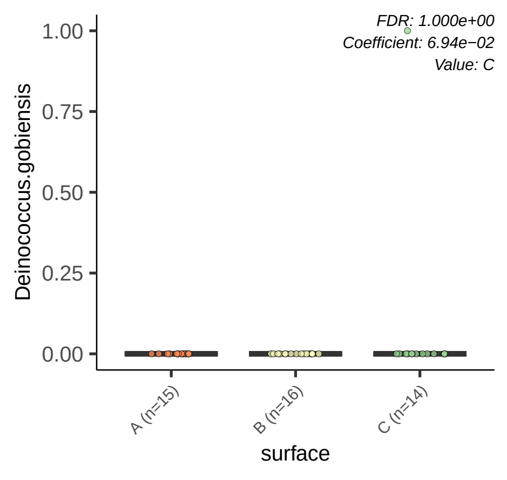
FDR: 1.000e+00
Coefficient: 6.94e−02
Value: C
Deinococcus.gobiensis
1.00
0.75
0.50
0.25
0.00
A (n=15)
B (n=16)
C (n=14)
surface

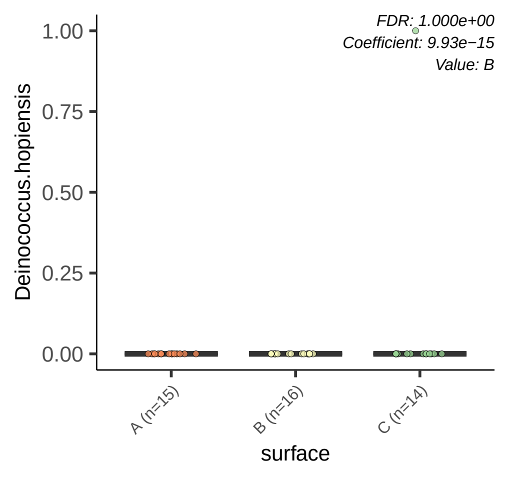
FDR: 1.000e+00
Coefficient: 9.93e−15
Value: B
Deinococcus.hopiensis
1.00
0.75
0.50
0.25
0.00
A (n=15)
B (n=16)
C (n=14)
surface

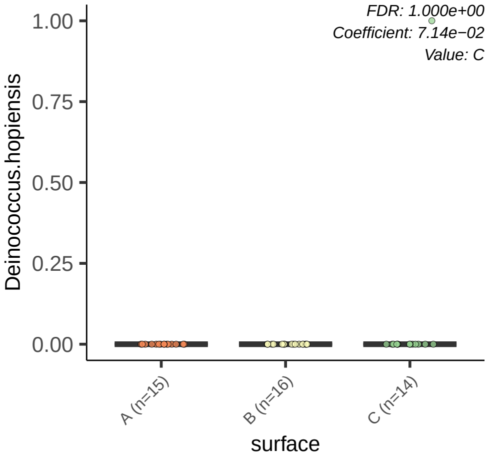

FDR: 1.000e+00
Coefficient: 7.14e−02
Value: C
Deinococcus.hopiensis
1.00
0.75
0.50
0.25
0.00
A (n=15)
B (n=16)
C (n=14)
surface

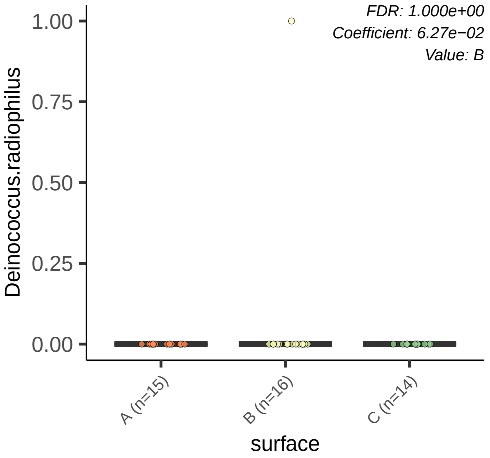
FDR: 1.000e+00
Coefficient: 6.27e−02
Value: B
Deinococcus.radiophilus
1.00
0.75
0.50
0.25
0.00
A (n=15)
B (n=16)
C (n=14)
surface

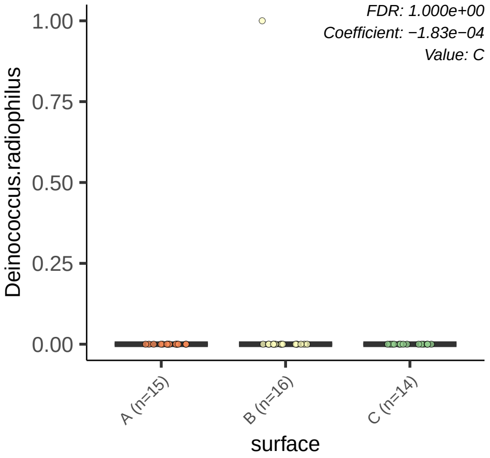

FDR: 1.000e+00
Coefficient: −1.83e−04
Value: C
Deinococcus.radiophilus
1.00
0.75
0.50
0.25
0.00
A (n=15)
B (n=16)
C (n=14)
surface

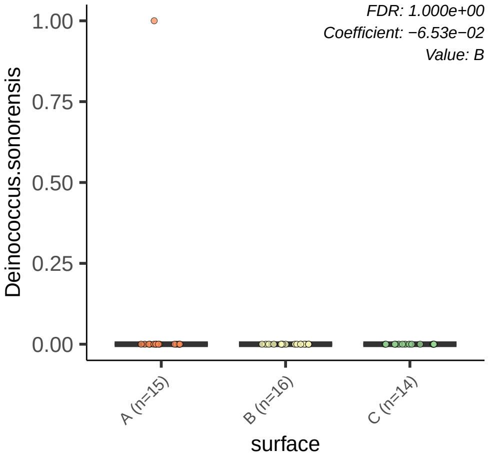

FDR: 1.000e+00
Coefficient: −6.53e−02
Value: B
Deinococcus.sonorensis
1.00
0.75
0.50
0.25
0.00
A (n=15)
B (n=16)
C (n=14)
surface

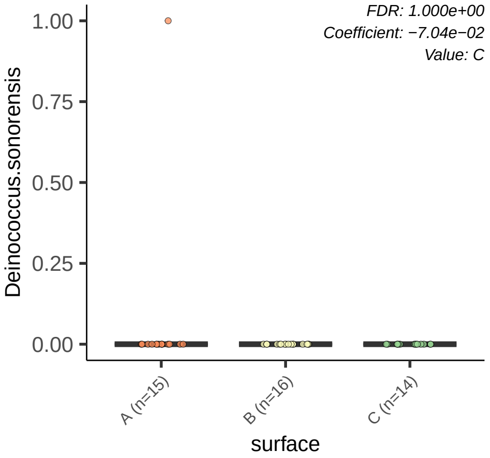
FDR: 1.000e+00
Coefficient: −7.04e−02
Value: C
Deinococcus.sonorensis
1.00
0.75
0.50
0.25
0.00
A (n=15)
B (n=16)
C (n=14)
surface

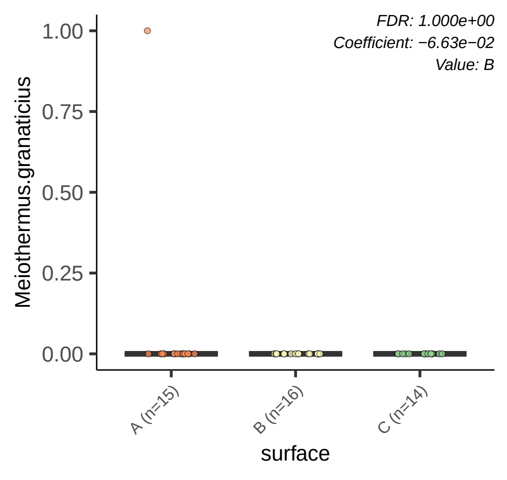
FDR: 1.000e+00
Coefficient: −6.63e−02
Value: B
Meiothermus.granaticius
1.00
0.75
0.50
0.25
0.00
A (n=15)
B (n=16)
C (n=14)
surface

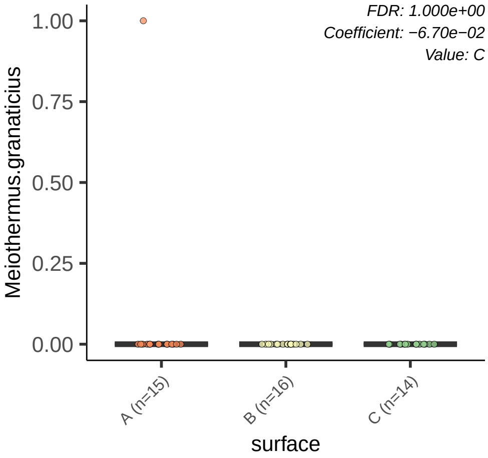
FDR: 1.000e+00
Coefficient: −6.70e−02
Value: C
Meiothermus.granaticius
1.00
0.75
0.50
0.25
0.00
A (n=15)
B (n=16)
C (n=14)
surface

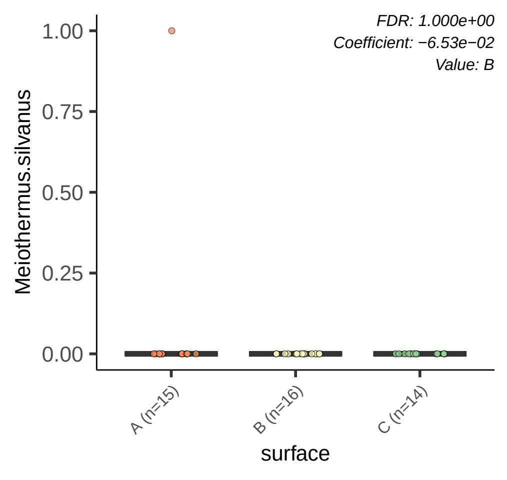
FDR: 1.000e+00
Coefficient: −6.53e−02
Value: B
Meiothermus.silvanus
1.00
0.75
0.50
0.25
0.00
A (n=15)
B (n=16)
C (n=14)
surface

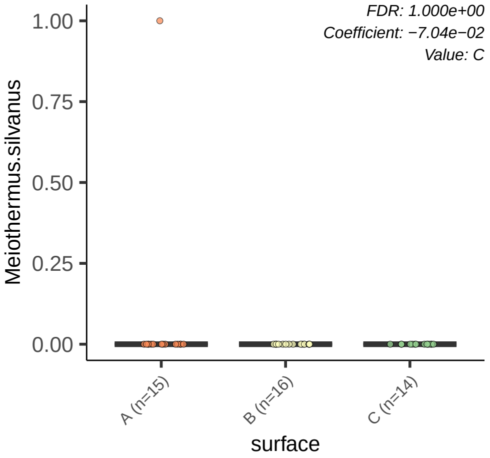
FDR: 1.000e+00
Coefficient: −7.04e−02
Value: C
1.00
0.75
0.50
0.25
0.00
Meiothermus.silvanus
A (n=15)
B (n=16)
C (n=14)
surface

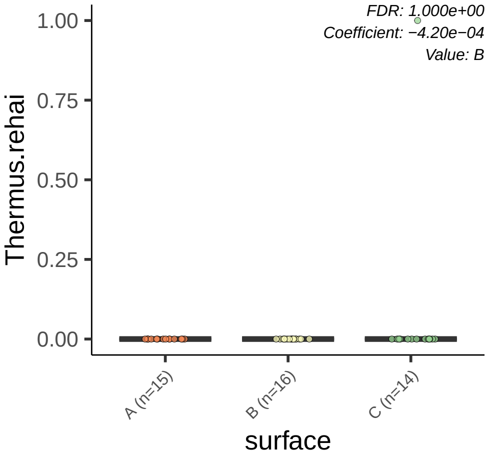
FDR: 1.000e+00
Coefficient: −4.20e−04
Value: B
Thermus.rehai
1.00
0.75
0.50
0.25
0.00
A (n=15)
B (n=16)
C (n=14)
surface

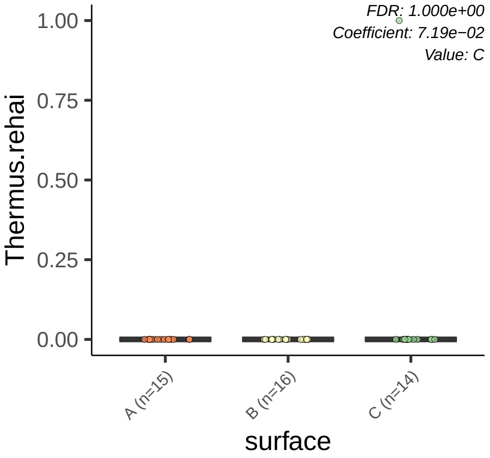
FDR: 1.000e+00
Coefficient: 7.19e−02
Value: C
Thermus.rehai
1.00
0.75
0.50
0.25
0.00
A (n=15)
B (n=16)
C (n=14)
surface

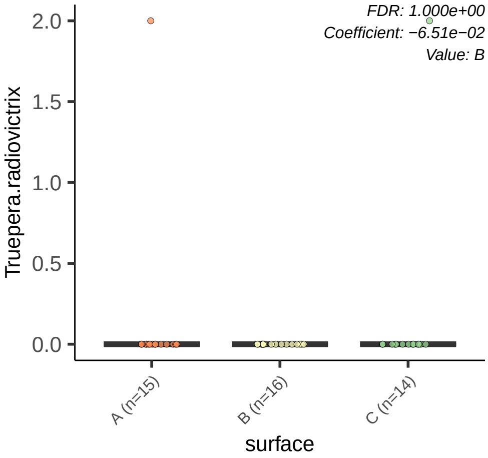

FDR: 1.000e+00
Coefficient: −6.51e−02
Value: B
Truepera.radiovictrix
2.0
1.5
1.0
0.5
0.0
A (n=15)
B (n=16)
C (n=14)
surface

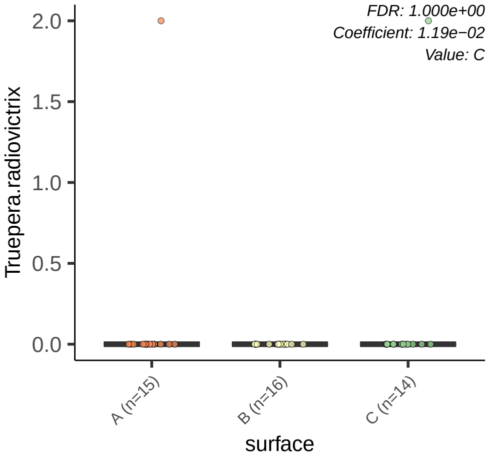
FDR: 1.000e+00
Coefficient: 1.19e−02
Value: C
Truepera.radiovictrix
2.0
1.5
1.0
0.5
0.0
A (n=15)
B (n=16)
C (n=14)
surface

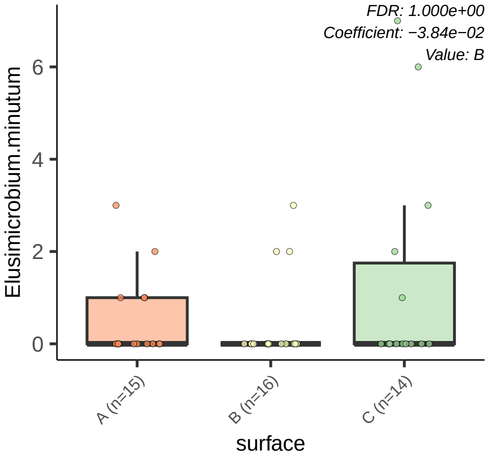
FDR: 1.000e+00
Coefficient: −3.84e−02
Value: B
Elusimicrobium.minutum
0
2
4
6
A (n=15)
B (n=16)
C (n=14)
surface

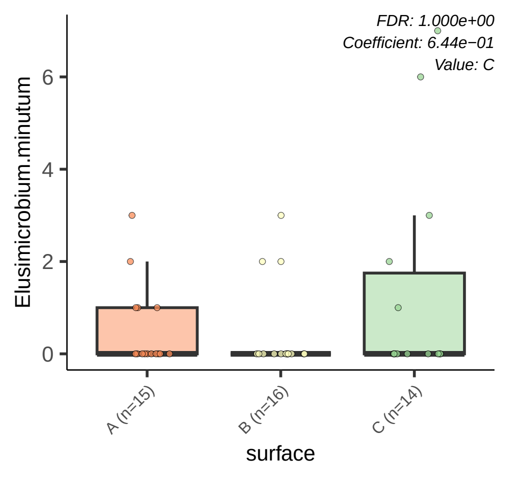

FDR: 1.000e+00
Coefficient: 6.44e−01
Value: C
Elusimicrobium.minutum
0
2
4
6
A (n=15)
B (n=16)
C (n=14)
surface

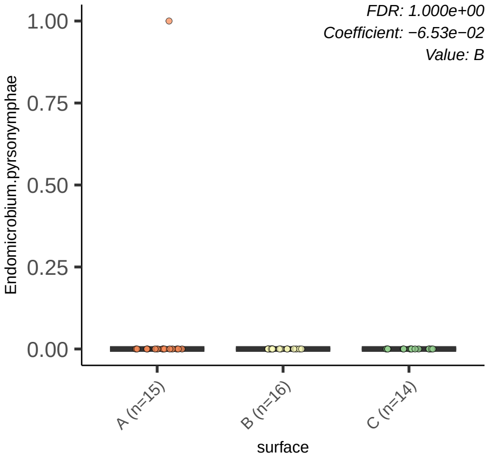

FDR: 1.000e+00
Coefficient: −6.53e−02
Value: B
1.00
0.75
0.50
0.25
0.00
Endomicrobium.pyrsonymphae
A (n=15)
B (n=16)
C (n=14)
surface

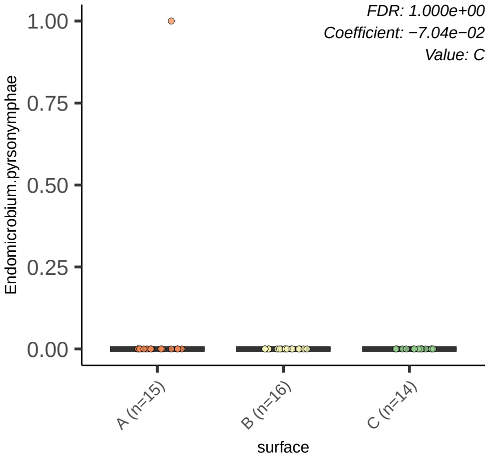
FDR: 1.000e+00
Coefficient: −7.04e−02
Value: C
Endomicrobium.pyrsonymphae
1.00
0.75
0.50
0.25
0.00
A (n=15)
B (n=16)
C (n=14)
surface

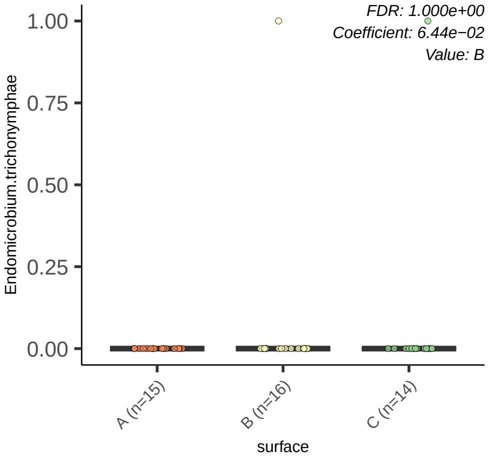

FDR: 1.000e+00
Coefficient: 6.44e−02
Value: B
Endomicrobium.trichonymphae
1.00
0.75
0.50
0.25
0.00
A (n=15)
B (n=16)
C (n=14)
surface

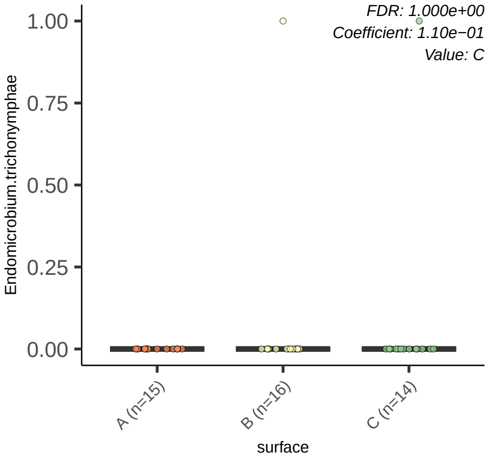
FDR: 1.000e+00
Coefficient: 1.10e−01
Value: C
Endomicrobium.trichonymphae
1.00
0.75
0.50
0.25
0.00
A (n=15)
B (n=16)
C (n=14)
surface

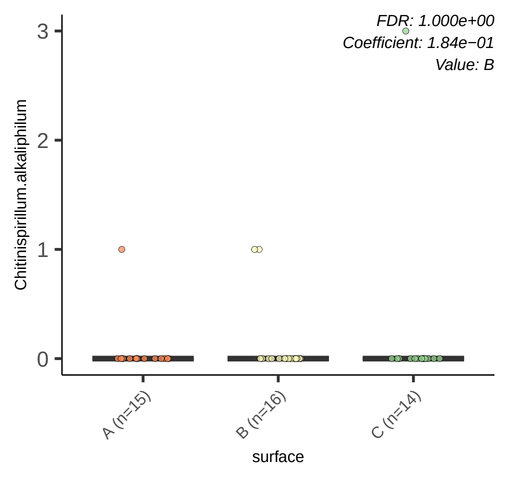

FDR: 1.000e+00
Coefficient: 1.84e−01
Value: B
Chitinispirillum.alkaliphilum
3
2
1
0
A (n=15)
B (n=16)
C (n=14)
surface

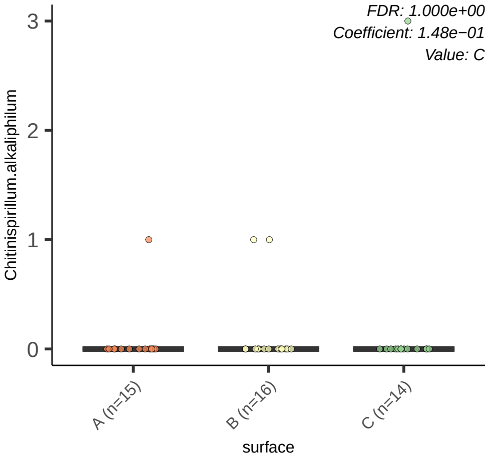
FDR: 1.000e+00
Coefficient: 1.48e−01
Value: C
Chitinispirillum.alkaliphilum
0
1
2
3
A (n=15)
B (n=16)
C (n=14)
surface

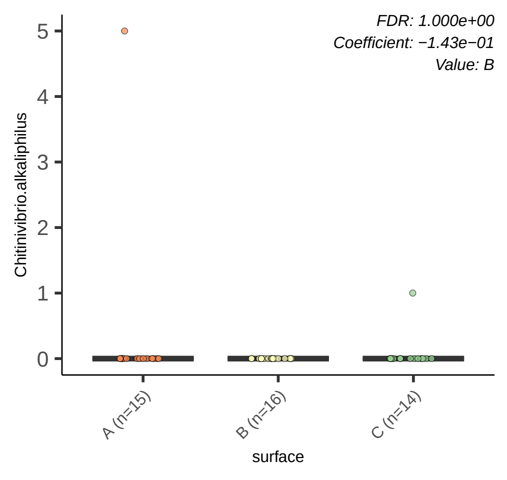
FDR: 1.000e+00
Coefficient: −1.43e−01
Value: B
Chitinivibrio.alkaliphilus
0
1
2
3
4
5
A (n=15)
B (n=16)
C (n=14)
surface

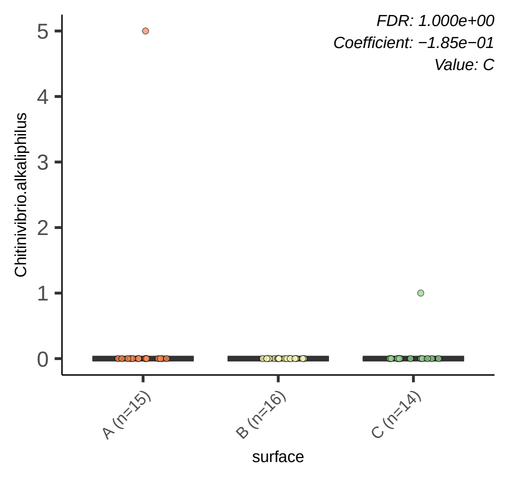

FDR: 1.000e+00
Coefficient: −1.85e−01
Value: C
Chitinivibrio.alkaliphilus
0
1
2
3
4
5
A (n=15)
B (n=16)
C (n=14)
surface

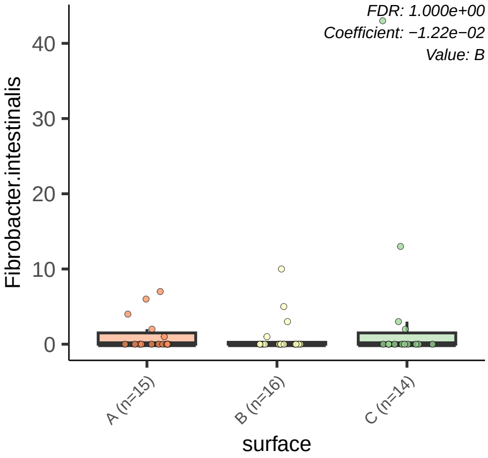

FDR: 1.000e+00
Coefficient: −1.22e−02
Value: B
Fibrobacter.intestinalis
0
10
20
30
40
A (n=15)
B (n=16)
C (n=14)
surface

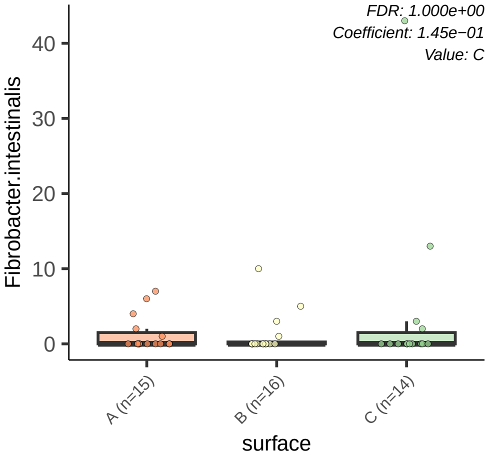

FDR: 1.000e+00
Coefficient: 1.45e−01
Value: C
Fibrobacter.intestinalis
0
10
20
30
40
A (n=15)
B (n=16)
C (n=14)
surface

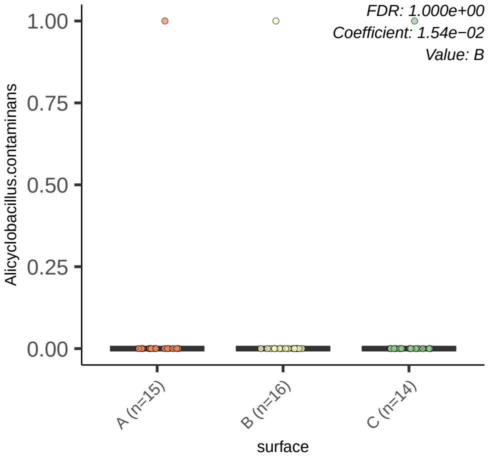

FDR: 1.000e+00
Coefficient: 1.54e−02
Value: B
1.00
0.75
0.50
0.25
0.00
Alicyclobacillus.contaminans
A (n=15)
B (n=16)
C (n=14)
surface

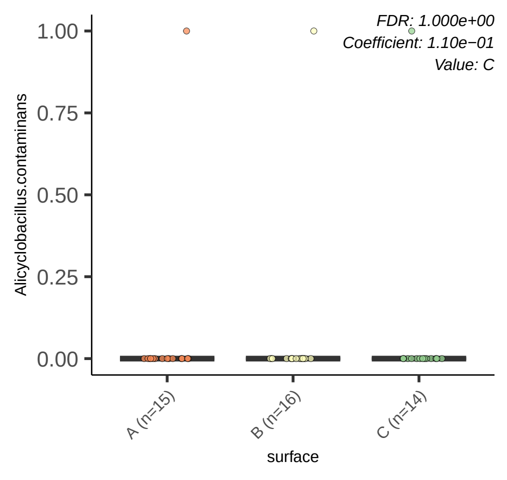
FDR: 1.000e+00
Coefficient: 1.10e−01
Value: C
1.00
0.75
0.50
0.25
0.00
Alicyclobacillus.contaminans
A (n=15)
B (n=16)
C (n=14)
surface

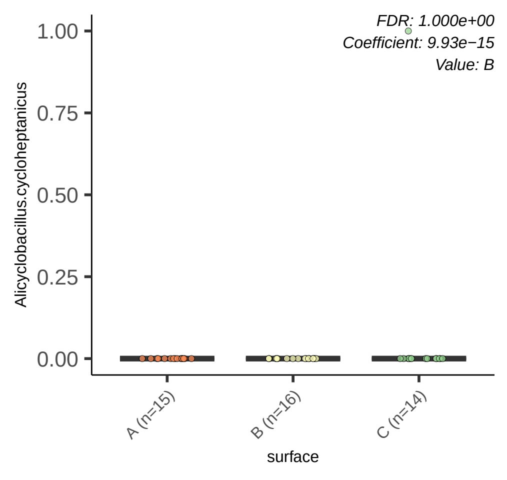

FDR: 1.000e+00
Coefficient: 9.93e−15
Value: B
Alicyclobacillus.cycloheptanicus
1.00
0.75
0.50
0.25
0.00
A (n=15)
B (n=16)
C (n=14)
surface

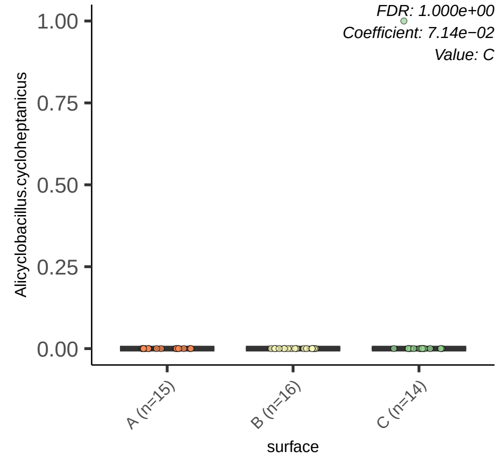

FDR: 1.000e+00
Coefficient: 7.14e−02
Value: C
1.00
0.75
0.50
0.25
0.00
Alicyclobacillus.cycloheptanicus
A (n=15)
B (n=16)
C (n=14)
surface

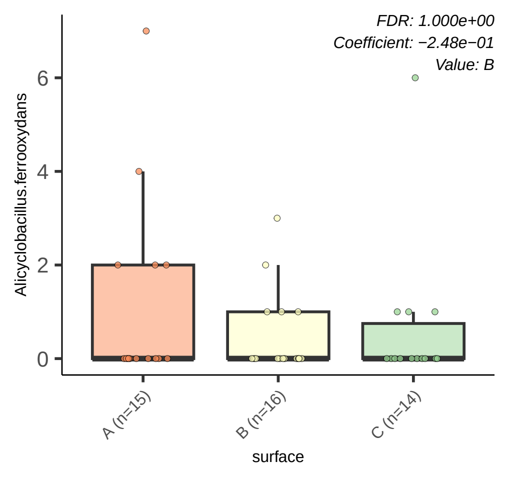

FDR: 1.000e+00
Coefficient: −2.48e−01
Value: B
Alicyclobacillus.ferrooxydans
0
2
4
6
A (n=15)
B (n=16)
C (n=14)
surface

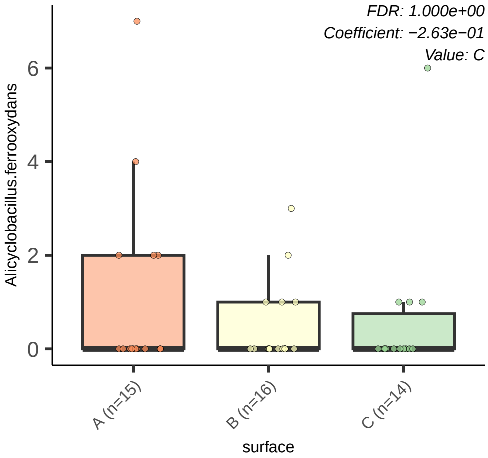
FDR: 1.000e+00
Coefficient: −2.63e−01
Value: C
Alicyclobacillus.ferrooxydans
0
2
4
6
A (n=15)
B (n=16)
C (n=14)
surface

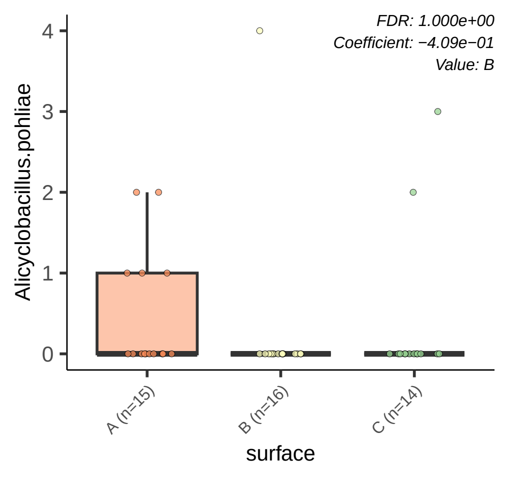
FDR: 1.000e+00
Coefficient: −4.09e−01
Value: B
Alicyclobacillus.pohliae
0
1
2
3
4
A (n=15)
B (n=16)
C (n=14)
surface

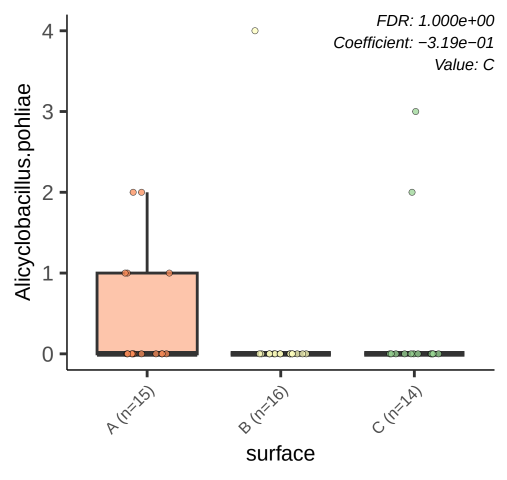
FDR: 1.000e+00
Coefficient: −3.19e−01
Value: C
Alicyclobacillus.pohliae
0
1
2
3
4
A (n=15)
B (n=16)
C (n=14)
surface

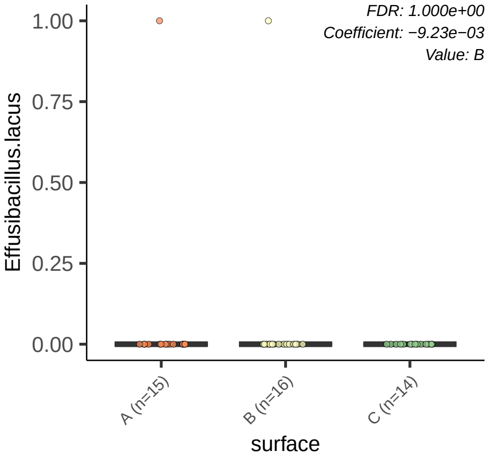
FDR: 1.000e+00
Coefficient: −9.23e−03
Value: B
Effusibacillus.lacus
1.00
0.75
0.50
0.25
0.00
A (n=15)
B (n=16)
C (n=14)
surface

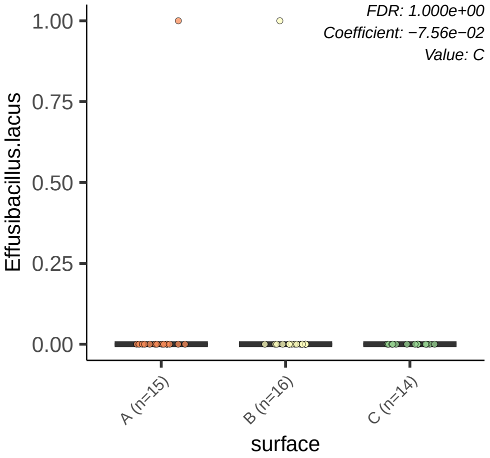
FDR: 1.000e+00
Coefficient: −7.56e−02
Value: C
Effusibacillus.lacus
1.00
0.75
0.50
0.25
0.00
A (n=15)
B (n=16)
C (n=14)
surface

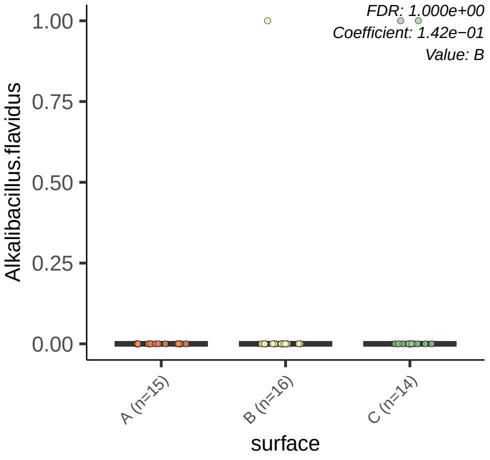
FDR: 1.000e+00
Coefficient: 1.42e−01
Value: B
1.00
0.75
0.50
0.25
0.00
Alkalibacillus.flavidus
A (n=15)
B (n=16)
C (n=14)
surface

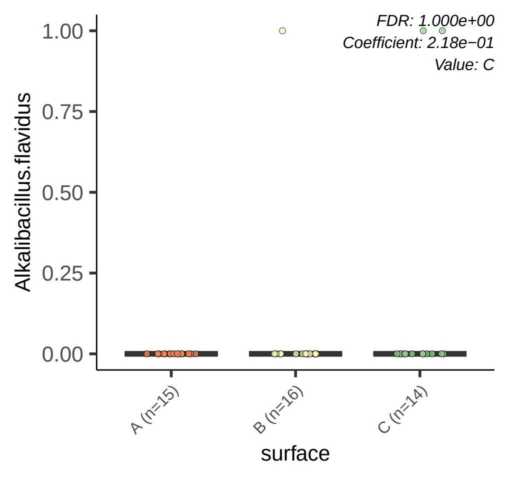

FDR: 1.000e+00
Coefficient: 2.18e−01
Value: C
Alkalibacillus.flavidus
1.00
0.75
0.50
0.25
0.00
A (n=15)
B (n=16)
C (n=14)
surface

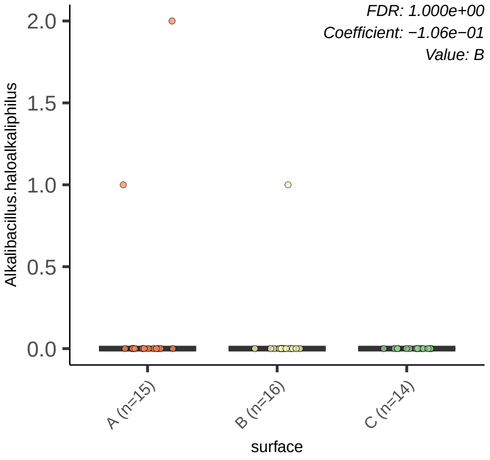
FDR: 1.000e+00
Coefficient: −1.06e−01
Value: B
Alkalibacillus.haloalkaliphilus
2.0
1.5
1.0
0.5
0.0
A (n=15)
B (n=16)
C (n=14)
surface

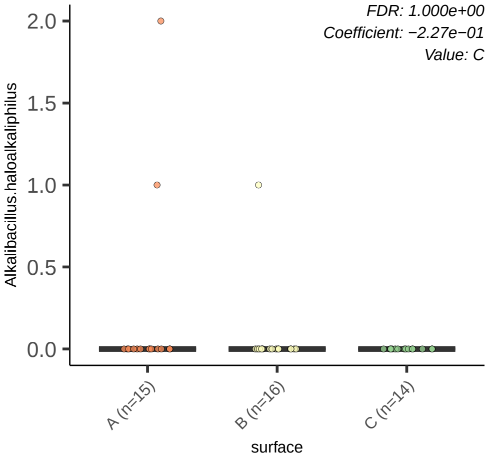

FDR: 1.000e+00
Coefficient: −2.27e−01
Value: C
Alkalibacillus.haloalkaliphilus
2.0
1.5
1.0
0.5
0.0
A (n=15)
B (n=16)
C (n=14)
surface

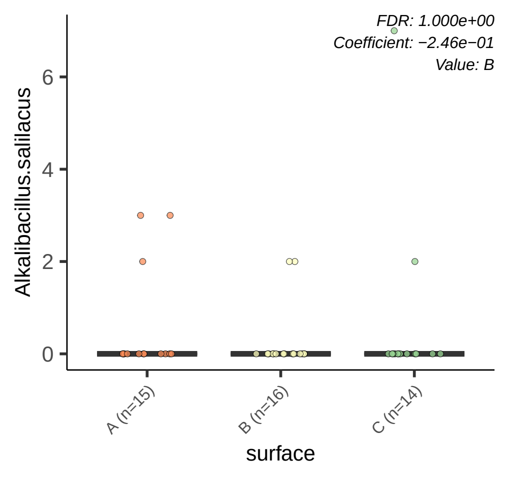

FDR: 1.000e+00
Coefficient: −2.46e−01
Value: B
Alkalibacillus.salilacus
0
2
4
6
A (n=15)
B (n=16)
C (n=14)
surface

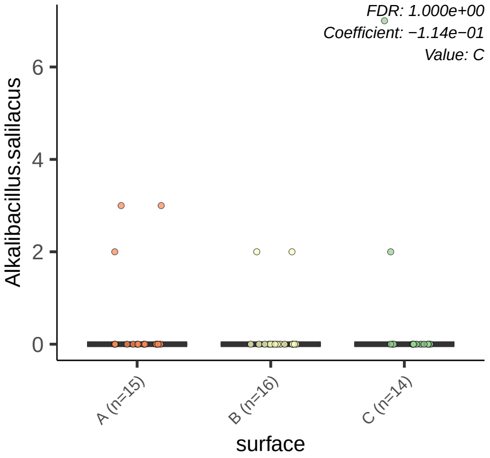

FDR: 1.000e+00
Coefficient: −1.14e−01
Value: C
Alkalibacillus.salilacus
0
2
4
6
A (n=15)
B (n=16)
C (n=14)
surface

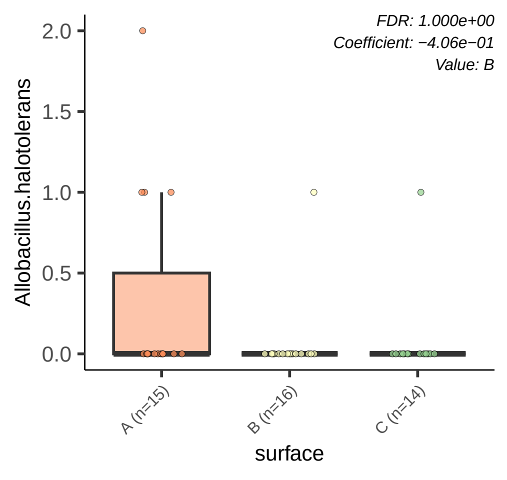

FDR: 1.000e+00
Coefficient: −4.06e−01
Value: B
Allobacillus.halotolerans
2.0
1.5
1.0
0.5
0.0
A (n=15)
B (n=16)
C (n=14)
surface

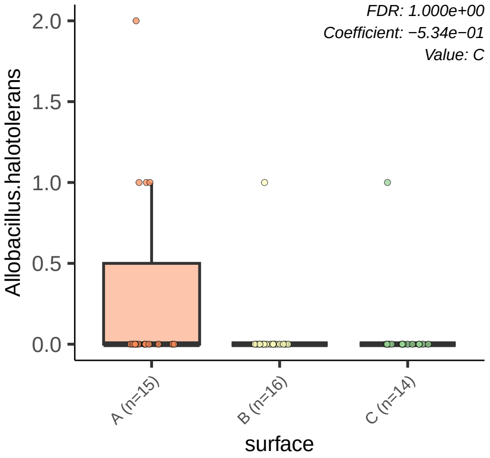
FDR: 1.000e+00
Coefficient: −5.34e−01
Value: C
Allobacillus.halotolerans
2.0
1.5
1.0
0.5
0.0
A (n=15)
B (n=16)
C (n=14)
surface

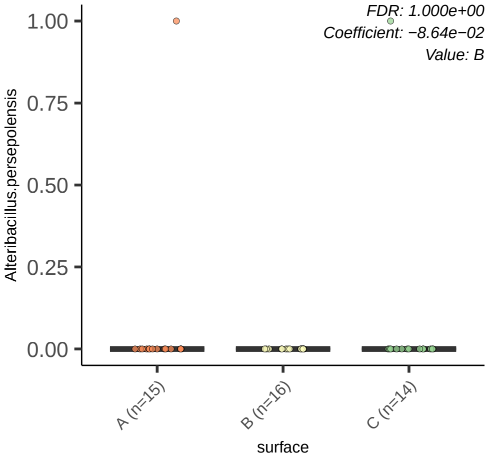
FDR: 1.000e+00
Coefficient: −8.64e−02
Value: B
Alteribacillus.persepolensis
1.00
0.75
0.50
0.25
0.00
A (n=15)
B (n=16)
C (n=14)
surface

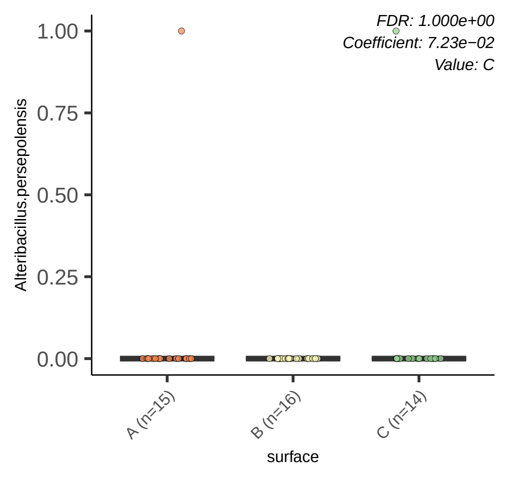

FDR: 1.000e+00
Coefficient: 7.23e−02
Value: C
Alteribacillus.persepolensis
1.00
0.75
0.50
0.25
0.00
A (n=15)
B (n=16)
C (n=14)
surface

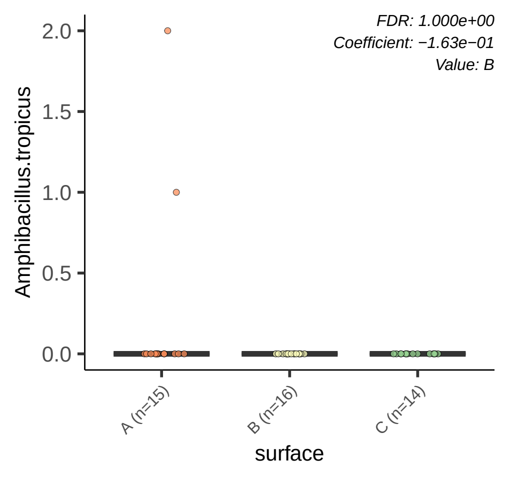

FDR: 1.000e+00
Coefficient: −1.63e−01
Value: B
Amphibacillus.tropicus
2.0
1.5
1.0
0.5
0.0
A (n=15)
B (n=16)
C (n=14)
surface

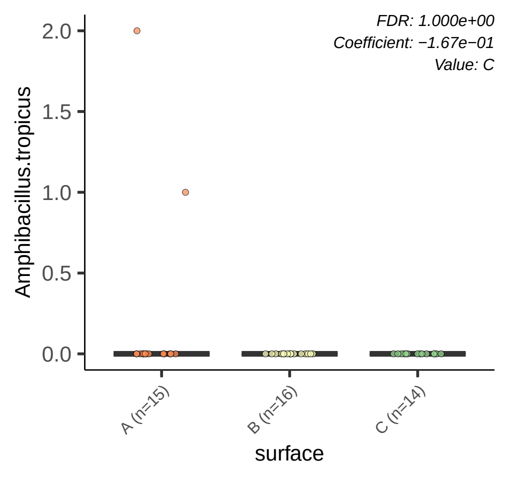
FDR: 1.000e+00
Coefficient: −1.67e−01
Value: C
Amphibacillus.tropicus
2.0
1.5
1.0
0.5
0.0
A (n=15)
B (n=16)
C (n=14)
surface

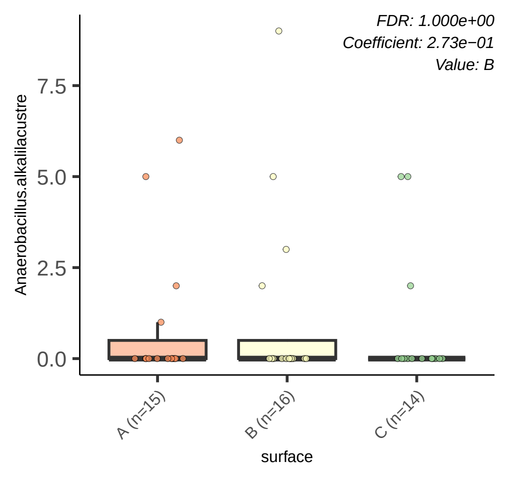
FDR: 1.000e+00
Coefficient: 2.73e−01
Value: B
Anaerobacillus.alkaliacustre
7.5
5.0
2.5
0.0
A (n=15)
B (n=16)
C (n=14)
surface

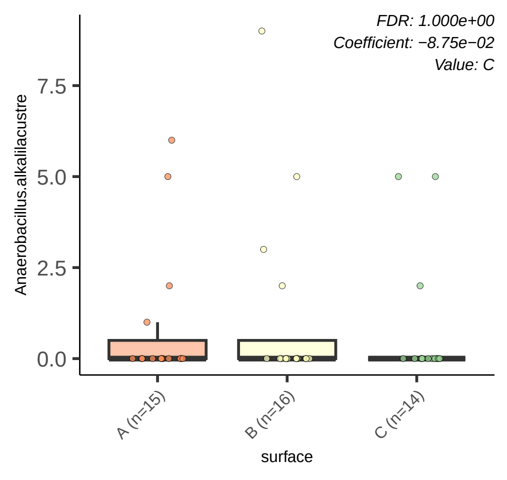
FDR: 1.000e+00
Coefficient: −8.75e−02
Value: C
Anaerobacillus.alkaliacustre
7.5
5.0
2.5
0.0
A (n=15)
B (n=16)
C (n=14)
surface

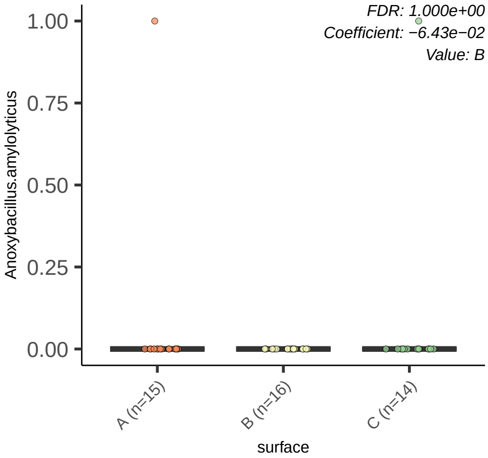
FDR: 1.000e+00
Coefficient: −6.43e−02
Value: B
Anoxybacillus.amylolyticus
1.00
0.75
0.50
0.25
0.00
A (n=15)
B (n=16)
C (n=14)
surface

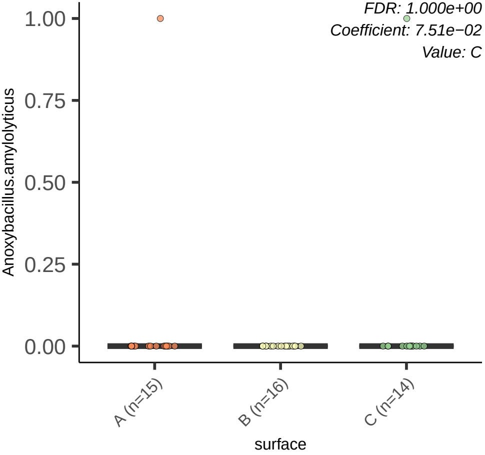
FDR: 1.000e+00
Coefficient: 7.51e−02
Value: C
1.00
0.75
0.50
0.25
0.00
Anoxybacillus.amylolyticus
A (n=15)
B (n=16)
C (n=14)
surface

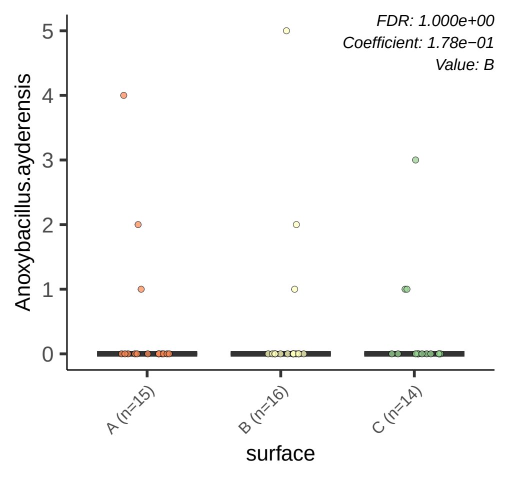

FDR: 1.000e+00
Coefficient: 1.78e−01
Value: B
Anoxybacillus.ayderensis
0
1
2
3
4
5
A (n=15)
B (n=16)
C (n=14)
surface

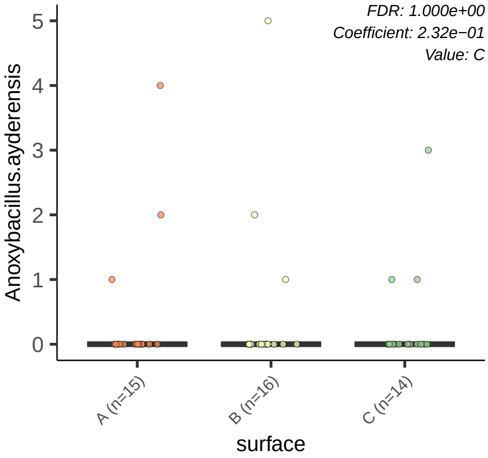
FDR: 1.000e+00
Coefficient: 2.32e−01
Value: C
Anoxybacillus.ayderensis
0
1
2
3
4
5
A (n=15)
B (n=16)
C (n=14)
surface

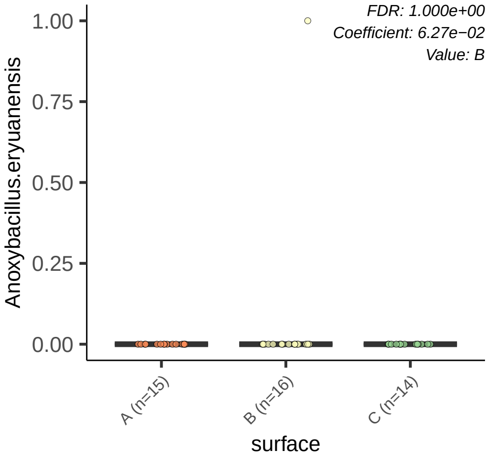
FDR: 1.000e+00
Coefficient: 6.27e−02
Value: B
Anoxybacillus.eryuanensis
1.00
0.75
0.50
0.25
0.00
A (n=15)
B (n=16)
C (n=14)
surface

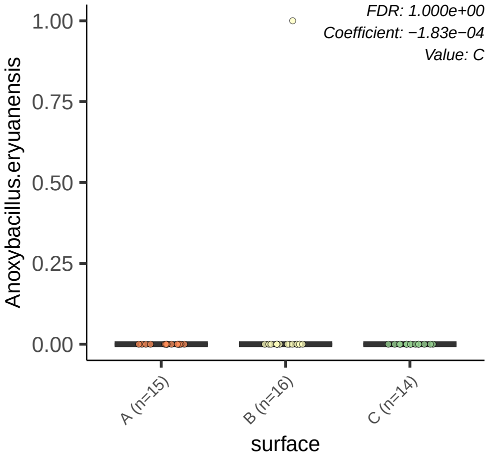

FDR: 1.000e+00
Coefficient: −1.83e−04
Value: C
Anoxybacillus.eryuanensis
1.00
0.75
0.50
0.25
0.00
A (n=15)
B (n=16)
C (n=14)
surface

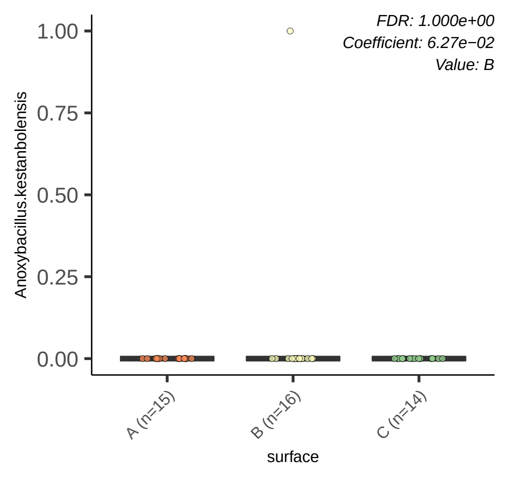
FDR: 1.000e+00
Coefficient: 6.27e−02
Value: B
1.00
0.75
0.50
0.25
0.00
Anoxybacillus.kestanbolensis
A (n=15)
B (n=16)
C (n=14)
surface

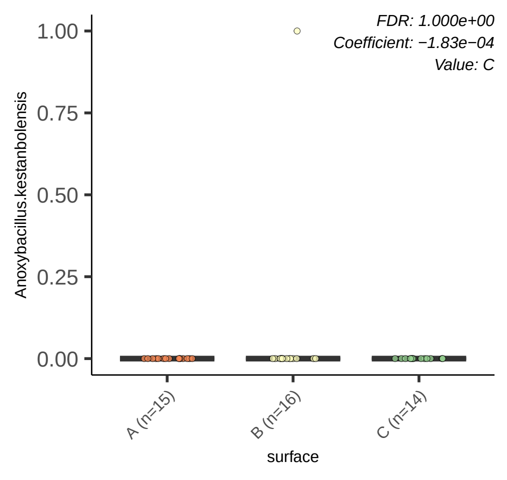
FDR: 1.000e+00
Coefficient: −1.83e−04
Value: C
1.00
0.75
0.50
0.25
0.00
Anoxybacillus.kestanbolensis
A (n=15)
B (n=16)
C (n=14)
surface

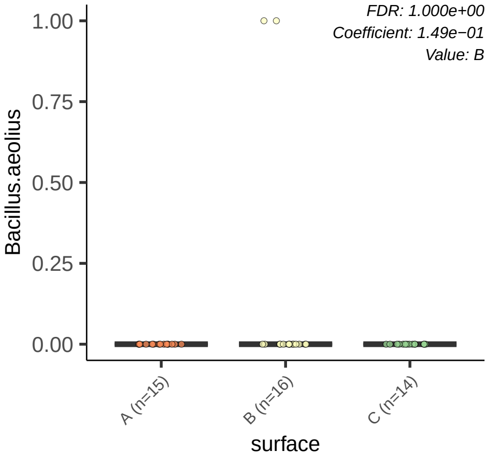
FDR: 1.000e+00
Coefficient: 1.49e−01
Value: B
1.00
0.75
0.50
0.25
0.00
Bacillus.aeolius
A (n=15)
B (n=16)
C (n=14)
surface

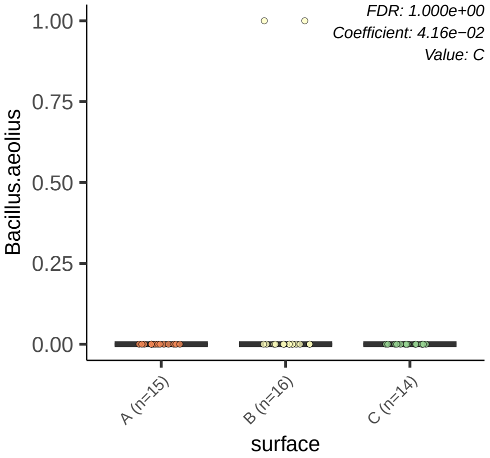
FDR: 1.000e+00
Coefficient: 4.16e−02
Value: C
1.00
0.75
0.50
0.25
0.00
Bacillus.aeolius
A (n=15)
B (n=16)
C (n=14)
surface

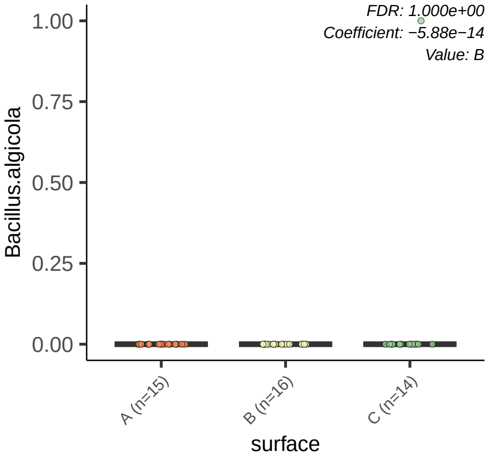
FDR: 1.000e+00
Coefficient: −5.88e−14
Value: B
Bacillus.algicola
1.00
0.75
0.50
0.25
0.00
A (n=15)
B (n=16)
C (n=14)
surface

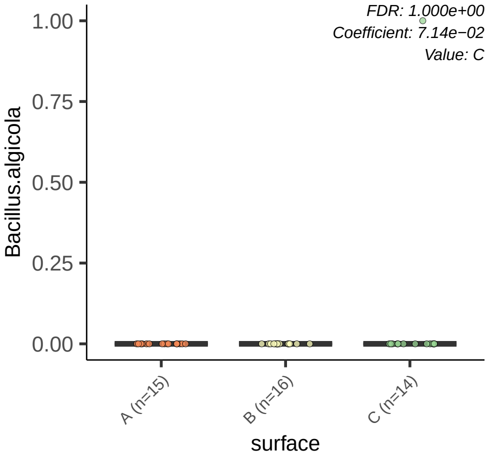

FDR: 1.000e+00
Coefficient: 7.14e−02
Value: C
1.00
0.75
0.50
0.25
0.00
Bacillus.algicola
A (n=15)
B (n=16)
C (n=14)
surface

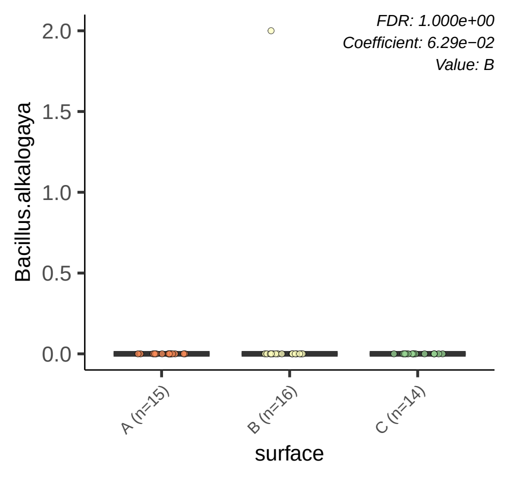
FDR: 1.000e+00
Coefficient: 6.29e−02
Value: B
Bacillus.alkalogaya
2.0
1.5
1.0
0.5
0.0
A (n=15)
B (n=16)
C (n=14)
surface

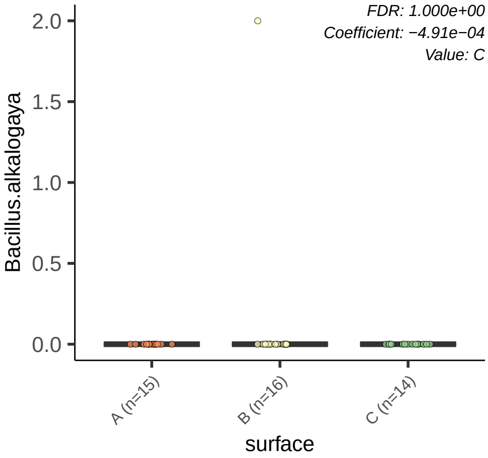
FDR: 1.000e+00
Coefficient: −4.91e−04
Value: C
Bacillus.alkalogaya
2.0
1.5
1.0
0.5
0.0
A (n=15)
B (n=16)
C (n=14)
surface

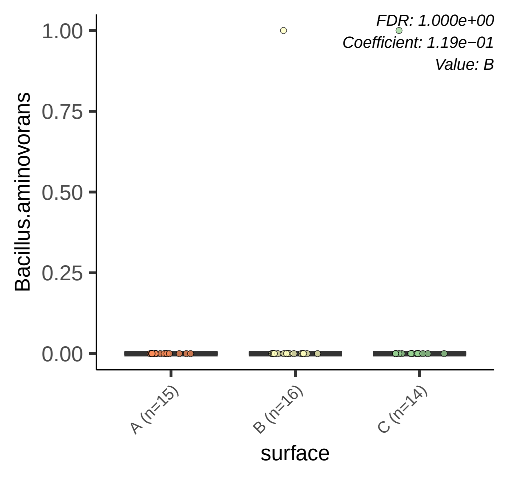
FDR: 1.000e+00
Coefficient: 1.19e−01
Value: B
Bacillus.aminovorans
1.00
0.75
0.50
0.25
0.00
A (n=15)
B (n=16)
C (n=14)
surface

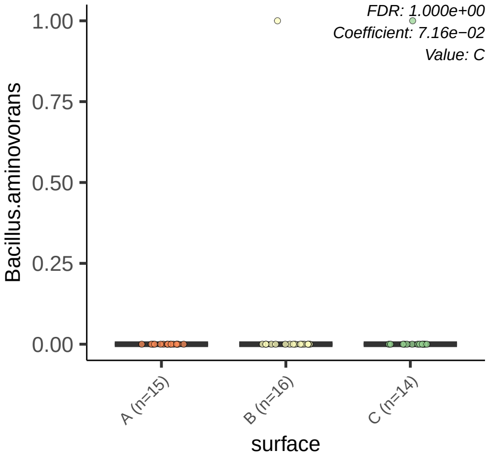

FDR: 1.000e+00
Coefficient: 7.16e−02
Value: C
Bacillus.aminovorans
1.00
0.75
0.50
0.25
0.00
A (n=15)
B (n=16)
C (n=14)
surface

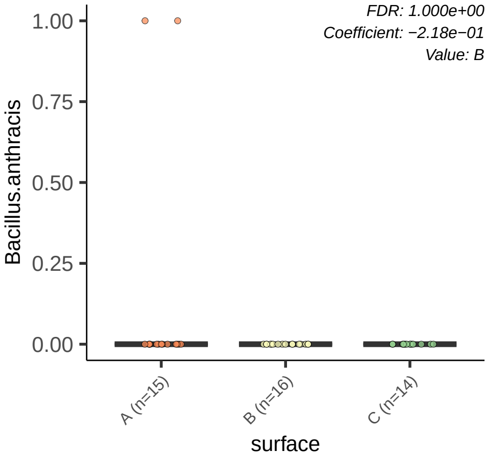
FDR: 1.000e+00
Coefficient: −2.18e−01
Value: B
Bacillus.anthracis
1.00
0.75
0.50
0.25
0.00
A (n=15)
B (n=16)
C (n=14)
surface

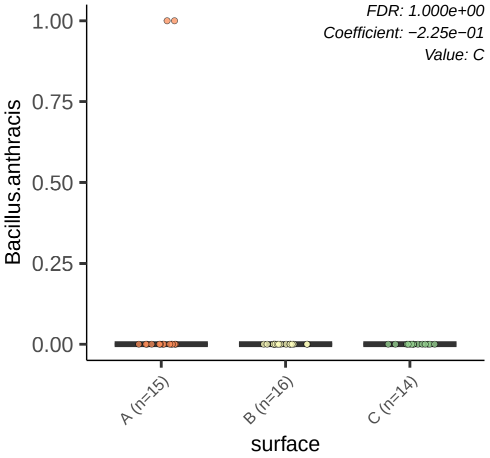
FDR: 1.000e+00
Coefficient: −2.25e−01
Value: C
Bacillus.anthracis
1.00
0.75
0.50
0.25
0.00
A (n=15)
B (n=16)
C (n=14)
surface

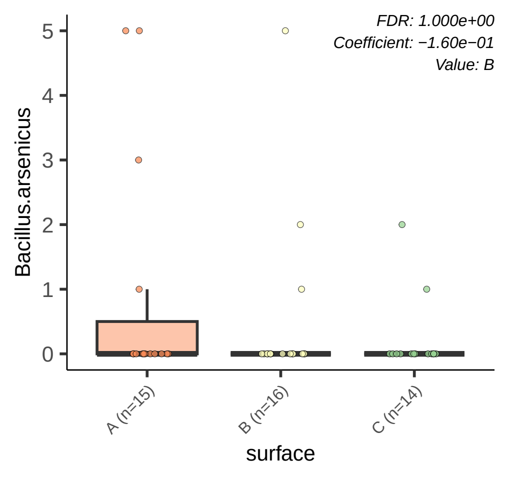

FDR: 1.000e+00
Coefficient: −1.60e−01
Value: B
Bacillus.arsenicus
0
1
2
3
4
5
A (n=15)
B (n=16)
C (n=14)
surface

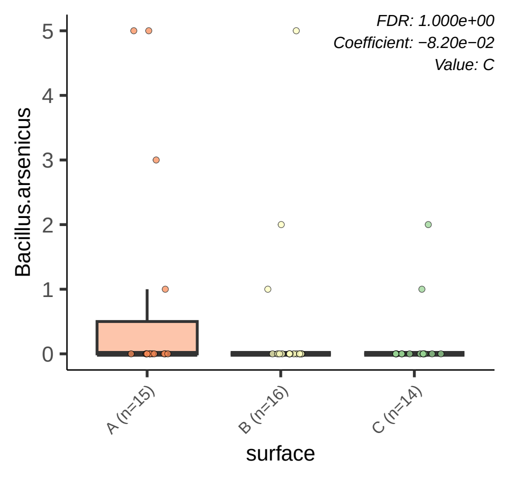

FDR: 1.000e+00
Coefficient: −8.20e−02
Value: C
Bacillus.arsenicus
0
1
2
3
4
5
A (n=15)
B (n=16)
C (n=14)
surface

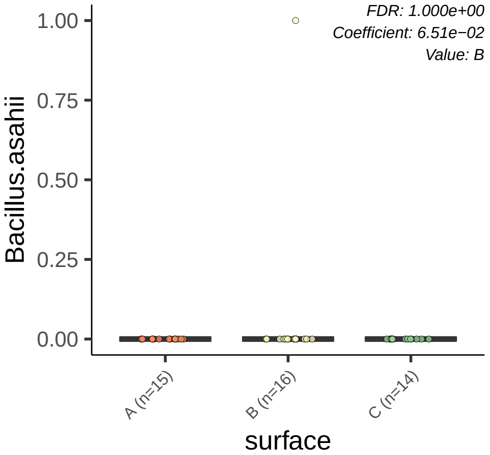
FDR: 1.000e+00
Coefficient: 6.51e−02
Value: B
Bacillus.asahii
1.00
0.75
0.50
0.25
0.00
A (n=15)
B (n=16)
C (n=14)
surface

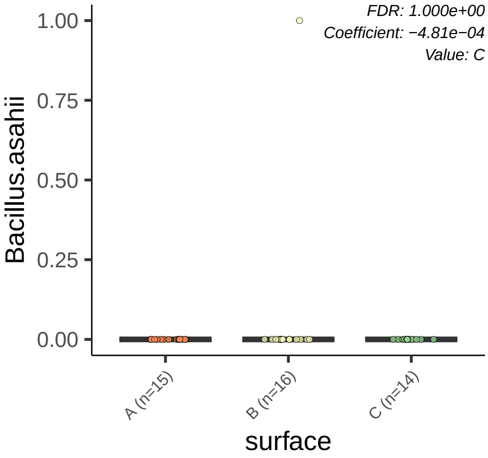

FDR: 1.000e+00
Coefficient: −4.81e−04
Value: C
Bacillus.asahii
1.00
0.75
0.50
0.25
0.00
A (n=15)
B (n=16)
C (n=14)
surface

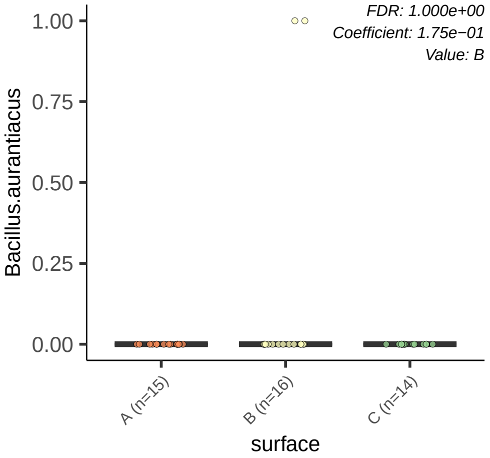
FDR: 1.000e+00
Coefficient: 1.75e−01
Value: B
Bacillus.aurantiacus
1.00
0.75
0.50
0.25
0.00
A (n=15)
B (n=16)
C (n=14)
surface

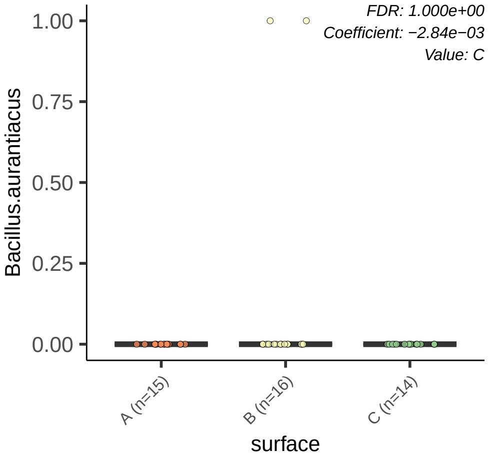

FDR: 1.000e+00
Coefficient: −2.84e−03
Value: C
1.00
0.75
0.50
0.25
0.00
Bacillus.aurantiacus
A (n=15)
B (n=16)
C (n=14)
surface

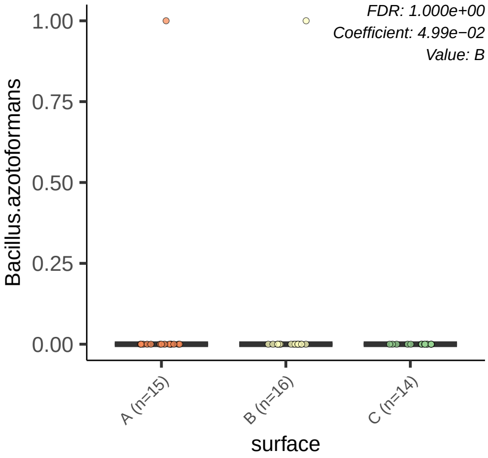
FDR: 1.000e+00
Coefficient: 4.99e−02
Value: B
1.00
0.75
0.50
0.25
0.00
Bacillus.azotoformans
A (n=15)
B (n=16)
C (n=14)
surface

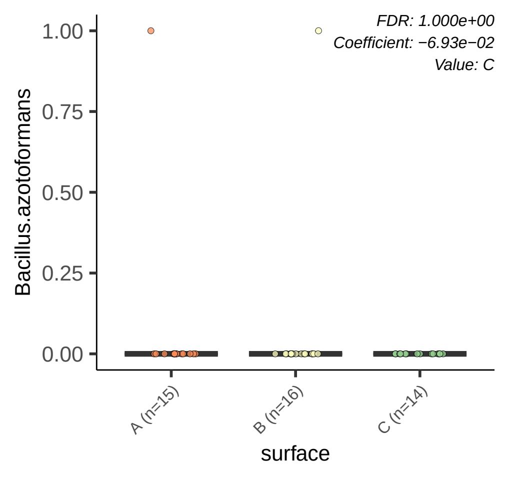
FDR: 1.000e+00
Coefficient: −6.93e−02
Value: C
Bacillus.azotoformans
1.00
0.75
0.50
0.25
0.00
A (n=15)
B (n=16)
C (n=14)
surface

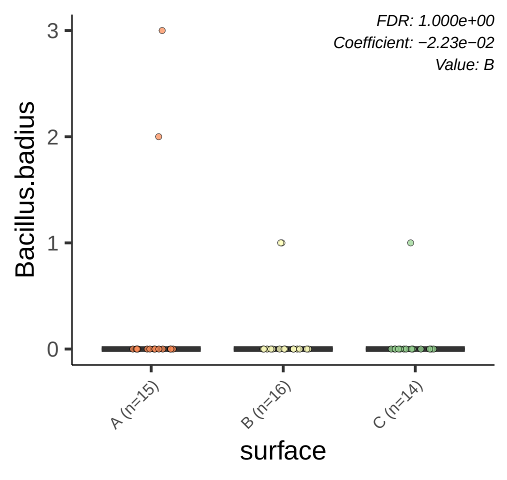

FDR: 1.000e+00
Coefficient: −2.23e−02
Value: B
Bacillus.badius
3
2
1
0
A (n=15)
B (n=16)
C (n=14)
surface

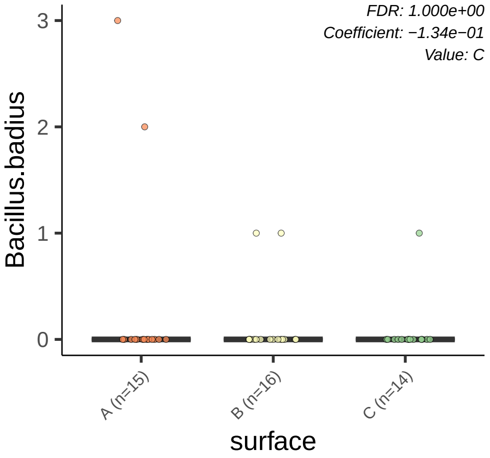

FDR: 1.000e+00
Coefficient: −1.34e−01
Value: C
Bacillus.badius
3
2
1
0
A (n=15)
B (n=16)
C (n=14)
surface

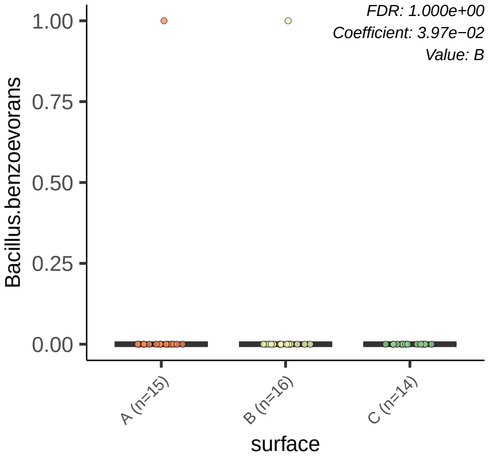
FDR: 1.000e+00
Coefficient: 3.97e−02
Value: B
Bacillus.benzoevorans
1.00
0.75
0.50
0.25
0.00
A (n=15)
B (n=16)
C (n=14)
surface

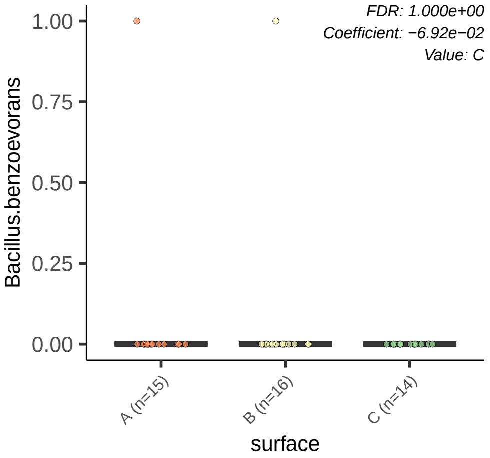
FDR: 1.000e+00
Coefficient: −6.92e−02
Value: C
Bacillus.benzoevorans
1.00
0.75
0.50
0.25
0.00
A (n=15)
B (n=16)
C (n=14)
surface

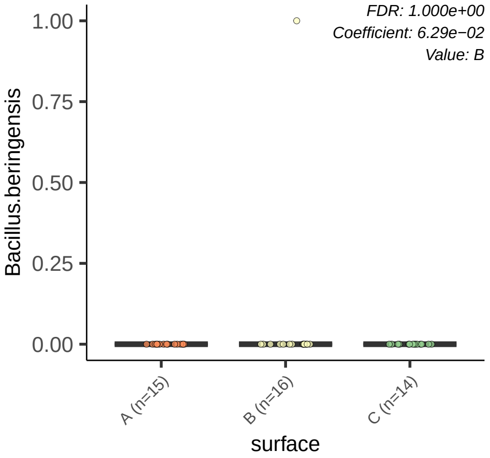
FDR: 1.000e+00
Coefficient: 6.29e−02
Value: B
Bacillus.beringensis
1.00
0.75
0.50
0.25
0.00
A (n=15)
B (n=16)
C (n=14)
surface

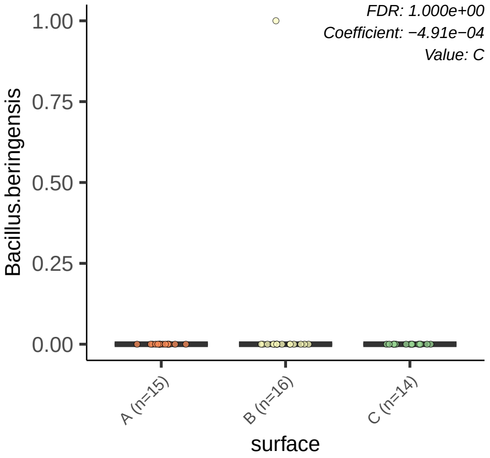
FDR: 1.000e+00
Coefficient: −4.91e−04
Value: C
Bacillus.beringensis
1.00
0.75
0.50
0.25
0.00
A (n=15)
B (n=16)
C (n=14)
surface

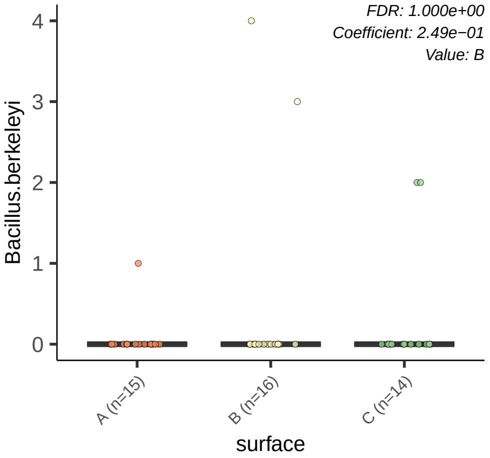
FDR: 1.000e+00
Coefficient: 2.49e−01
Value: B
Bacillus.berkeleyi
4
3
2
1
0
A (n=15)
B (n=16)
C (n=14)
surface

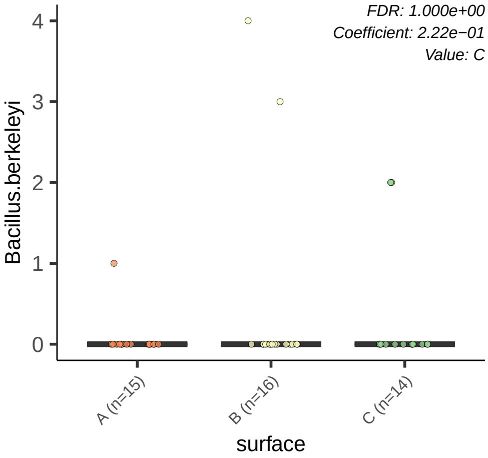
FDR: 1.000e+00
Coefficient: 2.22e−01
Value: C
Bacillus.berkeleyi
0
1
2
3
4
A (n=15)
B (n=16)
C (n=14)
surface

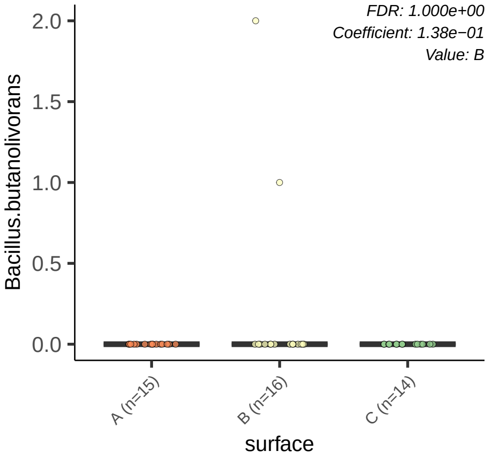
FDR: 1.000e+00
Coefficient: 1.38e−01
Value: B
Bacillus.butanolivorans
2.0
1.5
1.0
0.5
0.0
A (n=15)
B (n=16)
C (n=14)
surface

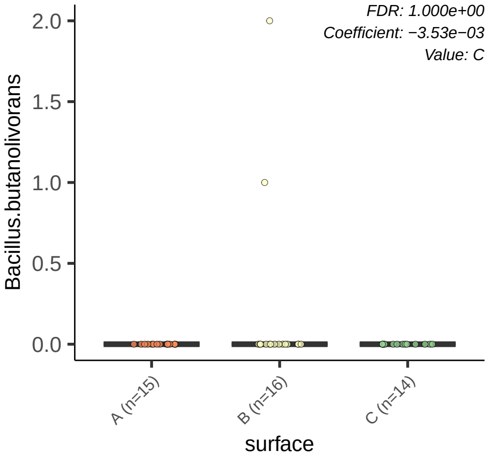

FDR: 1.000e+00
Coefficient: −3.53e−03
Value: C
2.0
1.5
1.0
0.5
0.0
Bacillus.butanolivorans
A (n=15)
B (n=16)
C (n=14)
surface

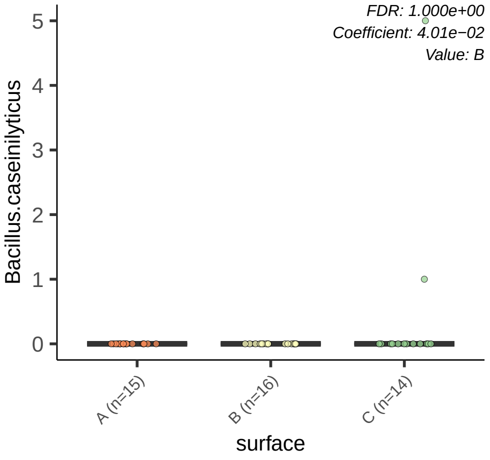

FDR: 1.000e+00
Coefficient: 4.01e−02
Value: B
Bacillus.caseinilyticus
0
1
2
3
4
5
A (n=15)
B (n=16)
C (n=14)
surface

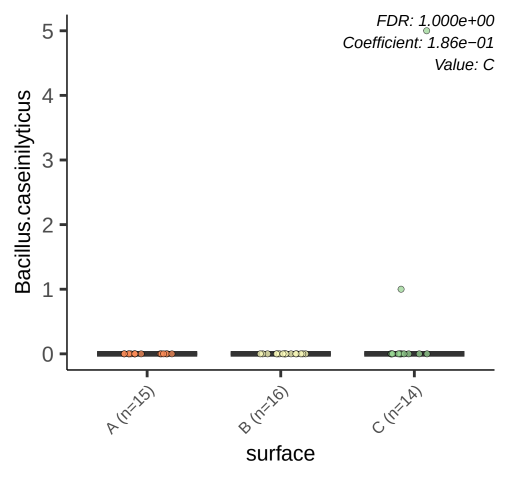
FDR: 1.000e+00
Coefficient: 1.86e−01
Value: C
Bacillus.caseinilyticus
0
1
2
3
4
5
A (n=15)
B (n=16)
C (n=14)
surface

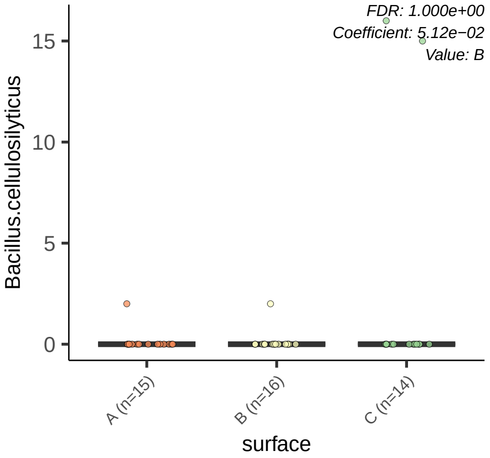
FDR: 1.000e+00
Coefficient: 5.12e−02
Value: B
Bacillus.cellulosilyticus
15
10
5
0
A (n=15)
B (n=16)
C (n=14)
surface

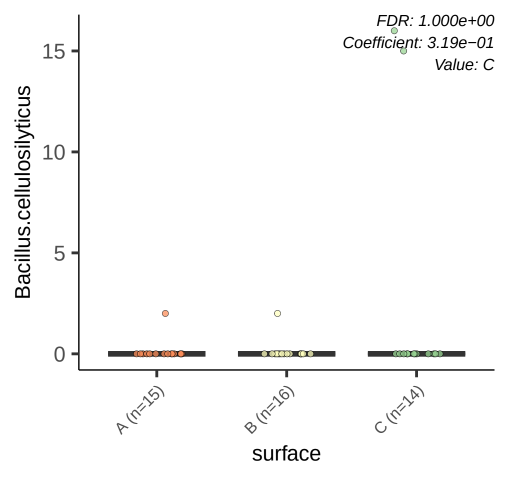
FDR: 1.000e+00
Coefficient: 3.19e−01
Value: C
Bacillus.cellulosilyticus
15
10
5
0
A (n=15)
B (n=16)
C (n=14)
surface

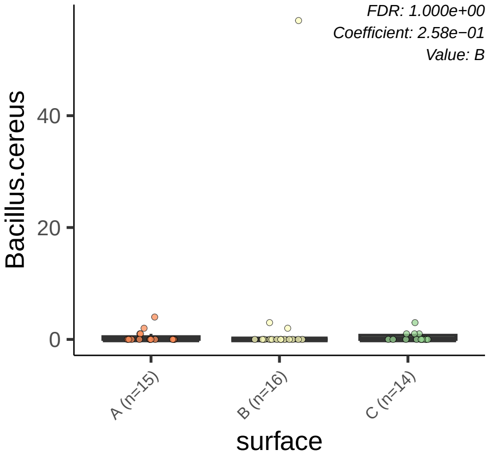

FDR: 1.000e+00
Coefficient: 2.58e−01
Value: B
Bacillus.cereus
0
20
40
A (n=15)
B (n=16)
C (n=14)
surface

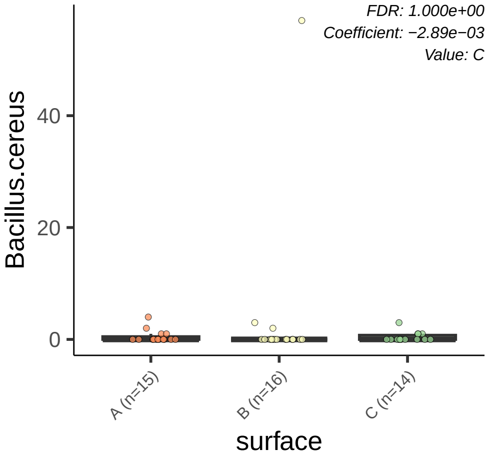

FDR: 1.000e+00
Coefficient: −2.89e−03
Value: C
Bacillus.cereus
40
20
0
A (n=15)
B (n=16)
C (n=14)
surface

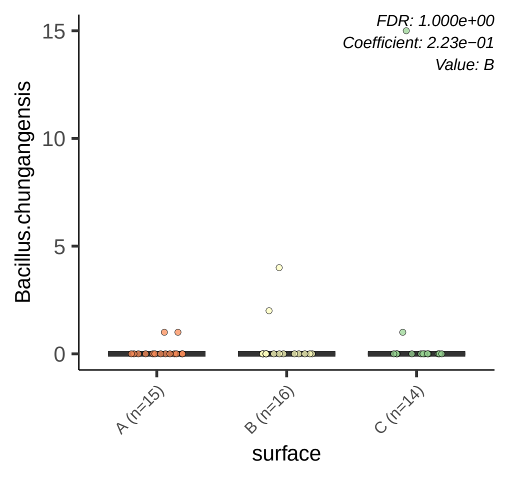
FDR: 1.000e+00
Coefficient: 2.23e−01
Value: B
Bacillus.chungangensis
15
10
5
0
A (n=15)
B (n=16)
C (n=14)
surface

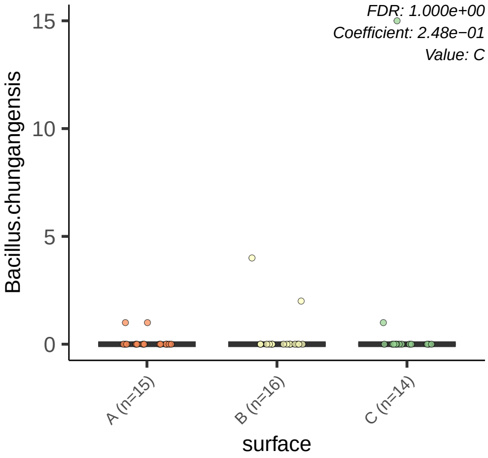

FDR: 1.000e+00
Coefficient: 2.48e−01
Value: C
15
10
5
0
Bacillus.chungangensis
A (n=15)
B (n=16)
C (n=14)
surface

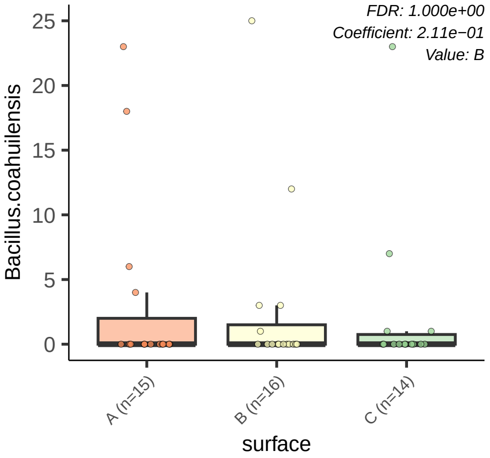
FDR: 1.000e+00
Coefficient: 2.11e−01
Value: B
Bacillus.coahuilensis
0
5
10
15
20
25
A (n=15)
B (n=16)
C (n=14)
surface

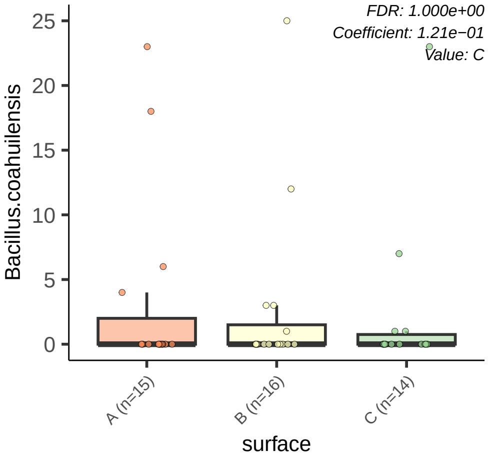
FDR: 1.000e+00
Coefficient: 1.21e−01
Value: C
Bacillus.coahuilensis
0
5
10
15
20
25
A (n=15)
B (n=16)
C (n=14)
surface

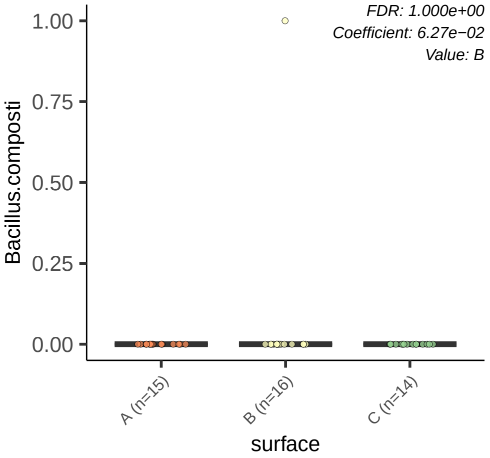

FDR: 1.000e+00
Coefficient: 6.27e−02
Value: B
Bacillus.composti
1.00
0.75
0.50
0.25
0.00
A (n=15)
B (n=16)
C (n=14)
surface

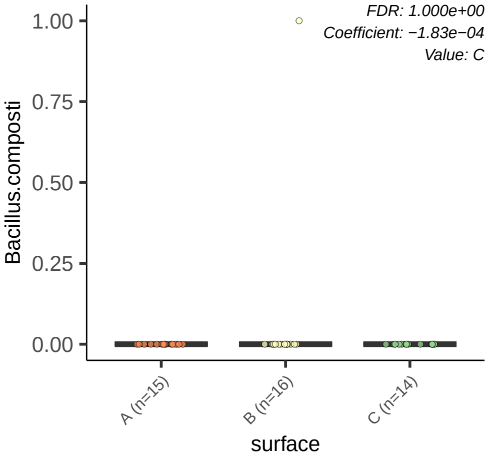
FDR: 1.000e+00
Coefficient: −1.83e−04
Value: C
Bacillus.composti
1.00
0.75
0.50
0.25
0.00
A (n=15)
B (n=16)
C (n=14)
surface

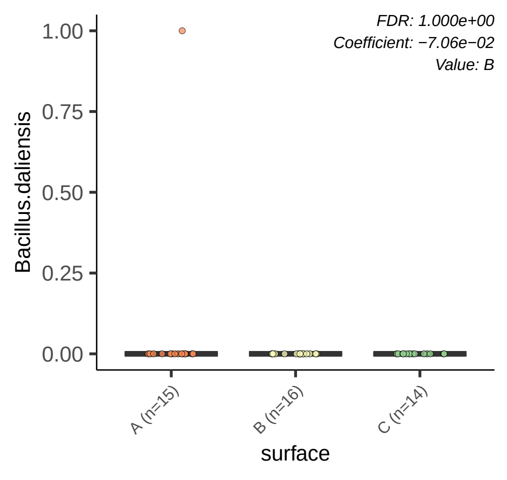
FDR: 1.000e+00
Coefficient: −7.06e−02
Value: B
Bacillus.daliensis
1.00
0.75
0.50
0.25
0.00
A (n=15)
B (n=16)
C (n=14)
surface

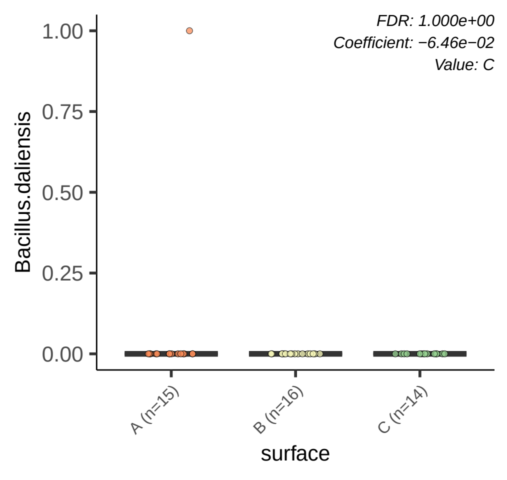
FDR: 1.000e+00
Coefficient: −6.46e−02
Value: C
Bacillus.daliensis
1.00
0.75
0.50
0.25
0.00
A (n=15)
B (n=16)
C (n=14)
surface

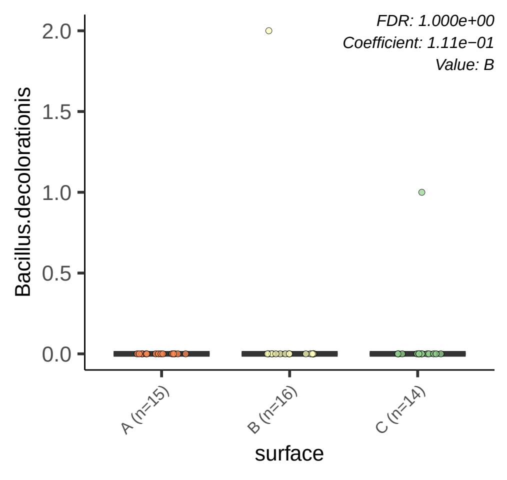
FDR: 1.000e+00
Coefficient: 1.11e−01
Value: B
Bacillus.decolorationis
2.0
1.5
1.0
0.5
0.0
A (n=15)
B (n=16)
C (n=14)
surface

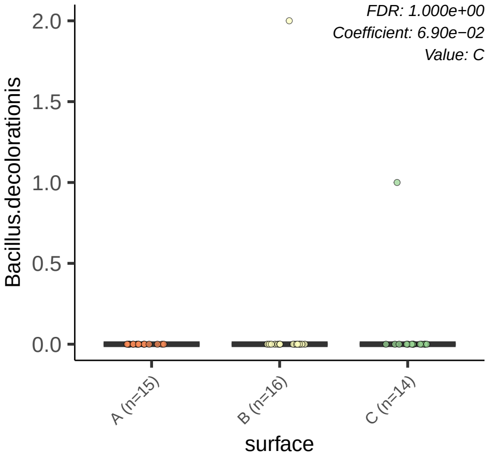

FDR: 1.000e+00
Coefficient: 6.90e−02
Value: C
Bacillus.decolorationis
2.0
1.5
1.0
0.5
0.0
A (n=15)
B (n=16)
C (n=14)
surface

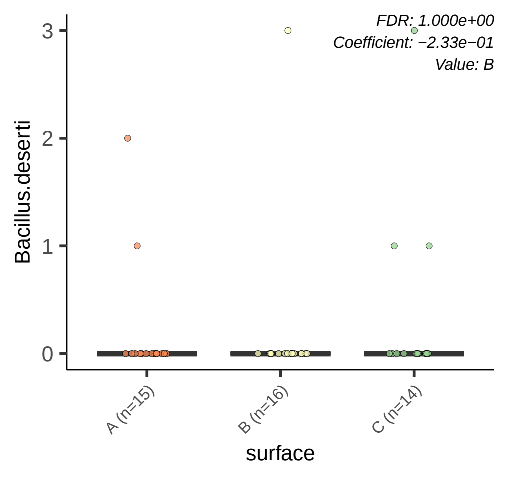
FDR: 1.000e+00
Coefficient: −2.33e−01
Value: B
Bacillus.deserti
3
2
1
0
A (n=15)
B (n=16)
C (n=14)
surface

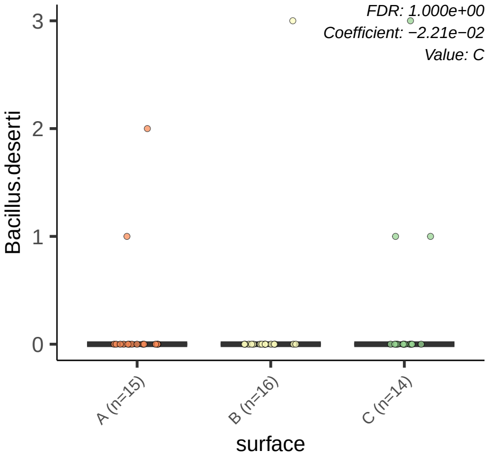

FDR: 1.000e+00
Coefficient: −2.21e−02
Value: C
Bacillus.deserti
3
2
1
0
A (n=15)
B (n=16)
C (n=14)
surface

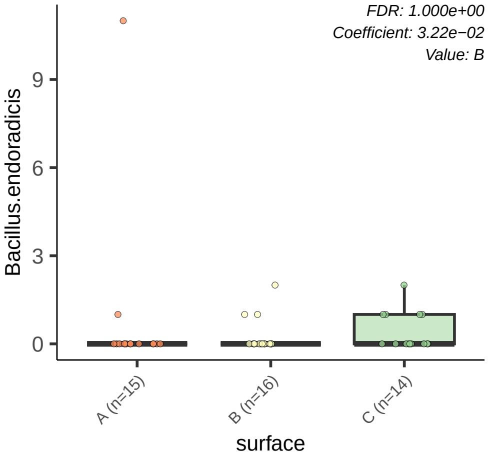
FDR: 1.000e+00
Coefficient: 3.22e−02
Value: B
Bacillus.endoradicis
0
3
6
9
A (n=15)
B (n=16)
C (n=14)
surface

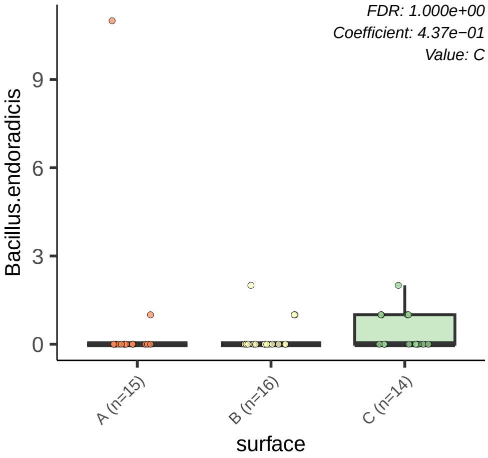

FDR: 1.000e+00
Coefficient: 4.37e−01
Value: C
Bacillus.endoradicis
0
3
6
9
A (n=15)
B (n=16)
C (n=14)
surface

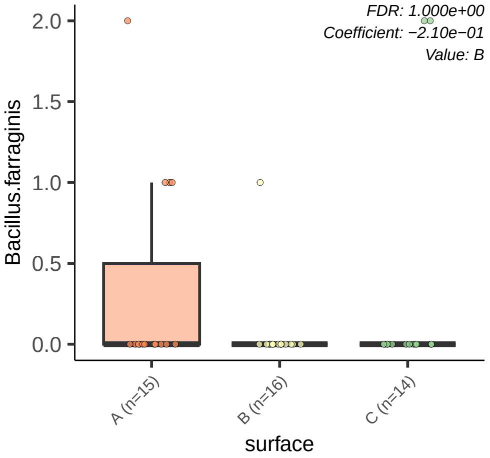
FDR: 1.000e+00
Coefficient: −2.10e−01
Value: B
Bacillus.farraginis
2.0
1.5
1.0
0.5
0.0
A (n=15)
B (n=16)
C (n=14)
surface

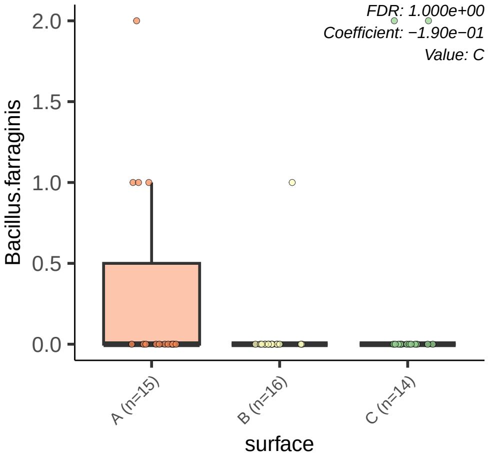

FDR: 1.000e+00
Coefficient: −1.90e−01
Value: C
Bacillus.farraginis
2.0
1.5
1.0
0.5
0.0
A (n=15)
B (n=16)
C (n=14)
surface

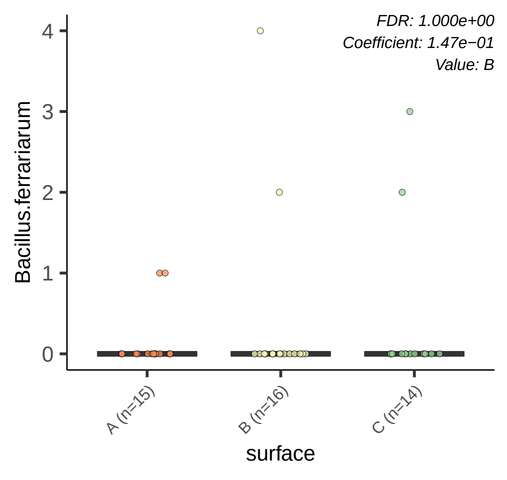

FDR: 1.000e+00
Coefficient: 1.47e−01
Value: B
Bacillus.ferrariarum
0
1
2
3
4
A (n=15)
B (n=16)
C (n=14)
surface

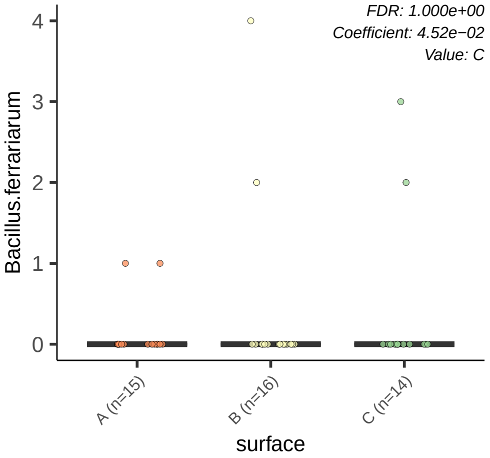
FDR: 1.000e+00
Coefficient: 4.52e−02
Value: C
Bacillus.ferrariarum
0
1
2
3
4
A (n=15)
B (n=16)
C (n=14)
surface

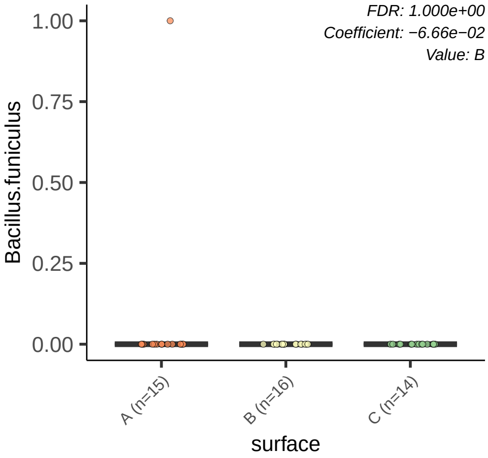

FDR: 1.000e+00
Coefficient: −6.66e−02
Value: B
Bacillus.funiculus
1.00
0.75
0.50
0.25
0.00
A (n=15)
B (n=16)
C (n=14)
surface

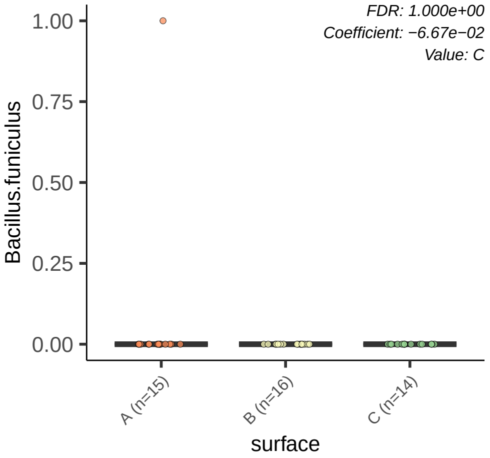
FDR: 1.000e+00
Coefficient: −6.67e−02
Value: C
Bacillus.funiculus
1.00
0.75
0.50
0.25
0.00
A (n=15)
B (n=16)
C (n=14)
surface

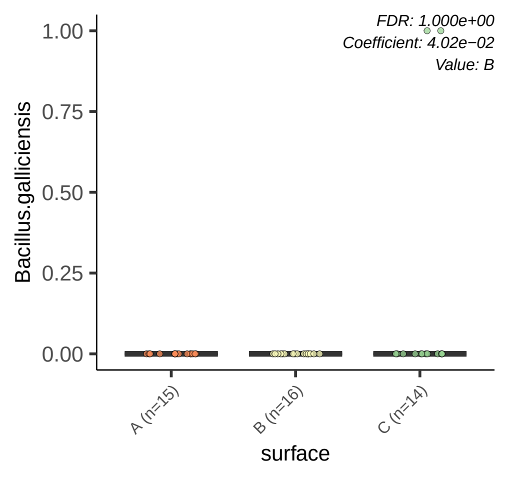

FDR: 1.000e+00
Coefficient: 4.02e−02
Value: B
1.00
0.75
0.50
0.25
0.00
Bacillus.galliciensis
A (n=15)
B (n=16)
C (n=14)
surface

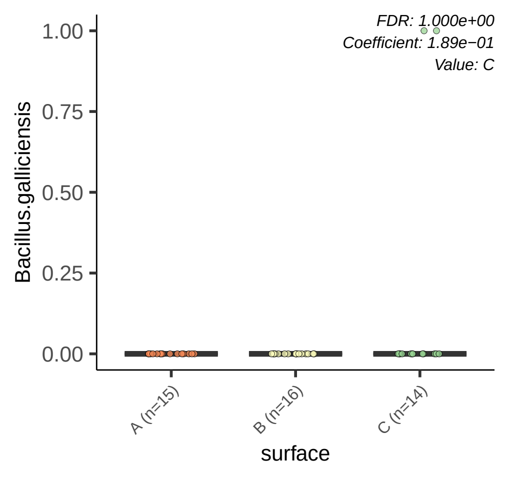
FDR: 1.000e+00
Coefficient: 1.89e−01
Value: C
Bacillus.galliciensis
1.00
0.75
0.50
0.25
0.00
A (n=15)
B (n=16)
C (n=14)
surface

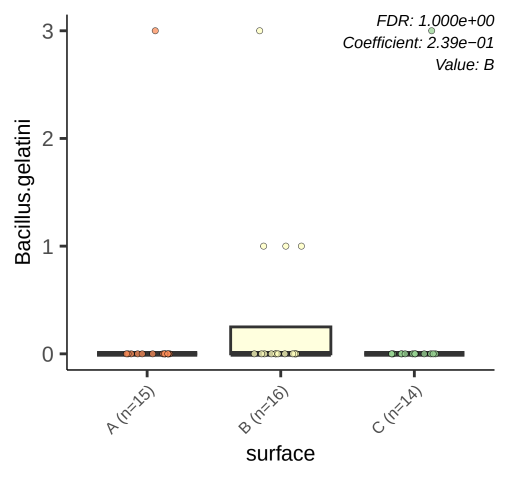
FDR: 1.000e+00
Coefficient: 2.39e−01
Value: B
Bacillus.gelatini
3
2
1
0
A (n=15)
B (n=16)
C (n=14)
surface

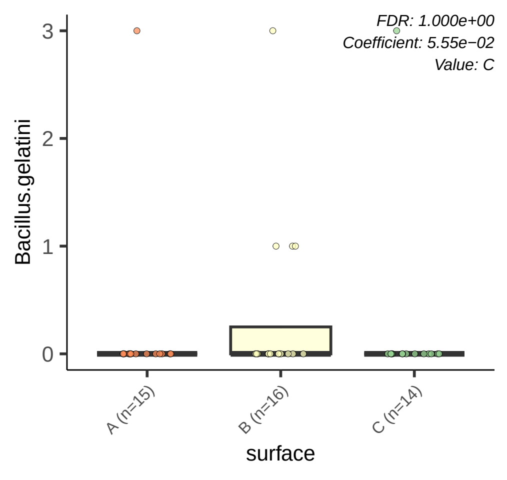

FDR: 1.000e+00
Coefficient: 5.55e−02
Value: C
Bacillus.gelatini
3
2
1
0
A (n=15)
B (n=16)
C (n=14)
surface

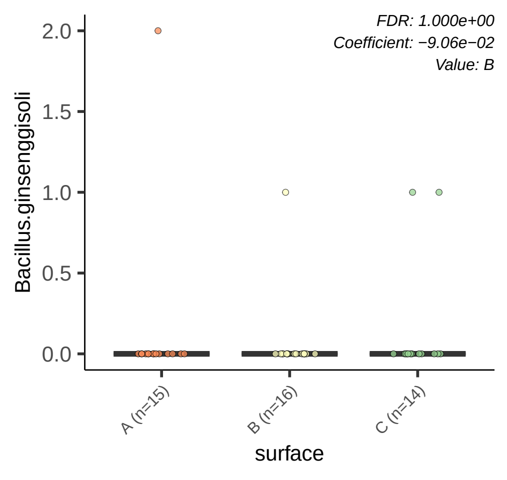
FDR: 1.000e+00
Coefficient: −9.06e−02
Value: B
Bacillus.ginsenggisoli
2.0
1.5
1.0
0.5
0.0
A (n=15)
B (n=16)
C (n=14)
surface

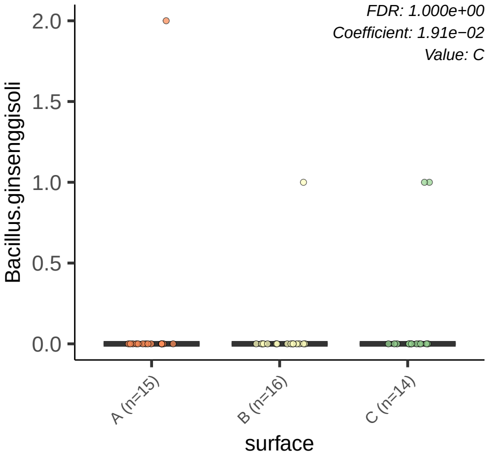
FDR: 1.000e+00
Coefficient: 1.91e−02
Value: C
Bacillus.ginsenggisoli
2.0
1.5
1.0
0.5
0.0
A (n=15)
B (n=16)
C (n=14)
surface

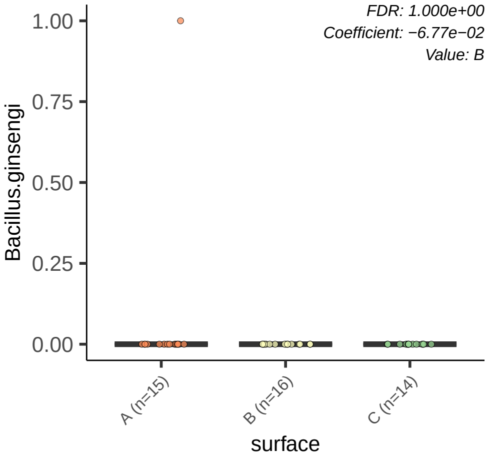
FDR: 1.000e+00
Coefficient: −6.77e−02
Value: B
Bacillus.ginsengi
1.00
0.75
0.50
0.25
0.00
A (n=15)
B (n=16)
C (n=14)
surface

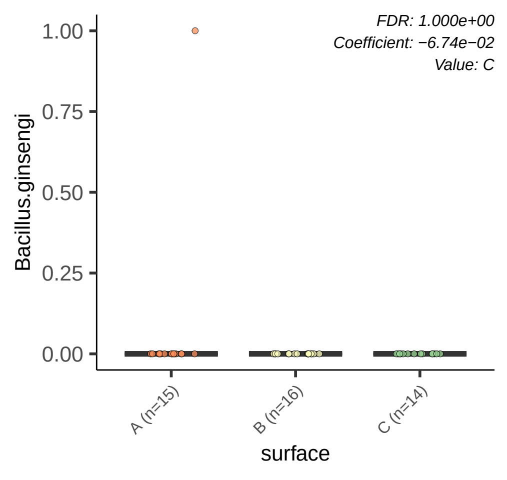

FDR: 1.000e+00
Coefficient: −6.74e−02
Value: C
Bacillus.ginsengi
1.00
0.75
0.50
0.25
0.00
A (n=15)
B (n=16)
C (n=14)
surface

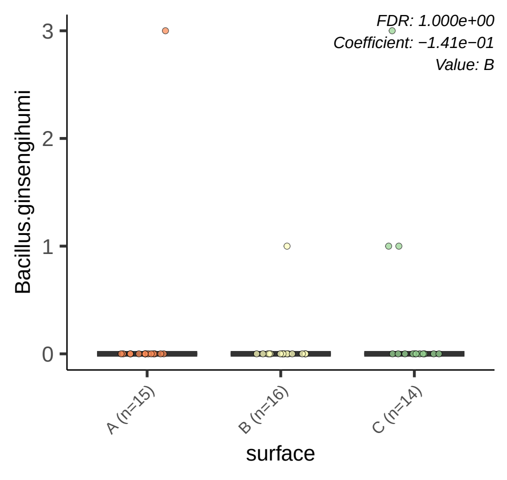
FDR: 1.000e+00
Coefficient: −1.41e−01
Value: B
Bacillus.ginsengihumi
3
2
1
0
A (n=15)
B (n=16)
C (n=14)
surface

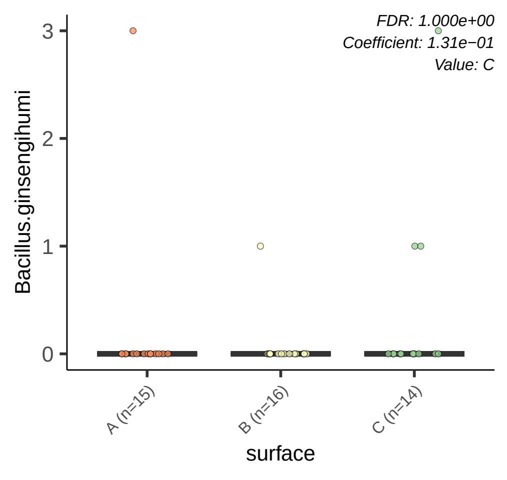
FDR: 1.000e+00
Coefficient: 1.31e−01
Value: C
Bacillus.ginsengihumi
3
2
1
0
A (n=15)
B (n=16)
C (n=14)
surface

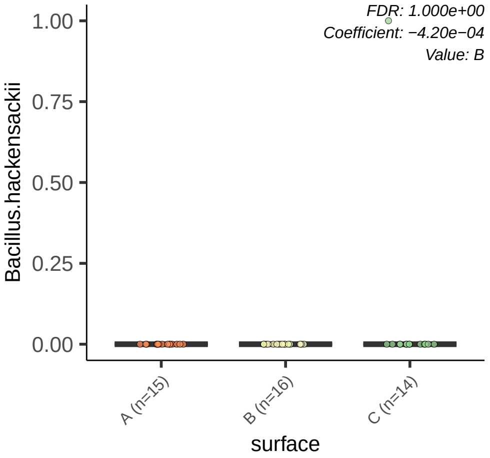
FDR: 1.000e+00
Coefficient: −4.20e−04
Value: B
Bacillus.hackensackii
1.00
0.75
0.50
0.25
0.00
A (n=15)
B (n=16)
C (n=14)
surface

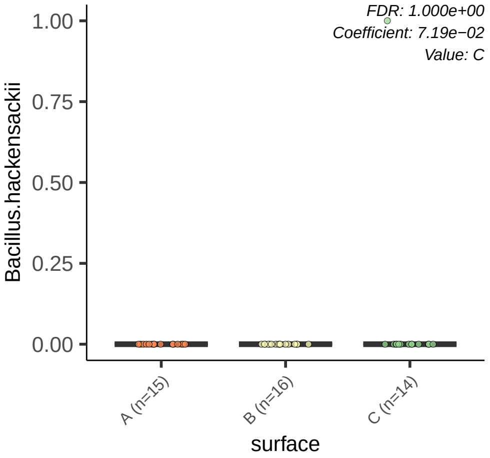
FDR: 1.000e+00
Coefficient: 7.19e−02
Value: C
1.00
0.75
0.50
0.25
0.00
Bacillus.hackensackii
A (n=15)
B (n=16)
C (n=14)
surface

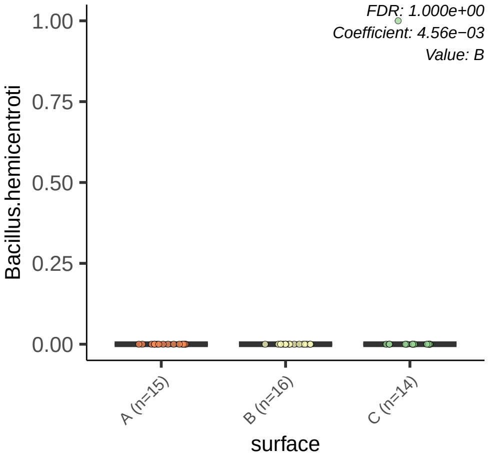
FDR: 1.000e+00
Coefficient: 4.56e−03
Value: B
Bacillus.hemicentroti
1.00
0.75
0.50
0.25
0.00
A (n=15)
B (n=16)
C (n=14)
surface

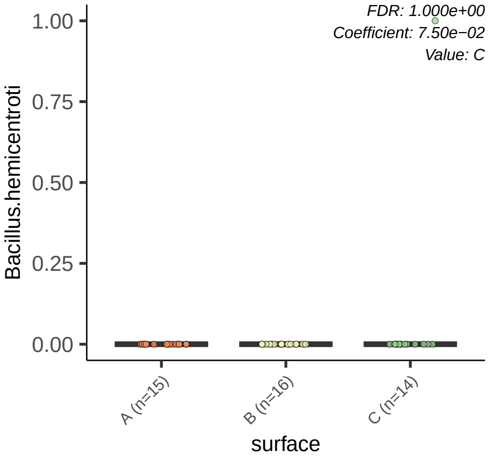

FDR: 1.000e+00
Coefficient: 7.50e−02
Value: C
Bacillus.hemicentroti
1.00
0.75
0.50
0.25
0.00
A (n=15)
B (n=16)
C (n=14)
surface

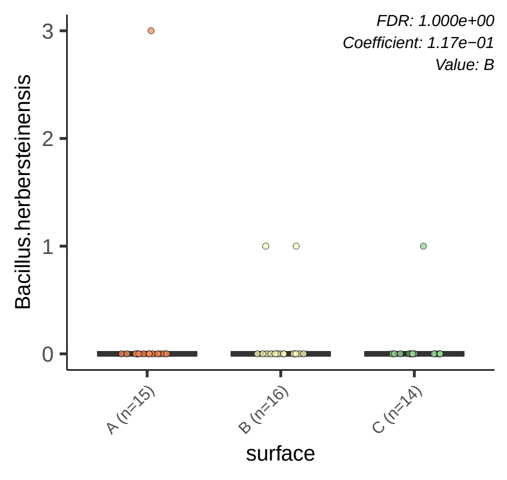
FDR: 1.000e+00
Coefficient: 1.17e−01
Value: B
Bacillus.herbersteinensis
3
2
1
0
A (n=15)
B (n=16)
C (n=14)
surface

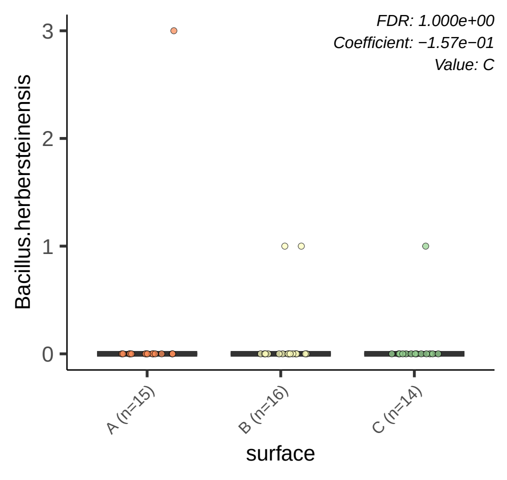
FDR: 1.000e+00
Coefficient: −1.57e−01
Value: C
Bacillus.herbersteinensis
0
1
2
3
A (n=15)
B (n=16)
C (n=14)
surface

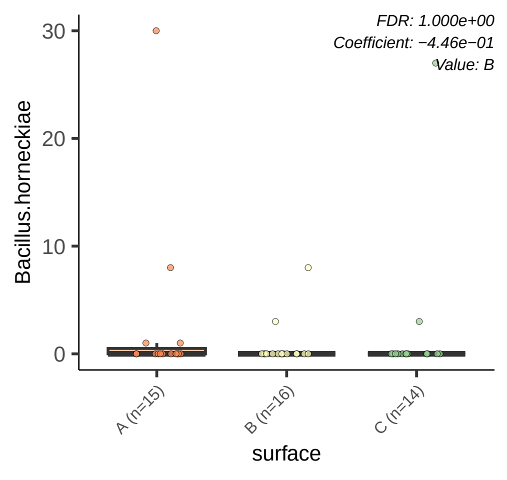
FDR: 1.000e+00
Coefficient: −4.46e−01
Value: B
Bacillus.horneckiae
30
20
10
0
A (n=15)
B (n=16)
C (n=14)
surface

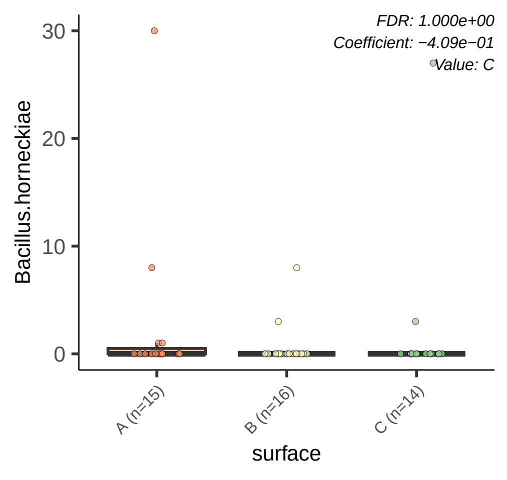
FDR: 1.000e+00
Coefficient: −4.09e−01
Value: C
Bacillus.horneckiae
30
20
10
0
A (n=15)
B (n=16)
C (n=14)
surface

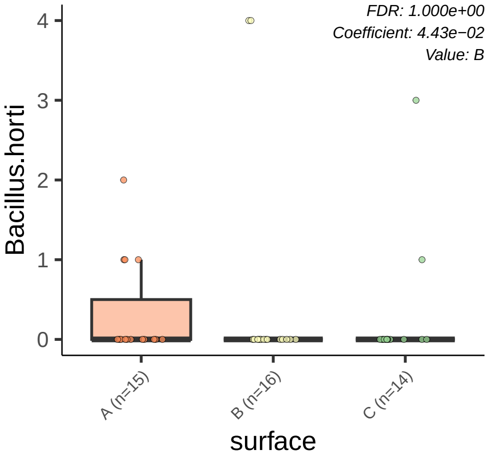

FDR: 1.000e+00
Coefficient: 4.43e−02
Value: B
Bacillus.horti
0
1
2
3
4
A (n=15)
B (n=16)
C (n=14)
surface

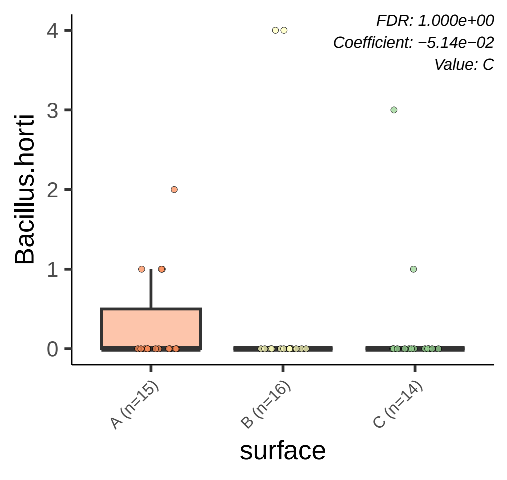
FDR: 1.000e+00
Coefficient: −5.14e−02
Value: C
Bacillus.horti
4
3
2
1
0
A (n=15)
B (n=16)
C (n=14)
surface

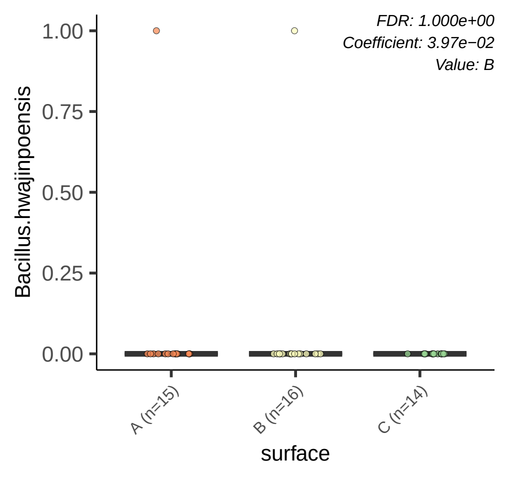

FDR: 1.000e+00
Coefficient: 3.97e−02
Value: B
Bacillus.hwajinpoensis
1.00
0.75
0.50
0.25
0.00
A (n=15)
B (n=16)
C (n=14)
surface

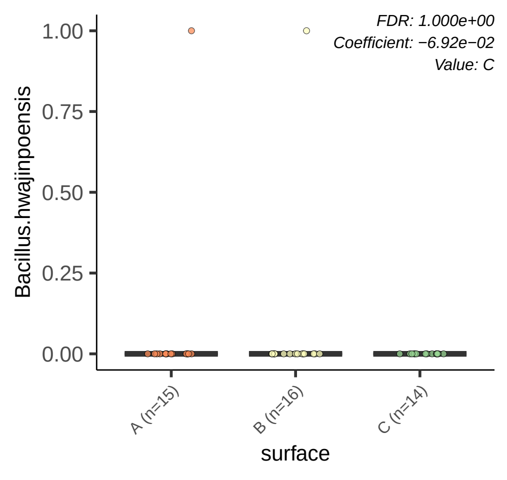

FDR: 1.000e+00
Coefficient: −6.92e−02
Value: C
Bacillus.hwajinpoensis
1.00
0.75
0.50
0.25
0.00
A (n=15)
B (n=16)
C (n=14)
surface

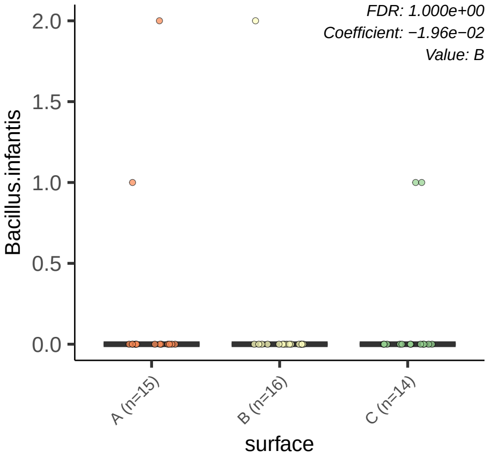
FDR: 1.000e+00
Coefficient: −1.96e−02
Value: B
Bacillus.infantis
2.0
1.5
1.0
0.5
0.0
A (n=15)
B (n=16)
C (n=14)
surface

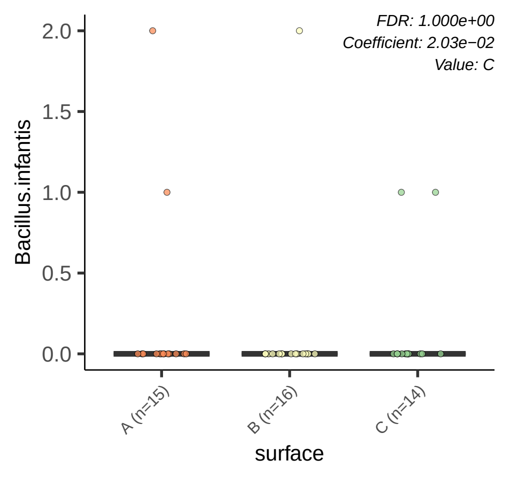

FDR: 1.000e+00
Coefficient: 2.03e−02
Value: C
Bacillus.infantis
2.0
1.5
1.0
0.5
0.0
A (n=15)
B (n=16)
C (n=14)
surface

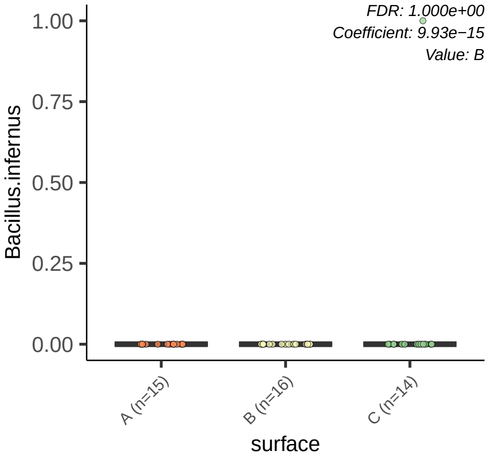

FDR: 1.000e+00
Coefficient: 9.93e−15
Value: B
Bacillus.infernus
1.00
0.75
0.50
0.25
0.00
A (n=15)
B (n=16)
C (n=14)
surface

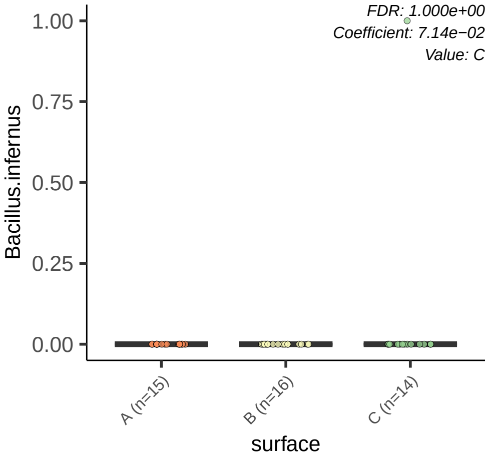

FDR: 1.000e+00
Coefficient: 7.14e−02
Value: C
1.00
0.75
0.50
0.25
0.00
Bacillus.infernus
A (n=15)
B (n=16)
C (n=14)
surface

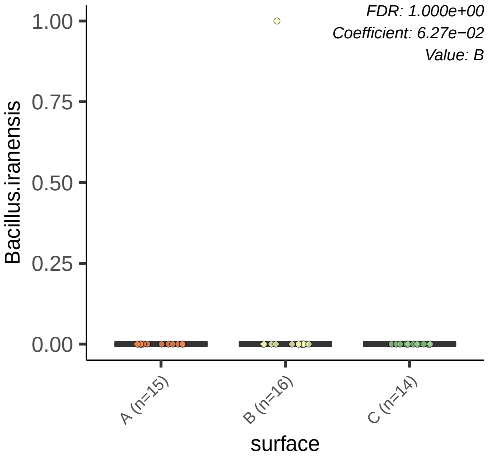
FDR: 1.000e+00
Coefficient: 6.27e−02
Value: B
Bacillus.iranensis
1.00
0.75
0.50
0.25
0.00
A (n=15)
B (n=16)
C (n=14)
surface

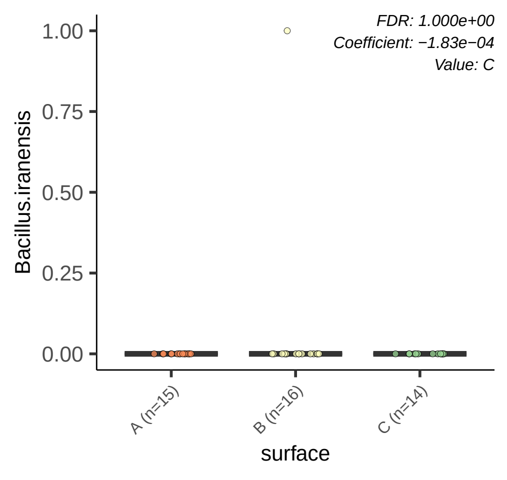

FDR: 1.000e+00
Coefficient: −1.83e−04
Value: C
Bacillus.iranensis
1.00
0.75
0.50
0.25
0.00
A (n=15)
B (n=16)
C (n=14)
surface

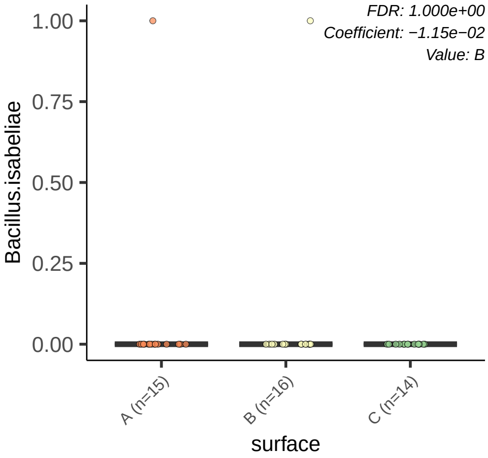

FDR: 1.000e+00
Coefficient: −1.15e−02
Value: B
1.00
0.75
0.50
0.25
0.00
Bacillus.isabeliae
A (n=15)
B (n=16)
C (n=14)
surface

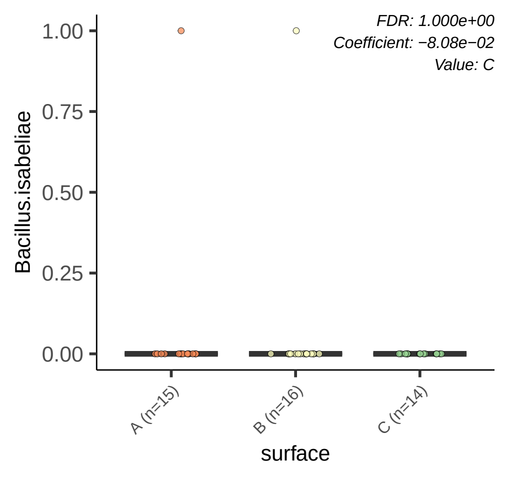
FDR: 1.000e+00
Coefficient: −8.08e−02
Value: C
Bacillus.isabeliae
1.00
0.75
0.50
0.25
0.00
A (n=15)
B (n=16)
C (n=14)
surface

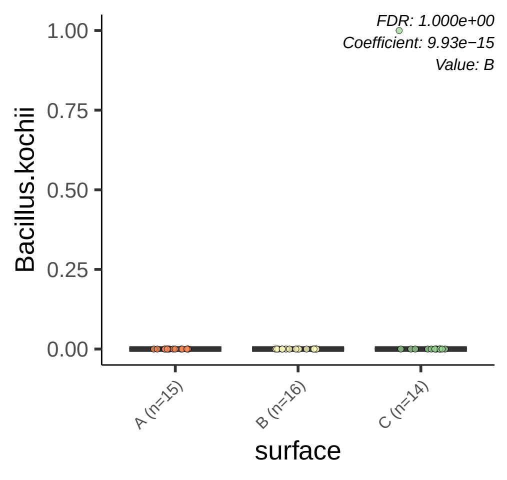
FDR: 1.000e+00
Coefficient: 9.93e−15
Value: B
1.00
0.75
0.50
0.25
0.00
Bacillus.kochii
A (n=15)
B (n=16)
C (n=14)
surface

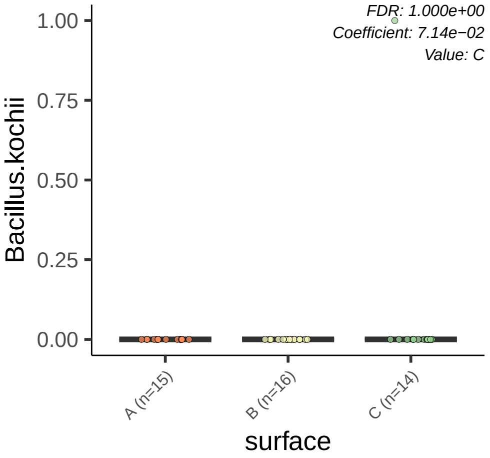
FDR: 1.000e+00
Coefficient: 7.14e−02
Value: C
Bacillus.kochii
1.00
0.75
0.50
0.25
0.00
A (n=15)
B (n=16)
C (n=14)
surface

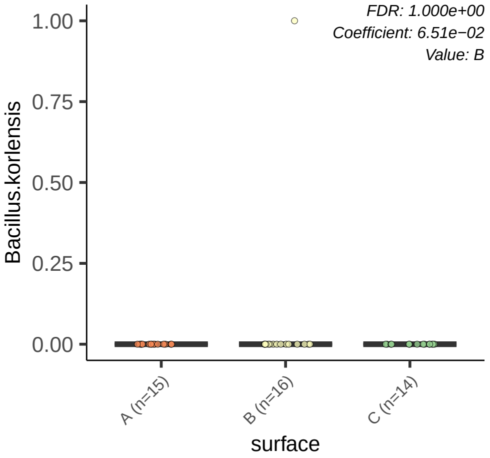

FDR: 1.000e+00
Coefficient: 6.51e−02
Value: B
Bacillus.korlensis
1.00
0.75
0.50
0.25
0.00
A (n=15)
B (n=16)
C (n=14)
surface

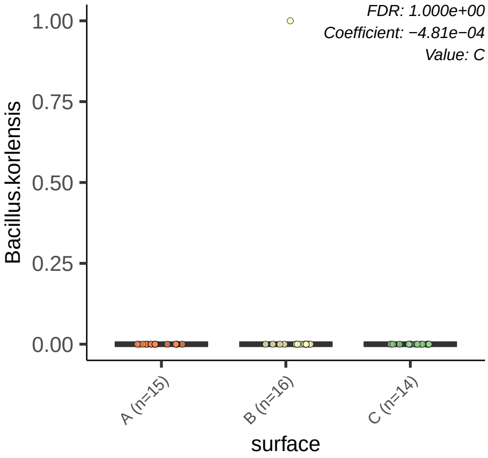

FDR: 1.000e+00
Coefficient: −4.81e−04
Value: C
Bacillus.korlensis
1.00
0.75
0.50
0.25
0.00
A (n=15)
B (n=16)
C (n=14)
surface

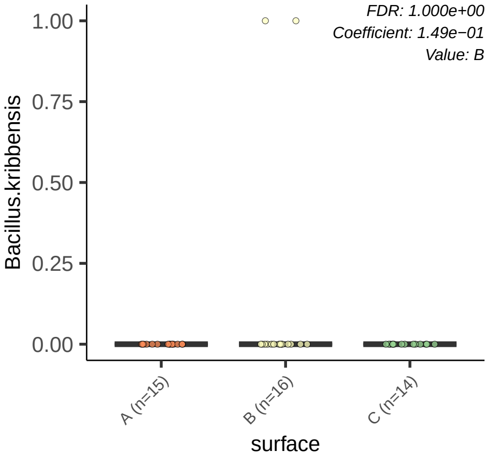

FDR: 1.000e+00
Coefficient: 1.49e−01
Value: B
1.00
0.75
0.50
0.25
0.00
Bacillus.kribbensis
A (n=15)
B (n=16)
C (n=14)
surface

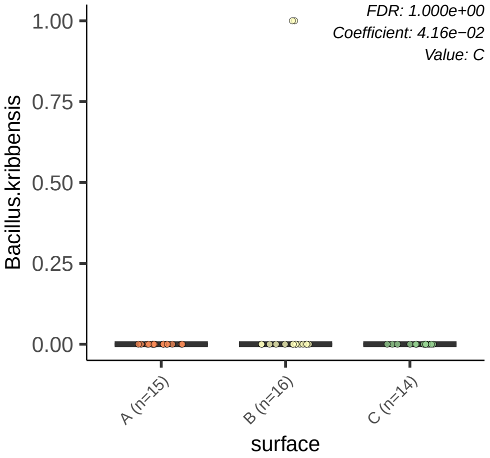

FDR: 1.000e+00
Coefficient: 4.16e−02
Value: C
Bacillus.kribbensis
1.00
0.75
0.50
0.25
0.00
A (n=15)
B (n=16)
C (n=14)
surface

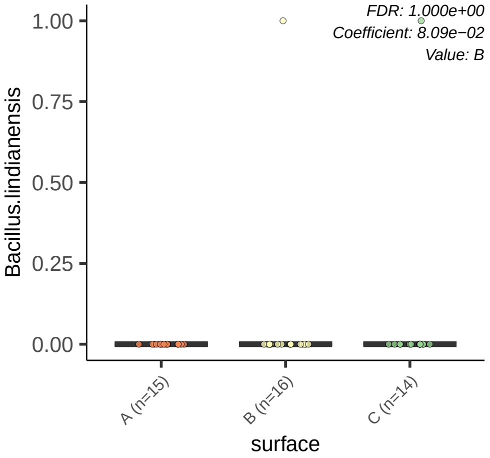
FDR: 1.000e+00
Coefficient: 8.09e−02
Value: B
Bacillus.lindianensis
1.00
0.75
0.50
0.25
0.00
A (n=15)
B (n=16)
C (n=14)
surface

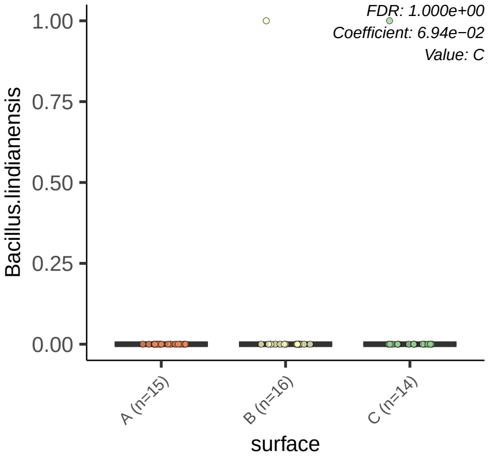
FDR: 1.000e+00
Coefficient: 6.94e−02
Value: C
Bacillus.lindianensis
1.00
0.75
0.50
0.25
0.00
A (n=15)
B (n=16)
C (n=14)
surface

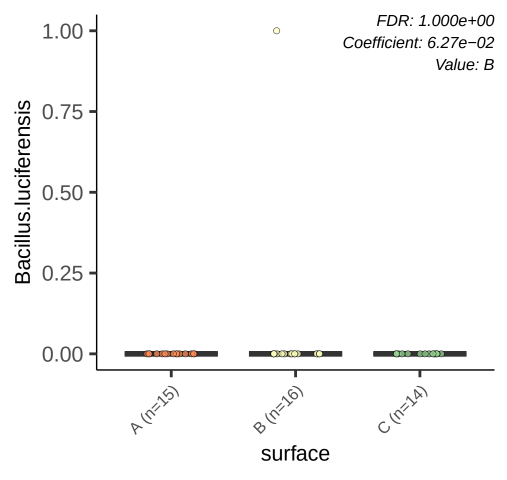

FDR: 1.000e+00
Coefficient: 6.27e−02
Value: B
Bacillus.luciferensis
1.00
0.75
0.50
0.25
0.00
A (n=15)
B (n=16)
C (n=14)
surface

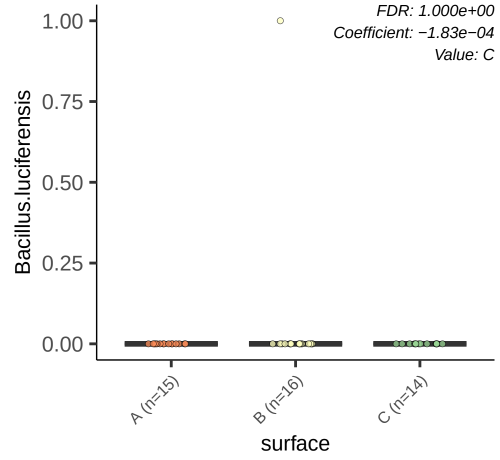

FDR: 1.000e+00
Coefficient: −1.83e−04
Value: C
Bacillus.luciferensis
1.00
0.75
0.50
0.25
0.00
A (n=15)
B (n=16)
C (n=14)
surface

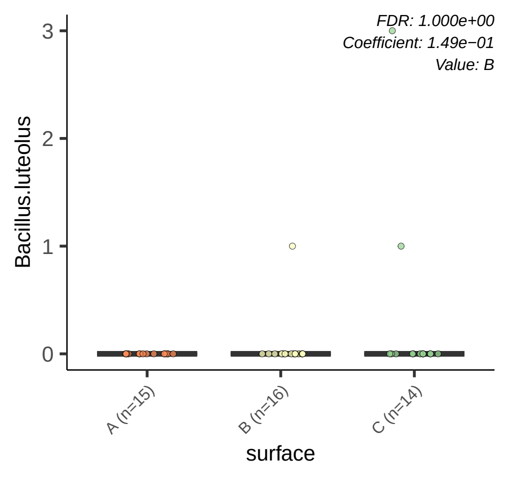

FDR: 1.000e+00
Coefficient: 1.49e−01
Value: B
Bacillus.luteolus
0
1
2
3
A (n=15)
B (n=16)
C (n=14)
surface

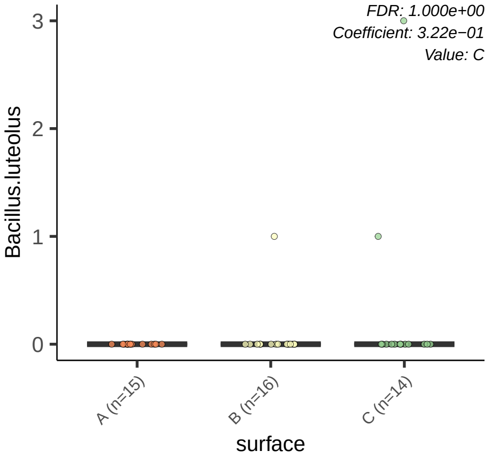

FDR: 1.000e+00
Coefficient: 3.22e−01
Value: C
Bacillus.luteolus
3
2
1
0
A (n=15)
B (n=16)
C (n=14)
surface

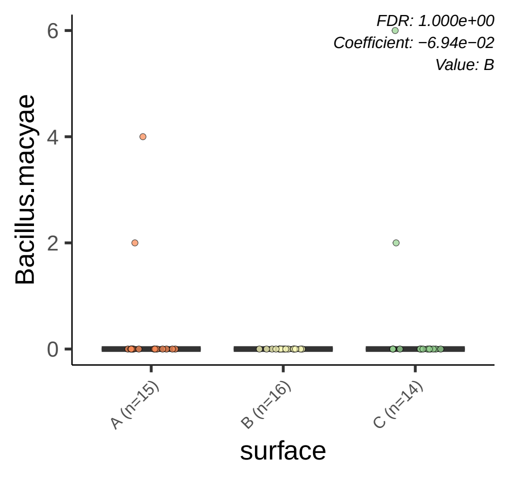

FDR: 1.000e+00
Coefficient: −6.94e−02
Value: B
Bacillus.macyae
0
2
4
6
A (n=15)
B (n=16)
C (n=14)
surface

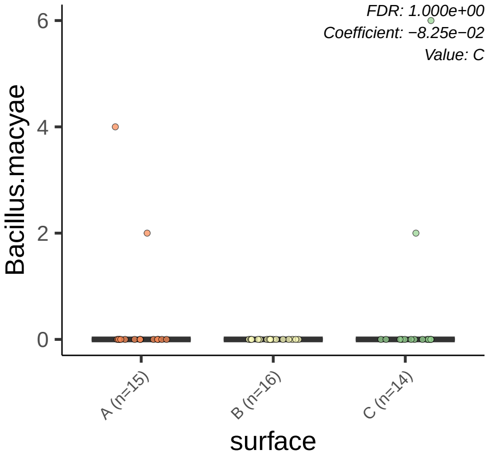
FDR: 1.000e+00
Coefficient: −8.25e−02
Value: C
Bacillus.macyae
6
4
2
0
A (n=15)
B (n=16)
C (n=14)
surface

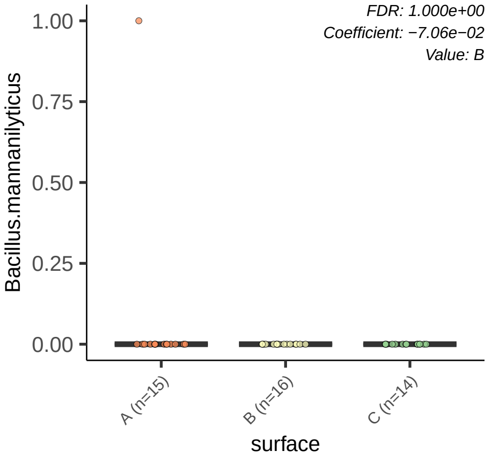
FDR: 1.000e+00
Coefficient: −7.06e−02
Value: B
Bacillus.mannanilyticus
1.00
0.75
0.50
0.25
0.00
A (n=15)
B (n=16)
C (n=14)
surface

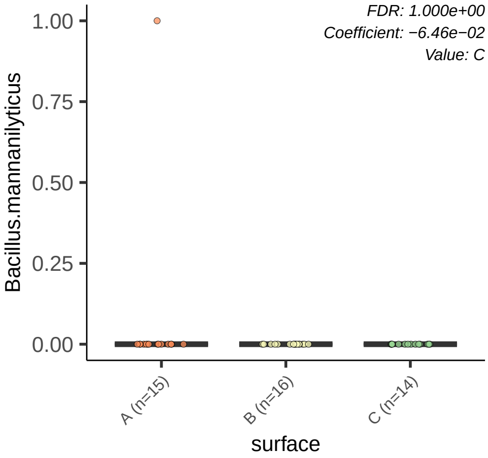

FDR: 1.000e+00
Coefficient: −6.46e−02
Value: C
Bacillus.mannanilyticus
1.00
0.75
0.50
0.25
0.00
A (n=15)
B (n=16)
C (n=14)
surface

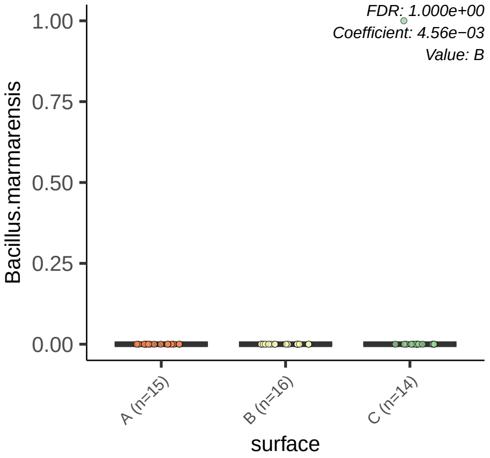
FDR: 1.000e+00
Coefficient: 4.56e−03
Value: B
Bacillus.marmarensis
1.00
0.75
0.50
0.25
0.00
A (n=15)
B (n=16)
C (n=14)
surface

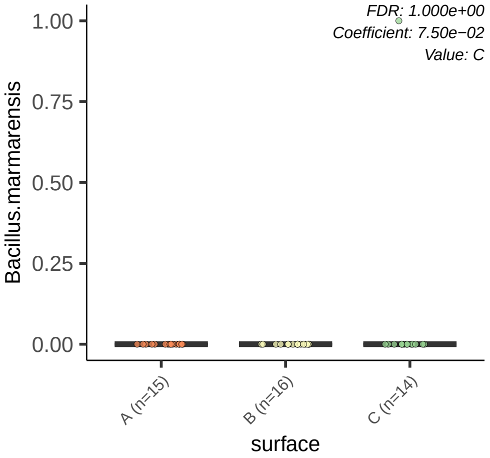

FDR: 1.000e+00
Coefficient: 7.50e−02
Value: C
Bacillus.marmarensis
1.00
0.75
0.50
0.25
0.00
A (n=15)
B (n=16)
C (n=14)
surface

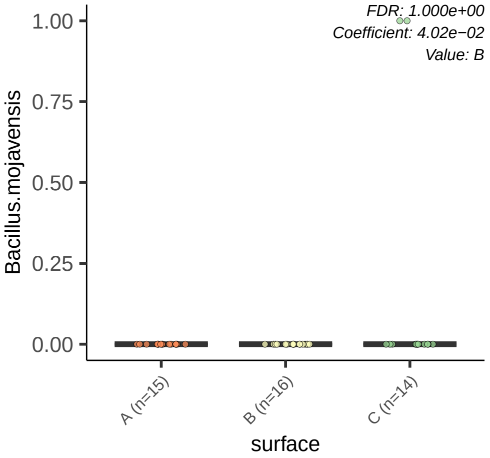
FDR: 1.000e+00
Coefficient: 4.02e−02
Value: B
Bacillus.mojavensis
1.00
0.75
0.50
0.25
0.00
A (n=15)
B (n=16)
C (n=14)
surface

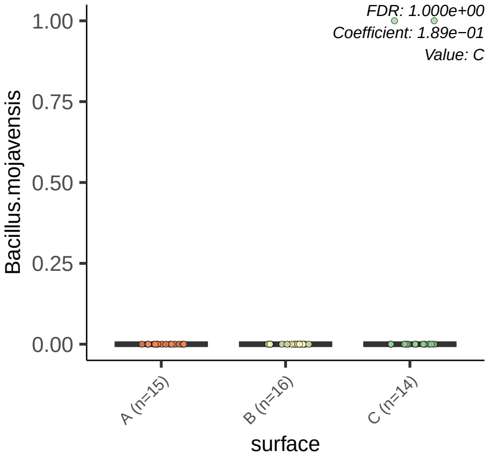
FDR: 1.000e+00
Coefficient: 1.89e−01
Value: C
Bacillus.mojavensis
1.00
0.75
0.50
0.25
0.00
A (n=15)
B (n=16)
C (n=14)
surface

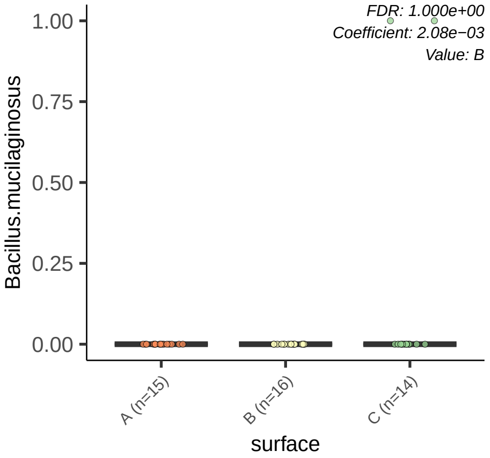
FDR: 1.000e+00
Coefficient: 2.08e−03
Value: B
1.00
0.75
0.50
0.25
0.00
Bacillus.mucilaginosus
A (n=15)
B (n=16)
C (n=14)
surface

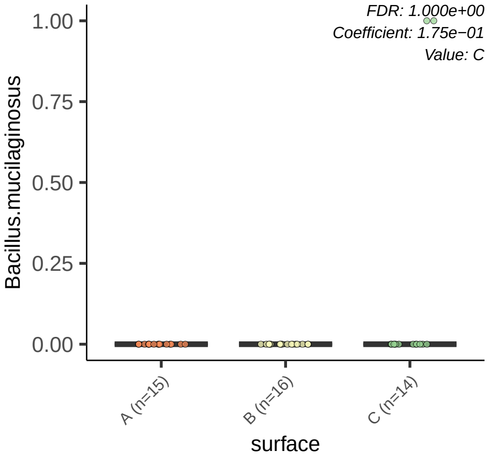

FDR: 1.000e+00
Coefficient: 1.75e−01
Value: C
Bacillus.mucilaginosus
1.00
0.75
0.50
0.25
0.00
A (n=15)
B (n=16)
C (n=14)
surface

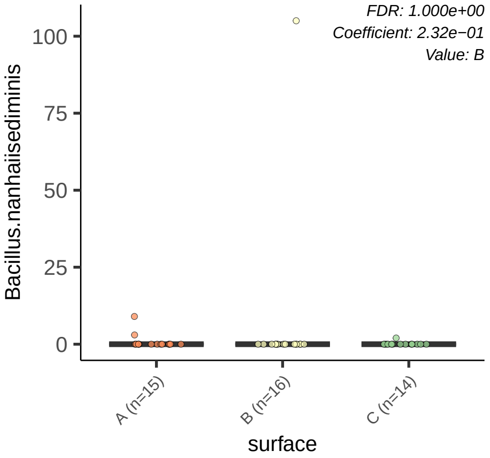

FDR: 1.000e+00
Coefficient: 2.32e−01
Value: B
Bacillus.nanhaisediminis
100
75
50
25
0
A (n=15)
B (n=16)
C (n=14)
surface

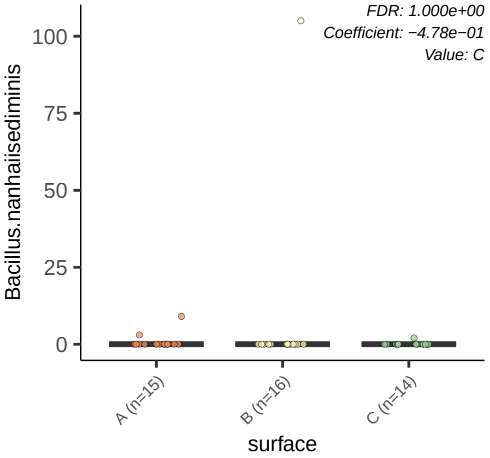
FDR: 1.000e+00
Coefficient: −4.78e−01
Value: C
Bacillus.nanhaisediminis
100
75
50
25
0
A (n=15)
B (n=16)
C (n=14)
surface

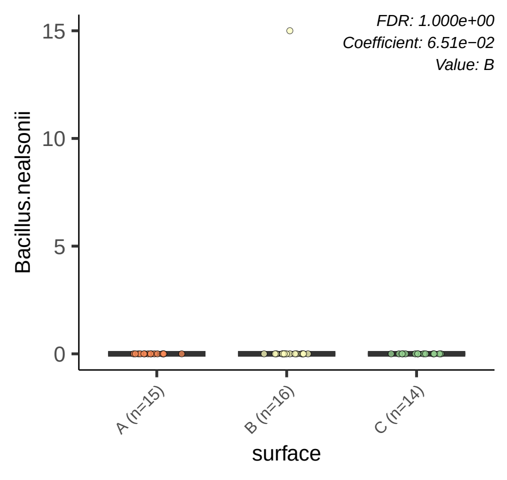
FDR: 1.000e+00
Coefficient: 6.51e−02
Value: B
Bacillus.nealsonii
15
10
5
0
A (n=15)
B (n=16)
C (n=14)
surface

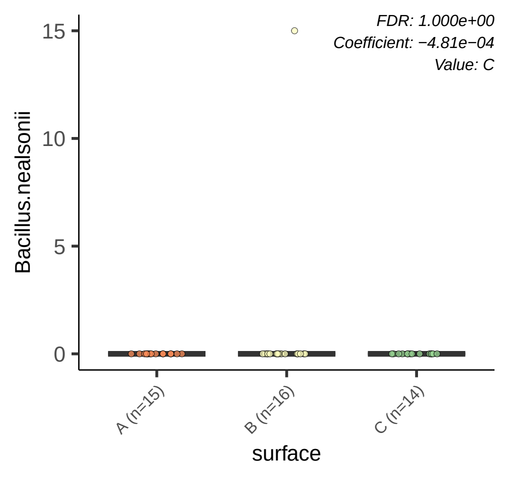

FDR: 1.000e+00
Coefficient: −4.81e−04
Value: C
Bacillus.nealsonii
15
10
5
0
A (n=15)
B (n=16)
C (n=14)
surface

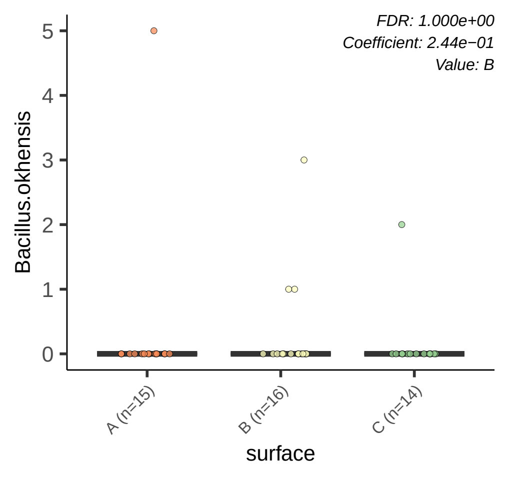

FDR: 1.000e+00
Coefficient: 2.44e−01
Value: B
Bacillus.okhensis
0
1
2
3
4
5
A (n=15)
B (n=16)
C (n=14)
surface

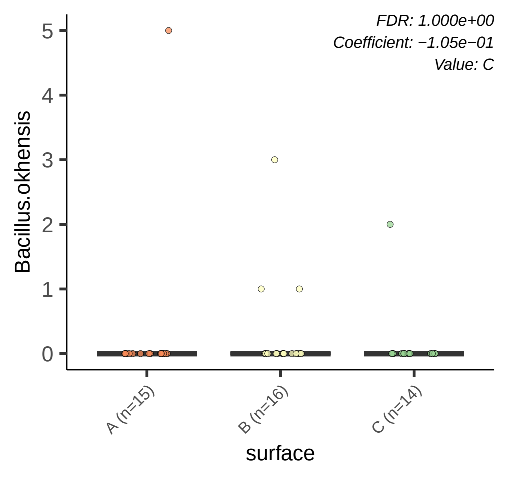
FDR: 1.000e+00
Coefficient: −1.05e−01
Value: C
Bacillus.okhensis
5
4
3
2
1
0
A (n=15)
B (n=16)
C (n=14)
surface

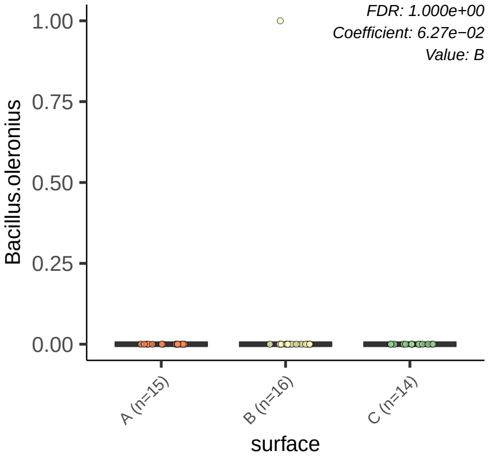
FDR: 1.000e+00
Coefficient: 6.27e−02
Value: B
Bacillus.oleronius
1.00
0.75
0.50
0.25
0.00
A (n=15)
B (n=16)
C (n=14)
surface

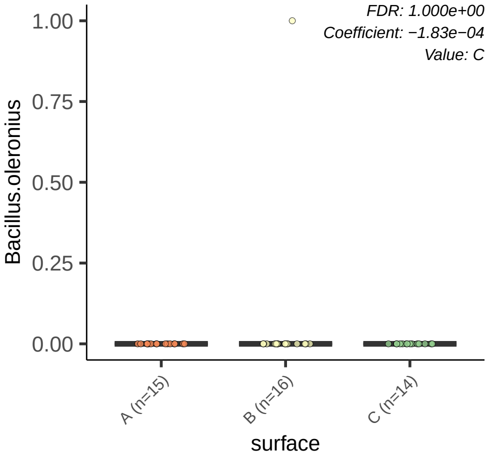
FDR: 1.000e+00
Coefficient: −1.83e−04
Value: C
Bacillus.oleronius
1.00
0.75
0.50
0.25
0.00
A (n=15)
B (n=16)
C (n=14)
surface

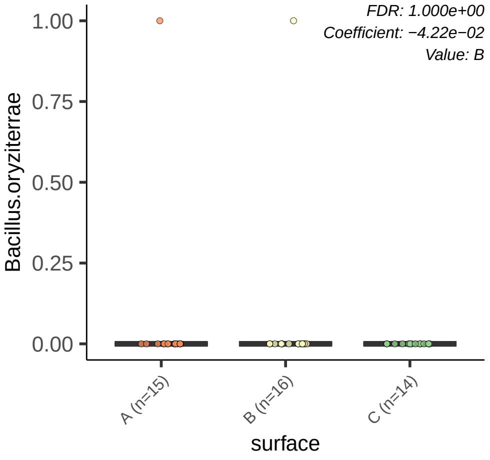

FDR: 1.000e+00
Coefficient: −4.22e−02
Value: B
Bacillus.oryziterrae
1.00
0.75
0.50
0.25
0.00
A (n=15)
B (n=16)
C (n=14)
surface

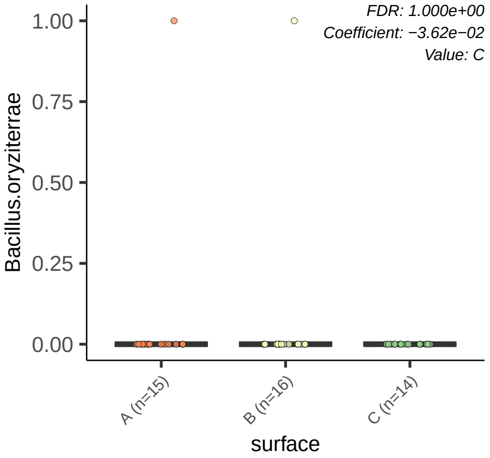
FDR: 1.000e+00
Coefficient: −3.62e−02
Value: C
Bacillus.oryziterrae
1.00
0.75
0.50
0.25
0.00
A (n=15)
B (n=16)
C (n=14)
surface

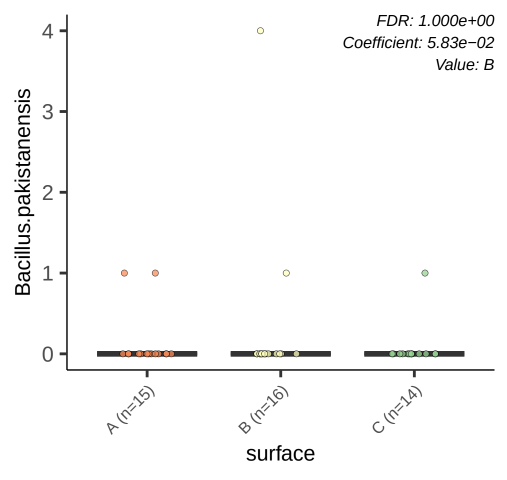
FDR: 1.000e+00
Coefficient: 5.83e−02
Value: B
Bacillus.pakistanensis
0
1
2
3
4
A (n=15)
B (n=16)
C (n=14)
surface

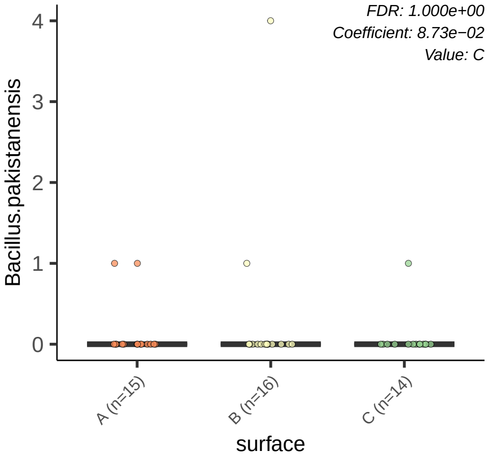
FDR: 1.000e+00
Coefficient: 8.73e−02
Value: C
Bacillus.pakistanensis
0
1
2
3
4
A (n=15)
B (n=16)
C (n=14)
surface

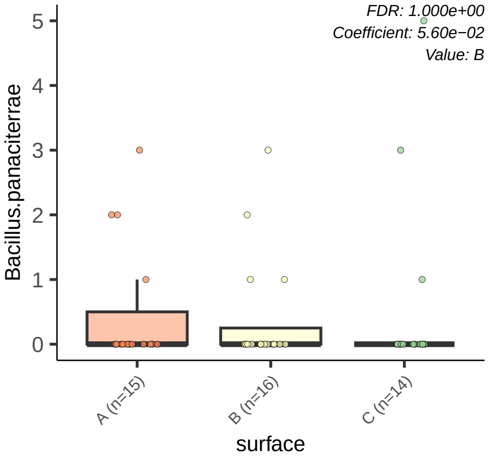
FDR: 1.000e+00
Coefficient: 5.60e−02
Value: B
Bacillus.panaciterrae
0
1
2
3
4
5
A (n=15)
B (n=16)
C (n=14)
surface

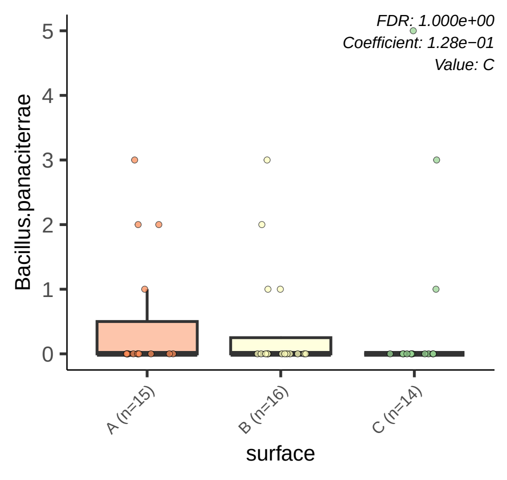
FDR: 1.000e+00
Coefficient: 1.28e−01
Value: C
Bacillus.panaciterrae
0
1
2
3
4
5
A (n=15)
B (n=16)
C (n=14)
surface

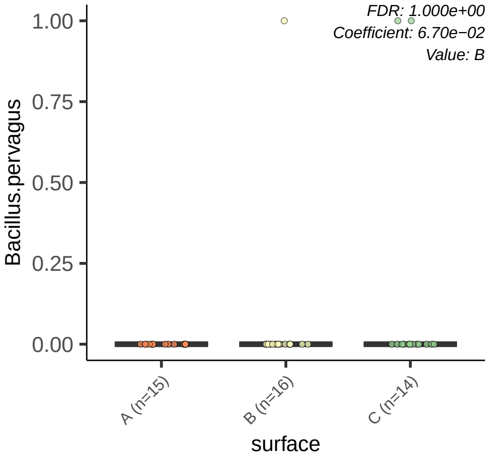

FDR: 1.000e+00
Coefficient: 6.70e−02
Value: B
Bacillus.pervagus
1.00
0.75
0.50
0.25
0.00
A (n=15)
B (n=16)
C (n=14)
surface

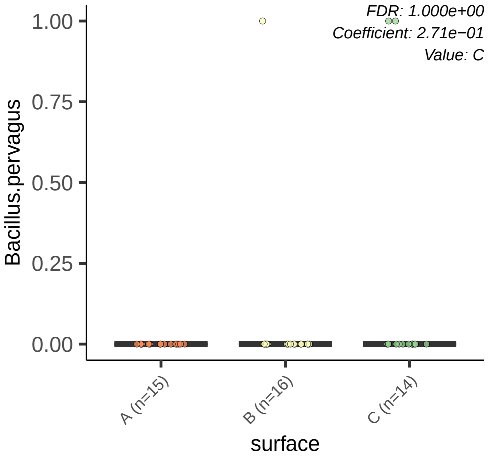

FDR: 1.000e+00
Coefficient: 2.71e−01
Value: C
Bacillus.pervagus
1.00
0.75
0.50
0.25
0.00
A (n=15)
B (n=16)
C (n=14)
surface

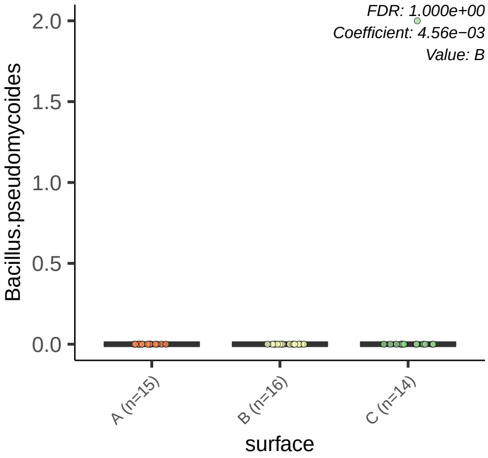
FDR: 1.000e+00
Coefficient: 4.56e−03
Value: B
Bacillus.pseudomycoides
2.0
1.5
1.0
0.5
0.0
A (n=15)
B (n=16)
C (n=14)
surface

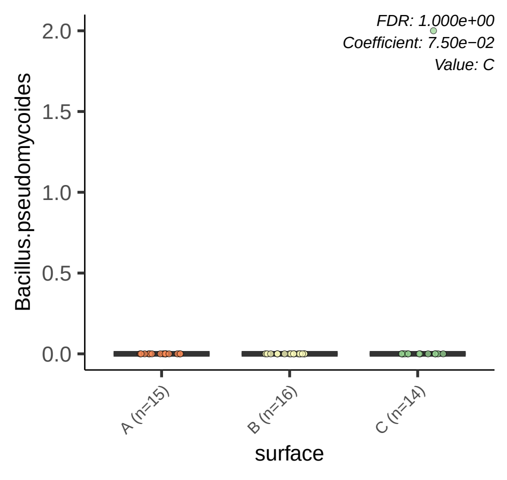

FDR: 1.000e+00
Coefficient: 7.50e−02
Value: C
Bacillus.pseudomycoides
2.0
1.5
1.0
0.5
0.0
A (n=15)
B (n=16)
C (n=14)
surface

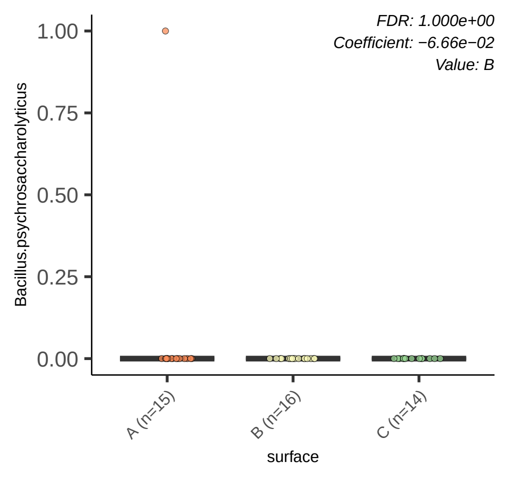
FDR: 1.000e+00
Coefficient: −6.66e−02
Value: B
Bacillus.psychrosaccharolyticus
1.00
0.75
0.50
0.25
0.00
A (n=15)
B (n=16)
C (n=14)
surface

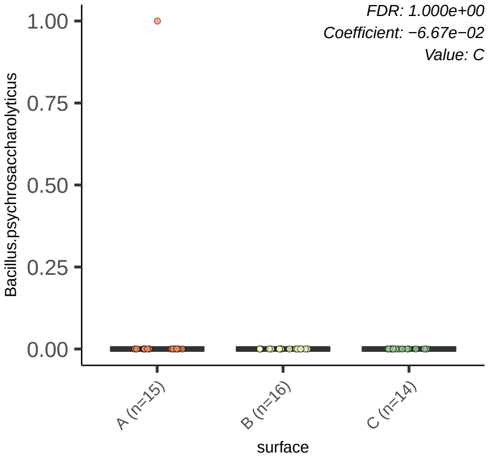
FDR: 1.000e+00
Coefficient: −6.67e−02
Value: C
1.00
0.75
0.50
0.25
0.00
Bacillus.psychrosaccharolyticus
A (n=15)
B (n=16)
C (n=14)
surface

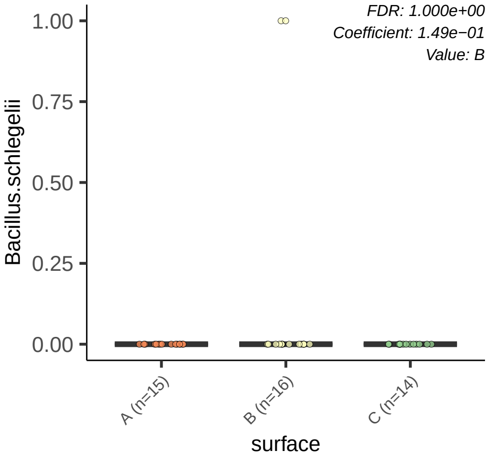
FDR: 1.000e+00
Coefficient: 1.49e−01
Value: B
Bacillus.schlegelii
1.00
0.75
0.50
0.25
0.00
A (n=15)
B (n=16)
C (n=14)
surface

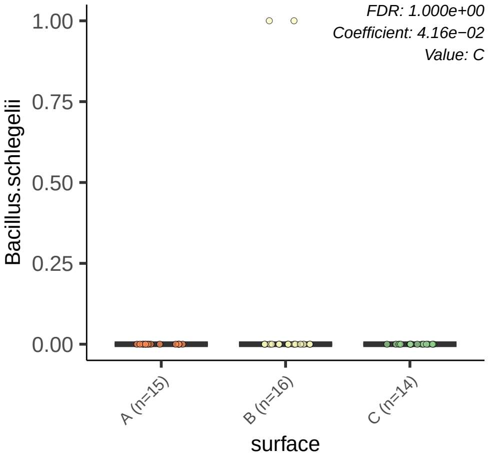
FDR: 1.000e+00
Coefficient: 4.16e−02
Value: C
Bacillus.schlegelii
1.00
0.75
0.50
0.25
0.00
A (n=15)
B (n=16)
C (n=14)
surface

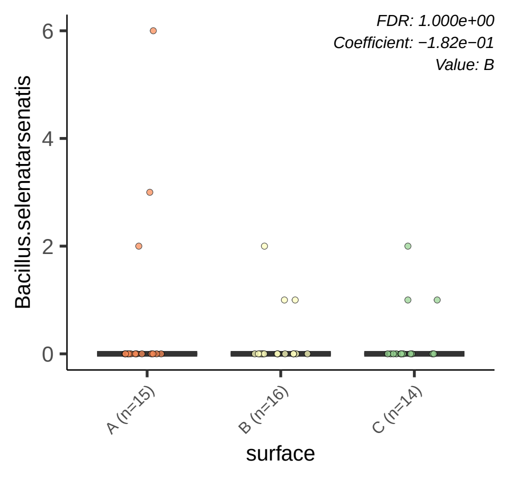
FDR: 1.000e+00
Coefficient: −1.82e−01
Value: B
Bacillus.selenatarsenatis
0
2
4
6
A (n=15)
B (n=16)
C (n=14)
surface

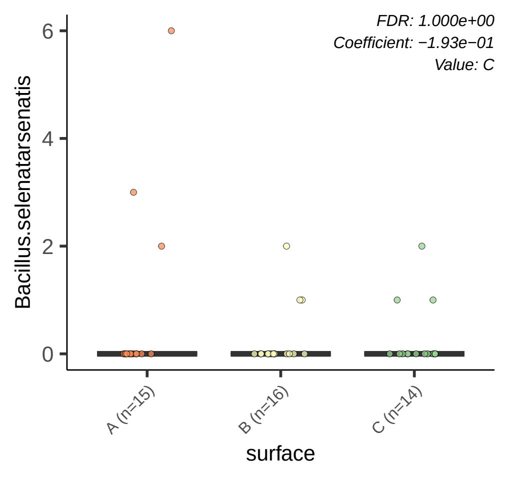

FDR: 1.000e+00
Coefficient: −1.93e−01
Value: C
Bacillus.selenatarsenatis
0
2
4
6
A (n=15)
B (n=16)
C (n=14)
surface

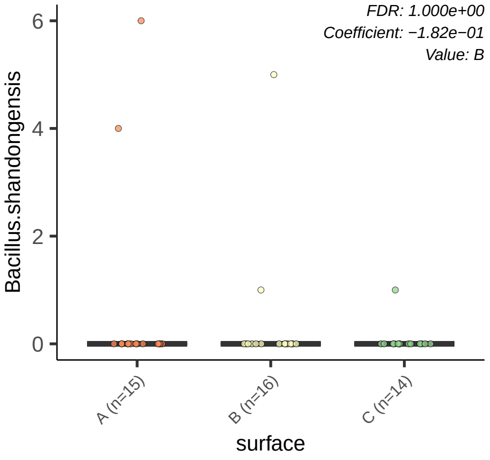
FDR: 1.000e+00
Coefficient: −1.82e−01
Value: B
Bacillus.shandongensis
0
2
4
6
A (n=15)
B (n=16)
C (n=14)
surface

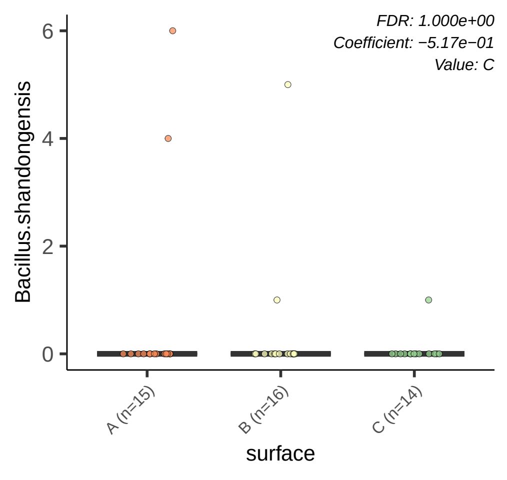
FDR: 1.000e+00
Coefficient: −5.17e−01
Value: C
Bacillus.shandongensis
6
4
2
0
A (n=15)
B (n=16)
C (n=14)
surface

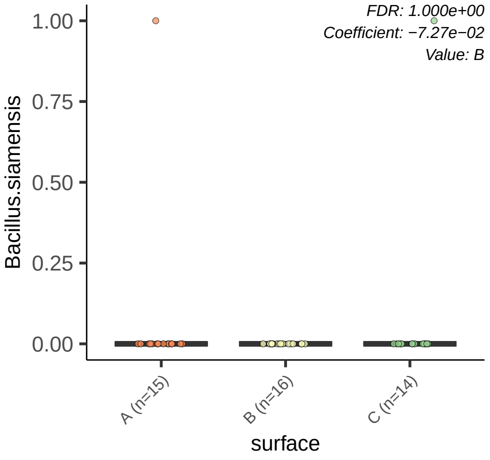
FDR: 1.000e+00
Coefficient: −7.27e−02
Value: B
Bacillus.siamensis
1.00
0.75
0.50
0.25
0.00
A (n=15)
B (n=16)
C (n=14)
surface

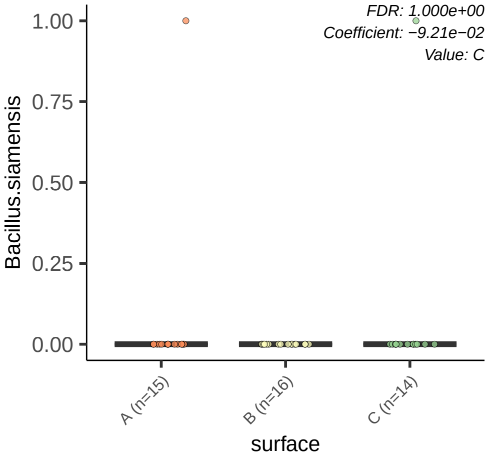

FDR: 1.000e+00
Coefficient: −9.21e−02
Value: C
1.00
0.75
0.50
0.25
0.00
Bacillus.siamensis
A (n=15)
B (n=16)
C (n=14)
surface

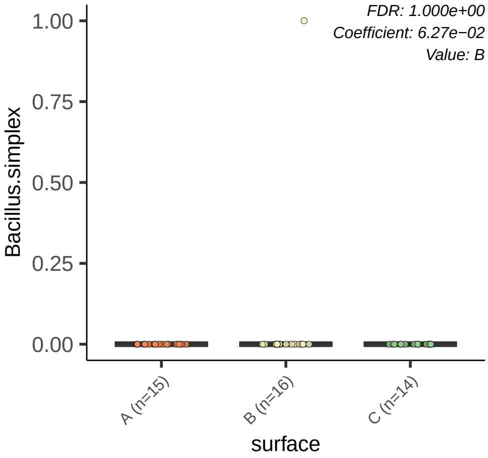
FDR: 1.000e+00
Coefficient: 6.27e−02
Value: B
1.00
0.75
0.50
0.25
0.00
Bacillus.simplex
A (n=15)
B (n=16)
C (n=14)
surface

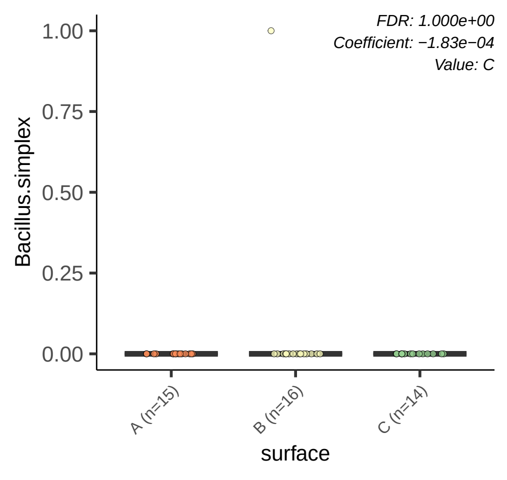
FDR: 1.000e+00
Coefficient: −1.83e−04
Value: C
Bacillus.simplex
1.00
0.75
0.50
0.25
0.00
A (n=15)
B (n=16)
C (n=14)
surface

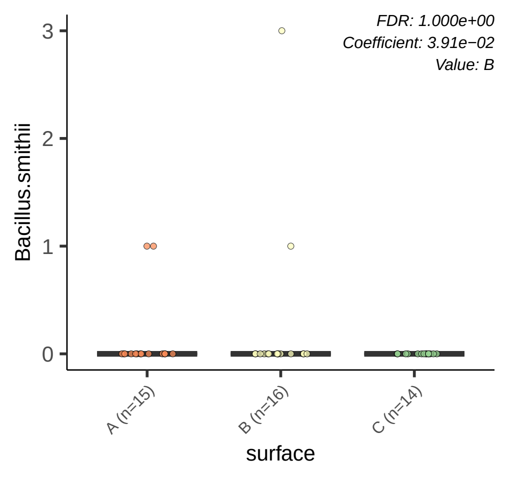
FDR: 1.000e+00
Coefficient: 3.91e−02
Value: B
Bacillus.smithii
0
1
2
3
A (n=15)
B (n=16)
C (n=14)
surface

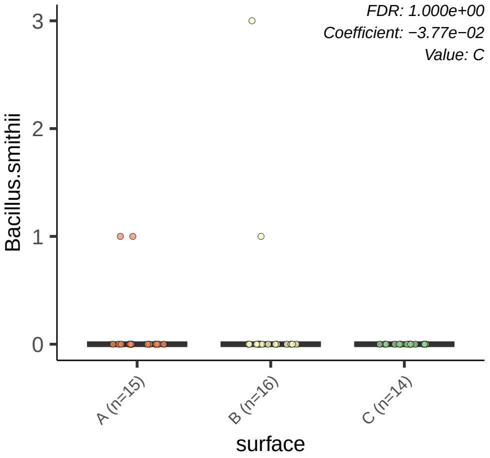
FDR: 1.000e+00
Coefficient: −3.77e−02
Value: C
Bacillus.smithii
3
2
1
0
A (n=15)
B (n=16)
C (n=14)
surface

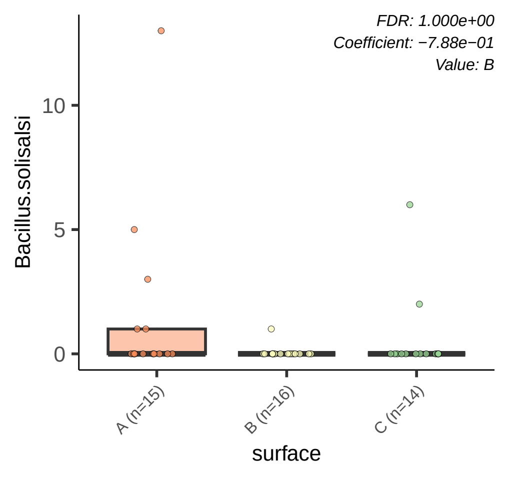

FDR: 1.000e+00
Coefficient: −7.88e−01
Value: B
Bacillus.solisalsi
10
5
0
A (n=15)
B (n=16)
C (n=14)
surface

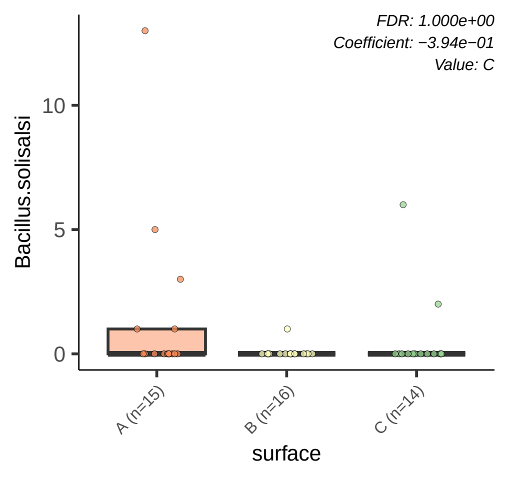

FDR: 1.000e+00
Coefficient: −3.94e−01
Value: C
Bacillus.solisalsi
10
5
0
A (n=15)
B (n=16)
C (n=14)
surface

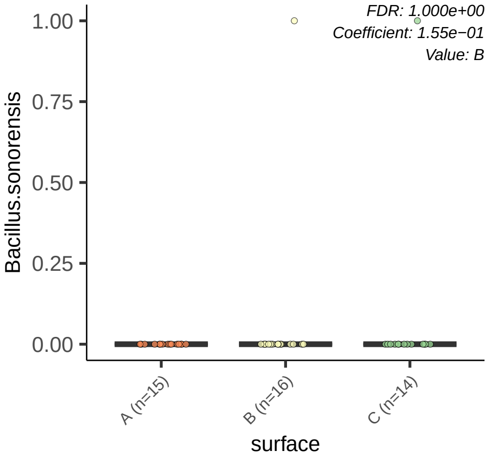

FDR: 1.000e+00
Coefficient: 1.55e−01
Value: B
Bacillus.sonorensis
1.00
0.75
0.50
0.25
0.00
A (n=15)
B (n=16)
C (n=14)
surface

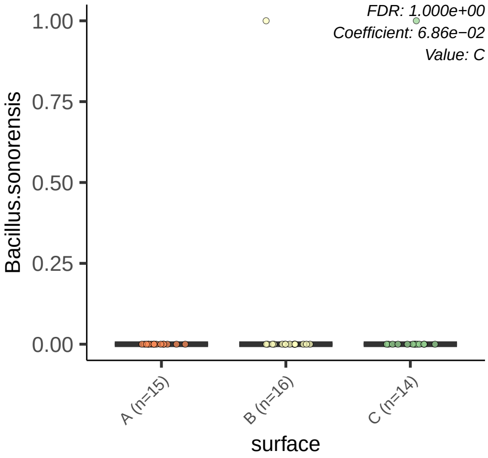

FDR: 1.000e+00
Coefficient: 6.86e−02
Value: C
Bacillus.sonorensis
1.00
0.75
0.50
0.25
0.00
A (n=15)
B (n=16)
C (n=14)
surface

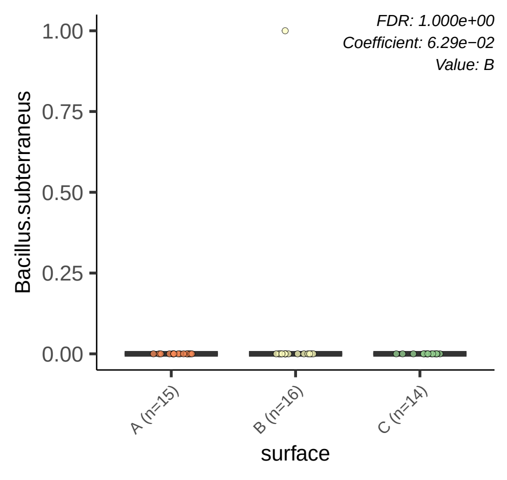
FDR: 1.000e+00
Coefficient: 6.29e−02
Value: B
Bacillus.subterraneus
1.00
0.75
0.50
0.25
0.00
A (n=15)
B (n=16)
C (n=14)
surface

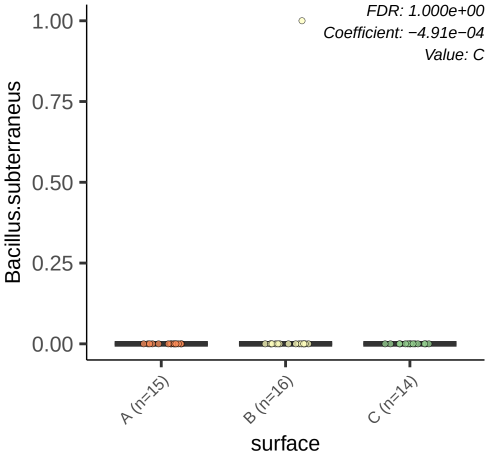
FDR: 1.000e+00
Coefficient: −4.91e−04
Value: C
Bacillus.subterraneus
1.00
0.75
0.50
0.25
0.00
A (n=15)
B (n=16)
C (n=14)
surface

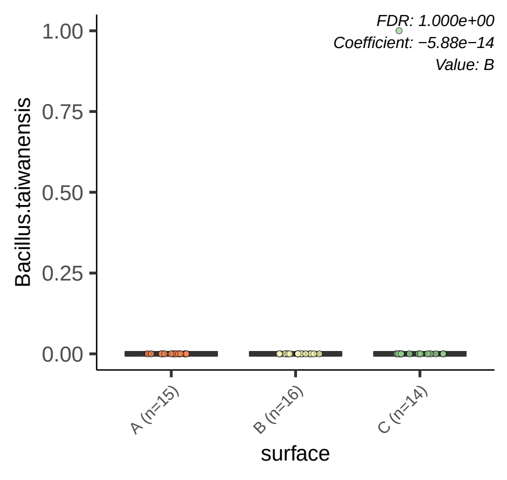
FDR: 1.000e+00
Coefficient: −5.88e−14
Value: B
Bacillus.taiwanensis
1.00
0.75
0.50
0.25
0.00
A (n=15)
B (n=16)
C (n=14)
surface

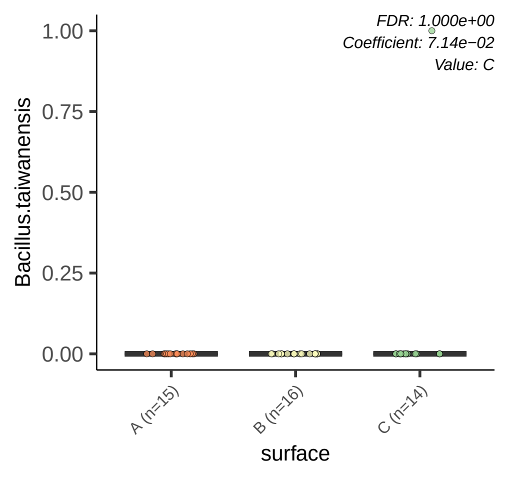
FDR: 1.000e+00
Coefficient: 7.14e−02
Value: C
Bacillus.taiwanensis
1.00
0.75
0.50
0.25
0.00
A (n=15)
B (n=16)
C (n=14)
surface

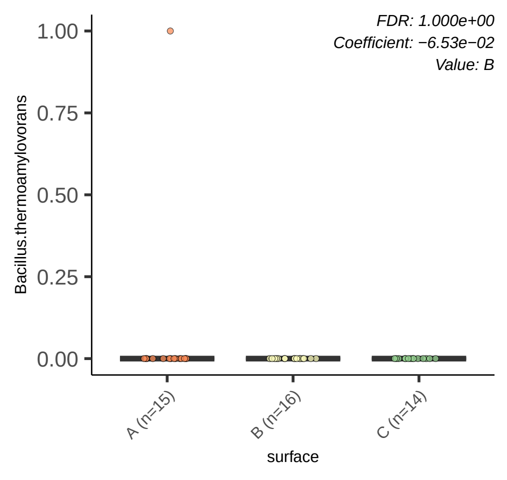
FDR: 1.000e+00
Coefficient: −6.53e−02
Value: B
Bacillus.thermoamylovorans
1.00
0.75
0.50
0.25
0.00
A (n=15)
B (n=16)
C (n=14)
surface

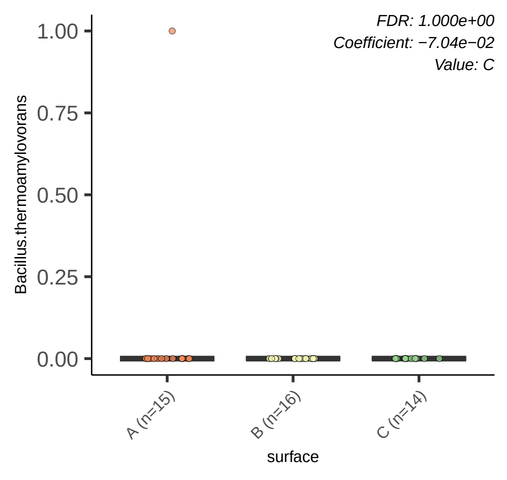
FDR: 1.000e+00
Coefficient: −7.04e−02
Value: C
Bacillus.thermoamylovorans
1.00
0.75
0.50
0.25
0.00
A (n=15)
B (n=16)
C (n=14)
surface

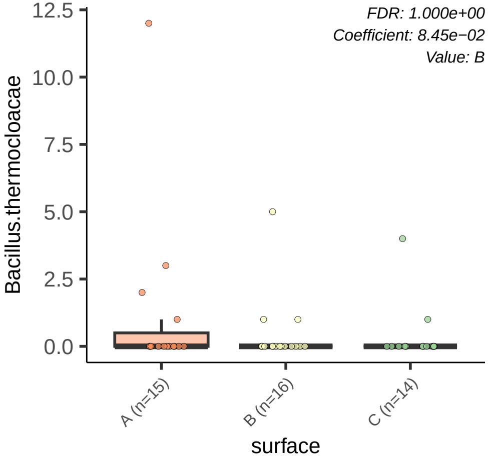
FDR: 1.000e+00
Coefficient: 8.45e−02
Value: B
12.5
10.0
7.5
5.0
2.5
0.0
Bacillus.thermocloacae
A (n=15)
B (n=16)
C (n=14)
surface

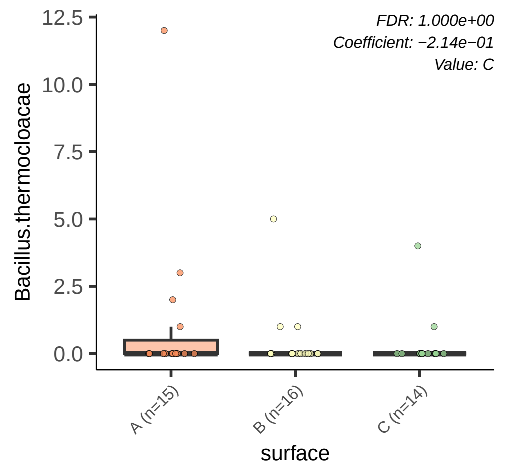
FDR: 1.000e+00
Coefficient: −2.14e−01
Value: C
Bacillus.thermocloacae
12.5
10.0
7.5
5.0
2.5
0.0
A (n=15)
B (n=16)
C (n=14)
surface

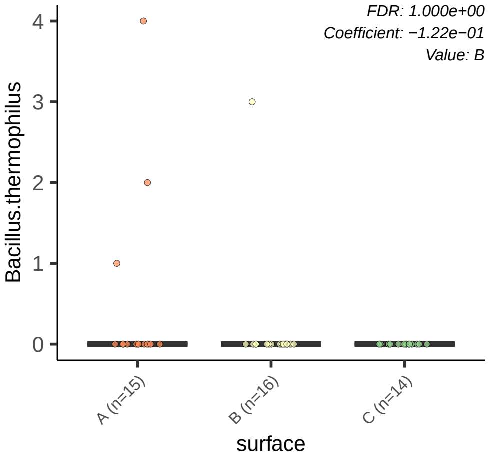

FDR: 1.000e+00
Coefficient: −1.22e−01
Value: B
Bacillus.thermophilus
4
3
2
1
0
A (n=15)
B (n=16)
C (n=14)
surface

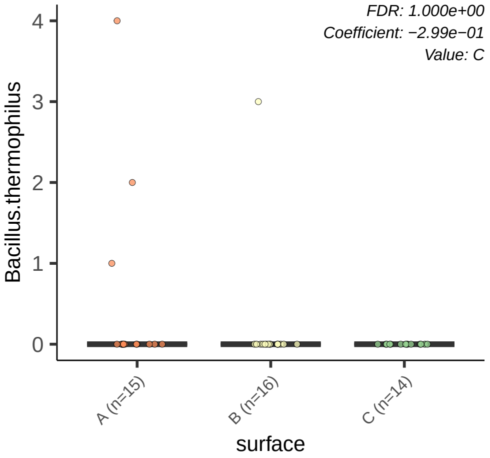

FDR: 1.000e+00
Coefficient: −2.99e−01
Value: C
Bacillus.thermophilus
4
3
2
1
0
A (n=15)
B (n=16)
C (n=14)
surface

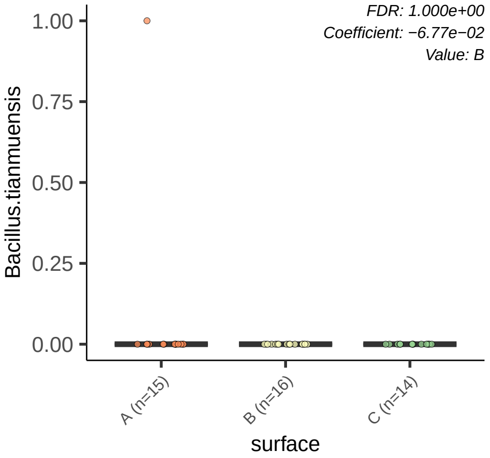
FDR: 1.000e+00
Coefficient: −6.77e−02
Value: B
Bacillus.tianmuensis
1.00
0.75
0.50
0.25
0.00
A (n=15)
B (n=16)
C (n=14)
surface

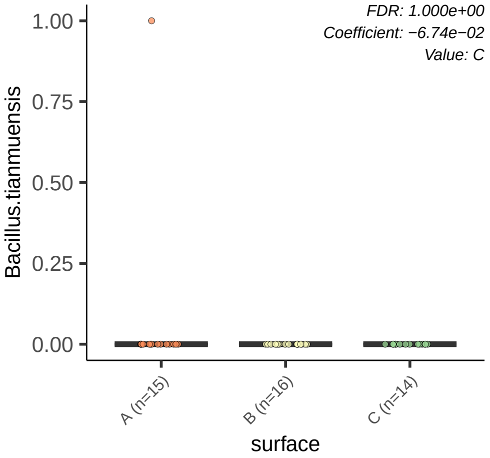
FDR: 1.000e+00
Coefficient: −6.74e−02
Value: C
Bacillus.tianmuensis
1.00
0.75
0.50
0.25
0.00
A (n=15)
B (n=16)
C (n=14)
surface

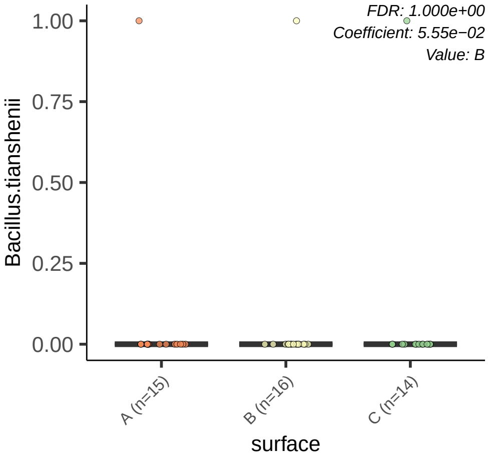
FDR: 1.000e+00
Coefficient: 5.55e−02
Value: B
Bacillus.tianshenii
1.00
0.75
0.50
0.25
0.00
A (n=15)
B (n=16)
C (n=14)
surface

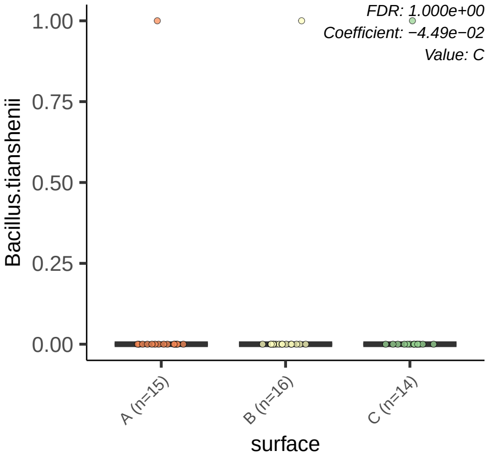
FDR: 1.000e+00
Coefficient: −4.49e−02
Value: C
Bacillus.tianshenii
1.00
0.75
0.50
0.25
0.00
A (n=15)
B (n=16)
C (n=14)
surface

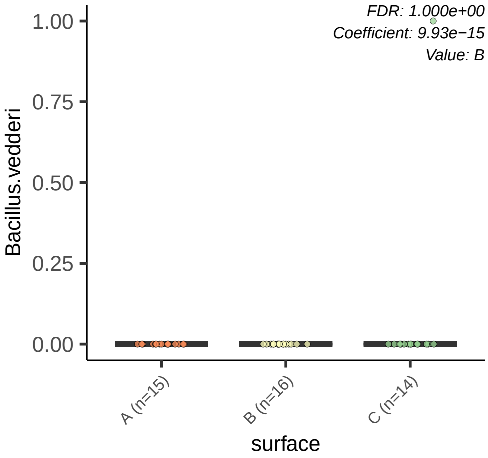
FDR: 1.000e+00
Coefficient: 9.93e−15
Value: B
Bacillus.vedderi
1.00
0.75
0.50
0.25
0.00
A (n=15)
B (n=16)
C (n=14)
surface

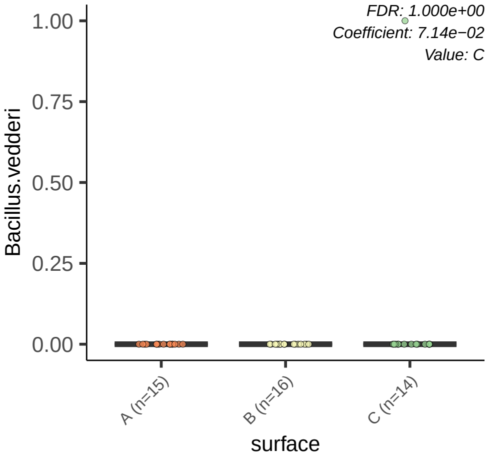

FDR: 1.000e+00
Coefficient: 7.14e−02
Value: C
1.00
0.75
0.50
0.25
0.00
Bacillus.vedderi
A (n=15)
B (n=16)
C (n=14)
surface

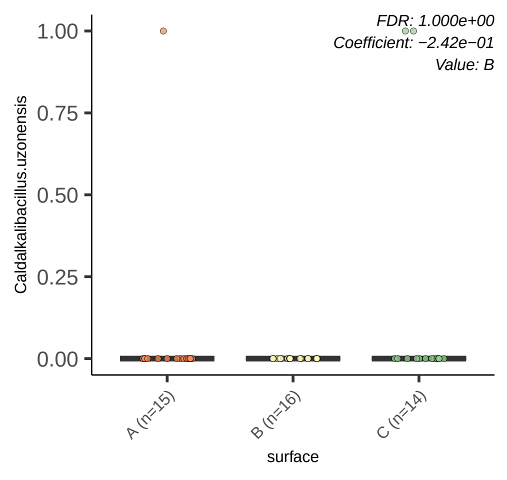
FDR: 1.000e+00
Coefficient: −2.42e−01
Value: B
1.00
0.75
0.50
0.25
0.00
Caldalkalibacillus.uzonensis
A (n=15)
B (n=16)
C (n=14)
surface

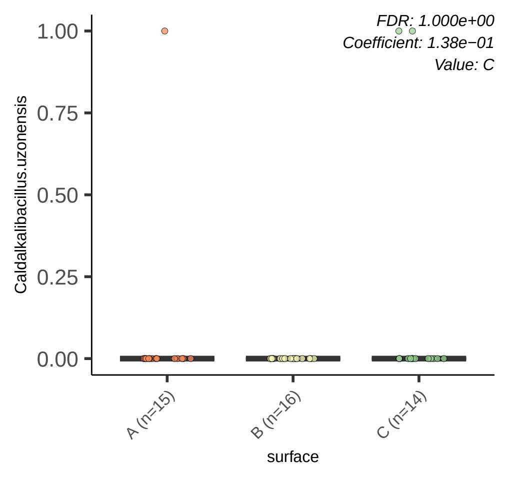
FDR: 1.000e+00
Coefficient: 1.38e−01
Value: C
Caldalkalibacillus.uzonensis
1.00
0.75
0.50
0.25
0.00
A (n=15)
B (n=16)
C (n=14)
surface

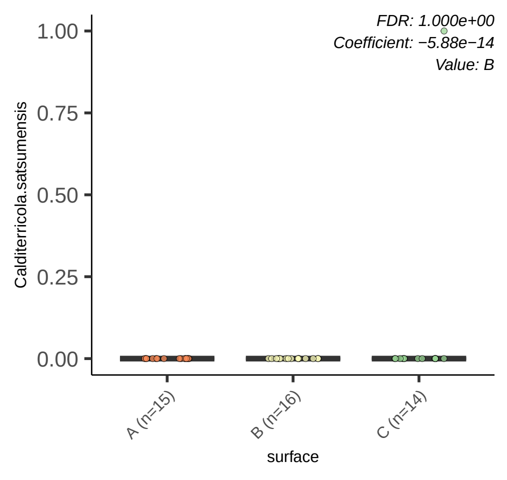
FDR: 1.000e+00
Coefficient: −5.88e−14
Value: B
1.00
0.75
0.50
0.25
0.00
Calditerricola.satsumensis
A (n=15)
B (n=16)
C (n=14)
surface

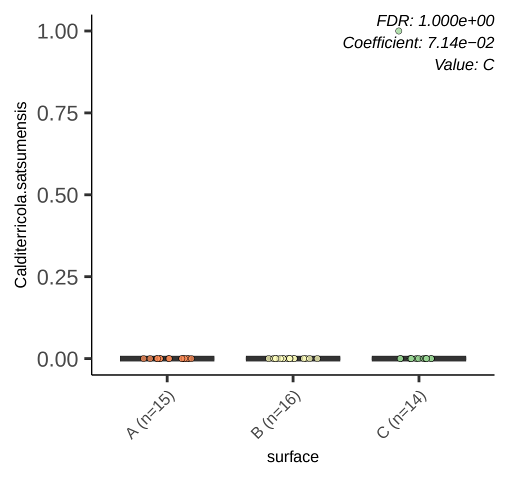
FDR: 1.000e+00
Coefficient: 7.14e−02
Value: C
Calditerricola.satsumensis
1.00
0.75
0.50
0.25
0.00
A (n=15)
B (n=16)
C (n=14)
surface

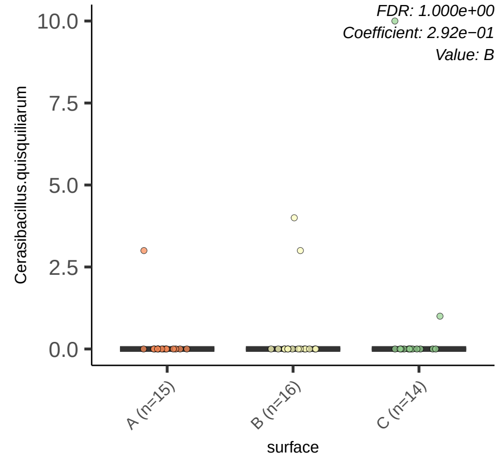
FDR: 1.000e+00
Coefficient: 2.92e−01
Value: B
10.0
7.5
5.0
2.5
0.0
Cerasibacillus.quisquiliarum
A (n=15)
B (n=16)
C (n=14)
surface

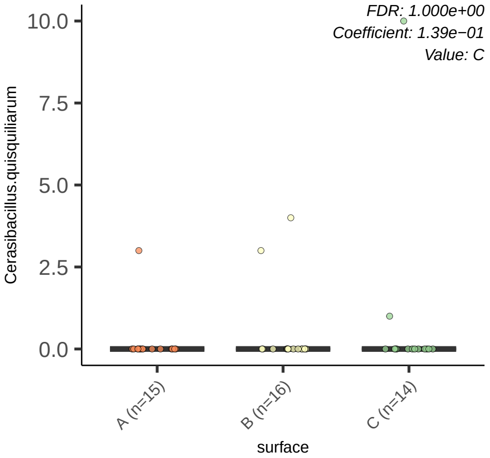
FDR: 1.000e+00
Coefficient: 1.39e−01
Value: C
10.0
7.5
5.0
2.5
0.0
Cerasibacillus.quisquiliarum
A (n=15)
B (n=16)
C (n=14)
surface

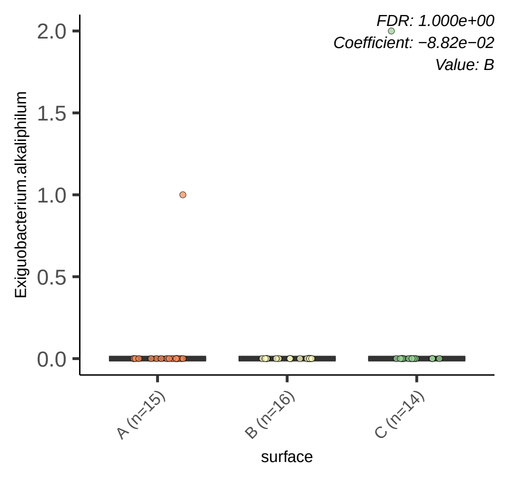
FDR: 1.000e+00
Coefficient: −8.82e−02
Value: B
Exiguobacterium.alkaliphilum
2.0
1.5
1.0
0.5
0.0
A (n=15)
B (n=16)
C (n=14)
surface

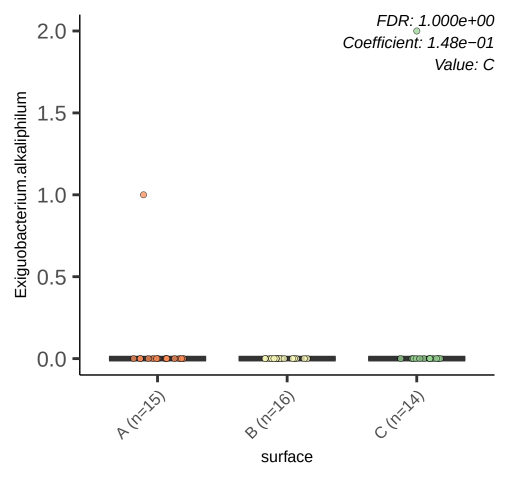

FDR: 1.000e+00
Coefficient: 1.48e−01
Value: C
Exiguobacterium.alkaliphilum
2.0
1.5
1.0
0.5
0.0
A (n=15)
B (n=16)
C (n=14)
surface

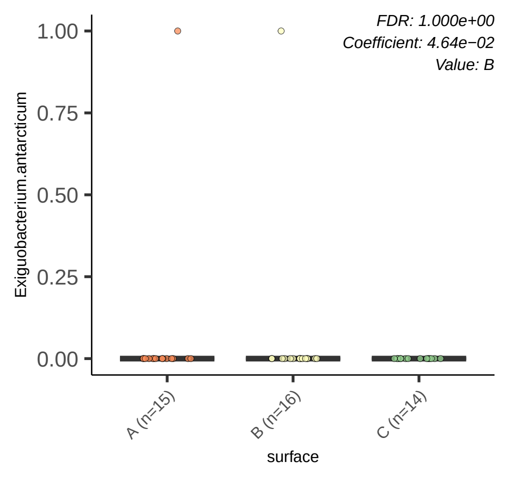

FDR: 1.000e+00
Coefficient: 4.64e−02
Value: B
Exiguobacterium.antarcticum
1.00
0.75
0.50
0.25
0.00
A (n=15)
B (n=16)
C (n=14)
surface

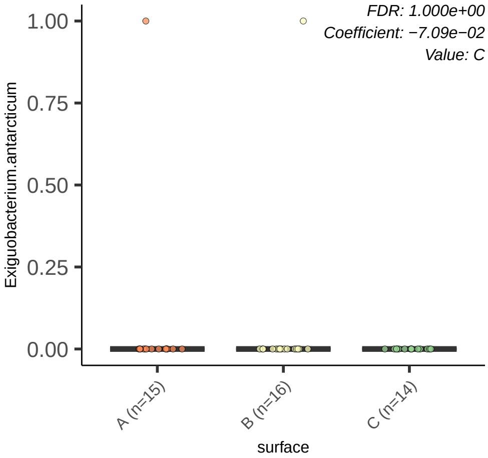

FDR: 1.000e+00
Coefficient: −7.09e−02
Value: C
1.00
0.75
0.50
0.25
0.00
Exiguobacterium.antarcticum
A (n=15)
B (n=16)
C (n=14)
surface

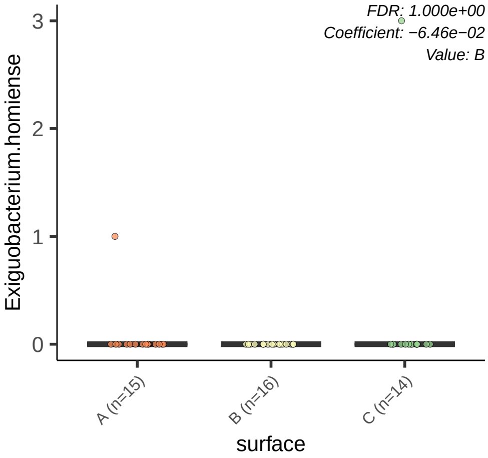

FDR: 1.000e+00
Coefficient: −6.46e−02
Value: B
Exiguobacterium.homiense
3
2
1
0
A (n=15)
B (n=16)
C (n=14)
surface

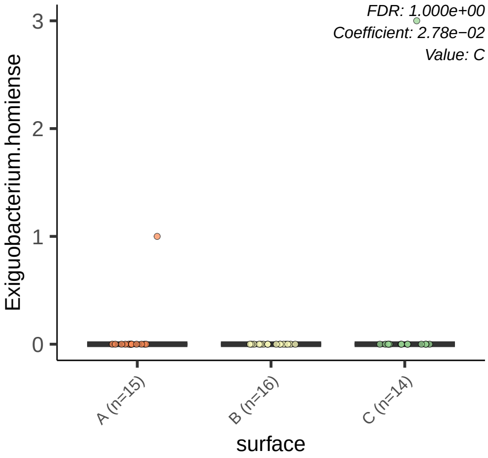

FDR: 1.000e+00
Coefficient: 2.78e−02
Value: C
Exiguobacterium.homiense
3
2
1
0
A (n=15)
B (n=16)
C (n=14)
surface

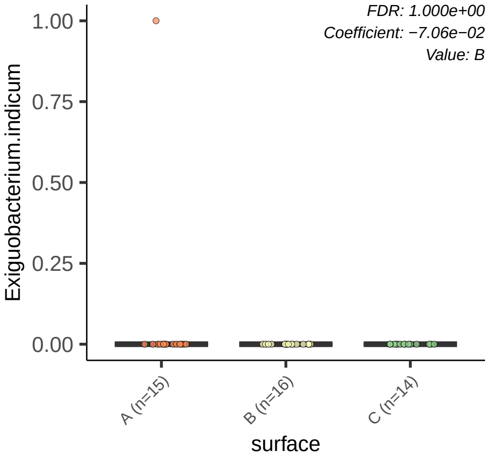

FDR: 1.000e+00
Coefficient: −7.06e−02
Value: B
Exiguobacterium.indicum
1.00
0.75
0.50
0.25
0.00
A (n=15)
B (n=16)
C (n=14)
surface

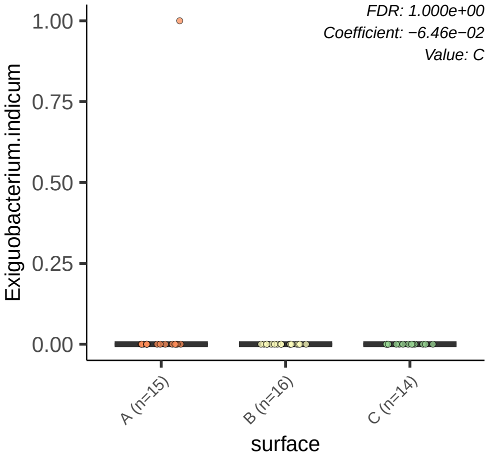
FDR: 1.000e+00
Coefficient: −6.46e−02
Value: C
Exiguobacterium.indicum
1.00
0.75
0.50
0.25
0.00
A (n=15)
B (n=16)
C (n=14)
surface

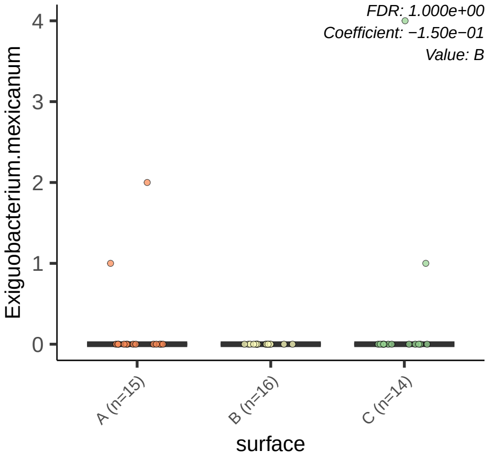
FDR: 1.000e+00
Coefficient: −1.50e−01
Value: B
Exiguobacterium.mexicanum
4
3
2
1
0
A (n=15)
B (n=16)
C (n=14)
surface

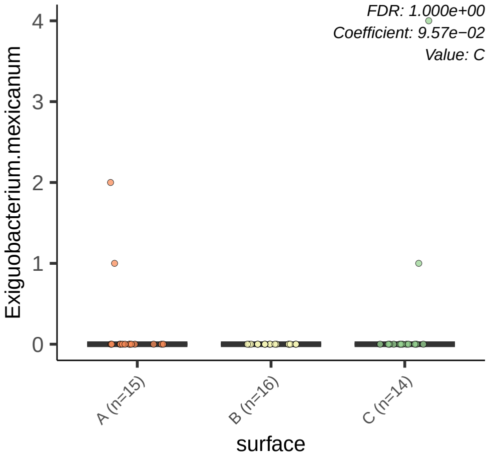

FDR: 1.000e+00
Coefficient: 9.57e−02
Value: C
Exiguobacterium.mexicanum
0
1
2
3
4
A (n=15)
B (n=16)
C (n=14)
surface

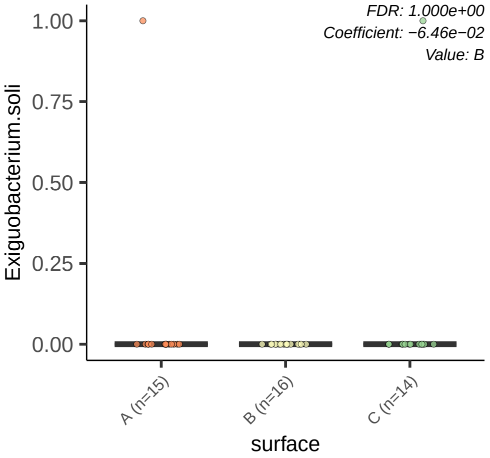

FDR: 1.000e+00
Coefficient: −6.46e−02
Value: B
Exiguobacterium.soli
1.00
0.75
0.50
0.25
0.00
A (n=15)
B (n=16)
C (n=14)
surface

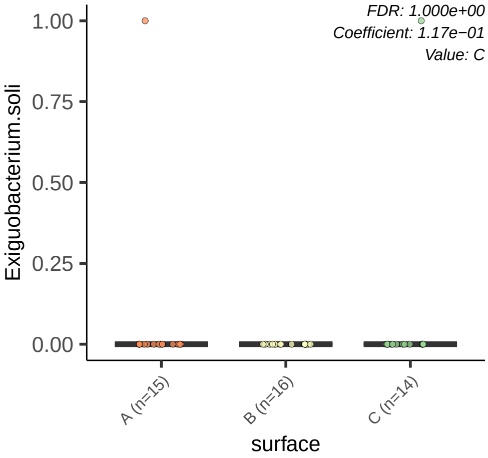

FDR: 1.000e+00
Coefficient: 1.17e−01
Value: C
Exiguobacterium.soli
1.00
0.75
0.50
0.25
0.00
A (n=15)
B (n=16)
C (n=14)
surface

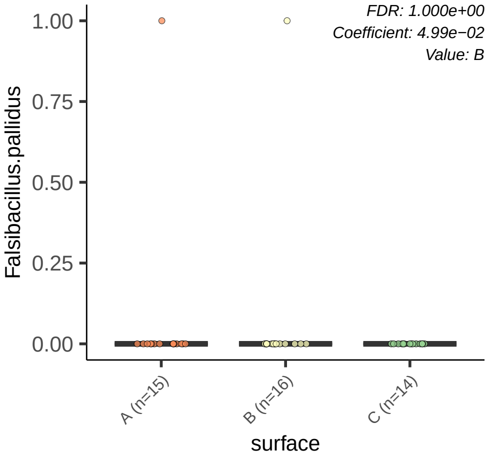
FDR: 1.000e+00
Coefficient: 4.99e−02
Value: B
Falsibacillus.pallidus
1.00
0.75
0.50
0.25
0.00
A (n=15)
B (n=16)
C (n=14)
surface

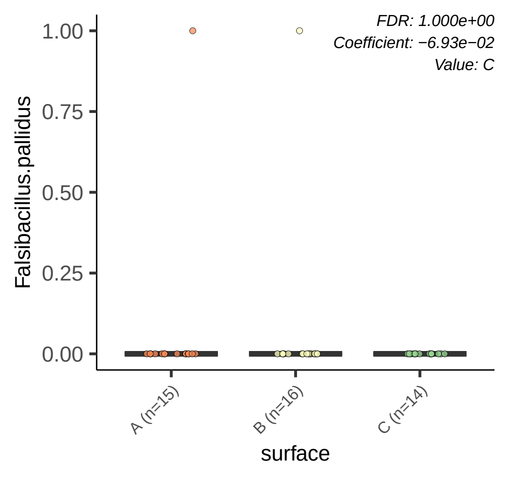

FDR: 1.000e+00
Coefficient: −6.93e−02
Value: C
Falsibacillus.pallidus
1.00
0.75
0.50
0.25
0.00
A (n=15)
B (n=16)
C (n=14)
surface

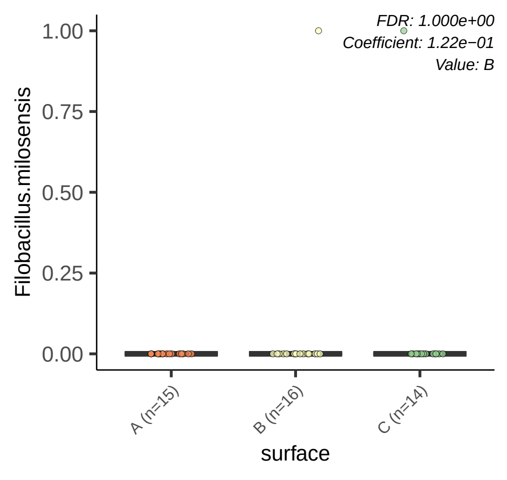
FDR: 1.000e+00
Coefficient: 1.22e−01
Value: B
Filobacillus.milosensis
1.00
0.75
0.50
0.25
0.00
A (n=15)
B (n=16)
C (n=14)
surface

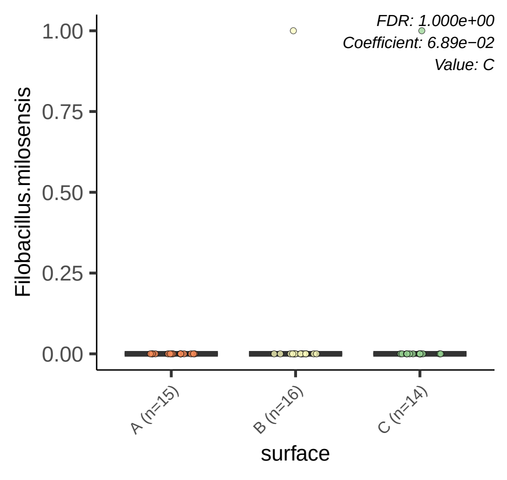

FDR: 1.000e+00
Coefficient: 6.89e−02
Value: C
Filobacillus.milosensis
1.00
0.75
0.50
0.25
0.00
A (n=15)
B (n=16)
C (n=14)
surface

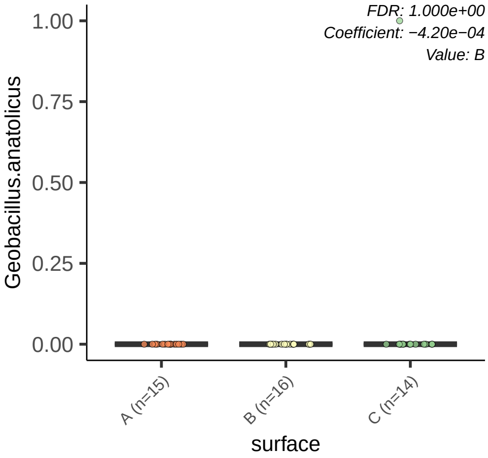

FDR: 1.000e+00
Coefficient: −4.20e−04
Value: B
Geobacillus.anatolicus
1.00
0.75
0.50
0.25
0.00
A (n=15)
B (n=16)
C (n=14)
surface

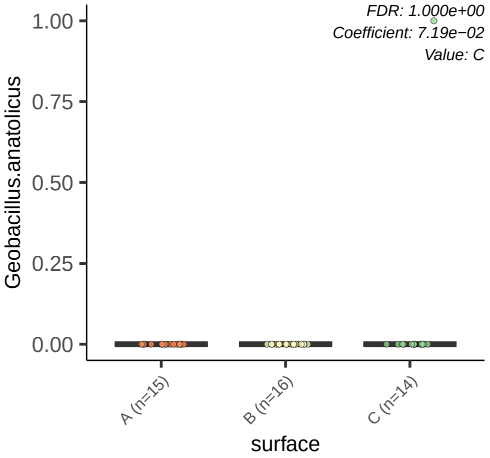
FDR: 1.000e+00
Coefficient: 7.19e−02
Value: C
Geobacillus.anatolicus
1.00
0.75
0.50
0.25
0.00
A (n=15)
B (n=16)
C (n=14)
surface

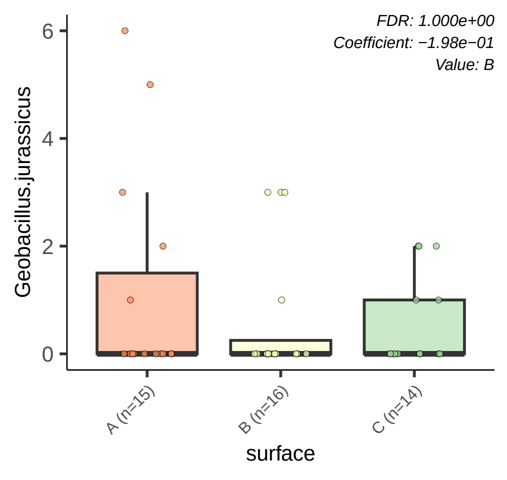
FDR: 1.000e+00
Coefficient: −1.98e−01
Value: B
Geobacillus.jurassicus
0
2
4
6
A (n=15)
B (n=16)
C (n=14)
surface

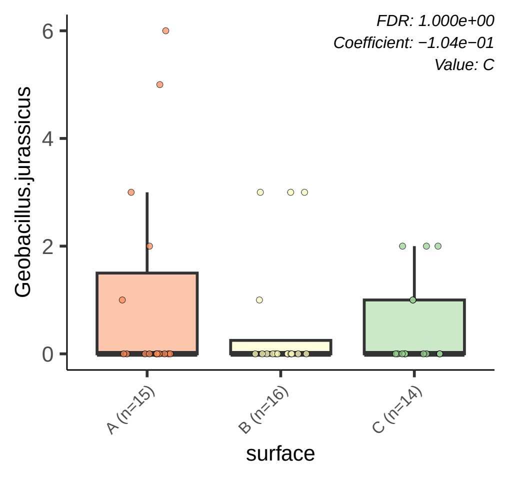
FDR: 1.000e+00
Coefficient: −1.04e−01
Value: C
Geobacillus.jurassicus
0
2
4
6
A (n=15)
B (n=16)
C (n=14)
surface

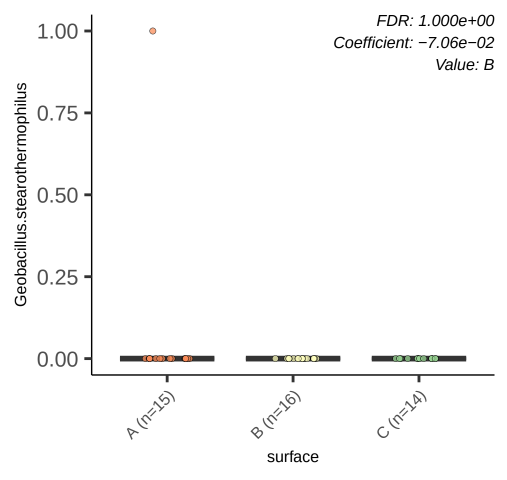

FDR: 1.000e+00
Coefficient: −7.06e−02
Value: B
Geobacillus.stearothermophilus
1.00
0.75
0.50
0.25
0.00
A (n=15)
B (n=16)
C (n=14)
surface

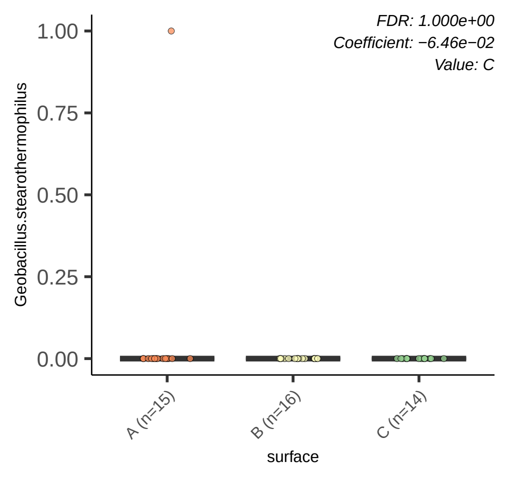
FDR: 1.000e+00
Coefficient: −6.46e−02
Value: C
Geobacillus.stearothermophilus
1.00
0.75
0.50
0.25
0.00
A (n=15)
B (n=16)
C (n=14)
surface

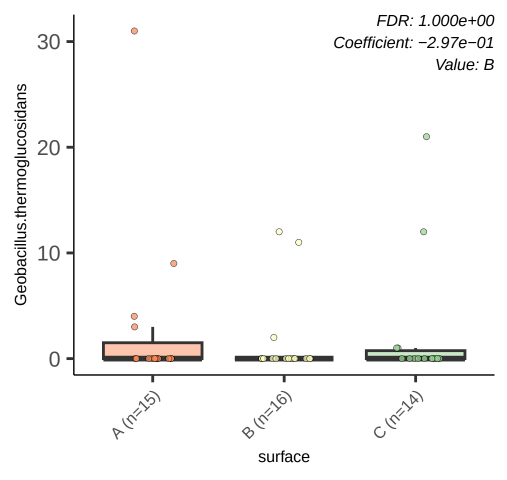
FDR: 1.000e+00
Coefficient: −2.97e−01
Value: B
Geobacillus.thermoglucosidans
30
20
10
0
A (n=15)
B (n=16)
C (n=14)
surface

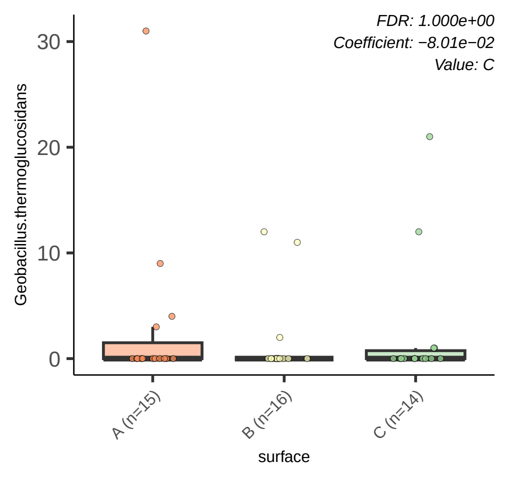

FDR: 1.000e+00
Coefficient: −8.01e−02
Value: C
Geobacillus.thermoglucosidans
30
20
10
0
A (n=15)
B (n=16)
C (n=14)
surface

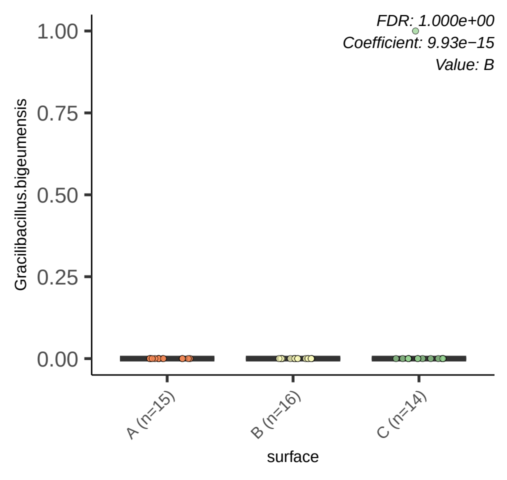
FDR: 1.000e+00
Coefficient: 9.93e−15
Value: B
Gracilibacillus.bigeumensis
1.00
0.75
0.50
0.25
0.00
A (n=15)
B (n=16)
C (n=14)
surface

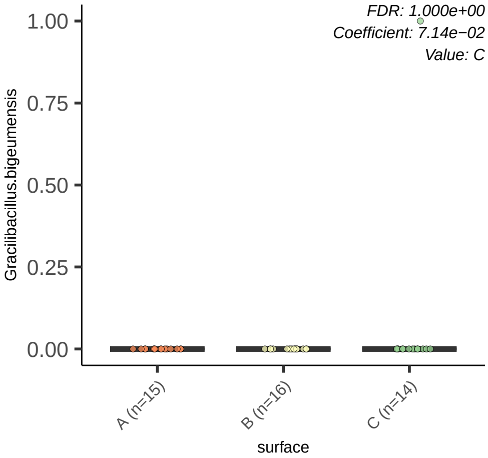
FDR: 1.000e+00
Coefficient: 7.14e−02
Value: C
1.00
0.75
0.50
0.25
0.00
Gracilibacillus.bigeumensis
A (n=15)
B (n=16)
C (n=14)
surface

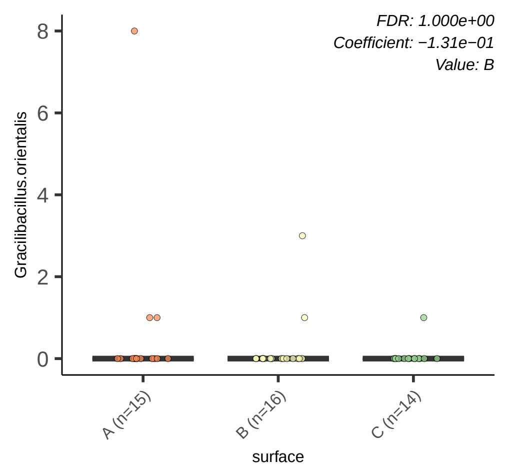

FDR: 1.000e+00
Coefficient: −1.31e−01
Value: B
Gracilibacillus.orientalis
0
2
4
6
8
A (n=15)
B (n=16)
C (n=14)
surface

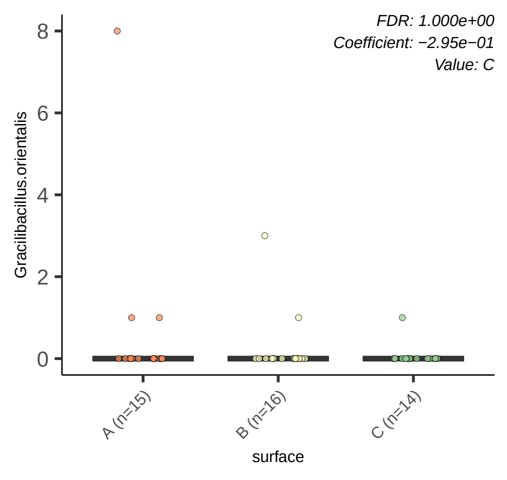

FDR: 1.000e+00
Coefficient: −2.95e−01
Value: C
Gracilibacillus.orientalis
8
6
4
2
0
A (n=15)
B (n=16)
C (n=14)
surface

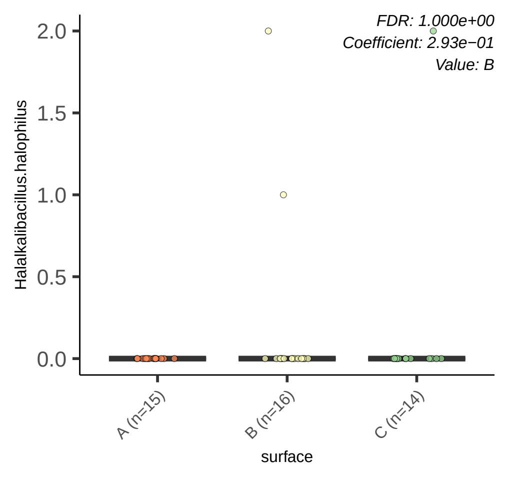
FDR: 1.000e+00
Coefficient: 2.93e−01
Value: B
Halalkalibacillus.halophilus
2.0
1.5
1.0
0.5
0.0
A (n=15)
B (n=16)
C (n=14)
surface

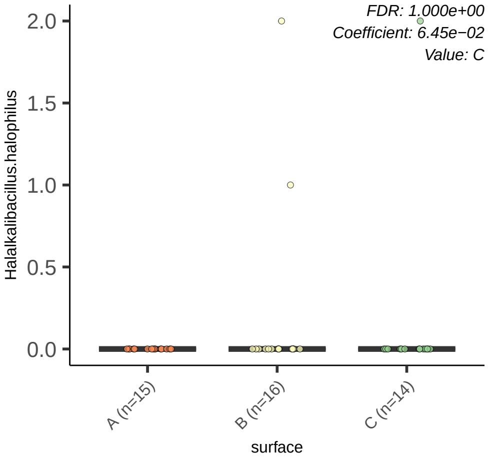

FDR: 1.000e+00
Coefficient: 6.45e−02
Value: C
Halalkalibacillus.halophilus
2.0
1.5
1.0
0.5
0.0
A (n=15)
B (n=16)
C (n=14)
surface

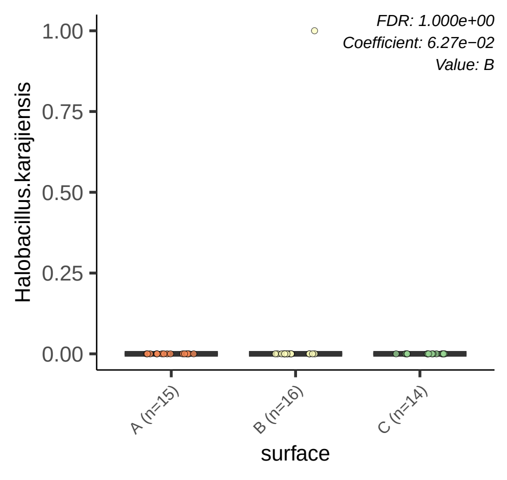
FDR: 1.000e+00
Coefficient: 6.27e−02
Value: B
Halobacillus.karajiensis
1.00
0.75
0.50
0.25
0.00
A (n=15)
B (n=16)
C (n=14)
surface

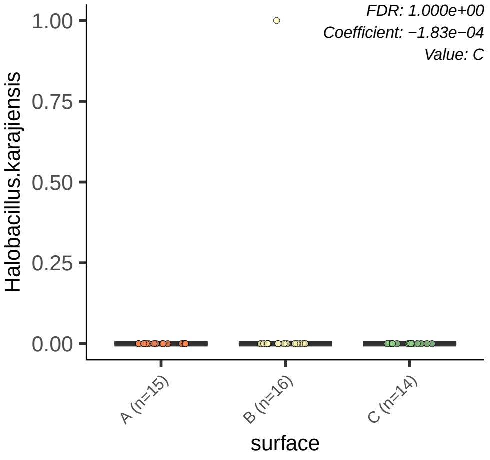

FDR: 1.000e+00
Coefficient: −1.83e−04
Value: C
1.00
0.75
0.50
0.25
0.00
Halobacillus.karajiensis
A (n=15)
B (n=16)
C (n=14)
surface

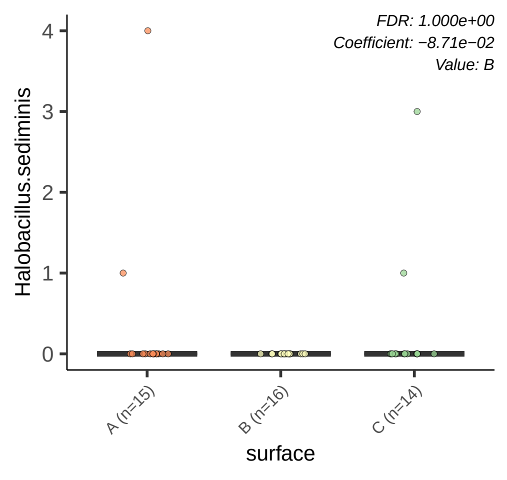
FDR: 1.000e+00
Coefficient: −8.71e−02
Value: B
Halobacillus.sedimінis
4
3
2
1
0
A (n=15)
B (n=16)
C (n=14)
surface

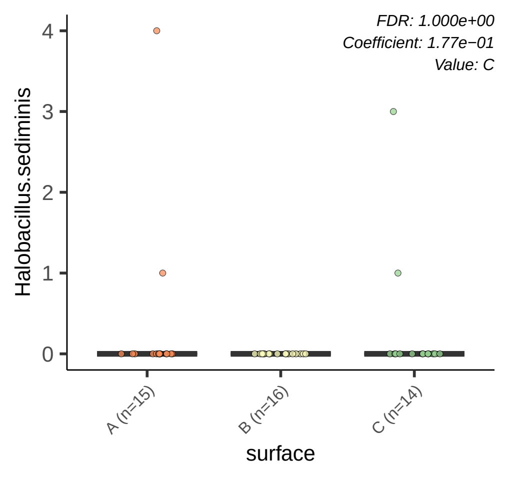

FDR: 1.000e+00
Coefficient: 1.77e−01
Value: C
Halobacillus.sedíminis
0
1
2
3
4
A (n=15)
B (n=16)
C (n=14)
surface

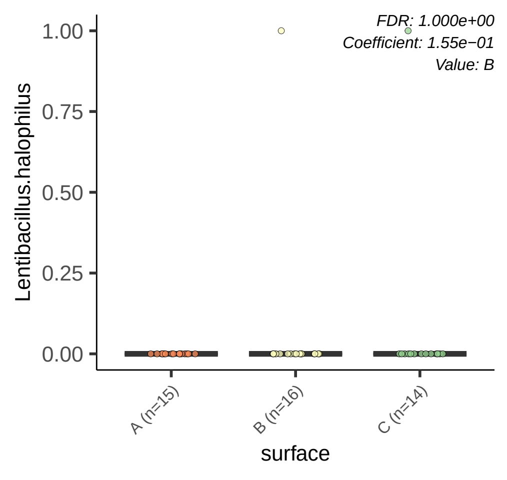
FDR: 1.000e+00
Coefficient: 1.55e−01
Value: B
Lentibacillus.halophilus
1.00
0.75
0.50
0.25
0.00
A (n=15)
B (n=16)
C (n=14)
surface

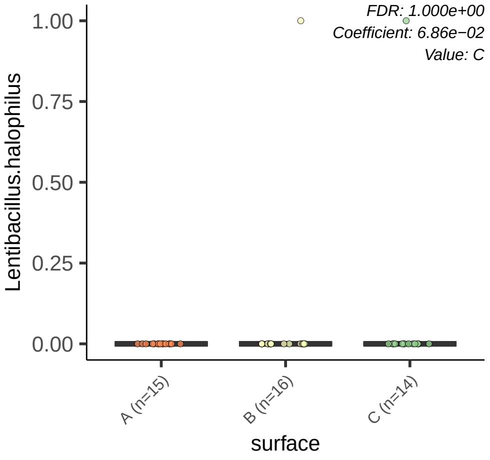

FDR: 1.000e+00
Coefficient: 6.86e−02
Value: C
Lentibacillus.halophilus
1.00
0.75
0.50
0.25
0.00
A (n=15)
B (n=16)
C (n=14)
surface

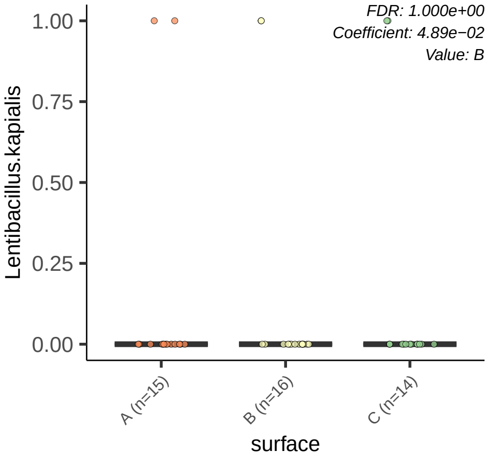
FDR: 1.000e+00
Coefficient: 4.89e−02
Value: B
Lentibacillus.kapialis
1.00
0.75
0.50
0.25
0.00
A (n=15)
B (n=16)
C (n=14)
surface

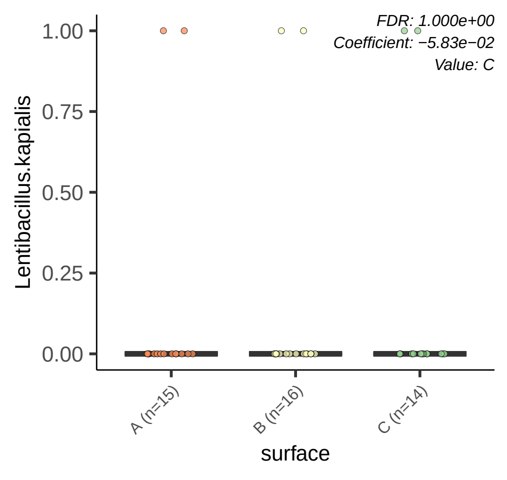
FDR: 1.000e+00
Coefficient: −5.83e−02
Value: C
Lentibacillus.kapialis
1.00
0.75
0.50
0.25
0.00
A (n=15)
B (n=16)
C (n=14)
surface

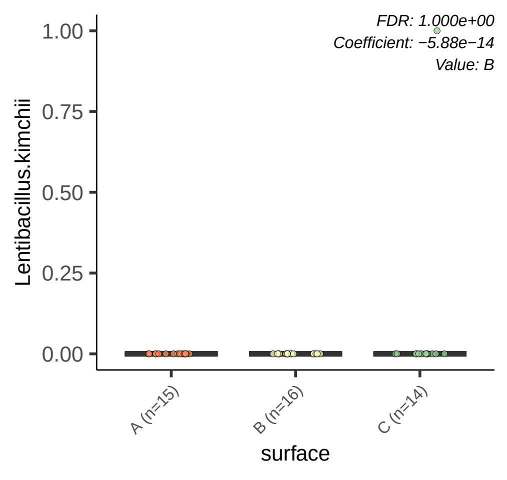
FDR: 1.000e+00
Coefficient: −5.88e−14
Value: B
Lentibacillus.kimchii
1.00
0.75
0.50
0.25
0.00
A (n=15)
B (n=16)
C (n=14)
surface

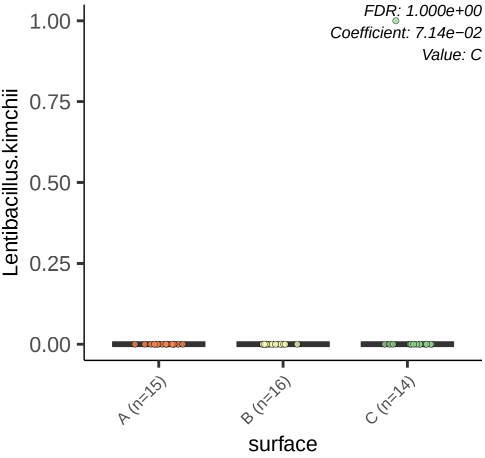
FDR: 1.000e+00
Coefficient: 7.14e−02
Value: C
Lentibacillus.kimchii
1.00
0.75
0.50
0.25
0.00
A (n=15)
B (n=16)
C (n=14)
surface

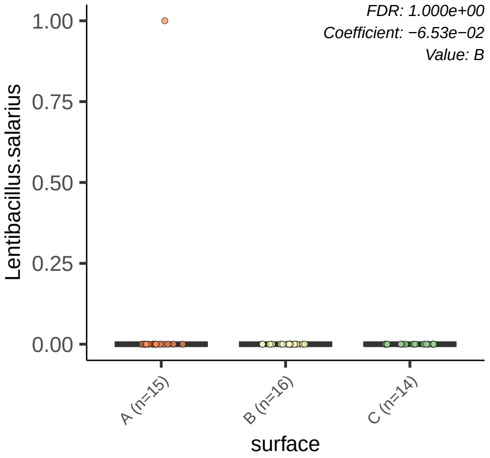
FDR: 1.000e+00
Coefficient: −6.53e−02
Value: B
Lentibacillus.salarius
1.00
0.75
0.50
0.25
0.00
A (n=15)
B (n=16)
C (n=14)
surface

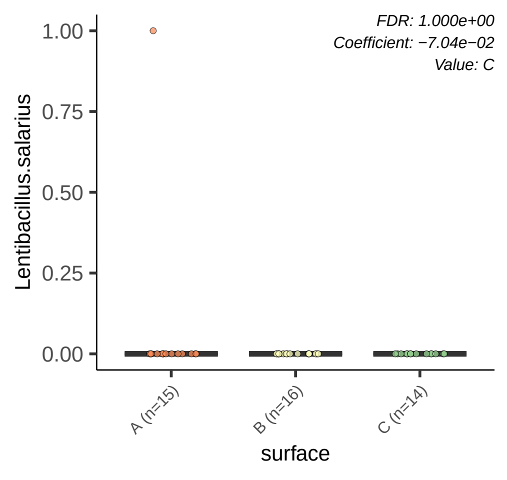
FDR: 1.000e+00
Coefficient: −7.04e−02
Value: C
Lentibacillus.salarius
1.00
0.75
0.50
0.25
0.00
A (n=15)
B (n=16)
C (n=14)
surface

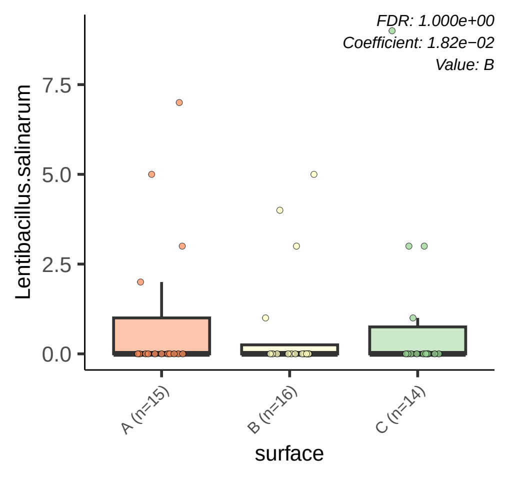

FDR: 1.000e+00
Coefficient: 1.82e−02
Value: B
Lentibacillus.salinarum
7.5
5.0
2.5
0.0
A (n=15)
B (n=16)
C (n=14)
surface

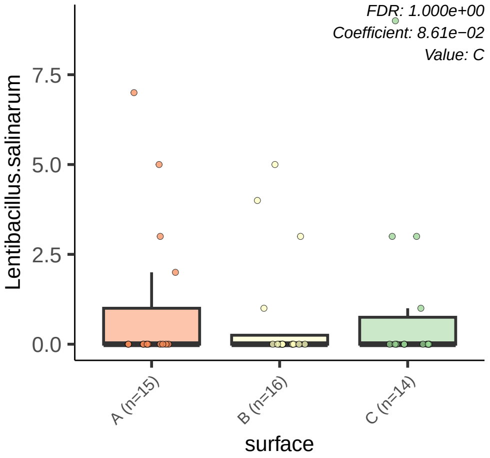
FDR: 1.000e+00
Coefficient: 8.61e−02
Value: C
Lentibacillus.salinarum
7.5
5.0
2.5
0.0
A (n=15)
B (n=16)
C (n=14)
surface

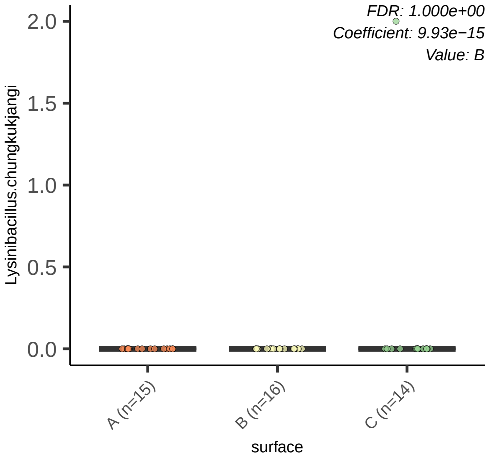

FDR: 1.000e+00
Coefficient: 9.93e−15
Value: B
Lysinibacillus.chungkukjangi
2.0
1.5
1.0
0.5
0.0
A (n=15)
B (n=16)
C (n=14)
surface

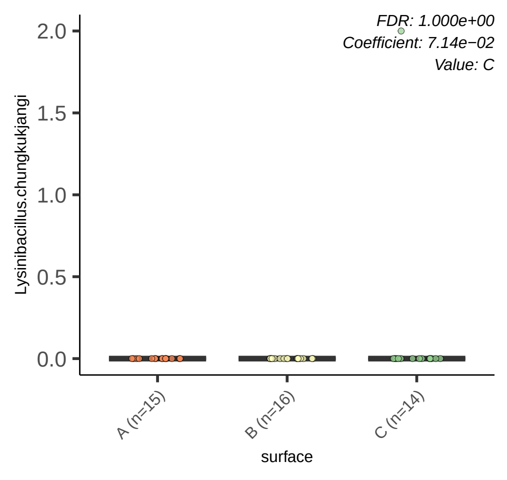

FDR: 1.000e+00
Coefficient: 7.14e−02
Value: C
Lysinibacillus.chungkukjangi
2.0
1.5
1.0
0.5
0.0
A (n=15)
B (n=16)
C (n=14)
surface

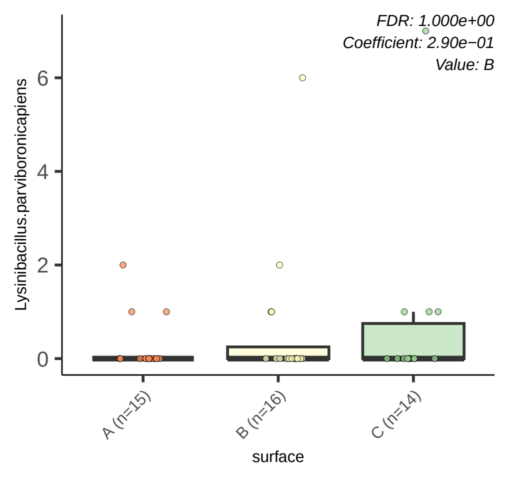
FDR: 1.000e+00
Coefficient: 2.90e−01
Value: B
Lysinibacillus.parviboronicapiens
0
2
4
6
A (n=15)
B (n=16)
C (n=14)
surface

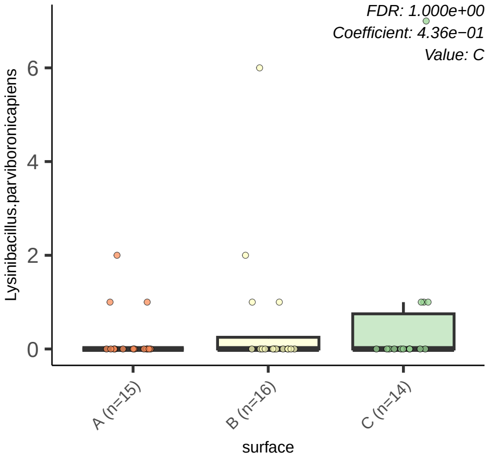
FDR: 1.000e+00
Coefficient: 4.36e−01
Value: C
Lysinibacillus.parviboronicapiens
0
2
4
6
A (n=15)
B (n=16)
C (n=14)
surface

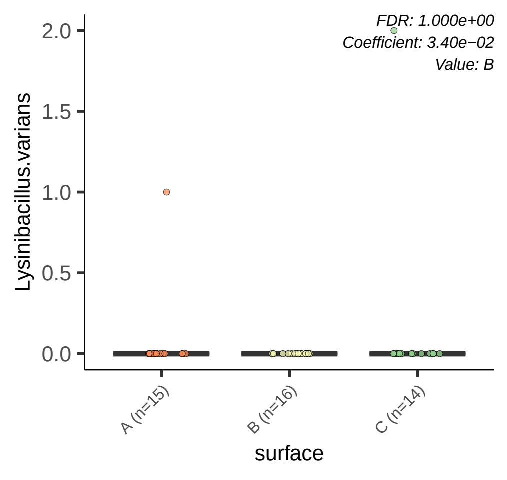
FDR: 1.000e+00
Coefficient: 3.40e−02
Value: B
Lysinibacillus.varians
2.0
1.5
1.0
0.5
0.0
A (n=15)
B (n=16)
C (n=14)
surface

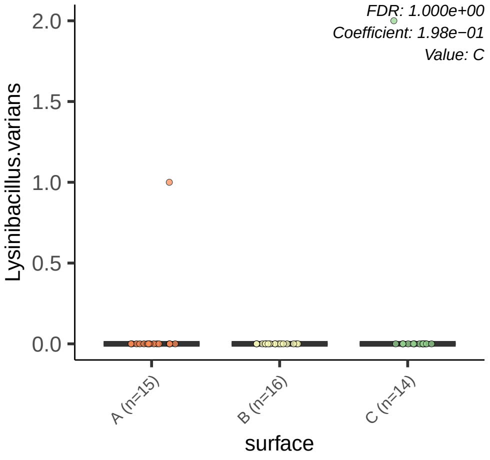

FDR: 1.000e+00
Coefficient: 1.98e−01
Value: C
Lysinibacillus.varians
2.0
1.5
1.0
0.5
0.0
A (n=15)
B (n=16)
C (n=14)
surface

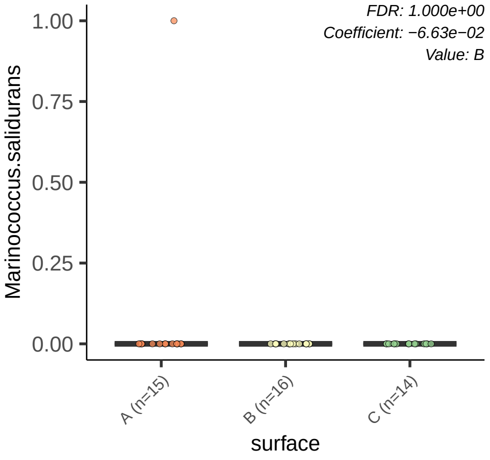
FDR: 1.000e+00
Coefficient: −6.63e−02
Value: B
Marinococcus.salidurans
1.00
0.75
0.50
0.25
0.00
A (n=15)
B (n=16)
C (n=14)
surface

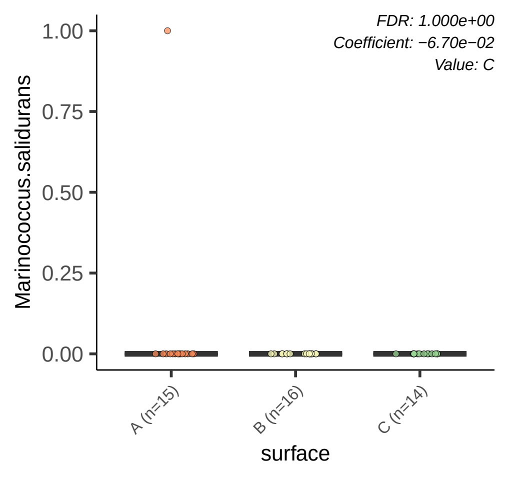
FDR: 1.000e+00
Coefficient: −6.70e−02
Value: C
Marinococcus.salidurans
1.00
0.75
0.50
0.25
0.00
A (n=15)
B (n=16)
C (n=14)
surface

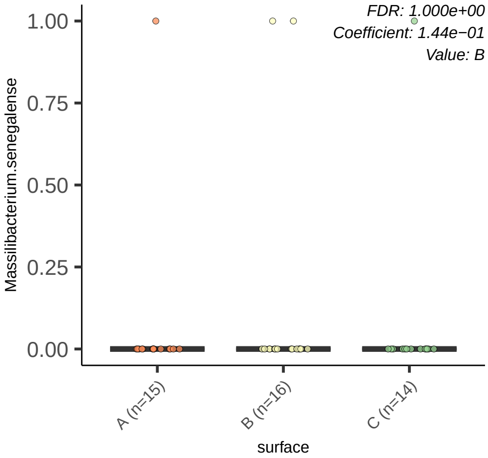
FDR: 1.000e+00
Coefficient: 1.44e−01
Value: B
Massilibacterium.senegalense
1.00
0.75
0.50
0.25
0.00
A (n=15)
B (n=16)
C (n=14)
surface

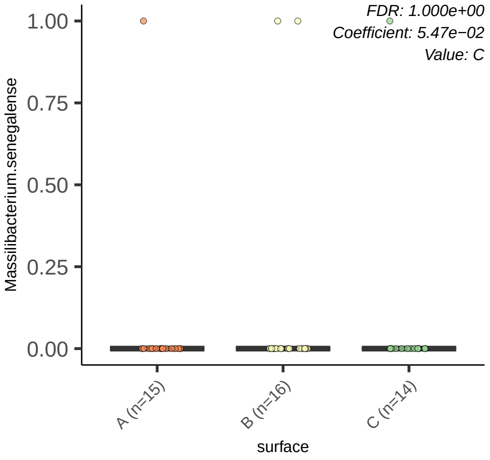

FDR: 1.000e+00
Coefficient: 5.47e−02
Value: C
1.00
0.75
0.50
0.25
0.00
Massilibacterium.senegalense
A (n=15)
B (n=16)
C (n=14)
surface

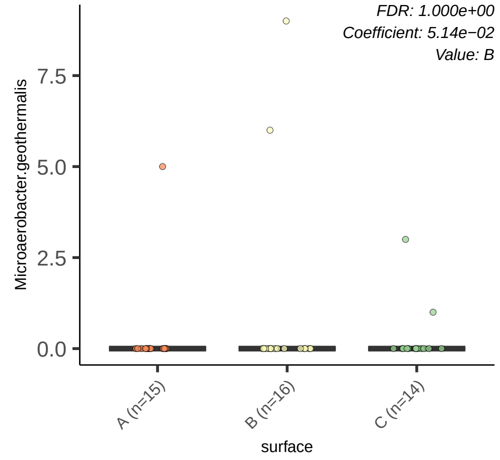
FDR: 1.000e+00
Coefficient: 5.14e−02
Value: B
Microaerobacter.geothermalis
7.5
5.0
2.5
0.0
A (n=15)
B (n=16)
C (n=14)
surface

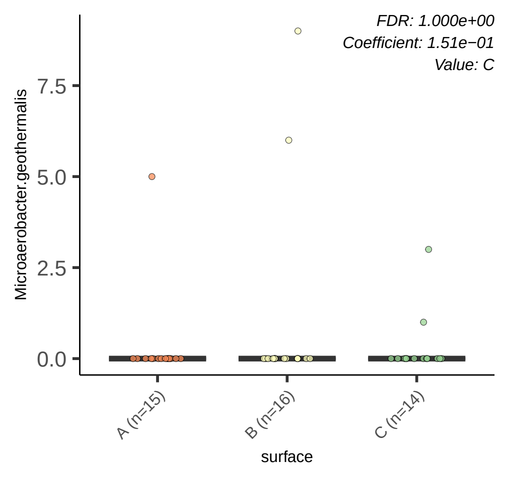
FDR: 1.000e+00
Coefficient: 1.51e−01
Value: C
Microaerobacter.geothermalis
7.5
5.0
2.5
0.0
A (n=15)
B (n=16)
C (n=14)
surface

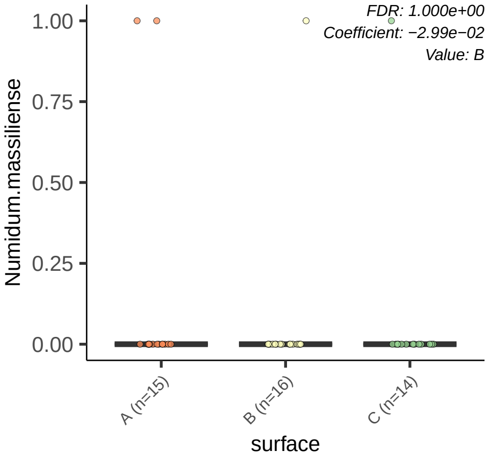
FDR: 1.000e+00
Coefficient: −2.99e−02
Value: B
Numidum.massiliense
1.00
0.75
0.50
0.25
0.00
A (n=15)
B (n=16)
C (n=14)
surface

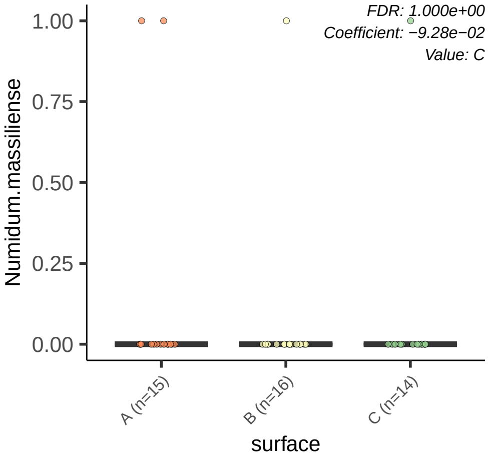

FDR: 1.000e+00
Coefficient: −9.28e−02
Value: C
Numidum.massiliense
1.00
0.75
0.50
0.25
0.00
A (n=15)
B (n=16)
C (n=14)
surface

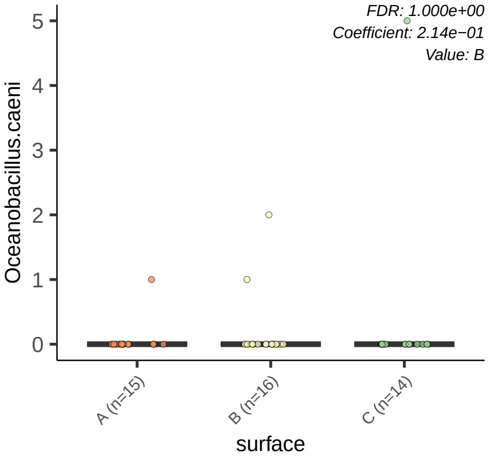
FDR: 1.000e+00
Coefficient: 2.14e−01
Value: B
Oceanobacillus.caeni
0
1
2
3
4
5
A (n=15)
B (n=16)
C (n=14)
surface

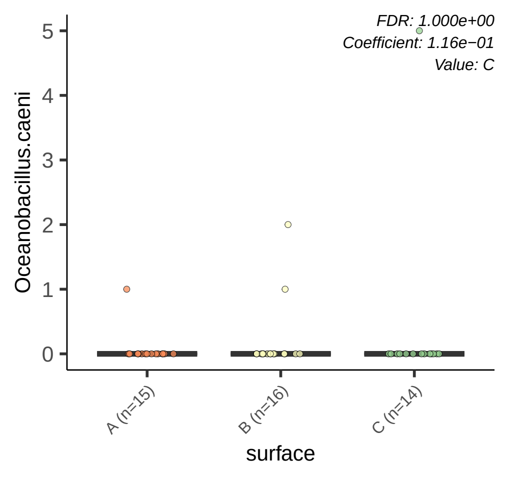
FDR: 1.000e+00
Coefficient: 1.16e−01
Value: C
Oceanobacillus.caeni
0
1
2
3
4
5
A (n=15)
B (n=16)
C (n=14)
surface

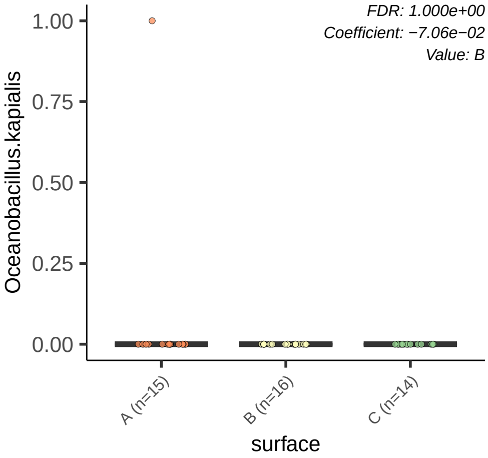
FDR: 1.000e+00
Coefficient: −7.06e−02
Value: B
Oceanobacillus.kapialis
1.00
0.75
0.50
0.25
0.00
A (n=15)
B (n=16)
C (n=14)
surface

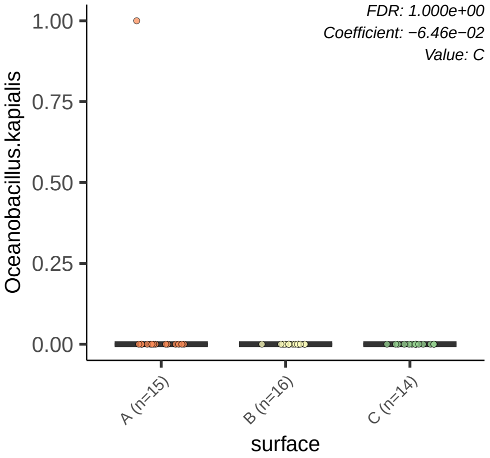
FDR: 1.000e+00
Coefficient: −6.46e−02
Value: C
Oceanobacillus.kapialis
1.00
0.75
0.50
0.25
0.00
A (n=15)
B (n=16)
C (n=14)
surface

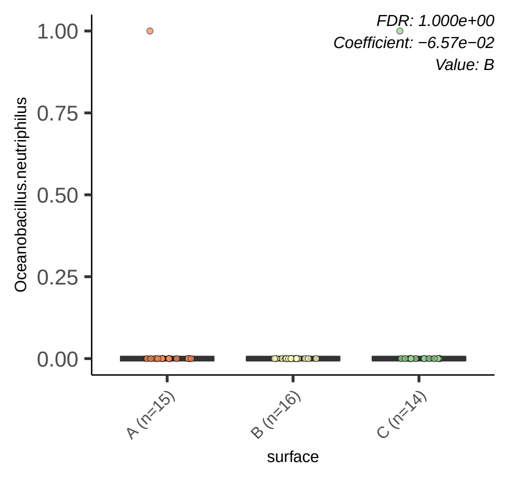
FDR: 1.000e+00
Coefficient: −6.57e−02
Value: B
Oceanobacillus.neutriphilus
1.00
0.75
0.50
0.25
0.00
A (n=15)
B (n=16)
C (n=14)
surface

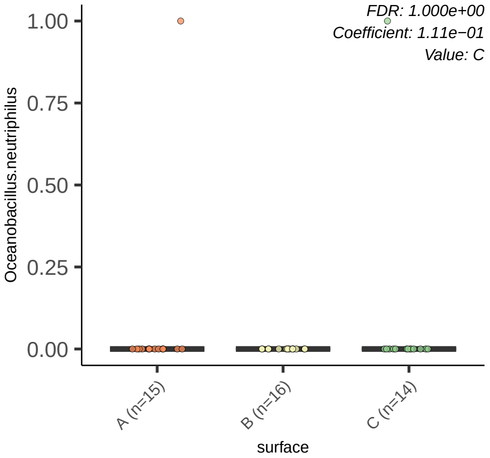
FDR: 1.000e+00
Coefficient: 1.11e−01
Value: C
1.00
0.75
0.50
0.25
0.00
Oceanobacillus.neutriphilus
A (n=15)
B (n=16)
C (n=14)
surface

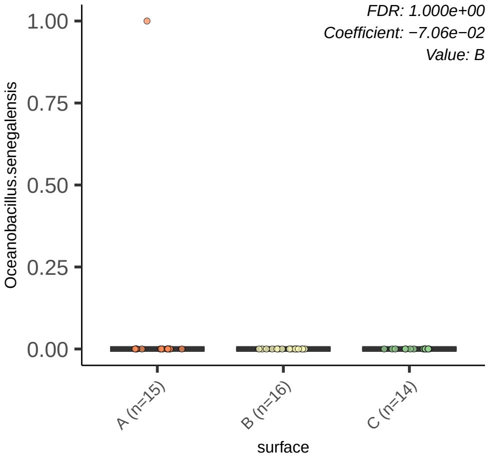
FDR: 1.000e+00
Coefficient: −7.06e−02
Value: B
1.00
0.75
0.50
0.25
0.00
Oceanobacillus.senegalensis
A (n=15)
B (n=16)
C (n=14)
surface

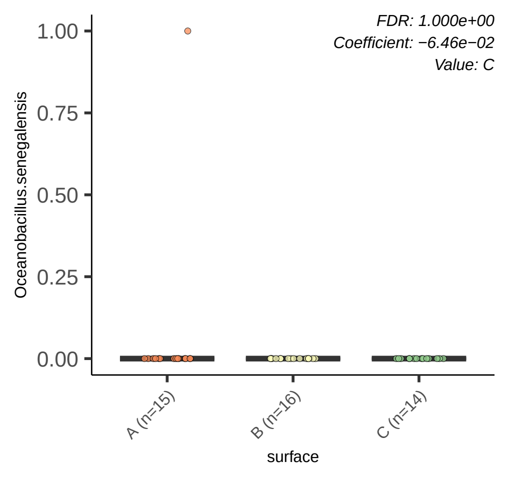

FDR: 1.000e+00
Coefficient: −6.46e−02
Value: C
1.00
0.75
0.50
0.25
0.00
Oceanobacillus.senegalensis
A (n=15)
B (n=16)
C (n=14)
surface

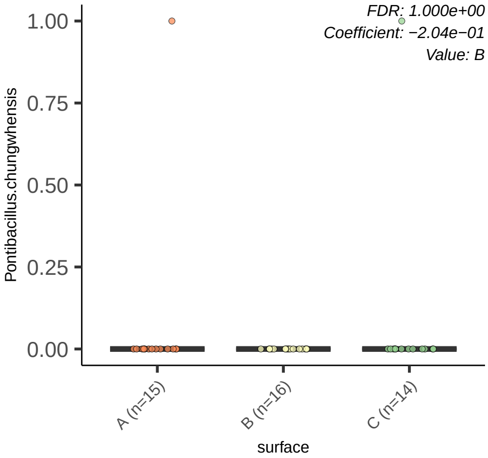

FDR: 1.000e+00
Coefficient: −2.04e−01
Value: B
Pontibacillus.chungwhensis
1.00
0.75
0.50
0.25
0.00
A (n=15)
B (n=16)
C (n=14)
surface

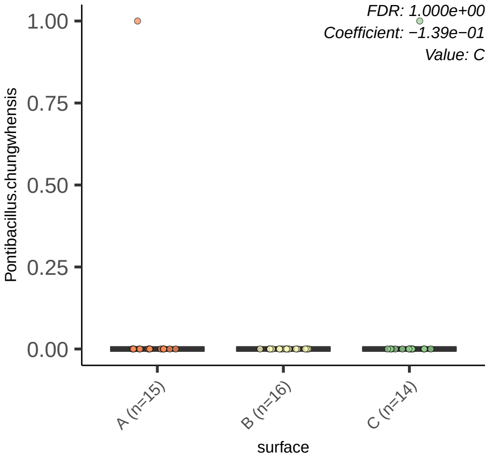
FDR: 1.000e+00
Coefficient: −1.39e−01
Value: C
Pontibacillus.chungwhensis
1.00
0.75
0.50
0.25
0.00
A (n=15)
B (n=16)
C (n=14)
surface

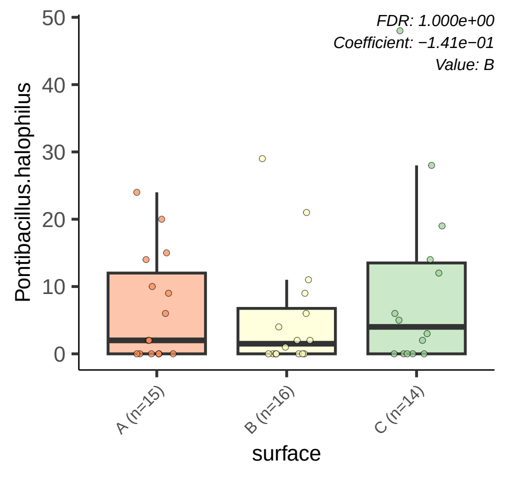
FDR: 1.000e+00
Coefficient: −1.41e−01
Value: B
Pontibacillus.halophilus
0
10
20
30
40
50
A (n=15)
B (n=16)
C (n=14)
surface

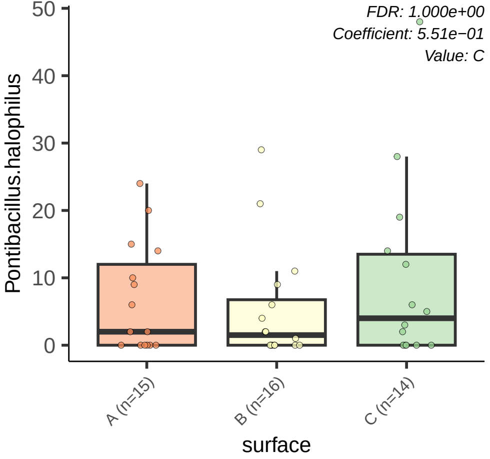

FDR: 1.000e+00
Coefficient: 5.51e−01
Value: C
Pontibacillus.halophilus
50
40
30
20
10
0
A (n=15)
B (n=16)
C (n=14)
surface

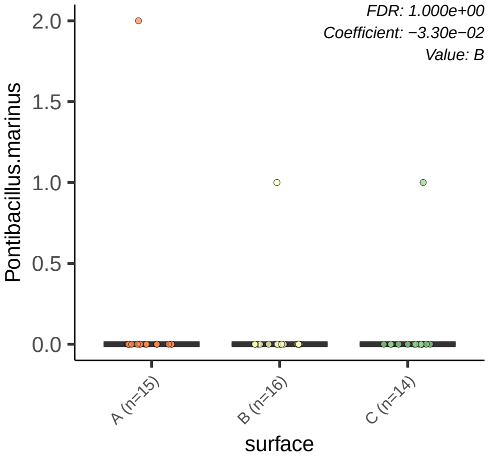
FDR: 1.000e+00
Coefficient: −3.30e−02
Value: B
Pontibacillus.marinus
2.0
1.5
1.0
0.5
0.0
A (n=15)
B (n=16)
C (n=14)
surface

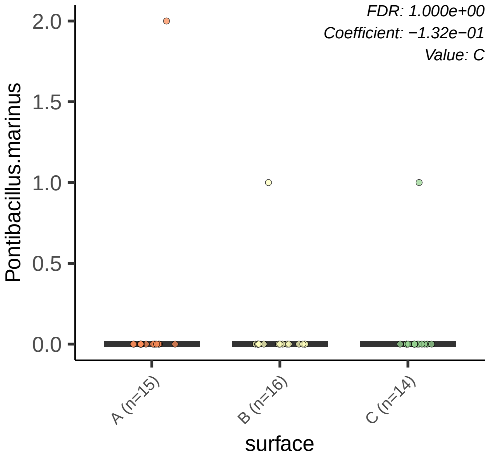

FDR: 1.000e+00
Coefficient: −1.32e−01
Value: C
Pontibacillus.marinus
2.0
1.5
1.0
0.5
0.0
A (n=15)
B (n=16)
C (n=14)
surface

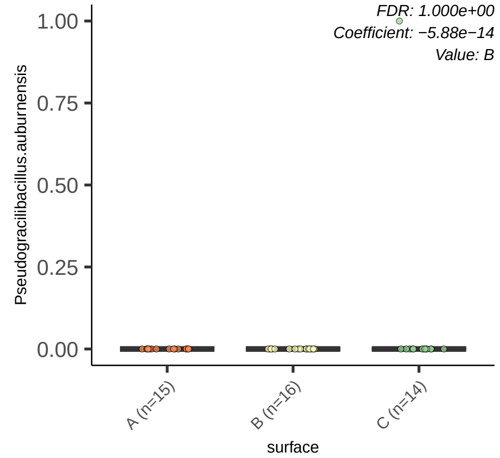
FDR: 1.000e+00
Coefficient: −5.88e−14
Value: B
1.00
0.75
0.50
0.25
0.00
Pseudogracilibacillus.auburnensis
A (n=15)
B (n=16)
C (n=14)
surface

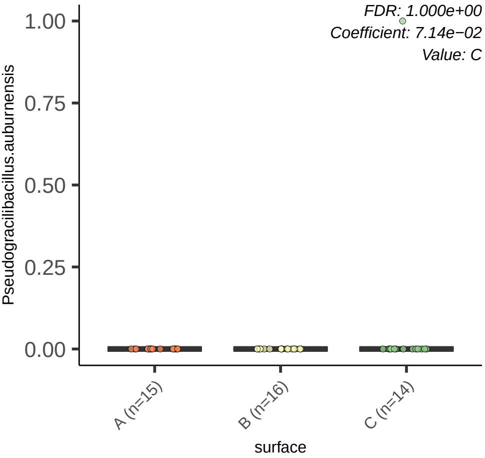
FDR: 1.000e+00
Coefficient: 7.14e−02
Value: C
1.00
0.75
0.50
0.25
0.00
Pseudogracilibacillus.auburnensis
A (n=15)
B (n=16)
C (n=14)
surface

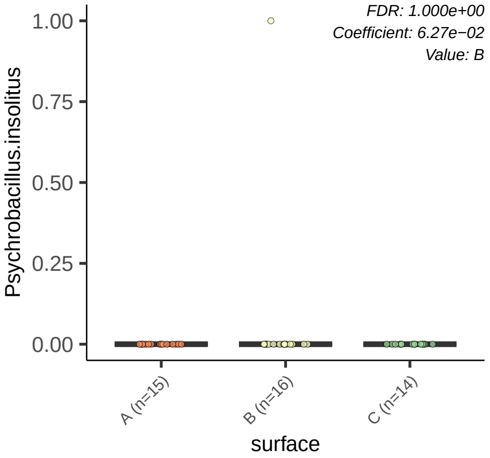
FDR: 1.000e+00
Coefficient: 6.27e−02
Value: B
Psychrobacillus.insolitus
1.00
0.75
0.50
0.25
0.00
A (n=15)
B (n=16)
C (n=14)
surface

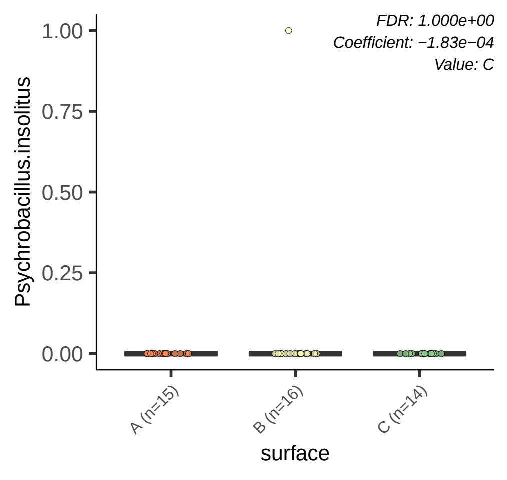
FDR: 1.000e+00
Coefficient: −1.83e−04
Value: C
Psychrobacillus.insolitus
1.00
0.75
0.50
0.25
0.00
A (n=15)
B (n=16)
C (n=14)
surface

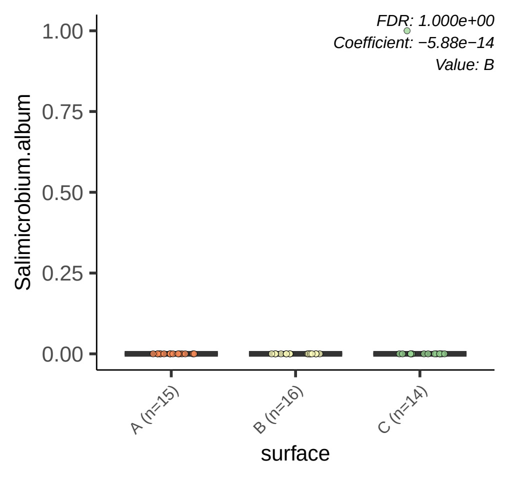
FDR: 1.000e+00
Coefficient: −5.88e−14
Value: B
Salimicrobium.album
1.00
0.75
0.50
0.25
0.00
A (n=15)
B (n=16)
C (n=14)
surface

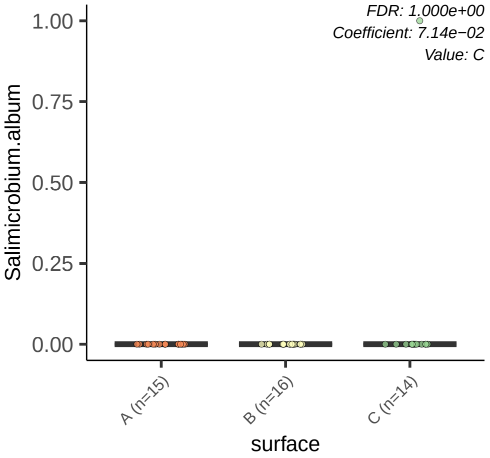
FDR: 1.000e+00
Coefficient: 7.14e−02
Value: C
Salimicrobium.album
1.00
0.75
0.50
0.25
0.00
A (n=15)
B (n=16)
C (n=14)
surface

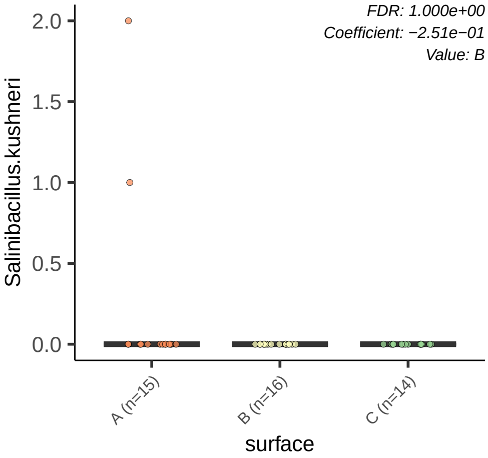
FDR: 1.000e+00
Coefficient: −2.51e−01
Value: B
Salinibacillus.kushneri
2.0
1.5
1.0
0.5
0.0
A (n=15)
B (n=16)
C (n=14)
surface

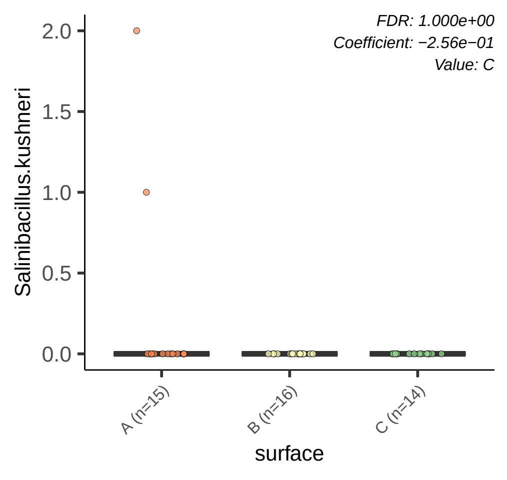

FDR: 1.000e+00
Coefficient: −2.56e−01
Value: C
Salinibacillus.kushneri
2.0
1.5
1.0
0.5
0.0
A (n=15)
B (n=16)
C (n=14)
surface

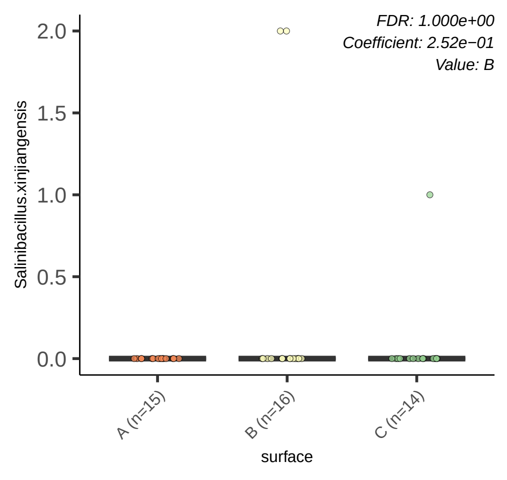
FDR: 1.000e+00
Coefficient: 2.52e−01
Value: B
Salinibacillus.xinjiangensis
2.0
1.5
1.0
0.5
0.0
A (n=15)
B (n=16)
C (n=14)
surface

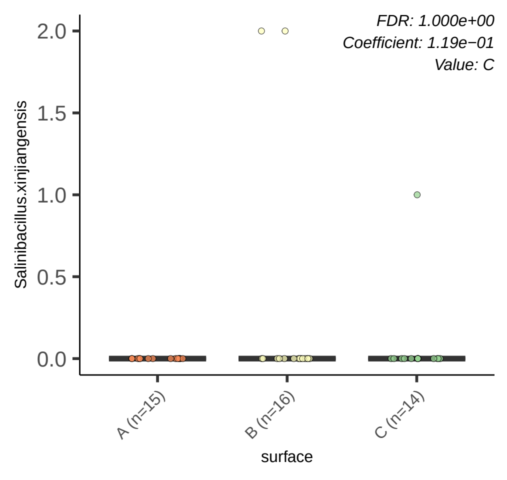

FDR: 1.000e+00
Coefficient: 1.19e−01
Value: C
Salinibacillus.xinjiangensis
2.0
1.5
1.0
0.5
0.0
A (n=15)
B (n=16)
C (n=14)
surface

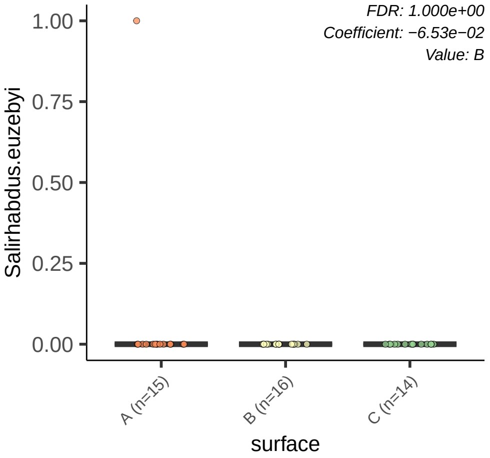

FDR: 1.000e+00
Coefficient: −6.53e−02
Value: B
Salirhabdus.euzebyi
1.00
0.75
0.50
0.25
0.00
A (n=15)
B (n=16)
C (n=14)
surface

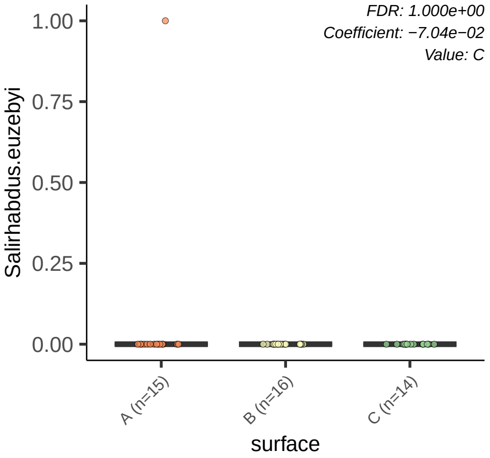
FDR: 1.000e+00
Coefficient: −7.04e−02
Value: C
Salirhabdus.euzebyi
1.00
0.75
0.50
0.25
0.00
A (n=15)
B (n=16)
C (n=14)
surface

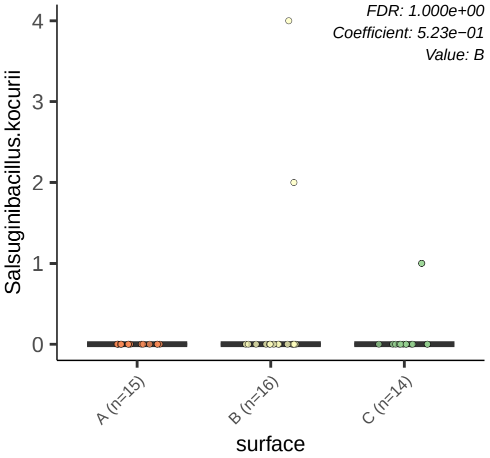

FDR: 1.000e+00
Coefficient: 5.23e−01
Value: B
Salsuginibacillus.kocurii
0
1
2
3
4
A (n=15)
B (n=16)
C (n=14)
surface

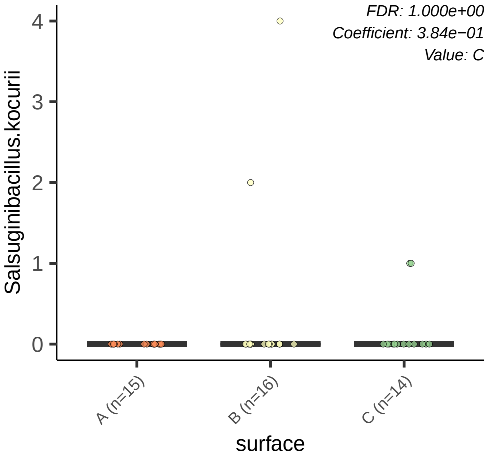

FDR: 1.000e+00
Coefficient: 3.84e−01
Value: C
Salsuginibacillus.kocurii
0
1
2
3
4
A (n=15)
B (n=16)
C (n=14)
surface

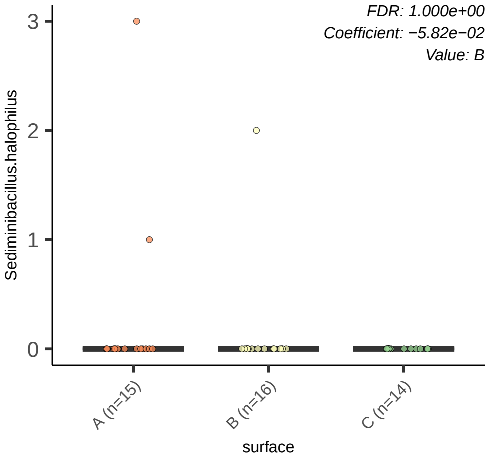

FDR: 1.000e+00
Coefficient: −5.82e−02
Value: B
Sediminibacillus.halophilus
3
2
1
0
A (n=15)
B (n=16)
C (n=14)
surface

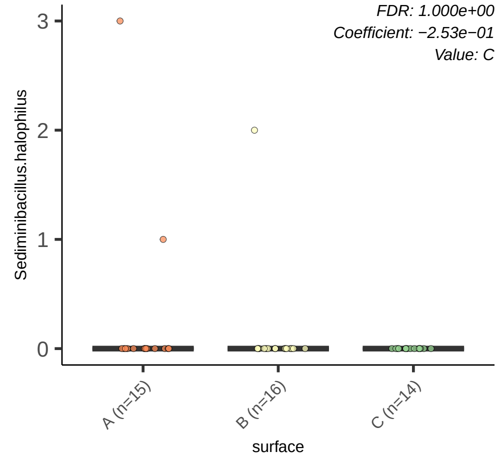
FDR: 1.000e+00
Coefficient: −2.53e−01
Value: C
Sediminibacillus.halophilus
3
2
1
0
A (n=15)
B (n=16)
C (n=14)
surface

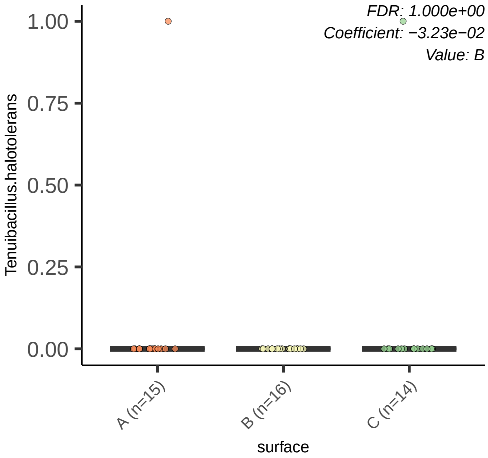

FDR: 1.000e+00
Coefficient: −3.23e−02
Value: B
1.00
0.75
0.50
0.25
0.00
Tenuibacillus.halotolerans
A (n=15)
B (n=16)
C (n=14)
surface

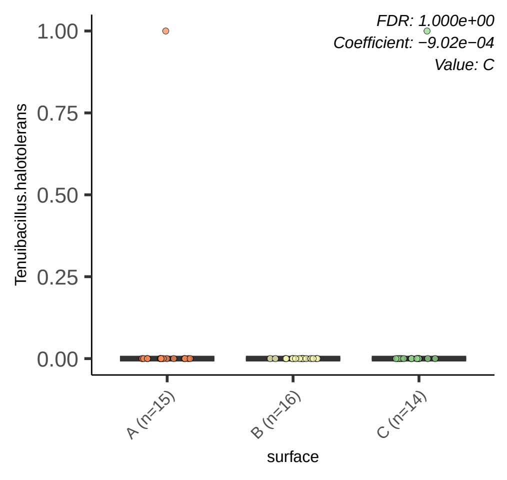
FDR: 1.000e+00
Coefficient: −9.02e−04
Value: C
Tenuibacillus.halotolerans
1.00
0.75
0.50
0.25
0.00
A (n=15)
B (n=16)
C (n=14)
surface

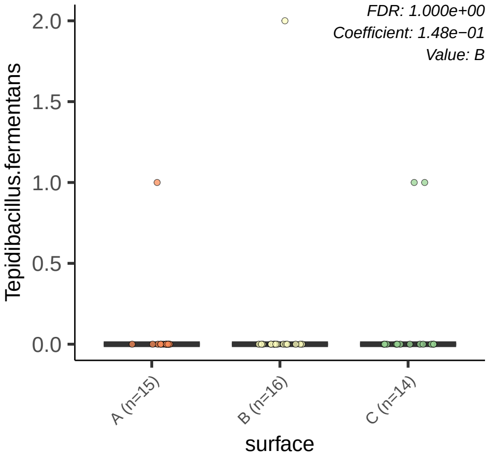
FDR: 1.000e+00
Coefficient: 1.48e−01
Value: B
Tepidibacillus.fermentans
2.0
1.5
1.0
0.5
0.0
A (n=15)
B (n=16)
C (n=14)
surface

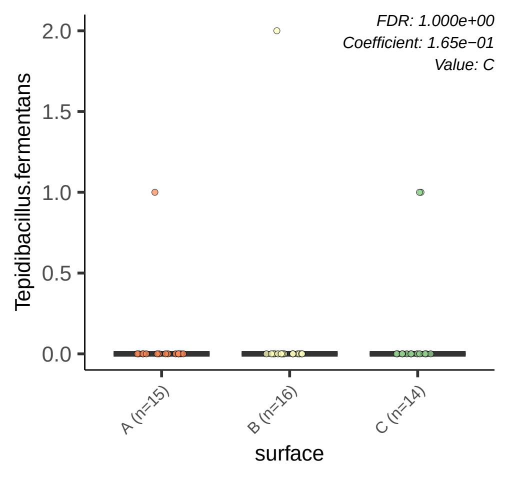
FDR: 1.000e+00
Coefficient: 1.65e−01
Value: C
Tepidibacillus.fermentans
2.0
1.5
1.0
0.5
0.0
A (n=15)
B (n=16)
C (n=14)
surface

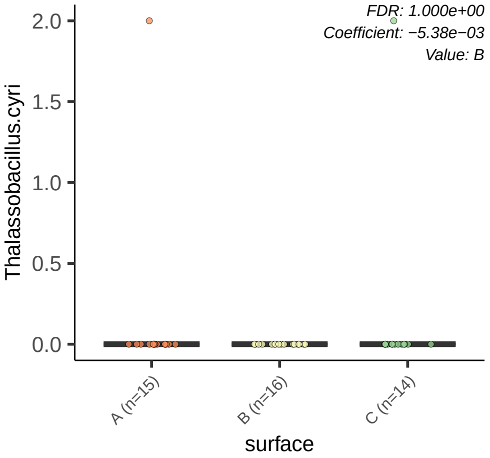
FDR: 1.000e+00
Coefficient: −5.38e−03
Value: B
Thalassobacillus.cyri
2.0
1.5
1.0
0.5
0.0
A (n=15)
B (n=16)
C (n=14)
surface

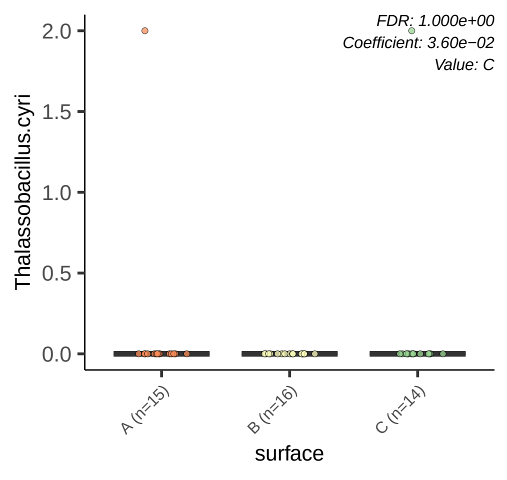
FDR: 1.000e+00
Coefficient: 3.60e−02
Value: C
Thalassobacillus.cyri
2.0
1.5
1.0
0.5
0.0
A (n=15)
B (n=16)
C (n=14)
surface

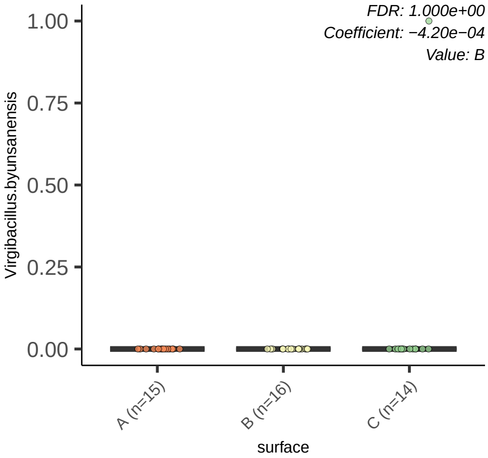
FDR: 1.000e+00
Coefficient: −4.20e−04
Value: B
1.00
0.75
0.50
0.25
0.00
Virgibacillus.byunsanensis
A (n=15)
B (n=16)
C (n=14)
surface

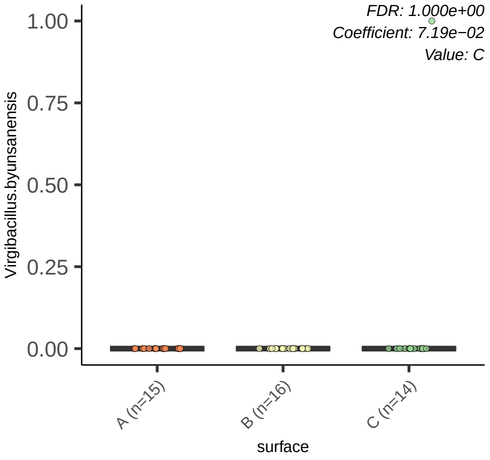
FDR: 1.000e+00
Coefficient: 7.19e−02
Value: C
Virgibacillus.byunsanensis
1.00
0.75
0.50
0.25
0.00
A (n=15)
B (n=16)
C (n=14)
surface

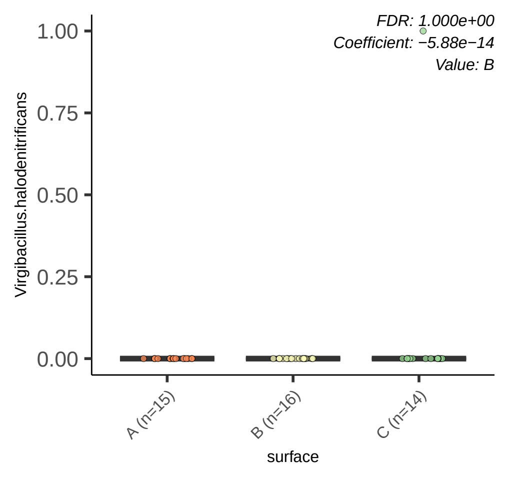
FDR: 1.000e+00
Coefficient: −5.88e−14
Value: B
Virgibacillus.halodenitrificans
1.00
0.75
0.50
0.25
0.00
A (n=15)
B (n=16)
C (n=14)
surface

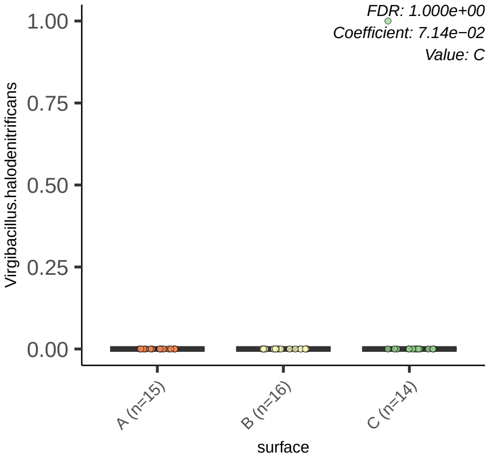

FDR: 1.000e+00
Coefficient: 7.14e−02
Value: C
1.00
0.75
0.50
0.25
0.00
Virgibacillus.halodenitrificans
A (n=15)
B (n=16)
C (n=14)
surface

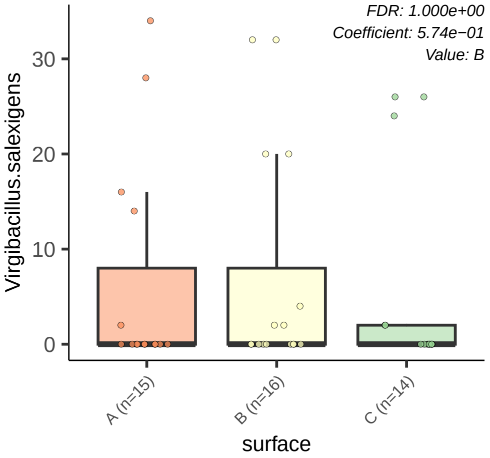

FDR: 1.000e+00
Coefficient: 5.74e−01
Value: B
Virgibacillus.salexigens
0
10
20
30
A (n=15)
B (n=16)
C (n=14)
surface

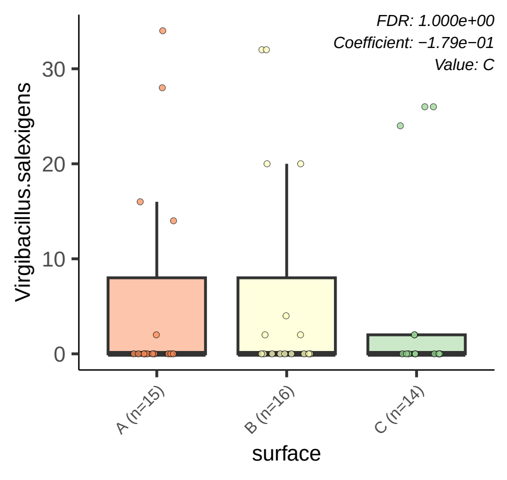

FDR: 1.000e+00
Coefficient: −1.79e−01
Value: C
Virgibacillus.salexigens
0
10
20
30
A (n=15)
B (n=16)
C (n=14)
surface

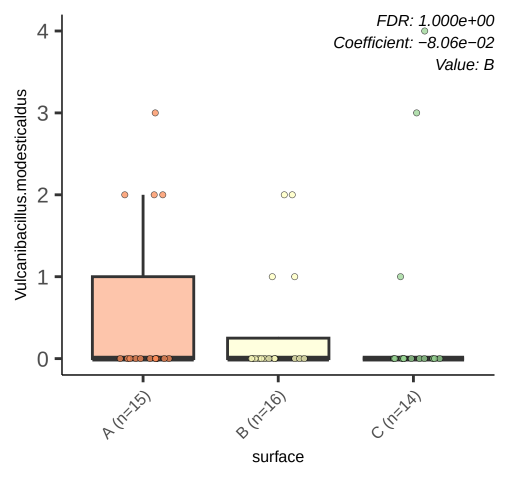
FDR: 1.000e+00
Coefficient: −8.06e−02
Value: B
Vulcanibacillus.modesticaldus
0
1
2
3
4
A (n=15)
B (n=16)
C (n=14)
surface

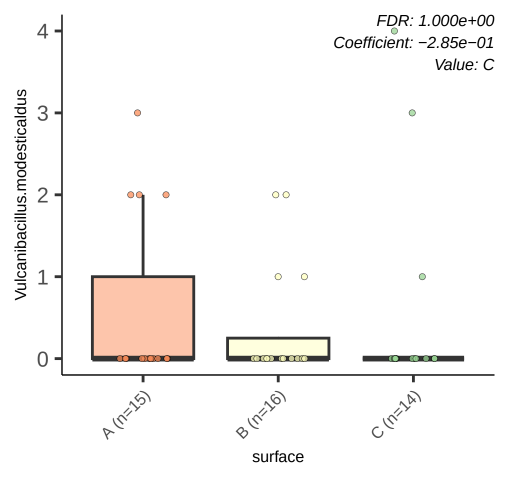

FDR: 1.000e+00
Coefficient: −2.85e−01
Value: C
Vulcanibacillus.modesticaldus
0
1
2
3
4
A (n=15)
B (n=16)
C (n=14)
surface

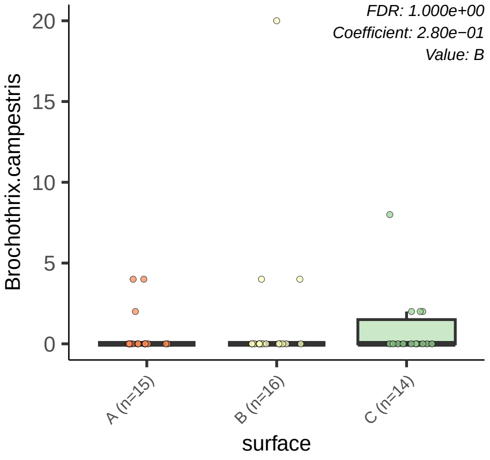

FDR: 1.000e+00
Coefficient: 2.80e−01
Value: B
Brochothrix.campestris
0
5
10
15
20
A (n=15)
B (n=16)
C (n=14)
surface

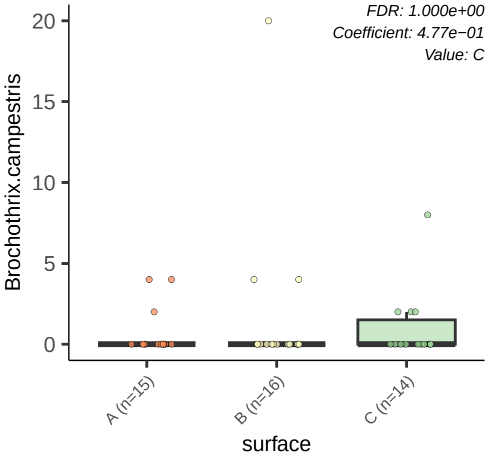

FDR: 1.000e+00
Coefficient: 4.77e−01
Value: C
Brochothrix.campestris
20
15
10
5
0
A (n=15)
B (n=16)
C (n=14)
surface

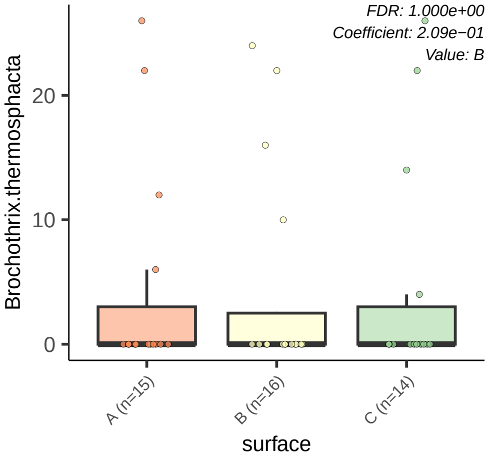

FDR: 1.000e+00
Coefficient: 2.09e−01
Value: B
Brochothrix.thermosphacta
20
10
0
A (n=15)
B (n=16)
C (n=14)
surface

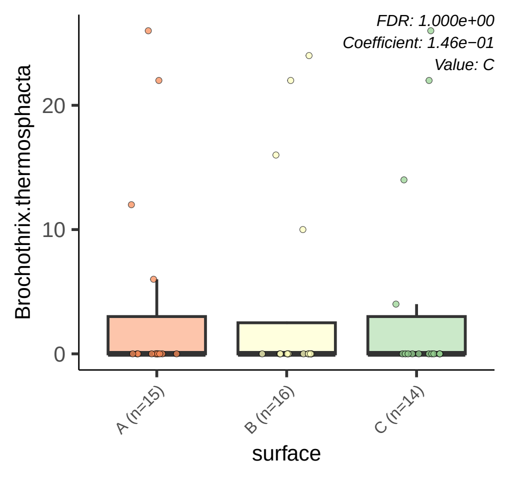
FDR: 1.000e+00
Coefficient: 1.46e−01
Value: C
Brochothrix.thermosphacta
20
10
0
A (n=15)
B (n=16)
C (n=14)
surface

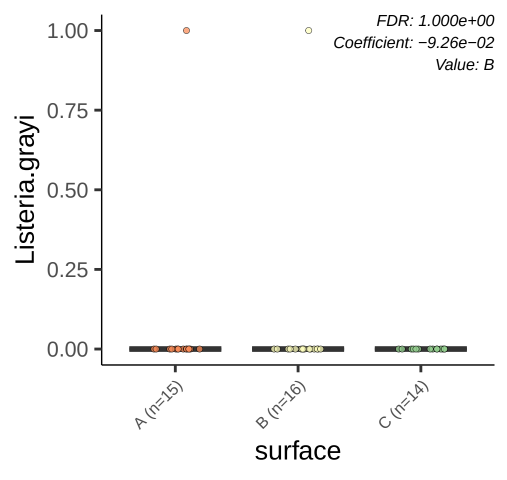

FDR: 1.000e+00
Coefficient: −9.26e−02
Value: B
Listeria.grayi
1.00
0.75
0.50
0.25
0.00
A (n=15)
B (n=16)
C (n=14)
surface

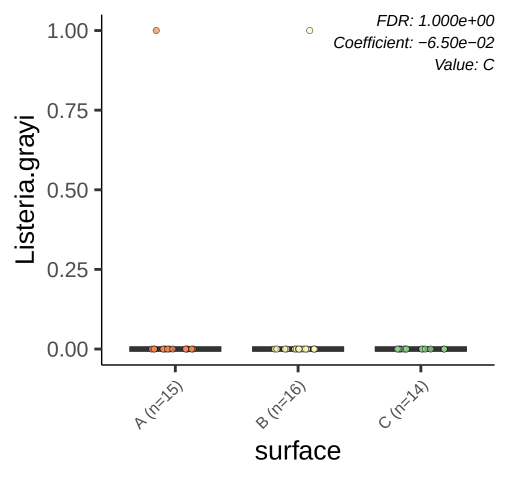
FDR: 1.000e+00
Coefficient: −6.50e−02
Value: C
Listeria.grayi
1.00
0.75
0.50
0.25
0.00
A (n=15)
B (n=16)
C (n=14)
surface

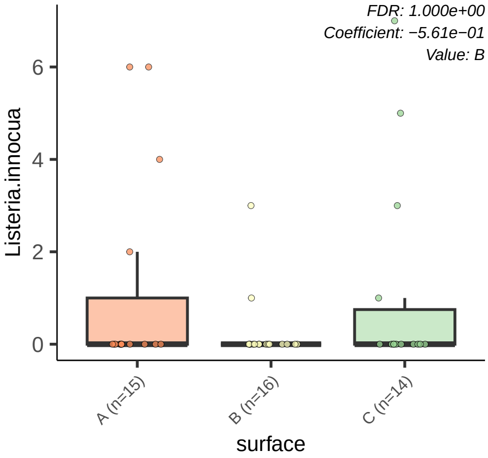
FDR: 1.000e+00
Coefficient: −5.61e−01
Value: B
Listeria.innocua
0
2
4
6
A (n=15)
B (n=16)
C (n=14)
surface

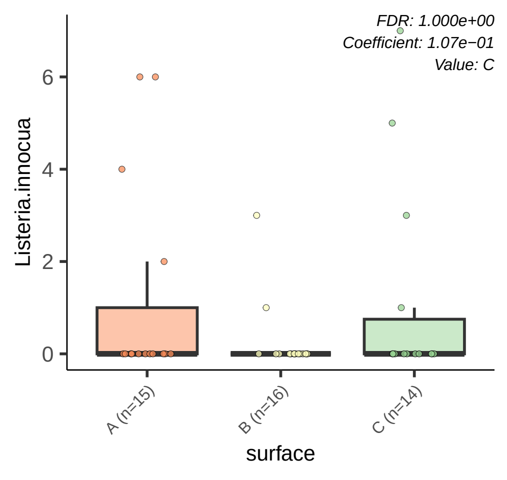
FDR: 1.000e+00
Coefficient: 1.07e−01
Value: C
Listeria.innocua
0
2
4
6
A (n=15)
B (n=16)
C (n=14)
surface

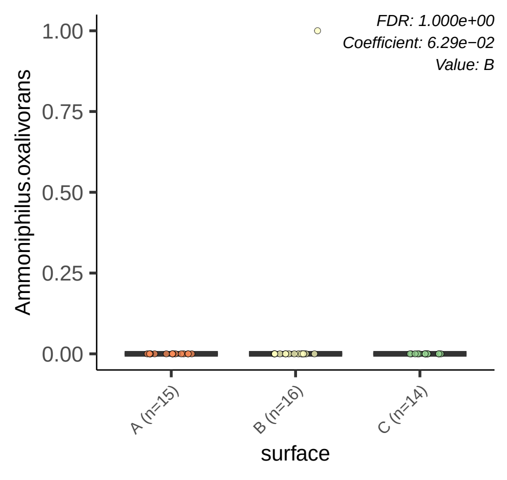
FDR: 1.000e+00
Coefficient: 6.29e−02
Value: B
Ammoniphilus.oxalivorans
1.00
0.75
0.50
0.25
0.00
A (n=15)
B (n=16)
C (n=14)
surface

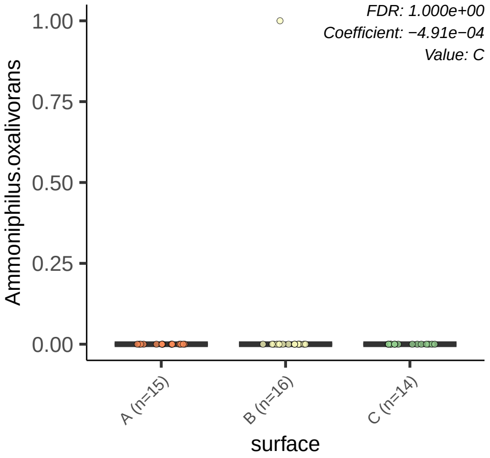
FDR: 1.000e+00
Coefficient: −4.91e−04
Value: C
Ammoniphilus.oxalivorans
1.00
0.75
0.50
0.25
0.00
A (n=15)
B (n=16)
C (n=14)
surface

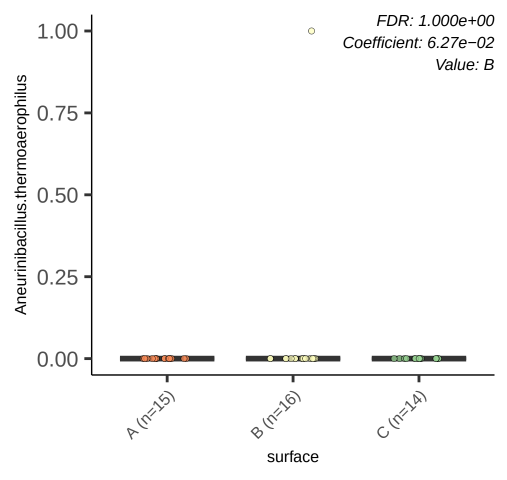
FDR: 1.000e+00
Coefficient: 6.27e−02
Value: B
Aneurinibacillus.thermoaerophilus
1.00
0.75
0.50
0.25
0.00
A (n=15)
B (n=16)
C (n=14)
surface

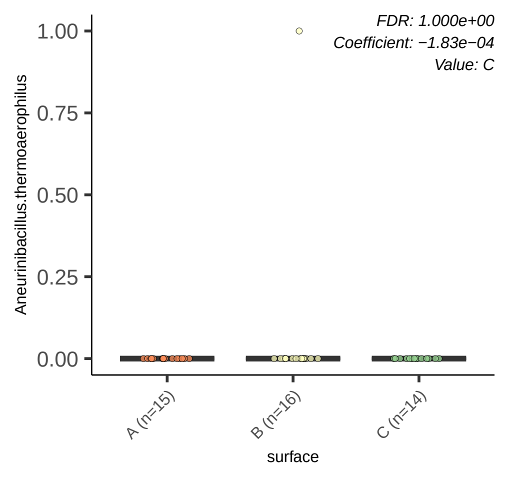
FDR: 1.000e+00
Coefficient: −1.83e−04
Value: C
Aneurinibacillus.thermoaerophilus
1.00
0.75
0.50
0.25
0.00
A (n=15)
B (n=16)
C (n=14)
surface

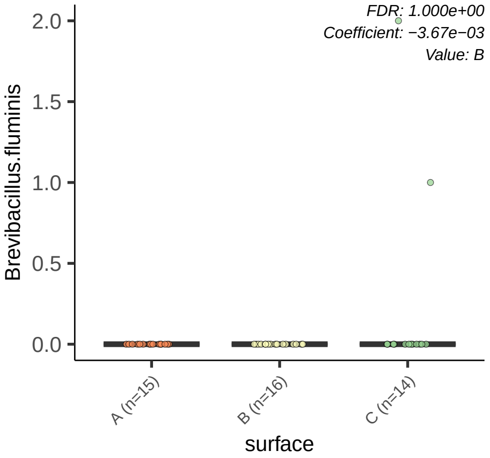
FDR: 1.000e+00
Coefficient: −3.67e−03
Value: B
Brevibacillus.fluminis
2.0
1.5
1.0
0.5
0.0
A (n=15)
B (n=16)
C (n=14)
surface

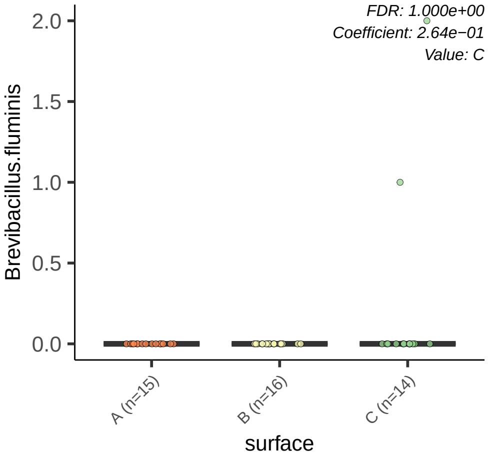
FDR: 1.000e+00
Coefficient: 2.64e−01
Value: C
Brevibacillus.fluminis
2.0
1.5
1.0
0.5
0.0
A (n=15)
B (n=16)
C (n=14)
surface

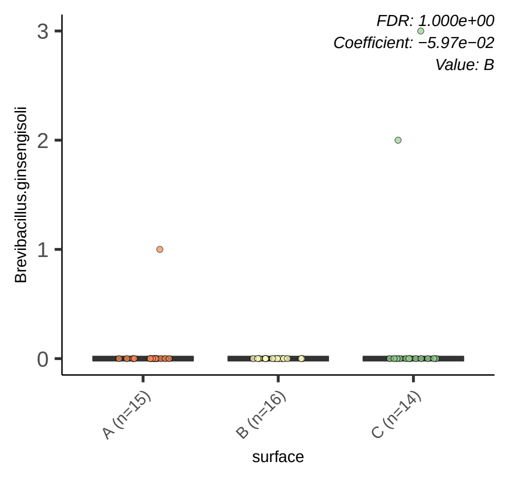
FDR: 1.000e+00
Coefficient: −5.97e−02
Value: B
Brevibacillus.ginsengisoli
3
2
1
0
A (n=15)
B (n=16)
C (n=14)
surface

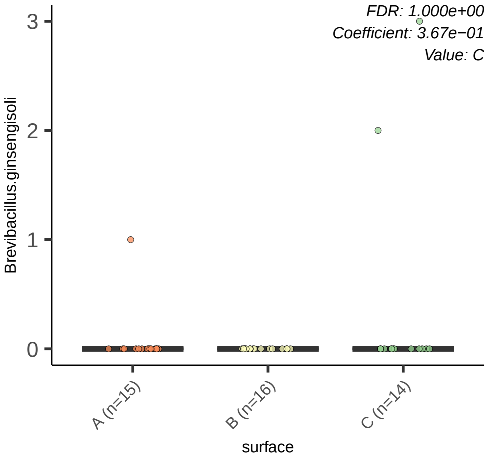
FDR: 1.000e+00
Coefficient: 3.67e−01
Value: C
Brevibacillus.ginsengisoli
3
2
1
0
A (n=15)
B (n=16)
C (n=14)
surface

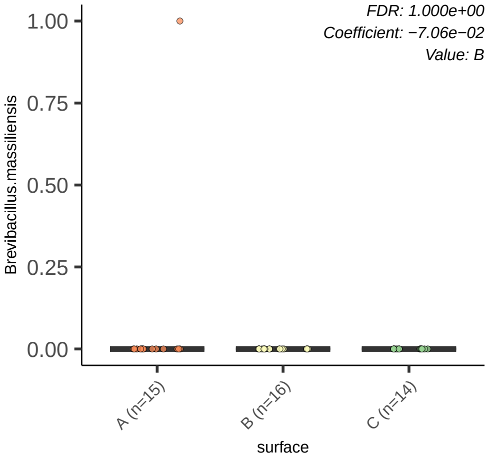
FDR: 1.000e+00
Coefficient: −7.06e−02
Value: B
Brevibacillus.massiliensis
1.00
0.75
0.50
0.25
0.00
A (n=15)
B (n=16)
C (n=14)
surface

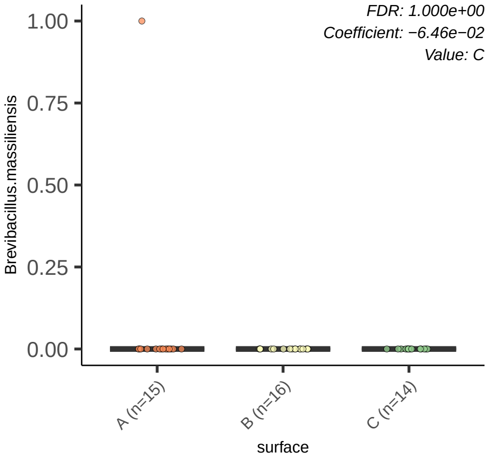
FDR: 1.000e+00
Coefficient: −6.46e−02
Value: C
Brevibacillus.massiliensis
1.00
0.75
0.50
0.25
0.00
A (n=15)
B (n=16)
C (n=14)
surface

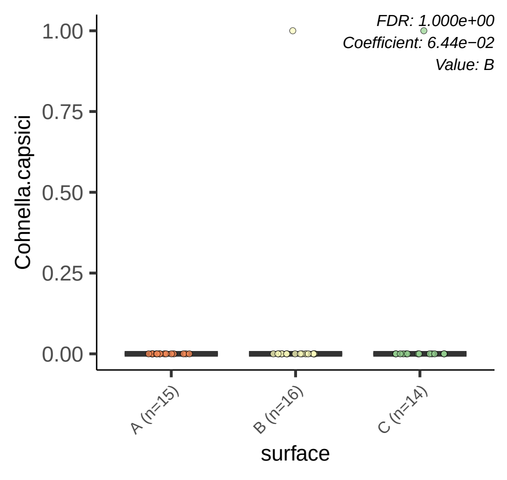
FDR: 1.000e+00
Coefficient: 6.44e−02
Value: B
Cohnella.capsici
1.00
0.75
0.50
0.25
0.00
A (n=15)
B (n=16)
C (n=14)
surface

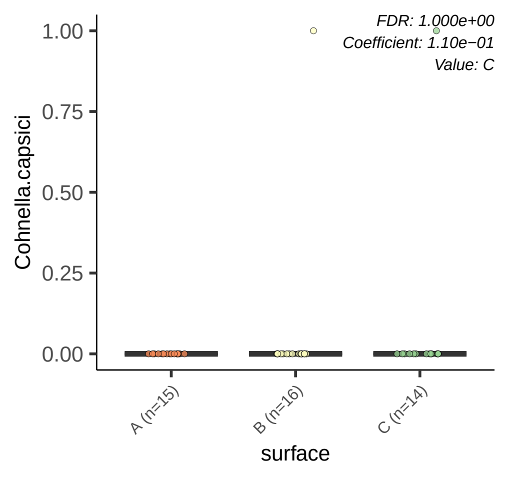

FDR: 1.000e+00
Coefficient: 1.10e−01
Value: C
Cohnella.capsici
1.00
0.75
0.50
0.25
0.00
A (n=15)
B (n=16)
C (n=14)
surface

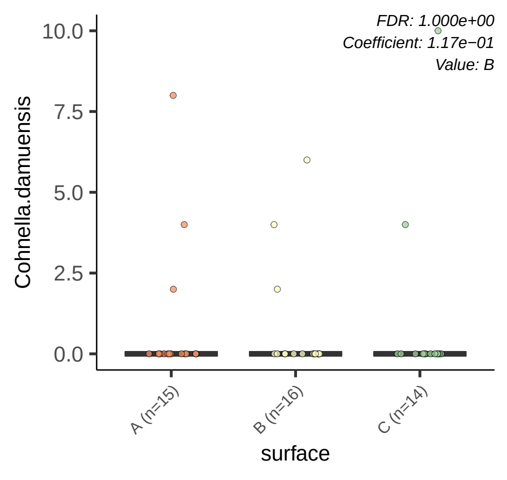
FDR: 1.000e+00
Coefficient: 1.17e−01
Value: B
Cohnella.damuensis
10.0
7.5
5.0
2.5
0.0
A (n=15)
B (n=16)
C (n=14)
surface

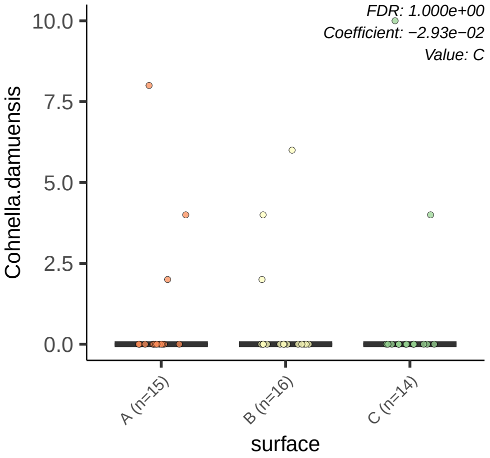

FDR: 1.000e+00
Coefficient: −2.93e−02
Value: C
10.0
7.5
5.0
2.5
0.0
Cohnella.damuensis
A (n=15)
B (n=16)
C (n=14)
surface

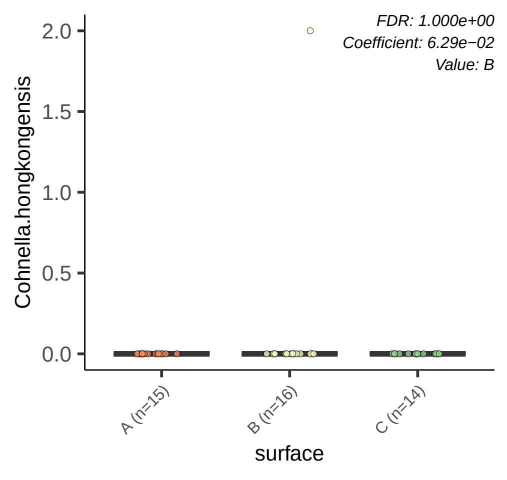
FDR: 1.000e+00
Coefficient: 6.29e−02
Value: B
Cohnella.hongkongensis
2.0
1.5
1.0
0.5
0.0
A (n=15)
B (n=16)
C (n=14)
surface

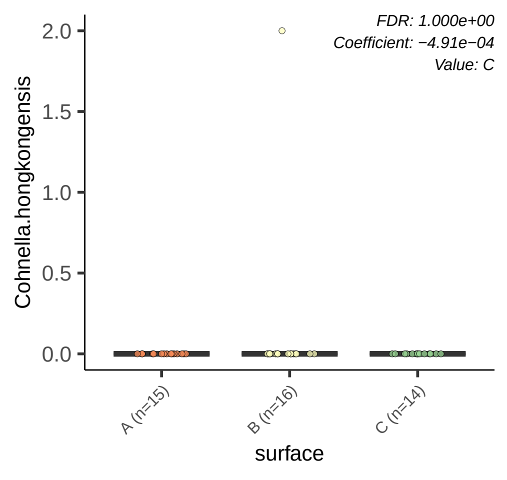

FDR: 1.000e+00
Coefficient: −4.91e−04
Value: C
Cohnella.hongkongensis
2.0
1.5
1.0
0.5
0.0
A (n=15)
B (n=16)
C (n=14)
surface

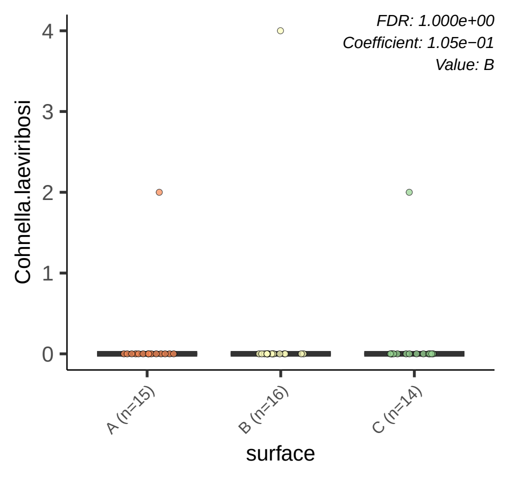
FDR: 1.000e+00
Coefficient: 1.05e−01
Value: B
Cohnella.laeviribosi
4
3
2
1
0
A (n=15)
B (n=16)
C (n=14)
surface

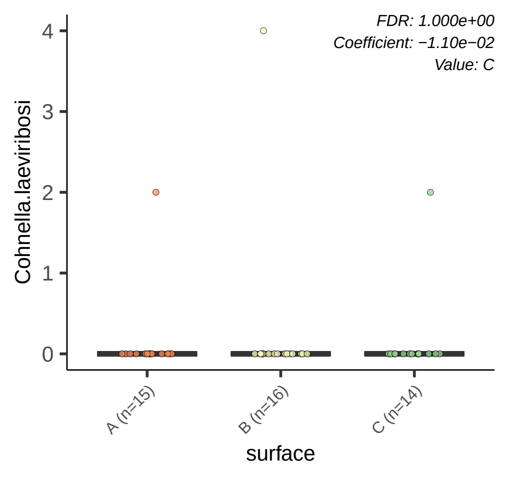
FDR: 1.000e+00
Coefficient: −1.10e−02
Value: C
Cohnella.laeviribosi
0
1
2
3
4
A (n=15)
B (n=16)
C (n=14)
surface

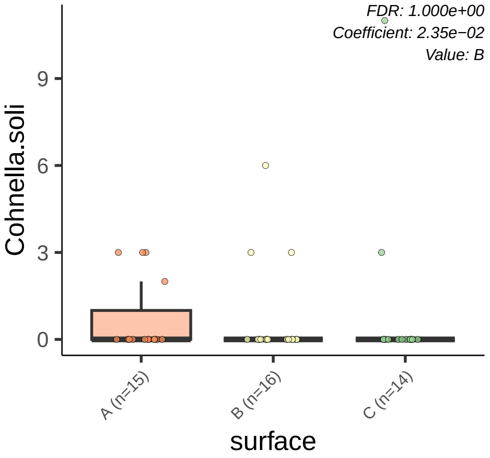
FDR: 1.000e+00
Coefficient: 2.35e−02
Value: B
Cohnella.soli
0
3
6
9
A (n=15)
B (n=16)
C (n=14)
surface

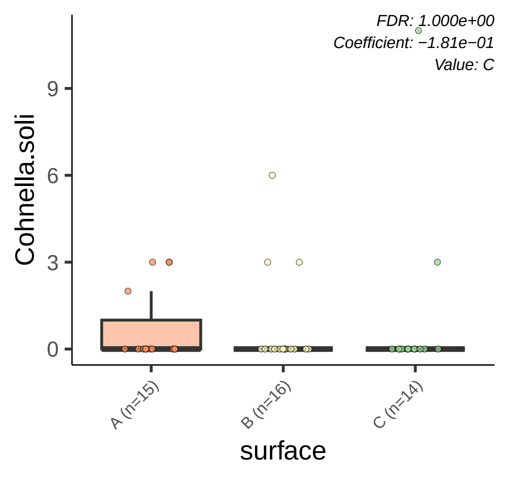
FDR: 1.000e+00
Coefficient: −1.81e−01
Value: C
Cohnella.soli
9
6
3
0
A (n=15)
B (n=16)
C (n=14)
surface

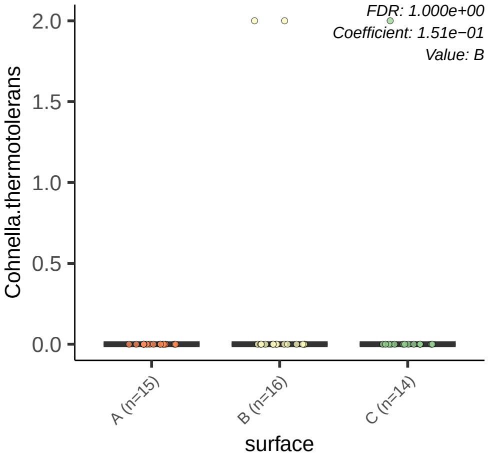
FDR: 1.000e+00
Coefficient: 1.51e−01
Value: B
2.0
1.5
1.0
0.5
0.0
Cohnella.thermotolerans
A (n=15)
B (n=16)
C (n=14)
surface

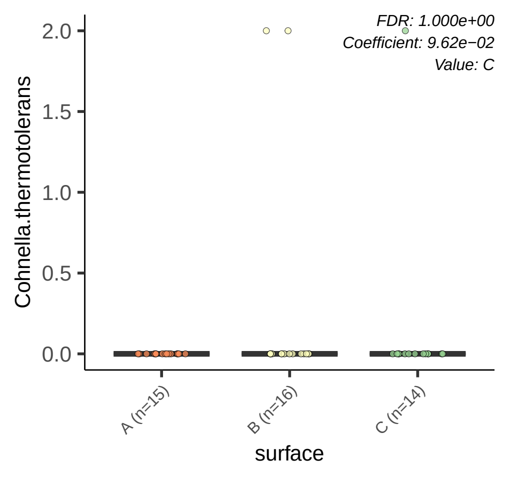

FDR: 1.000e+00
Coefficient: 9.62e−02
Value: C
Cohnella.thermotolerans
2.0
1.5
1.0
0.5
0.0
A (n=15)
B (n=16)
C (n=14)
surface

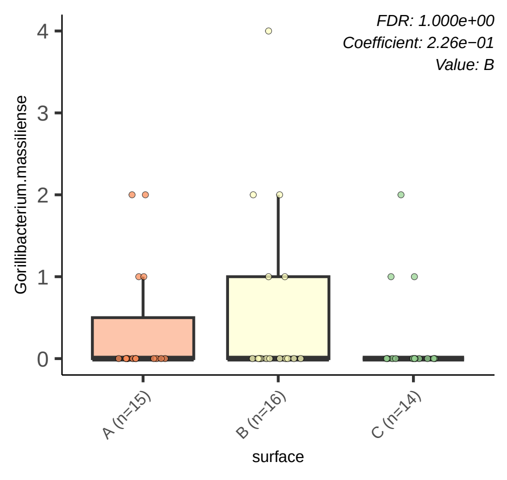

FDR: 1.000e+00
Coefficient: 2.26e−01
Value: B
Gorillibacterium.massiliense
0
1
2
3
4
A (n=15)
B (n=16)
C (n=14)
surface

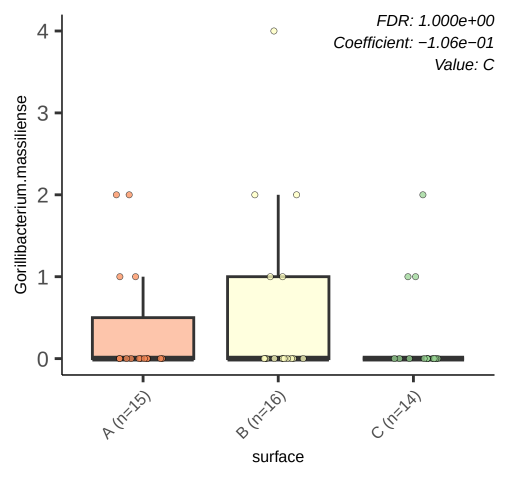

FDR: 1.000e+00
Coefficient: −1.06e−01
Value: C
Gorillibacterium.massiliense
0
1
2
3
4
A (n=15)
B (n=16)
C (n=14)
surface

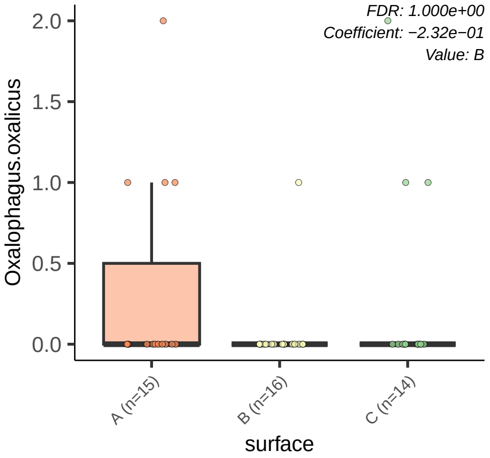
FDR: 1.000e+00
Coefficient: −2.32e−01
Value: B
Oxalophagus.oxalicus
2.0
1.5
1.0
0.5
0.0
A (n=15)
B (n=16)
C (n=14)
surface

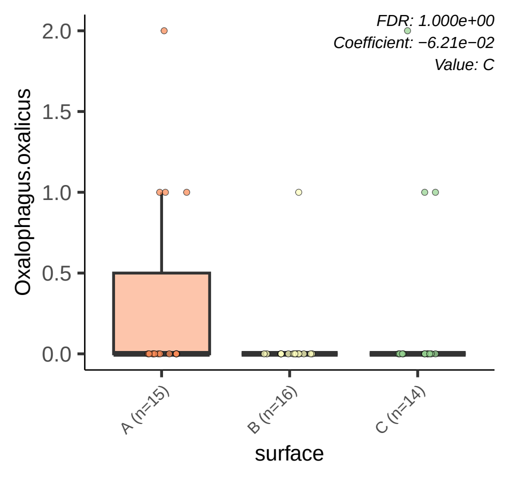

FDR: 1.000e+00
Coefficient: −6.21e−02
Value: C
Oxalophagus.oxalicus
2.0
1.5
1.0
0.5
0.0
A (n=15)
B (n=16)
C (n=14)
surface

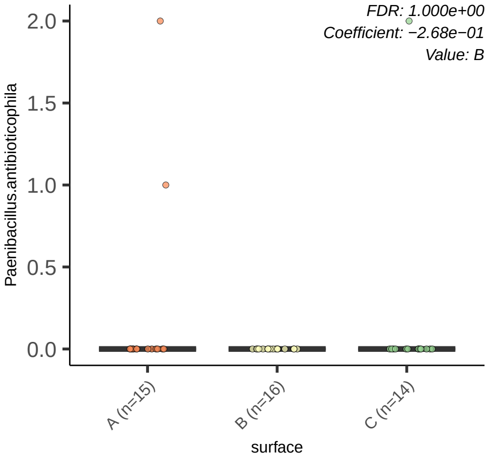
FDR: 1.000e+00
Coefficient: −2.68e−01
Value: B
Paenibacillus.antibioticophila
2.0
1.5
1.0
0.5
0.0
A (n=15)
B (n=16)
C (n=14)
surface

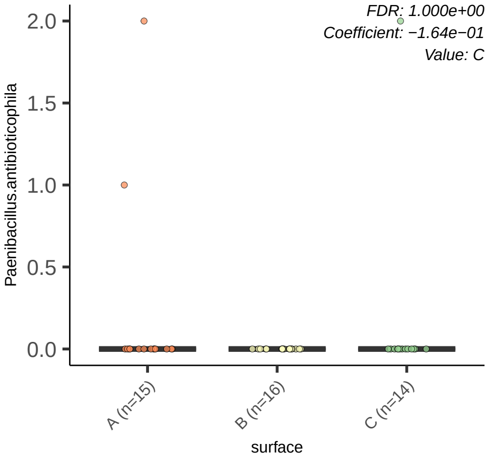

FDR: 1.000e+00
Coefficient: −1.64e−01
Value: C
Paenibacillus.antibioticophila
2.0
1.5
1.0
0.5
0.0
A (n=15)
B (n=16)
C (n=14)
surface

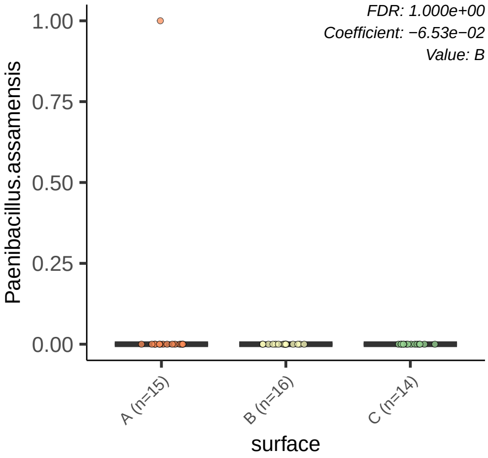

FDR: 1.000e+00
Coefficient: −6.53e−02
Value: B
Paenibacillus.assamensis
1.00
0.75
0.50
0.25
0.00
A (n=15)
B (n=16)
C (n=14)
surface

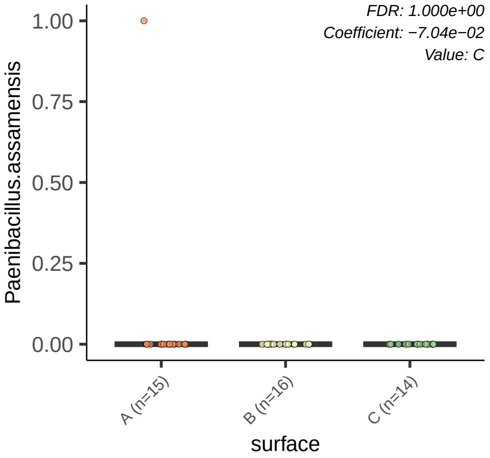
FDR: 1.000e+00
Coefficient: −7.04e−02
Value: C
Paenibacillus.assamensis
1.00
0.75
0.50
0.25
0.00
A (n=15)
B (n=16)
C (n=14)
surface

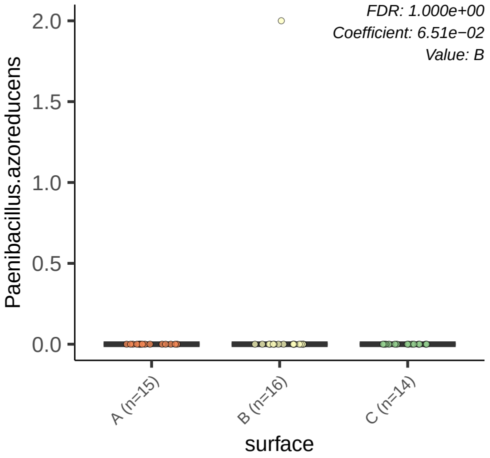

FDR: 1.000e+00
Coefficient: 6.51e−02
Value: B
Paenibacillus.azoreducens
2.0
1.5
1.0
0.5
0.0
A (n=15)
B (n=16)
C (n=14)
surface

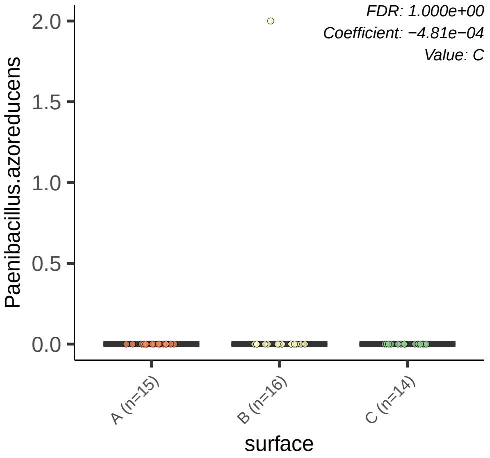
FDR: 1.000e+00
Coefficient: −4.81e−04
Value: C
Paenibacillus.azoreducens
2.0
1.5
1.0
0.5
0.0
A (n=15)
B (n=16)
C (n=14)
surface

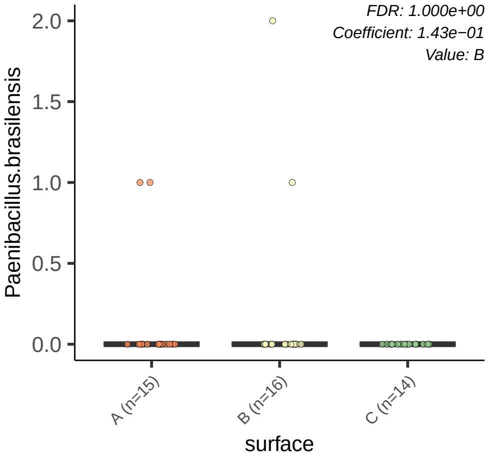

FDR: 1.000e+00
Coefficient: 1.43e−01
Value: B
Paenibacillus.brasilensis
2.0
1.5
1.0
0.5
0.0
A (n=15)
B (n=16)
C (n=14)
surface

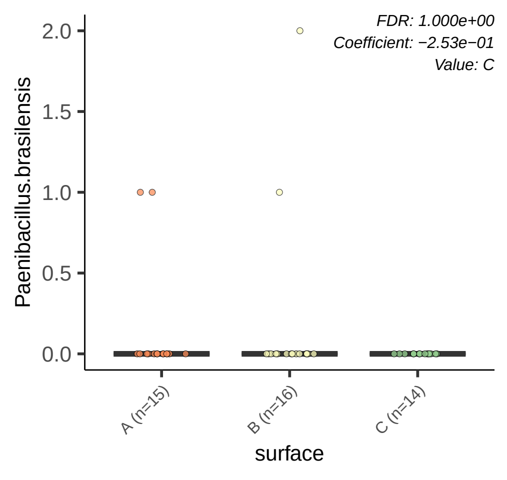
FDR: 1.000e+00
Coefficient: −2.53e−01
Value: C
Paenibacillus.brasilensis
2.0
1.5
1.0
0.5
0.0
A (n=15)
B (n=16)
C (n=14)
surface

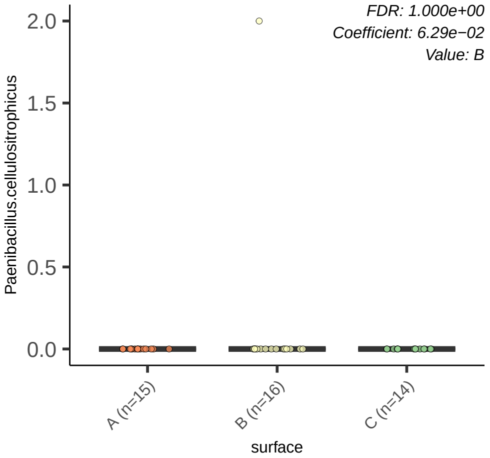
FDR: 1.000e+00
Coefficient: 6.29e−02
Value: B
Paenibacillus.cellulositrophicus
2.0
1.5
1.0
0.5
0.0
A (n=15)
B (n=16)
C (n=14)
surface

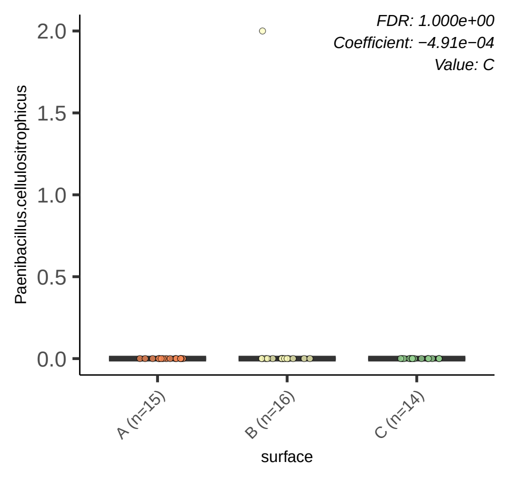
FDR: 1.000e+00
Coefficient: −4.91e−04
Value: C
Paenibacillus.cellulositrophicus
2.0
1.5
1.0
0.5
0.0
A (n=15)
B (n=16)
C (n=14)
surface

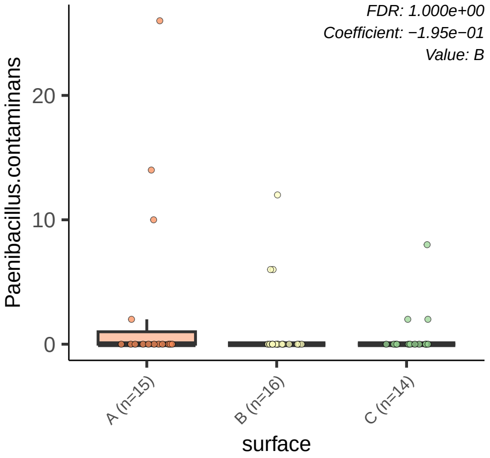
FDR: 1.000e+00
Coefficient: −1.95e−01
Value: B
Paenibacillus.contaminans
0
10
20
A (n=15)
B (n=16)
C (n=14)
surface

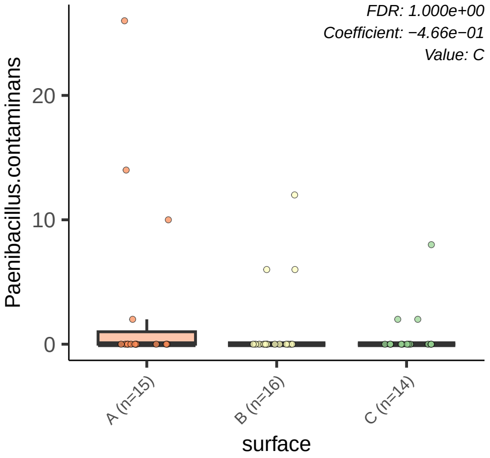
FDR: 1.000e+00
Coefficient: −4.66e−01
Value: C
Paenibacillus.contaminans
0
10
20
A (n=15)
B (n=16)
C (n=14)
surface

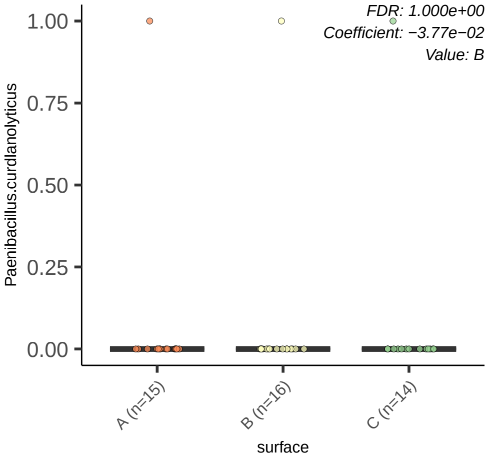

FDR: 1.000e+00
Coefficient: −3.77e−02
Value: B
1.00
0.75
0.50
0.25
0.00
Paenibacillus.curdlanolyticus
A (n=15)
B (n=16)
C (n=14)
surface

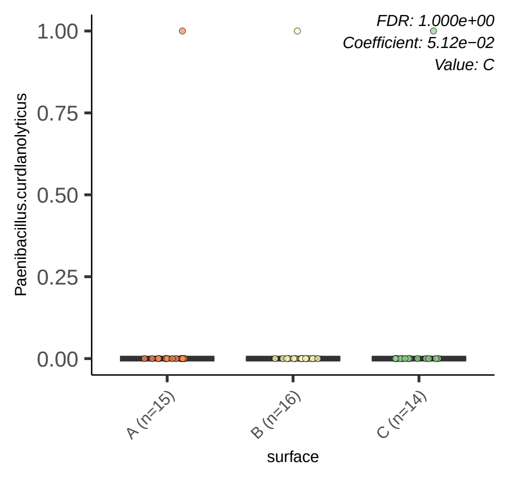
FDR: 1.000e+00
Coefficient: 5.12e−02
Value: C
Paenibacillus.curdlanolyticus
1.00
0.75
0.50
0.25
0.00
A (n=15)
B (n=16)
C (n=14)
surface

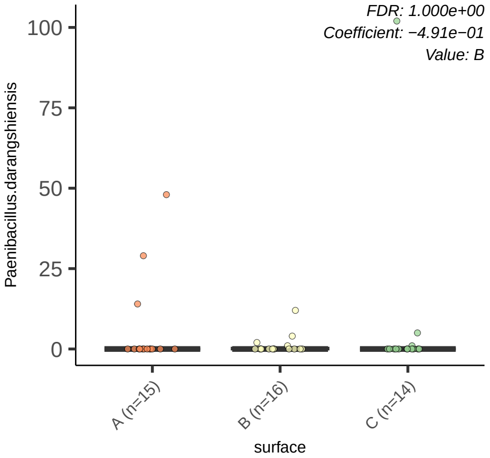
FDR: 1.000e+00
Coefficient: −4.91e−01
Value: B
Paenibacillus.darangshiensis
100
75
50
25
0
A (n=15)
B (n=16)
C (n=14)
surface

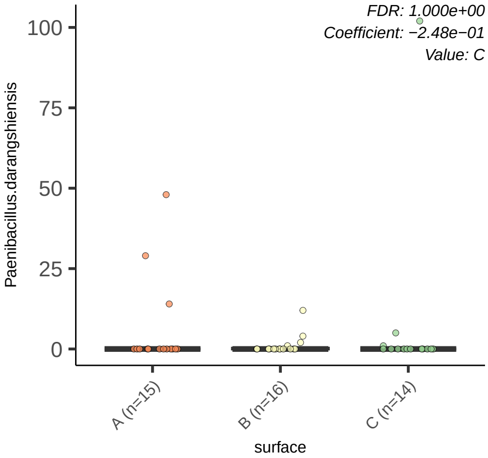
FDR: 1.000e+00
Coefficient: −2.48e−01
Value: C
Paenibacillus.darangshiensis
100
75
50
25
0
A (n=15)
B (n=16)
C (n=14)
surface

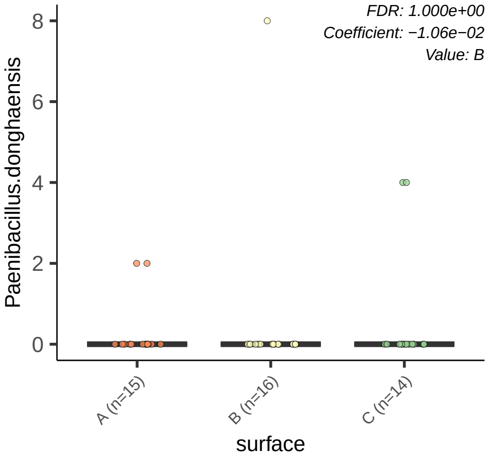
FDR: 1.000e+00
Coefficient: −1.06e−02
Value: B
Paenibacillus.donghaensis
8
6
4
2
0
A (n=15)
B (n=16)
C (n=14)
surface

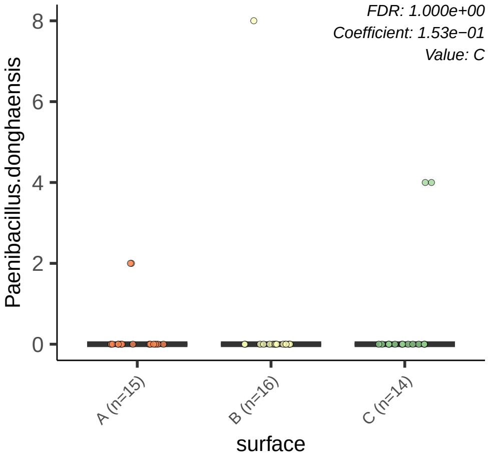

FDR: 1.000e+00
Coefficient: 1.53e−01
Value: C
Paenibacillus.donghaensis
0
2
4
6
8
A (n=15)
B (n=16)
C (n=14)
surface

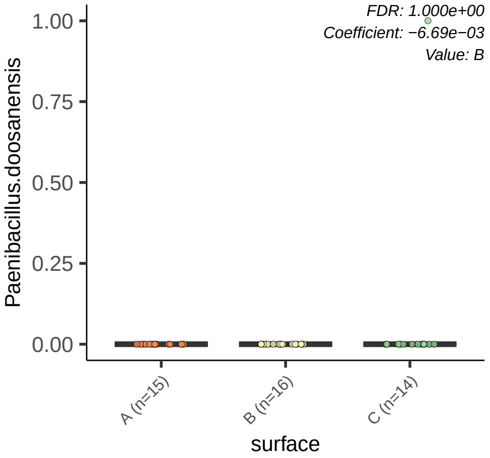
FDR: 1.000e+00
Coefficient: −6.69e−03
Value: B
Paenibacillus.doosanensis
1.00
0.75
0.50
0.25
0.00
A (n=15)
B (n=16)
C (n=14)
surface

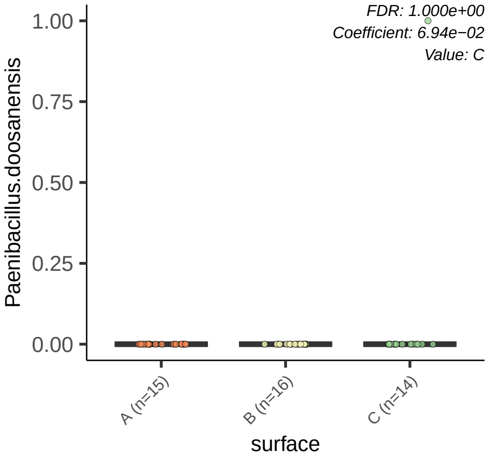

FDR: 1.000e+00
Coefficient: 6.94e−02
Value: C
Paenibacillus.doosanensis
1.00
0.75
0.50
0.25
0.00
A (n=15)
B (n=16)
C (n=14)
surface

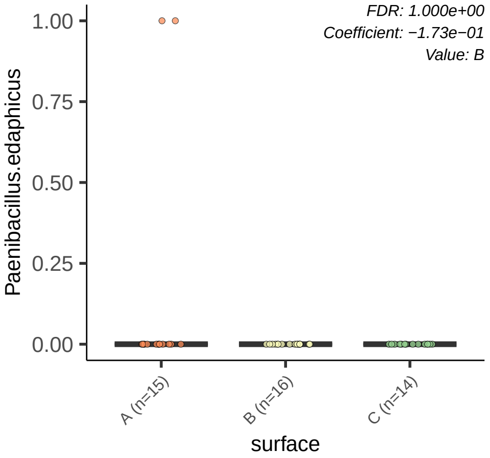
FDR: 1.000e+00
Coefficient: −1.73e−01
Value: B
Paenibacillus.edaphicus
1.00
0.75
0.50
0.25
0.00
A (n=15)
B (n=16)
C (n=14)
surface

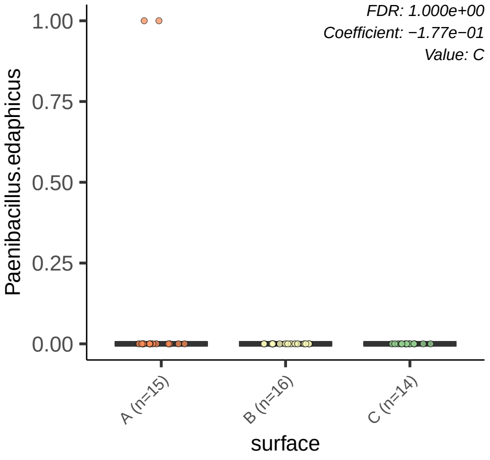
FDR: 1.000e+00
Coefficient: −1.77e−01
Value: C
Paenibacillus.edaphicus
1.00
0.75
0.50
0.25
0.00
A (n=15)
B (n=16)
C (n=14)
surface

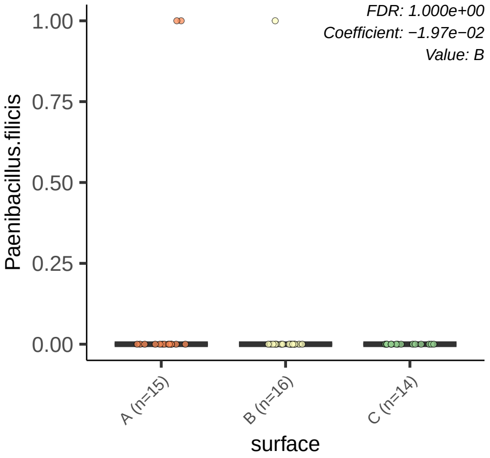
FDR: 1.000e+00
Coefficient: −1.97e−02
Value: B
Paenibacillus.filicis
1.00
0.75
0.50
0.25
0.00
A (n=15)
B (n=16)
C (n=14)
surface

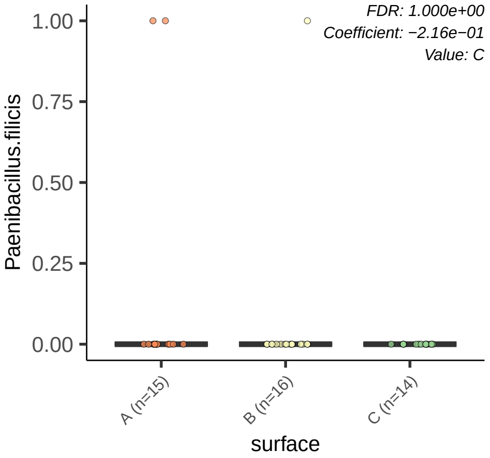
FDR: 1.000e+00
Coefficient: −2.16e−01
Value: C
Paenibacillus.filicis
1.00
0.75
0.50
0.25
0.00
A (n=15)
B (n=16)
C (n=14)
surface

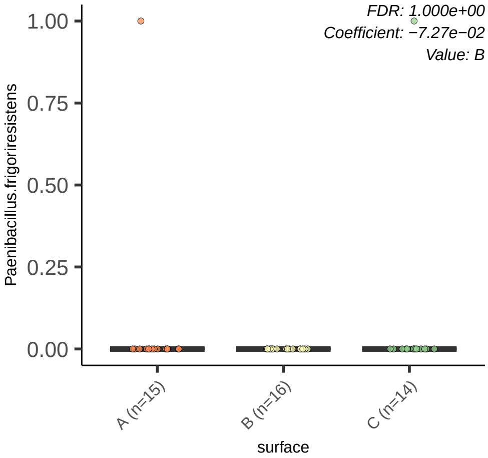
FDR: 1.000e+00
Coefficient: −7.27e−02
Value: B
1.00
0.75
0.50
0.25
0.00
Paenibacillus.frigoriresistens
A (n=15)
B (n=16)
C (n=14)
surface

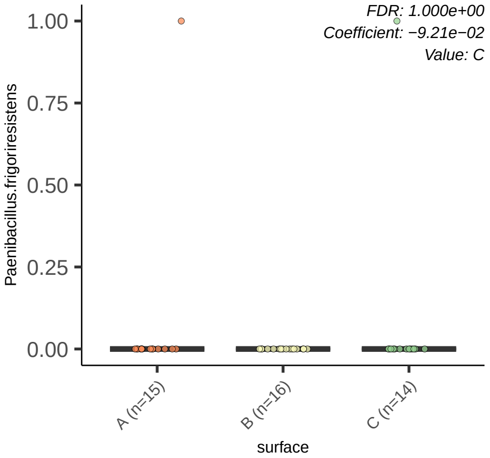
FDR: 1.000e+00
Coefficient: −9.21e−02
Value: C
Paenibacillus.frigoriresistens
1.00
0.75
0.50
0.25
0.00
A (n=15)
B (n=16)
C (n=14)
surface

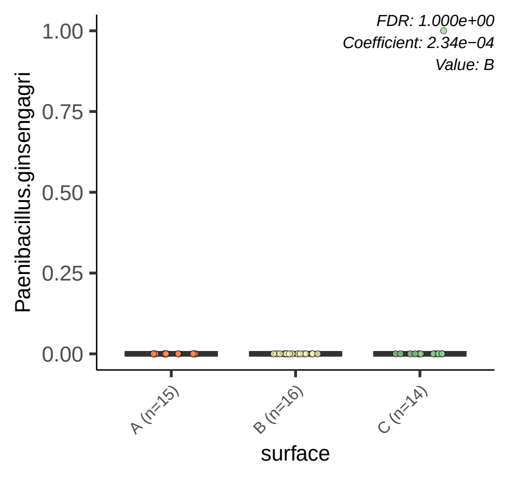
FDR: 1.000e+00
Coefficient: 2.34e−04
Value: B
Paenibacillus.ginsengagri
1.00
0.75
0.50
0.25
0.00
A (n=15)
B (n=16)
C (n=14)
surface

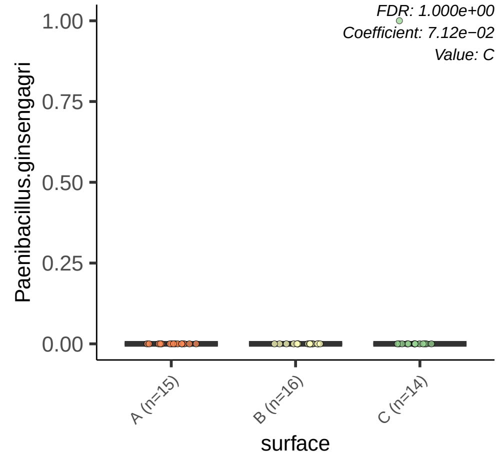
FDR: 1.000e+00
Coefficient: 7.12e−02
Value: C
Paenibacillus.ginsengagri
1.00
0.75
0.50
0.25
0.00
A (n=15)
B (n=16)
C (n=14)
surface

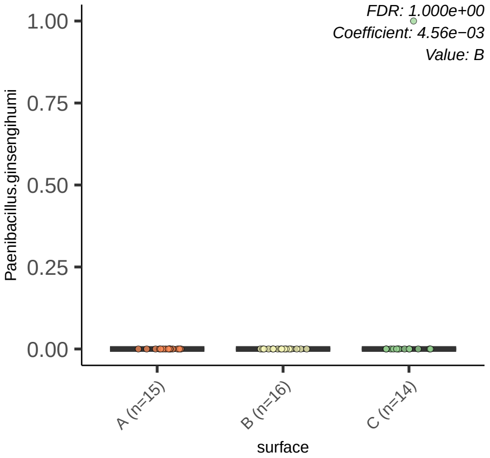

FDR: 1.000e+00
Coefficient: 4.56e−03
Value: B
1.00
0.75
0.50
0.25
0.00
Paenibacillus.ginsengihumi
A (n=15)
B (n=16)
C (n=14)
surface

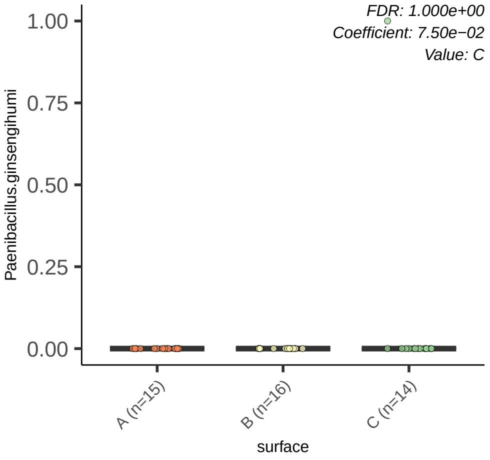
FDR: 1.000e+00
Coefficient: 7.50e−02
Value: C
1.00
0.75
0.50
0.25
0.00
Paenibacillus.ginsengihumi
A (n=15)
B (n=16)
C (n=14)
surface

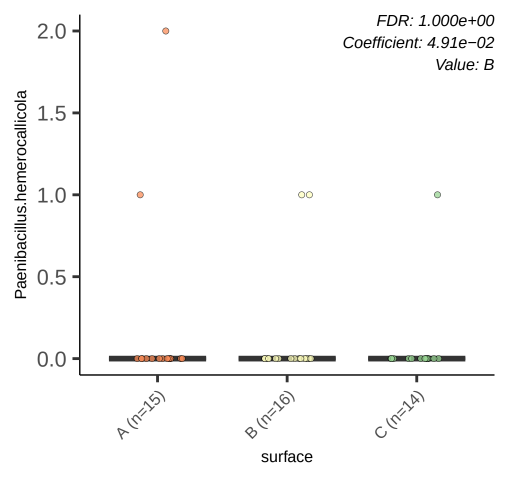
FDR: 1.000e+00
Coefficient: 4.91e−02
Value: B
Paenibacillus.hemerocallicola
2.0
1.5
1.0
0.5
0.0
A (n=15)
B (n=16)
C (n=14)
surface

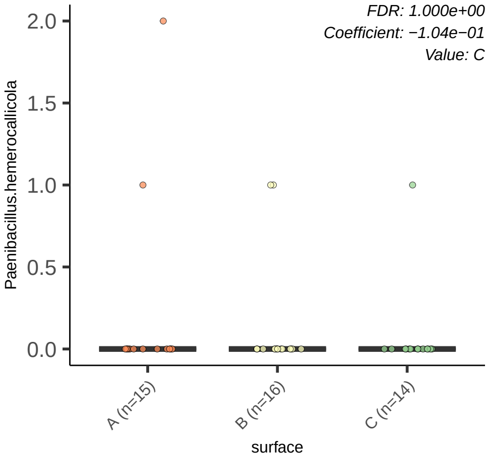
FDR: 1.000e+00
Coefficient: −1.04e−01
Value: C
Paenibacillus.hemerocallicola
2.0
1.5
1.0
0.5
0.0
A (n=15)
B (n=16)
C (n=14)
surface

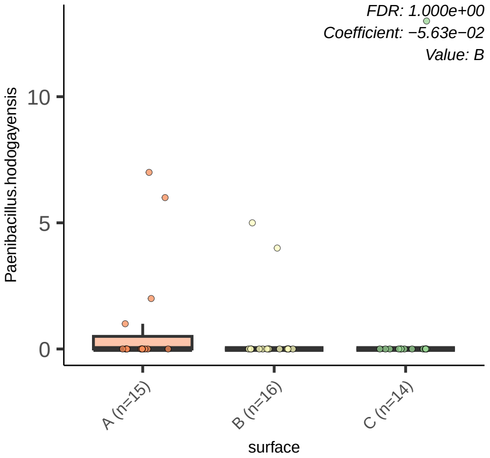
FDR: 1.000e+00
Coefficient: −5.63e−02
Value: B
Paenibacillus.hodogayensis
10
5
0
A (n=15)
B (n=16)
C (n=14)
surface

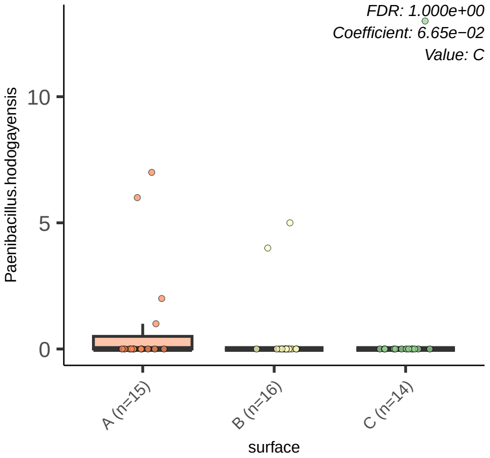
FDR: 1.000e+00
Coefficient: 6.65e−02
Value: C
Paenibacillus.hodogayensis
10
5
0
A (n=15)
B (n=16)
C (n=14)
surface

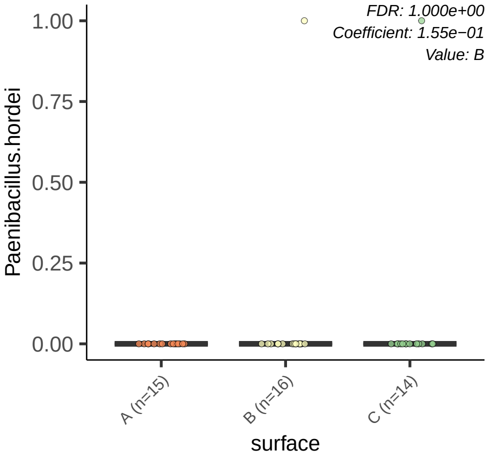
FDR: 1.000e+00
Coefficient: 1.55e−01
Value: B
Paenibacillus.hordei
1.00
0.75
0.50
0.25
0.00
A (n=15)
B (n=16)
C (n=14)
surface

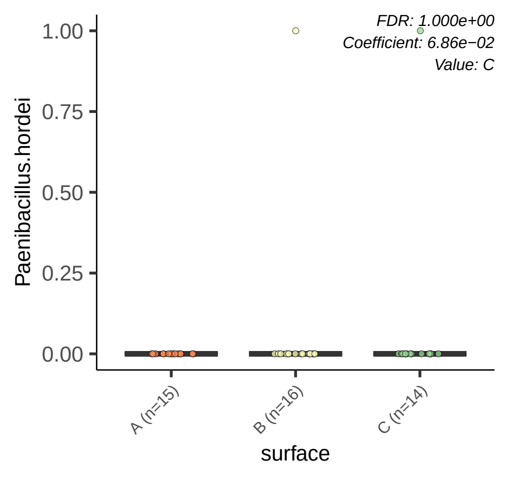
FDR: 1.000e+00
Coefficient: 6.86e−02
Value: C
Paenibacillus.hordei
1.00
0.75
0.50
0.25
0.00
A (n=15)
B (n=16)
C (n=14)
surface

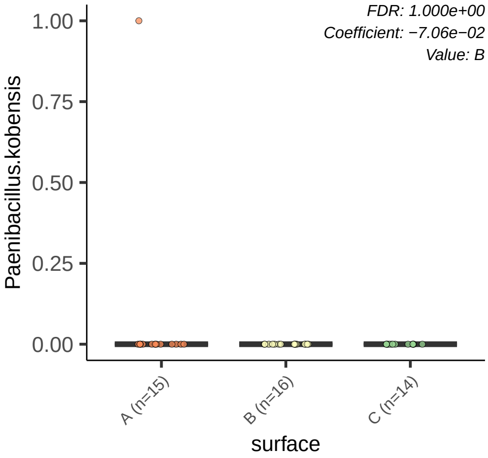

FDR: 1.000e+00
Coefficient: −7.06e−02
Value: B
Paenibacillus.kobensis
1.00
0.75
0.50
0.25
0.00
A (n=15)
B (n=16)
C (n=14)
surface

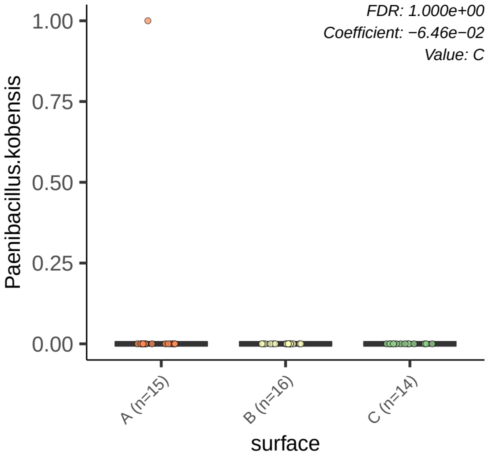
FDR: 1.000e+00
Coefficient: −6.46e−02
Value: C
Paenibacillus.kobensis
1.00
0.75
0.50
0.25
0.00
A (n=15)
B (n=16)
C (n=14)
surface

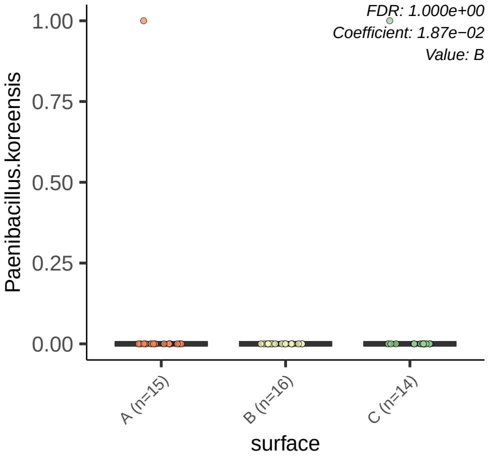
FDR: 1.000e+00
Coefficient: 1.87e−02
Value: B
Paenibacillus.koreensis
1.00
0.75
0.50
0.25
0.00
A (n=15)
B (n=16)
C (n=14)
surface

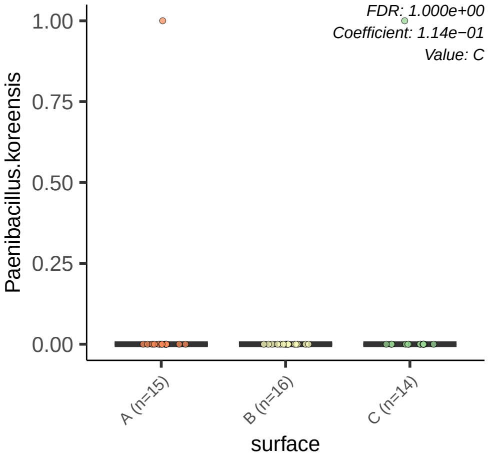
FDR: 1.000e+00
Coefficient: 1.14e−01
Value: C
Paenibacillus.koreensis
1.00
0.75
0.50
0.25
0.00
A (n=15)
B (n=16)
C (n=14)
surface

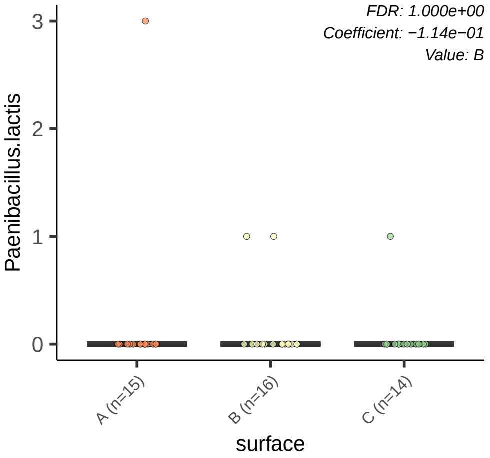
FDR: 1.000e+00
Coefficient: −1.14e−01
Value: B
Paenibacillus.lactis
3
2
1
0
A (n=15)
B (n=16)
C (n=14)
surface

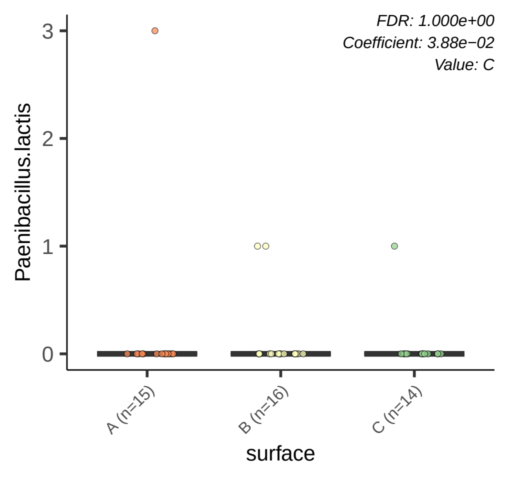
FDR: 1.000e+00
Coefficient: 3.88e−02
Value: C
Paenibacillus.lactis
3
2
1
0
A (n=15)
B (n=16)
C (n=14)
surface

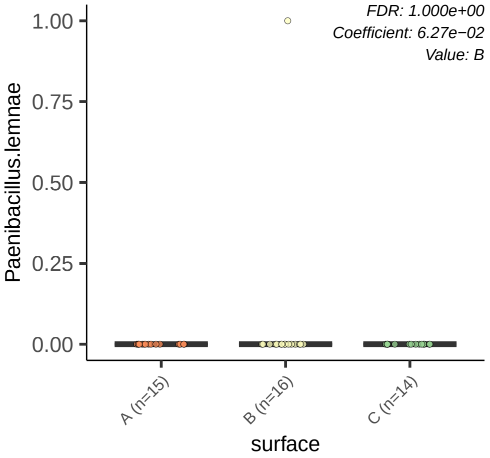
FDR: 1.000e+00
Coefficient: 6.27e−02
Value: B
Paenibacillus.lemnae
1.00
0.75
0.50
0.25
0.00
A (n=15)
B (n=16)
C (n=14)
surface

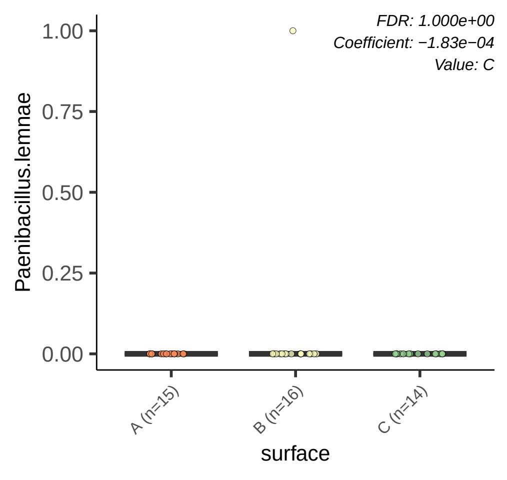
FDR: 1.000e+00
Coefficient: −1.83e−04
Value: C
Paenibacillus.lemnae
1.00
0.75
0.50
0.25
0.00
A (n=15)
B (n=16)
C (n=14)
surface

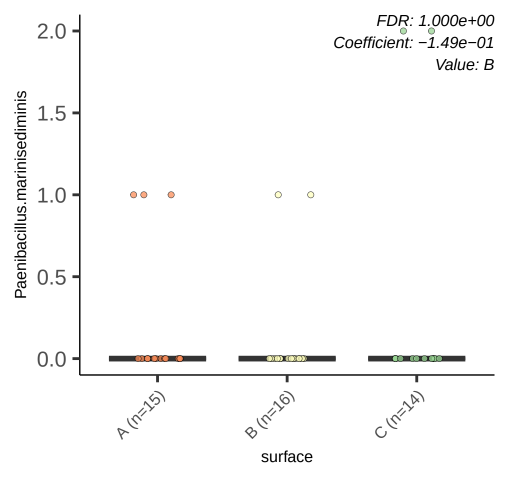
FDR: 1.000e+00
Coefficient: −1.49e−01
Value: B
Paenibacillus.marinisediminis
2.0
1.5
1.0
0.5
0.0
A (n=15)
B (n=16)
C (n=14)
surface

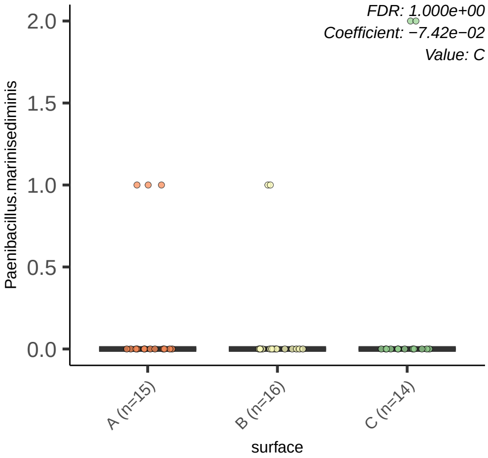
FDR: 1.000e+00
Coefficient: −7.42e−02
Value: C
Paenibacillus.marinisediminis
2.0
1.5
1.0
0.5
0.0
A (n=15)
B (n=16)
C (n=14)
surface

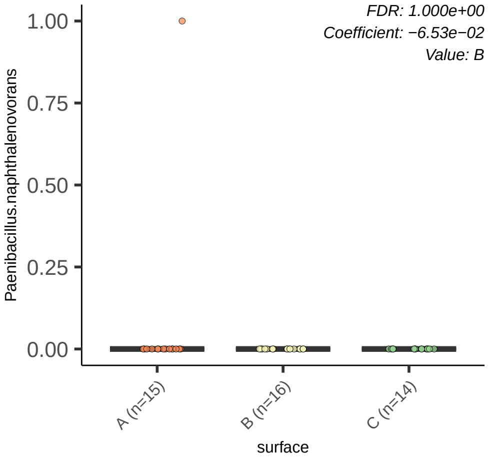
FDR: 1.000e+00
Coefficient: −6.53e−02
Value: B
Paenibacillus.naphthalenovorans
1.00
0.75
0.50
0.25
0.00
A (n=15)
B (n=16)
C (n=14)
surface

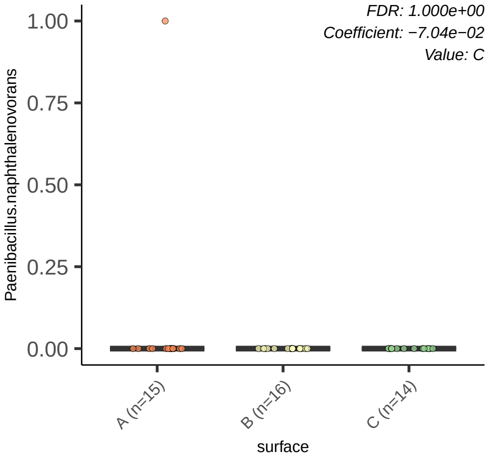
FDR: 1.000e+00
Coefficient: −7.04e−02
Value: C
Paenibacillus.naphthalenovorans
1.00
0.75
0.50
0.25
0.00
A (n=15)
B (n=16)
C (n=14)
surface

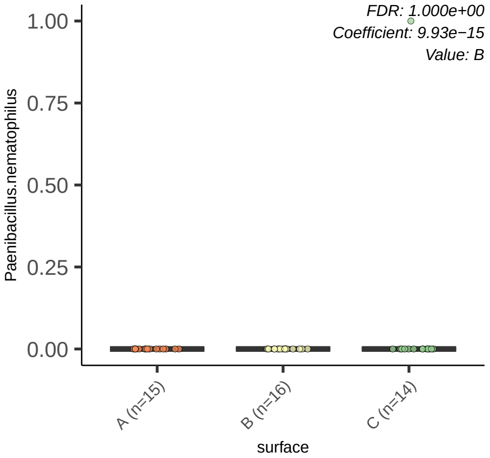
FDR: 1.000e+00
Coefficient: 9.93e−15
Value: B
1.00
0.75
0.50
0.25
0.00
Paenibacillus.nematophilus
A (n=15)
B (n=16)
C (n=14)
surface

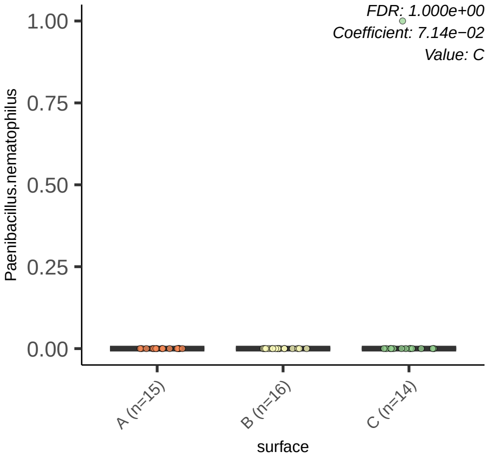
FDR: 1.000e+00
Coefficient: 7.14e−02
Value: C
1.00
0.75
0.50
0.25
0.00
Paenibacillus.nematophilus
A (n=15)
B (n=16)
C (n=14)
surface

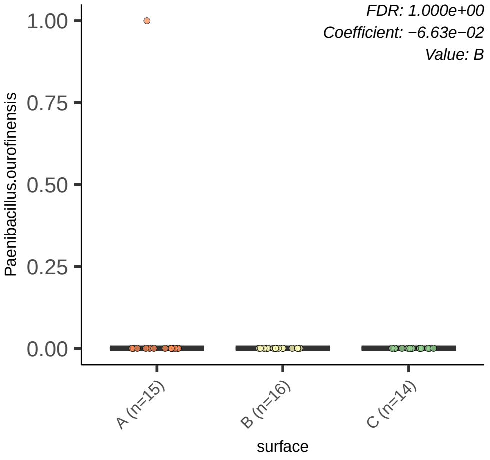
FDR: 1.000e+00
Coefficient: −6.63e−02
Value: B
Paenibacillus.ourofinensis
1.00
0.75
0.50
0.25
0.00
A (n=15)
B (n=16)
C (n=14)
surface

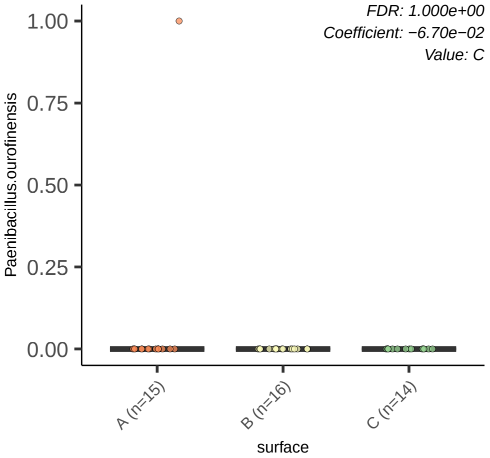

FDR: 1.000e+00
Coefficient: −6.70e−02
Value: C
Paenibacillus.ourofinensis
1.00
0.75
0.50
0.25
0.00
A (n=15)
B (n=16)
C (n=14)
surface

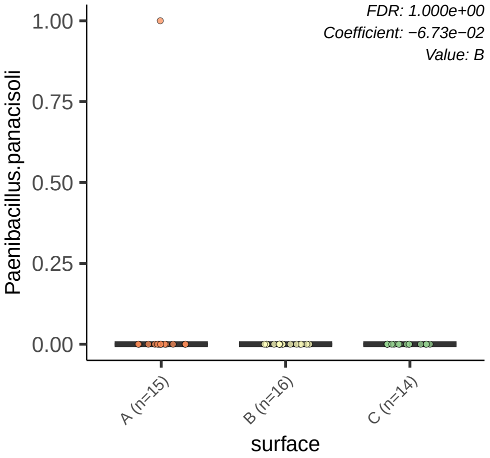

FDR: 1.000e+00
Coefficient: −6.73e−02
Value: B
Paenibacillus.panacisoli
1.00
0.75
0.50
0.25
0.00
A (n=15)
B (n=16)
C (n=14)
surface

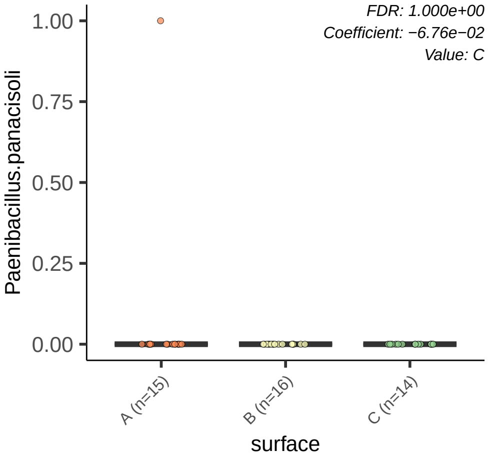
FDR: 1.000e+00
Coefficient: −6.76e−02
Value: C
Paenibacillus.panacisoli
1.00
0.75
0.50
0.25
0.00
A (n=15)
B (n=16)
C (n=14)
surface

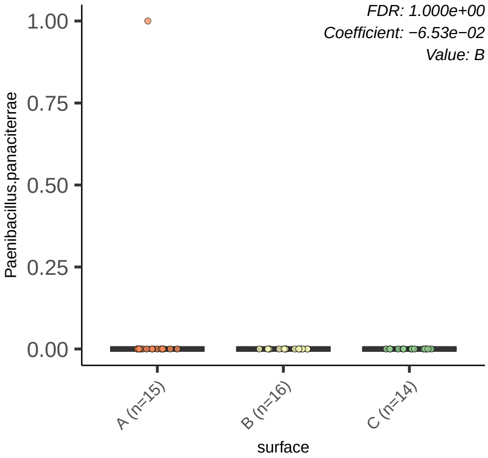

FDR: 1.000e+00
Coefficient: −6.53e−02
Value: B
1.00
0.75
0.50
0.25
0.00
Paenibacillus.panaciterrae
A (n=15)
B (n=16)
C (n=14)
surface

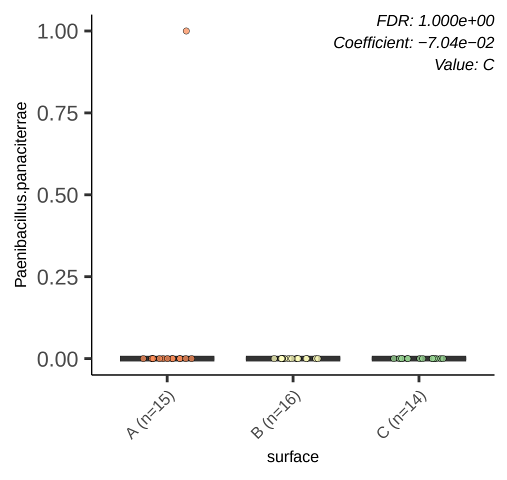

FDR: 1.000e+00
Coefficient: −7.04e−02
Value: C
Paenibacillus.panaciterrae
1.00
0.75
0.50
0.25
0.00
A (n=15)
B (n=16)
C (n=14)
surface

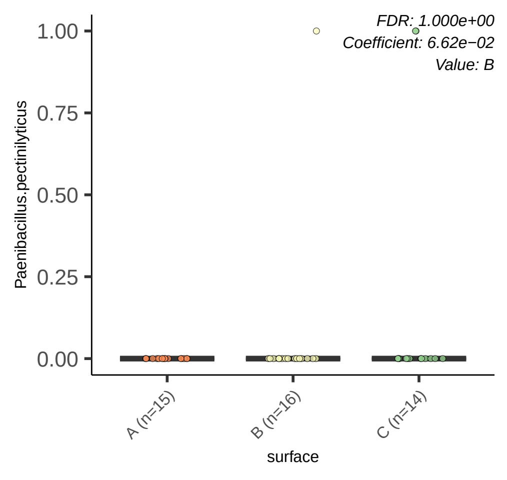
FDR: 1.000e+00
Coefficient: 6.62e−02
Value: B
1.00
0.75
0.50
0.25
0.00
Paenibacillus.pectinilyticus
A (n=15)
B (n=16)
C (n=14)
surface

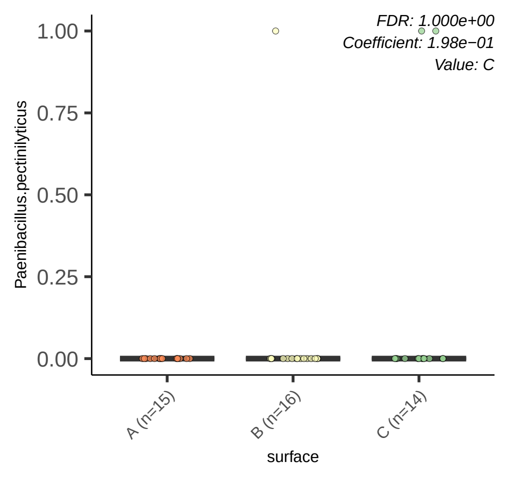
FDR: 1.000e+00
Coefficient: 1.98e−01
Value: C
1.00
0.75
0.50
0.25
0.00
Paenibacillus.pectinilyticus
A (n=15)
B (n=16)
C (n=14)
surface

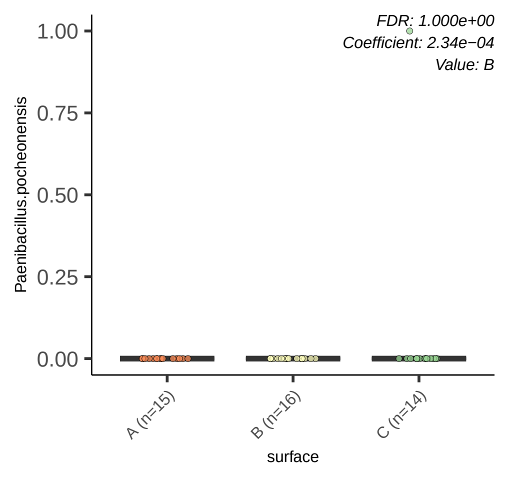
FDR: 1.000e+00
Coefficient: 2.34e−04
Value: B
Paenibacillus.pocheonensis
1.00
0.75
0.50
0.25
0.00
A (n=15)
B (n=16)
C (n=14)
surface

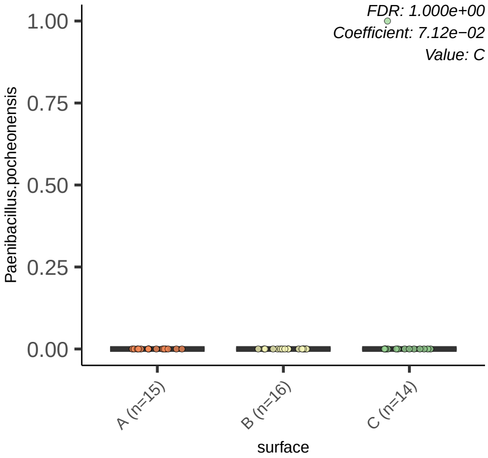
FDR: 1.000e+00
Coefficient: 7.12e−02
Value: C
Paenibacillus.pocheonensis
1.00
0.75
0.50
0.25
0.00
A (n=15)
B (n=16)
C (n=14)
surface

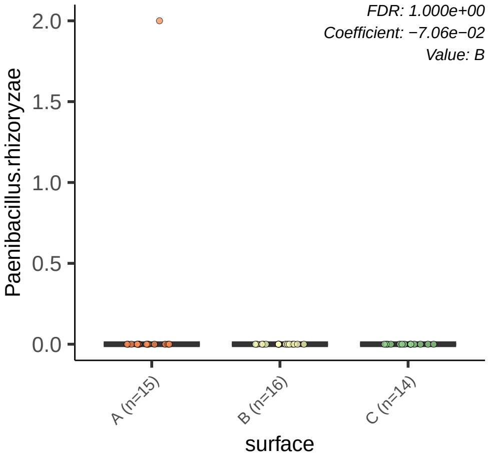
FDR: 1.000e+00
Coefficient: −7.06e−02
Value: B
Paenibacillus.rhizoryzae
2.0
1.5
1.0
0.5
0.0
A (n=15)
B (n=16)
C (n=14)
surface

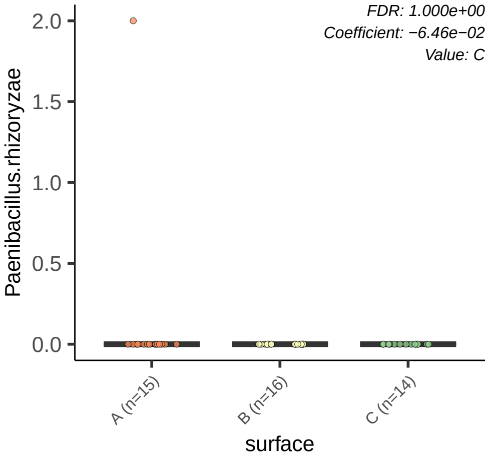

FDR: 1.000e+00
Coefficient: −6.46e−02
Value: C
2.0
1.5
1.0
0.5
0.0
Paenibacillus.rhizoryzae
A (n=15)
B (n=16)
C (n=14)
surface

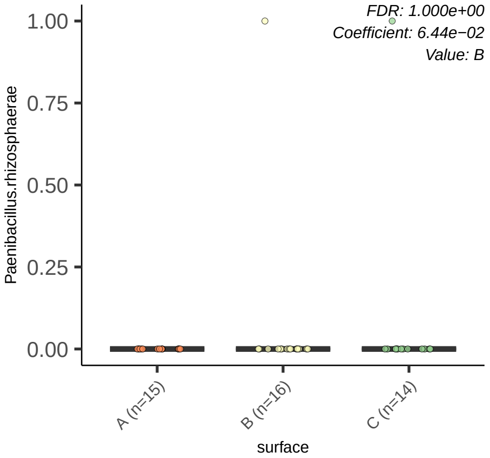

FDR: 1.000e+00
Coefficient: 6.44e−02
Value: B
Paenibacillus.rhizosphaerae
1.00
0.75
0.50
0.25
0.00
A (n=15)
B (n=16)
C (n=14)
surface

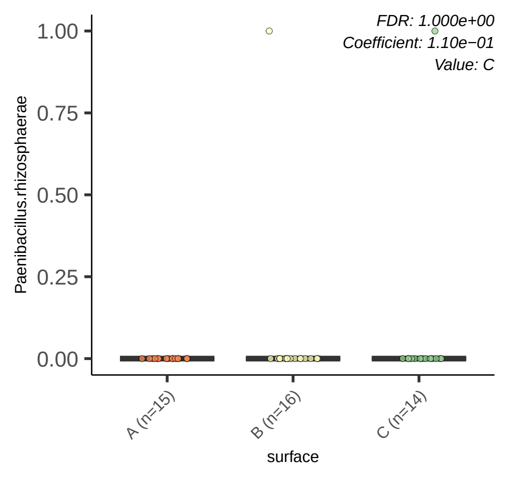
FDR: 1.000e+00
Coefficient: 1.10e−01
Value: C
Paenibacillus.rhizosphaerae
1.00
0.75
0.50
0.25
0.00
A (n=15)
B (n=16)
C (n=14)
surface

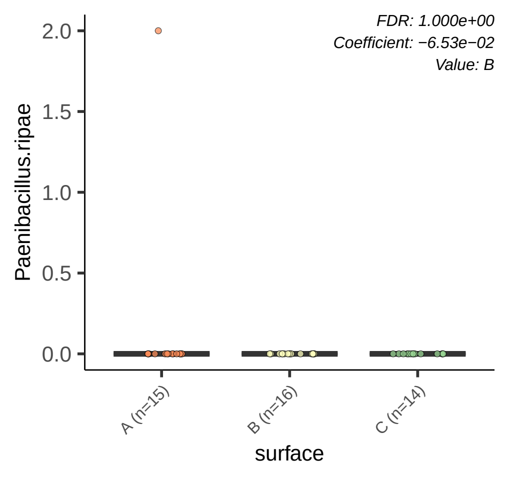
FDR: 1.000e+00
Coefficient: −6.53e−02
Value: B
Paenibacillus.ripae
2.0
1.5
1.0
0.5
0.0
A (n=15)
B (n=16)
C (n=14)
surface

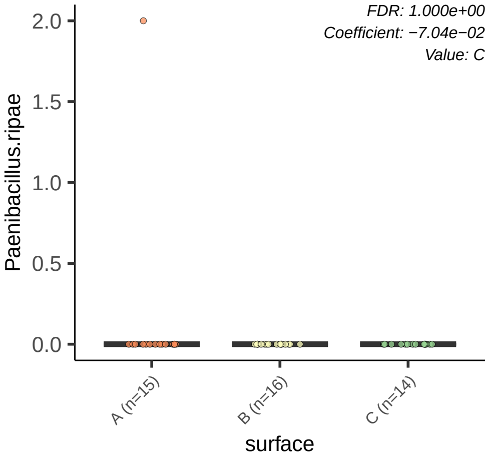
FDR: 1.000e+00
Coefficient: −7.04e−02
Value: C
Paenibacillus.ripae
2.0
1.5
1.0
0.5
0.0
A (n=15)
B (n=16)
C (n=14)
surface

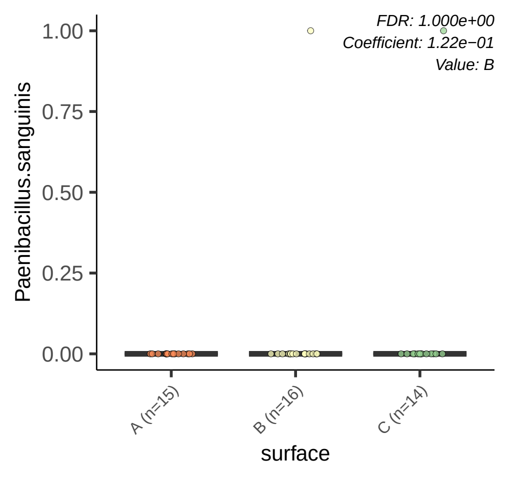

FDR: 1.000e+00
Coefficient: 1.22e−01
Value: B
Paenibacillus.sanguinis
1.00
0.75
0.50
0.25
0.00
A (n=15)
B (n=16)
C (n=14)
surface

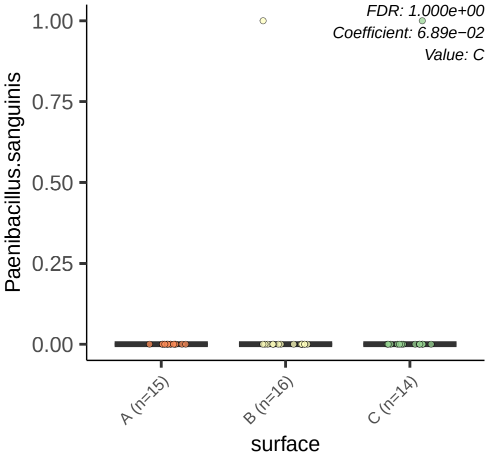
FDR: 1.000e+00
Coefficient: 6.89e−02
Value: C
Paenibacillus.sanguinis
1.00
0.75
0.50
0.25
0.00
A (n=15)
B (n=16)
C (n=14)
surface

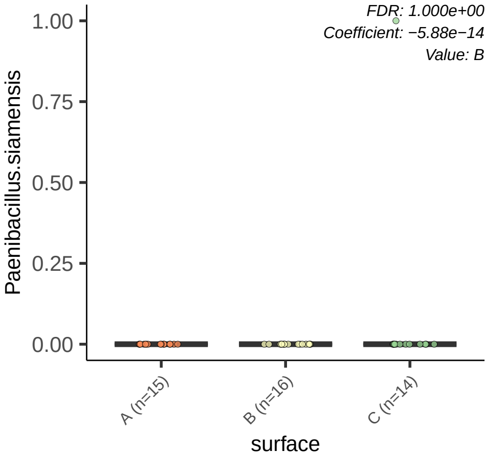
FDR: 1.000e+00
Coefficient: −5.88e−14
Value: B
Paenibacillus.siamensis
1.00
0.75
0.50
0.25
0.00
A (n=15)
B (n=16)
C (n=14)
surface

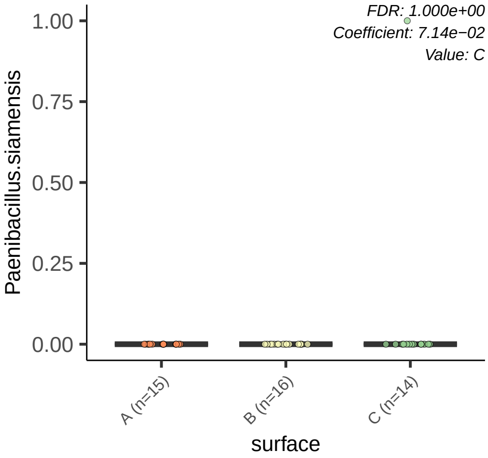

FDR: 1.000e+00
Coefficient: 7.14e−02
Value: C
Paenibacillus.siamensis
1.00
0.75
0.50
0.25
0.00
A (n=15)
B (n=16)
C (n=14)
surface

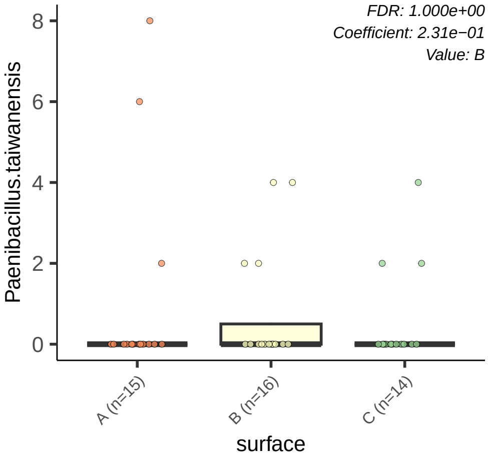

FDR: 1.000e+00
Coefficient: 2.31e−01
Value: B
Paenibacillus.taiwanensis
8
6
4
2
0
A (n=15)
B (n=16)
C (n=14)
surface

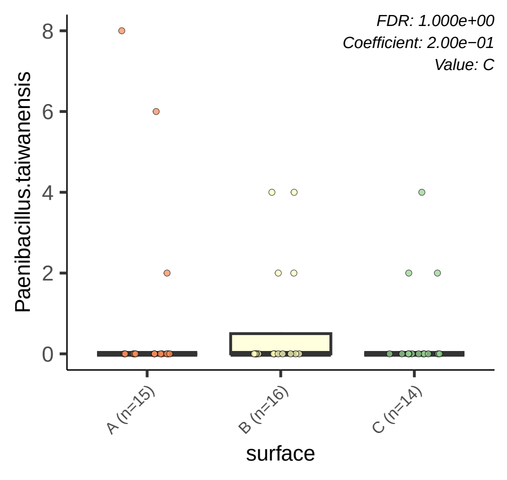

FDR: 1.000e+00
Coefficient: 2.00e−01
Value: C
Paenibacillus.taiwanensis
8
6
4
2
0
A (n=15)
B (n=16)
C (n=14)
surface

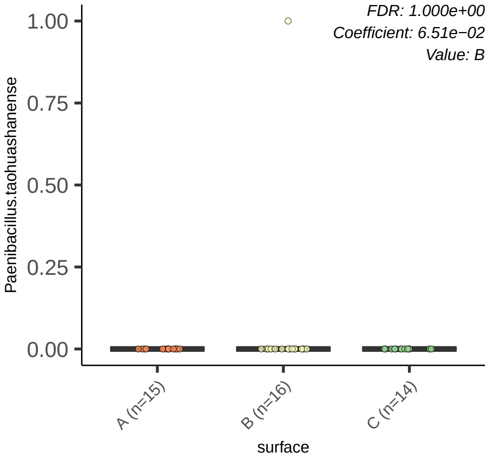

FDR: 1.000e+00
Coefficient: 6.51e−02
Value: B
Paenibacillus.taohuashanense
1.00
0.75
0.50
0.25
0.00
A (n=15)
B (n=16)
C (n=14)
surface

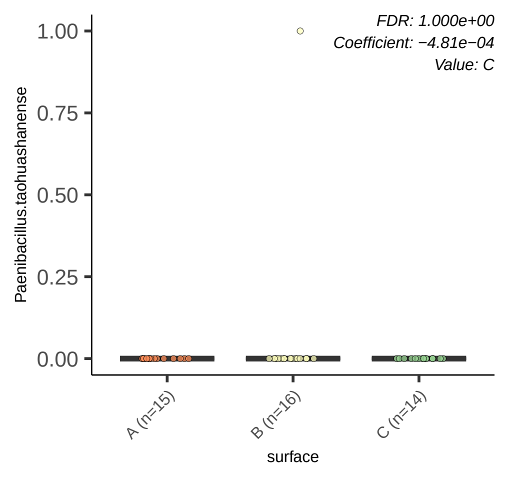
FDR: 1.000e+00
Coefficient: −4.81e−04
Value: C
Paenibacillus.taohuashanense
1.00
0.75
0.50
0.25
0.00
A (n=15)
B (n=16)
C (n=14)
surface

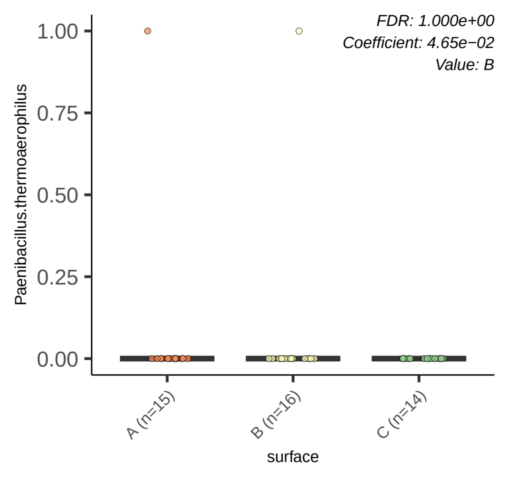

FDR: 1.000e+00
Coefficient: 4.65e−02
Value: B
Paenibacillus.thermoaerophilus
1.00
0.75
0.50
0.25
0.00
A (n=15)
B (n=16)
C (n=14)
surface

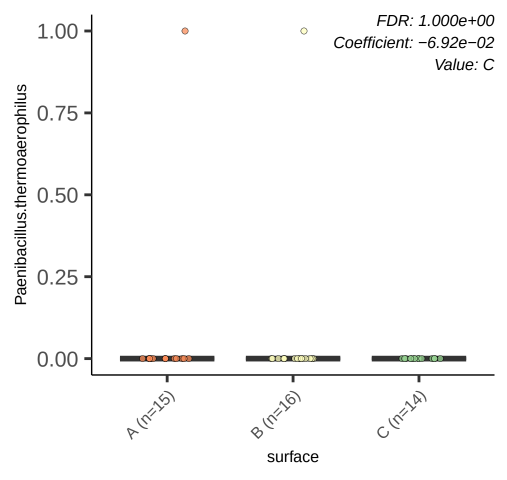
FDR: 1.000e+00
Coefficient: −6.92e−02
Value: C
Paenibacillus.thermoaerophilus
1.00
0.75
0.50
0.25
0.00
A (n=15)
B (n=16)
C (n=14)
surface

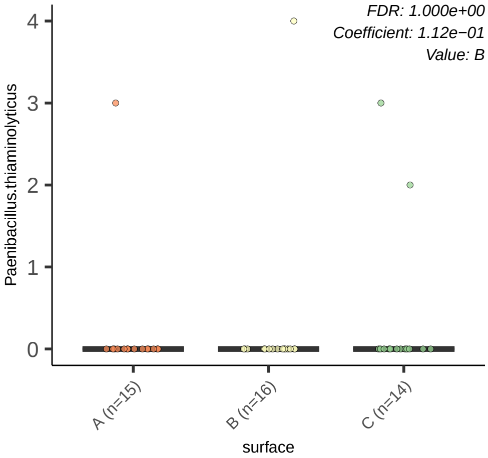
FDR: 1.000e+00
Coefficient: 1.12e−01
Value: B
Paenibacillus.thiaminolyticus
0
1
2
3
4
A (n=15)
B (n=16)
C (n=14)
surface

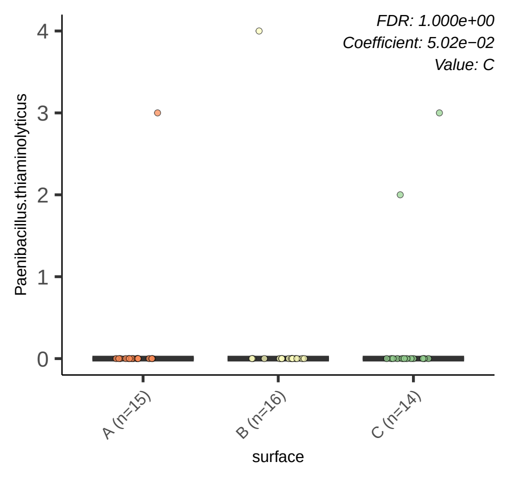

FDR: 1.000e+00
Coefficient: 5.02e−02
Value: C
Paenibacillus.thiaminolyticus
4
3
2
1
0
A (n=15)
B (n=16)
C (n=14)
surface

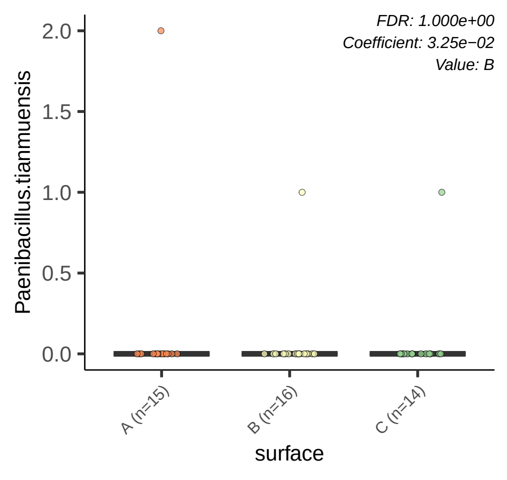

FDR: 1.000e+00
Coefficient: 3.25e−02
Value: B
Paenibacillus.tianmuensis
2.0
1.5
1.0
0.5
0.0
A (n=15)
B (n=16)
C (n=14)
surface

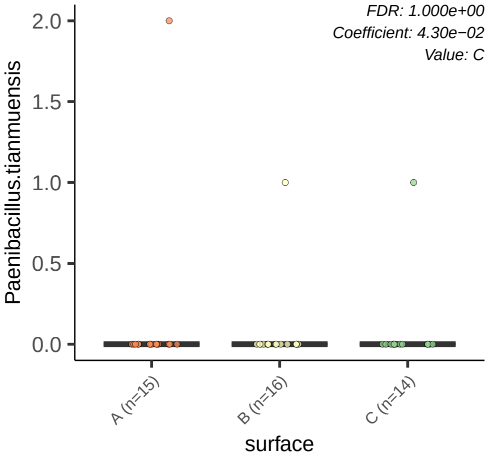

FDR: 1.000e+00
Coefficient: 4.30e−02
Value: C
Paenibacillus.tianmuensis
2.0
1.5
1.0
0.5
0.0
A (n=15)
B (n=16)
C (n=14)
surface

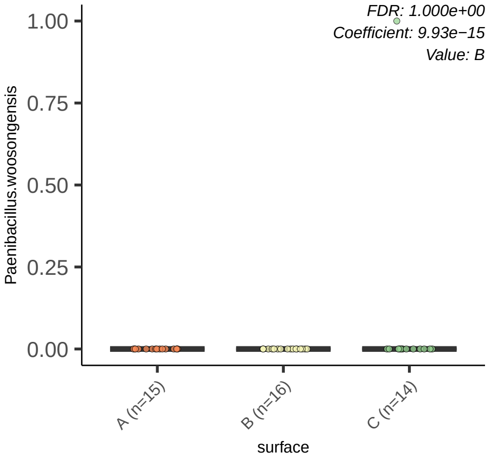
FDR: 1.000e+00
Coefficient: 9.93e−15
Value: B
Paenibacillus.woosongensis
1.00
0.75
0.50
0.25
0.00
A (n=15)
B (n=16)
C (n=14)
surface

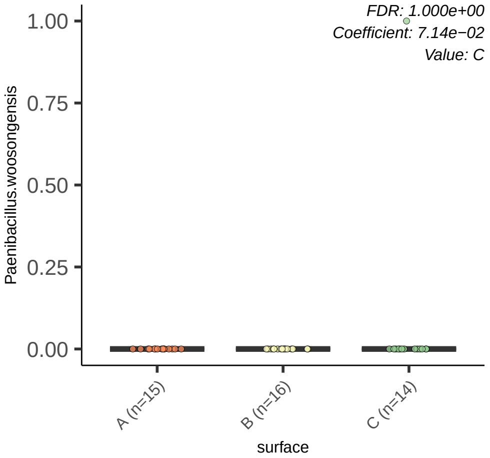

FDR: 1.000e+00
Coefficient: 7.14e−02
Value: C
1.00
0.75
0.50
0.25
0.00
Paenibacillus.woosongensis
A (n=15)
B (n=16)
C (n=14)
surface

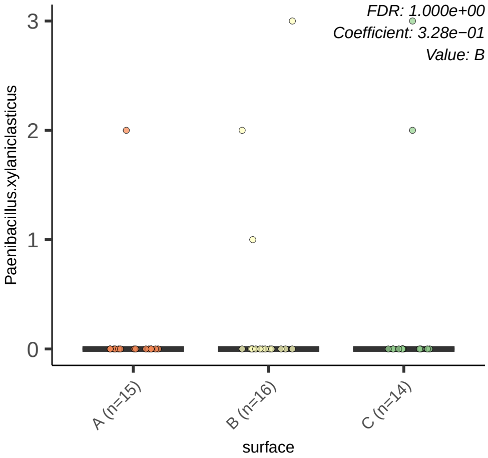
FDR: 1.000e+00
Coefficient: 3.28e−01
Value: B
Paenibacillus.xylaniclasticus
3
2
1
0
A (n=15)
B (n=16)
C (n=14)
surface

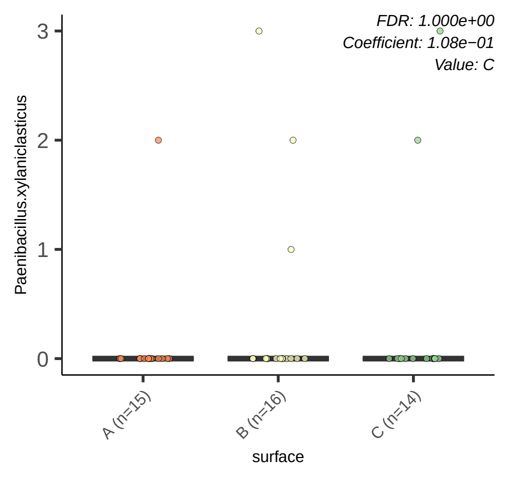
FDR: 1.000e+00
Coefficient: 1.08e−01
Value: C
Paenibacillus.xylaniclasticus
3
2
1
0
A (n=15)
B (n=16)
C (n=14)
surface

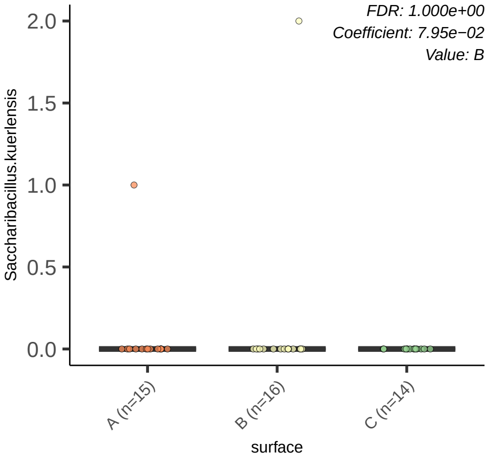
FDR: 1.000e+00
Coefficient: 7.95e−02
Value: B
Saccharibacillus.kuerlensis
2.0
1.5
1.0
0.5
0.0
A (n=15)
B (n=16)
C (n=14)
surface

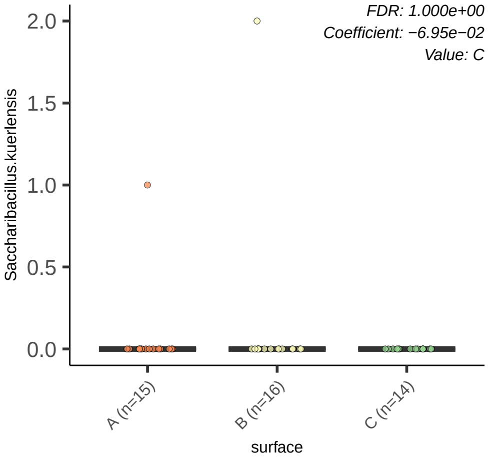

FDR: 1.000e+00
Coefficient: −6.95e−02
Value: C
Saccharibacillus.kuerlensis
2.0
1.5
1.0
0.5
0.0
A (n=15)
B (n=16)
C (n=14)
surface

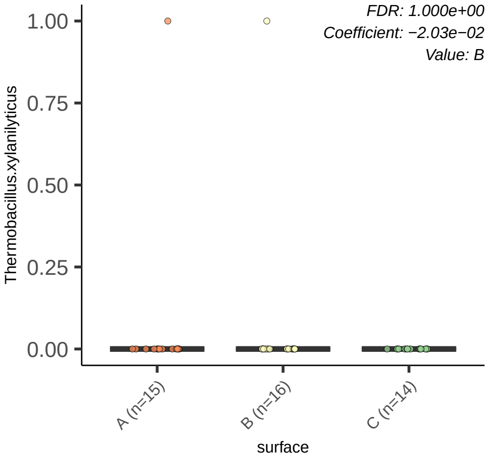

FDR: 1.000e+00
Coefficient: −2.03e−02
Value: B
1.00
0.75
0.50
0.25
0.00
Thermobacillus.xylanilyticus
A (n=15)
B (n=16)
C (n=14)
surface

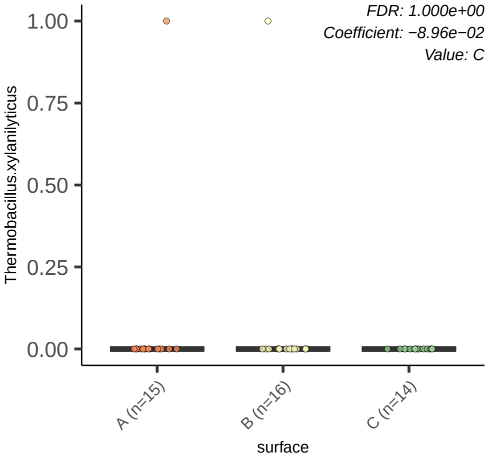
FDR: 1.000e+00
Coefficient: −8.96e−02
Value: C
Thermobacillus.xylanilyticus
1.00
0.75
0.50
0.25
0.00
A (n=15)
B (n=16)
C (n=14)
surface

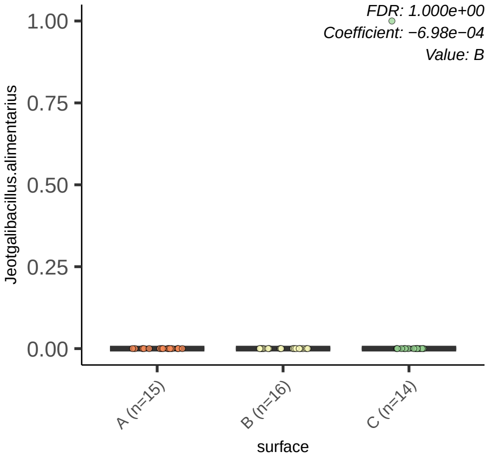

FDR: 1.000e+00
Coefficient: −6.98e−04
Value: B
Jeotgalibacillus.alimentarius
1.00
0.75
0.50
0.25
0.00
A (n=15)
B (n=16)
C (n=14)
surface

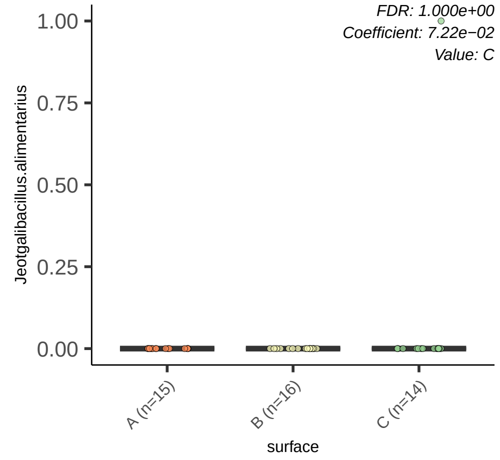
FDR: 1.000e+00
Coefficient: 7.22e−02
Value: C
1.00
0.75
0.50
0.25
0.00
Jeotgalibacillus.alimentarius
A (n=15)
B (n=16)
C (n=14)
surface

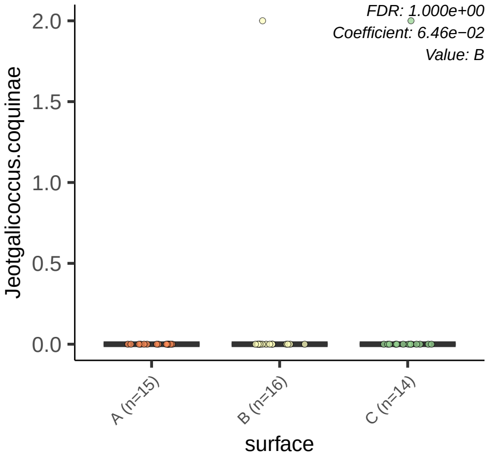

FDR: 1.000e+00
Coefficient: 6.46e−02
Value: B
Jeotgalicoccus.coquinae
2.0
1.5
1.0
0.5
0.0
A (n=15)
B (n=16)
C (n=14)
surface

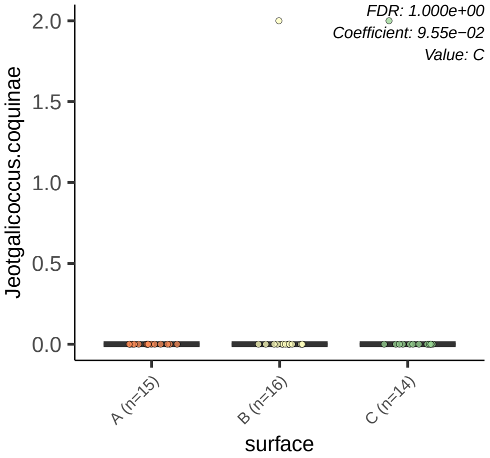

FDR: 1.000e+00
Coefficient: 9.55e−02
Value: C
Jeotgalicoccus.coquinae
2.0
1.5
1.0
0.5
0.0
A (n=15)
B (n=16)
C (n=14)
surface

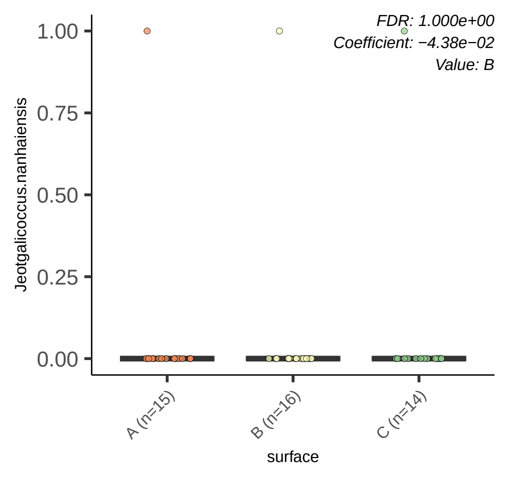
FDR: 1.000e+00
Coefficient: −4.38e−02
Value: B
Jeotgalicoccus.nanhaiensis
1.00
0.75
0.50
0.25
0.00
A (n=15)
B (n=16)
C (n=14)
surface

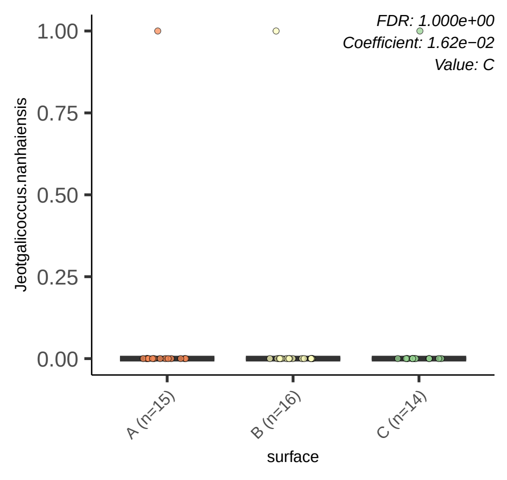

FDR: 1.000e+00
Coefficient: 1.62e−02
Value: C
Jeotgalicoccus.nanhaiensis
1.00
0.75
0.50
0.25
0.00
A (n=15)
B (n=16)
C (n=14)
surface

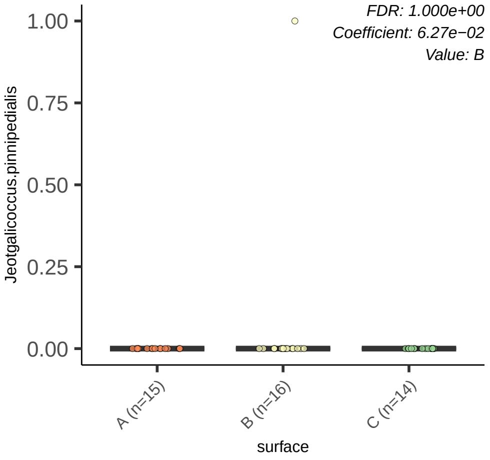
FDR: 1.000e+00
Coefficient: 6.27e−02
Value: B
Jeotgalicoccus.pinnipedialis
1.00
0.75
0.50
0.25
0.00
A (n=15)
B (n=16)
C (n=14)
surface

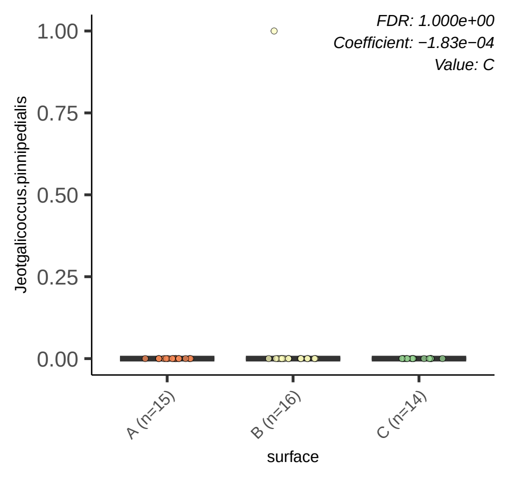
FDR: 1.000e+00
Coefficient: −1.83e−04
Value: C
Jeotgalicoccus.pinnipedialis
1.00
0.75
0.50
0.25
0.00
A (n=15)
B (n=16)
C (n=14)
surface

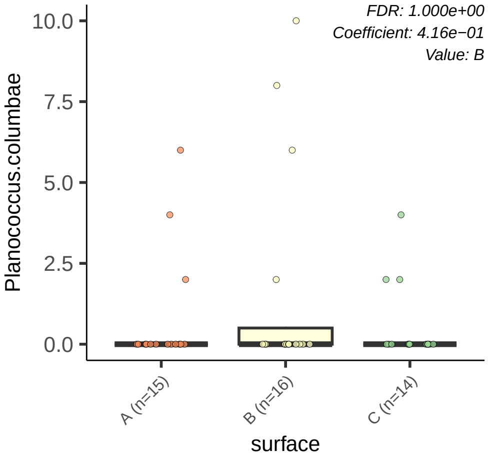

FDR: 1.000e+00
Coefficient: 4.16e−01
Value: B
Planococcus.columbae
10.0
7.5
5.0
2.5
0.0
A (n=15)
B (n=16)
C (n=14)
surface

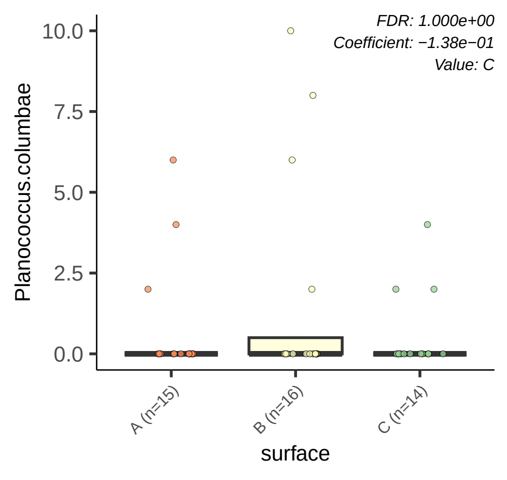

FDR: 1.000e+00
Coefficient: −1.38e−01
Value: C
Planococcus.columbae
10.0
7.5
5.0
2.5
0.0
A (n=15)
B (n=16)
C (n=14)
surface

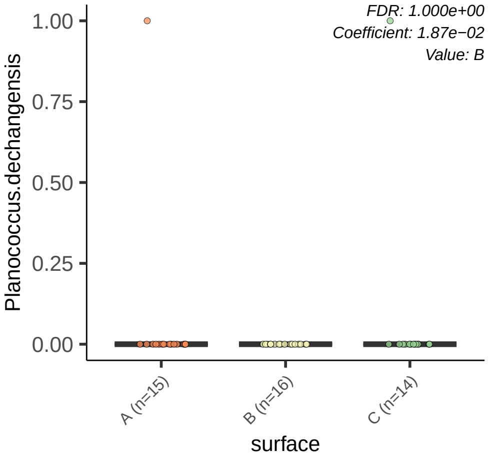

FDR: 1.000e+00
Coefficient: 1.87e−02
Value: B
Planococcus.dechangensis
1.00
0.75
0.50
0.25
0.00
A (n=15)
B (n=16)
C (n=14)
surface

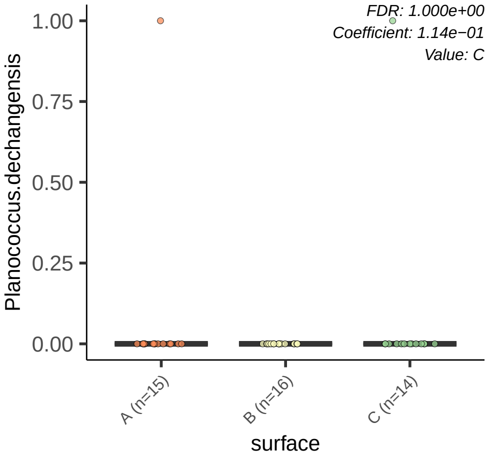

FDR: 1.000e+00
Coefficient: 1.14e−01
Value: C
Planococcus.dechangensis
1.00
0.75
0.50
0.25
0.00
A (n=15)
B (n=16)
C (n=14)
surface

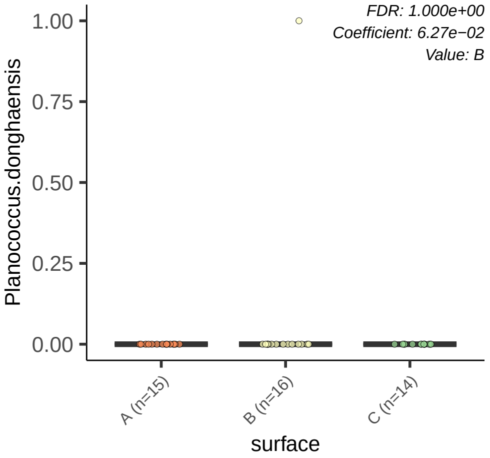
FDR: 1.000e+00
Coefficient: 6.27e−02
Value: B
Planococcus.donghaensis
1.00
0.75
0.50
0.25
0.00
A (n=15)
B (n=16)
C (n=14)
surface

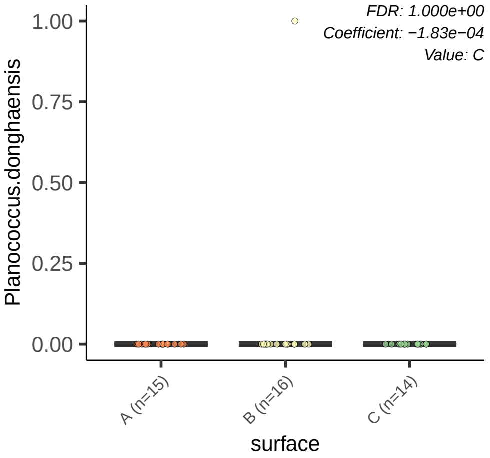
FDR: 1.000e+00
Coefficient: −1.83e−04
Value: C
Planococcus.donghaensis
1.00
0.75
0.50
0.25
0.00
A (n=15)
B (n=16)
C (n=14)
surface

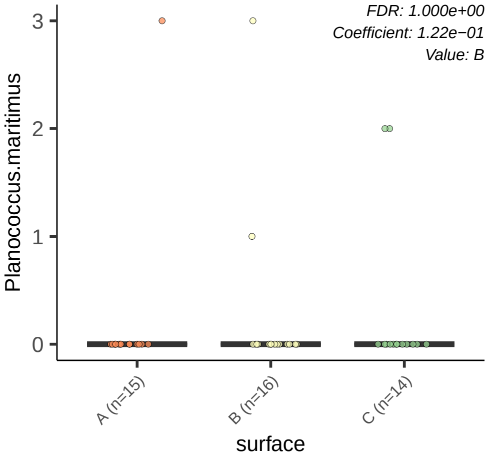

FDR: 1.000e+00
Coefficient: 1.22e−01
Value: B
Planococcus.maritimus
0
1
2
3
A (n=15)
B (n=16)
C (n=14)
surface

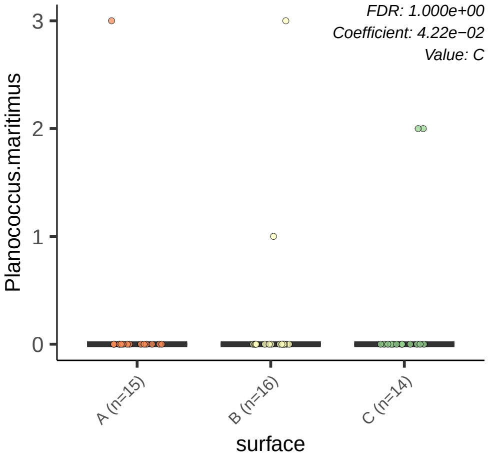

FDR: 1.000e+00
Coefficient: 4.22e−02
Value: C
Planococcus.maritimus
3
2
1
0
A (n=15)
B (n=16)
C (n=14)
surface

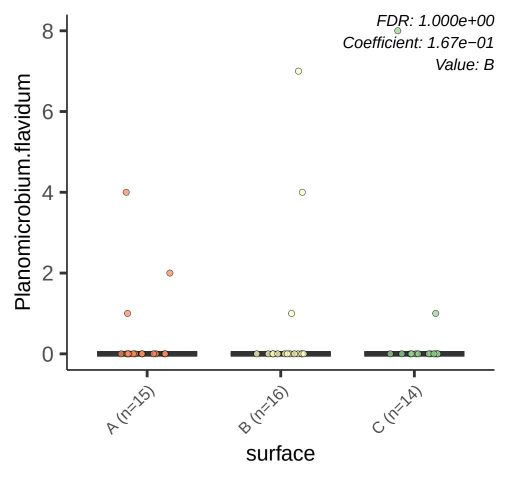

FDR: 1.000e+00
Coefficient: 1.67e−01
Value: B
Planomicrobium.flavidum
8
6
4
2
0
A (n=15)
B (n=16)
C (n=14)
surface

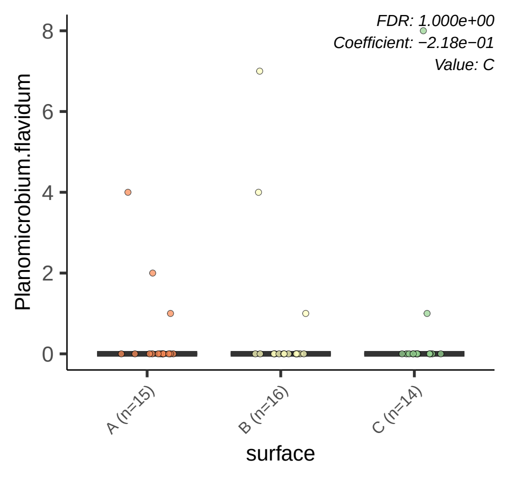
FDR: 1.000e+00
Coefficient: −2.18e−01
Value: C
Planomicrobium.flavidum
8
6
4
2
0
A (n=15)
B (n=16)
C (n=14)
surface

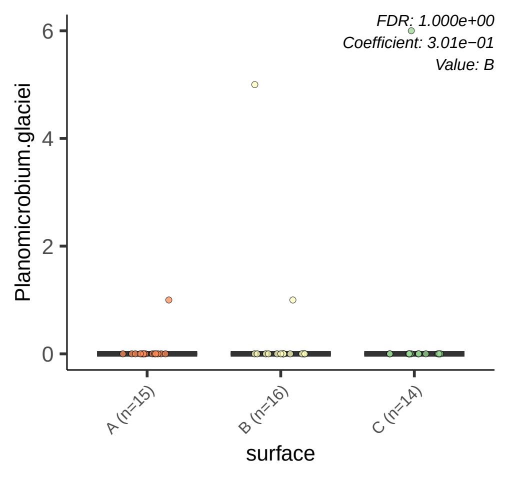
FDR: 1.000e+00
Coefficient: 3.01e−01
Value: B
Planomicrobium.glaciei
0
2
4
6
A (n=15)
B (n=16)
C (n=14)
surface

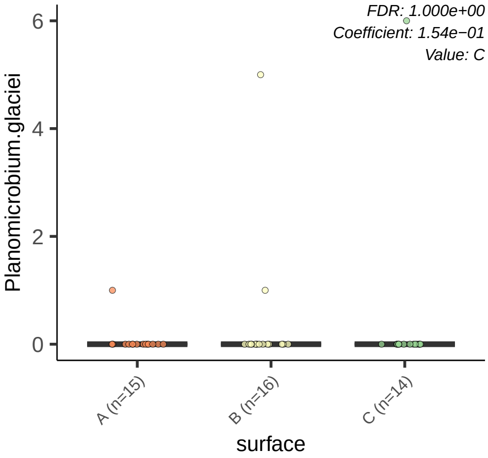
FDR: 1.000e+00
Coefficient: 1.54e−01
Value: C
Planomicrobium.glaciei
0
2
4
6
A (n=15)
B (n=16)
C (n=14)
surface

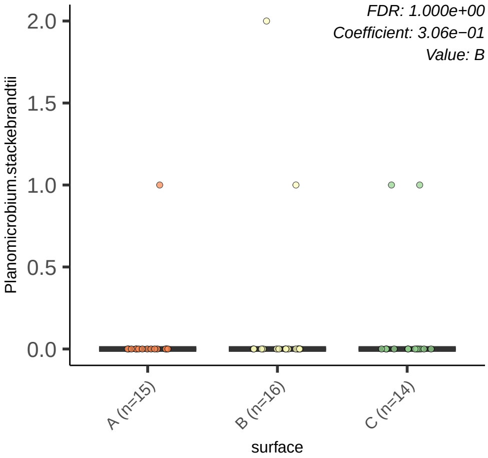

FDR: 1.000e+00
Coefficient: 3.06e−01
Value: B
Planomicrobium.stackebrandtii
2.0
1.5
1.0
0.5
0.0
A (n=15)
B (n=16)
C (n=14)
surface

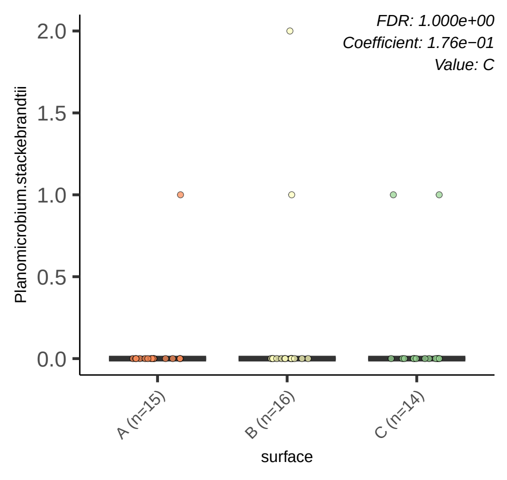

FDR: 1.000e+00
Coefficient: 1.76e−01
Value: C
Planomicrobium.stackebrandtii
2.0
1.5
1.0
0.5
0.0
A (n=15)
B (n=16)
C (n=14)
surface

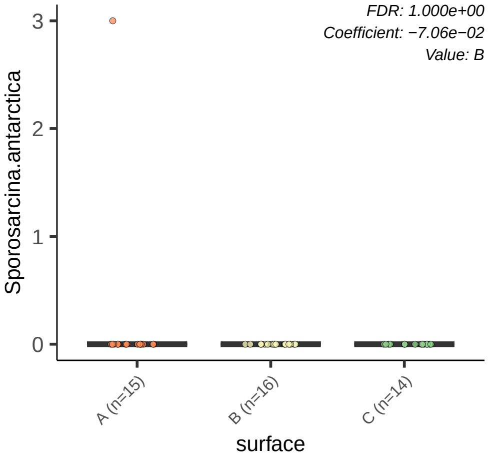
FDR: 1.000e+00
Coefficient: −7.06e−02
Value: B
Sporosarcina.antarctica
3
2
1
0
A (n=15)
B (n=16)
C (n=14)
surface

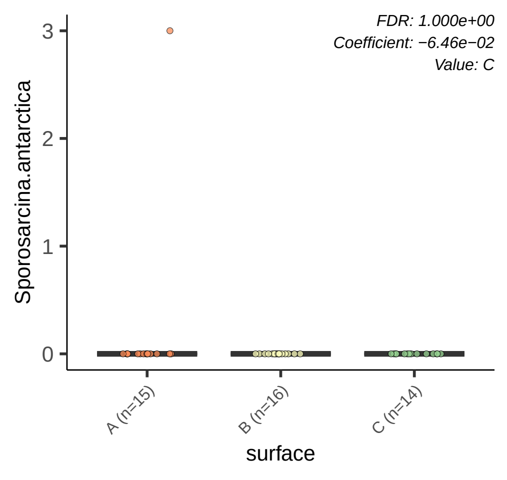

FDR: 1.000e+00
Coefficient: −6.46e−02
Value: C
Sporosarcina.antarctica
3
2
1
0
A (n=15)
B (n=16)
C (n=14)
surface

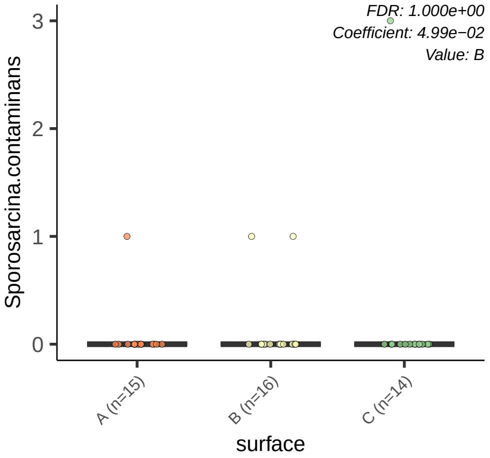
FDR: 1.000e+00
Coefficient: 4.99e−02
Value: B
Sporosarcina.contaminans
0
1
2
3
A (n=15)
B (n=16)
C (n=14)
surface

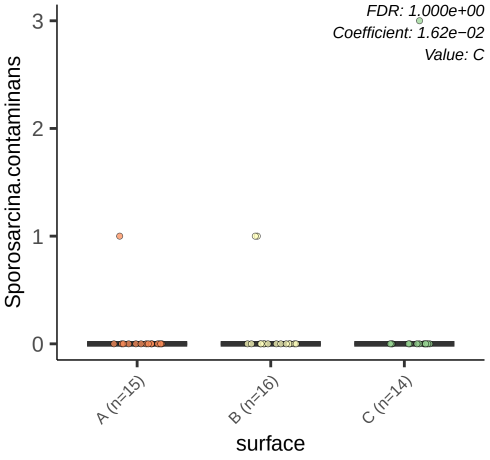

FDR: 1.000e+00
Coefficient: 1.62e−02
Value: C
Sporosarcina.contaminans
0
1
2
3
A (n=15)
B (n=16)
C (n=14)
surface

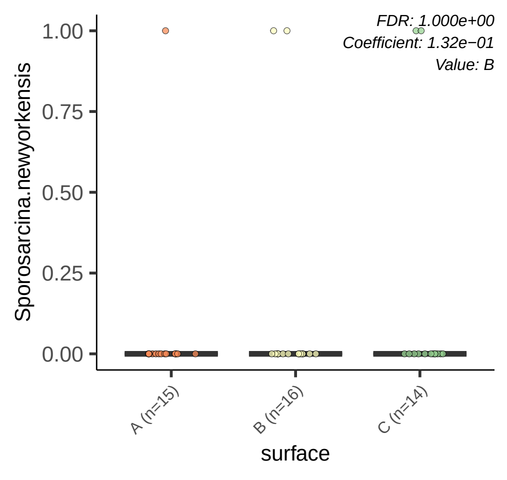

FDR: 1.000e+00
Coefficient: 1.32e−01
Value: B
Sporosarcina.newyorkensis
1.00
0.75
0.50
0.25
0.00
A (n=15)
B (n=16)
C (n=14)
surface

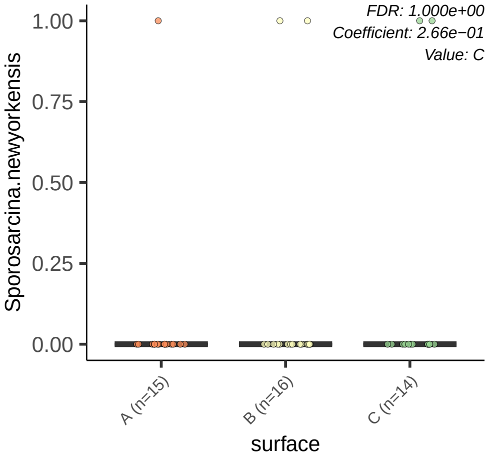

FDR: 1.000e+00
Coefficient: 2.66e−01
Value: C
Sporosarcina.newyorkensis
1.00
0.75
0.50
0.25
0.00
A (n=15)
B (n=16)
C (n=14)
surface

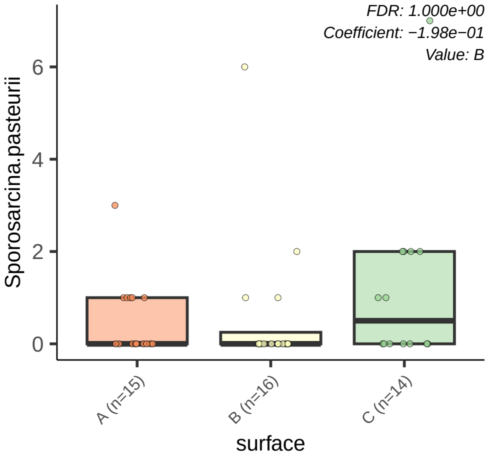
FDR: 1.000e+00
Coefficient: −1.98e−01
Value: B
Sporosarcina.pasteurii
0
2
4
6
A (n=15)
B (n=16)
C (n=14)
surface

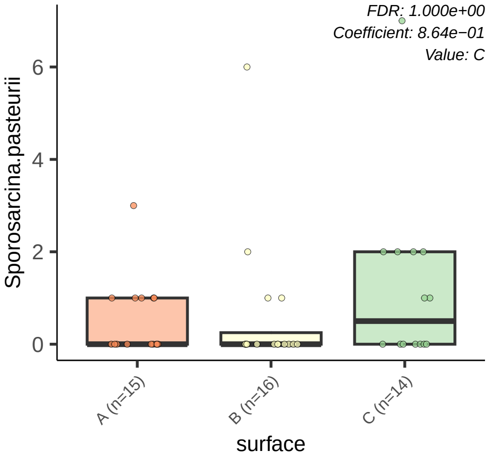

FDR: 1.000e+00
Coefficient: 8.64e−01
Value: C
Sporosarcina.pasteurii
0
2
4
6
A (n=15)
B (n=16)
C (n=14)
surface

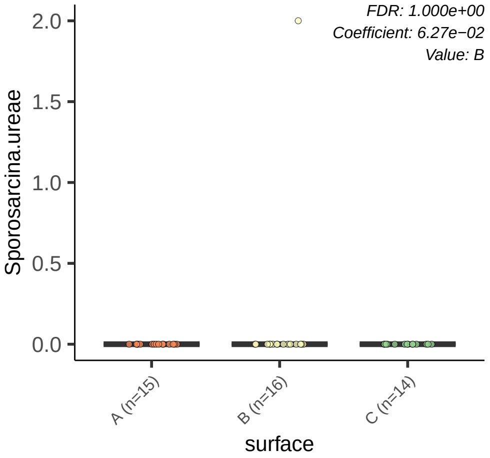

FDR: 1.000e+00
Coefficient: 6.27e−02
Value: B
2.0
1.5
1.0
0.5
0.0
Sporosarcina.ureae
A (n=15)
B (n=16)
C (n=14)
surface

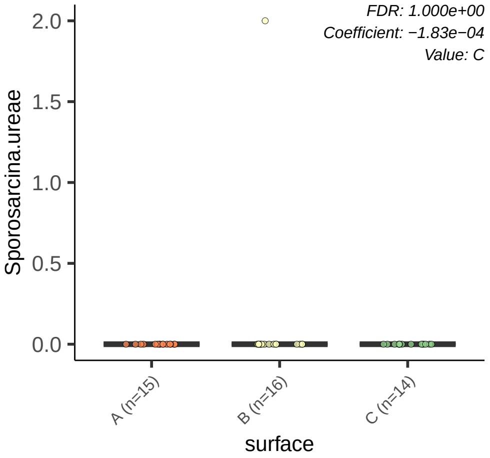
FDR: 1.000e+00
Coefficient: −1.83e−04
Value: C
Sporosarcina.ureae
2.0
1.5
1.0
0.5
0.0
A (n=15)
B (n=16)
C (n=14)
surface

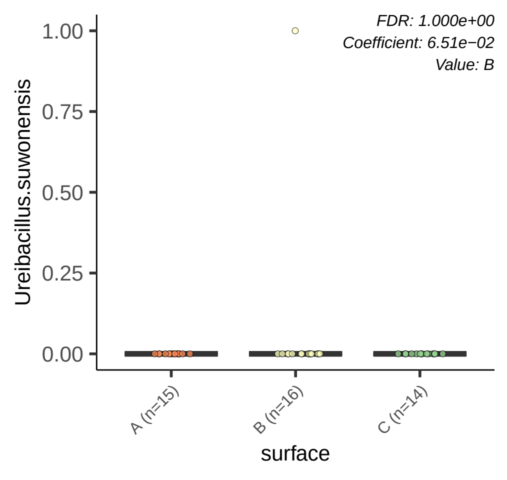
FDR: 1.000e+00
Coefficient: 6.51e−02
Value: B
1.00
0.75
0.50
0.25
0.00
Ureibacillus.suwonensis
A (n=15)
B (n=16)
C (n=14)
surface

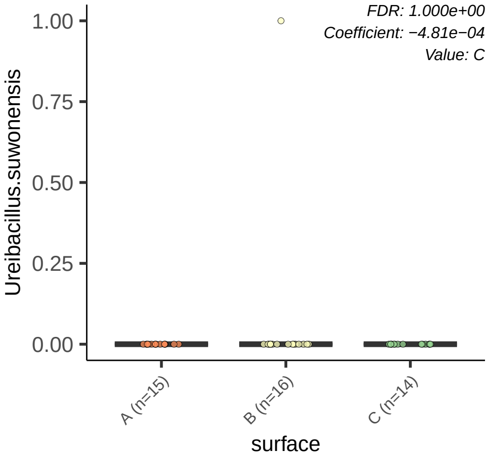
FDR: 1.000e+00
Coefficient: −4.81e−04
Value: C
Ureibacillus.suwonensis
1.00
0.75
0.50
0.25
0.00
A (n=15)
B (n=16)
C (n=14)
surface

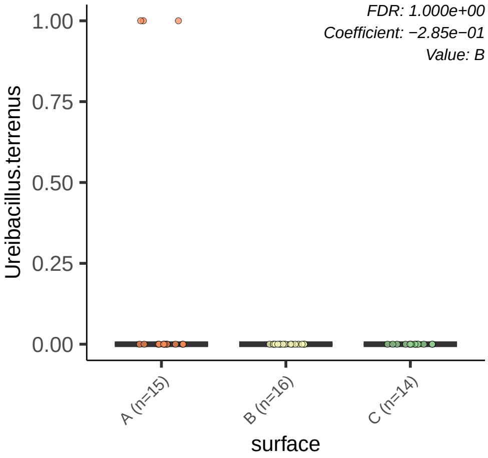

FDR: 1.000e+00
Coefficient: −2.85e−01
Value: B
Ureibacillus.terrenus
1.00
0.75
0.50
0.25
0.00
A (n=15)
B (n=16)
C (n=14)
surface

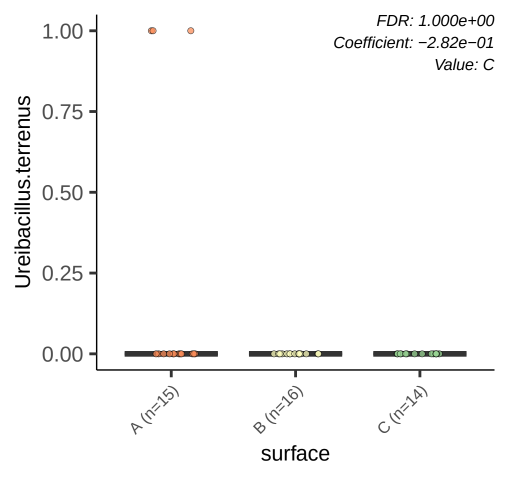

FDR: 1.000e+00
Coefficient: −2.82e−01
Value: C
Ureibacillus.terrenus
1.00
0.75
0.50
0.25
0.00
A (n=15)
B (n=16)
C (n=14)
surface

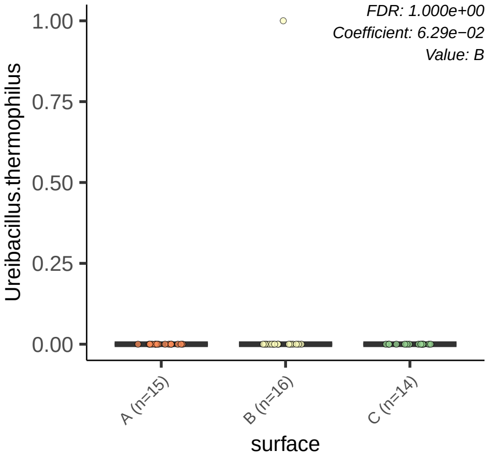

FDR: 1.000e+00
Coefficient: 6.29e−02
Value: B
Ureibacillus.thermophilus
1.00
0.75
0.50
0.25
0.00
A (n=15)
B (n=16)
C (n=14)
surface

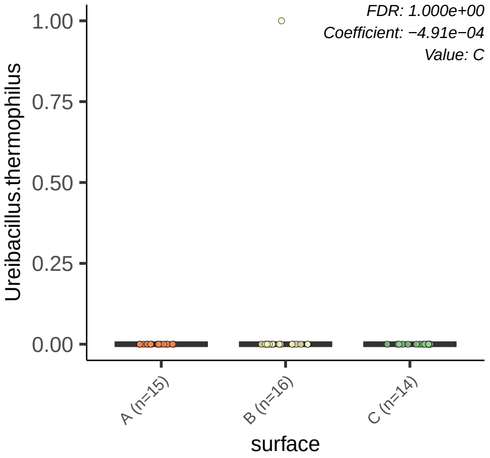
FDR: 1.000e+00
Coefficient: −4.91e−04
Value: C
Ureibacillus.thermophilus
1.00
0.75
0.50
0.25
0.00
A (n=15)
B (n=16)
C (n=14)
surface

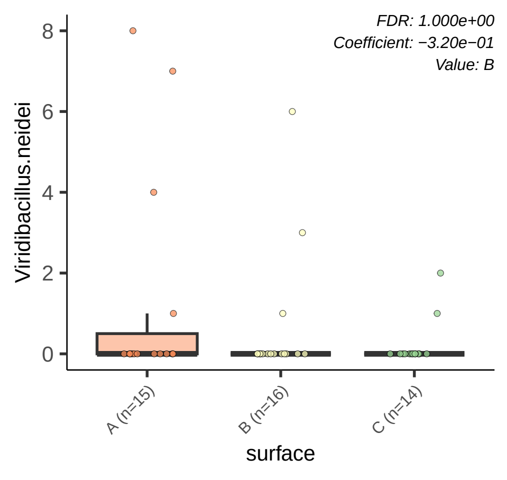
FDR: 1.000e+00
Coefficient: −3.20e−01
Value: B
Viridibacillus.neidei
0
2
4
6
8
A (n=15)
B (n=16)
C (n=14)
surface

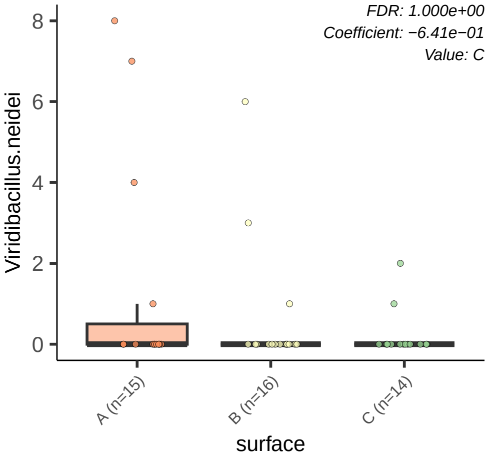

FDR: 1.000e+00
Coefficient: −6.41e−01
Value: C
Viridibacillus.neidei
8
6
4
2
0
A (n=15)
B (n=16)
C (n=14)
surface

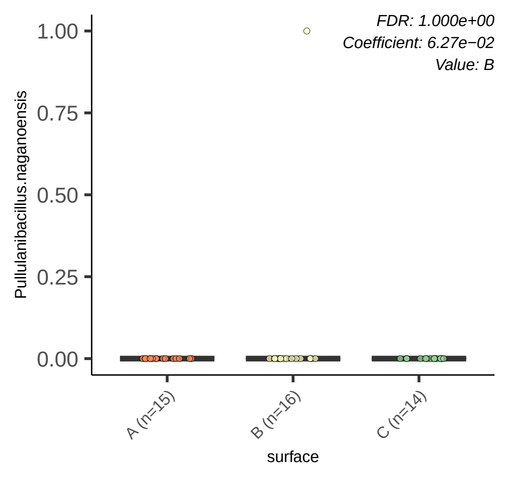
FDR: 1.000e+00
Coefficient: 6.27e−02
Value: B
Pullulanibacillus.naganoensis
1.00
0.75
0.50
0.25
0.00
A (n=15)
B (n=16)
C (n=14)
surface

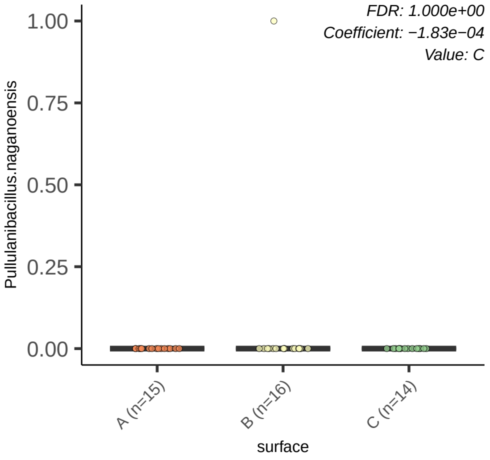

FDR: 1.000e+00
Coefficient: −1.83e−04
Value: C
1.00
0.75
0.50
0.25
0.00
Pullulanibacillus.naganoensis
A (n=15)
B (n=16)
C (n=14)
surface

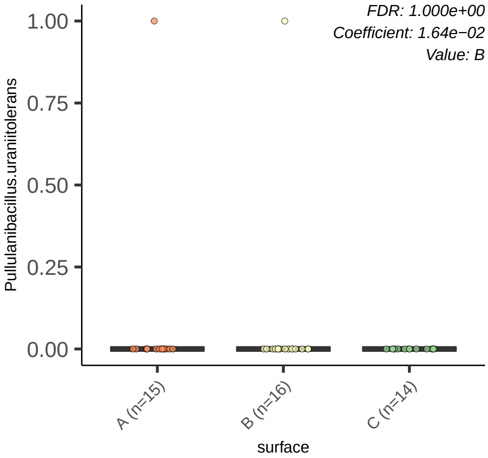
FDR: 1.000e+00
Coefficient: 1.64e−02
Value: B
Pullulanibacillus.uraniitolerans
1.00
0.75
0.50
0.25
0.00
A (n=15)
B (n=16)
C (n=14)
surface

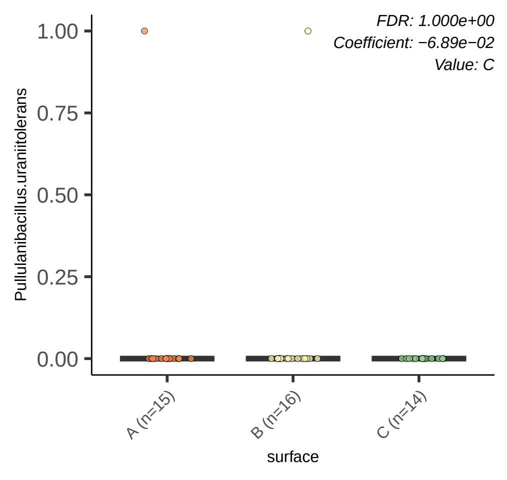
FDR: 1.000e+00
Coefficient: −6.89e−02
Value: C
1.00
0.75
0.50
0.25
0.00
Pullulanibacillus.uraniitolerans
A (n=15)
B (n=16)
C (n=14)
surface

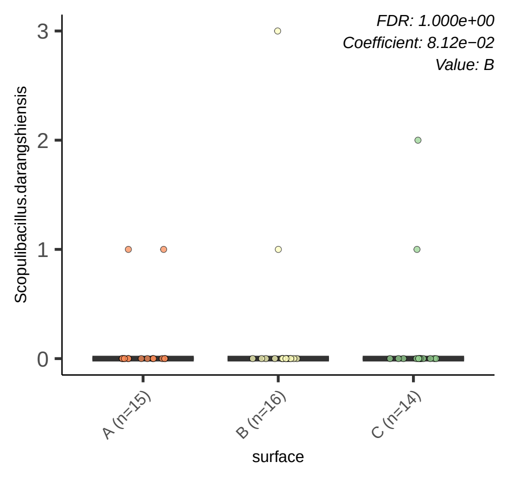
FDR: 1.000e+00
Coefficient: 8.12e−02
Value: B
Scopulibacillus.darangshiensis
3
2
1
0
A (n=15)
B (n=16)
C (n=14)
surface

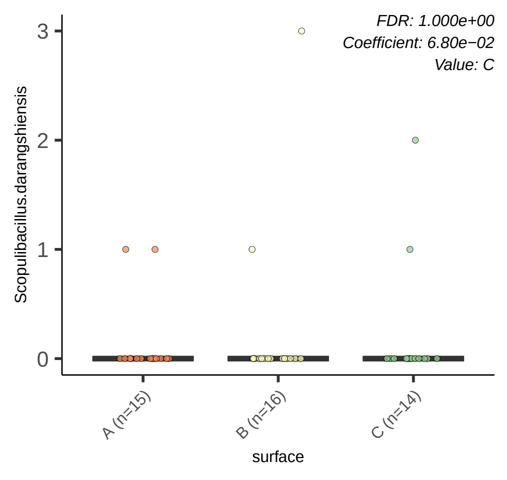
FDR: 1.000e+00
Coefficient: 6.80e−02
Value: C
Scopulibacillus.darangshiensis
3
2
1
0
A (n=15)
B (n=16)
C (n=14)
surface

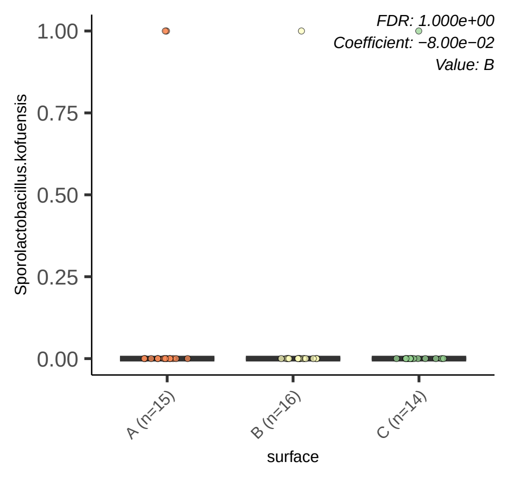

FDR: 1.000e+00
Coefficient: −8.00e−02
Value: B
Sporolactobacillus.kofuensis
1.00
0.75
0.50
0.25
0.00
A (n=15)
B (n=16)
C (n=14)
surface

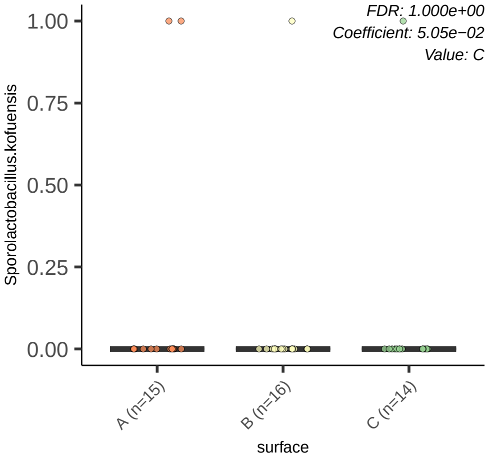
FDR: 1.000e+00
Coefficient: 5.05e−02
Value: C
1.00
0.75
0.50
0.25
0.00
Sporolactobacillus.kofuensis
A (n=15)
B (n=16)
C (n=14)
surface

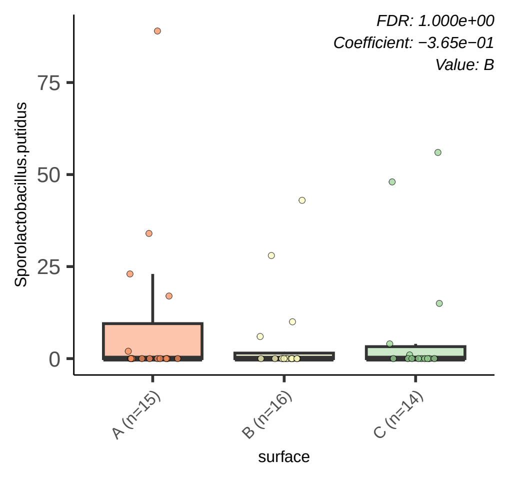
FDR: 1.000e+00
Coefficient: −3.65e−01
Value: B
Sporolactobacillus.putidus
75
50
25
0
A (n=15)
B (n=16)
C (n=14)
surface

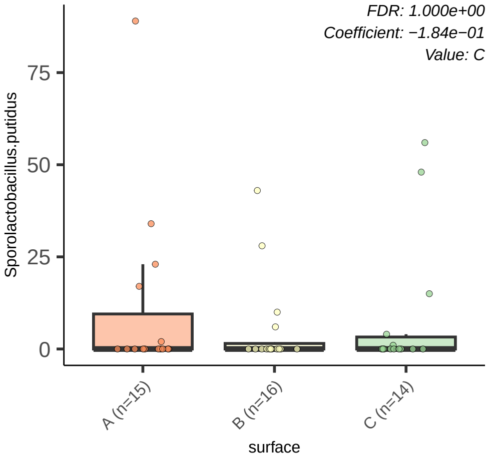
FDR: 1.000e+00
Coefficient: −1.84e−01
Value: C
Sporolactobacillus.putidus
75
50
25
0
A (n=15)
B (n=16)
C (n=14)
surface

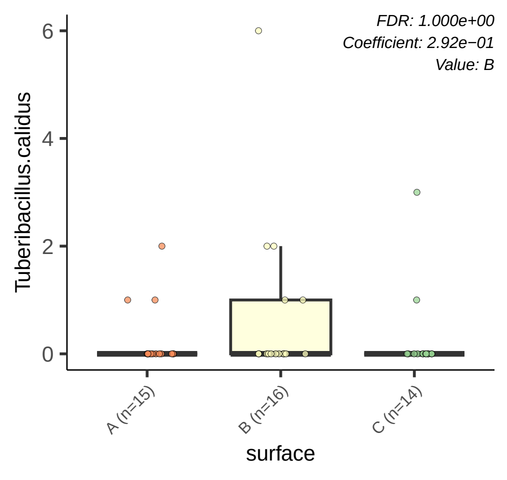
FDR: 1.000e+00
Coefficient: 2.92e−01
Value: B
Tuberibacillus.calidus
6
4
2
0
A (n=15)
B (n=16)
C (n=14)
surface

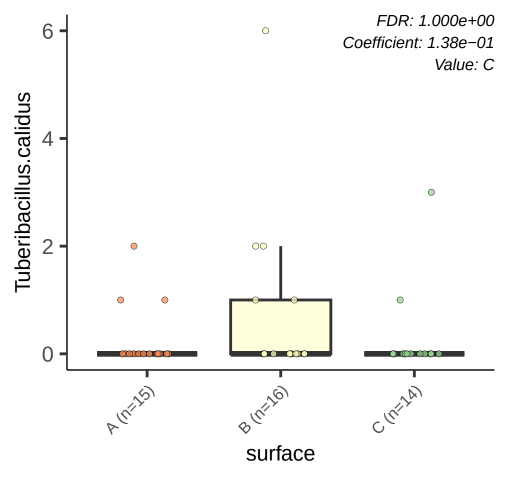
FDR: 1.000e+00
Coefficient: 1.38e−01
Value: C
Tuberibacillus.calidus
6
4
2
0
A (n=15)
B (n=16)
C (n=14)
surface

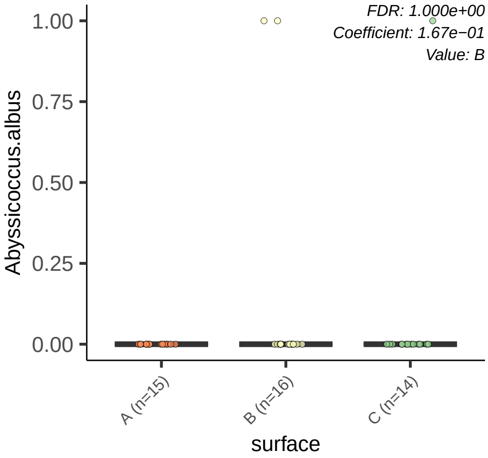

FDR: 1.000e+00
Coefficient: 1.67e−01
Value: B
Abyssicoccus.albus
1.00
0.75
0.50
0.25
0.00
A (n=15)
B (n=16)
C (n=14)
surface

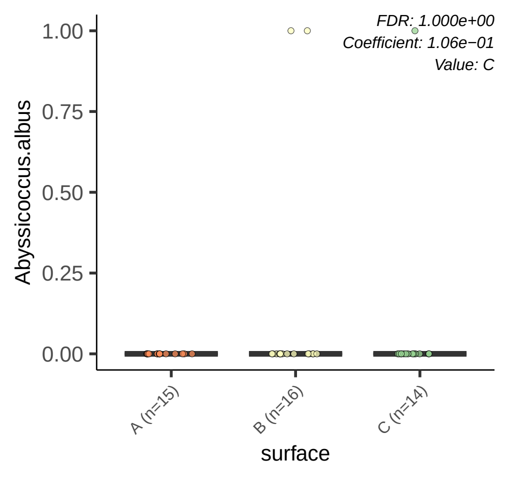
FDR: 1.000e+00
Coefficient: 1.06e−01
Value: C
Abyssicoccus.albus
1.00
0.75
0.50
0.25
0.00
A (n=15)
B (n=16)
C (n=14)
surface

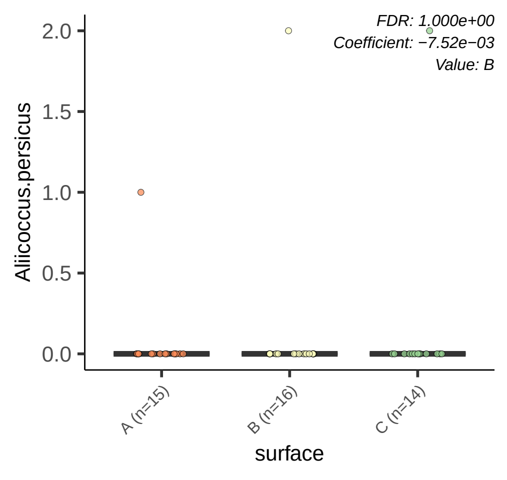

FDR: 1.000e+00
Coefficient: −7.52e−03
Value: B
Aliicoccus.persicus
2.0
1.5
1.0
0.5
0.0
A (n=15)
B (n=16)
C (n=14)
surface

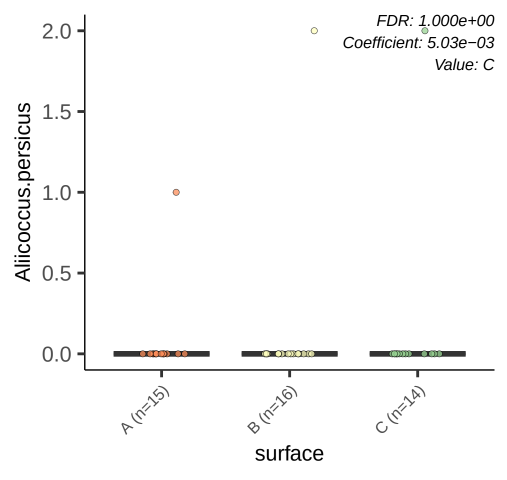
FDR: 1.000e+00
Coefficient: 5.03e−03
Value: C
Aliicoccus.persicus
2.0
1.5
1.0
0.5
0.0
A (n=15)
B (n=16)
C (n=14)
surface

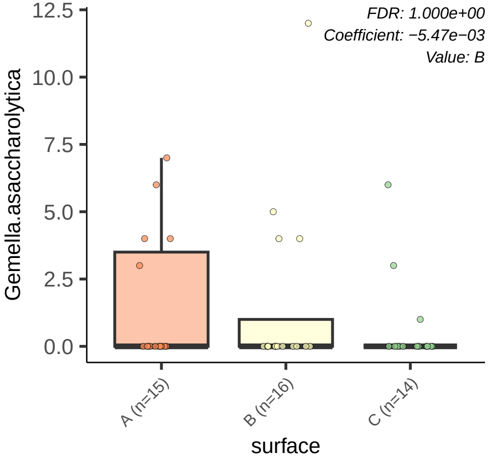

FDR: 1.000e+00
Coefficient: −5.47e−03
Value: B
Gemella.asaccharolytica
12.5
10.0
7.5
5.0
2.5
0.0
A (n=15)
B (n=16)
C (n=14)
surface

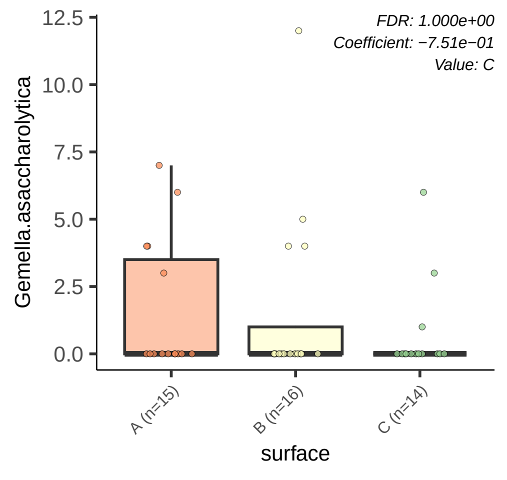
FDR: 1.000e+00
Coefficient: −7.51e−01
Value: C
Gemella.asaccharolytica
12.5
10.0
7.5
5.0
2.5
0.0
A (n=15)
B (n=16)
C (n=14)
surface

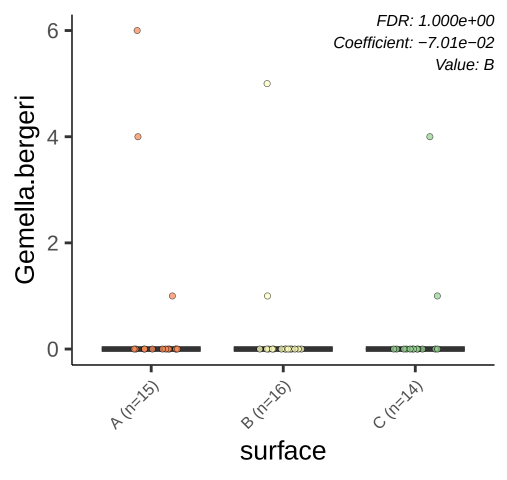

FDR: 1.000e+00
Coefficient: −7.01e−02
Value: B
Gemella.bergeri
6
4
2
0
A (n=15)
B (n=16)
C (n=14)
surface

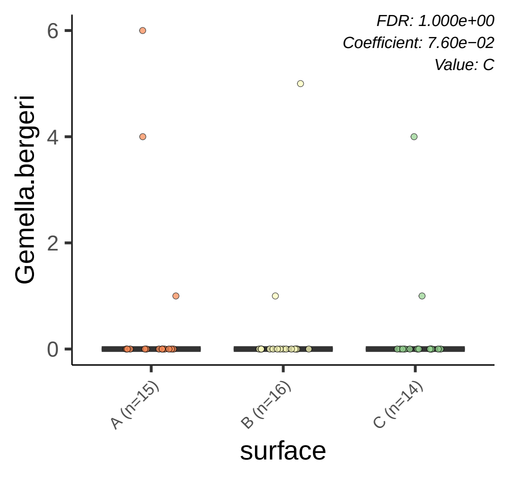
FDR: 1.000e+00
Coefficient: 7.60e−02
Value: C
Gemella.bergeri
6
4
2
0
A (n=15)
B (n=16)
C (n=14)
surface

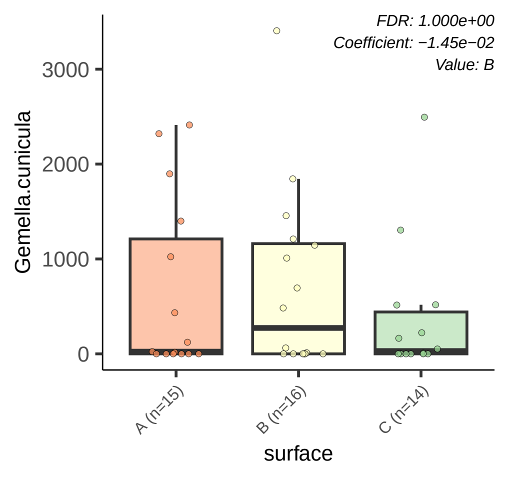

FDR: 1.000e+00
Coefficient: −1.45e−02
Value: B
3000
2000
1000
0
Gemella.cunicula
A (n=15)
B (n=16)
C (n=14)
surface

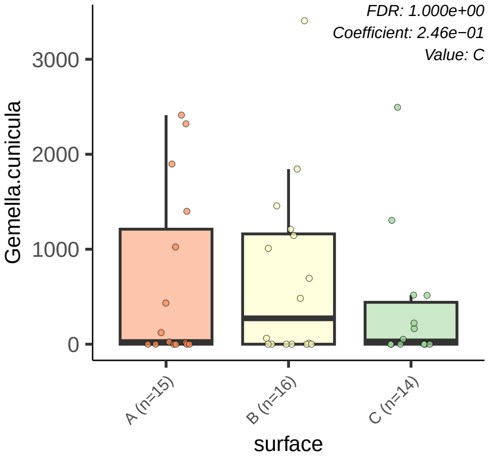

FDR: 1.000e+00
Coefficient: 2.46e−01
Value: C
Gemella.cunicula
3000
2000
1000
0
A (n=15)
B (n=16)
C (n=14)
surface

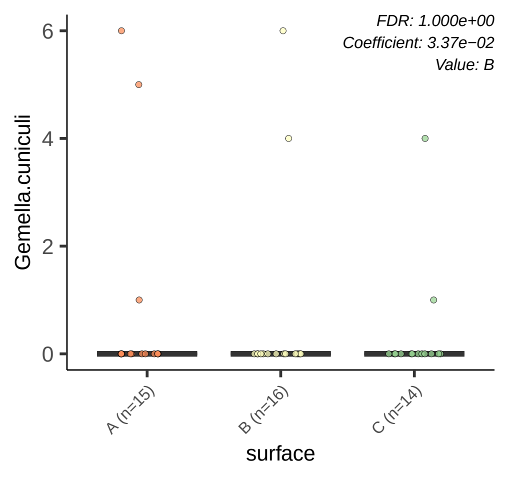
FDR: 1.000e+00
Coefficient: 3.37e−02
Value: B
Gemella.cuniculi
6
4
2
0
A (n=15)
B (n=16)
C (n=14)
surface

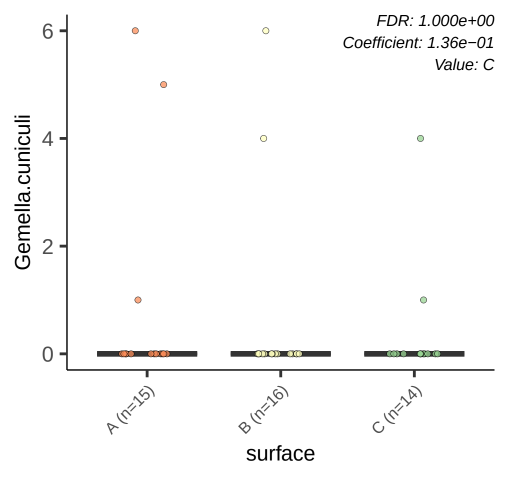
FDR: 1.000e+00
Coefficient: 1.36e−01
Value: C
Gemella.cuniculi
0
2
4
6
A (n=15)
B (n=16)
C (n=14)
surface

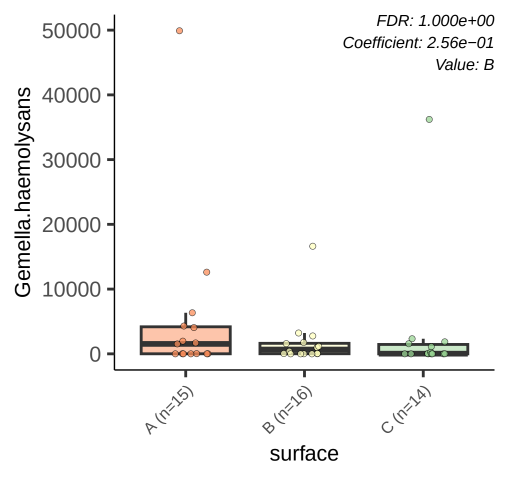
FDR: 1.000e+00
Coefficient: 2.56e−01
Value: B
Gemella.haemolysans
50000
40000
30000
20000
10000
0
A (n=15)
B (n=16)
C (n=14)
surface

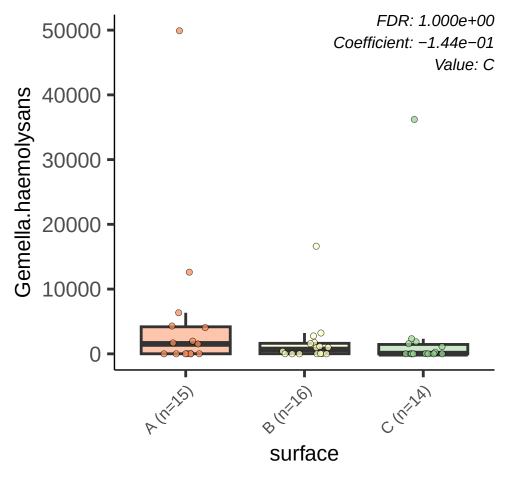
FDR: 1.000e+00
Coefficient: −1.44e−01
Value: C
Gemella.haemolysans
50000
40000
30000
20000
10000
0
A (n=15)
B (n=16)
C (n=14)
surface

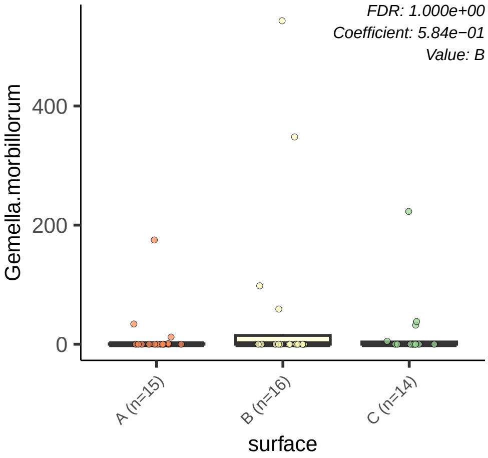

FDR: 1.000e+00
Coefficient: 5.84e−01
Value: B
Gemella.morbillorum
400
200
0
A (n=15)
B (n=16)
C (n=14)
surface

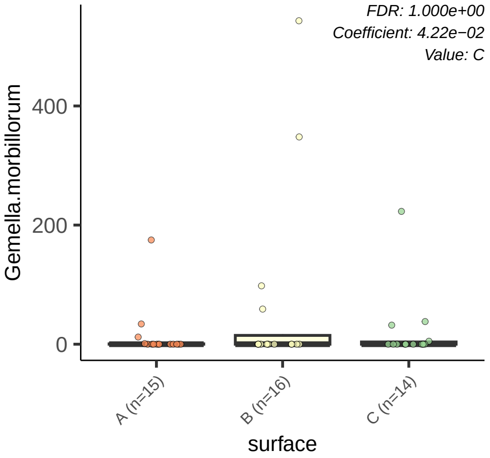
FDR: 1.000e+00
Coefficient: 4.22e−02
Value: C
Gemella.morbillorum
400
200
0
A (n=15)
B (n=16)
C (n=14)
surface

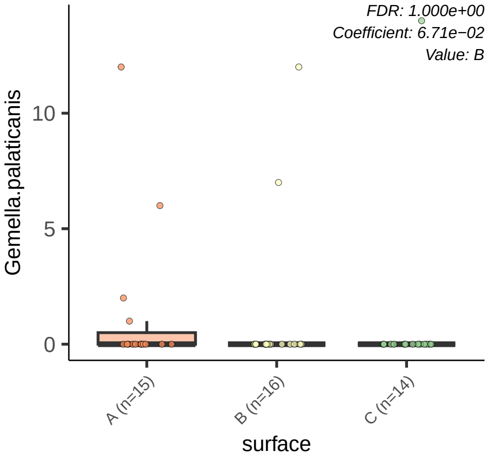
FDR: 1.000e+00
Coefficient: 6.71e−02
Value: B
Gemella.palaticanis
0
5
10
A (n=15)
B (n=16)
C (n=14)
surface

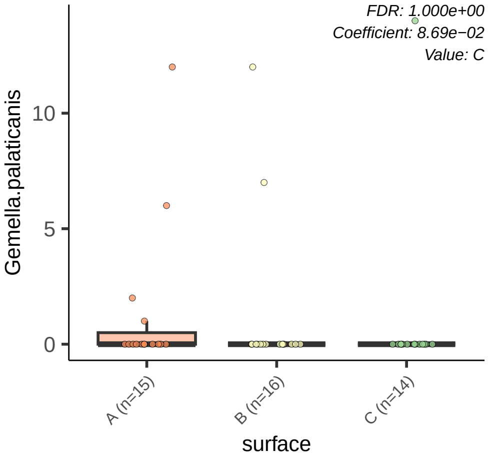
FDR: 1.000e+00
Coefficient: 8.69e−02
Value: C
Gemella.palaticanis
10
5
0
A (n=15)
B (n=16)
C (n=14)
surface

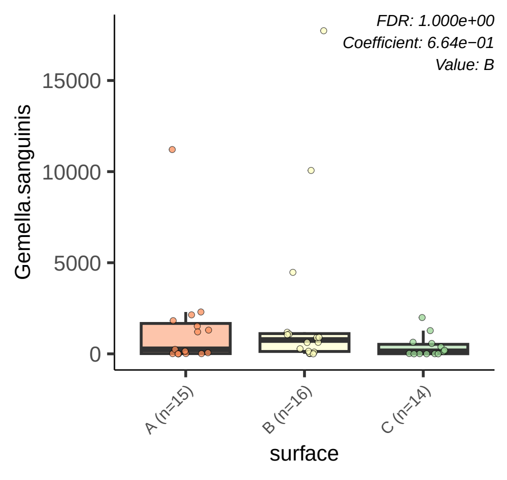
FDR: 1.000e+00
Coefficient: 6.64e−01
Value: B
Gemella.sanguinis
15000
10000
5000
0
A (n=15)
B (n=16)
C (n=14)
surface

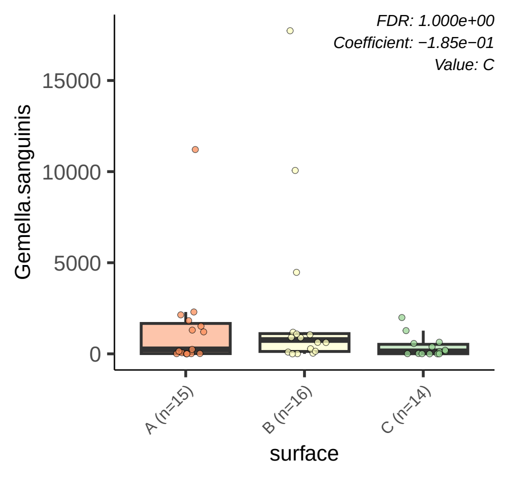
FDR: 1.000e+00
Coefficient: −1.85e−01
Value: C
Gemella.sanguinis
15000
10000
5000
0
A (n=15)
B (n=16)
C (n=14)
surface

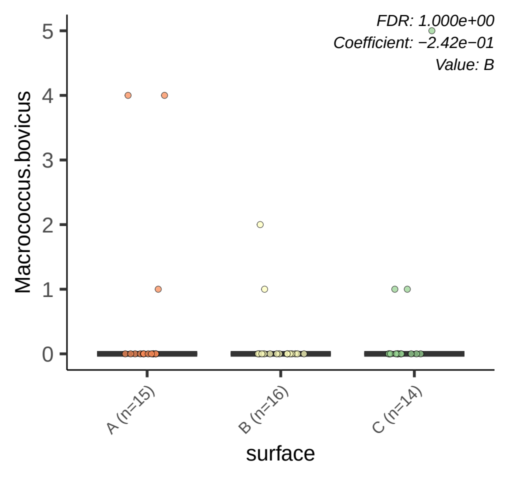

FDR: 1.000e+00
Coefficient: −2.42e−01
Value: B
Macrococcus.bovicus
0
1
2
3
4
5
A (n=15)
B (n=16)
C (n=14)
surface

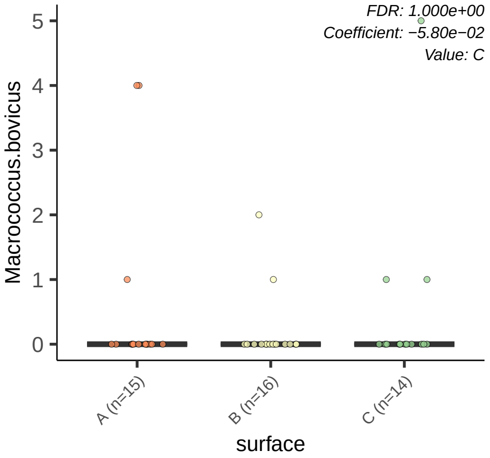

FDR: 1.000e+00
Coefficient: −5.80e−02
Value: C
Macrococcus.bovicus
0
1
2
3
4
5
A (n=15)
B (n=16)
C (n=14)
surface

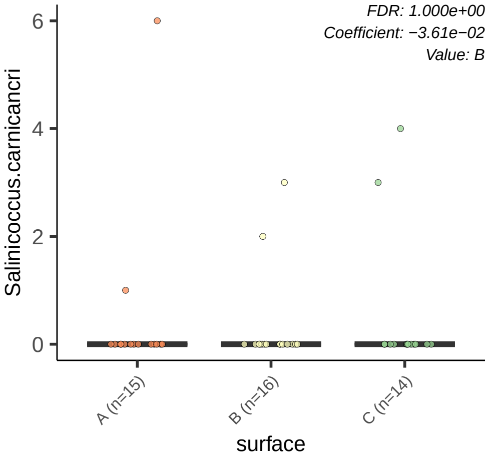

FDR: 1.000e+00
Coefficient: −3.61e−02
Value: B
Salinicoccus.carnicancri
6
4
2
0
A (n=15)
B (n=16)
C (n=14)
surface

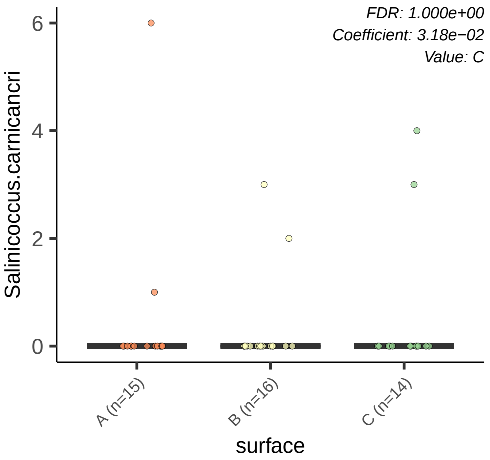
FDR: 1.000e+00
Coefficient: 3.18e−02
Value: C
Salinicoccus.carnicancri
0
2
4
6
A (n=15)
B (n=16)
C (n=14)
surface

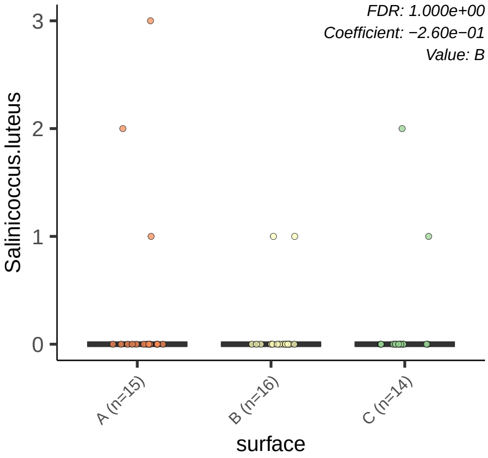
FDR: 1.000e+00
Coefficient: −2.60e−01
Value: B
Salinicoccus.luteus
3
2
1
0
A (n=15)
B (n=16)
C (n=14)
surface

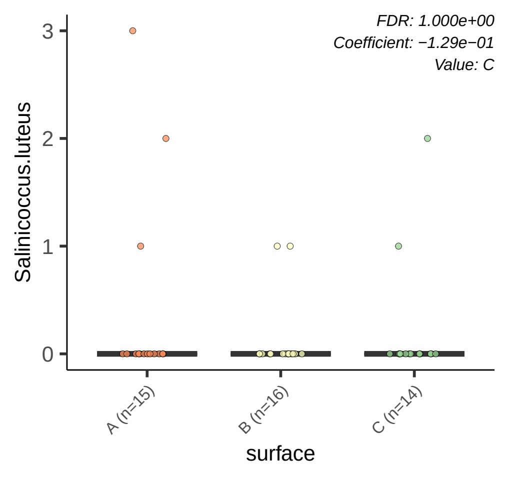

FDR: 1.000e+00
Coefficient: −1.29e−01
Value: C
Salinicoccus.luteus
0
1
2
3
A (n=15)
B (n=16)
C (n=14)
surface

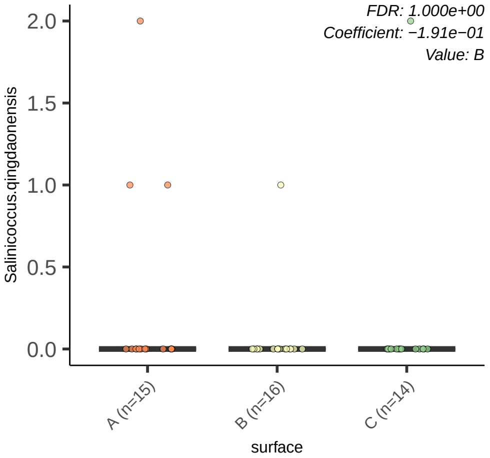
FDR: 1.000e+00
Coefficient: −1.91e−01
Value: B
Salinicoccus.qingdaonensis
2.0
1.5
1.0
0.5
0.0
A (n=15)
B (n=16)
C (n=14)
surface

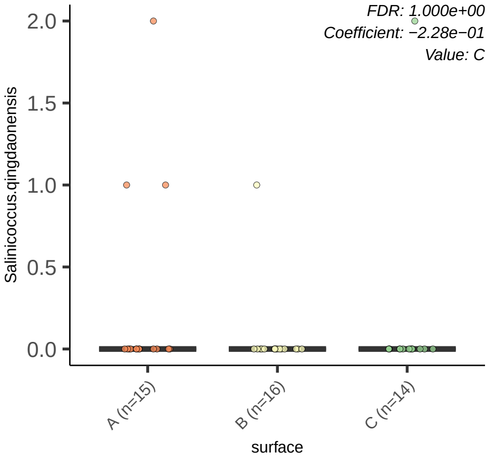
FDR: 1.000e+00
Coefficient: −2.28e−01
Value: C
Salinicoccus.qingdaonensis
2.0
1.5
1.0
0.5
0.0
A (n=15)
B (n=16)
C (n=14)
surface

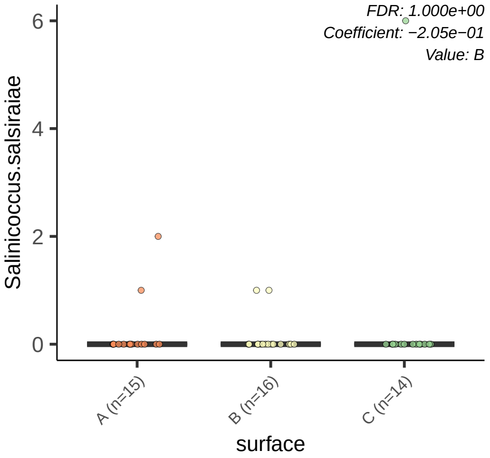
FDR: 1.000e+00
Coefficient: −2.05e−01
Value: B
Salinicoccus.salsiraiae
6
4
2
0
A (n=15)
B (n=16)
C (n=14)
surface

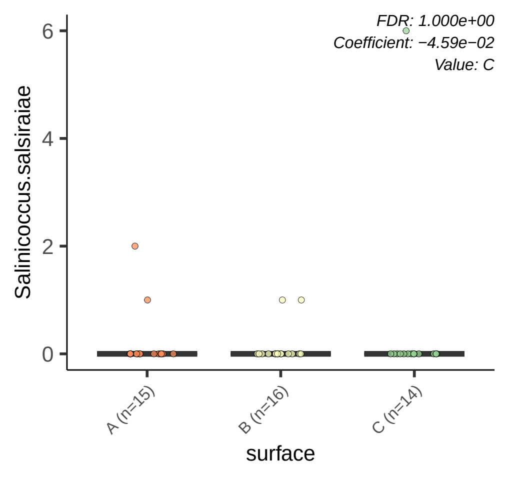

FDR: 1.000e+00
Coefficient: −4.59e−02
Value: C
Salinicoccus.salsiraiae
0
2
4
6
A (n=15)
B (n=16)
C (n=14)
surface

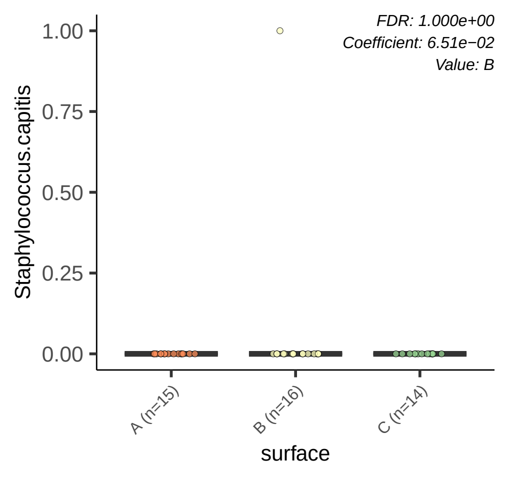

FDR: 1.000e+00
Coefficient: 6.51e−02
Value: B
Staphylococcus.capitis
1.00
0.75
0.50
0.25
0.00
A (n=15)
B (n=16)
C (n=14)
surface

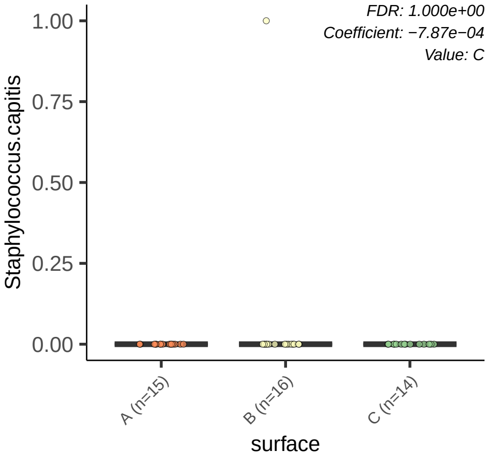
FDR: 1.000e+00
Coefficient: −7.87e−04
Value: C
Staphylococcus.capitis
1.00
0.75
0.50
0.25
0.00
A (n=15)
B (n=16)
C (n=14)
surface

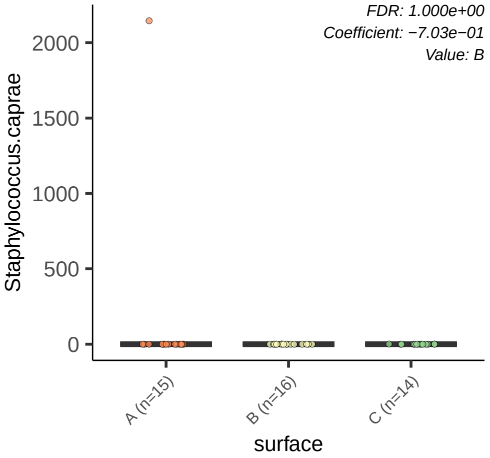

FDR: 1.000e+00
Coefficient: −7.03e−01
Value: B
Staphylococcus.caprae
2000
1500
1000
500
0
A (n=15)
B (n=16)
C (n=14)
surface

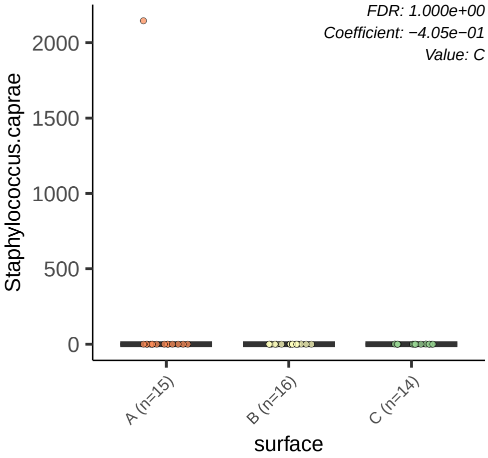

FDR: 1.000e+00
Coefficient: −4.05e−01
Value: C
Staphylococcus.caprae
2000
1500
1000
500
0
A (n=15)
B (n=16)
C (n=14)
surface

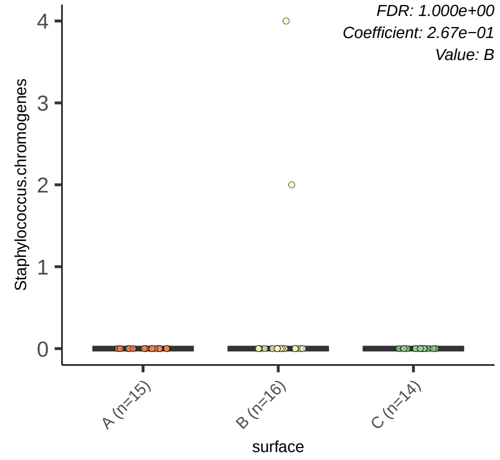

FDR: 1.000e+00
Coefficient: 2.67e−01
Value: B
Staphylococcus.chromogenes
0
1
2
3
4
A (n=15)
B (n=16)
C (n=14)
surface

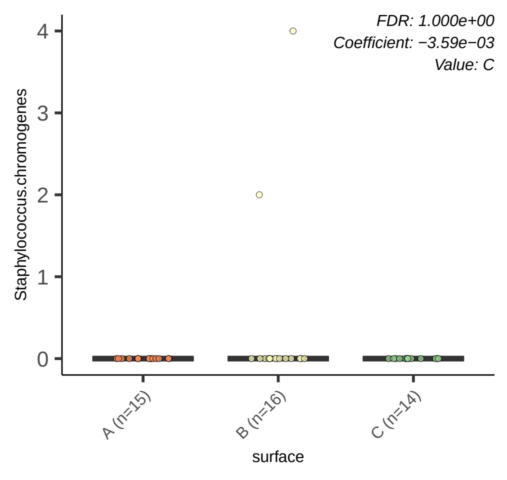
FDR: 1.000e+00
Coefficient: −3.59e−03
Value: C
Staphylococcus.chromogenes
0
1
2
3
4
A (n=15)
B (n=16)
C (n=14)
surface

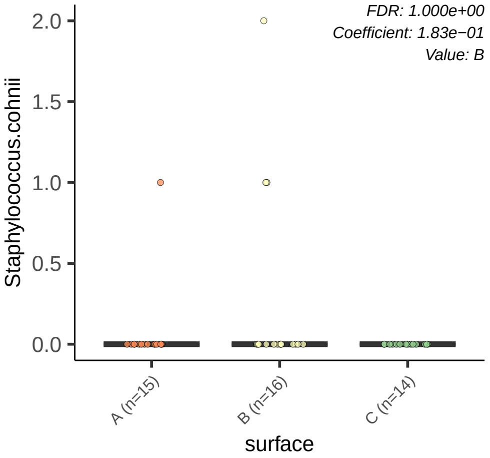
FDR: 1.000e+00
Coefficient: 1.83e−01
Value: B
Staphylococcus.cohnii
2.0
1.5
1.0
0.5
0.0
A (n=15)
B (n=16)
C (n=14)
surface

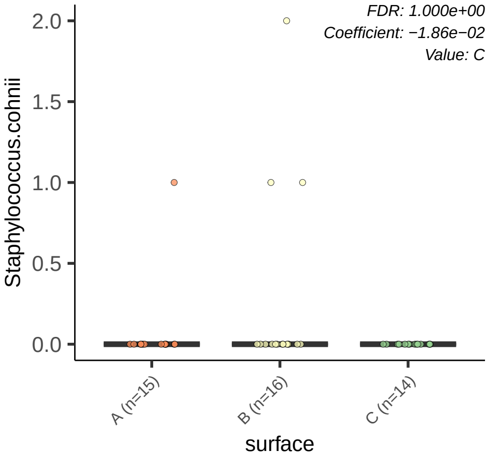
FDR: 1.000e+00
Coefficient: −1.86e−02
Value: C
Staphylococcus.cohnii
2.0
1.5
1.0
0.5
0.0
A (n=15)
B (n=16)
C (n=14)
surface

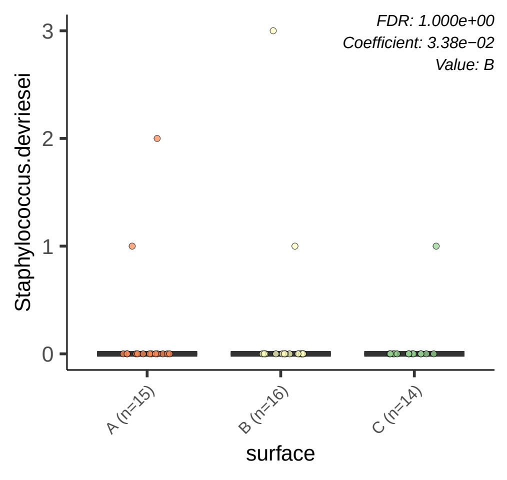

FDR: 1.000e+00
Coefficient: 3.38e−02
Value: B
Staphylococcus.devriesei
3
2
1
0
A (n=15)
B (n=16)
C (n=14)
surface

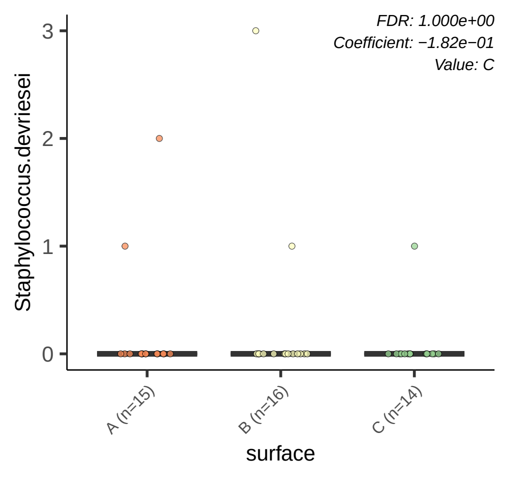
FDR: 1.000e+00
Coefficient: −1.82e−01
Value: C
Staphylococcus.devriesei
3
2
1
0
A (n=15)
B (n=16)
C (n=14)
surface

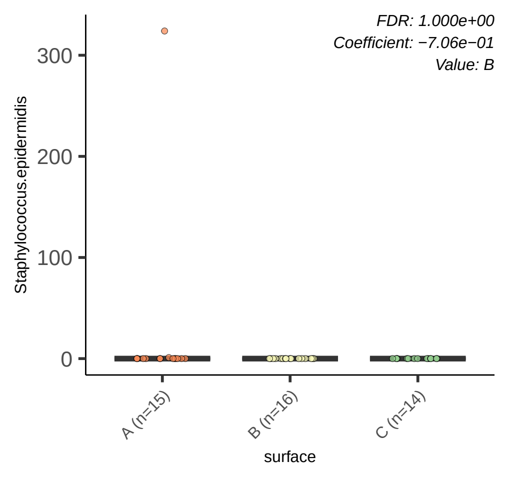
FDR: 1.000e+00
Coefficient: −7.06e−01
Value: B
Staphylococcus.epidermidis
300
200
100
0
A (n=15)
B (n=16)
C (n=14)
surface

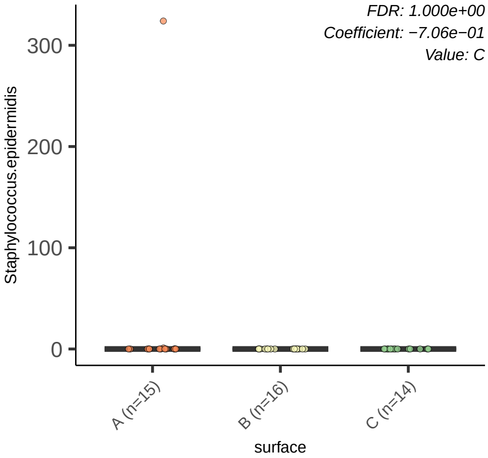
FDR: 1.000e+00
Coefficient: −7.06e−01
Value: C
Staphylococcus.epidermidis
300
200
100
0
A (n=15)
B (n=16)
C (n=14)
surface

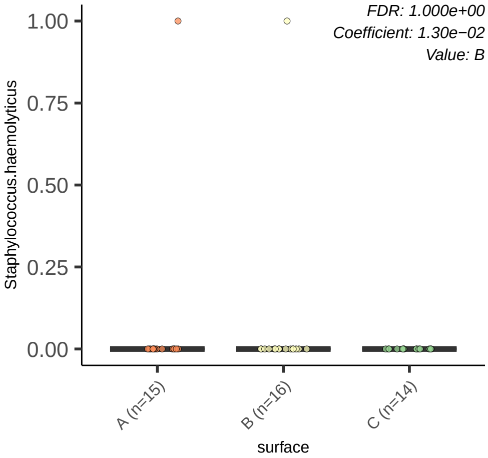

FDR: 1.000e+00
Coefficient: 1.30e−02
Value: B
Staphylococcus.haemolyticus
1.00
0.75
0.50
0.25
0.00
A (n=15)
B (n=16)
C (n=14)
surface

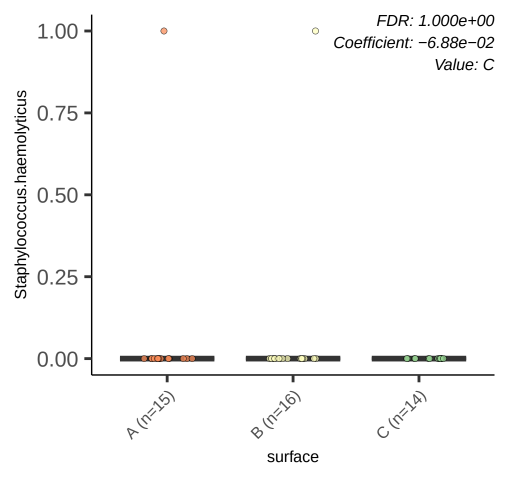
FDR: 1.000e+00
Coefficient: −6.88e−02
Value: C
Staphylococcus.haemolyticus
1.00
0.75
0.50
0.25
0.00
A (n=15)
B (n=16)
C (n=14)
surface

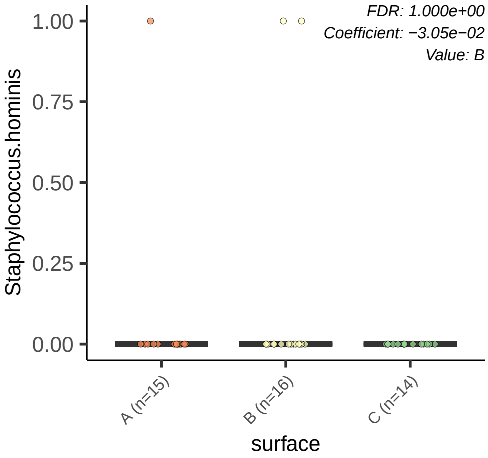

FDR: 1.000e+00
Coefficient: −3.05e−02
Value: B
1.00
0.75
0.50
0.25
0.00
Staphylococcus.hominis
A (n=15)
B (n=16)
C (n=14)
surface

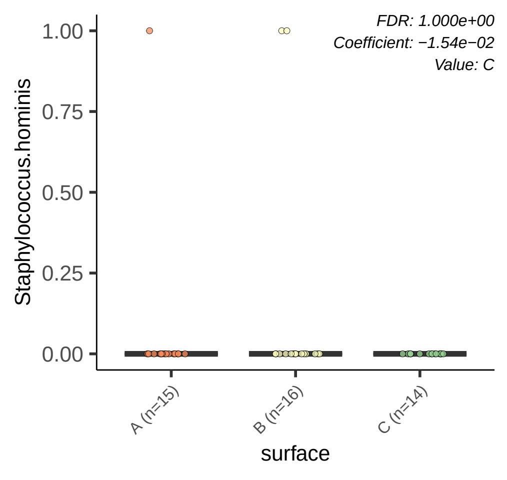
FDR: 1.000e+00
Coefficient: −1.54e−02
Value: C
Staphylococcus.hominis
1.00
0.75
0.50
0.25
0.00
A (n=15)
B (n=16)
C (n=14)
surface

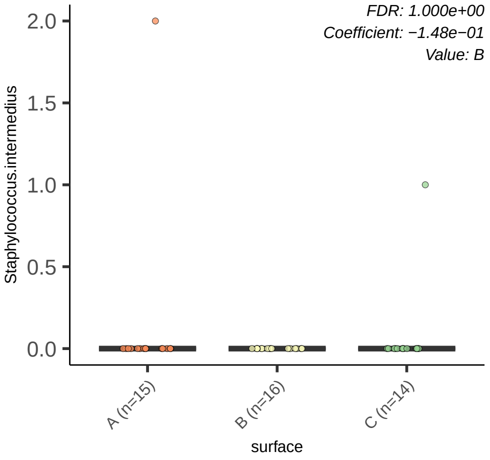

FDR: 1.000e+00
Coefficient: −1.48e−01
Value: B
Staphylococcus.intermedius
2.0
1.5
1.0
0.5
0.0
A (n=15)
B (n=16)
C (n=14)
surface

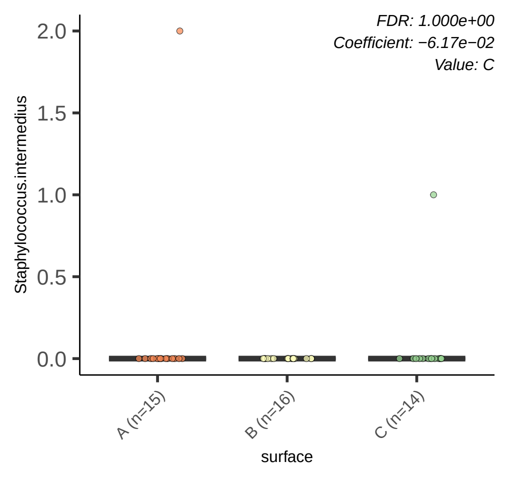
FDR: 1.000e+00
Coefficient: −6.17e−02
Value: C
Staphylococcus.intermedius
2.0
1.5
1.0
0.5
0.0
A (n=15)
B (n=16)
C (n=14)
surface

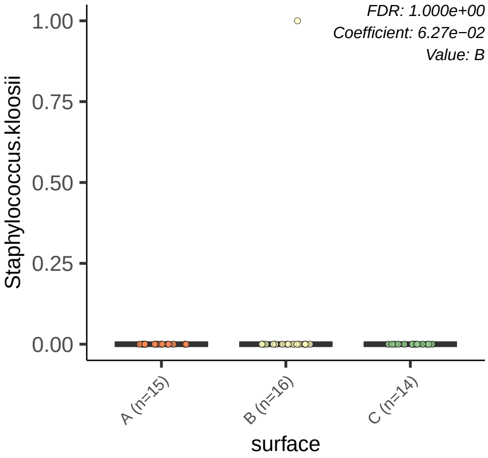

FDR: 1.000e+00
Coefficient: 6.27e−02
Value: B
Staphylococcus.kloosii
1.00
0.75
0.50
0.25
0.00
A (n=15)
B (n=16)
C (n=14)
surface

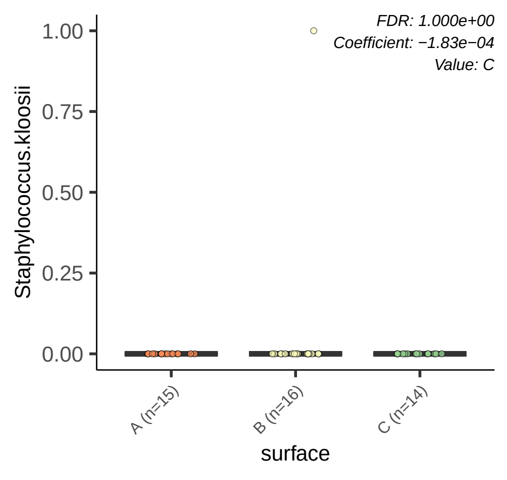
FDR: 1.000e+00
Coefficient: −1.83e−04
Value: C
Staphylococcus.kloosii
1.00
0.75
0.50
0.25
0.00
A (n=15)
B (n=16)
C (n=14)
surface

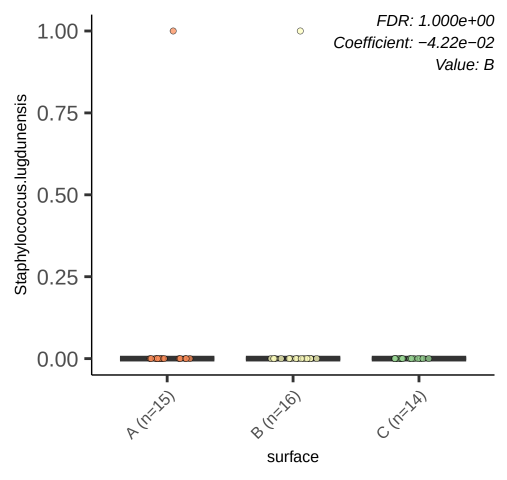

FDR: 1.000e+00
Coefficient: −4.22e−02
Value: B
1.00
0.75
0.50
0.25
0.00
Staphylococcus.lugdunensis
A (n=15)
B (n=16)
C (n=14)
surface

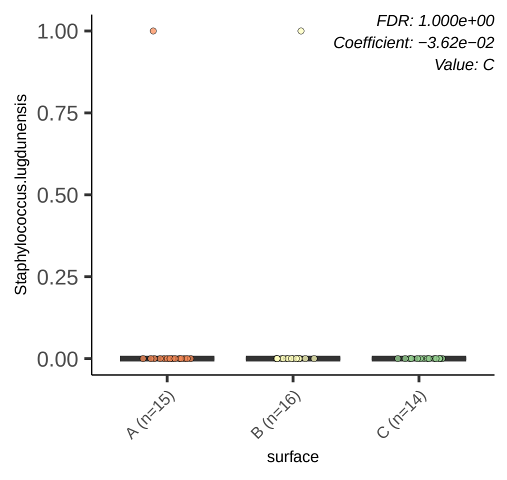
FDR: 1.000e+00
Coefficient: −3.62e−02
Value: C
1.00
0.75
0.50
0.25
0.00
Staphylococcus.lugdunensis
A (n=15)
B (n=16)
C (n=14)
surface

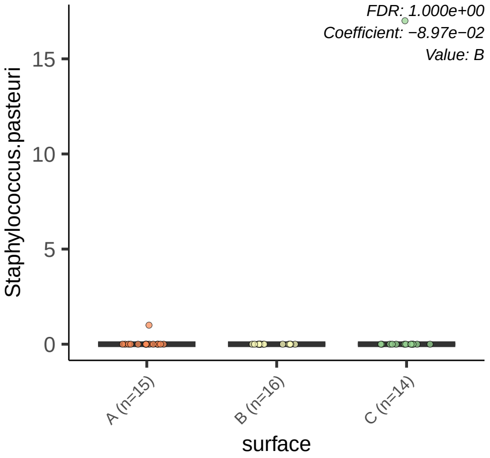

FDR: 1.000e+00
Coefficient: −8.97e−02
Value: B
Staphylococcus.pasteuri
15
10
5
0
A (n=15)
B (n=16)
C (n=14)
surface

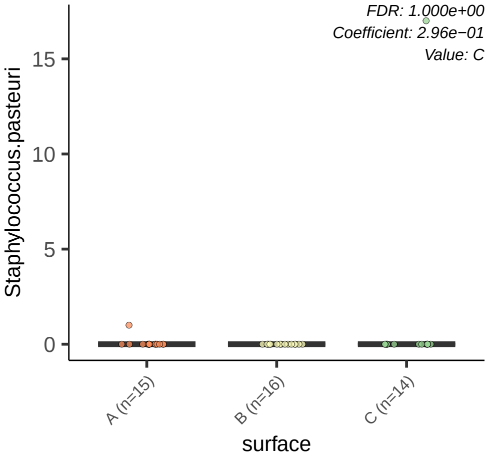

FDR: 1.000e+00
Coefficient: 2.96e−01
Value: C
Staphylococcus.pasteuri
15
10
5
0
A (n=15)
B (n=16)
C (n=14)
surface

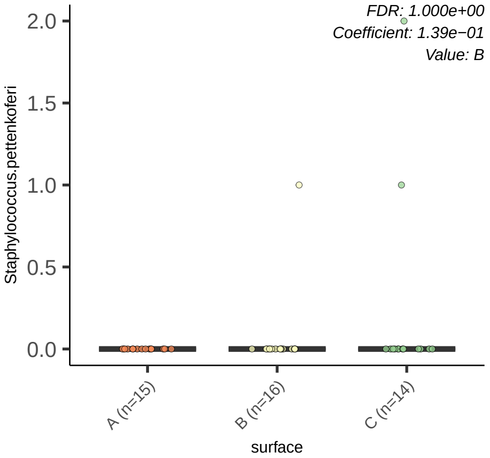
FDR: 1.000e+00
Coefficient: 1.39e−01
Value: B
Staphylococcus.pettenkoferi
2.0
1.5
1.0
0.5
0.0
A (n=15)
B (n=16)
C (n=14)
surface

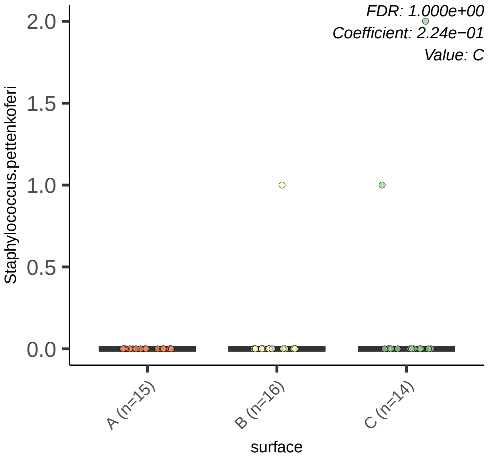
FDR: 1.000e+00
Coefficient: 2.24e−01
Value: C
Staphylococcus.pettenkoferi
2.0
1.5
1.0
0.5
0.0
A (n=15)
B (n=16)
C (n=14)
surface

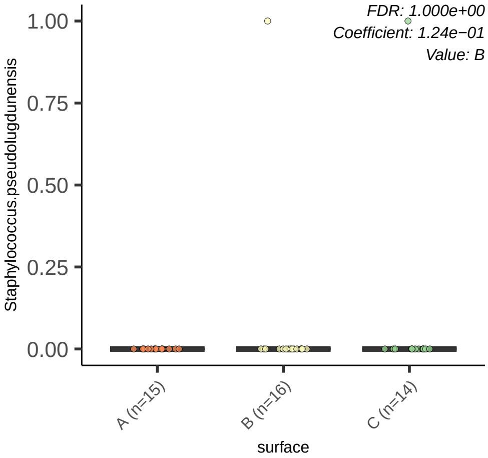

FDR: 1.000e+00
Coefficient: 1.24e−01
Value: B
1.00
0.75
0.50
0.25
0.00
Staphylococcus.pseudolugdunensis
A (n=15)
B (n=16)
C (n=14)
surface

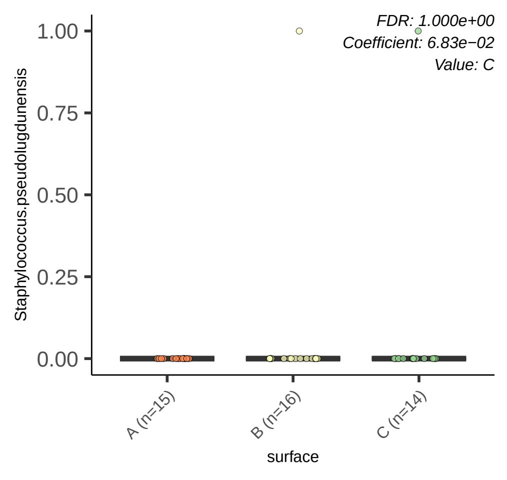
FDR: 1.000e+00
Coefficient: 6.83e−02
Value: C
Staphylococcus.pseudolugdunensis
1.00
0.75
0.50
0.25
0.00
A (n=15)
B (n=16)
C (n=14)
surface

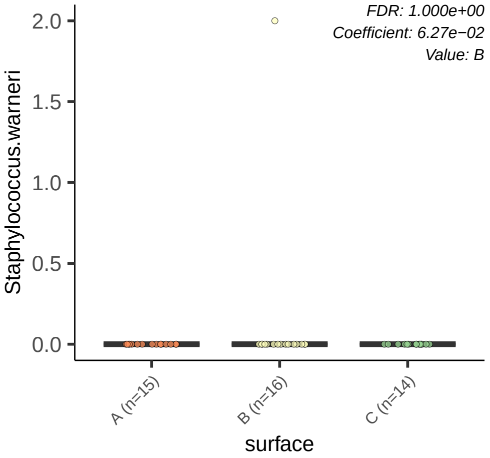

FDR: 1.000e+00
Coefficient: 6.27e−02
Value: B
Staphylococcus.warneri
2.0
1.5
1.0
0.5
0.0
A (n=15)
B (n=16)
C (n=14)
surface

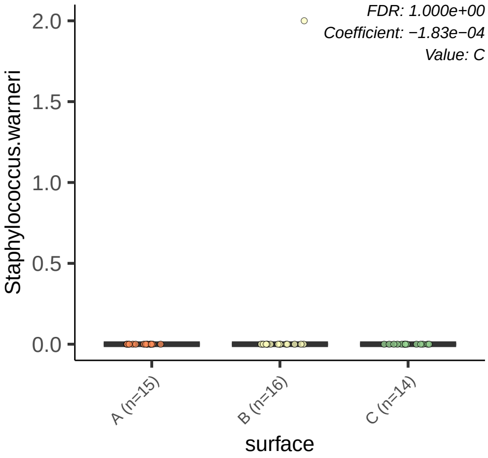

FDR: 1.000e+00
Coefficient: −1.83e−04
Value: C
Staphylococcus.warneri
2.0
1.5
1.0
0.5
0.0
A (n=15)
B (n=16)
C (n=14)
surface

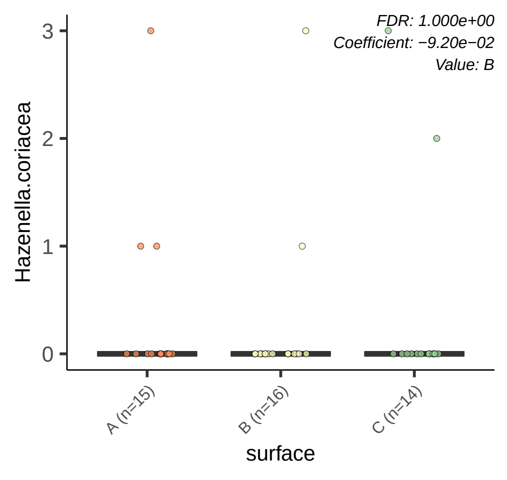
FDR: 1.000e+00
Coefficient: −9.20e−02
Value: B
Hazenella.coriacea
3
2
1
0
A (n=15)
B (n=16)
C (n=14)
surface

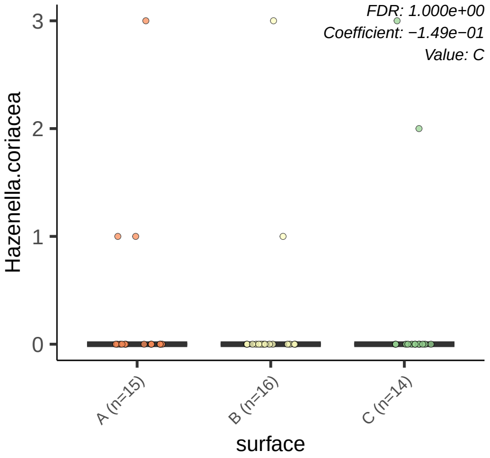
FDR: 1.000e+00
Coefficient: −1.49e−01
Value: C
Hazenella.coriacea
0
1
2
3
A (n=15)
B (n=16)
C (n=14)
surface

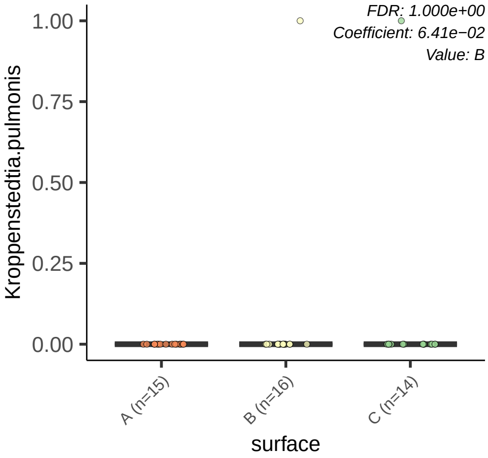

FDR: 1.000e+00
Coefficient: 6.41e−02
Value: B
Kroppenstedtia.pulmonis
1.00
0.75
0.50
0.25
0.00
A (n=15)
B (n=16)
C (n=14)
surface

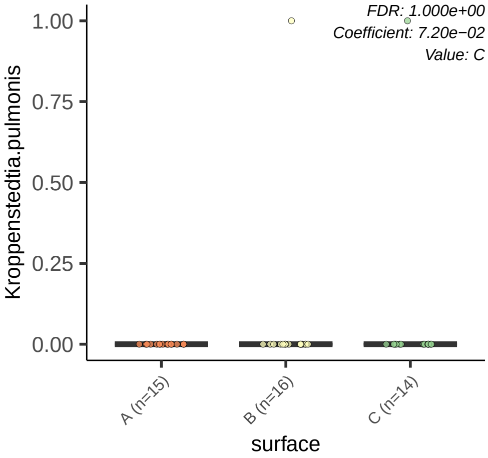

FDR: 1.000e+00
Coefficient: 7.20e−02
Value: C
Kroppenstedtia.pulmonis
1.00
0.75
0.50
0.25
0.00
A (n=15)
B (n=16)
C (n=14)
surface

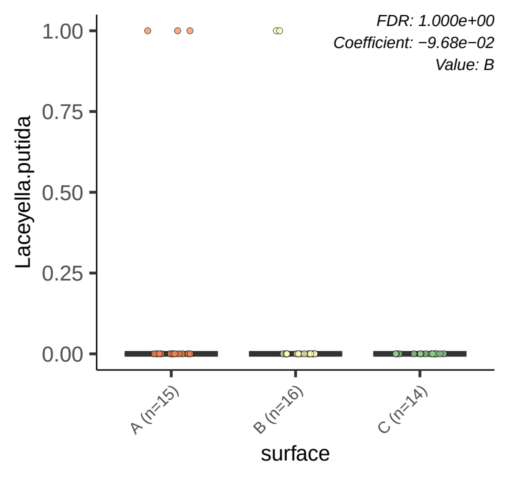

FDR: 1.000e+00
Coefficient: −9.68e−02
Value: B
Laceyella.putida
1.00
0.75
0.50
0.25
0.00
A (n=15)
B (n=16)
C (n=14)
surface

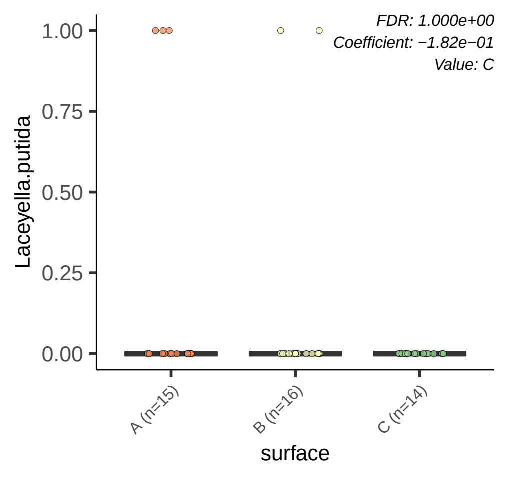

FDR: 1.000e+00
Coefficient: −1.82e−01
Value: C
Laceyella.putida
1.00
0.75
0.50
0.25
0.00
A (n=15)
B (n=16)
C (n=14)
surface

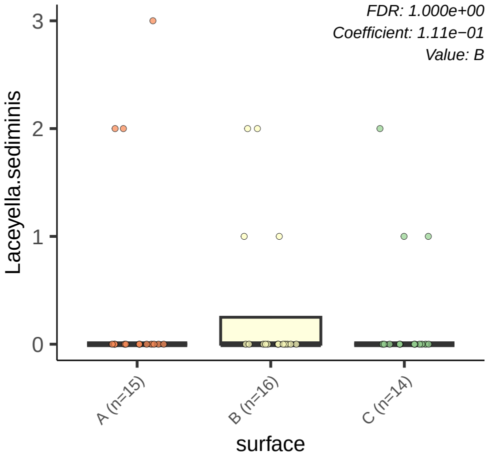
FDR: 1.000e+00
Coefficient: 1.11e−01
Value: B
Laceyella.sedimінis
3
2
1
0
A (n=15)
B (n=16)
C (n=14)
surface

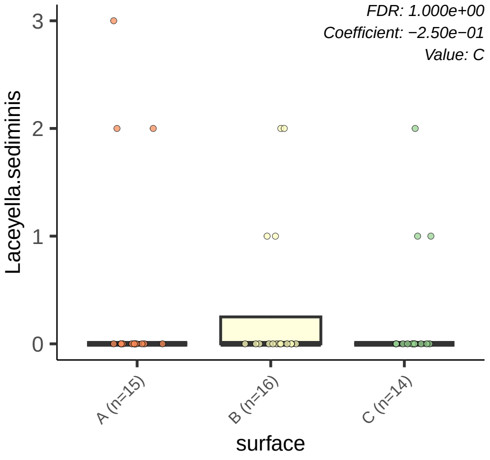
FDR: 1.000e+00
Coefficient: −2.50e−01
Value: C
Laceyella.sedimінis
3
2
1
0
A (n=15)
B (n=16)
C (n=14)
surface

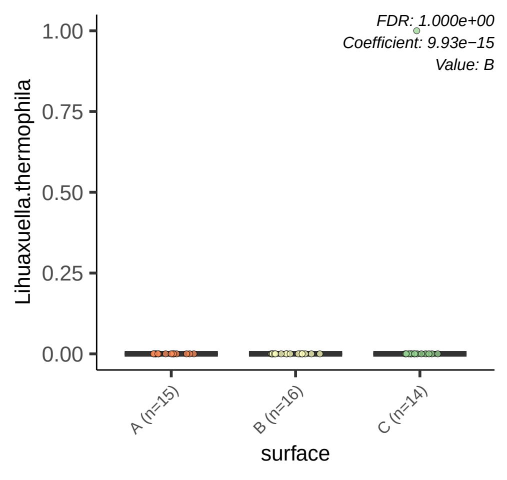

FDR: 1.000e+00
Coefficient: 9.93e−15
Value: B
Lihuaxuella.thermophila
1.00
0.75
0.50
0.25
0.00
A (n=15)
B (n=16)
C (n=14)
surface

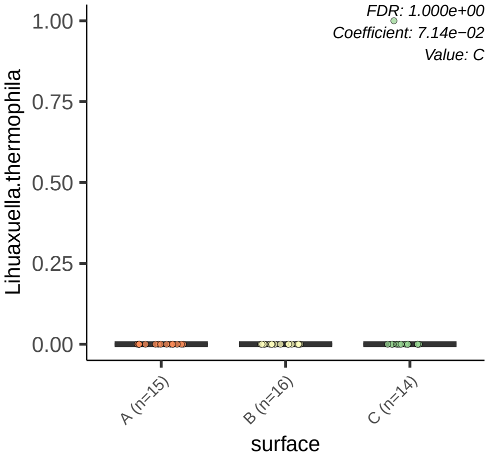
FDR: 1.000e+00
Coefficient: 7.14e−02
Value: C
Lihuaxuella.thermophila
1.00
0.75
0.50
0.25
0.00
A (n=15)
B (n=16)
C (n=14)
surface

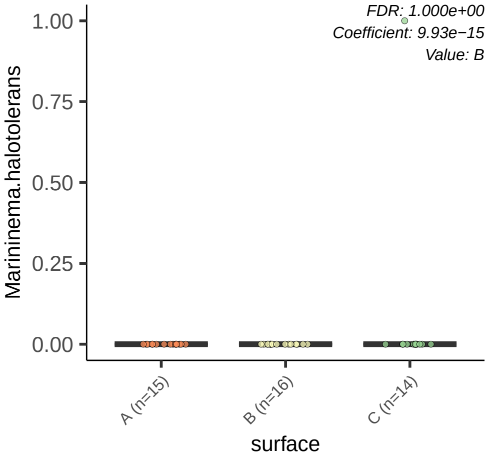
FDR: 1.000e+00
Coefficient: 9.93e−15
Value: B
Marininema.halotolerans
1.00
0.75
0.50
0.25
0.00
A (n=15)
B (n=16)
C (n=14)
surface

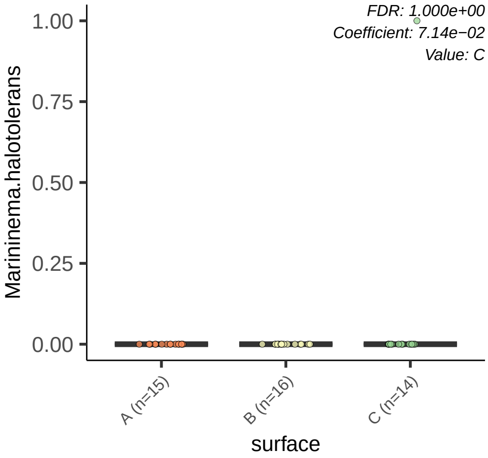
FDR: 1.000e+00
Coefficient: 7.14e−02
Value: C
Marininema.halotolerans
1.00
0.75
0.50
0.25
0.00
A (n=15)
B (n=16)
C (n=14)
surface

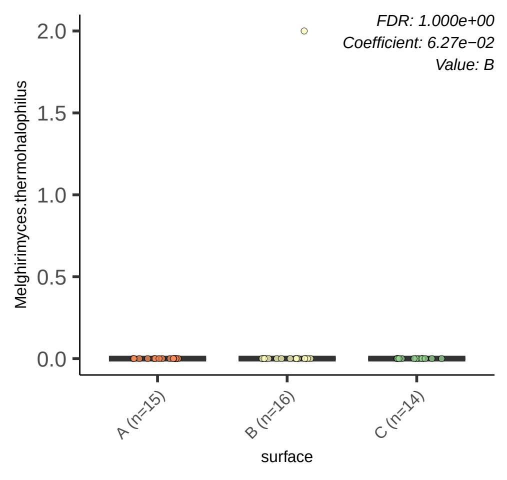
FDR: 1.000e+00
Coefficient: 6.27e−02
Value: B
2.0
1.5
1.0
0.5
0.0
Melghirimyces.thermohalophilus
A (n=15)
B (n=16)
C (n=14)
surface

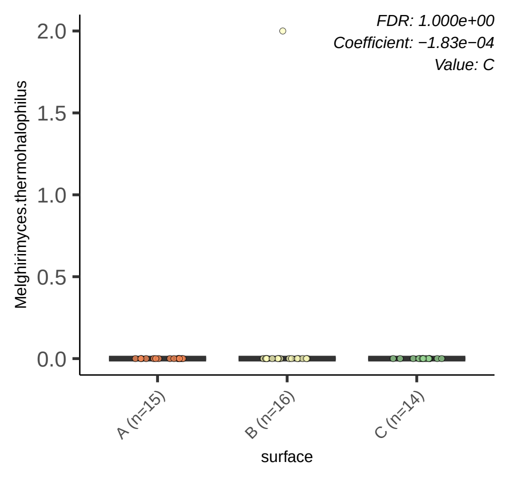
FDR: 1.000e+00
Coefficient: −1.83e−04
Value: C
Melghirimyces.thermohalophilus
2.0
1.5
1.0
0.5
0.0
A (n=15)
B (n=16)
C (n=14)
surface

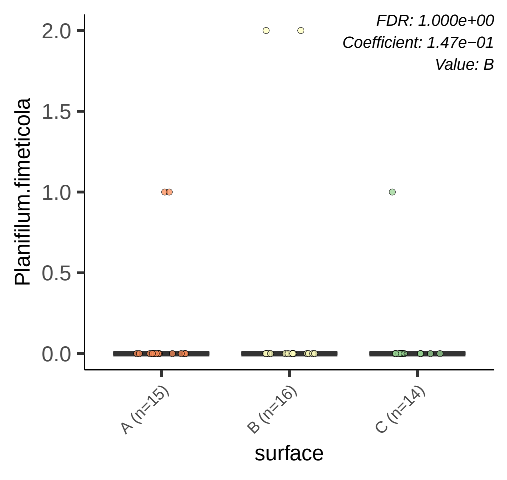

FDR: 1.000e+00
Coefficient: 1.47e−01
Value: B
Planifilum.fimeticola
2.0
1.5
1.0
0.5
0.0
A (n=15)
B (n=16)
C (n=14)
surface

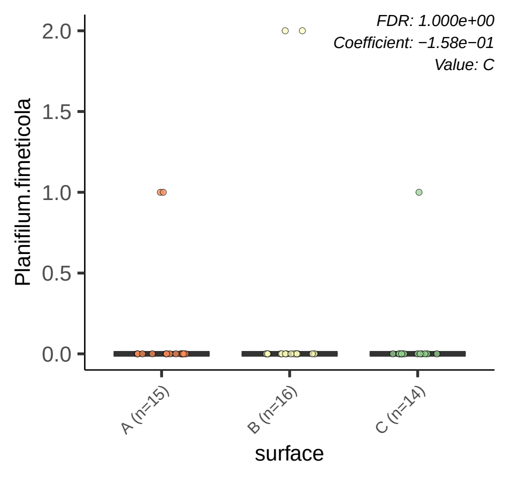
FDR: 1.000e+00
Coefficient: −1.58e−01
Value: C
Planifilum.fimeticola
2.0
1.5
1.0
0.5
0.0
A (n=15)
B (n=16)
C (n=14)
surface

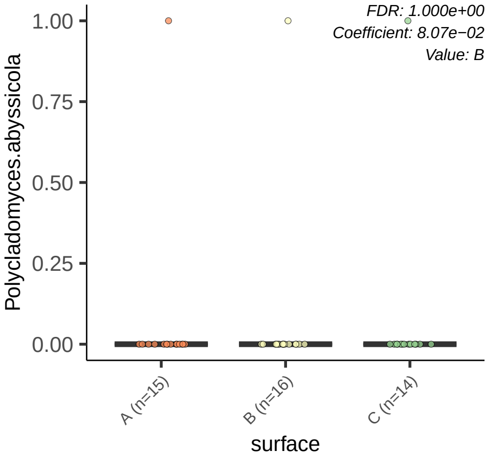
FDR: 1.000e+00
Coefficient: 8.07e−02
Value: B
Polycladomyces.abyssicola
1.00
0.75
0.50
0.25
0.00
A (n=15)
B (n=16)
C (n=14)
surface

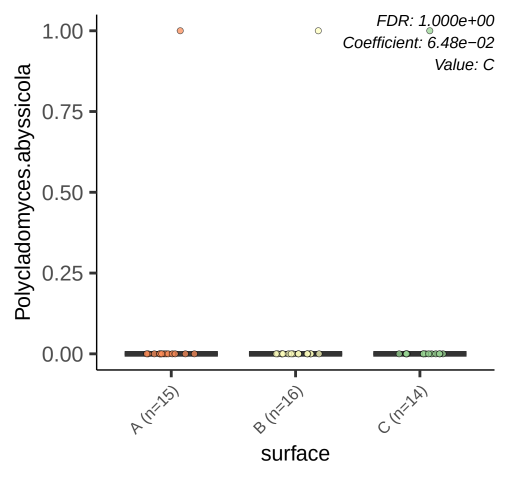
FDR: 1.000e+00
Coefficient: 6.48e−02
Value: C
Polycladomyces.abyssicola
1.00
0.75
0.50
0.25
0.00
A (n=15)
B (n=16)
C (n=14)
surface

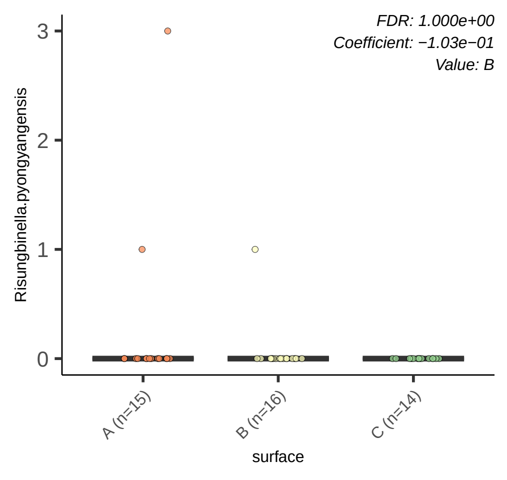
FDR: 1.000e+00
Coefficient: −1.03e−01
Value: B
Risungbinella.pyongyangensis
3
2
1
0
A (n=15)
B (n=16)
C (n=14)
surface

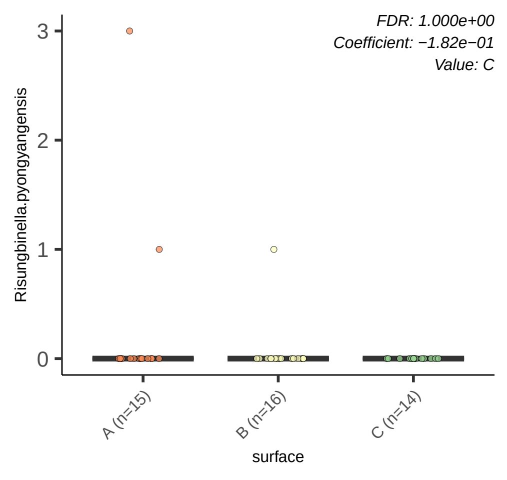
FDR: 1.000e+00
Coefficient: −1.82e−01
Value: C
Risungbinella.pyongyangensis
3
2
1
0
A (n=15)
B (n=16)
C (n=14)
surface

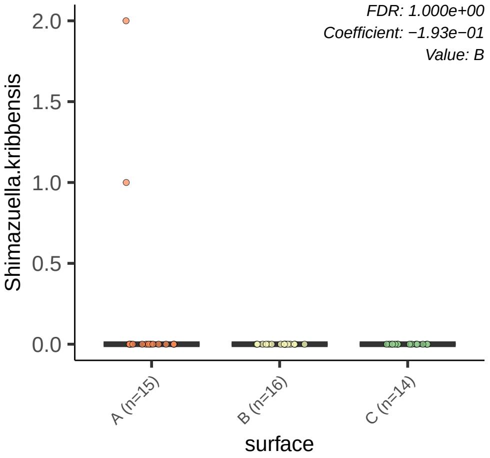
FDR: 1.000e+00
Coefficient: −1.93e−01
Value: B
Shimazuella.kribbensis
2.0
1.5
1.0
0.5
0.0
A (n=15)
B (n=16)
C (n=14)
surface

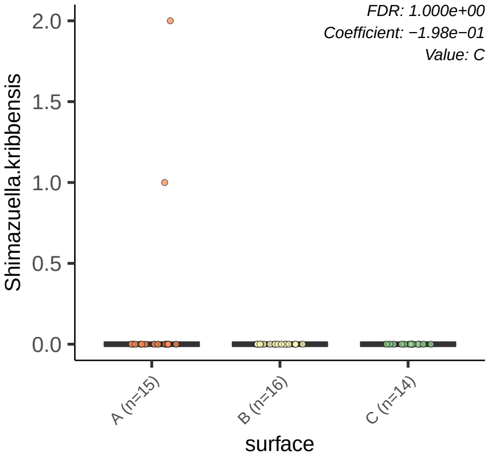

FDR: 1.000e+00
Coefficient: −1.98e−01
Value: C
Shimazuella.kribbensis
2.0
1.5
1.0
0.5
0.0
A (n=15)
B (n=16)
C (n=14)
surface

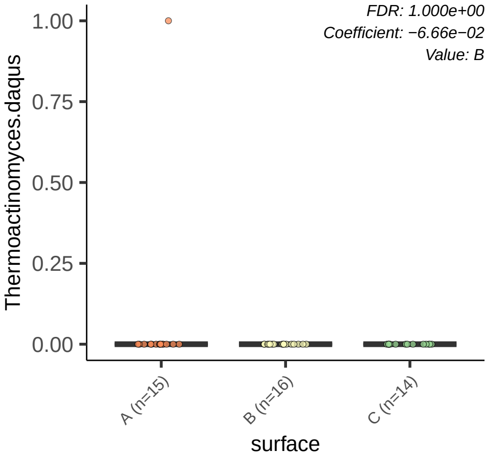

FDR: 1.000e+00
Coefficient: −6.66e−02
Value: B
Thermoactinomyces.daqus
1.00
0.75
0.50
0.25
0.00
A (n=15)
B (n=16)
C (n=14)
surface

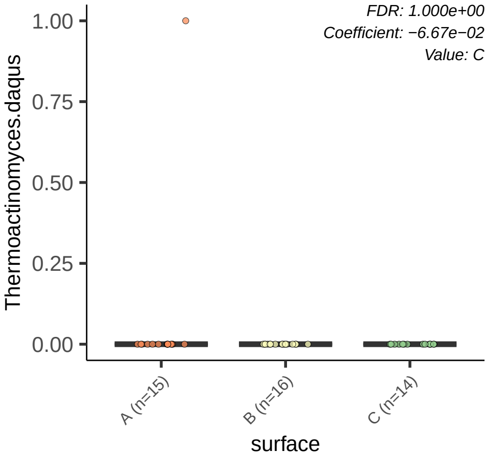

FDR: 1.000e+00
Coefficient: −6.67e−02
Value: C
Thermoactinomyces.daqus
1.00
0.75
0.50
0.25
0.00
A (n=15)
B (n=16)
C (n=14)
surface

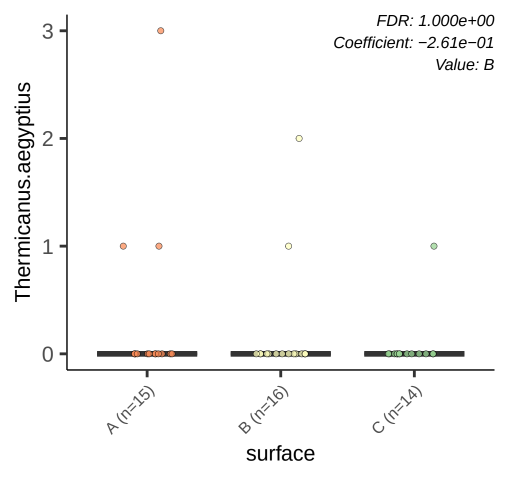

FDR: 1.000e+00
Coefficient: −2.61e−01
Value: B
Thermicanus.aegyptius
3
2
1
0
A (n=15)
B (n=16)
C (n=14)
surface

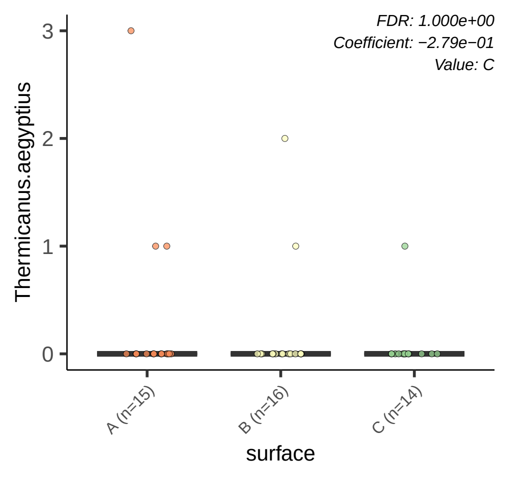

FDR: 1.000e+00
Coefficient: −2.79e−01
Value: C
Thermicanus.aegyptius
3
2
1
0
A (n=15)
B (n=16)
C (n=14)
surface

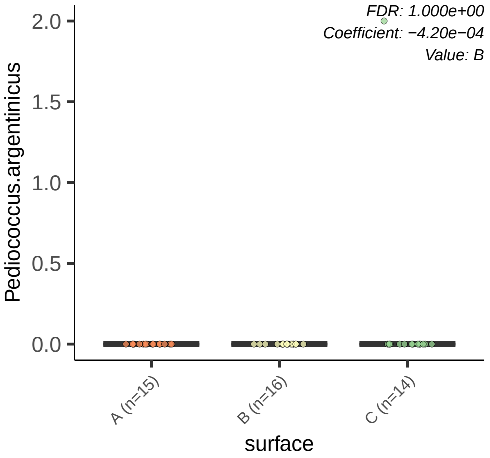
FDR: 1.000e+00
Coefficient: −4.20e−04
Value: B
Pediococcus.argentinicus
2.0
1.5
1.0
0.5
0.0
A (n=15)
B (n=16)
C (n=14)
surface

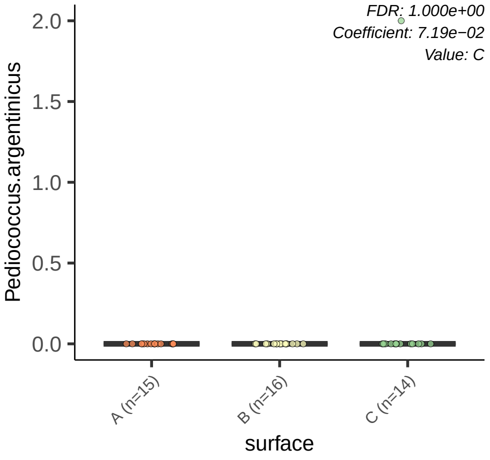
FDR: 1.000e+00
Coefficient: 7.19e−02
Value: C
Pediococcus.argentinicus
2.0
1.5
1.0
0.5
0.0
A (n=15)
B (n=16)
C (n=14)
surface

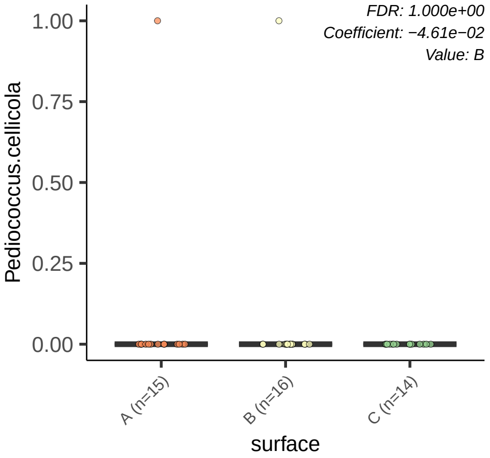

FDR: 1.000e+00
Coefficient: −4.61e−02
Value: B
Pediococcus.cellicola
1.00
0.75
0.50
0.25
0.00
A (n=15)
B (n=16)
C (n=14)
surface

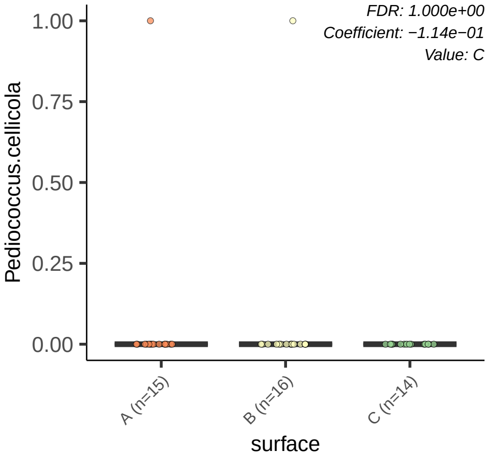

FDR: 1.000e+00
Coefficient: −1.14e−01
Value: C
Pediococcus.cellicola
1.00
0.75
0.50
0.25
0.00
A (n=15)
B (n=16)
C (n=14)
surface

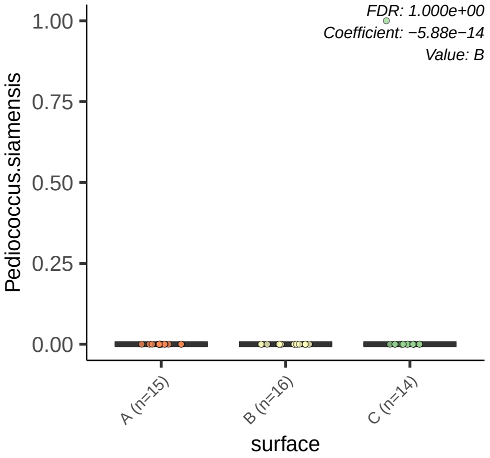

FDR: 1.000e+00
Coefficient: −5.88e−14
Value: B
Pediococcus.siamensis
1.00
0.75
0.50
0.25
0.00
A (n=15)
B (n=16)
C (n=14)
surface

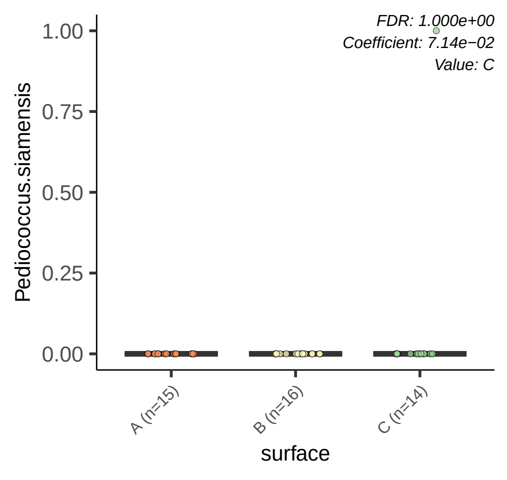
FDR: 1.000e+00
Coefficient: 7.14e−02
Value: C
Pediococcus.siamensis
1.00
0.75
0.50
0.25
0.00
A (n=15)
B (n=16)
C (n=14)
surface

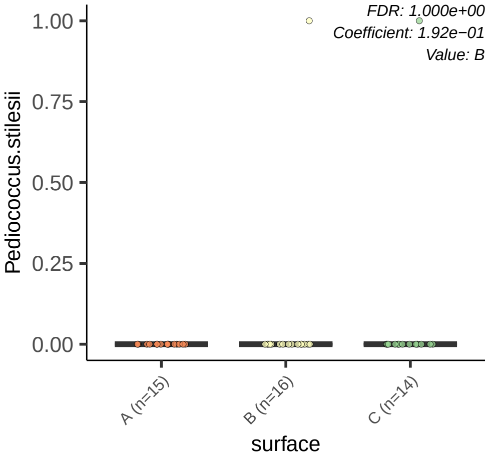
FDR: 1.000e+00
Coefficient: 1.92e−01
Value: B
Pediococcus.stilesii
1.00
0.75
0.50
0.25
0.00
A (n=15)
B (n=16)
C (n=14)
surface

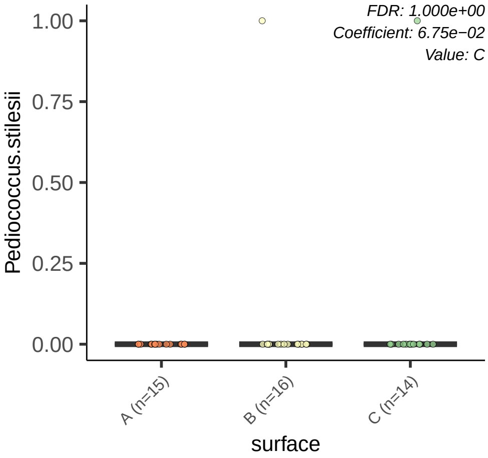

FDR: 1.000e+00
Coefficient: 6.75e−02
Value: C
Pediococcus.stilesii
1.00
0.75
0.50
0.25
0.00
A (n=15)
B (n=16)
C (n=14)
surface

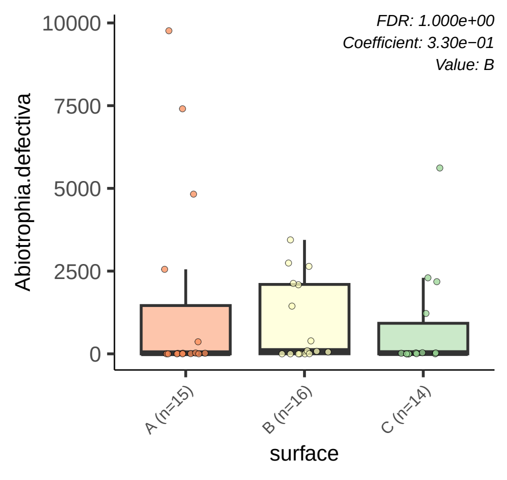
FDR: 1.000e+00
Coefficient: 3.30e−01
Value: B
Abiotrophia.defectiva
10000
7500
5000
2500
0
A (n=15)
B (n=16)
C (n=14)
surface

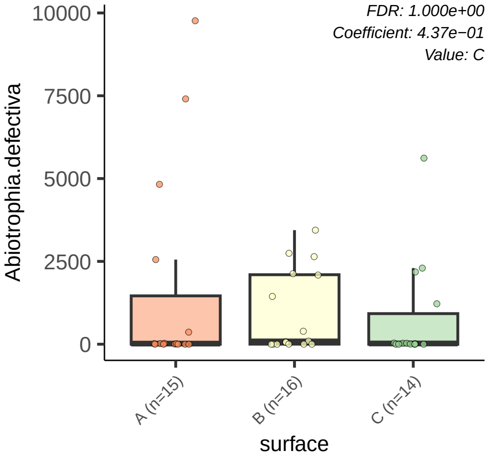
FDR: 1.000e+00
Coefficient: 4.37e−01
Value: C
10000
7500
5000
2500
0
Abiotrophia.defectiva
A (n=15)
B (n=16)
C (n=14)
surface

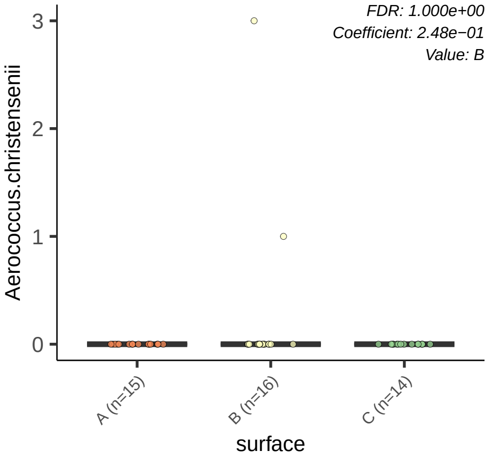
FDR: 1.000e+00
Coefficient: 2.48e−01
Value: B
Aerococcus.christensenii
3
2
1
0
A (n=15)
B (n=16)
C (n=14)
surface

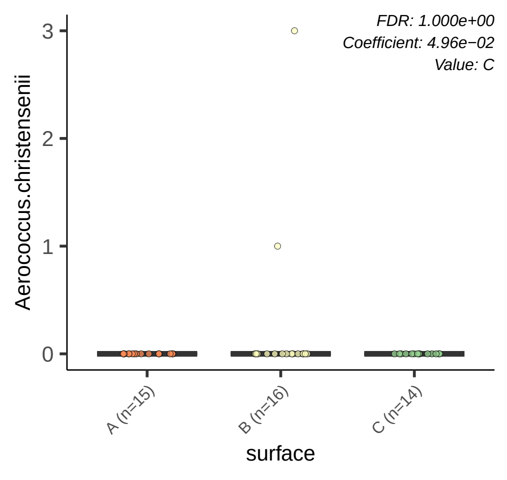
FDR: 1.000e+00
Coefficient: 4.96e−02
Value: C
Aerococcus.christensenii
0
1
2
3
A (n=15)
B (n=16)
C (n=14)
surface

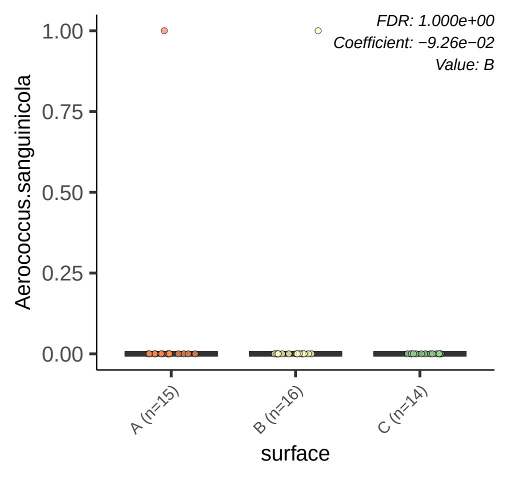
FDR: 1.000e+00
Coefficient: −9.26e−02
Value: B
Aerococcus.sanguinicola
1.00
0.75
0.50
0.25
0.00
A (n=15)
B (n=16)
C (n=14)
surface

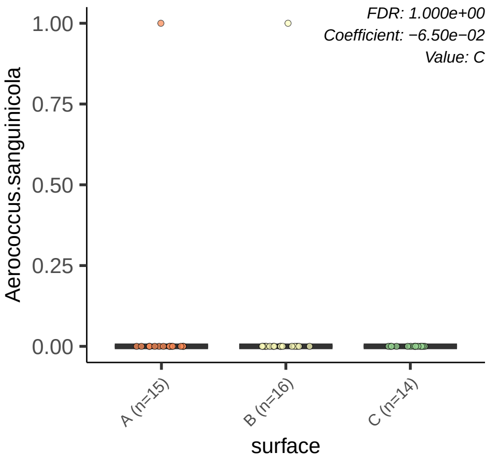

FDR: 1.000e+00
Coefficient: −6.50e−02
Value: C
Aerococcus.sanguinicola
1.00
0.75
0.50
0.25
0.00
A (n=15)
B (n=16)
C (n=14)
surface

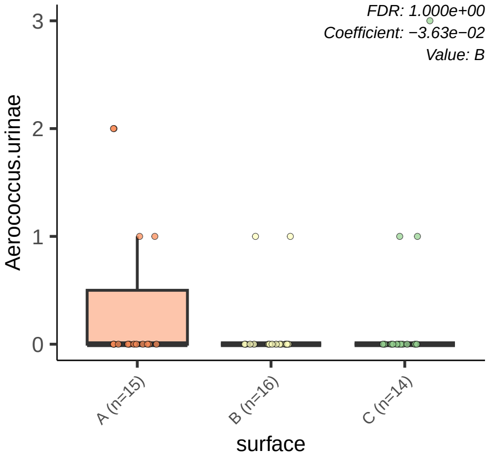
FDR: 1.000e+00
Coefficient: −3.63e−02
Value: B
Aerococcus.urinae
3
2
1
0
A (n=15)
B (n=16)
C (n=14)
surface

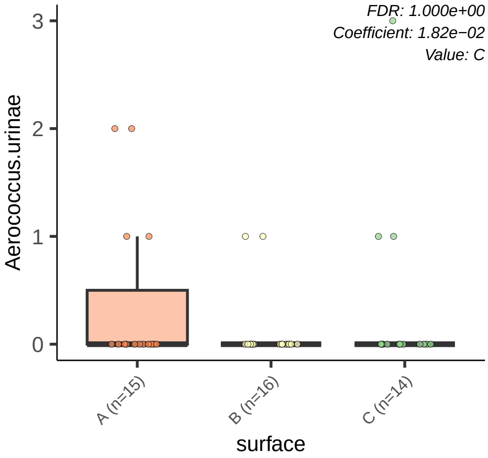
FDR: 1.000e+00
Coefficient: 1.82e−02
Value: C
Aerococcus.urinae
0
1
2
3
A (n=15)
B (n=16)
C (n=14)
surface

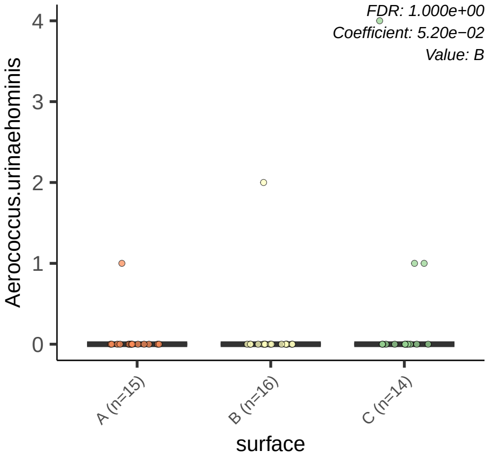

FDR: 1.000e+00
Coefficient: 5.20e−02
Value: B
Aerococcus.urinaehominis
0
1
2
3
4
A (n=15)
B (n=16)
C (n=14)
surface

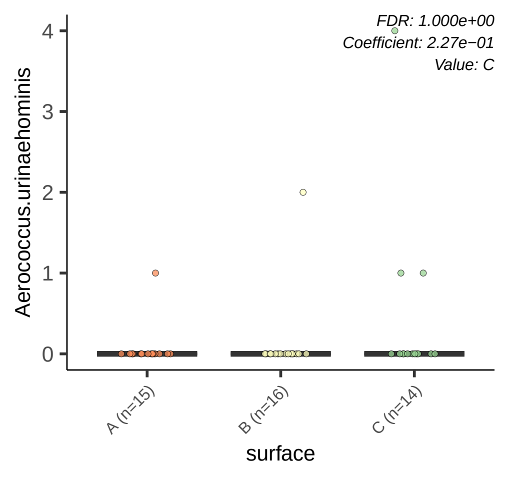

FDR: 1.000e+00
Coefficient: 2.27e−01
Value: C
Aerococcus.urinaehominis
0
1
2
3
4
A (n=15)
B (n=16)
C (n=14)
surface

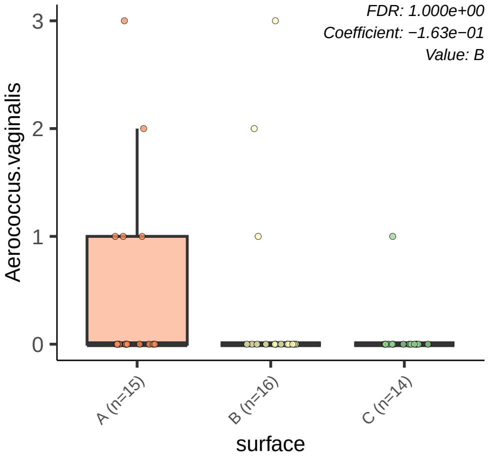

FDR: 1.000e+00
Coefficient: −1.63e−01
Value: B
Aerococcus.vaginalis
3
2
1
0
A (n=15)
B (n=16)
C (n=14)
surface

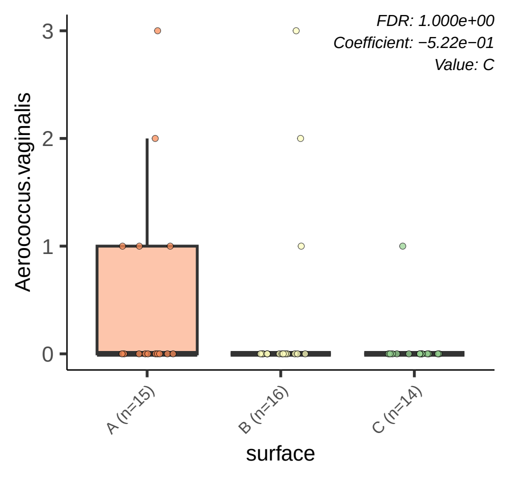
FDR: 1.000e+00
Coefficient: −5.22e−01
Value: C
Aerococcus.vaginalis
3
2
1
0
A (n=15)
B (n=16)
C (n=14)
surface

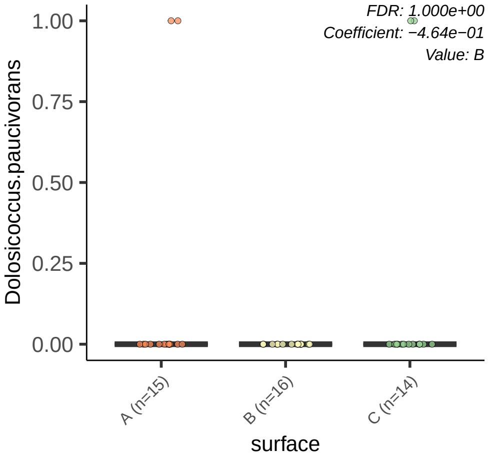
FDR: 1.000e+00
Coefficient: −4.64e−01
Value: B
Dolosicoccus.paucivorans
1.00
0.75
0.50
0.25
0.00
A (n=15)
B (n=16)
C (n=14)
surface

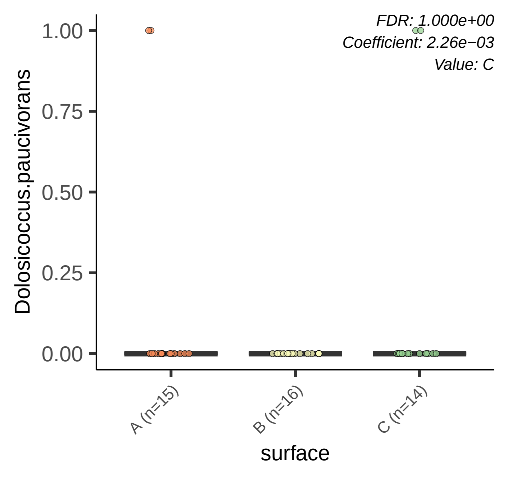
FDR: 1.000e+00
Coefficient: 2.26e−03
Value: C
Dolosicoccus.paucivorans
1.00
0.75
0.50
0.25
0.00
A (n=15)
B (n=16)
C (n=14)
surface

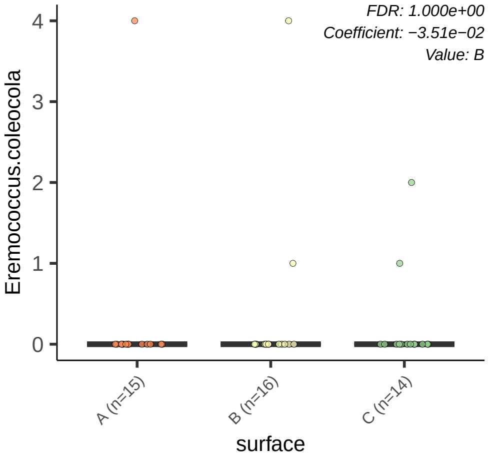

FDR: 1.000e+00
Coefficient: −3.51e−02
Value: B
Eremococcus.coleocola
4
3
2
1
0
A (n=15)
B (n=16)
C (n=14)
surface

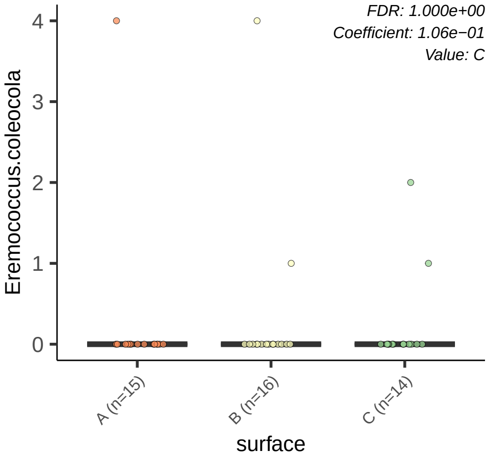

FDR: 1.000e+00
Coefficient: 1.06e−01
Value: C
Eremococcus.coleocola
0
1
2
3
4
A (n=15)
B (n=16)
C (n=14)
surface

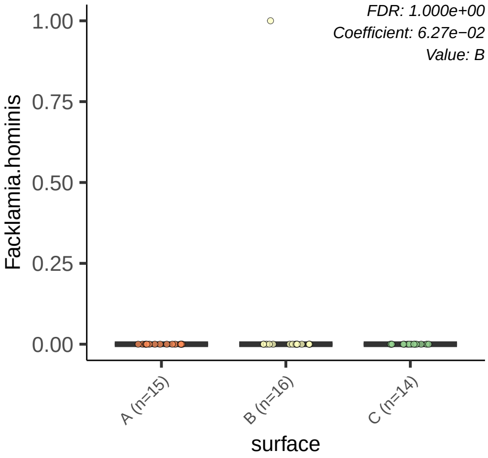

FDR: 1.000e+00
Coefficient: 6.27e−02
Value: B
Facklamia.hominis
1.00
0.75
0.50
0.25
0.00
A (n=15)
B (n=16)
C (n=14)
surface

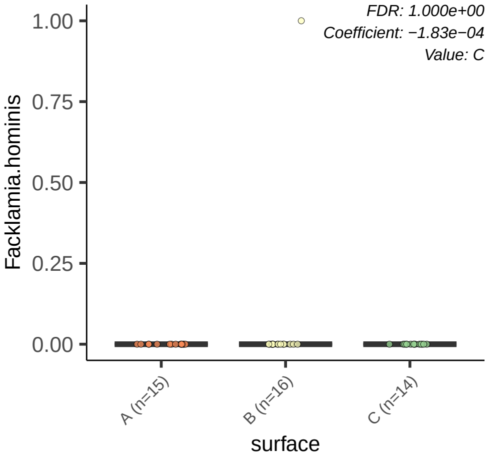
FDR: 1.000e+00
Coefficient: −1.83e−04
Value: C
Facklamia.hominis
1.00
0.75
0.50
0.25
0.00
A (n=15)
B (n=16)
C (n=14)
surface

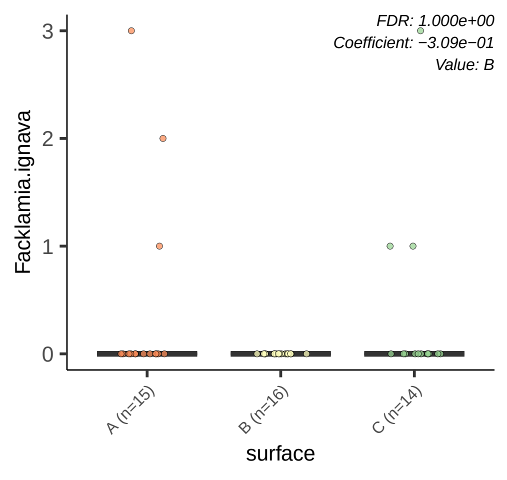
FDR: 1.000e+00
Coefficient: −3.09e−01
Value: B
Facklamia.ignava
3
2
1
0
A (n=15)
B (n=16)
C (n=14)
surface

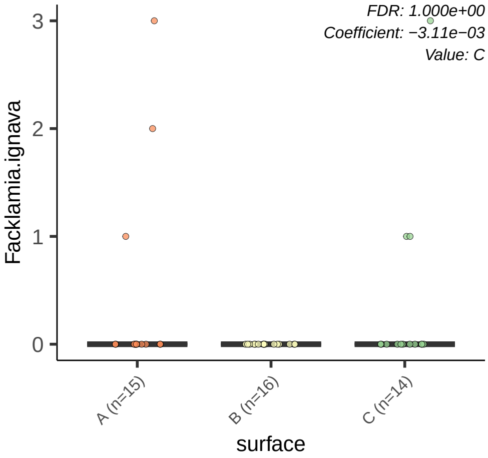

FDR: 1.000e+00
Coefficient: −3.11e−03
Value: C
Facklamia.ignava
3
2
1
0
A (n=15)
B (n=16)
C (n=14)
surface

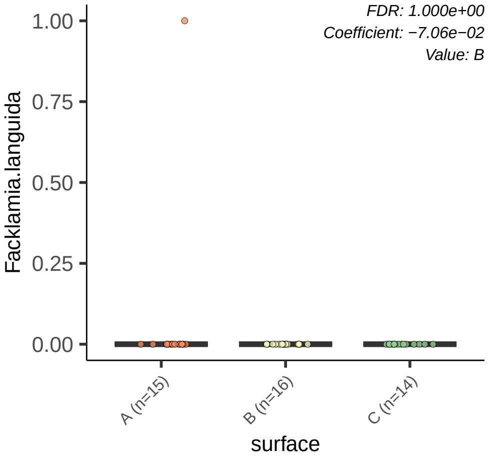
FDR: 1.000e+00
Coefficient: −7.06e−02
Value: B
Facklamia.languida
1.00
0.75
0.50
0.25
0.00
A (n=15)
B (n=16)
C (n=14)
surface

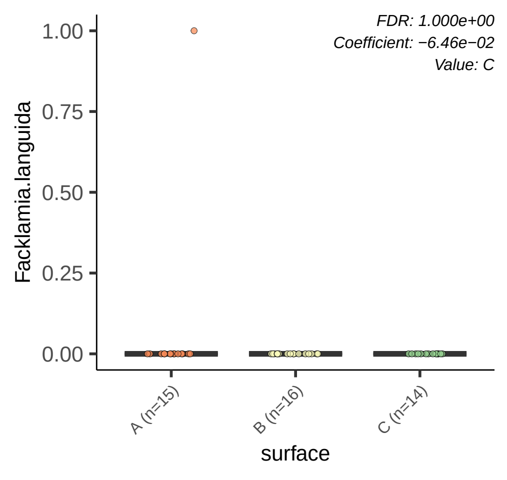
FDR: 1.000e+00
Coefficient: −6.46e−02
Value: C
Facklamia.languida
1.00
0.75
0.50
0.25
0.00
A (n=15)
B (n=16)
C (n=14)
surface

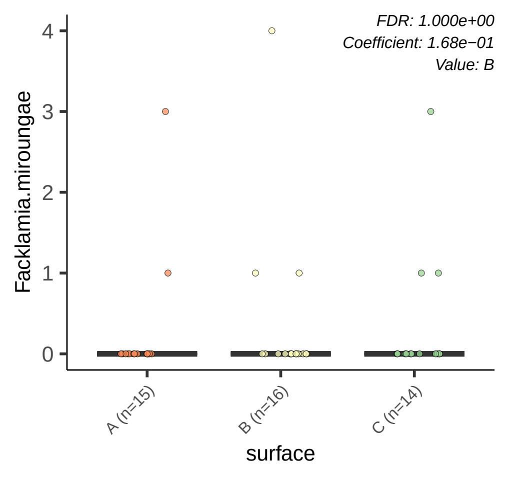
FDR: 1.000e+00
Coefficient: 1.68e−01
Value: B
Facklamia.miroungae
0
1
2
3
4
A (n=15)
B (n=16)
C (n=14)
surface

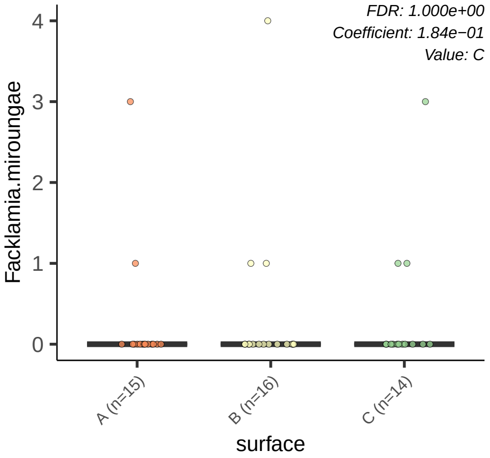
FDR: 1.000e+00
Coefficient: 1.84e−01
Value: C
Facklamia.miroungae
4
3
2
1
0
A (n=15)
B (n=16)
C (n=14)
surface

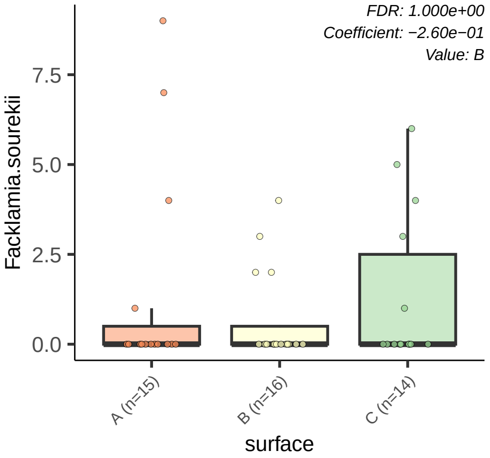

FDR: 1.000e+00
Coefficient: −2.60e−01
Value: B
Facklamia.sourekii
7.5
5.0
2.5
0.0
A (n=15)
B (n=16)
C (n=14)
surface

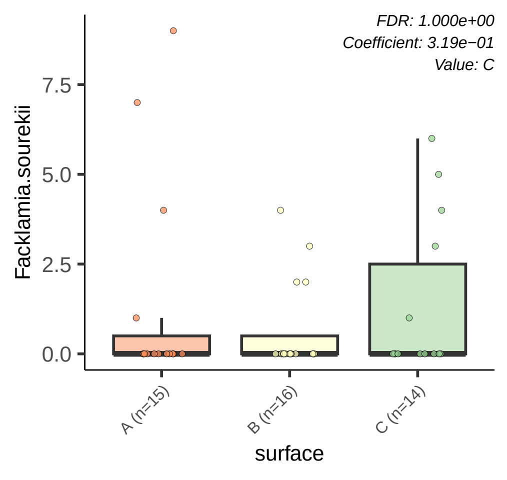

FDR: 1.000e+00
Coefficient: 3.19e−01
Value: C
Facklamia.sourekii
7.5
5.0
2.5
0.0
A (n=15)
B (n=16)
C (n=14)
surface

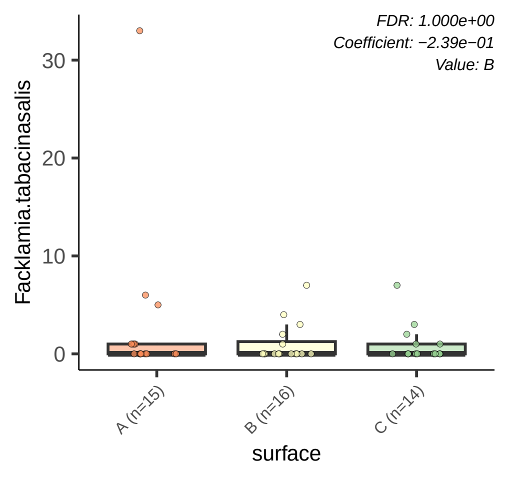
FDR: 1.000e+00
Coefficient: −2.39e−01
Value: B
Facklamia.tabacinasalis
30
20
10
0
A (n=15)
B (n=16)
C (n=14)
surface

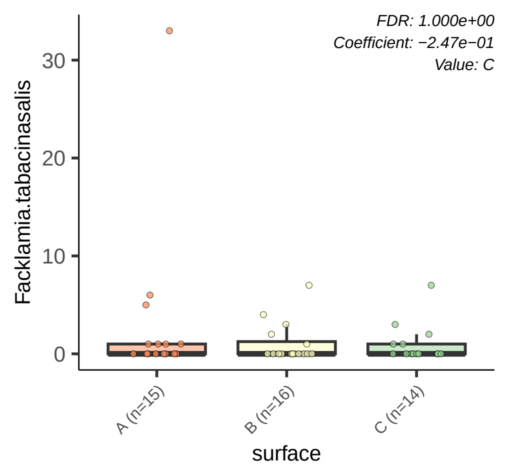

FDR: 1.000e+00
Coefficient: −2.47e−01
Value: C
Facklamia.tabacinasalis
30
20
10
0
A (n=15)
B (n=16)
C (n=14)
surface

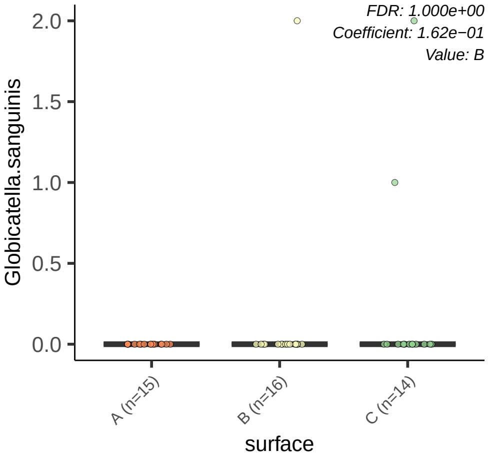
FDR: 1.000e+00
Coefficient: 1.62e−01
Value: B
Globicatella.sanguinis
2.0
1.5
1.0
0.5
0.0
A (n=15)
B (n=16)
C (n=14)
surface

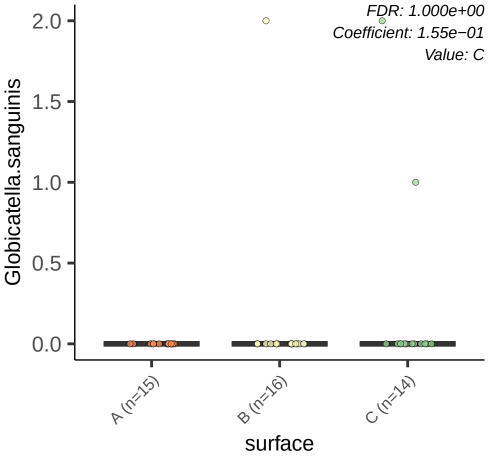

FDR: 1.000e+00
Coefficient: 1.55e−01
Value: C
Globicatella.sanguinis
2.0
1.5
1.0
0.5
0.0
A (n=15)
B (n=16)
C (n=14)
surface

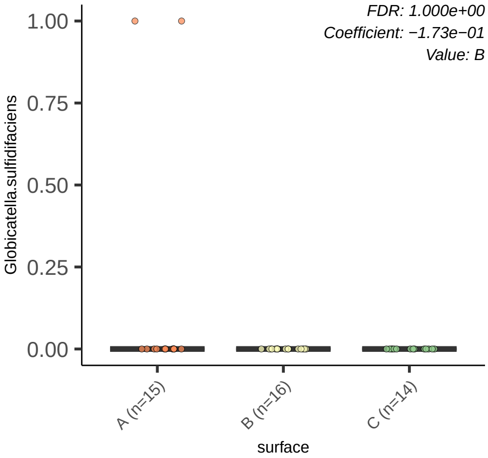
FDR: 1.000e+00
Coefficient: −1.73e−01
Value: B
Globicatella.sulfidifaciens
1.00
0.75
0.50
0.25
0.00
A (n=15)
B (n=16)
C (n=14)
surface

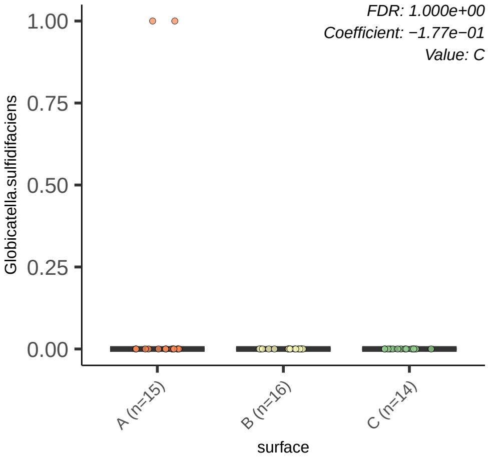

FDR: 1.000e+00
Coefficient: −1.77e−01
Value: C
1.00
0.75
0.50
0.25
0.00
Globicatella.sulfidifaciens
A (n=15)
B (n=16)
C (n=14)
surface

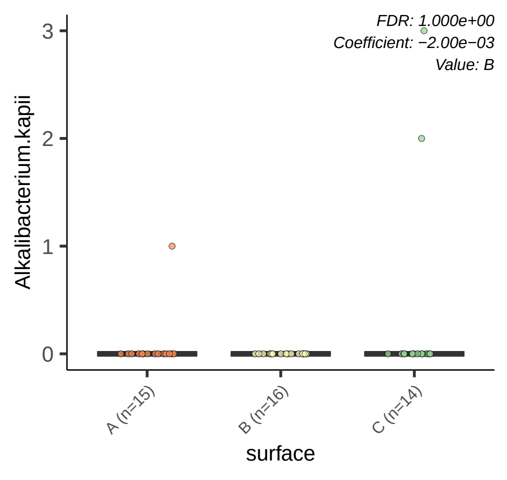
FDR: 1.000e+00
Coefficient: −2.00e−03
Value: B
Alkalibacterium.kapii
3
2
1
0
A (n=15)
B (n=16)
C (n=14)
surface

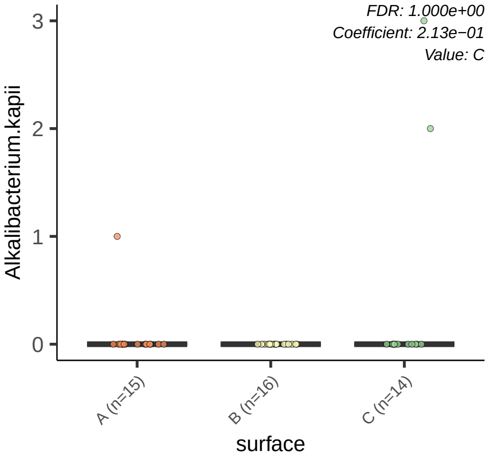
FDR: 1.000e+00
Coefficient: 2.13e−01
Value: C
Alkalibacterium.kapii
0
1
2
3
A (n=15)
B (n=16)
C (n=14)
surface

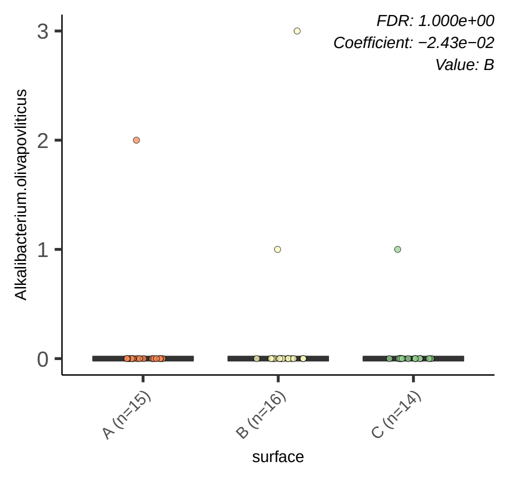
FDR: 1.000e+00
Coefficient: −2.43e−02
Value: B
Alkalibacterium.olivapovliticus
3
2
1
0
A (n=15)
B (n=16)
C (n=14)
surface

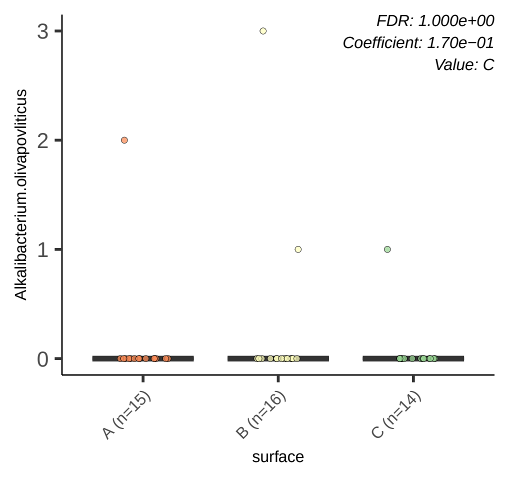
FDR: 1.000e+00
Coefficient: 1.70e−01
Value: C
Alkalibacterium.olivapovliticus
3
2
1
0
A (n=15)
B (n=16)
C (n=14)
surface

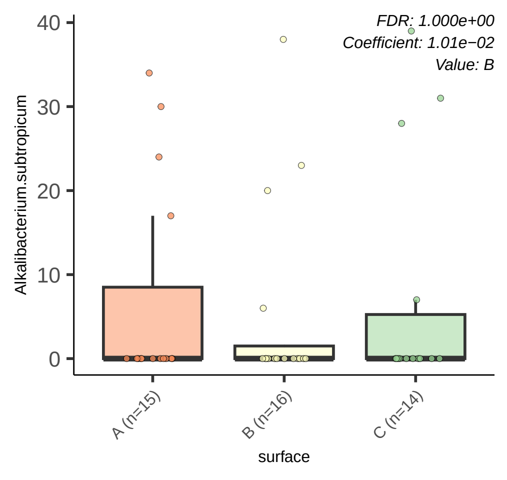

FDR: 1.000e+00
Coefficient: 1.01e−02
Value: B
40
30
20
10
0
Alkalibacterium.subtropicum
A (n=15)
B (n=16)
C (n=14)
surface

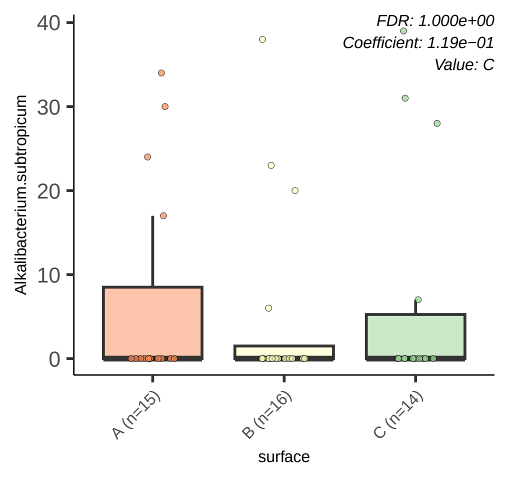

FDR: 1.000e+00
Coefficient: 1.19e−01
Value: C
Alkalibacterium.subtropicum
40
30
20
10
0
A (n=15)
B (n=16)
C (n=14)
surface

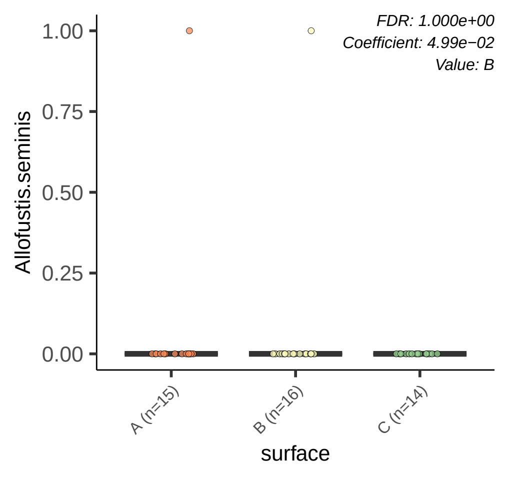
FDR: 1.000e+00
Coefficient: 4.99e−02
Value: B
Allofustis.seminis
1.00
0.75
0.50
0.25
0.00
A (n=15)
B (n=16)
C (n=14)
surface

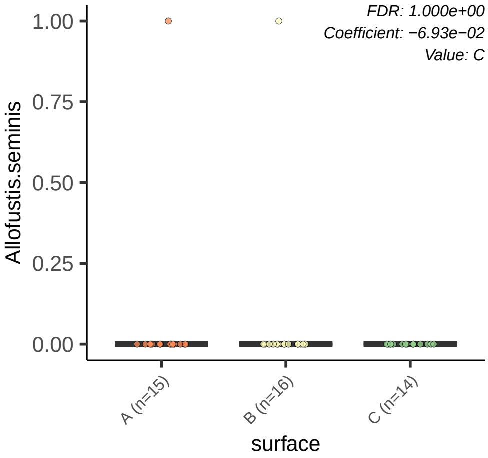
FDR: 1.000e+00
Coefficient: −6.93e−02
Value: C
Allofustis.seminis
1.00
0.75
0.50
0.25
0.00
A (n=15)
B (n=16)
C (n=14)
surface

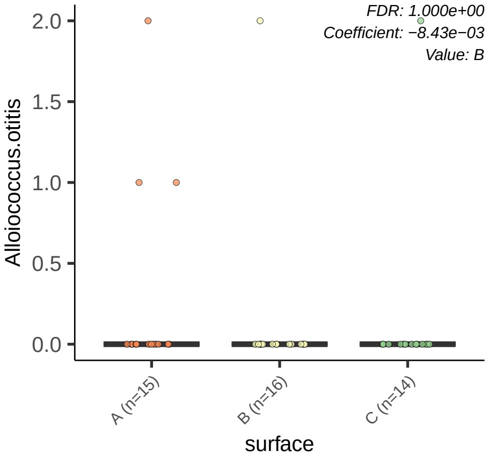

FDR: 1.000e+00
Coefficient: −8.43e−03
Value: B
Alloiococcus.otitis
2.0
1.5
1.0
0.5
0.0
A (n=15)
B (n=16)
C (n=14)
surface

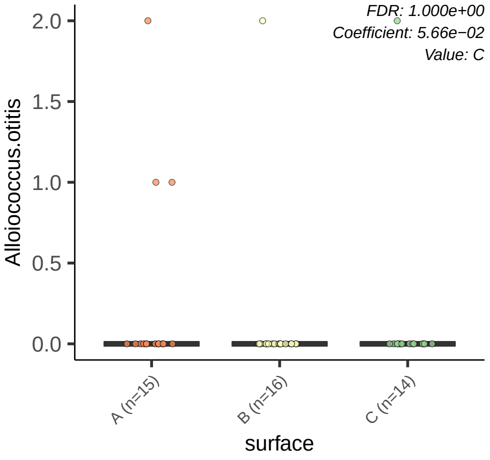
FDR: 1.000e+00
Coefficient: 5.66e−02
Value: C
Alloiococcus.otitis
2.0
1.5
1.0
0.5
0.0
A (n=15)
B (n=16)
C (n=14)
surface

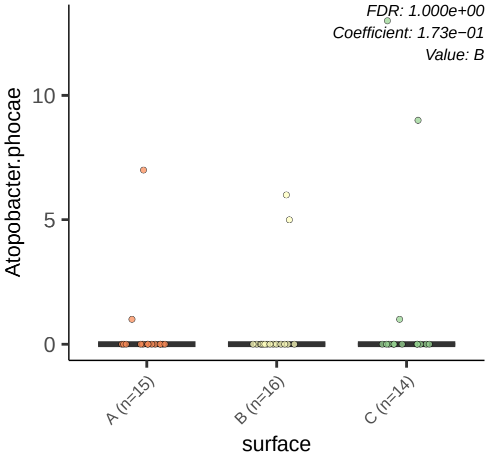

FDR: 1.000e+00
Coefficient: 1.73e−01
Value: B
Atopobacter.phocae
10
5
0
A (n=15)
B (n=16)
C (n=14)
surface

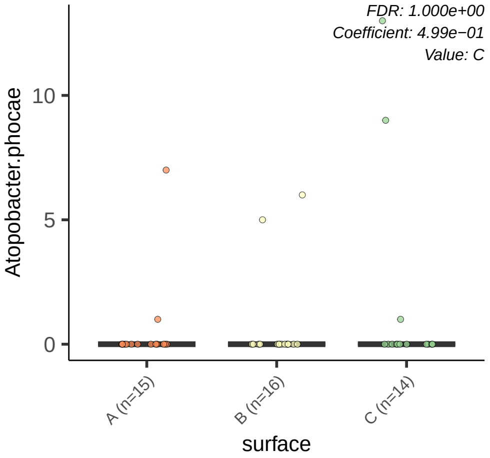
FDR: 1.000e+00
Coefficient: 4.99e−01
Value: C
Atopobacter.phocae
10
5
0
A (n=15)
B (n=16)
C (n=14)
surface

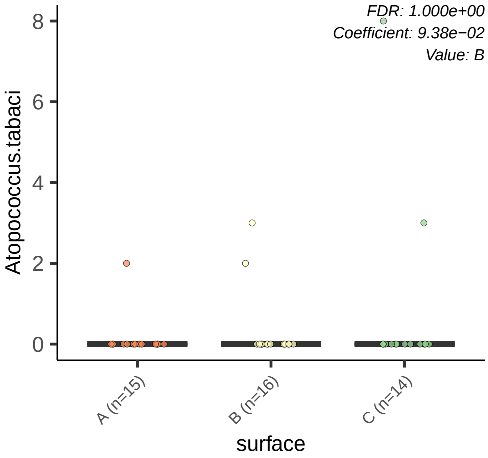

FDR: 1.000e+00
Coefficient: 9.38e−02
Value: B
Atopococcus.tabaci
8
6
4
2
0
A (n=15)
B (n=16)
C (n=14)
surface

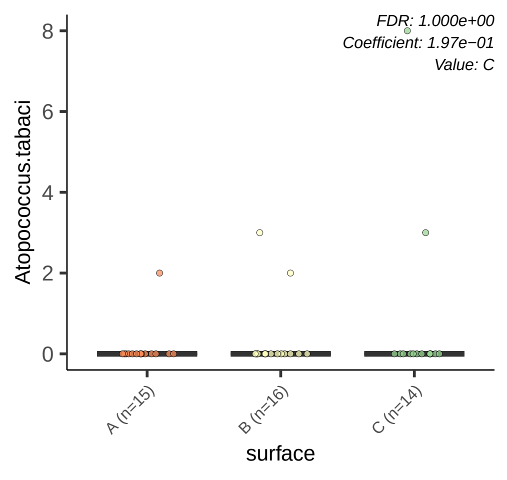
FDR: 1.000e+00
Coefficient: 1.97e−01
Value: C
Atopococcus.tabaci
8
6
4
2
0
A (n=15)
B (n=16)
C (n=14)
surface

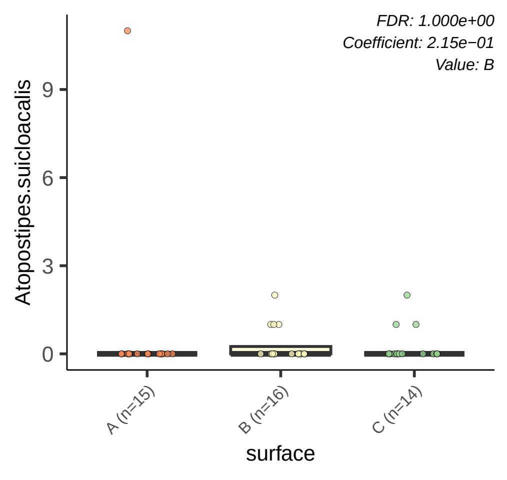
FDR: 1.000e+00
Coefficient: 2.15e−01
Value: B
Atopostipes.suicloacalis
0
3
6
9
A (n=15)
B (n=16)
C (n=14)
surface

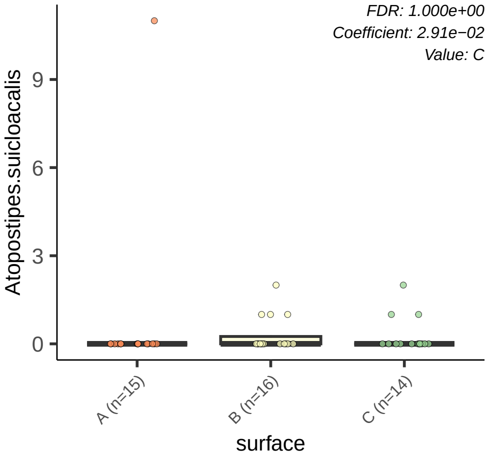
FDR: 1.000e+00
Coefficient: 2.91e−02
Value: C
Atopostipes.suicloacalis
0
3
6
9
A (n=15)
B (n=16)
C (n=14)
surface

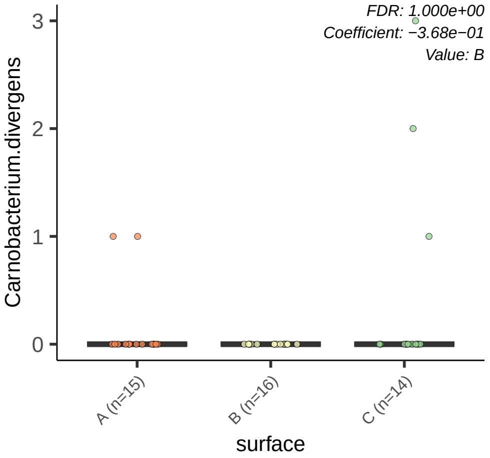

FDR: 1.000e+00
Coefficient: −3.68e−01
Value: B
Carnobacterium.divergens
3
2
1
0
A (n=15)
B (n=16)
C (n=14)
surface

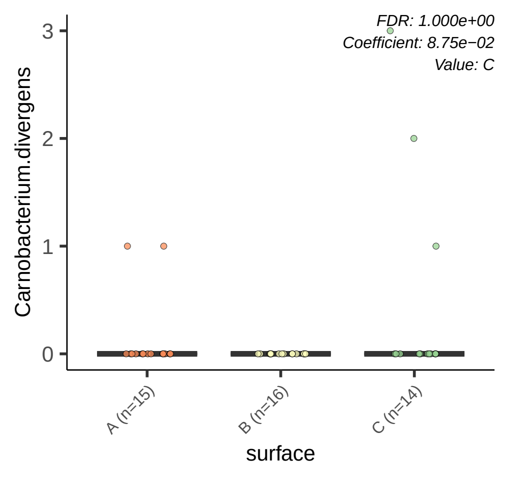
FDR: 1.000e+00
Coefficient: 8.75e−02
Value: C
Carnobacterium.divergens
0
1
2
3
A (n=15)
B (n=16)
C (n=14)
surface

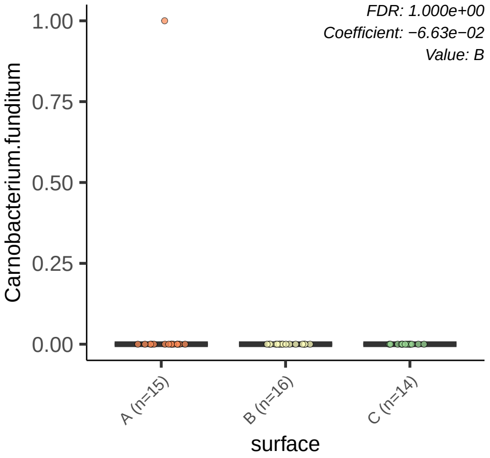
FDR: 1.000e+00
Coefficient: −6.63e−02
Value: B
Carnobacterium.funditum
1.00
0.75
0.50
0.25
0.00
A (n=15)
B (n=16)
C (n=14)
surface

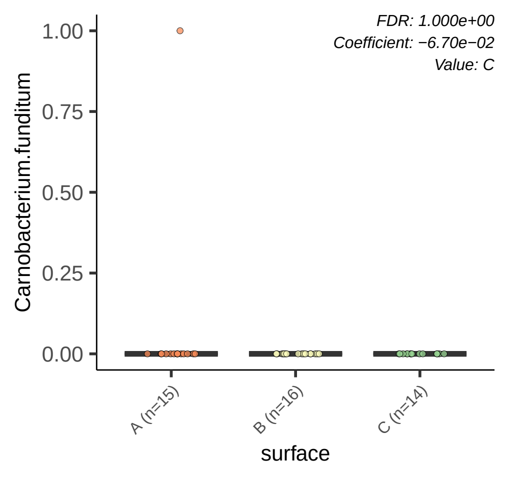
FDR: 1.000e+00
Coefficient: −6.70e−02
Value: C
Carnobacterium.funditum
1.00
0.75
0.50
0.25
0.00
A (n=15)
B (n=16)
C (n=14)
surface

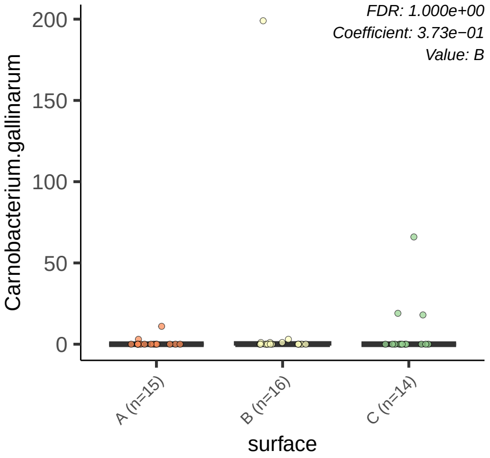
FDR: 1.000e+00
Coefficient: 3.73e−01
Value: B
Carnobacterium.gallinarum
200
150
100
50
0
A (n=15)
B (n=16)
C (n=14)
surface

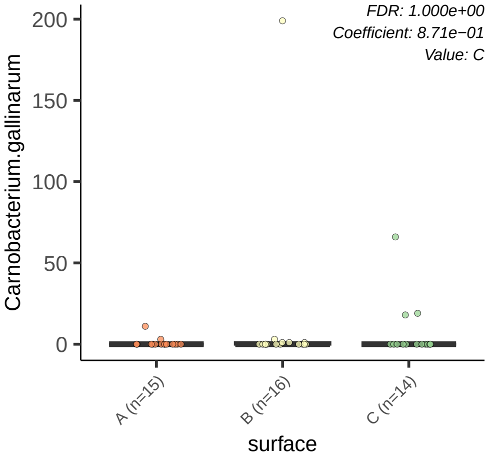
FDR: 1.000e+00
Coefficient: 8.71e−01
Value: C
Carnobacterium.gallinarum
200
150
100
50
0
A (n=15)
B (n=16)
C (n=14)
surface

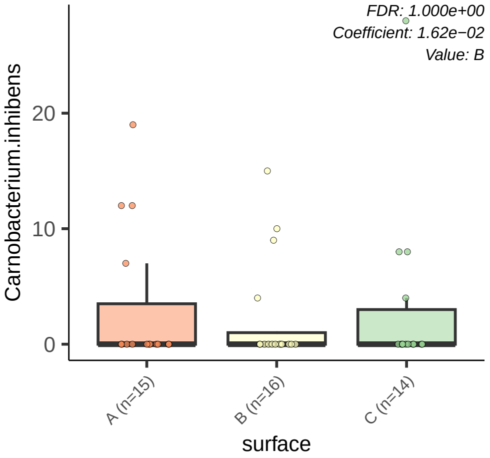

FDR: 1.000e+00
Coefficient: 1.62e−02
Value: B
Carnobacterium.inhibens
0
10
20
A (n=15)
B (n=16)
C (n=14)
surface

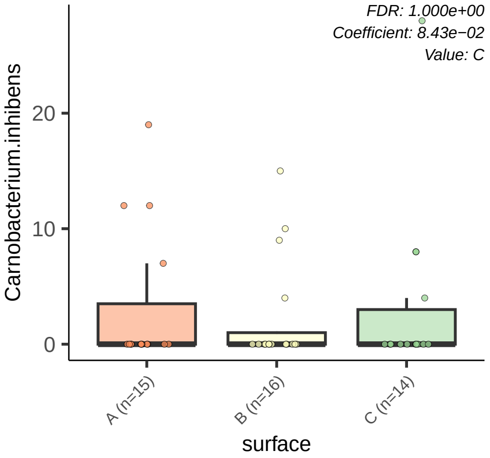

FDR: 1.000e+00
Coefficient: 8.43e−02
Value: C
Carnobacterium.inhibens
0
10
20
A (n=15)
B (n=16)
C (n=14)
surface

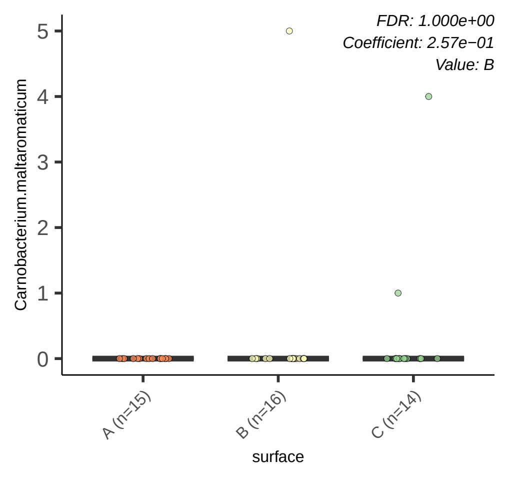

FDR: 1.000e+00
Coefficient: 2.57e−01
Value: B
Carnobacterium.maltaromaticum
0
1
2
3
4
5
A (n=15)
B (n=16)
C (n=14)
surface

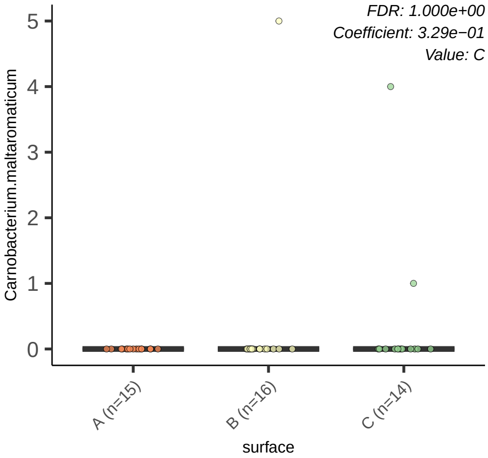

FDR: 1.000e+00
Coefficient: 3.29e−01
Value: C
Carnobacterium.maltaromaticum
5
4
3
2
1
0
A (n=15)
B (n=16)
C (n=14)
surface

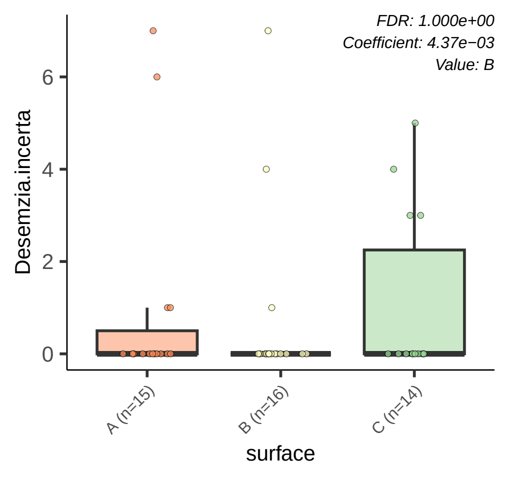

FDR: 1.000e+00
Coefficient: 4.37e−03
Value: B
Desemzia.incerta
0
2
4
6
A (n=15)
B (n=16)
C (n=14)
surface

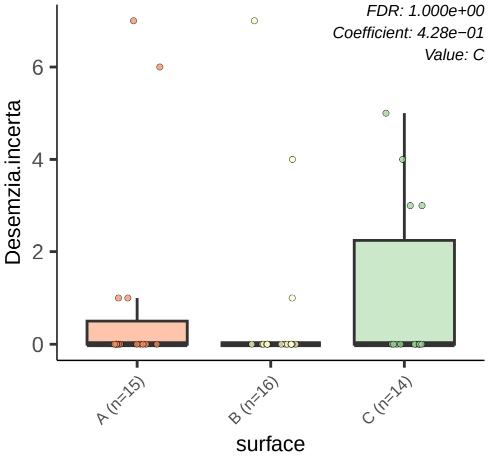
FDR: 1.000e+00
Coefficient: 4.28e−01
Value: C
Desemzia.incerta
0
2
4
6
A (n=15)
B (n=16)
C (n=14)
surface

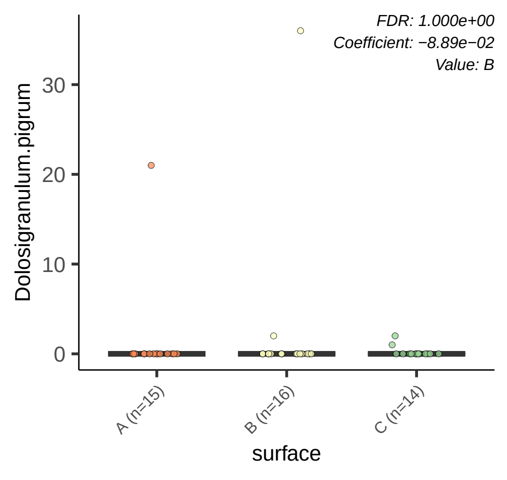
FDR: 1.000e+00
Coefficient: −8.89e−02
Value: B
Dolosigranulum.pigrum
0
10
20
30
A (n=15)
B (n=16)
C (n=14)
surface

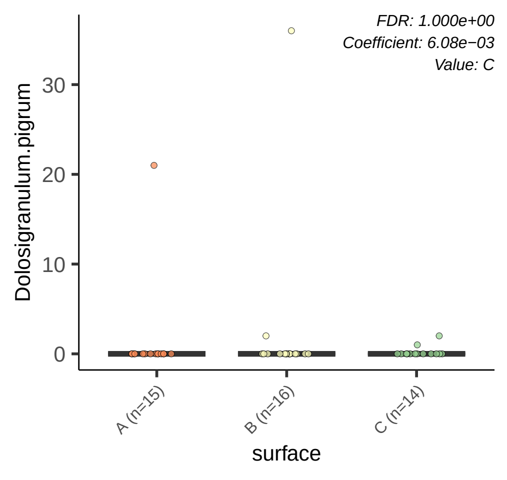
FDR: 1.000e+00
Coefficient: 6.08e−03
Value: C
Dolosigranulum.pigrum
0
10
20
30
A (n=15)
B (n=16)
C (n=14)
surface

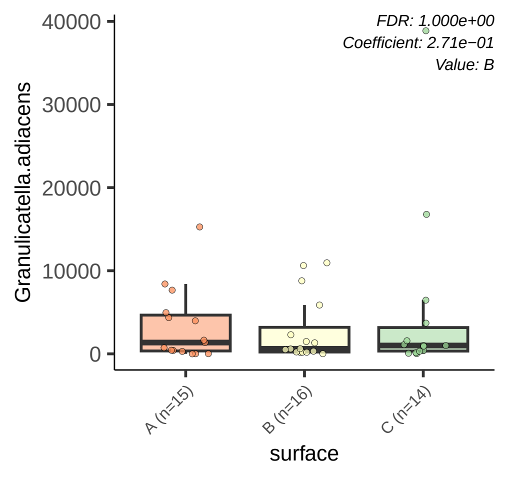
FDR: 1.000e+00
Coefficient: 2.71e−01
Value: B
40000
30000
20000
10000
0
Granulicatella.adiacens
A (n=15)
B (n=16)
C (n=14)
surface

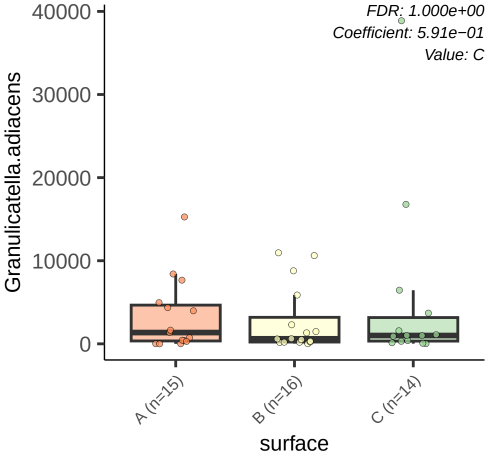

FDR: 1.000e+00
Coefficient: 5.91e−01
Value: C
40000
30000
20000
10000
0
Granulicatella.adiacens
A (n=15)
B (n=16)
C (n=14)
surface

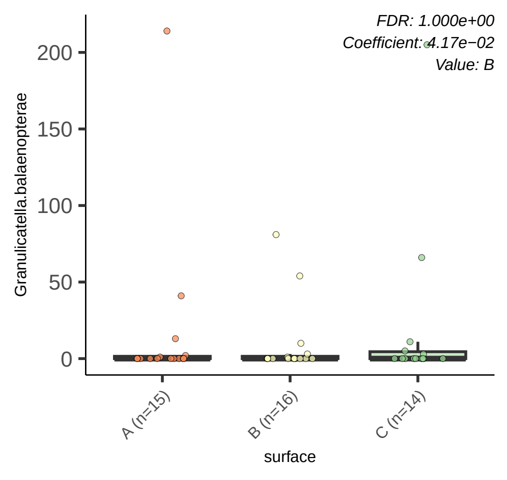

FDR: 1.000e+00
Coefficient: 4.17e−02
Value: B
Granulicatella.balaenopterae
200
150
100
50
0
A (n=15)
B (n=16)
C (n=14)
surface

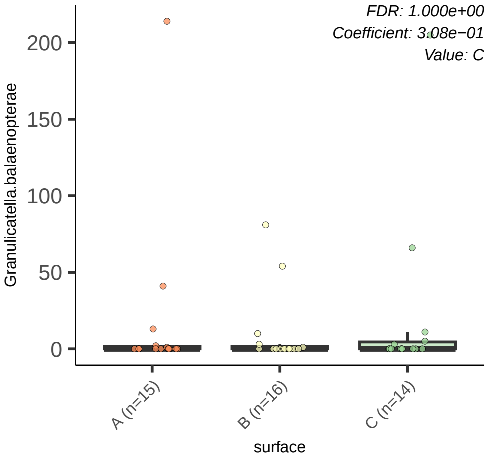
FDR: 1.000e+00
Coefficient: 3.08e−01
Value: C
Granulicatella.balaenopterae
200
150
100
50
0
A (n=15)
B (n=16)
C (n=14)
surface

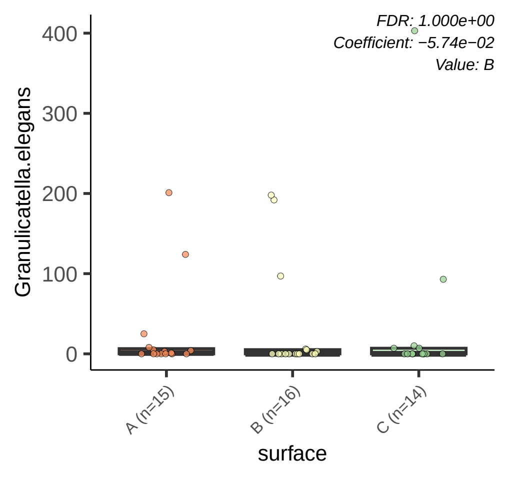

FDR: 1.000e+00
Coefficient: −5.74e−02
Value: B
Granulicatella.elegans
400
300
200
100
0
A (n=15)
B (n=16)
C (n=14)
surface

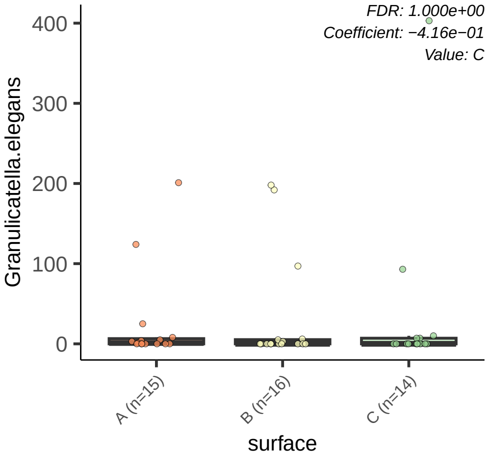

FDR: 1.000e+00
Coefficient: −4.16e−01
Value: C
Granulicatella.elegans
400
300
200
100
0
A (n=15)
B (n=16)
C (n=14)
surface

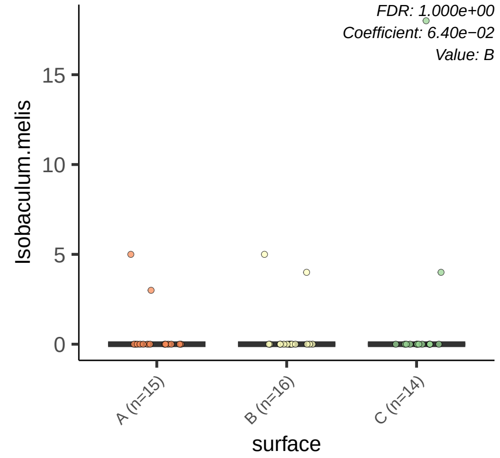

FDR: 1.000e+00
Coefficient: 6.40e−02
Value: B
Isobaculum.melis
15
10
5
0
A (n=15)
B (n=16)
C (n=14)
surface

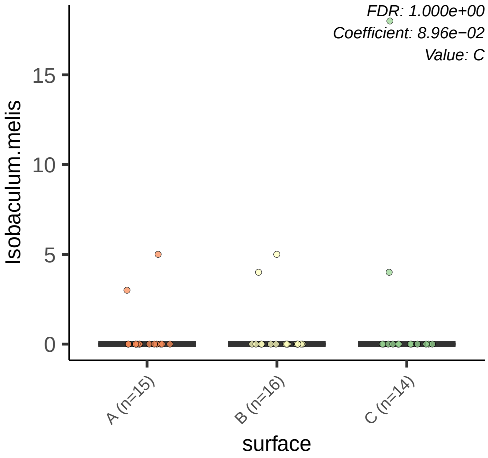
FDR: 1.000e+00
Coefficient: 8.96e−02
Value: C
Isobaculum.melis
15
10
5
0
A (n=15)
B (n=16)
C (n=14)
surface

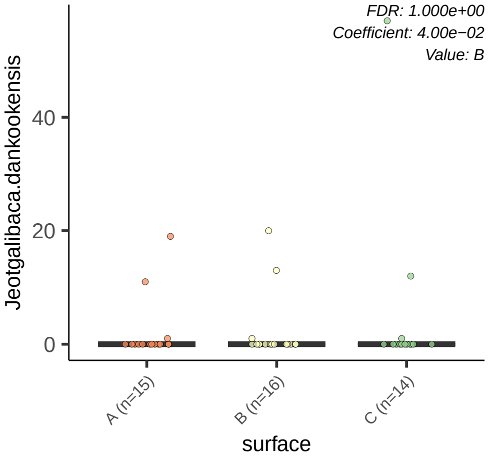

FDR: 1.000e+00
Coefficient: 4.00e−02
Value: B
Jeotgalibaca.dankookensis
40
20
0
A (n=15)
B (n=16)
C (n=14)
surface

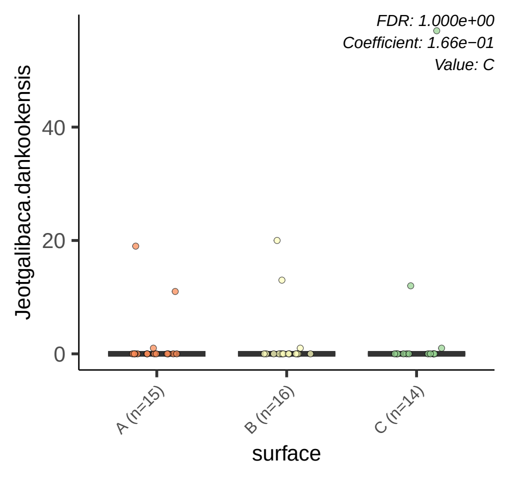
FDR: 1.000e+00
Coefficient: 1.66e−01
Value: C
Jeotgalibaca.dankookensis
40
20
0
A (n=15)
B (n=16)
C (n=14)
surface

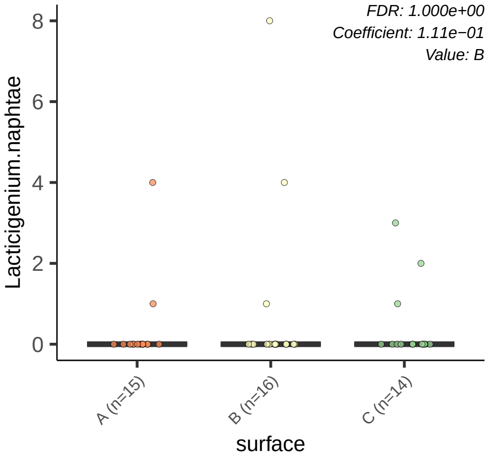

FDR: 1.000e+00
Coefficient: 1.11e−01
Value: B
Lacticigenium.naphtae
8
6
4
2
0
A (n=15)
B (n=16)
C (n=14)
surface

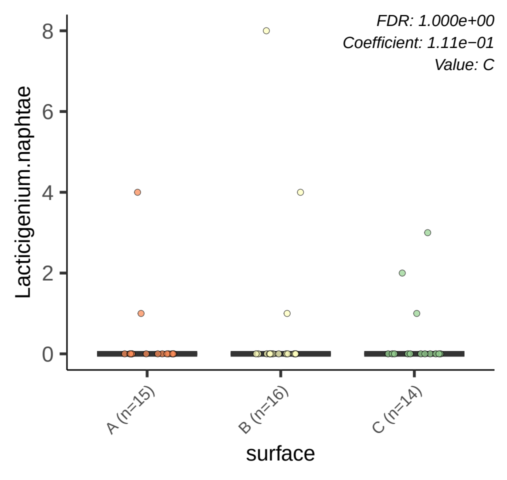
FDR: 1.000e+00
Coefficient: 1.11e−01
Value: C
Lacticigenium.naphtae
8
6
4
2
0
A (n=15)
B (n=16)
C (n=14)
surface

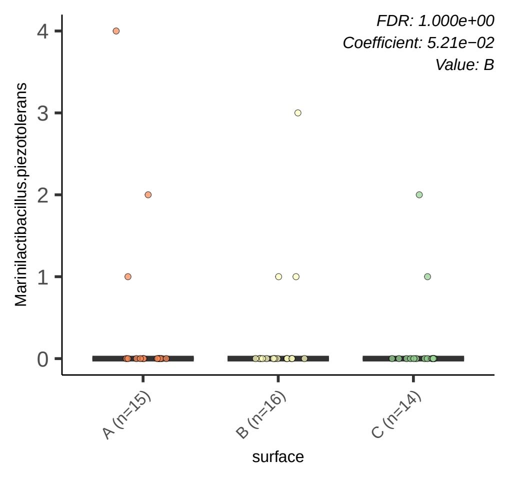
FDR: 1.000e+00
Coefficient: 5.21e−02
Value: B
Marinilactibacillus.piezotolerans
4
3
2
1
0
A (n=15)
B (n=16)
C (n=14)
surface

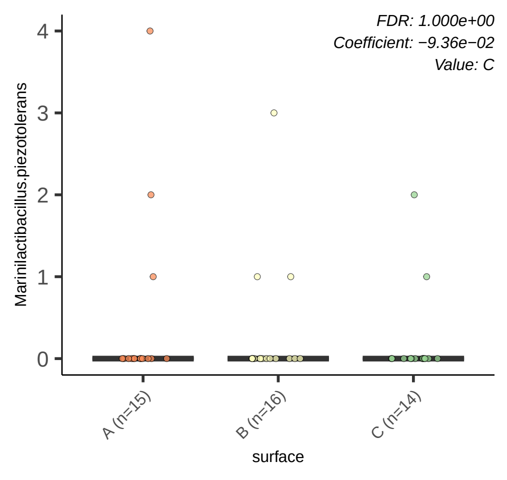

FDR: 1.000e+00
Coefficient: −9.36e−02
Value: C
Marinilactibacillus.piezotolerans
4
3
2
1
0
A (n=15)
B (n=16)
C (n=14)
surface

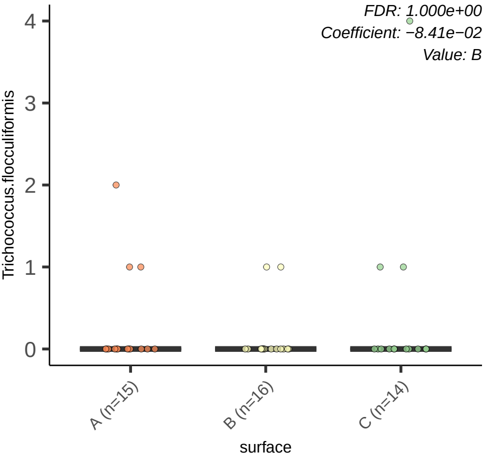

FDR: 1.000e+00
Coefficient: −8.41e−02
Value: B
Trichococcus.flocculiformis
0
1
2
3
4
A (n=15)
B (n=16)
C (n=14)
surface

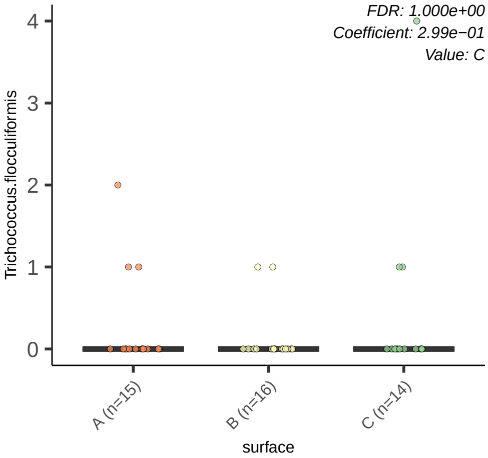
FDR: 1.000e+00
Coefficient: 2.99e−01
Value: C
Trichococcus.flocculiformis
0
1
2
3
4
A (n=15)
B (n=16)
C (n=14)
surface

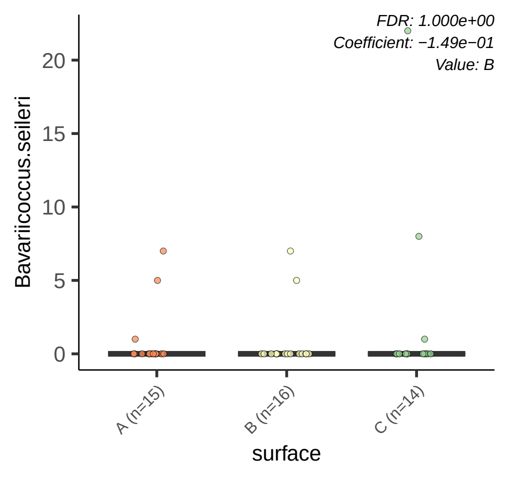
FDR: 1.000e+00
Coefficient: −1.49e−01
Value: B
Bavariicoccus.seileri
20
15
10
5
0
A (n=15)
B (n=16)
C (n=14)
surface

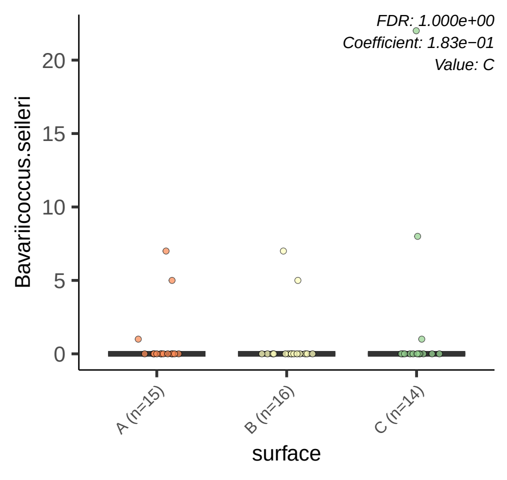
FDR: 1.000e+00
Coefficient: 1.83e−01
Value: C
Bavariicoccus.seileri
20
15
10
5
0
A (n=15)
B (n=16)
C (n=14)
surface

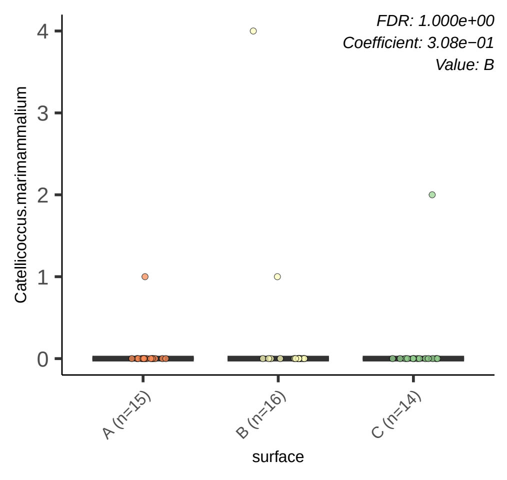

FDR: 1.000e+00
Coefficient: 3.08e−01
Value: B
Catellicoccus.marimammalium
4
3
2
1
0
A (n=15)
B (n=16)
C (n=14)
surface

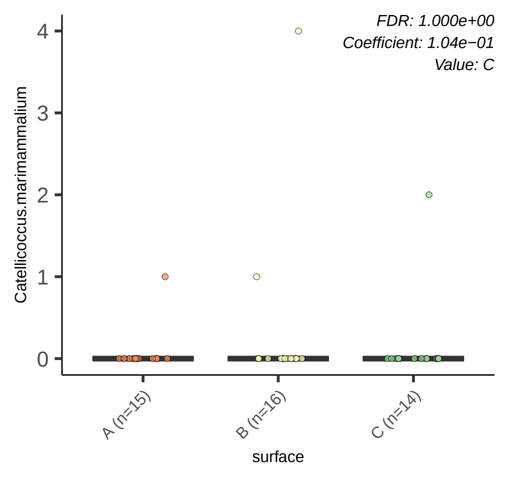
FDR: 1.000e+00
Coefficient: 1.04e−01
Value: C
Catellicoccus.marimammalium
0
1
2
3
4
A (n=15)
B (n=16)
C (n=14)
surface

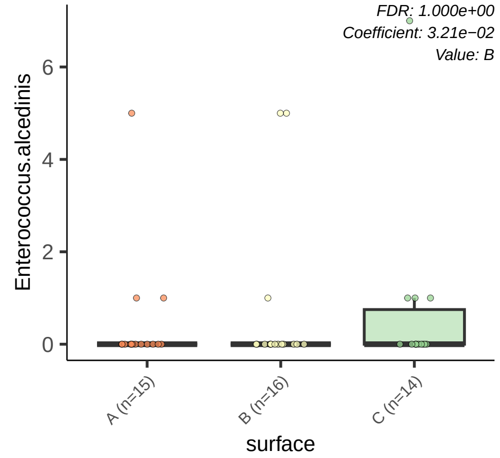

FDR: 1.000e+00
Coefficient: 3.21e−02
Value: B
Enterococcus.alcedinis
0
2
4
6
A (n=15)
B (n=16)
C (n=14)
surface

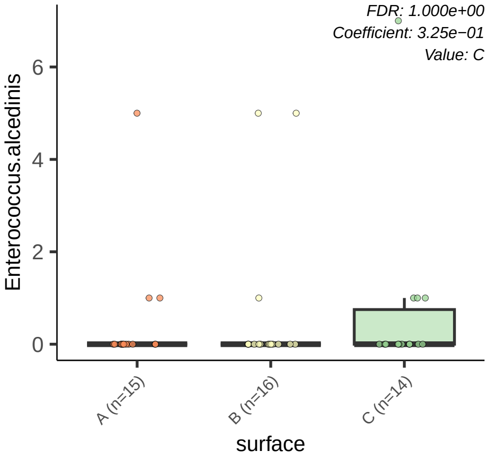

FDR: 1.000e+00
Coefficient: 3.25e−01
Value: C
Enterococcus.alcedinis
0
2
4
6
A (n=15)
B (n=16)
C (n=14)
surface

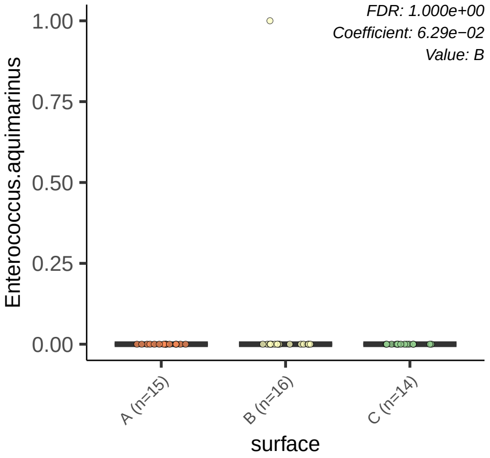
FDR: 1.000e+00
Coefficient: 6.29e−02
Value: B
Enterococcus.aquimarinus
1.00
0.75
0.50
0.25
0.00
A (n=15)
B (n=16)
C (n=14)
surface

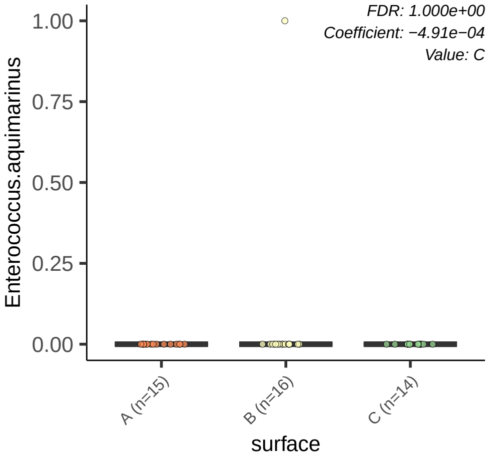

FDR: 1.000e+00
Coefficient: −4.91e−04
Value: C
Enterococcus.aquimarinus
1.00
0.75
0.50
0.25
0.00
A (n=15)
B (n=16)
C (n=14)
surface

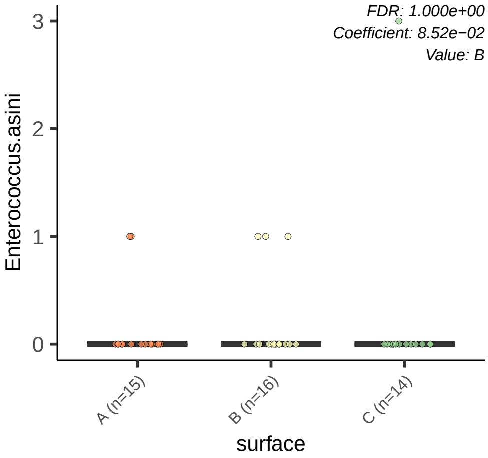
FDR: 1.000e+00
Coefficient: 8.52e−02
Value: B
Enterococcus.asini
3
2
1
0
A (n=15)
B (n=16)
C (n=14)
surface

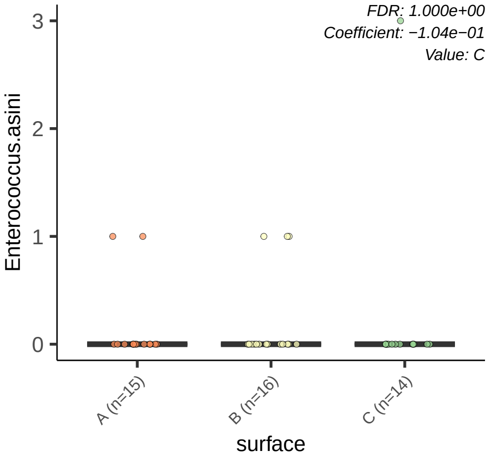
FDR: 1.000e+00
Coefficient: −1.04e−01
Value: C
Enterococcus.asini
0
1
2
3
A (n=15)
B (n=16)
C (n=14)
surface

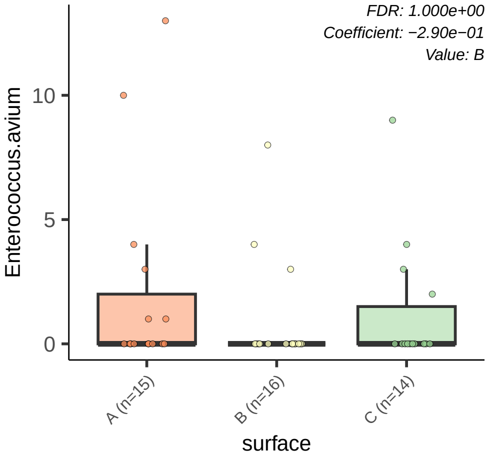

FDR: 1.000e+00
Coefficient: −2.90e−01
Value: B
Enterococcus.avium
10
5
0
A (n=15)
B (n=16)
C (n=14)
surface

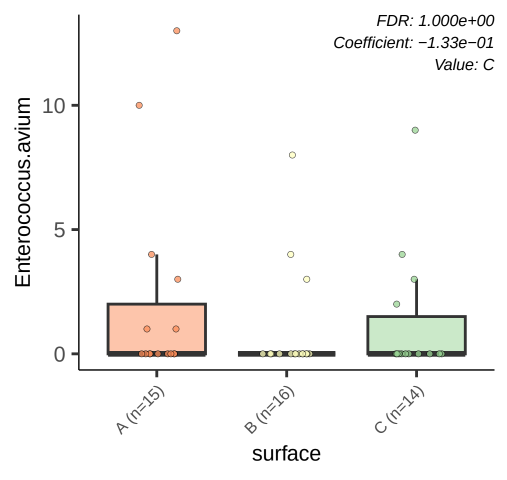

FDR: 1.000e+00
Coefficient: −1.33e−01
Value: C
Enterococcus.avium
10
5
0
A (n=15)
B (n=16)
C (n=14)
surface

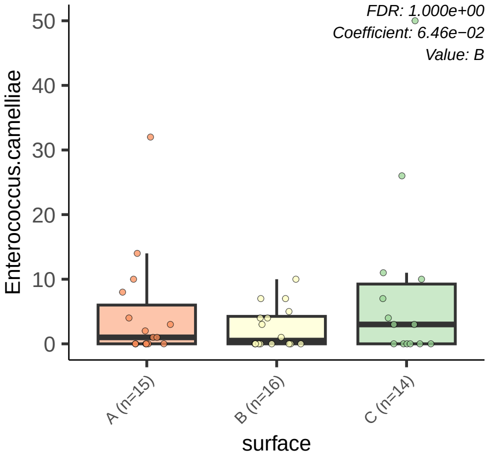

FDR: 1.000e+00
Coefficient: 6.46e−02
Value: B
Enterococcus.camelliae
0
10
20
30
40
50
A (n=15)
B (n=16)
C (n=14)
surface

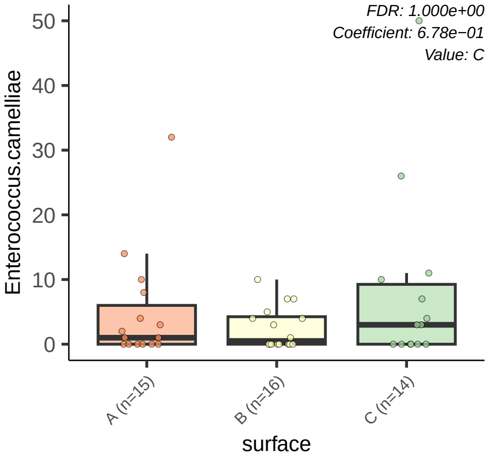

FDR: 1.000e+00
Coefficient: 6.78e−01
Value: C
Enterococcus.camelliae
0
10
20
30
40
50
A (n=15)
B (n=16)
C (n=14)
surface

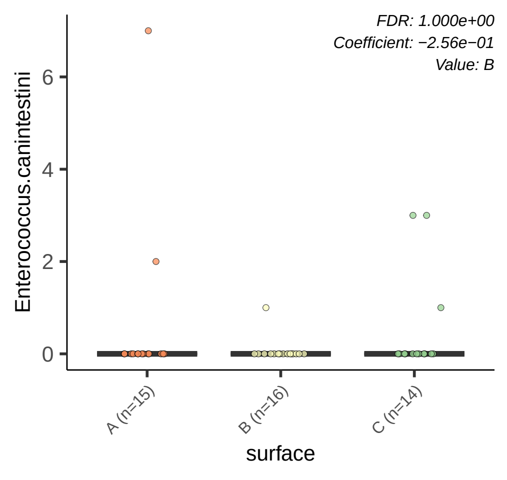

FDR: 1.000e+00
Coefficient: −2.56e−01
Value: B
Enterococcus.canintestini
0
2
4
6
A (n=15)
B (n=16)
C (n=14)
surface

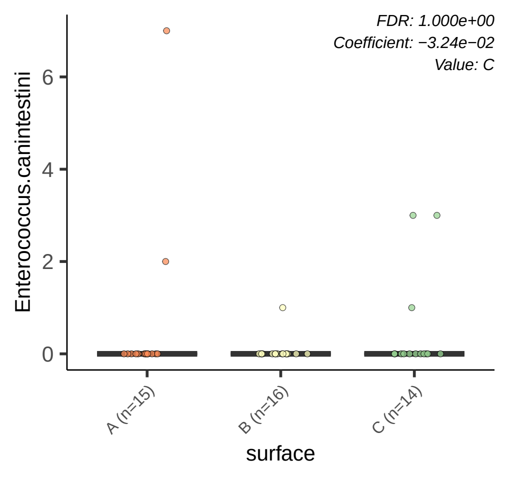

FDR: 1.000e+00
Coefficient: −3.24e−02
Value: C
Enterococcus.canintestini
0
2
4
6
A (n=15)
B (n=16)
C (n=14)
surface

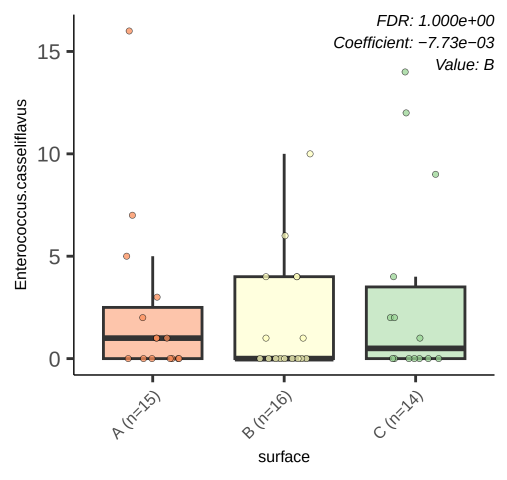

FDR: 1.000e+00
Coefficient: −7.73e−03
Value: B
Enterococcus.casseliflavus
0
5
10
15
A (n=15)
B (n=16)
C (n=14)
surface

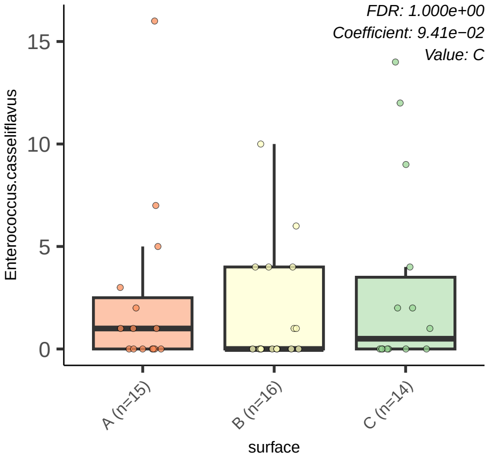
FDR: 1.000e+00
Coefficient: 9.41e−02
Value: C
Enterococcus.casseliflavus
15
10
5
0
A (n=15)
B (n=16)
C (n=14)
surface

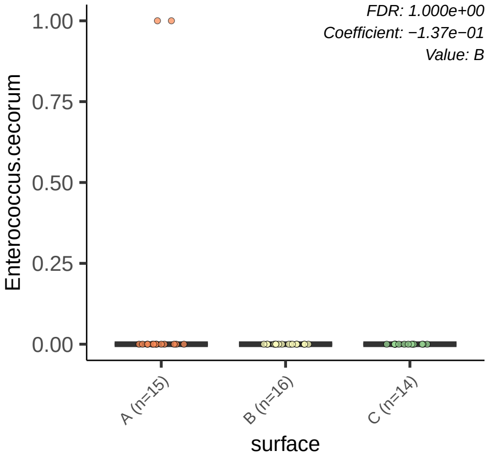
FDR: 1.000e+00
Coefficient: −1.37e−01
Value: B
Enterococcus.cecorum
1.00
0.75
0.50
0.25
0.00
A (n=15)
B (n=16)
C (n=14)
surface

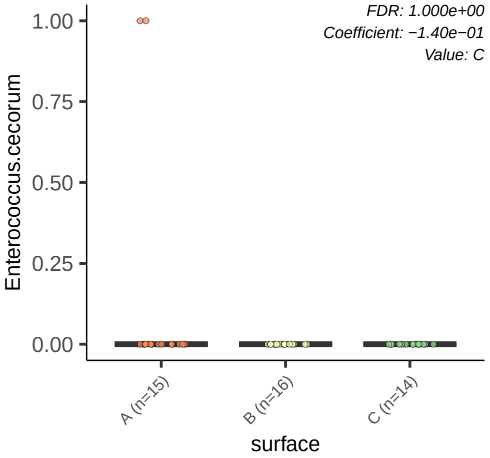

FDR: 1.000e+00
Coefficient: −1.40e−01
Value: C
Enterococcus.cecorum
1.00
0.75
0.50
0.25
0.00
A (n=15)
B (n=16)
C (n=14)
surface

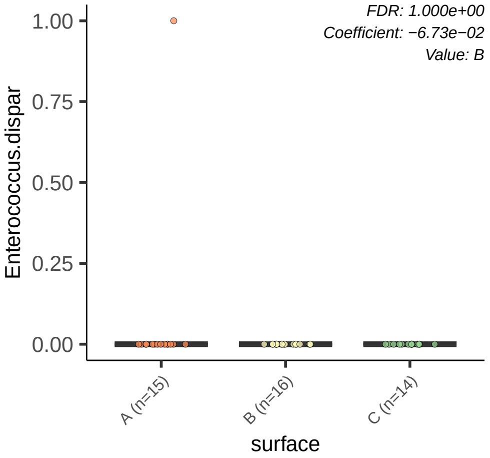
FDR: 1.000e+00
Coefficient: −6.73e−02
Value: B
Enterococcus.dispar
1.00
0.75
0.50
0.25
0.00
A (n=15)
B (n=16)
C (n=14)
surface

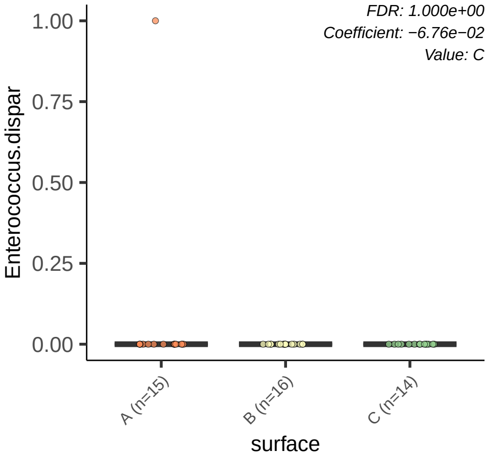

FDR: 1.000e+00
Coefficient: −6.76e−02
Value: C
Enterococcus.dispar
1.00
0.75
0.50
0.25
0.00
A (n=15)
B (n=16)
C (n=14)
surface

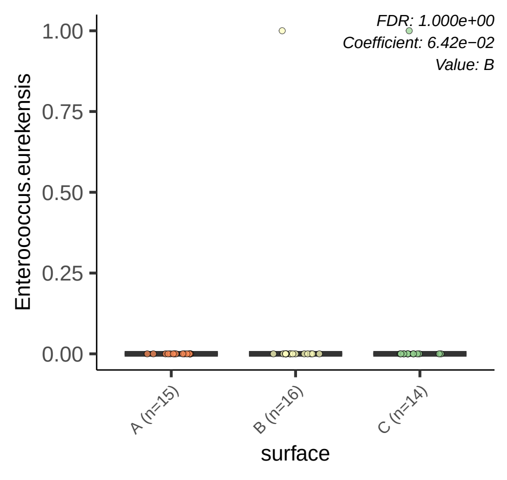
FDR: 1.000e+00
Coefficient: 6.42e−02
Value: B
Enterococcus.eurekensis
1.00
0.75
0.50
0.25
0.00
A (n=15)
B (n=16)
C (n=14)
surface

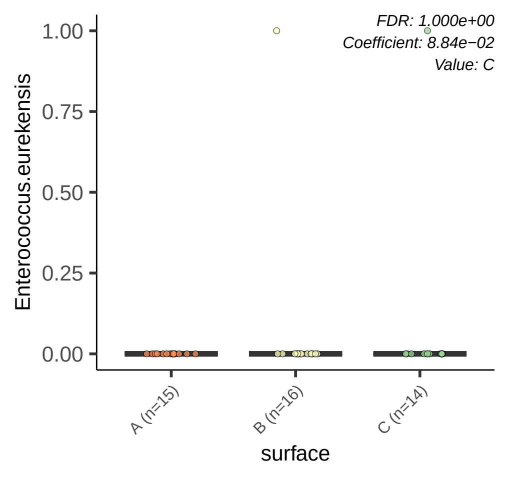
FDR: 1.000e+00
Coefficient: 8.84e−02
Value: C
Enterococcus.eurekensis
1.00
0.75
0.50
0.25
0.00
A (n=15)
B (n=16)
C (n=14)
surface

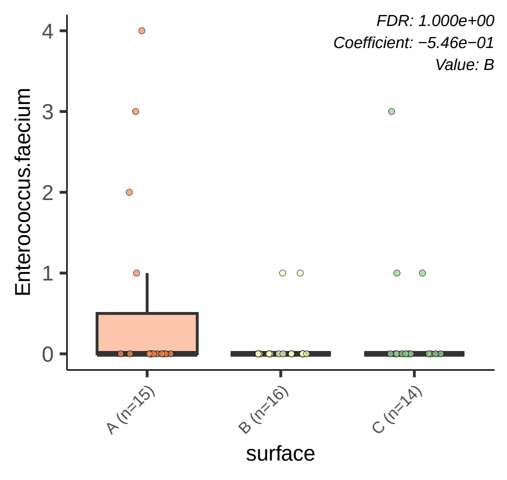
FDR: 1.000e+00
Coefficient: −5.46e−01
Value: B
Enterococcus.faecium
0
1
2
3
4
A (n=15)
B (n=16)
C (n=14)
surface

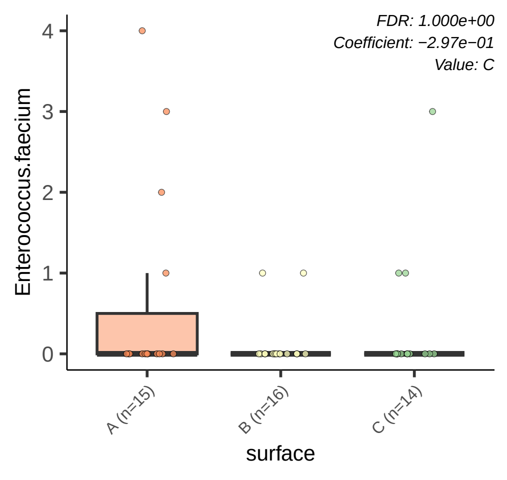
FDR: 1.000e+00
Coefficient: −2.97e−01
Value: C
Enterococcus.faecium
4
3
2
1
0
A (n=15)
B (n=16)
C (n=14)
surface

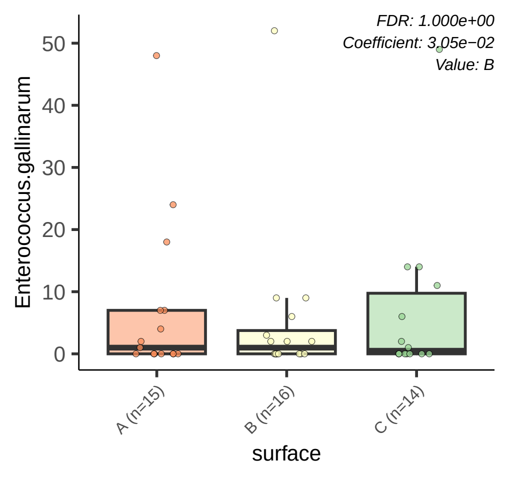
FDR: 1.000e+00
Coefficient: 3.05e−02
Value: B
Enterococcus.gallinarum
0
10
20
30
40
50
A (n=15)
B (n=16)
C (n=14)
surface

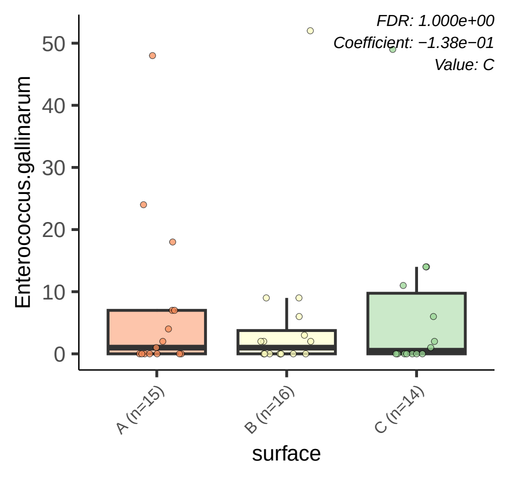
FDR: 1.000e+00
Coefficient: −1.38e−01
Value: C
Enterococcus.gallinarum
0
10
20
30
40
50
A (n=15)
B (n=16)
C (n=14)
surface

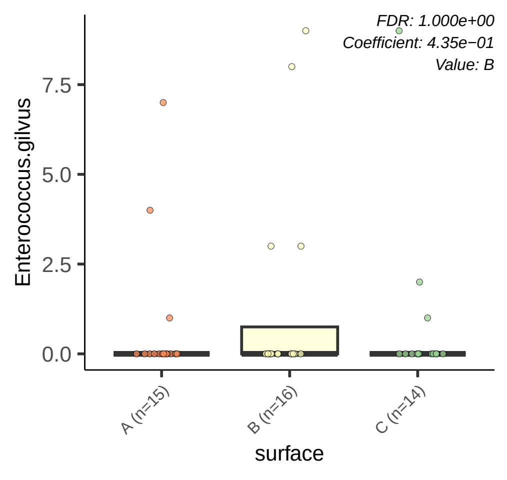
FDR: 1.000e+00
Coefficient: 4.35e−01
Value: B
Enterococcus.gilvus
7.5
5.0
2.5
0.0
A (n=15)
B (n=16)
C (n=14)
surface

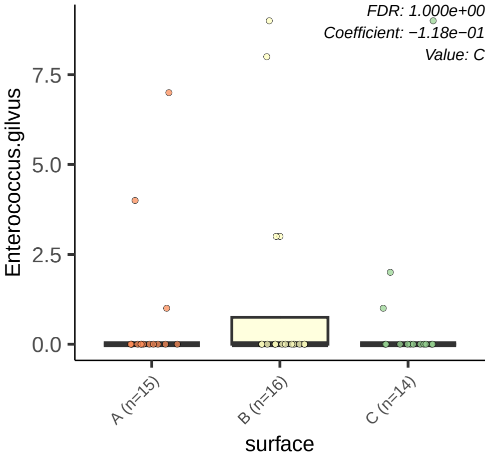

FDR: 1.000e+00
Coefficient: −1.18e−01
Value: C
Enterococcus.gilvus
7.5
5.0
2.5
0.0
A (n=15)
B (n=16)
C (n=14)
surface

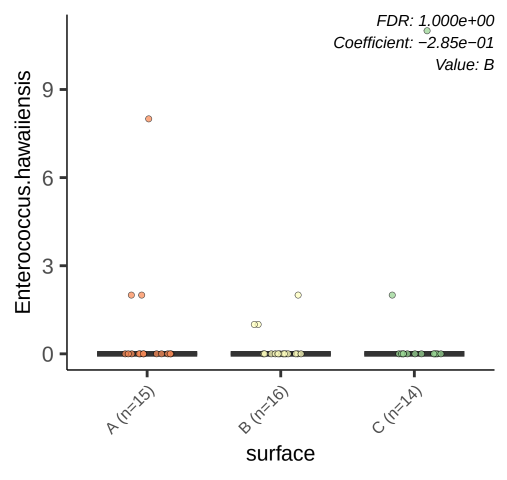

FDR: 1.000e+00
Coefficient: −2.85e−01
Value: B
Enterococcus.hawaiiensis
0
3
6
9
A (n=15)
B (n=16)
C (n=14)
surface

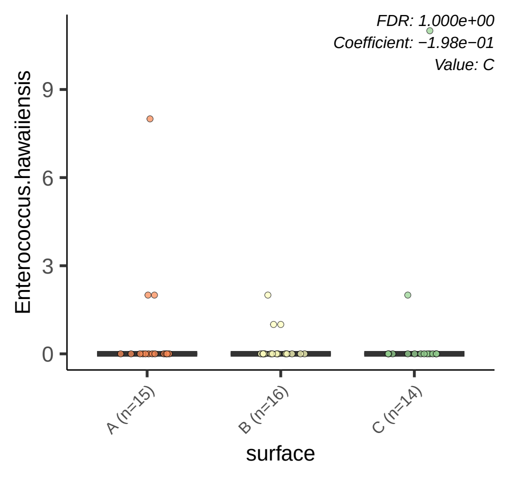
FDR: 1.000e+00
Coefficient: −1.98e−01
Value: C
Enterococcus.hawaiiensis
0
3
6
9
A (n=15)
B (n=16)
C (n=14)
surface

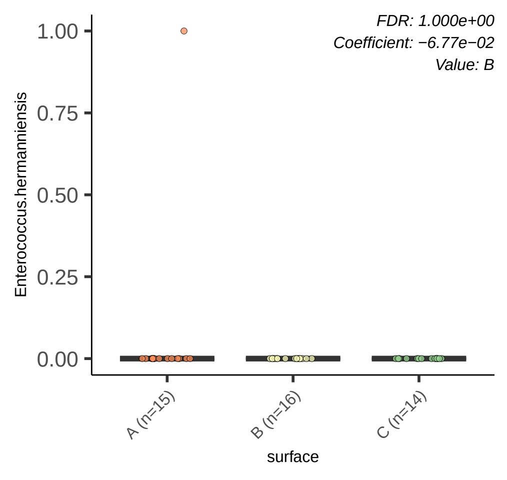
FDR: 1.000e+00
Coefficient: −6.77e−02
Value: B
1.00
0.75
0.50
0.25
0.00
Enterococcus.hermanniensis
A (n=15)
B (n=16)
C (n=14)
surface

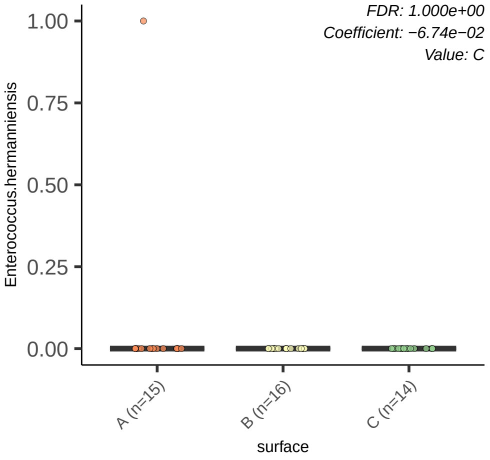
FDR: 1.000e+00
Coefficient: −6.74e−02
Value: C
Enterococcus.hermanniensis
1.00
0.75
0.50
0.25
0.00
A (n=15)
B (n=16)
C (n=14)
surface

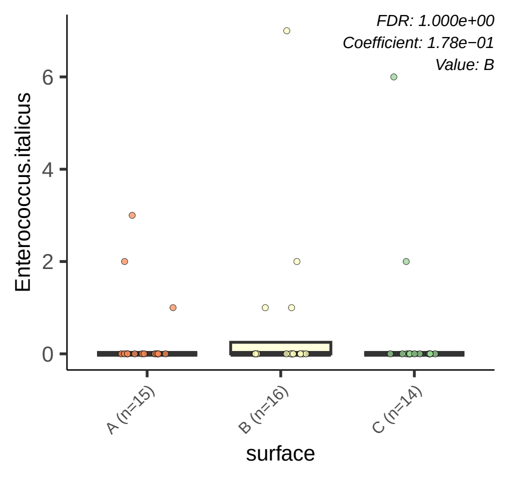

FDR: 1.000e+00
Coefficient: 1.78e−01
Value: B
Enterococcus.italicus
0
2
4
6
A (n=15)
B (n=16)
C (n=14)
surface

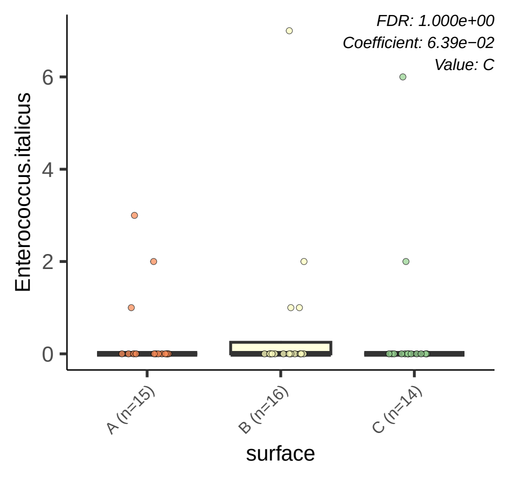

FDR: 1.000e+00
Coefficient: 6.39e−02
Value: C
Enterococcus.italicus
0
2
4
6
A (n=15)
B (n=16)
C (n=14)
surface

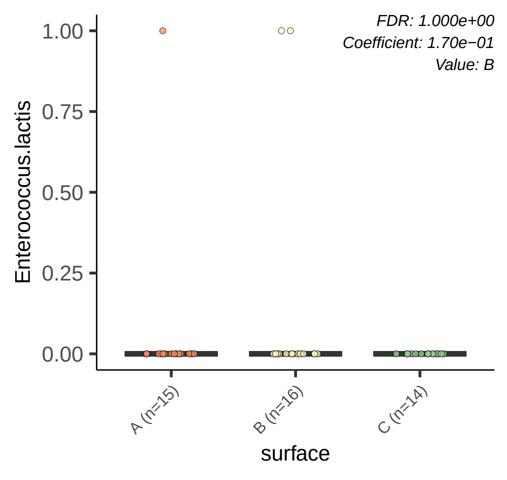
FDR: 1.000e+00
Coefficient: 1.70e−01
Value: B
Enterococcus.lactis
1.00
0.75
0.50
0.25
0.00
A (n=15)
B (n=16)
C (n=14)
surface

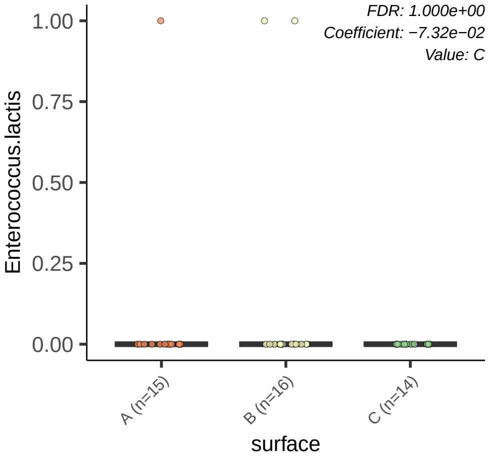
FDR: 1.000e+00
Coefficient: −7.32e−02
Value: C
Enterococcus.lactis
1.00
0.75
0.50
0.25
0.00
A (n=15)
B (n=16)
C (n=14)
surface

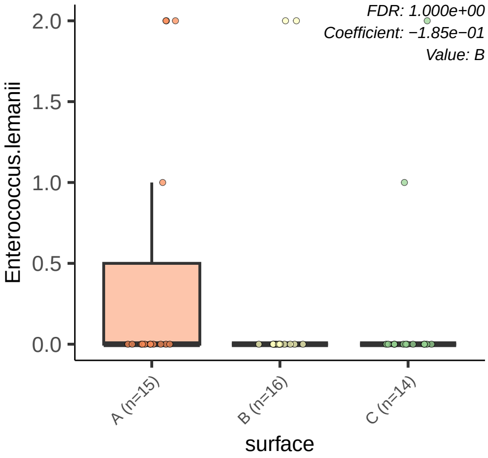
FDR: 1.000e+00
Coefficient: −1.85e−01
Value: B
Enterococcus.lemanii
2.0
1.5
1.0
0.5
0.0
A (n=15)
B (n=16)
C (n=14)
surface

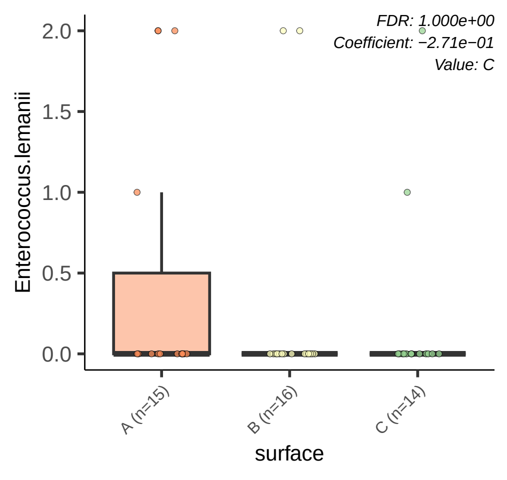

FDR: 1.000e+00
Coefficient: −2.71e−01
Value: C
Enterococcus.lemanii
2.0
1.5
1.0
0.5
0.0
A (n=15)
B (n=16)
C (n=14)
surface

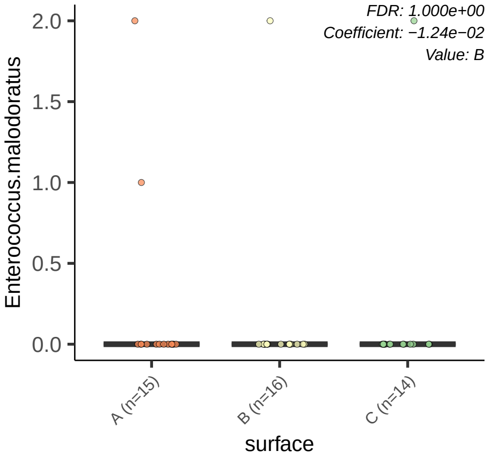

FDR: 1.000e+00
Coefficient: −1.24e−02
Value: B
Enterococcus.malodoratus
2.0
1.5
1.0
0.5
0.0
A (n=15)
B (n=16)
C (n=14)
surface

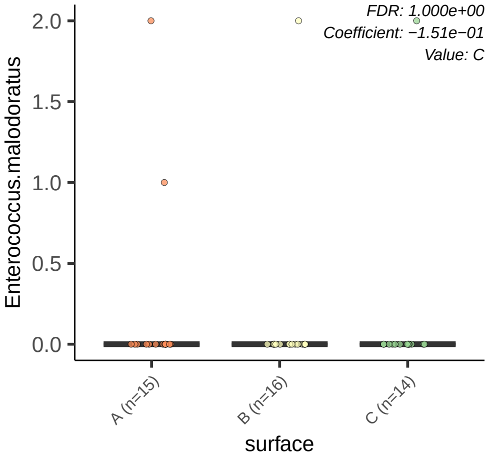

FDR: 1.000e+00
Coefficient: −1.51e−01
Value: C
Enterococcus.malodoratus
2.0
1.5
1.0
0.5
0.0
A (n=15)
B (n=16)
C (n=14)
surface

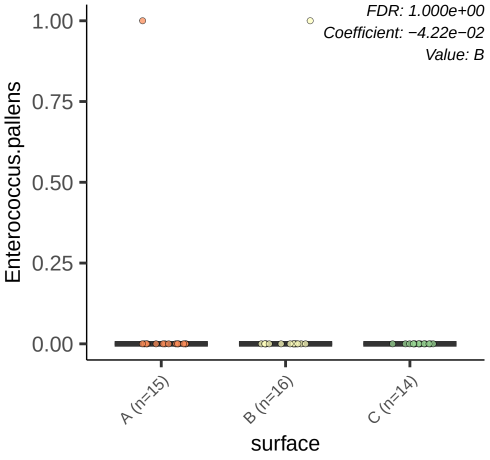
FDR: 1.000e+00
Coefficient: −4.22e−02
Value: B
Enterococcus.pallens
1.00
0.75
0.50
0.25
0.00
A (n=15)
B (n=16)
C (n=14)
surface

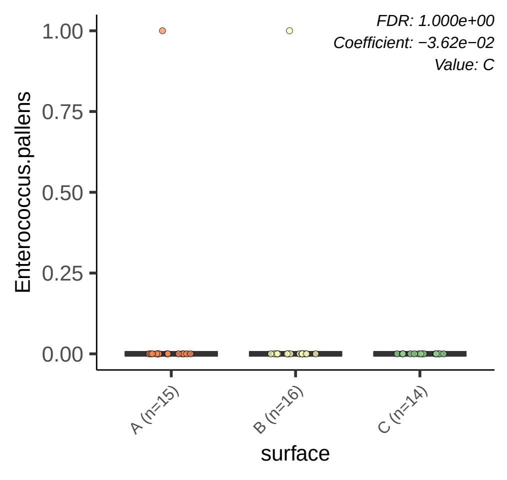
FDR: 1.000e+00
Coefficient: −3.62e−02
Value: C
1.00
0.75
0.50
0.25
0.00
Enterococcus.pallens
A (n=15)
B (n=16)
C (n=14)
surface

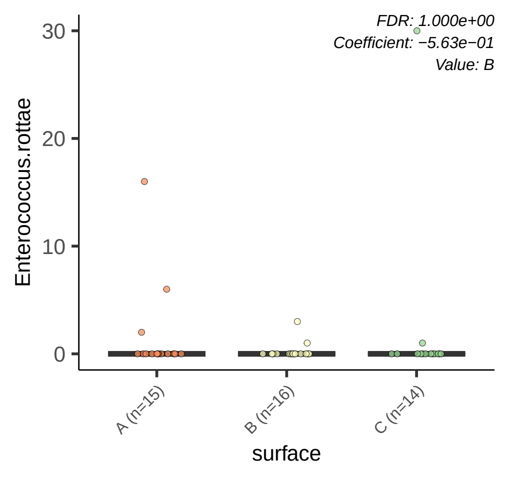
FDR: 1.000e+00
Coefficient: −5.63e−01
Value: B
Enterococcus.rottae
30
20
10
0
A (n=15)
B (n=16)
C (n=14)
surface

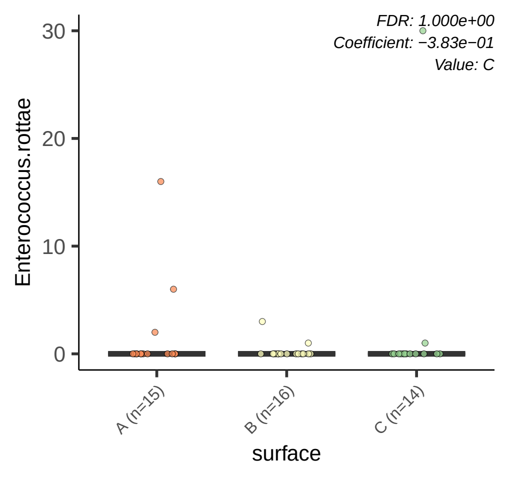
FDR: 1.000e+00
Coefficient: −3.83e−01
Value: C
Enterococcus.rottae
30
20
10
0
A (n=15)
B (n=16)
C (n=14)
surface

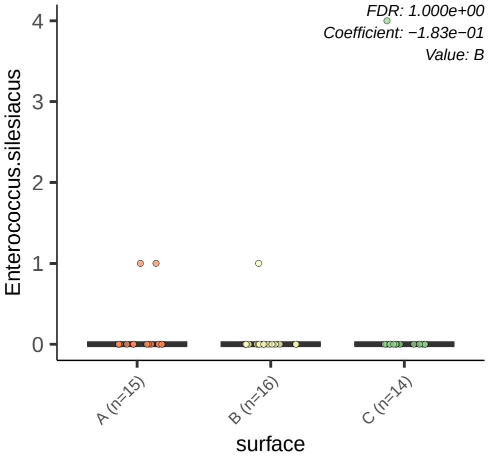

FDR: 1.000e+00
Coefficient: −1.83e−01
Value: B
Enterococcus.silesiacus
0
1
2
3
4
A (n=15)
B (n=16)
C (n=14)
surface

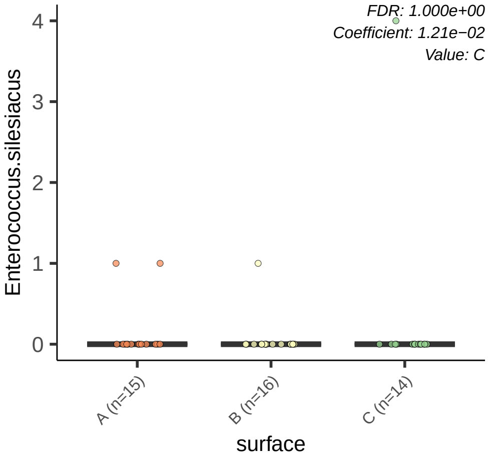

FDR: 1.000e+00
Coefficient: 1.21e−02
Value: C
Enterococcus.silesiacus
4
3
2
1
0
A (n=15)
B (n=16)
C (n=14)
surface

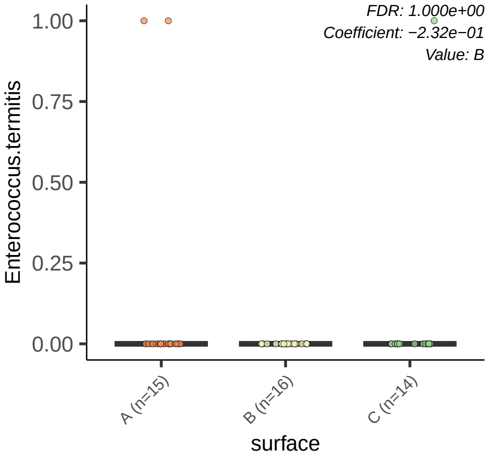

FDR: 1.000e+00
Coefficient: −2.32e−01
Value: B
Enterococcus.termitis
1.00
0.75
0.50
0.25
0.00
A (n=15)
B (n=16)
C (n=14)
surface

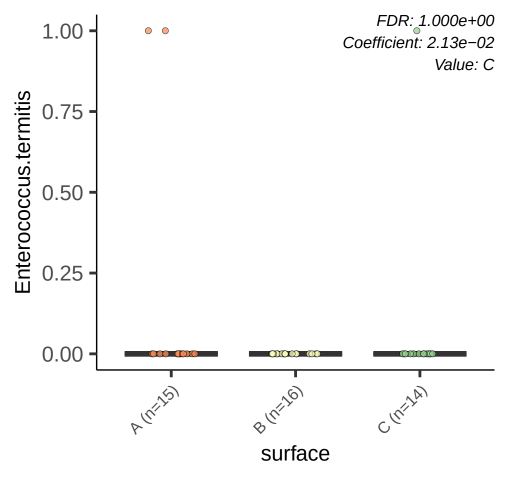

FDR: 1.000e+00
Coefficient: 2.13e−02
Value: C
Enterococcus.termitis
1.00
0.75
0.50
0.25
0.00
A (n=15)
B (n=16)
C (n=14)
surface

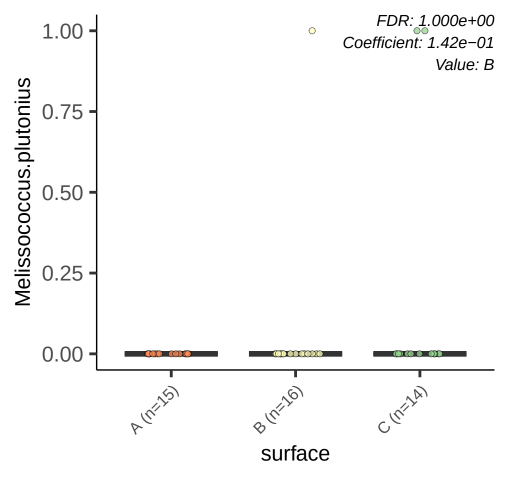

FDR: 1.000e+00
Coefficient: 1.42e−01
Value: B
Melissococcus.plutonius
1.00
0.75
0.50
0.25
0.00
A (n=15)
B (n=16)
C (n=14)
surface

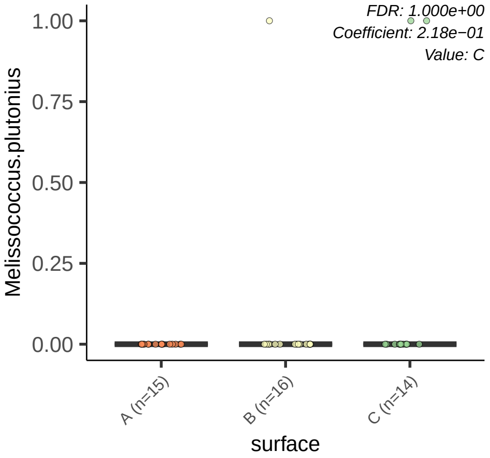
FDR: 1.000e+00
Coefficient: 2.18e−01
Value: C
Melissococcus.plutonius
1.00
0.75
0.50
0.25
0.00
A (n=15)
B (n=16)
C (n=14)
surface

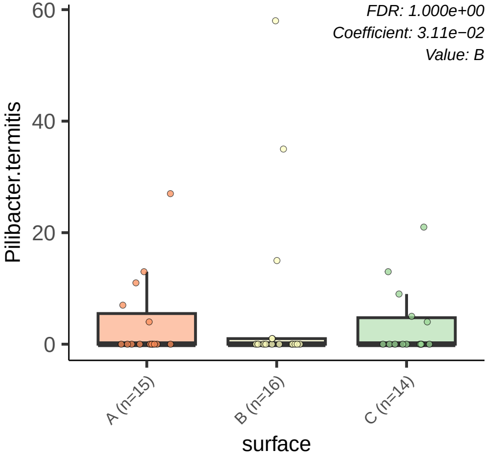
FDR: 1.000e+00
Coefficient: 3.11e−02
Value: B
Pilibacter.termitis
0
20
40
60
A (n=15)
B (n=16)
C (n=14)
surface

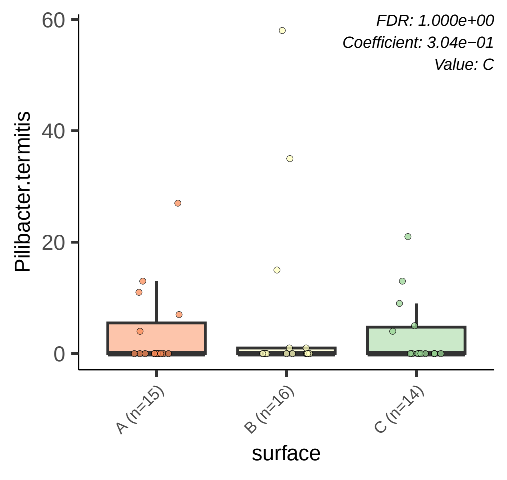
FDR: 1.000e+00
Coefficient: 3.04e−01
Value: C
Pilibacter.termitis
60
40
20
0
A (n=15)
B (n=16)
C (n=14)
surface

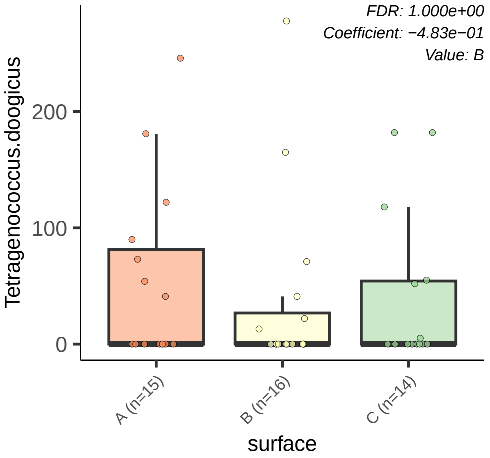
FDR: 1.000e+00
Coefficient: −4.83e−01
Value: B
Tetragenococcus.doogicus
200
100
0
A (n=15)
B (n=16)
C (n=14)
surface

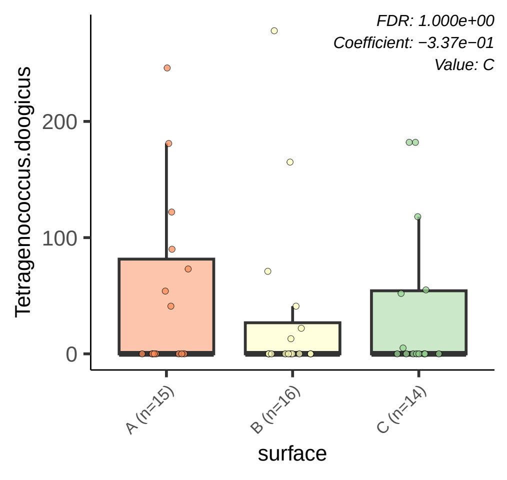
FDR: 1.000e+00
Coefficient: −3.37e−01
Value: C
Tetragenococcus.doogicus
200
100
0
A (n=15)
B (n=16)
C (n=14)
surface

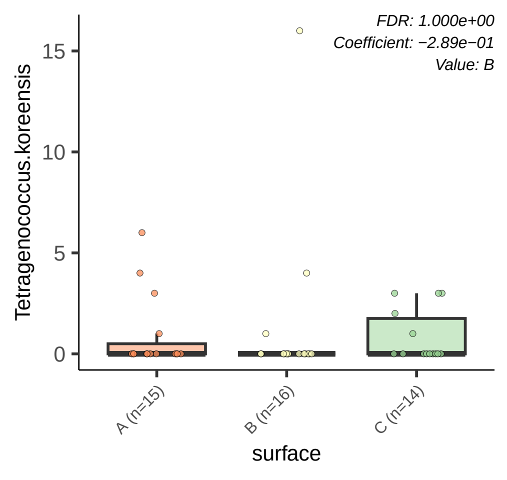

FDR: 1.000e+00
Coefficient: −2.89e−01
Value: B
Tetragenococcus.koreensis
0
5
10
15
A (n=15)
B (n=16)
C (n=14)
surface

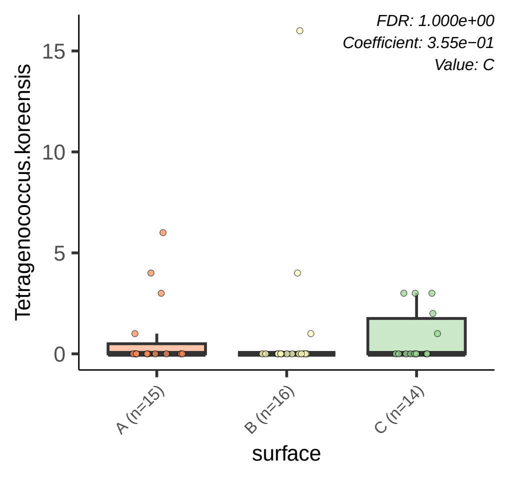

FDR: 1.000e+00
Coefficient: 3.55e−01
Value: C
Tetragenococcus.koreensis
0
5
10
15
A (n=15)
B (n=16)
C (n=14)
surface

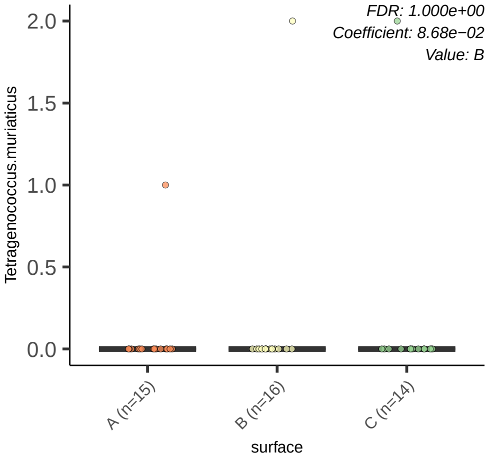

FDR: 1.000e+00
Coefficient: 8.68e−02
Value: B
Tetragenococcus.muriaticus
2.0
1.5
1.0
0.5
0.0
A (n=15)
B (n=16)
C (n=14)
surface

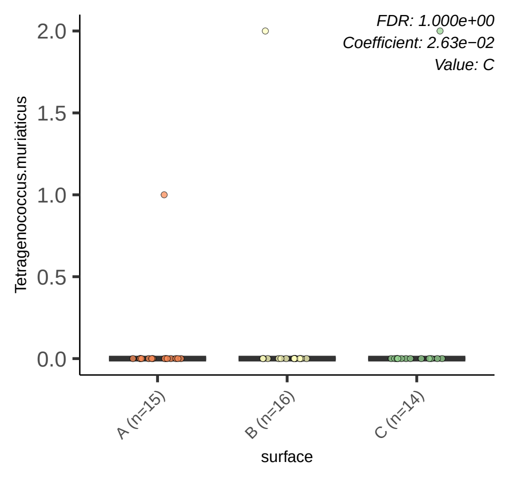
FDR: 1.000e+00
Coefficient: 2.63e−02
Value: C
Tetragenococcus.muriaticus
2.0
1.5
1.0
0.5
0.0
A (n=15)
B (n=16)
C (n=14)
surface

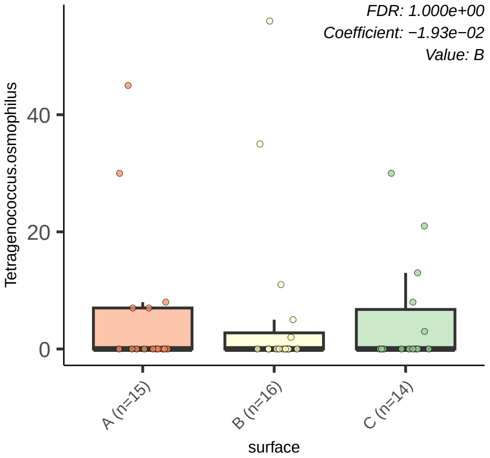

FDR: 1.000e+00
Coefficient: −1.93e−02
Value: B
Tetragenococcus.osmophilus
0
20
40
A (n=15)
B (n=16)
C (n=14)
surface

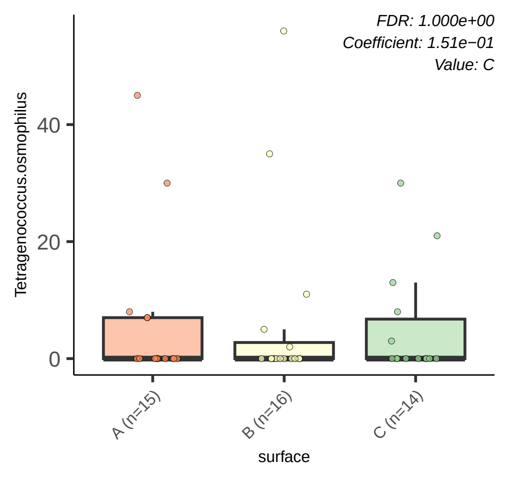

FDR: 1.000e+00
Coefficient: 1.51e−01
Value: C
Tetragenococcus.osmophilus
40
20
0
A (n=15)
B (n=16)
C (n=14)
surface

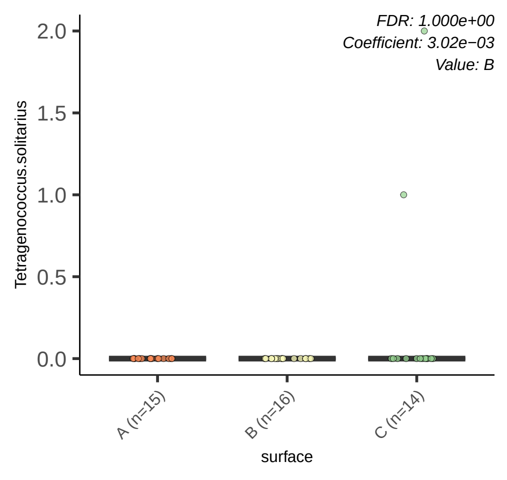
FDR: 1.000e+00
Coefficient: 3.02e−03
Value: B
Tetragenococcus.solitarius
2.0
1.5
1.0
0.5
0.0
A (n=15)
B (n=16)
C (n=14)
surface

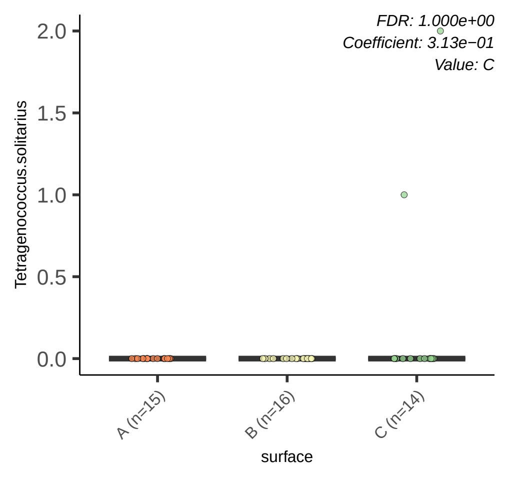
FDR: 1.000e+00
Coefficient: 3.13e−01
Value: C
Tetragenococcus.solitarius
2.0
1.5
1.0
0.5
0.0
A (n=15)
B (n=16)
C (n=14)
surface

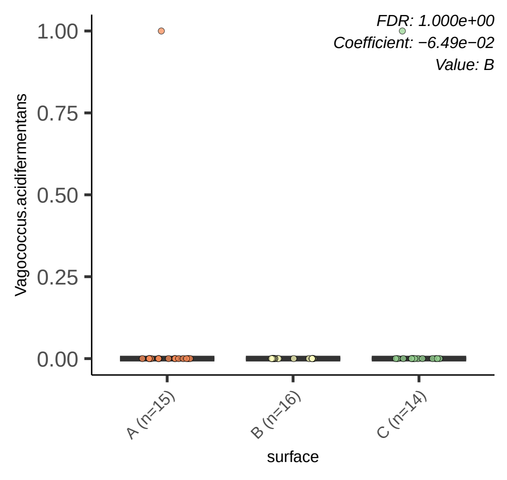

FDR: 1.000e+00
Coefficient: −6.49e−02
Value: B
1.00
0.75
0.50
0.25
0.00
Vagococcus.acidifermentans
A (n=15)
B (n=16)
C (n=14)
surface

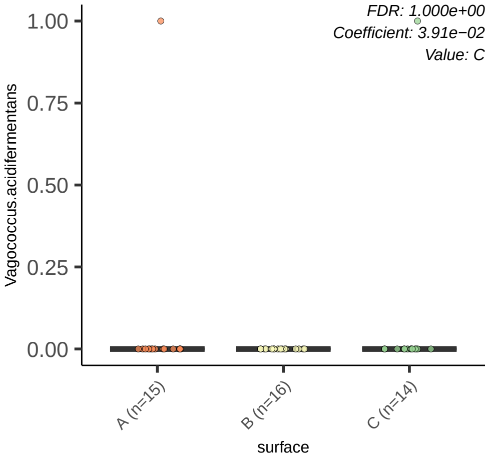
FDR: 1.000e+00
Coefficient: 3.91e−02
Value: C
1.00
0.75
0.50
0.25
0.00
Vagococcus.acidifermentans
A (n=15)
B (n=16)
C (n=14)
surface

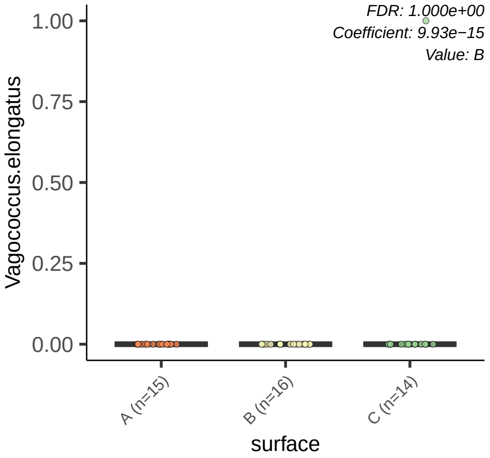

FDR: 1.000e+00
Coefficient: 9.93e−15
Value: B
1.00
0.75
0.50
0.25
0.00
Vagococcus.elongatus
A (n=15)
B (n=16)
C (n=14)
surface

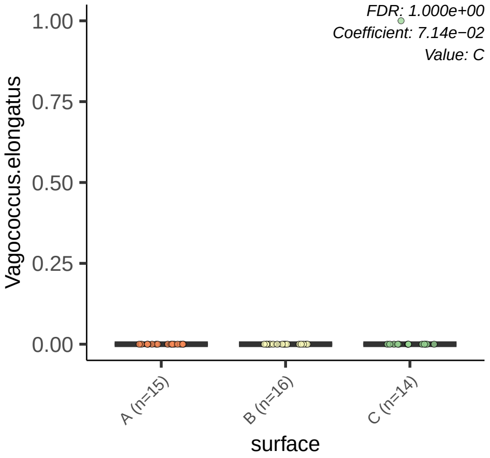

FDR: 1.000e+00
Coefficient: 7.14e−02
Value: C
Vagococcus.elongatus
1.00
0.75
0.50
0.25
0.00
A (n=15)
B (n=16)
C (n=14)
surface

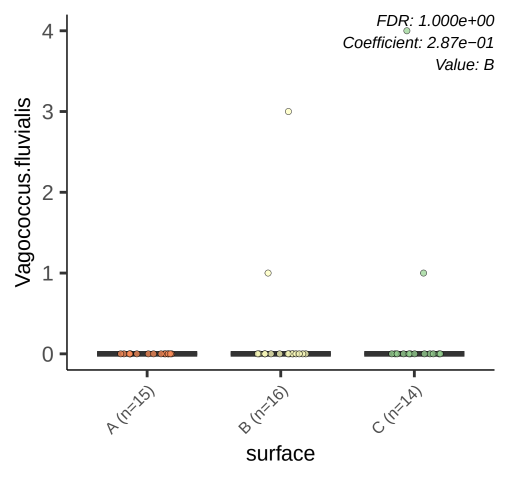
FDR: 1.000e+00
Coefficient: 2.87e−01
Value: B
Vagococcus.fluvialis
4
3
2
1
0
A (n=15)
B (n=16)
C (n=14)
surface

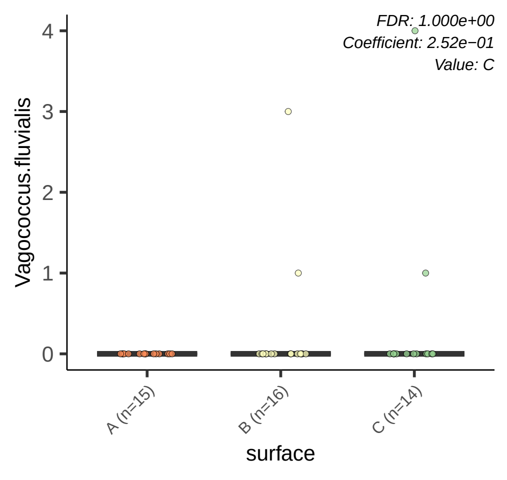

FDR: 1.000e+00
Coefficient: 2.52e−01
Value: C
Vagococcus.fluvialis
0
1
2
3
4
A (n=15)
B (n=16)
C (n=14)
surface

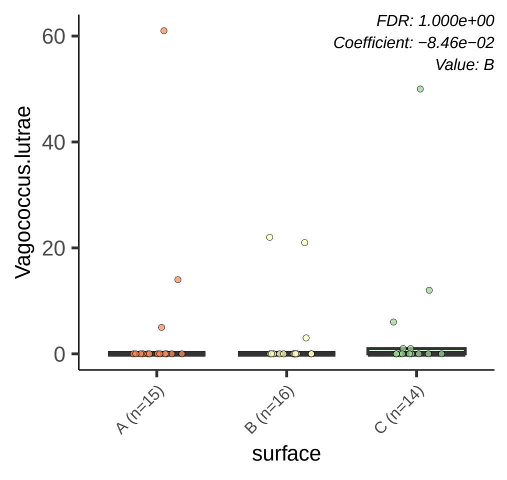
FDR: 1.000e+00
Coefficient: −8.46e−02
Value: B
60
40
20
0
Vagococcus.lutrae
A (n=15)
B (n=16)
C (n=14)
surface

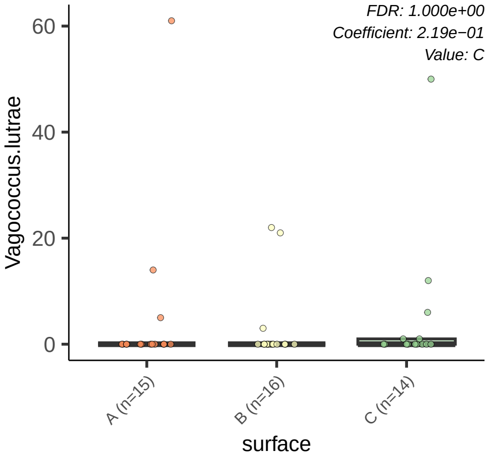

FDR: 1.000e+00
Coefficient: 2.19e−01
Value: C
Vagococcus.lutrae
0
20
40
60
A (n=15)
B (n=16)
C (n=14)
surface

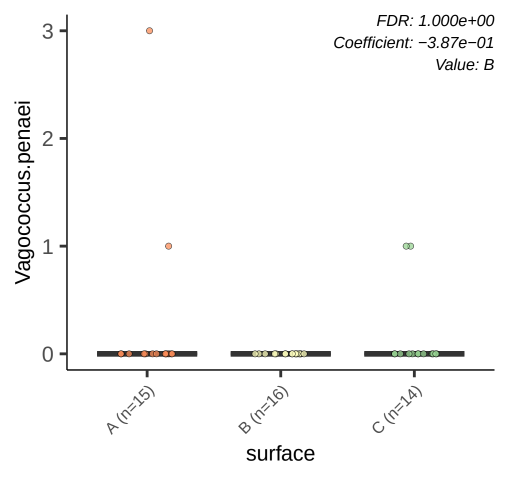

FDR: 1.000e+00
Coefficient: −3.87e−01
Value: B
Vagococcus.penaei
0
1
2
3
A (n=15)
B (n=16)
C (n=14)
surface

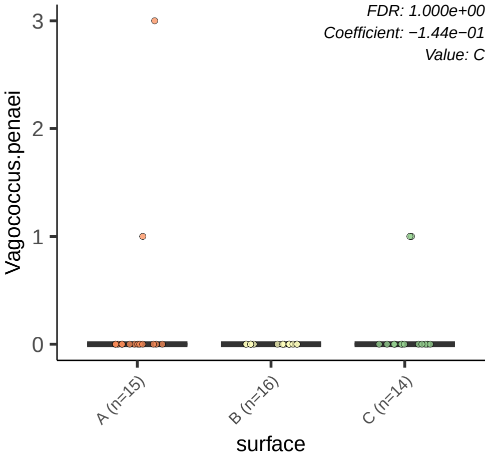

FDR: 1.000e+00
Coefficient: −1.44e−01
Value: C
Vagococcus.penaei
3
2
1
0
A (n=15)
B (n=16)
C (n=14)
surface

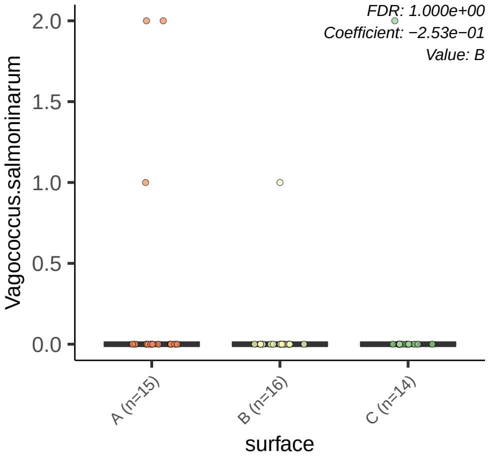

FDR: 1.000e+00
Coefficient: −2.53e−01
Value: B
Vagococcus.salmoninarum
2.0
1.5
1.0
0.5
0.0
A (n=15)
B (n=16)
C (n=14)
surface

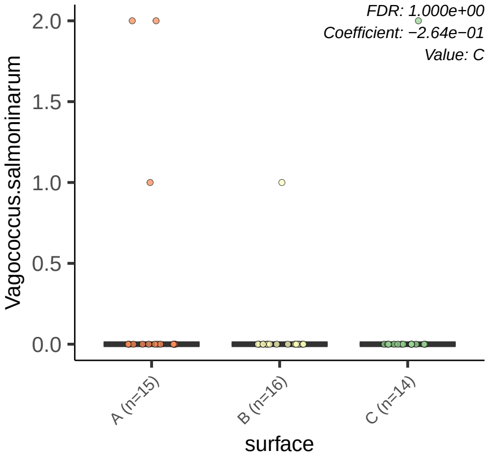
FDR: 1.000e+00
Coefficient: −2.64e−01
Value: C
Vagococcus.salmoninarum
2.0
1.5
1.0
0.5
0.0
A (n=15)
B (n=16)
C (n=14)
surface

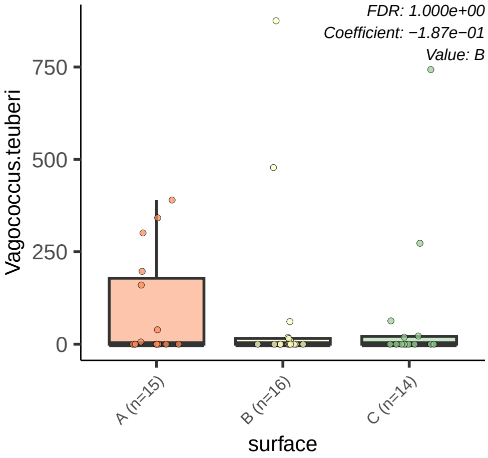

FDR: 1.000e+00
Coefficient: −1.87e−01
Value: B
Vagococcus.teuberi
750
500
250
0
A (n=15)
B (n=16)
C (n=14)
surface

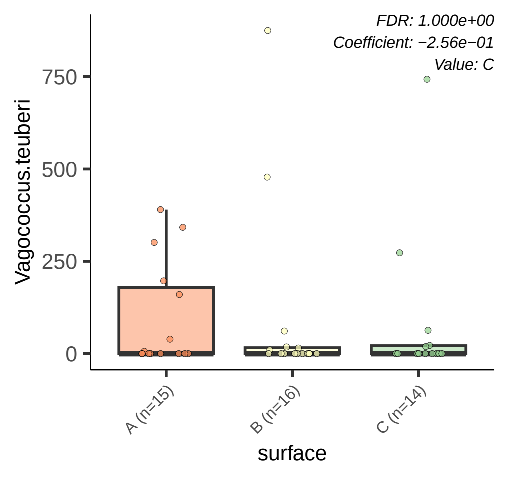
FDR: 1.000e+00
Coefficient: −2.56e−01
Value: C
Vagococcus.teuberi
750
500
250
0
A (n=15)
B (n=16)
C (n=14)
surface

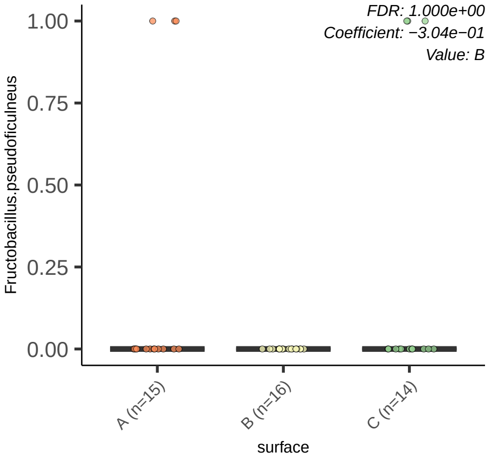
FDR: 1.000e+00
Coefficient: −3.04e−01
Value: B
1.00
0.75
0.50
0.25
0.00
Fructobacillus.pseudoficulneus
A (n=15)
B (n=16)
C (n=14)
surface

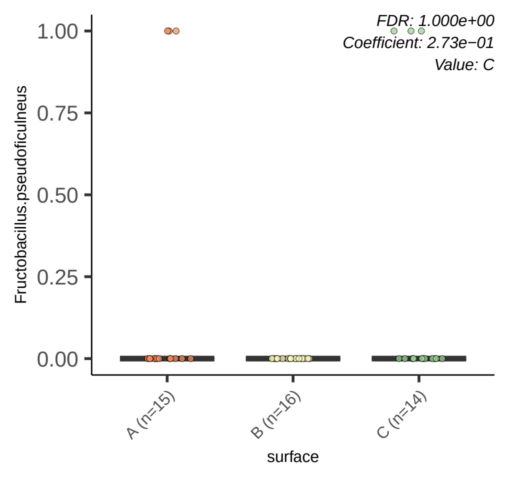
FDR: 1.000e+00
Coefficient: 2.73e−01
Value: C
Fructobacillus.pseudoficulneus
1.00
0.75
0.50
0.25
0.00
A (n=15)
B (n=16)
C (n=14)
surface

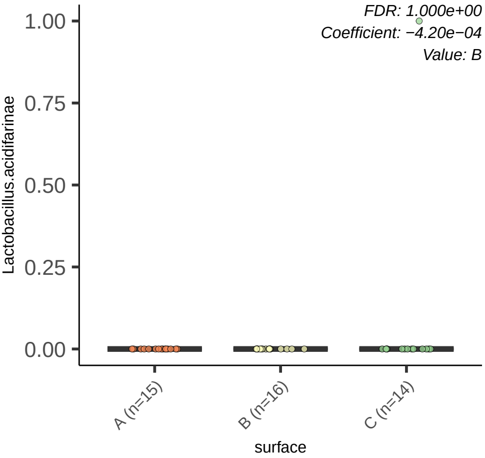

FDR: 1.000e+00
Coefficient: −4.20e−04
Value: B
Lactobacillus.acidifarinae
1.00
0.75
0.50
0.25
0.00
A (n=15)
B (n=16)
C (n=14)
surface

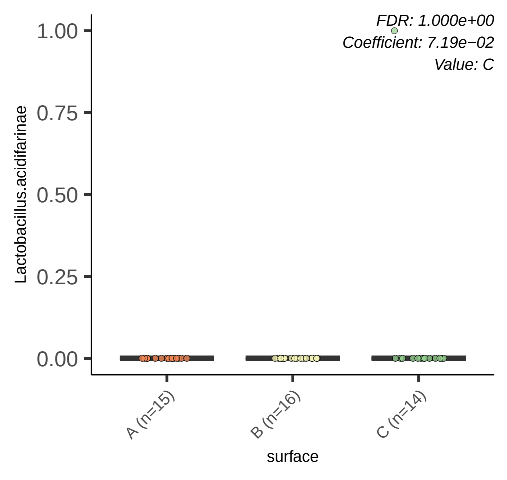
FDR: 1.000e+00
Coefficient: 7.19e−02
Value: C
Lactobacillus.acidifarinae
1.00
0.75
0.50
0.25
0.00
A (n=15)
B (n=16)
C (n=14)
surface

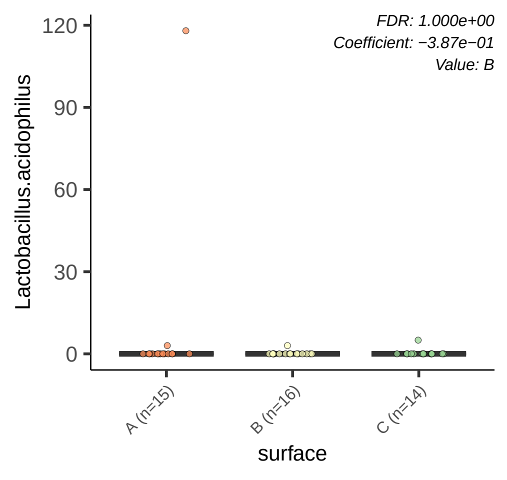

FDR: 1.000e+00
Coefficient: −3.87e−01
Value: B
120
90
60
30
0
Lactobacillus.acidophilus
A (n=15)
B (n=16)
C (n=14)
surface

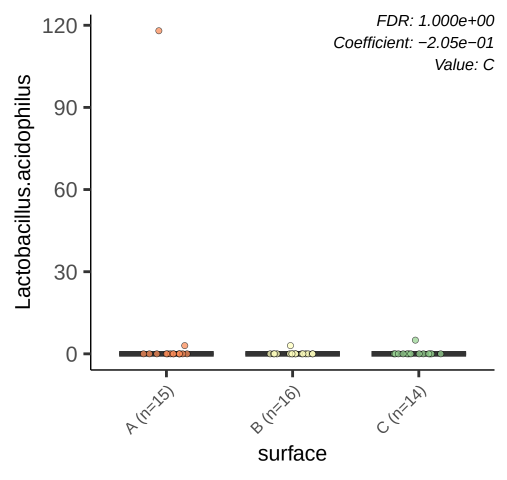
FDR: 1.000e+00
Coefficient: −2.05e−01
Value: C
Lactobacillus.acidophilus
120
90
60
30
0
A (n=15)
B (n=16)
C (n=14)
surface

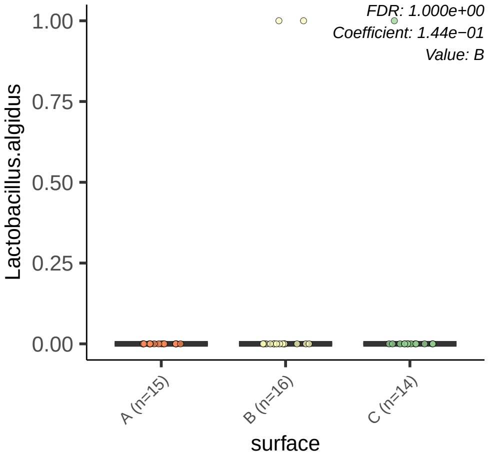

FDR: 1.000e+00
Coefficient: 1.44e−01
Value: B
Lactobacillus.algidus
1.00
0.75
0.50
0.25
0.00
A (n=15)
B (n=16)
C (n=14)
surface

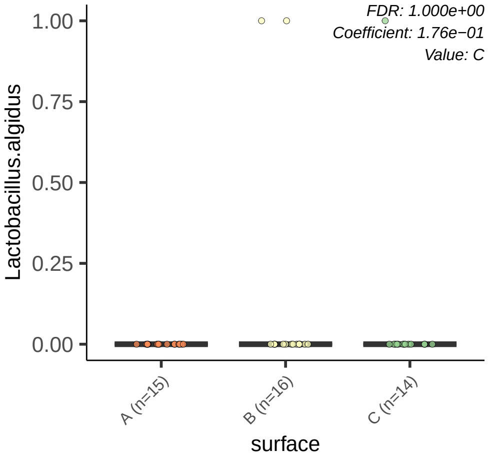

FDR: 1.000e+00
Coefficient: 1.76e−01
Value: C
Lactobacillus.algidus
1.00
0.75
0.50
0.25
0.00
A (n=15)
B (n=16)
C (n=14)
surface

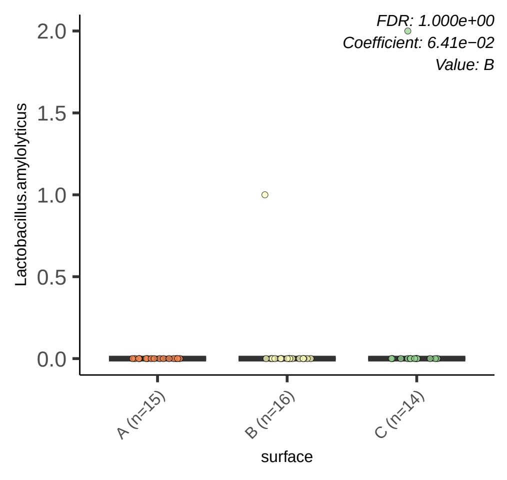

FDR: 1.000e+00
Coefficient: 6.41e−02
Value: B
Lactobacillus.amylolyticus
2.0
1.5
1.0
0.5
0.0
A (n=15)
B (n=16)
C (n=14)
surface

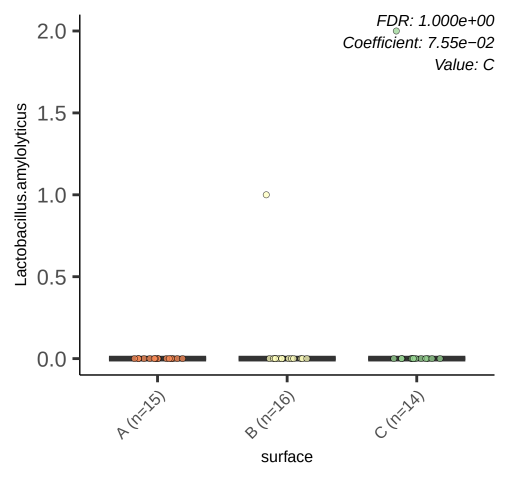

FDR: 1.000e+00
Coefficient: 7.55e−02
Value: C
Lactobacillus.amylolyticus
2.0
1.5
1.0
0.5
0.0
A (n=15)
B (n=16)
C (n=14)
surface

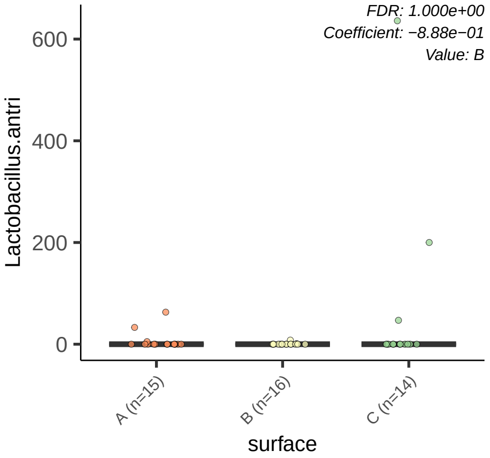

FDR: 1.000e+00
Coefficient: −8.88e−01
Value: B
Lactobacillus.antri
600
400
200
0
A (n=15)
B (n=16)
C (n=14)
surface

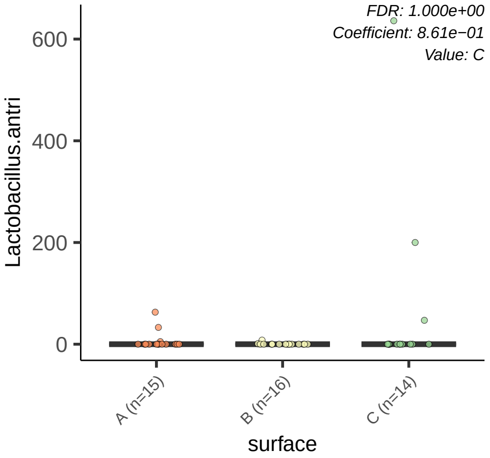
FDR: 1.000e+00
Coefficient: 8.61e−01
Value: C
Lactobacillus.antri
600
400
200
0
A (n=15)
B (n=16)
C (n=14)
surface

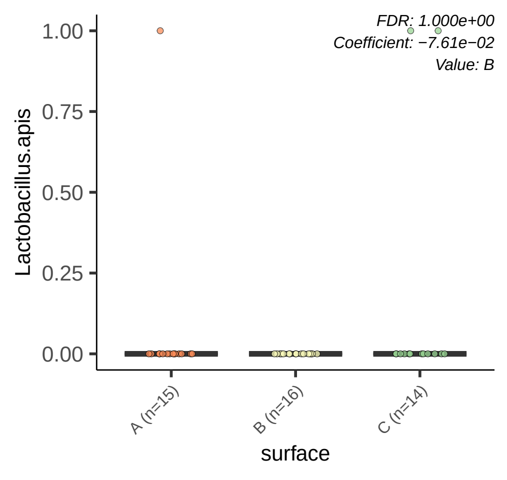
FDR: 1.000e+00
Coefficient: −7.61e−02
Value: B
Lactobacillus.apis
1.00
0.75
0.50
0.25
0.00
A (n=15)
B (n=16)
C (n=14)
surface

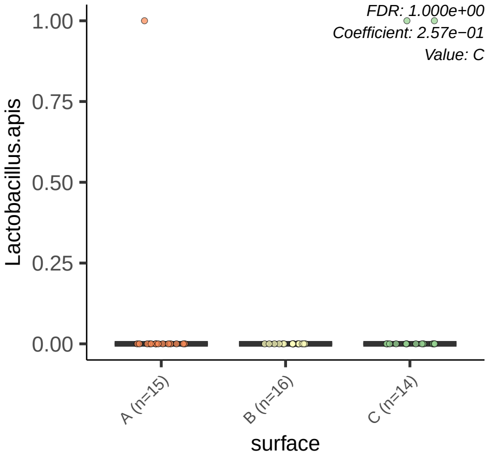

FDR: 1.000e+00
Coefficient: 2.57e−01
Value: C
1.00
0.75
0.50
0.25
0.00
Lactobacillus.apis
A (n=15)
B (n=16)
C (n=14)
surface

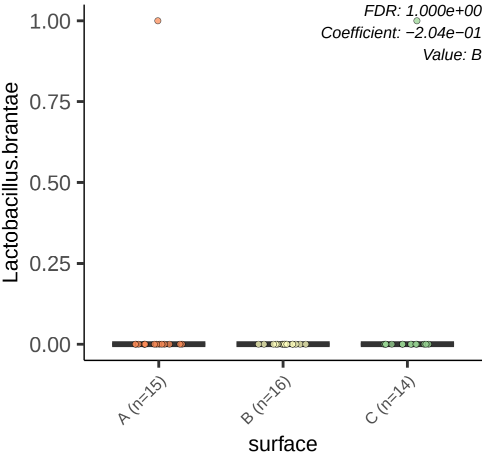
FDR: 1.000e+00
Coefficient: −2.04e−01
Value: B
Lactobacillus.brantae
1.00
0.75
0.50
0.25
0.00
A (n=15)
B (n=16)
C (n=14)
surface

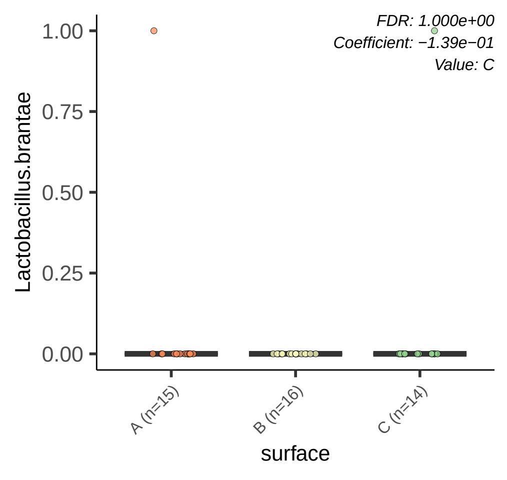
FDR: 1.000e+00
Coefficient: −1.39e−01
Value: C
Lactobacillus.brantae
1.00
0.75
0.50
0.25
0.00
A (n=15)
B (n=16)
C (n=14)
surface

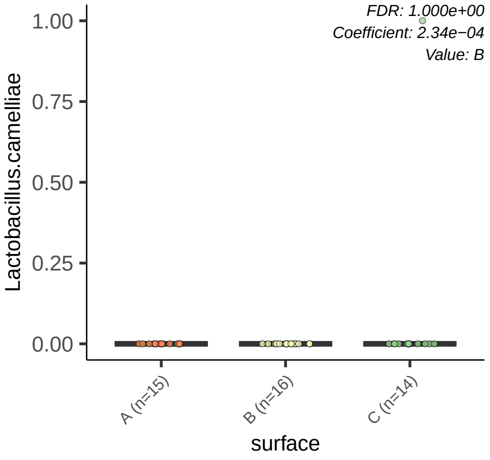
FDR: 1.000e+00
Coefficient: 2.34e−04
Value: B
Lactobacillus.camelliae
1.00
0.75
0.50
0.25
0.00
A (n=15)
B (n=16)
C (n=14)
surface

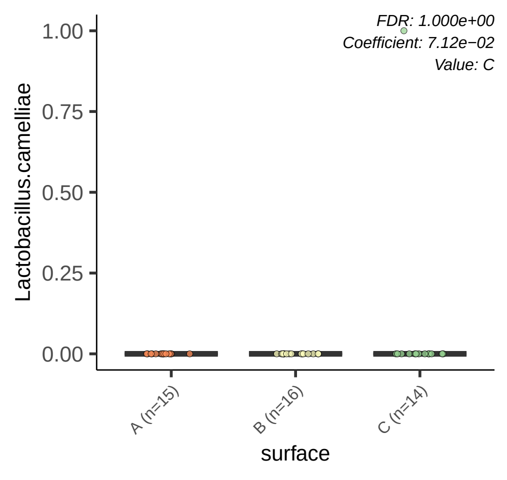
FDR: 1.000e+00
Coefficient: 7.12e−02
Value: C
Lactobacillus.camelliae
1.00
0.75
0.50
0.25
0.00
A (n=15)
B (n=16)
C (n=14)
surface

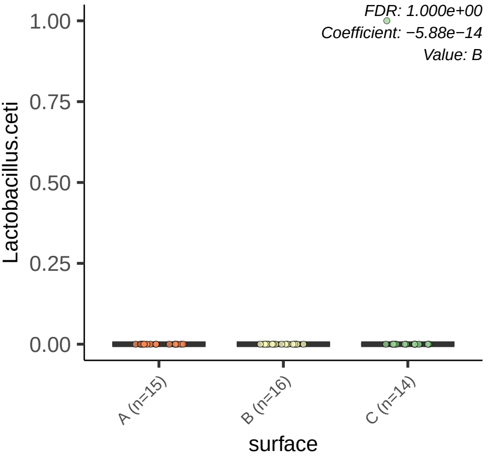
FDR: 1.000e+00
Coefficient: −5.88e−14
Value: B
Lactobacillus.ceti
1.00
0.75
0.50
0.25
0.00
A (n=15)
B (n=16)
C (n=14)
surface

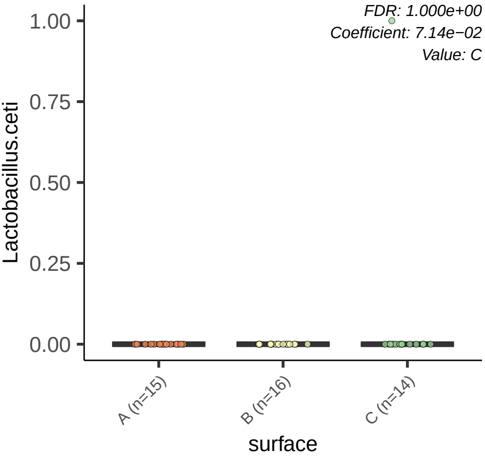
FDR: 1.000e+00
Coefficient: 7.14e−02
Value: C
Lactobacillus.ceti
1.00
0.75
0.50
0.25
0.00
A (n=15)
B (n=16)
C (n=14)
surface

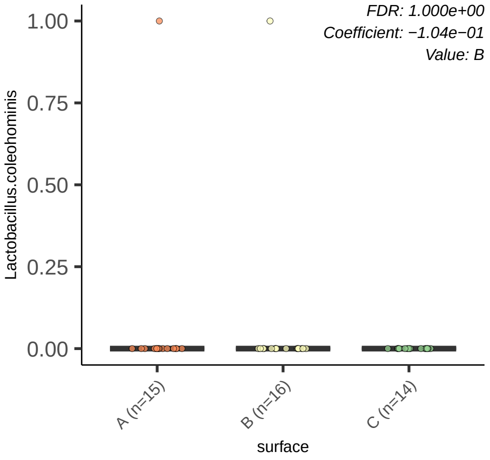

FDR: 1.000e+00
Coefficient: −1.04e−01
Value: B
1.00
0.75
0.50
0.25
0.00
Lactobacillus.coleohominis
A (n=15)
B (n=16)
C (n=14)
surface

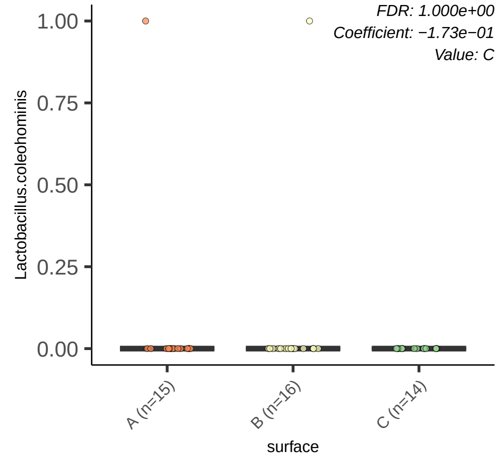
FDR: 1.000e+00
Coefficient: −1.73e−01
Value: C
1.00
0.75
0.50
0.25
0.00
Lactobacillus.coleohominis
A (n=15)
B (n=16)
C (n=14)
surface

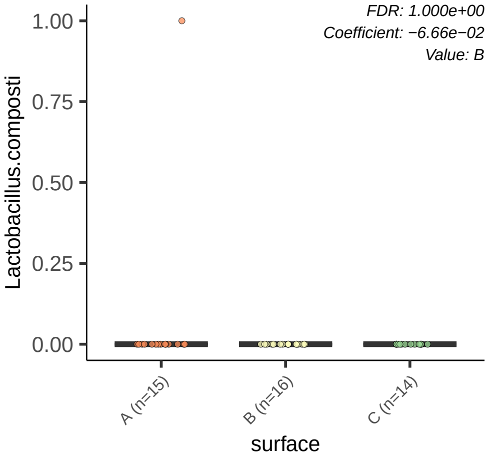

FDR: 1.000e+00
Coefficient: −6.66e−02
Value: B
Lactobacillus.composti
1.00
0.75
0.50
0.25
0.00
A (n=15)
B (n=16)
C (n=14)
surface

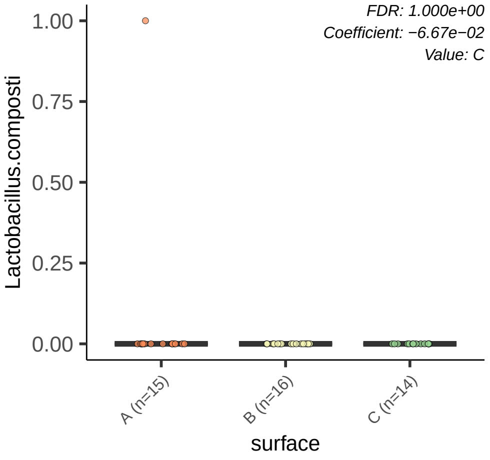

FDR: 1.000e+00
Coefficient: −6.67e−02
Value: C
Lactobacillus.composti
1.00
0.75
0.50
0.25
0.00
A (n=15)
B (n=16)
C (n=14)
surface

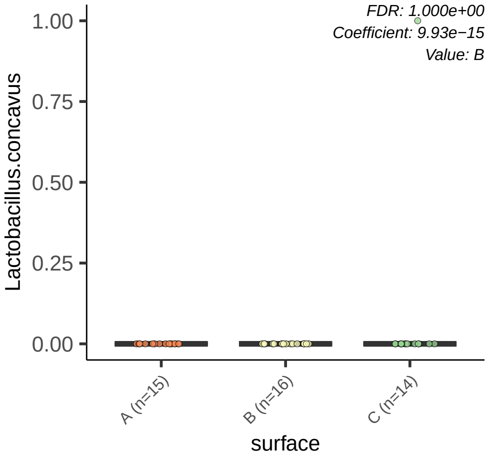
FDR: 1.000e+00
Coefficient: 9.93e−15
Value: B
Lactobacillus.concavus
1.00
0.75
0.50
0.25
0.00
A (n=15)
B (n=16)
C (n=14)
surface

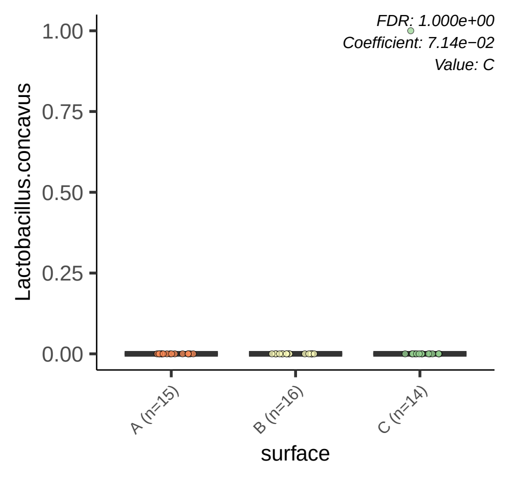
FDR: 1.000e+00
Coefficient: 7.14e−02
Value: C
Lactobacillus.concavus
1.00
0.75
0.50
0.25
0.00
A (n=15)
B (n=16)
C (n=14)
surface

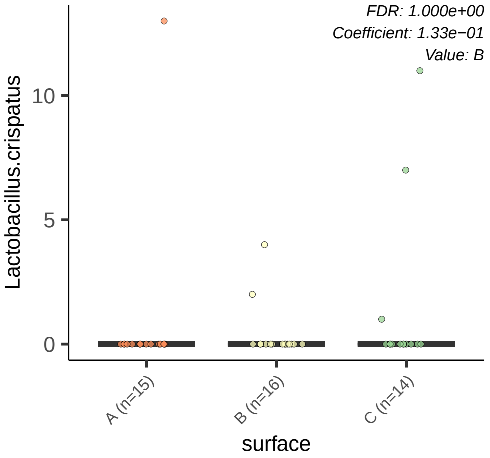

FDR: 1.000e+00
Coefficient: 1.33e−01
Value: B
Lactobacillus.crispatus
0
5
10
A (n=15)
B (n=16)
C (n=14)
surface

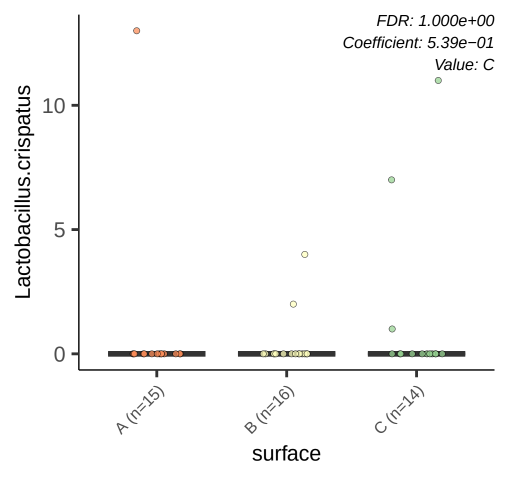
FDR: 1.000e+00
Coefficient: 5.39e−01
Value: C
Lactobacillus.crispatus
10
5
0
A (n=15)
B (n=16)
C (n=14)
surface

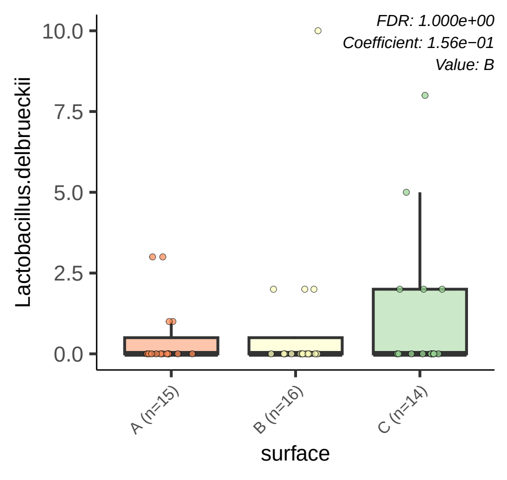
FDR: 1.000e+00
Coefficient: 1.56e−01
Value: B
10.0
7.5
5.0
2.5
0.0
Lactobacillus.delbrueckii
A (n=15)
B (n=16)
C (n=14)
surface

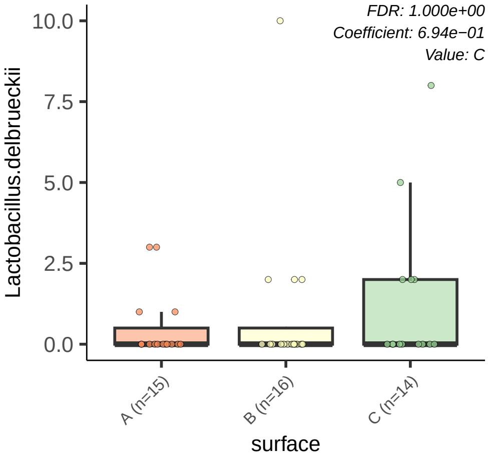

FDR: 1.000e+00
Coefficient: 6.94e−01
Value: C
Lactobacillus.delbrueckii
10.0
7.5
5.0
2.5
0.0
A (n=15)
B (n=16)
C (n=14)
surface

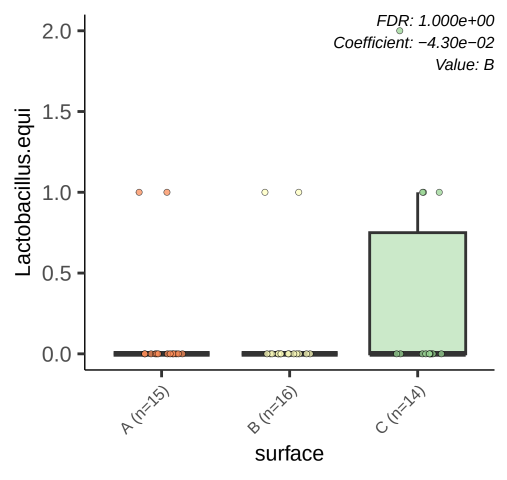
FDR: 1.000e+00
Coefficient: −4.30e−02
Value: B
Lactobacillus.equi
2.0
1.5
1.0
0.5
0.0
A (n=15)
B (n=16)
C (n=14)
surface

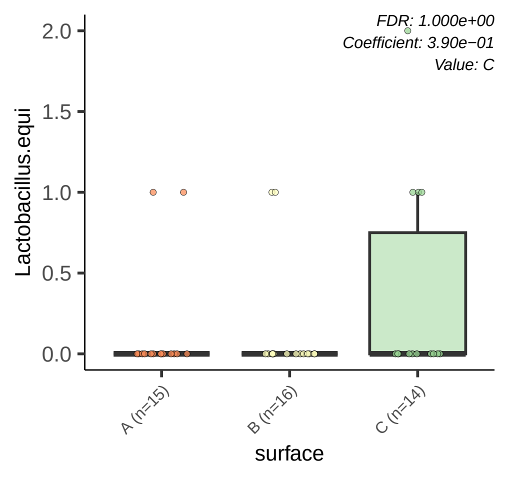

FDR: 1.000e+00
Coefficient: 3.90e−01
Value: C
Lactobacillus.equi
2.0
1.5
1.0
0.5
0.0
A (n=15)
B (n=16)
C (n=14)
surface

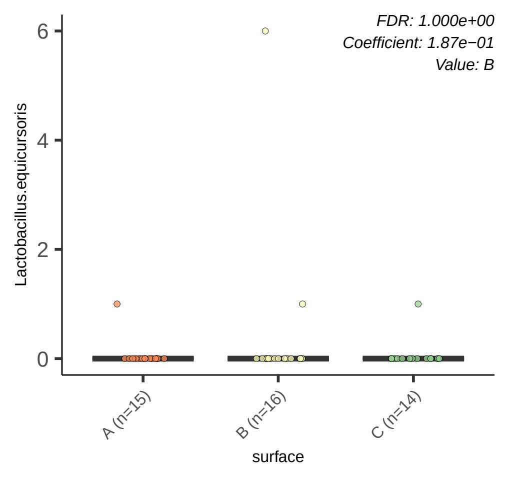
FDR: 1.000e+00
Coefficient: 1.87e−01
Value: B
Lactobacillus.equicursoris
6
4
2
0
A (n=15)
B (n=16)
C (n=14)
surface

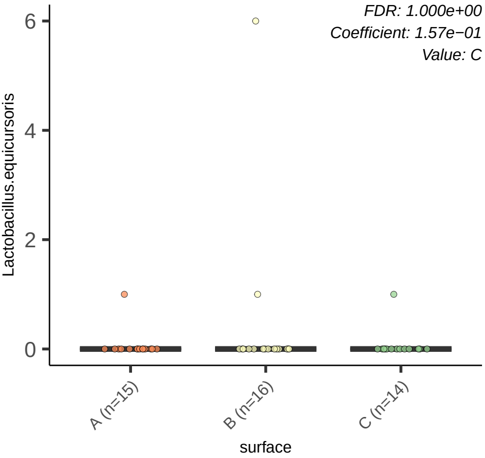

FDR: 1.000e+00
Coefficient: 1.57e−01
Value: C
Lactobacillus.equicursoris
0
2
4
6
A (n=15)
B (n=16)
C (n=14)
surface

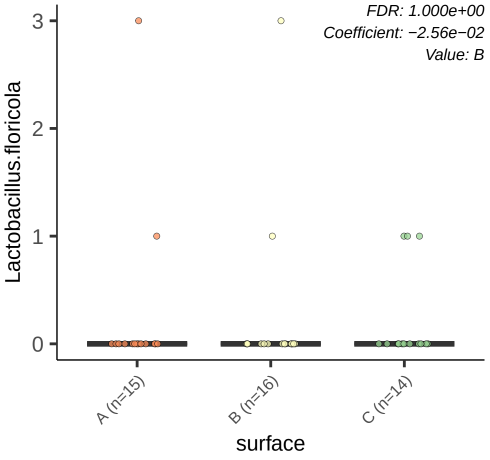

FDR: 1.000e+00
Coefficient: −2.56e−02
Value: B
Lactobacillus.floricola
0
1
2
3
A (n=15)
B (n=16)
C (n=14)
surface

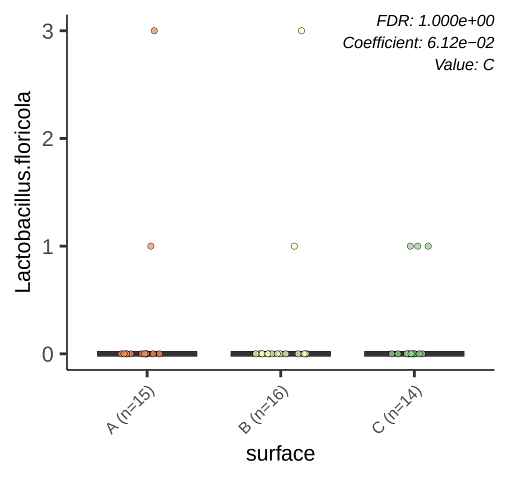

FDR: 1.000e+00
Coefficient: 6.12e−02
Value: C
Lactobacillus.floricola
3
2
1
0
A (n=15)
B (n=16)
C (n=14)
surface

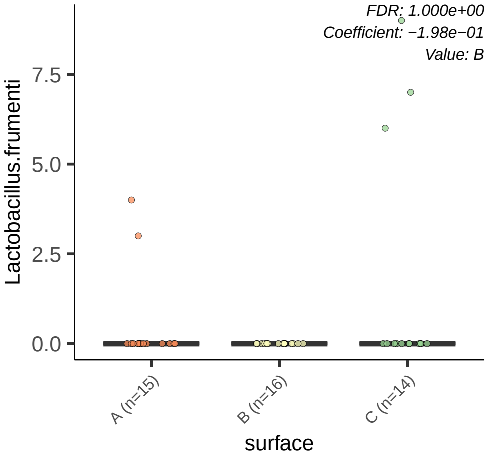
FDR: 1.000e+00
Coefficient: −1.98e−01
Value: B
Lactobacillus.frumenti
7.5
5.0
2.5
0.0
A (n=15)
B (n=16)
C (n=14)
surface

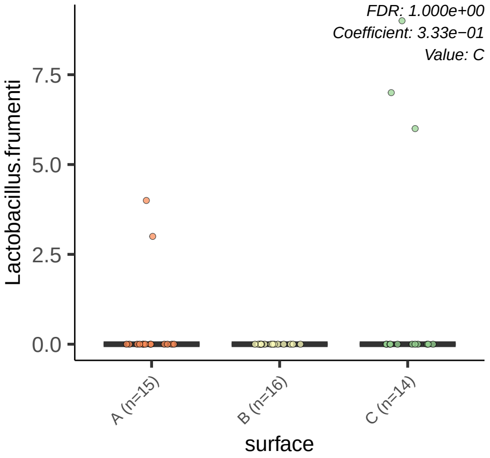
FDR: 1.000e+00
Coefficient: 3.33e−01
Value: C
Lactobacillus.frumenti
7.5
5.0
2.5
0.0
A (n=15)
B (n=16)
C (n=14)
surface

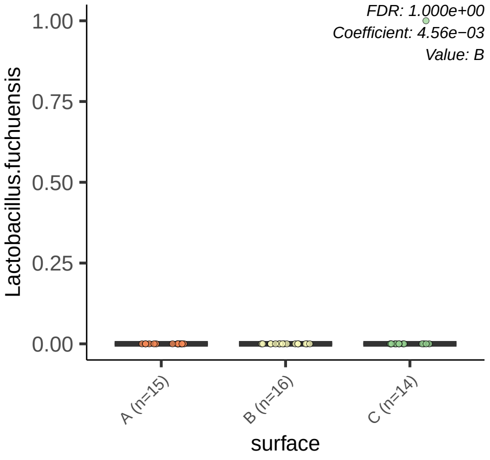
FDR: 1.000e+00
Coefficient: 4.56e−03
Value: B
1.00
0.75
0.50
0.25
0.00
Lactobacillus.fuchuensis
A (n=15)
B (n=16)
C (n=14)
surface

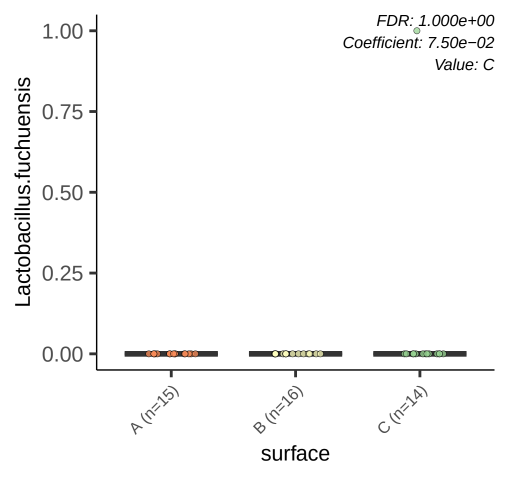

FDR: 1.000e+00
Coefficient: 7.50e−02
Value: C
Lactobacillus.fuchuensis
1.00
0.75
0.50
0.25
0.00
A (n=15)
B (n=16)
C (n=14)
surface

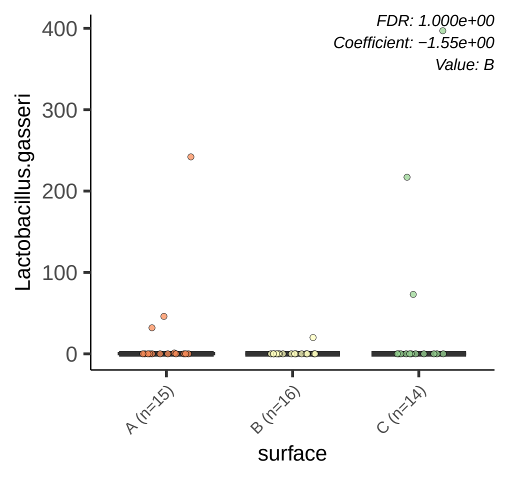

FDR: 1.000e+00
Coefficient: −1.55e+00
Value: B
Lactobacillus.gasseri
400
300
200
100
0
A (n=15)
B (n=16)
C (n=14)
surface

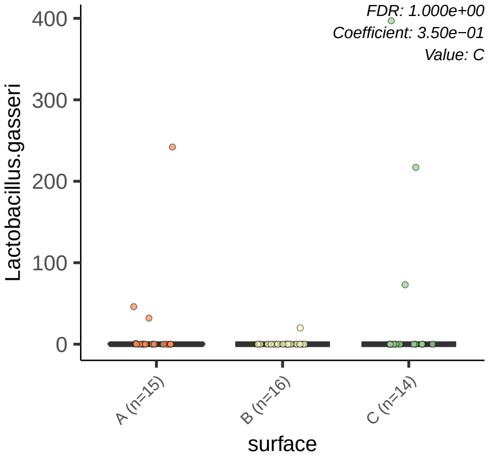

FDR: 1.000e+00
Coefficient: 3.50e−01
Value: C
Lactobacillus.gasseri
400
300
200
100
0
A (n=15)
B (n=16)
C (n=14)
surface

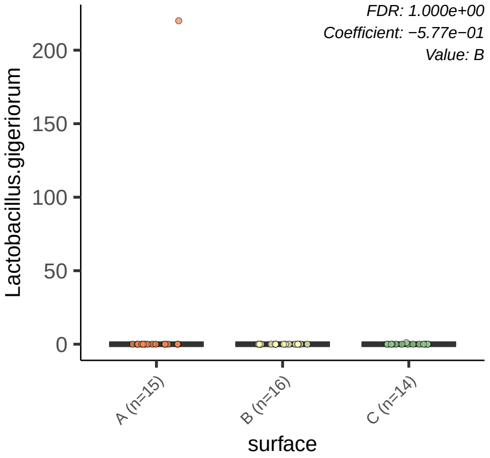
FDR: 1.000e+00
Coefficient: −5.77e−01
Value: B
Lactobacillus.gigeriorum
200
150
100
50
0
A (n=15)
B (n=16)
C (n=14)
surface

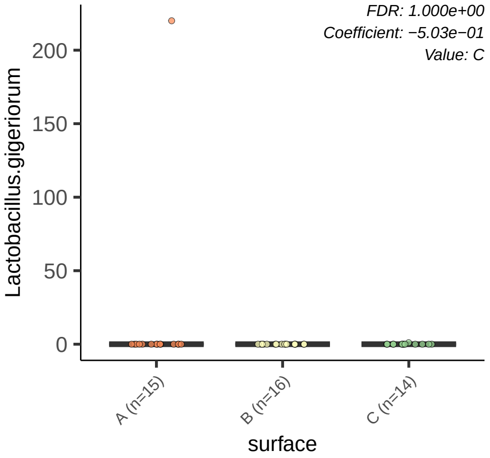
FDR: 1.000e+00
Coefficient: −5.03e−01
Value: C
Lactobacillus.gigeriorum
200
150
100
50
0
A (n=15)
B (n=16)
C (n=14)
surface

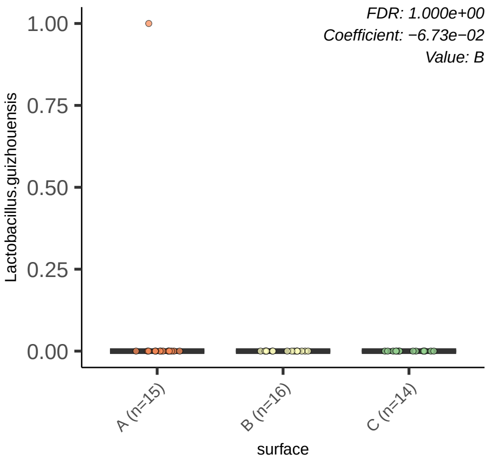

FDR: 1.000e+00
Coefficient: −6.73e−02
Value: B
Lactobacillus.guizhouensis
1.00
0.75
0.50
0.25
0.00
A (n=15)
B (n=16)
C (n=14)
surface

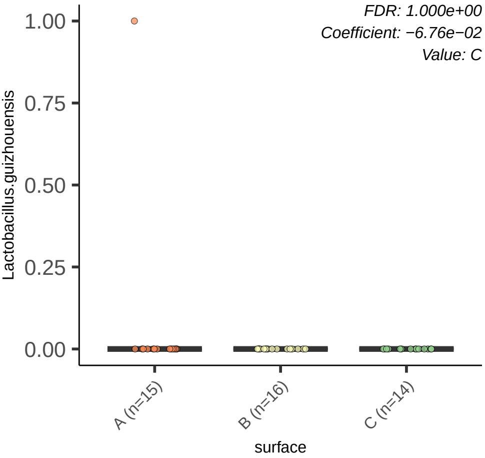
FDR: 1.000e+00
Coefficient: −6.76e−02
Value: C
Lactobacillus.guizhouensis
1.00
0.75
0.50
0.25
0.00
A (n=15)
B (n=16)
C (n=14)
surface

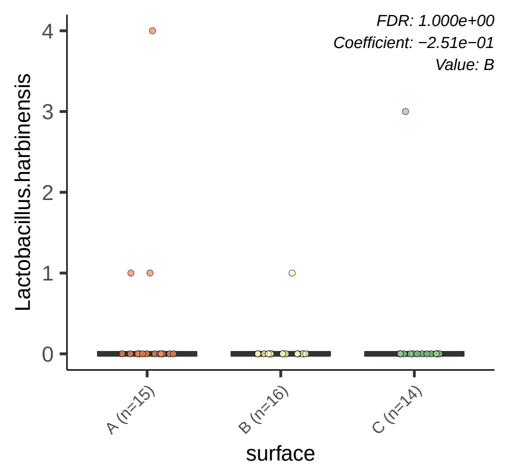
FDR: 1.000e+00
Coefficient: −2.51e−01
Value: B
Lactobacillus.harbinensis
0
1
2
3
4
A (n=15)
B (n=16)
C (n=14)
surface

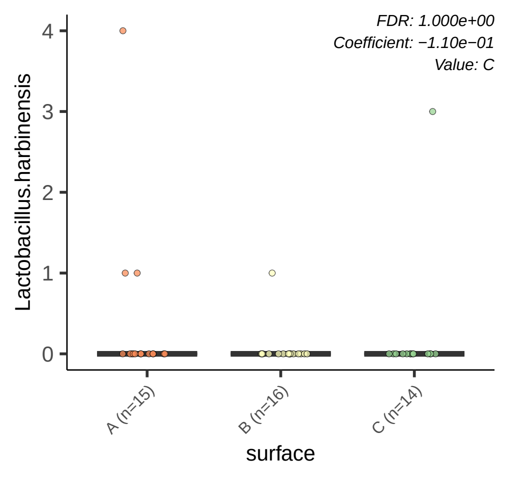

FDR: 1.000e+00
Coefficient: −1.10e−01
Value: C
Lactobacillus.harbinensis
4
3
2
1
0
A (n=15)
B (n=16)
C (n=14)
surface

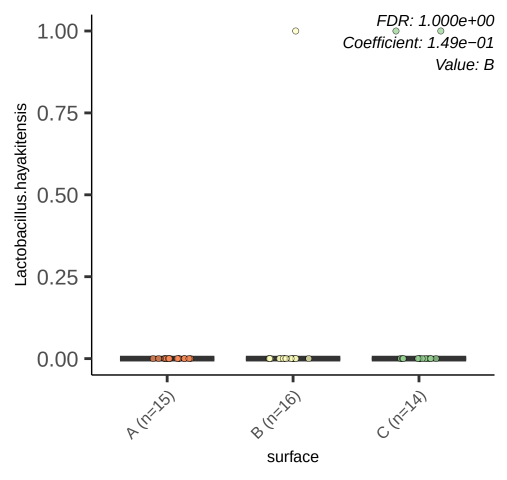

FDR: 1.000e+00
Coefficient: 1.49e−01
Value: B
1.00
0.75
0.50
0.25
0.00
Lactobacillus.hayakitensis
A (n=15)
B (n=16)
C (n=14)
surface

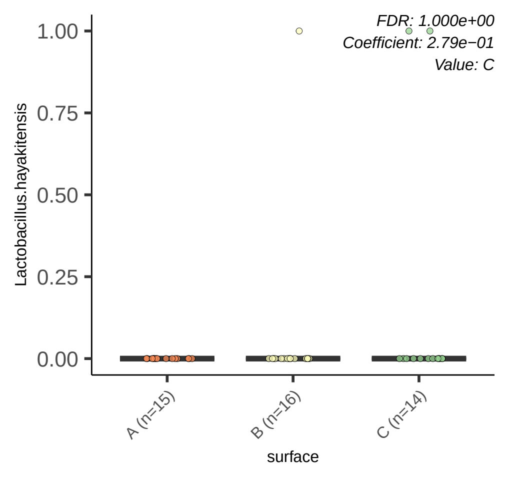

FDR: 1.000e+00
Coefficient: 2.79e−01
Value: C
1.00
0.75
0.50
0.25
0.00
Lactobacillus.hayakitensis
A (n=15)
B (n=16)
C (n=14)
surface

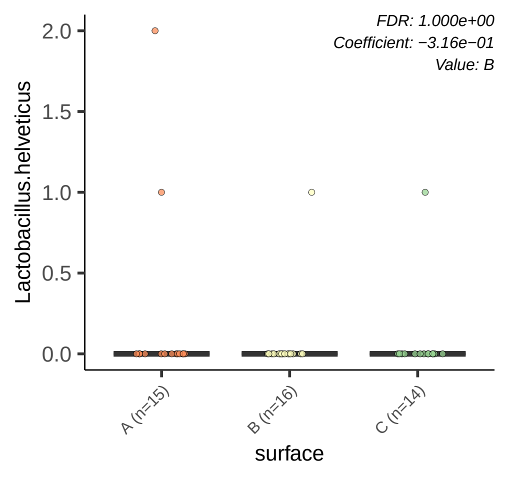

FDR: 1.000e+00
Coefficient: −3.16e−01
Value: B
Lactobacillus.helveticus
2.0
1.5
1.0
0.5
0.0
A (n=15)
B (n=16)
C (n=14)
surface

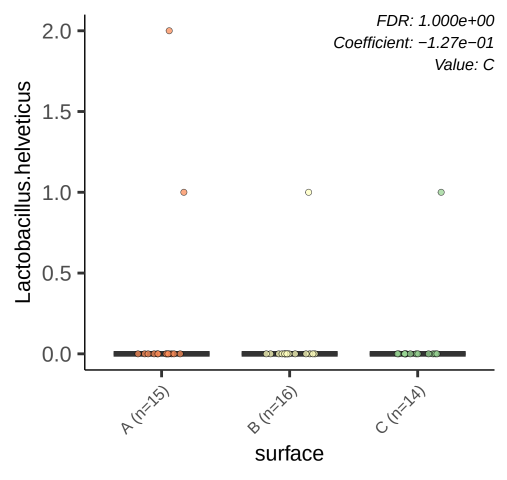

FDR: 1.000e+00
Coefficient: −1.27e−01
Value: C
Lactobacillus.helveticus
2.0
1.5
1.0
0.5
0.0
A (n=15)
B (n=16)
C (n=14)
surface

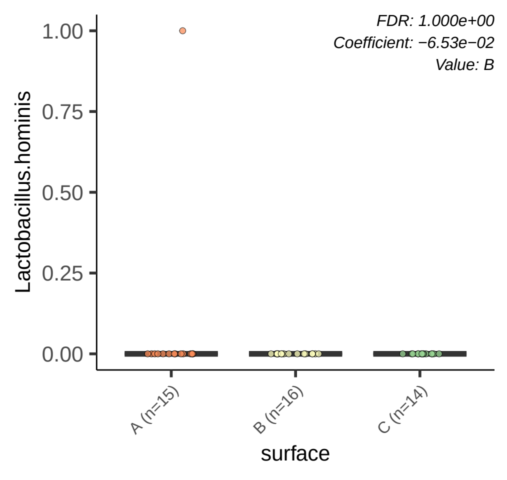

FDR: 1.000e+00
Coefficient: −6.53e−02
Value: B
Lactobacillus.hominis
1.00
0.75
0.50
0.25
0.00
A (n=15)
B (n=16)
C (n=14)
surface

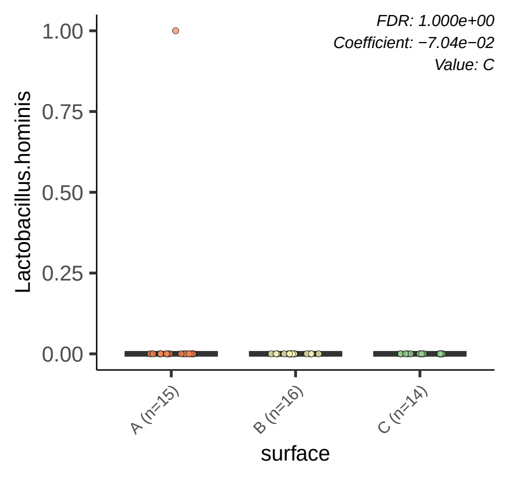

FDR: 1.000e+00
Coefficient: −7.04e−02
Value: C
Lactobacillus.hominis
1.00
0.75
0.50
0.25
0.00
A (n=15)
B (n=16)
C (n=14)
surface

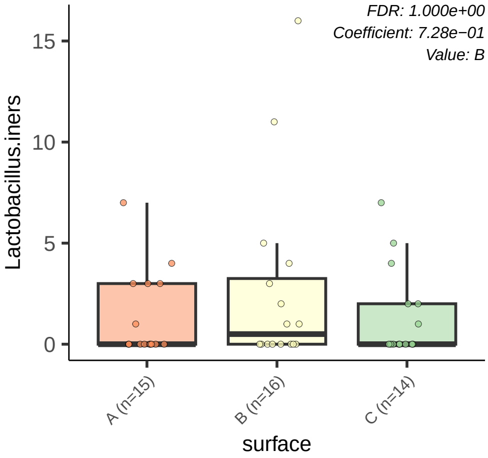

FDR: 1.000e+00
Coefficient: 7.28e−01
Value: B
Lactobacillus.iners
15
10
5
0
A (n=15)
B (n=16)
C (n=14)
surface

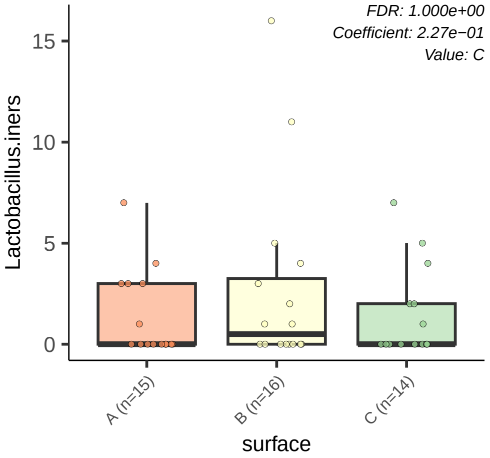
FDR: 1.000e+00
Coefficient: 2.27e−01
Value: C
Lactobacillus.iners
15
10
5
0
A (n=15)
B (n=16)
C (n=14)
surface

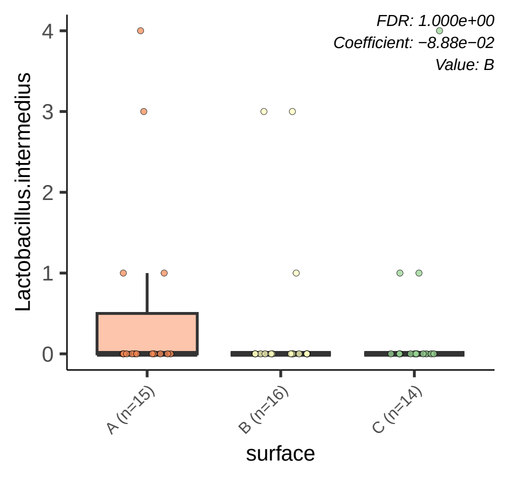

FDR: 1.000e+00
Coefficient: −8.88e−02
Value: B
Lactobacillus.intermedius
4
3
2
1
0
A (n=15)
B (n=16)
C (n=14)
surface

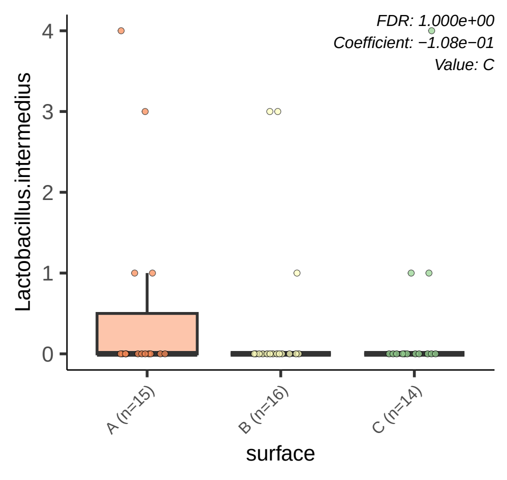

FDR: 1.000e+00
Coefficient: −1.08e−01
Value: C
Lactobacillus.intermedius
4
3
2
1
0
A (n=15)
B (n=16)
C (n=14)
surface

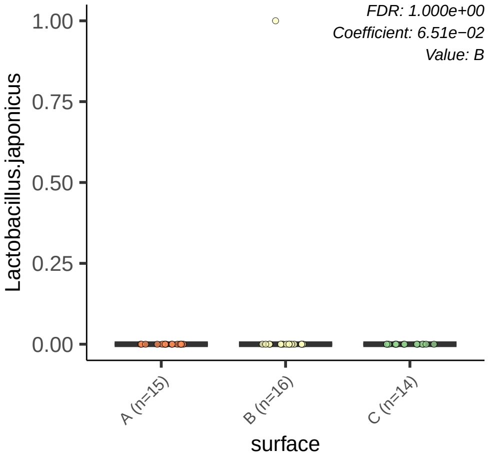

FDR: 1.000e+00
Coefficient: 6.51e−02
Value: B
Lactobacillus.japonicus
1.00
0.75
0.50
0.25
0.00
A (n=15)
B (n=16)
C (n=14)
surface

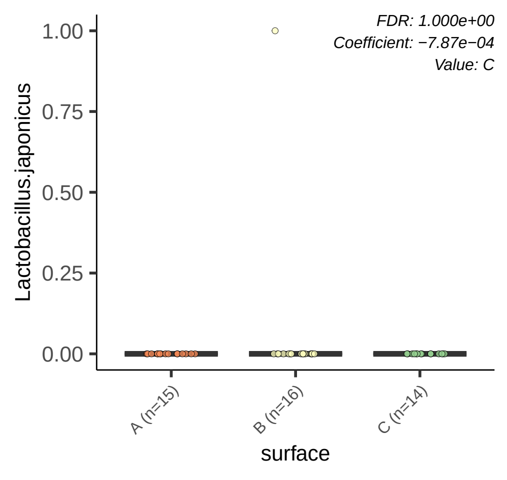
FDR: 1.000e+00
Coefficient: −7.87e−04
Value: C
1.00
0.75
0.50
0.25
0.00
Lactobacillus.japonicus
A (n=15)
B (n=16)
C (n=14)
surface

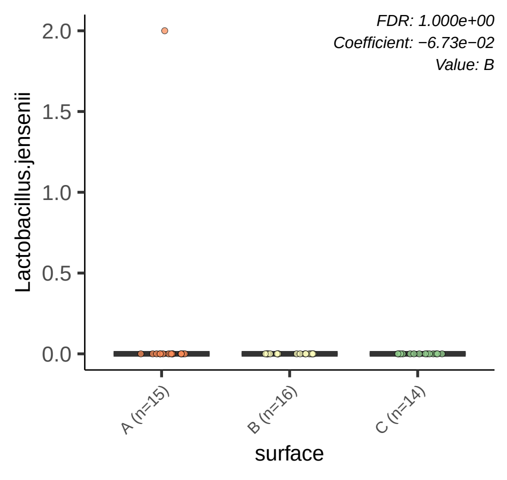

FDR: 1.000e+00
Coefficient: −6.73e−02
Value: B
Lactobacillus.jensenii
2.0
1.5
1.0
0.5
0.0
A (n=15)
B (n=16)
C (n=14)
surface

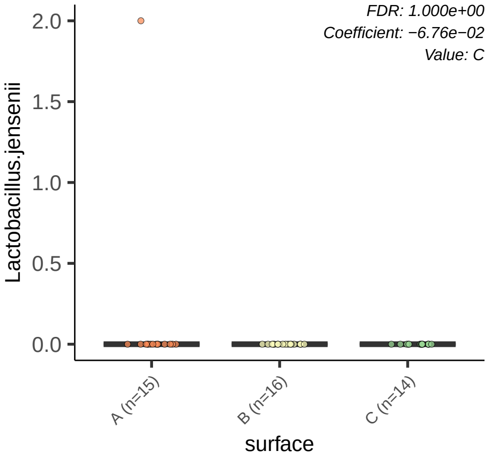
FDR: 1.000e+00
Coefficient: −6.76e−02
Value: C
Lactobacillus.jensenii
2.0
1.5
1.0
0.5
0.0
A (n=15)
B (n=16)
C (n=14)
surface

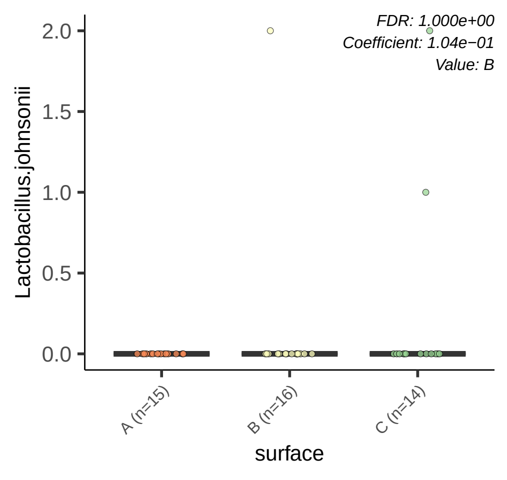
FDR: 1.000e+00
Coefficient: 1.04e−01
Value: B
Lactobacillus.johnsonii
2.0
1.5
1.0
0.5
0.0
A (n=15)
B (n=16)
C (n=14)
surface

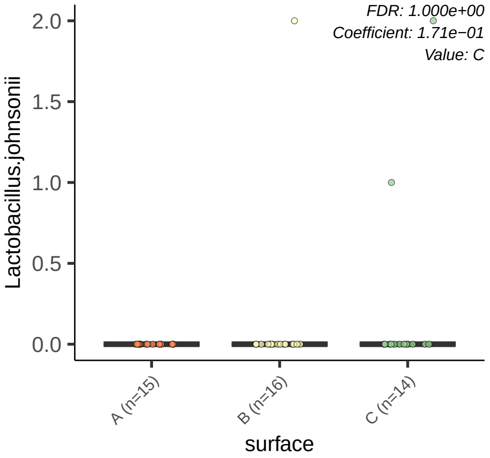
FDR: 1.000e+00
Coefficient: 1.71e−01
Value: C
Lactobacillus.johnsonii
2.0
1.5
1.0
0.5
0.0
A (n=15)
B (n=16)
C (n=14)
surface

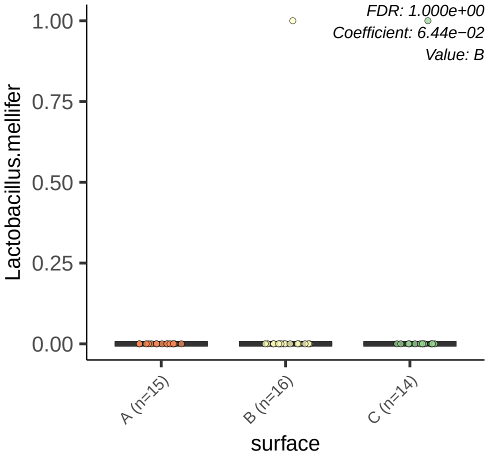
FDR: 1.000e+00
Coefficient: 6.44e−02
Value: B
Lactobacillus.mellifer
1.00
0.75
0.50
0.25
0.00
A (n=15)
B (n=16)
C (n=14)
surface

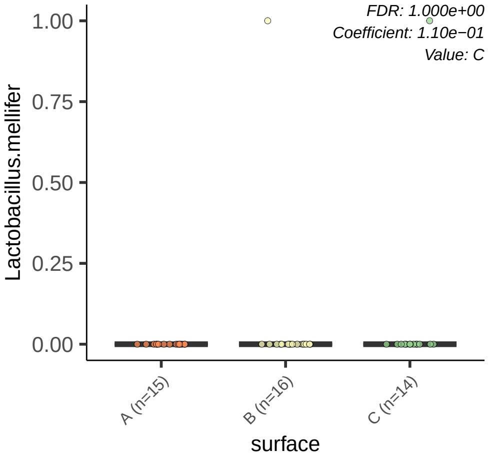

FDR: 1.000e+00
Coefficient: 1.10e−01
Value: C
Lactobacillus.mellifer
1.00
0.75
0.50
0.25
0.00
A (n=15)
B (n=16)
C (n=14)
surface

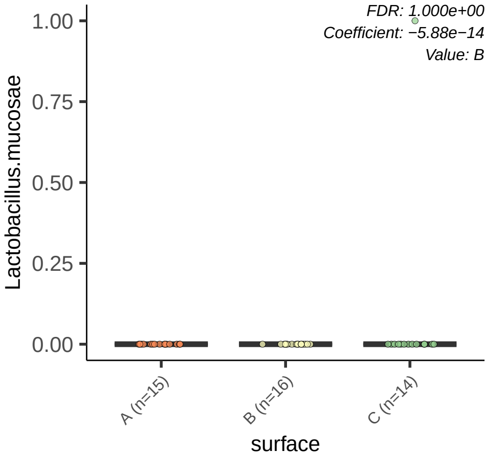

FDR: 1.000e+00
Coefficient: −5.88e−14
Value: B
Lactobacillus.mucosae
1.00
0.75
0.50
0.25
0.00
A (n=15)
B (n=16)
C (n=14)
surface

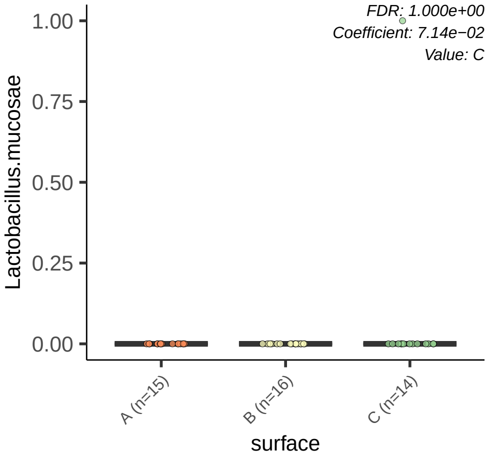

FDR: 1.000e+00
Coefficient: 7.14e−02
Value: C
Lactobacillus.mucosae
1.00
0.75
0.50
0.25
0.00
A (n=15)
B (n=16)
C (n=14)
surface

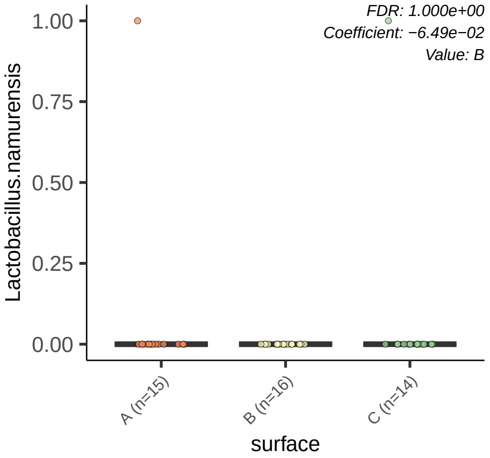
FDR: 1.000e+00
Coefficient: −6.49e−02
Value: B
Lactobacillus.namurensis
1.00
0.75
0.50
0.25
0.00
A (n=15)
B (n=16)
C (n=14)
surface

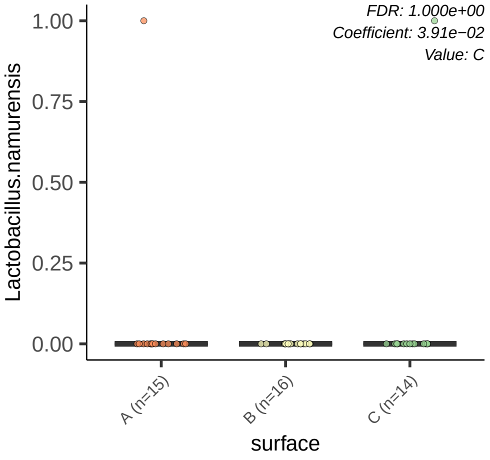
FDR: 1.000e+00
Coefficient: 3.91e−02
Value: C
Lactobacillus.namurensis
1.00
0.75
0.50
0.25
0.00
A (n=15)
B (n=16)
C (n=14)
surface

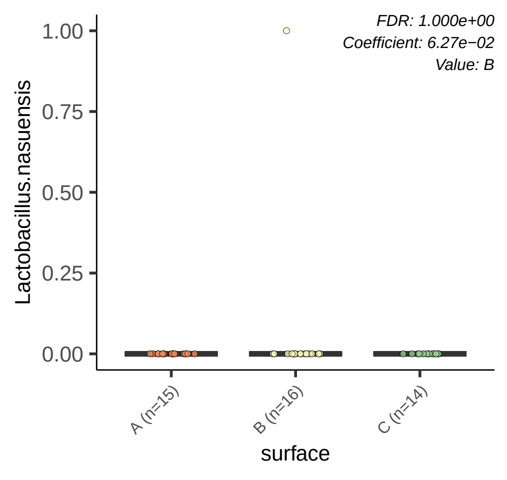
FDR: 1.000e+00
Coefficient: 6.27e−02
Value: B
Lactobacillus.nasuensis
1.00
0.75
0.50
0.25
0.00
A (n=15)
B (n=16)
C (n=14)
surface

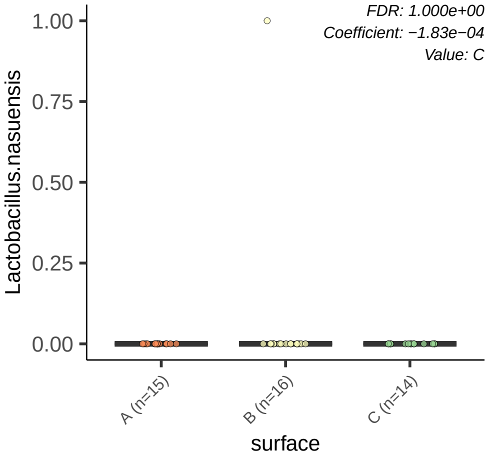
FDR: 1.000e+00
Coefficient: −1.83e−04
Value: C
Lactobacillus.nasuensis
1.00
0.75
0.50
0.25
0.00
A (n=15)
B (n=16)
C (n=14)
surface

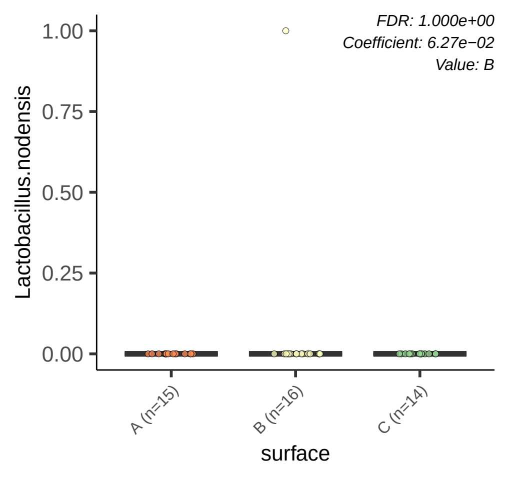
FDR: 1.000e+00
Coefficient: 6.27e−02
Value: B
Lactobacillus.nodensis
1.00
0.75
0.50
0.25
0.00
A (n=15)
B (n=16)
C (n=14)
surface

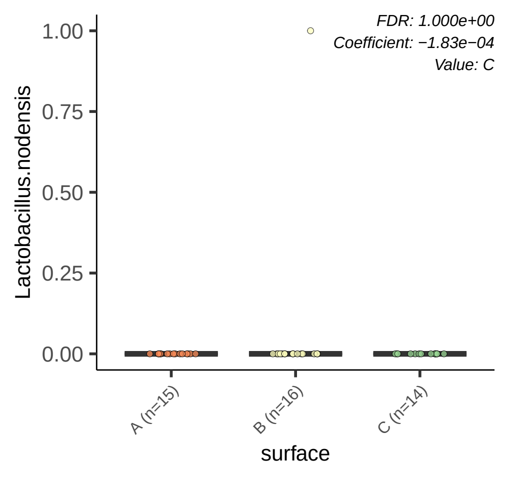
FDR: 1.000e+00
Coefficient: −1.83e−04
Value: C
Lactobacillus.nodensis
1.00
0.75
0.50
0.25
0.00
A (n=15)
B (n=16)
C (n=14)
surface

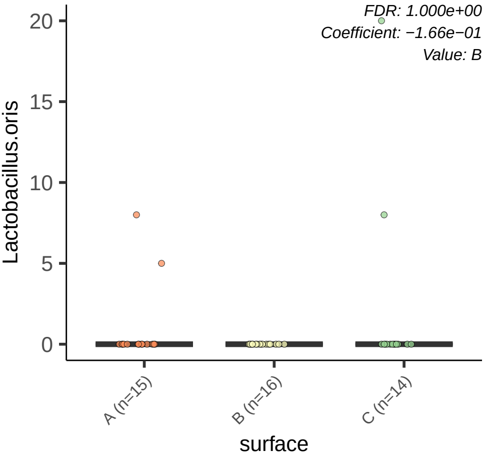

FDR: 1.000e+00
Coefficient: −1.66e−01
Value: B
Lactobacillus.oris
20
15
10
5
0
A (n=15)
B (n=16)
C (n=14)
surface

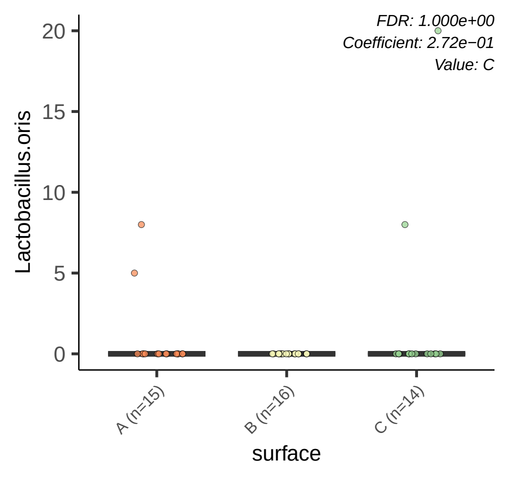
FDR: 1.000e+00
Coefficient: 2.72e−01
Value: C
Lactobacillus.oris
20
15
10
5
0
A (n=15)
B (n=16)
C (n=14)
surface

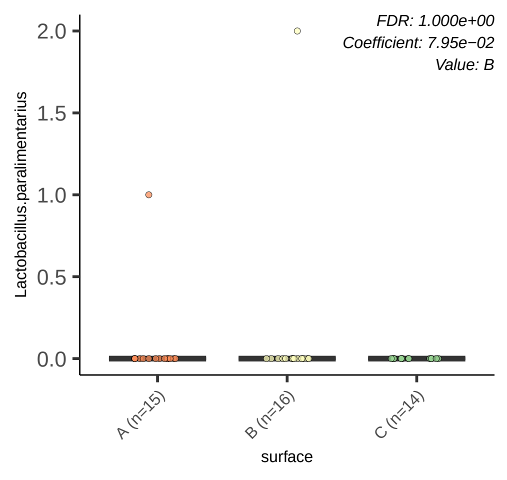
FDR: 1.000e+00
Coefficient: 7.95e−02
Value: B
Lactobacillus.paralimentarius
2.0
1.5
1.0
0.5
0.0
A (n=15)
B (n=16)
C (n=14)
surface

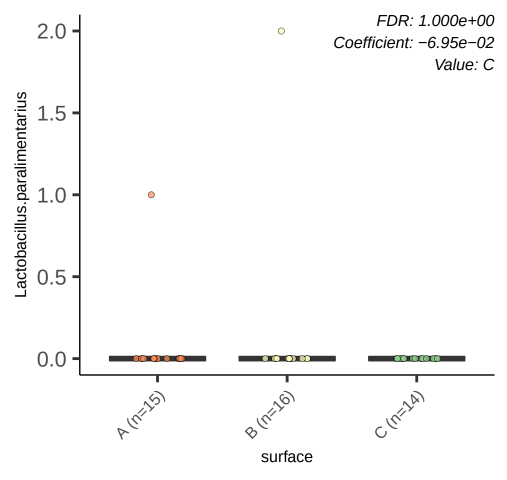

FDR: 1.000e+00
Coefficient: −6.95e−02
Value: C
Lactobacillus.paralimentarius
2.0
1.5
1.0
0.5
0.0
A (n=15)
B (n=16)
C (n=14)
surface

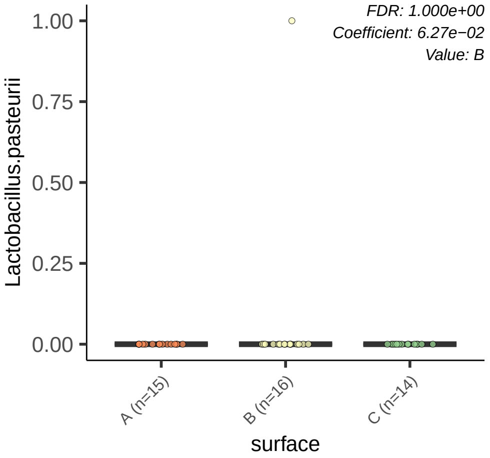

FDR: 1.000e+00
Coefficient: 6.27e−02
Value: B
Lactobacillus.pasteurii
1.00
0.75
0.50
0.25
0.00
A (n=15)
B (n=16)
C (n=14)
surface

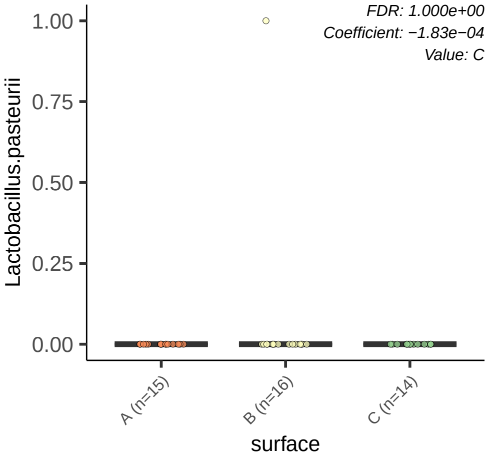
FDR: 1.000e+00
Coefficient: −1.83e−04
Value: C
Lactobacillus.pasteurii
1.00
0.75
0.50
0.25
0.00
A (n=15)
B (n=16)
C (n=14)
surface

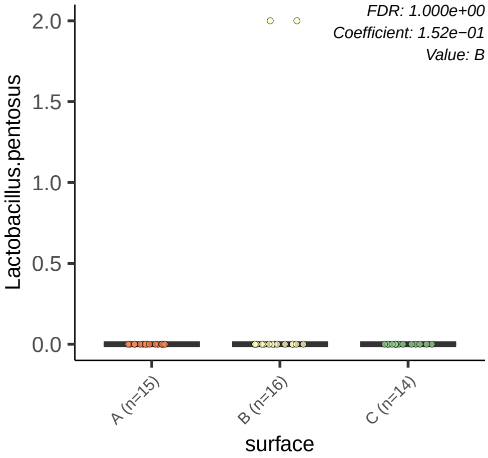

FDR: 1.000e+00
Coefficient: 1.52e−01
Value: B
Lactobacillus.pentosus
2.0
1.5
1.0
0.5
0.0
A (n=15)
B (n=16)
C (n=14)
surface

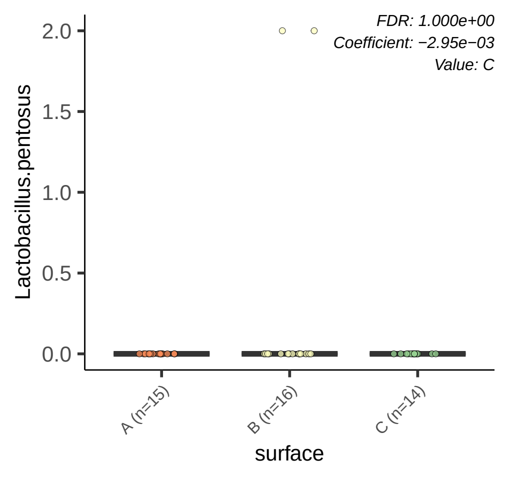
FDR: 1.000e+00
Coefficient: −2.95e−03
Value: C
Lactobacillus.pentosus
2.0
1.5
1.0
0.5
0.0
A (n=15)
B (n=16)
C (n=14)
surface

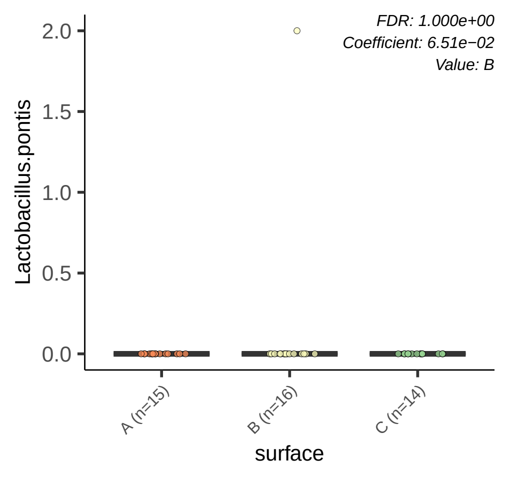
FDR: 1.000e+00
Coefficient: 6.51e−02
Value: B
Lactobacillus.pontis
2.0
1.5
1.0
0.5
0.0
A (n=15)
B (n=16)
C (n=14)
surface

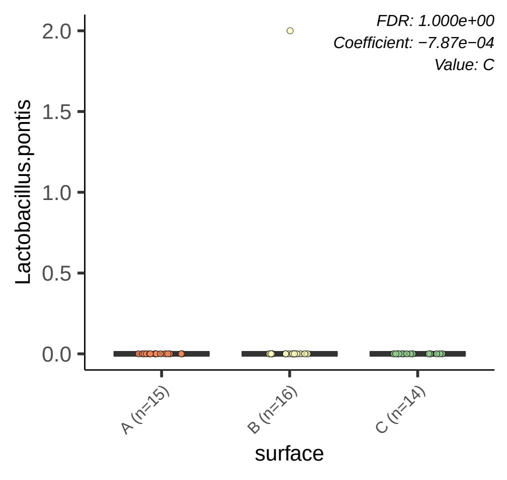
FDR: 1.000e+00
Coefficient: −7.87e−04
Value: C
Lactobacillus.pontis
2.0
1.5
1.0
0.5
0.0
A (n=15)
B (n=16)
C (n=14)
surface

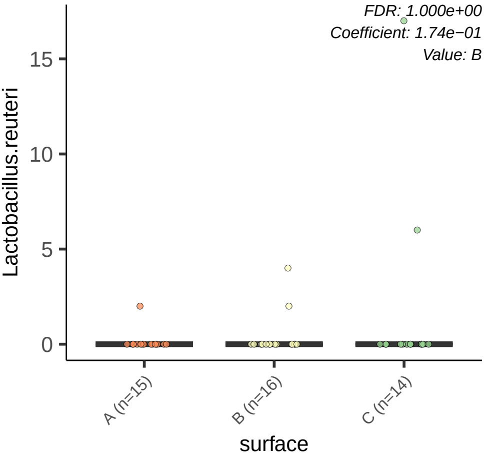

FDR: 1.000e+00
Coefficient: 1.74e−01
Value: B
Lactobacillus.reuteri
15
10
5
0
A (n=15)
B (n=16)
C (n=14)
surface

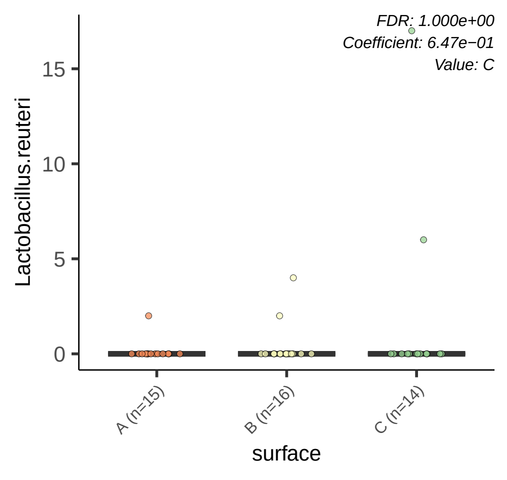

FDR: 1.000e+00
Coefficient: 6.47e−01
Value: C
Lactobacillus.reuteri
0
5
10
15
A (n=15)
B (n=16)
C (n=14)
surface

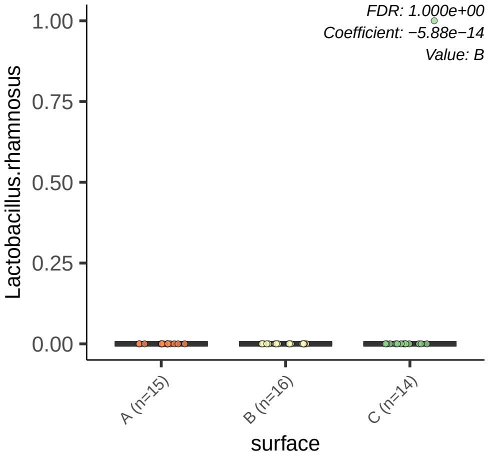
FDR: 1.000e+00
Coefficient: −5.88e−14
Value: B
Lactobacillus.rhamnosus
1.00
0.75
0.50
0.25
0.00
A (n=15)
B (n=16)
C (n=14)
surface

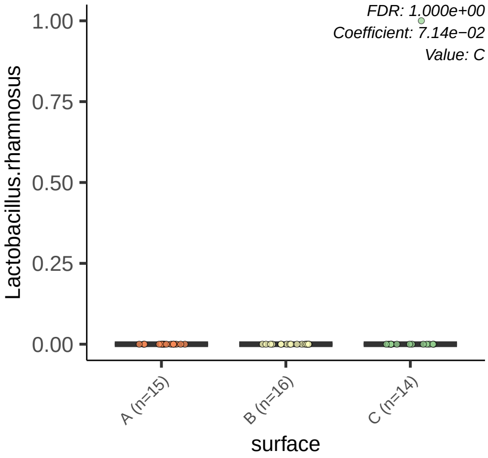

FDR: 1.000e+00
Coefficient: 7.14e−02
Value: C
Lactobacillus.rhamnosus
1.00
0.75
0.50
0.25
0.00
A (n=15)
B (n=16)
C (n=14)
surface

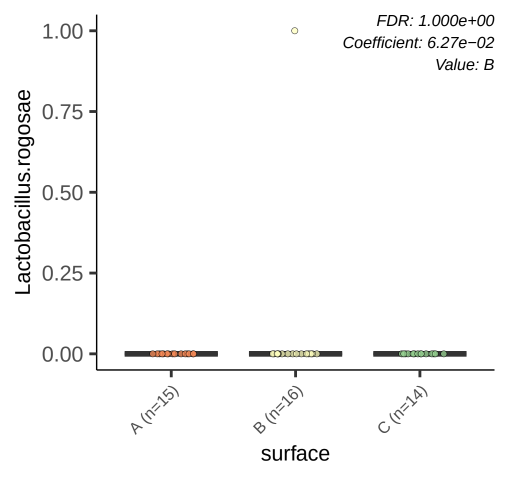
FDR: 1.000e+00
Coefficient: 6.27e−02
Value: B
Lactobacillus.rogosae
1.00
0.75
0.50
0.25
0.00
A (n=15)
B (n=16)
C (n=14)
surface

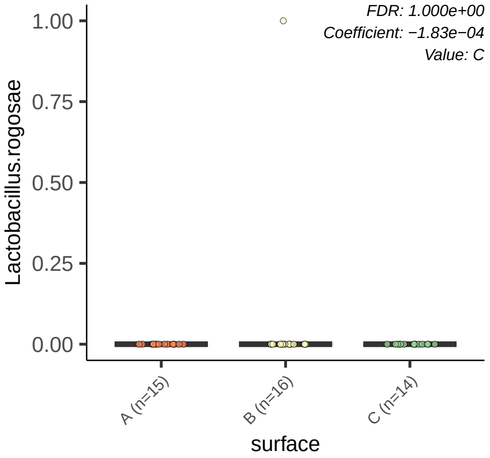
FDR: 1.000e+00
Coefficient: −1.83e−04
Value: C
Lactobacillus.rogosae
1.00
0.75
0.50
0.25
0.00
A (n=15)
B (n=16)
C (n=14)
surface

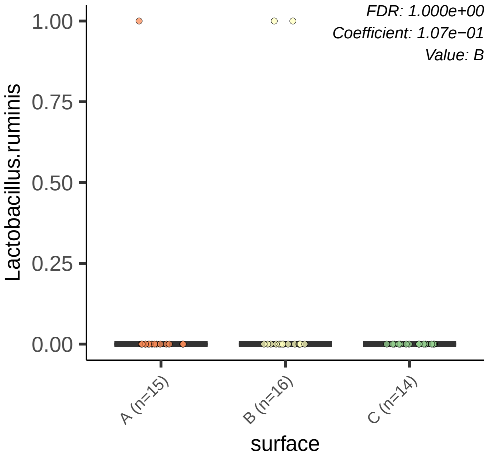
FDR: 1.000e+00
Coefficient: 1.07e−01
Value: B
Lactobacillus.ruminis
1.00
0.75
0.50
0.25
0.00
A (n=15)
B (n=16)
C (n=14)
surface

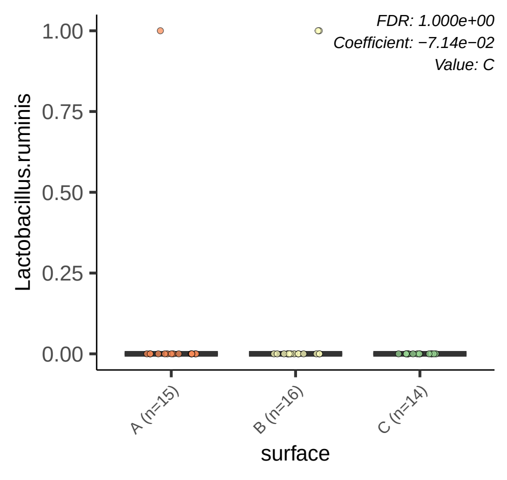
FDR: 1.000e+00
Coefficient: −7.14e−02
Value: C
Lactobacillus.ruminis
1.00
0.75
0.50
0.25
0.00
A (n=15)
B (n=16)
C (n=14)
surface

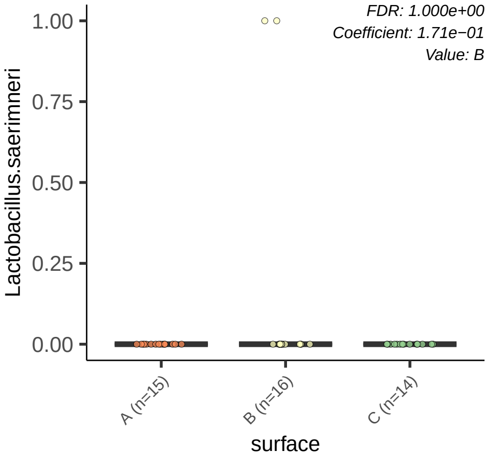
FDR: 1.000e+00
Coefficient: 1.71e−01
Value: B
Lactobacillus.saerimneri
1.00
0.75
0.50
0.25
0.00
A (n=15)
B (n=16)
C (n=14)
surface

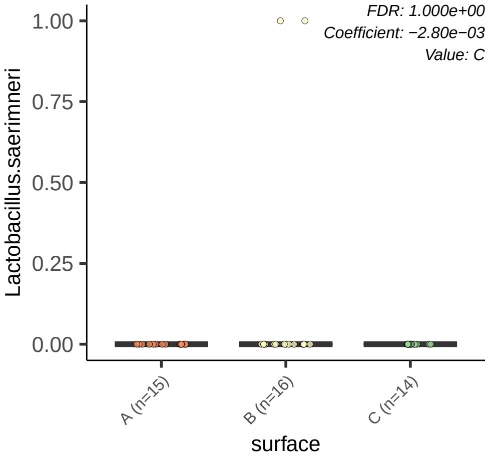
FDR: 1.000e+00
Coefficient: −2.80e−03
Value: C
Lactobacillus.saerimneri
1.00
0.75
0.50
0.25
0.00
A (n=15)
B (n=16)
C (n=14)
surface

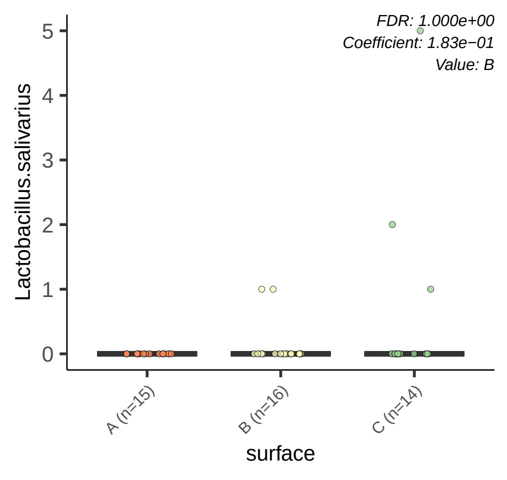
FDR: 1.000e+00
Coefficient: 1.83e−01
Value: B
Lactobacillus.salivarius
5
4
3
2
1
0
A (n=15)
B (n=16)
C (n=14)
surface

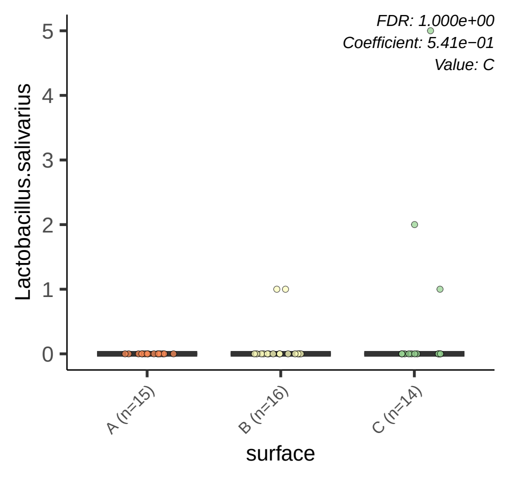
FDR: 1.000e+00
Coefficient: 5.41e−01
Value: C
Lactobacillus.salivarius
0
1
2
3
4
5
A (n=15)
B (n=16)
C (n=14)
surface

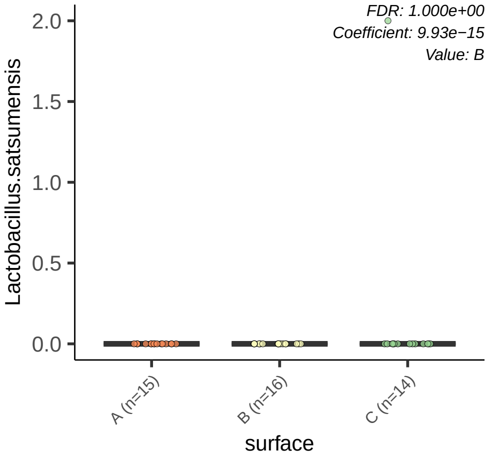

FDR: 1.000e+00
Coefficient: 9.93e−15
Value: B
Lactobacillus.satsumensis
2.0
1.5
1.0
0.5
0.0
A (n=15)
B (n=16)
C (n=14)
surface

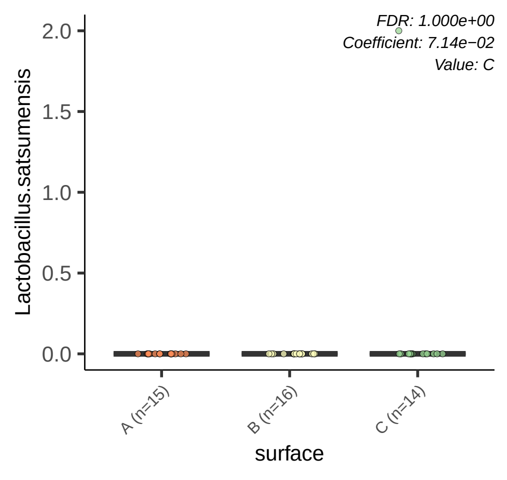
FDR: 1.000e+00
Coefficient: 7.14e−02
Value: C
Lactobacillus.satsumensis
2.0
1.5
1.0
0.5
0.0
A (n=15)
B (n=16)
C (n=14)
surface

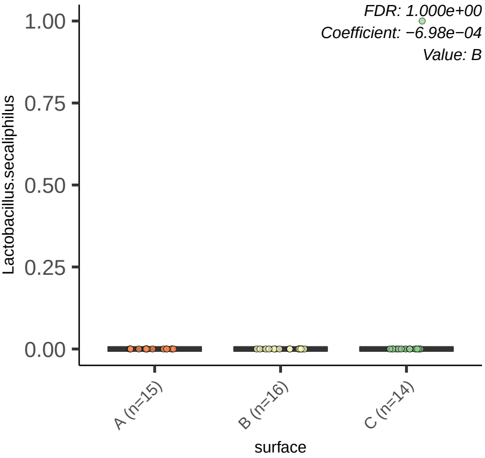
FDR: 1.000e+00
Coefficient: −6.98e−04
Value: B
1.00
0.75
0.50
0.25
0.00
Lactobacillus.secaliphilus
A (n=15)
B (n=16)
C (n=14)
surface

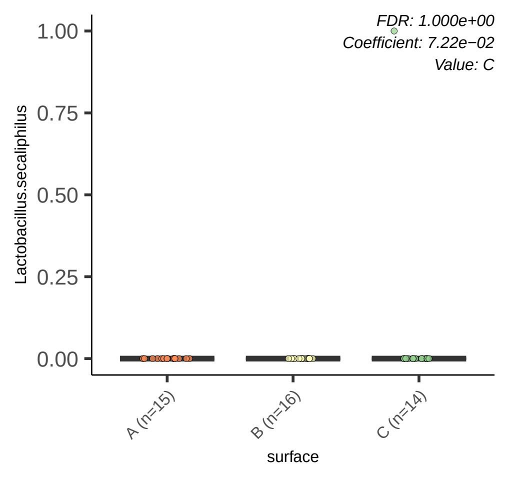
FDR: 1.000e+00
Coefficient: 7.22e−02
Value: C
Lactobacillus.secaliphilus
1.00
0.75
0.50
0.25
0.00
A (n=15)
B (n=16)
C (n=14)
surface

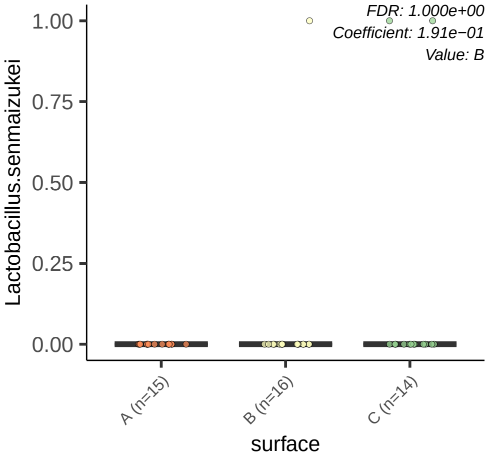

FDR: 1.000e+00
Coefficient: 1.91e−01
Value: B
1.00
0.75
0.50
0.25
0.00
Lactobacillus.senmaizukei
A (n=15)
B (n=16)
C (n=14)
surface

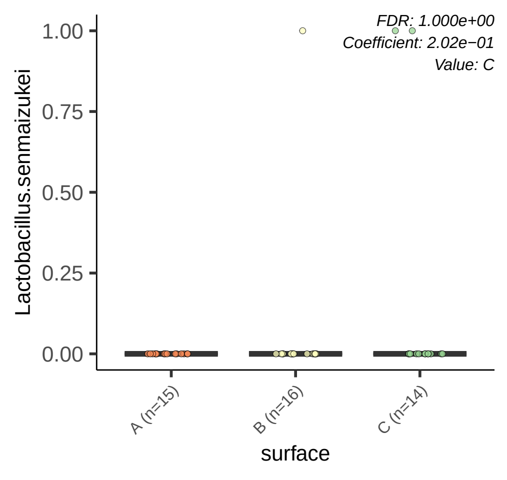
FDR: 1.000e+00
Coefficient: 2.02e−01
Value: C
Lactobacillus.senmaizukei
1.00
0.75
0.50
0.25
0.00
A (n=15)
B (n=16)
C (n=14)
surface

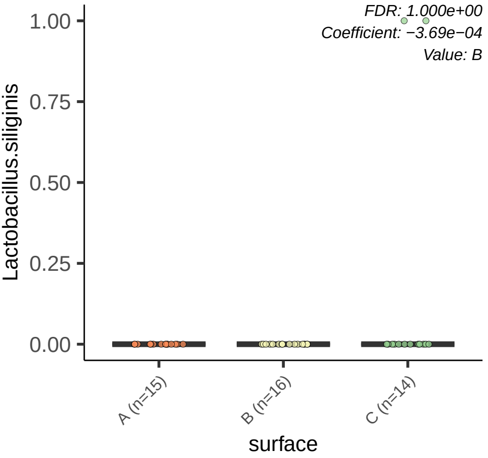

FDR: 1.000e+00
Coefficient: −3.69e−04
Value: B
Lactobacillus.siliginis
1.00
0.75
0.50
0.25
0.00
A (n=15)
B (n=16)
C (n=14)
surface

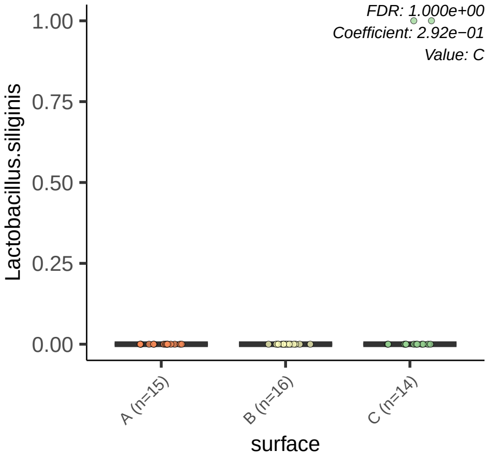

FDR: 1.000e+00
Coefficient: 2.92e−01
Value: C
Lactobacillus.siliginis
1.00
0.75
0.50
0.25
0.00
A (n=15)
B (n=16)
C (n=14)
surface

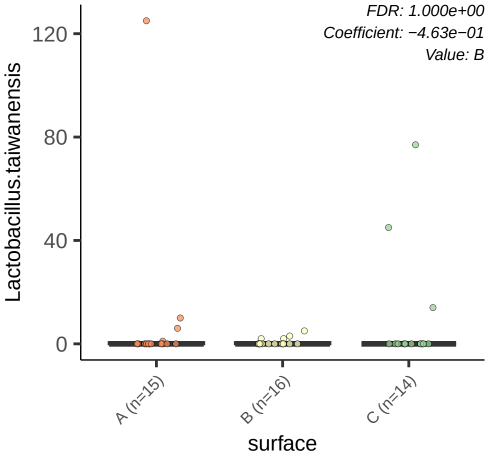

FDR: 1.000e+00
Coefficient: −4.63e−01
Value: B
Lactobacillus.taiwanensis
120
80
40
0
A (n=15)
B (n=16)
C (n=14)
surface

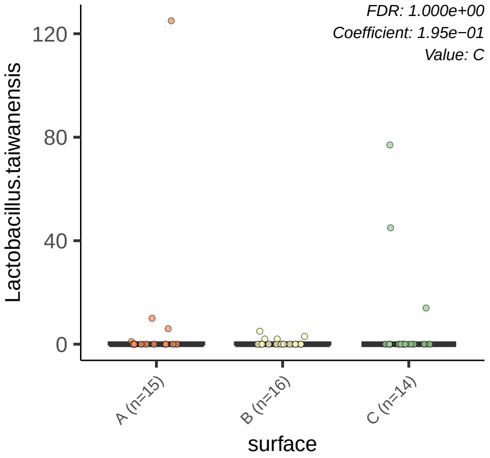

FDR: 1.000e+00
Coefficient: 1.95e−01
Value: C
Lactobacillus.taiwanensis
120
80
40
0
A (n=15)
B (n=16)
C (n=14)
surface

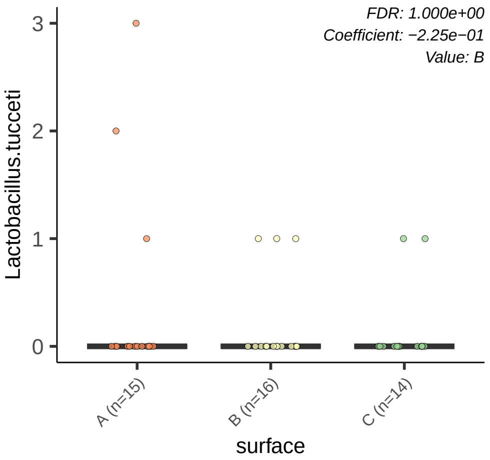
FDR: 1.000e+00
Coefficient: −2.25e−01
Value: B
Lactobacillus.tucceti
3
2
1
0
A (n=15)
B (n=16)
C (n=14)
surface

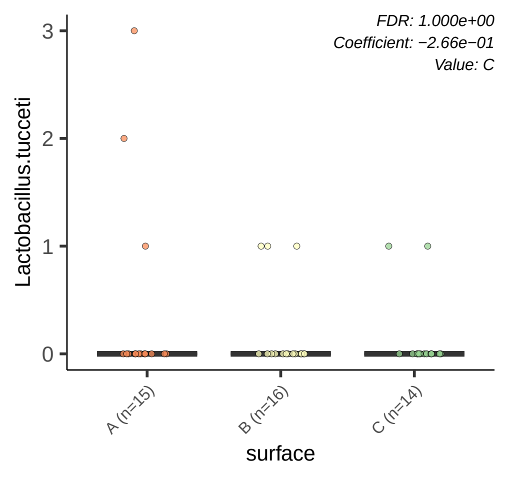

FDR: 1.000e+00
Coefficient: −2.66e−01
Value: C
Lactobacillus.tucceti
0
1
2
3
A (n=15)
B (n=16)
C (n=14)
surface

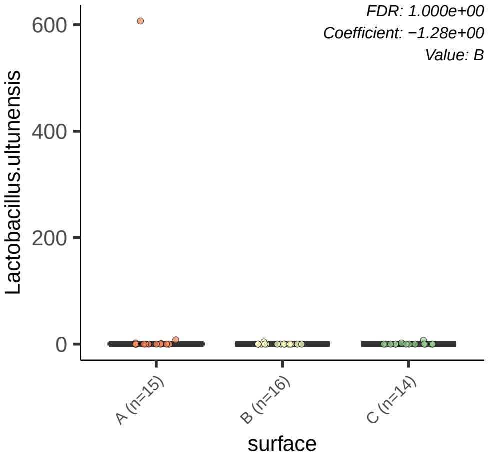
FDR: 1.000e+00
Coefficient: −1.28e+00
Value: B
Lactobacillus.ultunensis
600
400
200
0
A (n=15)
B (n=16)
C (n=14)
surface

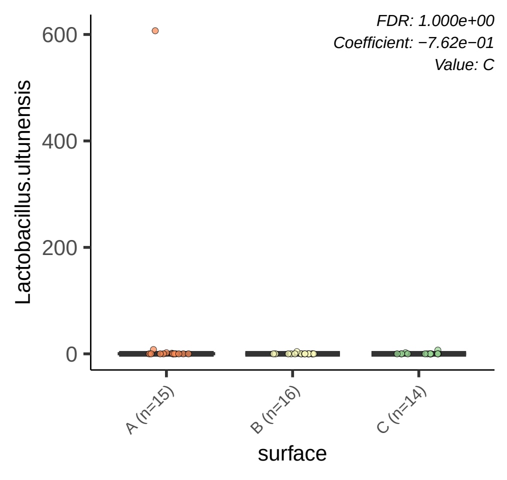
FDR: 1.000e+00
Coefficient: −7.62e−01
Value: C
Lactobacillus.ultunensis
600
400
200
0
A (n=15)
B (n=16)
C (n=14)
surface

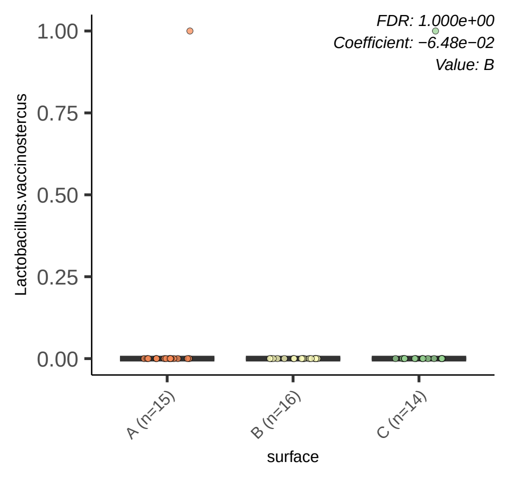

FDR: 1.000e+00
Coefficient: −6.48e−02
Value: B
1.00
0.75
0.50
0.25
0.00
Lactobacillus.vaccinostercus
A (n=15)
B (n=16)
C (n=14)
surface

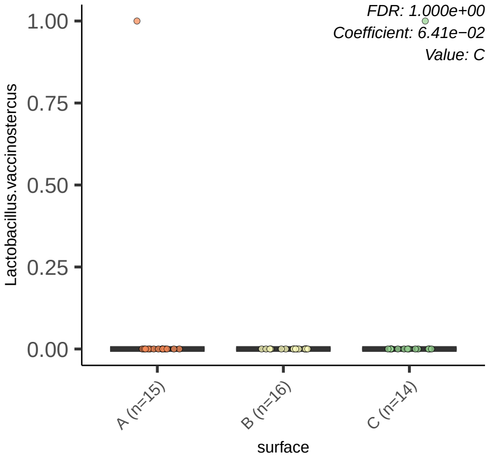
FDR: 1.000e+00
Coefficient: 6.41e−02
Value: C
Lactobacillus.vaccinostercus
1.00
0.75
0.50
0.25
0.00
A (n=15)
B (n=16)
C (n=14)
surface

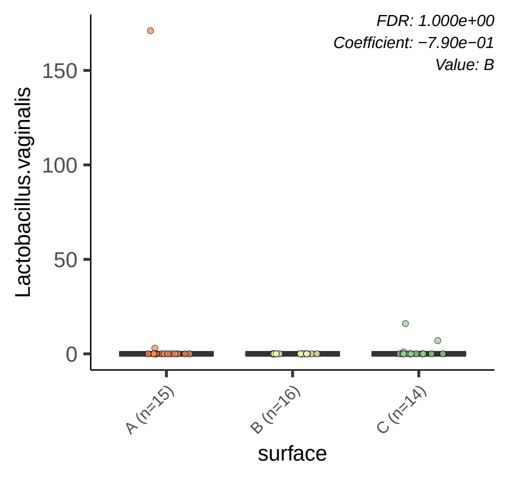
FDR: 1.000e+00
Coefficient: −7.90e−01
Value: B
Lactobacillus.vaginalis
150
100
50
0
A (n=15)
B (n=16)
C (n=14)
surface

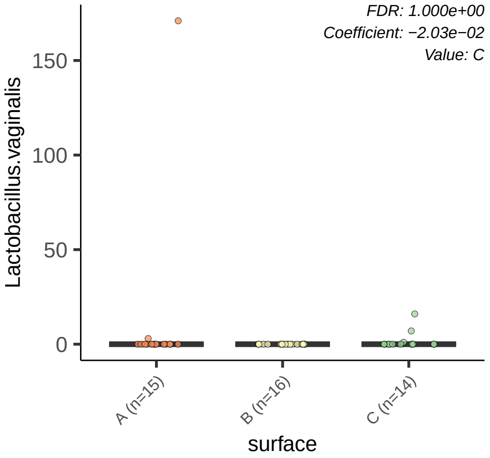
FDR: 1.000e+00
Coefficient: −2.03e−02
Value: C
Lactobacillus.vaginalis
150
100
50
0
A (n=15)
B (n=16)
C (n=14)
surface

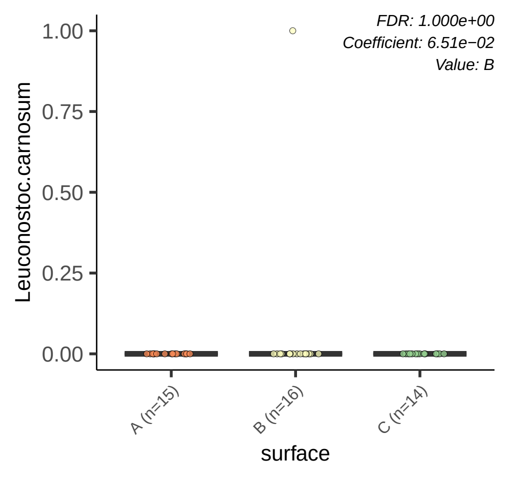

FDR: 1.000e+00
Coefficient: 6.51e−02
Value: B
Leuconostoc.carnosum
1.00
0.75
0.50
0.25
0.00
A (n=15)
B (n=16)
C (n=14)
surface

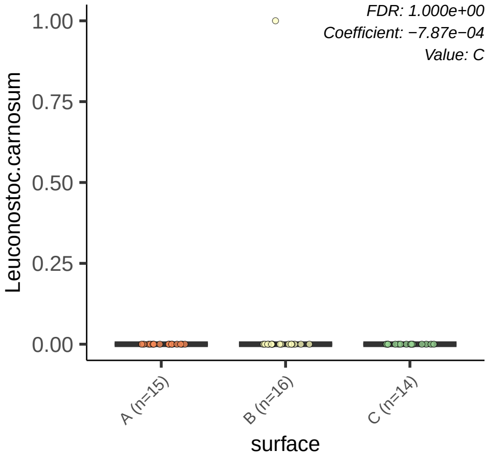
FDR: 1.000e+00
Coefficient: −7.87e−04
Value: C
Leuconostoc.carnosum
1.00
0.75
0.50
0.25
0.00
A (n=15)
B (n=16)
C (n=14)
surface

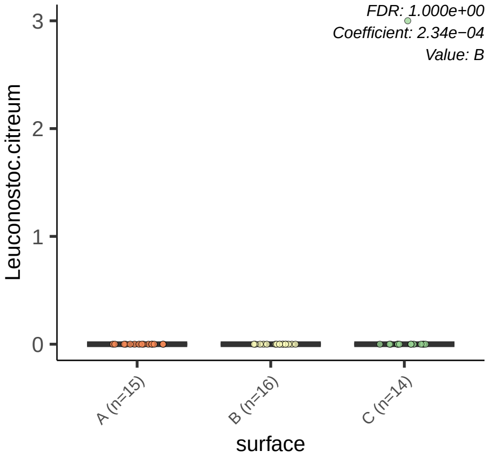

FDR: 1.000e+00
Coefficient: 2.34e−04
Value: B
Leuconostoc.citreum
3
2
1
0
A (n=15)
B (n=16)
C (n=14)
surface

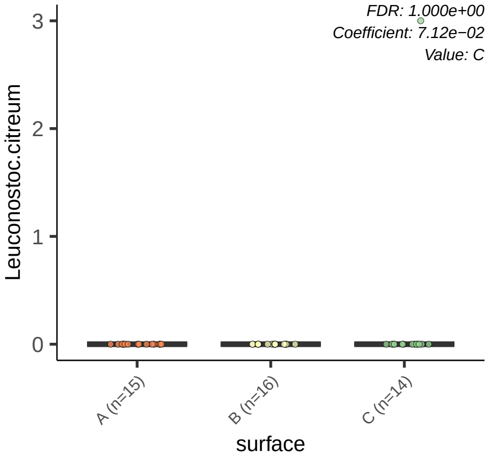
FDR: 1.000e+00
Coefficient: 7.12e−02
Value: C
Leuconostoc.citreum
3
2
1
0
A (n=15)
B (n=16)
C (n=14)
surface

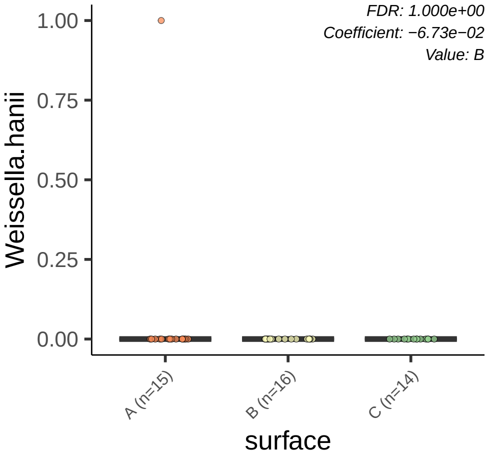

FDR: 1.000e+00
Coefficient: −6.73e−02
Value: B
1.00
0.75
0.50
0.25
0.00
Weissella.hanii
A (n=15)
B (n=16)
C (n=14)
surface

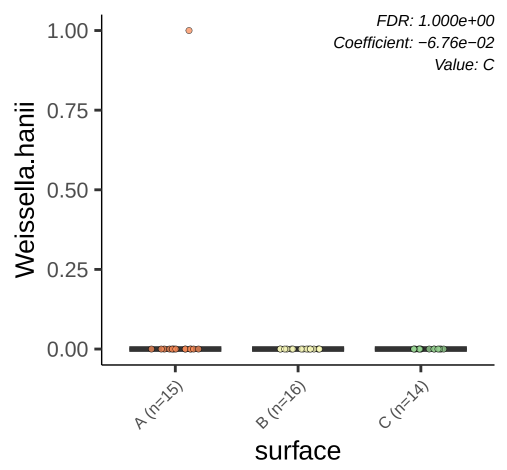

FDR: 1.000e+00
Coefficient: −6.76e−02
Value: C
Weissella.hanii
1.00
0.75
0.50
0.25
0.00
A (n=15)
B (n=16)
C (n=14)
surface

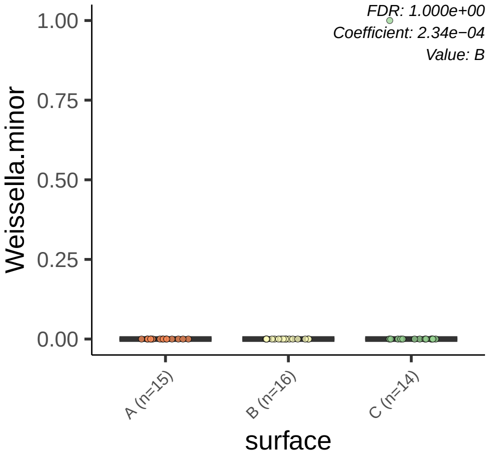

FDR: 1.000e+00
Coefficient: 2.34e−04
Value: B
Weissella.minor
1.00
0.75
0.50
0.25
0.00
A (n=15)
B (n=16)
C (n=14)
surface

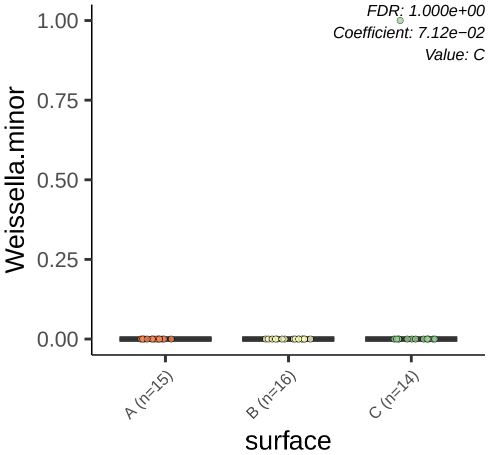
FDR: 1.000e+00
Coefficient: 7.12e−02
Value: C
Weissella.minor
1.00
0.75
0.50
0.25
0.00
A (n=15)
B (n=16)
C (n=14)
surface

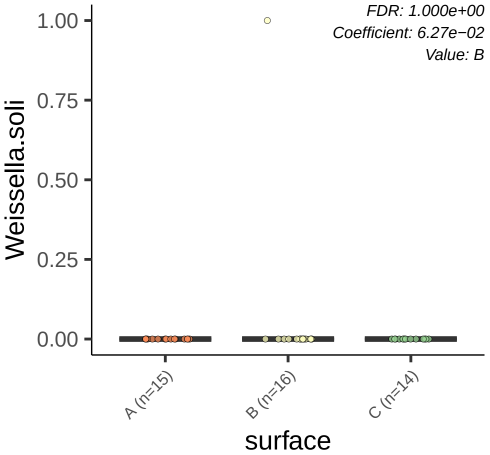
FDR: 1.000e+00
Coefficient: 6.27e−02
Value: B
Weissella.soli
1.00
0.75
0.50
0.25
0.00
A (n=15)
B (n=16)
C (n=14)
surface

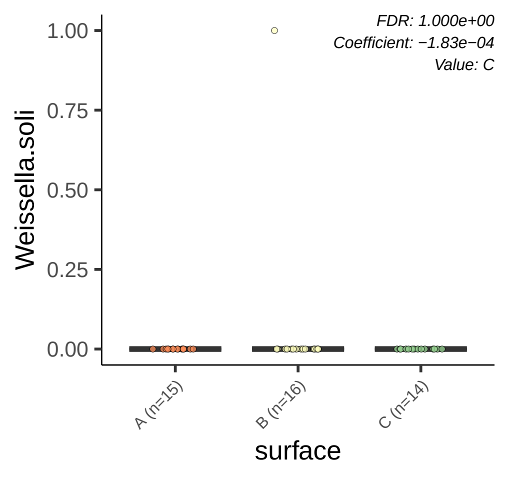
FDR: 1.000e+00
Coefficient: −1.83e−04
Value: C
1.00
0.75
0.50
0.25
0.00
Weissella.soli
A (n=15)
B (n=16)
C (n=14)
surface

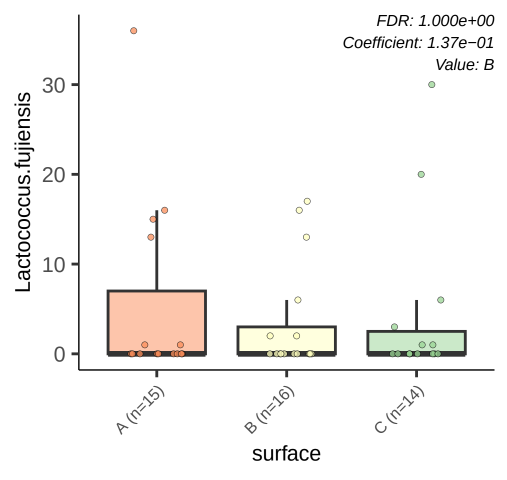
FDR: 1.000e+00
Coefficient: 1.37e−01
Value: B
Lactococcus.fujiensis
0
10
20
30
A (n=15)
B (n=16)
C (n=14)
surface

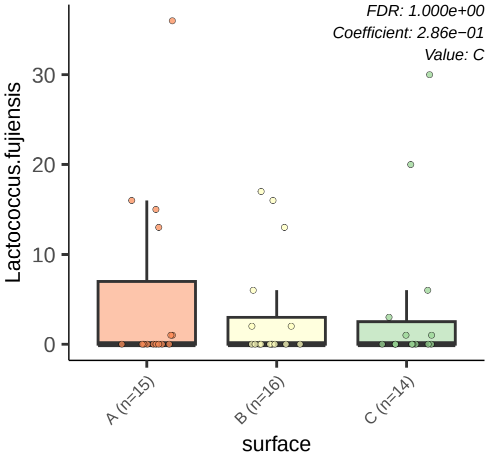
FDR: 1.000e+00
Coefficient: 2.86e−01
Value: C
Lactococcus.fujiensis
30
20
10
0
A (n=15)
B (n=16)
C (n=14)
surface

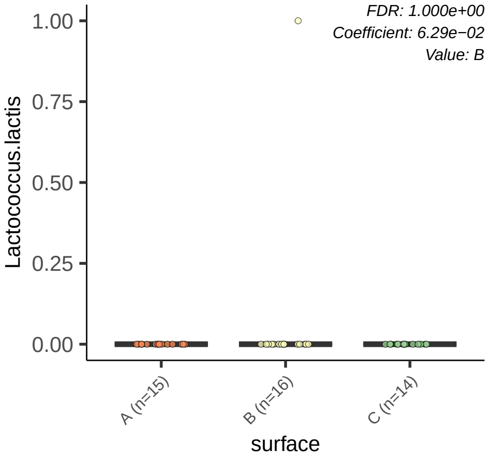

FDR: 1.000e+00
Coefficient: 6.29e−02
Value: B
Lactococcus.lactis
1.00
0.75
0.50
0.25
0.00
A (n=15)
B (n=16)
C (n=14)
surface

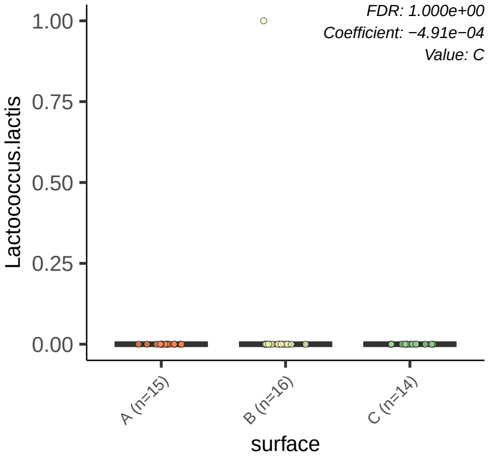

FDR: 1.000e+00
Coefficient: −4.91e−04
Value: C
1.00
0.75
0.50
0.25
0.00
Lactococcus.lactis
A (n=15)
B (n=16)
C (n=14)
surface

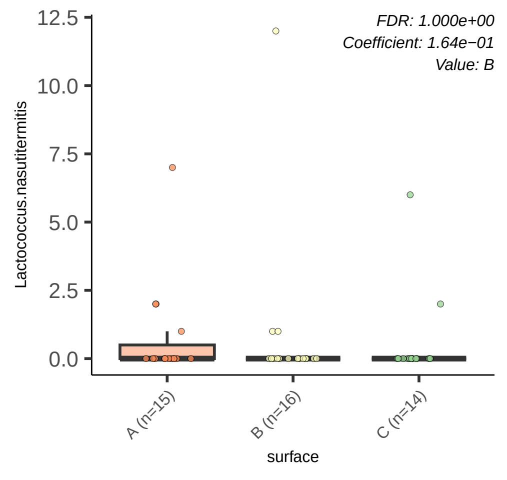
FDR: 1.000e+00
Coefficient: 1.64e−01
Value: B
12.5
10.0
7.5
5.0
2.5
0.0
Lactococcus.nasutitermitis
A (n=15)
B (n=16)
C (n=14)
surface

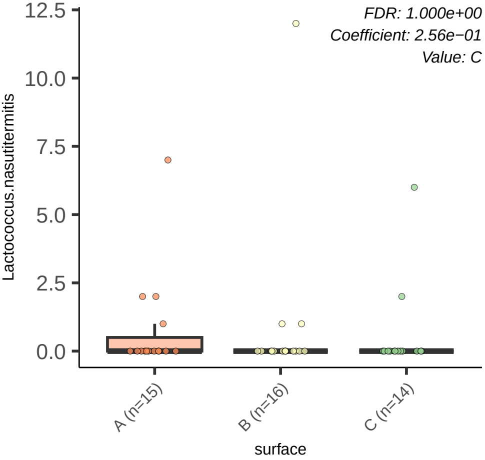

FDR: 1.000e+00
Coefficient: 2.56e−01
Value: C
Lactococcus.nasutitermitis
12.5
10.0
7.5
5.0
2.5
0.0
A (n=15)
B (n=16)
C (n=14)
surface

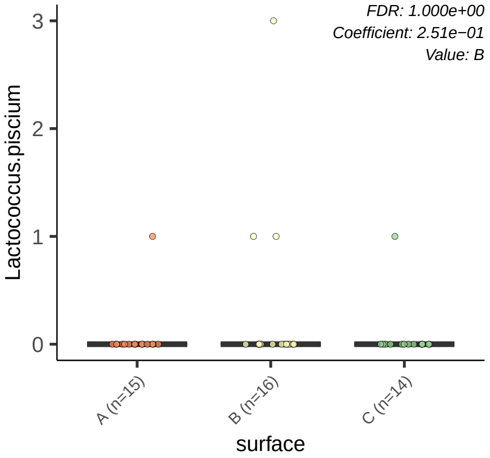
FDR: 1.000e+00
Coefficient: 2.51e−01
Value: B
Lactococcus.piscium
3
2
1
0
A (n=15)
B (n=16)
C (n=14)
surface

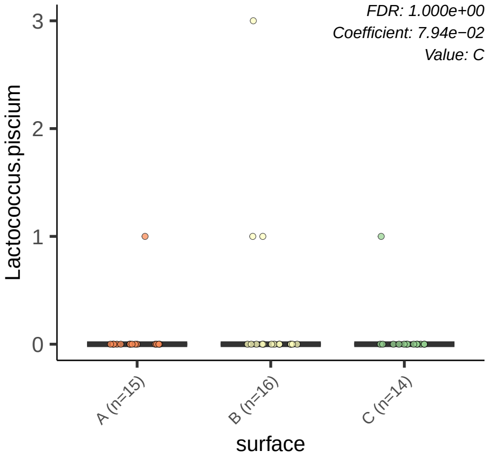
FDR: 1.000e+00
Coefficient: 7.94e−02
Value: C
Lactococcus.piscium
0
1
2
3
A (n=15)
B (n=16)
C (n=14)
surface

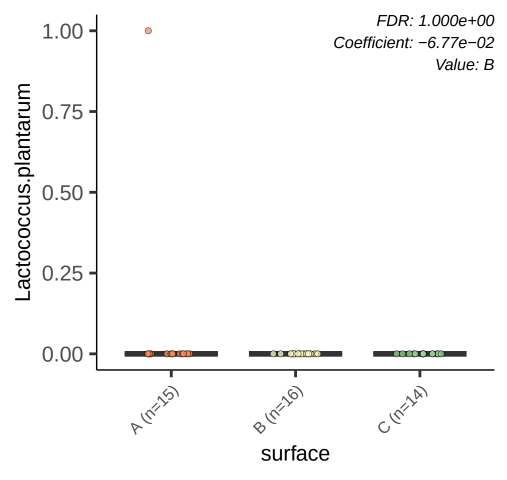

FDR: 1.000e+00
Coefficient: −6.77e−02
Value: B
1.00
0.75
0.50
0.25
0.00
Lactococcus.plantarum
A (n=15)
B (n=16)
C (n=14)
surface

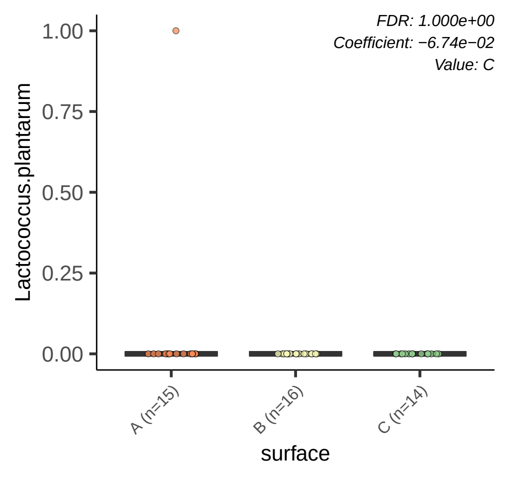
FDR: 1.000e+00
Coefficient: −6.74e−02
Value: C
Lactococcus.plantarum
1.00
0.75
0.50
0.25
0.00
A (n=15)
B (n=16)
C (n=14)
surface

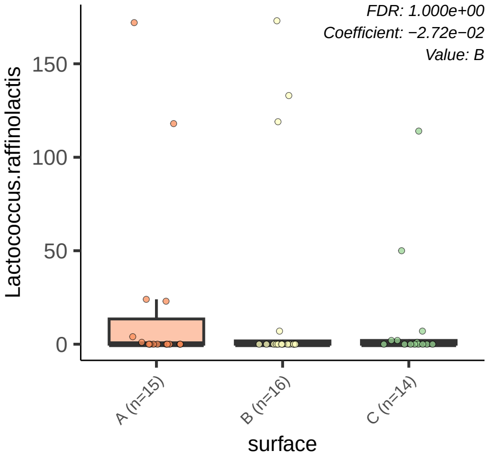

FDR: 1.000e+00
Coefficient: −2.72e−02
Value: B
Lactococcus.raffinolactis
150
100
50
0
A (n=15)
B (n=16)
C (n=14)
surface

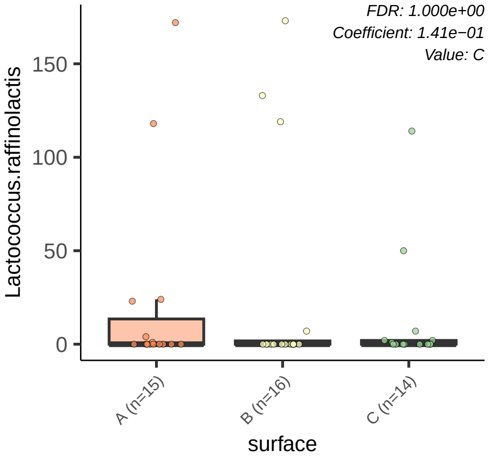
FDR: 1.000e+00
Coefficient: 1.41e−01
Value: C
Lactococcus.raffinolactis
150
100
50
0
A (n=15)
B (n=16)
C (n=14)
surface

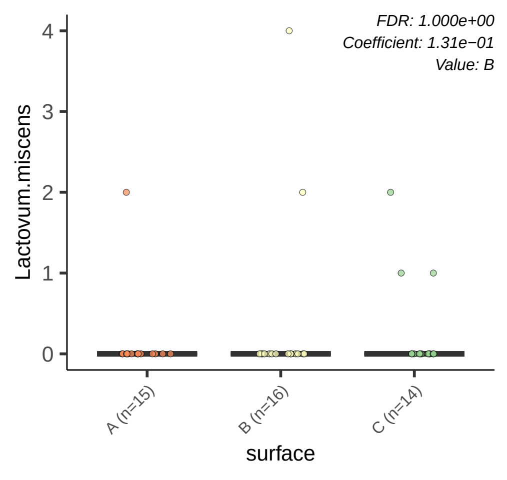

FDR: 1.000e+00
Coefficient: 1.31e−01
Value: B
Lactovum.miscens
4
3
2
1
0
A (n=15)
B (n=16)
C (n=14)
surface

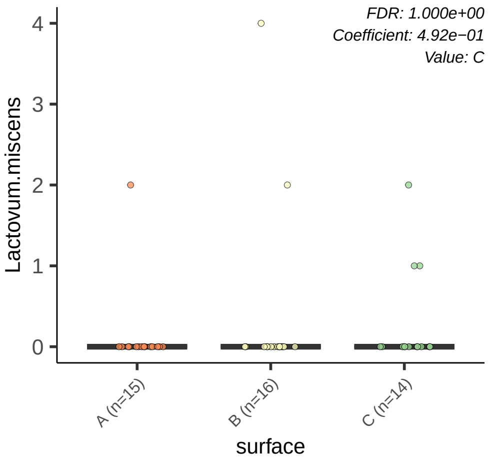

FDR: 1.000e+00
Coefficient: 4.92e−01
Value: C
Lactovum.miscens
0
1
2
3
4
A (n=15)
B (n=16)
C (n=14)
surface

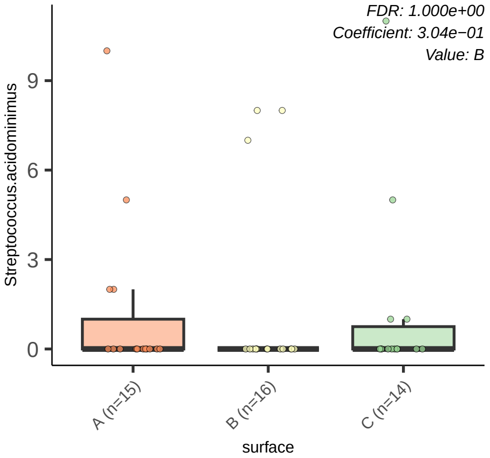

FDR: 1.000e+00
Coefficient: 3.04e−01
Value: B
Streptococcus.acidominimus
0
3
6
9
A (n=15)
B (n=16)
C (n=14)
surface

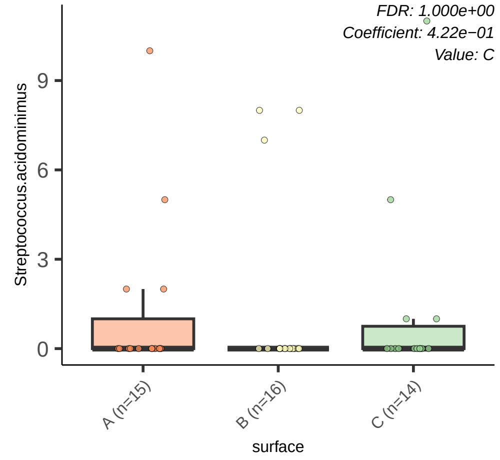
FDR: 1.000e+00
Coefficient: 4.22e−01
Value: C
Streptococcus.acidominimus
0
3
6
9
A (n=15)
B (n=16)
C (n=14)
surface

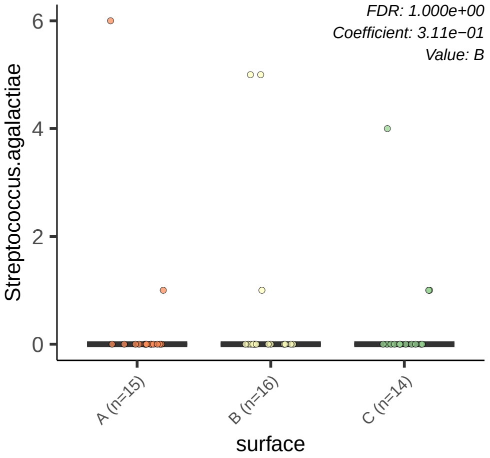

FDR: 1.000e+00
Coefficient: 3.11e−01
Value: B
Streptococcus.agalactiae
0
2
4
6
A (n=15)
B (n=16)
C (n=14)
surface

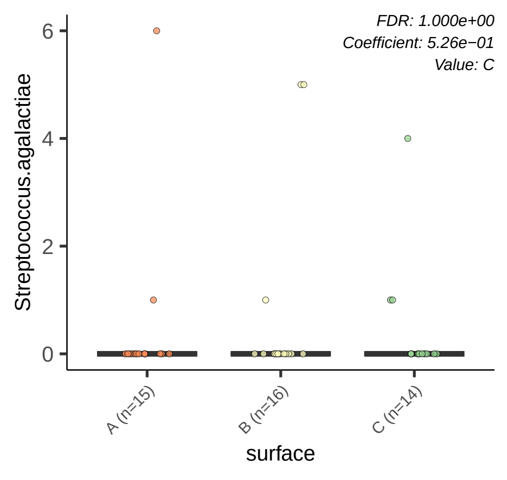
FDR: 1.000e+00
Coefficient: 5.26e−01
Value: C
Streptococcus.agalactiae
6
4
2
0
A (n=15)
B (n=16)
C (n=14)
surface

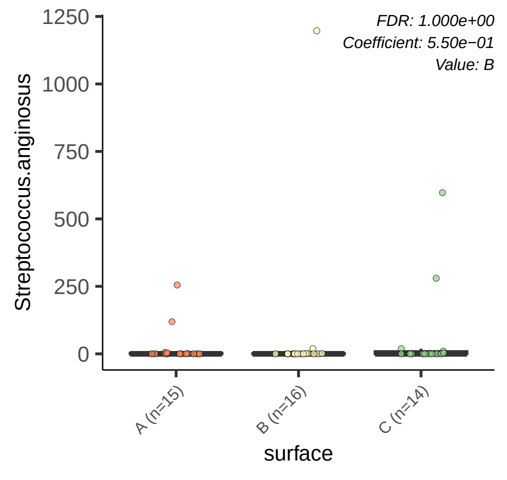

FDR: 1.000e+00
Coefficient: 5.50e−01
Value: B
Streptococcus.anginosus
1250
1000
750
500
250
0
A (n=15)
B (n=16)
C (n=14)
surface

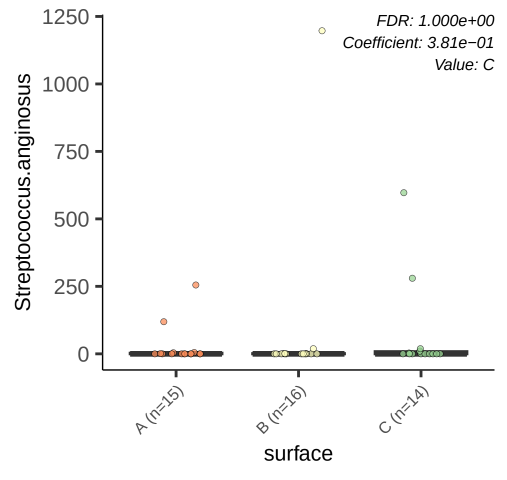
FDR: 1.000e+00
Coefficient: 3.81e−01
Value: C
Streptococcus.anginosus
1250
1000
750
500
250
0
A (n=15)
B (n=16)
C (n=14)
surface

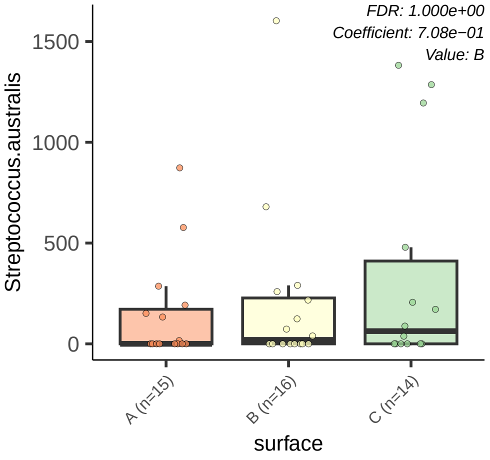

FDR: 1.000e+00
Coefficient: 7.08e−01
Value: B
Streptococcus.australis
1500
1000
500
0
A (n=15)
B (n=16)
C (n=14)
surface

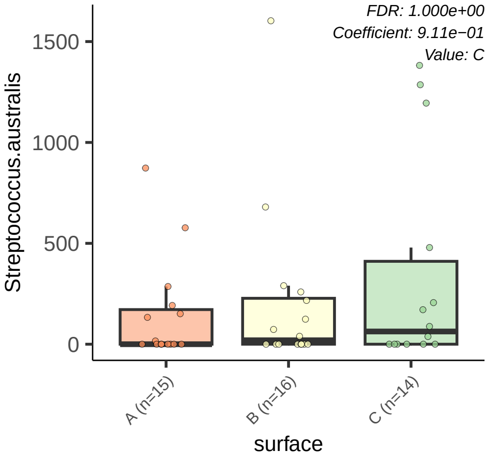
FDR: 1.000e+00
Coefficient: 9.11e−01
Value: C
Streptococcus.australis
1500
1000
500
0
A (n=15)
B (n=16)
C (n=14)
surface

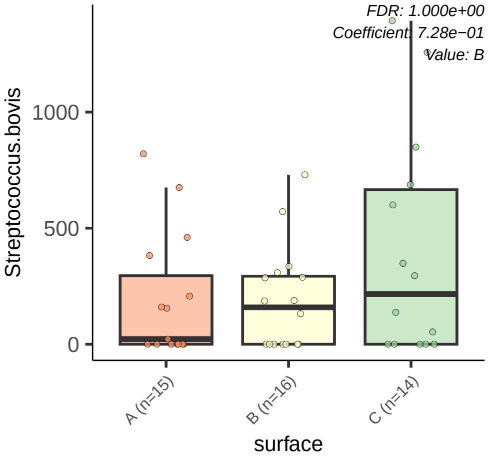

FDR: 1.000e+00
Coefficient: 7.28e−01
Value: B
Streptococcus.bovis
1000
500
0
A (n=15)
B (n=16)
C (n=14)
surface

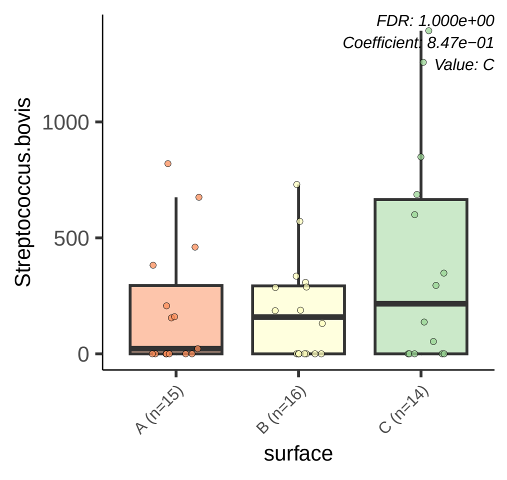

FDR: 1.000e+00
Coefficient: 8.47e−01
Value: C
Streptococcus.bovis
1000
500
0
A (n=15)
B (n=16)
C (n=14)
surface

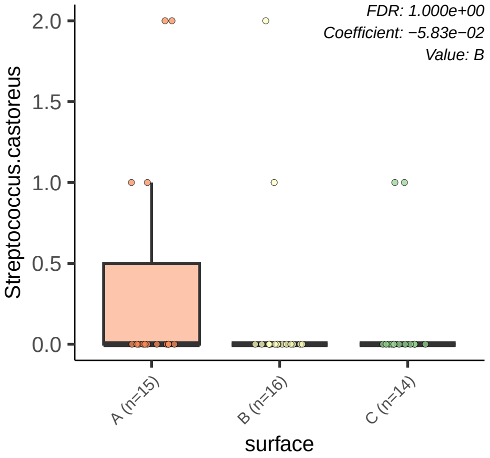
FDR: 1.000e+00
Coefficient: −5.83e−02
Value: B
Streptococcus.castoreus
2.0
1.5
1.0
0.5
0.0
A (n=15)
B (n=16)
C (n=14)
surface

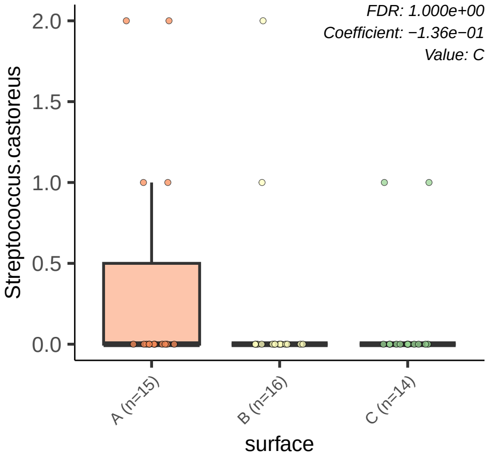

FDR: 1.000e+00
Coefficient: −1.36e−01
Value: C
Streptococcus.castoreus
2.0
1.5
1.0
0.5
0.0
A (n=15)
B (n=16)
C (n=14)
surface

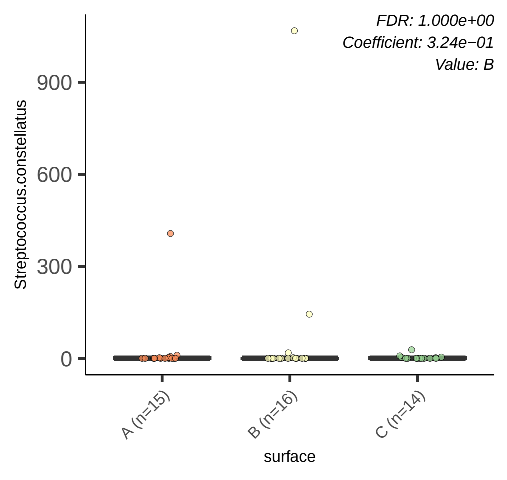

FDR: 1.000e+00
Coefficient: 3.24e−01
Value: B
900
600
300
0
Streptococcus.constellatus
A (n=15)
B (n=16)
C (n=14)
surface

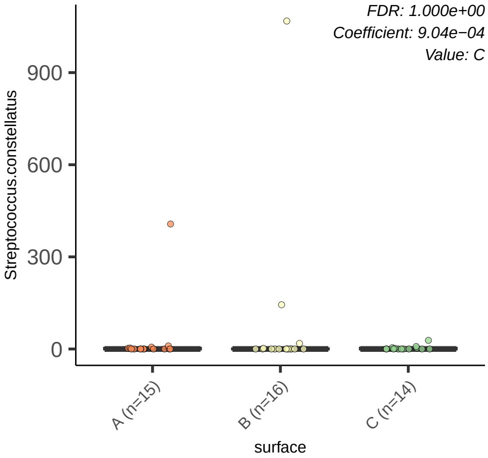
FDR: 1.000e+00
Coefficient: 9.04e−04
Value: C
Streptococcus.constellatus
900
600
300
0
A (n=15)
B (n=16)
C (n=14)
surface

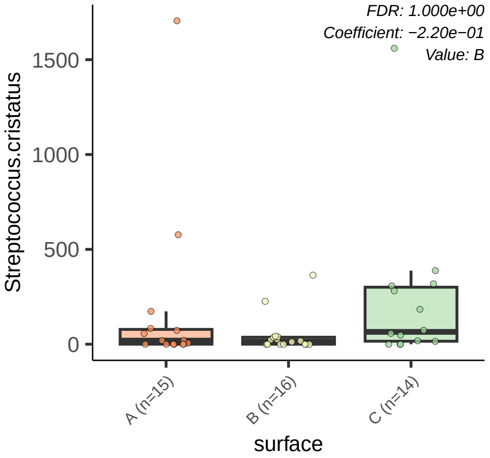

FDR: 1.000e+00
Coefficient: −2.20e−01
Value: B
Streptococcus.cristatus
1500
1000
500
0
A (n=15)
B (n=16)
C (n=14)
surface

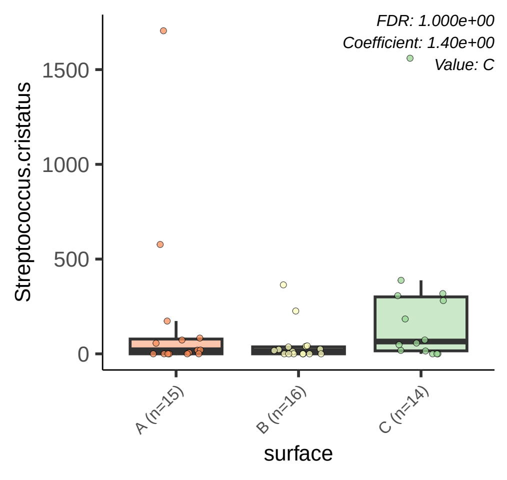

FDR: 1.000e+00
Coefficient: 1.40e+00
Value: C
Streptococcus.cristatus
1500
1000
500
0
A (n=15)
B (n=16)
C (n=14)
surface

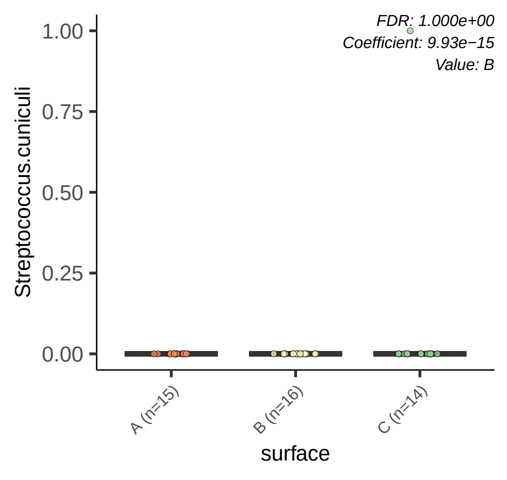

FDR: 1.000e+00
Coefficient: 9.93e−15
Value: B
Streptococcus.cuniculi
1.00
0.75
0.50
0.25
0.00
A (n=15)
B (n=16)
C (n=14)
surface

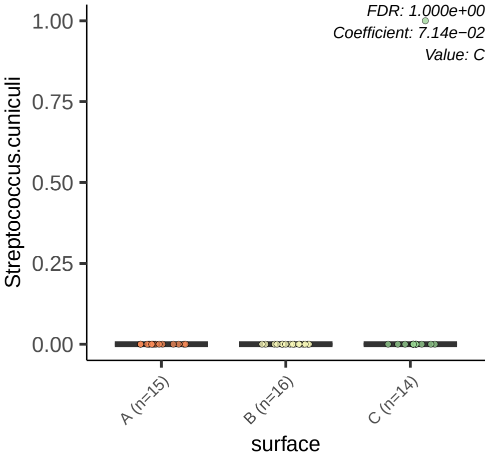

FDR: 1.000e+00
Coefficient: 7.14e−02
Value: C
Streptococcus.cuniculi
1.00
0.75
0.50
0.25
0.00
A (n=15)
B (n=16)
C (n=14)
surface

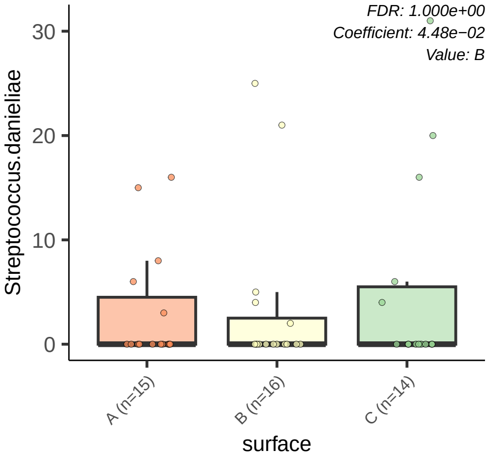
FDR: 1.000e+00
Coefficient: 4.48e−02
Value: B
Streptococcus.danieliae
30
20
10
0
A (n=15)
B (n=16)
C (n=14)
surface

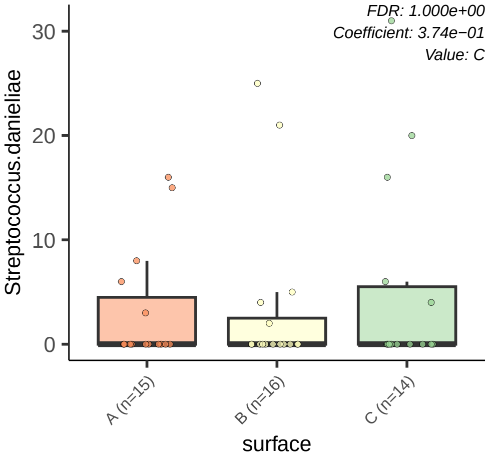

FDR: 1.000e+00
Coefficient: 3.74e−01
Value: C
Streptococcus.danieliae
30
20
10
0
A (n=15)
B (n=16)
C (n=14)
surface

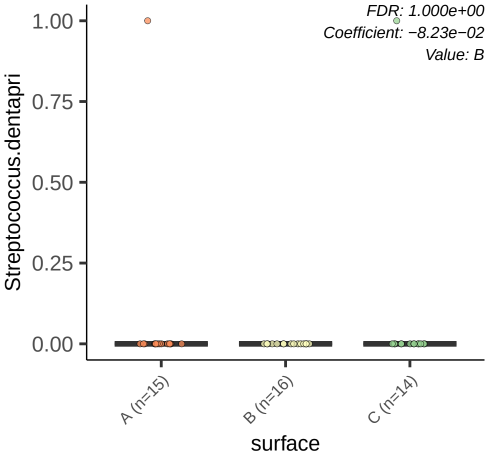

FDR: 1.000e+00
Coefficient: −8.23e−02
Value: B
Streptococcus.dentapri
1.00
0.75
0.50
0.25
0.00
A (n=15)
B (n=16)
C (n=14)
surface

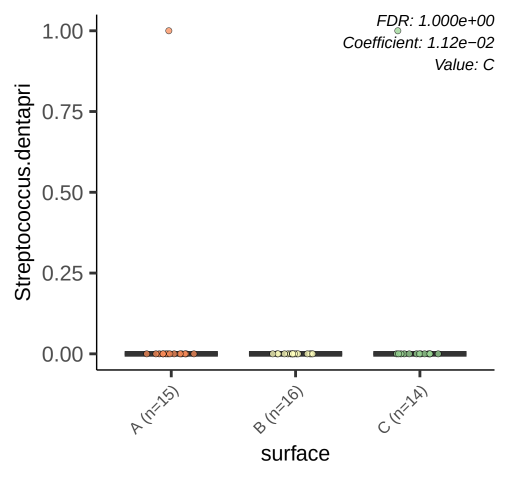
FDR: 1.000e+00
Coefficient: 1.12e−02
Value: C
Streptococcus.dentapri
1.00
0.75
0.50
0.25
0.00
A (n=15)
B (n=16)
C (n=14)
surface

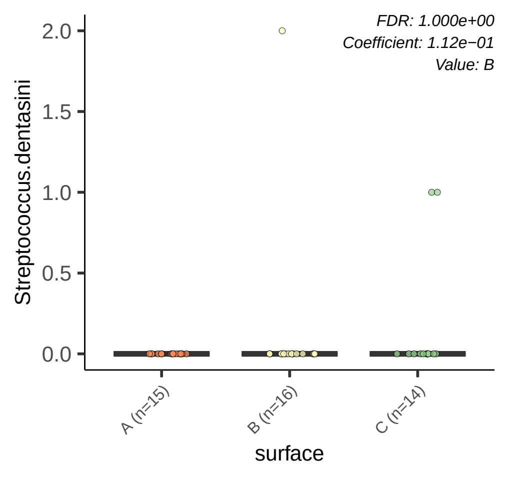
FDR: 1.000e+00
Coefficient: 1.12e−01
Value: B
Streptococcus.dentasini
2.0
1.5
1.0
0.5
0.0
A (n=15)
B (n=16)
C (n=14)
surface

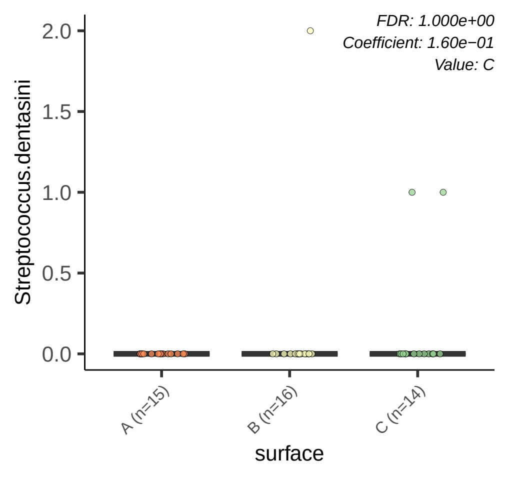
FDR: 1.000e+00
Coefficient: 1.60e−01
Value: C
Streptococcus.dentasini
2.0
1.5
1.0
0.5
0.0
A (n=15)
B (n=16)
C (n=14)
surface

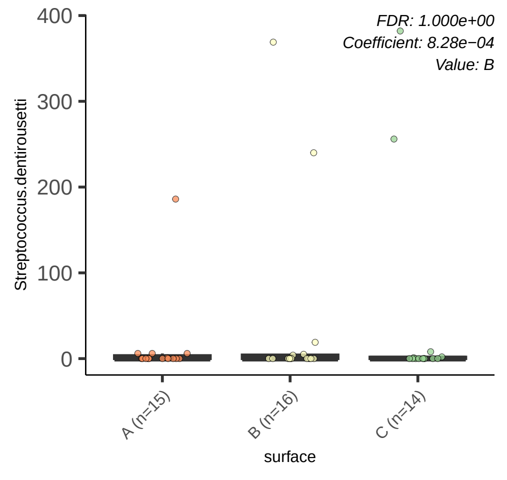
FDR: 1.000e+00
Coefficient: 8.28e−04
Value: B
400
300
200
100
0
Streptococcus.dentirousetti
A (n=15)
B (n=16)
C (n=14)
surface

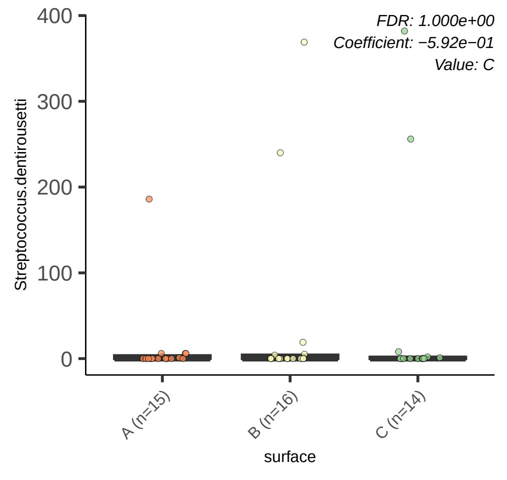
FDR: 1.000e+00
Coefficient: −5.92e−01
Value: C
Streptococcus.dentirousetti
400
300
200
100
0
A (n=15)
B (n=16)
C (n=14)
surface

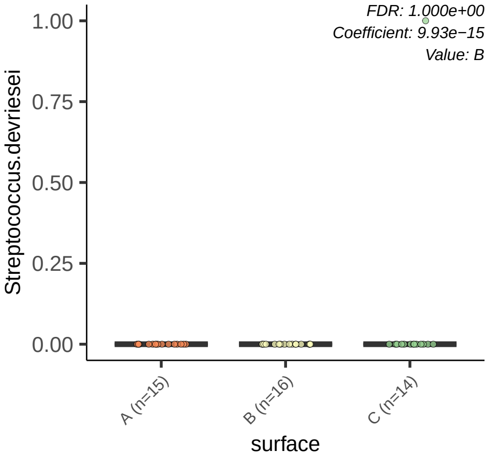
FDR: 1.000e+00
Coefficient: 9.93e−15
Value: B
Streptococcus.devriesei
1.00
0.75
0.50
0.25
0.00
A (n=15)
B (n=16)
C (n=14)
surface

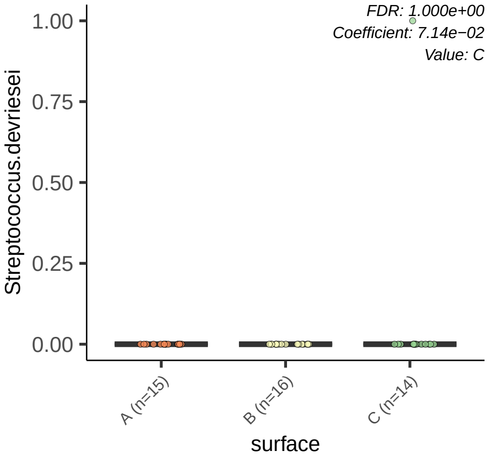
FDR: 1.000e+00
Coefficient: 7.14e−02
Value: C
Streptococcus.devriesei
1.00
0.75
0.50
0.25
0.00
A (n=15)
B (n=16)
C (n=14)
surface

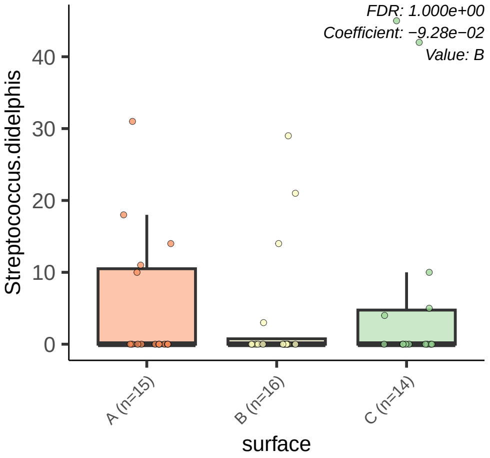
FDR: 1.000e+00
Coefficient: −9.28e−02
Value: B
Streptococcus.didelphis
0
10
20
30
40
A (n=15)
B (n=16)
C (n=14)
surface

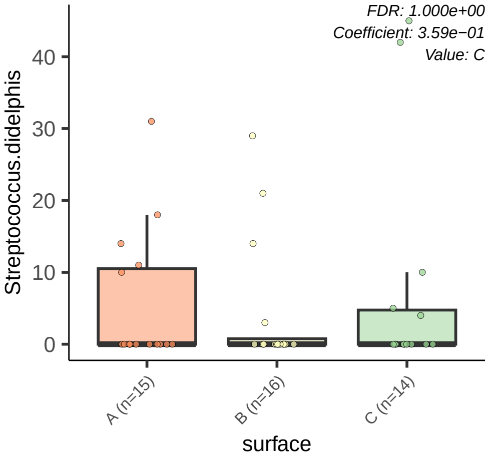
FDR: 1.000e+00
Coefficient: 3.59e−01
Value: C
Streptococcus.didelphis
0
10
20
30
40
A (n=15)
B (n=16)
C (n=14)
surface

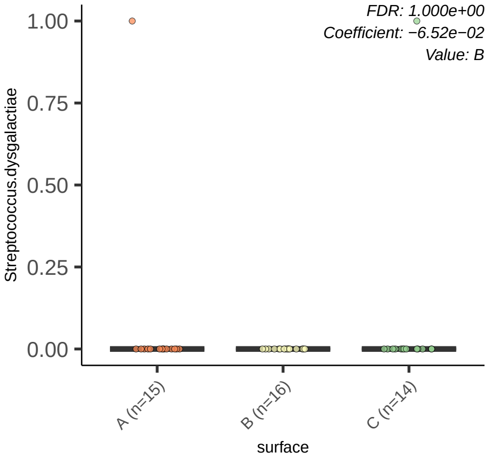
FDR: 1.000e+00
Coefficient: −6.52e−02
Value: B
Streptococcus.dysgalactiae
1.00
0.75
0.50
0.25
0.00
A (n=15)
B (n=16)
C (n=14)
surface

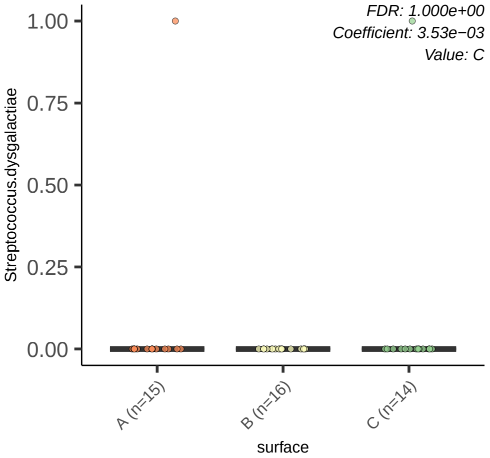
FDR: 1.000e+00
Coefficient: 3.53e−03
Value: C
Streptococcus.dysgalactiae
1.00
0.75
0.50
0.25
0.00
A (n=15)
B (n=16)
C (n=14)
surface

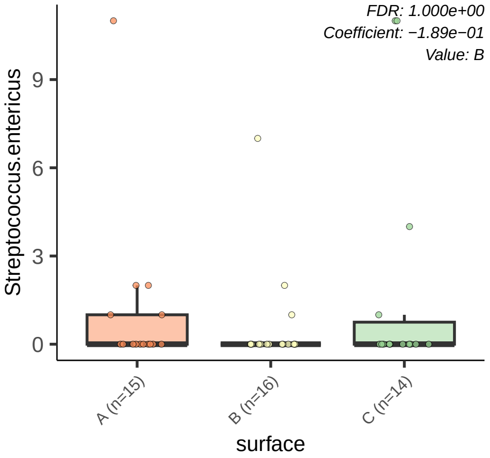
FDR: 1.000e+00
Coefficient: −1.89e−01
Value: B
Streptococcus.enterics
0
3
6
9
A (n=15)
B (n=16)
C (n=14)
surface

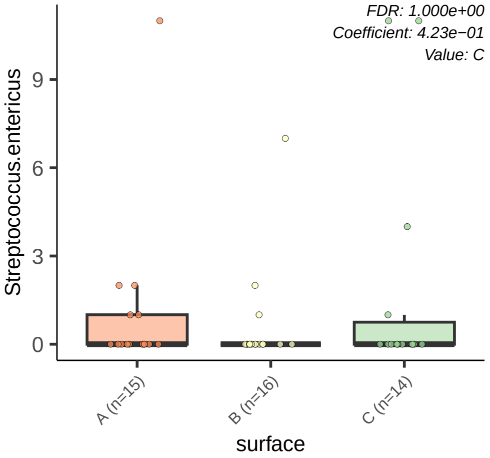
FDR: 1.000e+00
Coefficient: 4.23e−01
Value: C
Streptococcus.enterica
0
3
6
9
A (n=15)
B (n=16)
C (n=14)
surface

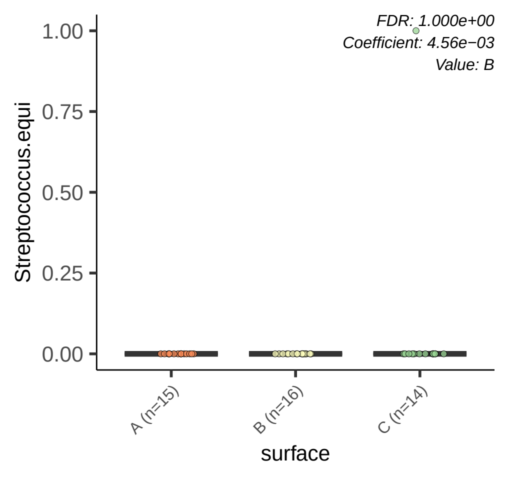
FDR: 1.000e+00
Coefficient: 4.56e−03
Value: B
Streptococcus.equi
1.00
0.75
0.50
0.25
0.00
A (n=15)
B (n=16)
C (n=14)
surface

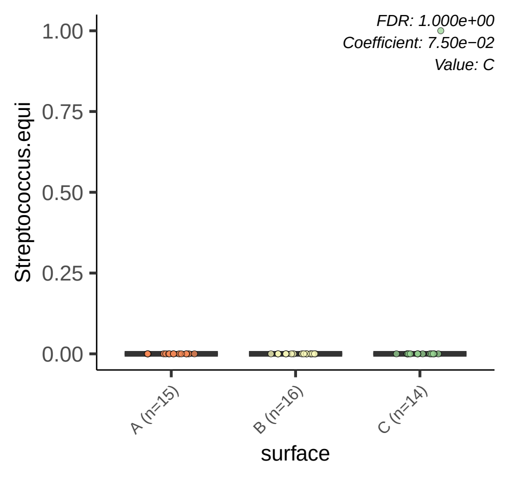
FDR: 1.000e+00
Coefficient: 7.50e−02
Value: C
Streptococcus.equi
1.00
0.75
0.50
0.25
0.00
A (n=15)
B (n=16)
C (n=14)
surface

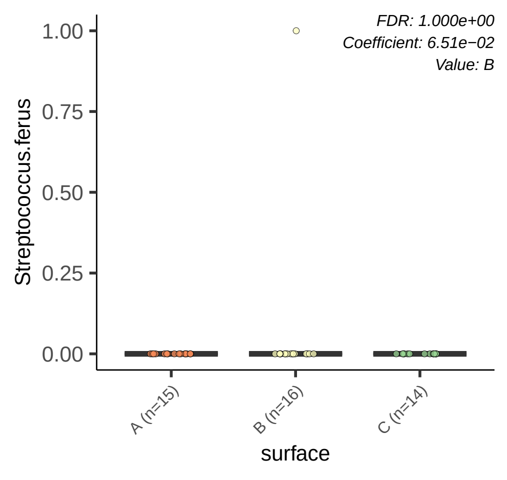
FDR: 1.000e+00
Coefficient: 6.51e−02
Value: B
Streptococcus.ferus
1.00
0.75
0.50
0.25
0.00
A (n=15)
B (n=16)
C (n=14)
surface

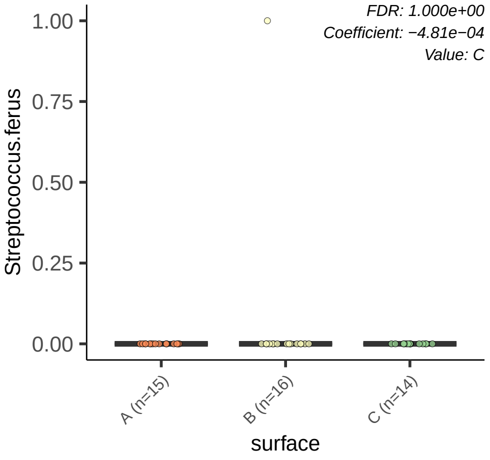
FDR: 1.000e+00
Coefficient: −4.81e−04
Value: C
Streptococcus.ferus
1.00
0.75
0.50
0.25
0.00
A (n=15)
B (n=16)
C (n=14)
surface

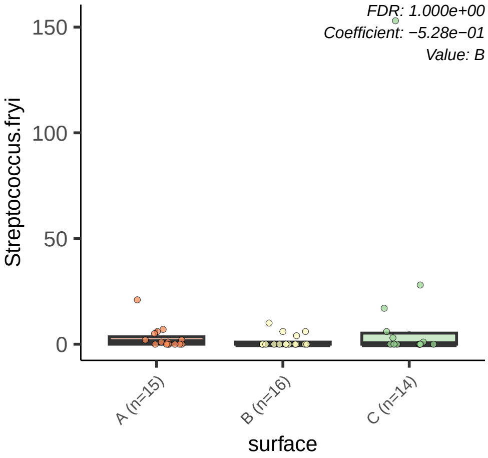

FDR: 1.000e+00
Coefficient: −5.28e−01
Value: B
Streptococcus.fryi
150
100
50
0
A (n=15)
B (n=16)
C (n=14)
surface

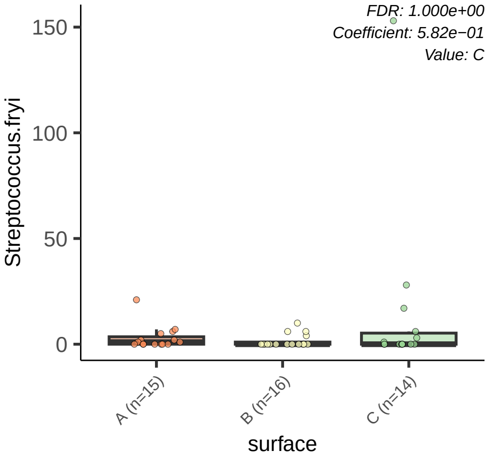

FDR: 1.000e+00
Coefficient: 5.82e−01
Value: C
Streptococcus.fryi
150
100
50
0
A (n=15)
B (n=16)
C (n=14)
surface

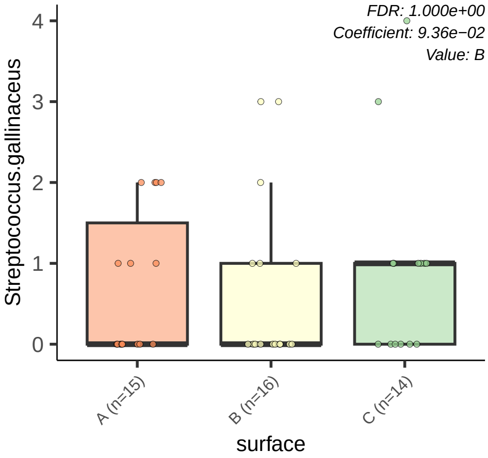
FDR: 1.000e+00
Coefficient: 9.36e−02
Value: B
Streptococcus.gallinaceus
0
1
2
3
4
A (n=15)
B (n=16)
C (n=14)
surface

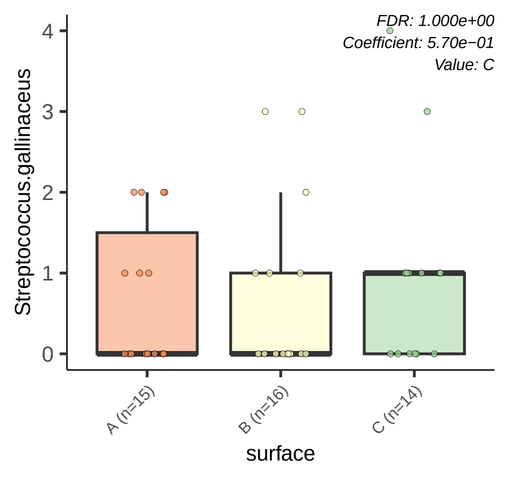
FDR: 1.000e+00
Coefficient: 5.70e−01
Value: C
Streptococcus.gallinaceus
0
1
2
3
4
A (n=15)
B (n=16)
C (n=14)
surface

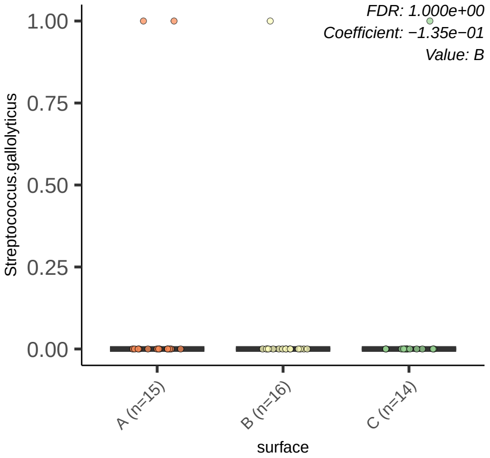
FDR: 1.000e+00
Coefficient: −1.35e−01
Value: B
Streptococcus.gallolyticus
1.00
0.75
0.50
0.25
0.00
A (n=15)
B (n=16)
C (n=14)
surface

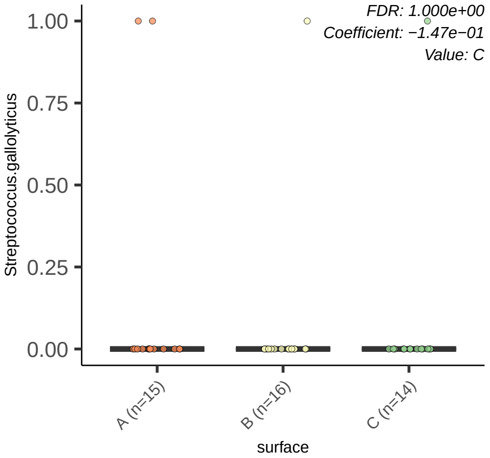

FDR: 1.000e+00
Coefficient: −1.47e−01
Value: C
Streptococcus.gallolyticus
1.00
0.75
0.50
0.25
0.00
A (n=15)
B (n=16)
C (n=14)
surface

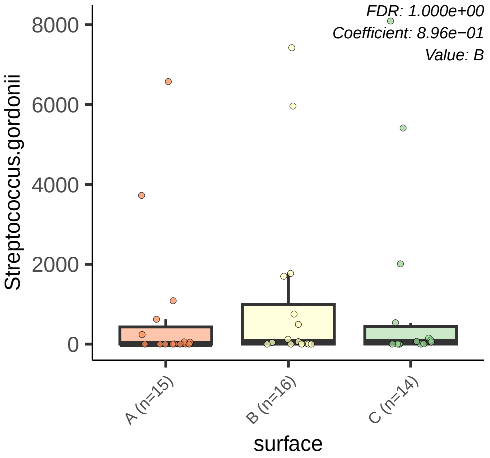
FDR: 1.000e+00
Coefficient: 8.96e−01
Value: B
Streptococcus.gordonii
8000
6000
4000
2000
0
A (n=15)
B (n=16)
C (n=14)
surface

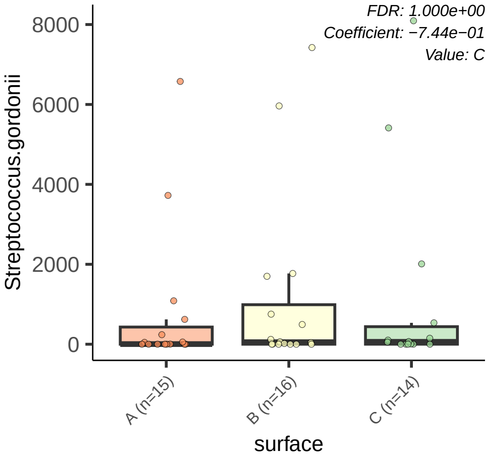
FDR: 1.000e+00
Coefficient: −7.44e−01
Value: C
Streptococcus.gordonii
8000
6000
4000
2000
0
A (n=15)
B (n=16)
C (n=14)
surface

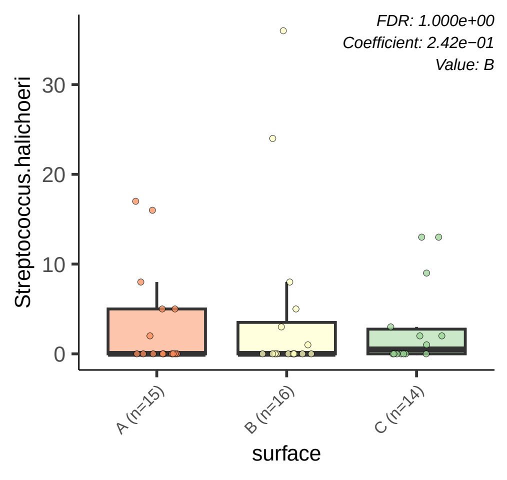
FDR: 1.000e+00
Coefficient: 2.42e−01
Value: B
Streptococcus.halichoeri
0
10
20
30
A (n=15)
B (n=16)
C (n=14)
surface

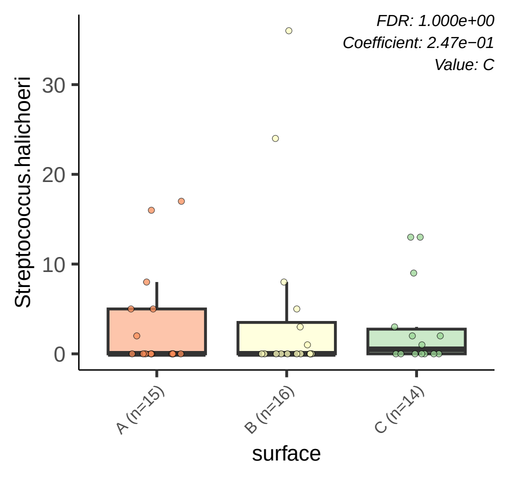
FDR: 1.000e+00
Coefficient: 2.47e−01
Value: C
Streptococcus.halichoeri
0
10
20
30
A (n=15)
B (n=16)
C (n=14)
surface

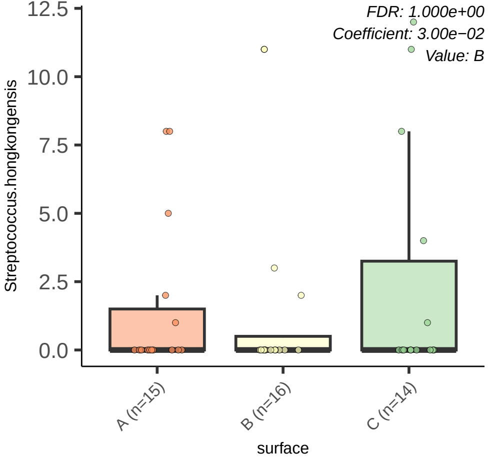

FDR: 1.000e+00
Coefficient: 3.00e−02
Value: B
Streptococcus.hongkongensis
12.5
10.0
7.5
5.0
2.5
0.0
A (n=15)
B (n=16)
C (n=14)
surface

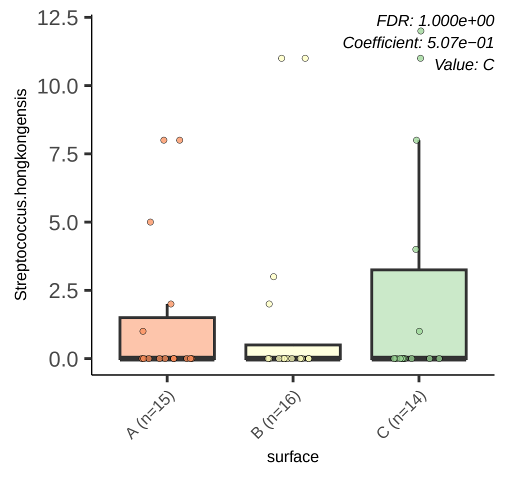
FDR: 1.000e+00
Coefficient: 5.07e−01
Value: C
12.5
10.0
7.5
5.0
2.5
0.0
Streptococcus.hongkongensis
A (n=15)
B (n=16)
C (n=14)
surface

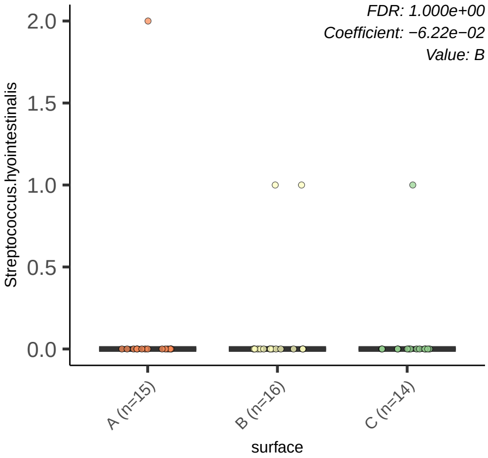
FDR: 1.000e+00
Coefficient: −6.22e−02
Value: B
Streptococcus.hyointestinalis
2.0
1.5
1.0
0.5
0.0
A (n=15)
B (n=16)
C (n=14)
surface

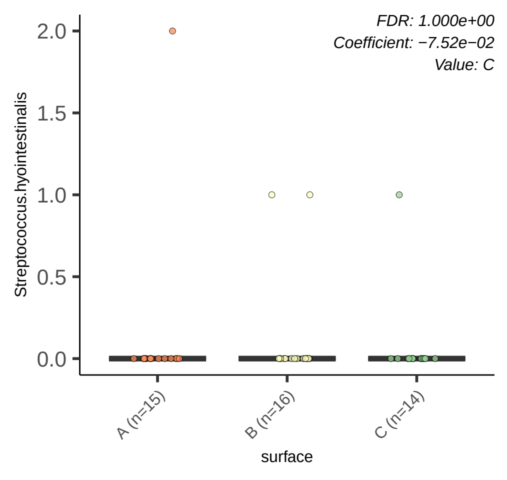
FDR: 1.000e+00
Coefficient: −7.52e−02
Value: C
Streptococcus.hyointestinalis
2.0
1.5
1.0
0.5
0.0
A (n=15)
B (n=16)
C (n=14)
surface

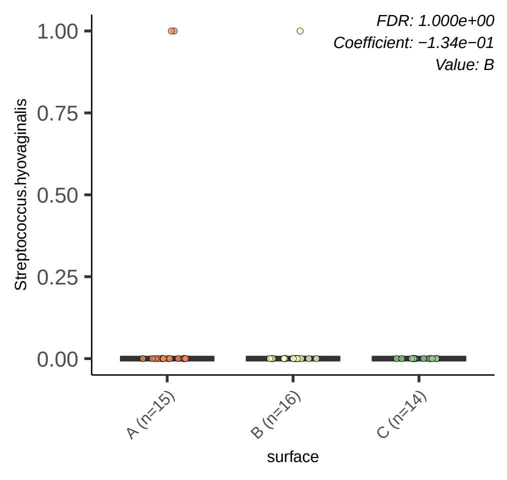
FDR: 1.000e+00
Coefficient: −1.34e−01
Value: B
Streptococcus.hyovaginalis
1.00
0.75
0.50
0.25
0.00
A (n=15)
B (n=16)
C (n=14)
surface

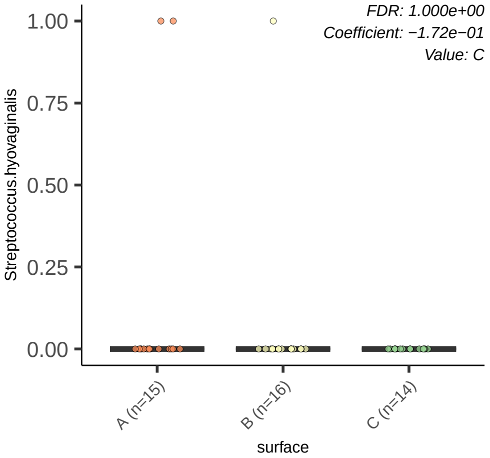
FDR: 1.000e+00
Coefficient: −1.72e−01
Value: C
Streptococcus.hyovaginalis
1.00
0.75
0.50
0.25
0.00
A (n=15)
B (n=16)
C (n=14)
surface

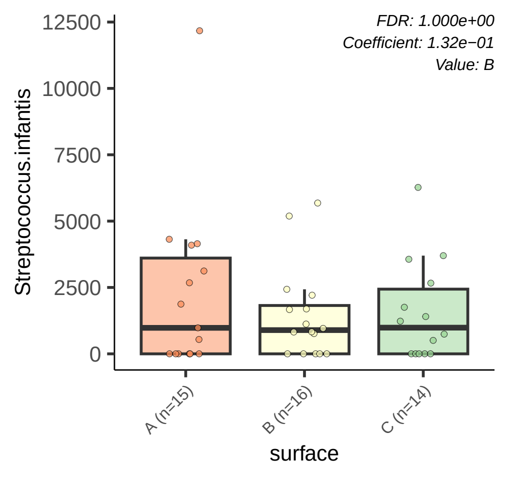
FDR: 1.000e+00
Coefficient: 1.32e−01
Value: B
Streptococcus.infantis
12500
10000
7500
5000
2500
0
A (n=15)
B (n=16)
C (n=14)
surface

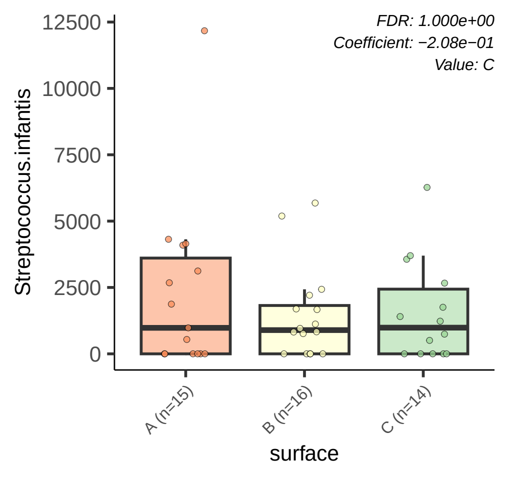

FDR: 1.000e+00
Coefficient: −2.08e−01
Value: C
Streptococcus.infantis
12500
10000
7500
5000
2500
0
A (n=15)
B (n=16)
C (n=14)
surface

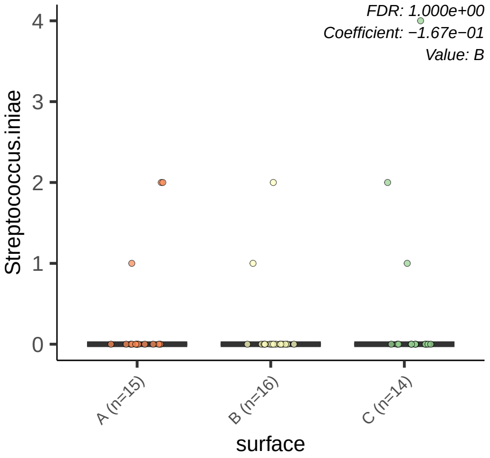

FDR: 1.000e+00
Coefficient: −1.67e−01
Value: B
Streptococcus.iniae
4
3
2
1
0
A (n=15)
B (n=16)
C (n=14)
surface

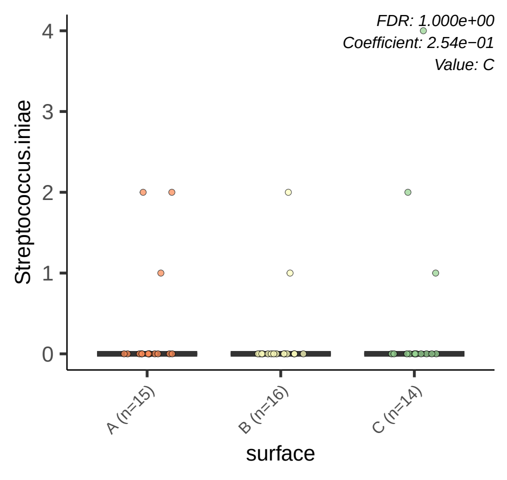
FDR: 1.000e+00
Coefficient: 2.54e−01
Value: C
Streptococcus.iniae
0
1
2
3
4
A (n=15)
B (n=16)
C (n=14)
surface

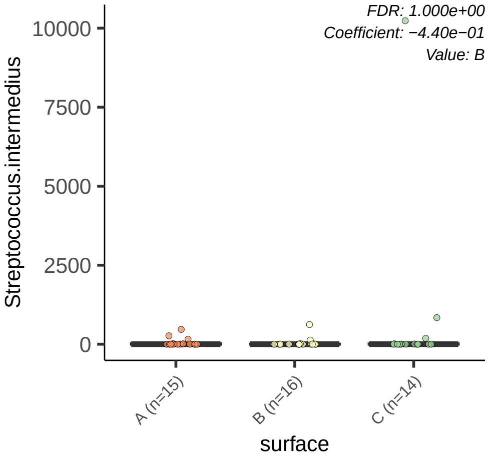

FDR: 1.000e+00
Coefficient: −4.40e−01
Value: B
Streptococcus.intermedius
10000
7500
5000
2500
0
A (n=15)
B (n=16)
C (n=14)
surface

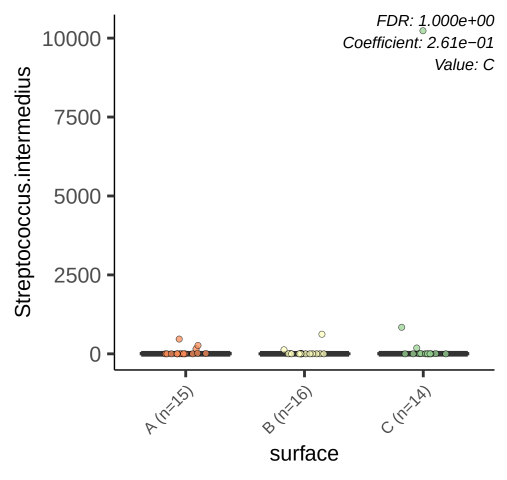

FDR: 1.000e+00
Coefficient: 2.61e−01
Value: C
Streptococcus.intermedius
10000
7500
5000
2500
0
A (n=15)
B (n=16)
C (n=14)
surface

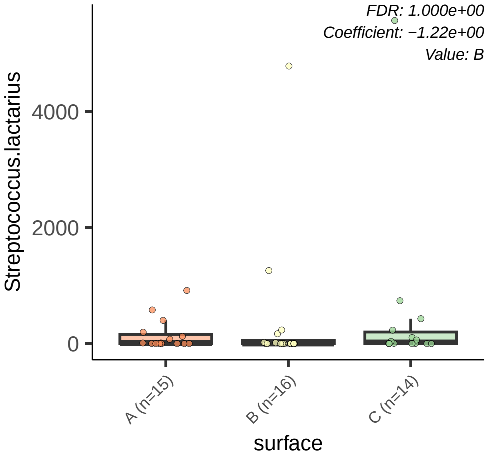
FDR: 1.000e+00
Coefficient: −1.22e+00
Value: B
Streptococcus.lactarius
4000
2000
0
A (n=15)
B (n=16)
C (n=14)
surface

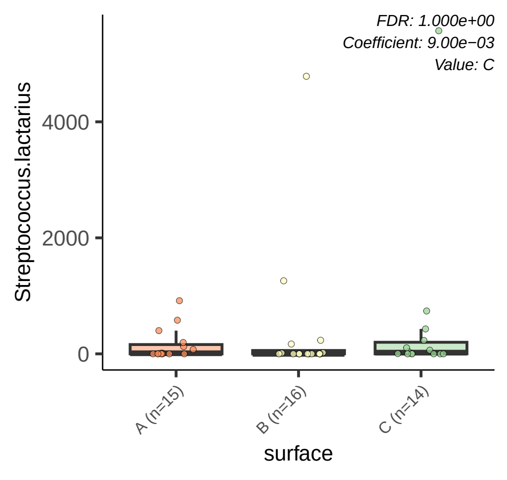

FDR: 1.000e+00
Coefficient: 9.00e−03
Value: C
Streptococcus.lactarius
4000
2000
0
A (n=15)
B (n=16)
C (n=14)
surface

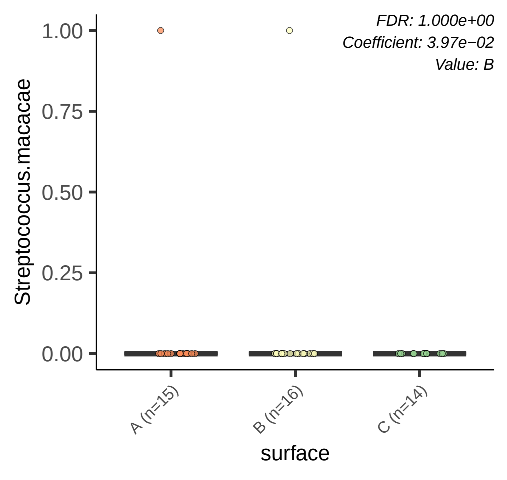
FDR: 1.000e+00
Coefficient: 3.97e−02
Value: B
Streptococcus.macacae
1.00
0.75
0.50
0.25
0.00
A (n=15)
B (n=16)
C (n=14)
surface

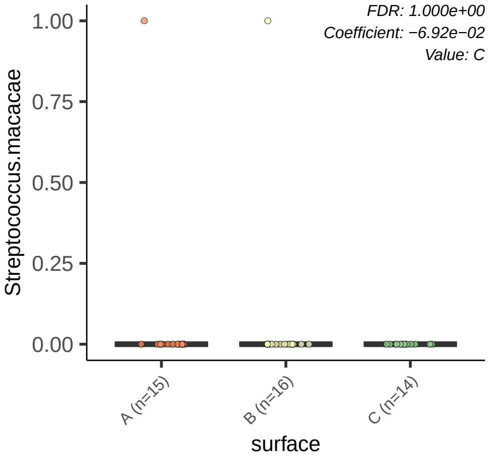

FDR: 1.000e+00
Coefficient: −6.92e−02
Value: C
Streptococcus.macacae
1.00
0.75
0.50
0.25
0.00
A (n=15)
B (n=16)
C (n=14)
surface

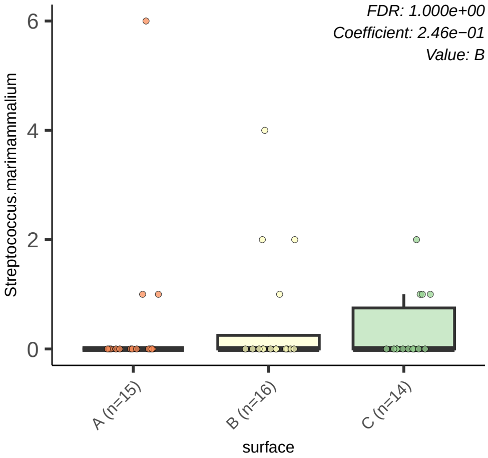
FDR: 1.000e+00
Coefficient: 2.46e−01
Value: B
Streptococcus.marimammalium
0
2
4
6
A (n=15)
B (n=16)
C (n=14)
surface

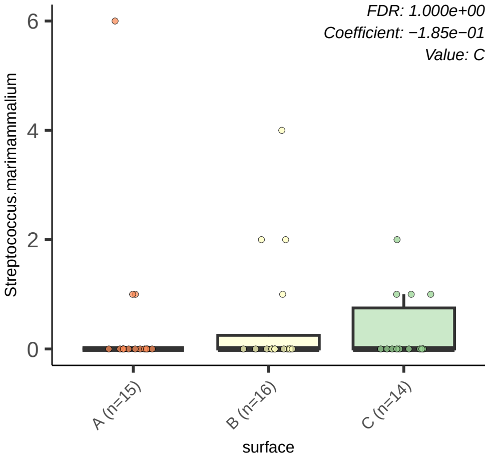

FDR: 1.000e+00
Coefficient: −1.85e−01
Value: C
Streptococcus.marimammalium
6
4
2
0
A (n=15)
B (n=16)
C (n=14)
surface

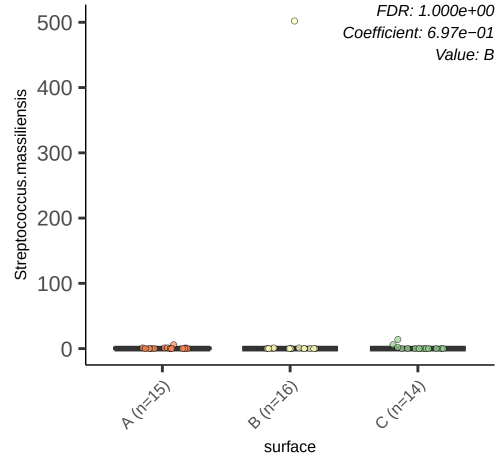

FDR: 1.000e+00
Coefficient: 6.97e−01
Value: B
Streptococcus.massiliensis
500
400
300
200
100
0
A (n=15)
B (n=16)
C (n=14)
surface

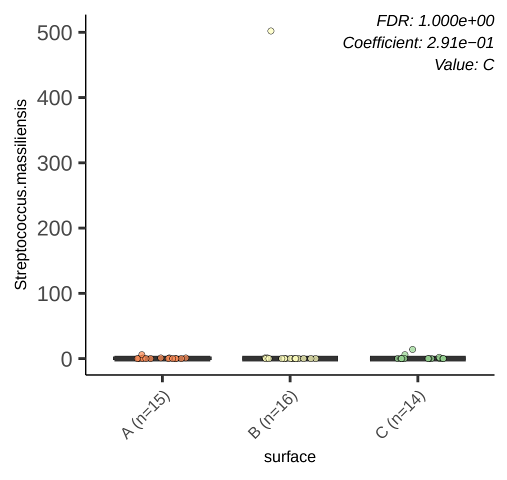

FDR: 1.000e+00
Coefficient: 2.91e−01
Value: C
Streptococcus.massiliensis
500
400
300
200
100
0
A (n=15)
B (n=16)
C (n=14)
surface

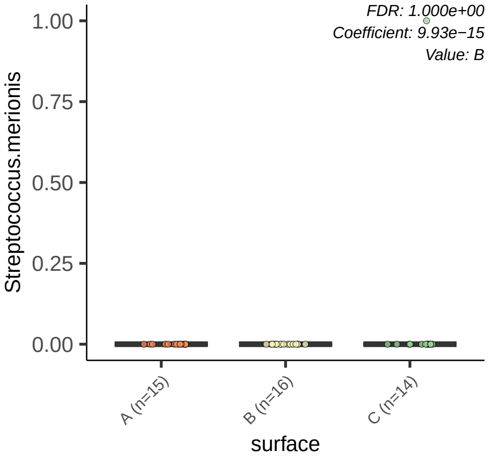
FDR: 1.000e+00
Coefficient: 9.93e−15
Value: B
Streptococcus.merionis
1.00
0.75
0.50
0.25
0.00
A (n=15)
B (n=16)
C (n=14)
surface

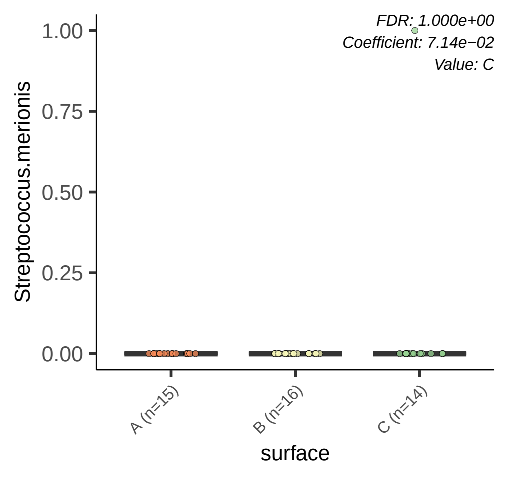
FDR: 1.000e+00
Coefficient: 7.14e−02
Value: C
Streptococcus.merionis
1.00
0.75
0.50
0.25
0.00
A (n=15)
B (n=16)
C (n=14)
surface

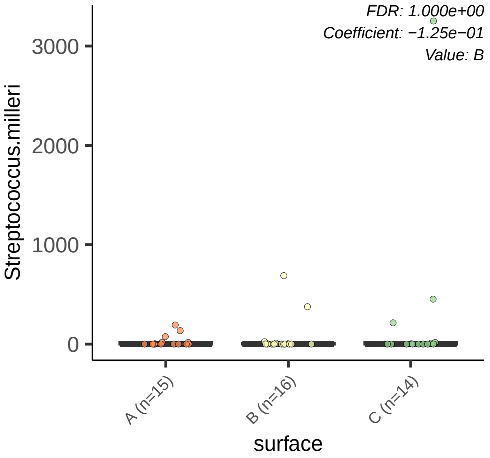
FDR: 1.000e+00
Coefficient: −1.25e−01
Value: B
Streptococcus.milleri
3000
2000
1000
0
A (n=15)
B (n=16)
C (n=14)
surface

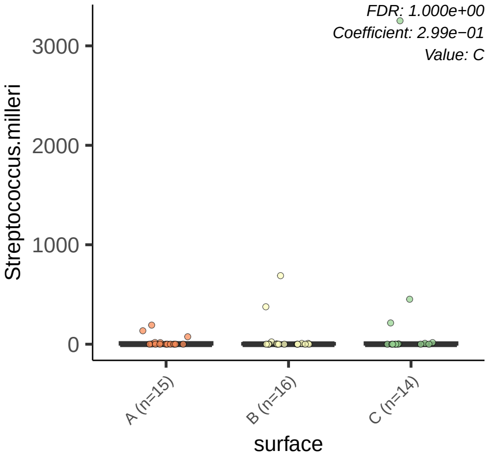
FDR: 1.000e+00
Coefficient: 2.99e−01
Value: C
Streptococcus.milleri
3000
2000
1000
0
A (n=15)
B (n=16)
C (n=14)
surface

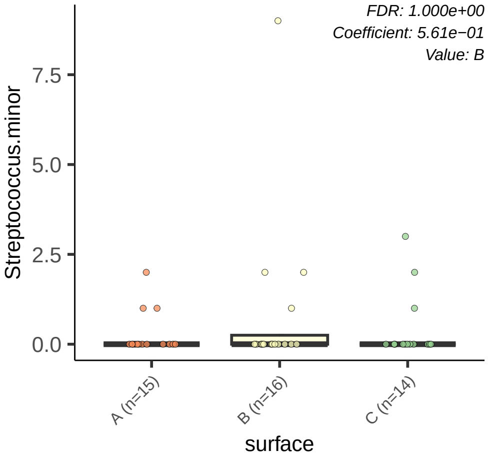
FDR: 1.000e+00
Coefficient: 5.61e−01
Value: B
Streptococcus.minor
7.5
5.0
2.5
0.0
A (n=15)
B (n=16)
C (n=14)
surface

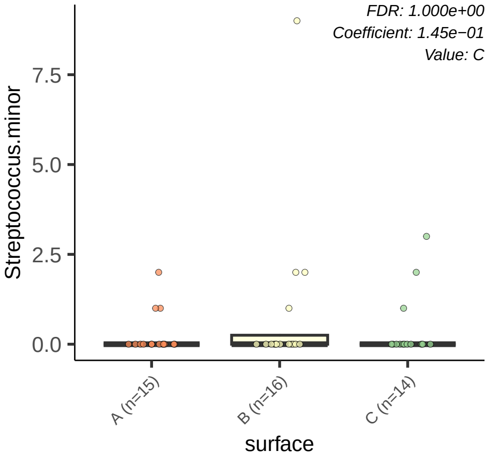
FDR: 1.000e+00
Coefficient: 1.45e−01
Value: C
Streptococcus.minor
7.5
5.0
2.5
0.0
A (n=15)
B (n=16)
C (n=14)
surface

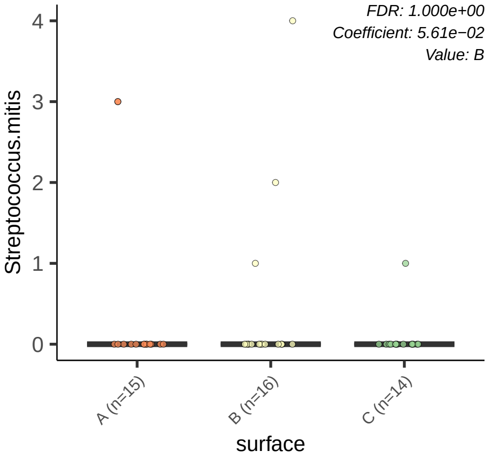
FDR: 1.000e+00
Coefficient: 5.61e−02
Value: B
Streptococcus.mitis
0
1
2
3
4
A (n=15)
B (n=16)
C (n=14)
surface

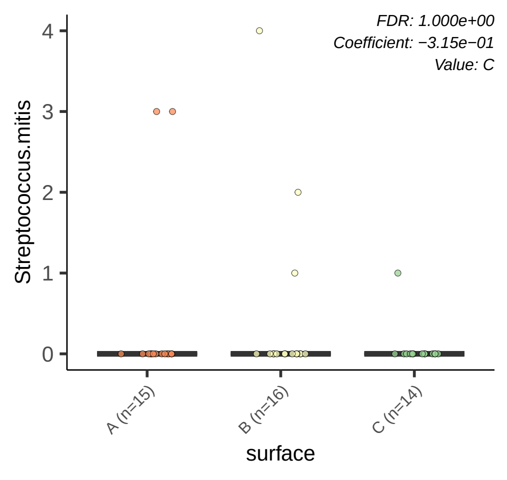

FDR: 1.000e+00
Coefficient: −3.15e−01
Value: C
Streptococcus.mitis
4
3
2
1
0
A (n=15)
B (n=16)
C (n=14)
surface

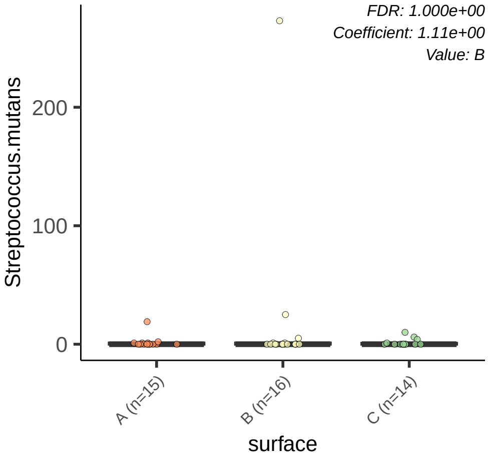

FDR: 1.000e+00
Coefficient: 1.11e+00
Value: B
Streptococcus.mutans
200
100
0
A (n=15)
B (n=16)
C (n=14)
surface

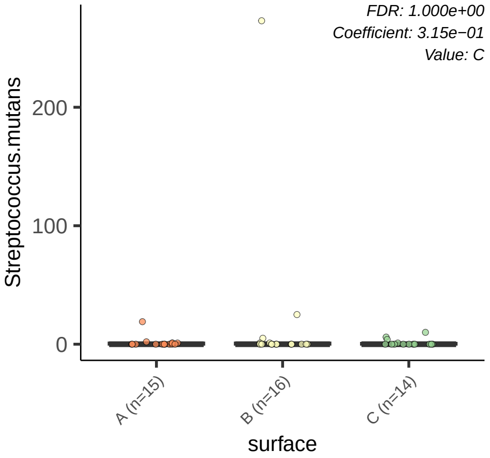
FDR: 1.000e+00
Coefficient: 3.15e−01
Value: C
Streptococcus.mutans
200
100
0
A (n=15)
B (n=16)
C (n=14)
surface

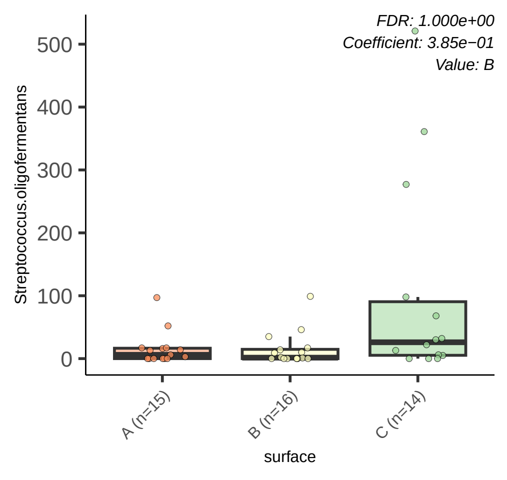
FDR: 1.000e+00
Coefficient: 3.85e−01
Value: B
Streptococcus.oligofermentans
500
400
300
200
100
0
A (n=15)
B (n=16)
C (n=14)
surface

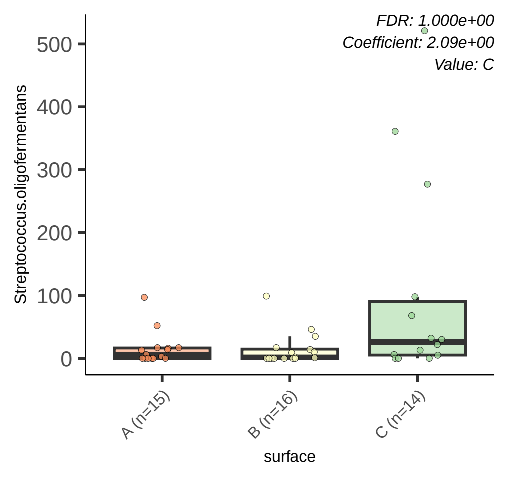

FDR: 1.000e+00
Coefficient: 2.09e+00
Value: C
Streptococcus.oligofermentans
0
100
200
300
400
500
A (n=15)
B (n=16)
C (n=14)
surface

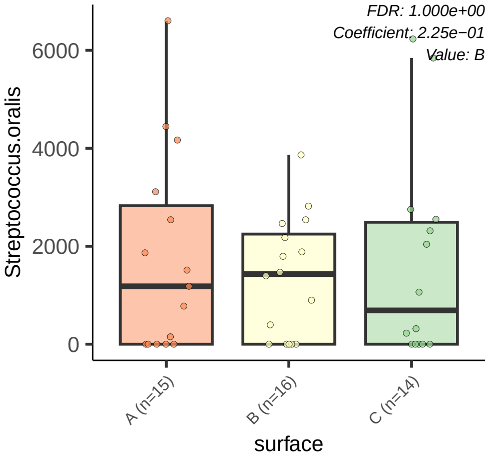
FDR: 1.000e+00
Coefficient: 2.25e−01
Value: B
Streptococcus.oralis
6000
4000
2000
0
A (n=15)
B (n=16)
C (n=14)
surface

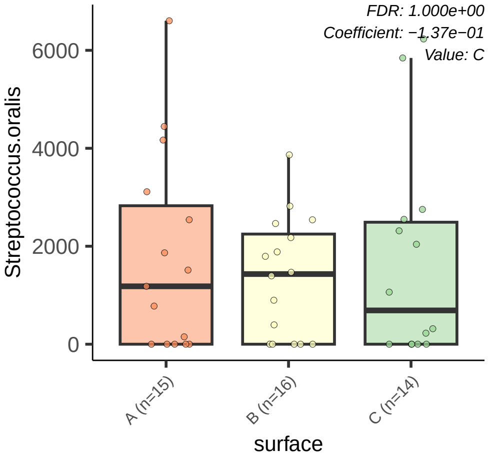
FDR: 1.000e+00
Coefficient: −1.37e−01
Value: C
Streptococcus.oralis
6000
4000
2000
0
A (n=15)
B (n=16)
C (n=14)
surface

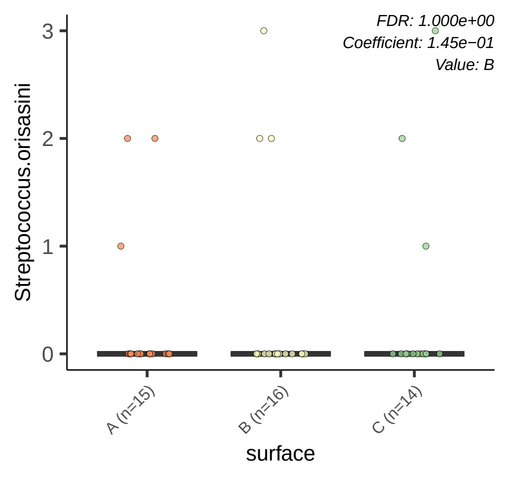
FDR: 1.000e+00
Coefficient: 1.45e−01
Value: B
Streptococcus.orisasini
0
1
2
3
A (n=15)
B (n=16)
C (n=14)
surface

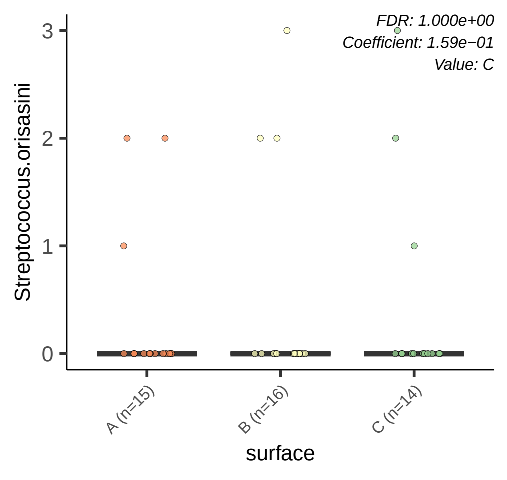
FDR: 1.000e+00
Coefficient: 1.59e−01
Value: C
Streptococcus.orisasini
0
1
2
3
A (n=15)
B (n=16)
C (n=14)
surface

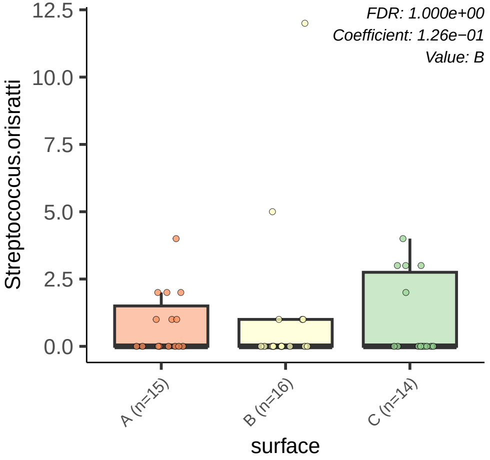

FDR: 1.000e+00
Coefficient: 1.26e−01
Value: B
12.5
10.0
7.5
5.0
2.5
0.0
Streptococcus.orisratti
A (n=15)
B (n=16)
C (n=14)
surface

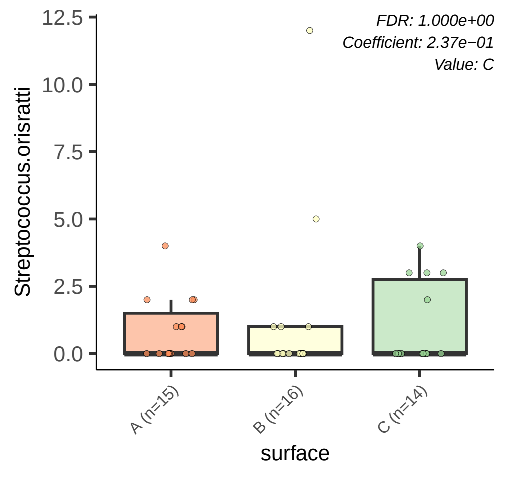

FDR: 1.000e+00
Coefficient: 2.37e−01
Value: C
Streptococcus.orisratti
12.5
10.0
7.5
5.0
2.5
0.0
A (n=15)
B (n=16)
C (n=14)
surface

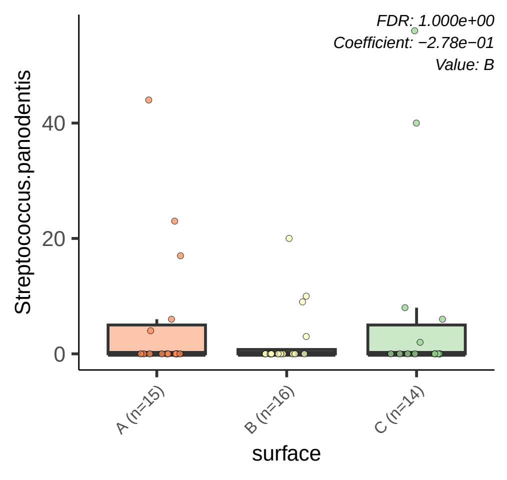

FDR: 1.000e+00
Coefficient: −2.78e−01
Value: B
Streptococcus.panodentis
0
20
40
A (n=15)
B (n=16)
C (n=14)
surface

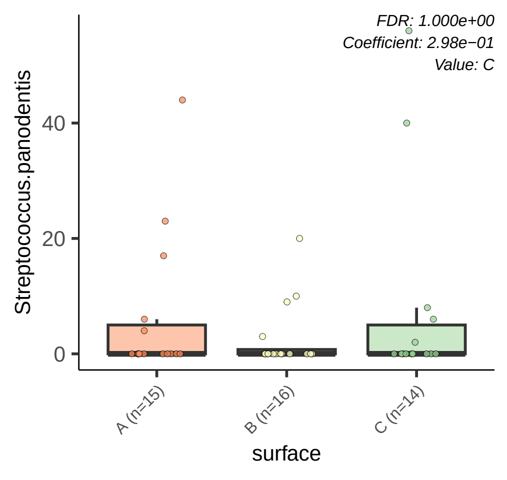

FDR: 1.000e+00
Coefficient: 2.98e−01
Value: C
Streptococcus.panodentis
0
20
40
A (n=15)
B (n=16)
C (n=14)
surface

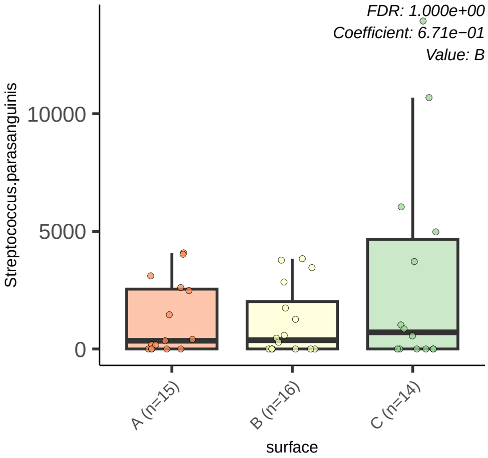

FDR: 1.000e+00
Coefficient: 6.71e−01
Value: B
Streptococcus.parasanguinis
10000
5000
0
A (n=15)
B (n=16)
C (n=14)
surface

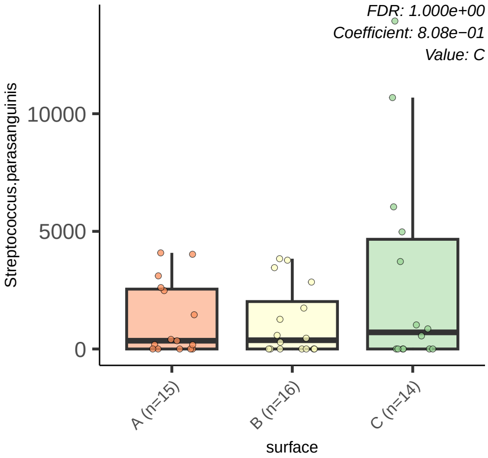
FDR: 1.000e+00
Coefficient: 8.08e−01
Value: C
Streptococcus.parasanguinis
10000
5000
0
A (n=15)
B (n=16)
C (n=14)
surface

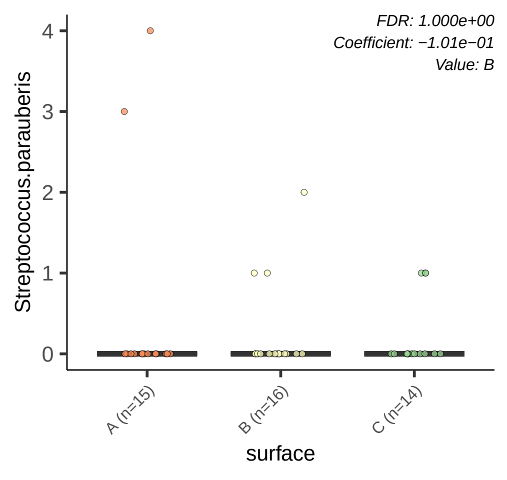
FDR: 1.000e+00
Coefficient: −1.01e−01
Value: B
Streptococcus.parauberis
4
3
2
1
0
A (n=15)
B (n=16)
C (n=14)
surface

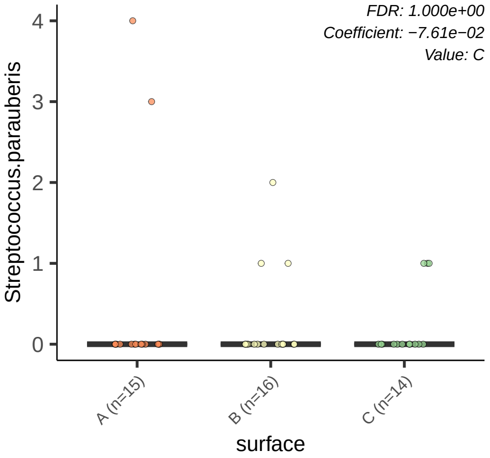

FDR: 1.000e+00
Coefficient: −7.61e−02
Value: C
Streptococcus.parauberis
0
1
2
3
4
A (n=15)
B (n=16)
C (n=14)
surface

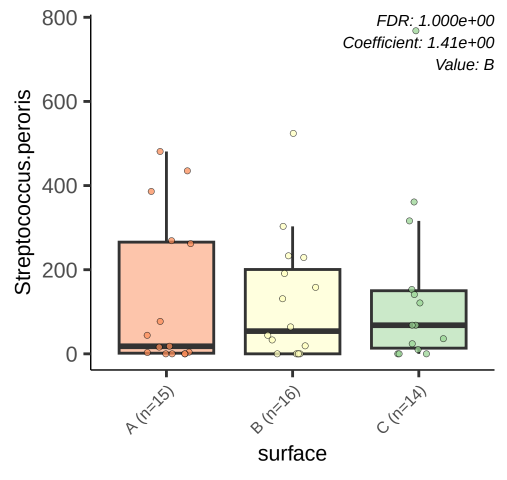

FDR: 1.000e+00
Coefficient: 1.41e+00
Value: B
800
600
400
200
0
Streptococcus.peroris
A (n=15)
B (n=16)
C (n=14)
surface

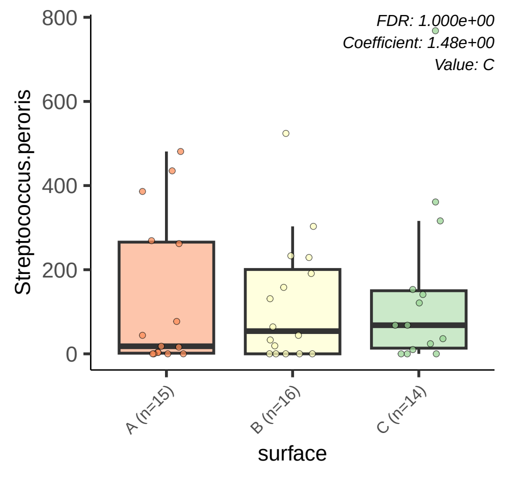

FDR: 1.000e+00
Coefficient: 1.48e+00
Value: C
Streptococcus.peroris
800
600
400
200
0
A (n=15)
B (n=16)
C (n=14)
surface

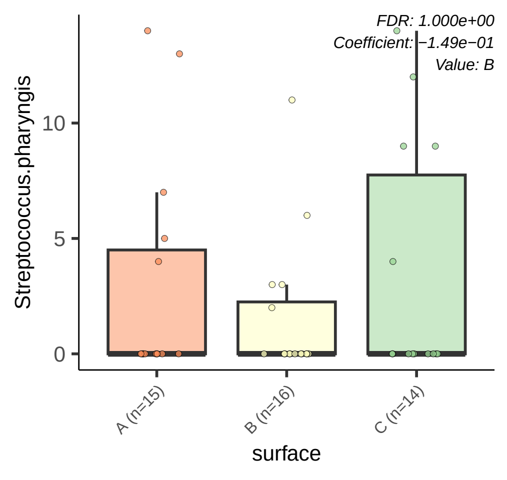

FDR: 1.000e+00
Coefficient: −1.49e−01
Value: B
Streptococcus.pharyngis
10
5
0
A (n=15)
B (n=16)
C (n=14)
surface

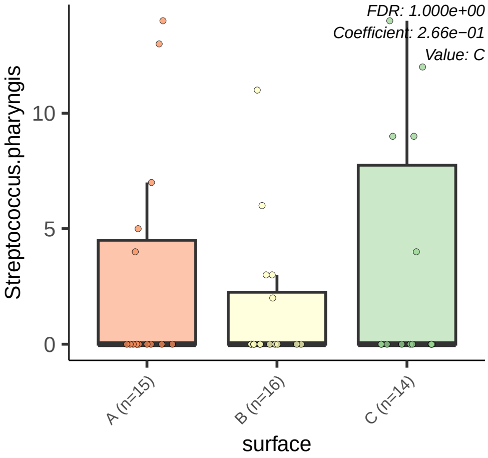

FDR: 1.000e+00
Coefficient: 2.66e−01
Value: C
Streptococcus.pharyngis
0
5
10
A (n=15)
B (n=16)
C (n=14)
surface

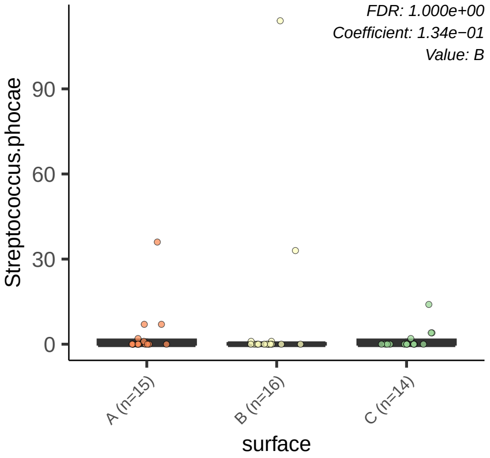

FDR: 1.000e+00
Coefficient: 1.34e−01
Value: B
Streptococcus.phocae
90
60
30
0
A (n=15)
B (n=16)
C (n=14)
surface

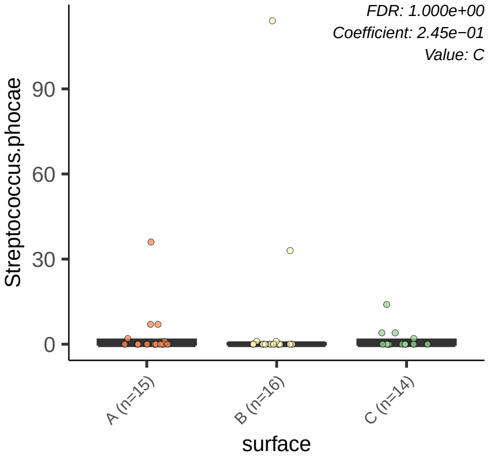
FDR: 1.000e+00
Coefficient: 2.45e−01
Value: C
Streptococcus.phocae
0
30
60
90
A (n=15)
B (n=16)
C (n=14)
surface

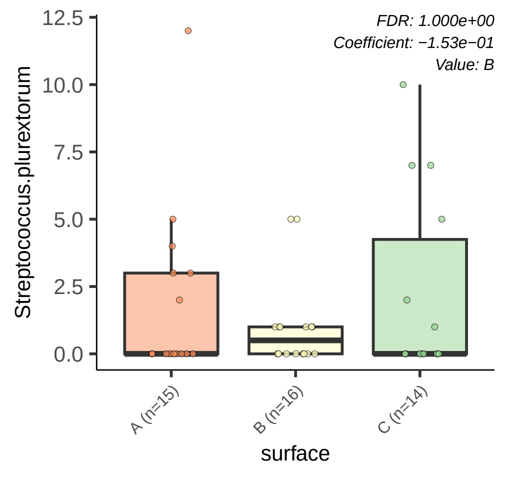
FDR: 1.000e+00
Coefficient: −1.53e−01
Value: B
Streptococcus.plurextorum
12.5
10.0
7.5
5.0
2.5
0.0
A (n=15)
B (n=16)
C (n=14)
surface

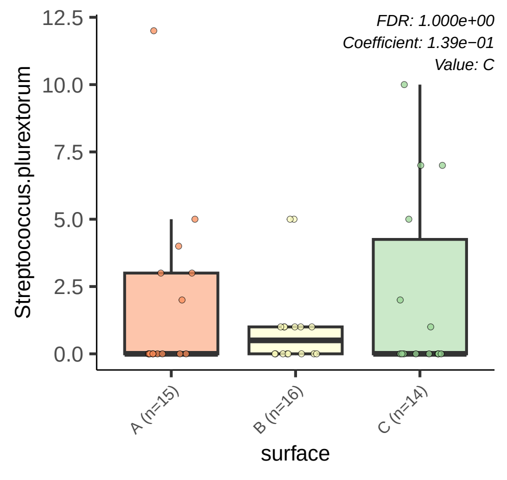

FDR: 1.000e+00
Coefficient: 1.39e−01
Value: C
Streptococcus.plurextorum
12.5
10.0
7.5
5.0
2.5
0.0
A (n=15)
B (n=16)
C (n=14)
surface

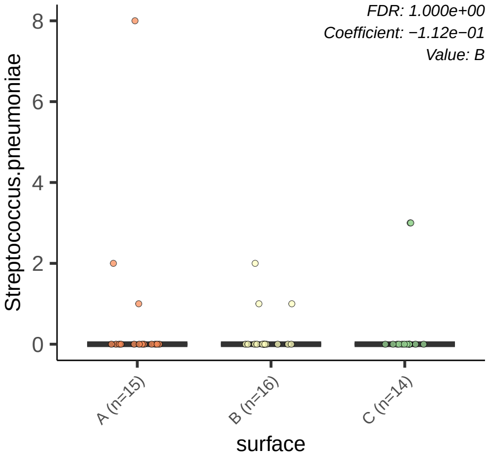

FDR: 1.000e+00
Coefficient: −1.12e−01
Value: B
Streptococcus.pneumoniae
8
6
4
2
0
A (n=15)
B (n=16)
C (n=14)
surface

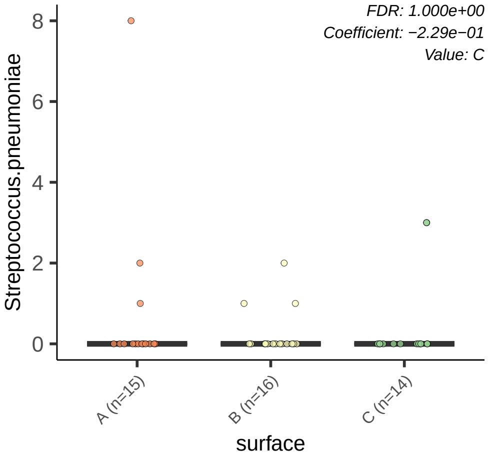

FDR: 1.000e+00
Coefficient: −2.29e−01
Value: C
Streptococcus.pneumoniae
8
6
4
2
0
A (n=15)
B (n=16)
C (n=14)
surface

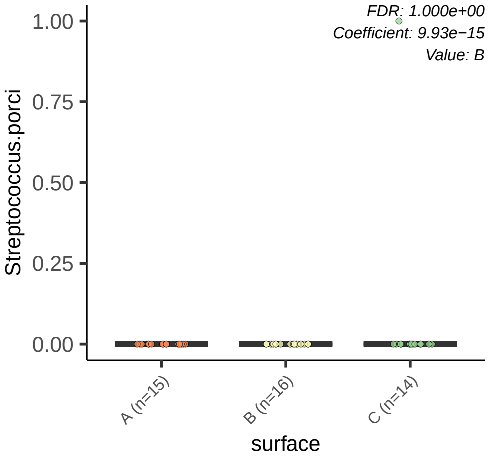
FDR: 1.000e+00
Coefficient: 9.93e−15
Value: B
Streptococcus.porci
1.00
0.75
0.50
0.25
0.00
A (n=15)
B (n=16)
C (n=14)
surface

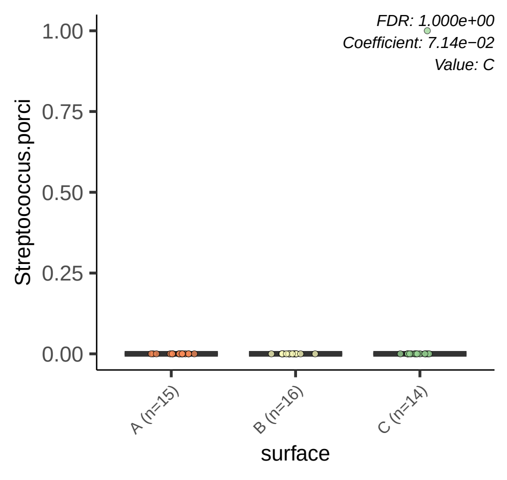
FDR: 1.000e+00
Coefficient: 7.14e−02
Value: C
Streptococcus.porci
1.00
0.75
0.50
0.25
0.00
A (n=15)
B (n=16)
C (n=14)
surface

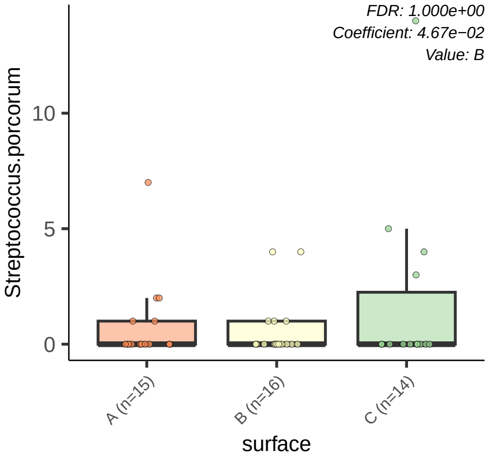
FDR: 1.000e+00
Coefficient: 4.67e−02
Value: B
Streptococcus.porcorum
10
5
0
A (n=15)
B (n=16)
C (n=14)
surface

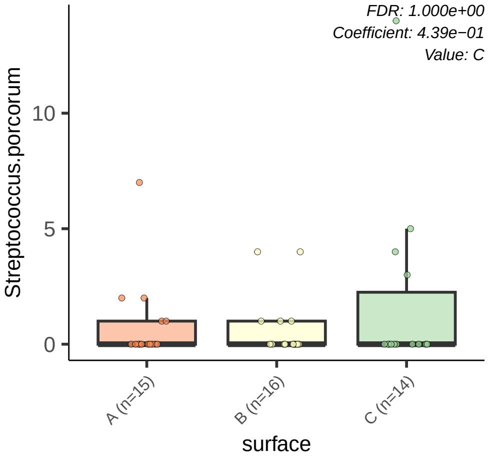
FDR: 1.000e+00
Coefficient: 4.39e−01
Value: C
Streptococcus.porcorum
10
5
0
A (n=15)
B (n=16)
C (n=14)
surface

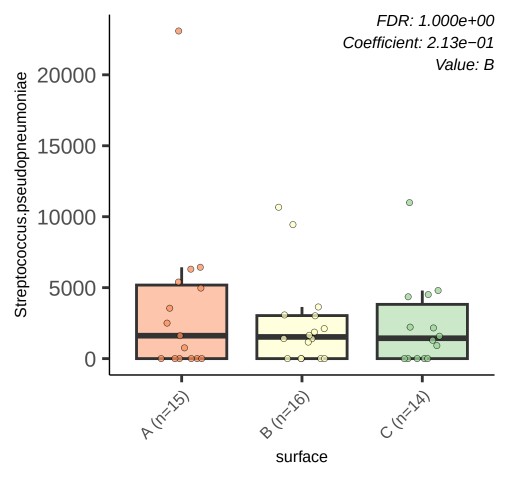

FDR: 1.000e+00
Coefficient: 2.13e−01
Value: B
Streptococcus.pseudopneumoniae
20000
15000
10000
5000
0
A (n=15)
B (n=16)
C (n=14)
surface

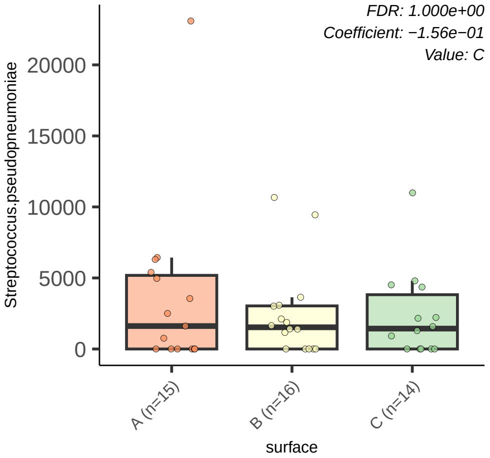

FDR: 1.000e+00
Coefficient: −1.56e−01
Value: C
Streptococcus.pseudopneumoniae
20000
15000
10000
5000
0
A (n=15)
B (n=16)
C (n=14)
surface

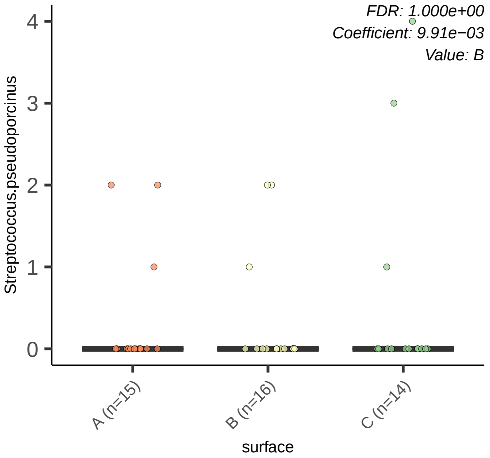
FDR: 1.000e+00
Coefficient: 9.91e−03
Value: B
Streptococcus.pseudoporcinus
4
3
2
1
0
A (n=15)
B (n=16)
C (n=14)
surface

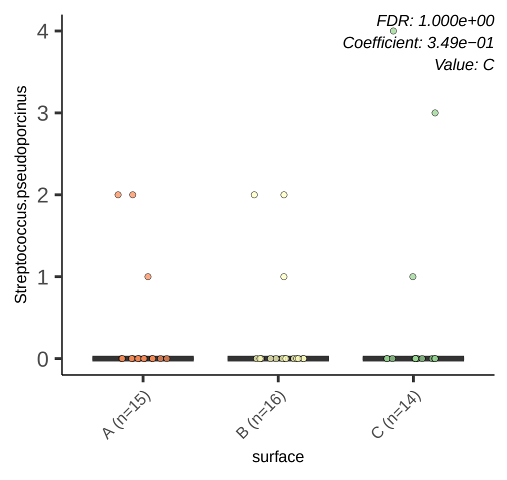
FDR: 1.000e+00
Coefficient: 3.49e−01
Value: C
Streptococcus.pseudoporcinus
4
3
2
1
0
A (n=15)
B (n=16)
C (n=14)
surface

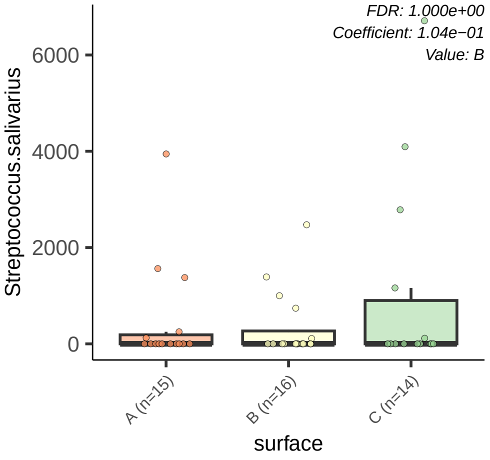
FDR: 1.000e+00
Coefficient: 1.04e−01
Value: B
6000
4000
2000
0
Streptococcus.salivarius
A (n=15)
B (n=16)
C (n=14)
surface

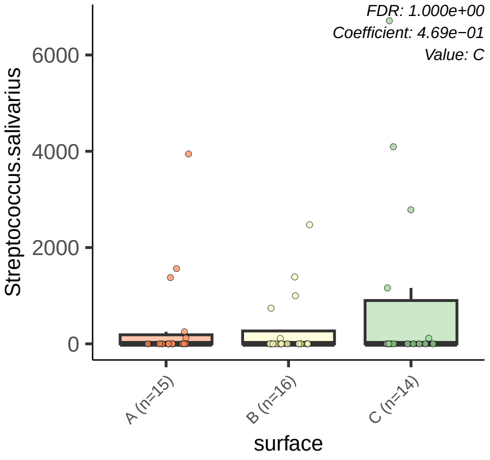
FDR: 1.000e+00
Coefficient: 4.69e−01
Value: C
Streptococcus.salivarius
6000
4000
2000
0
A (n=15)
B (n=16)
C (n=14)
surface

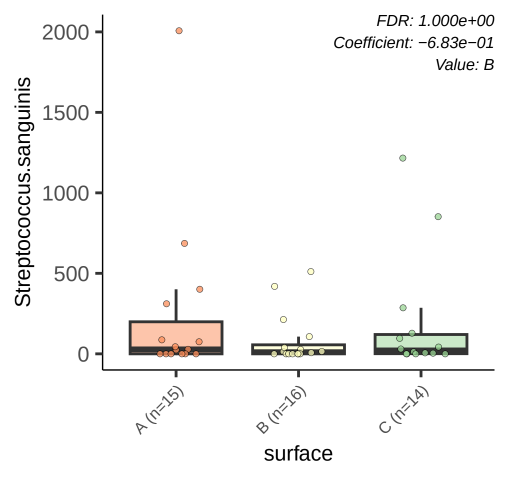
FDR: 1.000e+00
Coefficient: −6.83e−01
Value: B
Streptococcus.sanguinis
2000
1500
1000
500
0
A (n=15)
B (n=16)
C (n=14)
surface

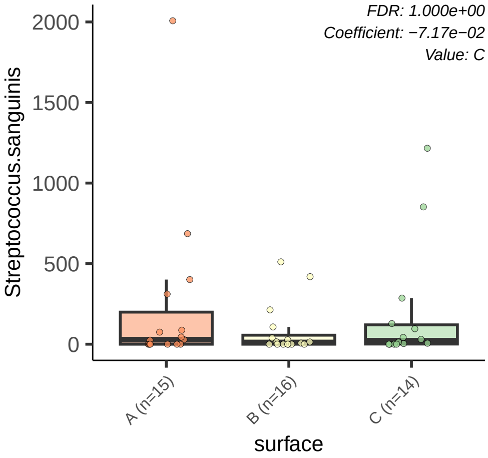
FDR: 1.000e+00
Coefficient: −7.17e−02
Value: C
Streptococcus.sanguinis
2000
1500
1000
500
0
A (n=15)
B (n=16)
C (n=14)
surface

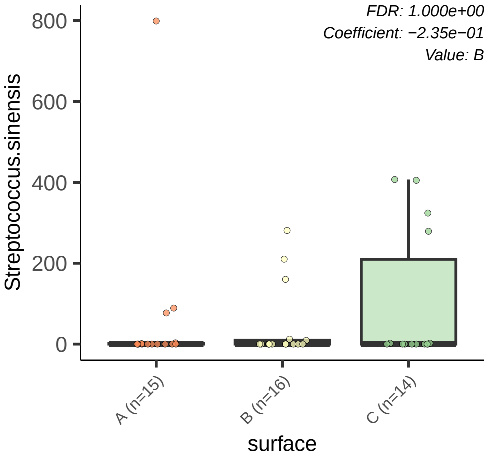

FDR: 1.000e+00
Coefficient: −2.35e−01
Value: B
Streptococcus.sinensis
800
600
400
200
0
A (n=15)
B (n=16)
C (n=14)
surface

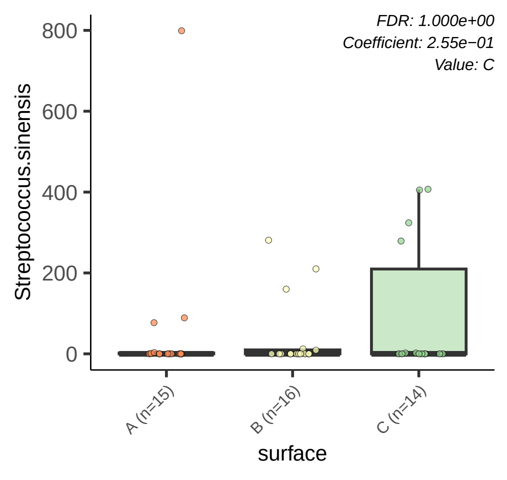
FDR: 1.000e+00
Coefficient: 2.55e−01
Value: C
Streptococcus.sinensis
800
600
400
200
0
A (n=15)
B (n=16)
C (n=14)
surface

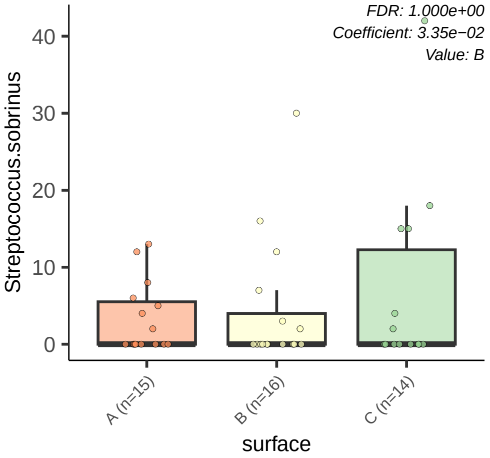
FDR: 1.000e+00
Coefficient: 3.35e−02
Value: B
Streptococcus.sobrinus
40
30
20
10
0
A (n=15)
B (n=16)
C (n=14)
surface

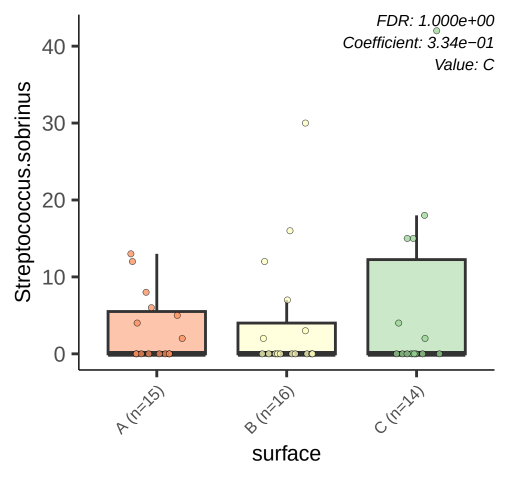

FDR: 1.000e+00
Coefficient: 3.34e−01
Value: C
Streptococcus.sobrinus
0
10
20
30
40
A (n=15)
B (n=16)
C (n=14)
surface

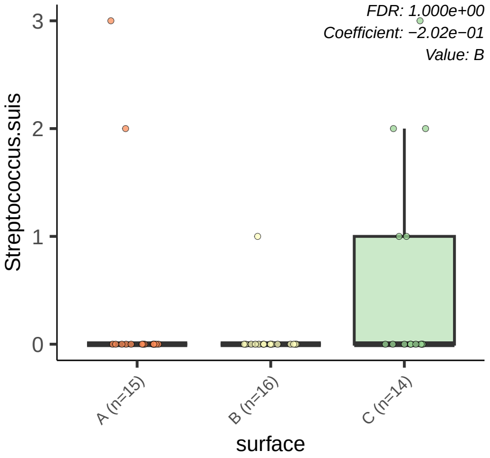
FDR: 1.000e+00
Coefficient: −2.02e−01
Value: B
Streptococcus.suis
3
2
1
0
A (n=15)
B (n=16)
C (n=14)
surface

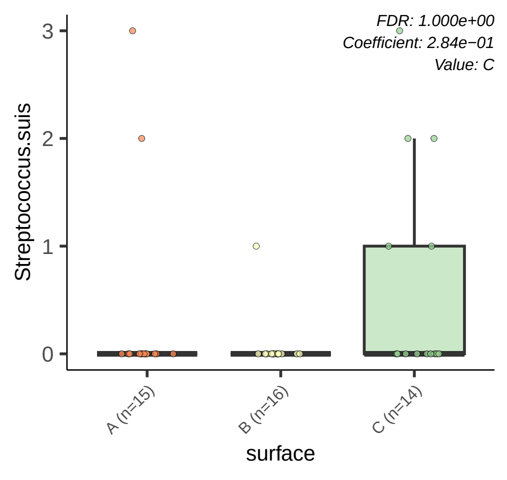
FDR: 1.000e+00
Coefficient: 2.84e−01
Value: C
Streptococcus.suis
3
2
1
0
A (n=15)
B (n=16)
C (n=14)
surface

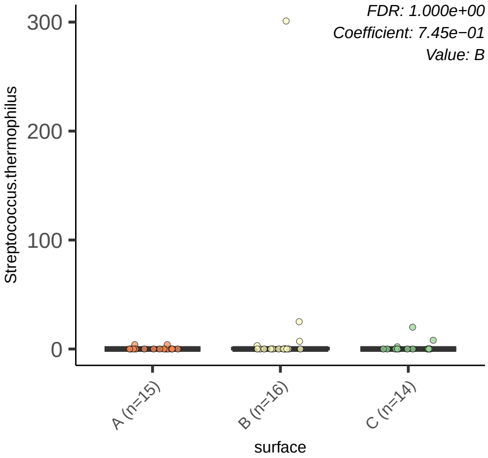

FDR: 1.000e+00
Coefficient: 7.45e−01
Value: B
Streptococcus.thermophilus
300
200
100
0
A (n=15)
B (n=16)
C (n=14)
surface

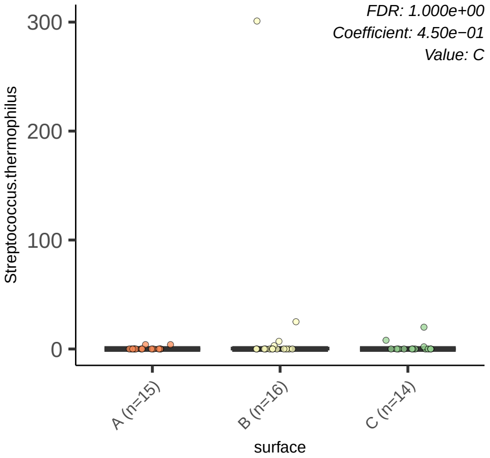
FDR: 1.000e+00
Coefficient: 4.50e−01
Value: C
Streptococcus.thermophilus
300
200
100
0
A (n=15)
B (n=16)
C (n=14)
surface

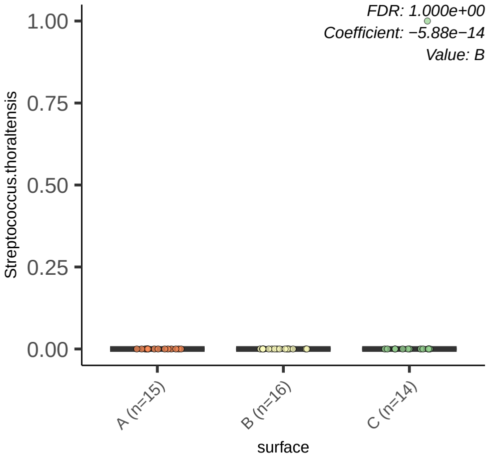
FDR: 1.000e+00
Coefficient: −5.88e−14
Value: B
Streptococcus.thoraltensis
1.00
0.75
0.50
0.25
0.00
A (n=15)
B (n=16)
C (n=14)
surface

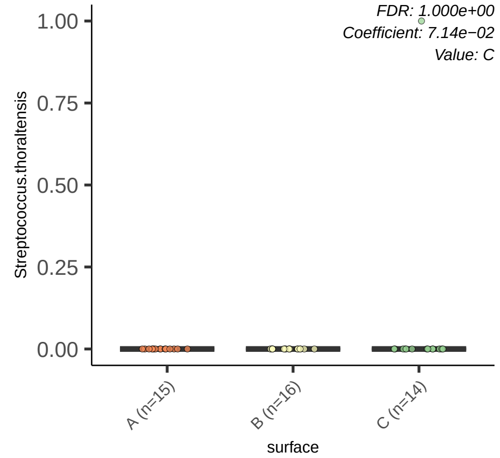
FDR: 1.000e+00
Coefficient: 7.14e−02
Value: C
1.00
0.75
0.50
0.25
0.00
Streptococcus.thoraltensis
A (n=15)
B (n=16)
C (n=14)
surface

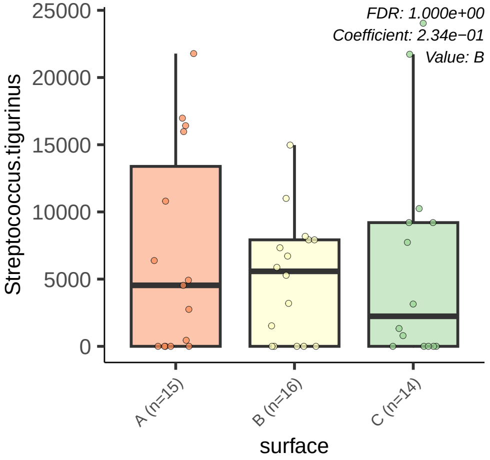
FDR: 1.000e+00
Coefficient: 2.34e−01
Value: B
Streptococcus.tigurinus
25000
20000
15000
10000
5000
0
A (n=15)
B (n=16)
C (n=14)
surface

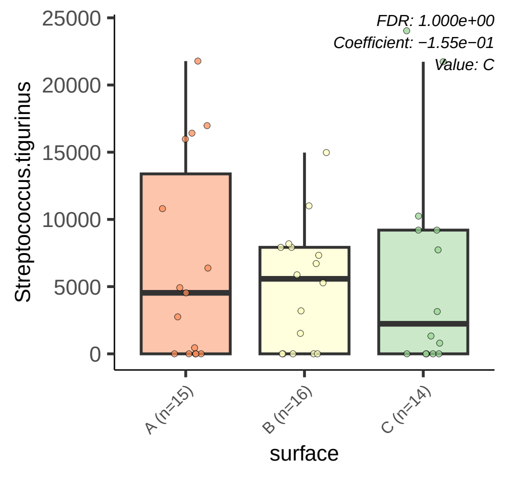
FDR: 1.000e+00
Coefficient: −1.55e−01
Value: C
25000
20000
15000
10000
5000
0
Streptococcus.tigurinus
A (n=15)
B (n=16)
C (n=14)
surface

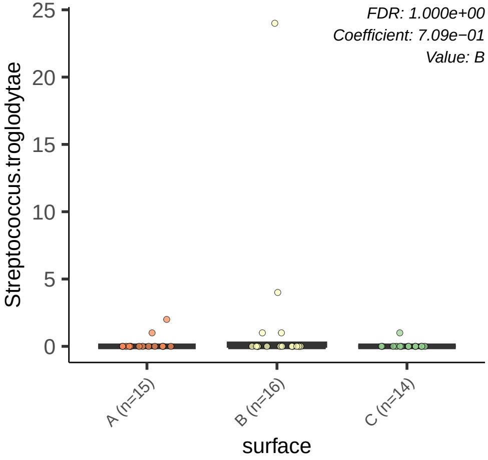
FDR: 1.000e+00
Coefficient: 7.09e−01
Value: B
Streptococcus.troglodytae
25
20
15
10
5
0
A (n=15)
B (n=16)
C (n=14)
surface

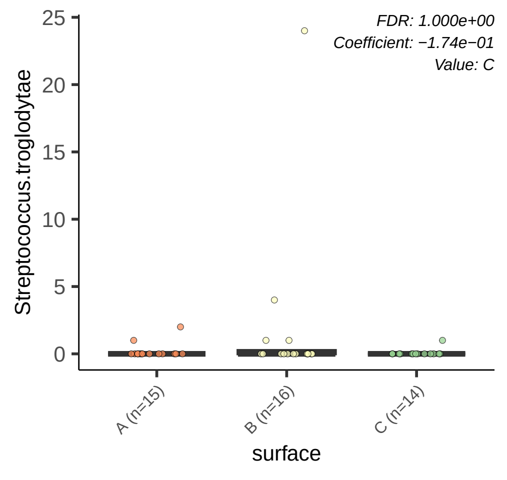
FDR: 1.000e+00
Coefficient: −1.74e−01
Value: C
Streptococcus.troglodytae
25
20
15
10
5
0
A (n=15)
B (n=16)
C (n=14)
surface

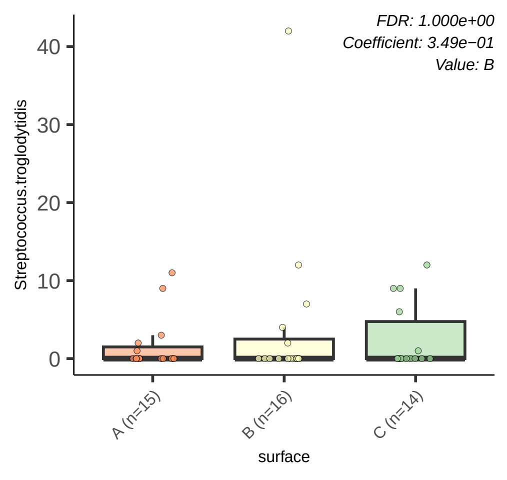

FDR: 1.000e+00
Coefficient: 3.49e−01
Value: B
Streptococcus.troglodytidis
40
30
20
10
0
A (n=15)
B (n=16)
C (n=14)
surface

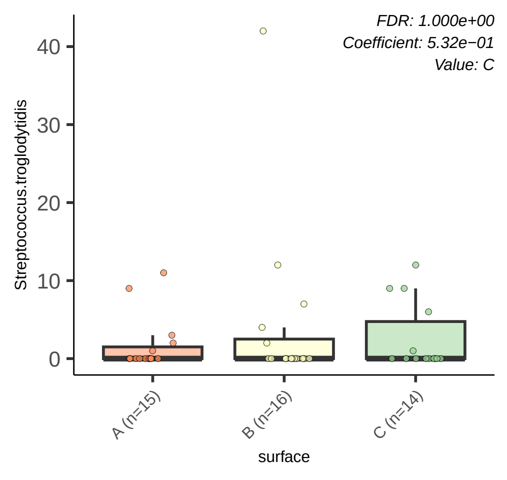
FDR: 1.000e+00
Coefficient: 5.32e−01
Value: C
Streptococcus.troglodytidis
40
30
20
10
0
A (n=15)
B (n=16)
C (n=14)
surface

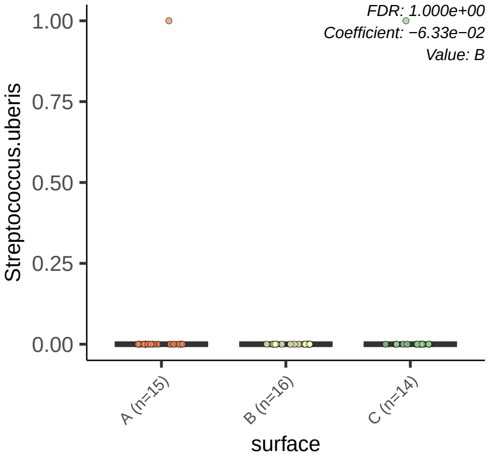
FDR: 1.000e+00
Coefficient: −6.33e−02
Value: B
Streptococcus.uberis
1.00
0.75
0.50
0.25
0.00
A (n=15)
B (n=16)
C (n=14)
surface

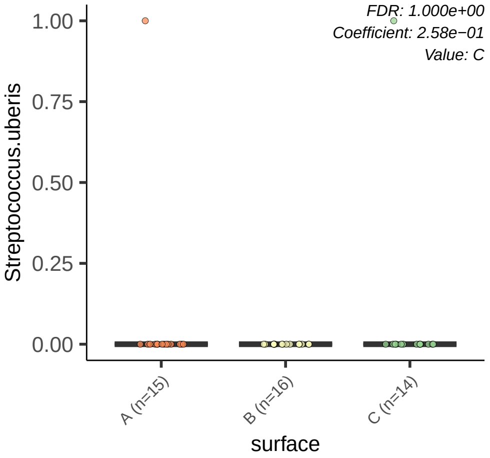
FDR: 1.000e+00
Coefficient: 2.58e−01
Value: C
Streptococcus.uberis
1.00
0.75
0.50
0.25
0.00
A (n=15)
B (n=16)
C (n=14)
surface

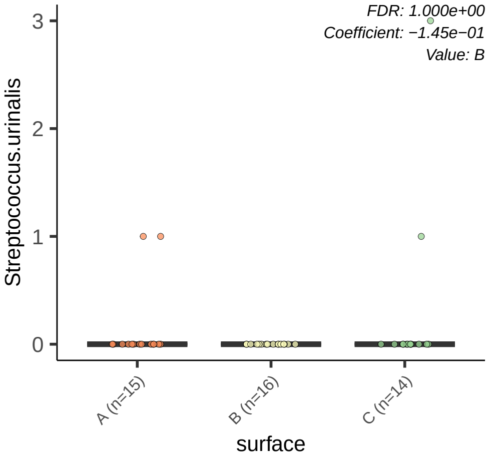

FDR: 1.000e+00
Coefficient: −1.45e−01
Value: B
Streptococcus.urinalis
3
2
1
0
A (n=15)
B (n=16)
C (n=14)
surface

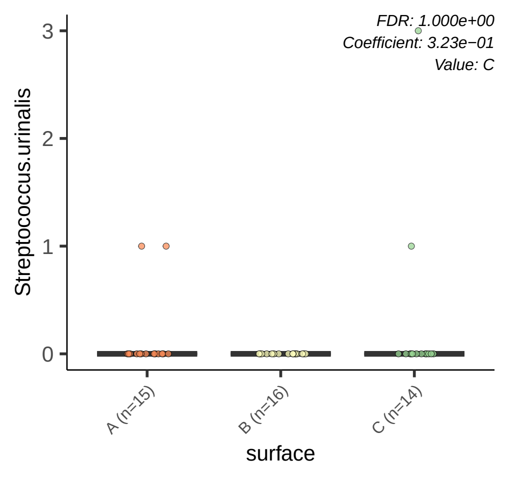
FDR: 1.000e+00
Coefficient: 3.23e−01
Value: C
Streptococcus.urinalis
3
2
1
0
A (n=15)
B (n=16)
C (n=14)
surface

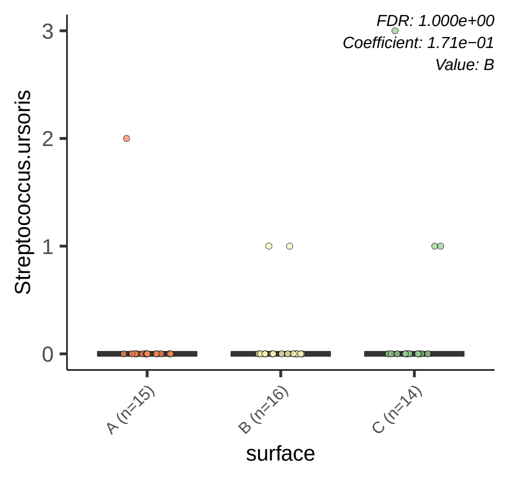

FDR: 1.000e+00
Coefficient: 1.71e−01
Value: B
Streptococcus.ursoris
0
1
2
3
A (n=15)
B (n=16)
C (n=14)
surface

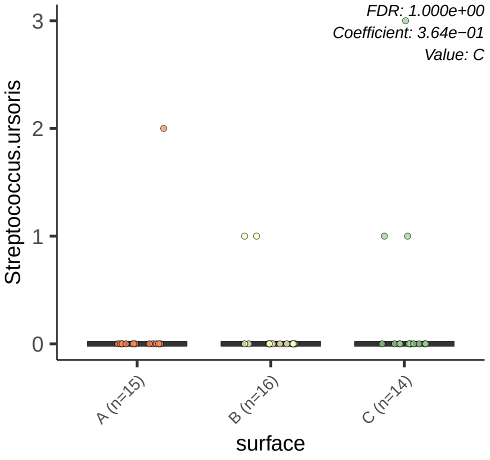
FDR: 1.000e+00
Coefficient: 3.64e−01
Value: C
Streptococcus.ursoris
3
2
1
0
A (n=15)
B (n=16)
C (n=14)
surface

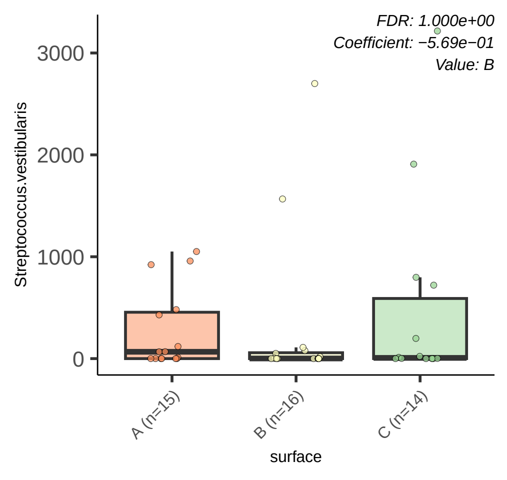
FDR: 1.000e+00
Coefficient: −5.69e−01
Value: B
3000
2000
1000
0
Streptococcus.vestibularis
A (n=15)
B (n=16)
C (n=14)
surface

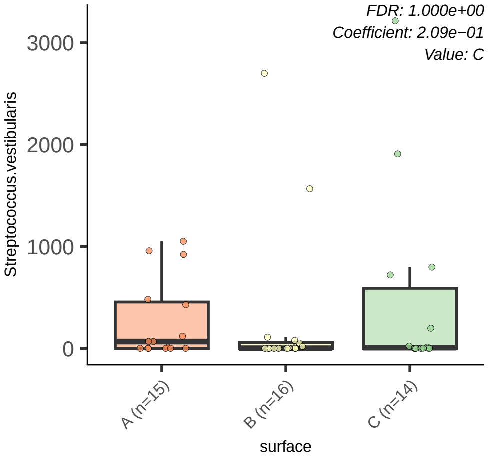

FDR: 1.000e+00
Coefficient: 2.09e−01
Value: C
Streptococcus.vestibularis
3000
2000
1000
0
A (n=15)
B (n=16)
C (n=14)
surface

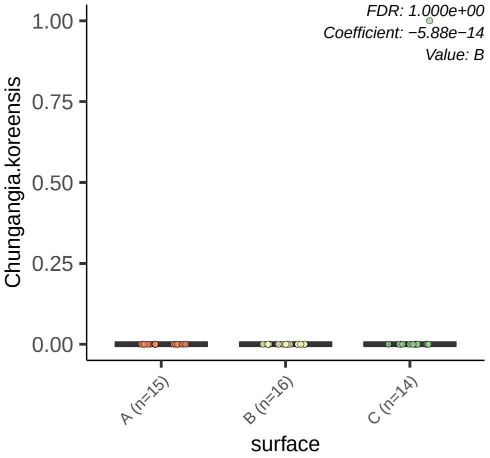
FDR: 1.000e+00
Coefficient: −5.88e−14
Value: B
Chungangia.koreensis
1.00
0.75
0.50
0.25
0.00
A (n=15)
B (n=16)
C (n=14)
surface

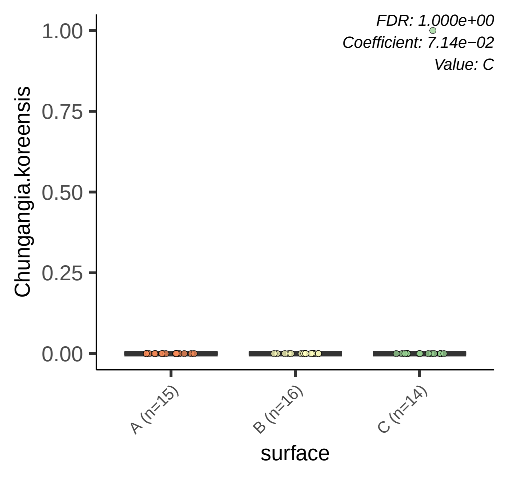
FDR: 1.000e+00
Coefficient: 7.14e−02
Value: C
Chungangia.koreensis
1.00
0.75
0.50
0.25
0.00
A (n=15)
B (n=16)
C (n=14)
surface

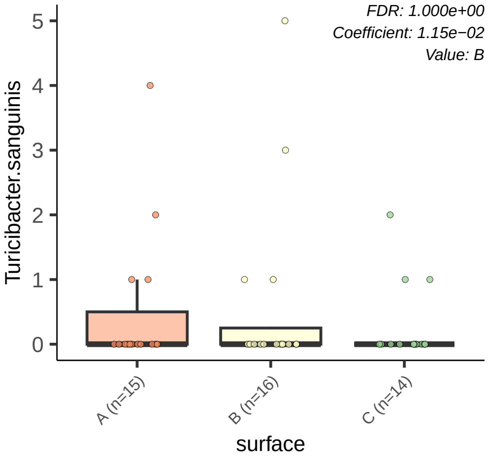

FDR: 1.000e+00
Coefficient: 1.15e−02
Value: B
Turicibacter.sanguinis
0
1
2
3
4
5
A (n=15)
B (n=16)
C (n=14)
surface

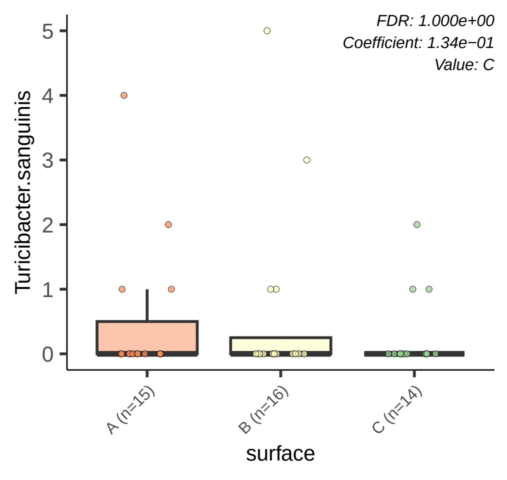
FDR: 1.000e+00
Coefficient: 1.34e−01
Value: C
Turicibacter.sanguinis
5
4
3
2
1
0
A (n=15)
B (n=16)
C (n=14)
surface

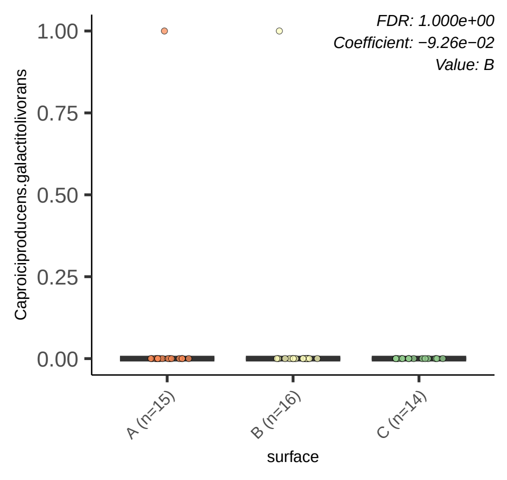
FDR: 1.000e+00
Coefficient: −9.26e−02
Value: B
Caproiciproducens.galactitolivorans
1.00
0.75
0.50
0.25
0.00
A (n=15)
B (n=16)
C (n=14)
surface

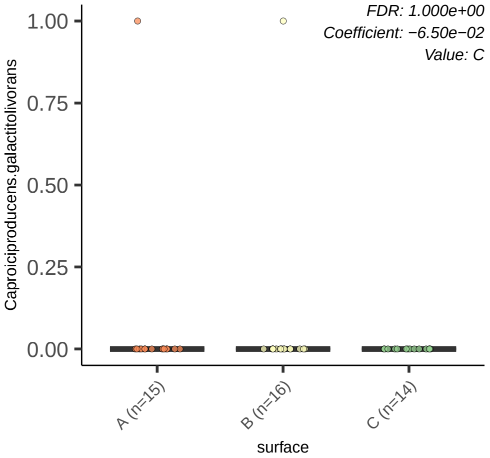

FDR: 1.000e+00
Coefficient: −6.50e−02
Value: C
1.00
0.75
0.50
0.25
0.00
Caproiciproducens.galactitolivorans
A (n=15)
B (n=16)
C (n=14)
surface

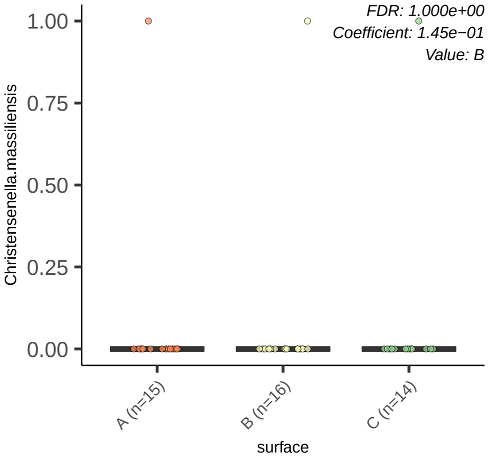
FDR: 1.000e+00
Coefficient: 1.45e−01
Value: B
1.00
0.75
0.50
0.25
0.00
Christensenella.massiliensis
A (n=15)
B (n=16)
C (n=14)
surface

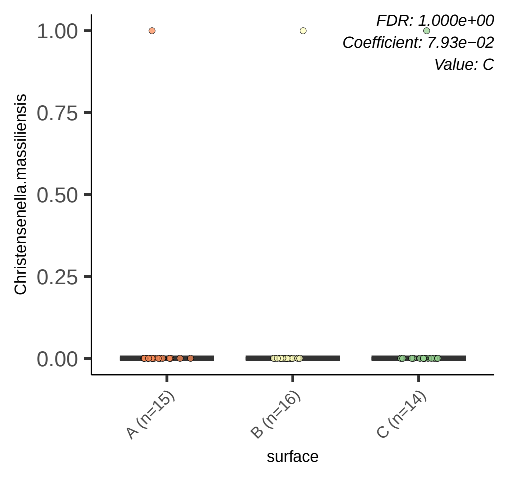
FDR: 1.000e+00
Coefficient: 7.93e−02
Value: C
1.00
0.75
0.50
0.25
0.00
Christensenella.massiliensis
A (n=15)
B (n=16)
C (n=14)
surface

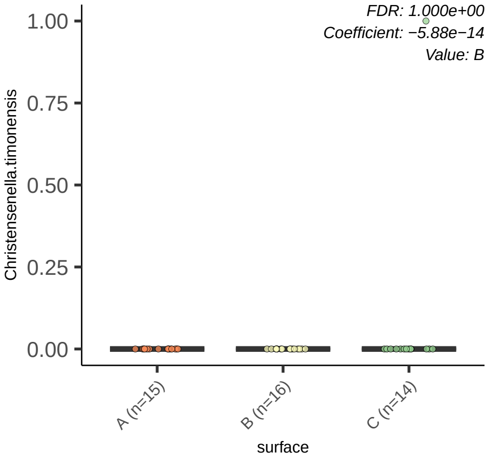
FDR: 1.000e+00
Coefficient: −5.88e−14
Value: B
Christensenella.timonensis
1.00
0.75
0.50
0.25
0.00
A (n=15)
B (n=16)
C (n=14)
surface

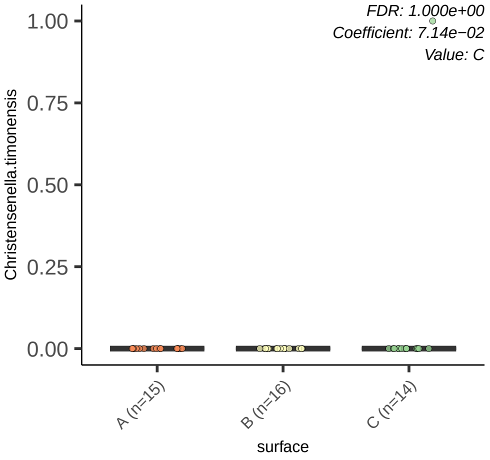
FDR: 1.000e+00
Coefficient: 7.14e−02
Value: C
1.00
0.75
0.50
0.25
0.00
Christensenella.timonensis
A (n=15)
B (n=16)
C (n=14)
surface

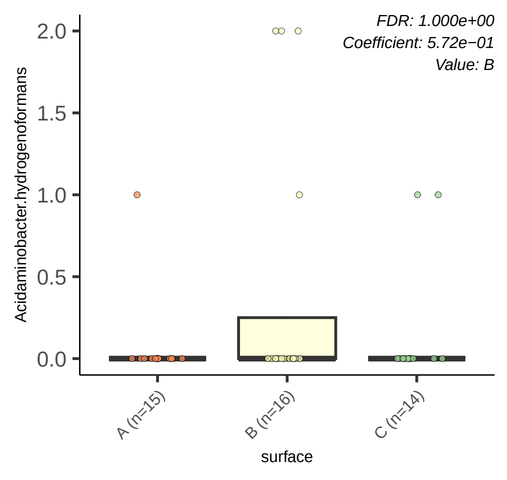
FDR: 1.000e+00
Coefficient: 5.72e−01
Value: B
Acidaminobacter.hydrogenoformans
2.0
1.5
1.0
0.5
0.0
A (n=15)
B (n=16)
C (n=14)
surface

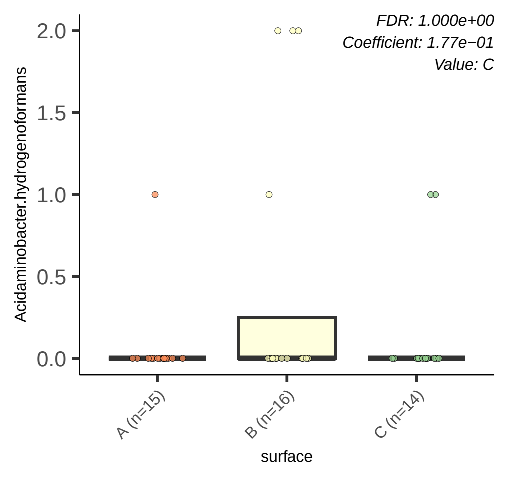
FDR: 1.000e+00
Coefficient: 1.77e−01
Value: C
Acidaminobacter.hydrogenoformans
2.0
1.5
1.0
0.5
0.0
A (n=15)
B (n=16)
C (n=14)
surface

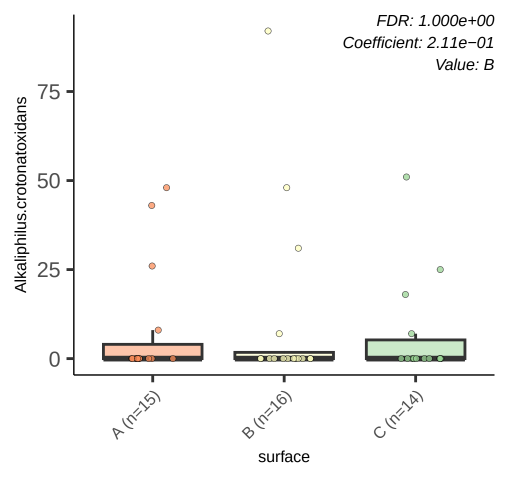
FDR: 1.000e+00
Coefficient: 2.11e−01
Value: B
Alkaliphilus.crotonatoxidans
0
25
50
75
A (n=15)
B (n=16)
C (n=14)
surface

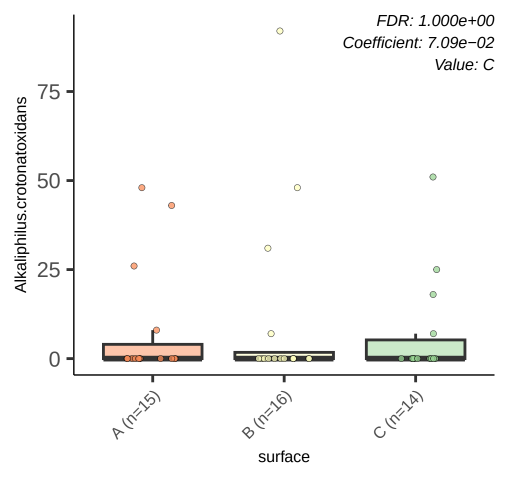
FDR: 1.000e+00
Coefficient: 7.09e−02
Value: C
Alkaliphilus.crotonatoxidans
0
25
50
75
A (n=15)
B (n=16)
C (n=14)
surface

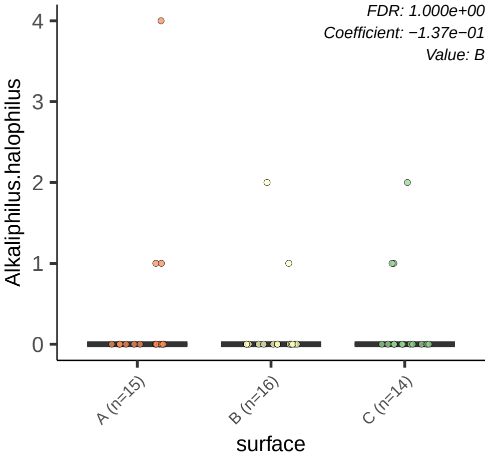
FDR: 1.000e+00
Coefficient: −1.37e−01
Value: B
Alkaliphilus.halophilus
0
1
2
3
4
A (n=15)
B (n=16)
C (n=14)
surface

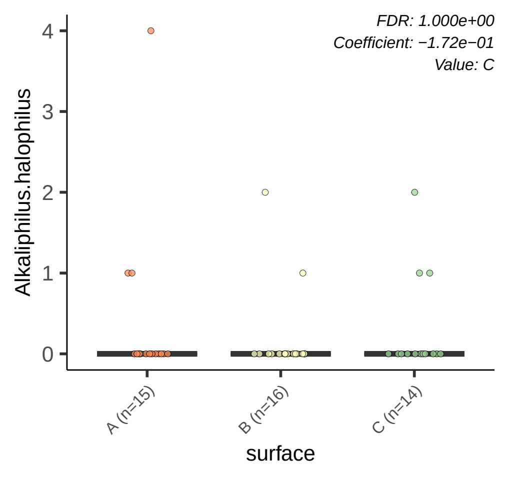
FDR: 1.000e+00
Coefficient: −1.72e−01
Value: C
Alkaliphilus.halophilus
4
3
2
1
0
A (n=15)
B (n=16)
C (n=14)
surface

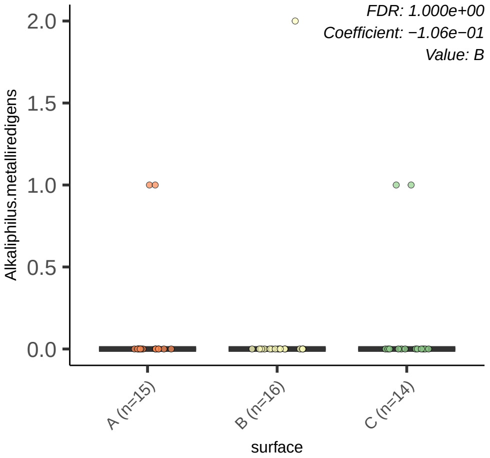
FDR: 1.000e+00
Coefficient: −1.06e−01
Value: B
Alkaliphilus.metalliredigens
2.0
1.5
1.0
0.5
0.0
A (n=15)
B (n=16)
C (n=14)
surface

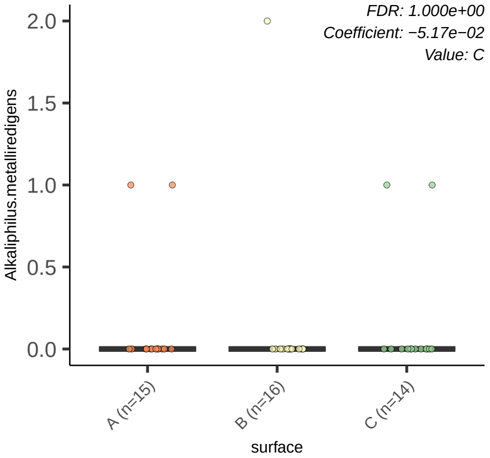
FDR: 1.000e+00
Coefficient: −5.17e−02
Value: C
Alkaliphilus.metalliredigens
2.0
1.5
1.0
0.5
0.0
A (n=15)
B (n=16)
C (n=14)
surface

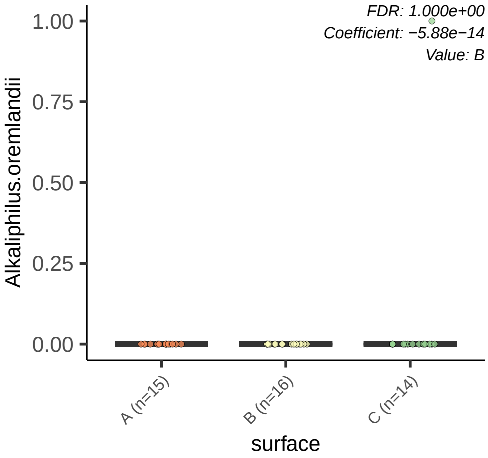

FDR: 1.000e+00
Coefficient: −5.88e−14
Value: B
1.00
0.75
0.50
0.25
0.00
Alkaliphilus.oremlandii
A (n=15)
B (n=16)
C (n=14)
surface

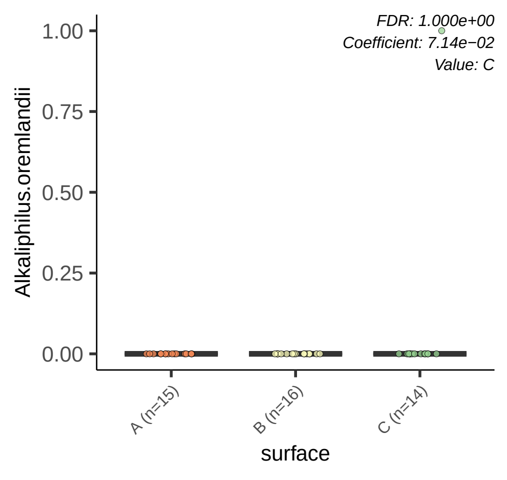
FDR: 1.000e+00
Coefficient: 7.14e−02
Value: C
Alkaliphilus.oremlandii
1.00
0.75
0.50
0.25
0.00
A (n=15)
B (n=16)
C (n=14)
surface

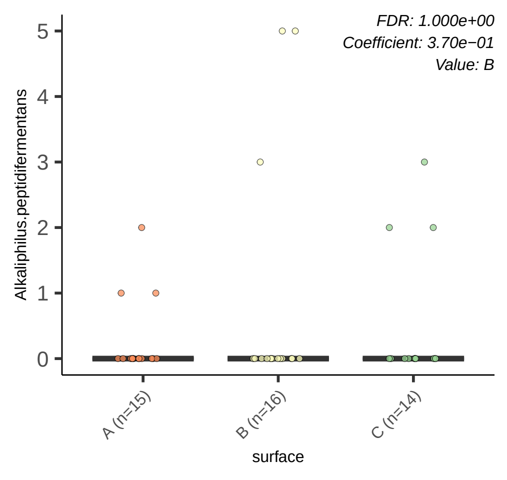
FDR: 1.000e+00
Coefficient: 3.70e−01
Value: B
Alkaliphilus.peptidifermentans
0
1
2
3
4
5
A (n=15)
B (n=16)
C (n=14)
surface

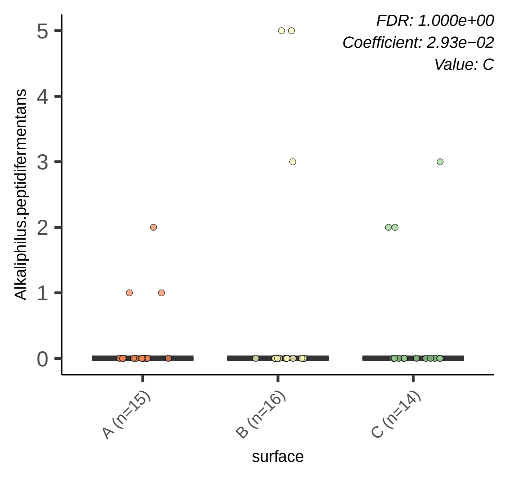
FDR: 1.000e+00
Coefficient: 2.93e−02
Value: C
Alkaliphilus.peptidifermentans
0
1
2
3
4
5
A (n=15)
B (n=16)
C (n=14)
surface

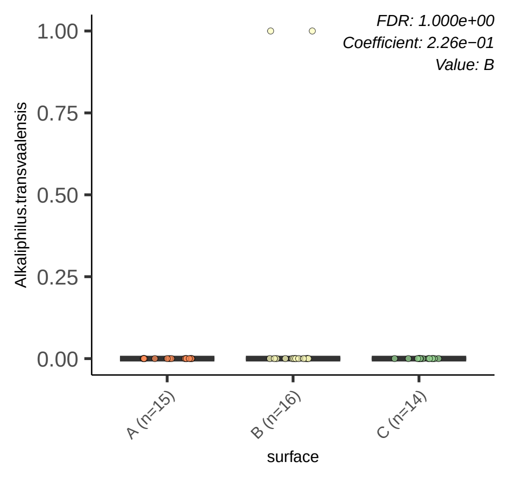

FDR: 1.000e+00
Coefficient: 2.26e−01
Value: B
1.00
0.75
0.50
0.25
0.00
Alkaliphilus.transvaalensis
A (n=15)
B (n=16)
C (n=14)
surface

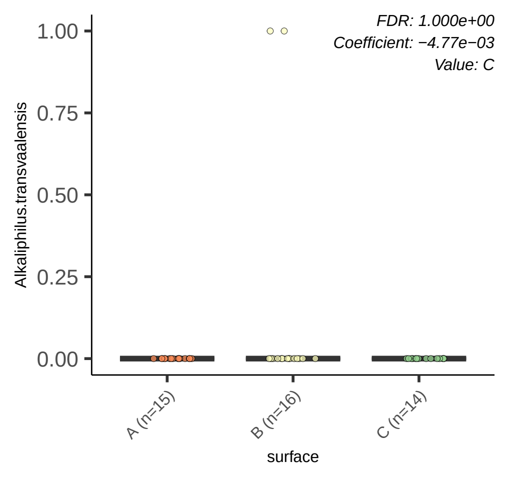

FDR: 1.000e+00
Coefficient: −4.77e−03
Value: C
1.00
0.75
0.50
0.25
0.00
Alkaliphilus.transvaalensis
A (n=15)
B (n=16)
C (n=14)
surface

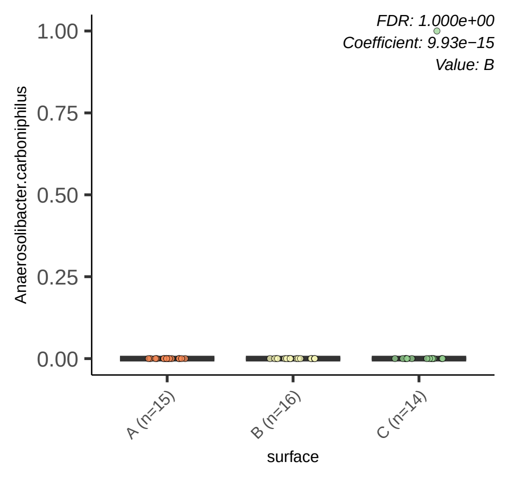

FDR: 1.000e+00
Coefficient: 9.93e−15
Value: B
1.00
0.75
0.50
0.25
0.00
Anaerosolibacter.carboniphilus
A (n=15)
B (n=16)
C (n=14)
surface

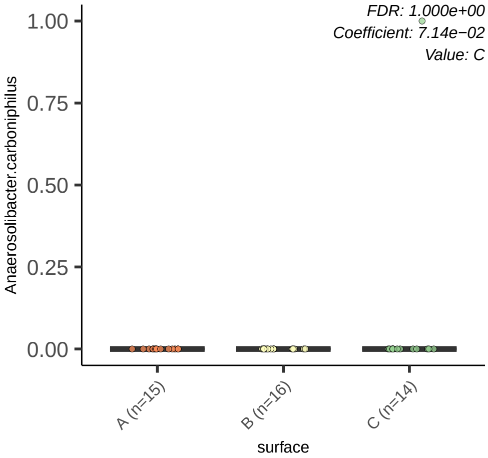

FDR: 1.000e+00
Coefficient: 7.14e−02
Value: C
Anaerosolibacter.carboniphilus
1.00
0.75
0.50
0.25
0.00
A (n=15)
B (n=16)
C (n=14)
surface

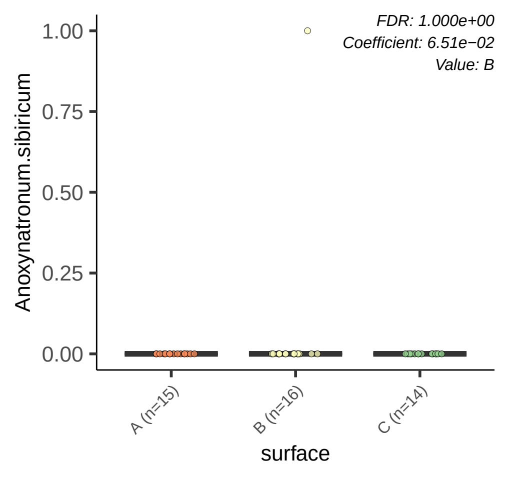
FDR: 1.000e+00
Coefficient: 6.51e−02
Value: B
Anoxynatronum.sibiricum
1.00
0.75
0.50
0.25
0.00
A (n=15)
B (n=16)
C (n=14)
surface

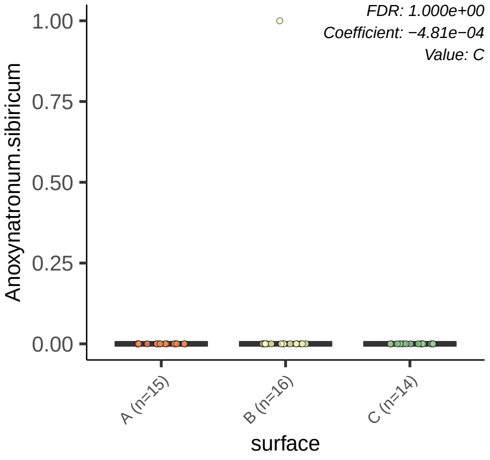
FDR: 1.000e+00
Coefficient: −4.81e−04
Value: C
Anoxynatronum.sibiricum
1.00
0.75
0.50
0.25
0.00
A (n=15)
B (n=16)
C (n=14)
surface

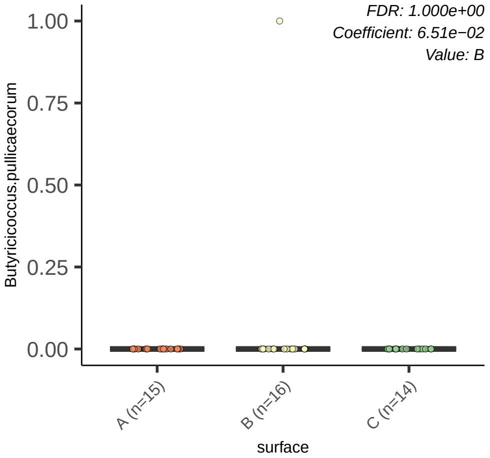
FDR: 1.000e+00
Coefficient: 6.51e−02
Value: B
Butyricicoccus.pulicaecorum
1.00
0.75
0.50
0.25
0.00
A (n=15)
B (n=16)
C (n=14)
surface

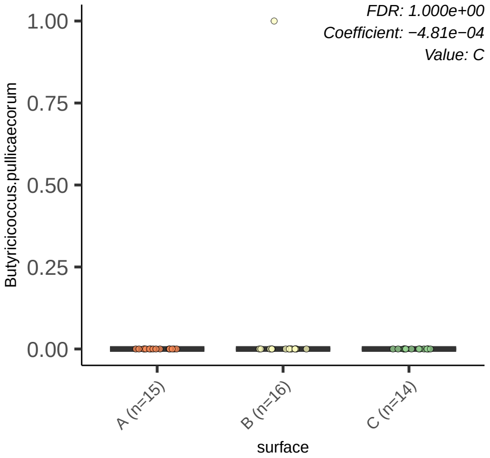

FDR: 1.000e+00
Coefficient: −4.81e−04
Value: C
Butyricicoccus.pullicaecorum
1.00
0.75
0.50
0.25
0.00
A (n=15)
B (n=16)
C (n=14)
surface

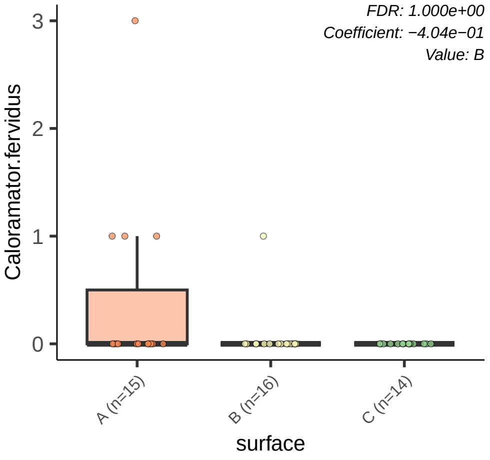
FDR: 1.000e+00
Coefficient: −4.04e−01
Value: B
Caloramator.fervidus
3
2
1
0
A (n=15)
B (n=16)
C (n=14)
surface

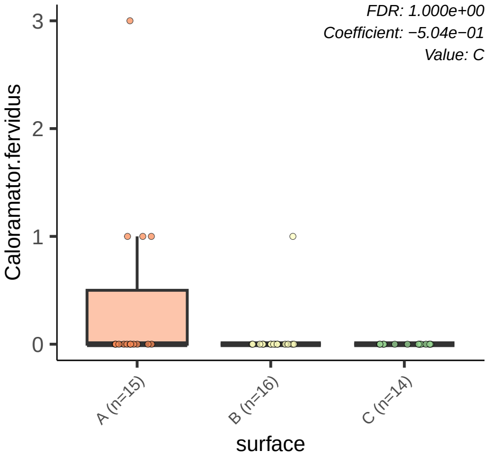

FDR: 1.000e+00
Coefficient: −5.04e−01
Value: C
Caloramator.fervidus
0
1
2
3
A (n=15)
B (n=16)
C (n=14)
surface

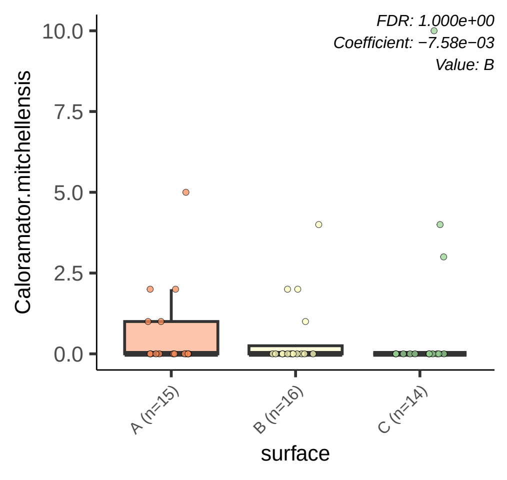

FDR: 1.000e+00
Coefficient: −7.58e−03
Value: B
Caloramator.mitchellensis
10.0
7.5
5.0
2.5
0.0
A (n=15)
B (n=16)
C (n=14)
surface

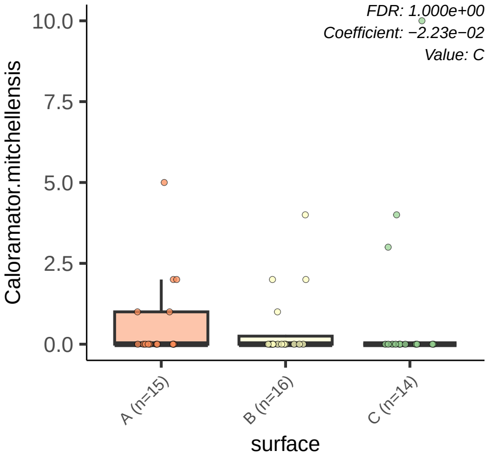

FDR: 1.000e+00
Coefficient: −2.23e−02
Value: C
Caloramator.mitchellensis
10.0
7.5
5.0
2.5
0.0
A (n=15)
B (n=16)
C (n=14)
surface

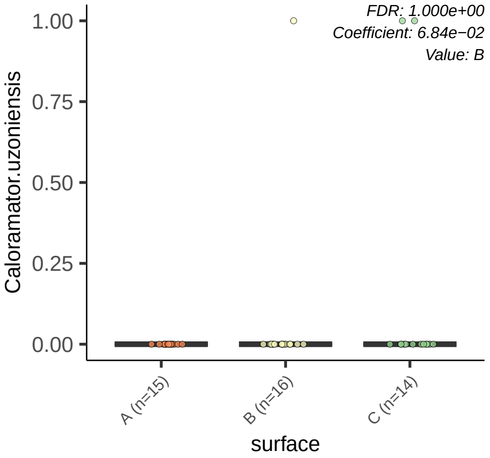
FDR: 1.000e+00
Coefficient: 6.84e−02
Value: B
Caloramator.uzoniensis
1.00
0.75
0.50
0.25
0.00
A (n=15)
B (n=16)
C (n=14)
surface

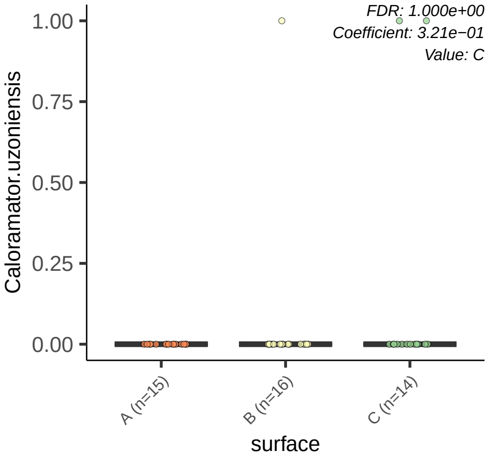

FDR: 1.000e+00
Coefficient: 3.21e−01
Value: C
1.00
0.75
0.50
0.25
0.00
Caloramator.uzoniensis
A (n=15)
B (n=16)
C (n=14)
surface

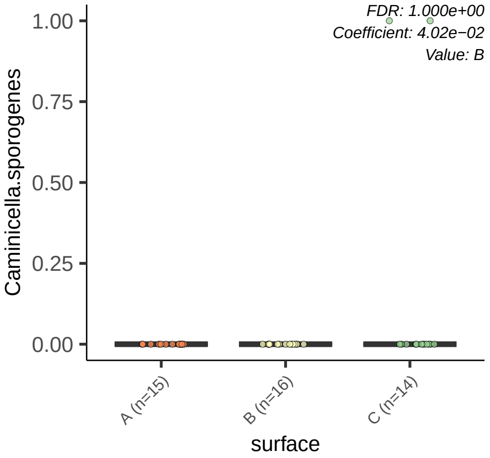

FDR: 1.000e+00
Coefficient: 4.02e−02
Value: B
Caminicella.sporogenes
1.00
0.75
0.50
0.25
0.00
A (n=15)
B (n=16)
C (n=14)
surface

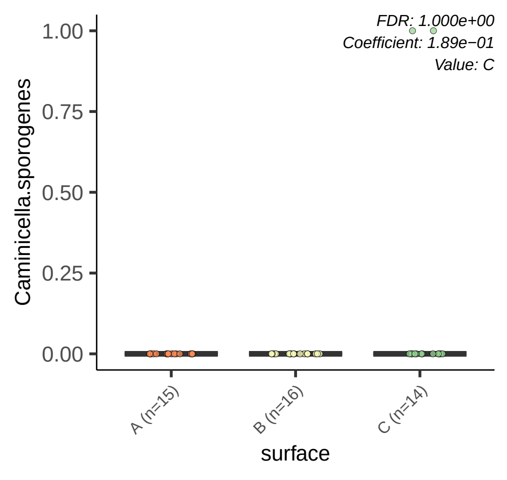

FDR: 1.000e+00
Coefficient: 1.89e−01
Value: C
Caminicella.sporogenes
1.00
0.75
0.50
0.25
0.00
A (n=15)
B (n=16)
C (n=14)
surface

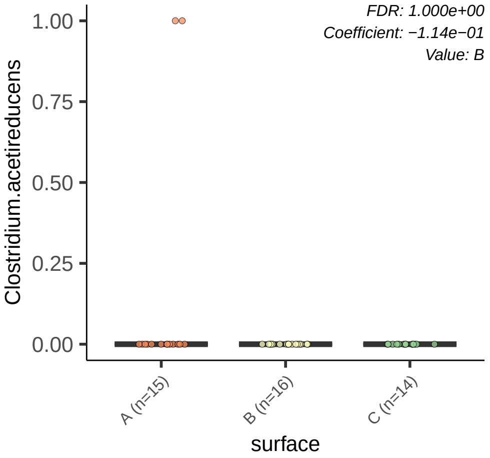
FDR: 1.000e+00
Coefficient: −1.14e−01
Value: B
1.00
0.75
0.50
0.25
0.00
Clostridium.acetireducens
A (n=15)
B (n=16)
C (n=14)
surface

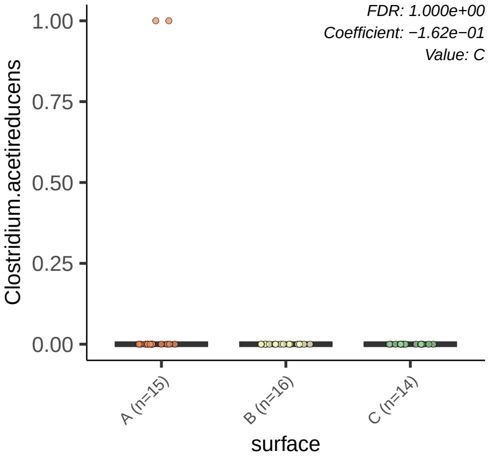
FDR: 1.000e+00
Coefficient: −1.62e−01
Value: C
Clostridium.acetireducens
1.00
0.75
0.50
0.25
0.00
A (n=15)
B (n=16)
C (n=14)
surface

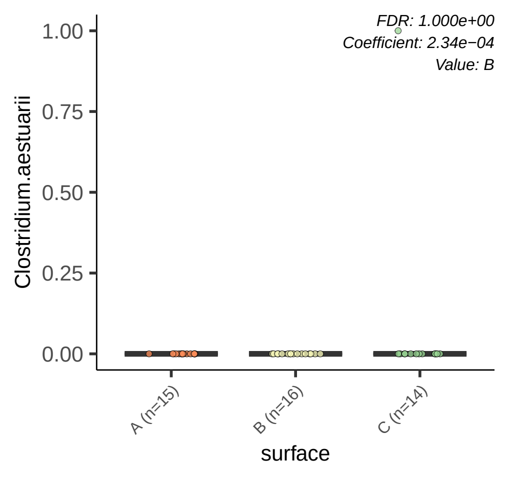
FDR: 1.000e+00
Coefficient: 2.34e−04
Value: B
1.00
0.75
0.50
0.25
0.00
Clostridium.aestuarii
A (n=15)
B (n=16)
C (n=14)
surface

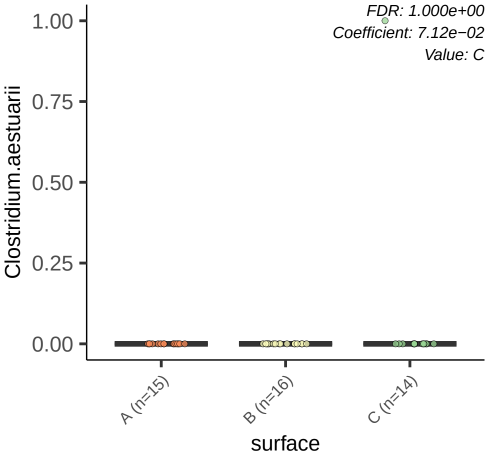
FDR: 1.000e+00
Coefficient: 7.12e−02
Value: C
Clostridium.aestuarii
1.00
0.75
0.50
0.25
0.00
A (n=15)
B (n=16)
C (n=14)
surface

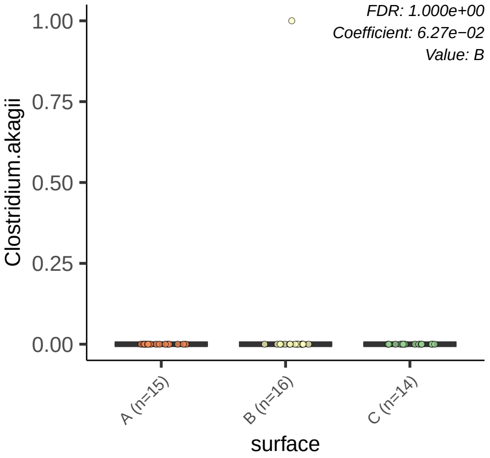

FDR: 1.000e+00
Coefficient: 6.27e−02
Value: B
Clostridium.akagii
1.00
0.75
0.50
0.25
0.00
A (n=15)
B (n=16)
C (n=14)
surface

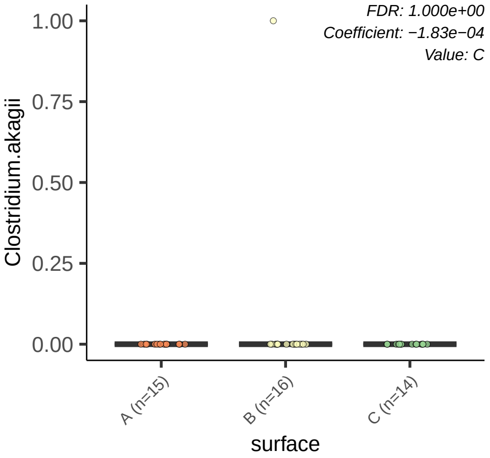
FDR: 1.000e+00
Coefficient: −1.83e−04
Value: C
Clostridium.akagii
1.00
0.75
0.50
0.25
0.00
A (n=15)
B (n=16)
C (n=14)
surface

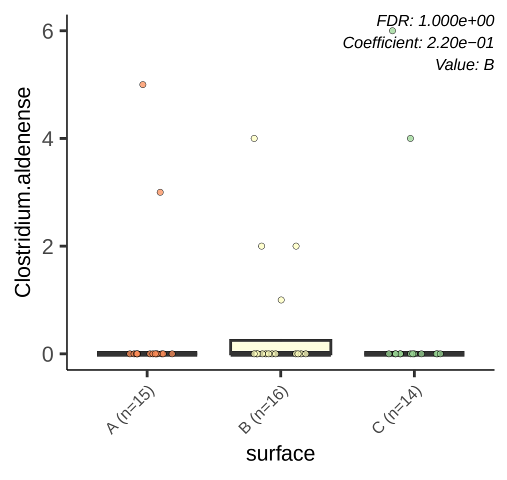
FDR: 1.000e+00
Coefficient: 2.20e−01
Value: B
Clostridium.aldenense
6
4
2
0
A (n=15)
B (n=16)
C (n=14)
surface

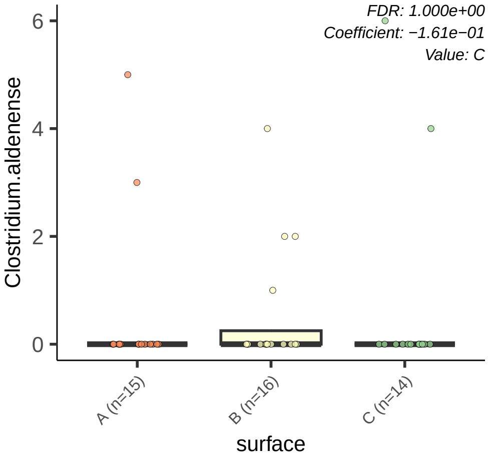
FDR: 1.000e+00
Coefficient: −1.61e−01
Value: C
Clostridium.aldenense
6
4
2
0
A (n=15)
B (n=16)
C (n=14)
surface

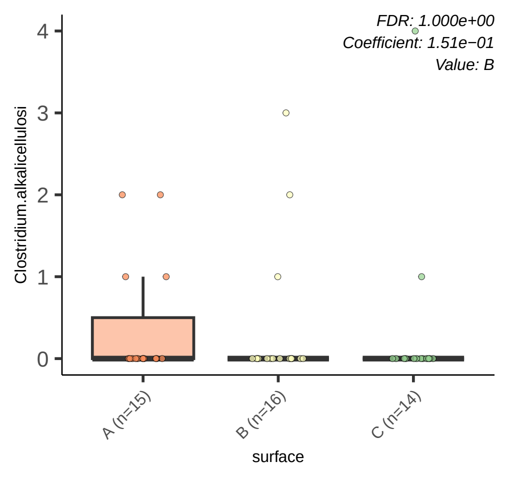

FDR: 1.000e+00
Coefficient: 1.51e−01
Value: B
Clostridium.alkalicellulosi
0
1
2
3
4
A (n=15)
B (n=16)
C (n=14)
surface

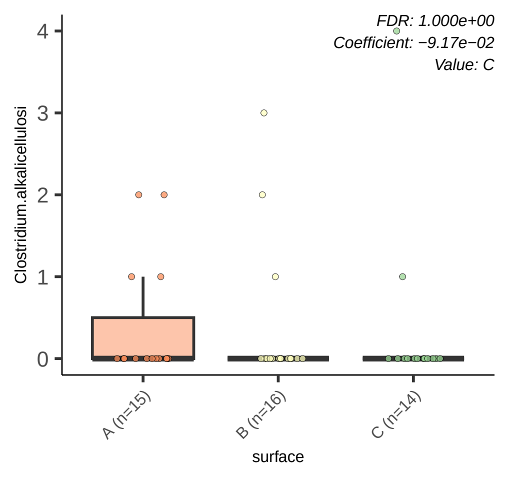
FDR: 1.000e+00
Coefficient: −9.17e−02
Value: C
Clostridium.alkalicellulosi
0
1
2
3
4
A (n=15)
B (n=16)
C (n=14)
surface

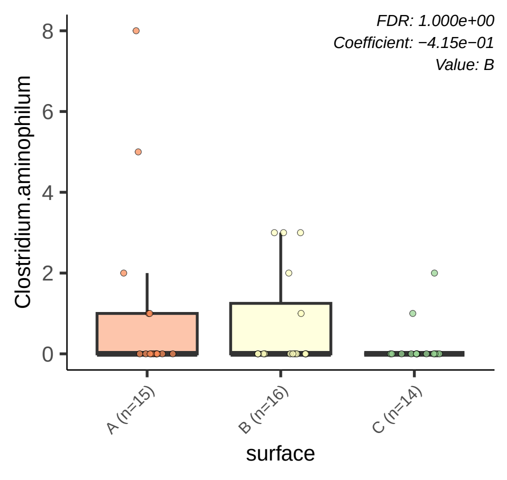
FDR: 1.000e+00
Coefficient: −4.15e−01
Value: B
Clostridium.aminophilum
8
6
4
2
0
A (n=15)
B (n=16)
C (n=14)
surface

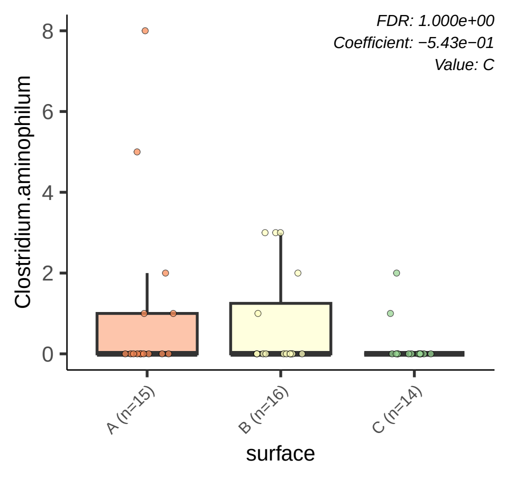

FDR: 1.000e+00
Coefficient: −5.43e−01
Value: C
Clostridium.aminophilum
8
6
4
2
0
A (n=15)
B (n=16)
C (n=14)
surface

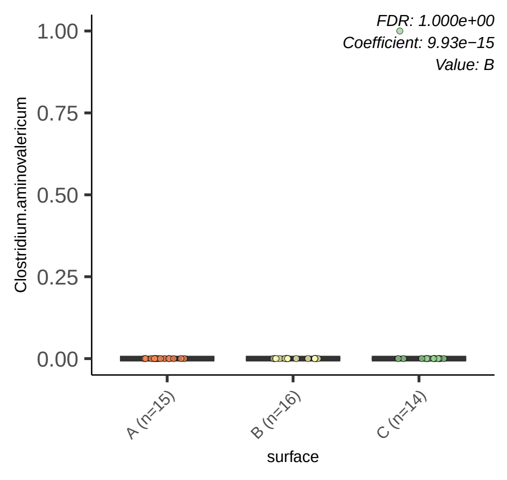

FDR: 1.000e+00
Coefficient: 9.93e−15
Value: B
1.00
0.75
0.50
0.25
0.00
Clostridium.aminovalericum
A (n=15)
B (n=16)
C (n=14)
surface

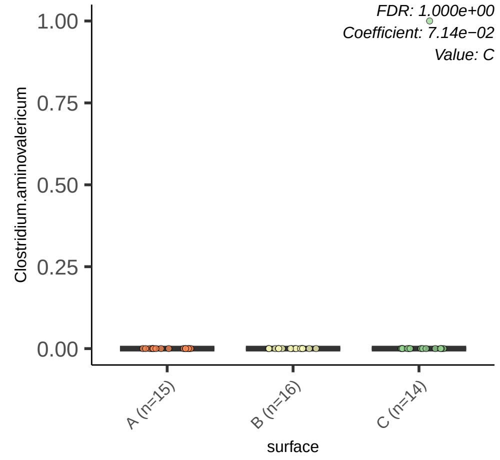
FDR: 1.000e+00
Coefficient: 7.14e−02
Value: C
Clostridium.aminovalericum
1.00
0.75
0.50
0.25
0.00
A (n=15)
B (n=16)
C (n=14)
surface

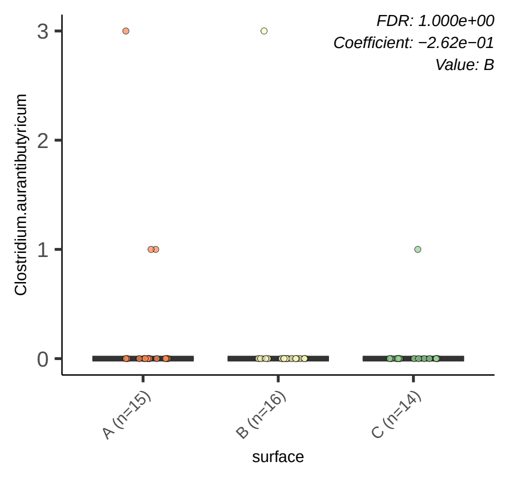
FDR: 1.000e+00
Coefficient: −2.62e−01
Value: B
Clostridium.aurantibutyricum
3
2
1
0
A (n=15)
B (n=16)
C (n=14)
surface

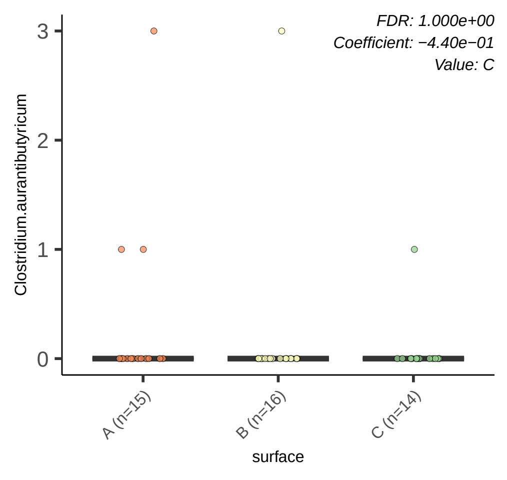
FDR: 1.000e+00
Coefficient: −4.40e−01
Value: C
Clostridium.aurantibutyricum
3
2
1
0
A (n=15)
B (n=16)
C (n=14)
surface

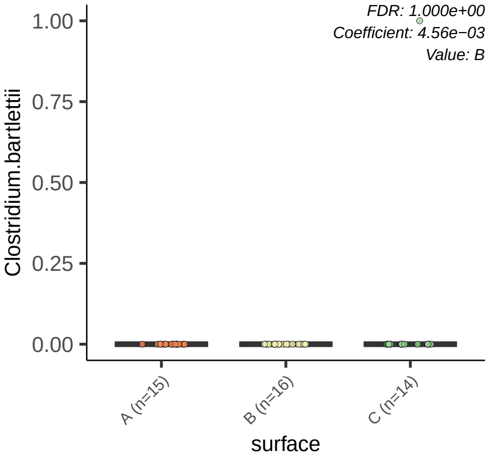
FDR: 1.000e+00
Coefficient: 4.56e−03
Value: B
Clostridium.bartlettii
1.00
0.75
0.50
0.25
0.00
A (n=15)
B (n=16)
C (n=14)
surface

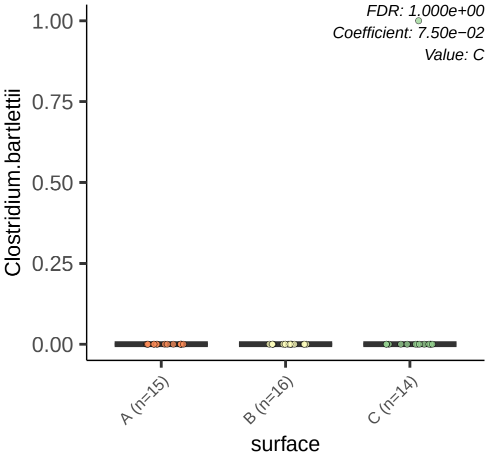

FDR: 1.000e+00
Coefficient: 7.50e−02
Value: C
Clostridium.bartlettii
1.00
0.75
0.50
0.25
0.00
A (n=15)
B (n=16)
C (n=14)
surface

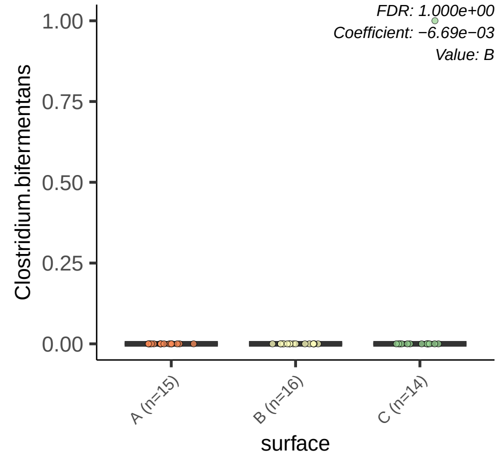
FDR: 1.000e+00
Coefficient: −6.69e−03
Value: B
Clostridium.bifermentans
1.00
0.75
0.50
0.25
0.00
A (n=15)
B (n=16)
C (n=14)
surface

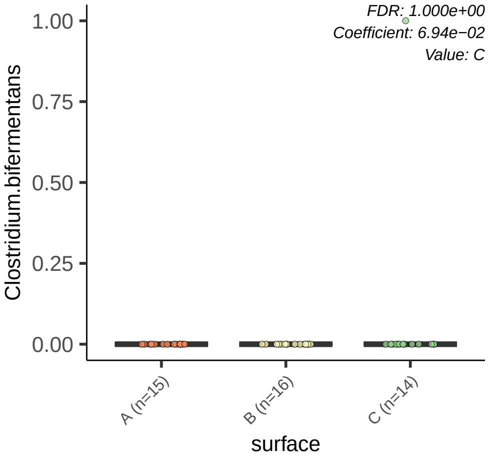

FDR: 1.000e+00
Coefficient: 6.94e−02
Value: C
Clostridium.bifermentans
1.00
0.75
0.50
0.25
0.00
A (n=15)
B (n=16)
C (n=14)
surface

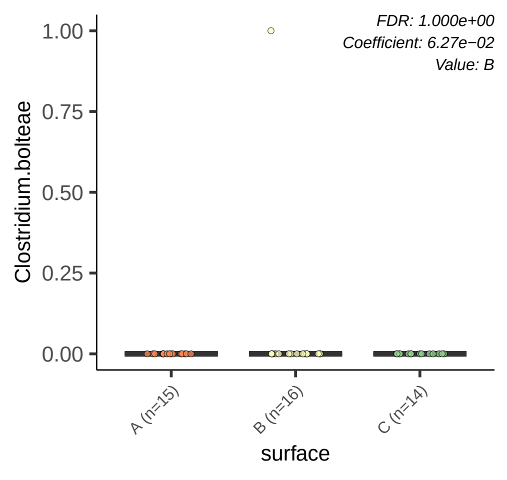
FDR: 1.000e+00
Coefficient: 6.27e−02
Value: B
Clostridium.bolteae
1.00
0.75
0.50
0.25
0.00
A (n=15)
B (n=16)
C (n=14)
surface

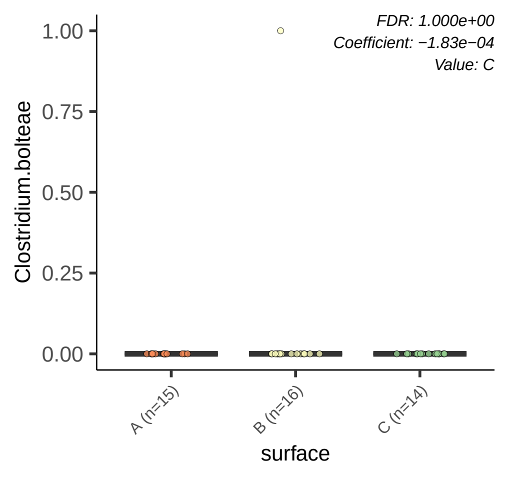
FDR: 1.000e+00
Coefficient: −1.83e−04
Value: C
Clostridium.bolteae
1.00
0.75
0.50
0.25
0.00
A (n=15)
B (n=16)
C (n=14)
surface

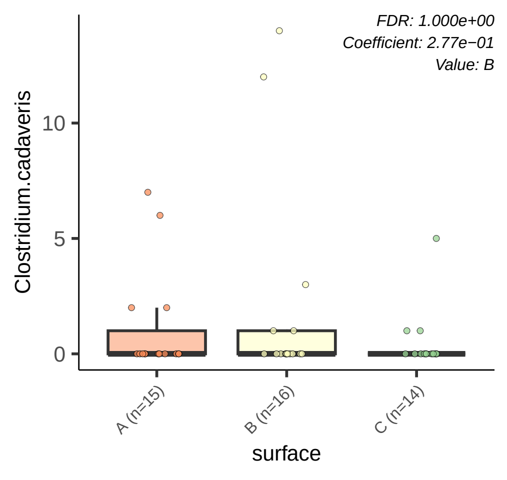
FDR: 1.000e+00
Coefficient: 2.77e−01
Value: B
Clostridium.cadaveris
10
5
0
A (n=15)
B (n=16)
C (n=14)
surface

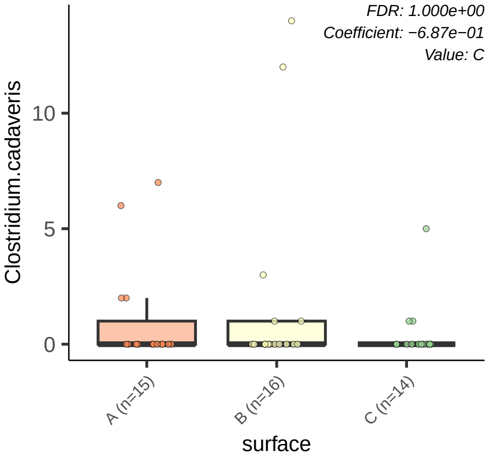

FDR: 1.000e+00
Coefficient: −6.87e−01
Value: C
Clostridium.cadaveris
10
5
0
A (n=15)
B (n=16)
C (n=14)
surface

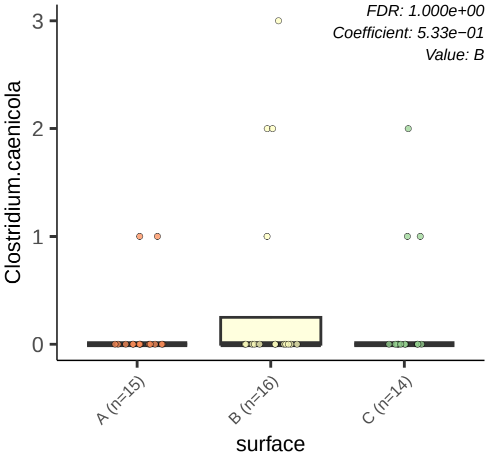
FDR: 1.000e+00
Coefficient: 5.33e−01
Value: B
Clostridium.caenicola
3
2
1
0
A (n=15)
B (n=16)
C (n=14)
surface

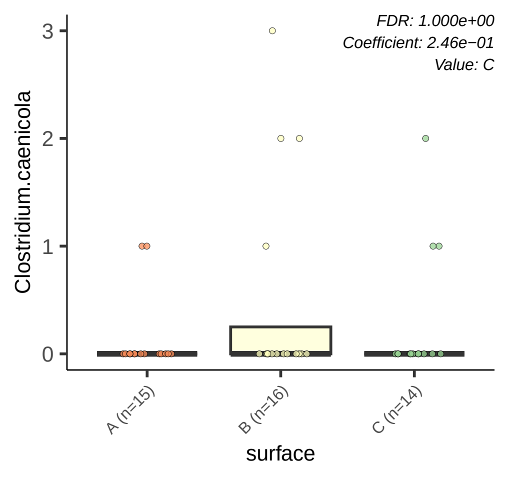
FDR: 1.000e+00
Coefficient: 2.46e−01
Value: C
Clostridium.caenicola
3
2
1
0
A (n=15)
B (n=16)
C (n=14)
surface

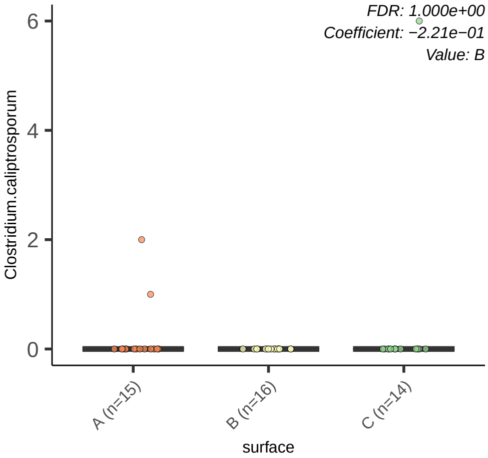

FDR: 1.000e+00
Coefficient: −2.21e−01
Value: B
Clostridium.caliptrosporum
0
2
4
6
A (n=15)
B (n=16)
C (n=14)
surface

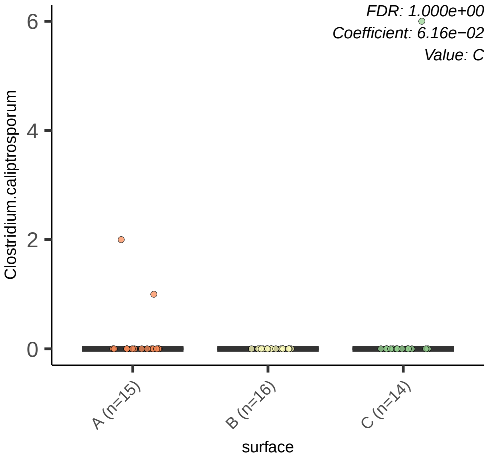
FDR: 1.000e+00
Coefficient: 6.16e−02
Value: C
Clostridium.caliptrosporum
0
2
4
6
A (n=15)
B (n=16)
C (n=14)
surface

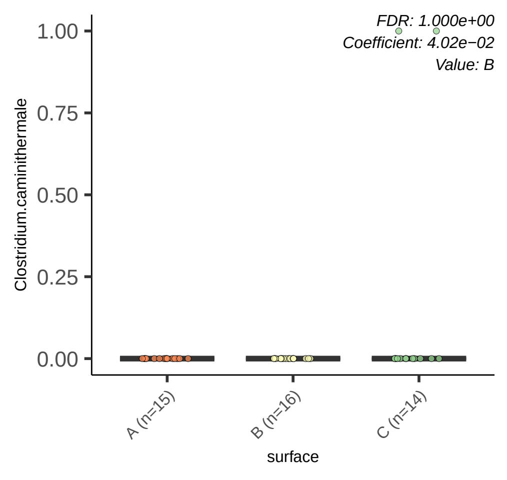
FDR: 1.000e+00
Coefficient: 4.02e−02
Value: B
Clostridium.caminithermale
1.00
0.75
0.50
0.25
0.00
A (n=15)
B (n=16)
C (n=14)
surface

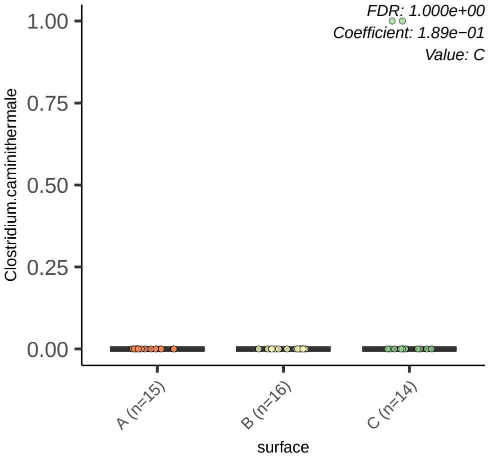
FDR: 1.000e+00
Coefficient: 1.89e−01
Value: C
1.00
0.75
0.50
0.25
0.00
Clostridium.caminithermale
A (n=15)
B (n=16)
C (n=14)
surface

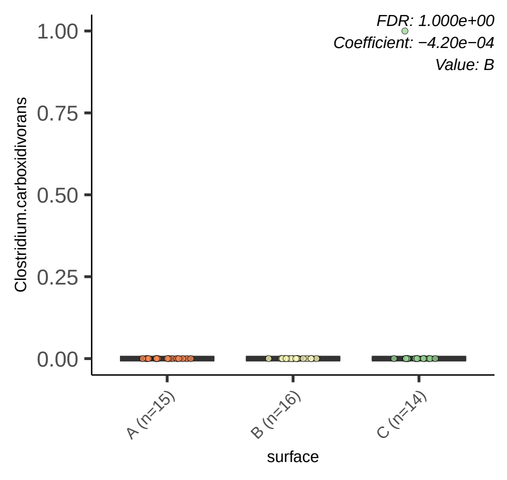
FDR: 1.000e+00
Coefficient: −4.20e−04
Value: B
1.00
0.75
0.50
0.25
0.00
Clostridium.carboxidivorans
A (n=15)
B (n=16)
C (n=14)
surface

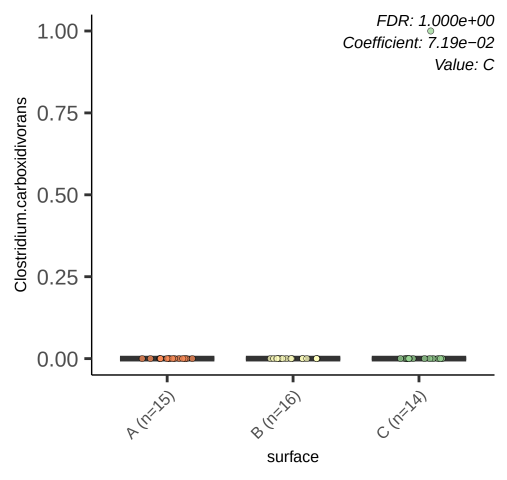
FDR: 1.000e+00
Coefficient: 7.19e−02
Value: C
Clostridium.carboxidivorans
1.00
0.75
0.50
0.25
0.00
A (n=15)
B (n=16)
C (n=14)
surface

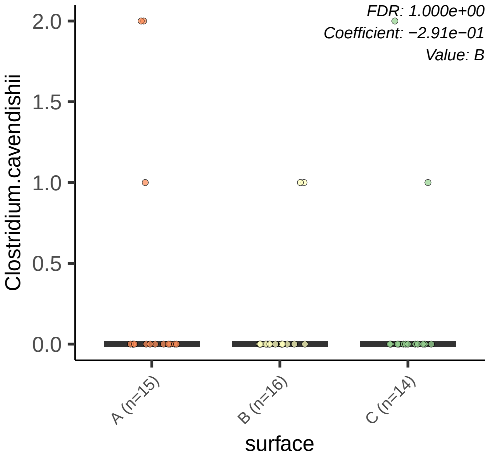

FDR: 1.000e+00
Coefficient: −2.91e−01
Value: B
Clostridium.cavendishii
2.0
1.5
1.0
0.5
0.0
A (n=15)
B (n=16)
C (n=14)
surface

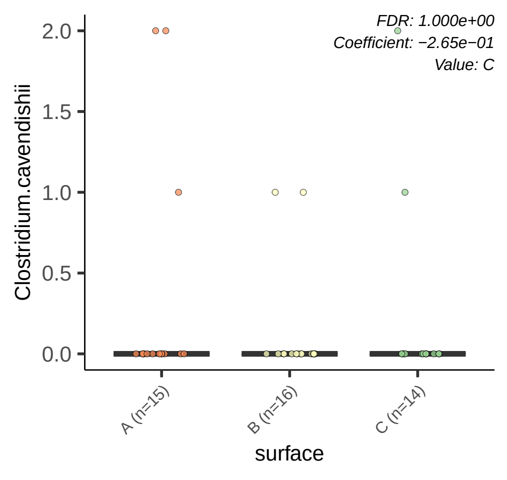
FDR: 1.000e+00
Coefficient: −2.65e−01
Value: C
Clostridium.cavendishii
2.0
1.5
1.0
0.5
0.0
A (n=15)
B (n=16)
C (n=14)
surface

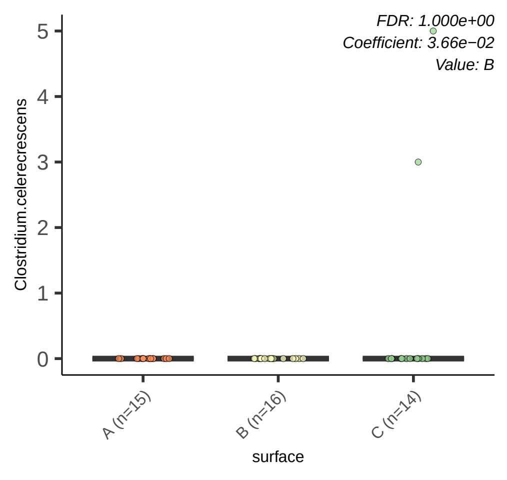

FDR: 1.000e+00
Coefficient: 3.66e−02
Value: B
Clostridium.celerecrescens
0
1
2
3
4
5
A (n=15)
B (n=16)
C (n=14)
surface

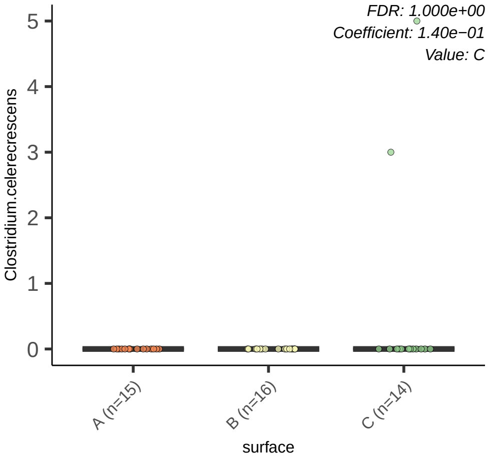

FDR: 1.000e+00
Coefficient: 1.40e−01
Value: C
Clostridium.celerecrescens
5
4
3
2
1
0
A (n=15)
B (n=16)
C (n=14)
surface

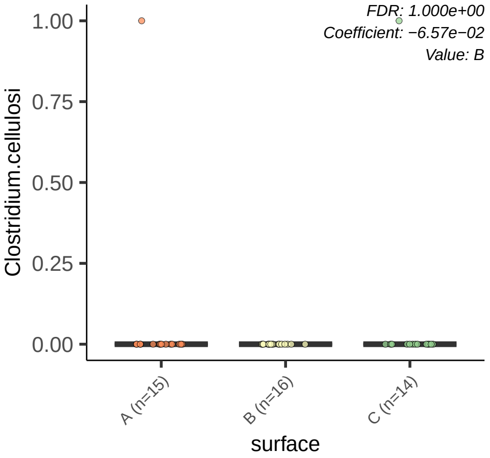

FDR: 1.000e+00
Coefficient: −6.57e−02
Value: B
Clostridium.cellulosi
1.00
0.75
0.50
0.25
0.00
A (n=15)
B (n=16)
C (n=14)
surface

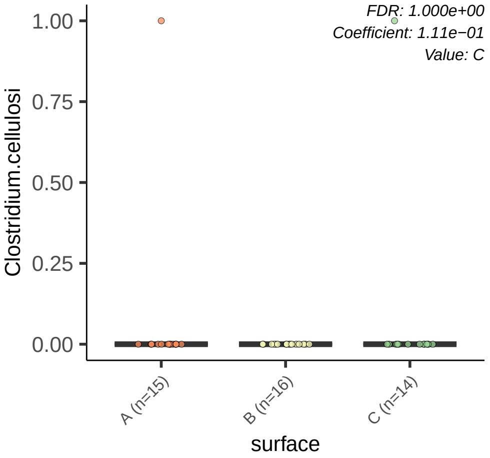
FDR: 1.000e+00
Coefficient: 1.11e−01
Value: C
Clostridium.cellulosi
1.00
0.75
0.50
0.25
0.00
A (n=15)
B (n=16)
C (n=14)
surface

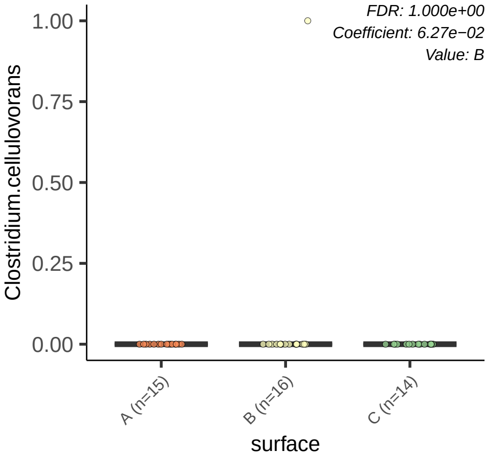

FDR: 1.000e+00
Coefficient: 6.27e−02
Value: B
Clostridium.cellulovorans
1.00
0.75
0.50
0.25
0.00
A (n=15)
B (n=16)
C (n=14)
surface

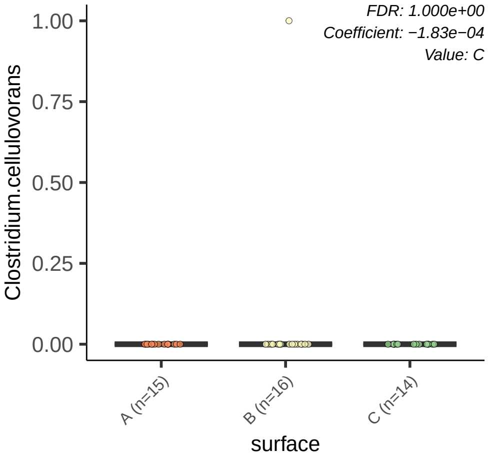
FDR: 1.000e+00
Coefficient: −1.83e−04
Value: C
Clostridium.cellulovorans
1.00
0.75
0.50
0.25
0.00
A (n=15)
B (n=16)
C (n=14)
surface

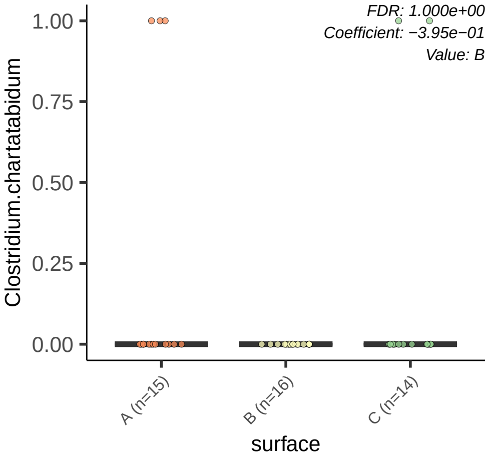
FDR: 1.000e+00
Coefficient: −3.95e−01
Value: B
Clostridium.chartatabidum
1.00
0.75
0.50
0.25
0.00
A (n=15)
B (n=16)
C (n=14)
surface

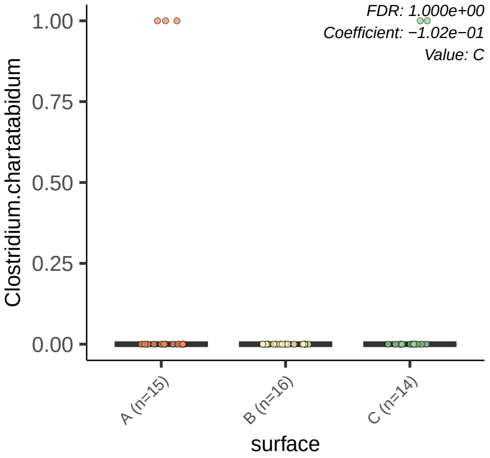
FDR: 1.000e+00
Coefficient: −1.02e−01
Value: C
Clostridium.chartatabidum
1.00
0.75
0.50
0.25
0.00
A (n=15)
B (n=16)
C (n=14)
surface

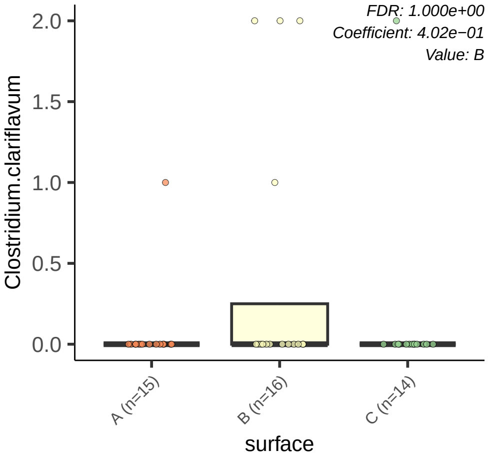

FDR: 1.000e+00
Coefficient: 4.02e−01
Value: B
Clostridium.clariflavum
2.0
1.5
1.0
0.5
0.0
A (n=15)
B (n=16)
C (n=14)
surface

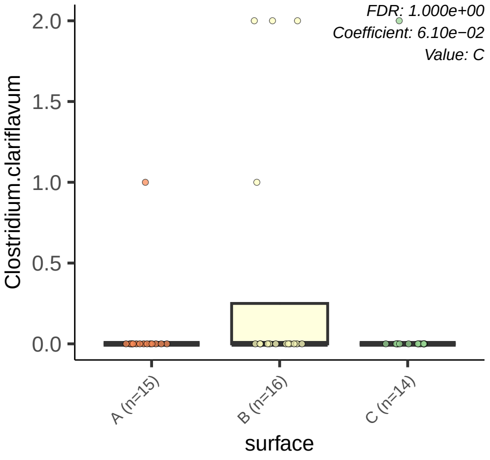

FDR: 1.000e+00
Coefficient: 6.10e−02
Value: C
Clostridium.clariflavum
2.0
1.5
1.0
0.5
0.0
A (n=15)
B (n=16)
C (n=14)
surface

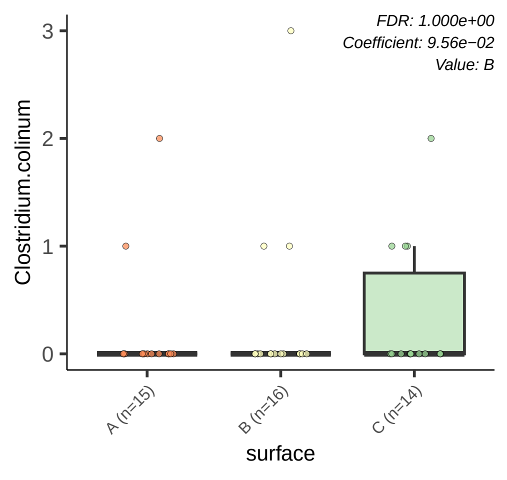

FDR: 1.000e+00
Coefficient: 9.56e−02
Value: B
Clostridium.colinum
0
1
2
3
A (n=15)
B (n=16)
C (n=14)
surface

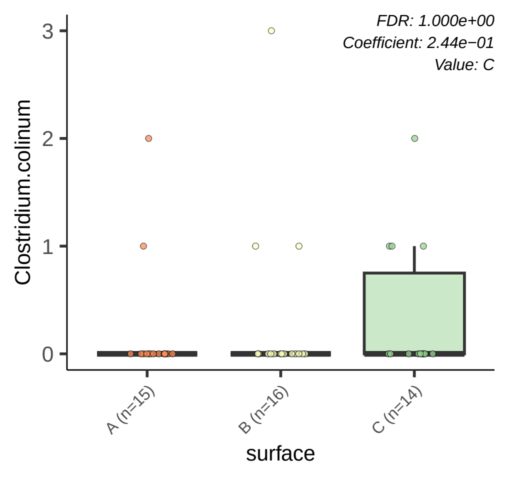
FDR: 1.000e+00
Coefficient: 2.44e−01
Value: C
Clostridium.colinum
3
2
1
0
A (n=15)
B (n=16)
C (n=14)
surface

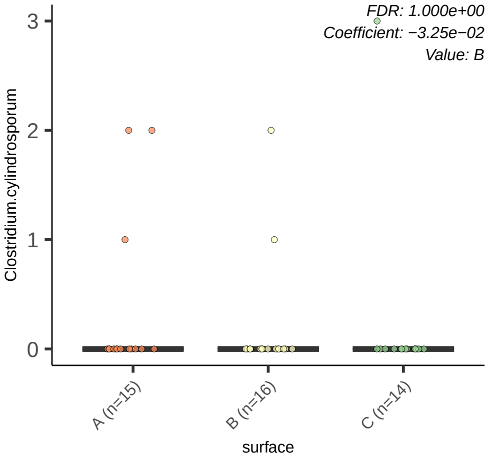
FDR: 1.000e+00
Coefficient: −3.25e−02
Value: B
Clostridium.cylindrosporum
3
2
1
0
A (n=15)
B (n=16)
C (n=14)
surface

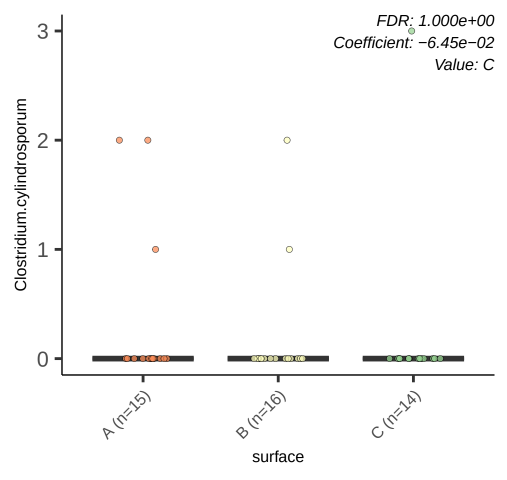

FDR: 1.000e+00
Coefficient: −6.45e−02
Value: C
Clostridium.cylindrosporum
3
2
1
0
A (n=15)
B (n=16)
C (n=14)
surface

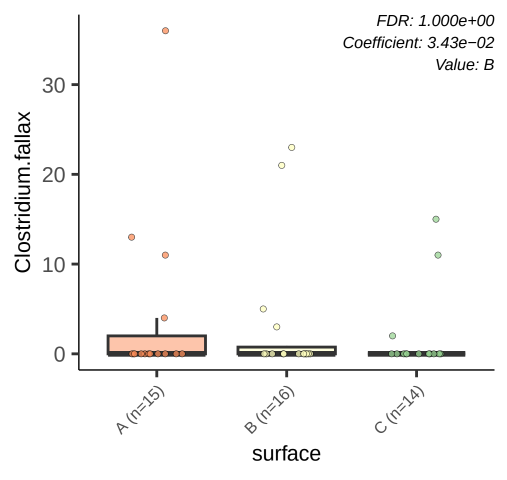

FDR: 1.000e+00
Coefficient: 3.43e−02
Value: B
Clostridium.fallax
0
10
20
30
A (n=15)
B (n=16)
C (n=14)
surface

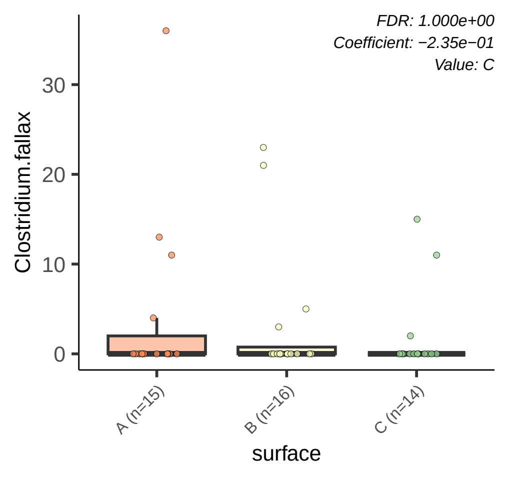

FDR: 1.000e+00
Coefficient: −2.35e−01
Value: C
Clostridium.fallax
30
20
10
0
A (n=15)
B (n=16)
C (n=14)
surface

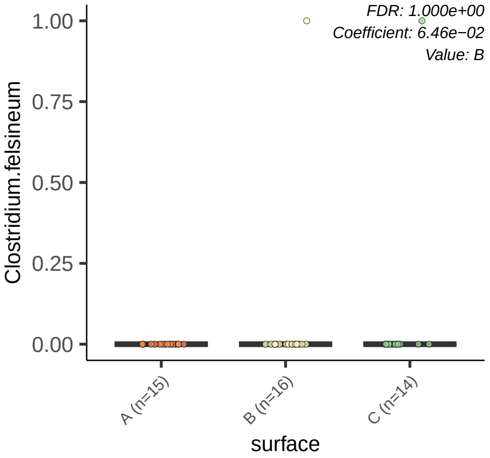
FDR: 1.000e+00
Coefficient: 6.46e−02
Value: B
Clostridium.felsineum
1.00
0.75
0.50
0.25
0.00
A (n=15)
B (n=16)
C (n=14)
surface

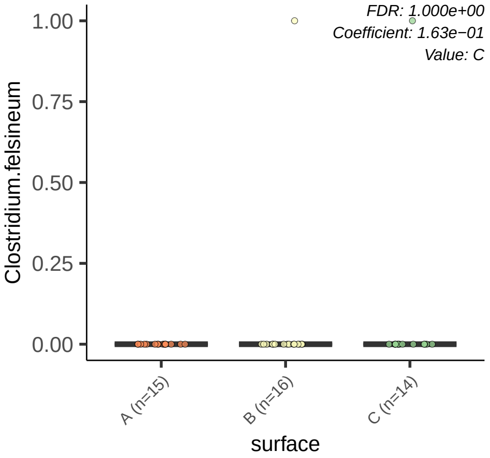
FDR: 1.000e+00
Coefficient: 1.63e−01
Value: C
Clostridium.felsineum
1.00
0.75
0.50
0.25
0.00
A (n=15)
B (n=16)
C (n=14)
surface

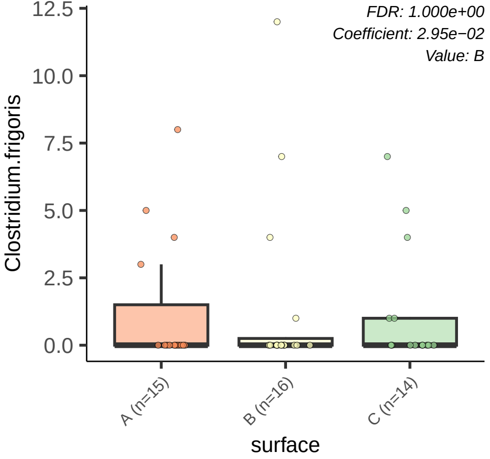

FDR: 1.000e+00
Coefficient: 2.95e−02
Value: B
Clostridium.frigoris
12.5
10.0
7.5
5.0
2.5
0.0
A (n=15)
B (n=16)
C (n=14)
surface

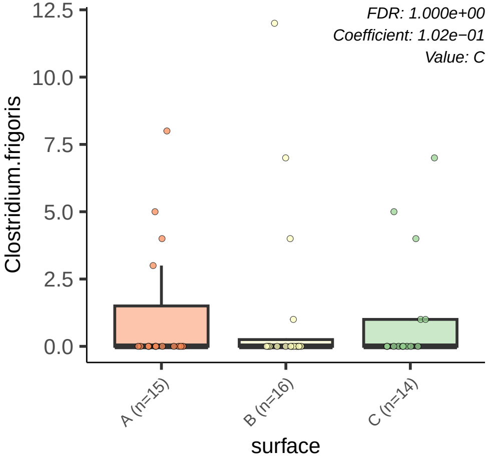
FDR: 1.000e+00
Coefficient: 1.02e−01
Value: C
Clostridium.frigoris
12.5
10.0
7.5
5.0
2.5
0.0
A (n=15)
B (n=16)
C (n=14)
surface

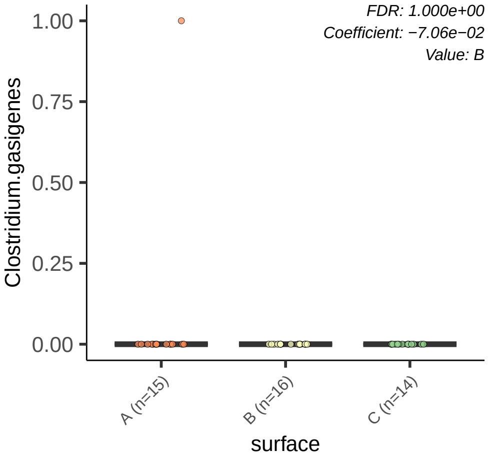
FDR: 1.000e+00
Coefficient: −7.06e−02
Value: B
Clostridium.gasigenes
1.00
0.75
0.50
0.25
0.00
A (n=15)
B (n=16)
C (n=14)
surface

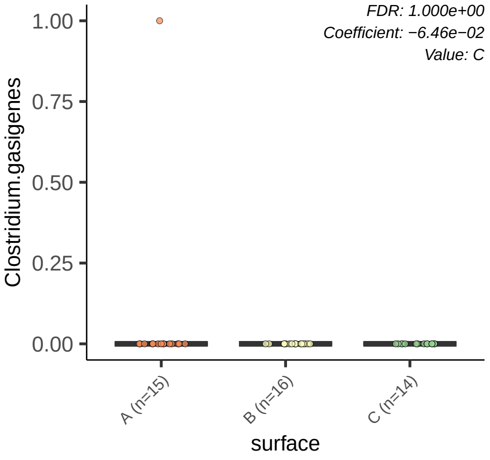
FDR: 1.000e+00
Coefficient: −6.46e−02
Value: C
Clostridium.gasigenes
1.00
0.75
0.50
0.25
0.00
A (n=15)
B (n=16)
C (n=14)
surface

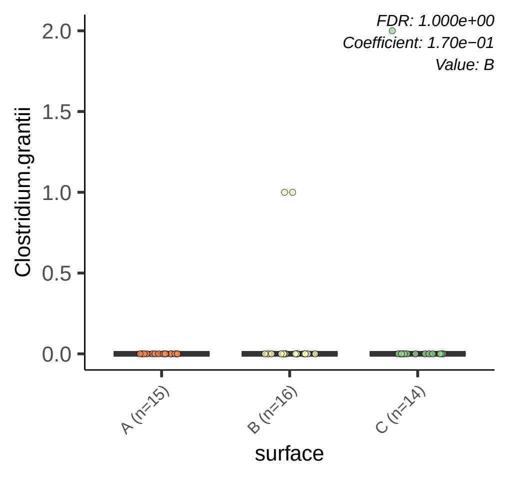
FDR: 1.000e+00
Coefficient: 1.70e−01
Value: B
Clostridium.grantii
2.0
1.5
1.0
0.5
0.0
A (n=15)
B (n=16)
C (n=14)
surface

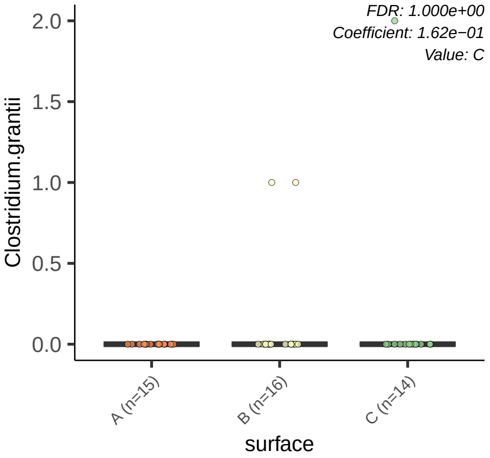
FDR: 1.000e+00
Coefficient: 1.62e−01
Value: C
2.0
1.5
1.0
0.5
0.0
Clostridium.grantii
A (n=15)
B (n=16)
C (n=14)
surface

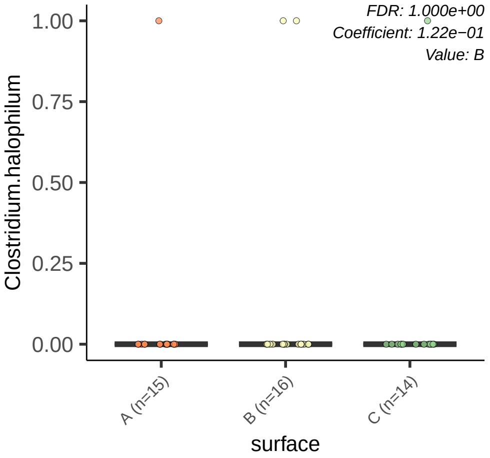
FDR: 1.000e+00
Coefficient: 1.22e−01
Value: B
Clostridium.halophilum
1.00
0.75
0.50
0.25
0.00
A (n=15)
B (n=16)
C (n=14)
surface

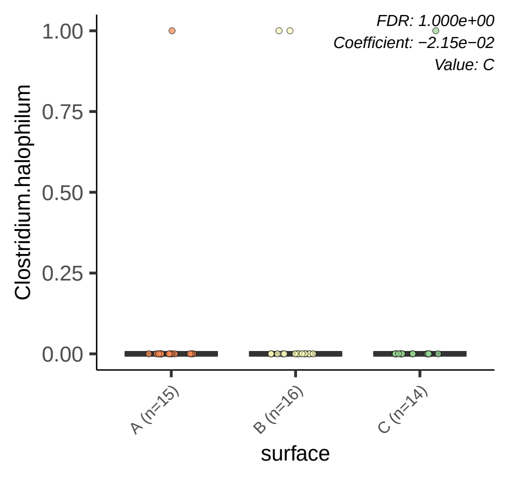
FDR: 1.000e+00
Coefficient: −2.15e−02
Value: C
Clostridium.halophilum
1.00
0.75
0.50
0.25
0.00
A (n=15)
B (n=16)
C (n=14)
surface

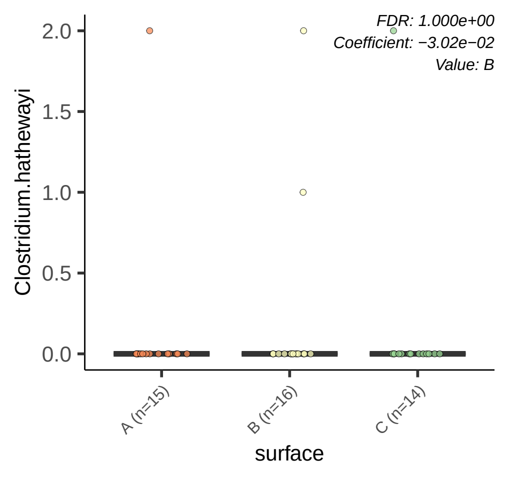
FDR: 1.000e+00
Coefficient: −3.02e−02
Value: B
Clostridium.hathewayi
2.0
1.5
1.0
0.5
0.0
A (n=15)
B (n=16)
C (n=14)
surface

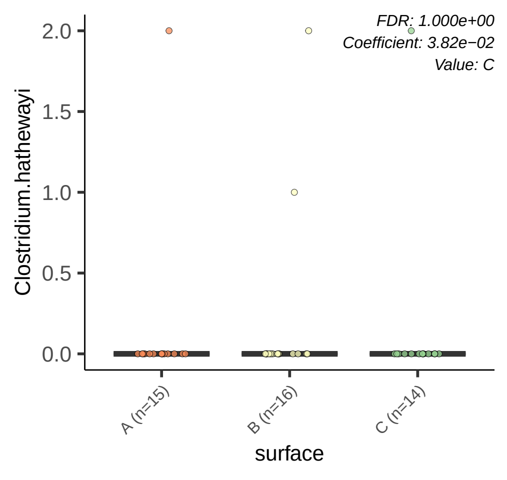
FDR: 1.000e+00
Coefficient: 3.82e−02
Value: C
Clostridium.hathewayi
2.0
1.5
1.0
0.5
0.0
A (n=15)
B (n=16)
C (n=14)
surface

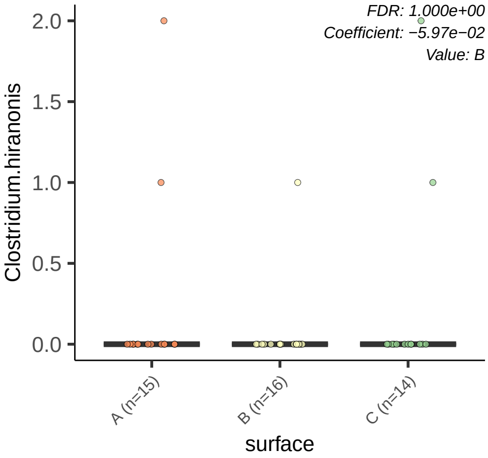
FDR: 1.000e+00
Coefficient: −5.97e−02
Value: B
Clostridium.hiranonis
2.0
1.5
1.0
0.5
0.0
A (n=15)
B (n=16)
C (n=14)
surface

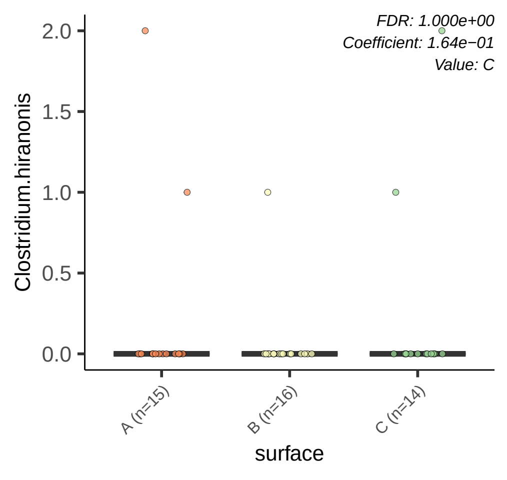
FDR: 1.000e+00
Coefficient: 1.64e−01
Value: C
Clostridium.hiranonis
2.0
1.5
1.0
0.5
0.0
A (n=15)
B (n=16)
C (n=14)
surface

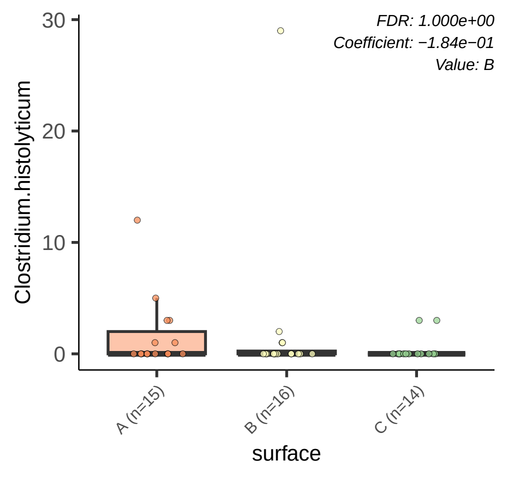
FDR: 1.000e+00
Coefficient: −1.84e−01
Value: B
Clostridium.histolyticum
0
10
20
30
A (n=15)
B (n=16)
C (n=14)
surface

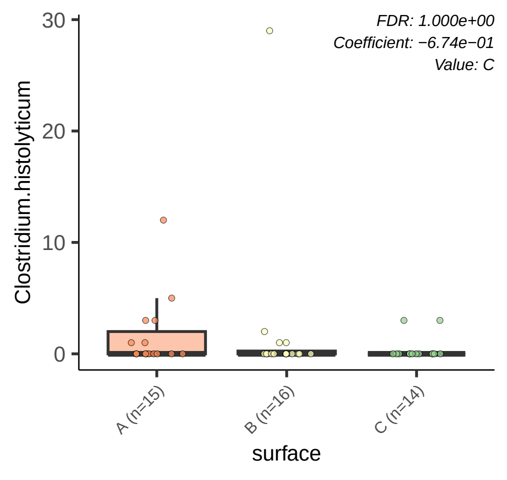
FDR: 1.000e+00
Coefficient: −6.74e−01
Value: C
Clostridium.histolyticum
30
20
10
0
A (n=15)
B (n=16)
C (n=14)
surface

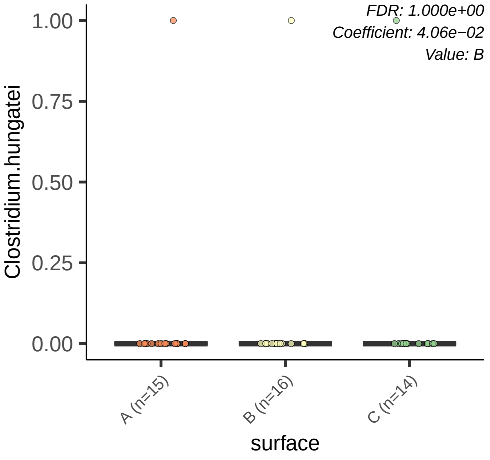
FDR: 1.000e+00
Coefficient: 4.06e−02
Value: B
Clostridium.hungatei
1.00
0.75
0.50
0.25
0.00
A (n=15)
B (n=16)
C (n=14)
surface

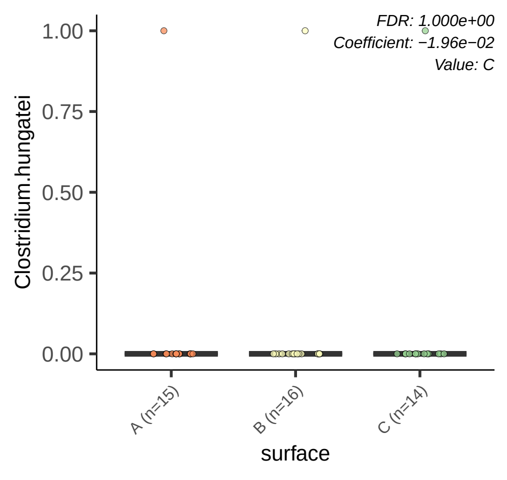
FDR: 1.000e+00
Coefficient: −1.96e−02
Value: C
Clostridium.hungatei
1.00
0.75
0.50
0.25
0.00
A (n=15)
B (n=16)
C (n=14)
surface

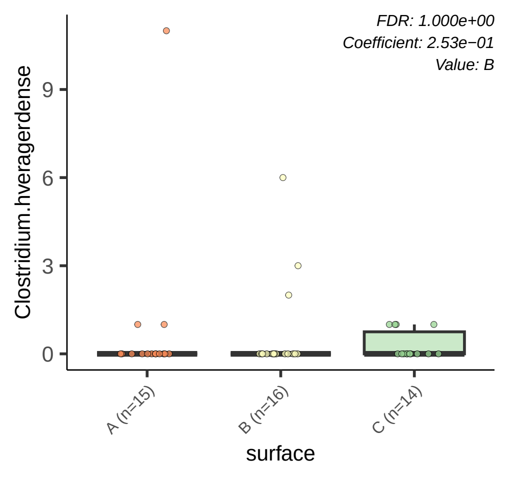
FDR: 1.000e+00
Coefficient: 2.53e−01
Value: B
Clostridium.hveragerdense
0
3
6
9
A (n=15)
B (n=16)
C (n=14)
surface

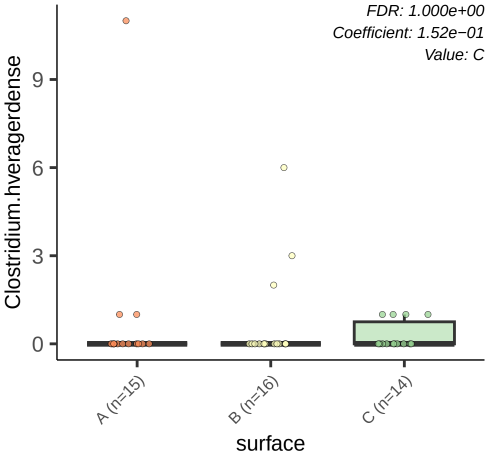
FDR: 1.000e+00
Coefficient: 1.52e−01
Value: C
Clostridium.hveragerdense
0
3
6
9
A (n=15)
B (n=16)
C (n=14)
surface

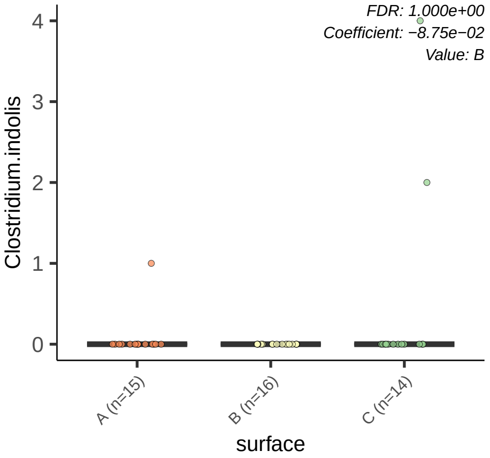
FDR: 1.000e+00
Coefficient: −8.75e−02
Value: B
Clostridium.indolis
0
1
2
3
4
A (n=15)
B (n=16)
C (n=14)
surface

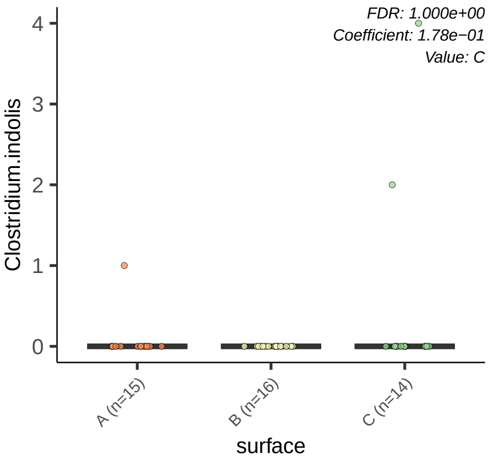
FDR: 1.000e+00
Coefficient: 1.78e−01
Value: C
Clostridium.indolis
4
3
2
1
0
A (n=15)
B (n=16)
C (n=14)
surface

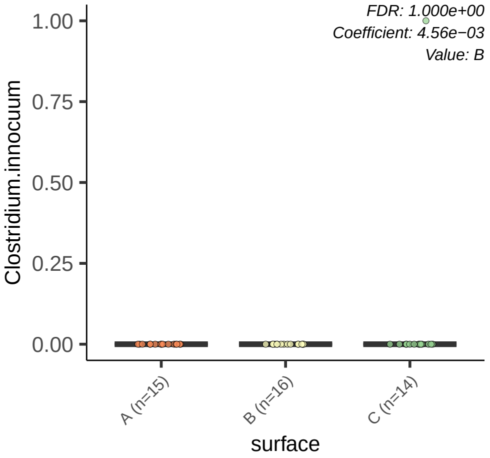
FDR: 1.000e+00
Coefficient: 4.56e−03
Value: B
Clostridium.innocuum
1.00
0.75
0.50
0.25
0.00
A (n=15)
B (n=16)
C (n=14)
surface

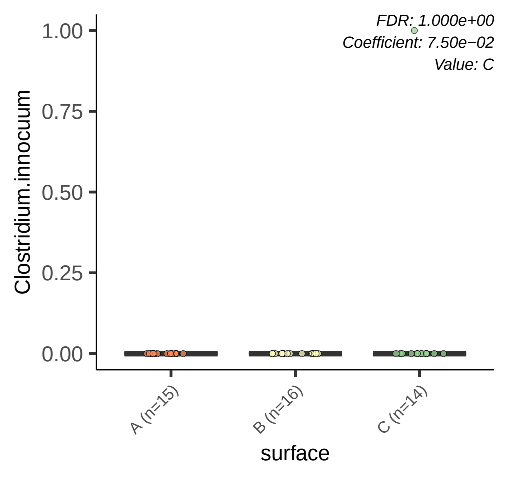
FDR: 1.000e+00
Coefficient: 7.50e−02
Value: C
1.00
0.75
0.50
0.25
0.00
Clostridium.innocuum
A (n=15)
B (n=16)
C (n=14)
surface

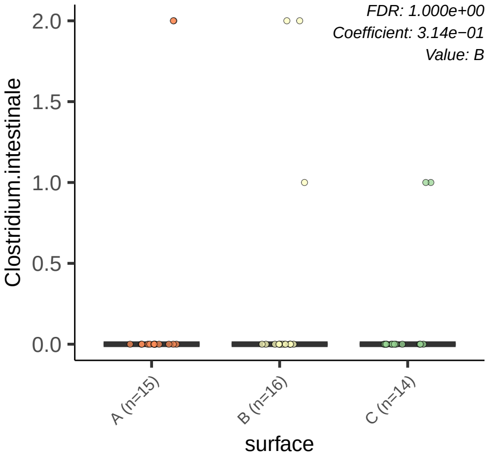
FDR: 1.000e+00
Coefficient: 3.14e−01
Value: B
Clostridium.intestinale
2.0
1.5
1.0
0.5
0.0
A (n=15)
B (n=16)
C (n=14)
surface

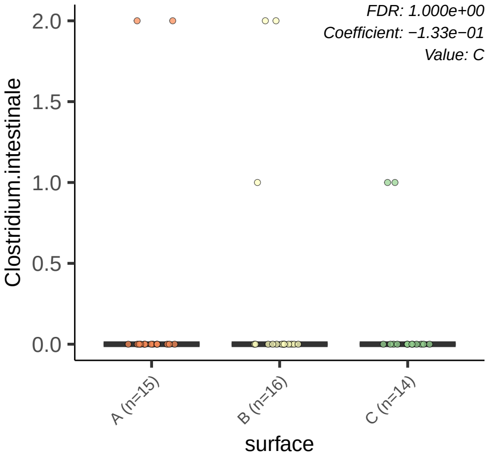
FDR: 1.000e+00
Coefficient: −1.33e−01
Value: C
Clostridium.intestinale
2.0
1.5
1.0
0.5
0.0
A (n=15)
B (n=16)
C (n=14)
surface

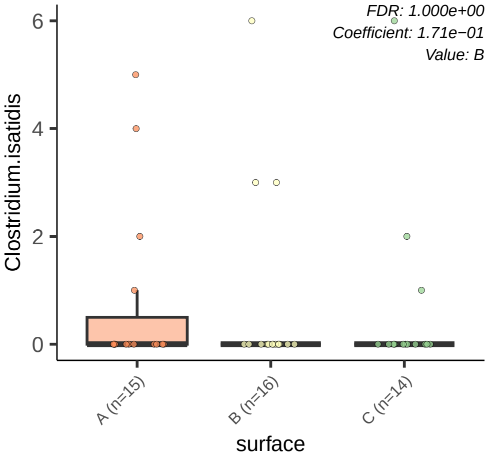
FDR: 1.000e+00
Coefficient: 1.71e−01
Value: B
Clostridium.isatidis
0
2
4
6
A (n=15)
B (n=16)
C (n=14)
surface

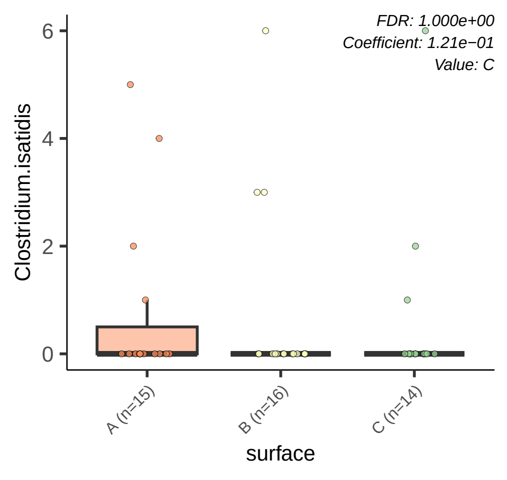
FDR: 1.000e+00
Coefficient: 1.21e−01
Value: C
Clostridium.isatidis
0
2
4
6
A (n=15)
B (n=16)
C (n=14)
surface

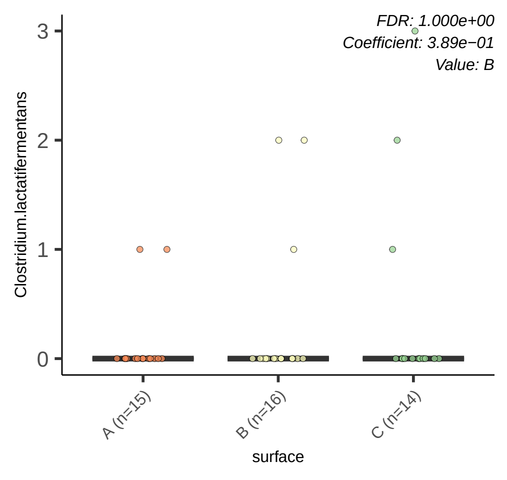
FDR: 1.000e+00
Coefficient: 3.89e−01
Value: B
Clostridium.lactatifermentans
0
1
2
3
A (n=15)
B (n=16)
C (n=14)
surface

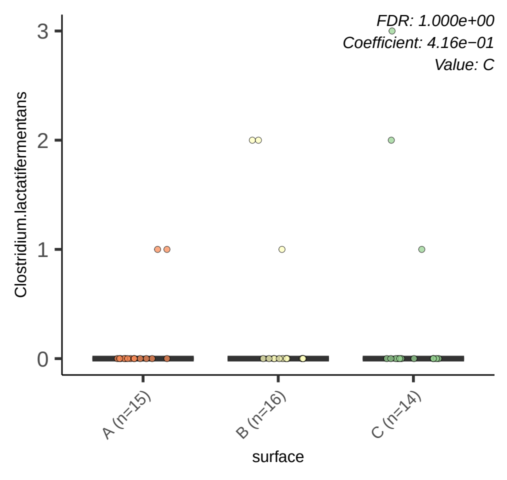

FDR: 1.000e+00
Coefficient: 4.16e−01
Value: C
Clostridium.lactatifermentans
3
2
1
0
A (n=15)
B (n=16)
C (n=14)
surface

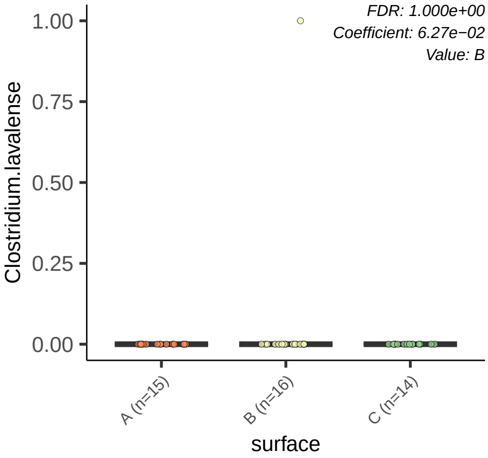

FDR: 1.000e+00
Coefficient: 6.27e−02
Value: B
Clostridium.lavalense
1.00
0.75
0.50
0.25
0.00
A (n=15)
B (n=16)
C (n=14)
surface

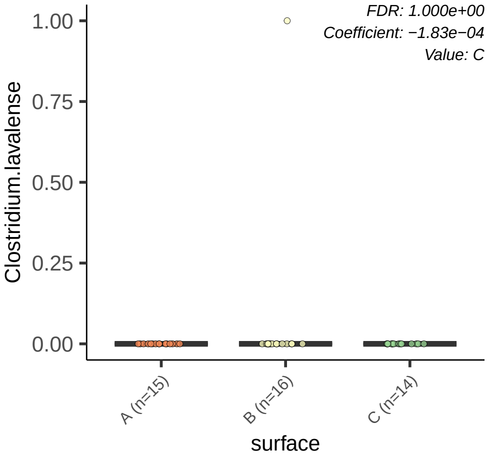
FDR: 1.000e+00
Coefficient: −1.83e−04
Value: C
Clostridium.lavalense
1.00
0.75
0.50
0.25
0.00
A (n=15)
B (n=16)
C (n=14)
surface

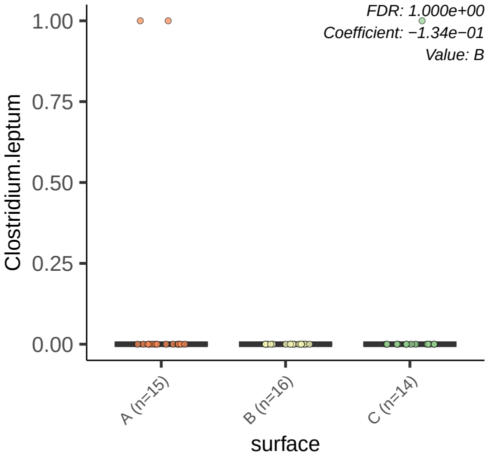

FDR: 1.000e+00
Coefficient: −1.34e−01
Value: B
1.00
0.75
0.50
0.25
0.00
Clostridium.leptum
A (n=15)
B (n=16)
C (n=14)
surface

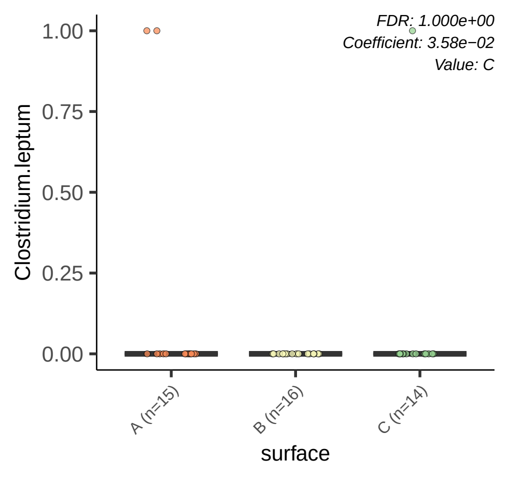
FDR: 1.000e+00
Coefficient: 3.58e−02
Value: C
Clostridium.leptum
1.00
0.75
0.50
0.25
0.00
A (n=15)
B (n=16)
C (n=14)
surface

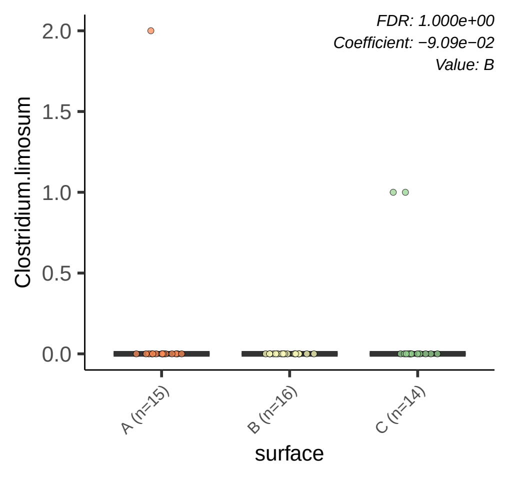
FDR: 1.000e+00
Coefficient: −9.09e−02
Value: B
Clostridium.limosum
2.0
1.5
1.0
0.5
0.0
A (n=15)
B (n=16)
C (n=14)
surface

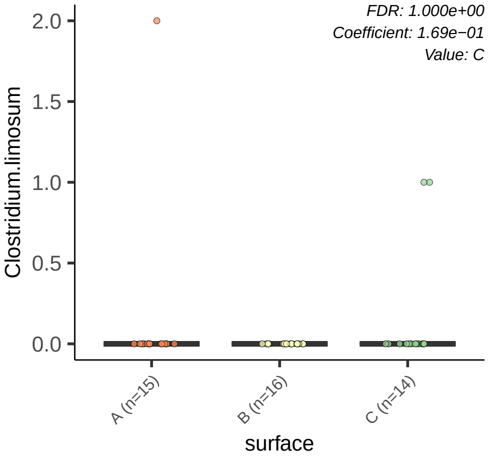
FDR: 1.000e+00
Coefficient: 1.69e−01
Value: C
Clostridium.limosum
2.0
1.5
1.0
0.5
0.0
A (n=15)
B (n=16)
C (n=14)
surface

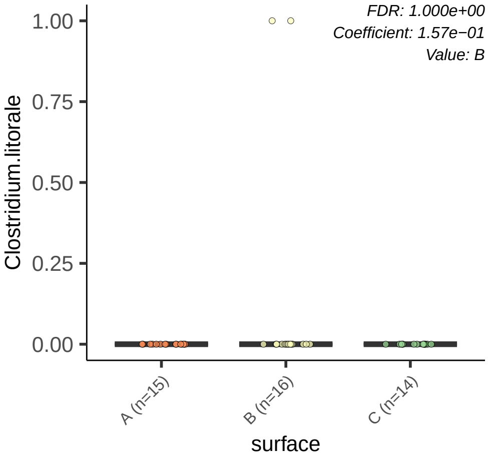
FDR: 1.000e+00
Coefficient: 1.57e−01
Value: B
Clostridium.litorale
1.00
0.75
0.50
0.25
0.00
A (n=15)
B (n=16)
C (n=14)
surface

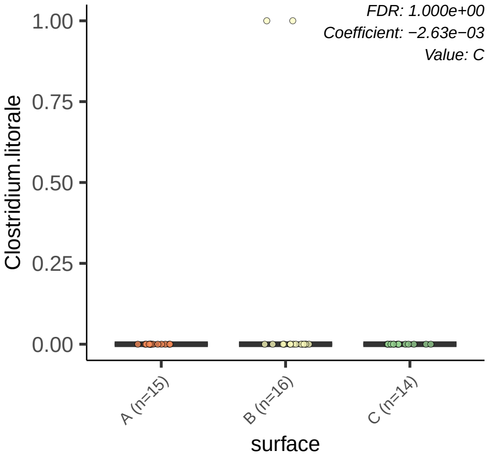

FDR: 1.000e+00
Coefficient: −2.63e−03
Value: C
Clostridium.litorale
1.00
0.75
0.50
0.25
0.00
A (n=15)
B (n=16)
C (n=14)
surface

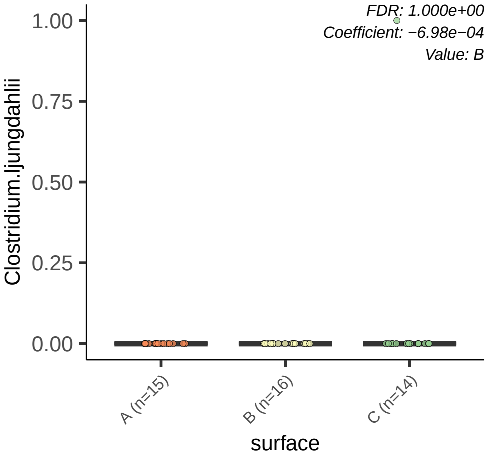
FDR: 1.000e+00
Coefficient: −6.98e−04
Value: B
1.00
0.75
0.50
0.25
0.00
Clostridium.ljungdahlii
A (n=15)
B (n=16)
C (n=14)
surface

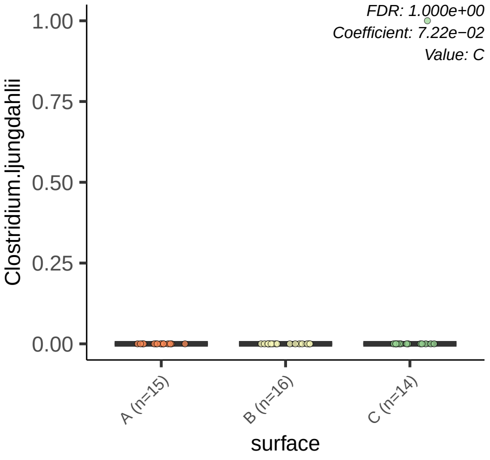

FDR: 1.000e+00
Coefficient: 7.22e−02
Value: C
Clostridium.ljungdahlii
1.00
0.75
0.50
0.25
0.00
A (n=15)
B (n=16)
C (n=14)
surface

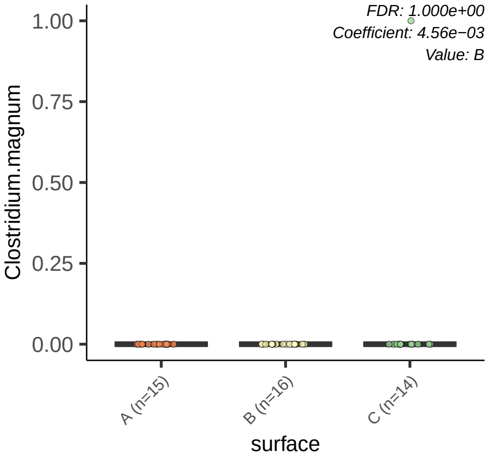

FDR: 1.000e+00
Coefficient: 4.56e−03
Value: B
1.00
0.75
0.50
0.25
0.00
Clostridium.magnum
A (n=15)
B (n=16)
C (n=14)
surface

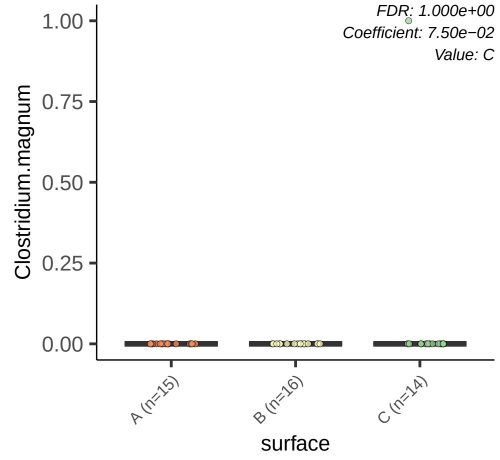

FDR: 1.000e+00
Coefficient: 7.50e−02
Value: C
Clostridium.magnum
1.00
0.75
0.50
0.25
0.00
A (n=15)
B (n=16)
C (n=14)
surface

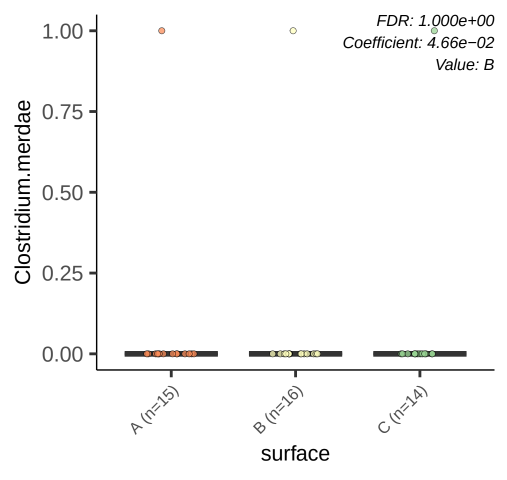
FDR: 1.000e+00
Coefficient: 4.66e−02
Value: B
Clostridium.merdae
1.00
0.75
0.50
0.25
0.00
A (n=15)
B (n=16)
C (n=14)
surface

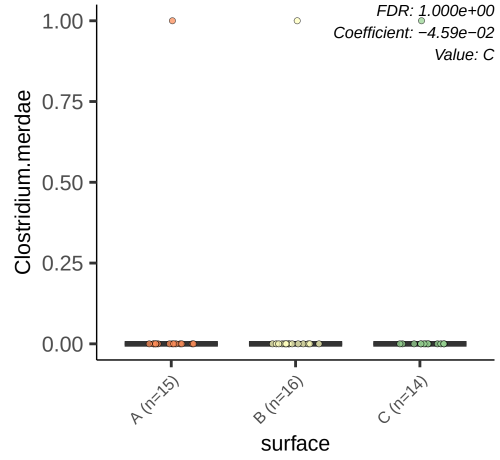
FDR: 1.000e+00
Coefficient: −4.59e−02
Value: C
Clostridium.merdae
1.00
0.75
0.50
0.25
0.00
A (n=15)
B (n=16)
C (n=14)
surface

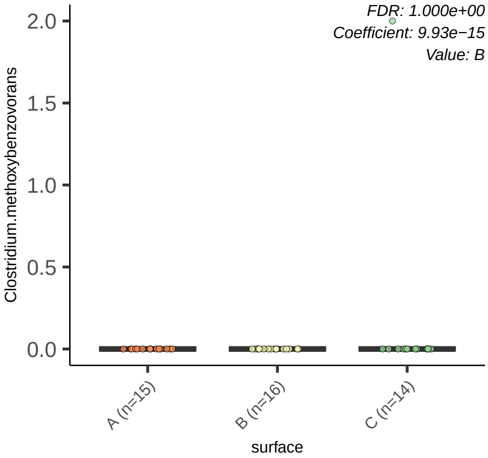

FDR: 1.000e+00
Coefficient: 9.93e−15
Value: B
Clostridium.methoxybenzovorans
2.0
1.5
1.0
0.5
0.0
A (n=15)
B (n=16)
C (n=14)
surface

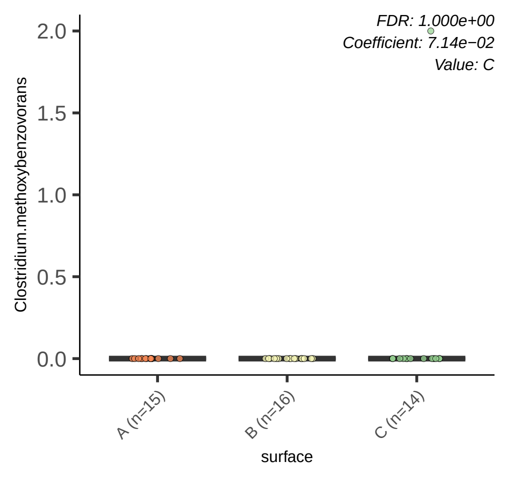
FDR: 1.000e+00
Coefficient: 7.14e−02
Value: C
Clostridium.methoxybenzovorans
2.0
1.5
1.0
0.5
0.0
A (n=15)
B (n=16)
C (n=14)
surface

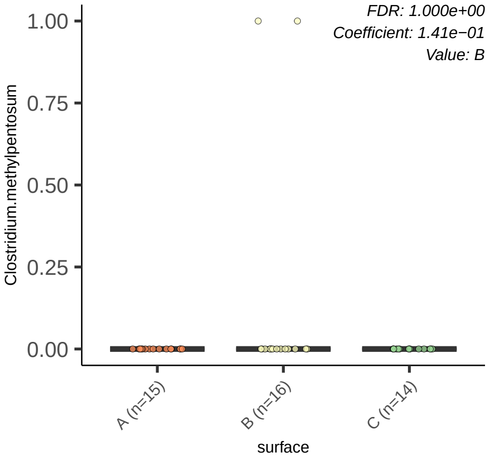

FDR: 1.000e+00
Coefficient: 1.41e−01
Value: B
Clostridium.methylpentosum
1.00
0.75
0.50
0.25
0.00
A (n=15)
B (n=16)
C (n=14)
surface

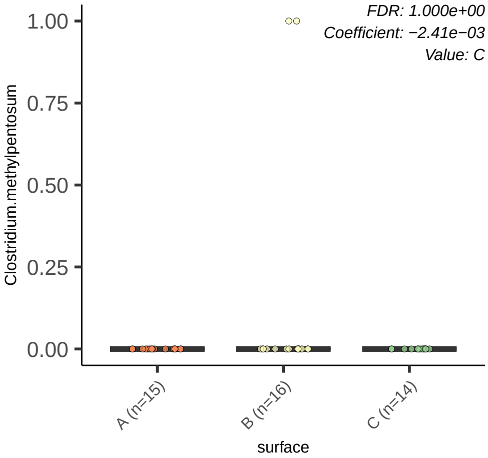

FDR: 1.000e+00
Coefficient: −2.41e−03
Value: C
1.00
0.75
0.50
0.25
0.00
Clostridium.methylpentosum
A (n=15)
B (n=16)
C (n=14)
surface

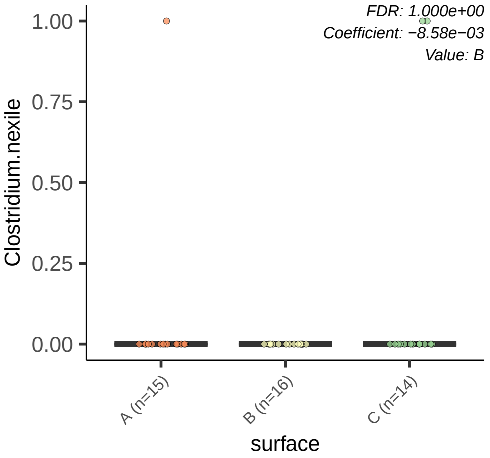

FDR: 1.000e+00
Coefficient: −8.58e−03
Value: B
Clostridium.nexile
1.00
0.75
0.50
0.25
0.00
A (n=15)
B (n=16)
C (n=14)
surface

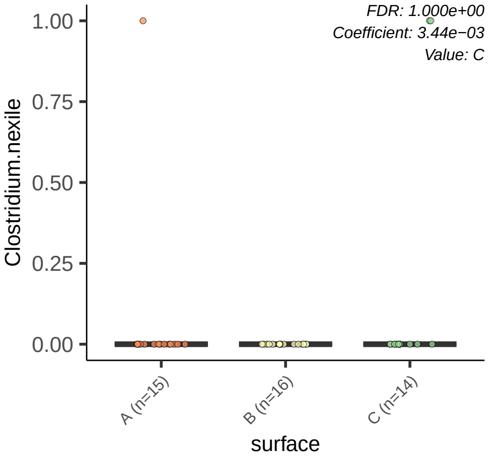

FDR: 1.000e+00
Coefficient: 3.44e−03
Value: C
Clostridium.nexile
1.00
0.75
0.50
0.25
0.00
A (n=15)
B (n=16)
C (n=14)
surface

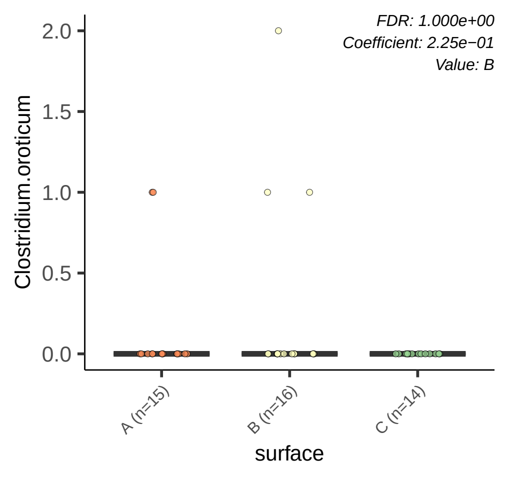
FDR: 1.000e+00
Coefficient: 2.25e−01
Value: B
Clostridium.oroticum
2.0
1.5
1.0
0.5
0.0
A (n=15)
B (n=16)
C (n=14)
surface

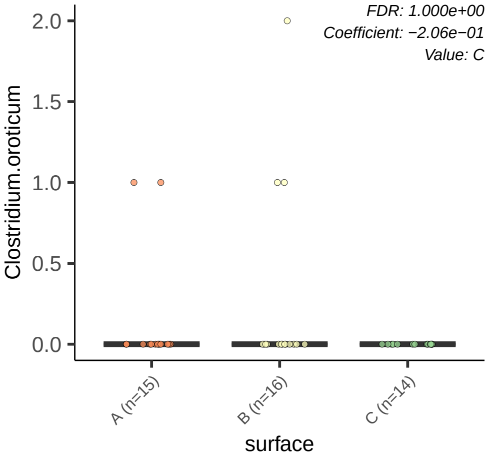

FDR: 1.000e+00
Coefficient: −2.06e−01
Value: C
2.0
1.5
1.0
0.5
0.0
Clostridium.oroticum
A (n=15)
B (n=16)
C (n=14)
surface

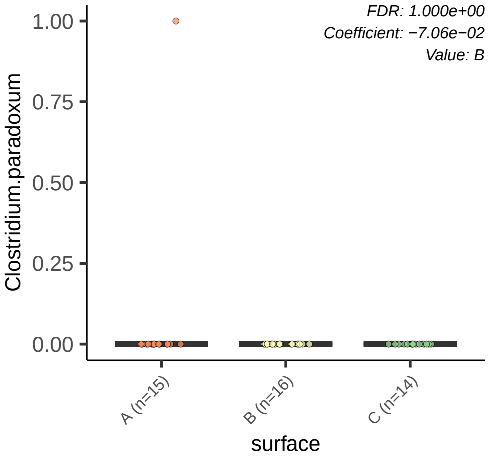
FDR: 1.000e+00
Coefficient: −7.06e−02
Value: B
Clostridium.paradoxum
1.00
0.75
0.50
0.25
0.00
A (n=15)
B (n=16)
C (n=14)
surface

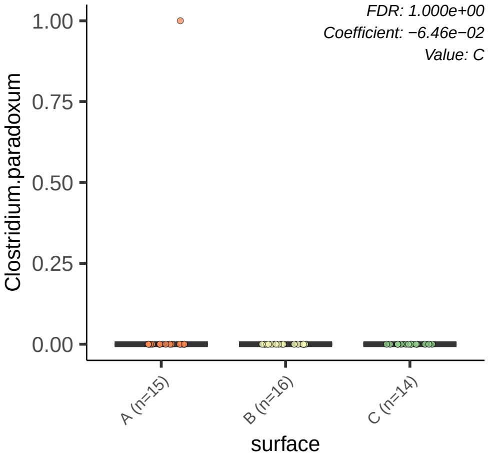

FDR: 1.000e+00
Coefficient: −6.46e−02
Value: C
Clostridium.paradoxum
1.00
0.75
0.50
0.25
0.00
A (n=15)
B (n=16)
C (n=14)
surface

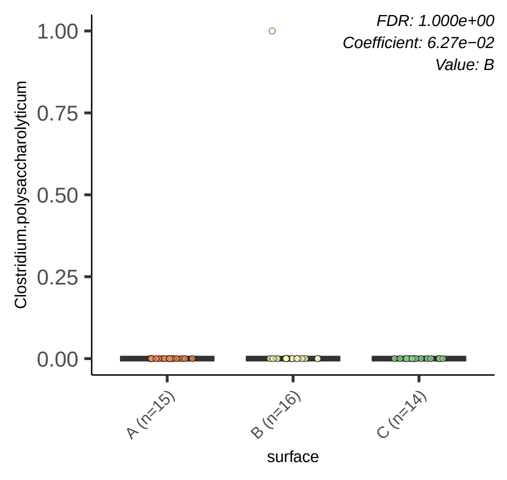
FDR: 1.000e+00
Coefficient: 6.27e−02
Value: B
Clostridium.polysaccharolyticum
1.00
0.75
0.50
0.25
0.00
A (n=15)
B (n=16)
C (n=14)
surface

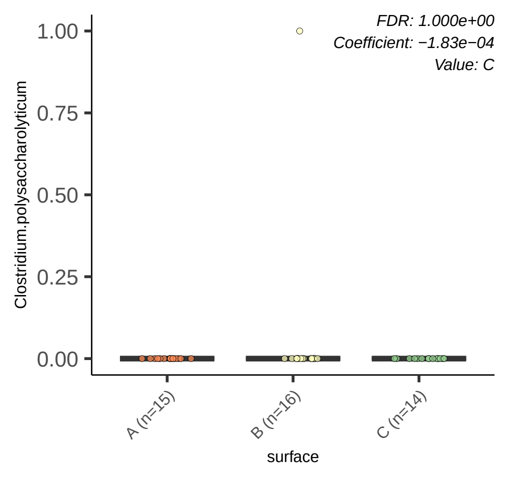

FDR: 1.000e+00
Coefficient: −1.83e−04
Value: C
Clostridium.polysaccharolyticum
1.00
0.75
0.50
0.25
0.00
A (n=15)
B (n=16)
C (n=14)
surface

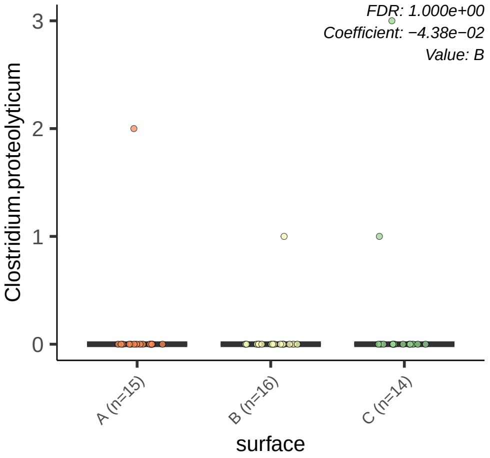

FDR: 1.000e+00
Coefficient: −4.38e−02
Value: B
Clostridium.proteolyticum
0
1
2
3
A (n=15)
B (n=16)
C (n=14)
surface

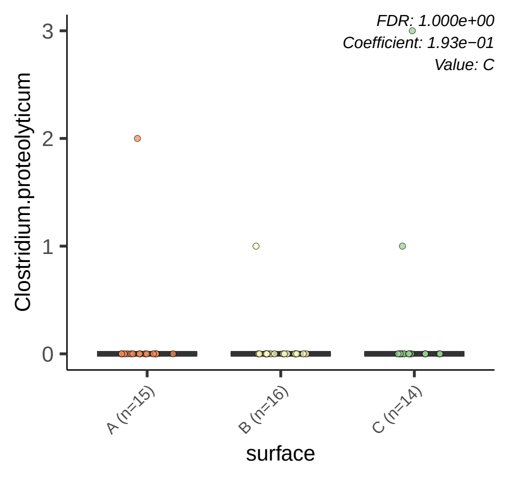
FDR: 1.000e+00
Coefficient: 1.93e−01
Value: C
Clostridium.proteolyticum
3
2
1
0
A (n=15)
B (n=16)
C (n=14)
surface

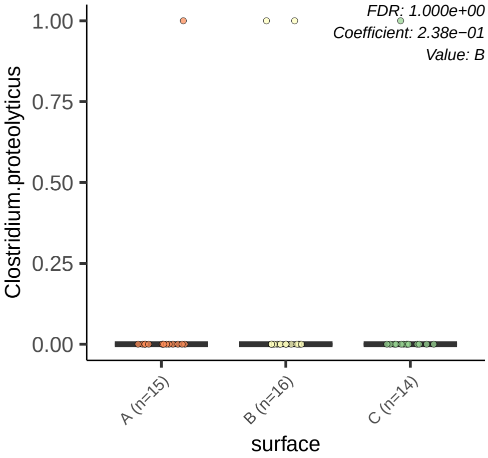

FDR: 1.000e+00
Coefficient: 2.38e−01
Value: B
Clostridium.proteolyticus
1.00
0.75
0.50
0.25
0.00
A (n=15)
B (n=16)
C (n=14)
surface

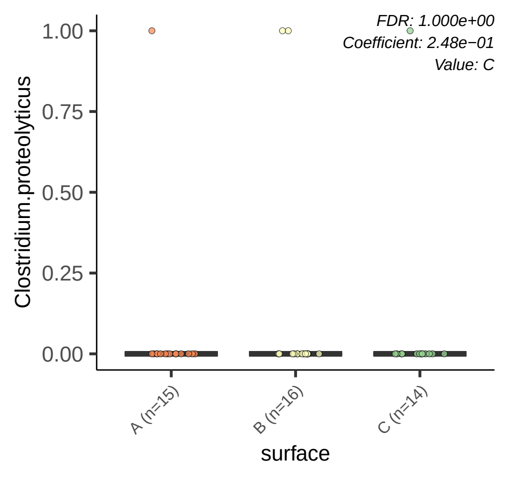
FDR: 1.000e+00
Coefficient: 2.48e−01
Value: C
Clostridium.proteolyticus
1.00
0.75
0.50
0.25
0.00
A (n=15)
B (n=16)
C (n=14)
surface

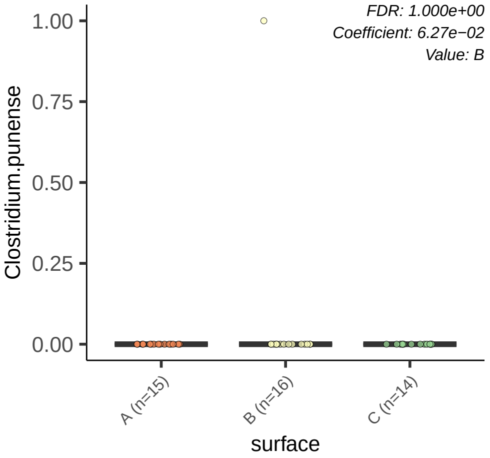

FDR: 1.000e+00
Coefficient: 6.27e−02
Value: B
Clostridium.punense
1.00
0.75
0.50
0.25
0.00
A (n=15)
B (n=16)
C (n=14)
surface

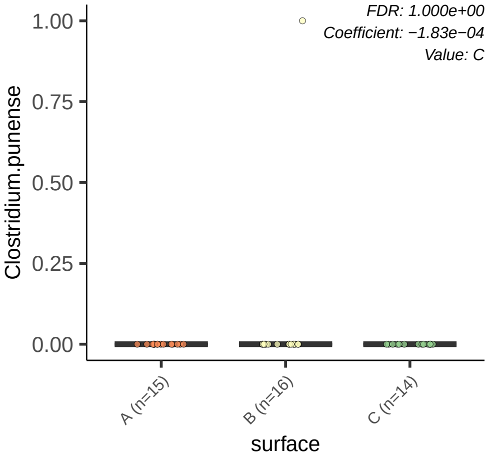
FDR: 1.000e+00
Coefficient: −1.83e−04
Value: C
1.00
0.75
0.50
0.25
0.00
Clostridium.punense
A (n=15)
B (n=16)
C (n=14)
surface

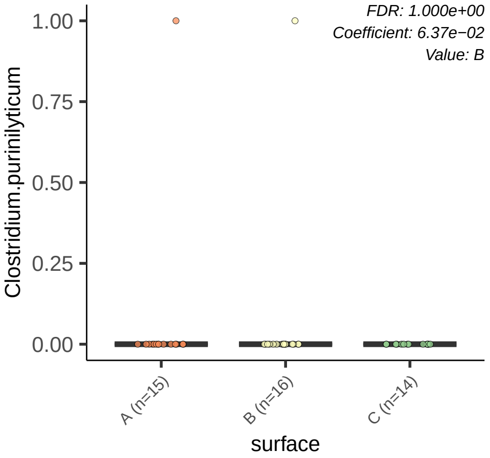

FDR: 1.000e+00
Coefficient: 6.37e−02
Value: B
Clostridium.purinilyticum
1.00
0.75
0.50
0.25
0.00
A (n=15)
B (n=16)
C (n=14)
surface

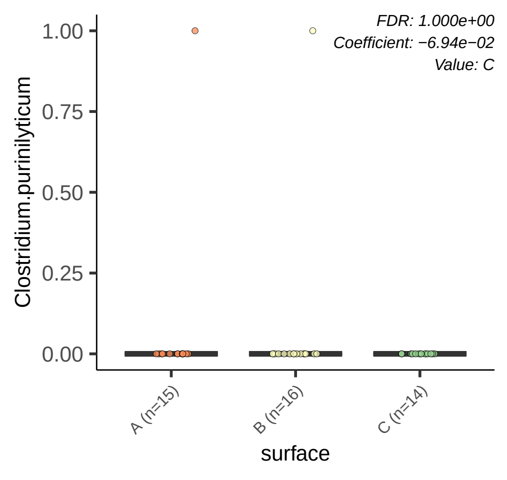

FDR: 1.000e+00
Coefficient: −6.94e−02
Value: C
Clostridium.purinilyticum
1.00
0.75
0.50
0.25
0.00
A (n=15)
B (n=16)
C (n=14)
surface

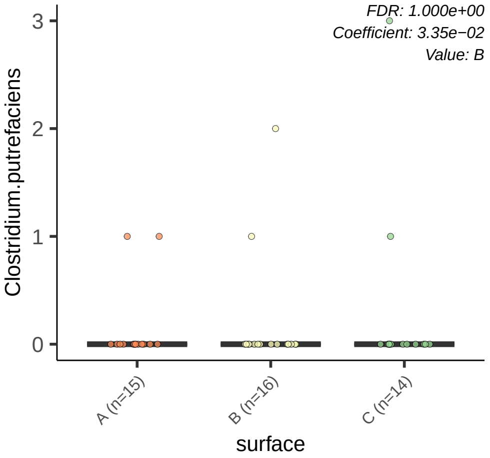

FDR: 1.000e+00
Coefficient: 3.35e−02
Value: B
Clostridium.putrefaciens
0
1
2
3
A (n=15)
B (n=16)
C (n=14)
surface

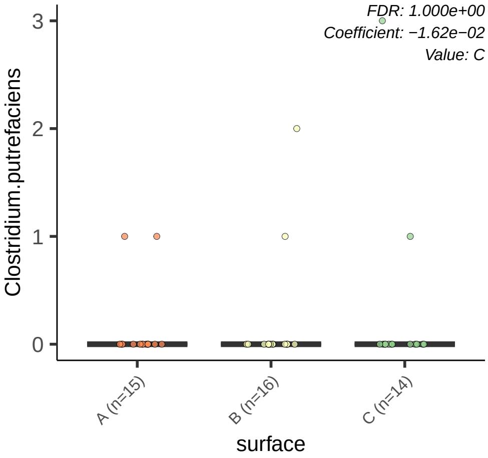
FDR: 1.000e+00
Coefficient: −1.62e−02
Value: C
Clostridium.putrefaciens
0
1
2
3
A (n=15)
B (n=16)
C (n=14)
surface

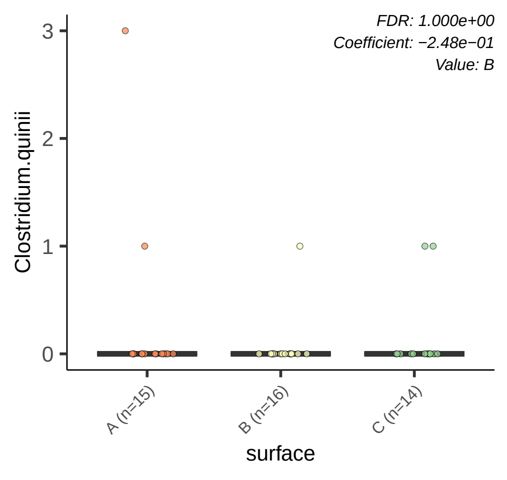
FDR: 1.000e+00
Coefficient: −2.48e−01
Value: B
Clostridium.quinii
3
2
1
0
A (n=15)
B (n=16)
C (n=14)
surface

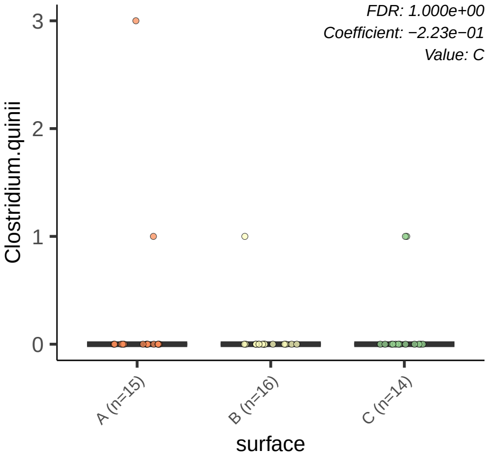

FDR: 1.000e+00
Coefficient: −2.23e−01
Value: C
Clostridium.quinii
3
2
1
0
A (n=15)
B (n=16)
C (n=14)
surface

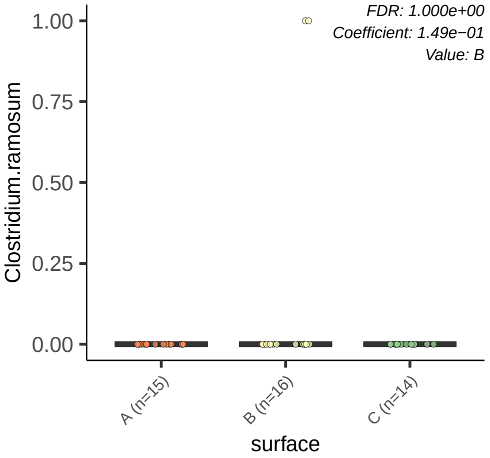
FDR: 1.000e+00
Coefficient: 1.49e−01
Value: B
Clostridium.ramosum
1.00
0.75
0.50
0.25
0.00
A (n=15)
B (n=16)
C (n=14)
surface

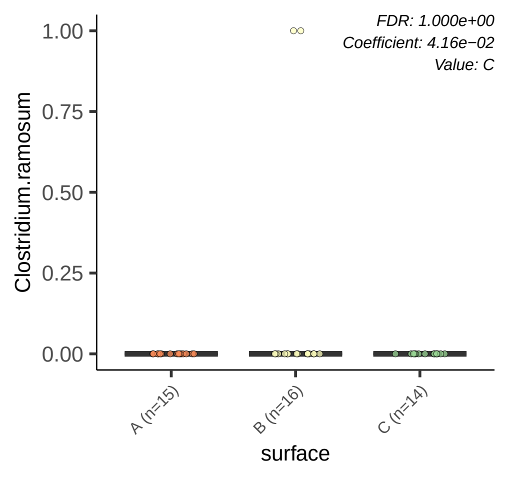

FDR: 1.000e+00
Coefficient: 4.16e−02
Value: C
Clostridium.ramosum
1.00
0.75
0.50
0.25
0.00
A (n=15)
B (n=16)
C (n=14)
surface

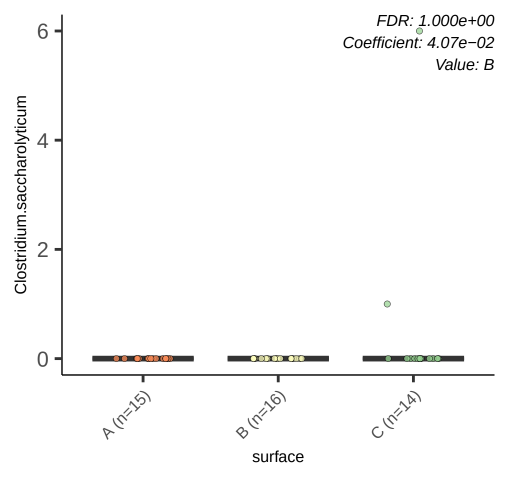

FDR: 1.000e+00
Coefficient: 4.07e−02
Value: B
Clostridium.saccharolyticum
0
2
4
6
A (n=15)
B (n=16)
C (n=14)
surface

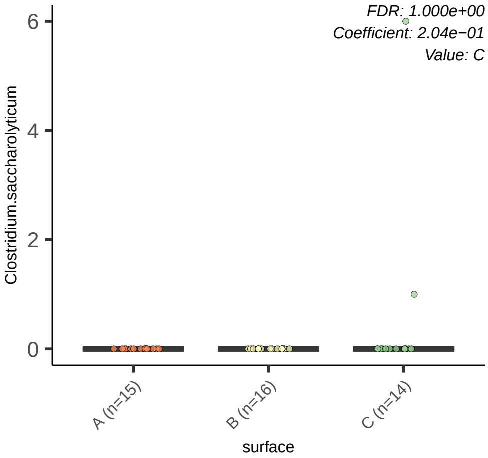

FDR: 1.000e+00
Coefficient: 2.04e−01
Value: C
Clostridium.saccharolyticum
0
2
4
6
A (n=15)
B (n=16)
C (n=14)
surface

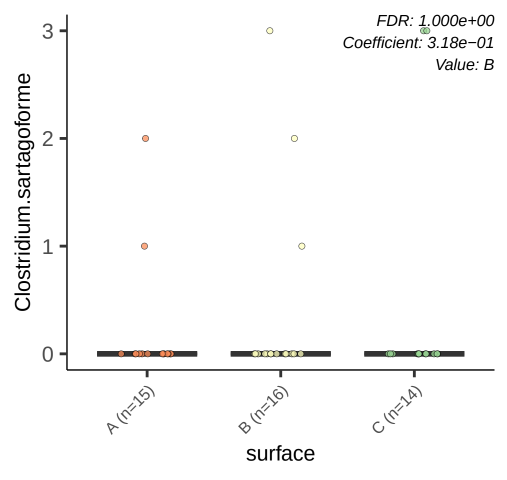

FDR: 1.000e+00
Coefficient: 3.18e−01
Value: B
Clostridium.sartagoforme
3
2
1
0
A (n=15)
B (n=16)
C (n=14)
surface

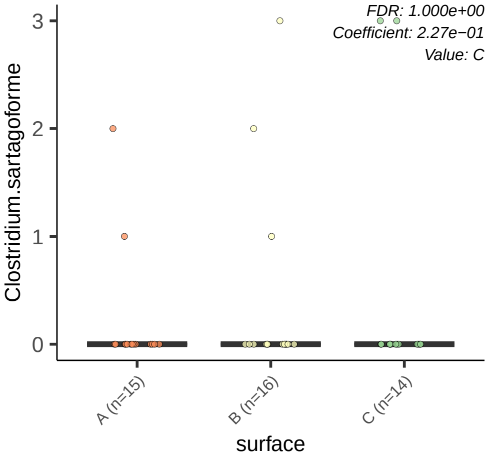
FDR: 1.000e+00
Coefficient: 2.27e−01
Value: C
Clostridium.sartagoforme
0
1
2
3
A (n=15)
B (n=16)
C (n=14)
surface

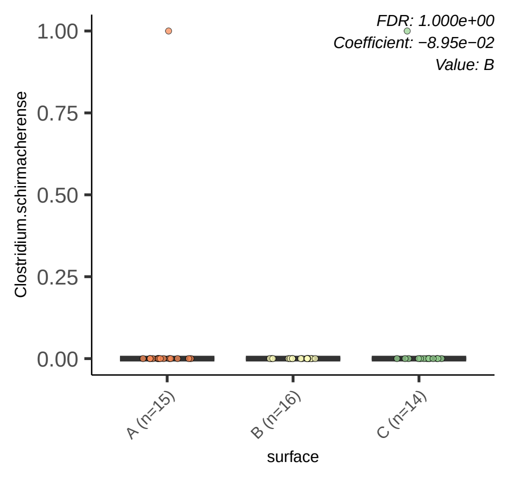
FDR: 1.000e+00
Coefficient: −8.95e−02
Value: B
Clostridium.schirmacherense
1.00
0.75
0.50
0.25
0.00
A (n=15)
B (n=16)
C (n=14)
surface

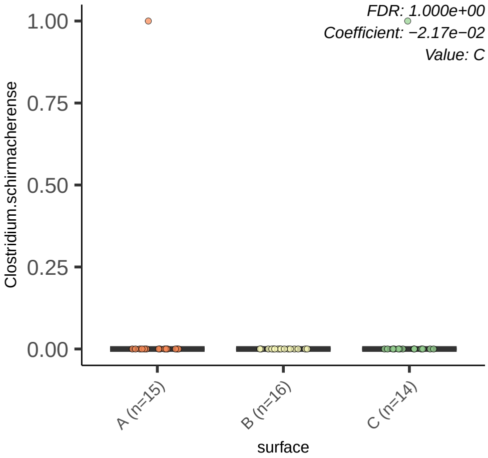
FDR: 1.000e+00
Coefficient: −2.17e−02
Value: C
1.00
0.75
0.50
0.25
0.00
Clostridium.schirmacherense
A (n=15)
B (n=16)
C (n=14)
surface

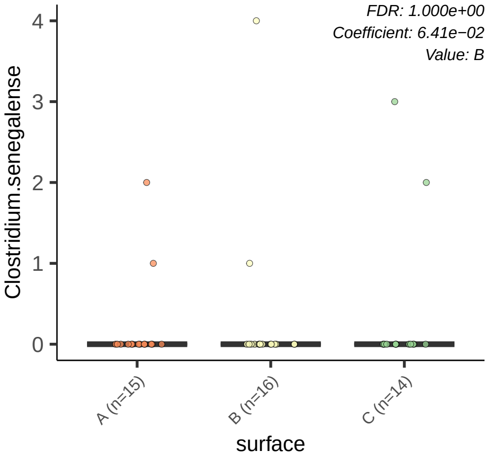
FDR: 1.000e+00
Coefficient: 6.41e−02
Value: B
Clostridium.senegalense
0
1
2
3
4
A (n=15)
B (n=16)
C (n=14)
surface

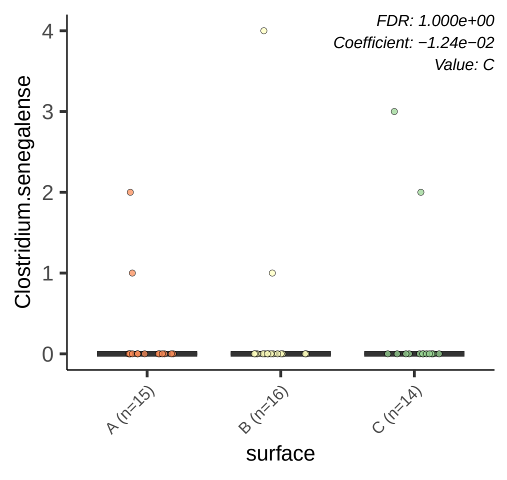
FDR: 1.000e+00
Coefficient: −1.24e−02
Value: C
Clostridium.senegalense
0
1
2
3
4
A (n=15)
B (n=16)
C (n=14)
surface

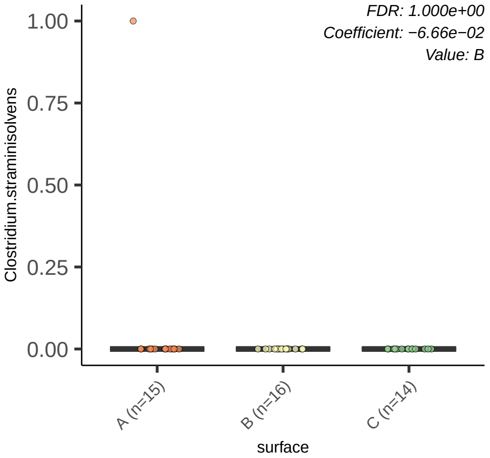
FDR: 1.000e+00
Coefficient: −6.66e−02
Value: B
1.00
0.75
0.50
0.25
0.00
Clostridium.straminisolvens
A (n=15)
B (n=16)
C (n=14)
surface

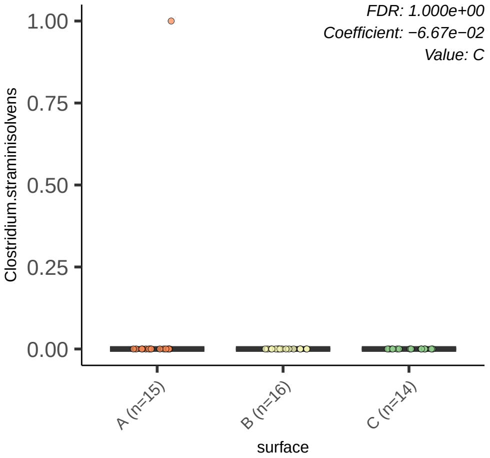
FDR: 1.000e+00
Coefficient: −6.67e−02
Value: C
1.00
0.75
0.50
0.25
0.00
Clostridium.straminisolvens
A (n=15)
B (n=16)
C (n=14)
surface

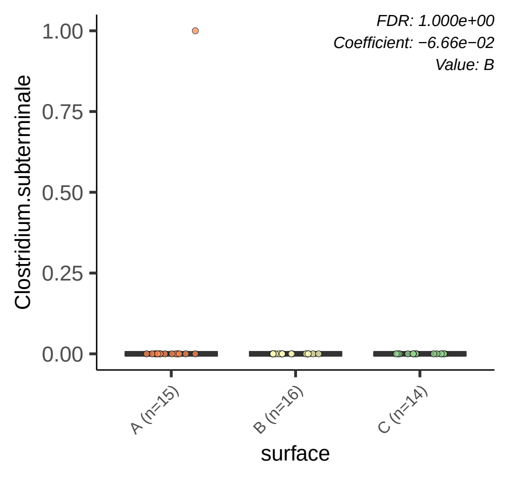

FDR: 1.000e+00
Coefficient: −6.66e−02
Value: B
Clostridium.subterminale
1.00
0.75
0.50
0.25
0.00
A (n=15)
B (n=16)
C (n=14)
surface

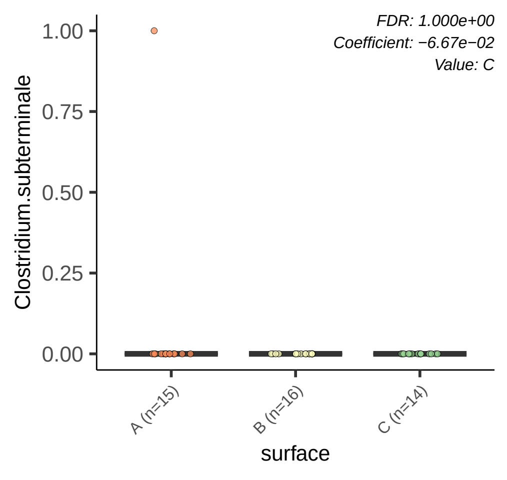
FDR: 1.000e+00
Coefficient: −6.67e−02
Value: C
Clostridium.subterminale
1.00
0.75
0.50
0.25
0.00
A (n=15)
B (n=16)
C (n=14)
surface

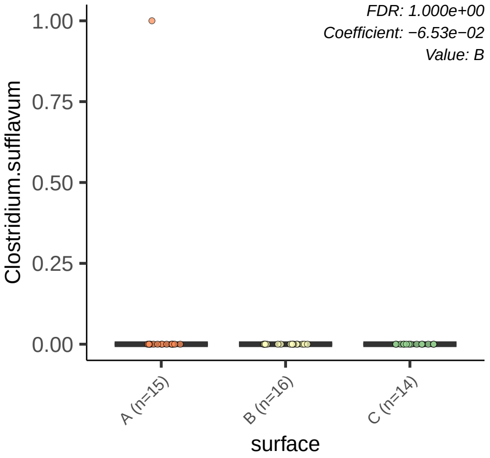

FDR: 1.000e+00
Coefficient: −6.53e−02
Value: B
Clostridium.sufflavum
1.00
0.75
0.50
0.25
0.00
A (n=15)
B (n=16)
C (n=14)
surface

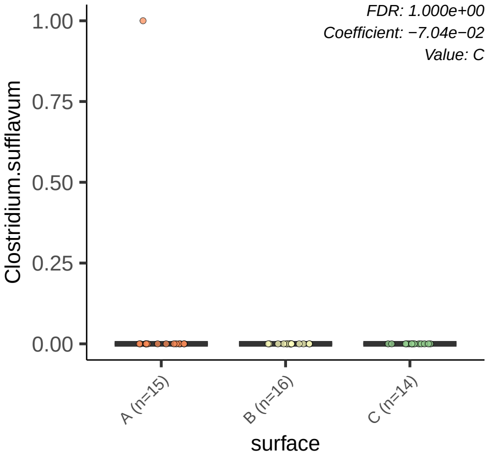

FDR: 1.000e+00
Coefficient: −7.04e−02
Value: C
Clostridium.sufflavum
1.00
0.75
0.50
0.25
0.00
A (n=15)
B (n=16)
C (n=14)
surface

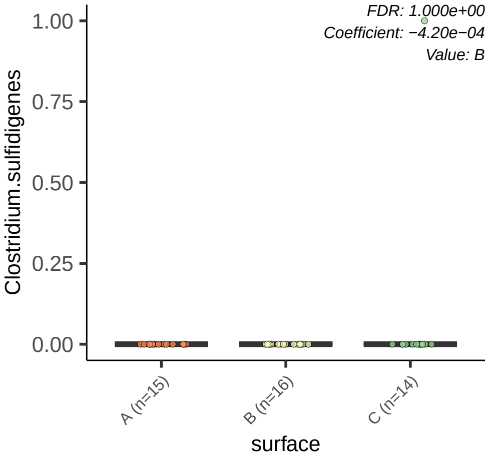
FDR: 1.000e+00
Coefficient: −4.20e−04
Value: B
Clostridium.sulfidigenes
1.00
0.75
0.50
0.25
0.00
A (n=15)
B (n=16)
C (n=14)
surface

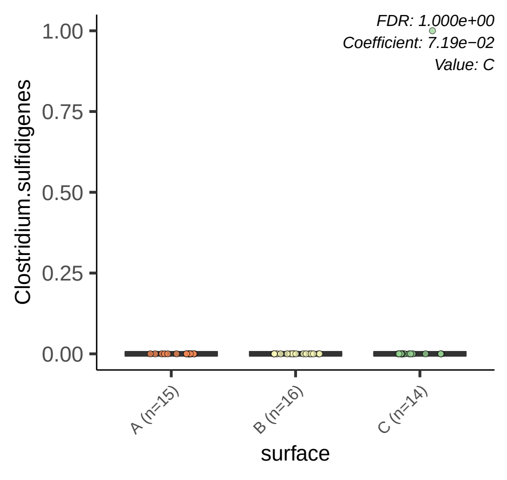
FDR: 1.000e+00
Coefficient: 7.19e−02
Value: C
Clostridium.sulfidigenes
1.00
0.75
0.50
0.25
0.00
A (n=15)
B (n=16)
C (n=14)
surface

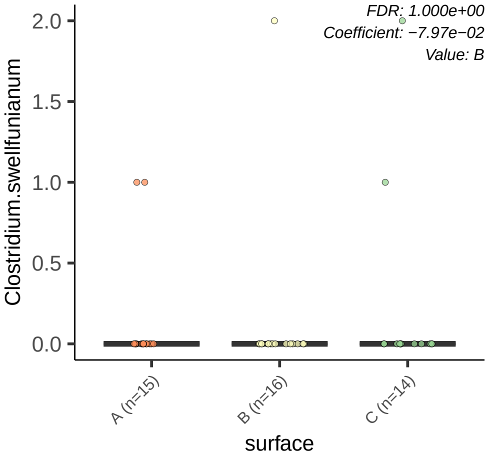
FDR: 1.000e+00
Coefficient: −7.97e−02
Value: B
Clostridium.swellfunianum
2.0
1.5
1.0
0.5
0.0
A (n=15)
B (n=16)
C (n=14)
surface

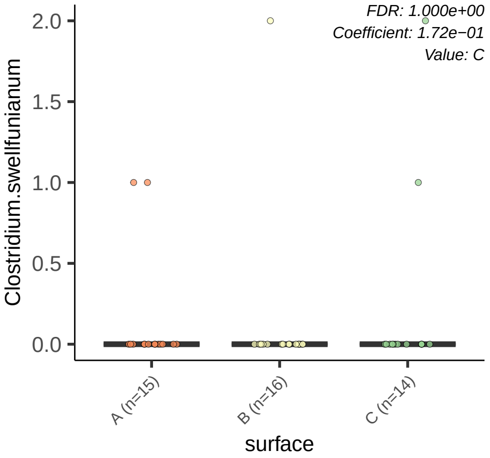
FDR: 1.000e+00
Coefficient: 1.72e−01
Value: C
Clostridium.swellfunianum
2.0
1.5
1.0
0.5
0.0
A (n=15)
B (n=16)
C (n=14)
surface

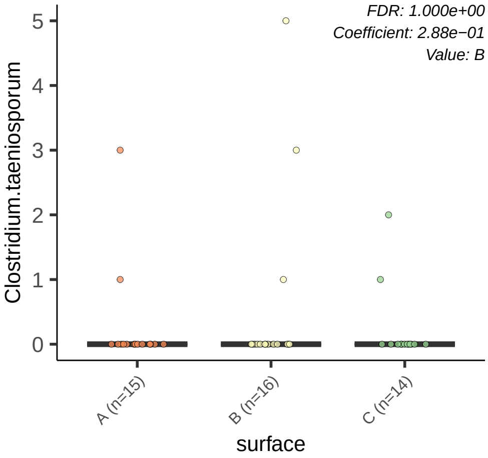
FDR: 1.000e+00
Coefficient: 2.88e−01
Value: B
Clostridium.taenioosporum
5
4
3
2
1
0
A (n=15)
B (n=16)
C (n=14)
surface

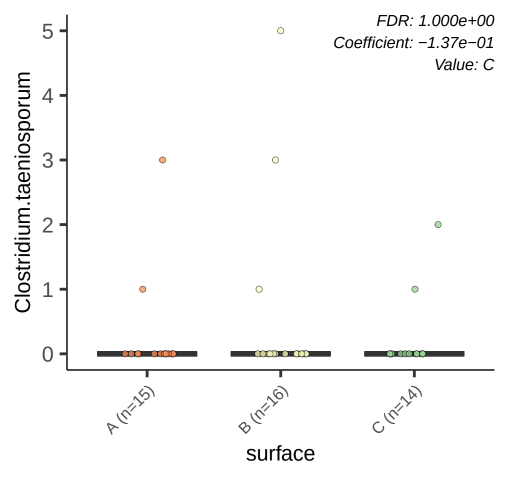

FDR: 1.000e+00
Coefficient: −1.37e−01
Value: C
Clostridium.taeniosporum
0
1
2
3
4
5
A (n=15)
B (n=16)
C (n=14)
surface

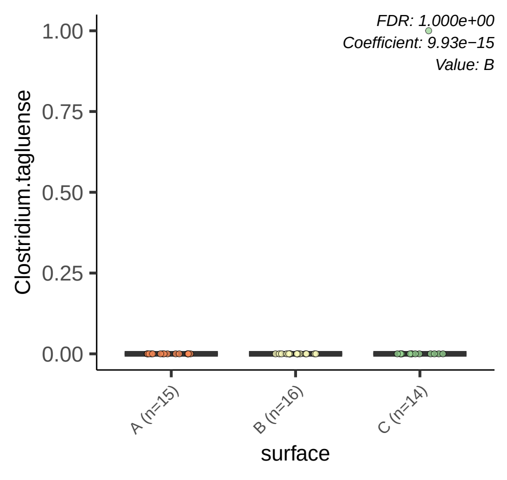
FDR: 1.000e+00
Coefficient: 9.93e−15
Value: B
Clostridium.tagluense
1.00
0.75
0.50
0.25
0.00
A (n=15)
B (n=16)
C (n=14)
surface

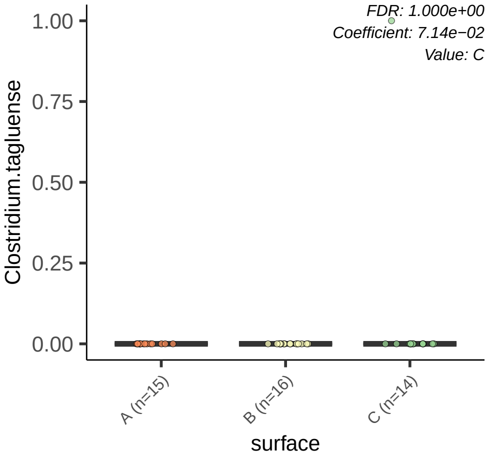
FDR: 1.000e+00
Coefficient: 7.14e−02
Value: C
1.00
0.75
0.50
0.25
0.00
Clostridium.tagluense
A (n=15)
B (n=16)
C (n=14)
surface

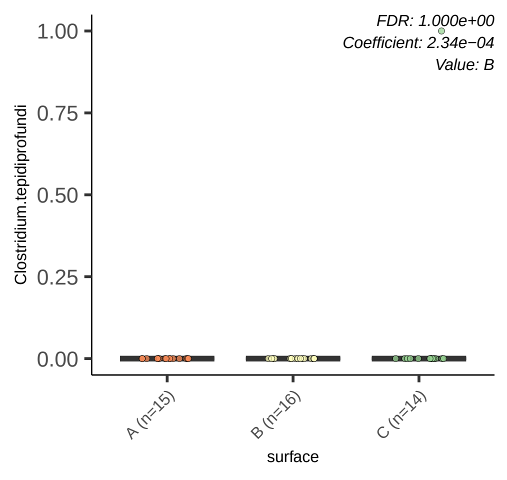

FDR: 1.000e+00
Coefficient: 2.34e−04
Value: B
1.00
0.75
0.50
0.25
0.00
Clostridium.tepidiprofundi
A (n=15)
B (n=16)
C (n=14)
surface

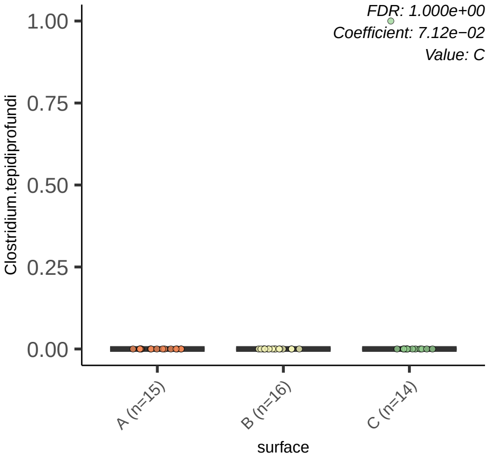

FDR: 1.000e+00
Coefficient: 7.12e−02
Value: C
1.00
0.75
0.50
0.25
0.00
Clostridium.tepidiprofundi
A (n=15)
B (n=16)
C (n=14)
surface

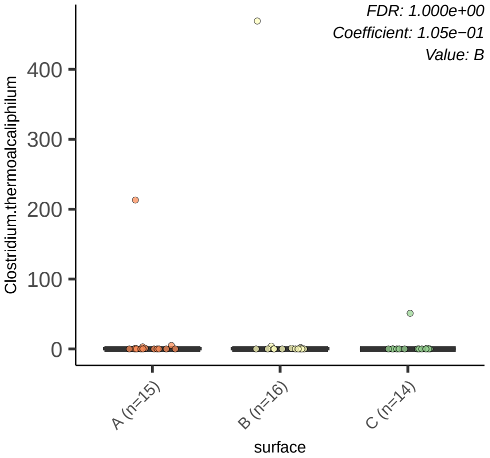
FDR: 1.000e+00
Coefficient: 1.05e−01
Value: B
Clostridium.thermoalcaliphilum
400
300
200
100
0
A (n=15)
B (n=16)
C (n=14)
surface

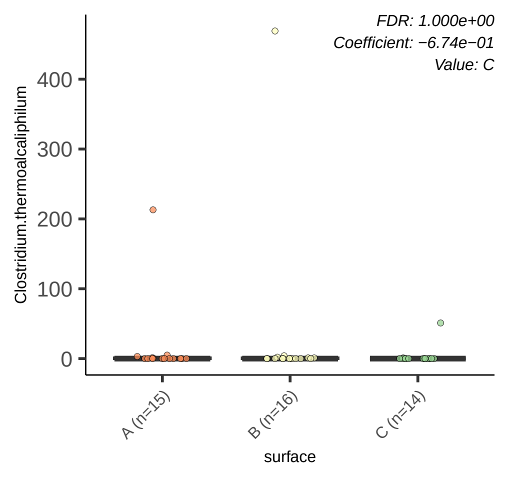

FDR: 1.000e+00
Coefficient: −6.74e−01
Value: C
Clostridium.thermoalcaliphilum
400
300
200
100
0
A (n=15)
B (n=16)
C (n=14)
surface

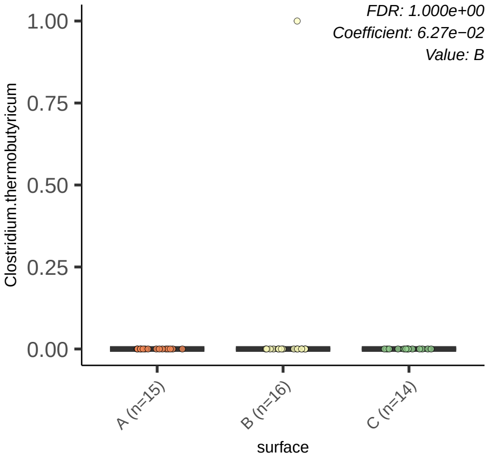
FDR: 1.000e+00
Coefficient: 6.27e−02
Value: B
1.00
0.75
0.50
0.25
0.00
Clostridium.thermobutyricum
A (n=15)
B (n=16)
C (n=14)
surface

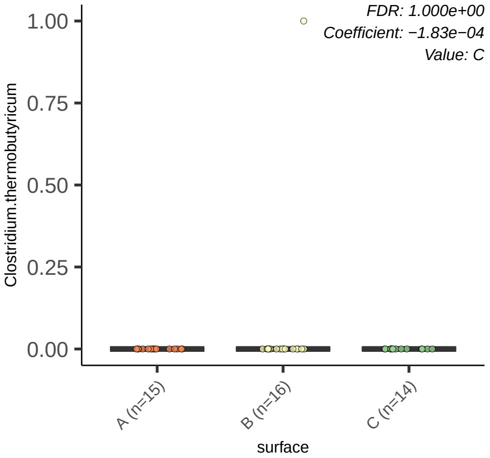
FDR: 1.000e+00
Coefficient: −1.83e−04
Value: C
1.00
0.75
0.50
0.25
0.00
Clostridium.thermobutyricum
A (n=15)
B (n=16)
C (n=14)
surface

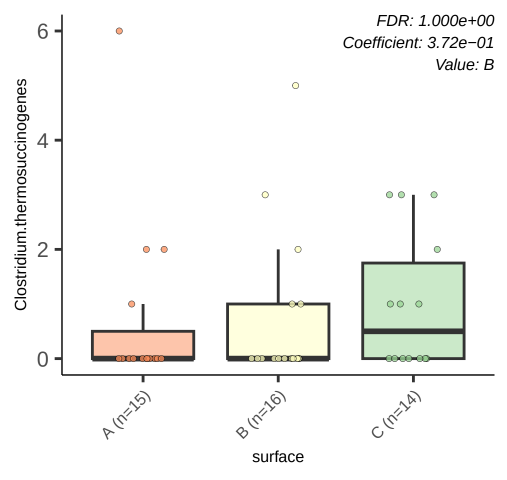
FDR: 1.000e+00
Coefficient: 3.72e−01
Value: B
Clostridium.thermosuccinogenes
0
2
4
6
A (n=15)
B (n=16)
C (n=14)
surface

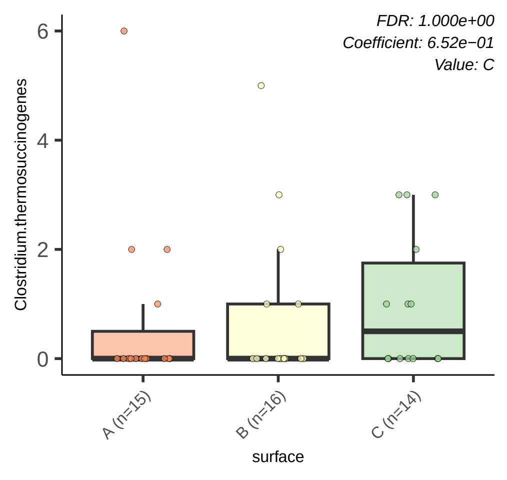

FDR: 1.000e+00
Coefficient: 6.52e−01
Value: C
Clostridium.thermosuccinogenes
0
2
4
6
A (n=15)
B (n=16)
C (n=14)
surface

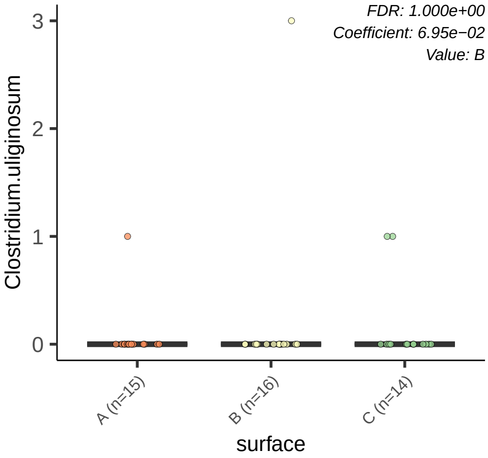

FDR: 1.000e+00
Coefficient: 6.95e−02
Value: B
Clostridium.uliginosum
3
2
1
0
A (n=15)
B (n=16)
C (n=14)
surface

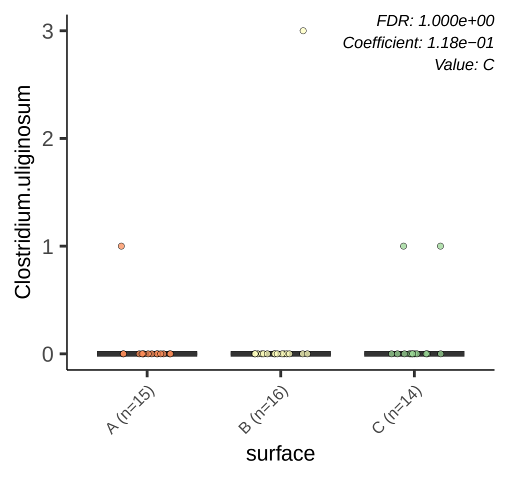
FDR: 1.000e+00
Coefficient: 1.18e−01
Value: C
Clostridium.uliginosum
3
2
1
0
A (n=15)
B (n=16)
C (n=14)
surface

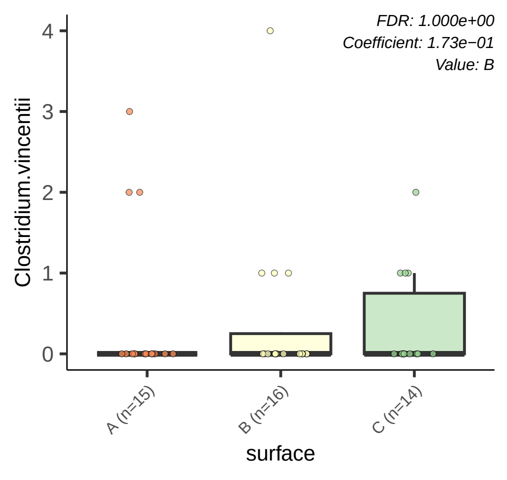
FDR: 1.000e+00
Coefficient: 1.73e−01
Value: B
Clostridium.vincentii
0
1
2
3
4
A (n=15)
B (n=16)
C (n=14)
surface

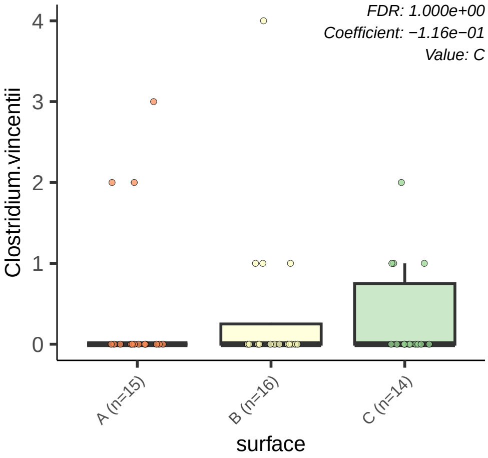

FDR: 1.000e+00
Coefficient: −1.16e−01
Value: C
Clostridium.vincentii
4
3
2
1
0
A (n=15)
B (n=16)
C (n=14)
surface

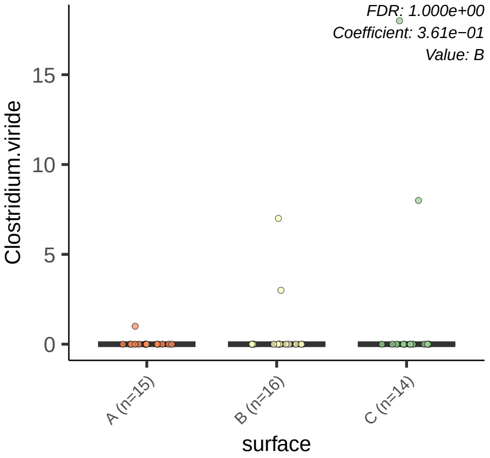
FDR: 1.000e+00
Coefficient: 3.61e−01
Value: B
Clostridium.viride
15
10
5
0
A (n=15)
B (n=16)
C (n=14)
surface

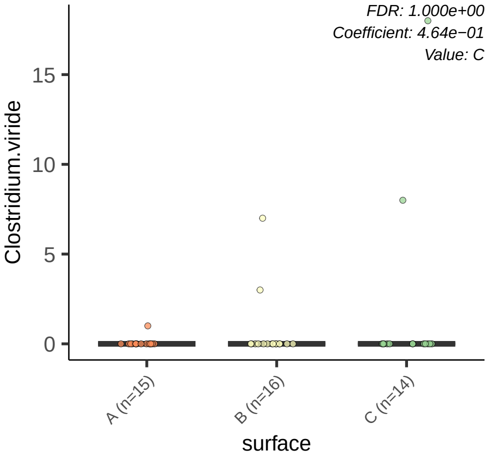

FDR: 1.000e+00
Coefficient: 4.64e−01
Value: C
Clostridium.viride
0
5
10
15
A (n=15)
B (n=16)
C (n=14)
surface

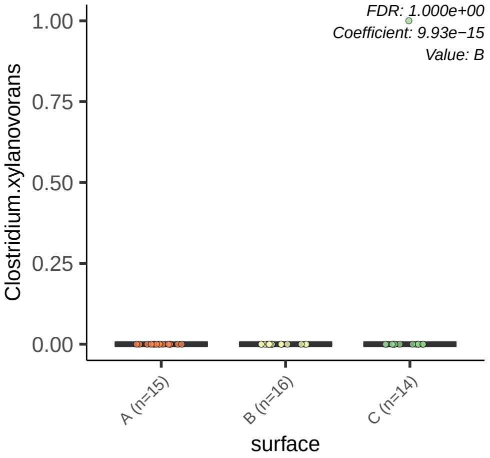
FDR: 1.000e+00
Coefficient: 9.93e−15
Value: B
Clostridium.xylanovorans
1.00
0.75
0.50
0.25
0.00
A (n=15)
B (n=16)
C (n=14)
surface

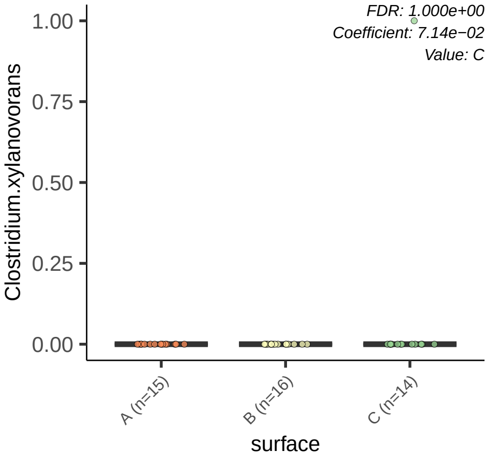
FDR: 1.000e+00
Coefficient: 7.14e−02
Value: C
Clostridium.xylanovorans
1.00
0.75
0.50
0.25
0.00
A (n=15)
B (n=16)
C (n=14)
surface

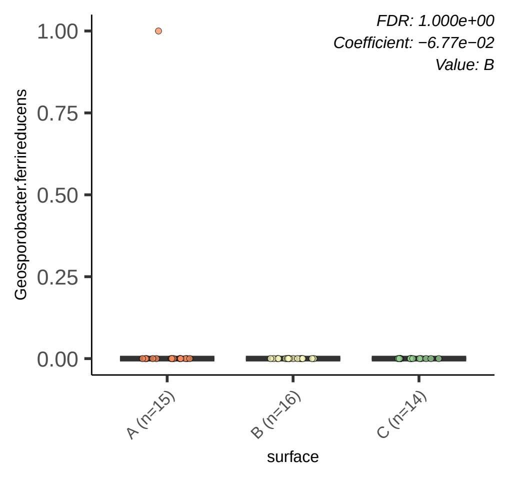

FDR: 1.000e+00
Coefficient: −6.77e−02
Value: B
Geosporobacter.ferrireducens
1.00
0.75
0.50
0.25
0.00
A (n=15)
B (n=16)
C (n=14)
surface

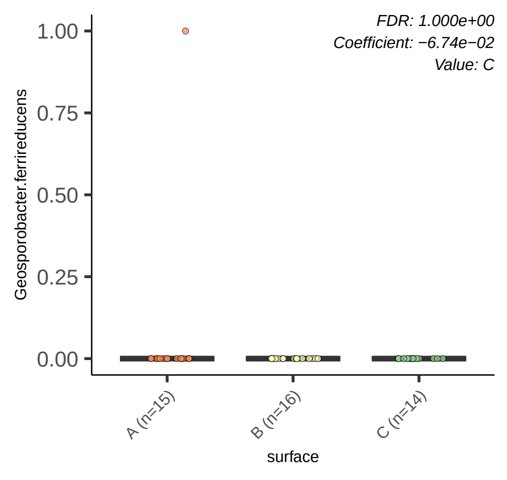

FDR: 1.000e+00
Coefficient: −6.74e−02
Value: C
Geosporobacter.ferrireducens
1.00
0.75
0.50
0.25
0.00
A (n=15)
B (n=16)
C (n=14)
surface

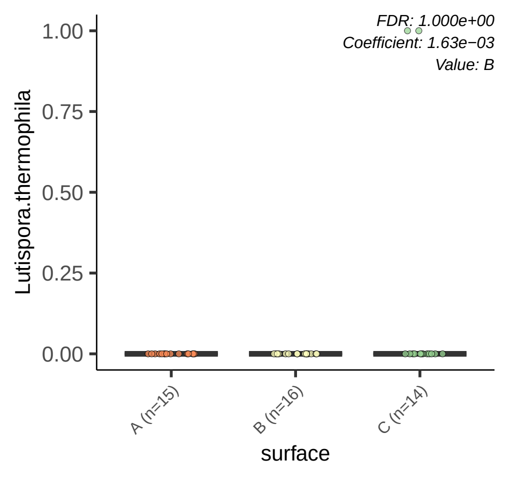
FDR: 1.000e+00
Coefficient: 1.63e−03
Value: B
Lutispora.thermophila
1.00
0.75
0.50
0.25
0.00
A (n=15)
B (n=16)
C (n=14)
surface

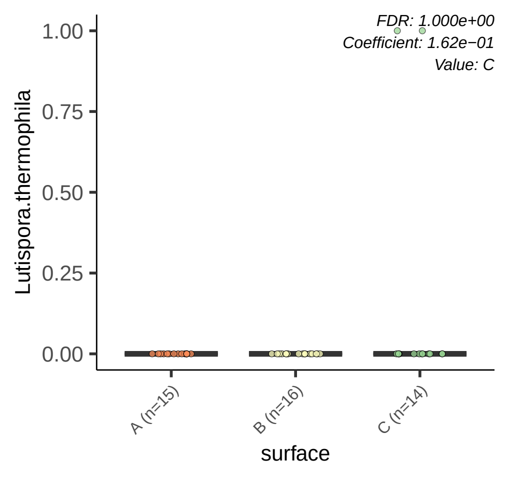
FDR: 1.000e+00
Coefficient: 1.62e−01
Value: C
Lutispora.thermophila
1.00
0.75
0.50
0.25
0.00
A (n=15)
B (n=16)
C (n=14)
surface

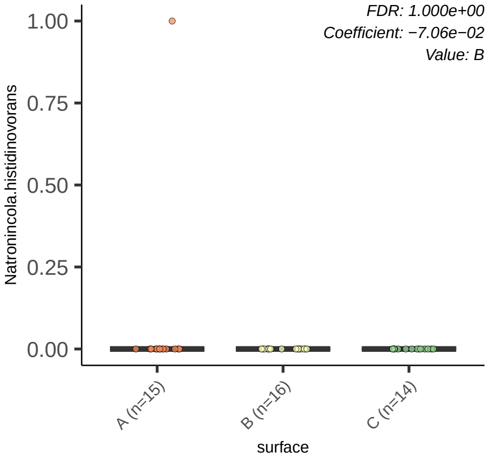
FDR: 1.000e+00
Coefficient: −7.06e−02
Value: B
Natronincola.histidinovorans
1.00
0.75
0.50
0.25
0.00
A (n=15)
B (n=16)
C (n=14)
surface

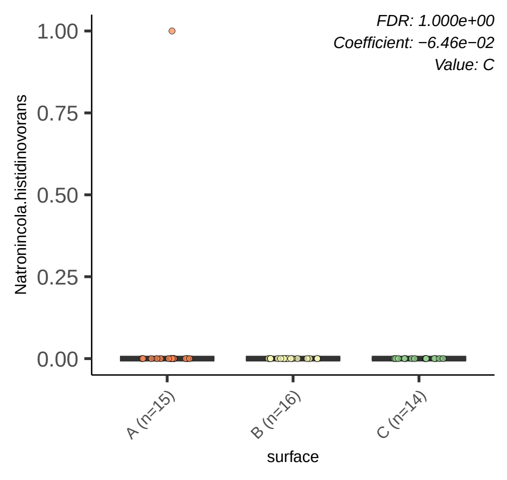
FDR: 1.000e+00
Coefficient: −6.46e−02
Value: C
Natronincola.histidinovorans
1.00
0.75
0.50
0.25
0.00
A (n=15)
B (n=16)
C (n=14)
surface

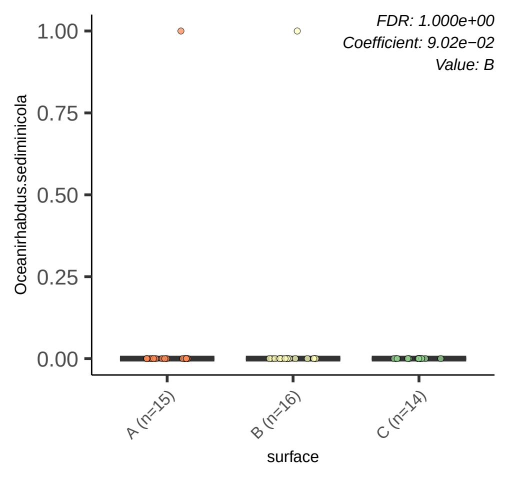

FDR: 1.000e+00
Coefficient: 9.02e−02
Value: B
1.00
0.75
0.50
0.25
0.00
Oceanirhabdus.sediminicola
A (n=15)
B (n=16)
C (n=14)
surface

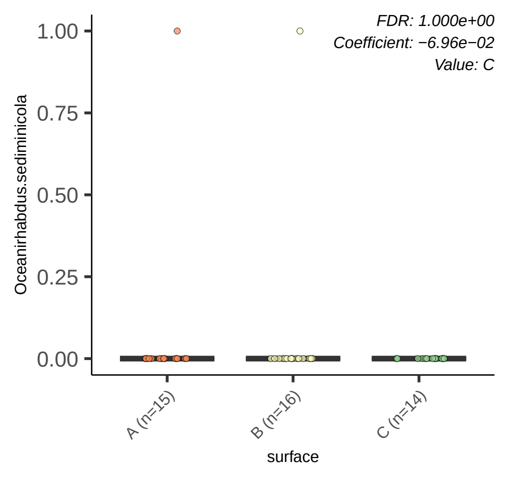
FDR: 1.000e+00
Coefficient: −6.96e−02
Value: C
1.00
0.75
0.50
0.25
0.00
Oceanirhabdus.sediminicola
A (n=15)
B (n=16)
C (n=14)
surface

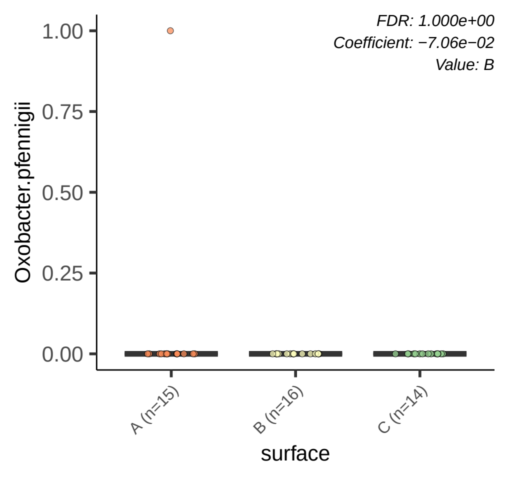

FDR: 1.000e+00
Coefficient: −7.06e−02
Value: B
Oxobacter.pfennigii
1.00
0.75
0.50
0.25
0.00
A (n=15)
B (n=16)
C (n=14)
surface

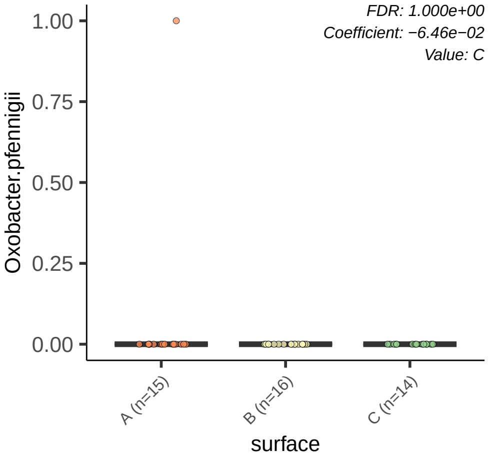
FDR: 1.000e+00
Coefficient: −6.46e−02
Value: C
Oxobacter.pfennigii
1.00
0.75
0.50
0.25
0.00
A (n=15)
B (n=16)
C (n=14)
surface

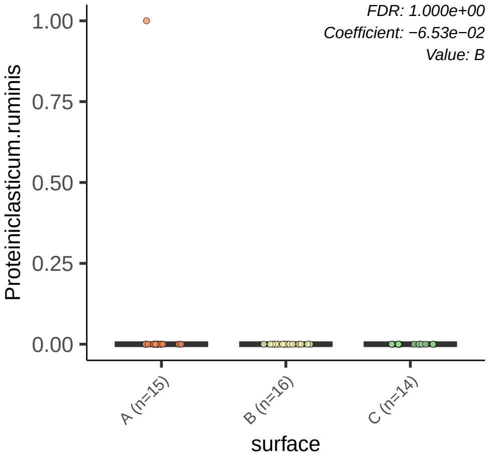

FDR: 1.000e+00
Coefficient: −6.53e−02
Value: B
Proteiniclasticum.ruminis
1.00
0.75
0.50
0.25
0.00
A (n=15)
B (n=16)
C (n=14)
surface

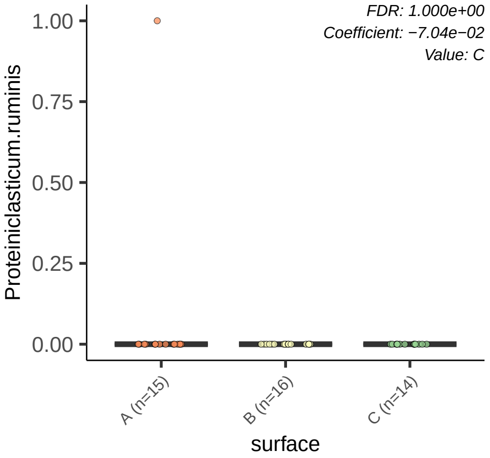
FDR: 1.000e+00
Coefficient: −7.04e−02
Value: C
Proteiniclasticum.ruminis
1.00
0.75
0.50
0.25
0.00
A (n=15)
B (n=16)
C (n=14)
surface

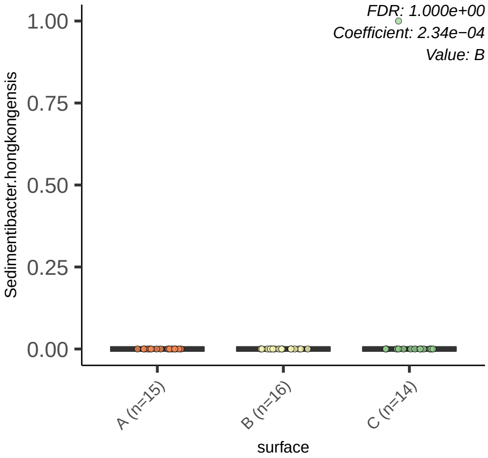
FDR: 1.000e+00
Coefficient: 2.34e−04
Value: B
1.00
0.75
0.50
0.25
0.00
Sedimentibacter.hongkongensis
A (n=15)
B (n=16)
C (n=14)
surface

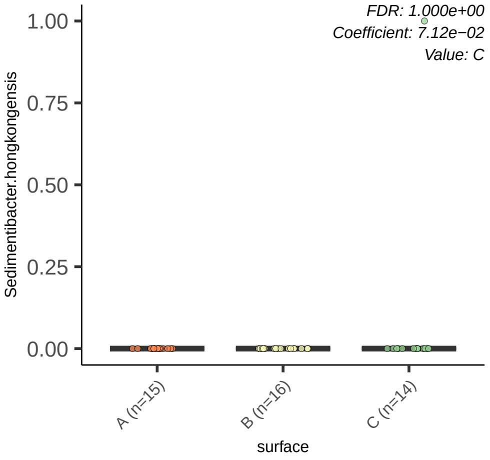
FDR: 1.000e+00
Coefficient: 7.12e−02
Value: C
Sedimentibacter.hongkongensis
1.00
0.75
0.50
0.25
0.00
A (n=15)
B (n=16)
C (n=14)
surface

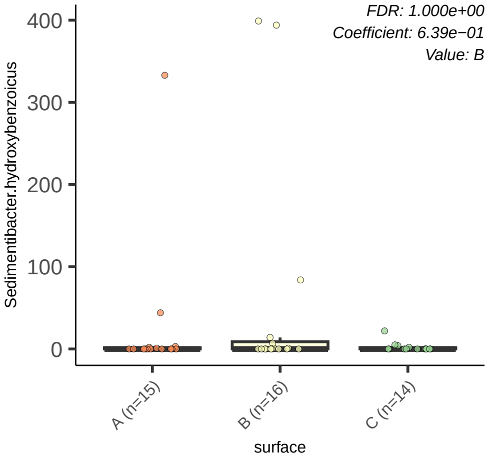

FDR: 1.000e+00
Coefficient: 6.39e−01
Value: B
Sedimentibacter.hydroxybenzoicus
400
300
200
100
0
A (n=15)
B (n=16)
C (n=14)
surface

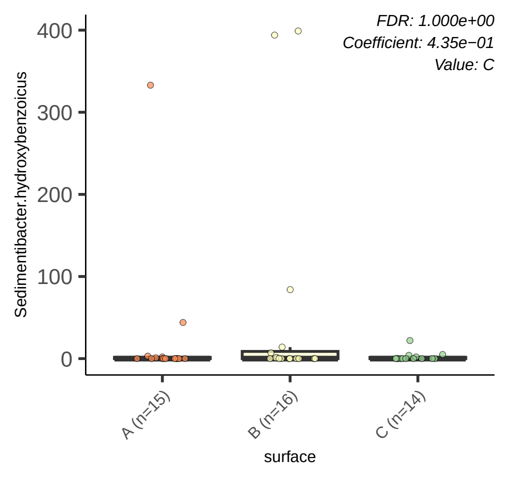
FDR: 1.000e+00
Coefficient: 4.35e−01
Value: C
Sedimentibacter.hydroxybenzoicus
400
300
200
100
0
A (n=15)
B (n=16)
C (n=14)
surface

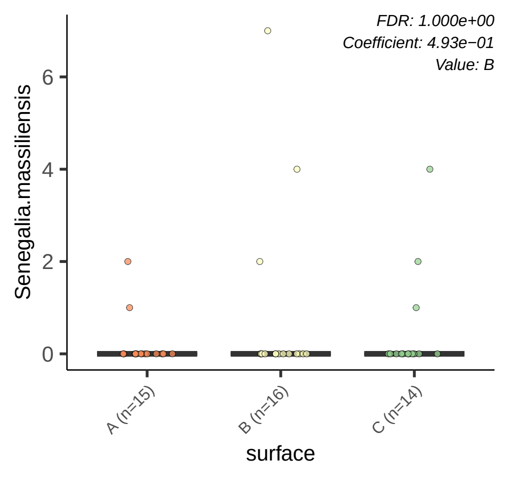

FDR: 1.000e+00
Coefficient: 4.93e−01
Value: B
Senegalia.massiliensis
0
2
4
6
A (n=15)
B (n=16)
C (n=14)
surface

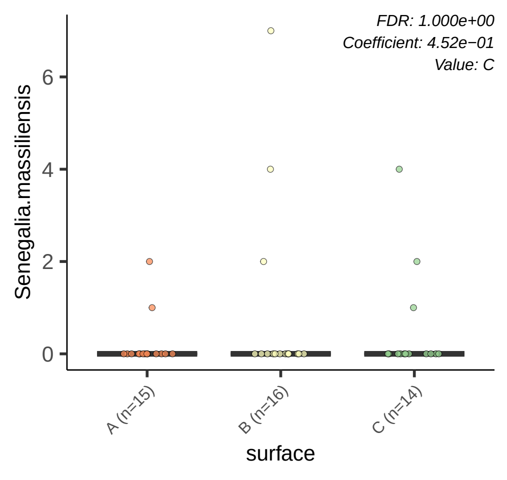

FDR: 1.000e+00
Coefficient: 4.52e−01
Value: C
Senegalia.massiliensis
0
2
4
6
A (n=15)
B (n=16)
C (n=14)
surface

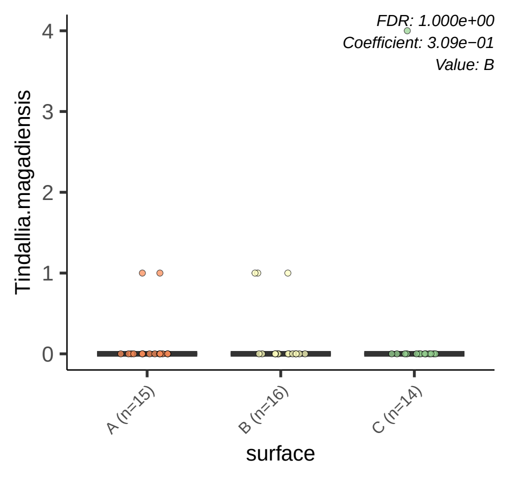

FDR: 1.000e+00
Coefficient: 3.09e−01
Value: B
Tindallia.magadiensis
0
1
2
3
4
A (n=15)
B (n=16)
C (n=14)
surface

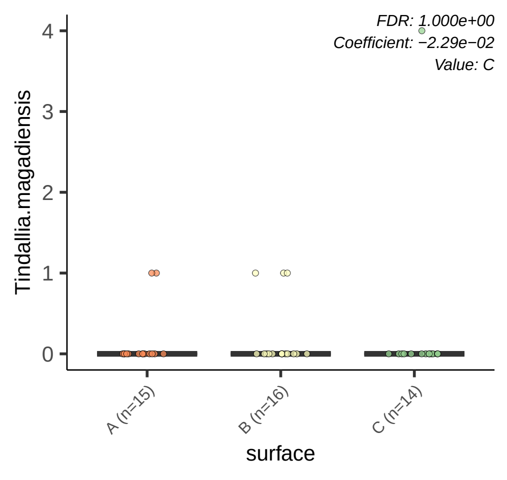
FDR: 1.000e+00
Coefficient: −2.29e−02
Value: C
Tindallia.magadiensis
0
1
2
3
4
A (n=15)
B (n=16)
C (n=14)
surface

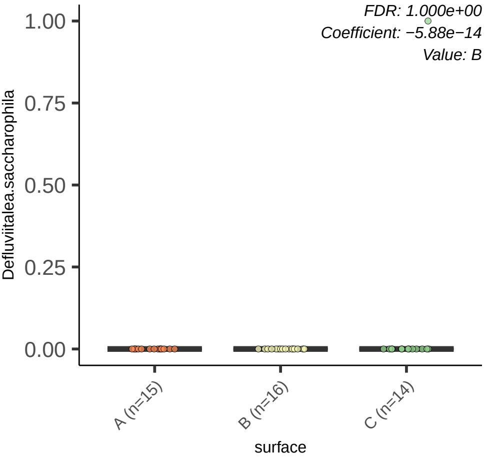

FDR: 1.000e+00
Coefficient: −5.88e−14
Value: B
Defluviitalea.saccharophila
1.00
0.75
0.50
0.25
0.00
A (n=15)
B (n=16)
C (n=14)
surface

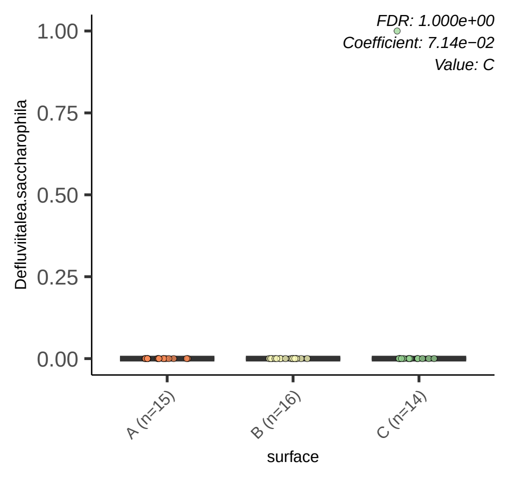

FDR: 1.000e+00
Coefficient: 7.14e−02
Value: C
1.00
0.75
0.50
0.25
0.00
Defluviitalea.saccharophila
A (n=15)
B (n=16)
C (n=14)
surface

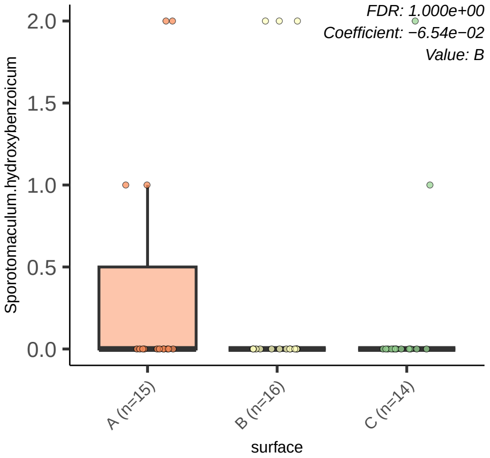
FDR: 1.000e+00
Coefficient: −6.54e−02
Value: B
Sporotomaculum.hydroxybenzoicum
2.0
1.5
1.0
0.5
0.0
A (n=15)
B (n=16)
C (n=14)
surface

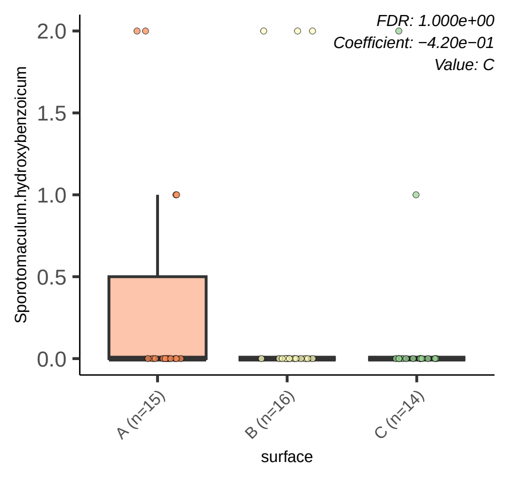

FDR: 1.000e+00
Coefficient: −4.20e−01
Value: C
Sporotomaculum.hydroxybenzoicum
2.0
1.5
1.0
0.5
0.0
A (n=15)
B (n=16)
C (n=14)
surface

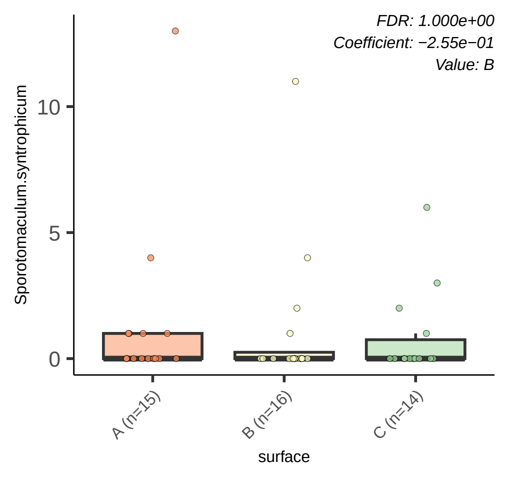

FDR: 1.000e+00
Coefficient: −2.55e−01
Value: B
Sporotomaculum.syntrophicum
10
5
0
A (n=15)
B (n=16)
C (n=14)
surface

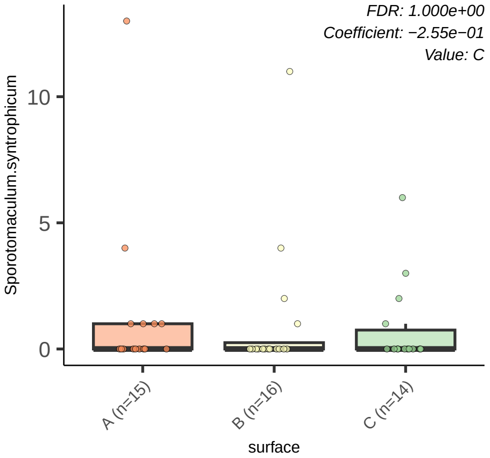

FDR: 1.000e+00
Coefficient: −2.55e−01
Value: C
Sporotomaculum.syntrophicum
10
5
0
A (n=15)
B (n=16)
C (n=14)
surface

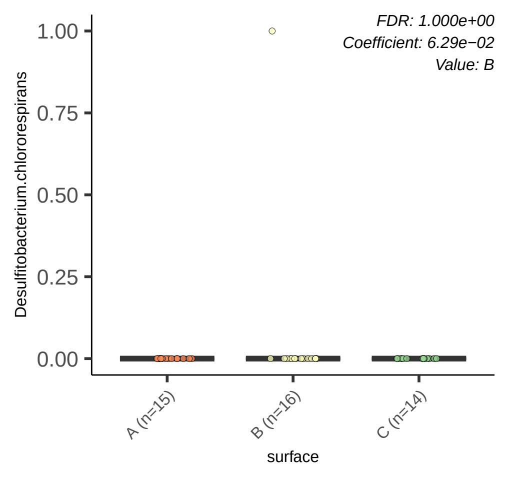
FDR: 1.000e+00
Coefficient: 6.29e−02
Value: B
1.00
0.75
0.50
0.25
0.00
Desulfitobacterium.chlororespirans
A (n=15)
B (n=16)
C (n=14)
surface

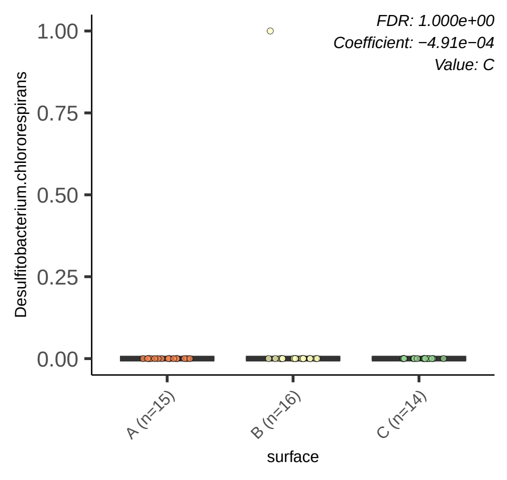
FDR: 1.000e+00
Coefficient: −4.91e−04
Value: C
1.00
0.75
0.50
0.25
0.00
Desulfitobacterium.chlororespirans
A (n=15)
B (n=16)
C (n=14)
surface

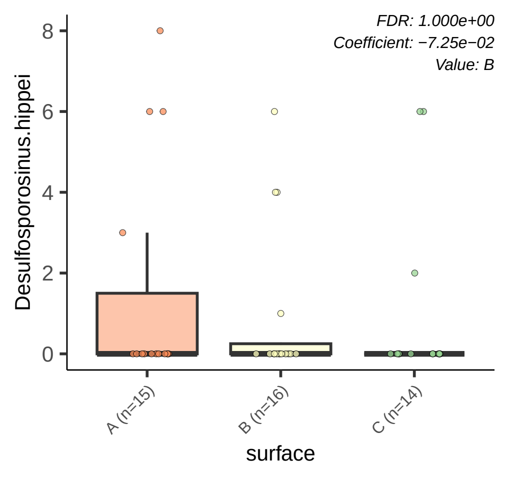
FDR: 1.000e+00
Coefficient: −7.25e−02
Value: B
Desulfosporosinus.hippei
8
6
4
2
0
A (n=15)
B (n=16)
C (n=14)
surface

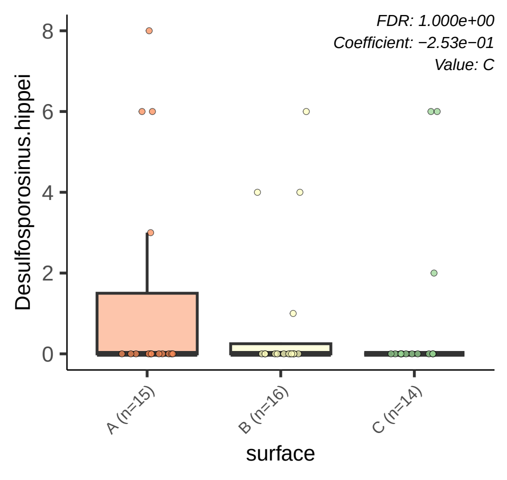
FDR: 1.000e+00
Coefficient: −2.53e−01
Value: C
Desulfosporosinus.hippei
0
2
4
6
8
A (n=15)
B (n=16)
C (n=14)
surface

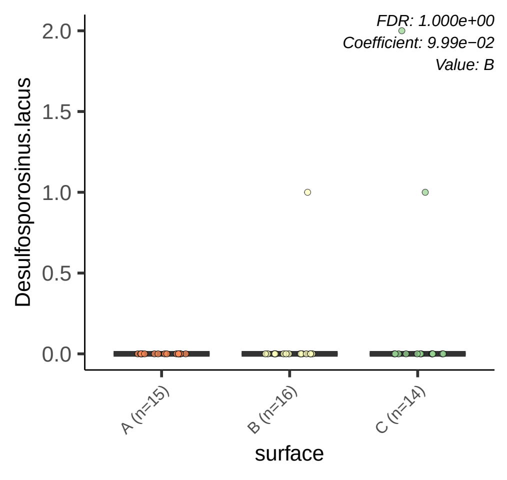

FDR: 1.000e+00
Coefficient: 9.99e−02
Value: B
Desulfosporosinus.lacus
2.0
1.5
1.0
0.5
0.0
A (n=15)
B (n=16)
C (n=14)
surface

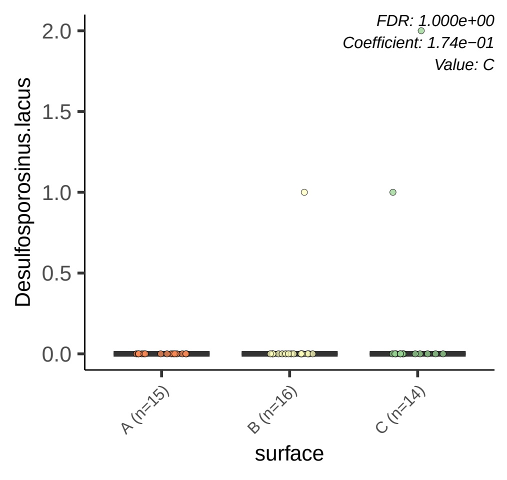

FDR: 1.000e+00
Coefficient: 1.74e−01
Value: C
Desulfosporosinus.lacus
2.0
1.5
1.0
0.5
0.0
A (n=15)
B (n=16)
C (n=14)
surface

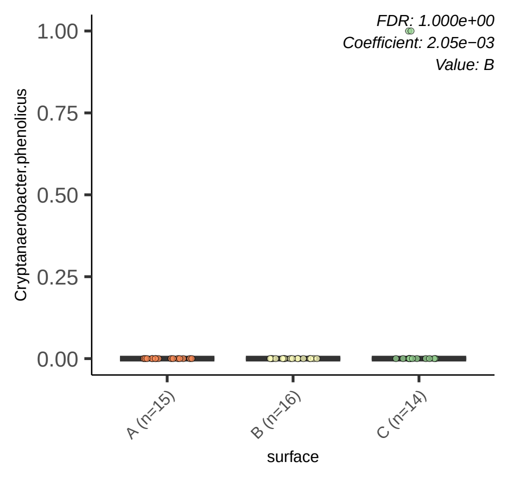

FDR: 1.000e+00
Coefficient: 2.05e−03
Value: B
1.00
0.75
0.50
0.25
0.00
Cryptanaerobacter.phenolicus
A (n=15)
B (n=16)
C (n=14)
surface

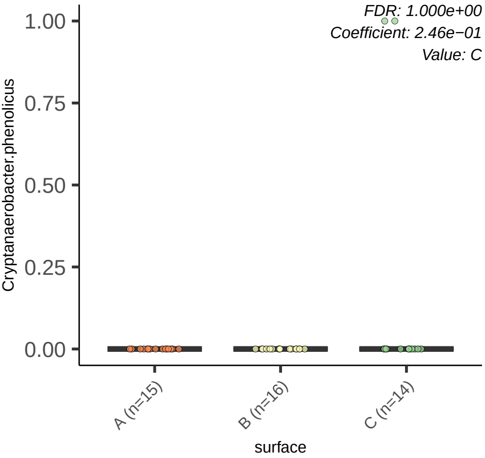
FDR: 1.000e+00
Coefficient: 2.46e−01
Value: C
Cryptanaerobacter.phenolicus
1.00
0.75
0.50
0.25
0.00
A (n=15)
B (n=16)
C (n=14)
surface

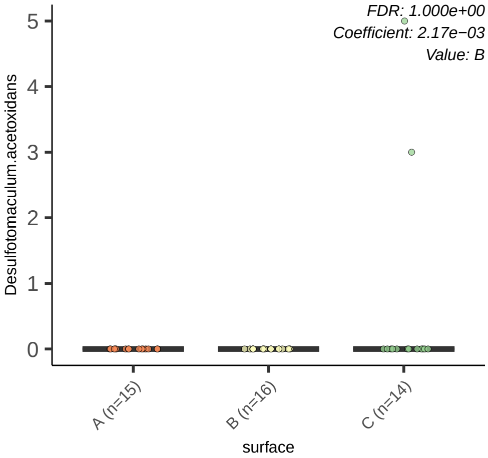
FDR: 1.000e+00
Coefficient: 2.17e−03
Value: B
Desulfotomaculum.acetoxidans
0
1
2
3
4
5
A (n=15)
B (n=16)
C (n=14)
surface

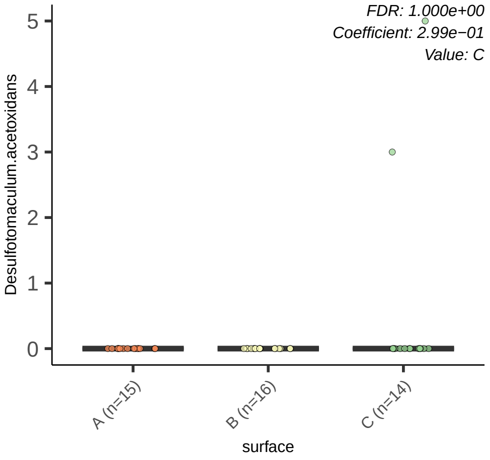

FDR: 1.000e+00
Coefficient: 2.99e−01
Value: C
Desulfotomaculum.acetoxidans
0
1
2
3
4
5
A (n=15)
B (n=16)
C (n=14)
surface

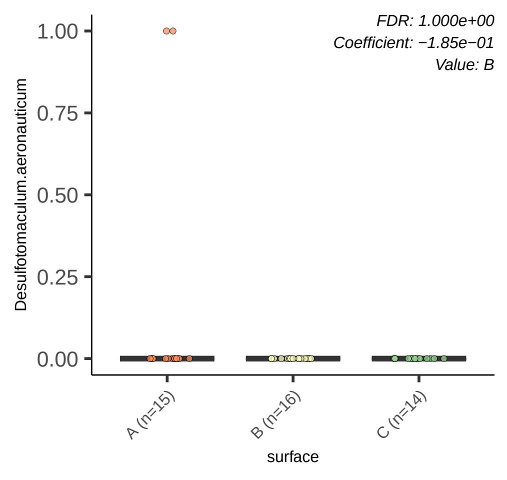
FDR: 1.000e+00
Coefficient: −1.85e−01
Value: B
Desulfotomaculum.aeronauticum
1.00
0.75
0.50
0.25
0.00
A (n=15)
B (n=16)
C (n=14)
surface

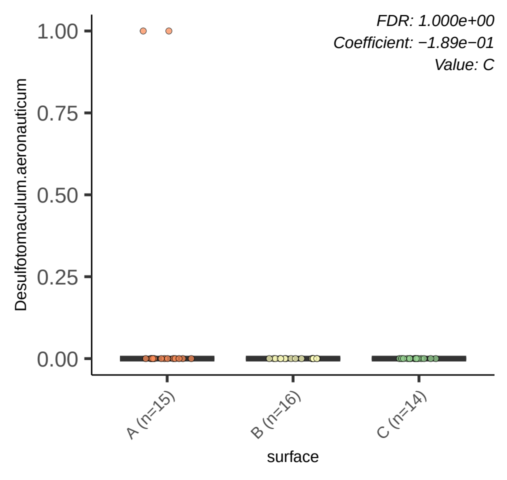

FDR: 1.000e+00
Coefficient: −1.89e−01
Value: C
Desulfotomaculum.aeronauticum
1.00
0.75
0.50
0.25
0.00
A (n=15)
B (n=16)
C (n=14)
surface

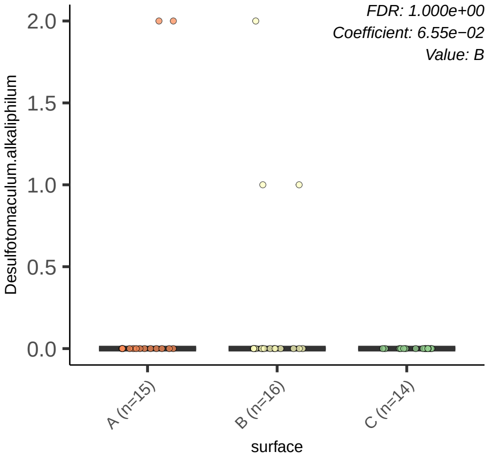
FDR: 1.000e+00
Coefficient: 6.55e−02
Value: B
Desulfotomaculum.alkaliphilum
2.0
1.5
1.0
0.5
0.0
A (n=15)
B (n=16)
C (n=14)
surface

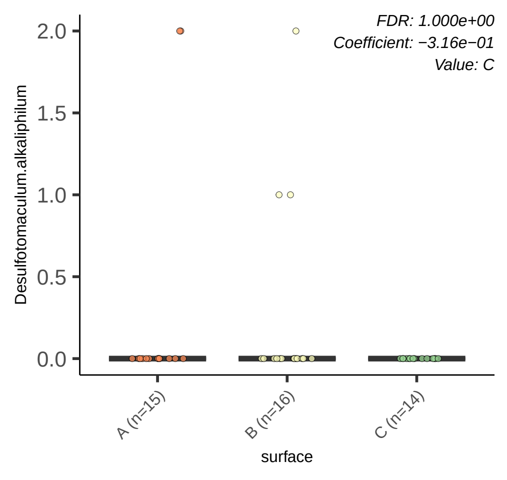
FDR: 1.000e+00
Coefficient: −3.16e−01
Value: C
Desulfotomaculum.alkaliphilum
2.0
1.5
1.0
0.5
0.0
A (n=15)
B (n=16)
C (n=14)
surface

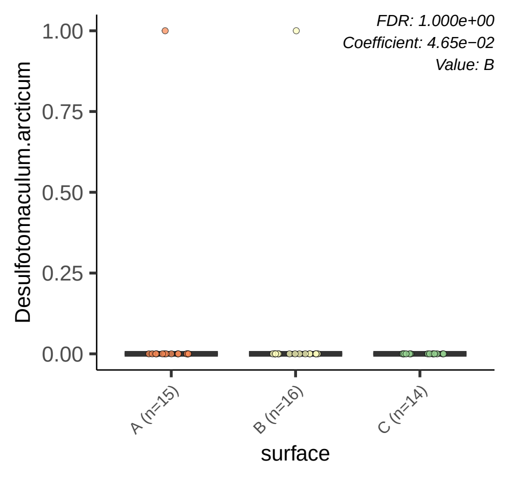
FDR: 1.000e+00
Coefficient: 4.65e−02
Value: B
Desulfotomaculum.arcticum
1.00
0.75
0.50
0.25
0.00
A (n=15)
B (n=16)
C (n=14)
surface

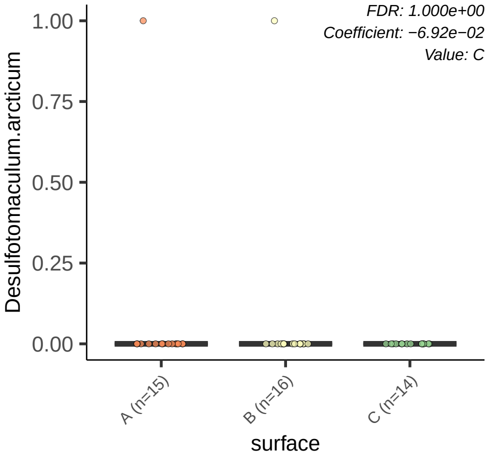
FDR: 1.000e+00
Coefficient: −6.92e−02
Value: C
Desulfotomaculum.arcticum
1.00
0.75
0.50
0.25
0.00
A (n=15)
B (n=16)
C (n=14)
surface

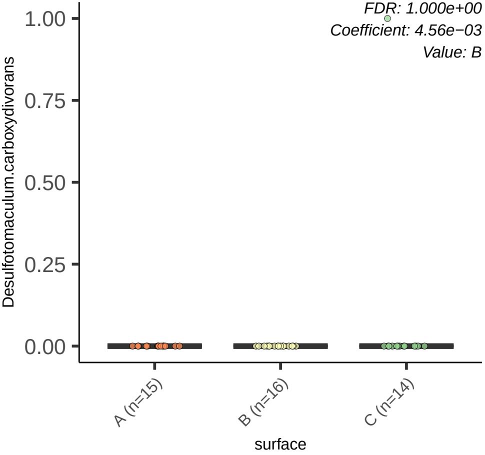
FDR: 1.000e+00
Coefficient: 4.56e−03
Value: B
Desulfotomaculum.carboxydivorans
1.00
0.75
0.50
0.25
0.00
A (n=15)
B (n=16)
C (n=14)
surface

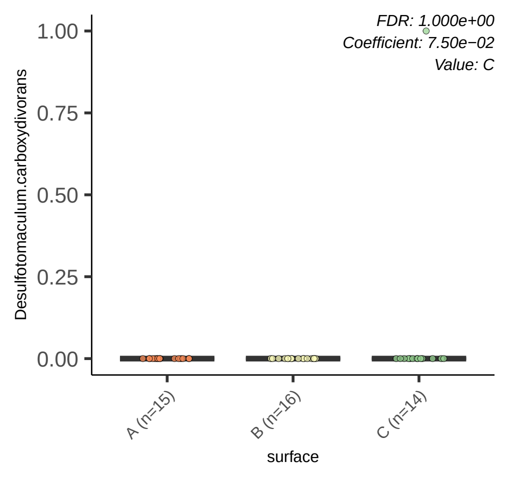

FDR: 1.000e+00
Coefficient: 7.50e−02
Value: C
Desulfotomaculum.carboxydivorans
1.00
0.75
0.50
0.25
0.00
A (n=15)
B (n=16)
C (n=14)
surface

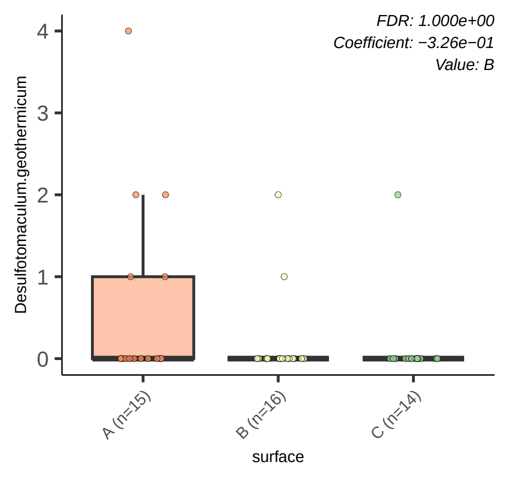

FDR: 1.000e+00
Coefficient: −3.26e−01
Value: B
Desulfotomaculum.geothermicum
4
3
2
1
0
A (n=15)
B (n=16)
C (n=14)
surface

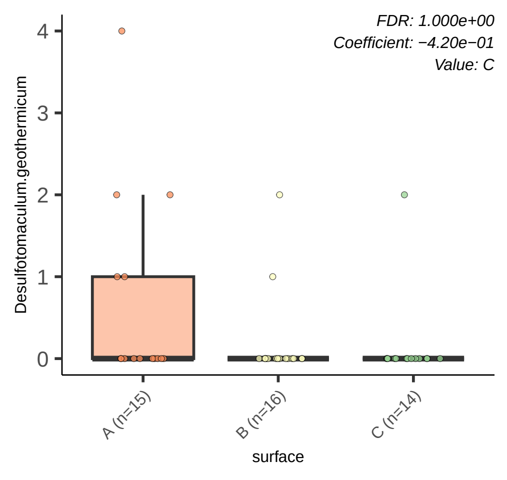

FDR: 1.000e+00
Coefficient: −4.20e−01
Value: C
Desulfotomaculum.geothermicum
0
1
2
3
4
A (n=15)
B (n=16)
C (n=14)
surface

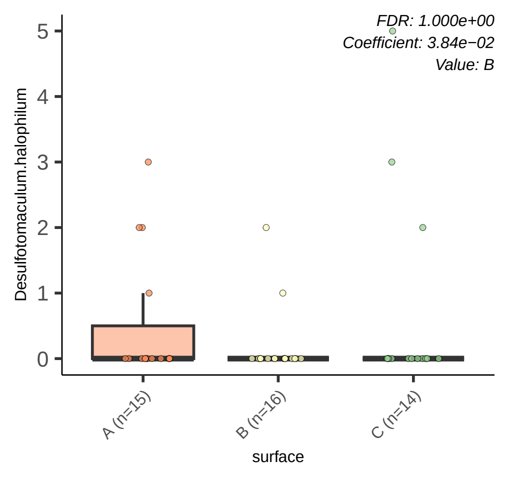
FDR: 1.000e+00
Coefficient: 3.84e−02
Value: B
Desulfotomaculum.halophilum
0
1
2
3
4
5
A (n=15)
B (n=16)
C (n=14)
surface

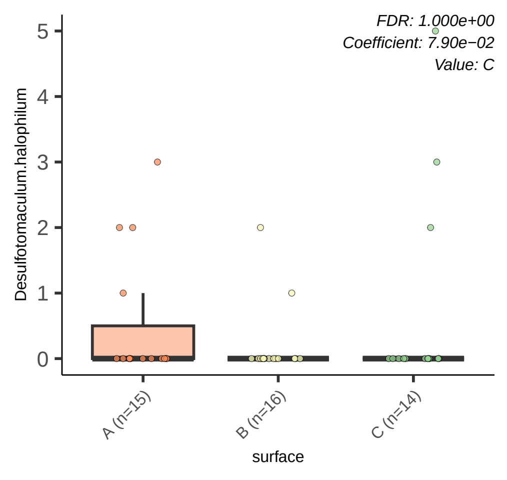
FDR: 1.000e+00
Coefficient: 7.90e−02
Value: C
Desulfotomaculum.halophilum
5
4
3
2
1
0
A (n=15)
B (n=16)
C (n=14)
surface

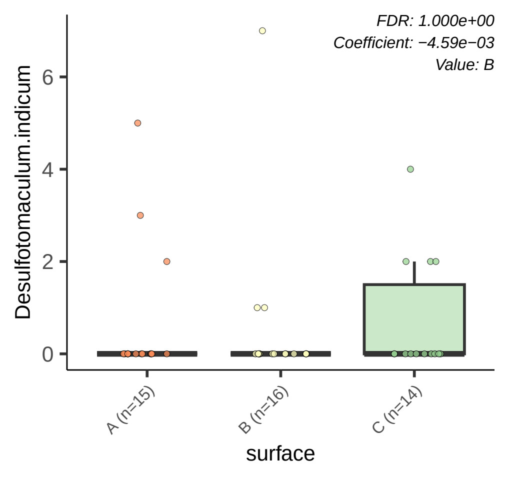

FDR: 1.000e+00
Coefficient: −4.59e−03
Value: B
Desulfotomaculum.indicum
0
2
4
6
A (n=15)
B (n=16)
C (n=14)
surface

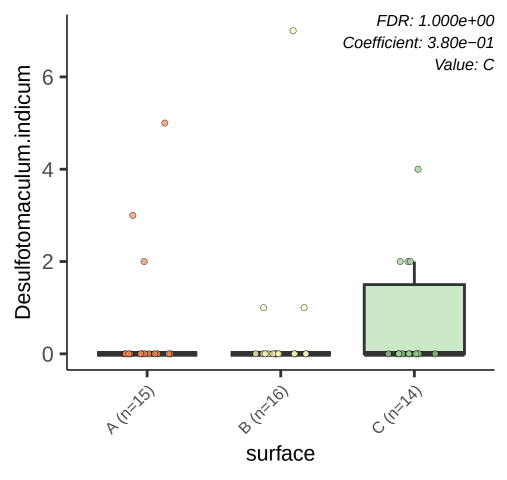
FDR: 1.000e+00
Coefficient: 3.80e−01
Value: C
Desulfotomaculum.indicum
0
2
4
6
A (n=15)
B (n=16)
C (n=14)
surface

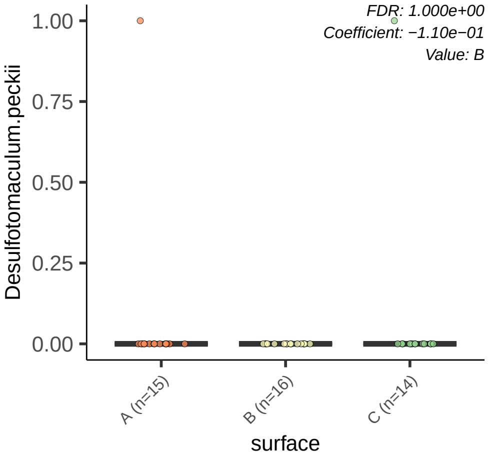
FDR: 1.000e+00
Coefficient: −1.10e−01
Value: B
Desulfotomaculum.peckii
1.00
0.75
0.50
0.25
0.00
A (n=15)
B (n=16)
C (n=14)
surface

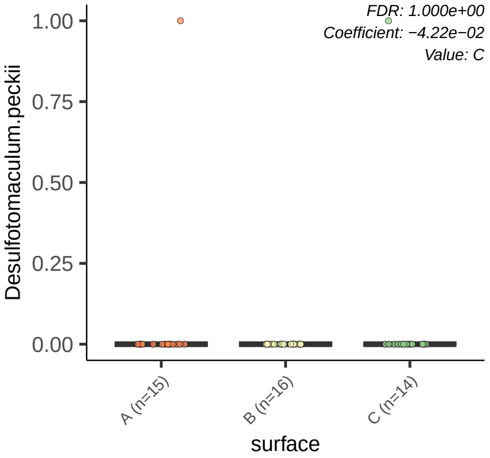
FDR: 1.000e+00
Coefficient: −4.22e−02
Value: C
Desulfotomaculum.peckii
1.00
0.75
0.50
0.25
0.00
A (n=15)
B (n=16)
C (n=14)
surface

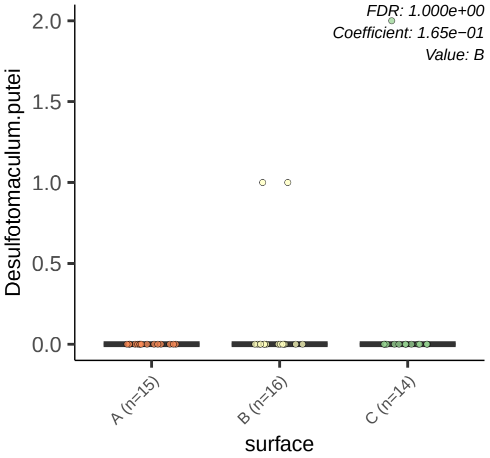
FDR: 1.000e+00
Coefficient: 1.65e−01
Value: B
Desulfotomaculum.putei
2.0
1.5
1.0
0.5
0.0
A (n=15)
B (n=16)
C (n=14)
surface

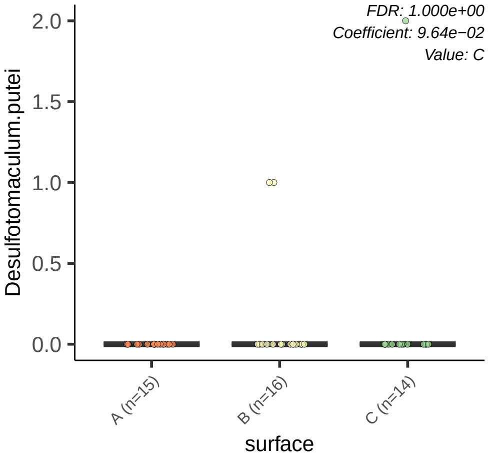
FDR: 1.000e+00
Coefficient: 9.64e−02
Value: C
Desulfotomaculum.putei
2.0
1.5
1.0
0.5
0.0
A (n=15)
B (n=16)
C (n=14)
surface

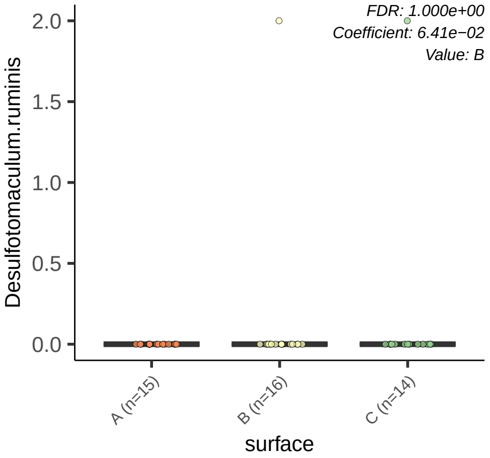
FDR: 1.000e+00
Coefficient: 6.41e−02
Value: B
Desulfotomaculum.ruminis
2.0
1.5
1.0
0.5
0.0
A (n=15)
B (n=16)
C (n=14)
surface

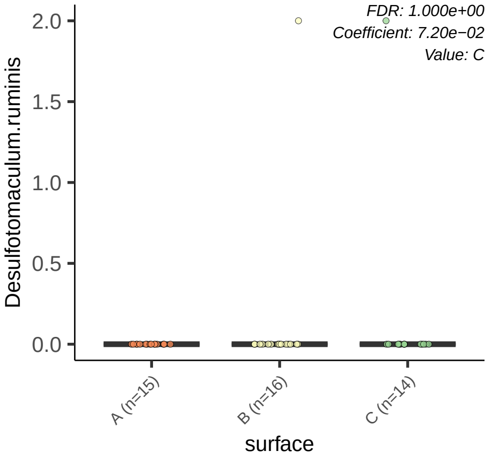
FDR: 1.000e+00
Coefficient: 7.20e−02
Value: C
Desulfotomaculum.ruminis
2.0
1.5
1.0
0.5
0.0
A (n=15)
B (n=16)
C (n=14)
surface

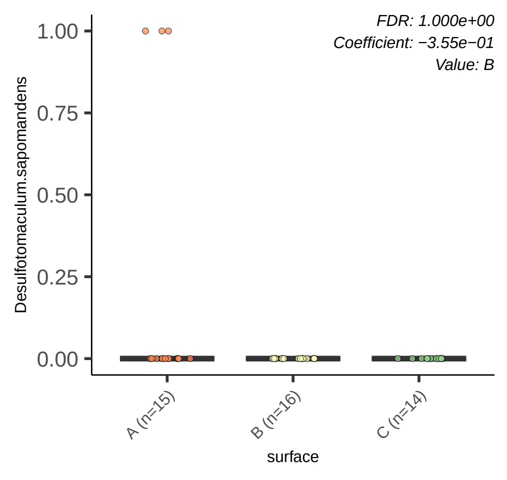
FDR: 1.000e+00
Coefficient: −3.55e−01
Value: B
Desulfotomaculum.sapomandens
1.00
0.75
0.50
0.25
0.00
A (n=15)
B (n=16)
C (n=14)
surface

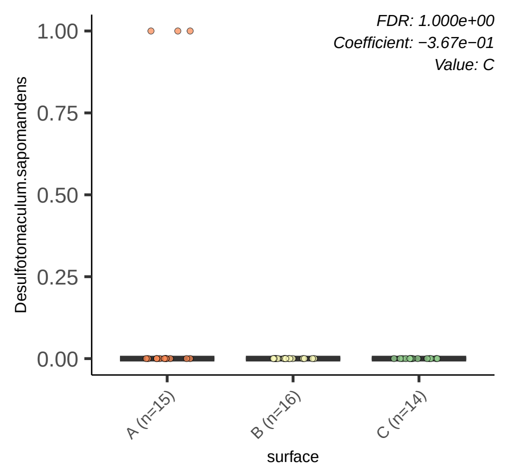

FDR: 1.000e+00
Coefficient: −3.67e−01
Value: C
Desulfotomaculum.sapomandens
1.00
0.75
0.50
0.25
0.00
A (n=15)
B (n=16)
C (n=14)
surface

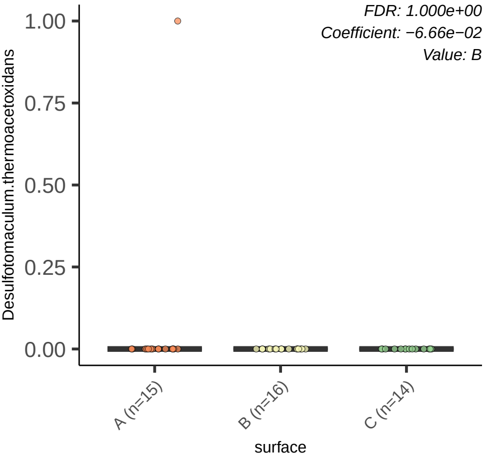
FDR: 1.000e+00
Coefficient: −6.66e−02
Value: B
Desulfotomaculum.thermoacetoxidans
1.00
0.75
0.50
0.25
0.00
A (n=15)
B (n=16)
C (n=14)
surface

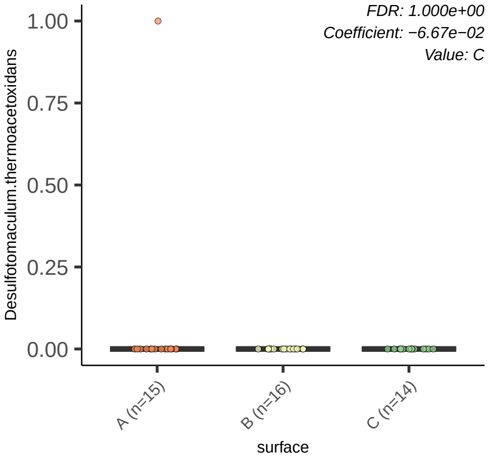

FDR: 1.000e+00
Coefficient: −6.67e−02
Value: C
1.00
0.75
0.50
0.25
0.00
Desulfotomaculum.thermoacetoxidans
A (n=15)
B (n=16)
C (n=14)
surface

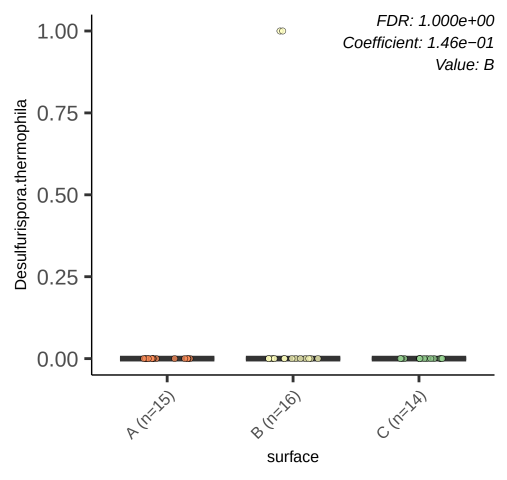

FDR: 1.000e+00
Coefficient: 1.46e−01
Value: B
1.00
0.75
0.50
0.25
0.00
Desulfurispora.thermophila
A (n=15)
B (n=16)
C (n=14)
surface

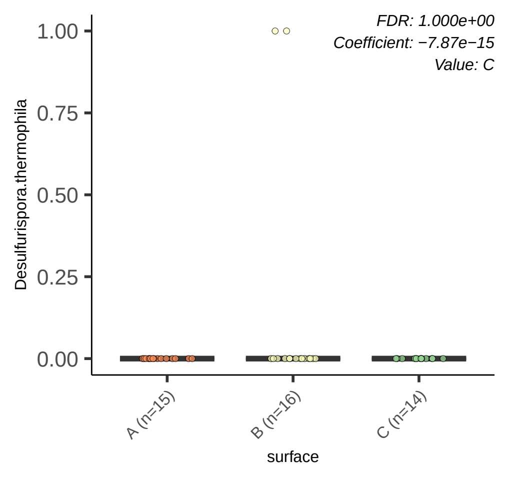
FDR: 1.000e+00
Coefficient: −7.87e−15
Value: C
Desulfurispora.thermophila
1.00
0.75
0.50
0.25
0.00
A (n=15)
B (n=16)
C (n=14)
surface

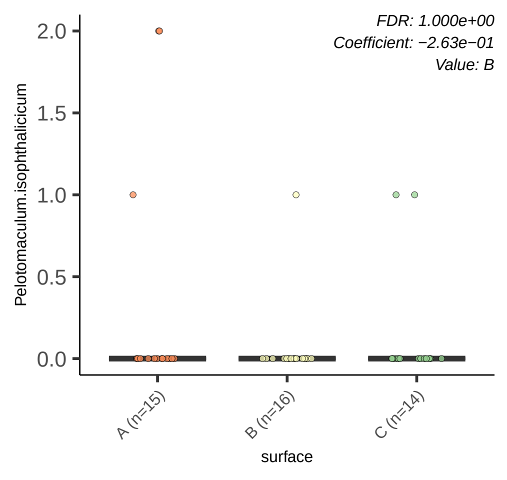
FDR: 1.000e+00
Coefficient: −2.63e−01
Value: B
Pelotomaculum.isophthalicicum
2.0
1.5
1.0
0.5
0.0
A (n=15)
B (n=16)
C (n=14)
surface

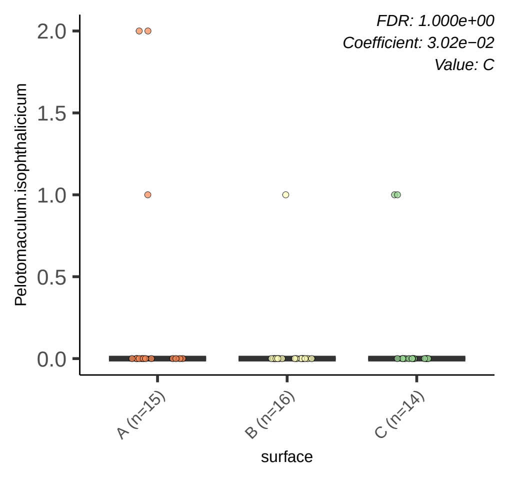

FDR: 1.000e+00
Coefficient: 3.02e−02
Value: C
Pelotomaculum.isophthalicicum
2.0
1.5
1.0
0.5
0.0
A (n=15)
B (n=16)
C (n=14)
surface

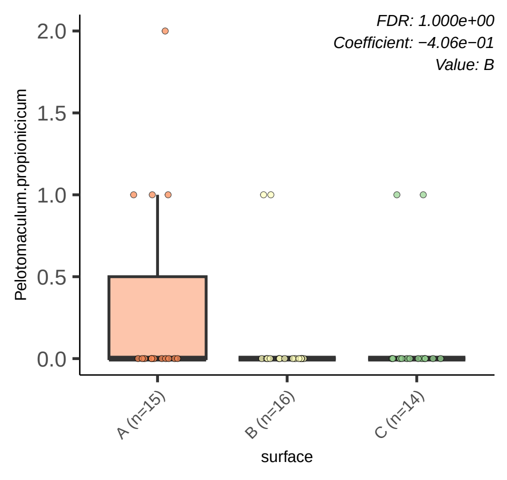
FDR: 1.000e+00
Coefficient: −4.06e−01
Value: B
Pelotomaculum.propionicicum
2.0
1.5
1.0
0.5
0.0
A (n=15)
B (n=16)
C (n=14)
surface

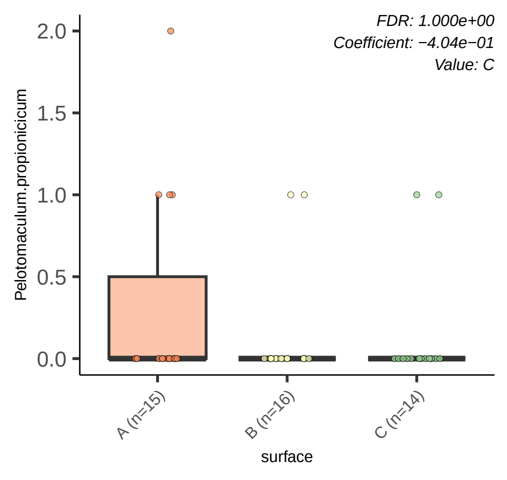
FDR: 1.000e+00
Coefficient: −4.04e−01
Value: C
Pelotomaculum.propionicicum
2.0
1.5
1.0
0.5
0.0
A (n=15)
B (n=16)
C (n=14)
surface

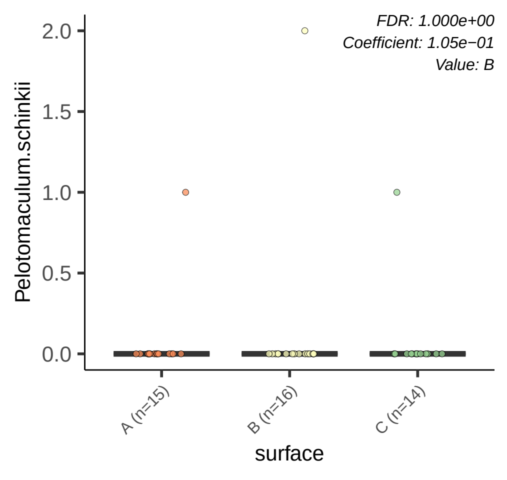
FDR: 1.000e+00
Coefficient: 1.05e−01
Value: B
Pelotomaculum.schinkii
2.0
1.5
1.0
0.5
0.0
A (n=15)
B (n=16)
C (n=14)
surface

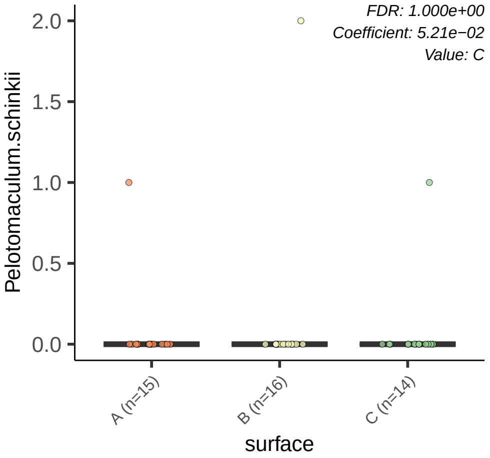
FDR: 1.000e+00
Coefficient: 5.21e−02
Value: C
Pelotomaculum.schinkii
2.0
1.5
1.0
0.5
0.0
A (n=15)
B (n=16)
C (n=14)
surface

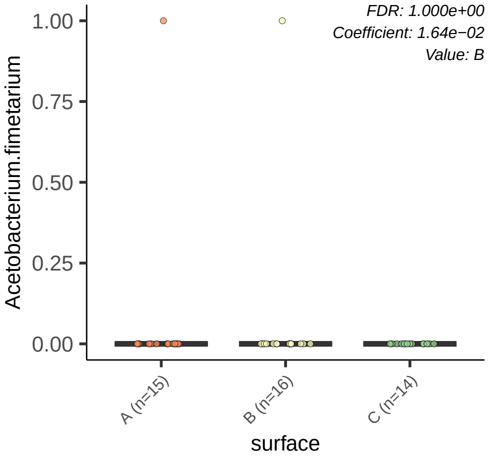
FDR: 1.000e+00
Coefficient: 1.64e−02
Value: B
Acetobacterium.fimetarium
1.00
0.75
0.50
0.25
0.00
A (n=15)
B (n=16)
C (n=14)
surface

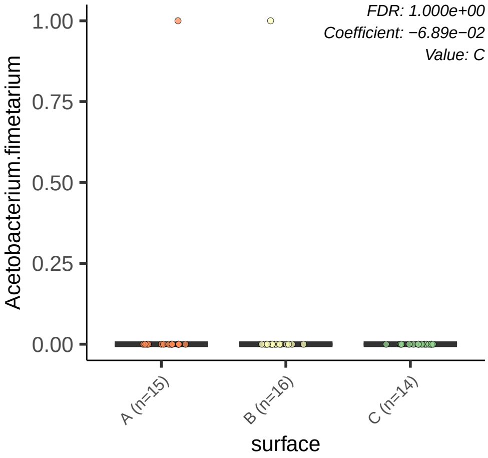

FDR: 1.000e+00
Coefficient: −6.89e−02
Value: C
Acetobacterium.fimetarium
1.00
0.75
0.50
0.25
0.00
A (n=15)
B (n=16)
C (n=14)
surface

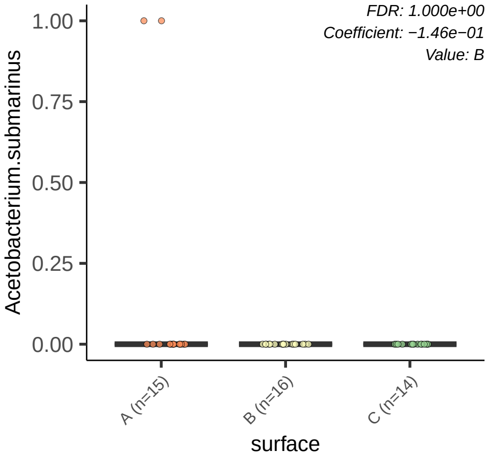

FDR: 1.000e+00
Coefficient: −1.46e−01
Value: B
Acetobacterium.submarinus
1.00
0.75
0.50
0.25
0.00
A (n=15)
B (n=16)
C (n=14)
surface

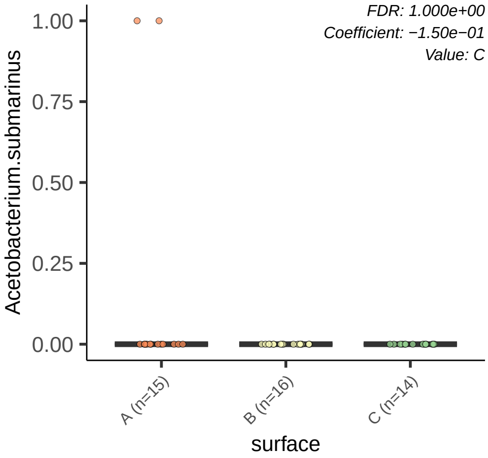

FDR: 1.000e+00
Coefficient: −1.50e−01
Value: C
Acetobacterium.submarinus
1.00
0.75
0.50
0.25
0.00
A (n=15)
B (n=16)
C (n=14)
surface

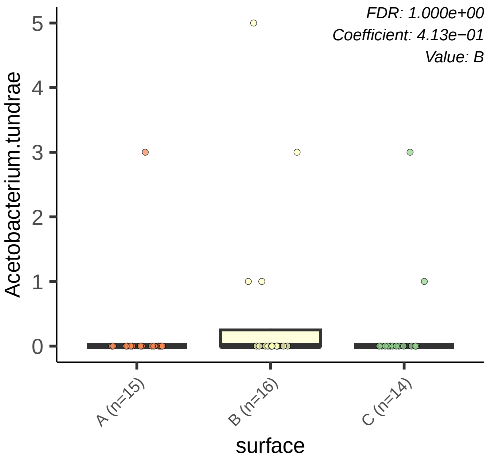
FDR: 1.000e+00
Coefficient: 4.13e−01
Value: B
Acetobacterium.tundrae
0
1
2
3
4
5
A (n=15)
B (n=16)
C (n=14)
surface

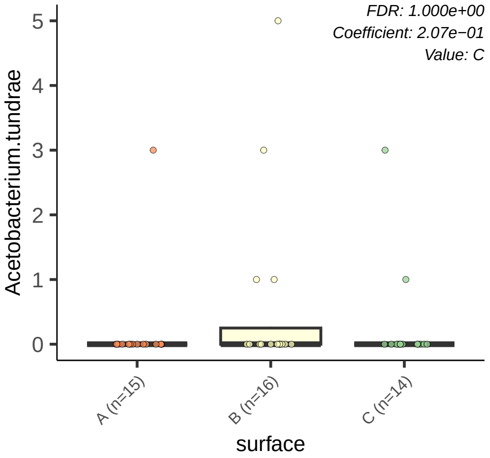

FDR: 1.000e+00
Coefficient: 2.07e−01
Value: C
Acetobacterium.tundrae
5
4
3
2
1
0
A (n=15)
B (n=16)
C (n=14)
surface

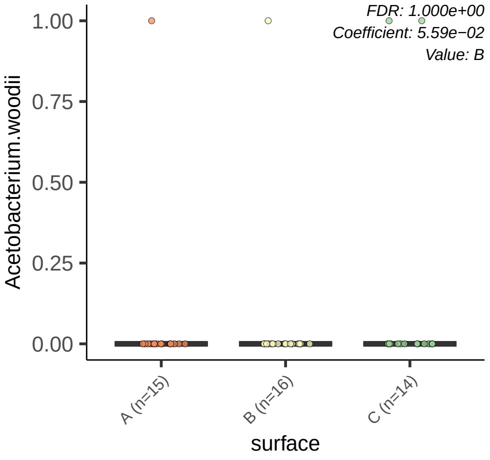
FDR: 1.000e+00
Coefficient: 5.59e−02
Value: B
Acetobacterium.woodii
1.00
0.75
0.50
0.25
0.00
A (n=15)
B (n=16)
C (n=14)
surface

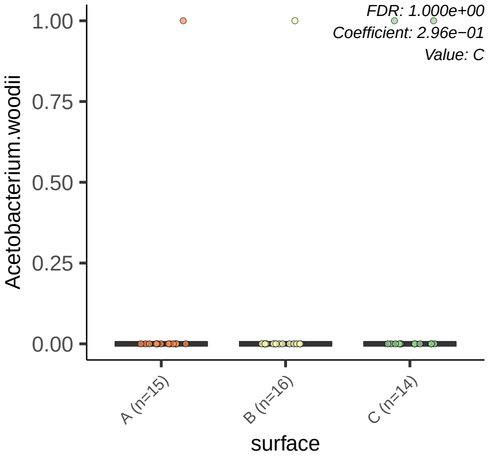
FDR: 1.000e+00
Coefficient: 2.96e−01
Value: C
Acetobacterium.woodii
1.00
0.75
0.50
0.25
0.00
A (n=15)
B (n=16)
C (n=14)
surface

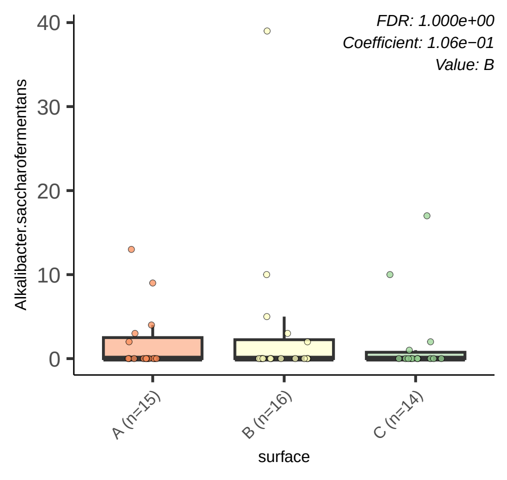

FDR: 1.000e+00
Coefficient: 1.06e−01
Value: B
Alkalibacter.saccharofermentans
0
10
20
30
40
A (n=15)
B (n=16)
C (n=14)
surface

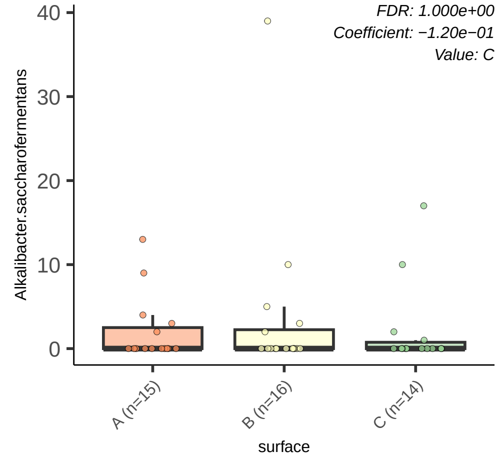
FDR: 1.000e+00
Coefficient: −1.20e−01
Value: C
Alkalibacter.saccharofermentans
40
30
20
10
0
A (n=15)
B (n=16)
C (n=14)
surface

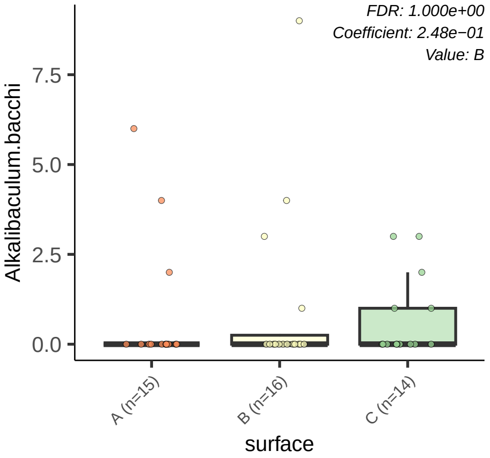
FDR: 1.000e+00
Coefficient: 2.48e−01
Value: B
Alkalibaculum.bacchi
7.5
5.0
2.5
0.0
A (n=15)
B (n=16)
C (n=14)
surface

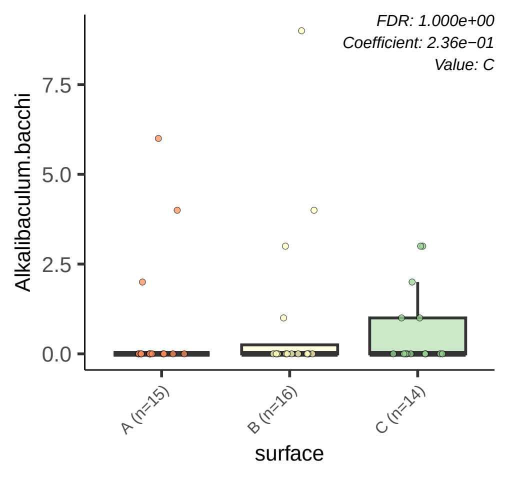

FDR: 1.000e+00
Coefficient: 2.36e−01
Value: C
Alkalibaculum.bacchi
7.5
5.0
2.5
0.0
A (n=15)
B (n=16)
C (n=14)
surface

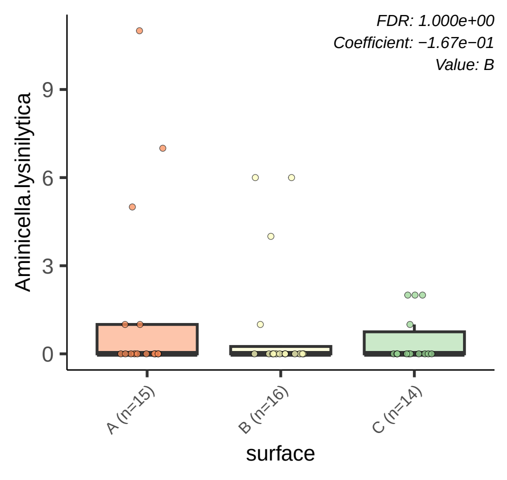

FDR: 1.000e+00
Coefficient: −1.67e−01
Value: B
Aminicella.lysinilytica
0
3
6
9
A (n=15)
B (n=16)
C (n=14)
surface

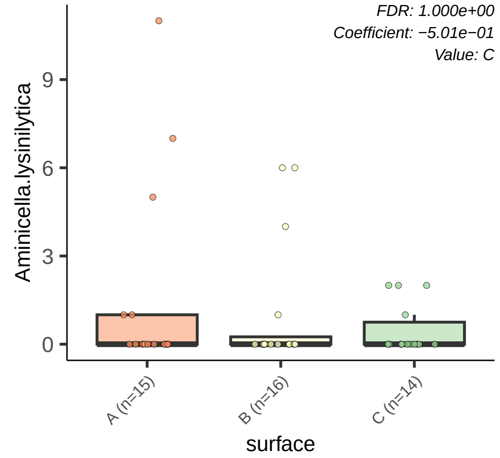
FDR: 1.000e+00
Coefficient: −5.01e−01
Value: C
Aminicella.lysinilytica
0
3
6
9
A (n=15)
B (n=16)
C (n=14)
surface

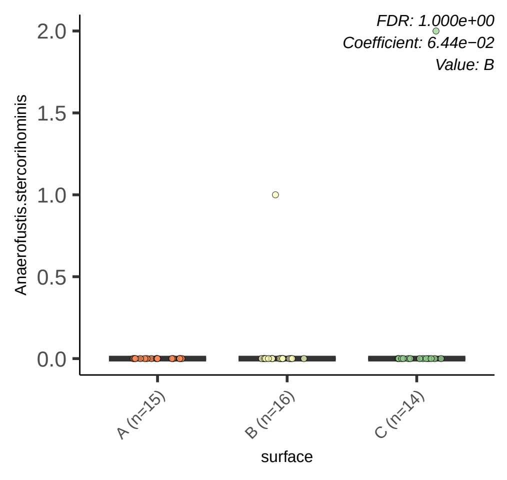

FDR: 1.000e+00
Coefficient: 6.44e−02
Value: B
Anaerofustis.stercorihominis
2.0
1.5
1.0
0.5
0.0
A (n=15)
B (n=16)
C (n=14)
surface

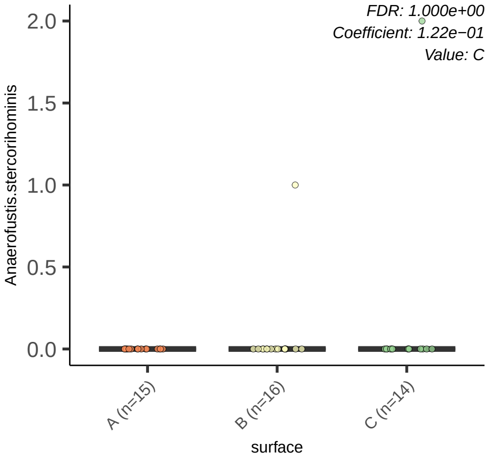
FDR: 1.000e+00
Coefficient: 1.22e−01
Value: C
Anaerofustis.stercorihominis
2.0
1.5
1.0
0.5
0.0
A (n=15)
B (n=16)
C (n=14)
surface

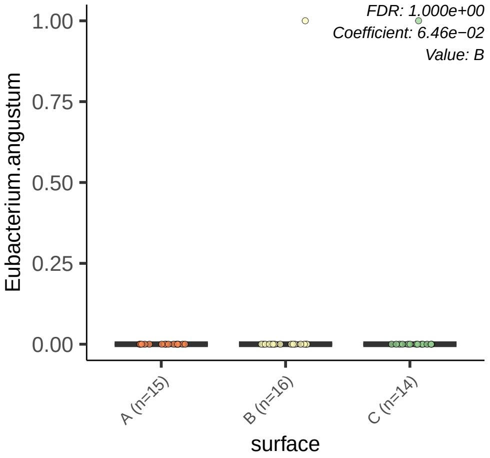
FDR: 1.000e+00
Coefficient: 6.46e−02
Value: B
Eubacterium.angustum
1.00
0.75
0.50
0.25
0.00
A (n=15)
B (n=16)
C (n=14)
surface

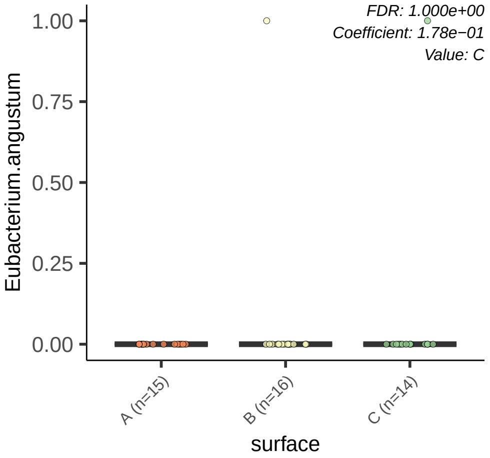
FDR: 1.000e+00
Coefficient: 1.78e−01
Value: C
Eubacterium.angustum
1.00
0.75
0.50
0.25
0.00
A (n=15)
B (n=16)
C (n=14)
surface

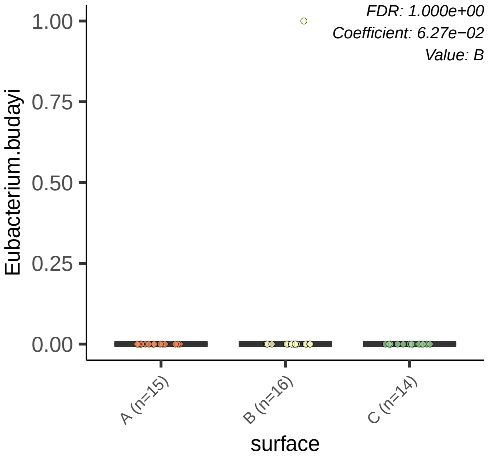
FDR: 1.000e+00
Coefficient: 6.27e−02
Value: B
Eubacterium.budayi
1.00
0.75
0.50
0.25
0.00
A (n=15)
B (n=16)
C (n=14)
surface

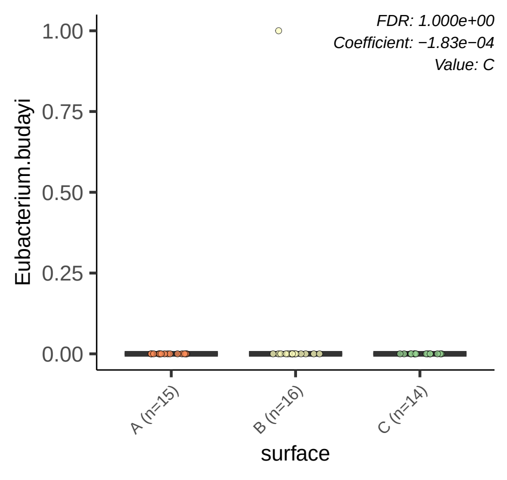

FDR: 1.000e+00
Coefficient: −1.83e−04
Value: C
Eubacterium.budayi
1.00
0.75
0.50
0.25
0.00
A (n=15)
B (n=16)
C (n=14)
surface

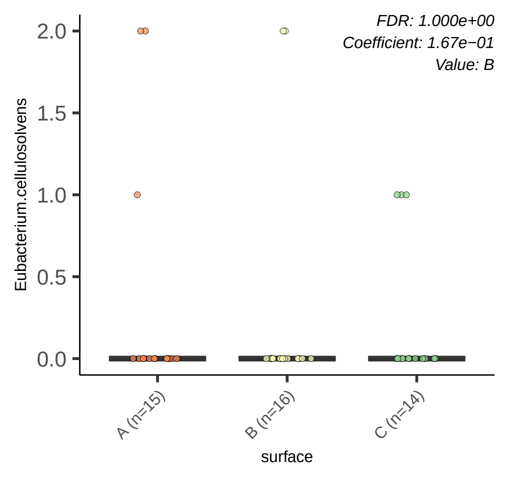
FDR: 1.000e+00
Coefficient: 1.67e−01
Value: B
Eubacterium.cellulosolvens
2.0
1.5
1.0
0.5
0.0
A (n=15)
B (n=16)
C (n=14)
surface

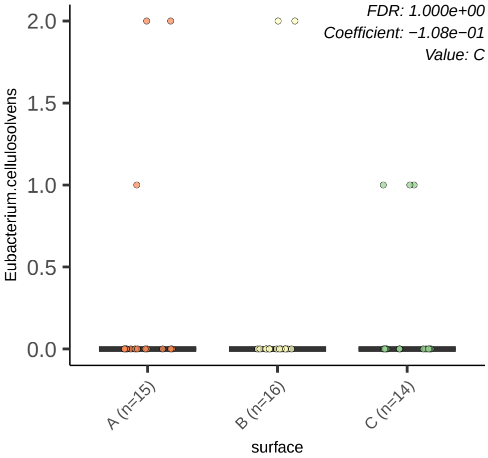

FDR: 1.000e+00
Coefficient: −1.08e−01
Value: C
Eubacterium.cellulosolvens
2.0
1.5
1.0
0.5
0.0
A (n=15)
B (n=16)
C (n=14)
surface

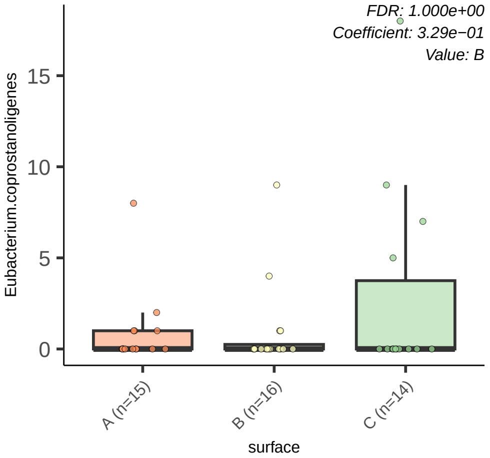

FDR: 1.000e+00
Coefficient: 3.29e−01
Value: B
Eubacterium.coprostanoligenes
0
5
10
15
A (n=15)
B (n=16)
C (n=14)
surface

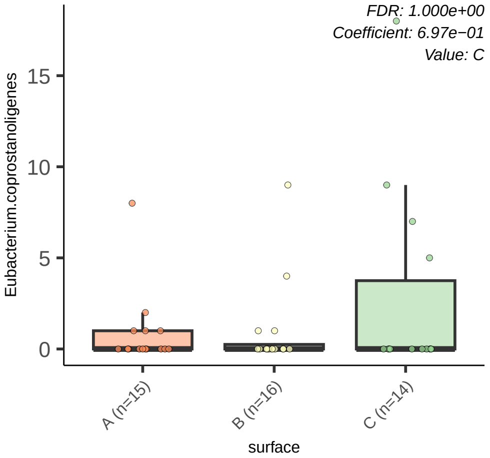
FDR: 1.000e+00
Coefficient: 6.97e−01
Value: C
Eubacterium.coprostanoligenes
0
5
10
15
A (n=15)
B (n=16)
C (n=14)
surface

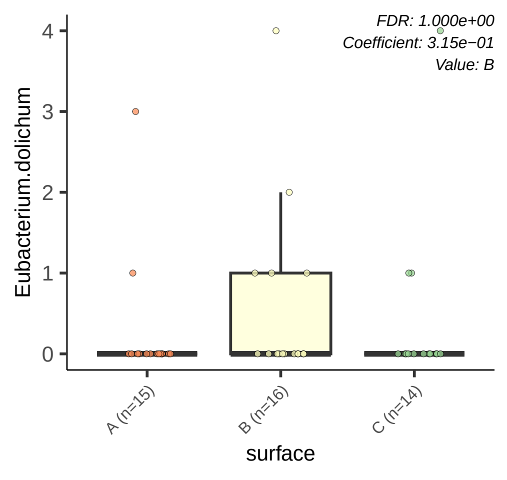
FDR: 1.000e+00
Coefficient: 3.15e−01
Value: B
Eubacterium.dolichum
0
1
2
3
4
A (n=15)
B (n=16)
C (n=14)
surface

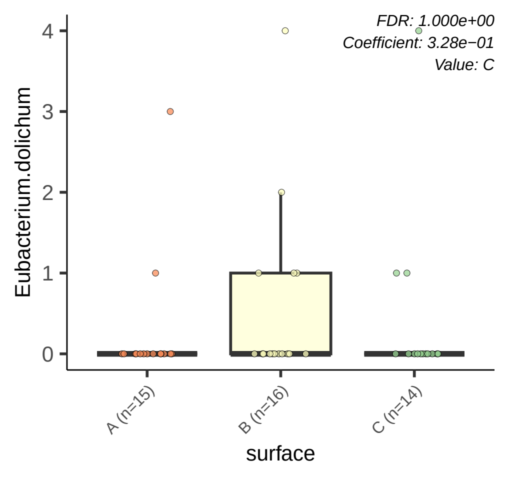

FDR: 1.000e+00
Coefficient: 3.28e−01
Value: C
Eubacterium.dolichum
0
1
2
3
4
A (n=15)
B (n=16)
C (n=14)
surface

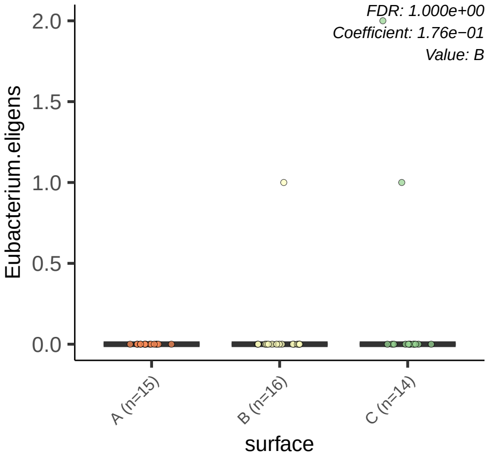

FDR: 1.000e+00
Coefficient: 1.76e−01
Value: B
Eubacterium.eligens
2.0
1.5
1.0
0.5
0.0
A (n=15)
B (n=16)
C (n=14)
surface

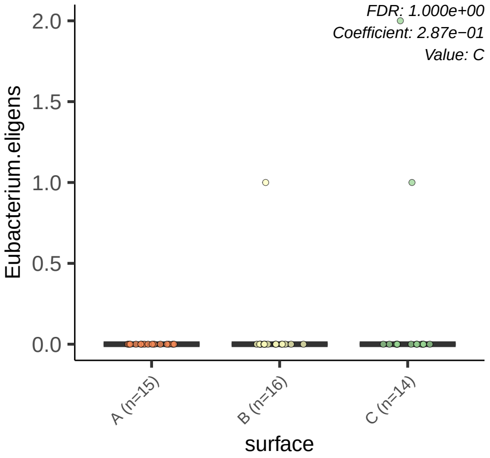

FDR: 1.000e+00
Coefficient: 2.87e−01
Value: C
Eubacterium.eligens
2.0
1.5
1.0
0.5
0.0
A (n=15)
B (n=16)
C (n=14)
surface

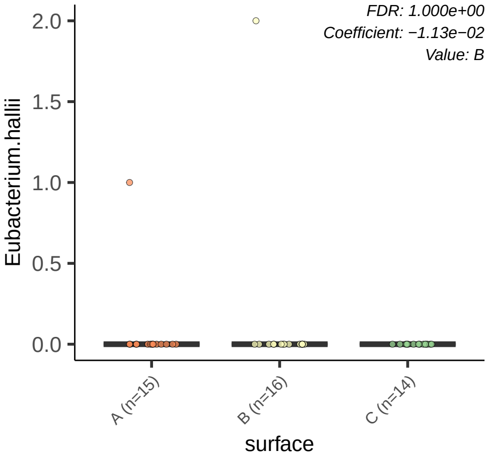
FDR: 1.000e+00
Coefficient: −1.13e−02
Value: B
Eubacterium.hallii
2.0
1.5
1.0
0.5
0.0
A (n=15)
B (n=16)
C (n=14)
surface

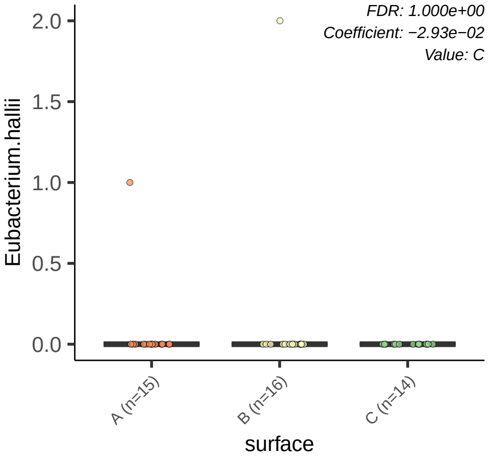

FDR: 1.000e+00
Coefficient: −2.93e−02
Value: C
Eubacterium.hallii
2.0
1.5
1.0
0.5
0.0
A (n=15)
B (n=16)
C (n=14)
surface

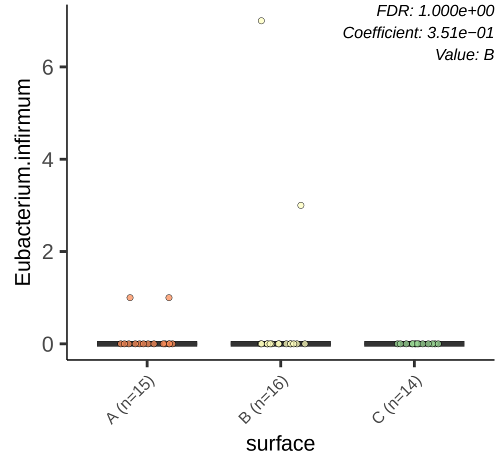
FDR: 1.000e+00
Coefficient: 3.51e−01
Value: B
Eubacterium.infirmum
0
2
4
6
A (n=15)
B (n=16)
C (n=14)
surface

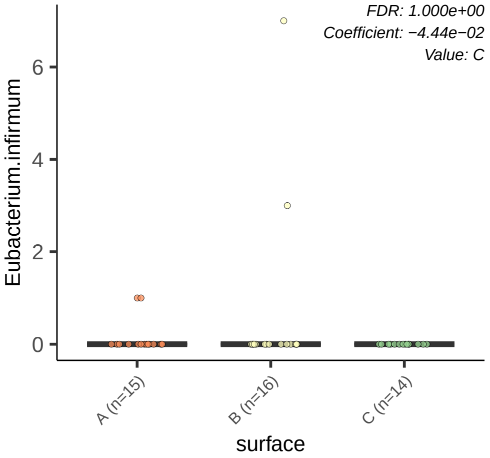
FDR: 1.000e+00
Coefficient: −4.44e−02
Value: C
Eubacterium.infirmum
0
2
4
6
A (n=15)
B (n=16)
C (n=14)
surface

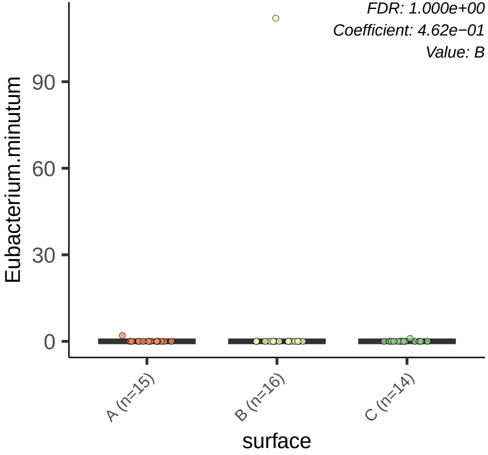
FDR: 1.000e+00
Coefficient: 4.62e−01
Value: B
Eubacterium.minutum
90
60
30
0
A (n=15)
B (n=16)
C (n=14)
surface

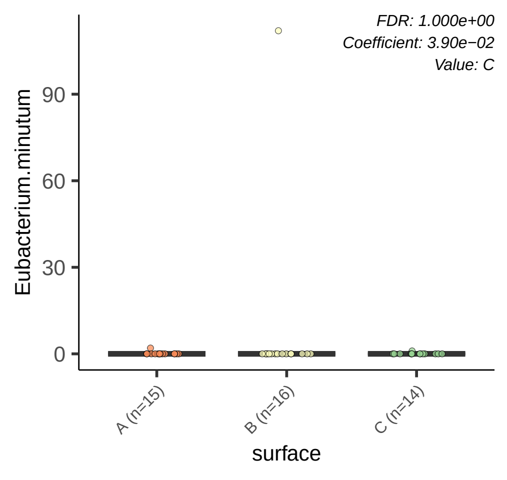
FDR: 1.000e+00
Coefficient: 3.90e−02
Value: C
Eubacterium.minutum
90
60
30
0
A (n=15)
B (n=16)
C (n=14)
surface

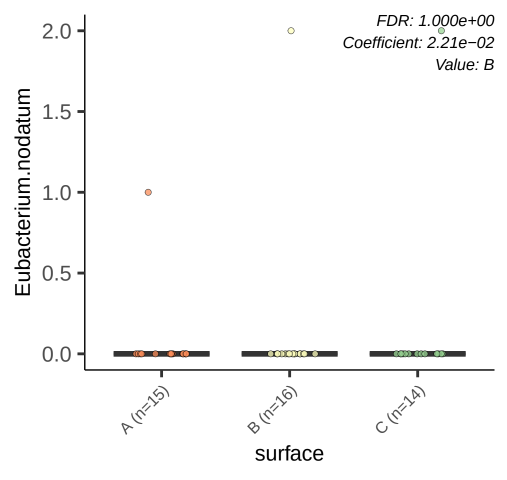
FDR: 1.000e+00
Coefficient: 2.21e−02
Value: B
Eubacterium.nodatum
2.0
1.5
1.0
0.5
0.0
A (n=15)
B (n=16)
C (n=14)
surface

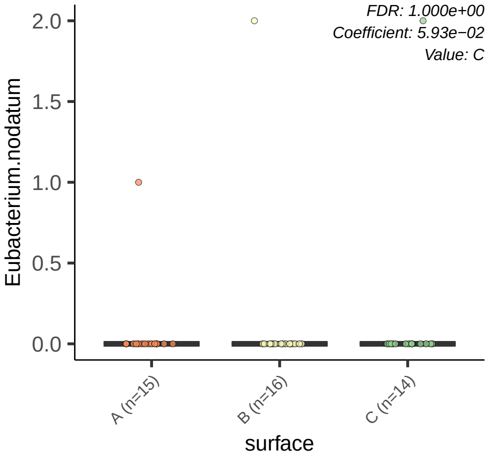
FDR: 1.000e+00
Coefficient: 5.93e−02
Value: C
Eubacterium.nodatum
2.0
1.5
1.0
0.5
0.0
A (n=15)
B (n=16)
C (n=14)
surface

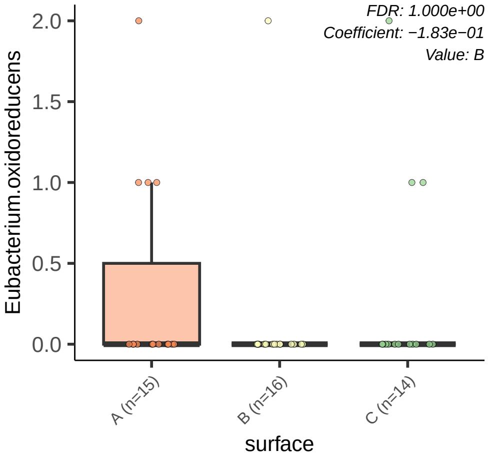
FDR: 1.000e+00
Coefficient: −1.83e−01
Value: B
Eubacterium.oxidoreducens
2.0
1.5
1.0
0.5
0.0
A (n=15)
B (n=16)
C (n=14)
surface

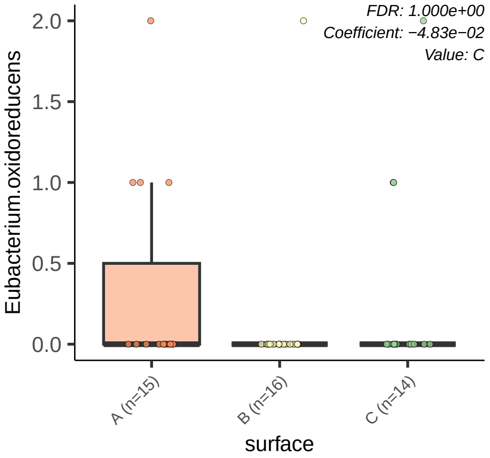

FDR: 1.000e+00
Coefficient: −4.83e−02
Value: C
Eubacterium.oxidoreducens
2.0
1.5
1.0
0.5
0.0
A (n=15)
B (n=16)
C (n=14)
surface

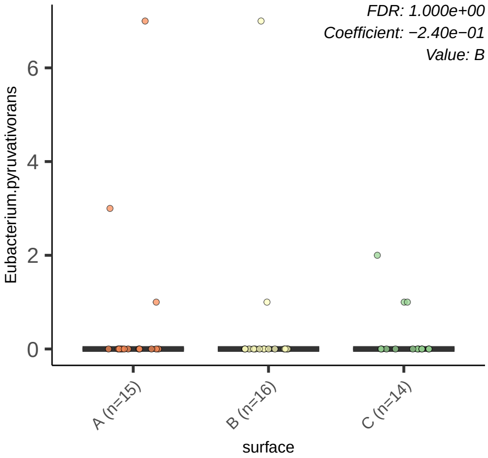
FDR: 1.000e+00
Coefficient: −2.40e−01
Value: B
Eubacterium.pyruvativorans
0
2
4
6
A (n=15)
B (n=16)
C (n=14)
surface

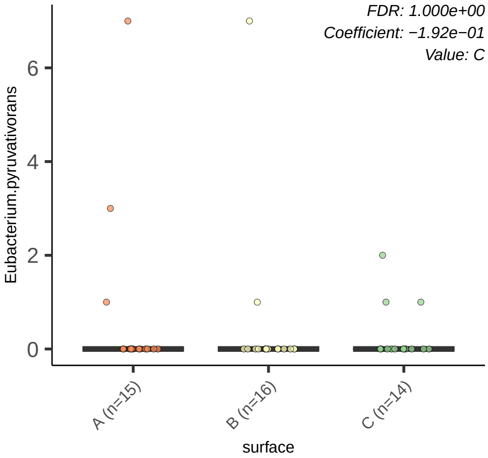
FDR: 1.000e+00
Coefficient: −1.92e−01
Value: C
Eubacterium.pyruvativorans
0
2
4
6
A (n=15)
B (n=16)
C (n=14)
surface

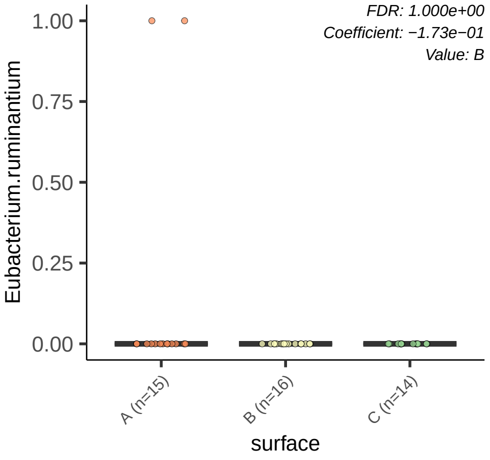
FDR: 1.000e+00
Coefficient: −1.73e−01
Value: B
Eubacterium.ruminantium
1.00
0.75
0.50
0.25
0.00
A (n=15)
B (n=16)
C (n=14)
surface

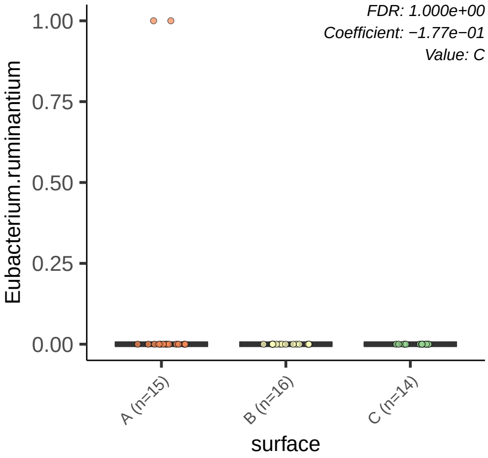
FDR: 1.000e+00
Coefficient: −1.77e−01
Value: C
Eubacterium.ruminantium
1.00
0.75
0.50
0.25
0.00
A (n=15)
B (n=16)
C (n=14)
surface

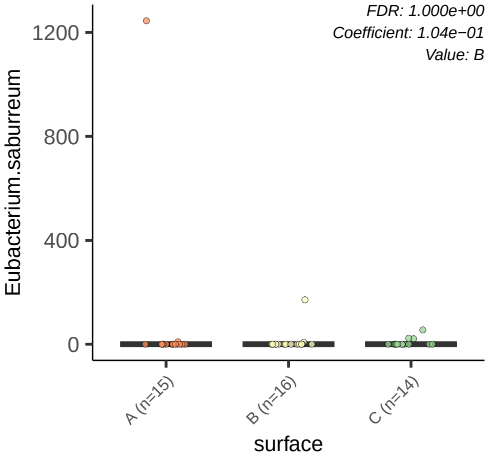

FDR: 1.000e+00
Coefficient: 1.04e−01
Value: B
Eubacterium.saburreum
1200
800
400
0
A (n=15)
B (n=16)
C (n=14)
surface

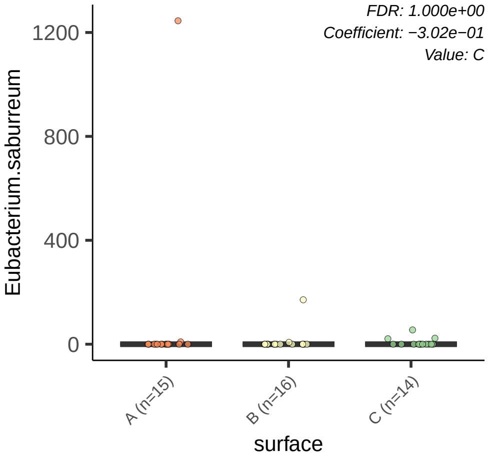

FDR: 1.000e+00
Coefficient: −3.02e−01
Value: C
Eubacterium.saburreum
1200
800
400
0
A (n=15)
B (n=16)
C (n=14)
surface

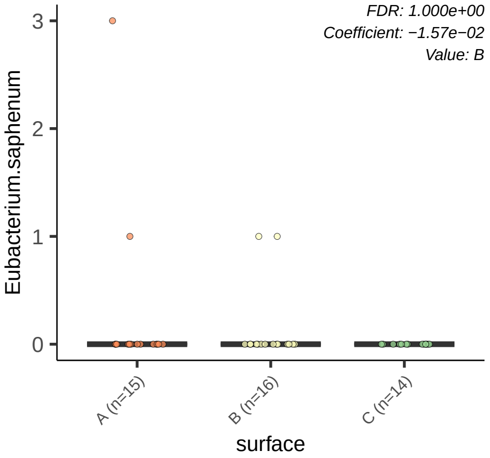

FDR: 1.000e+00
Coefficient: −1.57e−02
Value: B
Eubacterium.saphenum
0
1
2
3
A (n=15)
B (n=16)
C (n=14)
surface

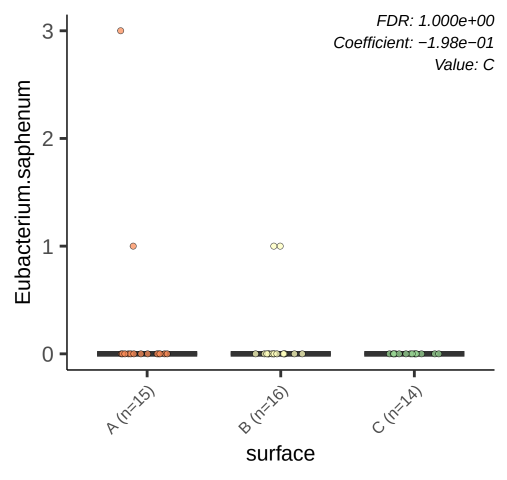
FDR: 1.000e+00
Coefficient: −1.98e−01
Value: C
Eubacterium.saphenum
0
1
2
3
A (n=15)
B (n=16)
C (n=14)
surface

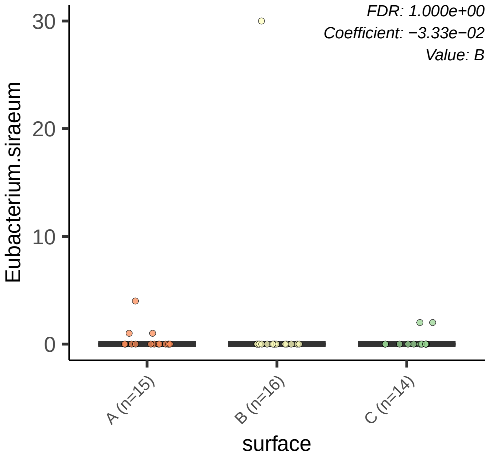

FDR: 1.000e+00
Coefficient: −3.33e−02
Value: B
Eubacterium.siraeum
30
20
10
0
A (n=15)
B (n=16)
C (n=14)
surface

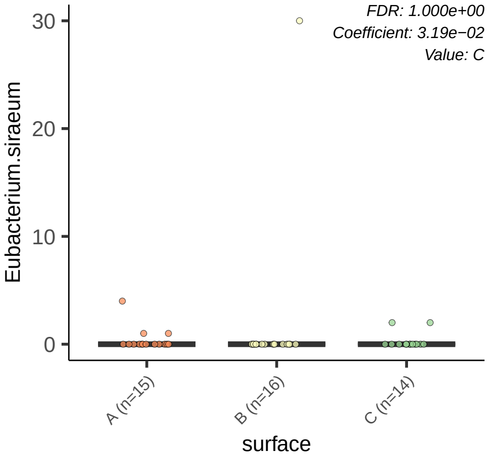

FDR: 1.000e+00
Coefficient: 3.19e−02
Value: C
Eubacterium.siraeum
30
20
10
0
A (n=15)
B (n=16)
C (n=14)
surface

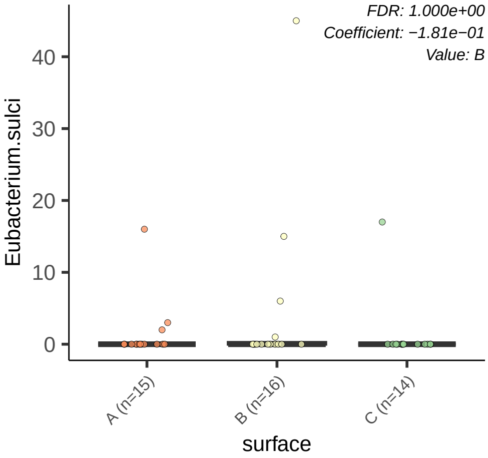

FDR: 1.000e+00
Coefficient: −1.81e−01
Value: B
Eubacterium.sulci
40
30
20
10
0
A (n=15)
B (n=16)
C (n=14)
surface

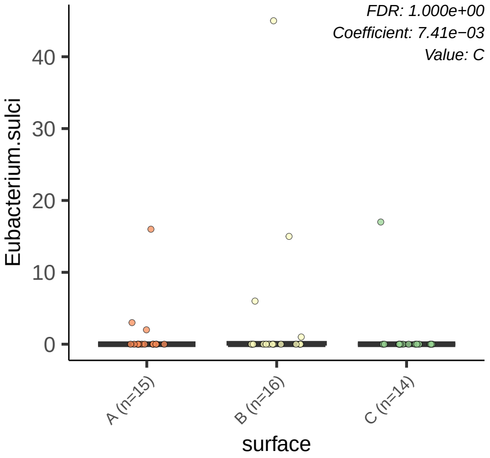
FDR: 1.000e+00
Coefficient: 7.41e−03
Value: C
Eubacterium.sulci
0
10
20
30
40
A (n=15)
B (n=16)
C (n=14)
surface

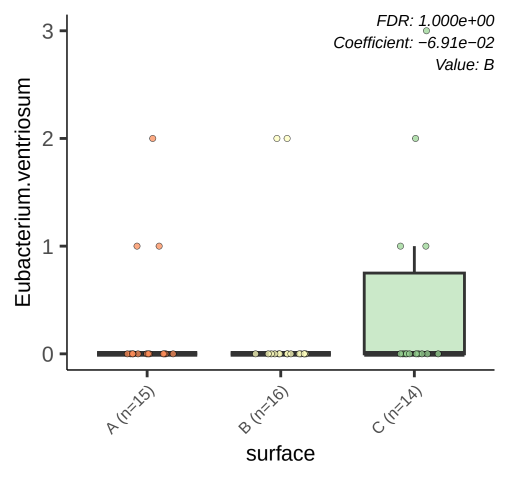
FDR: 1.000e+00
Coefficient: −6.91e−02
Value: B
Eubacterium.ventriosum
0
1
2
3
A (n=15)
B (n=16)
C (n=14)
surface

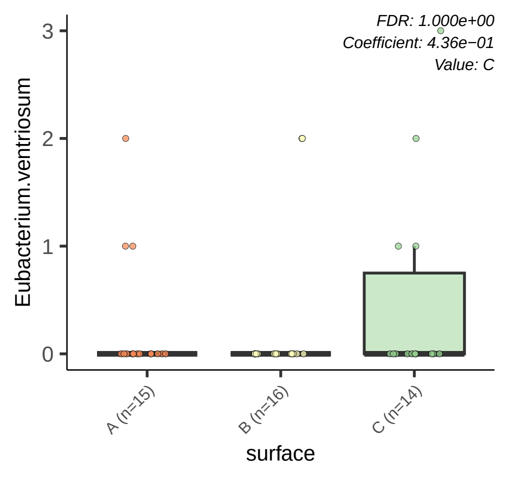

FDR: 1.000e+00
Coefficient: 4.36e−01
Value: C
Eubacterium.ventriosum
0
1
2
3
A (n=15)
B (n=16)
C (n=14)
surface

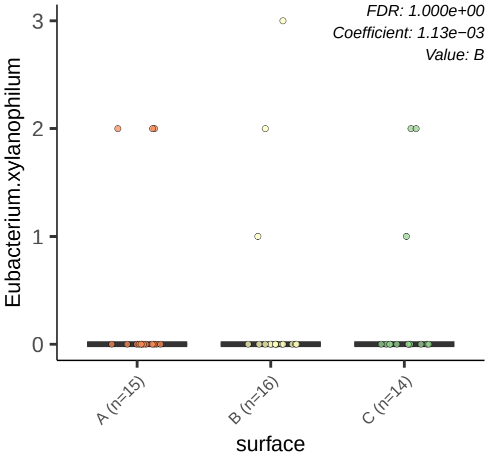

FDR: 1.000e+00
Coefficient: 1.13e−03
Value: B
Eubacterium.xylanophilum
3
2
1
0
A (n=15)
B (n=16)
C (n=14)
surface

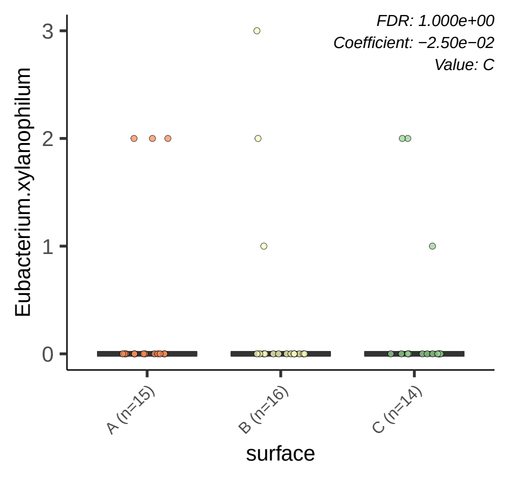

FDR: 1.000e+00
Coefficient: −2.50e−02
Value: C
Eubacterium.xylanophilum
3
2
1
0
A (n=15)
B (n=16)
C (n=14)
surface

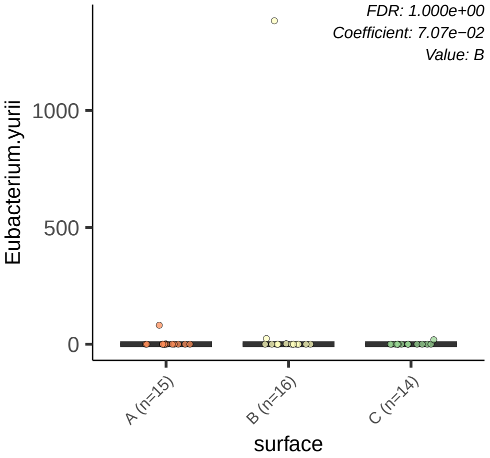
FDR: 1.000e+00
Coefficient: 7.07e−02
Value: B
Eubacterium.yurii
1000
500
0
A (n=15)
B (n=16)
C (n=14)
surface

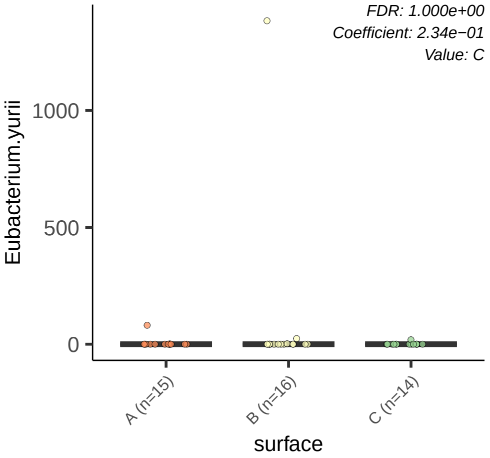
FDR: 1.000e+00
Coefficient: 2.34e−01
Value: C
Eubacterium.yurii
1000
500
0
A (n=15)
B (n=16)
C (n=14)
surface

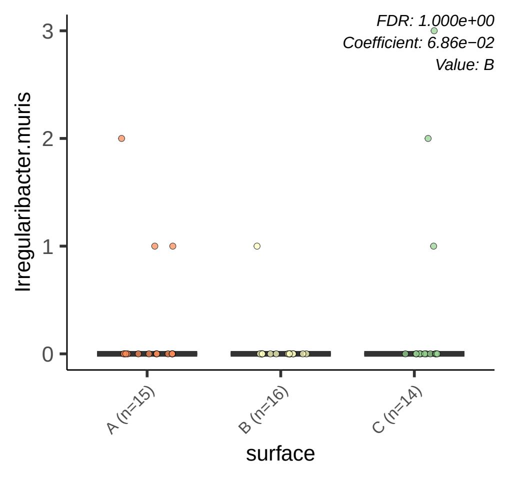
FDR: 1.000e+00
Coefficient: 6.86e−02
Value: B
Irregularibacter.muris
0
1
2
3
A (n=15)
B (n=16)
C (n=14)
surface

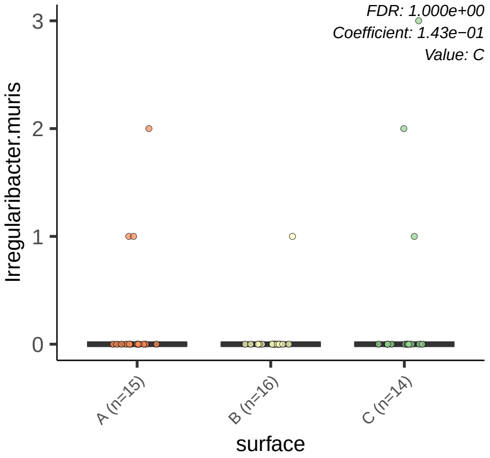

FDR: 1.000e+00
Coefficient: 1.43e−01
Value: C
Irregularibacter.muris
0
1
2
3
A (n=15)
B (n=16)
C (n=14)
surface

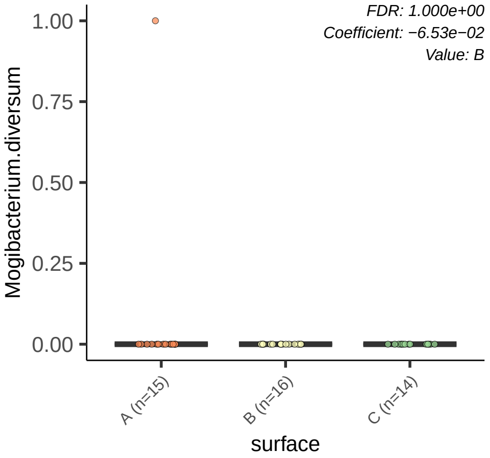
FDR: 1.000e+00
Coefficient: −6.53e−02
Value: B
Mogibacterium.diversum
1.00
0.75
0.50
0.25
0.00
A (n=15)
B (n=16)
C (n=14)
surface

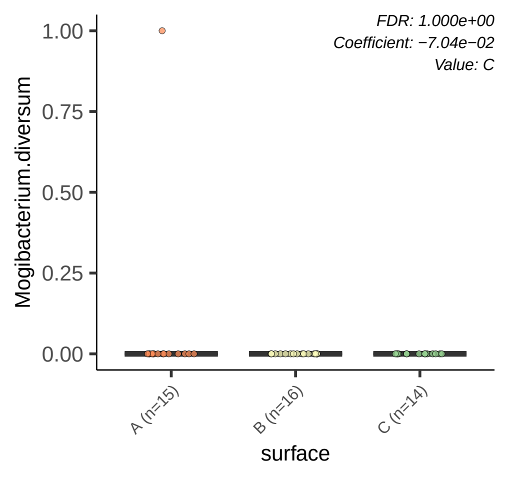

FDR: 1.000e+00
Coefficient: −7.04e−02
Value: C
Mogibacterium.diversum
1.00
0.75
0.50
0.25
0.00
A (n=15)
B (n=16)
C (n=14)
surface

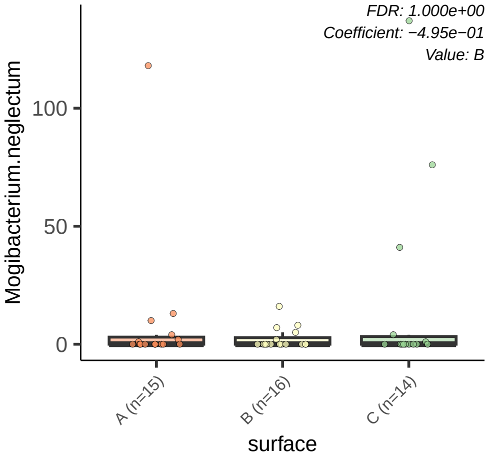

FDR: 1.000e+00
Coefficient: −4.95e−01
Value: B
Mogibacterium.neglectum
100
50
0
A (n=15)
B (n=16)
C (n=14)
surface

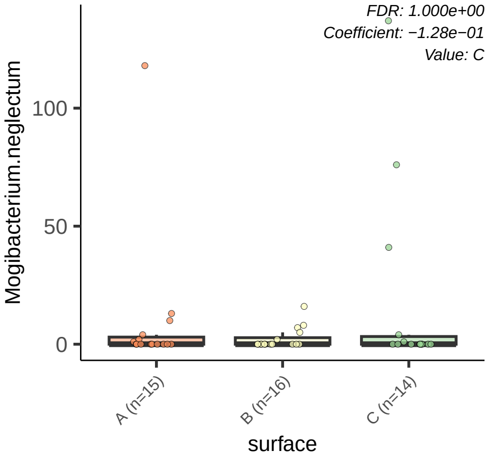
FDR: 1.000e+00
Coefficient: −1.28e−01
Value: C
Mogibacterium.neglectum
100
50
0
A (n=15)
B (n=16)
C (n=14)
surface

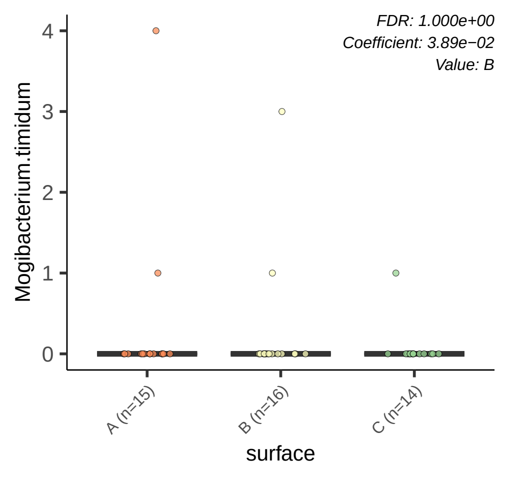

FDR: 1.000e+00
Coefficient: 3.89e−02
Value: B
Mogibacterium.timidum
4
3
2
1
0
A (n=15)
B (n=16)
C (n=14)
surface

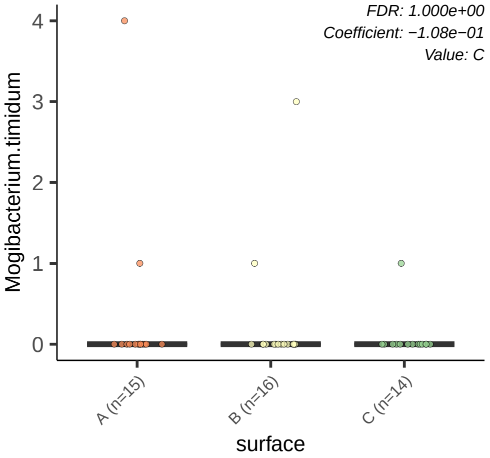
FDR: 1.000e+00
Coefficient: −1.08e−01
Value: C
Mogibacterium.timidum
4
3
2
1
0
A (n=15)
B (n=16)
C (n=14)
surface

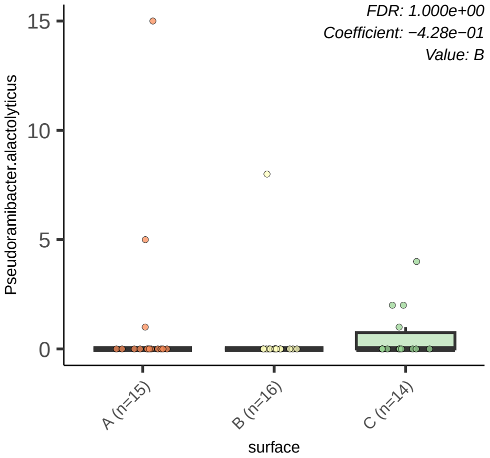

FDR: 1.000e+00
Coefficient: −4.28e−01
Value: B
Pseudoramibacter.alactolyticus
15
10
5
0
A (n=15)
B (n=16)
C (n=14)
surface

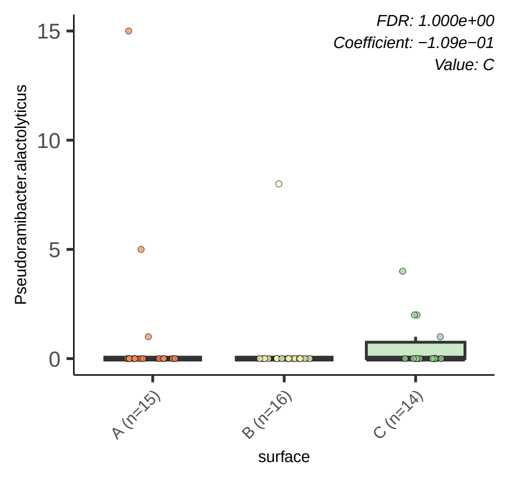

FDR: 1.000e+00
Coefficient: −1.09e−01
Value: C
Pseudoramibacter.alactolyticus
15
10
5
0
A (n=15)
B (n=16)
C (n=14)
surface

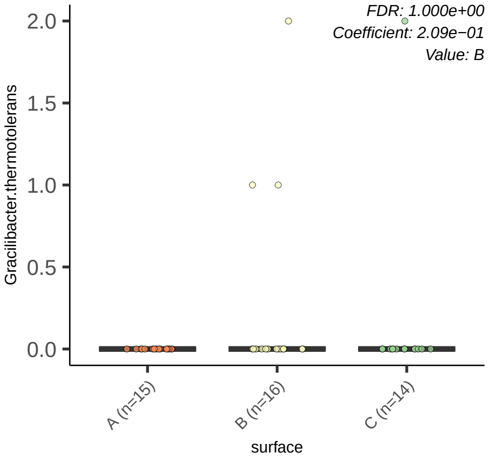
FDR: 1.000e+00
Coefficient: 2.09e−01
Value: B
Gracilibacter.thermotolerans
2.0
1.5
1.0
0.5
0.0
A (n=15)
B (n=16)
C (n=14)
surface

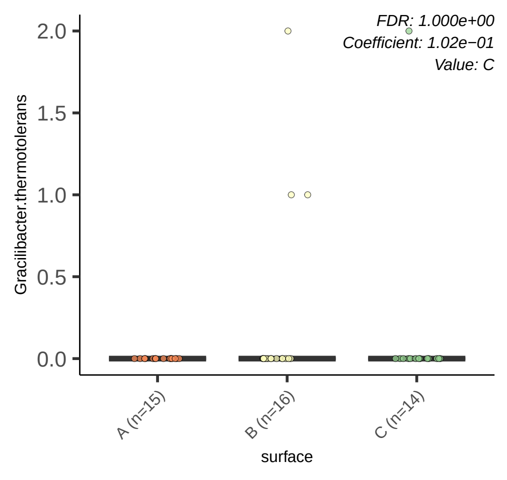
FDR: 1.000e+00
Coefficient: 1.02e−01
Value: C
Gracilibacter.thermotolerans
2.0
1.5
1.0
0.5
0.0
A (n=15)
B (n=16)
C (n=14)
surface

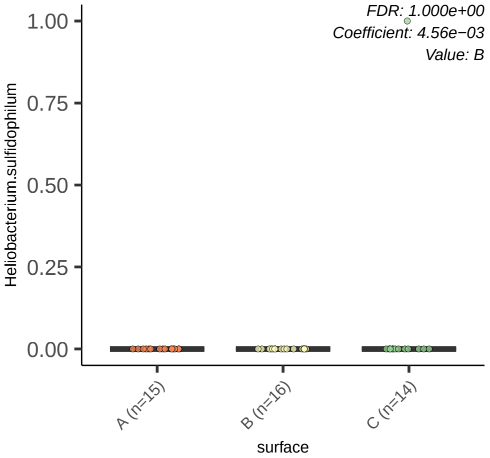
FDR: 1.000e+00
Coefficient: 4.56e−03
Value: B
1.00
0.75
0.50
0.25
0.00
Heliobacterium.sulfidophilum
A (n=15)
B (n=16)
C (n=14)
surface

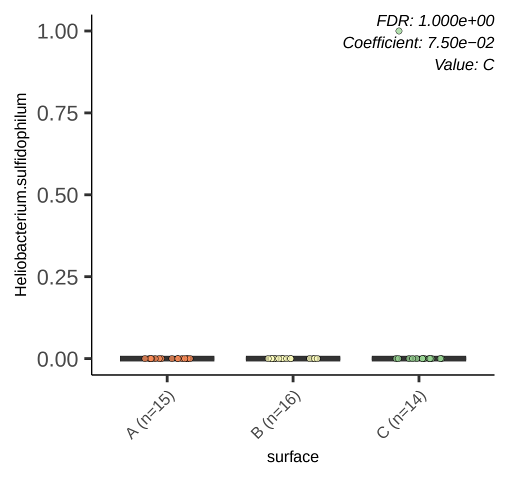
FDR: 1.000e+00
Coefficient: 7.50e−02
Value: C
Heliobacterium.sulfidophilum
1.00
0.75
0.50
0.25
0.00
A (n=15)
B (n=16)
C (n=14)
surface

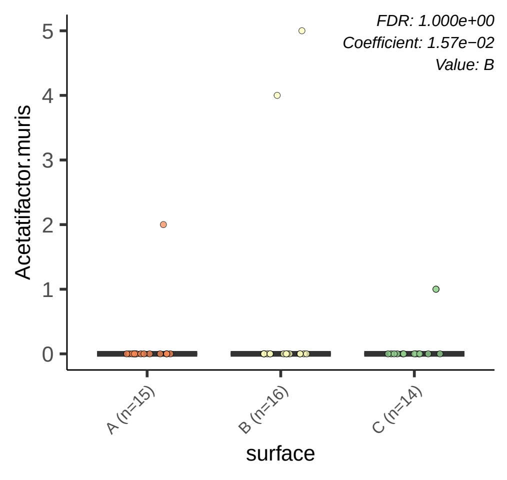
FDR: 1.000e+00
Coefficient: 1.57e−02
Value: B
Acetatifactor.muris
0
1
2
3
4
5
A (n=15)
B (n=16)
C (n=14)
surface

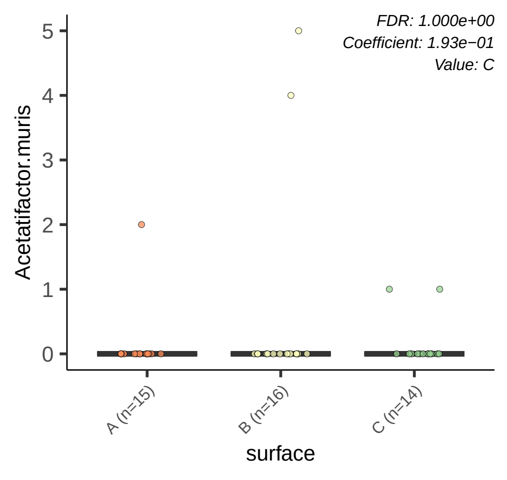

FDR: 1.000e+00
Coefficient: 1.93e−01
Value: C
Acetatifactor.muris
0
1
2
3
4
5
A (n=15)
B (n=16)
C (n=14)
surface

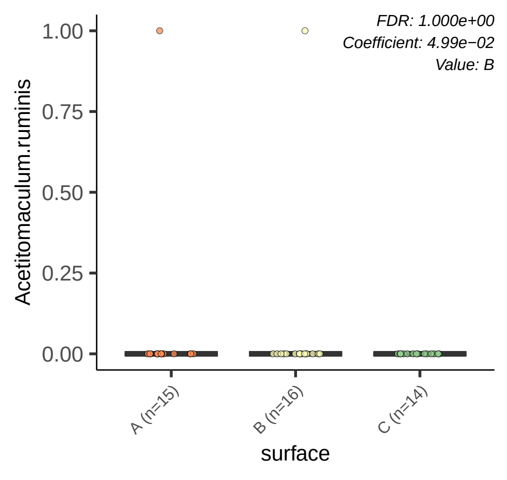
FDR: 1.000e+00
Coefficient: 4.99e−02
Value: B
Acetitomaculum.ruminis
1.00
0.75
0.50
0.25
0.00
A (n=15)
B (n=16)
C (n=14)
surface

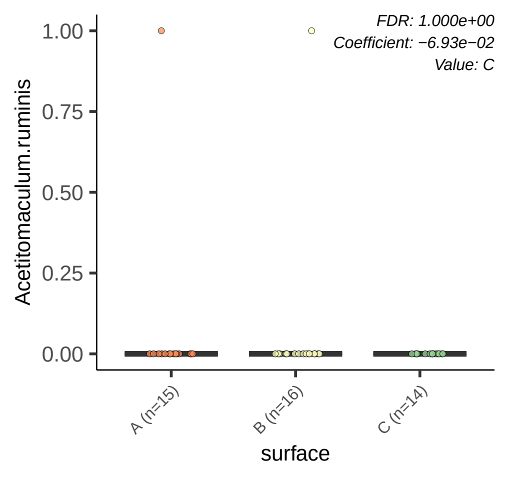

FDR: 1.000e+00
Coefficient: −6.93e−02
Value: C
Acetitomaculum.ruminis
1.00
0.75
0.50
0.25
0.00
A (n=15)
B (n=16)
C (n=14)
surface

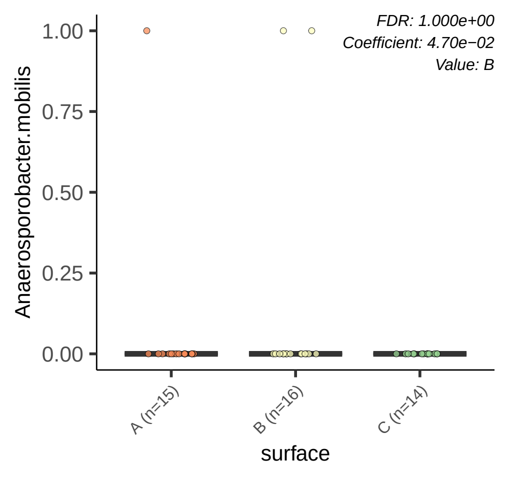

FDR: 1.000e+00
Coefficient: 4.70e−02
Value: B
Anaerosporobacter.mobilis
1.00
0.75
0.50
0.25
0.00
A (n=15)
B (n=16)
C (n=14)
surface

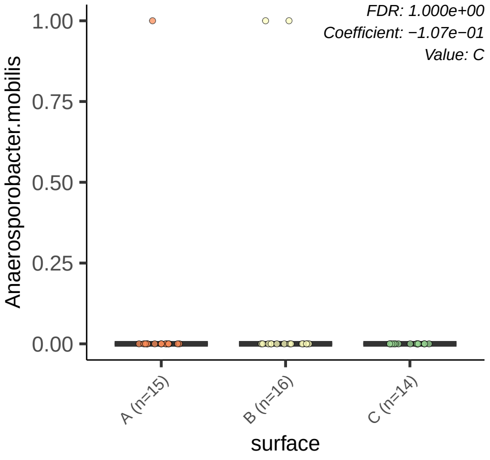
FDR: 1.000e+00
Coefficient: −1.07e−01
Value: C
Anaerosporobacter.mobilis
1.00
0.75
0.50
0.25
0.00
A (n=15)
B (n=16)
C (n=14)
surface

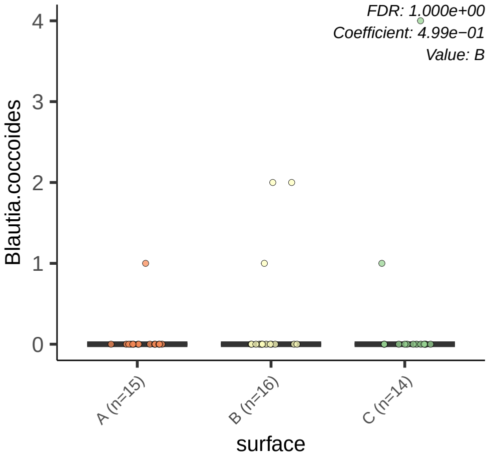
FDR: 1.000e+00
Coefficient: 4.99e−01
Value: B
Blautia.coccoides
0
1
2
3
4
A (n=15)
B (n=16)
C (n=14)
surface

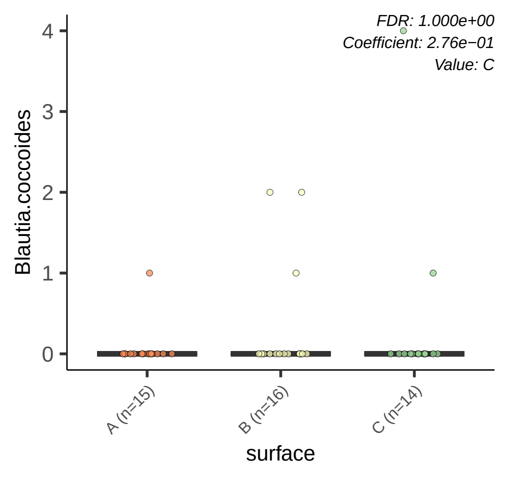
FDR: 1.000e+00
Coefficient: 2.76e−01
Value: C
Blautia.coccoides
0
1
2
3
4
A (n=15)
B (n=16)
C (n=14)
surface

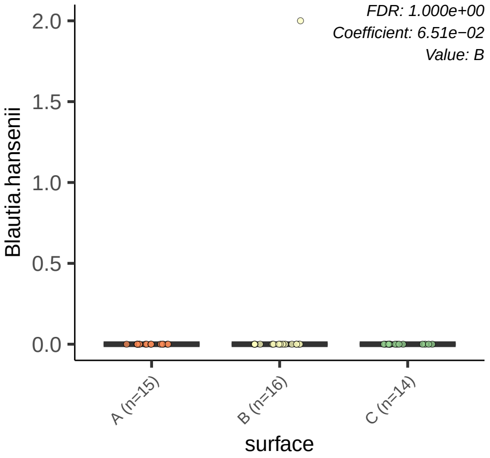
FDR: 1.000e+00
Coefficient: 6.51e−02
Value: B
Blautia.hansenii
2.0
1.5
1.0
0.5
0.0
A (n=15)
B (n=16)
C (n=14)
surface

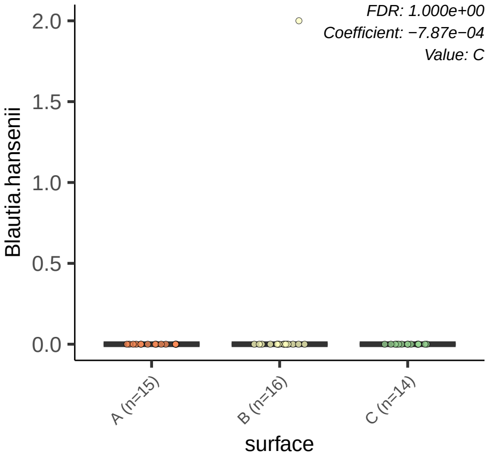

FDR: 1.000e+00
Coefficient: −7.87e−04
Value: C
Blautia.hansenii
2.0
1.5
1.0
0.5
0.0
A (n=15)
B (n=16)
C (n=14)
surface

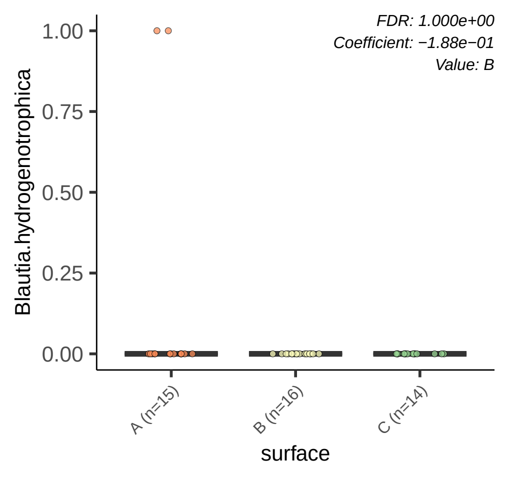
FDR: 1.000e+00
Coefficient: −1.88e−01
Value: B
Blautia.hydrogenotrophica
1.00
0.75
0.50
0.25
0.00
A (n=15)
B (n=16)
C (n=14)
surface

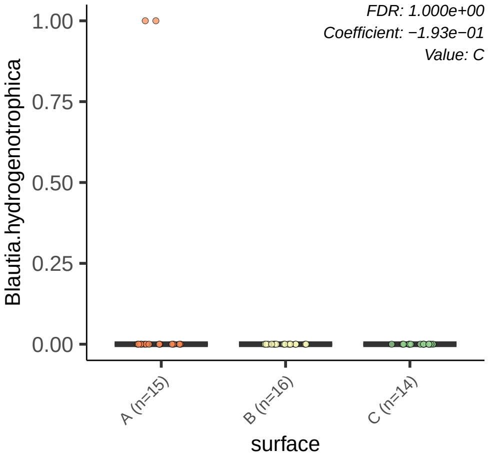
FDR: 1.000e+00
Coefficient: −1.93e−01
Value: C
Blautia.hydrogenotrophica
1.00
0.75
0.50
0.25
0.00
A (n=15)
B (n=16)
C (n=14)
surface

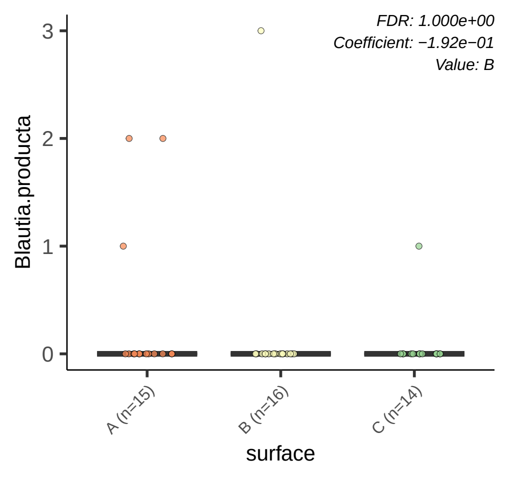

FDR: 1.000e+00
Coefficient: −1.92e−01
Value: B
Blautia.producta
3
2
1
0
A (n=15)
B (n=16)
C (n=14)
surface

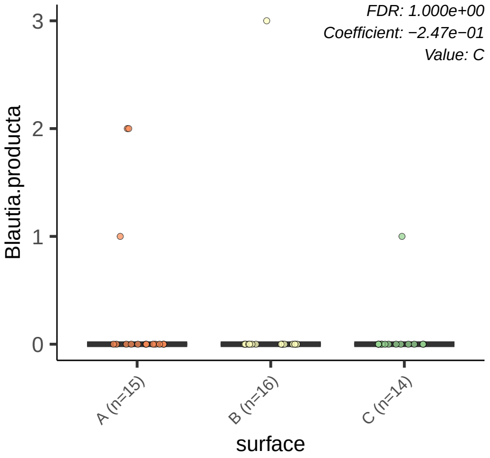
FDR: 1.000e+00
Coefficient: −2.47e−01
Value: C
Blautia.producta
3
2
1
0
A (n=15)
B (n=16)
C (n=14)
surface

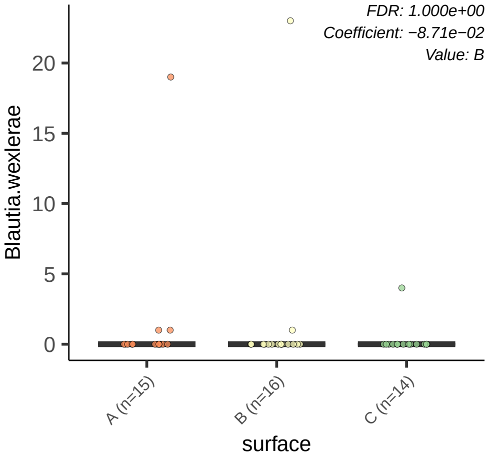

FDR: 1.000e+00
Coefficient: −8.71e−02
Value: B
Blautia.wexlerae
0
5
10
15
20
A (n=15)
B (n=16)
C (n=14)
surface

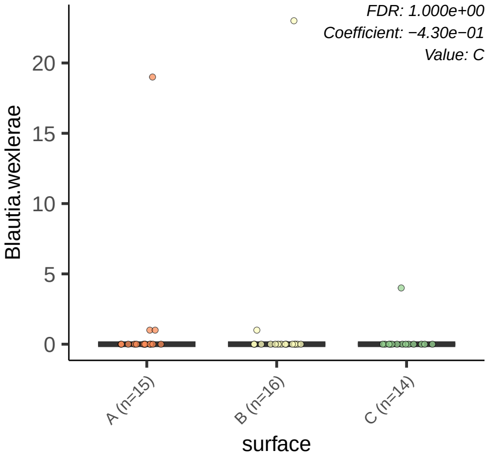

FDR: 1.000e+00
Coefficient: −4.30e−01
Value: C
Blautia.wexlerae
0
5
10
15
20
A (n=15)
B (n=16)
C (n=14)
surface

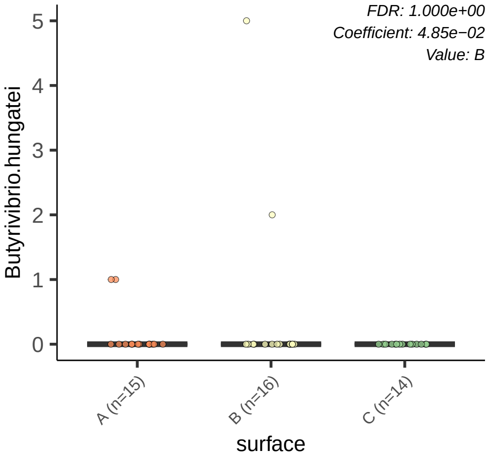
FDR: 1.000e+00
Coefficient: 4.85e−02
Value: B
Butyrivibrio.hungatei
0
1
2
3
4
5
A (n=15)
B (n=16)
C (n=14)
surface

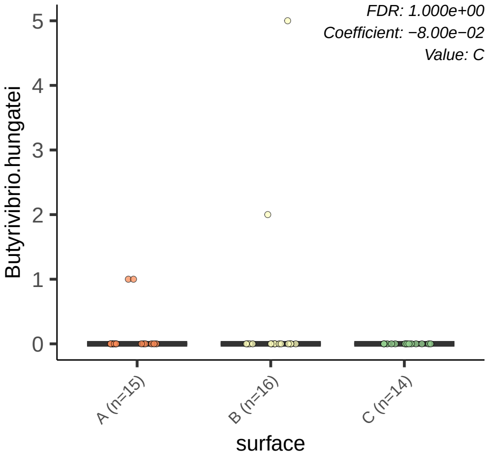

FDR: 1.000e+00
Coefficient: −8.00e−02
Value: C
Butyrivibrio.hungatei
0
1
2
3
4
5
A (n=15)
B (n=16)
C (n=14)
surface

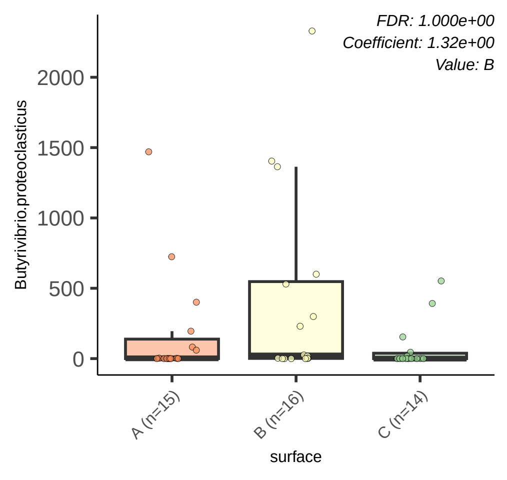
FDR: 1.000e+00
Coefficient: 1.32e+00
Value: B
Butyrivibrio.proteoclasticus
2000
1500
1000
500
0
A (n=15)
B (n=16)
C (n=14)
surface

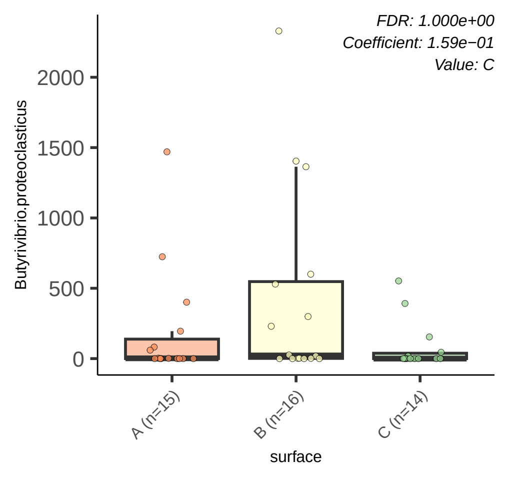

FDR: 1.000e+00
Coefficient: 1.59e−01
Value: C
Butyrivibrio.proteoclasticus
2000
1500
1000
500
0
A (n=15)
B (n=16)
C (n=14)
surface

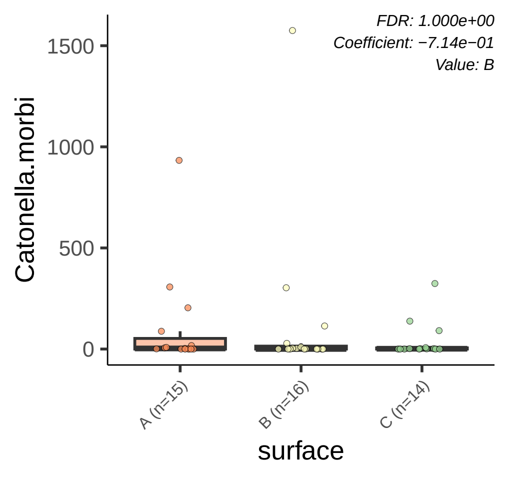
FDR: 1.000e+00
Coefficient: −7.14e−01
Value: B
Catonella.morbi
1500
1000
500
0
A (n=15)
B (n=16)
C (n=14)
surface

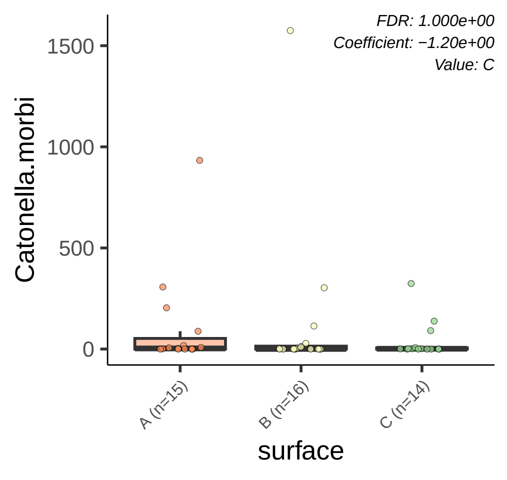
FDR: 1.000e+00
Coefficient: −1.20e+00
Value: C
Catonella.morbi
1500
1000
500
0
A (n=15)
B (n=16)
C (n=14)
surface

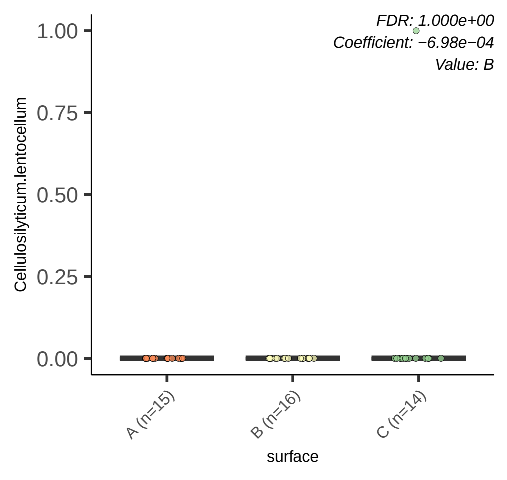
FDR: 1.000e+00
Coefficient: −6.98e−04
Value: B
1.00
0.75
0.50
0.25
0.00
Cellulosilyticum.lentocellum
A (n=15)
B (n=16)
C (n=14)
surface

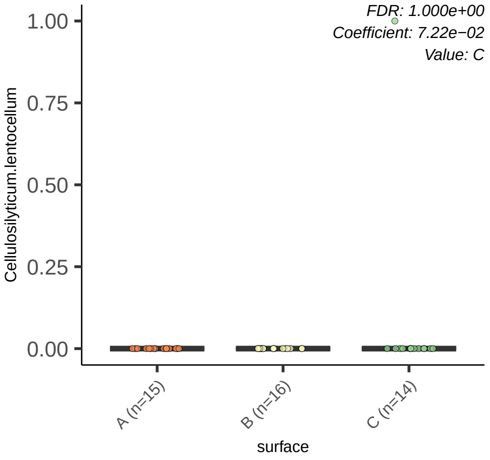
FDR: 1.000e+00
Coefficient: 7.22e−02
Value: C
1.00
0.75
0.50
0.25
0.00
Cellulosilyticum.lentocellum
A (n=15)
B (n=16)
C (n=14)
surface

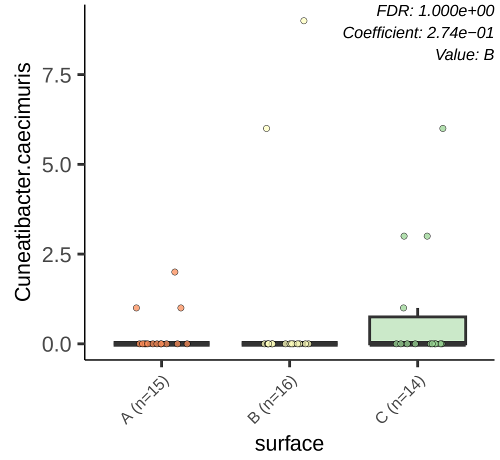

FDR: 1.000e+00
Coefficient: 2.74e−01
Value: B
Cuneatibacter.caecimuris
7.5
5.0
2.5
0.0
A (n=15)
B (n=16)
C (n=14)
surface

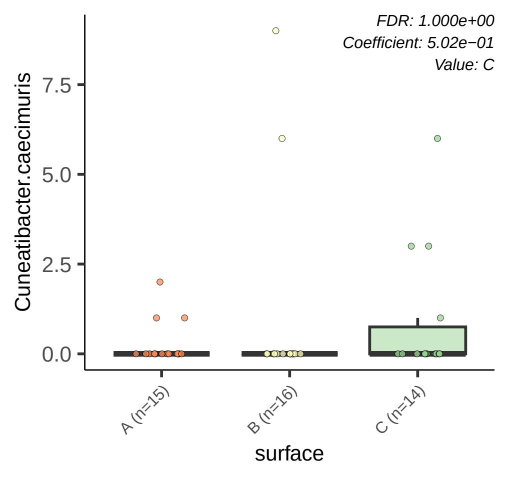

FDR: 1.000e+00
Coefficient: 5.02e−01
Value: C
Cuneatibacter.caecimuris
7.5
5.0
2.5
0.0
A (n=15)
B (n=16)
C (n=14)
surface

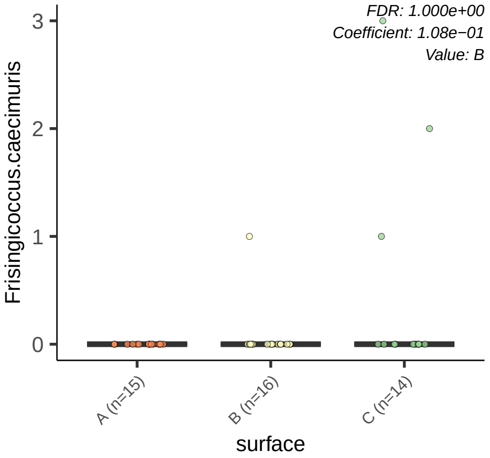
FDR: 1.000e+00
Coefficient: 1.08e−01
Value: B
Frisingicoccus.caecimuris
0
1
2
3
A (n=15)
B (n=16)
C (n=14)
surface

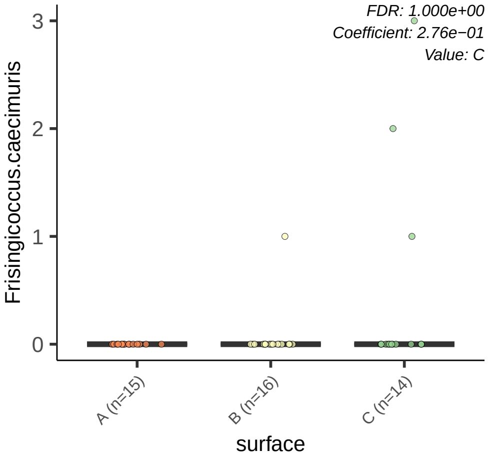

FDR: 1.000e+00
Coefficient: 2.76e−01
Value: C
Frisingicoccus.caecimuris
3
2
1
0
A (n=15)
B (n=16)
C (n=14)
surface

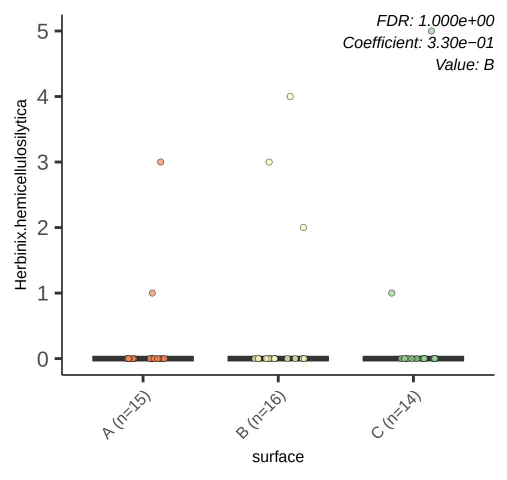
FDR: 1.000e+00
Coefficient: 3.30e−01
Value: B
Herbinix.hemicellulosilytica
0
1
2
3
4
5
A (n=15)
B (n=16)
C (n=14)
surface

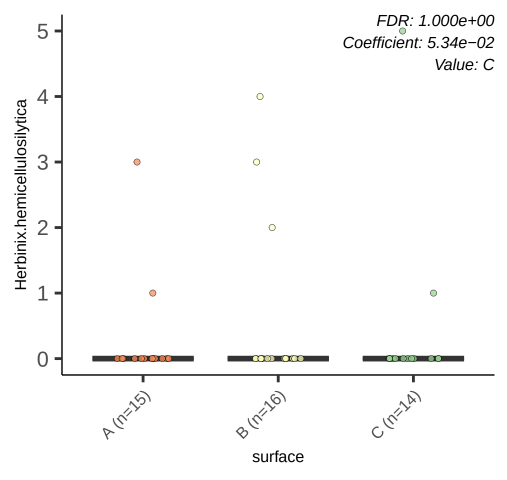
FDR: 1.000e+00
Coefficient: 5.34e−02
Value: C
Herbinix.hemicellulosilytica
0
1
2
3
4
5
A (n=15)
B (n=16)
C (n=14)
surface

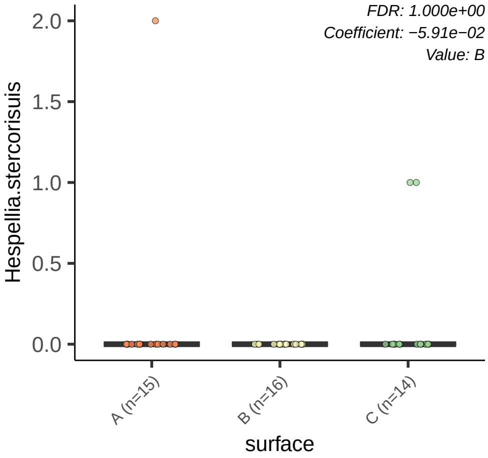
FDR: 1.000e+00
Coefficient: −5.91e−02
Value: B
Hespellia.stercorisuis
2.0
1.5
1.0
0.5
0.0
A (n=15)
B (n=16)
C (n=14)
surface

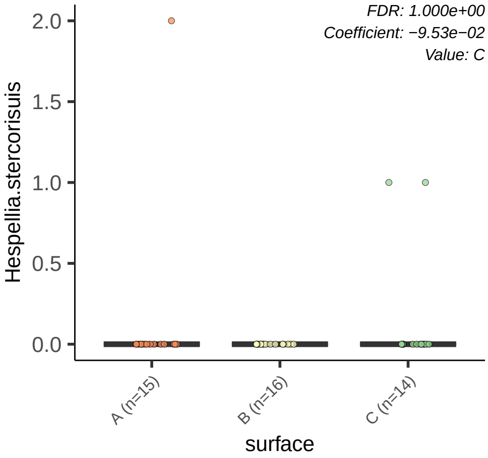

FDR: 1.000e+00
Coefficient: −9.53e−02
Value: C
Hespellia.stercorisuis
2.0
1.5
1.0
0.5
0.0
A (n=15)
B (n=16)
C (n=14)
surface

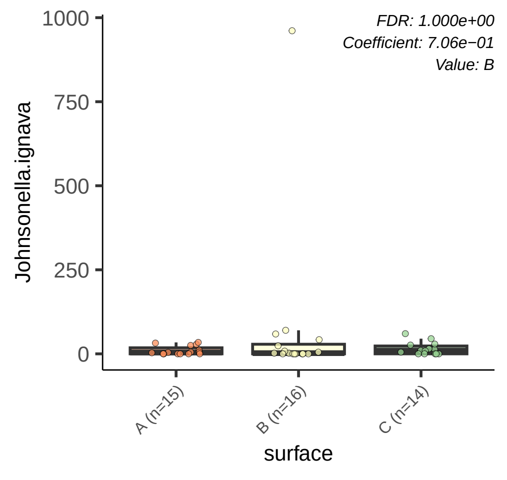
FDR: 1.000e+00
Coefficient: 7.06e−01
Value: B
Johnsonella.ignava
1000
750
500
250
0
A (n=15)
B (n=16)
C (n=14)
surface

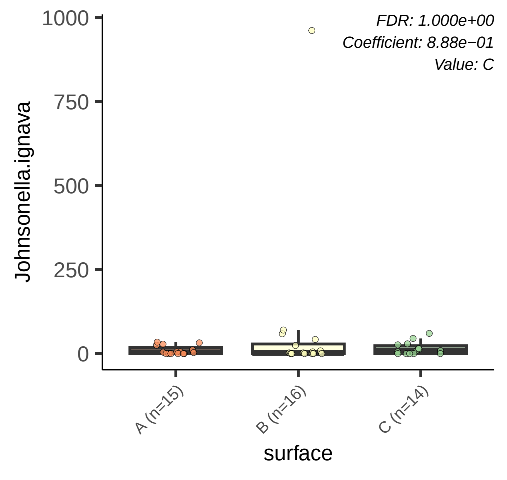
FDR: 1.000e+00
Coefficient: 8.88e−01
Value: C
Johnsonella.ignava
1000
750
500
250
0
A (n=15)
B (n=16)
C (n=14)
surface

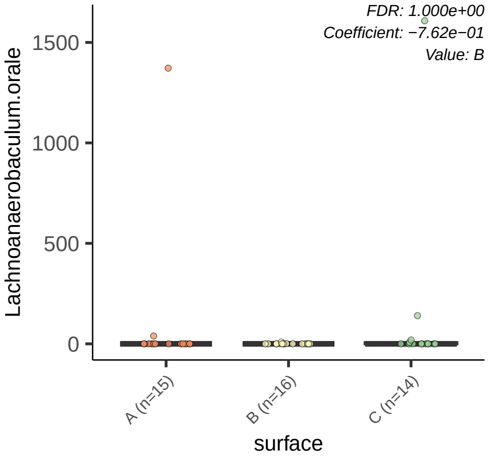

FDR: 1.000e+00
Coefficient: −7.62e−01
Value: B
Lachnoanaerobaculum.orale
1500
1000
500
0
A (n=15)
B (n=16)
C (n=14)
surface

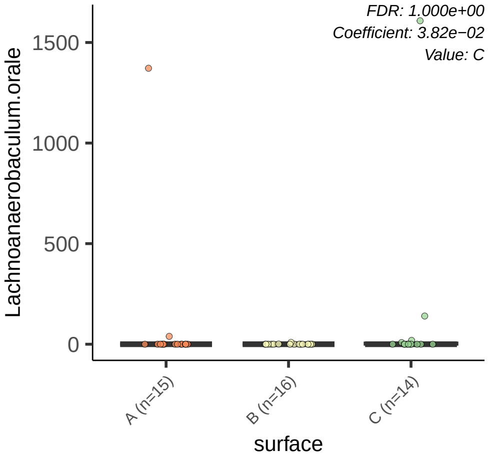

FDR: 1.000e+00
Coefficient: 3.82e−02
Value: C
1500
1000
500
0
Lachnoanaerobaculum.orale
A (n=15)
B (n=16)
C (n=14)
surface

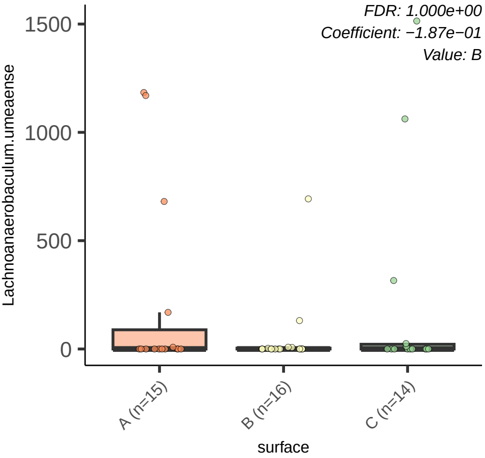
FDR: 1.000e+00
Coefficient: −1.87e−01
Value: B
Lachnoanaerobaculum.umeaense
1500
1000
500
0
A (n=15)
B (n=16)
C (n=14)
surface

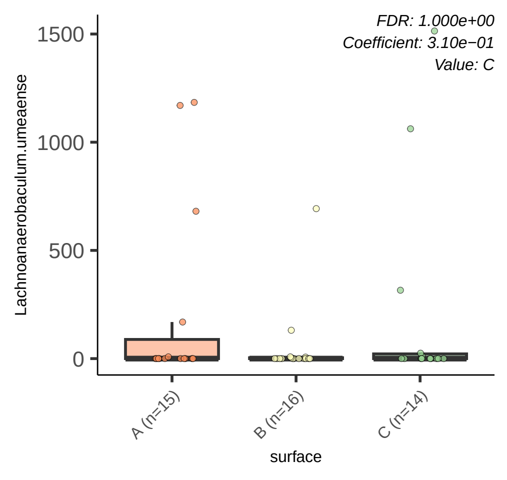

FDR: 1.000e+00
Coefficient: 3.10e−01
Value: C
Lachnoanaerobaculum.umeaense
1500
1000
500
0
A (n=15)
B (n=16)
C (n=14)
surface

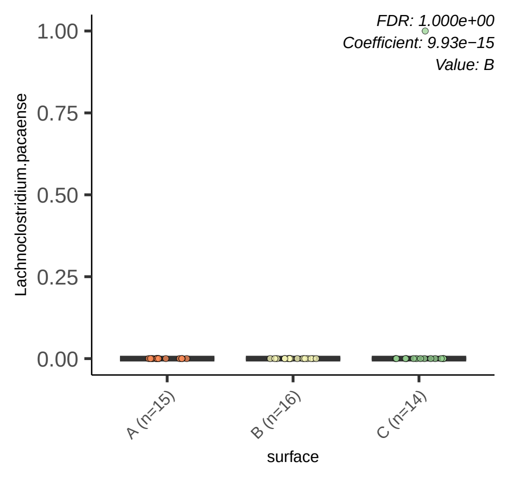
FDR: 1.000e+00
Coefficient: 9.93e−15
Value: B
Lachnoclostridium.pacaense
1.00
0.75
0.50
0.25
0.00
A (n=15)
B (n=16)
C (n=14)
surface

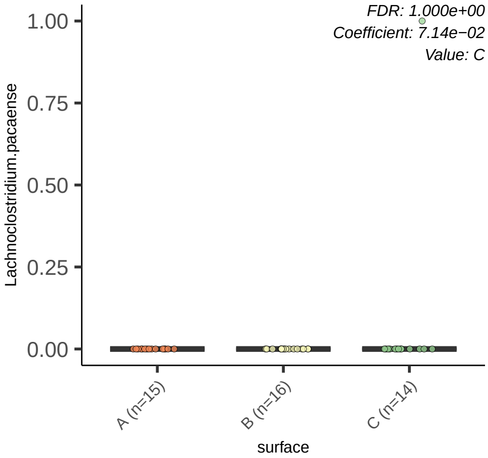
FDR: 1.000e+00
Coefficient: 7.14e−02
Value: C
Lachnoclostridium.pacaense
1.00
0.75
0.50
0.25
0.00
A (n=15)
B (n=16)
C (n=14)
surface

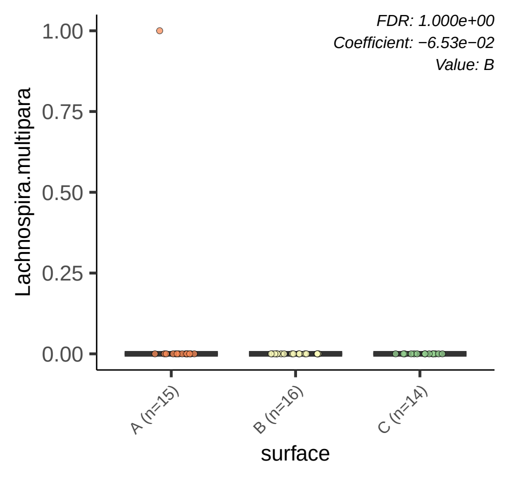

FDR: 1.000e+00
Coefficient: −6.53e−02
Value: B
Lachnospira.multipara
1.00
0.75
0.50
0.25
0.00
A (n=15)
B (n=16)
C (n=14)
surface

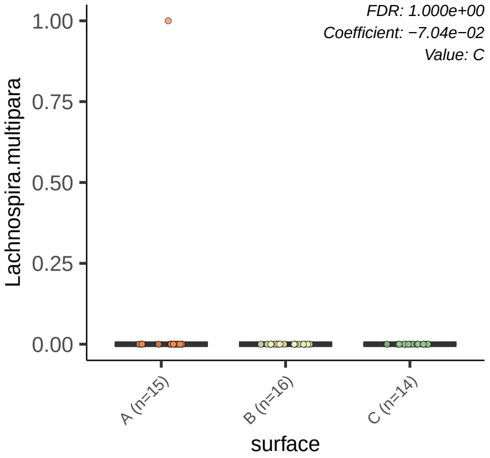

FDR: 1.000e+00
Coefficient: −7.04e−02
Value: C
Lachnospira.multipara
1.00
0.75
0.50
0.25
0.00
A (n=15)
B (n=16)
C (n=14)
surface

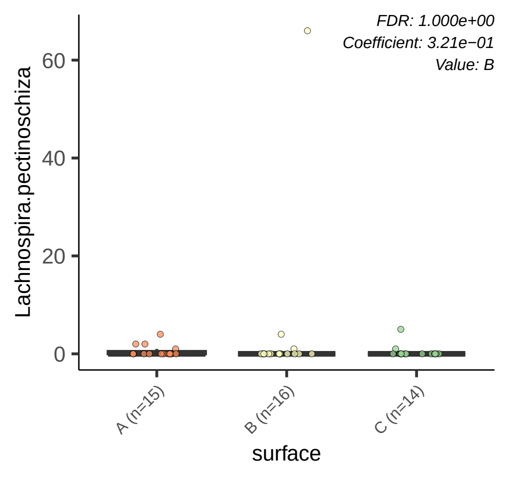
FDR: 1.000e+00
Coefficient: 3.21e−01
Value: B
Lachnospira.pectinoschiza
0
20
40
60
A (n=15)
B (n=16)
C (n=14)
surface

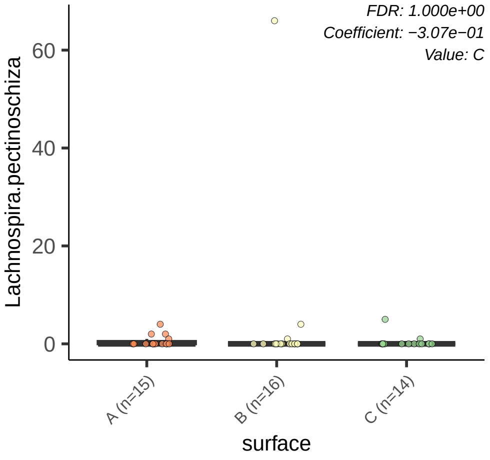
FDR: 1.000e+00
Coefficient: −3.07e−01
Value: C
Lachnospira.pectinoschiza
60
40
20
0
A (n=15)
B (n=16)
C (n=14)
surface

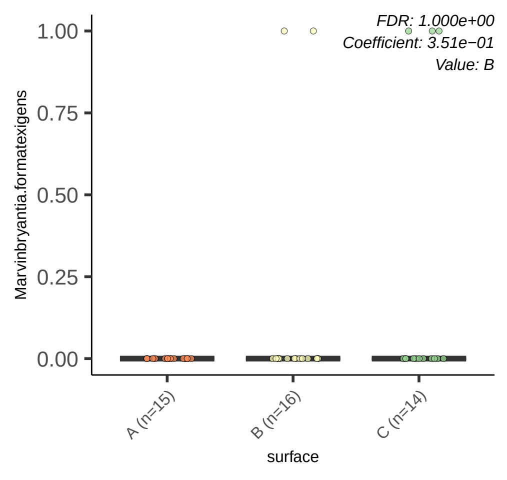
FDR: 1.000e+00
Coefficient: 3.51e−01
Value: B
Marvinbryantia.formatexigens
1.00
0.75
0.50
0.25
0.00
A (n=15)
B (n=16)
C (n=14)
surface

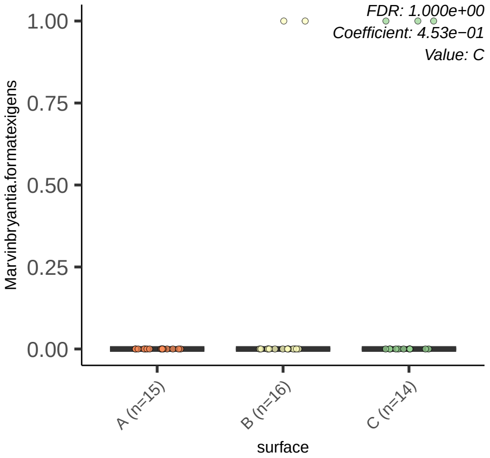
FDR: 1.000e+00
Coefficient: 4.53e−01
Value: C
1.00
0.75
0.50
0.25
0.00
Marvinbryantia.formatexigens
A (n=15)
B (n=16)
C (n=14)
surface

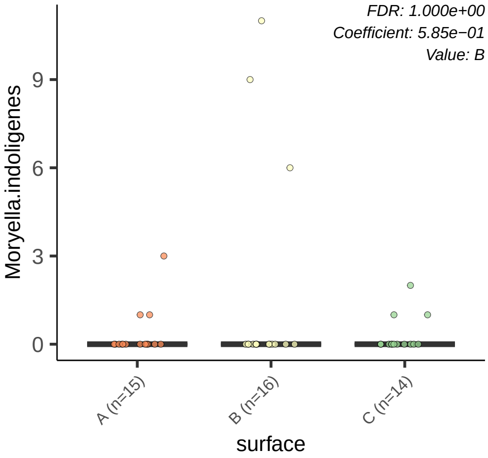

FDR: 1.000e+00
Coefficient: 5.85e−01
Value: B
Moryella.indoligenes
0
3
6
9
A (n=15)
B (n=16)
C (n=14)
surface

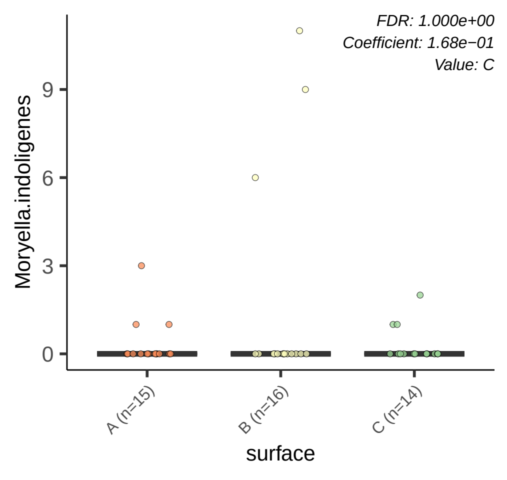

FDR: 1.000e+00
Coefficient: 1.68e−01
Value: C
Moryella.indoligenes
0
3
6
9
A (n=15)
B (n=16)
C (n=14)
surface

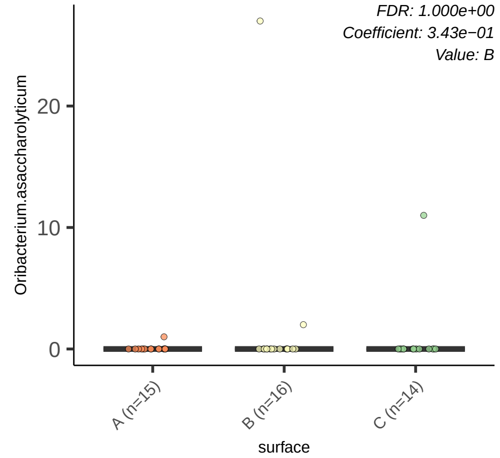
FDR: 1.000e+00
Coefficient: 3.43e−01
Value: B
Oribacterium.asaccharolyticum
20
10
0
A (n=15)
B (n=16)
C (n=14)
surface

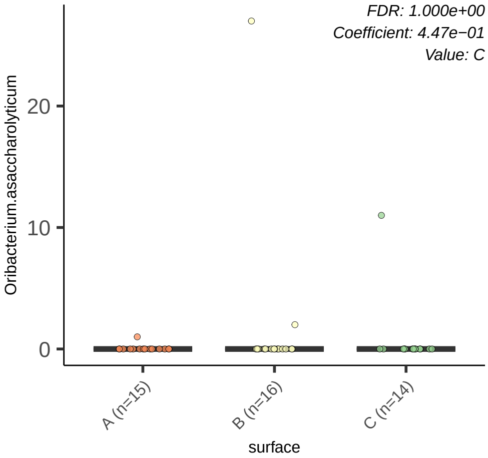

FDR: 1.000e+00
Coefficient: 4.47e−01
Value: C
Oribacterium.asaccharolyticum
20
10
0
A (n=15)
B (n=16)
C (n=14)
surface

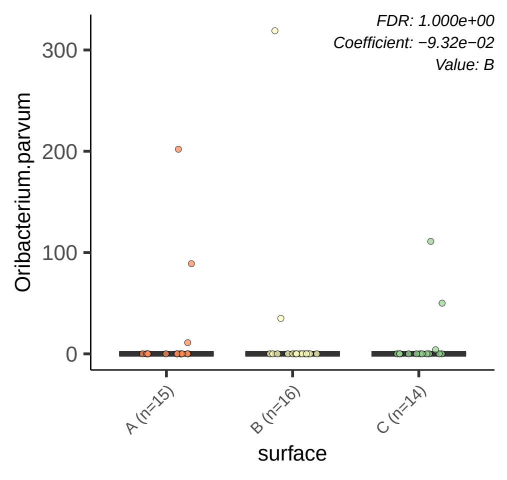
FDR: 1.000e+00
Coefficient: −9.32e−02
Value: B
Oribacterium.parvum
300
200
100
0
A (n=15)
B (n=16)
C (n=14)
surface

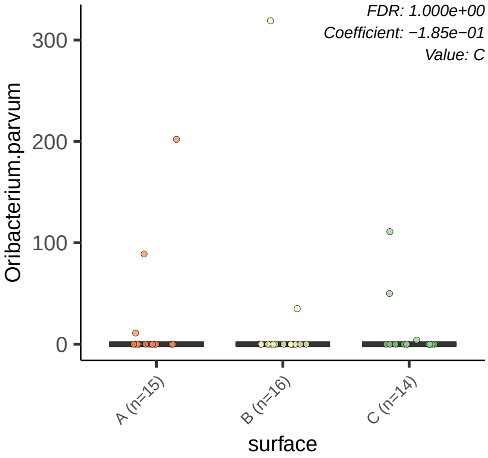
FDR: 1.000e+00
Coefficient: −1.85e−01
Value: C
Oribacterium.parvum
300
200
100
0
A (n=15)
B (n=16)
C (n=14)
surface

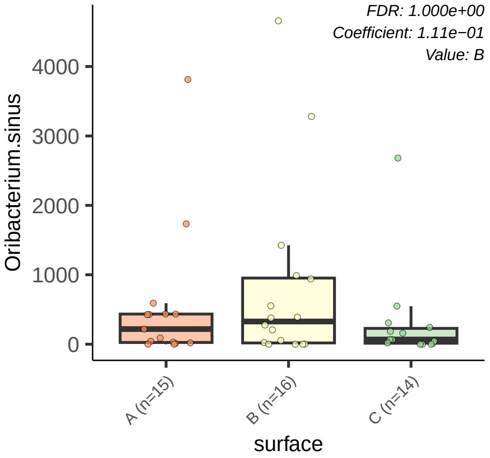

FDR: 1.000e+00
Coefficient: 1.11e−01
Value: B
Oribacterium.sinus
4000
3000
2000
1000
0
A (n=15)
B (n=16)
C (n=14)
surface

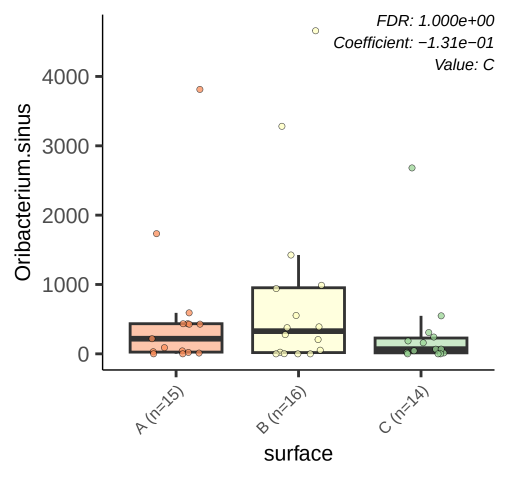
FDR: 1.000e+00
Coefficient: −1.31e−01
Value: C
Oribacterium.sinus
0
1000
2000
3000
4000
A (n=15)
B (n=16)
C (n=14)
surface

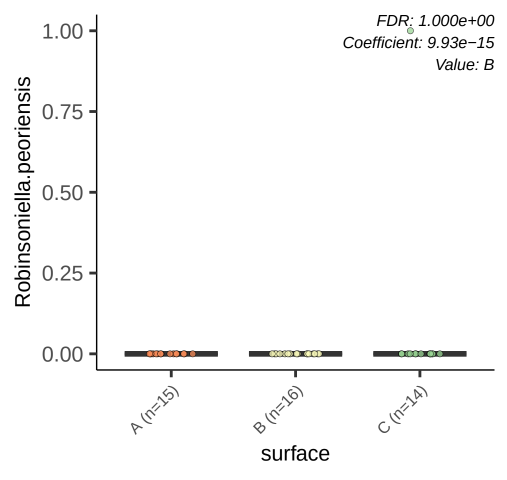

FDR: 1.000e+00
Coefficient: 9.93e−15
Value: B
Robinsoniella.peoriensis
1.00
0.75
0.50
0.25
0.00
A (n=15)
B (n=16)
C (n=14)
surface

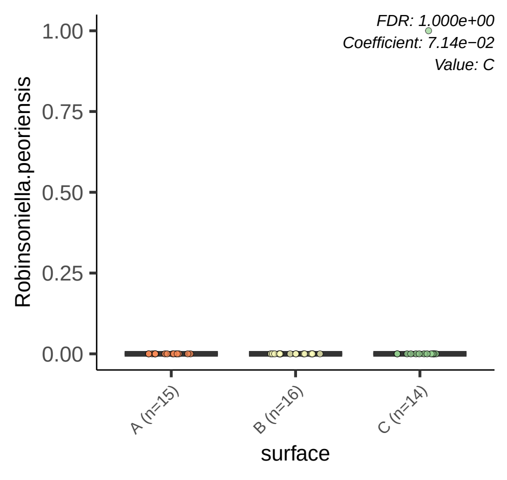

FDR: 1.000e+00
Coefficient: 7.14e−02
Value: C
Robinsoniella.peoriensis
1.00
0.75
0.50
0.25
0.00
A (n=15)
B (n=16)
C (n=14)
surface

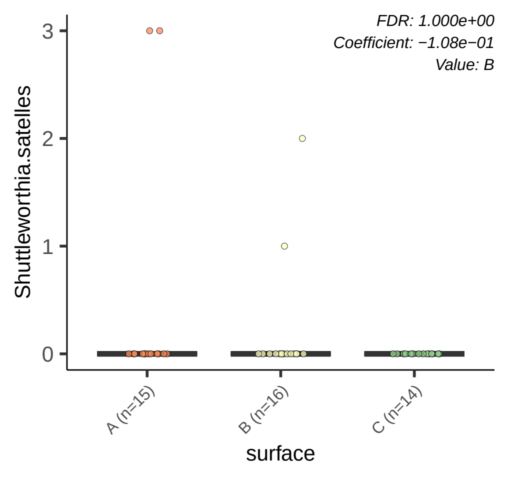
FDR: 1.000e+00
Coefficient: −1.08e−01
Value: B
Shuttleworthia.satelles
3
2
1
0
A (n=15)
B (n=16)
C (n=14)
surface

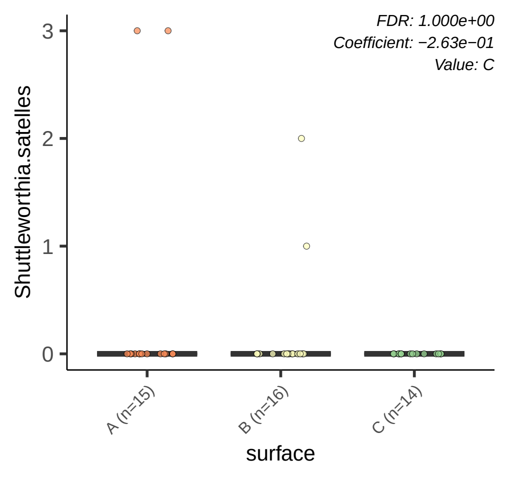

FDR: 1.000e+00
Coefficient: −2.63e−01
Value: C
Shuttleworthia.satelles
3
2
1
0
A (n=15)
B (n=16)
C (n=14)
surface

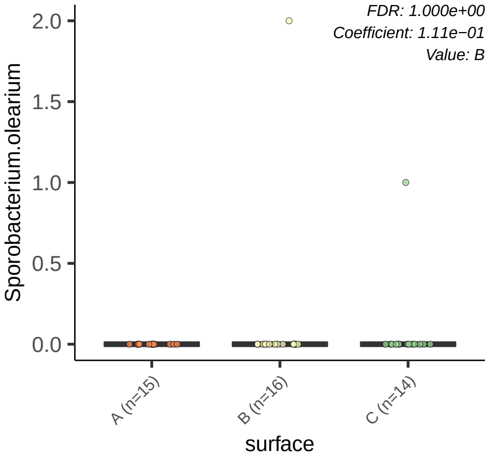

FDR: 1.000e+00
Coefficient: 1.11e−01
Value: B
Sporobacterium.olearium
2.0
1.5
1.0
0.5
0.0
A (n=15)
B (n=16)
C (n=14)
surface

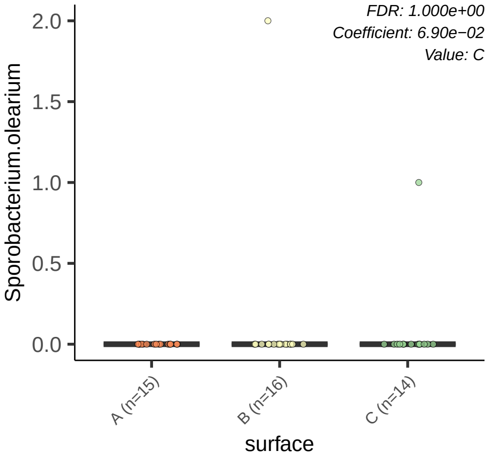

FDR: 1.000e+00
Coefficient: 6.90e−02
Value: C
Sporobacterium.olearium
2.0
1.5
1.0
0.5
0.0
A (n=15)
B (n=16)
C (n=14)
surface

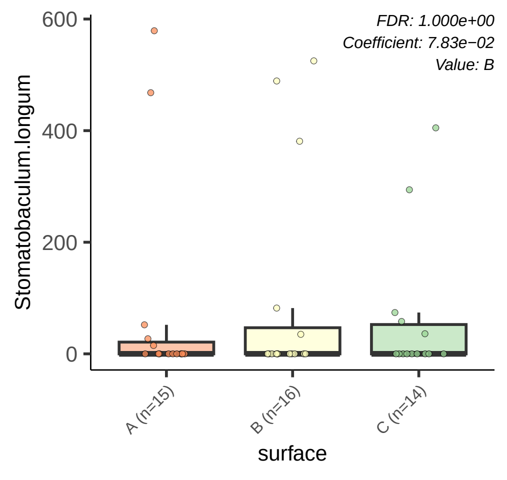

FDR: 1.000e+00
Coefficient: 7.83e−02
Value: B
600
400
200
0
Stomatobaculum.longum
A (n=15)
B (n=16)
C (n=14)
surface

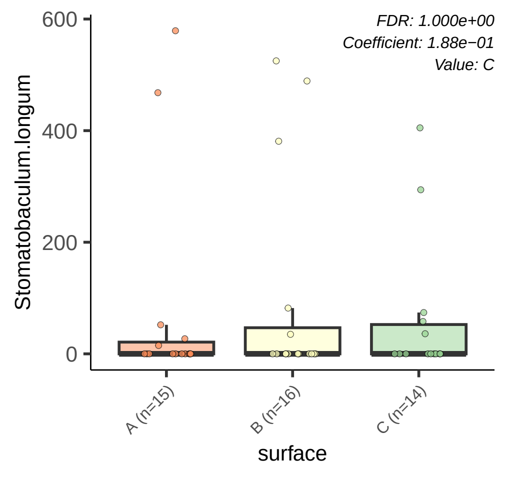
FDR: 1.000e+00
Coefficient: 1.88e−01
Value: C
Stomatobaculum.longum
600
400
200
0
A (n=15)
B (n=16)
C (n=14)
surface

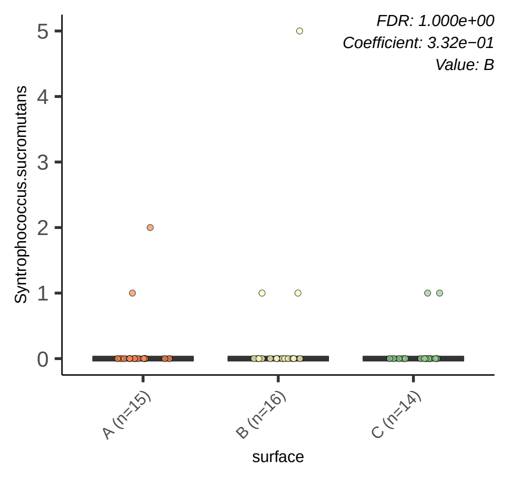

FDR: 1.000e+00
Coefficient: 3.32e−01
Value: B
Syntrophococcus.sucromutans
0
1
2
3
4
5
A (n=15)
B (n=16)
C (n=14)
surface

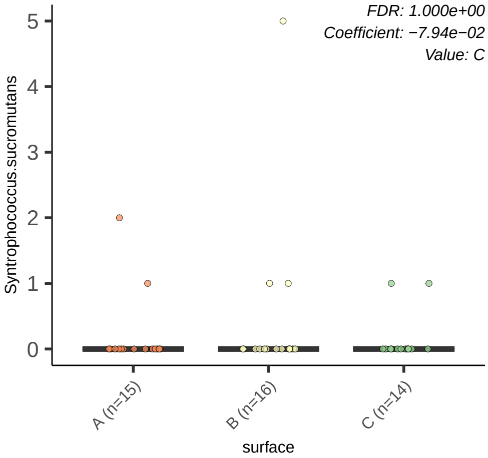

FDR: 1.000e+00
Coefficient: −7.94e−02
Value: C
Syntrophococcus.sucromutans
0
1
2
3
4
5
A (n=15)
B (n=16)
C (n=14)
surface

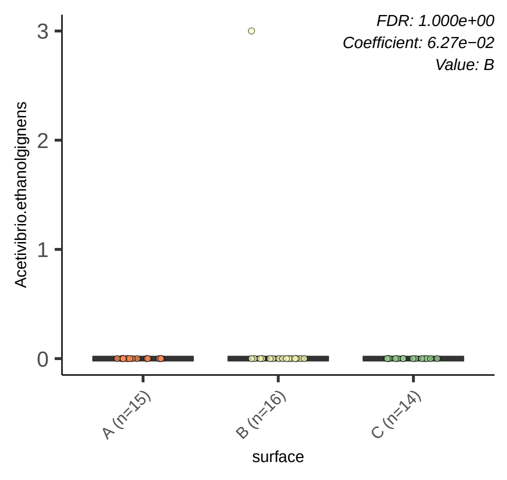

FDR: 1.000e+00
Coefficient: 6.27e−02
Value: B
Acetivibrio.ethanolgignens
3
2
1
0
A (n=15)
B (n=16)
C (n=14)
surface

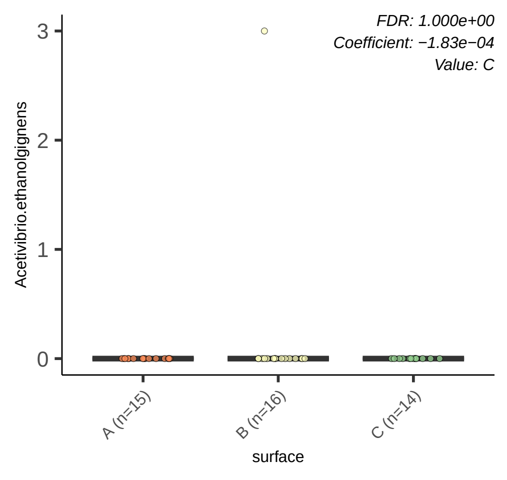
FDR: 1.000e+00
Coefficient: −1.83e−04
Value: C
Acetivibrio.ethanolgignens
3
2
1
0
A (n=15)
B (n=16)
C (n=14)
surface

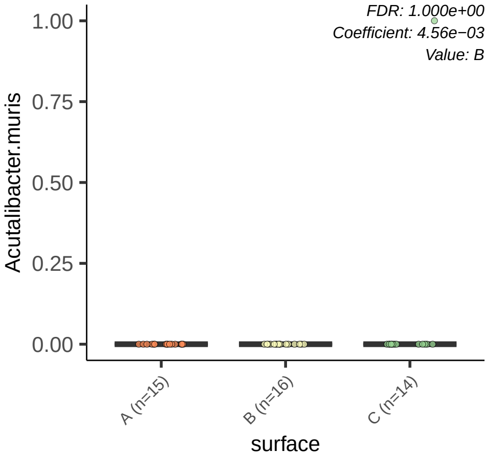
FDR: 1.000e+00
Coefficient: 4.56e−03
Value: B
Acutalibacter.muris
1.00
0.75
0.50
0.25
0.00
A (n=15)
B (n=16)
C (n=14)
surface

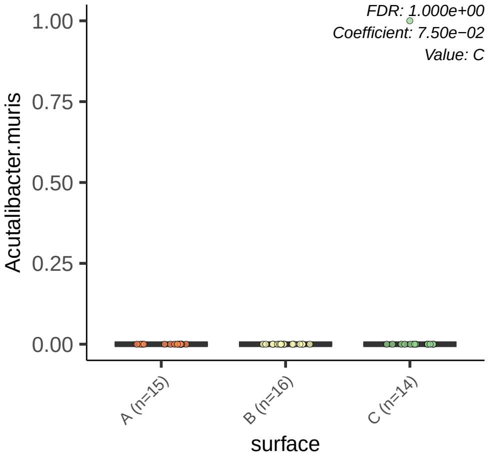
FDR: 1.000e+00
Coefficient: 7.50e−02
Value: C
Acutalibacter.muris
1.00
0.75
0.50
0.25
0.00
A (n=15)
B (n=16)
C (n=14)
surface

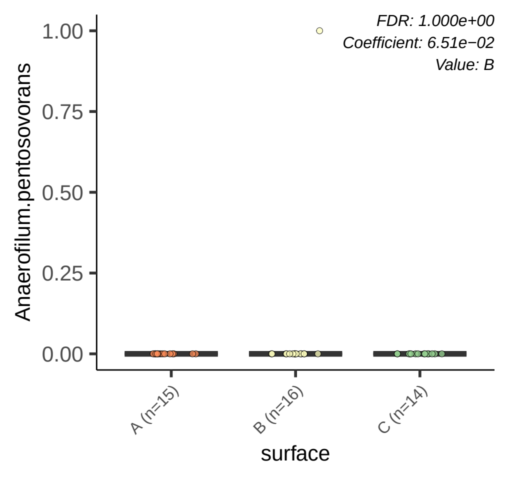
FDR: 1.000e+00
Coefficient: 6.51e−02
Value: B
Anaerofilum.pentosovorans
1.00
0.75
0.50
0.25
0.00
A (n=15)
B (n=16)
C (n=14)
surface

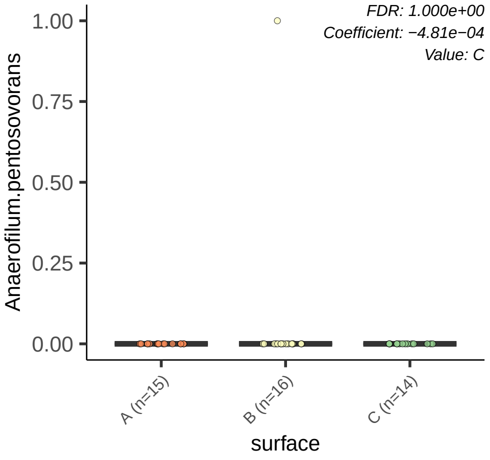
FDR: 1.000e+00
Coefficient: −4.81e−04
Value: C
Anaerofilum.pentosovorans
1.00
0.75
0.50
0.25
0.00
A (n=15)
B (n=16)
C (n=14)
surface

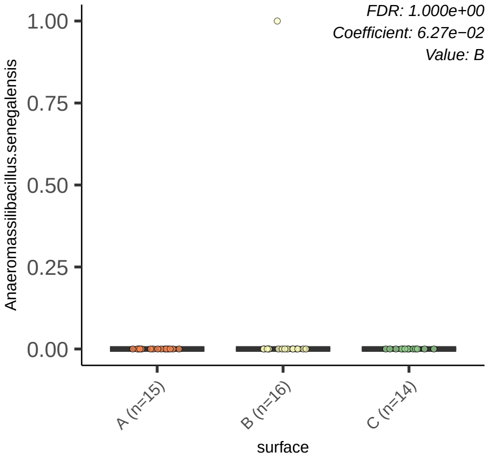
FDR: 1.000e+00
Coefficient: 6.27e−02
Value: B
Anaeromassilibacillus.senegalensis
1.00
0.75
0.50
0.25
0.00
A (n=15)
B (n=16)
C (n=14)
surface

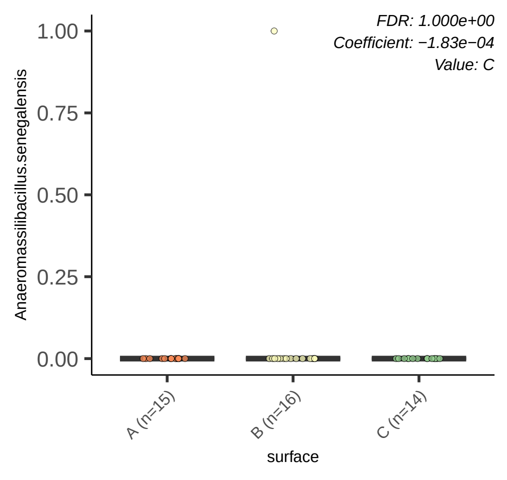

FDR: 1.000e+00
Coefficient: −1.83e−04
Value: C
Anaeromassilibacillus.senegalensis
1.00
0.75
0.50
0.25
0.00
A (n=15)
B (n=16)
C (n=14)
surface

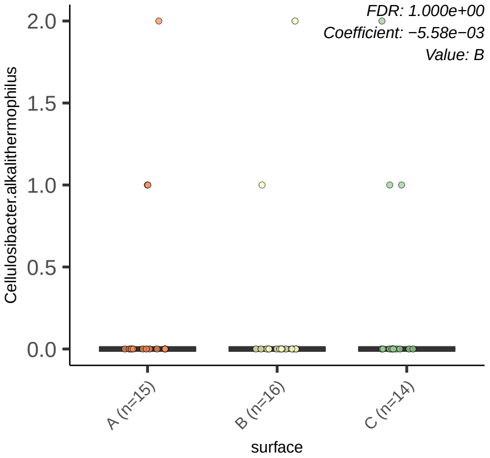
FDR: 1.000e+00
Coefficient: −5.58e−03
Value: B
Cellulosibacter.alkalithermophilus
2.0
1.5
1.0
0.5
0.0
A (n=15)
B (n=16)
C (n=14)
surface

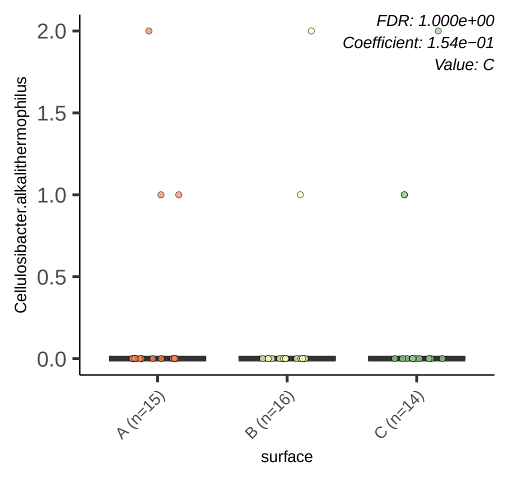
FDR: 1.000e+00
Coefficient: 1.54e−01
Value: C
Cellulosibacter.alkalithermophilus
2.0
1.5
1.0
0.5
0.0
A (n=15)
B (n=16)
C (n=14)
surface

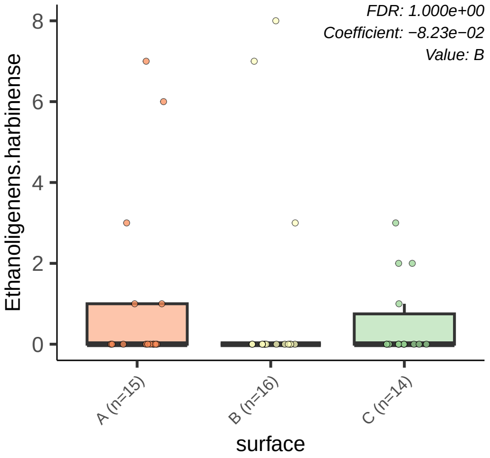

FDR: 1.000e+00
Coefficient: −8.23e−02
Value: B
Ethanoligenens.harbinense
8
6
4
2
0
A (n=15)
B (n=16)
C (n=14)
surface

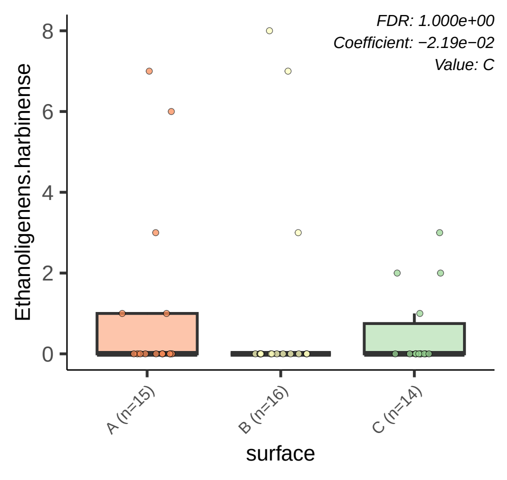
FDR: 1.000e+00
Coefficient: −2.19e−02
Value: C
Ethanoligenens.harbinense
0
2
4
6
8
A (n=15)
B (n=16)
C (n=14)
surface

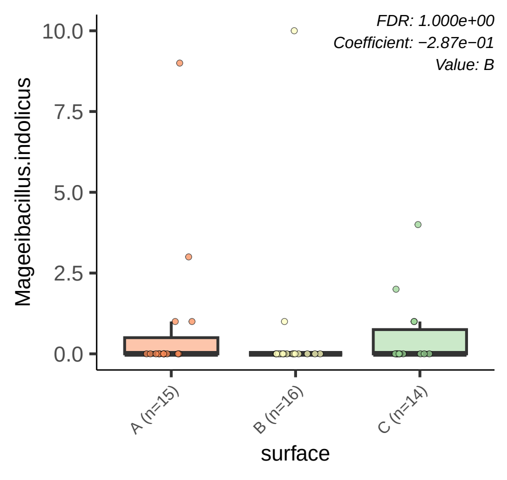

FDR: 1.000e+00
Coefficient: −2.87e−01
Value: B
Mageeibacillus.indolicus
10.0
7.5
5.0
2.5
0.0
A (n=15)
B (n=16)
C (n=14)
surface

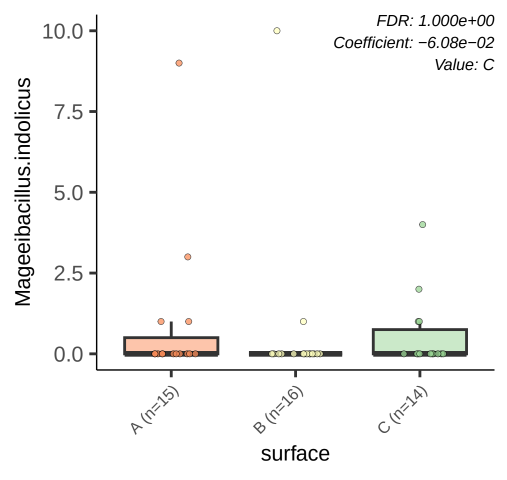
FDR: 1.000e+00
Coefficient: −6.08e−02
Value: C
Mageeibacillus.indolicus
10.0
7.5
5.0
2.5
0.0
A (n=15)
B (n=16)
C (n=14)
surface

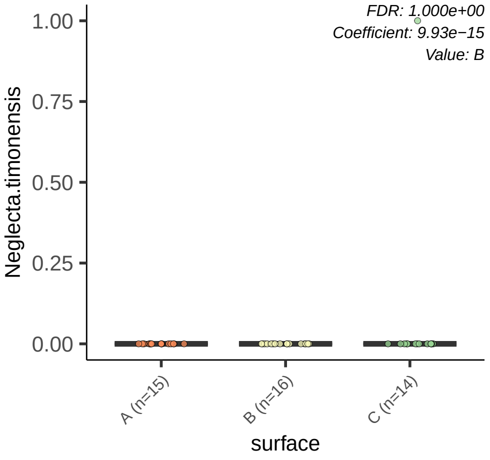
FDR: 1.000e+00
Coefficient: 9.93e−15
Value: B
Neglecta.timonensis
1.00
0.75
0.50
0.25
0.00
A (n=15)
B (n=16)
C (n=14)
surface

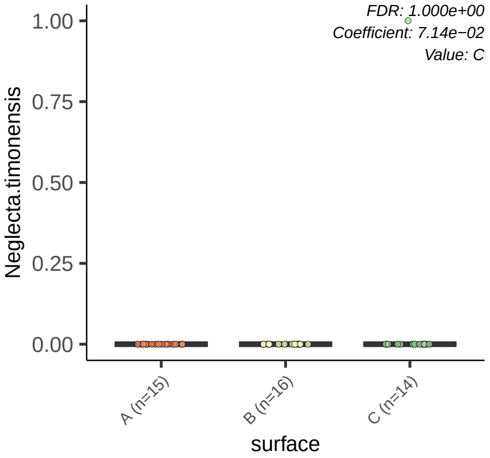
FDR: 1.000e+00
Coefficient: 7.14e−02
Value: C
Neglecta.timonensis
1.00
0.75
0.50
0.25
0.00
A (n=15)
B (n=16)
C (n=14)
surface

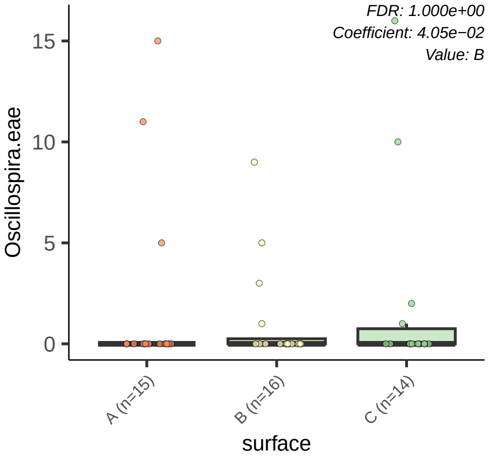
FDR: 1.000e+00
Coefficient: 4.05e−02
Value: B
Oscillospira.eae
15
10
5
0
A (n=15)
B (n=16)
C (n=14)
surface

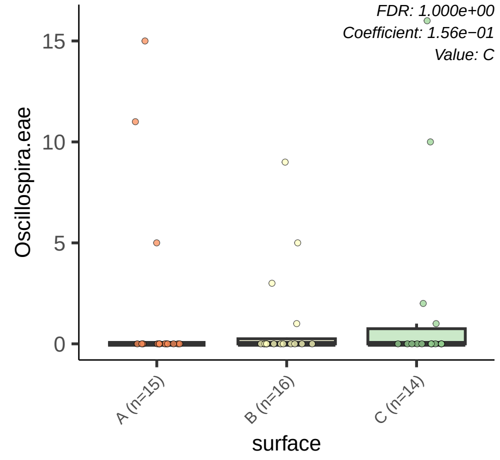

FDR: 1.000e+00
Coefficient: 1.56e−01
Value: C
Oscillospira.eae
15
10
5
0
A (n=15)
B (n=16)
C (n=14)
surface

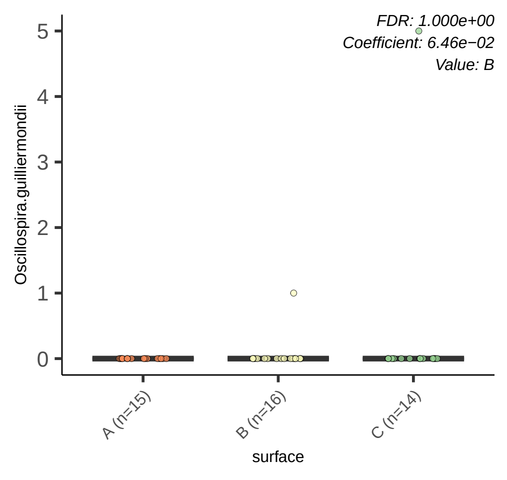

FDR: 1.000e+00
Coefficient: 6.46e−02
Value: B
Oscillospira.guilliermondii
5
4
3
2
1
0
A (n=15)
B (n=16)
C (n=14)
surface

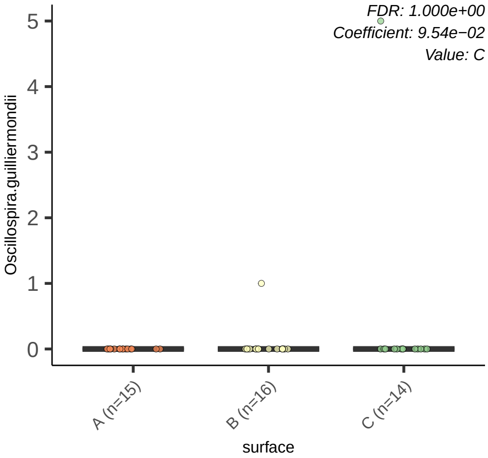

FDR: 1.000e+00
Coefficient: 9.54e−02
Value: C
Oscillospira.guilliermondii
0
1
2
3
4
5
A (n=15)
B (n=16)
C (n=14)
surface

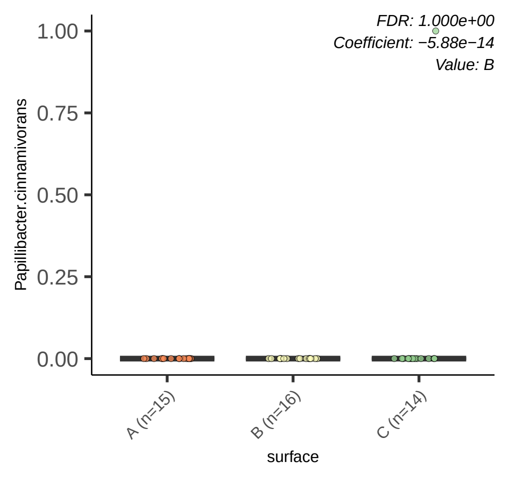

FDR: 1.000e+00
Coefficient: −5.88e−14
Value: B
1.00
0.75
0.50
0.25
0.00
Papillibacter.cinnamivorans
A (n=15)
B (n=16)
C (n=14)
surface

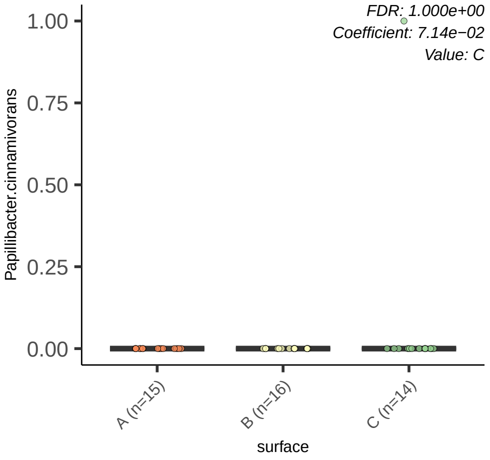

FDR: 1.000e+00
Coefficient: 7.14e−02
Value: C
1.00
0.75
0.50
0.25
0.00
Papillibacter.cinnamivorans
A (n=15)
B (n=16)
C (n=14)
surface

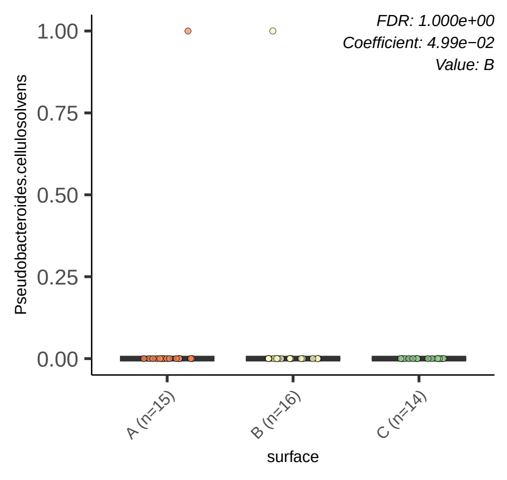
FDR: 1.000e+00
Coefficient: 4.99e−02
Value: B
Pseudobacteroides.cellulosolvens
1.00
0.75
0.50
0.25
0.00
A (n=15)
B (n=16)
C (n=14)
surface

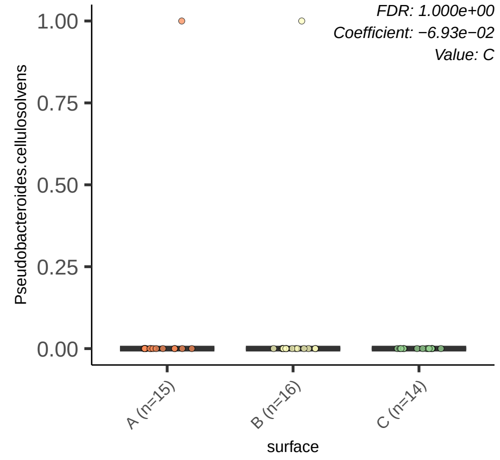
FDR: 1.000e+00
Coefficient: −6.93e−02
Value: C
Pseudobacteroides.cellulosolvens
1.00
0.75
0.50
0.25
0.00
A (n=15)
B (n=16)
C (n=14)
surface

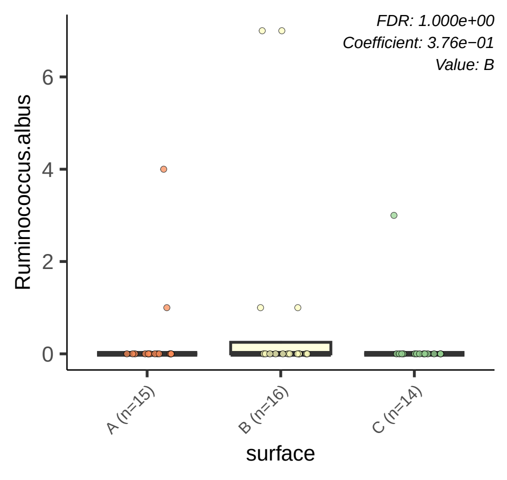
FDR: 1.000e+00
Coefficient: 3.76e−01
Value: B
Ruminococcus.albus
0
2
4
6
A (n=15)
B (n=16)
C (n=14)
surface

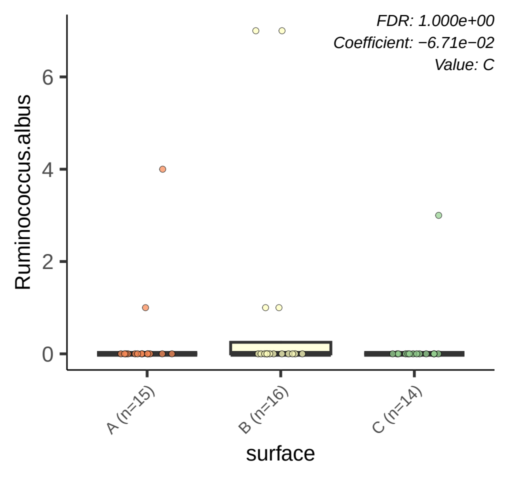
FDR: 1.000e+00
Coefficient: −6.71e−02
Value: C
Ruminococcus.albus
0
2
4
6
A (n=15)
B (n=16)
C (n=14)
surface

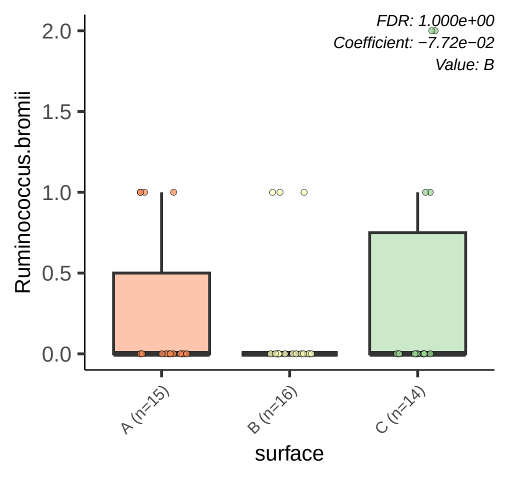

FDR: 1.000e+00
Coefficient: −7.72e−02
Value: B
Ruminococcus.bromii
2.0
1.5
1.0
0.5
0.0
A (n=15)
B (n=16)
C (n=14)
surface

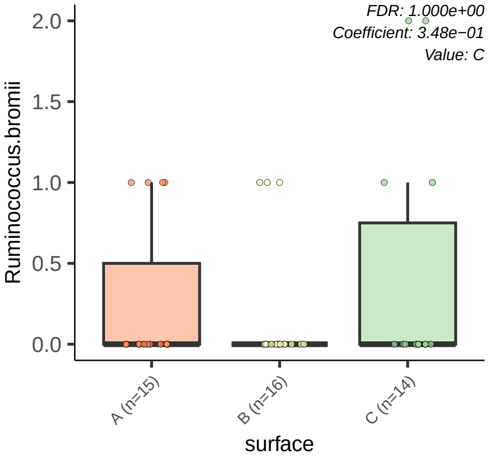
FDR: 1.000e+00
Coefficient: 3.48e−01
Value: C
Ruminococcus.bromii
2.0
1.5
1.0
0.5
0.0
A (n=15)
B (n=16)
C (n=14)
surface

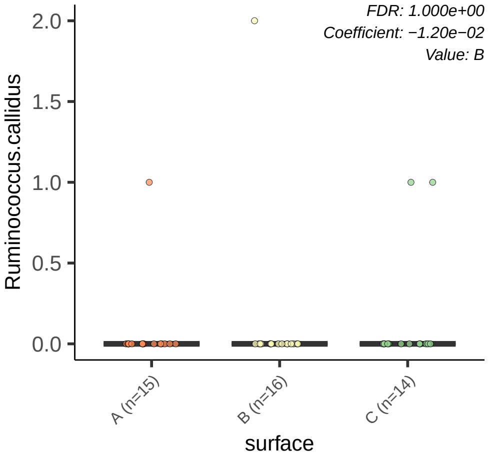
FDR: 1.000e+00
Coefficient: −1.20e−02
Value: B
Ruminococcus.callidus
2.0
1.5
1.0
0.5
0.0
A (n=15)
B (n=16)
C (n=14)
surface

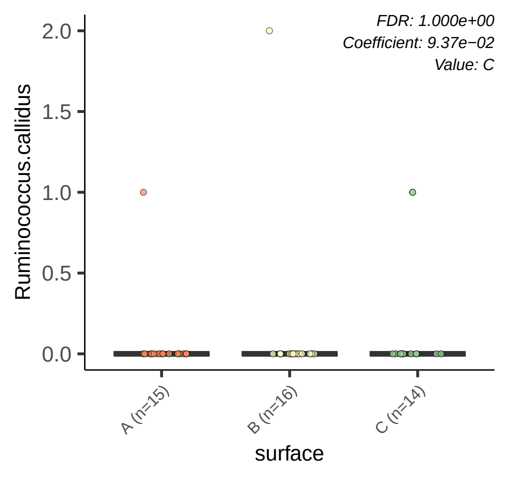
FDR: 1.000e+00
Coefficient: 9.37e−02
Value: C
Ruminococcus.callidus
2.0
1.5
1.0
0.5
0.0
A (n=15)
B (n=16)
C (n=14)
surface

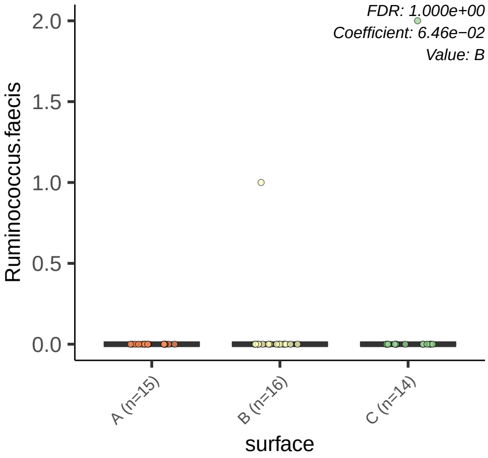

FDR: 1.000e+00
Coefficient: 6.46e−02
Value: B
Ruminococcus.faecis
2.0
1.5
1.0
0.5
0.0
A (n=15)
B (n=16)
C (n=14)
surface

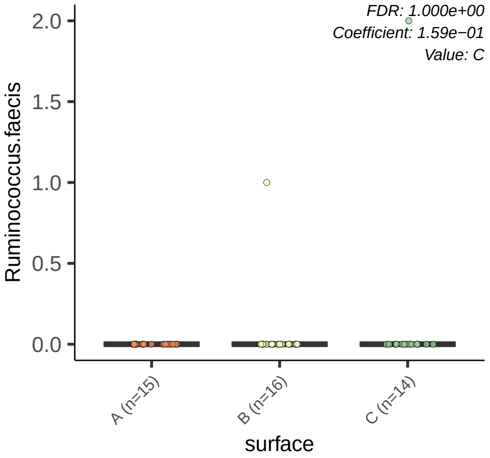

FDR: 1.000e+00
Coefficient: 1.59e−01
Value: C
Ruminococcus.faecis
2.0
1.5
1.0
0.5
0.0
A (n=15)
B (n=16)
C (n=14)
surface

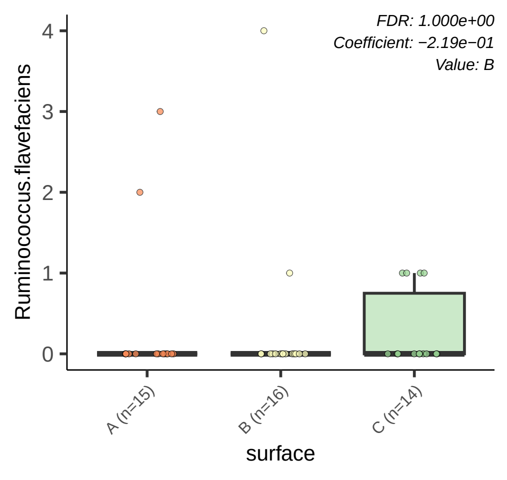
FDR: 1.000e+00
Coefficient: −2.19e−01
Value: B
Ruminococcus.flavefaciens
0
1
2
3
4
A (n=15)
B (n=16)
C (n=14)
surface

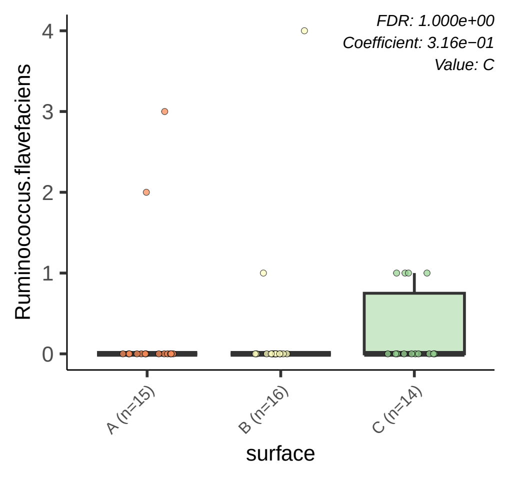
FDR: 1.000e+00
Coefficient: 3.16e−01
Value: C
Ruminococcus.flavefaciens
0
1
2
3
4
A (n=15)
B (n=16)
C (n=14)
surface

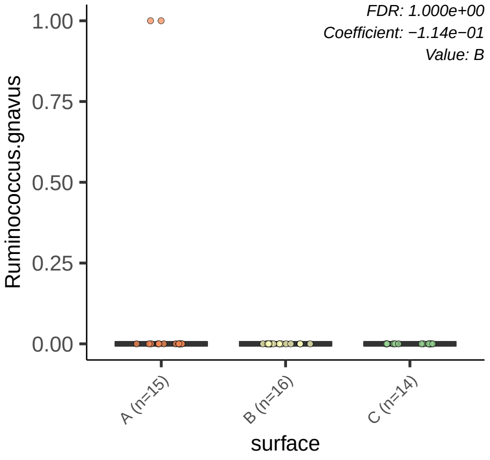
FDR: 1.000e+00
Coefficient: −1.14e−01
Value: B
Ruminococcus.gnavus
1.00
0.75
0.50
0.25
0.00
A (n=15)
B (n=16)
C (n=14)
surface

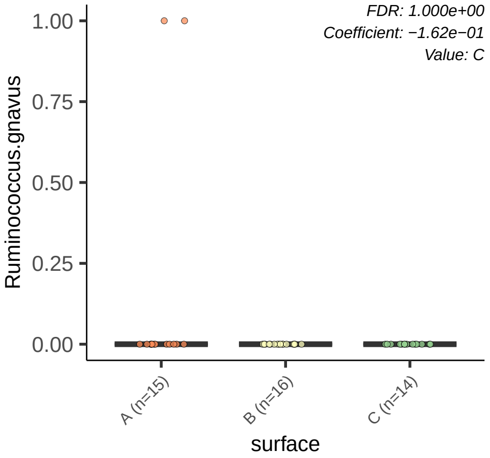
FDR: 1.000e+00
Coefficient: −1.62e−01
Value: C
Ruminococcus.gnavus
1.00
0.75
0.50
0.25
0.00
A (n=15)
B (n=16)
C (n=14)
surface

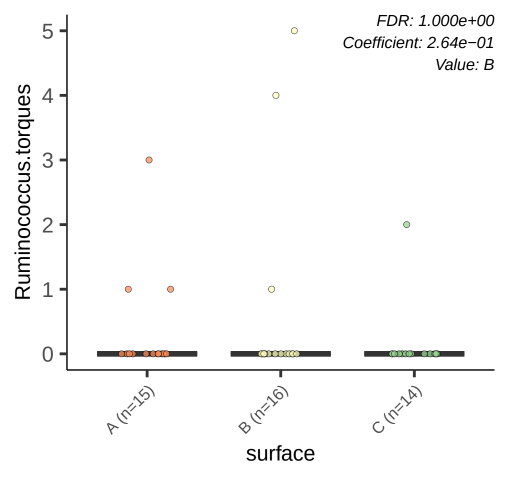
FDR: 1.000e+00
Coefficient: 2.64e−01
Value: B
Ruminococcus.torques
0
1
2
3
4
5
A (n=15)
B (n=16)
C (n=14)
surface

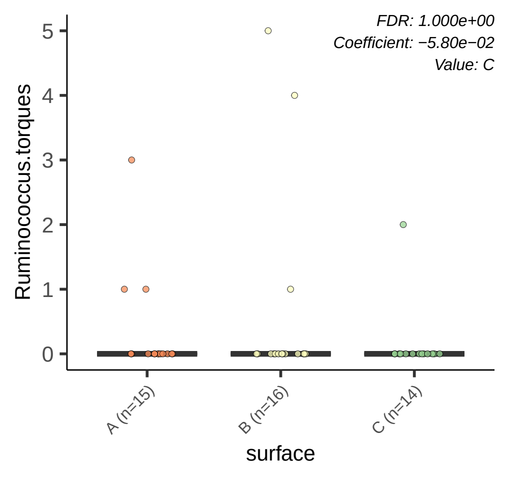

FDR: 1.000e+00
Coefficient: −5.80e−02
Value: C
Ruminococcus.torques
0
1
2
3
4
5
A (n=15)
B (n=16)
C (n=14)
surface

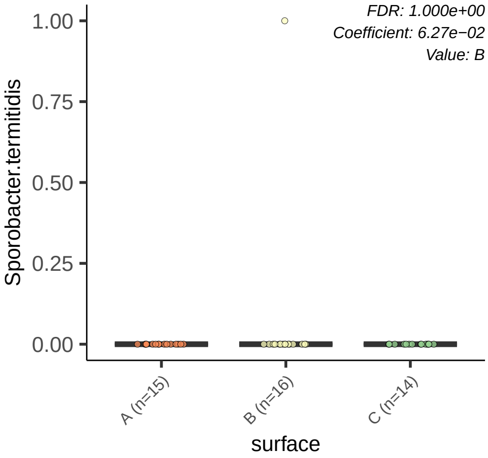
FDR: 1.000e+00
Coefficient: 6.27e−02
Value: B
Sporobacter.termitidis
1.00
0.75
0.50
0.25
0.00
A (n=15)
B (n=16)
C (n=14)
surface

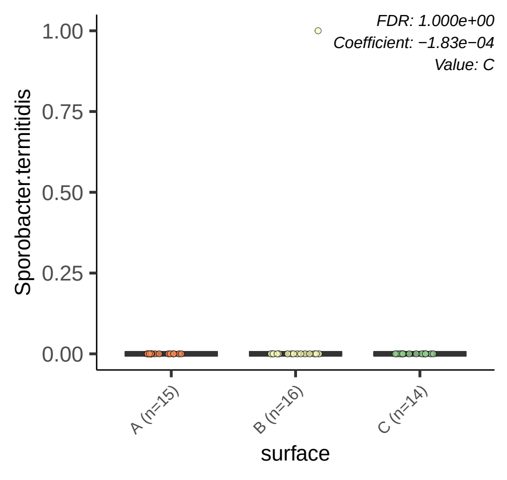

FDR: 1.000e+00
Coefficient: −1.83e−04
Value: C
Sporobacter.termitidis
1.00
0.75
0.50
0.25
0.00
A (n=15)
B (n=16)
C (n=14)
surface

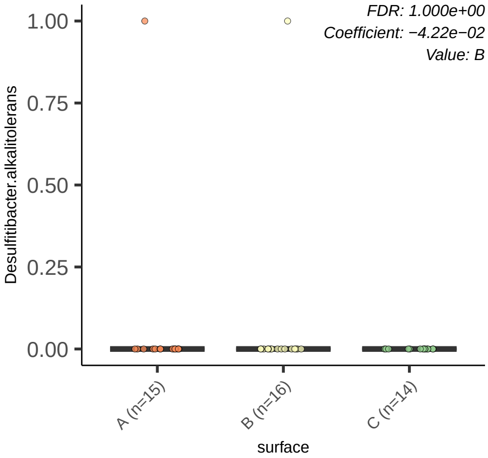
FDR: 1.000e+00
Coefficient: −4.22e−02
Value: B
Desulfitibacter.alkalitolerans
1.00
0.75
0.50
0.25
0.00
A (n=15)
B (n=16)
C (n=14)
surface

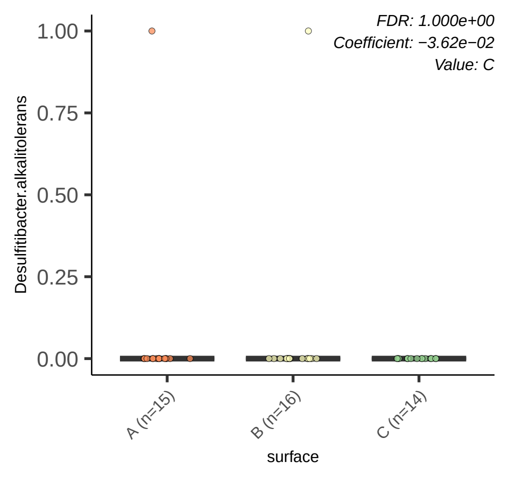

FDR: 1.000e+00
Coefficient: −3.62e−02
Value: C
1.00
0.75
0.50
0.25
0.00
Desulfitibacter.alkalitolerans
A (n=15)
B (n=16)
C (n=14)
surface

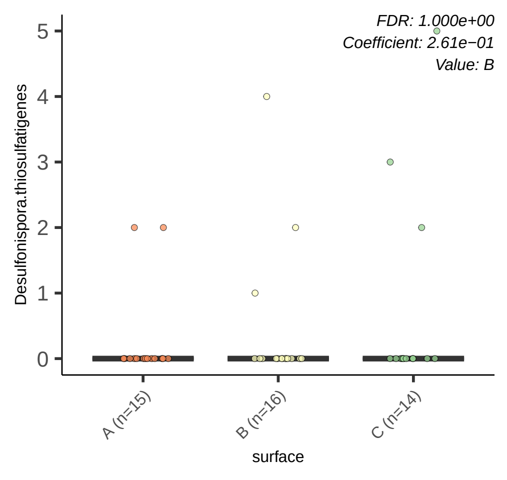
FDR: 1.000e+00
Coefficient: 2.61e−01
Value: B
Desulfonispora.thiosulfatigenes
0
1
2
3
4
5
A (n=15)
B (n=16)
C (n=14)
surface

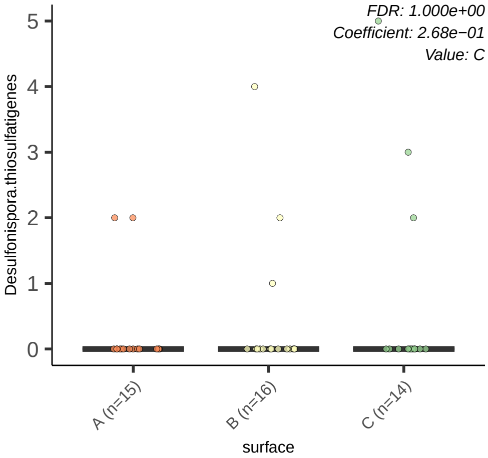

FDR: 1.000e+00
Coefficient: 2.68e−01
Value: C
Desulfonispora.thiosulfatigenes
0
1
2
3
4
5
A (n=15)
B (n=16)
C (n=14)
surface

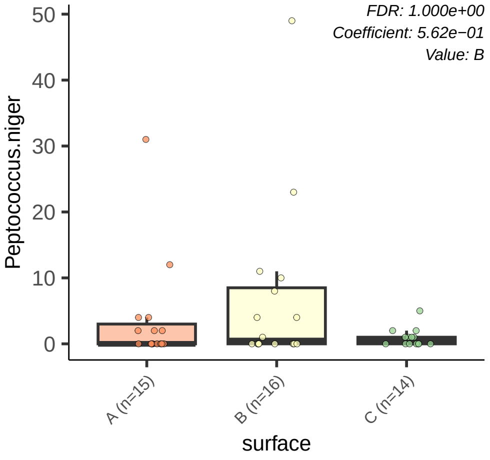
FDR: 1.000e+00
Coefficient: 5.62e−01
Value: B
Peptococcus.niger
0
10
20
30
40
50
A (n=15)
B (n=16)
C (n=14)
surface

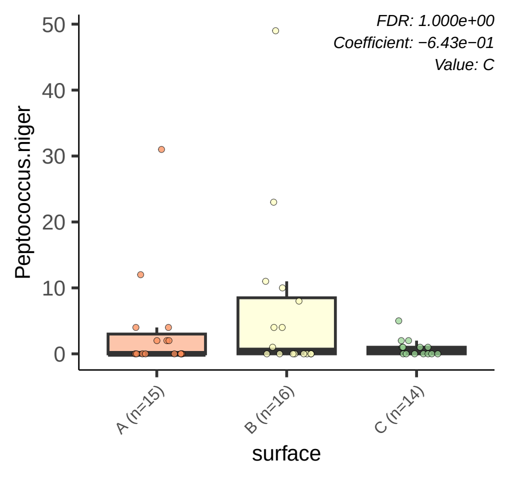
FDR: 1.000e+00
Coefficient: −6.43e−01
Value: C
Peptococcus.niger
50
40
30
20
10
0
A (n=15)
B (n=16)
C (n=14)
surface

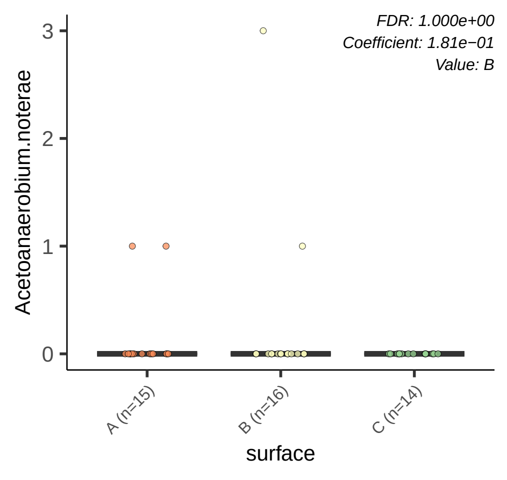

FDR: 1.000e+00
Coefficient: 1.81e−01
Value: B
Acetoanaerobium.noterae
3
2
1
0
A (n=15)
B (n=16)
C (n=14)
surface

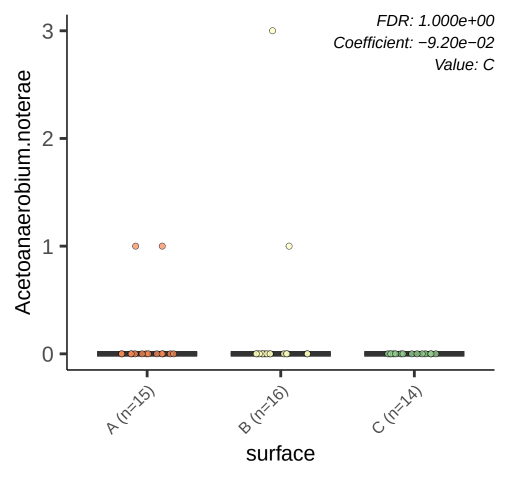
FDR: 1.000e+00
Coefficient: −9.20e−02
Value: C
Acetoanaerobium.noterae
3
2
1
0
A (n=15)
B (n=16)
C (n=14)
surface

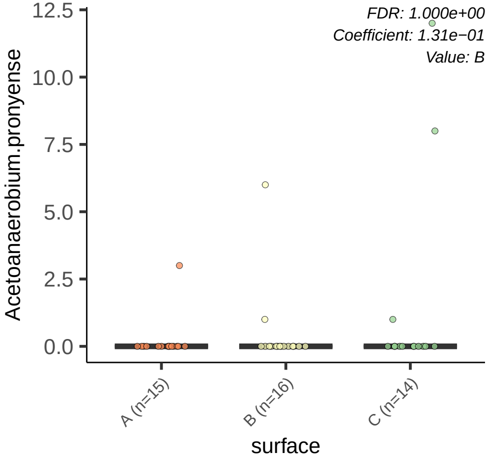

FDR: 1.000e+00
Coefficient: 1.31e−01
Value: B
Acetoanaerobium.pronyense
12.5
10.0
7.5
5.0
2.5
0.0
A (n=15)
B (n=16)
C (n=14)
surface

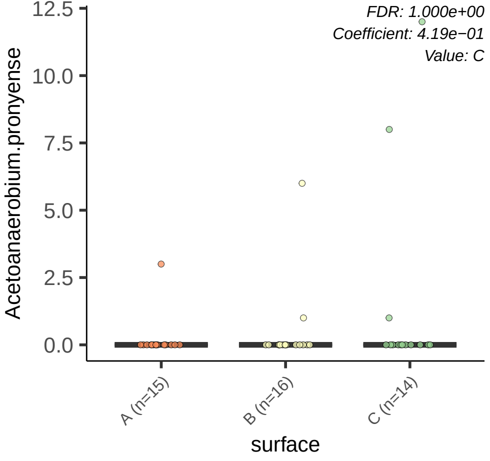

FDR: 1.000e+00
Coefficient: 4.19e−01
Value: C
Acetoanaerobium.pronyense
12.5
10.0
7.5
5.0
2.5
0.0
A (n=15)
B (n=16)
C (n=14)
surface

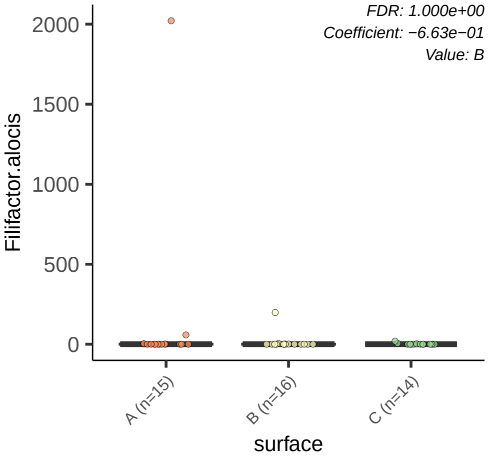

FDR: 1.000e+00
Coefficient: −6.63e−01
Value: B
Filifactor.alocis
2000
1500
1000
500
0
A (n=15)
B (n=16)
C (n=14)
surface

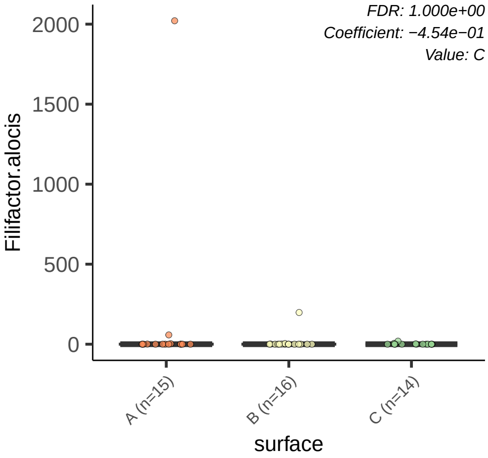

FDR: 1.000e+00
Coefficient: −4.54e−01
Value: C
Filifactor.alocis
2000
1500
1000
500
0
A (n=15)
B (n=16)
C (n=14)
surface

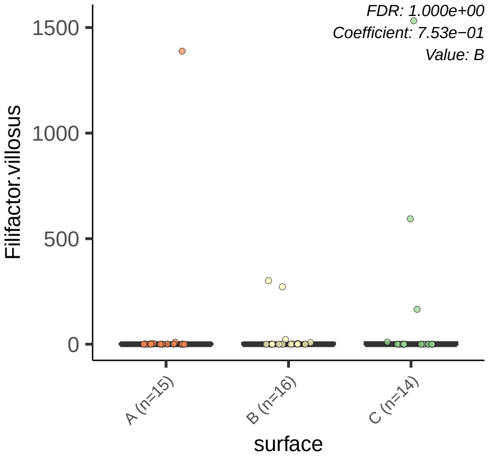

FDR: 1.000e+00
Coefficient: 7.53e−01
Value: B
1500
1000
500
0
Filifactor.villosus
A (n=15)
B (n=16)
C (n=14)
surface

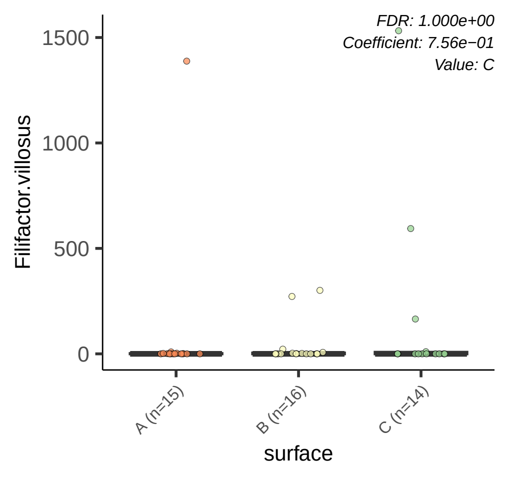
FDR: 1.000e+00
Coefficient: 7.56e−01
Value: C
Filifactor.villosus
1500
1000
500
0
A (n=15)
B (n=16)
C (n=14)
surface

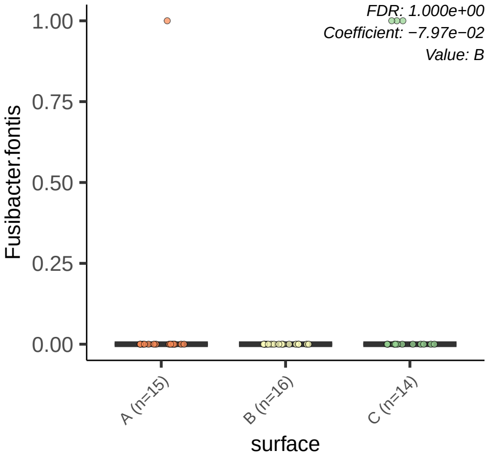
FDR: 1.000e+00
Coefficient: −7.97e−02
Value: B
Fusibacter.fontis
1.00
0.75
0.50
0.25
0.00
A (n=15)
B (n=16)
C (n=14)
surface

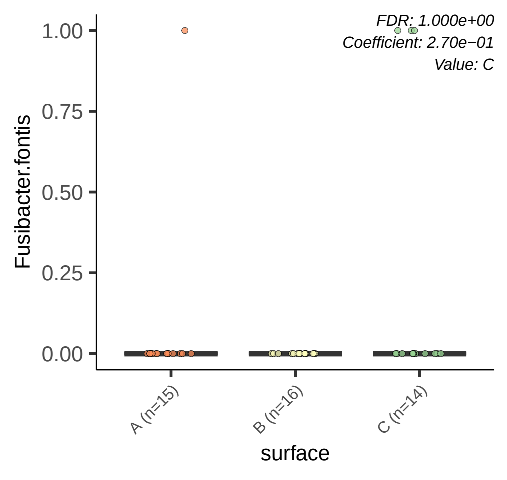
FDR: 1.000e+00
Coefficient: 2.70e−01
Value: C
Fusibacter.fontis
1.00
0.75
0.50
0.25
0.00
A (n=15)
B (n=16)
C (n=14)
surface

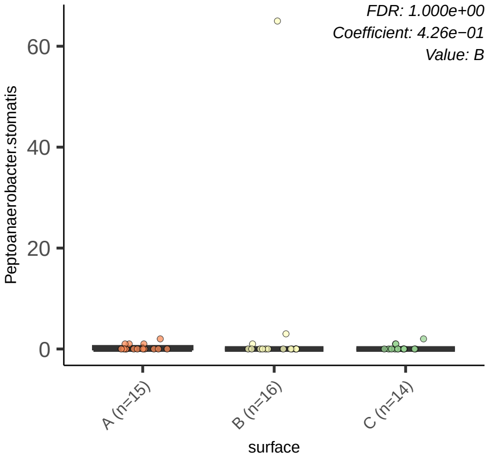
FDR: 1.000e+00
Coefficient: 4.26e−01
Value: B
60
40
20
0
Peptoanaerobacter.stomatis
A (n=15)
B (n=16)
C (n=14)
surface

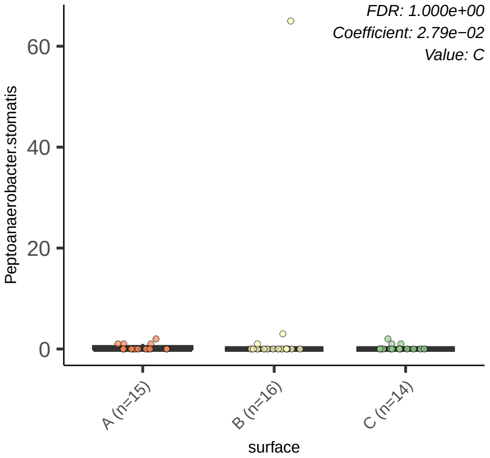
FDR: 1.000e+00
Coefficient: 2.79e−02
Value: C
60
40
20
0
Peptoanaerobacter.stomatis
A (n=15)
B (n=16)
C (n=14)
surface

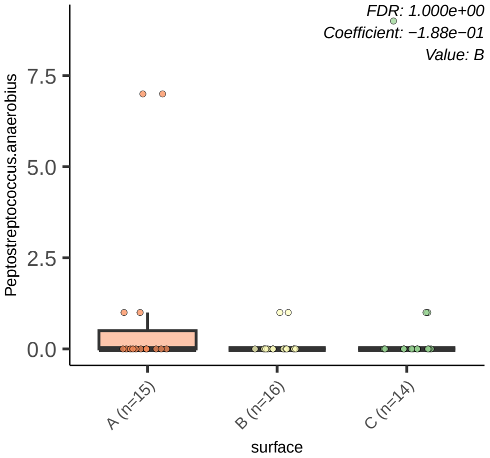

FDR: 1.000e+00
Coefficient: −1.88e−01
Value: B
Peptostreptococcus.anaerobius
7.5
5.0
2.5
0.0
A (n=15)
B (n=16)
C (n=14)
surface

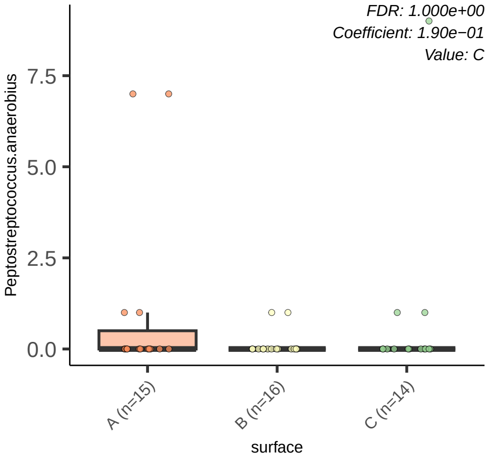
FDR: 1.000e+00
Coefficient: 1.90e−01
Value: C
Peptostreptococcus.anaerobius
7.5
5.0
2.5
0.0
A (n=15)
B (n=16)
C (n=14)
surface

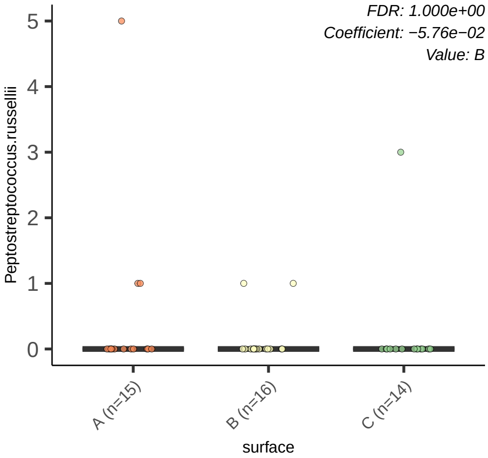
FDR: 1.000e+00
Coefficient: −5.76e−02
Value: B
Peptostreptococcus.russellii
0
1
2
3
4
5
A (n=15)
B (n=16)
C (n=14)
surface

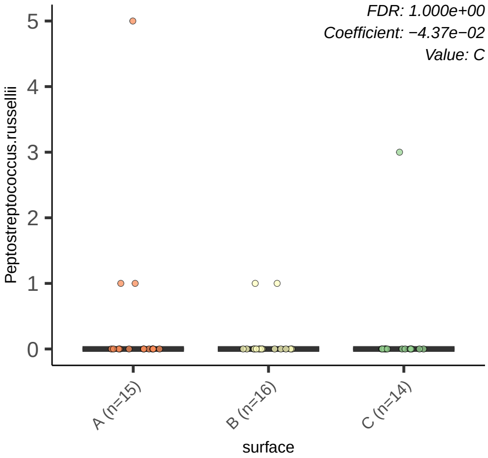
FDR: 1.000e+00
Coefficient: −4.37e−02
Value: C
Peptostreptococcus.russellii
0
1
2
3
4
5
A (n=15)
B (n=16)
C (n=14)
surface

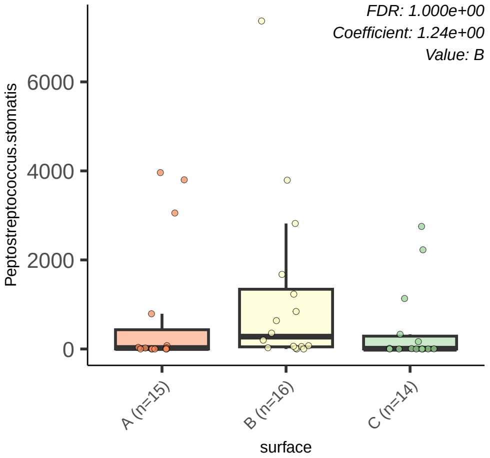
FDR: 1.000e+00
Coefficient: 1.24e+00
Value: B
6000
4000
2000
0
Peptostreptococcus.stomatis
A (n=15)
B (n=16)
C (n=14)
surface

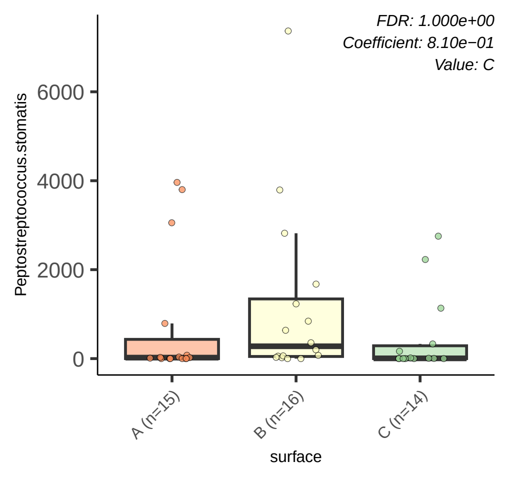
FDR: 1.000e+00
Coefficient: 8.10e−01
Value: C
Peptostreptococcus.stomatis
6000
4000
2000
0
A (n=15)
B (n=16)
C (n=14)
surface

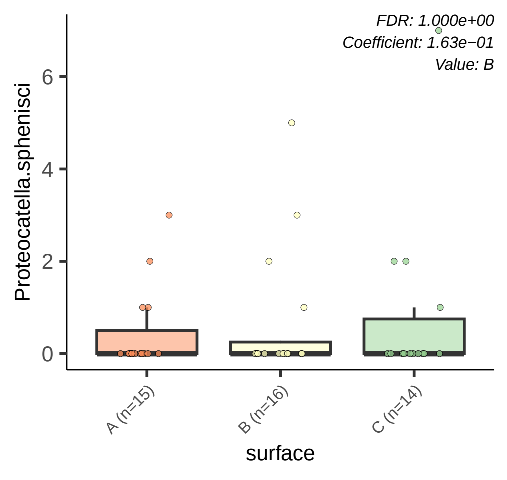
FDR: 1.000e+00
Coefficient: 1.63e−01
Value: B
Proteocatella.sphenisci
0
2
4
6
A (n=15)
B (n=16)
C (n=14)
surface

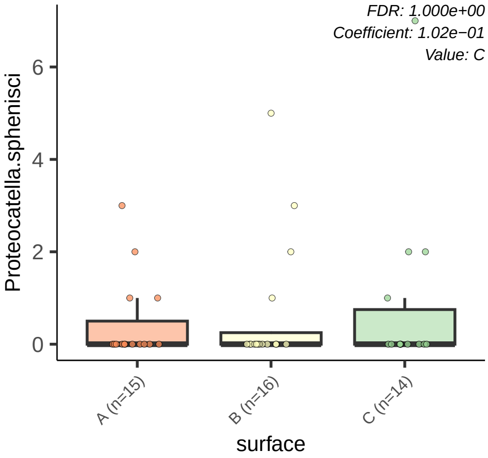
FDR: 1.000e+00
Coefficient: 1.02e−01
Value: C
Proteocatella.sphenisci
0
2
4
6
A (n=15)
B (n=16)
C (n=14)
surface

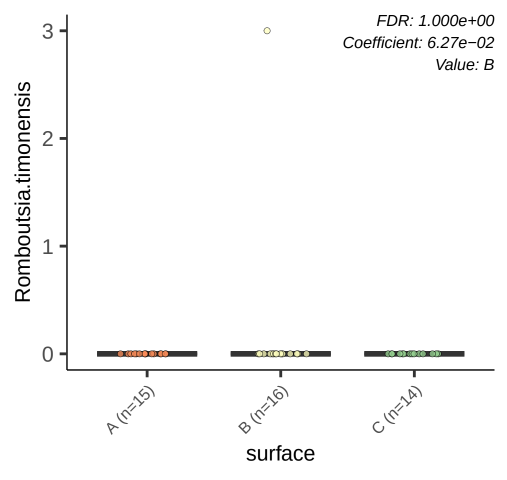

FDR: 1.000e+00
Coefficient: 6.27e−02
Value: B
Romboutsia.timonensis
3
2
1
0
A (n=15)
B (n=16)
C (n=14)
surface

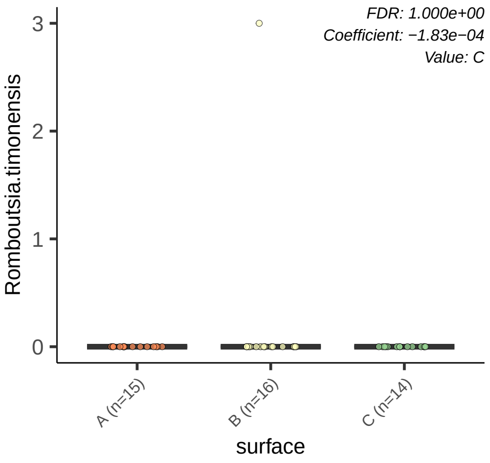

FDR: 1.000e+00
Coefficient: −1.83e−04
Value: C
Romboutsia.timonensis
3
2
1
0
A (n=15)
B (n=16)
C (n=14)
surface

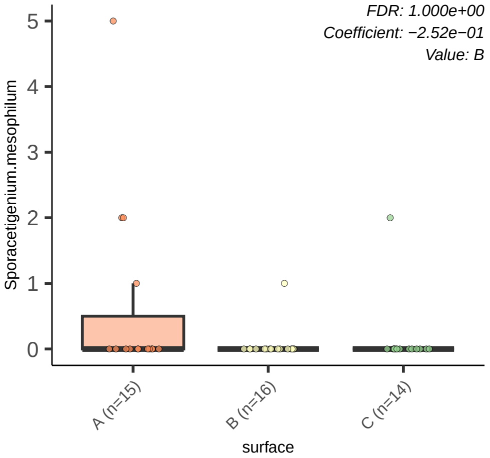

FDR: 1.000e+00
Coefficient: −2.52e−01
Value: B
Sporacetigenium.mesophilum
5
4
3
2
1
0
A (n=15)
B (n=16)
C (n=14)
surface

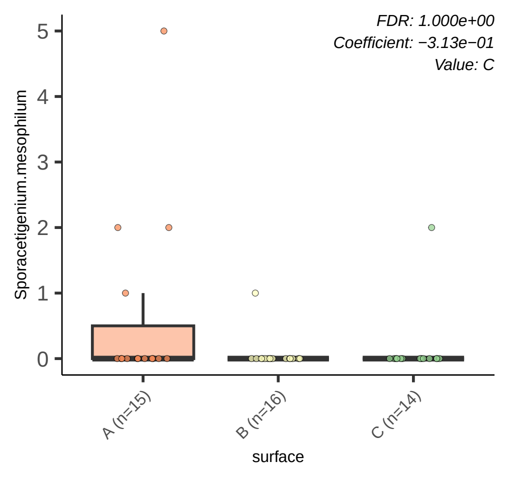

FDR: 1.000e+00
Coefficient: −3.13e−01
Value: C
Sporacetigenium.mesophilum
0
1
2
3
4
5
A (n=15)
B (n=16)
C (n=14)
surface

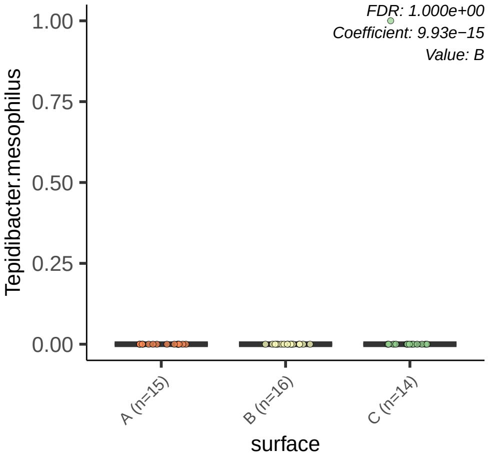

FDR: 1.000e+00
Coefficient: 9.93e−15
Value: B
Tepidibacter.mesophilus
1.00
0.75
0.50
0.25
0.00
A (n=15)
B (n=16)
C (n=14)
surface

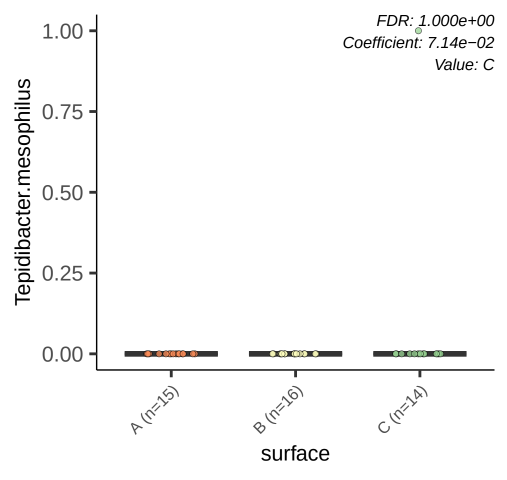

FDR: 1.000e+00
Coefficient: 7.14e−02
Value: C
Tepidibacter.mesophilus
1.00
0.75
0.50
0.25
0.00
A (n=15)
B (n=16)
C (n=14)
surface

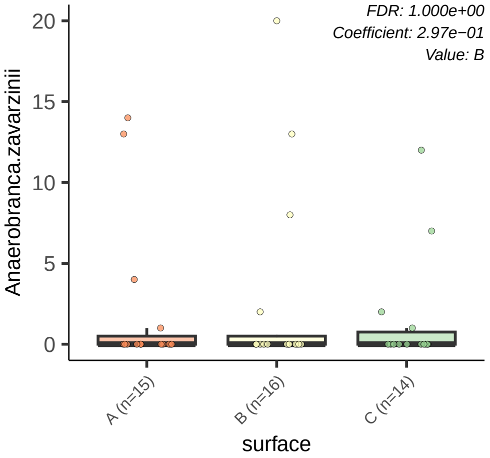
FDR: 1.000e+00
Coefficient: 2.97e−01
Value: B
Anaerobranca.zavarzinii
0
5
10
15
20
A (n=15)
B (n=16)
C (n=14)
surface

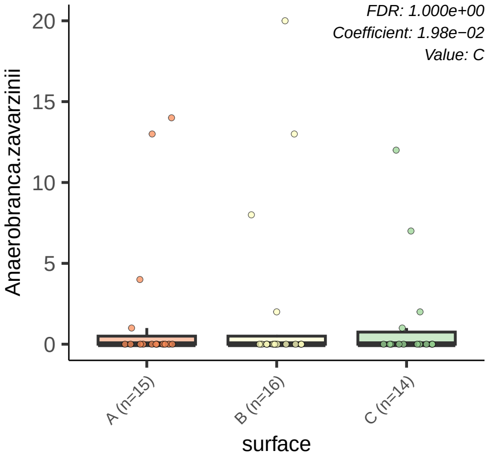
FDR: 1.000e+00
Coefficient: 1.98e−02
Value: C
Anaerobranca.zavarzinii
20
15
10
5
0
A (n=15)
B (n=16)
C (n=14)
surface

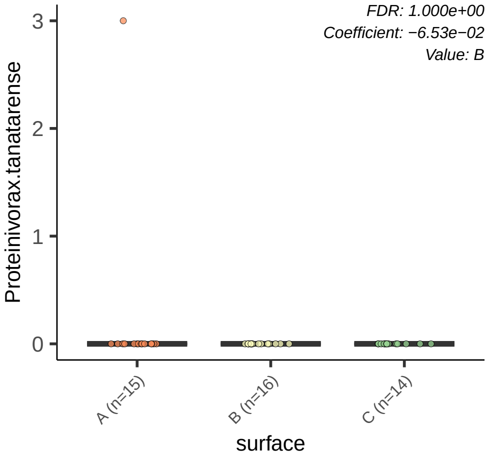
FDR: 1.000e+00
Coefficient: −6.53e−02
Value: B
Proteinivorax.tanatarense
3
2
1
0
A (n=15)
B (n=16)
C (n=14)
surface

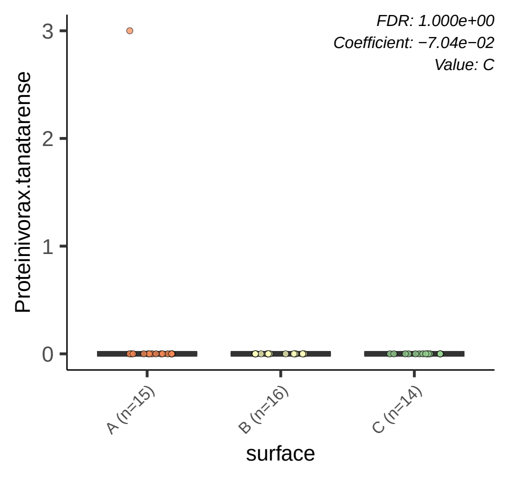
FDR: 1.000e+00
Coefficient: −7.04e−02
Value: C
Proteinivorax.tanatarense
3
2
1
0
A (n=15)
B (n=16)
C (n=14)
surface

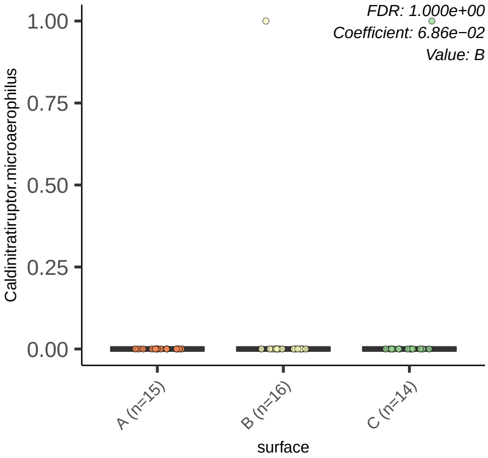
FDR: 1.000e+00
Coefficient: 6.86e−02
Value: B
Caldinitratiruptor.microaerophilus
1.00
0.75
0.50
0.25
0.00
A (n=15)
B (n=16)
C (n=14)
surface

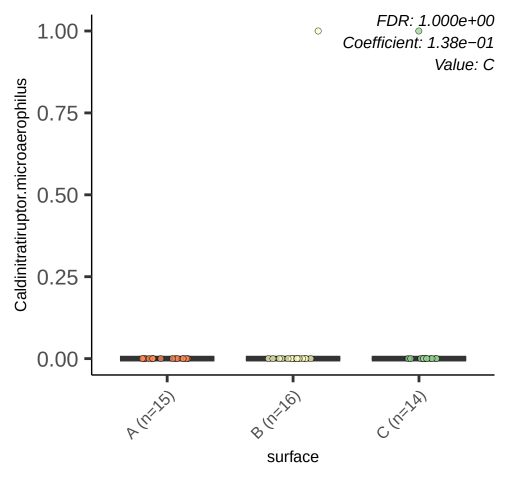
FDR: 1.000e+00
Coefficient: 1.38e−01
Value: C
Caldinitratiruptor.microaerophilus
1.00
0.75
0.50
0.25
0.00
A (n=15)
B (n=16)
C (n=14)
surface

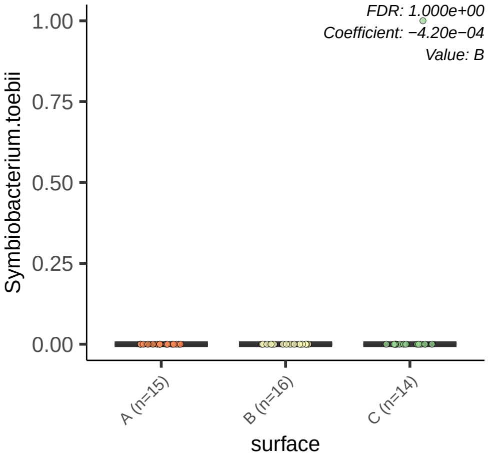
FDR: 1.000e+00
Coefficient: −4.20e−04
Value: B
Symbiobacterium.toebii
1.00
0.75
0.50
0.25
0.00
A (n=15)
B (n=16)
C (n=14)
surface

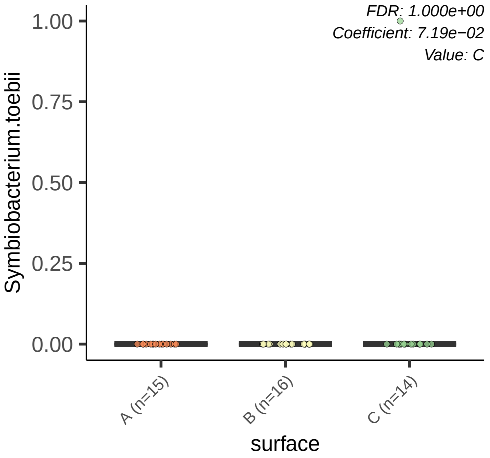
FDR: 1.000e+00
Coefficient: 7.19e−02
Value: C
Symbiobacterium.toebii
1.00
0.75
0.50
0.25
0.00
A (n=15)
B (n=16)
C (n=14)
surface

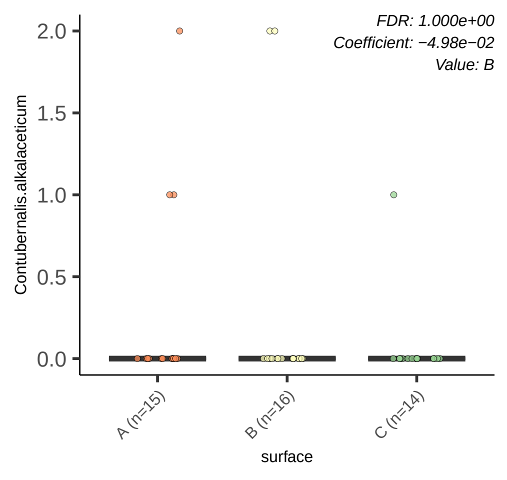
FDR: 1.000e+00
Coefficient: −4.98e−02
Value: B
Contubernalis.alkalaceticum
2.0
1.5
1.0
0.5
0.0
A (n=15)
B (n=16)
C (n=14)
surface

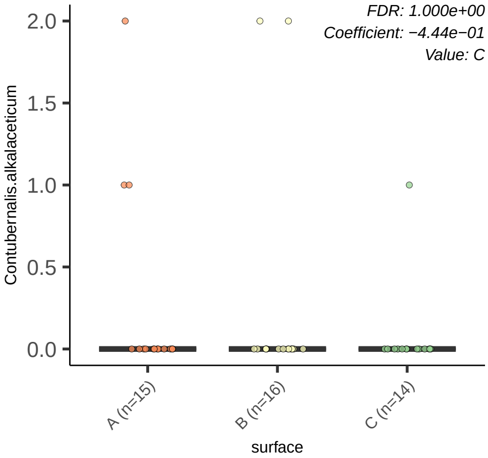
FDR: 1.000e+00
Coefficient: −4.44e−01
Value: C
Contubernalis.alkalaceticum
2.0
1.5
1.0
0.5
0.0
A (n=15)
B (n=16)
C (n=14)
surface

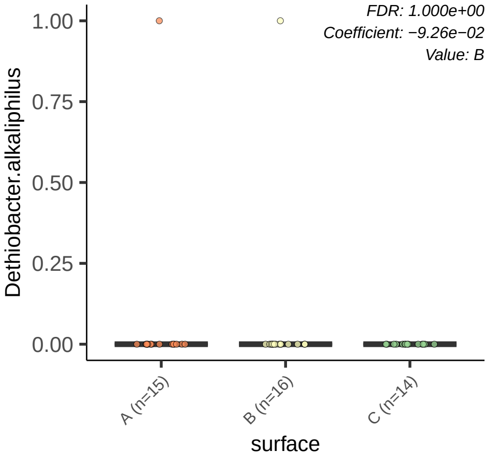
FDR: 1.000e+00
Coefficient: −9.26e−02
Value: B
Dethiobacter.alkaliphilus
1.00
0.75
0.50
0.25
0.00
A (n=15)
B (n=16)
C (n=14)
surface

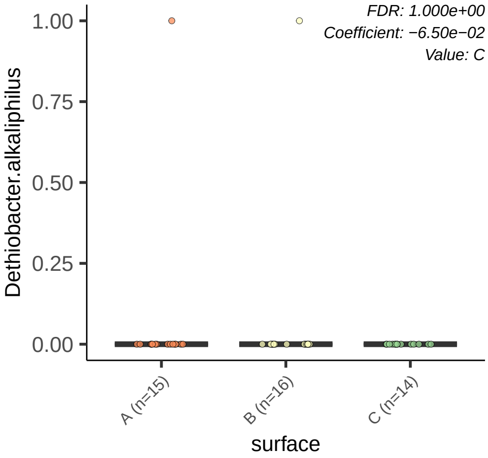

FDR: 1.000e+00
Coefficient: −6.50e−02
Value: C
Dethiobacter.alkaliphilus
1.00
0.75
0.50
0.25
0.00
A (n=15)
B (n=16)
C (n=14)
surface

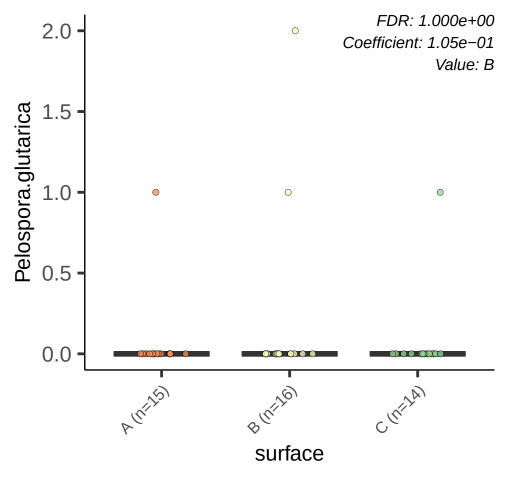
FDR: 1.000e+00
Coefficient: 1.05e−01
Value: B
Pelospora.glutarica
2.0
1.5
1.0
0.5
0.0
A (n=15)
B (n=16)
C (n=14)
surface

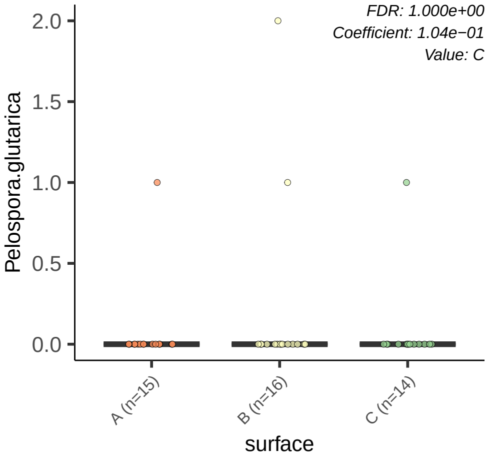
FDR: 1.000e+00
Coefficient: 1.04e−01
Value: C
Pelospora.glutarica
2.0
1.5
1.0
0.5
0.0
A (n=15)
B (n=16)
C (n=14)
surface

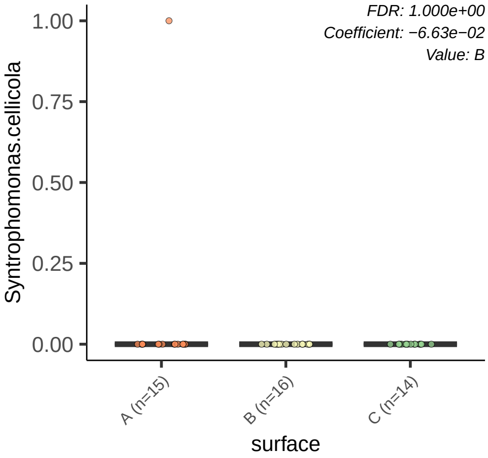
FDR: 1.000e+00
Coefficient: −6.63e−02
Value: B
Syntrophomonas.cellicola
1.00
0.75
0.50
0.25
0.00
A (n=15)
B (n=16)
C (n=14)
surface

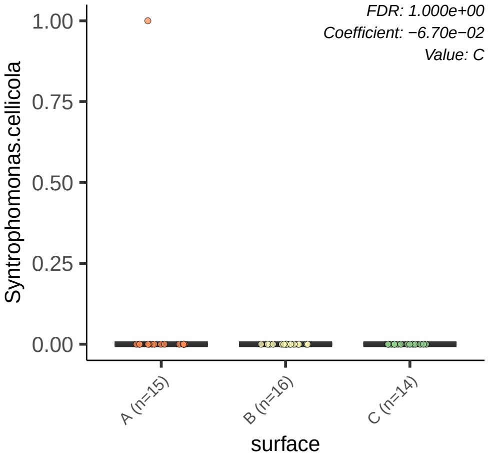
FDR: 1.000e+00
Coefficient: −6.70e−02
Value: C
Syntrophomonas.cellicola
1.00
0.75
0.50
0.25
0.00
A (n=15)
B (n=16)
C (n=14)
surface

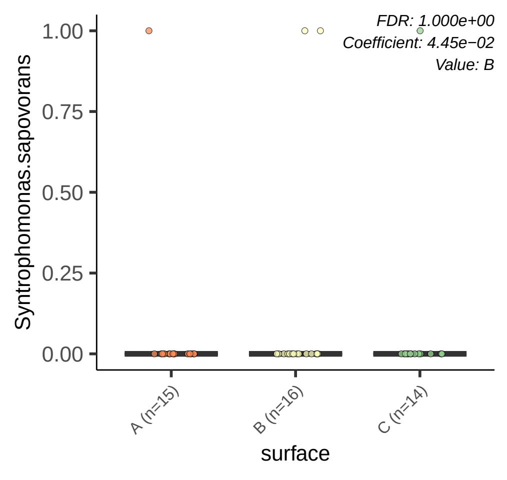

FDR: 1.000e+00
Coefficient: 4.45e−02
Value: B
Syntrophomonas.sapovorans
1.00
0.75
0.50
0.25
0.00
A (n=15)
B (n=16)
C (n=14)
surface

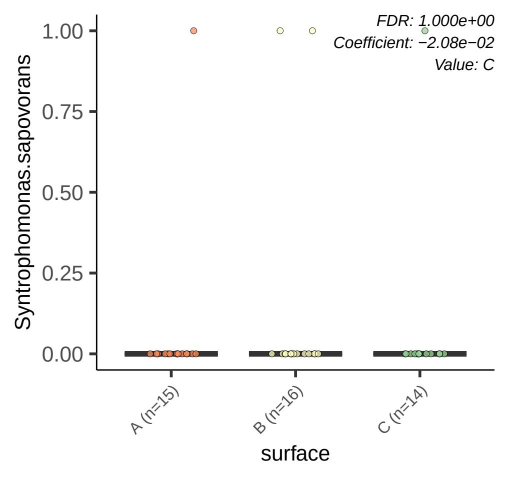

FDR: 1.000e+00
Coefficient: −2.08e−02
Value: C
Syntrophomonas.sapovorans
1.00
0.75
0.50
0.25
0.00
A (n=15)
B (n=16)
C (n=14)
surface

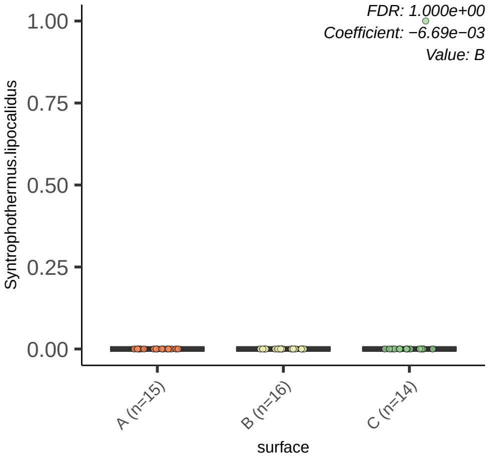

FDR: 1.000e+00
Coefficient: −6.69e−03
Value: B
Syntrophothermus.lipocalidus
1.00
0.75
0.50
0.25
0.00
A (n=15)
B (n=16)
C (n=14)
surface

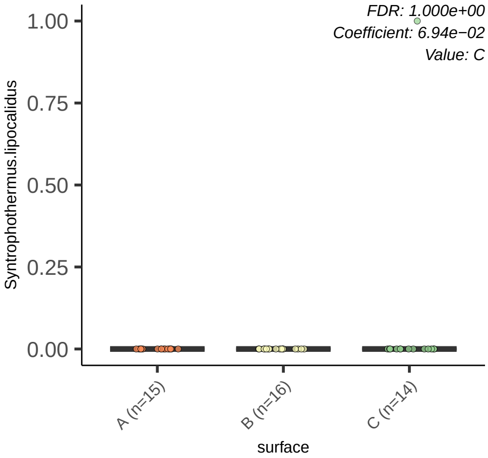
FDR: 1.000e+00
Coefficient: 6.94e−02
Value: C
Syntrophothermus.lipocalidus
1.00
0.75
0.50
0.25
0.00
A (n=15)
B (n=16)
C (n=14)
surface

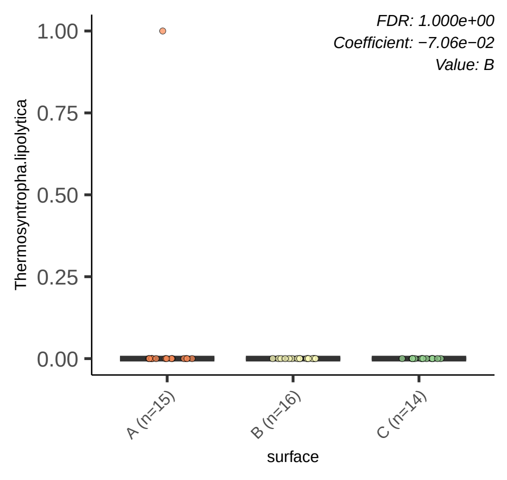
FDR: 1.000e+00
Coefficient: −7.06e−02
Value: B
Thermosyntropha.lipolytica
1.00
0.75
0.50
0.25
0.00
A (n=15)
B (n=16)
C (n=14)
surface

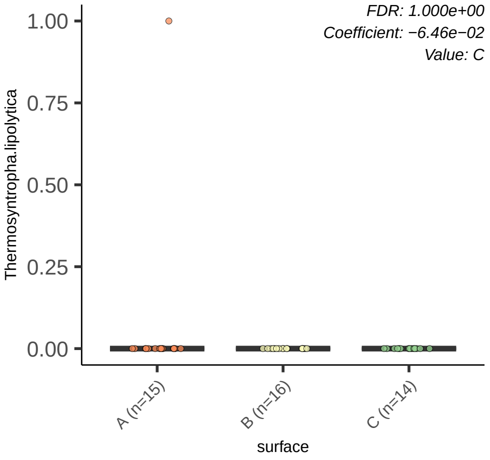
FDR: 1.000e+00
Coefficient: −6.46e−02
Value: C
Thermosyntropha.lipolytica
1.00
0.75
0.50
0.25
0.00
A (n=15)
B (n=16)
C (n=14)
surface

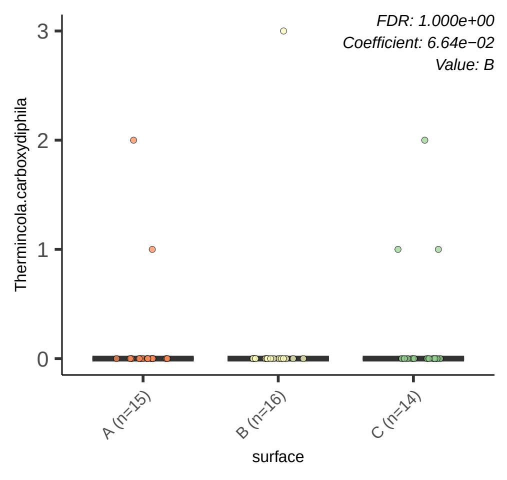
FDR: 1.000e+00
Coefficient: 6.64e−02
Value: B
Thermincola.carboxydiphila
3
2
1
0
A (n=15)
B (n=16)
C (n=14)
surface

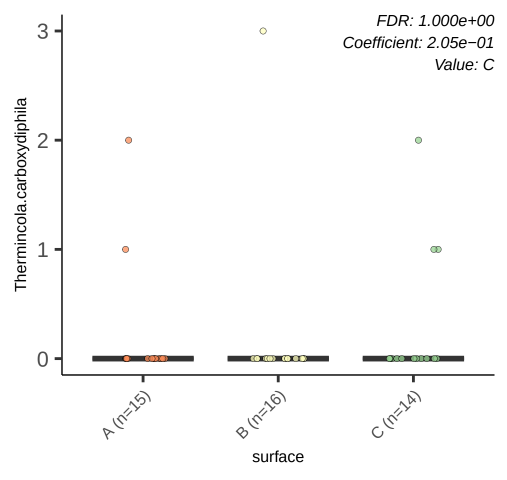
FDR: 1.000e+00
Coefficient: 2.05e−01
Value: C
Thermincola.carboxydiphila
3
2
1
0
A (n=15)
B (n=16)
C (n=14)
surface

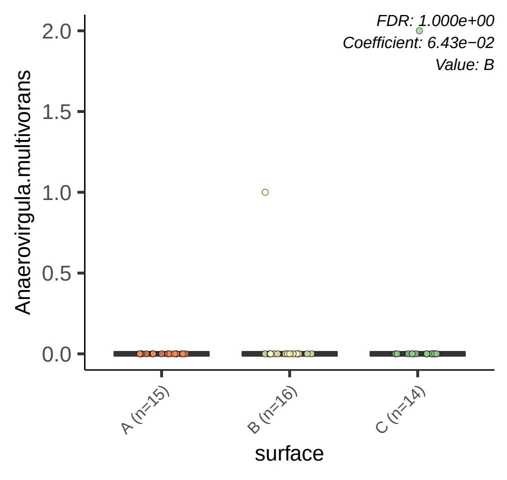
FDR: 1.000e+00
Coefficient: 6.43e−02
Value: B
Anaerovirgula.multivorans
2.0
1.5
1.0
0.5
0.0
A (n=15)
B (n=16)
C (n=14)
surface

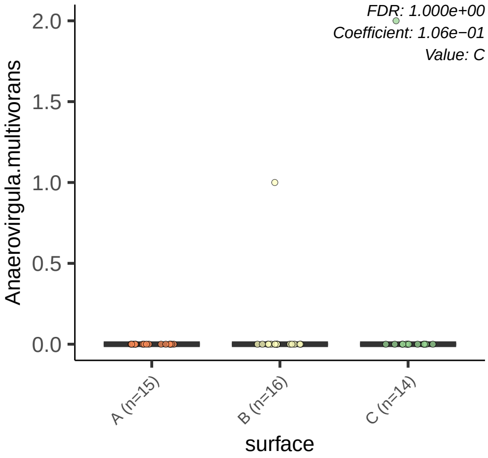
FDR: 1.000e+00
Coefficient: 1.06e−01
Value: C
Anaerovirgula.multivorans
2.0
1.5
1.0
0.5
0.0
A (n=15)
B (n=16)
C (n=14)
surface

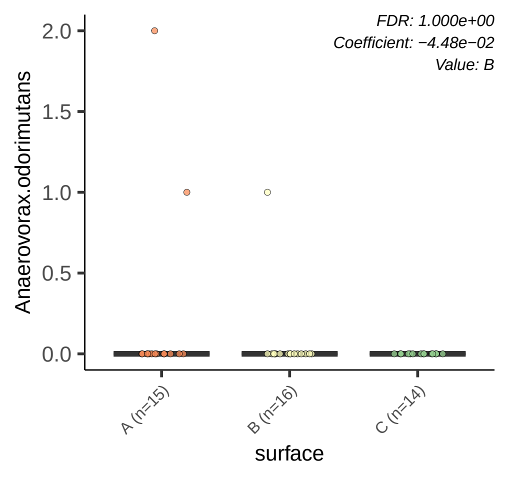

FDR: 1.000e+00
Coefficient: −4.48e−02
Value: B
Anaerovorax.odorimutans
2.0
1.5
1.0
0.5
0.0
A (n=15)
B (n=16)
C (n=14)
surface

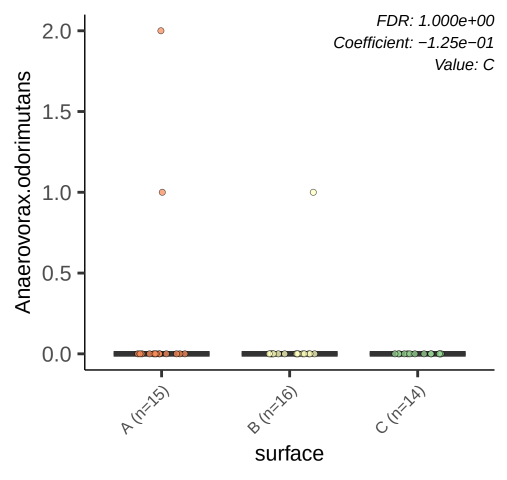
FDR: 1.000e+00
Coefficient: −1.25e−01
Value: C
Anaerovorax.odorimutans
2.0
1.5
1.0
0.5
0.0
A (n=15)
B (n=16)
C (n=14)
surface

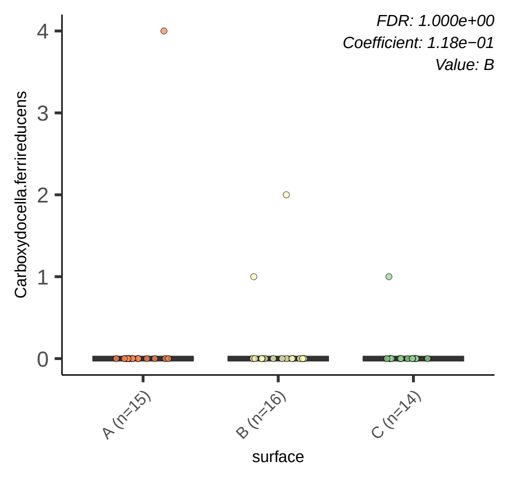
FDR: 1.000e+00
Coefficient: 1.18e−01
Value: B
Carboxydocella.ferrireducens
0
1
2
3
4
A (n=15)
B (n=16)
C (n=14)
surface

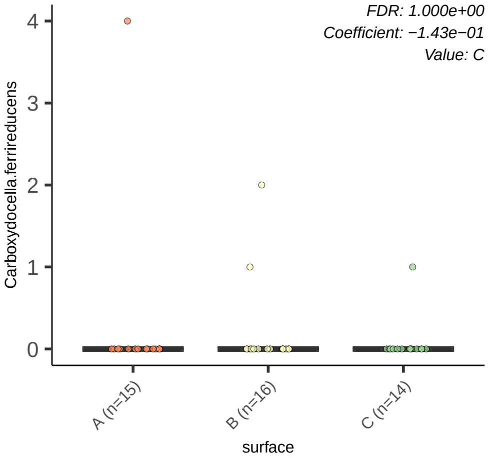
FDR: 1.000e+00
Coefficient: −1.43e−01
Value: C
Carboxydocella.ferrireducens
4
3
2
1
0
A (n=15)
B (n=16)
C (n=14)
surface

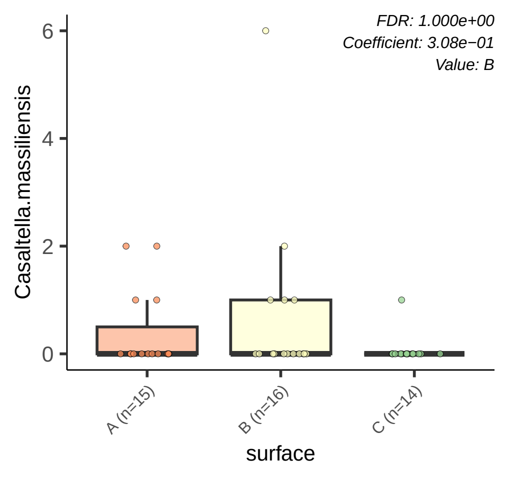

FDR: 1.000e+00
Coefficient: 3.08e−01
Value: B
Casaltella.massiliensis
6
4
2
0
A (n=15)
B (n=16)
C (n=14)
surface

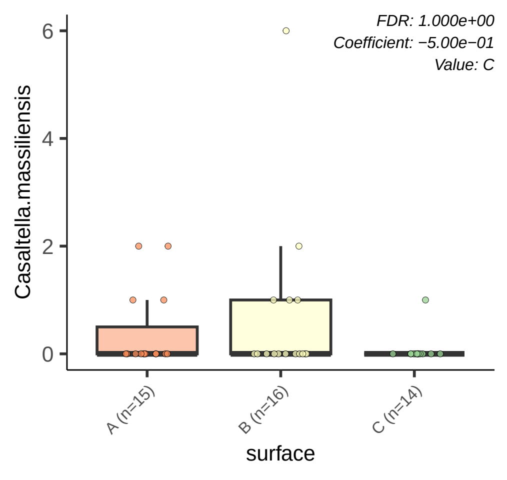

FDR: 1.000e+00
Coefficient: −5.00e−01
Value: C
Casaltella.massiliensis
0
2
4
6
A (n=15)
B (n=16)
C (n=14)
surface

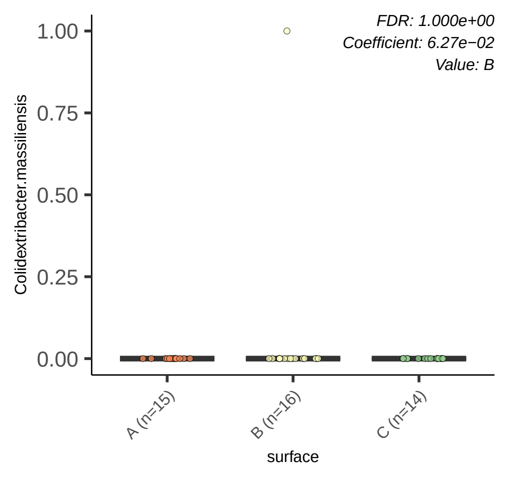

FDR: 1.000e+00
Coefficient: 6.27e−02
Value: B
1.00
0.75
0.50
0.25
0.00
Colidextribacter.massiliensis
A (n=15)
B (n=16)
C (n=14)
surface

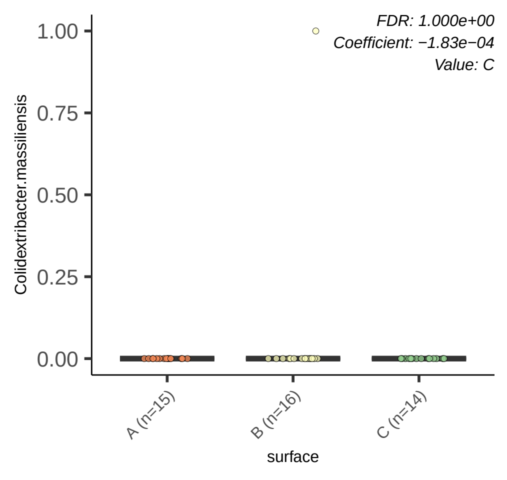
FDR: 1.000e+00
Coefficient: −1.83e−04
Value: C
1.00
0.75
0.50
0.25
0.00
Colidextribacter.massiliensis
A (n=15)
B (n=16)
C (n=14)
surface

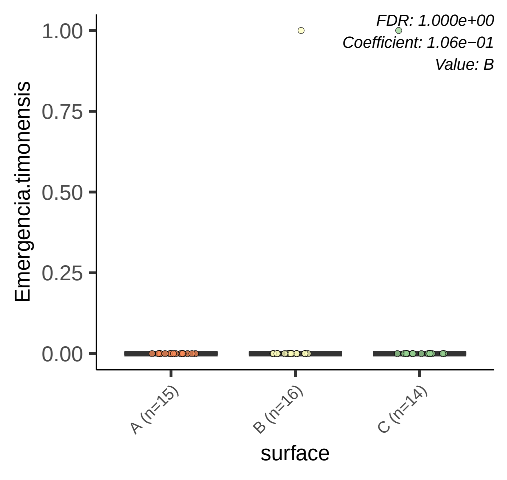
FDR: 1.000e+00
Coefficient: 1.06e−01
Value: B
Emergencia.timonensis
1.00
0.75
0.50
0.25
0.00
A (n=15)
B (n=16)
C (n=14)
surface

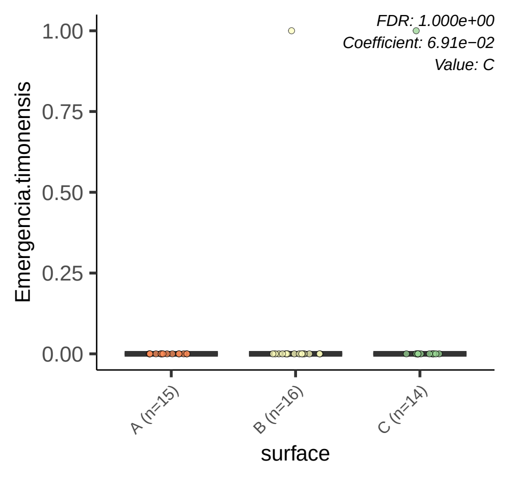
FDR: 1.000e+00
Coefficient: 6.91e−02
Value: C
Emergencia.timonensis
1.00
0.75
0.50
0.25
0.00
A (n=15)
B (n=16)
C (n=14)
surface

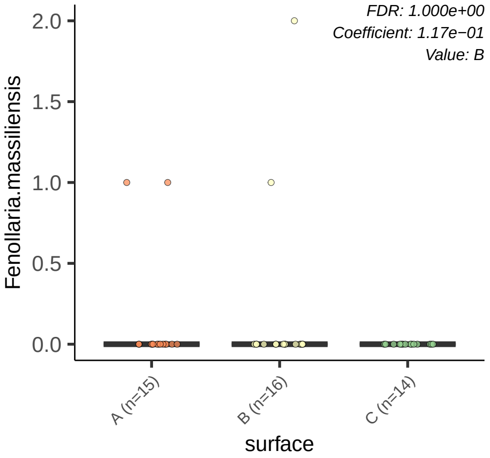

FDR: 1.000e+00
Coefficient: 1.17e−01
Value: B
Fenollaria.massiliensis
2.0
1.5
1.0
0.5
0.0
A (n=15)
B (n=16)
C (n=14)
surface

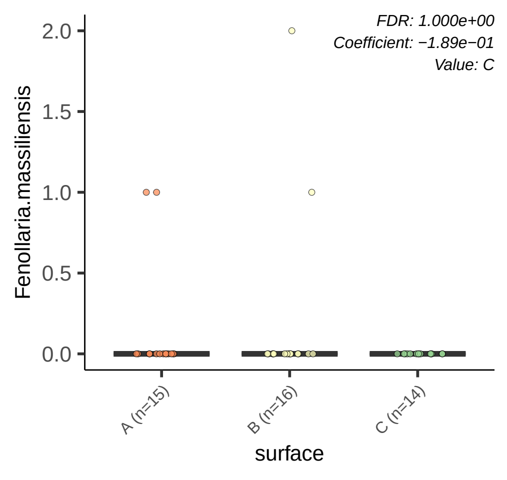

FDR: 1.000e+00
Coefficient: −1.89e−01
Value: C
Fenollaria.massiliensis
2.0
1.5
1.0
0.5
0.0
A (n=15)
B (n=16)
C (n=14)
surface

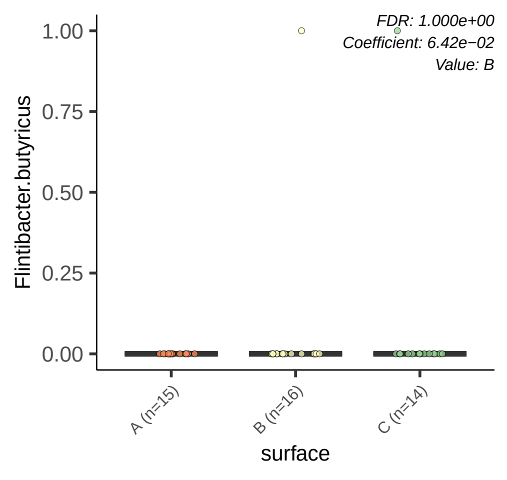
FDR: 1.000e+00
Coefficient: 6.42e−02
Value: B
Flintibacter.butyricus
1.00
0.75
0.50
0.25
0.00
A (n=15)
B (n=16)
C (n=14)
surface

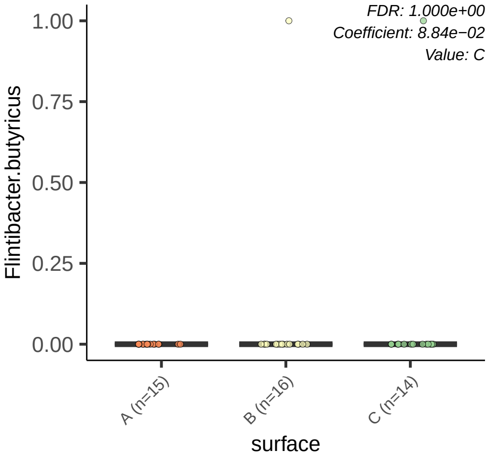

FDR: 1.000e+00
Coefficient: 8.84e−02
Value: C
1.00
0.75
0.50
0.25
0.00
Flintibacter.butyricus
A (n=15)
B (n=16)
C (n=14)
surface

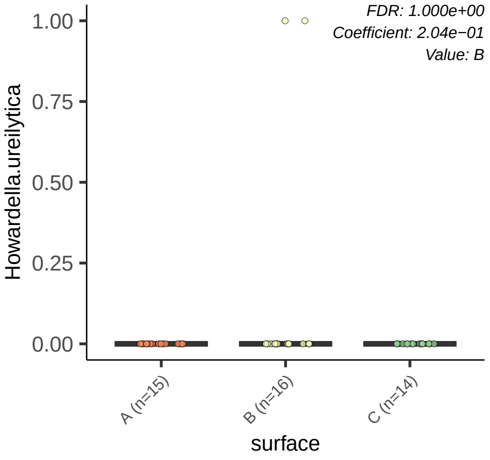
FDR: 1.000e+00
Coefficient: 2.04e−01
Value: B
Howardella.ureilytica
1.00
0.75
0.50
0.25
0.00
A (n=15)
B (n=16)
C (n=14)
surface

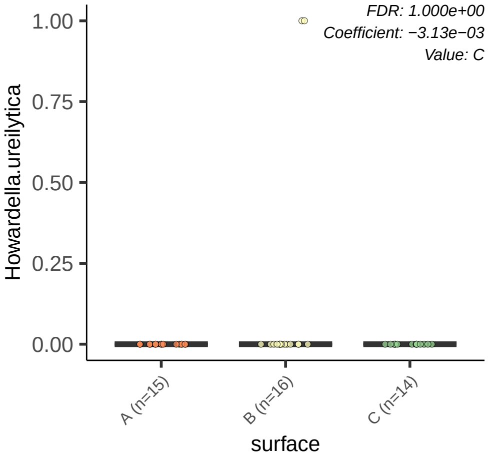
FDR: 1.000e+00
Coefficient: −3.13e−03
Value: C
Howardella.ureilytica
1.00
0.75
0.50
0.25
0.00
A (n=15)
B (n=16)
C (n=14)
surface

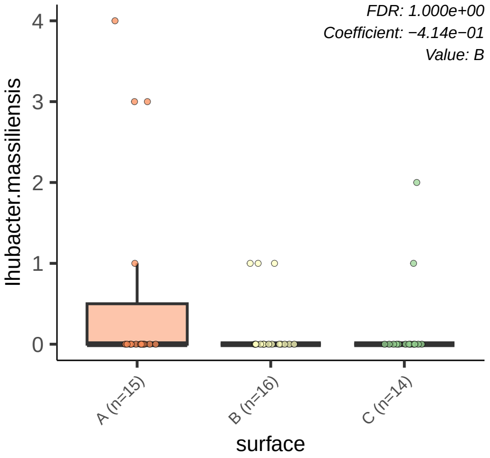

FDR: 1.000e+00
Coefficient: −4.14e−01
Value: B
Ihubacter.massiliensis
0
1
2
3
4
A (n=15)
B (n=16)
C (n=14)
surface

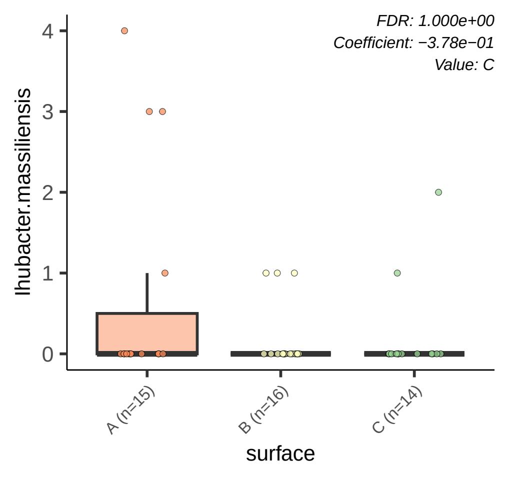
FDR: 1.000e+00
Coefficient: −3.78e−01
Value: C
Ihubacter.massiliensis
4
3
2
1
0
A (n=15)
B (n=16)
C (n=14)
surface

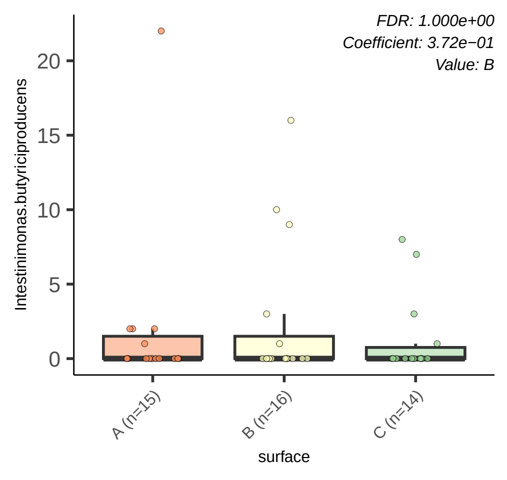
FDR: 1.000e+00
Coefficient: 3.72e−01
Value: B
Intestinimonas.butyriciproducens
0
5
10
15
20
A (n=15)
B (n=16)
C (n=14)
surface

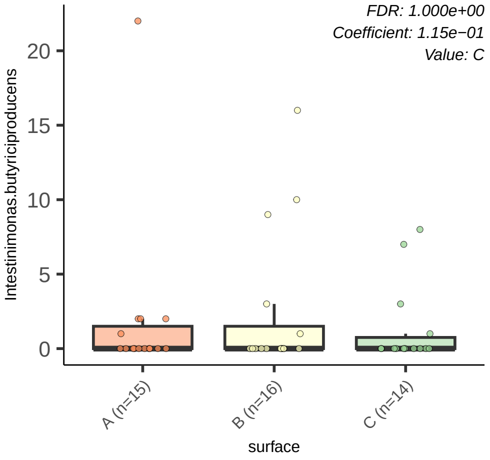
FDR: 1.000e+00
Coefficient: 1.15e−01
Value: C
Intestinimonas.butyriciproducens
0
5
10
15
20
A (n=15)
B (n=16)
C (n=14)
surface

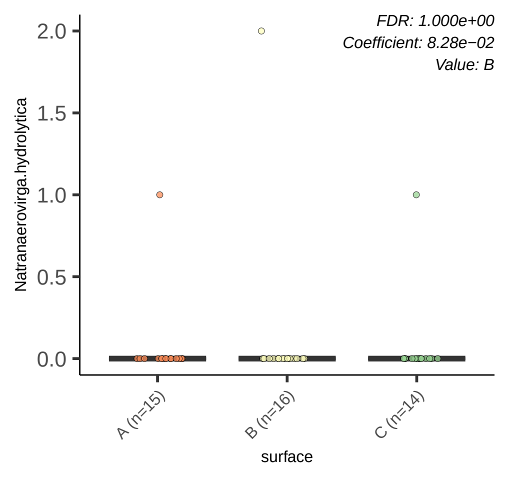

FDR: 1.000e+00
Coefficient: 8.28e−02
Value: B
Natranaerovirga.hydrolytica
2.0
1.5
1.0
0.5
0.0
A (n=15)
B (n=16)
C (n=14)
surface

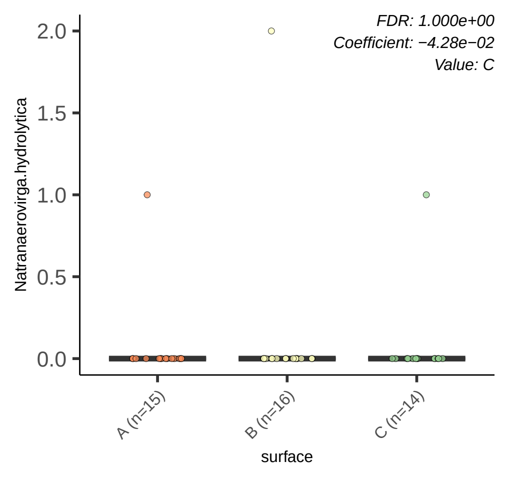

FDR: 1.000e+00
Coefficient: −4.28e−02
Value: C
Natranaerovirga.hydrolytica
2.0
1.5
1.0
0.5
0.0
A (n=15)
B (n=16)
C (n=14)
surface

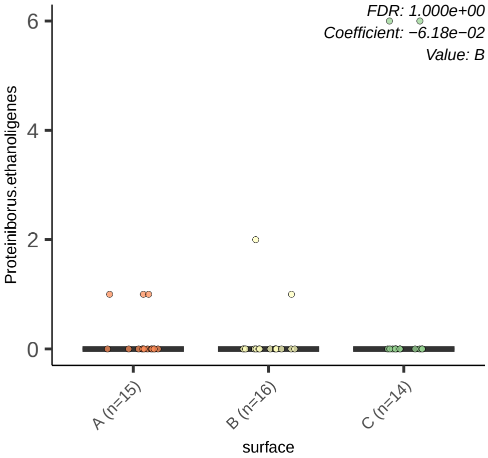
FDR: 1.000e+00
Coefficient: −6.18e−02
Value: B
Proteiniborus.ethanoligenes
0
2
4
6
A (n=15)
B (n=16)
C (n=14)
surface

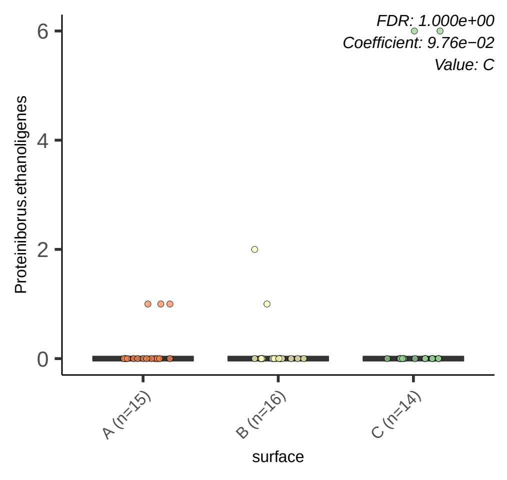
FDR: 1.000e+00
Coefficient: 9.76e−02
Value: C
Proteiniborus.ethanoligenes
0
2
4
6
A (n=15)
B (n=16)
C (n=14)
surface

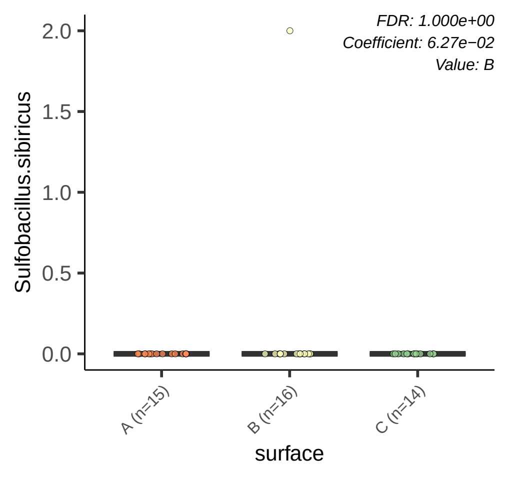
FDR: 1.000e+00
Coefficient: 6.27e−02
Value: B
Sulfobacillus.sibiricus
2.0
1.5
1.0
0.5
0.0
A (n=15)
B (n=16)
C (n=14)
surface

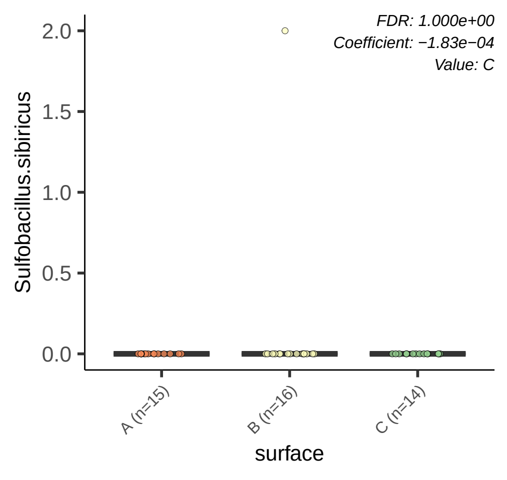
FDR: 1.000e+00
Coefficient: −1.83e−04
Value: C
Sulfobacillus.sibiricus
2.0
1.5
1.0
0.5
0.0
A (n=15)
B (n=16)
C (n=14)
surface

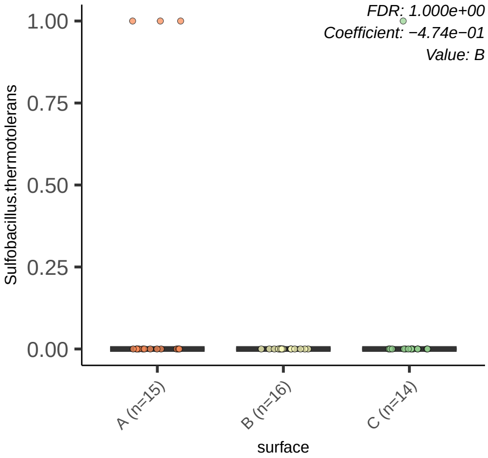
FDR: 1.000e+00
Coefficient: −4.74e−01
Value: B
1.00
0.75
0.50
0.25
0.00
Sulfobacillus.thermotolerans
A (n=15)
B (n=16)
C (n=14)
surface

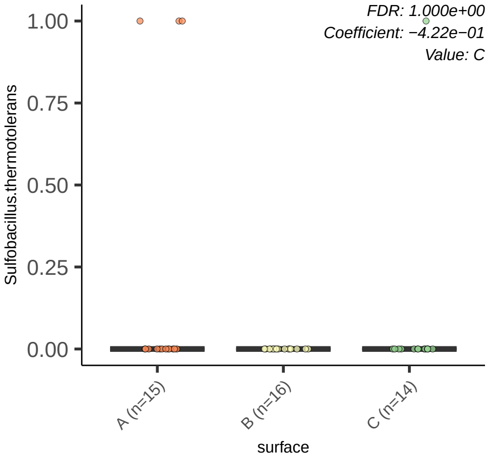

FDR: 1.000e+00
Coefficient: −4.22e−01
Value: C
Sulfobacillus.thermotolerans
1.00
0.75
0.50
0.25
0.00
A (n=15)
B (n=16)
C (n=14)
surface

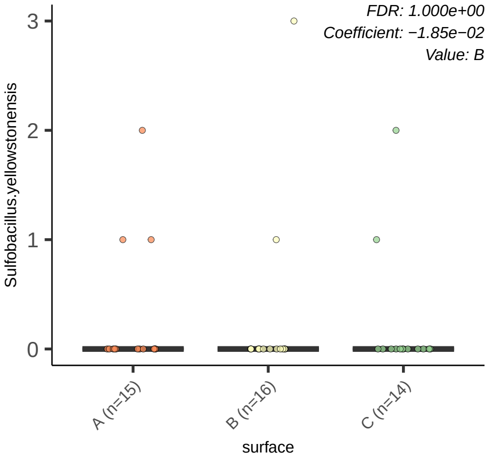
FDR: 1.000e+00
Coefficient: −1.85e−02
Value: B
Sulfobacillus.yellowstonensis
3
2
1
0
A (n=15)
B (n=16)
C (n=14)
surface

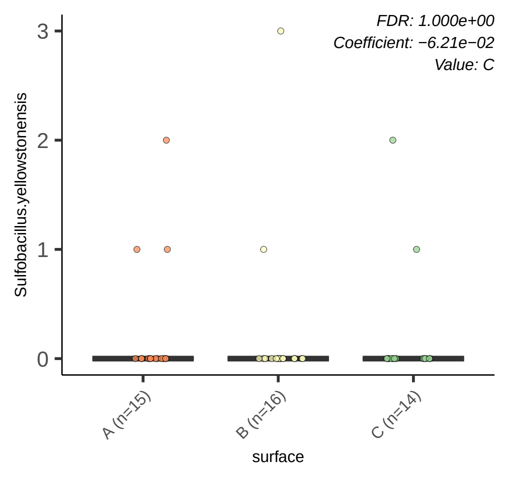
FDR: 1.000e+00
Coefficient: −6.21e−02
Value: C
Sulfobacillus.yellowstonensis
3
2
1
0
A (n=15)
B (n=16)
C (n=14)
surface

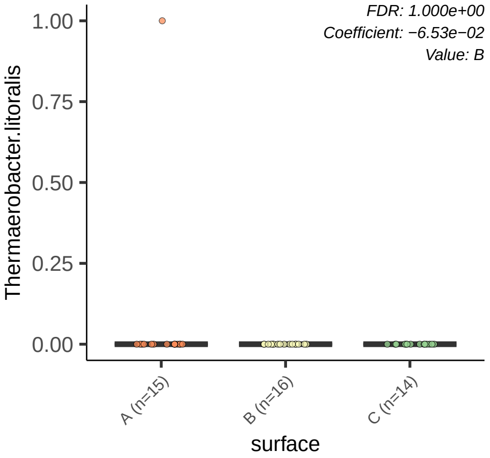
FDR: 1.000e+00
Coefficient: −6.53e−02
Value: B
Thermaerobacter.litoralis
1.00
0.75
0.50
0.25
0.00
A (n=15)
B (n=16)
C (n=14)
surface

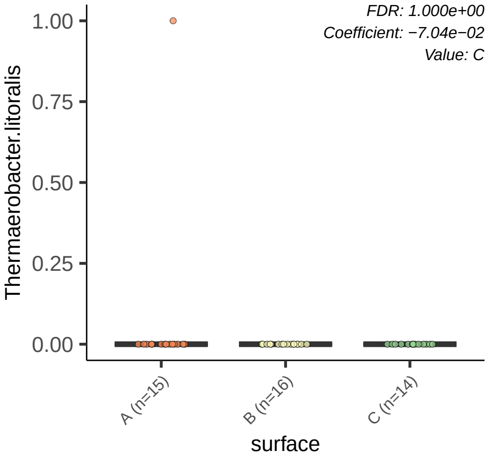
FDR: 1.000e+00
Coefficient: −7.04e−02
Value: C
Thermaerobacter.litoralis
1.00
0.75
0.50
0.25
0.00
A (n=15)
B (n=16)
C (n=14)
surface

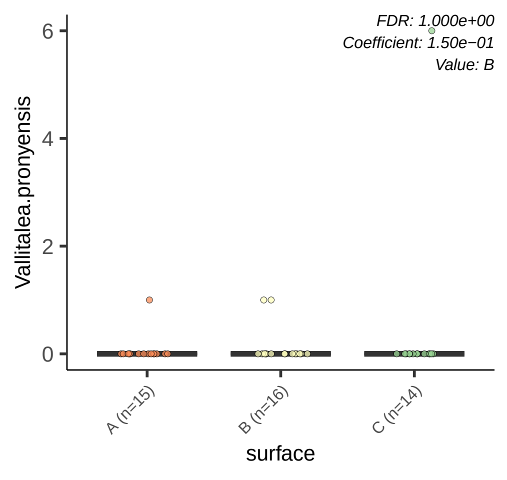
FDR: 1.000e+00
Coefficient: 1.50e−01
Value: B
Vallitalea.pronyensis
6
4
2
0
A (n=15)
B (n=16)
C (n=14)
surface

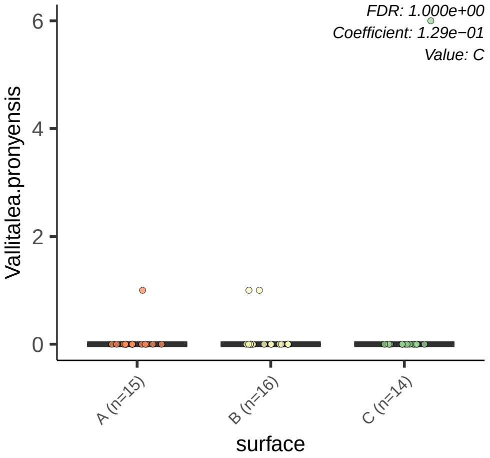
FDR: 1.000e+00
Coefficient: 1.29e−01
Value: C
Vallitalea.pronyensis
0
2
4
6
A (n=15)
B (n=16)
C (n=14)
surface

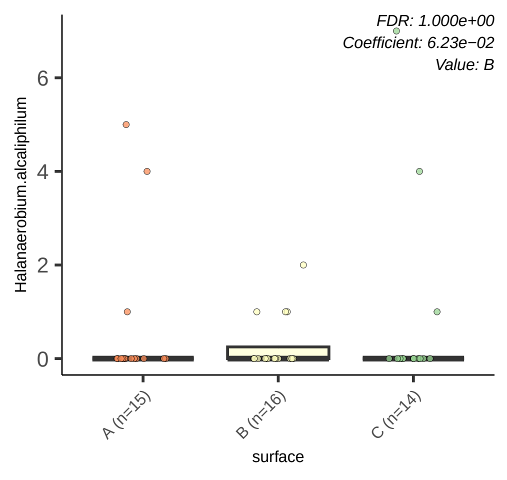
FDR: 1.000e+00
Coefficient: 6.23e−02
Value: B
Halanaerobium.alcaliphilum
0
2
4
6
A (n=15)
B (n=16)
C (n=14)
surface

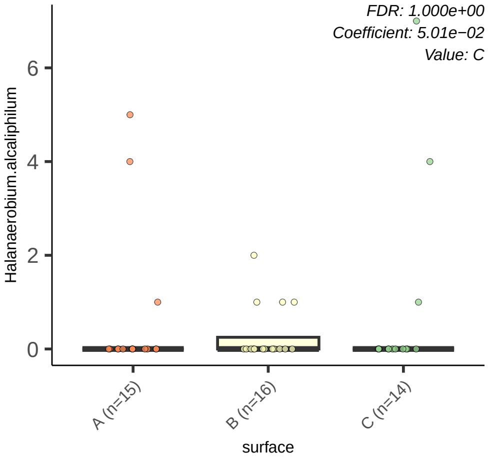

FDR: 1.000e+00
Coefficient: 5.01e−02
Value: C
Halanaerobium.alcaliphilum
0
2
4
6
A (n=15)
B (n=16)
C (n=14)
surface

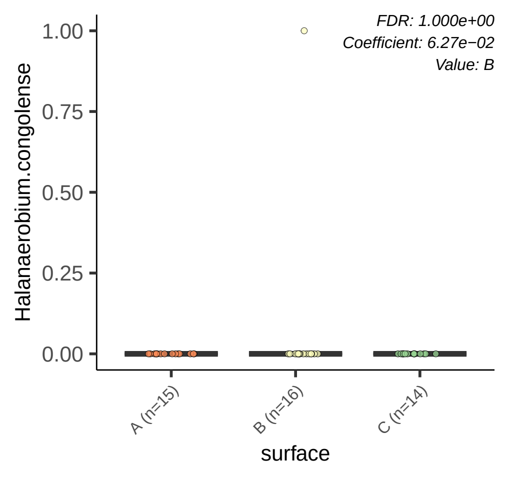

FDR: 1.000e+00
Coefficient: 6.27e−02
Value: B
Halanaerobium.congolense
1.00
0.75
0.50
0.25
0.00
A (n=15)
B (n=16)
C (n=14)
surface

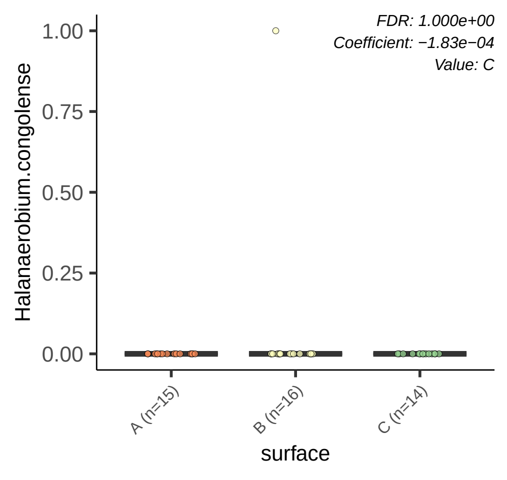

FDR: 1.000e+00
Coefficient: −1.83e−04
Value: C
Halanaerobium.congolense
1.00
0.75
0.50
0.25
0.00
A (n=15)
B (n=16)
C (n=14)
surface

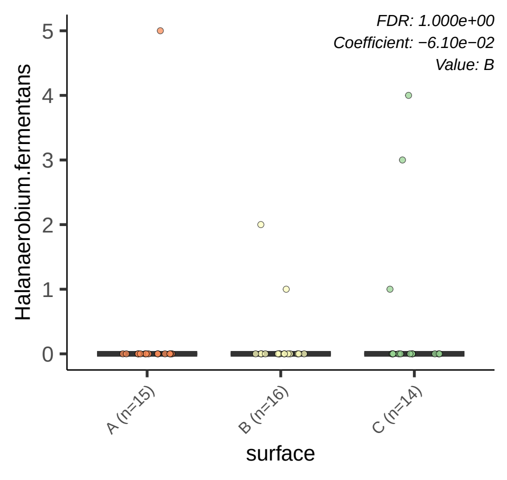
FDR: 1.000e+00
Coefficient: −6.10e−02
Value: B
Halanaerobium.fermentans
0
1
2
3
4
5
A (n=15)
B (n=16)
C (n=14)
surface

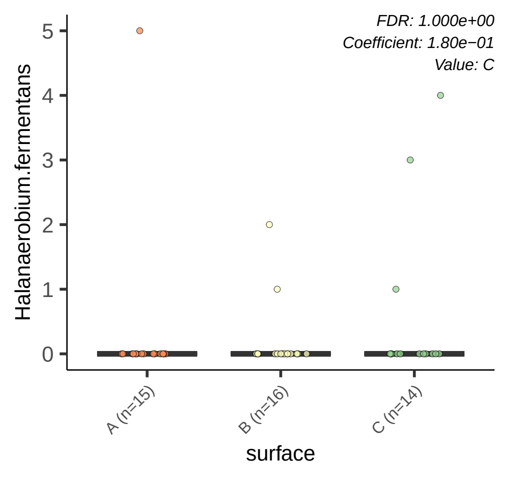

FDR: 1.000e+00
Coefficient: 1.80e−01
Value: C
Halanaerobium.fermentans
0
1
2
3
4
5
A (n=15)
B (n=16)
C (n=14)
surface

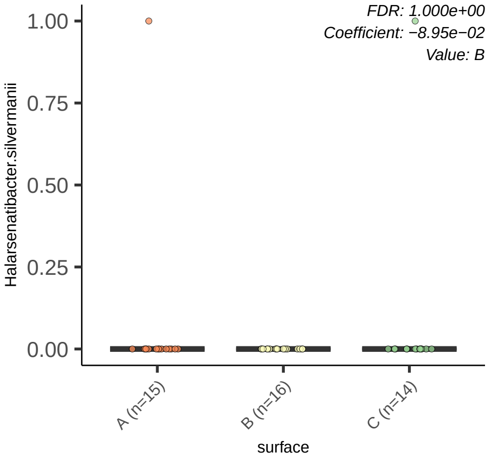

FDR: 1.000e+00
Coefficient: −8.95e−02
Value: B
Halarsenatibacter.silvermanii
1.00
0.75
0.50
0.25
0.00
A (n=15)
B (n=16)
C (n=14)
surface

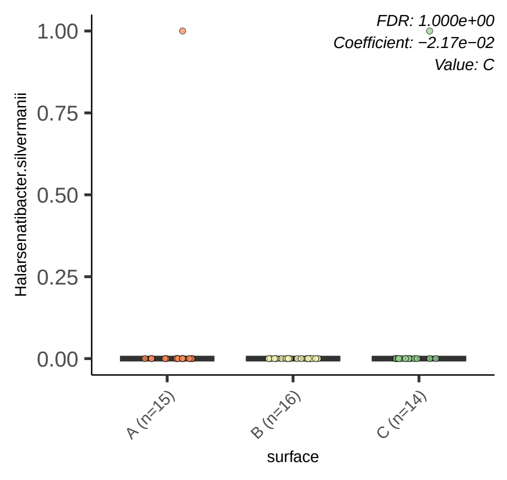
FDR: 1.000e+00
Coefficient: −2.17e−02
Value: C
Halarsenatibacter.silvermanii
1.00
0.75
0.50
0.25
0.00
A (n=15)
B (n=16)
C (n=14)
surface

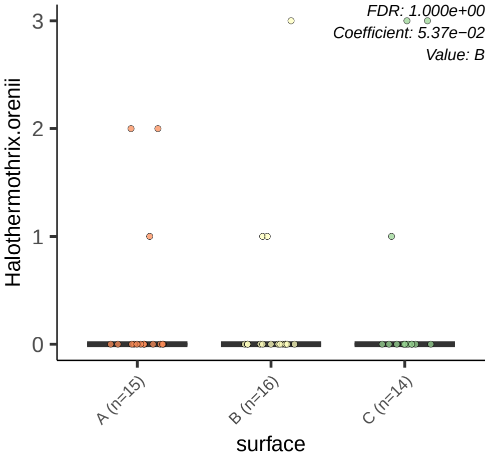

FDR: 1.000e+00
Coefficient: 5.37e−02
Value: B
Halothermothrix.orenii
3
2
1
0
A (n=15)
B (n=16)
C (n=14)
surface

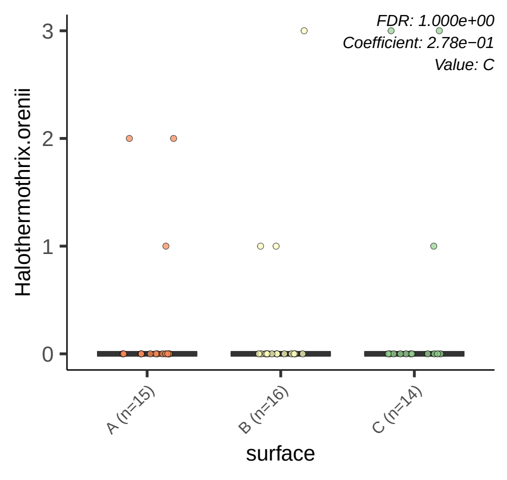
FDR: 1.000e+00
Coefficient: 2.78e−01
Value: C
Halothermothrix.orenii
3
2
1
0
A (n=15)
B (n=16)
C (n=14)
surface

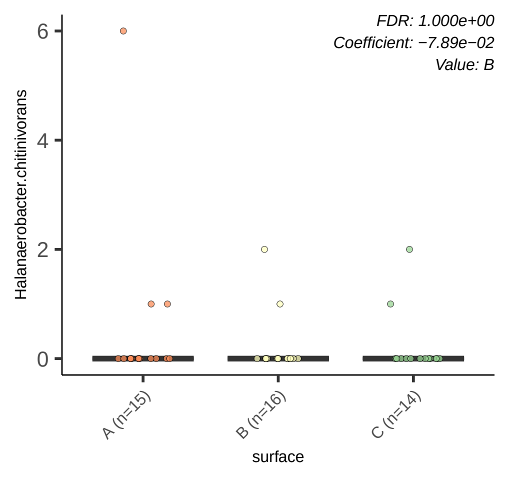

FDR: 1.000e+00
Coefficient: −7.89e−02
Value: B
Halanaerobacter.chitinivorans
0
2
4
6
A (n=15)
B (n=16)
C (n=14)
surface

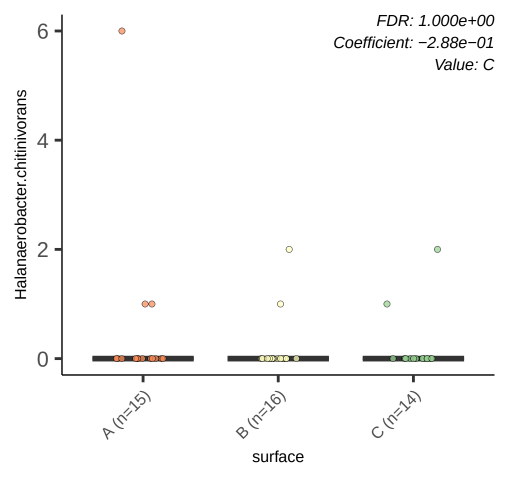
FDR: 1.000e+00
Coefficient: −2.88e−01
Value: C
Halanaerobacter.chitinivorans
0
2
4
6
A (n=15)
B (n=16)
C (n=14)
surface

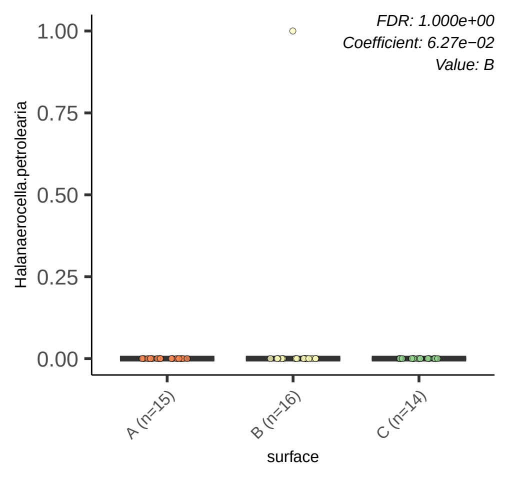
FDR: 1.000e+00
Coefficient: 6.27e−02
Value: B
Halanaerocella.petrolearia
1.00
0.75
0.50
0.25
0.00
A (n=15)
B (n=16)
C (n=14)
surface

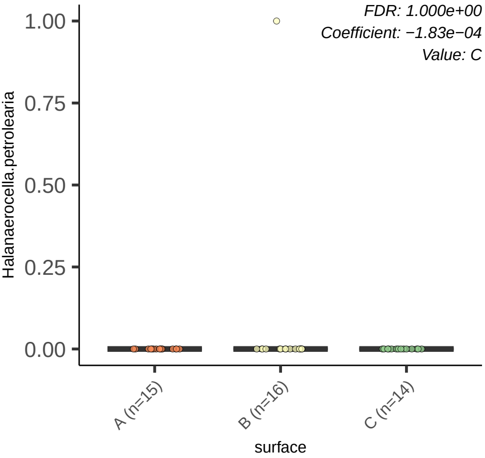

FDR: 1.000e+00
Coefficient: −1.83e−04
Value: C
1.00
0.75
0.50
0.25
0.00
Halanaerocella.petrolearia
A (n=15)
B (n=16)
C (n=14)
surface

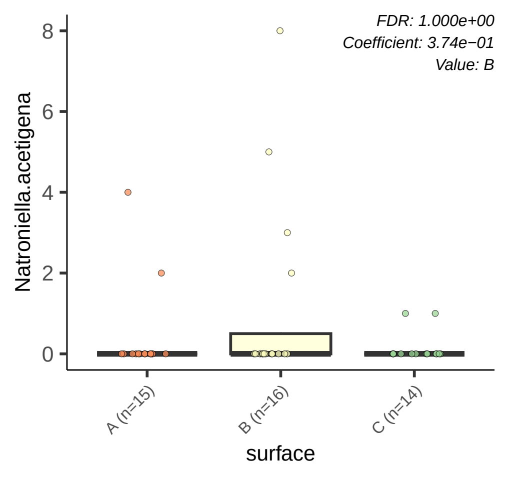
FDR: 1.000e+00
Coefficient: 3.74e−01
Value: B
Natroniella.acetigena
0
2
4
6
8
A (n=15)
B (n=16)
C (n=14)
surface

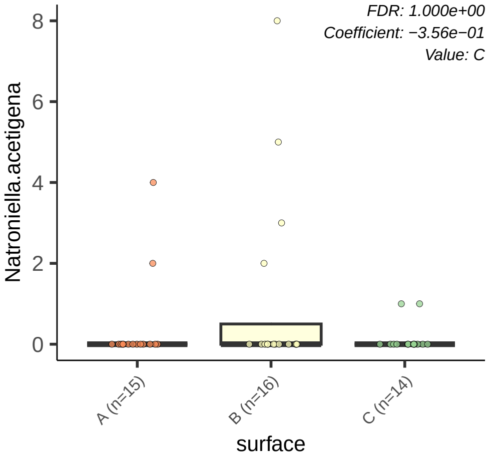
FDR: 1.000e+00
Coefficient: −3.56e−01
Value: C
Natroniella.acetigena
8
6
4
2
0
A (n=15)
B (n=16)
C (n=14)
surface

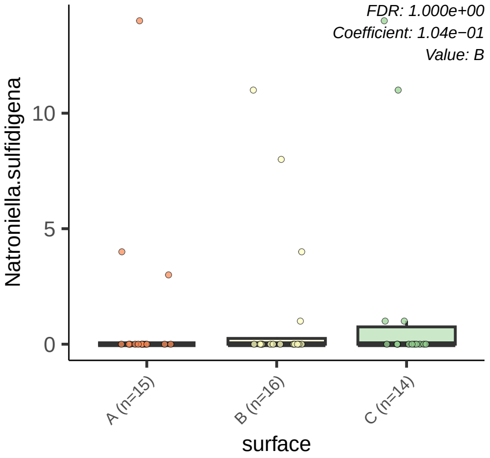
FDR: 1.000e+00
Coefficient: 1.04e−01
Value: B
Natroniella.sulfidigena
10
5
0
A (n=15)
B (n=16)
C (n=14)
surface

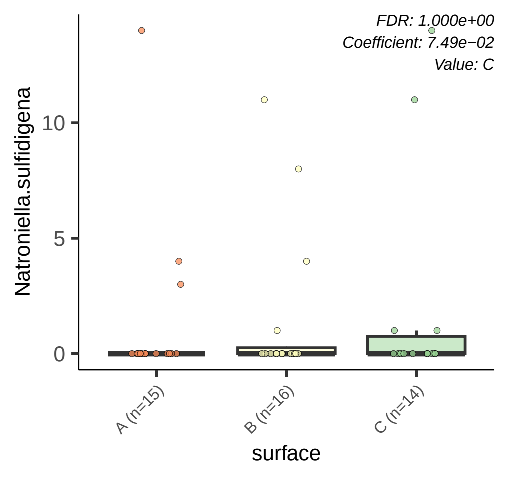
FDR: 1.000e+00
Coefficient: 7.49e−02
Value: C
Natroniella.sulfidigena
10
5
0
A (n=15)
B (n=16)
C (n=14)
surface

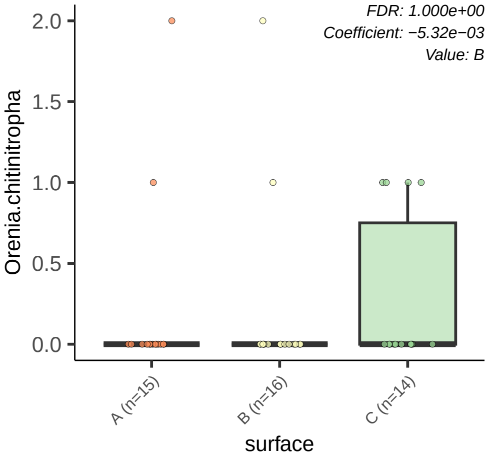
FDR: 1.000e+00
Coefficient: −5.32e−03
Value: B
Orenia.chitinitropha
2.0
1.5
1.0
0.5
0.0
A (n=15)
B (n=16)
C (n=14)
surface

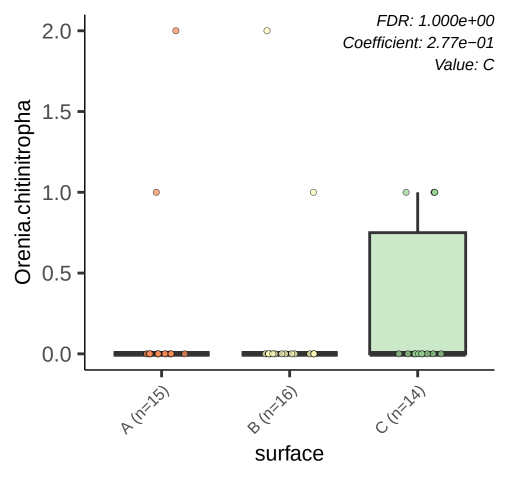

FDR: 1.000e+00
Coefficient: 2.77e−01
Value: C
Orenia.chitinitropha
2.0
1.5
1.0
0.5
0.0
A (n=15)
B (n=16)
C (n=14)
surface

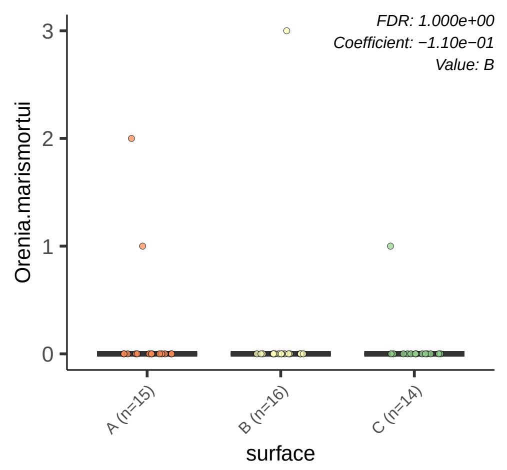

FDR: 1.000e+00
Coefficient: −1.10e−01
Value: B
Orenia.marismortui
3
2
1
0
A (n=15)
B (n=16)
C (n=14)
surface

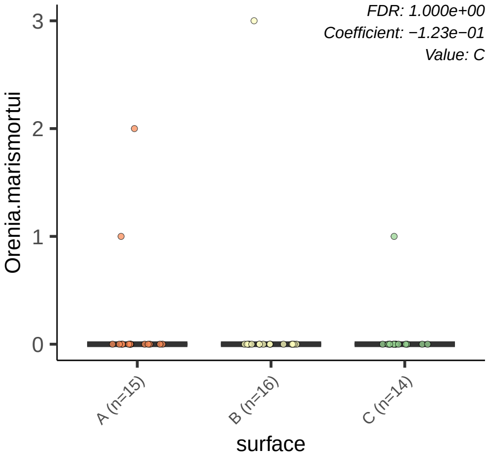
FDR: 1.000e+00
Coefficient: −1.23e−01
Value: C
Orenia.marismortui
0
1
2
3
A (n=15)
B (n=16)
C (n=14)
surface

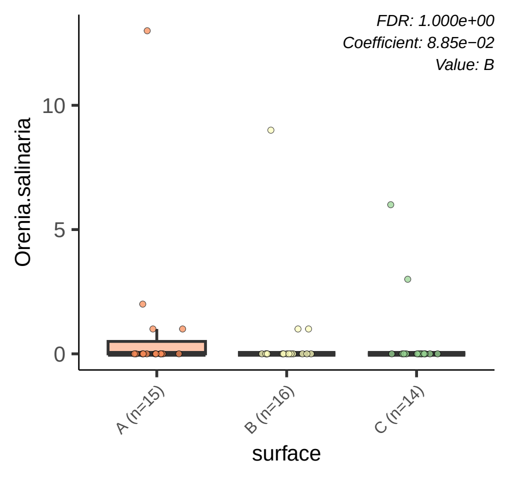

FDR: 1.000e+00
Coefficient: 8.85e−02
Value: B
Orenia.salinaria
10
5
0
A (n=15)
B (n=16)
C (n=14)
surface

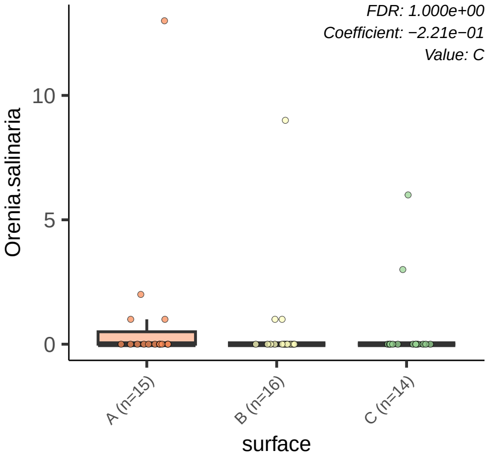
FDR: 1.000e+00
Coefficient: −2.21e−01
Value: C
Orenia.salinaria
0
5
10
A (n=15)
B (n=16)
C (n=14)
surface

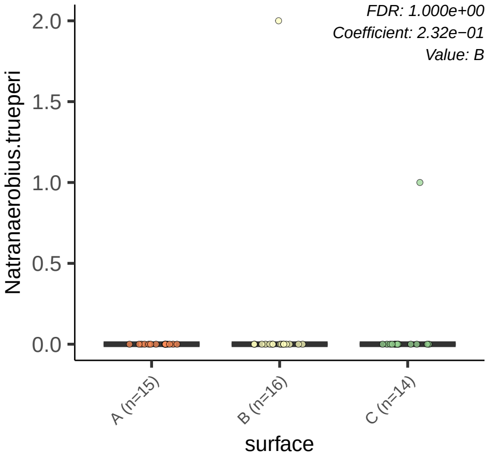
FDR: 1.000e+00
Coefficient: 2.32e−01
Value: B
Natranaerobius.trueperi
2.0
1.5
1.0
0.5
0.0
A (n=15)
B (n=16)
C (n=14)
surface

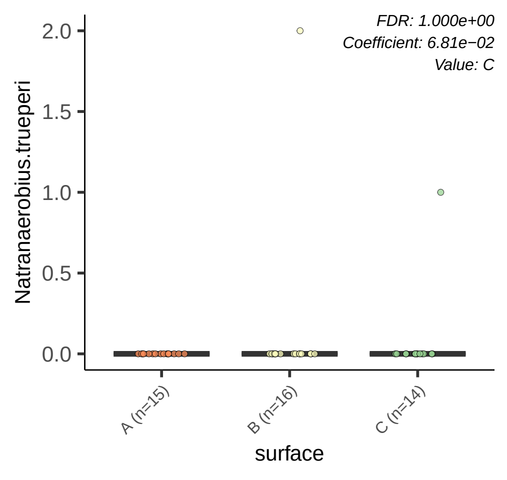
FDR: 1.000e+00
Coefficient: 6.81e−02
Value: C
Natranaerobius.trueperi
2.0
1.5
1.0
0.5
0.0
A (n=15)
B (n=16)
C (n=14)
surface

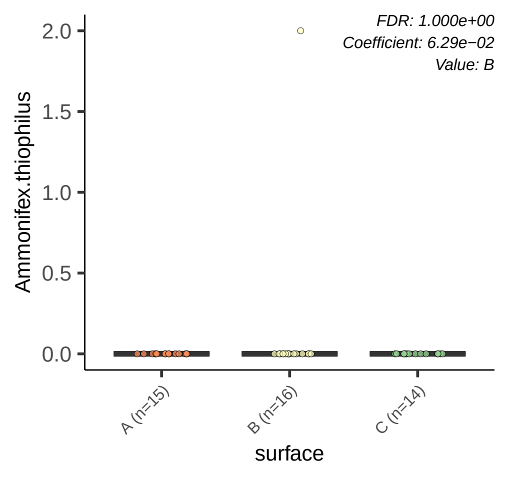
FDR: 1.000e+00
Coefficient: 6.29e−02
Value: B
Ammonifex.thiophilus
2.0
1.5
1.0
0.5
0.0
A (n=15)
B (n=16)
C (n=14)
surface

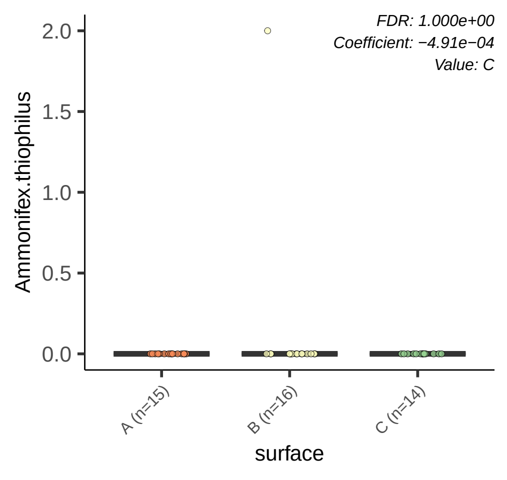

FDR: 1.000e+00
Coefficient: −4.91e−04
Value: C
Ammonifex.thiophilus
2.0
1.5
1.0
0.5
0.0
A (n=15)
B (n=16)
C (n=14)
surface

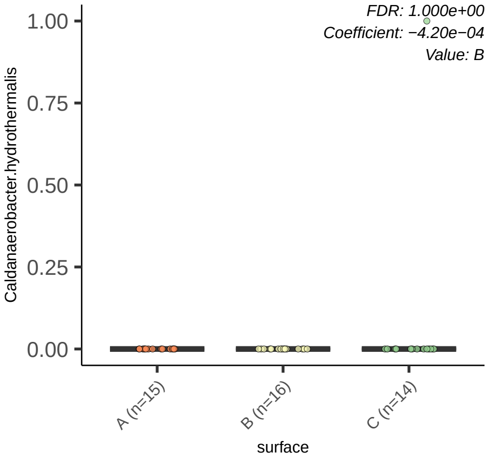
FDR: 1.000e+00
Coefficient: −4.20e−04
Value: B
Caldanaerobacter.hydrothermalis
1.00
0.75
0.50
0.25
0.00
A (n=15)
B (n=16)
C (n=14)
surface

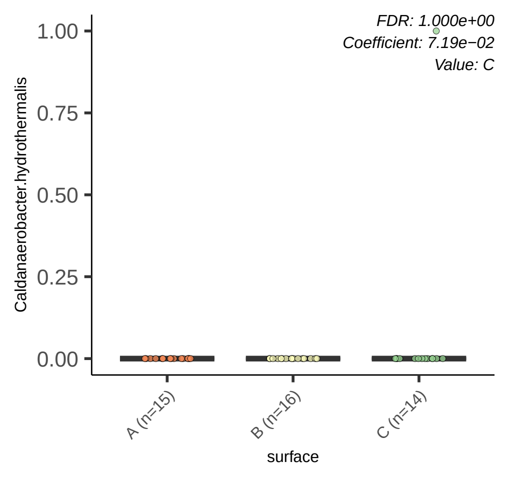

FDR: 1.000e+00
Coefficient: 7.19e−02
Value: C
1.00
0.75
0.50
0.25
0.00
Caldanaerobacter.hydrothermalis
A (n=15)
B (n=16)
C (n=14)
surface

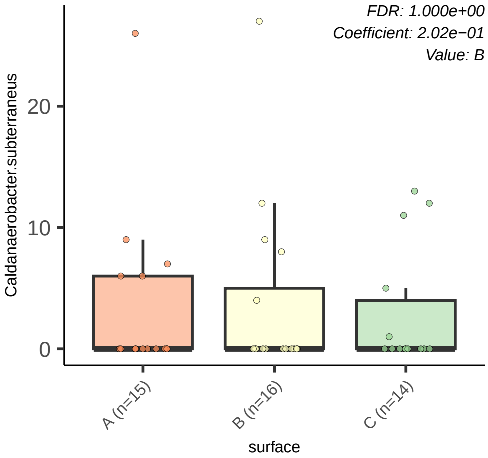

FDR: 1.000e+00
Coefficient: 2.02e−01
Value: B
Caldanaerobacter.subterraneus
20
10
0
A (n=15)
B (n=16)
C (n=14)
surface

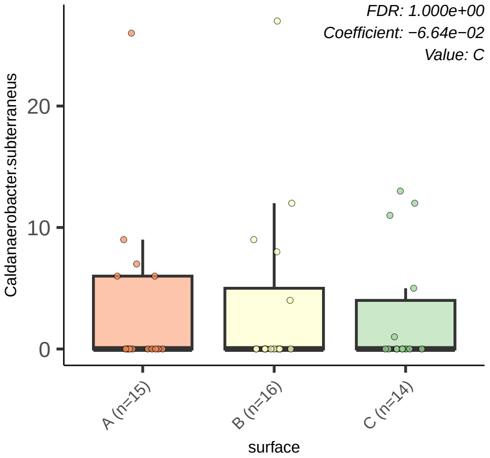

FDR: 1.000e+00
Coefficient: −6.64e−02
Value: C
Caldanaerobacter.subterraneus
20
10
0
A (n=15)
B (n=16)
C (n=14)
surface

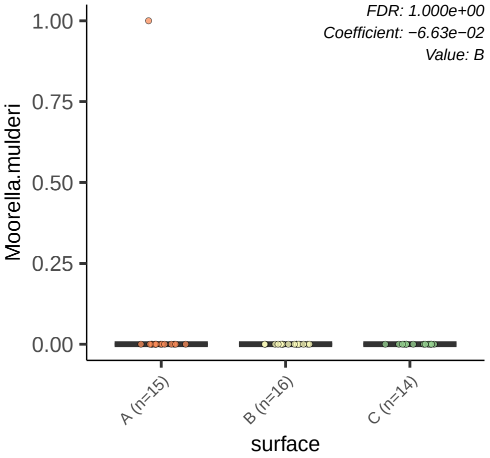
FDR: 1.000e+00
Coefficient: −6.63e−02
Value: B
Moorella.mulderi
1.00
0.75
0.50
0.25
0.00
A (n=15)
B (n=16)
C (n=14)
surface

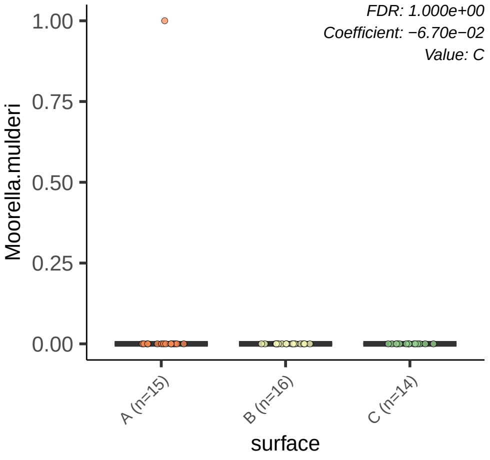
FDR: 1.000e+00
Coefficient: −6.70e−02
Value: C
Moorella.mulderi
1.00
0.75
0.50
0.25
0.00
A (n=15)
B (n=16)
C (n=14)
surface

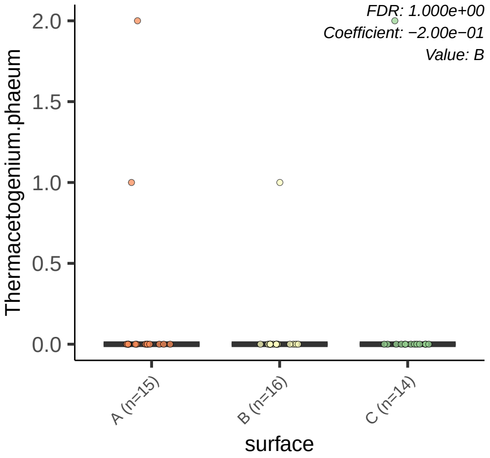
FDR: 1.000e+00
Coefficient: −2.00e−01
Value: B
Thermacetogenium.phaeum
2.0
1.5
1.0
0.5
0.0
A (n=15)
B (n=16)
C (n=14)
surface

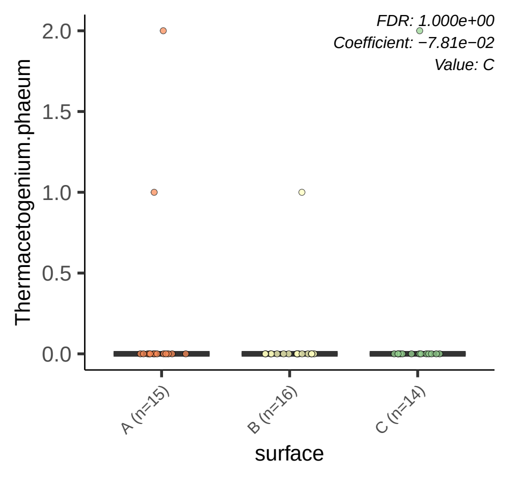

FDR: 1.000e+00
Coefficient: −7.81e−02
Value: C
Thermacetogenium.phaeum
2.0
1.5
1.0
0.5
0.0
A (n=15)
B (n=16)
C (n=14)
surface

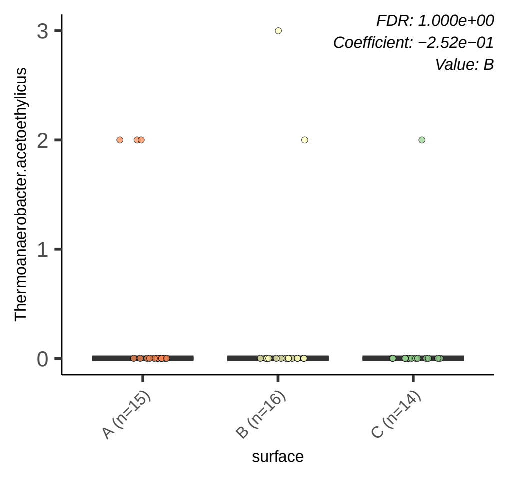
FDR: 1.000e+00
Coefficient: −2.52e−01
Value: B
Thermoanaerobacter.acetoethylicus
3
2
1
0
A (n=15)
B (n=16)
C (n=14)
surface

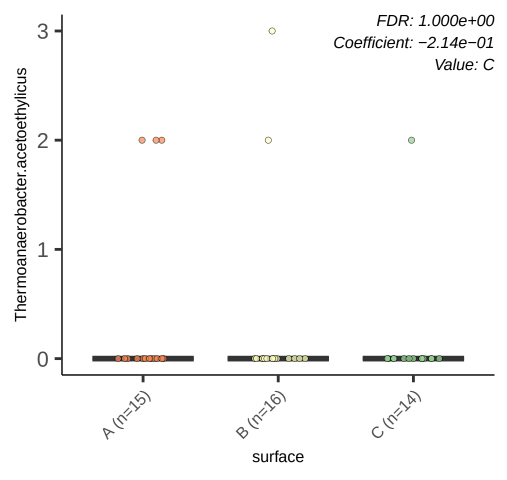

FDR: 1.000e+00
Coefficient: −2.14e−01
Value: C
Thermoanaerobacter.acetoethylicus
0
1
2
3
A (n=15)
B (n=16)
C (n=14)
surface

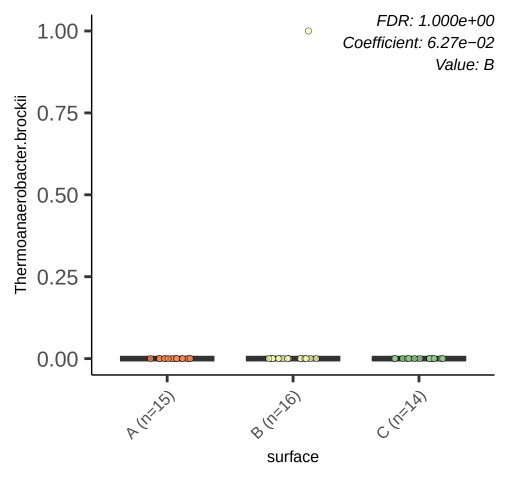
FDR: 1.000e+00
Coefficient: 6.27e−02
Value: B
1.00
0.75
0.50
0.25
0.00
Thermoanaerobacter.brockii
A (n=15)
B (n=16)
C (n=14)
surface

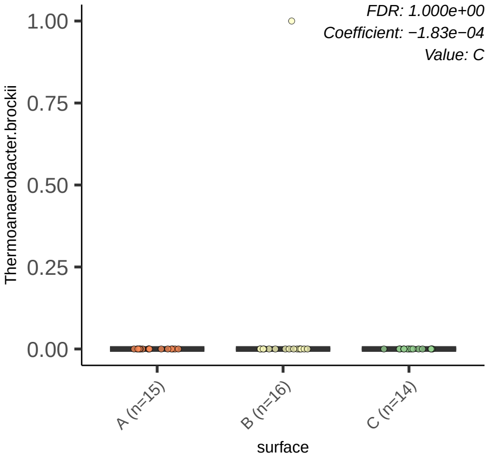
FDR: 1.000e+00
Coefficient: −1.83e−04
Value: C
1.00
0.75
0.50
0.25
0.00
Thermoanaerobacter.brockii
A (n=15)
B (n=16)
C (n=14)
surface

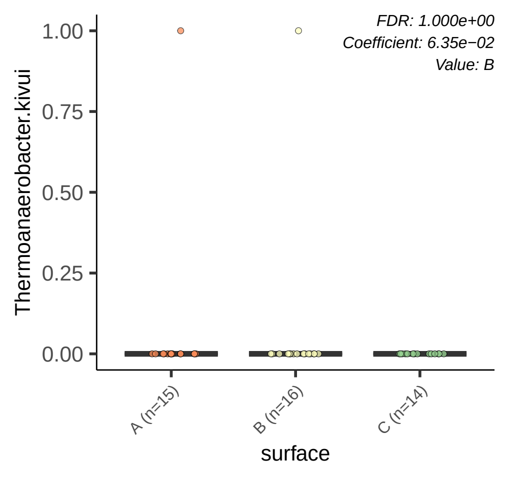

FDR: 1.000e+00
Coefficient: 6.35e−02
Value: B
Thermoanaerobacter.kivui
1.00
0.75
0.50
0.25
0.00
A (n=15)
B (n=16)
C (n=14)
surface

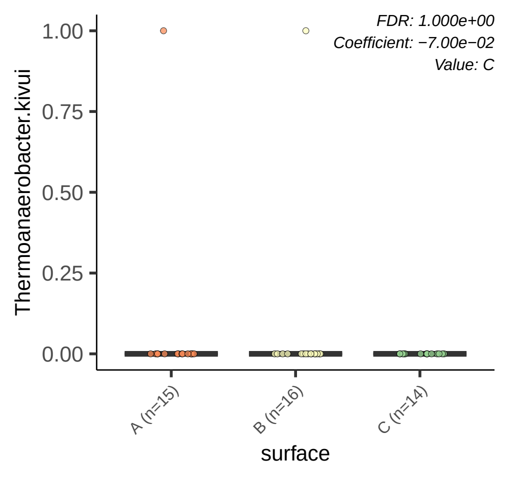
FDR: 1.000e+00
Coefficient: −7.00e−02
Value: C
Thermoanaerobacter.kivui
1.00
0.75
0.50
0.25
0.00
A (n=15)
B (n=16)
C (n=14)
surface

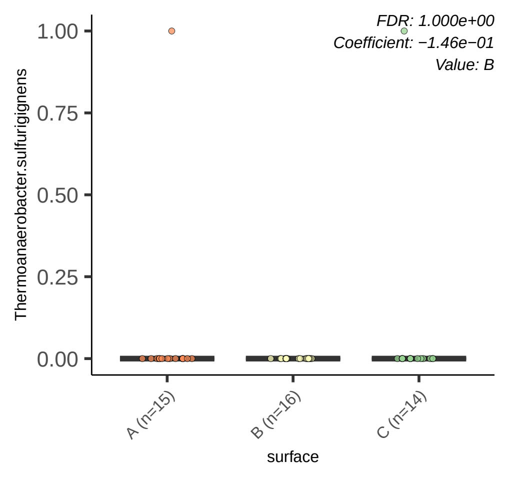
FDR: 1.000e+00
Coefficient: −1.46e−01
Value: B
Thermoanaerobacter.sulfurigignens
1.00
0.75
0.50
0.25
0.00
A (n=15)
B (n=16)
C (n=14)
surface

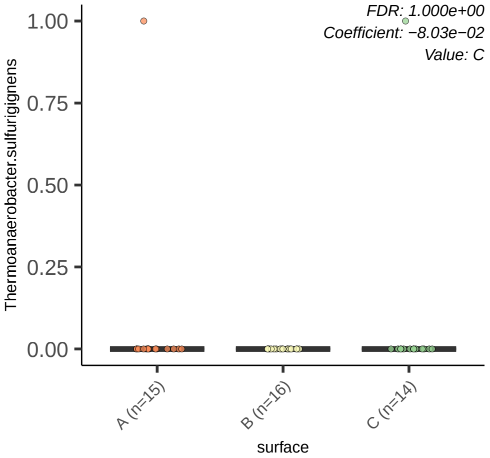

FDR: 1.000e+00
Coefficient: −8.03e−02
Value: C
Thermoanaerobacter.sulfurigignens
1.00
0.75
0.50
0.25
0.00
A (n=15)
B (n=16)
C (n=14)
surface

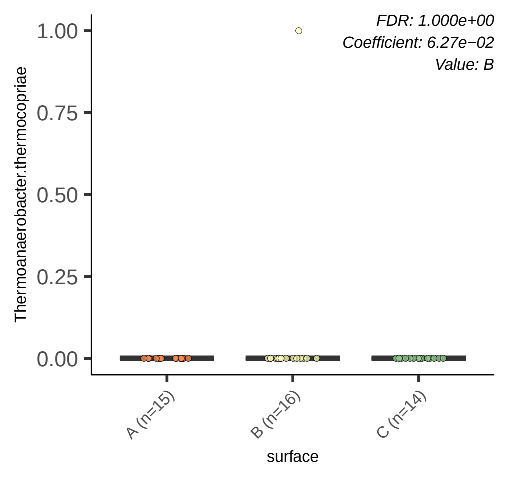

FDR: 1.000e+00
Coefficient: 6.27e−02
Value: B
Thermoanaerobacter.thermocopriae
1.00
0.75
0.50
0.25
0.00
A (n=15)
B (n=16)
C (n=14)
surface

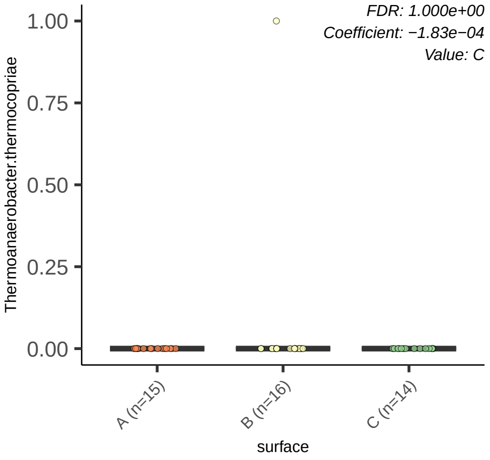
FDR: 1.000e+00
Coefficient: −1.83e−04
Value: C
Thermoanaerobacter.thermocopriae
1.00
0.75
0.50
0.25
0.00
A (n=15)
B (n=16)
C (n=14)
surface

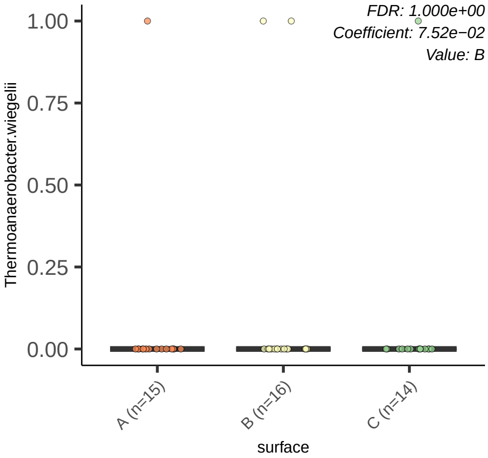
FDR: 1.000e+00
Coefficient: 7.52e−02
Value: B
Thermoanaerobacter.wiegelii
1.00
0.75
0.50
0.25
0.00
A (n=15)
B (n=16)
C (n=14)
surface

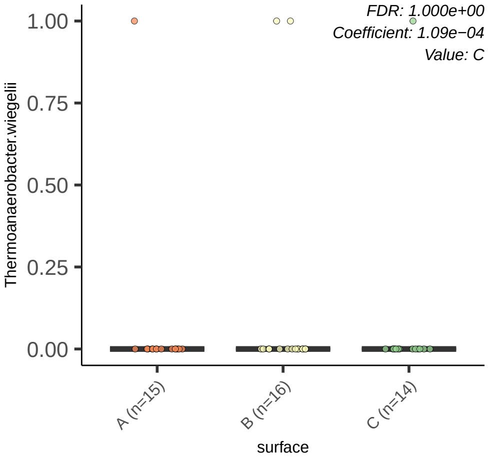
FDR: 1.000e+00
Coefficient: 1.09e−04
Value: C
1.00
0.75
0.50
0.25
0.00
Thermoanaerobacter.wiegelii
A (n=15)
B (n=16)
C (n=14)
surface

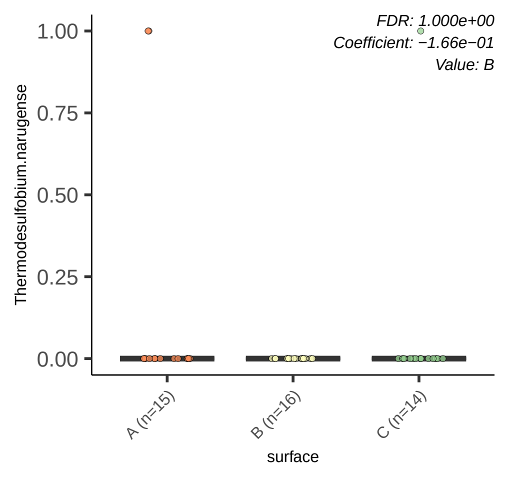
FDR: 1.000e+00
Coefficient: −1.66e−01
Value: B
Thermodesulfobium.narugense
1.00
0.75
0.50
0.25
0.00
A (n=15)
B (n=16)
C (n=14)
surface

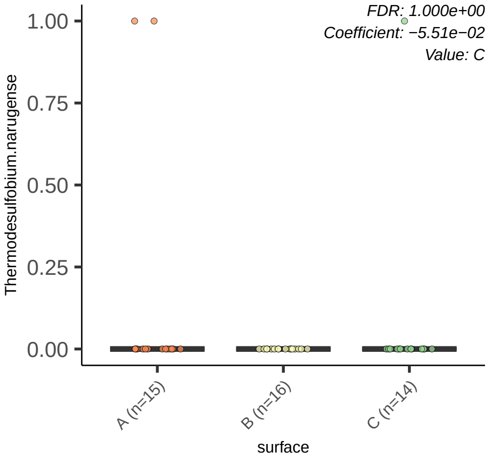

FDR: 1.000e+00
Coefficient: −5.51e−02
Value: C
1.00
0.75
0.50
0.25
0.00
Thermodesulfobium.narugense
A (n=15)
B (n=16)
C (n=14)
surface

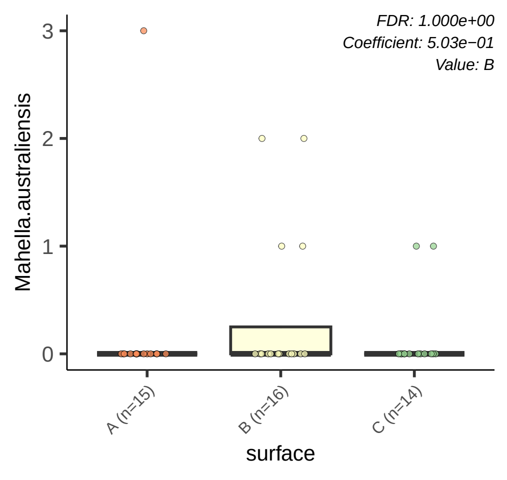

FDR: 1.000e+00
Coefficient: 5.03e−01
Value: B
Mahella.australiensis
0
1
2
3
A (n=15)
B (n=16)
C (n=14)
surface

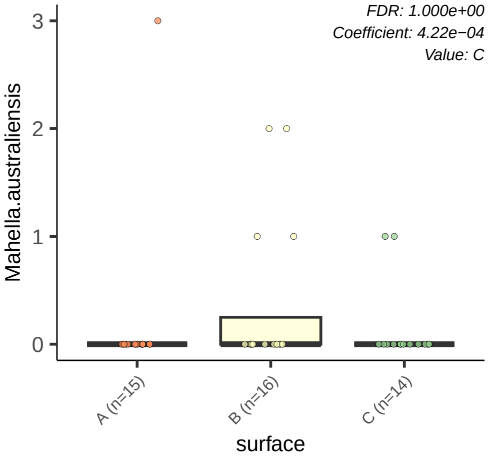

FDR: 1.000e+00
Coefficient: 4.22e−04
Value: C
Mahella.australiensis
3
2
1
0
A (n=15)
B (n=16)
C (n=14)
surface

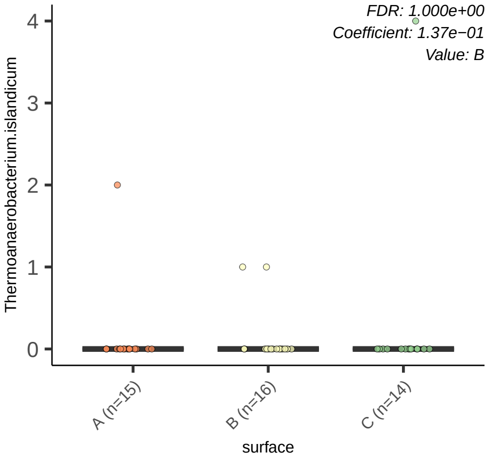

FDR: 1.000e+00
Coefficient: 1.37e−01
Value: B
Thermoanaerobacterium.islandicum
0
1
2
3
4
A (n=15)
B (n=16)
C (n=14)
surface

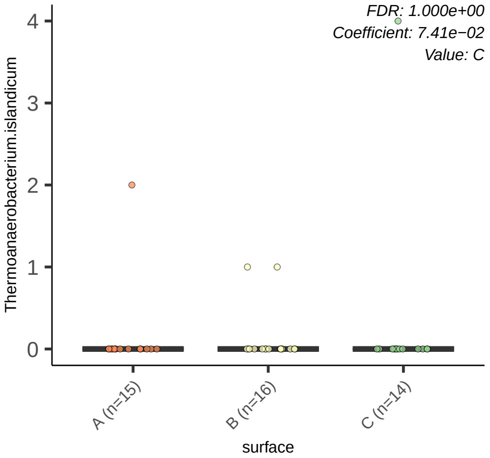

FDR: 1.000e+00
Coefficient: 7.41e−02
Value: C
Thermoanaerobacterium.islandicum
4
3
2
1
0
A (n=15)
B (n=16)
C (n=14)
surface

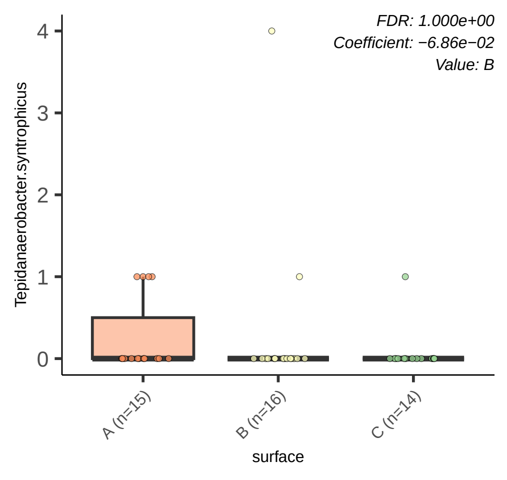

FDR: 1.000e+00
Coefficient: −6.86e−02
Value: B
Tepidanaerobacter.syntrophicus
4
3
2
1
0
A (n=15)
B (n=16)
C (n=14)
surface

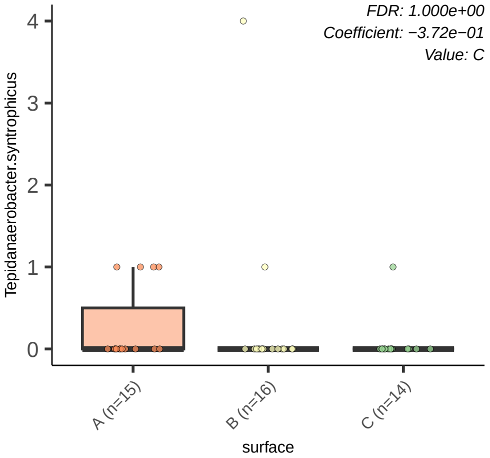

FDR: 1.000e+00
Coefficient: −3.72e−01
Value: C
Tepidanaerobacter.syntrophicus
4
3
2
1
0
A (n=15)
B (n=16)
C (n=14)
surface

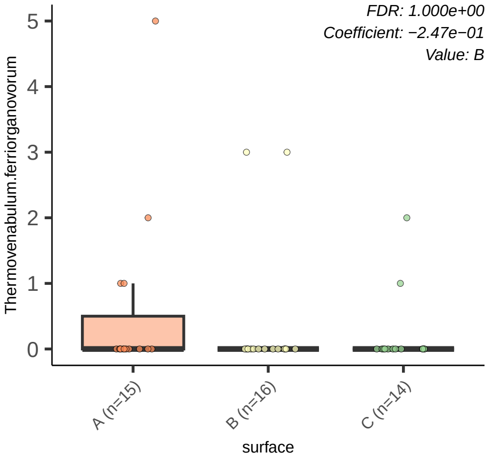
FDR: 1.000e+00
Coefficient: −2.47e−01
Value: B
Thermovenabulum.ferriorganovorum
0
1
2
3
4
5
A (n=15)
B (n=16)
C (n=14)
surface

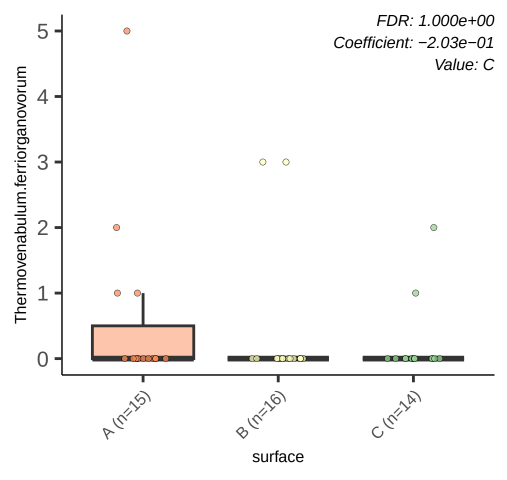

FDR: 1.000e+00
Coefficient: −2.03e−01
Value: C
Thermovenabulum.ferriorganovorum
0
1
2
3
4
5
A (n=15)
B (n=16)
C (n=14)
surface

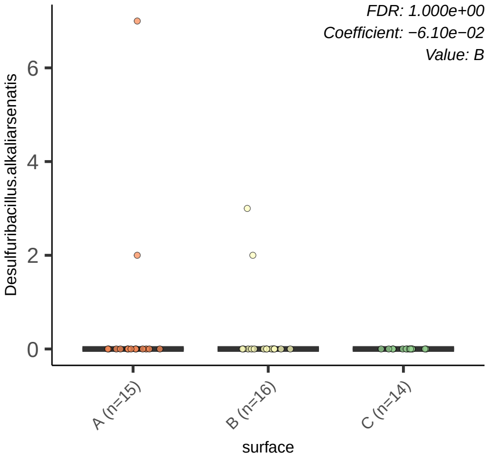

FDR: 1.000e+00
Coefficient: −6.10e−02
Value: B
Desulfuribacillus.alkaliarsenatis
0
2
4
6
A (n=15)
B (n=16)
C (n=14)
surface

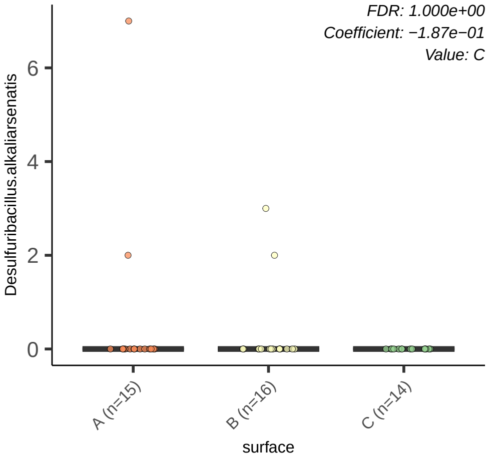
FDR: 1.000e+00
Coefficient: −1.87e−01
Value: C
Desulfuribacillus.alkaliarsenatis
0
2
4
6
A (n=15)
B (n=16)
C (n=14)
surface

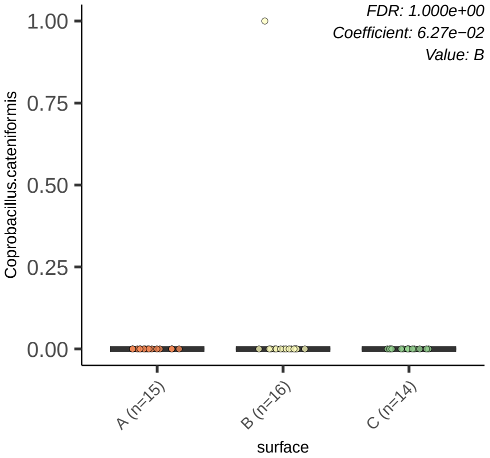

FDR: 1.000e+00
Coefficient: 6.27e−02
Value: B
Coprobacillus.cateniformis
1.00
0.75
0.50
0.25
0.00
A (n=15)
B (n=16)
C (n=14)
surface

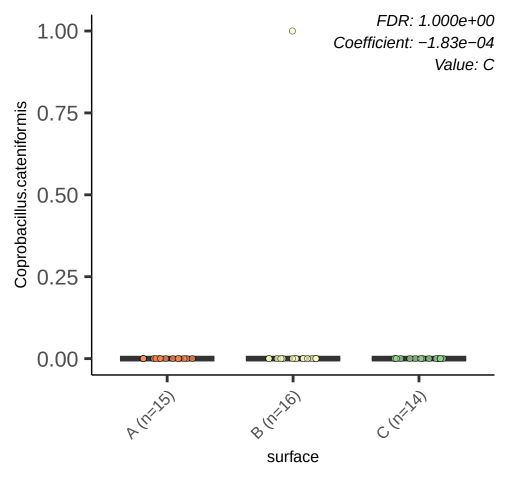
FDR: 1.000e+00
Coefficient: −1.83e−04
Value: C
1.00
0.75
0.50
0.25
0.00
Coprobacillus.cateniformis
A (n=15)
B (n=16)
C (n=14)
surface

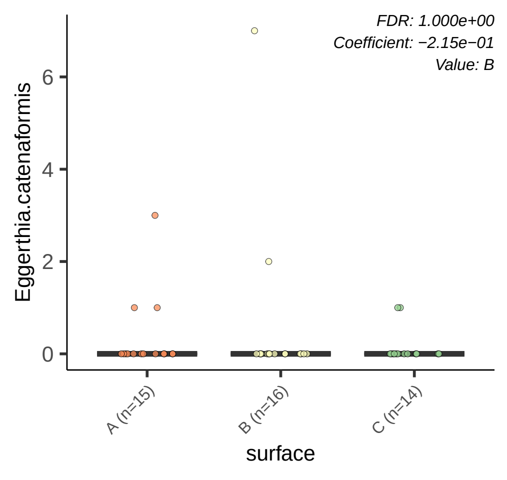
FDR: 1.000e+00
Coefficient: −2.15e−01
Value: B
Eggerthia.catenaformis
0
2
4
6
A (n=15)
B (n=16)
C (n=14)
surface

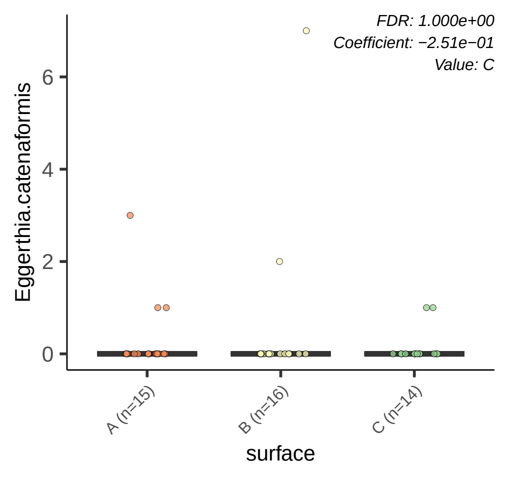
FDR: 1.000e+00
Coefficient: −2.51e−01
Value: C
Eggerthia.catenaformis
0
2
4
6
A (n=15)
B (n=16)
C (n=14)
surface

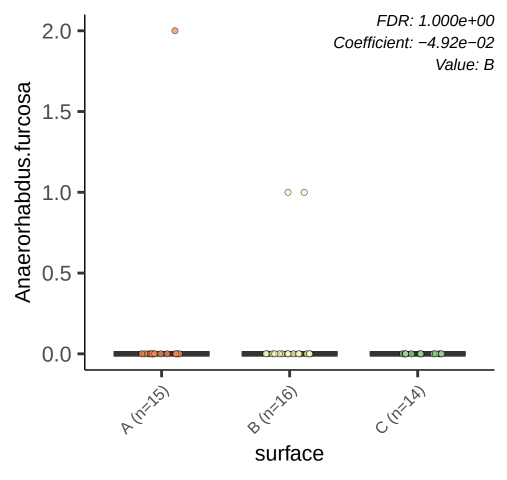
FDR: 1.000e+00
Coefficient: −4.92e−02
Value: B
Anaerorhabdus.furcosa
2.0
1.5
1.0
0.5
0.0
A (n=15)
B (n=16)
C (n=14)
surface

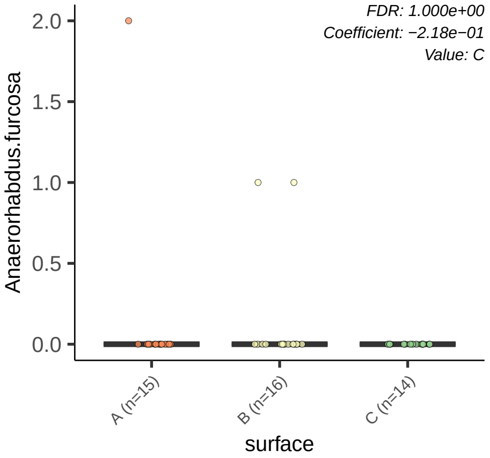

FDR: 1.000e+00
Coefficient: −2.18e−01
Value: C
Anaerorhabdus.furcosa
2.0
1.5
1.0
0.5
0.0
A (n=15)
B (n=16)
C (n=14)
surface

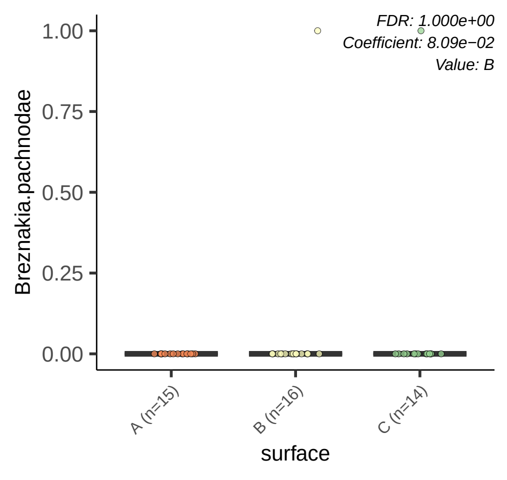
FDR: 1.000e+00
Coefficient: 8.09e−02
Value: B
Breznakia.pachnodae
1.00
0.75
0.50
0.25
0.00
A (n=15)
B (n=16)
C (n=14)
surface

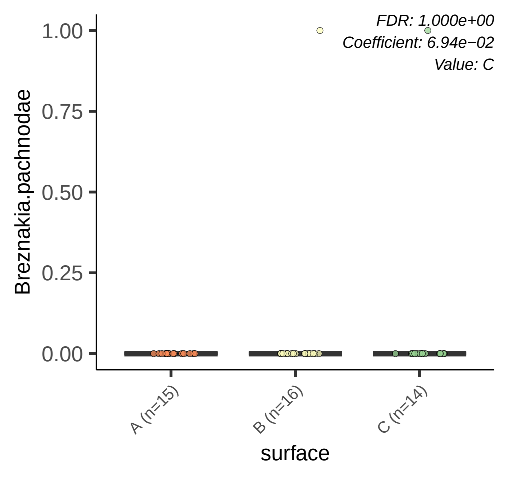

FDR: 1.000e+00
Coefficient: 6.94e−02
Value: C
Breznakia.pachnodae
1.00
0.75
0.50
0.25
0.00
A (n=15)
B (n=16)
C (n=14)
surface

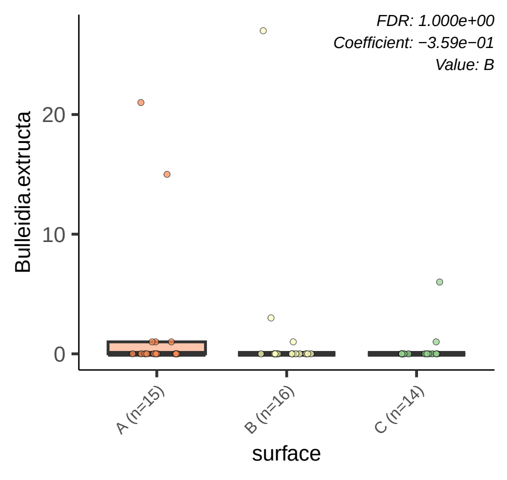
FDR: 1.000e+00
Coefficient: −3.59e−01
Value: B
Bulleidia.extructa
0
10
20
A (n=15)
B (n=16)
C (n=14)
surface

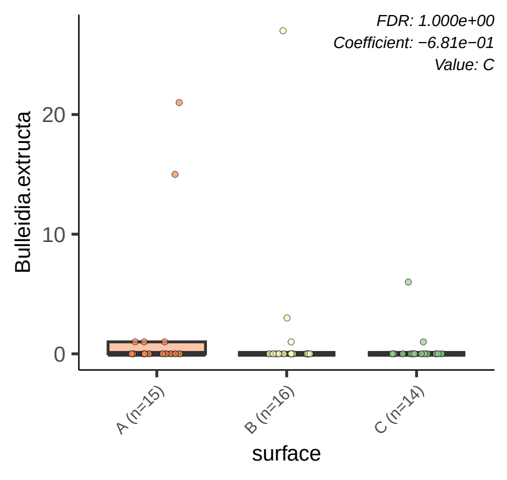

FDR: 1.000e+00
Coefficient: −6.81e−01
Value: C
Bulleidia.extructa
0
10
20
A (n=15)
B (n=16)
C (n=14)
surface

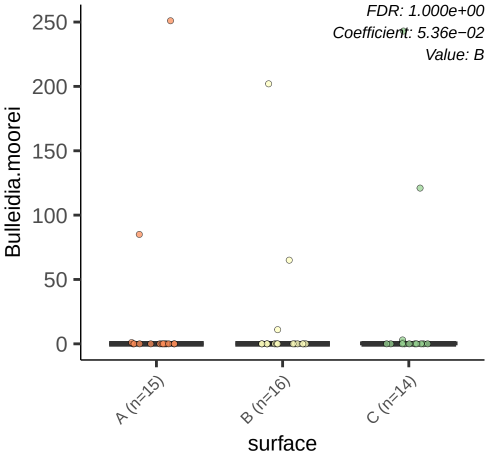
FDR: 1.000e+00
Coefficient: 5.36e−02
Value: B
Bulleidia.moorei
250
200
150
100
50
0
A (n=15)
B (n=16)
C (n=14)
surface

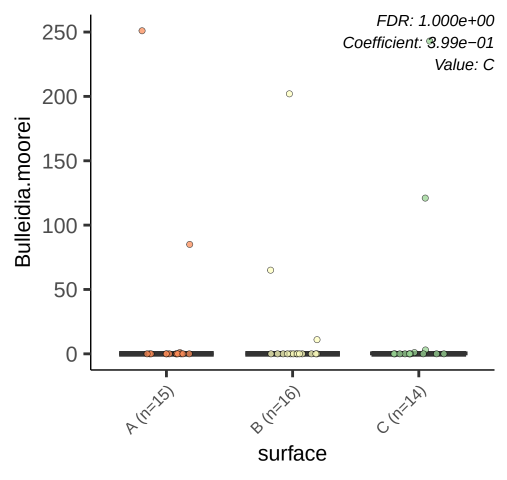

FDR: 1.000e+00
Coefficient: 3.99e−01
Value: C
Bulleidia.moorei
250
200
150
100
50
0
A (n=15)
B (n=16)
C (n=14)
surface

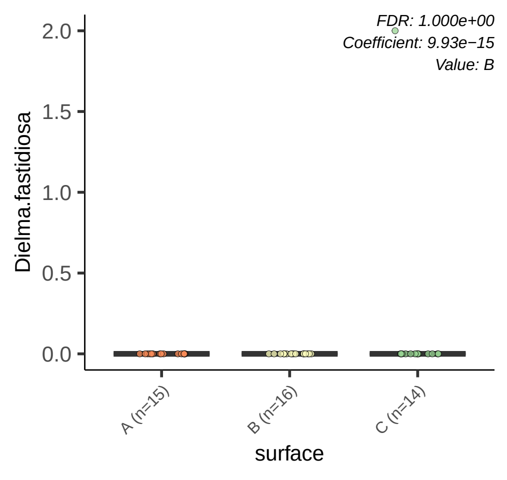

FDR: 1.000e+00
Coefficient: 9.93e−15
Value: B
Dielma.fastidiosa
2.0
1.5
1.0
0.5
0.0
A (n=15)
B (n=16)
C (n=14)
surface

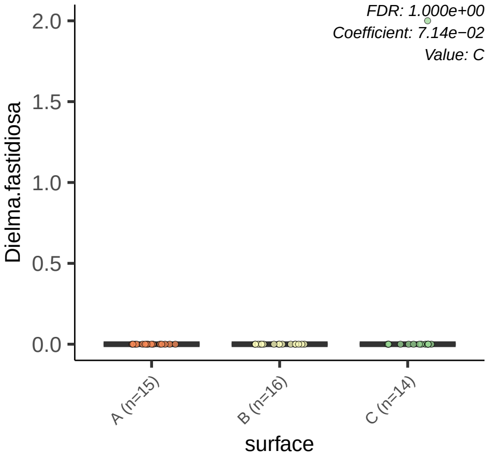

FDR: 1.000e+00
Coefficient: 7.14e−02
Value: C
2.0
1.5
1.0
0.5
0.0
Dielma.fastidiosa
A (n=15)
B (n=16)
C (n=14)
surface

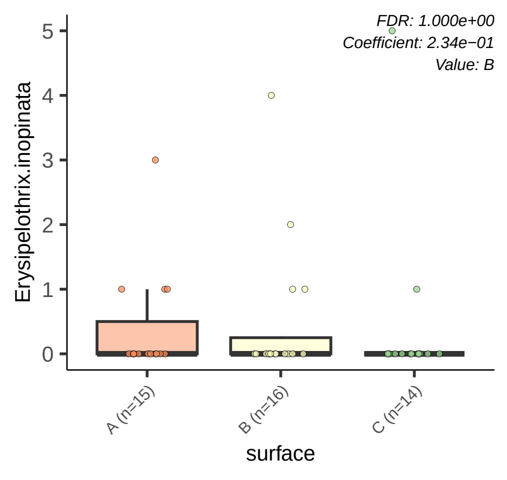

FDR: 1.000e+00
Coefficient: 2.34e−01
Value: B
Erysipelothrix.inopinata
0
1
2
3
4
5
A (n=15)
B (n=16)
C (n=14)
surface

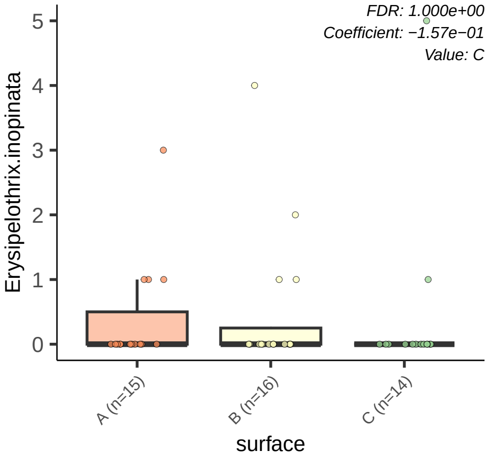
FDR: 1.000e+00
Coefficient: −1.57e−01
Value: C
Erysipelothrix.inopinata
0
1
2
3
4
5
A (n=15)
B (n=16)
C (n=14)
surface

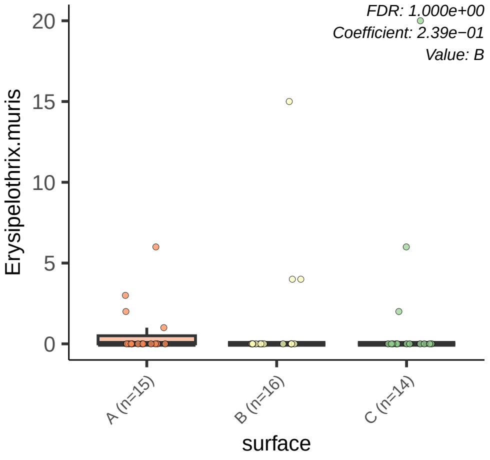
FDR: 1.000e+00
Coefficient: 2.39e−01
Value: B
Erysipelothrix.muris
20
15
10
5
0
A (n=15)
B (n=16)
C (n=14)
surface

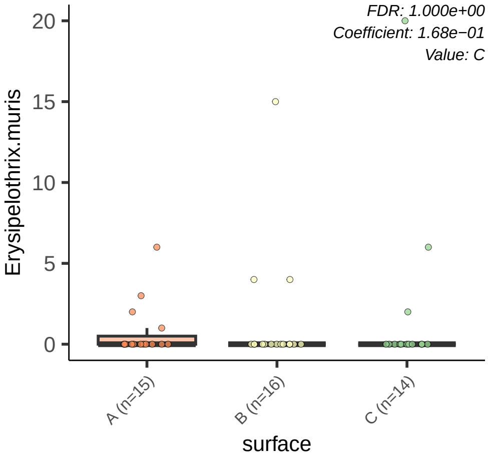

FDR: 1.000e+00
Coefficient: 1.68e−01
Value: C
Erysipelothrix.muris
0
5
10
15
20
A (n=15)
B (n=16)
C (n=14)
surface

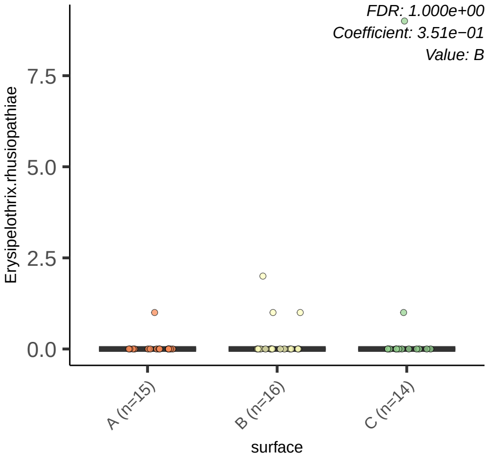
FDR: 1.000e+00
Coefficient: 3.51e−01
Value: B
Erysipelothrix.rhusiopathiae
0.0
2.5
5.0
7.5
A (n=15)
B (n=16)
C (n=14)
surface

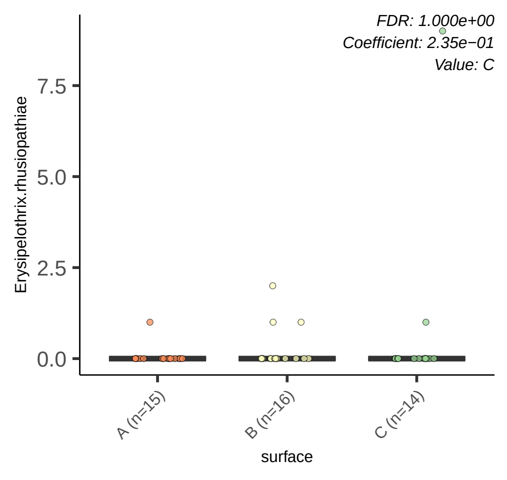
FDR: 1.000e+00
Coefficient: 2.35e−01
Value: C
7.5
5.0
2.5
0.0
Erysipelothrix.rhusiopathiae
A (n=15)
B (n=16)
C (n=14)
surface

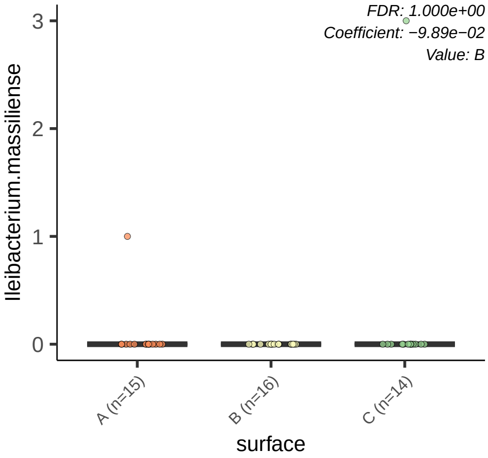
FDR: 1.000e+00
Coefficient: −9.89e−02
Value: B
Ileibacterium.massiliense
3
2
1
0
A (n=15)
B (n=16)
C (n=14)
surface

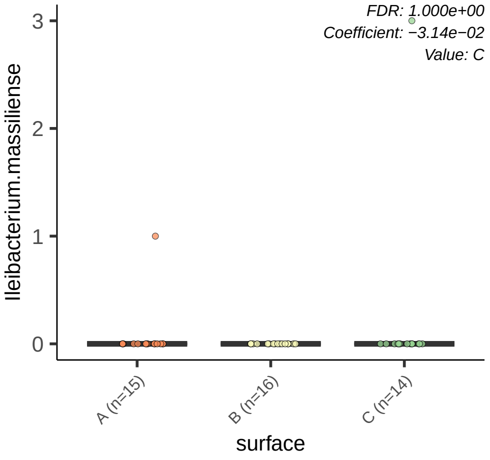

FDR: 1.000e+00
Coefficient: −3.14e−02
Value: C
Ileibacterium.massiliense
0
1
2
3
A (n=15)
B (n=16)
C (n=14)
surface

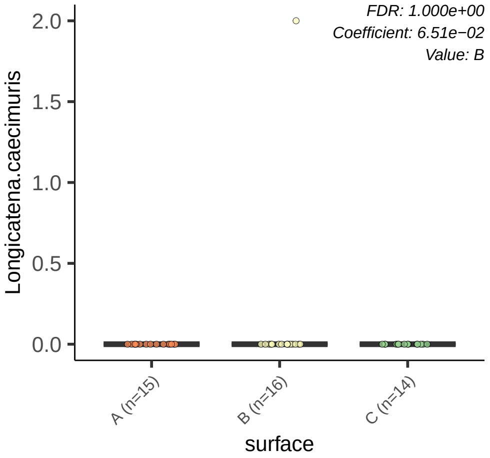
FDR: 1.000e+00
Coefficient: 6.51e−02
Value: B
Longicatena.caecimuris
2.0
1.5
1.0
0.5
0.0
A (n=15)
B (n=16)
C (n=14)
surface

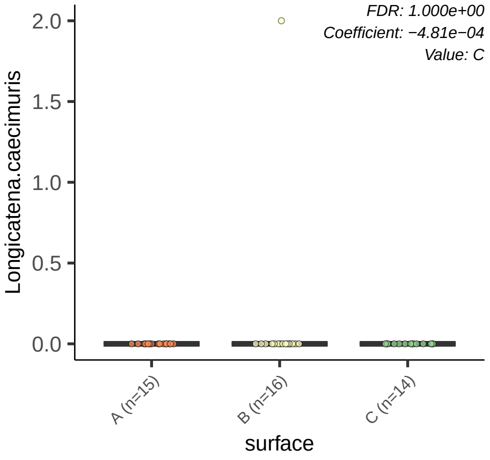
FDR: 1.000e+00
Coefficient: −4.81e−04
Value: C
Longicatena.caecimuris
2.0
1.5
1.0
0.5
0.0
A (n=15)
B (n=16)
C (n=14)
surface

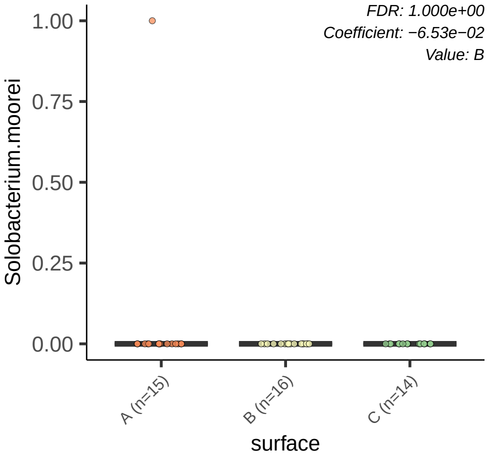
FDR: 1.000e+00
Coefficient: −6.53e−02
Value: B
Solobacterium.moorei
1.00
0.75
0.50
0.25
0.00
A (n=15)
B (n=16)
C (n=14)
surface

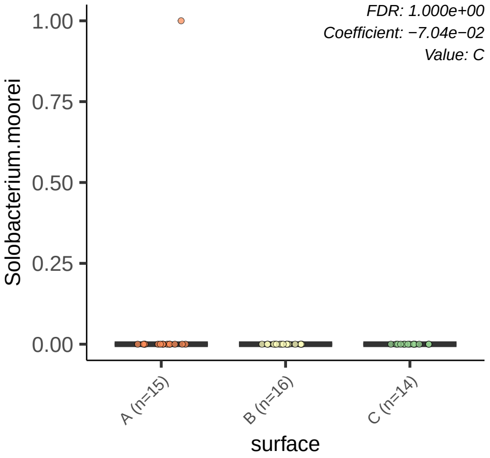

FDR: 1.000e+00
Coefficient: −7.04e−02
Value: C
Solobacterium.moorei
1.00
0.75
0.50
0.25
0.00
A (n=15)
B (n=16)
C (n=14)
surface

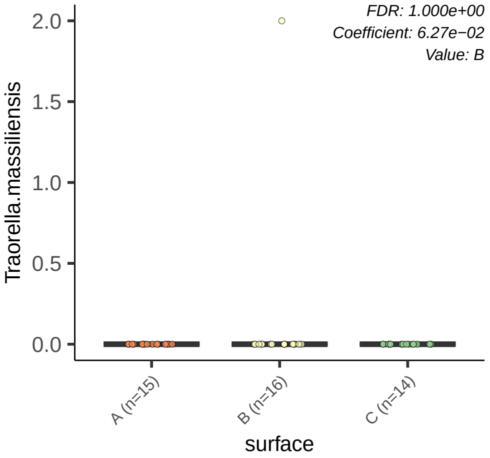
FDR: 1.000e+00
Coefficient: 6.27e−02
Value: B
2.0
1.5
1.0
0.5
0.0
Traorella.massiliensis
A (n=15)
B (n=16)
C (n=14)
surface

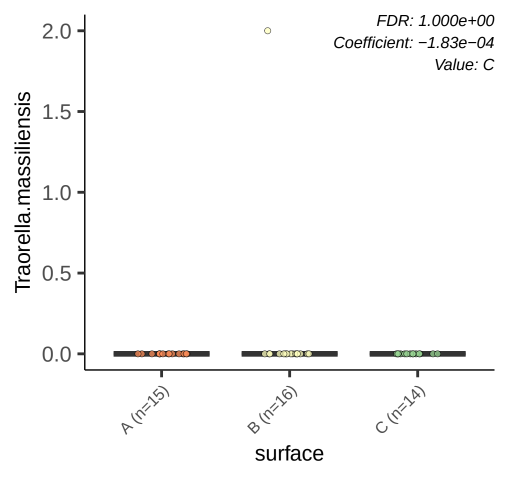

FDR: 1.000e+00
Coefficient: −1.83e−04
Value: C
Traorella.massiliensis
2.0
1.5
1.0
0.5
0.0
A (n=15)
B (n=16)
C (n=14)
surface

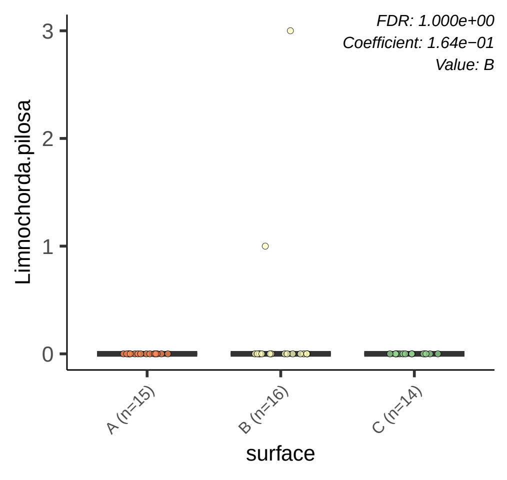
FDR: 1.000e+00
Coefficient: 1.64e−01
Value: B
Limnochorda.pilosa
3
2
1
0
A (n=15)
B (n=16)
C (n=14)
surface

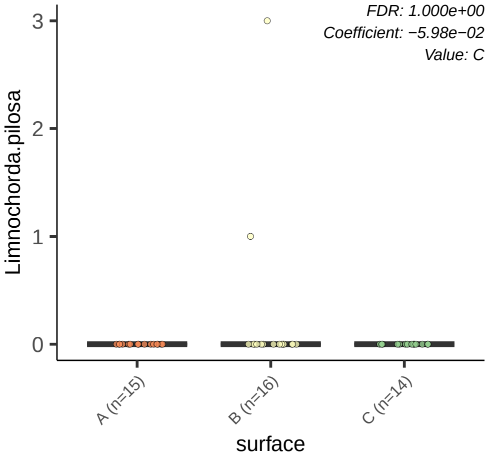
FDR: 1.000e+00
Coefficient: −5.98e−02
Value: C
Limnochorda.pilosa
3
2
1
0
A (n=15)
B (n=16)
C (n=14)
surface

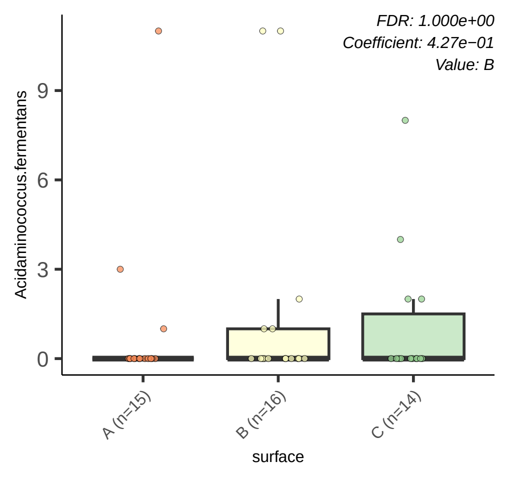
FDR: 1.000e+00
Coefficient: 4.27e−01
Value: B
Acidaminococcus.fermentans
9
6
3
0
A (n=15)
B (n=16)
C (n=14)
surface

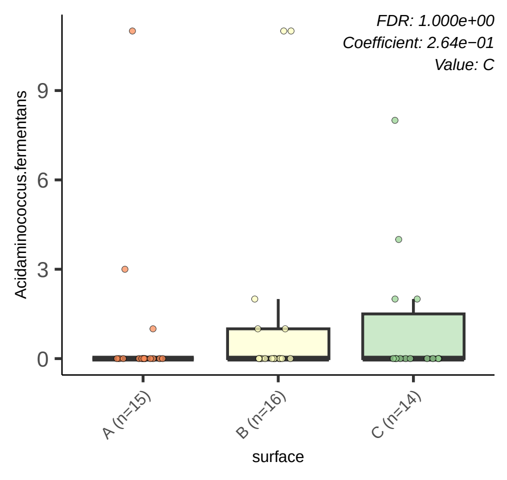

FDR: 1.000e+00
Coefficient: 2.64e−01
Value: C
Acidaminococcus.fermentans
0
3
6
9
A (n=15)
B (n=16)
C (n=14)
surface

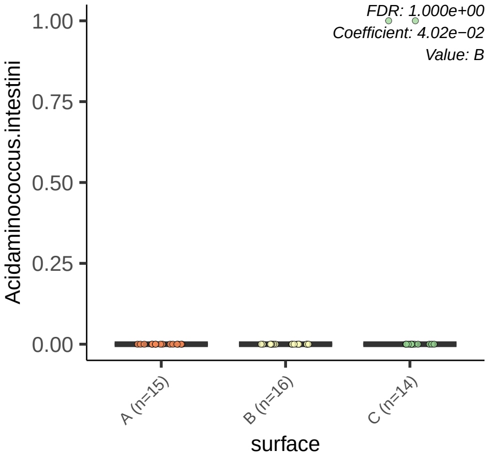

FDR: 1.000e+00
Coefficient: 4.02e−02
Value: B
1.00
0.75
0.50
0.25
0.00
Acidaminococcus.intestini
A (n=15)
B (n=16)
C (n=14)
surface

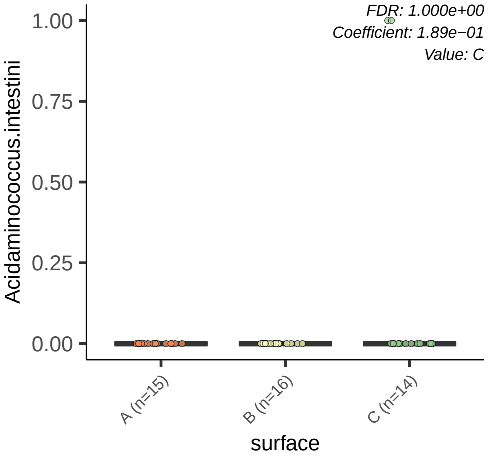

FDR: 1.000e+00
Coefficient: 1.89e−01
Value: C
Acidaminococcus.intestini
1.00
0.75
0.50
0.25
0.00
A (n=15)
B (n=16)
C (n=14)
surface

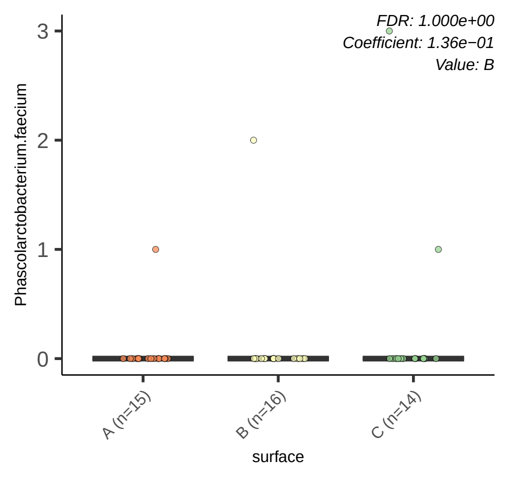

FDR: 1.000e+00
Coefficient: 1.36e−01
Value: B
Phascolarctobacterium.faecium
3
2
1
0
A (n=15)
B (n=16)
C (n=14)
surface

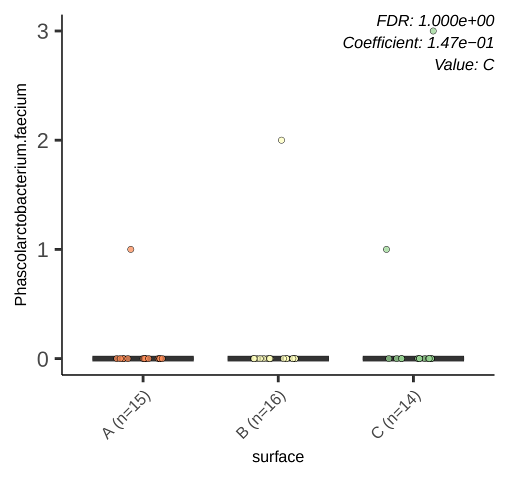
FDR: 1.000e+00
Coefficient: 1.47e−01
Value: C
Phascolarctobacterium.faecium
0
1
2
3
A (n=15)
B (n=16)
C (n=14)
surface

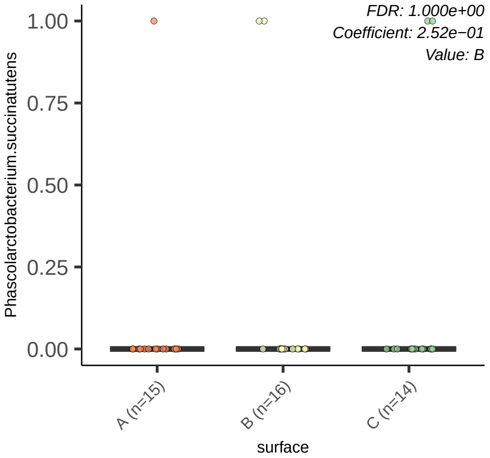
FDR: 1.000e+00
Coefficient: 2.52e−01
Value: B
Phascolarctobacterium.succinatutens
1.00
0.75
0.50
0.25
0.00
A (n=15)
B (n=16)
C (n=14)
surface

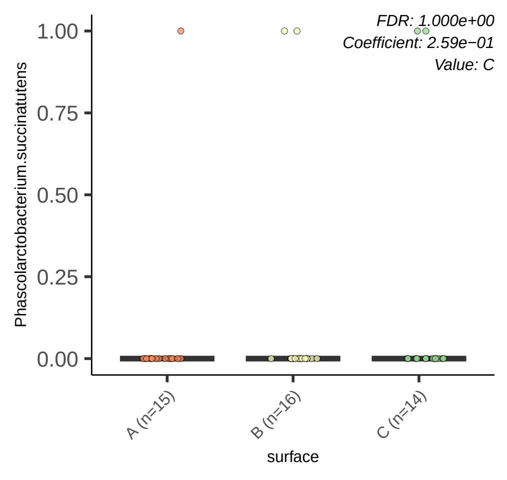
FDR: 1.000e+00
Coefficient: 2.59e−01
Value: C
Phascolarctobacterium.succinatutens
1.00
0.75
0.50
0.25
0.00
A (n=15)
B (n=16)
C (n=14)
surface

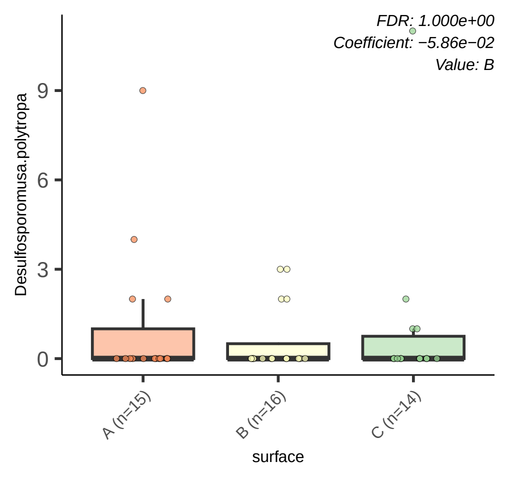

FDR: 1.000e+00
Coefficient: −5.86e−02
Value: B
Desulfosporomusa.polytropa
9
6
3
0
A (n=15)
B (n=16)
C (n=14)
surface

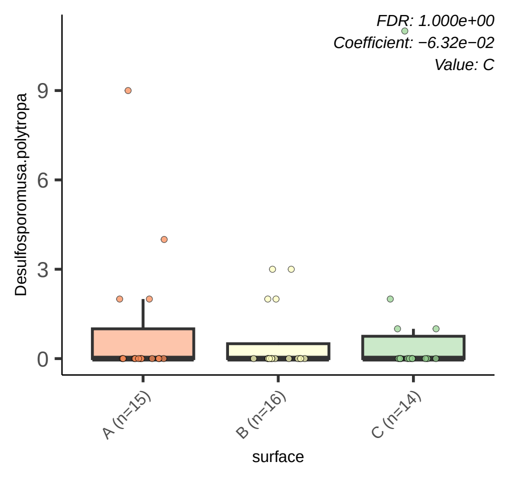

FDR: 1.000e+00
Coefficient: −6.32e−02
Value: C
Desulfosporomusa.polytropa
0
3
6
9
A (n=15)
B (n=16)
C (n=14)
surface

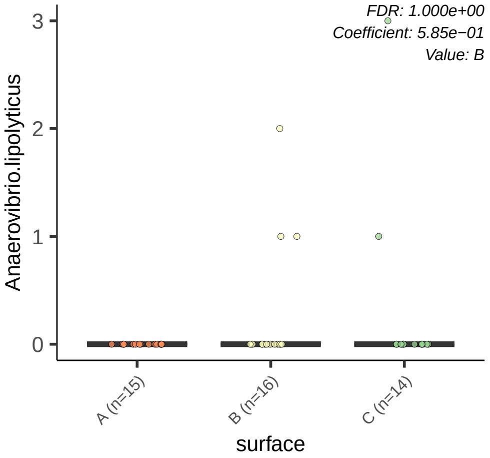

FDR: 1.000e+00
Coefficient: 5.85e−01
Value: B
Anaerovibrio.lipolyticus
3
2
1
0
A (n=15)
B (n=16)
C (n=14)
surface

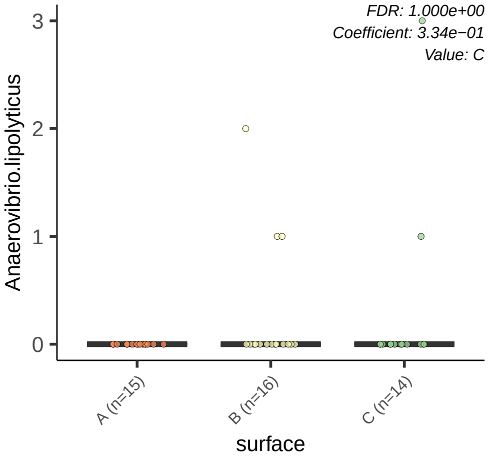

FDR: 1.000e+00
Coefficient: 3.34e−01
Value: C
Anaerovibrio.lipolyticus
3
2
1
0
A (n=15)
B (n=16)
C (n=14)
surface

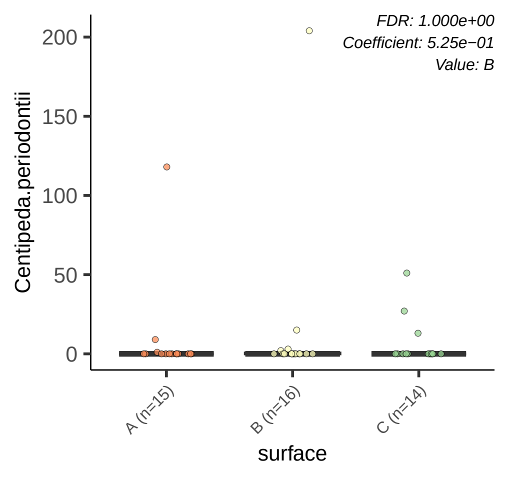

FDR: 1.000e+00
Coefficient: 5.25e−01
Value: B
Centipeda.periodontii
200
150
100
50
0
A (n=15)
B (n=16)
C (n=14)
surface

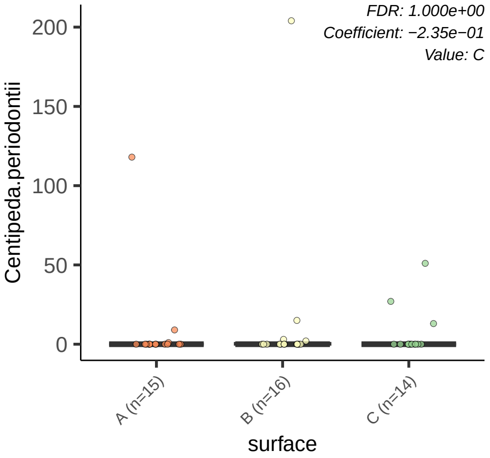
FDR: 1.000e+00
Coefficient: −2.35e−01
Value: C
Centipeda.periodontii
200
150
100
50
0
A (n=15)
B (n=16)
C (n=14)
surface

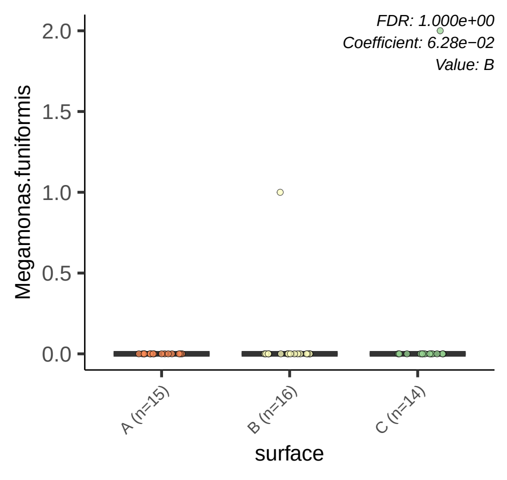

FDR: 1.000e+00
Coefficient: 6.28e−02
Value: B
Megamonas.funiformis
2.0
1.5
1.0
0.5
0.0
A (n=15)
B (n=16)
C (n=14)
surface

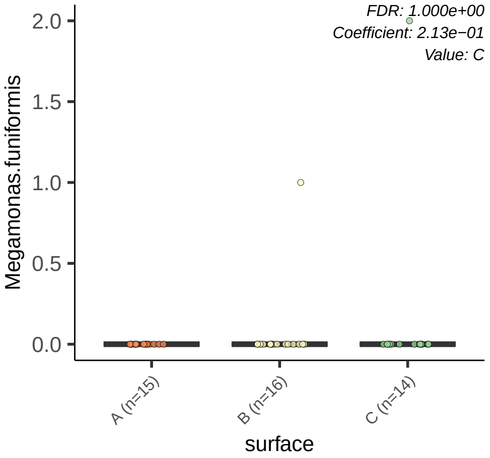
FDR: 1.000e+00
Coefficient: 2.13e−01
Value: C
Megamonas.funiformis
2.0
1.5
1.0
0.5
0.0
A (n=15)
B (n=16)
C (n=14)
surface

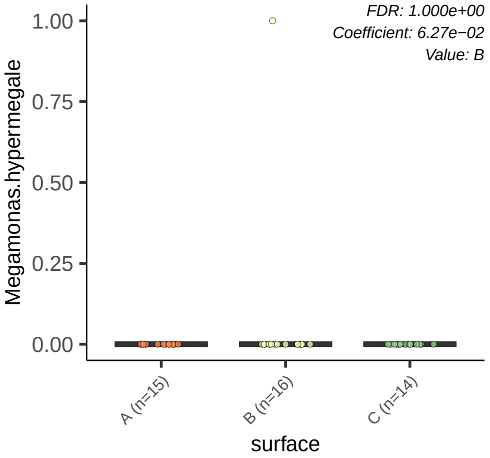
FDR: 1.000e+00
Coefficient: 6.27e−02
Value: B
Megamonas.hypermegale
1.00
0.75
0.50
0.25
0.00
A (n=15)
B (n=16)
C (n=14)
surface

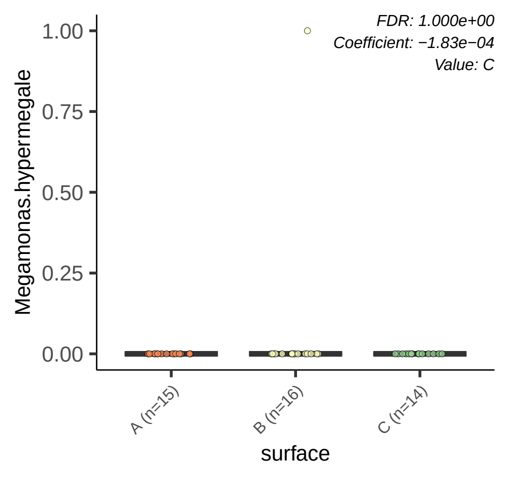
FDR: 1.000e+00
Coefficient: −1.83e−04
Value: C
Megamonas.hypermegale
1.00
0.75
0.50
0.25
0.00
A (n=15)
B (n=16)
C (n=14)
surface

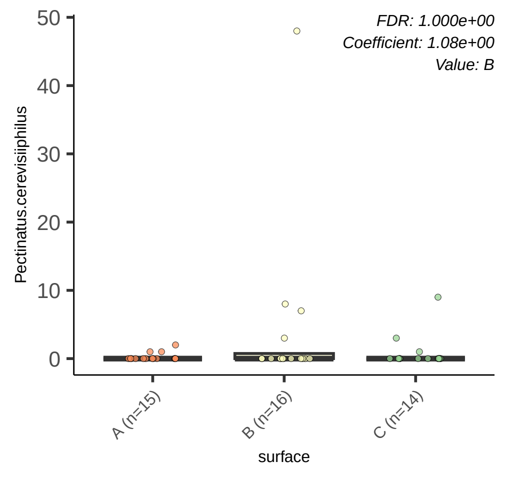

FDR: 1.000e+00
Coefficient: 1.08e+00
Value: B
50
40
30
20
10
0
Pectinatus.cerevisiiphilus
A (n=15)
B (n=16)
C (n=14)
surface

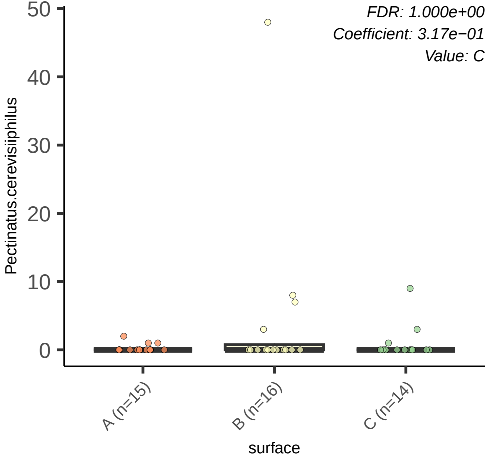

FDR: 1.000e+00
Coefficient: 3.17e−01
Value: C
Pectinatus.cerevisiiphilus
0
10
20
30
40
50
A (n=15)
B (n=16)
C (n=14)
surface

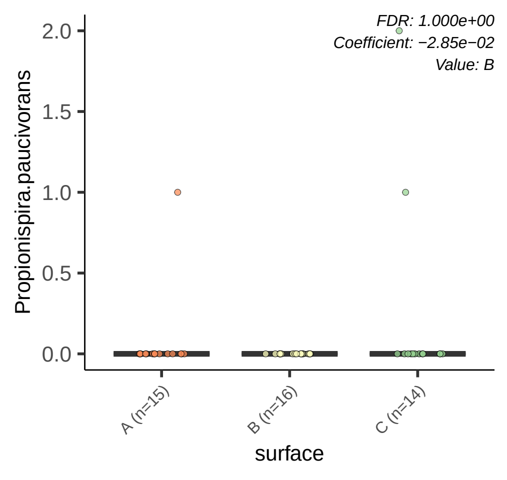

FDR: 1.000e+00
Coefficient: −2.85e−02
Value: B
Propionispira.paucivorans
2.0
1.5
1.0
0.5
0.0
A (n=15)
B (n=16)
C (n=14)
surface

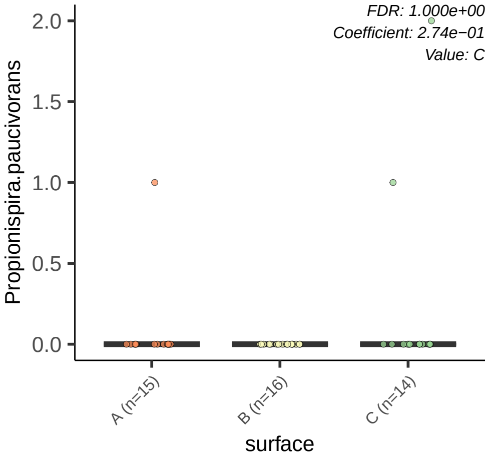
FDR: 1.000e+00
Coefficient: 2.74e−01
Value: C
Propionispira.paucivorans
2.0
1.5
1.0
0.5
0.0
A (n=15)
B (n=16)
C (n=14)
surface

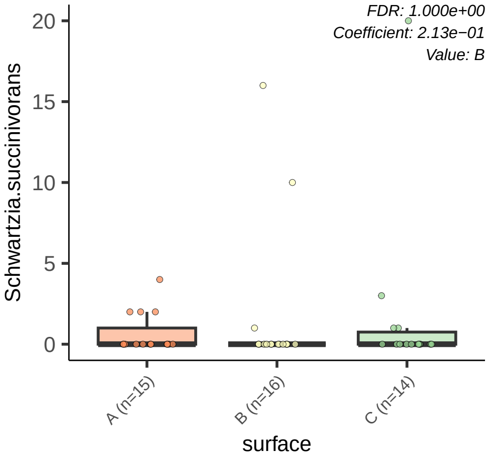

FDR: 1.000e+00
Coefficient: 2.13e−01
Value: B
Schwartzia.succinivorans
20
15
10
5
0
A (n=15)
B (n=16)
C (n=14)
surface

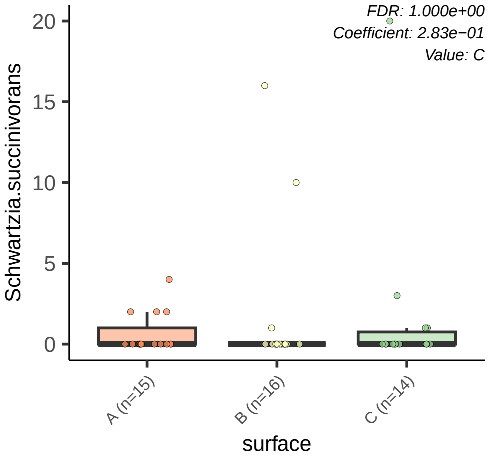
FDR: 1.000e+00
Coefficient: 2.83e−01
Value: C
Schwartzia.succinivorans
20
15
10
5
0
A (n=15)
B (n=16)
C (n=14)
surface

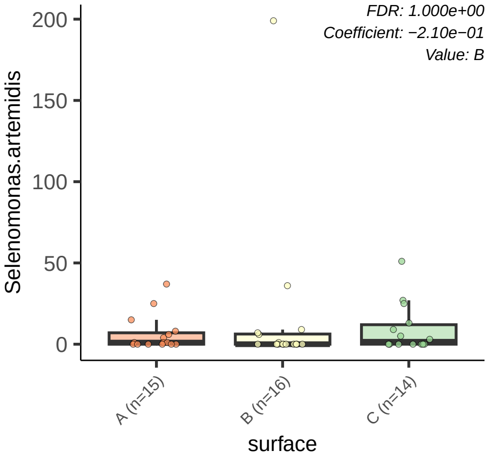

FDR: 1.000e+00
Coefficient: −2.10e−01
Value: B
Selenomonas.artemidis
200
150
100
50
0
A (n=15)
B (n=16)
C (n=14)
surface

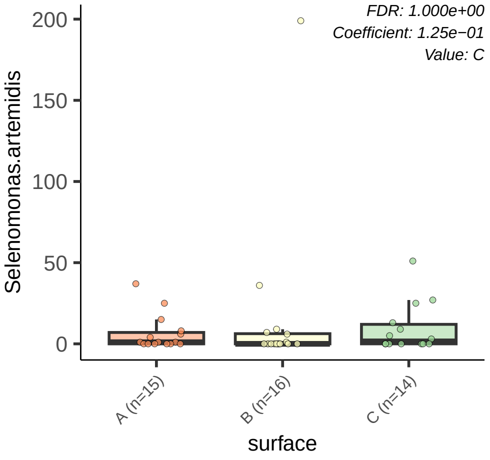
FDR: 1.000e+00
Coefficient: 1.25e−01
Value: C
Selenomonas.artemidis
200
150
100
50
0
A (n=15)
B (n=16)
C (n=14)
surface

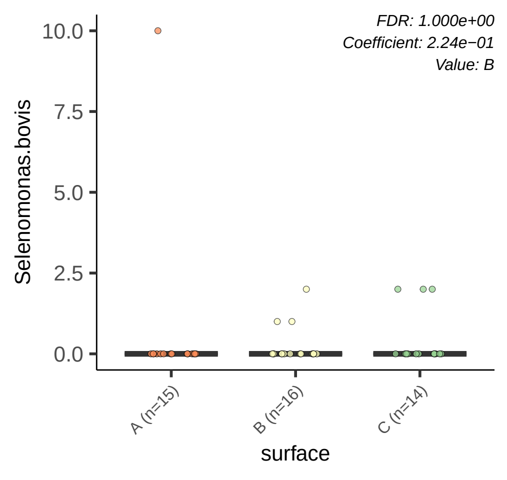
FDR: 1.000e+00
Coefficient: 2.24e−01
Value: B
Selenomonas.bovis
10.0
7.5
5.0
2.5
0.0
A (n=15)
B (n=16)
C (n=14)
surface

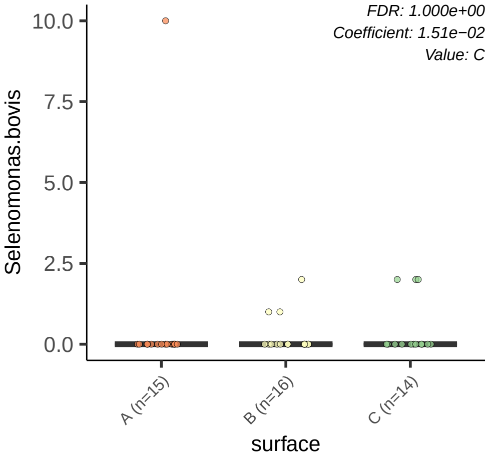

FDR: 1.000e+00
Coefficient: 1.51e−02
Value: C
Selenomonas.bovis
10.0
7.5
5.0
2.5
0.0
A (n=15)
B (n=16)
C (n=14)
surface

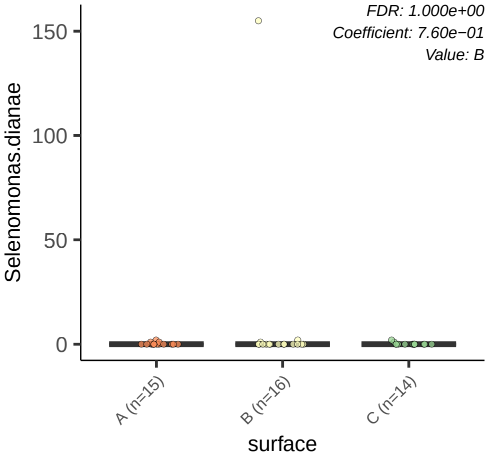

FDR: 1.000e+00
Coefficient: 7.60e−01
Value: B
150
100
50
0
Selenomonas.dianae
A (n=15)
B (n=16)
C (n=14)
surface

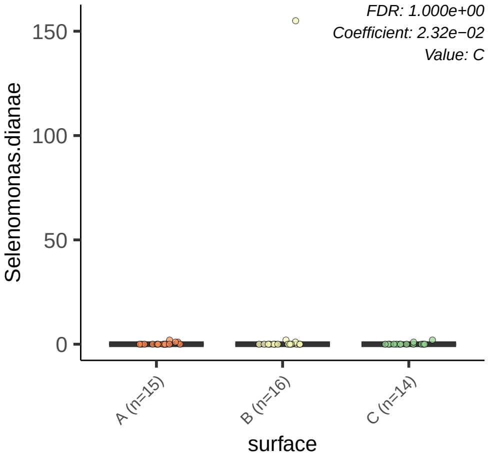

FDR: 1.000e+00
Coefficient: 2.32e−02
Value: C
Selenomonas.dianae
150
100
50
0
A (n=15)
B (n=16)
C (n=14)
surface

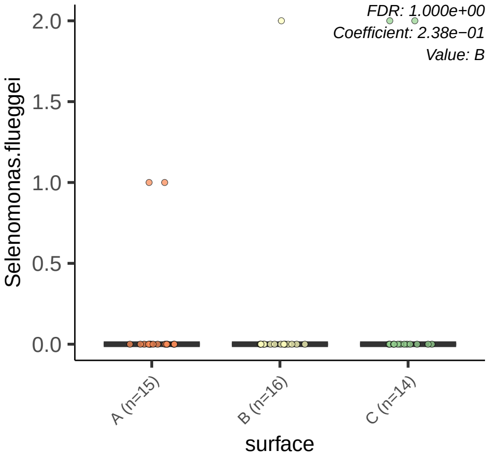
FDR: 1.000e+00
Coefficient: 2.38e−01
Value: B
Selenomonas.flueggei
2.0
1.5
1.0
0.5
0.0
A (n=15)
B (n=16)
C (n=14)
surface

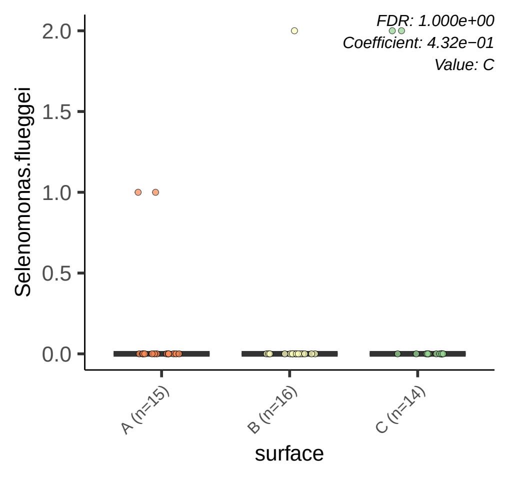
FDR: 1.000e+00
Coefficient: 4.32e−01
Value: C
Selenomonas.flueggei
2.0
1.5
1.0
0.5
0.0
A (n=15)
B (n=16)
C (n=14)
surface

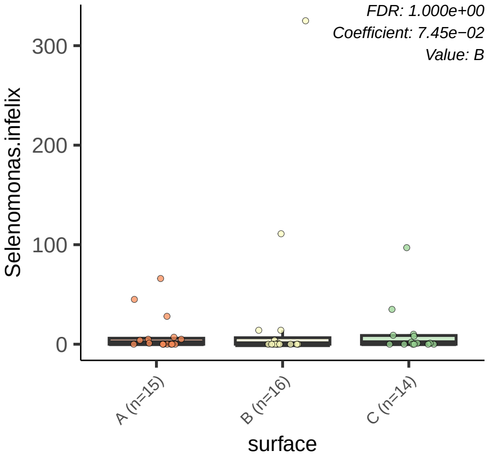
FDR: 1.000e+00
Coefficient: 7.45e−02
Value: B
Selenomonas.infelix
300
200
100
0
A (n=15)
B (n=16)
C (n=14)
surface

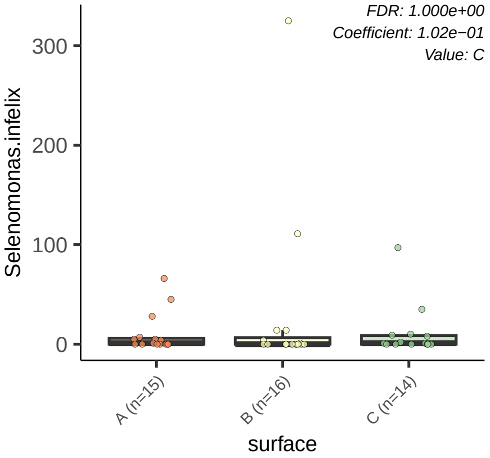

FDR: 1.000e+00
Coefficient: 1.02e−01
Value: C
Selenomonas.infelix
300
200
100
0
A (n=15)
B (n=16)
C (n=14)
surface

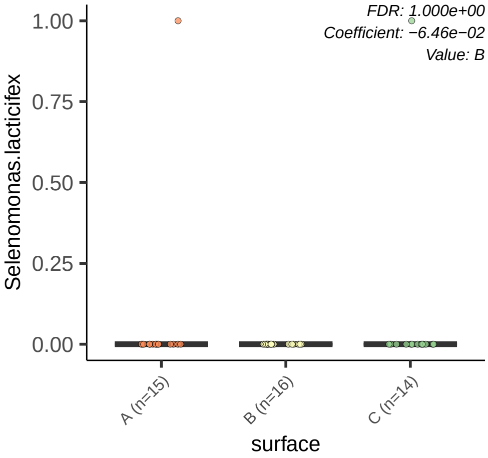

FDR: 1.000e+00
Coefficient: −6.46e−02
Value: B
Selenomonas.lacticifex
1.00
0.75
0.50
0.25
0.00
A (n=15)
B (n=16)
C (n=14)
surface

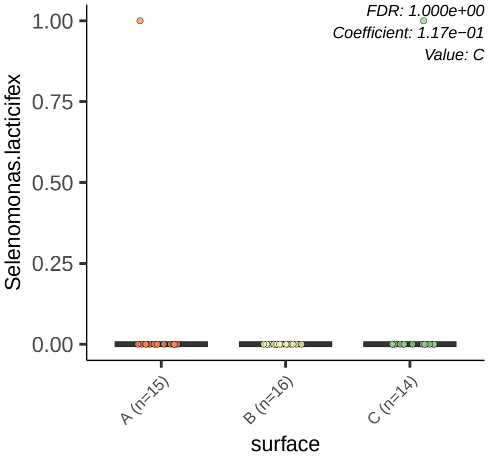

FDR: 1.000e+00
Coefficient: 1.17e−01
Value: C
Selenomonas.lacticifex
1.00
0.75
0.50
0.25
0.00
A (n=15)
B (n=16)
C (n=14)
surface

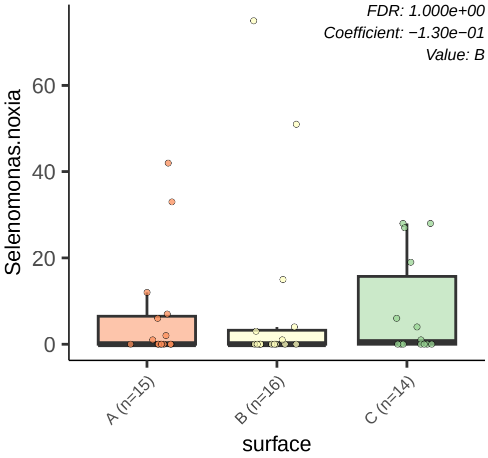
FDR: 1.000e+00
Coefficient: −1.30e−01
Value: B
Selenomonas.noxia
0
20
40
60
A (n=15)
B (n=16)
C (n=14)
surface

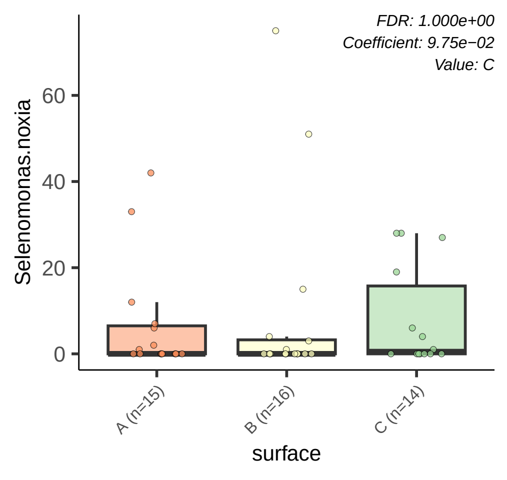
FDR: 1.000e+00
Coefficient: 9.75e−02
Value: C
Selenomonas.noxia
60
40
20
0
A (n=15)
B (n=16)
C (n=14)
surface

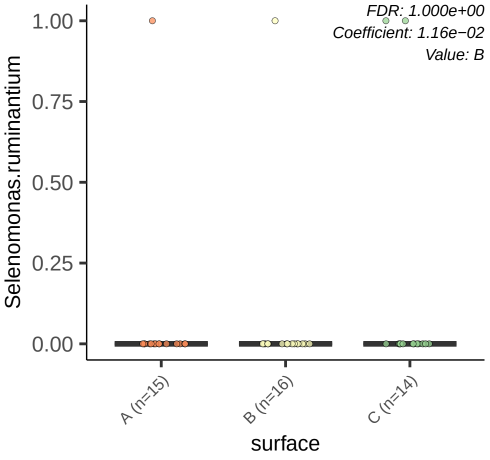
FDR: 1.000e+00
Coefficient: 1.16e−02
Value: B
Selenomonas.ruminantium
1.00
0.75
0.50
0.25
0.00
A (n=15)
B (n=16)
C (n=14)
surface

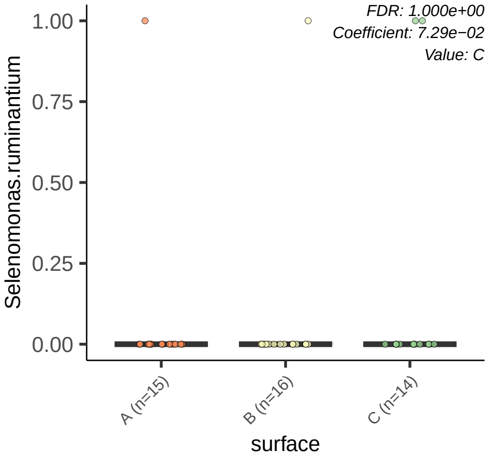

FDR: 1.000e+00
Coefficient: 7.29e−02
Value: C
Selenomonas.ruminantium
1.00
0.75
0.50
0.25
0.00
A (n=15)
B (n=16)
C (n=14)
surface

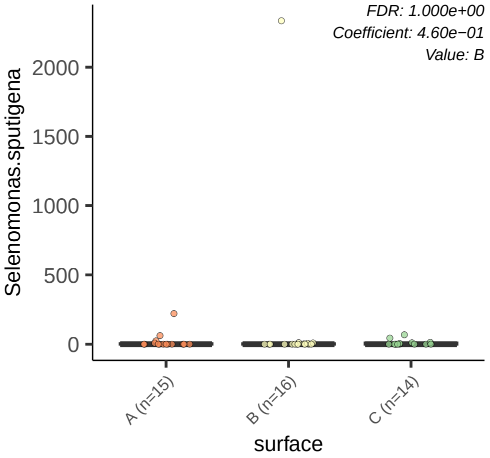

FDR: 1.000e+00
Coefficient: 4.60e−01
Value: B
Selenomonas.sputigena
0
500
1000
1500
2000
A (n=15)
B (n=16)
C (n=14)
surface

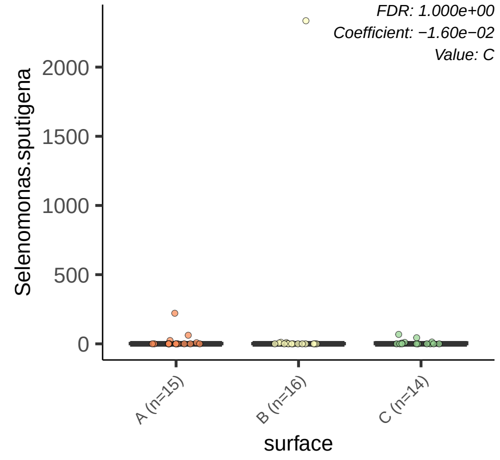

FDR: 1.000e+00
Coefficient: −1.60e−02
Value: C
Selenomonas.sputigena
2000
1500
1000
500
0
A (n=15)
B (n=16)
C (n=14)
surface

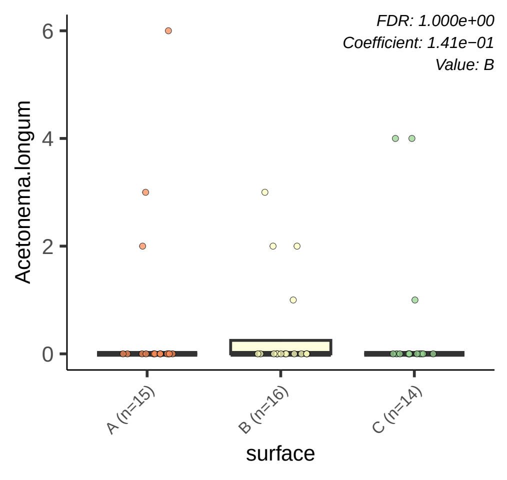

FDR: 1.000e+00
Coefficient: 1.41e−01
Value: B
Acetonema.longum
0
2
4
6
A (n=15)
B (n=16)
C (n=14)
surface

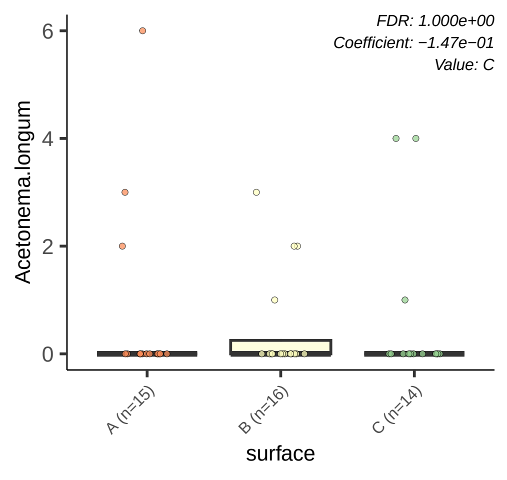

FDR: 1.000e+00
Coefficient: −1.47e−01
Value: C
Acetonema.longum
0
2
4
6
A (n=15)
B (n=16)
C (n=14)
surface

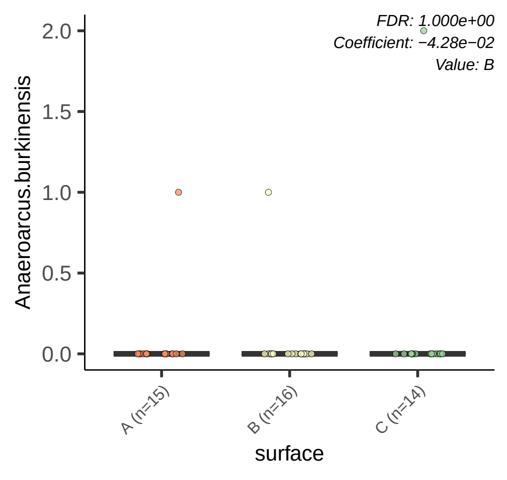
FDR: 1.000e+00
Coefficient: −4.28e−02
Value: B
Anaeroarcus.burkinensis
2.0
1.5
1.0
0.5
0.0
A (n=15)
B (n=16)
C (n=14)
surface

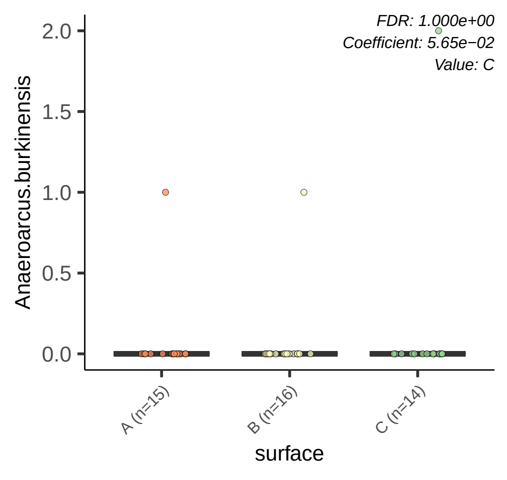
FDR: 1.000e+00
Coefficient: 5.65e−02
Value: C
Anaeroarcus.burkinensis
2.0
1.5
1.0
0.5
0.0
A (n=15)
B (n=16)
C (n=14)
surface

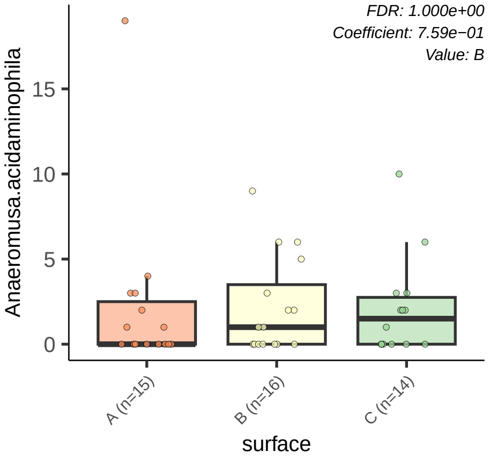
FDR: 1.000e+00
Coefficient: 7.59e−01
Value: B
Anaeromusa.acidaminophila
0
5
10
15
A (n=15)
B (n=16)
C (n=14)
surface

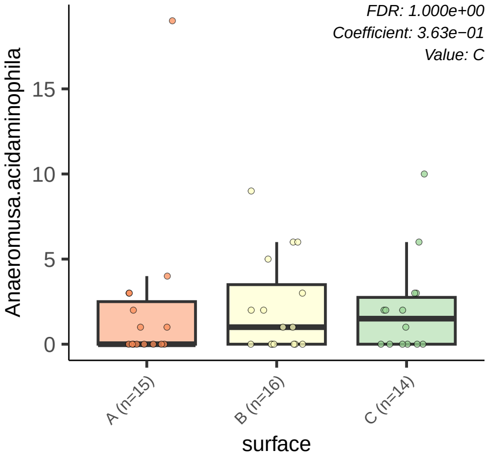

FDR: 1.000e+00
Coefficient: 3.63e−01
Value: C
Anaeromusa.acidaminophila
0
5
10
15
A (n=15)
B (n=16)
C (n=14)
surface

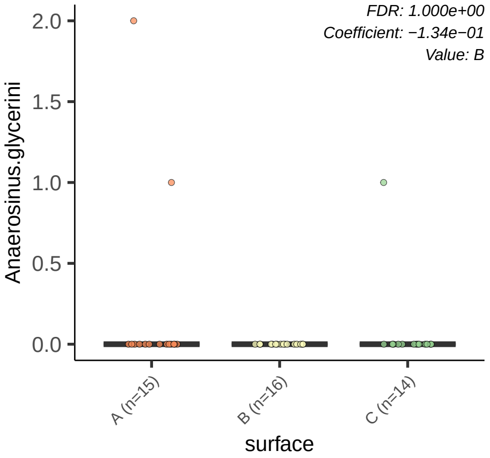
FDR: 1.000e+00
Coefficient: −1.34e−01
Value: B
Anaerosinus.glycerini
2.0
1.5
1.0
0.5
0.0
A (n=15)
B (n=16)
C (n=14)
surface

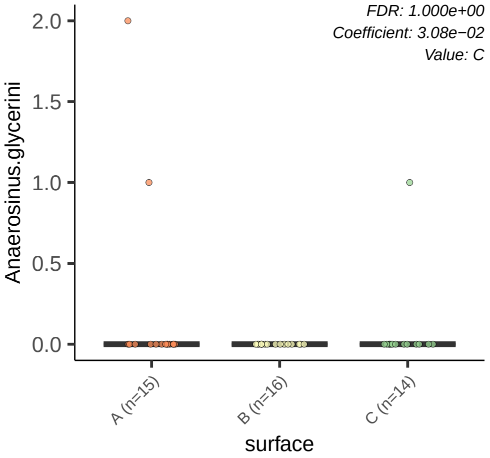
FDR: 1.000e+00
Coefficient: 3.08e−02
Value: C
Anaerosinus.glycerini
2.0
1.5
1.0
0.5
0.0
A (n=15)
B (n=16)
C (n=14)
surface

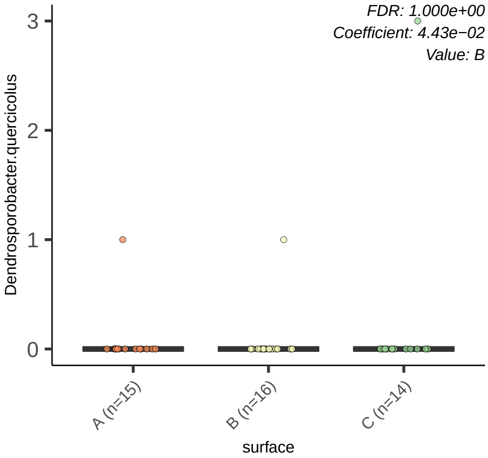

FDR: 1.000e+00
Coefficient: 4.43e−02
Value: B
Dendrosporobacter.quercicolus
3
2
1
0
A (n=15)
B (n=16)
C (n=14)
surface

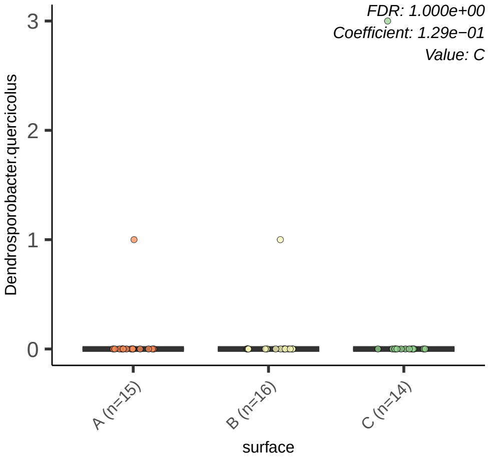
FDR: 1.000e+00
Coefficient: 1.29e−01
Value: C
Dendrosporobacter.quercicolus
3
2
1
0
A (n=15)
B (n=16)
C (n=14)
surface

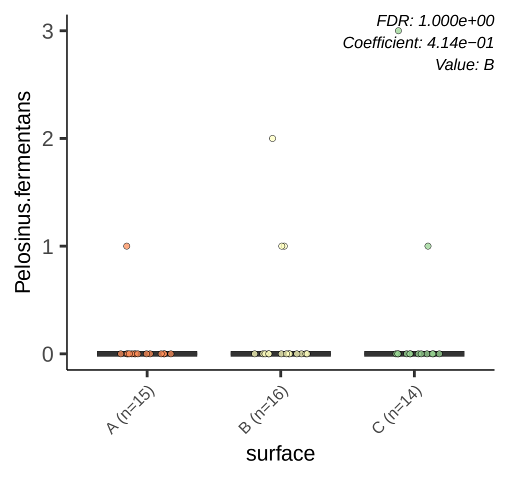
FDR: 1.000e+00
Coefficient: 4.14e−01
Value: B
Pelosinus.fermentans
3
2
1
0
A (n=15)
B (n=16)
C (n=14)
surface

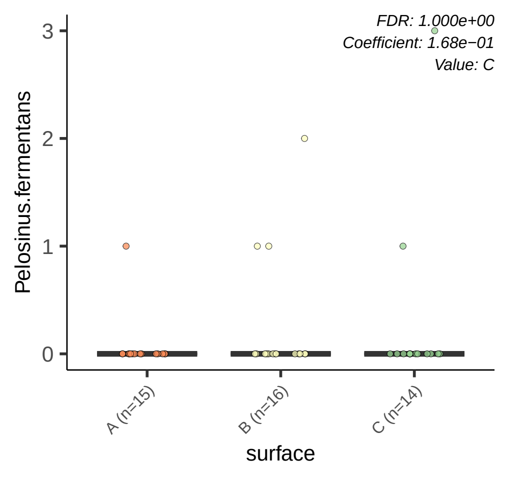

FDR: 1.000e+00
Coefficient: 1.68e−01
Value: C
Pelosinus.fermentans
0
1
2
3
A (n=15)
B (n=16)
C (n=14)
surface

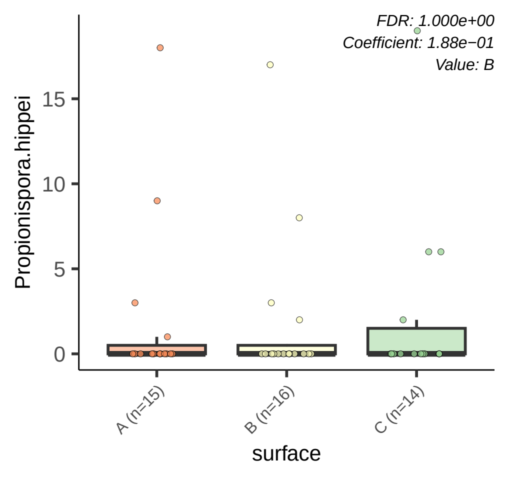
FDR: 1.000e+00
Coefficient: 1.88e−01
Value: B
Propionispora.hippei
0
5
10
15
A (n=15)
B (n=16)
C (n=14)
surface

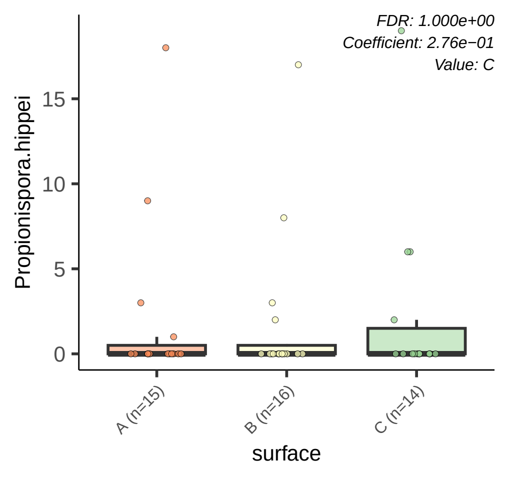
FDR: 1.000e+00
Coefficient: 2.76e−01
Value: C
Propionispora.hippei
0
5
10
15
A (n=15)
B (n=16)
C (n=14)
surface

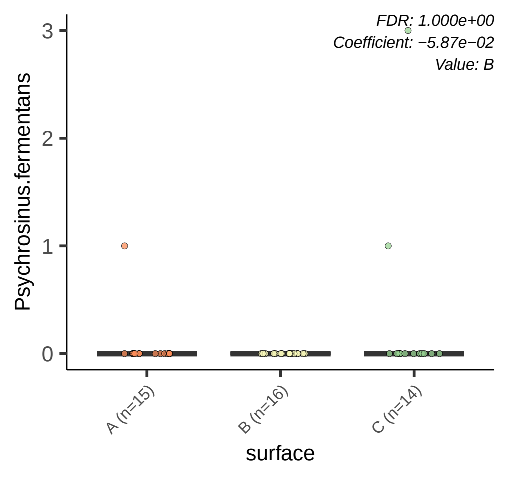
FDR: 1.000e+00
Coefficient: −5.87e−02
Value: B
Psychrosinus.fermentans
3
2
1
0
A (n=15)
B (n=16)
C (n=14)
surface

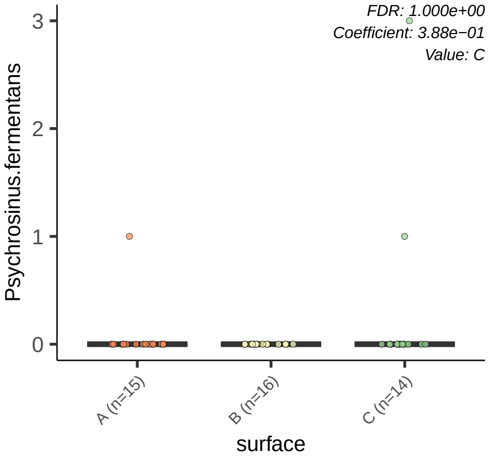
FDR: 1.000e+00
Coefficient: 3.88e−01
Value: C
Psychrosinus.fermentans
0
1
2
3
A (n=15)
B (n=16)
C (n=14)
surface

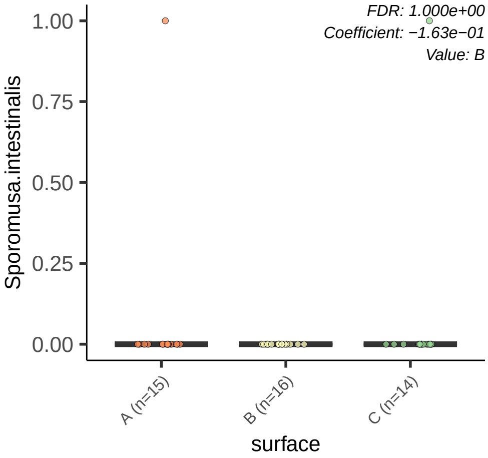

FDR: 1.000e+00
Coefficient: −1.63e−01
Value: B
Sporomusa.intestinalis
1.00
0.75
0.50
0.25
0.00
A (n=15)
B (n=16)
C (n=14)
surface

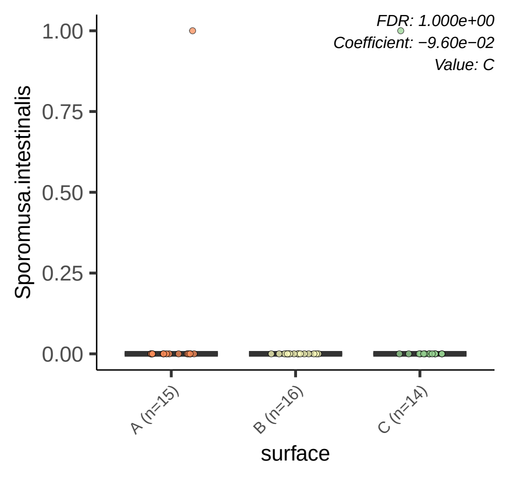
FDR: 1.000e+00
Coefficient: −9.60e−02
Value: C
Sporomusa.intestinalis
1.00
0.75
0.50
0.25
0.00
A (n=15)
B (n=16)
C (n=14)
surface

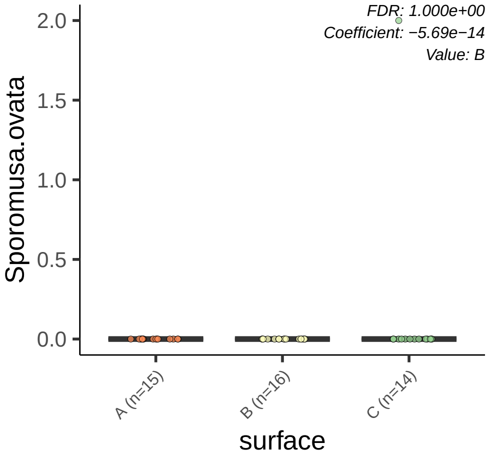
FDR: 1.000e+00
Coefficient: −5.69e−14
Value: B
Sporomusa.ovata
2.0
1.5
1.0
0.5
0.0
A (n=15)
B (n=16)
C (n=14)
surface

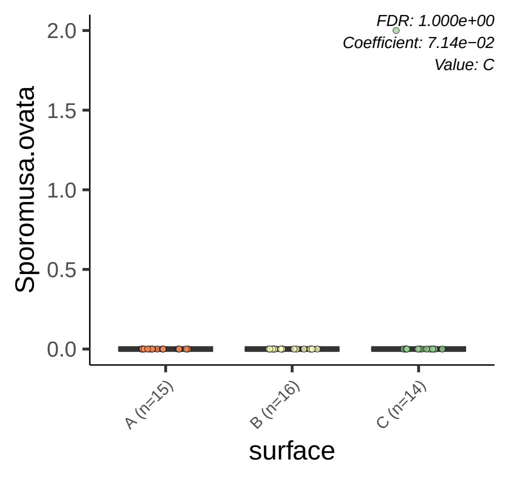
FDR: 1.000e+00
Coefficient: 7.14e−02
Value: C
Sporomusa.ovata
2.0
1.5
1.0
0.5
0.0
A (n=15)
B (n=16)
C (n=14)
surface

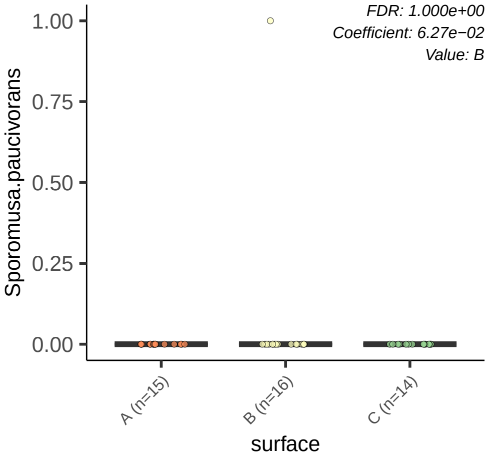
FDR: 1.000e+00
Coefficient: 6.27e−02
Value: B
Sporomusa.paucivorans
1.00
0.75
0.50
0.25
0.00
A (n=15)
B (n=16)
C (n=14)
surface

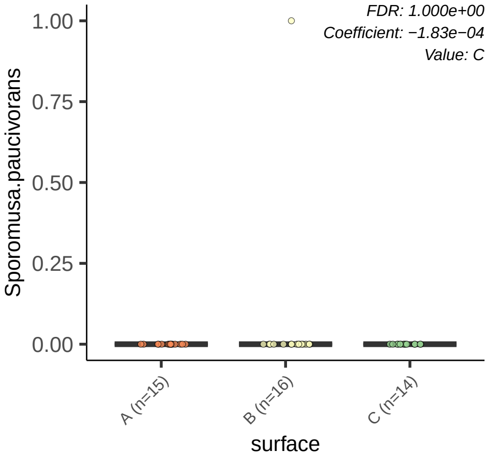
FDR: 1.000e+00
Coefficient: −1.83e−04
Value: C
Sporomusa.paucivorans
1.00
0.75
0.50
0.25
0.00
A (n=15)
B (n=16)
C (n=14)
surface

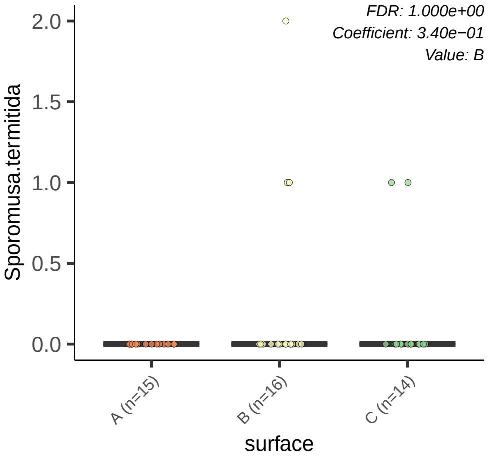
FDR: 1.000e+00
Coefficient: 3.40e−01
Value: B
Sporomusa.termitida
2.0
1.5
1.0
0.5
0.0
A (n=15)
B (n=16)
C (n=14)
surface

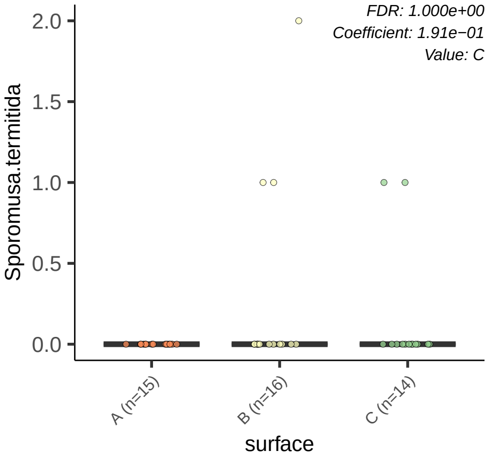

FDR: 1.000e+00
Coefficient: 1.91e−01
Value: C
Sporomusa.termitida
2.0
1.5
1.0
0.5
0.0
A (n=15)
B (n=16)
C (n=14)
surface

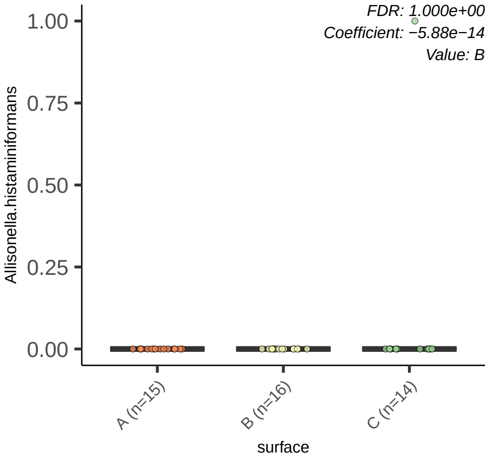
FDR: 1.000e+00
Coefficient: −5.88e−14
Value: B
1.00
0.75
0.50
0.25
0.00
Allisonella.histaminiformans
A (n=15)
B (n=16)
C (n=14)
surface

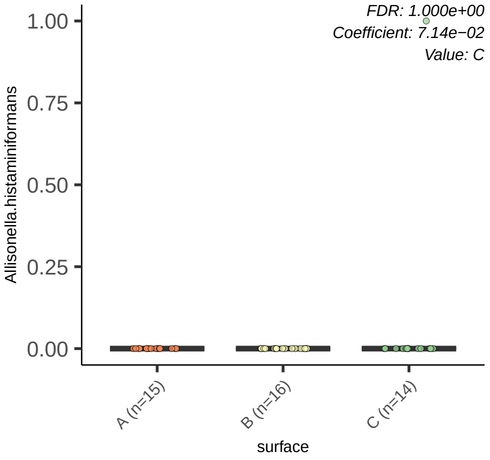

FDR: 1.000e+00
Coefficient: 7.14e−02
Value: C
Allisonella.histaminiformans
1.00
0.75
0.50
0.25
0.00
A (n=15)
B (n=16)
C (n=14)
surface

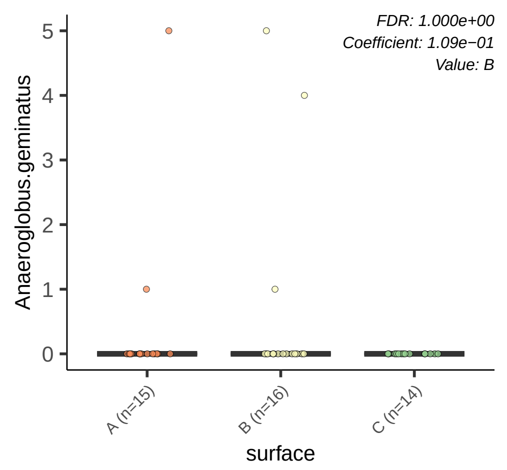

FDR: 1.000e+00
Coefficient: 1.09e−01
Value: B
Anaeroglobus.geminatus
0
1
2
3
4
5
A (n=15)
B (n=16)
C (n=14)
surface

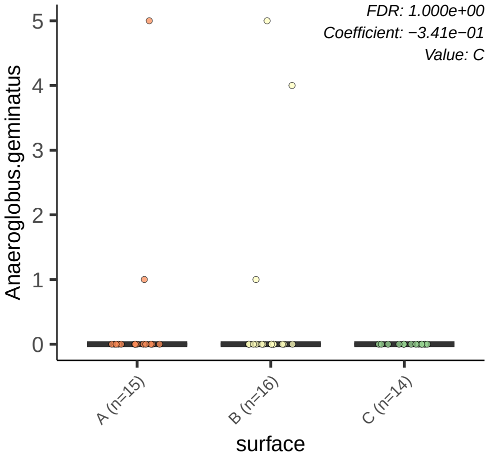
FDR: 1.000e+00
Coefficient: −3.41e−01
Value: C
Anaeroglobus.geminatus
0
1
2
3
4
5
A (n=15)
B (n=16)
C (n=14)
surface

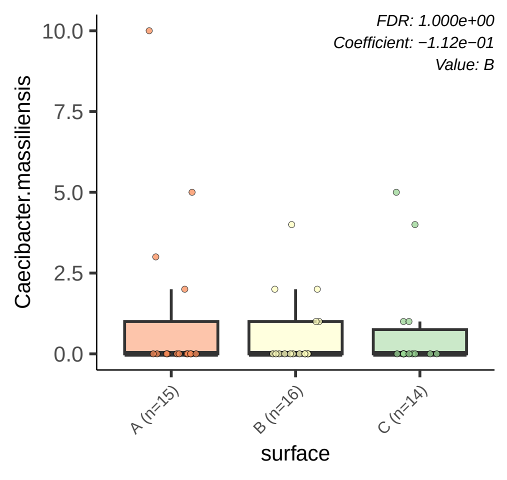
FDR: 1.000e+00
Coefficient: −1.12e−01
Value: B
Caecibacter.massiliensis
10.0
7.5
5.0
2.5
0.0
A (n=15)
B (n=16)
C (n=14)
surface

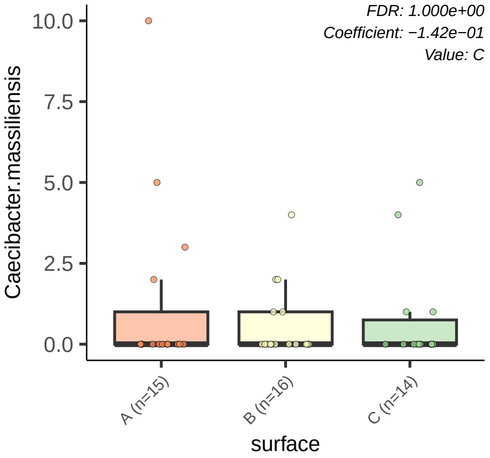

FDR: 1.000e+00
Coefficient: −1.42e−01
Value: C
Caecibacter.massiliensis
10.0
7.5
5.0
2.5
0.0
A (n=15)
B (n=16)
C (n=14)
surface

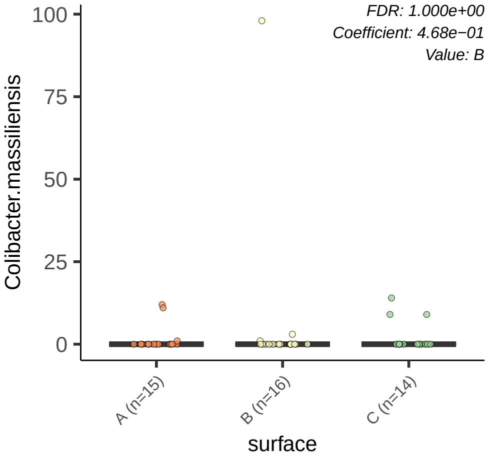
FDR: 1.000e+00
Coefficient: 4.68e−01
Value: B
100
75
50
25
0
Colibacter.massiliensis
A (n=15)
B (n=16)
C (n=14)
surface

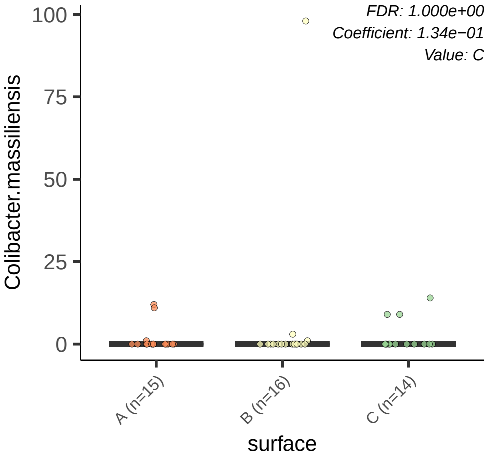

FDR: 1.000e+00
Coefficient: 1.34e−01
Value: C
Colibacter.massiliensis
100
75
50
25
0
A (n=15)
B (n=16)
C (n=14)
surface

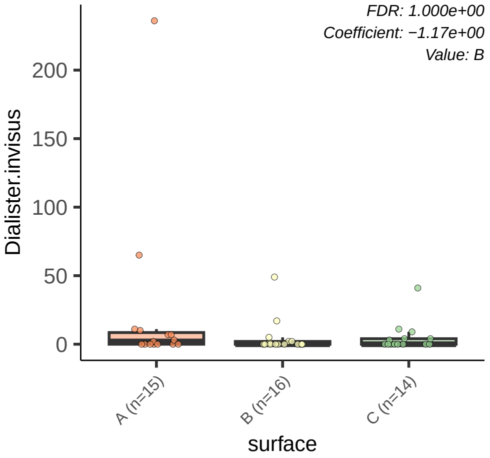
FDR: 1.000e+00
Coefficient: −1.17e+00
Value: B
Dialister.invisus
200
150
100
50
0
A (n=15)
B (n=16)
C (n=14)
surface

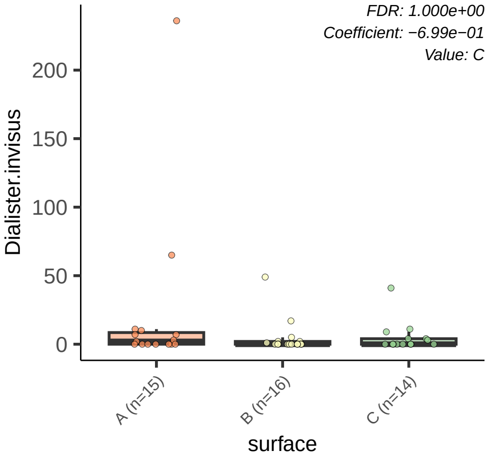
FDR: 1.000e+00
Coefficient: −6.99e−01
Value: C
Dialister.invisus
200
150
100
50
0
A (n=15)
B (n=16)
C (n=14)
surface

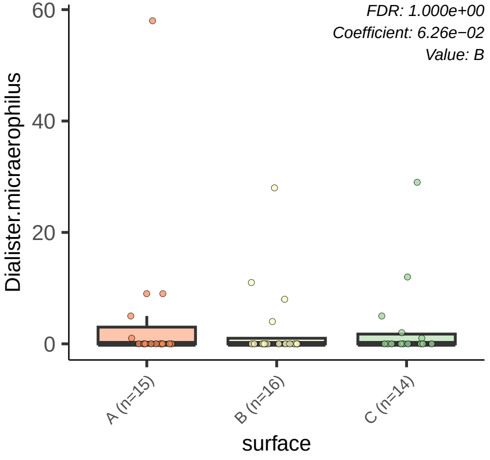
FDR: 1.000e+00
Coefficient: 6.26e−02
Value: B
Dialister.micraerophilus
0
20
40
60
A (n=15)
B (n=16)
C (n=14)
surface

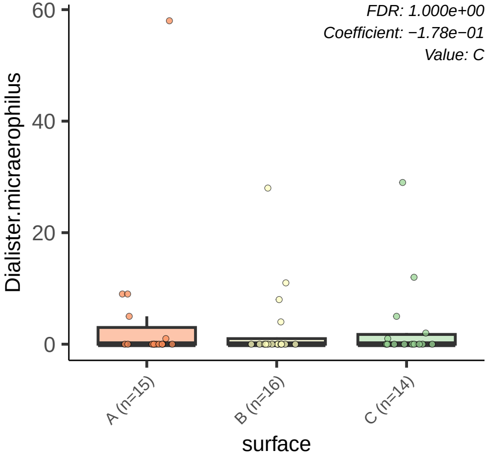
FDR: 1.000e+00
Coefficient: −1.78e−01
Value: C
Dialister.micraerophilus
0
20
40
60
A (n=15)
B (n=16)
C (n=14)
surface

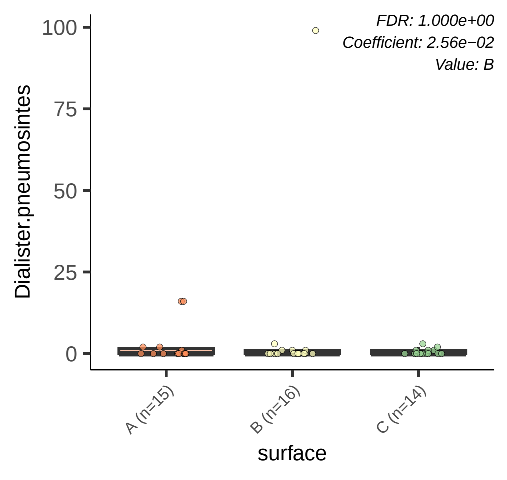

FDR: 1.000e+00
Coefficient: 2.56e−02
Value: B
Dialister.pneumosintes
100
75
50
25
0
A (n=15)
B (n=16)
C (n=14)
surface

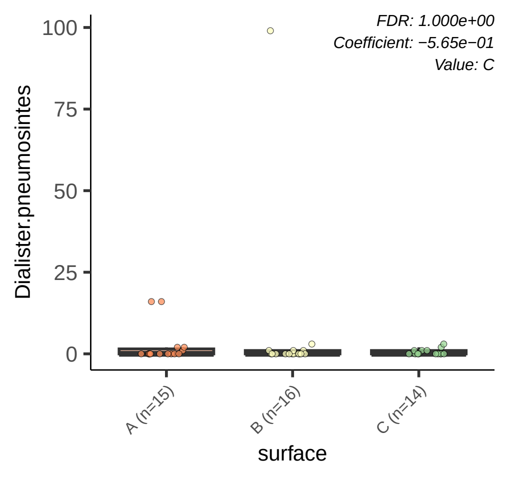
FDR: 1.000e+00
Coefficient: −5.65e−01
Value: C
Dialister.pneumosintes
100
75
50
25
0
A (n=15)
B (n=16)
C (n=14)
surface

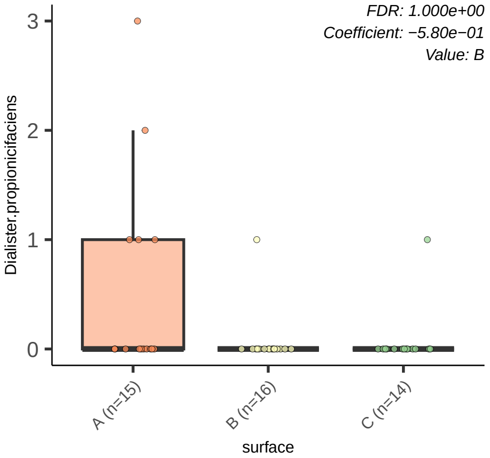
FDR: 1.000e+00
Coefficient: −5.80e−01
Value: B
Dialister.propionicifaciens
3
2
1
0
A (n=15)
B (n=16)
C (n=14)
surface

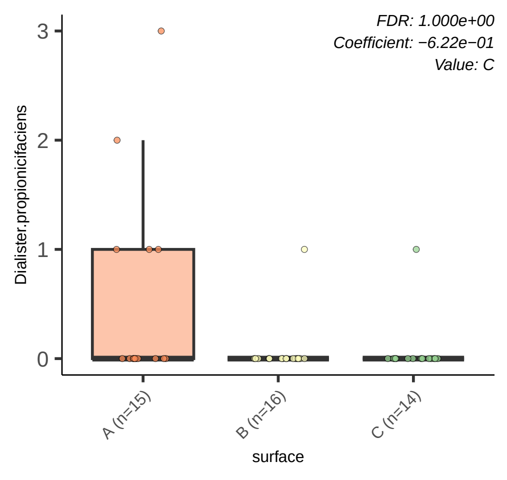
FDR: 1.000e+00
Coefficient: −6.22e−01
Value: C
Dialister.propionicifaciens
3
2
1
0
A (n=15)
B (n=16)
C (n=14)
surface

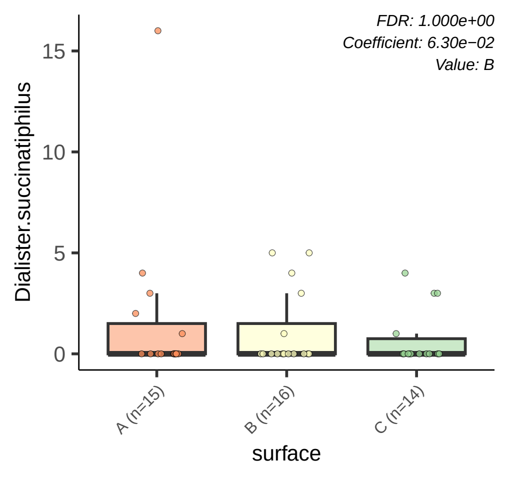

FDR: 1.000e+00
Coefficient: 6.30e−02
Value: B
Dialister.succinatiphilus
15
10
5
0
A (n=15)
B (n=16)
C (n=14)
surface

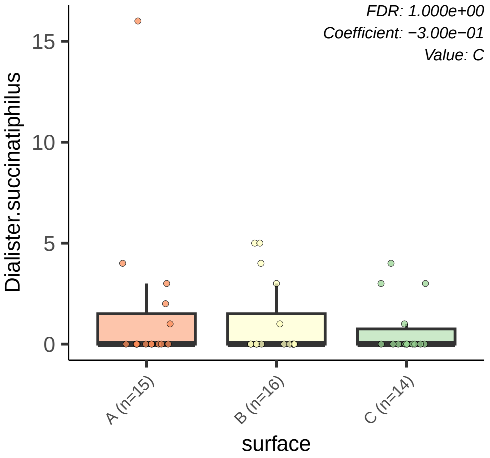

FDR: 1.000e+00
Coefficient: −3.00e−01
Value: C
Dialister.succinatiphilus
15
10
5
0
A (n=15)
B (n=16)
C (n=14)
surface

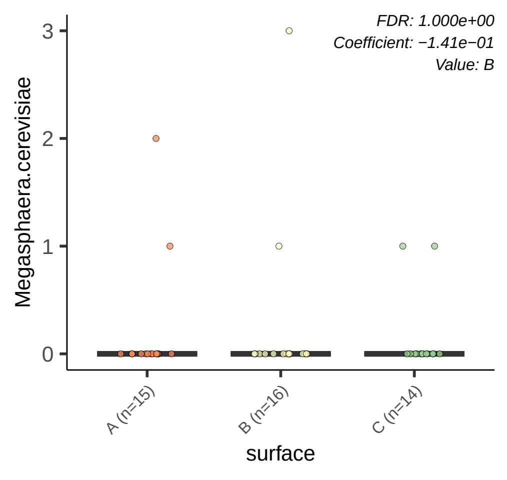
FDR: 1.000e+00
Coefficient: −1.41e−01
Value: B
Megasphaera.cerevisiae
3
2
1
0
A (n=15)
B (n=16)
C (n=14)
surface

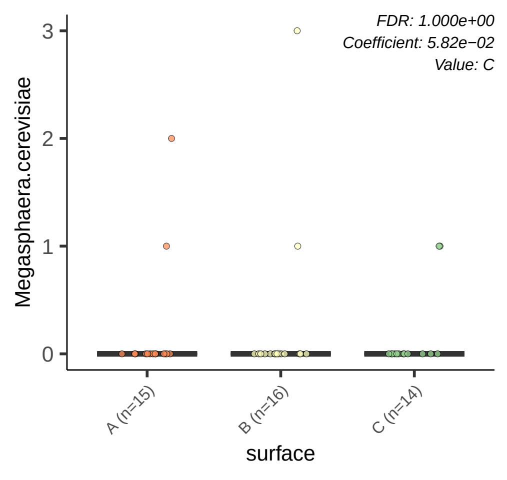
FDR: 1.000e+00
Coefficient: 5.82e−02
Value: C
Megasphaera.cerevisiae
3
2
1
0
A (n=15)
B (n=16)
C (n=14)
surface

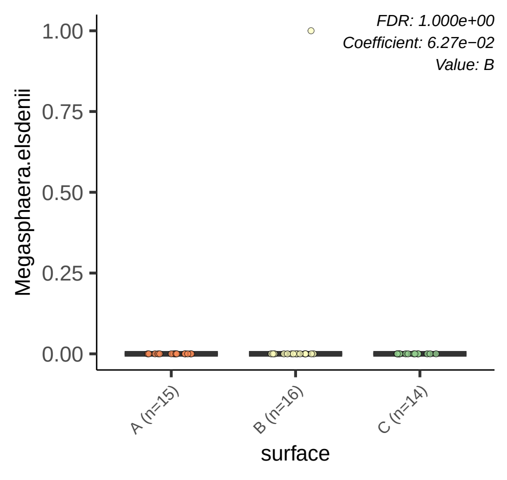

FDR: 1.000e+00
Coefficient: 6.27e−02
Value: B
Megasphaera.elsdenii
1.00
0.75
0.50
0.25
0.00
A (n=15)
B (n=16)
C (n=14)
surface

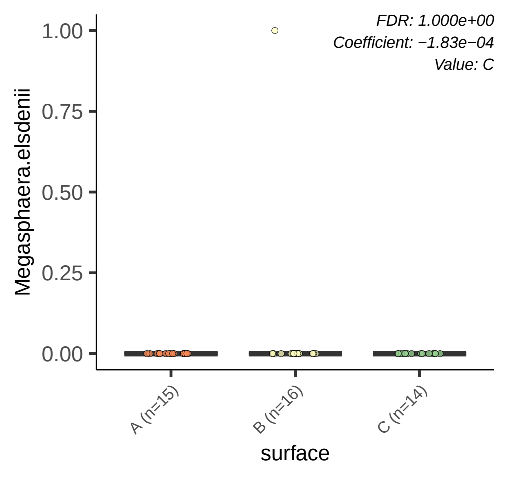
FDR: 1.000e+00
Coefficient: −1.83e−04
Value: C
Megasphaera.elsdenii
1.00
0.75
0.50
0.25
0.00
A (n=15)
B (n=16)
C (n=14)
surface

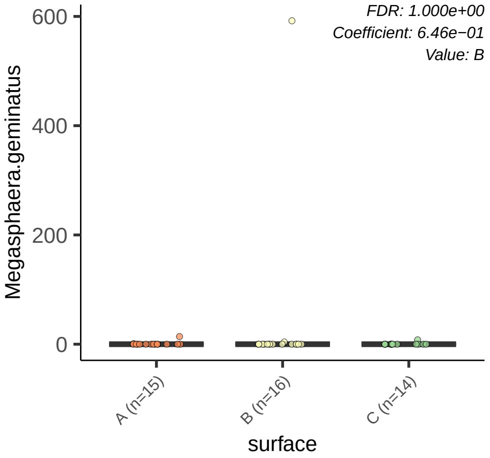

FDR: 1.000e+00
Coefficient: 6.46e−01
Value: B
600
400
200
0
Megasphaera.geminatus
A (n=15)
B (n=16)
C (n=14)
surface

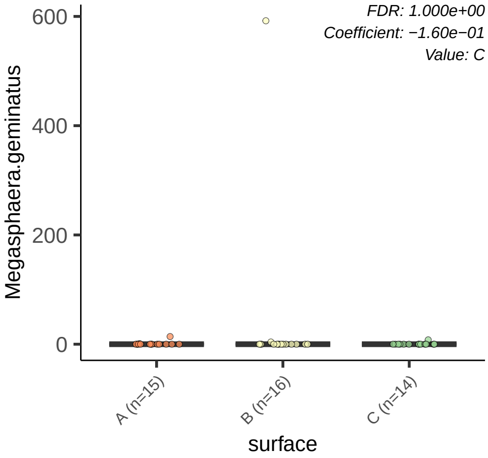

FDR: 1.000e+00
Coefficient: −1.60e−01
Value: C
600
400
200
0
Megasphaera.geminatus
A (n=15)
B (n=16)
C (n=14)
surface

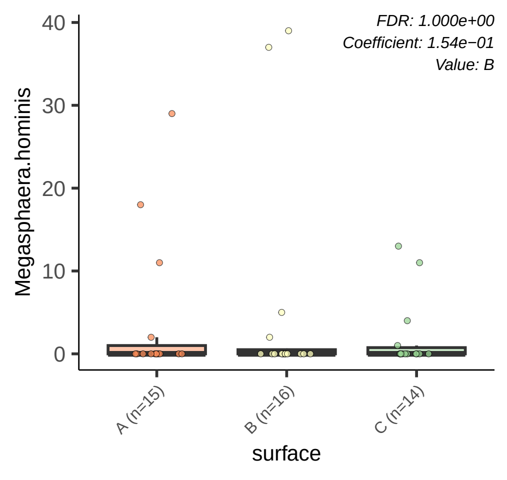

FDR: 1.000e+00
Coefficient: 1.54e−01
Value: B
Megasphaera.hominis
0
10
20
30
40
A (n=15)
B (n=16)
C (n=14)
surface

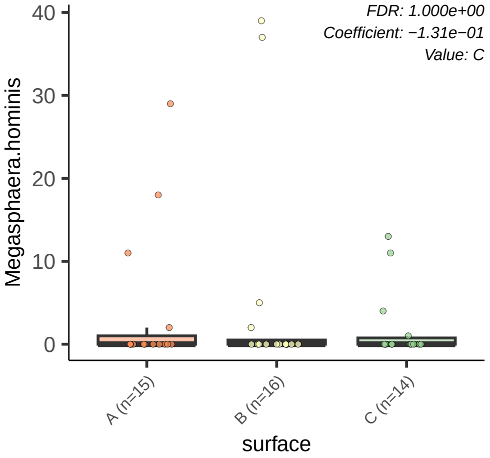

FDR: 1.000e+00
Coefficient: −1.31e−01
Value: C
Megasphaera.hominis
40
30
20
10
0
A (n=15)
B (n=16)
C (n=14)
surface

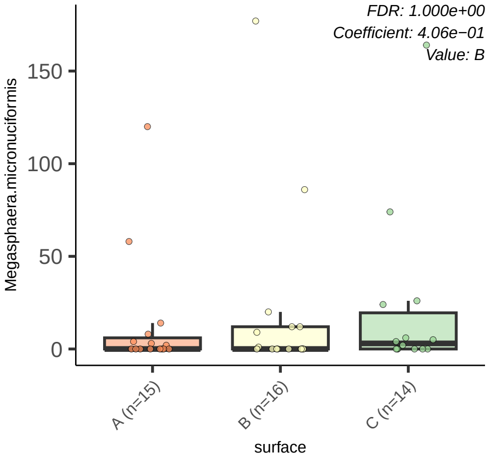

FDR: 1.000e+00
Coefficient: 4.06e−01
Value: B
Megasphaera.micronuciformis
150
100
50
0
A (n=15)
B (n=16)
C (n=14)
surface

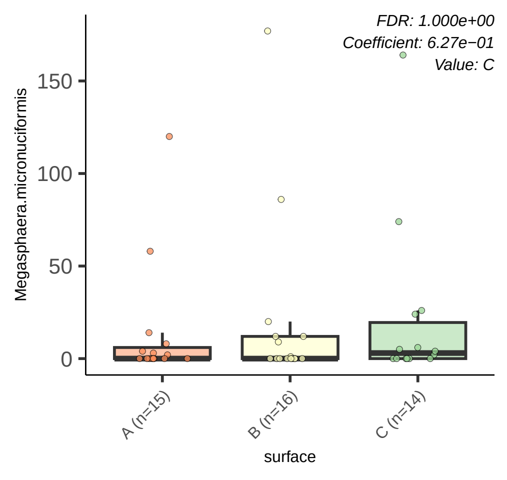

FDR: 1.000e+00
Coefficient: 6.27e−01
Value: C
Megasphaera.micronuciformis
150
100
50
0
A (n=15)
B (n=16)
C (n=14)
surface

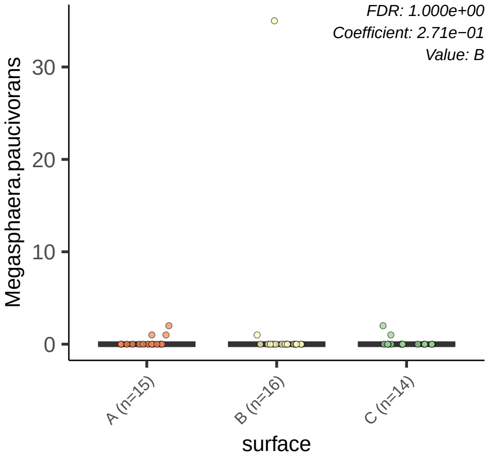

FDR: 1.000e+00
Coefficient: 2.71e−01
Value: B
Megasphaera.paucivorans
0
10
20
30
A (n=15)
B (n=16)
C (n=14)
surface

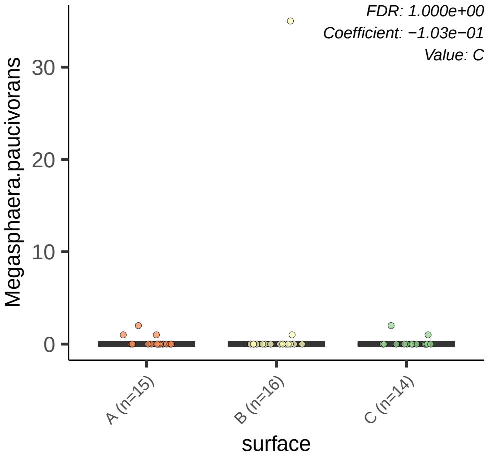

FDR: 1.000e+00
Coefficient: −1.03e−01
Value: C
Megasphaera.paucivorans
0
10
20
30
A (n=15)
B (n=16)
C (n=14)
surface

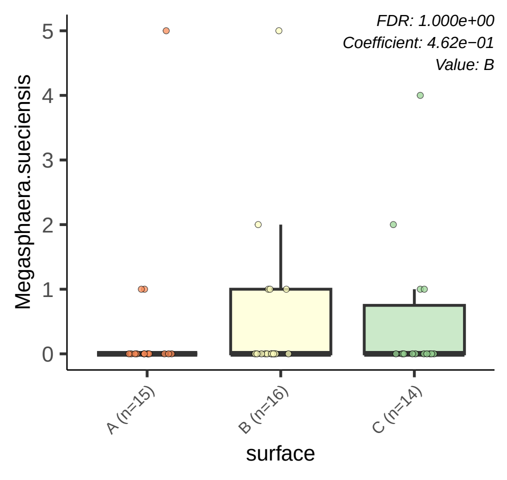
FDR: 1.000e+00
Coefficient: 4.62e−01
Value: B
Megasphaera.sueciensis
0
1
2
3
4
5
A (n=15)
B (n=16)
C (n=14)
surface

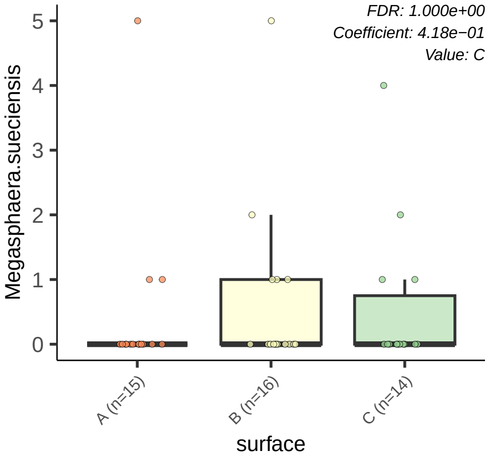

FDR: 1.000e+00
Coefficient: 4.18e−01
Value: C
Megasphaera.sueciensis
0
1
2
3
4
5
A (n=15)
B (n=16)
C (n=14)
surface

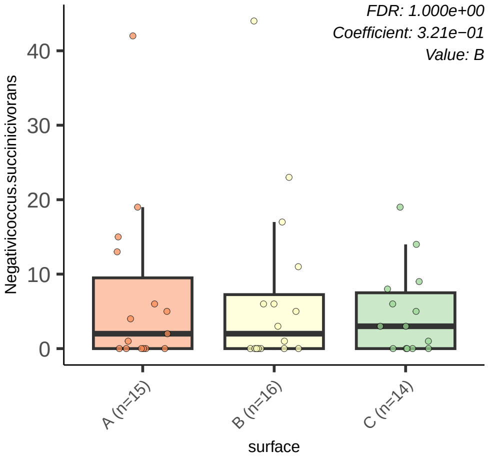

FDR: 1.000e+00
Coefficient: 3.21e−01
Value: B
Negativicoccus.succinicivorans
0
10
20
30
40
A (n=15)
B (n=16)
C (n=14)
surface

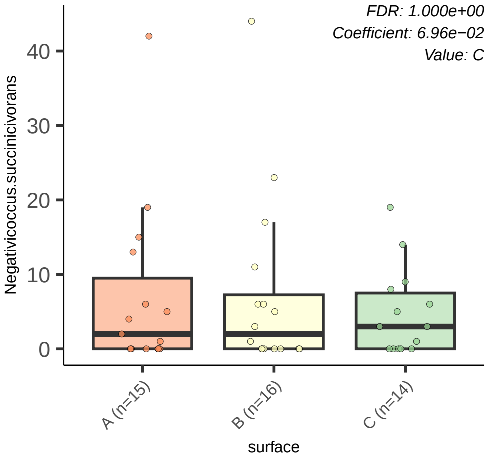

FDR: 1.000e+00
Coefficient: 6.96e−02
Value: C
Negativicoccus.succinicivorans
0
10
20
30
40
A (n=15)
B (n=16)
C (n=14)
surface

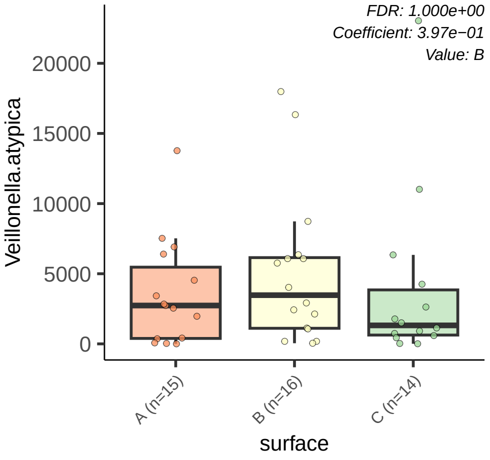
FDR: 1.000e+00
Coefficient: 3.97e−01
Value: B
Veillonella.atypica
20000
15000
10000
5000
0
A (n=15)
B (n=16)
C (n=14)
surface

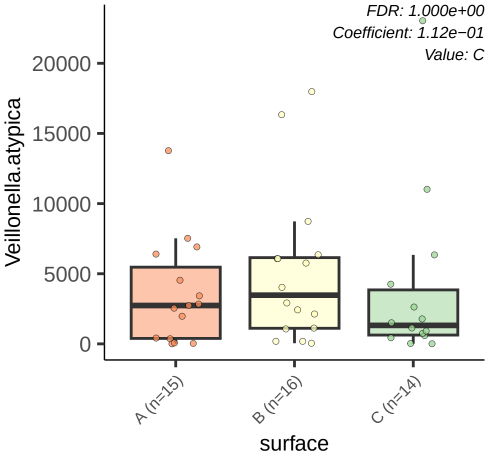
FDR: 1.000e+00
Coefficient: 1.12e−01
Value: C
Veillonella.atypica
20000
15000
10000
5000
0
A (n=15)
B (n=16)
C (n=14)
surface

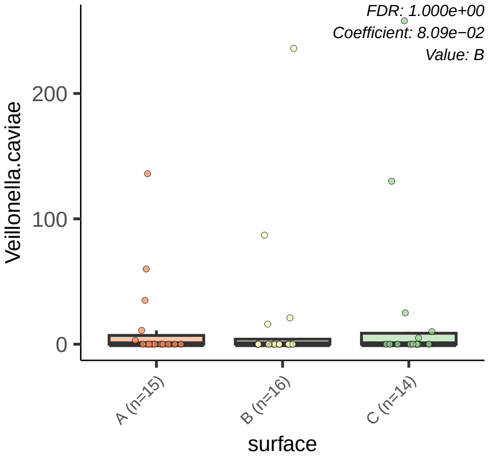

FDR: 1.000e+00
Coefficient: 8.09e−02
Value: B
Veillonella.caviae
200
100
0
A (n=15)
B (n=16)
C (n=14)
surface

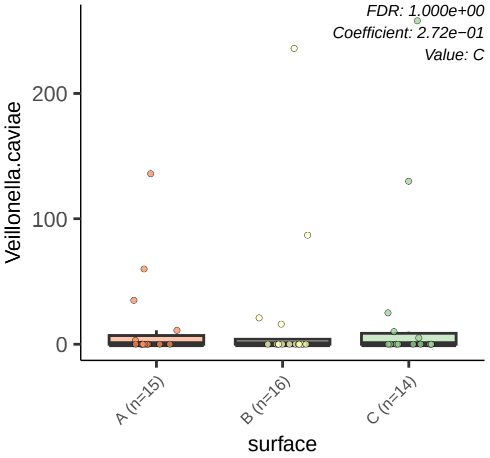
FDR: 1.000e+00
Coefficient: 2.72e−01
Value: C
Veillonella.caviae
200
100
0
A (n=15)
B (n=16)
C (n=14)
surface

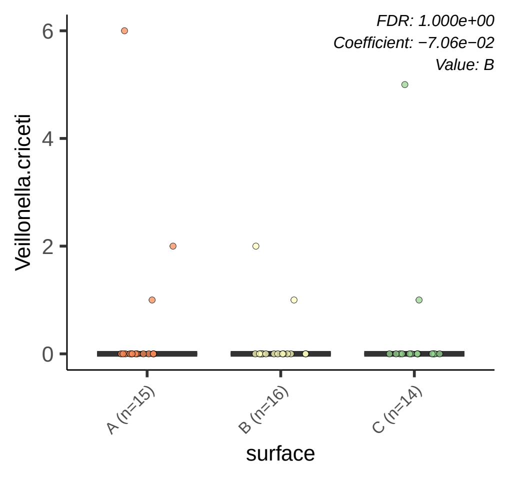
FDR: 1.000e+00
Coefficient: −7.06e−02
Value: B
Veillonella.criceti
6
4
2
0
A (n=15)
B (n=16)
C (n=14)
surface

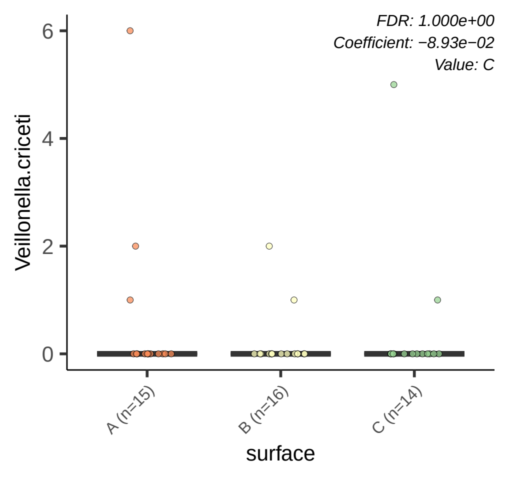

FDR: 1.000e+00
Coefficient: −8.93e−02
Value: C
Veillonella.criceti
0
2
4
6
A (n=15)
B (n=16)
C (n=14)
surface

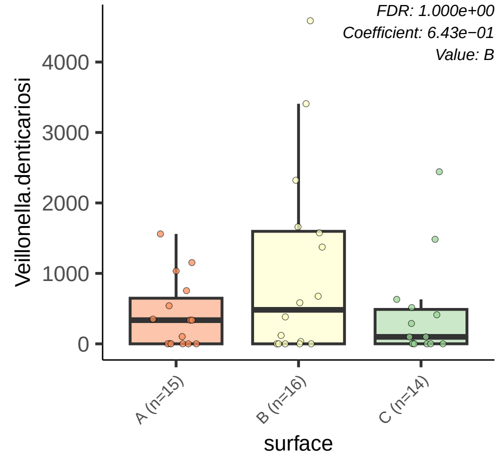

FDR: 1.000e+00
Coefficient: 6.43e−01
Value: B
Veillonella.denticariosi
0
1000
2000
3000
4000
A (n=15)
B (n=16)
C (n=14)
surface

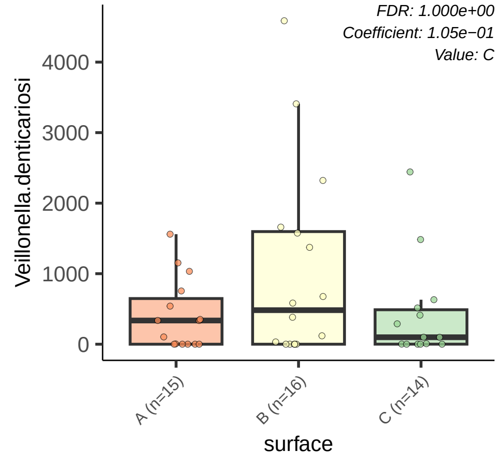

FDR: 1.000e+00
Coefficient: 1.05e−01
Value: C
4000
3000
2000
1000
0
Veillonella.denticariosi
A (n=15)
B (n=16)
C (n=14)
surface

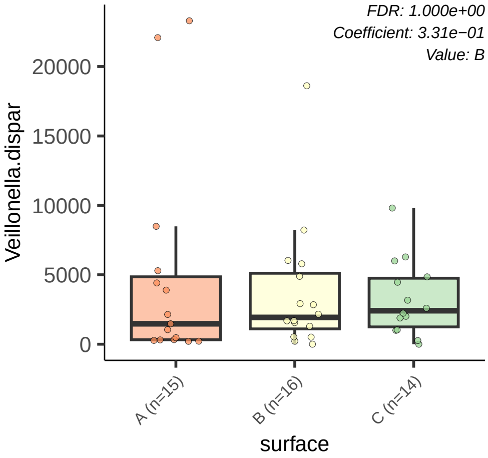
FDR: 1.000e+00
Coefficient: 3.31e−01
Value: B
Veillonella.dispar
20000
15000
10000
5000
0
A (n=15)
B (n=16)
C (n=14)
surface

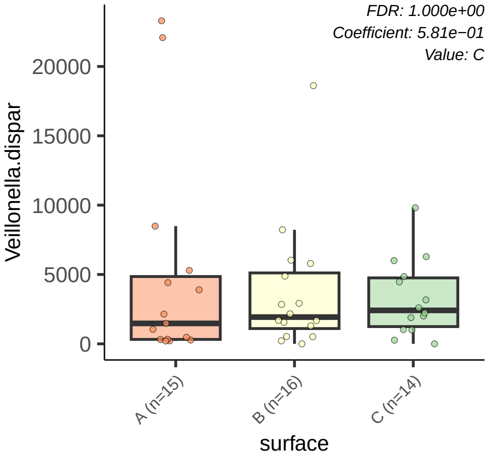

FDR: 1.000e+00
Coefficient: 5.81e−01
Value: C
Veillonella.dispar
20000
15000
10000
5000
0
A (n=15)
B (n=16)
C (n=14)
surface

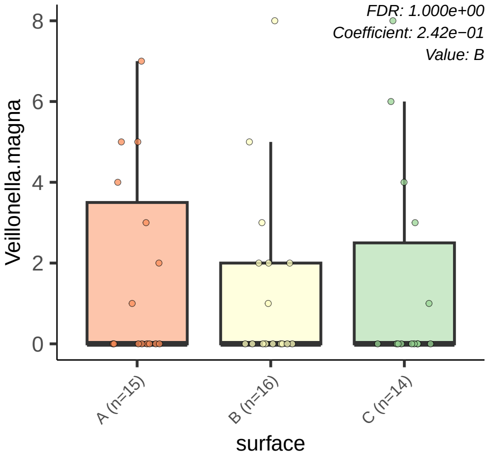

FDR: 1.000e+00
Coefficient: 2.42e−01
Value: B
Veillonella.magna
0
2
4
6
8
A (n=15)
B (n=16)
C (n=14)
surface

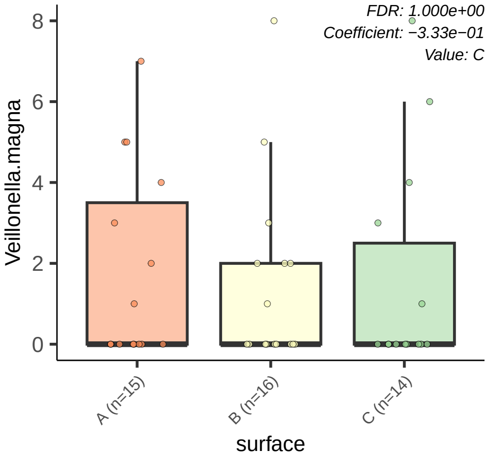

FDR: 1.000e+00
Coefficient: −3.33e−01
Value: C
Veillonella.magna
0
2
4
6
8
A (n=15)
B (n=16)
C (n=14)
surface

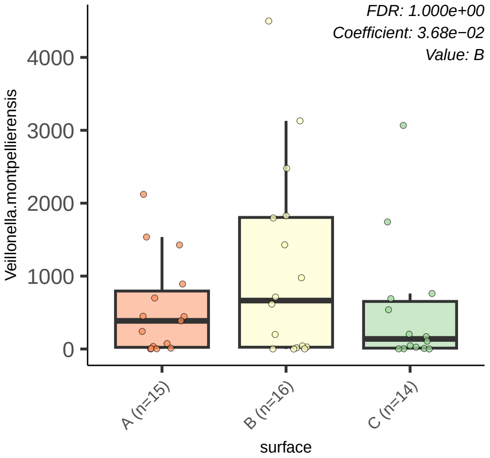
FDR: 1.000e+00
Coefficient: 3.68e−02
Value: B
Veillonella.montpellierensis
4000
3000
2000
1000
0
A (n=15)
B (n=16)
C (n=14)
surface

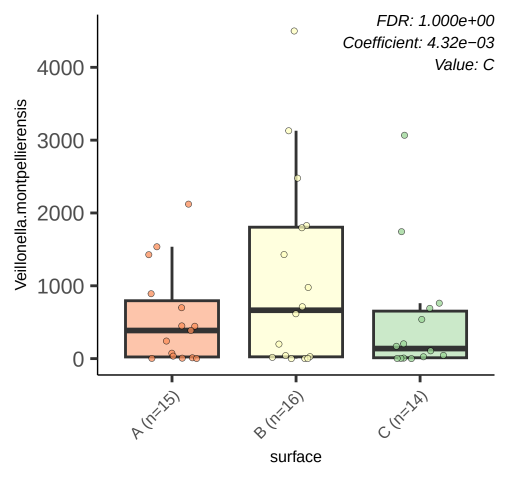

FDR: 1.000e+00
Coefficient: 4.32e−03
Value: C
Veillonella.montpellierensis
4000
3000
2000
1000
0
A (n=15)
B (n=16)
C (n=14)
surface

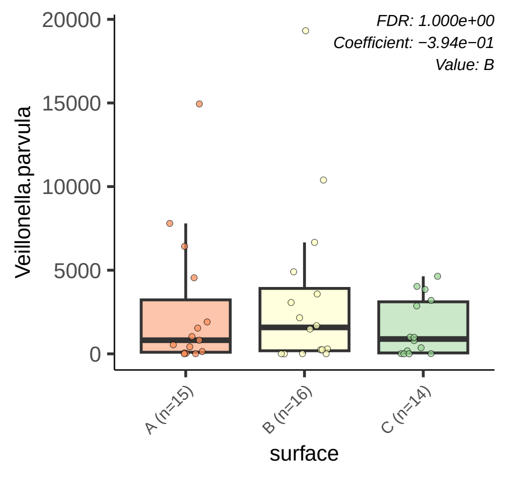

FDR: 1.000e+00
Coefficient: −3.94e−01
Value: B
20000
15000
10000
5000
0
Veillonella.parvula
A (n=15)
B (n=16)
C (n=14)
surface

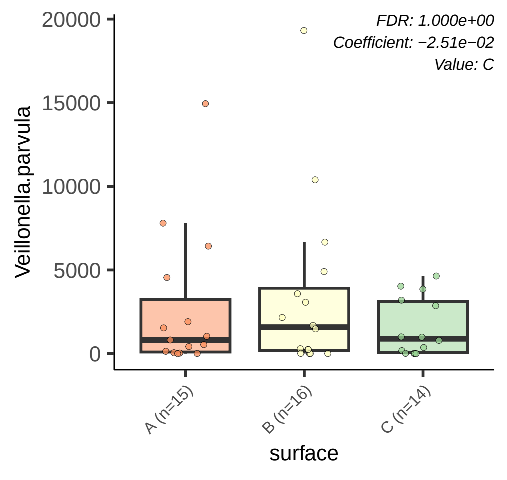
FDR: 1.000e+00
Coefficient: −2.51e−02
Value: C
Veillonella.parvula
20000
15000
10000
5000
0
A (n=15)
B (n=16)
C (n=14)
surface

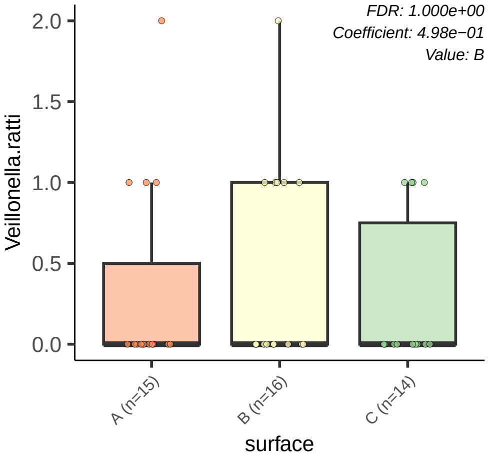
FDR: 1.000e+00
Coefficient: 4.98e−01
Value: B
Veillonella.ratti
2.0
1.5
1.0
0.5
0.0
A (n=15)
B (n=16)
C (n=14)
surface

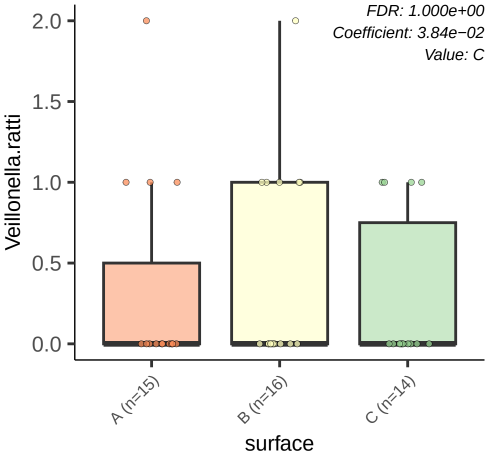
FDR: 1.000e+00
Coefficient: 3.84e−02
Value: C
Veillonella.ratti
2.0
1.5
1.0
0.5
0.0
A (n=15)
B (n=16)
C (n=14)
surface

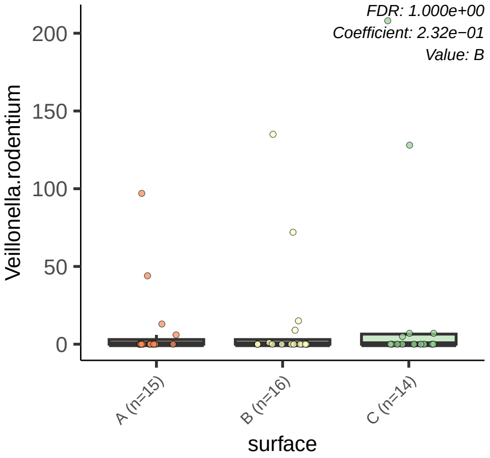

FDR: 1.000e+00
Coefficient: 2.32e−01
Value: B
Veillonella.rodentium
200
150
100
50
0
A (n=15)
B (n=16)
C (n=14)
surface

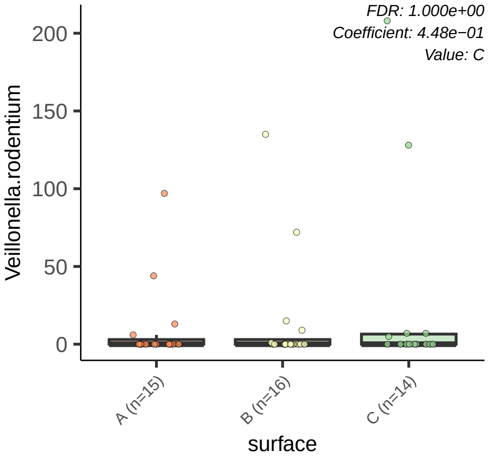
FDR: 1.000e+00
Coefficient: 4.48e−01
Value: C
Veillonella.rodentium
200
150
100
50
0
A (n=15)
B (n=16)
C (n=14)
surface

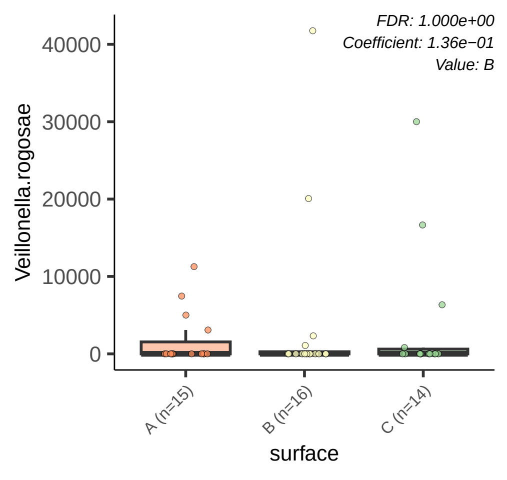
FDR: 1.000e+00
Coefficient: 1.36e−01
Value: B
Veillonella.rogosae
40000
30000
20000
10000
0
A (n=15)
B (n=16)
C (n=14)
surface

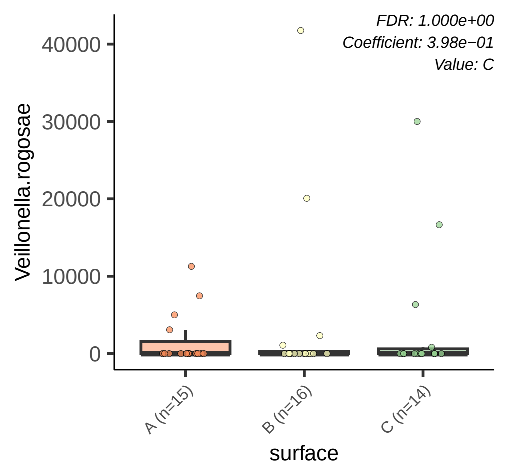

FDR: 1.000e+00
Coefficient: 3.98e−01
Value: C
Veillonella.rogosae
40000
30000
20000
10000
0
A (n=15)
B (n=16)
C (n=14)
surface

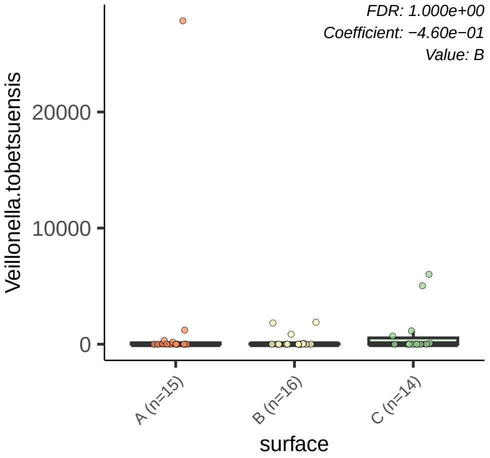

FDR: 1.000e+00
Coefficient: −4.60e−01
Value: B
Veillonella.tobetsuensis
20000
10000
0
A (n=15)
B (n=16)
C (n=14)
surface

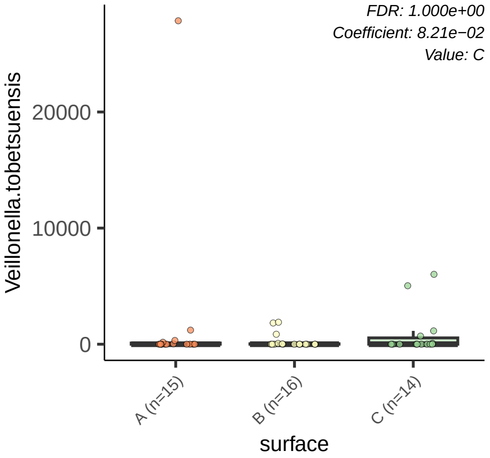

FDR: 1.000e+00
Coefficient: 8.21e−02
Value: C
Veillonella.tobetsuensis
20000
10000
0
A (n=15)
B (n=16)
C (n=14)
surface

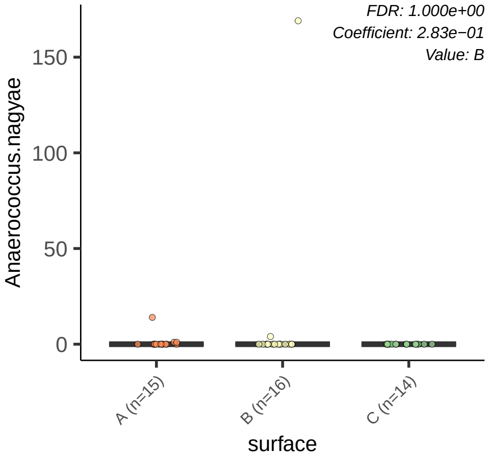
FDR: 1.000e+00
Coefficient: 2.83e−01
Value: B
Anaerococcus.nagyae
150
100
50
0
A (n=15)
B (n=16)
C (n=14)
surface

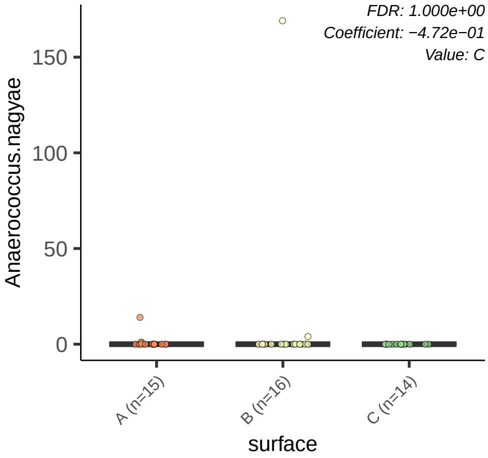

FDR: 1.000e+00
Coefficient: −4.72e−01
Value: C
Anaerococcus.nagyae
150
100
50
0
A (n=15)
B (n=16)
C (n=14)
surface

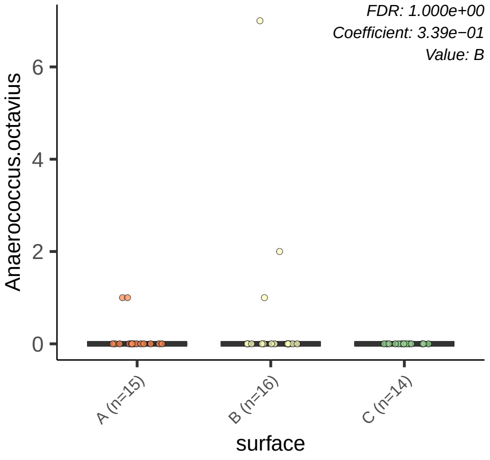

FDR: 1.000e+00
Coefficient: 3.39e−01
Value: B
Anaerococcus.octavius
0
2
4
6
A (n=15)
B (n=16)
C (n=14)
surface

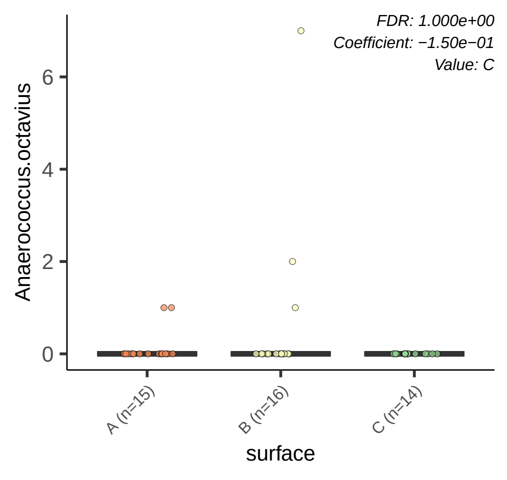
FDR: 1.000e+00
Coefficient: −1.50e−01
Value: C
Anaerococcus.octavius
0
2
4
6
A (n=15)
B (n=16)
C (n=14)
surface

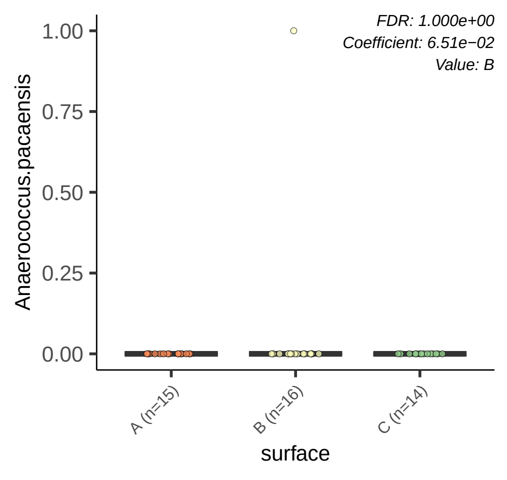

FDR: 1.000e+00
Coefficient: 6.51e−02
Value: B
Anaerococcus.pacaensis
1.00
0.75
0.50
0.25
0.00
A (n=15)
B (n=16)
C (n=14)
surface

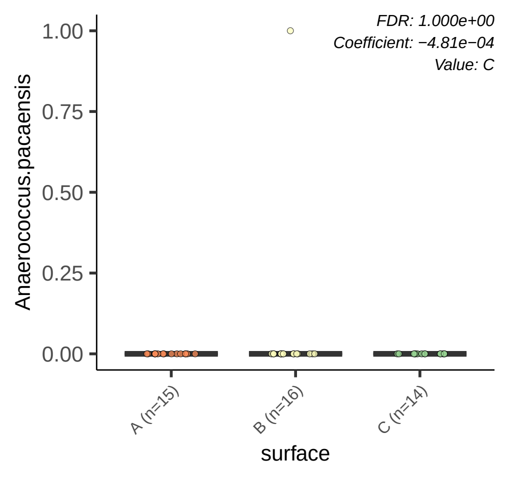

FDR: 1.000e+00
Coefficient: −4.81e−04
Value: C
Anaerococcus.pacaensis
1.00
0.75
0.50
0.25
0.00
A (n=15)
B (n=16)
C (n=14)
surface

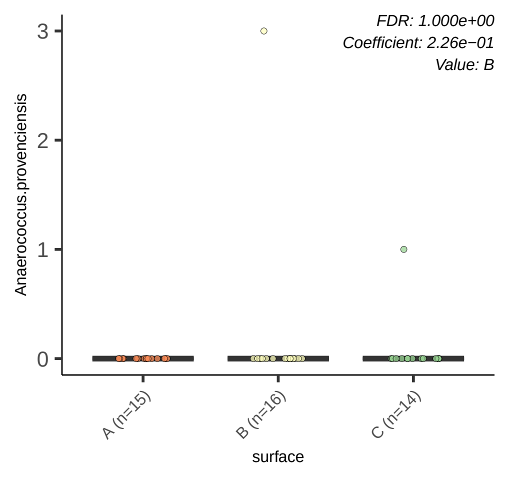

FDR: 1.000e+00
Coefficient: 2.26e−01
Value: B
3
2
1
0
Anaerococcus.provenciensis
A (n=15)
B (n=16)
C (n=14)
surface

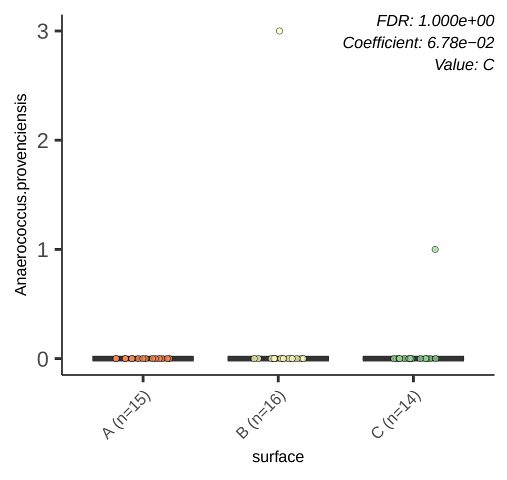
FDR: 1.000e+00
Coefficient: 6.78e−02
Value: C
Anaerococcus.provenciensis
3
2
1
0
A (n=15)
B (n=16)
C (n=14)
surface

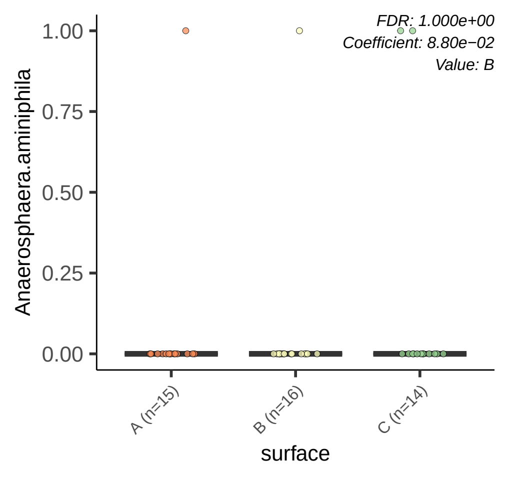
FDR: 1.000e+00
Coefficient: 8.80e−02
Value: B
Anaerosphaera.aminiphila
1.00
0.75
0.50
0.25
0.00
A (n=15)
B (n=16)
C (n=14)
surface

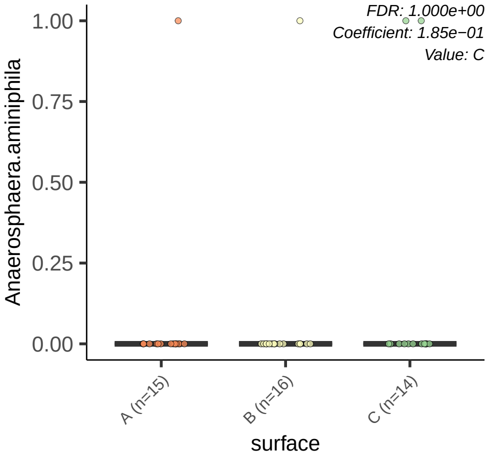

FDR: 1.000e+00
Coefficient: 1.85e−01
Value: C
Anaerosphaera.aminiphila
1.00
0.75
0.50
0.25
0.00
A (n=15)
B (n=16)
C (n=14)
surface

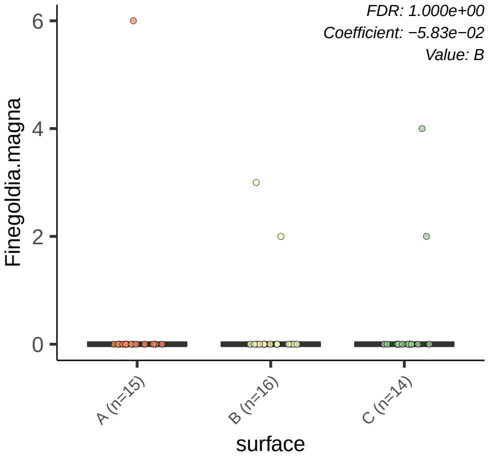
FDR: 1.000e+00
Coefficient: −5.83e−02
Value: B
Finegoldia.magna
6
4
2
0
A (n=15)
B (n=16)
C (n=14)
surface

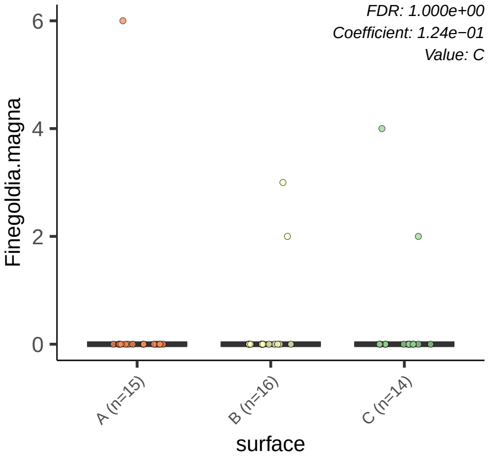

FDR: 1.000e+00
Coefficient: 1.24e−01
Value: C
Finegoldia.magna
6
4
2
0
A (n=15)
B (n=16)
C (n=14)
surface

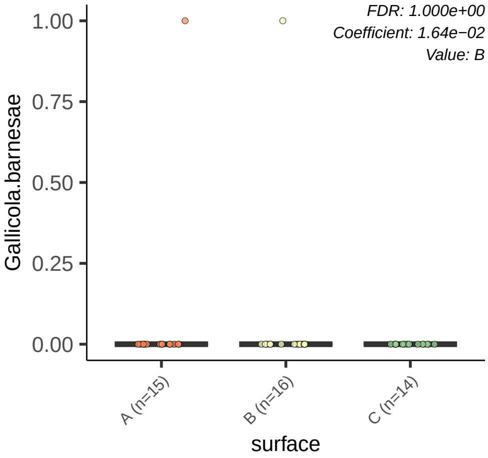

FDR: 1.000e+00
Coefficient: 1.64e−02
Value: B
Gallicola.barnesae
1.00
0.75
0.50
0.25
0.00
A (n=15)
B (n=16)
C (n=14)
surface

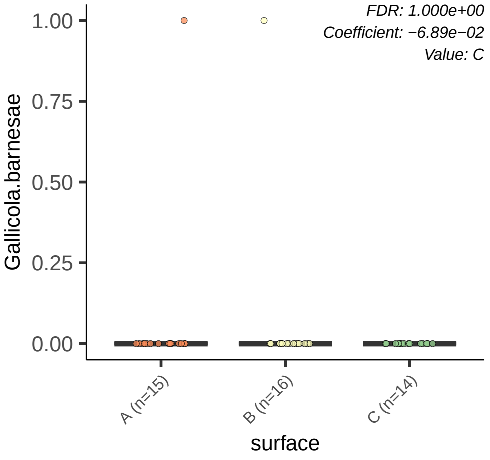
FDR: 1.000e+00
Coefficient: −6.89e−02
Value: C
Gallicola.barnesae
1.00
0.75
0.50
0.25
0.00
A (n=15)
B (n=16)
C (n=14)
surface

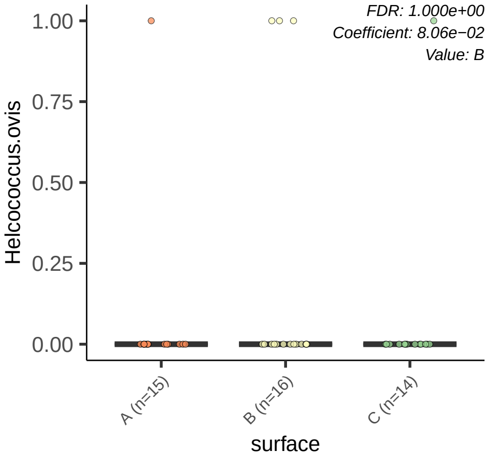
FDR: 1.000e+00
Coefficient: 8.06e−02
Value: B
Helcococcus.ovis
1.00
0.75
0.50
0.25
0.00
A (n=15)
B (n=16)
C (n=14)
surface

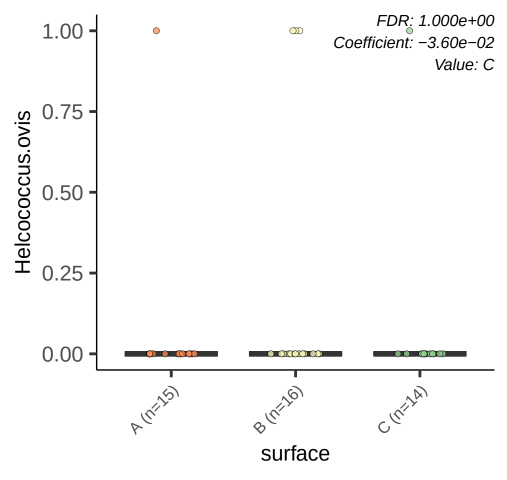

FDR: 1.000e+00
Coefficient: −3.60e−02
Value: C
Helcococcus.ovis
1.00
0.75
0.50
0.25
0.00
A (n=15)
B (n=16)
C (n=14)
surface

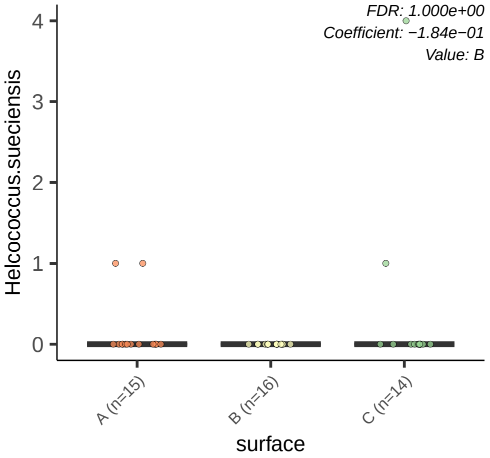
FDR: 1.000e+00
Coefficient: −1.84e−01
Value: B
Helcococcus.sueciensis
0
1
2
3
4
A (n=15)
B (n=16)
C (n=14)
surface

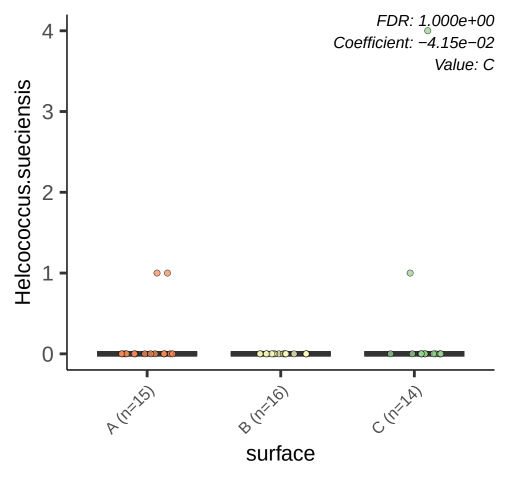
FDR: 1.000e+00
Coefficient: −4.15e−02
Value: C
Helcococcus.sueciensis
4
3
2
1
0
A (n=15)
B (n=16)
C (n=14)
surface

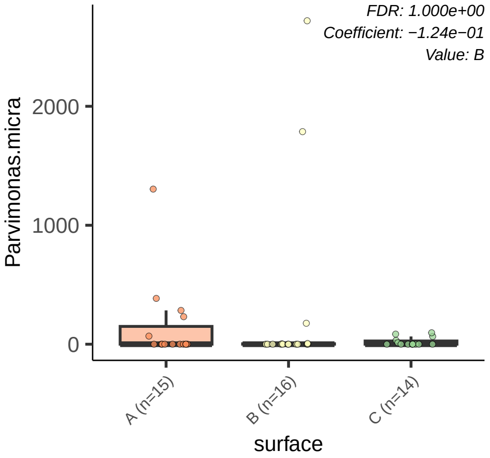

FDR: 1.000e+00
Coefficient: −1.24e−01
Value: B
Parvimonas.micra
2000
1000
0
A (n=15)
B (n=16)
C (n=14)
surface

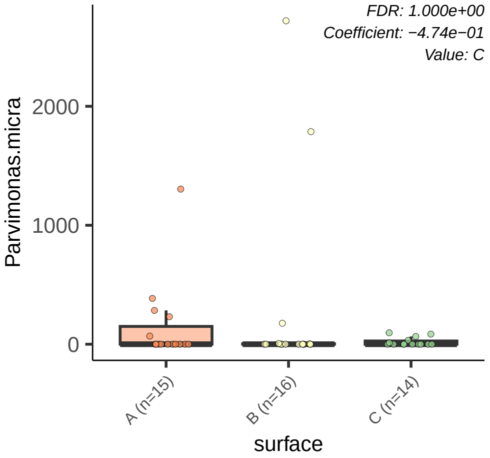
FDR: 1.000e+00
Coefficient: −4.74e−01
Value: C
Parvimonas.micra
2000
1000
0
A (n=15)
B (n=16)
C (n=14)
surface

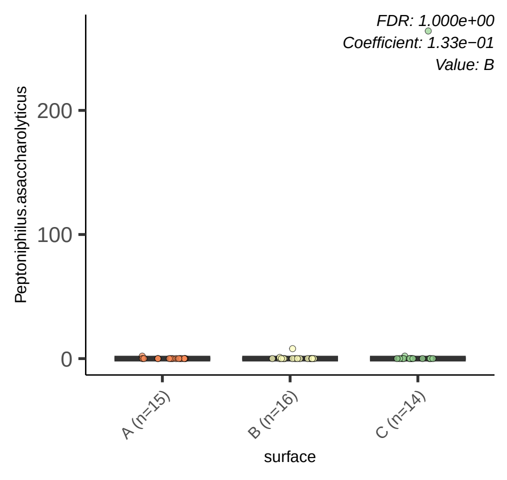
FDR: 1.000e+00
Coefficient: 1.33e−01
Value: B
Peptoniphilus.asaccharolyticus
200
100
0
A (n=15)
B (n=16)
C (n=14)
surface

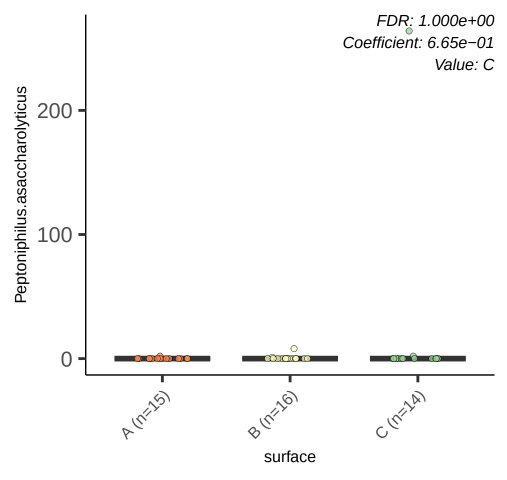

FDR: 1.000e+00
Coefficient: 6.65e−01
Value: C
Peptoniphilus.asaccharolyticus
200
100
0
A (n=15)
B (n=16)
C (n=14)
surface

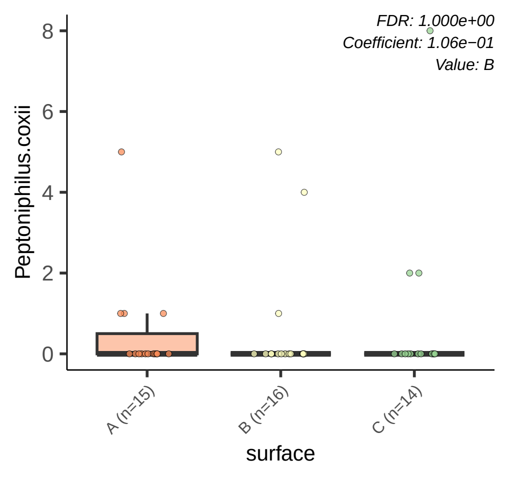

FDR: 1.000e+00
Coefficient: 1.06e−01
Value: B
Peptoniphilus.coxii
0
2
4
6
8
A (n=15)
B (n=16)
C (n=14)
surface

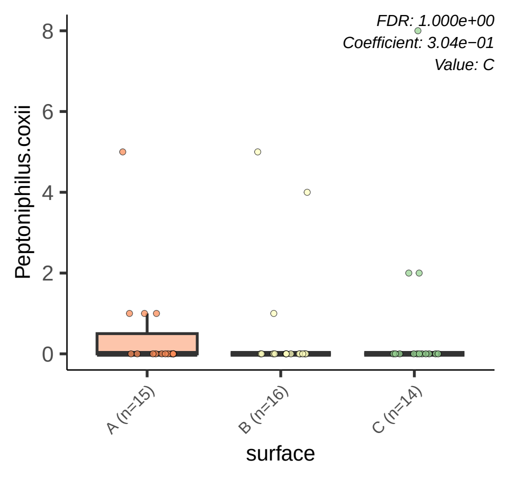
FDR: 1.000e+00
Coefficient: 3.04e−01
Value: C
Peptoniphilus.coxii
0
2
4
6
8
A (n=15)
B (n=16)
C (n=14)
surface

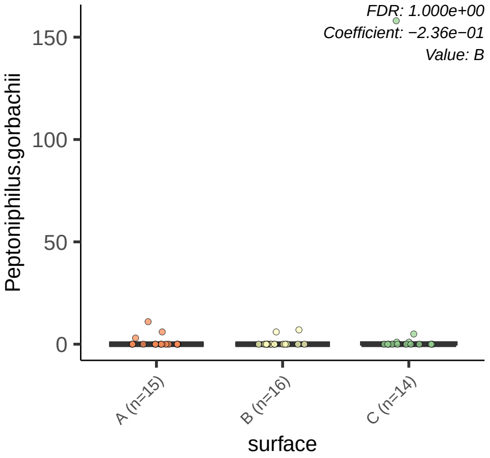
FDR: 1.000e+00
Coefficient: −2.36e−01
Value: B
Peptoniphilus.gorbachii
150
100
50
0
A (n=15)
B (n=16)
C (n=14)
surface

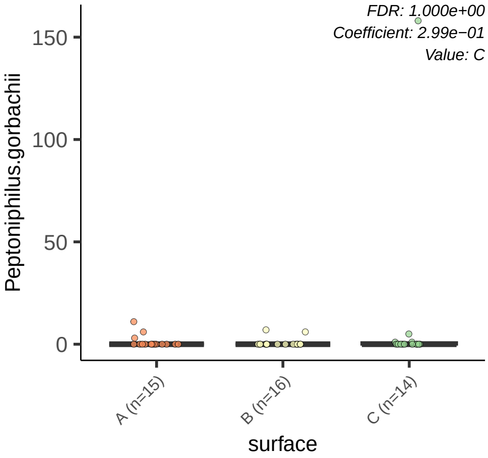

FDR: 1.000e+00
Coefficient: 2.99e−01
Value: C
150
100
50
0
Peptoniphilus.gorbachii
A (n=15)
B (n=16)
C (n=14)
surface

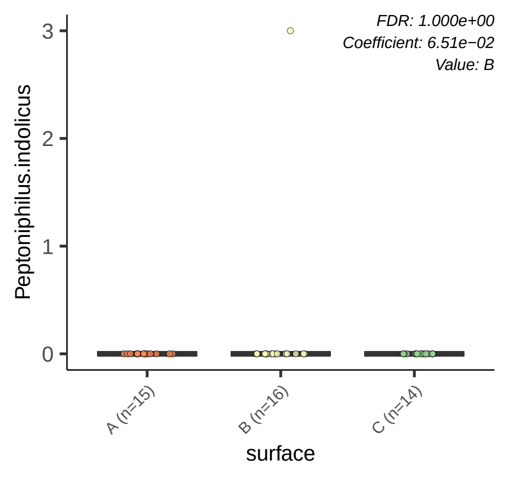
FDR: 1.000e+00
Coefficient: 6.51e−02
Value: B
Peptoniphilus.indolicus
3
2
1
0
A (n=15)
B (n=16)
C (n=14)
surface

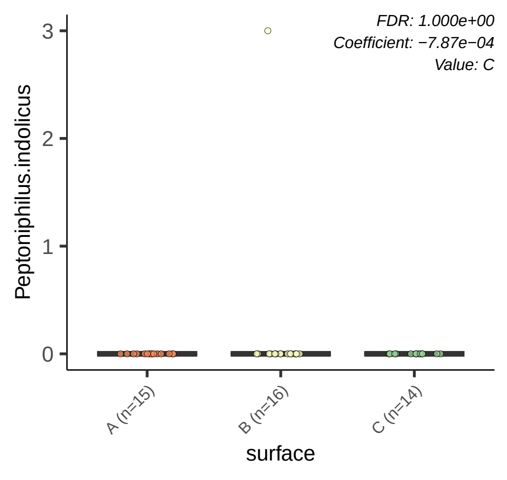

FDR: 1.000e+00
Coefficient: −7.87e−04
Value: C
Peptoniphilus.indolicus
0
1
2
3
A (n=15)
B (n=16)
C (n=14)
surface

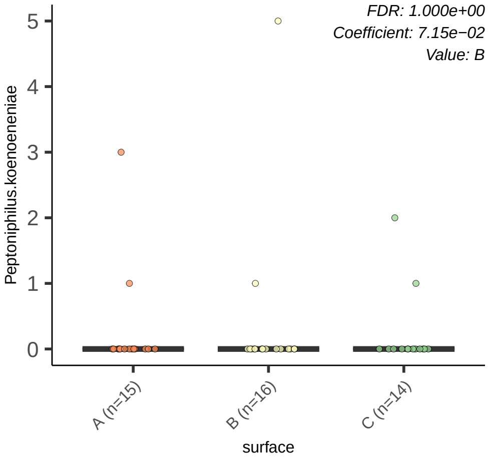

FDR: 1.000e+00
Coefficient: 7.15e−02
Value: B
Peptoniphilus.koenoeneniae
0
1
2
3
4
5
A (n=15)
B (n=16)
C (n=14)
surface

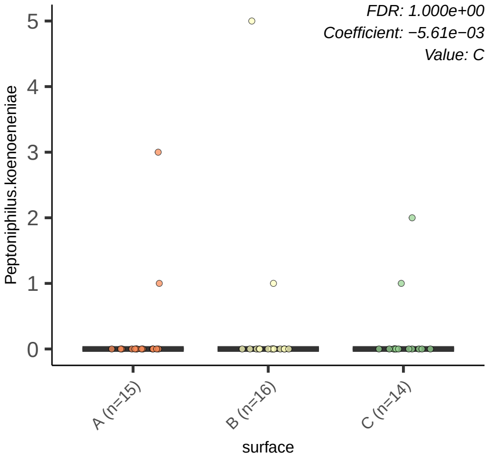
FDR: 1.000e+00
Coefficient: −5.61e−03
Value: C
Peptoniphilus.koenoeneniae
0
1
2
3
4
5
A (n=15)
B (n=16)
C (n=14)
surface

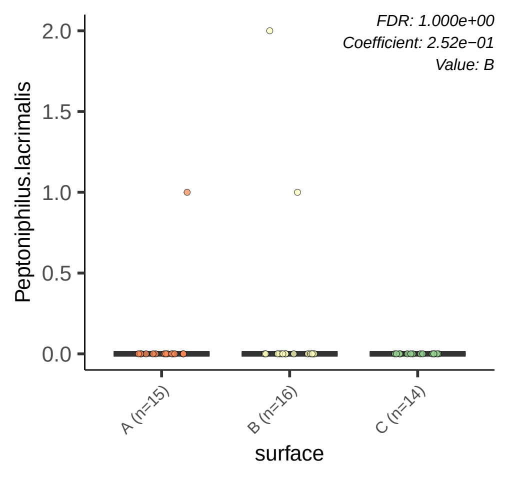
FDR: 1.000e+00
Coefficient: 2.52e−01
Value: B
Peptoniphilus.lacrimalis
2.0
1.5
1.0
0.5
0.0
A (n=15)
B (n=16)
C (n=14)
surface

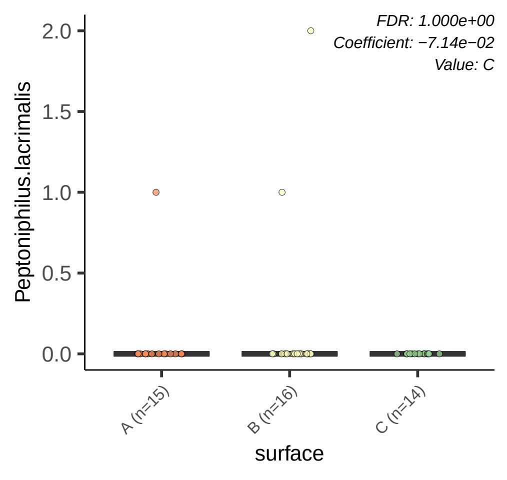
FDR: 1.000e+00
Coefficient: −7.14e−02
Value: C
Peptoniphilus.lacrimalis
2.0
1.5
1.0
0.5
0.0
A (n=15)
B (n=16)
C (n=14)
surface

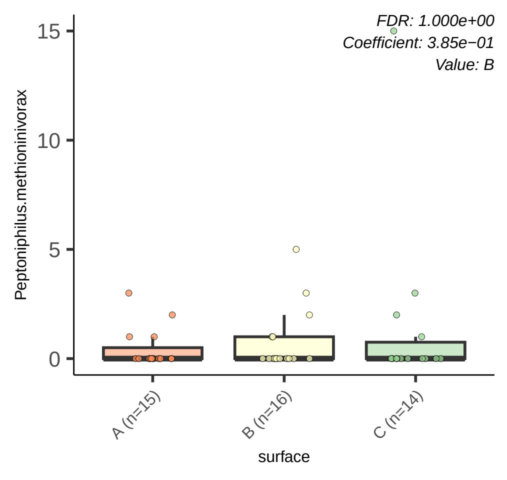
FDR: 1.000e+00
Coefficient: 3.85e−01
Value: B
Peptoniphilus.methioninivorax
0
5
10
15
A (n=15)
B (n=16)
C (n=14)
surface

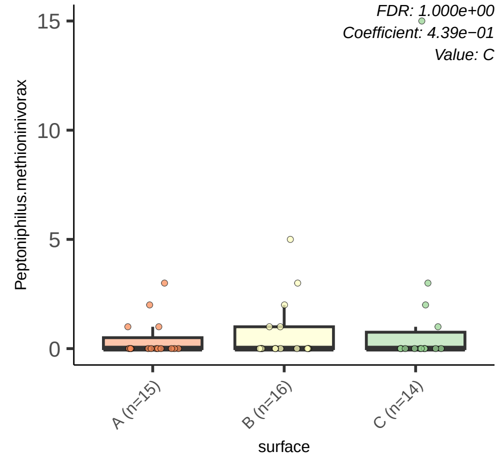

FDR: 1.000e+00
Coefficient: 4.39e−01
Value: C
Peptoniphilus.methioninivorax
15
10
5
0
A (n=15)
B (n=16)
C (n=14)
surface

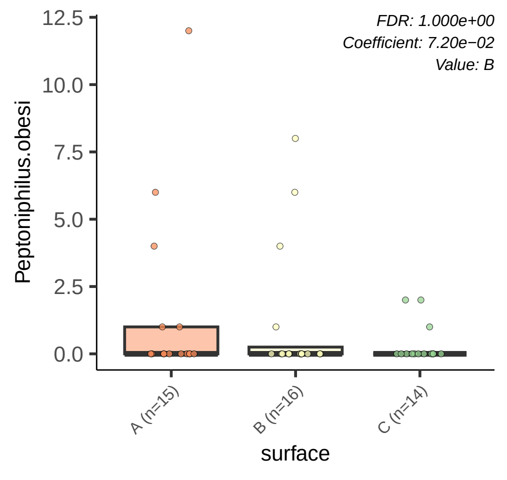

FDR: 1.000e+00
Coefficient: 7.20e−02
Value: B
Peptoniphilus.obesi
12.5
10.0
7.5
5.0
2.5
0.0
A (n=15)
B (n=16)
C (n=14)
surface

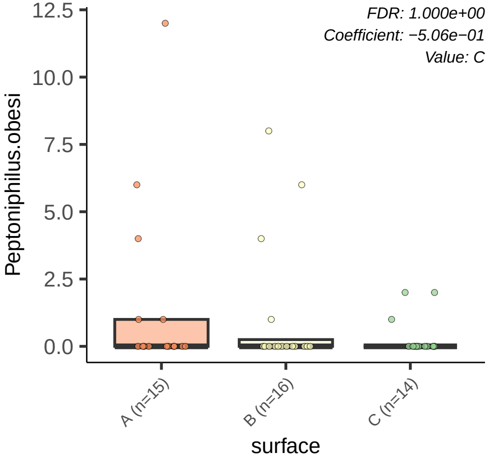
FDR: 1.000e+00
Coefficient: −5.06e−01
Value: C
Peptoniphilus.obesi
12.5
10.0
7.5
5.0
2.5
0.0
A (n=15)
B (n=16)
C (n=14)
surface

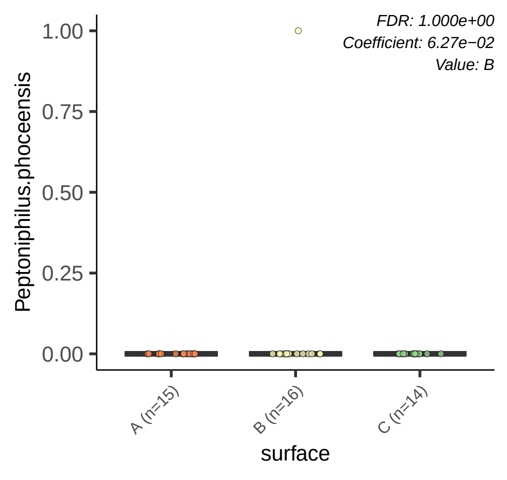
FDR: 1.000e+00
Coefficient: 6.27e−02
Value: B
Peptoniphilus.phoceensis
1.00
0.75
0.50
0.25
0.00
A (n=15)
B (n=16)
C (n=14)
surface

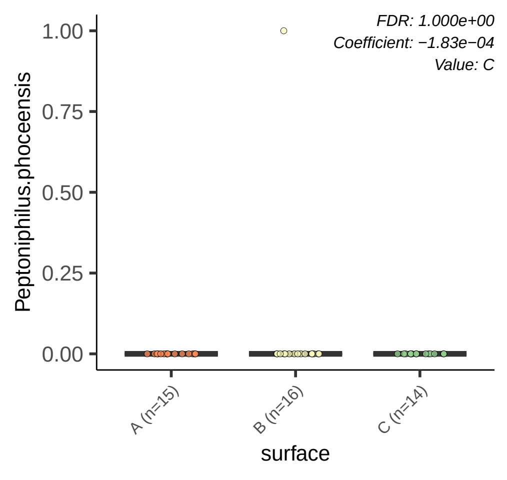
FDR: 1.000e+00
Coefficient: −1.83e−04
Value: C
Peptoniphilus.phoceensis
1.00
0.75
0.50
0.25
0.00
A (n=15)
B (n=16)
C (n=14)
surface

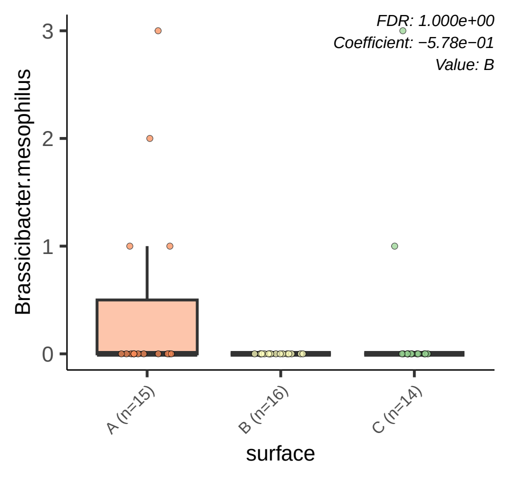

FDR: 1.000e+00
Coefficient: −5.78e−01
Value: B
Brassicibacter.mesophilus
0
1
2
3
A (n=15)
B (n=16)
C (n=14)
surface

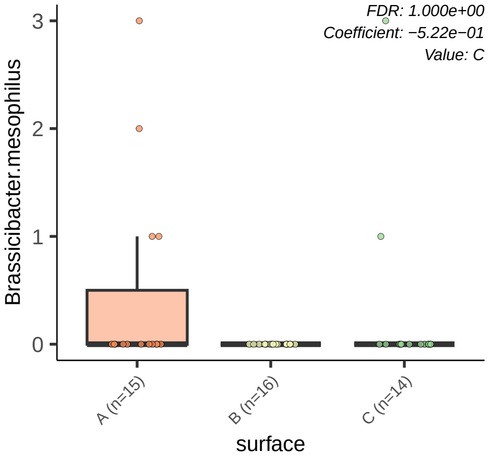
FDR: 1.000e+00
Coefficient: −5.22e−01
Value: C
Brassicibacter.mesophilus
0
1
2
3
A (n=15)
B (n=16)
C (n=14)
surface

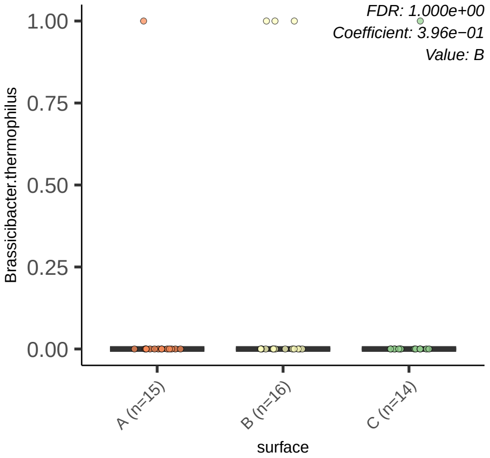
FDR: 1.000e+00
Coefficient: 3.96e−01
Value: B
1.00
0.75
0.50
0.25
0.00
Brassicibacter.thermophilus
A (n=15)
B (n=16)
C (n=14)
surface

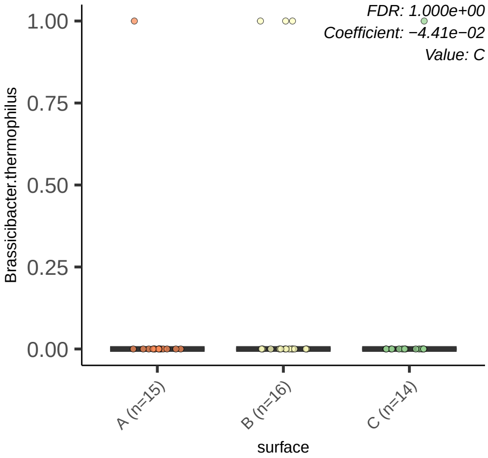
FDR: 1.000e+00
Coefficient: −4.41e−02
Value: C
Brassicibacter.thermophilus
1.00
0.75
0.50
0.25
0.00
A (n=15)
B (n=16)
C (n=14)
surface

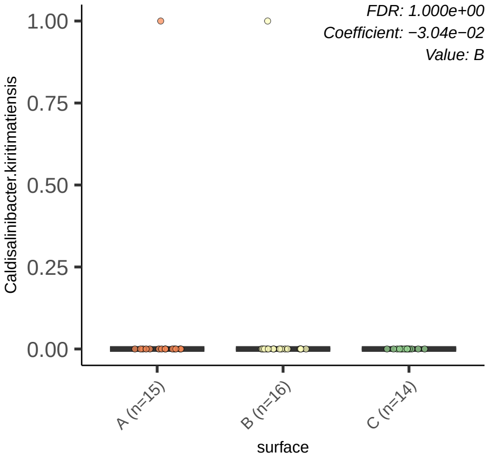
FDR: 1.000e+00
Coefficient: −3.04e−02
Value: B
Caldisalinibacter.kiritimatiensis
1.00
0.75
0.50
0.25
0.00
A (n=15)
B (n=16)
C (n=14)
surface

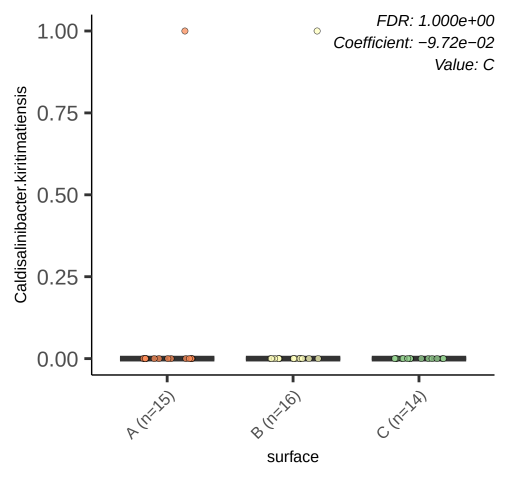
FDR: 1.000e+00
Coefficient: −9.72e−02
Value: C
Caldisalinibacter.kiritimatiensis
1.00
0.75
0.50
0.25
0.00
A (n=15)
B (n=16)
C (n=14)
surface

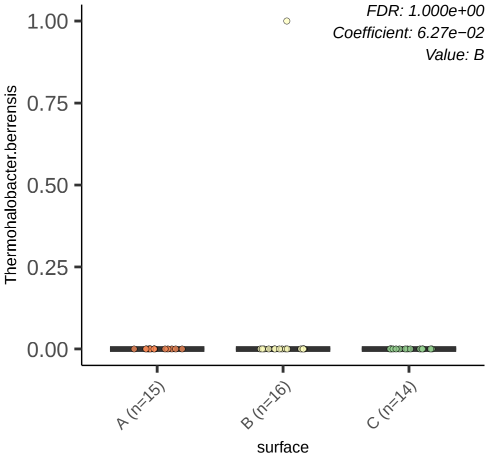
FDR: 1.000e+00
Coefficient: 6.27e−02
Value: B
1.00
0.75
0.50
0.25
0.00
Thermohalobacter.berrensis
A (n=15)
B (n=16)
C (n=14)
surface

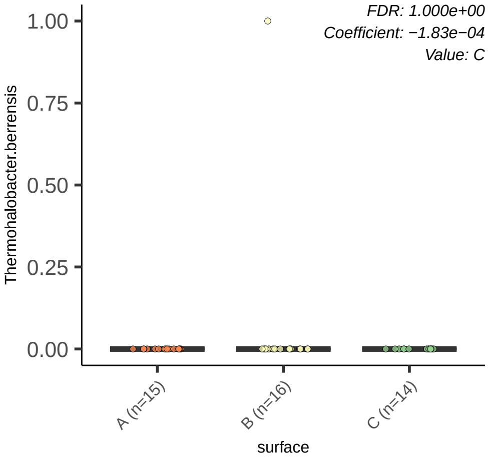
FDR: 1.000e+00
Coefficient: −1.83e−04
Value: C
1.00
0.75
0.50
0.25
0.00
Thermohalobacter.berrensis
A (n=15)
B (n=16)
C (n=14)
surface

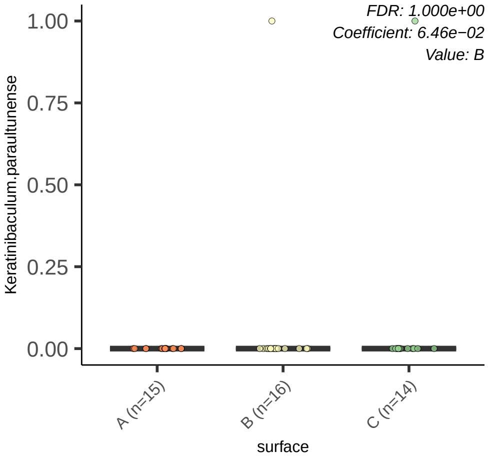

FDR: 1.000e+00
Coefficient: 6.46e−02
Value: B
Keratinibaculum.paraultunense
1.00
0.75
0.50
0.25
0.00
A (n=15)
B (n=16)
C (n=14)
surface

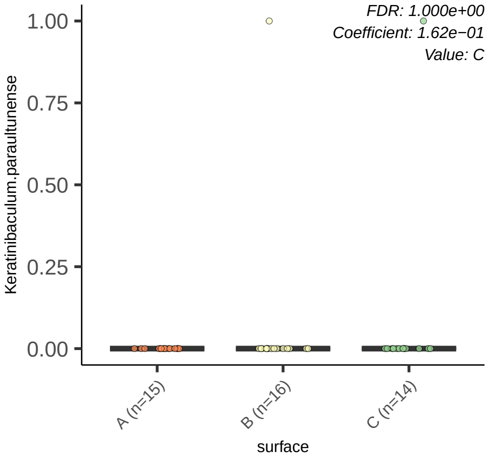
FDR: 1.000e+00
Coefficient: 1.62e−01
Value: C
Keratinibaculum.paraultunense
1.00
0.75
0.50
0.25
0.00
A (n=15)
B (n=16)
C (n=14)
surface

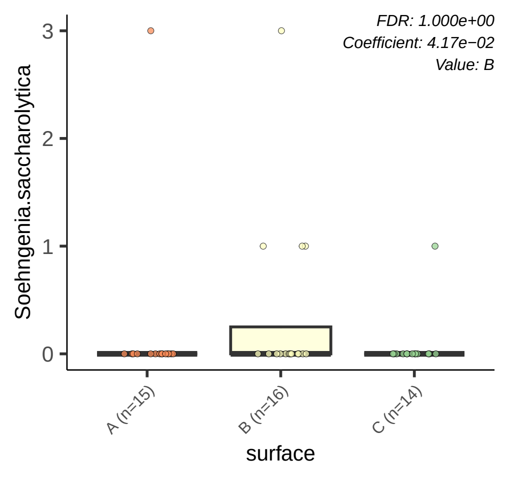
FDR: 1.000e+00
Coefficient: 4.17e−02
Value: B
Soehngenia.saccharolytica
3
2
1
0
A (n=15)
B (n=16)
C (n=14)
surface

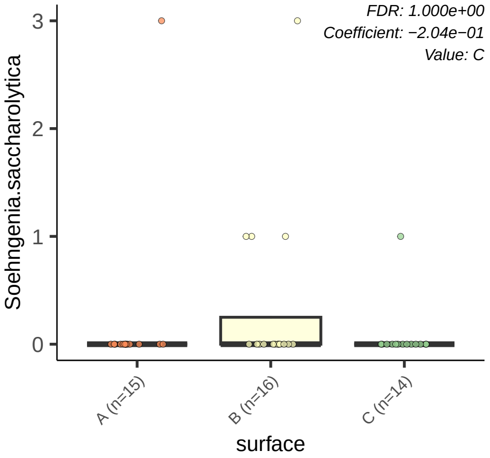
FDR: 1.000e+00
Coefficient: −2.04e−01
Value: C
Soehngenia.saccharolytica
3
2
1
0
A (n=15)
B (n=16)
C (n=14)
surface

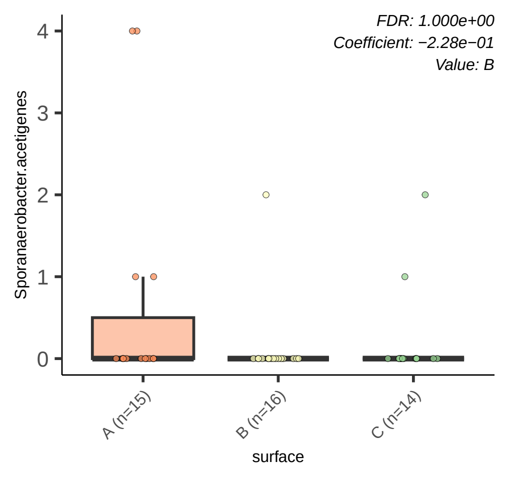

FDR: 1.000e+00
Coefficient: −2.28e−01
Value: B
Sporanaerobacter.acetigenes
0
1
2
3
4
A (n=15)
B (n=16)
C (n=14)
surface

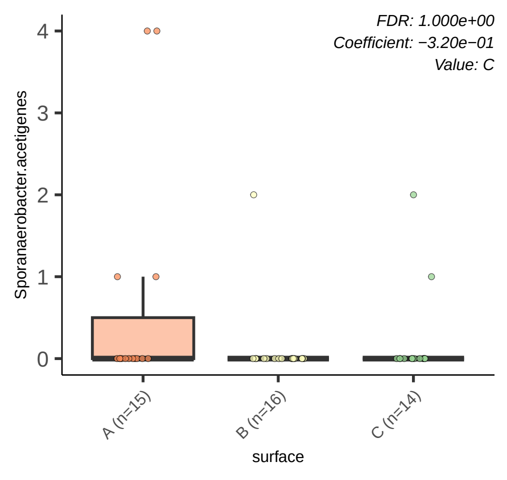

FDR: 1.000e+00
Coefficient: −3.20e−01
Value: C
Sporanaerobacter.acetigenes
4
3
2
1
0
A (n=15)
B (n=16)
C (n=14)
surface

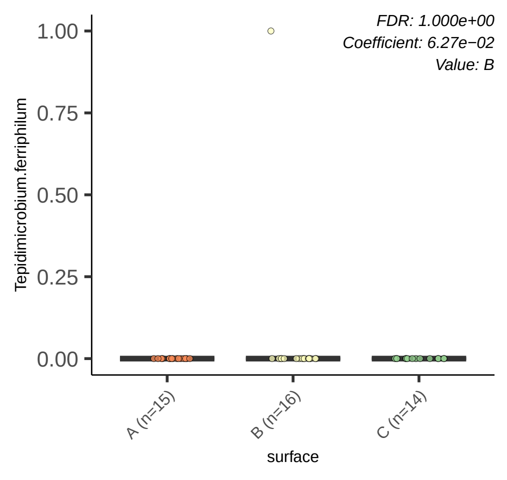
FDR: 1.000e+00
Coefficient: 6.27e−02
Value: B
1.00
0.75
0.50
0.25
0.00
Tepidimicrobium.ferriphilum
A (n=15)
B (n=16)
C (n=14)
surface

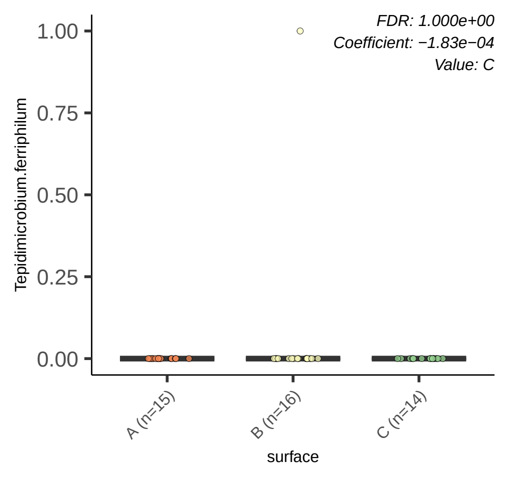
FDR: 1.000e+00
Coefficient: −1.83e−04
Value: C
Tepidimicrobium.ferriphilum
1.00
0.75
0.50
0.25
0.00
A (n=15)
B (n=16)
C (n=14)
surface

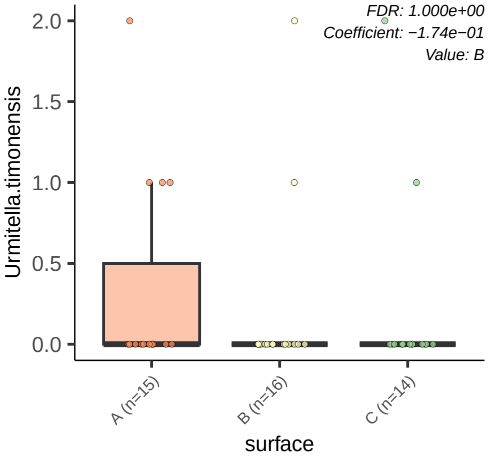
FDR: 1.000e+00
Coefficient: −1.74e−01
Value: B
Urmitella.timonensis
2.0
1.5
1.0
0.5
0.0
A (n=15)
B (n=16)
C (n=14)
surface

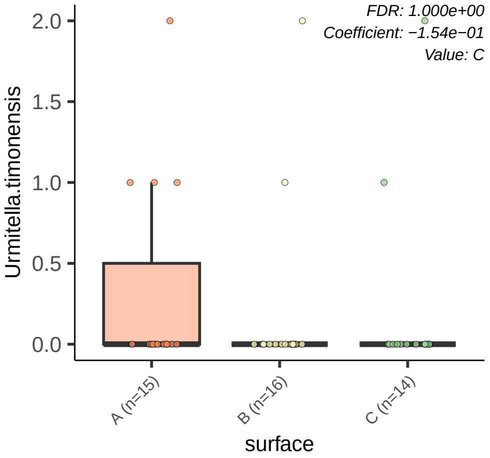
FDR: 1.000e+00
Coefficient: −1.54e−01
Value: C
Urmitella.timonensis
2.0
1.5
1.0
0.5
0.0
A (n=15)
B (n=16)
C (n=14)
surface

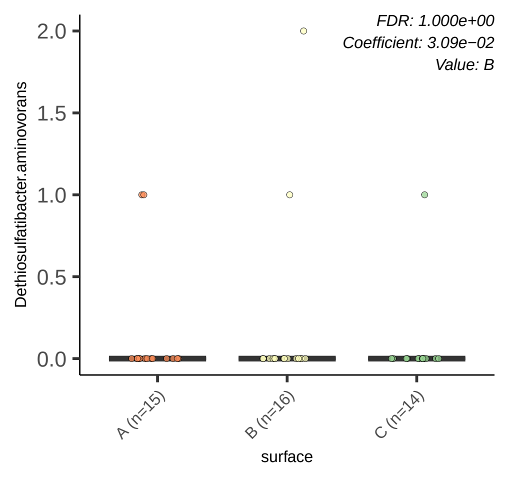
FDR: 1.000e+00
Coefficient: 3.09e−02
Value: B
Dethiosulfatibacter.aminovorans
2.0
1.5
1.0
0.5
0.0
A (n=15)
B (n=16)
C (n=14)
surface

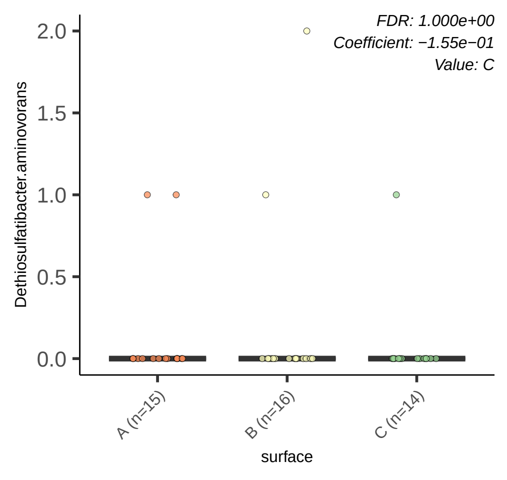
FDR: 1.000e+00
Coefficient: −1.55e−01
Value: C
Dethiosulfatibacter.aminovorans
2.0
1.5
1.0
0.5
0.0
A (n=15)
B (n=16)
C (n=14)
surface

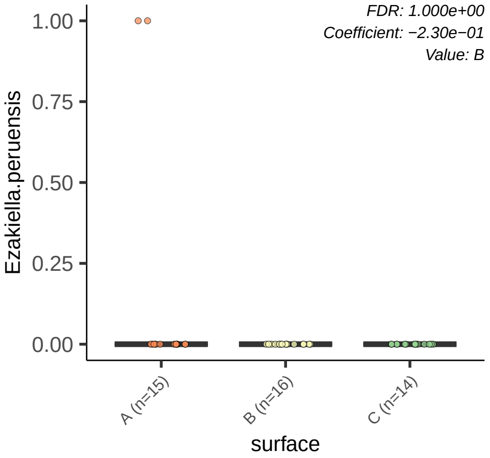

FDR: 1.000e+00
Coefficient: −2.30e−01
Value: B
Ezakiella.peruensis
1.00
0.75
0.50
0.25
0.00
A (n=15)
B (n=16)
C (n=14)
surface

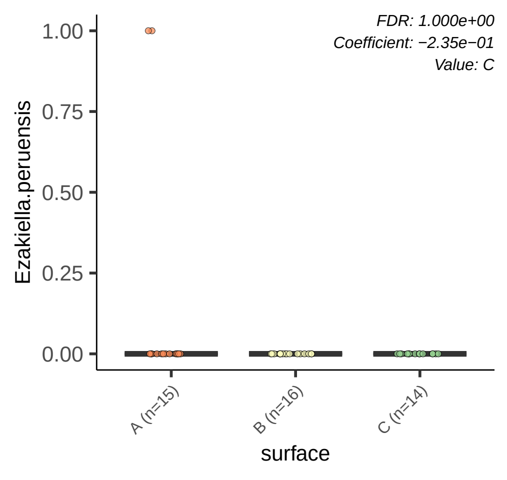
FDR: 1.000e+00
Coefficient: −2.35e−01
Value: C
Ezakiella.peruensis
1.00
0.75
0.50
0.25
0.00
A (n=15)
B (n=16)
C (n=14)
surface

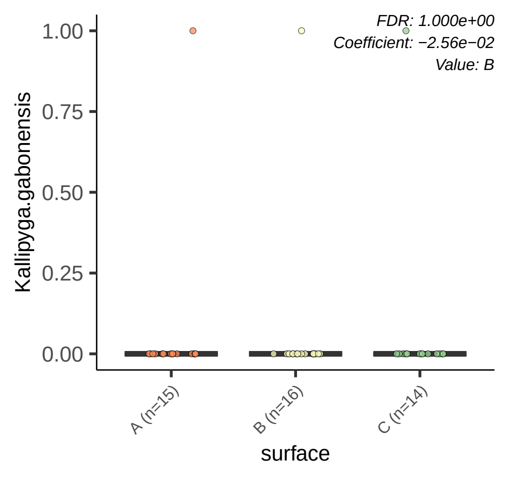
FDR: 1.000e+00
Coefficient: −2.56e−02
Value: B
Kallipyga.gabonensis
1.00
0.75
0.50
0.25
0.00
A (n=15)
B (n=16)
C (n=14)
surface

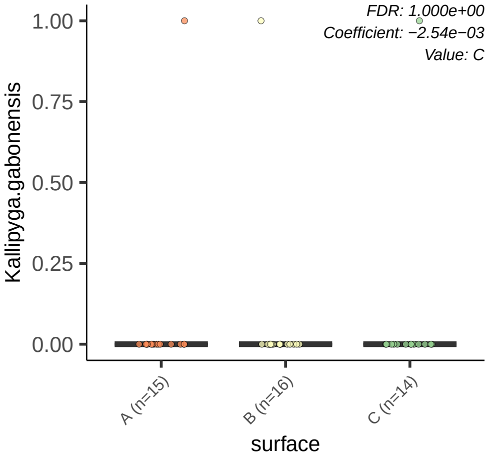

FDR: 1.000e+00
Coefficient: −2.54e−03
Value: C
Kallipyga.gabonensis
1.00
0.75
0.50
0.25
0.00
A (n=15)
B (n=16)
C (n=14)
surface

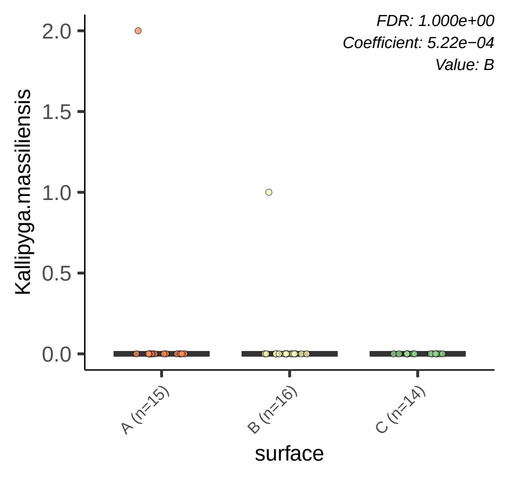
FDR: 1.000e+00
Coefficient: 5.22e−04
Value: B
Kallipyga.massiliensis
2.0
1.5
1.0
0.5
0.0
A (n=15)
B (n=16)
C (n=14)
surface

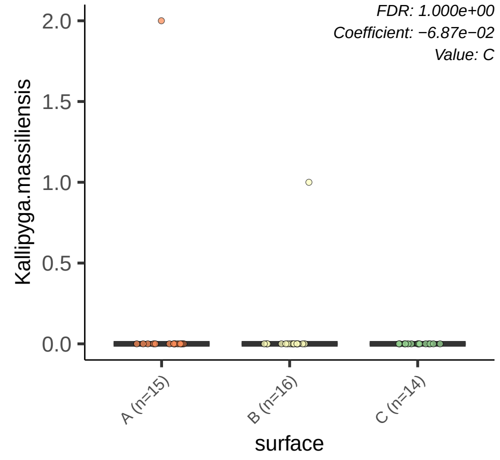
FDR: 1.000e+00
Coefficient: −6.87e−02
Value: C
Kallipyga.massiliensis
2.0
1.5
1.0
0.5
0.0
A (n=15)
B (n=16)
C (n=14)
surface

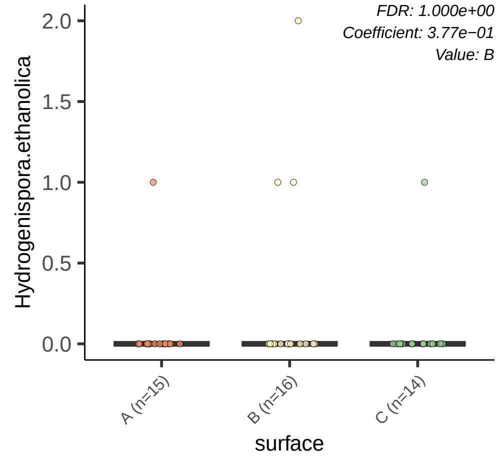
FDR: 1.000e+00
Coefficient: 3.77e−01
Value: B
Hydrogenispora.ethanolica
2.0
1.5
1.0
0.5
0.0
A (n=15)
B (n=16)
C (n=14)
surface

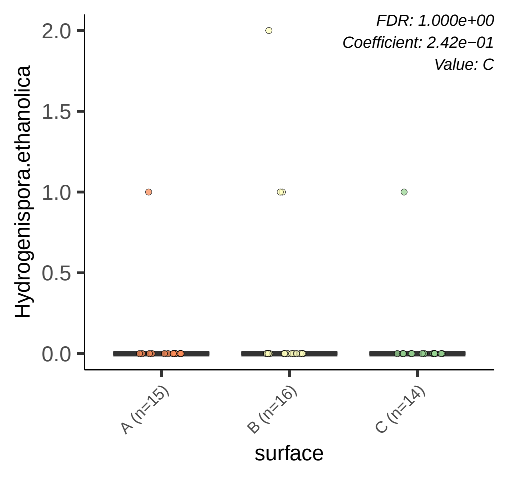
FDR: 1.000e+00
Coefficient: 2.42e−01
Value: C
Hydrogenispora.ethanolica
2.0
1.5
1.0
0.5
0.0
A (n=15)
B (n=16)
C (n=14)
surface

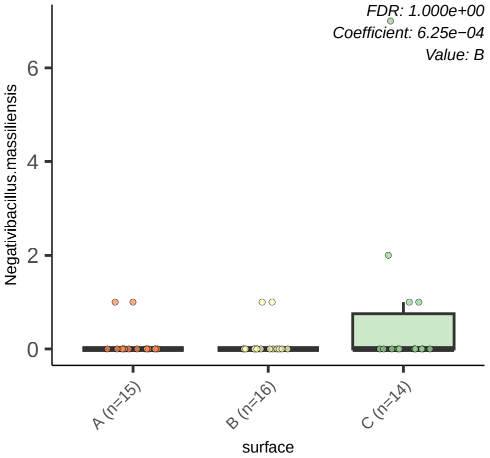
FDR: 1.000e+00
Coefficient: 6.25e−04
Value: B
Negativibacillus.massiliensis
0
2
4
6
A (n=15)
B (n=16)
C (n=14)
surface

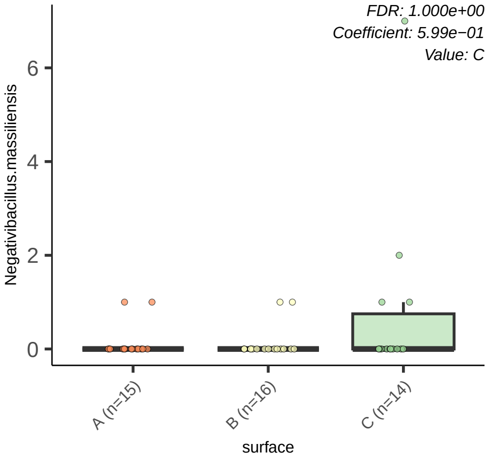

FDR: 1.000e+00
Coefficient: 5.99e−01
Value: C
Negativibacillus.massiliensis
0
2
4
6
A (n=15)
B (n=16)
C (n=14)
surface

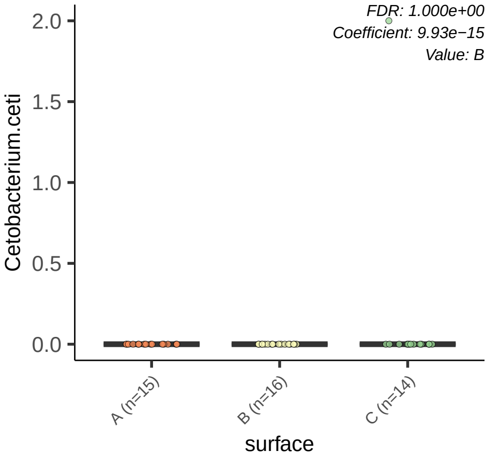

FDR: 1.000e+00
Coefficient: 9.93e−15
Value: B
Cetobacterium.ceti
2.0
1.5
1.0
0.5
0.0
A (n=15)
B (n=16)
C (n=14)
surface

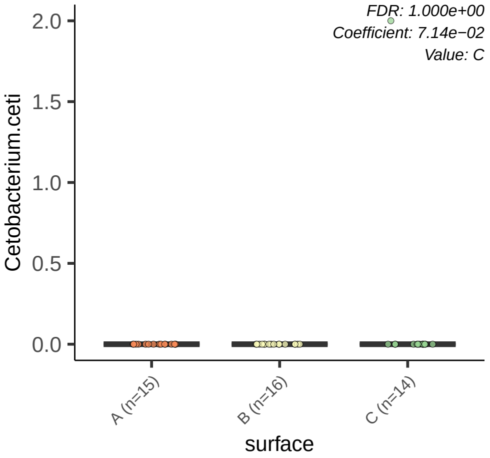
FDR: 1.000e+00
Coefficient: 7.14e−02
Value: C
Cetobacterium.ceti
2.0
1.5
1.0
0.5
0.0
A (n=15)
B (n=16)
C (n=14)
surface

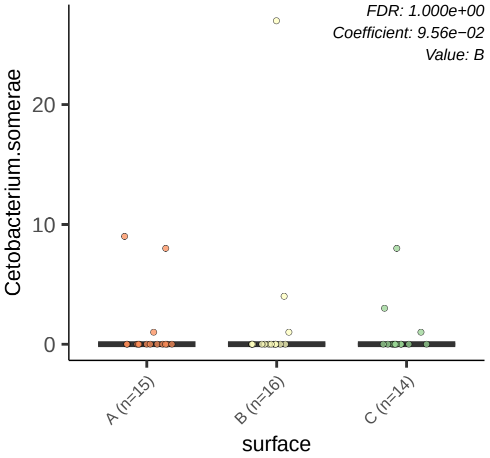

FDR: 1.000e+00
Coefficient: 9.56e−02
Value: B
Cetobacterium.somerae
20
10
0
A (n=15)
B (n=16)
C (n=14)
surface

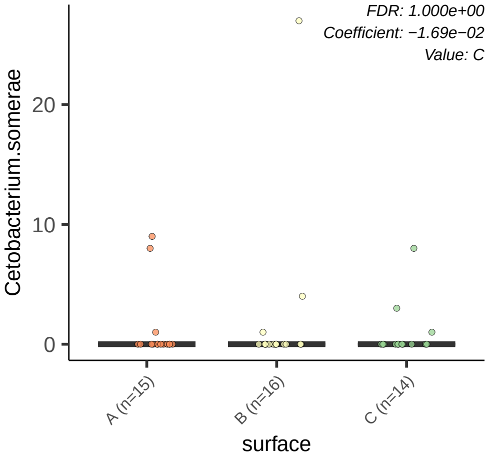

FDR: 1.000e+00
Coefficient: −1.69e−02
Value: C
Cetobacterium.somerae
0
10
20
A (n=15)
B (n=16)
C (n=14)
surface

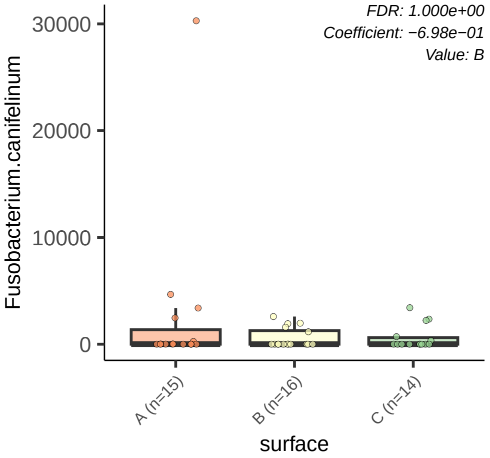
FDR: 1.000e+00
Coefficient: −6.98e−01
Value: B
Fusobacterium.canifelinum
30000
20000
10000
0
A (n=15)
B (n=16)
C (n=14)
surface

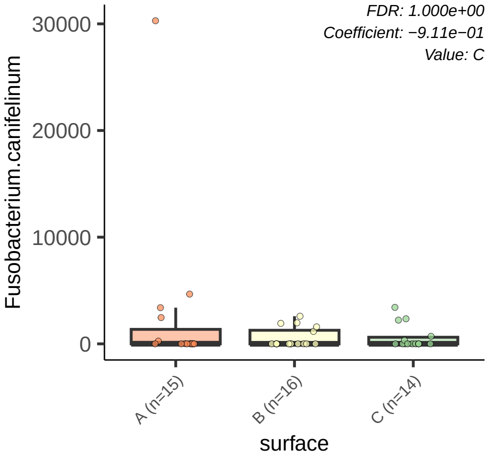
FDR: 1.000e+00
Coefficient: −9.11e−01
Value: C
Fusobacterium.canifelinum
30000
20000
10000
0
A (n=15)
B (n=16)
C (n=14)
surface

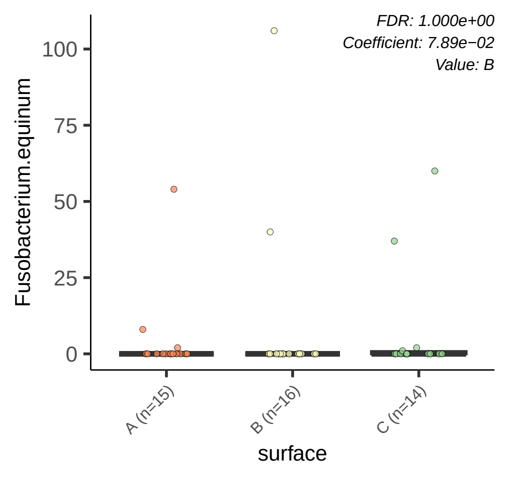
FDR: 1.000e+00
Coefficient: 7.89e−02
Value: B
Fusobacterium.equinum
100
75
50
25
0
A (n=15)
B (n=16)
C (n=14)
surface

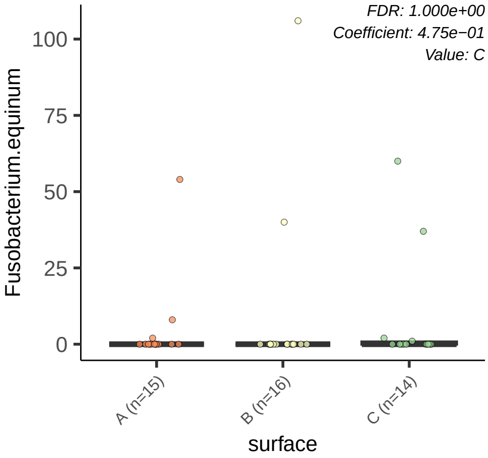
FDR: 1.000e+00
Coefficient: 4.75e−01
Value: C
Fusobacterium.equinum
100
75
50
25
0
A (n=15)
B (n=16)
C (n=14)
surface

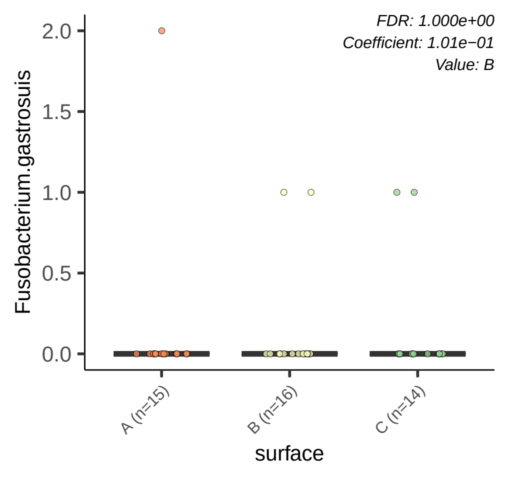

FDR: 1.000e+00
Coefficient: 1.01e−01
Value: B
2.0
1.5
1.0
0.5
0.0
Fusobacterium.gastrosuis
A (n=15)
B (n=16)
C (n=14)
surface

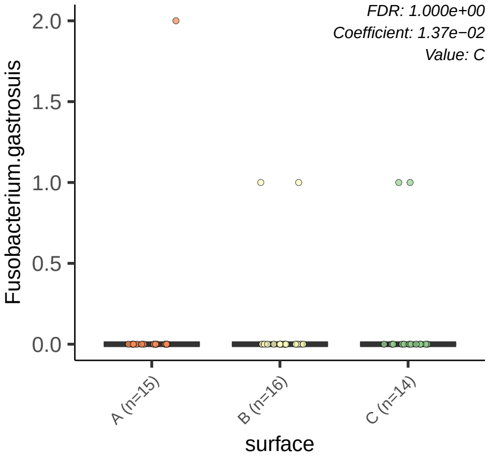

FDR: 1.000e+00
Coefficient: 1.37e−02
Value: C
Fusobacterium.gastrosuis
2.0
1.5
1.0
0.5
0.0
A (n=15)
B (n=16)
C (n=14)
surface

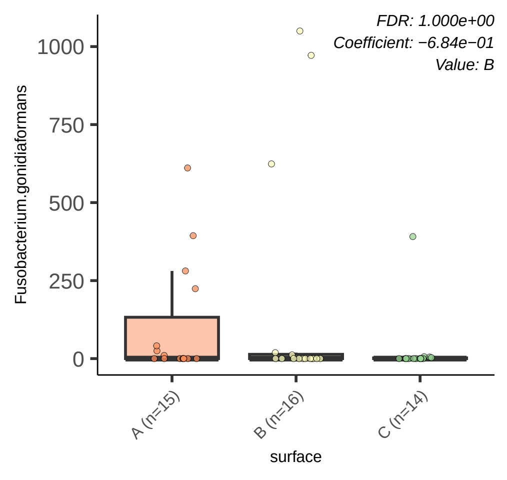

FDR: 1.000e+00
Coefficient: −6.84e−01
Value: B
Fusobacterium.gonidiaformans
1000
750
500
250
0
A (n=15)
B (n=16)
C (n=14)
surface

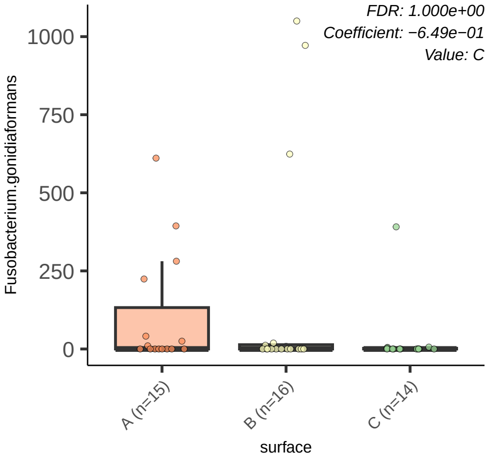

FDR: 1.000e+00
Coefficient: −6.49e−01
Value: C
1000
750
500
250
0
Fusobacterium.gonidiaformans
A (n=15)
B (n=16)
C (n=14)
surface

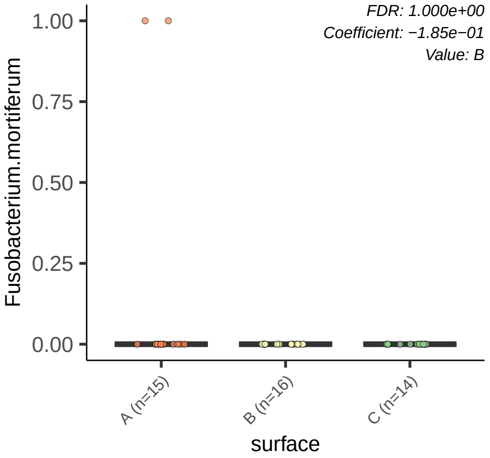
FDR: 1.000e+00
Coefficient: −1.85e−01
Value: B
Fusobacterium.mortiferum
1.00
0.75
0.50
0.25
0.00
A (n=15)
B (n=16)
C (n=14)
surface

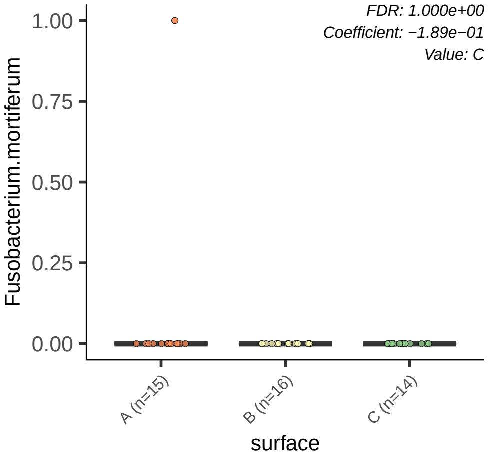
FDR: 1.000e+00
Coefficient: −1.89e−01
Value: C
Fusobacterium.mortiferum
1.00
0.75
0.50
0.25
0.00
A (n=15)
B (n=16)
C (n=14)
surface

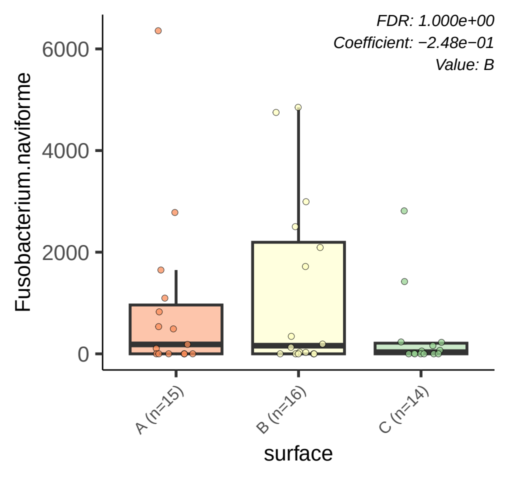

FDR: 1.000e+00
Coefficient: −2.48e−01
Value: B
Fusobacterium.naviforme
6000
4000
2000
0
A (n=15)
B (n=16)
C (n=14)
surface

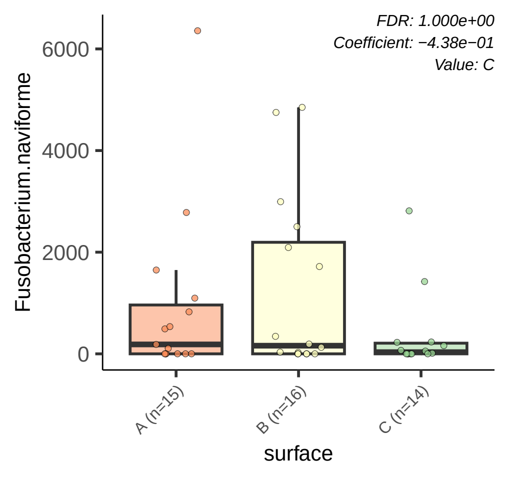
FDR: 1.000e+00
Coefficient: −4.38e−01
Value: C
Fusobacterium.naviforme
6000
4000
2000
0
A (n=15)
B (n=16)
C (n=14)
surface

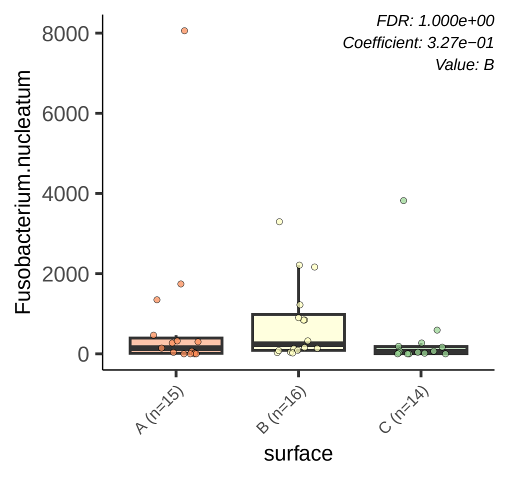

FDR: 1.000e+00
Coefficient: 3.27e−01
Value: B
Fusobacterium.nucleatum
8000
6000
4000
2000
0
A (n=15)
B (n=16)
C (n=14)
surface

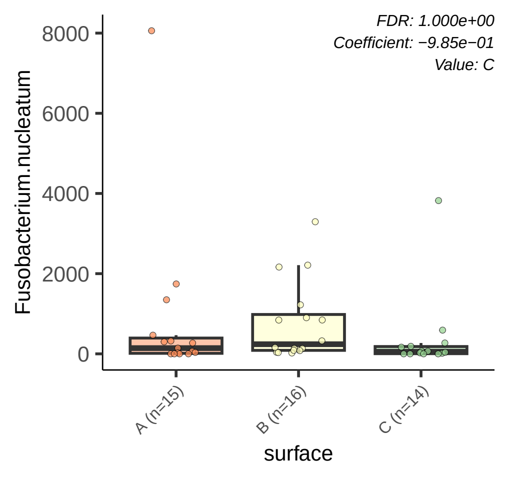

FDR: 1.000e+00
Coefficient: −9.85e−01
Value: C
Fusobacterium.nucleatum
8000
6000
4000
2000
0
A (n=15)
B (n=16)
C (n=14)
surface

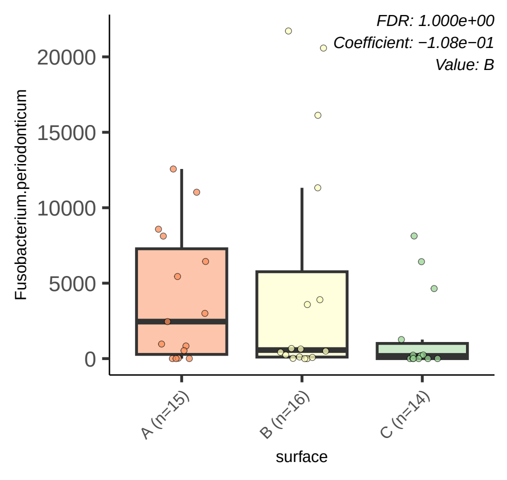

FDR: 1.000e+00
Coefficient: −1.08e−01
Value: B
Fusobacterium.periodonticum
20000
15000
10000
5000
0
A (n=15)
B (n=16)
C (n=14)
surface

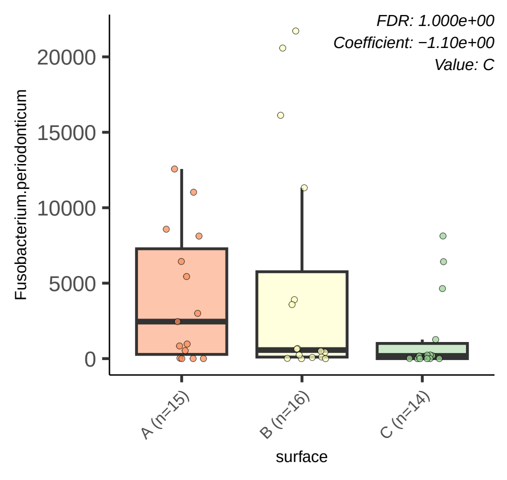

FDR: 1.000e+00
Coefficient: −1.10e+00
Value: C
Fusobacterium.periodonticum
20000
15000
10000
5000
0
A (n=15)
B (n=16)
C (n=14)
surface

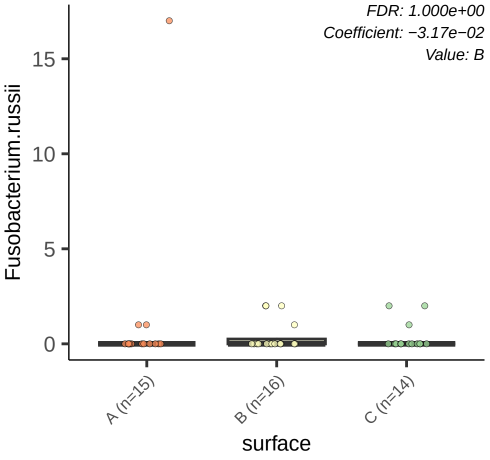

FDR: 1.000e+00
Coefficient: −3.17e−02
Value: B
Fusobacterium.russii
15
10
5
0
A (n=15)
B (n=16)
C (n=14)
surface

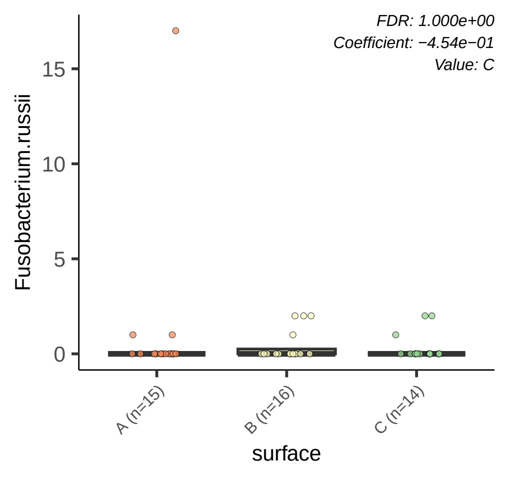

FDR: 1.000e+00
Coefficient: −4.54e−01
Value: C
Fusobacterium.russii
0
5
10
15
A (n=15)
B (n=16)
C (n=14)
surface

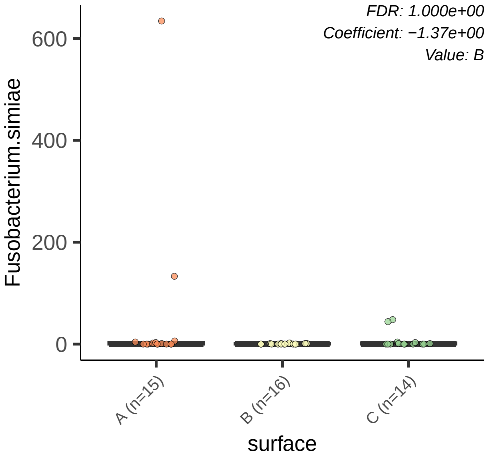
FDR: 1.000e+00
Coefficient: −1.37e+00
Value: B
Fusobacterium.simiae
600
400
200
0
A (n=15)
B (n=16)
C (n=14)
surface

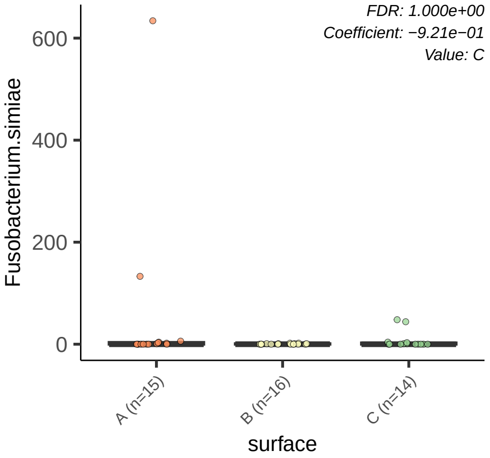
FDR: 1.000e+00
Coefficient: −9.21e−01
Value: C
Fusobacterium.simiae
600
400
200
0
A (n=15)
B (n=16)
C (n=14)
surface

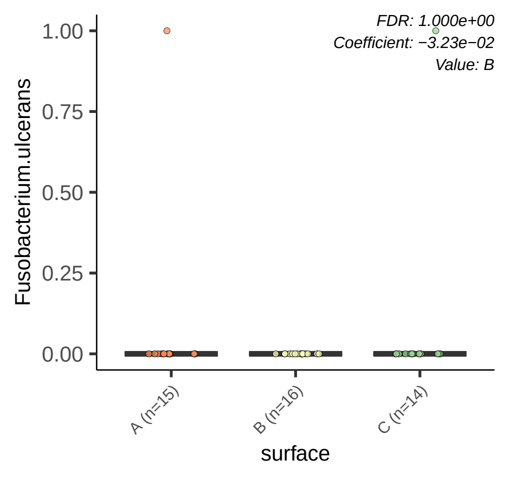

FDR: 1.000e+00
Coefficient: −3.23e−02
Value: B
Fusobacterium.ulcerans
1.00
0.75
0.50
0.25
0.00
A (n=15)
B (n=16)
C (n=14)
surface

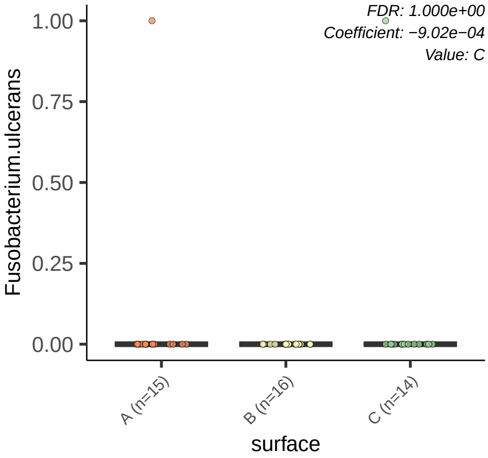

FDR: 1.000e+00
Coefficient: −9.02e−04
Value: C
Fusobacterium.ulcerans
1.00
0.75
0.50
0.25
0.00
A (n=15)
B (n=16)
C (n=14)
surface

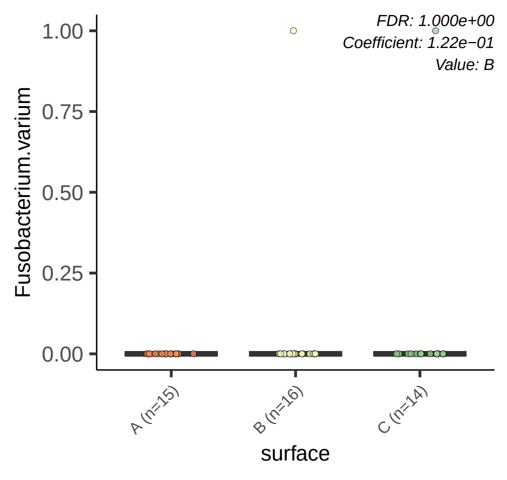
FDR: 1.000e+00
Coefficient: 1.22e−01
Value: B
Fusobacterium.varium
1.00
0.75
0.50
0.25
0.00
A (n=15)
B (n=16)
C (n=14)
surface

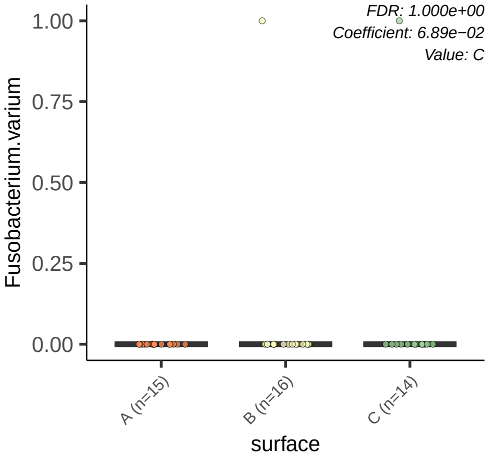
FDR: 1.000e+00
Coefficient: 6.89e−02
Value: C
Fusobacterium.varium
1.00
0.75
0.50
0.25
0.00
A (n=15)
B (n=16)
C (n=14)
surface

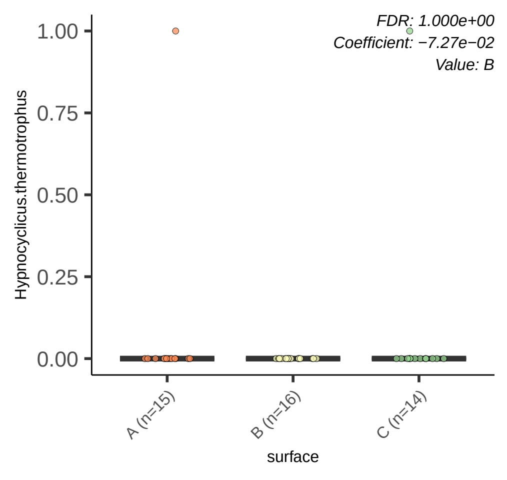

FDR: 1.000e+00
Coefficient: −7.27e−02
Value: B
Hypnocyclicus.thermotrophus
1.00
0.75
0.50
0.25
0.00
A (n=15)
B (n=16)
C (n=14)
surface

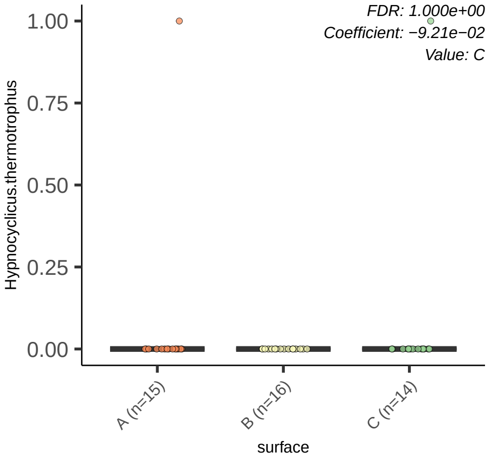
FDR: 1.000e+00
Coefficient: −9.21e−02
Value: C
Hypnocyclicus.thermotrophus
1.00
0.75
0.50
0.25
0.00
A (n=15)
B (n=16)
C (n=14)
surface

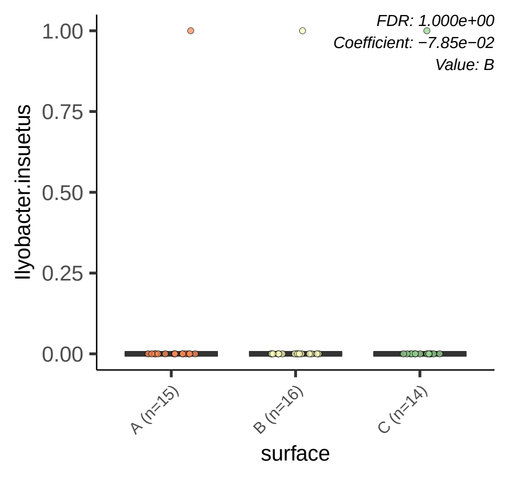
FDR: 1.000e+00
Coefficient: −7.85e−02
Value: B
1.00
0.75
0.50
0.25
0.00
Ilyobacter.insuetus
A (n=15)
B (n=16)
C (n=14)
surface

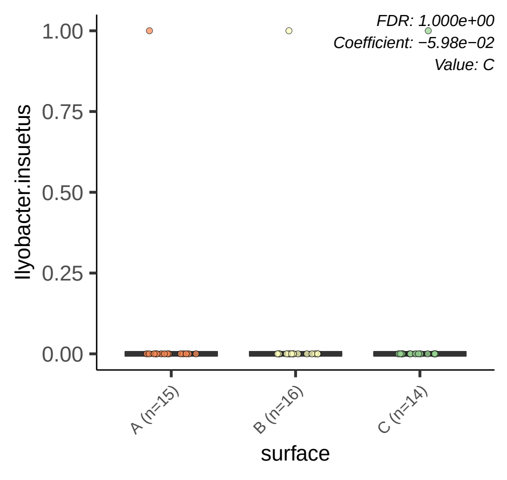
FDR: 1.000e+00
Coefficient: −5.98e−02
Value: C
Ilyobacter.insuetus
1.00
0.75
0.50
0.25
0.00
A (n=15)
B (n=16)
C (n=14)
surface

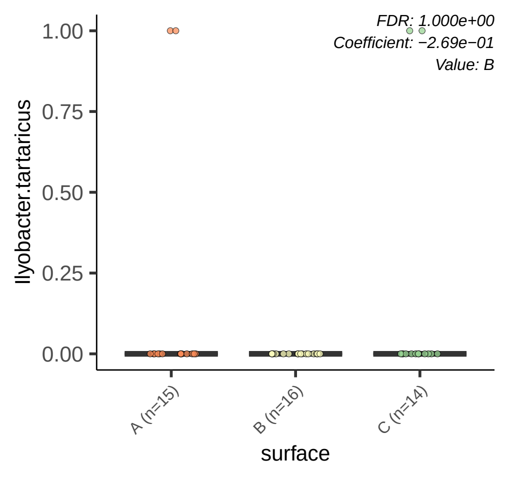
FDR: 1.000e+00
Coefficient: −2.69e−01
Value: B
Ilyobacter.tartaricus
1.00
0.75
0.50
0.25
0.00
A (n=15)
B (n=16)
C (n=14)
surface

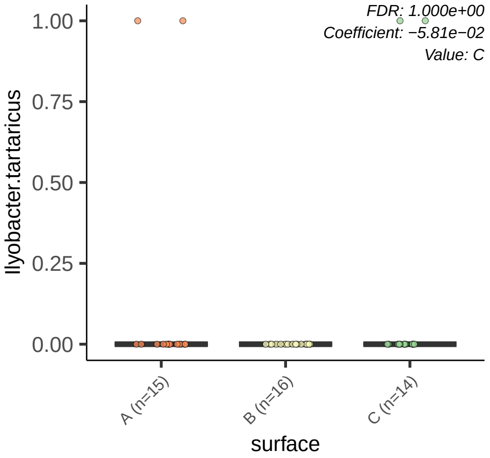

FDR: 1.000e+00
Coefficient: −5.81e−02
Value: C
Ilyobacter.tartaricus
1.00
0.75
0.50
0.25
0.00
A (n=15)
B (n=16)
C (n=14)
surface

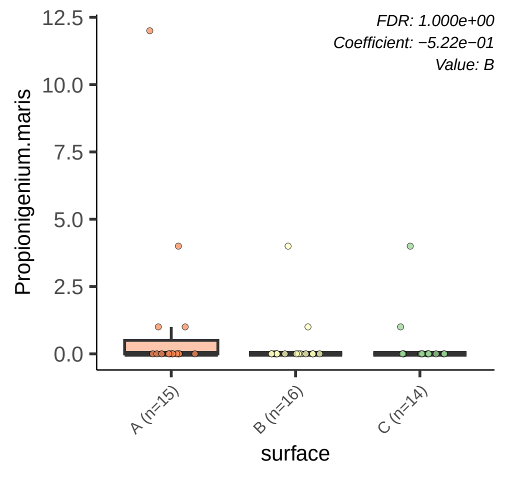
FDR: 1.000e+00
Coefficient: −5.22e−01
Value: B
Propionigenium.maris
12.5
10.0
7.5
5.0
2.5
0.0
A (n=15)
B (n=16)
C (n=14)
surface

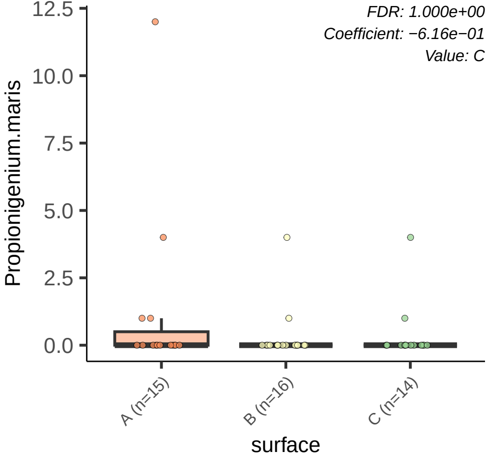
FDR: 1.000e+00
Coefficient: −6.16e−01
Value: C
Propionigenium.maris
12.5
10.0
7.5
5.0
2.5
0.0
A (n=15)
B (n=16)
C (n=14)
surface

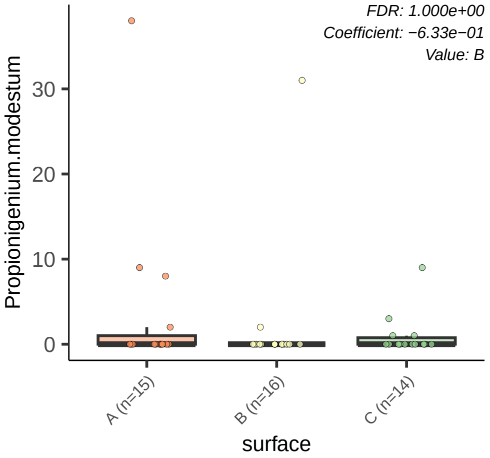

FDR: 1.000e+00
Coefficient: −6.33e−01
Value: B
Propionigenium.modestum
30
20
10
0
A (n=15)
B (n=16)
C (n=14)
surface

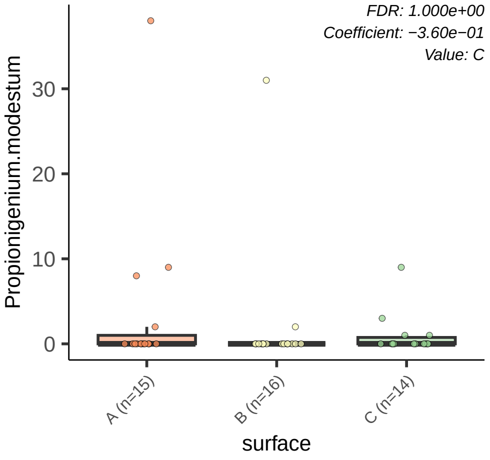
FDR: 1.000e+00
Coefficient: −3.60e−01
Value: C
Propionigenium.modestum
30
20
10
0
A (n=15)
B (n=16)
C (n=14)
surface

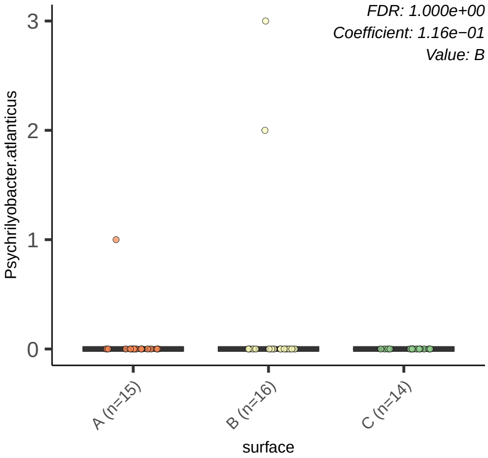

FDR: 1.000e+00
Coefficient: 1.16e−01
Value: B
Psychrilyobacter.atlanticus
3
2
1
0
A (n=15)
B (n=16)
C (n=14)
surface

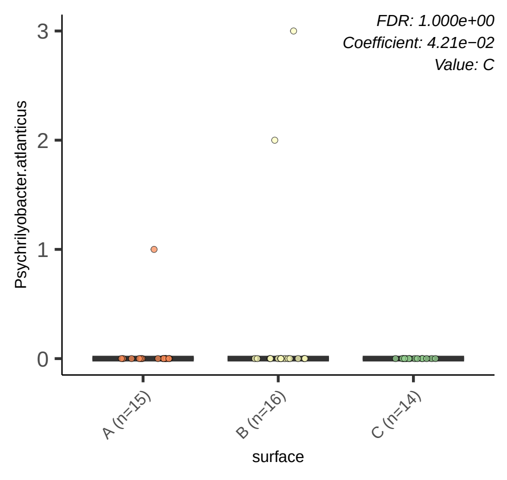

FDR: 1.000e+00
Coefficient: 4.21e−02
Value: C
Psychrilyobacter.atlanticus
0
1
2
3
A (n=15)
B (n=16)
C (n=14)
surface

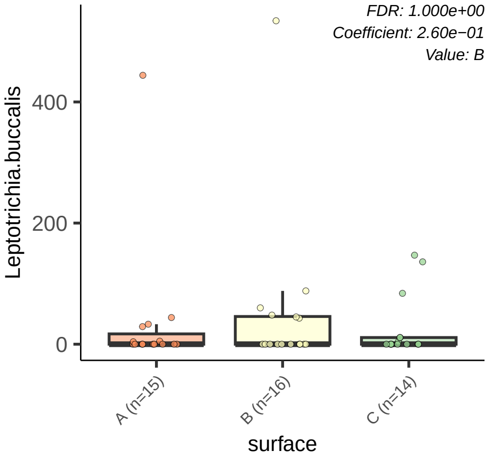
FDR: 1.000e+00
Coefficient: 2.60e−01
Value: B
Leptotrichia.buccalis
400
200
0
A (n=15)
B (n=16)
C (n=14)
surface

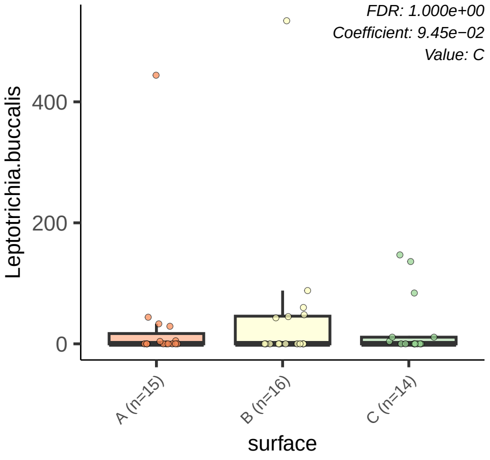
FDR: 1.000e+00
Coefficient: 9.45e−02
Value: C
Leptotrichia.buccalis
400
200
0
A (n=15)
B (n=16)
C (n=14)
surface

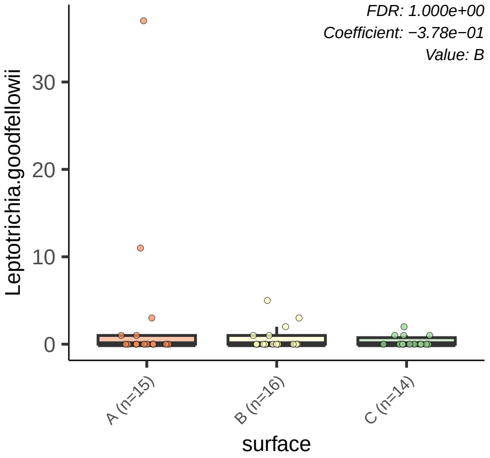

FDR: 1.000e+00
Coefficient: −3.78e−01
Value: B
Leptotrichia.goodfellowii
0
10
20
30
A (n=15)
B (n=16)
C (n=14)
surface

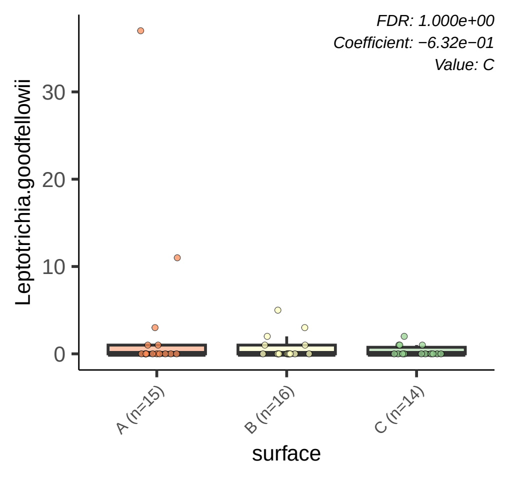

FDR: 1.000e+00
Coefficient: −6.32e−01
Value: C
Leptotrichia.goodfellowii
30
20
10
0
A (n=15)
B (n=16)
C (n=14)
surface

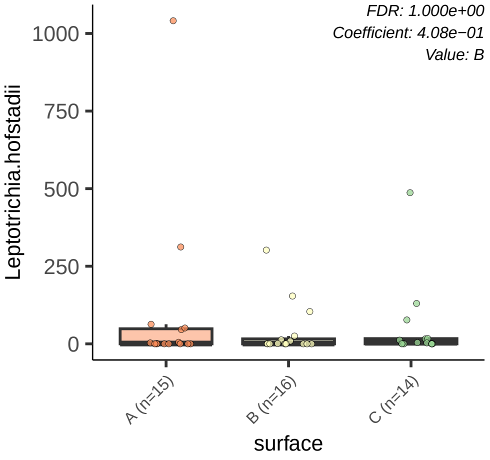

FDR: 1.000e+00
Coefficient: 4.08e−01
Value: B
Leptotrichia.hofstadii
1000
750
500
250
0
A (n=15)
B (n=16)
C (n=14)
surface

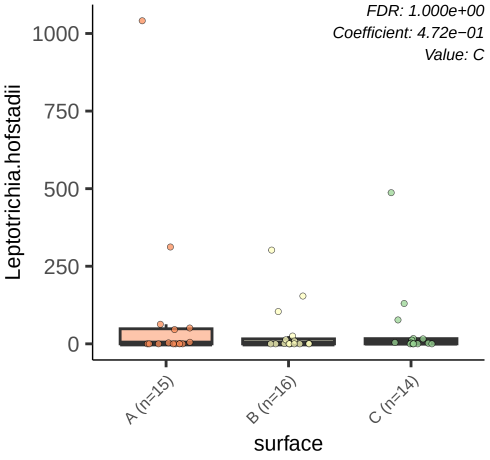

FDR: 1.000e+00
Coefficient: 4.72e−01
Value: C
Leptotrichia.hofstadii
1000
750
500
250
0
A (n=15)
B (n=16)
C (n=14)
surface

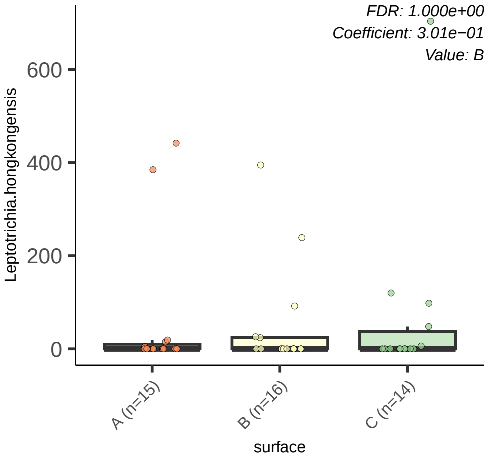
FDR: 1.000e+00
Coefficient: 3.01e−01
Value: B
Leptotrichia.hongkongensis
600
400
200
0
A (n=15)
B (n=16)
C (n=14)
surface

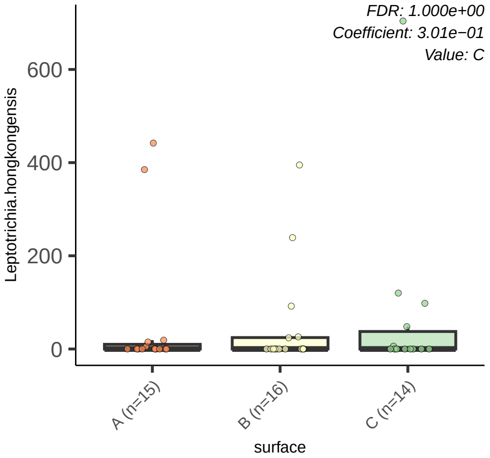
FDR: 1.000e+00
Coefficient: 3.01e−01
Value: C
Leptotrichia.hongkongensis
600
400
200
0
A (n=15)
B (n=16)
C (n=14)
surface

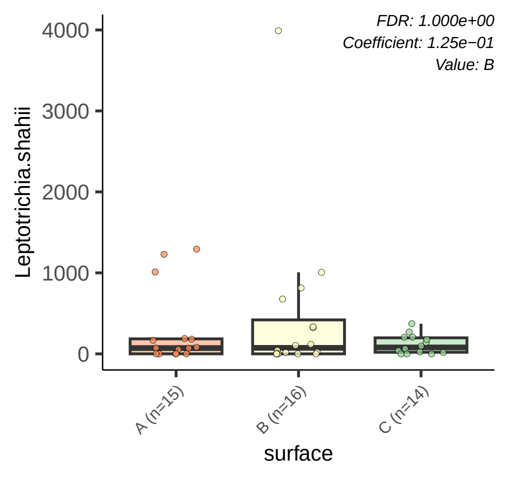
FDR: 1.000e+00
Coefficient: 1.25e−01
Value: B
Leptotrichia.shahii
4000
3000
2000
1000
0
A (n=15)
B (n=16)
C (n=14)
surface

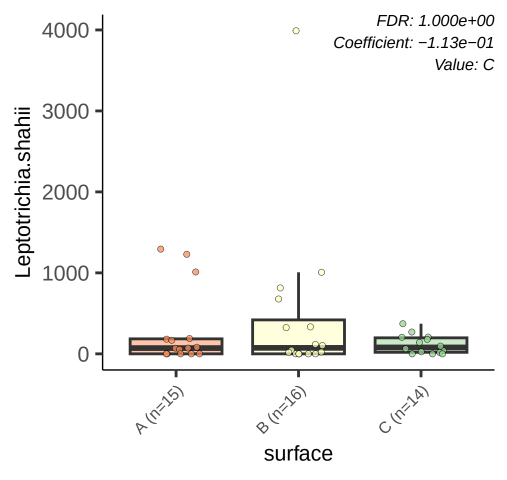

FDR: 1.000e+00
Coefficient: −1.13e−01
Value: C
Leptotrichia.shahii
4000
3000
2000
1000
0
A (n=15)
B (n=16)
C (n=14)
surface

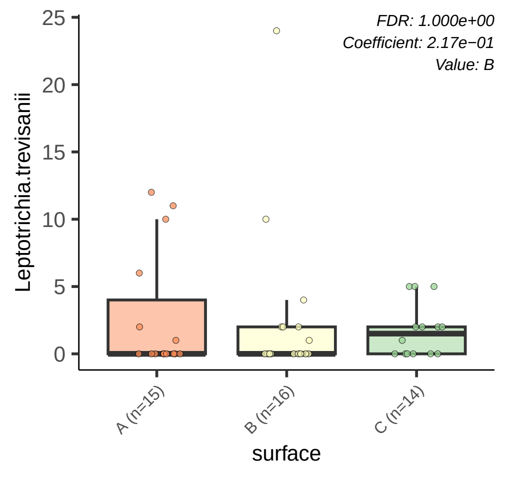

FDR: 1.000e+00
Coefficient: 2.17e−01
Value: B
Leptotrichia.trevisanii
0
5
10
15
20
25
A (n=15)
B (n=16)
C (n=14)
surface

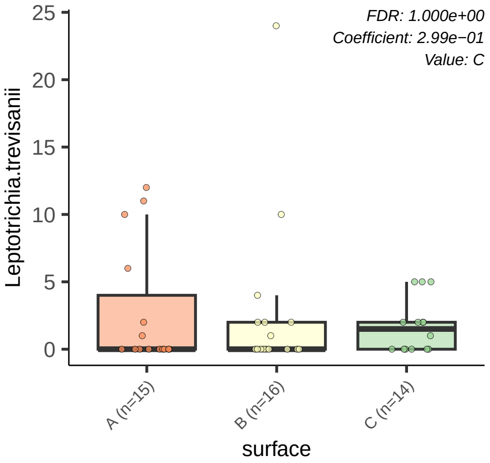
FDR: 1.000e+00
Coefficient: 2.99e−01
Value: C
Leptotrichia.trevisanii
25
20
15
10
5
0
A (n=15)
B (n=16)
C (n=14)
surface

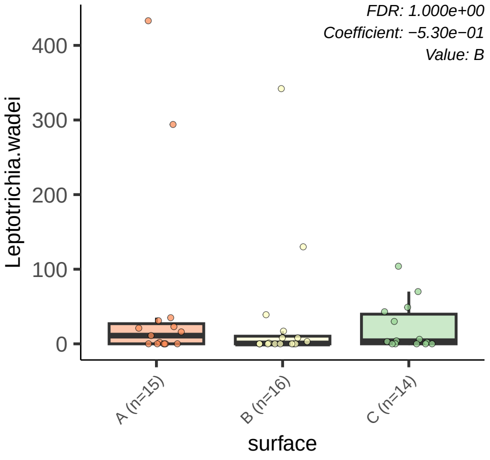
FDR: 1.000e+00
Coefficient: −5.30e−01
Value: B
Leptotrichia.wadei
400
300
200
100
0
A (n=15)
B (n=16)
C (n=14)
surface

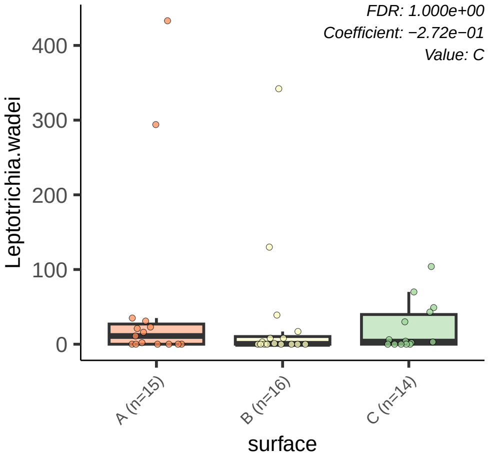

FDR: 1.000e+00
Coefficient: −2.72e−01
Value: C
400
300
200
100
0
Leptotrichia.wadei
A (n=15)
B (n=16)
C (n=14)
surface

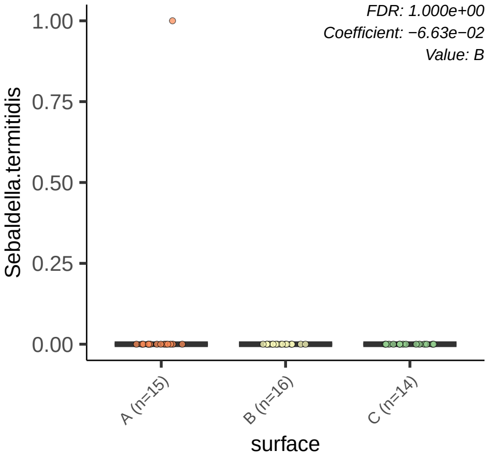
FDR: 1.000e+00
Coefficient: −6.63e−02
Value: B
Sebaldella.termitidis
1.00
0.75
0.50
0.25
0.00
A (n=15)
B (n=16)
C (n=14)
surface

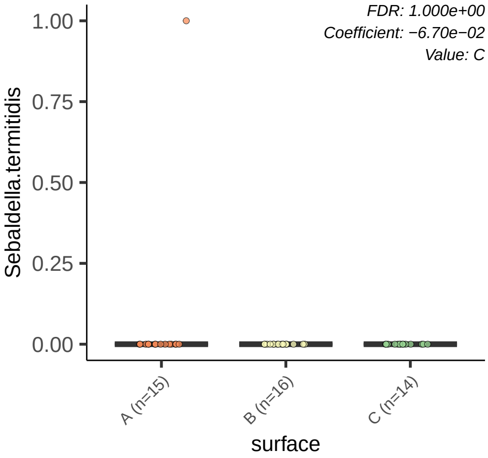
FDR: 1.000e+00
Coefficient: −6.70e−02
Value: C
Sebaldella.termitidis
1.00
0.75
0.50
0.25
0.00
A (n=15)
B (n=16)
C (n=14)
surface

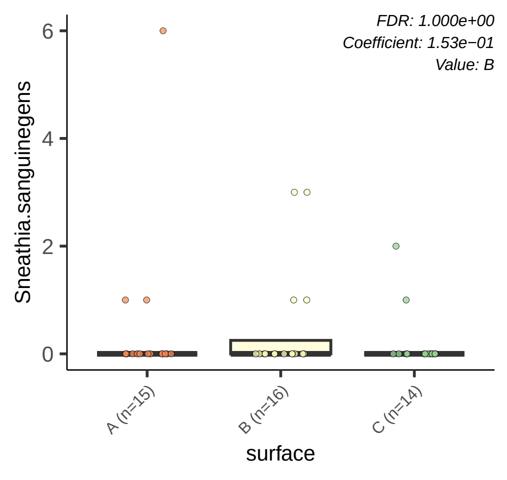
FDR: 1.000e+00
Coefficient: 1.53e−01
Value: B
Sneathia.sanguinegens
0
2
4
6
A (n=15)
B (n=16)
C (n=14)
surface

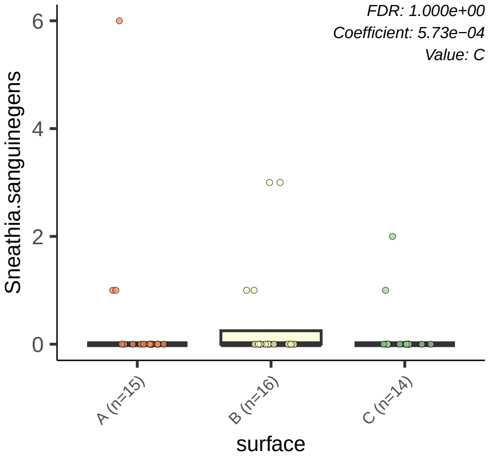
FDR: 1.000e+00
Coefficient: 5.73e−04
Value: C
Sneathia.sanguinegens
6
4
2
0
A (n=15)
B (n=16)
C (n=14)
surface

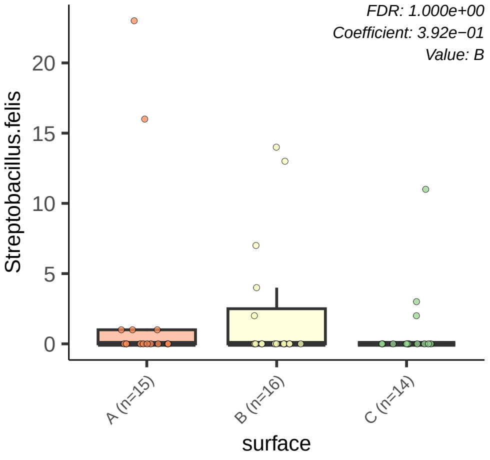

FDR: 1.000e+00
Coefficient: 3.92e−01
Value: B
Streptobacillus.felis
20
15
10
5
0
A (n=15)
B (n=16)
C (n=14)
surface

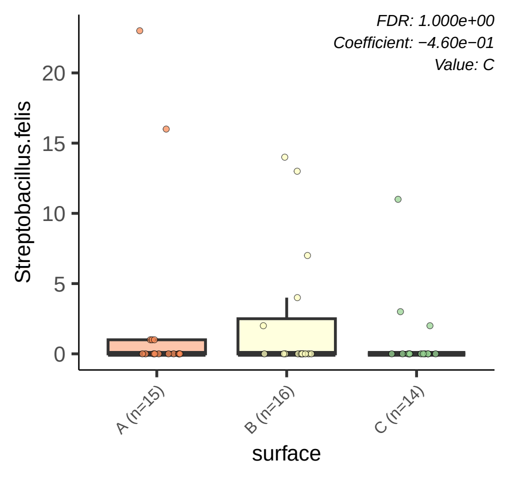

FDR: 1.000e+00
Coefficient: −4.60e−01
Value: C
Streptobacillus.felis
0
5
10
15
20
A (n=15)
B (n=16)
C (n=14)
surface

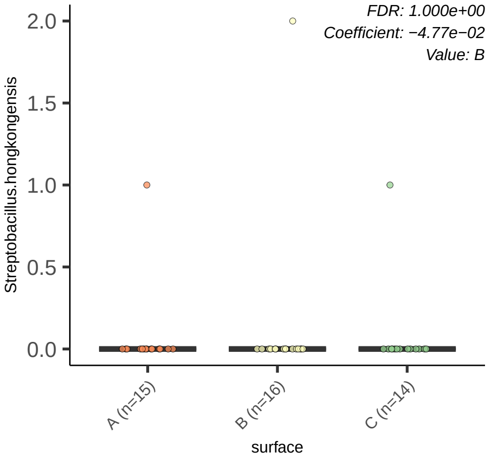

FDR: 1.000e+00
Coefficient: −4.77e−02
Value: B
2.0
1.5
1.0
0.5
0.0
Streptobacillus.hongkongensis
A (n=15)
B (n=16)
C (n=14)
surface

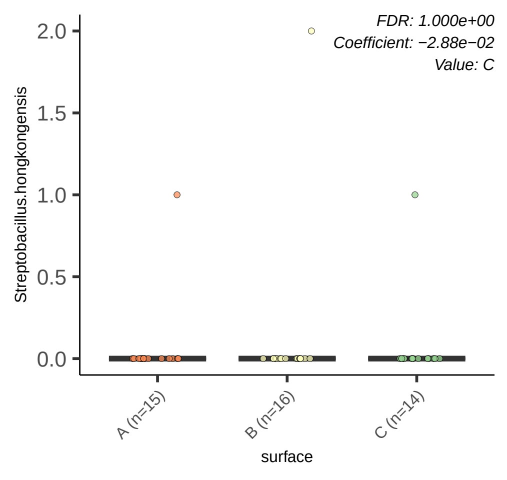
FDR: 1.000e+00
Coefficient: −2.88e−02
Value: C
2.0
1.5
1.0
0.5
0.0
Streptobacillus.hongkongensis
A (n=15)
B (n=16)
C (n=14)
surface

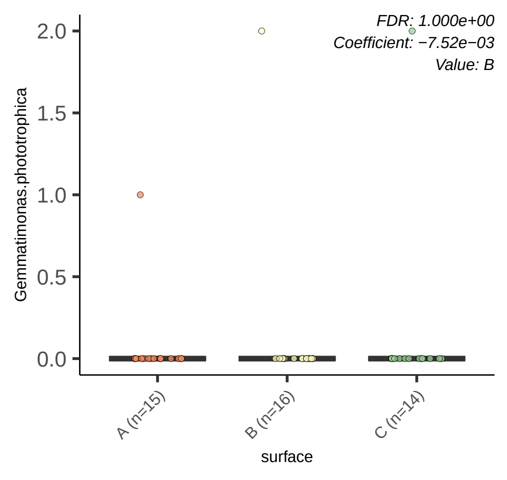
FDR: 1.000e+00
Coefficient: −7.52e−03
Value: B
Gemmatimonas.phototrophica
2.0
1.5
1.0
0.5
0.0
A (n=15)
B (n=16)
C (n=14)
surface

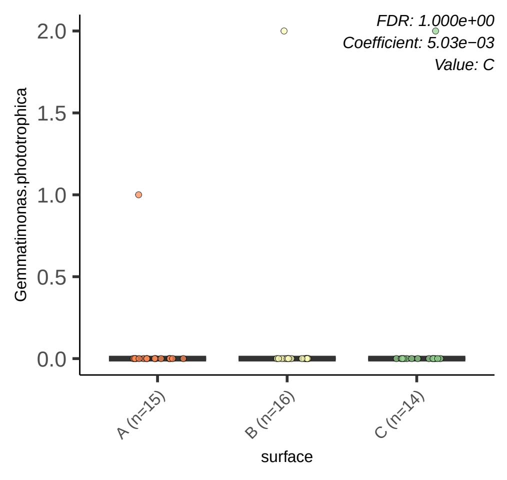
FDR: 1.000e+00
Coefficient: 5.03e−03
Value: C
Gemmatimonas.phototrophica
2.0
1.5
1.0
0.5
0.0
A (n=15)
B (n=16)
C (n=14)
surface

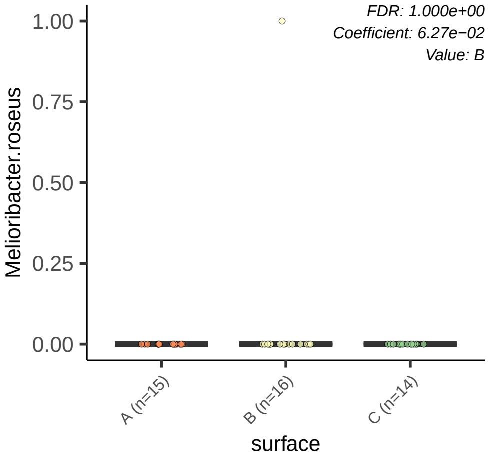

FDR: 1.000e+00
Coefficient: 6.27e−02
Value: B
Melioribacter.roseus
1.00
0.75
0.50
0.25
0.00
A (n=15)
B (n=16)
C (n=14)
surface

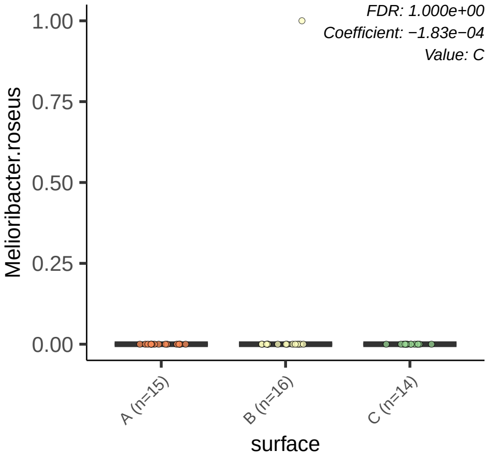

FDR: 1.000e+00
Coefficient: −1.83e−04
Value: C
Melioribacter.roseus
1.00
0.75
0.50
0.25
0.00
A (n=15)
B (n=16)
C (n=14)
surface

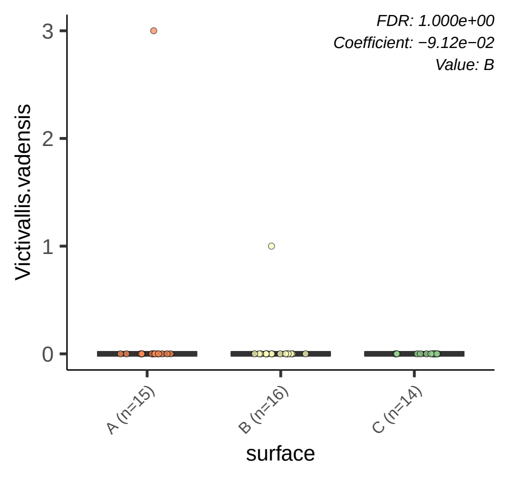
FDR: 1.000e+00
Coefficient: −9.12e−02
Value: B
Victivallis.vadensis
3
2
1
0
A (n=15)
B (n=16)
C (n=14)
surface

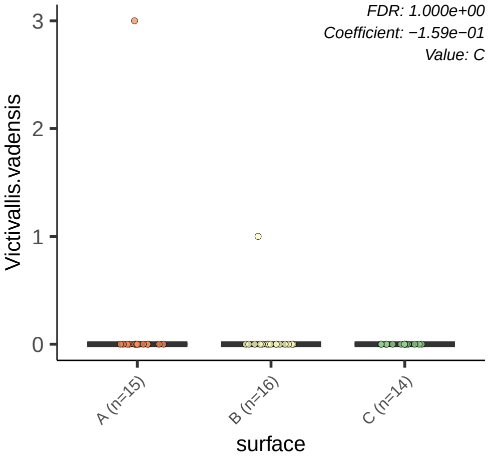

FDR: 1.000e+00
Coefficient: −1.59e−01
Value: C
Victivallis.vadensis
3
2
1
0
A (n=15)
B (n=16)
C (n=14)
surface

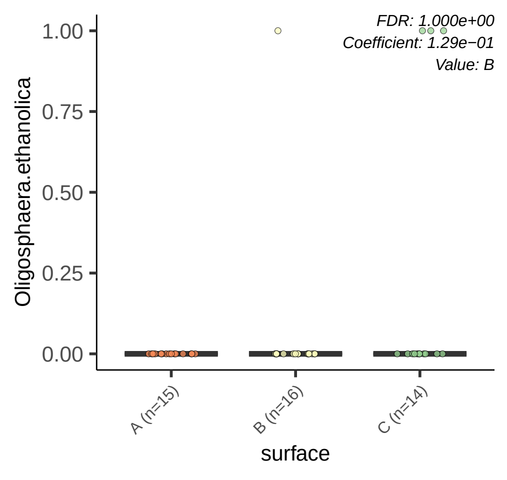
FDR: 1.000e+00
Coefficient: 1.29e−01
Value: B
Oligosphaera.ethanolica
1.00
0.75
0.50
0.25
0.00
A (n=15)
B (n=16)
C (n=14)
surface

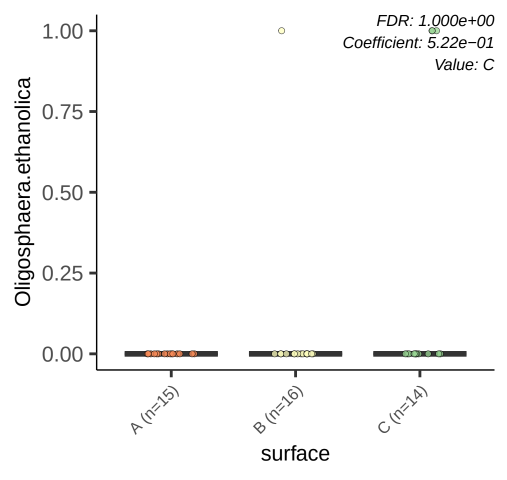

FDR: 1.000e+00
Coefficient: 5.22e−01
Value: C
Oligosphaera.ethanolica
1.00
0.75
0.50
0.25
0.00
A (n=15)
B (n=16)
C (n=14)
surface

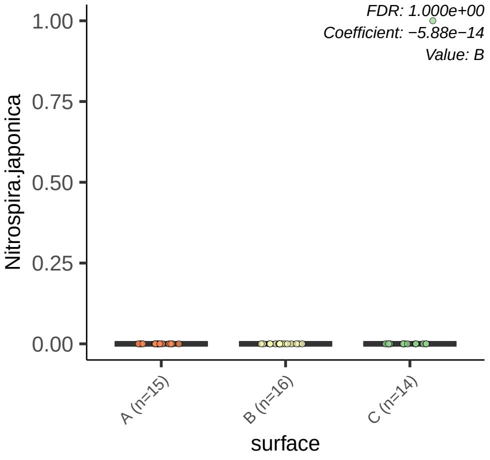
FDR: 1.000e+00
Coefficient: −5.88e−14
Value: B
1.00
0.75
0.50
0.25
0.00
Nitrospira.japonica
A (n=15)
B (n=16)
C (n=14)
surface

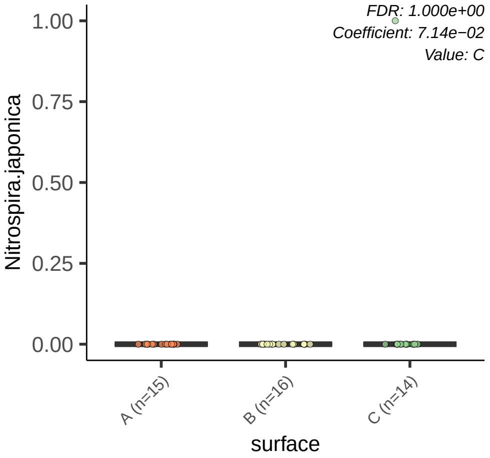
FDR: 1.000e+00
Coefficient: 7.14e−02
Value: C
Nitrospira.japonica
1.00
0.75
0.50
0.25
0.00
A (n=15)
B (n=16)
C (n=14)
surface

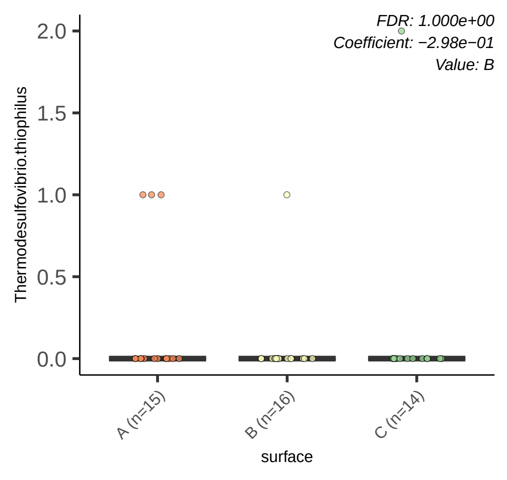

FDR: 1.000e+00
Coefficient: −2.98e−01
Value: B
Thermodesulfovibrio.thiophilus
2.0
1.5
1.0
0.5
0.0
A (n=15)
B (n=16)
C (n=14)
surface

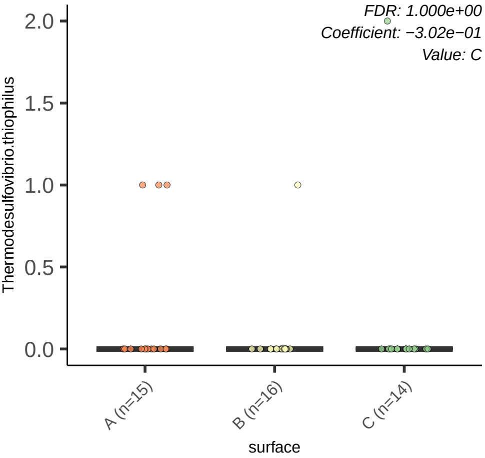

FDR: 1.000e+00
Coefficient: −3.02e−01
Value: C
2.0
1.5
1.0
0.5
0.0
Thermodesulfovibrio.thiophilus
A (n=15)
B (n=16)
C (n=14)
surface

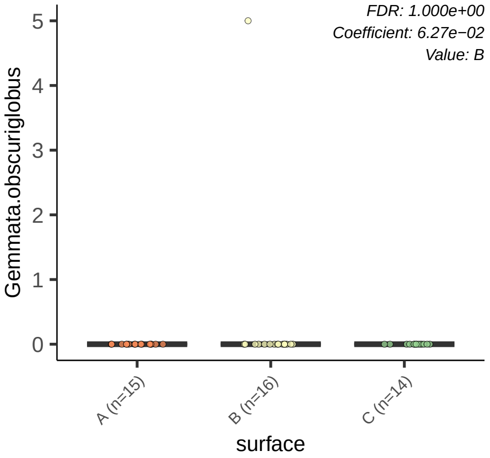

FDR: 1.000e+00
Coefficient: 6.27e−02
Value: B
Gemmata.obscuriglobus
0
1
2
3
4
5
A (n=15)
B (n=16)
C (n=14)
surface

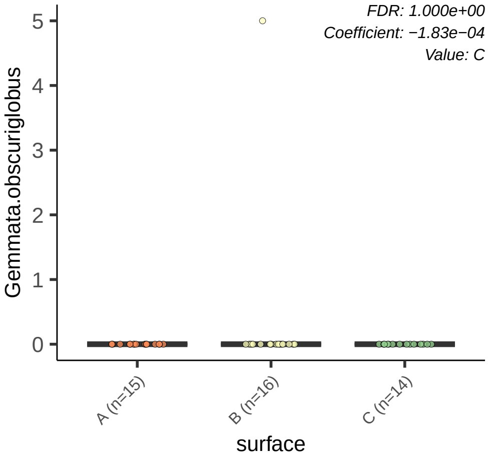
FDR: 1.000e+00
Coefficient: −1.83e−04
Value: C
Gemmata.obscuriglobus
0
1
2
3
4
5
A (n=15)
B (n=16)
C (n=14)
surface

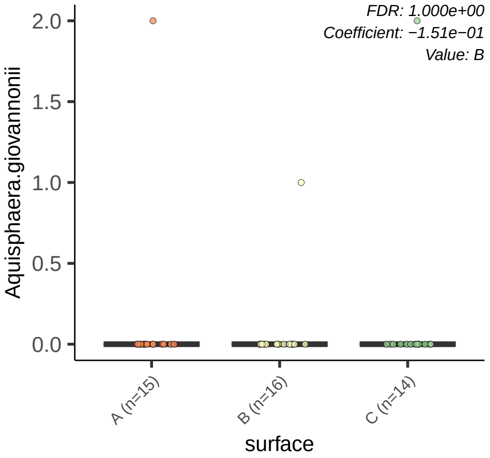
FDR: 1.000e+00
Coefficient: −1.51e−01
Value: B
Aquisphaera.giovannonii
2.0
1.5
1.0
0.5
0.0
A (n=15)
B (n=16)
C (n=14)
surface

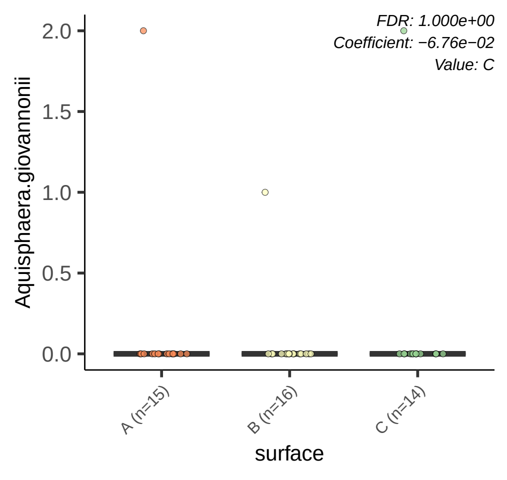
FDR: 1.000e+00
Coefficient: −6.76e−02
Value: C
Aquisphaera.giovannonii
2.0
1.5
1.0
0.5
0.0
A (n=15)
B (n=16)
C (n=14)
surface

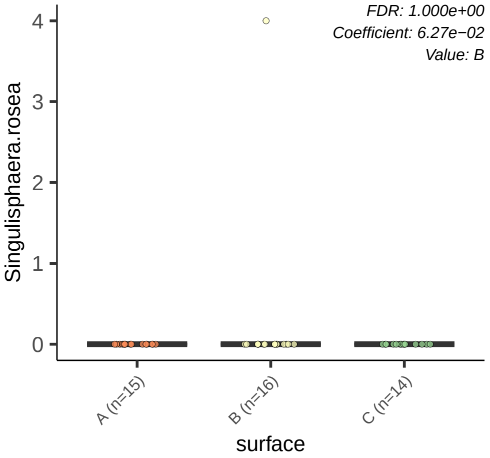

FDR: 1.000e+00
Coefficient: 6.27e−02
Value: B
Singulisphaera.rosea
4
3
2
1
0
A (n=15)
B (n=16)
C (n=14)
surface

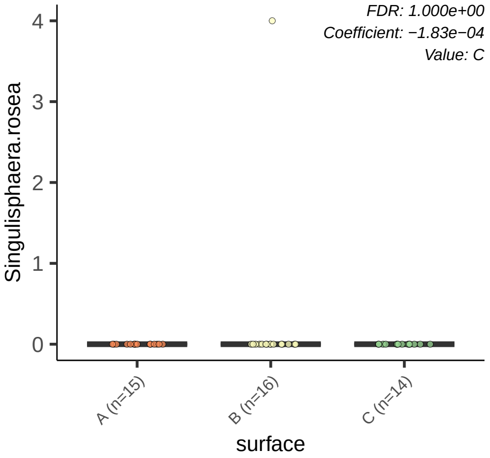
FDR: 1.000e+00
Coefficient: −1.83e−04
Value: C
Singulisphaera.rosea
0
1
2
3
4
A (n=15)
B (n=16)
C (n=14)
surface

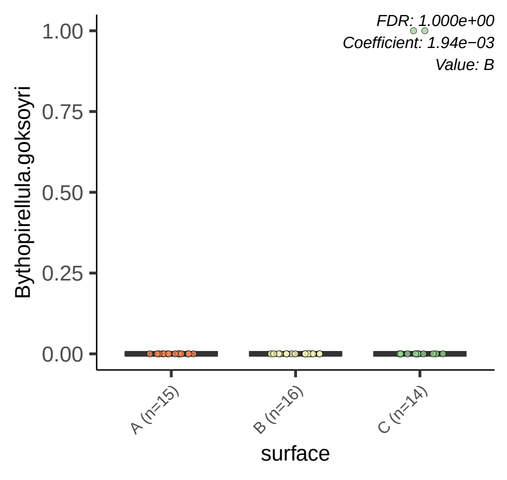

FDR: 1.000e+00
Coefficient: 1.94e−03
Value: B
Bythopirellula.goksoyri
1.00
0.75
0.50
0.25
0.00
A (n=15)
B (n=16)
C (n=14)
surface

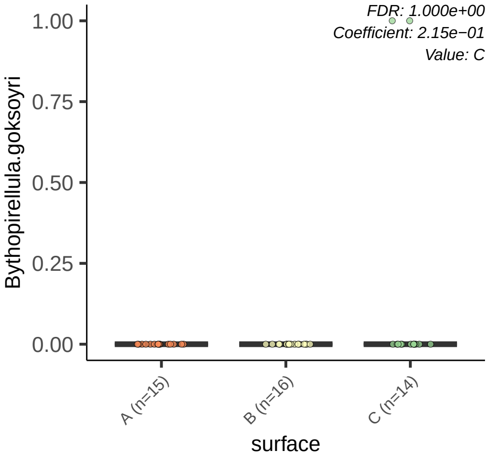

FDR: 1.000e+00
Coefficient: 2.15e−01
Value: C
Bythopirellula.goksoyri
1.00
0.75
0.50
0.25
0.00
A (n=15)
B (n=16)
C (n=14)
surface

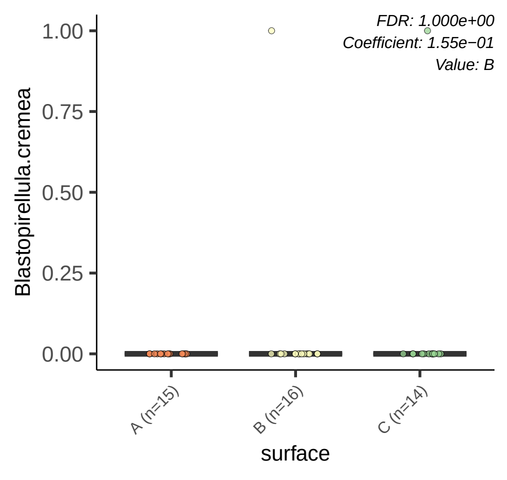
FDR: 1.000e+00
Coefficient: 1.55e−01
Value: B
Blastopirellula.cremea
1.00
0.75
0.50
0.25
0.00
A (n=15)
B (n=16)
C (n=14)
surface

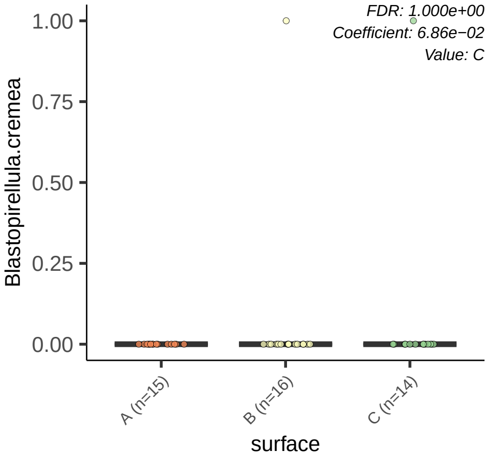
FDR: 1.000e+00
Coefficient: 6.86e−02
Value: C
Blastopirellula.cremea
1.00
0.75
0.50
0.25
0.00
A (n=15)
B (n=16)
C (n=14)
surface

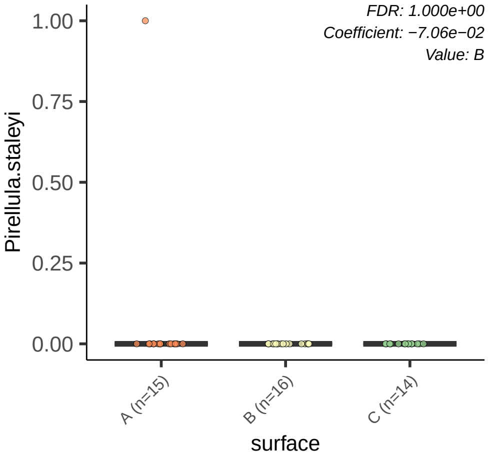
FDR: 1.000e+00
Coefficient: −7.06e−02
Value: B
Pirellula.staleyi
1.00
0.75
0.50
0.25
0.00
A (n=15)
B (n=16)
C (n=14)
surface

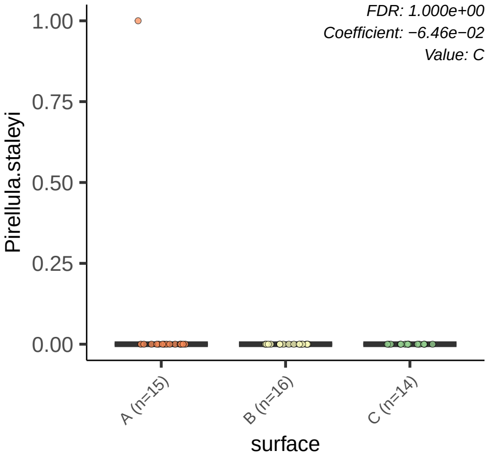
FDR: 1.000e+00
Coefficient: −6.46e−02
Value: C
Pirellula.staleyi
1.00
0.75
0.50
0.25
0.00
A (n=15)
B (n=16)
C (n=14)
surface

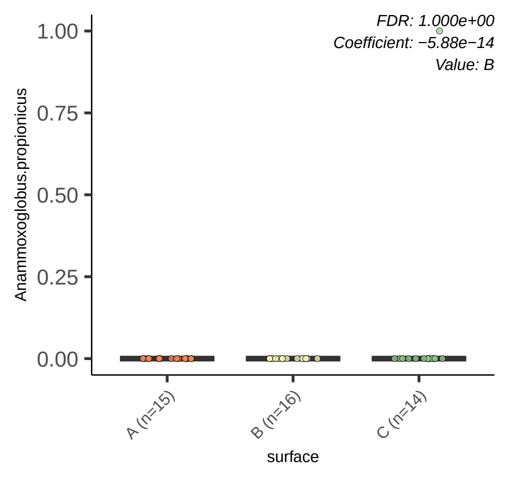
FDR: 1.000e+00
Coefficient: −5.88e−14
Value: B
Anammoxoglobus.propionicus
1.00
0.75
0.50
0.25
0.00
A (n=15)
B (n=16)
C (n=14)
surface

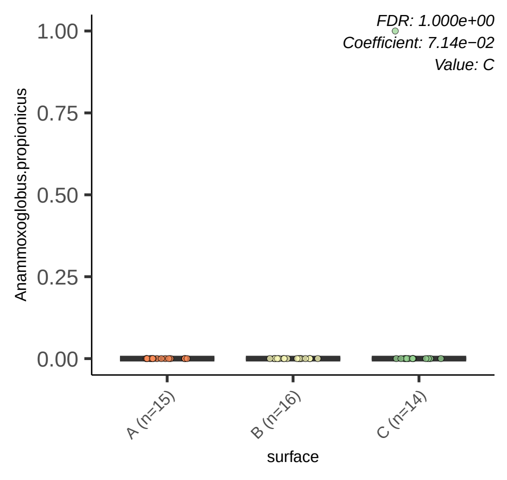
FDR: 1.000e+00
Coefficient: 7.14e−02
Value: C
1.00
0.75
0.50
0.25
0.00
Anammoxoglobus.propionicus
A (n=15)
B (n=16)
C (n=14)
surface

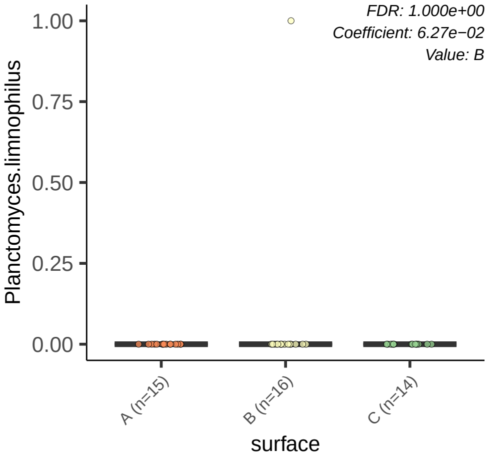
FDR: 1.000e+00
Coefficient: 6.27e−02
Value: B
Planctomyces.limnophilus
1.00
0.75
0.50
0.25
0.00
A (n=15)
B (n=16)
C (n=14)
surface

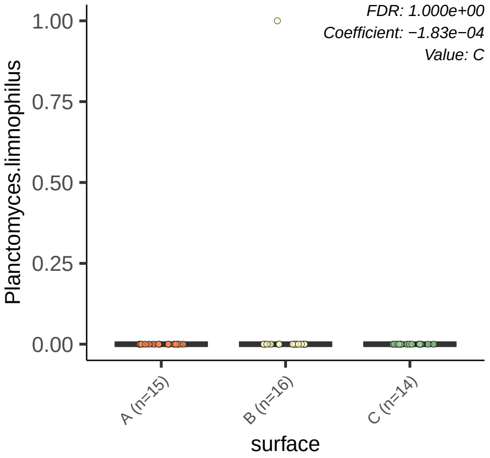
FDR: 1.000e+00
Coefficient: −1.83e−04
Value: C
Planctomyces.limnophilus
1.00
0.75
0.50
0.25
0.00
A (n=15)
B (n=16)
C (n=14)
surface

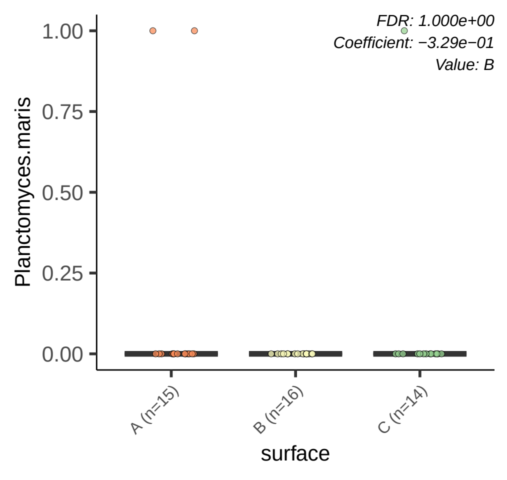
FDR: 1.000e+00
Coefficient: −3.29e−01
Value: B
Planctomyces.maris
1.00
0.75
0.50
0.25
0.00
A (n=15)
B (n=16)
C (n=14)
surface

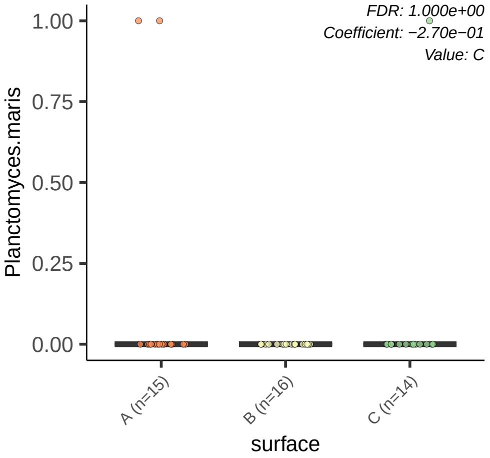
FDR: 1.000e+00
Coefficient: −2.70e−01
Value: C
Planctomyces.maris
1.00
0.75
0.50
0.25
0.00
A (n=15)
B (n=16)
C (n=14)
surface

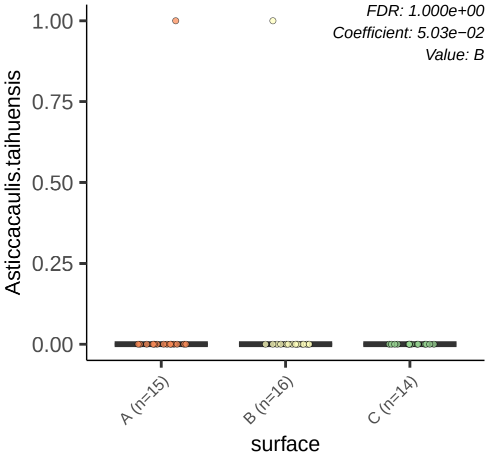
FDR: 1.000e+00
Coefficient: 5.03e−02
Value: B
Asticcacaulis.taihuensis
1.00
0.75
0.50
0.25
0.00
A (n=15)
B (n=16)
C (n=14)
surface

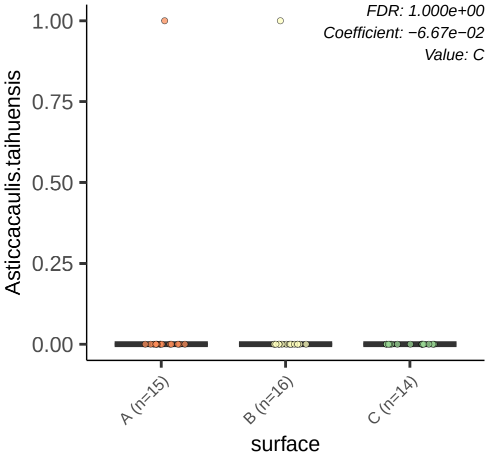
FDR: 1.000e+00
Coefficient: −6.67e−02
Value: C
1.00
0.75
0.50
0.25
0.00
Asticcacaulis.taihuensis
A (n=15)
B (n=16)
C (n=14)
surface

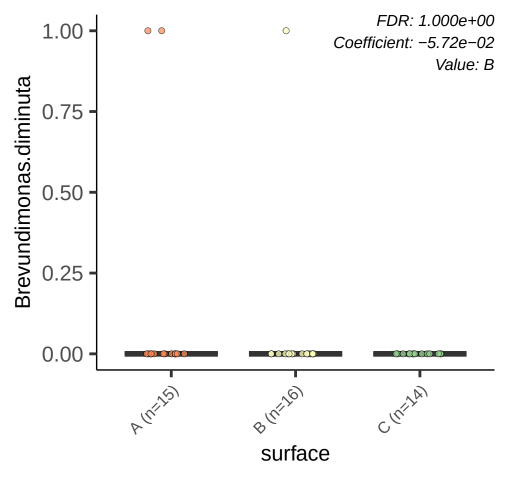
FDR: 1.000e+00
Coefficient: −5.72e−02
Value: B
Brevundimonas.diminuta
1.00
0.75
0.50
0.25
0.00
A (n=15)
B (n=16)
C (n=14)
surface

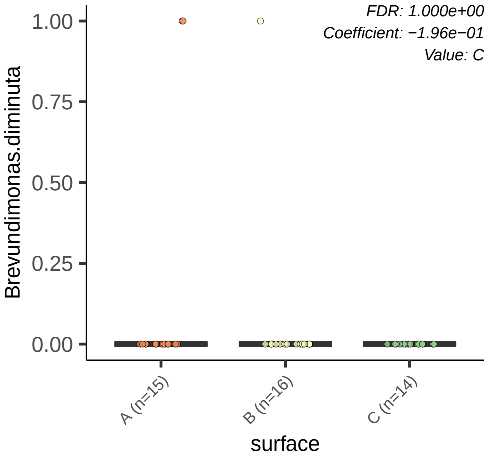
FDR: 1.000e+00
Coefficient: −1.96e−01
Value: C
Brevundimonas.diminuta
1.00
0.75
0.50
0.25
0.00
A (n=15)
B (n=16)
C (n=14)
surface

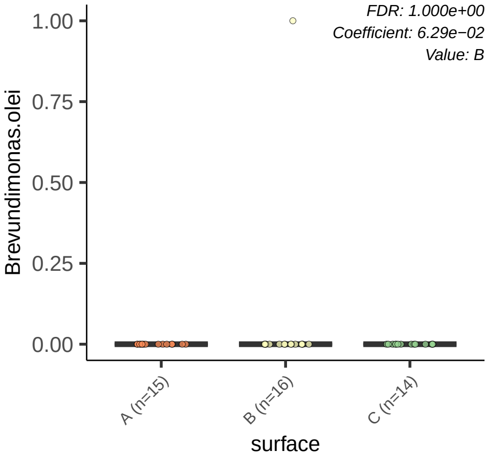

FDR: 1.000e+00
Coefficient: 6.29e−02
Value: B
Brevundimonas.olei
1.00
0.75
0.50
0.25
0.00
A (n=15)
B (n=16)
C (n=14)
surface

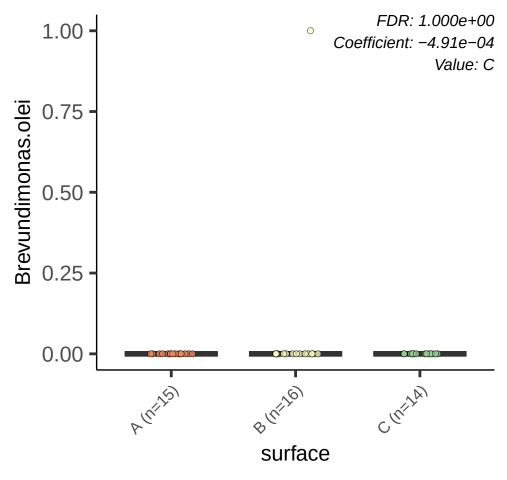
FDR: 1.000e+00
Coefficient: −4.91e−04
Value: C
Brevundimonas.olei
1.00
0.75
0.50
0.25
0.00
A (n=15)
B (n=16)
C (n=14)
surface

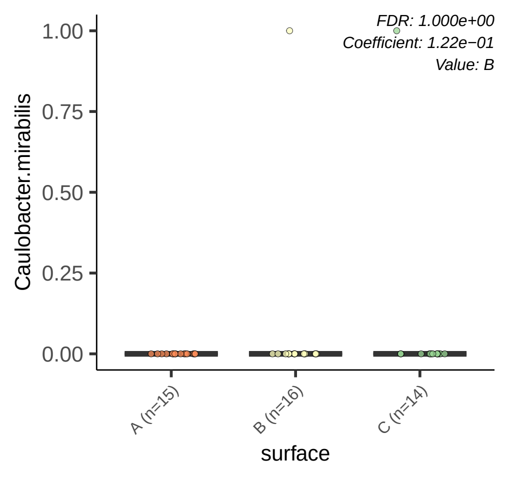
FDR: 1.000e+00
Coefficient: 1.22e−01
Value: B
Caulobacter.mirabilis
1.00
0.75
0.50
0.25
0.00
A (n=15)
B (n=16)
C (n=14)
surface

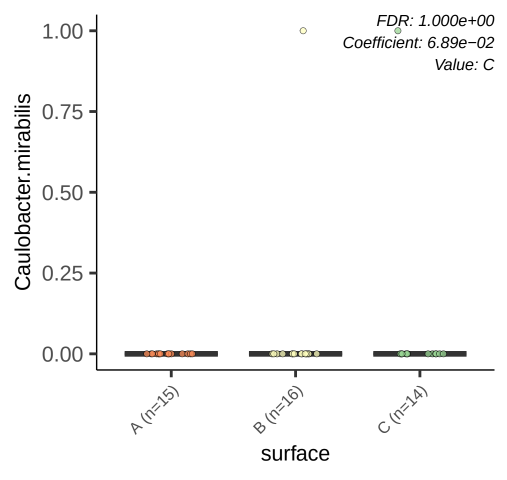
FDR: 1.000e+00
Coefficient: 6.89e−02
Value: C
1.00
0.75
0.50
0.25
0.00
Caulobacter.mirabilis
A (n=15)
B (n=16)
C (n=14)
surface

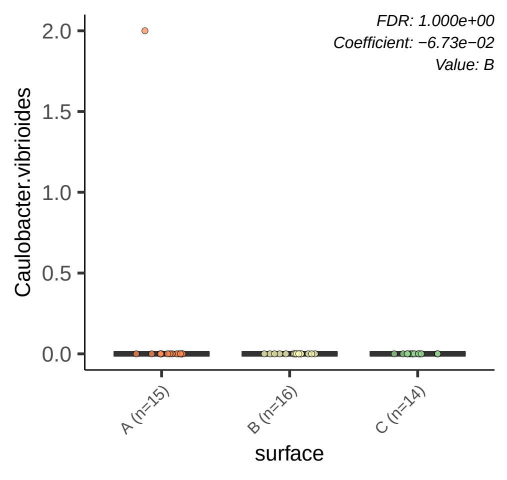
FDR: 1.000e+00
Coefficient: −6.73e−02
Value: B
Caulobacter.vibrioides
2.0
1.5
1.0
0.5
0.0
A (n=15)
B (n=16)
C (n=14)
surface

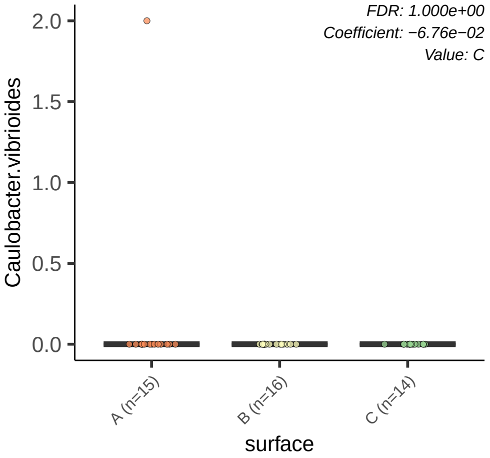
FDR: 1.000e+00
Coefficient: −6.76e−02
Value: C
Caulobacter.vibrioides
2.0
1.5
1.0
0.5
0.0
A (n=15)
B (n=16)
C (n=14)
surface

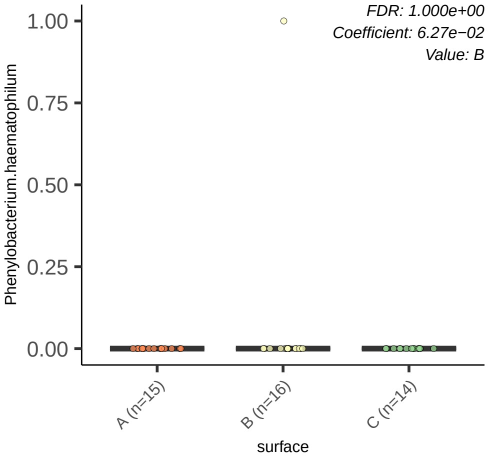
FDR: 1.000e+00
Coefficient: 6.27e−02
Value: B
Phenylobacterium.haematophilum
1.00
0.75
0.50
0.25
0.00
A (n=15)
B (n=16)
C (n=14)
surface

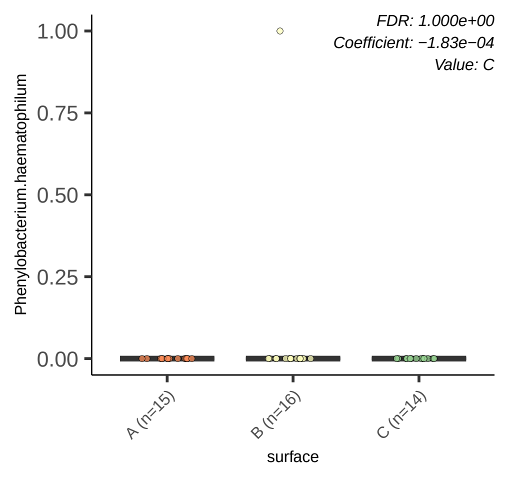
FDR: 1.000e+00
Coefficient: −1.83e−04
Value: C
Phenylobacterium.haematophilum
1.00
0.75
0.50
0.25
0.00
A (n=15)
B (n=16)
C (n=14)
surface

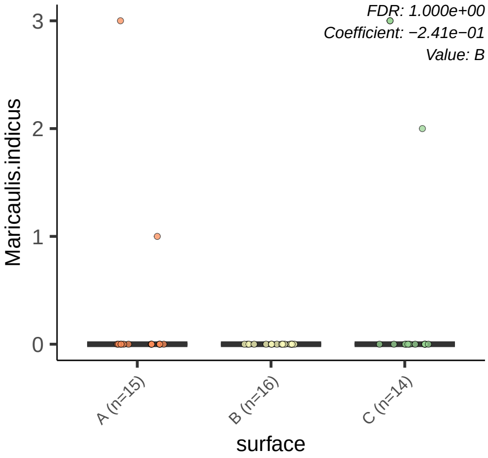

FDR: 1.000e+00
Coefficient: −2.41e−01
Value: B
Maricaulis.indicus
0
1
2
3
A (n=15)
B (n=16)
C (n=14)
surface

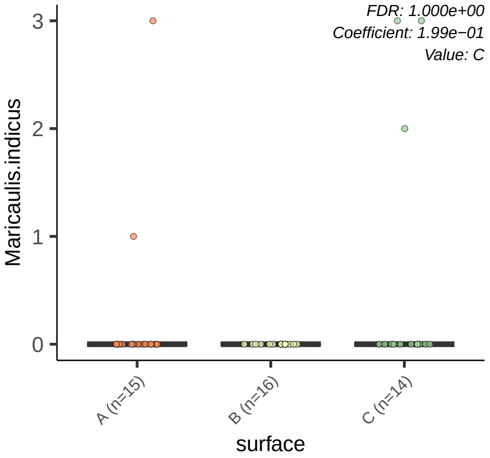
FDR: 1.000e+00
Coefficient: 1.99e−01
Value: C
Maricaulis.indicus
3
2
1
0
A (n=15)
B (n=16)
C (n=14)
surface

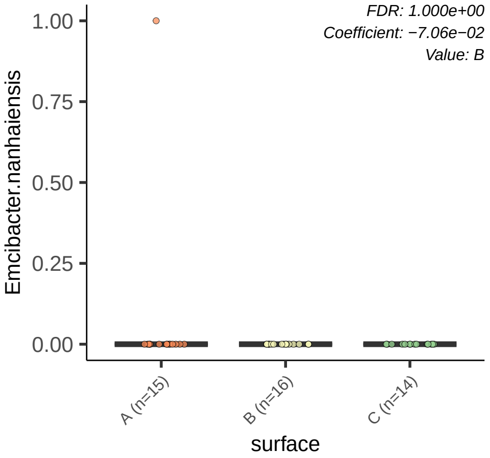
FDR: 1.000e+00
Coefficient: −7.06e−02
Value: B
Emcibacter.nanhaiensis
1.00
0.75
0.50
0.25
0.00
A (n=15)
B (n=16)
C (n=14)
surface

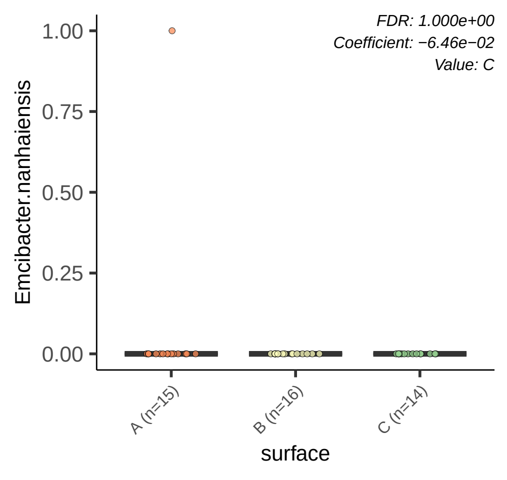
FDR: 1.000e+00
Coefficient: −6.46e−02
Value: C
1.00
0.75
0.50
0.25
0.00
Emcibacter.nanhaiensis
A (n=15)
B (n=16)
C (n=14)
surface

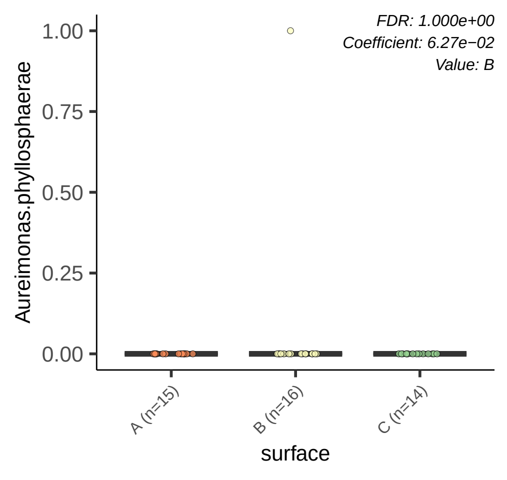

FDR: 1.000e+00
Coefficient: 6.27e−02
Value: B
Aureimonas.phyllosphaerae
1.00
0.75
0.50
0.25
0.00
A (n=15)
B (n=16)
C (n=14)
surface

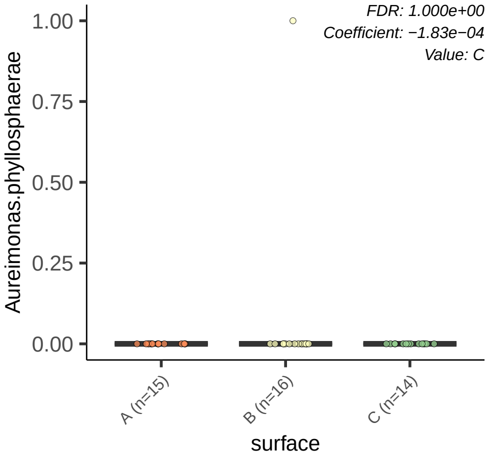
FDR: 1.000e+00
Coefficient: −1.83e−04
Value: C
Aureimonas.phyllosphaerae
1.00
0.75
0.50
0.25
0.00
A (n=15)
B (n=16)
C (n=14)
surface

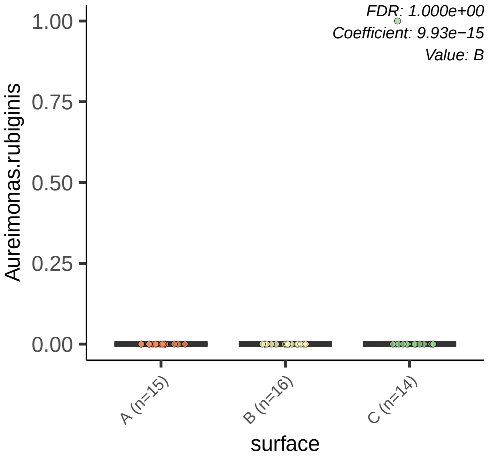
FDR: 1.000e+00
Coefficient: 9.93e−15
Value: B
Aureimonas.rubiginis
1.00
0.75
0.50
0.25
0.00
A (n=15)
B (n=16)
C (n=14)
surface

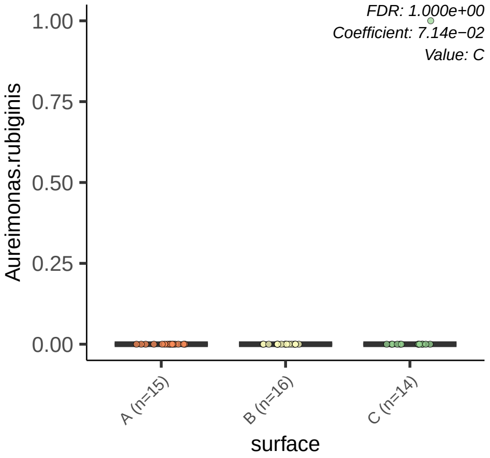
FDR: 1.000e+00
Coefficient: 7.14e−02
Value: C
Aureimonas.rubiginis
1.00
0.75
0.50
0.25
0.00
A (n=15)
B (n=16)
C (n=14)
surface

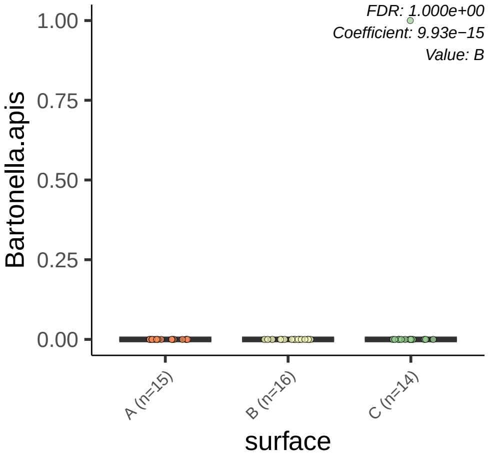

FDR: 1.000e+00
Coefficient: 9.93e−15
Value: B
Bartonella.apis
1.00
0.75
0.50
0.25
0.00
A (n=15)
B (n=16)
C (n=14)
surface

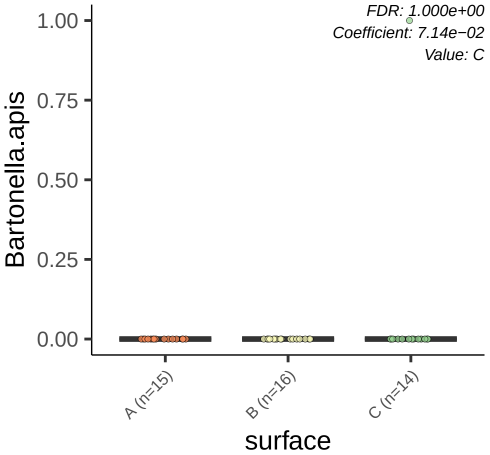

FDR: 1.000e+00
Coefficient: 7.14e−02
Value: C
Bartonella.apis
1.00
0.75
0.50
0.25
0.00
A (n=15)
B (n=16)
C (n=14)
surface

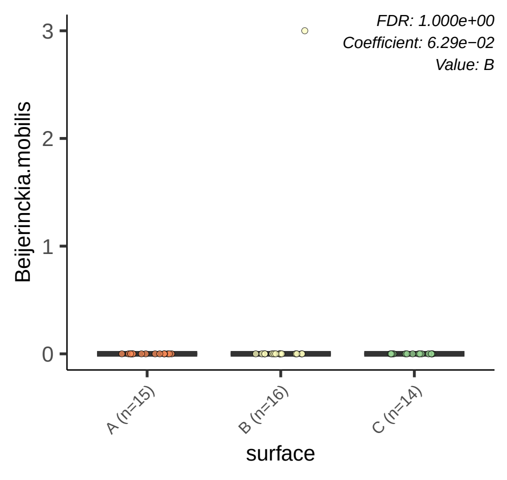

FDR: 1.000e+00
Coefficient: 6.29e−02
Value: B
Beijerinckia.mobilis
3
2
1
0
A (n=15)
B (n=16)
C (n=14)
surface

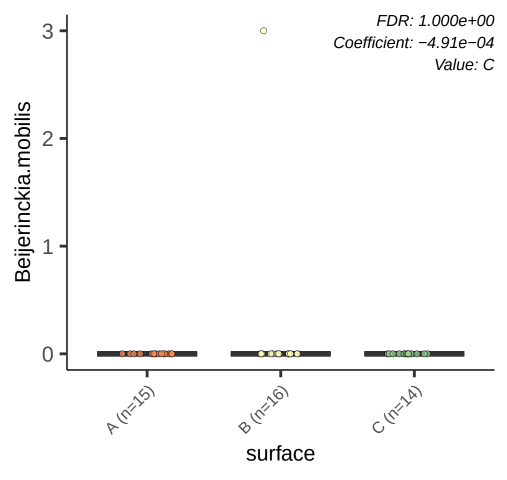
FDR: 1.000e+00
Coefficient: −4.91e−04
Value: C
Beijerinckia.mobilis
3
2
1
0
A (n=15)
B (n=16)
C (n=14)
surface

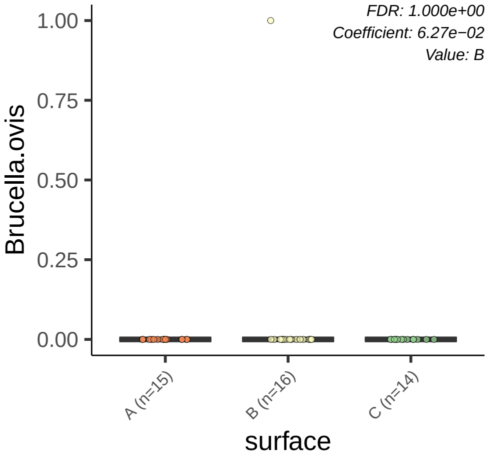
FDR: 1.000e+00
Coefficient: 6.27e−02
Value: B
Brucella.ovis
1.00
0.75
0.50
0.25
0.00
A (n=15)
B (n=16)
C (n=14)
surface

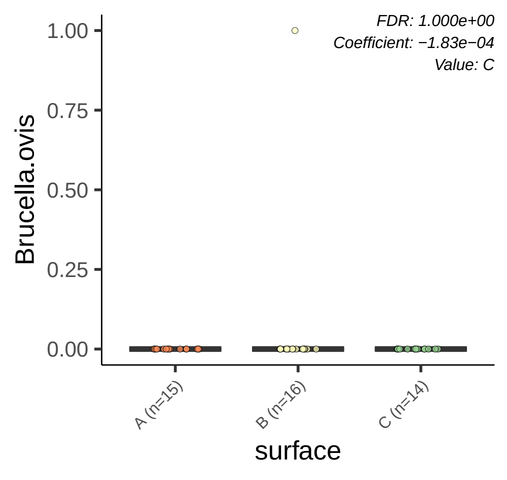
FDR: 1.000e+00
Coefficient: −1.83e−04
Value: C
Brucella.ovis
1.00
0.75
0.50
0.25
0.00
A (n=15)
B (n=16)
C (n=14)
surface

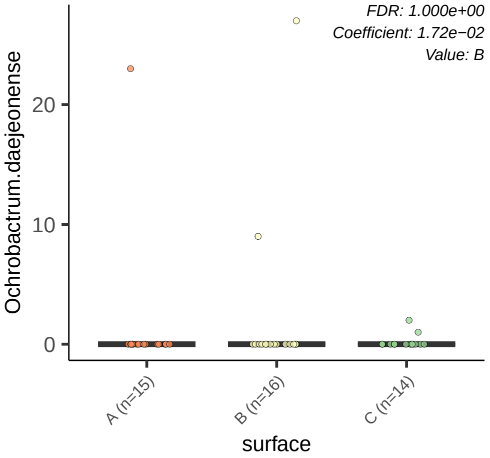

FDR: 1.000e+00
Coefficient: 1.72e−02
Value: B
Ochrobactrum.daejeonense
0
10
20
A (n=15)
B (n=16)
C (n=14)
surface

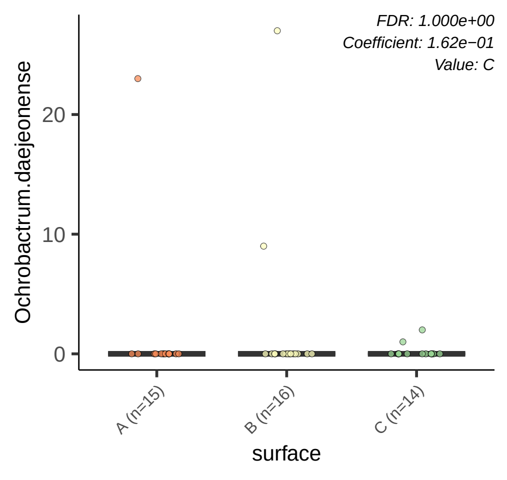
FDR: 1.000e+00
Coefficient: 1.62e−01
Value: C
Ochrobactrum.daejeonense
20
10
0
A (n=15)
B (n=16)
C (n=14)
surface

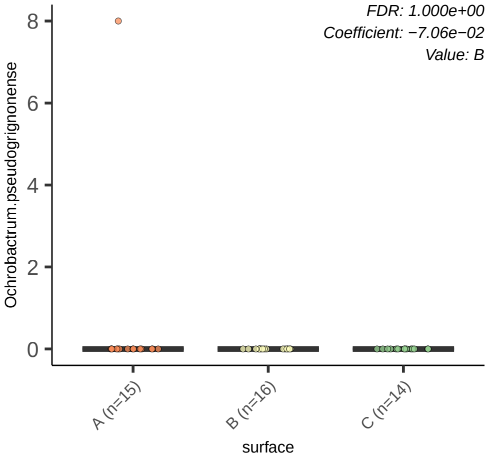
FDR: 1.000e+00
Coefficient: −7.06e−02
Value: B
Ochrobactrum.pseudogrignonense
0
2
4
6
8
A (n=15)
B (n=16)
C (n=14)
surface

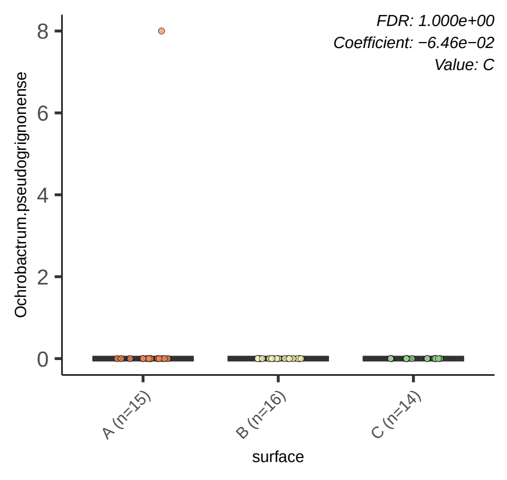
FDR: 1.000e+00
Coefficient: −6.46e−02
Value: C
Ochrobactrum.pseudogrignonense
0
2
4
6
8
A (n=15)
B (n=16)
C (n=14)
surface

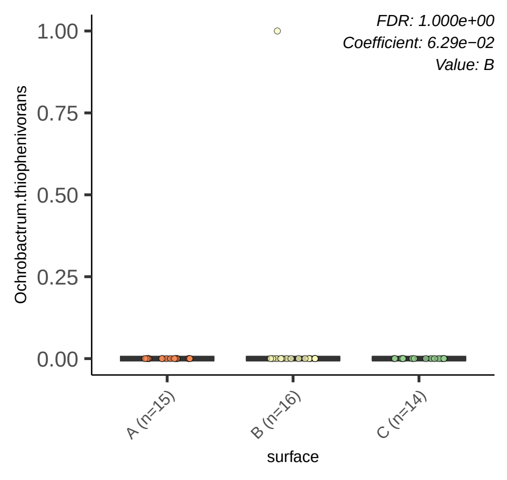

FDR: 1.000e+00
Coefficient: 6.29e−02
Value: B
1.00
0.75
0.50
0.25
0.00
Ochrobactrum.thiophenivorans
A (n=15)
B (n=16)
C (n=14)
surface

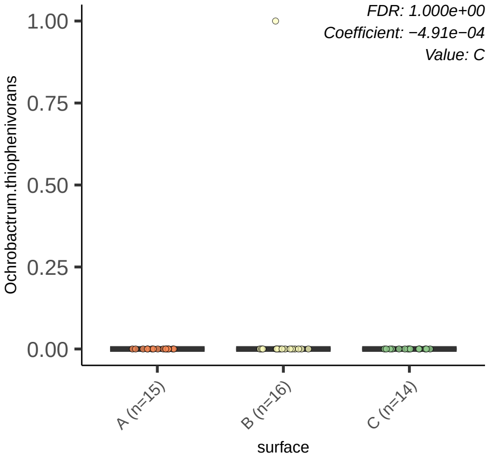

FDR: 1.000e+00
Coefficient: −4.91e−04
Value: C
Ochrobactrum.thiophenivorans
1.00
0.75
0.50
0.25
0.00
A (n=15)
B (n=16)
C (n=14)
surface

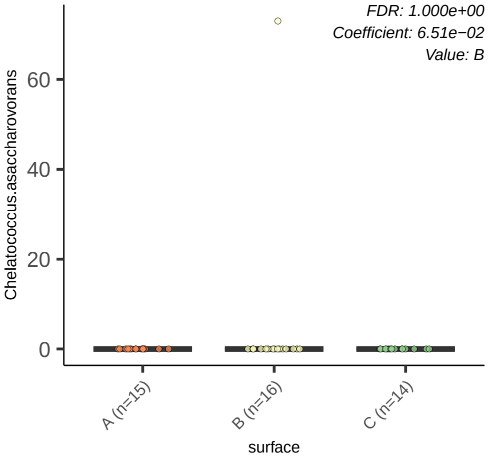
FDR: 1.000e+00
Coefficient: 6.51e−02
Value: B
Chelatococcus.asaccharovorans
0
20
40
60
A (n=15)
B (n=16)
C (n=14)
surface

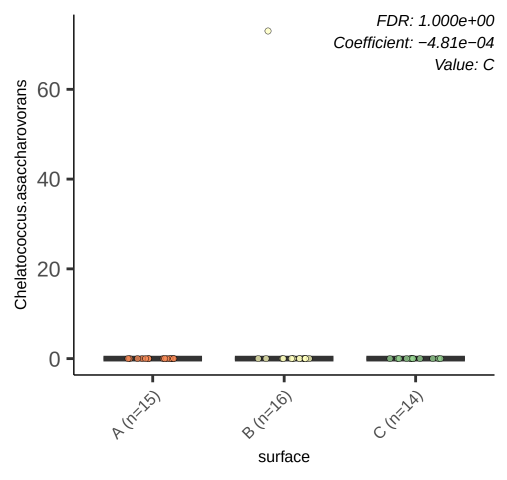

FDR: 1.000e+00
Coefficient: −4.81e−04
Value: C
Chelatococcus.asaccharovorans
0
20
40
60
A (n=15)
B (n=16)
C (n=14)
surface

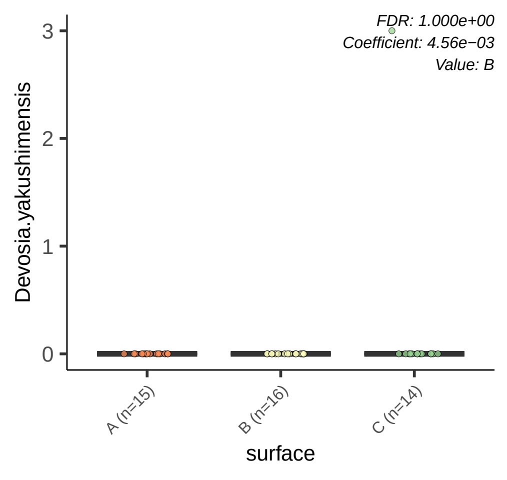
FDR: 1.000e+00
Coefficient: 4.56e−03
Value: B
Devosia.yakushimensis
3
2
1
0
A (n=15)
B (n=16)
C (n=14)
surface

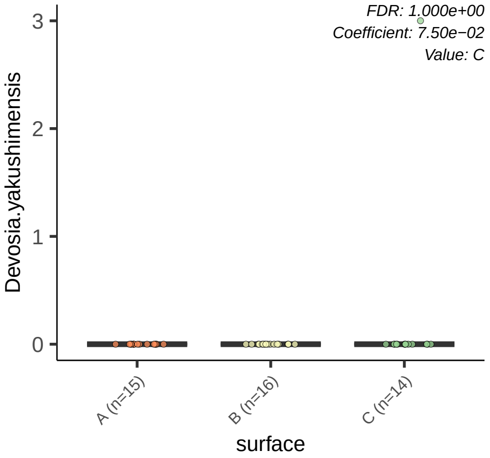

FDR: 1.000e+00
Coefficient: 7.50e−02
Value: C
Devosia.yakushimensis
3
2
1
0
A (n=15)
B (n=16)
C (n=14)
surface

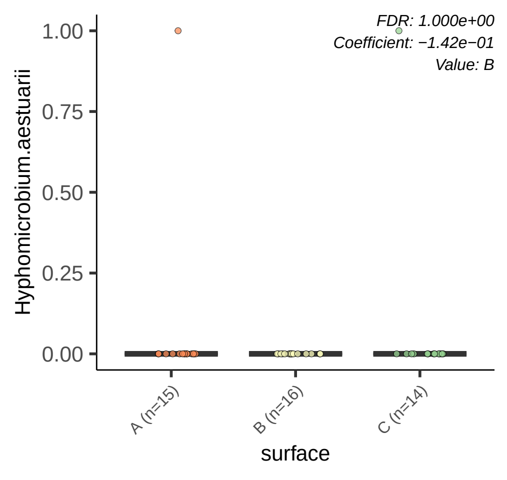
FDR: 1.000e+00
Coefficient: −1.42e−01
Value: B
Hyphomicrobium.aestuarii
1.00
0.75
0.50
0.25
0.00
A (n=15)
B (n=16)
C (n=14)
surface

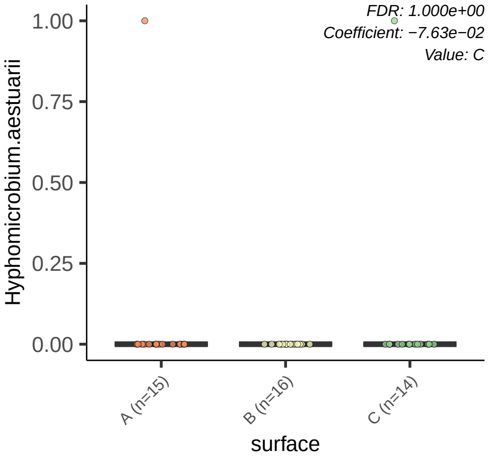

FDR: 1.000e+00
Coefficient: −7.63e−02
Value: C
Hyphomicrobium.aestuarii
1.00
0.75
0.50
0.25
0.00
A (n=15)
B (n=16)
C (n=14)
surface

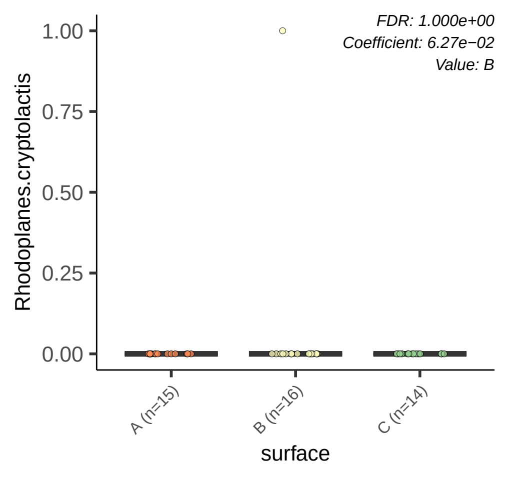
FDR: 1.000e+00
Coefficient: 6.27e−02
Value: B
Rhodoplanes.cryptolactis
1.00
0.75
0.50
0.25
0.00
A (n=15)
B (n=16)
C (n=14)
surface

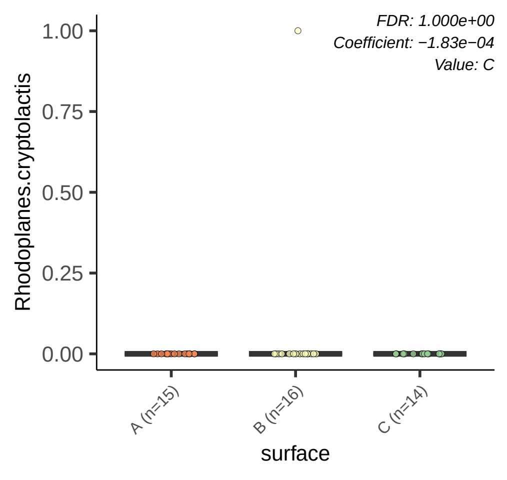
FDR: 1.000e+00
Coefficient: −1.83e−04
Value: C
Rhodoplanes.cryptolactis
1.00
0.75
0.50
0.25
0.00
A (n=15)
B (n=16)
C (n=14)
surface

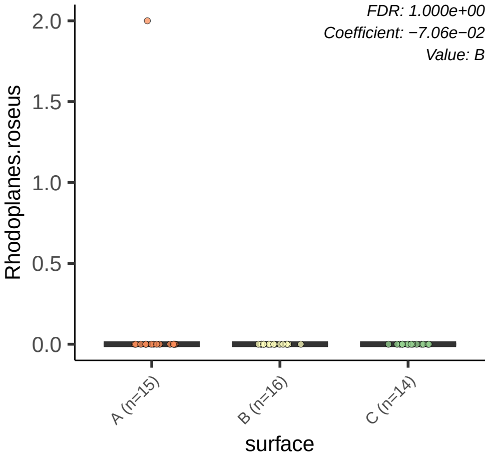

FDR: 1.000e+00
Coefficient: −7.06e−02
Value: B
Rhodoplanes.roseus
2.0
1.5
1.0
0.5
0.0
A (n=15)
B (n=16)
C (n=14)
surface

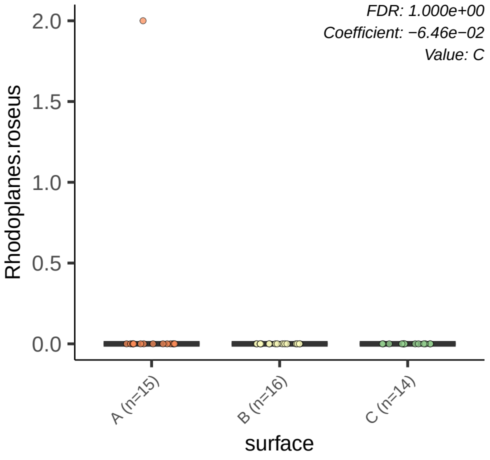
FDR: 1.000e+00
Coefficient: −6.46e−02
Value: C
Rhodoplanes.roseus
2.0
1.5
1.0
0.5
0.0
A (n=15)
B (n=16)
C (n=14)
surface

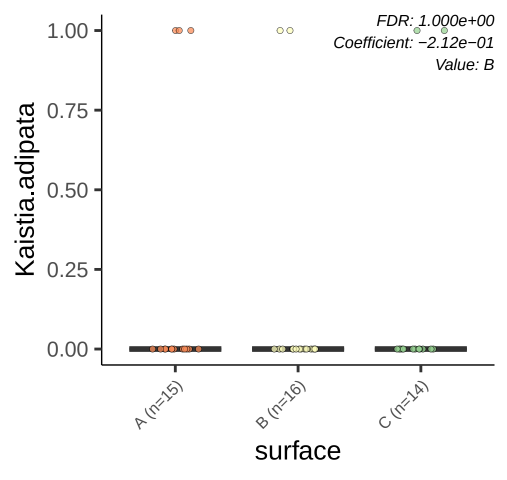

FDR: 1.000e+00
Coefficient: −2.12e−01
Value: B
Kaistia.adipata
1.00
0.75
0.50
0.25
0.00
A (n=15)
B (n=16)
C (n=14)
surface

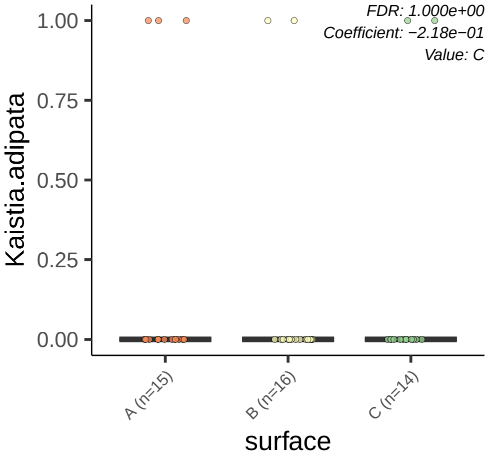
FDR: 1.000e+00
Coefficient: −2.18e−01
Value: C
Kaistia.adipata
1.00
0.75
0.50
0.25
0.00
A (n=15)
B (n=16)
C (n=14)
surface

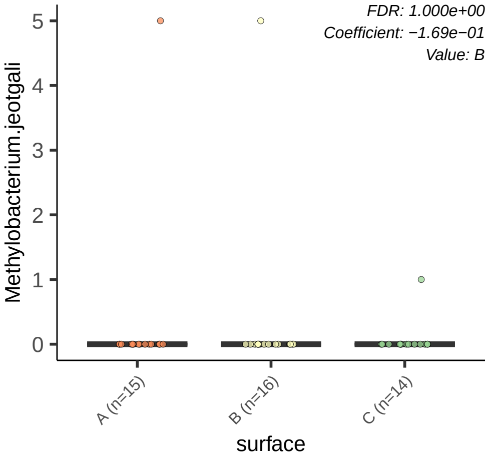

FDR: 1.000e+00
Coefficient: −1.69e−01
Value: B
Methylobacterium.jeotgali
5
4
3
2
1
0
A (n=15)
B (n=16)
C (n=14)
surface

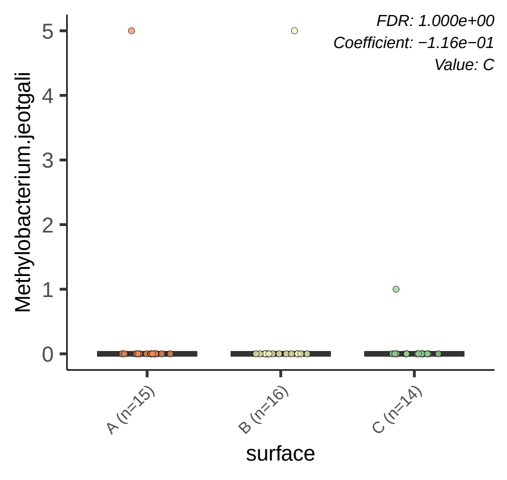
FDR: 1.000e+00
Coefficient: −1.16e−01
Value: C
Methylobacterium.jeotgali
0
1
2
3
4
5
A (n=15)
B (n=16)
C (n=14)
surface

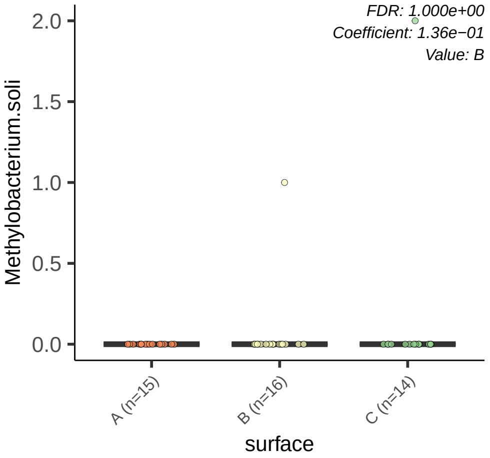
FDR: 1.000e+00
Coefficient: 1.36e−01
Value: B
Methylobacterium.soli
2.0
1.5
1.0
0.5
0.0
A (n=15)
B (n=16)
C (n=14)
surface

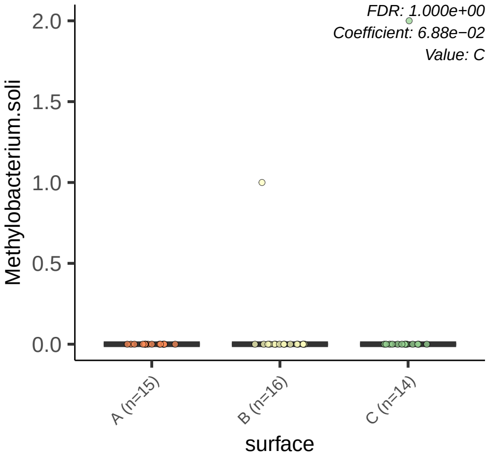

FDR: 1.000e+00
Coefficient: 6.88e−02
Value: C
Methylobacterium.soli
2.0
1.5
1.0
0.5
0.0
A (n=15)
B (n=16)
C (n=14)
surface

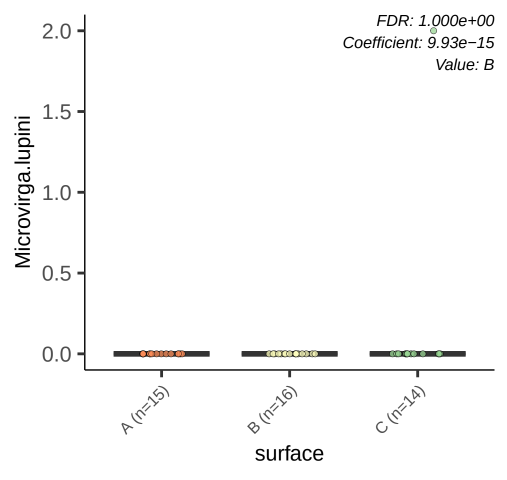
FDR: 1.000e+00
Coefficient: 9.93e−15
Value: B
Microvirga.lupini
2.0
1.5
1.0
0.5
0.0
A (n=15)
B (n=16)
C (n=14)
surface

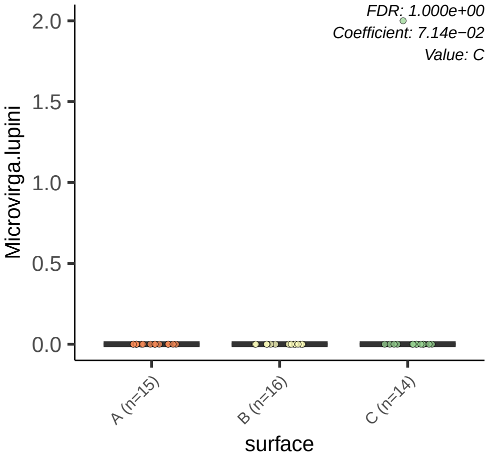
FDR: 1.000e+00
Coefficient: 7.14e−02
Value: C
Microvirga.lupini
2.0
1.5
1.0
0.5
0.0
A (n=15)
B (n=16)
C (n=14)
surface

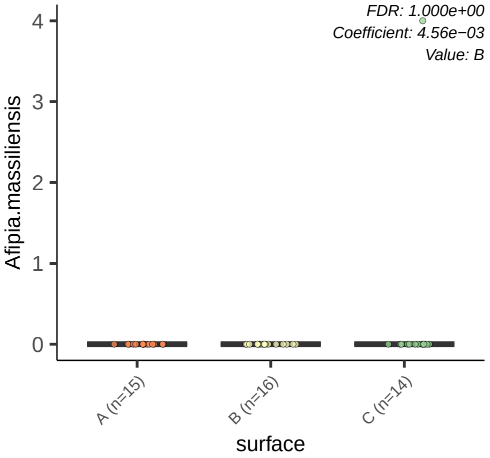
FDR: 1.000e+00
Coefficient: 4.56e−03
Value: B
Afipia.massiliensis
4
3
2
1
0
A (n=15)
B (n=16)
C (n=14)
surface

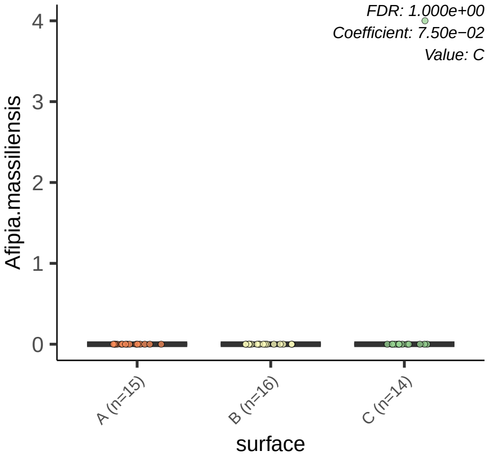
FDR: 1.000e+00
Coefficient: 7.50e−02
Value: C
Afipia.massiliensis
0
1
2
3
4
A (n=15)
B (n=16)
C (n=14)
surface

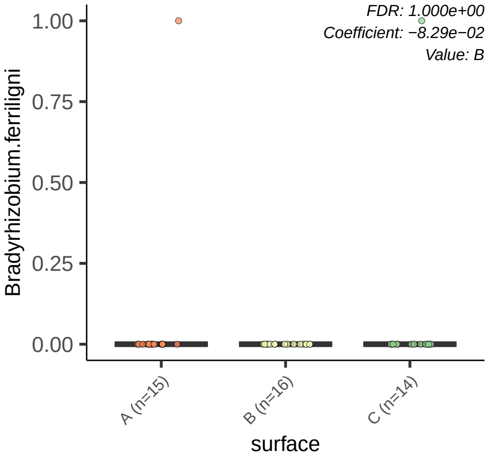

FDR: 1.000e+00
Coefficient: −8.29e−02
Value: B
Bradyrhizobium.ferriligni
1.00
0.75
0.50
0.25
0.00
A (n=15)
B (n=16)
C (n=14)
surface

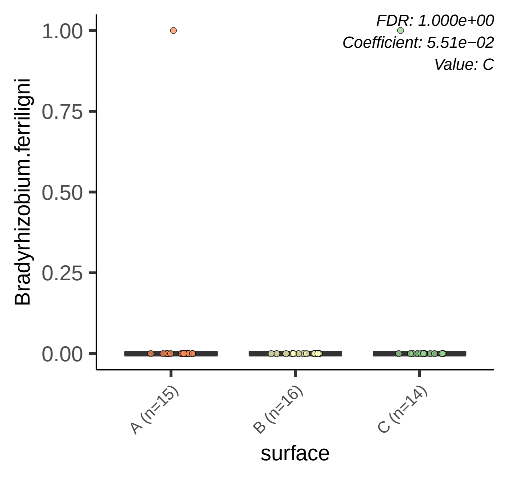
FDR: 1.000e+00
Coefficient: 5.51e−02
Value: C
Bradyrhizobium.ferriligni
1.00
0.75
0.50
0.25
0.00
A (n=15)
B (n=16)
C (n=14)
surface

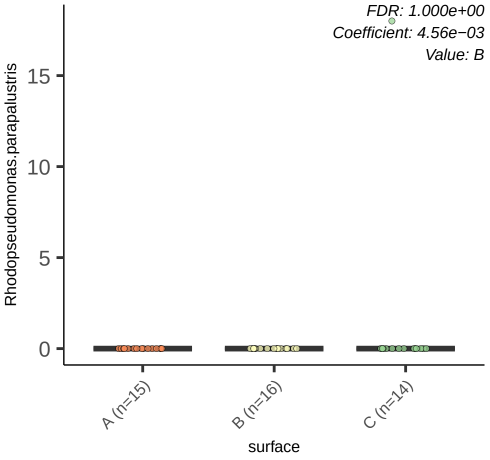

FDR: 1.000e+00
Coefficient: 4.56e−03
Value: B
Rhodopseudomonas.parapalustris
15
10
5
0
A (n=15)
B (n=16)
C (n=14)
surface

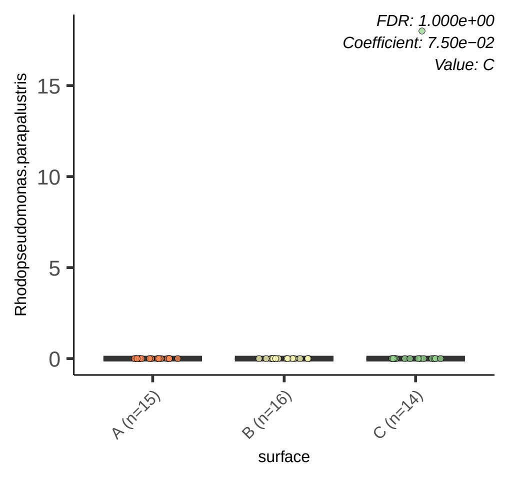
FDR: 1.000e+00
Coefficient: 7.50e−02
Value: C
Rhodopseudomonas.parapalustris
15
10
5
0
A (n=15)
B (n=16)
C (n=14)
surface

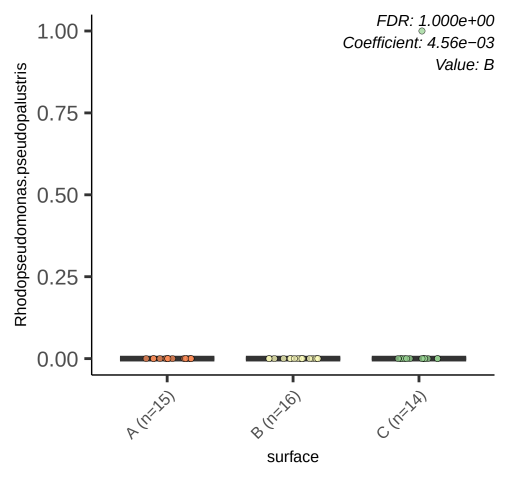
FDR: 1.000e+00
Coefficient: 4.56e−03
Value: B
Rhodopseudomonas.pseudopalustris
1.00
0.75
0.50
0.25
0.00
A (n=15)
B (n=16)
C (n=14)
surface

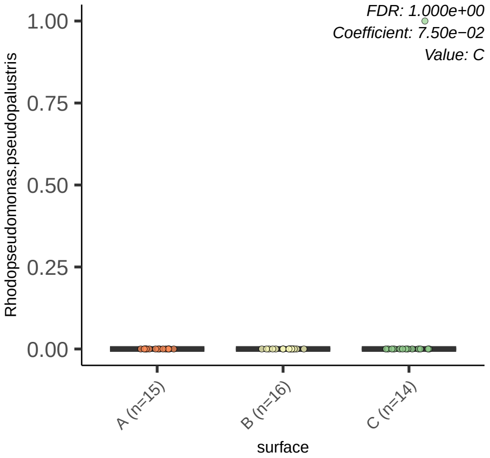

FDR: 1.000e+00
Coefficient: 7.50e−02
Value: C
1.00
0.75
0.50
0.25
0.00
Rhodopseudomonas.pseudopalustris
A (n=15)
B (n=16)
C (n=14)
surface

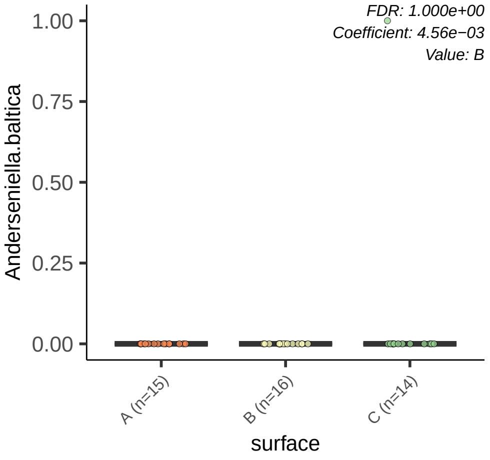
FDR: 1.000e+00
Coefficient: 4.56e−03
Value: B
Anderseniella.baltica
1.00
0.75
0.50
0.25
0.00
A (n=15)
B (n=16)
C (n=14)
surface

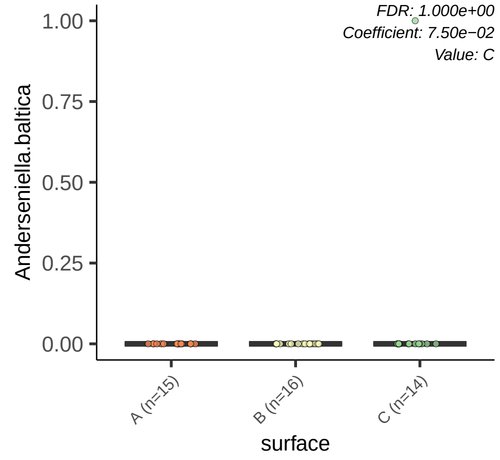

FDR: 1.000e+00
Coefficient: 7.50e−02
Value: C
Anderseniella.baltica
1.00
0.75
0.50
0.25
0.00
A (n=15)
B (n=16)
C (n=14)
surface

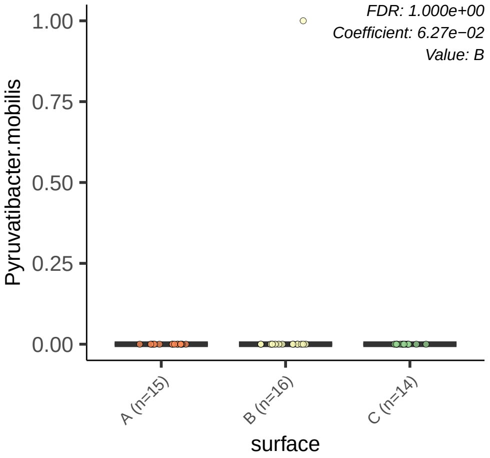
FDR: 1.000e+00
Coefficient: 6.27e−02
Value: B
1.00
0.75
0.50
0.25
0.00
Pyruvatibacter.mobilis
A (n=15)
B (n=16)
C (n=14)
surface

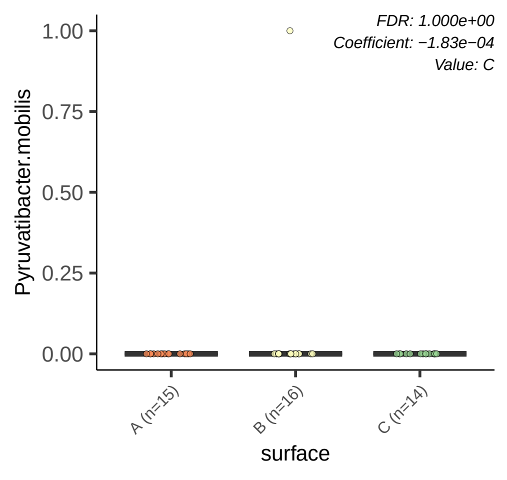
FDR: 1.000e+00
Coefficient: −1.83e−04
Value: C
Pyruvatibacter.mobilis
1.00
0.75
0.50
0.25
0.00
A (n=15)
B (n=16)
C (n=14)
surface

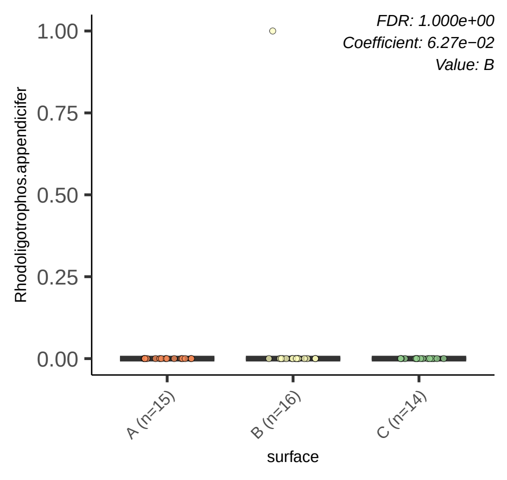
FDR: 1.000e+00
Coefficient: 6.27e−02
Value: B
Rhodoligotrophos.appendicifer
1.00
0.75
0.50
0.25
0.00
A (n=15)
B (n=16)
C (n=14)
surface

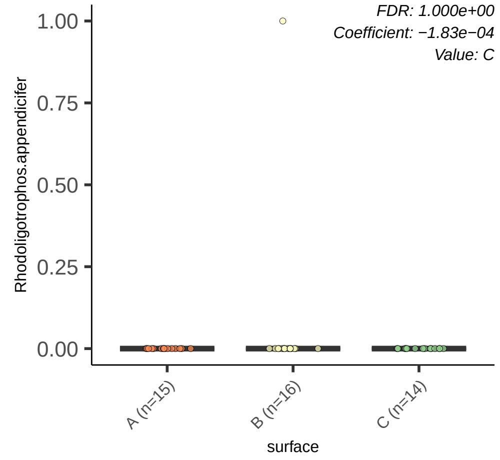
FDR: 1.000e+00
Coefficient: −1.83e−04
Value: C
1.00
0.75
0.50
0.25
0.00
Rhodoligotrophos.appendicifer
A (n=15)
B (n=16)
C (n=14)
surface

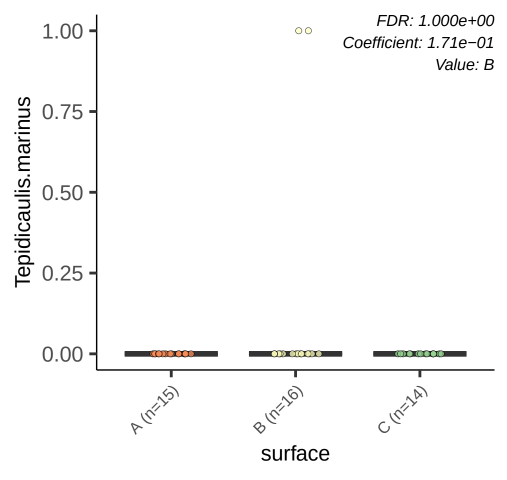
FDR: 1.000e+00
Coefficient: 1.71e−01
Value: B
Tepidicaulis.marinus
1.00
0.75
0.50
0.25
0.00
A (n=15)
B (n=16)
C (n=14)
surface

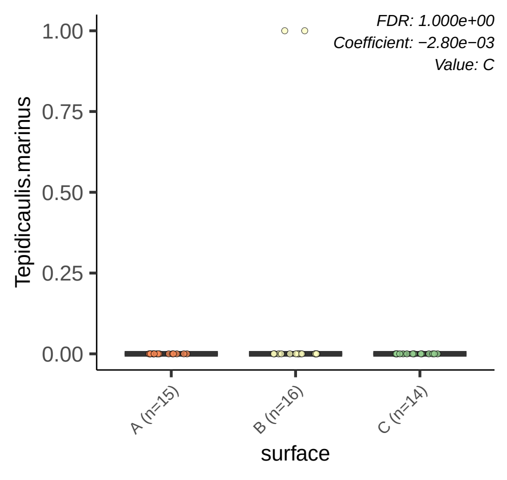
FDR: 1.000e+00
Coefficient: −2.80e−03
Value: C
Tepidicaulis.marinus
1.00
0.75
0.50
0.25
0.00
A (n=15)
B (n=16)
C (n=14)
surface

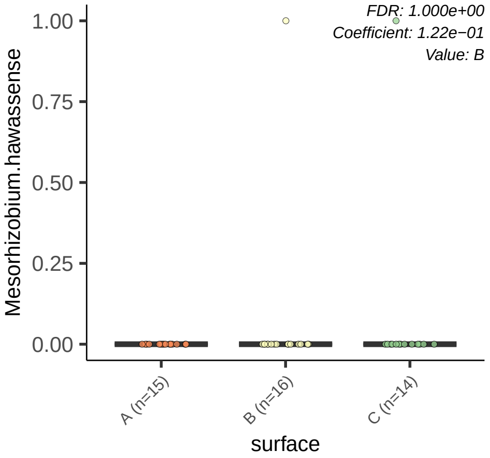
FDR: 1.000e+00
Coefficient: 1.22e−01
Value: B
Mesorhizobium.hawassense
1.00
0.75
0.50
0.25
0.00
A (n=15)
B (n=16)
C (n=14)
surface

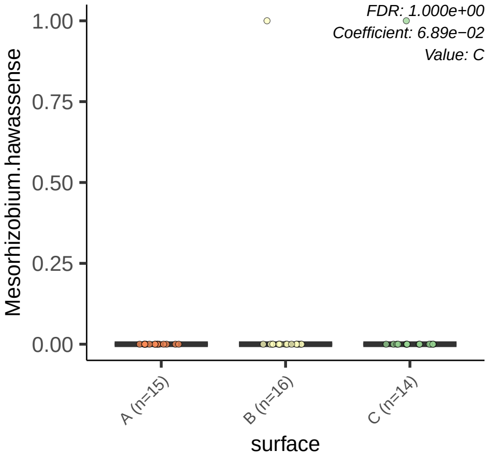
FDR: 1.000e+00
Coefficient: 6.89e−02
Value: C
Mesorhizobium.hawassense
1.00
0.75
0.50
0.25
0.00
A (n=15)
B (n=16)
C (n=14)
surface

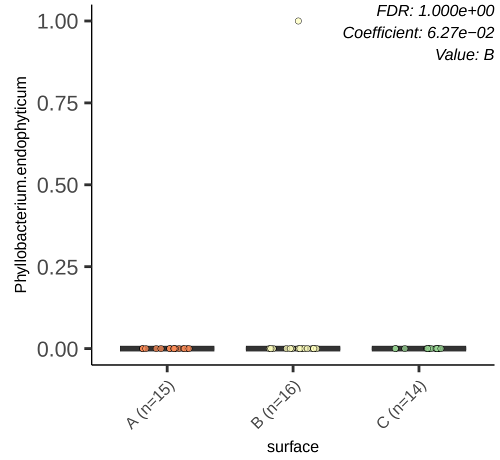
FDR: 1.000e+00
Coefficient: 6.27e−02
Value: B
Phyllobacterium.endophyticum
1.00
0.75
0.50
0.25
0.00
A (n=15)
B (n=16)
C (n=14)
surface

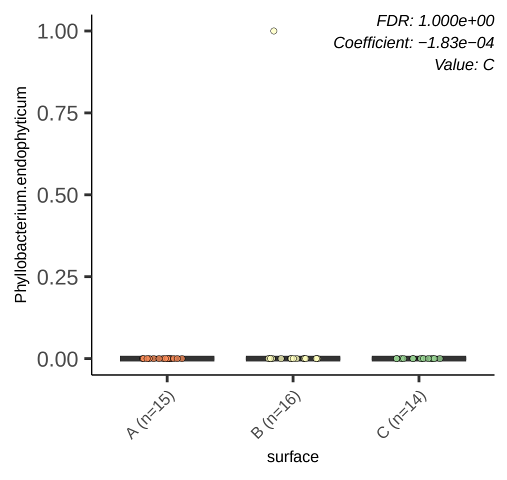
FDR: 1.000e+00
Coefficient: −1.83e−04
Value: C
Phyllobacterium.endophyticum
1.00
0.75
0.50
0.25
0.00
A (n=15)
B (n=16)
C (n=14)
surface

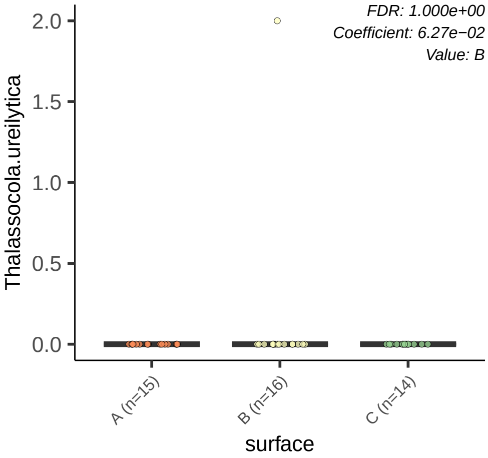

FDR: 1.000e+00
Coefficient: 6.27e−02
Value: B
Thalassocola.ureilytica
2.0
1.5
1.0
0.5
0.0
A (n=15)
B (n=16)
C (n=14)
surface

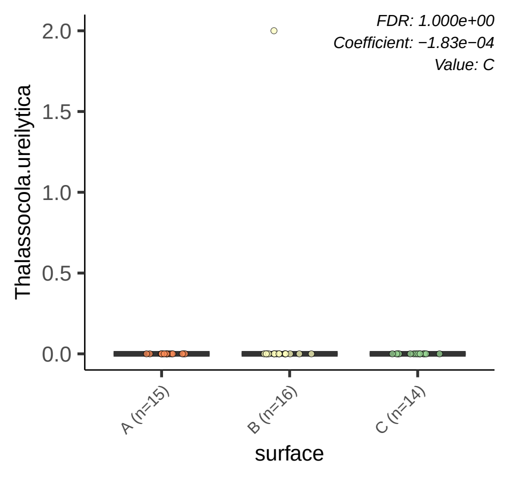
FDR: 1.000e+00
Coefficient: −1.83e−04
Value: C
Thalassocola.ureilytica
2.0
1.5
1.0
0.5
0.0
A (n=15)
B (n=16)
C (n=14)
surface

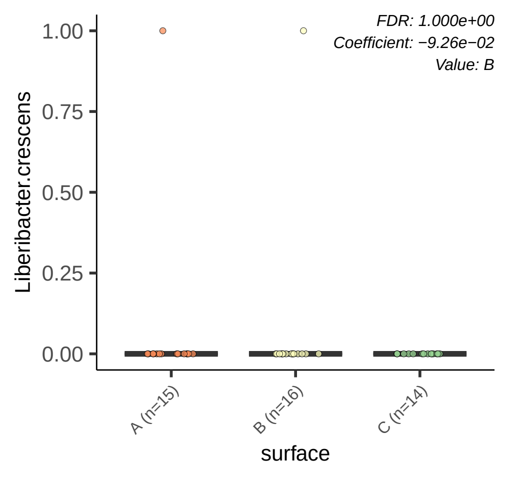

FDR: 1.000e+00
Coefficient: −9.26e−02
Value: B
Liberibacter.crescens
1.00
0.75
0.50
0.25
0.00
A (n=15)
B (n=16)
C (n=14)
surface

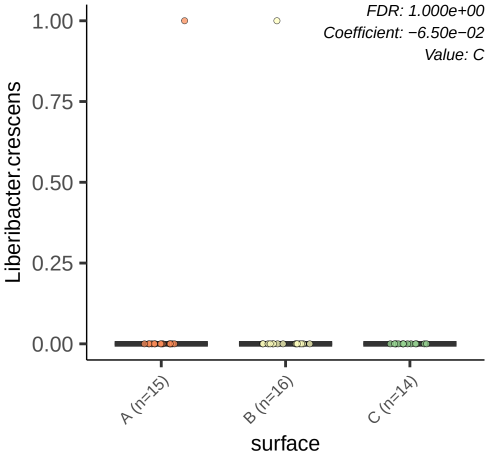
FDR: 1.000e+00
Coefficient: −6.50e−02
Value: C
Liberibacter.crescens
1.00
0.75
0.50
0.25
0.00
A (n=15)
B (n=16)
C (n=14)
surface

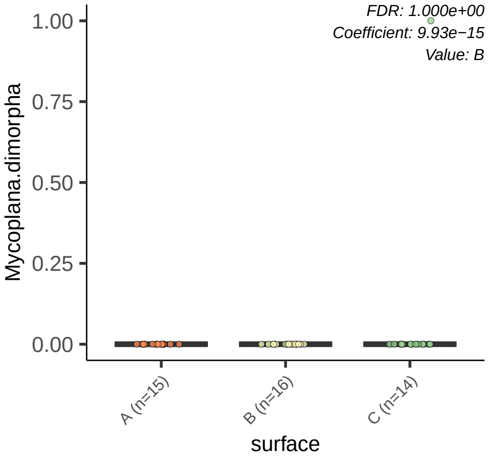

FDR: 1.000e+00
Coefficient: 9.93e−15
Value: B
Mycoplana.dimorpha
1.00
0.75
0.50
0.25
0.00
A (n=15)
B (n=16)
C (n=14)
surface

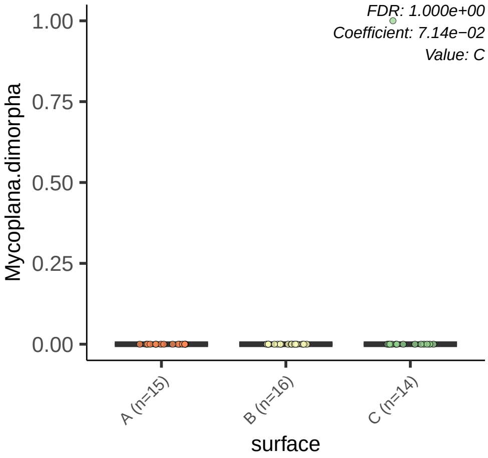
FDR: 1.000e+00
Coefficient: 7.14e−02
Value: C
Mycoplana.dimorpha
1.00
0.75
0.50
0.25
0.00
A (n=15)
B (n=16)
C (n=14)
surface

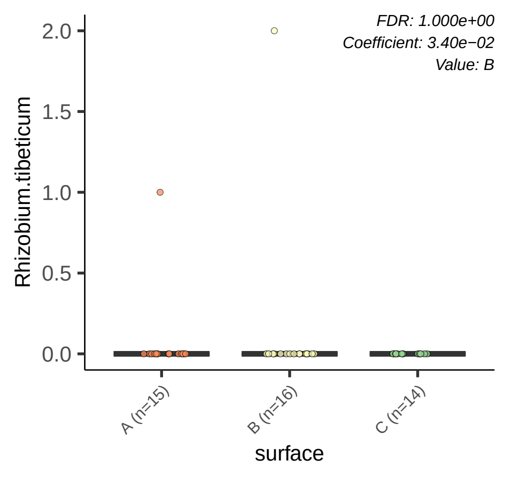
FDR: 1.000e+00
Coefficient: 3.40e−02
Value: B
Rhizobium.tibeticum
2.0
1.5
1.0
0.5
0.0
A (n=15)
B (n=16)
C (n=14)
surface

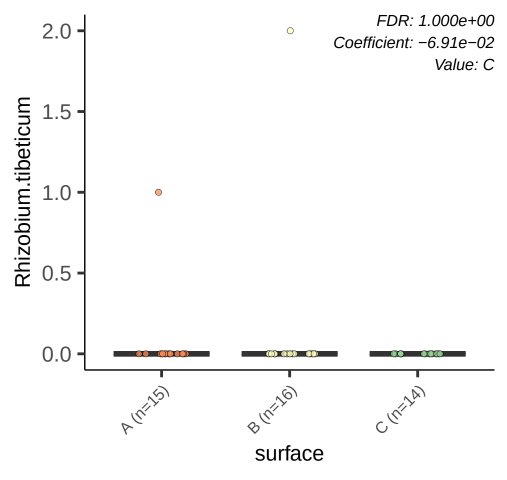
FDR: 1.000e+00
Coefficient: −6.91e−02
Value: C
Rhizobium.tibeticum
2.0
1.5
1.0
0.5
0.0
A (n=15)
B (n=16)
C (n=14)
surface

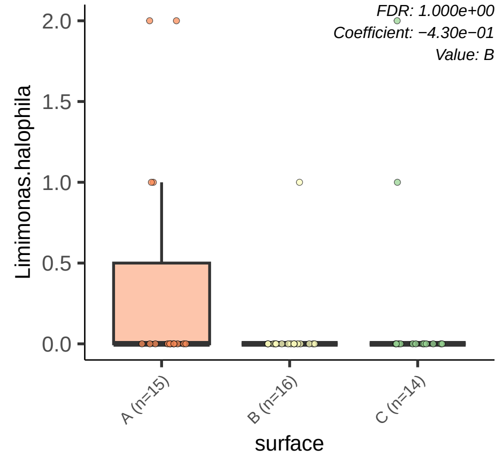

FDR: 1.000e+00
Coefficient: −4.30e−01
Value: B
Limimonas.halophila
2.0
1.5
1.0
0.5
0.0
A (n=15)
B (n=16)
C (n=14)
surface

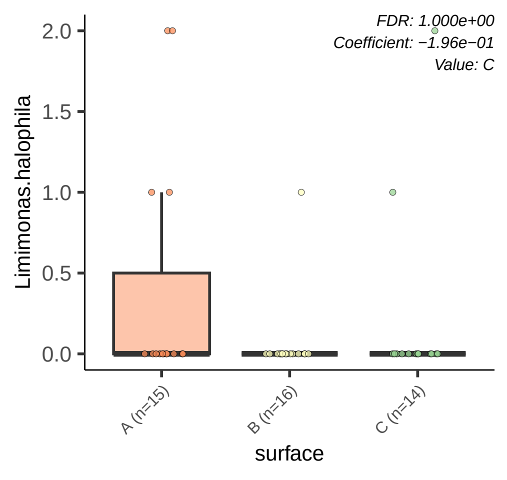

FDR: 1.000e+00
Coefficient: −1.96e−01
Value: C
Limimonas.halophila
2.0
1.5
1.0
0.5
0.0
A (n=15)
B (n=16)
C (n=14)
surface

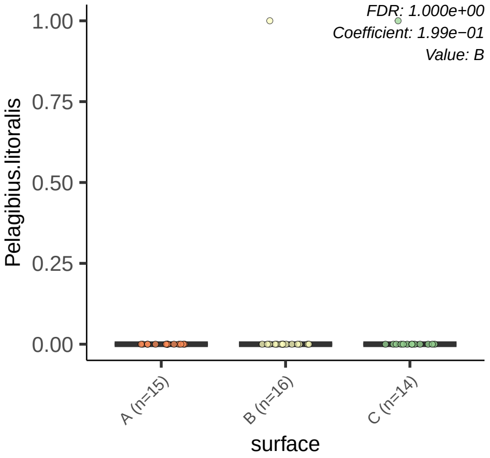

FDR: 1.000e+00
Coefficient: 1.99e−01
Value: B
Pelagibius.litoralis
1.00
0.75
0.50
0.25
0.00
A (n=15)
B (n=16)
C (n=14)
surface

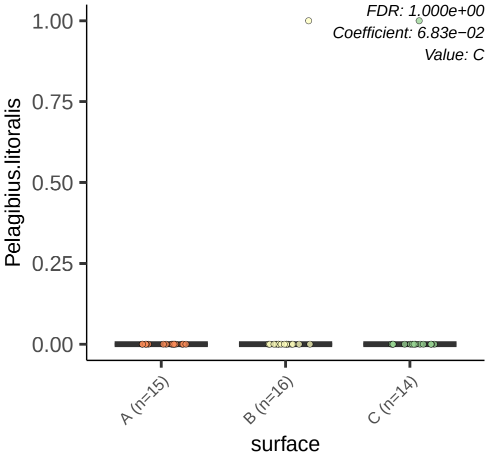
FDR: 1.000e+00
Coefficient: 6.83e−02
Value: C
Pelagibius.litoralis
1.00
0.75
0.50
0.25
0.00
A (n=15)
B (n=16)
C (n=14)
surface

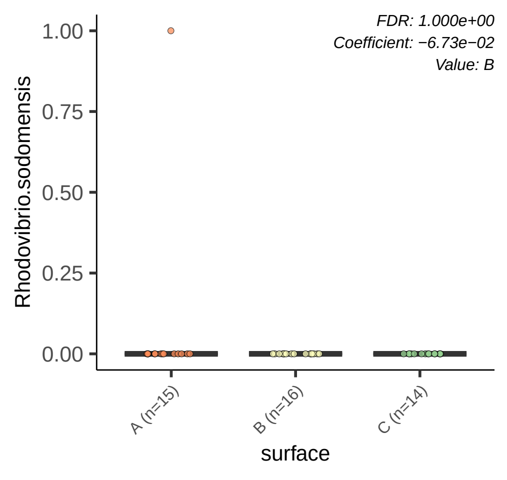

FDR: 1.000e+00
Coefficient: −6.73e−02
Value: B
Rhodovibrio.sodomensis
1.00
0.75
0.50
0.25
0.00
A (n=15)
B (n=16)
C (n=14)
surface

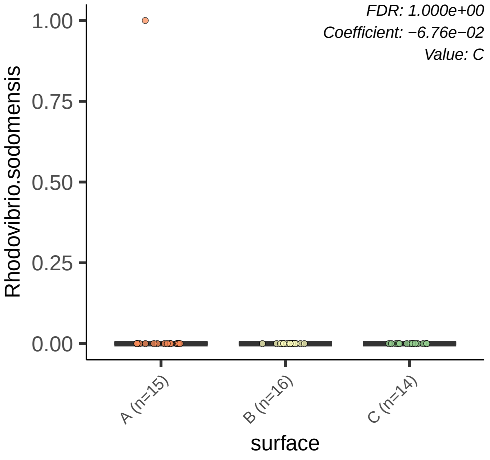

FDR: 1.000e+00
Coefficient: −6.76e−02
Value: C
Rhodovibrio.sodomensis
1.00
0.75
0.50
0.25
0.00
A (n=15)
B (n=16)
C (n=14)
surface

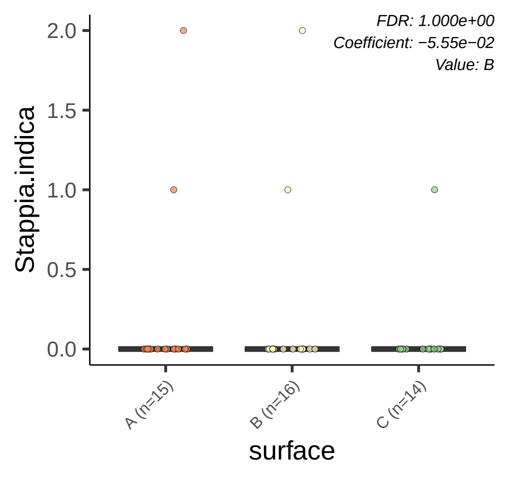
FDR: 1.000e+00
Coefficient: −5.55e−02
Value: B
Stappia.indica
2.0
1.5
1.0
0.5
0.0
A (n=15)
B (n=16)
C (n=14)
surface

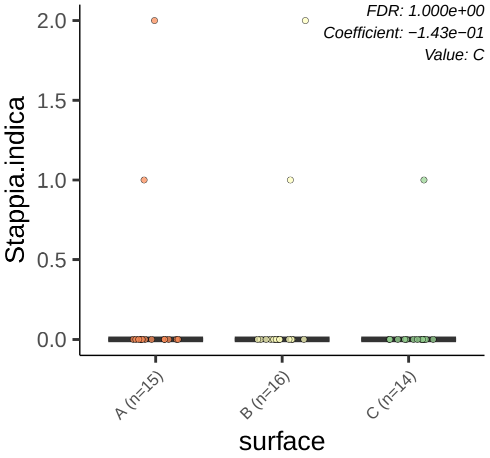
FDR: 1.000e+00
Coefficient: −1.43e−01
Value: C
Stappia.indica
2.0
1.5
1.0
0.5
0.0
A (n=15)
B (n=16)
C (n=14)
surface

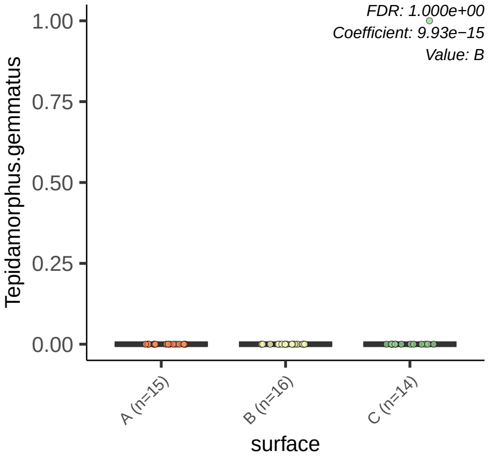

FDR: 1.000e+00
Coefficient: 9.93e−15
Value: B
Tepidamorphus.gemmatus
1.00
0.75
0.50
0.25
0.00
A (n=15)
B (n=16)
C (n=14)
surface

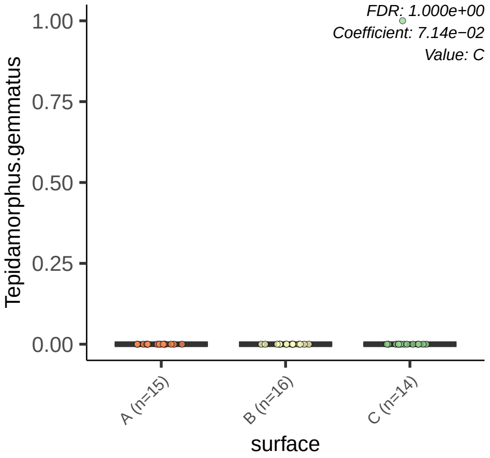
FDR: 1.000e+00
Coefficient: 7.14e−02
Value: C
1.00
0.75
0.50
0.25
0.00
Tepidamorphus.gemmatus
A (n=15)
B (n=16)
C (n=14)
surface

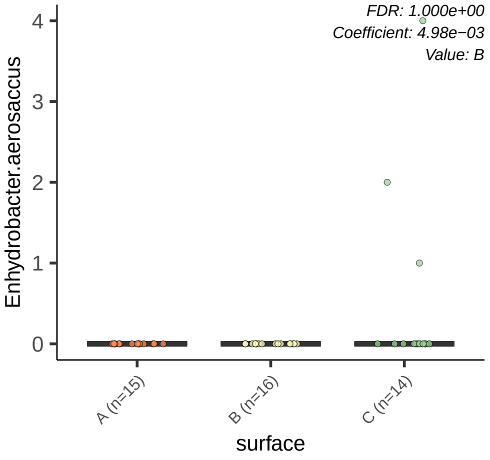

FDR: 1.000e+00
Coefficient: 4.98e−03
Value: B
Enhydrobacter.aerosaccus
4
3
2
1
0
A (n=15)
B (n=16)
C (n=14)
surface

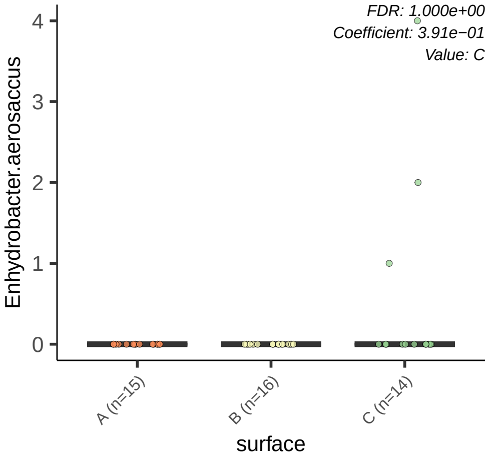

FDR: 1.000e+00
Coefficient: 3.91e−01
Value: C
Enhydrobacter.aerosaccus
0
1
2
3
4
A (n=15)
B (n=16)
C (n=14)
surface

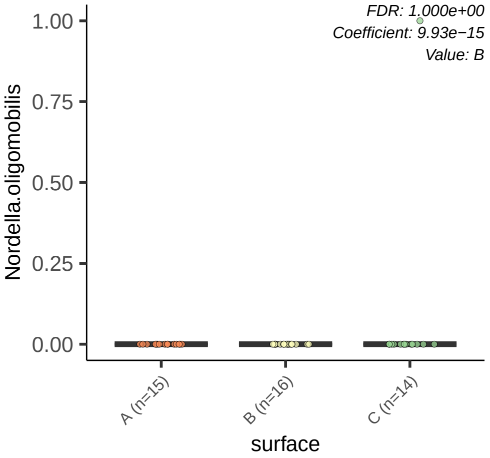

FDR: 1.000e+00
Coefficient: 9.93e−15
Value: B
Nordella.oligomobilis
1.00
0.75
0.50
0.25
0.00
A (n=15)
B (n=16)
C (n=14)
surface

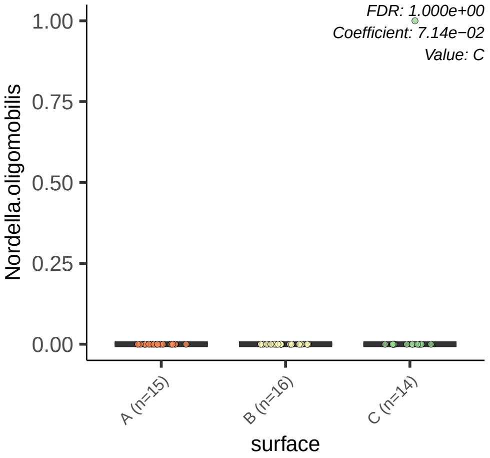
FDR: 1.000e+00
Coefficient: 7.14e−02
Value: C
Nordella.oligomobilis
1.00
0.75
0.50
0.25
0.00
A (n=15)
B (n=16)
C (n=14)
surface

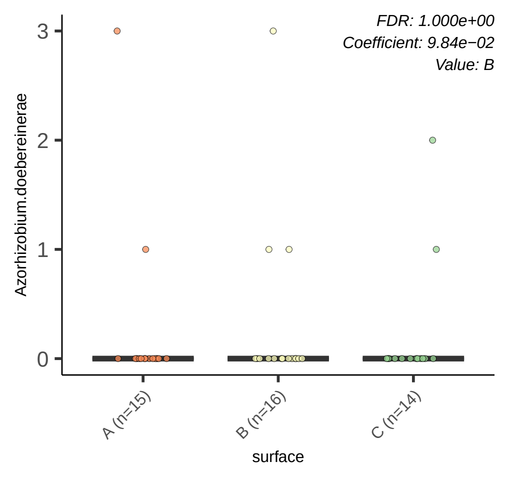
FDR: 1.000e+00
Coefficient: 9.84e−02
Value: B
Azorhizobium.doebereinerae
3
2
1
0
A (n=15)
B (n=16)
C (n=14)
surface

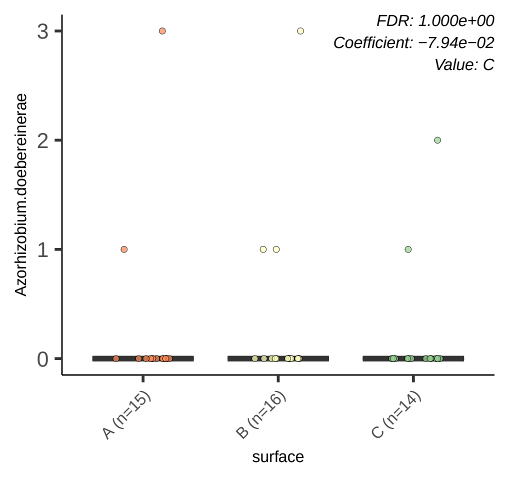

FDR: 1.000e+00
Coefficient: −7.94e−02
Value: C
Azorhizobium.doebereinerae
3
2
1
0
A (n=15)
B (n=16)
C (n=14)
surface

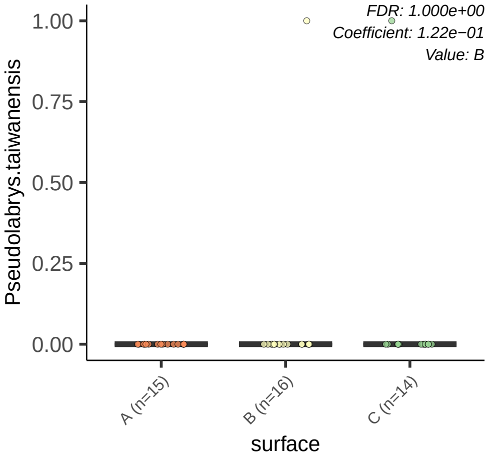
FDR: 1.000e+00
Coefficient: 1.22e−01
Value: B
Pseudolabrys.taiwanensis
1.00
0.75
0.50
0.25
0.00
A (n=15)
B (n=16)
C (n=14)
surface

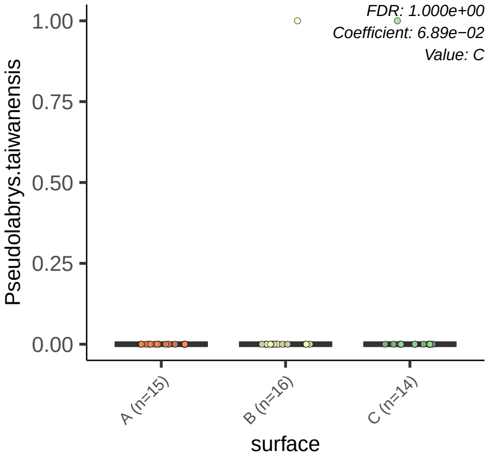
FDR: 1.000e+00
Coefficient: 6.89e−02
Value: C
Pseudolabrys.taiwanensis
1.00
0.75
0.50
0.25
0.00
A (n=15)
B (n=16)
C (n=14)
surface

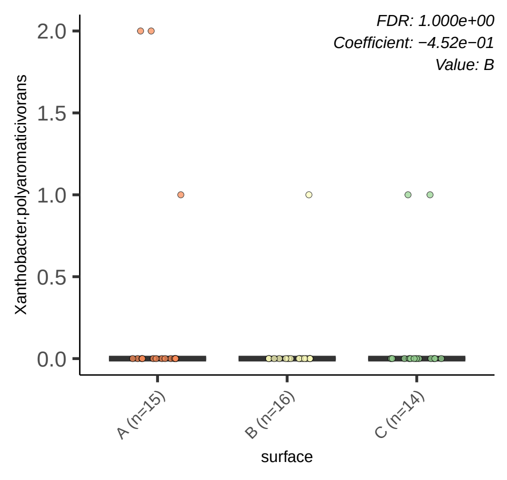
FDR: 1.000e+00
Coefficient: −4.52e−01
Value: B
Xanthobacter.polyaromaticivorans
2.0
1.5
1.0
0.5
0.0
A (n=15)
B (n=16)
C (n=14)
surface

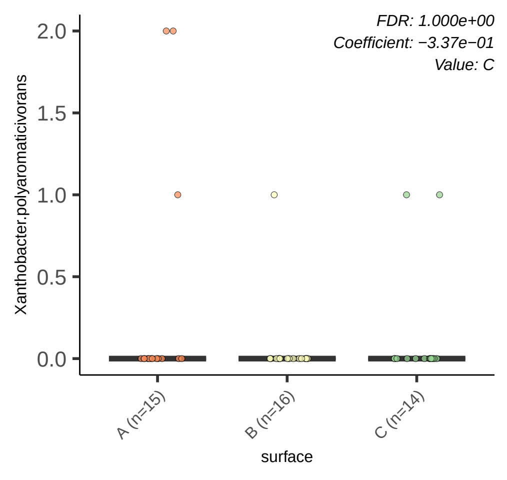

FDR: 1.000e+00
Coefficient: −3.37e−01
Value: C
Xanthobacter.polyaromaticivorans
2.0
1.5
1.0
0.5
0.0
A (n=15)
B (n=16)
C (n=14)
surface

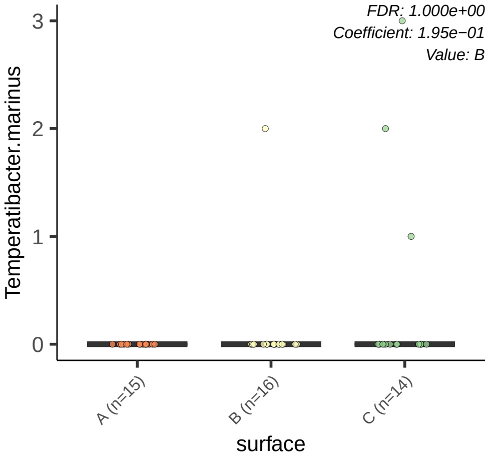
FDR: 1.000e+00
Coefficient: 1.95e−01
Value: B
Temperatibacter.marinus
3
2
1
0
A (n=15)
B (n=16)
C (n=14)
surface

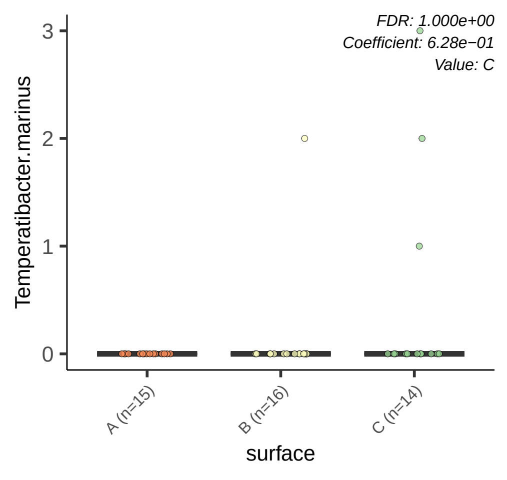
FDR: 1.000e+00
Coefficient: 6.28e−01
Value: C
Temperatibacter.marinus
0
1
2
3
A (n=15)
B (n=16)
C (n=14)
surface

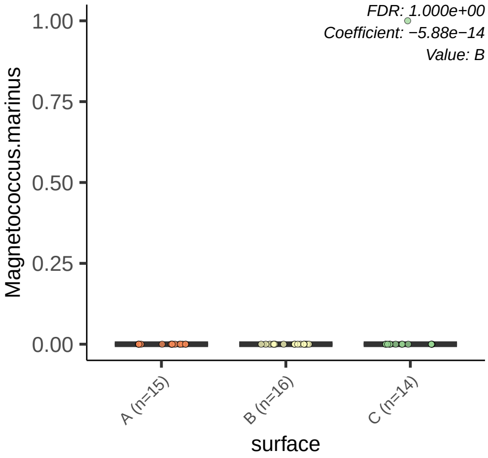
FDR: 1.000e+00
Coefficient: −5.88e−14
Value: B
Magnetococcus.marinus
1.00
0.75
0.50
0.25
0.00
A (n=15)
B (n=16)
C (n=14)
surface

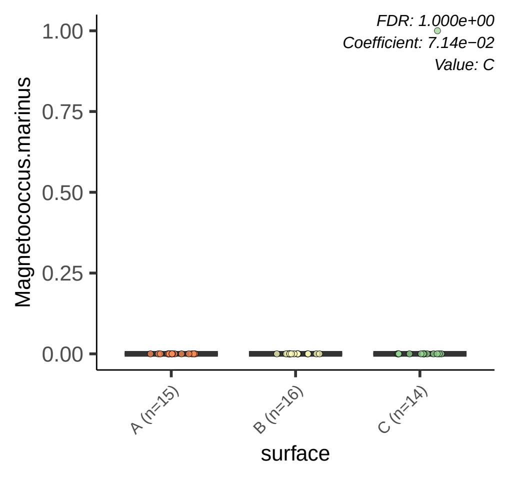
FDR: 1.000e+00
Coefficient: 7.14e−02
Value: C
Magnetococcus.marinus
1.00
0.75
0.50
0.25
0.00
A (n=15)
B (n=16)
C (n=14)
surface

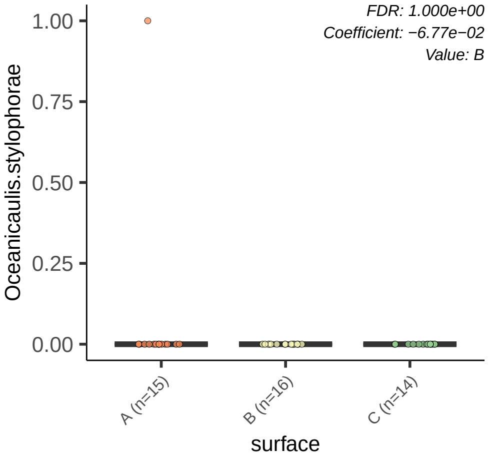

FDR: 1.000e+00
Coefficient: −6.77e−02
Value: B
Oceanicaulis.stylophorae
1.00
0.75
0.50
0.25
0.00
A (n=15)
B (n=16)
C (n=14)
surface

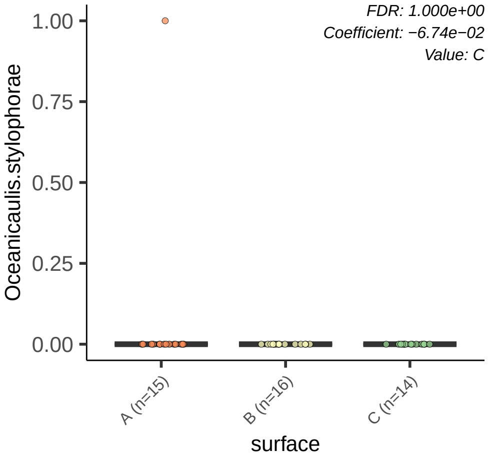
FDR: 1.000e+00
Coefficient: −6.74e−02
Value: C
Oceanicaulis.stylophorae
1.00
0.75
0.50
0.25
0.00
A (n=15)
B (n=16)
C (n=14)
surface

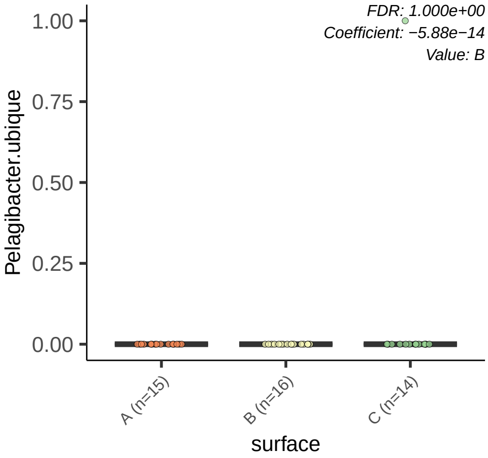

FDR: 1.000e+00
Coefficient: −5.88e−14
Value: B
Pelagibacter.ubique
1.00
0.75
0.50
0.25
0.00
A (n=15)
B (n=16)
C (n=14)
surface

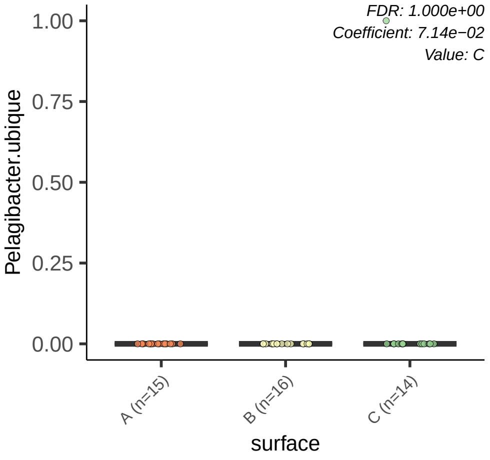

FDR: 1.000e+00
Coefficient: 7.14e−02
Value: C
1.00
0.75
0.50
0.25
0.00
Pelagibacter.ubique
A (n=15)
B (n=16)
C (n=14)
surface

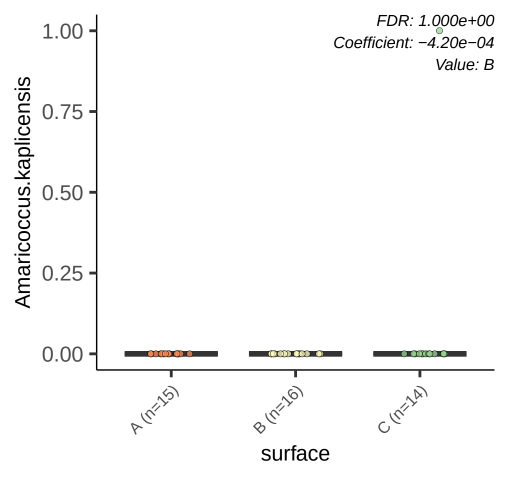
FDR: 1.000e+00
Coefficient: −4.20e−04
Value: B
Amaricoccus.kaplicensis
1.00
0.75
0.50
0.25
0.00
A (n=15)
B (n=16)
C (n=14)
surface

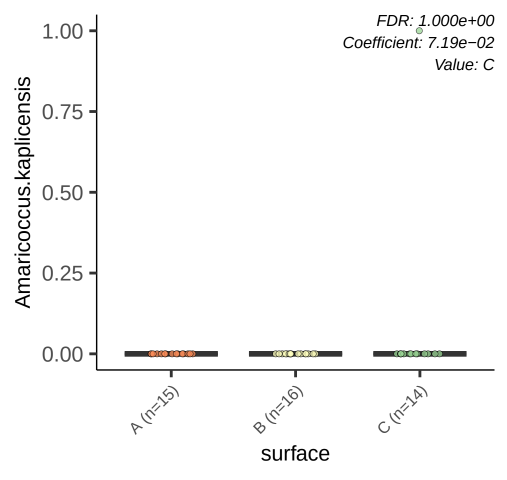

FDR: 1.000e+00
Coefficient: 7.19e−02
Value: C
Amaricoccus.kaplicensis
1.00
0.75
0.50
0.25
0.00
A (n=15)
B (n=16)
C (n=14)
surface

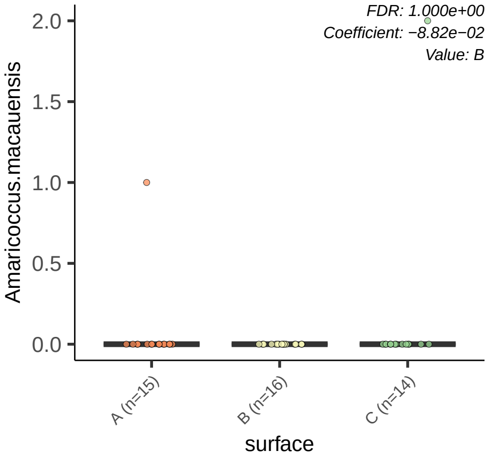
FDR: 1.000e+00
Coefficient: −8.82e−02
Value: B
Amaricoccus.macauensis
2.0
1.5
1.0
0.5
0.0
A (n=15)
B (n=16)
C (n=14)
surface

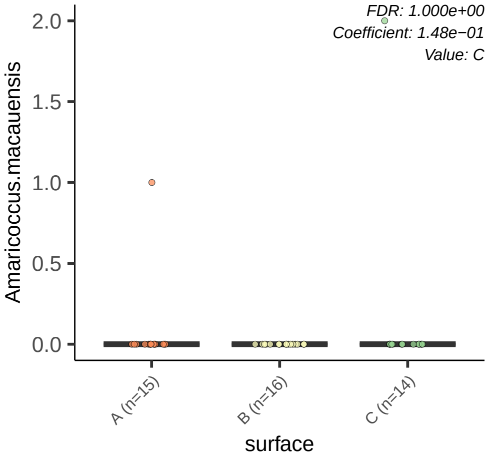
FDR: 1.000e+00
Coefficient: 1.48e−01
Value: C
Amaricoccus.macauensis
2.0
1.5
1.0
0.5
0.0
A (n=15)
B (n=16)
C (n=14)
surface

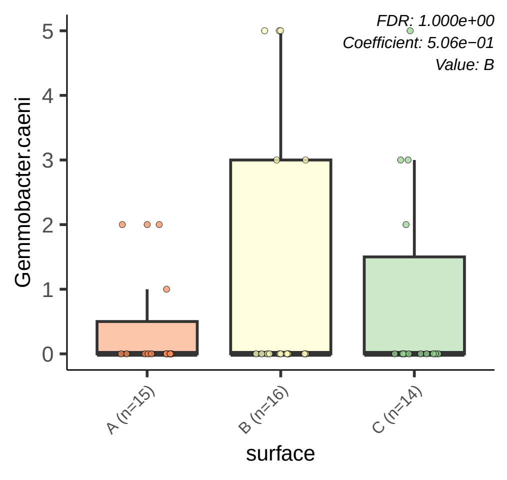

FDR: 1.000e+00
Coefficient: 5.06e−01
Value: B
Gemmobacter.caeni
0
1
2
3
4
5
A (n=15)
B (n=16)
C (n=14)
surface

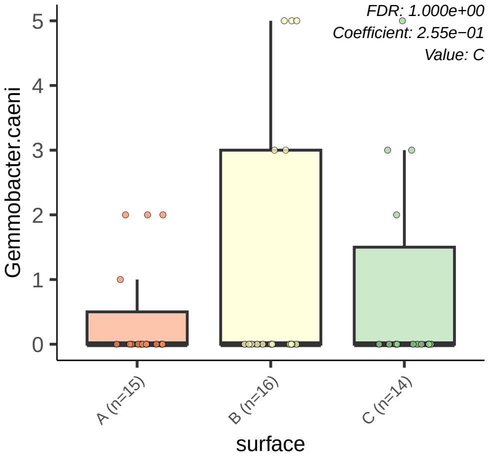
FDR: 1.000e+00
Coefficient: 2.55e−01
Value: C
Gemmobacter.caeni
0
1
2
3
4
5
A (n=15)
B (n=16)
C (n=14)
surface

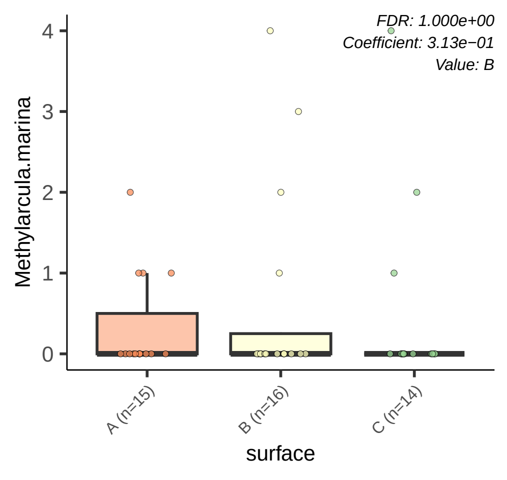
FDR: 1.000e+00
Coefficient: 3.13e−01
Value: B
Methylarcula.marina
0
1
2
3
4
A (n=15)
B (n=16)
C (n=14)
surface

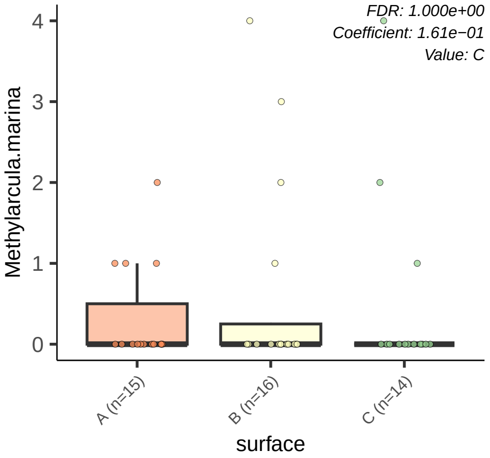

FDR: 1.000e+00
Coefficient: 1.61e−01
Value: C
Methylarcula.marina
0
1
2
3
4
A (n=15)
B (n=16)
C (n=14)
surface

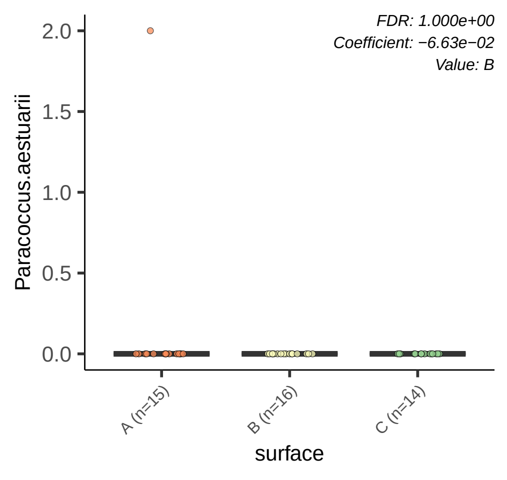
FDR: 1.000e+00
Coefficient: −6.63e−02
Value: B
Paracoccus.aestuarii
2.0
1.5
1.0
0.5
0.0
A (n=15)
B (n=16)
C (n=14)
surface

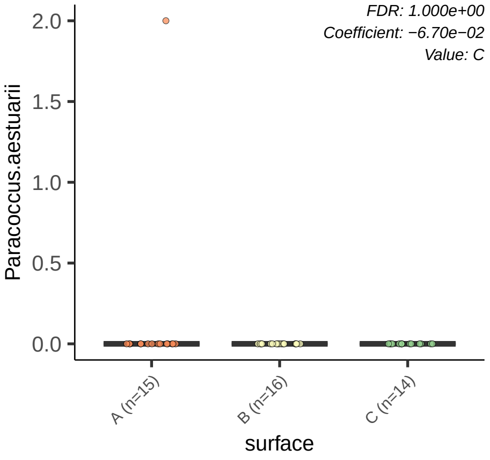

FDR: 1.000e+00
Coefficient: −6.70e−02
Value: C
Paracoccus.aestuarii
2.0
1.5
1.0
0.5
0.0
A (n=15)
B (n=16)
C (n=14)
surface

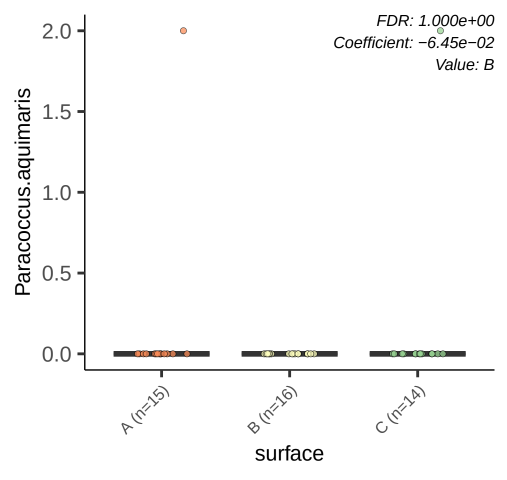

FDR: 1.000e+00
Coefficient: −6.45e−02
Value: B
Paracoccus.aquimaris
2.0
1.5
1.0
0.5
0.0
A (n=15)
B (n=16)
C (n=14)
surface

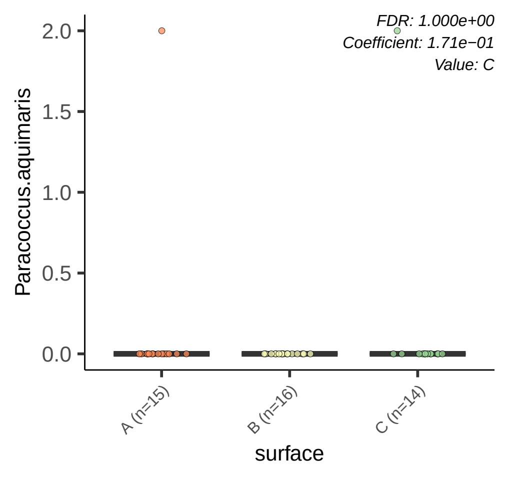
FDR: 1.000e+00
Coefficient: 1.71e−01
Value: C
Paracoccus.aquimaris
2.0
1.5
1.0
0.5
0.0
A (n=15)
B (n=16)
C (n=14)
surface

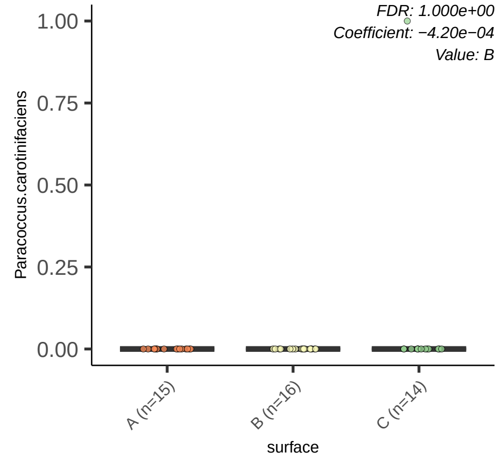

FDR: 1.000e+00
Coefficient: −4.20e−04
Value: B
1.00
0.75
0.50
0.25
0.00
Paracoccus.carotinifaciens
A (n=15)
B (n=16)
C (n=14)
surface

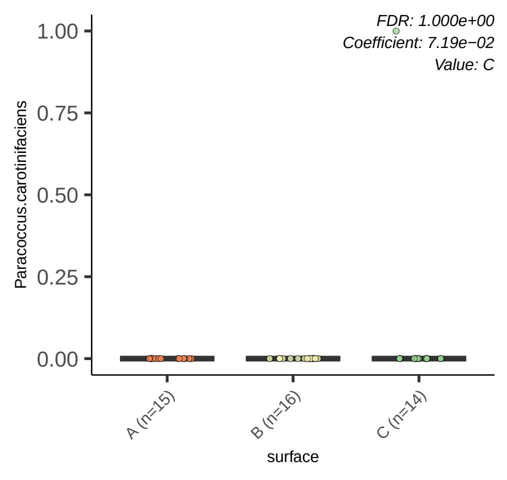
FDR: 1.000e+00
Coefficient: 7.19e−02
Value: C
1.00
0.75
0.50
0.25
0.00
Paracoccus.carotinifaciens
A (n=15)
B (n=16)
C (n=14)
surface

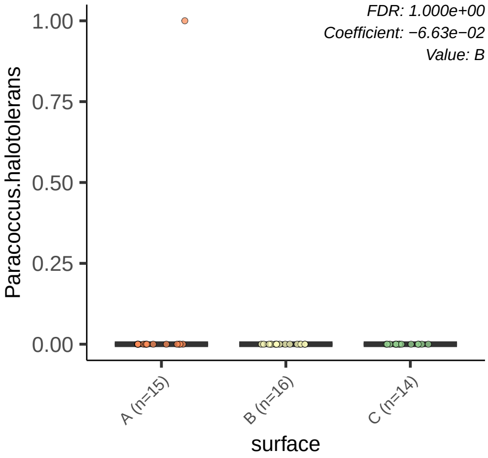

FDR: 1.000e+00
Coefficient: −6.63e−02
Value: B
Paracoccus.halotolerans
1.00
0.75
0.50
0.25
0.00
A (n=15)
B (n=16)
C (n=14)
surface

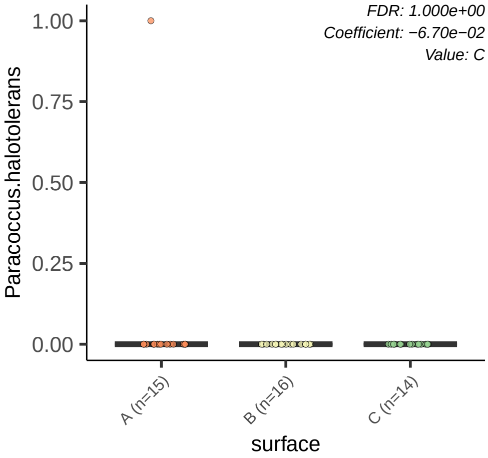

FDR: 1.000e+00
Coefficient: −6.70e−02
Value: C
Paracoccus.halotolerans
1.00
0.75
0.50
0.25
0.00
A (n=15)
B (n=16)
C (n=14)
surface

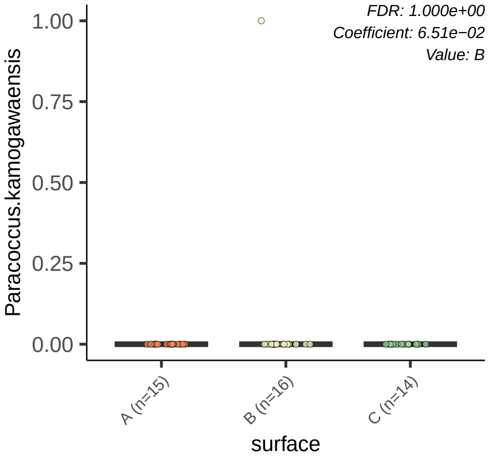
FDR: 1.000e+00
Coefficient: 6.51e−02
Value: B
Paracoccus.kamogawaensis
1.00
0.75
0.50
0.25
0.00
A (n=15)
B (n=16)
C (n=14)
surface

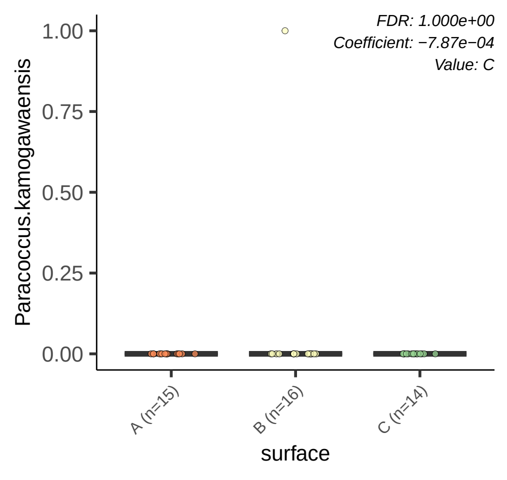

FDR: 1.000e+00
Coefficient: −7.87e−04
Value: C
Paracoccus.kamogawaensis
1.00
0.75
0.50
0.25
0.00
A (n=15)
B (n=16)
C (n=14)
surface

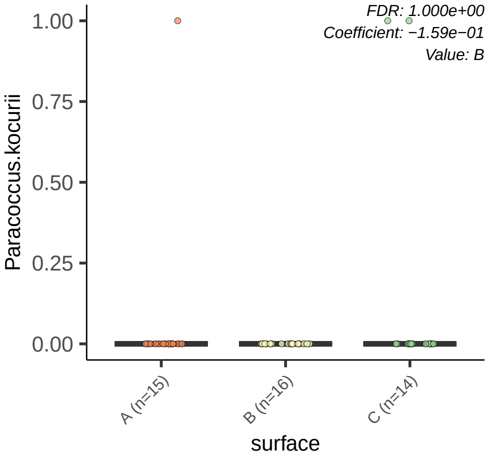
FDR: 1.000e+00
Coefficient: −1.59e−01
Value: B
Paracoccus.kocurii
1.00
0.75
0.50
0.25
0.00
A (n=15)
B (n=16)
C (n=14)
surface

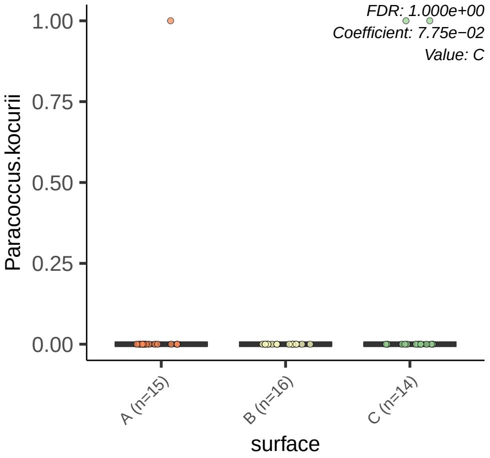

FDR: 1.000e+00
Coefficient: 7.75e−02
Value: C
Paracoccus.kocurii
1.00
0.75
0.50
0.25
0.00
A (n=15)
B (n=16)
C (n=14)
surface

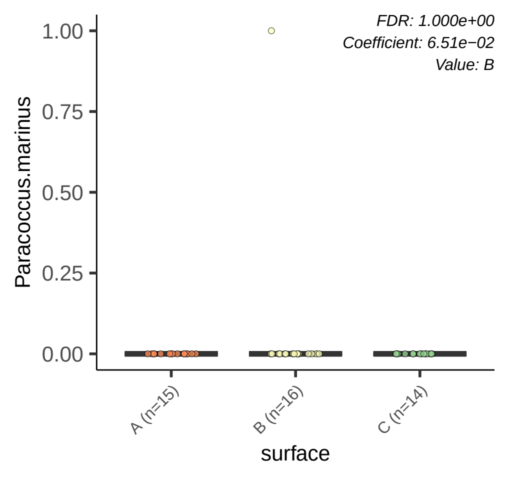

FDR: 1.000e+00
Coefficient: 6.51e−02
Value: B
Paracoccus.marinus
1.00
0.75
0.50
0.25
0.00
A (n=15)
B (n=16)
C (n=14)
surface

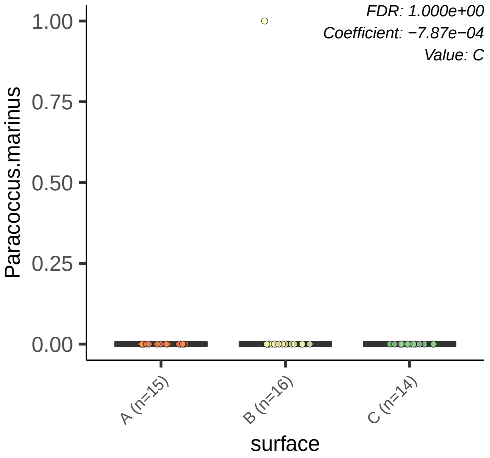

FDR: 1.000e+00
Coefficient: −7.87e−04
Value: C
Paracoccus.marinus
1.00
0.75
0.50
0.25
0.00
A (n=15)
B (n=16)
C (n=14)
surface

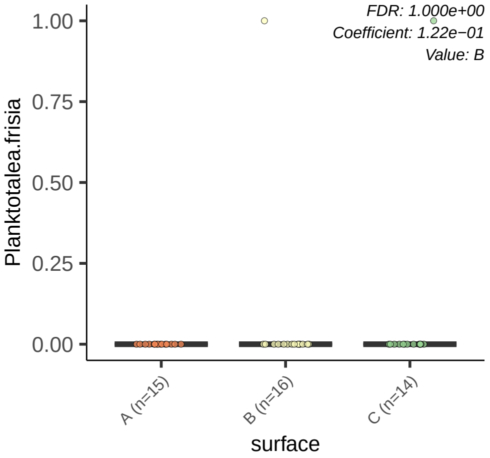
FDR: 1.000e+00
Coefficient: 1.22e−01
Value: B
Planktotalea.frisia
1.00
0.75
0.50
0.25
0.00
A (n=15)
B (n=16)
C (n=14)
surface

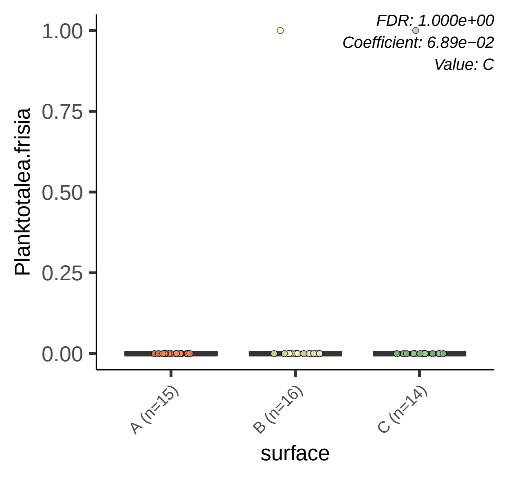

FDR: 1.000e+00
Coefficient: 6.89e−02
Value: C
1.00
0.75
0.50
0.25
0.00
Planktotalea.frisia
A (n=15)
B (n=16)
C (n=14)
surface

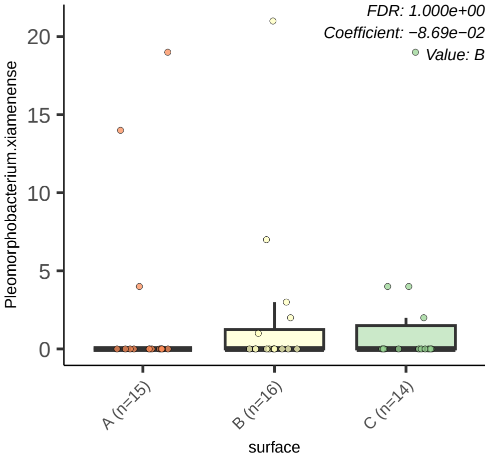
FDR: 1.000e+00
Coefficient: −8.69e−02
Value: B
Pleomorphobacterium.xiamenense
0
5
10
15
20
A (n=15)
B (n=16)
C (n=14)
surface

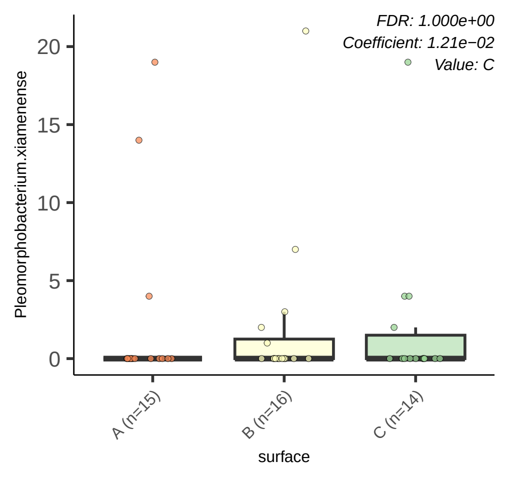
FDR: 1.000e+00
Coefficient: 1.21e−02
Value: C
Pleomorphobacterium.xiamenense
0
5
10
15
20
A (n=15)
B (n=16)
C (n=14)
surface

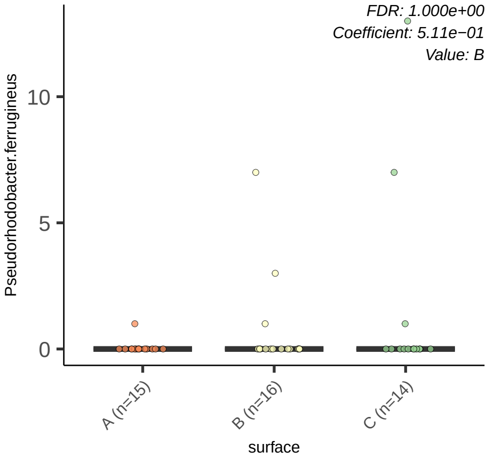

FDR: 1.000e+00
Coefficient: 5.11e−01
Value: B
Pseudorhodobacter.ferrugineus
10
5
0
A (n=15)
B (n=16)
C (n=14)
surface

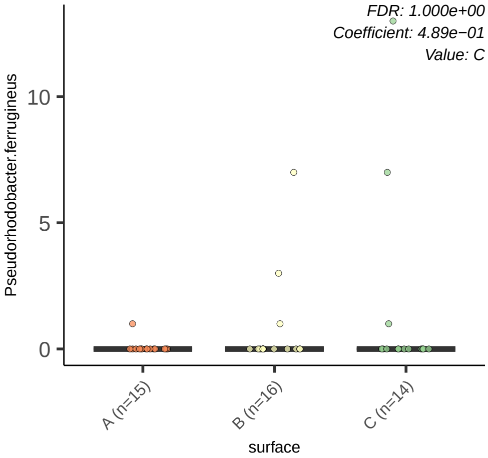
FDR: 1.000e+00
Coefficient: 4.89e−01
Value: C
Pseudorhodobacter.ferrugineus
0
5
10
A (n=15)
B (n=16)
C (n=14)
surface

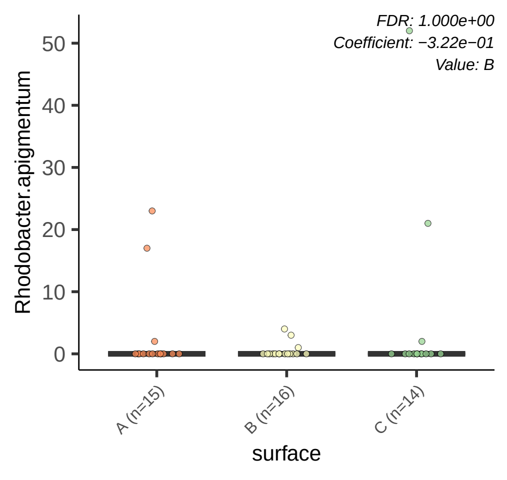

FDR: 1.000e+00
Coefficient: −3.22e−01
Value: B
Rhodobacter.apigmentum
50
40
30
20
10
0
A (n=15)
B (n=16)
C (n=14)
surface

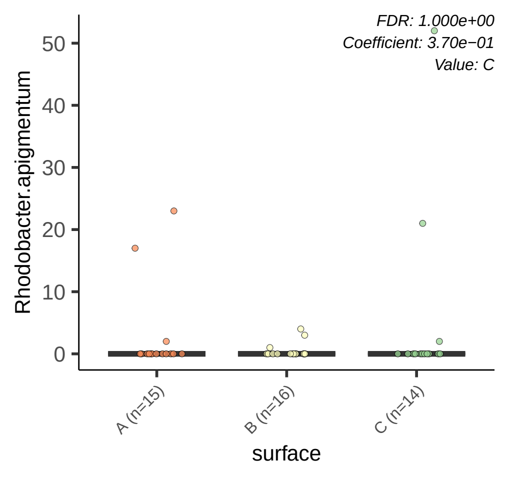
FDR: 1.000e+00
Coefficient: 3.70e−01
Value: C
Rhodobacter.apigmentum
0
10
20
30
40
50
A (n=15)
B (n=16)
C (n=14)
surface

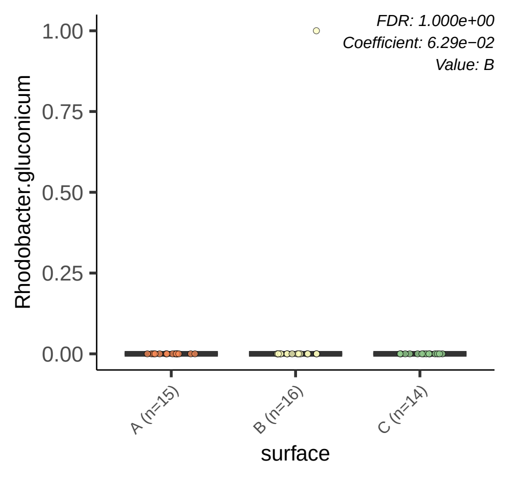

FDR: 1.000e+00
Coefficient: 6.29e−02
Value: B
Rhodobacter.gluconicum
1.00
0.75
0.50
0.25
0.00
A (n=15)
B (n=16)
C (n=14)
surface

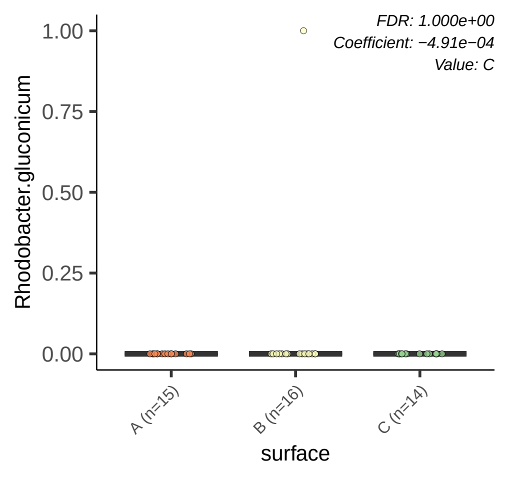

FDR: 1.000e+00
Coefficient: −4.91e−04
Value: C
Rhodobacter.gluconicum
1.00
0.75
0.50
0.25
0.00
A (n=15)
B (n=16)
C (n=14)
surface

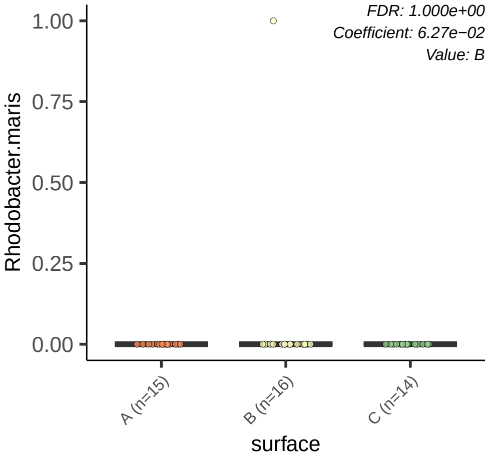
FDR: 1.000e+00
Coefficient: 6.27e−02
Value: B
Rhodobacter.maris
1.00
0.75
0.50
0.25
0.00
A (n=15)
B (n=16)
C (n=14)
surface

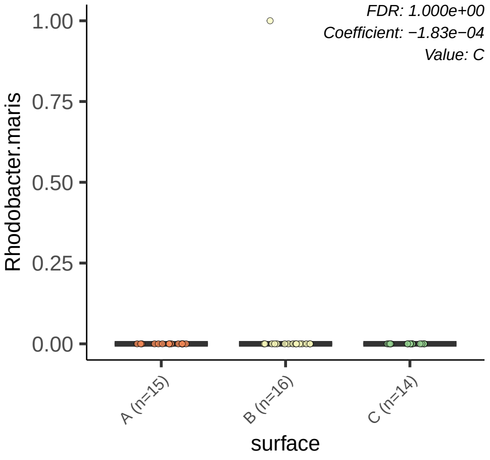
FDR: 1.000e+00
Coefficient: −1.83e−04
Value: C
Rhodobacter.maris
1.00
0.75
0.50
0.25
0.00
A (n=15)
B (n=16)
C (n=14)
surface

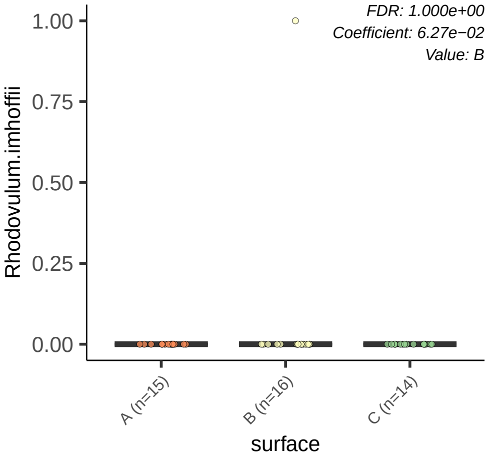
FDR: 1.000e+00
Coefficient: 6.27e−02
Value: B
Rhodovulum.imhoffii
1.00
0.75
0.50
0.25
0.00
A (n=15)
B (n=16)
C (n=14)
surface

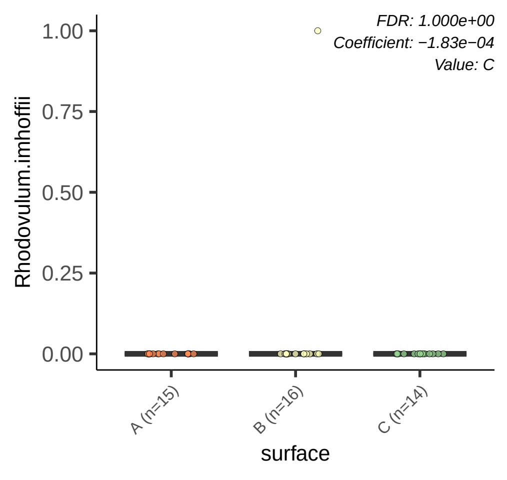

FDR: 1.000e+00
Coefficient: −1.83e−04
Value: C
Rhodovulum.imhoffii
1.00
0.75
0.50
0.25
0.00
A (n=15)
B (n=16)
C (n=14)
surface

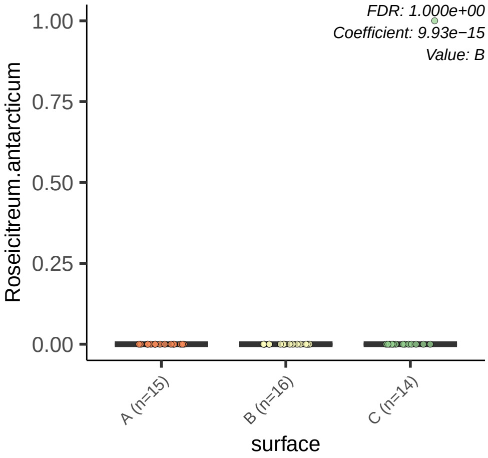

FDR: 1.000e+00
Coefficient: 9.93e−15
Value: B
Roseicitreum.antarcticum
1.00
0.75
0.50
0.25
0.00
A (n=15)
B (n=16)
C (n=14)
surface

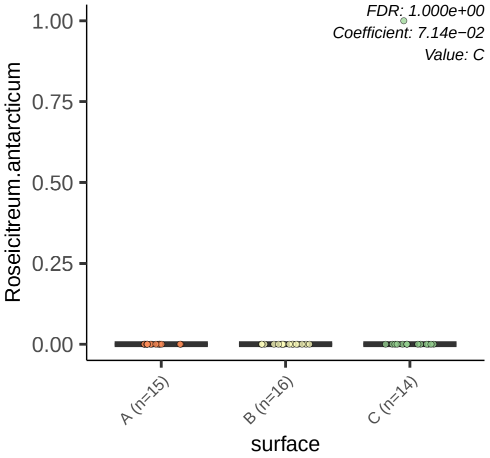
FDR: 1.000e+00
Coefficient: 7.14e−02
Value: C
Roseicitreum.antarcticum
1.00
0.75
0.50
0.25
0.00
A (n=15)
B (n=16)
C (n=14)
surface

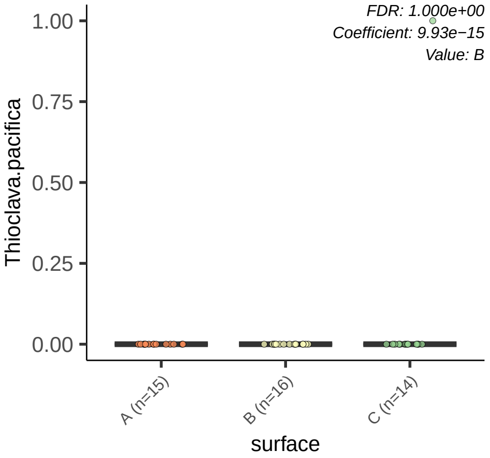
FDR: 1.000e+00
Coefficient: 9.93e−15
Value: B
1.00
0.75
0.50
0.25
0.00
Thioclava.pacifica
A (n=15)
B (n=16)
C (n=14)
surface

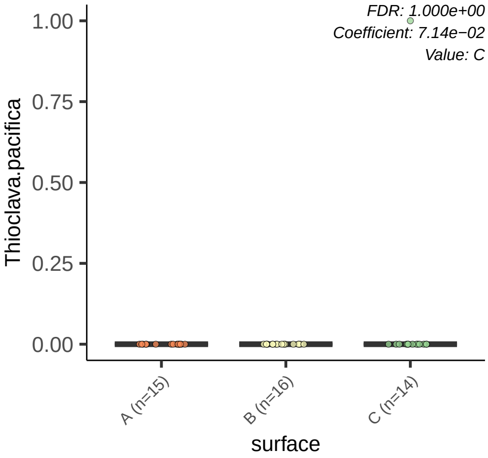

FDR: 1.000e+00
Coefficient: 7.14e−02
Value: C
1.00
0.75
0.50
0.25
0.00
Thioclava.pacifica
A (n=15)
B (n=16)
C (n=14)
surface

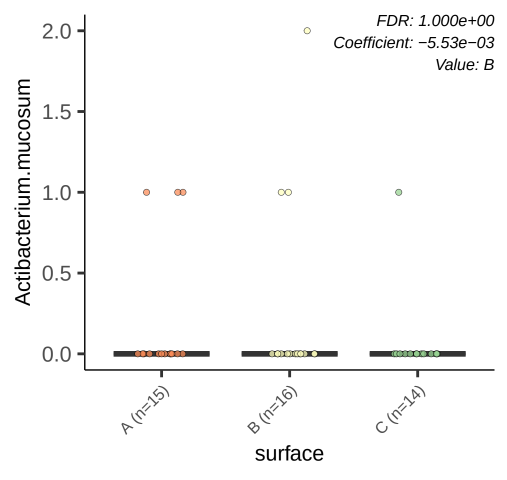
FDR: 1.000e+00
Coefficient: −5.53e−03
Value: B
Actibacterium.mucosum
2.0
1.5
1.0
0.5
0.0
A (n=15)
B (n=16)
C (n=14)
surface

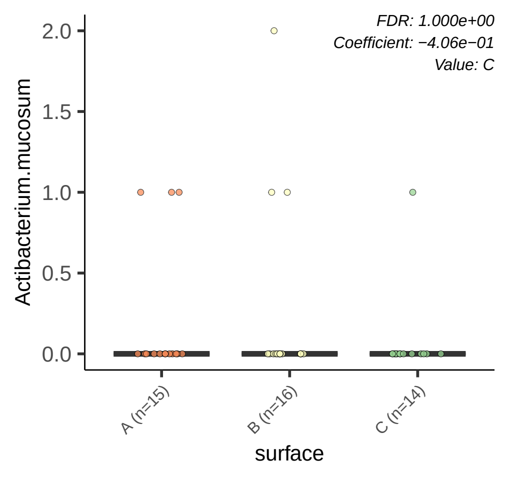
FDR: 1.000e+00
Coefficient: −4.06e−01
Value: C
Actibacterium.mucosum
2.0
1.5
1.0
0.5
0.0
A (n=15)
B (n=16)
C (n=14)
surface

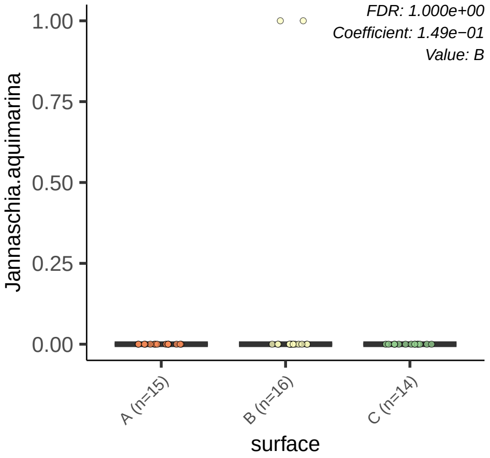

FDR: 1.000e+00
Coefficient: 1.49e−01
Value: B
Jannaschia.aquimarina
1.00
0.75
0.50
0.25
0.00
A (n=15)
B (n=16)
C (n=14)
surface

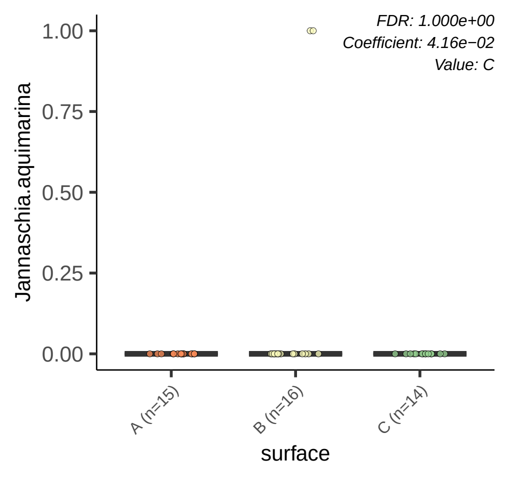
FDR: 1.000e+00
Coefficient: 4.16e−02
Value: C
Jannaschia.aquimarina
1.00
0.75
0.50
0.25
0.00
A (n=15)
B (n=16)
C (n=14)
surface

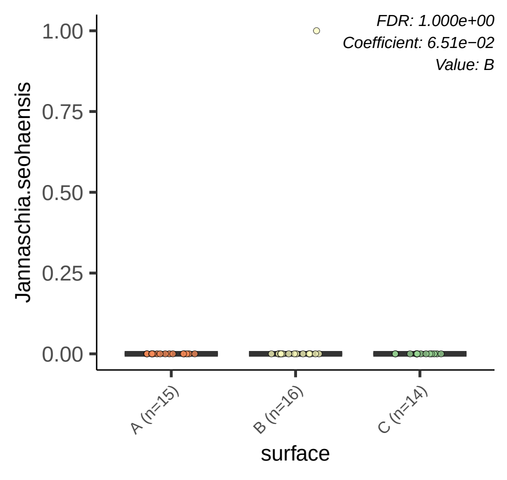

FDR: 1.000e+00
Coefficient: 6.51e−02
Value: B
Jannaschia.seohaensis
1.00
0.75
0.50
0.25
0.00
A (n=15)
B (n=16)
C (n=14)
surface

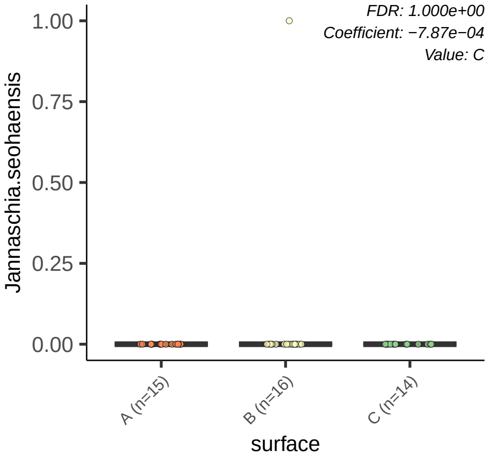
FDR: 1.000e+00
Coefficient: −7.87e−04
Value: C
Jannaschia.seohaensis
1.00
0.75
0.50
0.25
0.00
A (n=15)
B (n=16)
C (n=14)
surface

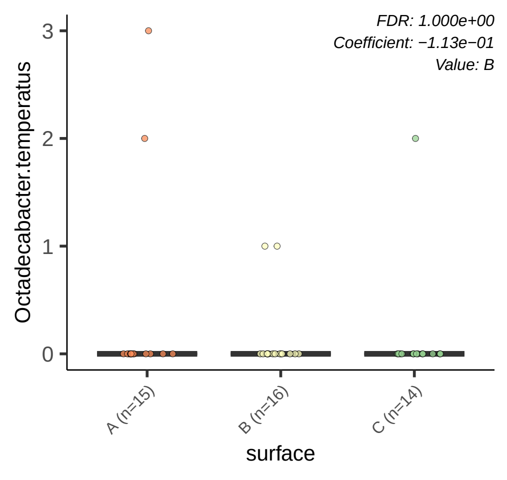

FDR: 1.000e+00
Coefficient: −1.13e−01
Value: B
Octadecabacter.temperatus
0
1
2
3
A (n=15)
B (n=16)
C (n=14)
surface

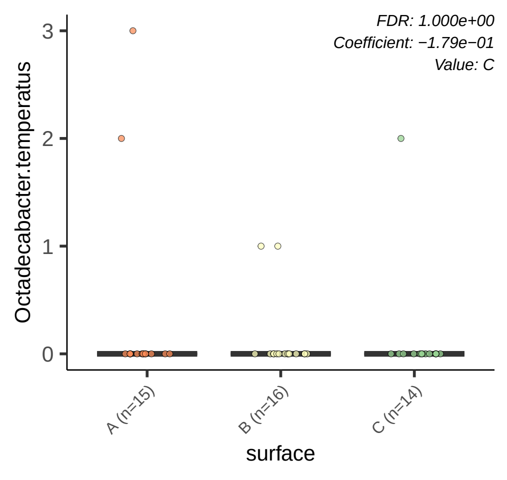
FDR: 1.000e+00
Coefficient: −1.79e−01
Value: C
Octadecabacter.temperatus
3
2
1
0
A (n=15)
B (n=16)
C (n=14)
surface

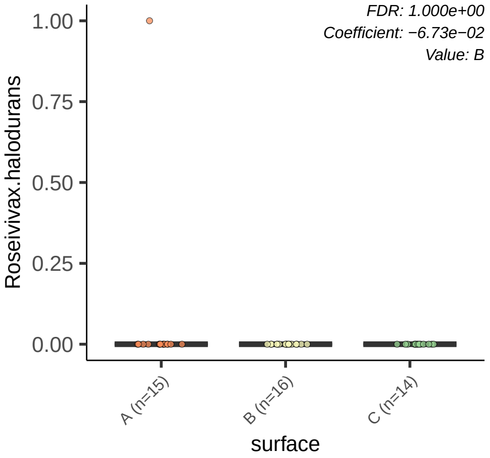

FDR: 1.000e+00
Coefficient: −6.73e−02
Value: B
Roseivivax.halodurans
1.00
0.75
0.50
0.25
0.00
A (n=15)
B (n=16)
C (n=14)
surface

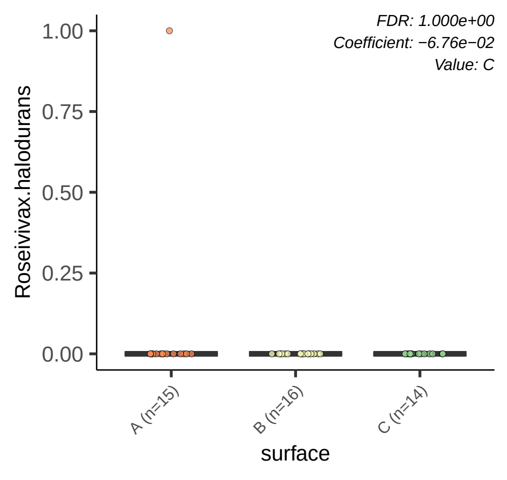
FDR: 1.000e+00
Coefficient: −6.76e−02
Value: C
Roseivivax.halodurans
1.00
0.75
0.50
0.25
0.00
A (n=15)
B (n=16)
C (n=14)
surface

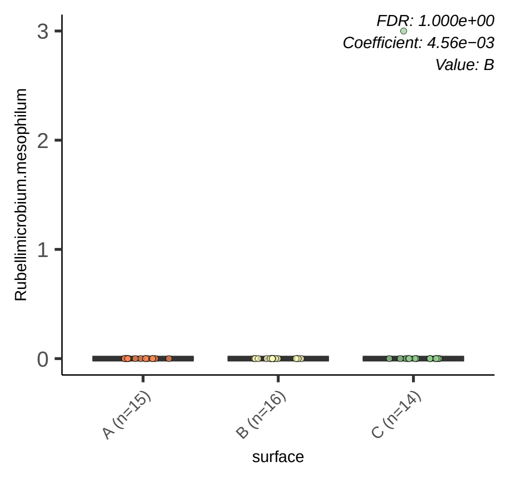
FDR: 1.000e+00
Coefficient: 4.56e−03
Value: B
Rubellimicrobium.mesophilum
3
2
1
0
A (n=15)
B (n=16)
C (n=14)
surface

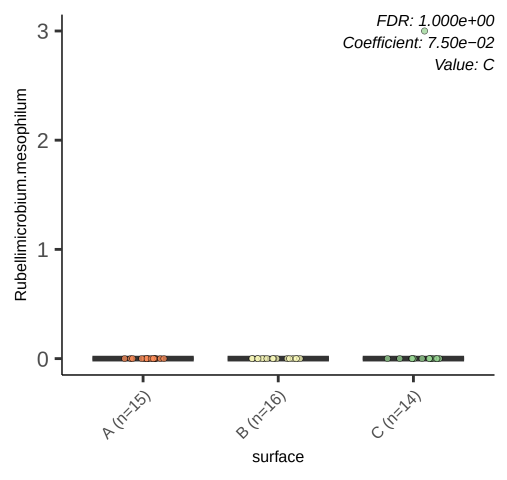

FDR: 1.000e+00
Coefficient: 7.50e−02
Value: C
3
2
1
0
Rubellimicrobium.mesophilum
A (n=15)
B (n=16)
C (n=14)
surface

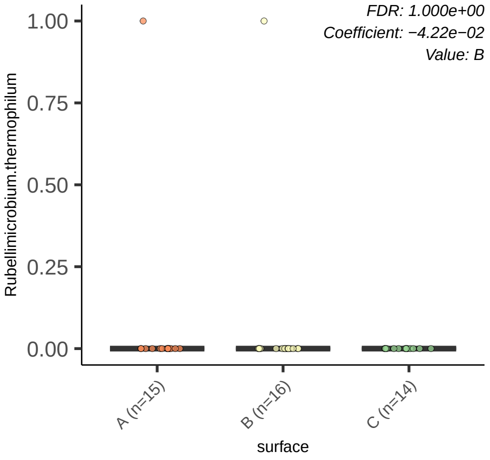
FDR: 1.000e+00
Coefficient: −4.22e−02
Value: B
Rubellimicrobium.thermophilum
1.00
0.75
0.50
0.25
0.00
A (n=15)
B (n=16)
C (n=14)
surface

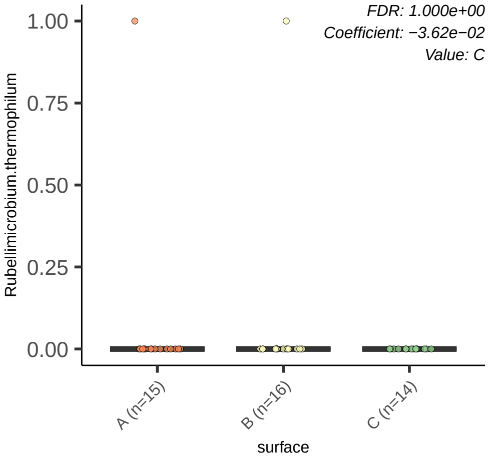
FDR: 1.000e+00
Coefficient: −3.62e−02
Value: C
Rubellimicrobium.thermophilum
1.00
0.75
0.50
0.25
0.00
A (n=15)
B (n=16)
C (n=14)
surface

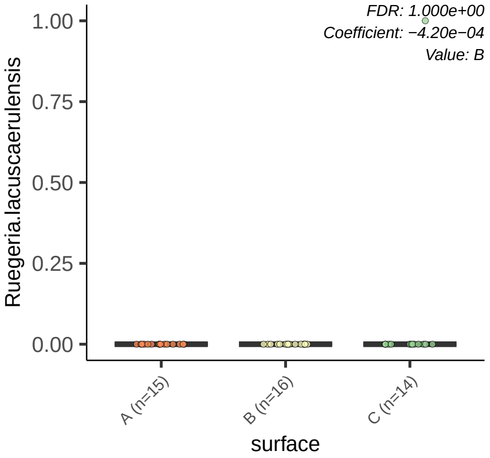

FDR: 1.000e+00
Coefficient: −4.20e−04
Value: B
Ruegeria.lacuscaerulensis
1.00
0.75
0.50
0.25
0.00
A (n=15)
B (n=16)
C (n=14)
surface

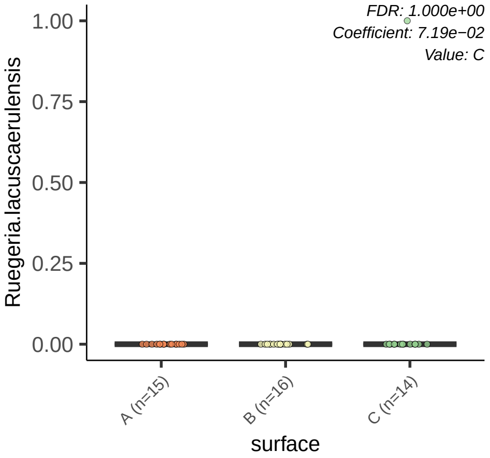
FDR: 1.000e+00
Coefficient: 7.19e−02
Value: C
Ruegeria.lacuscaerulensis
1.00
0.75
0.50
0.25
0.00
A (n=15)
B (n=16)
C (n=14)
surface

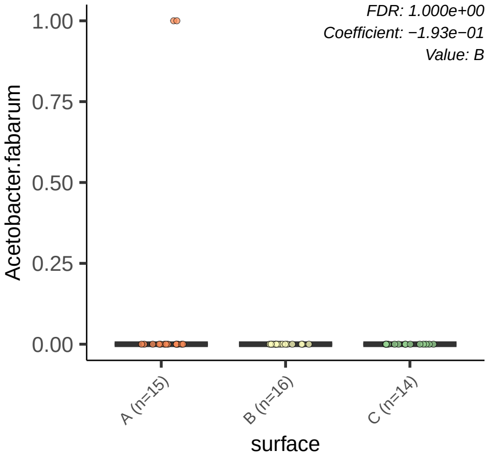

FDR: 1.000e+00
Coefficient: −1.93e−01
Value: B
Acetobacter.fabarum
1.00
0.75
0.50
0.25
0.00
A (n=15)
B (n=16)
C (n=14)
surface

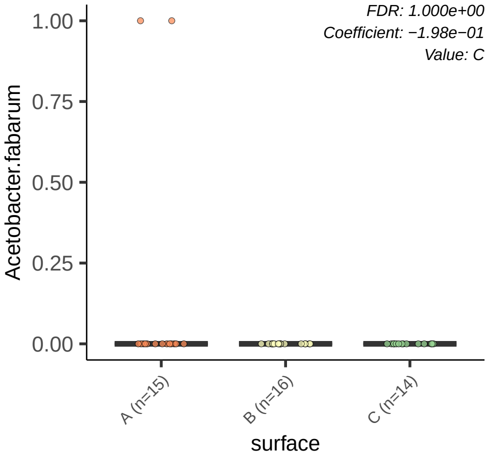

FDR: 1.000e+00
Coefficient: −1.98e−01
Value: C
Acetobacter.fabarum
1.00
0.75
0.50
0.25
0.00
A (n=15)
B (n=16)
C (n=14)
surface

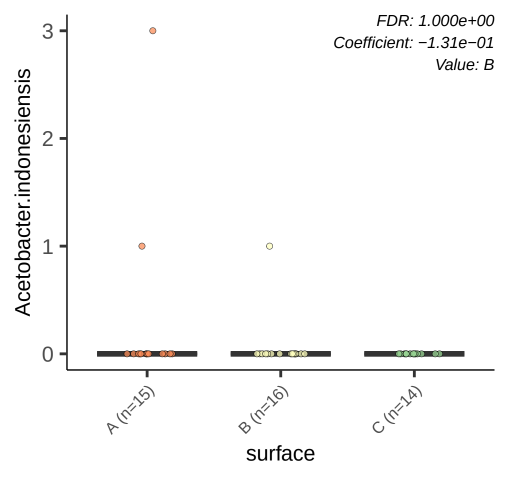
FDR: 1.000e+00
Coefficient: −1.31e−01
Value: B
Acetobacter.indonesiensis
0
1
2
3
A (n=15)
B (n=16)
C (n=14)
surface

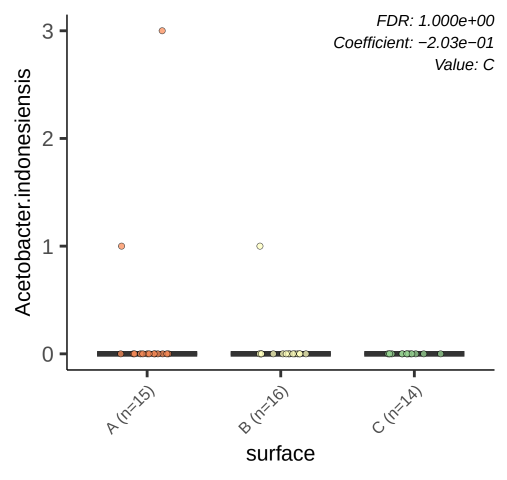

FDR: 1.000e+00
Coefficient: −2.03e−01
Value: C
Acetobacter.indonesiensis
3
2
1
0
A (n=15)
B (n=16)
C (n=14)
surface

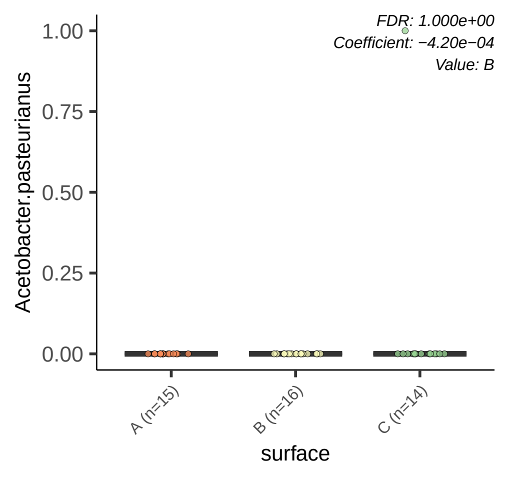
FDR: 1.000e+00
Coefficient: −4.20e−04
Value: B
Acetobacter.pasteurianus
1.00
0.75
0.50
0.25
0.00
A (n=15)
B (n=16)
C (n=14)
surface

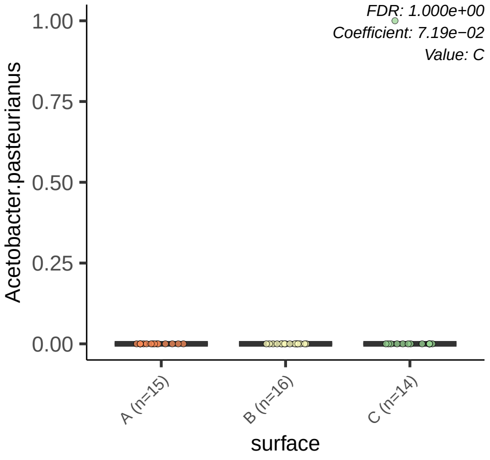
FDR: 1.000e+00
Coefficient: 7.19e−02
Value: C
Acetobacter.pasteurianus
1.00
0.75
0.50
0.25
0.00
A (n=15)
B (n=16)
C (n=14)
surface

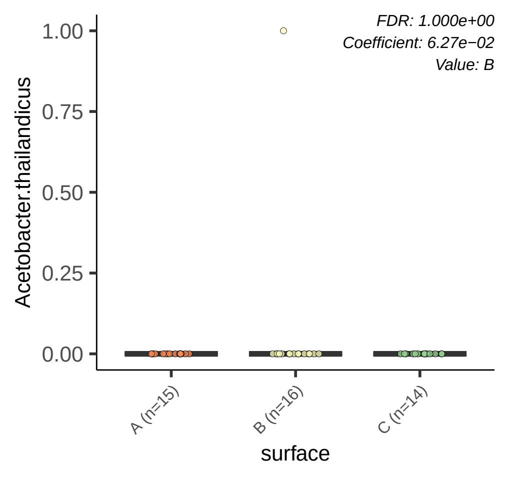
FDR: 1.000e+00
Coefficient: 6.27e−02
Value: B
Acetobacter.thailandicus
1.00
0.75
0.50
0.25
0.00
A (n=15)
B (n=16)
C (n=14)
surface

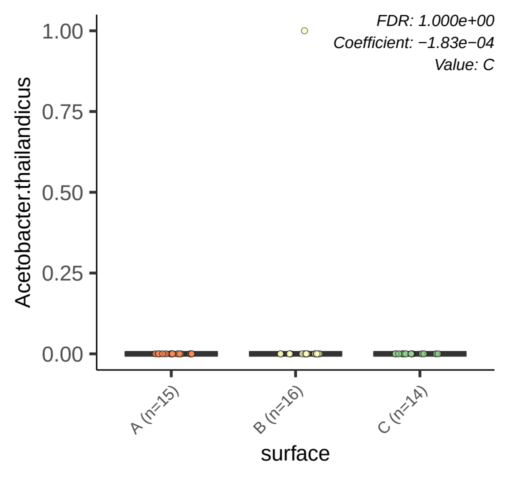

FDR: 1.000e+00
Coefficient: −1.83e−04
Value: C
Acetobacter.thailandicus
1.00
0.75
0.50
0.25
0.00
A (n=15)
B (n=16)
C (n=14)
surface

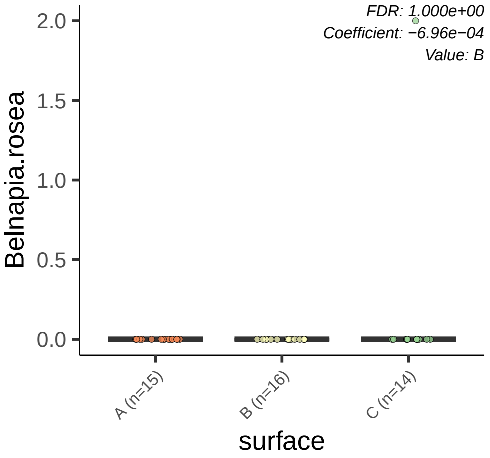
FDR: 1.000e+00
Coefficient: −6.96e−04
Value: B
Belnapia.rosea
2.0
1.5
1.0
0.5
0.0
A (n=15)
B (n=16)
C (n=14)
surface

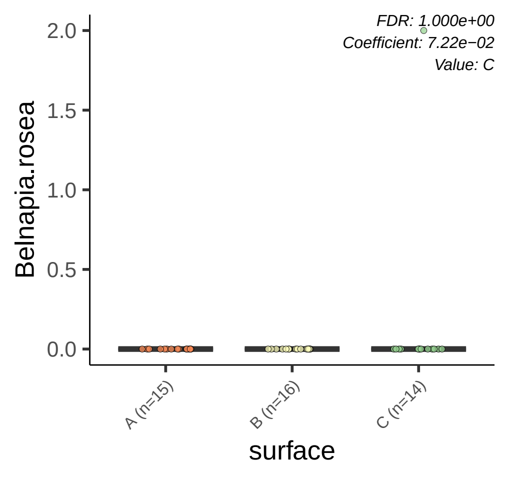

FDR: 1.000e+00
Coefficient: 7.22e−02
Value: C
Belnapia.rosea
2.0
1.5
1.0
0.5
0.0
A (n=15)
B (n=16)
C (n=14)
surface

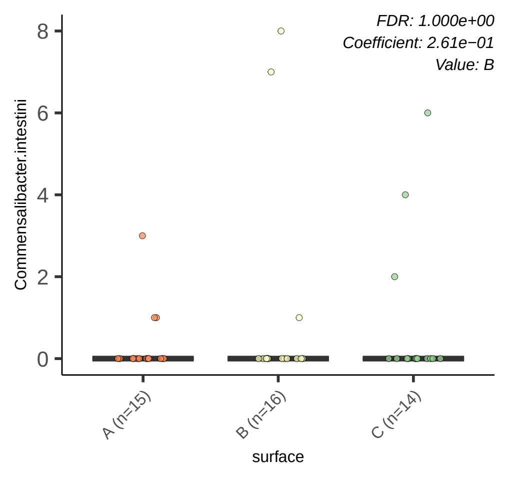

FDR: 1.000e+00
Coefficient: 2.61e−01
Value: B
Commensalibacter.intestini
8
6
4
2
0
A (n=15)
B (n=16)
C (n=14)
surface

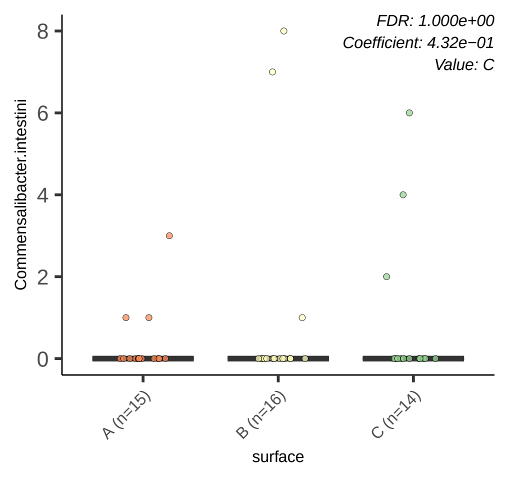
FDR: 1.000e+00
Coefficient: 4.32e−01
Value: C
Commensalibacter.intestini
8
6
4
2
0
A (n=15)
B (n=16)
C (n=14)
surface

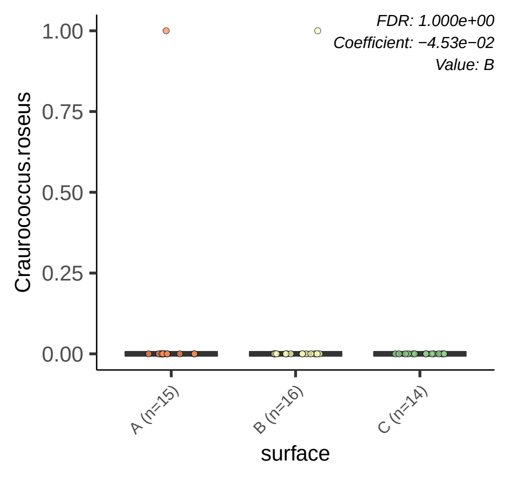

FDR: 1.000e+00
Coefficient: −4.53e−02
Value: B
Craurococcus.roseus
1.00
0.75
0.50
0.25
0.00
A (n=15)
B (n=16)
C (n=14)
surface

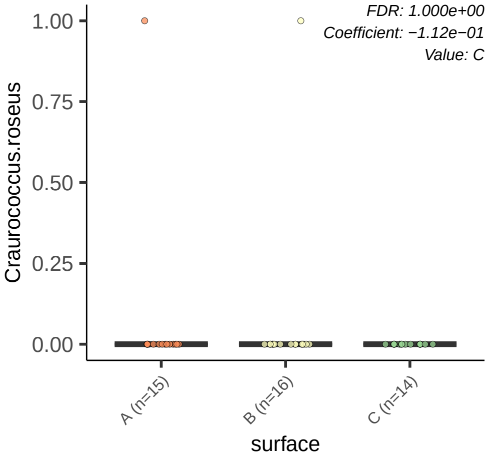

FDR: 1.000e+00
Coefficient: −1.12e−01
Value: C
Craurococcus.roseus
1.00
0.75
0.50
0.25
0.00
A (n=15)
B (n=16)
C (n=14)
surface

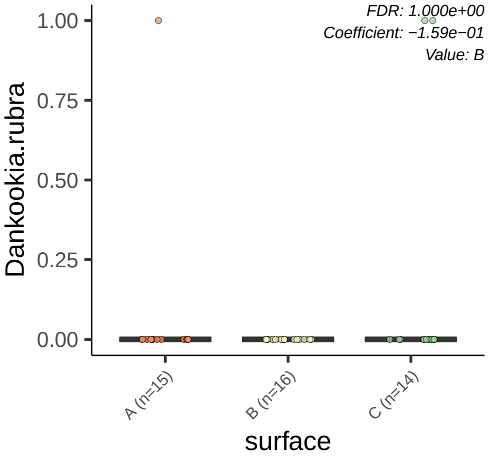
FDR: 1.000e+00
Coefficient: −1.59e−01
Value: B
Dankookia.rubra
1.00
0.75
0.50
0.25
0.00
A (n=15)
B (n=16)
C (n=14)
surface

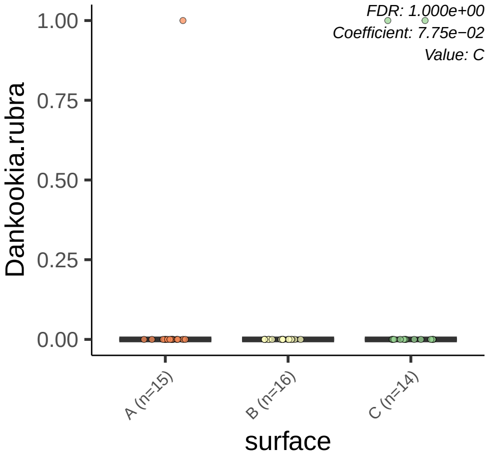

FDR: 1.000e+00
Coefficient: 7.75e−02
Value: C
Dankookia.rubra
1.00
0.75
0.50
0.25
0.00
A (n=15)
B (n=16)
C (n=14)
surface

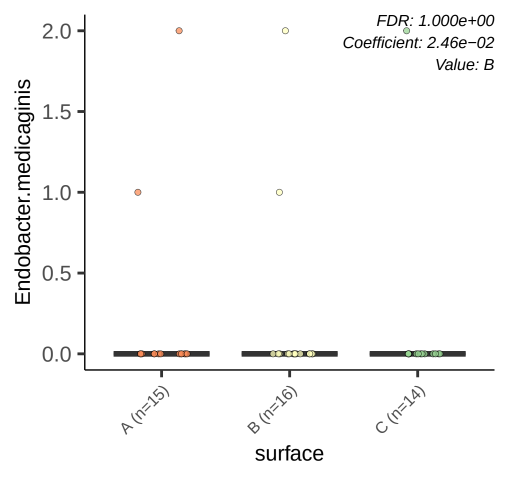
FDR: 1.000e+00
Coefficient: 2.46e−02
Value: B
Endobacter.medicaginis
2.0
1.5
1.0
0.5
0.0
A (n=15)
B (n=16)
C (n=14)
surface

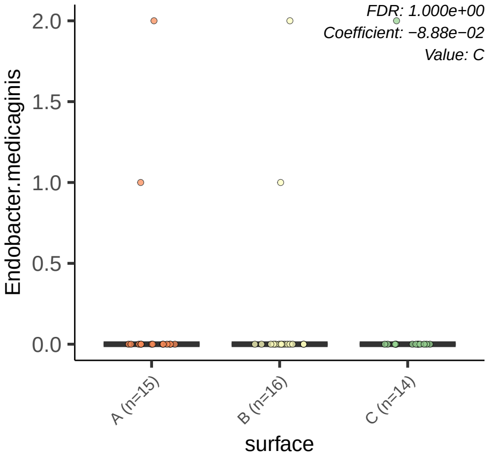

FDR: 1.000e+00
Coefficient: −8.88e−02
Value: C
Endobacter.medicaginis
2.0
1.5
1.0
0.5
0.0
A (n=15)
B (n=16)
C (n=14)
surface

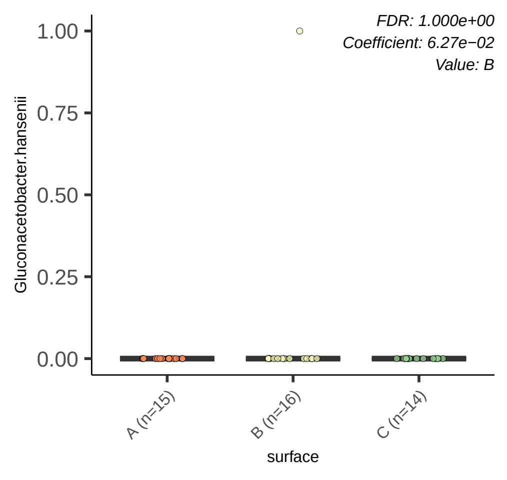
FDR: 1.000e+00
Coefficient: 6.27e−02
Value: B
Gluconacetobacter.hansenii
1.00
0.75
0.50
0.25
0.00
A (n=15)
B (n=16)
C (n=14)
surface

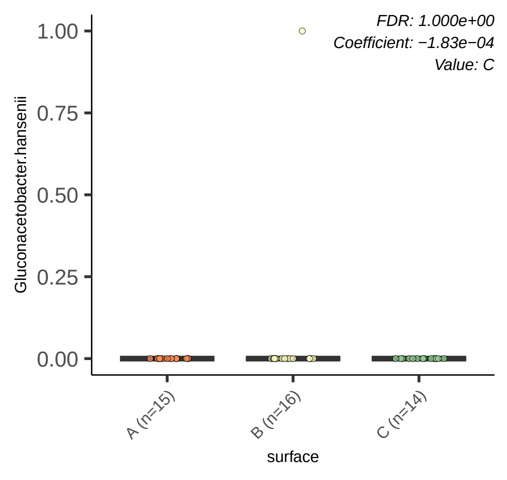
FDR: 1.000e+00
Coefficient: −1.83e−04
Value: C
Gluconacetobacter.hansenii
1.00
0.75
0.50
0.25
0.00
A (n=15)
B (n=16)
C (n=14)
surface

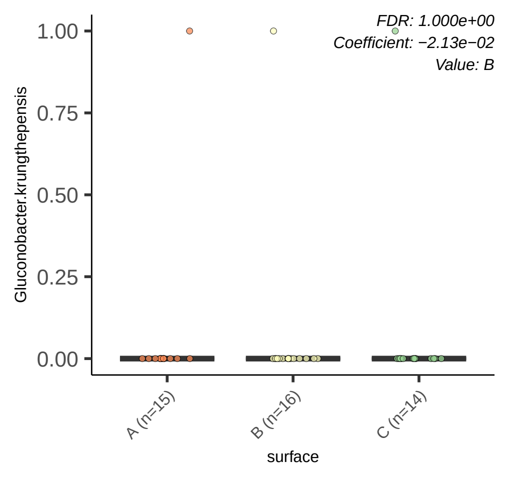

FDR: 1.000e+00
Coefficient: −2.13e−02
Value: B
1.00
0.75
0.50
0.25
0.00
Gluconobacter.krungthepensis
A (n=15)
B (n=16)
C (n=14)
surface

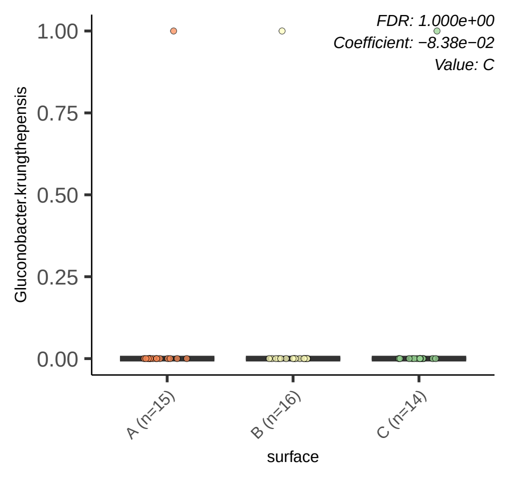
FDR: 1.000e+00
Coefficient: −8.38e−02
Value: C
1.00
0.75
0.50
0.25
0.00
Gluconobacter.krungthepensis
A (n=15)
B (n=16)
C (n=14)
surface

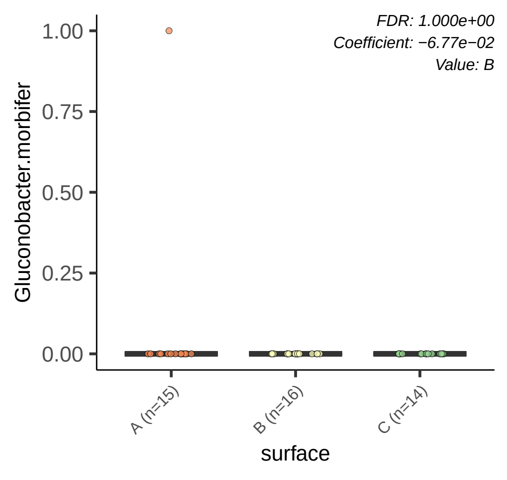
FDR: 1.000e+00
Coefficient: −6.77e−02
Value: B
Gluconobacter.morbifer
1.00
0.75
0.50
0.25
0.00
A (n=15)
B (n=16)
C (n=14)
surface

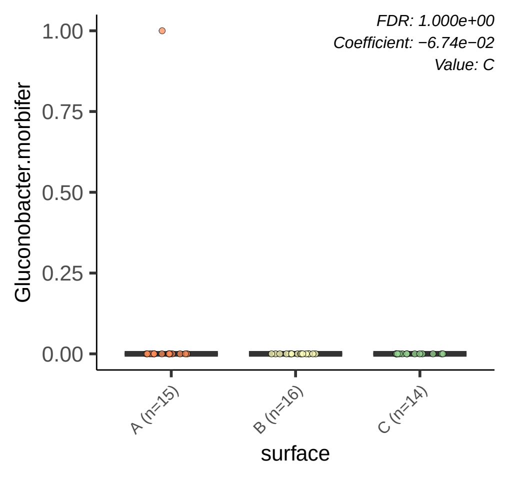
FDR: 1.000e+00
Coefficient: −6.74e−02
Value: C
Gluconobacter.morbifer
1.00
0.75
0.50
0.25
0.00
A (n=15)
B (n=16)
C (n=14)
surface

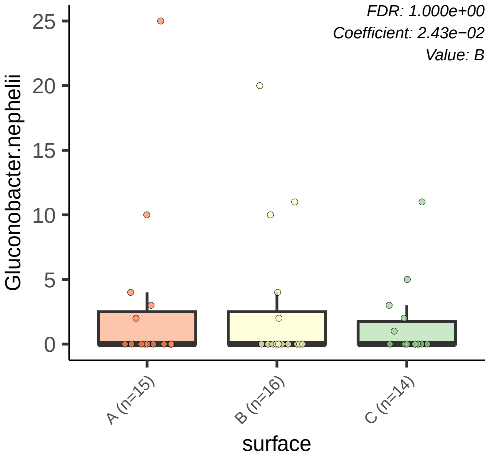

FDR: 1.000e+00
Coefficient: 2.43e−02
Value: B
Gluconobacter.nephelii
25
20
15
10
5
0
A (n=15)
B (n=16)
C (n=14)
surface

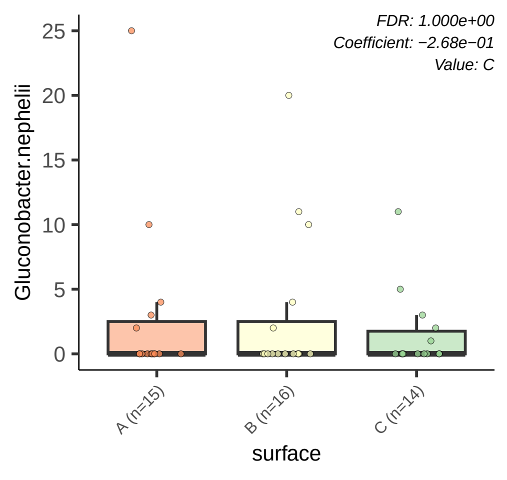
FDR: 1.000e+00
Coefficient: −2.68e−01
Value: C
Gluconobacter.nephelii
25
20
15
10
5
0
A (n=15)
B (n=16)
C (n=14)
surface

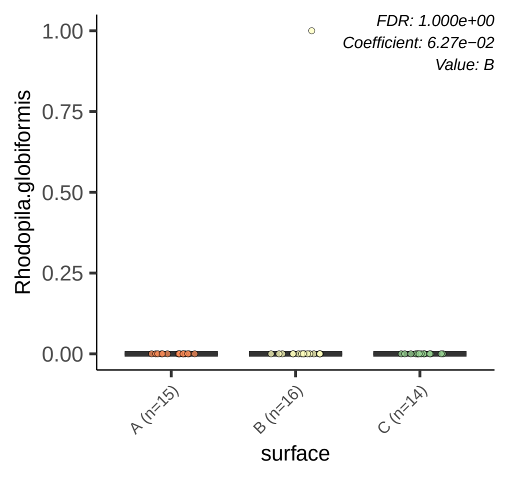

FDR: 1.000e+00
Coefficient: 6.27e−02
Value: B
Rhodopila.globiformis
1.00
0.75
0.50
0.25
0.00
A (n=15)
B (n=16)
C (n=14)
surface

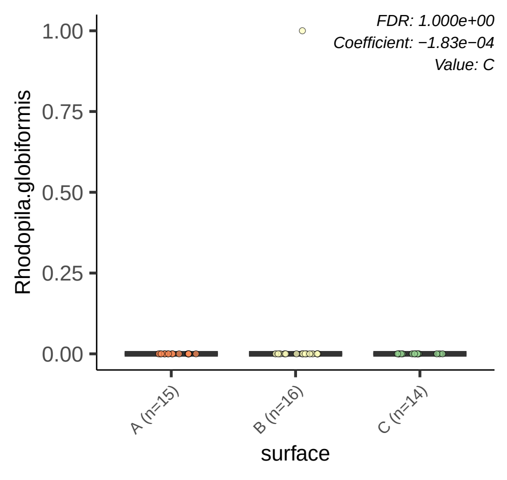

FDR: 1.000e+00
Coefficient: −1.83e−04
Value: C
Rhodopila.globiformis
1.00
0.75
0.50
0.25
0.00
A (n=15)
B (n=16)
C (n=14)
surface

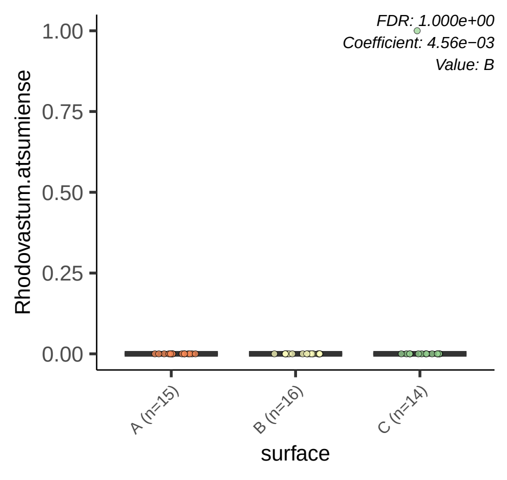
FDR: 1.000e+00
Coefficient: 4.56e−03
Value: B
Rhodovastum.atsumiense
1.00
0.75
0.50
0.25
0.00
A (n=15)
B (n=16)
C (n=14)
surface

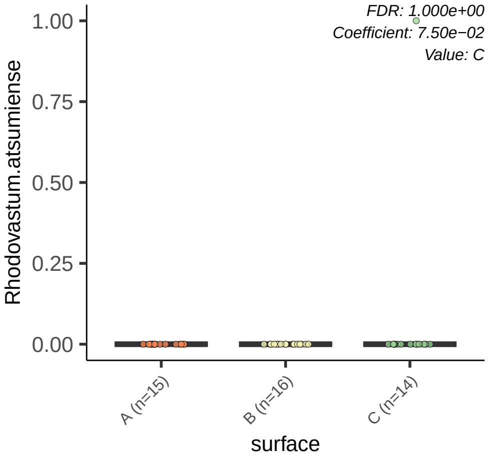

FDR: 1.000e+00
Coefficient: 7.50e−02
Value: C
Rhodovastum.atsumiense
1.00
0.75
0.50
0.25
0.00
A (n=15)
B (n=16)
C (n=14)
surface

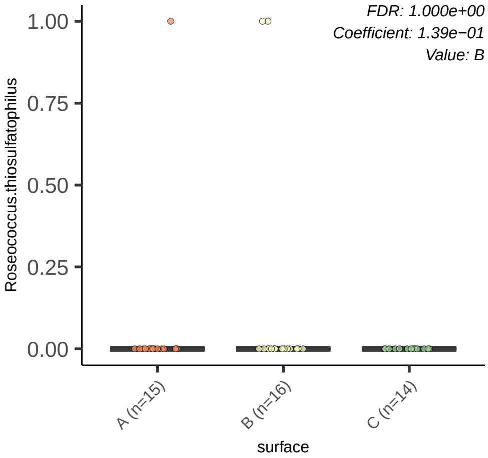
FDR: 1.000e+00
Coefficient: 1.39e−01
Value: B
Roseococcus.thiosulfatophilus
1.00
0.75
0.50
0.25
0.00
A (n=15)
B (n=16)
C (n=14)
surface

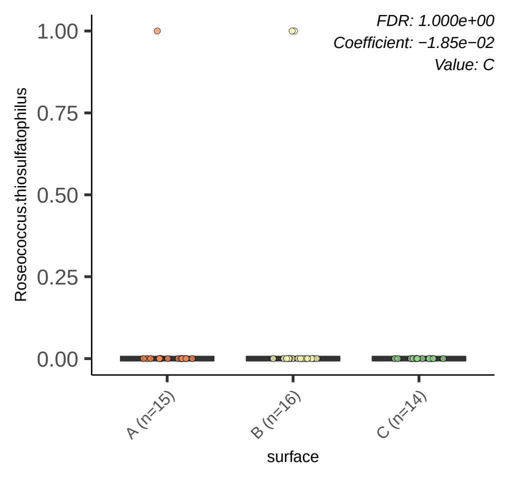
FDR: 1.000e+00
Coefficient: −1.85e−02
Value: C
Roseococcus.thiosulfatophilus
1.00
0.75
0.50
0.25
0.00
A (n=15)
B (n=16)
C (n=14)
surface

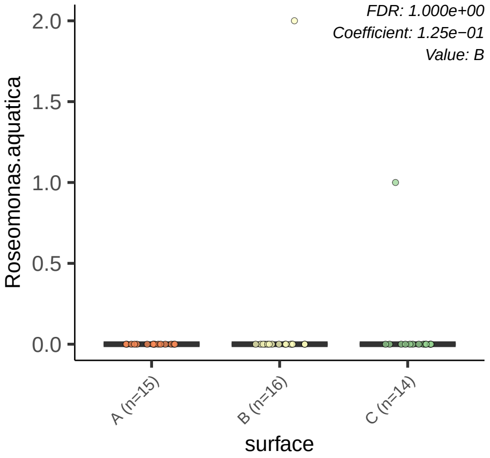
FDR: 1.000e+00
Coefficient: 1.25e−01
Value: B
Roseomonas.aquatica
2.0
1.5
1.0
0.5
0.0
A (n=15)
B (n=16)
C (n=14)
surface

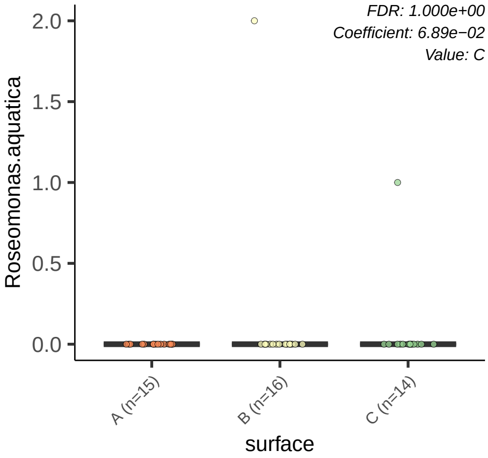

FDR: 1.000e+00
Coefficient: 6.89e−02
Value: C
Roseomonas.aquatica
2.0
1.5
1.0
0.5
0.0
A (n=15)
B (n=16)
C (n=14)
surface

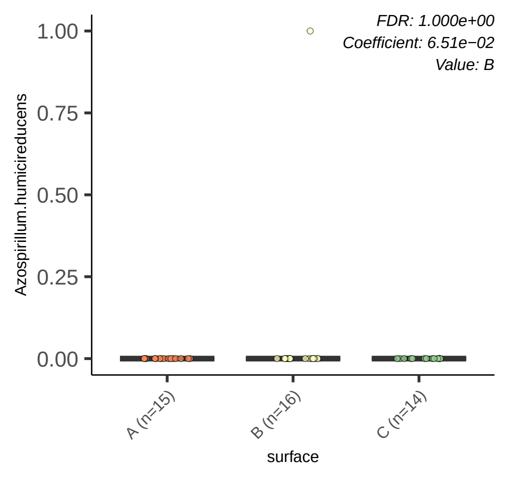
FDR: 1.000e+00
Coefficient: 6.51e−02
Value: B
1.00
0.75
0.50
0.25
0.00
Azospirillum.humicireducens
A (n=15)
B (n=16)
C (n=14)
surface

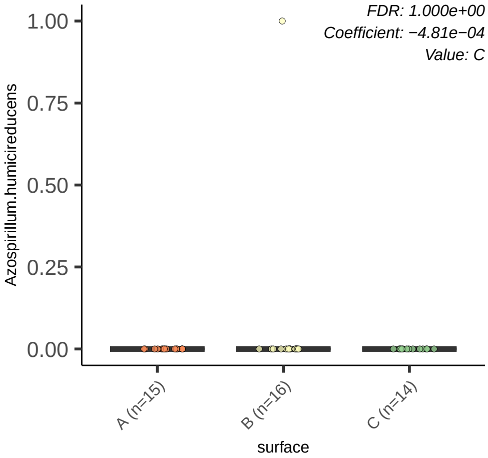

FDR: 1.000e+00
Coefficient: −4.81e−04
Value: C
Azospirillum.humicireducens
1.00
0.75
0.50
0.25
0.00
A (n=15)
B (n=16)
C (n=14)
surface

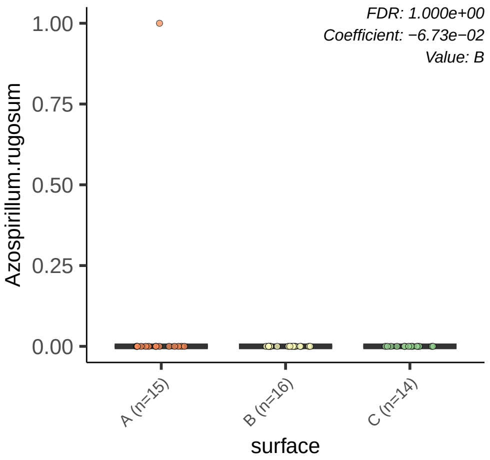
FDR: 1.000e+00
Coefficient: −6.73e−02
Value: B
Azospirillum.rugosum
1.00
0.75
0.50
0.25
0.00
A (n=15)
B (n=16)
C (n=14)
surface

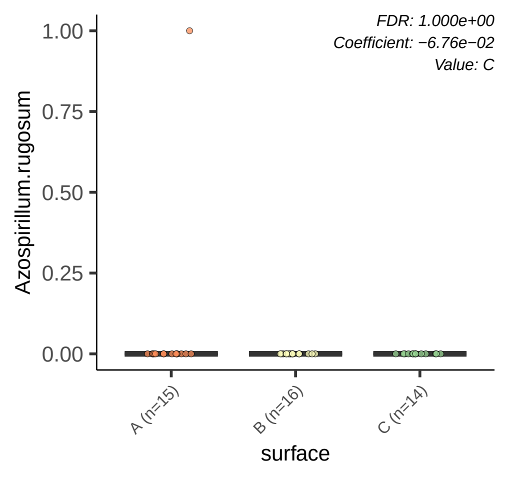
FDR: 1.000e+00
Coefficient: −6.76e−02
Value: C
Azospirillum.rugosum
1.00
0.75
0.50
0.25
0.00
A (n=15)
B (n=16)
C (n=14)
surface

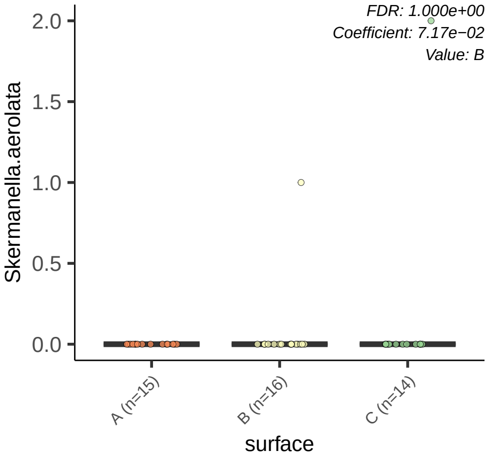
FDR: 1.000e+00
Coefficient: 7.17e−02
Value: B
Skermanella.aerolata
2.0
1.5
1.0
0.5
0.0
A (n=15)
B (n=16)
C (n=14)
surface

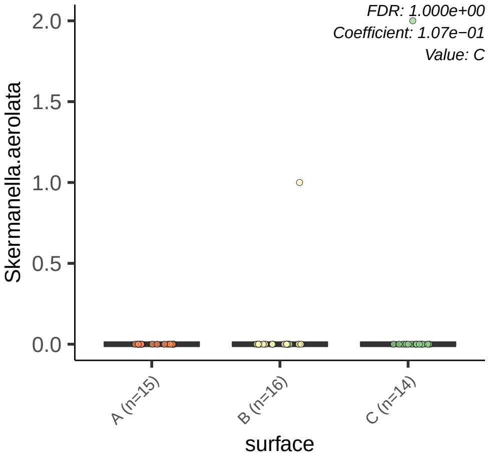
FDR: 1.000e+00
Coefficient: 1.07e−01
Value: C
Skermanella.aerolata
2.0
1.5
1.0
0.5
0.0
A (n=15)
B (n=16)
C (n=14)
surface

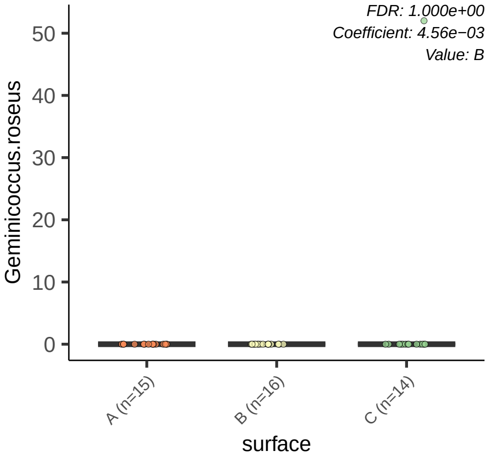
FDR: 1.000e+00
Coefficient: 4.56e−03
Value: B
Geminicoccus.roseus
50
40
30
20
10
0
A (n=15)
B (n=16)
C (n=14)
surface

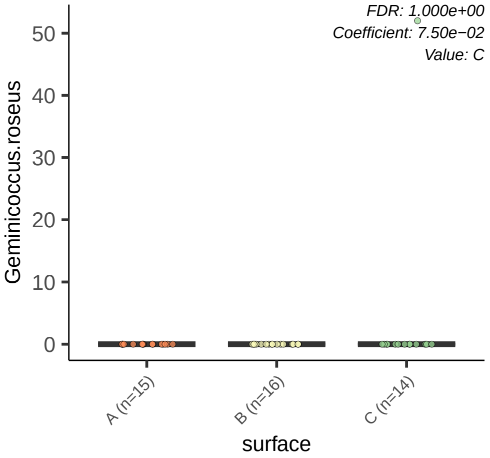

FDR: 1.000e+00
Coefficient: 7.50e−02
Value: C
Geminicoccus.roseus
50
40
30
20
10
0
A (n=15)
B (n=16)
C (n=14)
surface

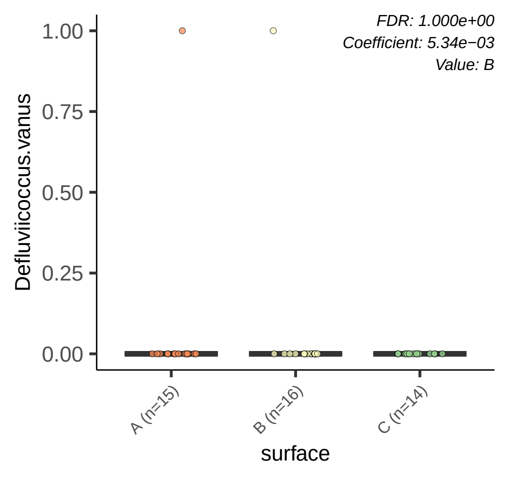

FDR: 1.000e+00
Coefficient: 5.34e−03
Value: B
Defluviicoccus.vanus
1.00
0.75
0.50
0.25
0.00
A (n=15)
B (n=16)
C (n=14)
surface

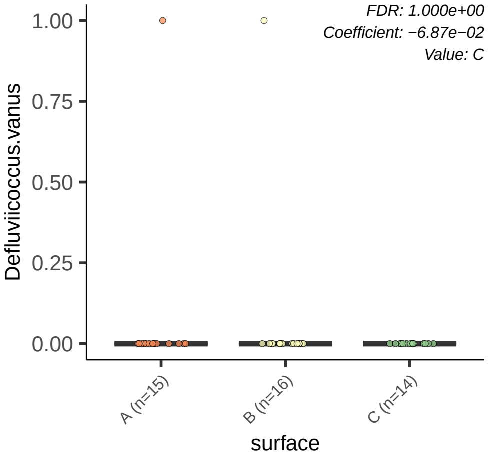
FDR: 1.000e+00
Coefficient: −6.87e−02
Value: C
Defluviicoccus.vanus
1.00
0.75
0.50
0.25
0.00
A (n=15)
B (n=16)
C (n=14)
surface

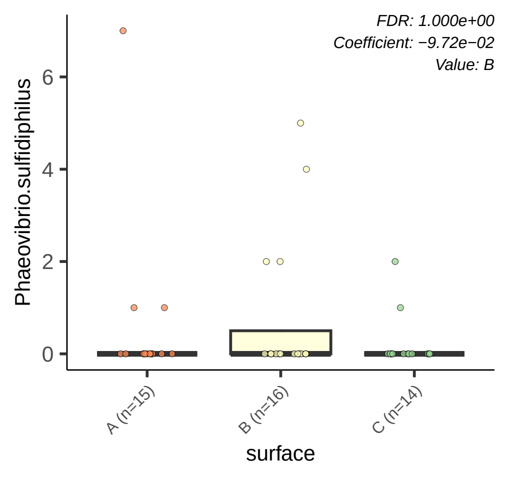
FDR: 1.000e+00
Coefficient: −9.72e−02
Value: B
Phaeovibrio.sulfidiphilus
0
2
4
6
A (n=15)
B (n=16)
C (n=14)
surface

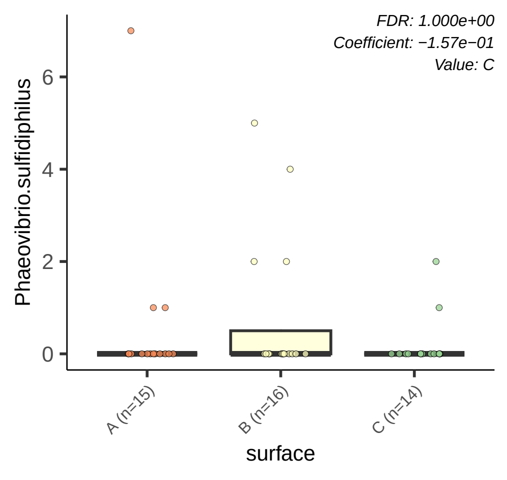

FDR: 1.000e+00
Coefficient: −1.57e−01
Value: C
Phaeovibrio.sulfidiphilus
0
2
4
6
A (n=15)
B (n=16)
C (n=14)
surface

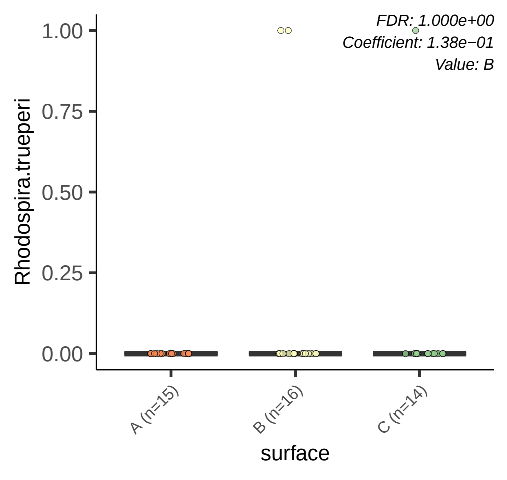

FDR: 1.000e+00
Coefficient: 1.38e−01
Value: B
Rhodospira.trueperi
1.00
0.75
0.50
0.25
0.00
A (n=15)
B (n=16)
C (n=14)
surface

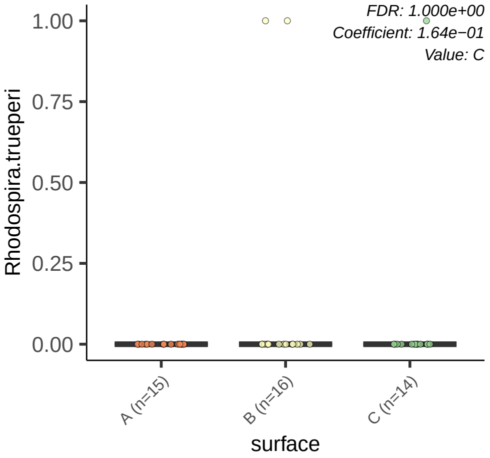

FDR: 1.000e+00
Coefficient: 1.64e−01
Value: C
Rhodospira.trueperi
1.00
0.75
0.50
0.25
0.00
A (n=15)
B (n=16)
C (n=14)
surface

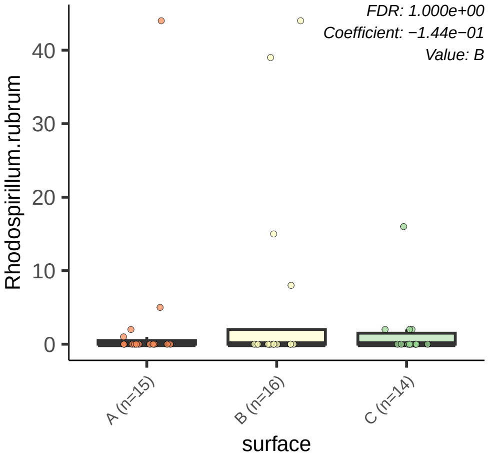

FDR: 1.000e+00
Coefficient: −1.44e−01
Value: B
Rhodospirillum.rubrum
40
30
20
10
0
A (n=15)
B (n=16)
C (n=14)
surface

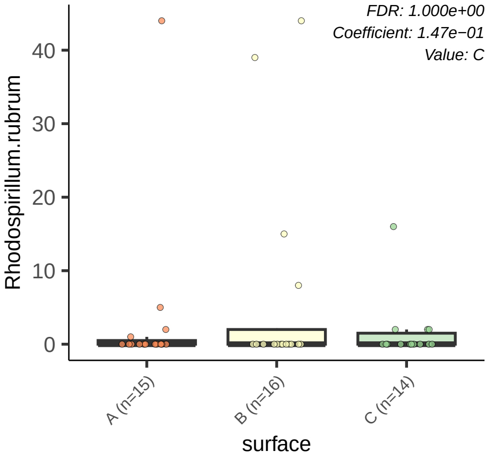
FDR: 1.000e+00
Coefficient: 1.47e−01
Value: C
Rhodospirillum.rubrum
40
30
20
10
0
A (n=15)
B (n=16)
C (n=14)
surface

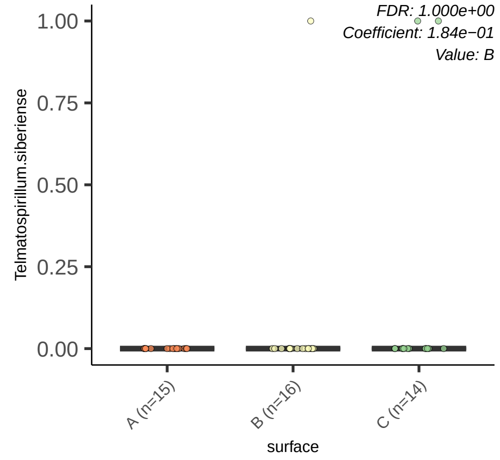
FDR: 1.000e+00
Coefficient: 1.84e−01
Value: B
Telmatospirillum.siberiense
1.00
0.75
0.50
0.25
0.00
A (n=15)
B (n=16)
C (n=14)
surface

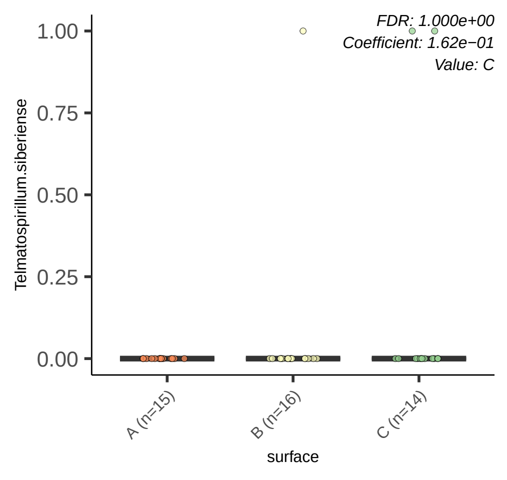

FDR: 1.000e+00
Coefficient: 1.62e−01
Value: C
1.00
0.75
0.50
0.25
0.00
Telmatospirillum.siberiense
A (n=15)
B (n=16)
C (n=14)
surface

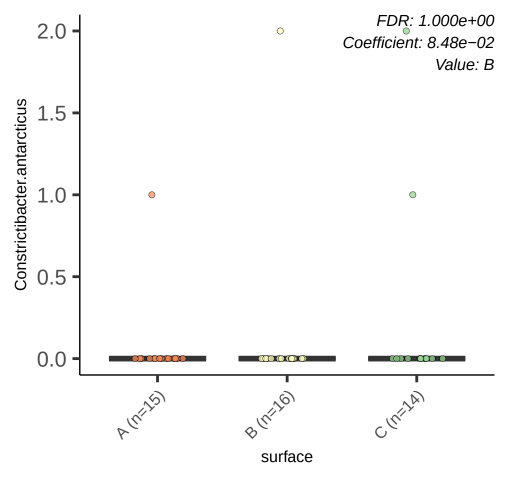
FDR: 1.000e+00
Coefficient: 8.48e−02
Value: B
Constrictibacter.antarcticus
2.0
1.5
1.0
0.5
0.0
A (n=15)
B (n=16)
C (n=14)
surface

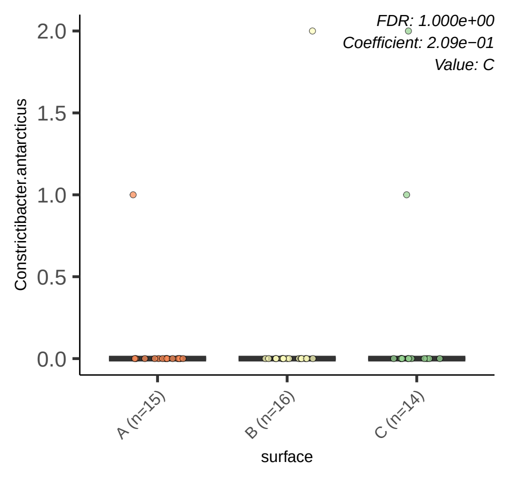

FDR: 1.000e+00
Coefficient: 2.09e−01
Value: C
Constrictibacter.antarcticus
2.0
1.5
1.0
0.5
0.0
A (n=15)
B (n=16)
C (n=14)
surface

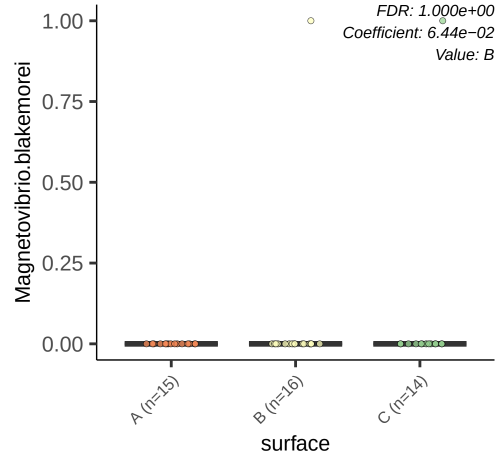
FDR: 1.000e+00
Coefficient: 6.44e−02
Value: B
Magnetovibrio.blakemorei
1.00
0.75
0.50
0.25
0.00
A (n=15)
B (n=16)
C (n=14)
surface

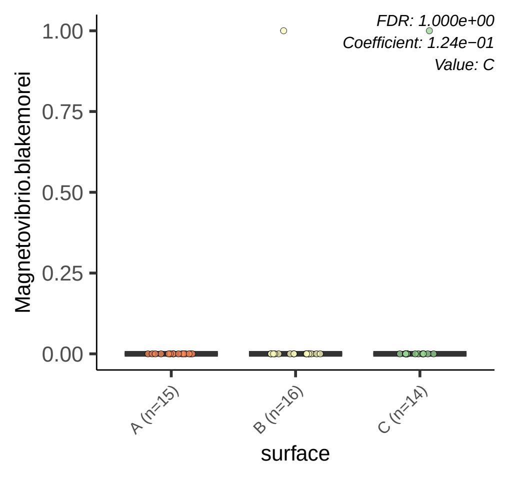
FDR: 1.000e+00
Coefficient: 1.24e−01
Value: C
Magnetovibrio.blakemorei
1.00
0.75
0.50
0.25
0.00
A (n=15)
B (n=16)
C (n=14)
surface

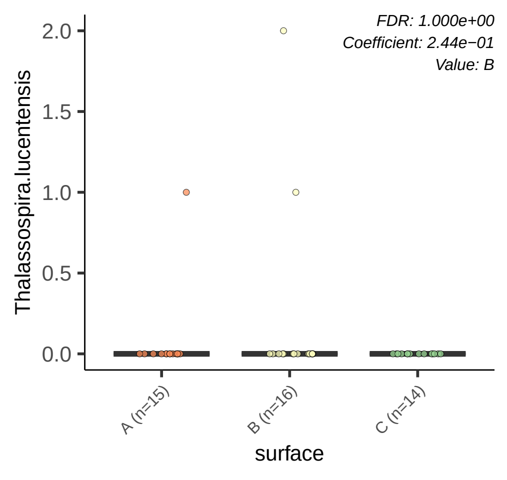
FDR: 1.000e+00
Coefficient: 2.44e−01
Value: B
Thalassospira.lucentensis
2.0
1.5
1.0
0.5
0.0
A (n=15)
B (n=16)
C (n=14)
surface

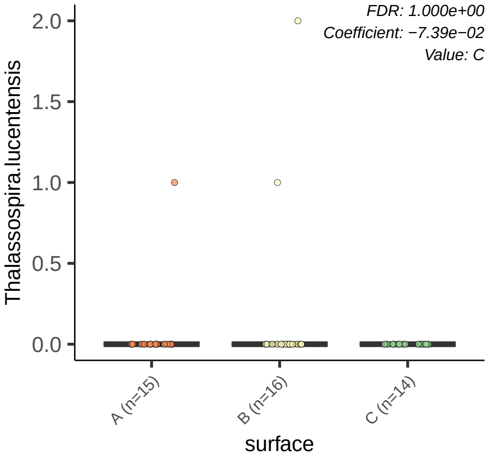
FDR: 1.000e+00
Coefficient: −7.39e−02
Value: C
Thalassospira.lucentensis
2.0
1.5
1.0
0.5
0.0
A (n=15)
B (n=16)
C (n=14)
surface

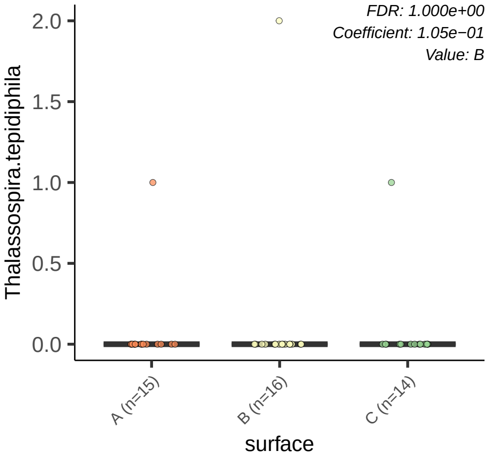
FDR: 1.000e+00
Coefficient: 1.05e−01
Value: B
Thalassospira.tepidiphila
2.0
1.5
1.0
0.5
0.0
A (n=15)
B (n=16)
C (n=14)
surface

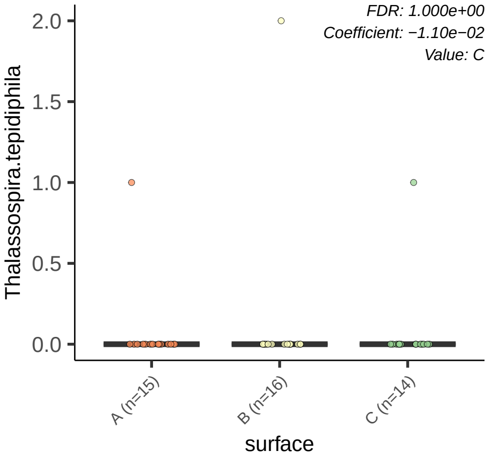
FDR: 1.000e+00
Coefficient: −1.10e−02
Value: C
Thalassospira.tepidiphila
2.0
1.5
1.0
0.5
0.0
A (n=15)
B (n=16)
C (n=14)
surface

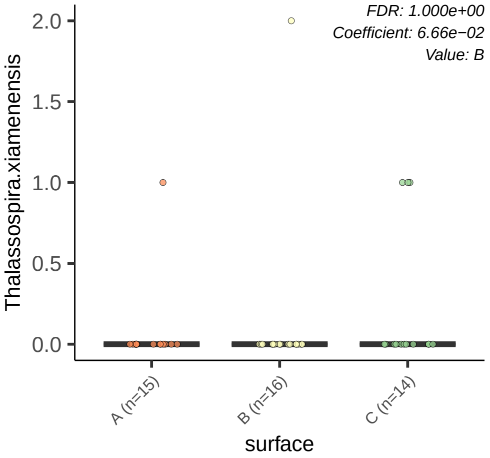
FDR: 1.000e+00
Coefficient: 6.66e−02
Value: B
Thalassospira.xiamenensis
2.0
1.5
1.0
0.5
0.0
A (n=15)
B (n=16)
C (n=14)
surface

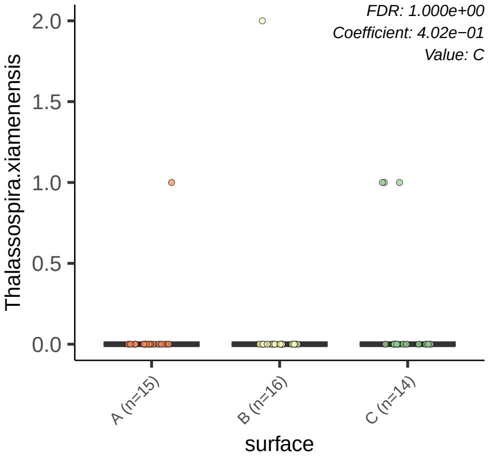

FDR: 1.000e+00
Coefficient: 4.02e−01
Value: C
Thalassospira.xiamenensis
2.0
1.5
1.0
0.5
0.0
A (n=15)
B (n=16)
C (n=14)
surface

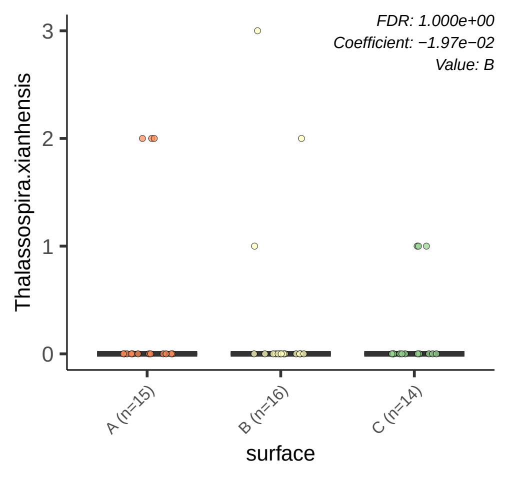

FDR: 1.000e+00
Coefficient: −1.97e−02
Value: B
Thalassospira.xianhensis
3
2
1
0
A (n=15)
B (n=16)
C (n=14)
surface

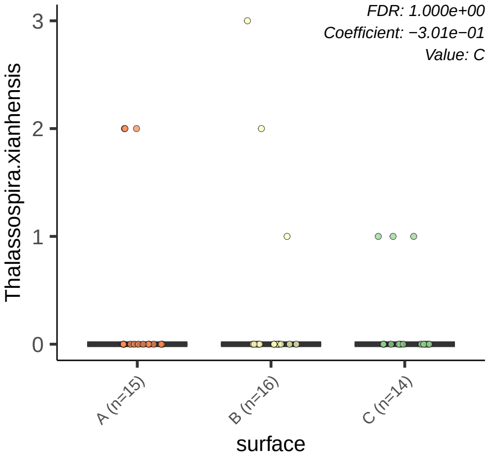
FDR: 1.000e+00
Coefficient: −3.01e−01
Value: C
Thalassospira.xianhensis
3
2
1
0
A (n=15)
B (n=16)
C (n=14)
surface

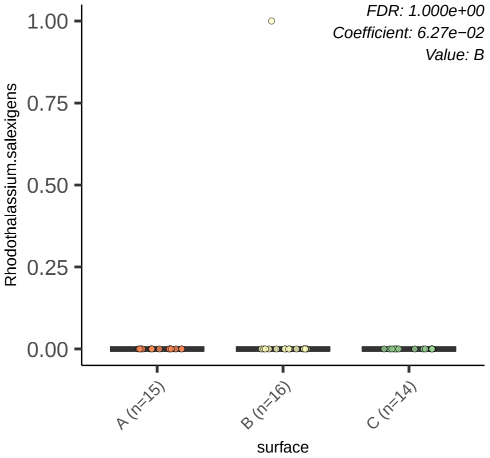
FDR: 1.000e+00
Coefficient: 6.27e−02
Value: B
Rhodothalassium.salexigens
1.00
0.75
0.50
0.25
0.00
A (n=15)
B (n=16)
C (n=14)
surface

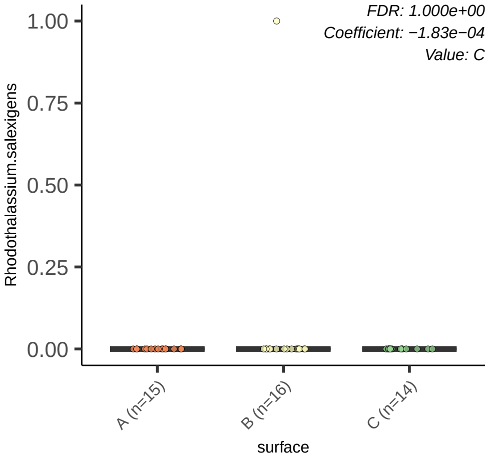

FDR: 1.000e+00
Coefficient: −1.83e−04
Value: C
Rhodothalassium.salexigens
1.00
0.75
0.50
0.25
0.00
A (n=15)
B (n=16)
C (n=14)
surface

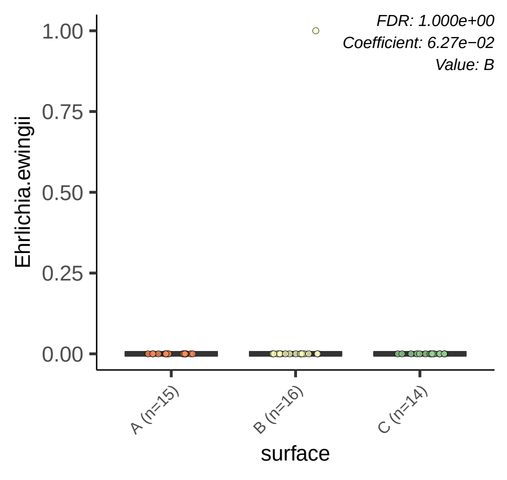
FDR: 1.000e+00
Coefficient: 6.27e−02
Value: B
Ehrlichia.ewingii
1.00
0.75
0.50
0.25
0.00
A (n=15)
B (n=16)
C (n=14)
surface

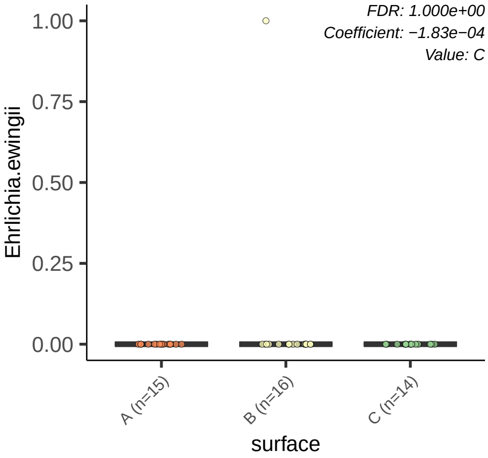
FDR: 1.000e+00
Coefficient: −1.83e−04
Value: C
Ehrlichia.ewingii
1.00
0.75
0.50
0.25
0.00
A (n=15)
B (n=16)
C (n=14)
surface

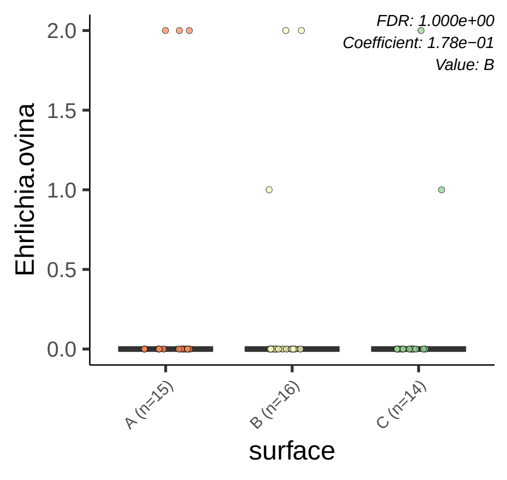
FDR: 1.000e+00
Coefficient: 1.78e−01
Value: B
Ehrlichia.ovina
2.0
1.5
1.0
0.5
0.0
A (n=15)
B (n=16)
C (n=14)
surface

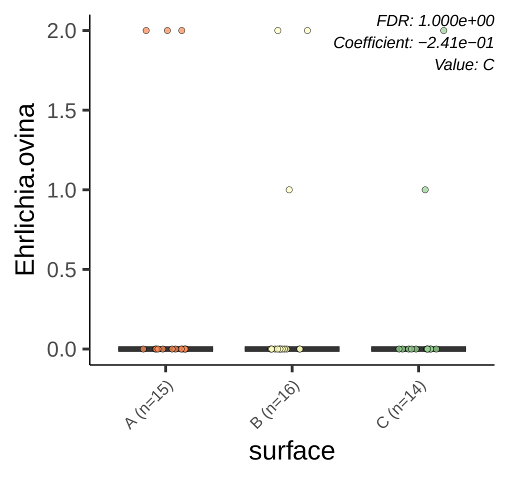

FDR: 1.000e+00
Coefficient: −2.41e−01
Value: C
Ehrlichia.ovina
2.0
1.5
1.0
0.5
0.0
A (n=15)
B (n=16)
C (n=14)
surface

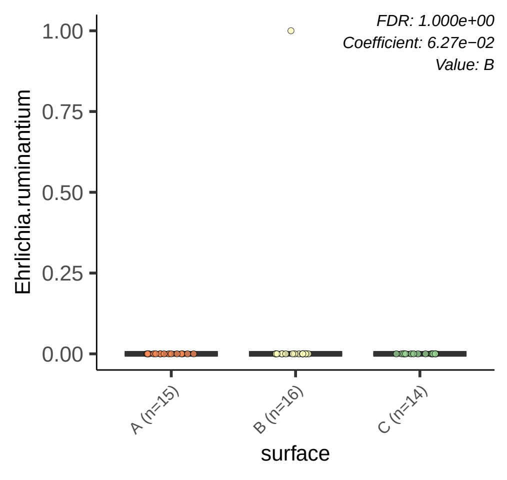
FDR: 1.000e+00
Coefficient: 6.27e−02
Value: B
1.00
0.75
0.50
0.25
0.00
Ehrlichia.ruminantium
A (n=15)
B (n=16)
C (n=14)
surface

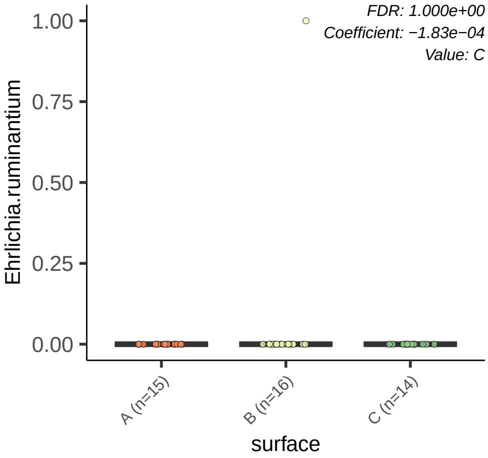

FDR: 1.000e+00
Coefficient: −1.83e−04
Value: C
1.00
0.75
0.50
0.25
0.00
Ehrlichia.ruminantium
A (n=15)
B (n=16)
C (n=14)
surface

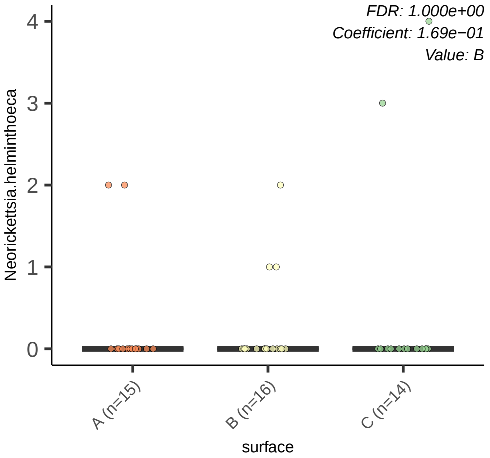
FDR: 1.000e+00
Coefficient: 1.69e−01
Value: B
Neorickettsia.helminthoeca
0
1
2
3
4
A (n=15)
B (n=16)
C (n=14)
surface

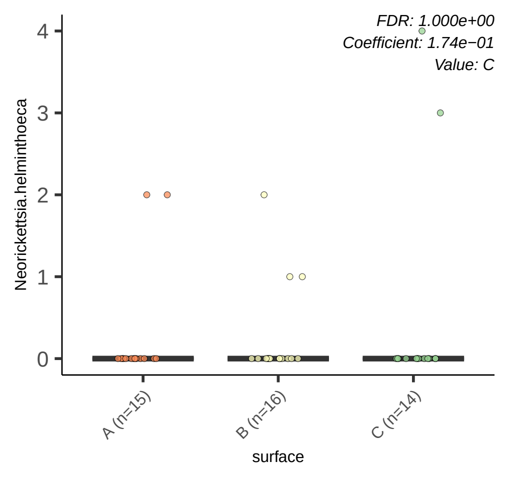

FDR: 1.000e+00
Coefficient: 1.74e−01
Value: C
Neorickettsia.helminthoeca
4
3
2
1
0
A (n=15)
B (n=16)
C (n=14)
surface

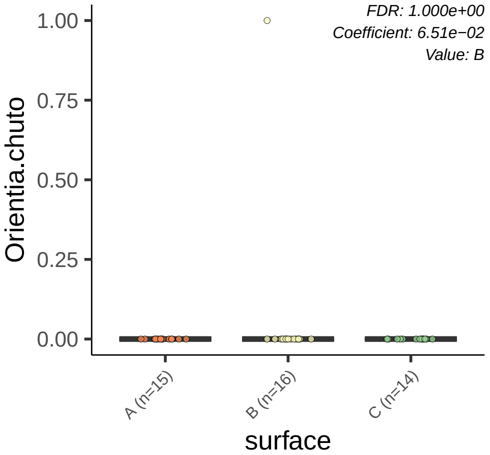

FDR: 1.000e+00
Coefficient: 6.51e−02
Value: B
1.00
0.75
0.50
0.25
0.00
Orientia.chuto
A (n=15)
B (n=16)
C (n=14)
surface

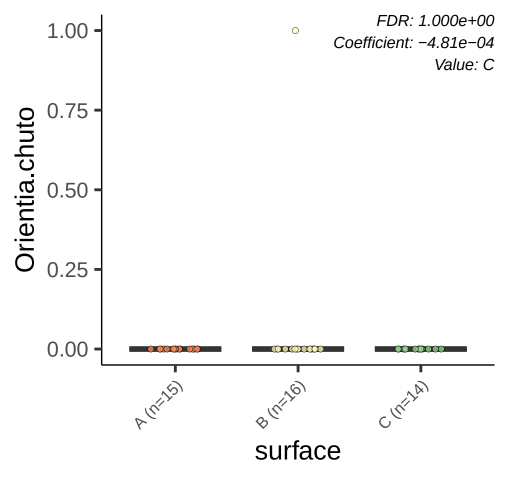
FDR: 1.000e+00
Coefficient: −4.81e−04
Value: C
Orientia.chuto
1.00
0.75
0.50
0.25
0.00
A (n=15)
B (n=16)
C (n=14)
surface

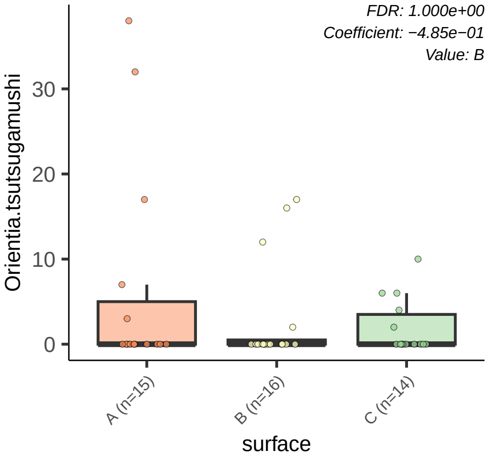

FDR: 1.000e+00
Coefficient: −4.85e−01
Value: B
Orientia.tsutsugamushi
30
20
10
0
A (n=15)
B (n=16)
C (n=14)
surface

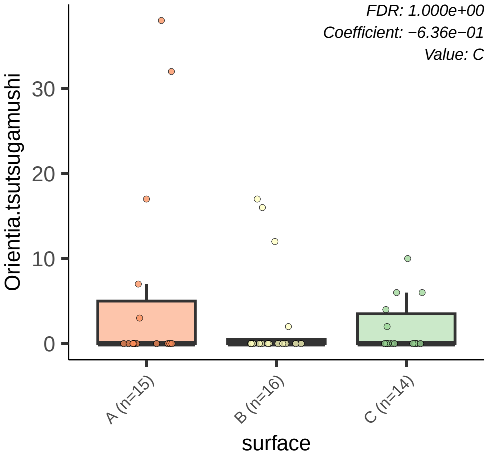
FDR: 1.000e+00
Coefficient: −6.36e−01
Value: C
Orientia.tsutsugamushi
30
20
10
0
A (n=15)
B (n=16)
C (n=14)
surface

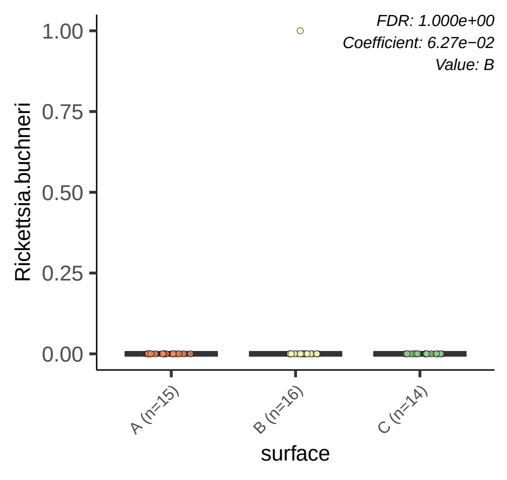

FDR: 1.000e+00
Coefficient: 6.27e−02
Value: B
Rickettsia.buchneri
1.00
0.75
0.50
0.25
0.00
A (n=15)
B (n=16)
C (n=14)
surface

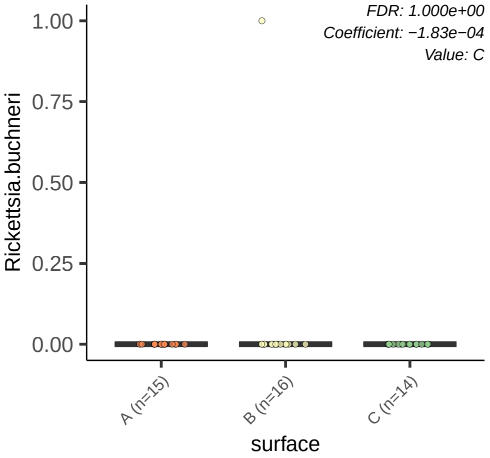
FDR: 1.000e+00
Coefficient: −1.83e−04
Value: C
Rickettsia.buchneri
1.00
0.75
0.50
0.25
0.00
A (n=15)
B (n=16)
C (n=14)
surface

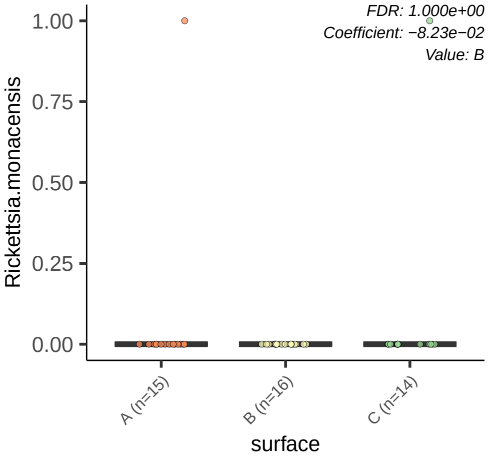
FDR: 1.000e+00
Coefficient: −8.23e−02
Value: B
Rickettsia.monacensis
1.00
0.75
0.50
0.25
0.00
A (n=15)
B (n=16)
C (n=14)
surface

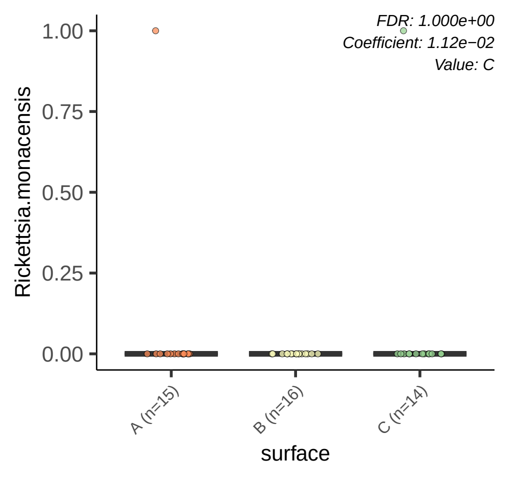
FDR: 1.000e+00
Coefficient: 1.12e−02
Value: C
Rickettsia.monacensis
1.00
0.75
0.50
0.25
0.00
A (n=15)
B (n=16)
C (n=14)
surface

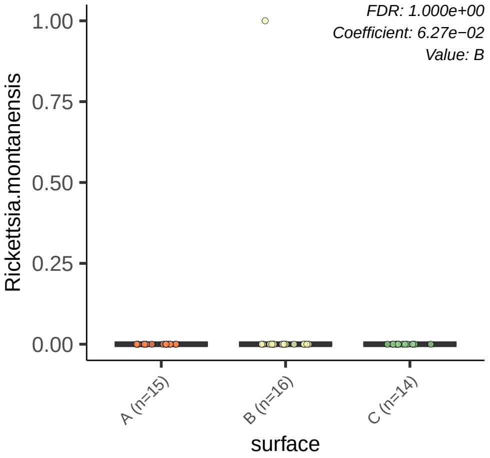
FDR: 1.000e+00
Coefficient: 6.27e−02
Value: B
Rickettsia.montanensis
1.00
0.75
0.50
0.25
0.00
A (n=15)
B (n=16)
C (n=14)
surface

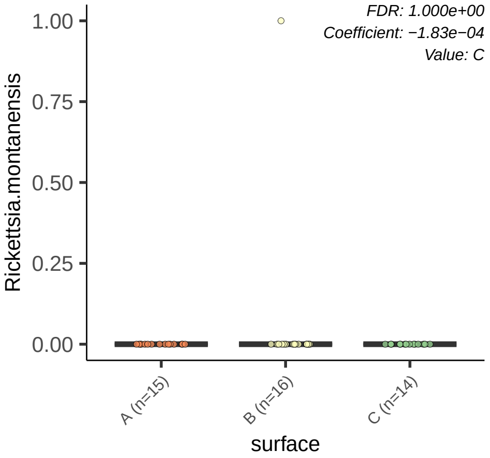
FDR: 1.000e+00
Coefficient: −1.83e−04
Value: C
Rickettsia.montanensis
1.00
0.75
0.50
0.25
0.00
A (n=15)
B (n=16)
C (n=14)
surface

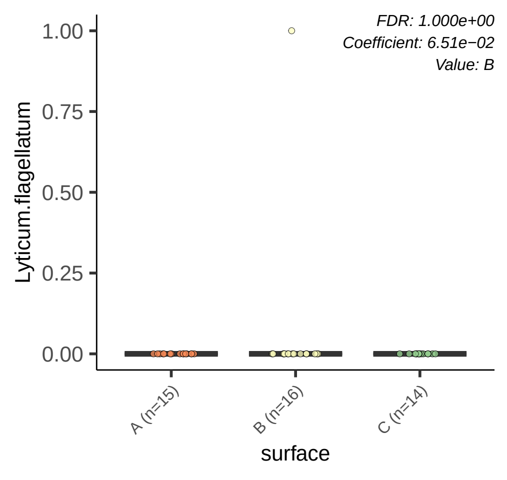
FDR: 1.000e+00
Coefficient: 6.51e−02
Value: B
Lyticum.flagellatum
1.00
0.75
0.50
0.25
0.00
A (n=15)
B (n=16)
C (n=14)
surface

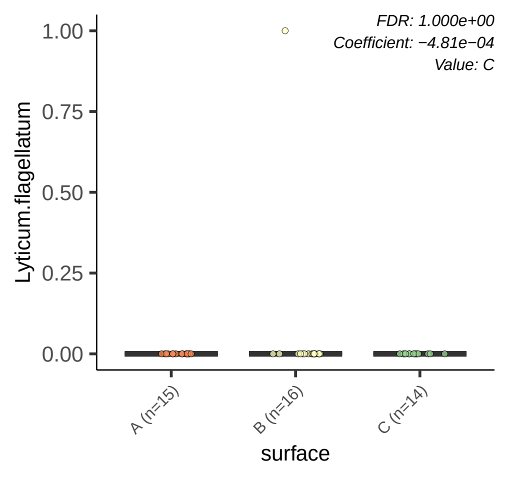
FDR: 1.000e+00
Coefficient: −4.81e−04
Value: C
Lyticum.flagellatum
1.00
0.75
0.50
0.25
0.00
A (n=15)
B (n=16)
C (n=14)
surface

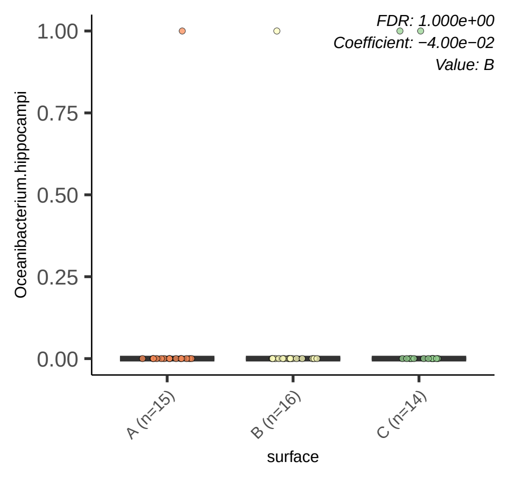

FDR: 1.000e+00
Coefficient: −4.00e−02
Value: B
Oceanibacterium.hippocampi
1.00
0.75
0.50
0.25
0.00
A (n=15)
B (n=16)
C (n=14)
surface

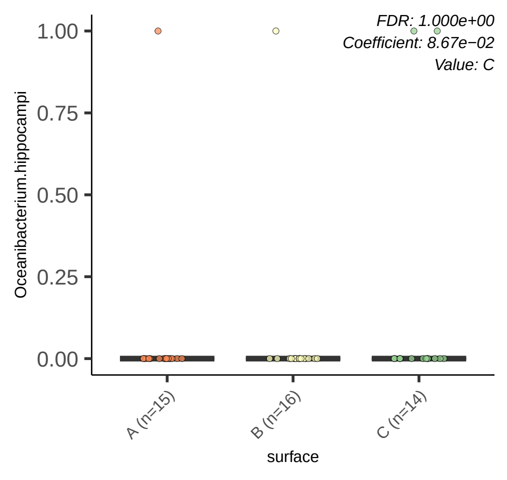

FDR: 1.000e+00
Coefficient: 8.67e−02
Value: C
Oceanibacterium.hippocampi
1.00
0.75
0.50
0.25
0.00
A (n=15)
B (n=16)
C (n=14)
surface

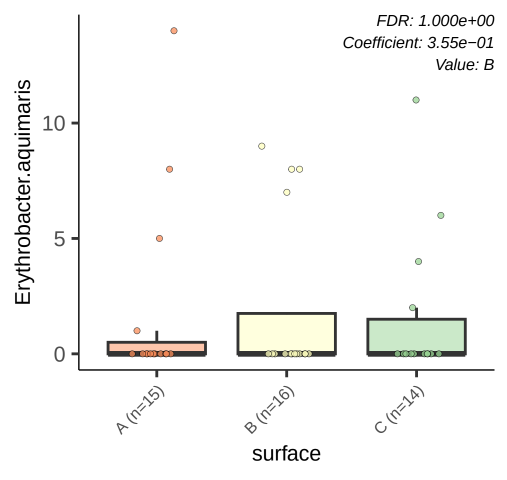

FDR: 1.000e+00
Coefficient: 3.55e−01
Value: B
Erythrobacter.aquimaris
0
5
10
A (n=15)
B (n=16)
C (n=14)
surface

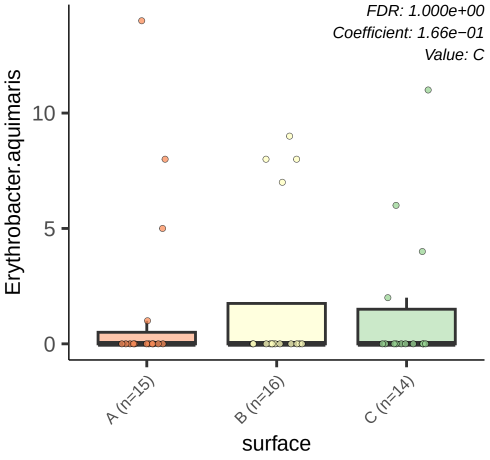
FDR: 1.000e+00
Coefficient: 1.66e−01
Value: C
Erythrobacter.aquimaris
0
5
10
A (n=15)
B (n=16)
C (n=14)
surface

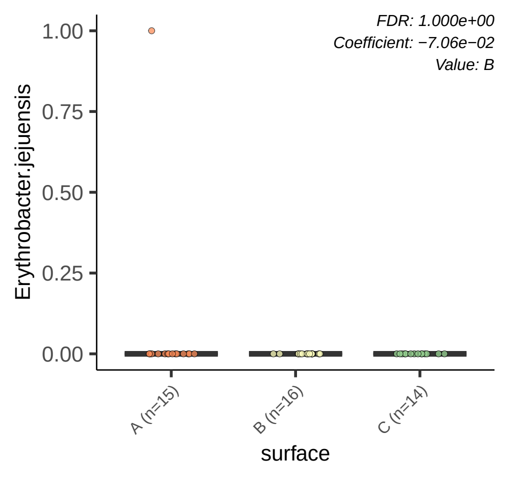
FDR: 1.000e+00
Coefficient: −7.06e−02
Value: B
Erythrobacter.jejuensis
1.00
0.75
0.50
0.25
0.00
A (n=15)
B (n=16)
C (n=14)
surface

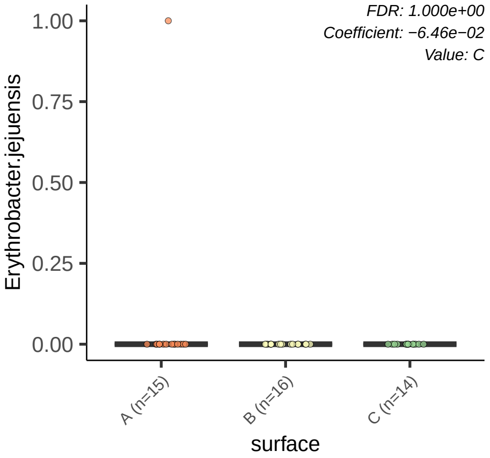

FDR: 1.000e+00
Coefficient: −6.46e−02
Value: C
Erythrobacter.jejuensis
1.00
0.75
0.50
0.25
0.00
A (n=15)
B (n=16)
C (n=14)
surface

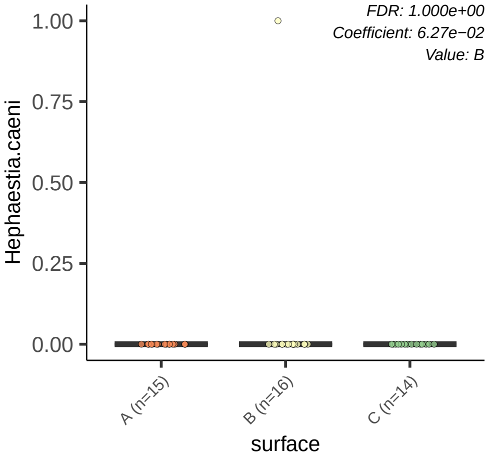
FDR: 1.000e+00
Coefficient: 6.27e−02
Value: B
Hephaestia.caeni
1.00
0.75
0.50
0.25
0.00
A (n=15)
B (n=16)
C (n=14)
surface

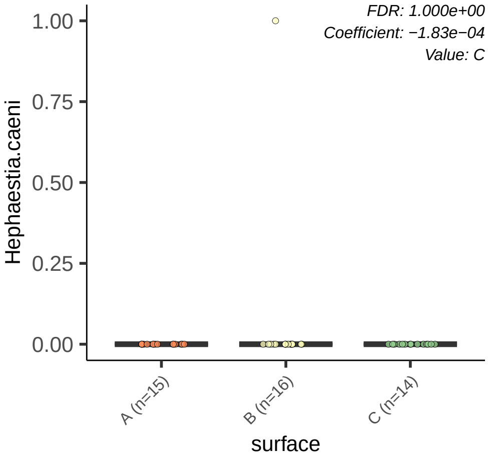
FDR: 1.000e+00
Coefficient: −1.83e−04
Value: C
Hephaestia.caeni
1.00
0.75
0.50
0.25
0.00
A (n=15)
B (n=16)
C (n=14)
surface

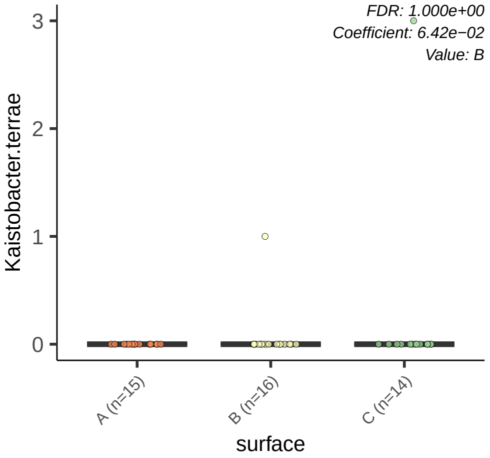

FDR: 1.000e+00
Coefficient: 6.42e−02
Value: B
Kaistobacter.terrae
3
2
1
0
A (n=15)
B (n=16)
C (n=14)
surface

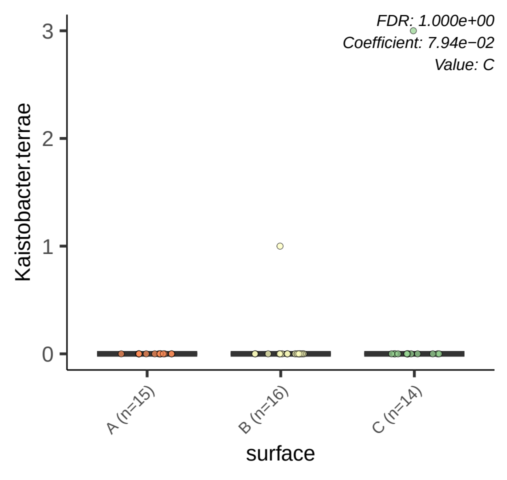
FDR: 1.000e+00
Coefficient: 7.94e−02
Value: C
Kaistobacter.terrae
0
1
2
3
A (n=15)
B (n=16)
C (n=14)
surface

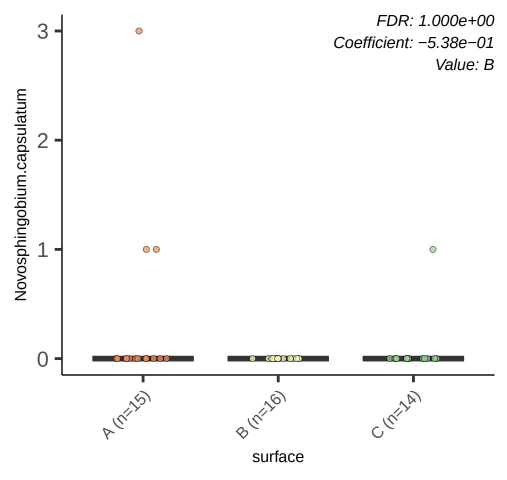
FDR: 1.000e+00
Coefficient: −5.38e−01
Value: B
Novosphingobium.capsulatum
3
2
1
0
A (n=15)
B (n=16)
C (n=14)
surface

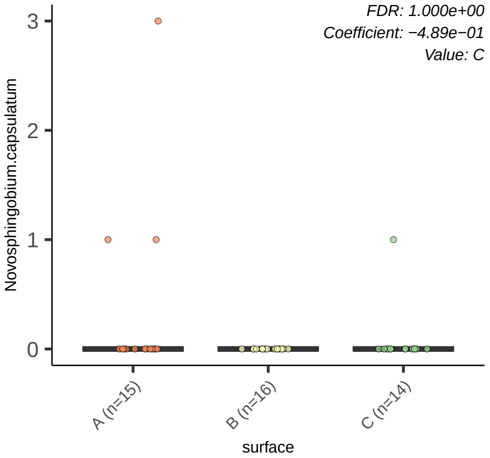
FDR: 1.000e+00
Coefficient: −4.89e−01
Value: C
Novosphingobium.capsulatum
3
2
1
0
A (n=15)
B (n=16)
C (n=14)
surface

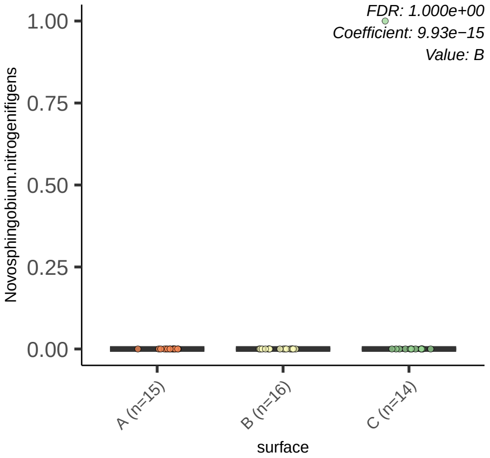
FDR: 1.000e+00
Coefficient: 9.93e−15
Value: B
Novosphingobium.nitrogenifigens
1.00
0.75
0.50
0.25
0.00
A (n=15)
B (n=16)
C (n=14)
surface

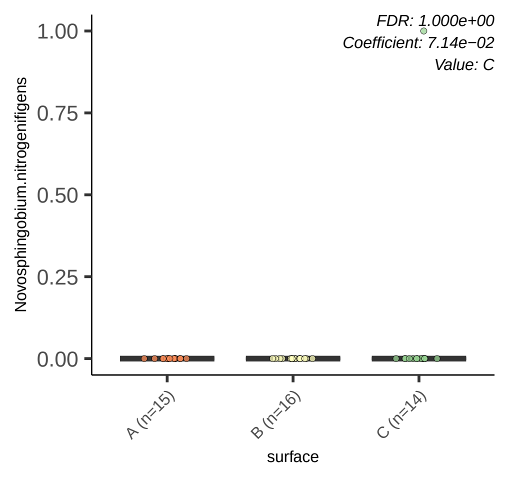
FDR: 1.000e+00
Coefficient: 7.14e−02
Value: C
Novosphingobium.nitrogenifigens
1.00
0.75
0.50
0.25
0.00
A (n=15)
B (n=16)
C (n=14)
surface

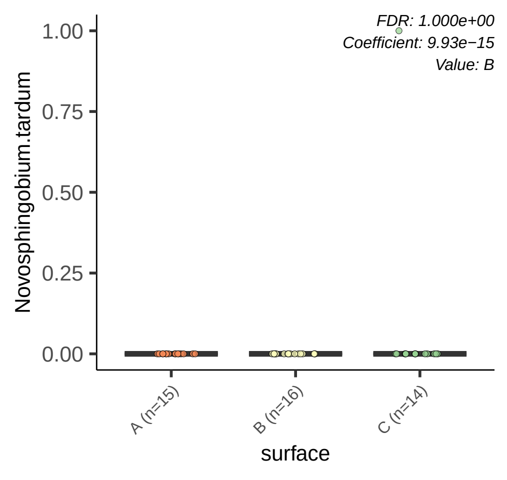
FDR: 1.000e+00
Coefficient: 9.93e−15
Value: B
Novosphingobium.tardum
1.00
0.75
0.50
0.25
0.00
A (n=15)
B (n=16)
C (n=14)
surface

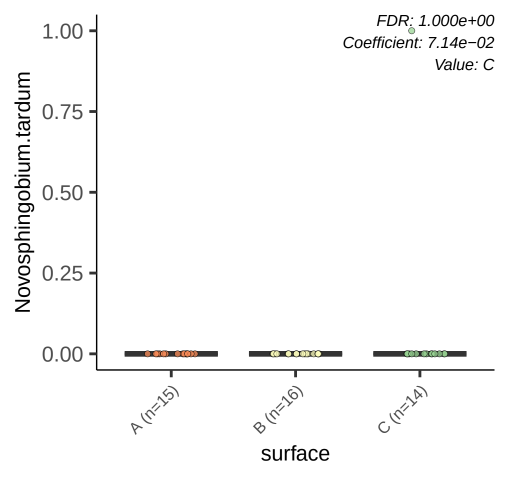

FDR: 1.000e+00
Coefficient: 7.14e−02
Value: C
Novosphingobium.tardum
1.00
0.75
0.50
0.25
0.00
A (n=15)
B (n=16)
C (n=14)
surface

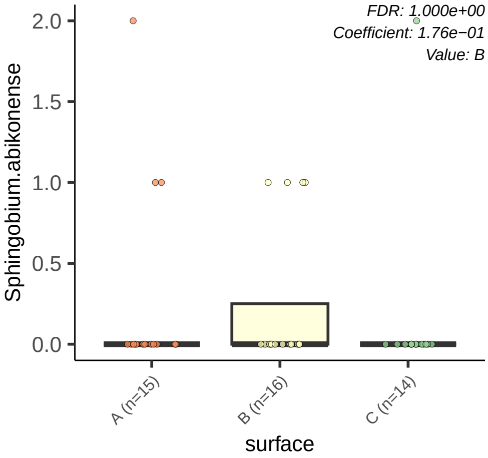

FDR: 1.000e+00
Coefficient: 1.76e−01
Value: B
Sphingobium.abikonense
2.0
1.5
1.0
0.5
0.0
A (n=15)
B (n=16)
C (n=14)
surface

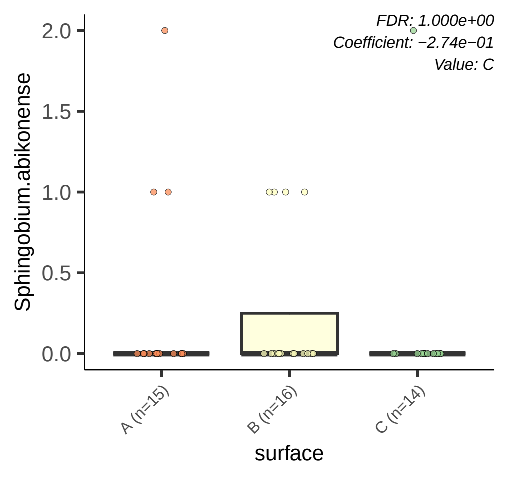

FDR: 1.000e+00
Coefficient: −2.74e−01
Value: C
Sphingobium.abikonense
2.0
1.5
1.0
0.5
0.0
A (n=15)
B (n=16)
C (n=14)
surface

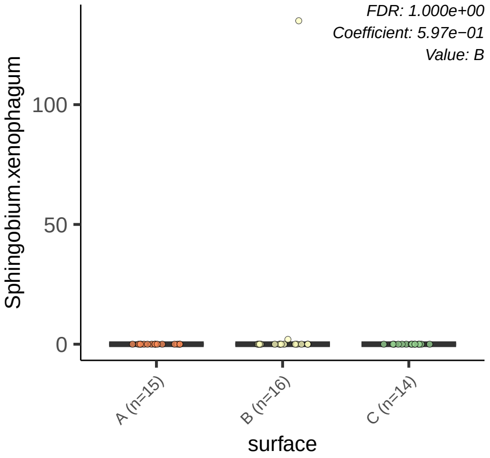
FDR: 1.000e+00
Coefficient: 5.97e−01
Value: B
Sphingobium.xenophagum
100
50
0
A (n=15)
B (n=16)
C (n=14)
surface

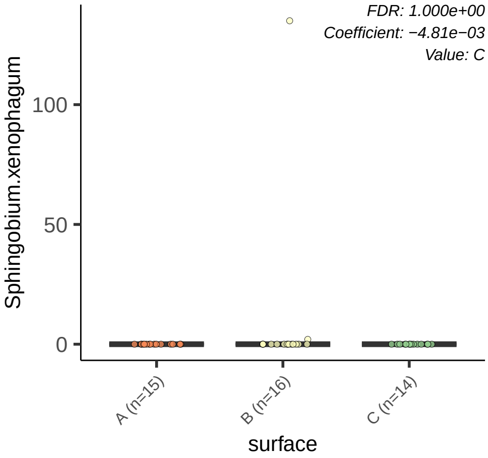
FDR: 1.000e+00
Coefficient: −4.81e−03
Value: C
Sphingobium.xenophagum
100
50
0
A (n=15)
B (n=16)
C (n=14)
surface

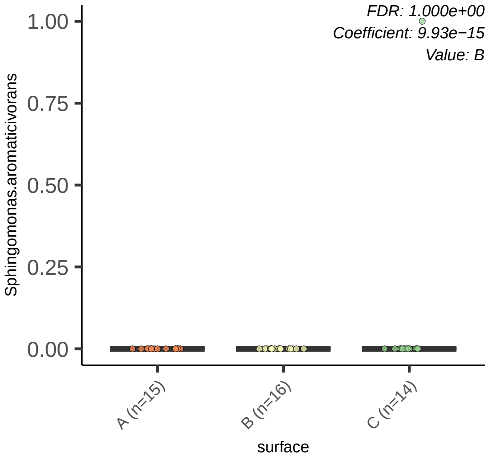
FDR: 1.000e+00
Coefficient: 9.93e−15
Value: B
1.00
0.75
0.50
0.25
0.00
Sphingomonas.aromaticivorans
A (n=15)
B (n=16)
C (n=14)
surface

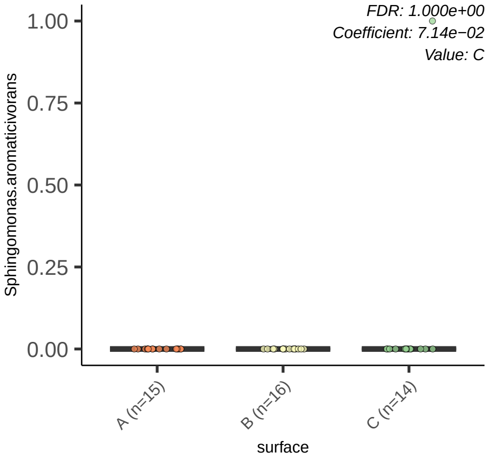
FDR: 1.000e+00
Coefficient: 7.14e−02
Value: C
Sphingomonas.aromaticivorans
1.00
0.75
0.50
0.25
0.00
A (n=15)
B (n=16)
C (n=14)
surface

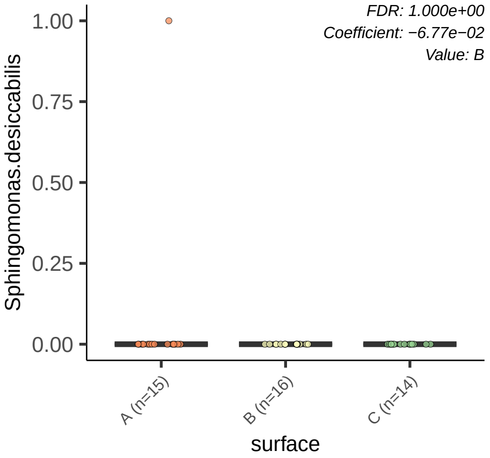

FDR: 1.000e+00
Coefficient: −6.77e−02
Value: B
Sphingomonas.desiccabilis
1.00
0.75
0.50
0.25
0.00
A (n=15)
B (n=16)
C (n=14)
surface

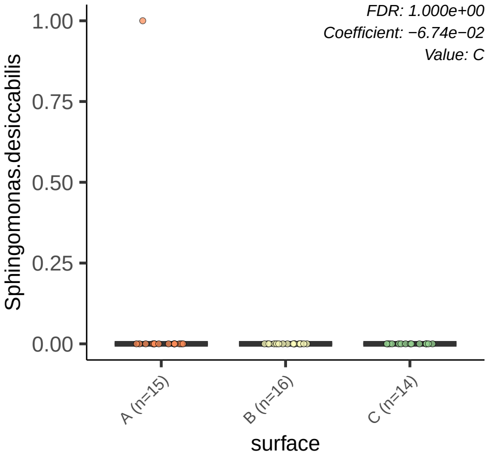
FDR: 1.000e+00
Coefficient: −6.74e−02
Value: C
Sphingomonas.desiccabilis
1.00
0.75
0.50
0.25
0.00
A (n=15)
B (n=16)
C (n=14)
surface

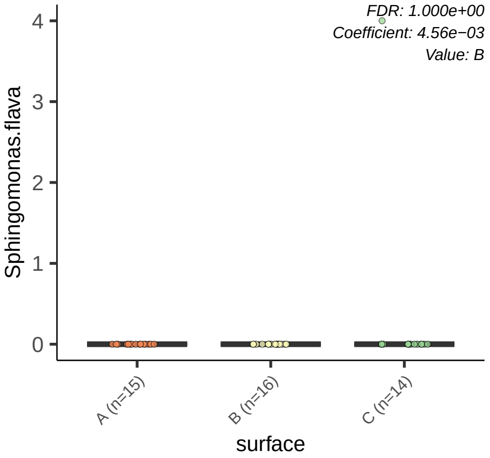

FDR: 1.000e+00
Coefficient: 4.56e−03
Value: B
Sphingomonas.flava
4
3
2
1
0
A (n=15)
B (n=16)
C (n=14)
surface

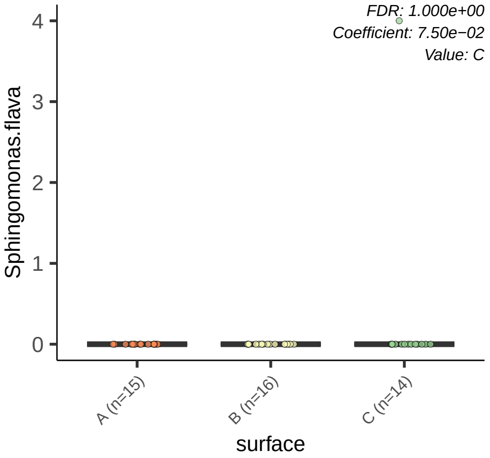

FDR: 1.000e+00
Coefficient: 7.50e−02
Value: C
Sphingomonas.flava
0
1
2
3
4
A (n=15)
B (n=16)
C (n=14)
surface

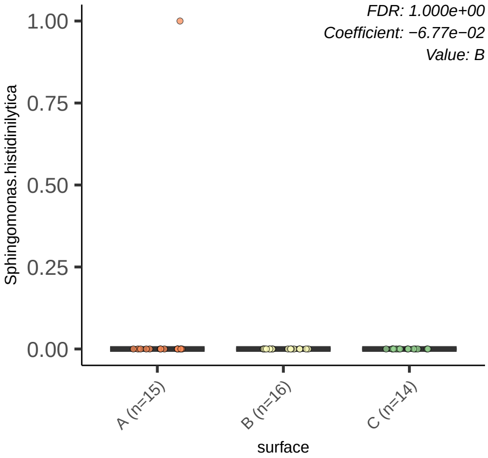

FDR: 1.000e+00
Coefficient: −6.77e−02
Value: B
Sphingomonas.histidinilytica
1.00
0.75
0.50
0.25
0.00
A (n=15)
B (n=16)
C (n=14)
surface

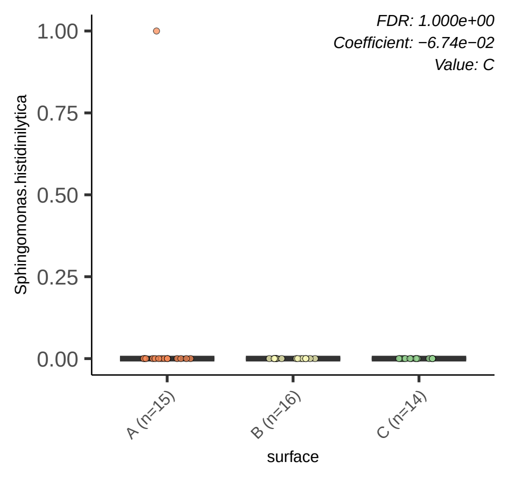

FDR: 1.000e+00
Coefficient: −6.74e−02
Value: C
Sphingomonas.histidinilytica
1.00
0.75
0.50
0.25
0.00
A (n=15)
B (n=16)
C (n=14)
surface

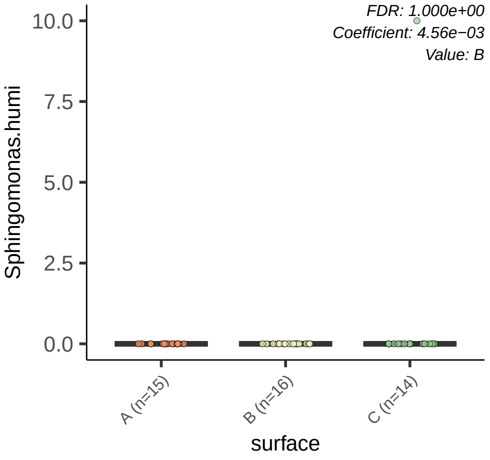

FDR: 1.000e+00
Coefficient: 4.56e−03
Value: B
10.0
7.5
5.0
2.5
0.0
Sphingomonas.humi
A (n=15)
B (n=16)
C (n=14)
surface

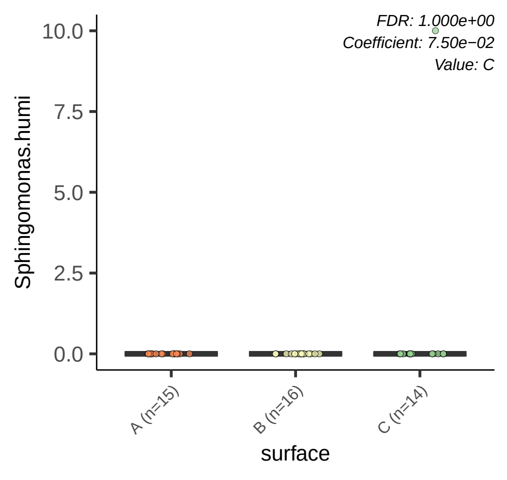

FDR: 1.000e+00
Coefficient: 7.50e−02
Value: C
Sphingomonas.humi
10.0
7.5
5.0
2.5
0.0
A (n=15)
B (n=16)
C (n=14)
surface

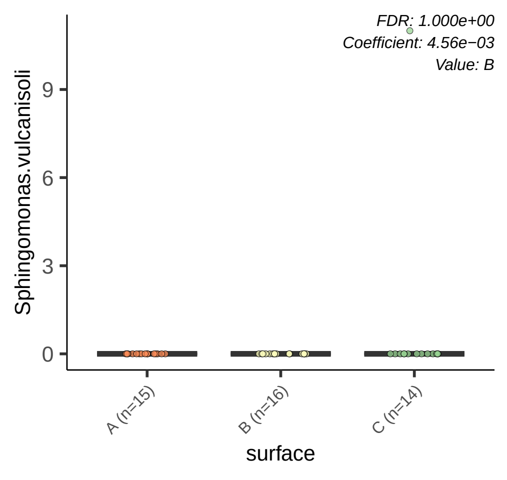
FDR: 1.000e+00
Coefficient: 4.56e−03
Value: B
Sphingomonas.vulcanisoli
0
3
6
9
A (n=15)
B (n=16)
C (n=14)
surface

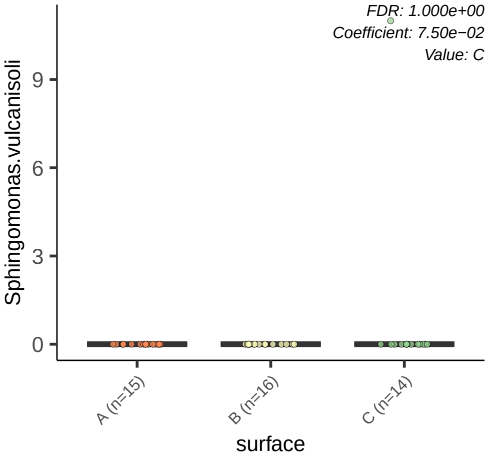
FDR: 1.000e+00
Coefficient: 7.50e−02
Value: C
Sphingomonas.vulcanisoli
9
6
3
0
A (n=15)
B (n=16)
C (n=14)
surface

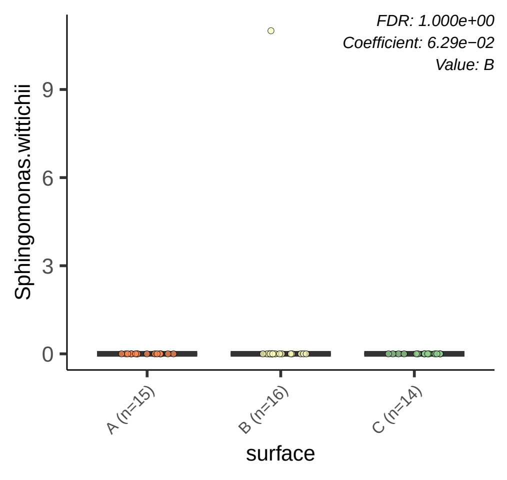
FDR: 1.000e+00
Coefficient: 6.29e−02
Value: B
Sphingomonas.wittichii
0
3
6
9
A (n=15)
B (n=16)
C (n=14)
surface

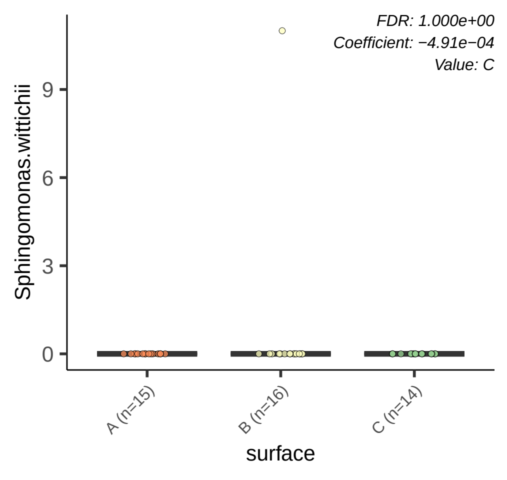
FDR: 1.000e+00
Coefficient: −4.91e−04
Value: C
Sphingomonas.wittichii
0
3
6
9
A (n=15)
B (n=16)
C (n=14)
surface

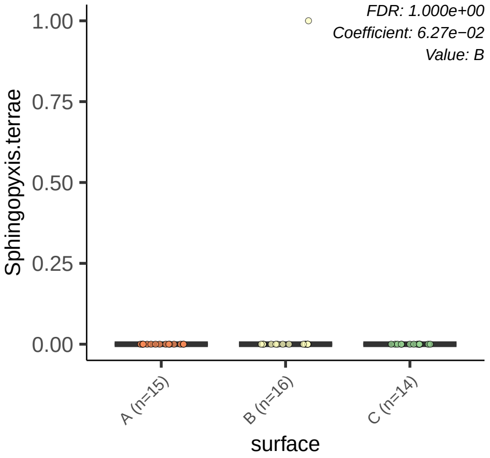

FDR: 1.000e+00
Coefficient: 6.27e−02
Value: B
Sphingopyxis.terrae
1.00
0.75
0.50
0.25
0.00
A (n=15)
B (n=16)
C (n=14)
surface

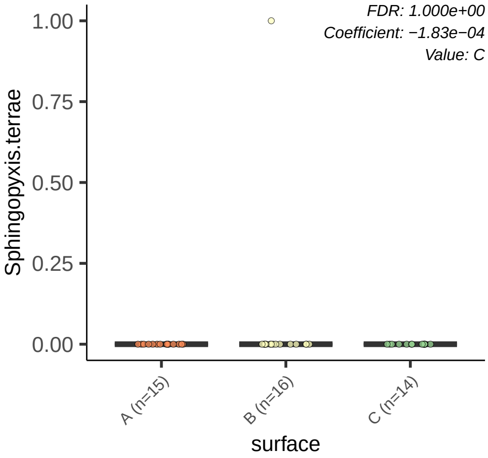

FDR: 1.000e+00
Coefficient: −1.83e−04
Value: C
Sphingopyxis.terrae
1.00
0.75
0.50
0.25
0.00
A (n=15)
B (n=16)
C (n=14)
surface

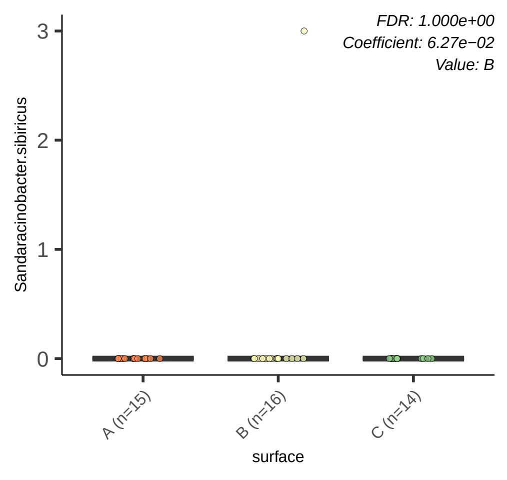

FDR: 1.000e+00
Coefficient: 6.27e−02
Value: B
Sandaracinobacter.sibiricus
3
2
1
0
A (n=15)
B (n=16)
C (n=14)
surface

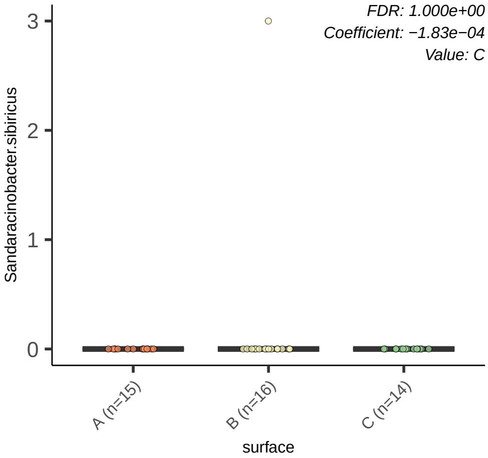

FDR: 1.000e+00
Coefficient: −1.83e−04
Value: C
Sandaracinobacter.sibiricus
3
2
1
0
A (n=15)
B (n=16)
C (n=14)
surface

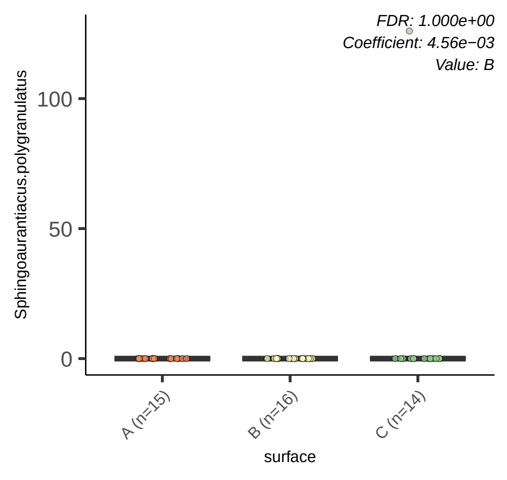

FDR: 1.000e+00
Coefficient: 4.56e−03
Value: B
Sphingoaurantiacus.polygranulatus
100
50
0
A (n=15)
B (n=16)
C (n=14)
surface

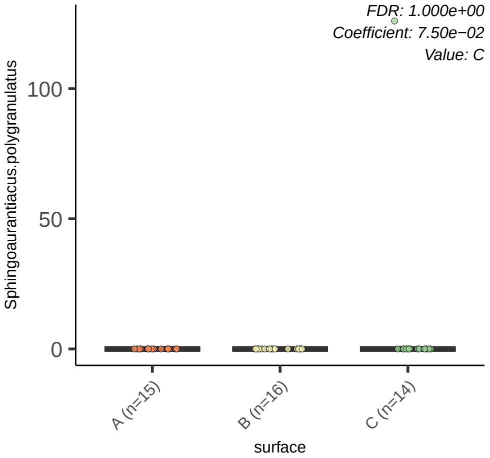

FDR: 1.000e+00
Coefficient: 7.50e−02
Value: C
Sphingoaurantiacus.polygranulatus
100
50
0
A (n=15)
B (n=16)
C (n=14)
surface

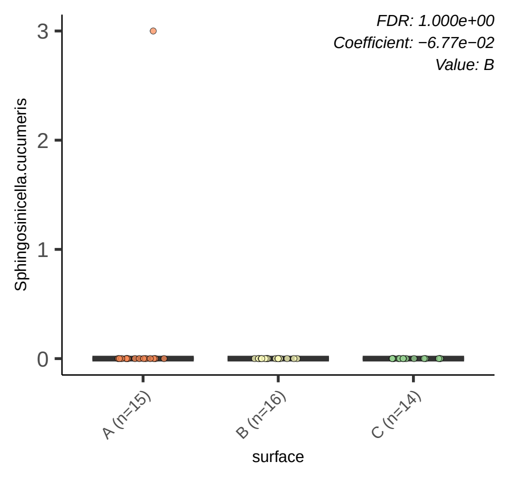
FDR: 1.000e+00
Coefficient: −6.77e−02
Value: B
Sphingosinicella.cucumeris
0
1
2
3
A (n=15)
B (n=16)
C (n=14)
surface

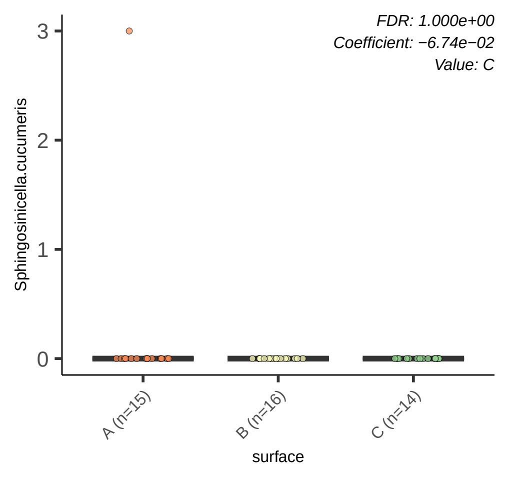
FDR: 1.000e+00
Coefficient: −6.74e−02
Value: C
Sphingosinicella.cucumeris
3
2
1
0
A (n=15)
B (n=16)
C (n=14)
surface

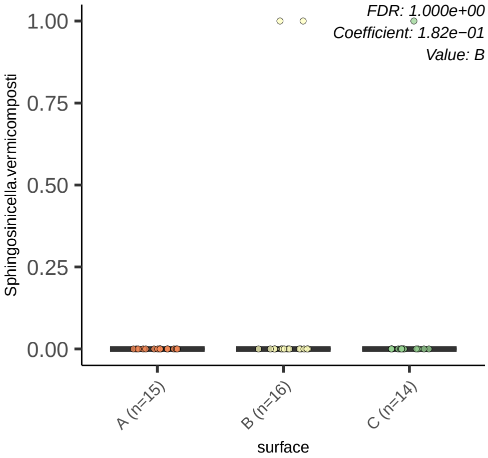
FDR: 1.000e+00
Coefficient: 1.82e−01
Value: B
1.00
0.75
0.50
0.25
0.00
Sphingosinicella.vermicomposti
A (n=15)
B (n=16)
C (n=14)
surface

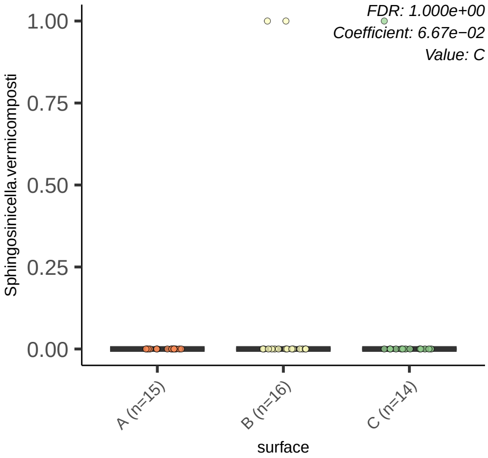

FDR: 1.000e+00
Coefficient: 6.67e−02
Value: C
Sphingosinicella.vermicomposti
1.00
0.75
0.50
0.25
0.00
A (n=15)
B (n=16)
C (n=14)
surface

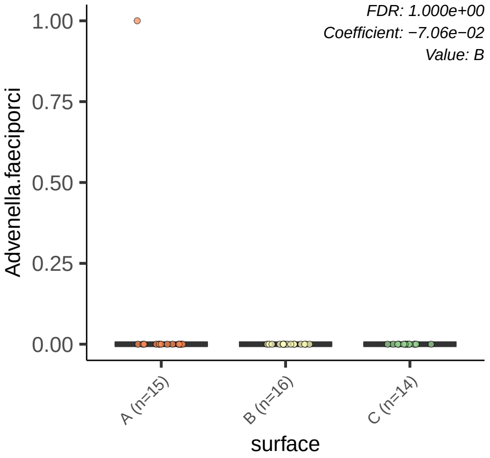

FDR: 1.000e+00
Coefficient: −7.06e−02
Value: B
Advenella.faeciporci
1.00
0.75
0.50
0.25
0.00
A (n=15)
B (n=16)
C (n=14)
surface

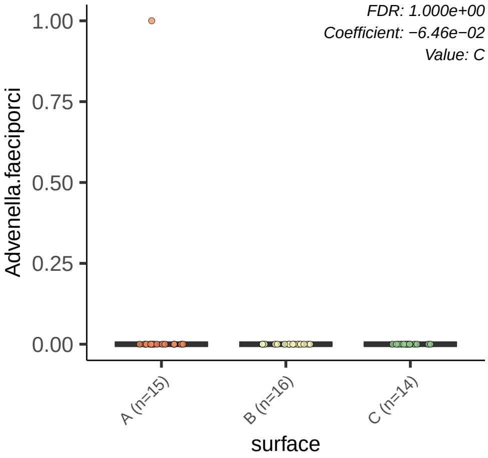

FDR: 1.000e+00
Coefficient: −6.46e−02
Value: C
Advenella.faeciporci
1.00
0.75
0.50
0.25
0.00
A (n=15)
B (n=16)
C (n=14)
surface

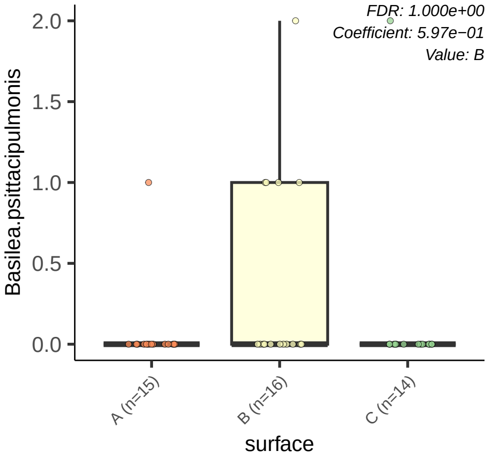
FDR: 1.000e+00
Coefficient: 5.97e−01
Value: B
Basilea.psittacipulmonis
2.0
1.5
1.0
0.5
0.0
A (n=15)
B (n=16)
C (n=14)
surface

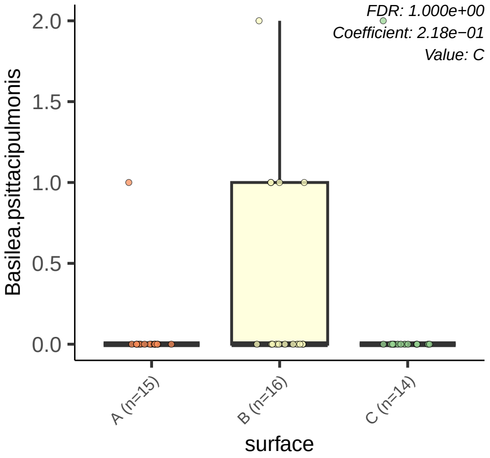
FDR: 1.000e+00
Coefficient: 2.18e−01
Value: C
Basilea.psittacipulmonis
2.0
1.5
1.0
0.5
0.0
A (n=15)
B (n=16)
C (n=14)
surface

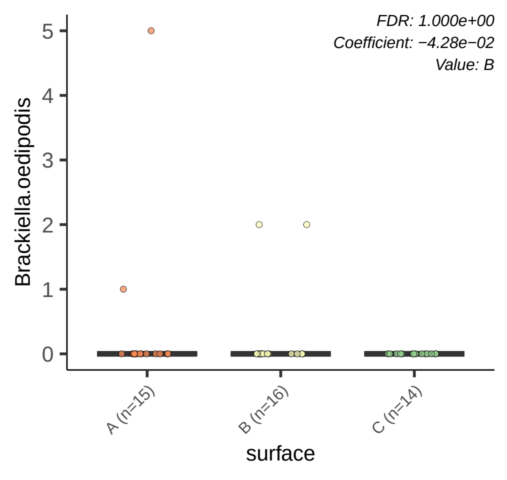
FDR: 1.000e+00
Coefficient: −4.28e−02
Value: B
Brackiella.oedipodis
0
1
2
3
4
5
A (n=15)
B (n=16)
C (n=14)
surface

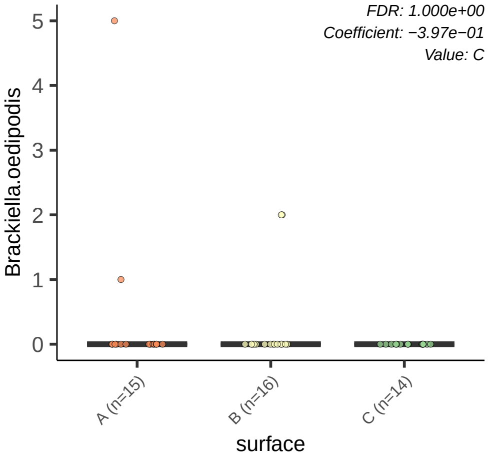
FDR: 1.000e+00
Coefficient: −3.97e−01
Value: C
Brackiella.oedipodis
0
1
2
3
4
5
A (n=15)
B (n=16)
C (n=14)
surface

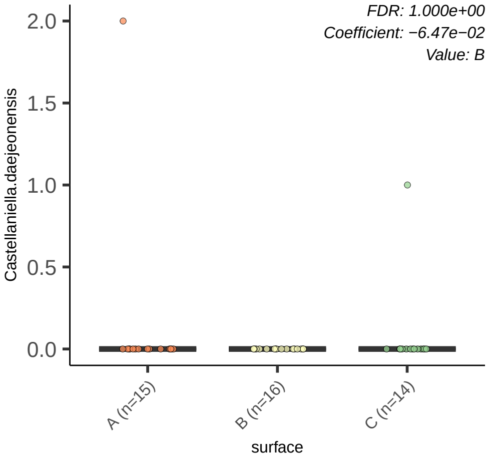

FDR: 1.000e+00
Coefficient: −6.47e−02
Value: B
Castellaniella.daejeonensis
2.0
1.5
1.0
0.5
0.0
A (n=15)
B (n=16)
C (n=14)
surface

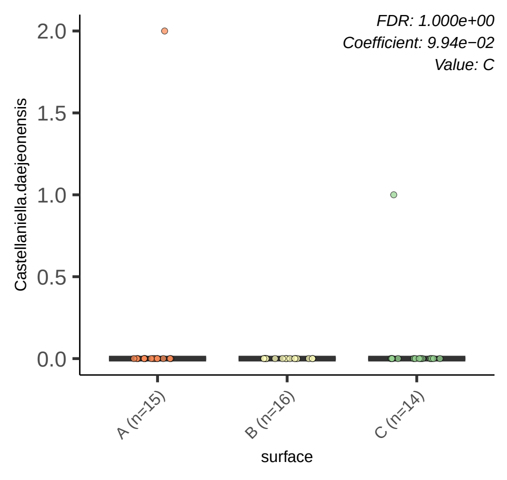
FDR: 1.000e+00
Coefficient: 9.94e−02
Value: C
Castellaniella.daejeonensis
2.0
1.5
1.0
0.5
0.0
A (n=15)
B (n=16)
C (n=14)
surface

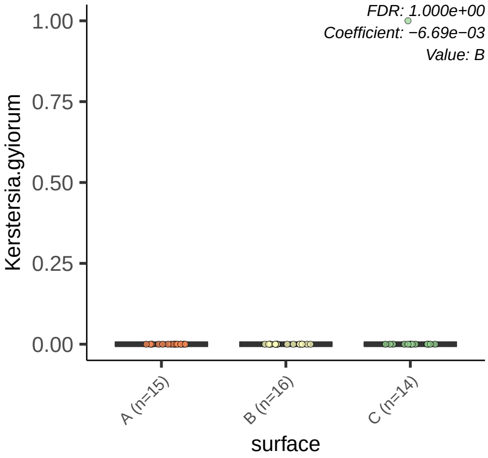
FDR: 1.000e+00
Coefficient: −6.69e−03
Value: B
1.00
0.75
0.50
0.25
0.00
Kerstersia.gyiorum
A (n=15)
B (n=16)
C (n=14)
surface

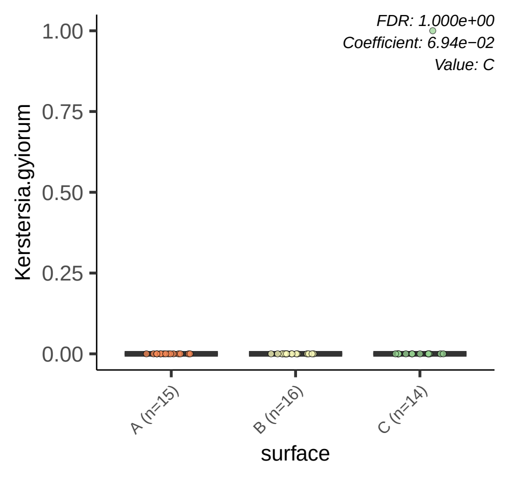
FDR: 1.000e+00
Coefficient: 6.94e−02
Value: C
Kerstersia.gyiorum
1.00
0.75
0.50
0.25
0.00
A (n=15)
B (n=16)
C (n=14)
surface

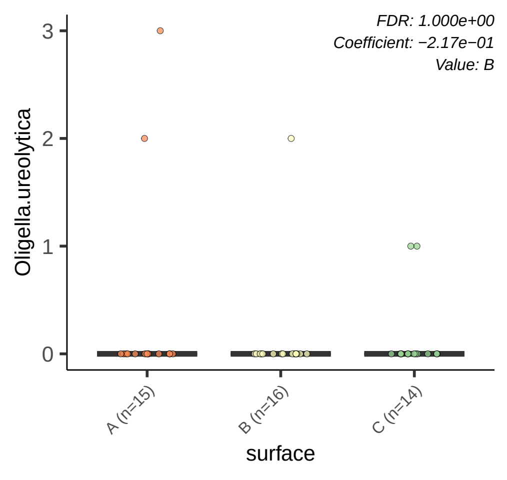

FDR: 1.000e+00
Coefficient: −2.17e−01
Value: B
Oligella.ureolytica
3
2
1
0
A (n=15)
B (n=16)
C (n=14)
surface

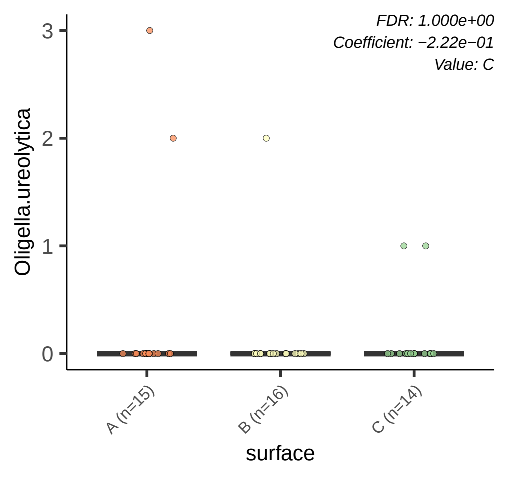

FDR: 1.000e+00
Coefficient: −2.22e−01
Value: C
Oligella.ureolytica
3
2
1
0
A (n=15)
B (n=16)
C (n=14)
surface

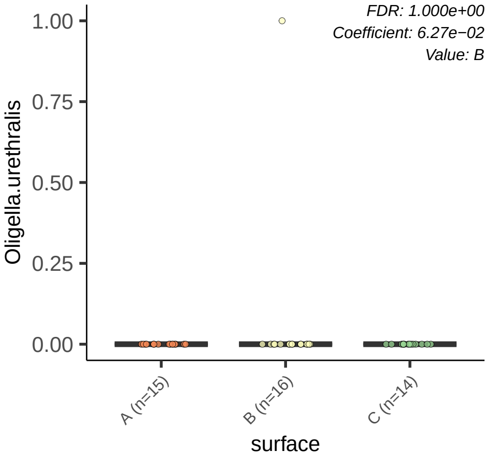
FDR: 1.000e+00
Coefficient: 6.27e−02
Value: B
Oligella.urethralis
1.00
0.75
0.50
0.25
0.00
A (n=15)
B (n=16)
C (n=14)
surface

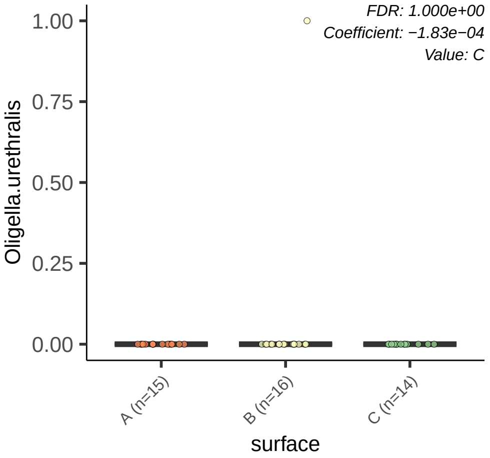
FDR: 1.000e+00
Coefficient: −1.83e−04
Value: C
Oligella.urethralis
1.00
0.75
0.50
0.25
0.00
A (n=15)
B (n=16)
C (n=14)
surface

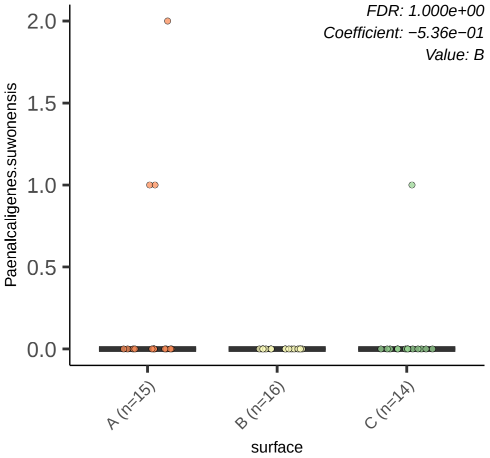

FDR: 1.000e+00
Coefficient: −5.36e−01
Value: B
Paenalcaligenes.suwonensis
2.0
1.5
1.0
0.5
0.0
A (n=15)
B (n=16)
C (n=14)
surface

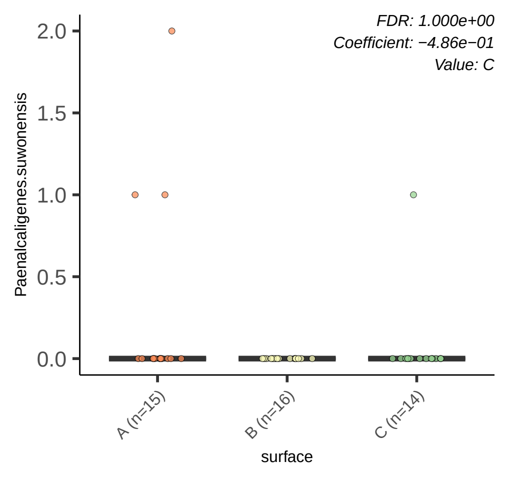
FDR: 1.000e+00
Coefficient: −4.86e−01
Value: C
Paenalcaligenes.suwonensis
2.0
1.5
1.0
0.5
0.0
A (n=15)
B (n=16)
C (n=14)
surface

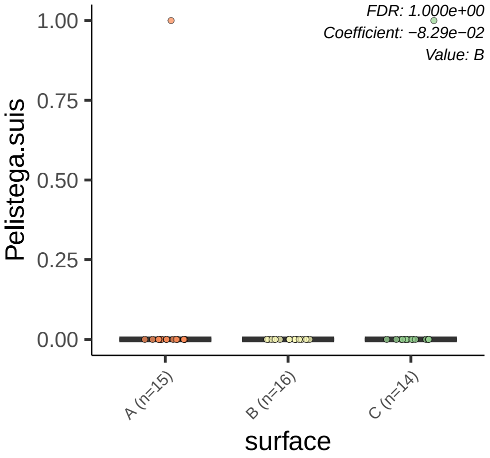
FDR: 1.000e+00
Coefficient: −8.29e−02
Value: B
Pelistega.suis
1.00
0.75
0.50
0.25
0.00
A (n=15)
B (n=16)
C (n=14)
surface

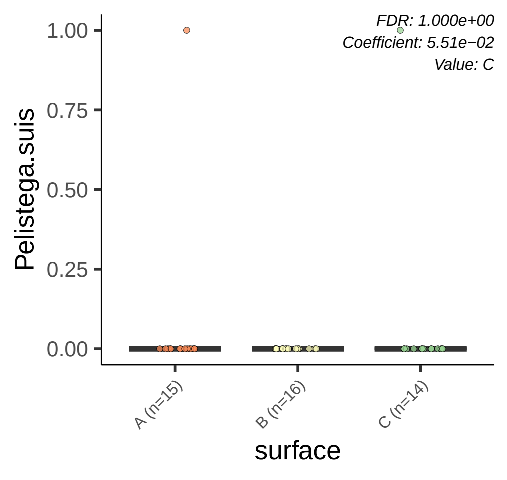

FDR: 1.000e+00
Coefficient: 5.51e−02
Value: C
Pelistega.suis
1.00
0.75
0.50
0.25
0.00
A (n=15)
B (n=16)
C (n=14)
surface

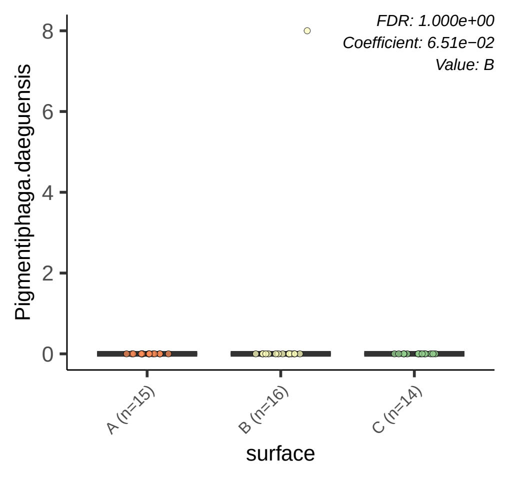

FDR: 1.000e+00
Coefficient: 6.51e−02
Value: B
Pigmentiphaga.daeguensis
8
6
4
2
0
A (n=15)
B (n=16)
C (n=14)
surface

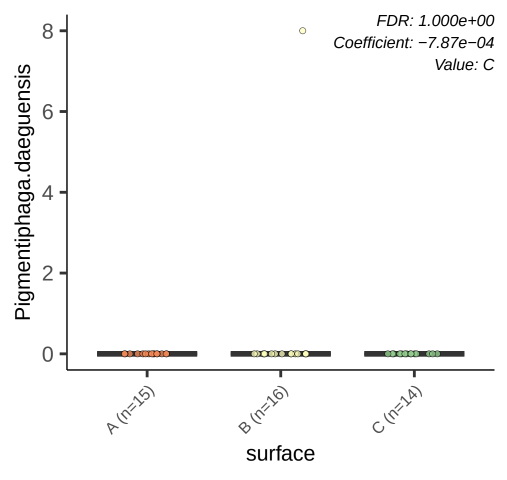

FDR: 1.000e+00
Coefficient: −7.87e−04
Value: C
Pigmentiphaga.daeguensis
8
6
4
2
0
A (n=15)
B (n=16)
C (n=14)
surface

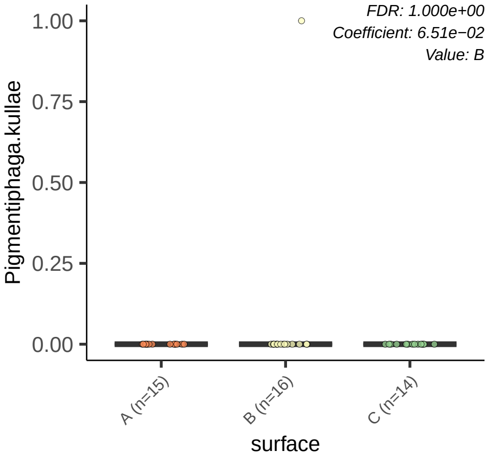

FDR: 1.000e+00
Coefficient: 6.51e−02
Value: B
Pigmentiphaga.kullae
1.00
0.75
0.50
0.25
0.00
A (n=15)
B (n=16)
C (n=14)
surface

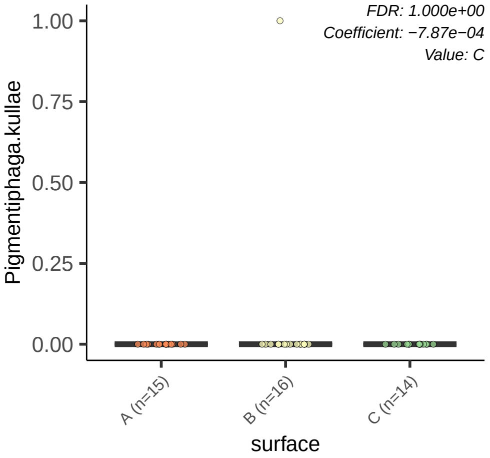

FDR: 1.000e+00
Coefficient: −7.87e−04
Value: C
Pigmentiphaga.kullae
1.00
0.75
0.50
0.25
0.00
A (n=15)
B (n=16)
C (n=14)
surface

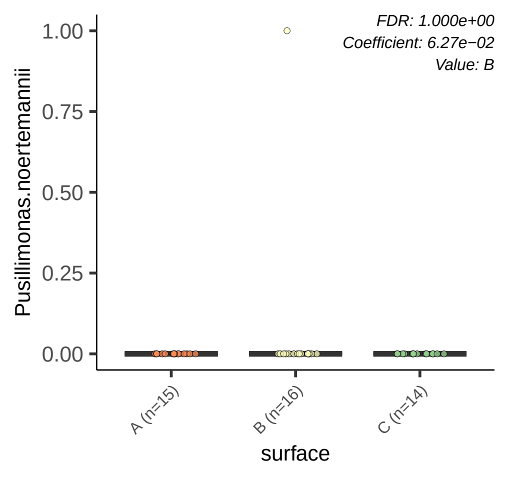
FDR: 1.000e+00
Coefficient: 6.27e−02
Value: B
Pusillimonas.noertemannii
1.00
0.75
0.50
0.25
0.00
A (n=15)
B (n=16)
C (n=14)
surface

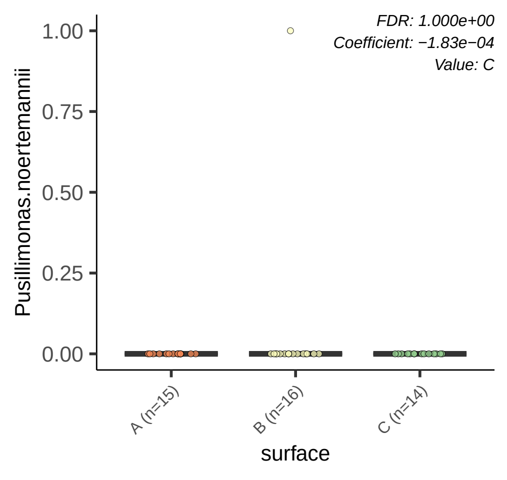
FDR: 1.000e+00
Coefficient: −1.83e−04
Value: C
Pusillimonas.noertemannii
1.00
0.75
0.50
0.25
0.00
A (n=15)
B (n=16)
C (n=14)
surface

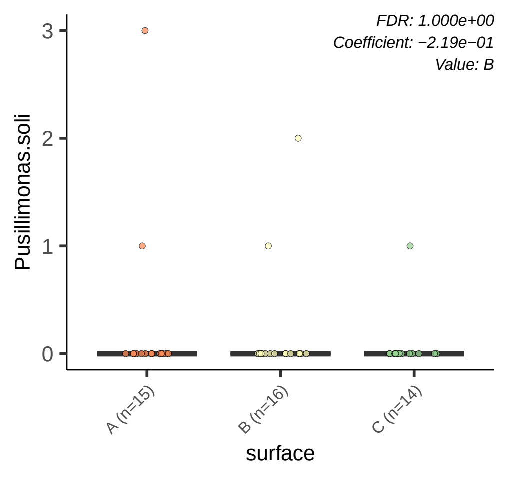

FDR: 1.000e+00
Coefficient: −2.19e−01
Value: B
Pusillimonas.soli
3
2
1
0
A (n=15)
B (n=16)
C (n=14)
surface

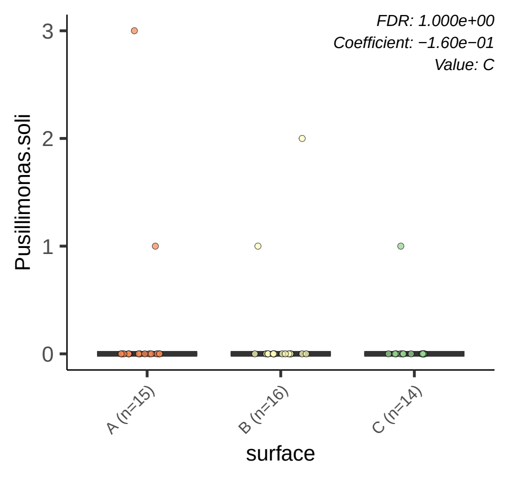
FDR: 1.000e+00
Coefficient: −1.60e−01
Value: C
Pusillimonas.soli
3
2
1
0
A (n=15)
B (n=16)
C (n=14)
surface

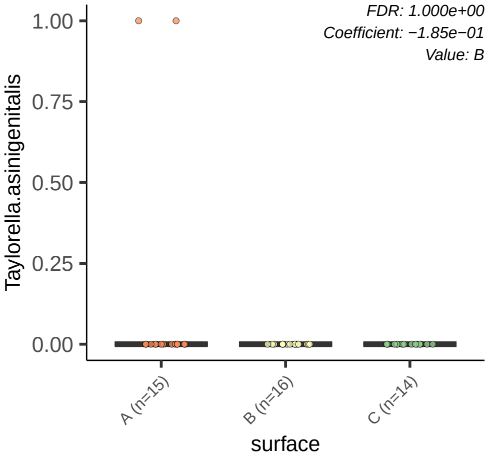
FDR: 1.000e+00
Coefficient: −1.85e−01
Value: B
Taylorella.asinigenitalis
1.00
0.75
0.50
0.25
0.00
A (n=15)
B (n=16)
C (n=14)
surface

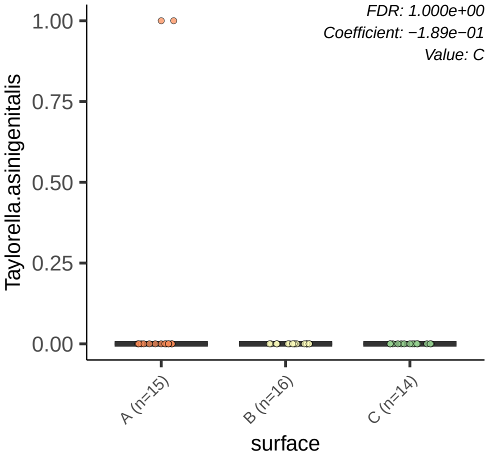

FDR: 1.000e+00
Coefficient: −1.89e−01
Value: C
Taylorella.asinigenitalis
1.00
0.75
0.50
0.25
0.00
A (n=15)
B (n=16)
C (n=14)
surface

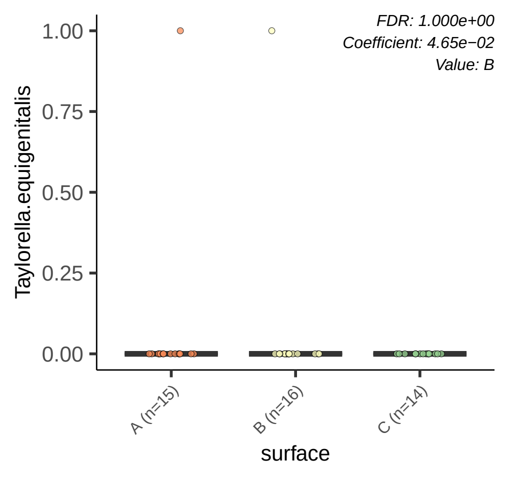

FDR: 1.000e+00
Coefficient: 4.65e−02
Value: B
Taylorella.equigenitalis
1.00
0.75
0.50
0.25
0.00
A (n=15)
B (n=16)
C (n=14)
surface

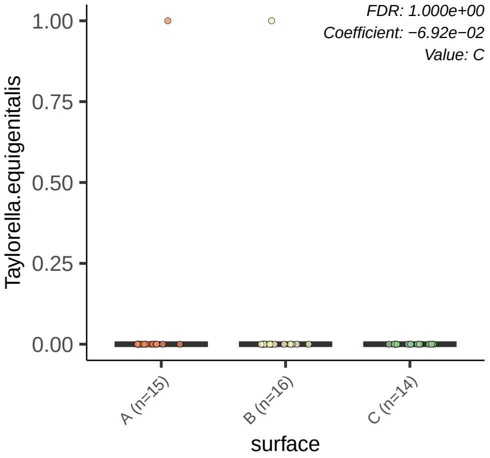

FDR: 1.000e+00
Coefficient: −6.92e−02
Value: C
Taylorella.equigenitalis
1.00
0.75
0.50
0.25
0.00
A (n=15)
B (n=16)
C (n=14)
surface

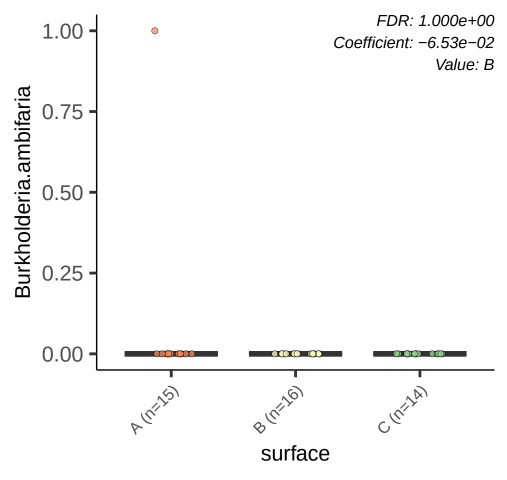
FDR: 1.000e+00
Coefficient: −6.53e−02
Value: B
Burkholderia.ambifaria
1.00
0.75
0.50
0.25
0.00
A (n=15)
B (n=16)
C (n=14)
surface

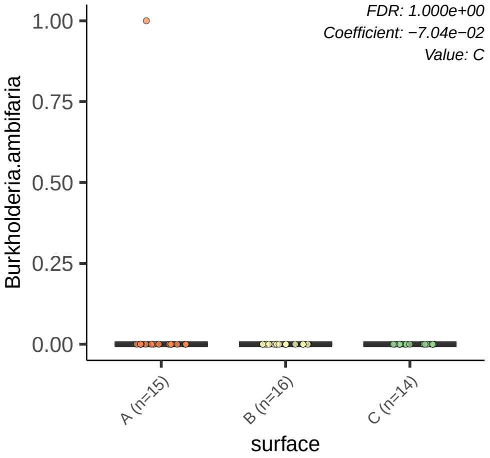
FDR: 1.000e+00
Coefficient: −7.04e−02
Value: C
Burkholderia.ambifaria
1.00
0.75
0.50
0.25
0.00
A (n=15)
B (n=16)
C (n=14)
surface

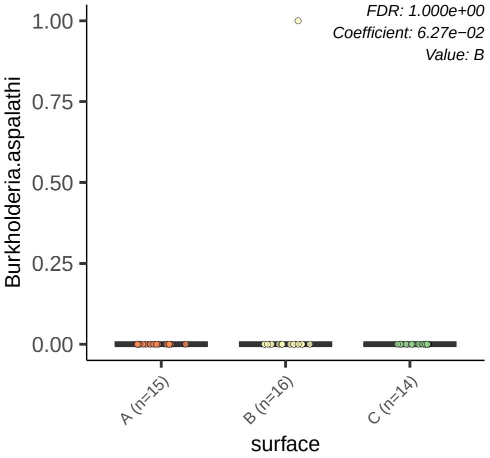

FDR: 1.000e+00
Coefficient: 6.27e−02
Value: B
Burkholderia.aspalathi
1.00
0.75
0.50
0.25
0.00
A (n=15)
B (n=16)
C (n=14)
surface

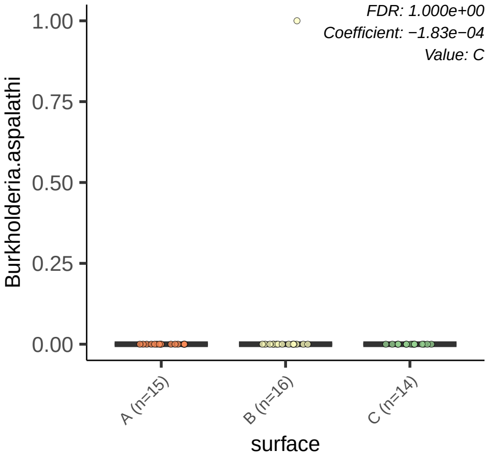

FDR: 1.000e+00
Coefficient: −1.83e−04
Value: C
Burkholderia.aspalathi
1.00
0.75
0.50
0.25
0.00
A (n=15)
B (n=16)
C (n=14)
surface

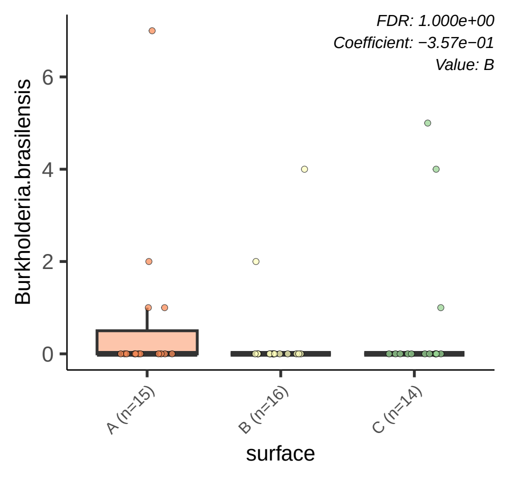
FDR: 1.000e+00
Coefficient: −3.57e−01
Value: B
Burkholderia.brasilensis
0
2
4
6
A (n=15)
B (n=16)
C (n=14)
surface

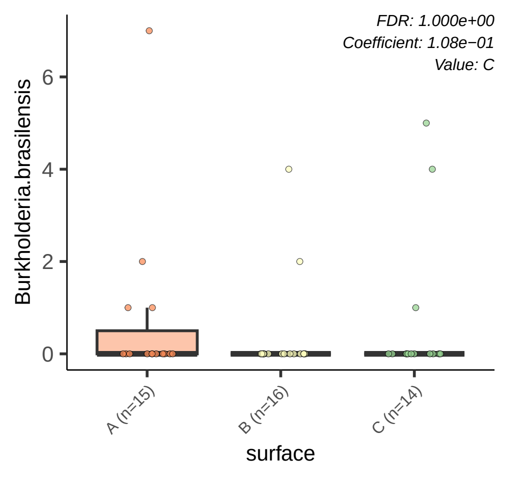
FDR: 1.000e+00
Coefficient: 1.08e−01
Value: C
Burkholderia.brasilensis
0
2
4
6
A (n=15)
B (n=16)
C (n=14)
surface

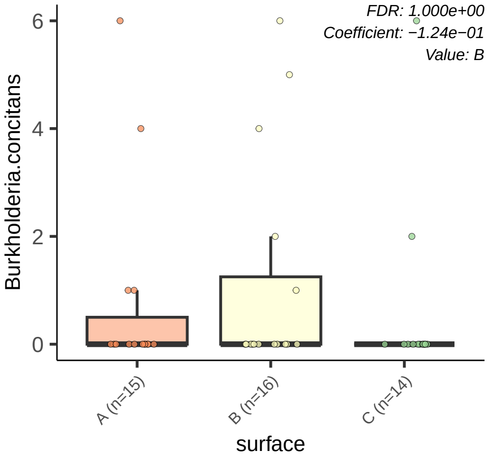
FDR: 1.000e+00
Coefficient: −1.24e−01
Value: B
Burkholderia.concitans
0
2
4
6
A (n=15)
B (n=16)
C (n=14)
surface

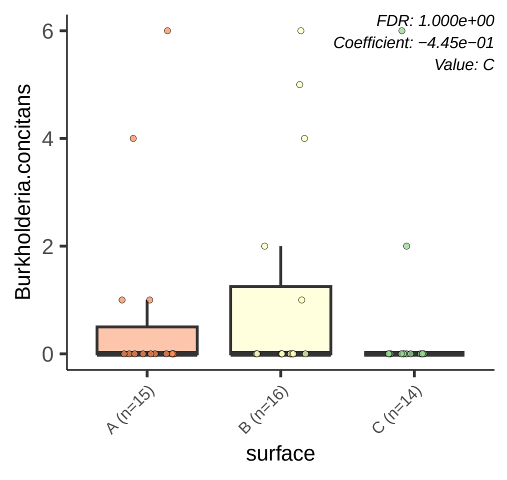
FDR: 1.000e+00
Coefficient: −4.45e−01
Value: C
Burkholderia.concitans
0
2
4
6
A (n=15)
B (n=16)
C (n=14)
surface

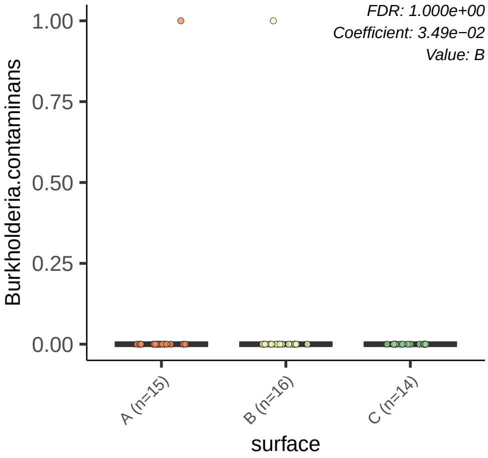
FDR: 1.000e+00
Coefficient: 3.49e−02
Value: B
Burkholderia.contaminans
1.00
0.75
0.50
0.25
0.00
A (n=15)
B (n=16)
C (n=14)
surface

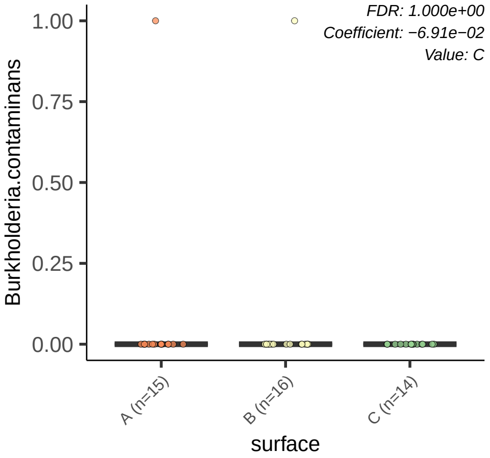
FDR: 1.000e+00
Coefficient: −6.91e−02
Value: C
Burkholderia.contaminans
1.00
0.75
0.50
0.25
0.00
A (n=15)
B (n=16)
C (n=14)
surface

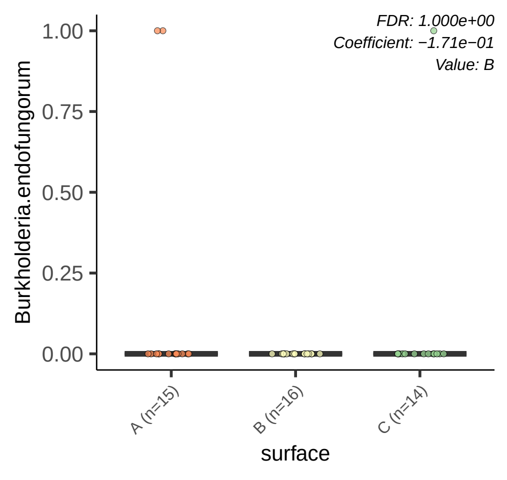
FDR: 1.000e+00
Coefficient: −1.71e−01
Value: B
Burkholderia.endofungorum
1.00
0.75
0.50
0.25
0.00
A (n=15)
B (n=16)
C (n=14)
surface

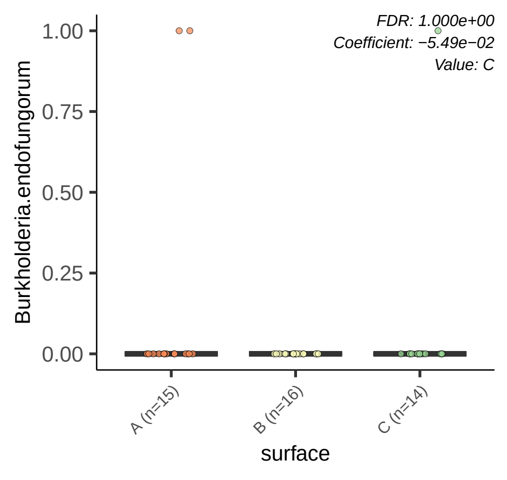

FDR: 1.000e+00
Coefficient: −5.49e−02
Value: C
Burkholderia.endofungorum
1.00
0.75
0.50
0.25
0.00
A (n=15)
B (n=16)
C (n=14)
surface

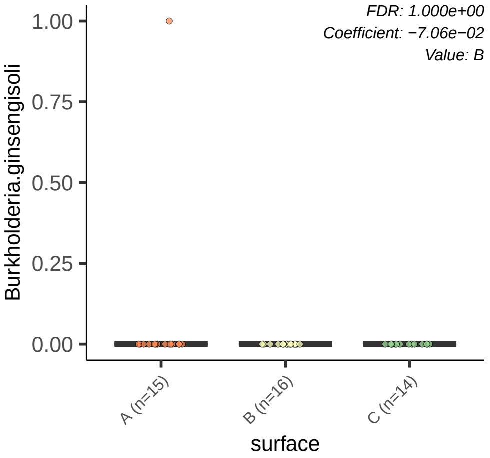

FDR: 1.000e+00
Coefficient: −7.06e−02
Value: B
Burkholderia.ginsengisoli
1.00
0.75
0.50
0.25
0.00
A (n=15)
B (n=16)
C (n=14)
surface

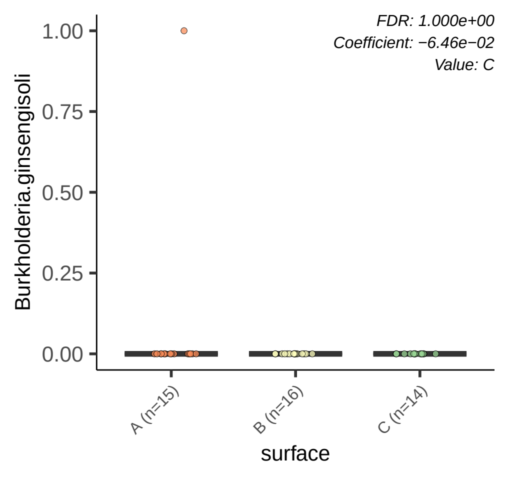

FDR: 1.000e+00
Coefficient: −6.46e−02
Value: C
Burkholderia.ginsengisoli
1.00
0.75
0.50
0.25
0.00
A (n=15)
B (n=16)
C (n=14)
surface

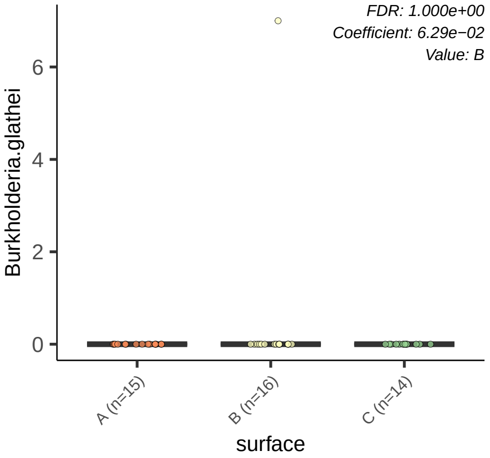
FDR: 1.000e+00
Coefficient: 6.29e−02
Value: B
Burkholderia.glathei
0
2
4
6
A (n=15)
B (n=16)
C (n=14)
surface

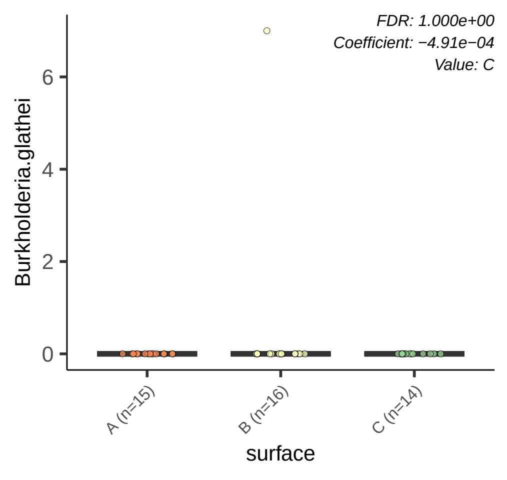
FDR: 1.000e+00
Coefficient: −4.91e−04
Value: C
Burkholderia.glathei
0
2
4
6
A (n=15)
B (n=16)
C (n=14)
surface

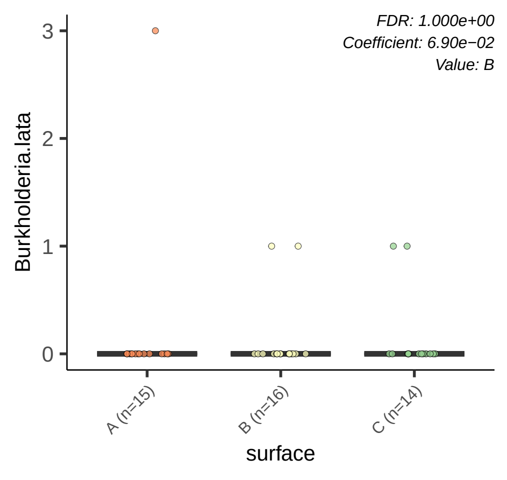
FDR: 1.000e+00
Coefficient: 6.90e−02
Value: B
Burkholderia.lata
3
2
1
0
A (n=15)
B (n=16)
C (n=14)
surface

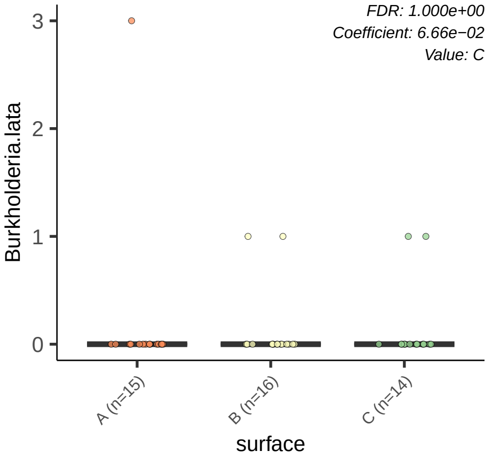

FDR: 1.000e+00
Coefficient: 6.66e−02
Value: C
Burkholderia.lata
3
2
1
0
A (n=15)
B (n=16)
C (n=14)
surface

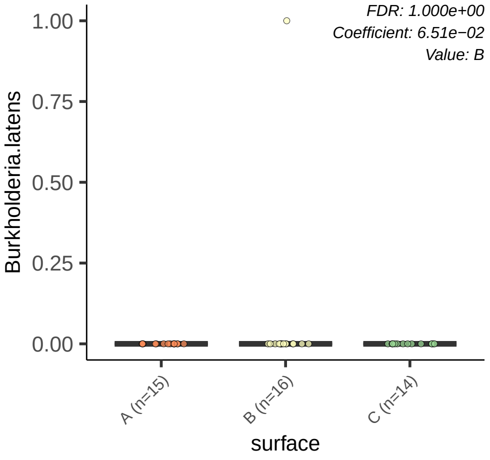
FDR: 1.000e+00
Coefficient: 6.51e−02
Value: B
1.00
0.75
0.50
0.25
0.00
Burkholderia.latens
A (n=15)
B (n=16)
C (n=14)
surface

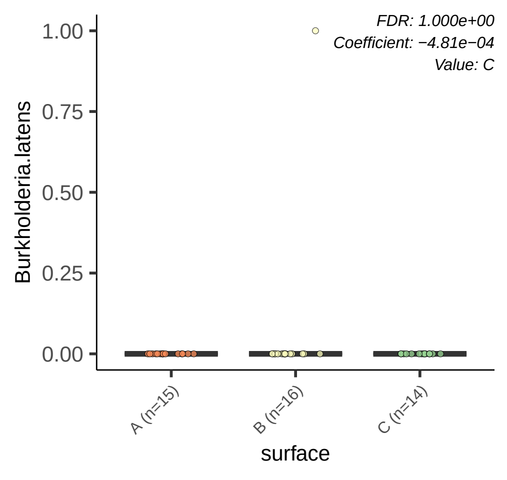

FDR: 1.000e+00
Coefficient: −4.81e−04
Value: C
Burkholderia.latens
1.00
0.75
0.50
0.25
0.00
A (n=15)
B (n=16)
C (n=14)
surface

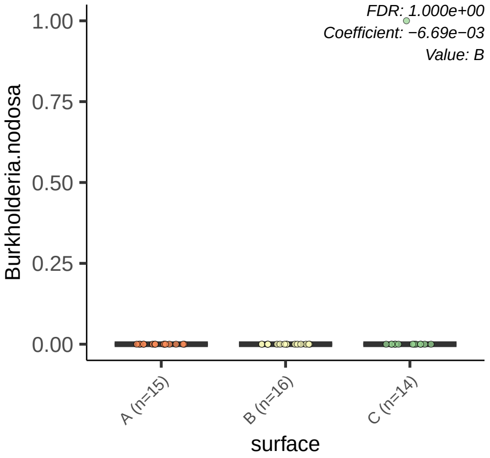
FDR: 1.000e+00
Coefficient: −6.69e−03
Value: B
Burkholderia.nodosa
1.00
0.75
0.50
0.25
0.00
A (n=15)
B (n=16)
C (n=14)
surface

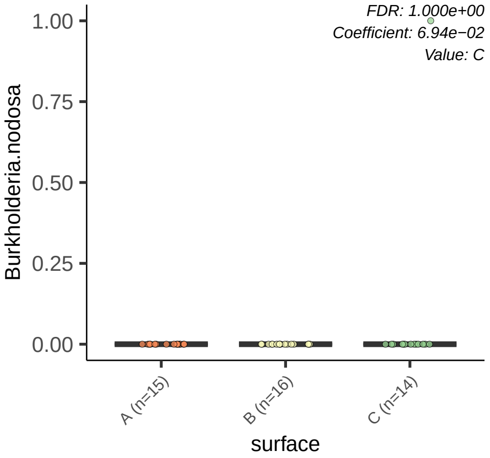
FDR: 1.000e+00
Coefficient: 6.94e−02
Value: C
Burkholderia.nodosa
1.00
0.75
0.50
0.25
0.00
A (n=15)
B (n=16)
C (n=14)
surface

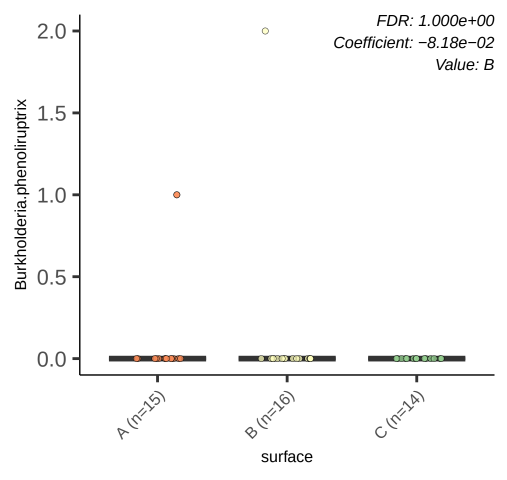
FDR: 1.000e+00
Coefficient: −8.18e−02
Value: B
Burkholderia.phenoliruptrix
2.0
1.5
1.0
0.5
0.0
A (n=15)
B (n=16)
C (n=14)
surface

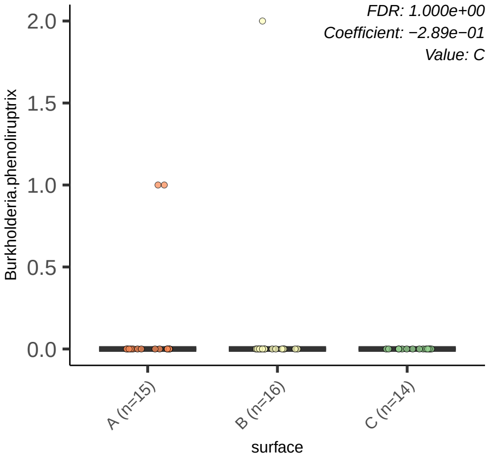
FDR: 1.000e+00
Coefficient: −2.89e−01
Value: C
Burkholderia.phenoliruptrix
2.0
1.5
1.0
0.5
0.0
A (n=15)
B (n=16)
C (n=14)
surface

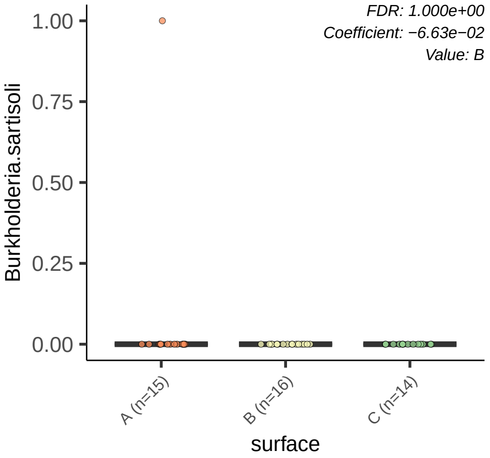
FDR: 1.000e+00
Coefficient: −6.63e−02
Value: B
Burkholderia.sartisoli
1.00
0.75
0.50
0.25
0.00
A (n=15)
B (n=16)
C (n=14)
surface

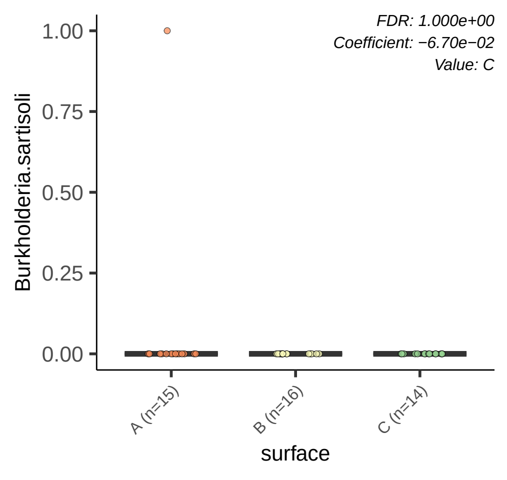

FDR: 1.000e+00
Coefficient: −6.70e−02
Value: C
Burkholderia.sartisoli
1.00
0.75
0.50
0.25
0.00
A (n=15)
B (n=16)
C (n=14)
surface

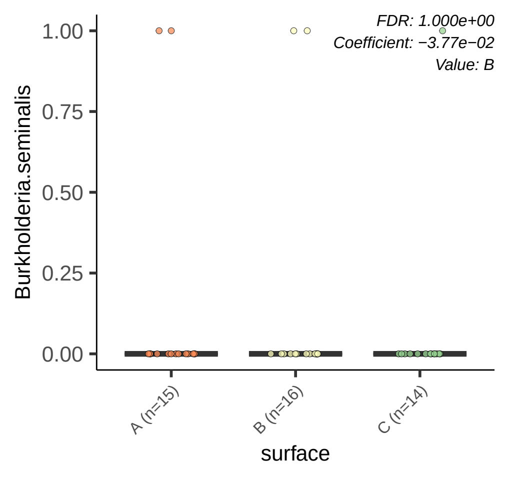
FDR: 1.000e+00
Coefficient: −3.77e−02
Value: B
Burkholderia.seminalis
1.00
0.75
0.50
0.25
0.00
A (n=15)
B (n=16)
C (n=14)
surface

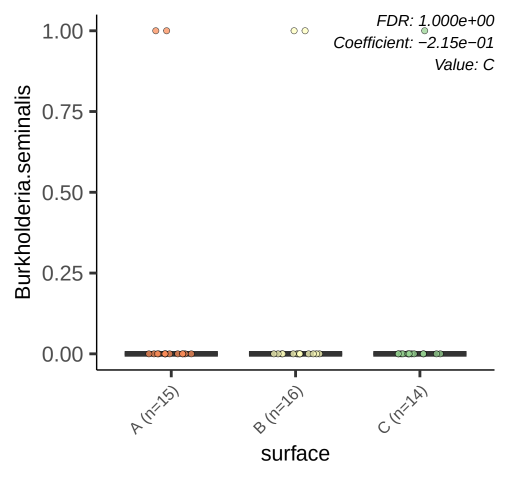

FDR: 1.000e+00
Coefficient: −2.15e−01
Value: C
1.00
0.75
0.50
0.25
0.00
Burkholderia.seminalis
A (n=15)
B (n=16)
C (n=14)
surface

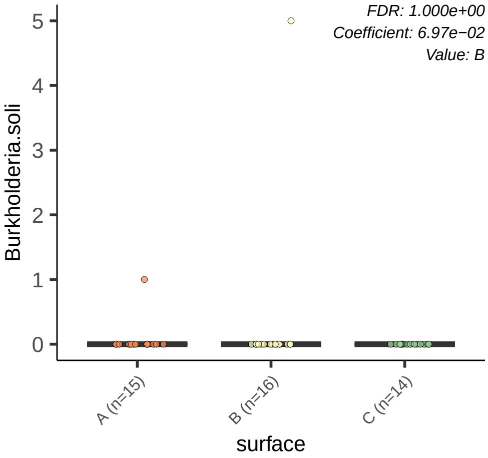
FDR: 1.000e+00
Coefficient: 6.97e−02
Value: B
Burkholderia.soli
0
1
2
3
4
5
A (n=15)
B (n=16)
C (n=14)
surface

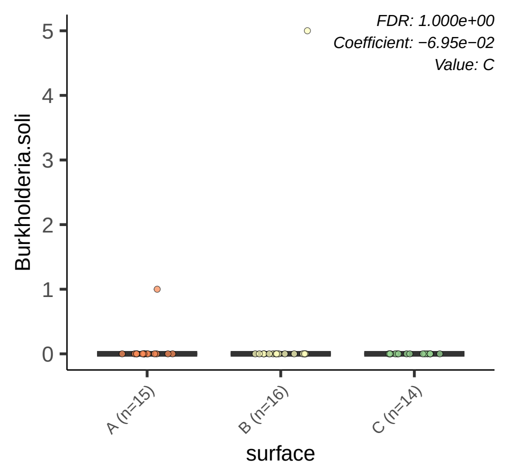

FDR: 1.000e+00
Coefficient: −6.95e−02
Value: C
Burkholderia.soli
0
1
2
3
4
5
A (n=15)
B (n=16)
C (n=14)
surface

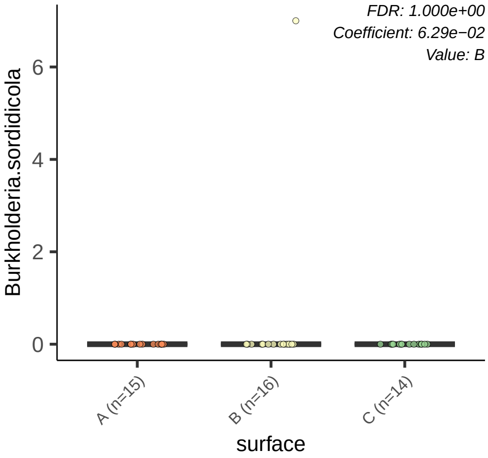
FDR: 1.000e+00
Coefficient: 6.29e−02
Value: B
Burkholderia.sordidicola
0
2
4
6
A (n=15)
B (n=16)
C (n=14)
surface

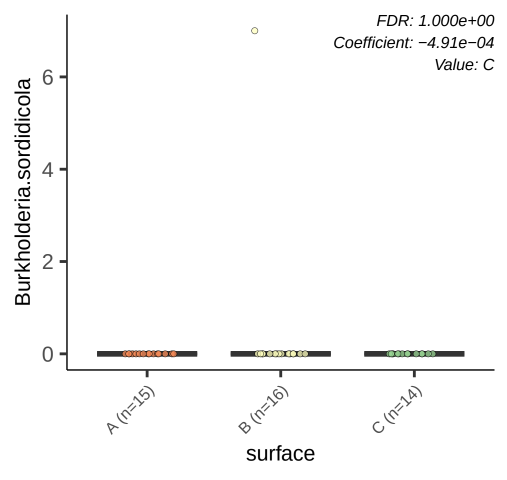
FDR: 1.000e+00
Coefficient: −4.91e−04
Value: C
Burkholderia.sordidicola
0
2
4
6
A (n=15)
B (n=16)
C (n=14)
surface

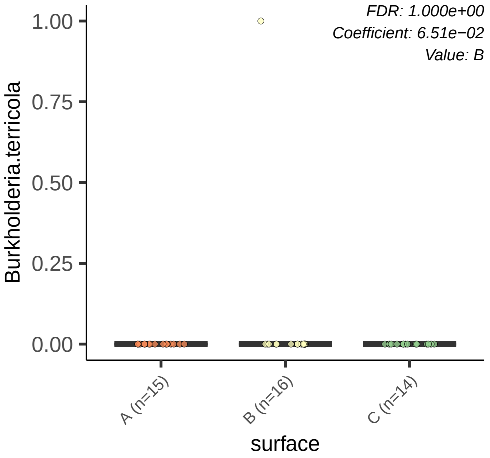
FDR: 1.000e+00
Coefficient: 6.51e−02
Value: B
Burkholderia.terricola
1.00
0.75
0.50
0.25
0.00
A (n=15)
B (n=16)
C (n=14)
surface

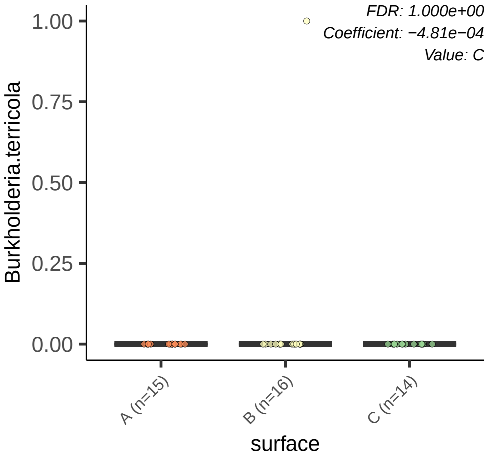
FDR: 1.000e+00
Coefficient: −4.81e−04
Value: C
Burkholderia.terricola
1.00
0.75
0.50
0.25
0.00
A (n=15)
B (n=16)
C (n=14)
surface

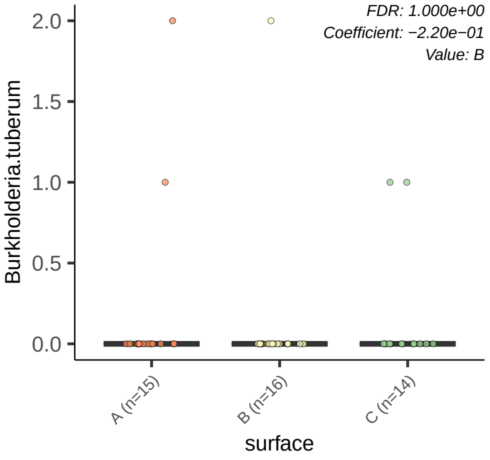
FDR: 1.000e+00
Coefficient: −2.20e−01
Value: B
Burkholderia.tuberum
2.0
1.5
1.0
0.5
0.0
A (n=15)
B (n=16)
C (n=14)
surface

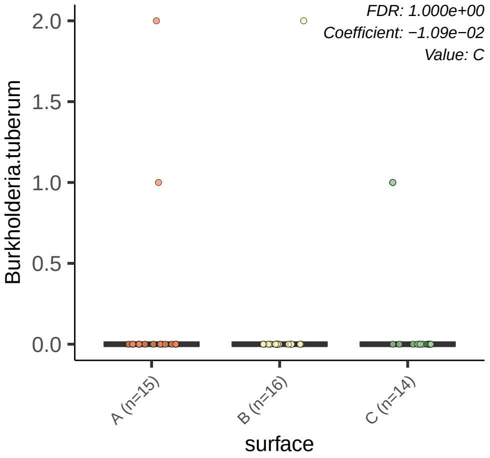
FDR: 1.000e+00
Coefficient: −1.09e−02
Value: C
Burkholderia.tuberum
2.0
1.5
1.0
0.5
0.0
A (n=15)
B (n=16)
C (n=14)
surface

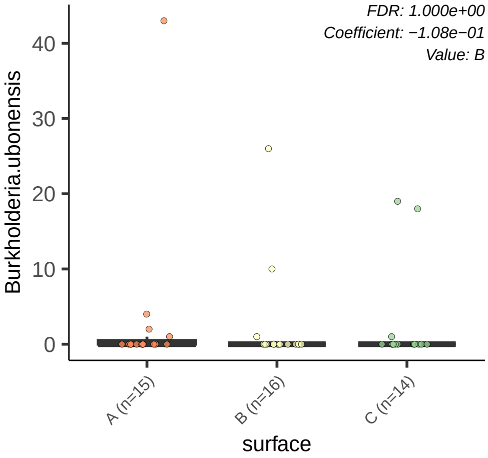
FDR: 1.000e+00
Coefficient: −1.08e−01
Value: B
Burkholderia.ubonensis
0
10
20
30
40
A (n=15)
B (n=16)
C (n=14)
surface

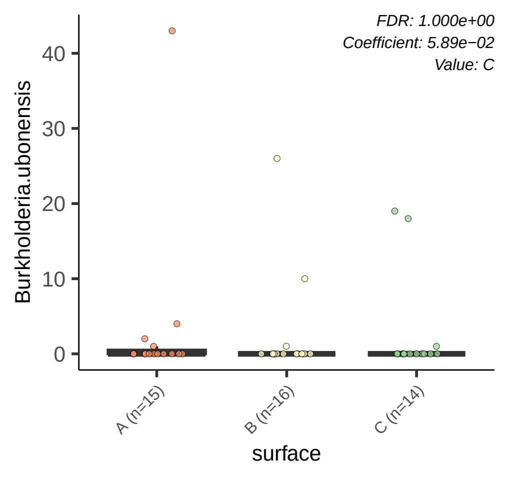

FDR: 1.000e+00
Coefficient: 5.89e−02
Value: C
Burkholderia.ubonensis
40
30
20
10
0
A (n=15)
B (n=16)
C (n=14)
surface

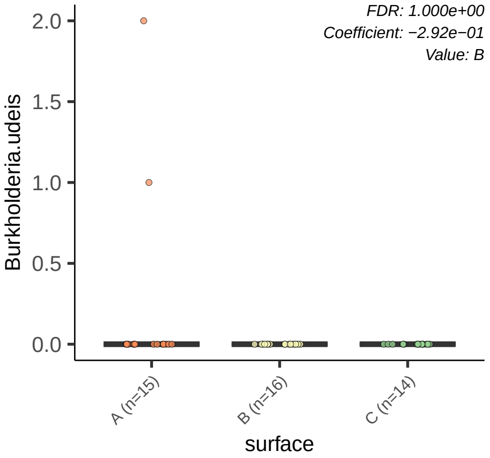
FDR: 1.000e+00
Coefficient: −2.92e−01
Value: B
Burkholderia.udeis
2.0
1.5
1.0
0.5
0.0
A (n=15)
B (n=16)
C (n=14)
surface

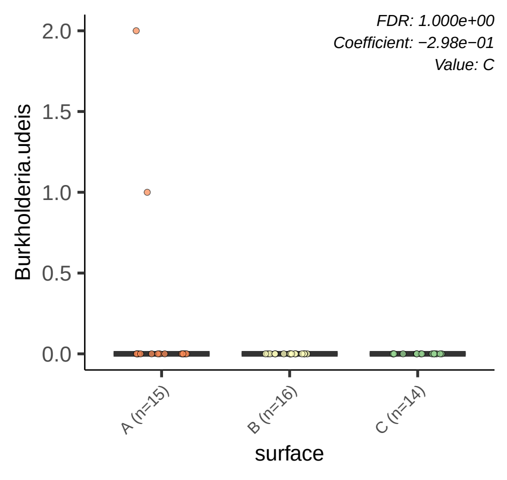
FDR: 1.000e+00
Coefficient: −2.98e−01
Value: C
Burkholderia.udeis
2.0
1.5
1.0
0.5
0.0
A (n=15)
B (n=16)
C (n=14)
surface

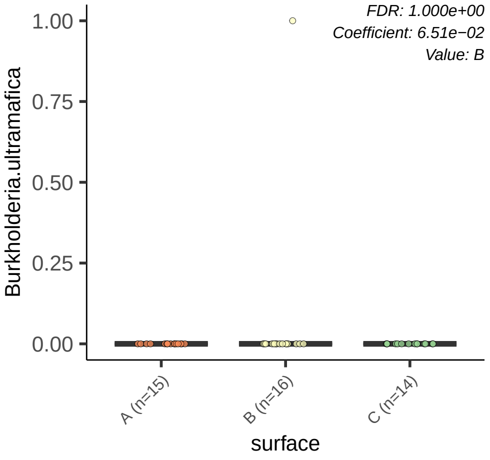

FDR: 1.000e+00
Coefficient: 6.51e−02
Value: B
Burkholderia.ultramafica
1.00
0.75
0.50
0.25
0.00
A (n=15)
B (n=16)
C (n=14)
surface

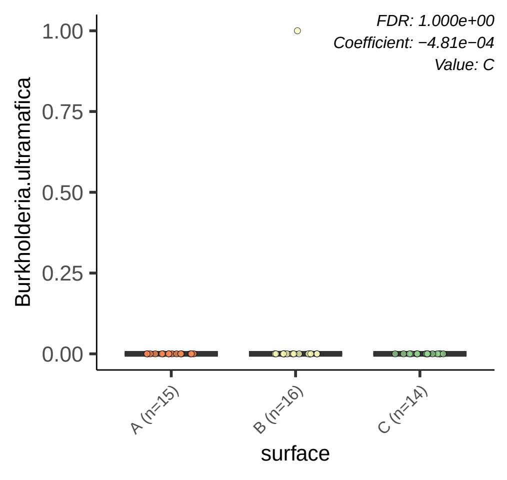
FDR: 1.000e+00
Coefficient: −4.81e−04
Value: C
Burkholderia.ultramafica
1.00
0.75
0.50
0.25
0.00
A (n=15)
B (n=16)
C (n=14)
surface

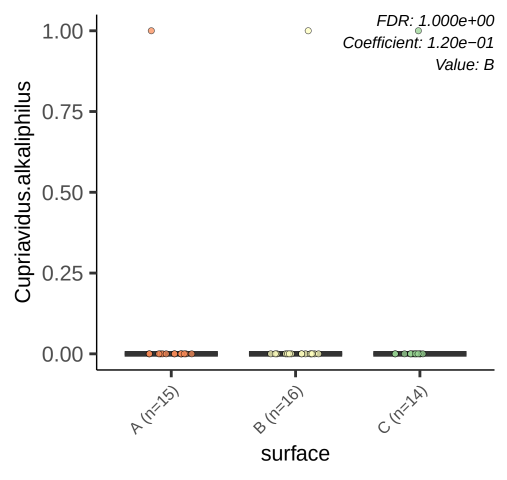
FDR: 1.000e+00
Coefficient: 1.20e−01
Value: B
Cupriavidus.alkaliphilus
1.00
0.75
0.50
0.25
0.00
A (n=15)
B (n=16)
C (n=14)
surface

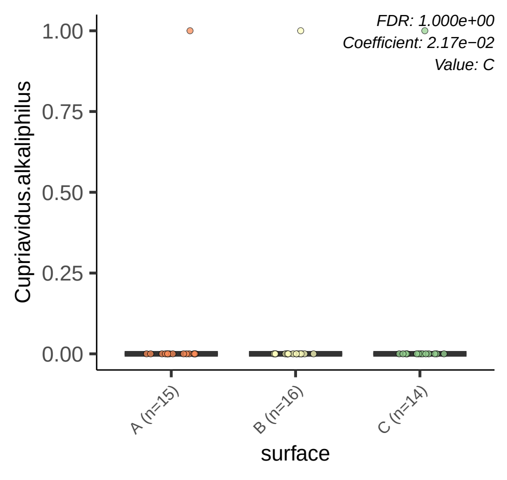
FDR: 1.000e+00
Coefficient: 2.17e−02
Value: C
Cupriavidus.alkaliphilus
1.00
0.75
0.50
0.25
0.00
A (n=15)
B (n=16)
C (n=14)
surface

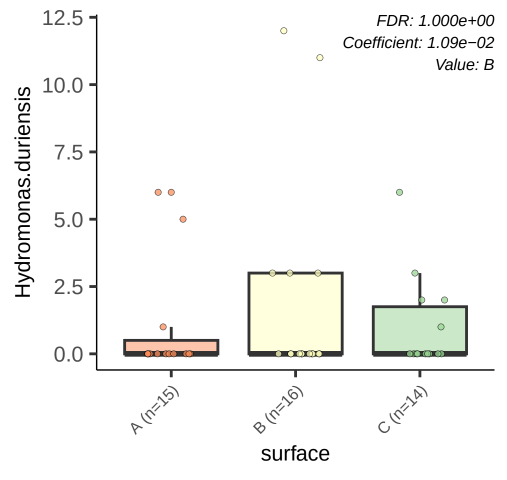

FDR: 1.000e+00
Coefficient: 1.09e−02
Value: B
Hydromonas.duriensis
12.5
10.0
7.5
5.0
2.5
0.0
A (n=15)
B (n=16)
C (n=14)
surface

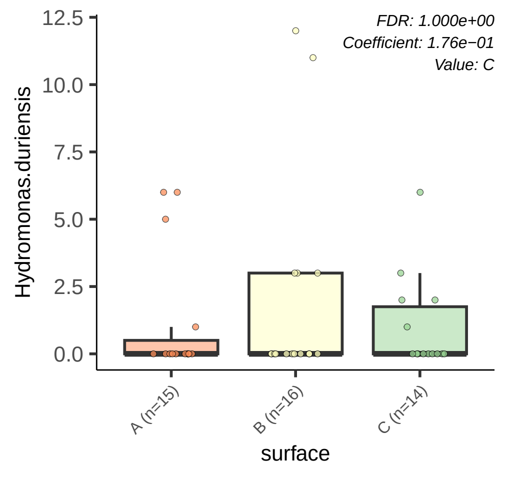

FDR: 1.000e+00
Coefficient: 1.76e−01
Value: C
Hydromonas.duriensis
0.0
2.5
5.0
7.5
10.0
12.5
A (n=15)
B (n=16)
C (n=14)
surface

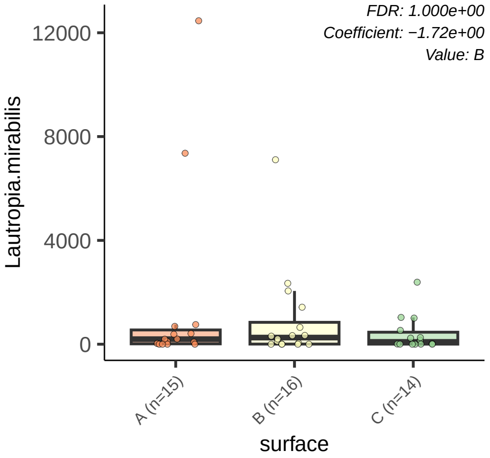

FDR: 1.000e+00
Coefficient: −1.72e+00
Value: B
Lautropia.mirabilis
12000
8000
4000
0
A (n=15)
B (n=16)
C (n=14)
surface

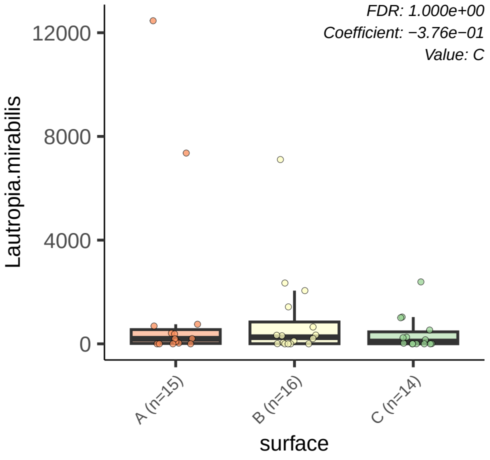
FDR: 1.000e+00
Coefficient: −3.76e−01
Value: C
Lautropia.mirabilis
12000
8000
4000
0
A (n=15)
B (n=16)
C (n=14)
surface

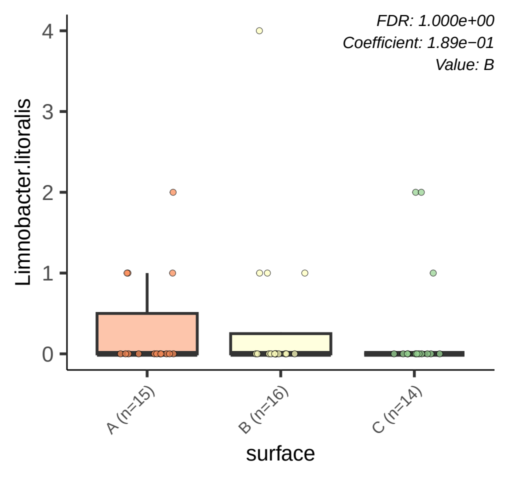

FDR: 1.000e+00
Coefficient: 1.89e−01
Value: B
Limnobacter.litoralis
4
3
2
1
0
A (n=15)
B (n=16)
C (n=14)
surface

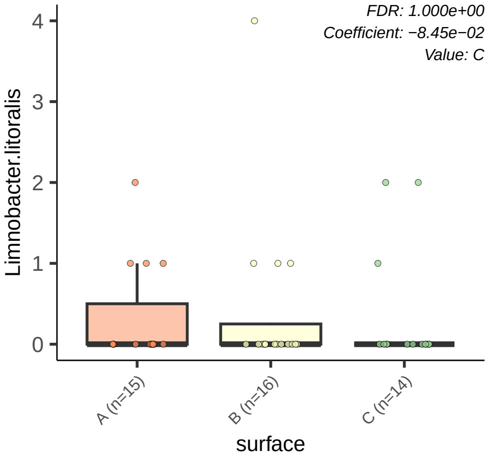

FDR: 1.000e+00
Coefficient: −8.45e−02
Value: C
Limnobacter.litoralis
0
1
2
3
4
A (n=15)
B (n=16)
C (n=14)
surface

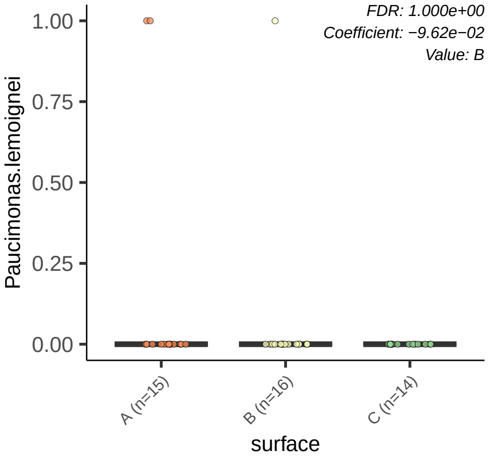
FDR: 1.000e+00
Coefficient: −9.62e−02
Value: B
Paucimonas.lemoignei
1.00
0.75
0.50
0.25
0.00
A (n=15)
B (n=16)
C (n=14)
surface

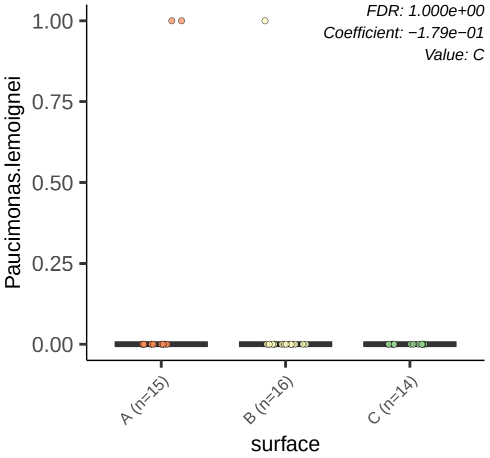

FDR: 1.000e+00
Coefficient: −1.79e−01
Value: C
Paucimonas.lemoignei
1.00
0.75
0.50
0.25
0.00
A (n=15)
B (n=16)
C (n=14)
surface

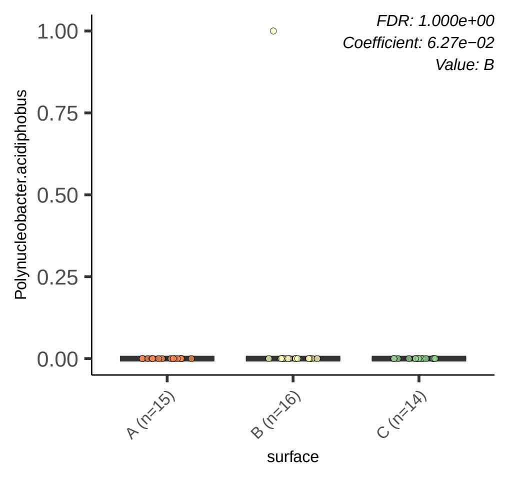
FDR: 1.000e+00
Coefficient: 6.27e−02
Value: B
Polynucleobacter.acidiphobus
1.00
0.75
0.50
0.25
0.00
A (n=15)
B (n=16)
C (n=14)
surface

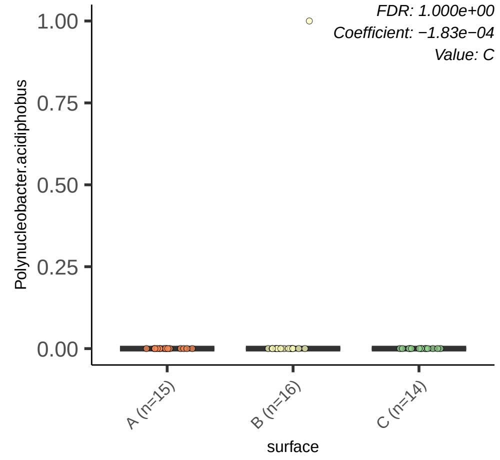
FDR: 1.000e+00
Coefficient: −1.83e−04
Value: C
Polynucleobacter.acidiphobus
1.00
0.75
0.50
0.25
0.00
A (n=15)
B (n=16)
C (n=14)
surface

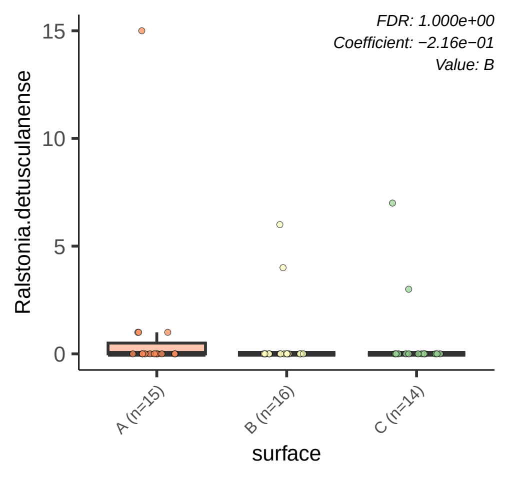

FDR: 1.000e+00
Coefficient: −2.16e−01
Value: B
15
10
5
0
Ralstonia.detusculanense
A (n=15)
B (n=16)
C (n=14)
surface

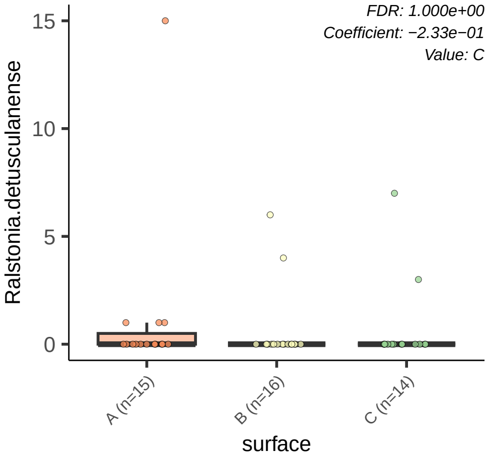

FDR: 1.000e+00
Coefficient: −2.33e−01
Value: C
15
10
5
0
Ralstonia.detusculanense
A (n=15)
B (n=16)
C (n=14)
surface

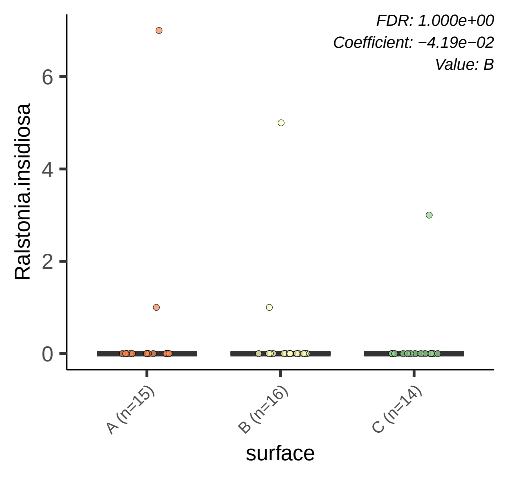
FDR: 1.000e+00
Coefficient: −4.19e−02
Value: B
Ralstonia.insidiosa
0
2
4
6
A (n=15)
B (n=16)
C (n=14)
surface

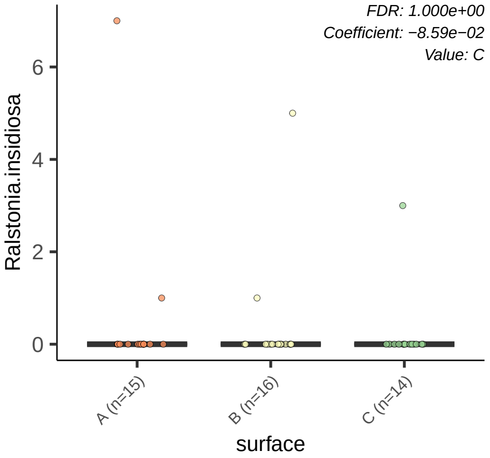
FDR: 1.000e+00
Coefficient: −8.59e−02
Value: C
Ralstonia.insidiosa
0
2
4
6
A (n=15)
B (n=16)
C (n=14)
surface

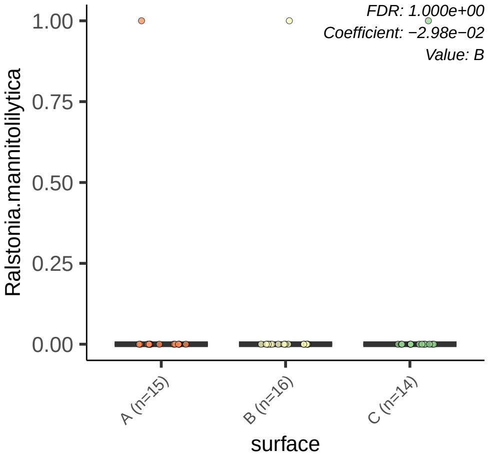
FDR: 1.000e+00
Coefficient: −2.98e−02
Value: B
Ralstonia.mannitolilytica
1.00
0.75
0.50
0.25
0.00
A (n=15)
B (n=16)
C (n=14)
surface

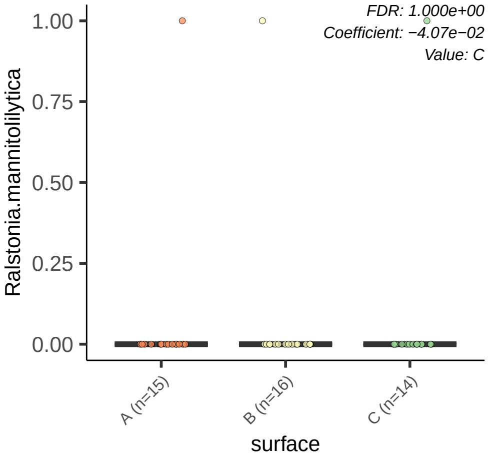
FDR: 1.000e+00
Coefficient: −4.07e−02
Value: C
Ralstonia.mannitolilytica
1.00
0.75
0.50
0.25
0.00
A (n=15)
B (n=16)
C (n=14)
surface

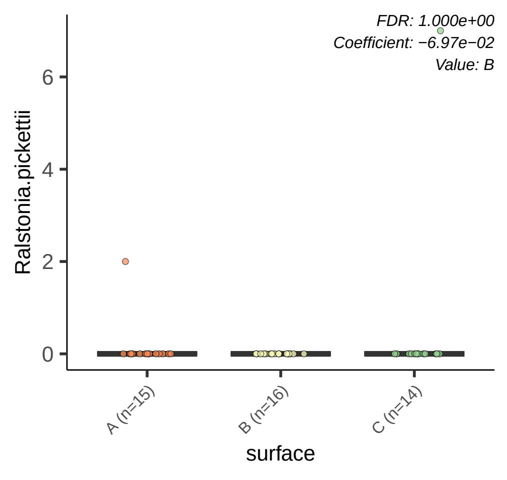
FDR: 1.000e+00
Coefficient: −6.97e−02
Value: B
Ralstonia.pickettii
0
2
4
6
A (n=15)
B (n=16)
C (n=14)
surface

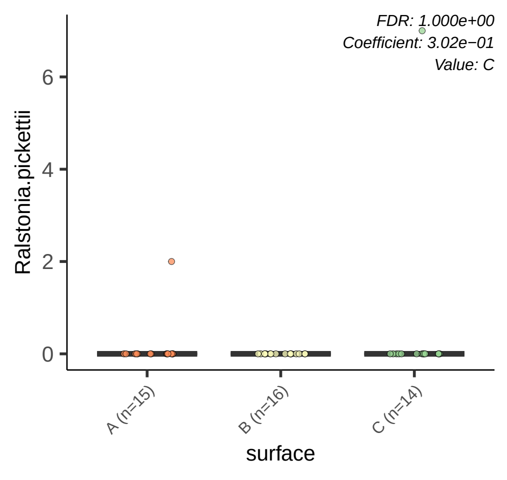
FDR: 1.000e+00
Coefficient: 3.02e−01
Value: C
Ralstonia.pickettii
0
2
4
6
A (n=15)
B (n=16)
C (n=14)
surface

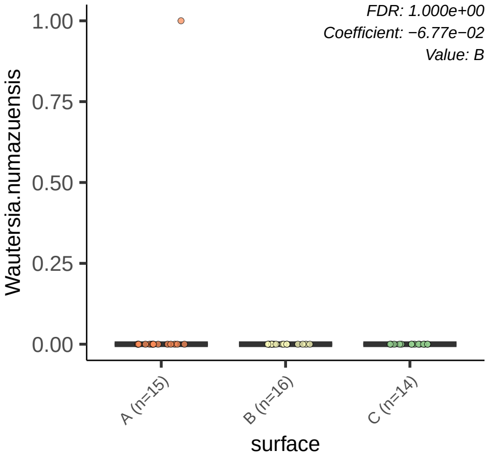

FDR: 1.000e+00
Coefficient: −6.77e−02
Value: B
Wautersia.numazuensis
1.00
0.75
0.50
0.25
0.00
A (n=15)
B (n=16)
C (n=14)
surface

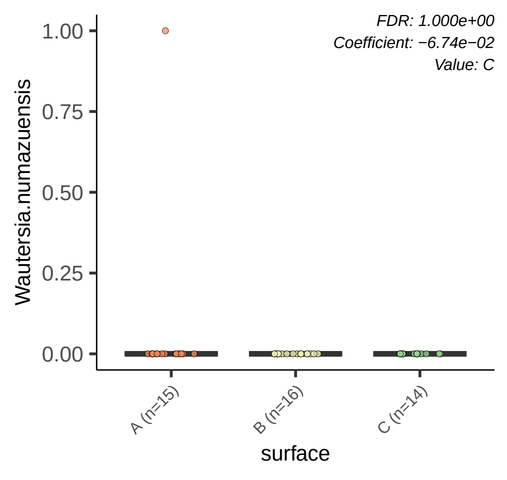

FDR: 1.000e+00
Coefficient: −6.74e−02
Value: C
Wautersia.numazuensis
1.00
0.75
0.50
0.25
0.00
A (n=15)
B (n=16)
C (n=14)
surface

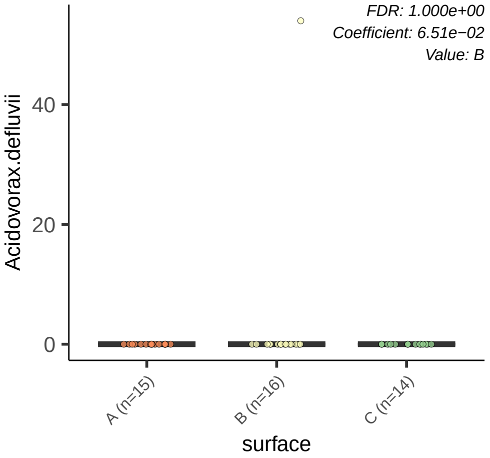
FDR: 1.000e+00
Coefficient: 6.51e−02
Value: B
Acidovorax.defluvii
40
20
0
A (n=15)
B (n=16)
C (n=14)
surface

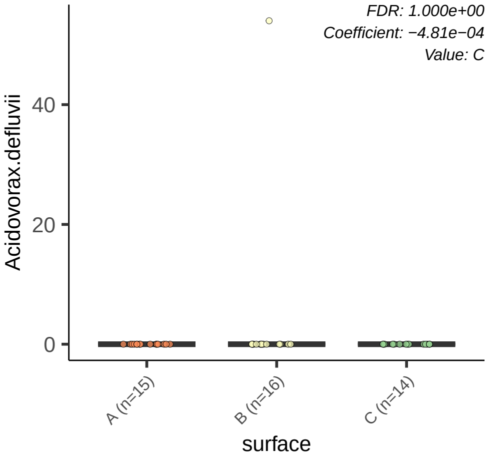
FDR: 1.000e+00
Coefficient: −4.81e−04
Value: C
Acidovorax.defluvii
40
20
0
A (n=15)
B (n=16)
C (n=14)
surface

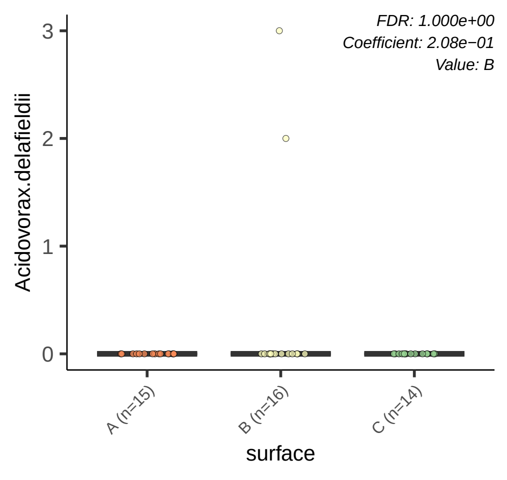

FDR: 1.000e+00
Coefficient: 2.08e−01
Value: B
Acidovorax.delafieldii
3
2
1
0
A (n=15)
B (n=16)
C (n=14)
surface

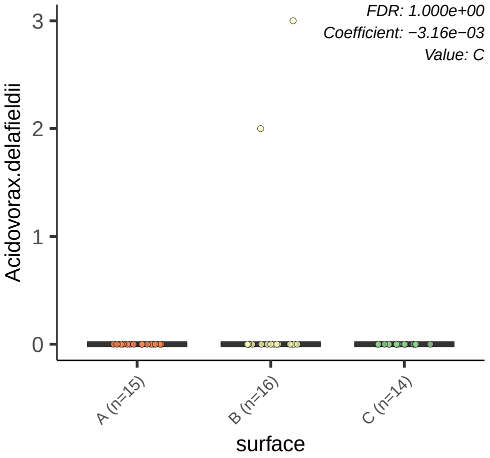

FDR: 1.000e+00
Coefficient: −3.16e−03
Value: C
3
2
1
0
Acidovorax.delafieldii
A (n=15)
B (n=16)
C (n=14)
surface

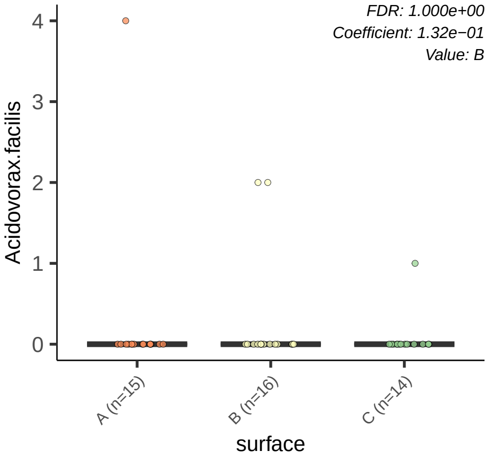
FDR: 1.000e+00
Coefficient: 1.32e−01
Value: B
Acidovorax.facilis
4
3
2
1
0
A (n=15)
B (n=16)
C (n=14)
surface

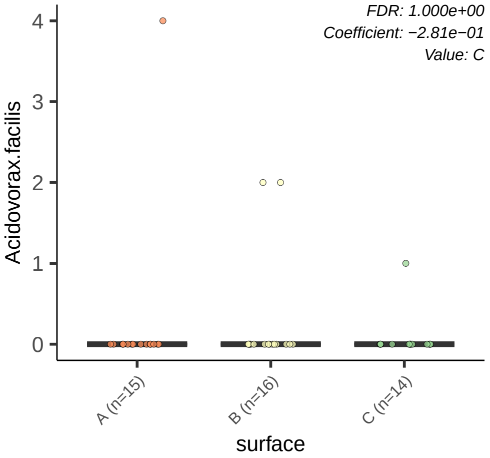
FDR: 1.000e+00
Coefficient: −2.81e−01
Value: C
Acidovorax.facilis
0
1
2
3
4
A (n=15)
B (n=16)
C (n=14)
surface

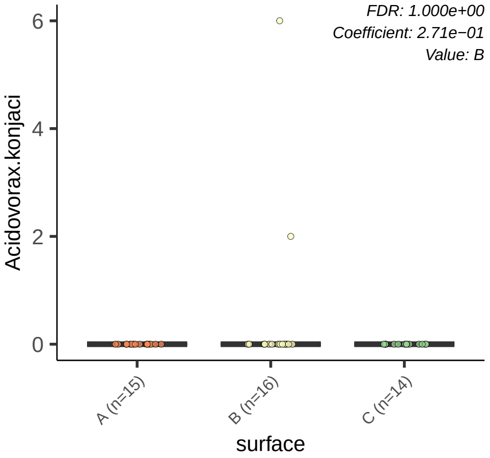
FDR: 1.000e+00
Coefficient: 2.71e−01
Value: B
Acidovorax.konjaci
6
4
2
0
A (n=15)
B (n=16)
C (n=14)
surface

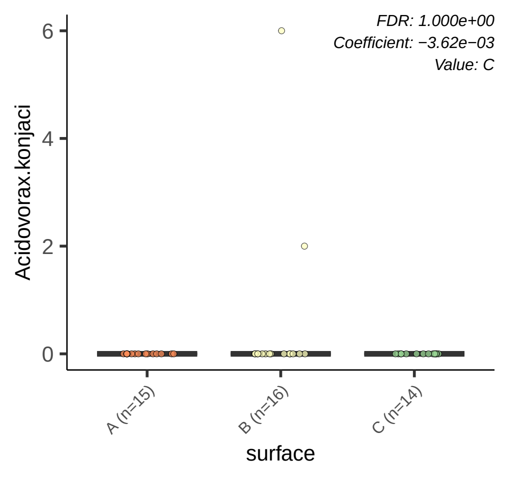
FDR: 1.000e+00
Coefficient: −3.62e−03
Value: C
Acidovorax.konjaci
6
4
2
0
A (n=15)
B (n=16)
C (n=14)
surface

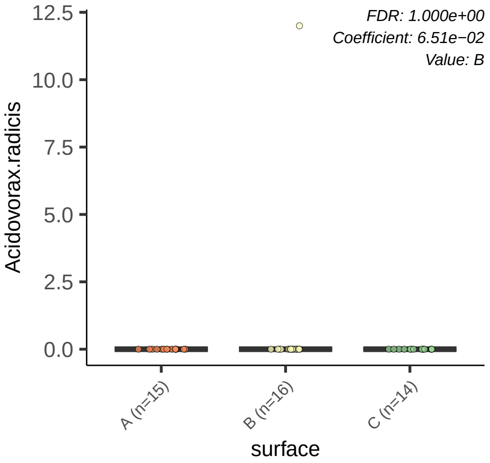

FDR: 1.000e+00
Coefficient: 6.51e−02
Value: B
Acidovorax.radicis
12.5
10.0
7.5
5.0
2.5
0.0
A (n=15)
B (n=16)
C (n=14)
surface

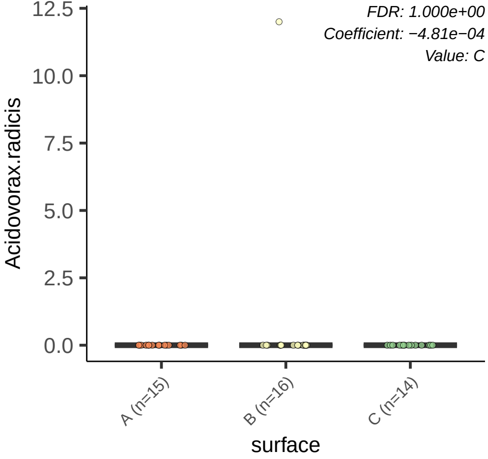

FDR: 1.000e+00
Coefficient: −4.81e−04
Value: C
Acidovorax.radicis
12.5
10.0
7.5
5.0
2.5
0.0
A (n=15)
B (n=16)
C (n=14)
surface

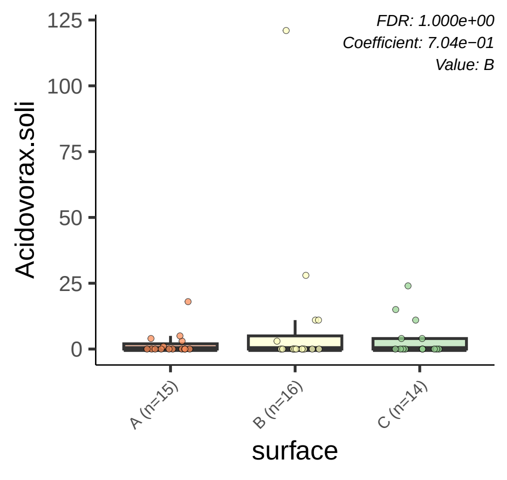
FDR: 1.000e+00
Coefficient: 7.04e−01
Value: B
Acidovorax.soli
125
100
75
50
25
0
A (n=15)
B (n=16)
C (n=14)
surface

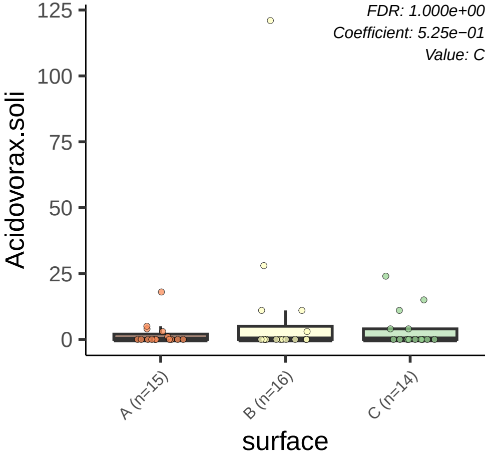

FDR: 1.000e+00
Coefficient: 5.25e−01
Value: C
Acidovorax.soli
125
100
75
50
25
0
A (n=15)
B (n=16)
C (n=14)
surface

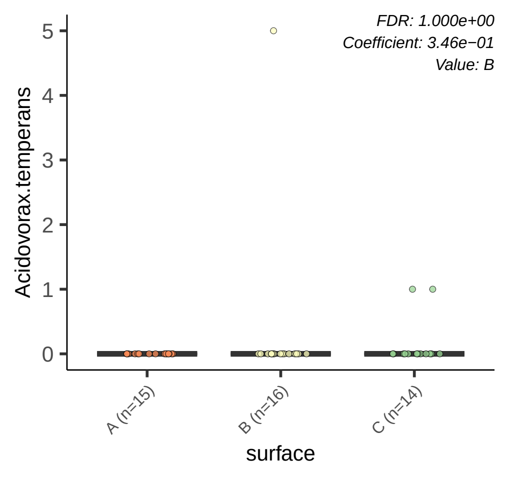

FDR: 1.000e+00
Coefficient: 3.46e−01
Value: B
Acidovorax.temperans
0
1
2
3
4
5
A (n=15)
B (n=16)
C (n=14)
surface

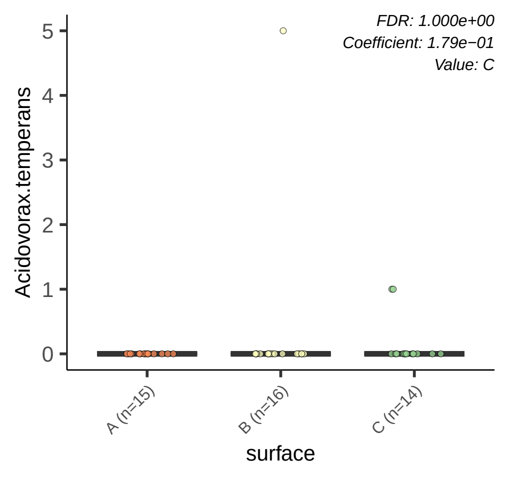
FDR: 1.000e+00
Coefficient: 1.79e−01
Value: C
Acidovorax.temperans
0
1
2
3
4
5
A (n=15)
B (n=16)
C (n=14)
surface

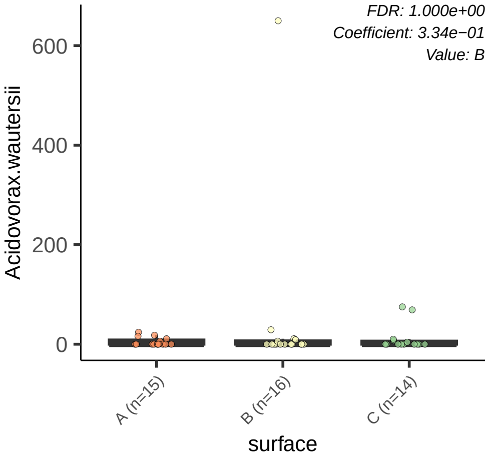

FDR: 1.000e+00
Coefficient: 3.34e−01
Value: B
Acidovorax.wautersii
600
400
200
0
A (n=15)
B (n=16)
C (n=14)
surface

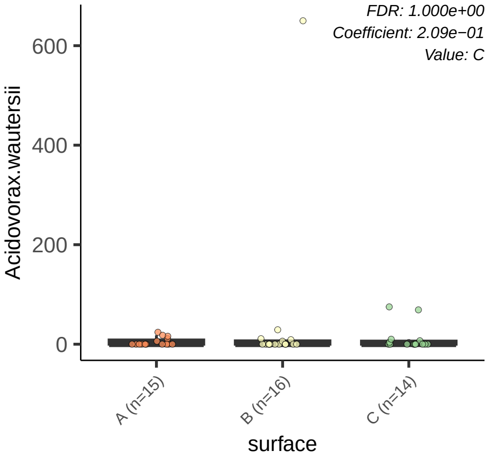
FDR: 1.000e+00
Coefficient: 2.09e−01
Value: C
Acidovorax.wautersii
600
400
200
0
A (n=15)
B (n=16)
C (n=14)
surface

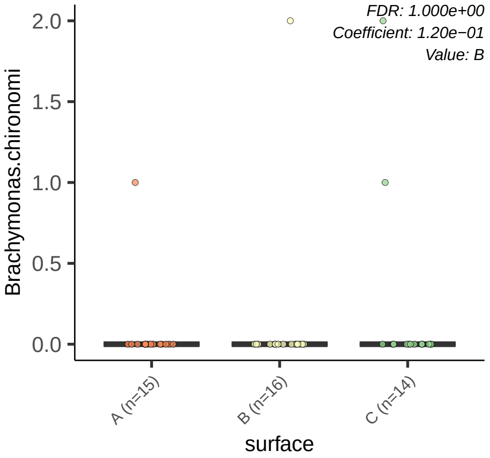

FDR: 1.000e+00
Coefficient: 1.20e−01
Value: B
Brachymonas.chironomi
2.0
1.5
1.0
0.5
0.0
A (n=15)
B (n=16)
C (n=14)
surface

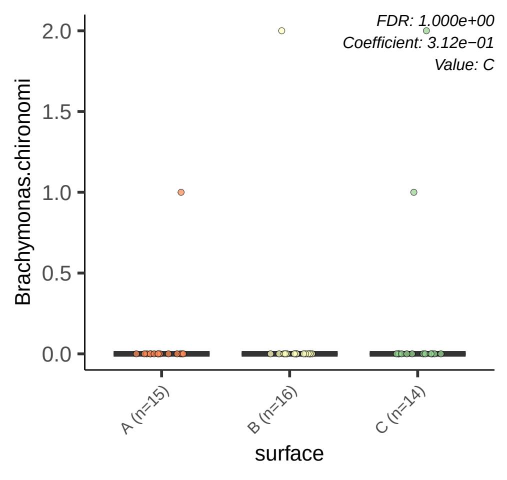
FDR: 1.000e+00
Coefficient: 3.12e−01
Value: C
Brachymonas.chironomi
2.0
1.5
1.0
0.5
0.0
A (n=15)
B (n=16)
C (n=14)
surface

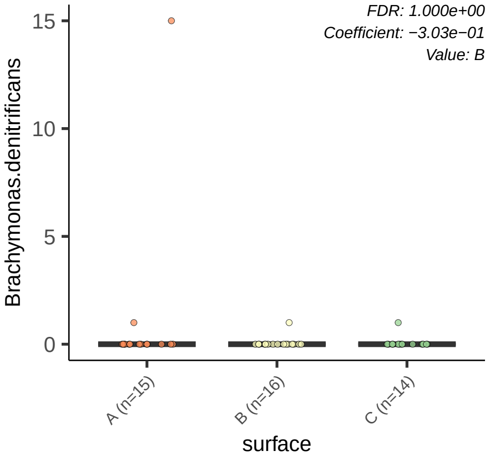
FDR: 1.000e+00
Coefficient: −3.03e−01
Value: B
Brachymonas.denitrificans
15
10
5
0
A (n=15)
B (n=16)
C (n=14)
surface

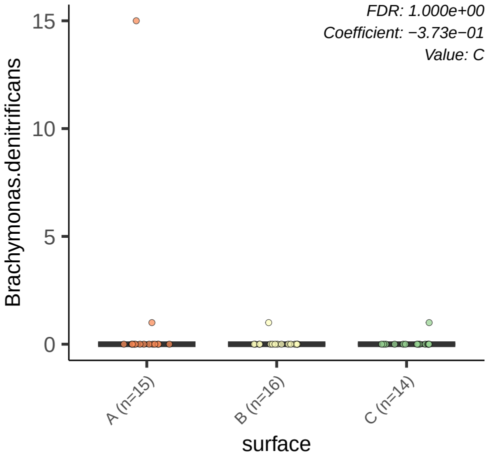

FDR: 1.000e+00
Coefficient: −3.73e−01
Value: C
Brachymonas.denitrificans
15
10
5
0
A (n=15)
B (n=16)
C (n=14)
surface

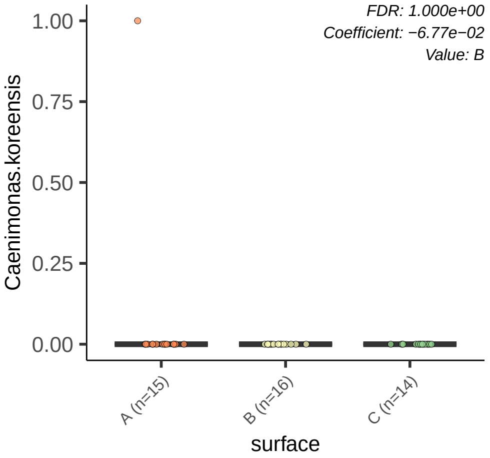
FDR: 1.000e+00
Coefficient: −6.77e−02
Value: B
Caenimonas.koreensis
1.00
0.75
0.50
0.25
0.00
A (n=15)
B (n=16)
C (n=14)
surface

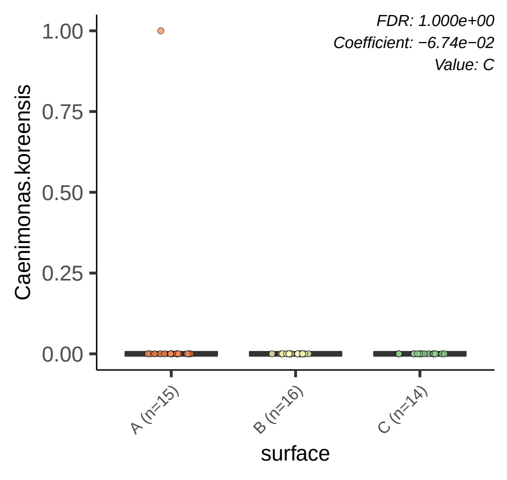

FDR: 1.000e+00
Coefficient: −6.74e−02
Value: C
Caenimonas.koreensis
1.00
0.75
0.50
0.25
0.00
A (n=15)
B (n=16)
C (n=14)
surface

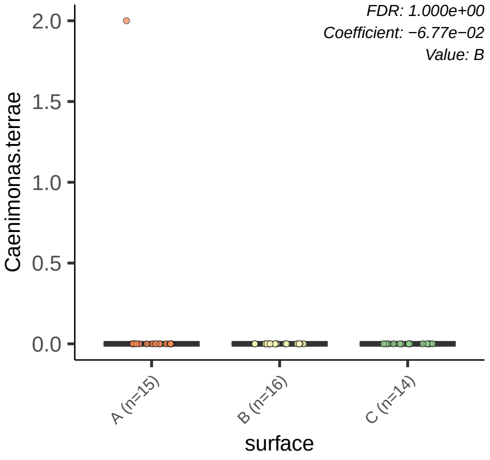

FDR: 1.000e+00
Coefficient: −6.77e−02
Value: B
Caenimonas.terrae
2.0
1.5
1.0
0.5
0.0
A (n=15)
B (n=16)
C (n=14)
surface

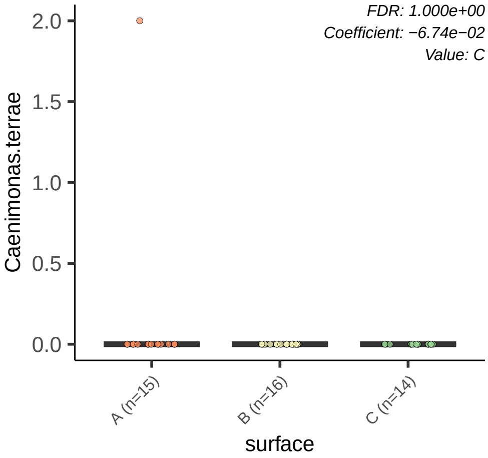
FDR: 1.000e+00
Coefficient: −6.74e−02
Value: C
Caenimonas.terrae
2.0
1.5
1.0
0.5
0.0
A (n=15)
B (n=16)
C (n=14)
surface

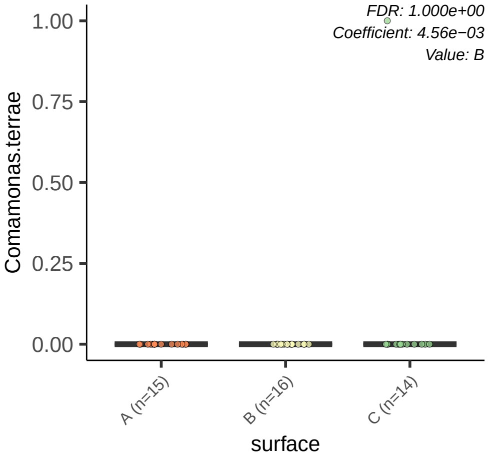
FDR: 1.000e+00
Coefficient: 4.56e−03
Value: B
Comamonas.terrae
1.00
0.75
0.50
0.25
0.00
A (n=15)
B (n=16)
C (n=14)
surface

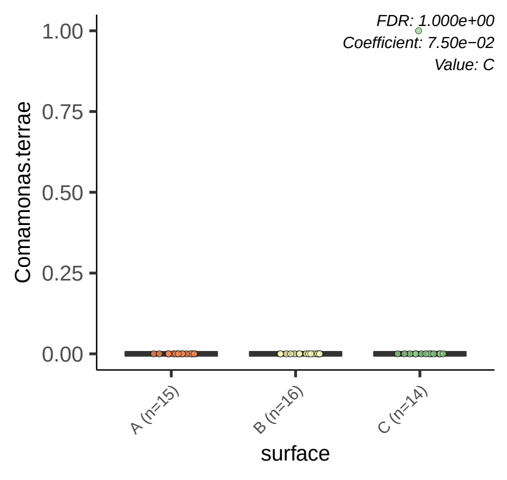
FDR: 1.000e+00
Coefficient: 7.50e−02
Value: C
Comamonas.terrae
1.00
0.75
0.50
0.25
0.00
A (n=15)
B (n=16)
C (n=14)
surface

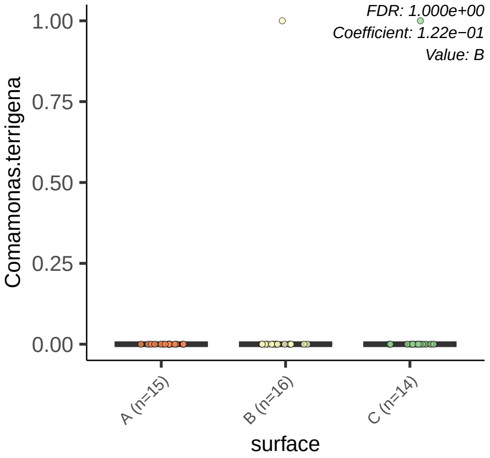
FDR: 1.000e+00
Coefficient: 1.22e−01
Value: B
Comamonas.terrigena
1.00
0.75
0.50
0.25
0.00
A (n=15)
B (n=16)
C (n=14)
surface

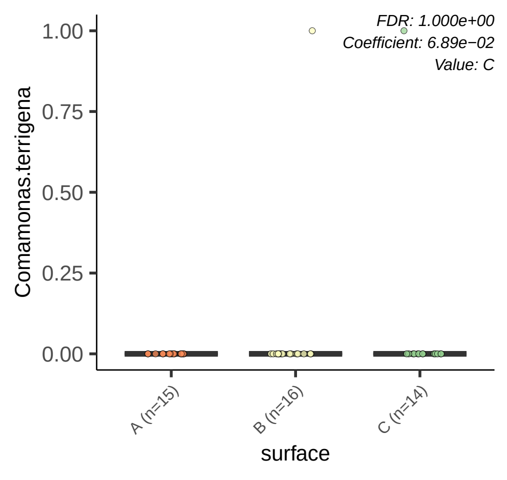
FDR: 1.000e+00
Coefficient: 6.89e−02
Value: C
Comamonas.terrigena
1.00
0.75
0.50
0.25
0.00
A (n=15)
B (n=16)
C (n=14)
surface

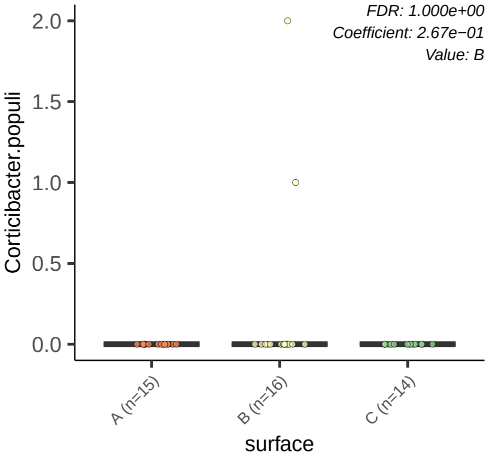
FDR: 1.000e+00
Coefficient: 2.67e−01
Value: B
Corticibacter.populi
2.0
1.5
1.0
0.5
0.0
A (n=15)
B (n=16)
C (n=14)
surface

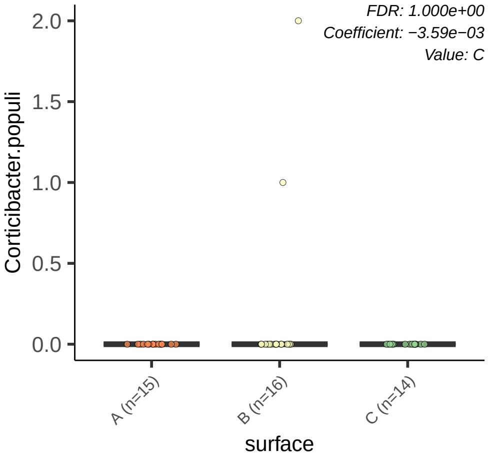

FDR: 1.000e+00
Coefficient: −3.59e−03
Value: C
Corticibacter.populi
2.0
1.5
1.0
0.5
0.0
A (n=15)
B (n=16)
C (n=14)
surface

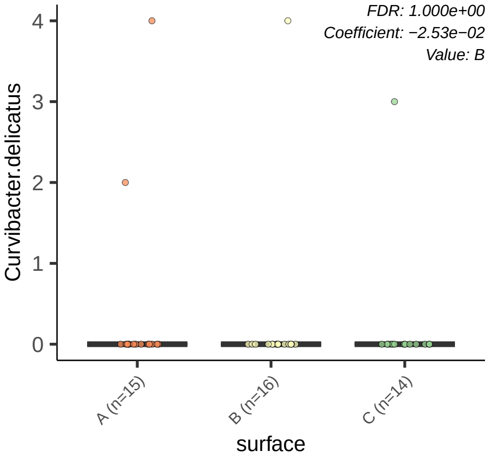
FDR: 1.000e+00
Coefficient: −2.53e−02
Value: B
Curvibacter.delicatus
0
1
2
3
4
A (n=15)
B (n=16)
C (n=14)
surface

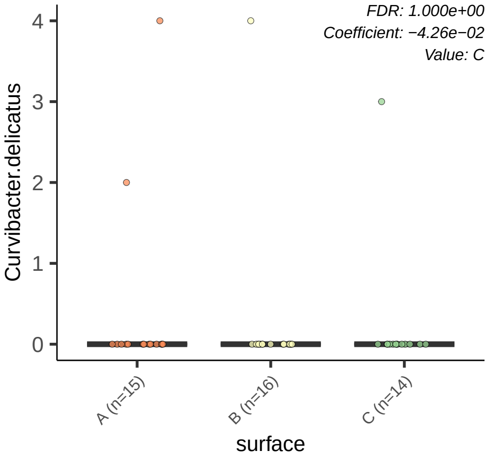
FDR: 1.000e+00
Coefficient: −4.26e−02
Value: C
Curvibacter.delicatus
4
3
2
1
0
A (n=15)
B (n=16)
C (n=14)
surface

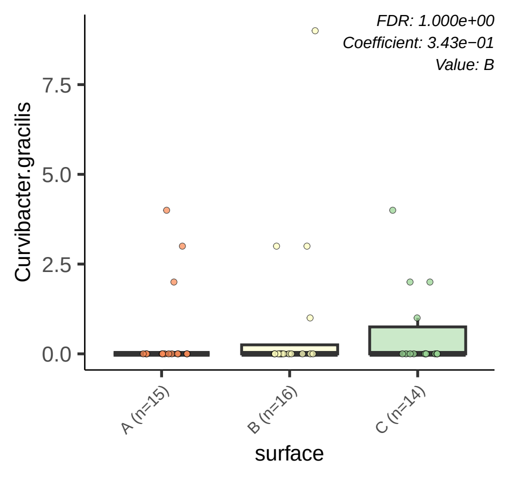

FDR: 1.000e+00
Coefficient: 3.43e−01
Value: B
Curvibacter.gracilis
7.5
5.0
2.5
0.0
A (n=15)
B (n=16)
C (n=14)
surface

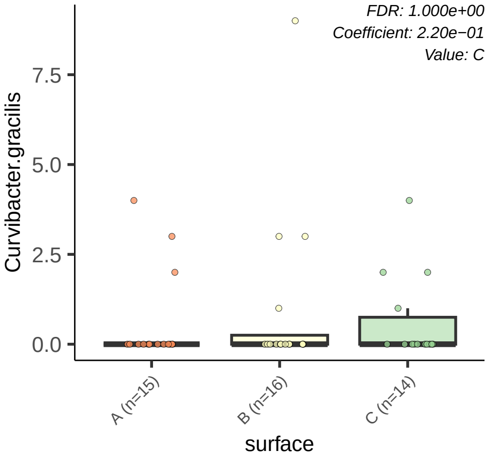
FDR: 1.000e+00
Coefficient: 2.20e−01
Value: C
Curvibacter.gracilis
7.5
5.0
2.5
0.0
A (n=15)
B (n=16)
C (n=14)
surface

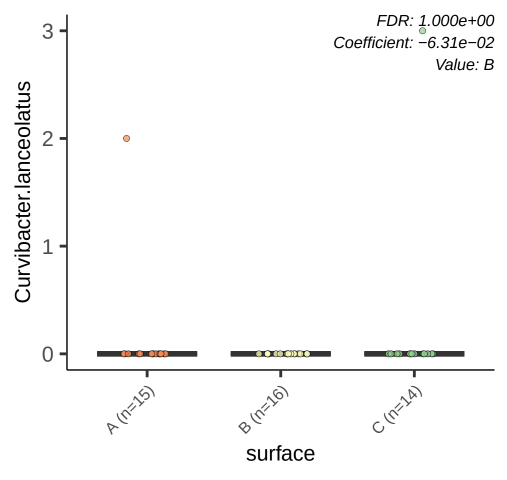
FDR: 1.000e+00
Coefficient: −6.31e−02
Value: B
Curvibacter.lanceolatus
3
2
1
0
A (n=15)
B (n=16)
C (n=14)
surface

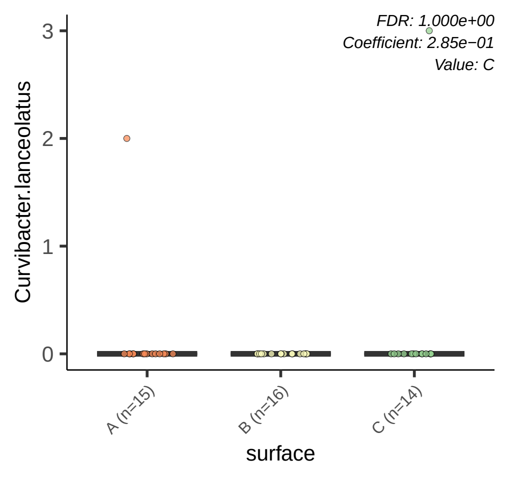

FDR: 1.000e+00
Coefficient: 2.85e−01
Value: C
Curvibacter.lanceolatus
3
2
1
0
A (n=15)
B (n=16)
C (n=14)
surface

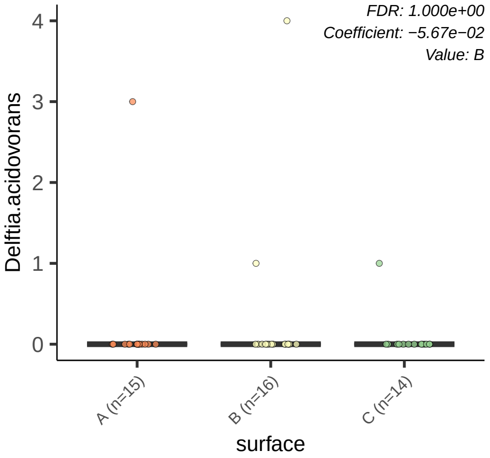
FDR: 1.000e+00
Coefficient: −5.67e−02
Value: B
Delftia.acidovorans
0
1
2
3
4
A (n=15)
B (n=16)
C (n=14)
surface

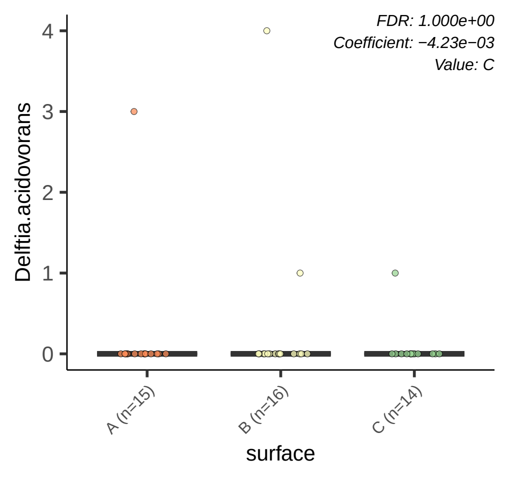
FDR: 1.000e+00
Coefficient: −4.23e−03
Value: C
Delftia.acidovorans
0
1
2
3
4
A (n=15)
B (n=16)
C (n=14)
surface

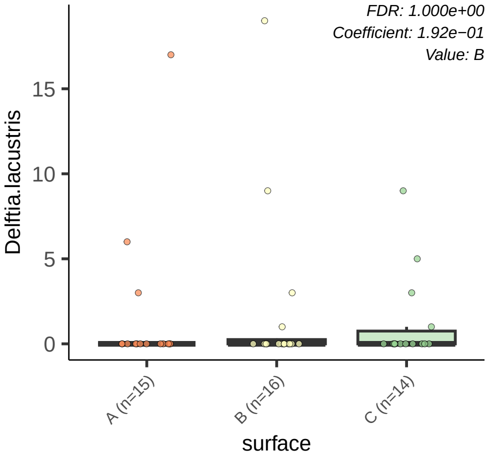

FDR: 1.000e+00
Coefficient: 1.92e−01
Value: B
Delftia.lacustris
15
10
5
0
A (n=15)
B (n=16)
C (n=14)
surface

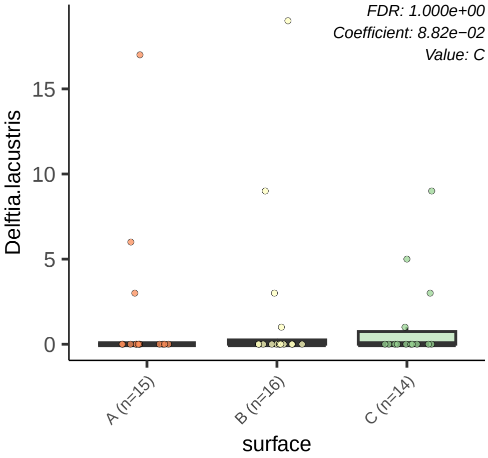
FDR: 1.000e+00
Coefficient: 8.82e−02
Value: C
Delftia.lacustris
15
10
5
0
A (n=15)
B (n=16)
C (n=14)
surface

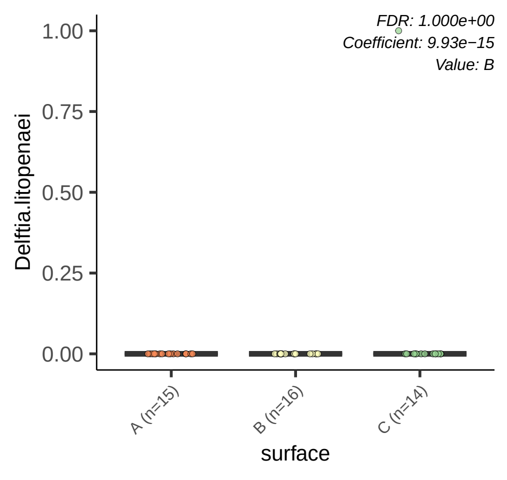

FDR: 1.000e+00
Coefficient: 9.93e−15
Value: B
Delftia.litopenaei
1.00
0.75
0.50
0.25
0.00
A (n=15)
B (n=16)
C (n=14)
surface

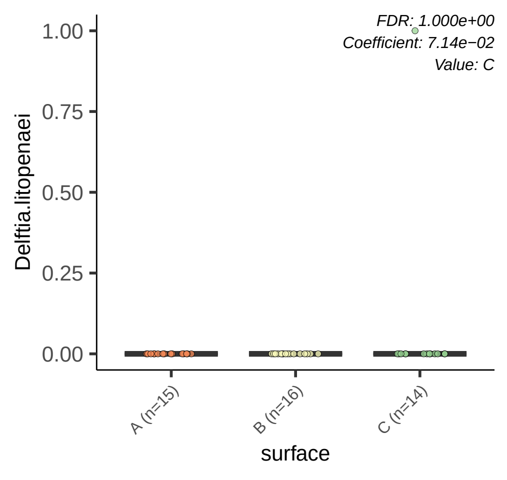
FDR: 1.000e+00
Coefficient: 7.14e−02
Value: C
1.00
0.75
0.50
0.25
0.00
Delftia.litopenaei
A (n=15)
B (n=16)
C (n=14)
surface

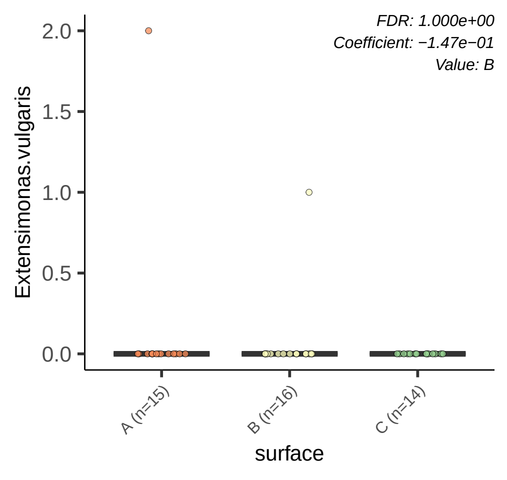

FDR: 1.000e+00
Coefficient: −1.47e−01
Value: B
Extensimonas.vulgaris
2.0
1.5
1.0
0.5
0.0
A (n=15)
B (n=16)
C (n=14)
surface

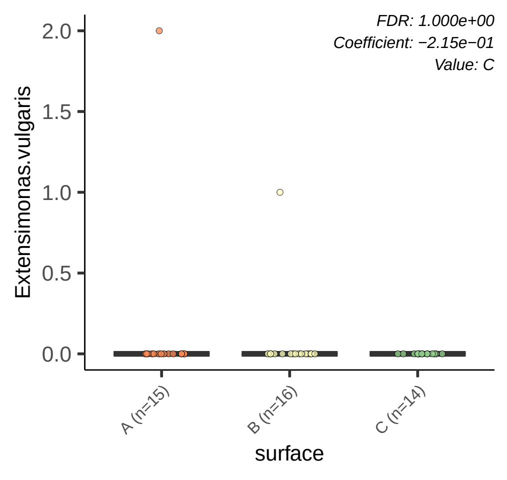

FDR: 1.000e+00
Coefficient: −2.15e−01
Value: C
Extensimonas.vulgaris
2.0
1.5
1.0
0.5
0.0
A (n=15)
B (n=16)
C (n=14)
surface

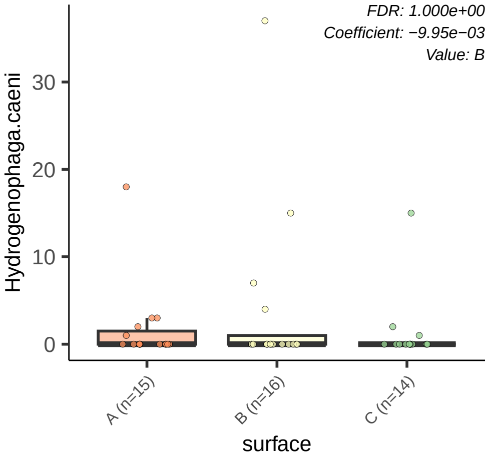
FDR: 1.000e+00
Coefficient: −9.95e−03
Value: B
Hydrogenophaga.caeni
0
10
20
30
A (n=15)
B (n=16)
C (n=14)
surface

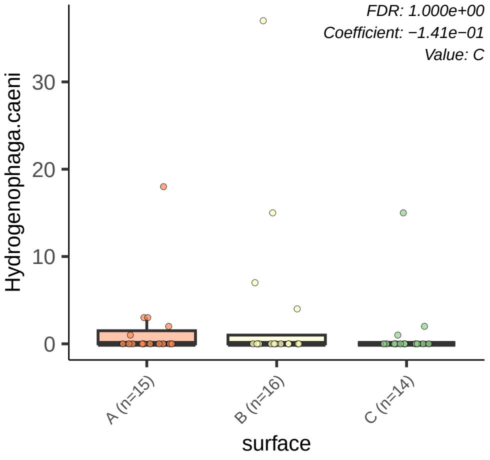

FDR: 1.000e+00
Coefficient: −1.41e−01
Value: C
Hydrogenophaga.caeni
0
10
20
30
A (n=15)
B (n=16)
C (n=14)
surface

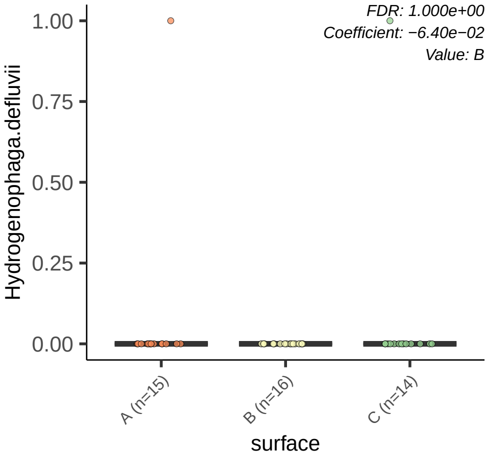
FDR: 1.000e+00
Coefficient: −6.40e−02
Value: B
Hydrogenophaga.defluvii
1.00
0.75
0.50
0.25
0.00
A (n=15)
B (n=16)
C (n=14)
surface

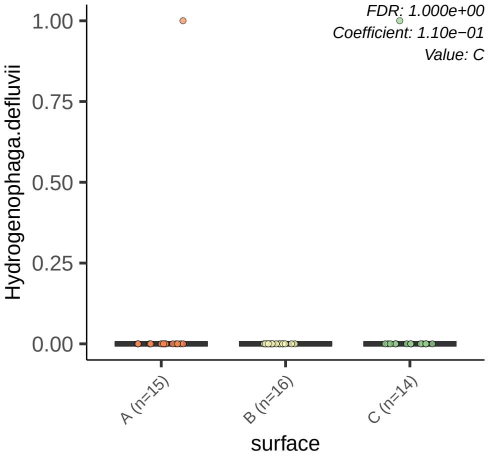
FDR: 1.000e+00
Coefficient: 1.10e−01
Value: C
Hydrogenophaga.defluvii
1.00
0.75
0.50
0.25
0.00
A (n=15)
B (n=16)
C (n=14)
surface

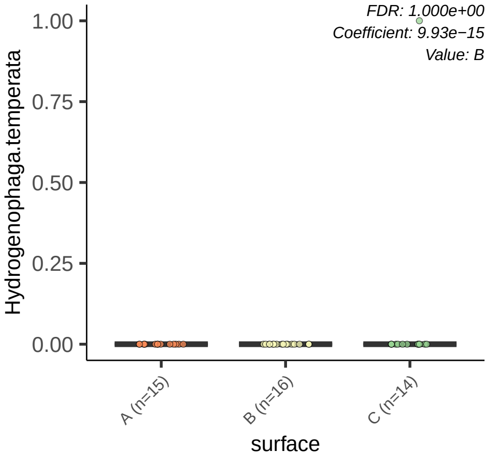

FDR: 1.000e+00
Coefficient: 9.93e−15
Value: B
Hydrogenophaga.temperata
1.00
0.75
0.50
0.25
0.00
A (n=15)
B (n=16)
C (n=14)
surface

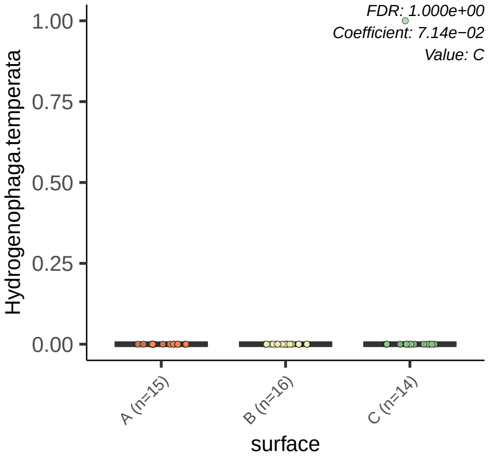

FDR: 1.000e+00
Coefficient: 7.14e−02
Value: C
Hydrogenophaga.temperata
1.00
0.75
0.50
0.25
0.00
A (n=15)
B (n=16)
C (n=14)
surface

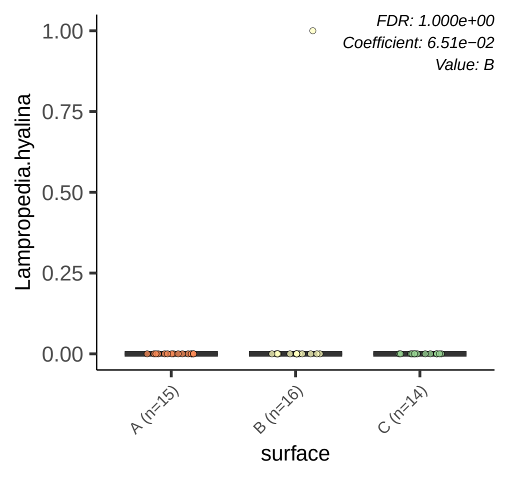
FDR: 1.000e+00
Coefficient: 6.51e−02
Value: B
Lampropedia.hyalina
1.00
0.75
0.50
0.25
0.00
A (n=15)
B (n=16)
C (n=14)
surface

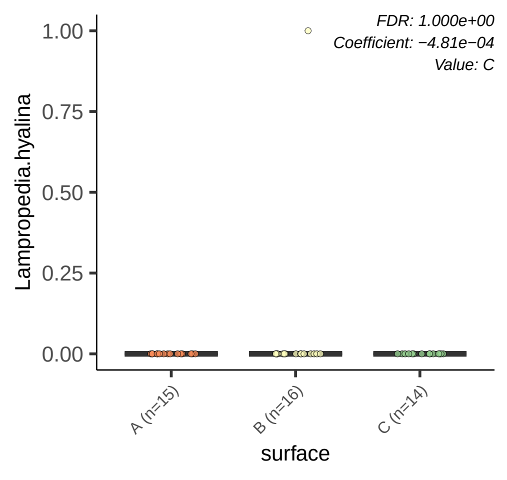
FDR: 1.000e+00
Coefficient: −4.81e−04
Value: C
Lampropedia.hyalina
1.00
0.75
0.50
0.25
0.00
A (n=15)
B (n=16)
C (n=14)
surface

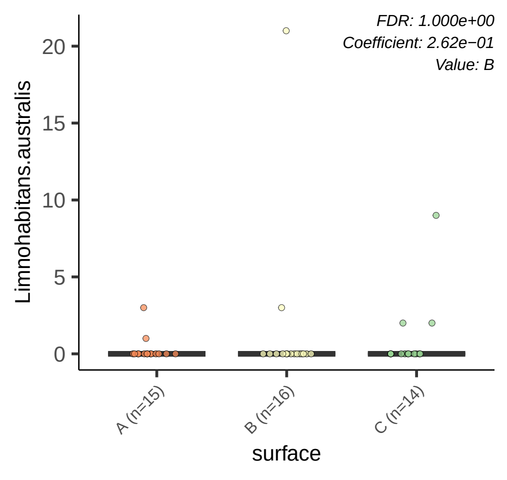

FDR: 1.000e+00
Coefficient: 2.62e−01
Value: B
Limnohabitans.australis
20
15
10
5
0
A (n=15)
B (n=16)
C (n=14)
surface

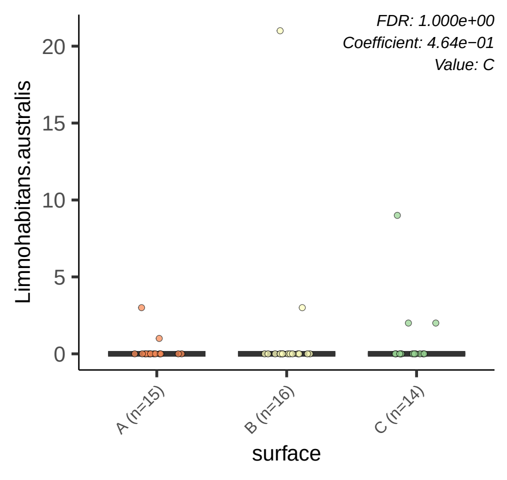
FDR: 1.000e+00
Coefficient: 4.64e−01
Value: C
Limnohabitans.australis
0
5
10
15
20
A (n=15)
B (n=16)
C (n=14)
surface

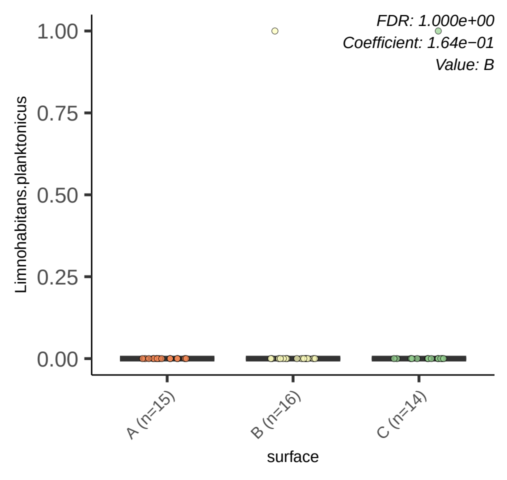
FDR: 1.000e+00
Coefficient: 1.64e−01
Value: B
Limnohabitans.planktonicus
1.00
0.75
0.50
0.25
0.00
A (n=15)
B (n=16)
C (n=14)
surface

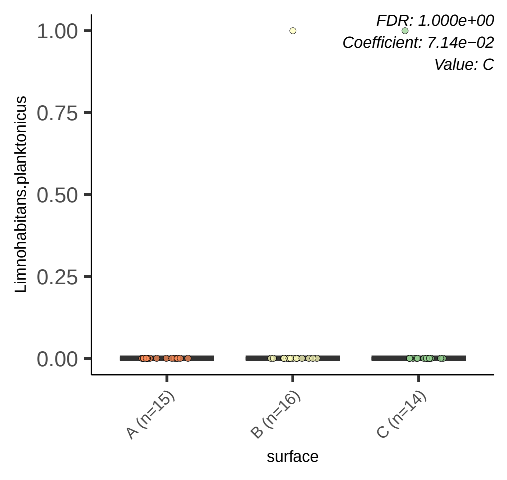
FDR: 1.000e+00
Coefficient: 7.14e−02
Value: C
1.00
0.75
0.50
0.25
0.00
Limnohabitans.planktonicus
A (n=15)
B (n=16)
C (n=14)
surface

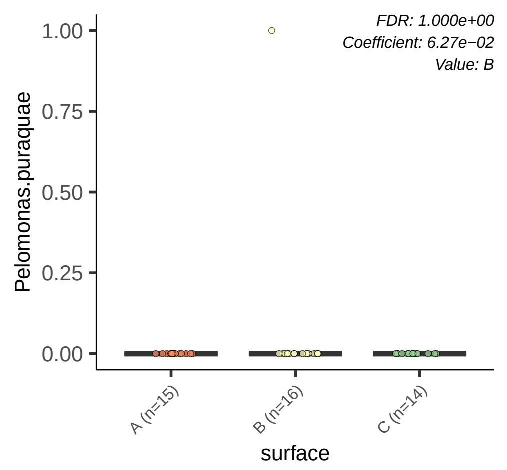

FDR: 1.000e+00
Coefficient: 6.27e−02
Value: B
Pelomonas.puraquae
1.00
0.75
0.50
0.25
0.00
A (n=15)
B (n=16)
C (n=14)
surface

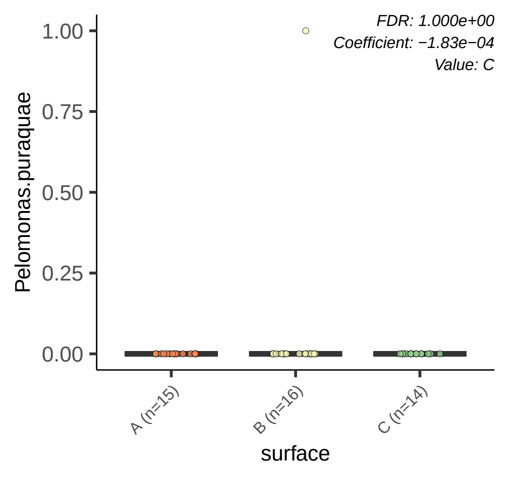
FDR: 1.000e+00
Coefficient: −1.83e−04
Value: C
Pelomonas.puraquae
1.00
0.75
0.50
0.25
0.00
A (n=15)
B (n=16)
C (n=14)
surface

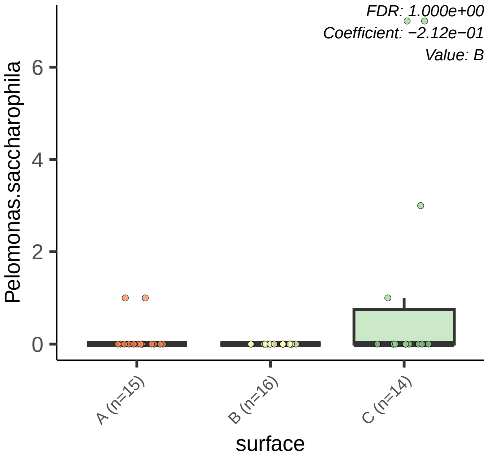

FDR: 1.000e+00
Coefficient: −2.12e−01
Value: B
Pelomonas.saccharophila
0
2
4
6
A (n=15)
B (n=16)
C (n=14)
surface

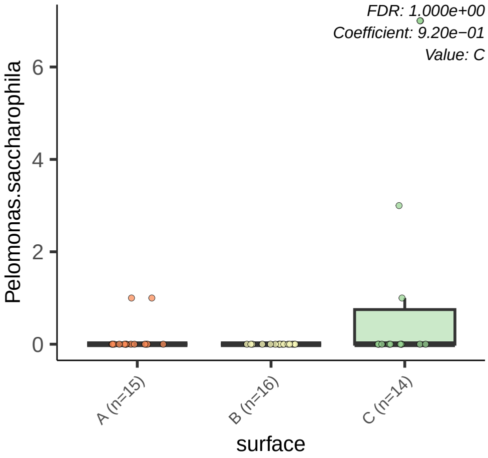

FDR: 1.000e+00
Coefficient: 9.20e−01
Value: C
Pelomonas.saccharophila
0
2
4
6
A (n=15)
B (n=16)
C (n=14)
surface

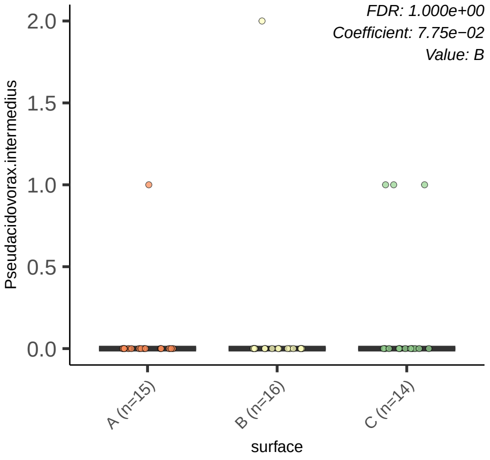
FDR: 1.000e+00
Coefficient: 7.75e−02
Value: B
Pseudacidovorax.intermedius
2.0
1.5
1.0
0.5
0.0
A (n=15)
B (n=16)
C (n=14)
surface

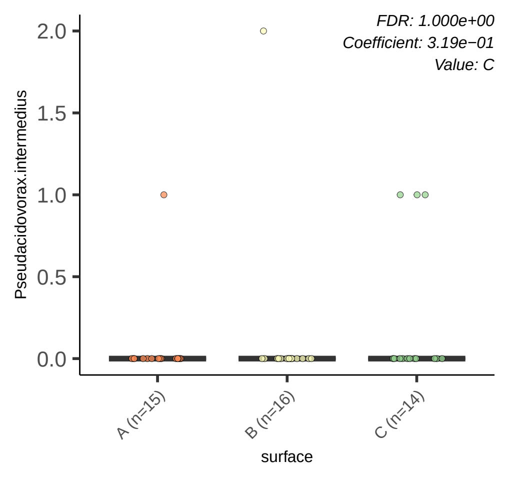
FDR: 1.000e+00
Coefficient: 3.19e−01
Value: C
Pseudacidovorax.intermedius
2.0
1.5
1.0
0.5
0.0
A (n=15)
B (n=16)
C (n=14)
surface

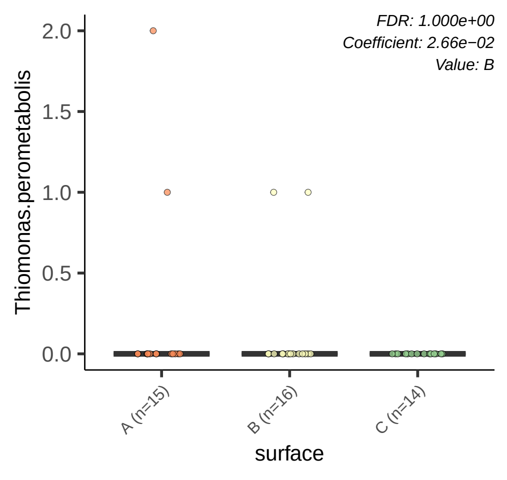
FDR: 1.000e+00
Coefficient: 2.66e−02
Value: B
Thiomonas.perometabolis
2.0
1.5
1.0
0.5
0.0
A (n=15)
B (n=16)
C (n=14)
surface

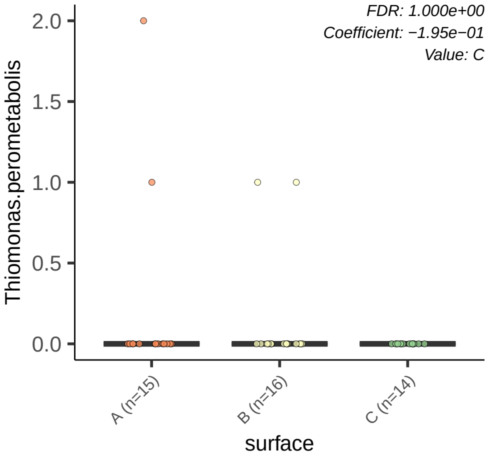
FDR: 1.000e+00
Coefficient: −1.95e−01
Value: C
Thiomonas.perometabolis
2.0
1.5
1.0
0.5
0.0
A (n=15)
B (n=16)
C (n=14)
surface

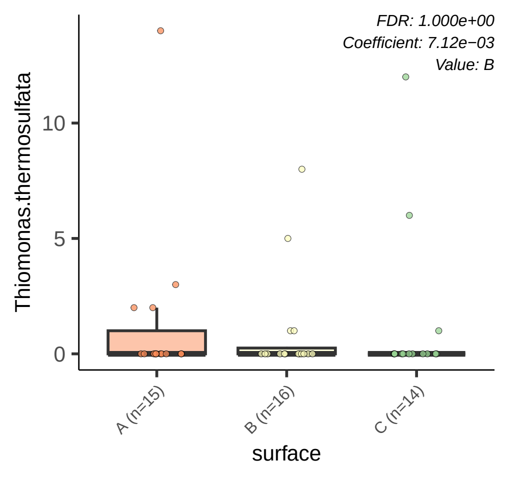
FDR: 1.000e+00
Coefficient: 7.12e−03
Value: B
Thiomonas.thermosulfata
10
5
0
A (n=15)
B (n=16)
C (n=14)
surface

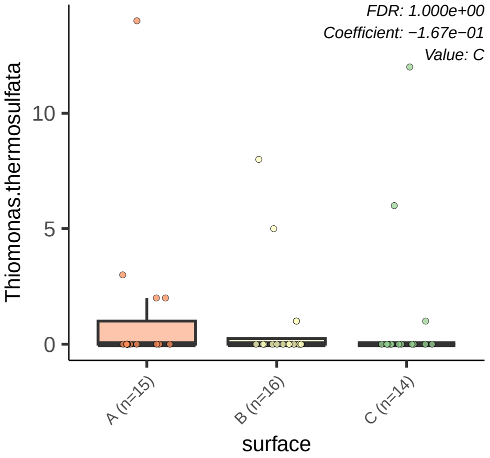
FDR: 1.000e+00
Coefficient: −1.67e−01
Value: C
Thiomonas.thermosulfata
10
5
0
A (n=15)
B (n=16)
C (n=14)
surface

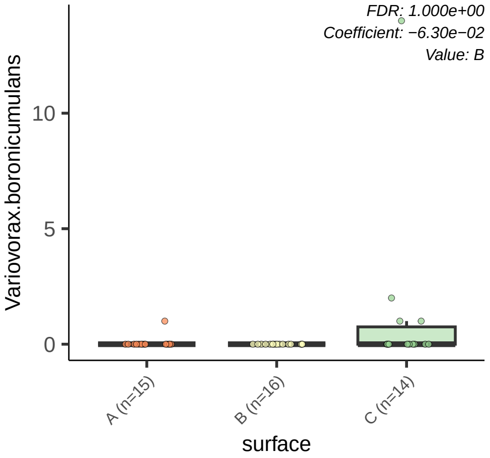

FDR: 1.000e+00
Coefficient: −6.30e−02
Value: B
Variovorax.boronicumulans
0
5
10
A (n=15)
B (n=16)
C (n=14)
surface

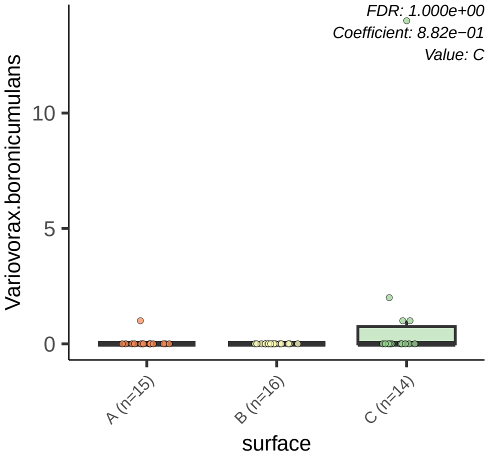
FDR: 1.000e+00
Coefficient: 8.82e−01
Value: C
Variovorax.boronicumulans
10
5
0
A (n=15)
B (n=16)
C (n=14)
surface

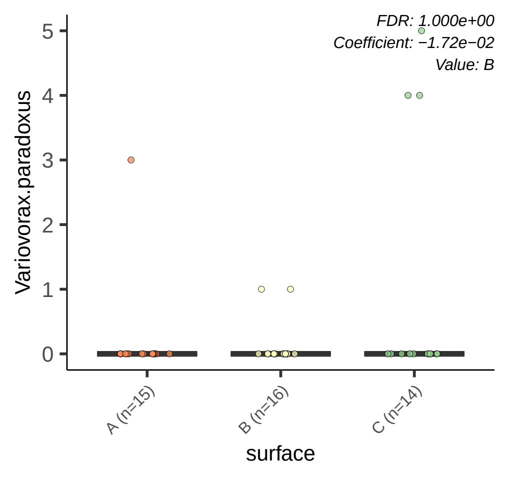
FDR: 1.000e+00
Coefficient: −1.72e−02
Value: B
Variovorax.paradoxus
0
1
2
3
4
5
A (n=15)
B (n=16)
C (n=14)
surface

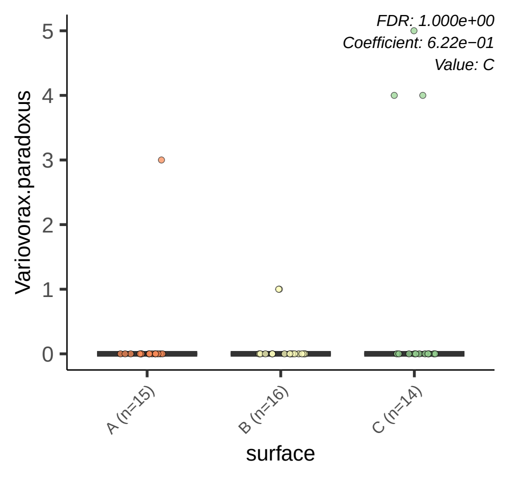
FDR: 1.000e+00
Coefficient: 6.22e−01
Value: C
Variovorax.paradoxus
0
1
2
3
4
5
A (n=15)
B (n=16)
C (n=14)
surface

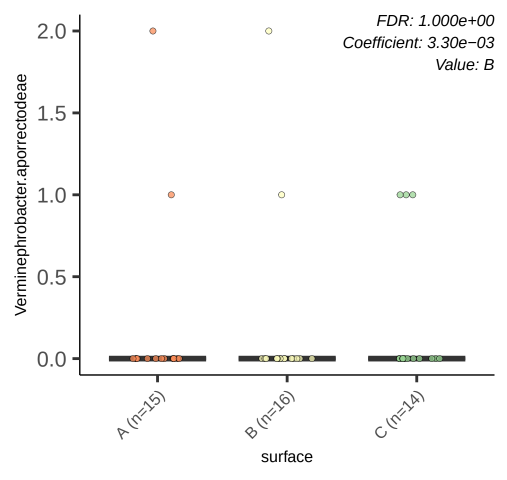

FDR: 1.000e+00
Coefficient: 3.30e−03
Value: B
Verminephrobacter.aporrectodeae
2.0
1.5
1.0
0.5
0.0
A (n=15)
B (n=16)
C (n=14)
surface

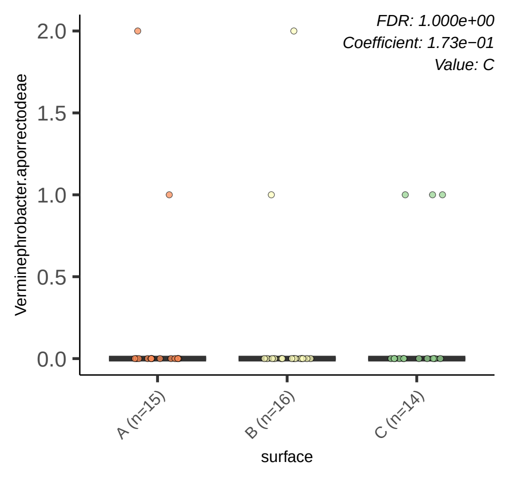
FDR: 1.000e+00
Coefficient: 1.73e−01
Value: C
Verminephrobacter.aporrectodeae
2.0
1.5
1.0
0.5
0.0
A (n=15)
B (n=16)
C (n=14)
surface

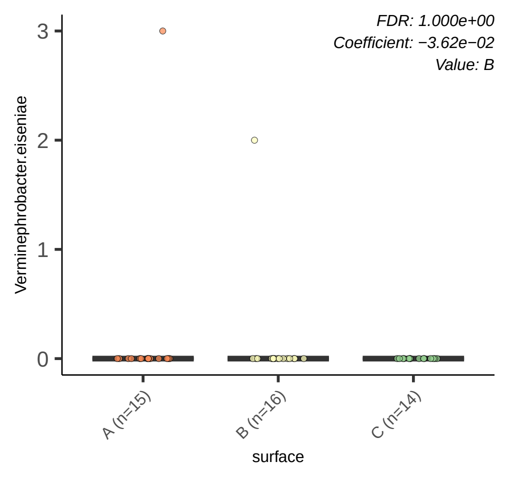
FDR: 1.000e+00
Coefficient: −3.62e−02
Value: B
Verminephrobacter.eiseniae
3
2
1
0
A (n=15)
B (n=16)
C (n=14)
surface

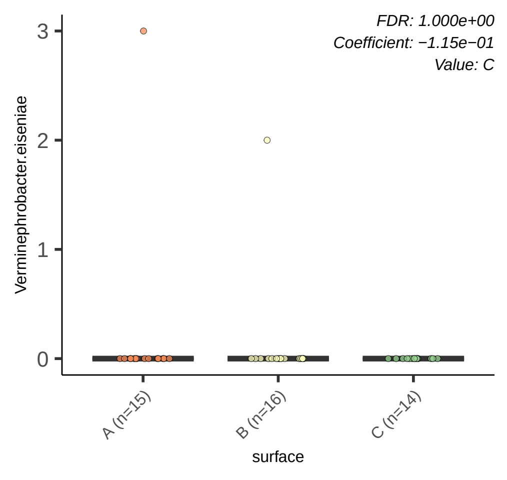

FDR: 1.000e+00
Coefficient: −1.15e−01
Value: C
Verminephrobacter.eiseniae
3
2
1
0
A (n=15)
B (n=16)
C (n=14)
surface

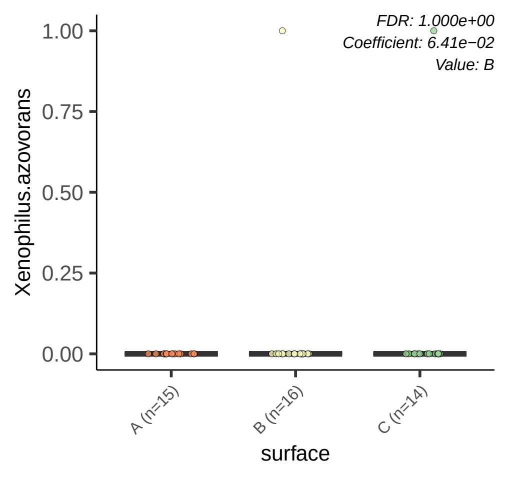
FDR: 1.000e+00
Coefficient: 6.41e−02
Value: B
Xenophilus.azovorans
1.00
0.75
0.50
0.25
0.00
A (n=15)
B (n=16)
C (n=14)
surface

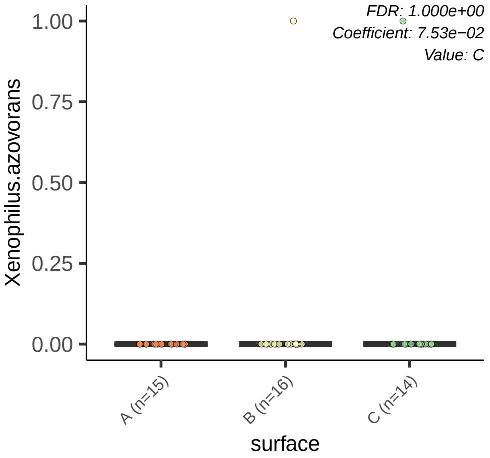

FDR: 1.000e+00
Coefficient: 7.53e−02
Value: C
Xenophilus.azovorans
1.00
0.75
0.50
0.25
0.00
A (n=15)
B (n=16)
C (n=14)
surface

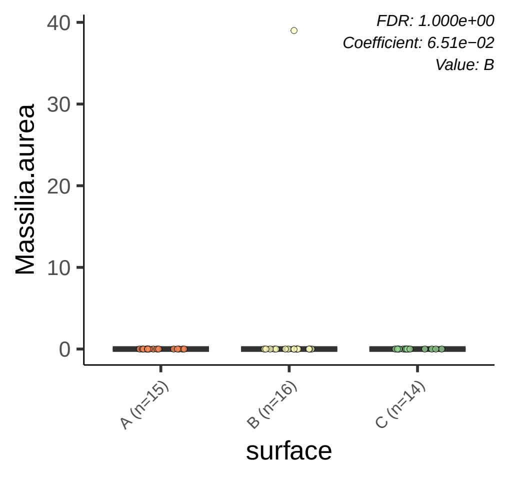

FDR: 1.000e+00
Coefficient: 6.51e−02
Value: B
Massilia.aurea
40
30
20
10
0
A (n=15)
B (n=16)
C (n=14)
surface

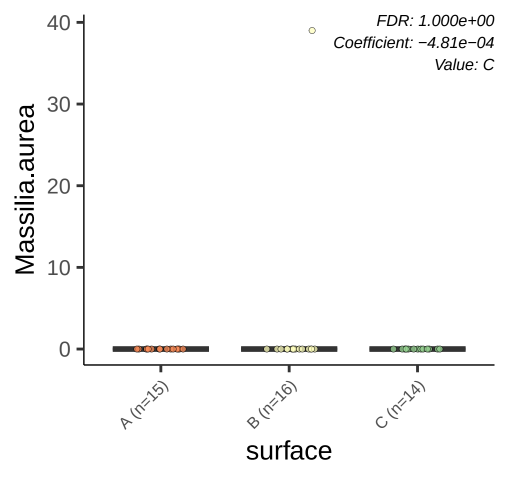
FDR: 1.000e+00
Coefficient: −4.81e−04
Value: C
Massilia.aurea
40
30
20
10
0
A (n=15)
B (n=16)
C (n=14)
surface

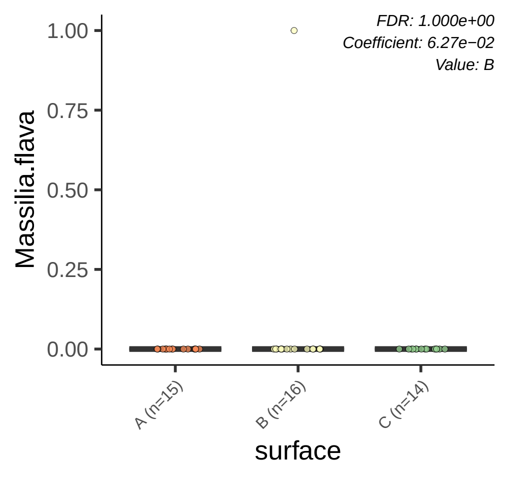

FDR: 1.000e+00
Coefficient: 6.27e−02
Value: B
Massilia.flava
1.00
0.75
0.50
0.25
0.00
A (n=15)
B (n=16)
C (n=14)
surface

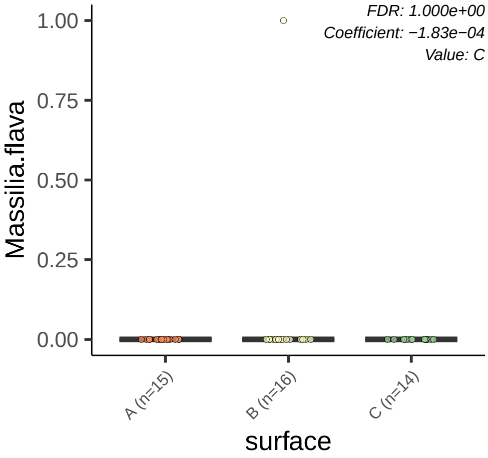
FDR: 1.000e+00
Coefficient: −1.83e−04
Value: C
Massilia.flava
1.00
0.75
0.50
0.25
0.00
A (n=15)
B (n=16)
C (n=14)
surface

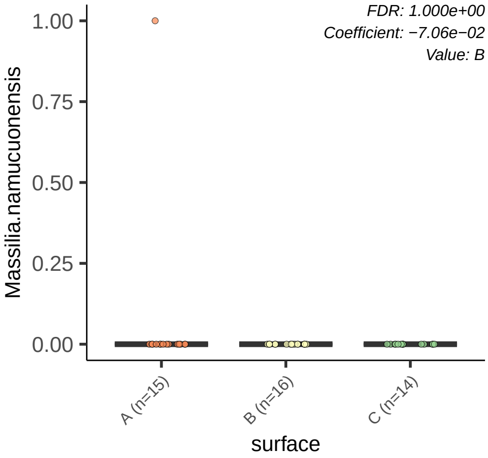
FDR: 1.000e+00
Coefficient: −7.06e−02
Value: B
Massilia.namucuonensis
1.00
0.75
0.50
0.25
0.00
A (n=15)
B (n=16)
C (n=14)
surface

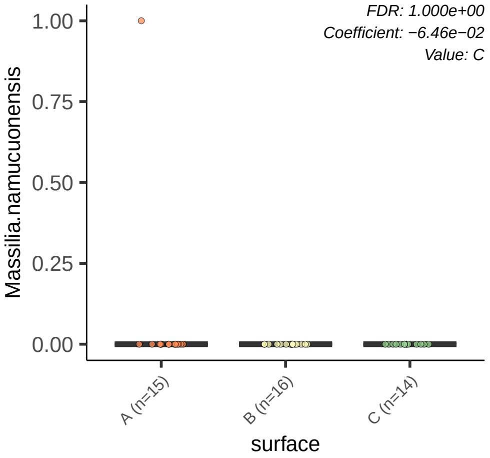
FDR: 1.000e+00
Coefficient: −6.46e−02
Value: C
Massilia.namucuonensis
1.00
0.75
0.50
0.25
0.00
A (n=15)
B (n=16)
C (n=14)
surface

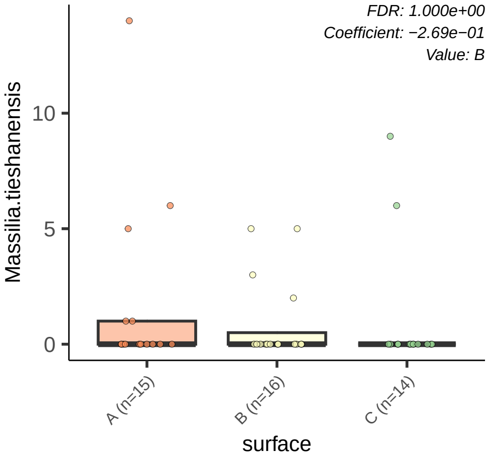

FDR: 1.000e+00
Coefficient: −2.69e−01
Value: B
Massilia.tieshanensis
10
5
0
A (n=15)
B (n=16)
C (n=14)
surface

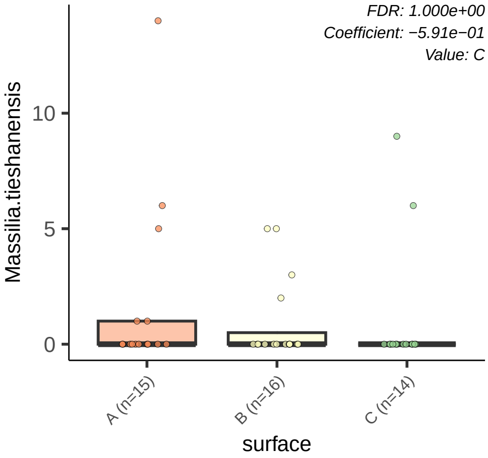

FDR: 1.000e+00
Coefficient: −5.91e−01
Value: C
Massilia.tieshanensis
10
5
0
A (n=15)
B (n=16)
C (n=14)
surface

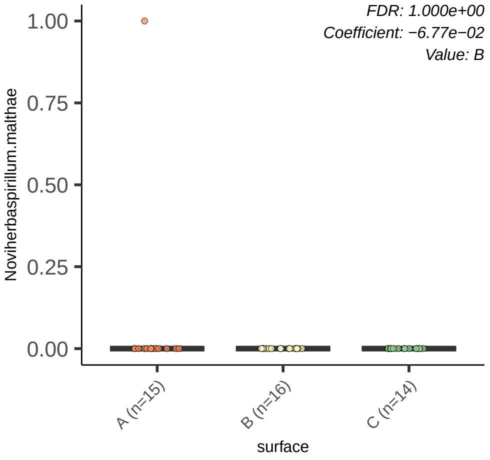
FDR: 1.000e+00
Coefficient: −6.77e−02
Value: B
Noviherbaspirillum.malthae
1.00
0.75
0.50
0.25
0.00
A (n=15)
B (n=16)
C (n=14)
surface

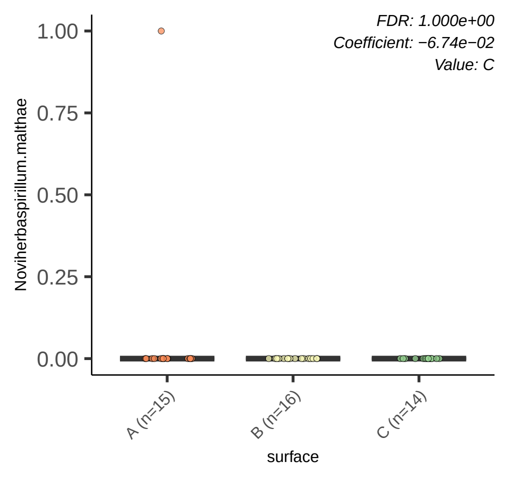
FDR: 1.000e+00
Coefficient: −6.74e−02
Value: C
1.00
0.75
0.50
0.25
0.00
Noviherbaspirillum.malthae
A (n=15)
B (n=16)
C (n=14)
surface

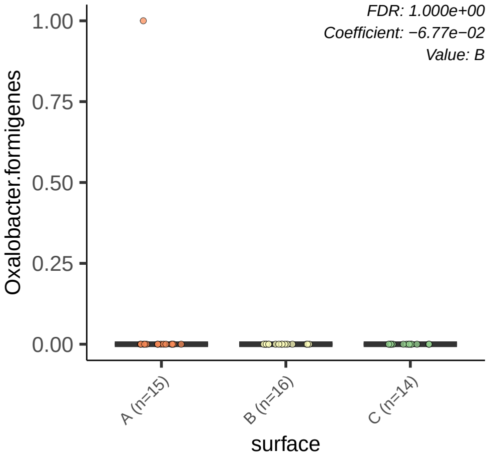
FDR: 1.000e+00
Coefficient: −6.77e−02
Value: B
1.00
0.75
0.50
0.25
0.00
Oxalobacter.formigenes
A (n=15)
B (n=16)
C (n=14)
surface

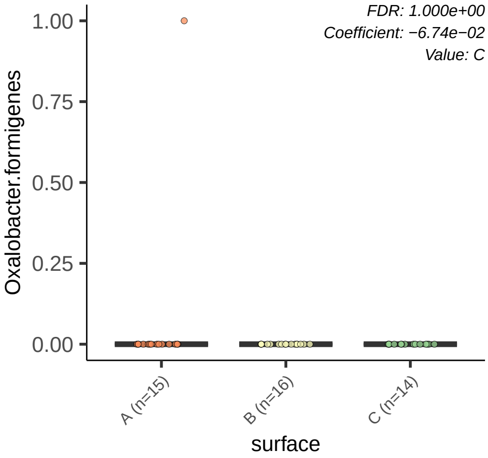

FDR: 1.000e+00
Coefficient: −6.74e−02
Value: C
Oxalobacter.formigenes
1.00
0.75
0.50
0.25
0.00
A (n=15)
B (n=16)
C (n=14)
surface

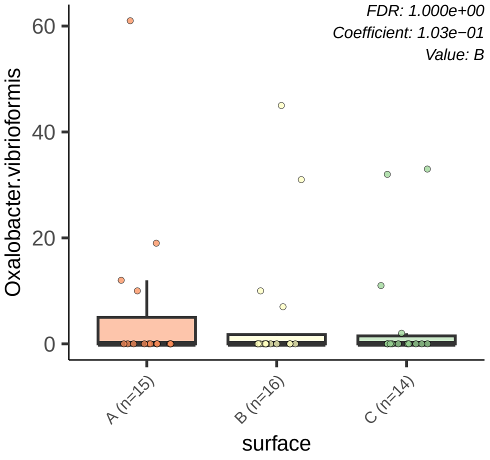
FDR: 1.000e+00
Coefficient: 1.03e−01
Value: B
Oxalobacter.vibrioformis
60
40
20
0
A (n=15)
B (n=16)
C (n=14)
surface

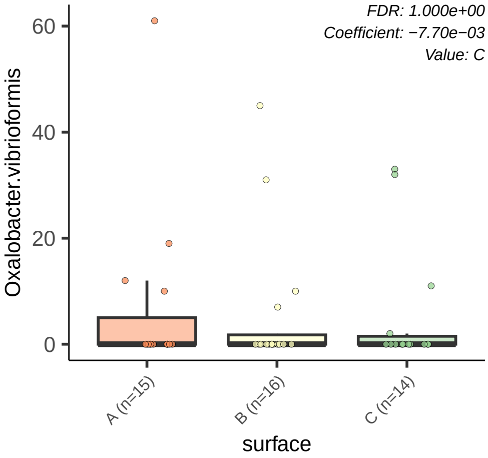
FDR: 1.000e+00
Coefficient: −7.70e−03
Value: C
Oxalobacter.vibrioformis
60
40
20
0
A (n=15)
B (n=16)
C (n=14)
surface

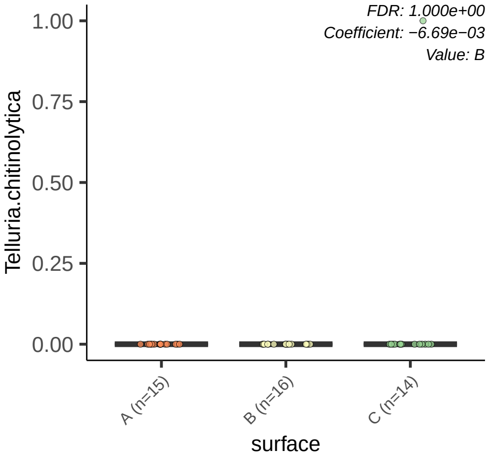

FDR: 1.000e+00
Coefficient: −6.69e−03
Value: B
Telluria.chitinolytica
1.00
0.75
0.50
0.25
0.00
A (n=15)
B (n=16)
C (n=14)
surface

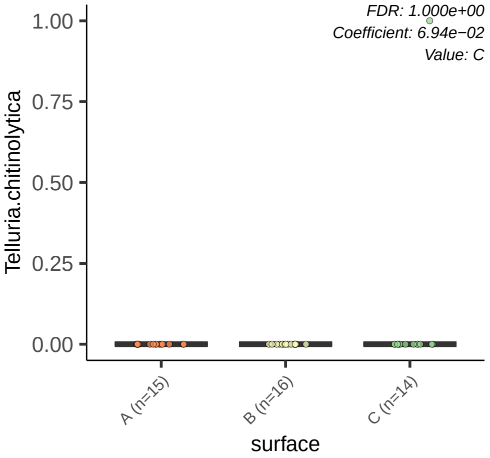
FDR: 1.000e+00
Coefficient: 6.94e−02
Value: C
Telluria.chitinolytica
1.00
0.75
0.50
0.25
0.00
A (n=15)
B (n=16)
C (n=14)
surface

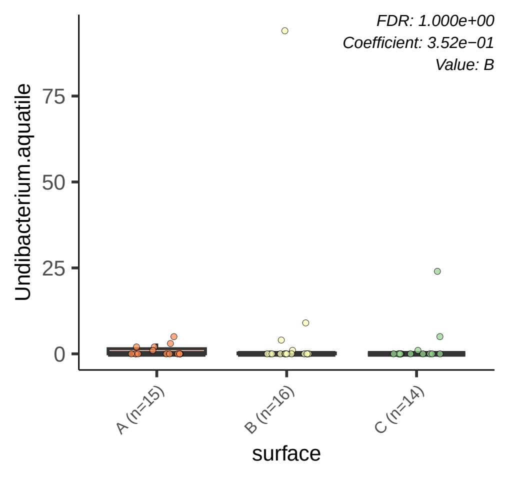

FDR: 1.000e+00
Coefficient: 3.52e−01
Value: B
Undibacterium.aquatile
75
50
25
0
A (n=15)
B (n=16)
C (n=14)
surface

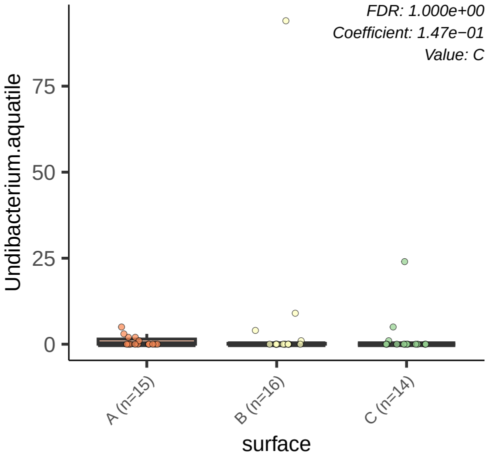

FDR: 1.000e+00
Coefficient: 1.47e−01
Value: C
Undibacterium.aquatile
75
50
25
0
A (n=15)
B (n=16)
C (n=14)
surface

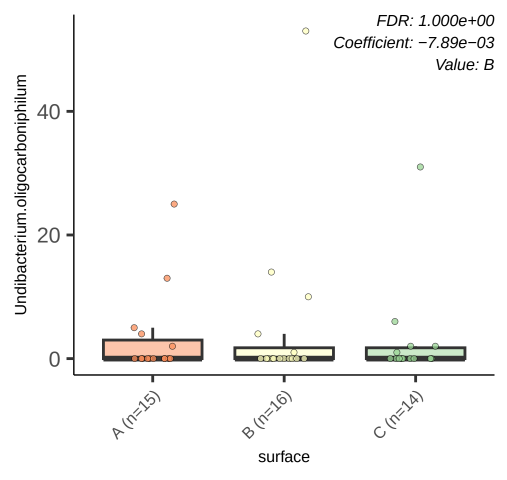
FDR: 1.000e+00
Coefficient: −7.89e−03
Value: B
Undibacterium.oligocarboniphilum
0
20
40
A (n=15)
B (n=16)
C (n=14)
surface

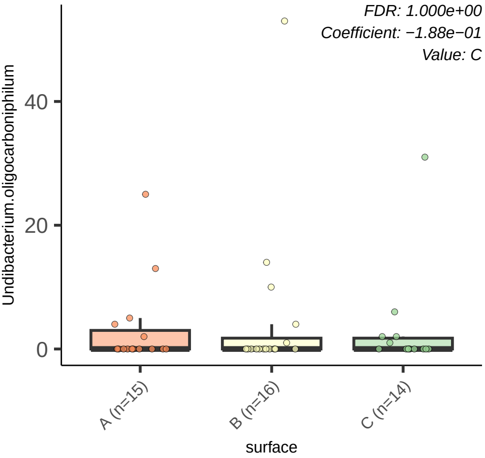
FDR: 1.000e+00
Coefficient: −1.88e−01
Value: C
Undibacterium.oligocarboniphilum
0
20
40
A (n=15)
B (n=16)
C (n=14)
surface

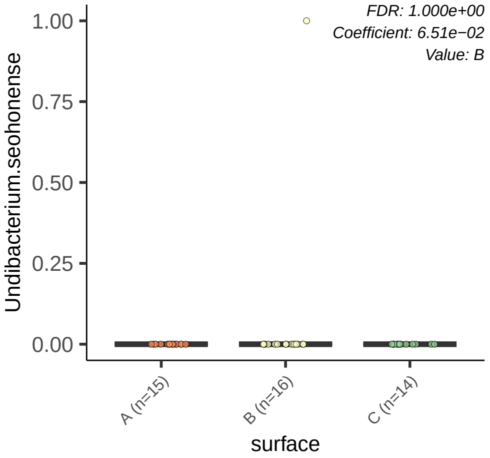

FDR: 1.000e+00
Coefficient: 6.51e−02
Value: B
Undibacterium.seohonense
1.00
0.75
0.50
0.25
0.00
A (n=15)
B (n=16)
C (n=14)
surface

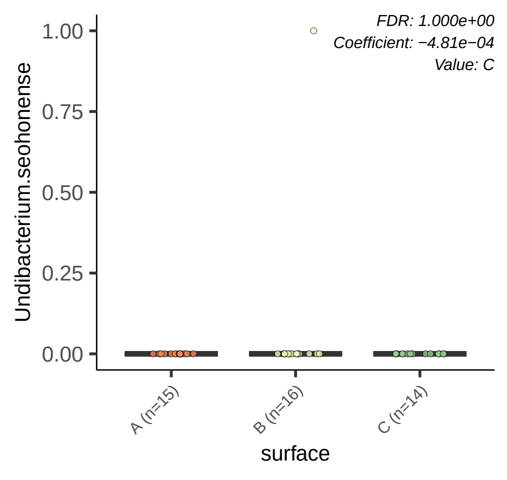
FDR: 1.000e+00
Coefficient: −4.81e−04
Value: C
Undibacterium.seohonense
1.00
0.75
0.50
0.25
0.00
A (n=15)
B (n=16)
C (n=14)
surface

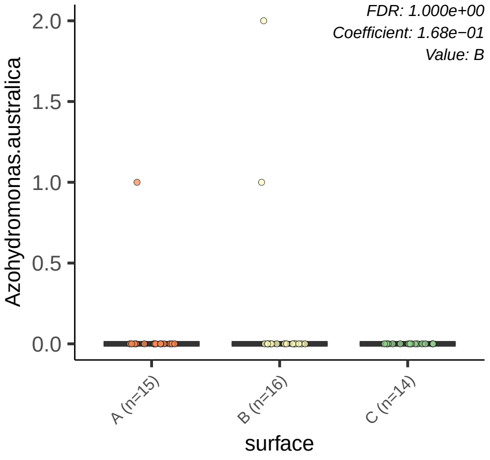
FDR: 1.000e+00
Coefficient: 1.68e−01
Value: B
Azohydromonas.australica
2.0
1.5
1.0
0.5
0.0
A (n=15)
B (n=16)
C (n=14)
surface

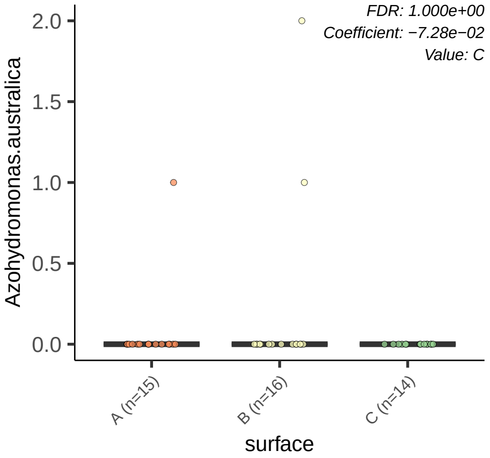
FDR: 1.000e+00
Coefficient: −7.28e−02
Value: C
Azohydromonas.australica
2.0
1.5
1.0
0.5
0.0
A (n=15)
B (n=16)
C (n=14)
surface

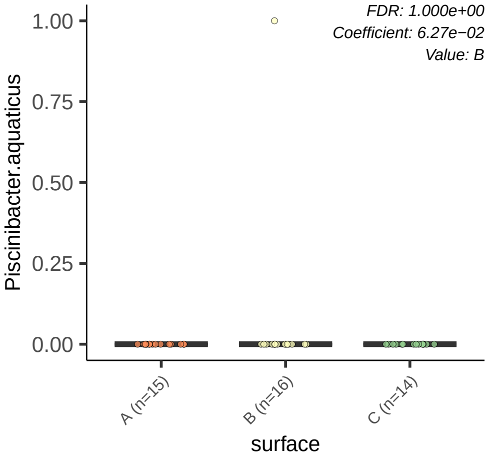
FDR: 1.000e+00
Coefficient: 6.27e−02
Value: B
Piscinibacter.aquaticus
1.00
0.75
0.50
0.25
0.00
A (n=15)
B (n=16)
C (n=14)
surface

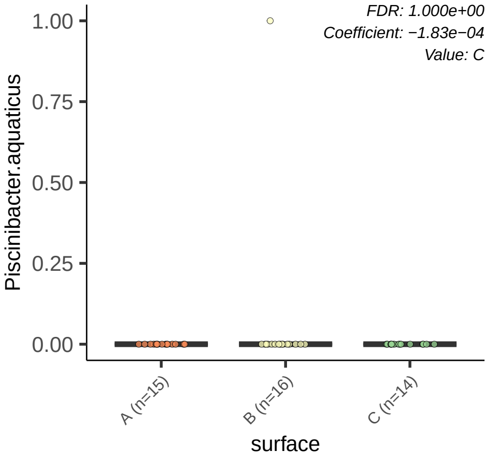

FDR: 1.000e+00
Coefficient: −1.83e−04
Value: C
Piscinibacter.aquaticus
1.00
0.75
0.50
0.25
0.00
A (n=15)
B (n=16)
C (n=14)
surface

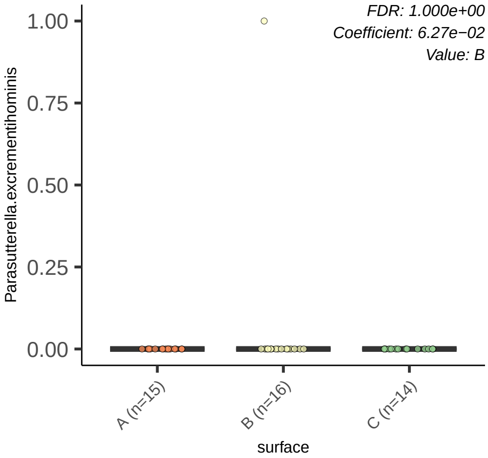

FDR: 1.000e+00
Coefficient: 6.27e−02
Value: B
Parasutterella.excrementihominis
1.00
0.75
0.50
0.25
0.00
A (n=15)
B (n=16)
C (n=14)
surface

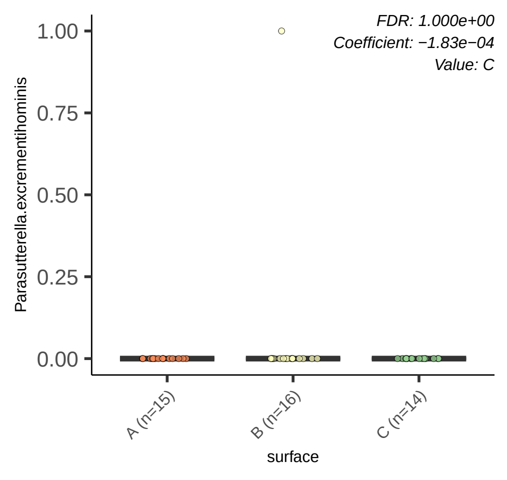

FDR: 1.000e+00
Coefficient: −1.83e−04
Value: C
Parasutterella.excrementihominis
1.00
0.75
0.50
0.25
0.00
A (n=15)
B (n=16)
C (n=14)
surface

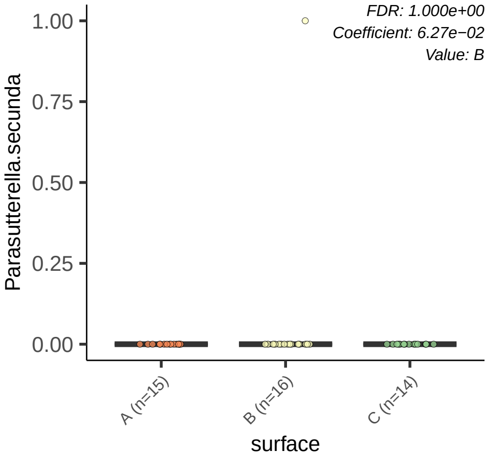

FDR: 1.000e+00
Coefficient: 6.27e−02
Value: B
Parasutterella.secunda
1.00
0.75
0.50
0.25
0.00
A (n=15)
B (n=16)
C (n=14)
surface

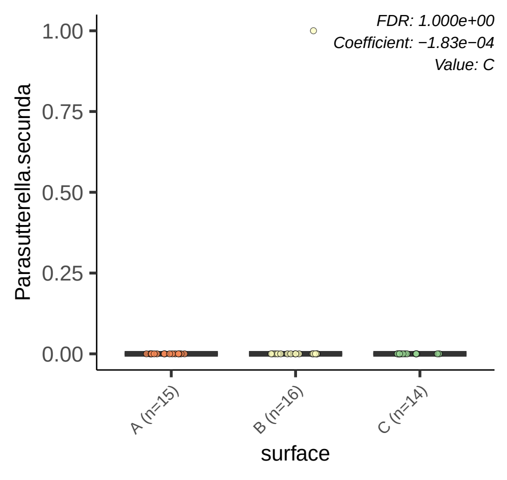

FDR: 1.000e+00
Coefficient: −1.83e−04
Value: C
Parasutterella.secunda
1.00
0.75
0.50
0.25
0.00
A (n=15)
B (n=16)
C (n=14)
surface

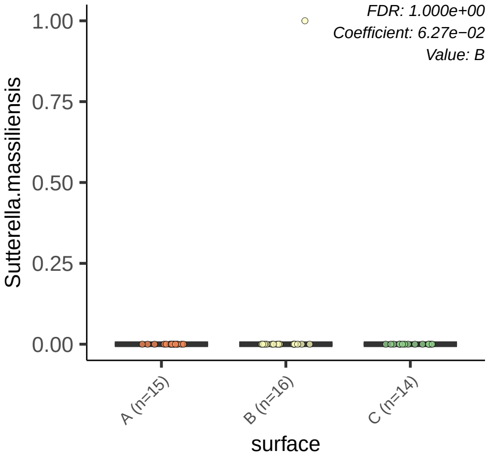

FDR: 1.000e+00
Coefficient: 6.27e−02
Value: B
Sutterella.massiliensis
1.00
0.75
0.50
0.25
0.00
A (n=15)
B (n=16)
C (n=14)
surface

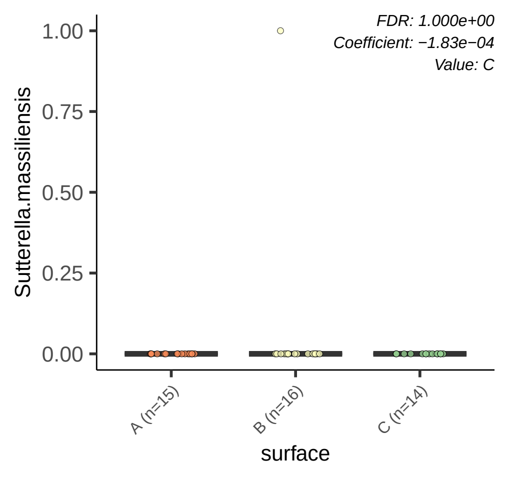
FDR: 1.000e+00
Coefficient: −1.83e−04
Value: C
Sutterella.massiliensis
1.00
0.75
0.50
0.25
0.00
A (n=15)
B (n=16)
C (n=14)
surface

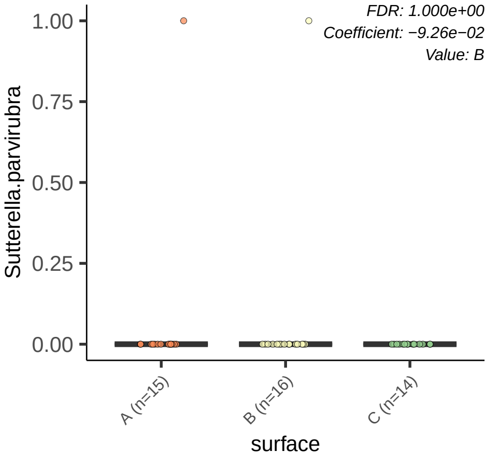
FDR: 1.000e+00
Coefficient: −9.26e−02
Value: B
Sutterella.parvirubra
1.00
0.75
0.50
0.25
0.00
A (n=15)
B (n=16)
C (n=14)
surface

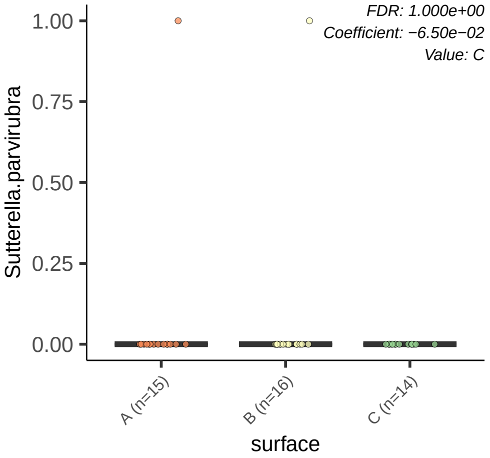

FDR: 1.000e+00
Coefficient: −6.50e−02
Value: C
Sutterella.parvirubra
1.00
0.75
0.50
0.25
0.00
A (n=15)
B (n=16)
C (n=14)
surface

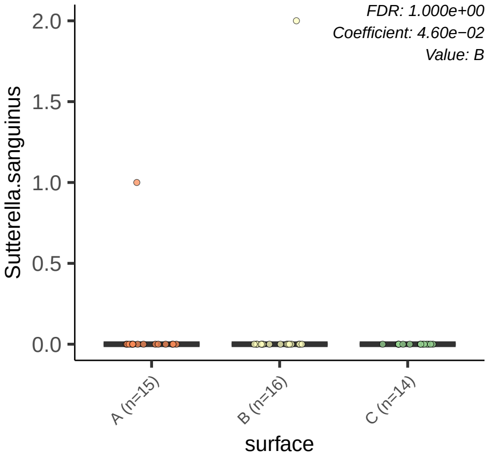

FDR: 1.000e+00
Coefficient: 4.60e−02
Value: B
Sutterella.sanguinus
2.0
1.5
1.0
0.5
0.0
A (n=15)
B (n=16)
C (n=14)
surface

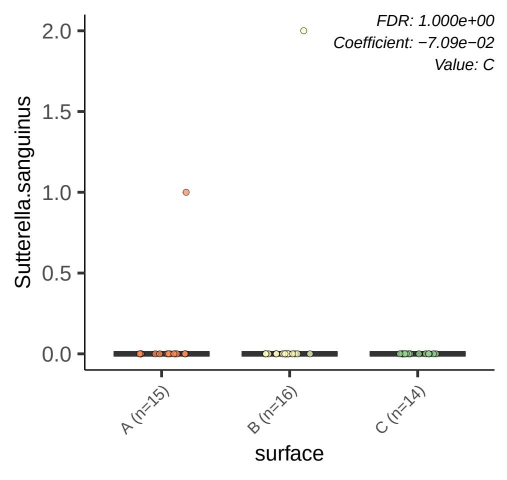
FDR: 1.000e+00
Coefficient: −7.09e−02
Value: C
Sutterella.sanguinus
2.0
1.5
1.0
0.5
0.0
A (n=15)
B (n=16)
C (n=14)
surface

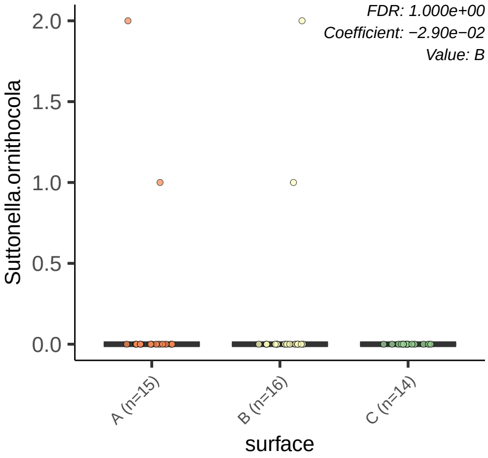

FDR: 1.000e+00
Coefficient: −2.90e−02
Value: B
Suttonella.ornithocola
2.0
1.5
1.0
0.5
0.0
A (n=15)
B (n=16)
C (n=14)
surface

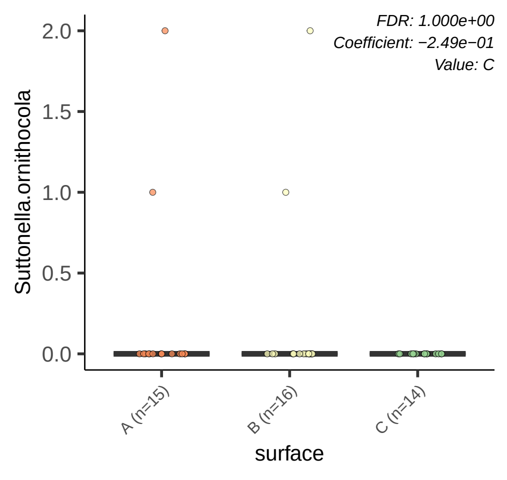

FDR: 1.000e+00
Coefficient: −2.49e−01
Value: C
Suttonella.ornithocola
2.0
1.5
1.0
0.5
0.0
A (n=15)
B (n=16)
C (n=14)
surface

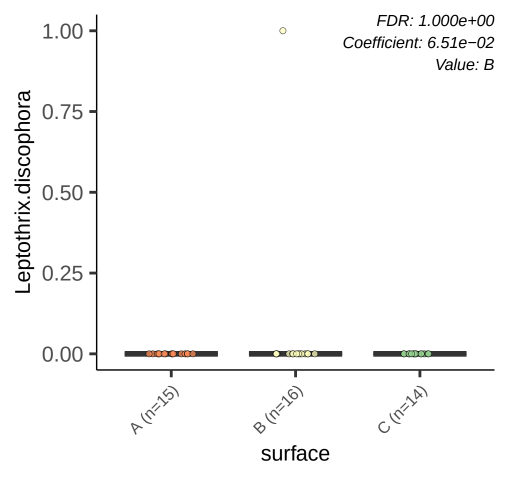

FDR: 1.000e+00
Coefficient: 6.51e−02
Value: B
Leptothrix.discophora
1.00
0.75
0.50
0.25
0.00
A (n=15)
B (n=16)
C (n=14)
surface

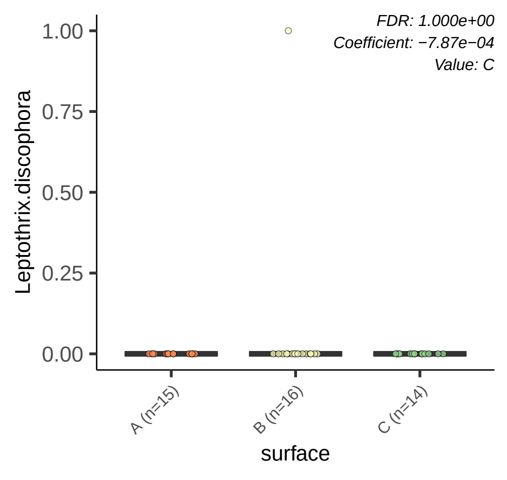

FDR: 1.000e+00
Coefficient: −7.87e−04
Value: C
Leptothrix.discophora
1.00
0.75
0.50
0.25
0.00
A (n=15)
B (n=16)
C (n=14)
surface

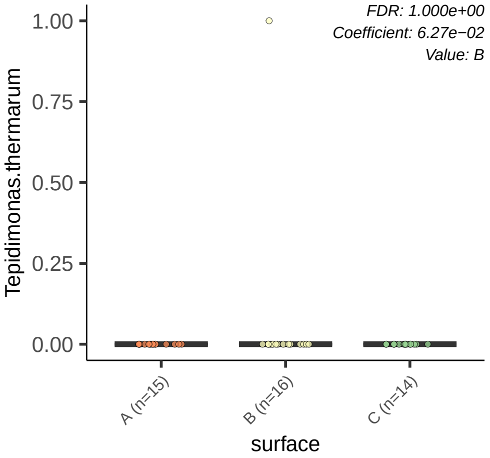
FDR: 1.000e+00
Coefficient: 6.27e−02
Value: B
Tepidimonas.thermarum
1.00
0.75
0.50
0.25
0.00
A (n=15)
B (n=16)
C (n=14)
surface

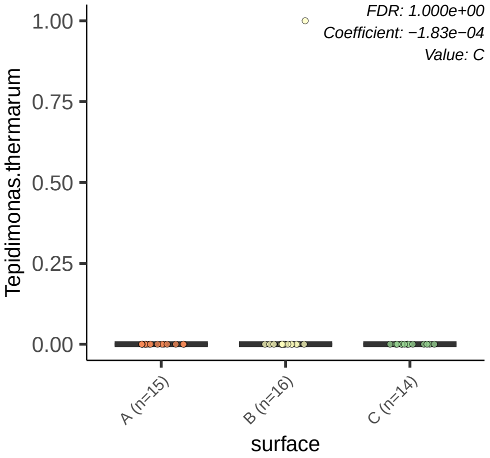
FDR: 1.000e+00
Coefficient: −1.83e−04
Value: C
Tepidimonas.thermarum
1.00
0.75
0.50
0.25
0.00
A (n=15)
B (n=16)
C (n=14)
surface

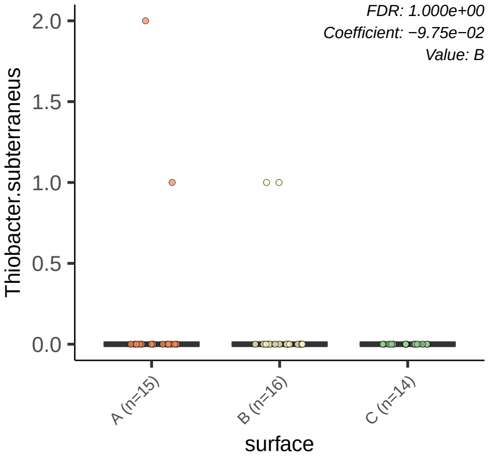
FDR: 1.000e+00
Coefficient: −9.75e−02
Value: B
Thiobacter.subterraneus
2.0
1.5
1.0
0.5
0.0
A (n=15)
B (n=16)
C (n=14)
surface

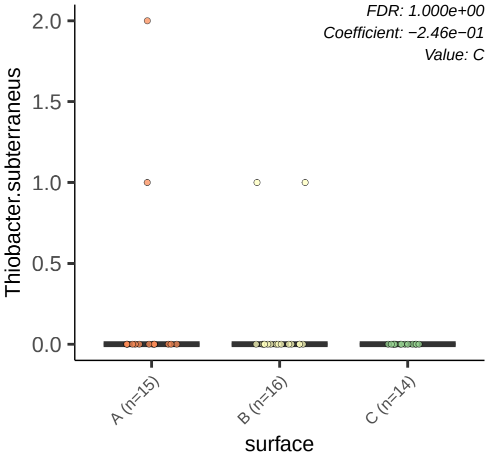

FDR: 1.000e+00
Coefficient: −2.46e−01
Value: C
Thiobacter.subterraneus
2.0
1.5
1.0
0.5
0.0
A (n=15)
B (n=16)
C (n=14)
surface

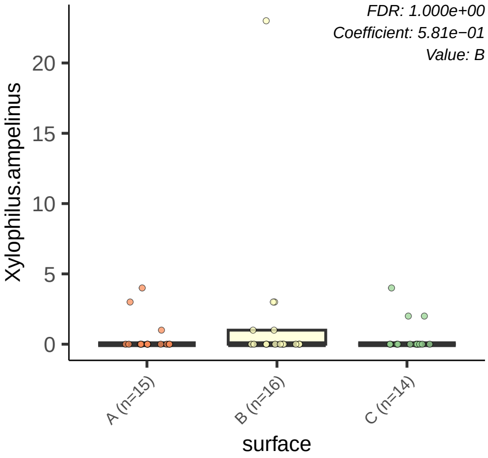

FDR: 1.000e+00
Coefficient: 5.81e−01
Value: B
Xylophilus.ampelinus
0
5
10
15
20
A (n=15)
B (n=16)
C (n=14)
surface

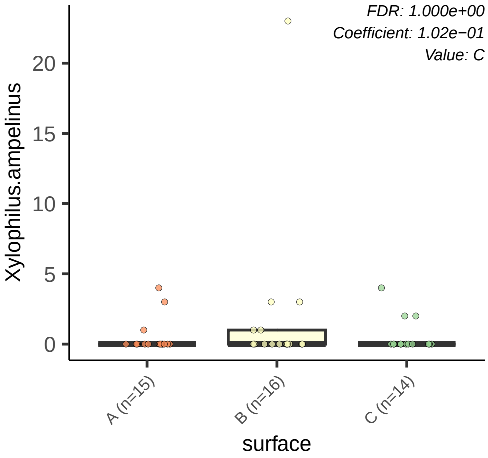

FDR: 1.000e+00
Coefficient: 1.02e−01
Value: C
Xylophilus.ampelinus
0
5
10
15
20
A (n=15)
B (n=16)
C (n=14)
surface

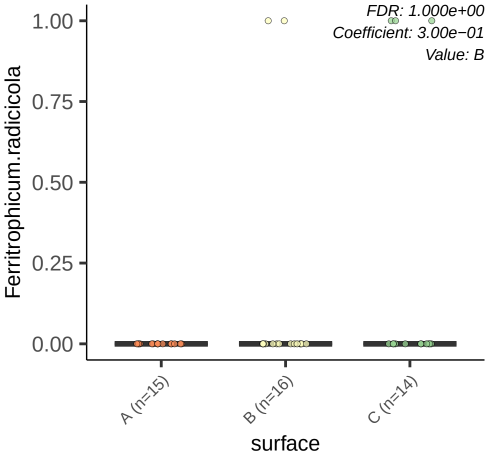
FDR: 1.000e+00
Coefficient: 3.00e−01
Value: B
1.00
0.75
0.50
0.25
0.00
Ferritrophicum.radicicola
A (n=15)
B (n=16)
C (n=14)
surface

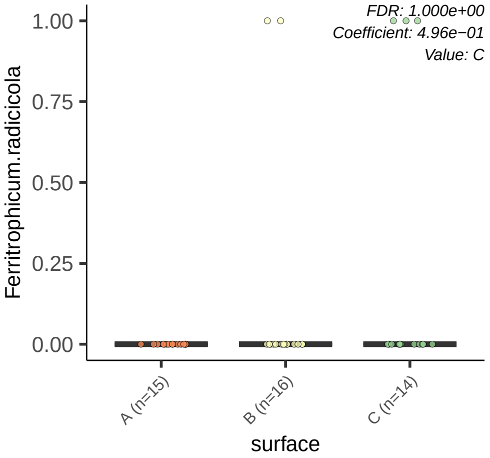

FDR: 1.000e+00
Coefficient: 4.96e−01
Value: C
Ferritrophicum.radicicola
1.00
0.75
0.50
0.25
0.00
A (n=15)
B (n=16)
C (n=14)
surface

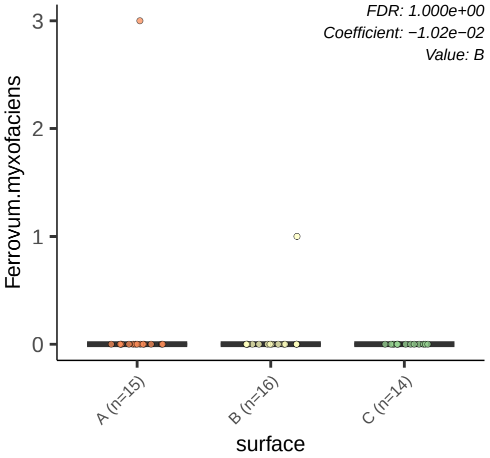
FDR: 1.000e+00
Coefficient: −1.02e−02
Value: B
Ferrovum.myxofaciens
0
1
2
3
A (n=15)
B (n=16)
C (n=14)
surface

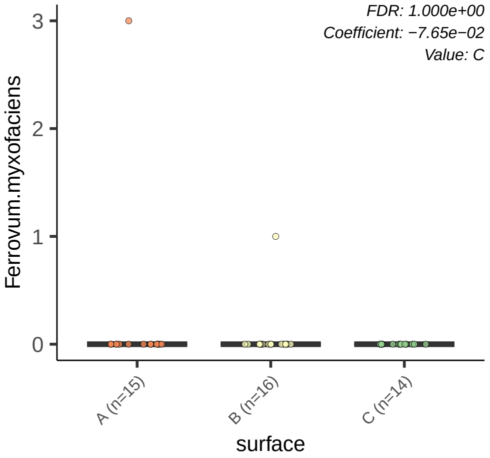

FDR: 1.000e+00
Coefficient: −7.65e−02
Value: C
Ferrovum.myxofaciens
3
2
1
0
A (n=15)
B (n=16)
C (n=14)
surface

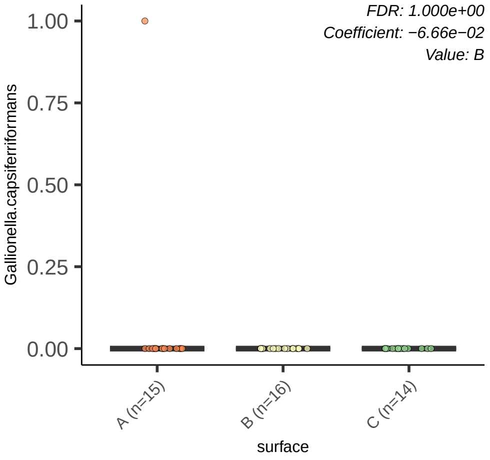

FDR: 1.000e+00
Coefficient: −6.66e−02
Value: B
Gallionella.capsiferriformans
1.00
0.75
0.50
0.25
0.00
A (n=15)
B (n=16)
C (n=14)
surface

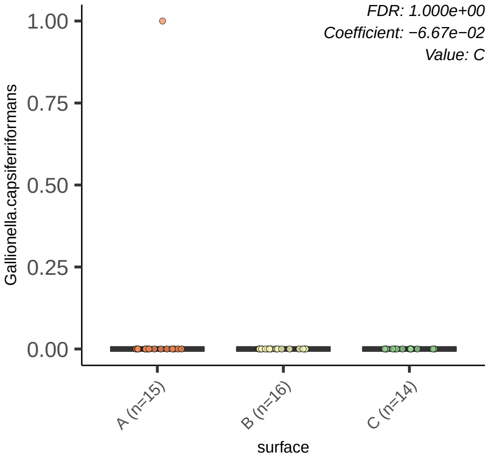

FDR: 1.000e+00
Coefficient: −6.67e−02
Value: C
Gallionella.capsiferriformans
1.00
0.75
0.50
0.25
0.00
A (n=15)
B (n=16)
C (n=14)
surface

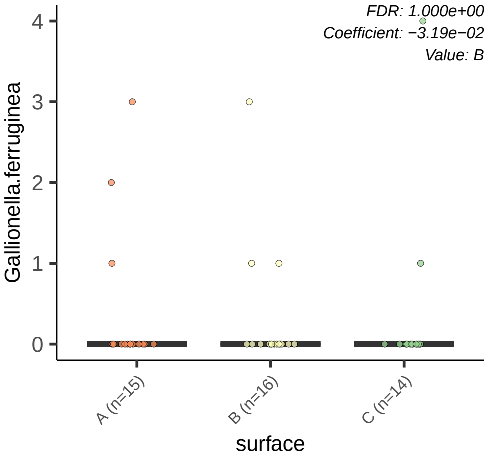
FDR: 1.000e+00
Coefficient: −3.19e−02
Value: B
Gallionella.ferruginea
4
3
2
1
0
A (n=15)
B (n=16)
C (n=14)
surface

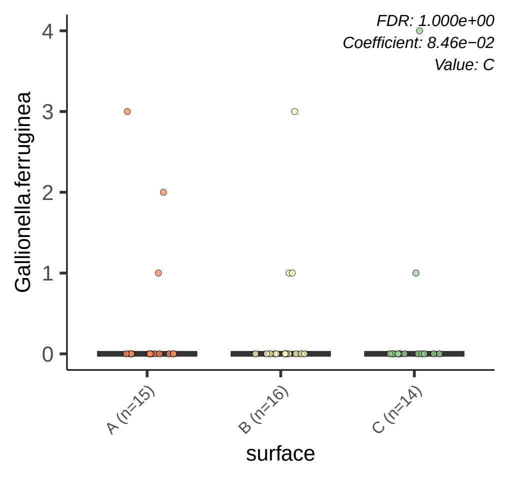
FDR: 1.000e+00
Coefficient: 8.46e−02
Value: C
Gallionella.ferruginea
0
1
2
3
4
A (n=15)
B (n=16)
C (n=14)
surface

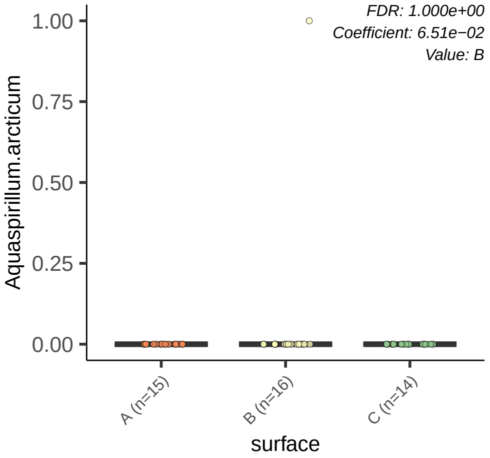

FDR: 1.000e+00
Coefficient: 6.51e−02
Value: B
Aquaspirillum.arcticum
1.00
0.75
0.50
0.25
0.00
A (n=15)
B (n=16)
C (n=14)
surface

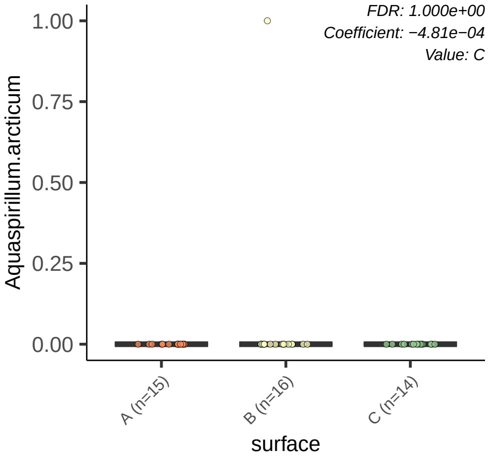
FDR: 1.000e+00
Coefficient: −4.81e−04
Value: C
1.00
0.75
0.50
0.25
0.00
Aquaspirillum.arcticum
A (n=15)
B (n=16)
C (n=14)
surface

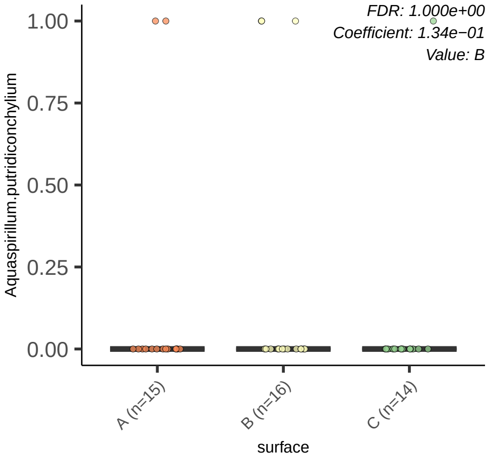

FDR: 1.000e+00
Coefficient: 1.34e−01
Value: B
Aquaspirillum.putridiconchylium
1.00
0.75
0.50
0.25
0.00
A (n=15)
B (n=16)
C (n=14)
surface

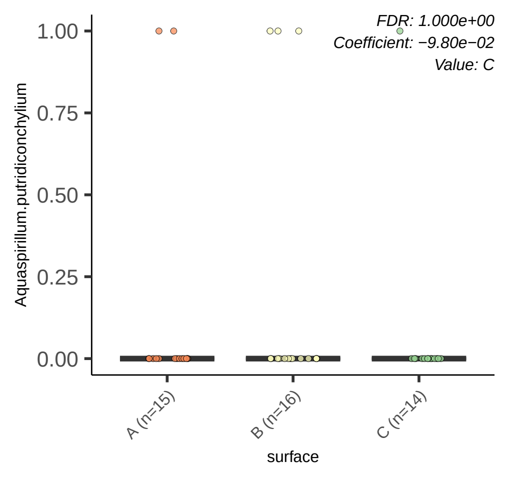
FDR: 1.000e+00
Coefficient: −9.80e−02
Value: C
Aquaspirillum.putridiconchylium
1.00
0.75
0.50
0.25
0.00
A (n=15)
B (n=16)
C (n=14)
surface

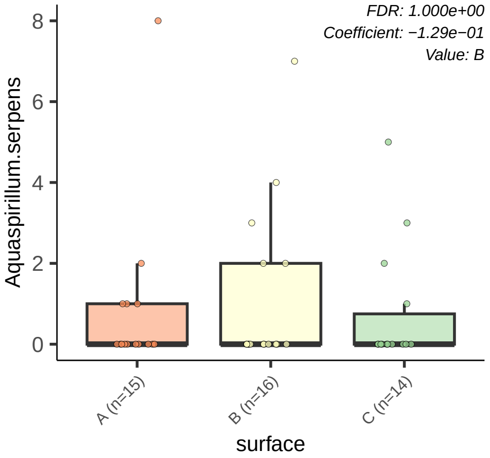

FDR: 1.000e+00
Coefficient: −1.29e−01
Value: B
Aquaspirillum.serpens
0
2
4
6
8
A (n=15)
B (n=16)
C (n=14)
surface

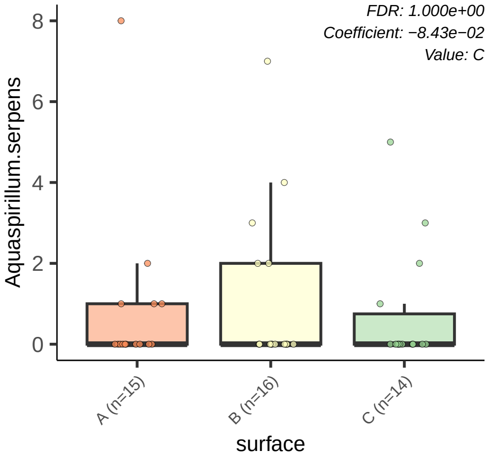
FDR: 1.000e+00
Coefficient: −8.43e−02
Value: C
Aquaspirillum.serpens
0
2
4
6
8
A (n=15)
B (n=16)
C (n=14)
surface

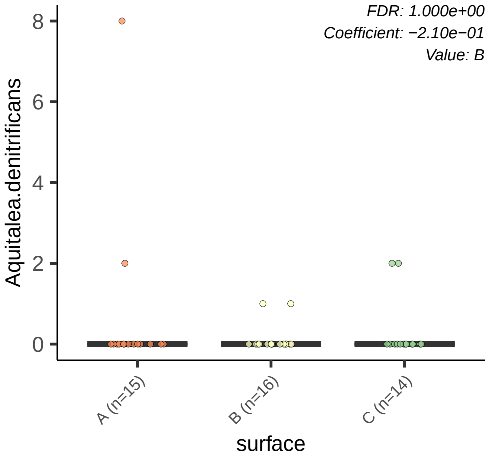
FDR: 1.000e+00
Coefficient: −2.10e−01
Value: B
Aquitalea.denitrificans
8
6
4
2
0
A (n=15)
B (n=16)
C (n=14)
surface

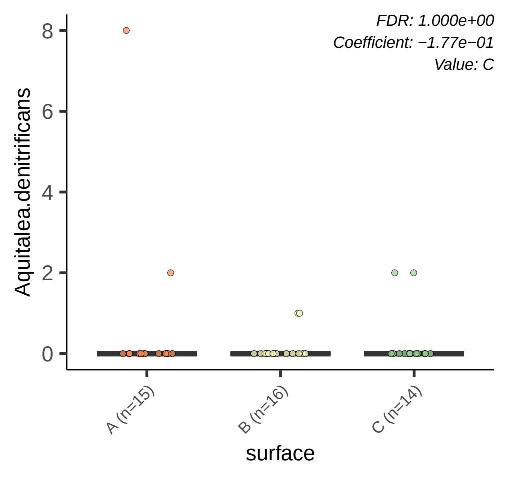

FDR: 1.000e+00
Coefficient: −1.77e−01
Value: C
Aquitalea.denitrificans
8
6
4
2
0
A (n=15)
B (n=16)
C (n=14)
surface

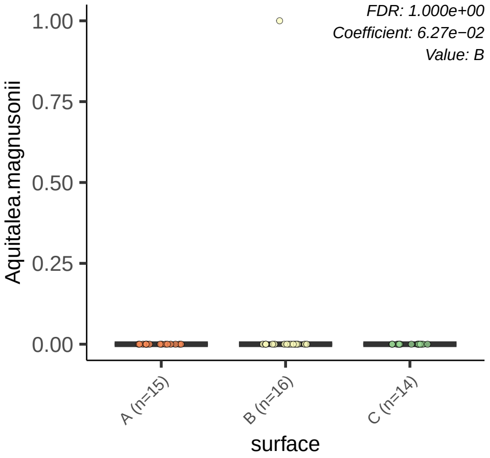
FDR: 1.000e+00
Coefficient: 6.27e−02
Value: B
Aquitalea.magnusonii
1.00
0.75
0.50
0.25
0.00
A (n=15)
B (n=16)
C (n=14)
surface

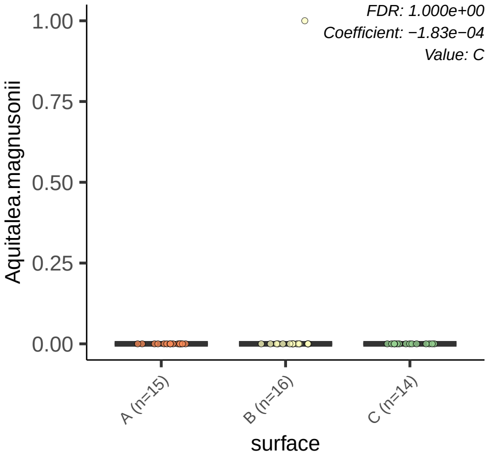

FDR: 1.000e+00
Coefficient: −1.83e−04
Value: C
Aquitalea.magnusonii
1.00
0.75
0.50
0.25
0.00
A (n=15)
B (n=16)
C (n=14)
surface

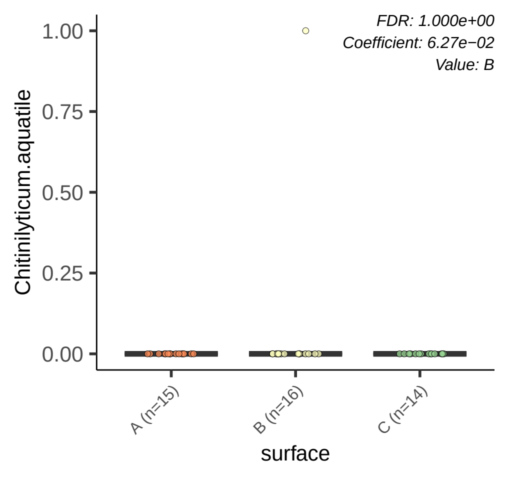

FDR: 1.000e+00
Coefficient: 6.27e−02
Value: B
Chitinilyticum.aquatile
1.00
0.75
0.50
0.25
0.00
A (n=15)
B (n=16)
C (n=14)
surface

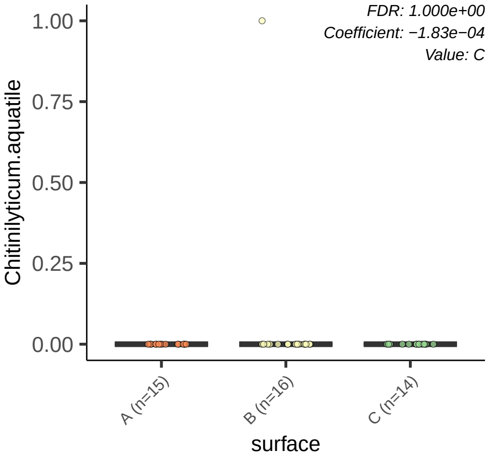

FDR: 1.000e+00
Coefficient: −1.83e−04
Value: C
1.00
0.75
0.50
0.25
0.00
Chitinilyticum.aquatile
A (n=15)
B (n=16)
C (n=14)
surface

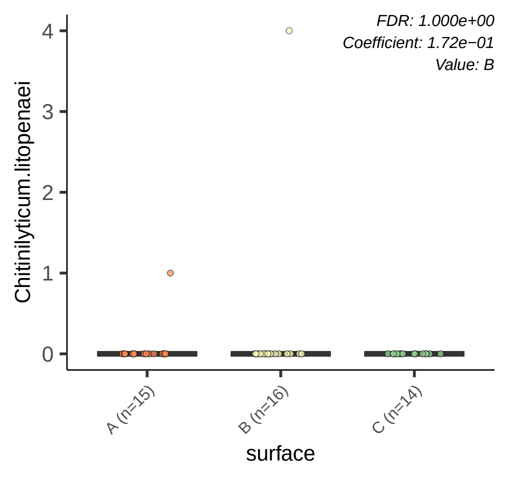
FDR: 1.000e+00
Coefficient: 1.72e−01
Value: B
Chitinilyticum.litopenaei
4
3
2
1
0
A (n=15)
B (n=16)
C (n=14)
surface

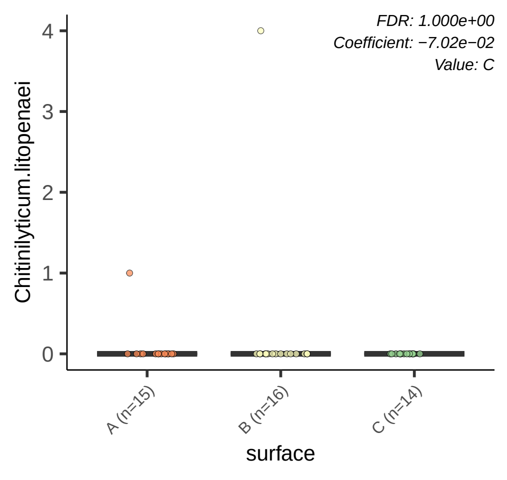
FDR: 1.000e+00
Coefficient: −7.02e−02
Value: C
Chitinilyticum.litopenaei
4
3
2
1
0
A (n=15)
B (n=16)
C (n=14)
surface

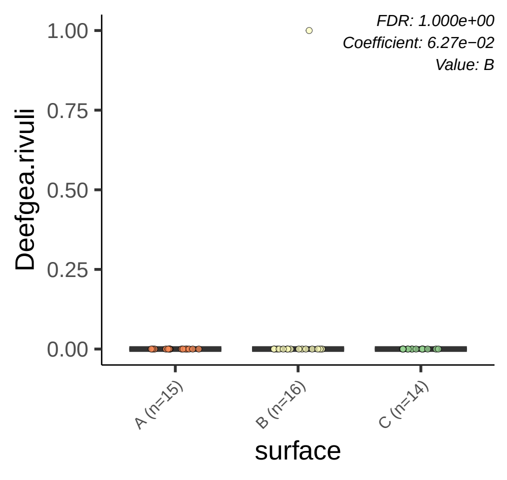
FDR: 1.000e+00
Coefficient: 6.27e−02
Value: B
1.00
0.75
0.50
0.25
0.00
Deefgea.rivuli
A (n=15)
B (n=16)
C (n=14)
surface

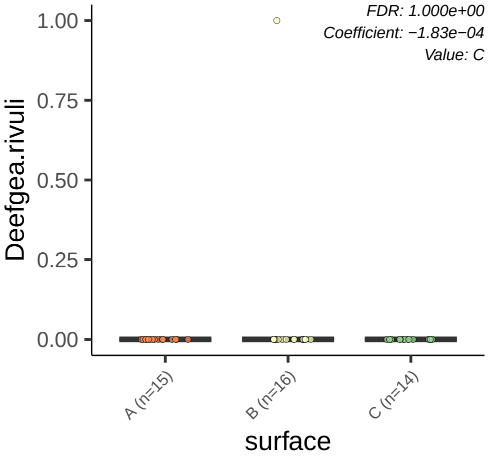

FDR: 1.000e+00
Coefficient: −1.83e−04
Value: C
Deefgea.rivuli
1.00
0.75
0.50
0.25
0.00
A (n=15)
B (n=16)
C (n=14)
surface

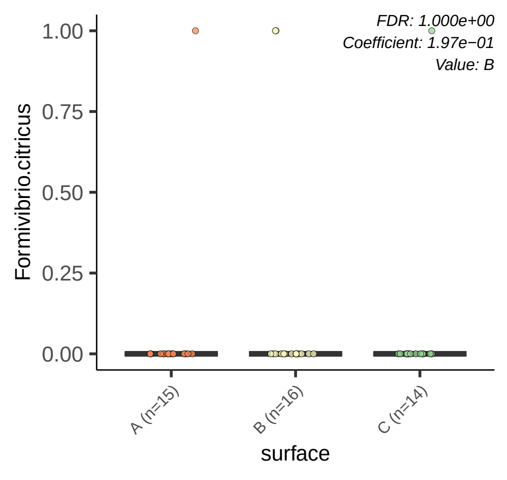

FDR: 1.000e+00
Coefficient: 1.97e−01
Value: B
Formivibrio.citricus
1.00
0.75
0.50
0.25
0.00
A (n=15)
B (n=16)
C (n=14)
surface

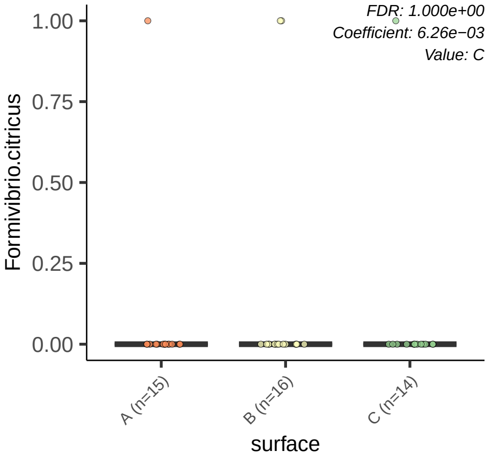

FDR: 1.000e+00
Coefficient: 6.26e−03
Value: C
Formivibrio.citricus
1.00
0.75
0.50
0.25
0.00
A (n=15)
B (n=16)
C (n=14)
surface

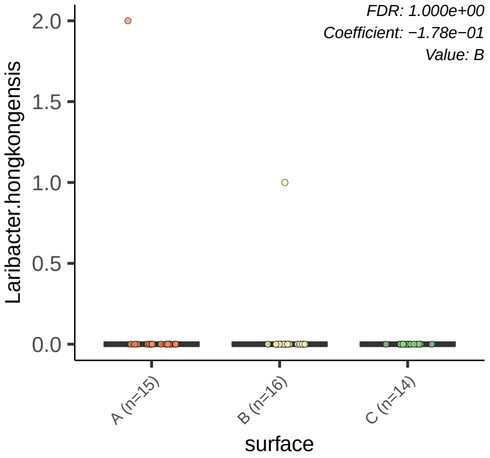
FDR: 1.000e+00
Coefficient: −1.78e−01
Value: B
Laribacter.hongkongensis
2.0
1.5
1.0
0.5
0.0
A (n=15)
B (n=16)
C (n=14)
surface

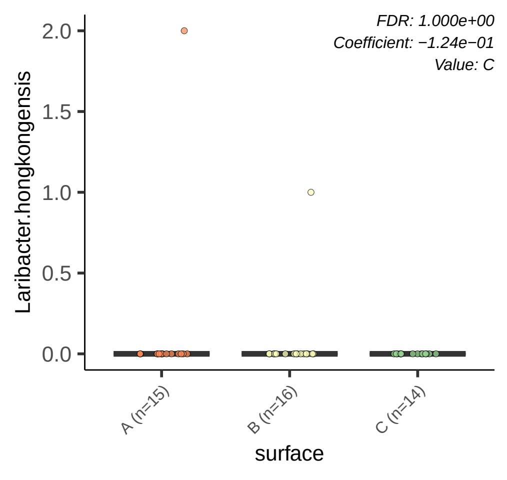
FDR: 1.000e+00
Coefficient: −1.24e−01
Value: C
Laribacter.hongkongensis
2.0
1.5
1.0
0.5
0.0
A (n=15)
B (n=16)
C (n=14)
surface

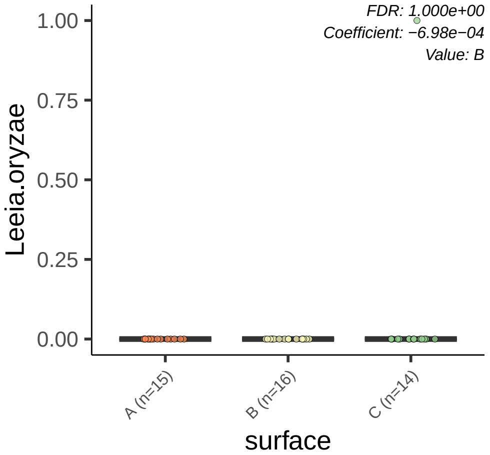
FDR: 1.000e+00
Coefficient: −6.98e−04
Value: B
1.00
0.75
0.50
0.25
0.00
Leeia.oryzae
A (n=15)
B (n=16)
C (n=14)
surface

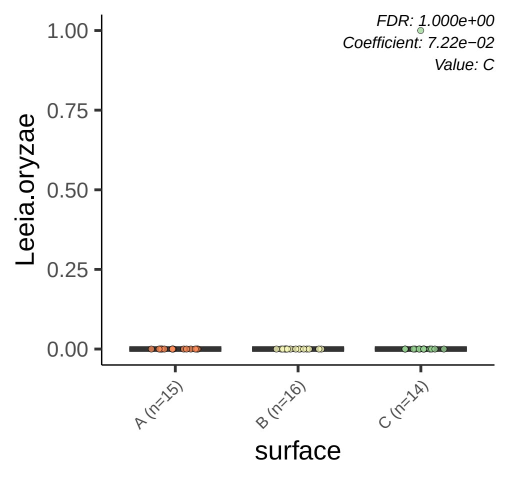

FDR: 1.000e+00
Coefficient: 7.22e−02
Value: C
1.00
0.75
0.50
0.25
0.00
Leeia.oryzae
A (n=15)
B (n=16)
C (n=14)
surface

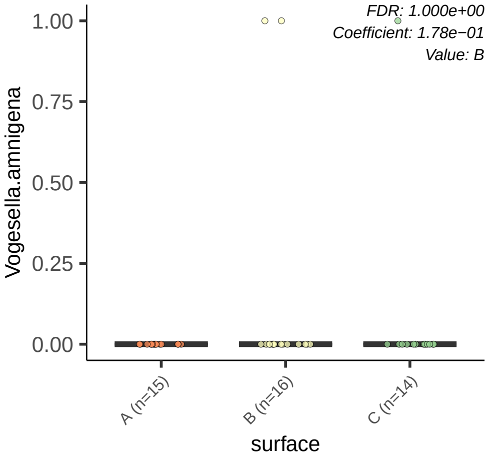
FDR: 1.000e+00
Coefficient: 1.78e−01
Value: B
Vogesella.amnigena
1.00
0.75
0.50
0.25
0.00
A (n=15)
B (n=16)
C (n=14)
surface

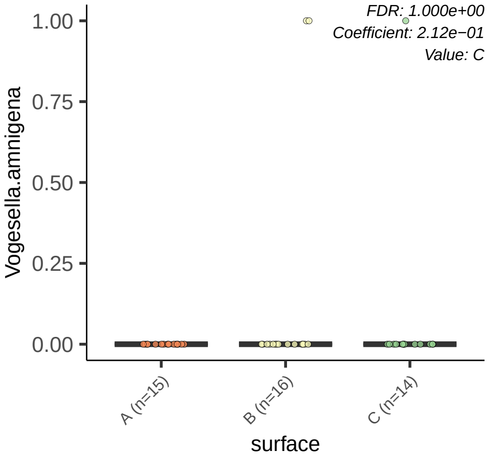
FDR: 1.000e+00
Coefficient: 2.12e−01
Value: C
Vogesella.amnigena
1.00
0.75
0.50
0.25
0.00
A (n=15)
B (n=16)
C (n=14)
surface

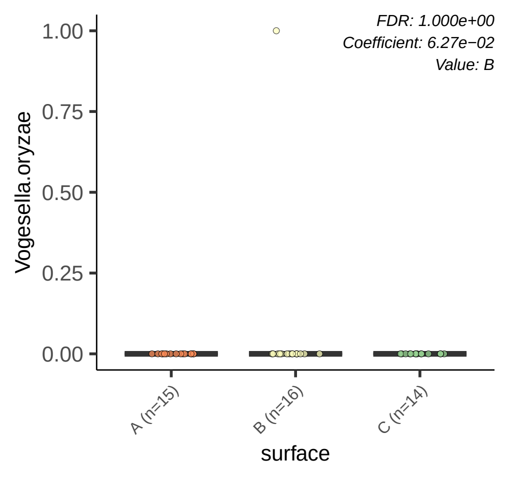
FDR: 1.000e+00
Coefficient: 6.27e−02
Value: B
Vogesella.oryzae
1.00
0.75
0.50
0.25
0.00
A (n=15)
B (n=16)
C (n=14)
surface

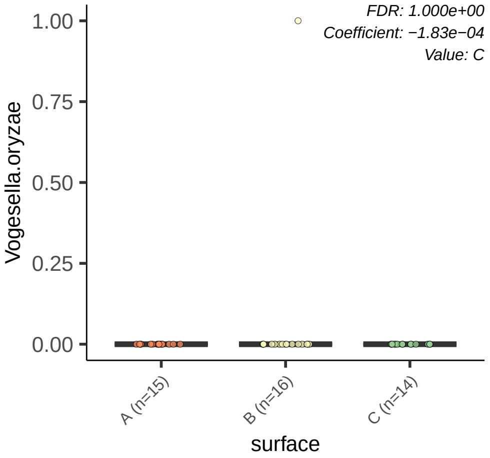

FDR: 1.000e+00
Coefficient: −1.83e−04
Value: C
Vogesella.oryzae
1.00
0.75
0.50
0.25
0.00
A (n=15)
B (n=16)
C (n=14)
surface

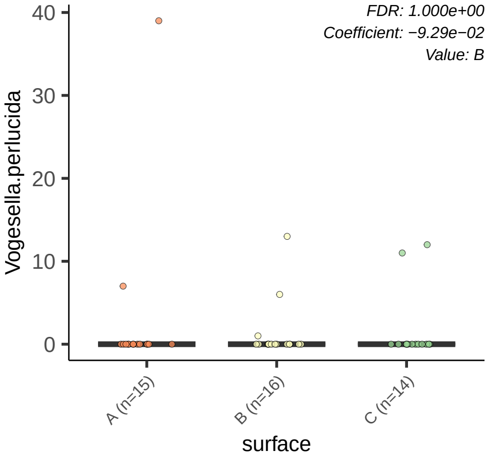

FDR: 1.000e+00
Coefficient: −9.29e−02
Value: B
Vogesella.perlucida
40
30
20
10
0
A (n=15)
B (n=16)
C (n=14)
surface

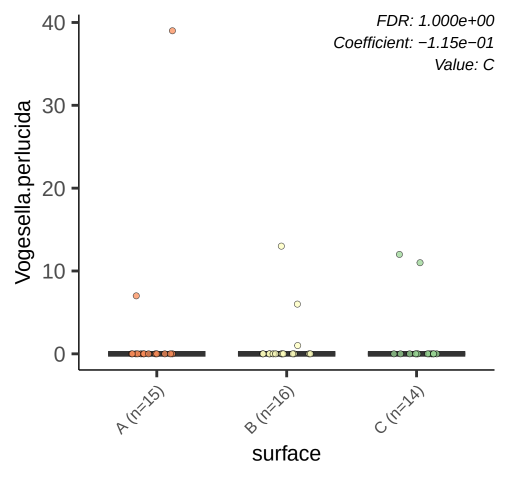
FDR: 1.000e+00
Coefficient: −1.15e−01
Value: C
Vogesella.perlucida
40
30
20
10
0
A (n=15)
B (n=16)
C (n=14)
surface

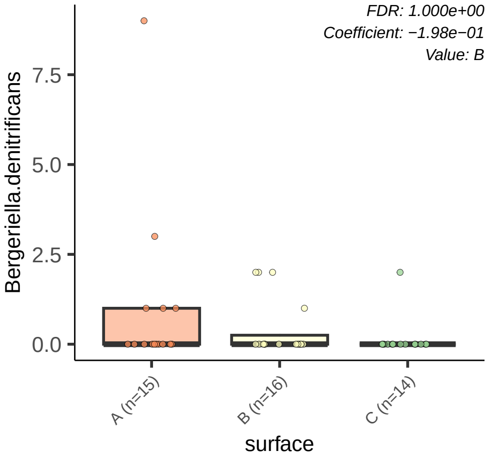

FDR: 1.000e+00
Coefficient: −1.98e−01
Value: B
Bergeriella.denitrificans
7.5
5.0
2.5
0.0
A (n=15)
B (n=16)
C (n=14)
surface

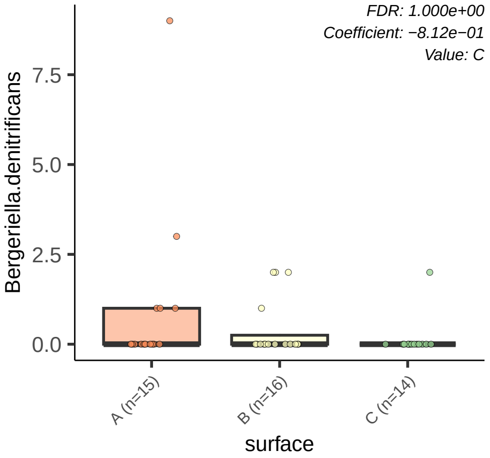

FDR: 1.000e+00
Coefficient: −8.12e−01
Value: C
Bergeriella.denitrificans
0.0
2.5
5.0
7.5
A (n=15)
B (n=16)
C (n=14)
surface

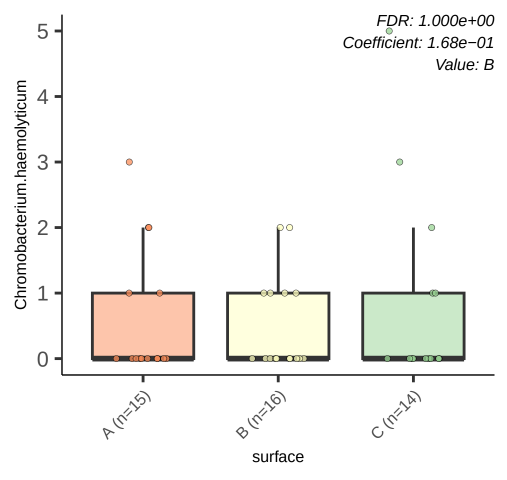

FDR: 1.000e+00
Coefficient: 1.68e−01
Value: B
Chromobacterium.haemolyticum
0
1
2
3
4
5
A (n=15)
B (n=16)
C (n=14)
surface

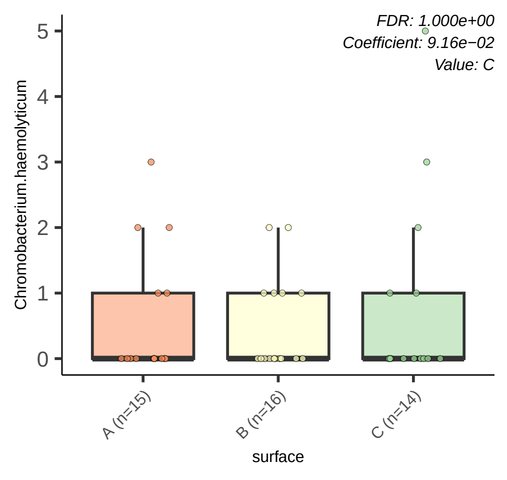
FDR: 1.000e+00
Coefficient: 9.16e−02
Value: C
Chromobacterium.haemolyticum
0
1
2
3
4
5
A (n=15)
B (n=16)
C (n=14)
surface

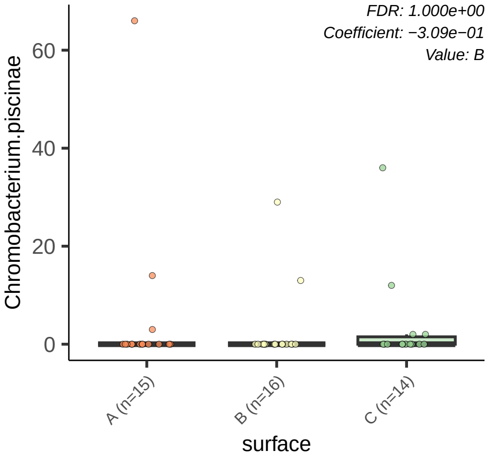

FDR: 1.000e+00
Coefficient: −3.09e−01
Value: B
Chromobacterium.piscinae
0
20
40
60
A (n=15)
B (n=16)
C (n=14)
surface

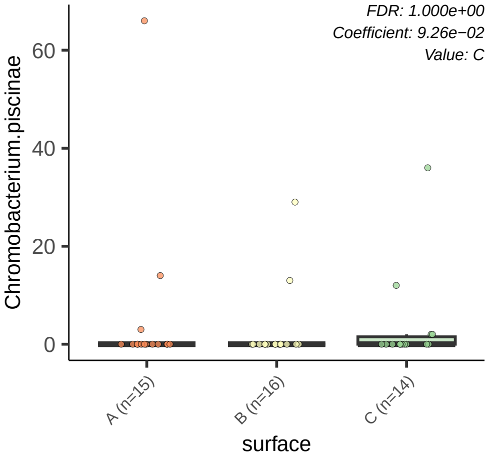
FDR: 1.000e+00
Coefficient: 9.26e−02
Value: C
Chromobacterium.piscinae
0
20
40
60
A (n=15)
B (n=16)
C (n=14)
surface

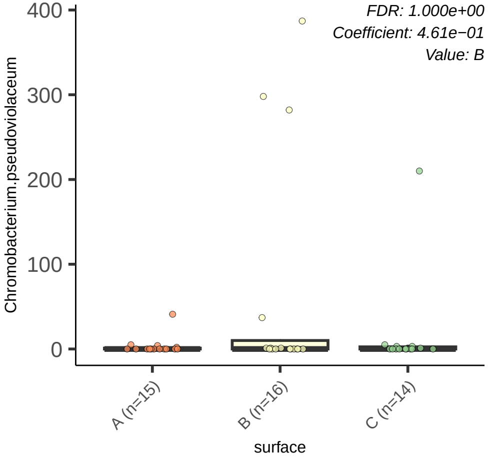
FDR: 1.000e+00
Coefficient: 4.61e−01
Value: B
Chromobacterium.pseudoviolaceum
400
300
200
100
0
A (n=15)
B (n=16)
C (n=14)
surface

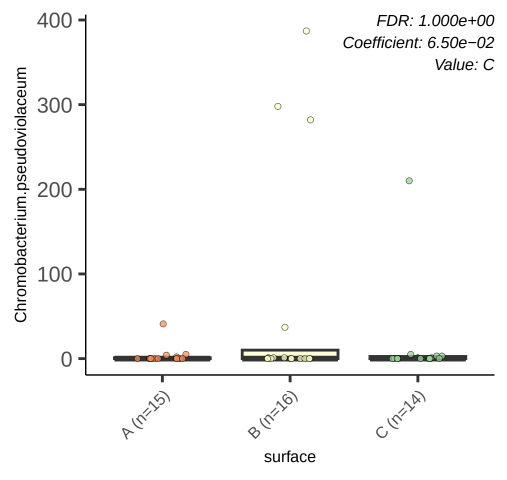

FDR: 1.000e+00
Coefficient: 6.50e−02
Value: C
400
300
200
100
0
Chromobacterium.pseudoviolaceum
A (n=15)
B (n=16)
C (n=14)
surface

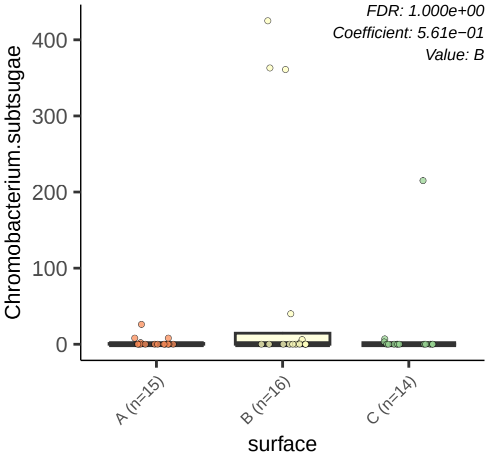
FDR: 1.000e+00
Coefficient: 5.61e−01
Value: B
Chromobacterium.subtsugae
400
300
200
100
0
A (n=15)
B (n=16)
C (n=14)
surface

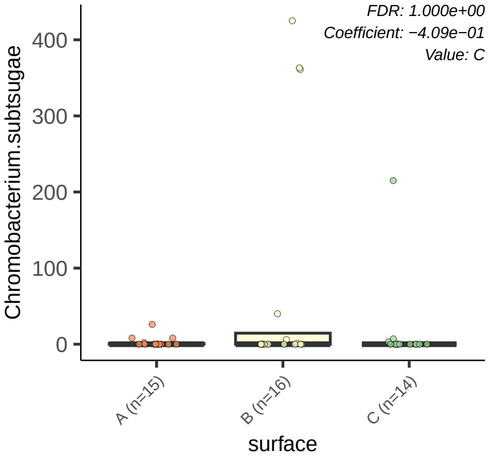

FDR: 1.000e+00
Coefficient: −4.09e−01
Value: C
Chromobacterium.subtsugae
400
300
200
100
0
A (n=15)
B (n=16)
C (n=14)
surface

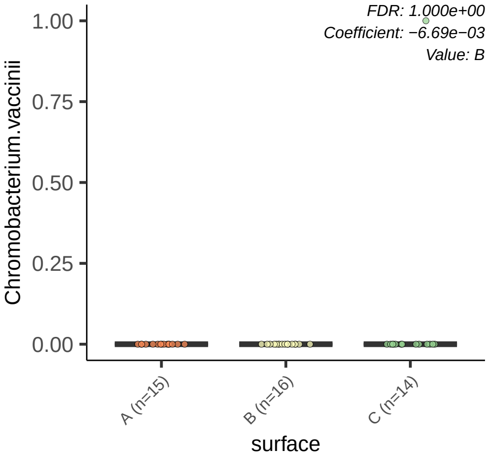

FDR: 1.000e+00
Coefficient: −6.69e−03
Value: B
Chromobacterium.vaccinii
1.00
0.75
0.50
0.25
0.00
A (n=15)
B (n=16)
C (n=14)
surface

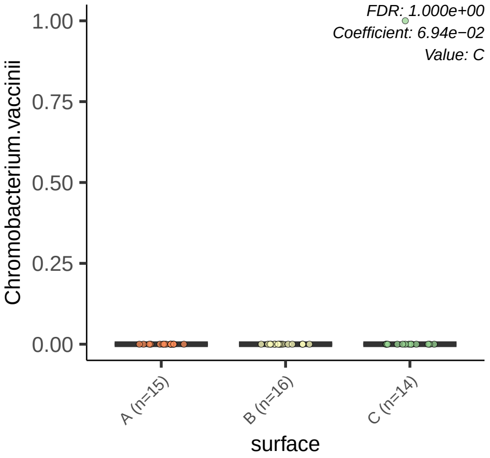

FDR: 1.000e+00
Coefficient: 6.94e−02
Value: C
Chromobacterium.vaccinii
1.00
0.75
0.50
0.25
0.00
A (n=15)
B (n=16)
C (n=14)
surface

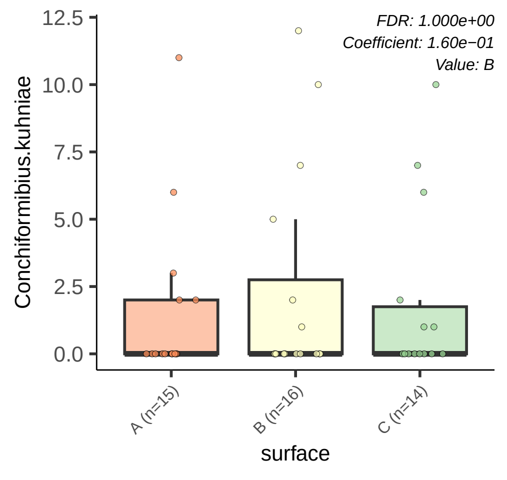
FDR: 1.000e+00
Coefficient: 1.60e−01
Value: B
Conchiformibius.kuhniae
12.5
10.0
7.5
5.0
2.5
0.0
A (n=15)
B (n=16)
C (n=14)
surface

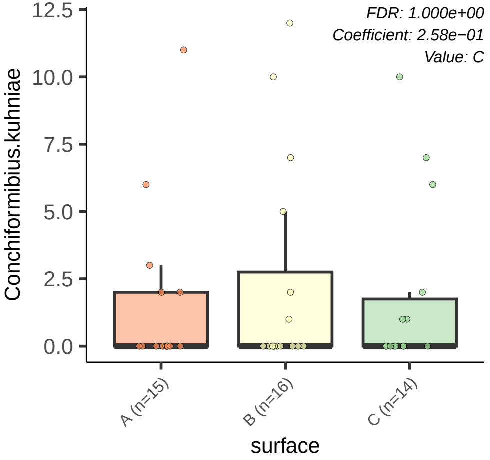

FDR: 1.000e+00
Coefficient: 2.58e−01
Value: C
Conchiformibius.kuhniae
12.5
10.0
7.5
5.0
2.5
0.0
A (n=15)
B (n=16)
C (n=14)
surface

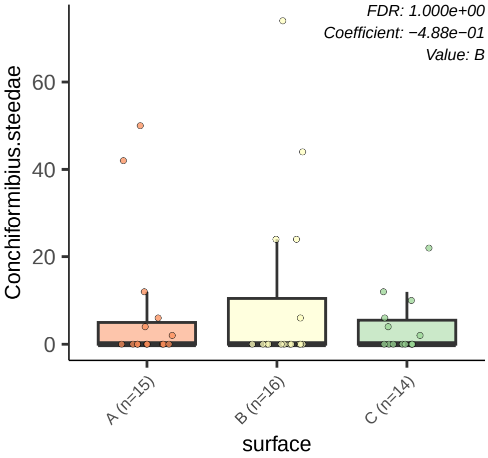
FDR: 1.000e+00
Coefficient: −4.88e−01
Value: B
Conchiformibius.steedae
0
20
40
60
A (n=15)
B (n=16)
C (n=14)
surface

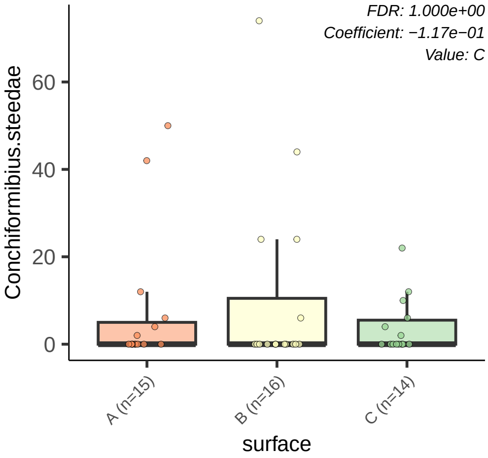

FDR: 1.000e+00
Coefficient: −1.17e−01
Value: C
Conchiformibius.steedae
60
40
20
0
A (n=15)
B (n=16)
C (n=14)
surface

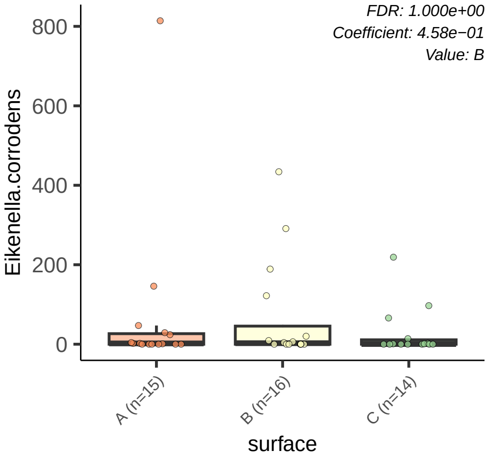

FDR: 1.000e+00
Coefficient: 4.58e−01
Value: B
Eikenella.corrodens
800
600
400
200
0
A (n=15)
B (n=16)
C (n=14)
surface

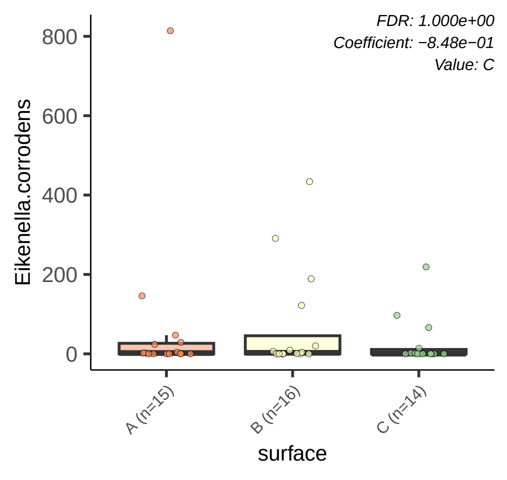

FDR: 1.000e+00
Coefficient: −8.48e−01
Value: C
Eikenella.corrodens
0
200
400
600
800
A (n=15)
B (n=16)
C (n=14)
surface

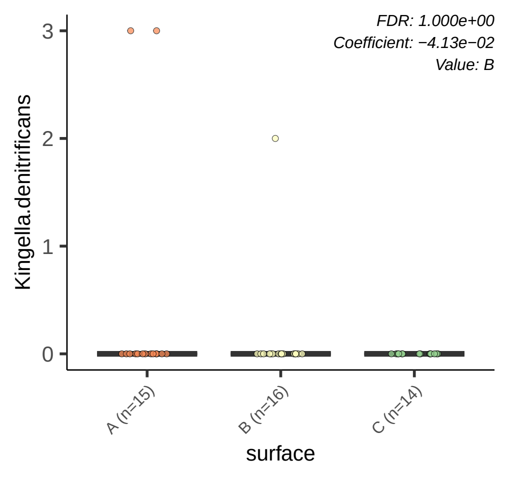

FDR: 1.000e+00
Coefficient: −4.13e−02
Value: B
Kingella.denitrificans
3
2
1
0
A (n=15)
B (n=16)
C (n=14)
surface

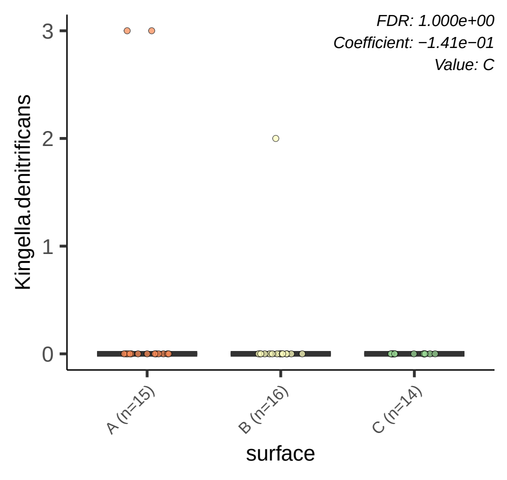
FDR: 1.000e+00
Coefficient: −1.41e−01
Value: C
Kingella.denitrificans
3
2
1
0
A (n=15)
B (n=16)
C (n=14)
surface

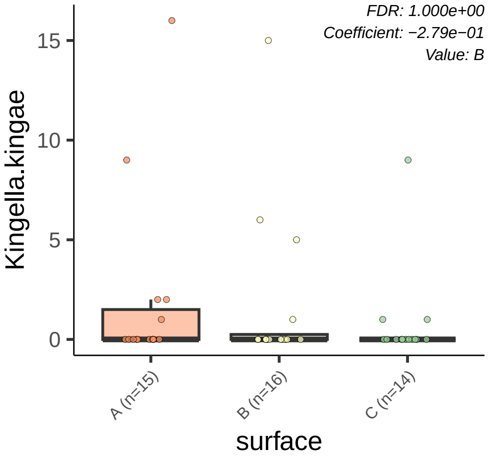
FDR: 1.000e+00
Coefficient: −2.79e−01
Value: B
Kingella.kingae
15
10
5
0
A (n=15)
B (n=16)
C (n=14)
surface

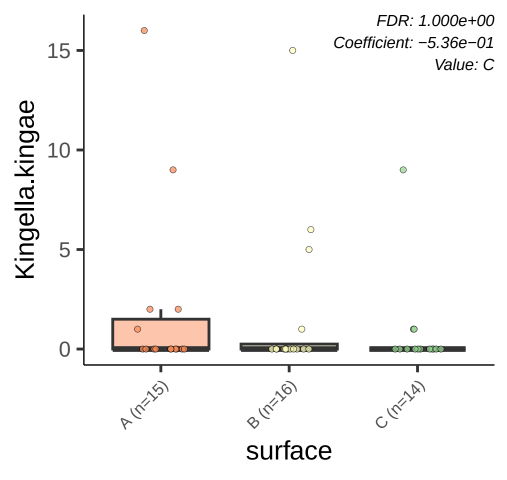
FDR: 1.000e+00
Coefficient: −5.36e−01
Value: C
Kingella.kingae
0
5
10
15
A (n=15)
B (n=16)
C (n=14)
surface

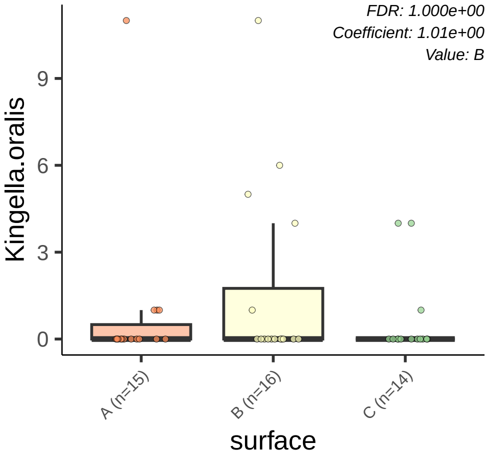
FDR: 1.000e+00
Coefficient: 1.01e+00
Value: B
Kingella.oralis
0
3
6
9
A (n=15)
B (n=16)
C (n=14)
surface

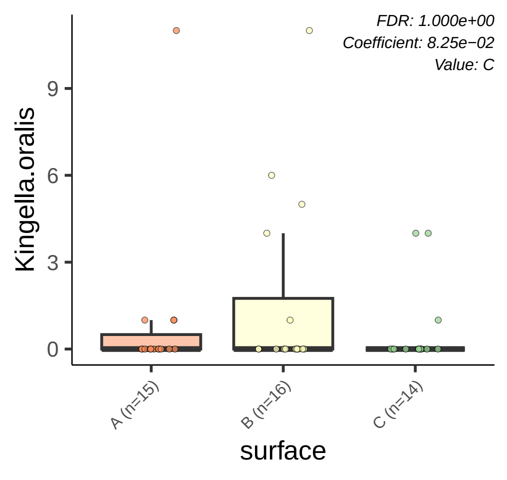
FDR: 1.000e+00
Coefficient: 8.25e−02
Value: C
Kingella.oralis
0
3
6
9
A (n=15)
B (n=16)
C (n=14)
surface

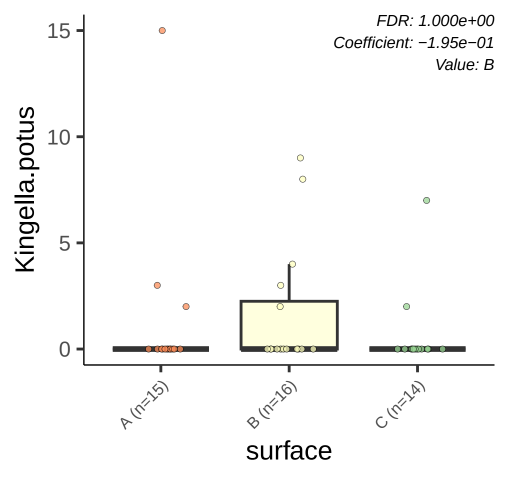
FDR: 1.000e+00
Coefficient: −1.95e−01
Value: B
Kingella.potus
15
10
5
0
A (n=15)
B (n=16)
C (n=14)
surface

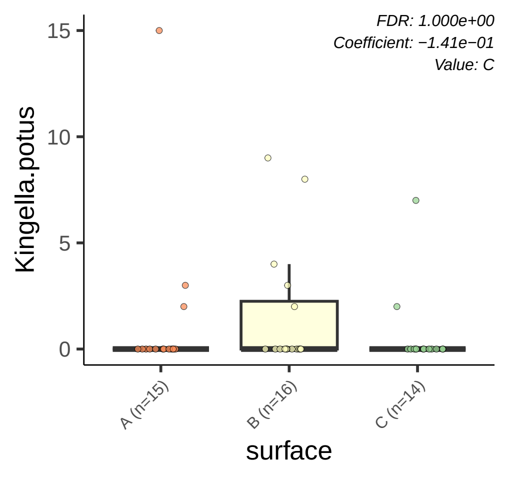
FDR: 1.000e+00
Coefficient: −1.41e−01
Value: C
Kingella.potus
15
10
5
0
A (n=15)
B (n=16)
C (n=14)
surface

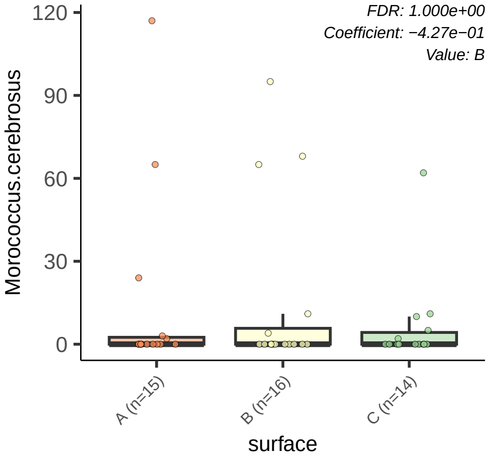

FDR: 1.000e+00
Coefficient: −4.27e−01
Value: B
Morococcus.cerebrosus
120
90
60
30
0
A (n=15)
B (n=16)
C (n=14)
surface

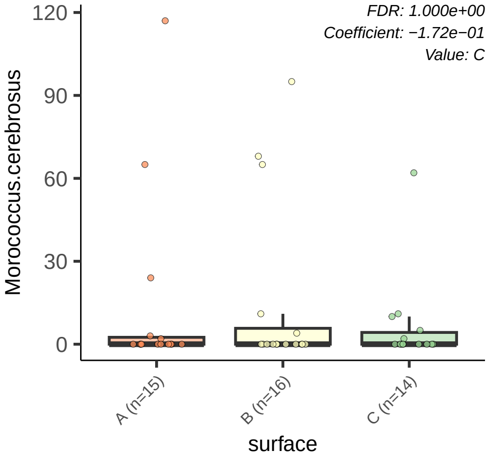

FDR: 1.000e+00
Coefficient: −1.72e−01
Value: C
120
90
60
30
0
Morococcus.cerebrosus
A (n=15)
B (n=16)
C (n=14)
surface

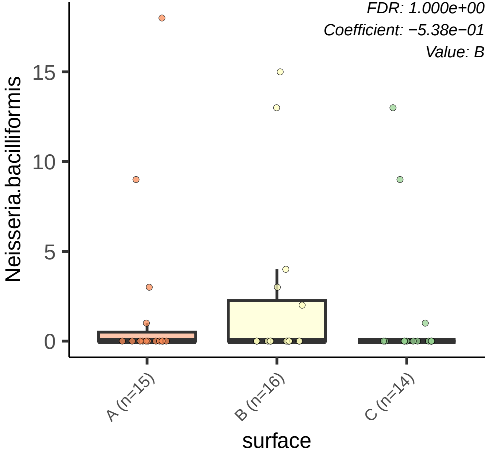

FDR: 1.000e+00
Coefficient: −5.38e−01
Value: B
Neisseria.bacilliformis
0
5
10
15
A (n=15)
B (n=16)
C (n=14)
surface

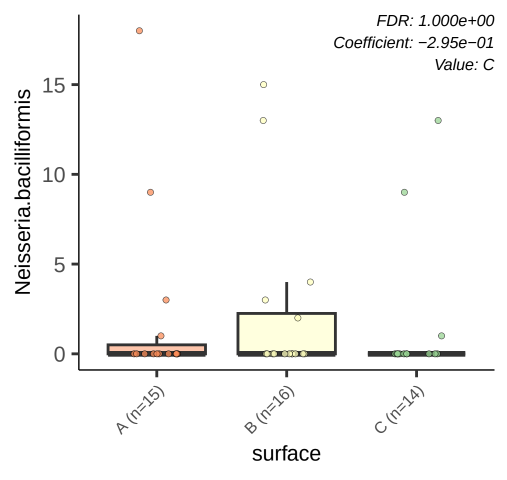

FDR: 1.000e+00
Coefficient: −2.95e−01
Value: C
Neisseria.bacilliformis
0
5
10
15
A (n=15)
B (n=16)
C (n=14)
surface

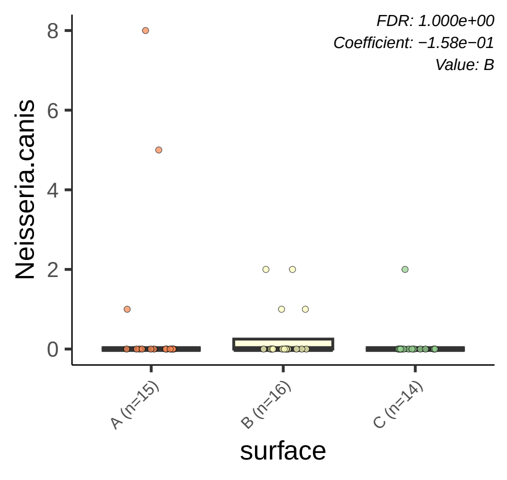
FDR: 1.000e+00
Coefficient: −1.58e−01
Value: B
Neisseria.canis
8
6
4
2
0
A (n=15)
B (n=16)
C (n=14)
surface

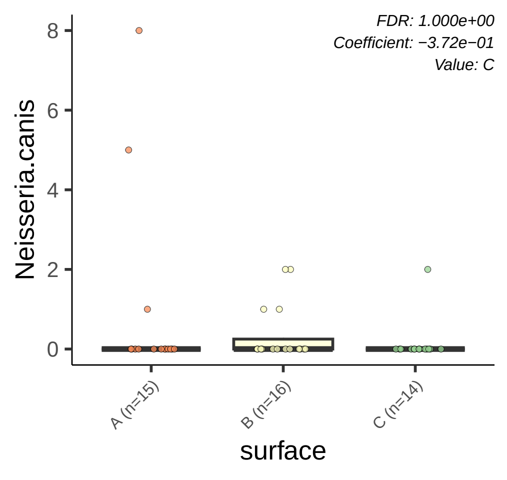
FDR: 1.000e+00
Coefficient: −3.72e−01
Value: C
Neisseria.canis
0
2
4
6
8
A (n=15)
B (n=16)
C (n=14)
surface

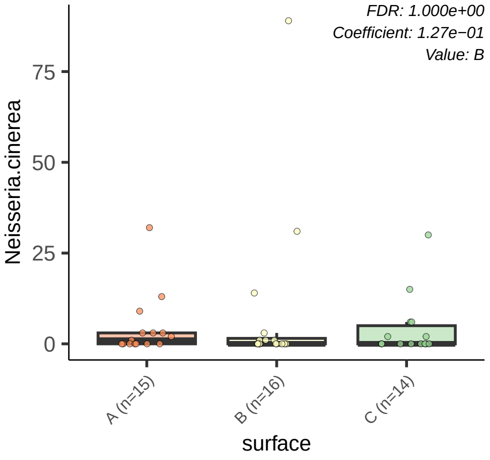
FDR: 1.000e+00
Coefficient: 1.27e−01
Value: B
Neisseria.cinerea
75
50
25
0
A (n=15)
B (n=16)
C (n=14)
surface

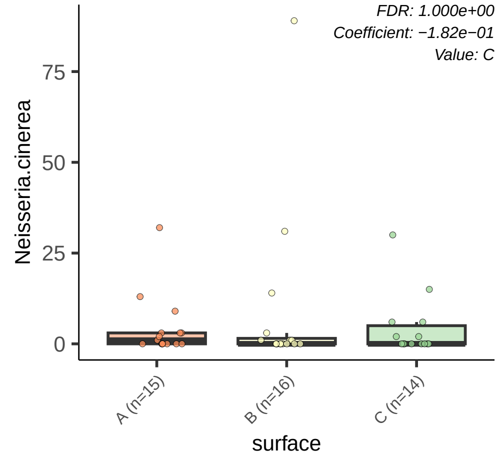
FDR: 1.000e+00
Coefficient: −1.82e−01
Value: C
Neisseria.cinerea
75
50
25
0
A (n=15)
B (n=16)
C (n=14)
surface

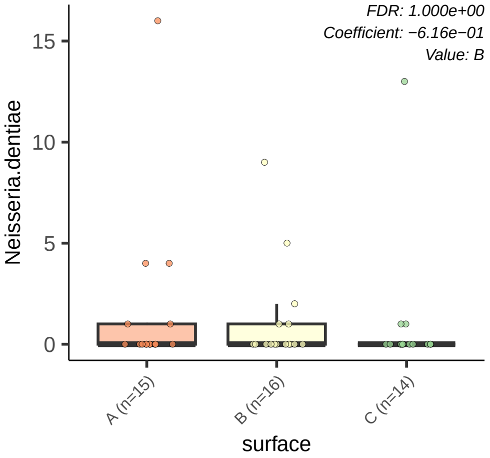

FDR: 1.000e+00
Coefficient: −6.16e−01
Value: B
Neisseria.dentiae
15
10
5
0
A (n=15)
B (n=16)
C (n=14)
surface

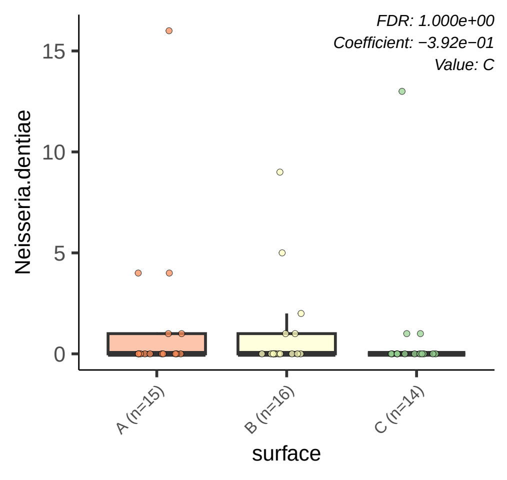

FDR: 1.000e+00
Coefficient: −3.92e−01
Value: C
Neisseria.dentiae
0
5
10
15
A (n=15)
B (n=16)
C (n=14)
surface

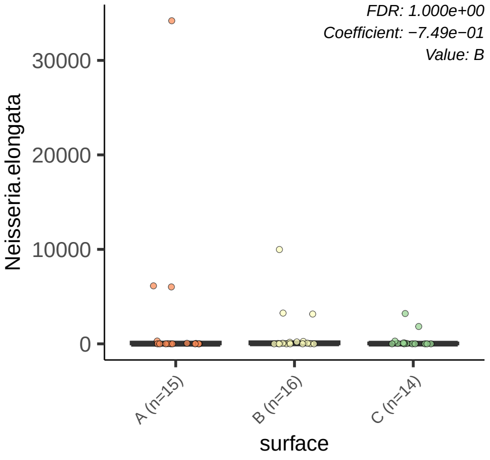
FDR: 1.000e+00
Coefficient: −7.49e−01
Value: B
Neisseria.elongata
30000
20000
10000
0
A (n=15)
B (n=16)
C (n=14)
surface

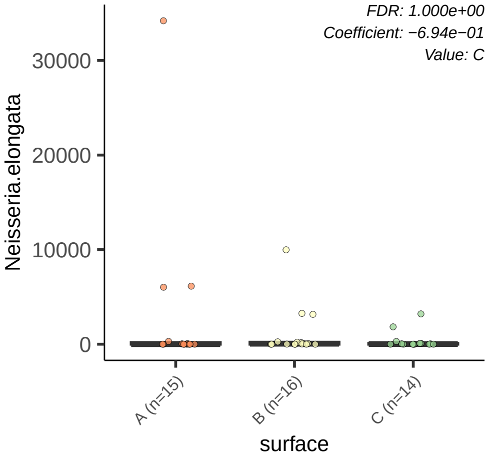
FDR: 1.000e+00
Coefficient: −6.94e−01
Value: C
Neisseria.elongata
30000
20000
10000
0
A (n=15)
B (n=16)
C (n=14)
surface

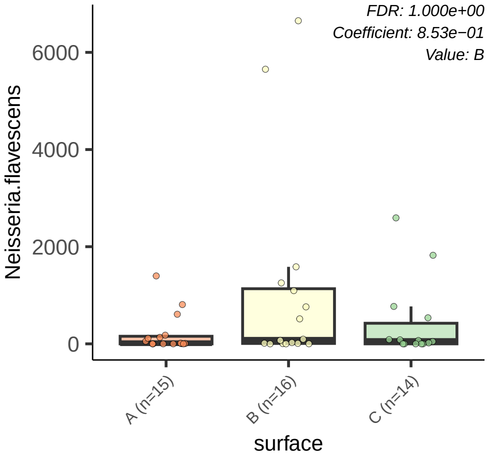
FDR: 1.000e+00
Coefficient: 8.53e−01
Value: B
Neisseria.flavescens
6000
4000
2000
0
A (n=15)
B (n=16)
C (n=14)
surface

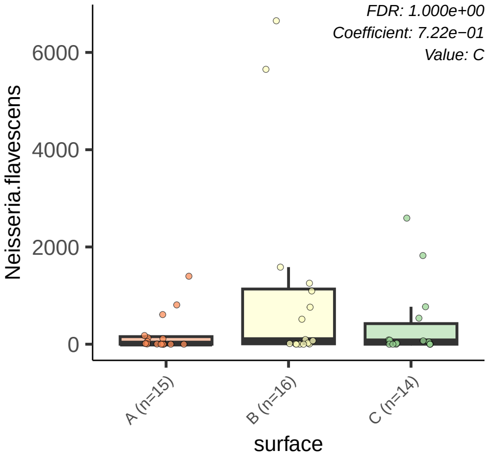

FDR: 1.000e+00
Coefficient: 7.22e−01
Value: C
Neisseria.flavescens
6000
4000
2000
0
A (n=15)
B (n=16)
C (n=14)
surface

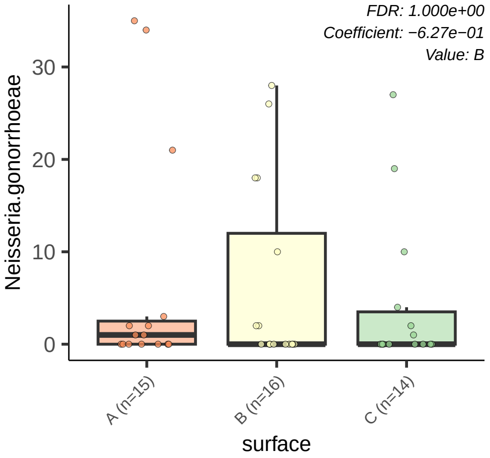

FDR: 1.000e+00
Coefficient: −6.27e−01
Value: B
Neisseria.gonorrhoeae
0
10
20
30
A (n=15)
B (n=16)
C (n=14)
surface

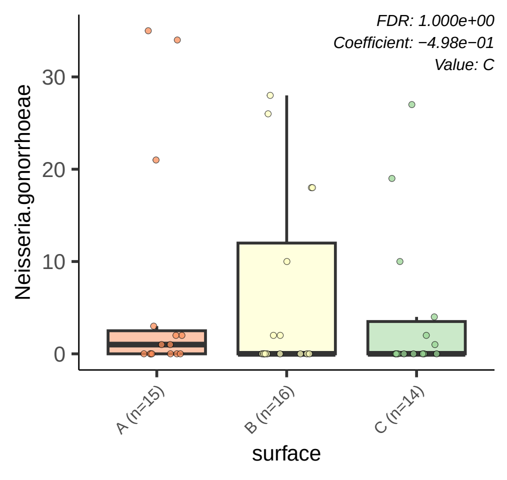
FDR: 1.000e+00
Coefficient: −4.98e−01
Value: C
Neisseria.gonorrhoeae
30
20
10
0
A (n=15)
B (n=16)
C (n=14)
surface

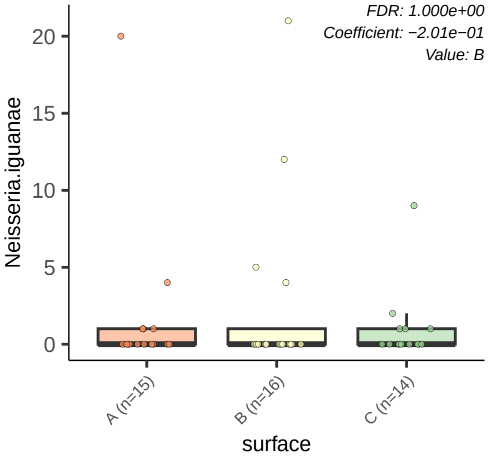

FDR: 1.000e+00
Coefficient: −2.01e−01
Value: B
Neisseria.iguanae
0
5
10
15
20
A (n=15)
B (n=16)
C (n=14)
surface

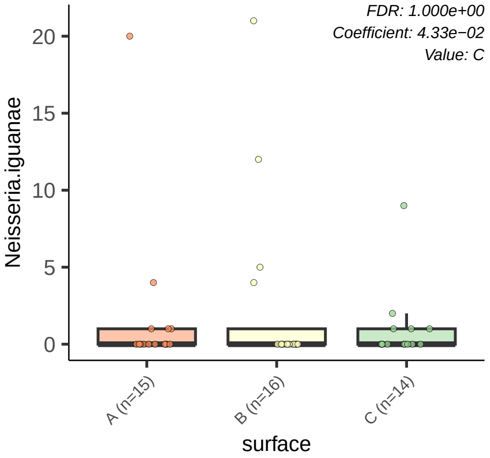

FDR: 1.000e+00
Coefficient: 4.33e−02
Value: C
Neisseria.iguanae
0
5
10
15
20
A (n=15)
B (n=16)
C (n=14)
surface

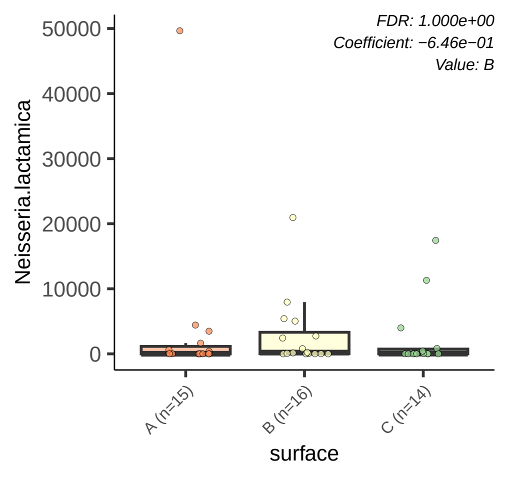

FDR: 1.000e+00
Coefficient: −6.46e−01
Value: B
Neisseria.lactamica
50000
40000
30000
20000
10000
0
A (n=15)
B (n=16)
C (n=14)
surface

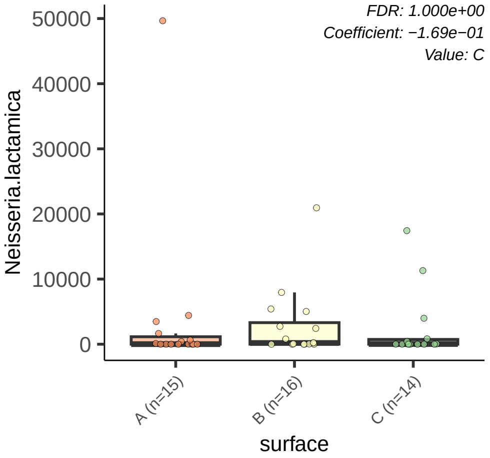
FDR: 1.000e+00
Coefficient: −1.69e−01
Value: C
Neisseria.lactamica
50000
40000
30000
20000
10000
0
A (n=15)
B (n=16)
C (n=14)
surface

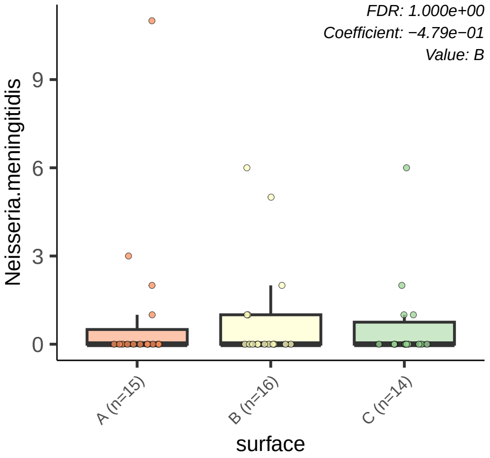
FDR: 1.000e+00
Coefficient: −4.79e−01
Value: B
Neisseria.meningitidis
0
3
6
9
A (n=15)
B (n=16)
C (n=14)
surface

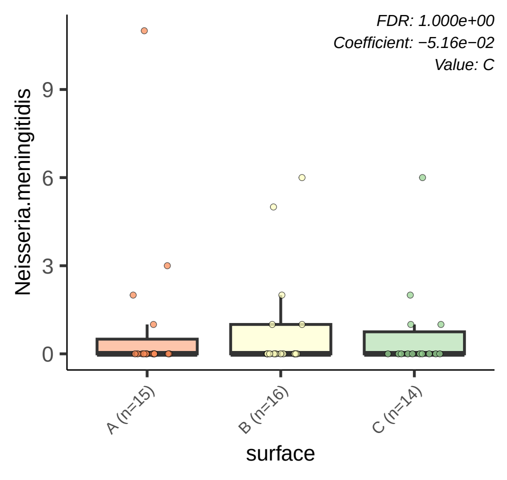

FDR: 1.000e+00
Coefficient: −5.16e−02
Value: C
Neisseria.meningitidis
0
3
6
9
A (n=15)
B (n=16)
C (n=14)
surface

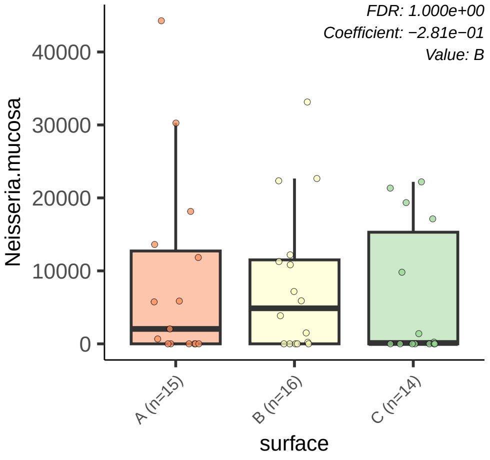
FDR: 1.000e+00
Coefficient: −2.81e−01
Value: B
Neisseria.mucosa
40000
30000
20000
10000
0
A (n=15)
B (n=16)
C (n=14)
surface

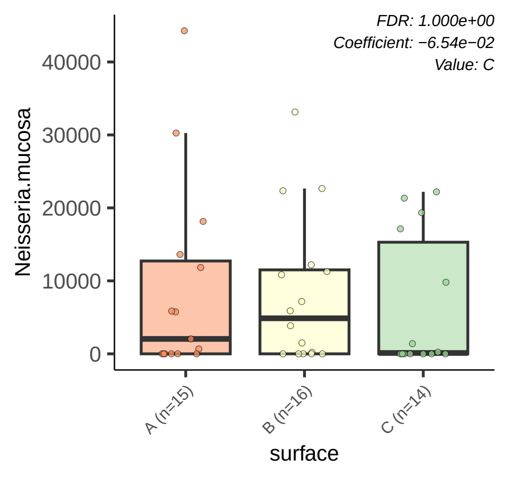
FDR: 1.000e+00
Coefficient: −6.54e−02
Value: C
Neisseria.mucosa
40000
30000
20000
10000
0
A (n=15)
B (n=16)
C (n=14)
surface

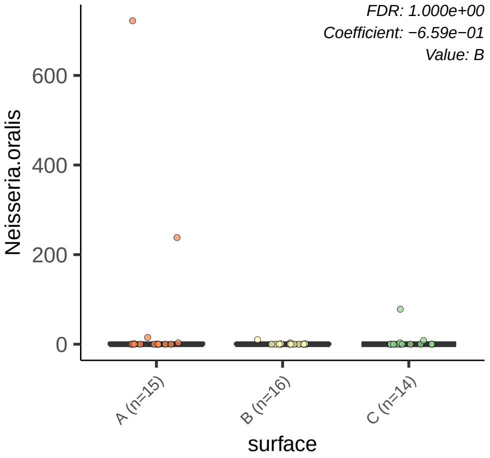

FDR: 1.000e+00
Coefficient: −6.59e−01
Value: B
Neisseria.oralis
600
400
200
0
A (n=15)
B (n=16)
C (n=14)
surface

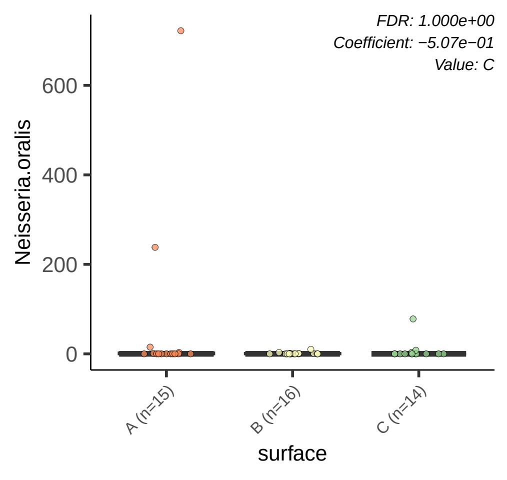
FDR: 1.000e+00
Coefficient: −5.07e−01
Value: C
Neisseria.oralis
600
400
200
0
A (n=15)
B (n=16)
C (n=14)
surface

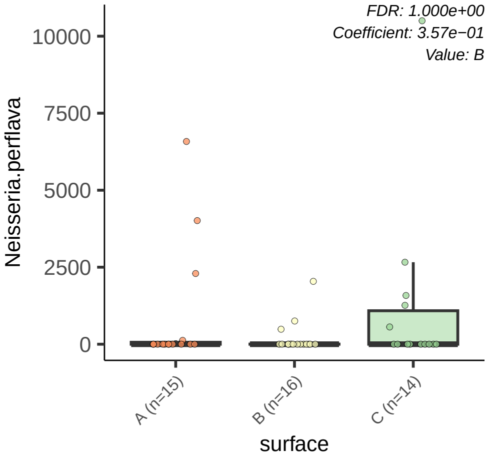

FDR: 1.000e+00
Coefficient: 3.57e−01
Value: B
10000
7500
5000
2500
0
Neisseria.perflava
A (n=15)
B (n=16)
C (n=14)
surface

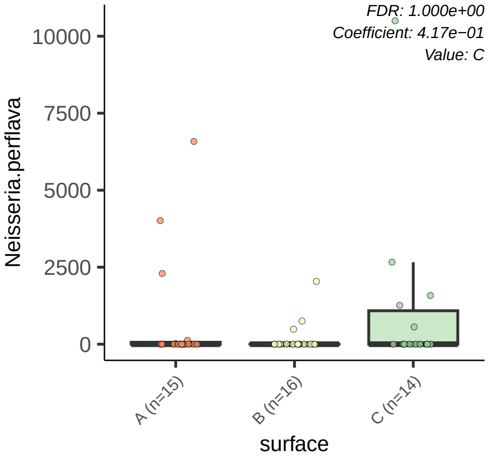

FDR: 1.000e+00
Coefficient: 4.17e−01
Value: C
Neisseria.perflava
10000
7500
5000
2500
0
A (n=15)
B (n=16)
C (n=14)
surface

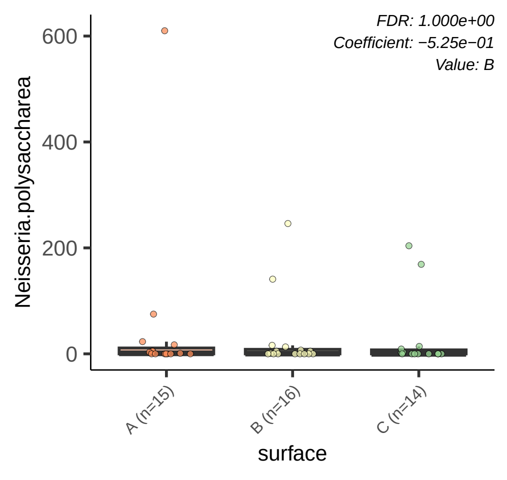

FDR: 1.000e+00
Coefficient: −5.25e−01
Value: B
Neisseria.polysaccharea
600
400
200
0
A (n=15)
B (n=16)
C (n=14)
surface

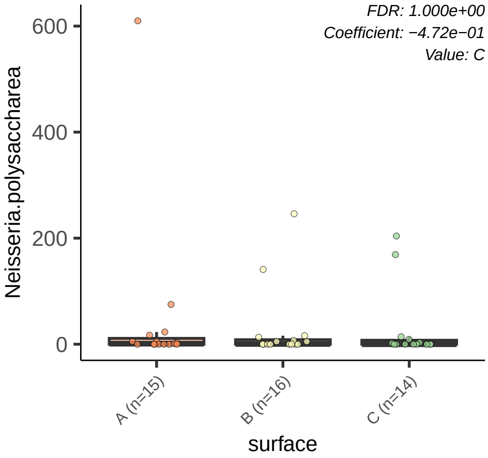

FDR: 1.000e+00
Coefficient: −4.72e−01
Value: C
Neisseria.polysaccharea
600
400
200
0
A (n=15)
B (n=16)
C (n=14)
surface

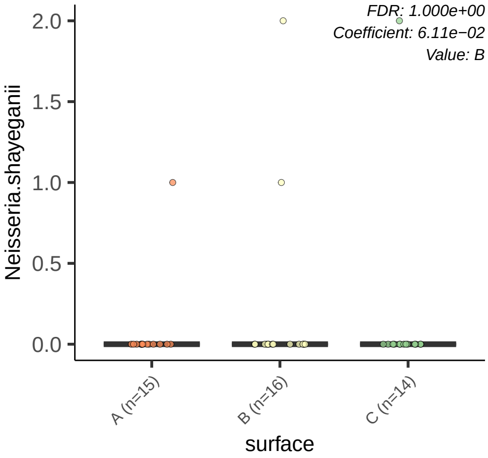

FDR: 1.000e+00
Coefficient: 6.11e−02
Value: B
Neisseria.shayeganii
2.0
1.5
1.0
0.5
0.0
A (n=15)
B (n=16)
C (n=14)
surface

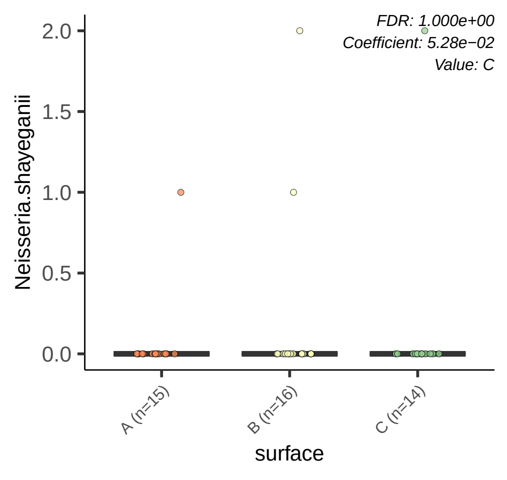

FDR: 1.000e+00
Coefficient: 5.28e−02
Value: C
Neisseria.shayeganii
2.0
1.5
1.0
0.5
0.0
A (n=15)
B (n=16)
C (n=14)
surface

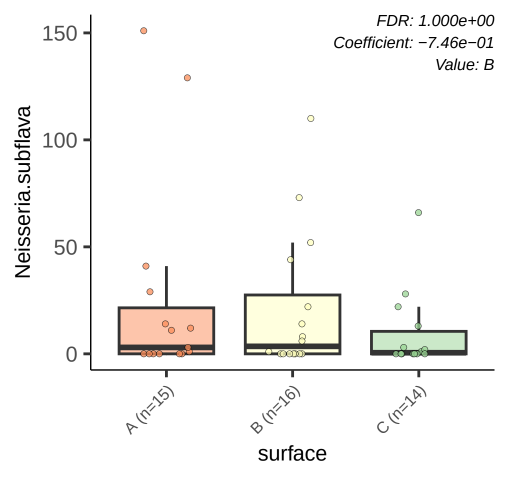

FDR: 1.000e+00
Coefficient: −7.46e−01
Value: B
Neisseria.subflava
150
100
50
0
A (n=15)
B (n=16)
C (n=14)
surface

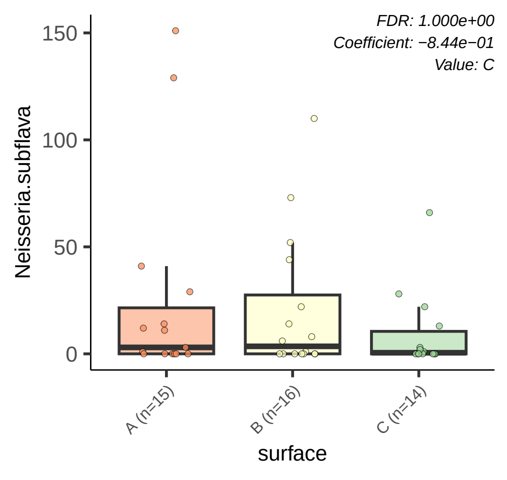

FDR: 1.000e+00
Coefficient: −8.44e−01
Value: C
Neisseria.subflava
150
100
50
0
A (n=15)
B (n=16)
C (n=14)
surface

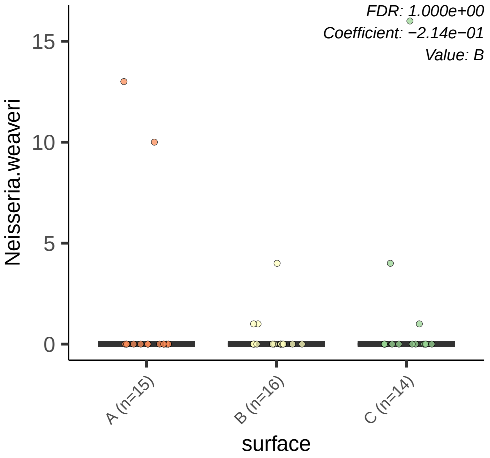
FDR: 1.000e+00
Coefficient: −2.14e−01
Value: B
Neisseria.weaveri
15
10
5
0
A (n=15)
B (n=16)
C (n=14)
surface

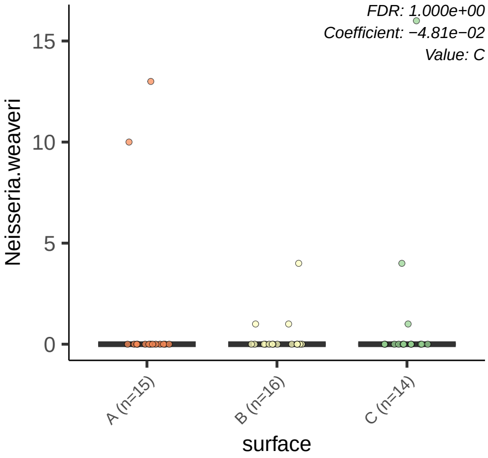
FDR: 1.000e+00
Coefficient: −4.81e−02
Value: C
Neisseria.weaveri
15
10
5
0
A (n=15)
B (n=16)
C (n=14)
surface

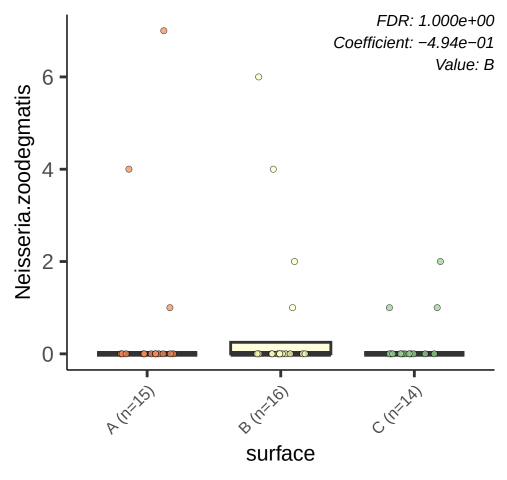

FDR: 1.000e+00
Coefficient: −4.94e−01
Value: B
Neisseria.zoodegmatis
0
2
4
6
A (n=15)
B (n=16)
C (n=14)
surface

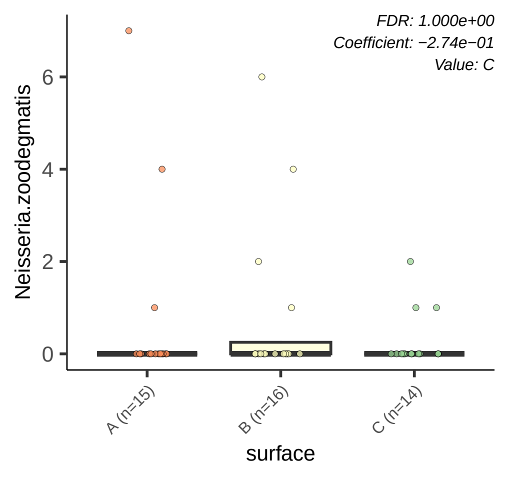
FDR: 1.000e+00
Coefficient: −2.74e−01
Value: C
Neisseria.zoodegmatis
0
2
4
6
A (n=15)
B (n=16)
C (n=14)
surface

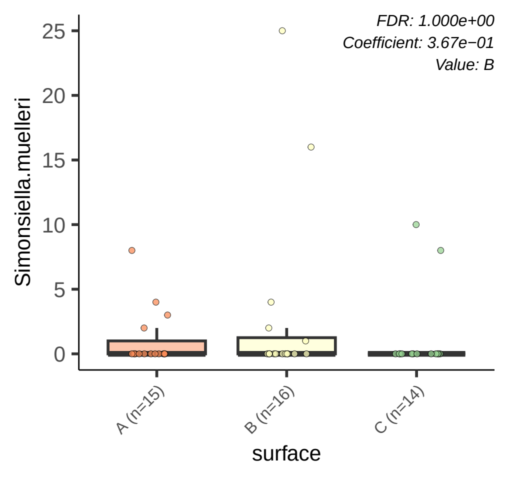
FDR: 1.000e+00
Coefficient: 3.67e−01
Value: B
Simonsiella.muelleri
0
5
10
15
20
25
A (n=15)
B (n=16)
C (n=14)
surface

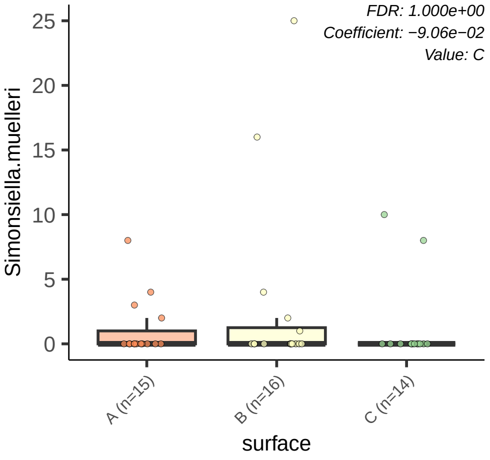

FDR: 1.000e+00
Coefficient: −9.06e−02
Value: C
Simonsiella.muelleri
25
20
15
10
5
0
A (n=15)
B (n=16)
C (n=14)
surface

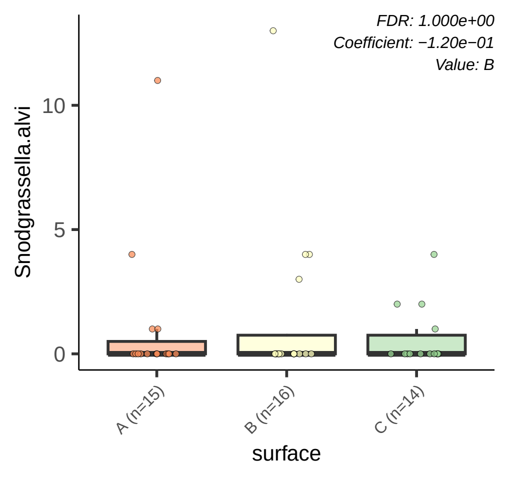

FDR: 1.000e+00
Coefficient: −1.20e−01
Value: B
Snodgrassella.alvi
10
5
0
A (n=15)
B (n=16)
C (n=14)
surface

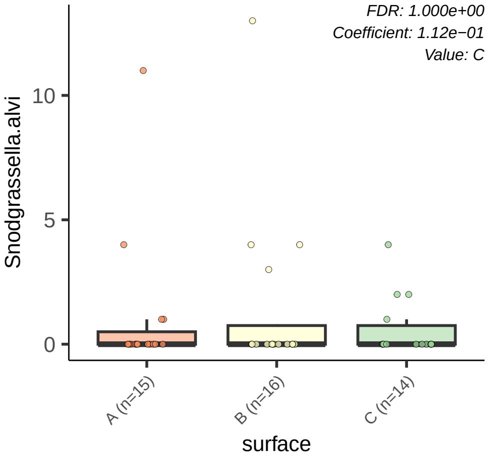
FDR: 1.000e+00
Coefficient: 1.12e−01
Value: C
Snodgrassella.alvi
10
5
0
A (n=15)
B (n=16)
C (n=14)
surface

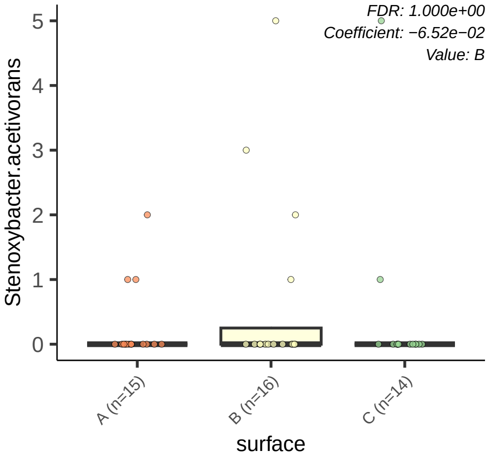
FDR: 1.000e+00
Coefficient: −6.52e−02
Value: B
Stenoxybacter.acetivorans
0
1
2
3
4
5
A (n=15)
B (n=16)
C (n=14)
surface

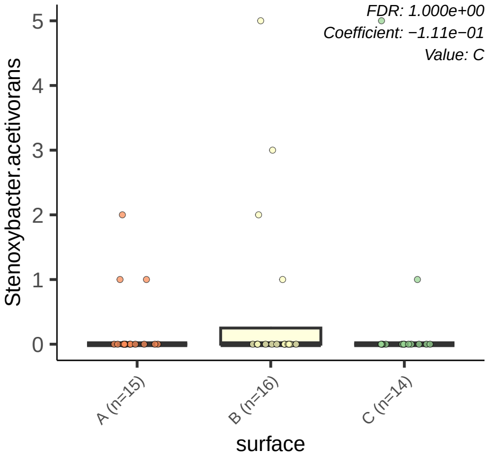
FDR: 1.000e+00
Coefficient: −1.11e−01
Value: C
Stenoxybacter.acetivorans
5
4
3
2
1
0
A (n=15)
B (n=16)
C (n=14)
surface

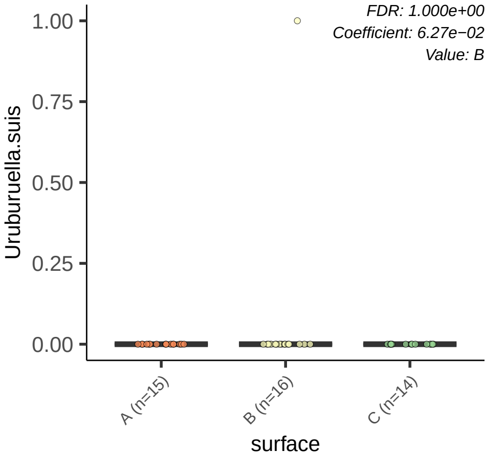

FDR: 1.000e+00
Coefficient: 6.27e−02
Value: B
1.00
0.75
0.50
0.25
0.00
Uruburuella.suis
A (n=15)
B (n=16)
C (n=14)
surface

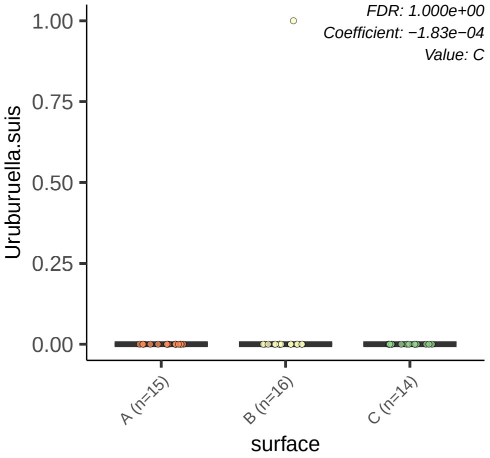

FDR: 1.000e+00
Coefficient: −1.83e−04
Value: C
Uruburuella.suis
1.00
0.75
0.50
0.25
0.00
A (n=15)
B (n=16)
C (n=14)
surface

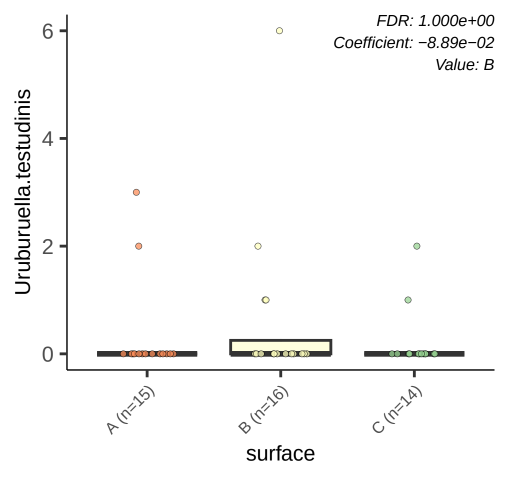

FDR: 1.000e+00
Coefficient: −8.89e−02
Value: B
Uruburuella.testudinis
0
2
4
6
A (n=15)
B (n=16)
C (n=14)
surface

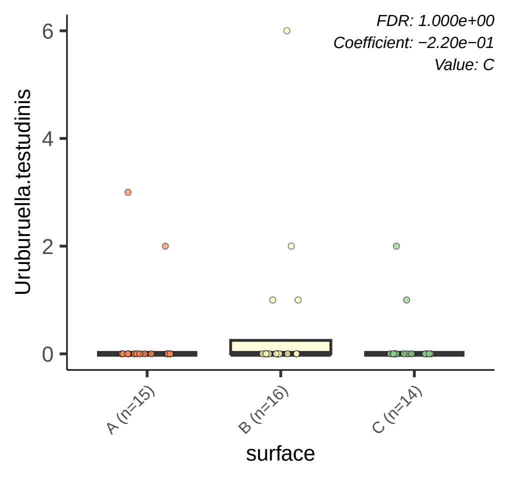

FDR: 1.000e+00
Coefficient: −2.20e−01
Value: C
Uruburuella.testudinis
6
4
2
0
A (n=15)
B (n=16)
C (n=14)
surface

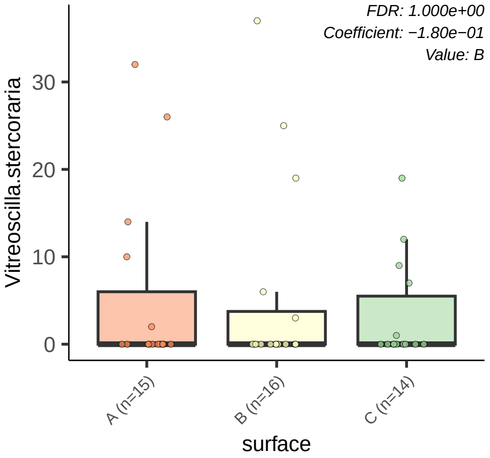

FDR: 1.000e+00
Coefficient: −1.80e−01
Value: B
Vitreoscilla.stercoraria
0
10
20
30
A (n=15)
B (n=16)
C (n=14)
surface

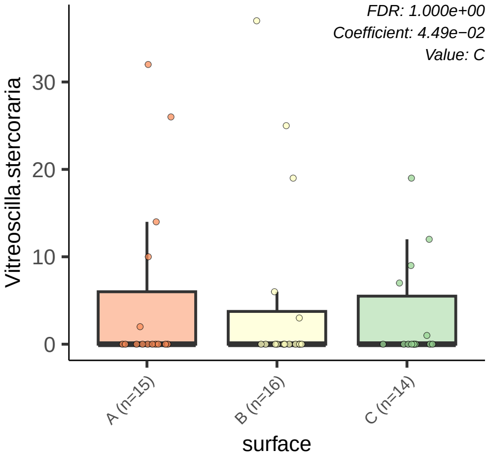

FDR: 1.000e+00
Coefficient: 4.49e−02
Value: C
Vitreoscilla.stercoraria
0
10
20
30
A (n=15)
B (n=16)
C (n=14)
surface

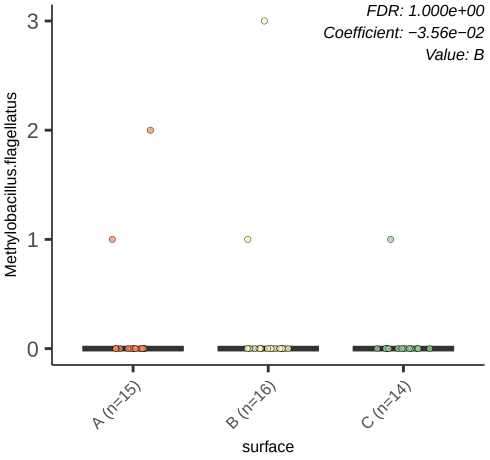
FDR: 1.000e+00
Coefficient: −3.56e−02
Value: B
Methylobacillus.flagellatus
3
2
1
0
A (n=15)
B (n=16)
C (n=14)
surface

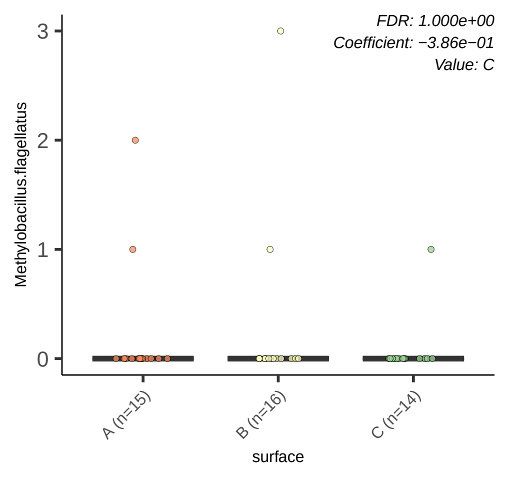
FDR: 1.000e+00
Coefficient: −3.86e−01
Value: C
Methylobacillus.flagellatus
0
1
2
3
A (n=15)
B (n=16)
C (n=14)
surface

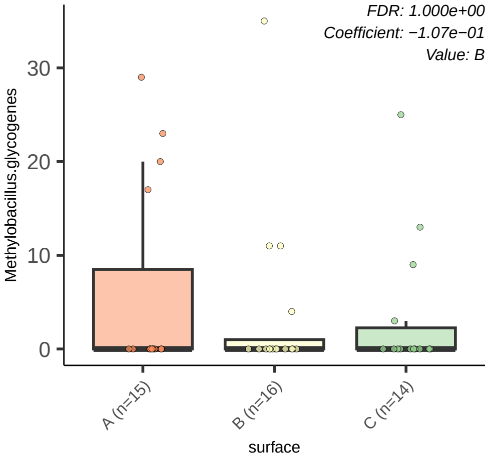
FDR: 1.000e+00
Coefficient: −1.07e−01
Value: B
Methylobacillus.glycogenes
30
20
10
0
A (n=15)
B (n=16)
C (n=14)
surface

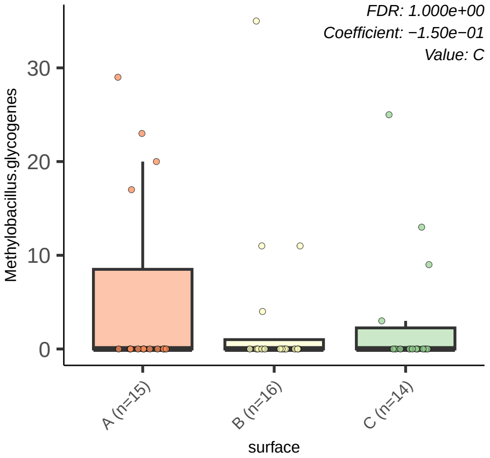

FDR: 1.000e+00
Coefficient: −1.50e−01
Value: C
Methylobacillus.glycogenes
0
10
20
30
A (n=15)
B (n=16)
C (n=14)
surface

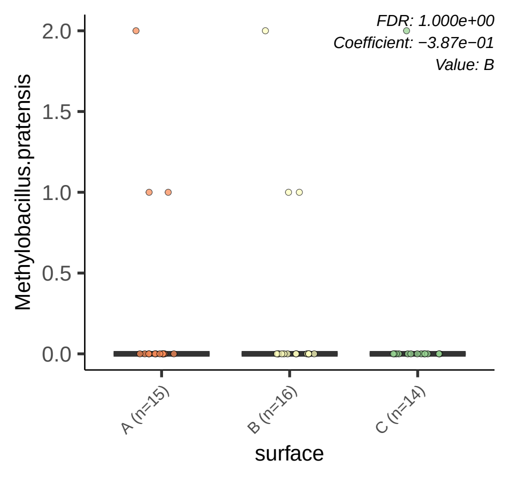
FDR: 1.000e+00
Coefficient: −3.87e−01
Value: B
Methylobacillus.pratensis
2.0
1.5
1.0
0.5
0.0
A (n=15)
B (n=16)
C (n=14)
surface

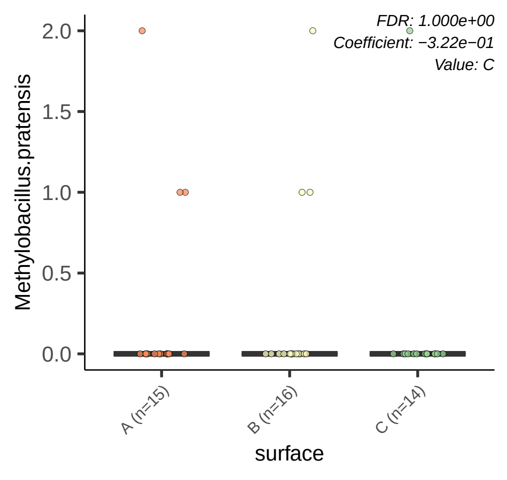
FDR: 1.000e+00
Coefficient: −3.22e−01
Value: C
Methylobacillus.pratensis
2.0
1.5
1.0
0.5
0.0
A (n=15)
B (n=16)
C (n=14)
surface

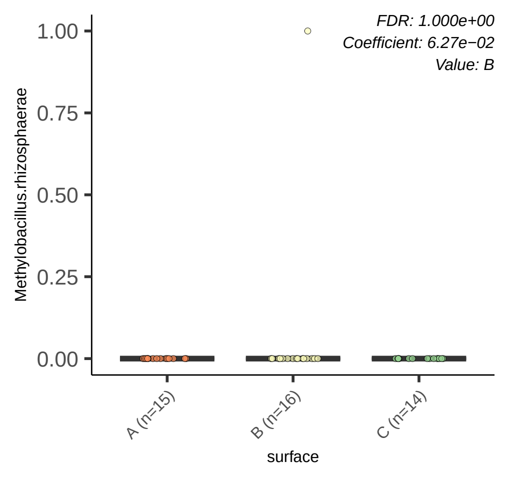
FDR: 1.000e+00
Coefficient: 6.27e−02
Value: B
Methylobacillus.rhizosphaerae
1.00
0.75
0.50
0.25
0.00
A (n=15)
B (n=16)
C (n=14)
surface

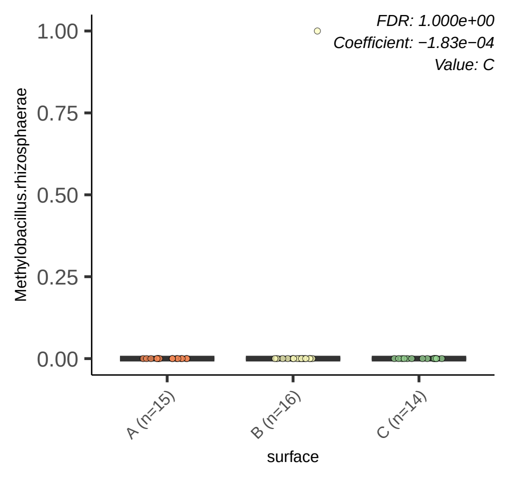
FDR: 1.000e+00
Coefficient: −1.83e−04
Value: C
Methylobacillus.rhizosphaerae
1.00
0.75
0.50
0.25
0.00
A (n=15)
B (n=16)
C (n=14)
surface

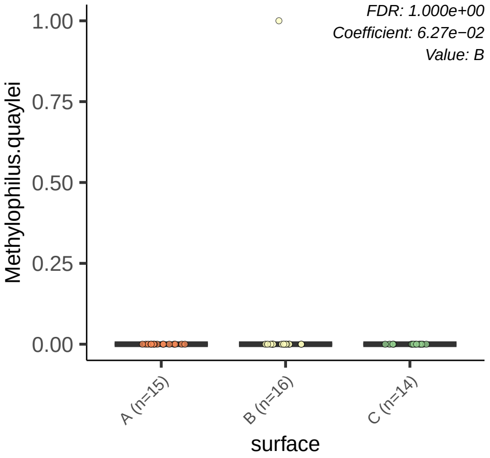
FDR: 1.000e+00
Coefficient: 6.27e−02
Value: B
Methylophilus.quaylei
1.00
0.75
0.50
0.25
0.00
A (n=15)
B (n=16)
C (n=14)
surface

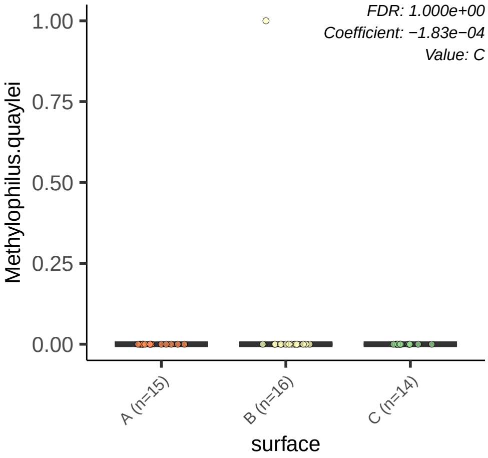
FDR: 1.000e+00
Coefficient: −1.83e−04
Value: C
Methylophilus.quaylei
1.00
0.75
0.50
0.25
0.00
A (n=15)
B (n=16)
C (n=14)
surface

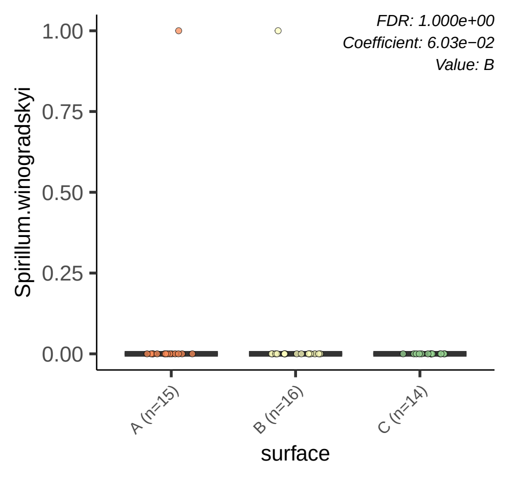

FDR: 1.000e+00
Coefficient: 6.03e−02
Value: B
Spirillum.winogradskyi
1.00
0.75
0.50
0.25
0.00
A (n=15)
B (n=16)
C (n=14)
surface

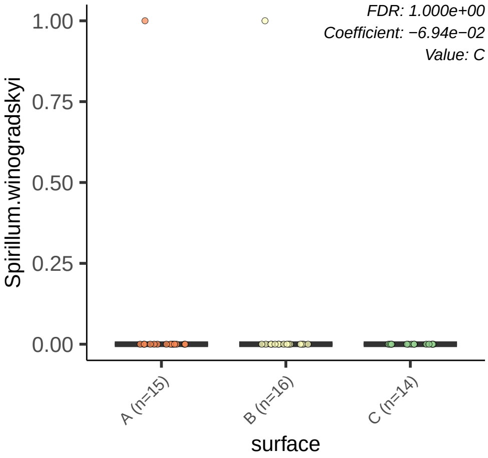

FDR: 1.000e+00
Coefficient: −6.94e−02
Value: C
Spirillum.winogradskyi
1.00
0.75
0.50
0.25
0.00
A (n=15)
B (n=16)
C (n=14)
surface

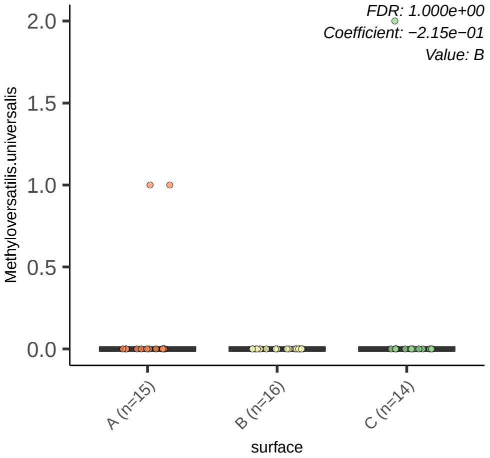
FDR: 1.000e+00
Coefficient: −2.15e−01
Value: B
Methyloversatilis.universalis
2.0
1.5
1.0
0.5
0.0
A (n=15)
B (n=16)
C (n=14)
surface

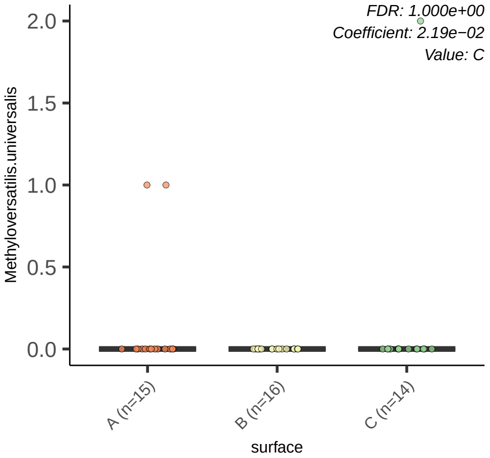
FDR: 1.000e+00
Coefficient: 2.19e−02
Value: C
Methyloversatilis.universalis
2.0
1.5
1.0
0.5
0.0
A (n=15)
B (n=16)
C (n=14)
surface

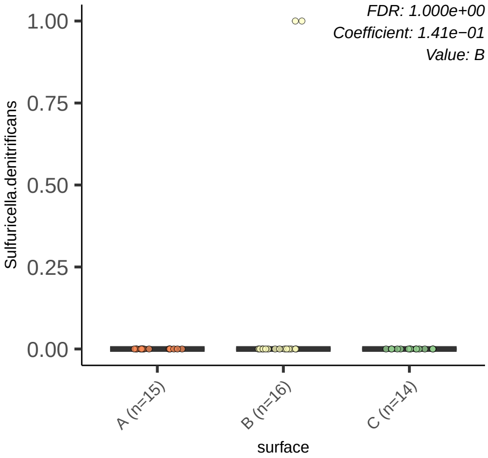

FDR: 1.000e+00
Coefficient: 1.41e−01
Value: B
1.00
0.75
0.50
0.25
0.00
Sulfuricella.denitrificans
A (n=15)
B (n=16)
C (n=14)
surface

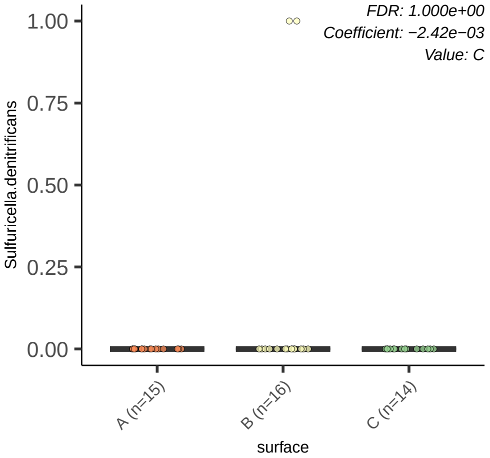

FDR: 1.000e+00
Coefficient: −2.42e−03
Value: C
Sulfuricella.denitrificans
1.00
0.75
0.50
0.25
0.00
A (n=15)
B (n=16)
C (n=14)
surface

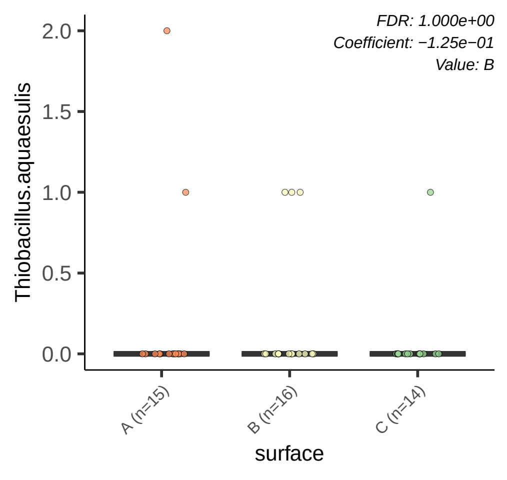
FDR: 1.000e+00
Coefficient: −1.25e−01
Value: B
Thiobacillus.aquaesulis
2.0
1.5
1.0
0.5
0.0
A (n=15)
B (n=16)
C (n=14)
surface

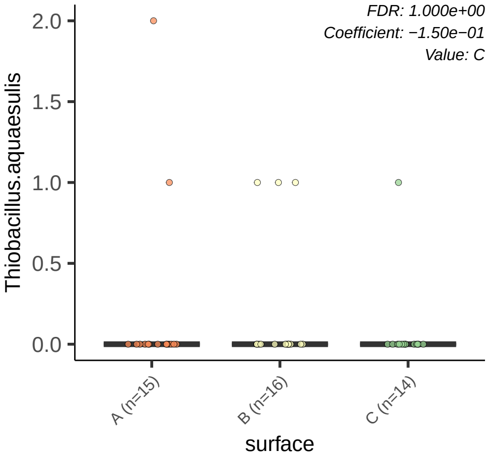
FDR: 1.000e+00
Coefficient: −1.50e−01
Value: C
Thiobacillus.aquaesulis
2.0
1.5
1.0
0.5
0.0
A (n=15)
B (n=16)
C (n=14)
surface

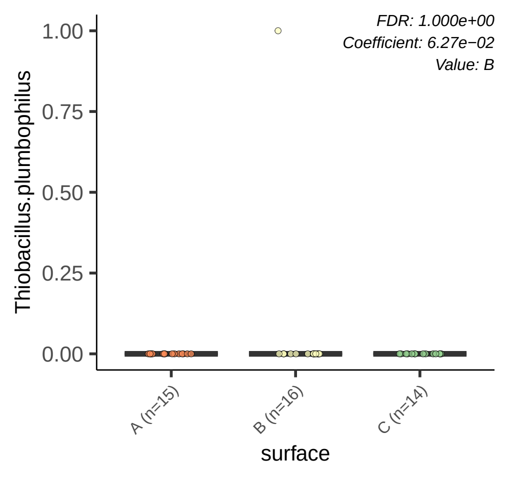

FDR: 1.000e+00
Coefficient: 6.27e−02
Value: B
Thiobacillus.plumbophilus
1.00
0.75
0.50
0.25
0.00
A (n=15)
B (n=16)
C (n=14)
surface

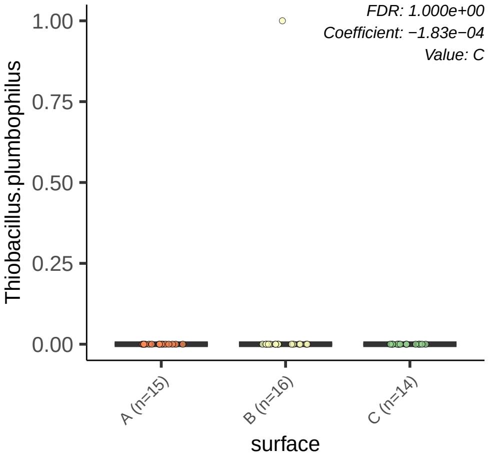
FDR: 1.000e+00
Coefficient: −1.83e−04
Value: C
Thiobacillus.plumbophilus
1.00
0.75
0.50
0.25
0.00
A (n=15)
B (n=16)
C (n=14)
surface

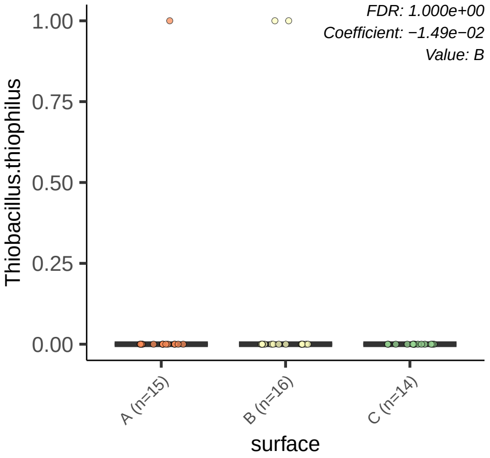
FDR: 1.000e+00
Coefficient: −1.49e−02
Value: B
Thiobacillus.thiophilus
1.00
0.75
0.50
0.25
0.00
A (n=15)
B (n=16)
C (n=14)
surface

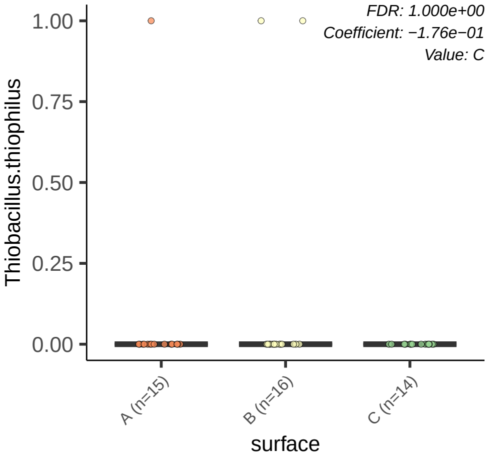

FDR: 1.000e+00
Coefficient: −1.76e−01
Value: C
Thiobacillus.thiophilus
1.00
0.75
0.50
0.25
0.00
A (n=15)
B (n=16)
C (n=14)
surface

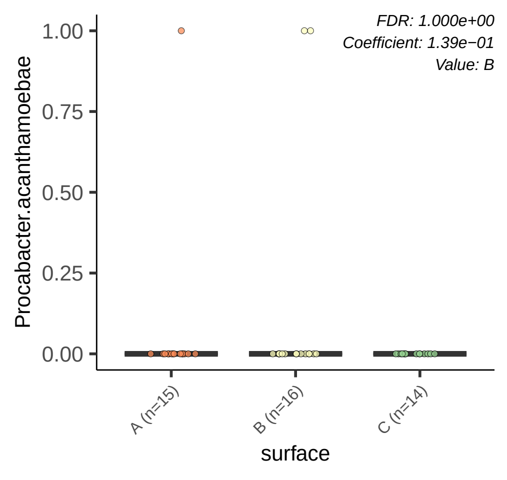
FDR: 1.000e+00
Coefficient: 1.39e−01
Value: B
Procabacter.acanthamoebae
1.00
0.75
0.50
0.25
0.00
A (n=15)
B (n=16)
C (n=14)
surface

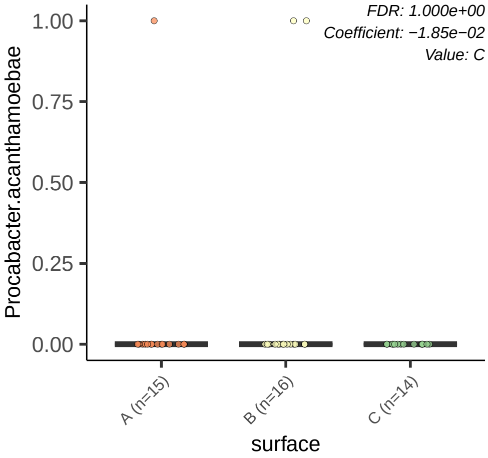
FDR: 1.000e+00
Coefficient: −1.85e−02
Value: C
Procabacter.acanthamoebae
1.00
0.75
0.50
0.25
0.00
A (n=15)
B (n=16)
C (n=14)
surface

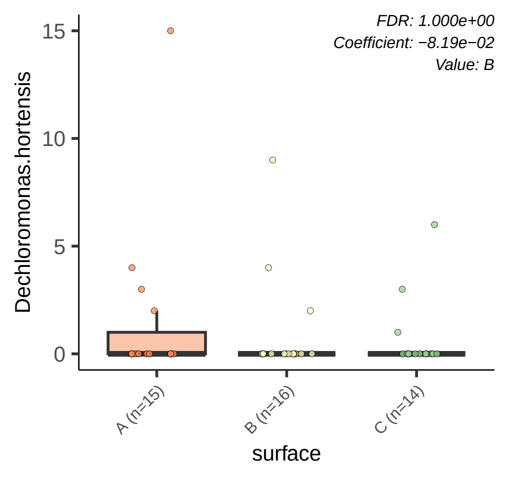
FDR: 1.000e+00
Coefficient: −8.19e−02
Value: B
Dechloromonas.hortensis
15
10
5
0
A (n=15)
B (n=16)
C (n=14)
surface

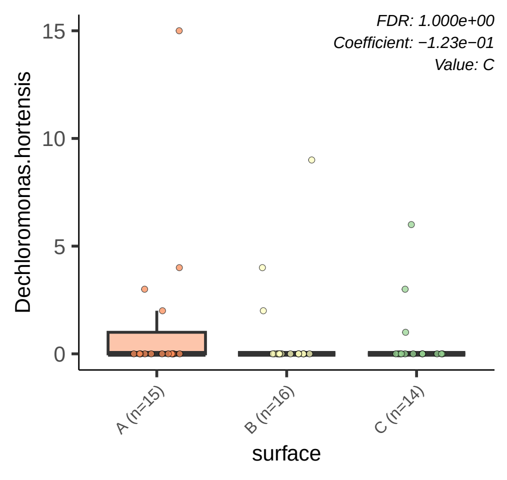
FDR: 1.000e+00
Coefficient: −1.23e−01
Value: C
Dechloromonas.hortensis
15
10
5
0
A (n=15)
B (n=16)
C (n=14)
surface

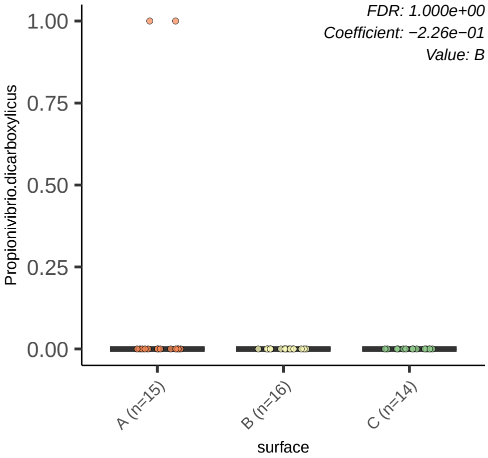

FDR: 1.000e+00
Coefficient: −2.26e−01
Value: B
Propionivibrio.dicarboxylicus
1.00
0.75
0.50
0.25
0.00
A (n=15)
B (n=16)
C (n=14)
surface

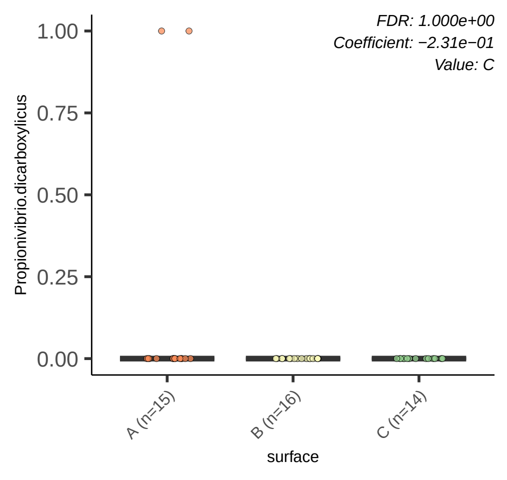
FDR: 1.000e+00
Coefficient: −2.31e−01
Value: C
Propionivibrio.dicarboxylicus
1.00
0.75
0.50
0.25
0.00
A (n=15)
B (n=16)
C (n=14)
surface

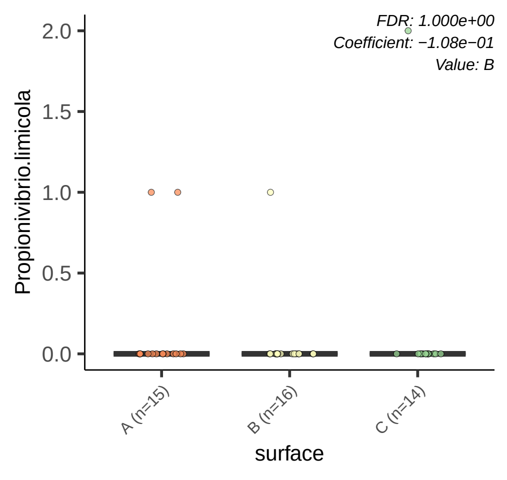
FDR: 1.000e+00
Coefficient: −1.08e−01
Value: B
Propionivibrio.limicola
2.0
1.5
1.0
0.5
0.0
A (n=15)
B (n=16)
C (n=14)
surface

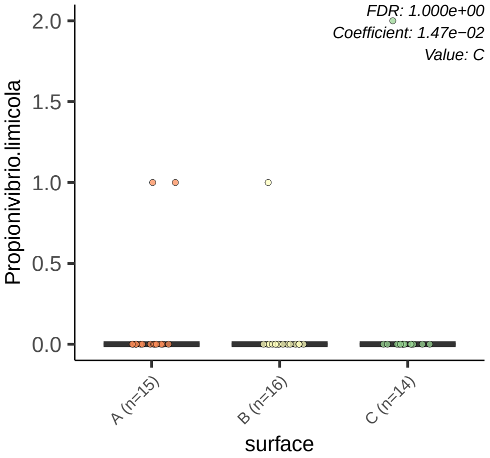

FDR: 1.000e+00
Coefficient: 1.47e−02
Value: C
Propionivibrio.limicola
2.0
1.5
1.0
0.5
0.0
A (n=15)
B (n=16)
C (n=14)
surface

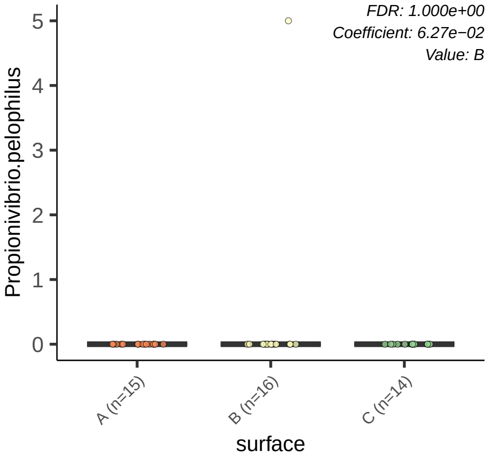

FDR: 1.000e+00
Coefficient: 6.27e−02
Value: B
Propionivibrio.pelophilus
0
1
2
3
4
5
A (n=15)
B (n=16)
C (n=14)
surface

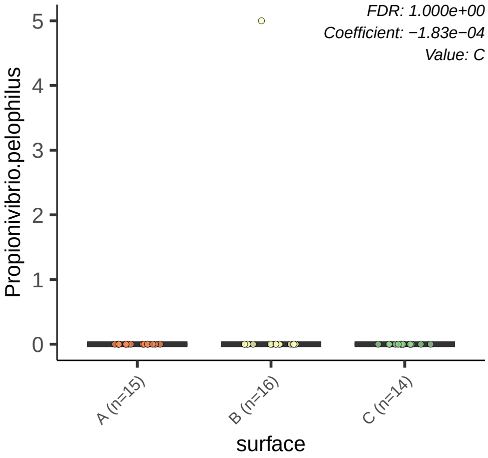
FDR: 1.000e+00
Coefficient: −1.83e−04
Value: C
Propionivibrio.pelophilus
0
1
2
3
4
5
A (n=15)
B (n=16)
C (n=14)
surface

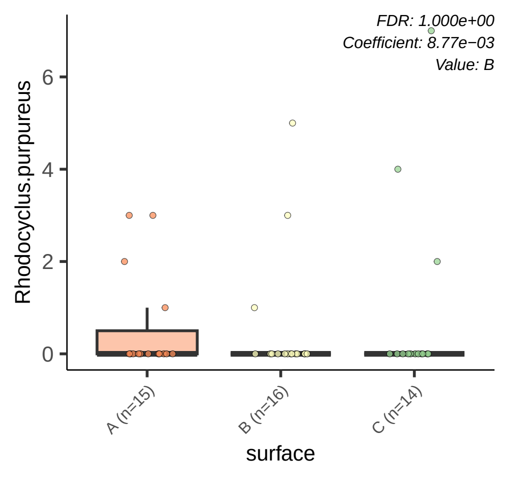

FDR: 1.000e+00
Coefficient: 8.77e−03
Value: B
Rhodocyclus.purpureus
0
2
4
6
A (n=15)
B (n=16)
C (n=14)
surface

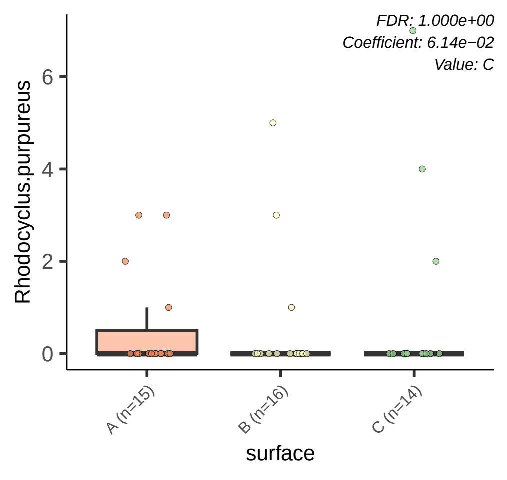
FDR: 1.000e+00
Coefficient: 6.14e−02
Value: C
Rhodocyclus.purpureus
0
2
4
6
A (n=15)
B (n=16)
C (n=14)
surface

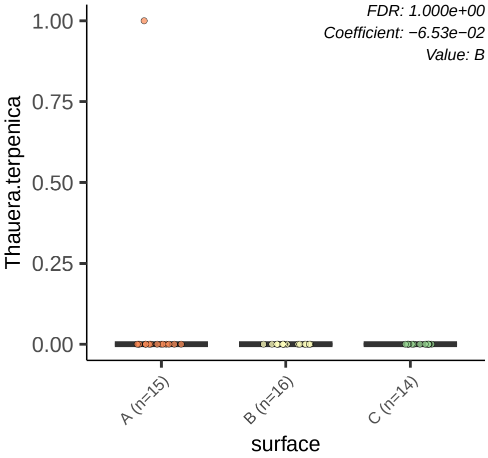

FDR: 1.000e+00
Coefficient: −6.53e−02
Value: B
Thauera.terpenica
1.00
0.75
0.50
0.25
0.00
A (n=15)
B (n=16)
C (n=14)
surface

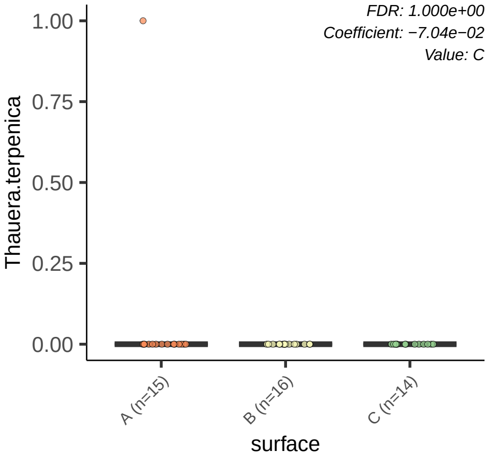

FDR: 1.000e+00
Coefficient: −7.04e−02
Value: C
1.00
0.75
0.50
0.25
0.00
Thauera.terpenica
A (n=15)
B (n=16)
C (n=14)
surface

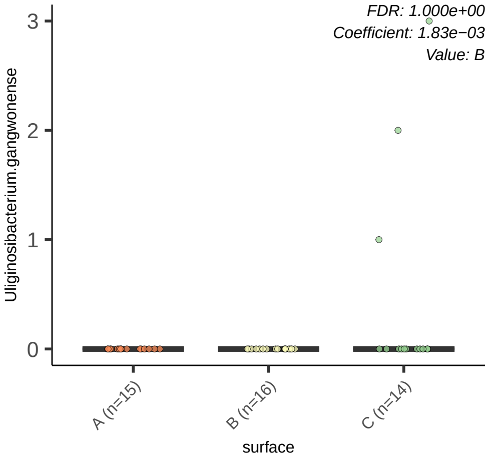
FDR: 1.000e+00
Coefficient: 1.83e−03
Value: B
Uliginosibacterium.gangwonense
3
2
1
0
A (n=15)
B (n=16)
C (n=14)
surface

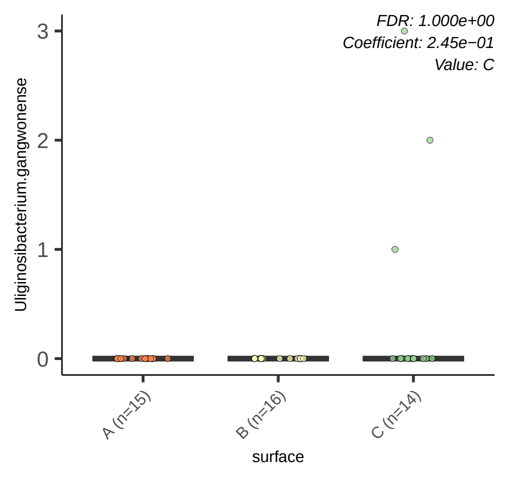
FDR: 1.000e+00
Coefficient: 2.45e−01
Value: C
Uliginosibacterium.gangwonense
3
2
1
0
A (n=15)
B (n=16)
C (n=14)
surface

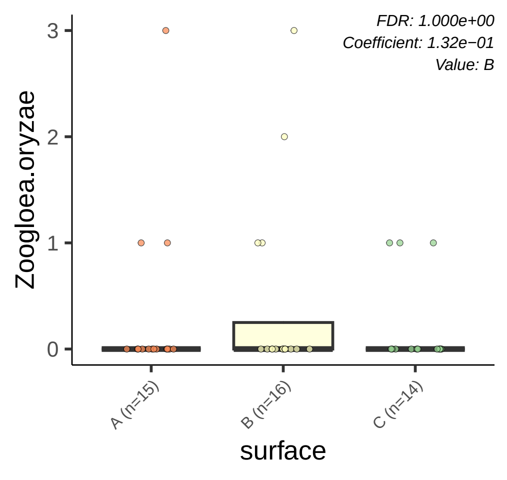
FDR: 1.000e+00
Coefficient: 1.32e−01
Value: B
Zoogloea.oryzae
3
2
1
0
A (n=15)
B (n=16)
C (n=14)
surface

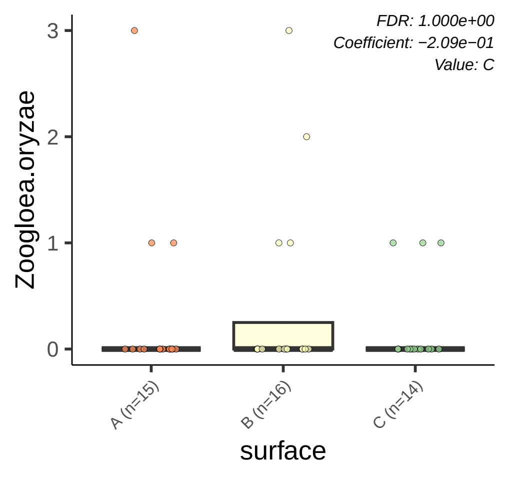
FDR: 1.000e+00
Coefficient: −2.09e−01
Value: C
Zoogloea.oryzae
3
2
1
0
A (n=15)
B (n=16)
C (n=14)
surface

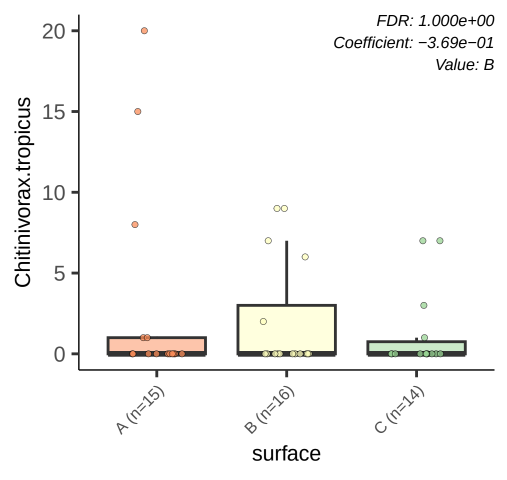

FDR: 1.000e+00
Coefficient: −3.69e−01
Value: B
Chitinivorax.tropicus
20
15
10
5
0
A (n=15)
B (n=16)
C (n=14)
surface

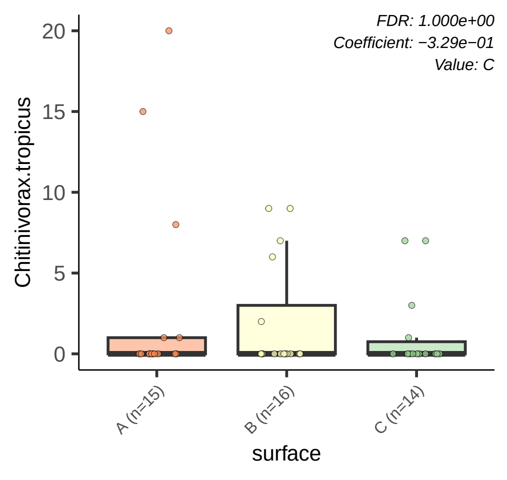

FDR: 1.000e+00
Coefficient: −3.29e−01
Value: C
Chitinivorax.tropicus
0
5
10
15
20
A (n=15)
B (n=16)
C (n=14)
surface

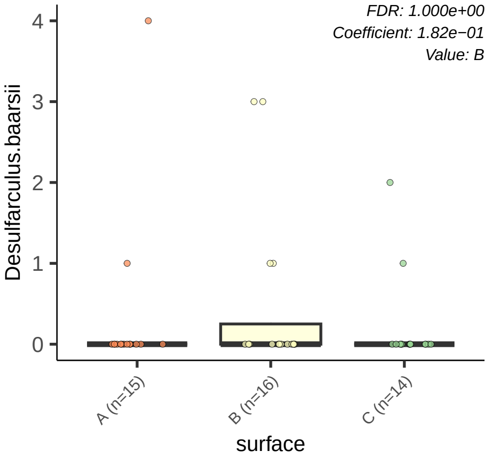

FDR: 1.000e+00
Coefficient: 1.82e−01
Value: B
Desulfarculus.baarsii
0
1
2
3
4
A (n=15)
B (n=16)
C (n=14)
surface

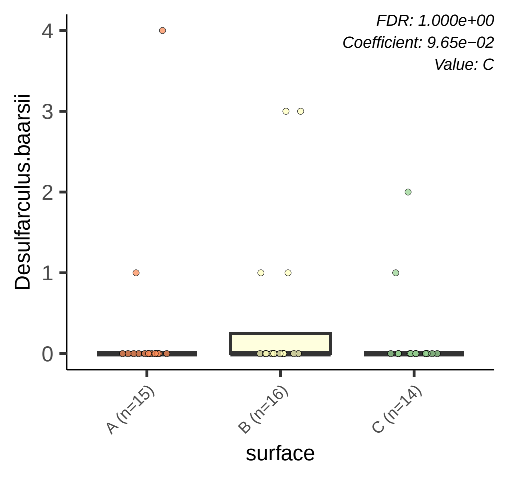

FDR: 1.000e+00
Coefficient: 9.65e−02
Value: C
Desulfarculus.baarsii
4
3
2
1
0
A (n=15)
B (n=16)
C (n=14)
surface

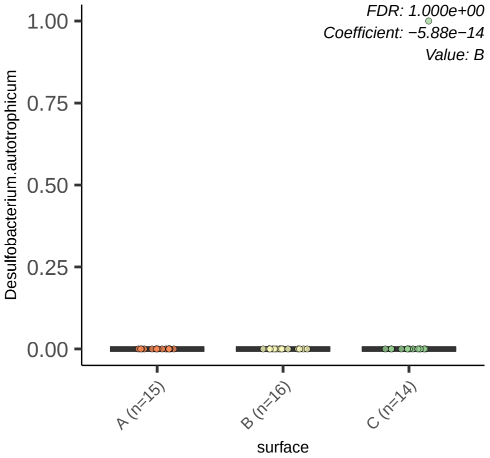

FDR: 1.000e+00
Coefficient: −5.88e−14
Value: B
1.00
0.75
0.50
0.25
0.00
Desulfobacterium.autotrophicum
A (n=15)
B (n=16)
C (n=14)
surface

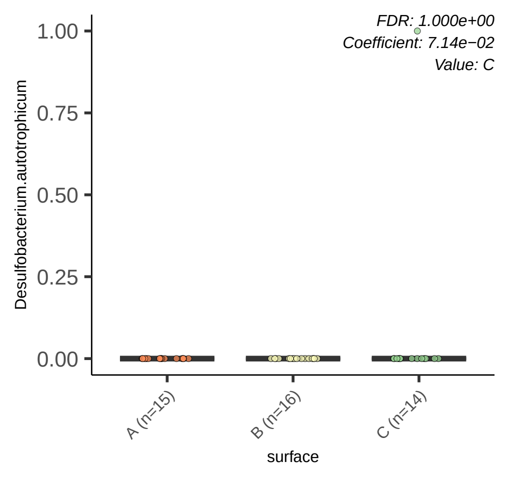

FDR: 1.000e+00
Coefficient: 7.14e−02
Value: C
Desulfobacterium.autotrophicum
1.00
0.75
0.50
0.25
0.00
A (n=15)
B (n=16)
C (n=14)
surface

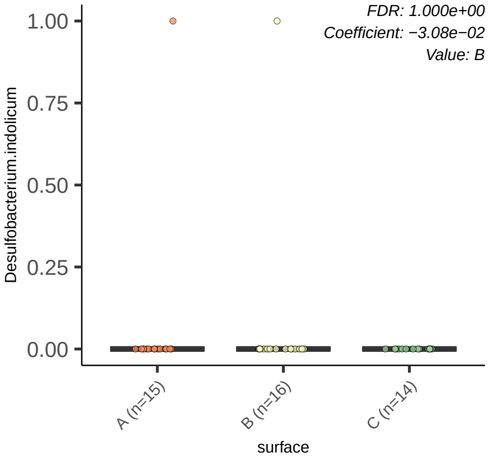

FDR: 1.000e+00
Coefficient: −3.08e−02
Value: B
Desulfobacterium.indolicum
1.00
0.75
0.50
0.25
0.00
A (n=15)
B (n=16)
C (n=14)
surface

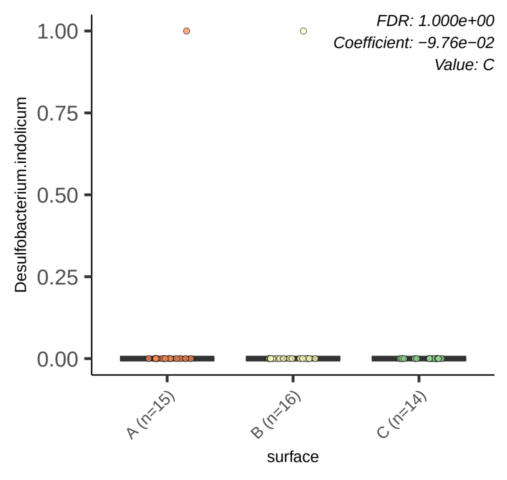
FDR: 1.000e+00
Coefficient: −9.76e−02
Value: C
Desulfobacterium.indolicum
1.00
0.75
0.50
0.25
0.00
A (n=15)
B (n=16)
C (n=14)
surface

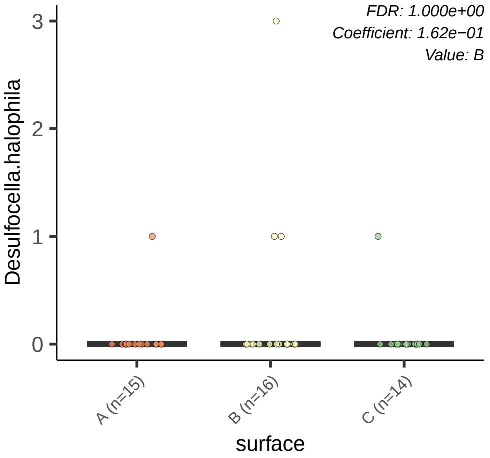

FDR: 1.000e+00
Coefficient: 1.62e−01
Value: B
Desulfocella.halophila
0
1
2
3
A (n=15)
B (n=16)
C (n=14)
surface

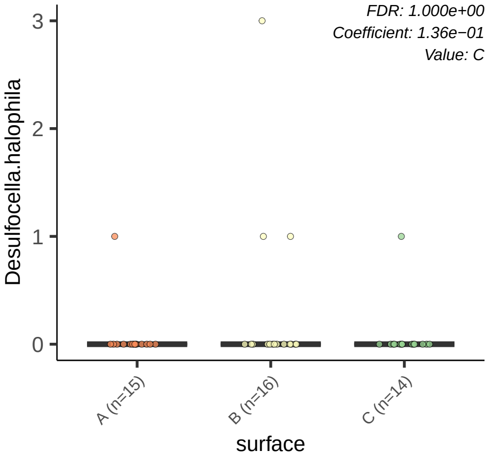
FDR: 1.000e+00
Coefficient: 1.36e−01
Value: C
Desulfocella.halophila
3
2
1
0
A (n=15)
B (n=16)
C (n=14)
surface

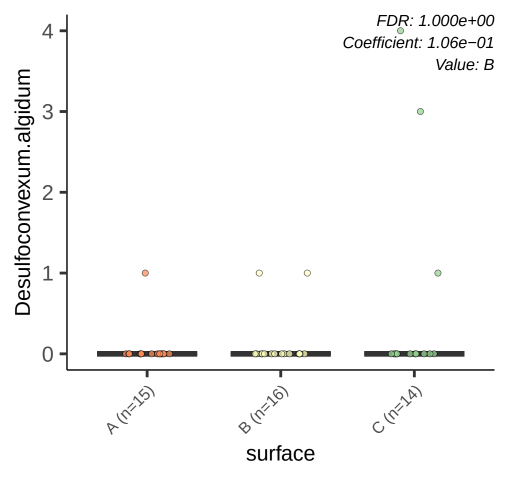
FDR: 1.000e+00
Coefficient: 1.06e−01
Value: B
Desulfoconvexum.algidum
0
1
2
3
4
A (n=15)
B (n=16)
C (n=14)
surface

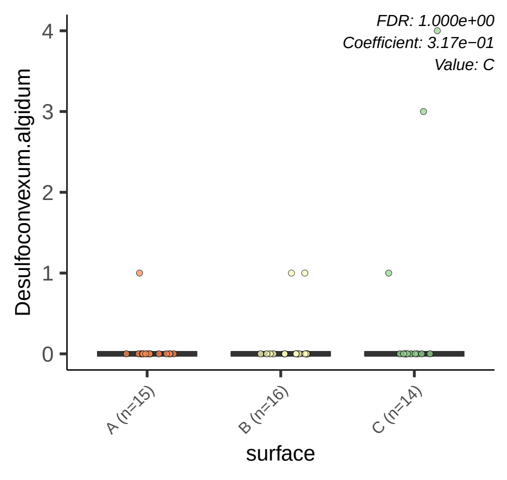
FDR: 1.000e+00
Coefficient: 3.17e−01
Value: C
Desulfoconvexum.algidum
0
1
2
3
4
A (n=15)
B (n=16)
C (n=14)
surface

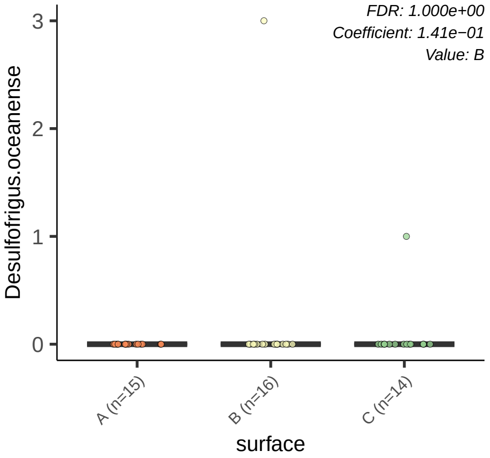

FDR: 1.000e+00
Coefficient: 1.41e−01
Value: B
Desulfofrigus.oceanense
3
2
1
0
A (n=15)
B (n=16)
C (n=14)
surface

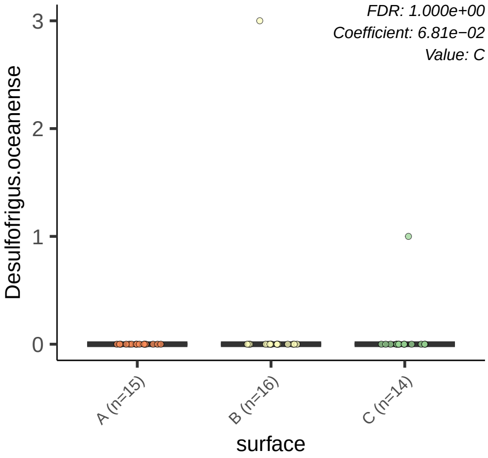

FDR: 1.000e+00
Coefficient: 6.81e−02
Value: C
Desulfofrigus.oceanense
3
2
1
0
A (n=15)
B (n=16)
C (n=14)
surface

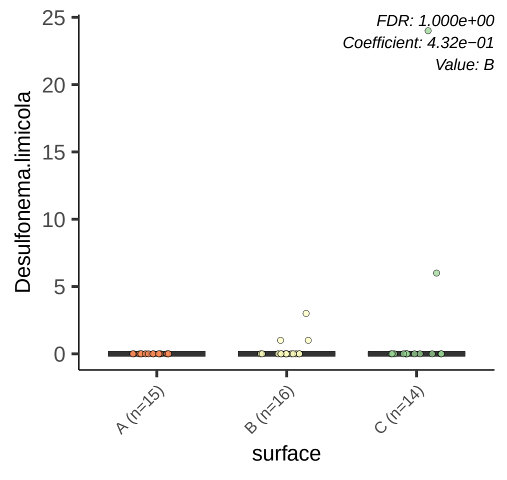

FDR: 1.000e+00
Coefficient: 4.32e−01
Value: B
Desulfonema.limicola
25
20
15
10
5
0
A (n=15)
B (n=16)
C (n=14)
surface

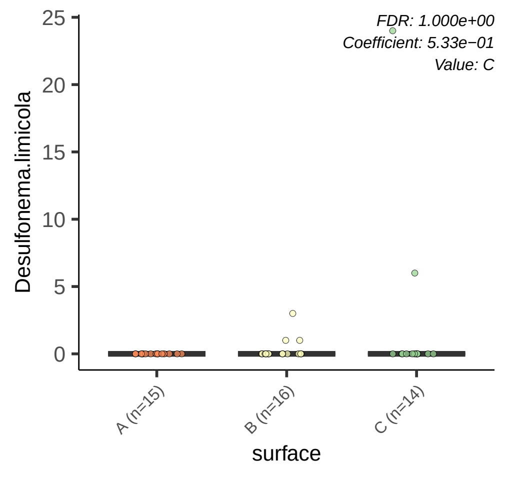
FDR: 1.000e+00
Coefficient: 5.33e−01
Value: C
Desulfonema.limicola
25
20
15
10
5
0
A (n=15)
B (n=16)
C (n=14)
surface

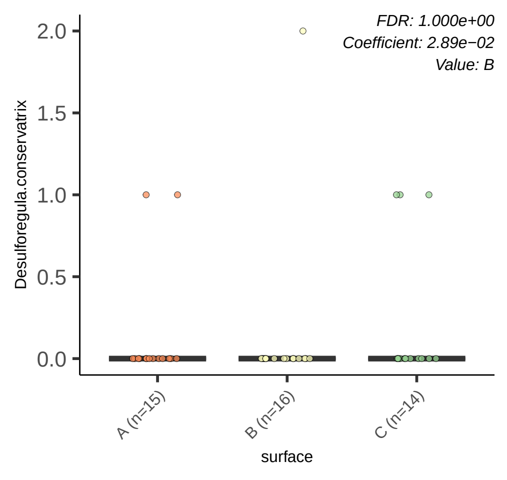

FDR: 1.000e+00
Coefficient: 2.89e−02
Value: B
Desulforegula.conservatrix
2.0
1.5
1.0
0.5
0.0
A (n=15)
B (n=16)
C (n=14)
surface

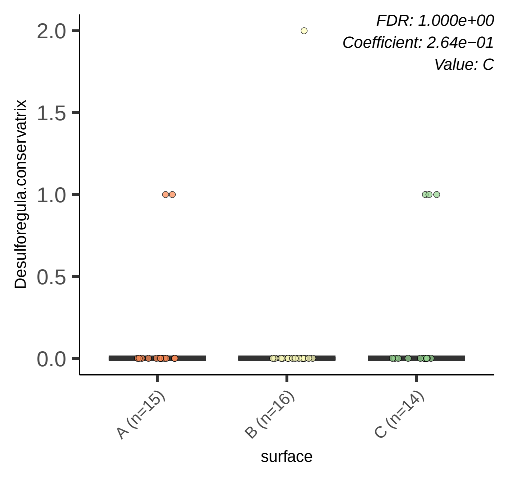

FDR: 1.000e+00
Coefficient: 2.64e−01
Value: C
Desulforegula.conservatrix
2.0
1.5
1.0
0.5
0.0
A (n=15)
B (n=16)
C (n=14)
surface

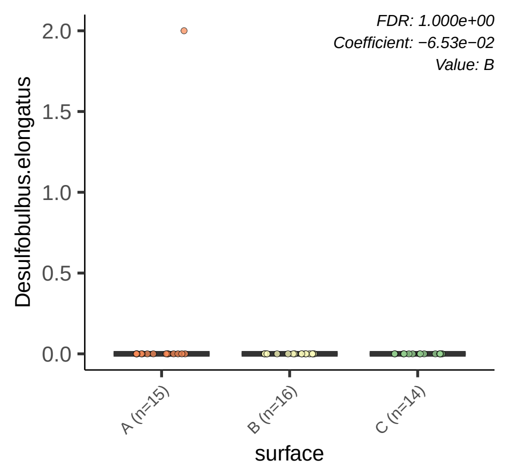
FDR: 1.000e+00
Coefficient: −6.53e−02
Value: B
Desulfobulbus.elongatus
2.0
1.5
1.0
0.5
0.0
A (n=15)
B (n=16)
C (n=14)
surface

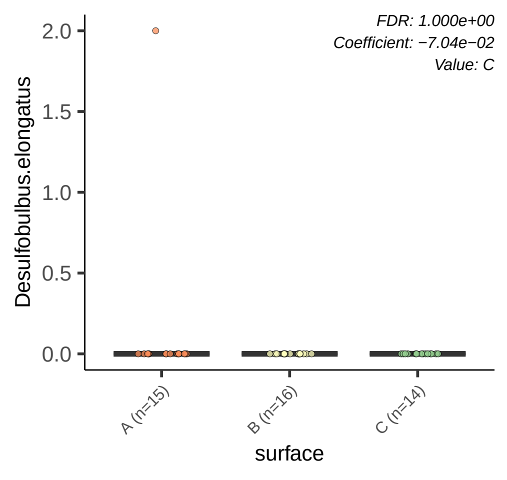
FDR: 1.000e+00
Coefficient: −7.04e−02
Value: C
Desulfobulbus.elongatus
2.0
1.5
1.0
0.5
0.0
A (n=15)
B (n=16)
C (n=14)
surface

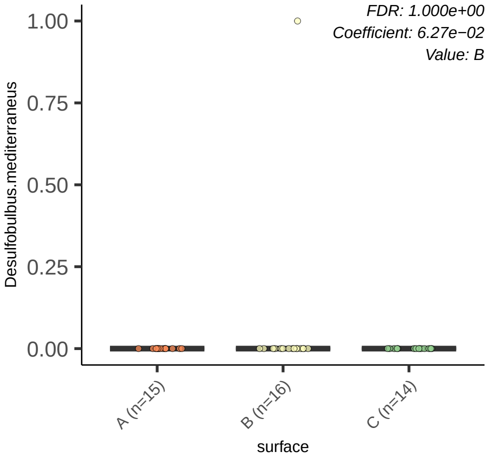
FDR: 1.000e+00
Coefficient: 6.27e−02
Value: B
1.00
0.75
0.50
0.25
0.00
Desulfobulbus.mediterraneus
A (n=15)
B (n=16)
C (n=14)
surface

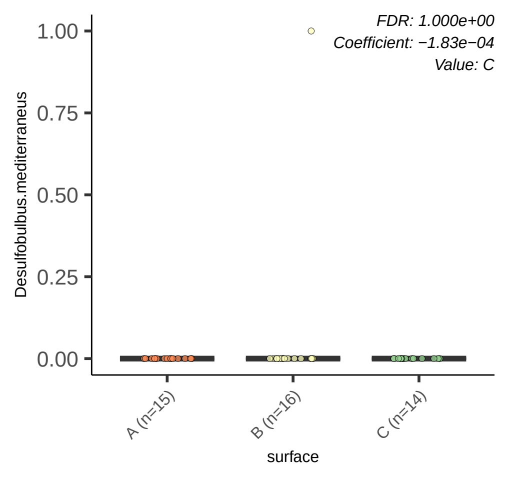
FDR: 1.000e+00
Coefficient: −1.83e−04
Value: C
Desulfobulbus.mediterraneus
1.00
0.75
0.50
0.25
0.00
A (n=15)
B (n=16)
C (n=14)
surface

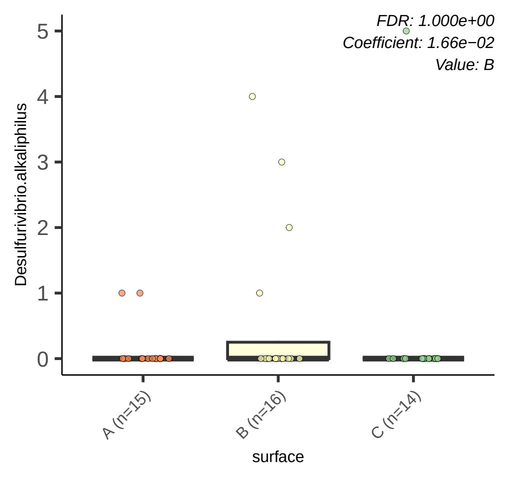

FDR: 1.000e+00
Coefficient: 1.66e−02
Value: B
Desulfurivibrio.alkaliphilus
0
1
2
3
4
5
A (n=15)
B (n=16)
C (n=14)
surface

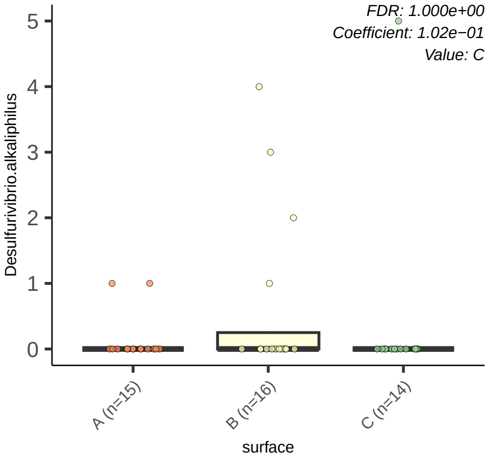

FDR: 1.000e+00
Coefficient: 1.02e−01
Value: C
Desulfurivibrio.alkaliphilus
0
1
2
3
4
5
A (n=15)
B (n=16)
C (n=14)
surface

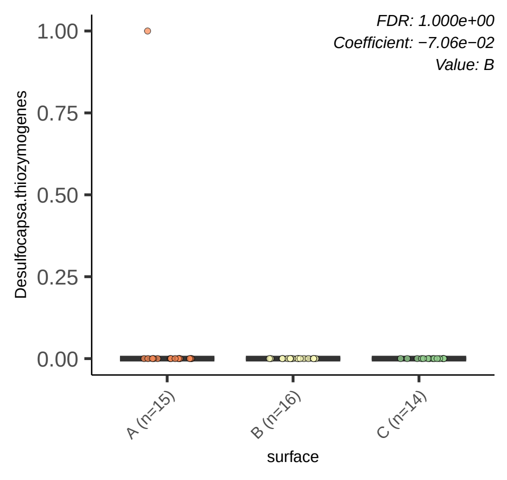
FDR: 1.000e+00
Coefficient: −7.06e−02
Value: B
1.00
0.75
0.50
0.25
0.00
Desulfocapsa.thiozymogenes
A (n=15)
B (n=16)
C (n=14)
surface

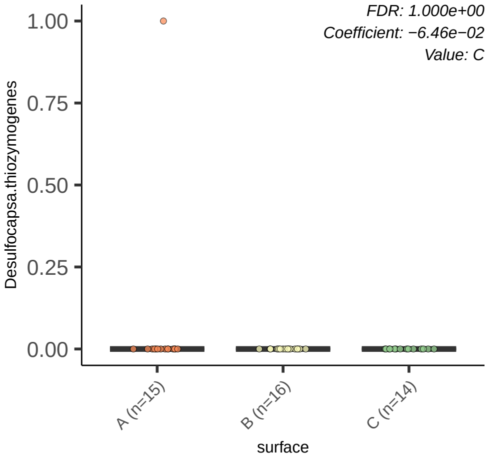
FDR: 1.000e+00
Coefficient: −6.46e−02
Value: C
Desulfocapsa.thiozymogenes
1.00
0.75
0.50
0.25
0.00
A (n=15)
B (n=16)
C (n=14)
surface

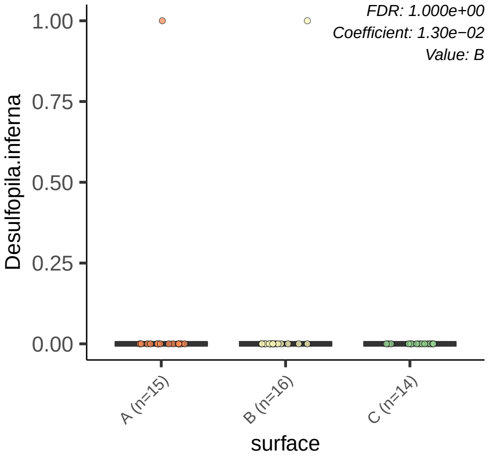
FDR: 1.000e+00
Coefficient: 1.30e−02
Value: B
Desulfopila.inferna
1.00
0.75
0.50
0.25
0.00
A (n=15)
B (n=16)
C (n=14)
surface

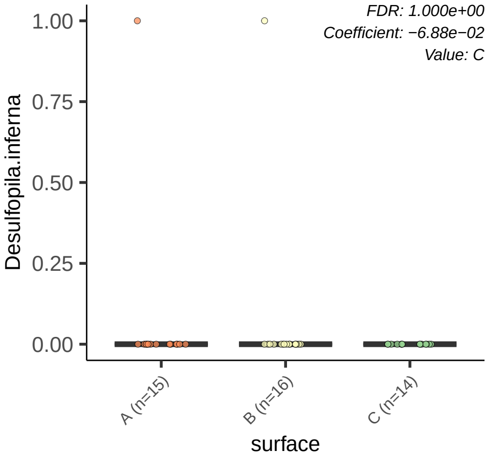
FDR: 1.000e+00
Coefficient: −6.88e−02
Value: C
Desulfopila.inferna
1.00
0.75
0.50
0.25
0.00
A (n=15)
B (n=16)
C (n=14)
surface

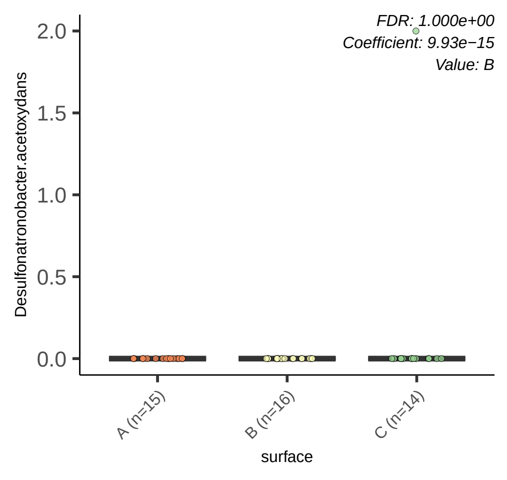
FDR: 1.000e+00
Coefficient: 9.93e−15
Value: B
2.0
1.5
1.0
0.5
0.0
Desulfonatronobacter.acetoxydans
A (n=15)
B (n=16)
C (n=14)
surface

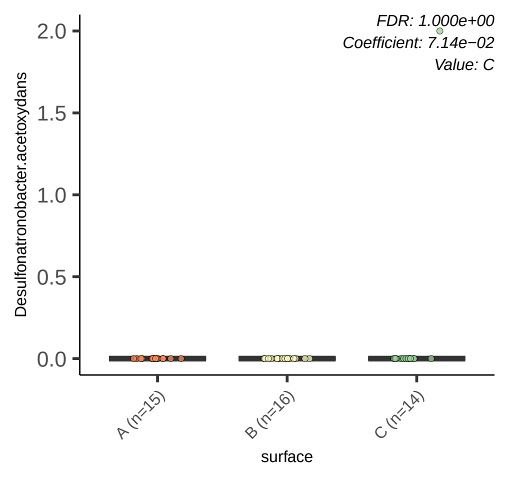
FDR: 1.000e+00
Coefficient: 7.14e−02
Value: C
Desulfonatronobacter.acetoxydans
2.0
1.5
1.0
0.5
0.0
A (n=15)
B (n=16)
C (n=14)
surface

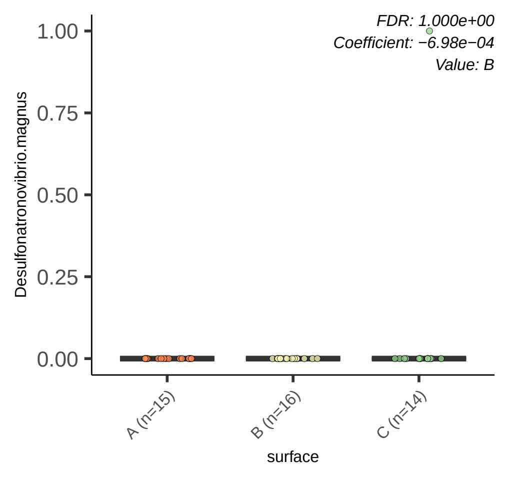
FDR: 1.000e+00
Coefficient: −6.98e−04
Value: B
Desulfonatronovibrio.magnus
1.00
0.75
0.50
0.25
0.00
A (n=15)
B (n=16)
C (n=14)
surface

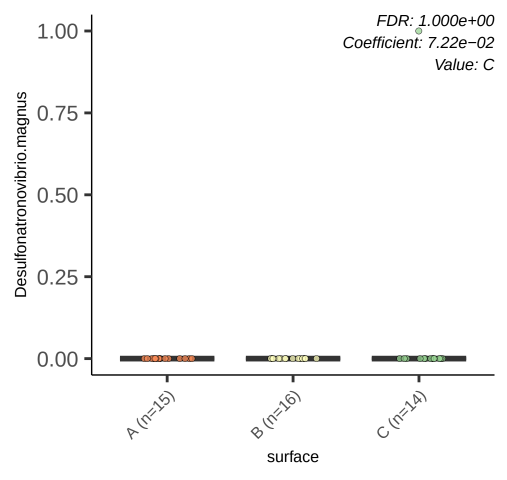
FDR: 1.000e+00
Coefficient: 7.22e−02
Value: C
Desulfonatronovibrio.magnus
1.00
0.75
0.50
0.25
0.00
A (n=15)
B (n=16)
C (n=14)
surface

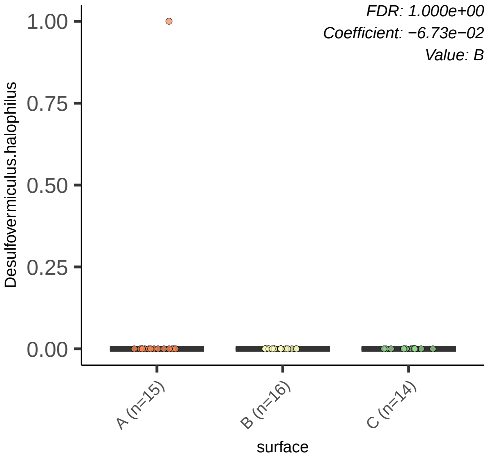
FDR: 1.000e+00
Coefficient: −6.73e−02
Value: B
Desulfovermiculus.halophilus
1.00
0.75
0.50
0.25
0.00
A (n=15)
B (n=16)
C (n=14)
surface

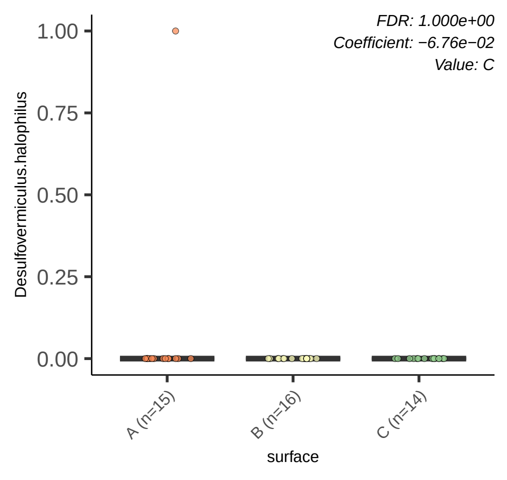

FDR: 1.000e+00
Coefficient: −6.76e−02
Value: C
Desulfovermiculus.halophilus
1.00
0.75
0.50
0.25
0.00
A (n=15)
B (n=16)
C (n=14)
surface

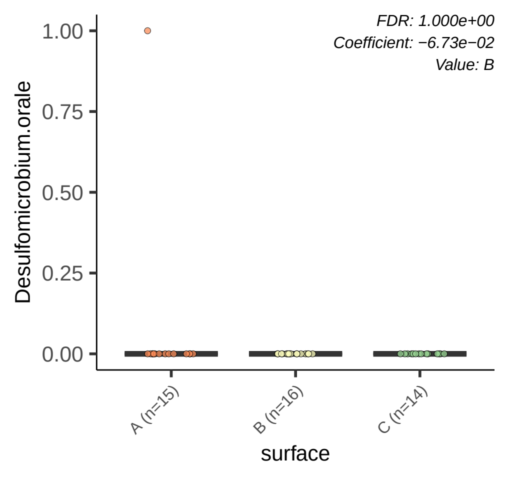
FDR: 1.000e+00
Coefficient: −6.73e−02
Value: B
Desulfomicrobium.orale
1.00
0.75
0.50
0.25
0.00
A (n=15)
B (n=16)
C (n=14)
surface

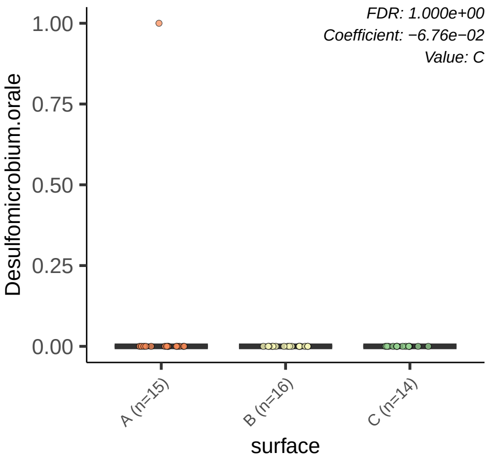
FDR: 1.000e+00
Coefficient: −6.76e−02
Value: C
Desulfomicrobium.orale
1.00
0.75
0.50
0.25
0.00
A (n=15)
B (n=16)
C (n=14)
surface

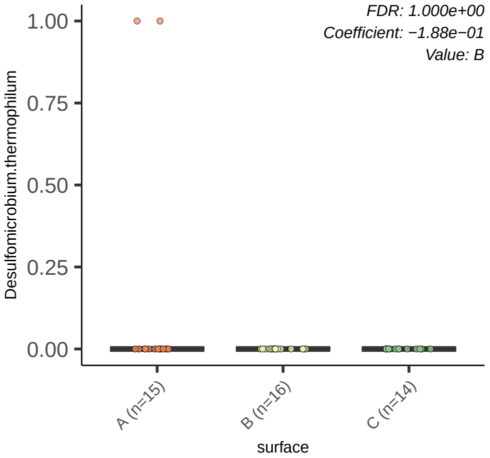
FDR: 1.000e+00
Coefficient: −1.88e−01
Value: B
1.00
0.75
0.50
0.25
0.00
Desulfomicrobium.thermophilum
A (n=15)
B (n=16)
C (n=14)
surface

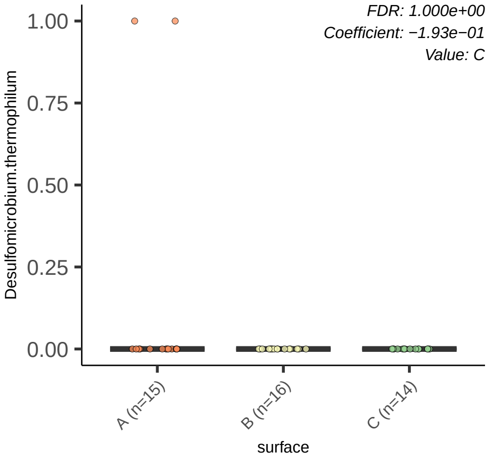

FDR: 1.000e+00
Coefficient: −1.93e−01
Value: C
1.00
0.75
0.50
0.25
0.00
Desulfomicrobium.thermophilum
A (n=15)
B (n=16)
C (n=14)
surface

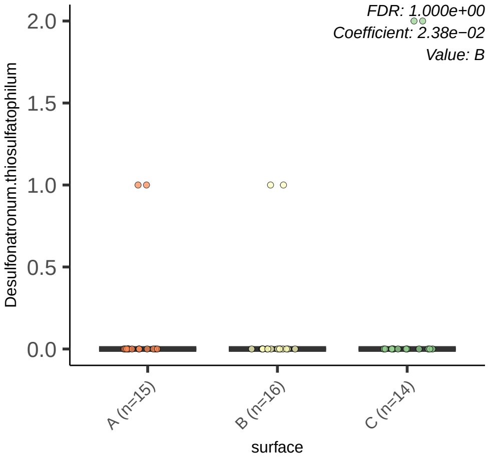
FDR: 1.000e+00
Coefficient: 2.38e−02
Value: B
Desulfonatronum.thiosulfatophilum
2.0
1.5
1.0
0.5
0.0
A (n=15)
B (n=16)
C (n=14)
surface

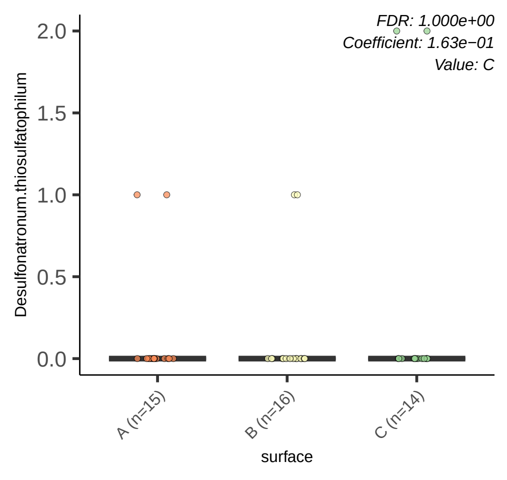
FDR: 1.000e+00
Coefficient: 1.63e−01
Value: C
Desulfonatronum.thiosulfatophilum
2.0
1.5
1.0
0.5
0.0
A (n=15)
B (n=16)
C (n=14)
surface

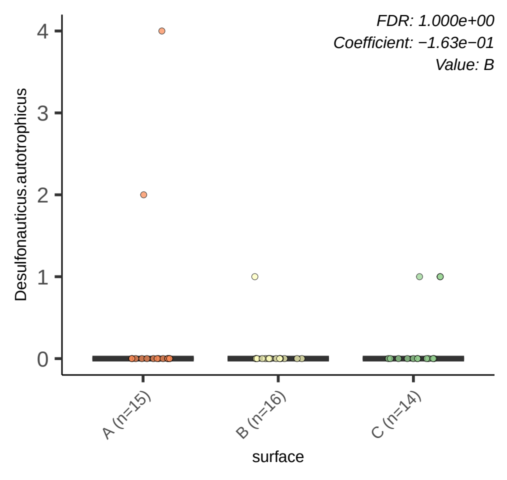
FDR: 1.000e+00
Coefficient: −1.63e−01
Value: B
Desulfonauticus.autotrophicus
4
3
2
1
0
A (n=15)
B (n=16)
C (n=14)
surface

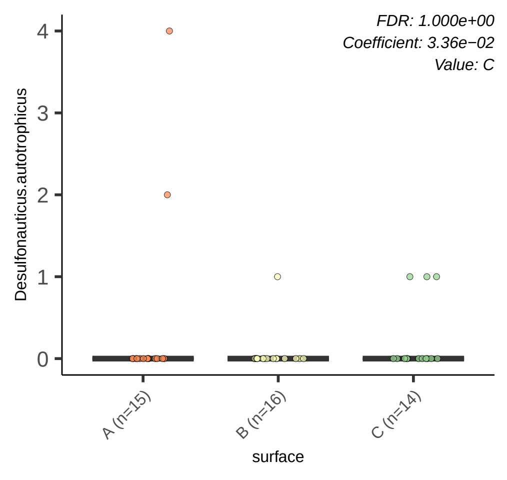

FDR: 1.000e+00
Coefficient: 3.36e−02
Value: C
Desulfonauticus.autotrophicus
0
1
2
3
4
A (n=15)
B (n=16)
C (n=14)
surface

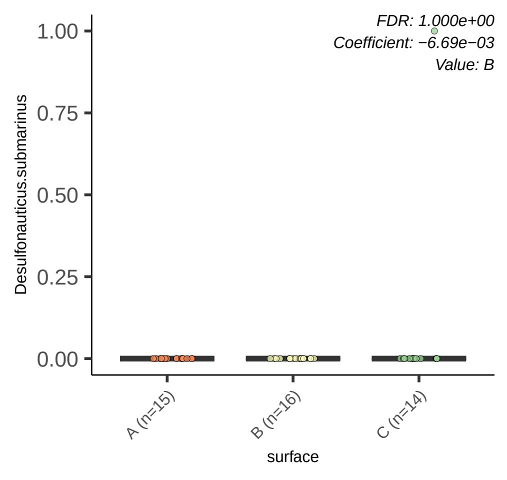
FDR: 1.000e+00
Coefficient: −6.69e−03
Value: B
Desulfonauticus.submarinus
1.00
0.75
0.50
0.25
0.00
A (n=15)
B (n=16)
C (n=14)
surface

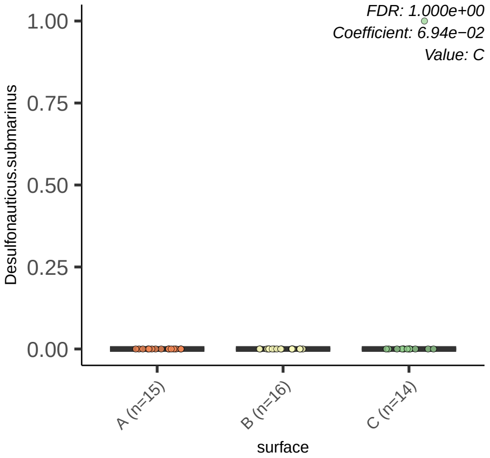

FDR: 1.000e+00
Coefficient: 6.94e−02
Value: C
1.00
0.75
0.50
0.25
0.00
Desulfonauticus.submarinus
A (n=15)
B (n=16)
C (n=14)
surface

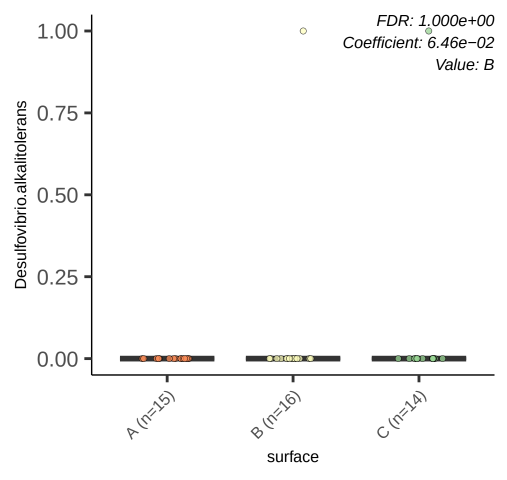
FDR: 1.000e+00
Coefficient: 6.46e−02
Value: B
1.00
0.75
0.50
0.25
0.00
Desulfovibrio.alkalitolerans
A (n=15)
B (n=16)
C (n=14)
surface

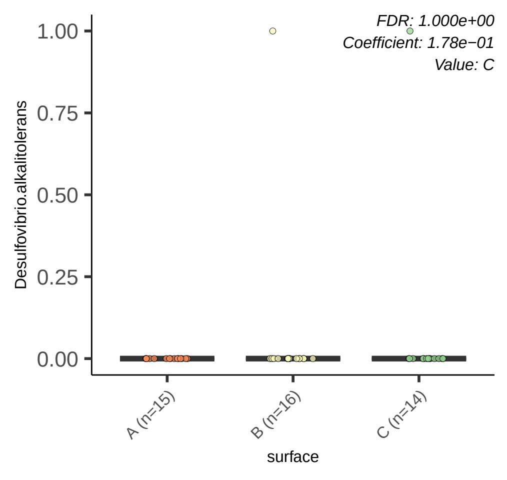
FDR: 1.000e+00
Coefficient: 1.78e−01
Value: C
1.00
0.75
0.50
0.25
0.00
Desulfovibrio.alkalitolerans
A (n=15)
B (n=16)
C (n=14)
surface

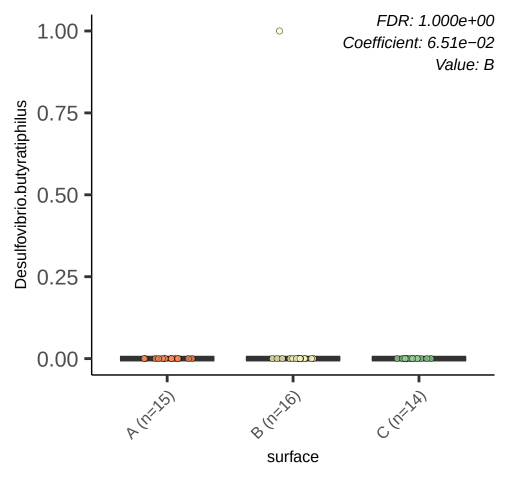
FDR: 1.000e+00
Coefficient: 6.51e−02
Value: B
Desulfovibrio.butyratiphilus
1.00
0.75
0.50
0.25
0.00
A (n=15)
B (n=16)
C (n=14)
surface

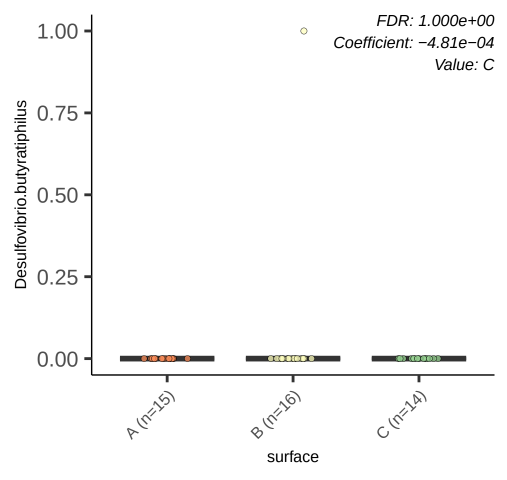

FDR: 1.000e+00
Coefficient: −4.81e−04
Value: C
1.00
0.75
0.50
0.25
0.00
Desulfovibrio.butyratiphilus
A (n=15)
B (n=16)
C (n=14)
surface

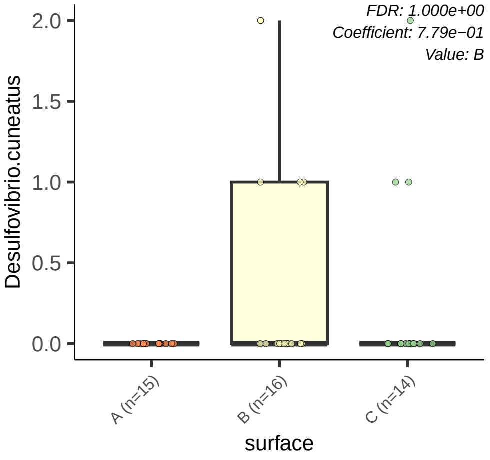

FDR: 1.000e+00
Coefficient: 7.79e−01
Value: B
Desulfovibrio.cuneatus
2.0
1.5
1.0
0.5
0.0
A (n=15)
B (n=16)
C (n=14)
surface

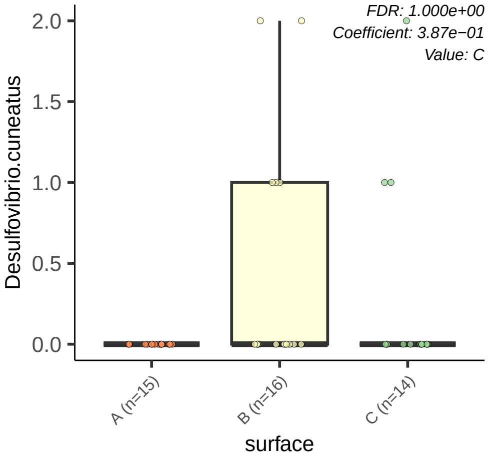

FDR: 1.000e+00
Coefficient: 3.87e−01
Value: C
Desulfovibrio.cuneatus
2.0
1.5
1.0
0.5
0.0
A (n=15)
B (n=16)
C (n=14)
surface

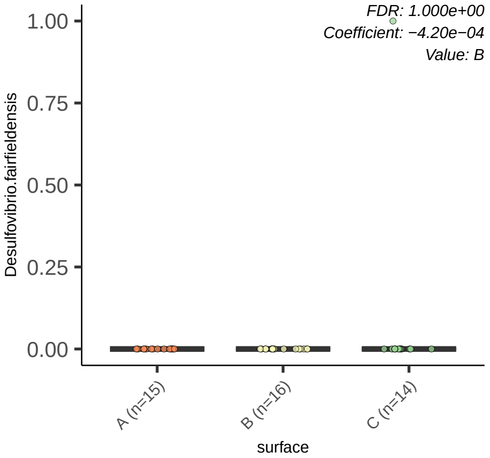

FDR: 1.000e+00
Coefficient: −4.20e−04
Value: B
1.00
0.75
0.50
0.25
0.00
Desulfovibrio.fairfieldensis
A (n=15)
B (n=16)
C (n=14)
surface

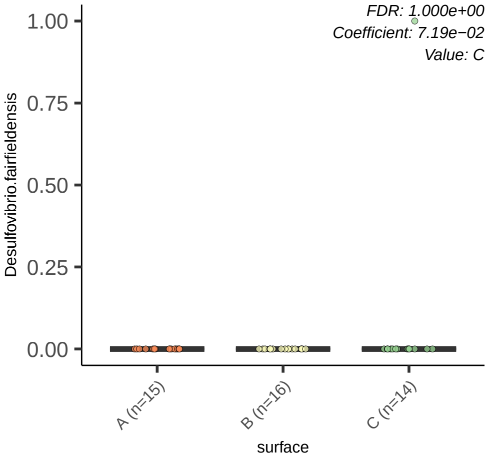

FDR: 1.000e+00
Coefficient: 7.19e−02
Value: C
1.00
0.75
0.50
0.25
0.00
Desulfovibrio.fairfieldensis
A (n=15)
B (n=16)
C (n=14)
surface

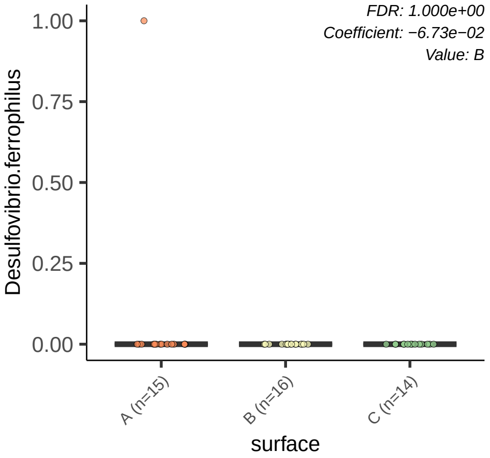

FDR: 1.000e+00
Coefficient: −6.73e−02
Value: B
Desulfovibrio.ferrophilus
1.00
0.75
0.50
0.25
0.00
A (n=15)
B (n=16)
C (n=14)
surface

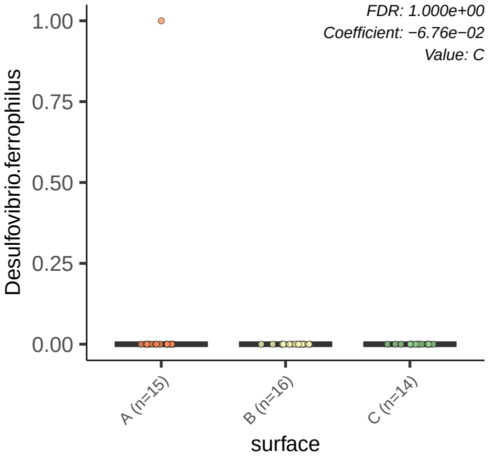
FDR: 1.000e+00
Coefficient: −6.76e−02
Value: C
Desulfovibrio.ferrophilus
1.00
0.75
0.50
0.25
0.00
A (n=15)
B (n=16)
C (n=14)
surface

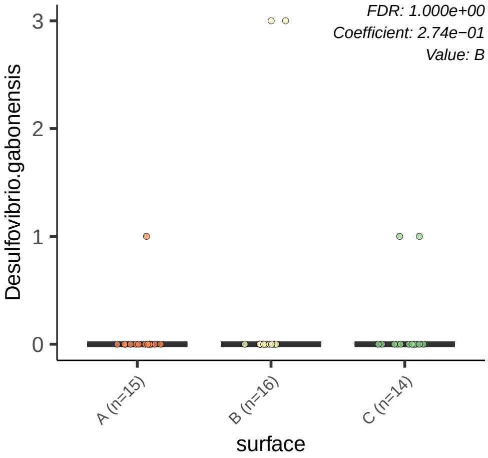

FDR: 1.000e+00
Coefficient: 2.74e−01
Value: B
Desulfovibrio.gabonensis
3
2
1
0
A (n=15)
B (n=16)
C (n=14)
surface

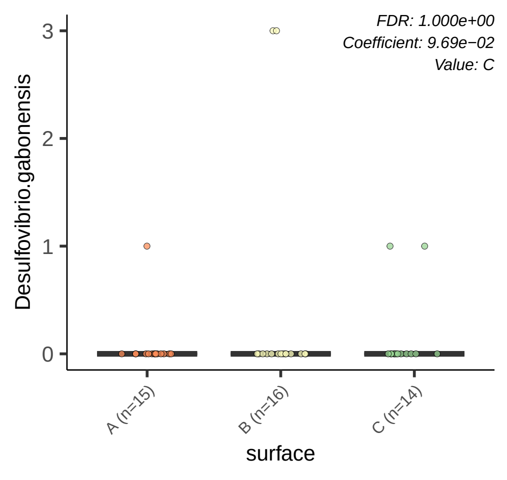

FDR: 1.000e+00
Coefficient: 9.69e−02
Value: C
Desulfovibrio.gabonensis
3
2
1
0
A (n=15)
B (n=16)
C (n=14)
surface

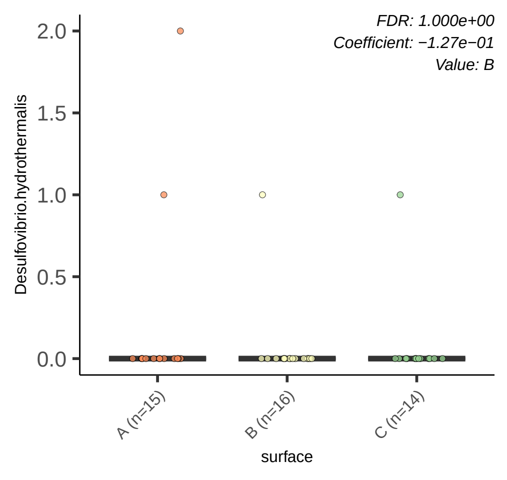

FDR: 1.000e+00
Coefficient: −1.27e−01
Value: B
Desulfovibrio.hydrothermalis
2.0
1.5
1.0
0.5
0.0
A (n=15)
B (n=16)
C (n=14)
surface

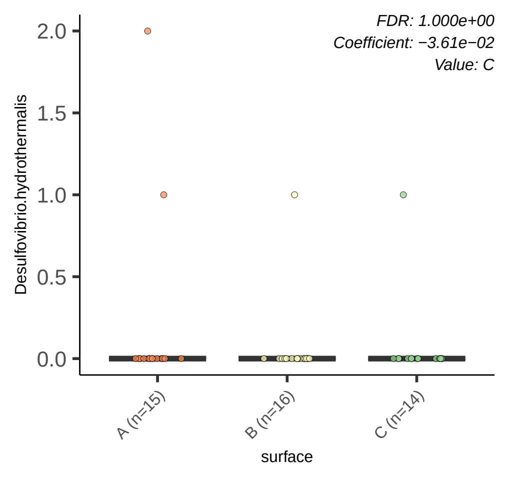
FDR: 1.000e+00
Coefficient: −3.61e−02
Value: C
Desulfovibrio.hydrothermalis
2.0
1.5
1.0
0.5
0.0
A (n=15)
B (n=16)
C (n=14)
surface

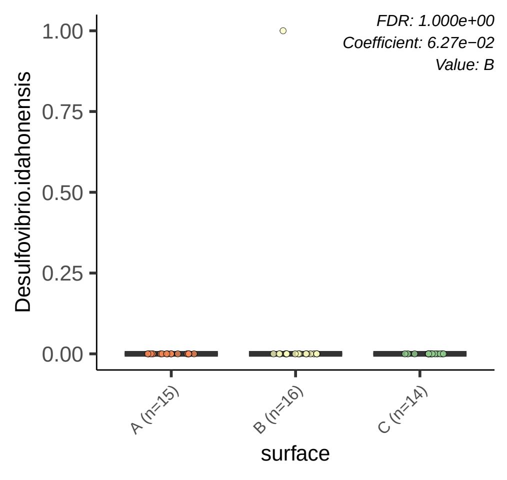
FDR: 1.000e+00
Coefficient: 6.27e−02
Value: B
Desulfovibrio.idahonensis
1.00
0.75
0.50
0.25
0.00
A (n=15)
B (n=16)
C (n=14)
surface

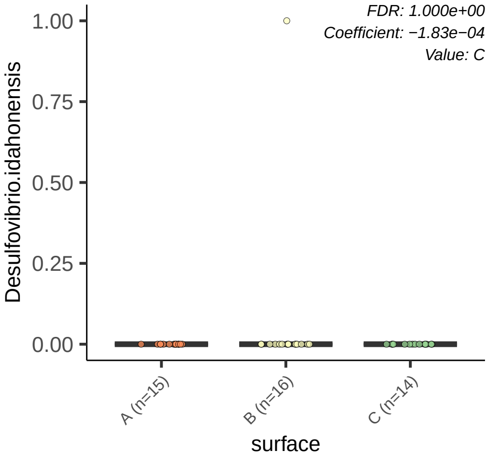
FDR: 1.000e+00
Coefficient: −1.83e−04
Value: C
Desulfovibrio.idahonensis
1.00
0.75
0.50
0.25
0.00
A (n=15)
B (n=16)
C (n=14)
surface

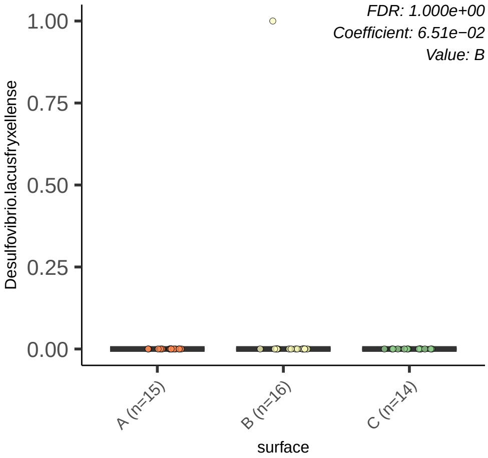
FDR: 1.000e+00
Coefficient: 6.51e−02
Value: B
1.00
0.75
0.50
0.25
0.00
Desulfovibrio.lacusfryxellense
A (n=15)
B (n=16)
C (n=14)
surface

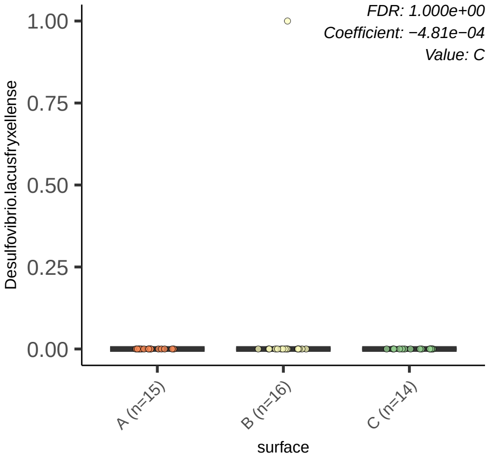
FDR: 1.000e+00
Coefficient: −4.81e−04
Value: C
1.00
0.75
0.50
0.25
0.00
Desulfovibrio.lacusfryxellense
A (n=15)
B (n=16)
C (n=14)
surface

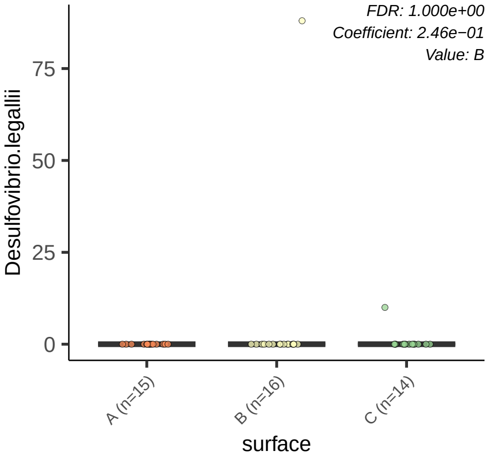

FDR: 1.000e+00
Coefficient: 2.46e−01
Value: B
Desulfovibrio.legallii
75
50
25
0
A (n=15)
B (n=16)
C (n=14)
surface

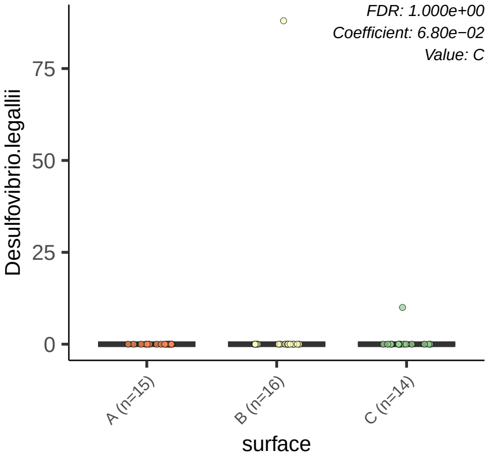

FDR: 1.000e+00
Coefficient: 6.80e−02
Value: C
Desulfovibrio.legallii
0
25
50
75
A (n=15)
B (n=16)
C (n=14)
surface

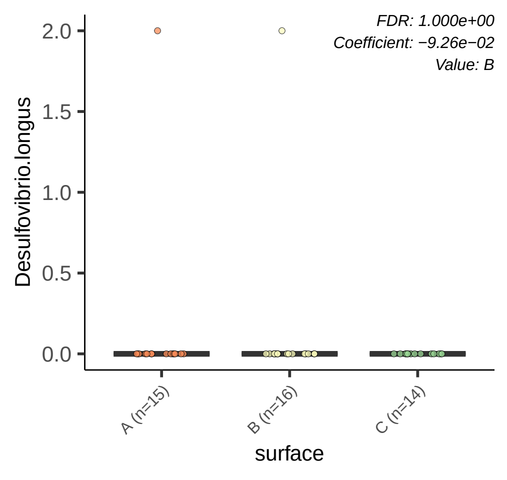
FDR: 1.000e+00
Coefficient: −9.26e−02
Value: B
Desulfovibrio.longus
2.0
1.5
1.0
0.5
0.0
A (n=15)
B (n=16)
C (n=14)
surface

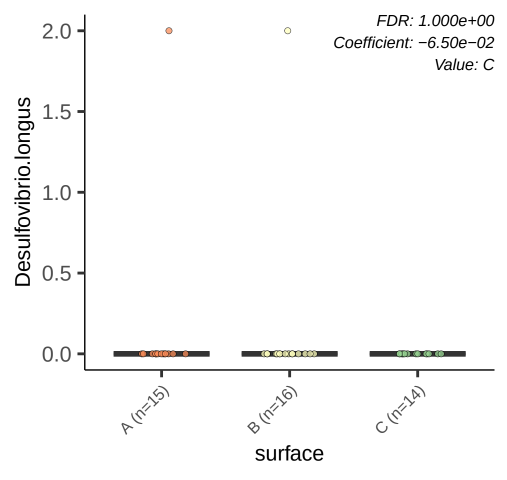

FDR: 1.000e+00
Coefficient: −6.50e−02
Value: C
Desulfovibrio.longus
2.0
1.5
1.0
0.5
0.0
A (n=15)
B (n=16)
C (n=14)
surface

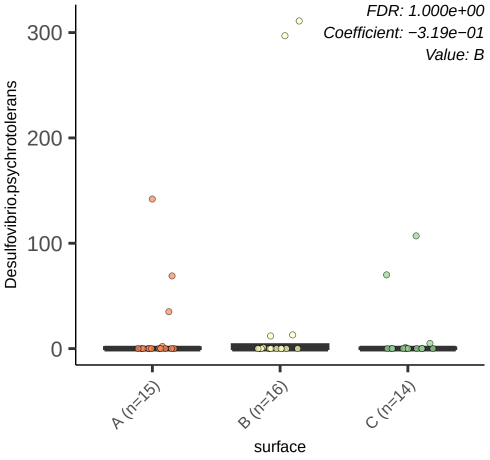
FDR: 1.000e+00
Coefficient: −3.19e−01
Value: B
Desulfovibrio.psychrotolerans
300
200
100
0
A (n=15)
B (n=16)
C (n=14)
surface

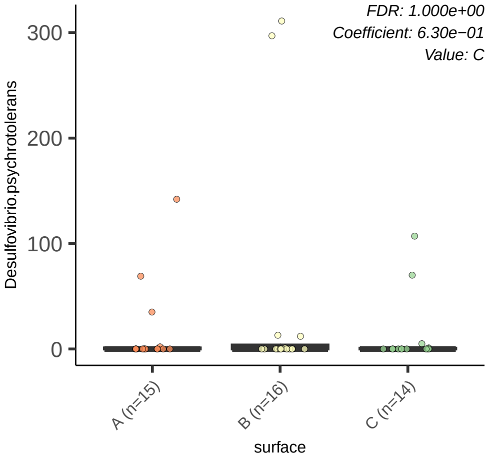

FDR: 1.000e+00
Coefficient: 6.30e−01
Value: C
Desulfovibrio.psychrotolerans
300
200
100
0
A (n=15)
B (n=16)
C (n=14)
surface

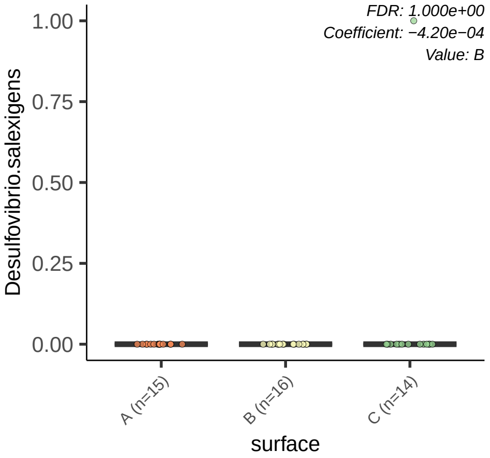
FDR: 1.000e+00
Coefficient: −4.20e−04
Value: B
Desulfovibrio.salexigens
1.00
0.75
0.50
0.25
0.00
A (n=15)
B (n=16)
C (n=14)
surface

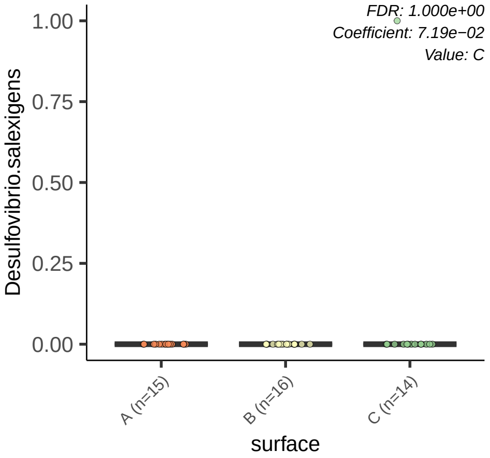
FDR: 1.000e+00
Coefficient: 7.19e−02
Value: C
Desulfovibrio.salexigens
1.00
0.75
0.50
0.25
0.00
A (n=15)
B (n=16)
C (n=14)
surface

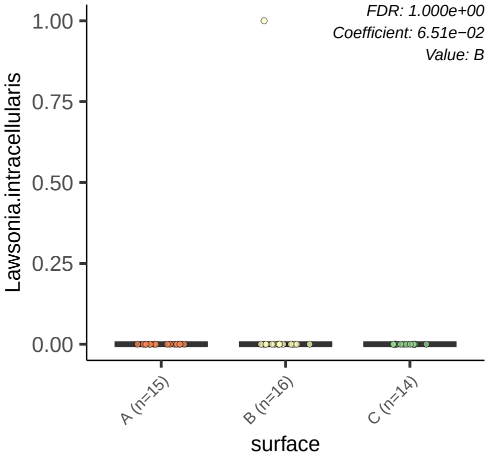

FDR: 1.000e+00
Coefficient: 6.51e−02
Value: B
Lawsonia.intracellularis
1.00
0.75
0.50
0.25
0.00
A (n=15)
B (n=16)
C (n=14)
surface

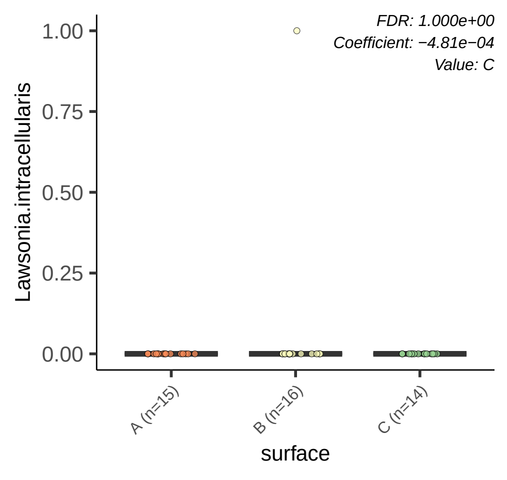

FDR: 1.000e+00
Coefficient: −4.81e−04
Value: C
Lawsonia.intracellularis
1.00
0.75
0.50
0.25
0.00
A (n=15)
B (n=16)
C (n=14)
surface

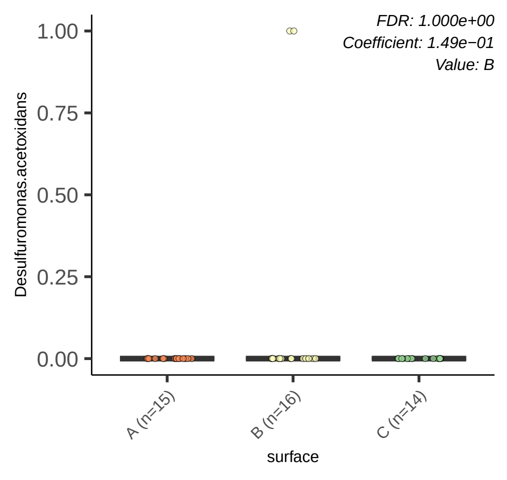

FDR: 1.000e+00
Coefficient: 1.49e−01
Value: B
Desulfuromonas.acetoxidans
1.00
0.75
0.50
0.25
0.00
A (n=15)
B (n=16)
C (n=14)
surface

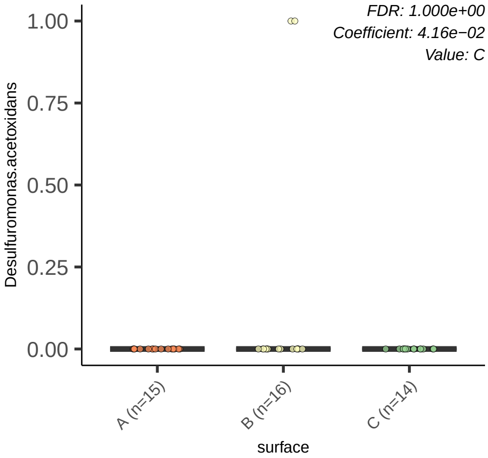

FDR: 1.000e+00
Coefficient: 4.16e−02
Value: C
1.00
0.75
0.50
0.25
0.00
Desulfuromonas.acetoxidans
A (n=15)
B (n=16)
C (n=14)
surface

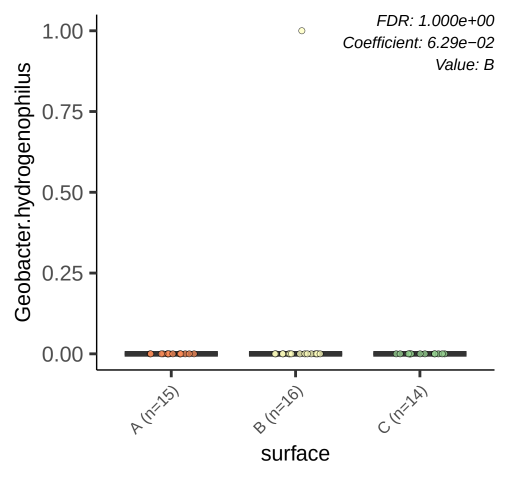
FDR: 1.000e+00
Coefficient: 6.29e−02
Value: B
Geobacter.hydrogenophilus
1.00
0.75
0.50
0.25
0.00
A (n=15)
B (n=16)
C (n=14)
surface

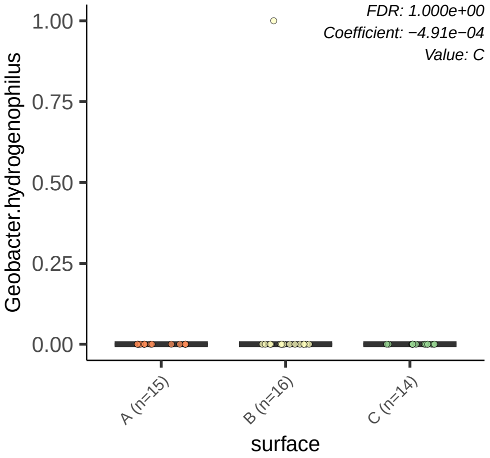
FDR: 1.000e+00
Coefficient: −4.91e−04
Value: C
Geobacter.hydrogenophilus
1.00
0.75
0.50
0.25
0.00
A (n=15)
B (n=16)
C (n=14)
surface

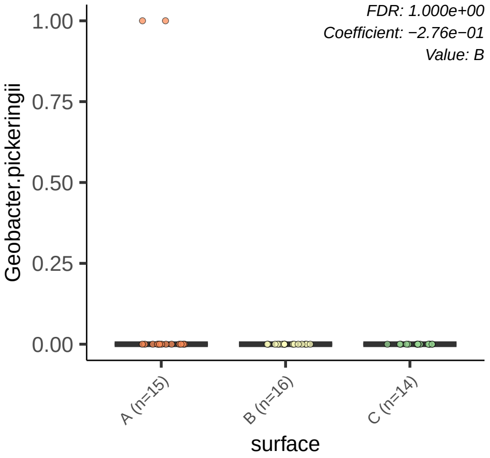
FDR: 1.000e+00
Coefficient: −2.76e−01
Value: B
Geobacter.pickeringii
1.00
0.75
0.50
0.25
0.00
A (n=15)
B (n=16)
C (n=14)
surface

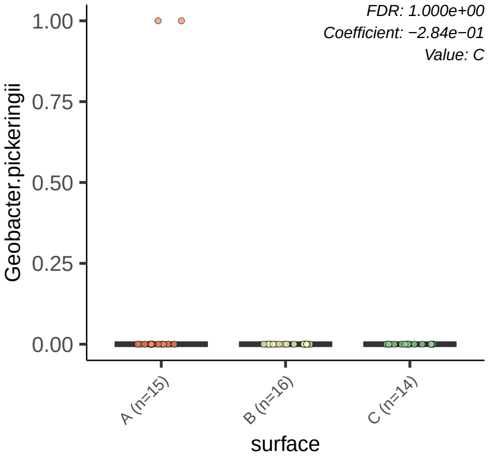

FDR: 1.000e+00
Coefficient: −2.84e−01
Value: C
1.00
0.75
0.50
0.25
0.00
Geobacter.pickeringii
A (n=15)
B (n=16)
C (n=14)
surface

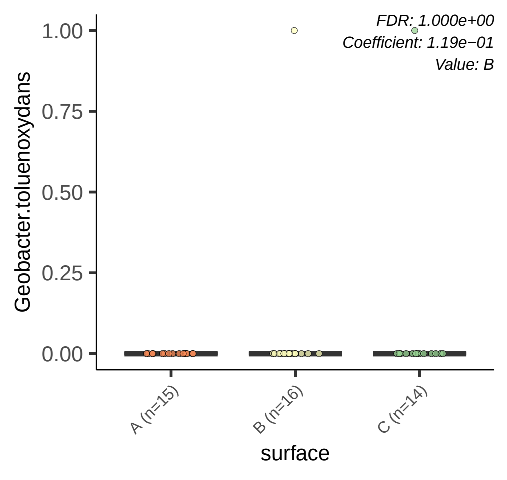
FDR: 1.000e+00
Coefficient: 1.19e−01
Value: B
Geobacter.toluenoxydans
1.00
0.75
0.50
0.25
0.00
A (n=15)
B (n=16)
C (n=14)
surface

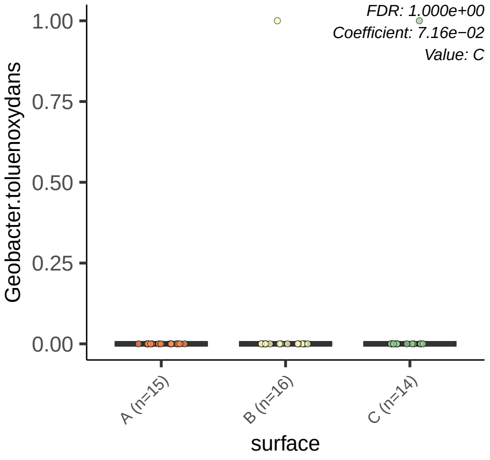

FDR: 1.000e+00
Coefficient: 7.16e−02
Value: C
Geobacter.toluenoxydans
1.00
0.75
0.50
0.25
0.00
A (n=15)
B (n=16)
C (n=14)
surface

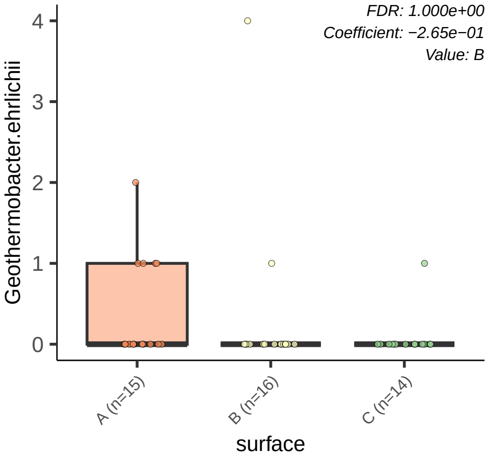
FDR: 1.000e+00
Coefficient: −2.65e−01
Value: B
Geothermobacter.ehrlichii
0
1
2
3
4
A (n=15)
B (n=16)
C (n=14)
surface

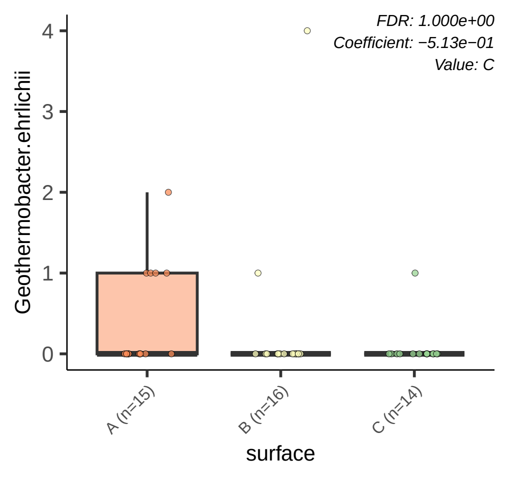
FDR: 1.000e+00
Coefficient: −5.13e−01
Value: C
Geothermobacter.ehrlichii
0
1
2
3
4
A (n=15)
B (n=16)
C (n=14)
surface

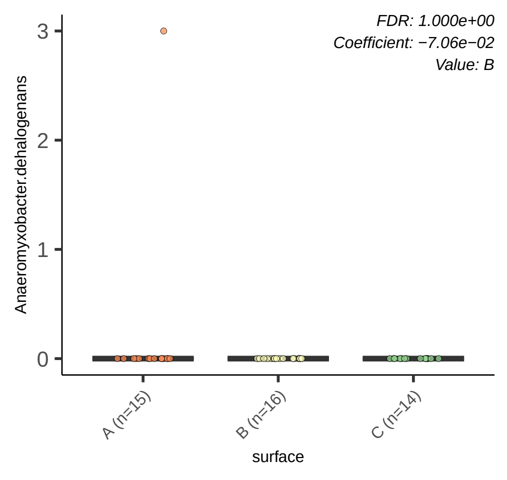

FDR: 1.000e+00
Coefficient: −7.06e−02
Value: B
Anaeromyxobacter.dehalogenans
3
2
1
0
A (n=15)
B (n=16)
C (n=14)
surface

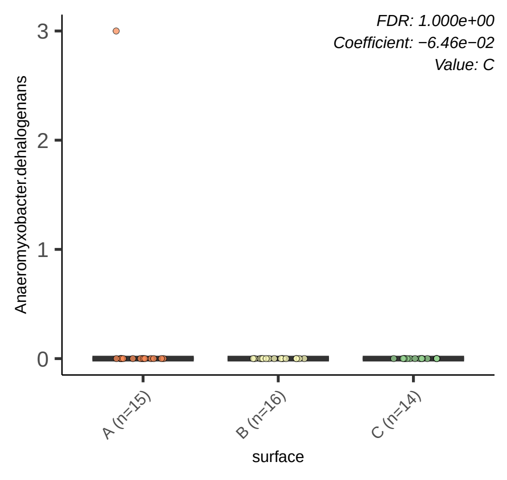
FDR: 1.000e+00
Coefficient: −6.46e−02
Value: C
Anaeromyxobacter.dehalogenans
3
2
1
0
A (n=15)
B (n=16)
C (n=14)
surface

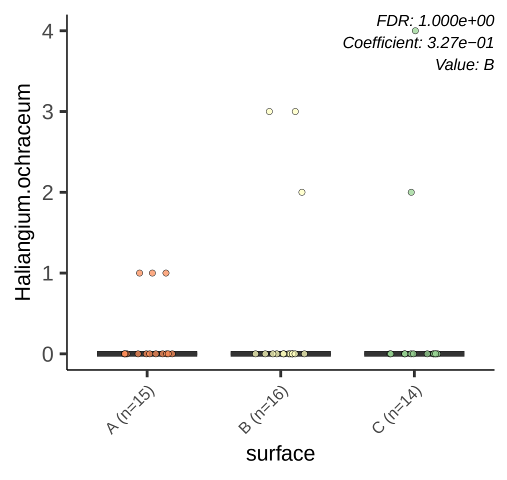
FDR: 1.000e+00
Coefficient: 3.27e−01
Value: B
Haliangium.ochraceum
0
1
2
3
4
A (n=15)
B (n=16)
C (n=14)
surface

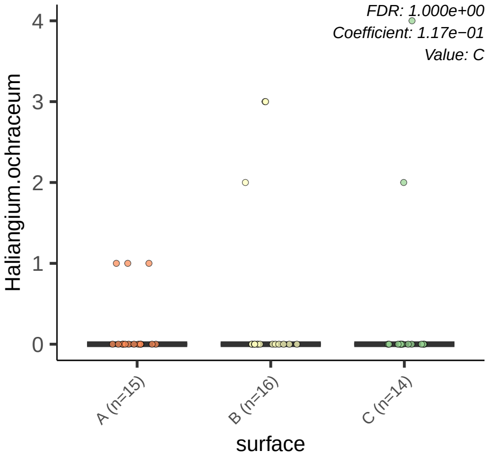

FDR: 1.000e+00
Coefficient: 1.17e−01
Value: C
Haliangium.ochraceum
0
1
2
3
4
A (n=15)
B (n=16)
C (n=14)
surface

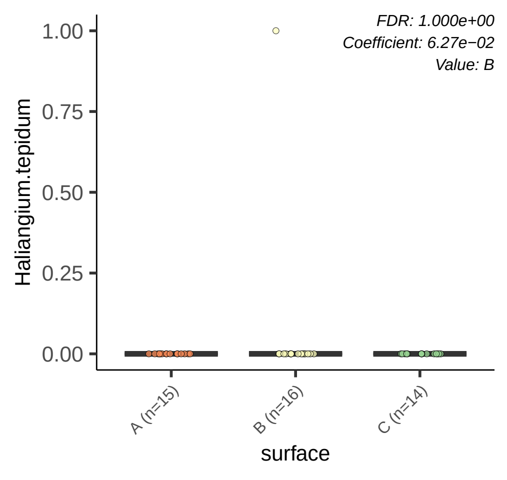
FDR: 1.000e+00
Coefficient: 6.27e−02
Value: B
1.00
0.75
0.50
0.25
0.00
Haliangium.tepidum
A (n=15)
B (n=16)
C (n=14)
surface

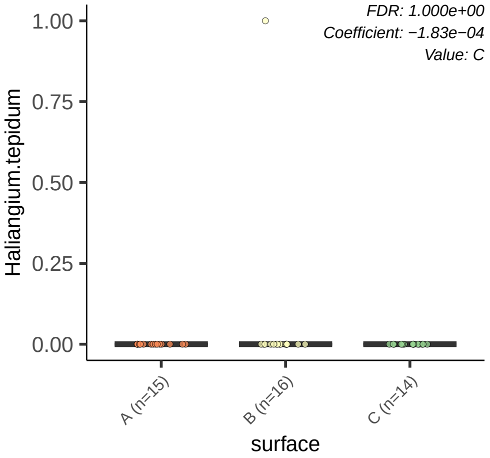
FDR: 1.000e+00
Coefficient: −1.83e−04
Value: C
Haliangium.tepidum
1.00
0.75
0.50
0.25
0.00
A (n=15)
B (n=16)
C (n=14)
surface

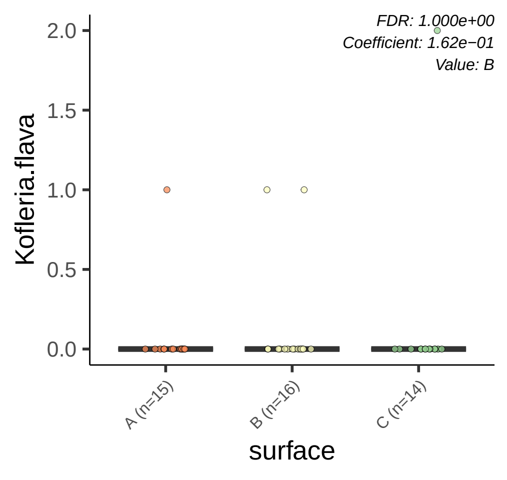
FDR: 1.000e+00
Coefficient: 1.62e−01
Value: B
Kofleria.flava
2.0
1.5
1.0
0.5
0.0
A (n=15)
B (n=16)
C (n=14)
surface

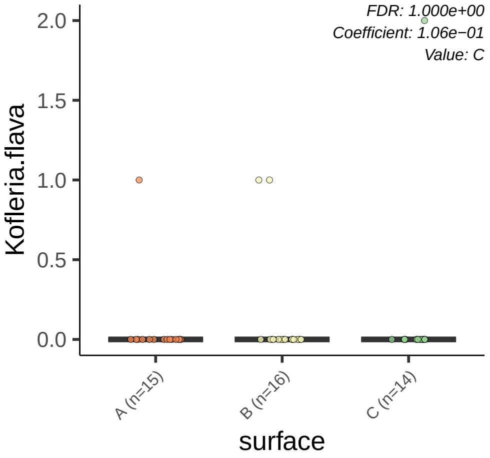

FDR: 1.000e+00
Coefficient: 1.06e−01
Value: C
Kofleria.flava
2.0
1.5
1.0
0.5
0.0
A (n=15)
B (n=16)
C (n=14)
surface

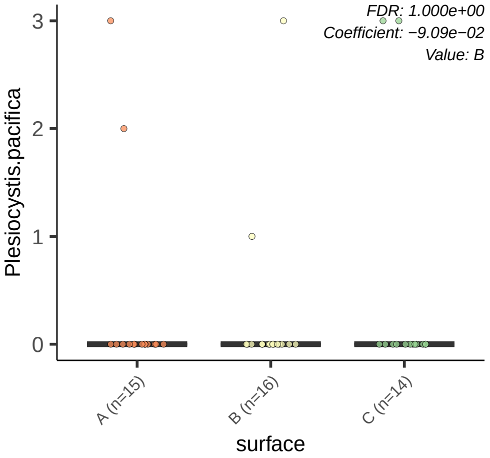
FDR: 1.000e+00
Coefficient: −9.09e−02
Value: B
Plesiocystis.pacifica
3
2
1
0
A (n=15)
B (n=16)
C (n=14)
surface

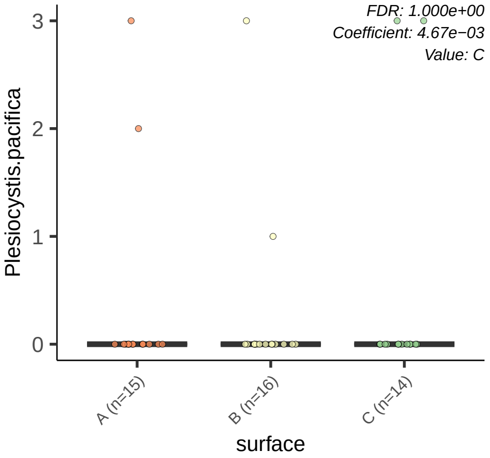
FDR: 1.000e+00
Coefficient: 4.67e−03
Value: C
Plesiocystis.pacifica
0
1
2
3
A (n=15)
B (n=16)
C (n=14)
surface

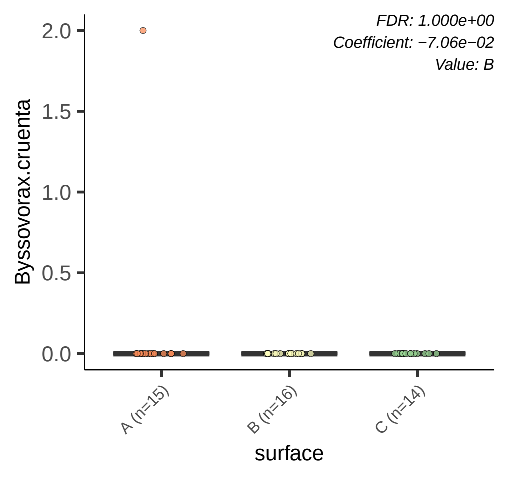

FDR: 1.000e+00
Coefficient: −7.06e−02
Value: B
Byssovorax.cruenta
2.0
1.5
1.0
0.5
0.0
A (n=15)
B (n=16)
C (n=14)
surface

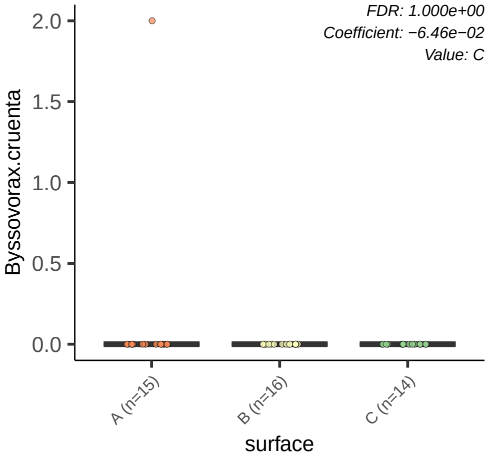
FDR: 1.000e+00
Coefficient: −6.46e−02
Value: C
Byssovorax.cruenta
2.0
1.5
1.0
0.5
0.0
A (n=15)
B (n=16)
C (n=14)
surface

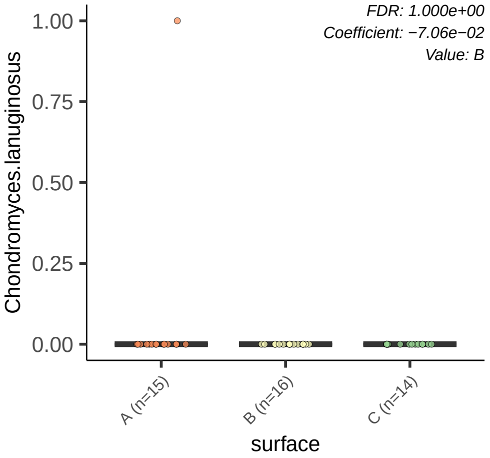
FDR: 1.000e+00
Coefficient: −7.06e−02
Value: B
Chondromyces.lanuginosus
1.00
0.75
0.50
0.25
0.00
A (n=15)
B (n=16)
C (n=14)
surface

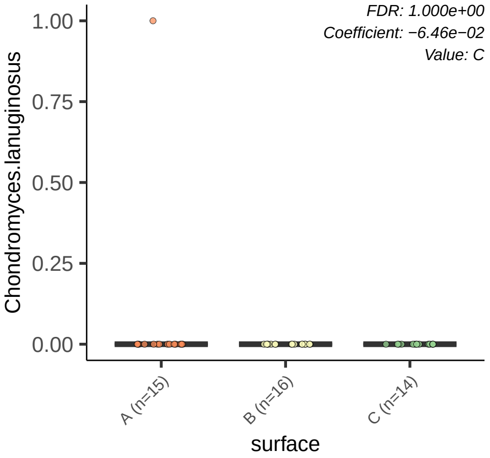
FDR: 1.000e+00
Coefficient: −6.46e−02
Value: C
Chondromyces.lanuginosus
1.00
0.75
0.50
0.25
0.00
A (n=15)
B (n=16)
C (n=14)
surface

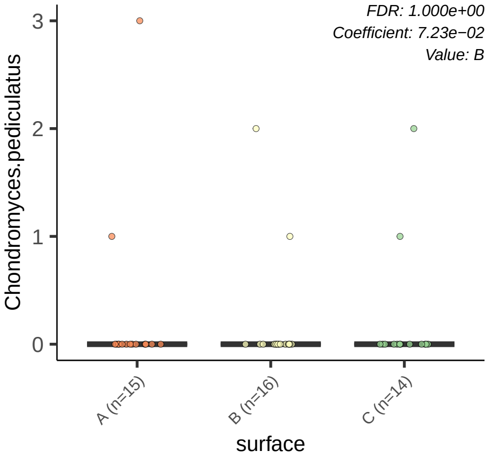

FDR: 1.000e+00
Coefficient: 7.23e−02
Value: B
Chondromyces.pediculatus
0
1
2
3
A (n=15)
B (n=16)
C (n=14)
surface

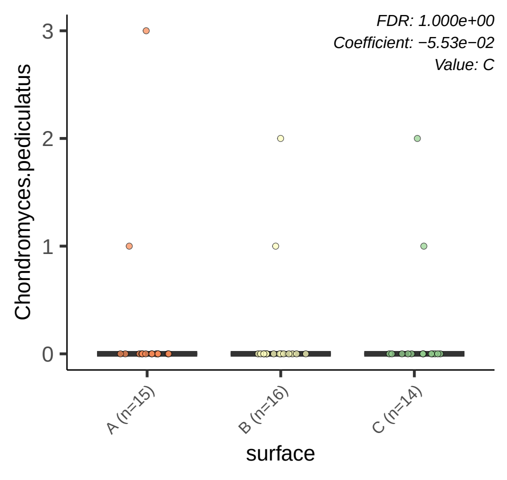
FDR: 1.000e+00
Coefficient: −5.53e−02
Value: C
Chondromyces.pediculatus
0
1
2
3
A (n=15)
B (n=16)
C (n=14)
surface

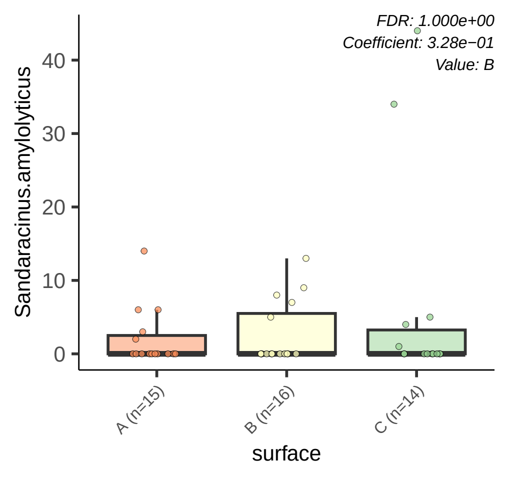

FDR: 1.000e+00
Coefficient: 3.28e−01
Value: B
Sandaracinus.amylolyticus
0
10
20
30
40
A (n=15)
B (n=16)
C (n=14)
surface

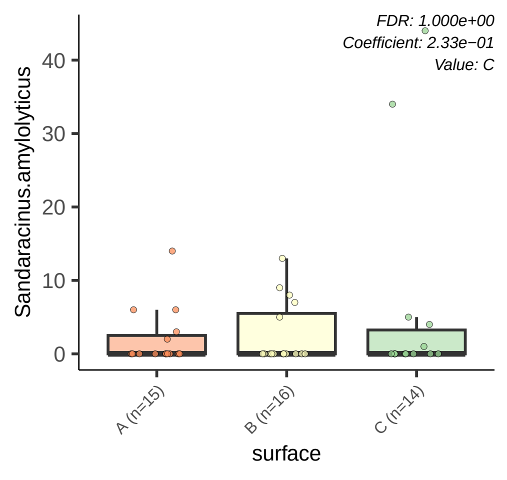
FDR: 1.000e+00
Coefficient: 2.33e−01
Value: C
Sandaracinus.amylolyticus
40
30
20
10
0
A (n=15)
B (n=16)
C (n=14)
surface

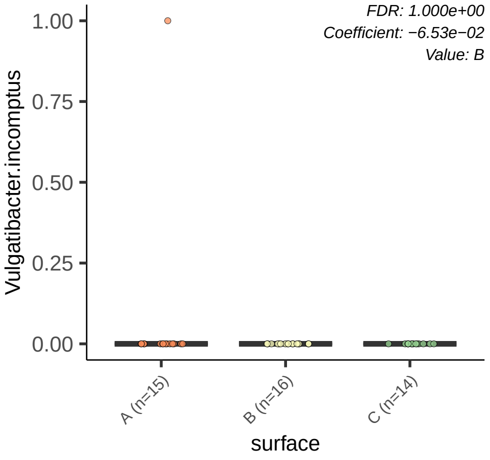
FDR: 1.000e+00
Coefficient: −6.53e−02
Value: B
Vulgatibacter.incomptus
1.00
0.75
0.50
0.25
0.00
A (n=15)
B (n=16)
C (n=14)
surface

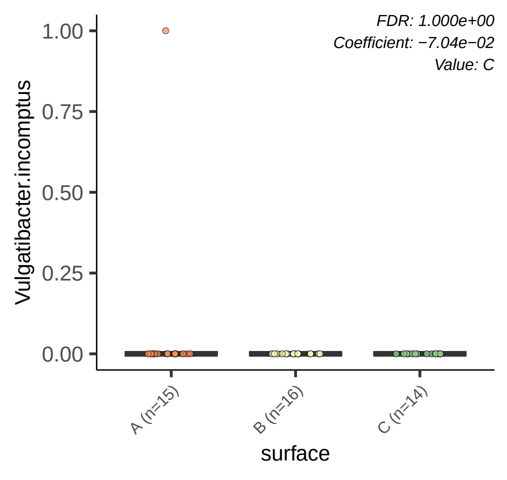

FDR: 1.000e+00
Coefficient: −7.04e−02
Value: C
Vulgatibacter.incomptus
1.00
0.75
0.50
0.25
0.00
A (n=15)
B (n=16)
C (n=14)
surface

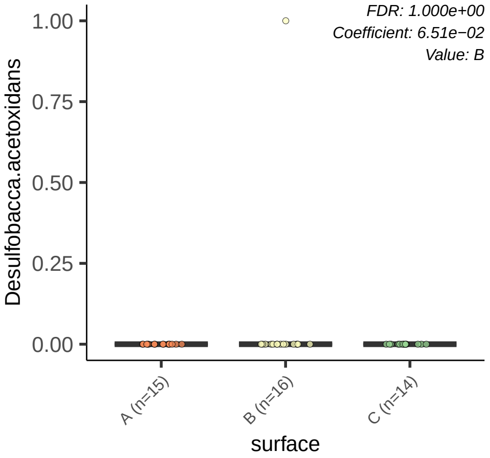

FDR: 1.000e+00
Coefficient: 6.51e−02
Value: B
Desulfobacca.acetoxidans
1.00
0.75
0.50
0.25
0.00
A (n=15)
B (n=16)
C (n=14)
surface

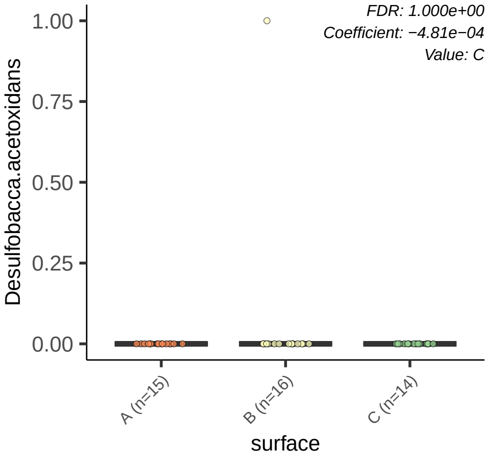
FDR: 1.000e+00
Coefficient: −4.81e−04
Value: C
Desulfobacca.acetoxidans
1.00
0.75
0.50
0.25
0.00
A (n=15)
B (n=16)
C (n=14)
surface

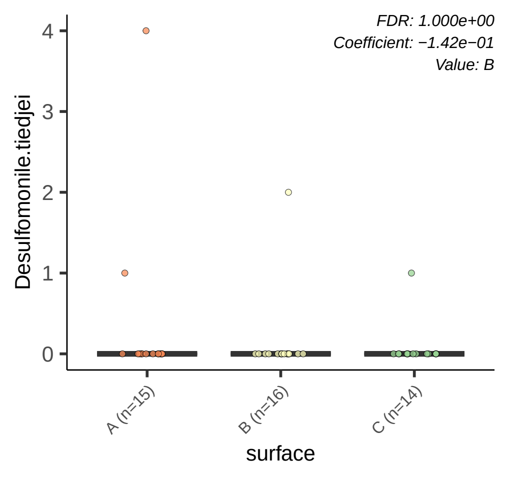
FDR: 1.000e+00
Coefficient: −1.42e−01
Value: B
Desulfomonile.tiedjei
0
1
2
3
4
A (n=15)
B (n=16)
C (n=14)
surface

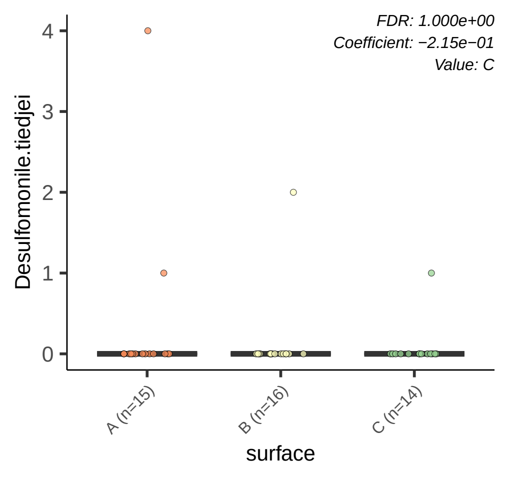
FDR: 1.000e+00
Coefficient: −2.15e−01
Value: C
Desulfomonile.tiedjei
4
3
2
1
0
A (n=15)
B (n=16)
C (n=14)
surface

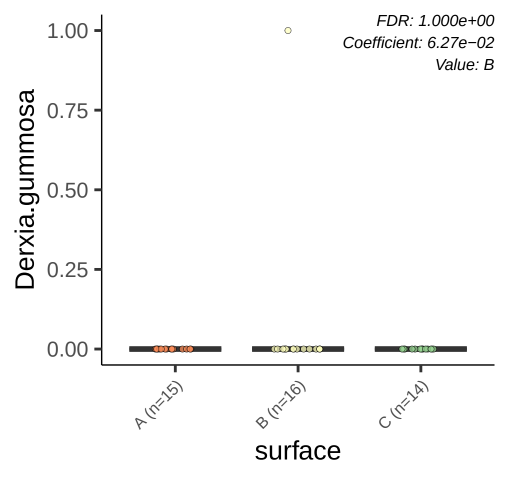
FDR: 1.000e+00
Coefficient: 6.27e−02
Value: B
Derxia.gummosa
1.00
0.75
0.50
0.25
0.00
A (n=15)
B (n=16)
C (n=14)
surface

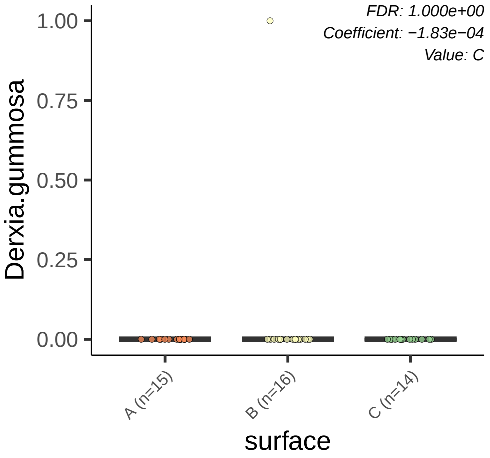
FDR: 1.000e+00
Coefficient: −1.83e−04
Value: C
Derxia.gummosa
1.00
0.75
0.50
0.25
0.00
A (n=15)
B (n=16)
C (n=14)
surface

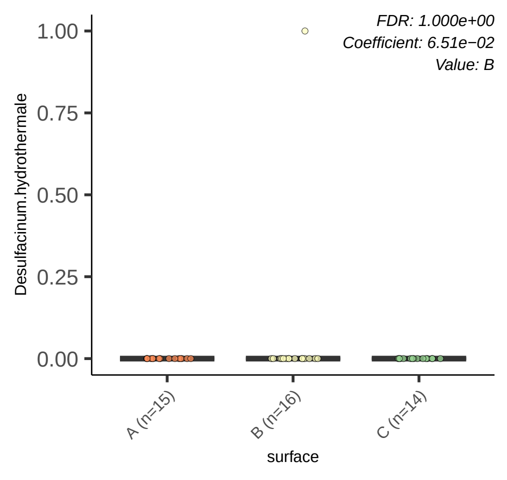

FDR: 1.000e+00
Coefficient: 6.51e−02
Value: B
1.00
0.75
0.50
0.25
0.00
Desulfacinum.hydrothermale
A (n=15)
B (n=16)
C (n=14)
surface

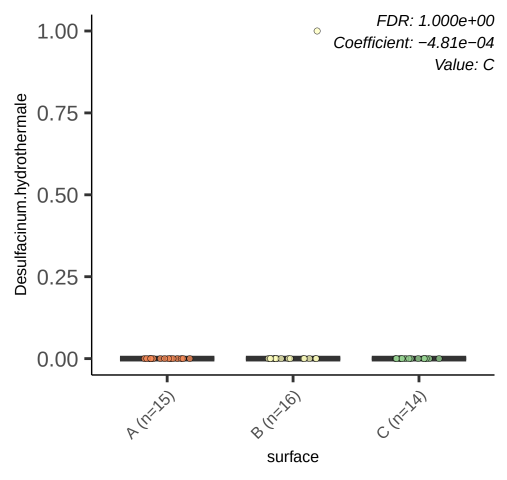
FDR: 1.000e+00
Coefficient: −4.81e−04
Value: C
Desulfacinum.hydrothermale
1.00
0.75
0.50
0.25
0.00
A (n=15)
B (n=16)
C (n=14)
surface

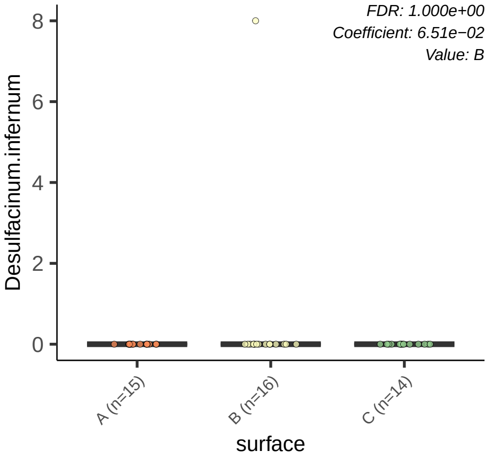

FDR: 1.000e+00
Coefficient: 6.51e−02
Value: B
Desulfacinum.infernum
8
6
4
2
0
A (n=15)
B (n=16)
C (n=14)
surface

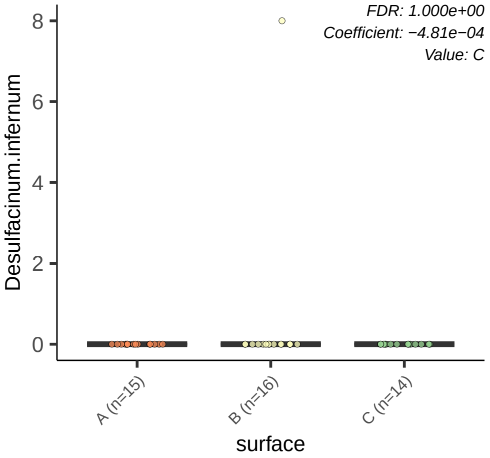

FDR: 1.000e+00
Coefficient: −4.81e−04
Value: C
Desulfacinum.infernum
8
6
4
2
0
A (n=15)
B (n=16)
C (n=14)
surface

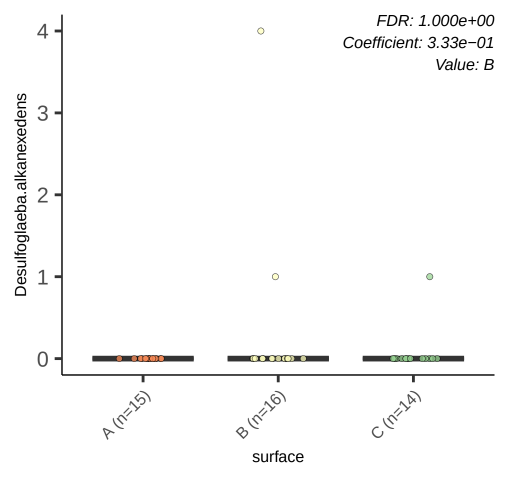

FDR: 1.000e+00
Coefficient: 3.33e−01
Value: B
Desulfoglaeba.alkanexedens
4
3
2
1
0
A (n=15)
B (n=16)
C (n=14)
surface

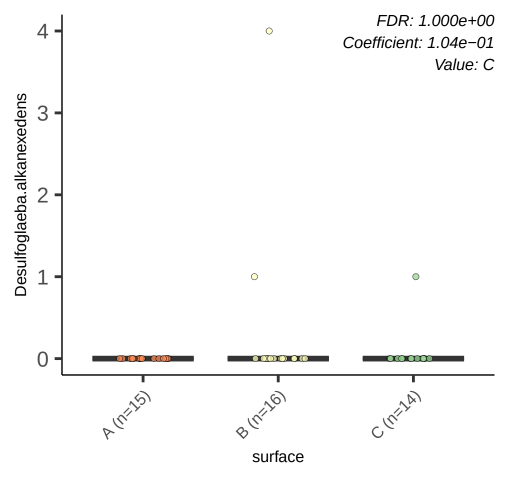

FDR: 1.000e+00
Coefficient: 1.04e−01
Value: C
Desulfoglaeba.alkanexedens
0
1
2
3
4
A (n=15)
B (n=16)
C (n=14)
surface

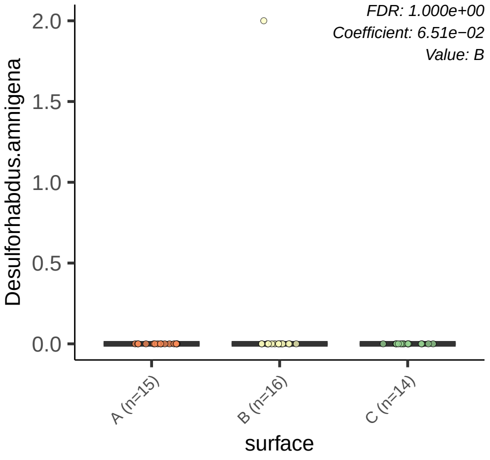
FDR: 1.000e+00
Coefficient: 6.51e−02
Value: B
Desulforhabdus.amnigena
2.0
1.5
1.0
0.5
0.0
A (n=15)
B (n=16)
C (n=14)
surface

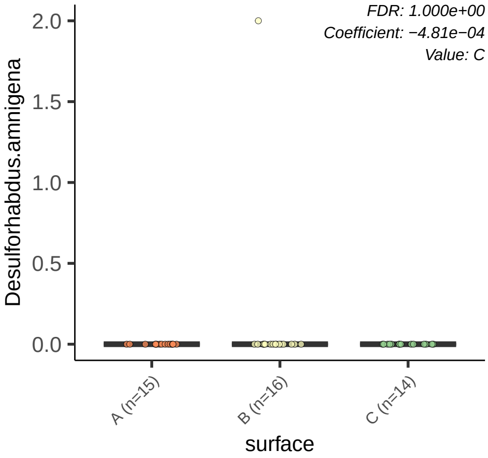

FDR: 1.000e+00
Coefficient: −4.81e−04
Value: C
Desulforhabdus.amnigena
2.0
1.5
1.0
0.5
0.0
A (n=15)
B (n=16)
C (n=14)
surface

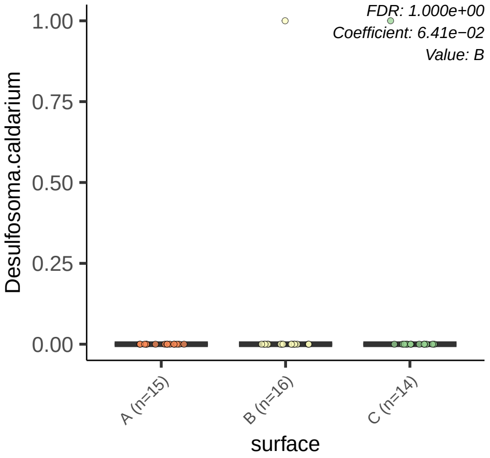
FDR: 1.000e+00
Coefficient: 6.41e−02
Value: B
Desulfosoma.caldarium
1.00
0.75
0.50
0.25
0.00
A (n=15)
B (n=16)
C (n=14)
surface

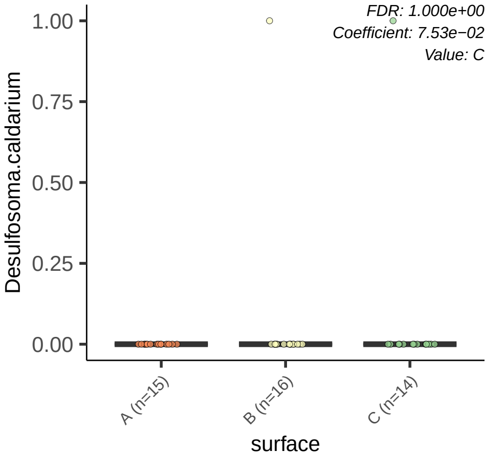
FDR: 1.000e+00
Coefficient: 7.53e−02
Value: C
Desulfosoma.caldarium
1.00
0.75
0.50
0.25
0.00
A (n=15)
B (n=16)
C (n=14)
surface

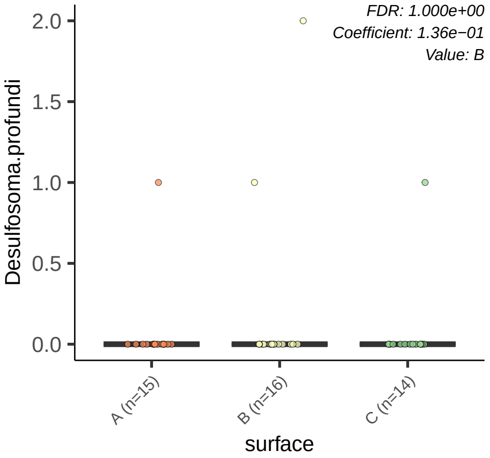
FDR: 1.000e+00
Coefficient: 1.36e−01
Value: B
Desulfosoma.profundi
2.0
1.5
1.0
0.5
0.0
A (n=15)
B (n=16)
C (n=14)
surface

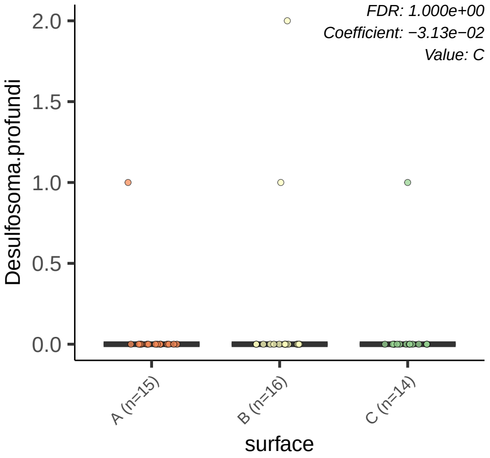

FDR: 1.000e+00
Coefficient: −3.13e−02
Value: C
Desulfosoma.profundi
2.0
1.5
1.0
0.5
0.0
A (n=15)
B (n=16)
C (n=14)
surface

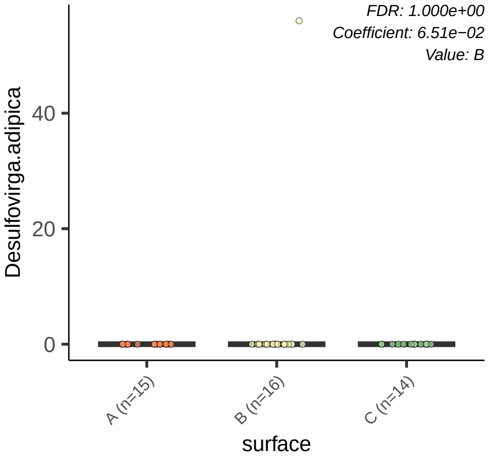

FDR: 1.000e+00
Coefficient: 6.51e−02
Value: B
Desulfovirga.adipica
40
20
0
A (n=15)
B (n=16)
C (n=14)
surface

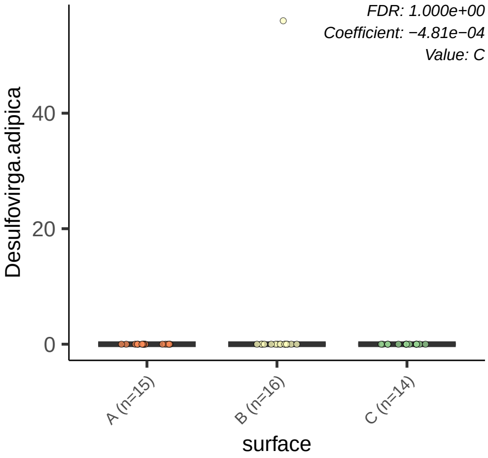

FDR: 1.000e+00
Coefficient: −4.81e−04
Value: C
Desulfovirga.adipica
40
20
0
A (n=15)
B (n=16)
C (n=14)
surface

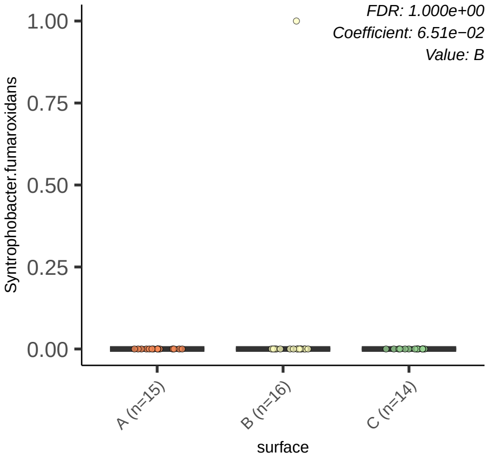
FDR: 1.000e+00
Coefficient: 6.51e−02
Value: B
1.00
0.75
0.50
0.25
0.00
Syntrophobacter.fumaroxidans
A (n=15)
B (n=16)
C (n=14)
surface

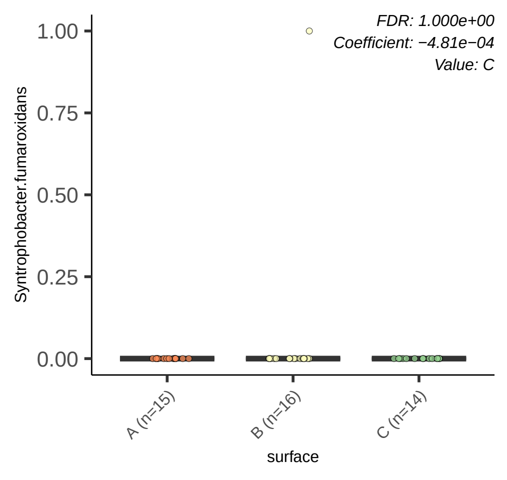
FDR: 1.000e+00
Coefficient: −4.81e−04
Value: C
Syntrophobacter.fumaroxidans
1.00
0.75
0.50
0.25
0.00
A (n=15)
B (n=16)
C (n=14)
surface

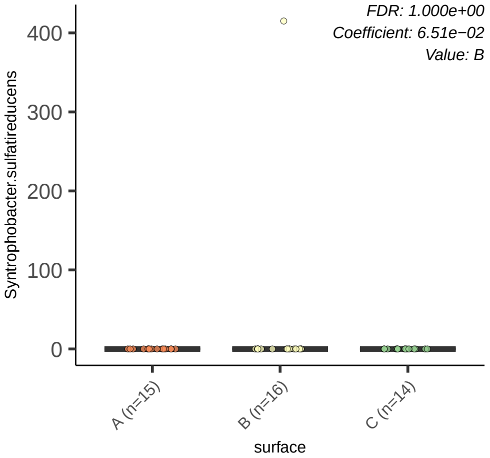
FDR: 1.000e+00
Coefficient: 6.51e−02
Value: B
Syntrophobacter.sulfatireducens
400
300
200
100
0
A (n=15)
B (n=16)
C (n=14)
surface

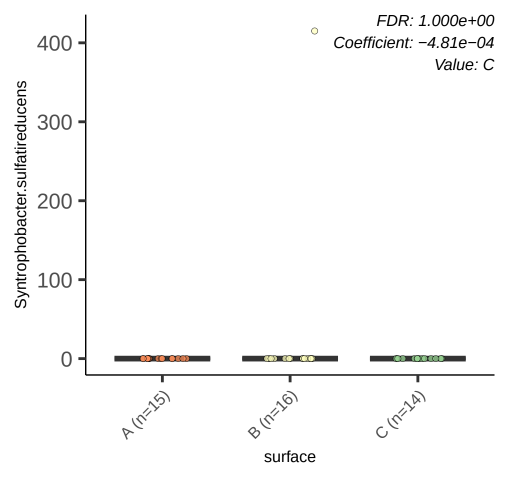
FDR: 1.000e+00
Coefficient: −4.81e−04
Value: C
Syntrophobacter.sulfatireducens
400
300
200
100
0
A (n=15)
B (n=16)
C (n=14)
surface

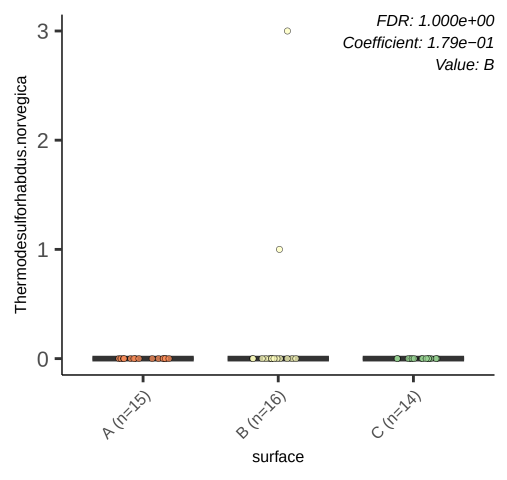
FDR: 1.000e+00
Coefficient: 1.79e−01
Value: B
Thermodesulforhabdus.norvegica
3
2
1
0
A (n=15)
B (n=16)
C (n=14)
surface

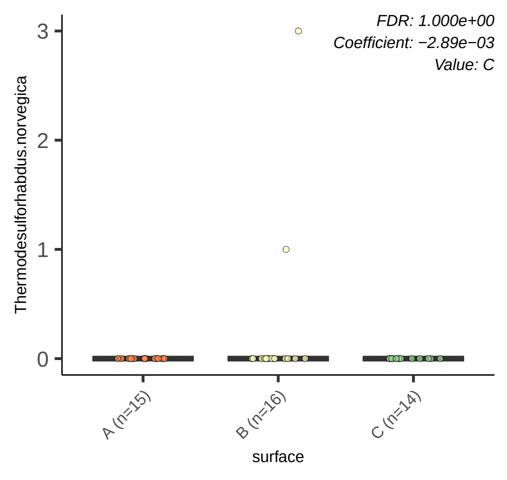
FDR: 1.000e+00
Coefficient: −2.89e−03
Value: C
Thermodesulforhabdus.norvegica
3
2
1
0
A (n=15)
B (n=16)
C (n=14)
surface

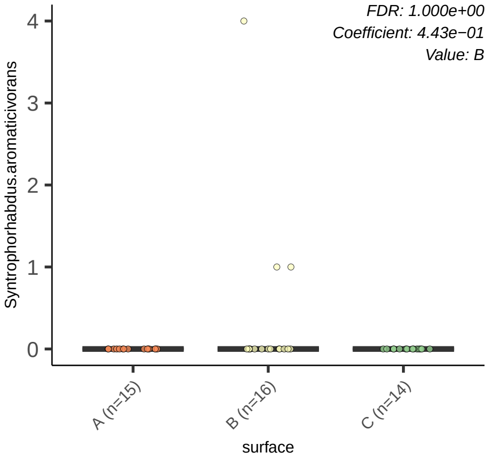
FDR: 1.000e+00
Coefficient: 4.43e−01
Value: B
Syntrophorhabdus.aromaticivorans
0
1
2
3
4
A (n=15)
B (n=16)
C (n=14)
surface

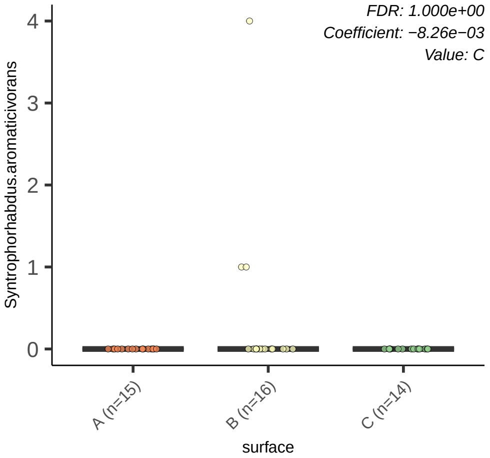
FDR: 1.000e+00
Coefficient: −8.26e−03
Value: C
Syntrophorhabdus.aromaticivorans
0
1
2
3
4
A (n=15)
B (n=16)
C (n=14)
surface

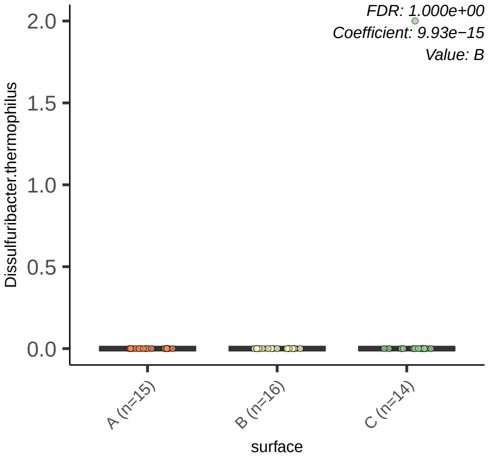
FDR: 1.000e+00
Coefficient: 9.93e−15
Value: B
Dissulfuribacter.thermophilus
2.0
1.5
1.0
0.5
0.0
A (n=15)
B (n=16)
C (n=14)
surface

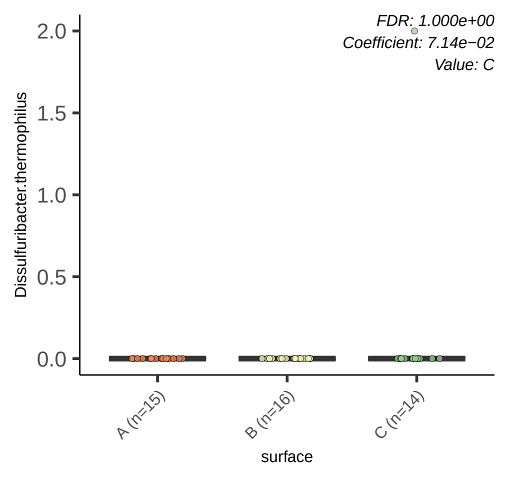
FDR: 1.000e+00
Coefficient: 7.14e−02
Value: C
Dissulfuribacter.thermophilus
2.0
1.5
1.0
0.5
0.0
A (n=15)
B (n=16)
C (n=14)
surface

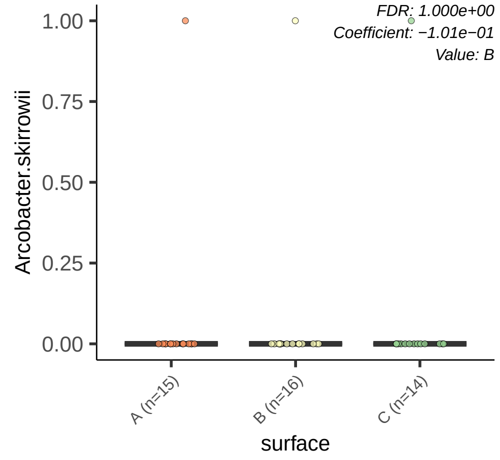

FDR: 1.000e+00
Coefficient: −1.01e−01
Value: B
Arcobacter.skirrowii
1.00
0.75
0.50
0.25
0.00
A (n=15)
B (n=16)
C (n=14)
surface

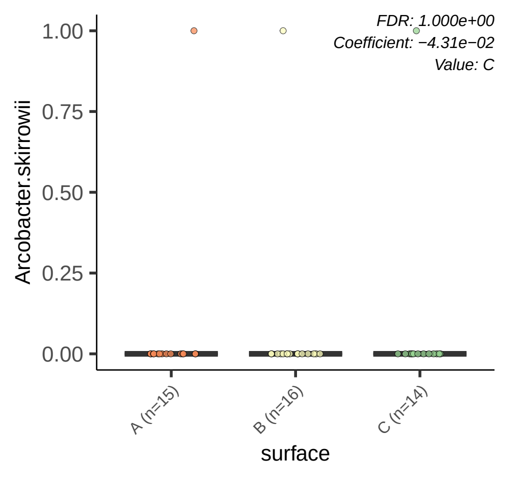

FDR: 1.000e+00
Coefficient: −4.31e−02
Value: C
Arcobacter.skirrowii
1.00
0.75
0.50
0.25
0.00
A (n=15)
B (n=16)
C (n=14)
surface

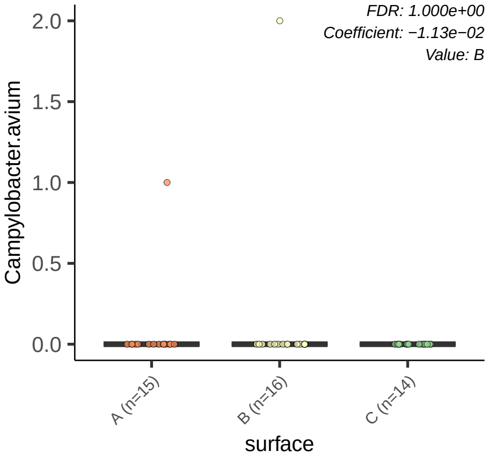

FDR: 1.000e+00
Coefficient: −1.13e−02
Value: B
Campylobacter.avium
2.0
1.5
1.0
0.5
0.0
A (n=15)
B (n=16)
C (n=14)
surface

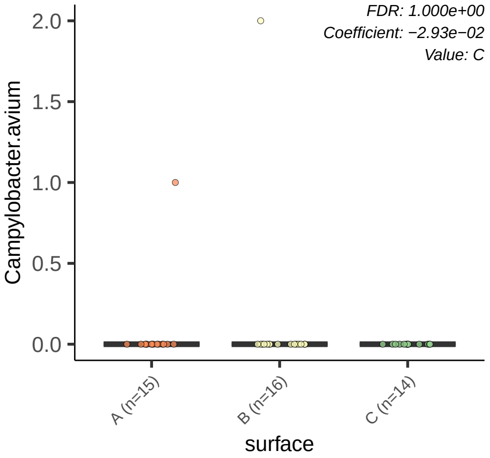
FDR: 1.000e+00
Coefficient: −2.93e−02
Value: C
Campylobacter.avium
2.0
1.5
1.0
0.5
0.0
A (n=15)
B (n=16)
C (n=14)
surface

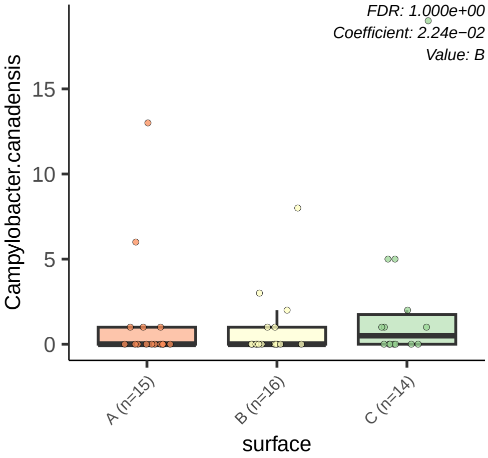
FDR: 1.000e+00
Coefficient: 2.24e−02
Value: B
Campylobacter.canadensis
15
10
5
0
A (n=15)
B (n=16)
C (n=14)
surface

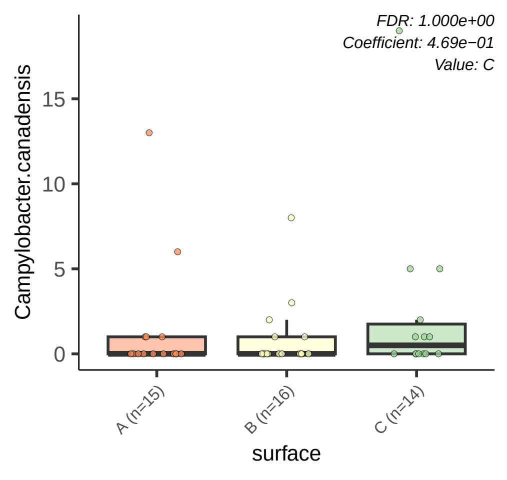

FDR: 1.000e+00
Coefficient: 4.69e−01
Value: C
Campylobacter.canadensis
15
10
5
0
A (n=15)
B (n=16)
C (n=14)
surface

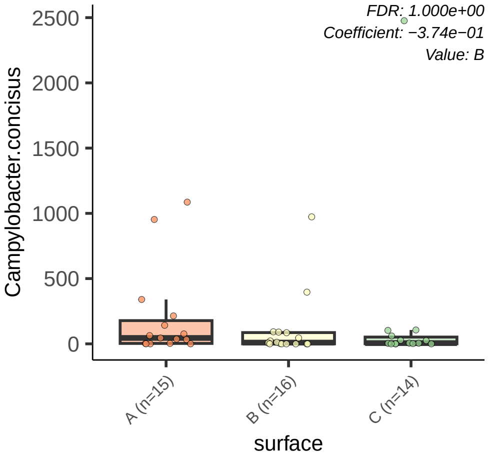

FDR: 1.000e+00
Coefficient: −3.74e−01
Value: B
Campylobacter.concisus
2500
2000
1500
1000
500
0
A (n=15)
B (n=16)
C (n=14)
surface

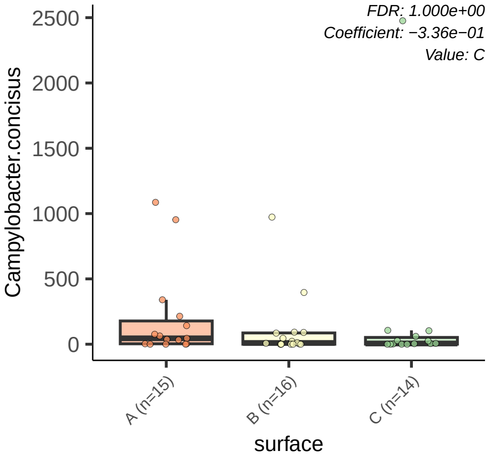
FDR: 1.000e+00
Coefficient: −3.36e−01
Value: C
Campylobacter.concisus
2500
2000
1500
1000
500
0
A (n=15)
B (n=16)
C (n=14)
surface

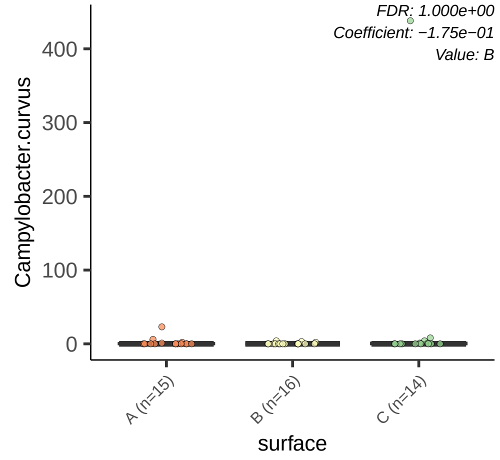
FDR: 1.000e+00
Coefficient: −1.75e−01
Value: B
Campylobacter.curvus
400
300
200
100
0
A (n=15)
B (n=16)
C (n=14)
surface

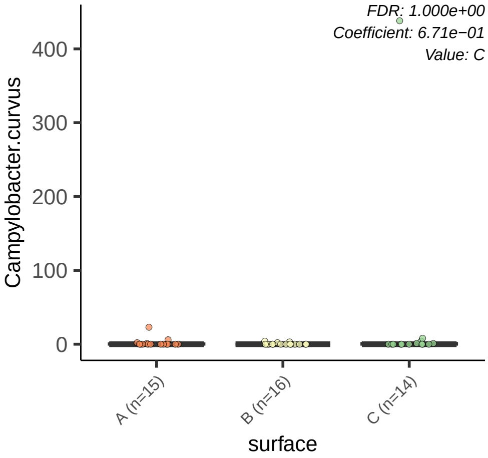

FDR: 1.000e+00
Coefficient: 6.71e−01
Value: C
Campylobacter.curvus
400
300
200
100
0
A (n=15)
B (n=16)
C (n=14)
surface

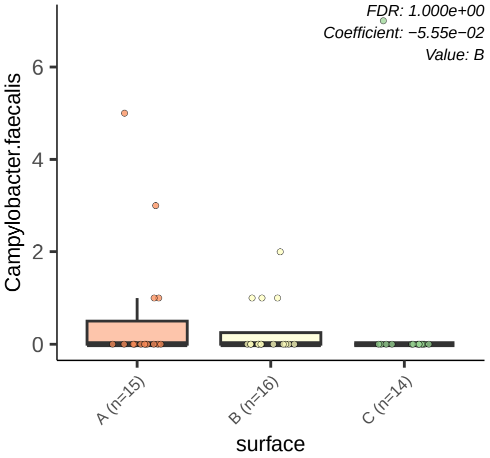

FDR: 1.000e+00
Coefficient: −5.55e−02
Value: B
Campylobacter.faecalis
0
2
4
6
A (n=15)
B (n=16)
C (n=14)
surface

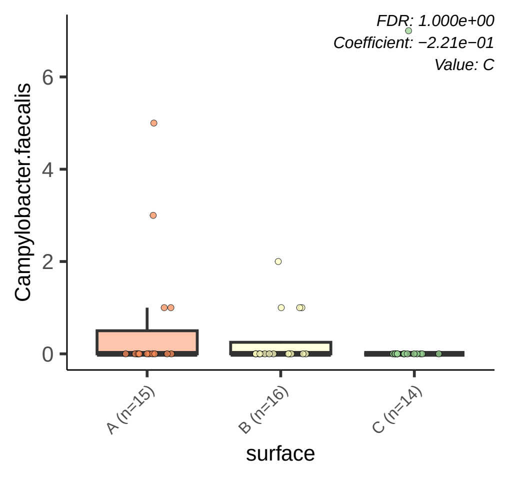
FDR: 1.000e+00
Coefficient: −2.21e−01
Value: C
Campylobacter.faecalis
6
4
2
0
A (n=15)
B (n=16)
C (n=14)
surface

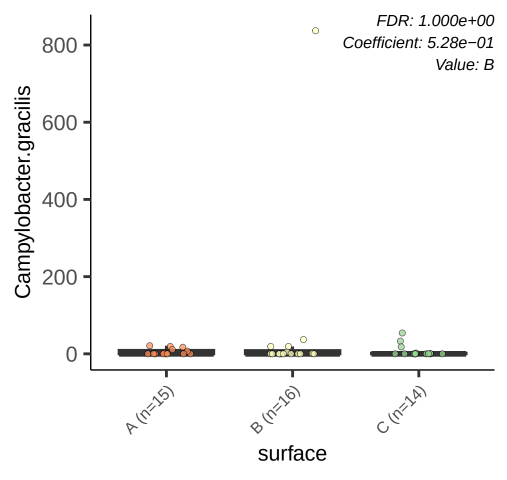
FDR: 1.000e+00
Coefficient: 5.28e−01
Value: B
Campylobacter.gracilis
800
600
400
200
0
A (n=15)
B (n=16)
C (n=14)
surface

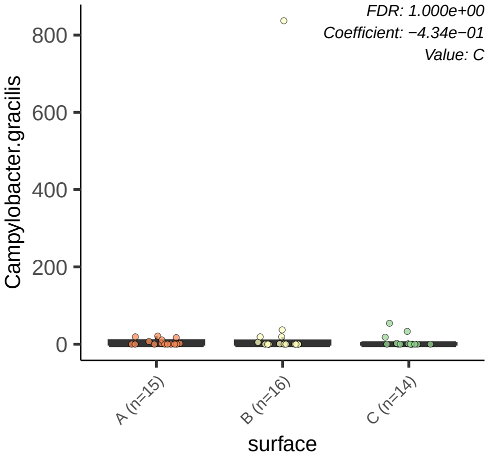
FDR: 1.000e+00
Coefficient: −4.34e−01
Value: C
Campylobacter.gracilis
800
600
400
200
0
A (n=15)
B (n=16)
C (n=14)
surface

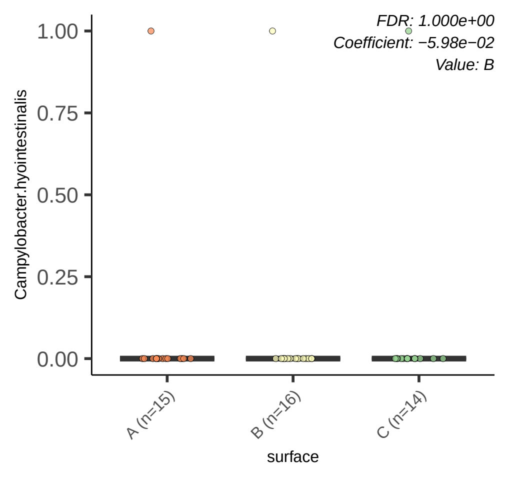

FDR: 1.000e+00
Coefficient: −5.98e−02
Value: B
Campylobacter.hyointestinalis
1.00
0.75
0.50
0.25
0.00
A (n=15)
B (n=16)
C (n=14)
surface

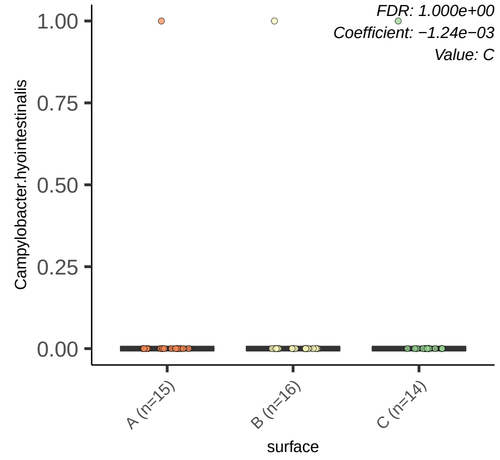
FDR: 1.000e+00
Coefficient: −1.24e−03
Value: C
Campylobacter.hyointestinalis
1.00
0.75
0.50
0.25
0.00
A (n=15)
B (n=16)
C (n=14)
surface

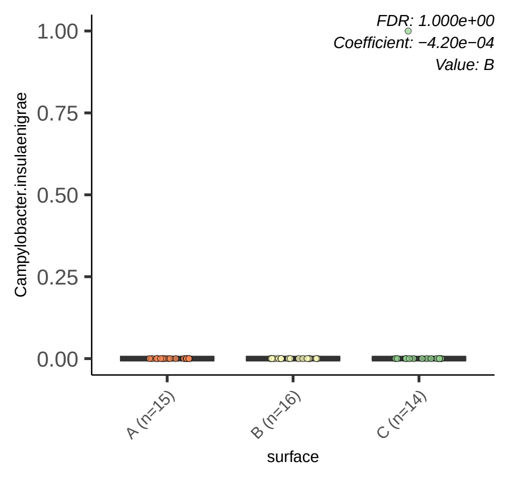
FDR: 1.000e+00
Coefficient: −4.20e−04
Value: B
1.00
0.75
0.50
0.25
0.00
Campylobacter.insulaenigrae
A (n=15)
B (n=16)
C (n=14)
surface

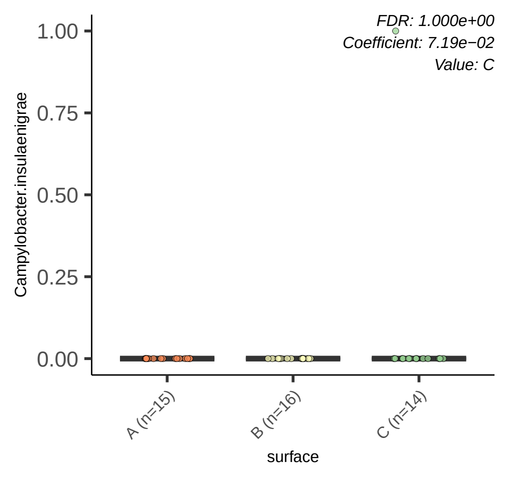

FDR: 1.000e+00
Coefficient: 7.19e−02
Value: C
1.00
0.75
0.50
0.25
0.00
Campylobacter.insulaenigrae
A (n=15)
B (n=16)
C (n=14)
surface

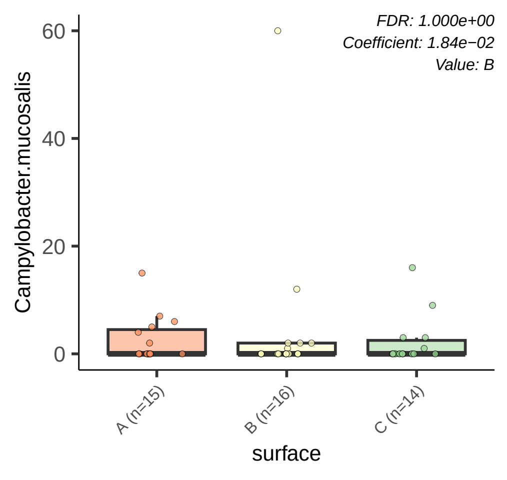

FDR: 1.000e+00
Coefficient: 1.84e−02
Value: B
Campylobacter.mucosalis
60
40
20
0
A (n=15)
B (n=16)
C (n=14)
surface

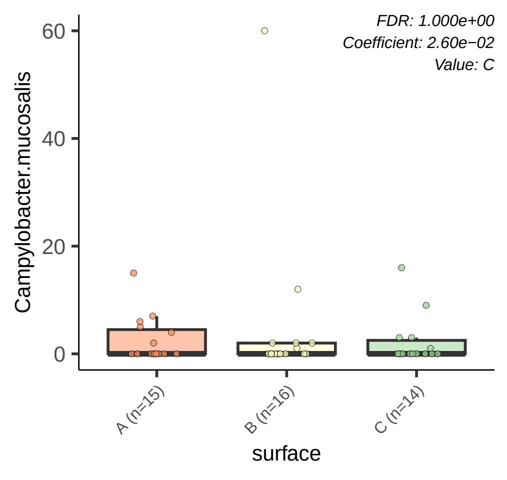
FDR: 1.000e+00
Coefficient: 2.60e−02
Value: C
Campylobacter.mucosalis
60
40
20
0
A (n=15)
B (n=16)
C (n=14)
surface

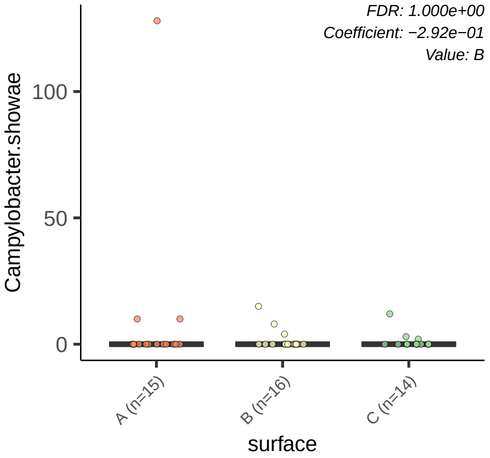

FDR: 1.000e+00
Coefficient: −2.92e−01
Value: B
Campylobacter.showae
100
50
0
A (n=15)
B (n=16)
C (n=14)
surface

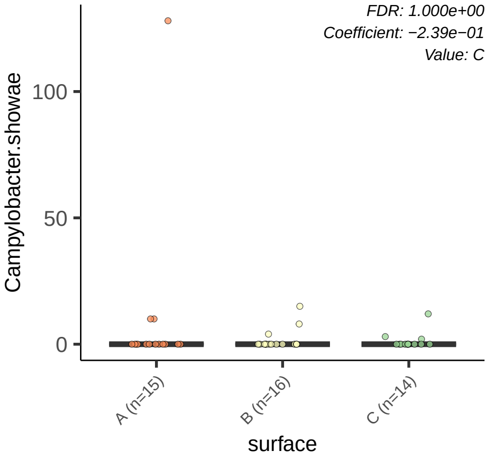

FDR: 1.000e+00
Coefficient: −2.39e−01
Value: C
Campylobacter.showae
100
50
0
A (n=15)
B (n=16)
C (n=14)
surface

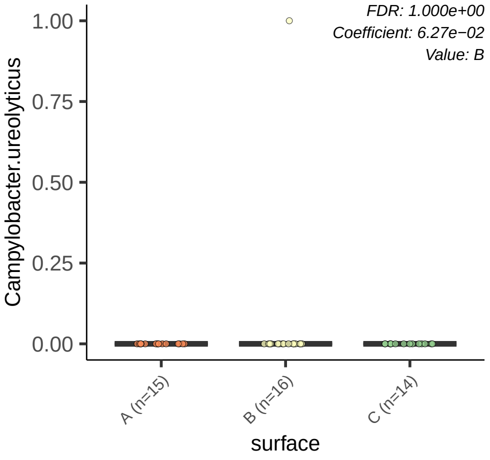

FDR: 1.000e+00
Coefficient: 6.27e−02
Value: B
Campylobacter.ureolyticus
1.00
0.75
0.50
0.25
0.00
A (n=15)
B (n=16)
C (n=14)
surface

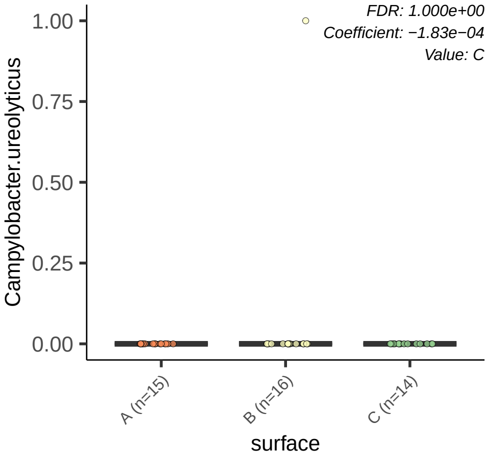
FDR: 1.000e+00
Coefficient: −1.83e−04
Value: C
Campylobacter.ureolyticus
1.00
0.75
0.50
0.25
0.00
A (n=15)
B (n=16)
C (n=14)
surface

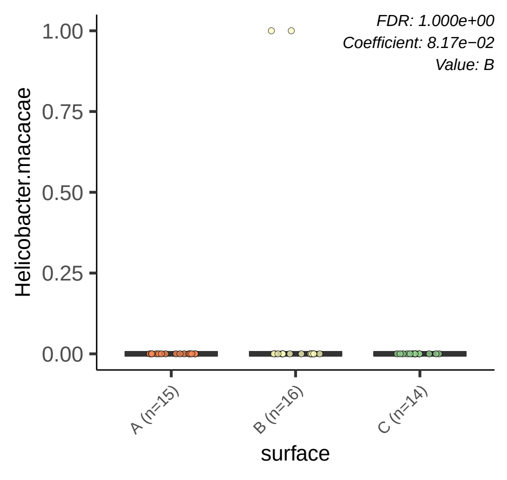
FDR: 1.000e+00
Coefficient: 8.17e−02
Value: B
Helicobacter.macacae
1.00
0.75
0.50
0.25
0.00
A (n=15)
B (n=16)
C (n=14)
surface

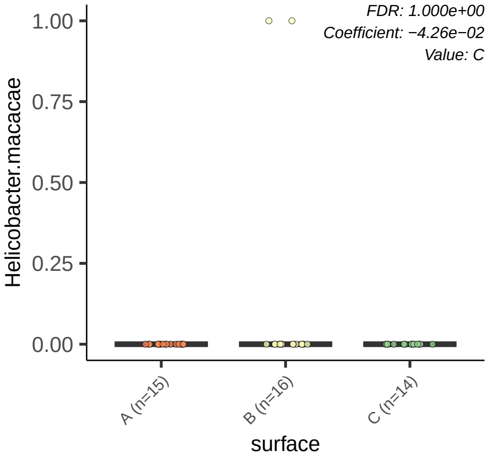

FDR: 1.000e+00
Coefficient: −4.26e−02
Value: C
Helicobacter.macacae
1.00
0.75
0.50
0.25
0.00
A (n=15)
B (n=16)
C (n=14)
surface

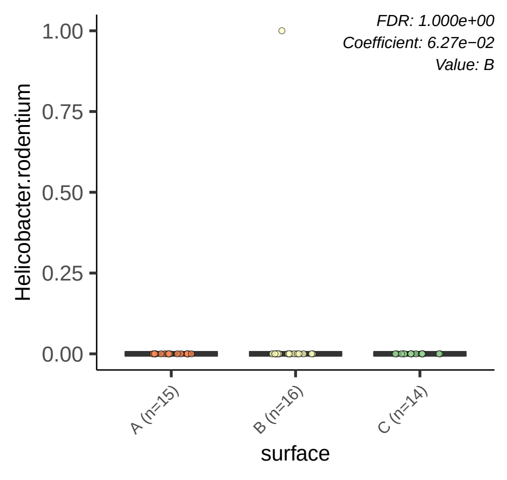
FDR: 1.000e+00
Coefficient: 6.27e−02
Value: B
Helicobacter.rodentium
1.00
0.75
0.50
0.25
0.00
A (n=15)
B (n=16)
C (n=14)
surface

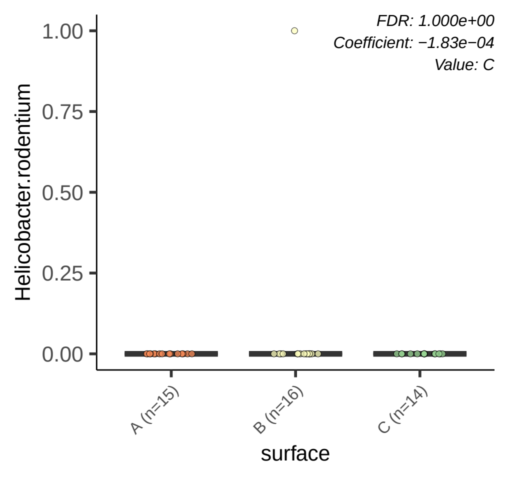
FDR: 1.000e+00
Coefficient: −1.83e−04
Value: C
Helicobacter.rodentium
1.00
0.75
0.50
0.25
0.00
A (n=15)
B (n=16)
C (n=14)
surface

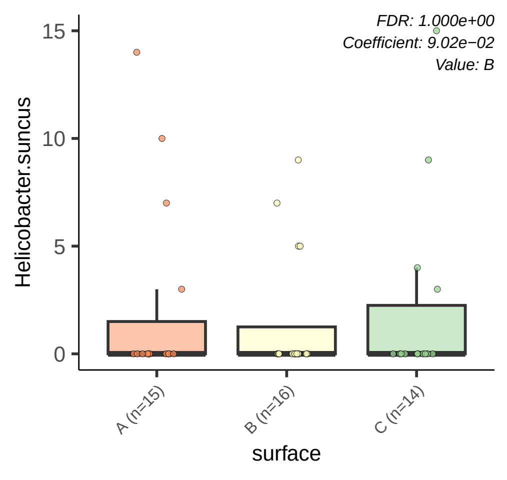
FDR: 1.000e+00
Coefficient: 9.02e−02
Value: B
Helicobacter.suncus
15
10
5
0
A (n=15)
B (n=16)
C (n=14)
surface

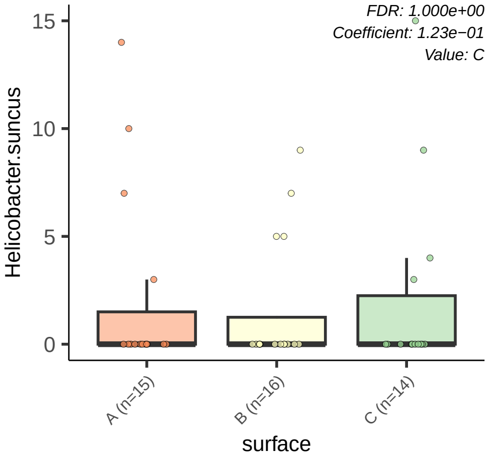

FDR: 1.000e+00
Coefficient: 1.23e−01
Value: C
Helicobacter.suncus
15
10
5
0
A (n=15)
B (n=16)
C (n=14)
surface

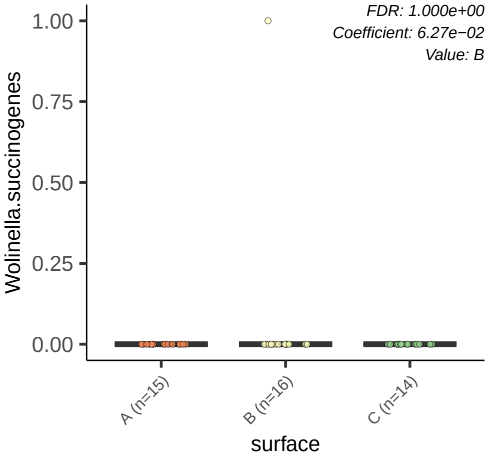

FDR: 1.000e+00
Coefficient: 6.27e−02
Value: B
Wolinella.succinogenes
1.00
0.75
0.50
0.25
0.00
A (n=15)
B (n=16)
C (n=14)
surface

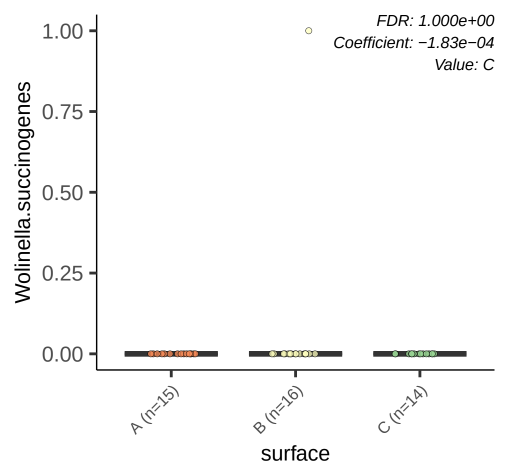

FDR: 1.000e+00
Coefficient: −1.83e−04
Value: C
Wolinella.succinogenes
1.00
0.75
0.50
0.25
0.00
A (n=15)
B (n=16)
C (n=14)
surface

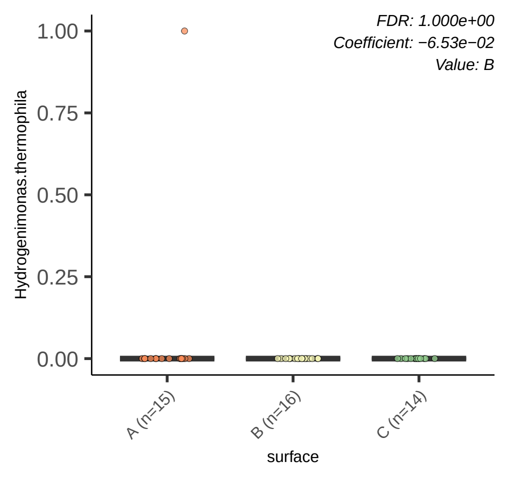
FDR: 1.000e+00
Coefficient: −6.53e−02
Value: B
Hydrogenimonas.thermophila
1.00
0.75
0.50
0.25
0.00
A (n=15)
B (n=16)
C (n=14)
surface

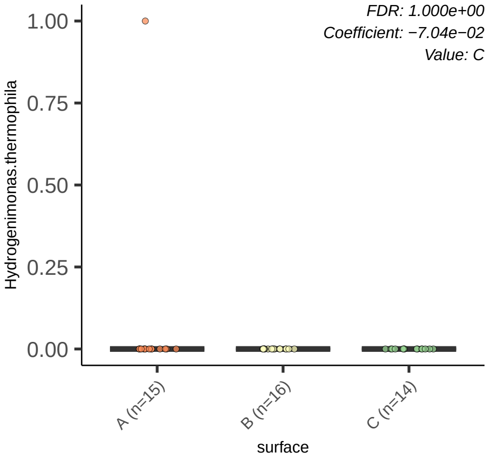
FDR: 1.000e+00
Coefficient: −7.04e−02
Value: C
Hydrogenimonas.thermophila
1.00
0.75
0.50
0.25
0.00
A (n=15)
B (n=16)
C (n=14)
surface

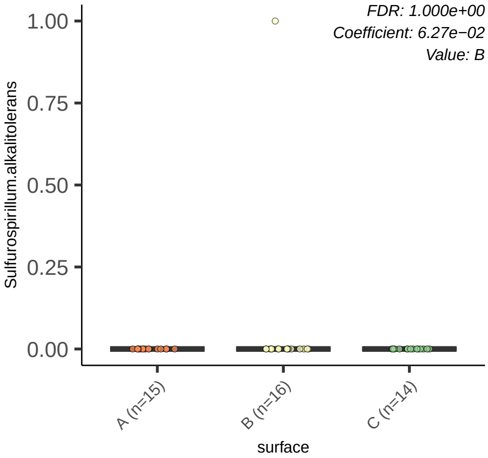
FDR: 1.000e+00
Coefficient: 6.27e−02
Value: B
1.00
0.75
0.50
0.25
0.00
Sulfurospirillum.alkalitolerans
A (n=15)
B (n=16)
C (n=14)
surface

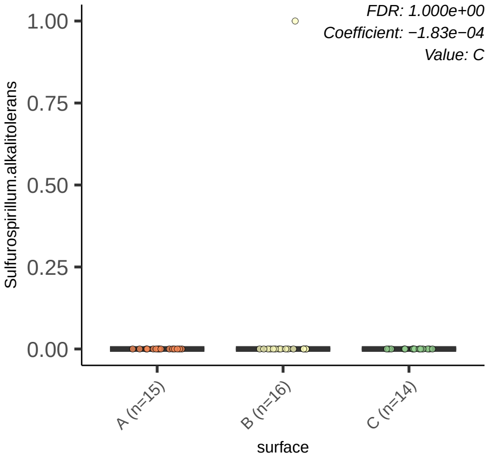
FDR: 1.000e+00
Coefficient: −1.83e−04
Value: C
Sulfurospirillum.alkalitolerans
1.00
0.75
0.50
0.25
0.00
A (n=15)
B (n=16)
C (n=14)
surface

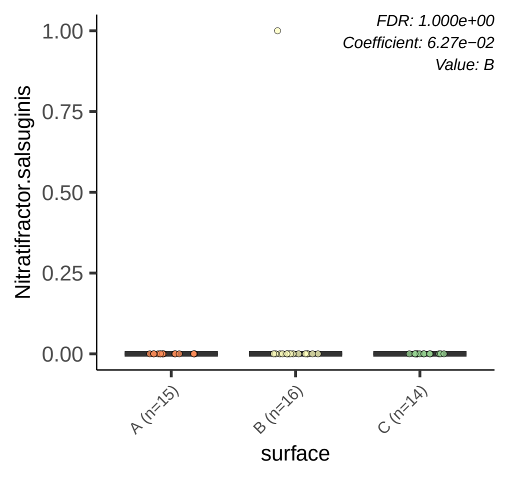
FDR: 1.000e+00
Coefficient: 6.27e−02
Value: B
Nitratifractor.salsuginis
1.00
0.75
0.50
0.25
0.00
A (n=15)
B (n=16)
C (n=14)
surface

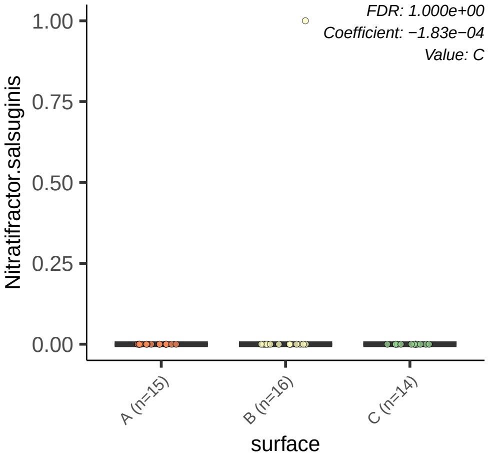
FDR: 1.000e+00
Coefficient: −1.83e−04
Value: C
Nitratifractor.salsuginis
1.00
0.75
0.50
0.25
0.00
A (n=15)
B (n=16)
C (n=14)
surface

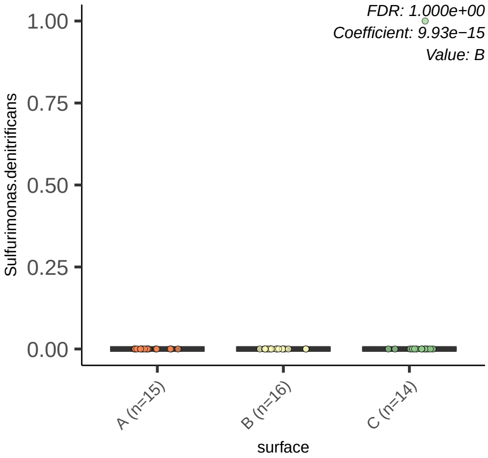
FDR: 1.000e+00
Coefficient: 9.93e−15
Value: B
Sulfurimonas.denitrificans
1.00
0.75
0.50
0.25
0.00
A (n=15)
B (n=16)
C (n=14)
surface

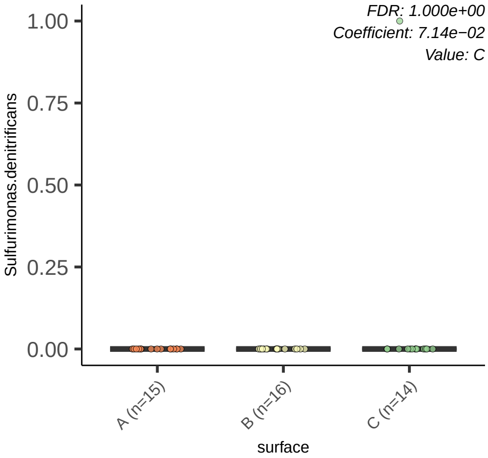
FDR: 1.000e+00
Coefficient: 7.14e−02
Value: C
1.00
0.75
0.50
0.25
0.00
Sulfurimonas.denitrificans
A (n=15)
B (n=16)
C (n=14)
surface

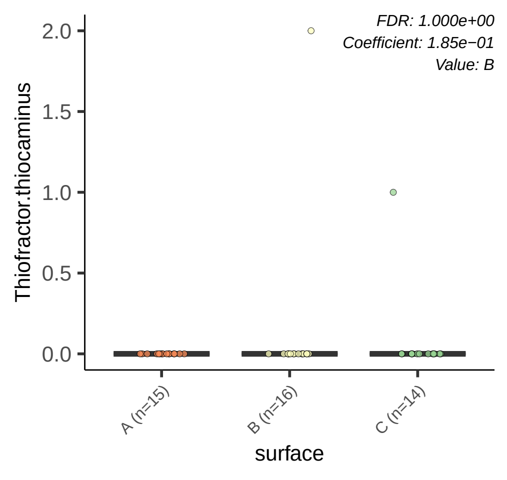
FDR: 1.000e+00
Coefficient: 1.85e−01
Value: B
Thiofractor.thiocaminus
2.0
1.5
1.0
0.5
0.0
A (n=15)
B (n=16)
C (n=14)
surface

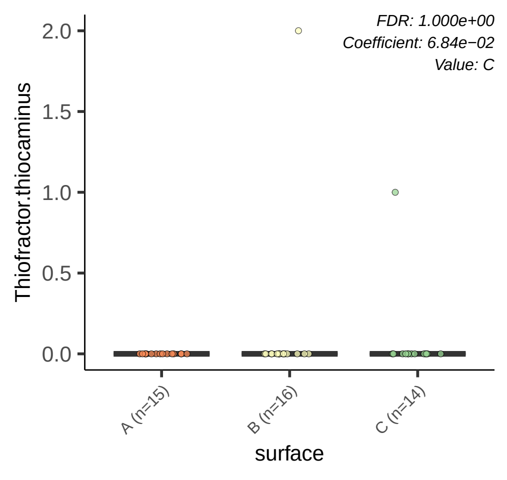

FDR: 1.000e+00
Coefficient: 6.84e−02
Value: C
Thiofractor.thiocaminus
2.0
1.5
1.0
0.5
0.0
A (n=15)
B (n=16)
C (n=14)
surface

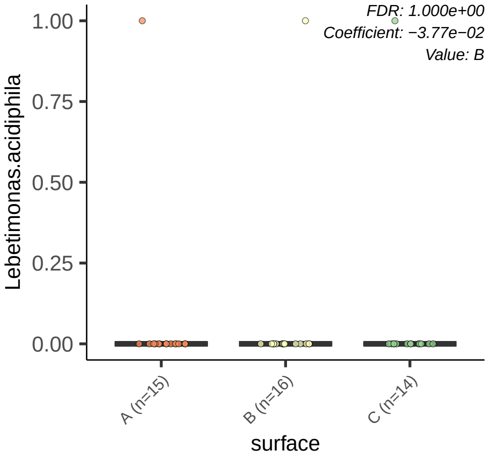

FDR: 1.000e+00
Coefficient: −3.77e−02
Value: B
Lebetimonas.acidiphila
1.00
0.75
0.50
0.25
0.00
A (n=15)
B (n=16)
C (n=14)
surface

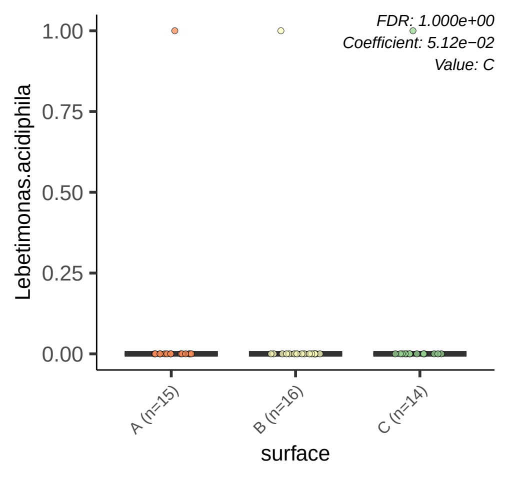

FDR: 1.000e+00
Coefficient: 5.12e−02
Value: C
Lebetimonas.acidiphila
1.00
0.75
0.50
0.25
0.00
A (n=15)
B (n=16)
C (n=14)
surface

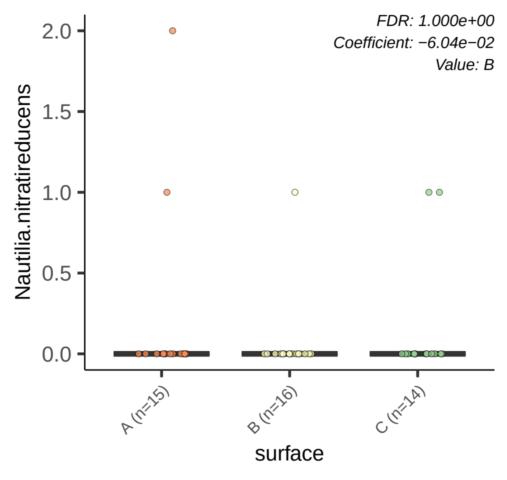

FDR: 1.000e+00
Coefficient: −6.04e−02
Value: B
Nautilia.nitratireducens
2.0
1.5
1.0
0.5
0.0
A (n=15)
B (n=16)
C (n=14)
surface

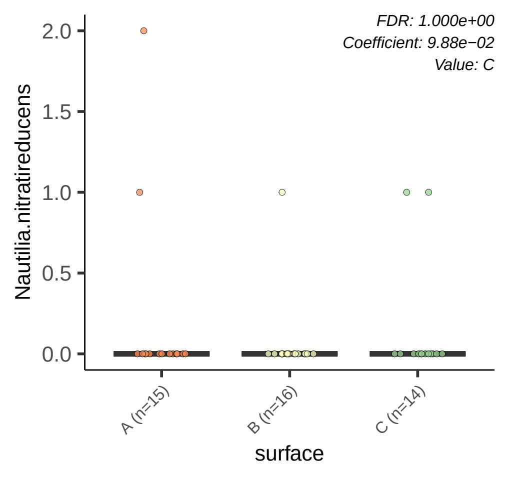
FDR: 1.000e+00
Coefficient: 9.88e−02
Value: C
Nautilia.nitratireducens
2.0
1.5
1.0
0.5
0.0
A (n=15)
B (n=16)
C (n=14)
surface

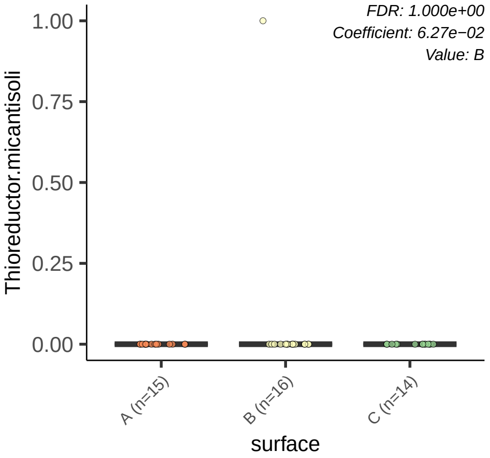

FDR: 1.000e+00
Coefficient: 6.27e−02
Value: B
Thioreductor.micantisoli
1.00
0.75
0.50
0.25
0.00
A (n=15)
B (n=16)
C (n=14)
surface

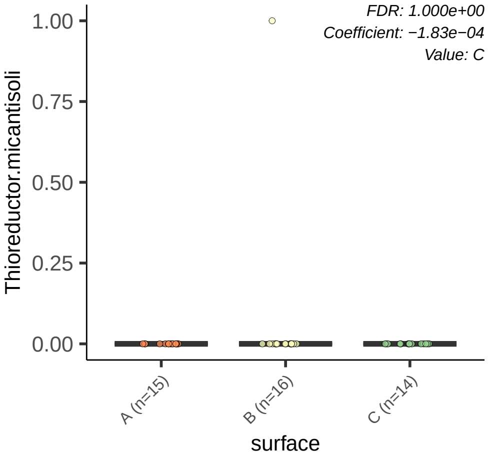
FDR: 1.000e+00
Coefficient: −1.83e−04
Value: C
Thioreductor.micantisoli
1.00
0.75
0.50
0.25
0.00
A (n=15)
B (n=16)
C (n=14)
surface

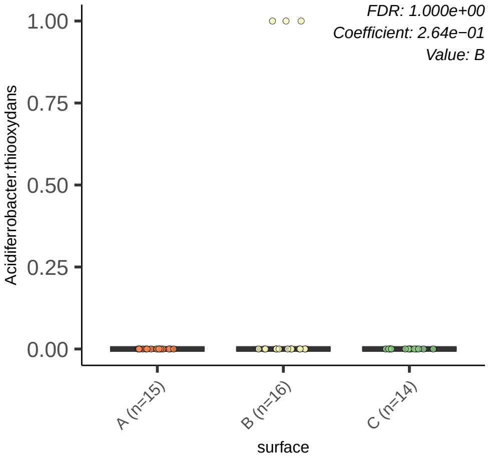

FDR: 1.000e+00
Coefficient: 2.64e−01
Value: B
1.00
0.75
0.50
0.25
0.00
Acidiferrobacter.thiooxydans
A (n=15)
B (n=16)
C (n=14)
surface

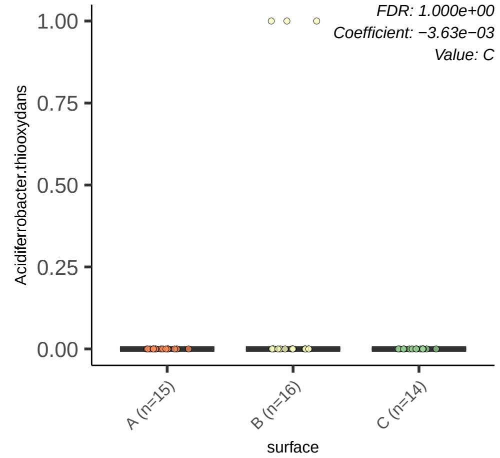
FDR: 1.000e+00
Coefficient: −3.63e−03
Value: C
1.00
0.75
0.50
0.25
0.00
Acidiferrobacter.thiooxydans
A (n=15)
B (n=16)
C (n=14)
surface

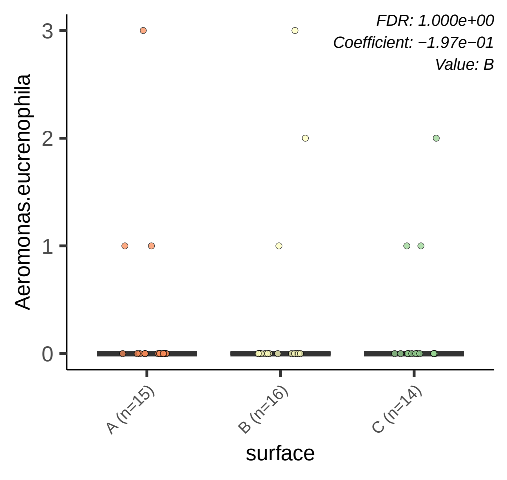
FDR: 1.000e+00
Coefficient: −1.97e−01
Value: B
Aeromonas.eucrenophila
3
2
1
0
A (n=15)
B (n=16)
C (n=14)
surface

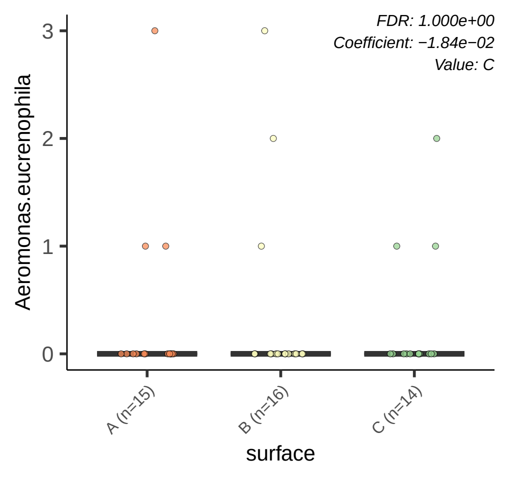

FDR: 1.000e+00
Coefficient: −1.84e−02
Value: C
Aeromonas.eucrenophila
3
2
1
0
A (n=15)
B (n=16)
C (n=14)
surface

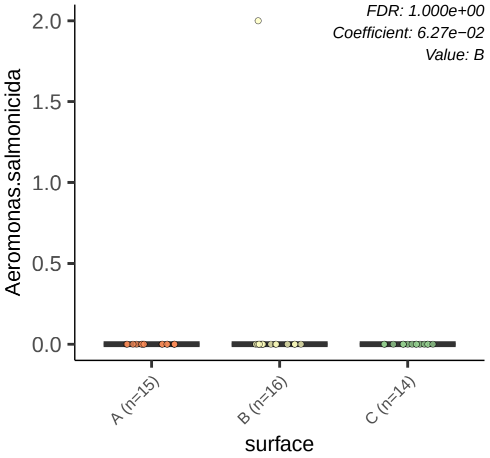
FDR: 1.000e+00
Coefficient: 6.27e−02
Value: B
Aeromonas.salmonicida
2.0
1.5
1.0
0.5
0.0
A (n=15)
B (n=16)
C (n=14)
surface

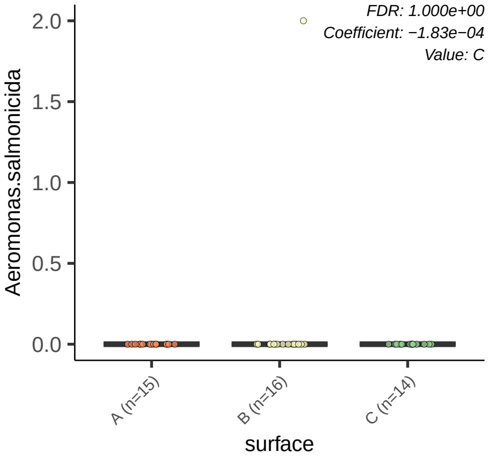
FDR: 1.000e+00
Coefficient: −1.83e−04
Value: C
Aeromonas.salmonicida
2.0
1.5
1.0
0.5
0.0
A (n=15)
B (n=16)
C (n=14)
surface

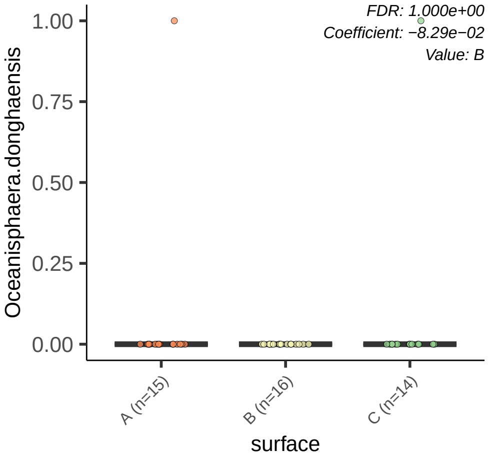
FDR: 1.000e+00
Coefficient: −8.29e−02
Value: B
Oceanisphaera.donghaensis
1.00
0.75
0.50
0.25
0.00
A (n=15)
B (n=16)
C (n=14)
surface

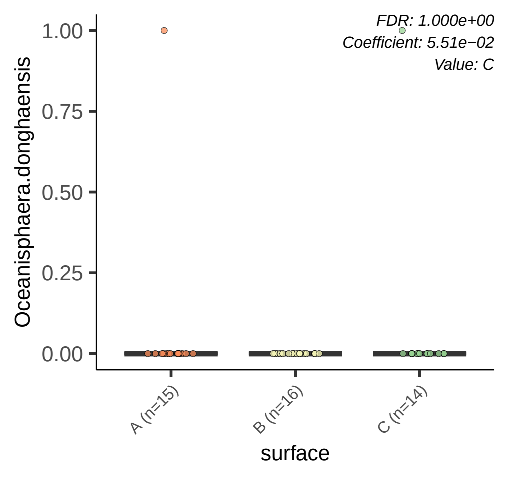

FDR: 1.000e+00
Coefficient: 5.51e−02
Value: C
Oceanisphaera.donghaensis
1.00
0.75
0.50
0.25
0.00
A (n=15)
B (n=16)
C (n=14)
surface

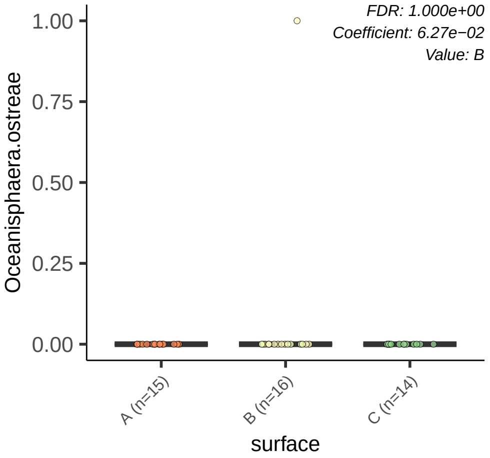
FDR: 1.000e+00
Coefficient: 6.27e−02
Value: B
Oceanisphaera.ostreae
1.00
0.75
0.50
0.25
0.00
A (n=15)
B (n=16)
C (n=14)
surface

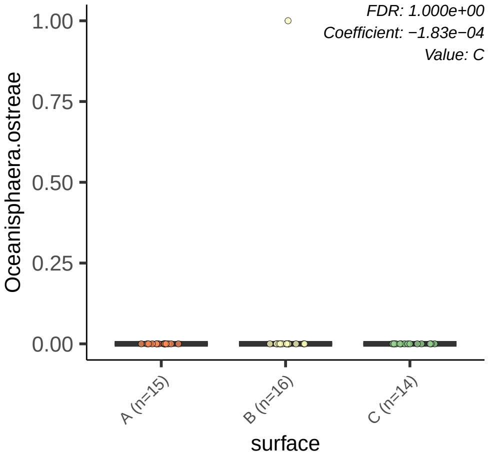
FDR: 1.000e+00
Coefficient: −1.83e−04
Value: C
Oceanisphaera.ostreae
1.00
0.75
0.50
0.25
0.00
A (n=15)
B (n=16)
C (n=14)
surface

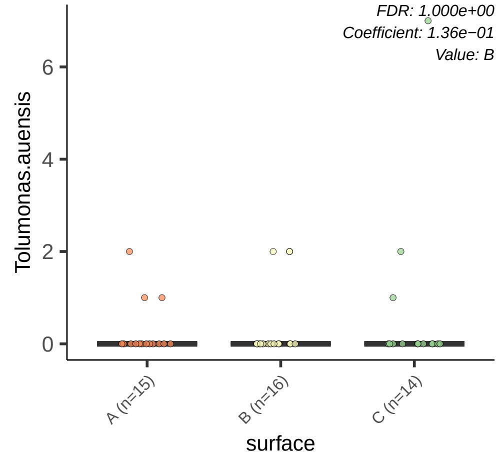
FDR: 1.000e+00
Coefficient: 1.36e−01
Value: B
Tolumonas.auensis
0
2
4
6
A (n=15)
B (n=16)
C (n=14)
surface

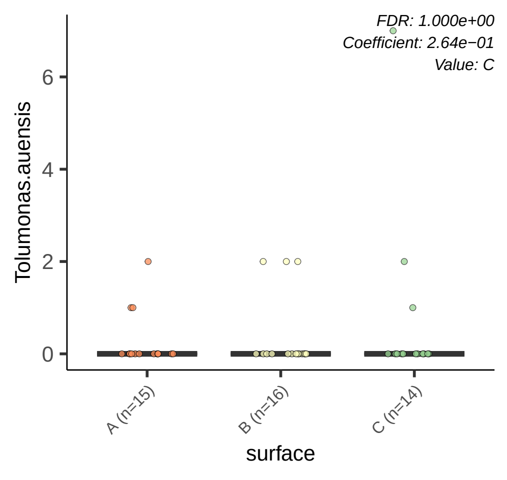

FDR: 1.000e+00
Coefficient: 2.64e−01
Value: C
Tolumonas.auensis
0
2
4
6
A (n=15)
B (n=16)
C (n=14)
surface

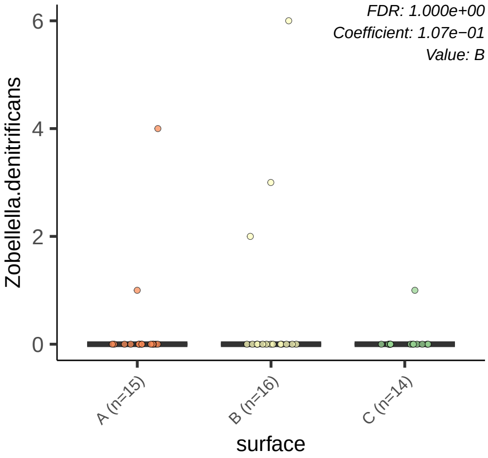

FDR: 1.000e+00
Coefficient: 1.07e−01
Value: B
Zobellella.denitrificans
0
2
4
6
A (n=15)
B (n=16)
C (n=14)
surface

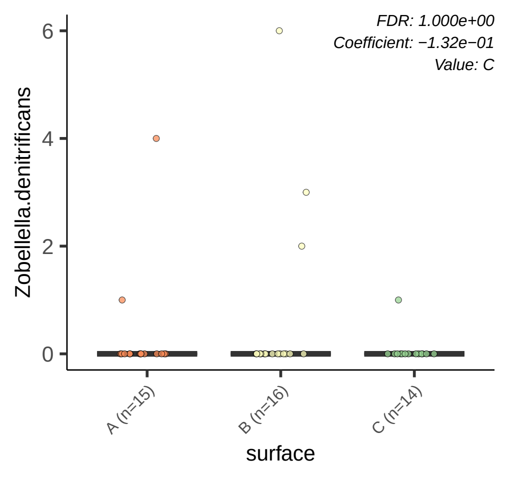

FDR: 1.000e+00
Coefficient: −1.32e−01
Value: C
Zobellella.denitrificans
0
2
4
6
A (n=15)
B (n=16)
C (n=14)
surface

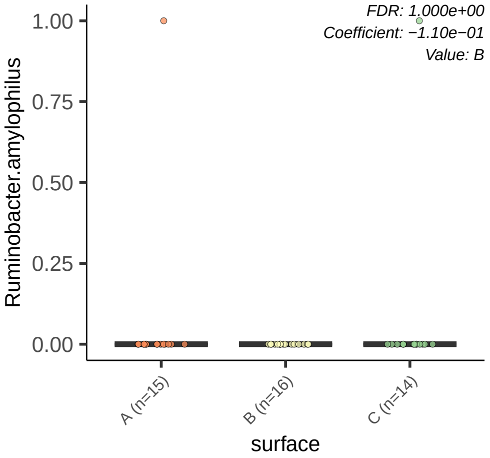

FDR: 1.000e+00
Coefficient: −1.10e−01
Value: B
Ruminobacter.amylophilus
1.00
0.75
0.50
0.25
0.00
A (n=15)
B (n=16)
C (n=14)
surface

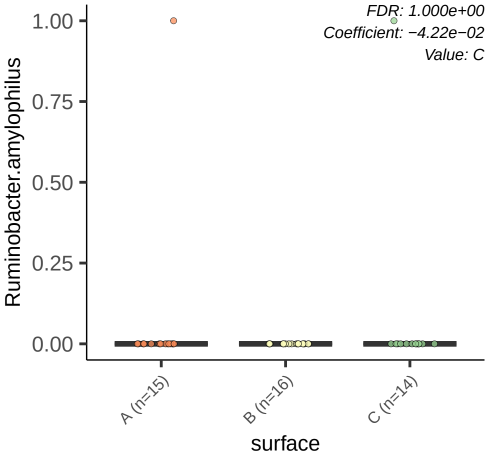
FDR: 1.000e+00
Coefficient: −4.22e−02
Value: C
Ruminobacter.amylophilus
1.00
0.75
0.50
0.25
0.00
A (n=15)
B (n=16)
C (n=14)
surface

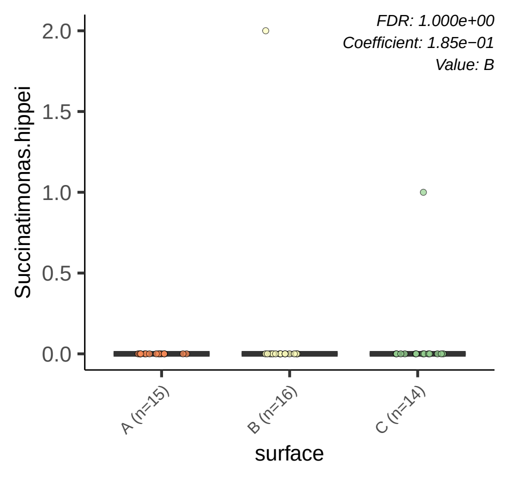

FDR: 1.000e+00
Coefficient: 1.85e−01
Value: B
Succinatimonas.hippei
2.0
1.5
1.0
0.5
0.0
A (n=15)
B (n=16)
C (n=14)
surface

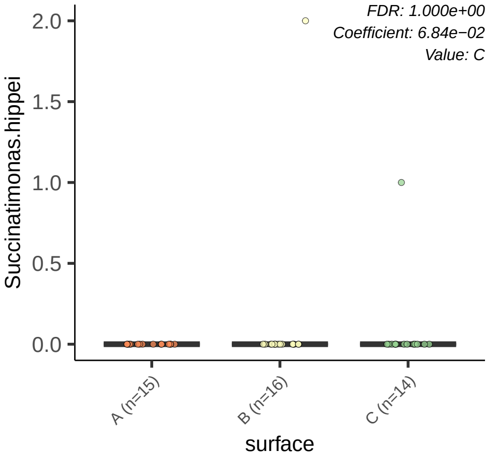
FDR: 1.000e+00
Coefficient: 6.84e−02
Value: C
Succinatimonas.hippei
2.0
1.5
1.0
0.5
0.0
A (n=15)
B (n=16)
C (n=14)
surface

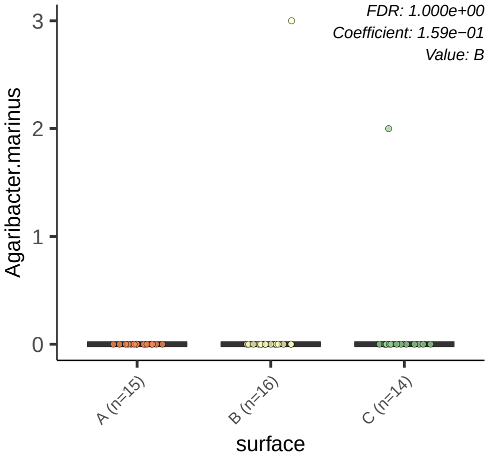

FDR: 1.000e+00
Coefficient: 1.59e−01
Value: B
Agaribacter.marinus
0
1
2
3
A (n=15)
B (n=16)
C (n=14)
surface

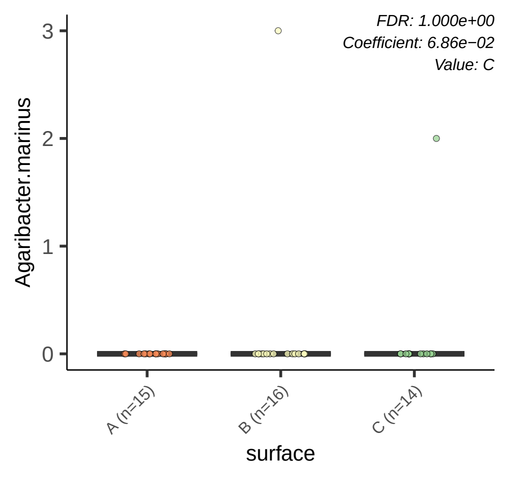
FDR: 1.000e+00
Coefficient: 6.86e−02
Value: C
Agaribacter.marinus
0
1
2
3
A (n=15)
B (n=16)
C (n=14)
surface

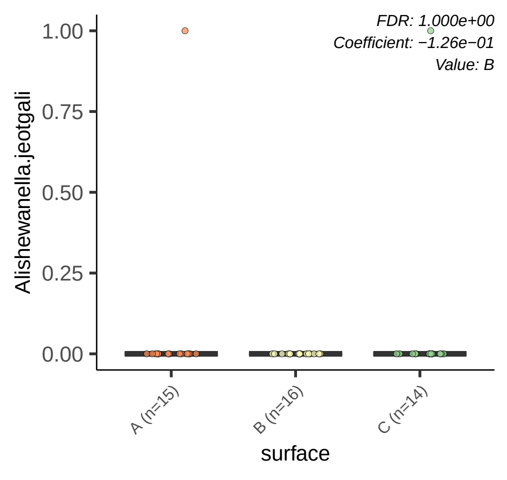

FDR: 1.000e+00
Coefficient: −1.26e−01
Value: B
Alishewanella.jeotgali
1.00
0.75
0.50
0.25
0.00
A (n=15)
B (n=16)
C (n=14)
surface

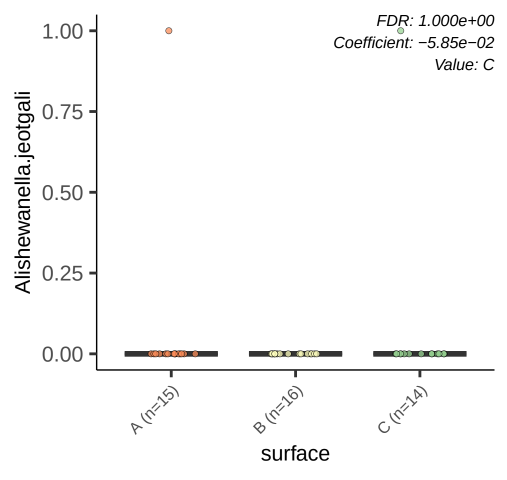
FDR: 1.000e+00
Coefficient: −5.85e−02
Value: C
Alishewanella.jeotgali
1.00
0.75
0.50
0.25
0.00
A (n=15)
B (n=16)
C (n=14)
surface

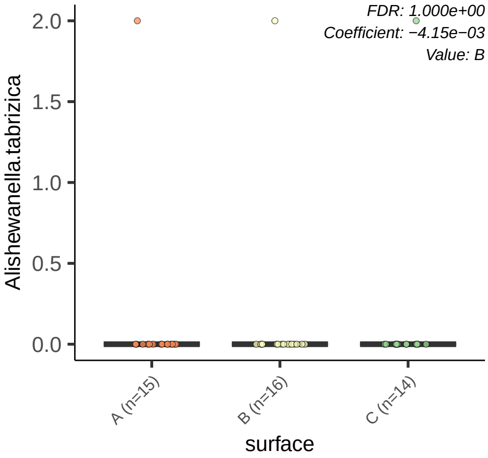

FDR: 1.000e+00
Coefficient: −4.15e−03
Value: B
Alishewanella.tabrizica
2.0
1.5
1.0
0.5
0.0
A (n=15)
B (n=16)
C (n=14)
surface

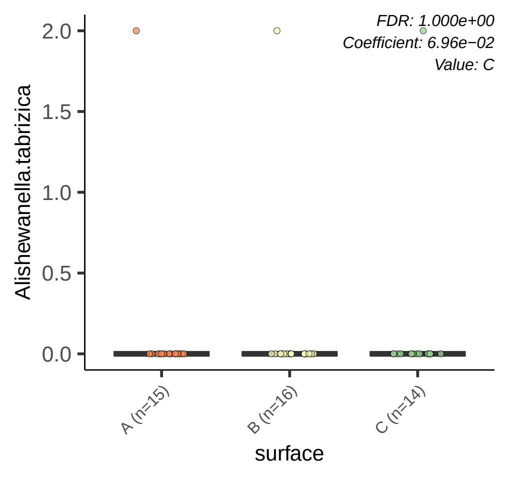
FDR: 1.000e+00
Coefficient: 6.96e−02
Value: C
Alishewanella.tabrizica
2.0
1.5
1.0
0.5
0.0
A (n=15)
B (n=16)
C (n=14)
surface

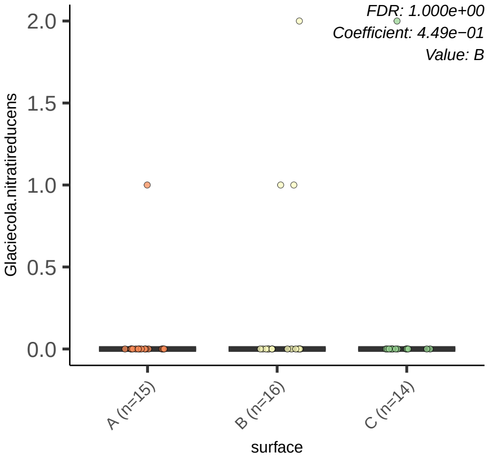
FDR: 1.000e+00
Coefficient: 4.49e−01
Value: B
Glaciecola.nitratireducens
2.0
1.5
1.0
0.5
0.0
A (n=15)
B (n=16)
C (n=14)
surface

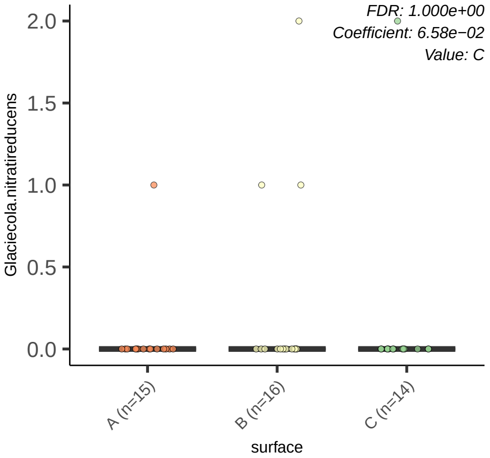

FDR: 1.000e+00
Coefficient: 6.58e−02
Value: C
Glaciecola.nitratireducens
2.0
1.5
1.0
0.5
0.0
A (n=15)
B (n=16)
C (n=14)
surface

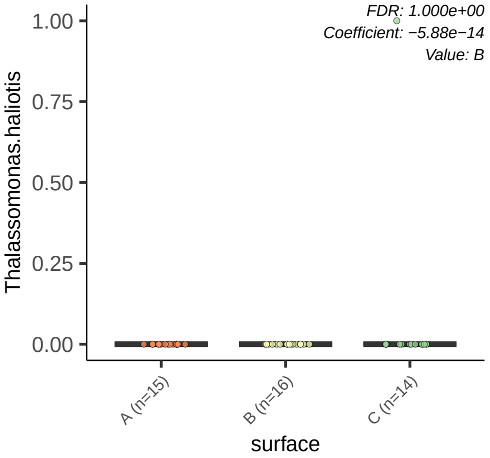
FDR: 1.000e+00
Coefficient: −5.88e−14
Value: B
1.00
0.75
0.50
0.25
0.00
Thalassomonas.haliotis
A (n=15)
B (n=16)
C (n=14)
surface

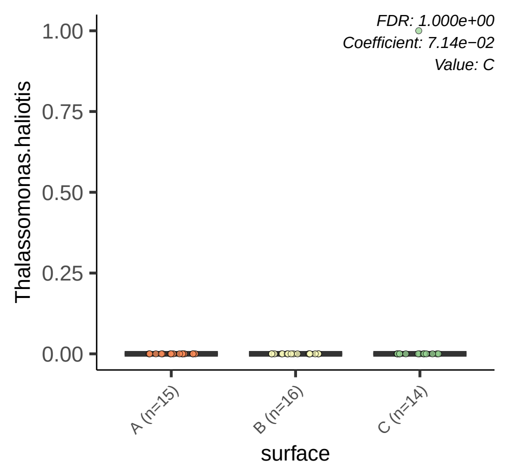
FDR: 1.000e+00
Coefficient: 7.14e−02
Value: C
Thalassomonas.haliotis
1.00
0.75
0.50
0.25
0.00
A (n=15)
B (n=16)
C (n=14)
surface

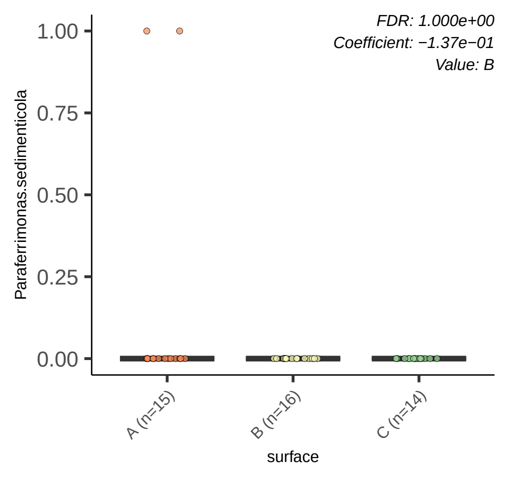

FDR: 1.000e+00
Coefficient: −1.37e−01
Value: B
1.00
0.75
0.50
0.25
0.00
Paraferrimonas.sedimenticola
A (n=15)
B (n=16)
C (n=14)
surface

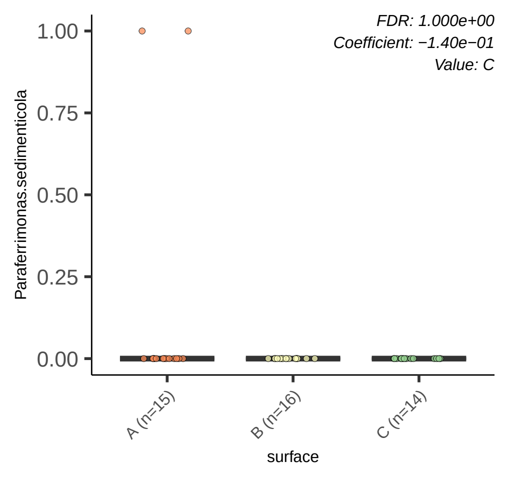
FDR: 1.000e+00
Coefficient: −1.40e−01
Value: C
Paraferrimonas.sedimenticola
1.00
0.75
0.50
0.25
0.00
A (n=15)
B (n=16)
C (n=14)
surface

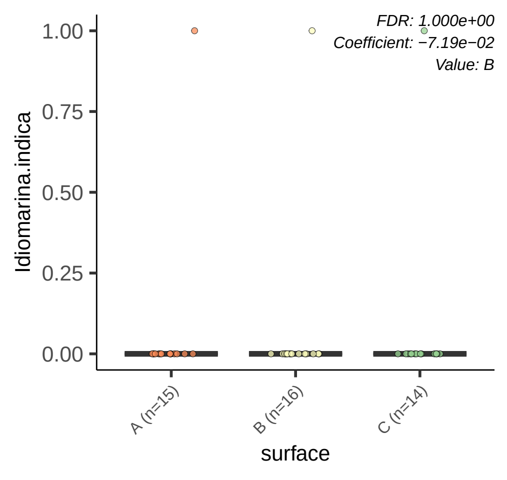
FDR: 1.000e+00
Coefficient: −7.19e−02
Value: B
1.00
0.75
0.50
0.25
0.00
Idiomarina.indica
A (n=15)
B (n=16)
C (n=14)
surface

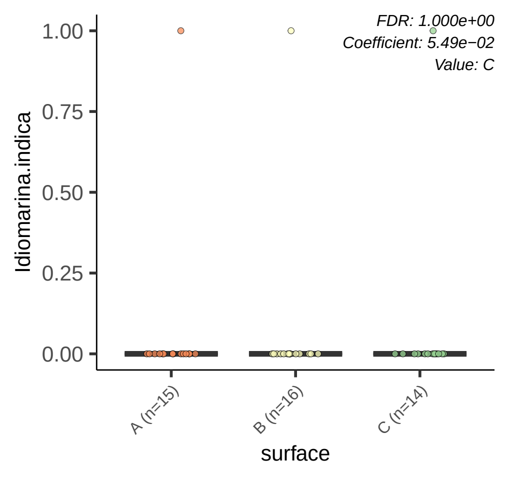
FDR: 1.000e+00
Coefficient: 5.49e−02
Value: C
1.00
0.75
0.50
0.25
0.00
Idiomarina.indica
A (n=15)
B (n=16)
C (n=14)
surface

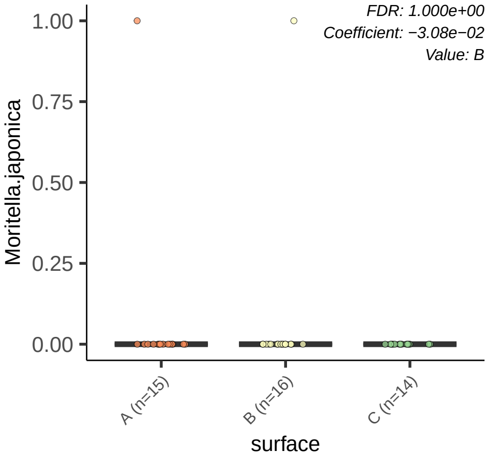

FDR: 1.000e+00
Coefficient: −3.08e−02
Value: B
Moritella.japonica
1.00
0.75
0.50
0.25
0.00
A (n=15)
B (n=16)
C (n=14)
surface

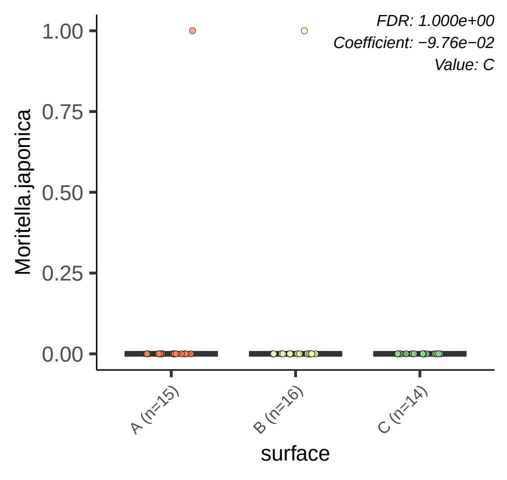

FDR: 1.000e+00
Coefficient: −9.76e−02
Value: C
Moritella.japonica
1.00
0.75
0.50
0.25
0.00
A (n=15)
B (n=16)
C (n=14)
surface

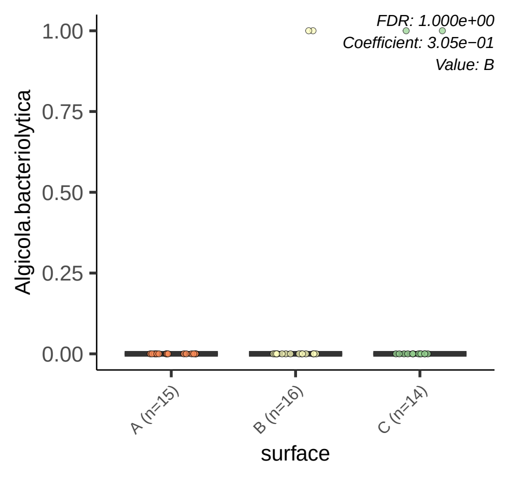

FDR: 1.000e+00
Coefficient: 3.05e−01
Value: B
Algicola.bacteriolytica
1.00
0.75
0.50
0.25
0.00
A (n=15)
B (n=16)
C (n=14)
surface

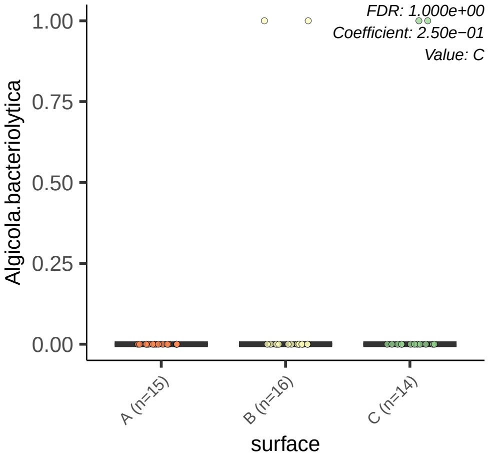

FDR: 1.000e+00
Coefficient: 2.50e−01
Value: C
Algicola.bacteriolytica
1.00
0.75
0.50
0.25
0.00
A (n=15)
B (n=16)
C (n=14)
surface

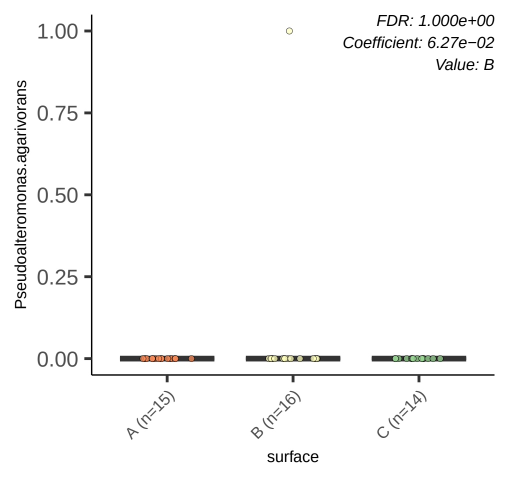
FDR: 1.000e+00
Coefficient: 6.27e−02
Value: B
1.00
0.75
0.50
0.25
0.00
Pseudoalteromonas.agarivorans
A (n=15)
B (n=16)
C (n=14)
surface

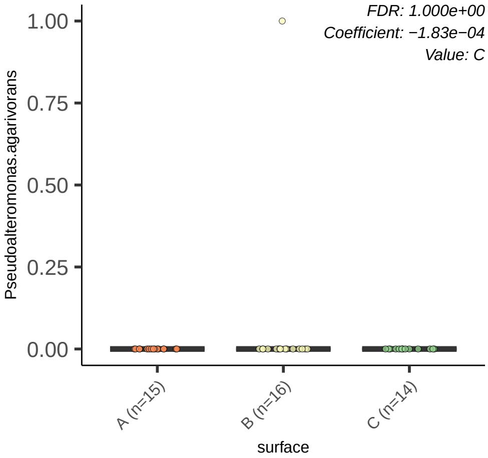
FDR: 1.000e+00
Coefficient: −1.83e−04
Value: C
Pseudoalteromonas.agarivorans
1.00
0.75
0.50
0.25
0.00
A (n=15)
B (n=16)
C (n=14)
surface

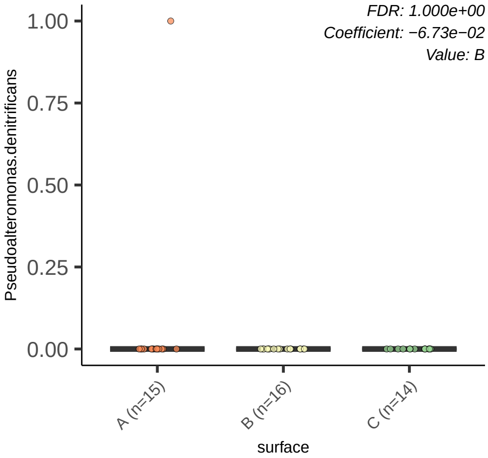
FDR: 1.000e+00
Coefficient: −6.73e−02
Value: B
Pseudoalteromonas.denitrificans
1.00
0.75
0.50
0.25
0.00
A (n=15)
B (n=16)
C (n=14)
surface

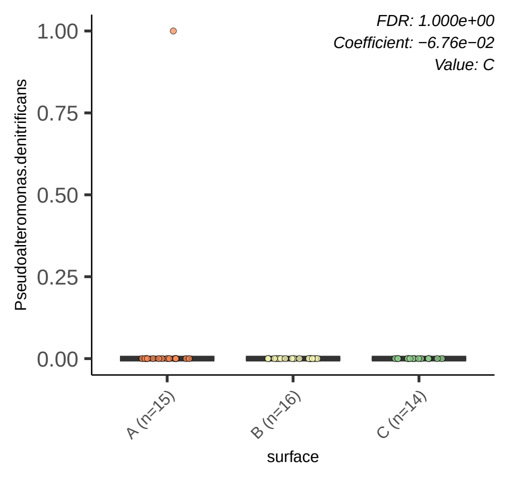
FDR: 1.000e+00
Coefficient: −6.76e−02
Value: C
1.00
0.75
0.50
0.25
0.00
Pseudoalteromonas.denitrificans
A (n=15)
B (n=16)
C (n=14)
surface

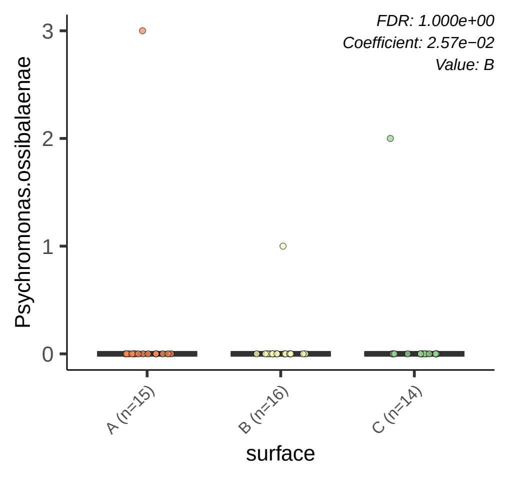

FDR: 1.000e+00
Coefficient: 2.57e−02
Value: B
Psychromonas.ossibalaenae
3
2
1
0
A (n=15)
B (n=16)
C (n=14)
surface

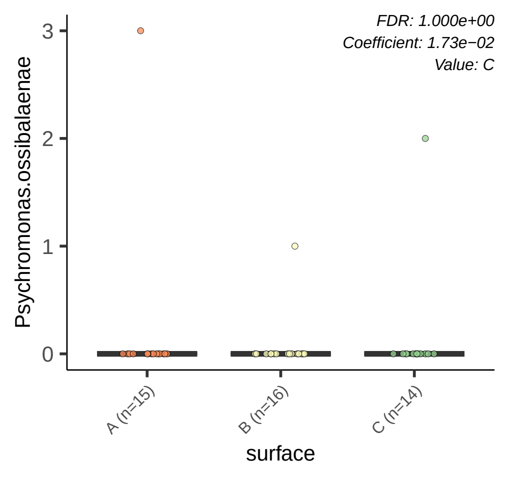
FDR: 1.000e+00
Coefficient: 1.73e−02
Value: C
Psychromonas.ossibalaenae
3
2
1
0
A (n=15)
B (n=16)
C (n=14)
surface

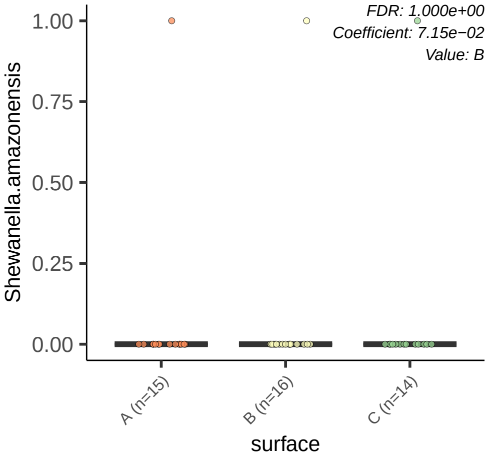
FDR: 1.000e+00
Coefficient: 7.15e−02
Value: B
Shewanella.amazonensis
1.00
0.75
0.50
0.25
0.00
A (n=15)
B (n=16)
C (n=14)
surface

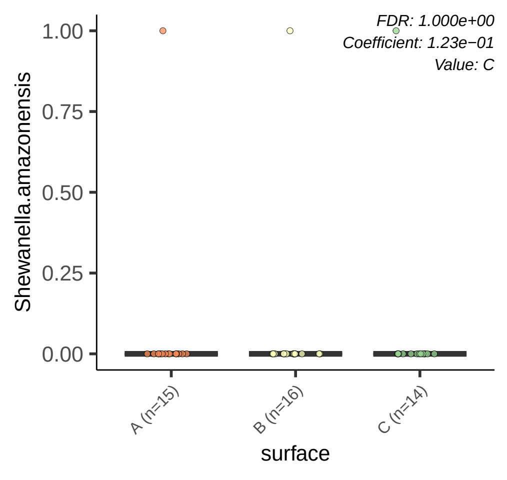

FDR: 1.000e+00
Coefficient: 1.23e−01
Value: C
Shewanella.amazonensis
1.00
0.75
0.50
0.25
0.00
A (n=15)
B (n=16)
C (n=14)
surface

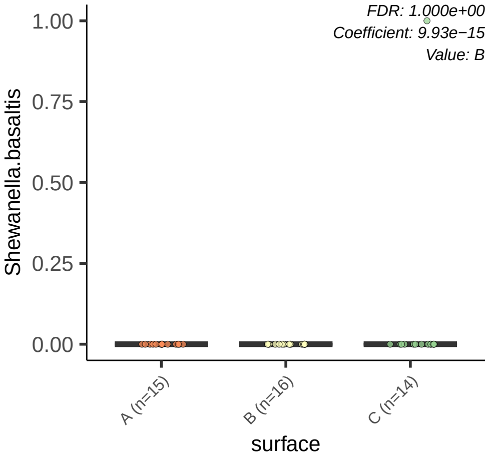
FDR: 1.000e+00
Coefficient: 9.93e−15
Value: B
Shewanella.basaltis
1.00
0.75
0.50
0.25
0.00
A (n=15)
B (n=16)
C (n=14)
surface

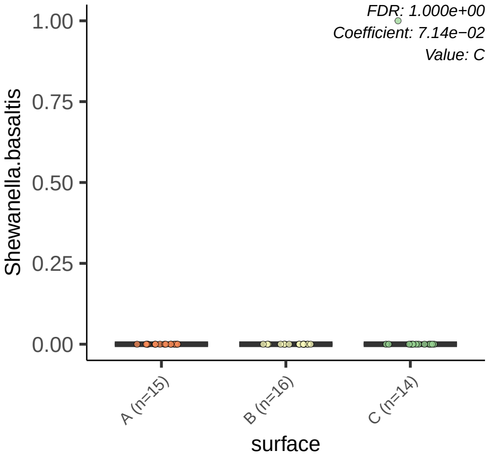
FDR: 1.000e+00
Coefficient: 7.14e−02
Value: C
1.00
0.75
0.50
0.25
0.00
Shewanella.basaltis
A (n=15)
B (n=16)
C (n=14)
surface

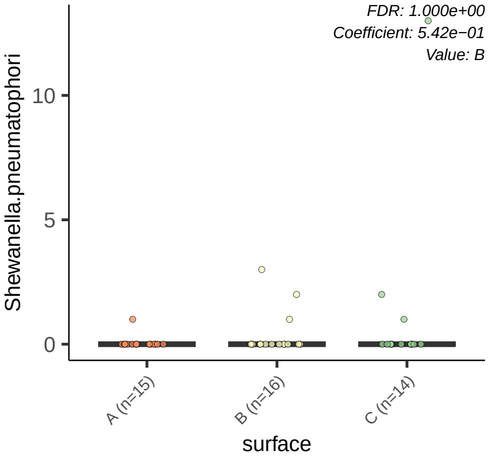
FDR: 1.000e+00
Coefficient: 5.42e−01
Value: B
Shewanella.pneumatophori
10
5
0
A (n=15)
B (n=16)
C (n=14)
surface

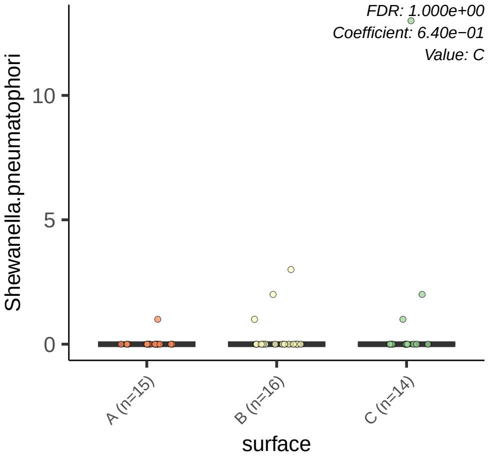
FDR: 1.000e+00
Coefficient: 6.40e−01
Value: C
Shewanella.pneumatophori
10
5
0
A (n=15)
B (n=16)
C (n=14)
surface

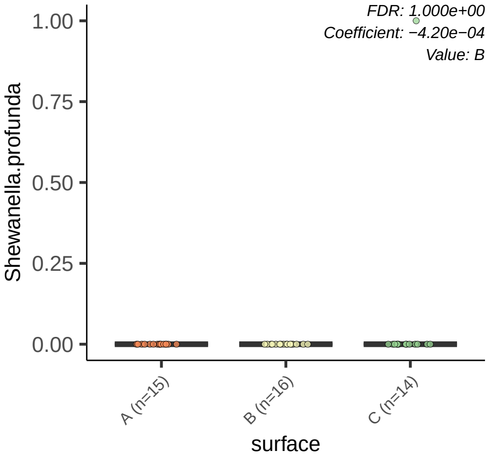
FDR: 1.000e+00
Coefficient: −4.20e−04
Value: B
Shewanella.profunda
1.00
0.75
0.50
0.25
0.00
A (n=15)
B (n=16)
C (n=14)
surface

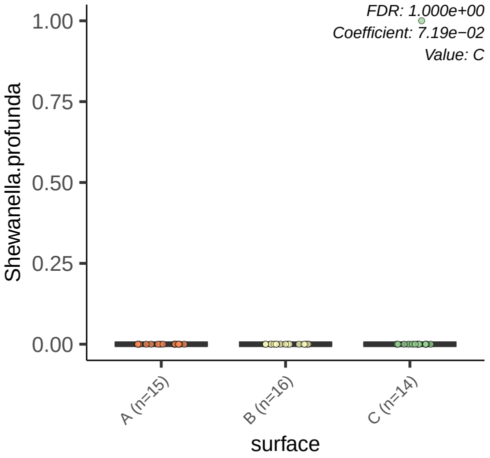
FDR: 1.000e+00
Coefficient: 7.19e−02
Value: C
Shewanella.profunda
1.00
0.75
0.50
0.25
0.00
A (n=15)
B (n=16)
C (n=14)
surface

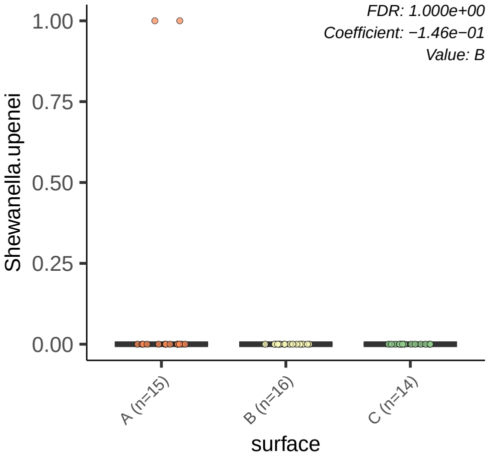
FDR: 1.000e+00
Coefficient: −1.46e−01
Value: B
Shewanella.upenei
1.00
0.75
0.50
0.25
0.00
A (n=15)
B (n=16)
C (n=14)
surface

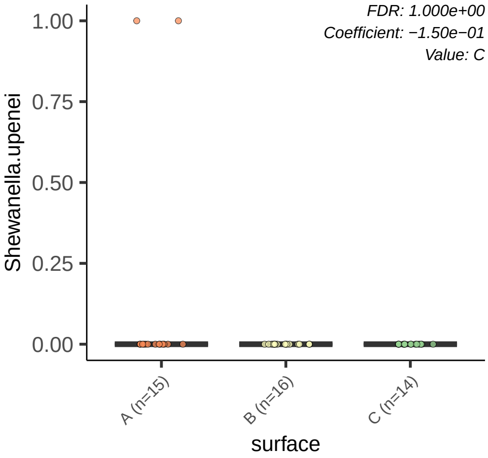
FDR: 1.000e+00
Coefficient: −1.50e−01
Value: C
Shewanella.upenei
1.00
0.75
0.50
0.25
0.00
A (n=15)
B (n=16)
C (n=14)
surface

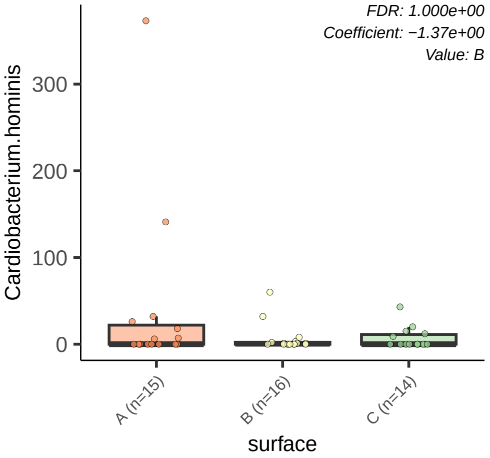
FDR: 1.000e+00
Coefficient: −1.37e+00
Value: B
Cardiobacterium.hominis
300
200
100
0
A (n=15)
B (n=16)
C (n=14)
surface

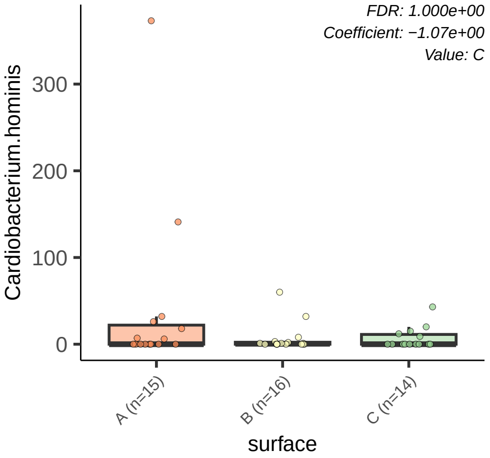
FDR: 1.000e+00
Coefficient: −1.07e+00
Value: C
Cardiobacterium.hominis
300
200
100
0
A (n=15)
B (n=16)
C (n=14)
surface

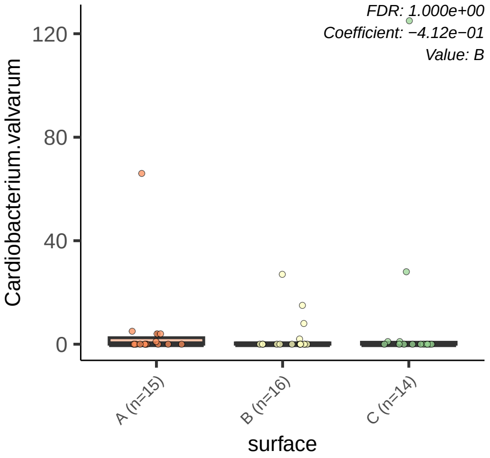
FDR: 1.000e+00
Coefficient: −4.12e−01
Value: B
Cardiobacterium.valvarum
120
80
40
0
A (n=15)
B (n=16)
C (n=14)
surface

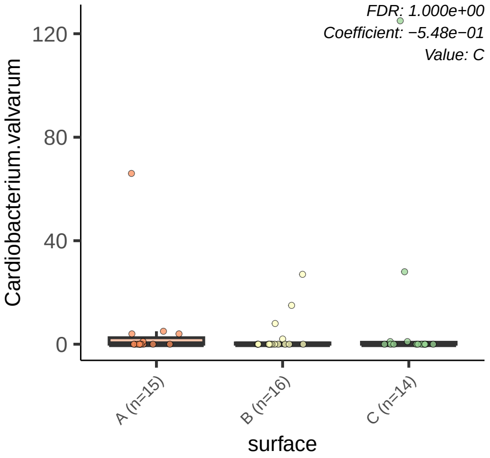

FDR: 1.000e+00
Coefficient: −5.48e−01
Value: C
Cardiobacterium.valvarum
120
80
40
0
A (n=15)
B (n=16)
C (n=14)
surface

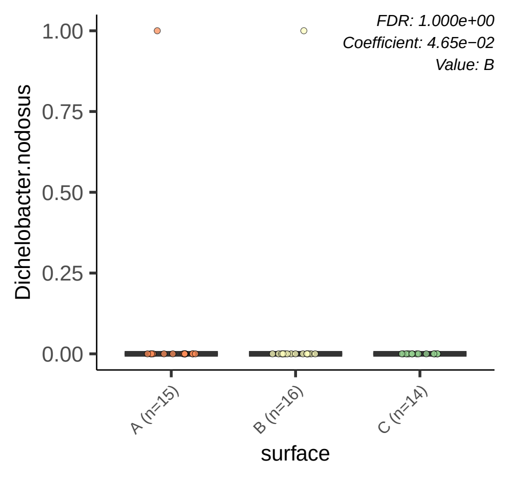

FDR: 1.000e+00
Coefficient: 4.65e−02
Value: B
Dichelobacter.nodosus
1.00
0.75
0.50
0.25
0.00
A (n=15)
B (n=16)
C (n=14)
surface

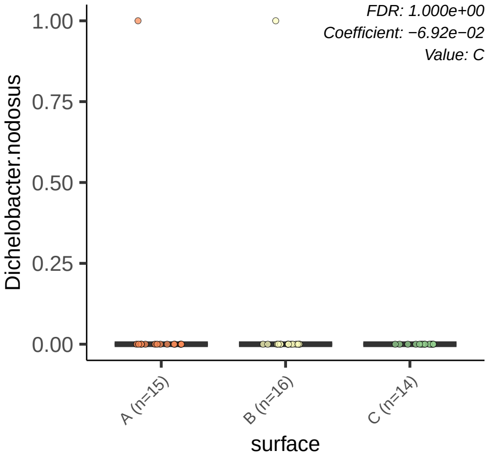
FDR: 1.000e+00
Coefficient: −6.92e−02
Value: C
Dichelobacter.nodosus
1.00
0.75
0.50
0.25
0.00
A (n=15)
B (n=16)
C (n=14)
surface

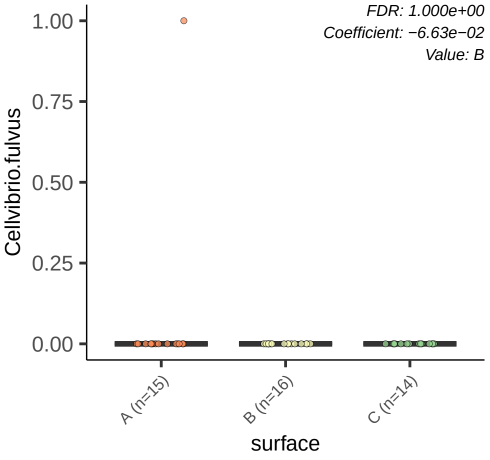

FDR: 1.000e+00
Coefficient: −6.63e−02
Value: B
1.00
0.75
0.50
0.25
0.00
Cellvibrio.fulvus
A (n=15)
B (n=16)
C (n=14)
surface

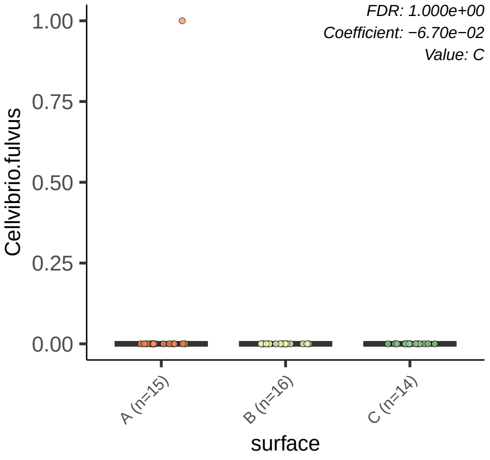
FDR: 1.000e+00
Coefficient: −6.70e−02
Value: C
Cellvibrio.fulvus
1.00
0.75
0.50
0.25
0.00
A (n=15)
B (n=16)
C (n=14)
surface

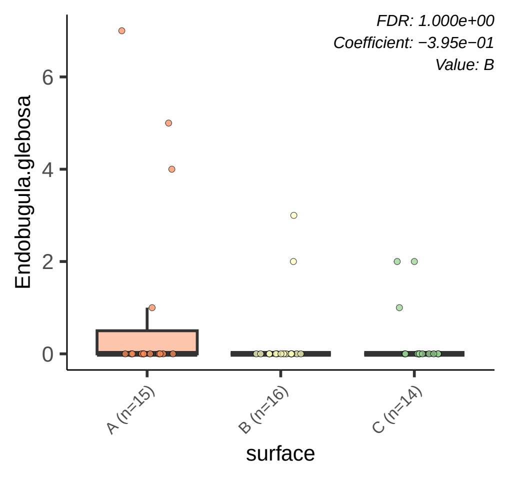

FDR: 1.000e+00
Coefficient: −3.95e−01
Value: B
Endobugula.glebosa
0
2
4
6
A (n=15)
B (n=16)
C (n=14)
surface

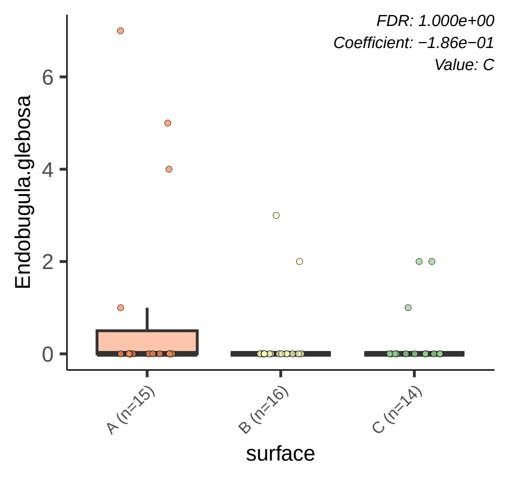
FDR: 1.000e+00
Coefficient: −1.86e−01
Value: C
Endobugula.glebosa
0
2
4
6
A (n=15)
B (n=16)
C (n=14)
surface

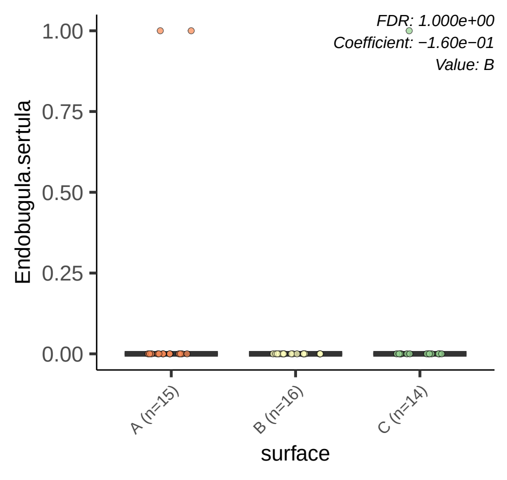

FDR: 1.000e+00
Coefficient: −1.60e−01
Value: B
Endobugula.sertula
1.00
0.75
0.50
0.25
0.00
A (n=15)
B (n=16)
C (n=14)
surface

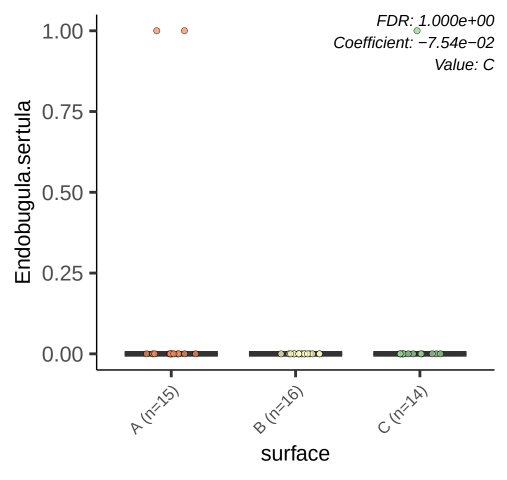

FDR: 1.000e+00
Coefficient: −7.54e−02
Value: C
1.00
0.75
0.50
0.25
0.00
Endobugula.sertula
A (n=15)
B (n=16)
C (n=14)
surface

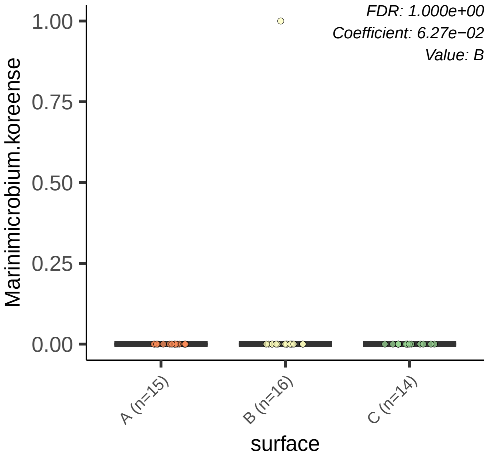

FDR: 1.000e+00
Coefficient: 6.27e−02
Value: B
Marinimicrobium.koreense
1.00
0.75
0.50
0.25
0.00
A (n=15)
B (n=16)
C (n=14)
surface

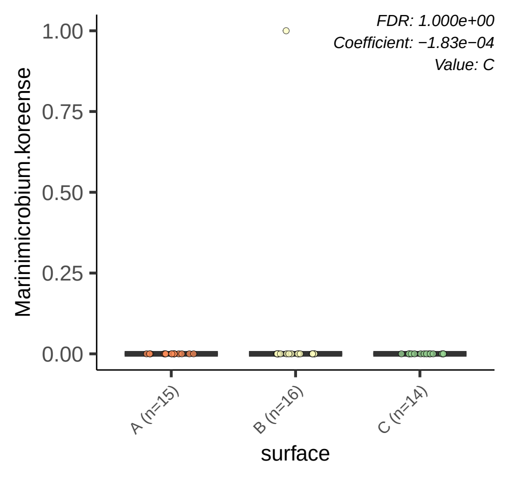
FDR: 1.000e+00
Coefficient: −1.83e−04
Value: C
Marinimicrobium.koreense
1.00
0.75
0.50
0.25
0.00
A (n=15)
B (n=16)
C (n=14)
surface

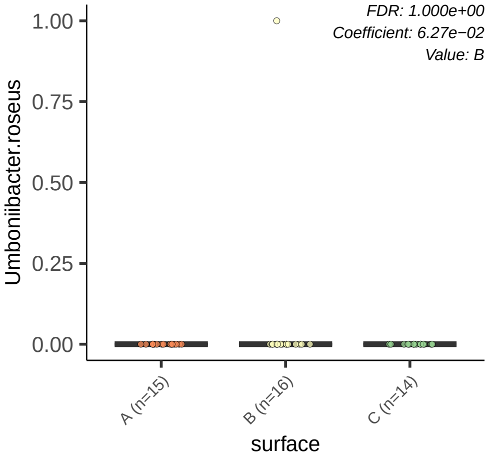
FDR: 1.000e+00
Coefficient: 6.27e−02
Value: B
Umboniibacter.roseus
1.00
0.75
0.50
0.25
0.00
A (n=15)
B (n=16)
C (n=14)
surface

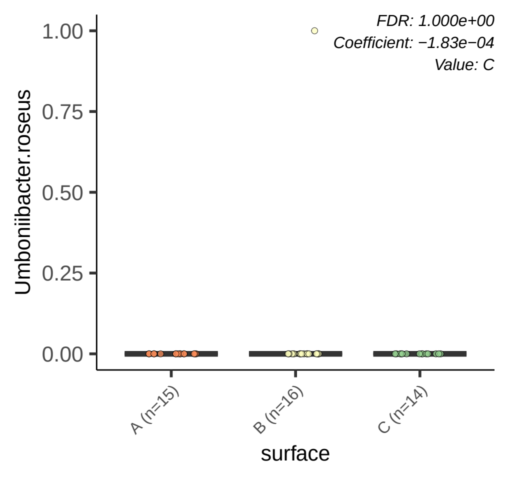

FDR: 1.000e+00
Coefficient: −1.83e−04
Value: C
Umboniibacter.roseus
1.00
0.75
0.50
0.25
0.00
A (n=15)
B (n=16)
C (n=14)
surface

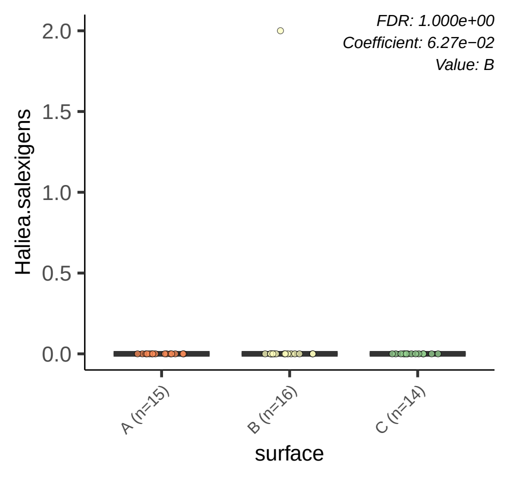
FDR: 1.000e+00
Coefficient: 6.27e−02
Value: B
Haliea.salexigens
2.0
1.5
1.0
0.5
0.0
A (n=15)
B (n=16)
C (n=14)
surface

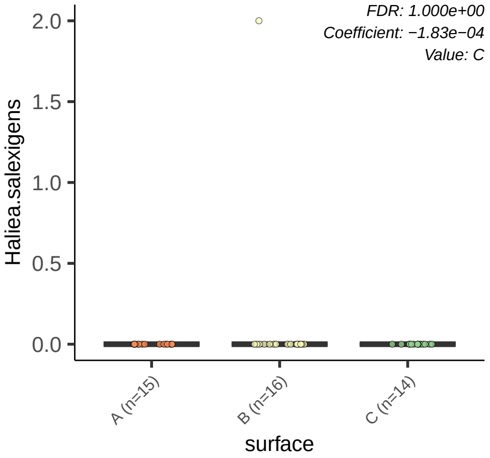
FDR: 1.000e+00
Coefficient: −1.83e−04
Value: C
Haliea.salexigens
2.0
1.5
1.0
0.5
0.0
A (n=15)
B (n=16)
C (n=14)
surface

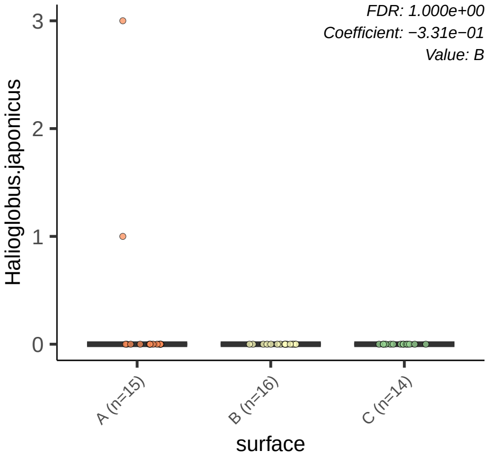

FDR: 1.000e+00
Coefficient: −3.31e−01
Value: B
Halioglobus.japonicus
3
2
1
0
A (n=15)
B (n=16)
C (n=14)
surface

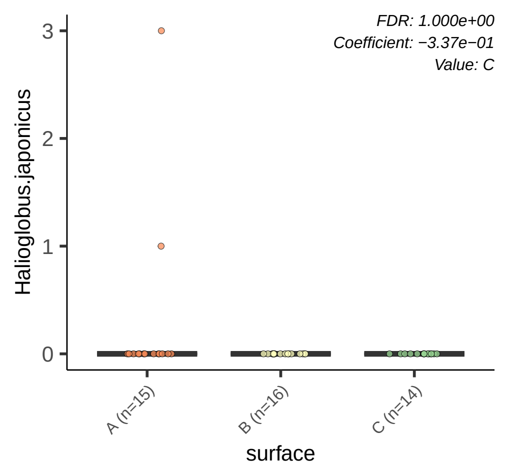

FDR: 1.000e+00
Coefficient: −3.37e−01
Value: C
Halioglobus.japonicus
3
2
1
0
A (n=15)
B (n=16)
C (n=14)
surface

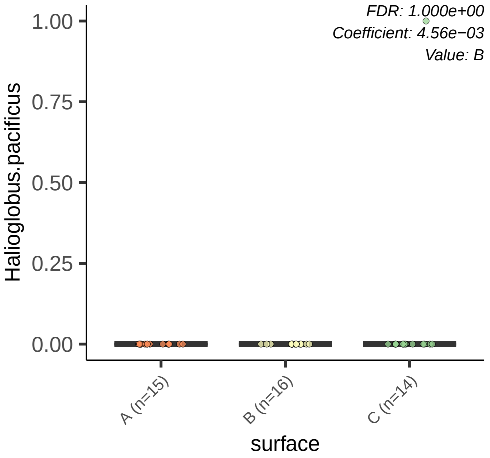
FDR: 1.000e+00
Coefficient: 4.56e−03
Value: B
Halioglobus.pacificus
1.00
0.75
0.50
0.25
0.00
A (n=15)
B (n=16)
C (n=14)
surface

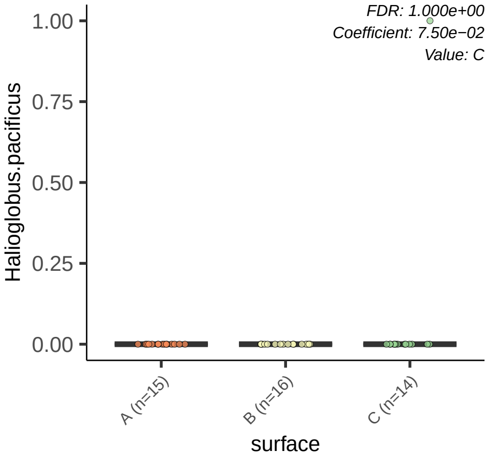

FDR: 1.000e+00
Coefficient: 7.50e−02
Value: C
Halioglobus.pacificus
1.00
0.75
0.50
0.25
0.00
A (n=15)
B (n=16)
C (n=14)
surface

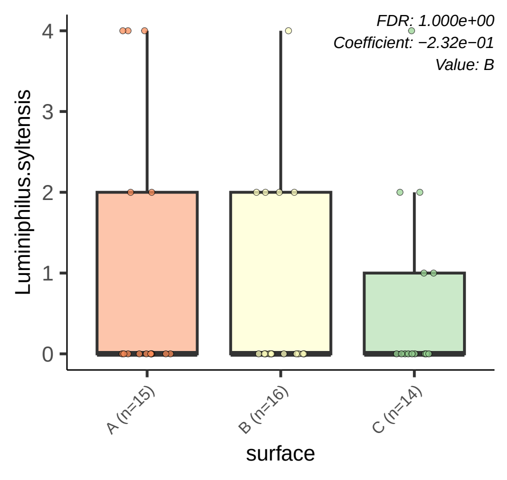

FDR: 1.000e+00
Coefficient: −2.32e−01
Value: B
Luminiphilus.syltensis
0
1
2
3
4
A (n=15)
B (n=16)
C (n=14)
surface

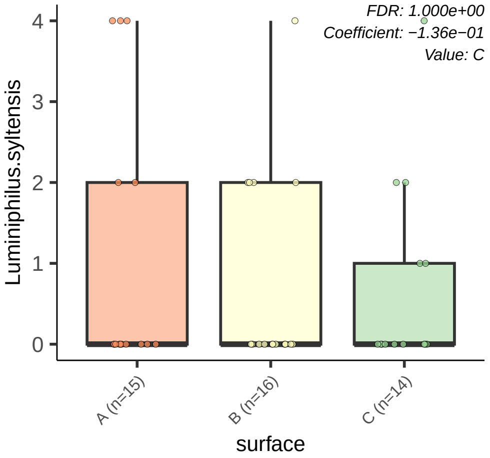
FDR: 1.000e+00
Coefficient: −1.36e−01
Value: C
Luminiphilus.syltensis
0
1
2
3
4
A (n=15)
B (n=16)
C (n=14)
surface

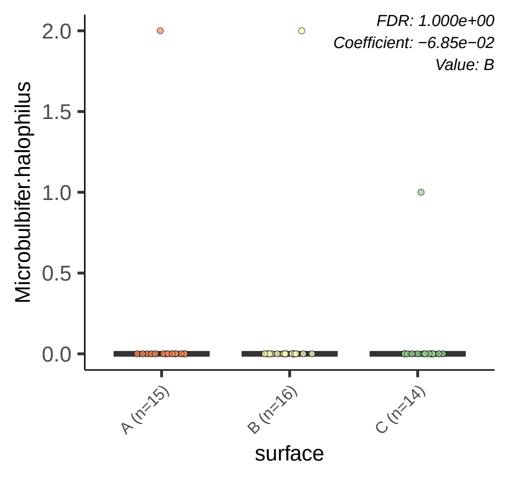
FDR: 1.000e+00
Coefficient: −6.85e−02
Value: B
Microbulbifer.halophilus
2.0
1.5
1.0
0.5
0.0
A (n=15)
B (n=16)
C (n=14)
surface

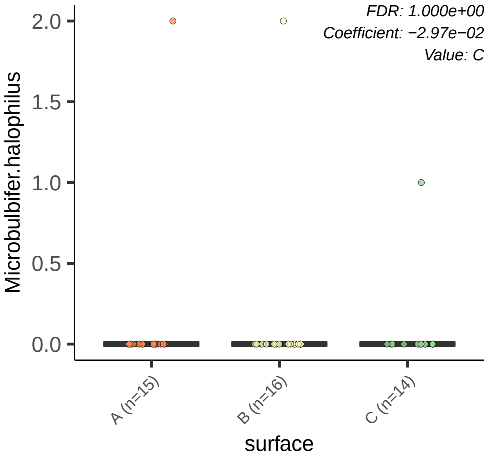
FDR: 1.000e+00
Coefficient: −2.97e−02
Value: C
Microbulbifer.halophilus
2.0
1.5
1.0
0.5
0.0
A (n=15)
B (n=16)
C (n=14)
surface

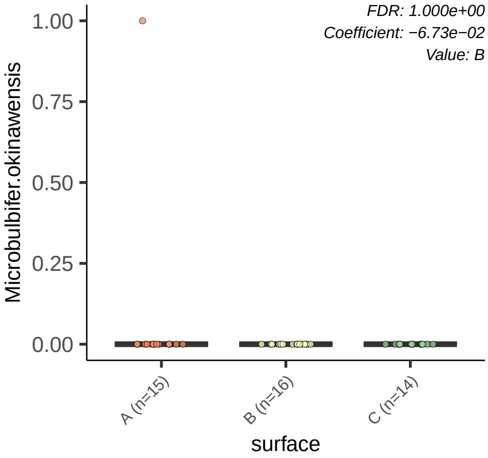

FDR: 1.000e+00
Coefficient: −6.73e−02
Value: B
Microbulbifer.okinawensis
1.00
0.75
0.50
0.25
0.00
A (n=15)
B (n=16)
C (n=14)
surface

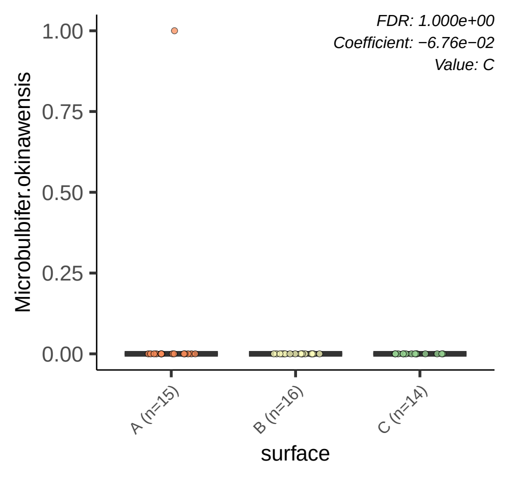
FDR: 1.000e+00
Coefficient: −6.76e−02
Value: C
Microbulbifer.okinawensis
1.00
0.75
0.50
0.25
0.00
A (n=15)
B (n=16)
C (n=14)
surface

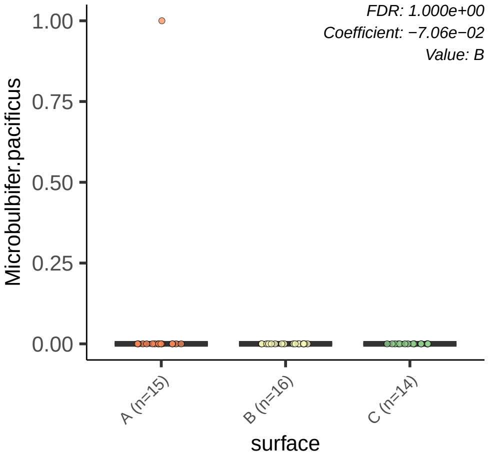

FDR: 1.000e+00
Coefficient: −7.06e−02
Value: B
Microbulbifer.pacificus
1.00
0.75
0.50
0.25
0.00
A (n=15)
B (n=16)
C (n=14)
surface

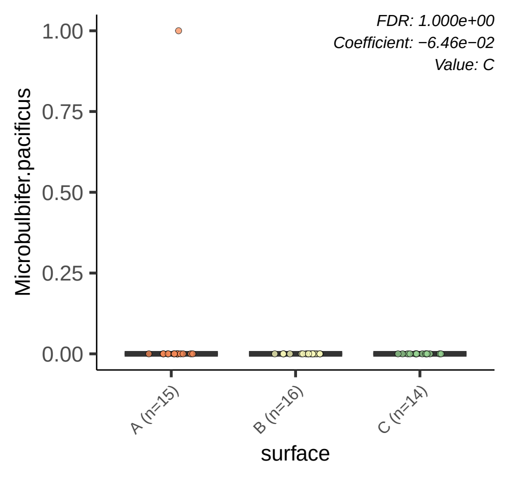
FDR: 1.000e+00
Coefficient: −6.46e−02
Value: C
Microbulbifer.pacificus
1.00
0.75
0.50
0.25
0.00
A (n=15)
B (n=16)
C (n=14)
surface

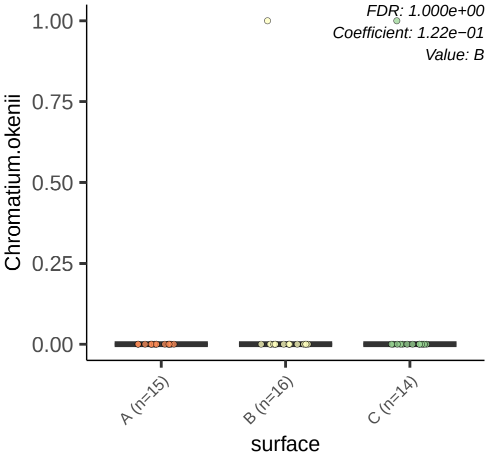

FDR: 1.000e+00
Coefficient: 1.22e−01
Value: B
Chromatium.okenii
1.00
0.75
0.50
0.25
0.00
A (n=15)
B (n=16)
C (n=14)
surface

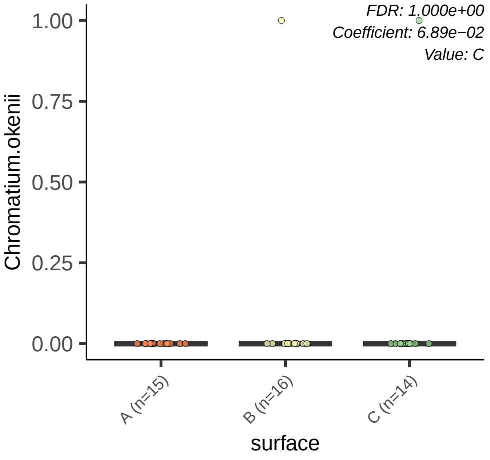
FDR: 1.000e+00
Coefficient: 6.89e−02
Value: C
Chromatium.okenii
1.00
0.75
0.50
0.25
0.00
A (n=15)
B (n=16)
C (n=14)
surface

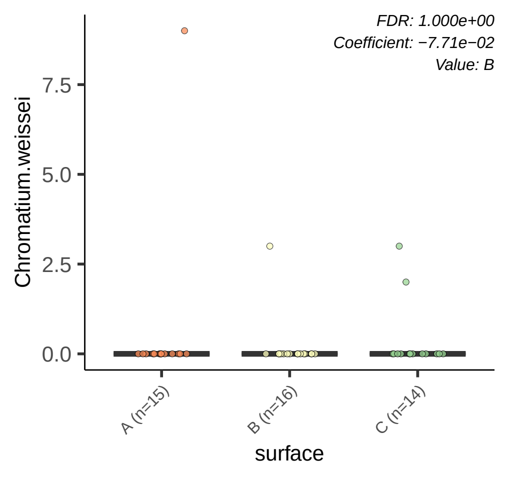
FDR: 1.000e+00
Coefficient: −7.71e−02
Value: B
Chromatium.weissei
7.5
5.0
2.5
0.0
A (n=15)
B (n=16)
C (n=14)
surface

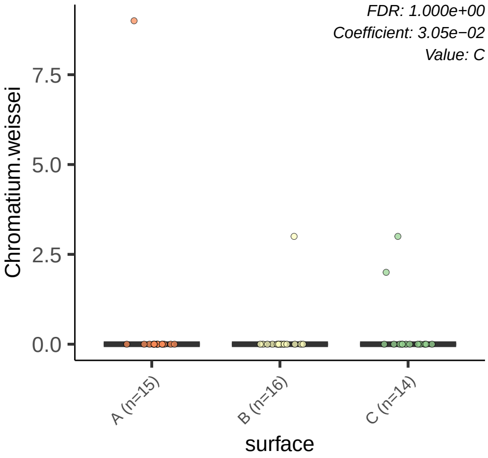

FDR: 1.000e+00
Coefficient: 3.05e−02
Value: C
Chromatium.weissei
7.5
5.0
2.5
0.0
A (n=15)
B (n=16)
C (n=14)
surface

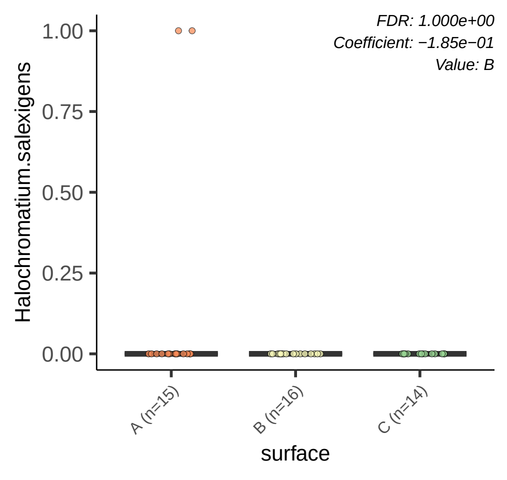

FDR: 1.000e+00
Coefficient: −1.85e−01
Value: B
Halochromatium.salexigens
1.00
0.75
0.50
0.25
0.00
A (n=15)
B (n=16)
C (n=14)
surface

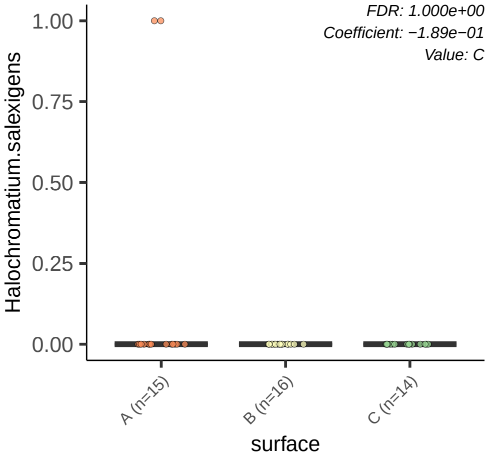
FDR: 1.000e+00
Coefficient: −1.89e−01
Value: C
Halochromatium.salexigens
1.00
0.75
0.50
0.25
0.00
A (n=15)
B (n=16)
C (n=14)
surface

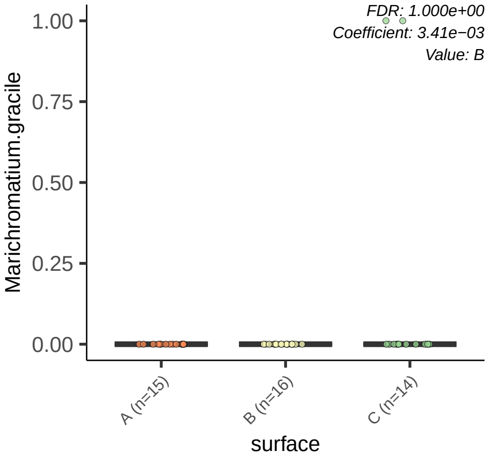
FDR: 1.000e+00
Coefficient: 3.41e−03
Value: B
Marichromatium.gracile
1.00
0.75
0.50
0.25
0.00
A (n=15)
B (n=16)
C (n=14)
surface

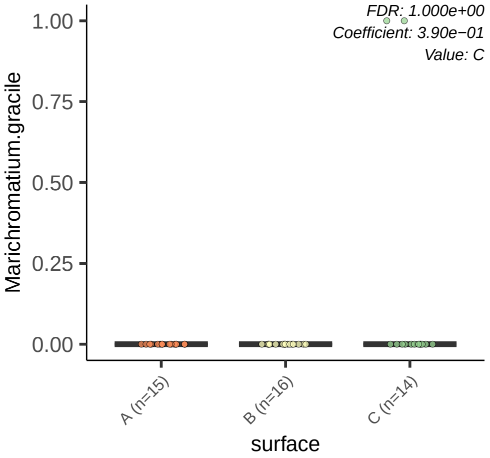
FDR: 1.000e+00
Coefficient: 3.90e−01
Value: C
Marichromatium.gracile
1.00
0.75
0.50
0.25
0.00
A (n=15)
B (n=16)
C (n=14)
surface

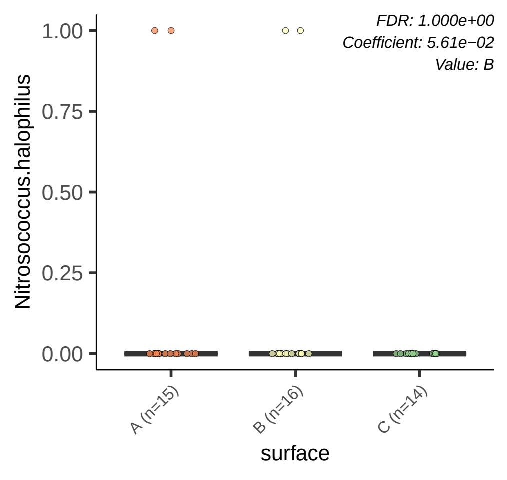
FDR: 1.000e+00
Coefficient: 5.61e−02
Value: B
Nitrosococcus.halophilus
1.00
0.75
0.50
0.25
0.00
A (n=15)
B (n=16)
C (n=14)
surface

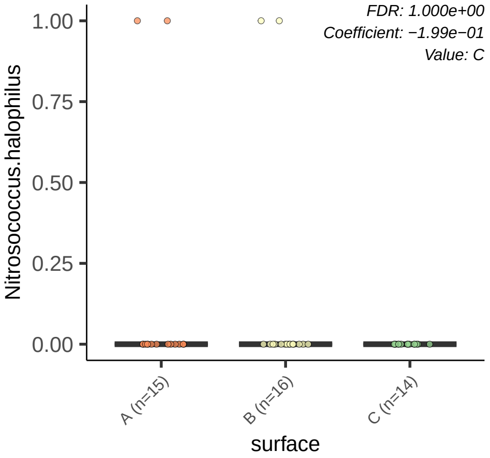
FDR: 1.000e+00
Coefficient: −1.99e−01
Value: C
Nitrosococcus.halophilus
1.00
0.75
0.50
0.25
0.00
A (n=15)
B (n=16)
C (n=14)
surface

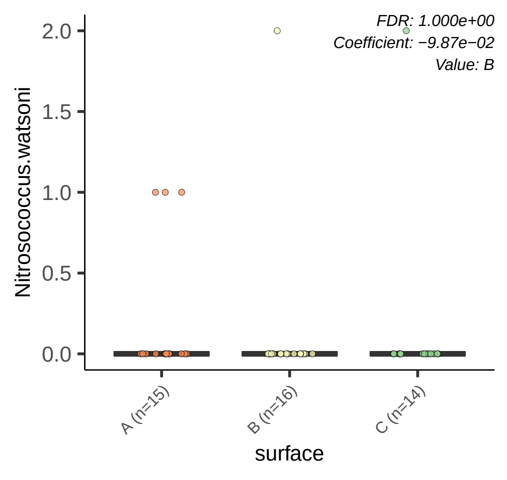
FDR: 1.000e+00
Coefficient: −9.87e−02
Value: B
Nitrosococcus.watsoni
2.0
1.5
1.0
0.5
0.0
A (n=15)
B (n=16)
C (n=14)
surface

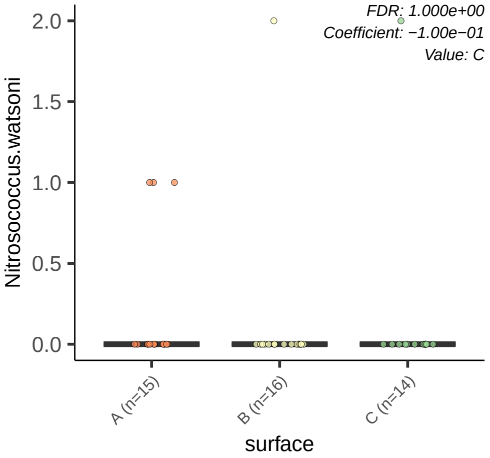
FDR: 1.000e+00
Coefficient: −1.00e−01
Value: C
Nitrosococcus.watsoni
2.0
1.5
1.0
0.5
0.0
A (n=15)
B (n=16)
C (n=14)
surface

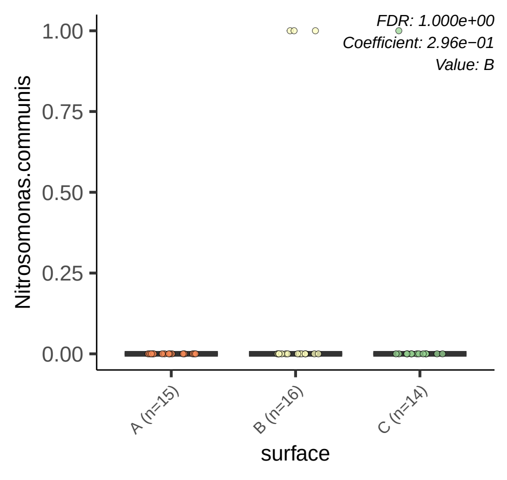
FDR: 1.000e+00
Coefficient: 2.96e−01
Value: B
Nitrosomonas.communis
1.00
0.75
0.50
0.25
0.00
A (n=15)
B (n=16)
C (n=14)
surface

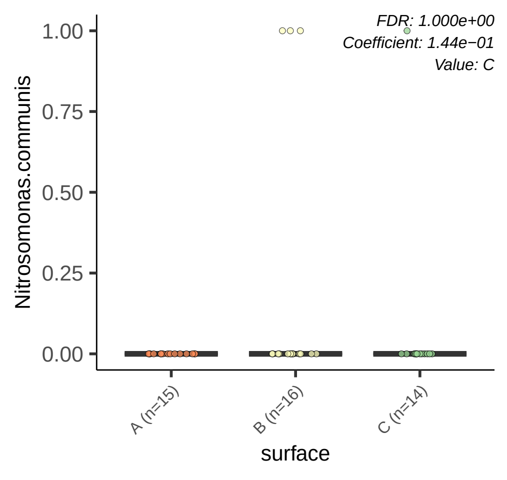
FDR: 1.000e+00
Coefficient: 1.44e−01
Value: C
Nitrosomonas.communis
1.00
0.75
0.50
0.25
0.00
A (n=15)
B (n=16)
C (n=14)
surface

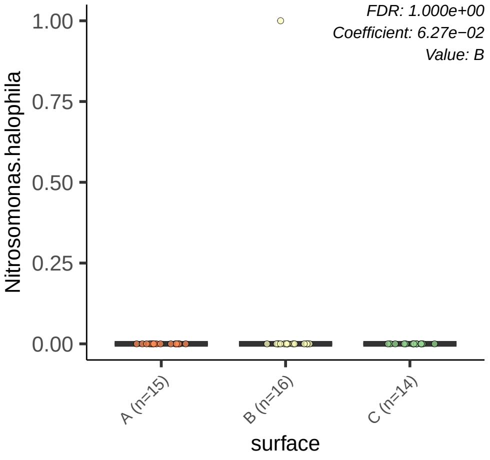
FDR: 1.000e+00
Coefficient: 6.27e−02
Value: B
Nitrosomonas.halophila
1.00
0.75
0.50
0.25
0.00
A (n=15)
B (n=16)
C (n=14)
surface

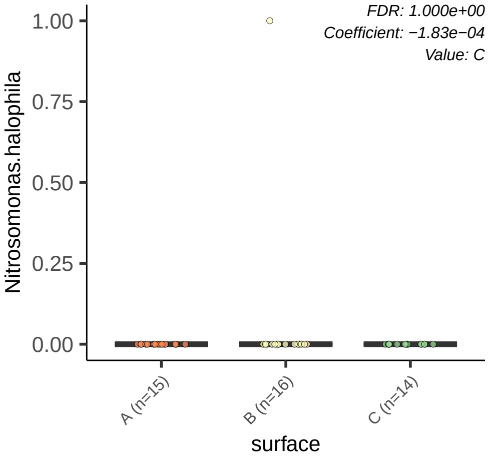

FDR: 1.000e+00
Coefficient: −1.83e−04
Value: C
Nitrosomonas.halophila
1.00
0.75
0.50
0.25
0.00
A (n=15)
B (n=16)
C (n=14)
surface

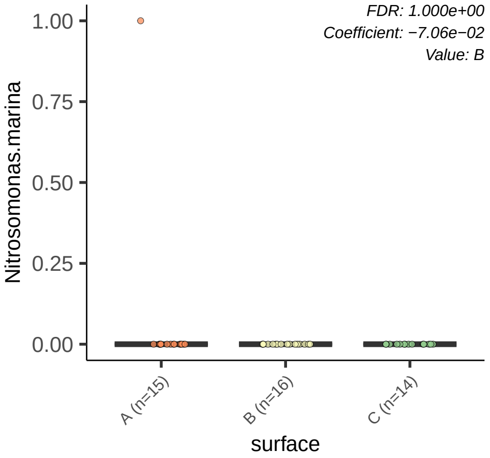

FDR: 1.000e+00
Coefficient: −7.06e−02
Value: B
Nitrosomonas.marina
1.00
0.75
0.50
0.25
0.00
A (n=15)
B (n=16)
C (n=14)
surface

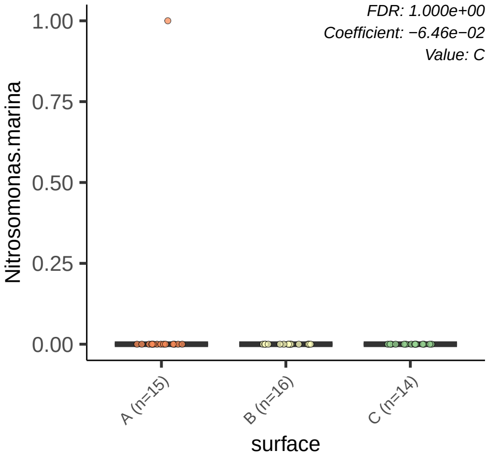

FDR: 1.000e+00
Coefficient: −6.46e−02
Value: C
Nitrosomonas.marina
1.00
0.75
0.50
0.25
0.00
A (n=15)
B (n=16)
C (n=14)
surface

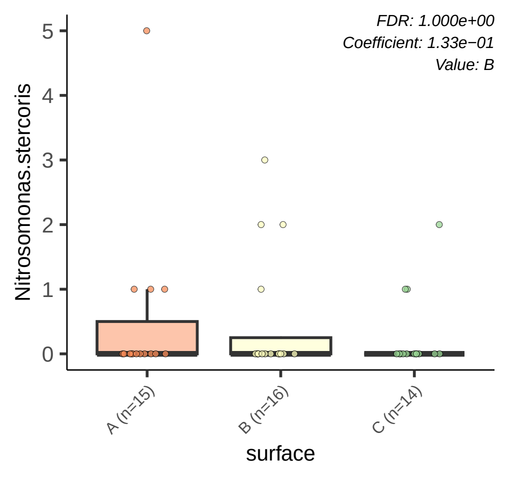
FDR: 1.000e+00
Coefficient: 1.33e−01
Value: B
Nitrosomonas.stercoris
0
1
2
3
4
5
A (n=15)
B (n=16)
C (n=14)
surface

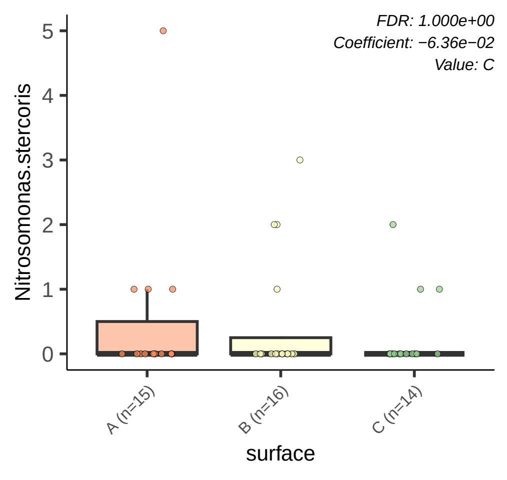
FDR: 1.000e+00
Coefficient: −6.36e−02
Value: C
Nitrosomonas.stercoris
0
1
2
3
4
5
A (n=15)
B (n=16)
C (n=14)
surface

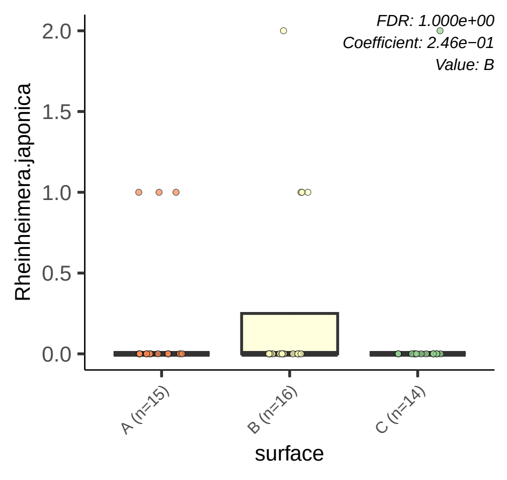
FDR: 1.000e+00
Coefficient: 2.46e−01
Value: B
Rheinheimera.japonica
2.0
1.5
1.0
0.5
0.0
A (n=15)
B (n=16)
C (n=14)
surface

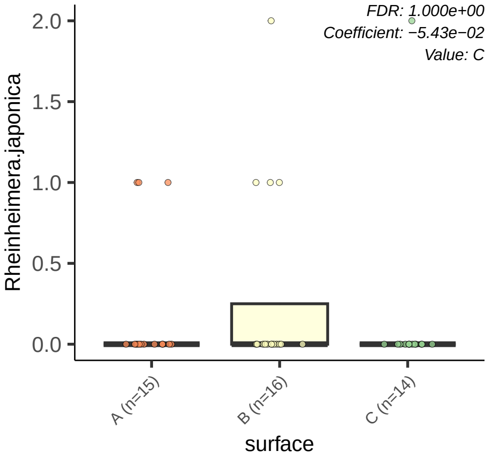
FDR: 1.000e+00
Coefficient: −5.43e−02
Value: C
Rheinheimera.japonica
2.0
1.5
1.0
0.5
0.0
A (n=15)
B (n=16)
C (n=14)
surface

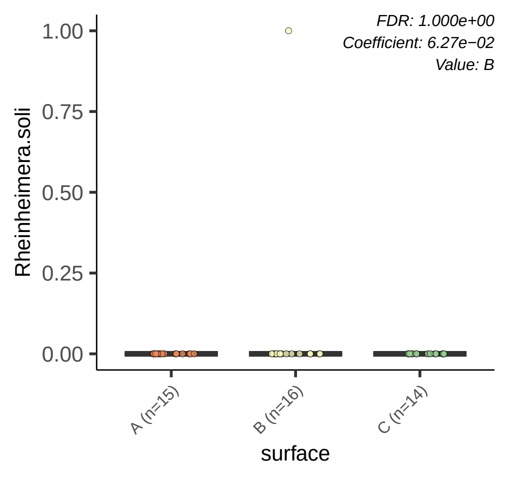
FDR: 1.000e+00
Coefficient: 6.27e−02
Value: B
Rheinheimera.soli
1.00
0.75
0.50
0.25
0.00
A (n=15)
B (n=16)
C (n=14)
surface

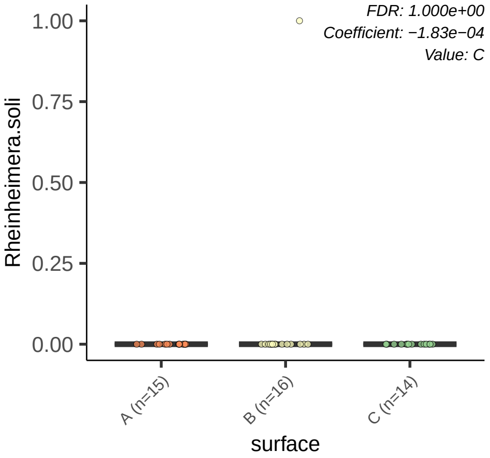
FDR: 1.000e+00
Coefficient: −1.83e−04
Value: C
Rheinheimera.soli
1.00
0.75
0.50
0.25
0.00
A (n=15)
B (n=16)
C (n=14)
surface

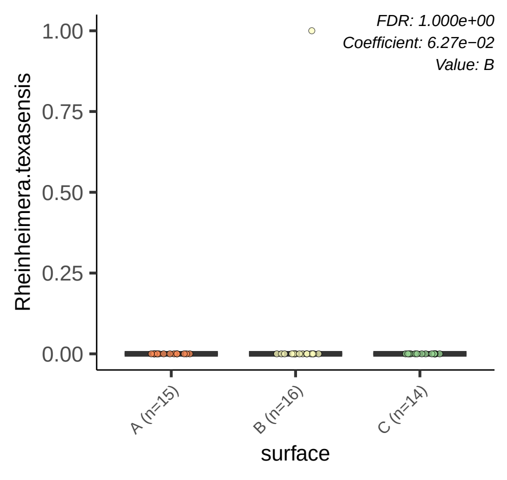
FDR: 1.000e+00
Coefficient: 6.27e−02
Value: B
Rheinheimera.texasensis
1.00
0.75
0.50
0.25
0.00
A (n=15)
B (n=16)
C (n=14)
surface

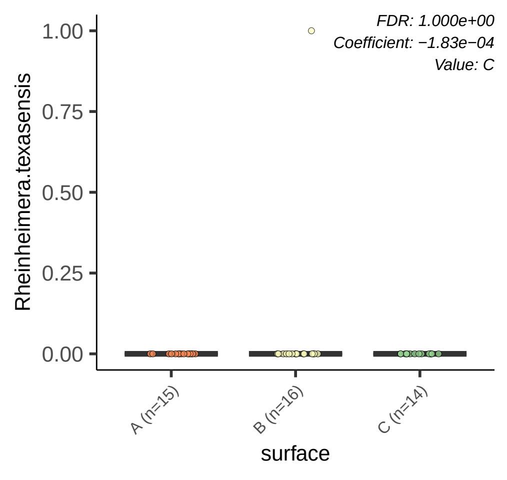

FDR: 1.000e+00
Coefficient: −1.83e−04
Value: C
Rheinheimera.texasensis
1.00
0.75
0.50
0.25
0.00
A (n=15)
B (n=16)
C (n=14)
surface

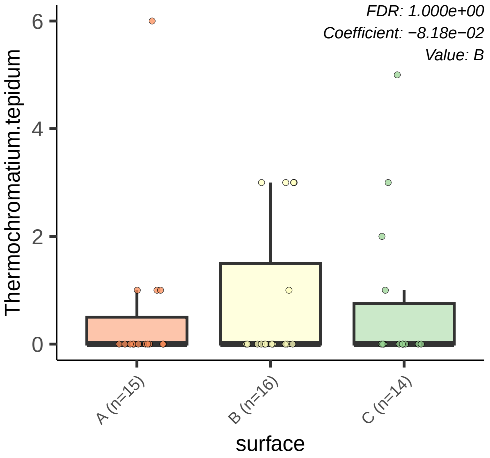
FDR: 1.000e+00
Coefficient: −8.18e−02
Value: B
Thermochromatium.tepidum
0
2
4
6
A (n=15)
B (n=16)
C (n=14)
surface

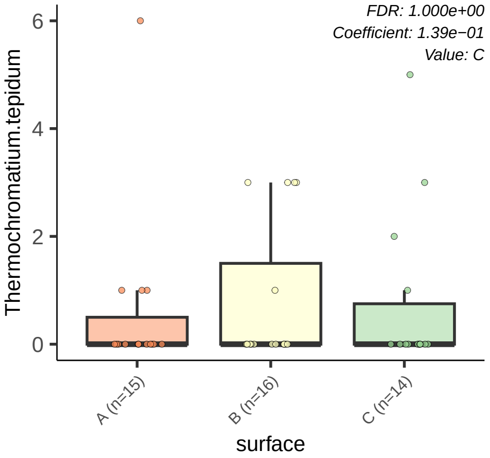
FDR: 1.000e+00
Coefficient: 1.39e−01
Value: C
Thermochromatium.tepidum
0
2
4
6
A (n=15)
B (n=16)
C (n=14)
surface

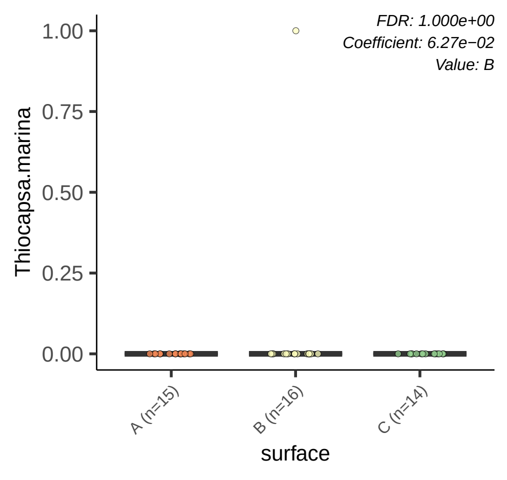
FDR: 1.000e+00
Coefficient: 6.27e−02
Value: B
Thiocapsa.marina
1.00
0.75
0.50
0.25
0.00
A (n=15)
B (n=16)
C (n=14)
surface

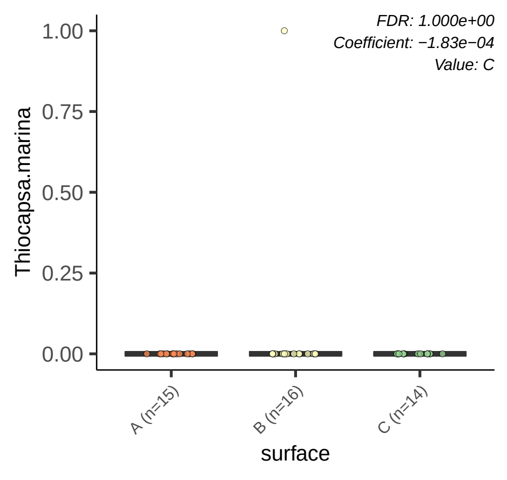

FDR: 1.000e+00
Coefficient: −1.83e−04
Value: C
Thiocapsa.marina
1.00
0.75
0.50
0.25
0.00
A (n=15)
B (n=16)
C (n=14)
surface

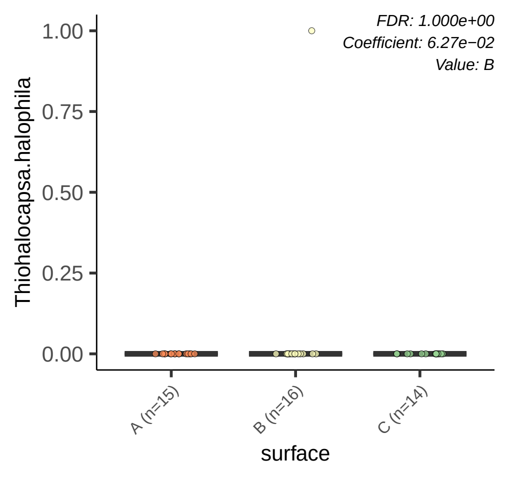
FDR: 1.000e+00
Coefficient: 6.27e−02
Value: B
Thiohalocapsa.halophila
1.00
0.75
0.50
0.25
0.00
A (n=15)
B (n=16)
C (n=14)
surface

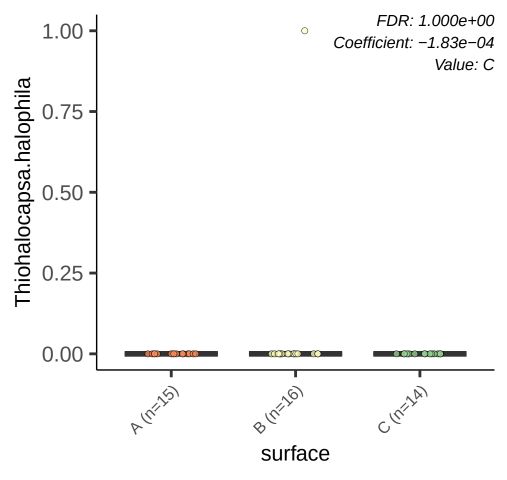
FDR: 1.000e+00
Coefficient: −1.83e−04
Value: C
Thiohalocapsa.halophila
1.00
0.75
0.50
0.25
0.00
A (n=15)
B (n=16)
C (n=14)
surface

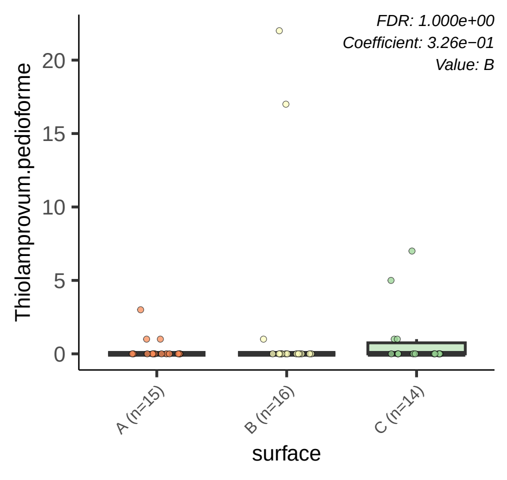

FDR: 1.000e+00
Coefficient: 3.26e−01
Value: B
Thiolamprovum.pedioforme
20
15
10
5
0
A (n=15)
B (n=16)
C (n=14)
surface

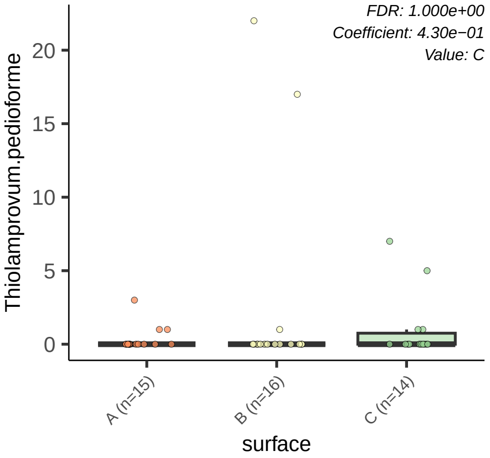

FDR: 1.000e+00
Coefficient: 4.30e−01
Value: C
Thiolamprovum.pedioforme
0
5
10
15
20
A (n=15)
B (n=16)
C (n=14)
surface

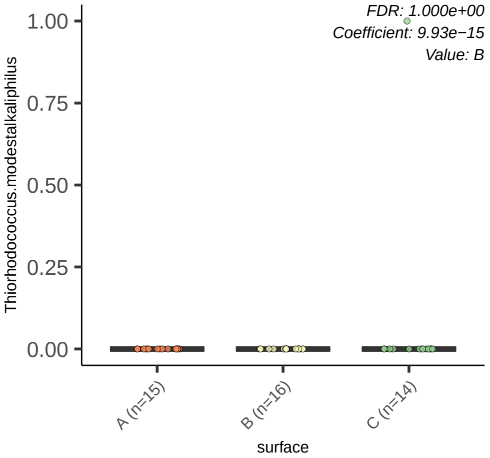
FDR: 1.000e+00
Coefficient: 9.93e−15
Value: B
Thiorhodococcus.modestalkaliphilus
1.00
0.75
0.50
0.25
0.00
A (n=15)
B (n=16)
C (n=14)
surface

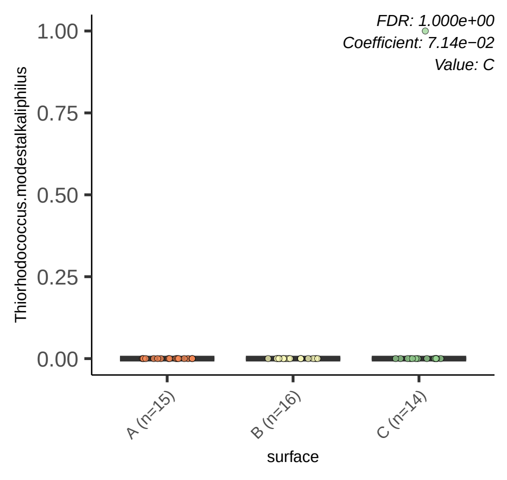

FDR: 1.000e+00
Coefficient: 7.14e−02
Value: C
Thiorhodococcus.modestalkaliphilus
1.00
0.75
0.50
0.25
0.00
A (n=15)
B (n=16)
C (n=14)
surface

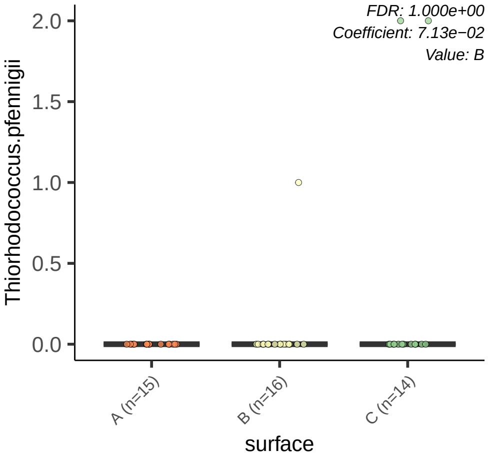
FDR: 1.000e+00
Coefficient: 7.13e−02
Value: B
Thiorhodococcus.pfennigii
2.0
1.5
1.0
0.5
0.0
A (n=15)
B (n=16)
C (n=14)
surface

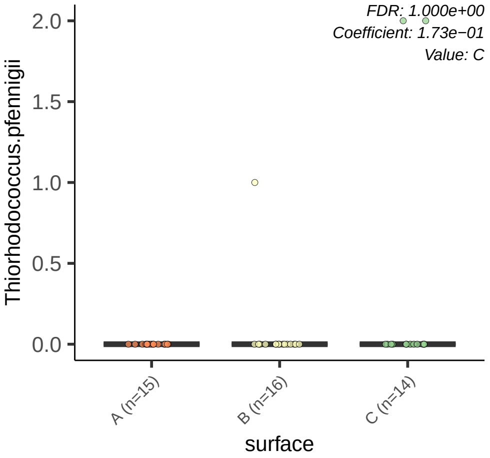
FDR: 1.000e+00
Coefficient: 1.73e−01
Value: C
Thiorhodococcus.pfennigii
2.0
1.5
1.0
0.5
0.0
A (n=15)
B (n=16)
C (n=14)
surface

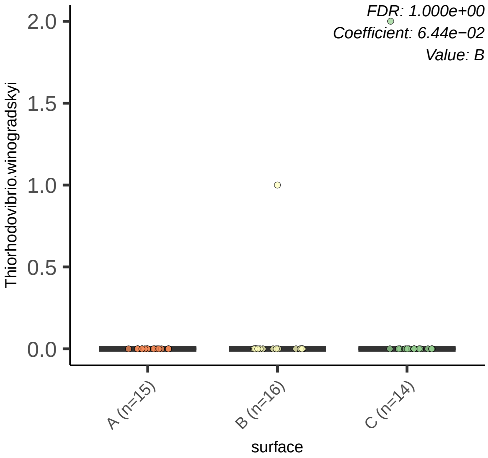

FDR: 1.000e+00
Coefficient: 6.44e−02
Value: B
Thiorhodovibrio.winogradskyi
2.0
1.5
1.0
0.5
0.0
A (n=15)
B (n=16)
C (n=14)
surface

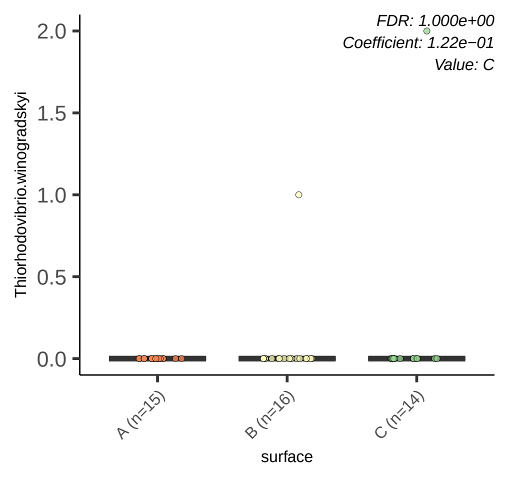

FDR: 1.000e+00
Coefficient: 1.22e−01
Value: C
Thiorhodovibrio.winogradskyi
2.0
1.5
1.0
0.5
0.0
A (n=15)
B (n=16)
C (n=14)
surface

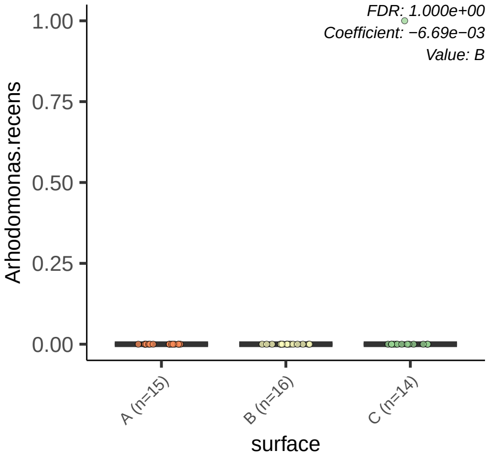
FDR: 1.000e+00
Coefficient: −6.69e−03
Value: B
Arhodomonas.recens
1.00
0.75
0.50
0.25
0.00
A (n=15)
B (n=16)
C (n=14)
surface

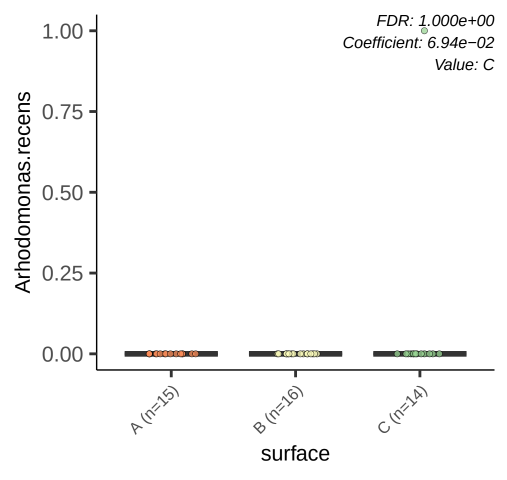
FDR: 1.000e+00
Coefficient: 6.94e−02
Value: C
Arhodomonas.recens
1.00
0.75
0.50
0.25
0.00
A (n=15)
B (n=16)
C (n=14)
surface

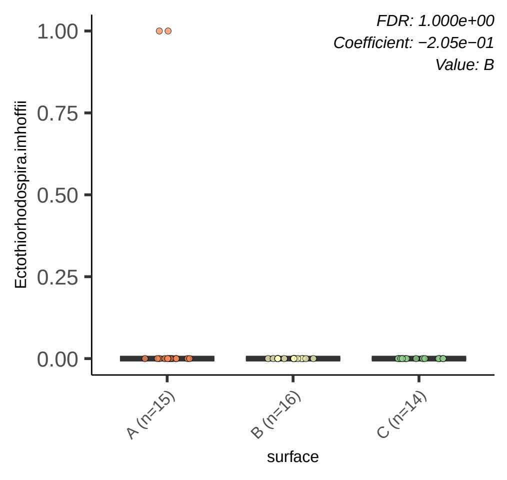

FDR: 1.000e+00
Coefficient: −2.05e−01
Value: B
1.00
0.75
0.50
0.25
0.00
Ectothiorhodospira.imhoffii
A (n=15)
B (n=16)
C (n=14)
surface

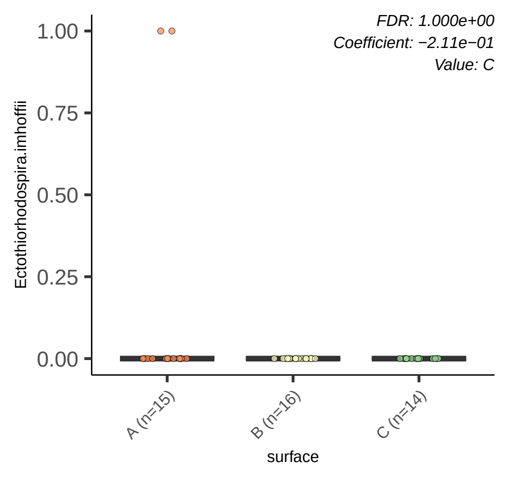

FDR: 1.000e+00
Coefficient: −2.11e−01
Value: C
1.00
0.75
0.50
0.25
0.00
Ectothiorhodospira.imhoffii
A (n=15)
B (n=16)
C (n=14)
surface

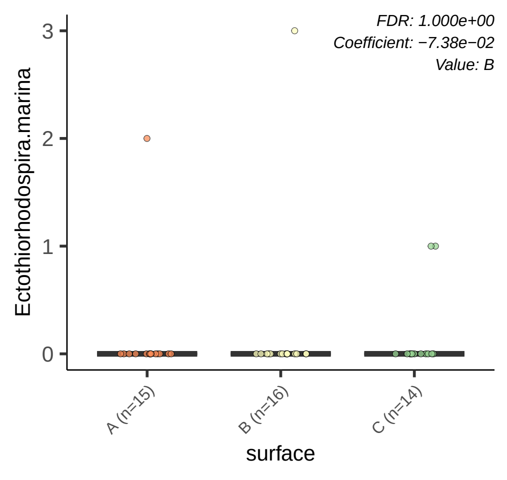

FDR: 1.000e+00
Coefficient: −7.38e−02
Value: B
Ectothiorhodospira.marina
0
1
2
3
A (n=15)
B (n=16)
C (n=14)
surface

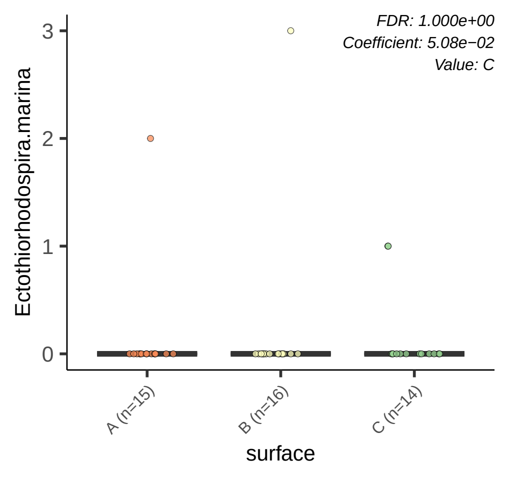
FDR: 1.000e+00
Coefficient: 5.08e−02
Value: C
Ectothiorhodospira.marina
0
1
2
3
A (n=15)
B (n=16)
C (n=14)
surface

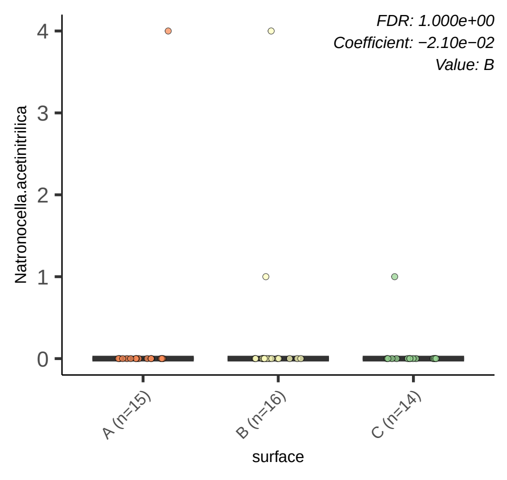
FDR: 1.000e+00
Coefficient: −2.10e−02
Value: B
Natronocella.acetinitrilica
4
3
2
1
0
A (n=15)
B (n=16)
C (n=14)
surface

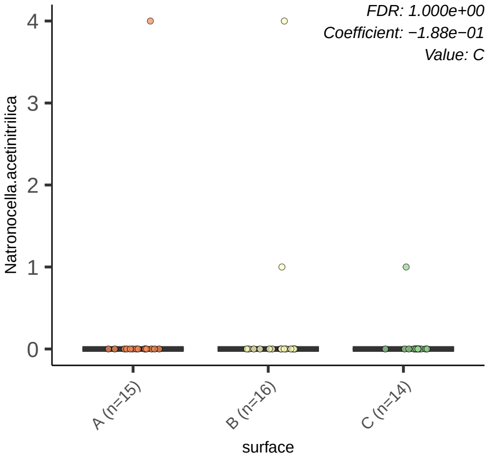
FDR: 1.000e+00
Coefficient: −1.88e−01
Value: C
Natronocella.acetinitrilica
0
1
2
3
4
A (n=15)
B (n=16)
C (n=14)
surface

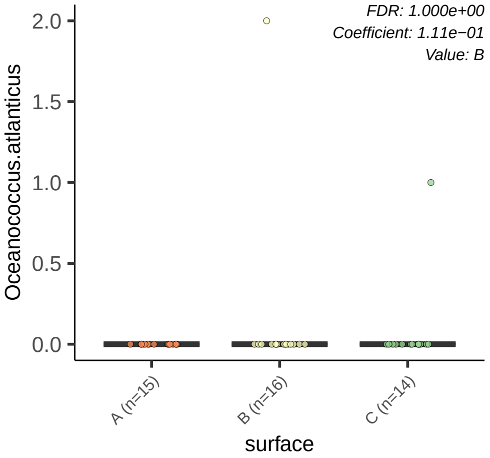

FDR: 1.000e+00
Coefficient: 1.11e−01
Value: B
Oceanococcus.atlanticus
2.0
1.5
1.0
0.5
0.0
A (n=15)
B (n=16)
C (n=14)
surface

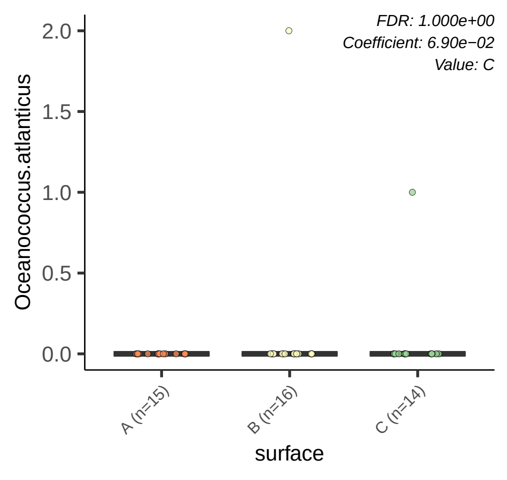

FDR: 1.000e+00
Coefficient: 6.90e−02
Value: C
Oceanococcus.atlanticus
2.0
1.5
1.0
0.5
0.0
A (n=15)
B (n=16)
C (n=14)
surface

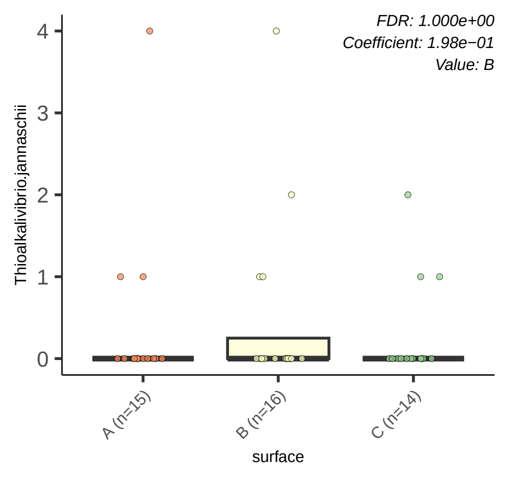
FDR: 1.000e+00
Coefficient: 1.98e−01
Value: B
Thioalkalivibrio.jannaschii
0
1
2
3
4
A (n=15)
B (n=16)
C (n=14)
surface

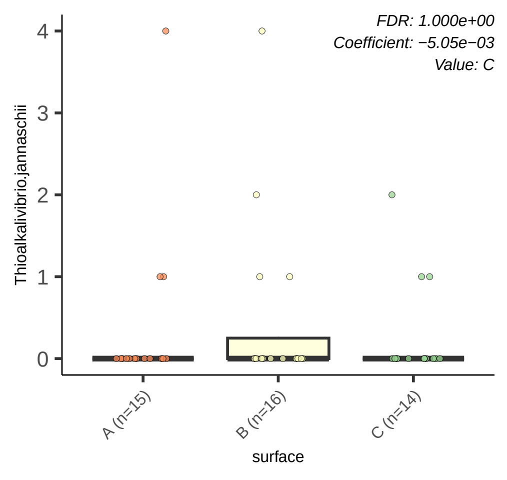

FDR: 1.000e+00
Coefficient: −5.05e−03
Value: C
Thioalkalivibrio.jannaschii
4
3
2
1
0
A (n=15)
B (n=16)
C (n=14)
surface

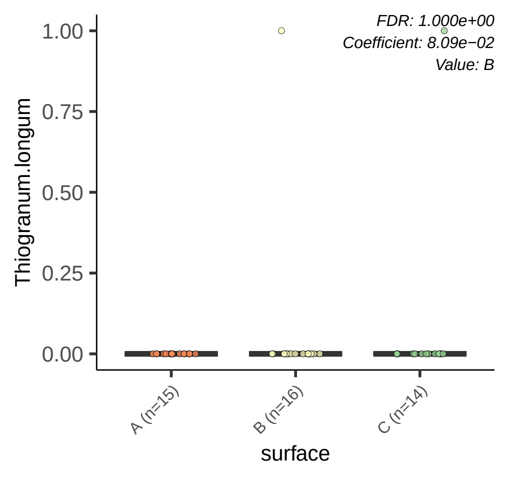

FDR: 1.000e+00
Coefficient: 8.09e−02
Value: B
Thiogranum.longum
1.00
0.75
0.50
0.25
0.00
A (n=15)
B (n=16)
C (n=14)
surface

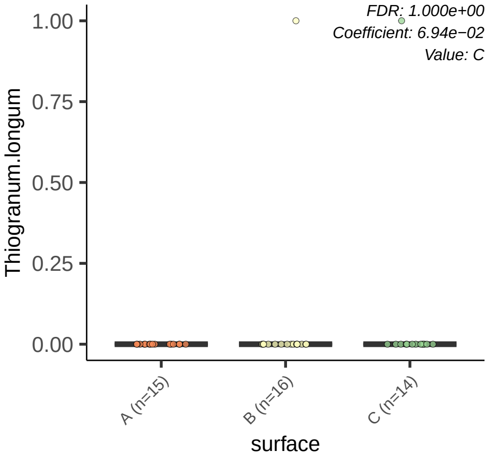
FDR: 1.000e+00
Coefficient: 6.94e−02
Value: C
Thiogranum.longum
1.00
0.75
0.50
0.25
0.00
A (n=15)
B (n=16)
C (n=14)
surface

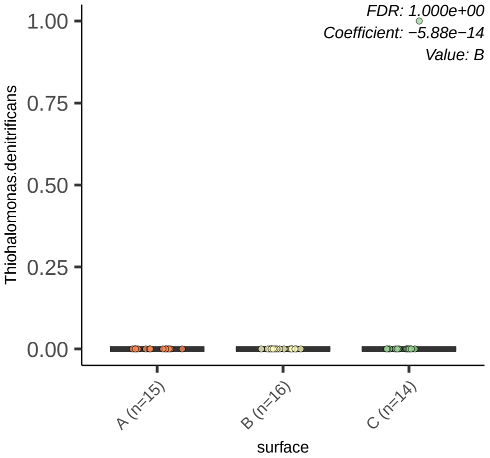

FDR: 1.000e+00
Coefficient: −5.88e−14
Value: B
Thiohalomonas.denitrificans
1.00
0.75
0.50
0.25
0.00
A (n=15)
B (n=16)
C (n=14)
surface

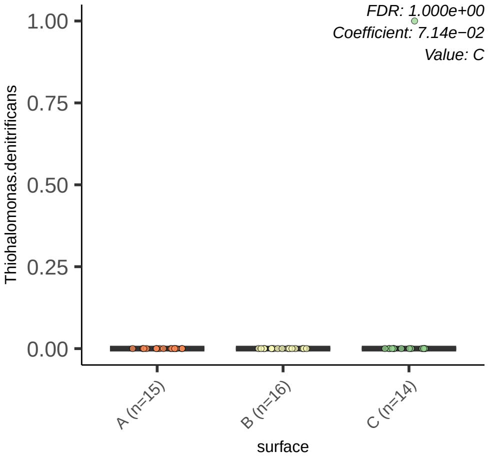
FDR: 1.000e+00
Coefficient: 7.14e−02
Value: C
1.00
0.75
0.50
0.25
0.00
Thiohalomonas.denitrificans
A (n=15)
B (n=16)
C (n=14)
surface

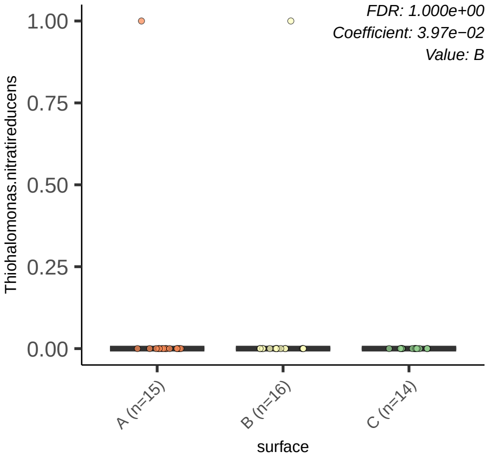

FDR: 1.000e+00
Coefficient: 3.97e−02
Value: B
Thiohalomonas.nitratireducens
1.00
0.75
0.50
0.25
0.00
A (n=15)
B (n=16)
C (n=14)
surface

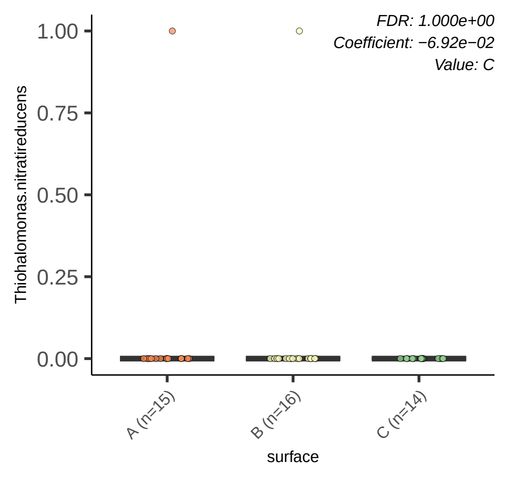
FDR: 1.000e+00
Coefficient: −6.92e−02
Value: C
1.00
0.75
0.50
0.25
0.00
Thiohalomonas.nitratireducens
A (n=15)
B (n=16)
C (n=14)
surface

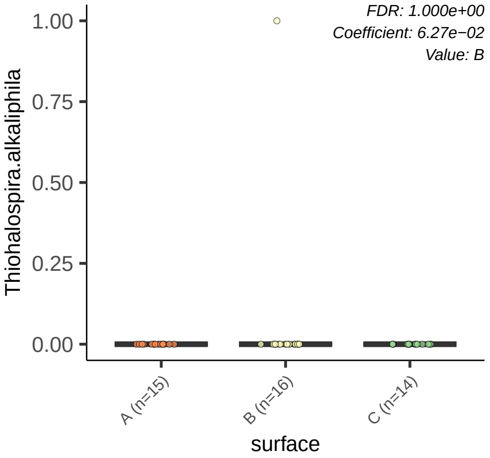

FDR: 1.000e+00
Coefficient: 6.27e−02
Value: B
Thiohalospira.alkaliphila
1.00
0.75
0.50
0.25
0.00
A (n=15)
B (n=16)
C (n=14)
surface

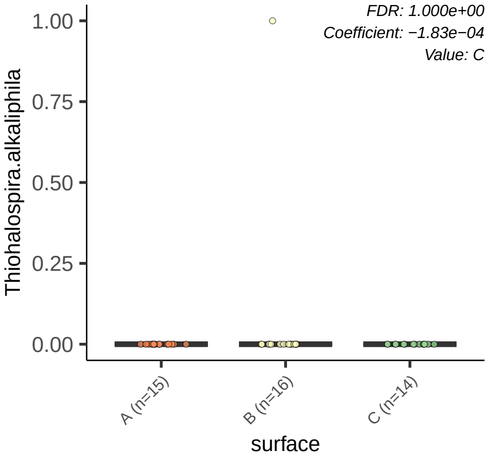
FDR: 1.000e+00
Coefficient: −1.83e−04
Value: C
Thiohalospira.alkaliphila
1.00
0.75
0.50
0.25
0.00
A (n=15)
B (n=16)
C (n=14)
surface

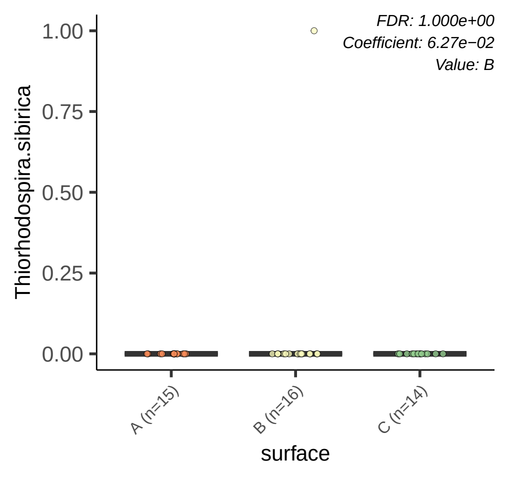
FDR: 1.000e+00
Coefficient: 6.27e−02
Value: B
Thiorhodospira.sibirica
1.00
0.75
0.50
0.25
0.00
A (n=15)
B (n=16)
C (n=14)
surface

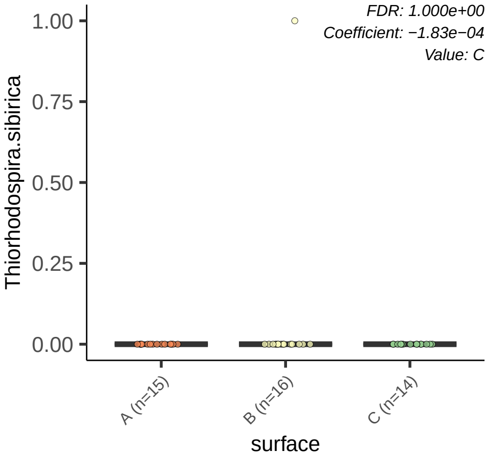
FDR: 1.000e+00
Coefficient: −1.83e−04
Value: C
Thiorhodospira.sibirica
1.00
0.75
0.50
0.25
0.00
A (n=15)
B (n=16)
C (n=14)
surface

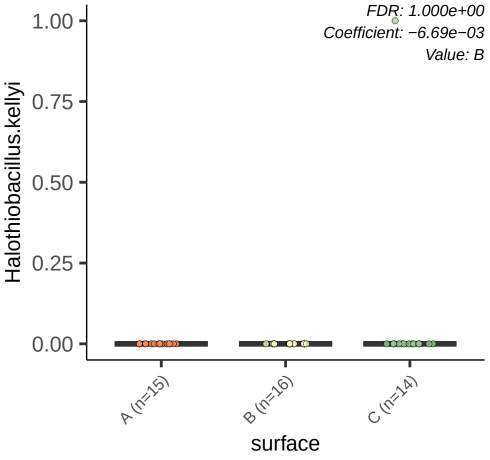
FDR: 1.000e+00
Coefficient: −6.69e−03
Value: B
Halothiobacillus.kellyi
1.00
0.75
0.50
0.25
0.00
A (n=15)
B (n=16)
C (n=14)
surface

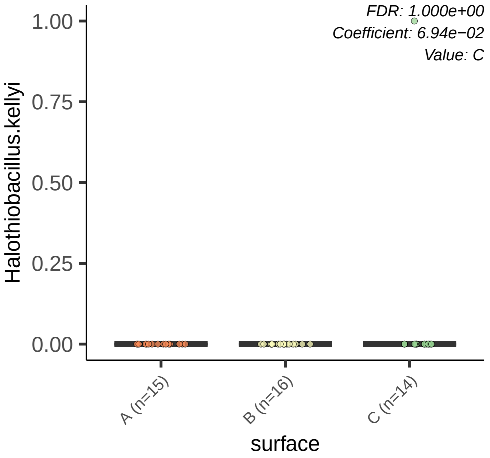
FDR: 1.000e+00
Coefficient: 6.94e−02
Value: C
Halothiobacillus.kellyi
1.00
0.75
0.50
0.25
0.00
A (n=15)
B (n=16)
C (n=14)
surface

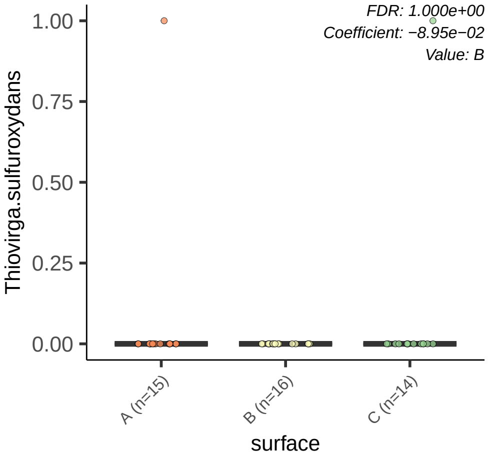

FDR: 1.000e+00
Coefficient: −8.95e−02
Value: B
Thiovirga.sulfuroxydans
1.00
0.75
0.50
0.25
0.00
A (n=15)
B (n=16)
C (n=14)
surface

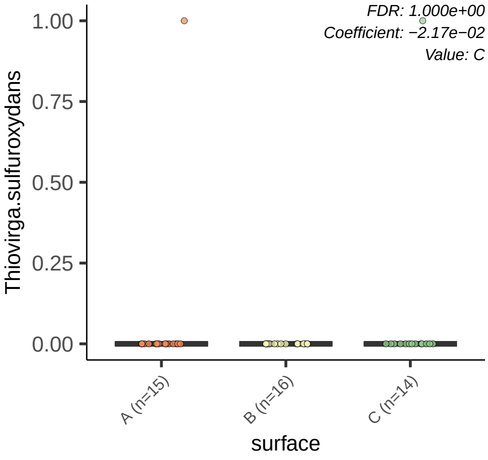

FDR: 1.000e+00
Coefficient: −2.17e−02
Value: C
Thiovirga.sulfuroxydans
1.00
0.75
0.50
0.25
0.00
A (n=15)
B (n=16)
C (n=14)
surface

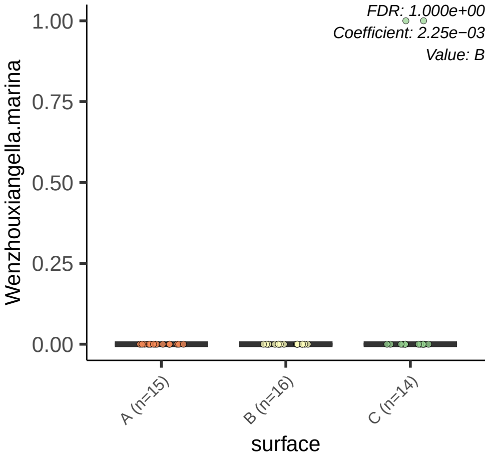
FDR: 1.000e+00
Coefficient: 2.25e−03
Value: B
Wenzhouxiangella.marina
1.00
0.75
0.50
0.25
0.00
A (n=15)
B (n=16)
C (n=14)
surface

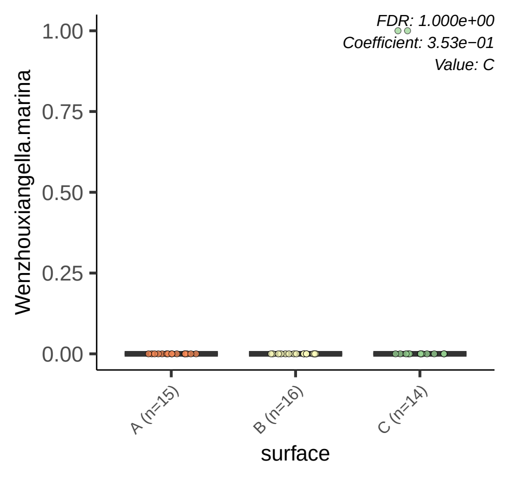

FDR: 1.000e+00
Coefficient: 3.53e−01
Value: C
Wenzhouxiangella.marina
1.00
0.75
0.50
0.25
0.00
A (n=15)
B (n=16)
C (n=14)
surface

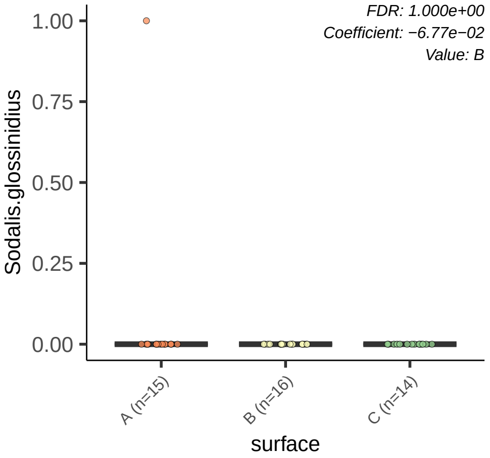
FDR: 1.000e+00
Coefficient: −6.77e−02
Value: B
Sodalis.glossinidius
1.00
0.75
0.50
0.25
0.00
A (n=15)
B (n=16)
C (n=14)
surface

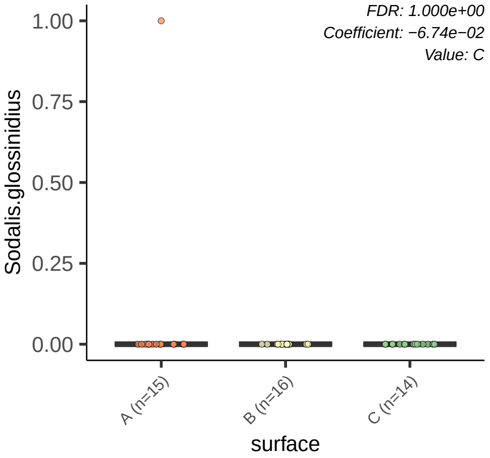
FDR: 1.000e+00
Coefficient: −6.74e−02
Value: C
Sodalis.glossinidius
1.00
0.75
0.50
0.25
0.00
A (n=15)
B (n=16)
C (n=14)
surface

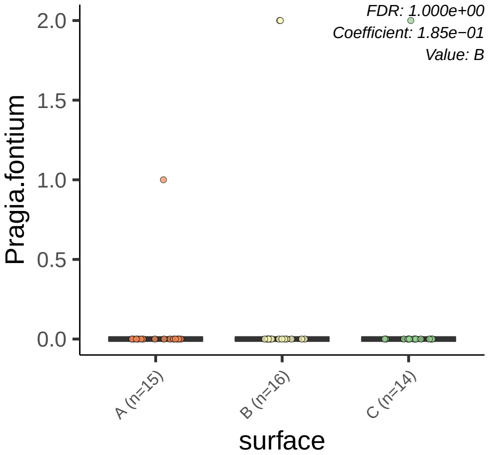

FDR: 1.000e+00
Coefficient: 1.85e−01
Value: B
Pragia.fontium
2.0
1.5
1.0
0.5
0.0
A (n=15)
B (n=16)
C (n=14)
surface

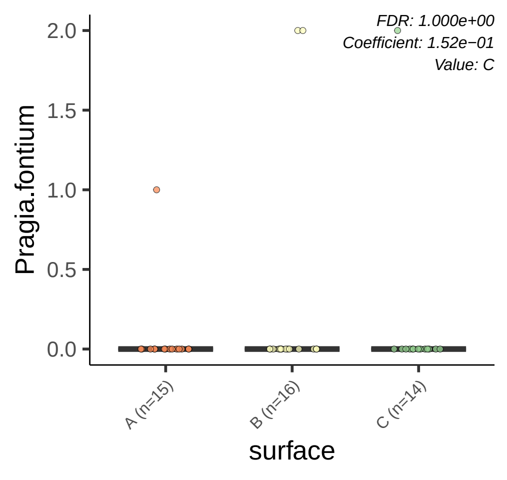
FDR: 1.000e+00
Coefficient: 1.52e−01
Value: C
Pragia.fontium
2.0
1.5
1.0
0.5
0.0
A (n=15)
B (n=16)
C (n=14)
surface

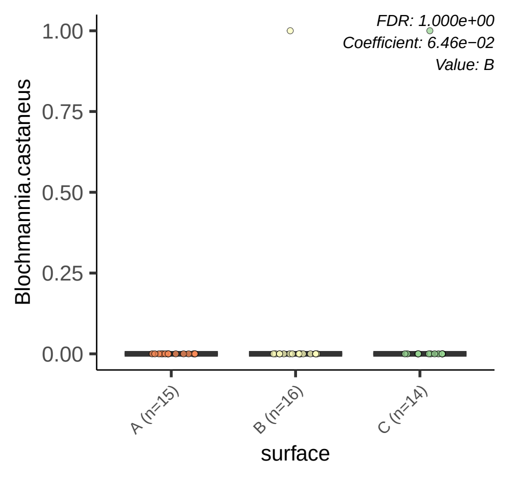

FDR: 1.000e+00
Coefficient: 6.46e−02
Value: B
Blochmannia.castaneus
1.00
0.75
0.50
0.25
0.00
A (n=15)
B (n=16)
C (n=14)
surface

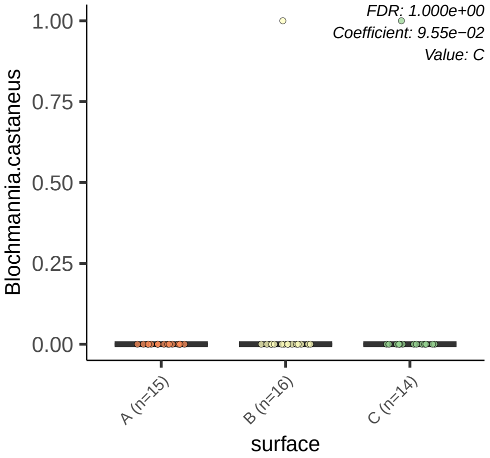

FDR: 1.000e+00
Coefficient: 9.55e−02
Value: C
Blochmannia.castaneus
1.00
0.75
0.50
0.25
0.00
A (n=15)
B (n=16)
C (n=14)
surface

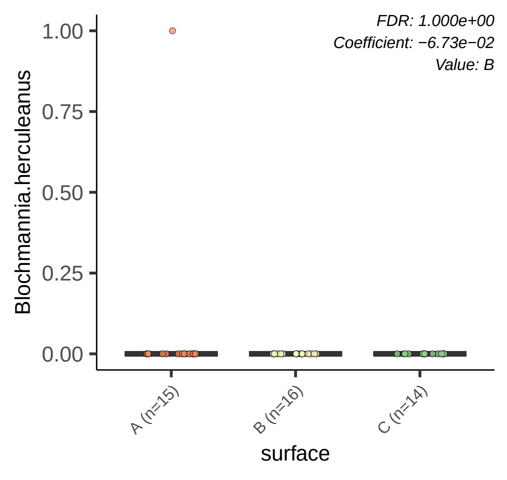

FDR: 1.000e+00
Coefficient: −6.73e−02
Value: B
Blochmannia.herculeanus
1.00
0.75
0.50
0.25
0.00
A (n=15)
B (n=16)
C (n=14)
surface

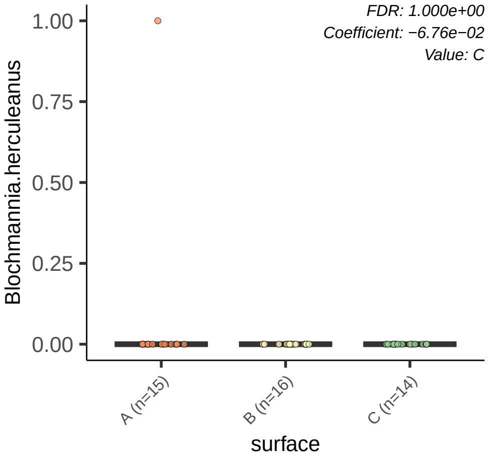

FDR: 1.000e+00
Coefficient: −6.76e−02
Value: C
Blochmannia.herculeanus
1.00
0.75
0.50
0.25
0.00
A (n=15)
B (n=16)
C (n=14)
surface

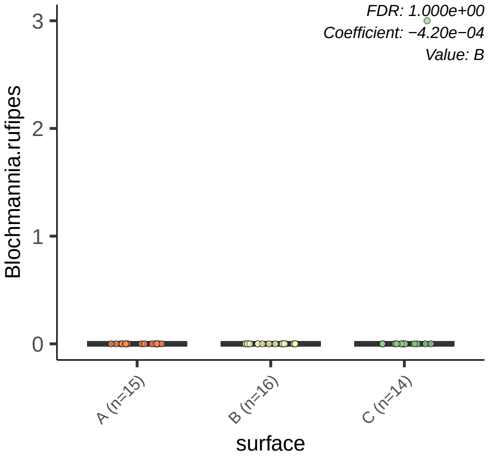
FDR: 1.000e+00
Coefficient: −4.20e−04
Value: B
Blochmannia.rufipes
3
2
1
0
A (n=15)
B (n=16)
C (n=14)
surface

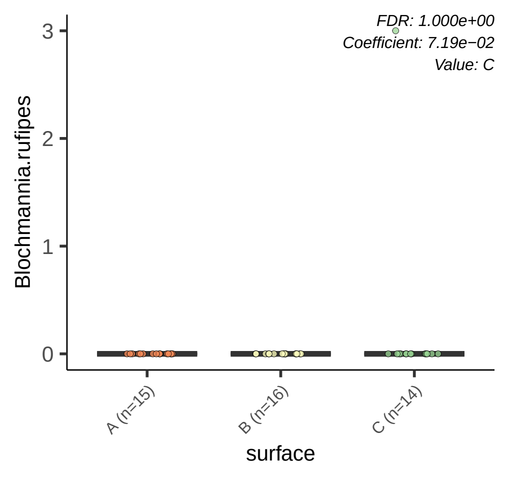
FDR: 1.000e+00
Coefficient: 7.19e−02
Value: C
Blochmannia.rufipes
0
1
2
3
A (n=15)
B (n=16)
C (n=14)
surface

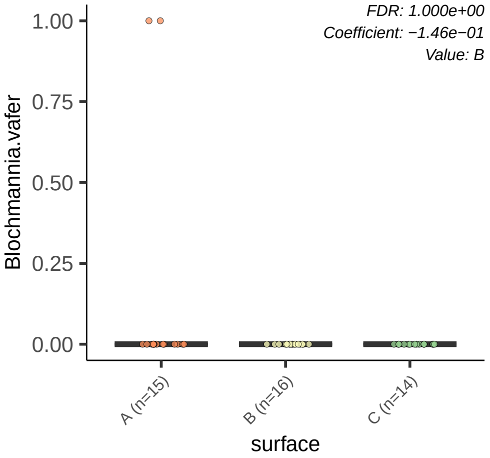

FDR: 1.000e+00
Coefficient: −1.46e−01
Value: B
Blochmannia.vafer
1.00
0.75
0.50
0.25
0.00
A (n=15)
B (n=16)
C (n=14)
surface

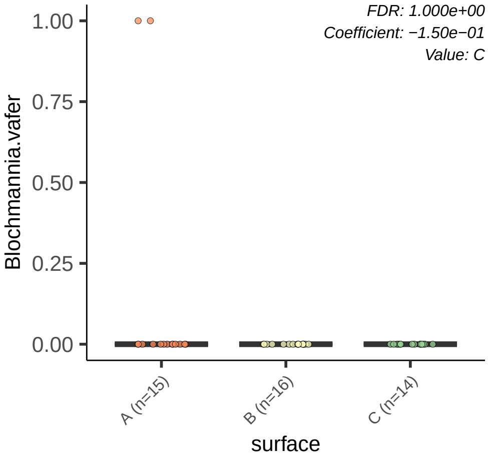
FDR: 1.000e+00
Coefficient: −1.50e−01
Value: C
Blochmannia.vafer
1.00
0.75
0.50
0.25
0.00
A (n=15)
B (n=16)
C (n=14)
surface

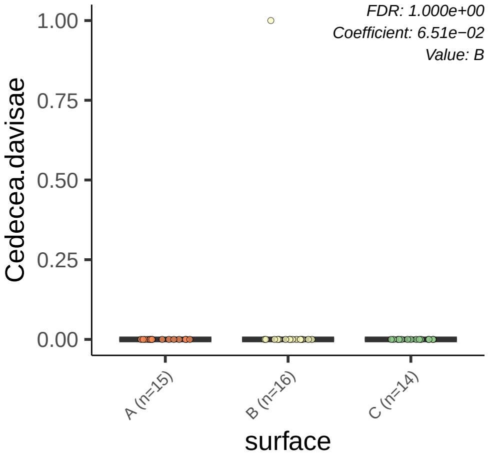
FDR: 1.000e+00
Coefficient: 6.51e−02
Value: B
Cedecea.davisae
1.00
0.75
0.50
0.25
0.00
A (n=15)
B (n=16)
C (n=14)
surface

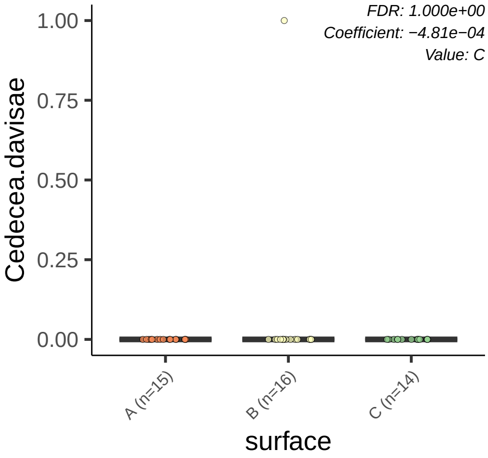

FDR: 1.000e+00
Coefficient: −4.81e−04
Value: C
Cedecea.davisae
1.00
0.75
0.50
0.25
0.00
A (n=15)
B (n=16)
C (n=14)
surface

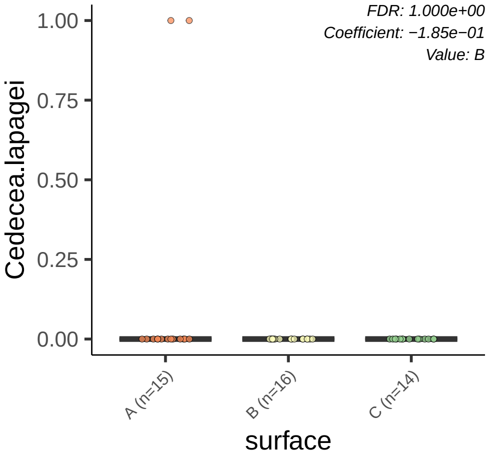
FDR: 1.000e+00
Coefficient: −1.85e−01
Value: B
Cedecea.lapagei
1.00
0.75
0.50
0.25
0.00
A (n=15)
B (n=16)
C (n=14)
surface

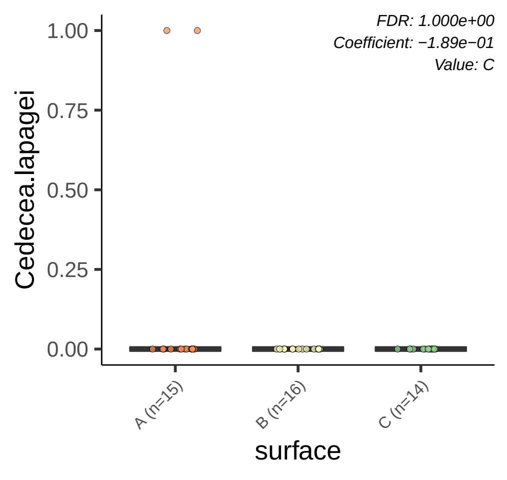

FDR: 1.000e+00
Coefficient: −1.89e−01
Value: C
Cedecea.lapagei
1.00
0.75
0.50
0.25
0.00
A (n=15)
B (n=16)
C (n=14)
surface

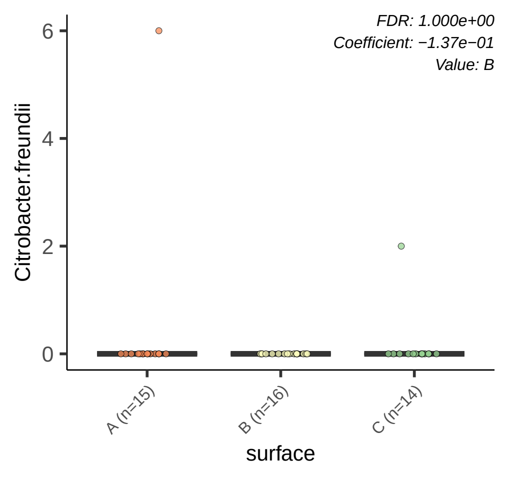
FDR: 1.000e+00
Coefficient: −1.37e−01
Value: B
Citrobacter.freundii
6
4
2
0
A (n=15)
B (n=16)
C (n=14)
surface

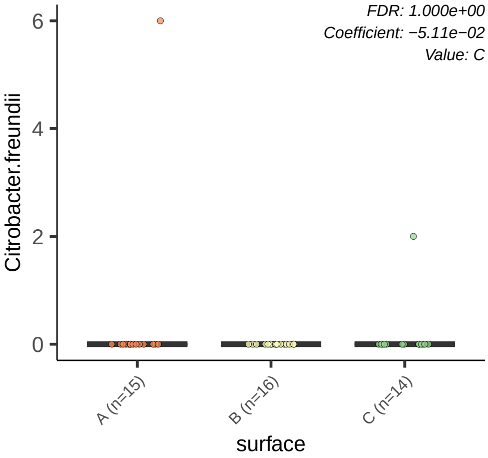
FDR: 1.000e+00
Coefficient: −5.11e−02
Value: C
Citrobacter.freundii
0
2
4
6
A (n=15)
B (n=16)
C (n=14)
surface

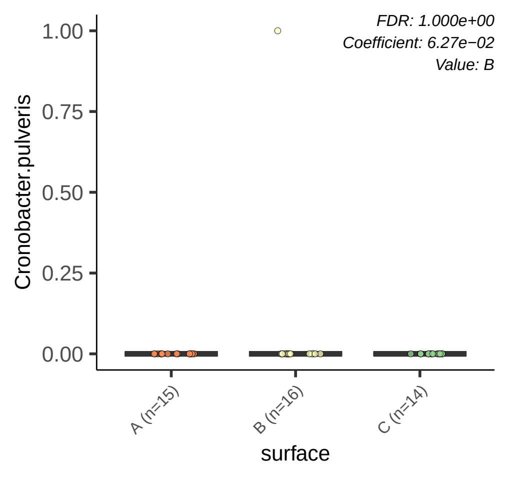
FDR: 1.000e+00
Coefficient: 6.27e−02
Value: B
Cronobacter.pulveris
1.00
0.75
0.50
0.25
0.00
A (n=15)
B (n=16)
C (n=14)
surface

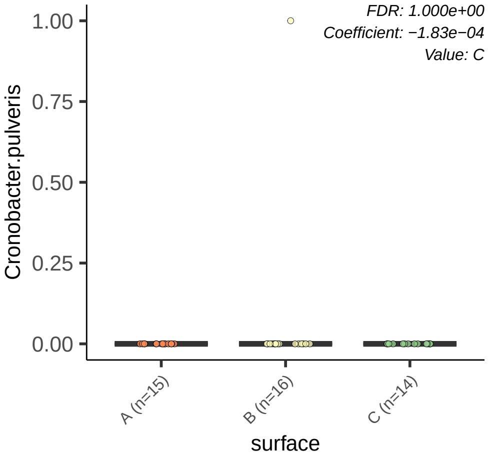
FDR: 1.000e+00
Coefficient: −1.83e−04
Value: C
Cronobacter.pulveris
1.00
0.75
0.50
0.25
0.00
A (n=15)
B (n=16)
C (n=14)
surface

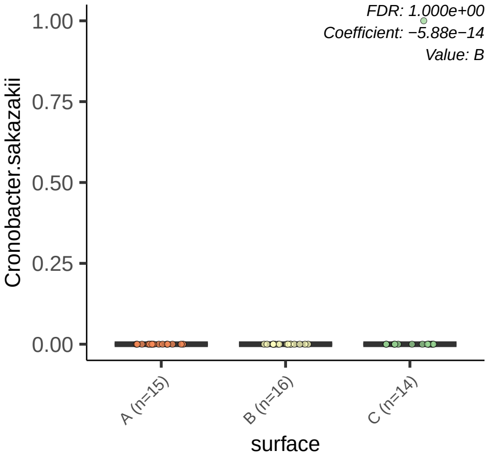

FDR: 1.000e+00
Coefficient: −5.88e−14
Value: B
Cronobacter.sakazakii
1.00
0.75
0.50
0.25
0.00
A (n=15)
B (n=16)
C (n=14)
surface

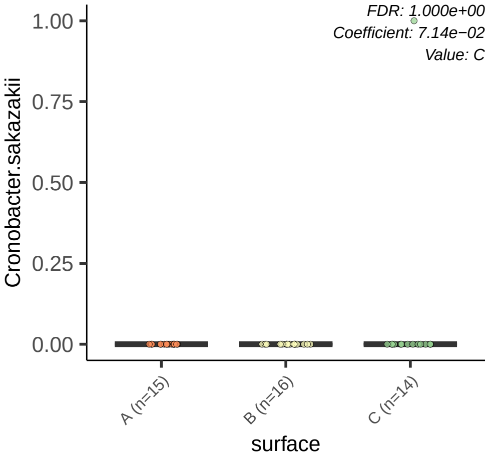

FDR: 1.000e+00
Coefficient: 7.14e−02
Value: C
Cronobacter.sakazakii
1.00
0.75
0.50
0.25
0.00
A (n=15)
B (n=16)
C (n=14)
surface

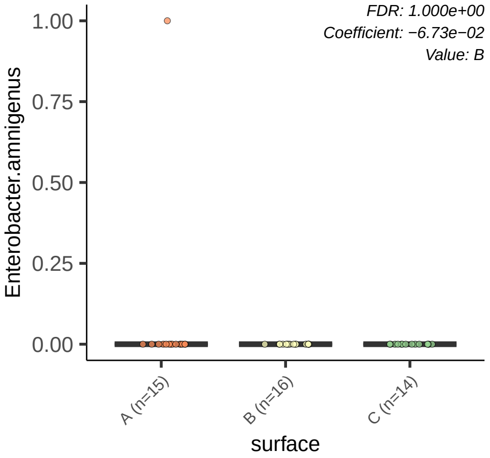
FDR: 1.000e+00
Coefficient: −6.73e−02
Value: B
Enterobacter.amnigenus
1.00
0.75
0.50
0.25
0.00
A (n=15)
B (n=16)
C (n=14)
surface

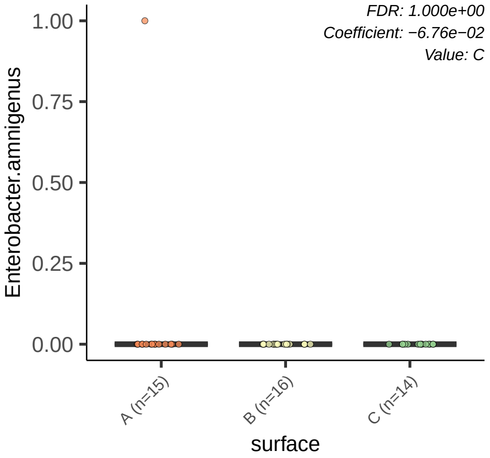

FDR: 1.000e+00
Coefficient: −6.76e−02
Value: C
1.00
0.75
0.50
0.25
0.00
Enterobacter.amnigenus
A (n=15)
B (n=16)
C (n=14)
surface

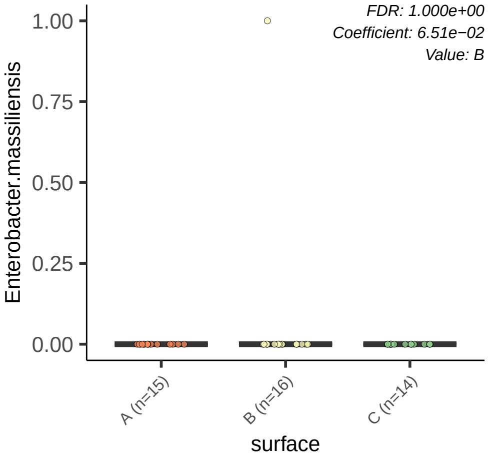
FDR: 1.000e+00
Coefficient: 6.51e−02
Value: B
Enterobacter.massiliensis
1.00
0.75
0.50
0.25
0.00
A (n=15)
B (n=16)
C (n=14)
surface

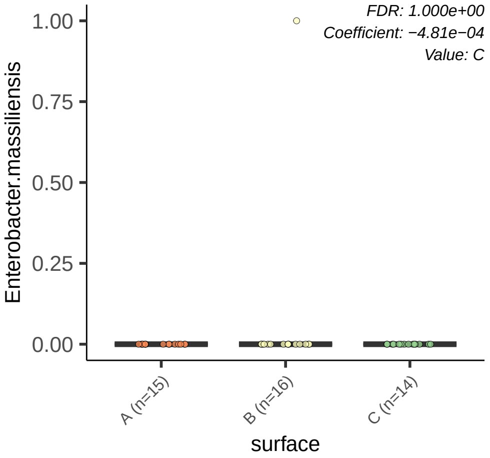

FDR: 1.000e+00
Coefficient: −4.81e−04
Value: C
Enterobacter.massiliensis
1.00
0.75
0.50
0.25
0.00
A (n=15)
B (n=16)
C (n=14)
surface

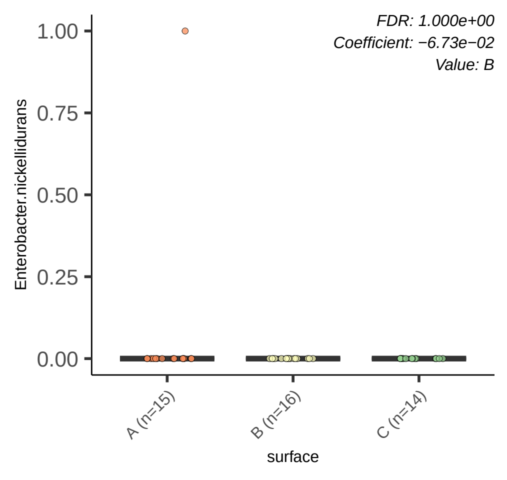
FDR: 1.000e+00
Coefficient: −6.73e−02
Value: B
Enterobacter.nickellidurans
1.00
0.75
0.50
0.25
0.00
A (n=15)
B (n=16)
C (n=14)
surface

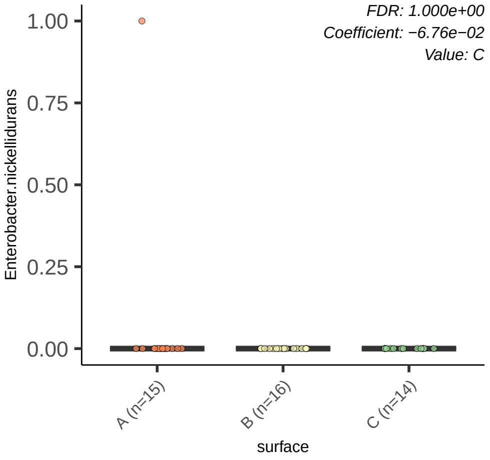
FDR: 1.000e+00
Coefficient: −6.76e−02
Value: C
1.00
0.75
0.50
0.25
0.00
Enterobacter.nickellidurans
A (n=15)
B (n=16)
C (n=14)
surface

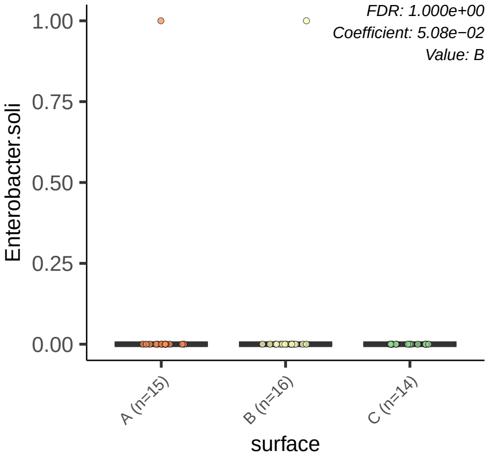

FDR: 1.000e+00
Coefficient: 5.08e−02
Value: B
1.00
0.75
0.50
0.25
0.00
Enterobacter.soli
A (n=15)
B (n=16)
C (n=14)
surface

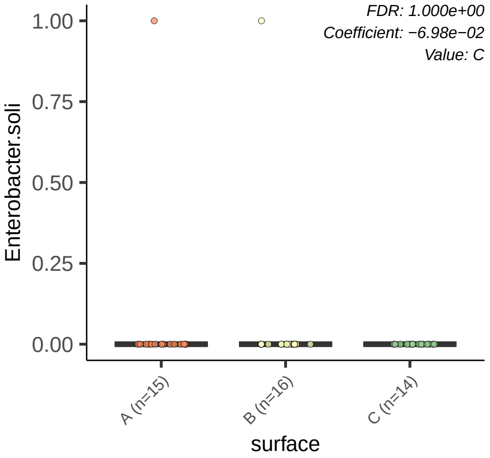
FDR: 1.000e+00
Coefficient: −6.98e−02
Value: C
Enterobacter.soli
1.00
0.75
0.50
0.25
0.00
A (n=15)
B (n=16)
C (n=14)
surface

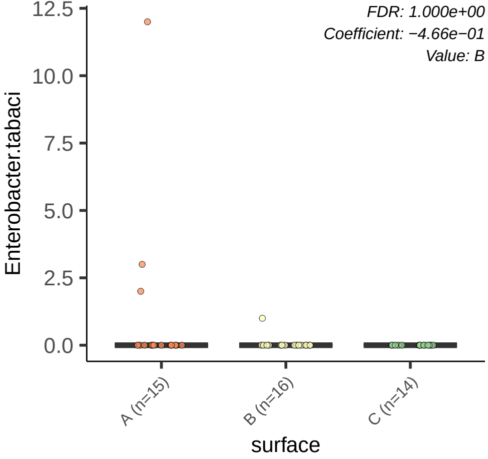
FDR: 1.000e+00
Coefficient: −4.66e−01
Value: B
Enterobacter.tabaci
12.5
10.0
7.5
5.0
2.5
0.0
A (n=15)
B (n=16)
C (n=14)
surface

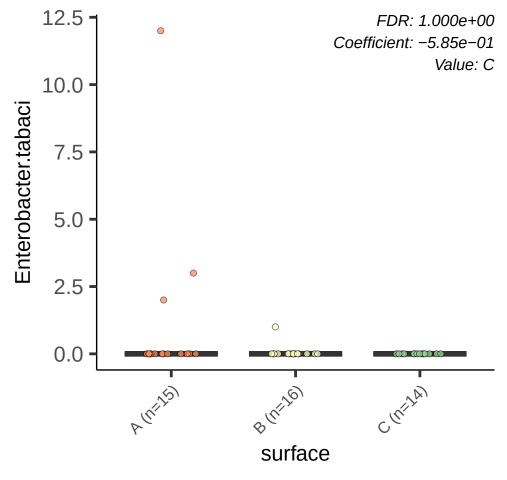
FDR: 1.000e+00
Coefficient: −5.85e−01
Value: C
Enterobacter.tabaci
12.5
10.0
7.5
5.0
2.5
0.0
A (n=15)
B (n=16)
C (n=14)
surface

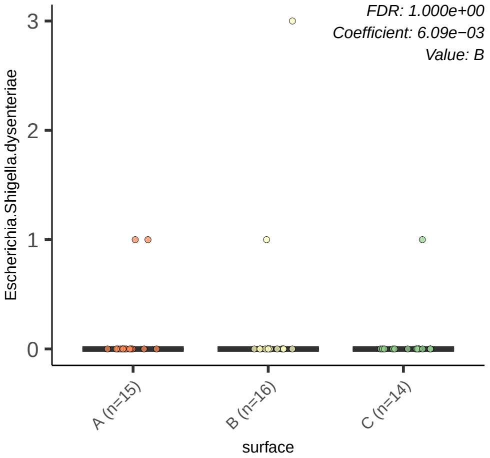

FDR: 1.000e+00
Coefficient: 6.09e−03
Value: B
Escherichia.Shigella.dysenteriae
3
2
1
0
A (n=15)
B (n=16)
C (n=14)
surface

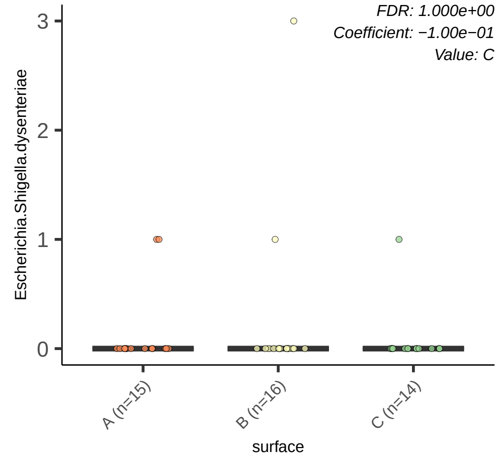
FDR: 1.000e+00
Coefficient: −1.00e−01
Value: C
Escherichia.Shigella.dysenteriae
3
2
1
0
A (n=15)
B (n=16)
C (n=14)
surface

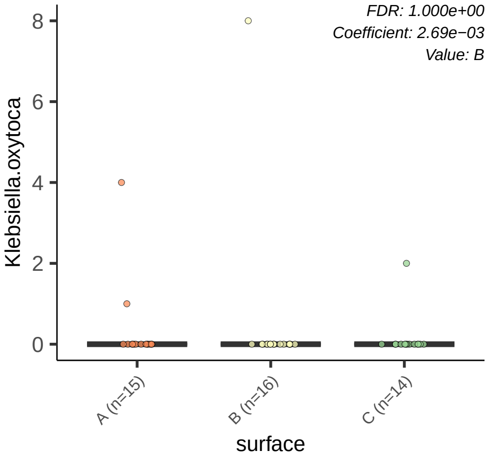
FDR: 1.000e+00
Coefficient: 2.69e−03
Value: B
Klebsiella.oxytoca
8
6
4
2
0
A (n=15)
B (n=16)
C (n=14)
surface

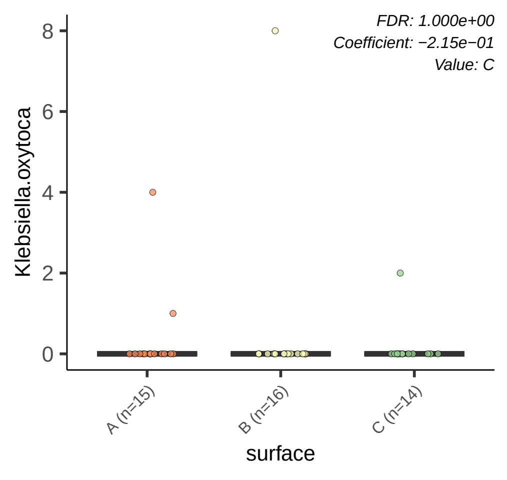
FDR: 1.000e+00
Coefficient: −2.15e−01
Value: C
Klebsiella.oxytoca
0
2
4
6
8
A (n=15)
B (n=16)
C (n=14)
surface

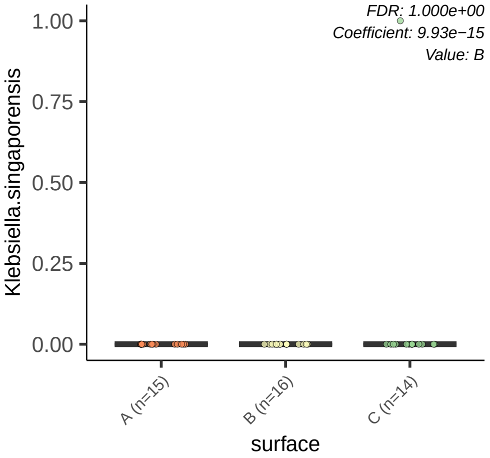
FDR: 1.000e+00
Coefficient: 9.93e−15
Value: B
Klebsiella.singaporensis
1.00
0.75
0.50
0.25
0.00
A (n=15)
B (n=16)
C (n=14)
surface

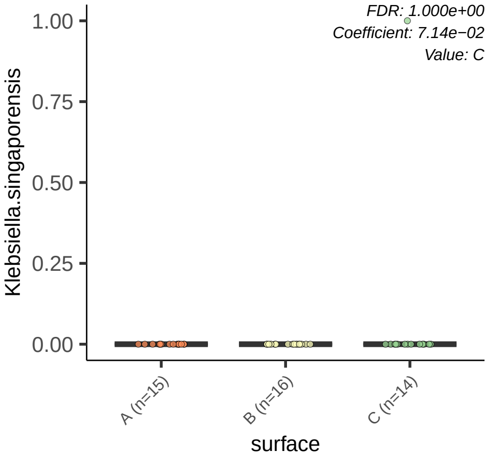
FDR: 1.000e+00
Coefficient: 7.14e−02
Value: C
Klebsiella.singaporensis
1.00
0.75
0.50
0.25
0.00
A (n=15)
B (n=16)
C (n=14)
surface

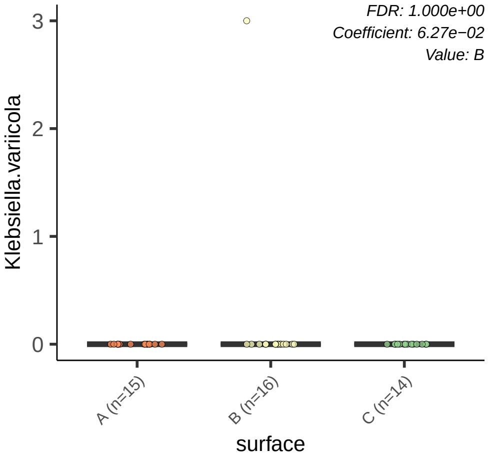
FDR: 1.000e+00
Coefficient: 6.27e−02
Value: B
Klebsiella.variicola
3
2
1
0
A (n=15)
B (n=16)
C (n=14)
surface

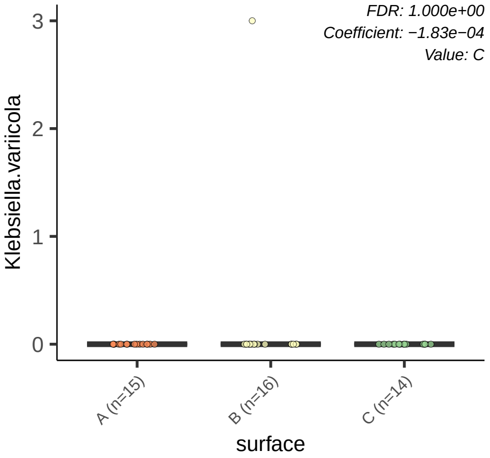
FDR: 1.000e+00
Coefficient: −1.83e−04
Value: C
Klebsiella.variicola
0
1
2
3
A (n=15)
B (n=16)
C (n=14)
surface

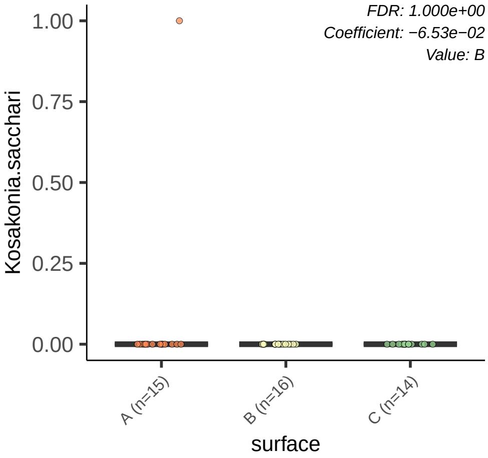
FDR: 1.000e+00
Coefficient: −6.53e−02
Value: B
Kosakonia.sacchari
1.00
0.75
0.50
0.25
0.00
A (n=15)
B (n=16)
C (n=14)
surface

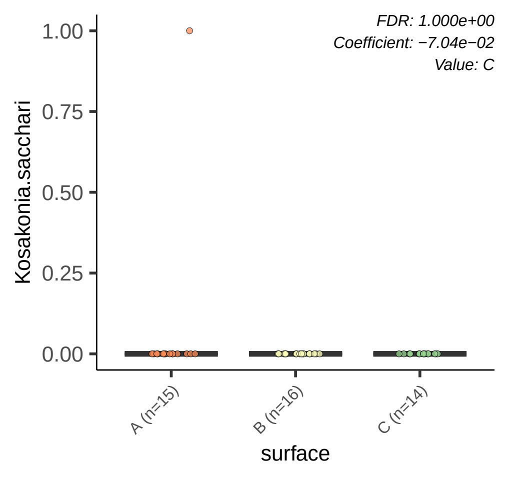
FDR: 1.000e+00
Coefficient: −7.04e−02
Value: C
1.00
0.75
0.50
0.25
0.00
Kosakonia.sacchari
A (n=15)
B (n=16)
C (n=14)
surface

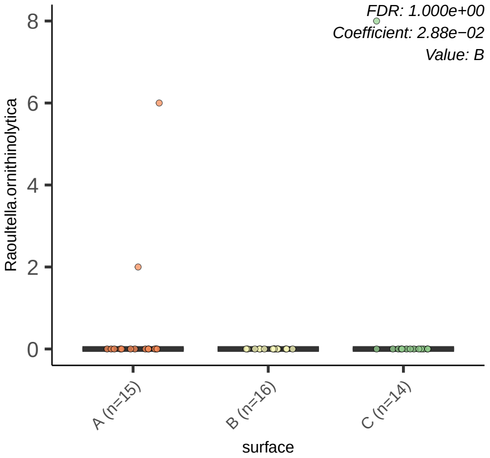
FDR: 1.000e+00
Coefficient: 2.88e−02
Value: B
Raoultella.ornithinolytica
8
6
4
2
0
A (n=15)
B (n=16)
C (n=14)
surface

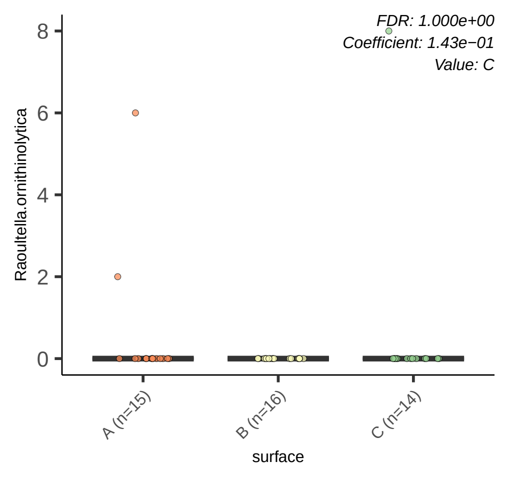
FDR: 1.000e+00
Coefficient: 1.43e−01
Value: C
Raoultella.ornithinolytica
8
6
4
2
0
A (n=15)
B (n=16)
C (n=14)
surface

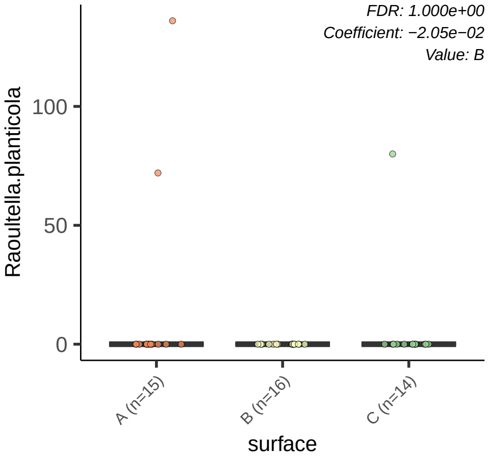

FDR: 1.000e+00
Coefficient: −2.05e−02
Value: B
Raoultella.planticola
100
50
0
A (n=15)
B (n=16)
C (n=14)
surface

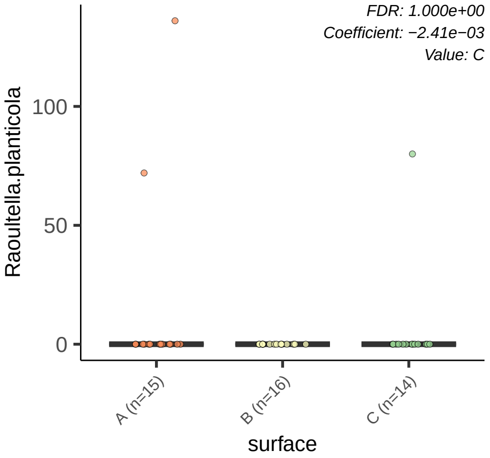

FDR: 1.000e+00
Coefficient: −2.41e−03
Value: C
Raoultella.planticola
100
50
0
A (n=15)
B (n=16)
C (n=14)
surface

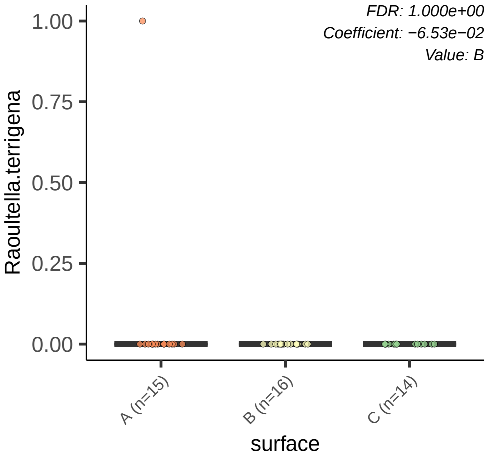
FDR: 1.000e+00
Coefficient: −6.53e−02
Value: B
Raoultella.terrigena
1.00
0.75
0.50
0.25
0.00
A (n=15)
B (n=16)
C (n=14)
surface

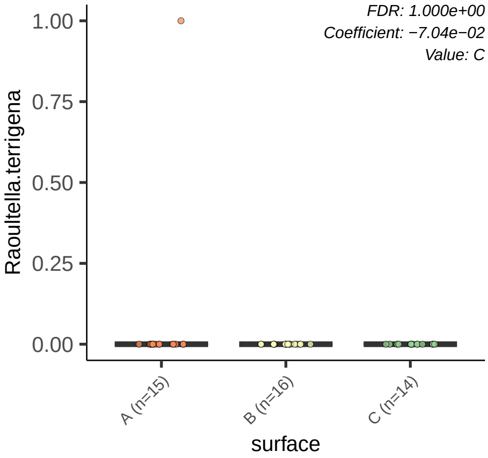
FDR: 1.000e+00
Coefficient: −7.04e−02
Value: C
Raoultella.terrigena
1.00
0.75
0.50
0.25
0.00
A (n=15)
B (n=16)
C (n=14)
surface

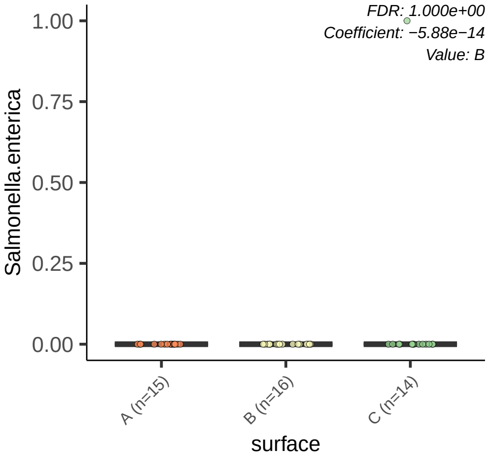
FDR: 1.000e+00
Coefficient: −5.88e−14
Value: B
Salmonella.enterica
1.00
0.75
0.50
0.25
0.00
A (n=15)
B (n=16)
C (n=14)
surface

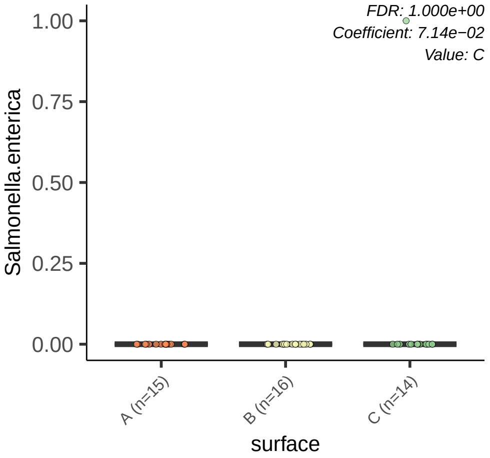
FDR: 1.000e+00
Coefficient: 7.14e−02
Value: C
Salmonella.enterica
1.00
0.75
0.50
0.25
0.00
A (n=15)
B (n=16)
C (n=14)
surface

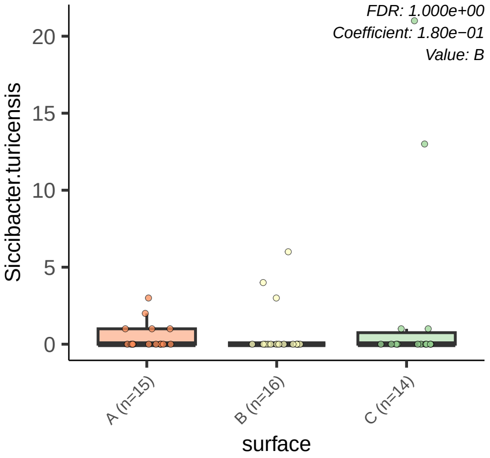

FDR: 1.000e+00
Coefficient: 1.80e−01
Value: B
Siccibacter.turicensis
0
5
10
15
20
A (n=15)
B (n=16)
C (n=14)
surface

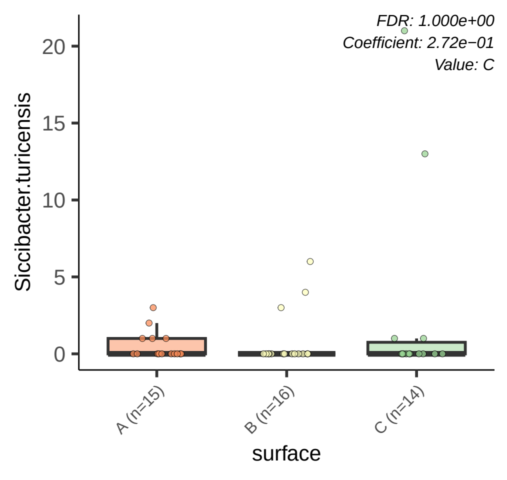
FDR: 1.000e+00
Coefficient: 2.72e−01
Value: C
Siccibacter.turicensis
0
5
10
15
20
A (n=15)
B (n=16)
C (n=14)
surface

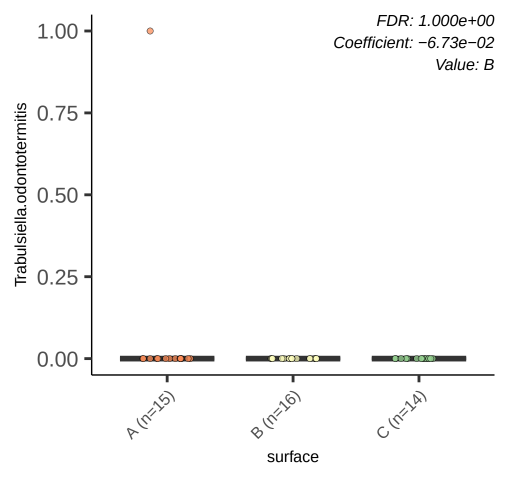
FDR: 1.000e+00
Coefficient: −6.73e−02
Value: B
Trabulsiella.odontotermitis
1.00
0.75
0.50
0.25
0.00
A (n=15)
B (n=16)
C (n=14)
surface

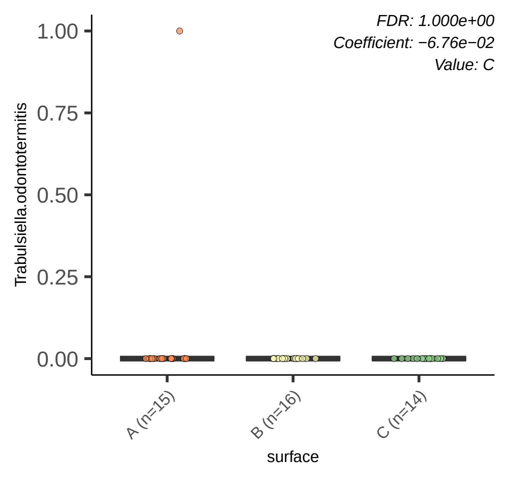
FDR: 1.000e+00
Coefficient: −6.76e−02
Value: C
1.00
0.75
0.50
0.25
0.00
Trabulsiella.odontotermitis
A (n=15)
B (n=16)
C (n=14)
surface

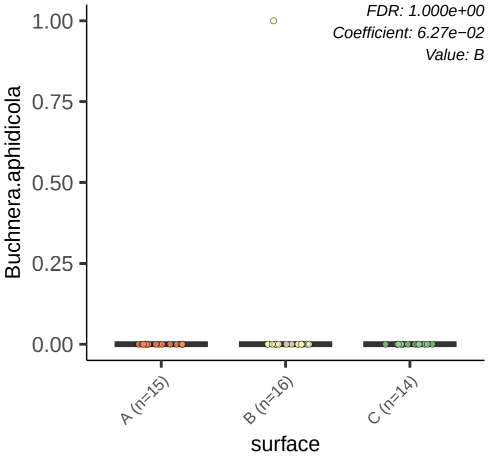

FDR: 1.000e+00
Coefficient: 6.27e−02
Value: B
Buchnera.aphidicola
1.00
0.75
0.50
0.25
0.00
A (n=15)
B (n=16)
C (n=14)
surface

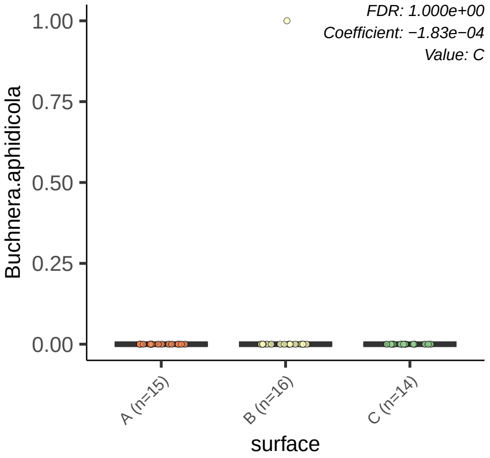
FDR: 1.000e+00
Coefficient: −1.83e−04
Value: C
Buchnera.aphidicola
1.00
0.75
0.50
0.25
0.00
A (n=15)
B (n=16)
C (n=14)
surface

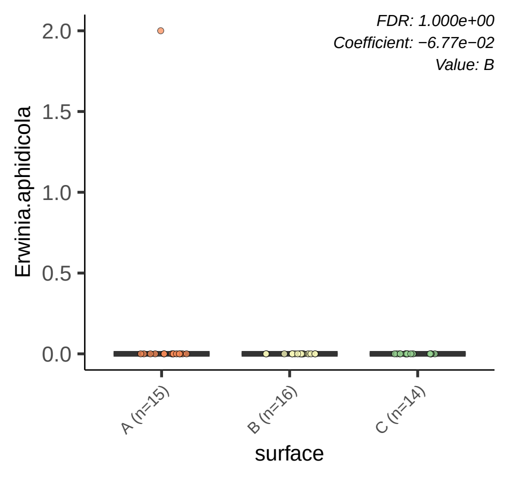

FDR: 1.000e+00
Coefficient: −6.77e−02
Value: B
Erwinia.aphidicola
2.0
1.5
1.0
0.5
0.0
A (n=15)
B (n=16)
C (n=14)
surface

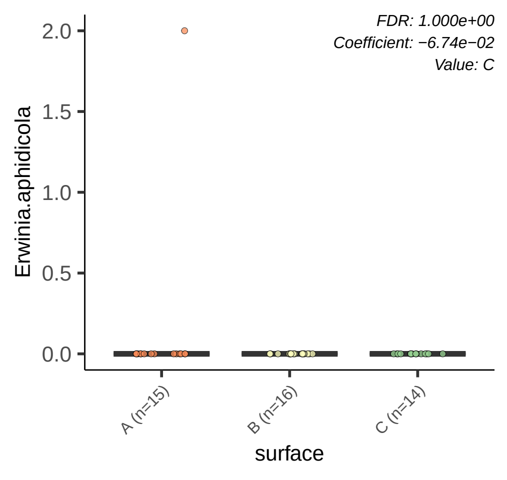
FDR: 1.000e+00
Coefficient: −6.74e−02
Value: C
Erwinia.aphidicola
2.0
1.5
1.0
0.5
0.0
A (n=15)
B (n=16)
C (n=14)
surface

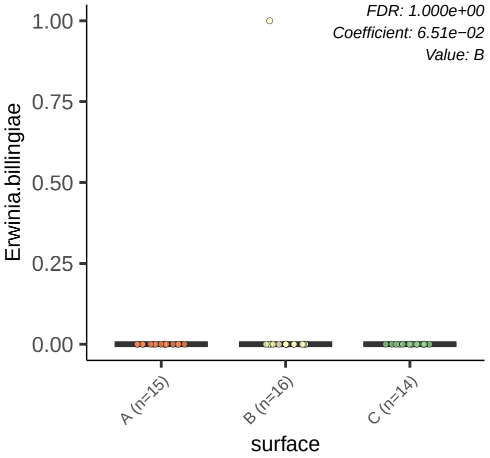
FDR: 1.000e+00
Coefficient: 6.51e−02
Value: B
Erwinia.billingiae
1.00
0.75
0.50
0.25
0.00
A (n=15)
B (n=16)
C (n=14)
surface

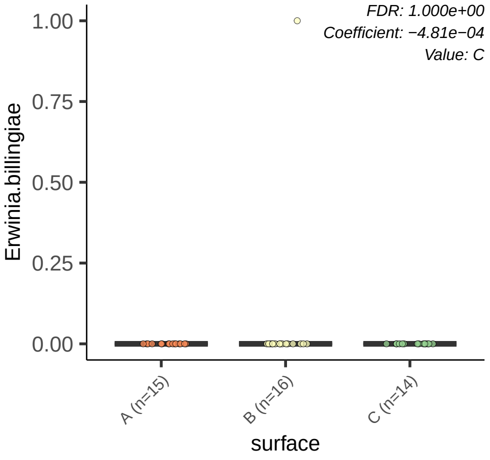
FDR: 1.000e+00
Coefficient: −4.81e−04
Value: C
Erwinia.billingiae
1.00
0.75
0.50
0.25
0.00
A (n=15)
B (n=16)
C (n=14)
surface

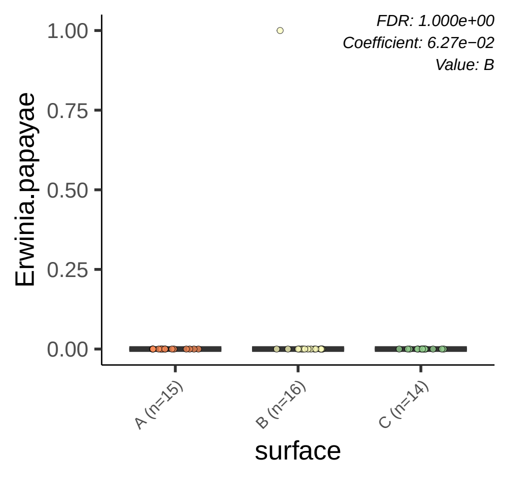
FDR: 1.000e+00
Coefficient: 6.27e−02
Value: B
Erwinia.papayae
1.00
0.75
0.50
0.25
0.00
A (n=15)
B (n=16)
C (n=14)
surface

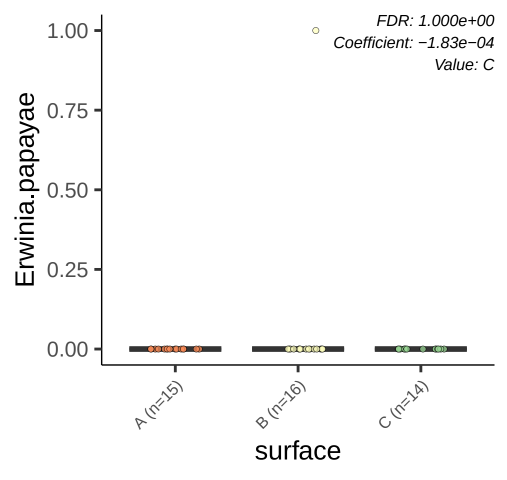
FDR: 1.000e+00
Coefficient: −1.83e−04
Value: C
Erwinia.papayae
1.00
0.75
0.50
0.25
0.00
A (n=15)
B (n=16)
C (n=14)
surface

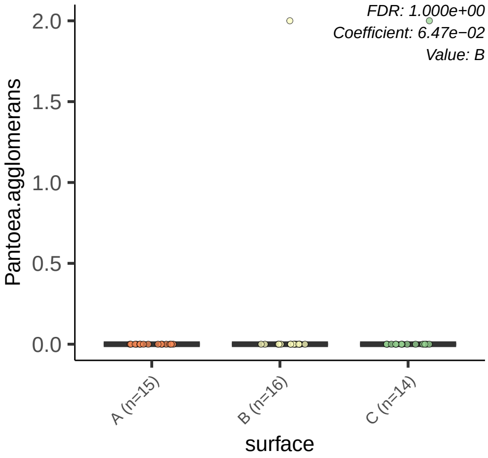

FDR: 1.000e+00
Coefficient: 6.47e−02
Value: B
Pantoea.agglomerans
2.0
1.5
1.0
0.5
0.0
A (n=15)
B (n=16)
C (n=14)
surface

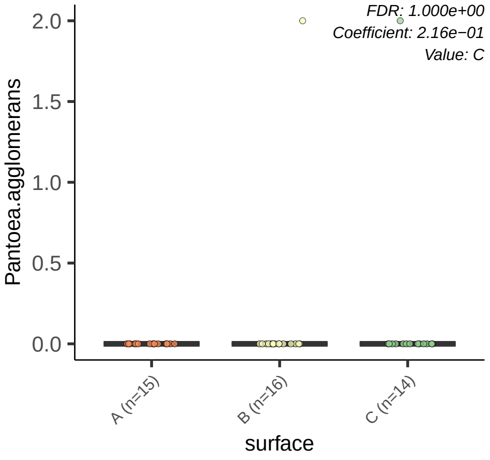
FDR: 1.000e+00
Coefficient: 2.16e−01
Value: C
Pantoea.agglomerans
2.0
1.5
1.0
0.5
0.0
A (n=15)
B (n=16)
C (n=14)
surface

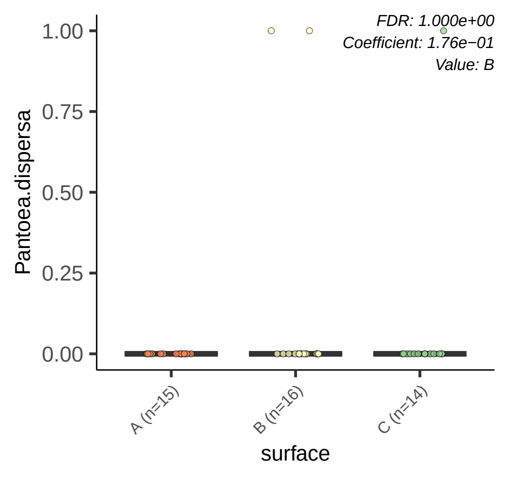

FDR: 1.000e+00
Coefficient: 1.76e−01
Value: B
1.00
0.75
0.50
0.25
0.00
Pantoea.dispersa
A (n=15)
B (n=16)
C (n=14)
surface

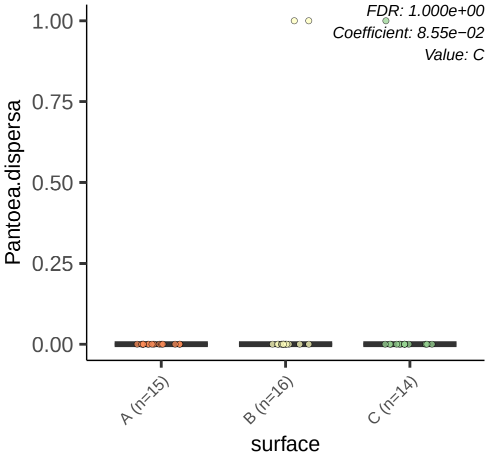
FDR: 1.000e+00
Coefficient: 8.55e−02
Value: C
Pantoea.dispersa
1.00
0.75
0.50
0.25
0.00
A (n=15)
B (n=16)
C (n=14)
surface

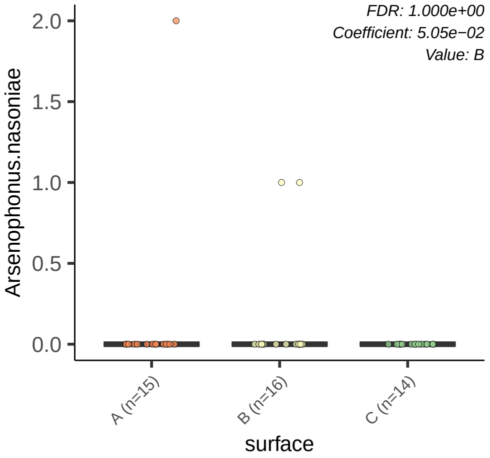
FDR: 1.000e+00
Coefficient: 5.05e−02
Value: B
Arsenophonus.nasoniae
2.0
1.5
1.0
0.5
0.0
A (n=15)
B (n=16)
C (n=14)
surface

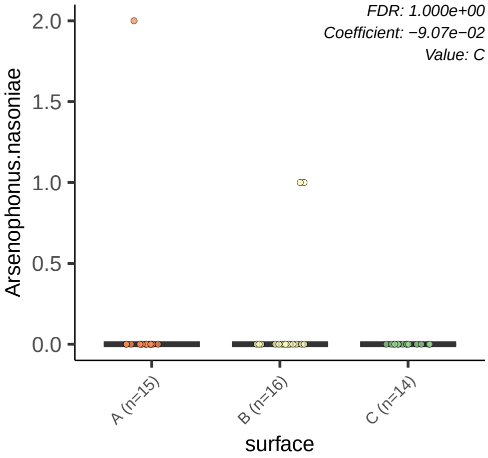
FDR: 1.000e+00
Coefficient: −9.07e−02
Value: C
Arsenophonus.nasoniae
2.0
1.5
1.0
0.5
0.0
A (n=15)
B (n=16)
C (n=14)
surface

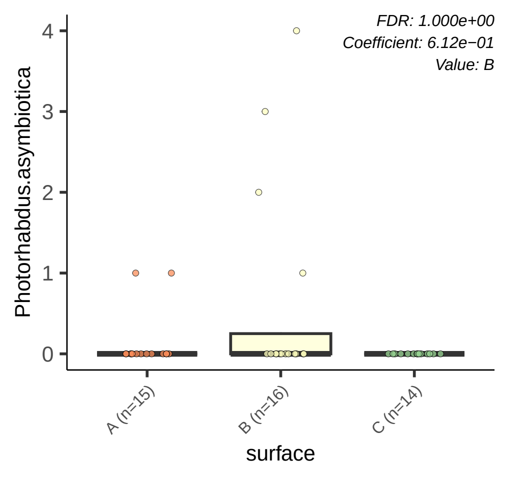
FDR: 1.000e+00
Coefficient: 6.12e−01
Value: B
Photorhabdus.asymbiotica
4
3
2
1
0
A (n=15)
B (n=16)
C (n=14)
surface

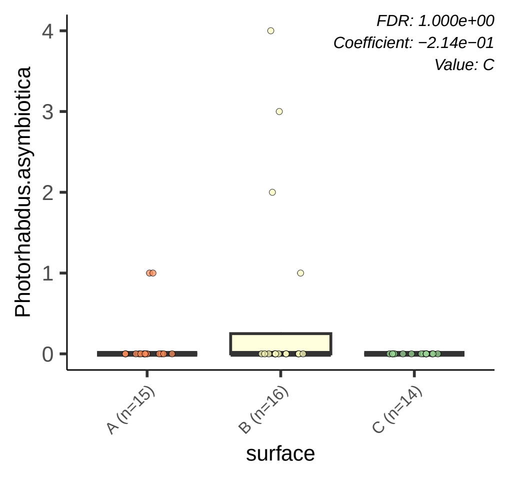
FDR: 1.000e+00
Coefficient: −2.14e−01
Value: C
Photorhabdus.asymbiotica
0
1
2
3
4
A (n=15)
B (n=16)
C (n=14)
surface

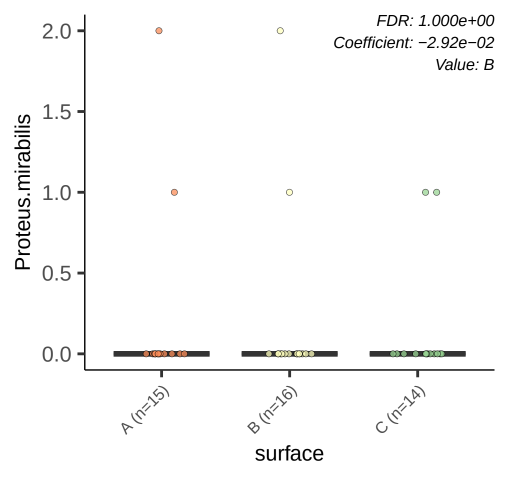
FDR: 1.000e+00
Coefficient: −2.92e−02
Value: B
Proteus.mirabilis
2.0
1.5
1.0
0.5
0.0
A (n=15)
B (n=16)
C (n=14)
surface

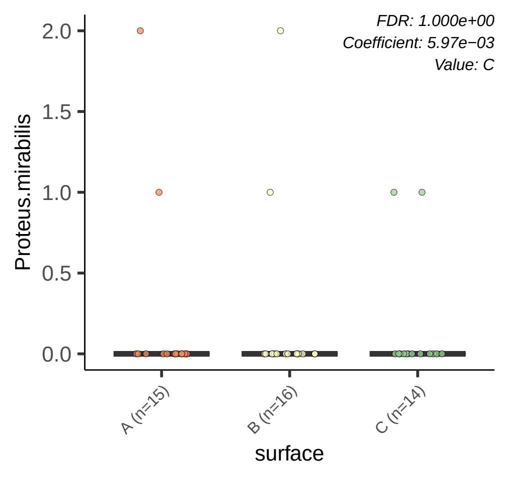
FDR: 1.000e+00
Coefficient: 5.97e−03
Value: C
Proteus.mirabilis
2.0
1.5
1.0
0.5
0.0
A (n=15)
B (n=16)
C (n=14)
surface

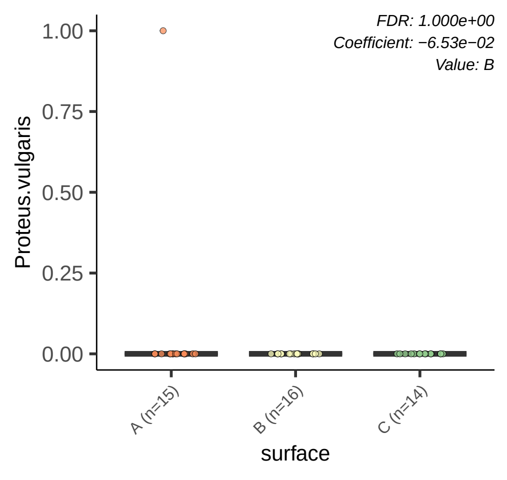
FDR: 1.000e+00
Coefficient: −6.53e−02
Value: B
Proteus.vulgaris
1.00
0.75
0.50
0.25
0.00
A (n=15)
B (n=16)
C (n=14)
surface

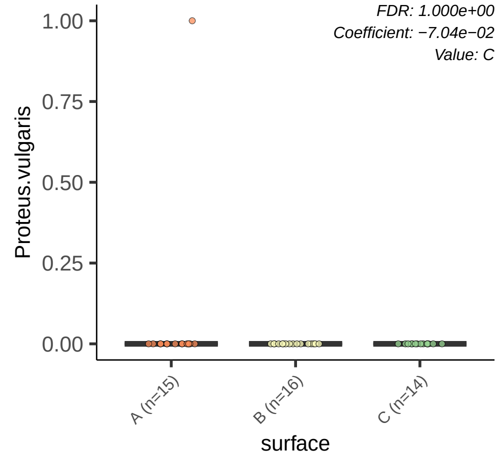
FDR: 1.000e+00
Coefficient: −7.04e−02
Value: C
Proteus.vulgaris
1.00
0.75
0.50
0.25
0.00
A (n=15)
B (n=16)
C (n=14)
surface

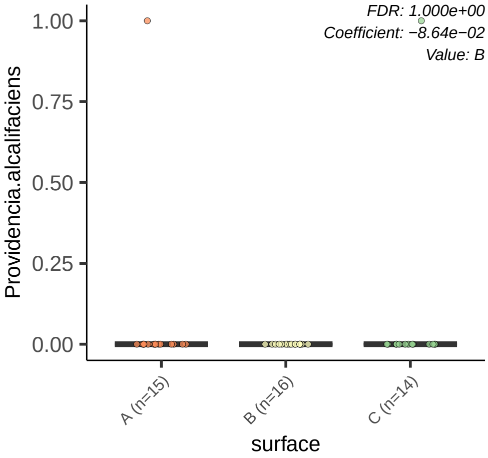
FDR: 1.000e+00
Coefficient: −8.64e−02
Value: B
Providencia.alcalifaciens
1.00
0.75
0.50
0.25
0.00
A (n=15)
B (n=16)
C (n=14)
surface

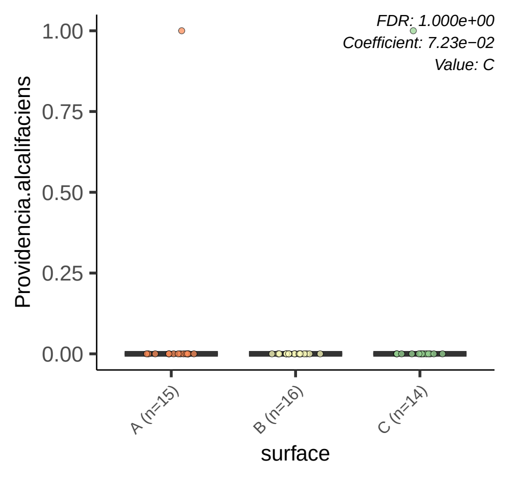
FDR: 1.000e+00
Coefficient: 7.23e−02
Value: C
Providencia.alcalifaciens
1.00
0.75
0.50
0.25
0.00
A (n=15)
B (n=16)
C (n=14)
surface

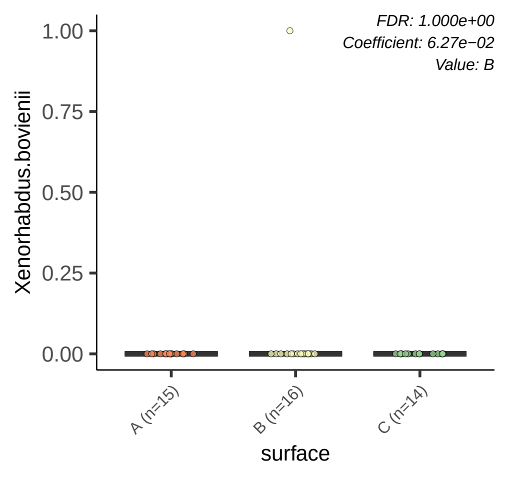

FDR: 1.000e+00
Coefficient: 6.27e−02
Value: B
Xenorhabdus.bovienii
1.00
0.75
0.50
0.25
0.00
A (n=15)
B (n=16)
C (n=14)
surface

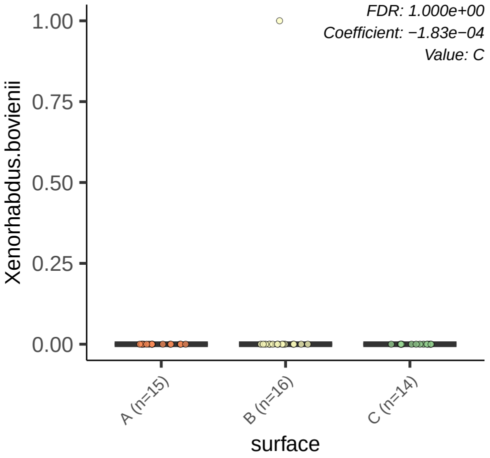

FDR: 1.000e+00
Coefficient: −1.83e−04
Value: C
Xenorhabdus.bovienii
1.00
0.75
0.50
0.25
0.00
A (n=15)
B (n=16)
C (n=14)
surface

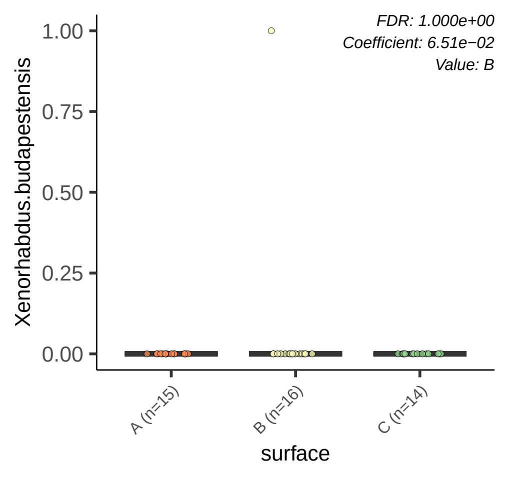
FDR: 1.000e+00
Coefficient: 6.51e−02
Value: B
Xenorhabdus.budapestensis
1.00
0.75
0.50
0.25
0.00
A (n=15)
B (n=16)
C (n=14)
surface

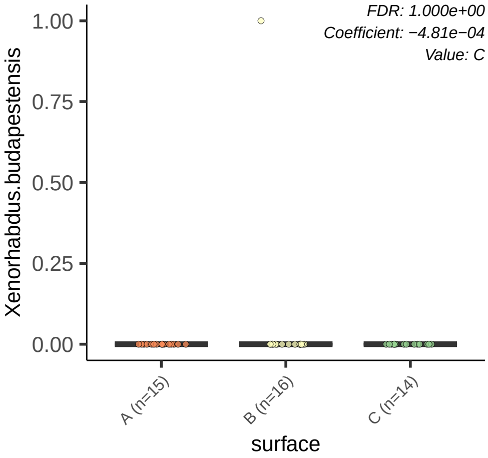
FDR: 1.000e+00
Coefficient: −4.81e−04
Value: C
Xenorhabdus.budapestensis
1.00
0.75
0.50
0.25
0.00
A (n=15)
B (n=16)
C (n=14)
surface

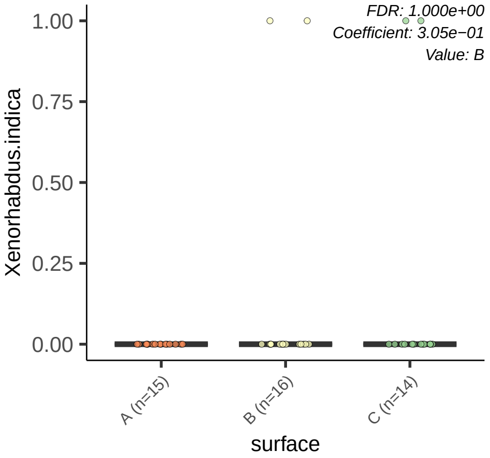
FDR: 1.000e+00
Coefficient: 3.05e−01
Value: B
Xenorhabdus.indica
1.00
0.75
0.50
0.25
0.00
A (n=15)
B (n=16)
C (n=14)
surface

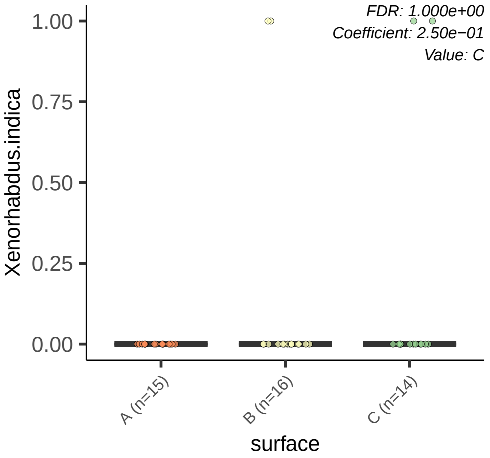

FDR: 1.000e+00
Coefficient: 2.50e−01
Value: C
Xenorhabdus.indica
1.00
0.75
0.50
0.25
0.00
A (n=15)
B (n=16)
C (n=14)
surface

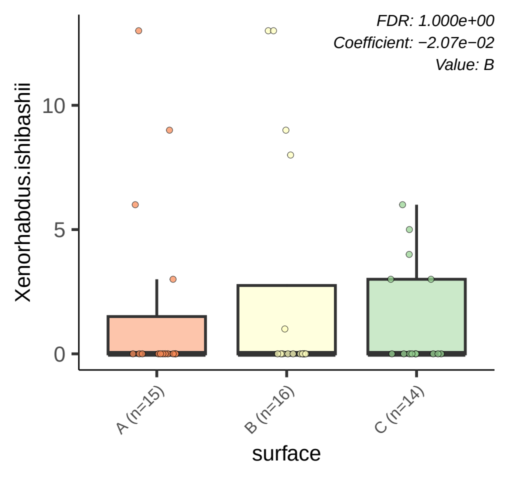

FDR: 1.000e+00
Coefficient: −2.07e−02
Value: B
Xenorhabdus.ishibashii
0
5
10
A (n=15)
B (n=16)
C (n=14)
surface

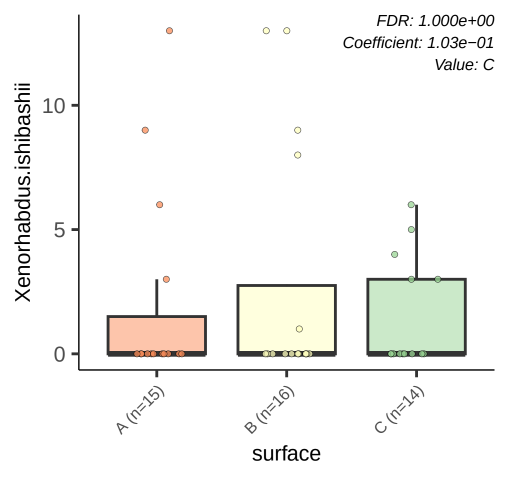
FDR: 1.000e+00
Coefficient: 1.03e−01
Value: C
Xenorhabdus.ishibashii
0
5
10
A (n=15)
B (n=16)
C (n=14)
surface

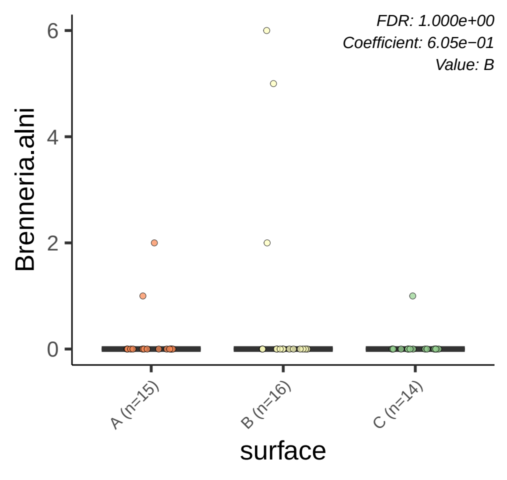
FDR: 1.000e+00
Coefficient: 6.05e−01
Value: B
Brenneria.alni
0
2
4
6
A (n=15)
B (n=16)
C (n=14)
surface

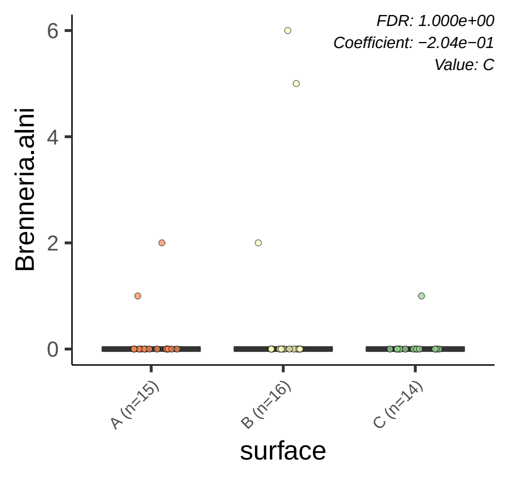

FDR: 1.000e+00
Coefficient: −2.04e−01
Value: C
Brenneria.alni
0
2
4
6
A (n=15)
B (n=16)
C (n=14)
surface

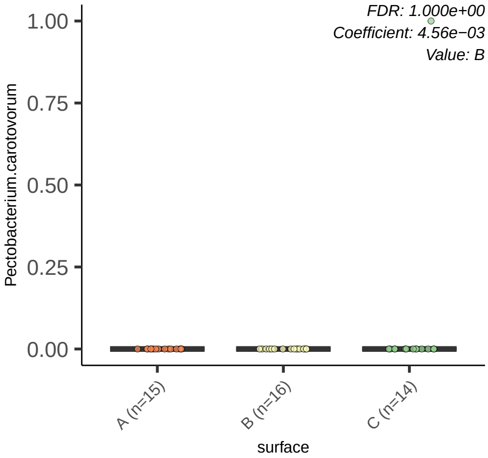
FDR: 1.000e+00
Coefficient: 4.56e−03
Value: B
Pectobacterium.carotovorum
1.00
0.75
0.50
0.25
0.00
A (n=15)
B (n=16)
C (n=14)
surface

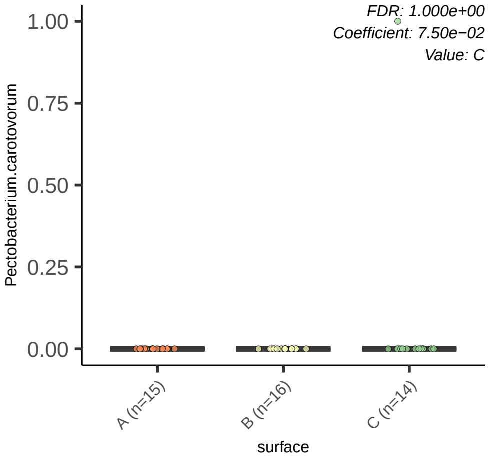

FDR: 1.000e+00
Coefficient: 7.50e−02
Value: C
Pectobacterium.carotovorum
1.00
0.75
0.50
0.25
0.00
A (n=15)
B (n=16)
C (n=14)
surface

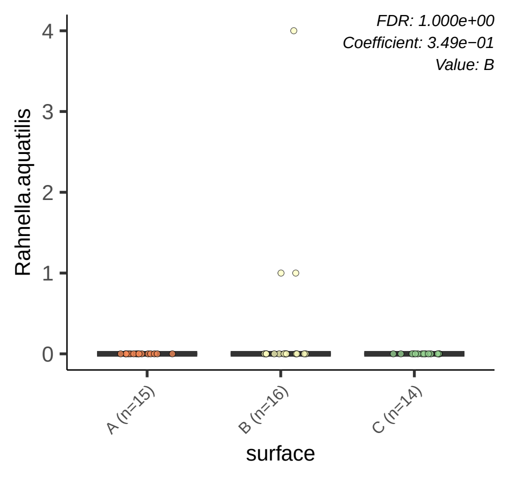
FDR: 1.000e+00
Coefficient: 3.49e−01
Value: B
Rahnella.aquatilis
0
1
2
3
4
A (n=15)
B (n=16)
C (n=14)
surface

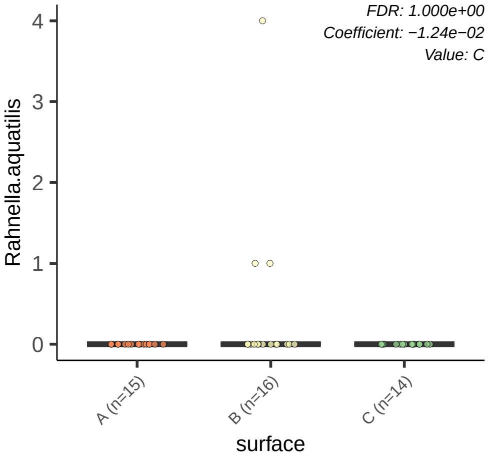

FDR: 1.000e+00
Coefficient: −1.24e−02
Value: C
Rahnella.aquatilis
0
1
2
3
4
A (n=15)
B (n=16)
C (n=14)
surface

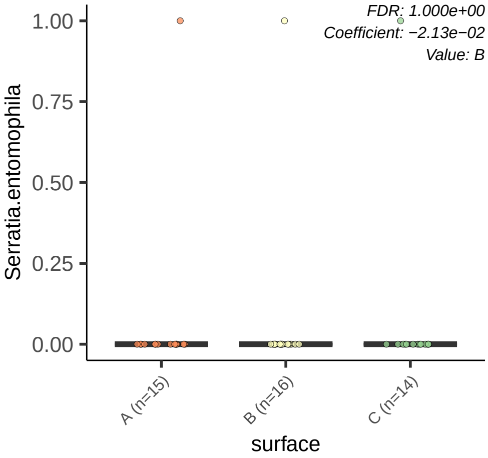

FDR: 1.000e+00
Coefficient: −2.13e−02
Value: B
Serratia.entomophila
1.00
0.75
0.50
0.25
0.00
A (n=15)
B (n=16)
C (n=14)
surface

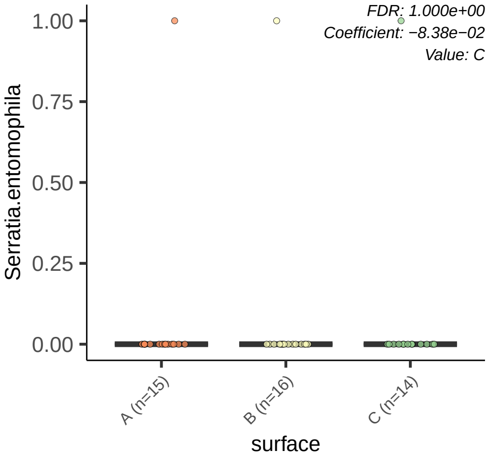

FDR: 1.000e+00
Coefficient: −8.38e−02
Value: C
Serratia.entomophila
1.00
0.75
0.50
0.25
0.00
A (n=15)
B (n=16)
C (n=14)
surface

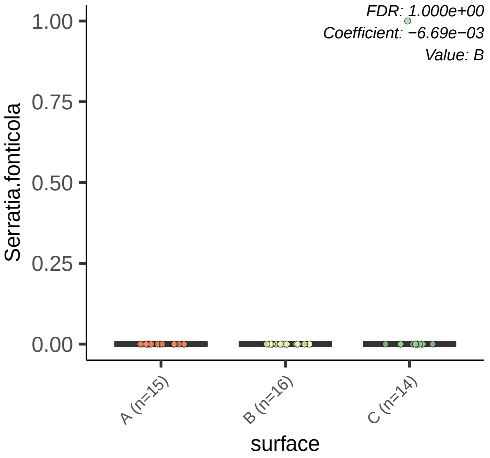
FDR: 1.000e+00
Coefficient: −6.69e−03
Value: B
Serratia.fonticola
1.00
0.75
0.50
0.25
0.00
A (n=15)
B (n=16)
C (n=14)
surface

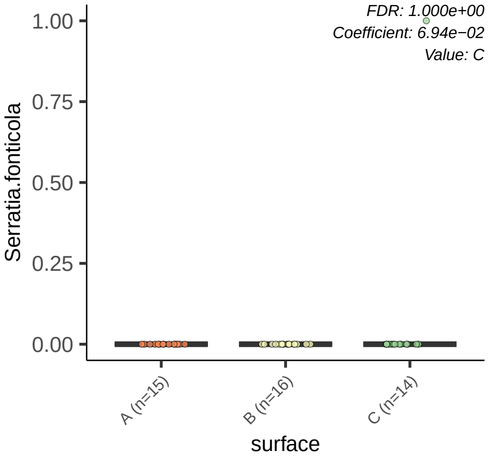

FDR: 1.000e+00
Coefficient: 6.94e−02
Value: C
Serratia.fonticola
1.00
0.75
0.50
0.25
0.00
A (n=15)
B (n=16)
C (n=14)
surface

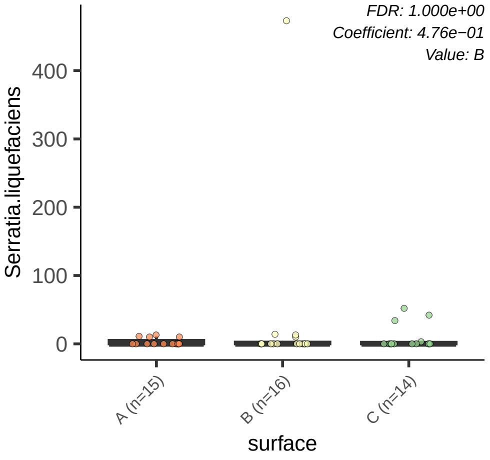

FDR: 1.000e+00
Coefficient: 4.76e−01
Value: B
Serratia.liquefaciens
400
300
200
100
0
A (n=15)
B (n=16)
C (n=14)
surface

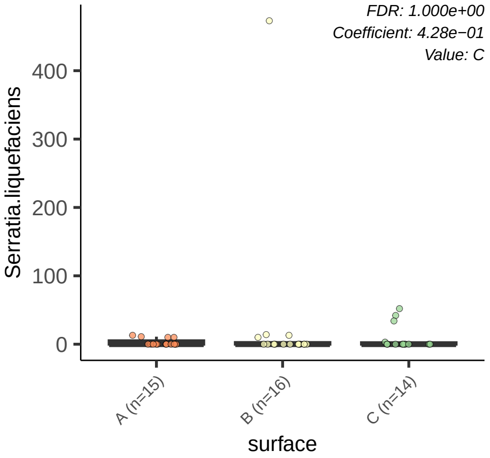

FDR: 1.000e+00
Coefficient: 4.28e−01
Value: C
Serratia.liquefaciens
0
100
200
300
400
A (n=15)
B (n=16)
C (n=14)
surface

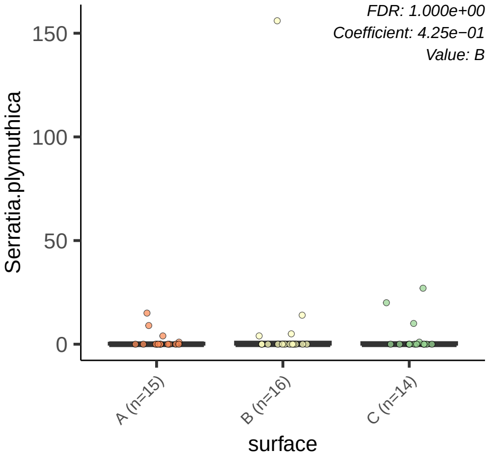

FDR: 1.000e+00
Coefficient: 4.25e−01
Value: B
Serratia.plymuthica
150
100
50
0
A (n=15)
B (n=16)
C (n=14)
surface

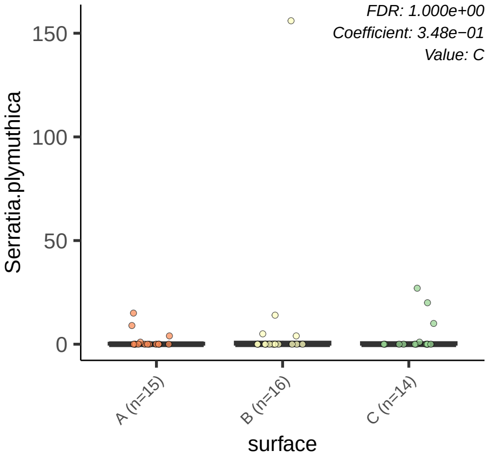
FDR: 1.000e+00
Coefficient: 3.48e−01
Value: C
150
100
50
0
Serratia.plymuthica
A (n=15)
B (n=16)
C (n=14)
surface

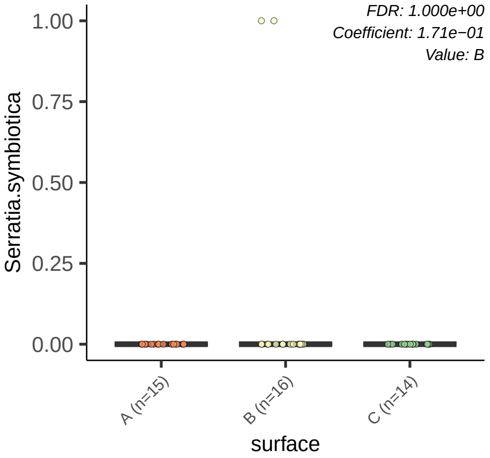

FDR: 1.000e+00
Coefficient: 1.71e−01
Value: B
1.00
0.75
0.50
0.25
0.00
Serratia.symbiotica
A (n=15)
B (n=16)
C (n=14)
surface

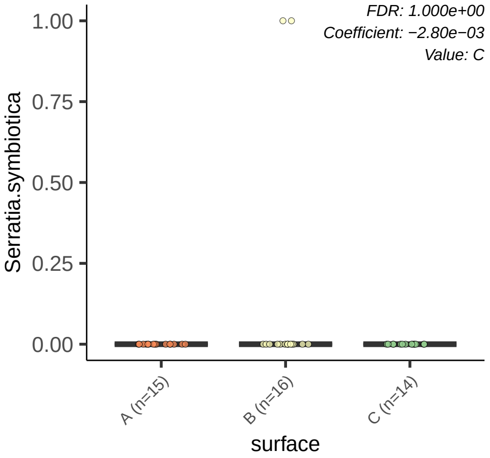
FDR: 1.000e+00
Coefficient: −2.80e−03
Value: C
Serratia.symbiotica
1.00
0.75
0.50
0.25
0.00
A (n=15)
B (n=16)
C (n=14)
surface

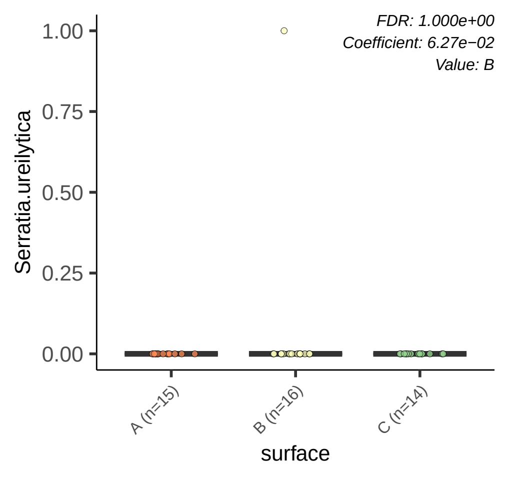

FDR: 1.000e+00
Coefficient: 6.27e−02
Value: B
Serratia.ureilytica
1.00
0.75
0.50
0.25
0.00
A (n=15)
B (n=16)
C (n=14)
surface

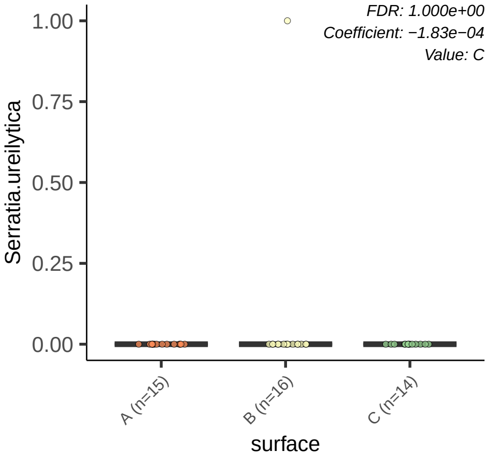

FDR: 1.000e+00
Coefficient: −1.83e−04
Value: C
Serratia.ureilytica
1.00
0.75
0.50
0.25
0.00
A (n=15)
B (n=16)
C (n=14)
surface

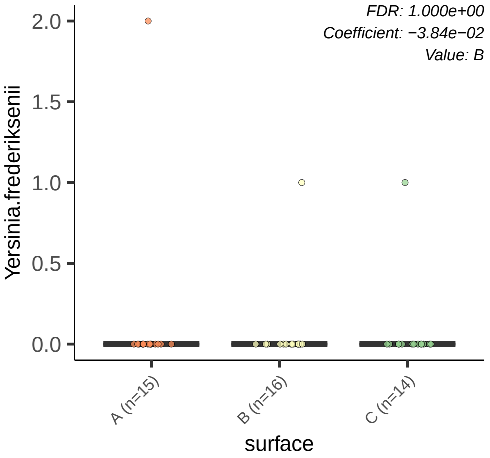
FDR: 1.000e+00
Coefficient: −3.84e−02
Value: B
Yersinia.frederiksenii
2.0
1.5
1.0
0.5
0.0
A (n=15)
B (n=16)
C (n=14)
surface

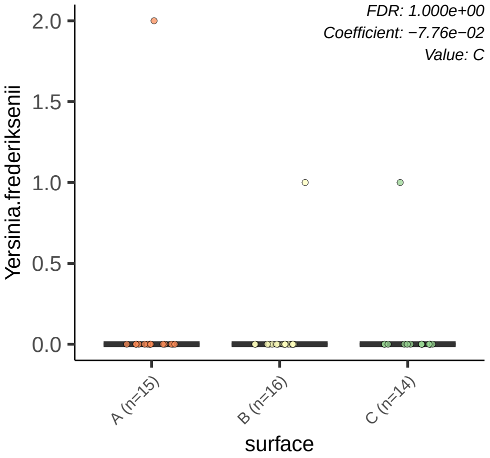
FDR: 1.000e+00
Coefficient: −7.76e−02
Value: C
Yersinia.frederiksenii
2.0
1.5
1.0
0.5
0.0
A (n=15)
B (n=16)
C (n=14)
surface

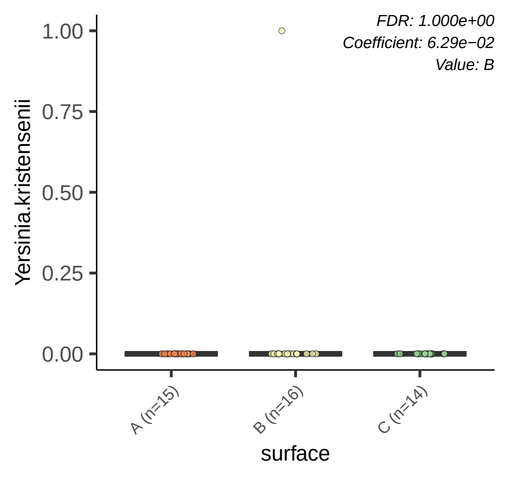
FDR: 1.000e+00
Coefficient: 6.29e−02
Value: B
Yersinia.kristensenii
1.00
0.75
0.50
0.25
0.00
A (n=15)
B (n=16)
C (n=14)
surface

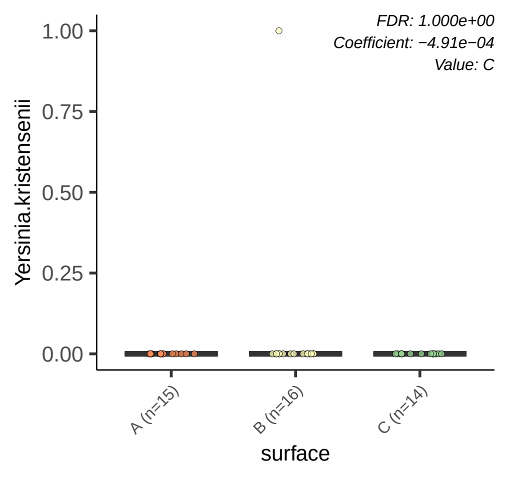
FDR: 1.000e+00
Coefficient: −4.91e−04
Value: C
Yersinia.kristensenii
1.00
0.75
0.50
0.25
0.00
A (n=15)
B (n=16)
C (n=14)
surface

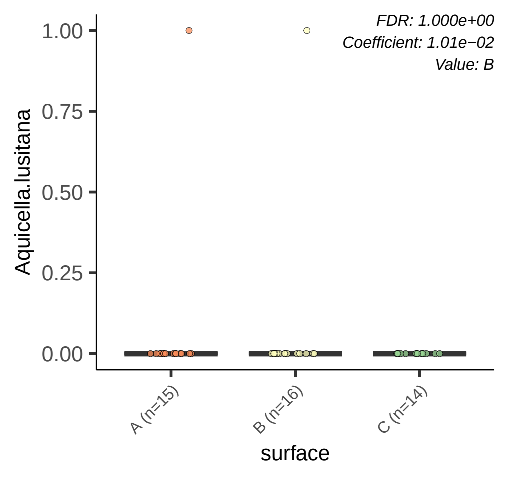
FDR: 1.000e+00
Coefficient: 1.01e−02
Value: B
Aquicella.lusitana
1.00
0.75
0.50
0.25
0.00
A (n=15)
B (n=16)
C (n=14)
surface

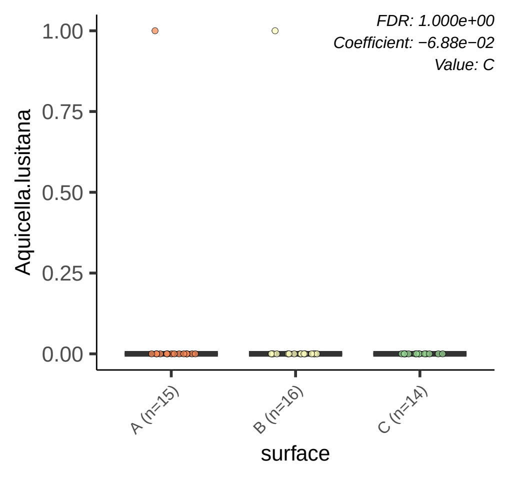

FDR: 1.000e+00
Coefficient: −6.88e−02
Value: C
1.00
0.75
0.50
0.25
0.00
Aquicella.lusitana
A (n=15)
B (n=16)
C (n=14)
surface

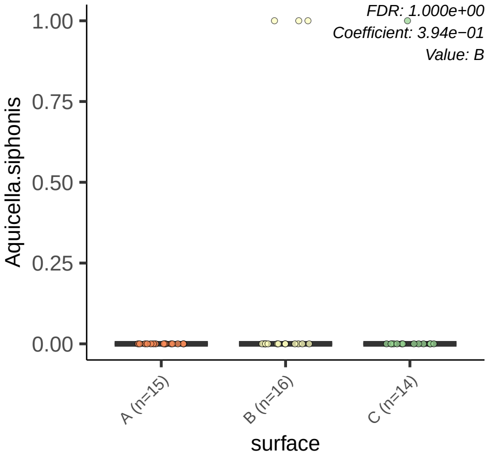
FDR: 1.000e+00
Coefficient: 3.94e−01
Value: B
1.00
0.75
0.50
0.25
0.00
Aquicella.siphonis
A (n=15)
B (n=16)
C (n=14)
surface

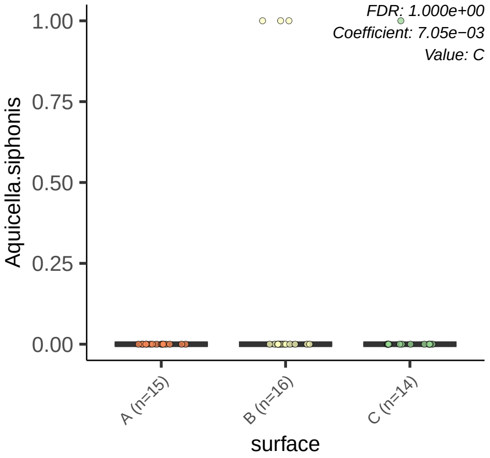
FDR: 1.000e+00
Coefficient: 7.05e−03
Value: C
Aquicella.siphonis
1.00
0.75
0.50
0.25
0.00
A (n=15)
B (n=16)
C (n=14)
surface

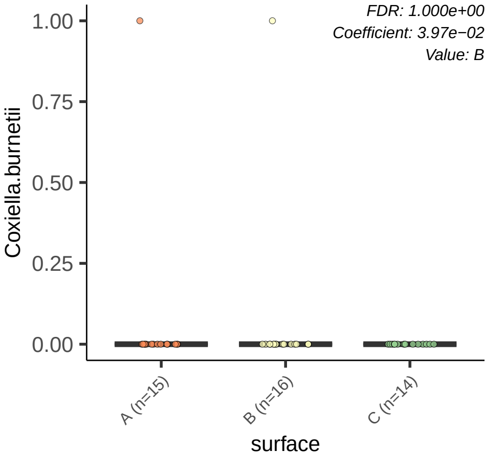

FDR: 1.000e+00
Coefficient: 3.97e−02
Value: B
Coxiella.burnetii
1.00
0.75
0.50
0.25
0.00
A (n=15)
B (n=16)
C (n=14)
surface

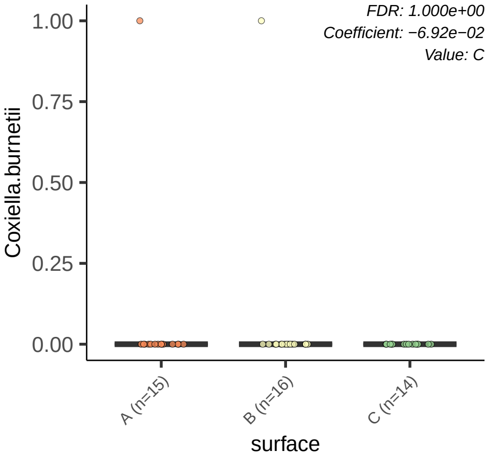
FDR: 1.000e+00
Coefficient: −6.92e−02
Value: C
Coxiella.burnetii
1.00
0.75
0.50
0.25
0.00
A (n=15)
B (n=16)
C (n=14)
surface

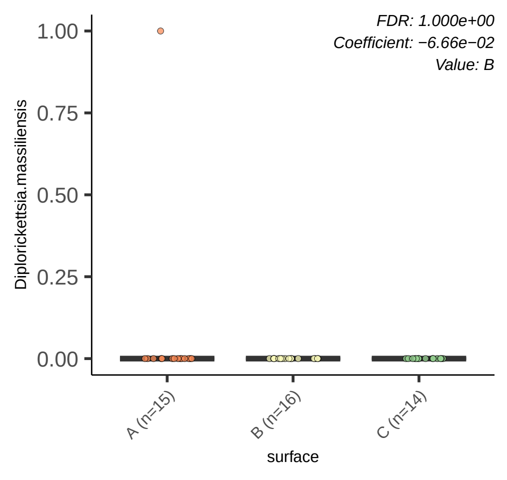

FDR: 1.000e+00
Coefficient: −6.66e−02
Value: B
1.00
0.75
0.50
0.25
0.00
Diplorickettsia.massiliensis
A (n=15)
B (n=16)
C (n=14)
surface

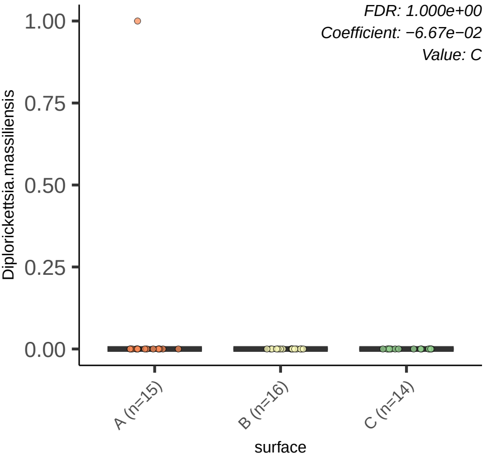
FDR: 1.000e+00
Coefficient: −6.67e−02
Value: C
1.00
0.75
0.50
0.25
0.00
Diploricketsia.massiliensis
A (n=15)
B (n=16)
C (n=14)
surface

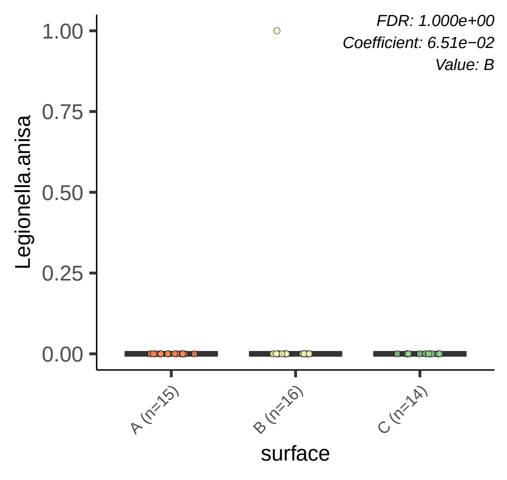
FDR: 1.000e+00
Coefficient: 6.51e−02
Value: B
Legionella.anisa
1.00
0.75
0.50
0.25
0.00
A (n=15)
B (n=16)
C (n=14)
surface

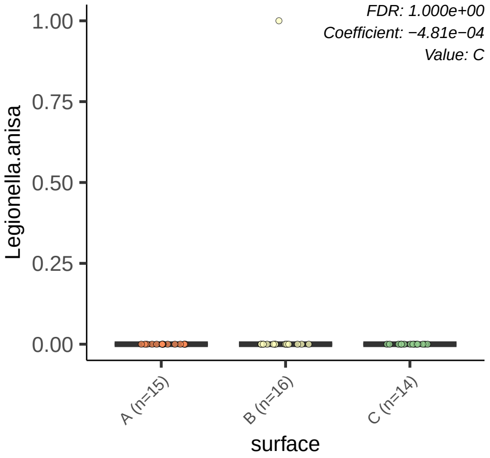
FDR: 1.000e+00
Coefficient: −4.81e−04
Value: C
Legionella.anisa
1.00
0.75
0.50
0.25
0.00
A (n=15)
B (n=16)
C (n=14)
surface

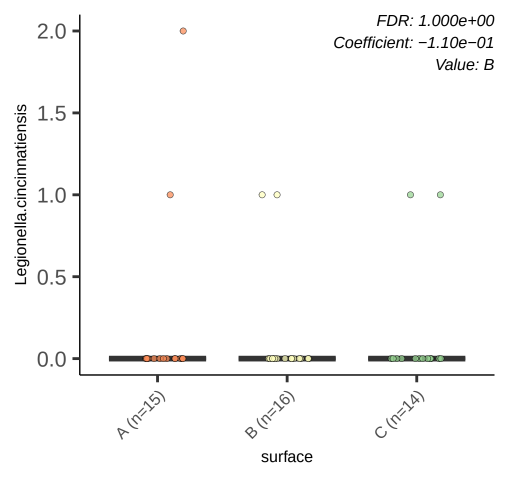
FDR: 1.000e+00
Coefficient: −1.10e−01
Value: B
Legionella.cincinnatiensis
2.0
1.5
1.0
0.5
0.0
A (n=15)
B (n=16)
C (n=14)
surface

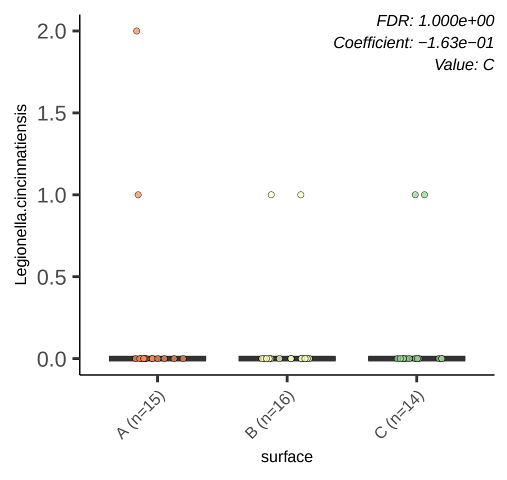
FDR: 1.000e+00
Coefficient: −1.63e−01
Value: C
Legionella.cincinnatiensis
2.0
1.5
1.0
0.5
0.0
A (n=15)
B (n=16)
C (n=14)
surface

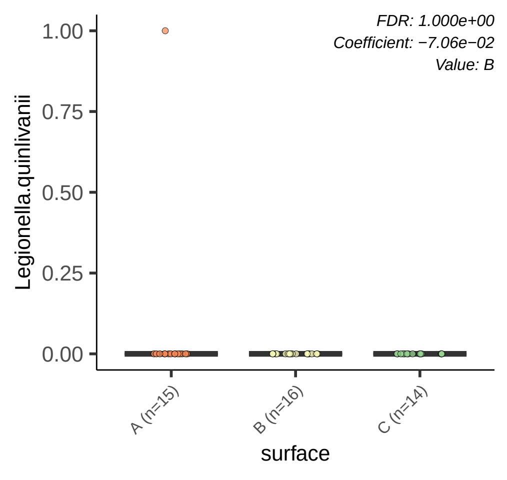
FDR: 1.000e+00
Coefficient: −7.06e−02
Value: B
Legionella.quinlivanii
1.00
0.75
0.50
0.25
0.00
A (n=15)
B (n=16)
C (n=14)
surface

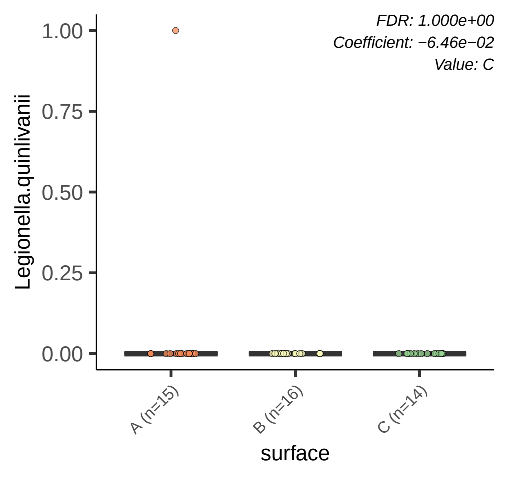
FDR: 1.000e+00
Coefficient: −6.46e−02
Value: C
Legionella.quinlivanii
1.00
0.75
0.50
0.25
0.00
A (n=15)
B (n=16)
C (n=14)
surface

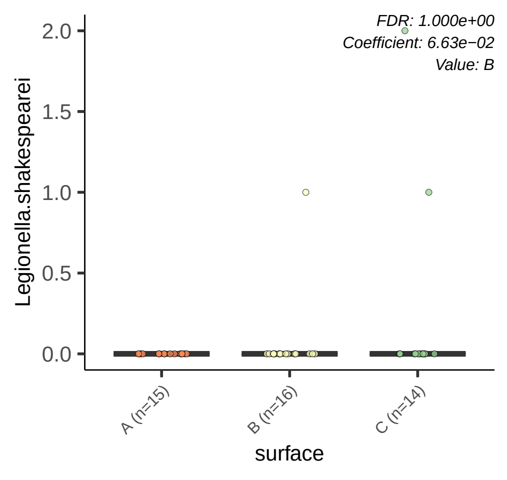

FDR: 1.000e+00
Coefficient: 6.63e−02
Value: B
Legionella.shakespearei
2.0
1.5
1.0
0.5
0.0
A (n=15)
B (n=16)
C (n=14)
surface

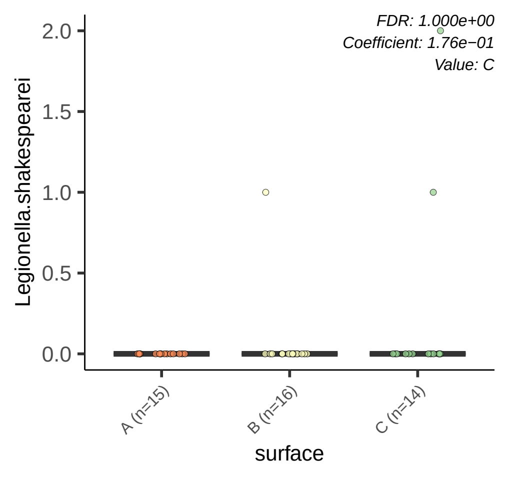
FDR: 1.000e+00
Coefficient: 1.76e−01
Value: C
Legionella.shakespearei
2.0
1.5
1.0
0.5
0.0
A (n=15)
B (n=16)
C (n=14)
surface

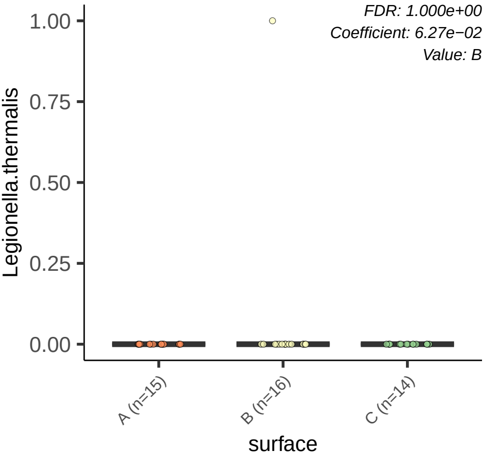
FDR: 1.000e+00
Coefficient: 6.27e−02
Value: B
Legionella.thermalis
1.00
0.75
0.50
0.25
0.00
A (n=15)
B (n=16)
C (n=14)
surface

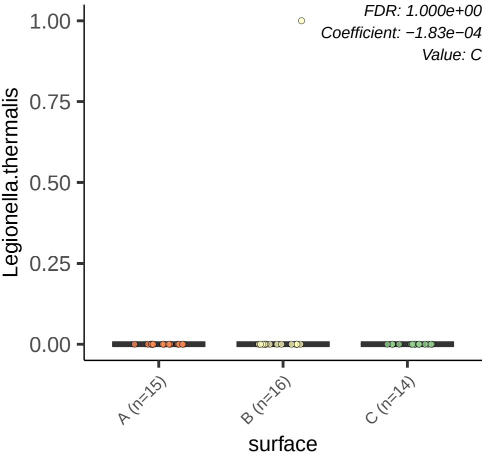
FDR: 1.000e+00
Coefficient: −1.83e−04
Value: C
1.00
0.75
0.50
0.25
0.00
Legionella.thermalis
A (n=15)
B (n=16)
C (n=14)
surface

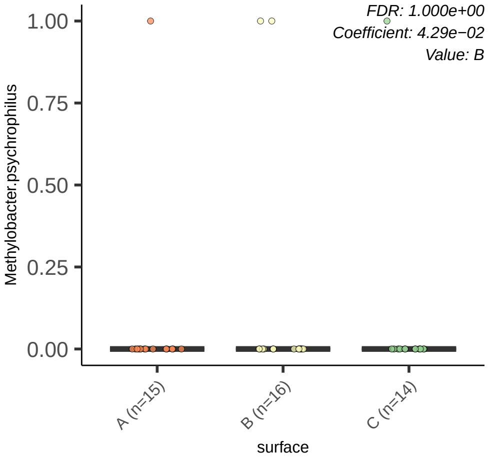

FDR: 1.000e+00
Coefficient: 4.29e−02
Value: B
Methylobacter.psychrophilus
1.00
0.75
0.50
0.25
0.00
A (n=15)
B (n=16)
C (n=14)
surface

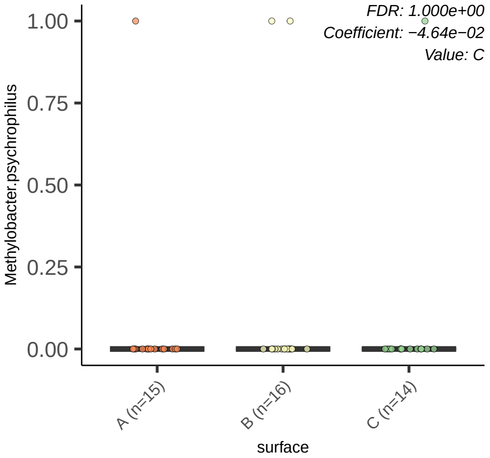
FDR: 1.000e+00
Coefficient: −4.64e−02
Value: C
Methylobacter.psychrophilus
1.00
0.75
0.50
0.25
0.00
A (n=15)
B (n=16)
C (n=14)
surface

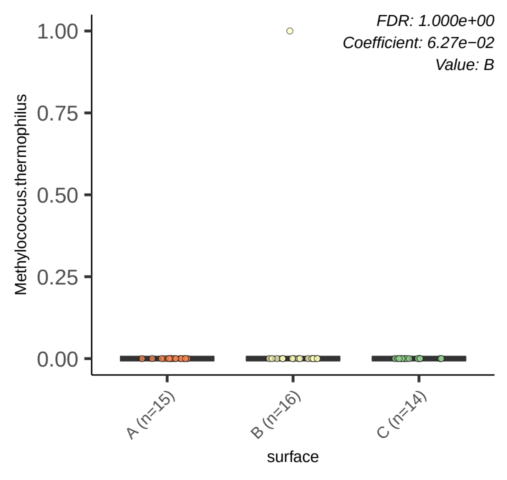

FDR: 1.000e+00
Coefficient: 6.27e−02
Value: B
Methylococcus.thermophilus
1.00
0.75
0.50
0.25
0.00
A (n=15)
B (n=16)
C (n=14)
surface

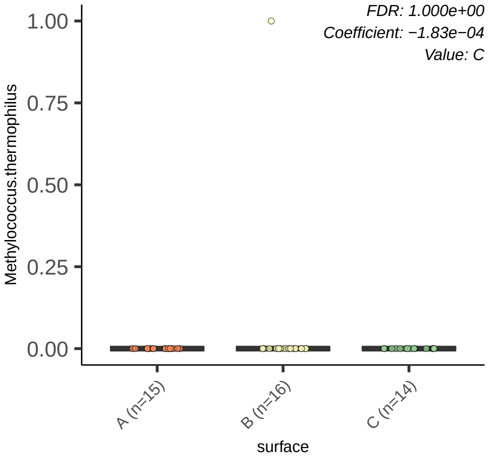
FDR: 1.000e+00
Coefficient: −1.83e−04
Value: C
Methylococcus.thermophilus
1.00
0.75
0.50
0.25
0.00
A (n=15)
B (n=16)
C (n=14)
surface

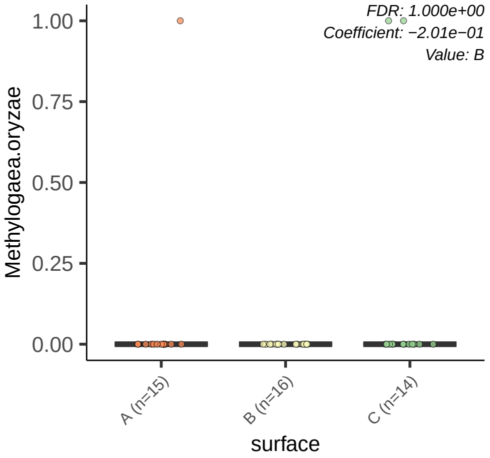

FDR: 1.000e+00
Coefficient: −2.01e−01
Value: B
Methylogaea.oryzae
1.00
0.75
0.50
0.25
0.00
A (n=15)
B (n=16)
C (n=14)
surface

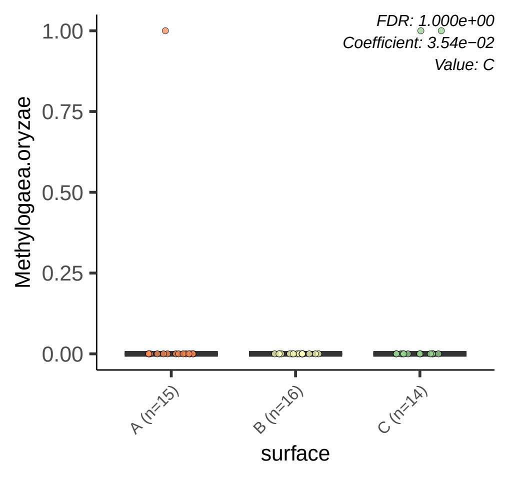
FDR: 1.000e+00
Coefficient: 3.54e−02
Value: C
Methylogaea.oryzae
1.00
0.75
0.50
0.25
0.00
A (n=15)
B (n=16)
C (n=14)
surface

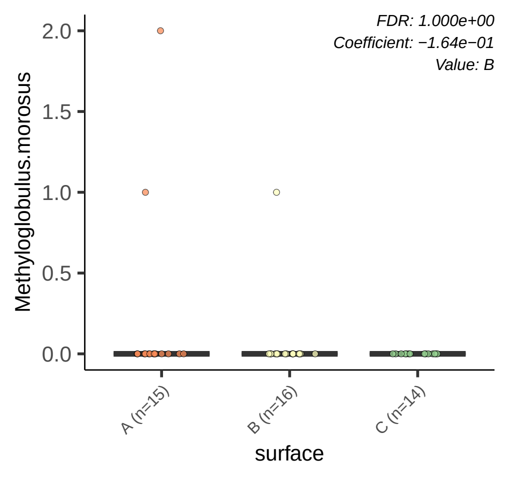

FDR: 1.000e+00
Coefficient: −1.64e−01
Value: B
Methyloglobulus.morosus
2.0
1.5
1.0
0.5
0.0
A (n=15)
B (n=16)
C (n=14)
surface

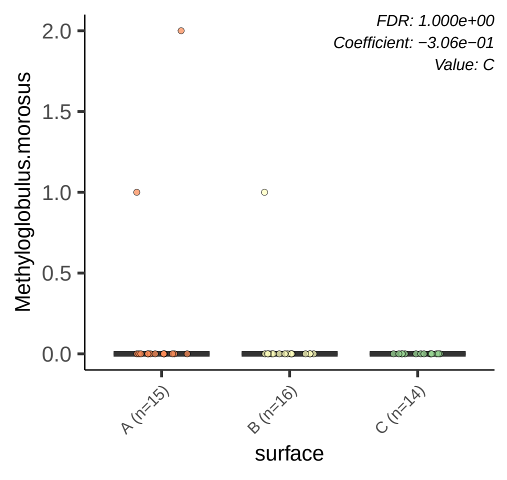
FDR: 1.000e+00
Coefficient: −3.06e−01
Value: C
Methyloglobulus.morosus
2.0
1.5
1.0
0.5
0.0
A (n=15)
B (n=16)
C (n=14)
surface

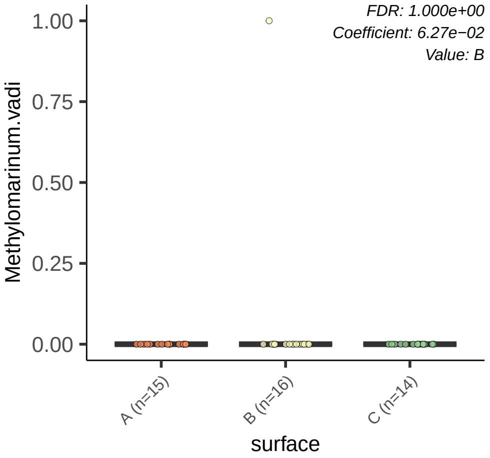
FDR: 1.000e+00
Coefficient: 6.27e−02
Value: B
Methylomarinum.vadi
1.00
0.75
0.50
0.25
0.00
A (n=15)
B (n=16)
C (n=14)
surface

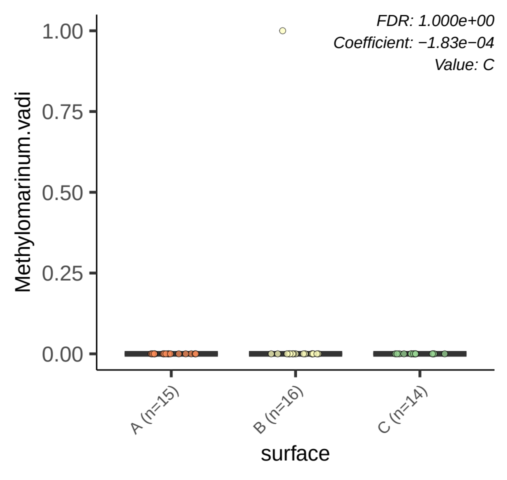
FDR: 1.000e+00
Coefficient: −1.83e−04
Value: C
Methylomarinum.vadi
1.00
0.75
0.50
0.25
0.00
A (n=15)
B (n=16)
C (n=14)
surface

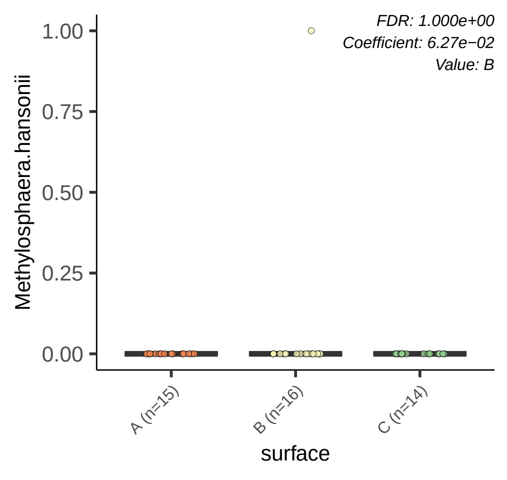

FDR: 1.000e+00
Coefficient: 6.27e−02
Value: B
Methylosphaera.hansonii
1.00
0.75
0.50
0.25
0.00
A (n=15)
B (n=16)
C (n=14)
surface

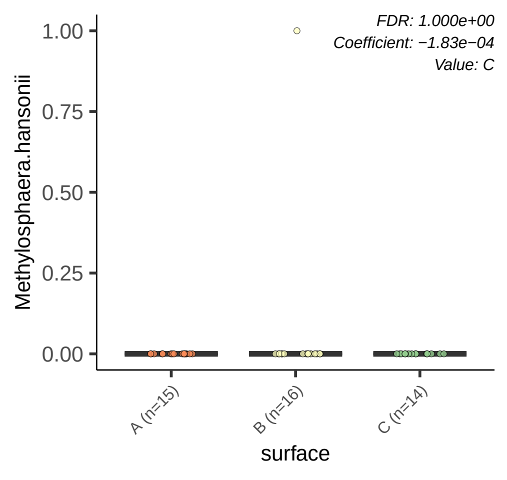
FDR: 1.000e+00
Coefficient: −1.83e−04
Value: C
Methylosphaera.hansonii
1.00
0.75
0.50
0.25
0.00
A (n=15)
B (n=16)
C (n=14)
surface

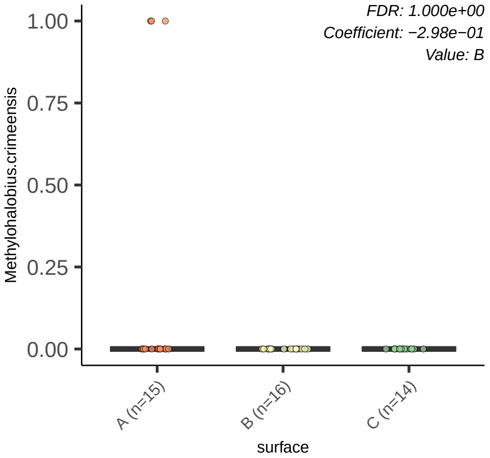
FDR: 1.000e+00
Coefficient: −2.98e−01
Value: B
Methylohalobius.crimeensis
1.00
0.75
0.50
0.25
0.00
A (n=15)
B (n=16)
C (n=14)
surface

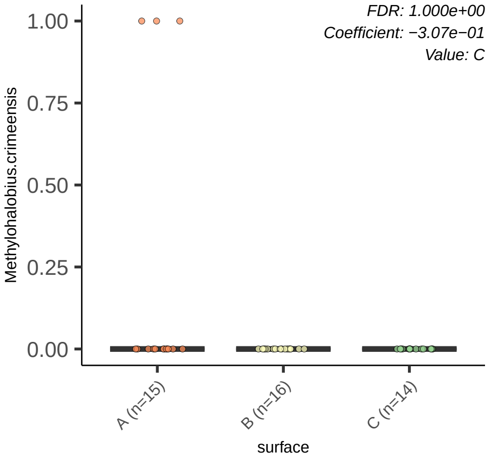

FDR: 1.000e+00
Coefficient: −3.07e−01
Value: C
1.00
0.75
0.50
0.25
0.00
Methylohalobius.crimeensis
A (n=15)
B (n=16)
C (n=14)
surface

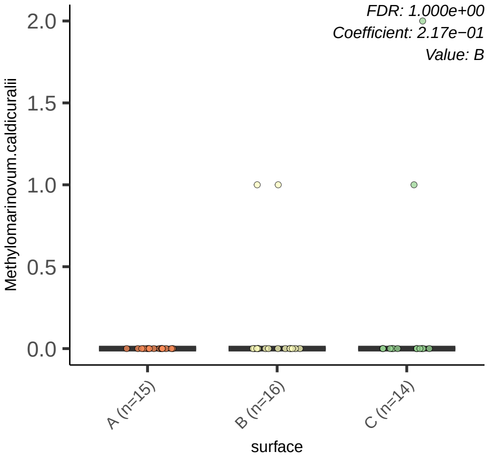
FDR: 1.000e+00
Coefficient: 2.17e−01
Value: B
Methylomarinovum.caldicuralii
2.0
1.5
1.0
0.5
0.0
A (n=15)
B (n=16)
C (n=14)
surface

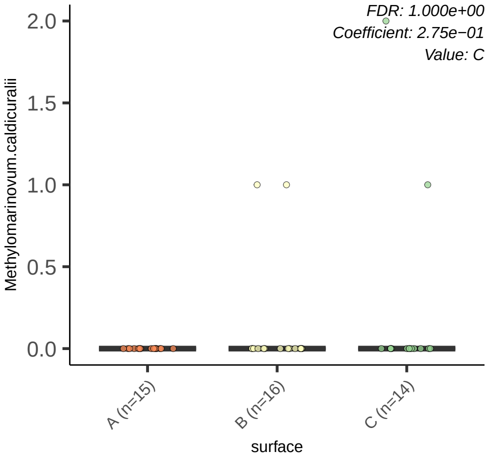

FDR: 1.000e+00
Coefficient: 2.75e−01
Value: C
Methylomarinovum.caldicuralii
2.0
1.5
1.0
0.5
0.0
A (n=15)
B (n=16)
C (n=14)
surface

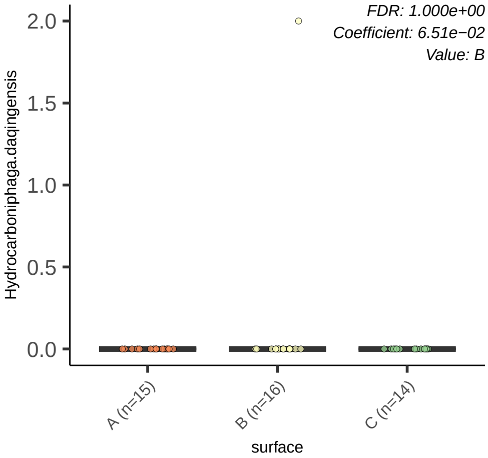
FDR: 1.000e+00
Coefficient: 6.51e−02
Value: B
Hydrocarboniphaga.daqingensis
2.0
1.5
1.0
0.5
0.0
A (n=15)
B (n=16)
C (n=14)
surface

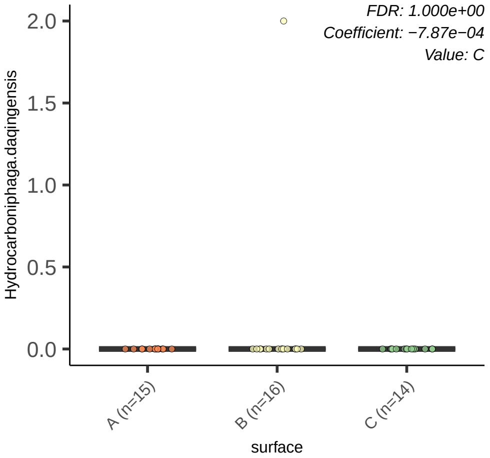

FDR: 1.000e+00
Coefficient: −7.87e−04
Value: C
Hydrocarboniphaga.daqingensis
2.0
1.5
1.0
0.5
0.0
A (n=15)
B (n=16)
C (n=14)
surface

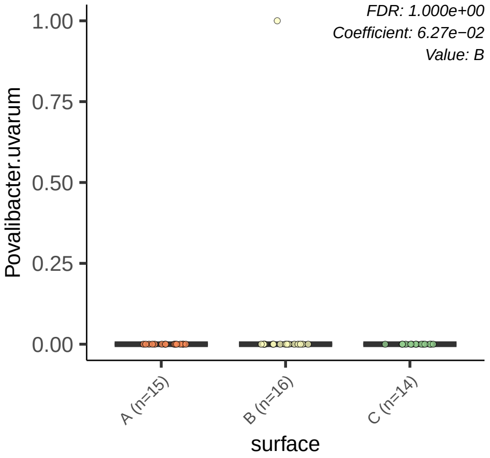
FDR: 1.000e+00
Coefficient: 6.27e−02
Value: B
Povalibacter.uvarum
1.00
0.75
0.50
0.25
0.00
A (n=15)
B (n=16)
C (n=14)
surface

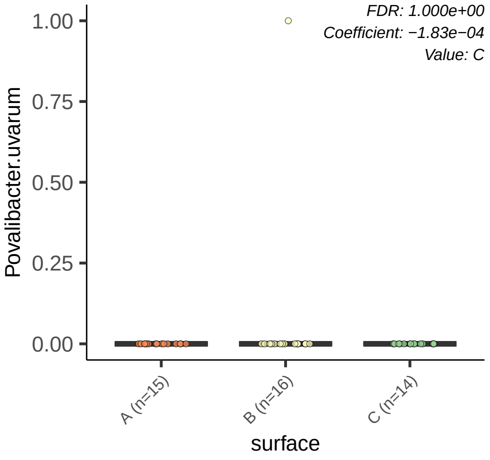

FDR: 1.000e+00
Coefficient: −1.83e−04
Value: C
Povalibacter.uvarum
1.00
0.75
0.50
0.25
0.00
A (n=15)
B (n=16)
C (n=14)
surface

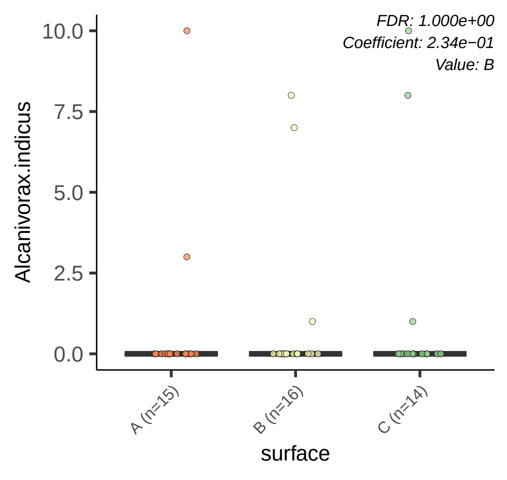
FDR: 1.000e+00
Coefficient: 2.34e−01
Value: B
Alcanivorax.indicus
10.0
7.5
5.0
2.5
0.0
A (n=15)
B (n=16)
C (n=14)
surface

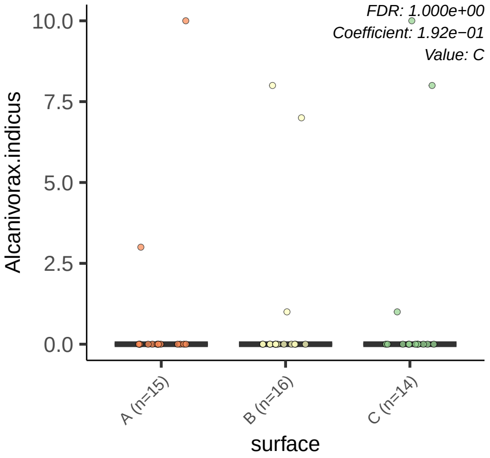
FDR: 1.000e+00
Coefficient: 1.92e−01
Value: C
Alcanivorax.indicus
10.0
7.5
5.0
2.5
0.0
A (n=15)
B (n=16)
C (n=14)
surface

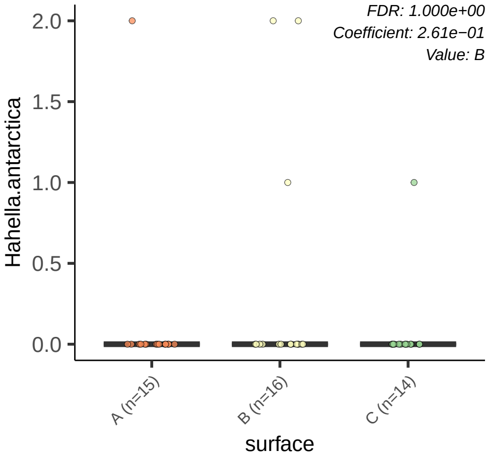
FDR: 1.000e+00
Coefficient: 2.61e−01
Value: B
Hahella.antarctica
2.0
1.5
1.0
0.5
0.0
A (n=15)
B (n=16)
C (n=14)
surface

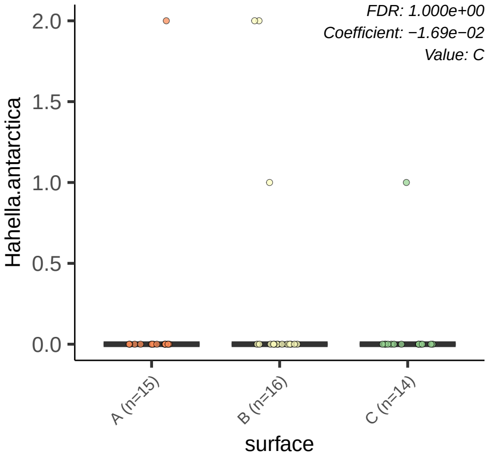

FDR: 1.000e+00
Coefficient: −1.69e−02
Value: C
Hahella.antarctica
2.0
1.5
1.0
0.5
0.0
A (n=15)
B (n=16)
C (n=14)
surface

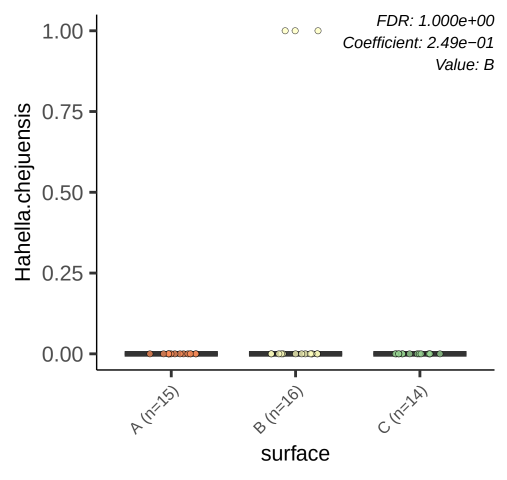

FDR: 1.000e+00
Coefficient: 2.49e−01
Value: B
Hahella.chejuensis
1.00
0.75
0.50
0.25
0.00
A (n=15)
B (n=16)
C (n=14)
surface

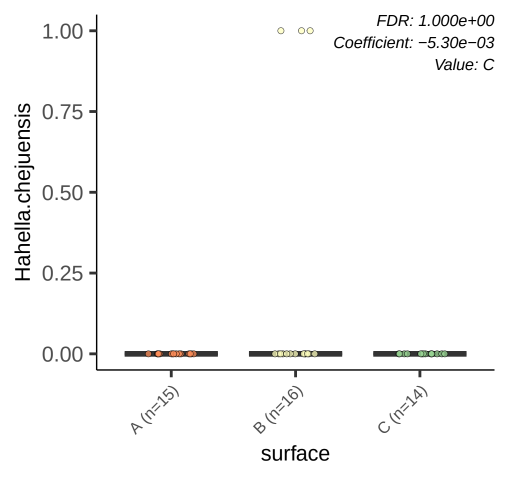

FDR: 1.000e+00
Coefficient: −5.30e−03
Value: C
Hahella.chejuensis
1.00
0.75
0.50
0.25
0.00
A (n=15)
B (n=16)
C (n=14)
surface

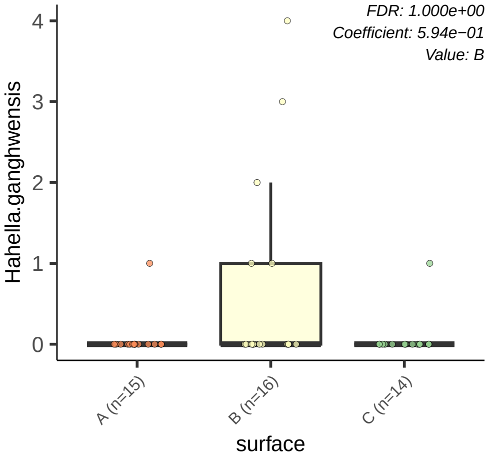
FDR: 1.000e+00
Coefficient: 5.94e−01
Value: B
Hahella.ganghwensis
4
3
2
1
0
A (n=15)
B (n=16)
C (n=14)
surface

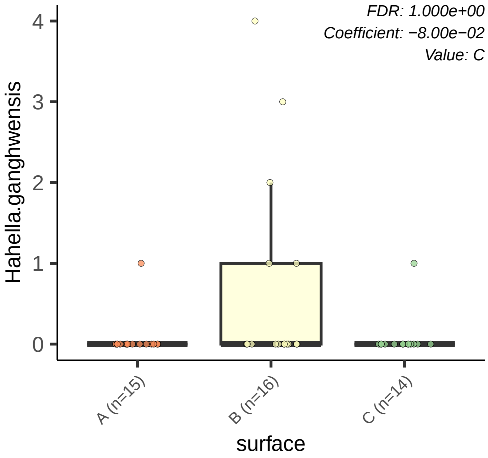

FDR: 1.000e+00
Coefficient: −8.00e−02
Value: C
Hahella.ganghwensis
0
1
2
3
4
A (n=15)
B (n=16)
C (n=14)
surface

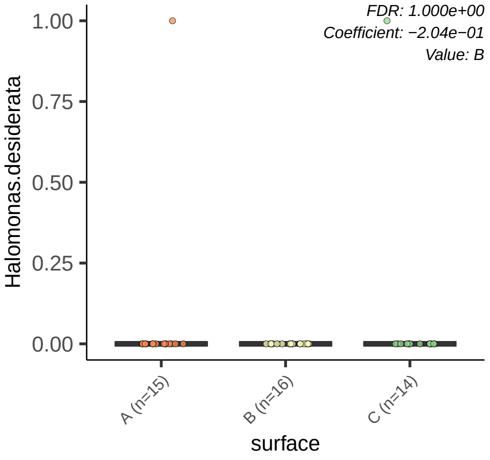
FDR: 1.000e+00
Coefficient: −2.04e−01
Value: B
Halomonas.desiderata
1.00
0.75
0.50
0.25
0.00
A (n=15)
B (n=16)
C (n=14)
surface

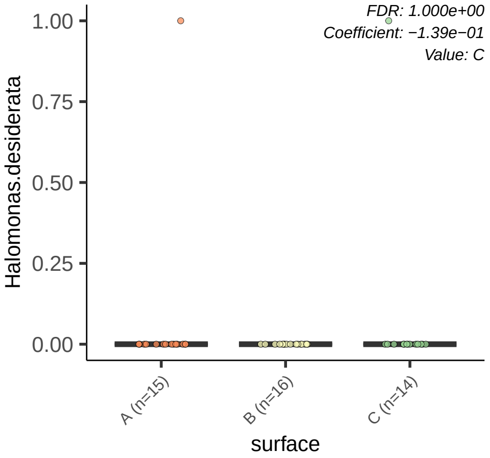
FDR: 1.000e+00
Coefficient: −1.39e−01
Value: C
1.00
0.75
0.50
0.25
0.00
Halomonas.desiderata
A (n=15)
B (n=16)
C (n=14)
surface

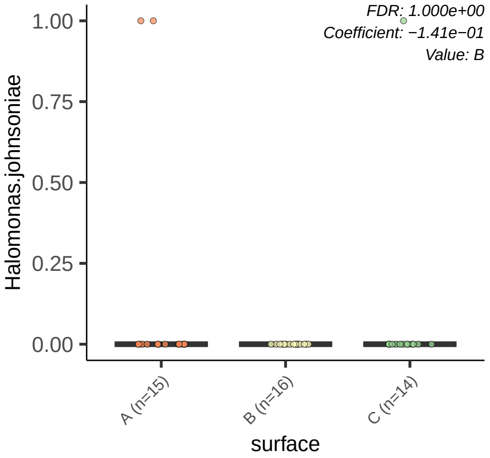

FDR: 1.000e+00
Coefficient: −1.41e−01
Value: B
1.00
0.75
0.50
0.25
0.00
Halomonas.johnsoniae
A (n=15)
B (n=16)
C (n=14)
surface

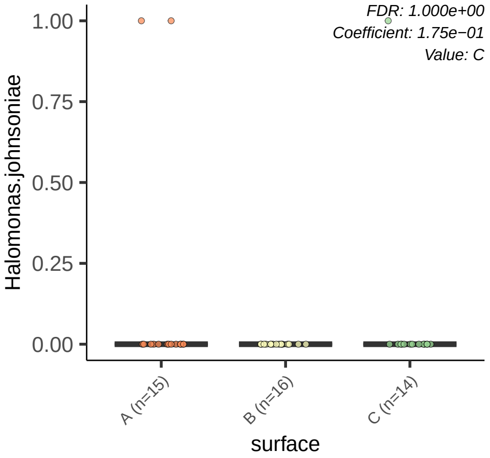

FDR: 1.000e+00
Coefficient: 1.75e−01
Value: C
Halomonas.johnsoniae
1.00
0.75
0.50
0.25
0.00
A (n=15)
B (n=16)
C (n=14)
surface

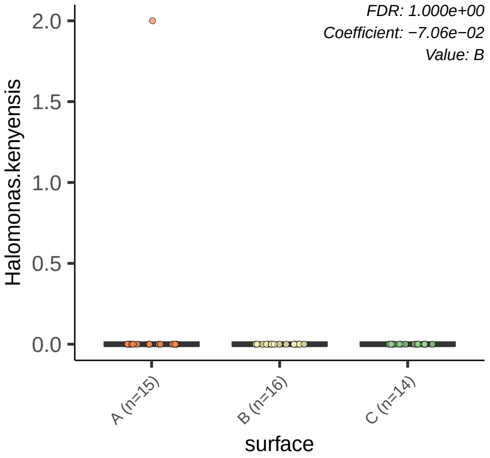
FDR: 1.000e+00
Coefficient: −7.06e−02
Value: B
Halomonas.kenyensis
2.0
1.5
1.0
0.5
0.0
A (n=15)
B (n=16)
C (n=14)
surface

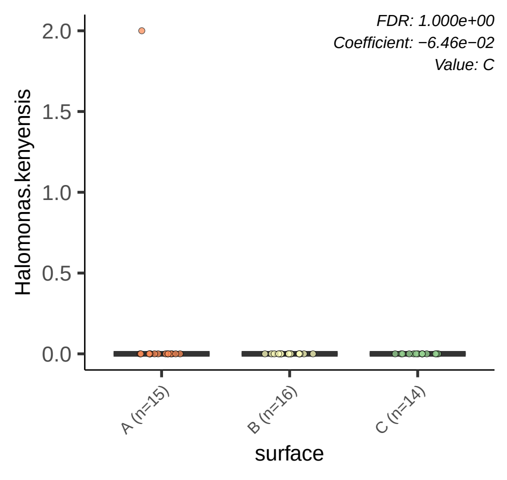
FDR: 1.000e+00
Coefficient: −6.46e−02
Value: C
Halomonas.kenyensis
2.0
1.5
1.0
0.5
0.0
A (n=15)
B (n=16)
C (n=14)
surface

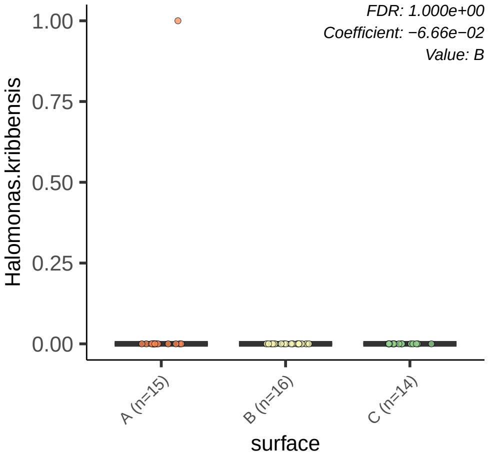

FDR: 1.000e+00
Coefficient: −6.66e−02
Value: B
Halomonas.kribbensis
1.00
0.75
0.50
0.25
0.00
A (n=15)
B (n=16)
C (n=14)
surface

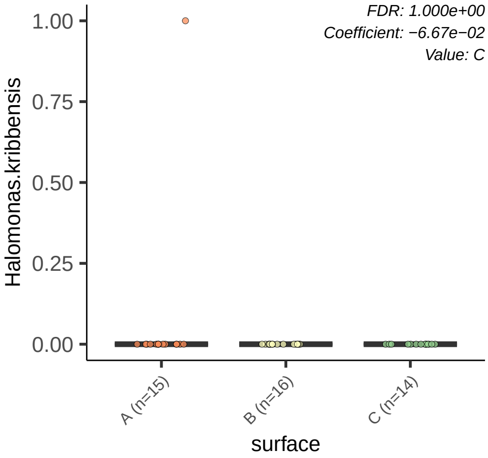

FDR: 1.000e+00
Coefficient: −6.67e−02
Value: C
Halomonas.kribbensis
1.00
0.75
0.50
0.25
0.00
A (n=15)
B (n=16)
C (n=14)
surface

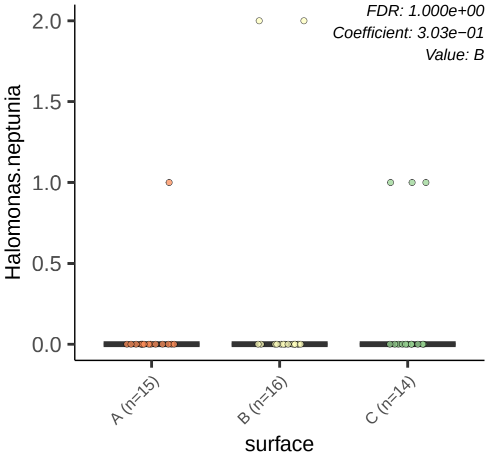

FDR: 1.000e+00
Coefficient: 3.03e−01
Value: B
Halomonas.neptunia
2.0
1.5
1.0
0.5
0.0
A (n=15)
B (n=16)
C (n=14)
surface

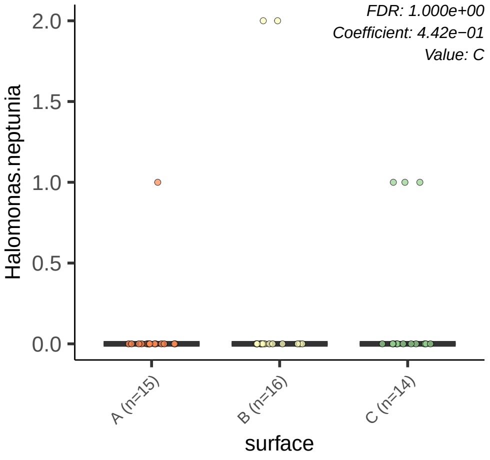
FDR: 1.000e+00
Coefficient: 4.42e−01
Value: C
Halomonas.neptunia
2.0
1.5
1.0
0.5
0.0
A (n=15)
B (n=16)
C (n=14)
surface

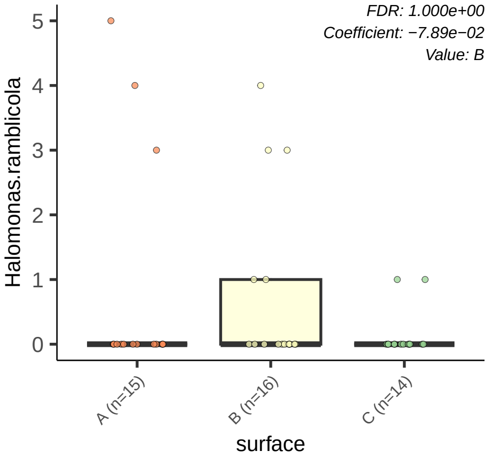

FDR: 1.000e+00
Coefficient: −7.89e−02
Value: B
Halomonas.ramblicola
5
4
3
2
1
0
A (n=15)
B (n=16)
C (n=14)
surface

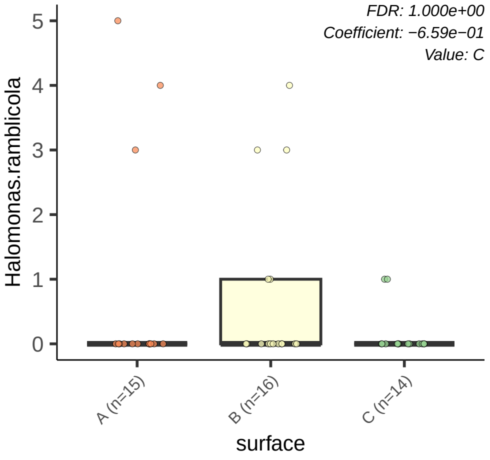
FDR: 1.000e+00
Coefficient: −6.59e−01
Value: C
Halomonas.ramblicola
0
1
2
3
4
5
A (n=15)
B (n=16)
C (n=14)
surface

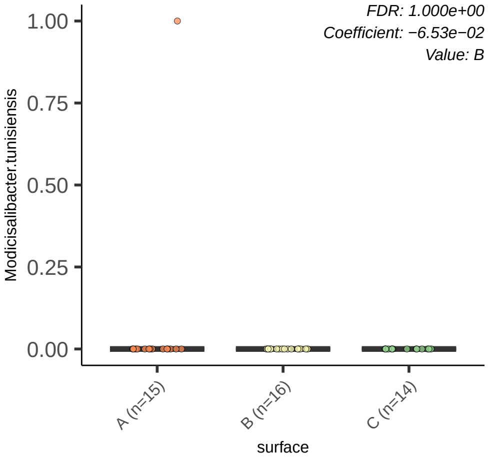

FDR: 1.000e+00
Coefficient: −6.53e−02
Value: B
Modicisalibacter.tunisiensis
1.00
0.75
0.50
0.25
0.00
A (n=15)
B (n=16)
C (n=14)
surface

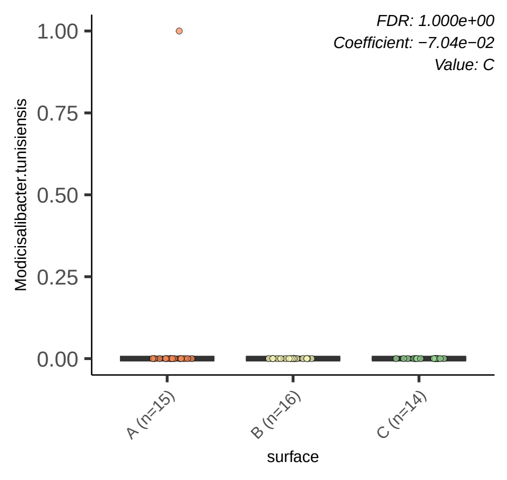
FDR: 1.000e+00
Coefficient: −7.04e−02
Value: C
Modicisalibacter.tunisiensis
1.00
0.75
0.50
0.25
0.00
A (n=15)
B (n=16)
C (n=14)
surface

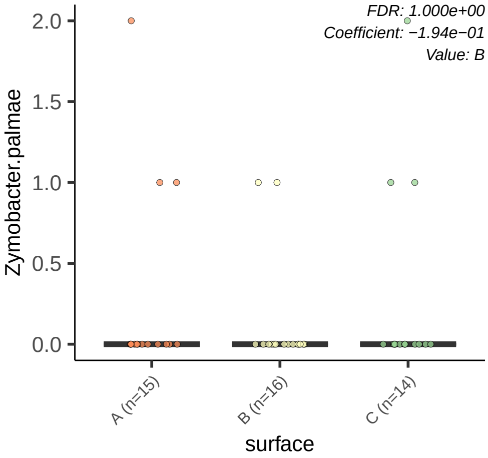

FDR: 1.000e+00
Coefficient: −1.94e−01
Value: B
Zymobacter.palmae
2.0
1.5
1.0
0.5
0.0
A (n=15)
B (n=16)
C (n=14)
surface

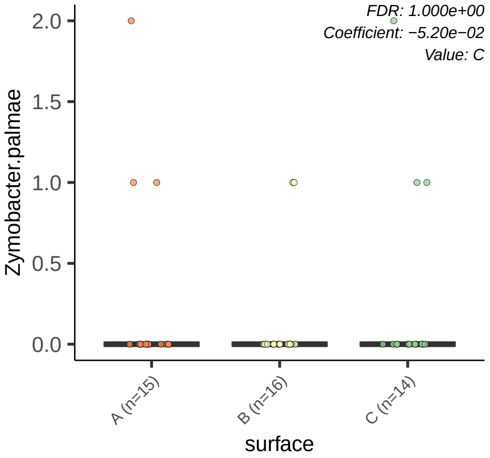
FDR: 1.000e+00
Coefficient: −5.20e−02
Value: C
Zymobacter.palmae
2.0
1.5
1.0
0.5
0.0
A (n=15)
B (n=16)
C (n=14)
surface

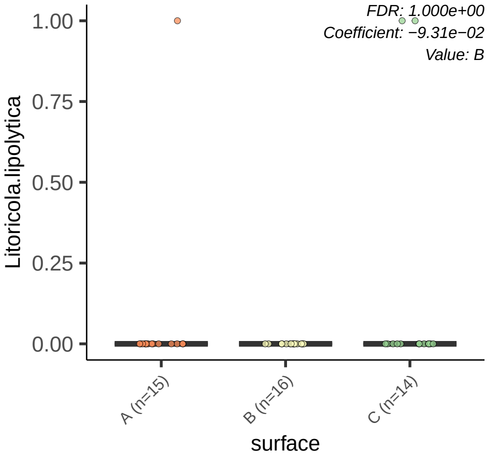

FDR: 1.000e+00
Coefficient: −9.31e−02
Value: B
Litoricola.lipolytica
1.00
0.75
0.50
0.25
0.00
A (n=15)
B (n=16)
C (n=14)
surface

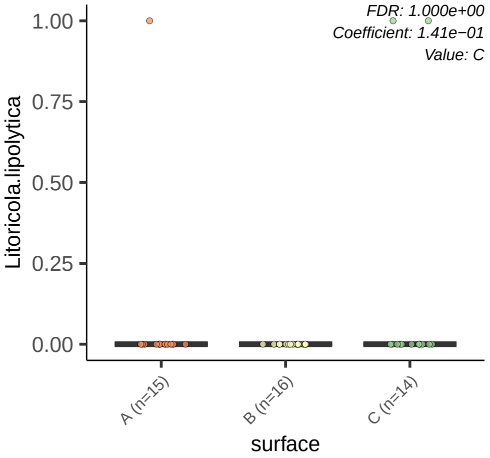
FDR: 1.000e+00
Coefficient: 1.41e−01
Value: C
Litoricola.lipolytica
1.00
0.75
0.50
0.25
0.00
A (n=15)
B (n=16)
C (n=14)
surface

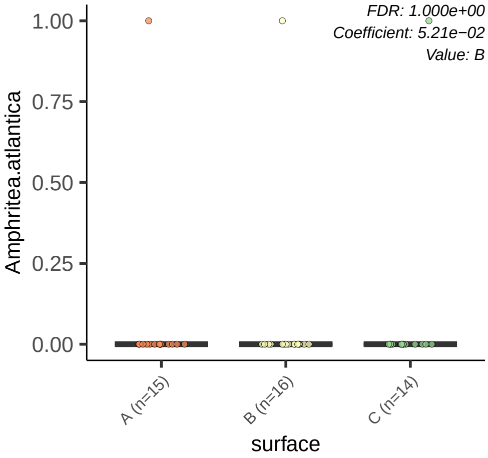
FDR: 1.000e+00
Coefficient: 5.21e−02
Value: B
Amphritea.atlantica
1.00
0.75
0.50
0.25
0.00
A (n=15)
B (n=16)
C (n=14)
surface

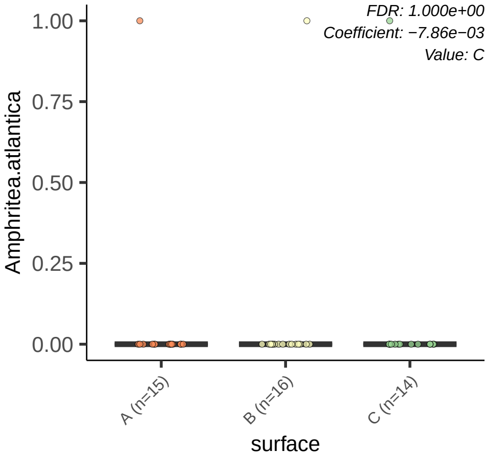
FDR: 1.000e+00
Coefficient: −7.86e−03
Value: C
Amphritea.atlantica
1.00
0.75
0.50
0.25
0.00
A (n=15)
B (n=16)
C (n=14)
surface

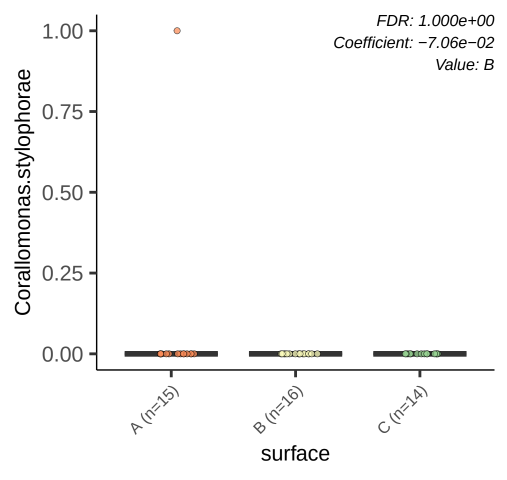
FDR: 1.000e+00
Coefficient: −7.06e−02
Value: B
Corallomonas.stylophorae
1.00
0.75
0.50
0.25
0.00
A (n=15)
B (n=16)
C (n=14)
surface

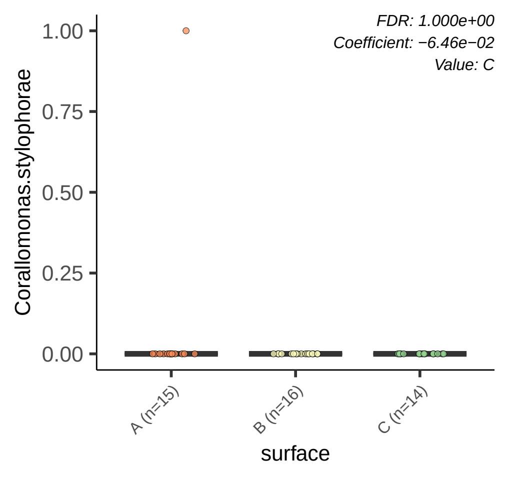
FDR: 1.000e+00
Coefficient: −6.46e−02
Value: C
Corallomonas.stylophorae
1.00
0.75
0.50
0.25
0.00
A (n=15)
B (n=16)
C (n=14)
surface

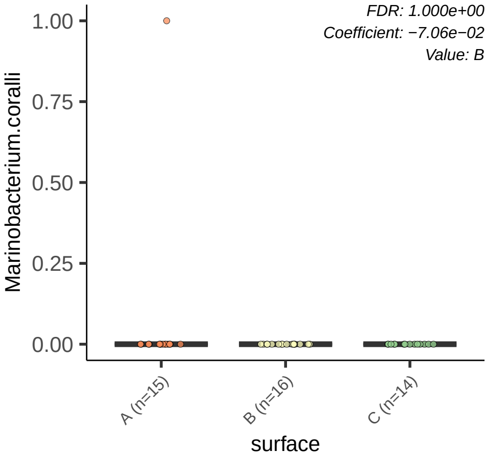

FDR: 1.000e+00
Coefficient: −7.06e−02
Value: B
Marinobacterium.coralli
1.00
0.75
0.50
0.25
0.00
A (n=15)
B (n=16)
C (n=14)
surface

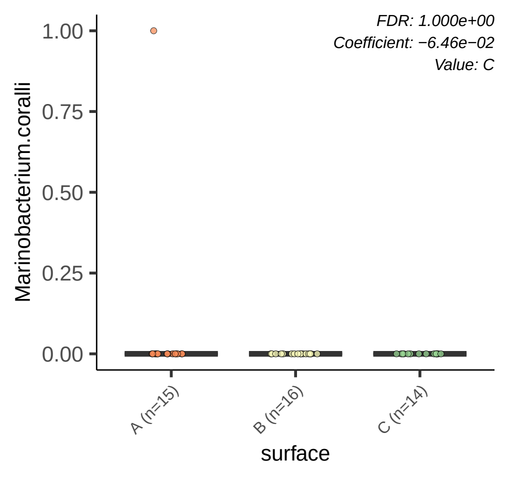
FDR: 1.000e+00
Coefficient: −6.46e−02
Value: C
Marinobacterium.coralli
1.00
0.75
0.50
0.25
0.00
A (n=15)
B (n=16)
C (n=14)
surface

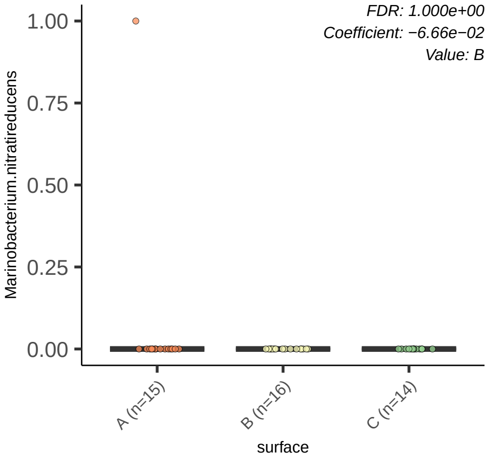
FDR: 1.000e+00
Coefficient: −6.66e−02
Value: B
1.00
0.75
0.50
0.25
0.00
Marinobacterium.nitratireducens
A (n=15)
B (n=16)
C (n=14)
surface

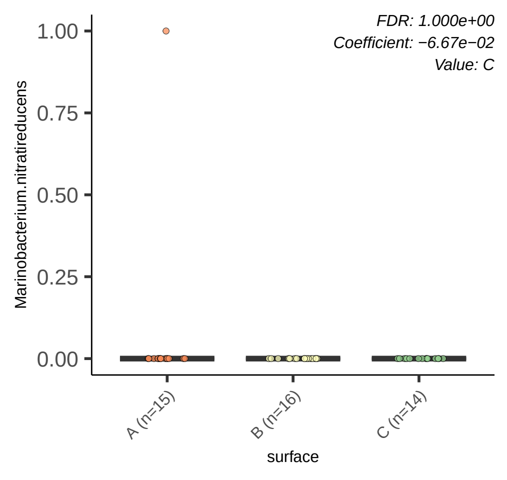
FDR: 1.000e+00
Coefficient: −6.67e−02
Value: C
Marinobacterium.nitratireducens
1.00
0.75
0.50
0.25
0.00
A (n=15)
B (n=16)
C (n=14)
surface

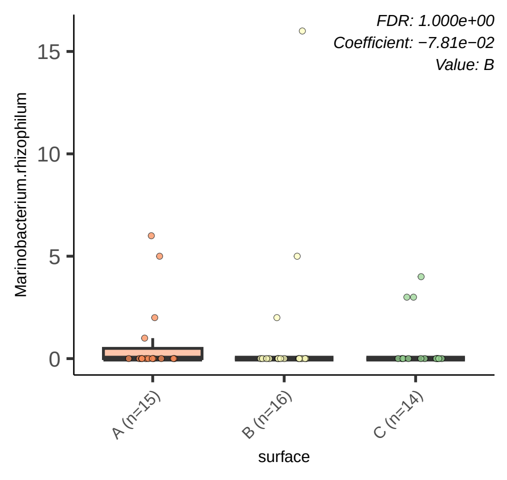

FDR: 1.000e+00
Coefficient: −7.81e−02
Value: B
Marinobacterium.rhizophilum
0
5
10
15
A (n=15)
B (n=16)
C (n=14)
surface

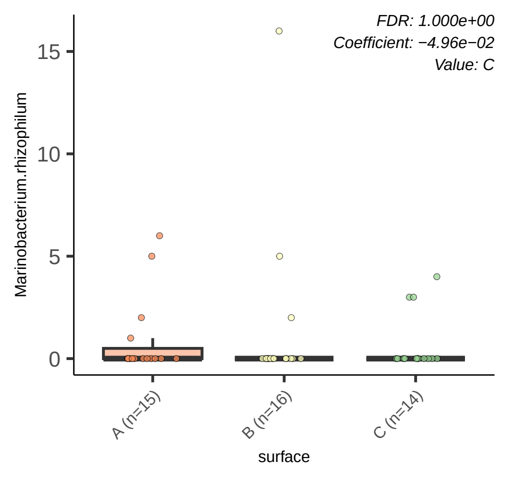

FDR: 1.000e+00
Coefficient: −4.96e−02
Value: C
15
10
5
0
Marinobacterium.rhizophilum
A (n=15)
B (n=16)
C (n=14)
surface

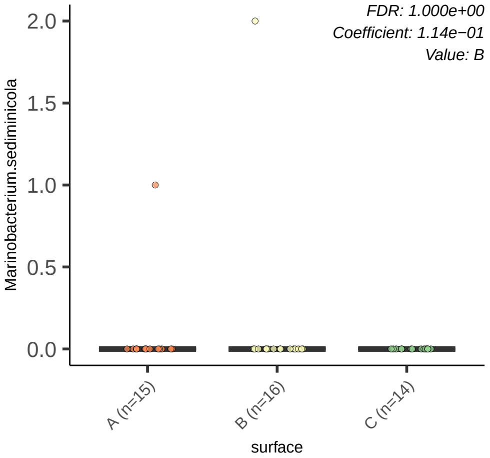
FDR: 1.000e+00
Coefficient: 1.14e−01
Value: B
Marinobacterium.sedimenicola
2.0
1.5
1.0
0.5
0.0
A (n=15)
B (n=16)
C (n=14)
surface

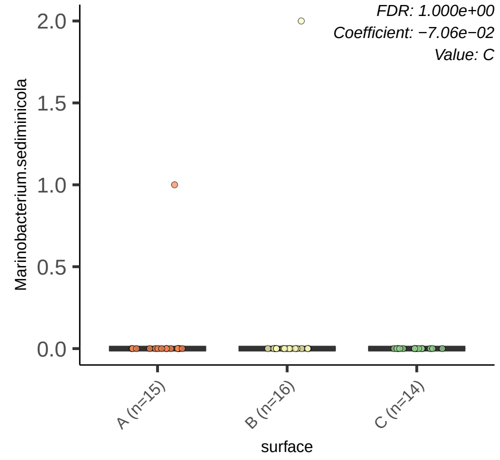

FDR: 1.000e+00
Coefficient: −7.06e−02
Value: C
Marinobacterium.sedimenicola
2.0
1.5
1.0
0.5
0.0
A (n=15)
B (n=16)
C (n=14)
surface

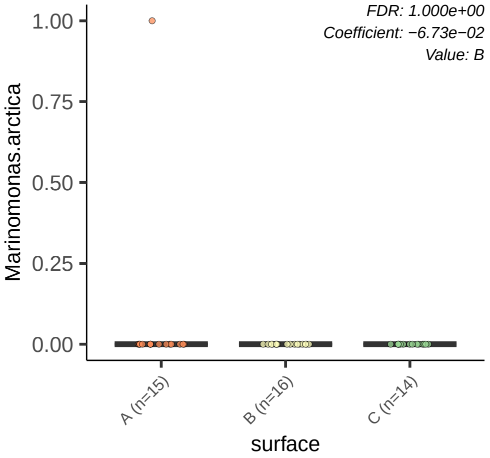
FDR: 1.000e+00
Coefficient: −6.73e−02
Value: B
Marinomonas.arctica
1.00
0.75
0.50
0.25
0.00
A (n=15)
B (n=16)
C (n=14)
surface

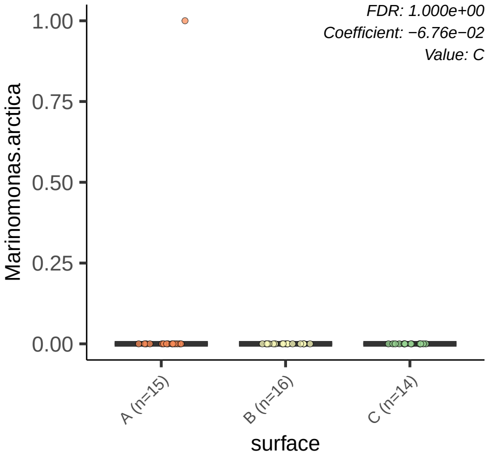

FDR: 1.000e+00
Coefficient: −6.76e−02
Value: C
Marinomonas.arctica
1.00
0.75
0.50
0.25
0.00
A (n=15)
B (n=16)
C (n=14)
surface

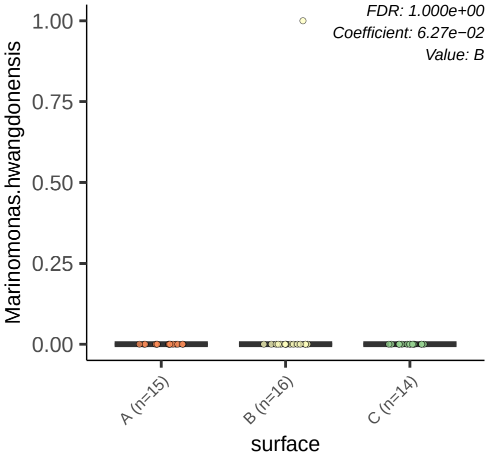
FDR: 1.000e+00
Coefficient: 6.27e−02
Value: B
Marinomonas.hwangdonensis
1.00
0.75
0.50
0.25
0.00
A (n=15)
B (n=16)
C (n=14)
surface

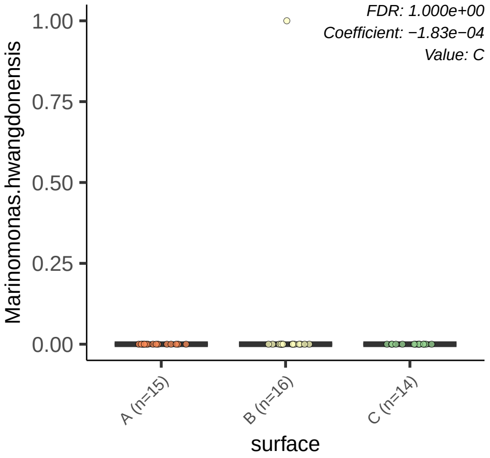
FDR: 1.000e+00
Coefficient: −1.83e−04
Value: C
Marinomonas.hwangdonensis
1.00
0.75
0.50
0.25
0.00
A (n=15)
B (n=16)
C (n=14)
surface

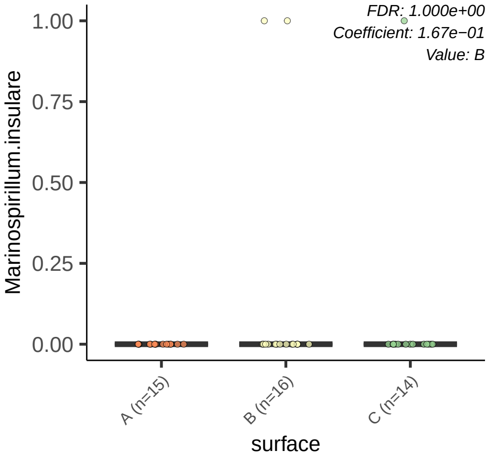
FDR: 1.000e+00
Coefficient: 1.67e−01
Value: B
Marinospirillum.insulare
1.00
0.75
0.50
0.25
0.00
A (n=15)
B (n=16)
C (n=14)
surface

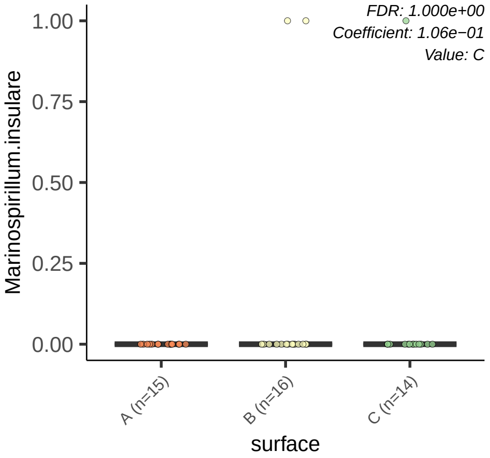
FDR: 1.000e+00
Coefficient: 1.06e−01
Value: C
Marinospirillum.insulare
1.00
0.75
0.50
0.25
0.00
A (n=15)
B (n=16)
C (n=14)
surface

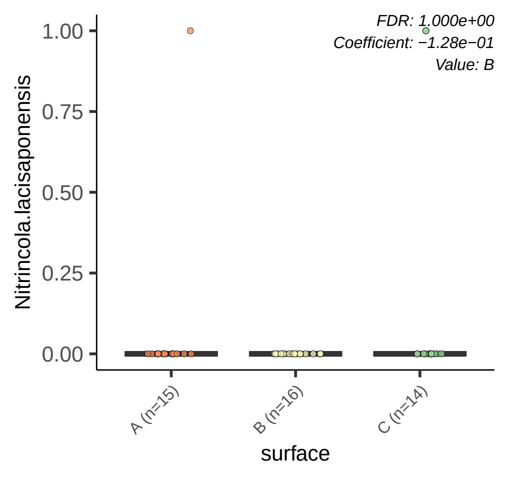
FDR: 1.000e+00
Coefficient: −1.28e−01
Value: B
Nitrincola.lacisaponensis
1.00
0.75
0.50
0.25
0.00
A (n=15)
B (n=16)
C (n=14)
surface

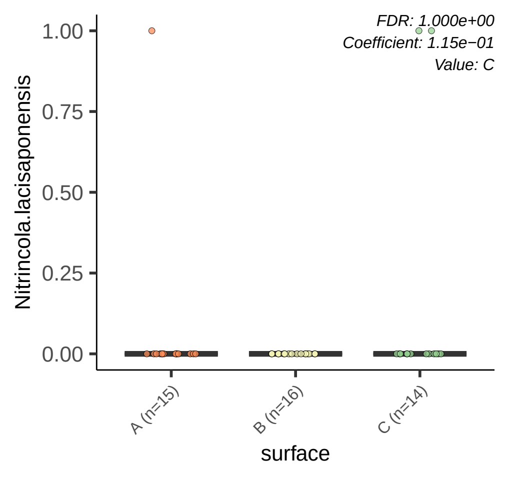
FDR: 1.000e+00
Coefficient: 1.15e−01
Value: C
Nitrincola.lacisaponensis
1.00
0.75
0.50
0.25
0.00
A (n=15)
B (n=16)
C (n=14)
surface

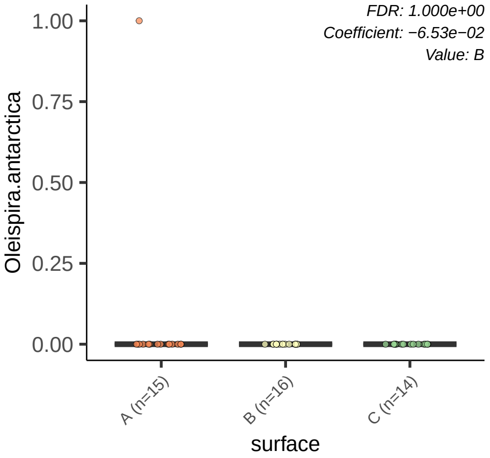
FDR: 1.000e+00
Coefficient: −6.53e−02
Value: B
Oleispira.antarctica
1.00
0.75
0.50
0.25
0.00
A (n=15)
B (n=16)
C (n=14)
surface

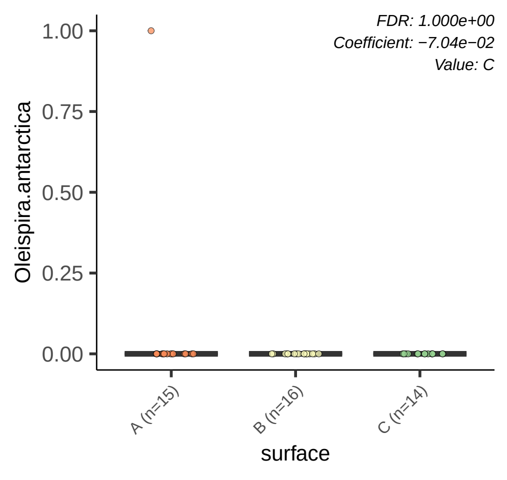

FDR: 1.000e+00
Coefficient: −7.04e−02
Value: C
Oleispira.antarctica
1.00
0.75
0.50
0.25
0.00
A (n=15)
B (n=16)
C (n=14)
surface

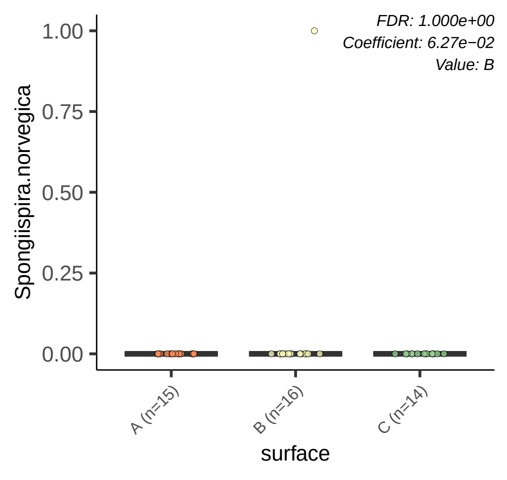

FDR: 1.000e+00
Coefficient: 6.27e−02
Value: B
Spongiispira.norvegica
1.00
0.75
0.50
0.25
0.00
A (n=15)
B (n=16)
C (n=14)
surface

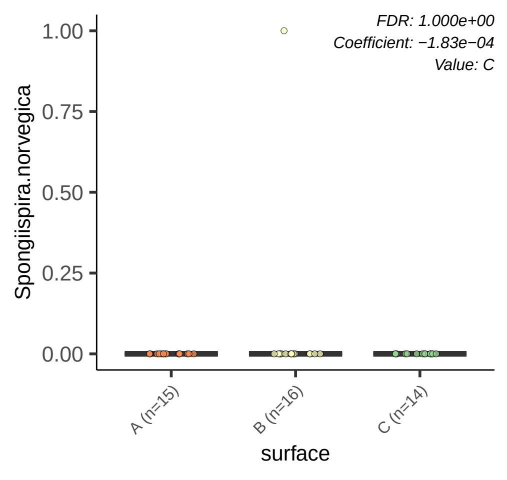
FDR: 1.000e+00
Coefficient: −1.83e−04
Value: C
Spongiispira.norvegica
1.00
0.75
0.50
0.25
0.00
A (n=15)
B (n=16)
C (n=14)
surface

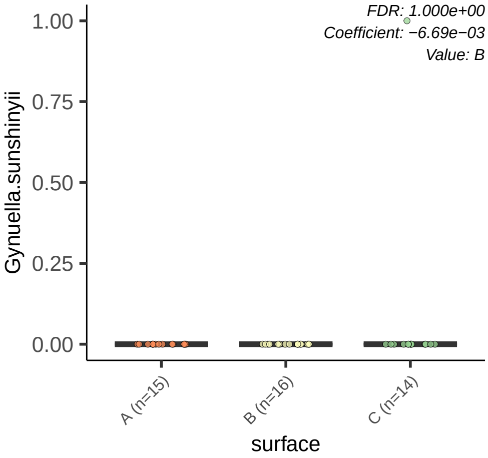

FDR: 1.000e+00
Coefficient: −6.69e−03
Value: B
Gynuella.sunshinyii
1.00
0.75
0.50
0.25
0.00
A (n=15)
B (n=16)
C (n=14)
surface

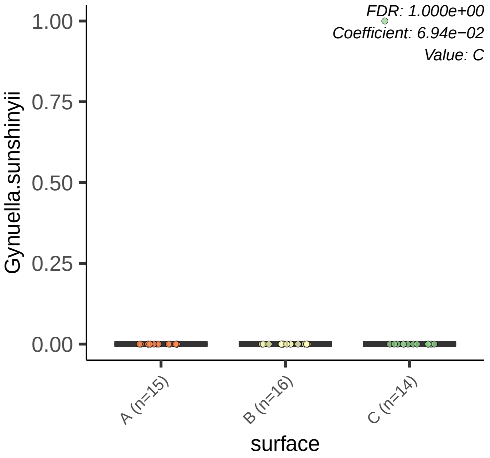

FDR: 1.000e+00
Coefficient: 6.94e−02
Value: C
1.00
0.75
0.50
0.25
0.00
Gynuella.sunshinyii
A (n=15)
B (n=16)
C (n=14)
surface

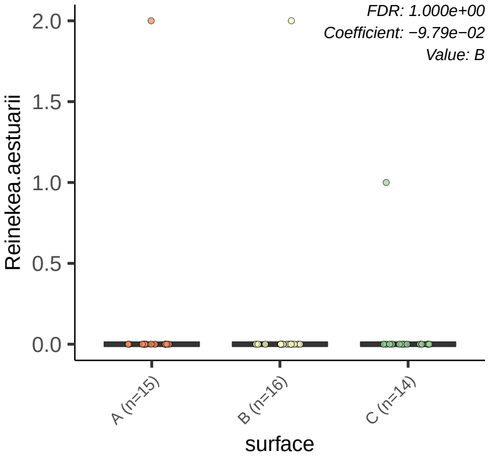

FDR: 1.000e+00
Coefficient: −9.79e−02
Value: B
Reinekea.aestuarii
2.0
1.5
1.0
0.5
0.0
A (n=15)
B (n=16)
C (n=14)
surface

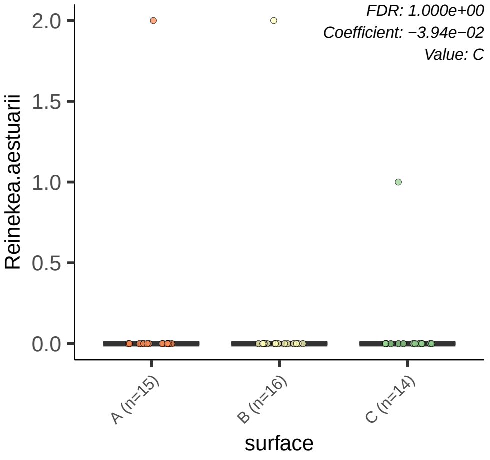
FDR: 1.000e+00
Coefficient: −3.94e−02
Value: C
Reinekea.aestuarii
2.0
1.5
1.0
0.5
0.0
A (n=15)
B (n=16)
C (n=14)
surface

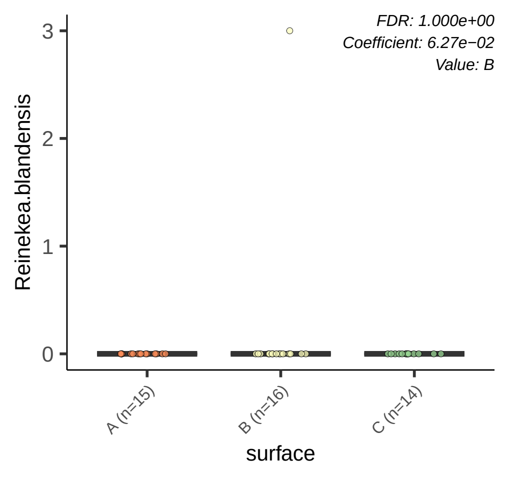
FDR: 1.000e+00
Coefficient: 6.27e−02
Value: B
Reinekea.blandensis
3
2
1
0
A (n=15)
B (n=16)
C (n=14)
surface

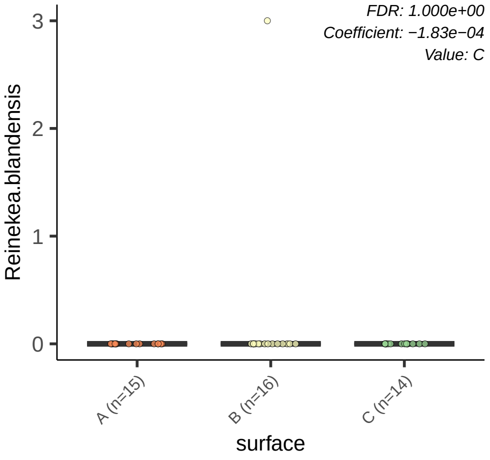
FDR: 1.000e+00
Coefficient: −1.83e−04
Value: C
Reinekea.blandensis
3
2
1
0
A (n=15)
B (n=16)
C (n=14)
surface

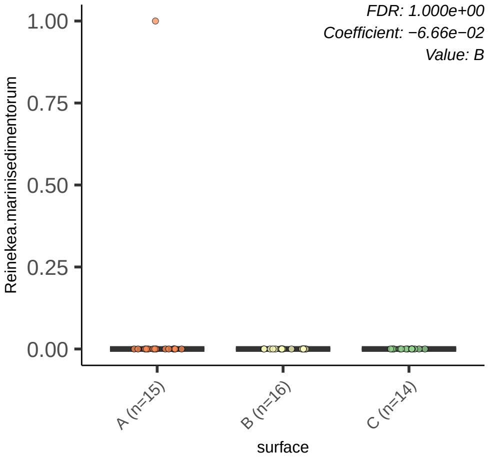
FDR: 1.000e+00
Coefficient: −6.66e−02
Value: B
1.00
0.75
0.50
0.25
0.00
Reinekea.marinisedimentorum
A (n=15)
B (n=16)
C (n=14)
surface

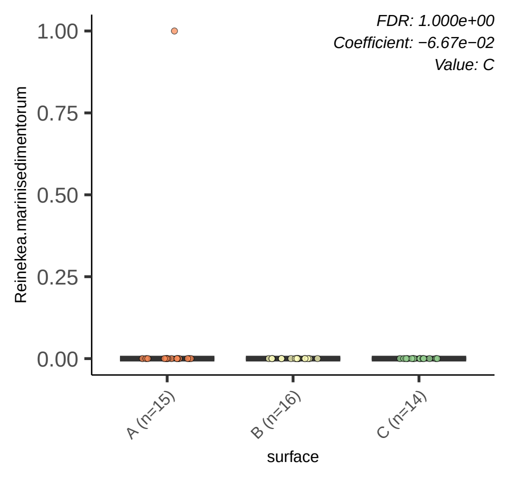

FDR: 1.000e+00
Coefficient: −6.67e−02
Value: C
1.00
0.75
0.50
0.25
0.00
Reinekea.marinisedimentorum
A (n=15)
B (n=16)
C (n=14)
surface

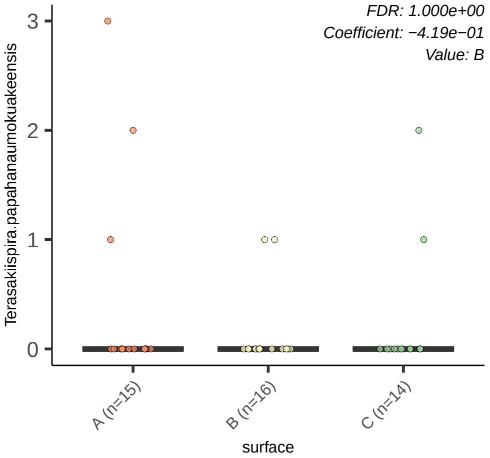

FDR: 1.000e+00
Coefficient: −4.19e−01
Value: B
Terasakiispira.papahanaumokuakeensis
3
2
1
0
A (n=15)
B (n=16)
C (n=14)
surface

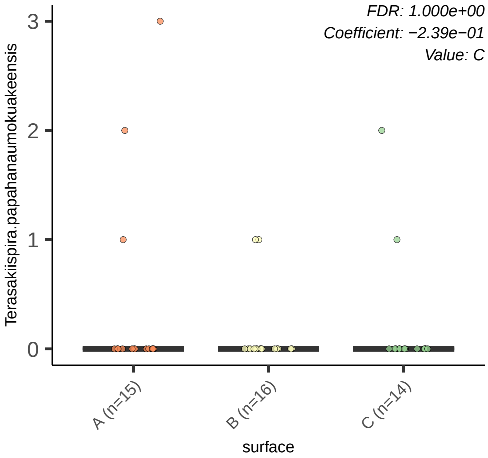
FDR: 1.000e+00
Coefficient: −2.39e−01
Value: C
Terasakiispira.papahanaumokuakeensis
3
2
1
0
A (n=15)
B (n=16)
C (n=14)
surface

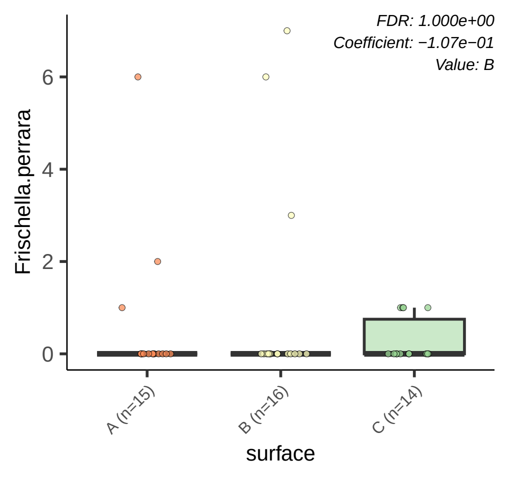
FDR: 1.000e+00
Coefficient: −1.07e−01
Value: B
Frischella.perrara
0
2
4
6
A (n=15)
B (n=16)
C (n=14)
surface

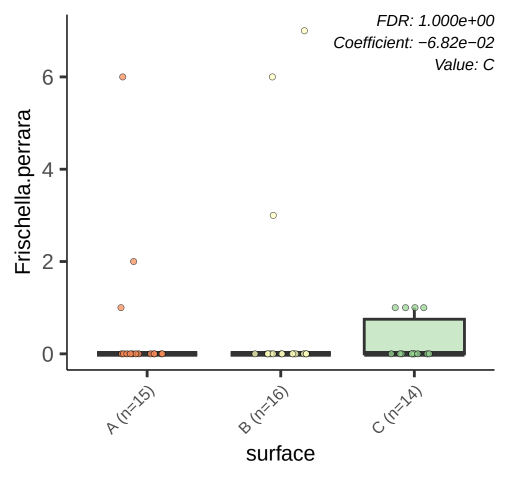

FDR: 1.000e+00
Coefficient: −6.82e−02
Value: C
Frischella.perrara
0
2
4
6
A (n=15)
B (n=16)
C (n=14)
surface

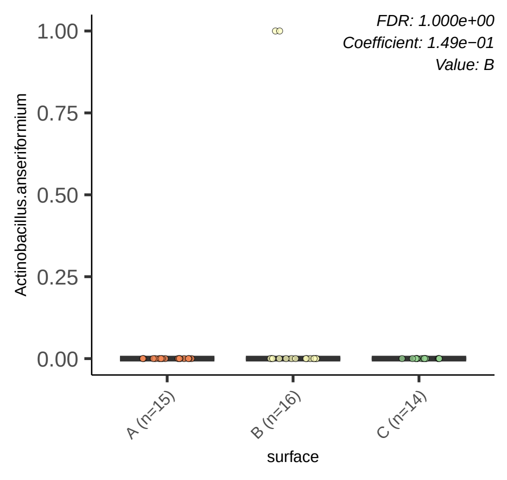
FDR: 1.000e+00
Coefficient: 1.49e−01
Value: B
1.00
0.75
0.50
0.25
0.00
Actinobacillus.anseriformium
A (n=15)
B (n=16)
C (n=14)
surface

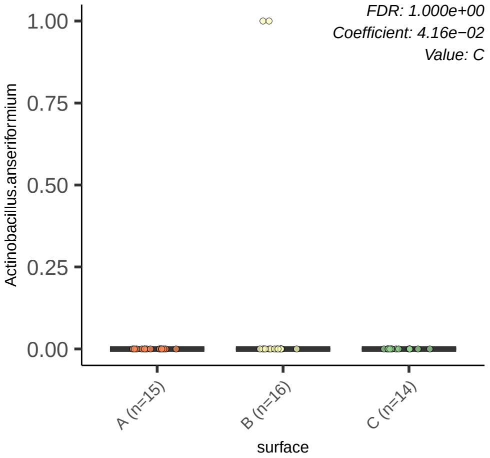

FDR: 1.000e+00
Coefficient: 4.16e−02
Value: C
1.00
0.75
0.50
0.25
0.00
Actinobacillus.anseriformium
A (n=15)
B (n=16)
C (n=14)
surface

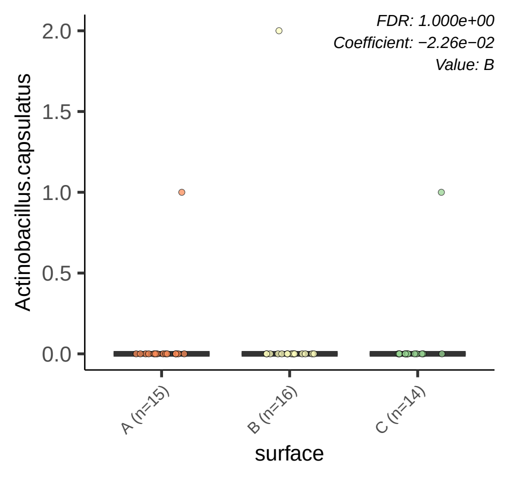

FDR: 1.000e+00
Coefficient: −2.26e−02
Value: B
2.0
1.5
1.0
0.5
0.0
Actinobacillus.capsulatus
A (n=15)
B (n=16)
C (n=14)
surface

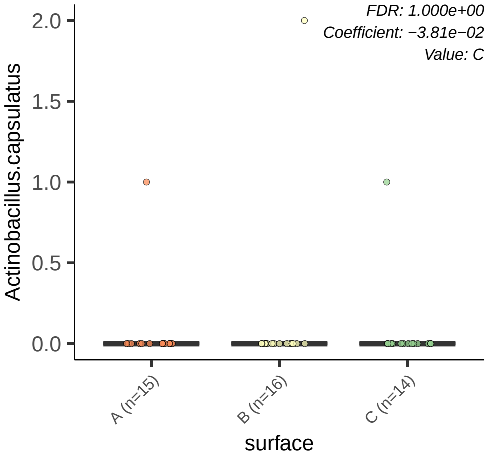
FDR: 1.000e+00
Coefficient: −3.81e−02
Value: C
Actinobacillus.capsulatus
2.0
1.5
1.0
0.5
0.0
A (n=15)
B (n=16)
C (n=14)
surface

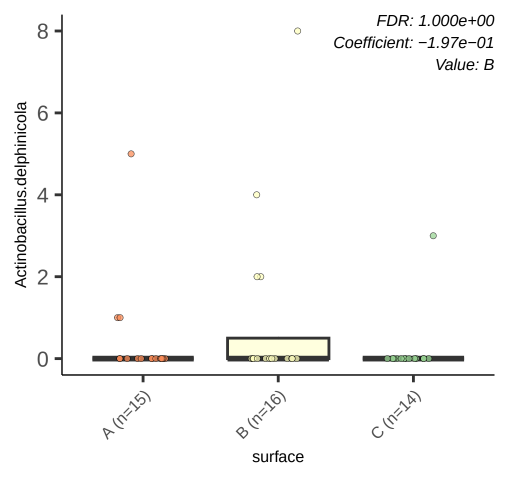
FDR: 1.000e+00
Coefficient: −1.97e−01
Value: B
Actinobacillus.delphinicola
0
2
4
6
8
A (n=15)
B (n=16)
C (n=14)
surface

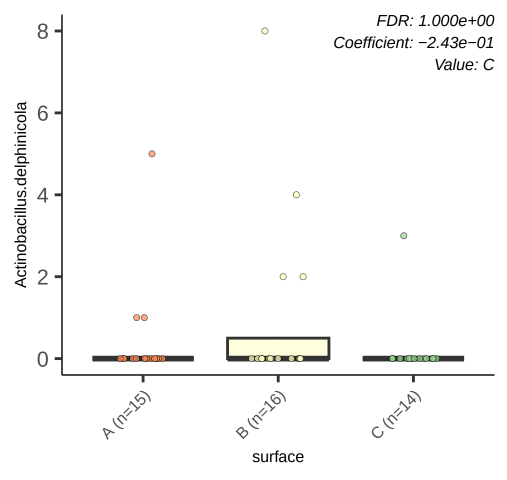
FDR: 1.000e+00
Coefficient: −2.43e−01
Value: C
Actinobacillus.delphinicola
8
6
4
2
0
A (n=15)
B (n=16)
C (n=14)
surface

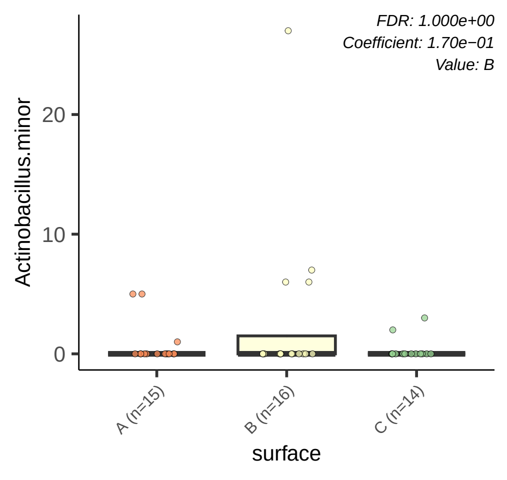
FDR: 1.000e+00
Coefficient: 1.70e−01
Value: B
Actinobacillus.minor
0
10
20
A (n=15)
B (n=16)
C (n=14)
surface

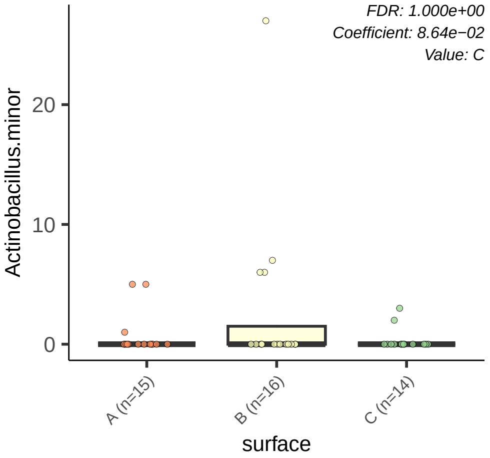

FDR: 1.000e+00
Coefficient: 8.64e−02
Value: C
Actinobacillus.minor
0
10
20
A (n=15)
B (n=16)
C (n=14)
surface

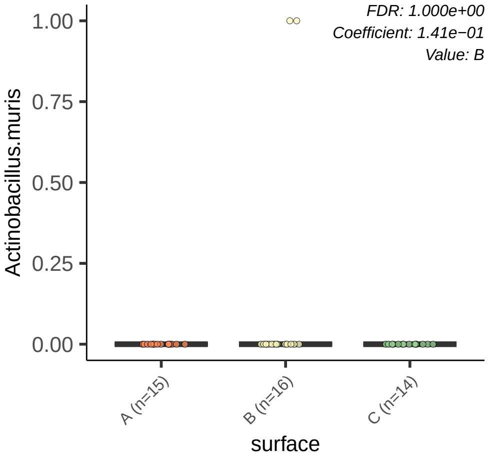
FDR: 1.000e+00
Coefficient: 1.41e−01
Value: B
Actinobacillus.muris
1.00
0.75
0.50
0.25
0.00
A (n=15)
B (n=16)
C (n=14)
surface

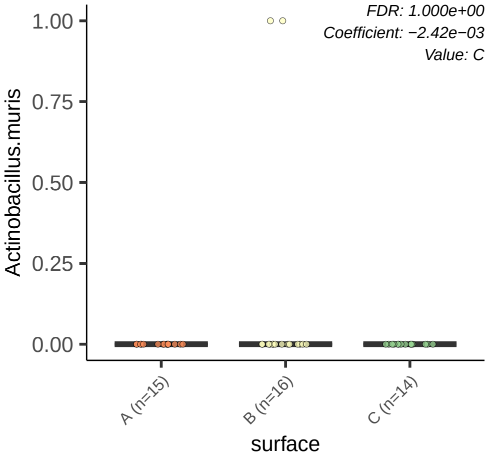

FDR: 1.000e+00
Coefficient: −2.42e−03
Value: C
Actinobacillus.muris
1.00
0.75
0.50
0.25
0.00
A (n=15)
B (n=16)
C (n=14)
surface

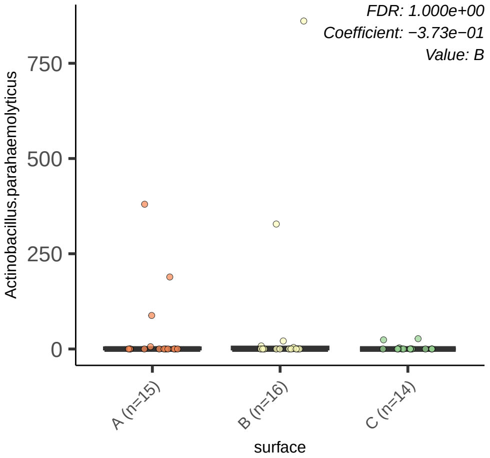

FDR: 1.000e+00
Coefficient: −3.73e−01
Value: B
Actinobacillus.parahaemolyticus
750
500
250
0
A (n=15)
B (n=16)
C (n=14)
surface

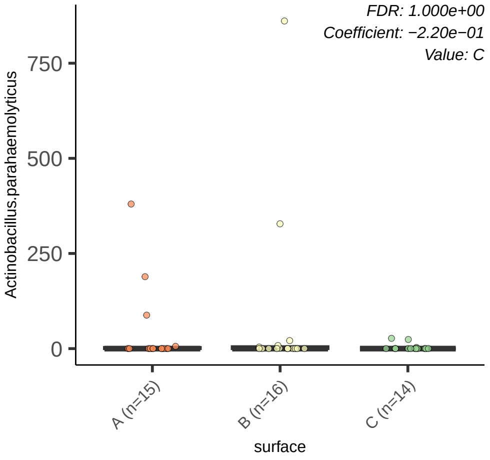

FDR: 1.000e+00
Coefficient: −2.20e−01
Value: C
Actinobacillus.parahaemolyticus
750
500
250
0
A (n=15)
B (n=16)
C (n=14)
surface

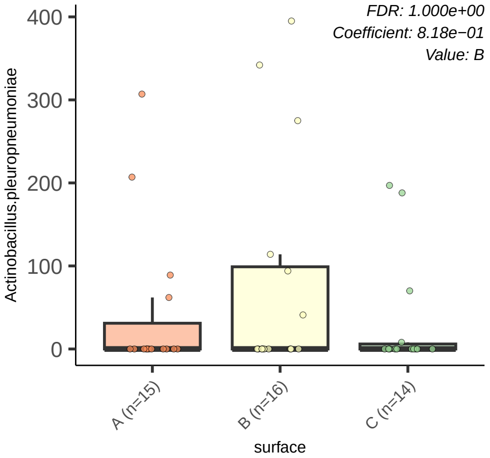
FDR: 1.000e+00
Coefficient: 8.18e−01
Value: B
Actinobacillus.pleuropneumoniae
400
300
200
100
0
A (n=15)
B (n=16)
C (n=14)
surface

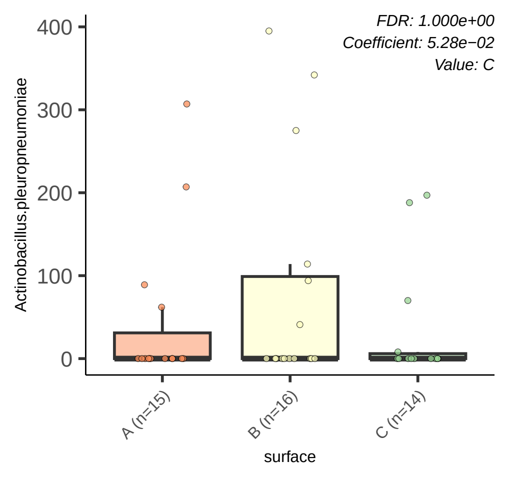

FDR: 1.000e+00
Coefficient: 5.28e−02
Value: C
Actinobacillus.pleuropneumoniae
400
300
200
100
0
A (n=15)
B (n=16)
C (n=14)
surface

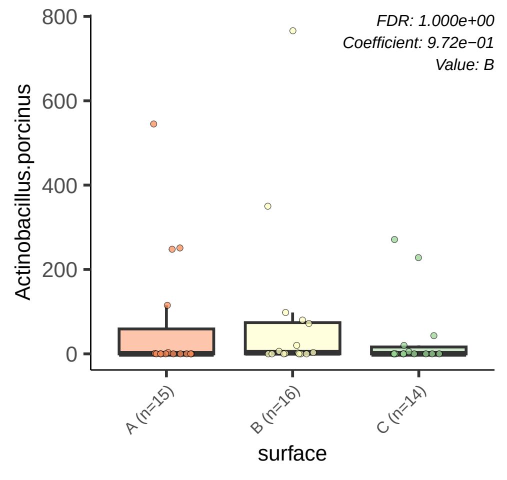
FDR: 1.000e+00
Coefficient: 9.72e−01
Value: B
800
600
400
200
0
Actinobacillus.porcinus
A (n=15)
B (n=16)
C (n=14)
surface

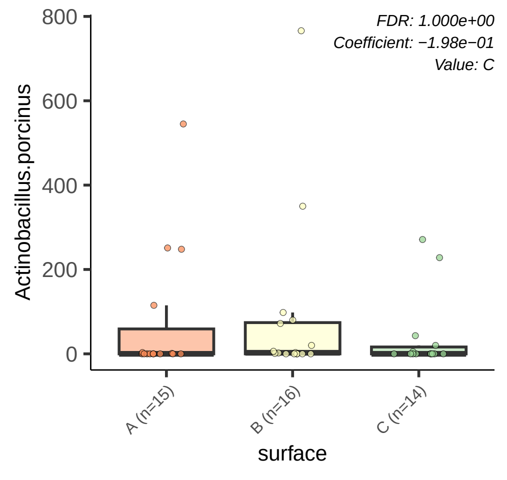
FDR: 1.000e+00
Coefficient: −1.98e−01
Value: C
Actinobacillus.porcinus
0
200
400
600
800
A (n=15)
B (n=16)
C (n=14)
surface

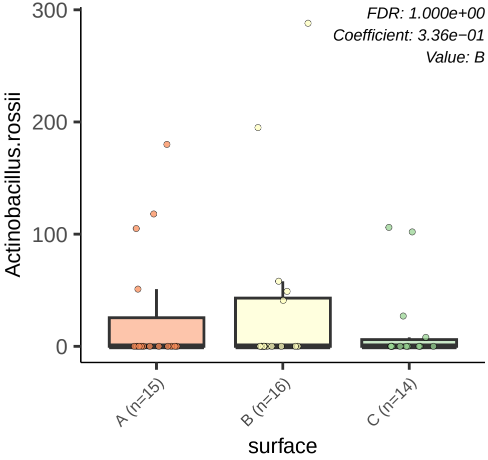

FDR: 1.000e+00
Coefficient: 3.36e−01
Value: B
Actinobacillus.rossii
300
200
100
0
A (n=15)
B (n=16)
C (n=14)
surface

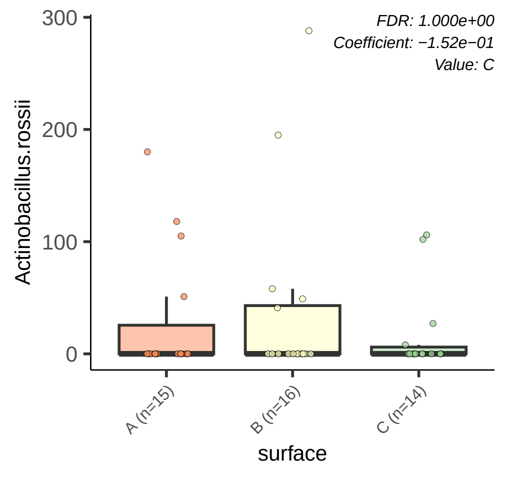
FDR: 1.000e+00
Coefficient: −1.52e−01
Value: C
Actinobacillus.rossii
300
200
100
0
A (n=15)
B (n=16)
C (n=14)
surface

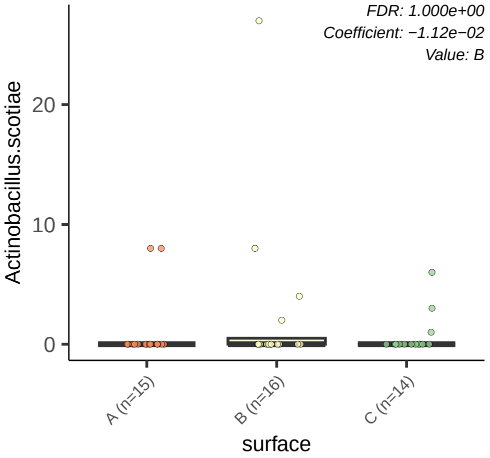

FDR: 1.000e+00
Coefficient: −1.12e−02
Value: B
Actinobacillus.scotiae
20
10
0
A (n=15)
B (n=16)
C (n=14)
surface

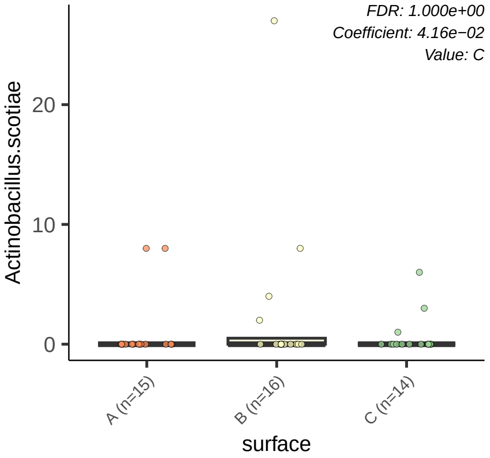
FDR: 1.000e+00
Coefficient: 4.16e−02
Value: C
Actinobacillus.scotiae
0
10
20
A (n=15)
B (n=16)
C (n=14)
surface

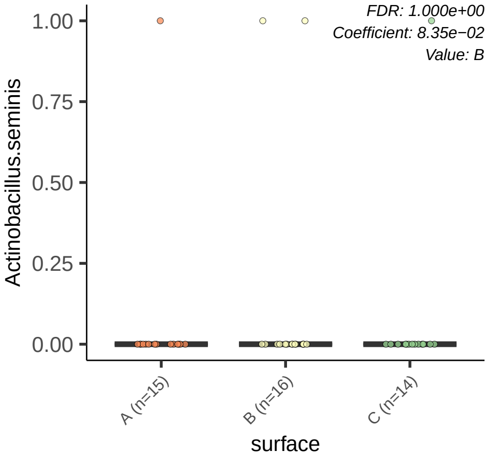
FDR: 1.000e+00
Coefficient: 8.35e−02
Value: B
Actinobacillus.seminis
1.00
0.75
0.50
0.25
0.00
A (n=15)
B (n=16)
C (n=14)
surface

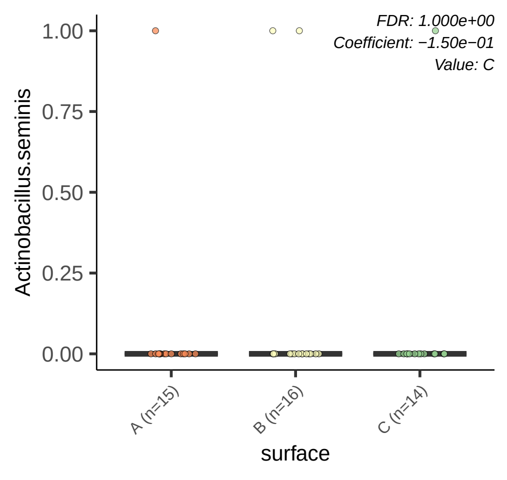
FDR: 1.000e+00
Coefficient: −1.50e−01
Value: C
Actinobacillus.seminis
1.00
0.75
0.50
0.25
0.00
A (n=15)
B (n=16)
C (n=14)
surface

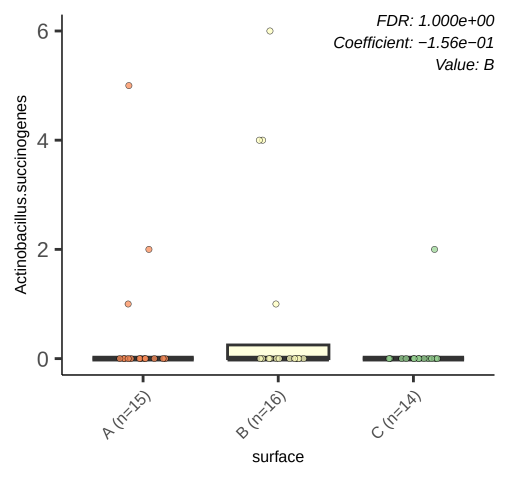

FDR: 1.000e+00
Coefficient: −1.56e−01
Value: B
Actinobacillus.succinogenes
0
2
4
6
A (n=15)
B (n=16)
C (n=14)
surface

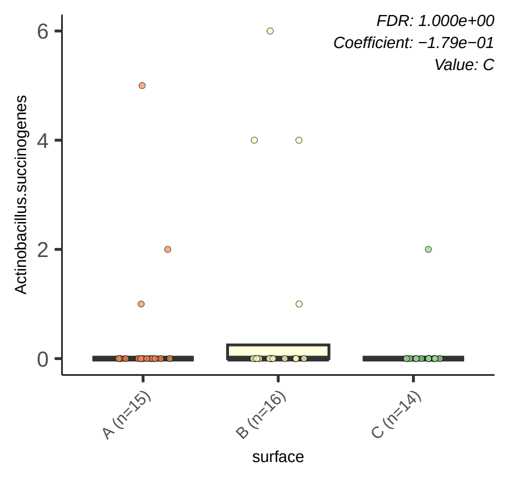
FDR: 1.000e+00
Coefficient: −1.79e−01
Value: C
Actinobacillus.succinogenes
0
2
4
6
A (n=15)
B (n=16)
C (n=14)
surface

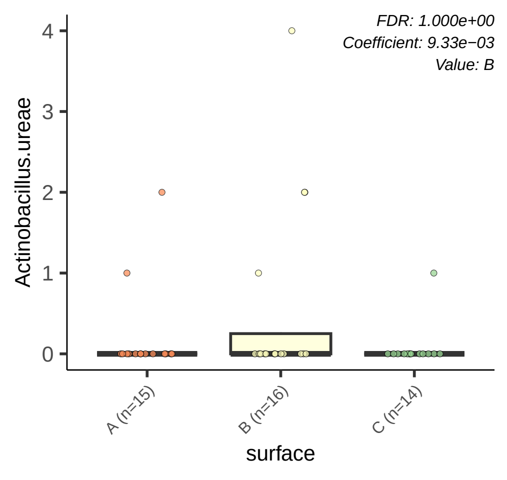
FDR: 1.000e+00
Coefficient: 9.33e−03
Value: B
Actinobacillus.ureae
0
1
2
3
4
A (n=15)
B (n=16)
C (n=14)
surface

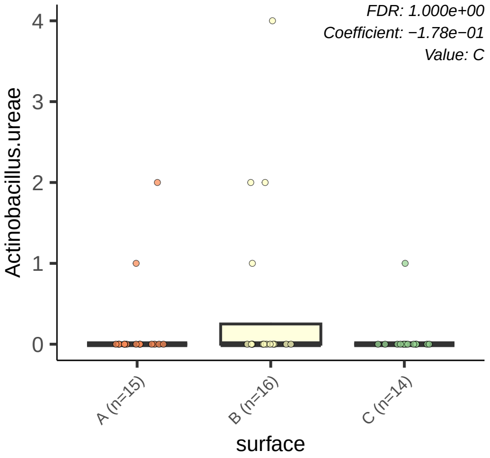

FDR: 1.000e+00
Coefficient: −1.78e−01
Value: C
Actinobacillus.ureae
0
1
2
3
4
A (n=15)
B (n=16)
C (n=14)
surface

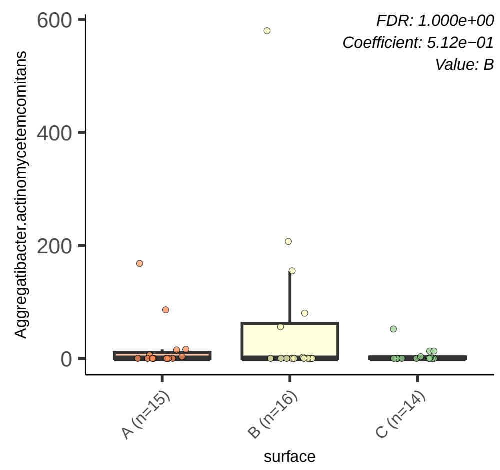

FDR: 1.000e+00
Coefficient: 5.12e−01
Value: B
600
400
200
0
Aggregatibacter.actinomycetemcomitans
A (n=15)
B (n=16)
C (n=14)
surface

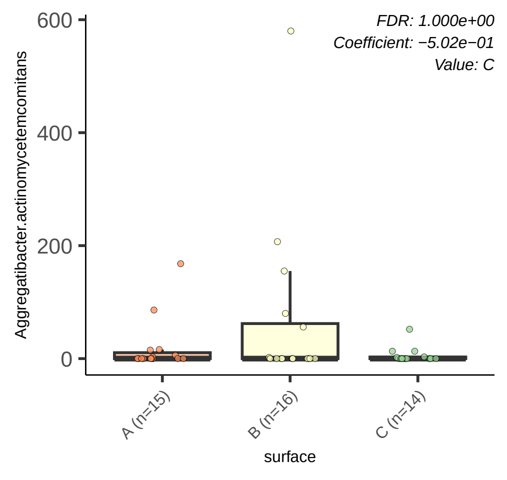
FDR: 1.000e+00
Coefficient: −5.02e−01
Value: C
Aggregatibacter.actinomycetemcomitans
600
400
200
0
A (n=15)
B (n=16)
C (n=14)
surface

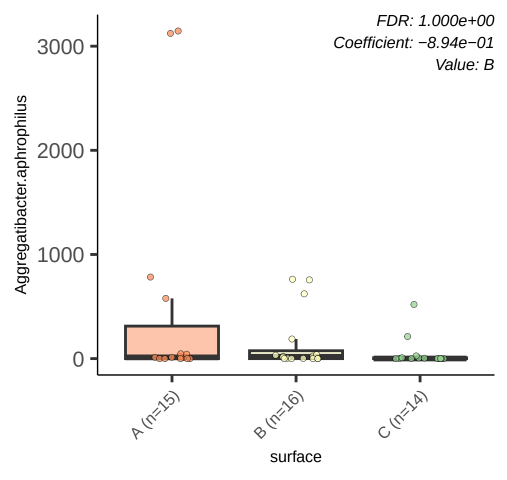

FDR: 1.000e+00
Coefficient: −8.94e−01
Value: B
Aggregatibacter.aphrophilus
3000
2000
1000
0
A (n=15)
B (n=16)
C (n=14)
surface

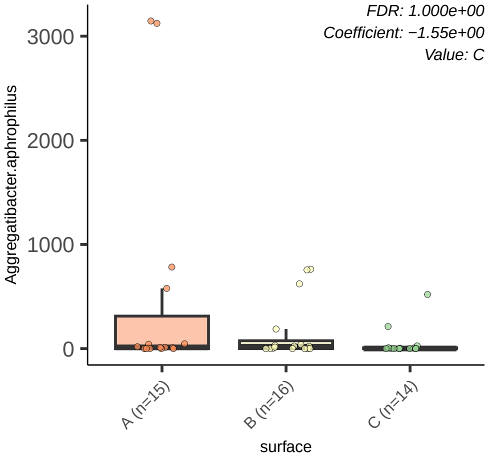
FDR: 1.000e+00
Coefficient: −1.55e+00
Value: C
Aggregatibacter.aphrophilus
3000
2000
1000
0
A (n=15)
B (n=16)
C (n=14)
surface

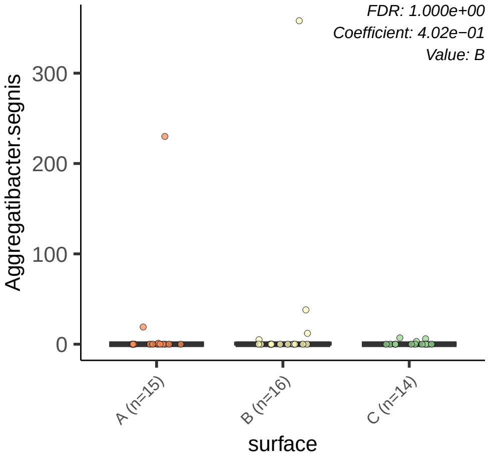

FDR: 1.000e+00
Coefficient: 4.02e−01
Value: B
Aggregatibacter.segnis
0
100
200
300
A (n=15)
B (n=16)
C (n=14)
surface

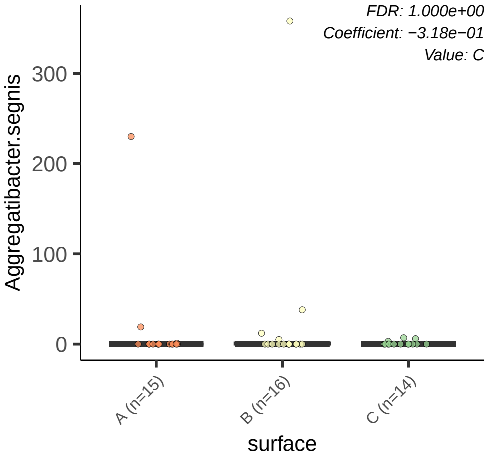
FDR: 1.000e+00
Coefficient: −3.18e−01
Value: C
Aggregatibacter.segnis
300
200
100
0
A (n=15)
B (n=16)
C (n=14)
surface

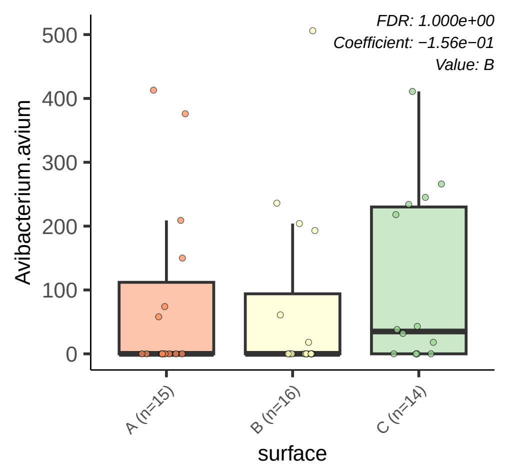

FDR: 1.000e+00
Coefficient: −1.56e−01
Value: B
Avibacterium.avium
0
100
200
300
400
500
A (n=15)
B (n=16)
C (n=14)
surface

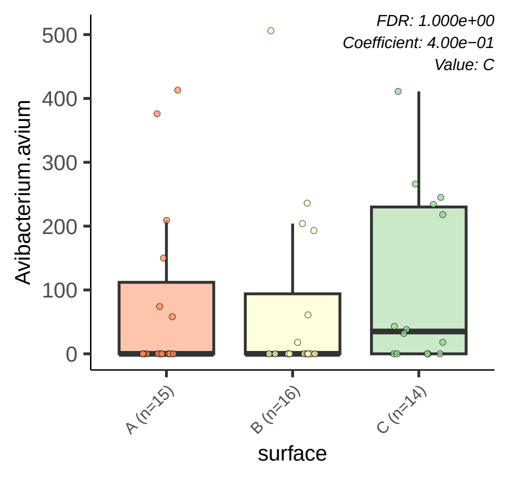
FDR: 1.000e+00
Coefficient: 4.00e−01
Value: C
Avibacterium.avium
500
400
300
200
100
0
A (n=15)
B (n=16)
C (n=14)
surface

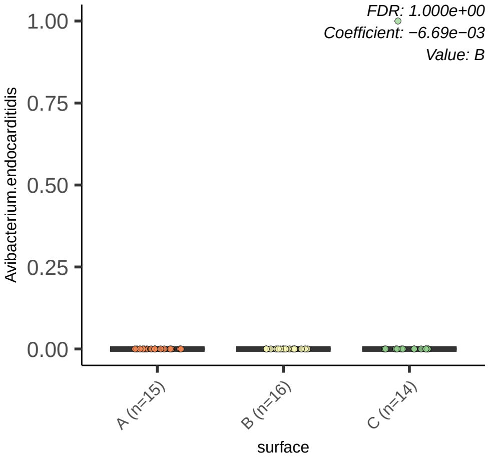
FDR: 1.000e+00
Coefficient: −6.69e−03
Value: B
Avibacterium.endocarditidis
1.00
0.75
0.50
0.25
0.00
A (n=15)
B (n=16)
C (n=14)
surface

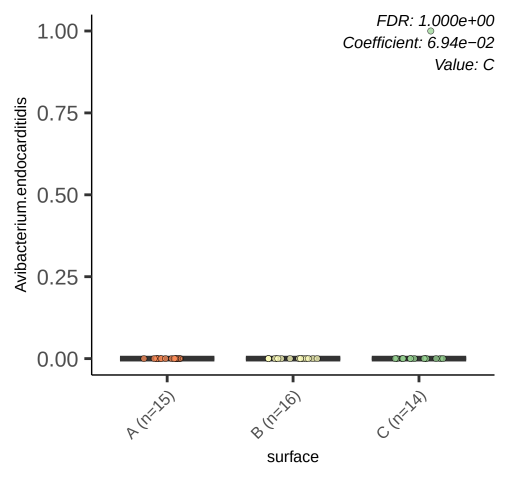
FDR: 1.000e+00
Coefficient: 6.94e−02
Value: C
Avibacterium.endocarditidis
1.00
0.75
0.50
0.25
0.00
A (n=15)
B (n=16)
C (n=14)
surface

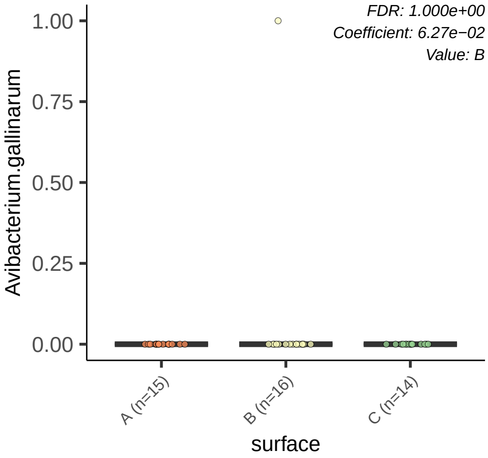
FDR: 1.000e+00
Coefficient: 6.27e−02
Value: B
Avibacterium.gallinarum
1.00
0.75
0.50
0.25
0.00
A (n=15)
B (n=16)
C (n=14)
surface

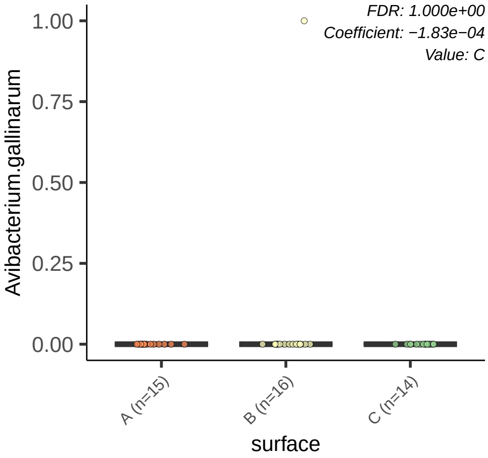
FDR: 1.000e+00
Coefficient: −1.83e−04
Value: C
Avibacterium.gallinarum
1.00
0.75
0.50
0.25
0.00
A (n=15)
B (n=16)
C (n=14)
surface

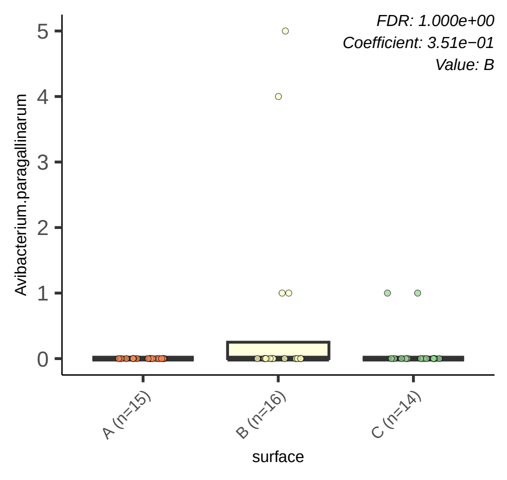

FDR: 1.000e+00
Coefficient: 3.51e−01
Value: B
Avibacterium.paragallinarum
5
4
3
2
1
0
A (n=15)
B (n=16)
C (n=14)
surface

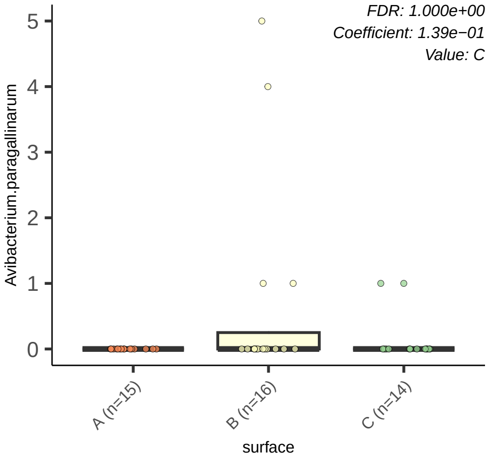
FDR: 1.000e+00
Coefficient: 1.39e−01
Value: C
Avibacterium.paragallinarum
0
1
2
3
4
5
A (n=15)
B (n=16)
C (n=14)
surface

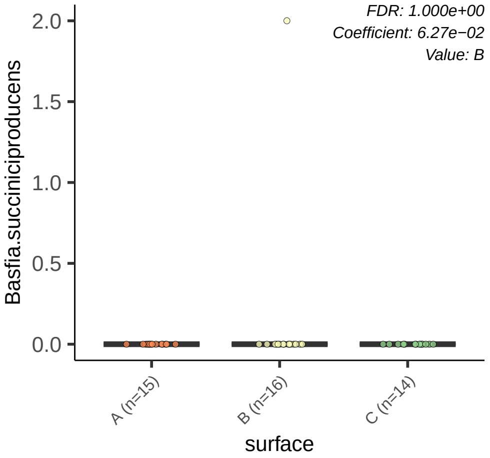
FDR: 1.000e+00
Coefficient: 6.27e−02
Value: B
Basfia.succiniciproducens
2.0
1.5
1.0
0.5
0.0
A (n=15)
B (n=16)
C (n=14)
surface

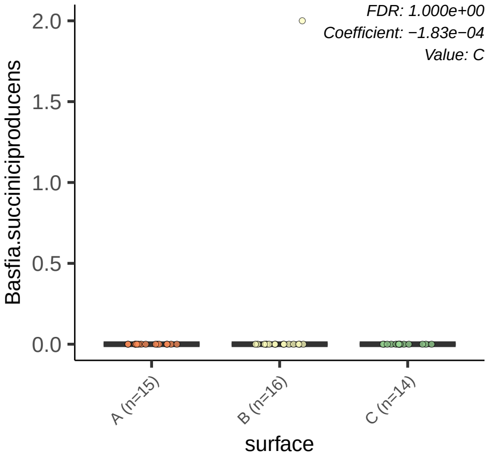
FDR: 1.000e+00
Coefficient: −1.83e−04
Value: C
Basfia.succiniciproducens
2.0
1.5
1.0
0.5
0.0
A (n=15)
B (n=16)
C (n=14)
surface

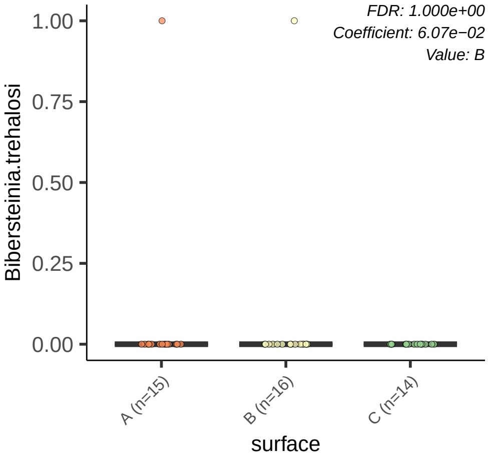

FDR: 1.000e+00
Coefficient: 6.07e−02
Value: B
1.00
0.75
0.50
0.25
0.00
Bibersteinia.trehalosi
A (n=15)
B (n=16)
C (n=14)
surface

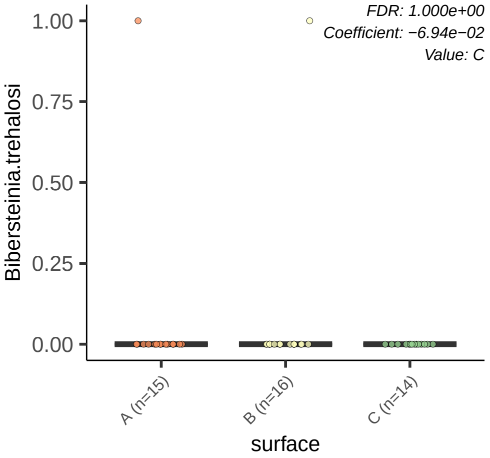
FDR: 1.000e+00
Coefficient: −6.94e−02
Value: C
Bibersteinia.trehalosi
1.00
0.75
0.50
0.25
0.00
A (n=15)
B (n=16)
C (n=14)
surface

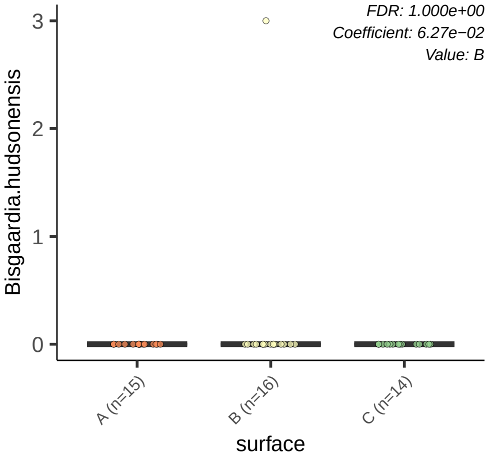
FDR: 1.000e+00
Coefficient: 6.27e−02
Value: B
Bisgaardia.hudsonensis
3
2
1
0
A (n=15)
B (n=16)
C (n=14)
surface

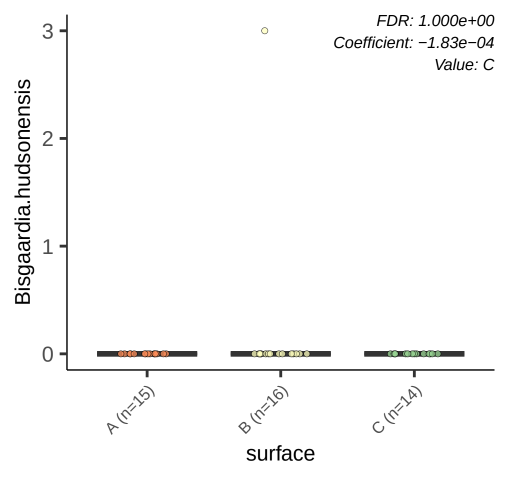

FDR: 1.000e+00
Coefficient: −1.83e−04
Value: C
Bisgaardia.hudsonensis
3
2
1
0
A (n=15)
B (n=16)
C (n=14)
surface

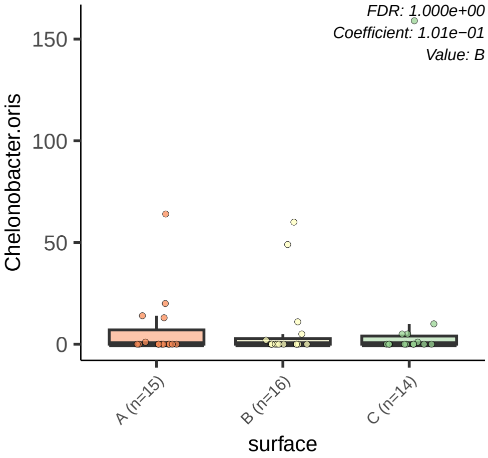
FDR: 1.000e+00
Coefficient: 1.01e−01
Value: B
Chelonobacter.oris
150
100
50
0
A (n=15)
B (n=16)
C (n=14)
surface

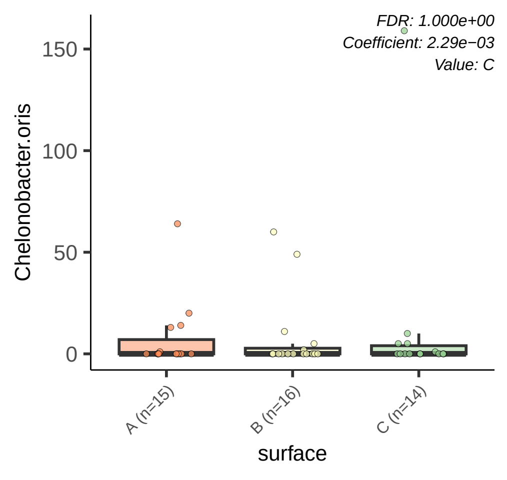
FDR: 1.000e+00
Coefficient: 2.29e−03
Value: C
Chelonobacter.oris
150
100
50
0
A (n=15)
B (n=16)
C (n=14)
surface

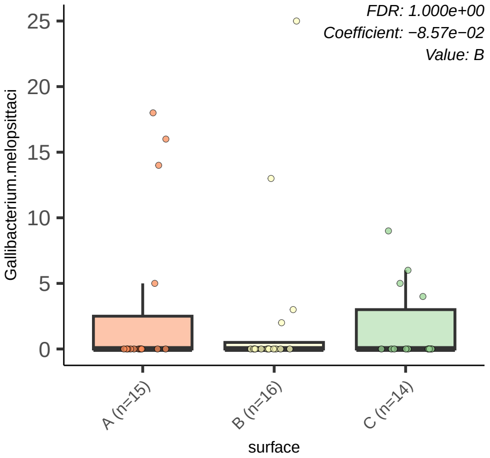
FDR: 1.000e+00
Coefficient: −8.57e−02
Value: B
Gallibacterium.melopsittaci
0
5
10
15
20
25
A (n=15)
B (n=16)
C (n=14)
surface

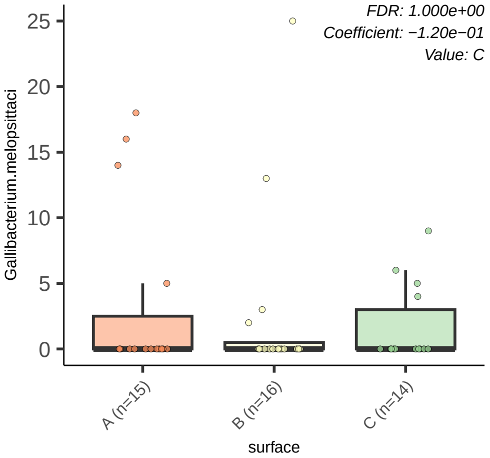
FDR: 1.000e+00
Coefficient: −1.20e−01
Value: C
Gallibacterium.melopsittaci
25
20
15
10
5
0
A (n=15)
B (n=16)
C (n=14)
surface

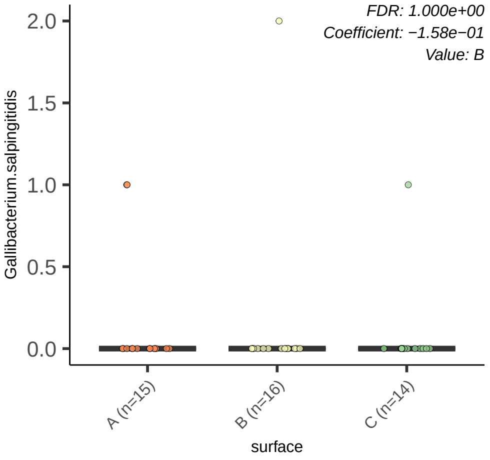

FDR: 1.000e+00
Coefficient: −1.58e−01
Value: B
Gallibacterium.salpingitidis
2.0
1.5
1.0
0.5
0.0
A (n=15)
B (n=16)
C (n=14)
surface

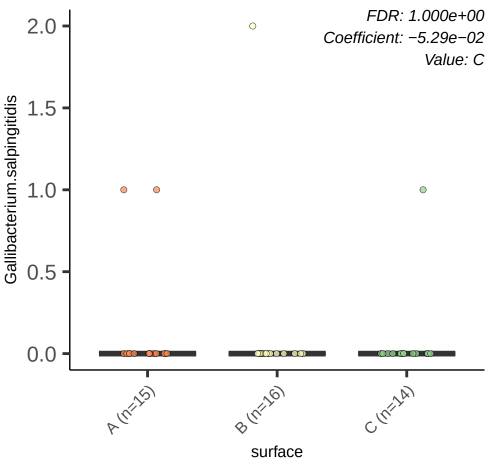
FDR: 1.000e+00
Coefficient: −5.29e−02
Value: C
Gallibacterium.salpingitidis
2.0
1.5
1.0
0.5
0.0
A (n=15)
B (n=16)
C (n=14)
surface

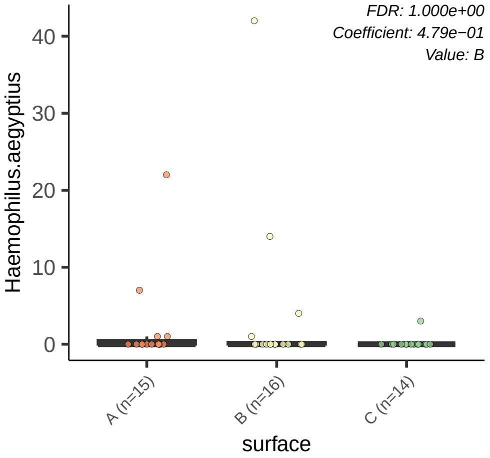
FDR: 1.000e+00
Coefficient: 4.79e−01
Value: B
Haemophilus.aegyptius
0
10
20
30
40
A (n=15)
B (n=16)
C (n=14)
surface

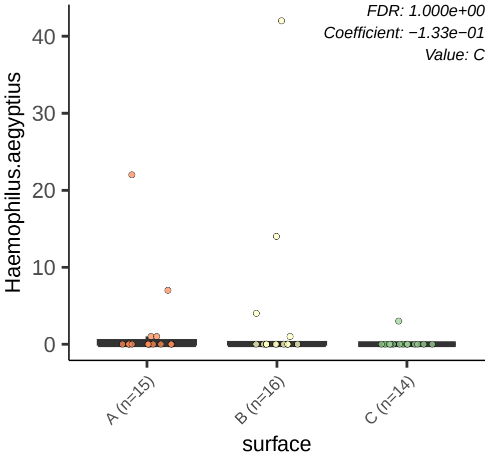

FDR: 1.000e+00
Coefficient: −1.33e−01
Value: C
Haemophilus.aegyptius
40
30
20
10
0
A (n=15)
B (n=16)
C (n=14)
surface

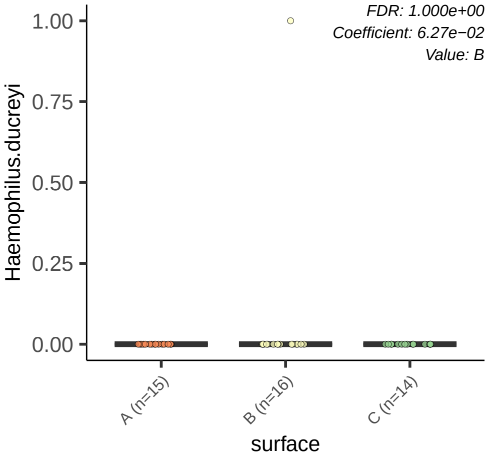

FDR: 1.000e+00
Coefficient: 6.27e−02
Value: B
Haemophilus.ducreyi
1.00
0.75
0.50
0.25
0.00
A (n=15)
B (n=16)
C (n=14)
surface

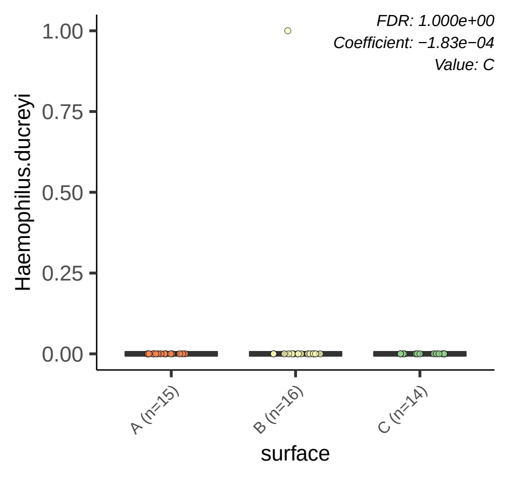

FDR: 1.000e+00
Coefficient: −1.83e−04
Value: C
Haemophilus.ducreyi
1.00
0.75
0.50
0.25
0.00
A (n=15)
B (n=16)
C (n=14)
surface

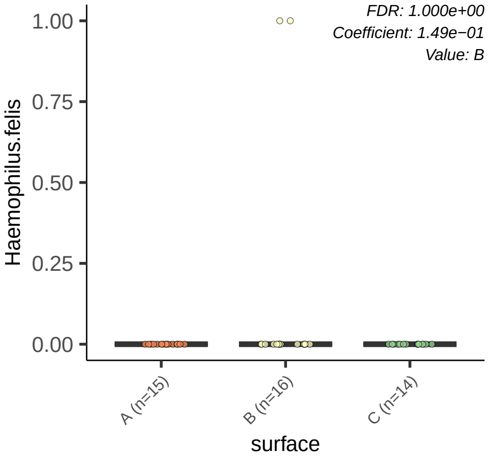
FDR: 1.000e+00
Coefficient: 1.49e−01
Value: B
Haemophilus.felis
1.00
0.75
0.50
0.25
0.00
A (n=15)
B (n=16)
C (n=14)
surface

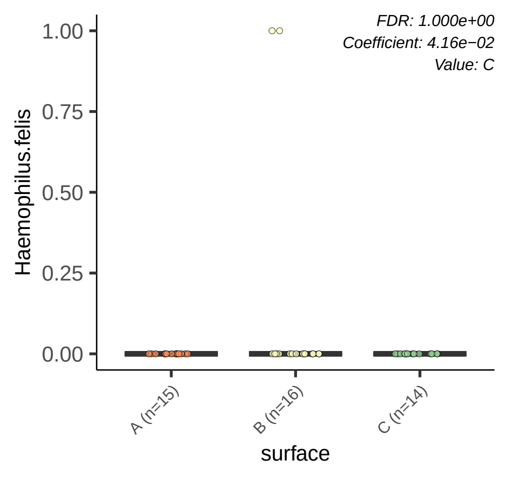
FDR: 1.000e+00
Coefficient: 4.16e−02
Value: C
Haemophilus.felis
1.00
0.75
0.50
0.25
0.00
A (n=15)
B (n=16)
C (n=14)
surface

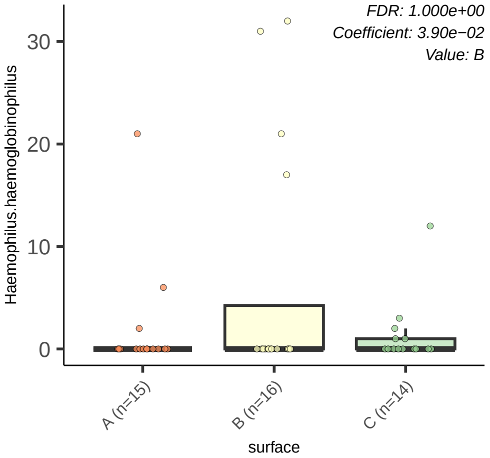
FDR: 1.000e+00
Coefficient: 3.90e−02
Value: B
Haemophilus.haemoglobinophilus
30
20
10
0
A (n=15)
B (n=16)
C (n=14)
surface

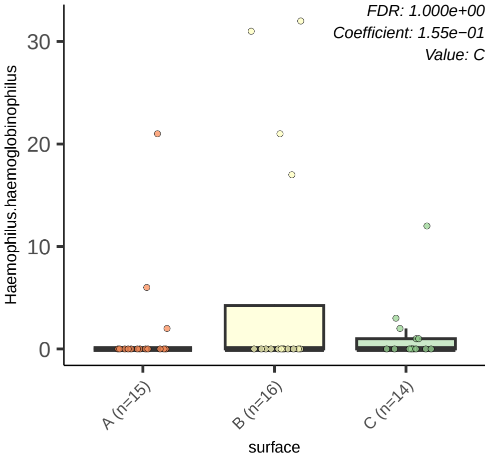
FDR: 1.000e+00
Coefficient: 1.55e−01
Value: C
Haemophilus.haemoglobinophilus
30
20
10
0
A (n=15)
B (n=16)
C (n=14)
surface

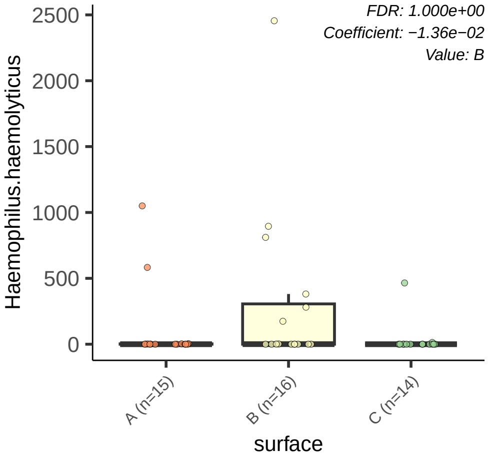

FDR: 1.000e+00
Coefficient: −1.36e−02
Value: B
Haemophilus.haemolyticus
2500
2000
1500
1000
500
0
A (n=15)
B (n=16)
C (n=14)
surface

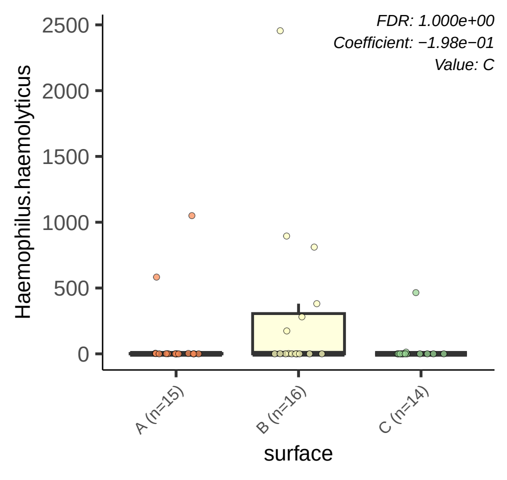
FDR: 1.000e+00
Coefficient: −1.98e−01
Value: C
Haemophilus.haemolyticus
2500
2000
1500
1000
500
0
A (n=15)
B (n=16)
C (n=14)
surface

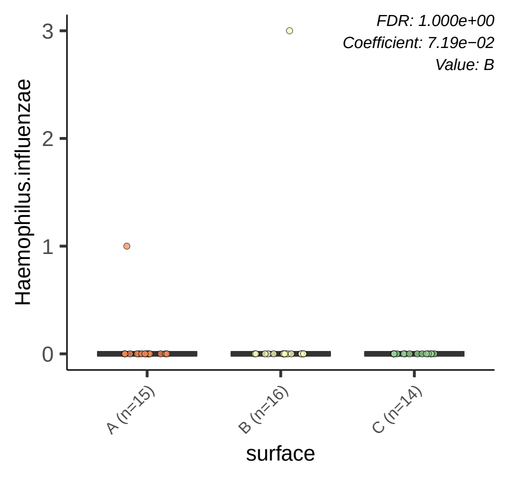
FDR: 1.000e+00
Coefficient: 7.19e−02
Value: B
Haemophilus.influenzae
3
2
1
0
A (n=15)
B (n=16)
C (n=14)
surface

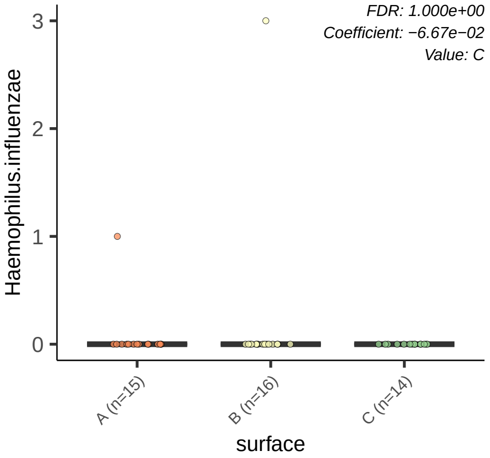
FDR: 1.000e+00
Coefficient: −6.67e−02
Value: C
Haemophilus.influenzae
3
2
1
0
A (n=15)
B (n=16)
C (n=14)
surface

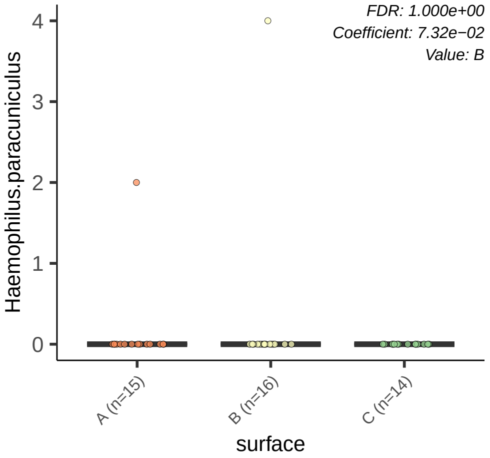
FDR: 1.000e+00
Coefficient: 7.32e−02
Value: B
Haemophilus.paracuniculus
4
3
2
1
0
A (n=15)
B (n=16)
C (n=14)
surface

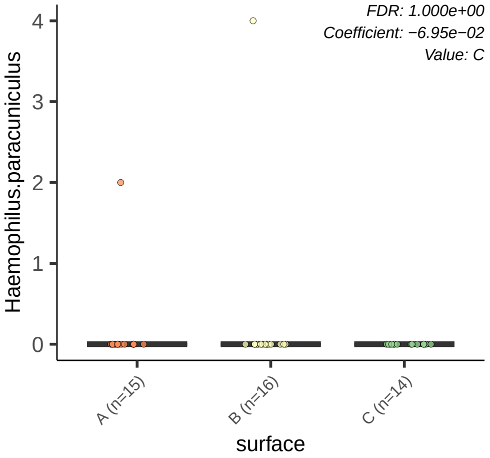
FDR: 1.000e+00
Coefficient: −6.95e−02
Value: C
Haemophilus.paracuniculus
0
1
2
3
4
A (n=15)
B (n=16)
C (n=14)
surface

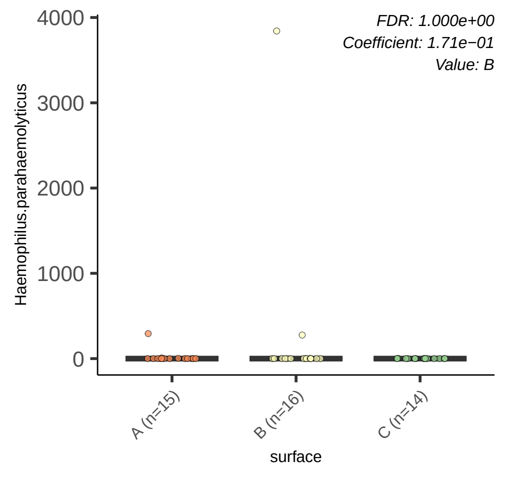
FDR: 1.000e+00
Coefficient: 1.71e−01
Value: B
4000
3000
2000
1000
0
Haemophilus.parahaemolyticus
A (n=15)
B (n=16)
C (n=14)
surface

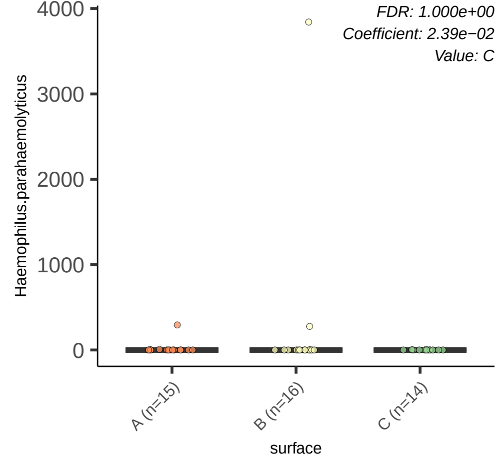

FDR: 1.000e+00
Coefficient: 2.39e−02
Value: C
Haemophilus.parahaemolyticus
4000
3000
2000
1000
0
A (n=15)
B (n=16)
C (n=14)
surface

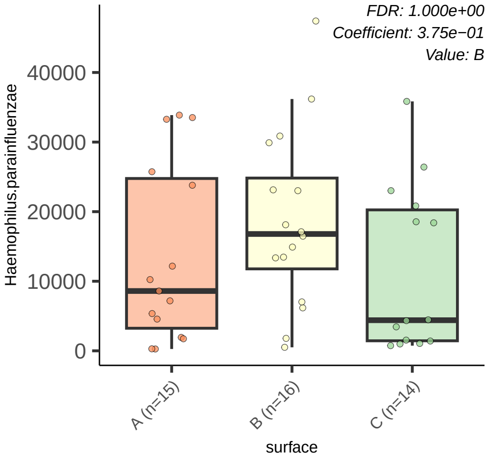

FDR: 1.000e+00
Coefficient: 3.75e−01
Value: B
Haemophilus.parainfluenzae
40000
30000
20000
10000
0
A (n=15)
B (n=16)
C (n=14)
surface

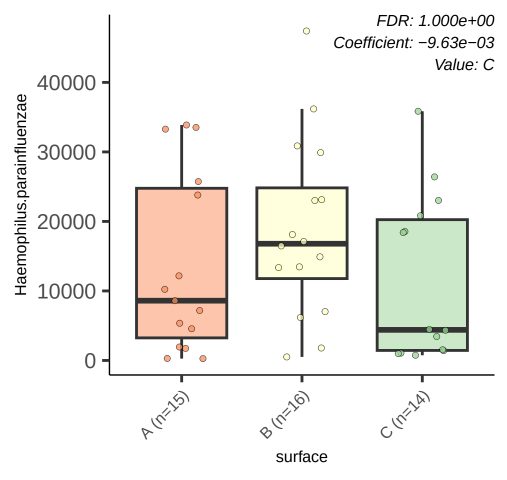
FDR: 1.000e+00
Coefficient: −9.63e−03
Value: C
Haemophilus.parainfluenzae
40000
30000
20000
10000
0
A (n=15)
B (n=16)
C (n=14)
surface

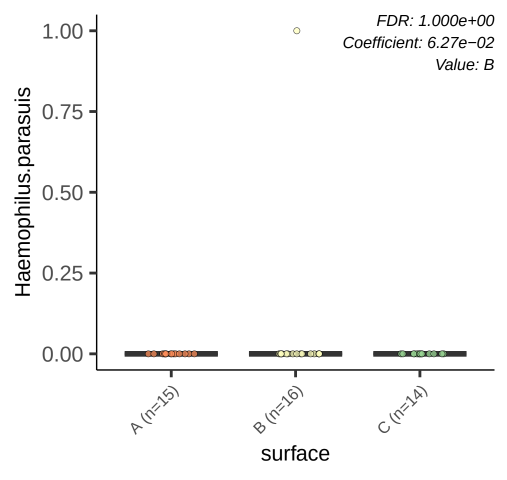
FDR: 1.000e+00
Coefficient: 6.27e−02
Value: B
Haemophilus.parasuis
1.00
0.75
0.50
0.25
0.00
A (n=15)
B (n=16)
C (n=14)
surface

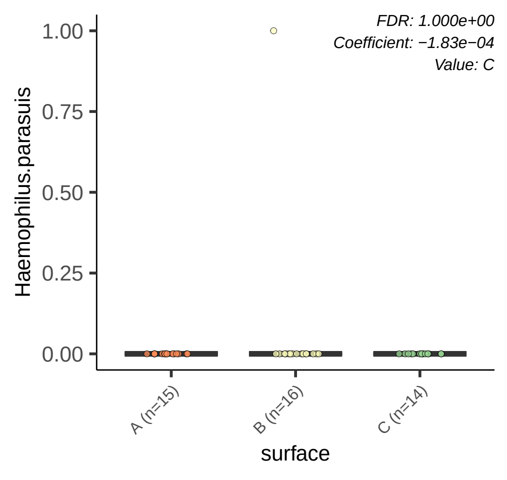

FDR: 1.000e+00
Coefficient: −1.83e−04
Value: C
Haemophilus.parasuis
1.00
0.75
0.50
0.25
0.00
A (n=15)
B (n=16)
C (n=14)
surface

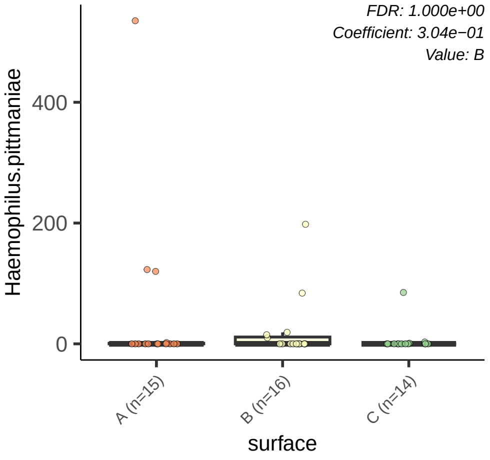
FDR: 1.000e+00
Coefficient: 3.04e−01
Value: B
Haemophilus.pittmaniae
400
200
0
A (n=15)
B (n=16)
C (n=14)
surface

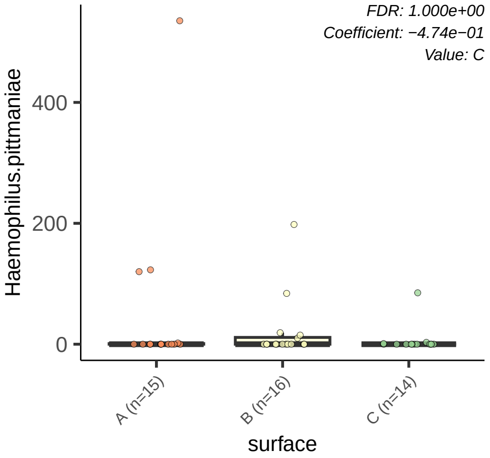

FDR: 1.000e+00
Coefficient: −4.74e−01
Value: C
Haemophilus.pittmaniae
400
200
0
A (n=15)
B (n=16)
C (n=14)
surface

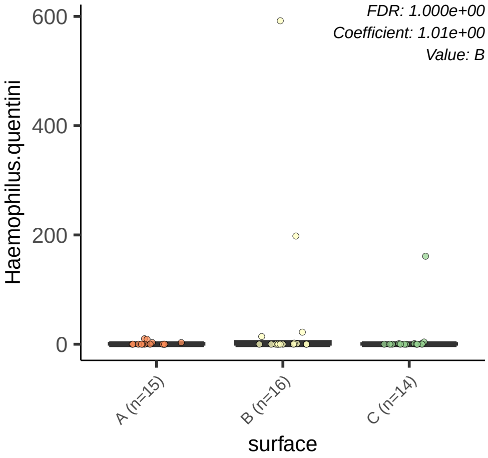
FDR: 1.000e+00
Coefficient: 1.01e+00
Value: B
Haemophilus.quentini
600
400
200
0
A (n=15)
B (n=16)
C (n=14)
surface

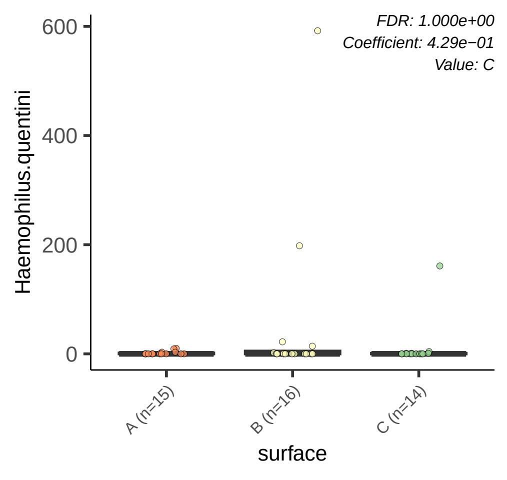

FDR: 1.000e+00
Coefficient: 4.29e−01
Value: C
Haemophilus.quentini
600
400
200
0
A (n=15)
B (n=16)
C (n=14)
surface

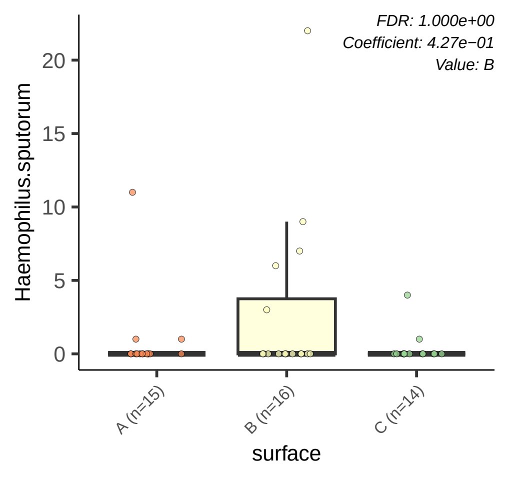
FDR: 1.000e+00
Coefficient: 4.27e−01
Value: B
Haemophilus.sputorum
0
5
10
15
20
A (n=15)
B (n=16)
C (n=14)
surface

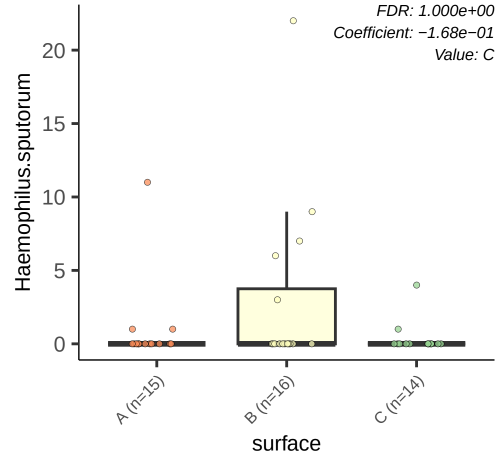
FDR: 1.000e+00
Coefficient: −1.68e−01
Value: C
Haemophilus.sputorum
0
5
10
15
20
A (n=15)
B (n=16)
C (n=14)
surface

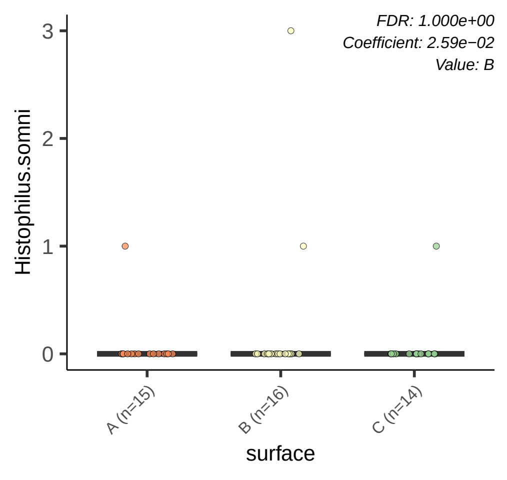

FDR: 1.000e+00
Coefficient: 2.59e−02
Value: B
Histophilus.somni
3
2
1
0
A (n=15)
B (n=16)
C (n=14)
surface

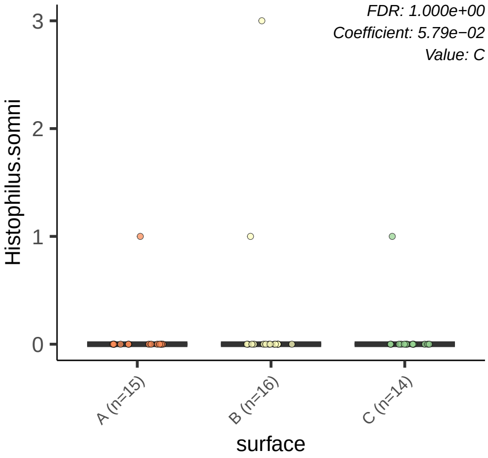
FDR: 1.000e+00
Coefficient: 5.79e−02
Value: C
Histophilus.somni
0
1
2
3
A (n=15)
B (n=16)
C (n=14)
surface

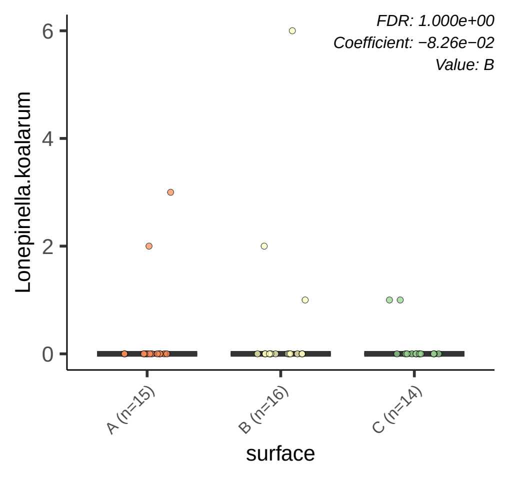
FDR: 1.000e+00
Coefficient: −8.26e−02
Value: B
Lonepinella.koalarum
0
2
4
6
A (n=15)
B (n=16)
C (n=14)
surface

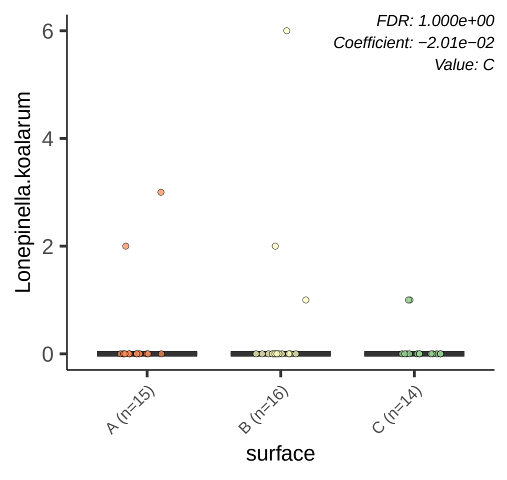
FDR: 1.000e+00
Coefficient: −2.01e−02
Value: C
Lonepinella.koalarum
0
2
4
6
A (n=15)
B (n=16)
C (n=14)
surface

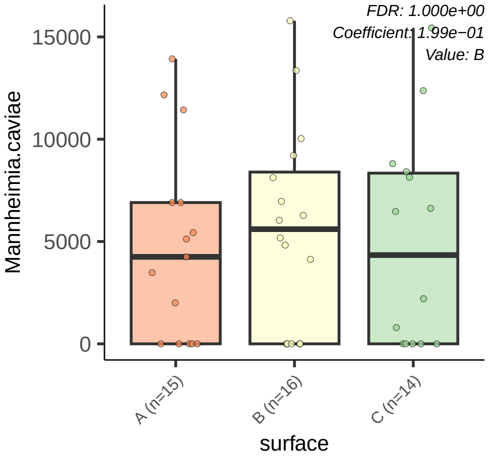

FDR: 1.000e+00
Coefficient: 1.99e−01
Value: B
Mannheimia.caviae
15000
10000
5000
0
A (n=15)
B (n=16)
C (n=14)
surface

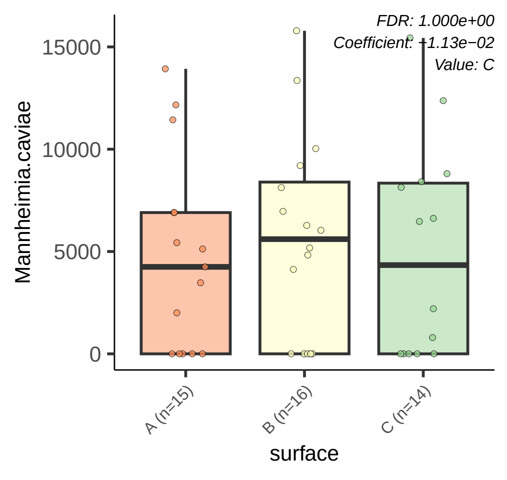
FDR: 1.000e+00
Coefficient: -1.13e-02
Value: C
Mannheimia.caviae
15000
10000
5000
0
A (n=15)
B (n=16)
C (n=14)
surface

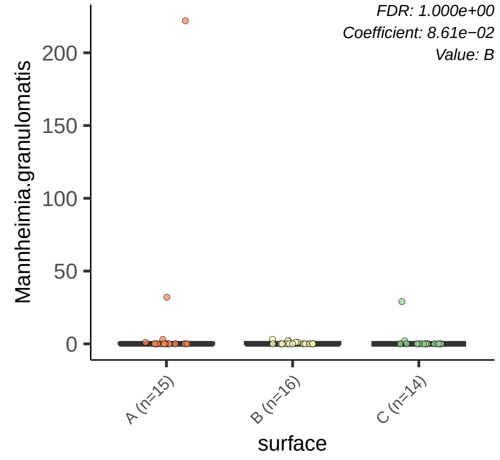
FDR: 1.000e+00
Coefficient: 8.61e−02
Value: B
Mannheimia.granulomatis
200
150
100
50
0
A (n=15)
B (n=16)
C (n=14)
surface

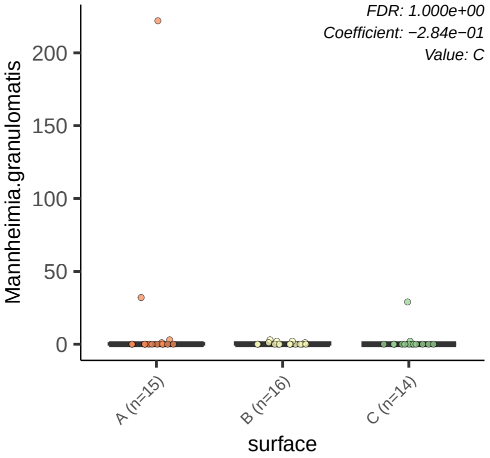
FDR: 1.000e+00
Coefficient: −2.84e−01
Value: C
Mannheimia.granulomatis
200
150
100
50
0
A (n=15)
B (n=16)
C (n=14)
surface

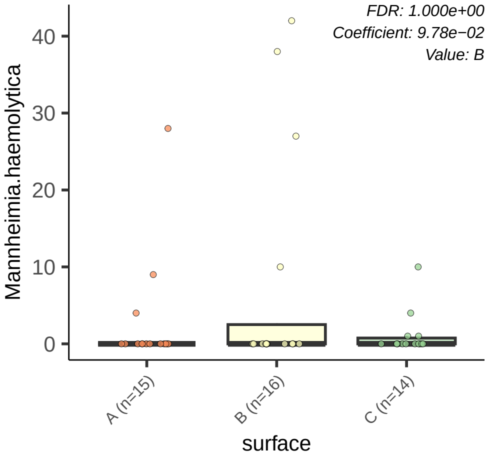
FDR: 1.000e+00
Coefficient: 9.78e−02
Value: B
Mannheimia.haemolytica
0
10
20
30
40
A (n=15)
B (n=16)
C (n=14)
surface

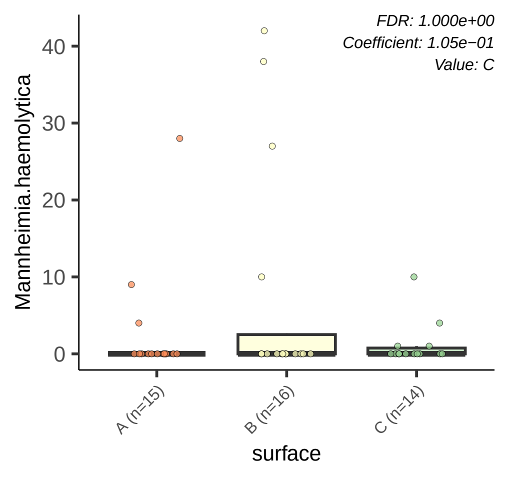
FDR: 1.000e+00
Coefficient: 1.05e−01
Value: C
Mannheimia.haemolytica
0
10
20
30
40
A (n=15)
B (n=16)
C (n=14)
surface

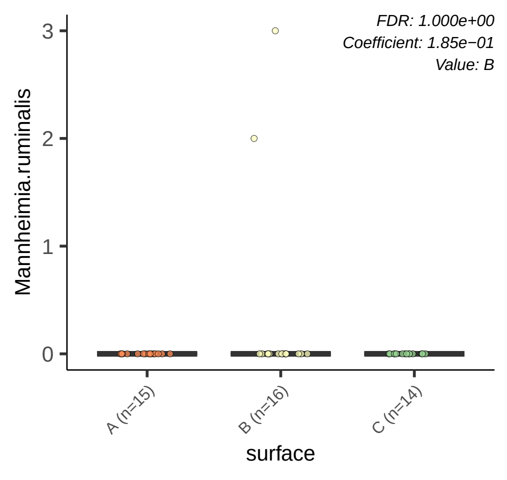
FDR: 1.000e+00
Coefficient: 1.85e−01
Value: B
Mannheimia.ruminalis
3
2
1
0
A (n=15)
B (n=16)
C (n=14)
surface

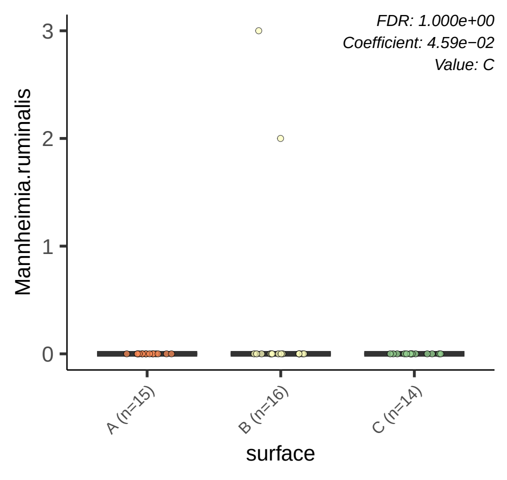
FDR: 1.000e+00
Coefficient: 4.59e−02
Value: C
Mannheimia.ruminalis
3
2
1
0
A (n=15)
B (n=16)
C (n=14)
surface

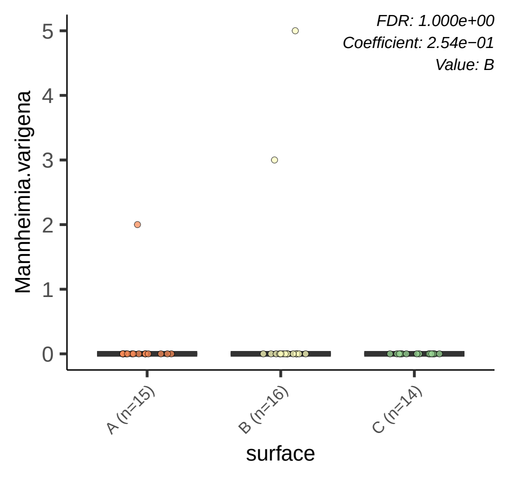
FDR: 1.000e+00
Coefficient: 2.54e−01
Value: B
Mannheimia.varigena
0
1
2
3
4
5
A (n=15)
B (n=16)
C (n=14)
surface

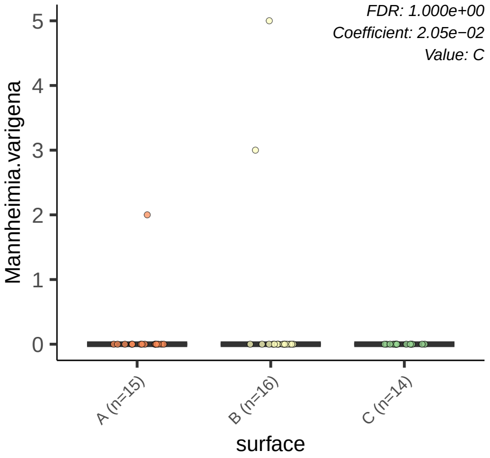

FDR: 1.000e+00
Coefficient: 2.05e−02
Value: C
Mannheimia.varigena
0
1
2
3
4
5
A (n=15)
B (n=16)
C (n=14)
surface

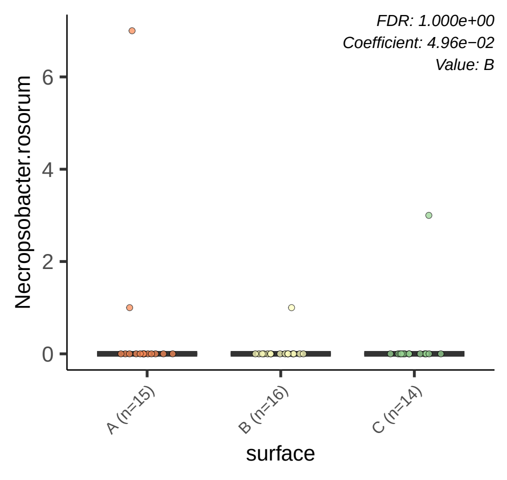
FDR: 1.000e+00
Coefficient: 4.96e−02
Value: B
Necropsobacter.rosorum
0
2
4
6
A (n=15)
B (n=16)
C (n=14)
surface

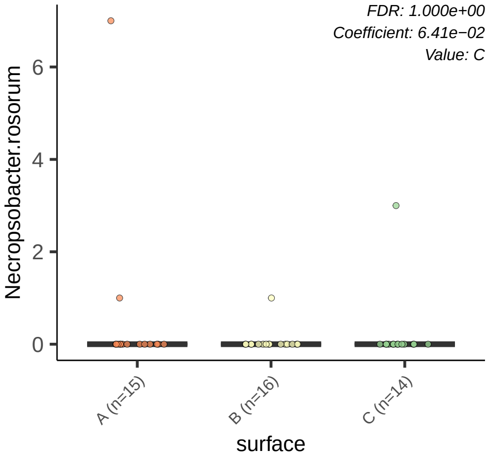

FDR: 1.000e+00
Coefficient: 6.41e−02
Value: C
Necropsobacter.rosorum
0
2
4
6
A (n=15)
B (n=16)
C (n=14)
surface

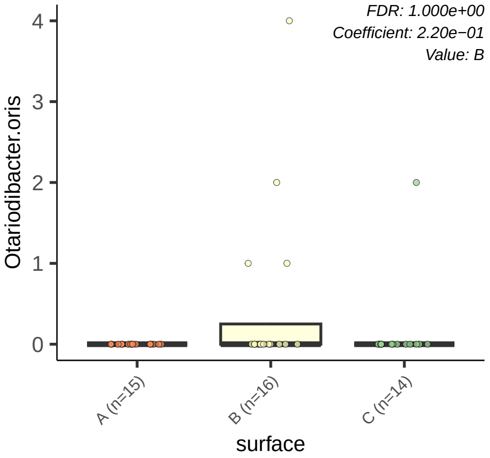

FDR: 1.000e+00
Coefficient: 2.20e−01
Value: B
Otariodibacter.oris
0
1
2
3
4
A (n=15)
B (n=16)
C (n=14)
surface

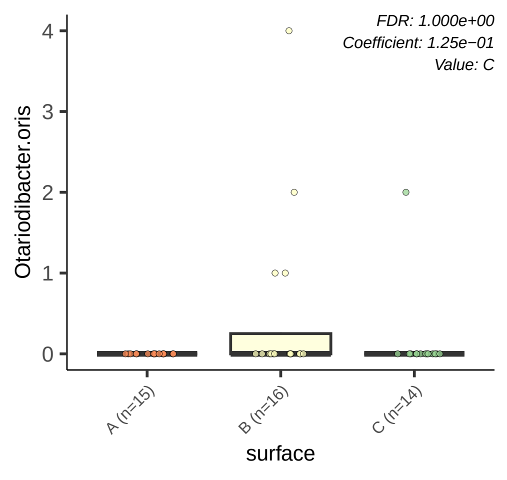
FDR: 1.000e+00
Coefficient: 1.25e−01
Value: C
Otariodibacter.oris
0
1
2
3
4
A (n=15)
B (n=16)
C (n=14)
surface

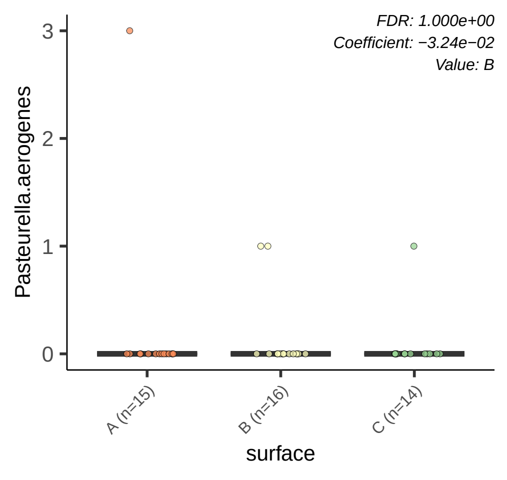
FDR: 1.000e+00
Coefficient: −3.24e−02
Value: B
Pasteurella.aerogenes
3
2
1
0
A (n=15)
B (n=16)
C (n=14)
surface

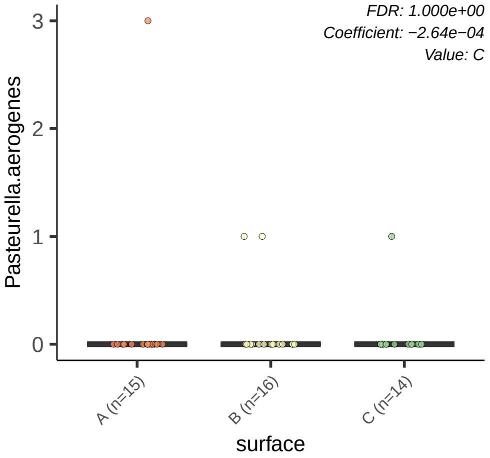
FDR: 1.000e+00
Coefficient: −2.64e−04
Value: C
Pasteurella.aerogenes
0
1
2
3
A (n=15)
B (n=16)
C (n=14)
surface

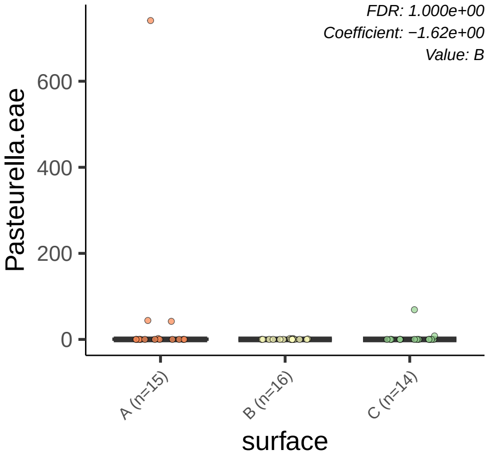

FDR: 1.000e+00
Coefficient: −1.62e+00
Value: B
Pasteurella.eae
600
400
200
0
A (n=15)
B (n=16)
C (n=14)
surface

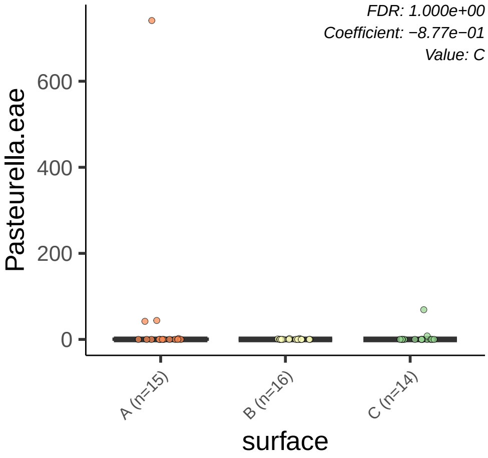

FDR: 1.000e+00
Coefficient: −8.77e−01
Value: C
Pasteurella.eae
600
400
200
0
A (n=15)
B (n=16)
C (n=14)
surface

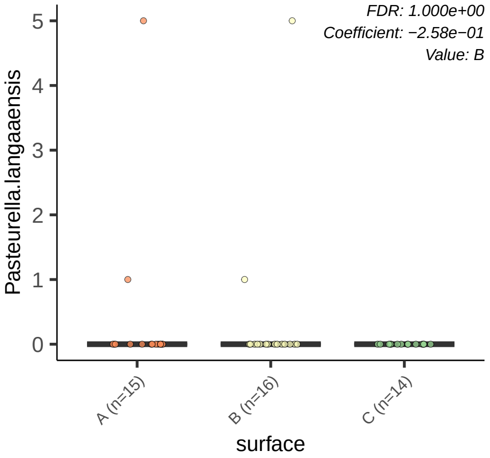
FDR: 1.000e+00
Coefficient: −2.58e−01
Value: B
Pasteurella.langaaensis
0
1
2
3
4
5
A (n=15)
B (n=16)
C (n=14)
surface

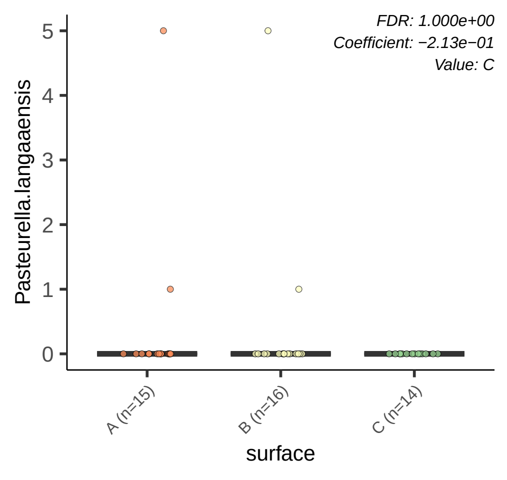
FDR: 1.000e+00
Coefficient: −2.13e−01
Value: C
Pasteurella.langaaensis
0
1
2
3
4
5
A (n=15)
B (n=16)
C (n=14)
surface

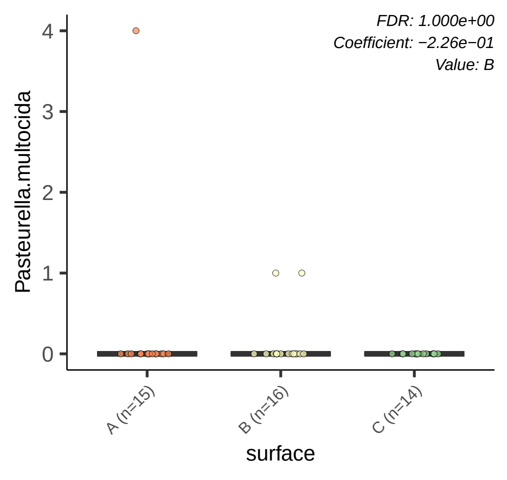
FDR: 1.000e+00
Coefficient: −2.26e−01
Value: B
Pasteurella.multocida
0
1
2
3
4
A (n=15)
B (n=16)
C (n=14)
surface

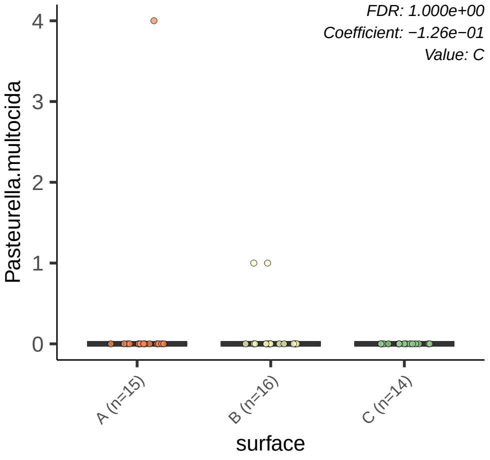
FDR: 1.000e+00
Coefficient: −1.26e−01
Value: C
Pasteurella.multocida
0
1
2
3
4
A (n=15)
B (n=16)
C (n=14)
surface

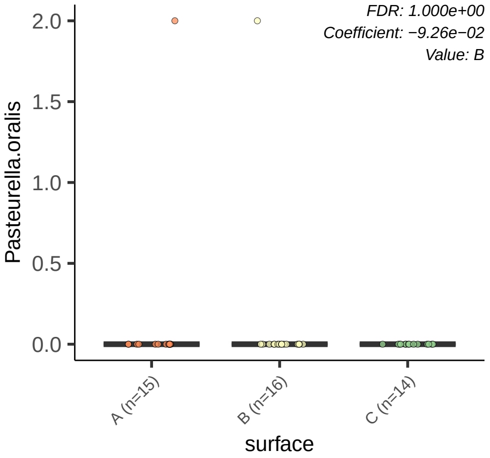

FDR: 1.000e+00
Coefficient: −9.26e−02
Value: B
Pasteurella.oralis
2.0
1.5
1.0
0.5
0.0
A (n=15)
B (n=16)
C (n=14)
surface

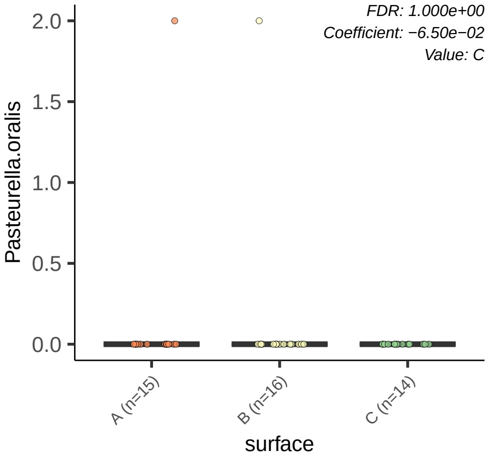

FDR: 1.000e+00
Coefficient: −6.50e−02
Value: C
Pasteurella.oralis
2.0
1.5
1.0
0.5
0.0
A (n=15)
B (n=16)
C (n=14)
surface

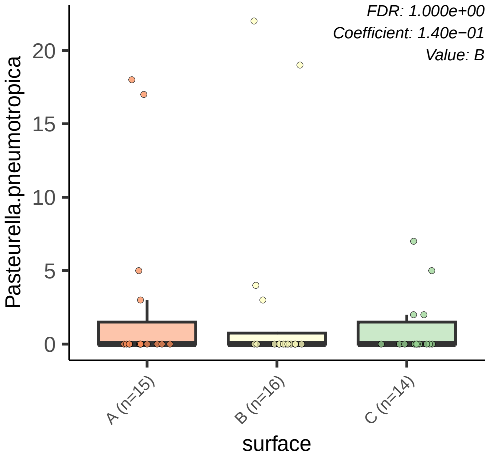

FDR: 1.000e+00
Coefficient: 1.40e−01
Value: B
Pasteurella.pneumotropica
0
5
10
15
20
A (n=15)
B (n=16)
C (n=14)
surface

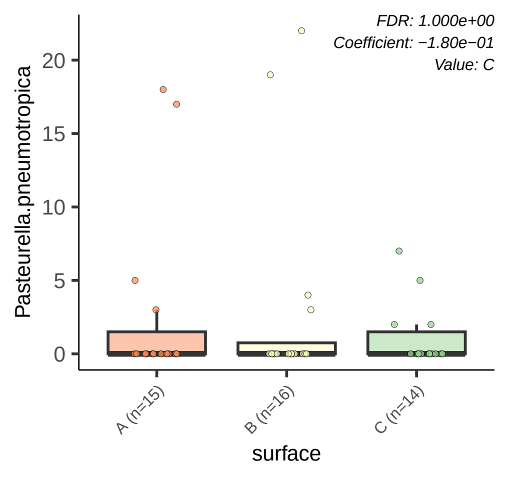
FDR: 1.000e+00
Coefficient: −1.80e−01
Value: C
Pasteurella.pneumotropica
0
5
10
15
20
A (n=15)
B (n=16)
C (n=14)
surface

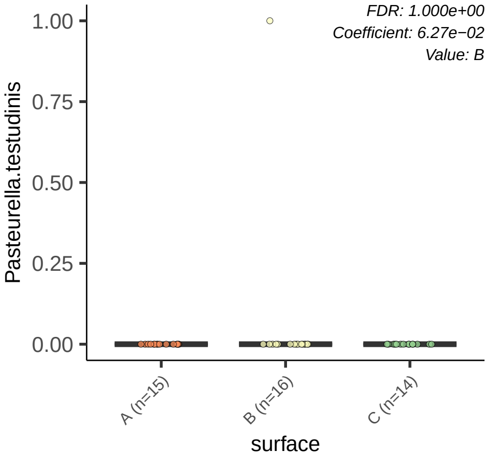
FDR: 1.000e+00
Coefficient: 6.27e−02
Value: B
Pasteurella.testudinis
1.00
0.75
0.50
0.25
0.00
A (n=15)
B (n=16)
C (n=14)
surface

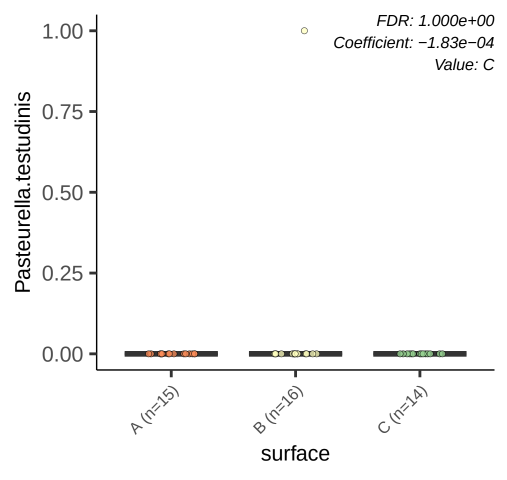
FDR: 1.000e+00
Coefficient: −1.83e−04
Value: C
Pasteurella.testudinis
1.00
0.75
0.50
0.25
0.00
A (n=15)
B (n=16)
C (n=14)
surface

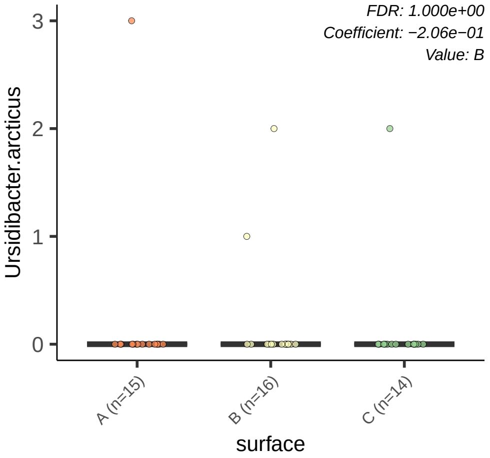
FDR: 1.000e+00
Coefficient: −2.06e−01
Value: B
Ursidibacter.arcticus
3
2
1
0
A (n=15)
B (n=16)
C (n=14)
surface

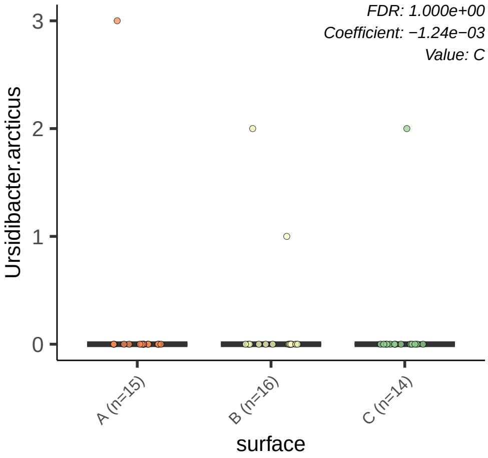

FDR: 1.000e+00
Coefficient: −1.24e−03
Value: C
Ursidibacter.arcticus
3
2
1
0
A (n=15)
B (n=16)
C (n=14)
surface

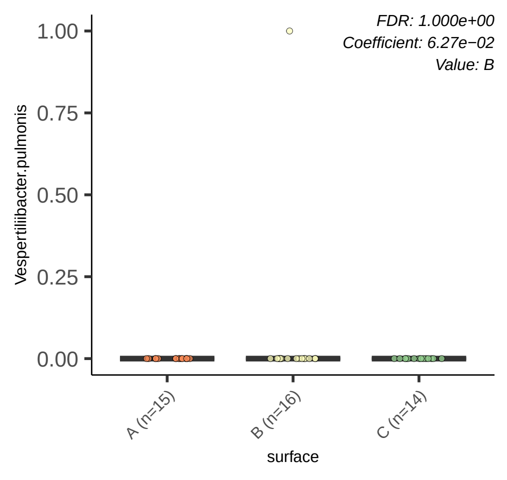
FDR: 1.000e+00
Coefficient: 6.27e−02
Value: B
1.00
0.75
0.50
0.25
0.00
Vespertiliibacter.pulmonis
A (n=15)
B (n=16)
C (n=14)
surface

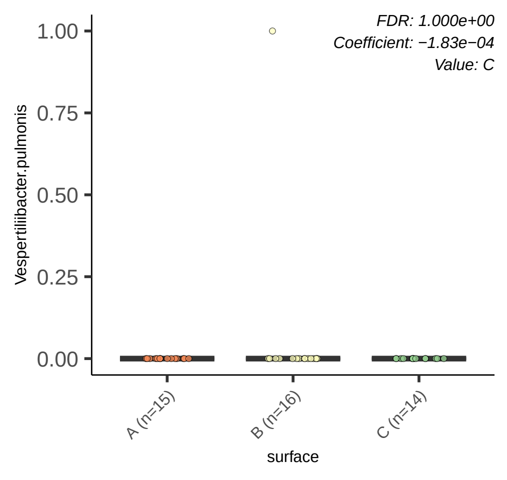
FDR: 1.000e+00
Coefficient: −1.83e−04
Value: C
1.00
0.75
0.50
0.25
0.00
Vespertiliibacter.pulmonis
A (n=15)
B (n=16)
C (n=14)
surface

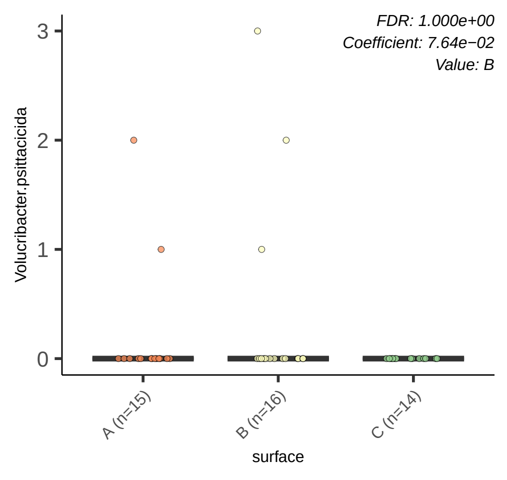
FDR: 1.000e+00
Coefficient: 7.64e−02
Value: B
Volucribacter.psittacicida
0
1
2
3
A (n=15)
B (n=16)
C (n=14)
surface

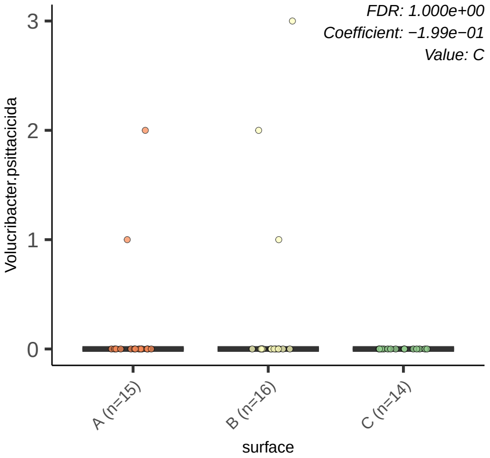
FDR: 1.000e+00
Coefficient: −1.99e−01
Value: C
Volucribacter.psittacicida
3
2
1
0
A (n=15)
B (n=16)
C (n=14)
surface

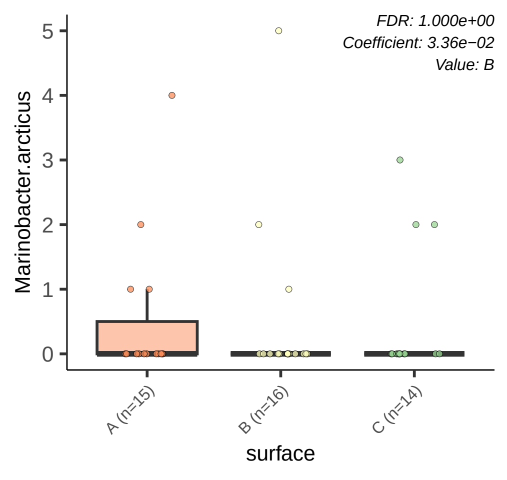
FDR: 1.000e+00
Coefficient: 3.36e−02
Value: B
Marinobacter.arcticus
0
1
2
3
4
5
A (n=15)
B (n=16)
C (n=14)
surface

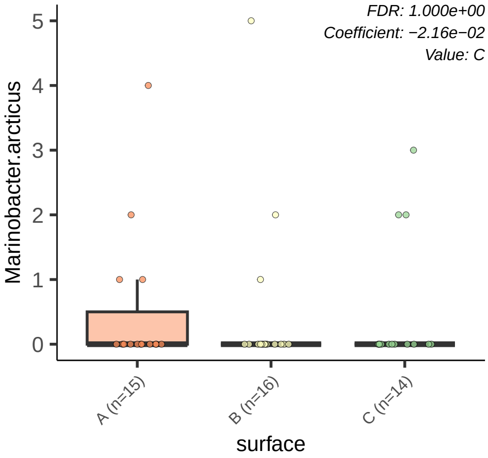
FDR: 1.000e+00
Coefficient: −2.16e−02
Value: C
Marinobacter.arcticus
0
1
2
3
4
5
A (n=15)
B (n=16)
C (n=14)
surface

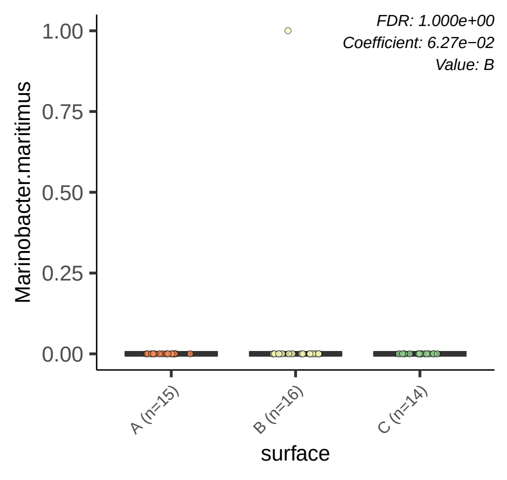
FDR: 1.000e+00
Coefficient: 6.27e−02
Value: B
Marinobacter.maritimus
1.00
0.75
0.50
0.25
0.00
A (n=15)
B (n=16)
C (n=14)
surface

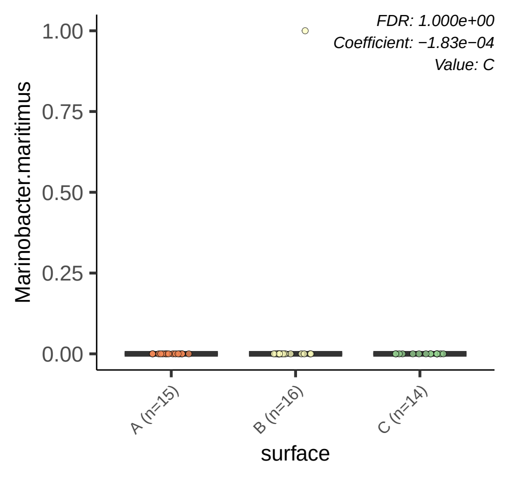
FDR: 1.000e+00
Coefficient: −1.83e−04
Value: C
Marinobacter.maritimus
1.00
0.75
0.50
0.25
0.00
A (n=15)
B (n=16)
C (n=14)
surface

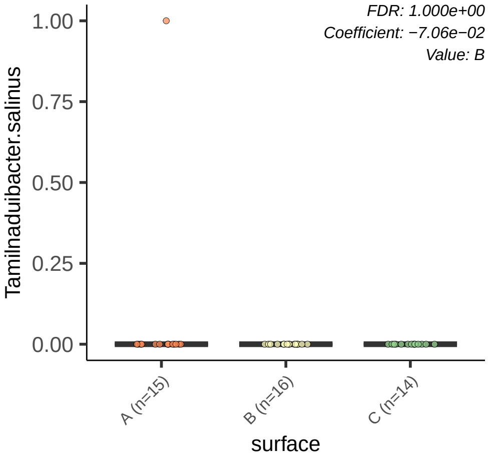
FDR: 1.000e+00
Coefficient: −7.06e−02
Value: B
Tamilnaduibacter.salinus
1.00
0.75
0.50
0.25
0.00
A (n=15)
B (n=16)
C (n=14)
surface

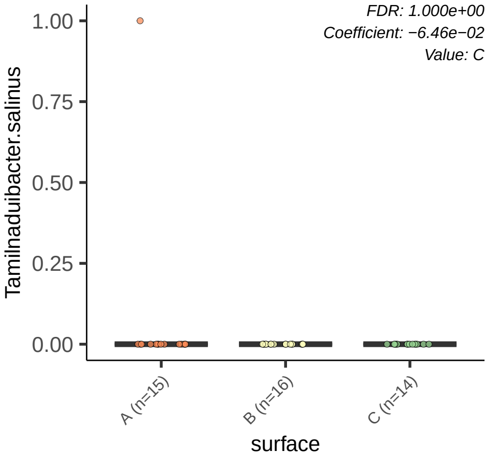
FDR: 1.000e+00
Coefficient: −6.46e−02
Value: C
Tamilnaduibacter.salinus
1.00
0.75
0.50
0.25
0.00
A (n=15)
B (n=16)
C (n=14)
surface

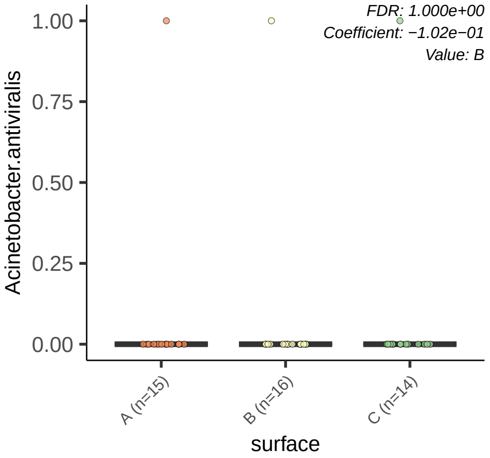

FDR: 1.000e+00
Coefficient: −1.02e−01
Value: B
Acinetobacter.antiviralis
1.00
0.75
0.50
0.25
0.00
A (n=15)
B (n=16)
C (n=14)
surface

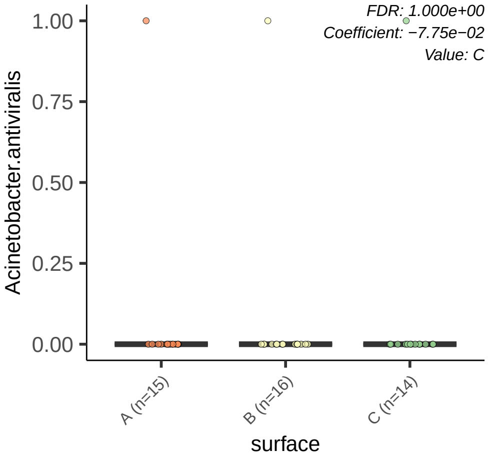
FDR: 1.000e+00
Coefficient: −7.75e−02
Value: C
Acinetobacter.antiviralis
1.00
0.75
0.50
0.25
0.00
A (n=15)
B (n=16)
C (n=14)
surface

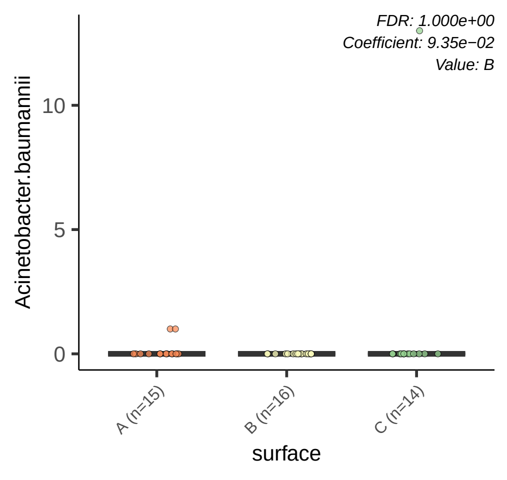
FDR: 1.000e+00
Coefficient: 9.35e−02
Value: B
Acinetobacter.baumannii
10
5
0
A (n=15)
B (n=16)
C (n=14)
surface

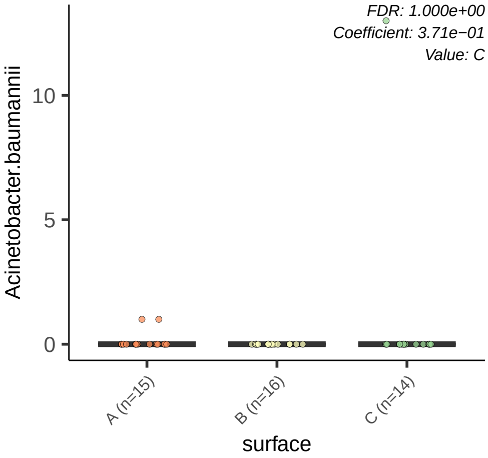
FDR: 1.000e+00
Coefficient: 3.71e−01
Value: C
Acinetobacter.baumannii
0
5
10
A (n=15)
B (n=16)
C (n=14)
surface

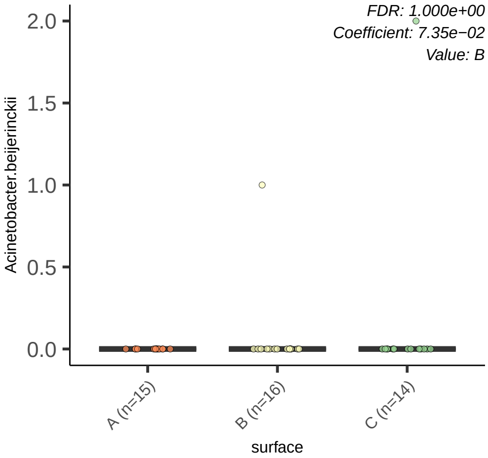

FDR: 1.000e+00
Coefficient: 7.35e−02
Value: B
Acinetobacter.beijerinckii
2.0
1.5
1.0
0.5
0.0
A (n=15)
B (n=16)
C (n=14)
surface

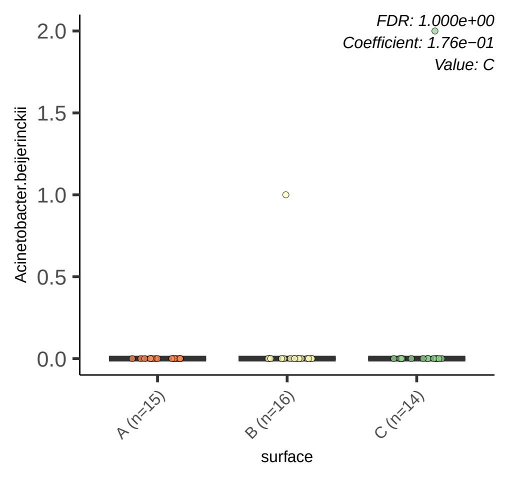
FDR: 1.000e+00
Coefficient: 1.76e−01
Value: C
Acinetobacter.beijerinckii
2.0
1.5
1.0
0.5
0.0
A (n=15)
B (n=16)
C (n=14)
surface

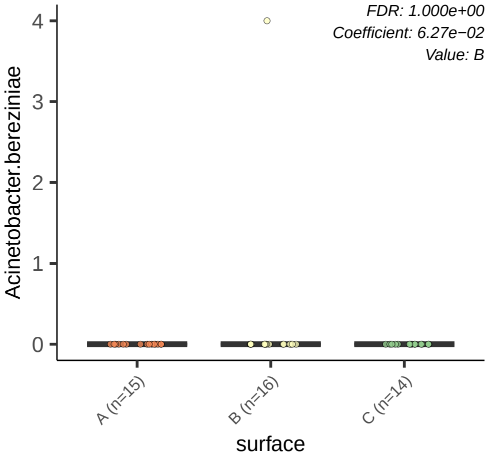

FDR: 1.000e+00
Coefficient: 6.27e−02
Value: B
Acinetobacter.bereziniae
4
3
2
1
0
A (n=15)
B (n=16)
C (n=14)
surface

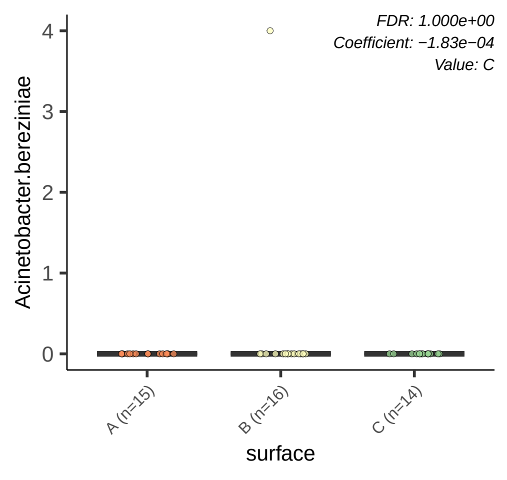
FDR: 1.000e+00
Coefficient: −1.83e−04
Value: C
Acinetobacter.bereziniae
0
1
2
3
4
A (n=15)
B (n=16)
C (n=14)
surface

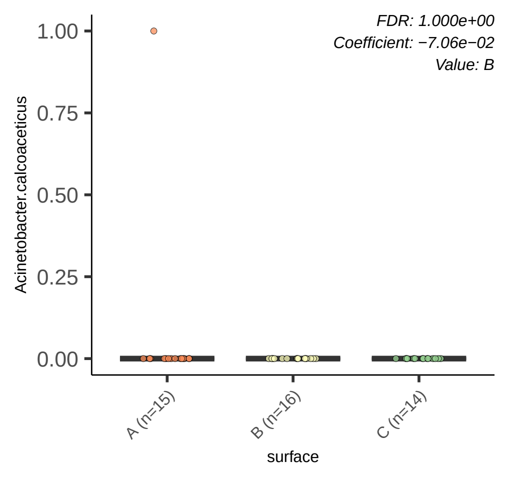

FDR: 1.000e+00
Coefficient: −7.06e−02
Value: B
1.00
0.75
0.50
0.25
0.00
Acinetobacter.calcoaceticus
A (n=15)
B (n=16)
C (n=14)
surface

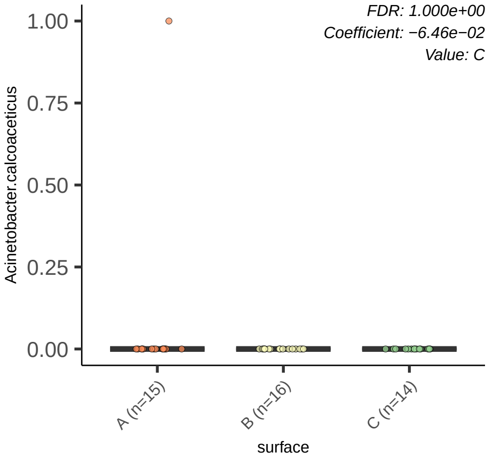
FDR: 1.000e+00
Coefficient: −6.46e−02
Value: C
Acinetobacter.calcoaceticus
1.00
0.75
0.50
0.25
0.00
A (n=15)
B (n=16)
C (n=14)
surface

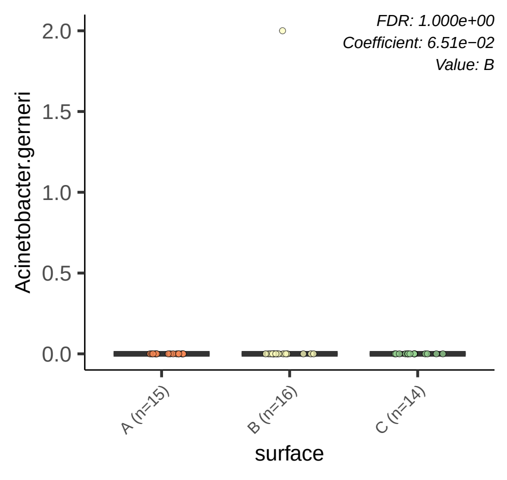
FDR: 1.000e+00
Coefficient: 6.51e−02
Value: B
Acinetobacter.gerneri
2.0
1.5
1.0
0.5
0.0
A (n=15)
B (n=16)
C (n=14)
surface

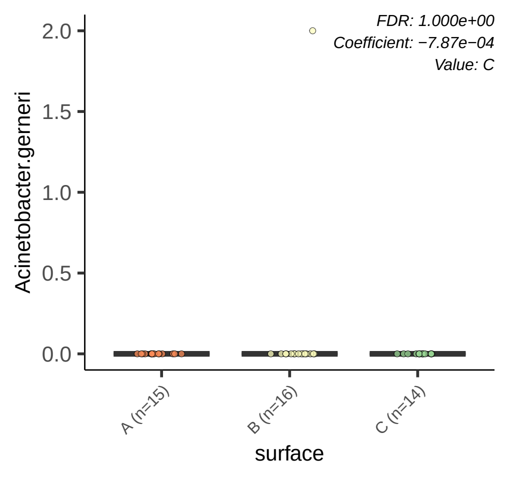
FDR: 1.000e+00
Coefficient: −7.87e−04
Value: C
Acinetobacter.gerneri
2.0
1.5
1.0
0.5
0.0
A (n=15)
B (n=16)
C (n=14)
surface

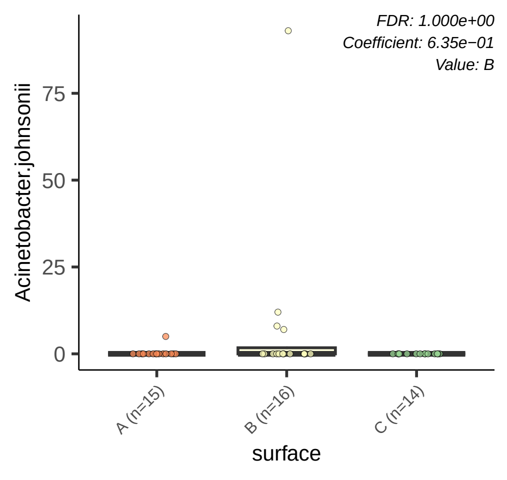
FDR: 1.000e+00
Coefficient: 6.35e−01
Value: B
Acinetobacter.johnsonii
75
50
25
0
A (n=15)
B (n=16)
C (n=14)
surface

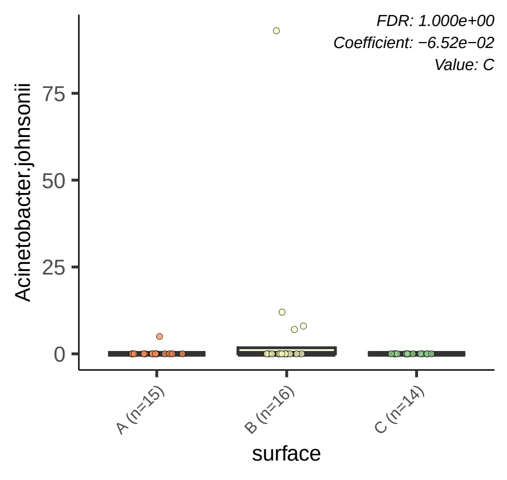

FDR: 1.000e+00
Coefficient: −6.52e−02
Value: C
Acinetobacter.johnsonii
75
50
25
0
A (n=15)
B (n=16)
C (n=14)
surface

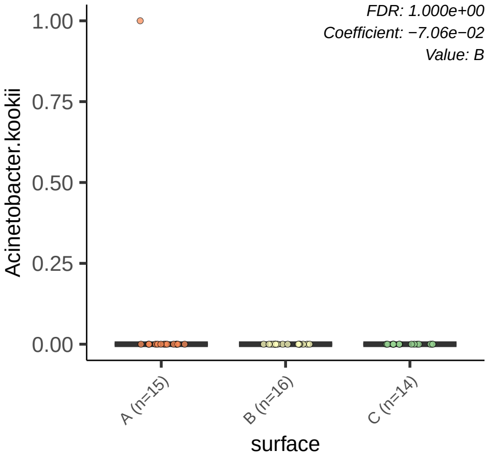
FDR: 1.000e+00
Coefficient: −7.06e−02
Value: B
1.00
0.75
0.50
0.25
0.00
Acinetobacter.kookii
A (n=15)
B (n=16)
C (n=14)
surface

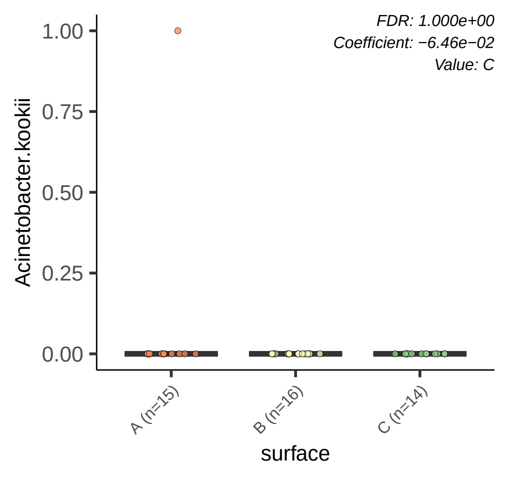

FDR: 1.000e+00
Coefficient: −6.46e−02
Value: C
Acinetobacter.kookii
1.00
0.75
0.50
0.25
0.00
A (n=15)
B (n=16)
C (n=14)
surface

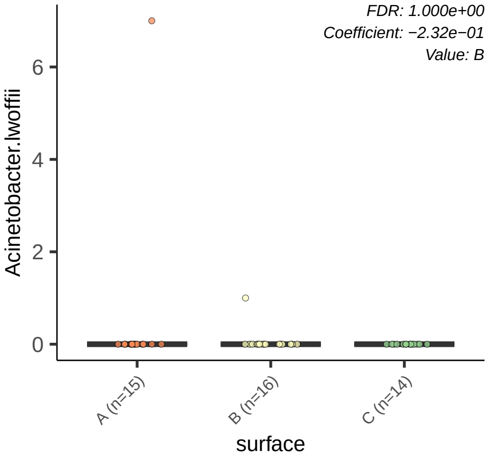
FDR: 1.000e+00
Coefficient: −2.32e−01
Value: B
Acinetobacter.lwoffii
0
2
4
6
A (n=15)
B (n=16)
C (n=14)
surface

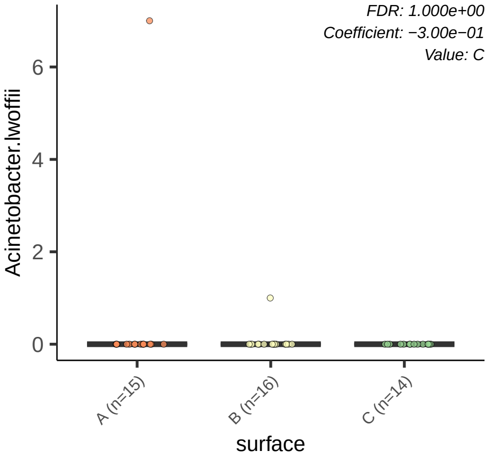
FDR: 1.000e+00
Coefficient: −3.00e−01
Value: C
Acinetobacter.lwoffii
0
2
4
6
A (n=15)
B (n=16)
C (n=14)
surface

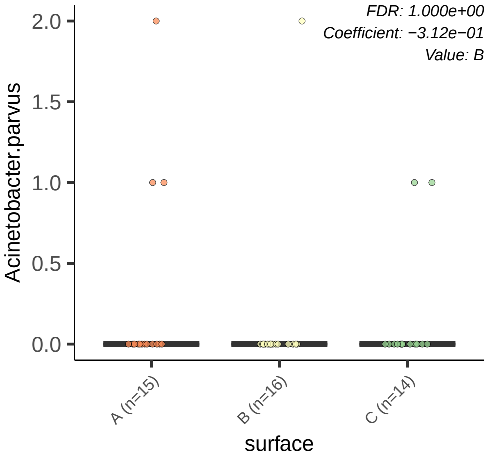
FDR: 1.000e+00
Coefficient: −3.12e−01
Value: B
Acinetobacter.parvus
2.0
1.5
1.0
0.5
0.0
A (n=15)
B (n=16)
C (n=14)
surface

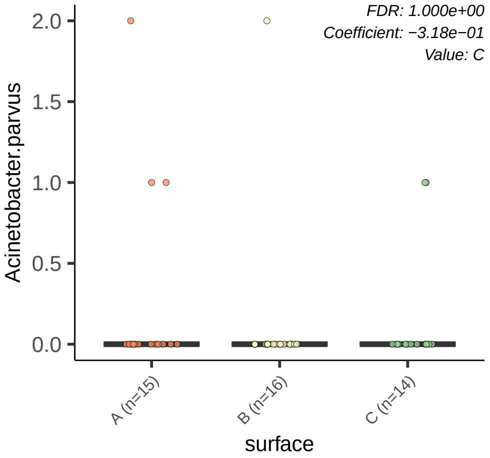

FDR: 1.000e+00
Coefficient: −3.18e−01
Value: C
Acinetobacter.parvus
2.0
1.5
1.0
0.5
0.0
A (n=15)
B (n=16)
C (n=14)
surface

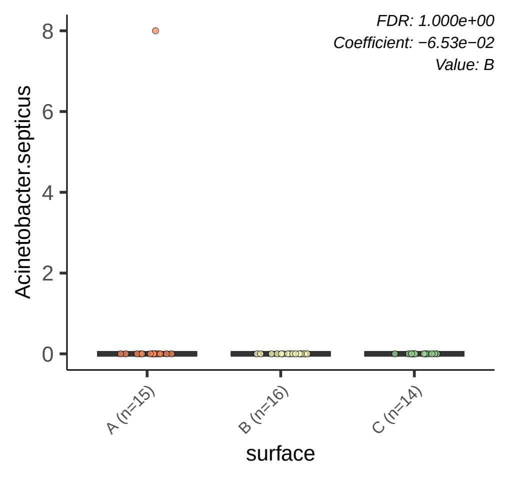
FDR: 1.000e+00
Coefficient: −6.53e−02
Value: B
Acinetobacter.septicus
8
6
4
2
0
A (n=15)
B (n=16)
C (n=14)
surface

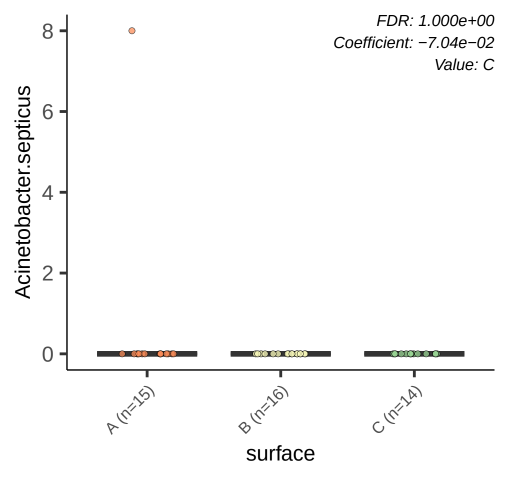
FDR: 1.000e+00
Coefficient: −7.04e−02
Value: C
Acinetobacter.septicus
8
6
4
2
0
A (n=15)
B (n=16)
C (n=14)
surface

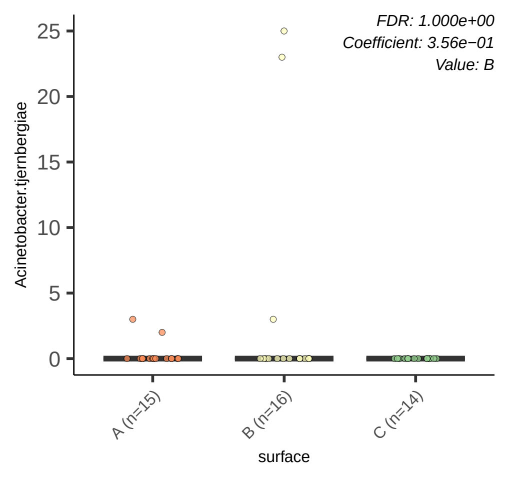
FDR: 1.000e+00
Coefficient: 3.56e−01
Value: B
Acinetobacter.tjernbergiae
0
5
10
15
20
25
A (n=15)
B (n=16)
C (n=14)
surface

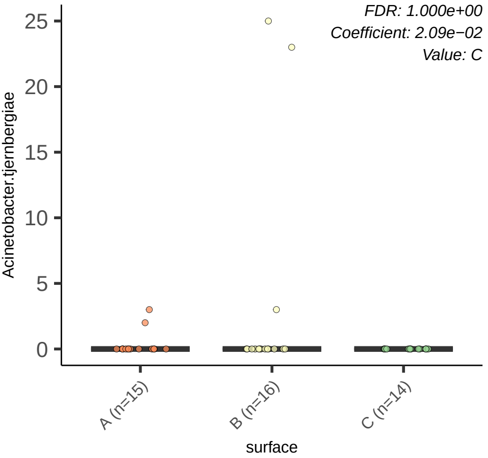
FDR: 1.000e+00
Coefficient: 2.09e−02
Value: C
Acinetobacter.tjernbergiae
25
20
15
10
5
0
A (n=15)
B (n=16)
C (n=14)
surface

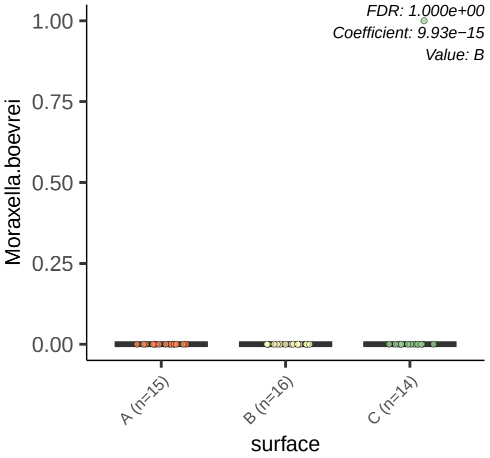

FDR: 1.000e+00
Coefficient: 9.93e−15
Value: B
1.00
0.75
0.50
0.25
0.00
Moraxella.boevrei
A (n=15)
B (n=16)
C (n=14)
surface

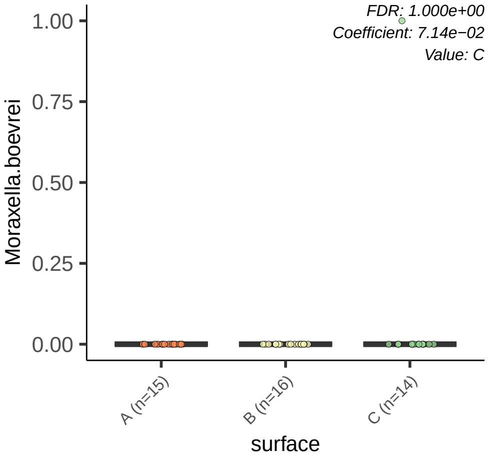

FDR: 1.000e+00
Coefficient: 7.14e−02
Value: C
Moraxella.boevrei
1.00
0.75
0.50
0.25
0.00
A (n=15)
B (n=16)
C (n=14)
surface

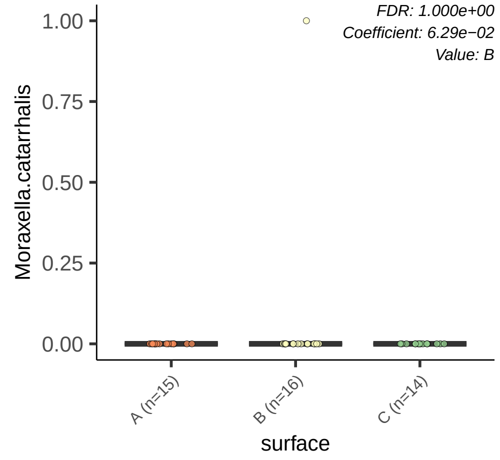

FDR: 1.000e+00
Coefficient: 6.29e−02
Value: B
Moraxella.catarrhalis
1.00
0.75
0.50
0.25
0.00
A (n=15)
B (n=16)
C (n=14)
surface

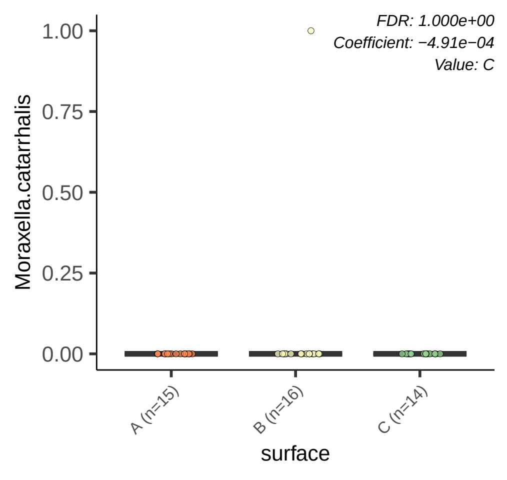
FDR: 1.000e+00
Coefficient: −4.91e−04
Value: C
Moraxella.catarrhalis
1.00
0.75
0.50
0.25
0.00
A (n=15)
B (n=16)
C (n=14)
surface

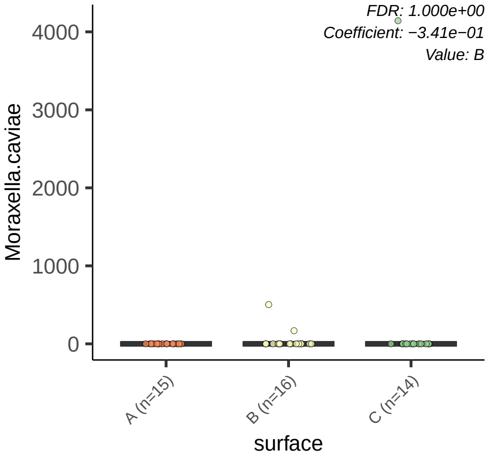
FDR: 1.000e+00
Coefficient: −3.41e−01
Value: B
4000
3000
2000
1000
0
Moraxella.caviae
A (n=15)
B (n=16)
C (n=14)
surface

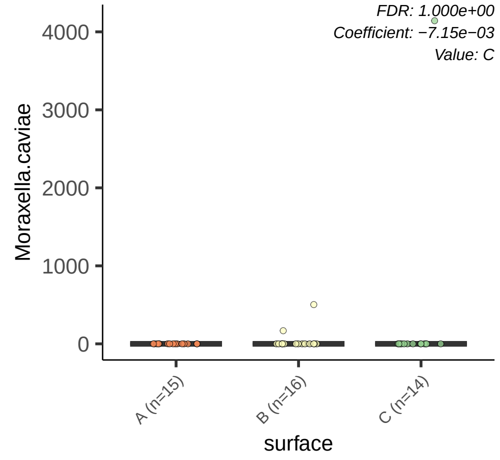
FDR: 1.000e+00
Coefficient: −7.15e−03
Value: C
Moraxella.caviae
4000
3000
2000
1000
0
A (n=15)
B (n=16)
C (n=14)
surface

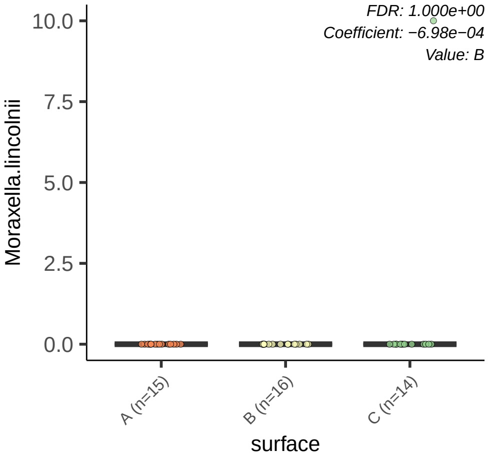
FDR: 1.000e+00
Coefficient: −6.98e−04
Value: B
Moraxella.lincolnii
10.0
7.5
5.0
2.5
0.0
A (n=15)
B (n=16)
C (n=14)
surface

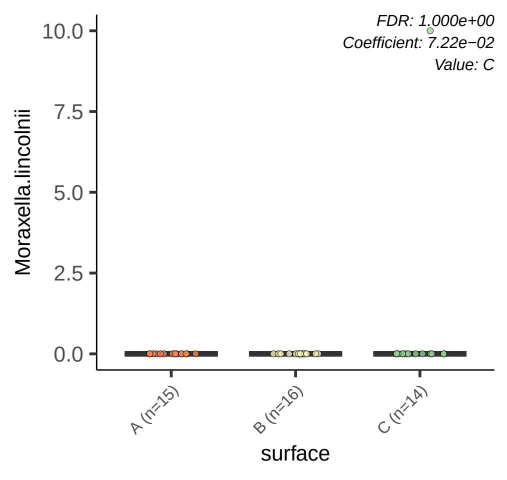
FDR: 1.000e+00
Coefficient: 7.22e−02
Value: C
Moraxella.lincolnii
10.0
7.5
5.0
2.5
0.0
A (n=15)
B (n=16)
C (n=14)
surface

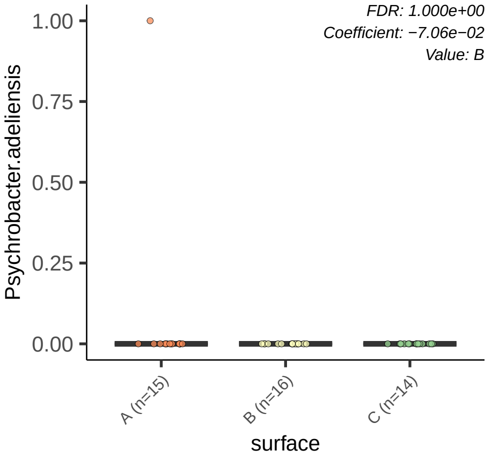
FDR: 1.000e+00
Coefficient: −7.06e−02
Value: B
Psychrobacter.adeliensis
1.00
0.75
0.50
0.25
0.00
A (n=15)
B (n=16)
C (n=14)
surface

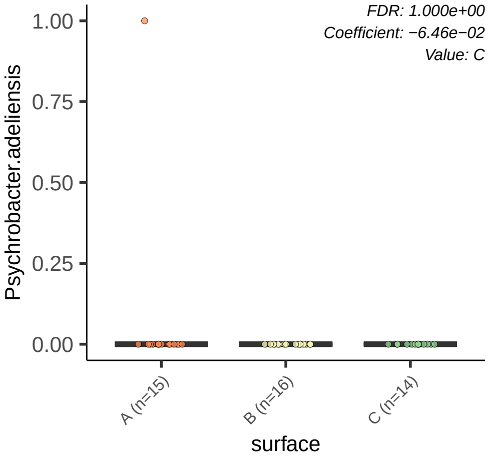
FDR: 1.000e+00
Coefficient: −6.46e−02
Value: C
Psychrobacter.adeliensis
1.00
0.75
0.50
0.25
0.00
A (n=15)
B (n=16)
C (n=14)
surface

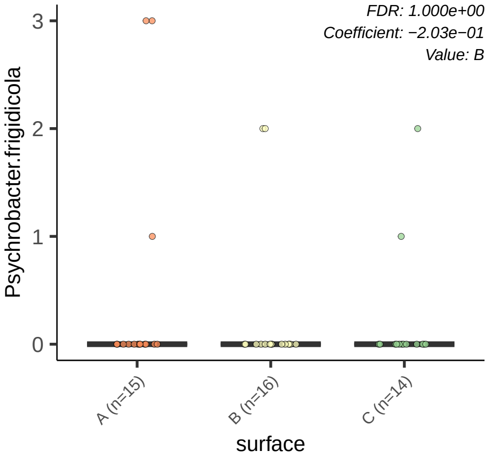
FDR: 1.000e+00
Coefficient: −2.03e−01
Value: B
Psychrobacter.frigidicola
3
2
1
0
A (n=15)
B (n=16)
C (n=14)
surface

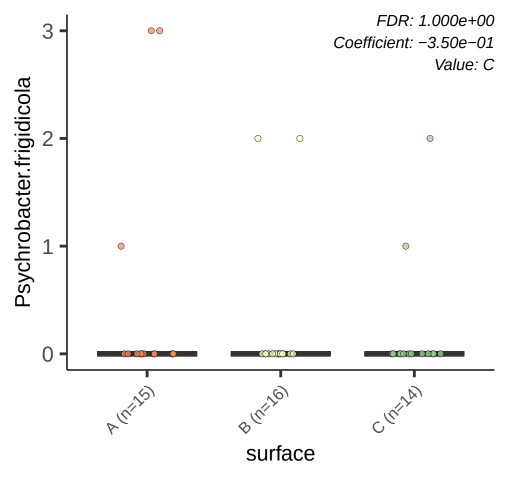

FDR: 1.000e+00
Coefficient: −3.50e−01
Value: C
Psychrobacter.frigidicola
3
2
1
0
A (n=15)
B (n=16)
C (n=14)
surface

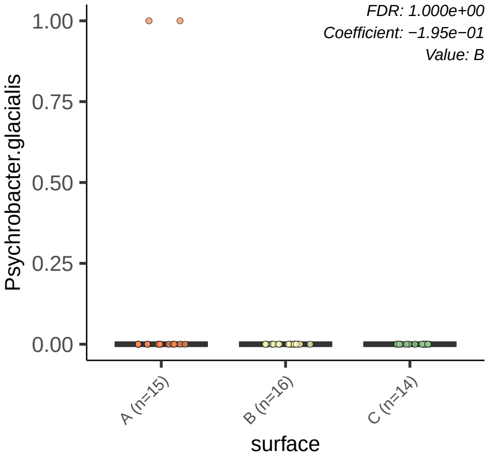

FDR: 1.000e+00
Coefficient: −1.95e−01
Value: B
Psychrobacter.glacialis
1.00
0.75
0.50
0.25
0.00
A (n=15)
B (n=16)
C (n=14)
surface

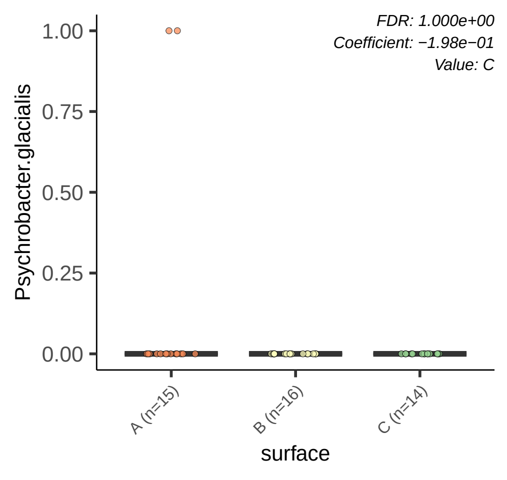
FDR: 1.000e+00
Coefficient: −1.98e−01
Value: C
Psychrobacter.glacialis
1.00
0.75
0.50
0.25
0.00
A (n=15)
B (n=16)
C (n=14)
surface

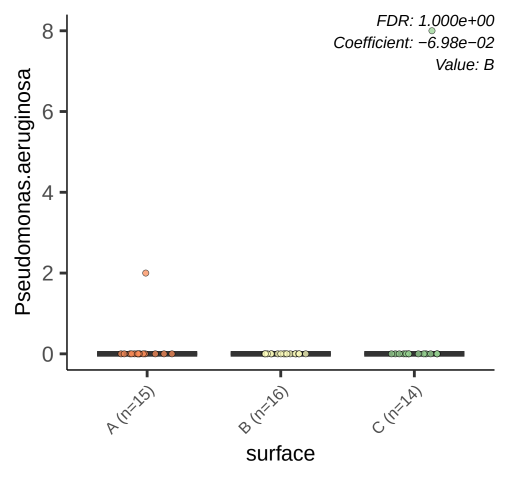
FDR: 1.000e+00
Coefficient: −6.98e−02
Value: B
Pseudomonas.aeruginosa
0
2
4
6
8
A (n=15)
B (n=16)
C (n=14)
surface

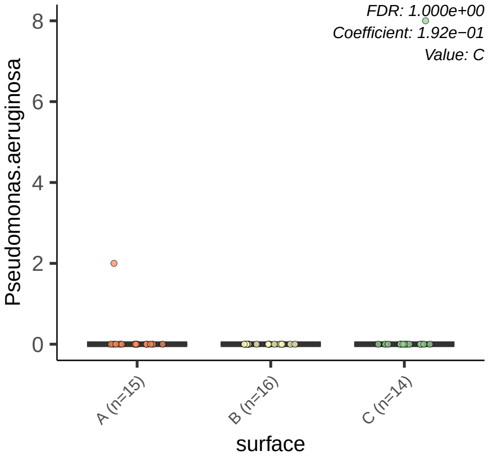
FDR: 1.000e+00
Coefficient: 1.92e−01
Value: C
Pseudomonas.aeruginosa
0
2
4
6
8
A (n=15)
B (n=16)
C (n=14)
surface

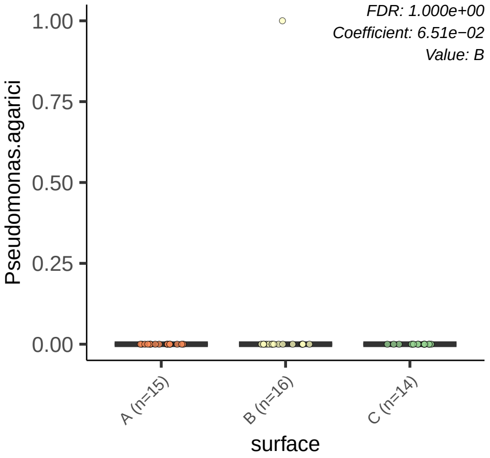

FDR: 1.000e+00
Coefficient: 6.51e−02
Value: B
Pseudomonas.agarici
1.00
0.75
0.50
0.25
0.00
A (n=15)
B (n=16)
C (n=14)
surface

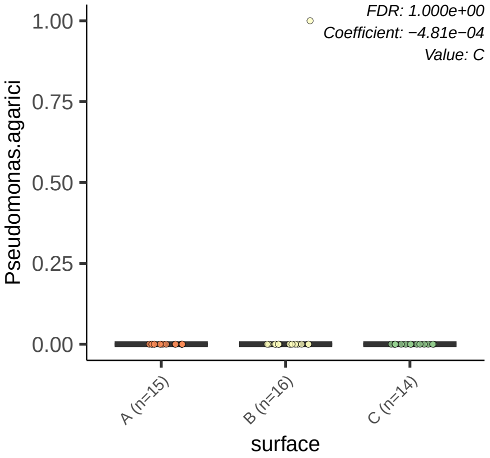

FDR: 1.000e+00
Coefficient: −4.81e−04
Value: C
Pseudomonas.agarici
1.00
0.75
0.50
0.25
0.00
A (n=15)
B (n=16)
C (n=14)
surface

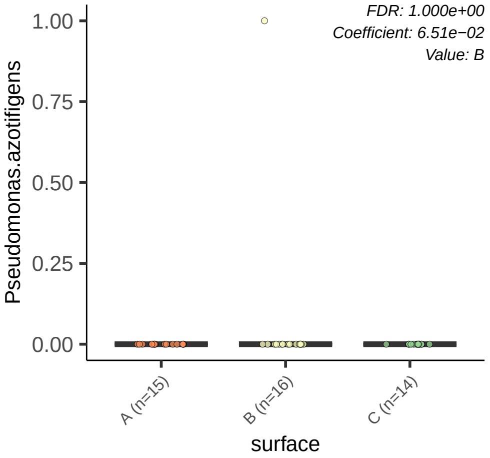

FDR: 1.000e+00
Coefficient: 6.51e−02
Value: B
Pseudomonas.azotifigens
1.00
0.75
0.50
0.25
0.00
A (n=15)
B (n=16)
C (n=14)
surface

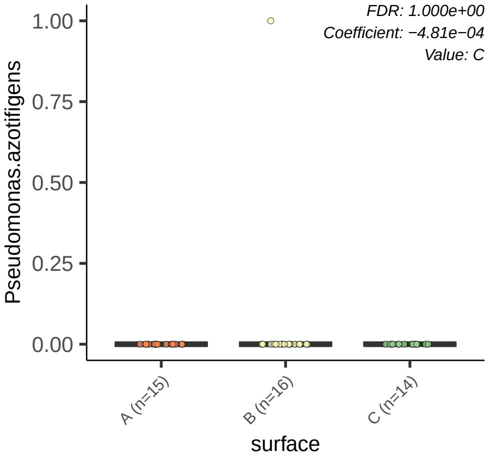
FDR: 1.000e+00
Coefficient: −4.81e−04
Value: C
Pseudomonas.azotifigens
1.00
0.75
0.50
0.25
0.00
A (n=15)
B (n=16)
C (n=14)
surface

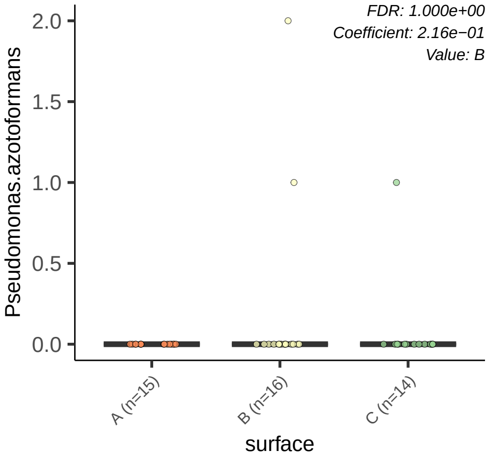
FDR: 1.000e+00
Coefficient: 2.16e−01
Value: B
Pseudomonas.azotoformans
2.0
1.5
1.0
0.5
0.0
A (n=15)
B (n=16)
C (n=14)
surface

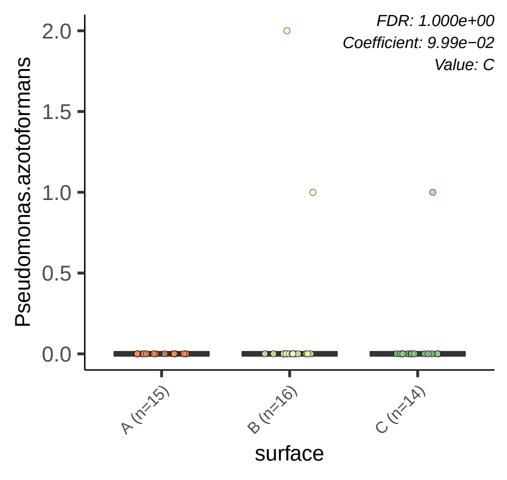
FDR: 1.000e+00
Coefficient: 9.99e−02
Value: C
Pseudomonas.azotoformans
2.0
1.5
1.0
0.5
0.0
A (n=15)
B (n=16)
C (n=14)
surface

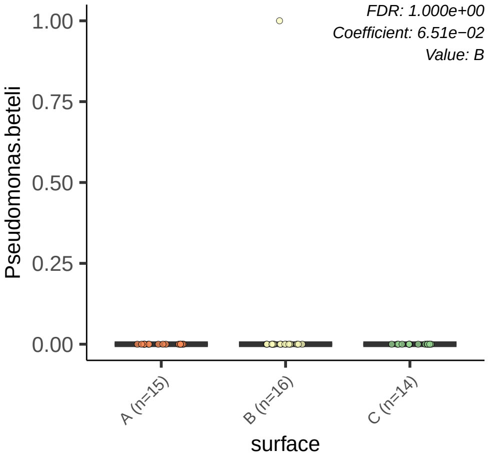

FDR: 1.000e+00
Coefficient: 6.51e−02
Value: B
Pseudomonas.beteli
1.00
0.75
0.50
0.25
0.00
A (n=15)
B (n=16)
C (n=14)
surface

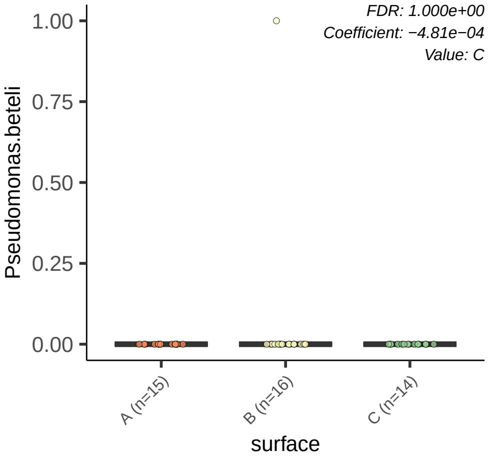
FDR: 1.000e+00
Coefficient: −4.81e−04
Value: C
Pseudomonas.beteli
1.00
0.75
0.50
0.25
0.00
A (n=15)
B (n=16)
C (n=14)
surface

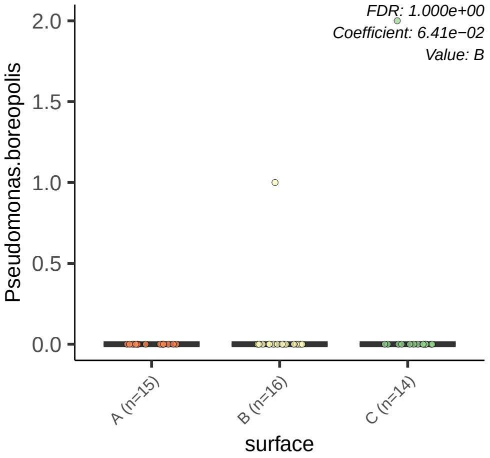
FDR: 1.000e+00
Coefficient: 6.41e−02
Value: B
Pseudomonas.boreopolis
2.0
1.5
1.0
0.5
0.0
A (n=15)
B (n=16)
C (n=14)
surface

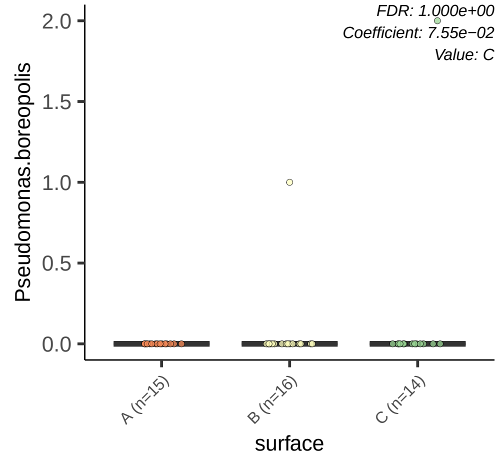

FDR: 1.000e+00
Coefficient: 7.55e−02
Value: C
Pseudomonas.boreopolis
2.0
1.5
1.0
0.5
0.0
A (n=15)
B (n=16)
C (n=14)
surface

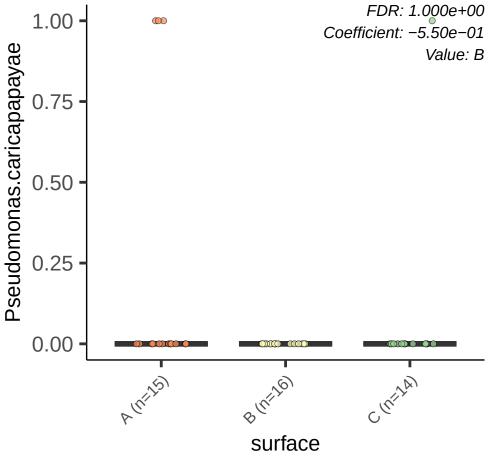
FDR: 1.000e+00
Coefficient: −5.50e−01
Value: B
Pseudomonas.caricapapayae
1.00
0.75
0.50
0.25
0.00
A (n=15)
B (n=16)
C (n=14)
surface

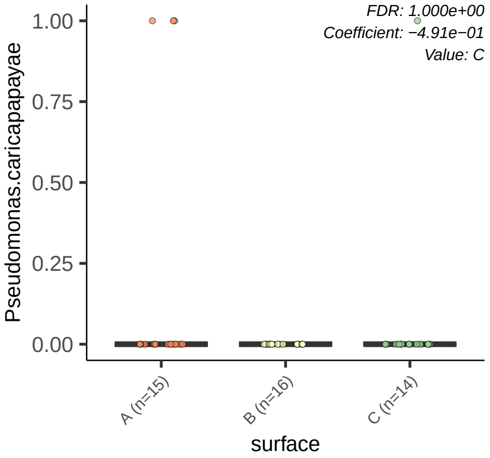
FDR: 1.000e+00
Coefficient: −4.91e−01
Value: C
Pseudomonas.caricapapayae
1.00
0.75
0.50
0.25
0.00
A (n=15)
B (n=16)
C (n=14)
surface

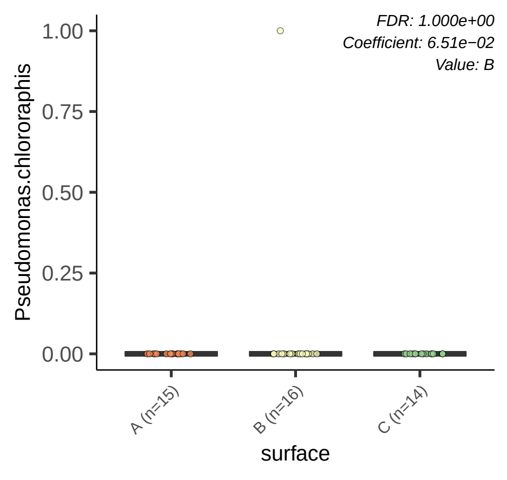

FDR: 1.000e+00
Coefficient: 6.51e−02
Value: B
Pseudomonas.chlororaphis
1.00
0.75
0.50
0.25
0.00
A (n=15)
B (n=16)
C (n=14)
surface

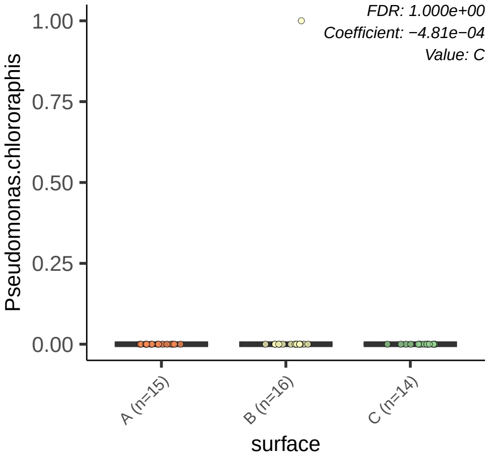

FDR: 1.000e+00
Coefficient: −4.81e−04
Value: C
Pseudomonas.chlororaphis
1.00
0.75
0.50
0.25
0.00
A (n=15)
B (n=16)
C (n=14)
surface

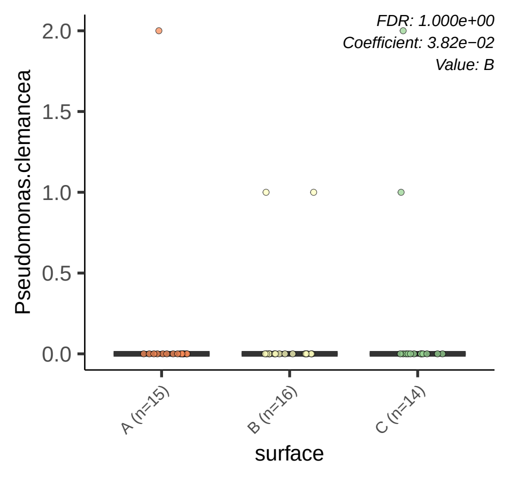
FDR: 1.000e+00
Coefficient: 3.82e−02
Value: B
Pseudomonas.clemancea
2.0
1.5
1.0
0.5
0.0
A (n=15)
B (n=16)
C (n=14)
surface

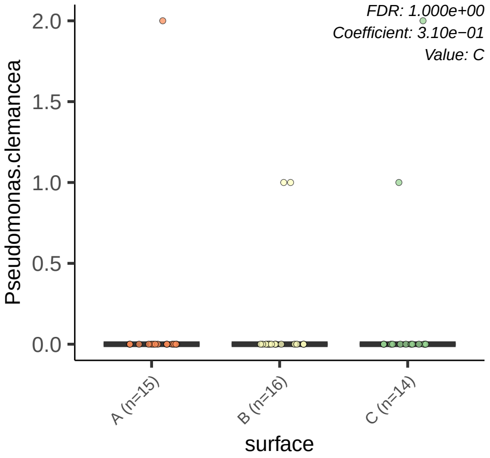
FDR: 1.000e+00
Coefficient: 3.10e−01
Value: C
Pseudomonas.clemancea
2.0
1.5
1.0
0.5
0.0
A (n=15)
B (n=16)
C (n=14)
surface

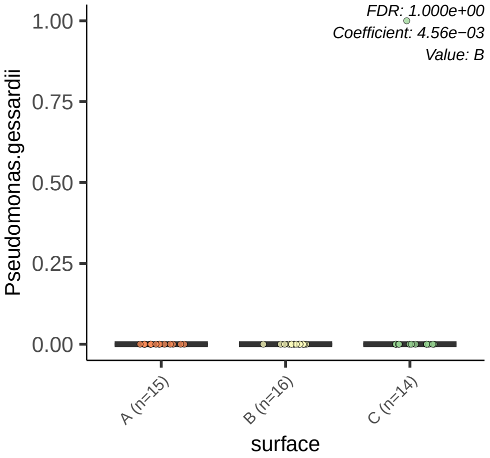
FDR: 1.000e+00
Coefficient: 4.56e−03
Value: B
Pseudomonas.gessardii
1.00
0.75
0.50
0.25
0.00
A (n=15)
B (n=16)
C (n=14)
surface

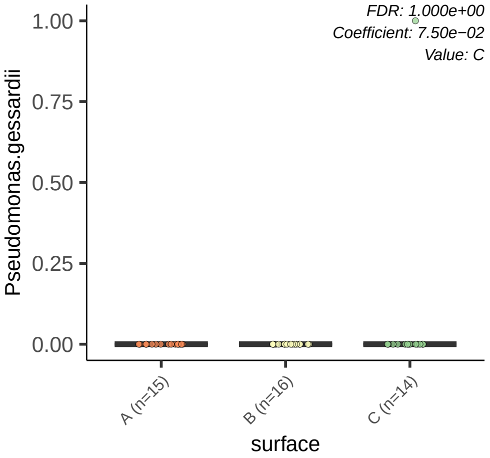

FDR: 1.000e+00
Coefficient: 7.50e−02
Value: C
1.00
0.75
0.50
0.25
0.00
Pseudomonas.gessardii
A (n=15)
B (n=16)
C (n=14)
surface

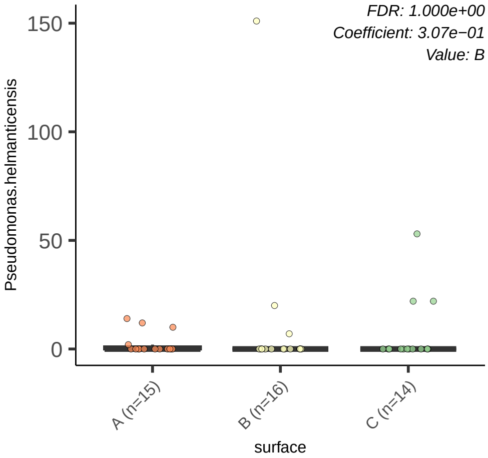
FDR: 1.000e+00
Coefficient: 3.07e−01
Value: B
150
100
50
0
Pseudomonas.helmanticensis
A (n=15)
B (n=16)
C (n=14)
surface

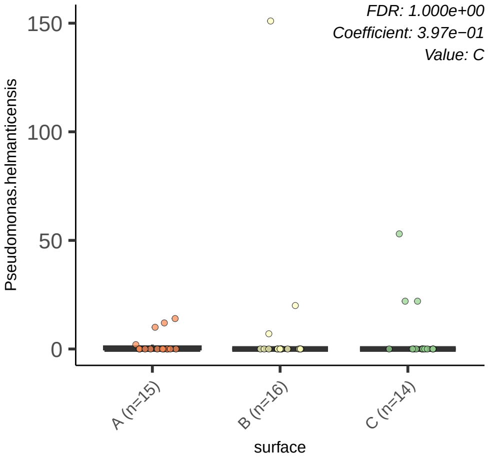

FDR: 1.000e+00
Coefficient: 3.97e−01
Value: C
Pseudomonas.helmanticensis
150
100
50
0
A (n=15)
B (n=16)
C (n=14)
surface

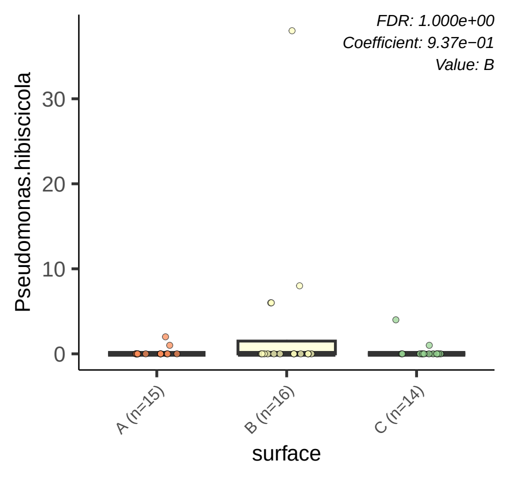

FDR: 1.000e+00
Coefficient: 9.37e−01
Value: B
Pseudomonas.hibiscicola
30
20
10
0
A (n=15)
B (n=16)
C (n=14)
surface

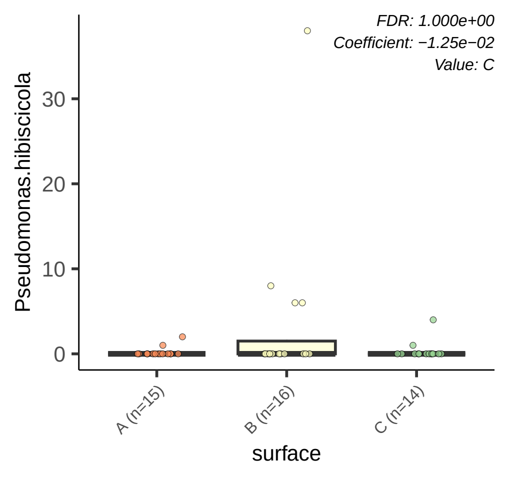
FDR: 1.000e+00
Coefficient: −1.25e−02
Value: C
Pseudomonas.hibiscicola
30
20
10
0
A (n=15)
B (n=16)
C (n=14)
surface

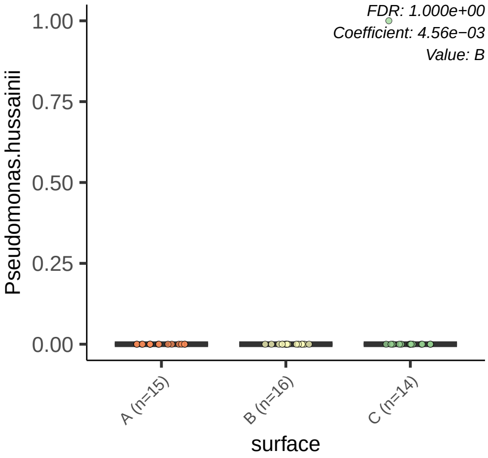

FDR: 1.000e+00
Coefficient: 4.56e−03
Value: B
Pseudomonas.hussainii
1.00
0.75
0.50
0.25
0.00
A (n=15)
B (n=16)
C (n=14)
surface

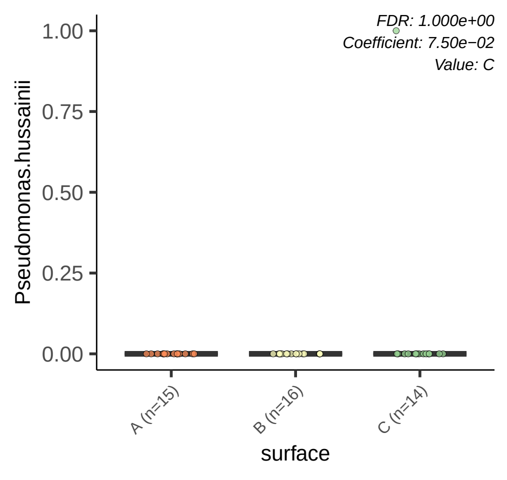

FDR: 1.000e+00
Coefficient: 7.50e−02
Value: C
Pseudomonas.hussainii
1.00
0.75
0.50
0.25
0.00
A (n=15)
B (n=16)
C (n=14)
surface

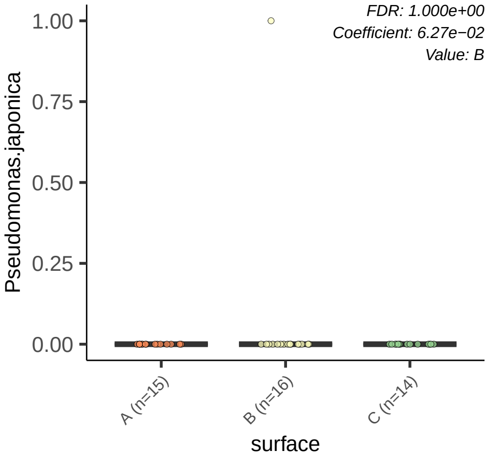
FDR: 1.000e+00
Coefficient: 6.27e−02
Value: B
Pseudomonas.japonica
1.00
0.75
0.50
0.25
0.00
A (n=15)
B (n=16)
C (n=14)
surface

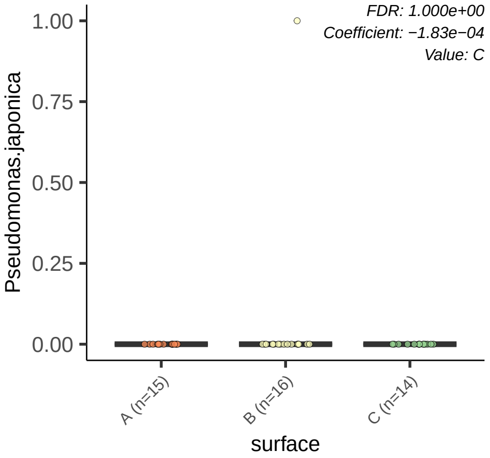

FDR: 1.000e+00
Coefficient: −1.83e−04
Value: C
Pseudomonas.japonica
1.00
0.75
0.50
0.25
0.00
A (n=15)
B (n=16)
C (n=14)
surface

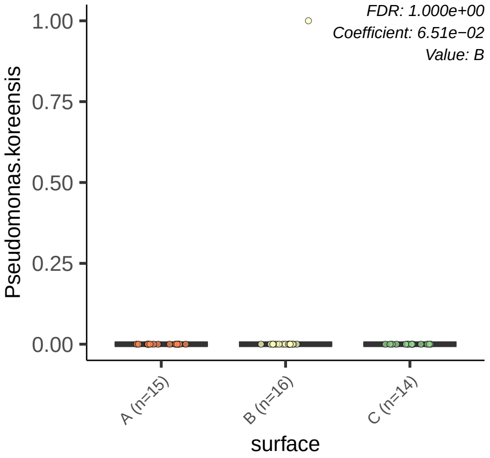

FDR: 1.000e+00
Coefficient: 6.51e−02
Value: B
Pseudomonas.koreensis
1.00
0.75
0.50
0.25
0.00
A (n=15)
B (n=16)
C (n=14)
surface

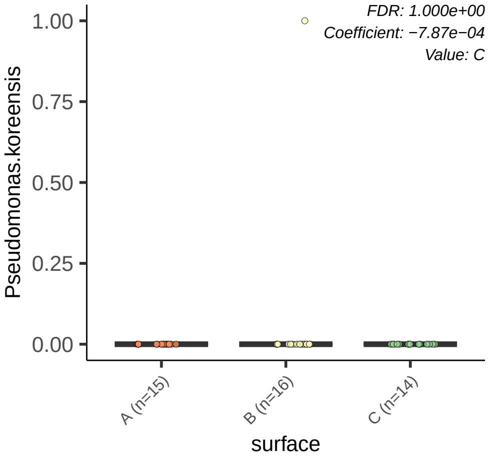
FDR: 1.000e+00
Coefficient: −7.87e−04
Value: C
Pseudomonas.koreensis
1.00
0.75
0.50
0.25
0.00
A (n=15)
B (n=16)
C (n=14)
surface

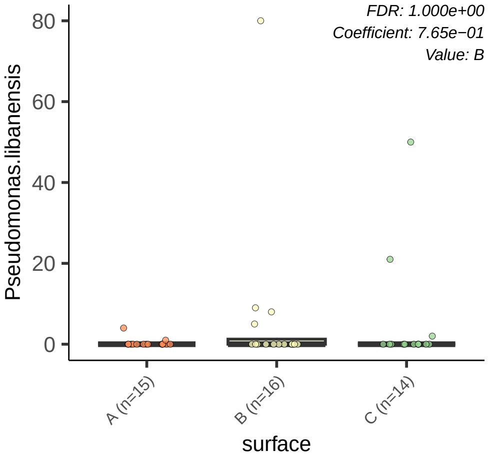
FDR: 1.000e+00
Coefficient: 7.65e−01
Value: B
Pseudomonas.libanensis
80
60
40
20
0
A (n=15)
B (n=16)
C (n=14)
surface

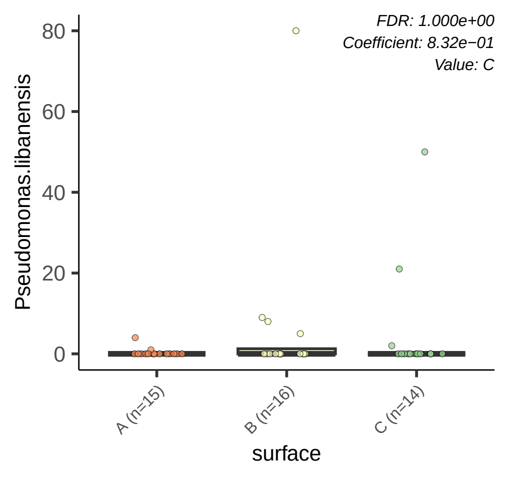
FDR: 1.000e+00
Coefficient: 8.32e−01
Value: C
Pseudomonas.libanensis
0
20
40
60
80
A (n=15)
B (n=16)
C (n=14)
surface

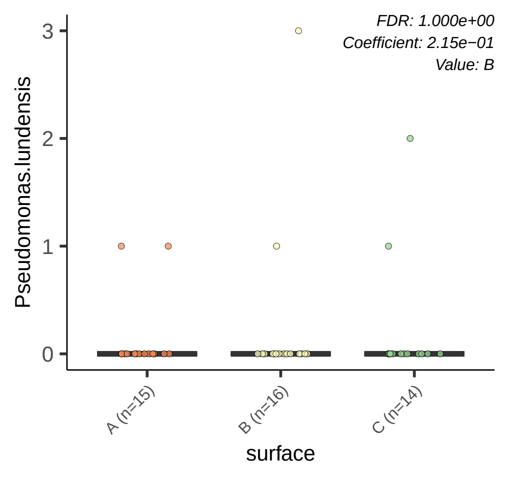

FDR: 1.000e+00
Coefficient: 2.15e−01
Value: B
Pseudomonas.lundensis
3
2
1
0
A (n=15)
B (n=16)
C (n=14)
surface

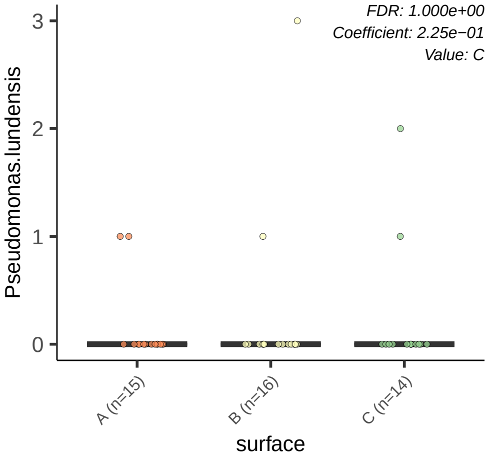
FDR: 1.000e+00
Coefficient: 2.25e−01
Value: C
Pseudomonas.lundensis
0
1
2
3
A (n=15)
B (n=16)
C (n=14)
surface

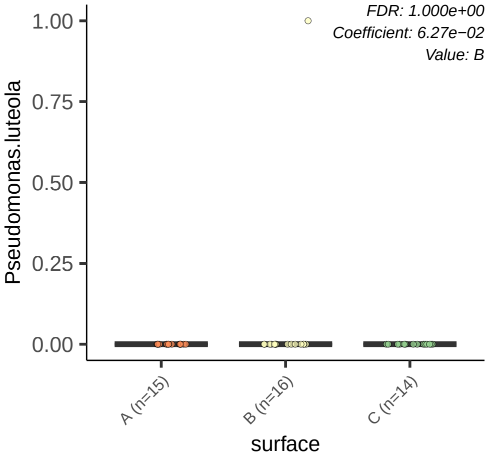

FDR: 1.000e+00
Coefficient: 6.27e−02
Value: B
Pseudomonas.luteola
1.00
0.75
0.50
0.25
0.00
A (n=15)
B (n=16)
C (n=14)
surface

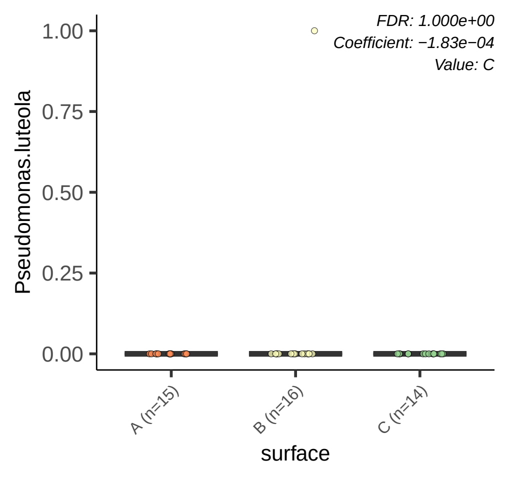

FDR: 1.000e+00
Coefficient: −1.83e−04
Value: C
Pseudomonas.luteola
1.00
0.75
0.50
0.25
0.00
A (n=15)
B (n=16)
C (n=14)
surface

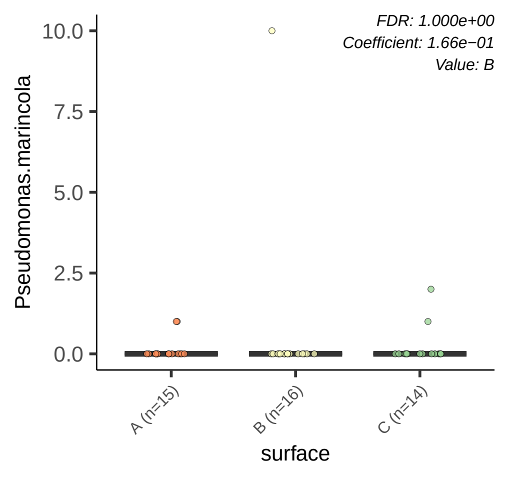
FDR: 1.000e+00
Coefficient: 1.66e−01
Value: B
10.0
7.5
5.0
2.5
0.0
Pseudomonas.marincola
A (n=15)
B (n=16)
C (n=14)
surface

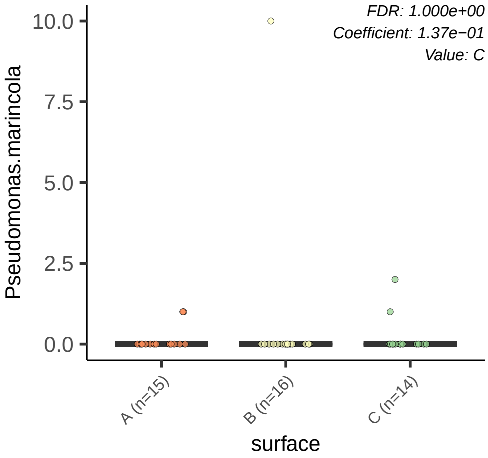
FDR: 1.000e+00
Coefficient: 1.37e−01
Value: C
Pseudomonas.marincola
10.0
7.5
5.0
2.5
0.0
A (n=15)
B (n=16)
C (n=14)
surface

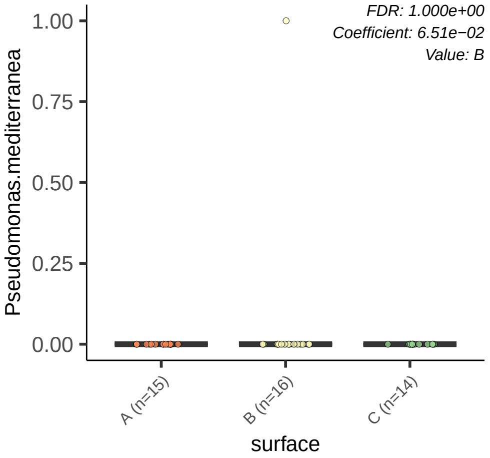

FDR: 1.000e+00
Coefficient: 6.51e−02
Value: B
Pseudomonas.mediterranea
1.00
0.75
0.50
0.25
0.00
A (n=15)
B (n=16)
C (n=14)
surface

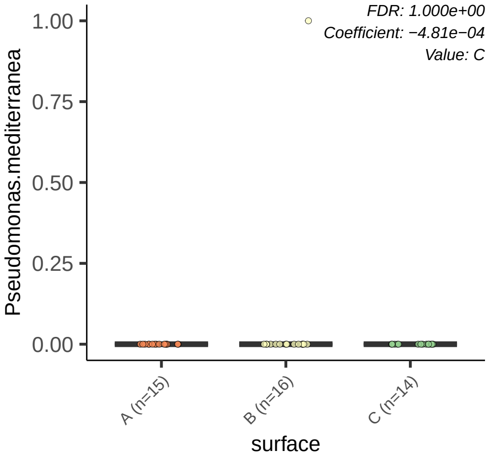

FDR: 1.000e+00
Coefficient: −4.81e−04
Value: C
Pseudomonas.mediterranea
1.00
0.75
0.50
0.25
0.00
A (n=15)
B (n=16)
C (n=14)
surface

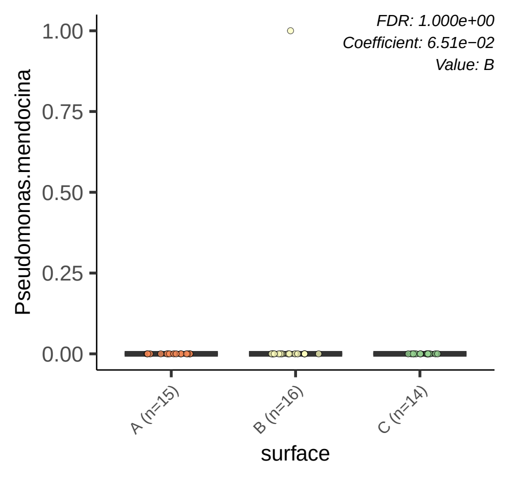
FDR: 1.000e+00
Coefficient: 6.51e−02
Value: B
Pseudomonas.mendocina
1.00
0.75
0.50
0.25
0.00
A (n=15)
B (n=16)
C (n=14)
surface

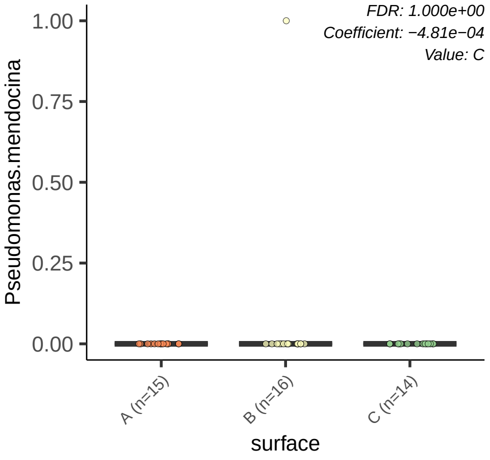

FDR: 1.000e+00
Coefficient: −4.81e−04
Value: C
Pseudomonas.mendocina
1.00
0.75
0.50
0.25
0.00
A (n=15)
B (n=16)
C (n=14)
surface

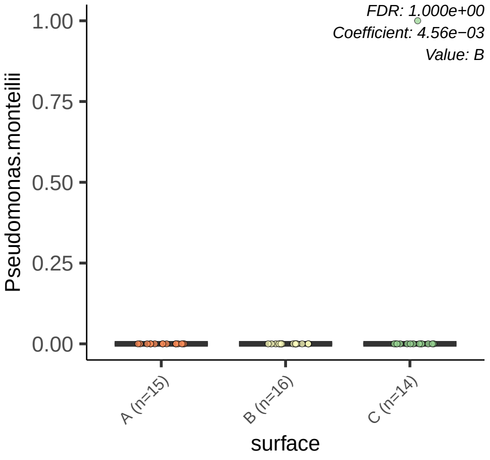
FDR: 1.000e+00
Coefficient: 4.56e−03
Value: B
Pseudomonas.monteilii
1.00
0.75
0.50
0.25
0.00
A (n=15)
B (n=16)
C (n=14)
surface

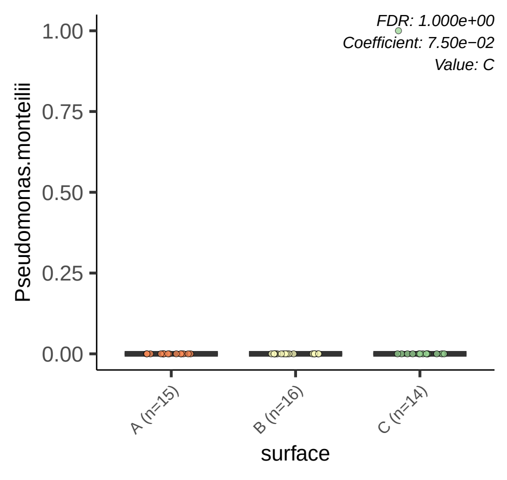

FDR: 1.000e+00
Coefficient: 7.50e−02
Value: C
Pseudomonas.monteilii
1.00
0.75
0.50
0.25
0.00
A (n=15)
B (n=16)
C (n=14)
surface

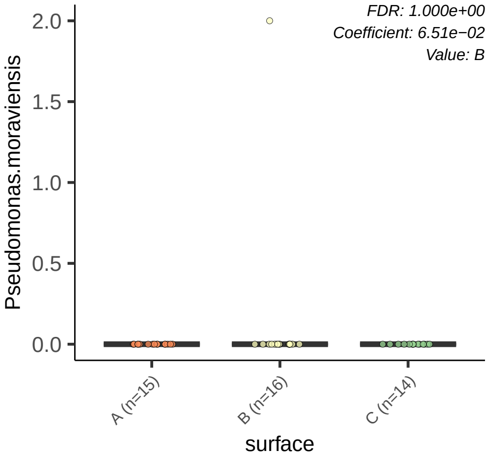
FDR: 1.000e+00
Coefficient: 6.51e−02
Value: B
Pseudomonas.moraviensis
2.0
1.5
1.0
0.5
0.0
A (n=15)
B (n=16)
C (n=14)
surface

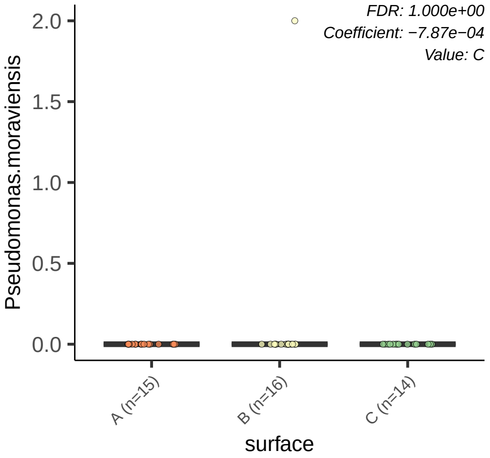
FDR: 1.000e+00
Coefficient: −7.87e−04
Value: C
Pseudomonas.moraviensis
2.0
1.5
1.0
0.5
0.0
A (n=15)
B (n=16)
C (n=14)
surface

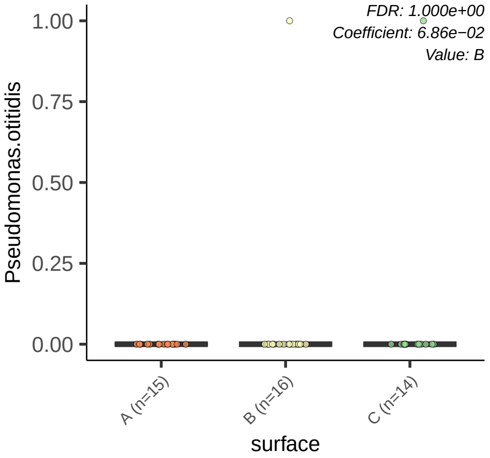
FDR: 1.000e+00
Coefficient: 6.86e−02
Value: B
Pseudomonas.otitidis
1.00
0.75
0.50
0.25
0.00
A (n=15)
B (n=16)
C (n=14)
surface

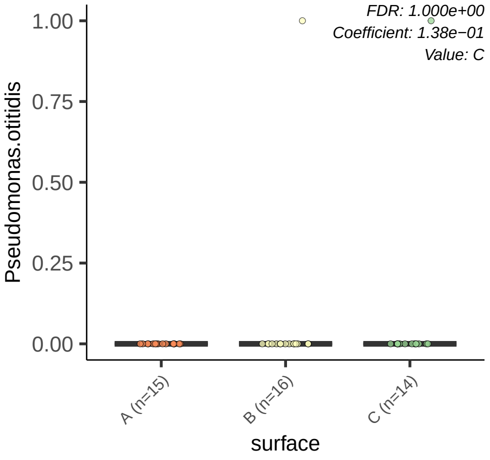
FDR: 1.000e+00
Coefficient: 1.38e−01
Value: C
Pseudomonas.otitidis
1.00
0.75
0.50
0.25
0.00
A (n=15)
B (n=16)
C (n=14)
surface

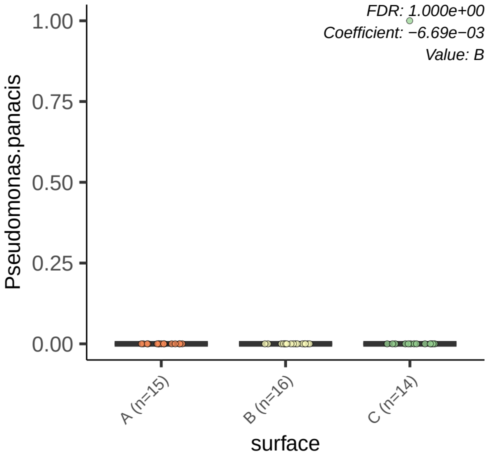

FDR: 1.000e+00
Coefficient: −6.69e−03
Value: B
Pseudomonas.panacis
1.00
0.75
0.50
0.25
0.00
A (n=15)
B (n=16)
C (n=14)
surface

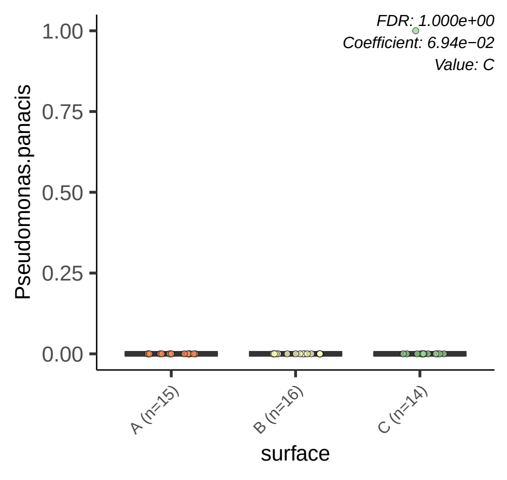
FDR: 1.000e+00
Coefficient: 6.94e−02
Value: C
Pseudomonas.panacis
1.00
0.75
0.50
0.25
0.00
A (n=15)
B (n=16)
C (n=14)
surface

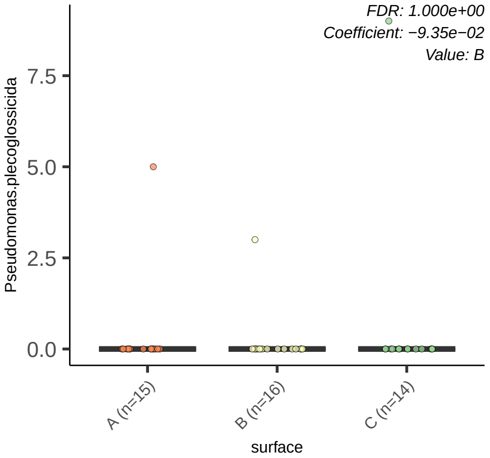
FDR: 1.000e+00
Coefficient: −9.35e−02
Value: B
Pseudomonas.plecoglossicida
7.5
5.0
2.5
0.0
A (n=15)
B (n=16)
C (n=14)
surface

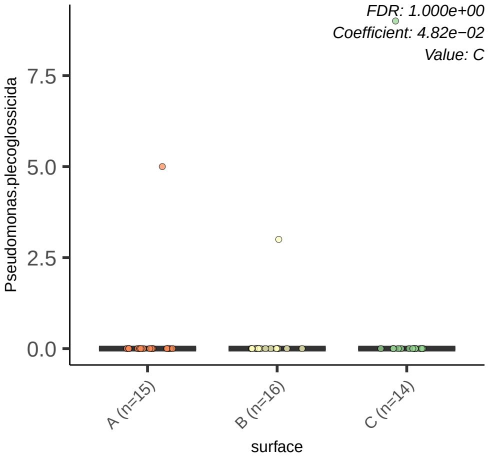

FDR: 1.000e+00
Coefficient: 4.82e−02
Value: C
Pseudomonas.plecoglossicida
7.5
5.0
2.5
0.0
A (n=15)
B (n=16)
C (n=14)
surface

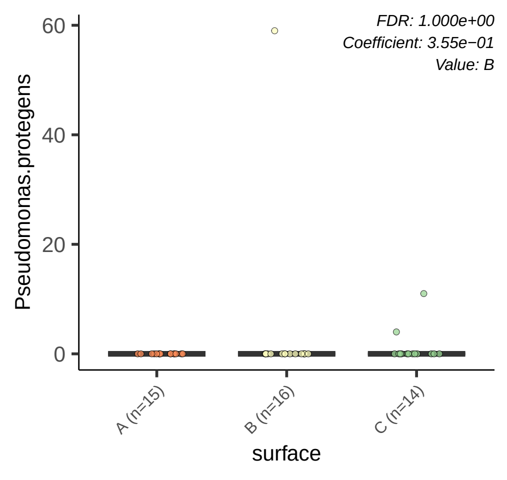

FDR: 1.000e+00
Coefficient: 3.55e−01
Value: B
Pseudomonas.protegens
60
40
20
0
A (n=15)
B (n=16)
C (n=14)
surface

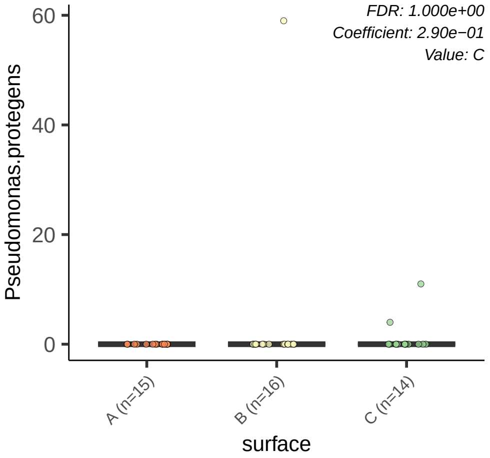
FDR: 1.000e+00
Coefficient: 2.90e−01
Value: C
Pseudomonas.protegens
60
40
20
0
A (n=15)
B (n=16)
C (n=14)
surface

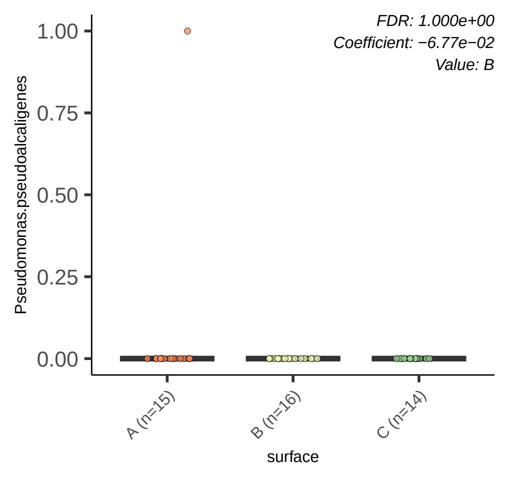

FDR: 1.000e+00
Coefficient: −6.77e−02
Value: B
1.00
0.75
0.50
0.25
0.00
Pseudomonas.pseudoalcaligenes
A (n=15)
B (n=16)
C (n=14)
surface

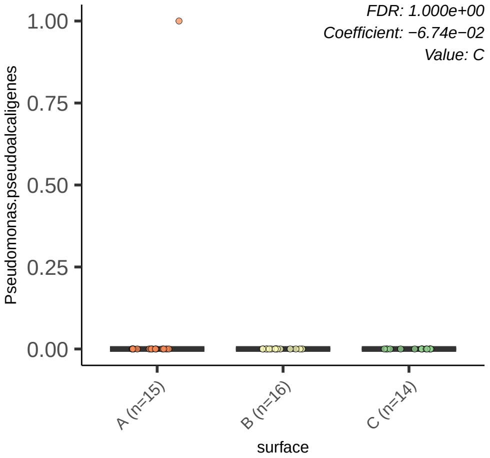
FDR: 1.000e+00
Coefficient: −6.74e−02
Value: C
Pseudomonas.pseudoalcaligenes
1.00
0.75
0.50
0.25
0.00
A (n=15)
B (n=16)
C (n=14)
surface

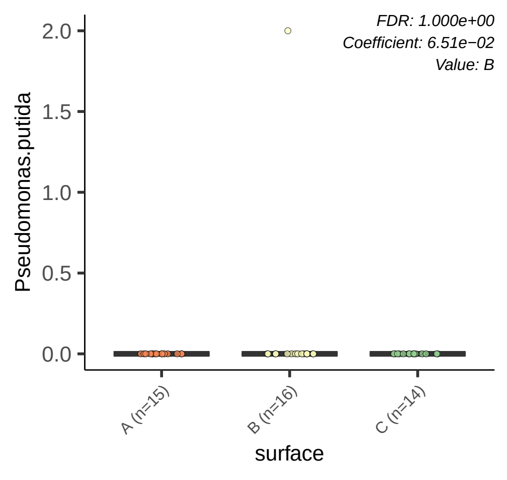
FDR: 1.000e+00
Coefficient: 6.51e−02
Value: B
Pseudomonas.putida
2.0
1.5
1.0
0.5
0.0
A (n=15)
B (n=16)
C (n=14)
surface

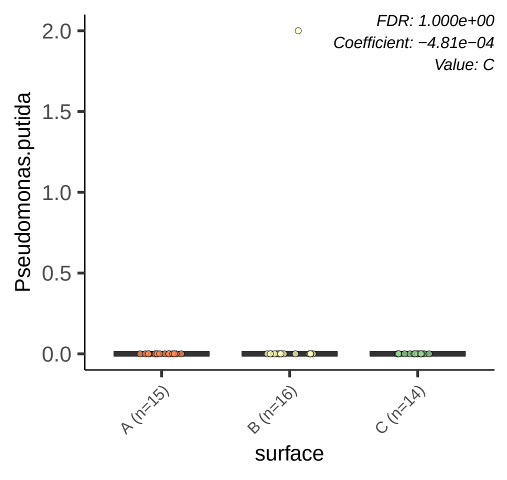

FDR: 1.000e+00
Coefficient: −4.81e−04
Value: C
Pseudomonas.putida
2.0
1.5
1.0
0.5
0.0
A (n=15)
B (n=16)
C (n=14)
surface

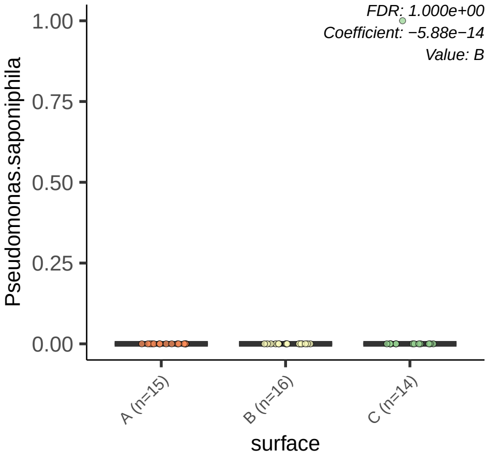
FDR: 1.000e+00
Coefficient: −5.88e−14
Value: B
Pseudomonas.saponiphila
1.00
0.75
0.50
0.25
0.00
A (n=15)
B (n=16)
C (n=14)
surface

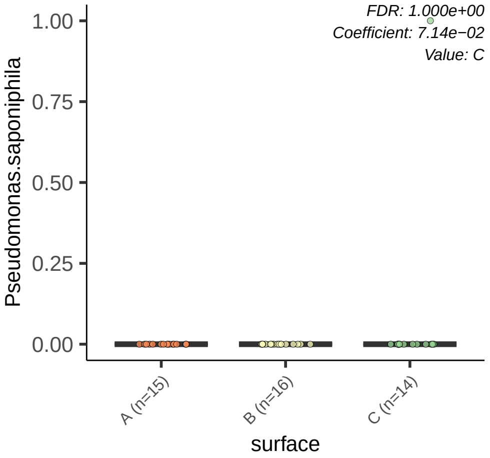

FDR: 1.000e+00
Coefficient: 7.14e−02
Value: C
Pseudomonas.saponiphila
1.00
0.75
0.50
0.25
0.00
A (n=15)
B (n=16)
C (n=14)
surface

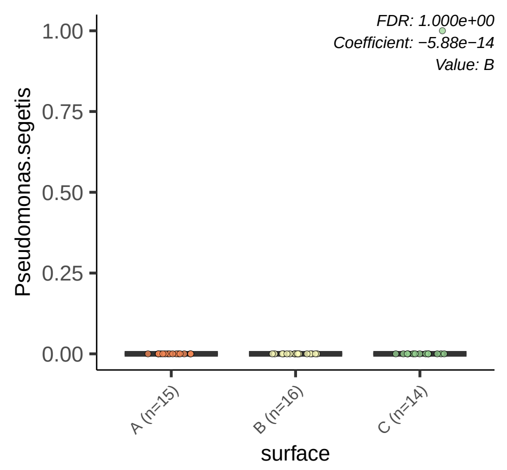

FDR: 1.000e+00
Coefficient: −5.88e−14
Value: B
Pseudomonas.segetis
1.00
0.75
0.50
0.25
0.00
A (n=15)
B (n=16)
C (n=14)
surface

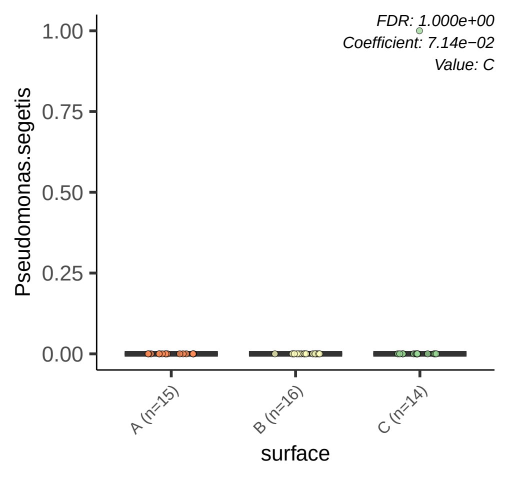
FDR: 1.000e+00
Coefficient: 7.14e−02
Value: C
Pseudomonas.segetis
1.00
0.75
0.50
0.25
0.00
A (n=15)
B (n=16)
C (n=14)
surface

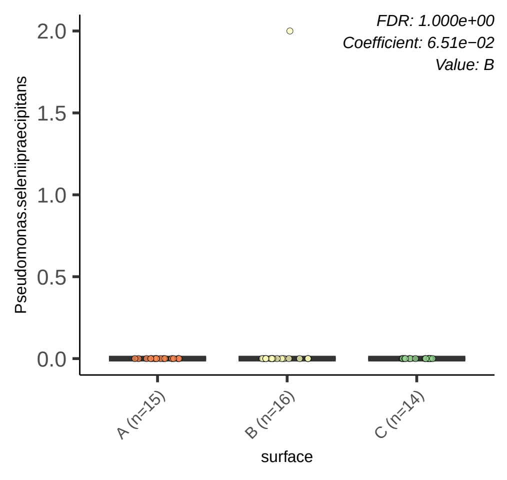
FDR: 1.000e+00
Coefficient: 6.51e−02
Value: B
Pseudomonas.seleniipraecipitans
2.0
1.5
1.0
0.5
0.0
A (n=15)
B (n=16)
C (n=14)
surface

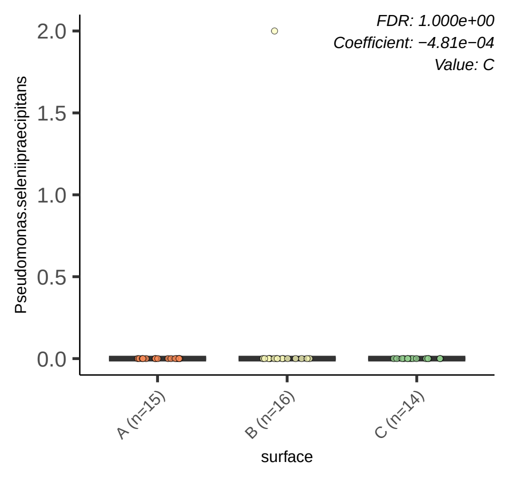

FDR: 1.000e+00
Coefficient: −4.81e−04
Value: C
Pseudomonas.seleniipraecipitans
2.0
1.5
1.0
0.5
0.0
A (n=15)
B (n=16)
C (n=14)
surface

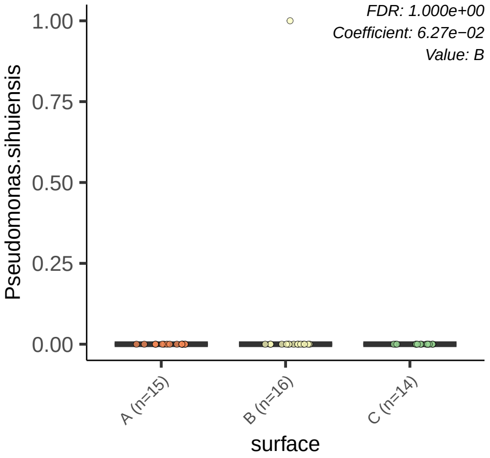
FDR: 1.000e+00
Coefficient: 6.27e−02
Value: B
Pseudomonas.sihuiensis
1.00
0.75
0.50
0.25
0.00
A (n=15)
B (n=16)
C (n=14)
surface

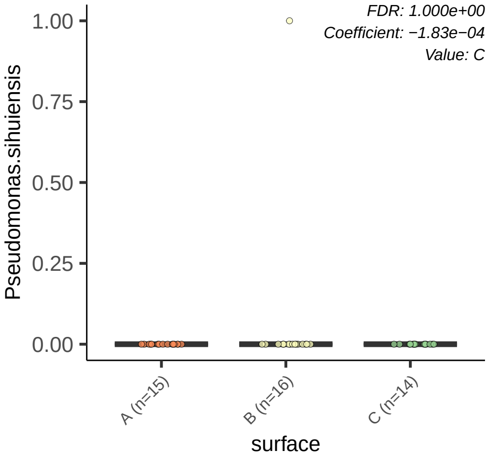
FDR: 1.000e+00
Coefficient: −1.83e−04
Value: C
Pseudomonas.sihuiensis
1.00
0.75
0.50
0.25
0.00
A (n=15)
B (n=16)
C (n=14)
surface

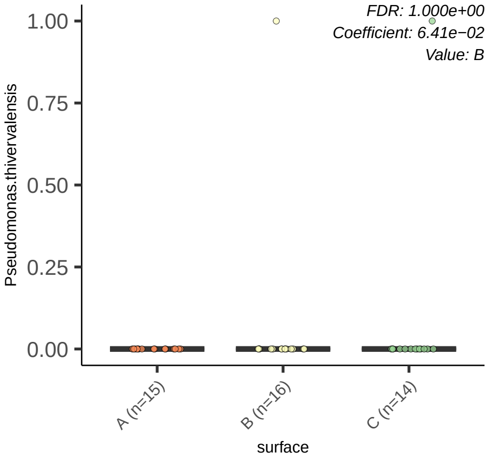
FDR: 1.000e+00
Coefficient: 6.41e−02
Value: B
Pseudomonas.thivervalensis
1.00
0.75
0.50
0.25
0.00
A (n=15)
B (n=16)
C (n=14)
surface

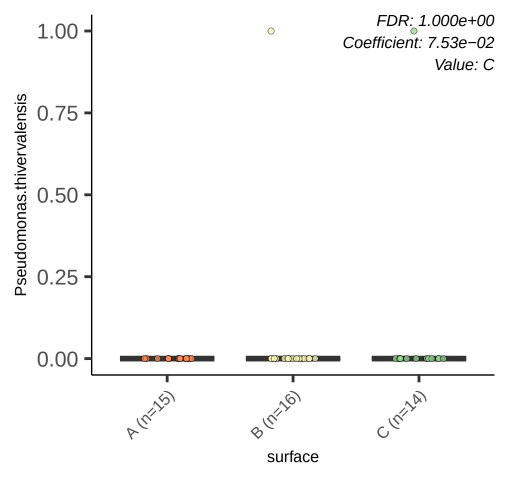

FDR: 1.000e+00
Coefficient: 7.53e−02
Value: C
Pseudomonas.thivervalensis
1.00
0.75
0.50
0.25
0.00
A (n=15)
B (n=16)
C (n=14)
surface

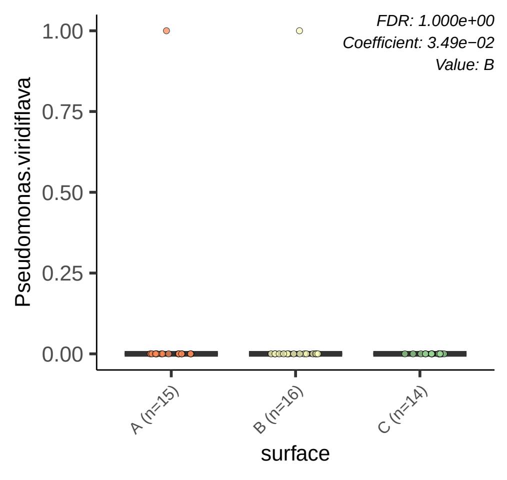

FDR: 1.000e+00
Coefficient: 3.49e−02
Value: B
Pseudomonas.viridiflava
1.00
0.75
0.50
0.25
0.00
A (n=15)
B (n=16)
C (n=14)
surface

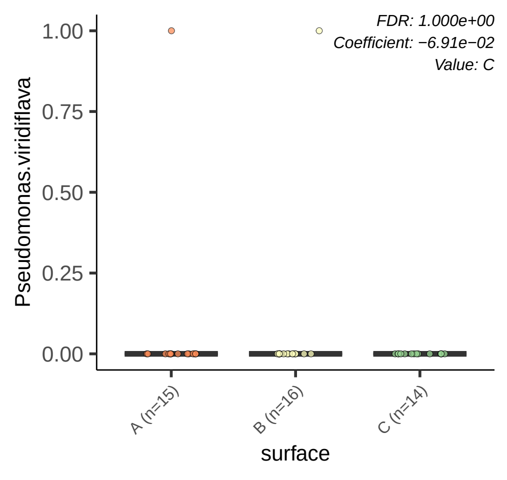

FDR: 1.000e+00
Coefficient: −6.91e−02
Value: C
Pseudomonas.viridiflava
1.00
0.75
0.50
0.25
0.00
A (n=15)
B (n=16)
C (n=14)
surface

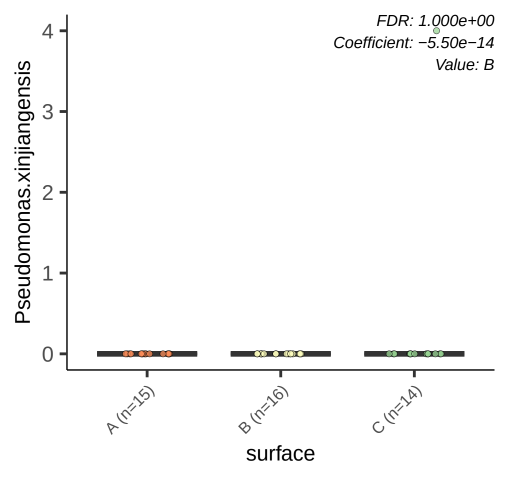
FDR: 1.000e+00
Coefficient: −5.50e−14
Value: B
Pseudomonas.xinjiangensis
0
1
2
3
4
A (n=15)
B (n=16)
C (n=14)
surface

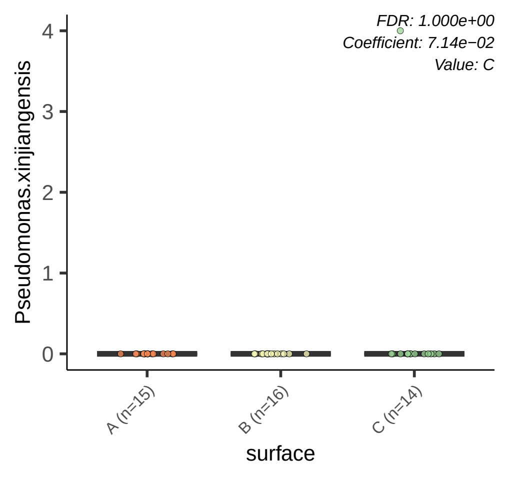

FDR: 1.000e+00
Coefficient: 7.14e−02
Value: C
Pseudomonas.xinjiangensis
0
1
2
3
4
A (n=15)
B (n=16)
C (n=14)
surface

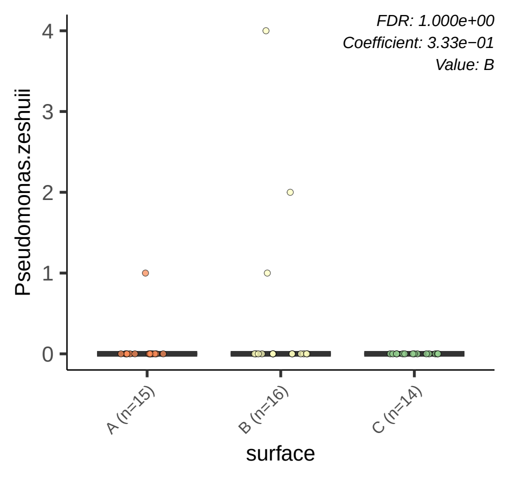
FDR: 1.000e+00
Coefficient: 3.33e−01
Value: B
Pseudomonas.zeshuii
0
1
2
3
4
A (n=15)
B (n=16)
C (n=14)
surface

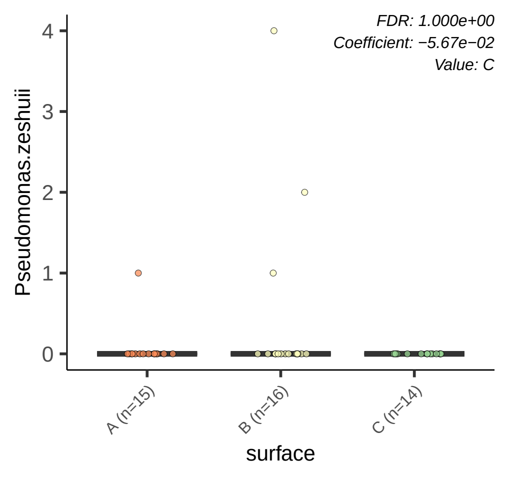
FDR: 1.000e+00
Coefficient: −5.67e−02
Value: C
Pseudomonas.zeshuii
0
1
2
3
4
A (n=15)
B (n=16)
C (n=14)
surface

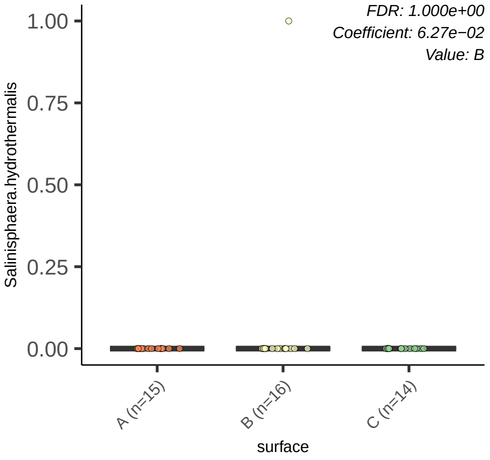
FDR: 1.000e+00
Coefficient: 6.27e−02
Value: B
1.00
0.75
0.50
0.25
0.00
Salinisphaera.hydrothermalis
A (n=15)
B (n=16)
C (n=14)
surface

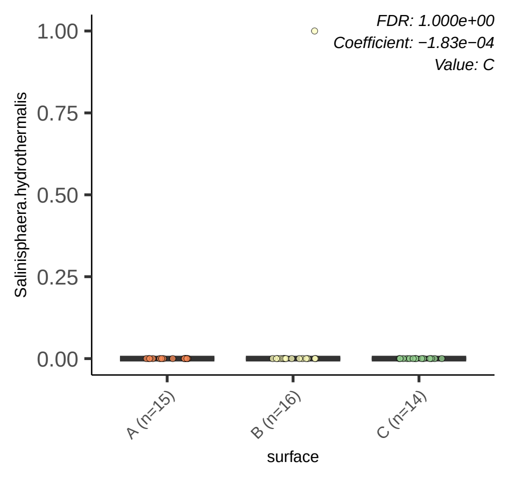
FDR: 1.000e+00
Coefficient: −1.83e−04
Value: C
1.00
0.75
0.50
0.25
0.00
Salinisphaera.hydrothermalis
A (n=15)
B (n=16)
C (n=14)
surface

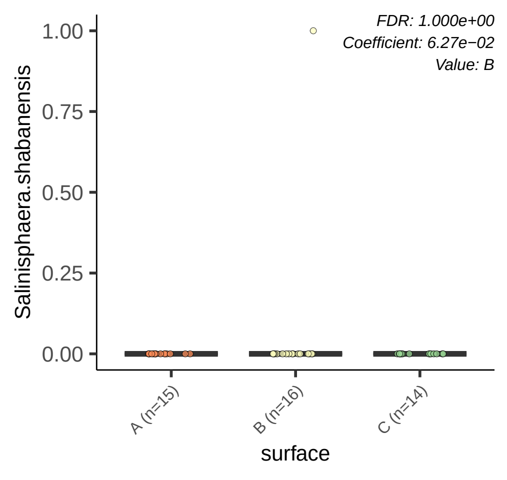

FDR: 1.000e+00
Coefficient: 6.27e−02
Value: B
Salinisphaera.shabanensis
1.00
0.75
0.50
0.25
0.00
A (n=15)
B (n=16)
C (n=14)
surface

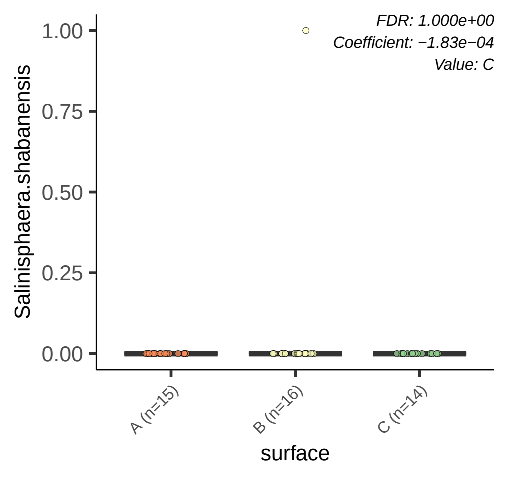

FDR: 1.000e+00
Coefficient: −1.83e−04
Value: C
Salinisphaera.shabanensis
1.00
0.75
0.50
0.25
0.00
A (n=15)
B (n=16)
C (n=14)
surface

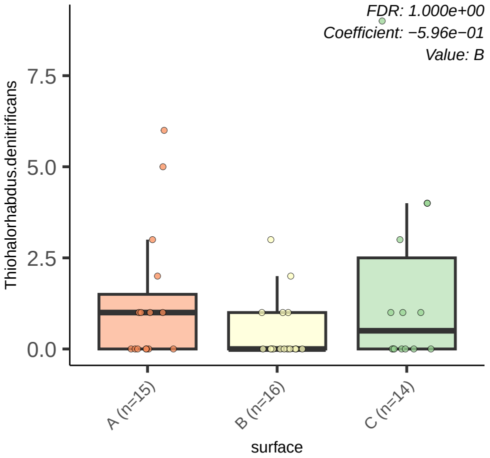
FDR: 1.000e+00
Coefficient: −5.96e−01
Value: B
Thiohalorhabdus.denitrificans
7.5
5.0
2.5
0.0
A (n=15)
B (n=16)
C (n=14)
surface

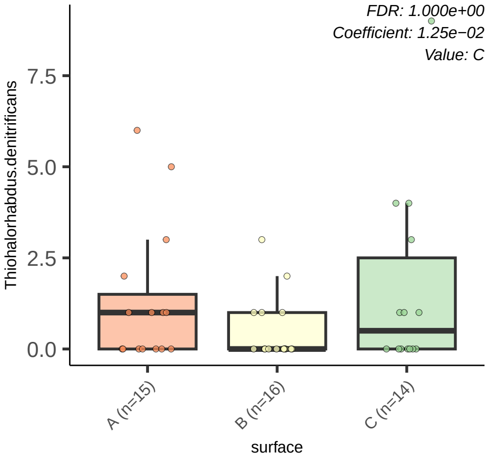

FDR: 1.000e+00
Coefficient: 1.25e−02
Value: C
Thiohalorhabdus.denitrificans
7.5
5.0
2.5
0.0
A (n=15)
B (n=16)
C (n=14)
surface

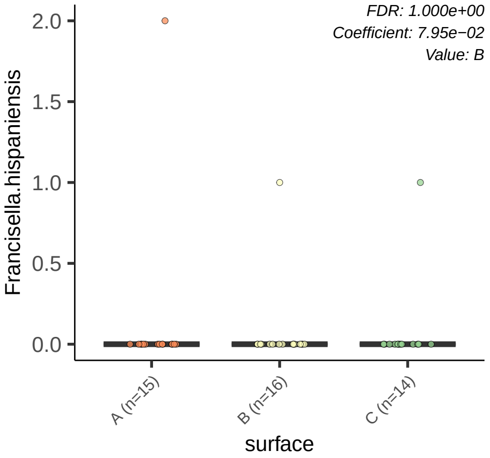

FDR: 1.000e+00
Coefficient: 7.95e−02
Value: B
Francisella.hispaniensis
2.0
1.5
1.0
0.5
0.0
A (n=15)
B (n=16)
C (n=14)
surface

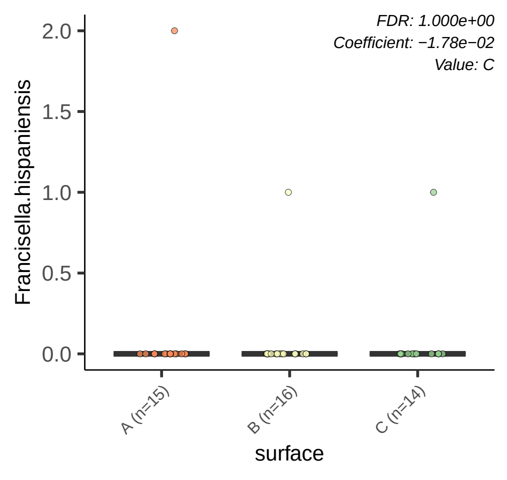

FDR: 1.000e+00
Coefficient: −1.78e−02
Value: C
Francisella.hispaniensis
2.0
1.5
1.0
0.5
0.0
A (n=15)
B (n=16)
C (n=14)
surface

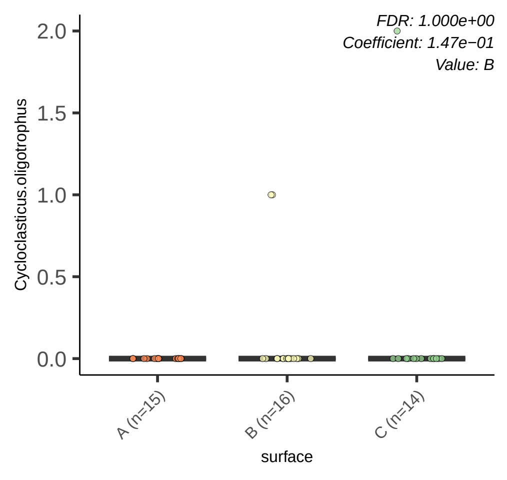
FDR: 1.000e+00
Coefficient: 1.47e−01
Value: B
2.0
1.5
1.0
0.5
0.0
Cycloclasticus.oligotrophus
A (n=15)
B (n=16)
C (n=14)
surface

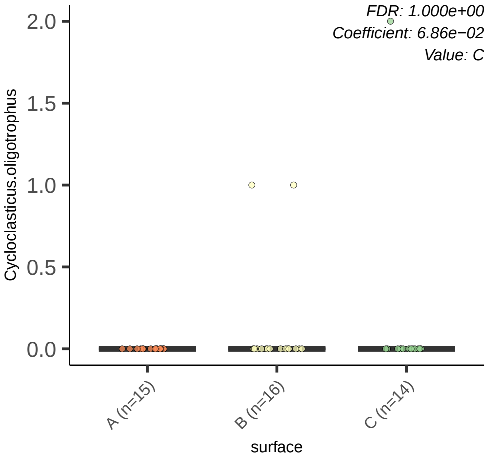

FDR: 1.000e+00
Coefficient: 6.86e−02
Value: C
2.0
1.5
1.0
0.5
0.0
Cycloclasticus.oligotrophus
A (n=15)
B (n=16)
C (n=14)
surface

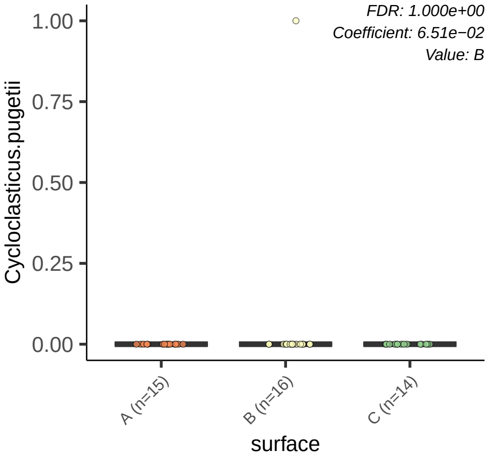
FDR: 1.000e+00
Coefficient: 6.51e−02
Value: B
Cycloclasticus.pugetii
1.00
0.75
0.50
0.25
0.00
A (n=15)
B (n=16)
C (n=14)
surface

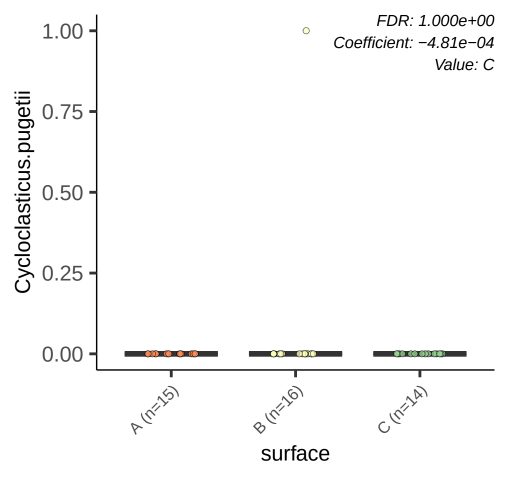
FDR: 1.000e+00
Coefficient: −4.81e−04
Value: C
Cycloclasticus.pugetii
1.00
0.75
0.50
0.25
0.00
A (n=15)
B (n=16)
C (n=14)
surface

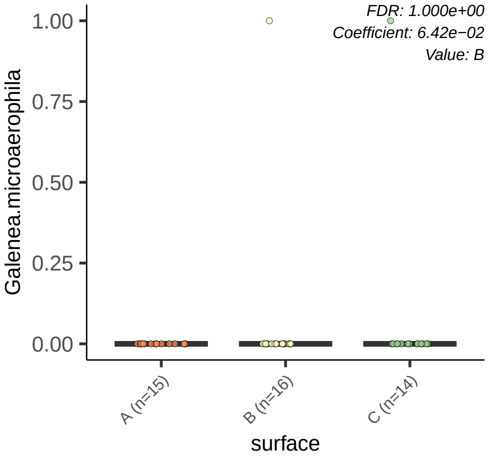
FDR: 1.000e+00
Coefficient: 6.42e−02
Value: B
Galenea.microaerophila
1.00
0.75
0.50
0.25
0.00
A (n=15)
B (n=16)
C (n=14)
surface

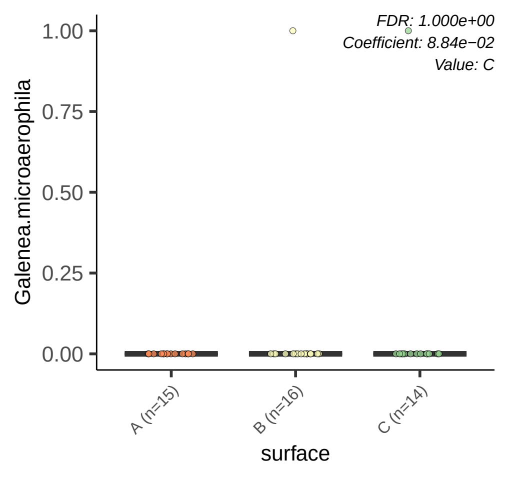
FDR: 1.000e+00
Coefficient: 8.84e−02
Value: C
Galenea.microaerophila
1.00
0.75
0.50
0.25
0.00
A (n=15)
B (n=16)
C (n=14)
surface

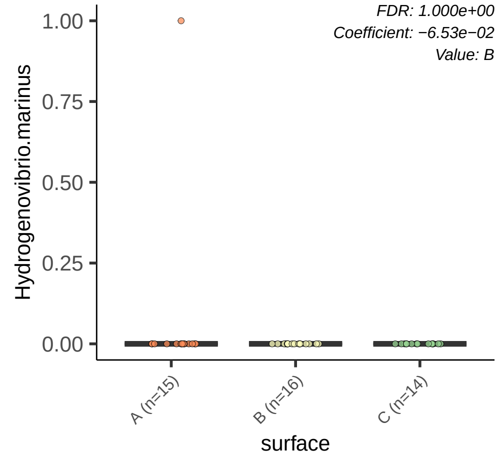

FDR: 1.000e+00
Coefficient: −6.53e−02
Value: B
Hydrogenovibrio.marinus
1.00
0.75
0.50
0.25
0.00
A (n=15)
B (n=16)
C (n=14)
surface

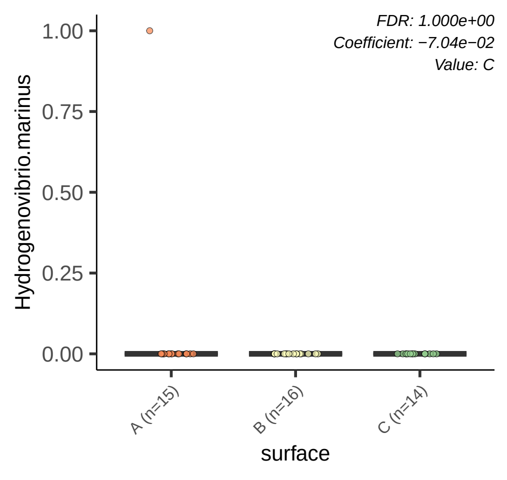
FDR: 1.000e+00
Coefficient: −7.04e−02
Value: C
Hydrogenovibrio.marinus
1.00
0.75
0.50
0.25
0.00
A (n=15)
B (n=16)
C (n=14)
surface

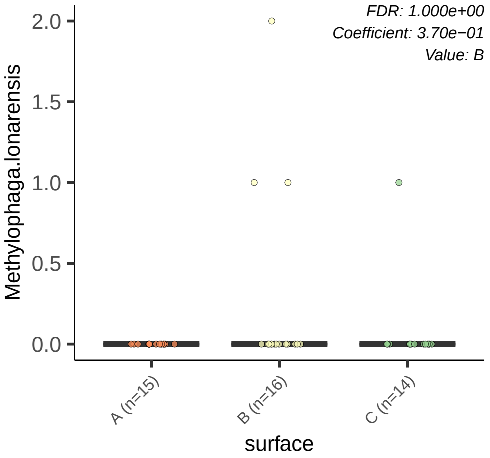
FDR: 1.000e+00
Coefficient: 3.70e−01
Value: B
Methylophaga.lonarensis
2.0
1.5
1.0
0.5
0.0
A (n=15)
B (n=16)
C (n=14)
surface

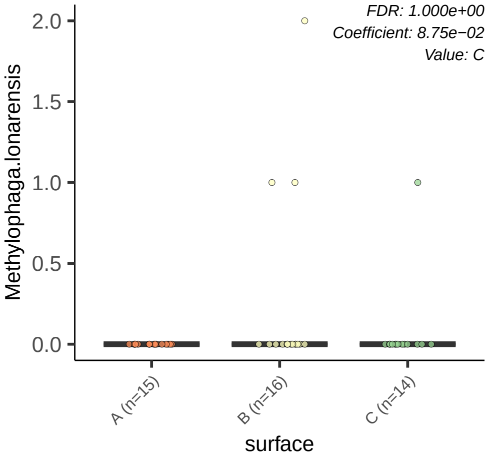
FDR: 1.000e+00
Coefficient: 8.75e−02
Value: C
Methylophaga.lonarensis
2.0
1.5
1.0
0.5
0.0
A (n=15)
B (n=16)
C (n=14)
surface

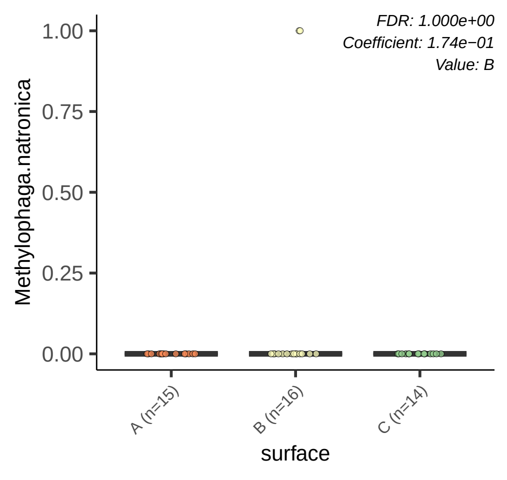
FDR: 1.000e+00
Coefficient: 1.74e−01
Value: B
Methylophaga.natronica
1.00
0.75
0.50
0.25
0.00
A (n=15)
B (n=16)
C (n=14)
surface

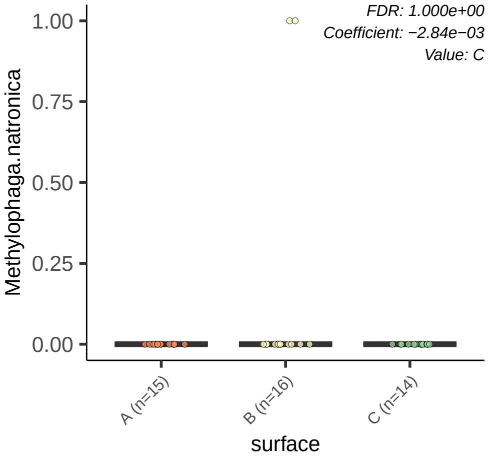
FDR: 1.000e+00
Coefficient: −2.84e−03
Value: C
Methylophaga.natronica
1.00
0.75
0.50
0.25
0.00
A (n=15)
B (n=16)
C (n=14)
surface

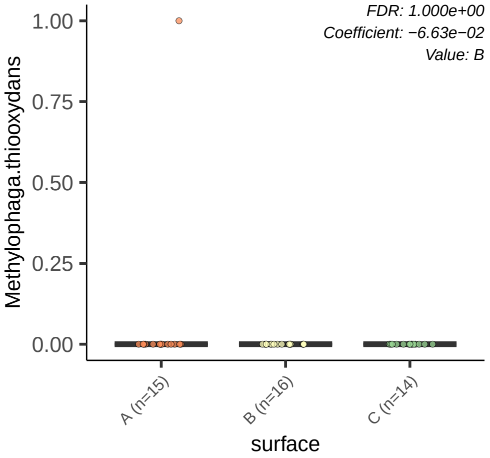
FDR: 1.000e+00
Coefficient: −6.63e−02
Value: B
Methylophaga.thiooxydans
1.00
0.75
0.50
0.25
0.00
A (n=15)
B (n=16)
C (n=14)
surface

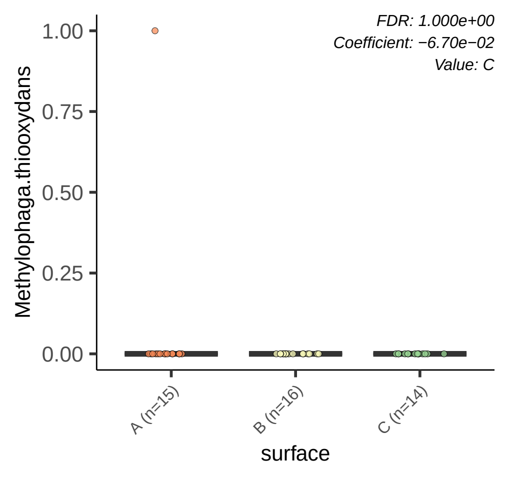

FDR: 1.000e+00
Coefficient: −6.70e−02
Value: C
Methylophaga.thiooxydans
1.00
0.75
0.50
0.25
0.00
A (n=15)
B (n=16)
C (n=14)
surface

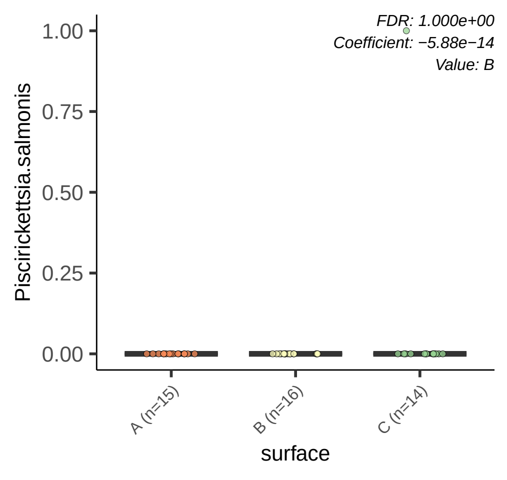

FDR: 1.000e+00
Coefficient: −5.88e−14
Value: B
Piscirickettsia.salmonis
1.00
0.75
0.50
0.25
0.00
A (n=15)
B (n=16)
C (n=14)
surface

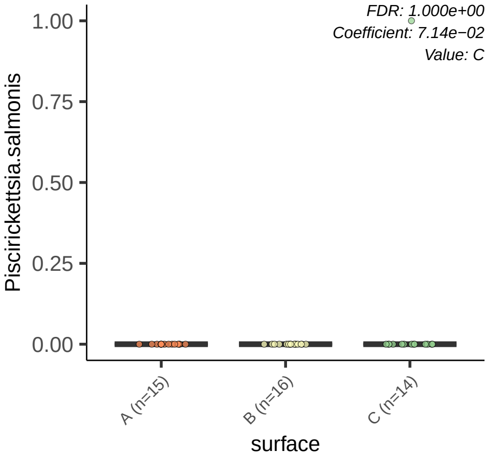
FDR: 1.000e+00
Coefficient: 7.14e−02
Value: C
Piscirickettsia.salmonis
1.00
0.75
0.50
0.25
0.00
A (n=15)
B (n=16)
C (n=14)
surface

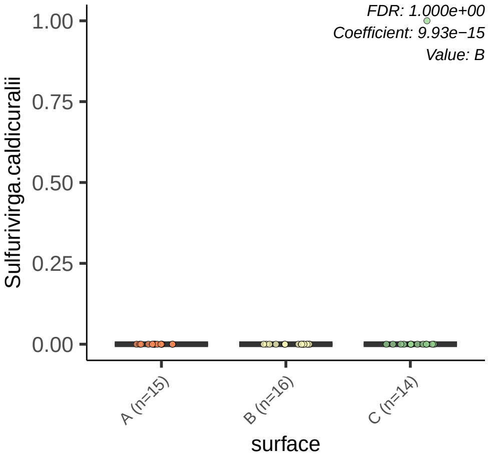

FDR: 1.000e+00
Coefficient: 9.93e−15
Value: B
Sulfurivirga.caldicuralii
1.00
0.75
0.50
0.25
0.00
A (n=15)
B (n=16)
C (n=14)
surface

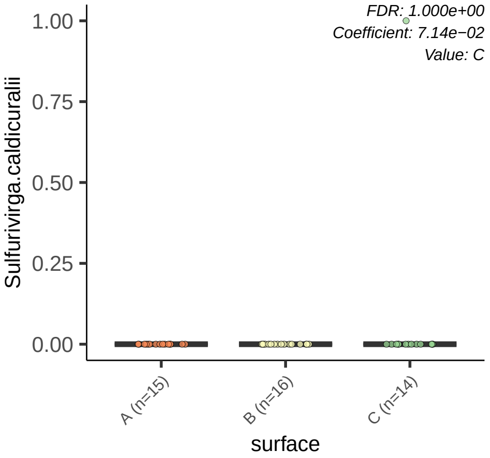
FDR: 1.000e+00
Coefficient: 7.14e−02
Value: C
Sulfurivirga.caldicuralii
1.00
0.75
0.50
0.25
0.00
A (n=15)
B (n=16)
C (n=14)
surface

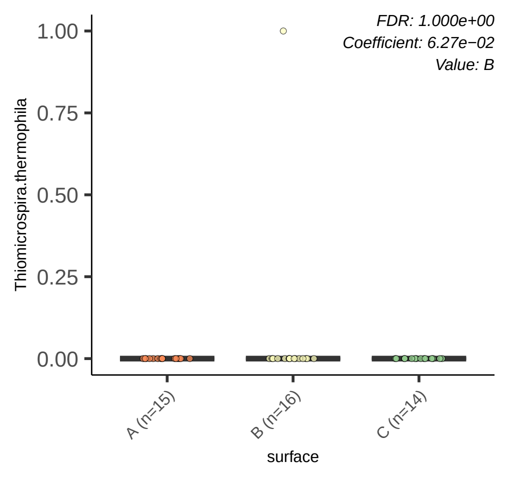
FDR: 1.000e+00
Coefficient: 6.27e−02
Value: B
Thiomicrospira.thermophila
1.00
0.75
0.50
0.25
0.00
A (n=15)
B (n=16)
C (n=14)
surface

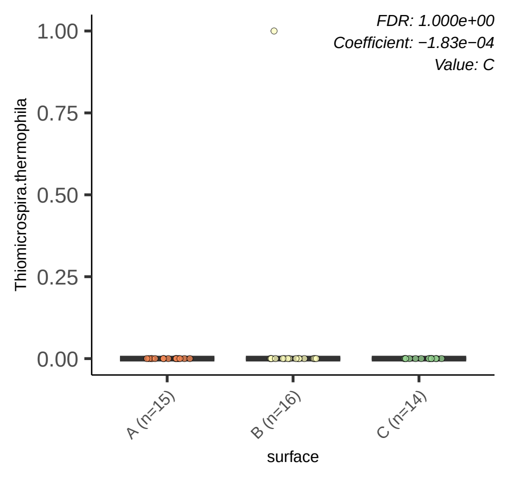
FDR: 1.000e+00
Coefficient: −1.83e−04
Value: C
Thiomicrospira.thermophila
1.00
0.75
0.50
0.25
0.00
A (n=15)
B (n=16)
C (n=14)
surface

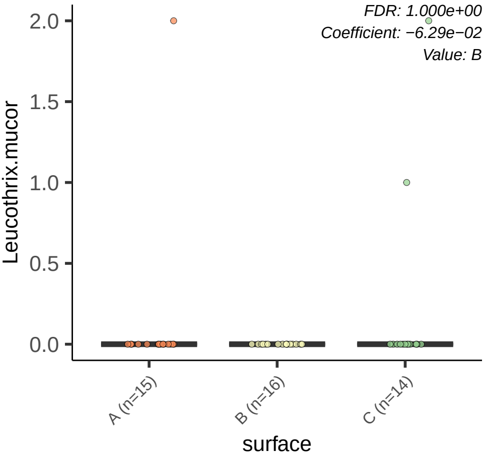

FDR: 1.000e+00
Coefficient: −6.29e−02
Value: B
Leucothrix.mucor
2.0
1.5
1.0
0.5
0.0
A (n=15)
B (n=16)
C (n=14)
surface

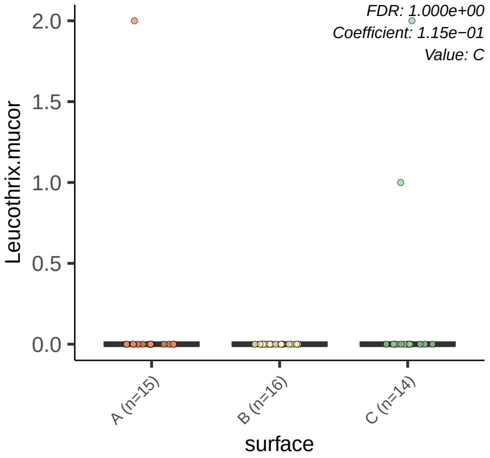
FDR: 1.000e+00
Coefficient: 1.15e−01
Value: C
Leucothrix.mucor
2.0
1.5
1.0
0.5
0.0
A (n=15)
B (n=16)
C (n=14)
surface

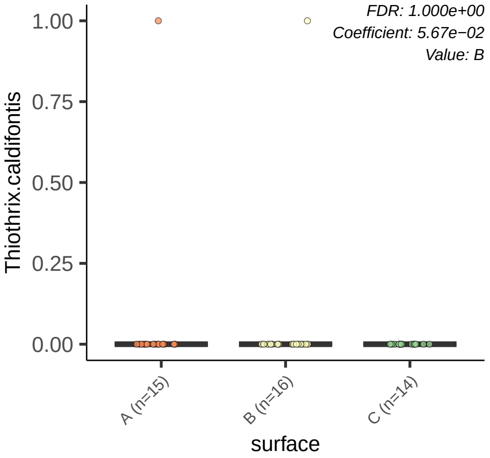

FDR: 1.000e+00
Coefficient: 5.67e−02
Value: B
Thiothrix.caldifontis
1.00
0.75
0.50
0.25
0.00
A (n=15)
B (n=16)
C (n=14)
surface

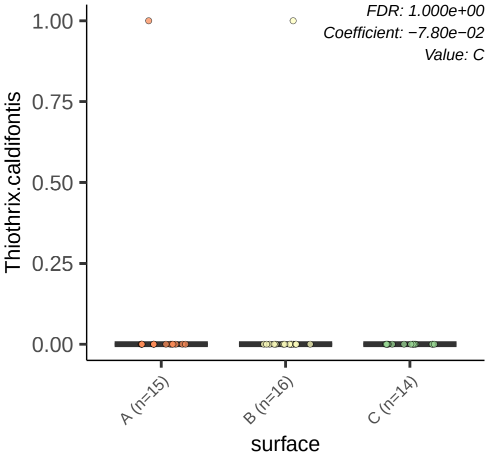

FDR: 1.000e+00
Coefficient: −7.80e−02
Value: C
Thiothrix.caldifontis
1.00
0.75
0.50
0.25
0.00
A (n=15)
B (n=16)
C (n=14)
surface

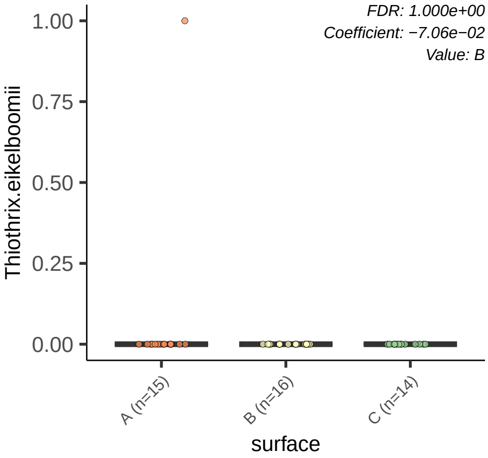
FDR: 1.000e+00
Coefficient: −7.06e−02
Value: B
Thiothrix.eikelboomii
1.00
0.75
0.50
0.25
0.00
A (n=15)
B (n=16)
C (n=14)
surface

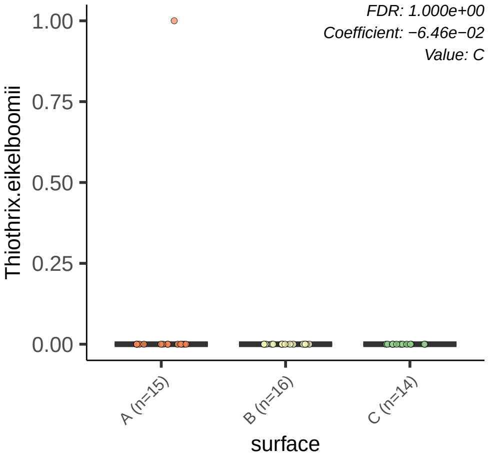
FDR: 1.000e+00
Coefficient: −6.46e−02
Value: C
Thiothrix.eikelboomii
1.00
0.75
0.50
0.25
0.00
A (n=15)
B (n=16)
C (n=14)
surface

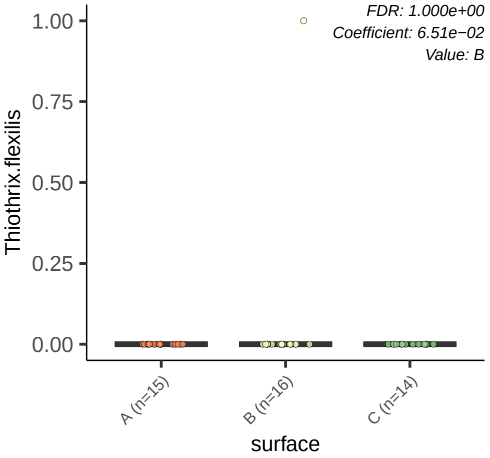

FDR: 1.000e+00
Coefficient: 6.51e−02
Value: B
Thiothrix.flexilis
1.00
0.75
0.50
0.25
0.00
A (n=15)
B (n=16)
C (n=14)
surface

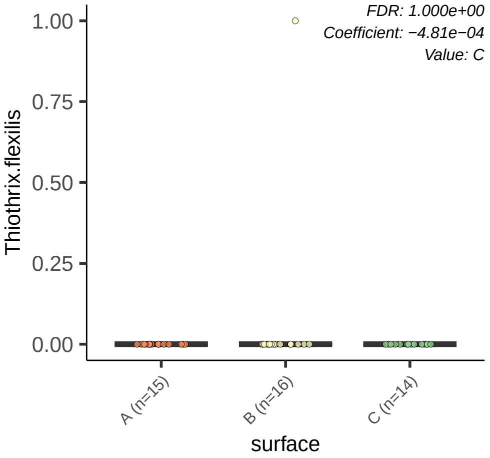

FDR: 1.000e+00
Coefficient: −4.81e−04
Value: C
Thiothrix.flexilis
1.00
0.75
0.50
0.25
0.00
A (n=15)
B (n=16)
C (n=14)
surface

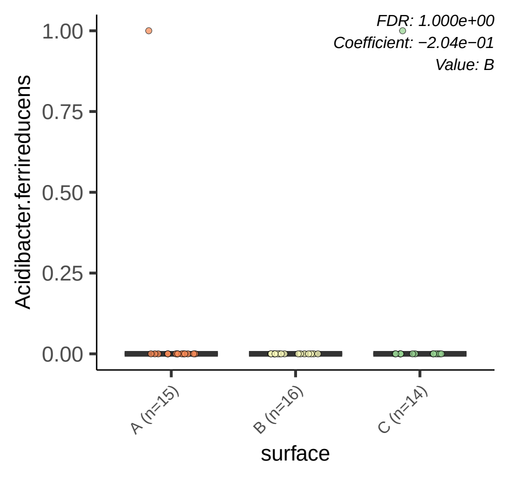
FDR: 1.000e+00
Coefficient: −2.04e−01
Value: B
Acidibacter.ferrireducens
1.00
0.75
0.50
0.25
0.00
A (n=15)
B (n=16)
C (n=14)
surface

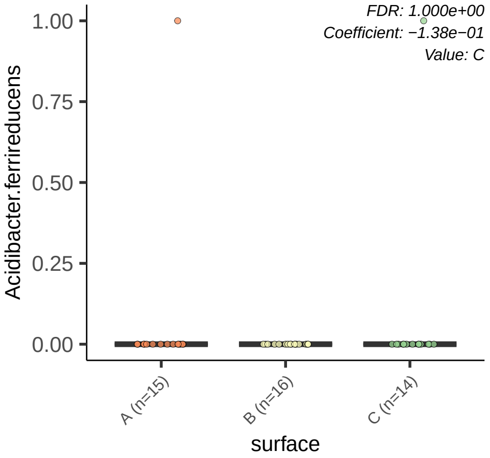
FDR: 1.000e+00
Coefficient: −1.38e−01
Value: C
1.00
0.75
0.50
0.25
0.00
Acidibacter.ferrireducens
A (n=15)
B (n=16)
C (n=14)
surface

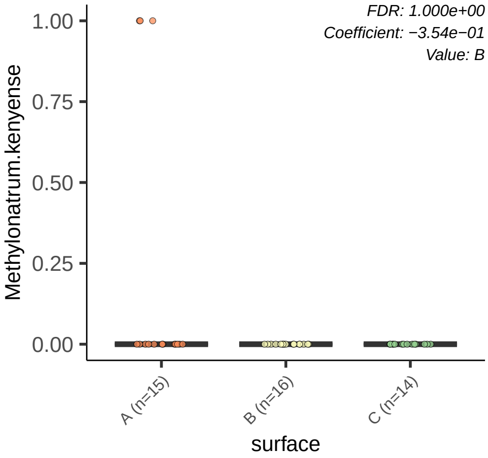
FDR: 1.000e+00
Coefficient: −3.54e−01
Value: B
Methylonatrum.kenyense
1.00
0.75
0.50
0.25
0.00
A (n=15)
B (n=16)
C (n=14)
surface

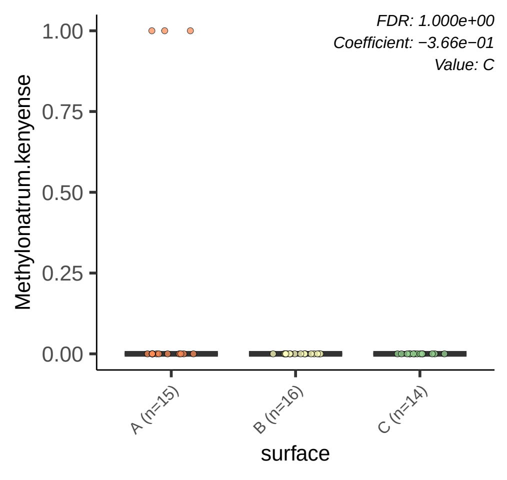

FDR: 1.000e+00
Coefficient: −3.66e−01
Value: C
Methylonatrum.kenyense
1.00
0.75
0.50
0.25
0.00
A (n=15)
B (n=16)
C (n=14)
surface

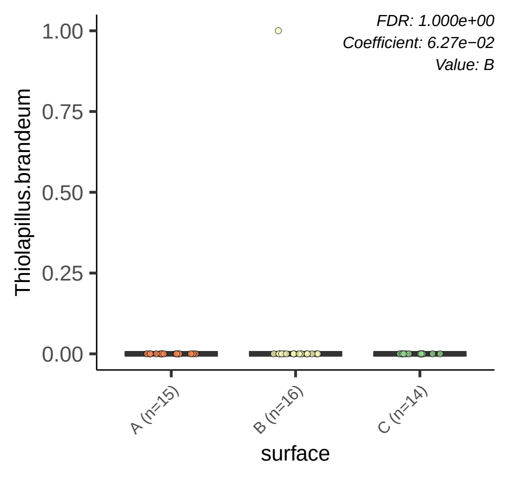
FDR: 1.000e+00
Coefficient: 6.27e−02
Value: B
Thiolapillus.brandeum
1.00
0.75
0.50
0.25
0.00
A (n=15)
B (n=16)
C (n=14)
surface

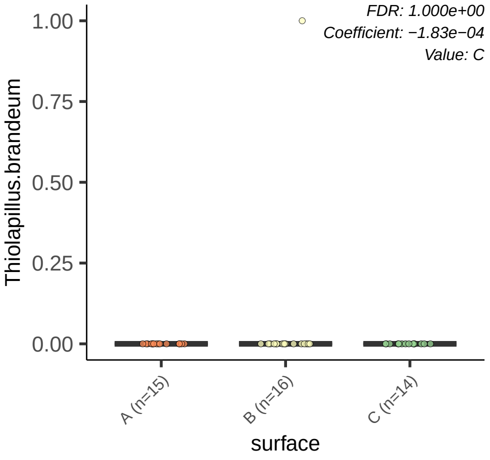
FDR: 1.000e+00
Coefficient: −1.83e−04
Value: C
Thiolapillus.brandeum
1.00
0.75
0.50
0.25
0.00
A (n=15)
B (n=16)
C (n=14)
surface

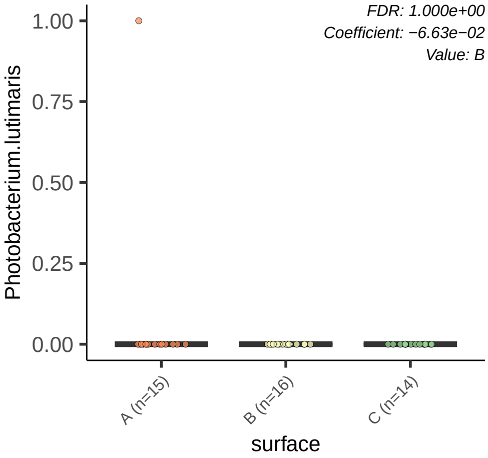
FDR: 1.000e+00
Coefficient: −6.63e−02
Value: B
Photobacterium.lutimaris
1.00
0.75
0.50
0.25
0.00
A (n=15)
B (n=16)
C (n=14)
surface

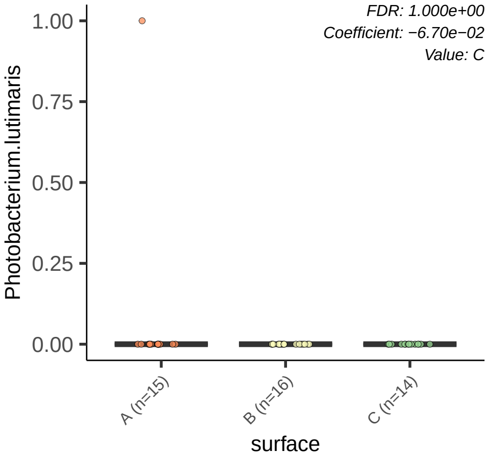

FDR: 1.000e+00
Coefficient: −6.70e−02
Value: C
Photobacterium.lutimaris
1.00
0.75
0.50
0.25
0.00
A (n=15)
B (n=16)
C (n=14)
surface

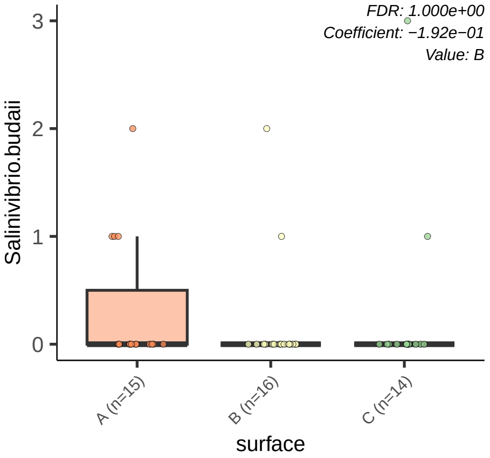
FDR: 1.000e+00
Coefficient: −1.92e−01
Value: B
Salinivibrio.budaii
0
1
2
3
A (n=15)
B (n=16)
C (n=14)
surface

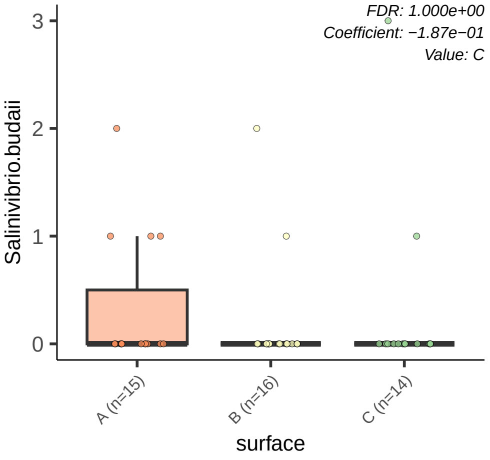
FDR: 1.000e+00
Coefficient: −1.87e−01
Value: C
Salinivibrio.budaii
0
1
2
3
A (n=15)
B (n=16)
C (n=14)
surface

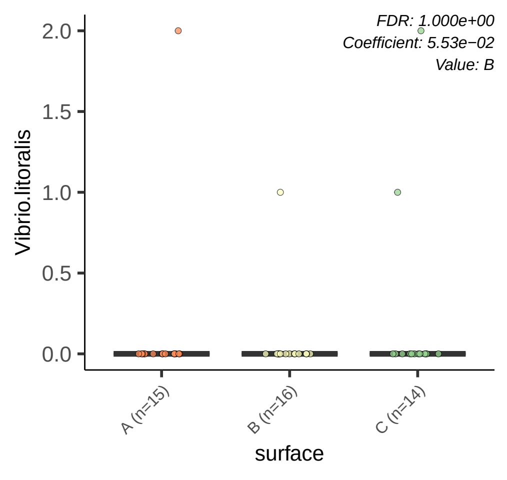

FDR: 1.000e+00
Coefficient: 5.53e−02
Value: B
Vibrio.litoralis
2.0
1.5
1.0
0.5
0.0
A (n=15)
B (n=16)
C (n=14)
surface

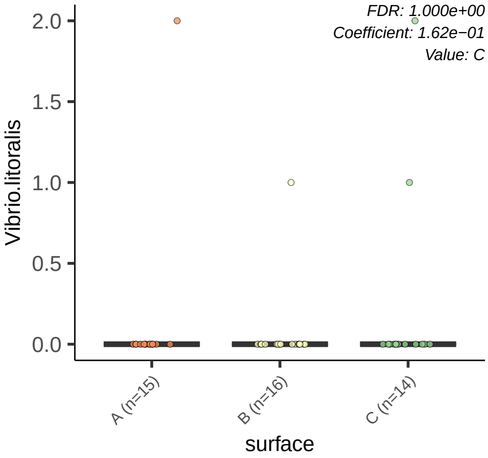

FDR: 1.000e+00
Coefficient: 1.62e−01
Value: C
Vibrio.litoralis
2.0
1.5
1.0
0.5
0.0
A (n=15)
B (n=16)
C (n=14)
surface

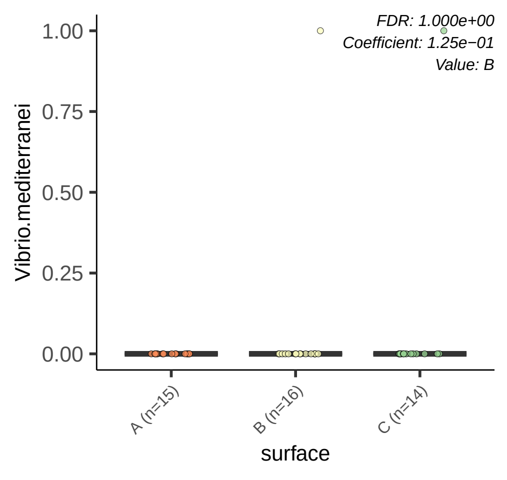
FDR: 1.000e+00
Coefficient: 1.25e−01
Value: B
Vibrio.mediterranei
1.00
0.75
0.50
0.25
0.00
A (n=15)
B (n=16)
C (n=14)
surface

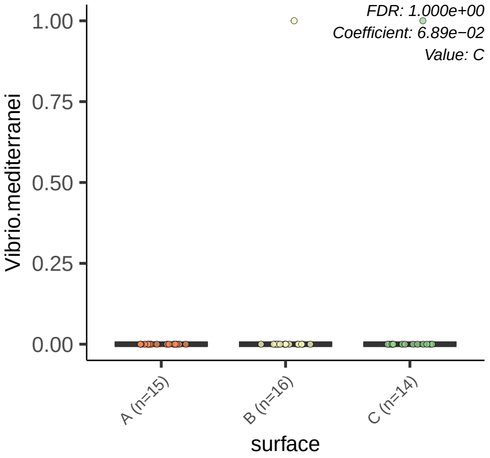

FDR: 1.000e+00
Coefficient: 6.89e−02
Value: C
Vibrio.mediterranei
1.00
0.75
0.50
0.25
0.00
A (n=15)
B (n=16)
C (n=14)
surface

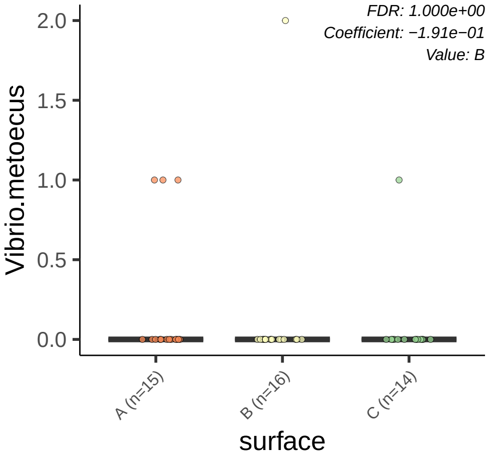
FDR: 1.000e+00
Coefficient: −1.91e−01
Value: B
Vibrio.metoecus
2.0
1.5
1.0
0.5
0.0
A (n=15)
B (n=16)
C (n=14)
surface

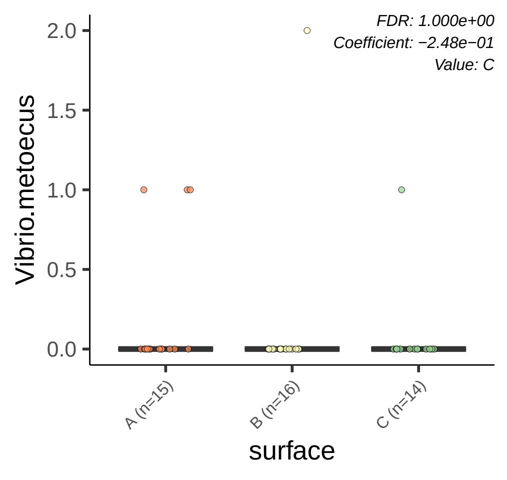

FDR: 1.000e+00
Coefficient: −2.48e−01
Value: C
Vibrio.metoecus
2.0
1.5
1.0
0.5
0.0
A (n=15)
B (n=16)
C (n=14)
surface

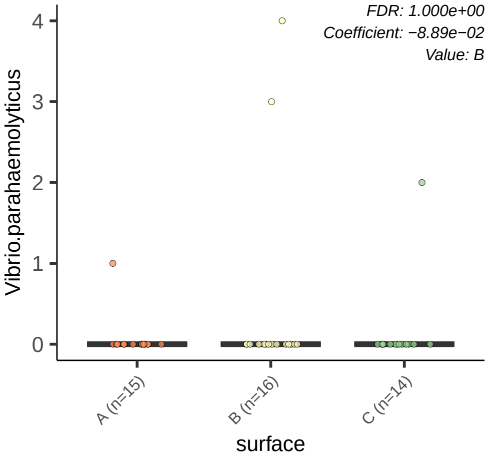
FDR: 1.000e+00
Coefficient: −8.89e−02
Value: B
Vibrio.parahaemolyticus
0
1
2
3
4
A (n=15)
B (n=16)
C (n=14)
surface

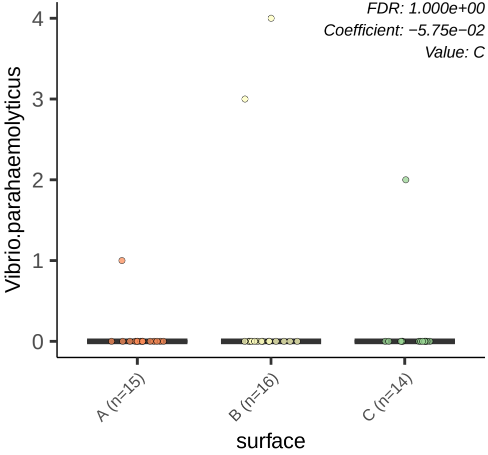
FDR: 1.000e+00
Coefficient: −5.75e−02
Value: C
Vibrio.parahaemolyticus
0
1
2
3
4
A (n=15)
B (n=16)
C (n=14)
surface

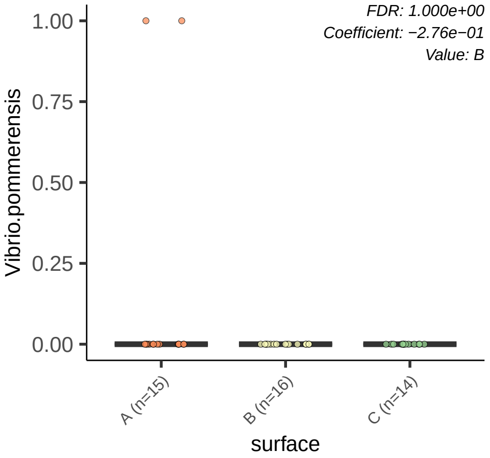

FDR: 1.000e+00
Coefficient: −2.76e−01
Value: B
Vibrio.pommerensis
1.00
0.75
0.50
0.25
0.00
A (n=15)
B (n=16)
C (n=14)
surface

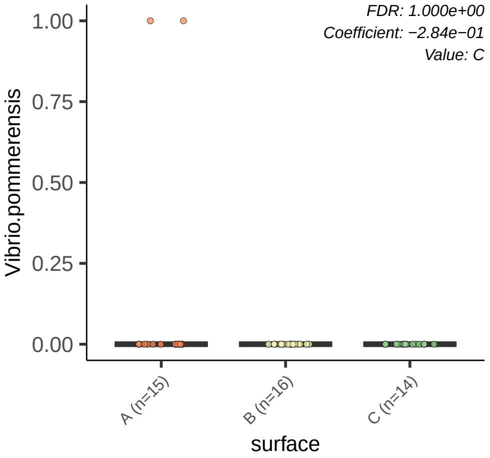

FDR: 1.000e+00
Coefficient: −2.84e−01
Value: C
Vibrio.pommerensis
1.00
0.75
0.50
0.25
0.00
A (n=15)
B (n=16)
C (n=14)
surface

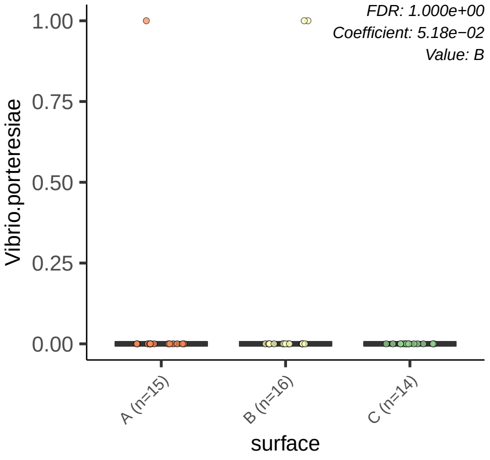

FDR: 1.000e+00
Coefficient: 5.18e−02
Value: B
Vibrio.porteresiae
1.00
0.75
0.50
0.25
0.00
A (n=15)
B (n=16)
C (n=14)
surface

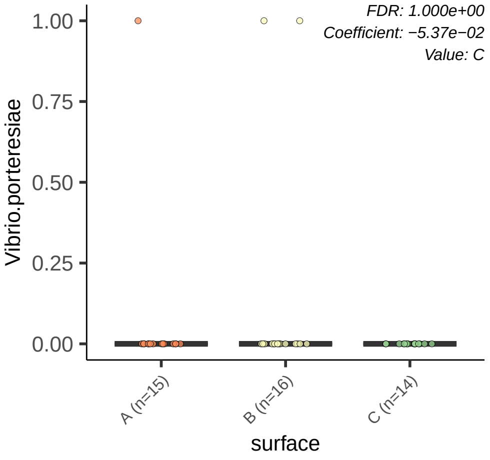
FDR: 1.000e+00
Coefficient: −5.37e−02
Value: C
Vibrio.porteresiae
1.00
0.75
0.50
0.25
0.00
A (n=15)
B (n=16)
C (n=14)
surface

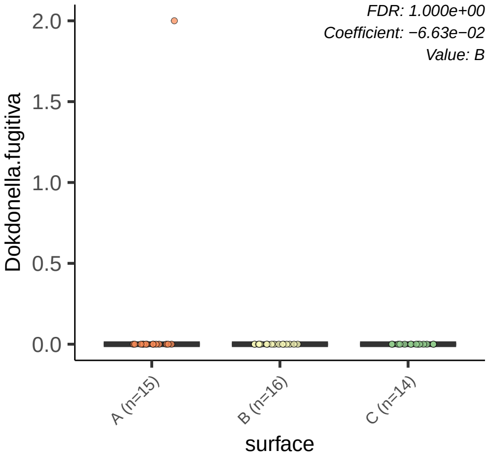
FDR: 1.000e+00
Coefficient: −6.63e−02
Value: B
Dokdonella.fugitiva
2.0
1.5
1.0
0.5
0.0
A (n=15)
B (n=16)
C (n=14)
surface

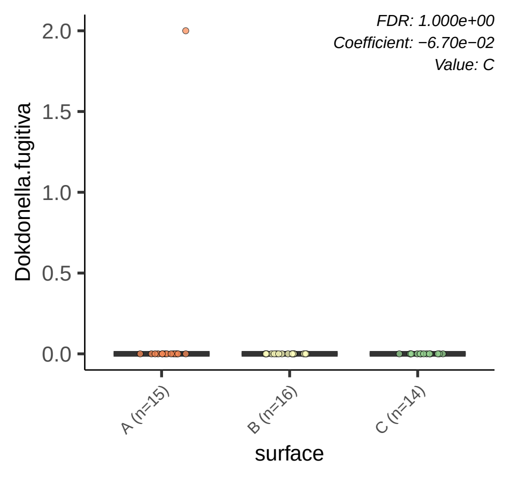

FDR: 1.000e+00
Coefficient: −6.70e−02
Value: C
Dokdonella.fugitiva
2.0
1.5
1.0
0.5
0.0
A (n=15)
B (n=16)
C (n=14)
surface

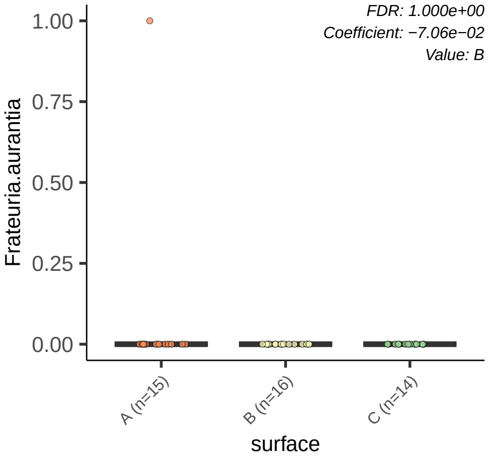
FDR: 1.000e+00
Coefficient: −7.06e−02
Value: B
Frateuria.aurantia
1.00
0.75
0.50
0.25
0.00
A (n=15)
B (n=16)
C (n=14)
surface

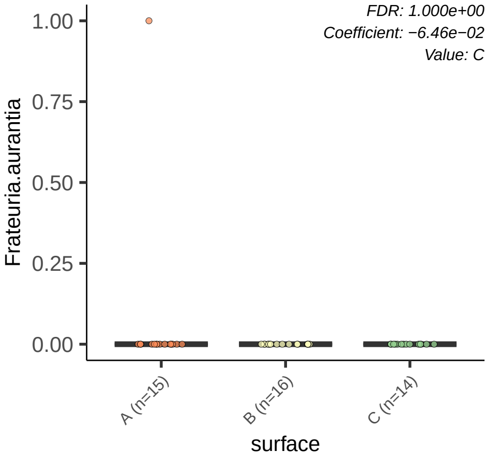
FDR: 1.000e+00
Coefficient: −6.46e−02
Value: C
Frateuria.aurantia
1.00
0.75
0.50
0.25
0.00
A (n=15)
B (n=16)
C (n=14)
surface

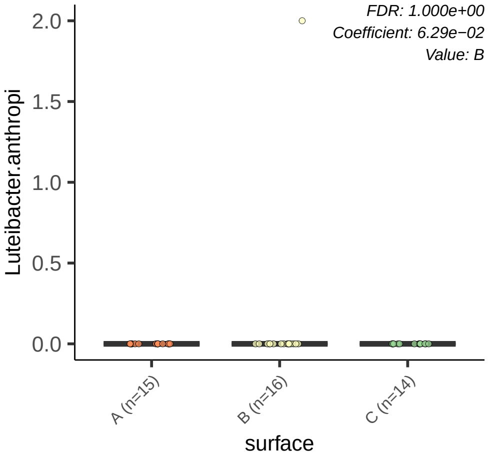

FDR: 1.000e+00
Coefficient: 6.29e−02
Value: B
Luteibacter.anthropi
2.0
1.5
1.0
0.5
0.0
A (n=15)
B (n=16)
C (n=14)
surface

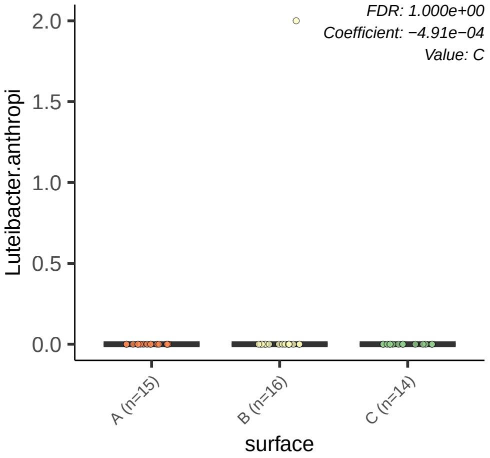

FDR: 1.000e+00
Coefficient: −4.91e−04
Value: C
Luteibacter.anthropi
2.0
1.5
1.0
0.5
0.0
A (n=15)
B (n=16)
C (n=14)
surface

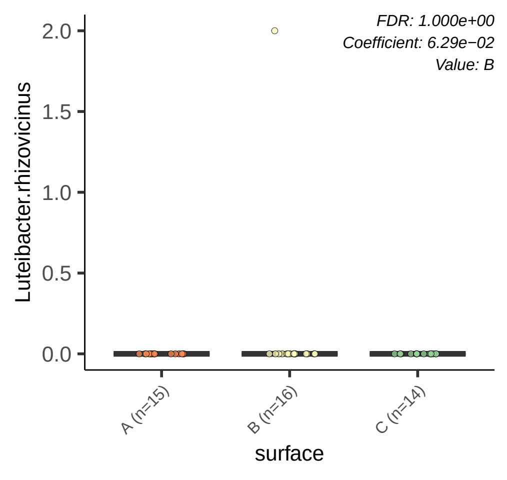

FDR: 1.000e+00
Coefficient: 6.29e−02
Value: B
Luteibacter.rhizovicinus
2.0
1.5
1.0
0.5
0.0
A (n=15)
B (n=16)
C (n=14)
surface

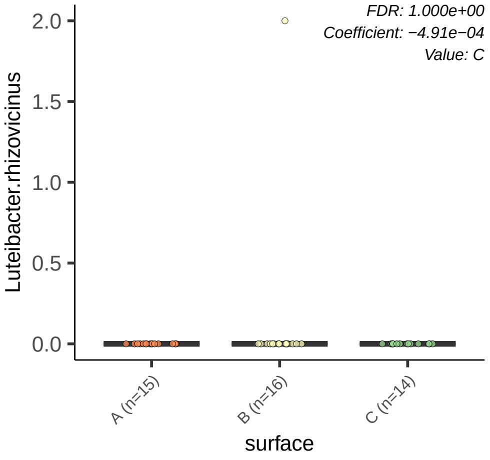
FDR: 1.000e+00
Coefficient: −4.91e−04
Value: C
Luteibacter.rhizovicinus
2.0
1.5
1.0
0.5
0.0
A (n=15)
B (n=16)
C (n=14)
surface

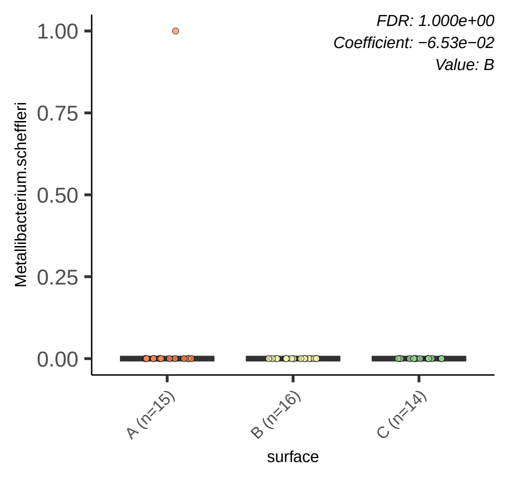
FDR: 1.000e+00
Coefficient: −6.53e−02
Value: B
Metallibacterium.scheffleri
1.00
0.75
0.50
0.25
0.00
A (n=15)
B (n=16)
C (n=14)
surface

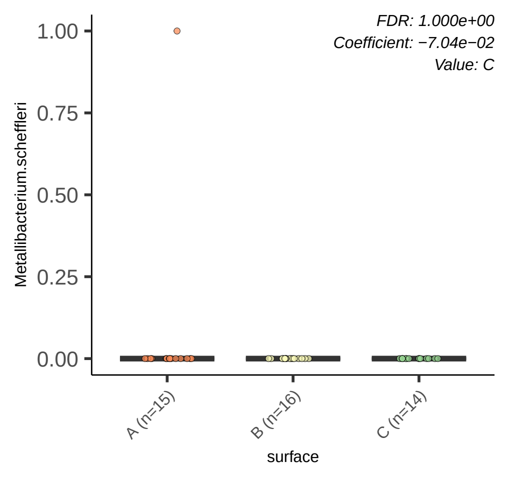

FDR: 1.000e+00
Coefficient: −7.04e−02
Value: C
1.00
0.75
0.50
0.25
0.00
Metallibacterium.scheffleri
A (n=15)
B (n=16)
C (n=14)
surface

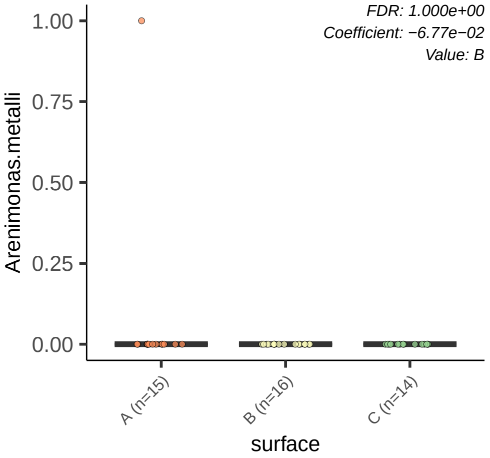
FDR: 1.000e+00
Coefficient: −6.77e−02
Value: B
Arenimonas.metalli
1.00
0.75
0.50
0.25
0.00
A (n=15)
B (n=16)
C (n=14)
surface

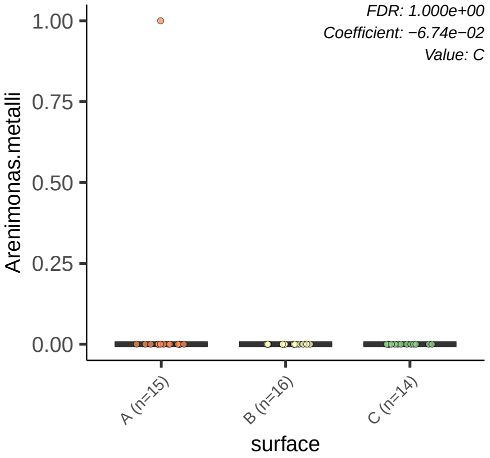
FDR: 1.000e+00
Coefficient: −6.74e−02
Value: C
Arenimonas.metalli
1.00
0.75
0.50
0.25
0.00
A (n=15)
B (n=16)
C (n=14)
surface

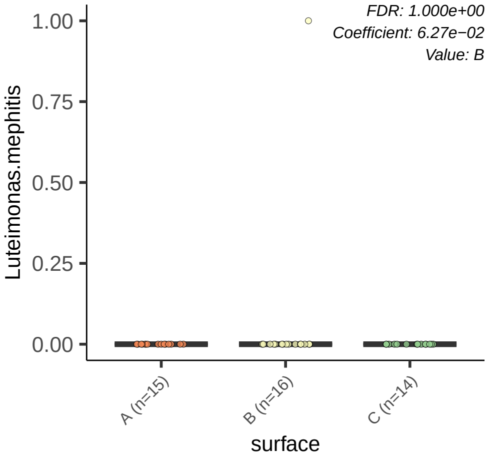
FDR: 1.000e+00
Coefficient: 6.27e−02
Value: B
Luteimonas.mephitis
1.00
0.75
0.50
0.25
0.00
A (n=15)
B (n=16)
C (n=14)
surface

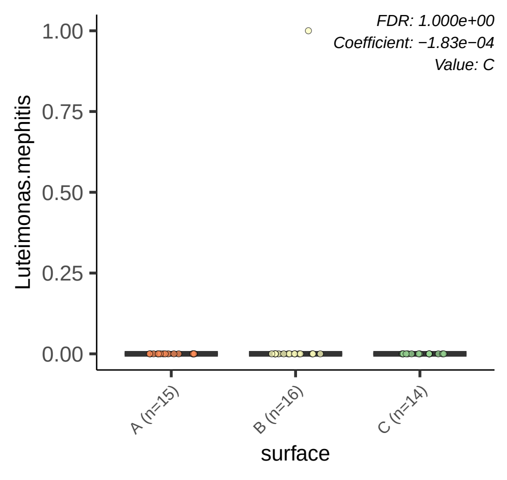

FDR: 1.000e+00
Coefficient: −1.83e−04
Value: C
Luteimonas.mephitis
1.00
0.75
0.50
0.25
0.00
A (n=15)
B (n=16)
C (n=14)
surface

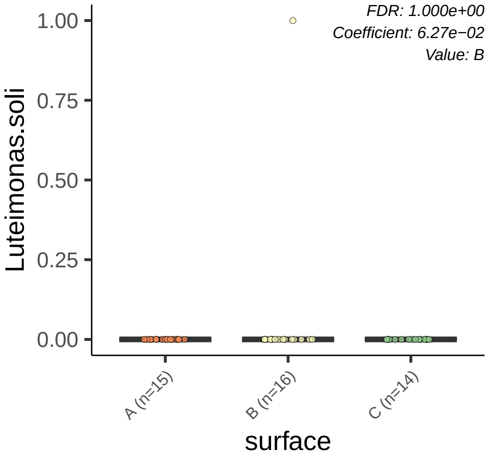
FDR: 1.000e+00
Coefficient: 6.27e−02
Value: B
Luteimonas.soli
1.00
0.75
0.50
0.25
0.00
A (n=15)
B (n=16)
C (n=14)
surface

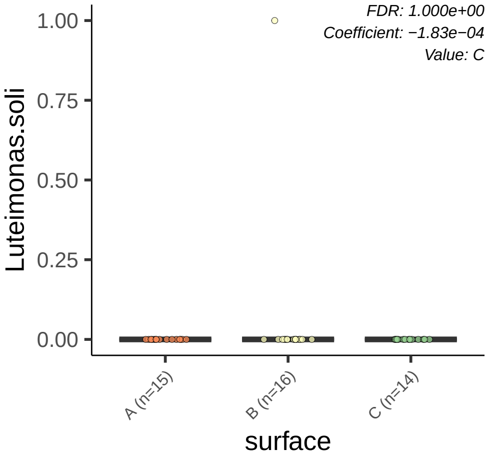

FDR: 1.000e+00
Coefficient: −1.83e−04
Value: C
Luteimonas.soli
1.00
0.75
0.50
0.25
0.00
A (n=15)
B (n=16)
C (n=14)
surface

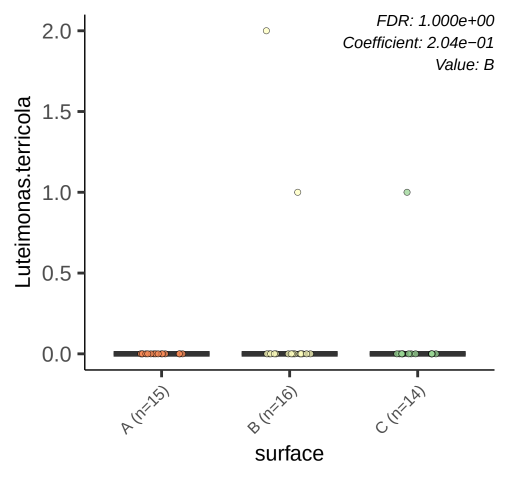
FDR: 1.000e+00
Coefficient: 2.04e−01
Value: B
Luteimonas.terricola
2.0
1.5
1.0
0.5
0.0
A (n=15)
B (n=16)
C (n=14)
surface

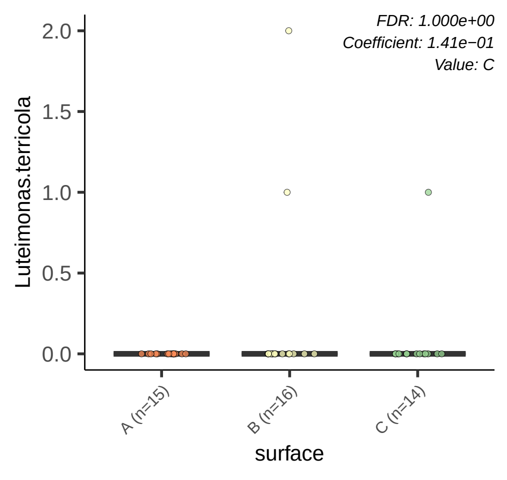
FDR: 1.000e+00
Coefficient: 1.41e−01
Value: C
Luteimonas.terricola
2.0
1.5
1.0
0.5
0.0
A (n=15)
B (n=16)
C (n=14)
surface

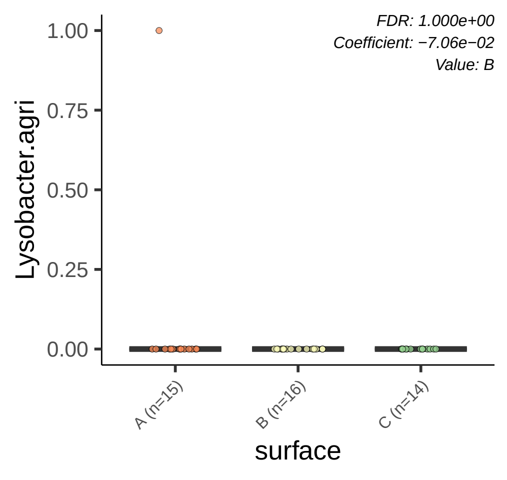
FDR: 1.000e+00
Coefficient: −7.06e−02
Value: B
Lysobacter.agri
1.00
0.75
0.50
0.25
0.00
A (n=15)
B (n=16)
C (n=14)
surface

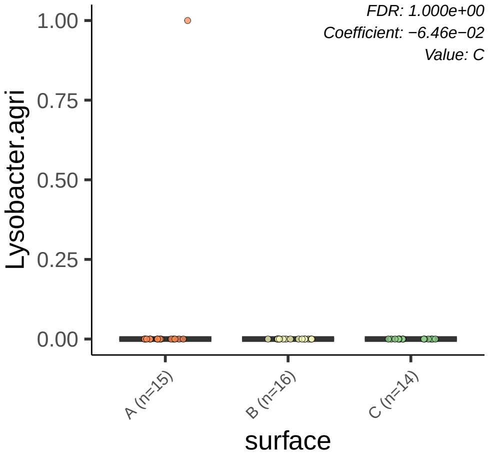

FDR: 1.000e+00
Coefficient: −6.46e−02
Value: C
Lysobacter.agri
1.00
0.75
0.50
0.25
0.00
A (n=15)
B (n=16)
C (n=14)
surface

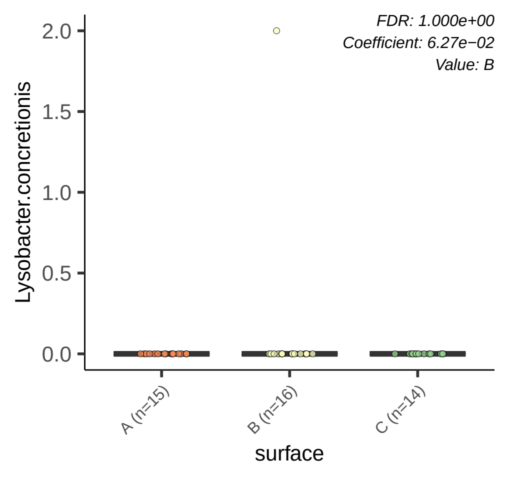
FDR: 1.000e+00
Coefficient: 6.27e−02
Value: B
Lysobacter.concretionis
2.0
1.5
1.0
0.5
0.0
A (n=15)
B (n=16)
C (n=14)
surface

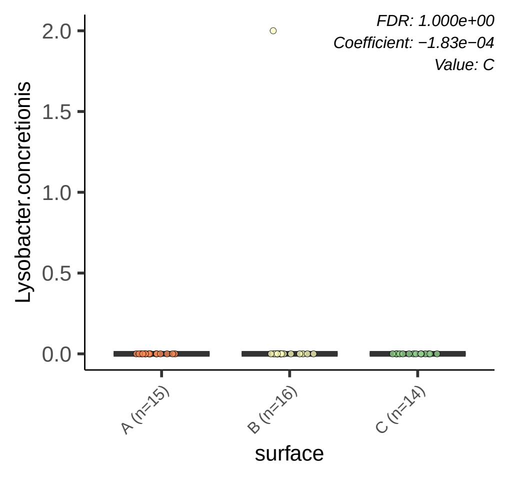

FDR: 1.000e+00
Coefficient: −1.83e−04
Value: C
Lysobacter.concretionis
2.0
1.5
1.0
0.5
0.0
A (n=15)
B (n=16)
C (n=14)
surface

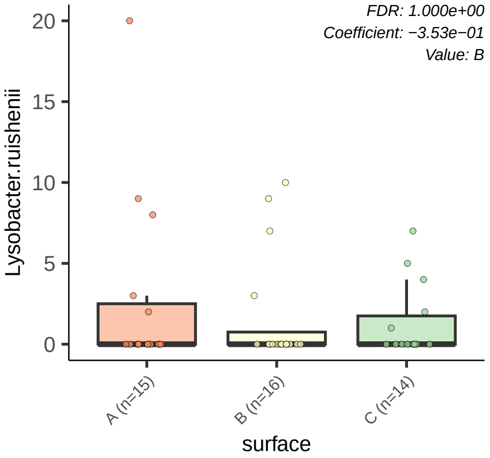

FDR: 1.000e+00
Coefficient: −3.53e−01
Value: B
Lysobacter.ruishenii
20
15
10
5
0
A (n=15)
B (n=16)
C (n=14)
surface

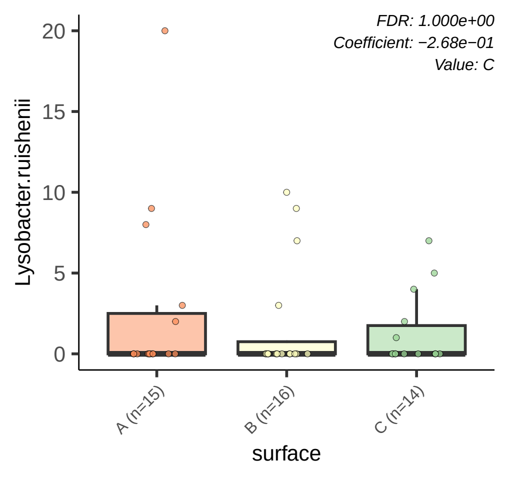

FDR: 1.000e+00
Coefficient: −2.68e−01
Value: C
Lysobacter.ruishenii
20
15
10
5
0
A (n=15)
B (n=16)
C (n=14)
surface

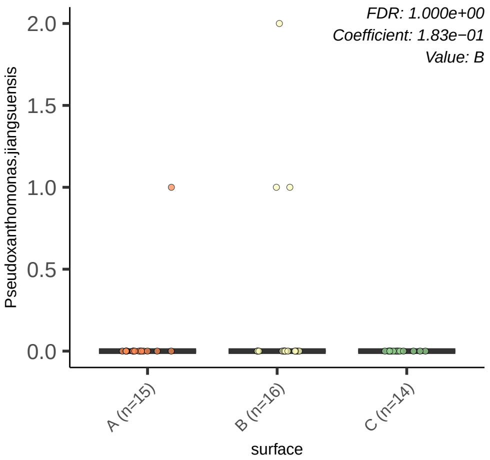
FDR: 1.000e+00
Coefficient: 1.83e−01
Value: B
Pseudoxanthomonas.jiangsuensis
2.0
1.5
1.0
0.5
0.0
A (n=15)
B (n=16)
C (n=14)
surface

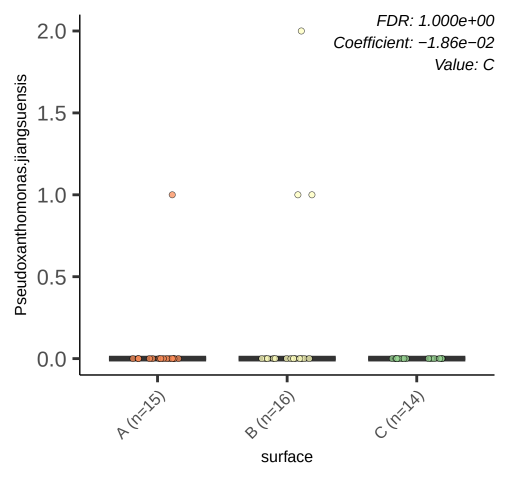

FDR: 1.000e+00
Coefficient: −1.86e−02
Value: C
Pseudoxanthomonas.jiangsuensis
2.0
1.5
1.0
0.5
0.0
A (n=15)
B (n=16)
C (n=14)
surface

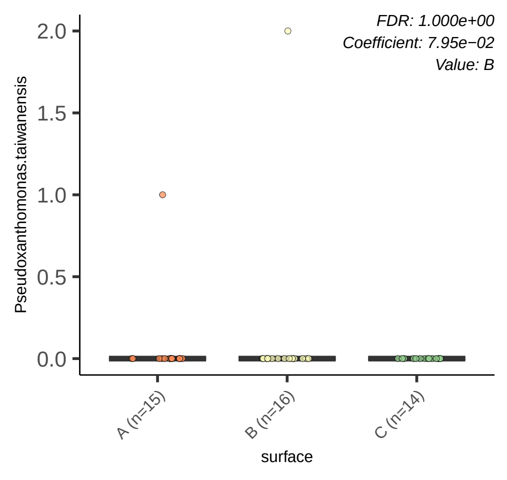

FDR: 1.000e+00
Coefficient: 7.95e−02
Value: B
Pseudoxanthomonas.taiwanensis
2.0
1.5
1.0
0.5
0.0
A (n=15)
B (n=16)
C (n=14)
surface

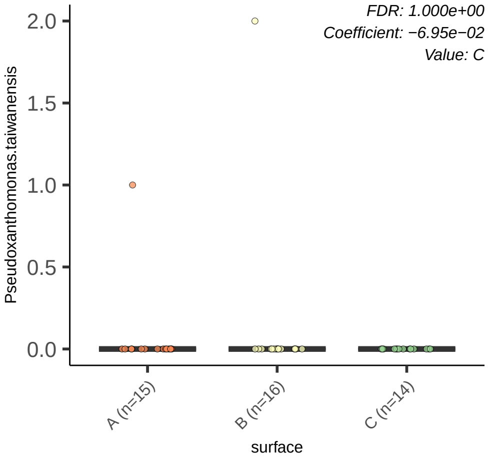
FDR: 1.000e+00
Coefficient: −6.95e−02
Value: C
Pseudoxanthomonas.taiwanensis
2.0
1.5
1.0
0.5
0.0
A (n=15)
B (n=16)
C (n=14)
surface

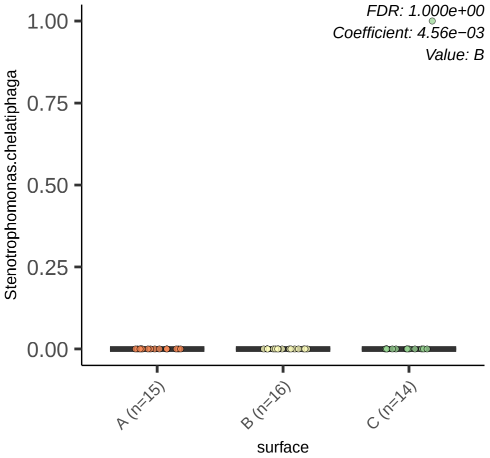

FDR: 1.000e+00
Coefficient: 4.56e−03
Value: B
Stenotrophomonas.chelatiphaga
1.00
0.75
0.50
0.25
0.00
A (n=15)
B (n=16)
C (n=14)
surface

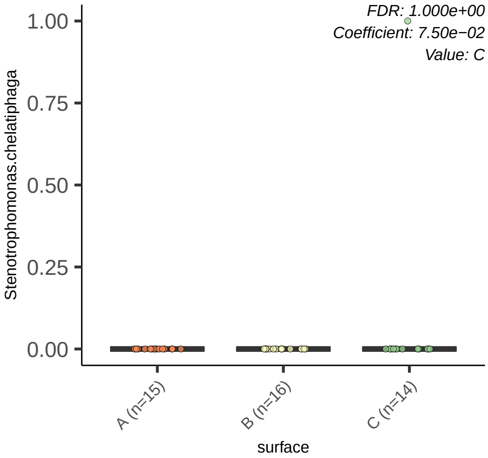
FDR: 1.000e+00
Coefficient: 7.50e−02
Value: C
1.00
0.75
0.50
0.25
0.00
Stenotrophomonas.chelatiphaga
A (n=15)
B (n=16)
C (n=14)
surface

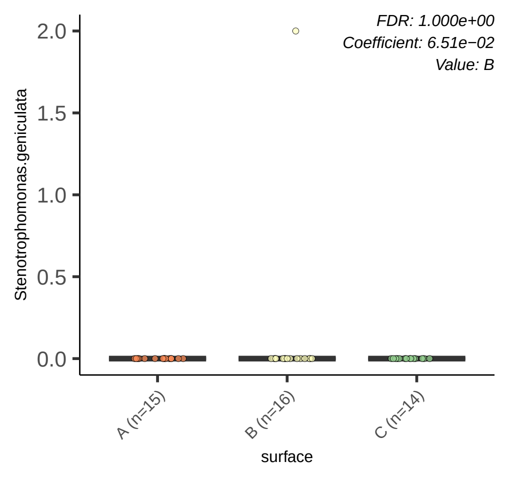
FDR: 1.000e+00
Coefficient: 6.51e−02
Value: B
Stenotrophomonas.geniculata
2.0
1.5
1.0
0.5
0.0
A (n=15)
B (n=16)
C (n=14)
surface

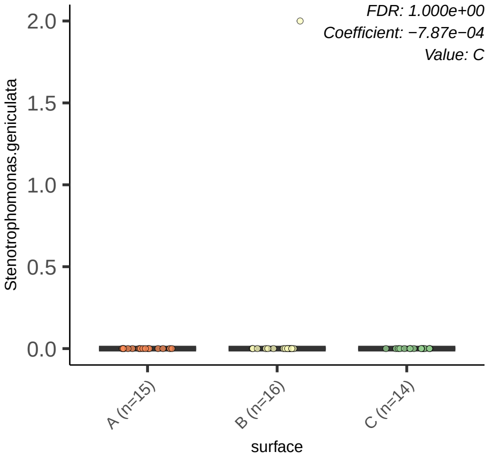

FDR: 1.000e+00
Coefficient: −7.87e−04
Value: C
Stenotrophomonas.geniculata
2.0
1.5
1.0
0.5
0.0
A (n=15)
B (n=16)
C (n=14)
surface

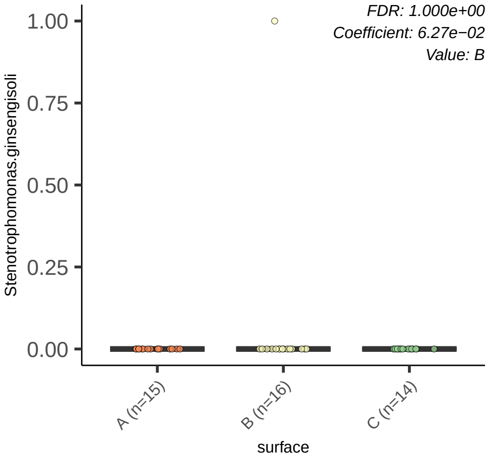

FDR: 1.000e+00
Coefficient: 6.27e−02
Value: B
Stenotrophomonas.ginsengisoli
1.00
0.75
0.50
0.25
0.00
A (n=15)
B (n=16)
C (n=14)
surface

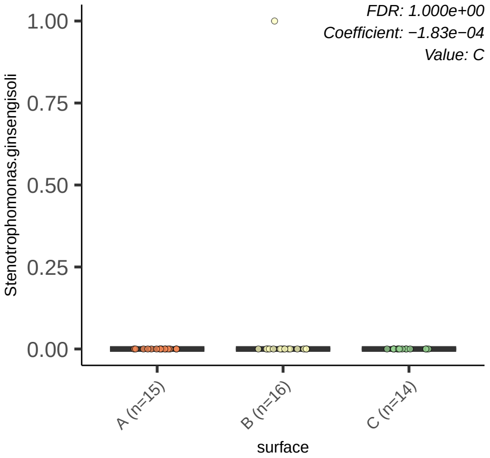
FDR: 1.000e+00
Coefficient: −1.83e−04
Value: C
1.00
0.75
0.50
0.25
0.00
Stenotrophomonas.ginsengisoli
A (n=15)
B (n=16)
C (n=14)
surface

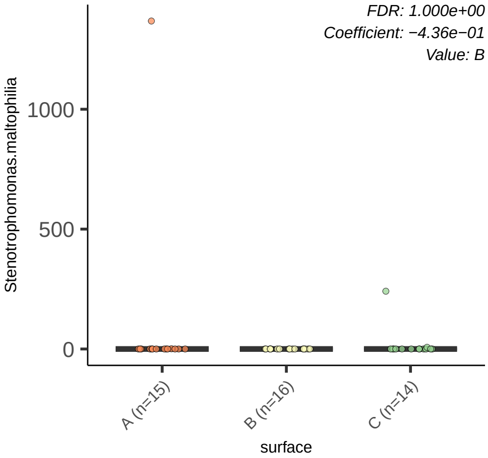
FDR: 1.000e+00
Coefficient: −4.36e−01
Value: B
Stenotrophomonas.maltophilia
1000
500
0
A (n=15)
B (n=16)
C (n=14)
surface

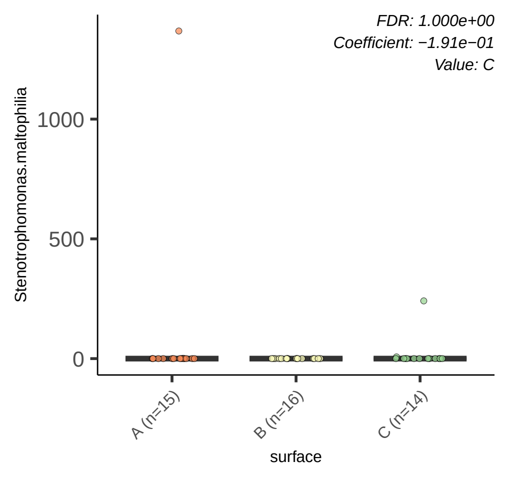

FDR: 1.000e+00
Coefficient: −1.91e−01
Value: C
Stenotrophomonas.maltophilia
1000
500
0
A (n=15)
B (n=16)
C (n=14)
surface

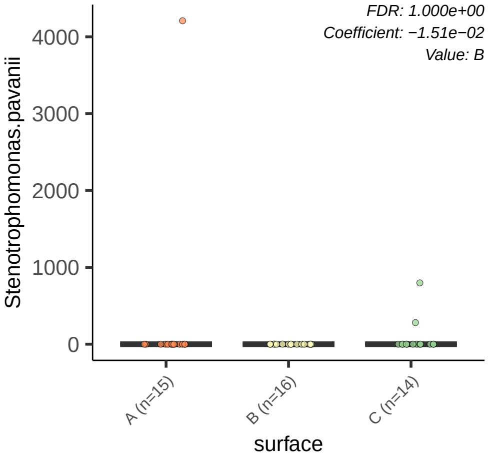

FDR: 1.000e+00
Coefficient: −1.51e−02
Value: B
Stenotrophomonas.pavanii
4000
3000
2000
1000
0
A (n=15)
B (n=16)
C (n=14)
surface

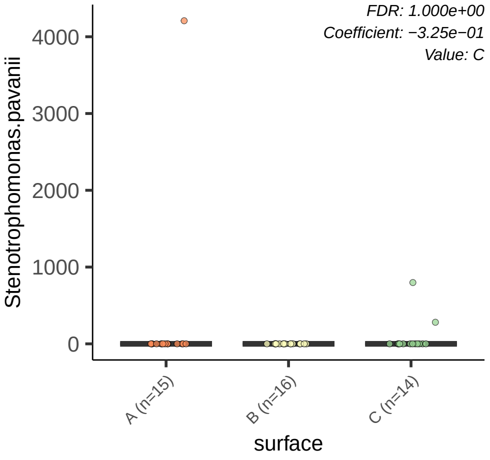

FDR: 1.000e+00
Coefficient: −3.25e−01
Value: C
Stenotrophomonas.pavanii
4000
3000
2000
1000
0
A (n=15)
B (n=16)
C (n=14)
surface

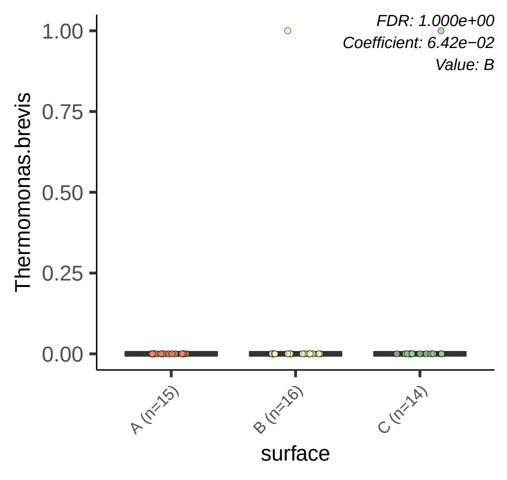

FDR: 1.000e+00
Coefficient: 6.42e−02
Value: B
Thermomonas.brevis
1.00
0.75
0.50
0.25
0.00
A (n=15)
B (n=16)
C (n=14)
surface

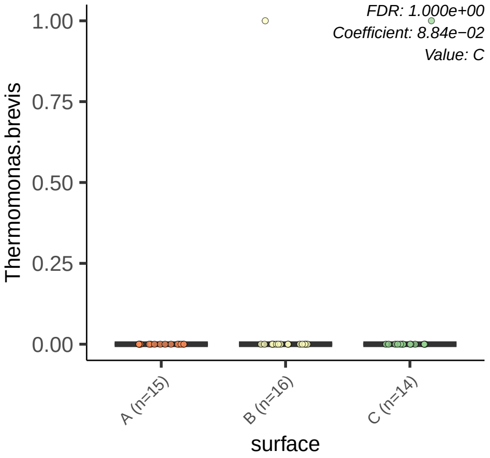

FDR: 1.000e+00
Coefficient: 8.84e−02
Value: C
Thermomonas.brevis
1.00
0.75
0.50
0.25
0.00
A (n=15)
B (n=16)
C (n=14)
surface

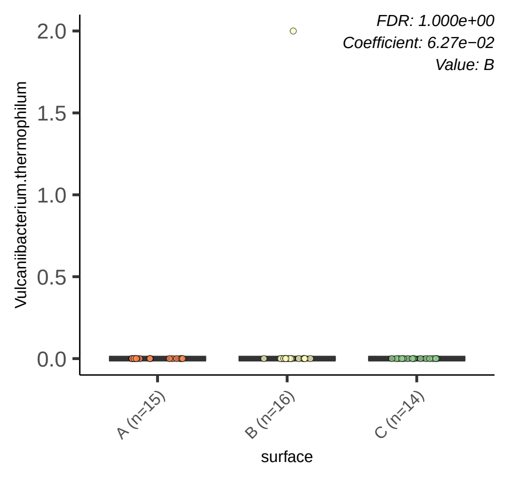

FDR: 1.000e+00
Coefficient: 6.27e−02
Value: B
Vulcaniibacterium.thermophilum
2.0
1.5
1.0
0.5
0.0
A (n=15)
B (n=16)
C (n=14)
surface

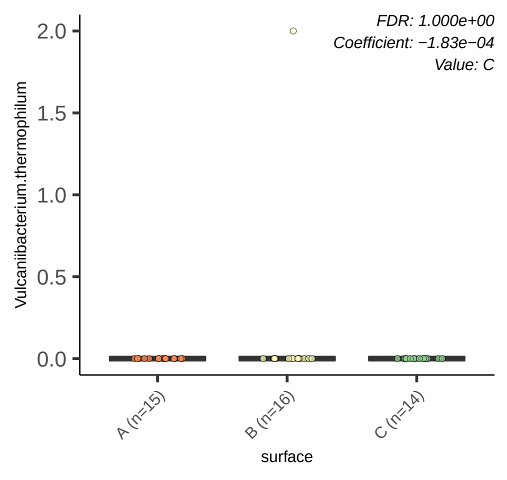

FDR: 1.000e+00
Coefficient: −1.83e−04
Value: C
Vulcaniibacterium.thermophilum
2.0
1.5
1.0
0.5
0.0
A (n=15)
B (n=16)
C (n=14)
surface

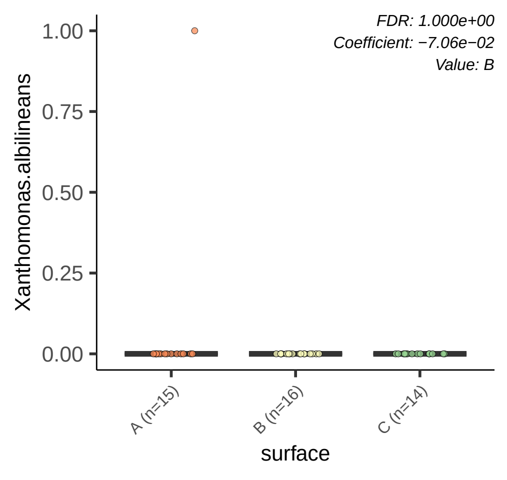
FDR: 1.000e+00
Coefficient: −7.06e−02
Value: B
Xanthomonas.albilineans
1.00
0.75
0.50
0.25
0.00
A (n=15)
B (n=16)
C (n=14)
surface

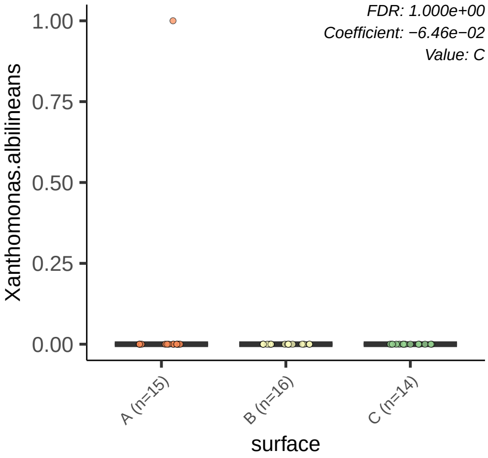

FDR: 1.000e+00
Coefficient: −6.46e−02
Value: C
Xanthomonas.albilineans
1.00
0.75
0.50
0.25
0.00
A (n=15)
B (n=16)
C (n=14)
surface

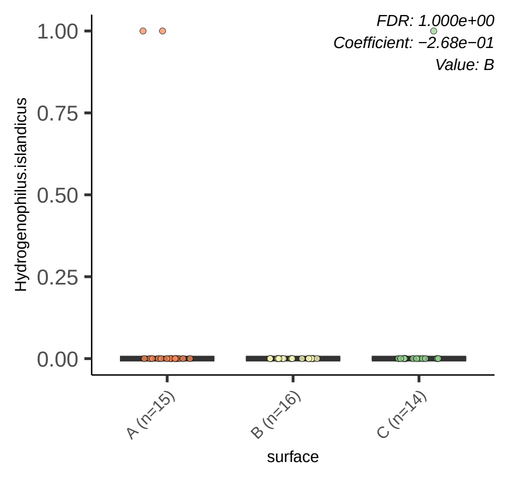

FDR: 1.000e+00
Coefficient: −2.68e−01
Value: B
1.00
0.75
0.50
0.25
0.00
Hydrogenophilus.islandicus
A (n=15)
B (n=16)
C (n=14)
surface

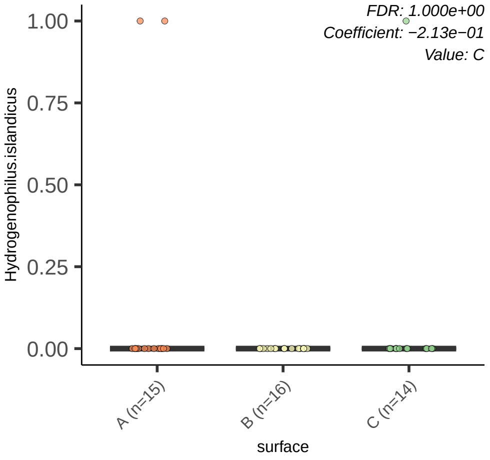

FDR: 1.000e+00
Coefficient: −2.13e−01
Value: C
Hydrogenophilus.islandicus
1.00
0.75
0.50
0.25
0.00
A (n=15)
B (n=16)
C (n=14)
surface

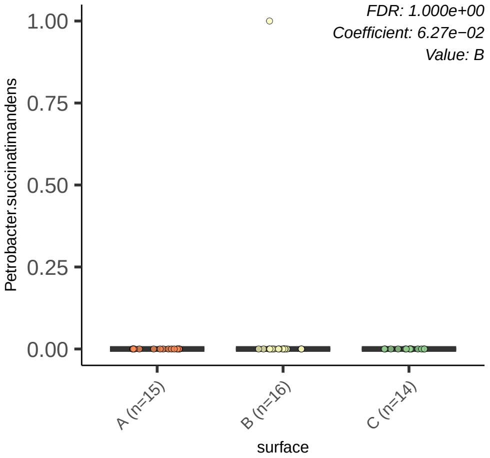

FDR: 1.000e+00
Coefficient: 6.27e−02
Value: B
1.00
0.75
0.50
0.25
0.00
Petrobacter.succinatimandens
A (n=15)
B (n=16)
C (n=14)
surface

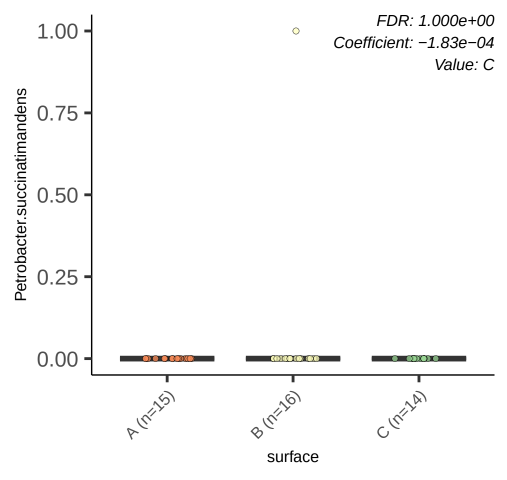
FDR: 1.000e+00
Coefficient: −1.83e−04
Value: C
1.00
0.75
0.50
0.25
0.00
Petrobacter.succinatimandens
A (n=15)
B (n=16)
C (n=14)
surface

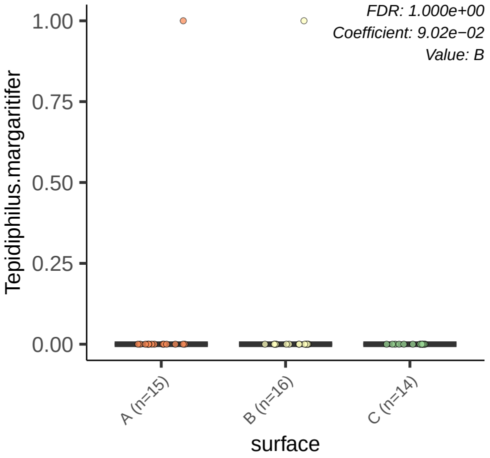
FDR: 1.000e+00
Coefficient: 9.02e−02
Value: B
Tepidiphilus.margaritifer
1.00
0.75
0.50
0.25
0.00
A (n=15)
B (n=16)
C (n=14)
surface

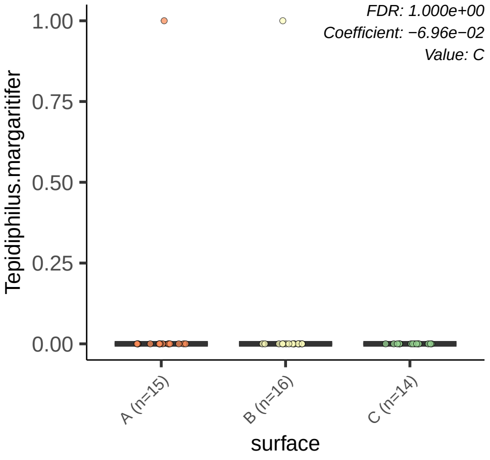
FDR: 1.000e+00
Coefficient: −6.96e−02
Value: C
Tepidiphilus.margaritifer
1.00
0.75
0.50
0.25
0.00
A (n=15)
B (n=16)
C (n=14)
surface

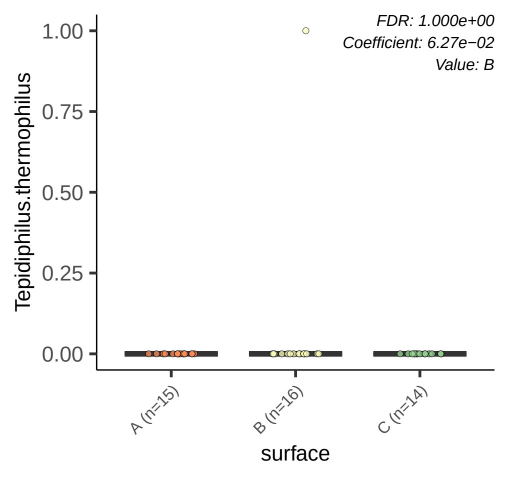
FDR: 1.000e+00
Coefficient: 6.27e−02
Value: B
Tepidiphilus.thermophilus
1.00
0.75
0.50
0.25
0.00
A (n=15)
B (n=16)
C (n=14)
surface

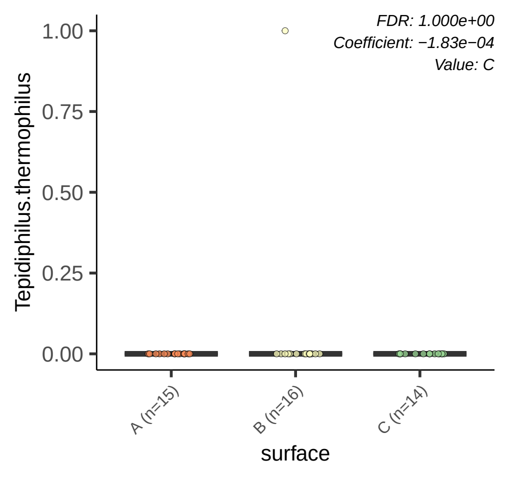

FDR: 1.000e+00
Coefficient: −1.83e−04
Value: C
Tepidiphilus.thermophilus
1.00
0.75
0.50
0.25
0.00
A (n=15)
B (n=16)
C (n=14)
surface

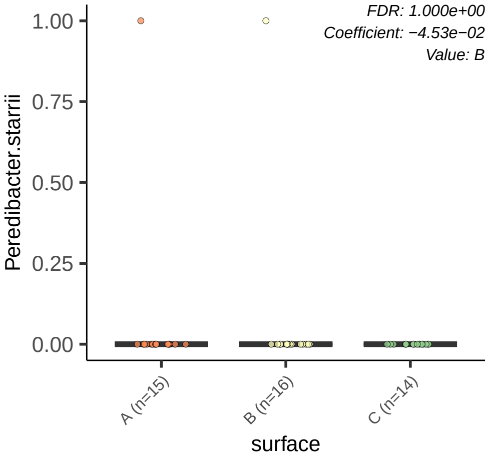

FDR: 1.000e+00
Coefficient: −4.53e−02
Value: B
Peredibacter.starrii
1.00
0.75
0.50
0.25
0.00
A (n=15)
B (n=16)
C (n=14)
surface

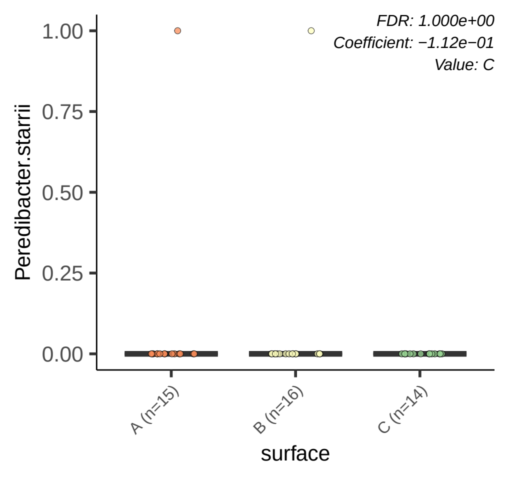
FDR: 1.000e+00
Coefficient: −1.12e−01
Value: C
1.00
0.75
0.50
0.25
0.00
Peredibacter.starrii
A (n=15)
B (n=16)
C (n=14)
surface

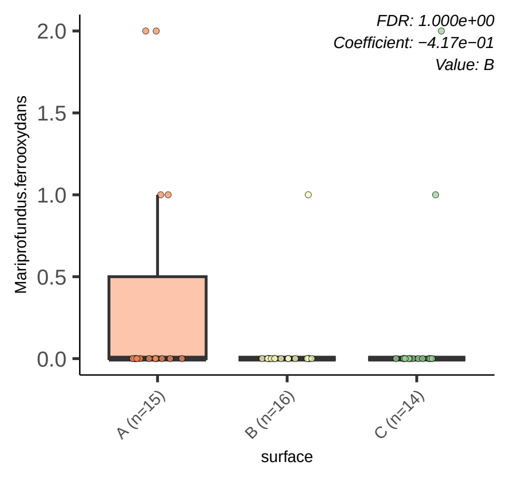
FDR: 1.000e+00
Coefficient: −4.17e−01
Value: B
Mariprofundus.ferrooxydans
2.0
1.5
1.0
0.5
0.0
A (n=15)
B (n=16)
C (n=14)
surface

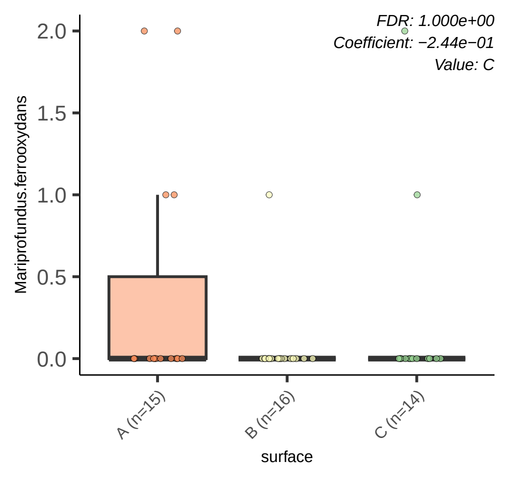
FDR: 1.000e+00
Coefficient: −2.44e−01
Value: C
Mariprofundus.ferrooxydans
2.0
1.5
1.0
0.5
0.0
A (n=15)
B (n=16)
C (n=14)
surface

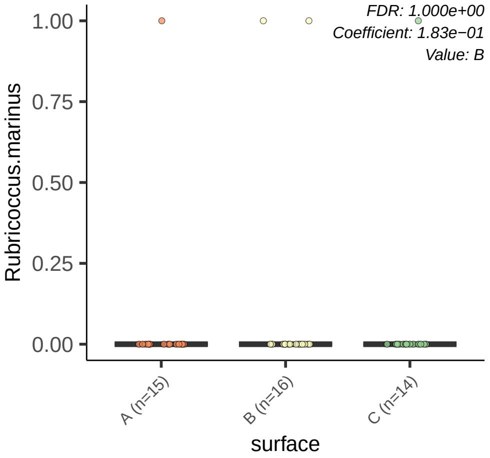
FDR: 1.000e+00
Coefficient: 1.83e−01
Value: B
Rubricoccus.marinus
1.00
0.75
0.50
0.25
0.00
A (n=15)
B (n=16)
C (n=14)
surface

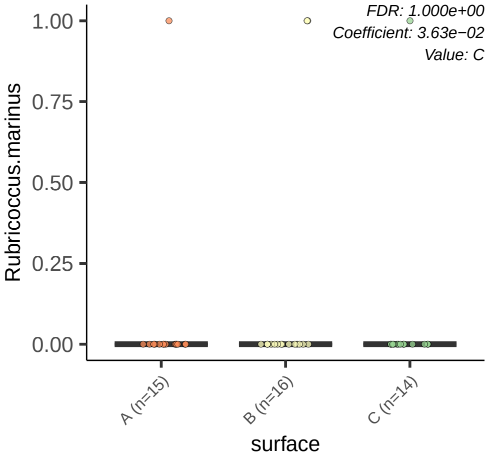

FDR: 1.000e+00
Coefficient: 3.63e−02
Value: C
Rubricoccus.marinus
1.00
0.75
0.50
0.25
0.00
A (n=15)
B (n=16)
C (n=14)
surface

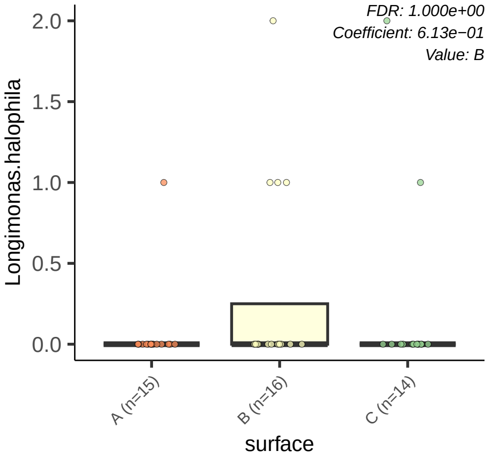
FDR: 1.000e+00
Coefficient: 6.13e−01
Value: B
Longimonas.halophila
2.0
1.5
1.0
0.5
0.0
A (n=15)
B (n=16)
C (n=14)
surface

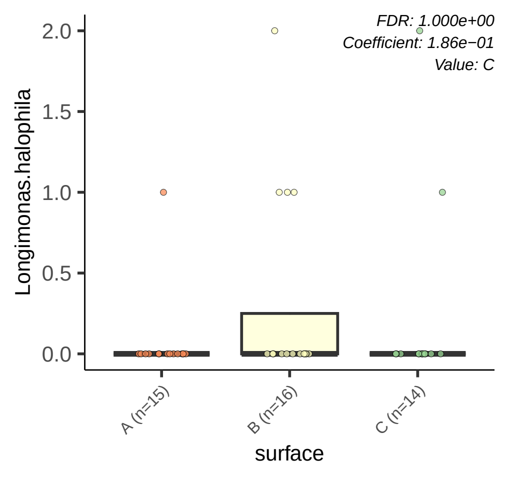

FDR: 1.000e+00
Coefficient: 1.86e−01
Value: C
Longimonas.halophila
2.0
1.5
1.0
0.5
0.0
A (n=15)
B (n=16)
C (n=14)
surface

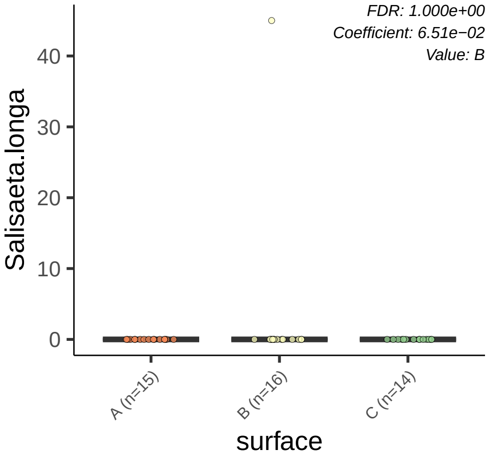

FDR: 1.000e+00
Coefficient: 6.51e−02
Value: B
Salisaeta.longa
40
30
20
10
0
A (n=15)
B (n=16)
C (n=14)
surface

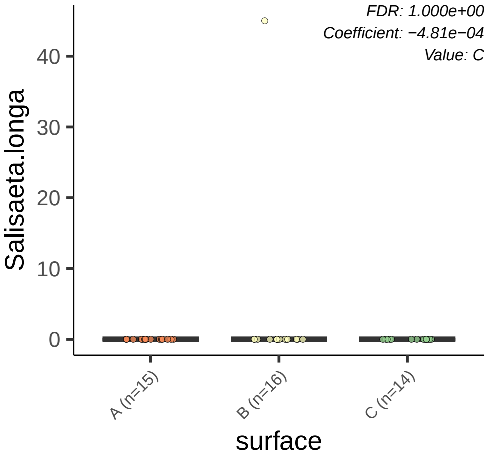
FDR: 1.000e+00
Coefficient: −4.81e−04
Value: C
Salisaeta.longa
0
10
20
30
40
A (n=15)
B (n=16)
C (n=14)
surface

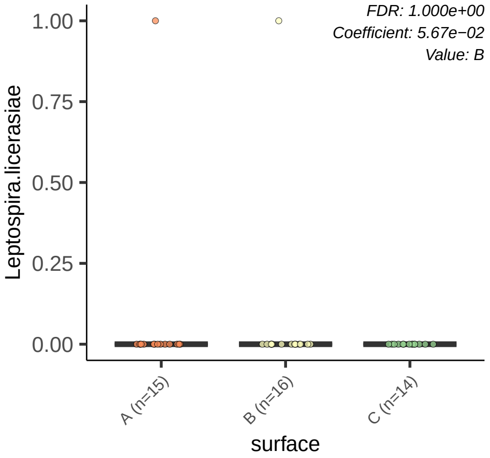

FDR: 1.000e+00
Coefficient: 5.67e−02
Value: B
Leptospira.licerasiae
1.00
0.75
0.50
0.25
0.00
A (n=15)
B (n=16)
C (n=14)
surface

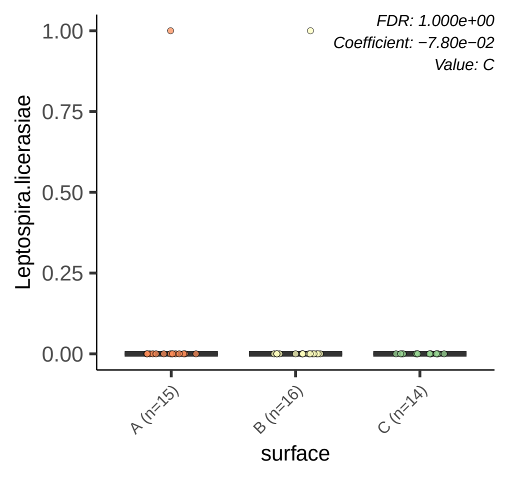

FDR: 1.000e+00
Coefficient: −7.80e−02
Value: C
Leptospira.licerasiae
1.00
0.75
0.50
0.25
0.00
A (n=15)
B (n=16)
C (n=14)
surface

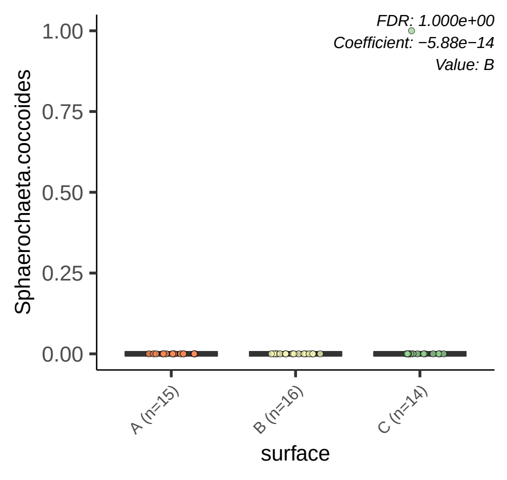

FDR: 1.000e+00
Coefficient: −5.88e−14
Value: B
Sphaerochaeta.coccoides
1.00
0.75
0.50
0.25
0.00
A (n=15)
B (n=16)
C (n=14)
surface

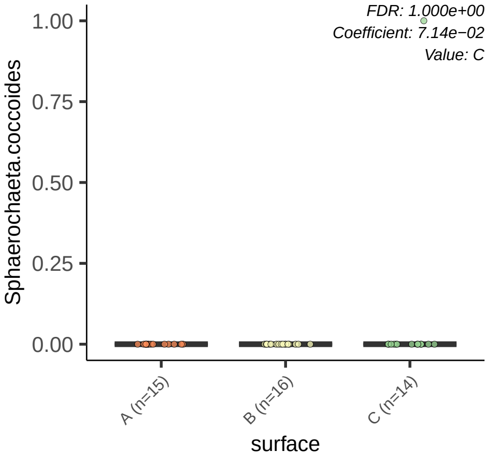
FDR: 1.000e+00
Coefficient: 7.14e−02
Value: C
Sphaerochaeta.coccoides
1.00
0.75
0.50
0.25
0.00
A (n=15)
B (n=16)
C (n=14)
surface

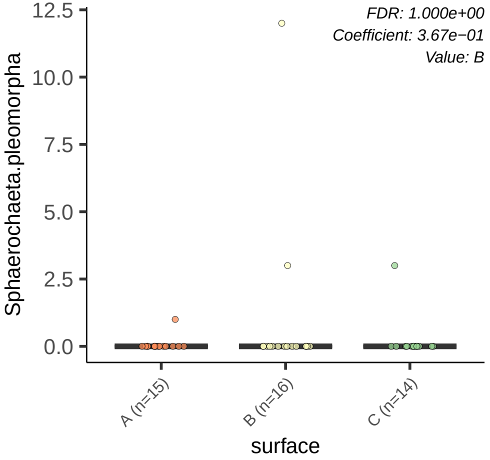

FDR: 1.000e+00
Coefficient: 3.67e−01
Value: B
Sphaerochaeta.pleomorpha
12.5
10.0
7.5
5.0
2.5
0.0
A (n=15)
B (n=16)
C (n=14)
surface

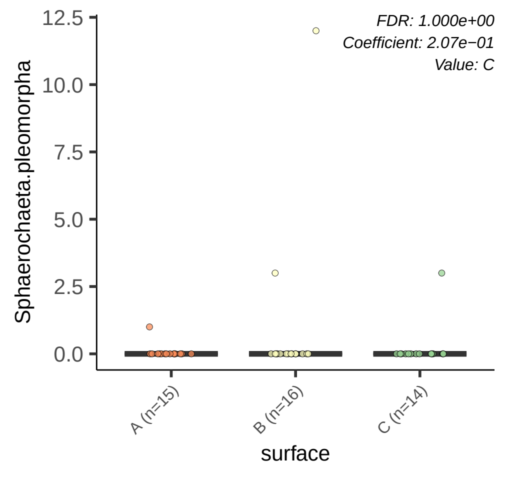

FDR: 1.000e+00
Coefficient: 2.07e−01
Value: C
Sphaerochaeta.pleomorpha
12.5
10.0
7.5
5.0
2.5
0.0
A (n=15)
B (n=16)
C (n=14)
surface

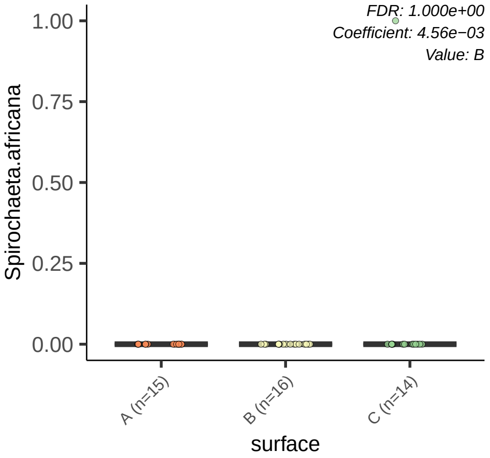

FDR: 1.000e+00
Coefficient: 4.56e−03
Value: B
Spirochaeta.africana
1.00
0.75
0.50
0.25
0.00
A (n=15)
B (n=16)
C (n=14)
surface

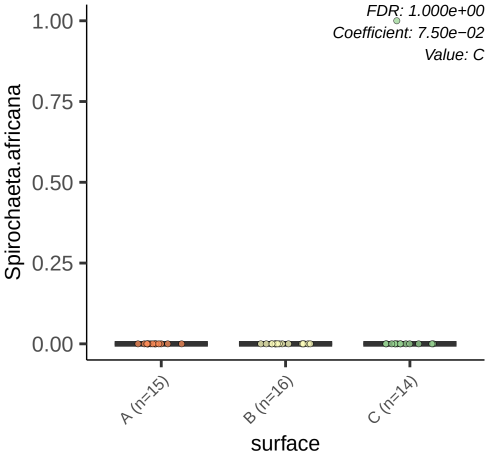
FDR: 1.000e+00
Coefficient: 7.50e−02
Value: C
Spirochaeta.africana
1.00
0.75
0.50
0.25
0.00
A (n=15)
B (n=16)
C (n=14)
surface

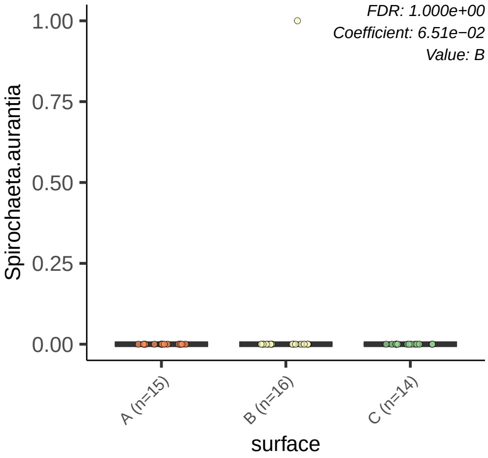
FDR: 1.000e+00
Coefficient: 6.51e−02
Value: B
Spirochaeta.aurantia
1.00
0.75
0.50
0.25
0.00
A (n=15)
B (n=16)
C (n=14)
surface

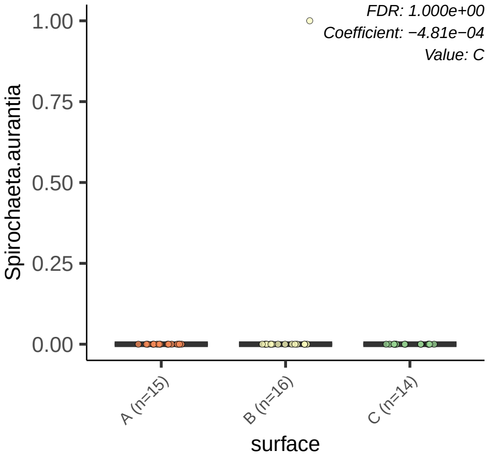
FDR: 1.000e+00
Coefficient: −4.81e−04
Value: C
Spirochaeta.aurantia
1.00
0.75
0.50
0.25
0.00
A (n=15)
B (n=16)
C (n=14)
surface

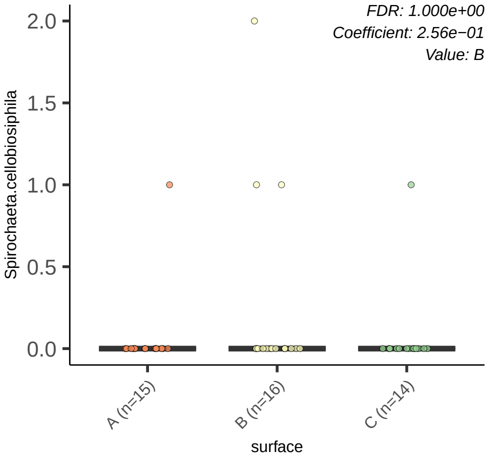
FDR: 1.000e+00
Coefficient: 2.56e−01
Value: B
Spirochaeta.cellobiosiphila
2.0
1.5
1.0
0.5
0.0
A (n=15)
B (n=16)
C (n=14)
surface

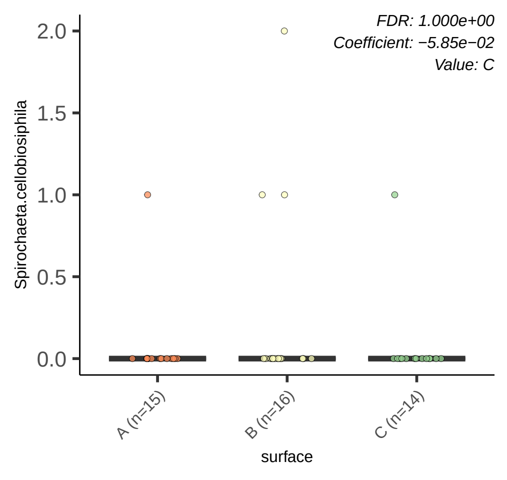

FDR: 1.000e+00
Coefficient: −5.85e−02
Value: C
Spirochaeta.cellobiosiphila
2.0
1.5
1.0
0.5
0.0
A (n=15)
B (n=16)
C (n=14)
surface

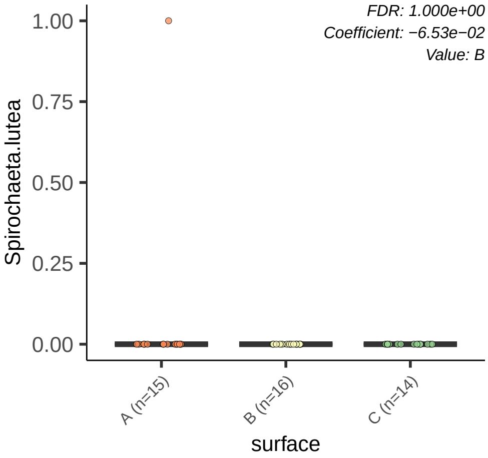

FDR: 1.000e+00
Coefficient: −6.53e−02
Value: B
Spirochaeta.lutea
1.00
0.75
0.50
0.25
0.00
A (n=15)
B (n=16)
C (n=14)
surface

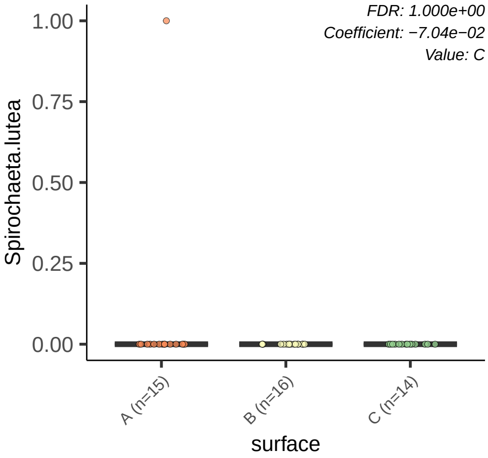
FDR: 1.000e+00
Coefficient: −7.04e−02
Value: C
Spirochaeta.lutea
1.00
0.75
0.50
0.25
0.00
A (n=15)
B (n=16)
C (n=14)
surface

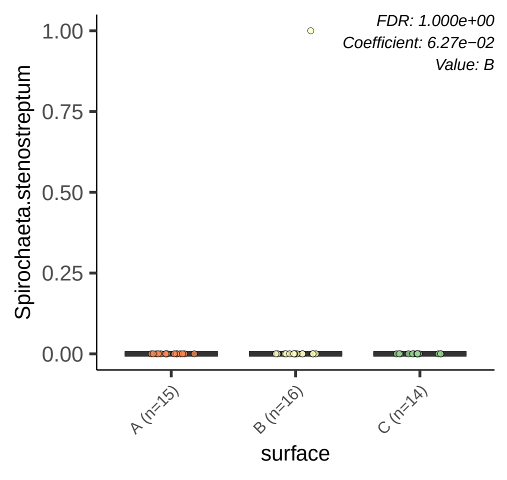

FDR: 1.000e+00
Coefficient: 6.27e−02
Value: B
Spirochaeta.stenostreptum
1.00
0.75
0.50
0.25
0.00
A (n=15)
B (n=16)
C (n=14)
surface

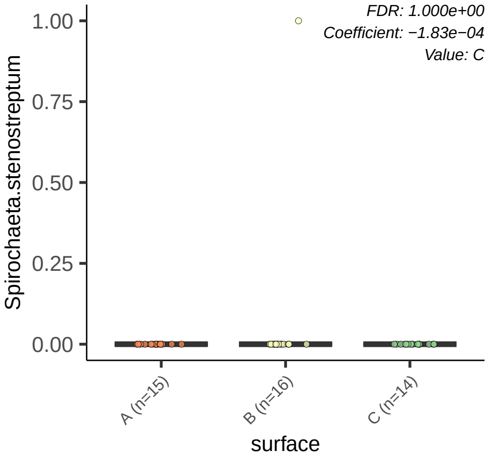

FDR: 1.000e+00
Coefficient: −1.83e−04
Value: C
Spirochaeta.stenostreptum
1.00
0.75
0.50
0.25
0.00
A (n=15)
B (n=16)
C (n=14)
surface

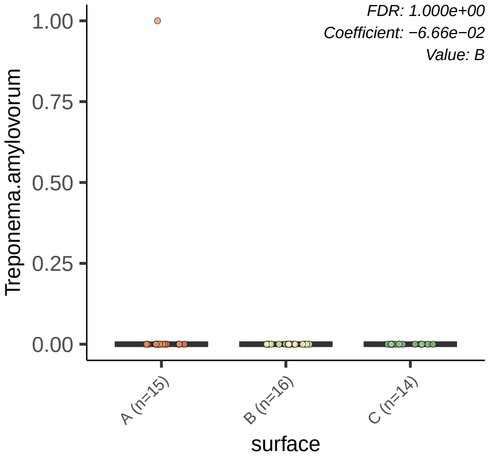

FDR: 1.000e+00
Coefficient: −6.66e−02
Value: B
Treponema.amylovorum
1.00
0.75
0.50
0.25
0.00
A (n=15)
B (n=16)
C (n=14)
surface

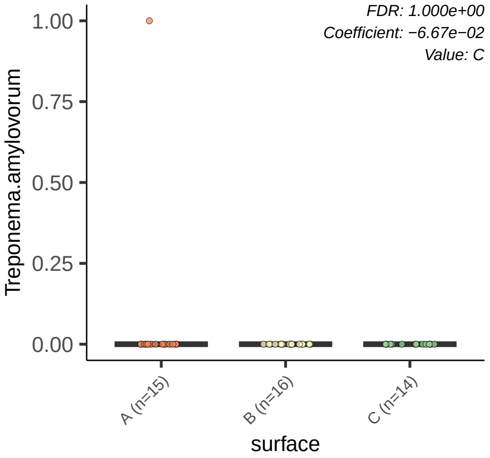
FDR: 1.000e+00
Coefficient: −6.67e−02
Value: C
Treponema.amylovorum
1.00
0.75
0.50
0.25
0.00
A (n=15)
B (n=16)
C (n=14)
surface

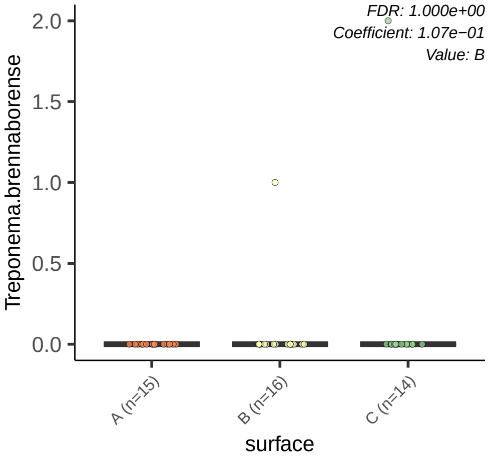

FDR: 1.000e+00
Coefficient: 1.07e−01
Value: B
Treponema.brennaborense
2.0
1.5
1.0
0.5
0.0
A (n=15)
B (n=16)
C (n=14)
surface

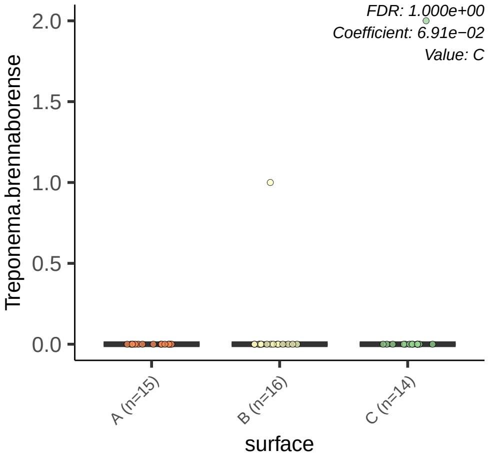

FDR: 1.000e+00
Coefficient: 6.91e−02
Value: C
Treponema.brennaborense
2.0
1.5
1.0
0.5
0.0
A (n=15)
B (n=16)
C (n=14)
surface

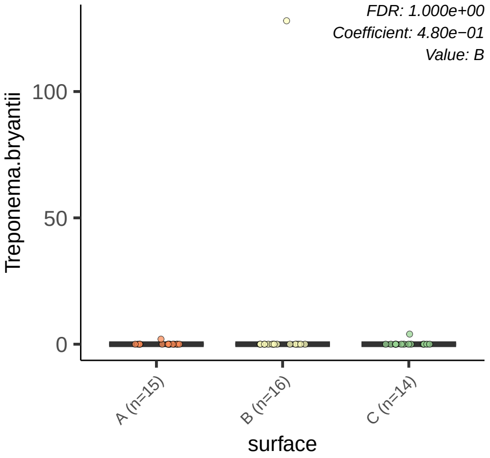
FDR: 1.000e+00
Coefficient: 4.80e−01
Value: B
Treponema.bryantii
100
50
0
A (n=15)
B (n=16)
C (n=14)
surface

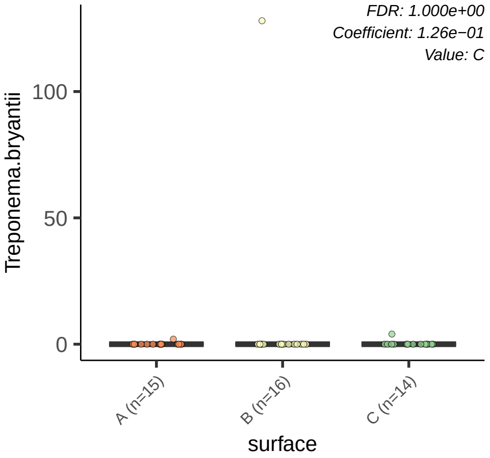
FDR: 1.000e+00
Coefficient: 1.26e−01
Value: C
Treponema.bryantii
100
50
0
A (n=15)
B (n=16)
C (n=14)
surface

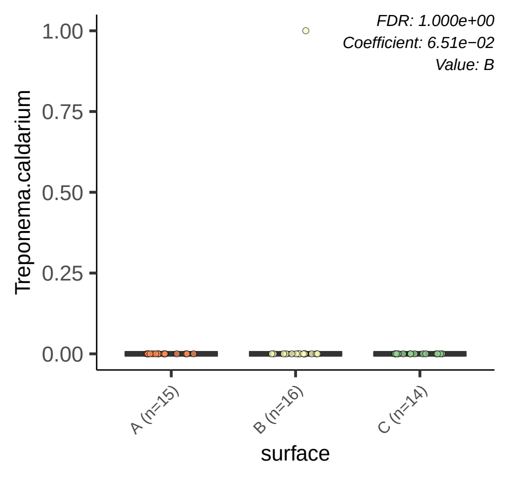

FDR: 1.000e+00
Coefficient: 6.51e−02
Value: B
Treponema.caldarium
1.00
0.75
0.50
0.25
0.00
A (n=15)
B (n=16)
C (n=14)
surface

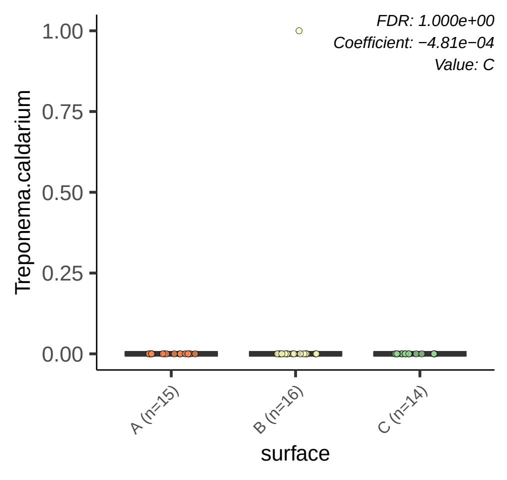
FDR: 1.000e+00
Coefficient: −4.81e−04
Value: C
Treponema.caldarium
1.00
0.75
0.50
0.25
0.00
A (n=15)
B (n=16)
C (n=14)
surface

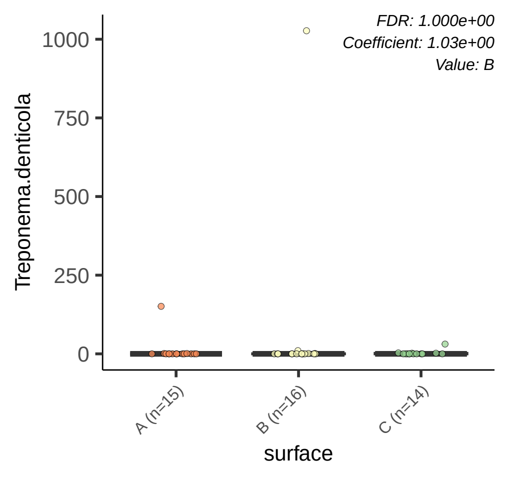

FDR: 1.000e+00
Coefficient: 1.03e+00
Value: B
1000
750
500
250
0
Treponema.denticola
A (n=15)
B (n=16)
C (n=14)
surface

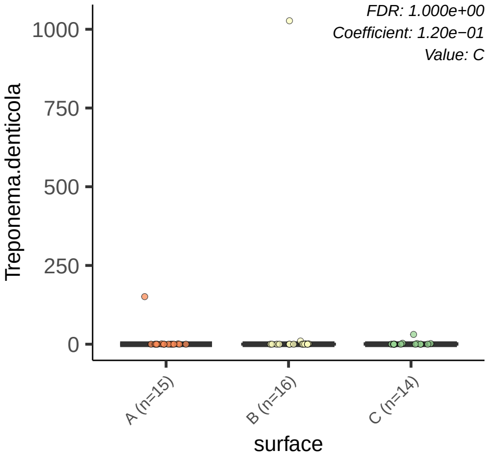

FDR: 1.000e+00
Coefficient: 1.20e−01
Value: C
Treponema.denticola
1000
750
500
250
0
A (n=15)
B (n=16)
C (n=14)
surface

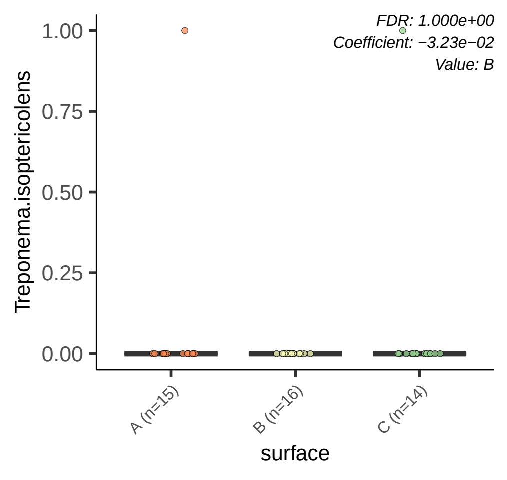

FDR: 1.000e+00
Coefficient: −3.23e−02
Value: B
Treponema.isoptericolens
1.00
0.75
0.50
0.25
0.00
A (n=15)
B (n=16)
C (n=14)
surface

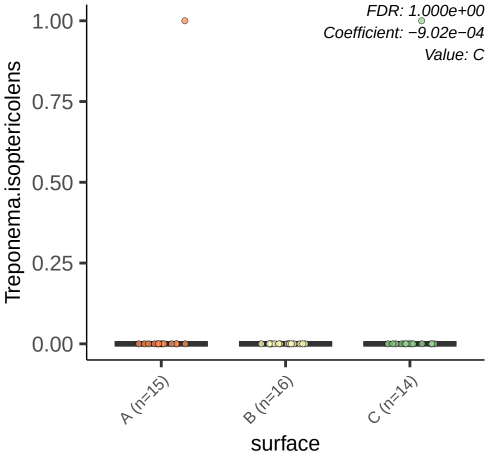

FDR: 1.000e+00
Coefficient: −9.02e−04
Value: C
Treponema.isoptericolens
1.00
0.75
0.50
0.25
0.00
A (n=15)
B (n=16)
C (n=14)
surface

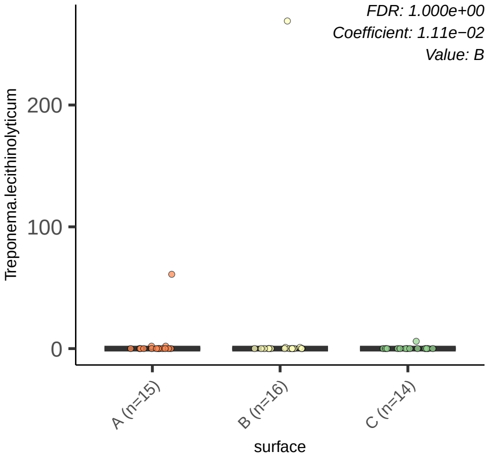

FDR: 1.000e+00
Coefficient: 1.11e−02
Value: B
Treponema.lecithinolyticum
200
100
0
A (n=15)
B (n=16)
C (n=14)
surface

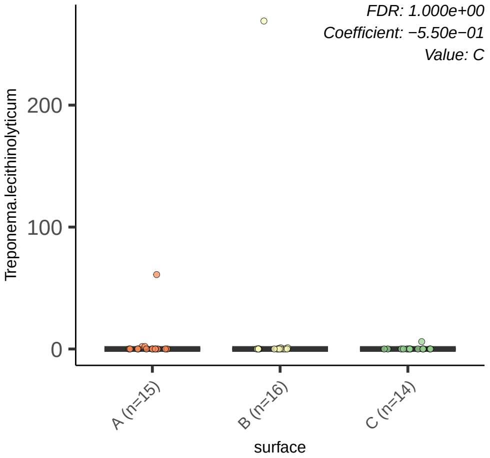
FDR: 1.000e+00
Coefficient: −5.50e−01
Value: C
Treponema.lecithinolyticum
200
100
0
A (n=15)
B (n=16)
C (n=14)
surface

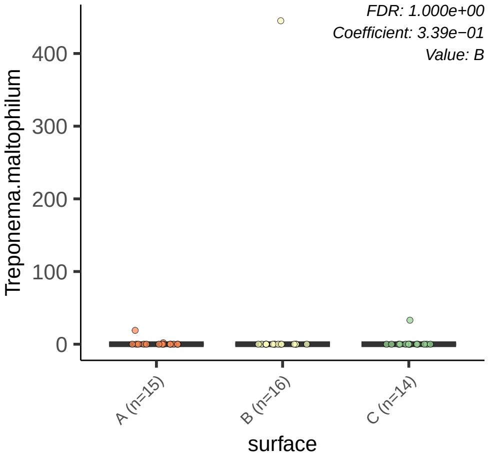
FDR: 1.000e+00
Coefficient: 3.39e−01
Value: B
Treponema.maltophilum
400
300
200
100
0
A (n=15)
B (n=16)
C (n=14)
surface

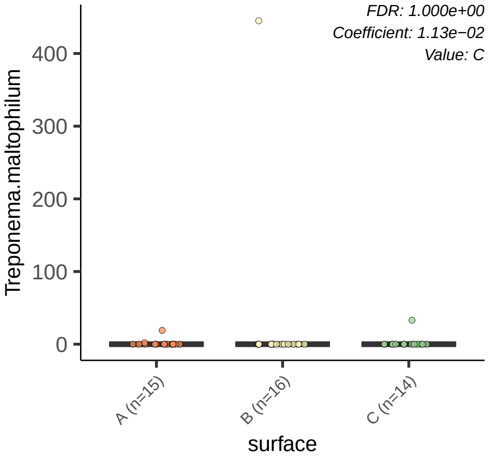
FDR: 1.000e+00
Coefficient: 1.13e−02
Value: C
Treponema.maltophilum
400
300
200
100
0
A (n=15)
B (n=16)
C (n=14)
surface

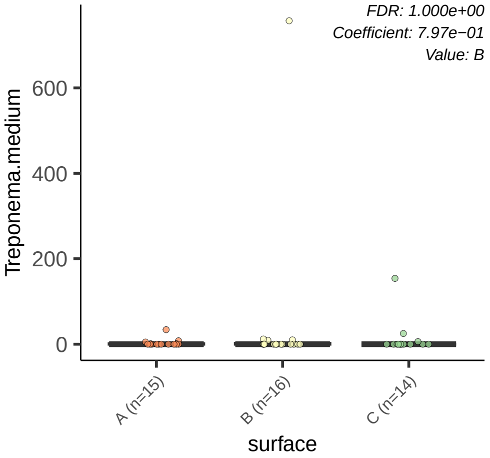

FDR: 1.000e+00
Coefficient: 7.97e−01
Value: B
Treponema.medium
600
400
200
0
A (n=15)
B (n=16)
C (n=14)
surface

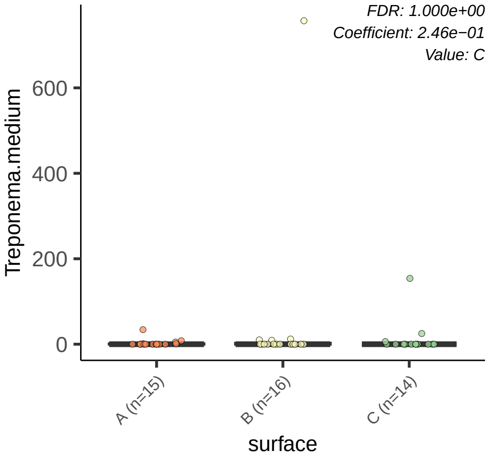

FDR: 1.000e+00
Coefficient: 2.46e−01
Value: C
Treponema.medium
600
400
200
0
A (n=15)
B (n=16)
C (n=14)
surface

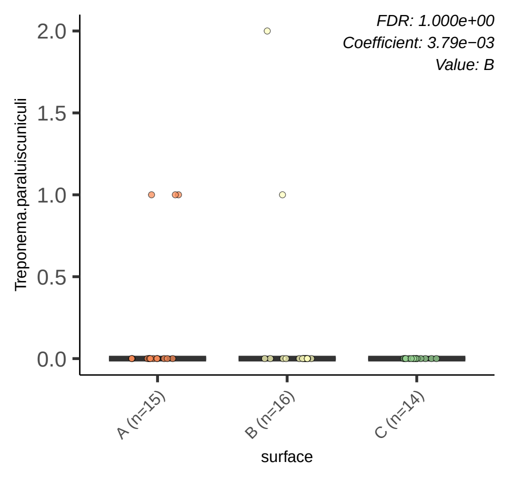
FDR: 1.000e+00
Coefficient: 3.79e−03
Value: B
Treponema.paraluiscuniculi
2.0
1.5
1.0
0.5
0.0
A (n=15)
B (n=16)
C (n=14)
surface

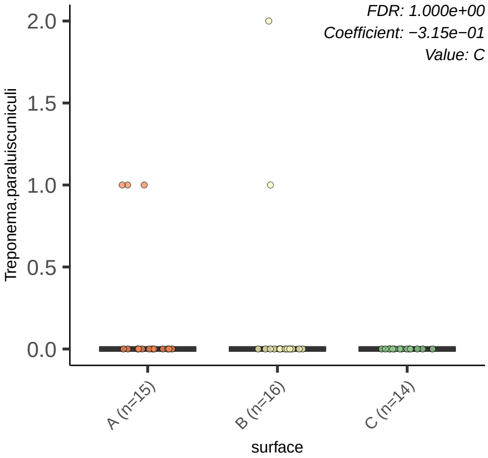

FDR: 1.000e+00
Coefficient: −3.15e−01
Value: C
Treponema.paraluiscuniculi
2.0
1.5
1.0
0.5
0.0
A (n=15)
B (n=16)
C (n=14)
surface

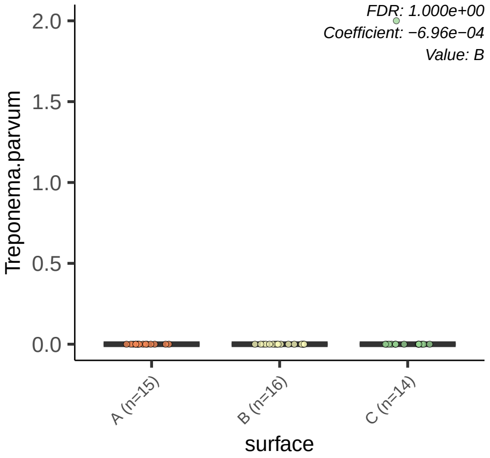
FDR: 1.000e+00
Coefficient: −6.96e−04
Value: B
Treponema.parvum
2.0
1.5
1.0
0.5
0.0
A (n=15)
B (n=16)
C (n=14)
surface

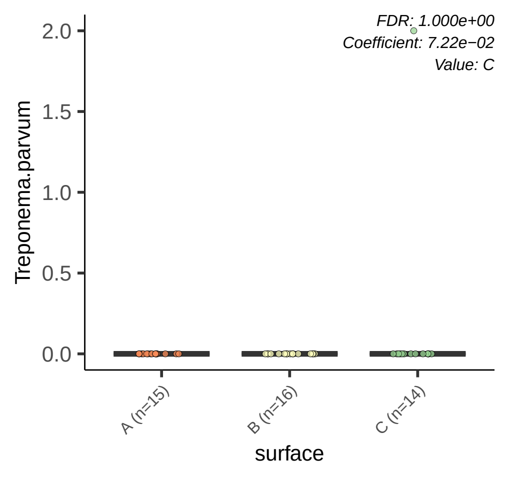
FDR: 1.000e+00
Coefficient: 7.22e−02
Value: C
Treponema.parvum
2.0
1.5
1.0
0.5
0.0
A (n=15)
B (n=16)
C (n=14)
surface

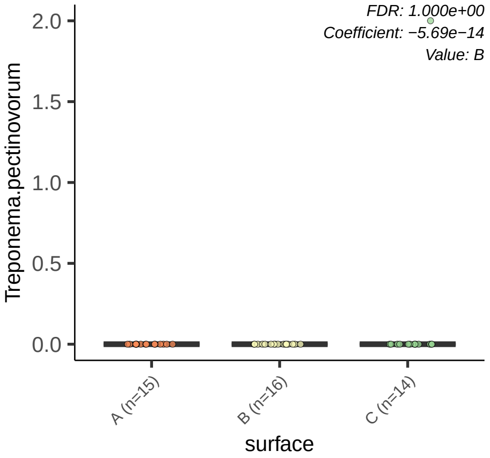
FDR: 1.000e+00
Coefficient: −5.69e−14
Value: B
Treponema.pectinovorum
2.0
1.5
1.0
0.5
0.0
A (n=15)
B (n=16)
C (n=14)
surface

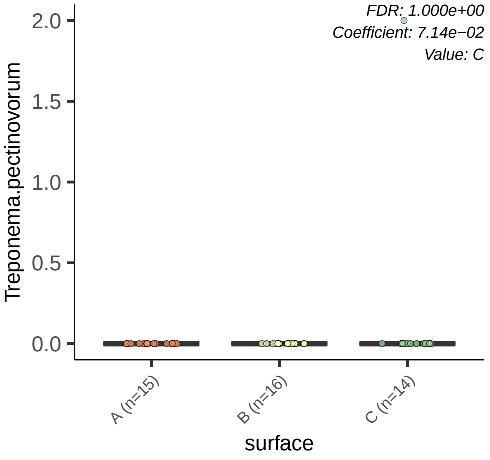

FDR: 1.000e+00
Coefficient: 7.14e−02
Value: C
2.0
1.5
1.0
0.5
0.0
Treponema.pectinovorum
A (n=15)
B (n=16)
C (n=14)
surface

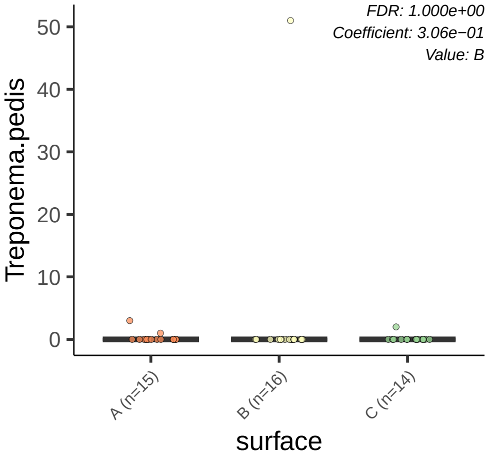

FDR: 1.000e+00
Coefficient: 3.06e−01
Value: B
Treponema.pedis
50
40
30
20
10
0
A (n=15)
B (n=16)
C (n=14)
surface

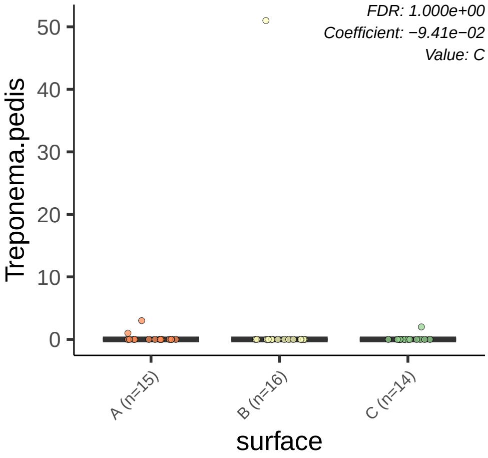
FDR: 1.000e+00
Coefficient: −9.41e−02
Value: C
Treponema.pedis
50
40
30
20
10
0
A (n=15)
B (n=16)
C (n=14)
surface

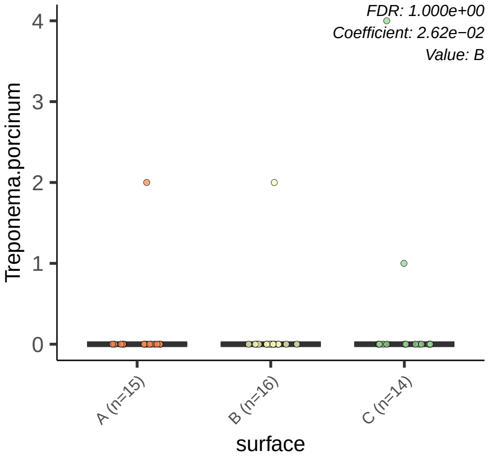

FDR: 1.000e+00
Coefficient: 2.62e−02
Value: B
Treponema.porcinum
0
1
2
3
4
A (n=15)
B (n=16)
C (n=14)
surface

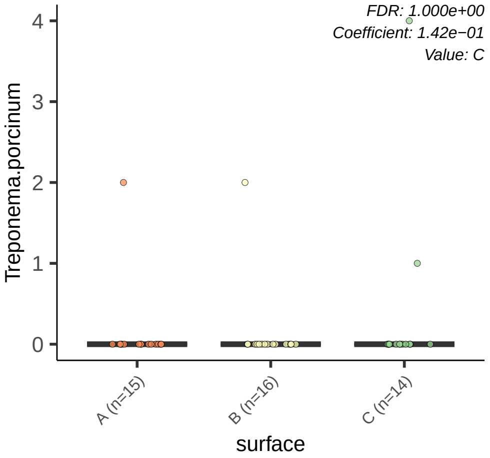
FDR: 1.000e+00
Coefficient: 1.42e−01
Value: C
Treponema.porcinum
0
1
2
3
4
A (n=15)
B (n=16)
C (n=14)
surface

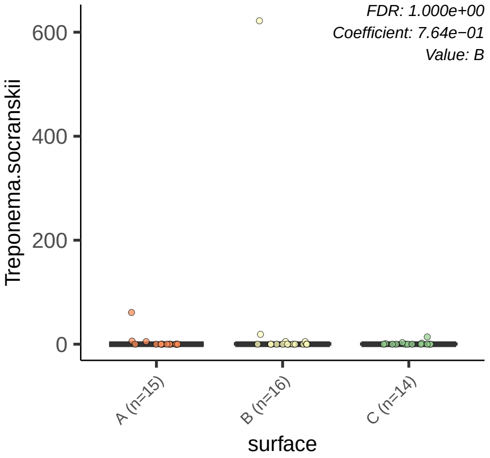

FDR: 1.000e+00
Coefficient: 7.64e−01
Value: B
600
400
200
0
Treponema.socranskii
A (n=15)
B (n=16)
C (n=14)
surface

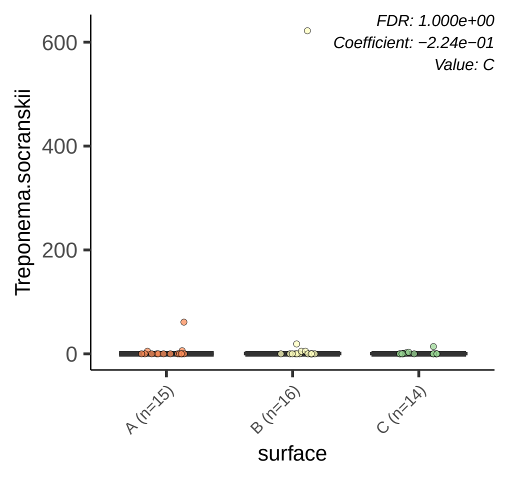
FDR: 1.000e+00
Coefficient: −2.24e−01
Value: C
Treponema.socranskii
600
400
200
0
A (n=15)
B (n=16)
C (n=14)
surface

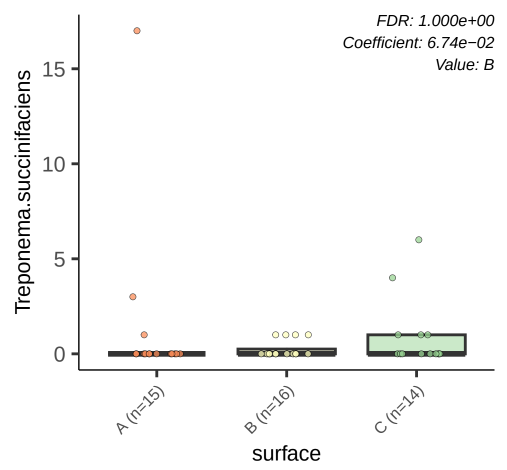
FDR: 1.000e+00
Coefficient: 6.74e−02
Value: B
Treponema.succinifaciens
0
5
10
15
A (n=15)
B (n=16)
C (n=14)
surface

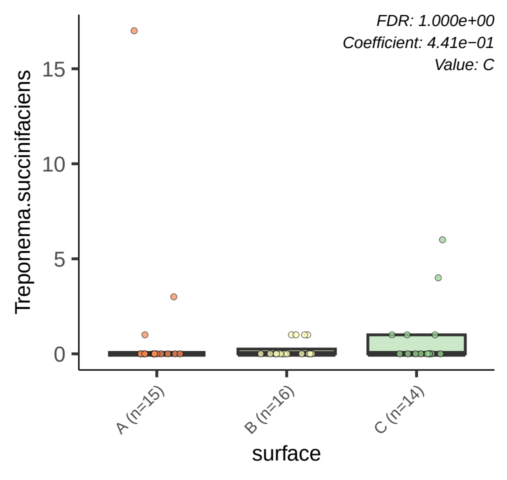
FDR: 1.000e+00
Coefficient: 4.41e−01
Value: C
Treponema.succinifaciens
15
10
5
0
A (n=15)
B (n=16)
C (n=14)
surface

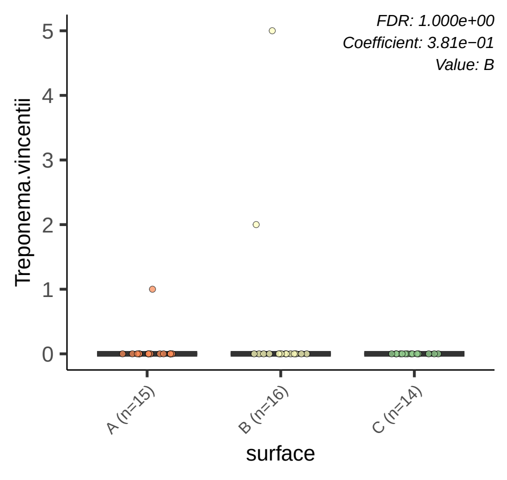

FDR: 1.000e+00
Coefficient: 3.81e−01
Value: B
Treponema.vincentii
0
1
2
3
4
5
A (n=15)
B (n=16)
C (n=14)
surface

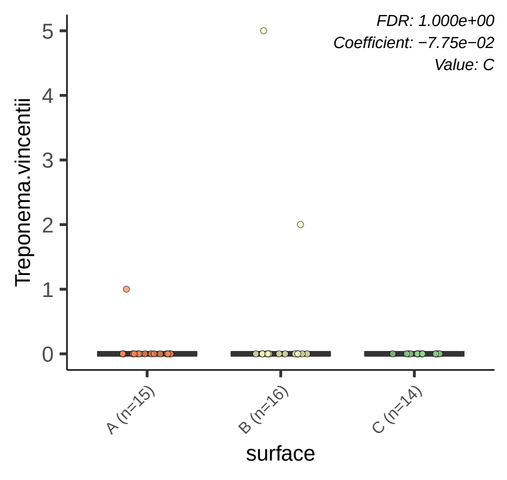
FDR: 1.000e+00
Coefficient: −7.75e−02
Value: C
Treponema.vincentii
5
4
3
2
1
0
A (n=15)
B (n=16)
C (n=14)
surface

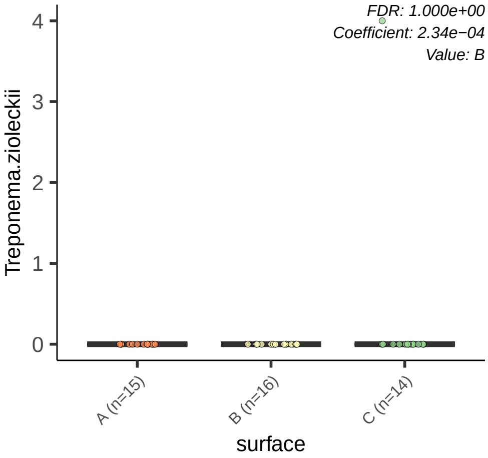
FDR: 1.000e+00
Coefficient: 2.34e−04
Value: B
Treponema.ziolecki
0
1
2
3
4
A (n=15)
B (n=16)
C (n=14)
surface

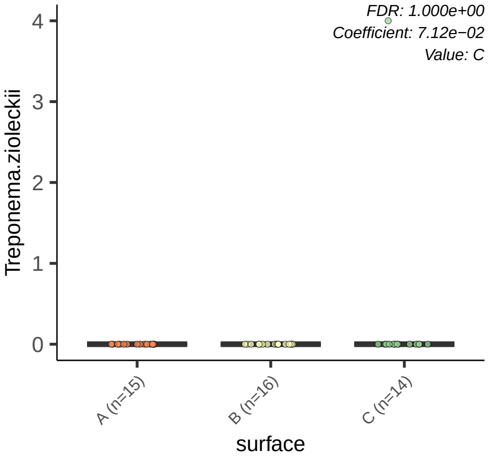

FDR: 1.000e+00
Coefficient: 7.12e−02
Value: C
Treponema.zioleckii
4
3
2
1
0
A (n=15)
B (n=16)
C (n=14)
surface

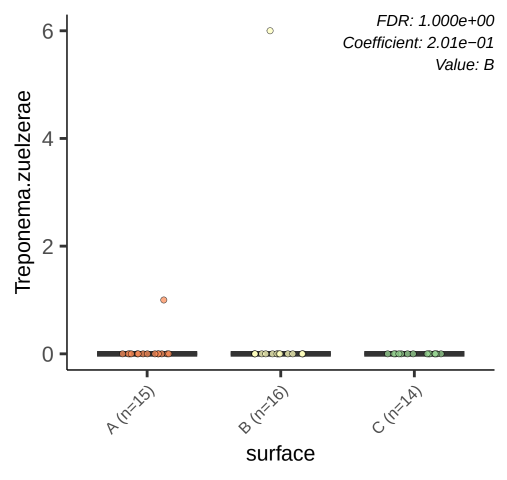

FDR: 1.000e+00
Coefficient: 2.01e−01
Value: B
Treponema.zuelzerae
6
4
2
0
A (n=15)
B (n=16)
C (n=14)
surface

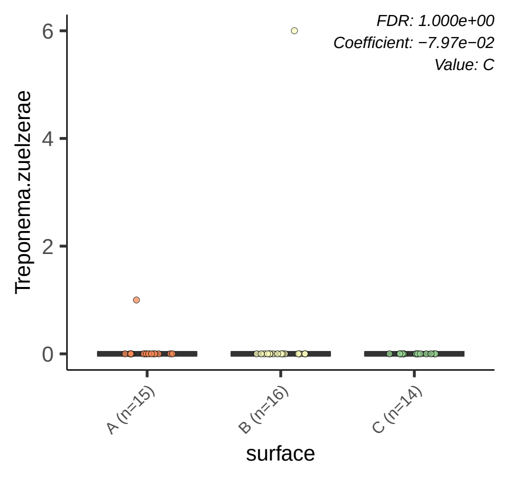
FDR: 1.000e+00
Coefficient: −7.97e−02
Value: C
Treponema.zuelzerae
6
4
2
0
A (n=15)
B (n=16)
C (n=14)
surface

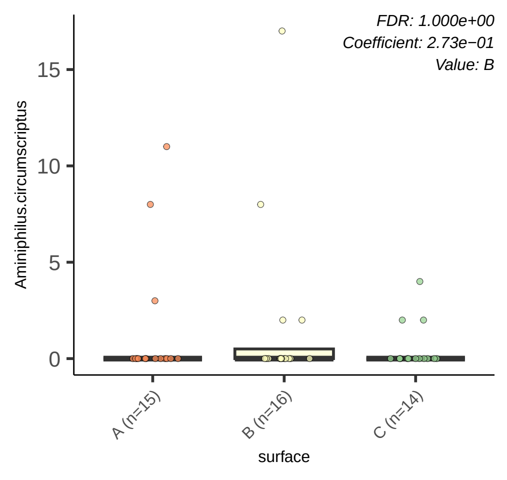
FDR: 1.000e+00
Coefficient: 2.73e−01
Value: B
Aminiphilus.circumscriptus
0
5
10
15
A (n=15)
B (n=16)
C (n=14)
surface

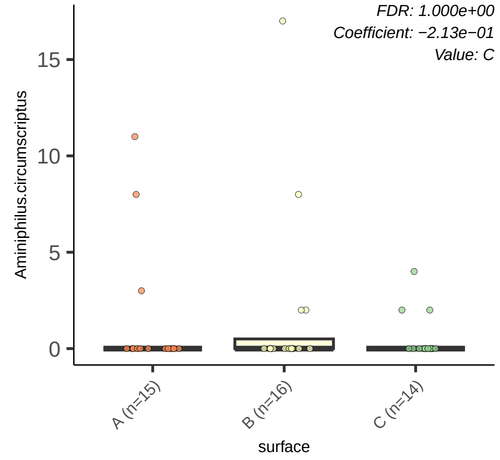

FDR: 1.000e+00
Coefficient: −2.13e−01
Value: C
Aminiphilus.circumscriptus
15
10
5
0
A (n=15)
B (n=16)
C (n=14)
surface

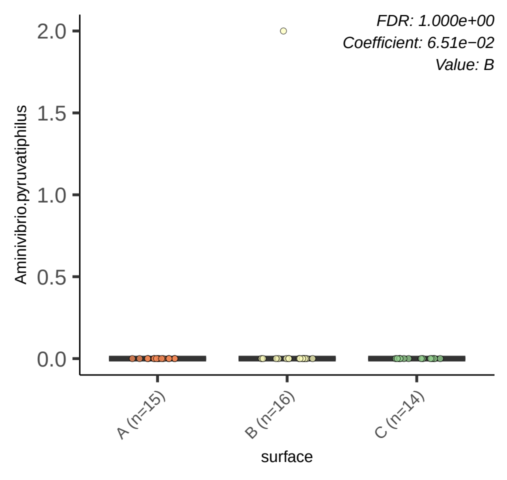

FDR: 1.000e+00
Coefficient: 6.51e−02
Value: B
Aminivibrio.pyruvatiphilus
2.0
1.5
1.0
0.5
0.0
A (n=15)
B (n=16)
C (n=14)
surface

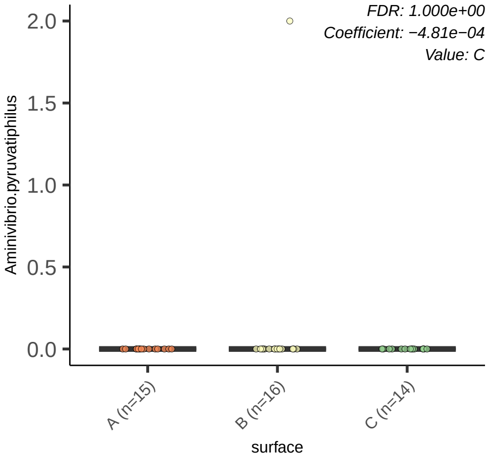
FDR: 1.000e+00
Coefficient: −4.81e−04
Value: C
Aminivibrio.pyruvatiphilus
2.0
1.5
1.0
0.5
0.0
A (n=15)
B (n=16)
C (n=14)
surface

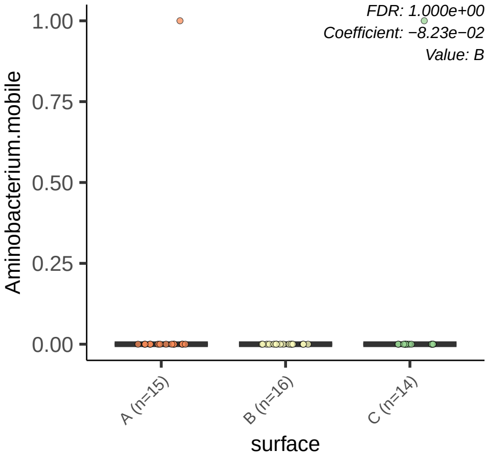
FDR: 1.000e+00
Coefficient: −8.23e−02
Value: B
Aminobacterium.mobile
1.00
0.75
0.50
0.25
0.00
A (n=15)
B (n=16)
C (n=14)
surface

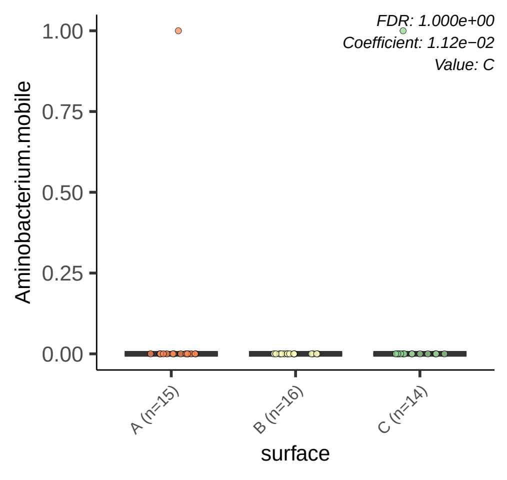

FDR: 1.000e+00
Coefficient: 1.12e−02
Value: C
Aminobacterium.mobile
1.00
0.75
0.50
0.25
0.00
A (n=15)
B (n=16)
C (n=14)
surface

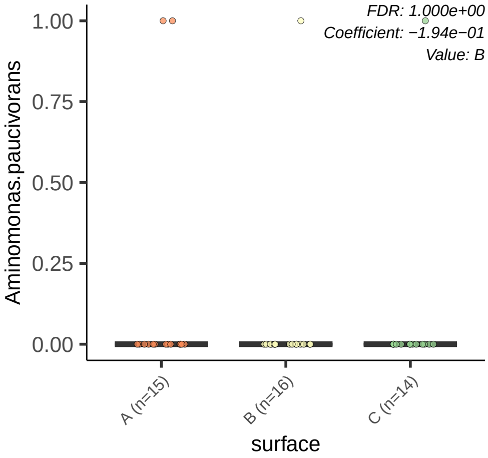

FDR: 1.000e+00
Coefficient: −1.94e−01
Value: B
Aminomonas.paucivorans
1.00
0.75
0.50
0.25
0.00
A (n=15)
B (n=16)
C (n=14)
surface

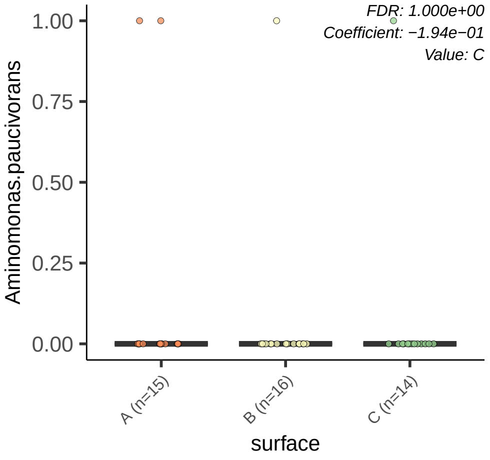

FDR: 1.000e+00
Coefficient: −1.94e−01
Value: C
Aminomonas.paucivorans
1.00
0.75
0.50
0.25
0.00
A (n=15)
B (n=16)
C (n=14)
surface

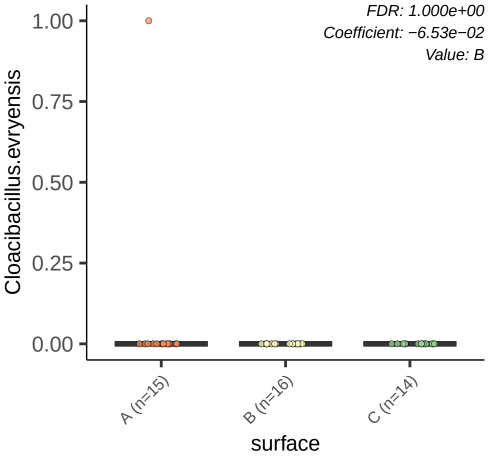
FDR: 1.000e+00
Coefficient: −6.53e−02
Value: B
Cloacibacillus.evryensis
1.00
0.75
0.50
0.25
0.00
A (n=15)
B (n=16)
C (n=14)
surface

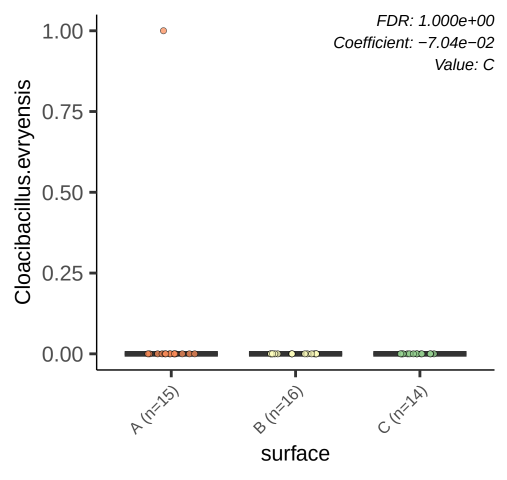
FDR: 1.000e+00
Coefficient: −7.04e−02
Value: C
Cloacibacillus.evryensis
1.00
0.75
0.50
0.25
0.00
A (n=15)
B (n=16)
C (n=14)
surface

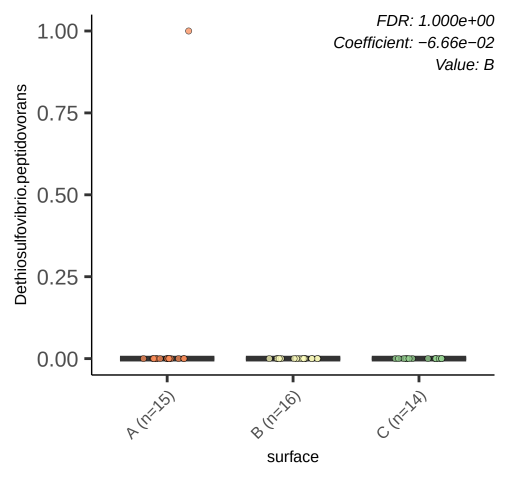
FDR: 1.000e+00
Coefficient: −6.66e−02
Value: B
1.00
0.75
0.50
0.25
0.00
Dethiosulfovibrio.peptidovorans
A (n=15)
B (n=16)
C (n=14)
surface

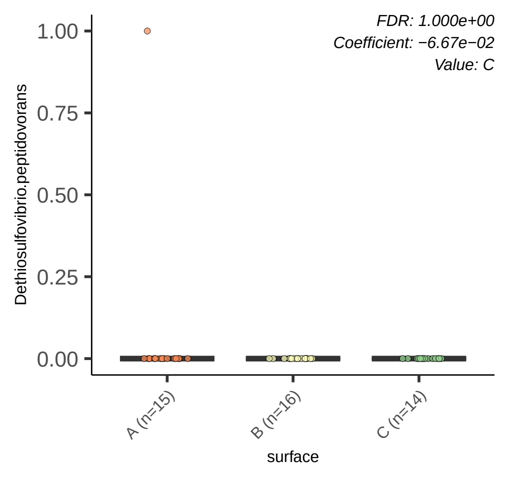

FDR: 1.000e+00
Coefficient: −6.67e−02
Value: C
Dethiosulfovibrio.peptidovorans
1.00
0.75
0.50
0.25
0.00
A (n=15)
B (n=16)
C (n=14)
surface

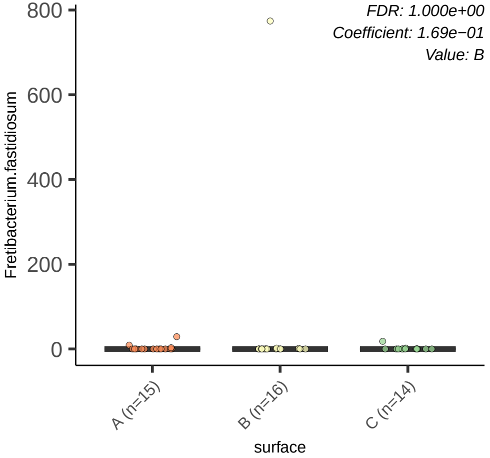
FDR: 1.000e+00
Coefficient: 1.69e−01
Value: B
Fretibacterium.fastidiosum
800
600
400
200
0
A (n=15)
B (n=16)
C (n=14)
surface

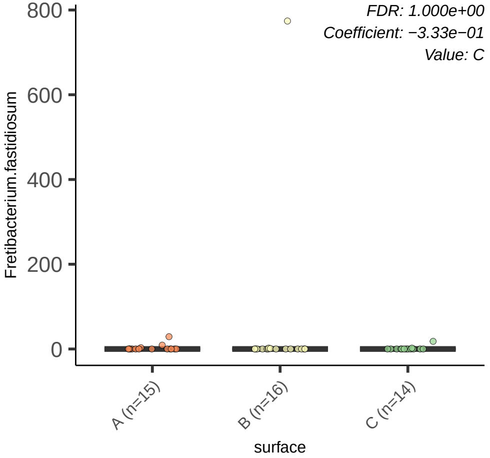
FDR: 1.000e+00
Coefficient: −3.33e−01
Value: C
800
600
400
200
0
Fretibacterium.fastidiosum
A (n=15)
B (n=16)
C (n=14)
surface

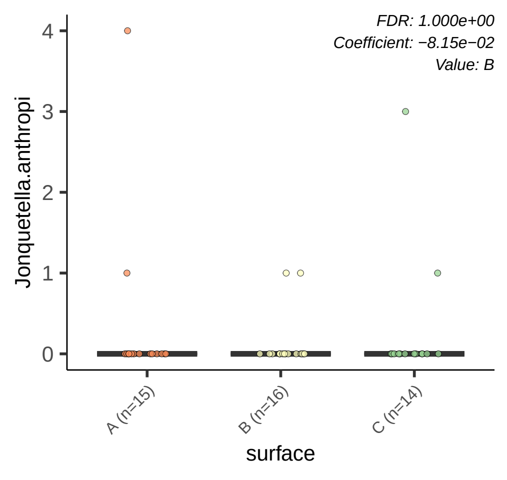
FDR: 1.000e+00
Coefficient: −8.15e−02
Value: B
Jonquetella.anthropi
0
1
2
3
4
A (n=15)
B (n=16)
C (n=14)
surface

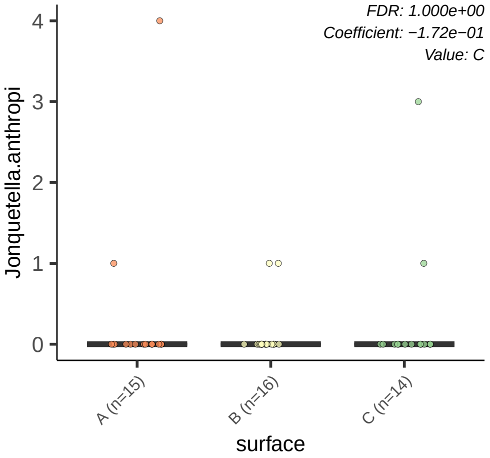

FDR: 1.000e+00
Coefficient: −1.72e−01
Value: C
Jonquetella.anthropi
4
3
2
1
0
A (n=15)
B (n=16)
C (n=14)
surface

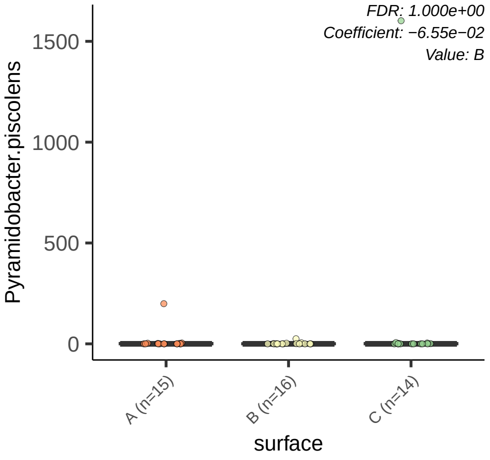
FDR: 1.000e+00
Coefficient: −6.55e−02
Value: B
Pyramidobacter.piscolens
1500
1000
500
0
A (n=15)
B (n=16)
C (n=14)
surface

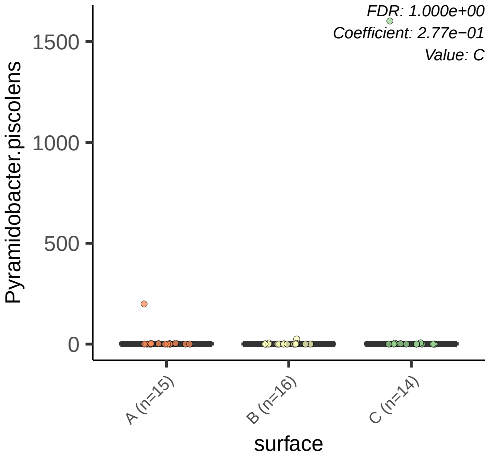
FDR: 1.000e+00
Coefficient: 2.77e−01
Value: C
Pyramidobacter.piscolens
1500
1000
500
0
A (n=15)
B (n=16)
C (n=14)
surface

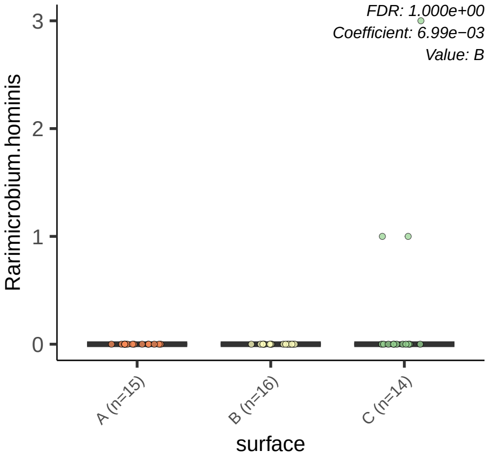

FDR: 1.000e+00
Coefficient: 6.99e−03
Value: B
Rarimicrobium.hominis
3
2
1
0
A (n=15)
B (n=16)
C (n=14)
surface

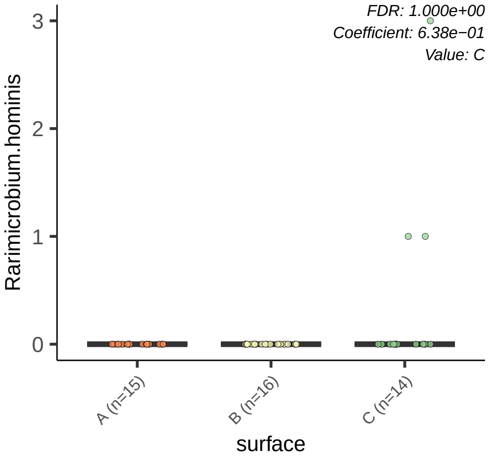

FDR: 1.000e+00
Coefficient: 6.38e−01
Value: C
Rarimicrobium.hominis
3
2
1
0
A (n=15)
B (n=16)
C (n=14)
surface

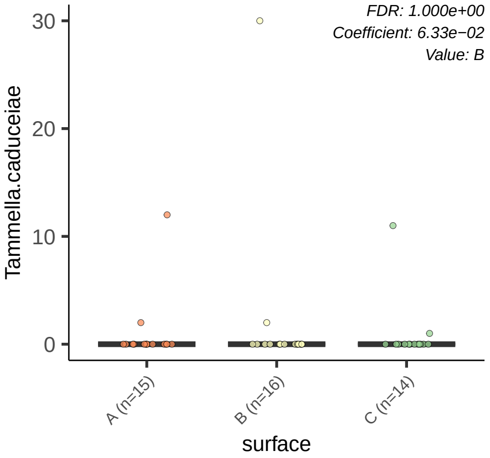
FDR: 1.000e+00
Coefficient: 6.33e−02
Value: B
Tammella.caduceiae
30
20
10
0
A (n=15)
B (n=16)
C (n=14)
surface

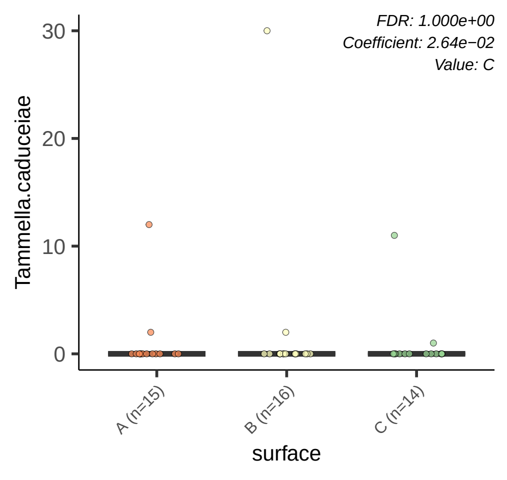
FDR: 1.000e+00
Coefficient: 2.64e−02
Value: C
Tammella.caduceiae
30
20
10
0
A (n=15)
B (n=16)
C (n=14)
surface

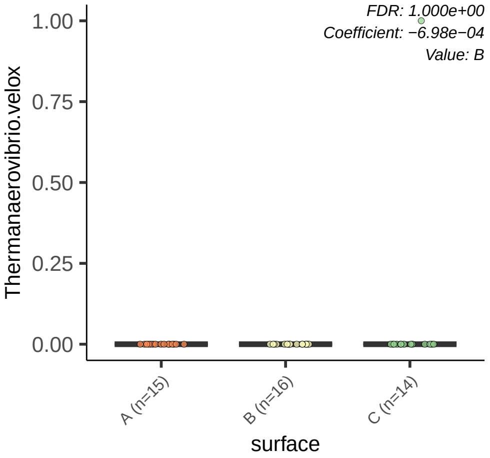
FDR: 1.000e+00
Coefficient: −6.98e−04
Value: B
Thermanaerovibrio.velox
1.00
0.75
0.50
0.25
0.00
A (n=15)
B (n=16)
C (n=14)
surface

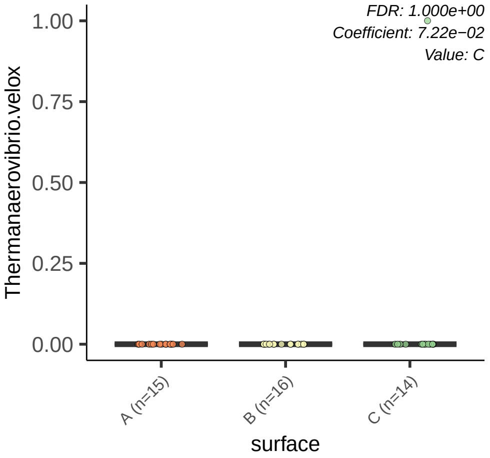

FDR: 1.000e+00
Coefficient: 7.22e−02
Value: C
Thermanaerovibrio.velox
1.00
0.75
0.50
0.25
0.00
A (n=15)
B (n=16)
C (n=14)
surface

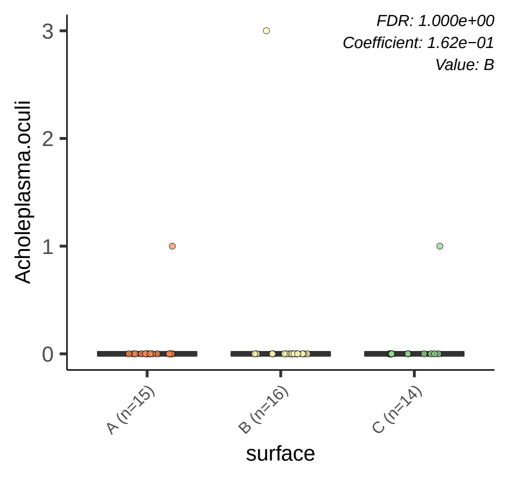
FDR: 1.000e+00
Coefficient: 1.62e−01
Value: B
Acholeplasma.oculi
3
2
1
0
A (n=15)
B (n=16)
C (n=14)
surface

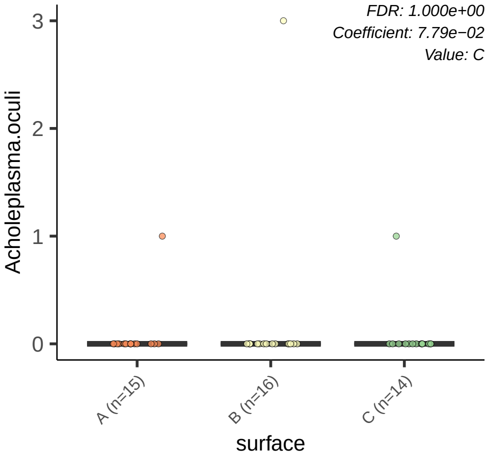
FDR: 1.000e+00
Coefficient: 7.79e−02
Value: C
Acholeplasma.oculi
3
2
1
0
A (n=15)
B (n=16)
C (n=14)
surface

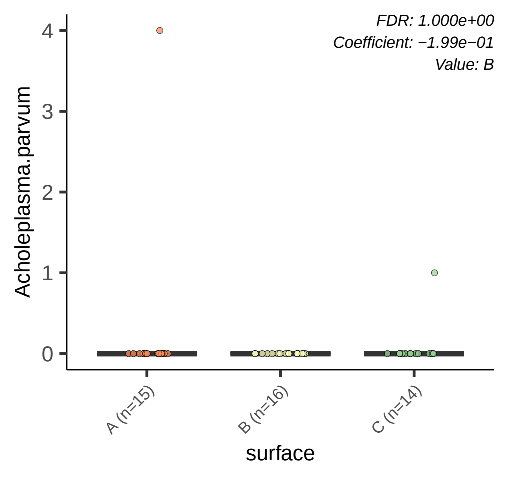
FDR: 1.000e+00
Coefficient: −1.99e−01
Value: B
Acholeplasma.parvum
4
3
2
1
0
A (n=15)
B (n=16)
C (n=14)
surface

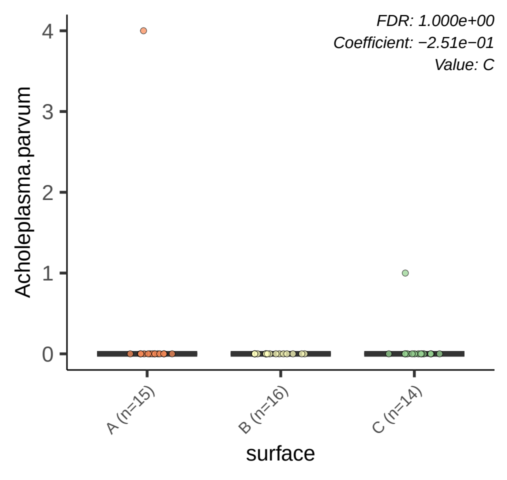

FDR: 1.000e+00
Coefficient: −2.51e−01
Value: C
Acholeplasma.parvum
0
1
2
3
4
A (n=15)
B (n=16)
C (n=14)
surface

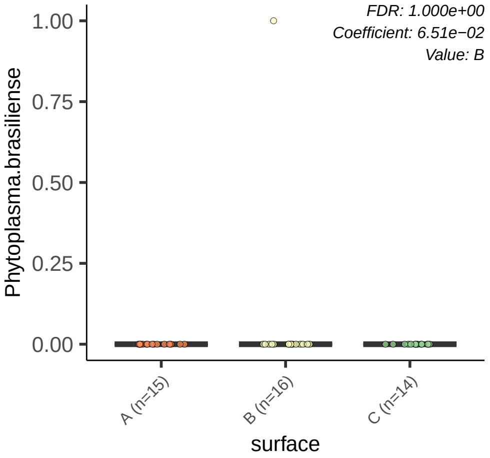

FDR: 1.000e+00
Coefficient: 6.51e−02
Value: B
Phytoplasma.brasiliense
1.00
0.75
0.50
0.25
0.00
A (n=15)
B (n=16)
C (n=14)
surface

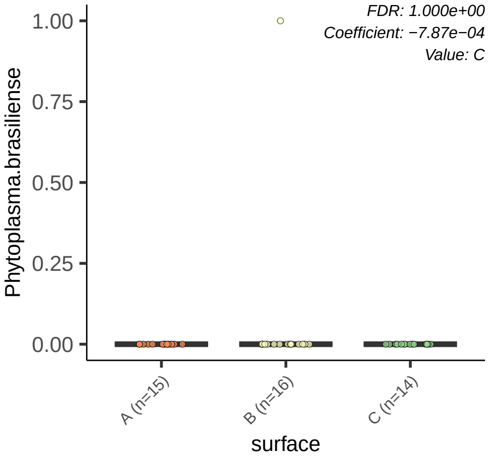

FDR: 1.000e+00
Coefficient: −7.87e−04
Value: C
Phytoplasma.brasiliense
1.00
0.75
0.50
0.25
0.00
A (n=15)
B (n=16)
C (n=14)
surface

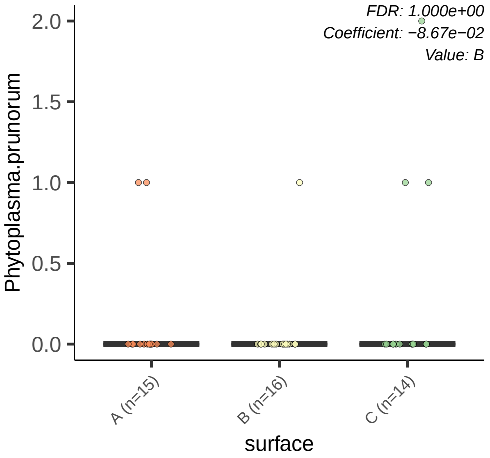

FDR: 1.000e+00
Coefficient: −8.67e−02
Value: B
Phytoplasma.prunorum
2.0
1.5
1.0
0.5
0.0
A (n=15)
B (n=16)
C (n=14)
surface

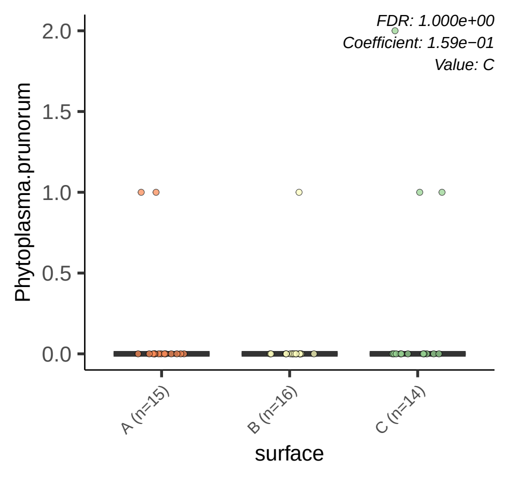

FDR: 1.000e+00
Coefficient: 1.59e−01
Value: C
Phytoplasma.prunorum
2.0
1.5
1.0
0.5
0.0
A (n=15)
B (n=16)
C (n=14)
surface

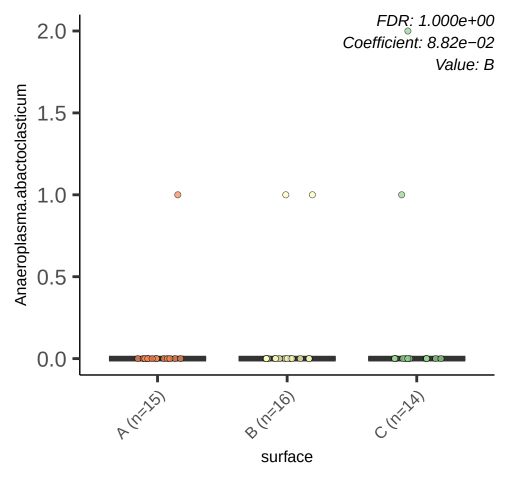

FDR: 1.000e+00
Coefficient: 8.82e−02
Value: B
Anaeroplasma.abactoclasticum
2.0
1.5
1.0
0.5
0.0
A (n=15)
B (n=16)
C (n=14)
surface

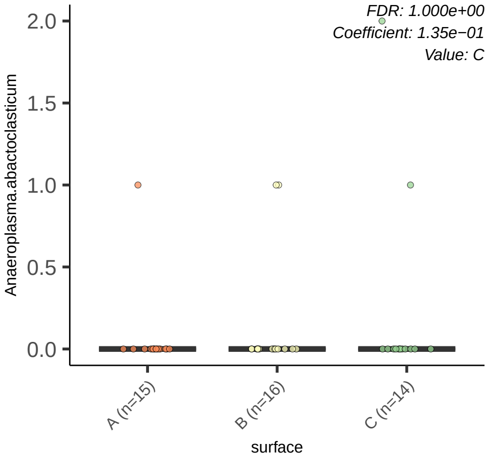
FDR: 1.000e+00
Coefficient: 1.35e−01
Value: C
Anaeroplasma.abactoclasticum
2.0
1.5
1.0
0.5
0.0
A (n=15)
B (n=16)
C (n=14)
surface

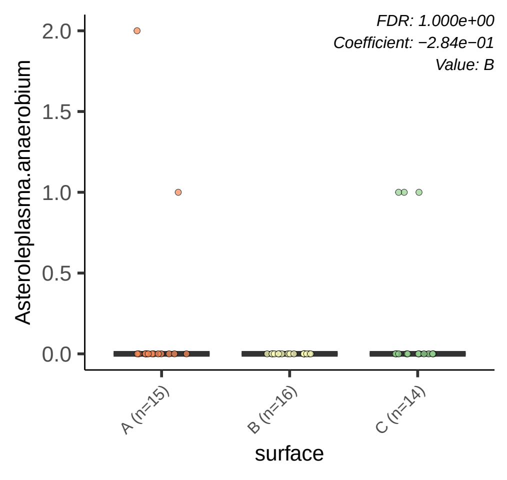
FDR: 1.000e+00
Coefficient: −2.84e−01
Value: B
Asteroleplasma.anaerobium
2.0
1.5
1.0
0.5
0.0
A (n=15)
B (n=16)
C (n=14)
surface

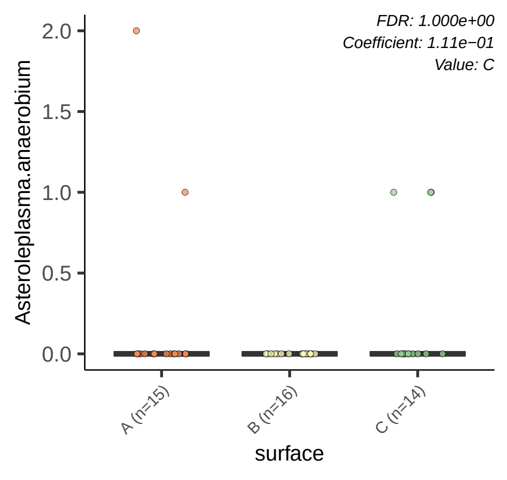
FDR: 1.000e+00
Coefficient: 1.11e−01
Value: C
Asteroleplasma.anaerobium
2.0
1.5
1.0
0.5
0.0
A (n=15)
B (n=16)
C (n=14)
surface

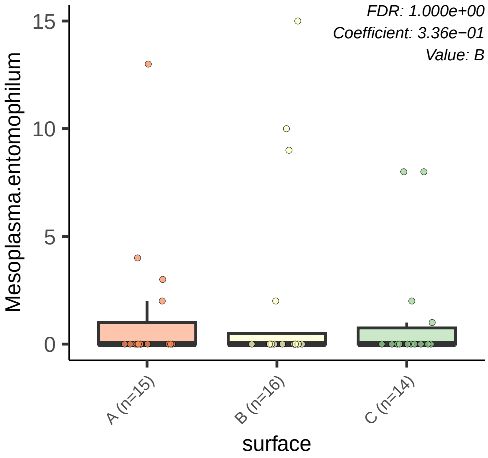

FDR: 1.000e+00
Coefficient: 3.36e−01
Value: B
Mesoplasma.entomophilum
15
10
5
0
A (n=15)
B (n=16)
C (n=14)
surface

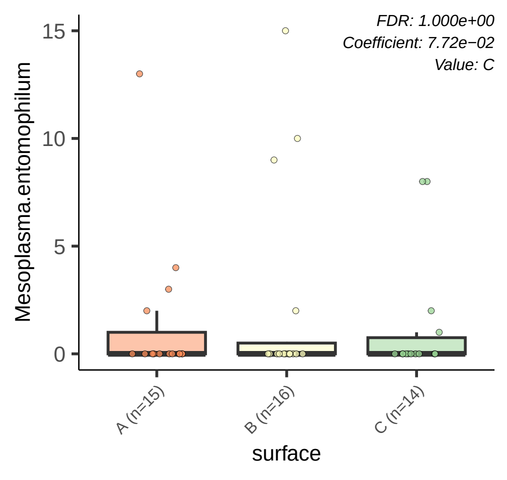

FDR: 1.000e+00
Coefficient: 7.72e−02
Value: C
Mesoplasma.entomophilum
15
10
5
0
A (n=15)
B (n=16)
C (n=14)
surface

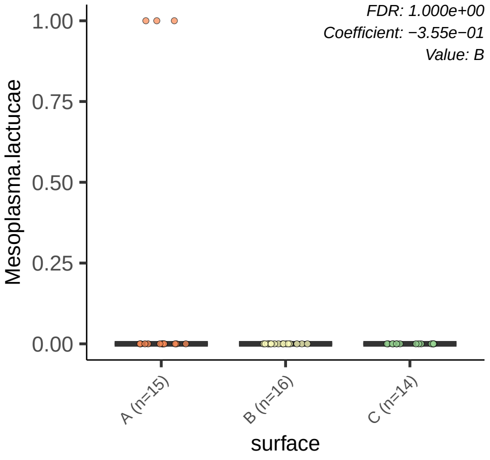
FDR: 1.000e+00
Coefficient: −3.55e−01
Value: B
Mesoplasma.lactucae
1.00
0.75
0.50
0.25
0.00
A (n=15)
B (n=16)
C (n=14)
surface

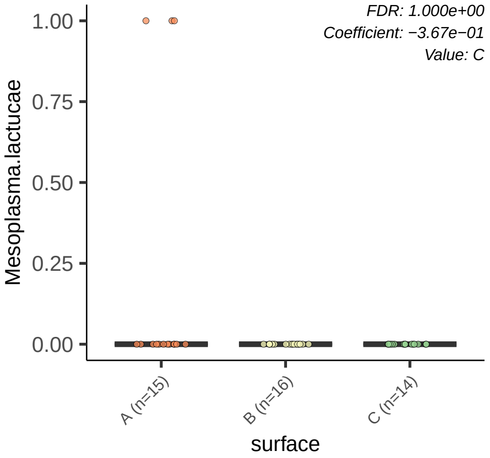
FDR: 1.000e+00
Coefficient: −3.67e−01
Value: C
Mesoplasma.lactucae
1.00
0.75
0.50
0.25
0.00
A (n=15)
B (n=16)
C (n=14)
surface

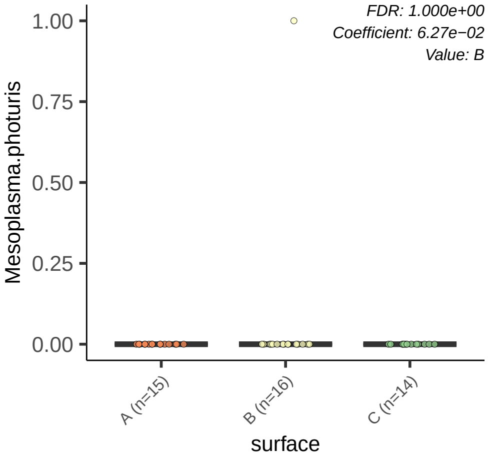

FDR: 1.000e+00
Coefficient: 6.27e−02
Value: B
Mesoplasma.photuris
1.00
0.75
0.50
0.25
0.00
A (n=15)
B (n=16)
C (n=14)
surface

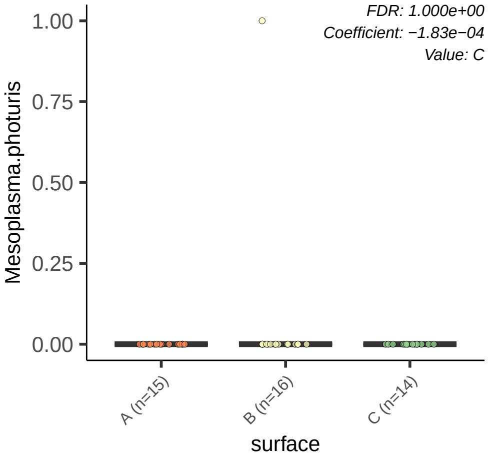

FDR: 1.000e+00
Coefficient: −1.83e−04
Value: C
Mesoplasma.photuris
1.00
0.75
0.50
0.25
0.00
A (n=15)
B (n=16)
C (n=14)
surface

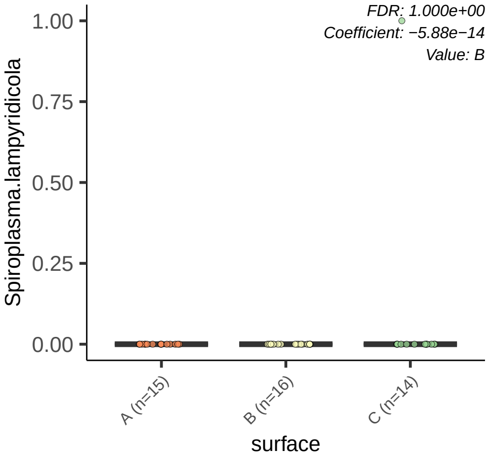

FDR: 1.000e+00
Coefficient: −5.88e−14
Value: B
Spiroplasma.lampyridicola
1.00
0.75
0.50
0.25
0.00
A (n=15)
B (n=16)
C (n=14)
surface

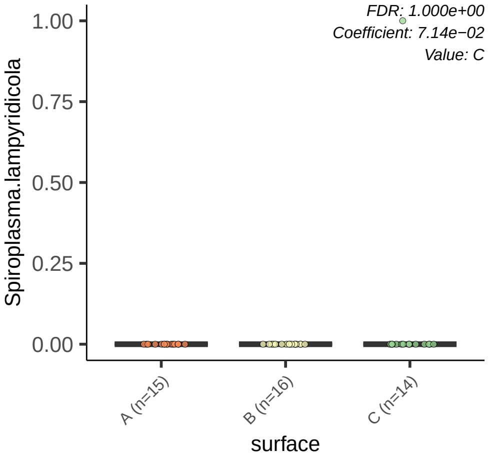

FDR: 1.000e+00
Coefficient: 7.14e−02
Value: C
Spiroplasma.lampyridicola
1.00
0.75
0.50
0.25
0.00
A (n=15)
B (n=16)
C (n=14)
surface

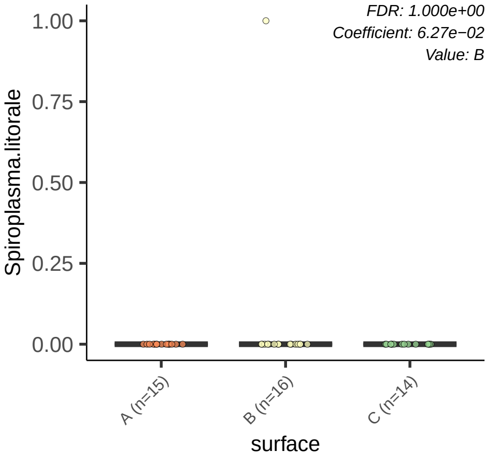

FDR: 1.000e+00
Coefficient: 6.27e−02
Value: B
Spiroplasma.litorale
1.00
0.75
0.50
0.25
0.00
A (n=15)
B (n=16)
C (n=14)
surface

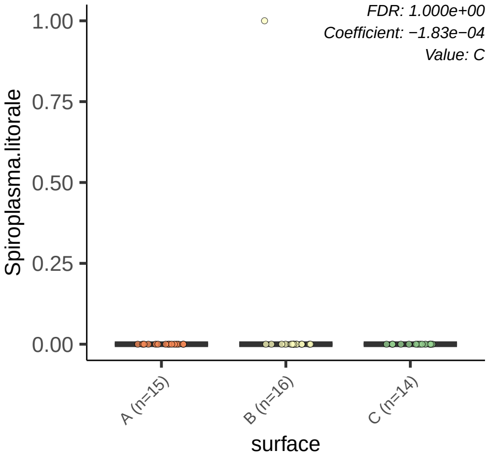

FDR: 1.000e+00
Coefficient: −1.83e−04
Value: C
Spiroplasma.litorale
1.00
0.75
0.50
0.25
0.00
A (n=15)
B (n=16)
C (n=14)
surface

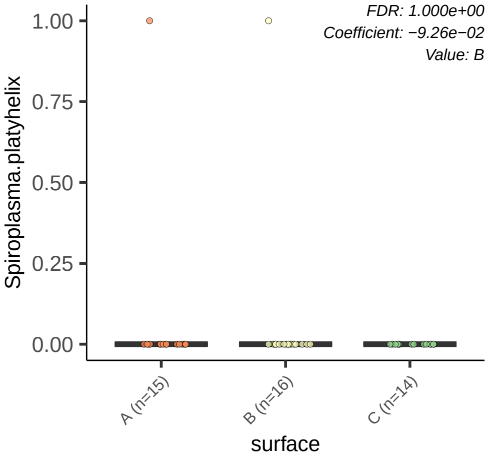
FDR: 1.000e+00
Coefficient: −9.26e−02
Value: B
Spiroplasma.platyhelix
1.00
0.75
0.50
0.25
0.00
A (n=15)
B (n=16)
C (n=14)
surface

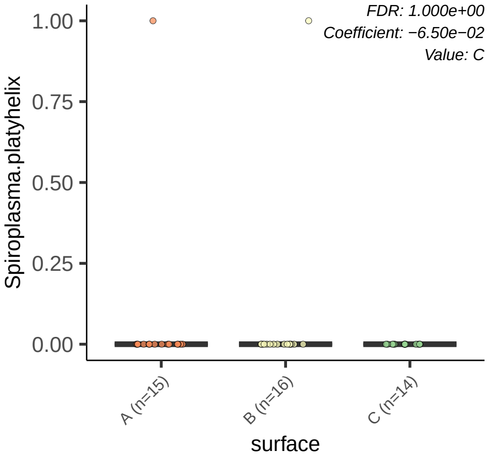

FDR: 1.000e+00
Coefficient: −6.50e−02
Value: C
Spiroplasma.platyhelix
1.00
0.75
0.50
0.25
0.00
A (n=15)
B (n=16)
C (n=14)
surface

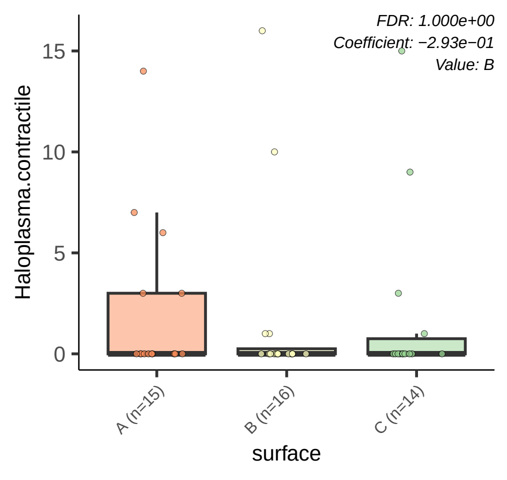
FDR: 1.000e+00
Coefficient: −2.93e−01
Value: B
Haloplasma.contractile
0
5
10
15
A (n=15)
B (n=16)
C (n=14)
surface

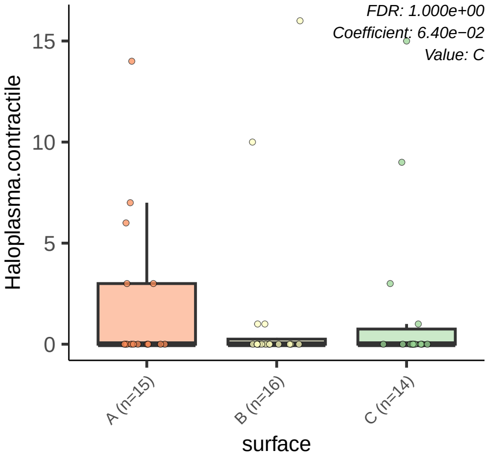
FDR: 1.000e+00
Coefficient: 6.40e−02
Value: C
Haloplasma.contractile
15
10
5
0
A (n=15)
B (n=16)
C (n=14)
surface

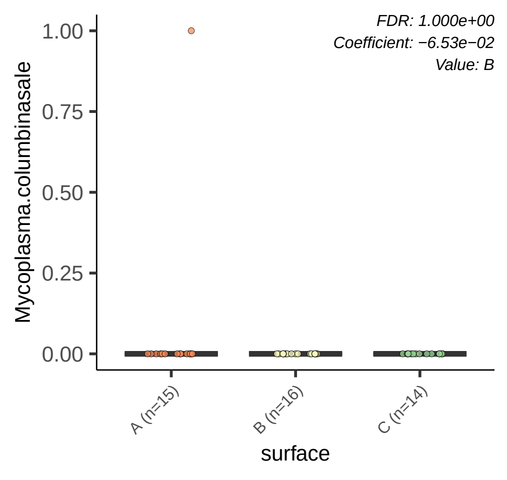
FDR: 1.000e+00
Coefficient: −6.53e−02
Value: B
Mycoplasma.columbinasale
1.00
0.75
0.50
0.25
0.00
A (n=15)
B (n=16)
C (n=14)
surface

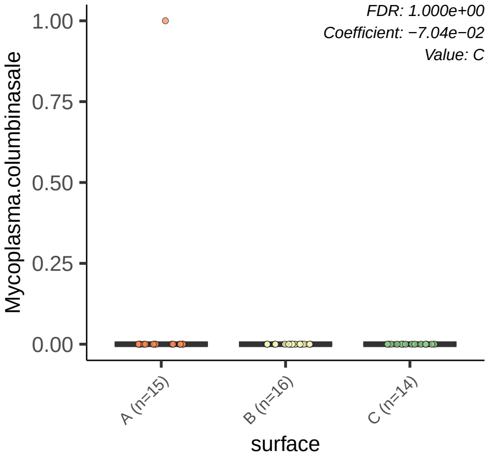

FDR: 1.000e+00
Coefficient: −7.04e−02
Value: C
Mycoplasma.columbinasale
1.00
0.75
0.50
0.25
0.00
A (n=15)
B (n=16)
C (n=14)
surface

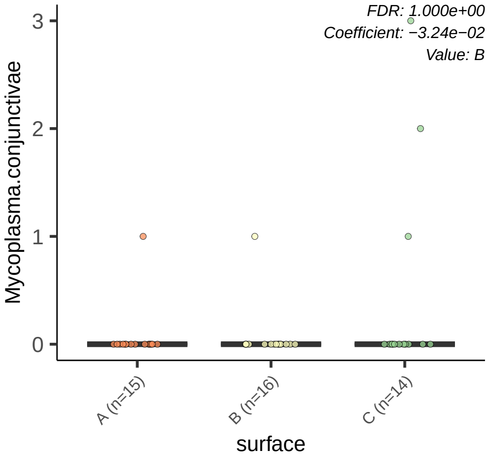
FDR: 1.000e+00
Coefficient: −3.24e−02
Value: B
Mycoplasma.conjunctivae
3
2
1
0
A (n=15)
B (n=16)
C (n=14)
surface

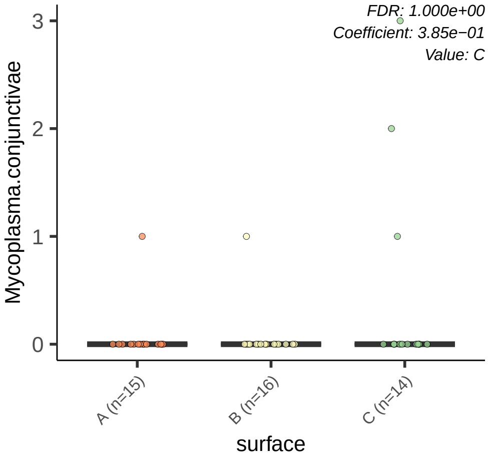

FDR: 1.000e+00
Coefficient: 3.85e−01
Value: C
Mycoplasma.conjunctivae
3
2
1
0
A (n=15)
B (n=16)
C (n=14)
surface

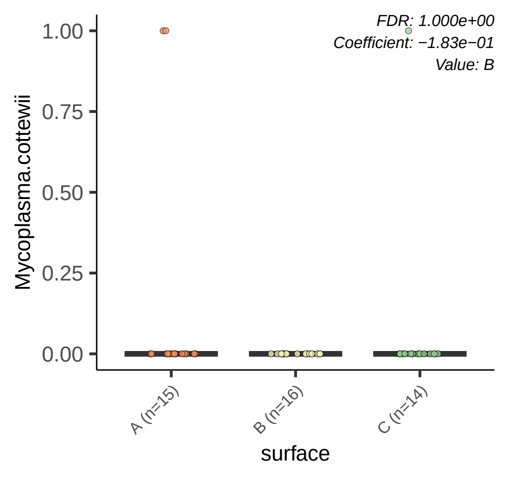
FDR: 1.000e+00
Coefficient: −1.83e−01
Value: B
Mycoplasma.cottewii
1.00
0.75
0.50
0.25
0.00
A (n=15)
B (n=16)
C (n=14)
surface

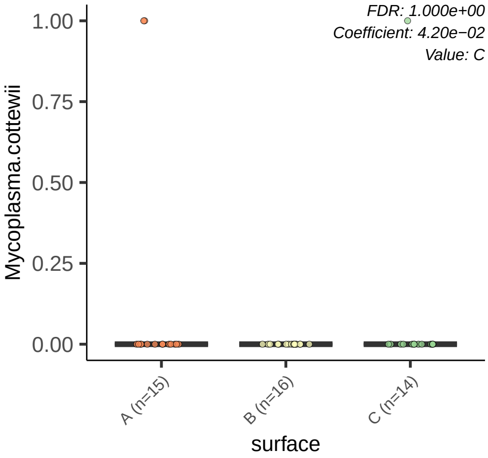
FDR: 1.000e+00
Coefficient: 4.20e−02
Value: C
Mycoplasma.cottewii
1.00
0.75
0.50
0.25
0.00
A (n=15)
B (n=16)
C (n=14)
surface

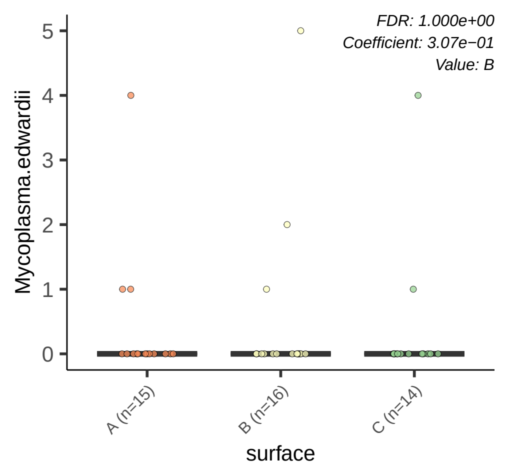
FDR: 1.000e+00
Coefficient: 3.07e−01
Value: B
Mycoplasma.edwardii
0
1
2
3
4
5
A (n=15)
B (n=16)
C (n=14)
surface

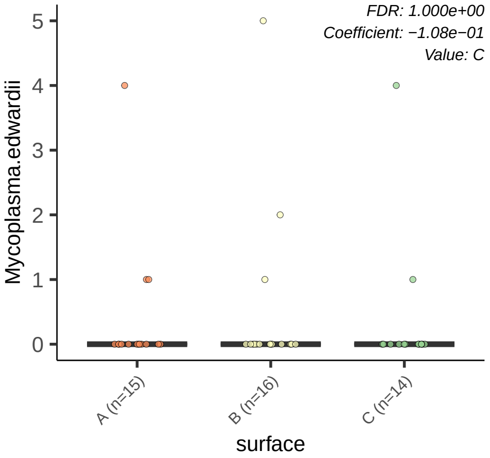
FDR: 1.000e+00
Coefficient: −1.08e−01
Value: C
Mycoplasma.edwardii
0
1
2
3
4
5
A (n=15)
B (n=16)
C (n=14)
surface

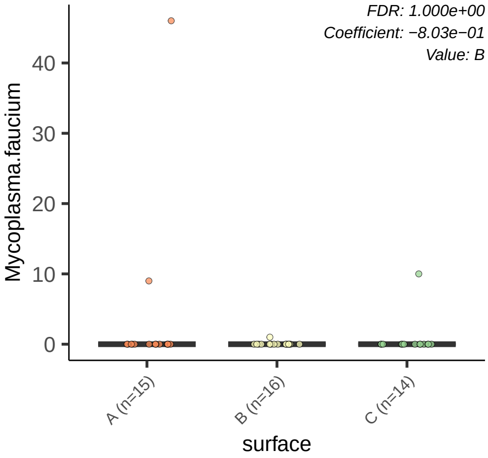
FDR: 1.000e+00
Coefficient: −8.03e−01
Value: B
Mycoplasma.faucium
0
10
20
30
40
A (n=15)
B (n=16)
C (n=14)
surface

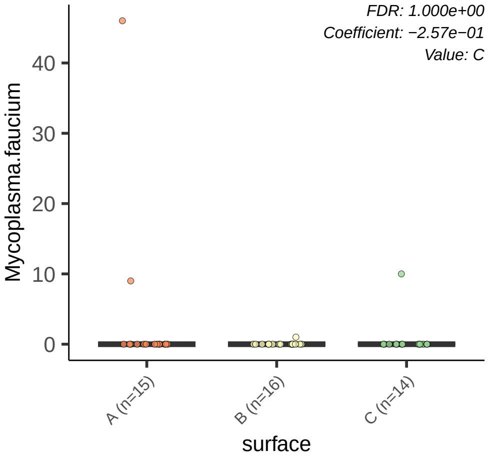
FDR: 1.000e+00
Coefficient: −2.57e−01
Value: C
Mycoplasma.faucium
40
30
20
10
0
A (n=15)
B (n=16)
C (n=14)
surface

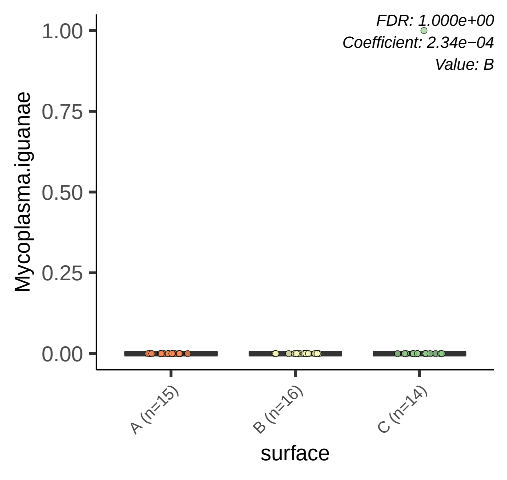

FDR: 1.000e+00
Coefficient: 2.34e−04
Value: B
Mycoplasma.iguanae
1.00
0.75
0.50
0.25
0.00
A (n=15)
B (n=16)
C (n=14)
surface

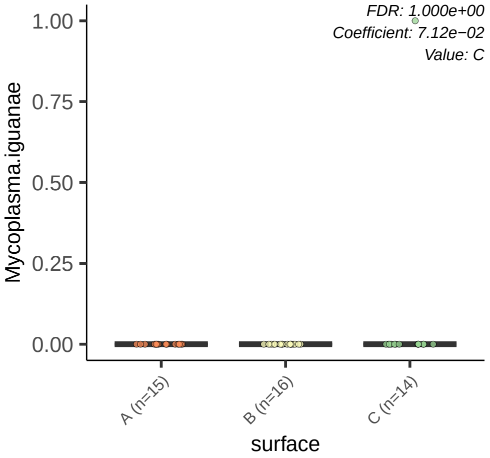
FDR: 1.000e+00
Coefficient: 7.12e−02
Value: C
Mycoplasma.iguanae
1.00
0.75
0.50
0.25
0.00
A (n=15)
B (n=16)
C (n=14)
surface

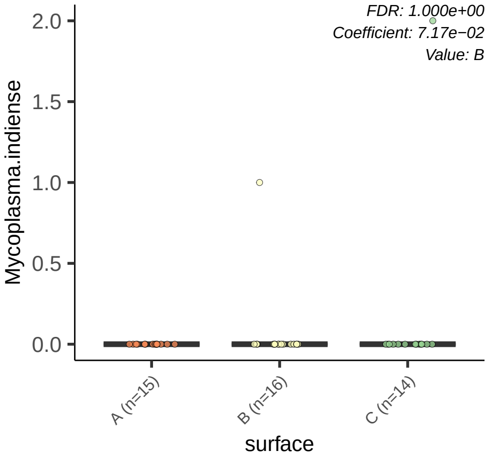

FDR: 1.000e+00
Coefficient: 7.17e−02
Value: B
Mycoplasma.indiense
2.0
1.5
1.0
0.5
0.0
A (n=15)
B (n=16)
C (n=14)
surface

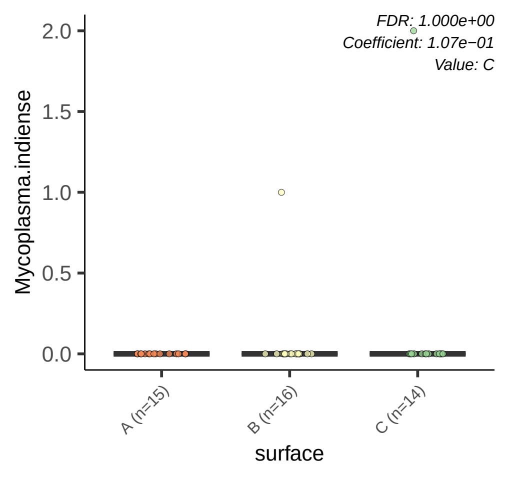
FDR: 1.000e+00
Coefficient: 1.07e−01
Value: C
Mycoplasma.indiense
2.0
1.5
1.0
0.5
0.0
A (n=15)
B (n=16)
C (n=14)
surface

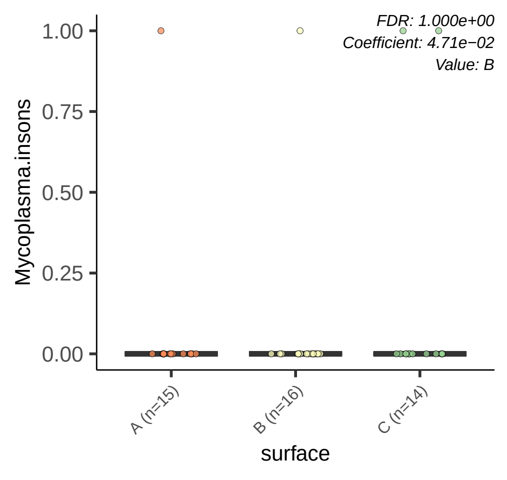
FDR: 1.000e+00
Coefficient: 4.71e−02
Value: B
Mycoplasma.insons
1.00
0.75
0.50
0.25
0.00
A (n=15)
B (n=16)
C (n=14)
surface

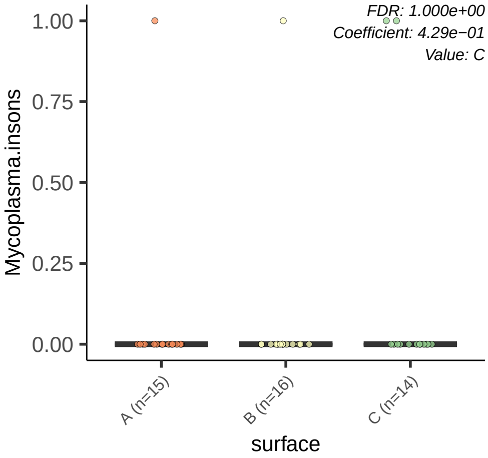
FDR: 1.000e+00
Coefficient: 4.29e−01
Value: C
Mycoplasma.insons
1.00
0.75
0.50
0.25
0.00
A (n=15)
B (n=16)
C (n=14)
surface

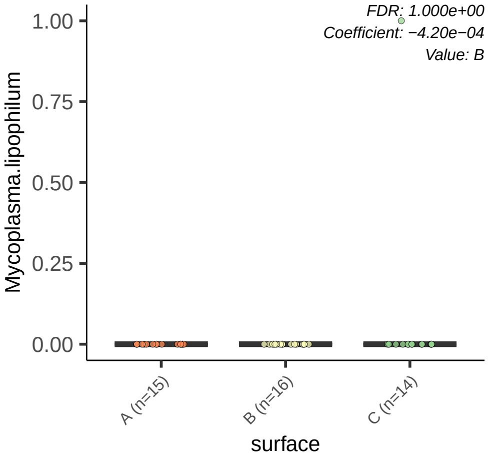
FDR: 1.000e+00
Coefficient: −4.20e−04
Value: B
Mycoplasma.lipophilum
1.00
0.75
0.50
0.25
0.00
A (n=15)
B (n=16)
C (n=14)
surface

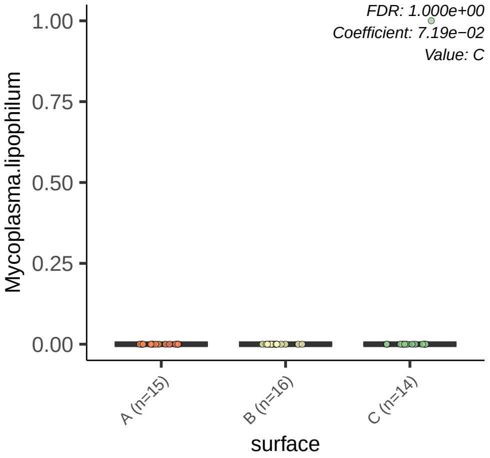

FDR: 1.000e+00
Coefficient: 7.19e−02
Value: C
Mycoplasma.lipophilum
1.00
0.75
0.50
0.25
0.00
A (n=15)
B (n=16)
C (n=14)
surface

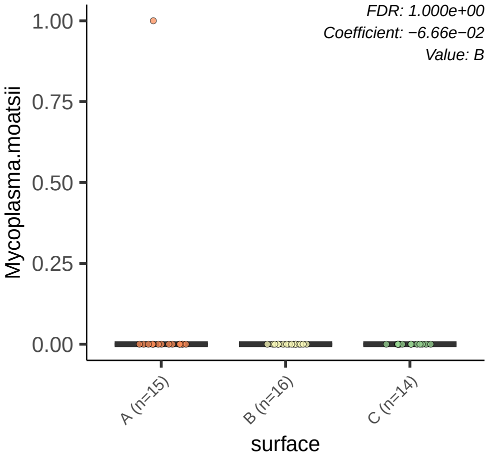
FDR: 1.000e+00
Coefficient: −6.66e−02
Value: B
Mycoplasma.moatsii
1.00
0.75
0.50
0.25
0.00
A (n=15)
B (n=16)
C (n=14)
surface

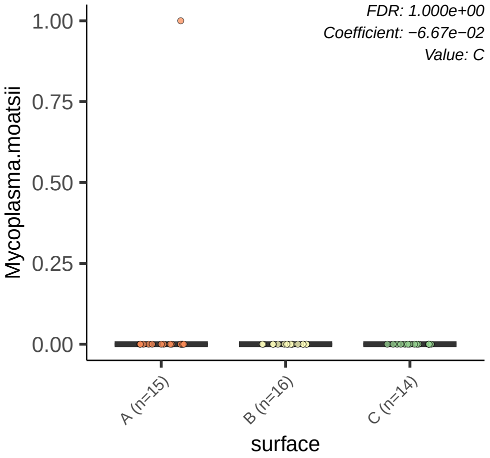

FDR: 1.000e+00
Coefficient: −6.67e−02
Value: C
Mycoplasma.moatsii
1.00
0.75
0.50
0.25
0.00
A (n=15)
B (n=16)
C (n=14)
surface

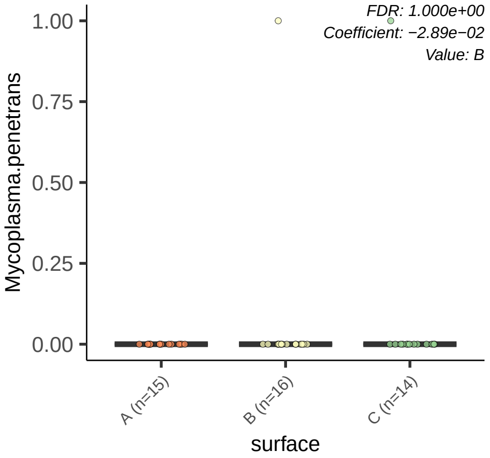
FDR: 1.000e+00
Coefficient: −2.89e−02
Value: B
Mycoplasma.penetrans
1.00
0.75
0.50
0.25
0.00
A (n=15)
B (n=16)
C (n=14)
surface

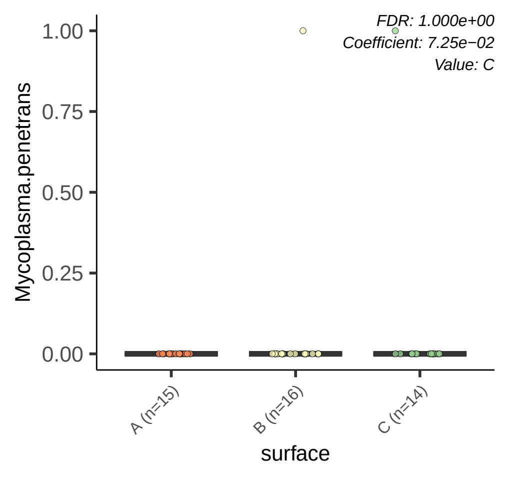

FDR: 1.000e+00
Coefficient: 7.25e−02
Value: C
Mycoplasma.penetrans
1.00
0.75
0.50
0.25
0.00
A (n=15)
B (n=16)
C (n=14)
surface

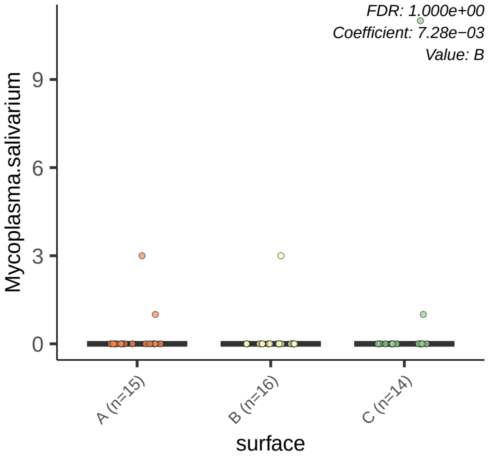

FDR: 1.000e+00
Coefficient: 7.28e−03
Value: B
Mycoplasma.salivarium
0
3
6
9
A (n=15)
B (n=16)
C (n=14)
surface

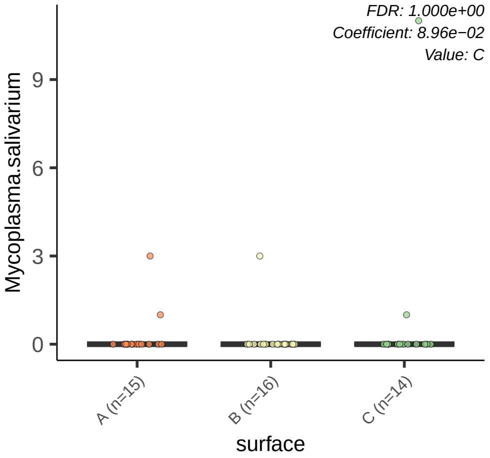
FDR: 1.000e+00
Coefficient: 8.96e−02
Value: C
Mycoplasma.salivarium
9
6
3
0
A (n=15)
B (n=16)
C (n=14)
surface

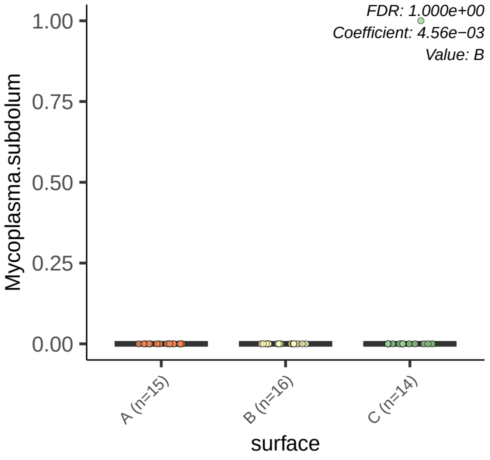
FDR: 1.000e+00
Coefficient: 4.56e−03
Value: B
Mycoplasma.subdolum
1.00
0.75
0.50
0.25
0.00
A (n=15)
B (n=16)
C (n=14)
surface

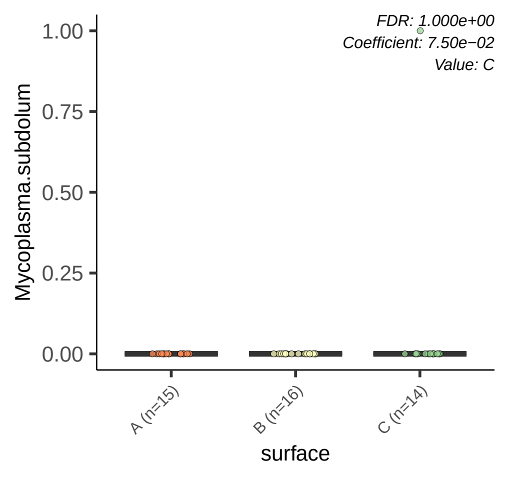
FDR: 1.000e+00
Coefficient: 7.50e−02
Value: C
Mycoplasma.subdolum
1.00
0.75
0.50
0.25
0.00
A (n=15)
B (n=16)
C (n=14)
surface

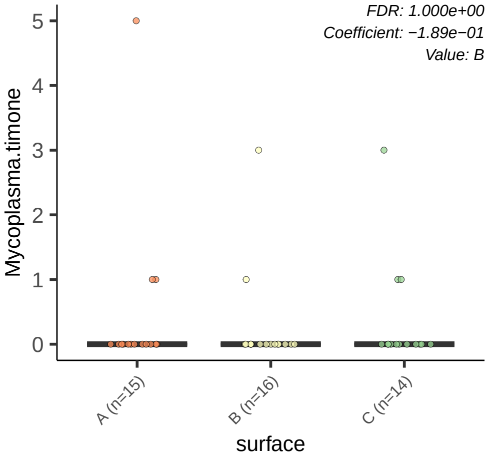

FDR: 1.000e+00
Coefficient: −1.89e−01
Value: B
Mycoplasma.timone
0
1
2
3
4
5
A (n=15)
B (n=16)
C (n=14)
surface

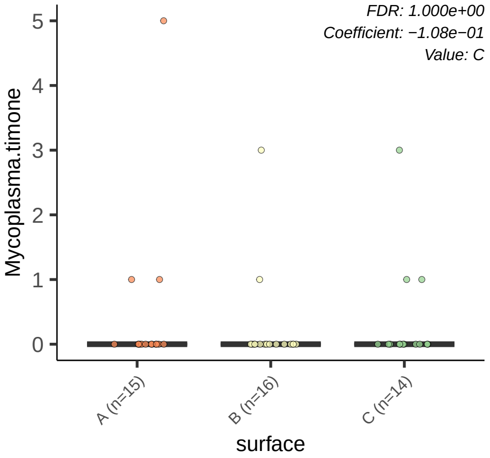

FDR: 1.000e+00
Coefficient: −1.08e−01
Value: C
Mycoplasma.timone
0
1
2
3
4
5
A (n=15)
B (n=16)
C (n=14)
surface

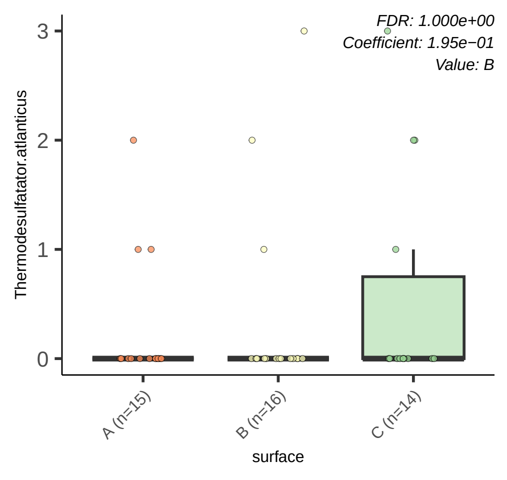
FDR: 1.000e+00
Coefficient: 1.95e−01
Value: B
Thermodesulfatator.atlanticus
0
1
2
3
A (n=15)
B (n=16)
C (n=14)
surface

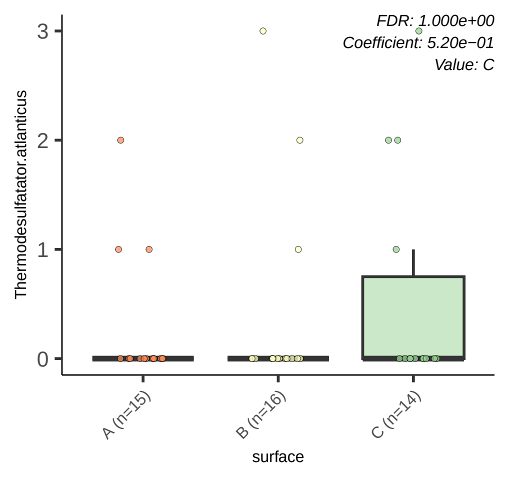
FDR: 1.000e+00
Coefficient: 5.20e−01
Value: C
Thermodesulfatator.atlanticus
0
1
2
3
A (n=15)
B (n=16)
C (n=14)
surface

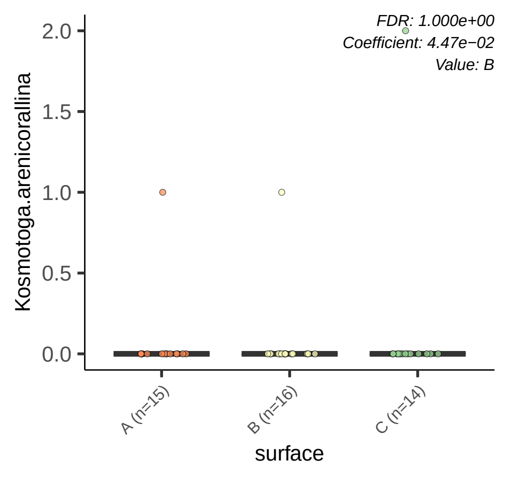
FDR: 1.000e+00
Coefficient: 4.47e−02
Value: B
Kosmotoga.arenicorallina
2.0
1.5
1.0
0.5
0.0
A (n=15)
B (n=16)
C (n=14)
surface

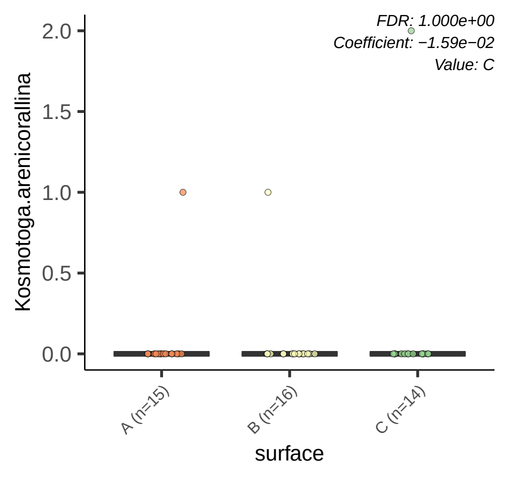
FDR: 1.000e+00
Coefficient: −1.59e−02
Value: C
Kosmotoga.arenicorallina
2.0
1.5
1.0
0.5
0.0
A (n=15)
B (n=16)
C (n=14)
surface

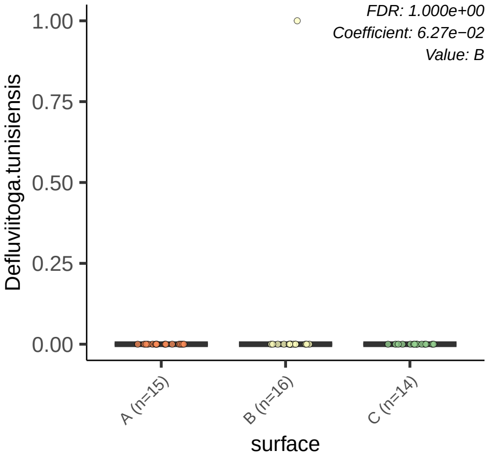

FDR: 1.000e+00
Coefficient: 6.27e−02
Value: B
Defluviitoga.tunisiensis
1.00
0.75
0.50
0.25
0.00
A (n=15)
B (n=16)
C (n=14)
surface

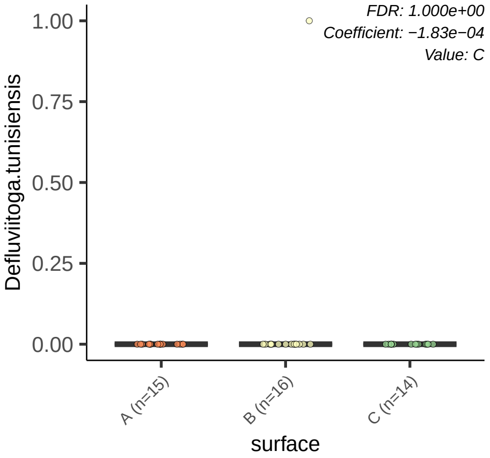

FDR: 1.000e+00
Coefficient: −1.83e−04
Value: C
Defluviitoga.tunisiensis
1.00
0.75
0.50
0.25
0.00
A (n=15)
B (n=16)
C (n=14)
surface

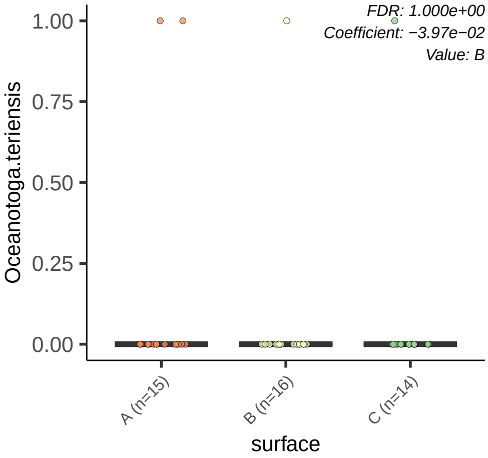

FDR: 1.000e+00
Coefficient: −3.97e−02
Value: B
Oceanotoga.teriensis
1.00
0.75
0.50
0.25
0.00
A (n=15)
B (n=16)
C (n=14)
surface

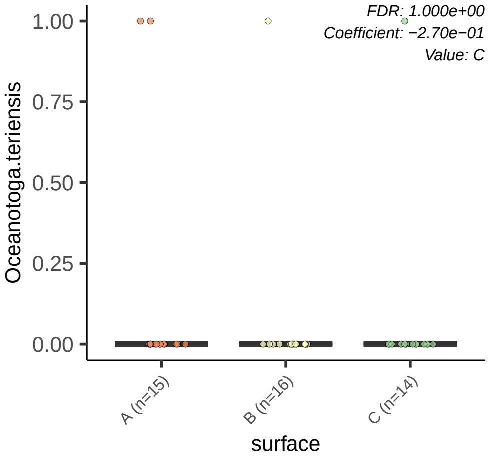

FDR: 1.000e+00
Coefficient: −2.70e−01
Value: C
Oceanotoga.teriensis
1.00
0.75
0.50
0.25
0.00
A (n=15)
B (n=16)
C (n=14)
surface

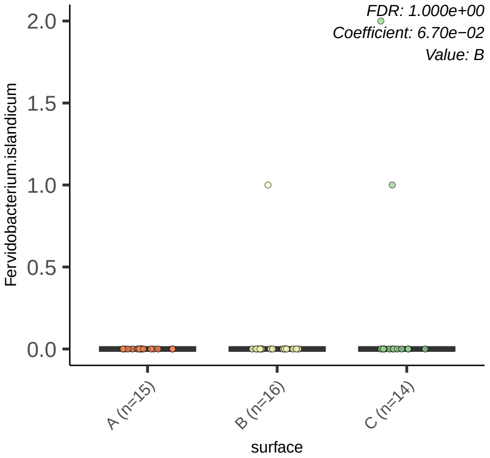
FDR: 1.000e+00
Coefficient: 6.70e−02
Value: B
Fervidobacterium.islandicum
2.0
1.5
1.0
0.5
0.0
A (n=15)
B (n=16)
C (n=14)
surface

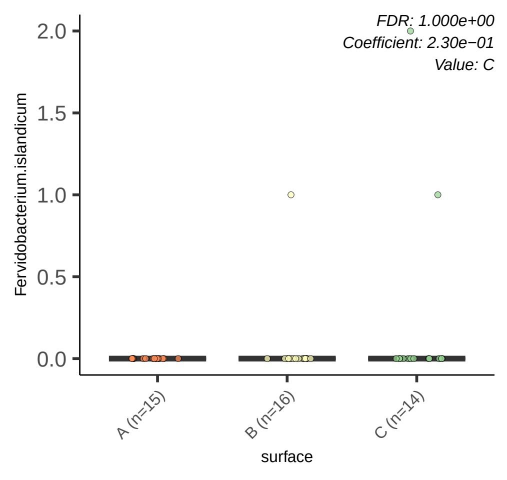
FDR: 1.000e+00
Coefficient: 2.30e−01
Value: C
Fervidobacterium.islandicum
2.0
1.5
1.0
0.5
0.0
A (n=15)
B (n=16)
C (n=14)
surface

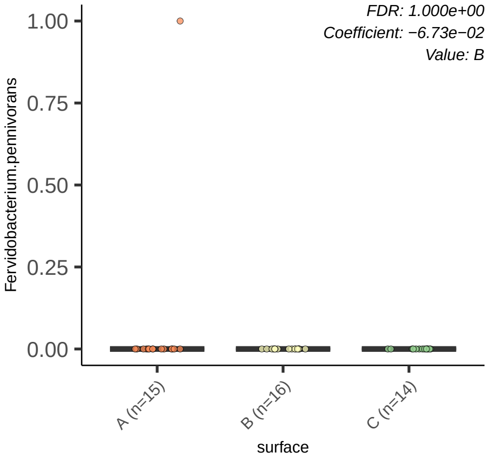
FDR: 1.000e+00
Coefficient: −6.73e−02
Value: B
Fervidobacterium.pennivorans
1.00
0.75
0.50
0.25
0.00
A (n=15)
B (n=16)
C (n=14)
surface

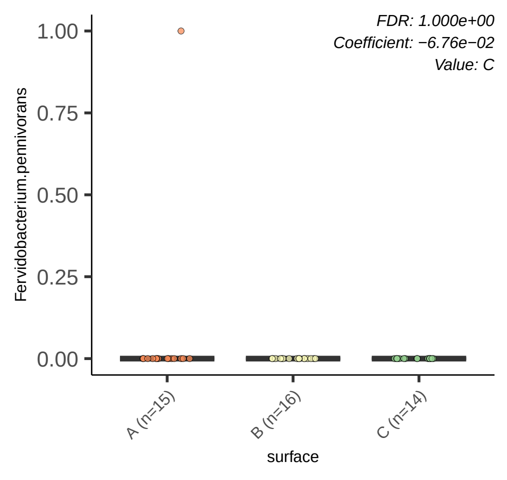
FDR: 1.000e+00
Coefficient: −6.76e−02
Value: C
Fervidobacterium.pennivorans
1.00
0.75
0.50
0.25
0.00
A (n=15)
B (n=16)
C (n=14)
surface

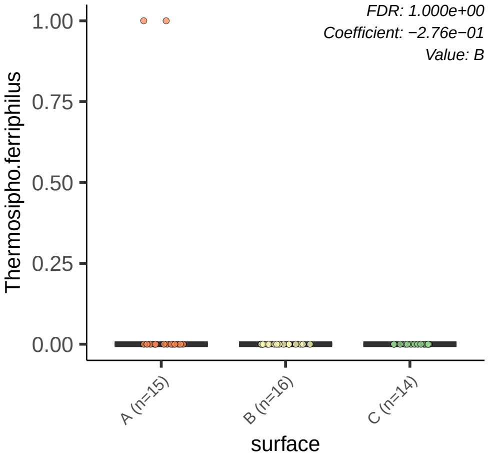

FDR: 1.000e+00
Coefficient: −2.76e−01
Value: B
Thermosipho.ferriphilus
1.00
0.75
0.50
0.25
0.00
A (n=15)
B (n=16)
C (n=14)
surface

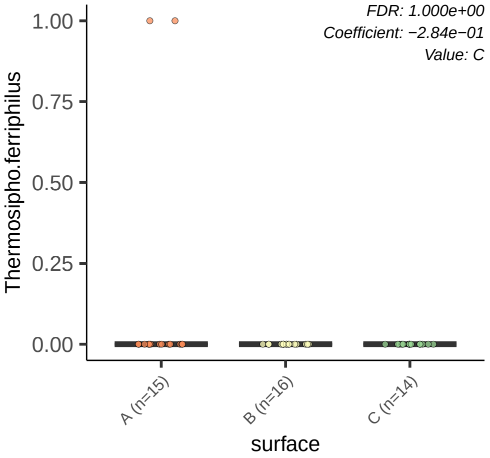
FDR: 1.000e+00
Coefficient: −2.84e−01
Value: C
Thermosipho.ferriphilus
1.00
0.75
0.50
0.25
0.00
A (n=15)
B (n=16)
C (n=14)
surface

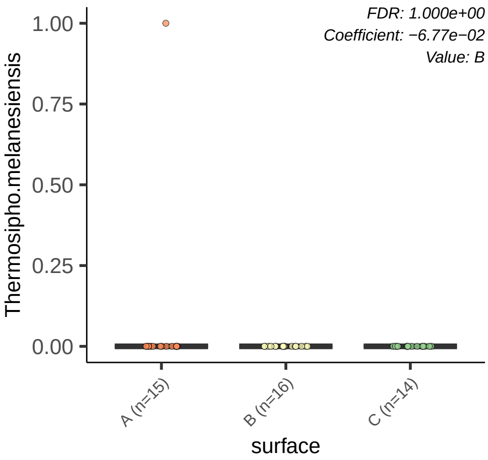
FDR: 1.000e+00
Coefficient: −6.77e−02
Value: B
Thermosipho.melanesiensis
1.00
0.75
0.50
0.25
0.00
A (n=15)
B (n=16)
C (n=14)
surface

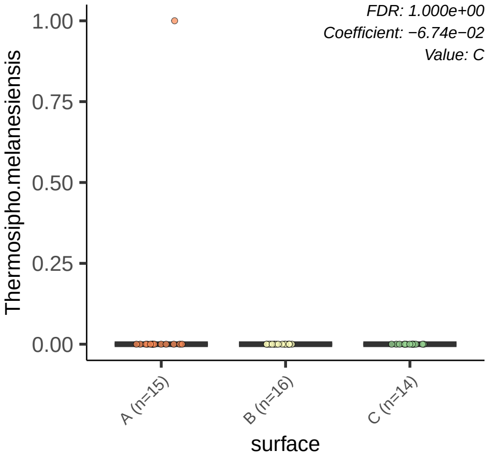

FDR: 1.000e+00
Coefficient: −6.74e−02
Value: C
Thermosipho.melanesiensis
1.00
0.75
0.50
0.25
0.00
A (n=15)
B (n=16)
C (n=14)
surface

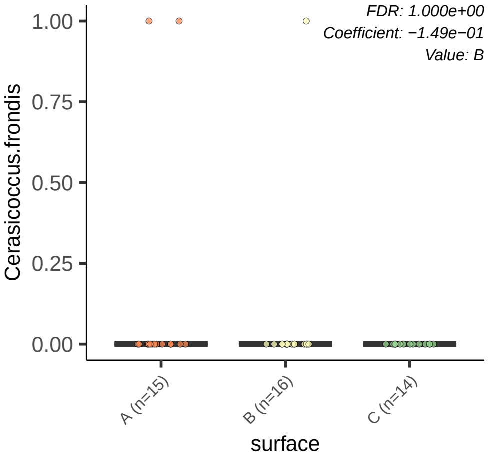

FDR: 1.000e+00
Coefficient: −1.49e−01
Value: B
Cerasicoccus.frondis
1.00
0.75
0.50
0.25
0.00
A (n=15)
B (n=16)
C (n=14)
surface

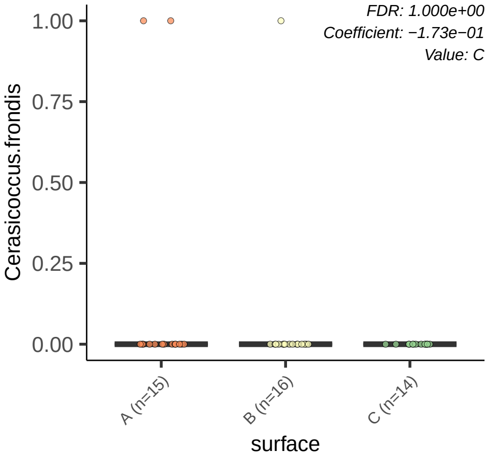

FDR: 1.000e+00
Coefficient: −1.73e−01
Value: C
Cerasicoccus.frondis
1.00
0.75
0.50
0.25
0.00
A (n=15)
B (n=16)
C (n=14)
surface

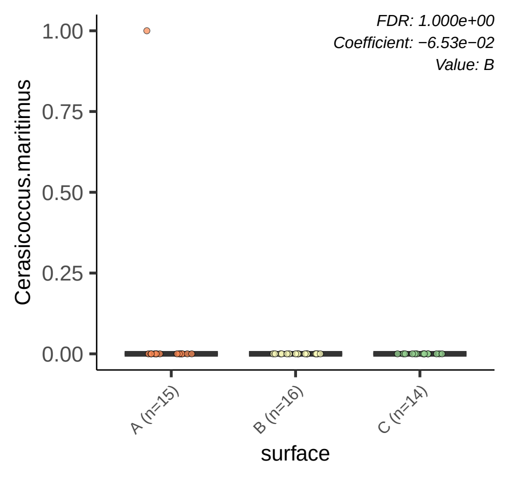

FDR: 1.000e+00
Coefficient: −6.53e−02
Value: B
Cerasicoccus.maritimus
1.00
0.75
0.50
0.25
0.00
A (n=15)
B (n=16)
C (n=14)
surface

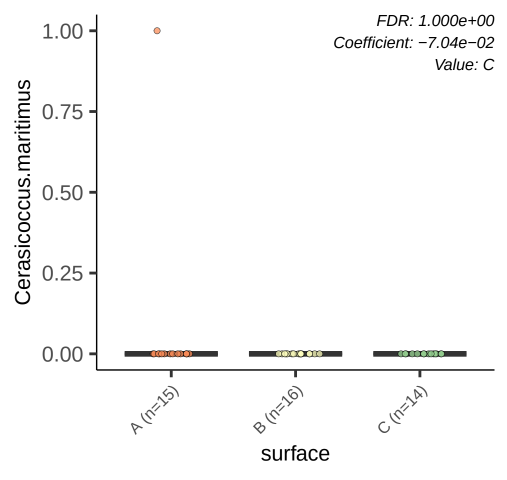
FDR: 1.000e+00
Coefficient: −7.04e−02
Value: C
Cerasicoccus.maritimus
1.00
0.75
0.50
0.25
0.00
A (n=15)
B (n=16)
C (n=14)
surface

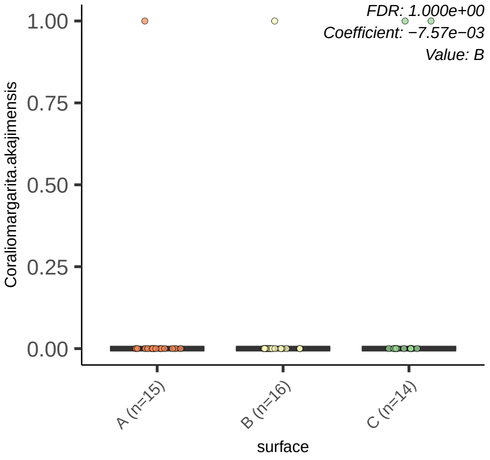
FDR: 1.000e+00
Coefficient: −7.57e−03
Value: B
Coraliomargarita.akajimensis
1.00
0.75
0.50
0.25
0.00
A (n=15)
B (n=16)
C (n=14)
surface

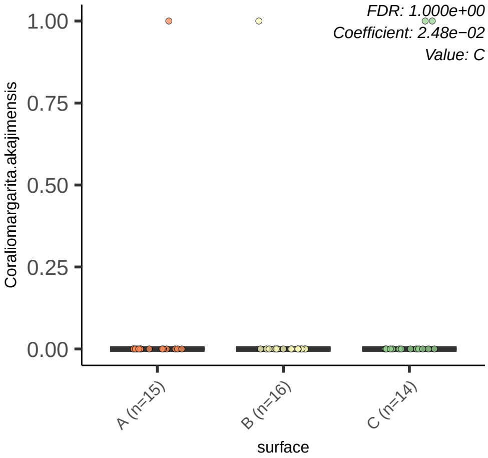
FDR: 1.000e+00
Coefficient: 2.48e−02
Value: C
Coraliomargarita.akajimensis
1.00
0.75
0.50
0.25
0.00
A (n=15)
B (n=16)
C (n=14)
surface

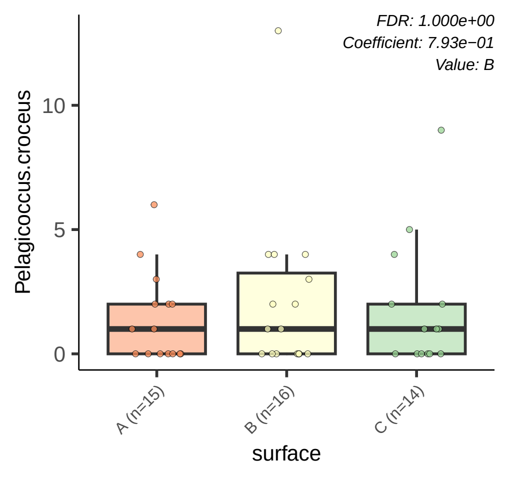
FDR: 1.000e+00
Coefficient: 7.93e−01
Value: B
Pelagicoccus.croceus
10
5
0
A (n=15)
B (n=16)
C (n=14)
surface

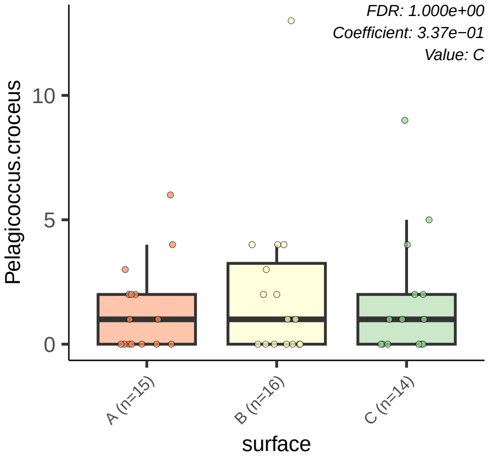
FDR: 1.000e+00
Coefficient: 3.37e−01
Value: C
Pelagicoccus.croceus
0
5
10
A (n=15)
B (n=16)
C (n=14)
surface

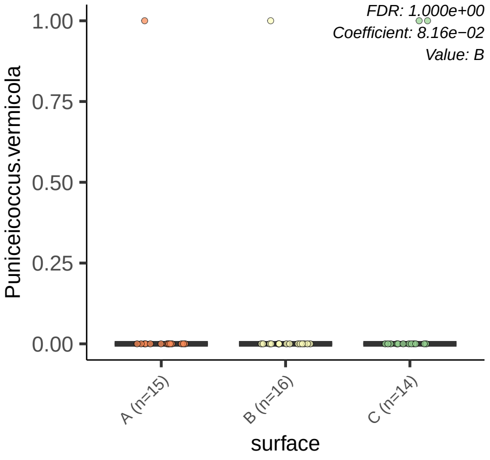

FDR: 1.000e+00
Coefficient: 8.16e−02
Value: B
Puniceicoccus.vermicola
1.00
0.75
0.50
0.25
0.00
A (n=15)
B (n=16)
C (n=14)
surface

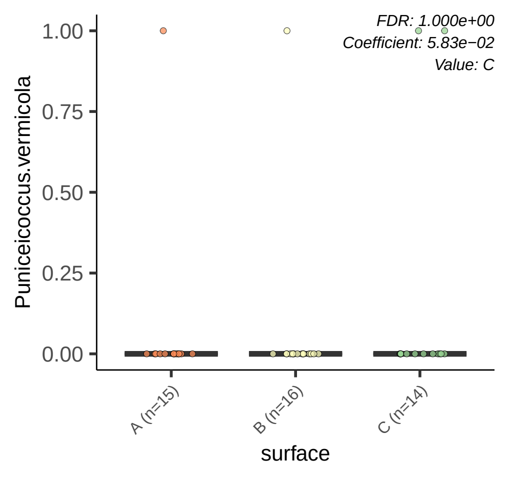

FDR: 1.000e+00
Coefficient: 5.83e−02
Value: C
Puniceicoccus.vermicola
1.00
0.75
0.50
0.25
0.00
A (n=15)
B (n=16)
C (n=14)
surface

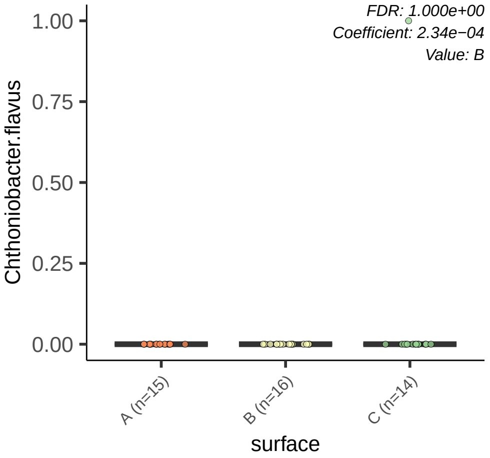
FDR: 1.000e+00
Coefficient: 2.34e−04
Value: B
1.00
0.75
0.50
0.25
0.00
Chthoniobacter.flavus
A (n=15)
B (n=16)
C (n=14)
surface

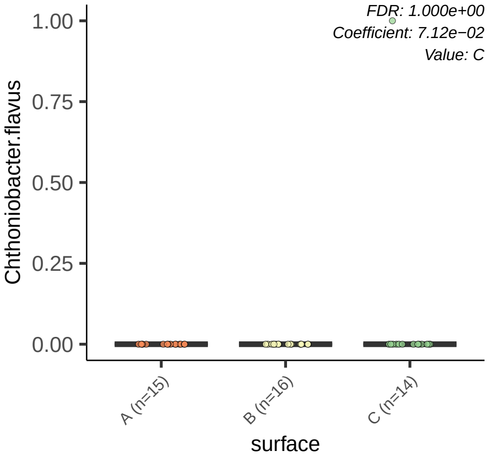

FDR: 1.000e+00
Coefficient: 7.12e−02
Value: C
Chthoniobacter.flavus
1.00
0.75
0.50
0.25
0.00
A (n=15)
B (n=16)
C (n=14)
surface

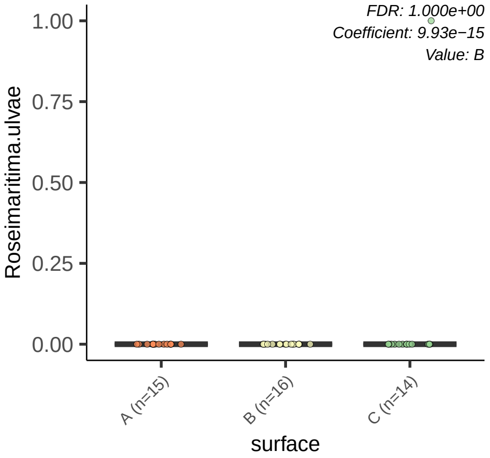

FDR: 1.000e+00
Coefficient: 9.93e−15
Value: B
Roseimaritima.ulvae
1.00
0.75
0.50
0.25
0.00
A (n=15)
B (n=16)
C (n=14)
surface

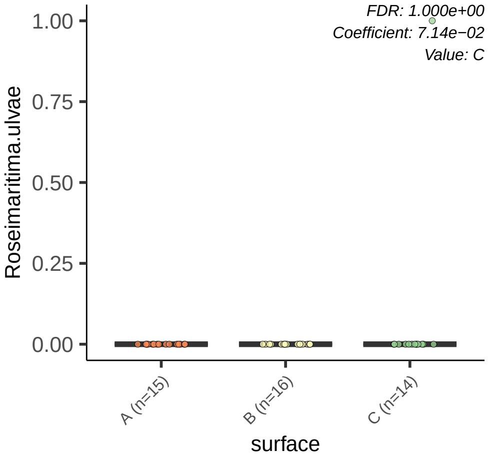

FDR: 1.000e+00
Coefficient: 7.14e−02
Value: C
Roseimaritima.ulvae
1.00
0.75
0.50
0.25
0.00
A (n=15)
B (n=16)
C (n=14)
surface

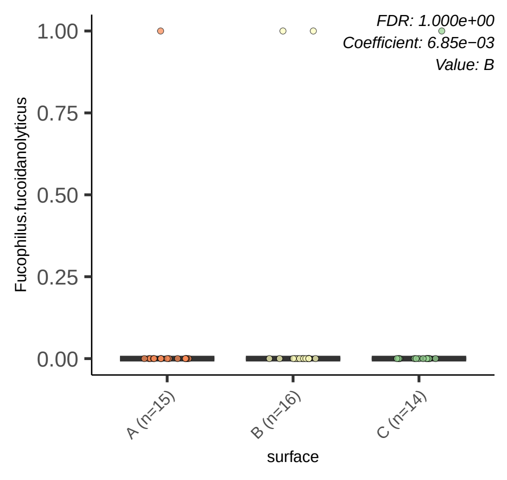
FDR: 1.000e+00
Coefficient: 6.85e−03
Value: B
Fucophilus.fucoidanolyticus
1.00
0.75
0.50
0.25
0.00
A (n=15)
B (n=16)
C (n=14)
surface

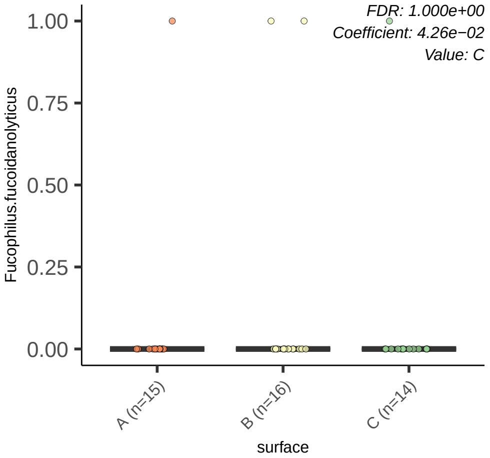
FDR: 1.000e+00
Coefficient: 4.26e−02
Value: C
Fucophilus.fucoidanolyticus
1.00
0.75
0.50
0.25
0.00
A (n=15)
B (n=16)
C (n=14)
surface

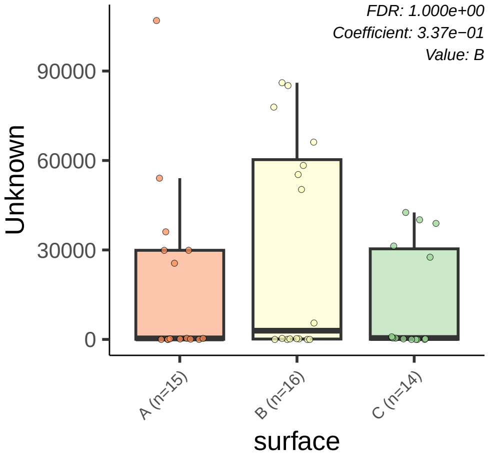
FDR: 1.000e+00
Coefficient: 3.37e−01
Value: B
Unknown
90000
60000
30000
0
A (n=15)
B (n=16)
C (n=14)
surface

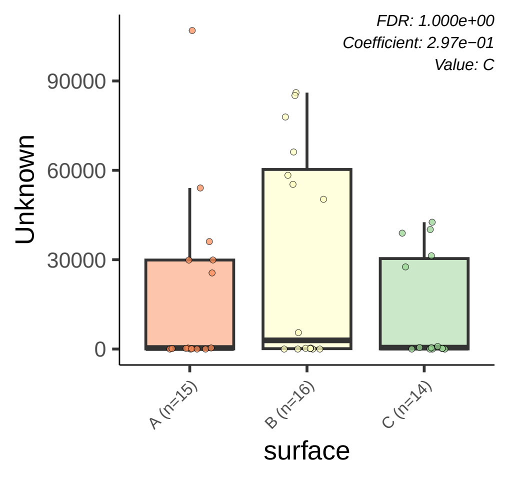

FDR: 1.000e+00
Coefficient: 2.97e−01
Value: C
90000
60000
30000
0
Unknown
A (n=15)
B (n=16)
C (n=14)
surface

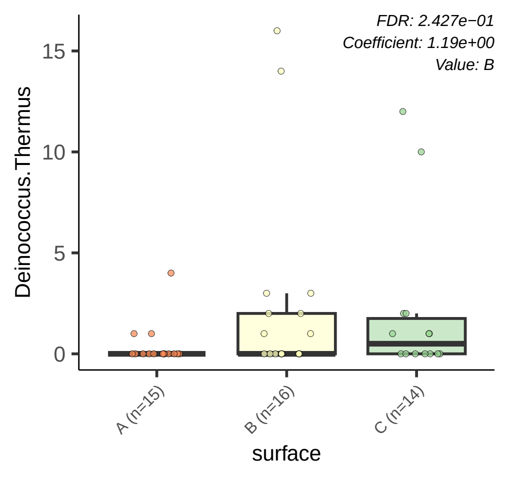
FDR: 2.427e−01
Coefficient: 1.19e+00
Value: B
Deinococcus.Thermus
15
10
5
0
A (n=15)
B (n=16)
C (n=14)
surface

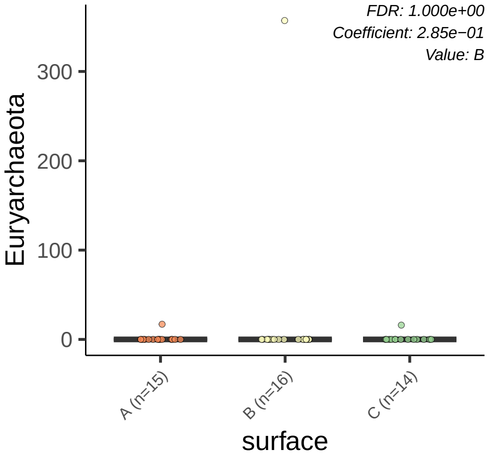

FDR: 1.000e+00
Coefficient: 2.85e−01
Value: B
Euryarchaeota
300
200
100
0
A (n=15)
B (n=16)
C (n=14)
surface

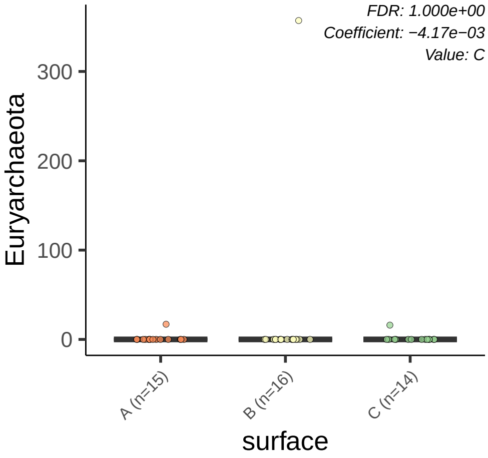
FDR: 1.000e+00
Coefficient: −4.17e−03
Value: C
Euryarchaeota
300
200
100
0
A (n=15)
B (n=16)
C (n=14)
surface

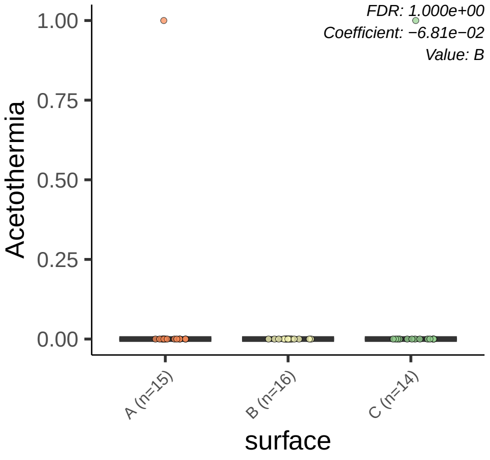
FDR: 1.000e+00
Coefficient: −6.81e−02
Value: B
Acetothermia
1.00
0.75
0.50
0.25
0.00
A (n=15)
B (n=16)
C (n=14)
surface

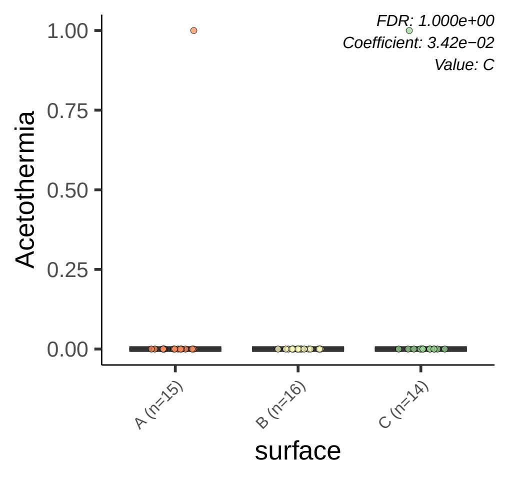
FDR: 1.000e+00
Coefficient: 3.42e−02
Value: C
Acetothermia
1.00
0.75
0.50
0.25
0.00
A (n=15)
B (n=16)
C (n=14)
surface

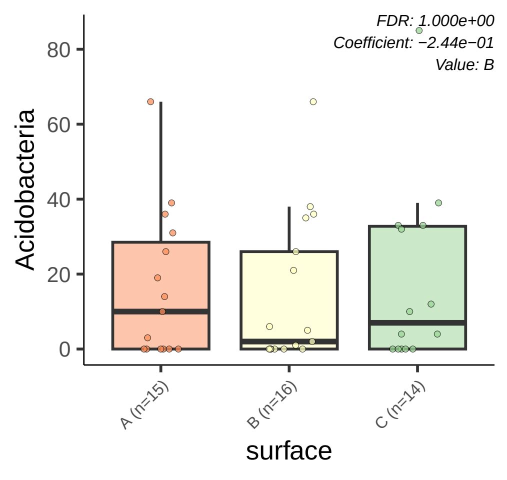
FDR: 1.000e+00
Coefficient: −2.44e−01
Value: B
Acidobacteria
0
20
40
60
80
A (n=15)
B (n=16)
C (n=14)
surface

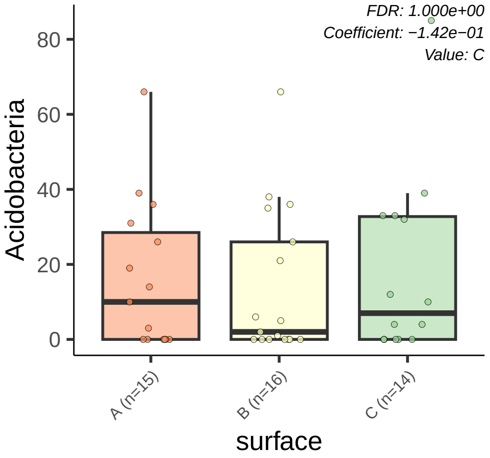

FDR: 1.000e+00
Coefficient: −1.42e−01
Value: C
Acidobacteria
0
20
40
60
80
A (n=15)
B (n=16)
C (n=14)
surface

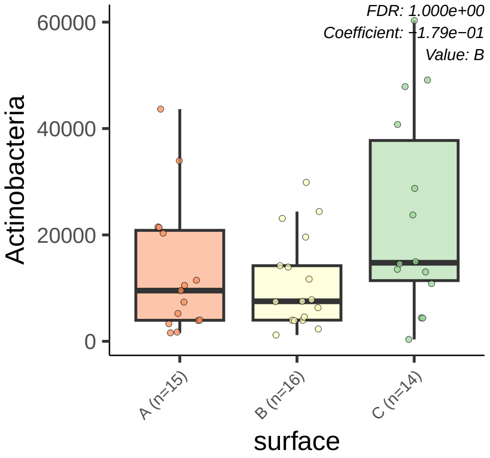
FDR: 1.000e+00
Coefficient: −1.79e−01
Value: B
Actinobacteria
60000
40000
20000
0
A (n=15)
B (n=16)
C (n=14)
surface

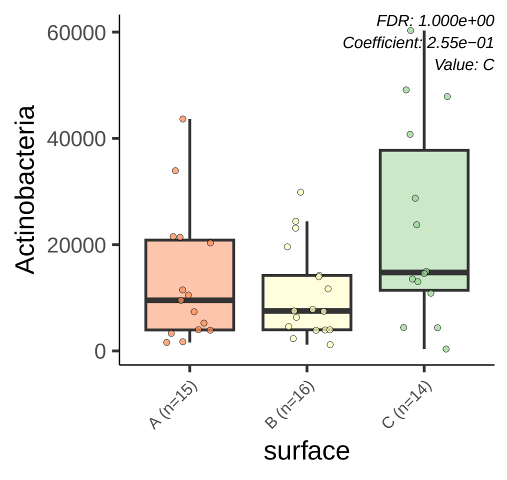

FDR: 1.000e+00
Coefficient: 2.55e−01
Value: C
Actinobacteria
60000
40000
20000
0
A (n=15)
B (n=16)
C (n=14)
surface

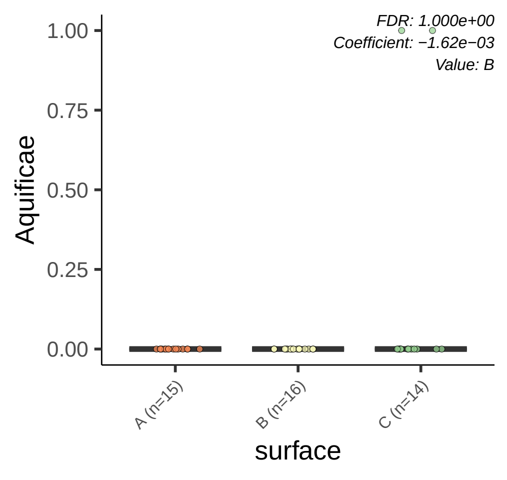
FDR: 1.000e+00
Coefficient: −1.62e−03
Value: B
Aquificae
1.00
0.75
0.50
0.25
0.00
A (n=15)
B (n=16)
C (n=14)
surface

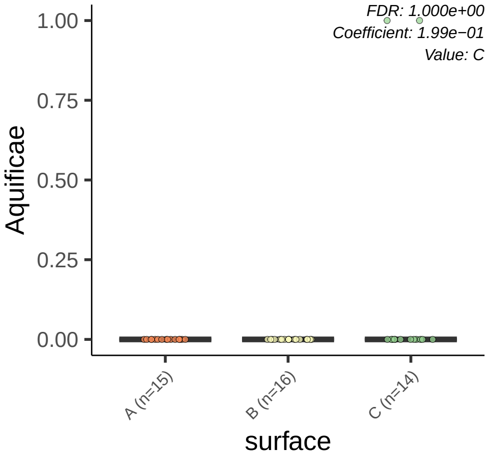
FDR: 1.000e+00
Coefficient: 1.99e−01
Value: C
Aquificae
1.00
0.75
0.50
0.25
0.00
A (n=15)
B (n=16)
C (n=14)
surface

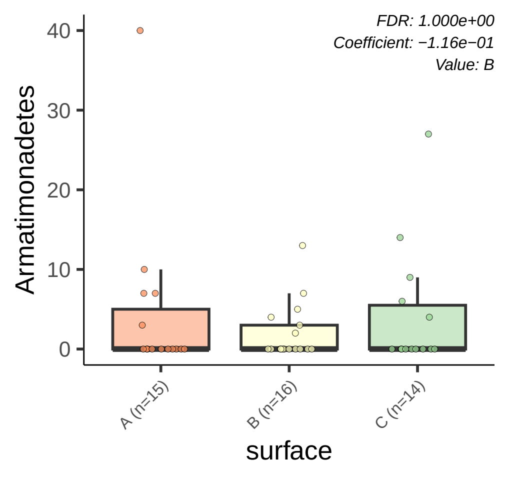

FDR: 1.000e+00
Coefficient: −1.16e−01
Value: B
Armatimonadetes
40
30
20
10
0
A (n=15)
B (n=16)
C (n=14)
surface

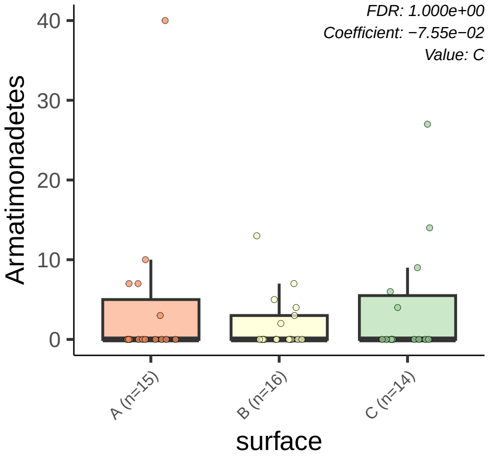

FDR: 1.000e+00
Coefficient: −7.55e−02
Value: C
Armatimonadetes
40
30
20
10
0
A (n=15)
B (n=16)
C (n=14)
surface

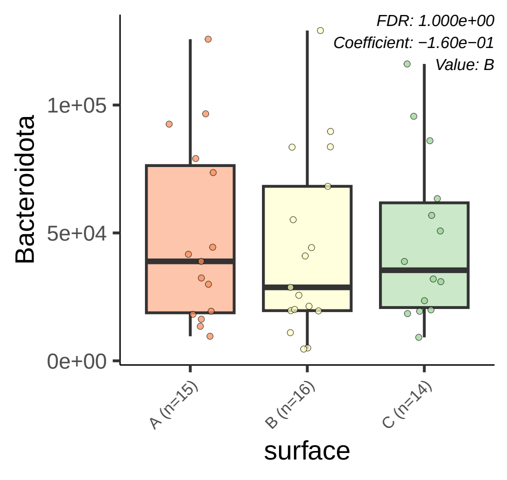
FDR: 1.000e+00
Coefficient: −1.60e−01
Value: B
Bacteroidota
1e+05
5e+04
0e+00
A (n=15)
B (n=16)
C (n=14)
surface

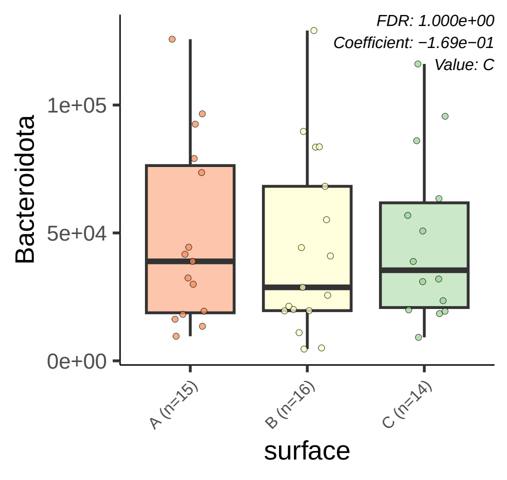

FDR: 1.000e+00
Coefficient: −1.69e−01
Value: C
Bacteroidota
1e+05
5e+04
0e+00
A (n=15)
B (n=16)
C (n=14)
surface

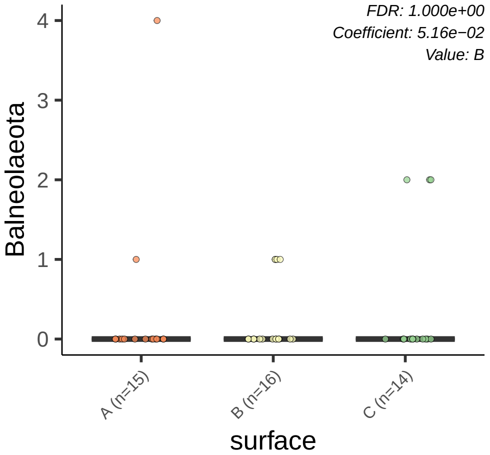
FDR: 1.000e+00
Coefficient: 5.16e−02
Value: B
Balneolaeota
0
1
2
3
4
A (n=15)
B (n=16)
C (n=14)
surface

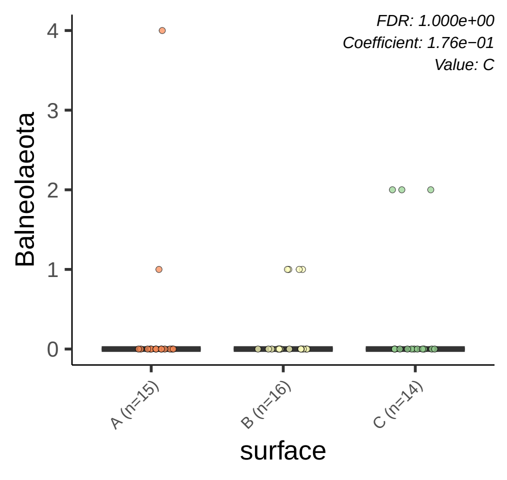
FDR: 1.000e+00
Coefficient: 1.76e−01
Value: C
Balneolaeota
0
1
2
3
4
A (n=15)
B (n=16)
C (n=14)
surface

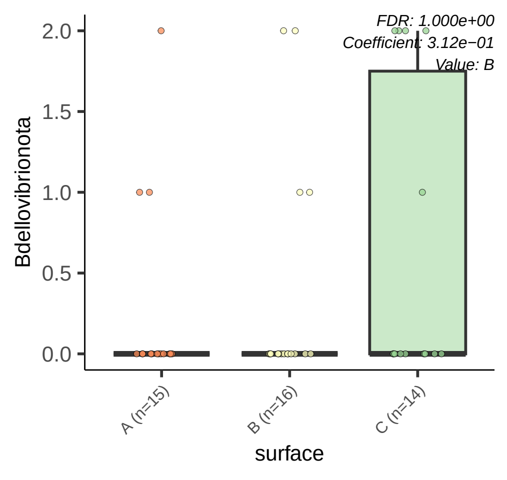

FDR: 1.000e+00
Coefficient: 3.12e−01
Value: B
Bdellovibrionota
2.0
1.5
1.0
0.5
0.0
A (n=15)
B (n=16)
C (n=14)
surface

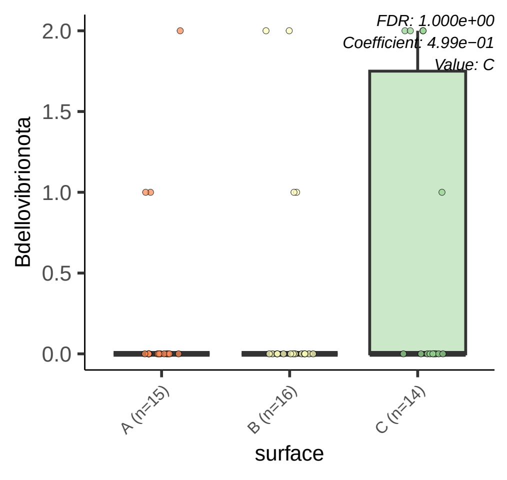
FDR: 1.000e+00
Coefficient: 4.99e−01
Value: C
Bdellovibrionota
2.0
1.5
1.0
0.5
0.0
A (n=15)
B (n=16)
C (n=14)
surface

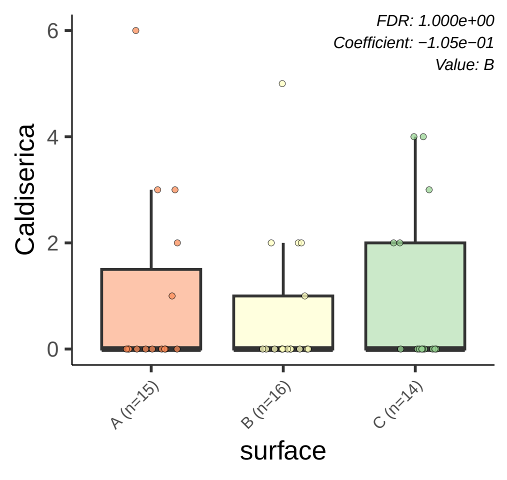

FDR: 1.000e+00
Coefficient: −1.05e−01
Value: B
Caldiserica
6
4
2
0
A (n=15)
B (n=16)
C (n=14)
surface

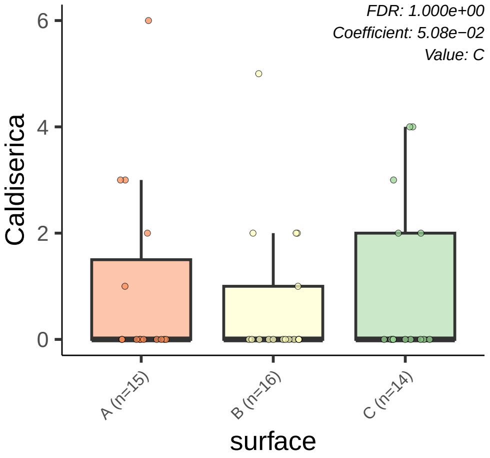

FDR: 1.000e+00
Coefficient: 5.08e−02
Value: C
Caldiserica
6
4
2
0
A (n=15)
B (n=16)
C (n=14)
surface

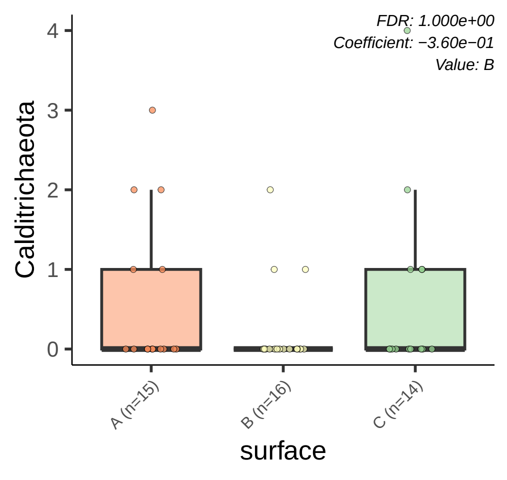
FDR: 1.000e+00
Coefficient: −3.60e−01
Value: B
Calditrichaeota
0
1
2
3
4
A (n=15)
B (n=16)
C (n=14)
surface

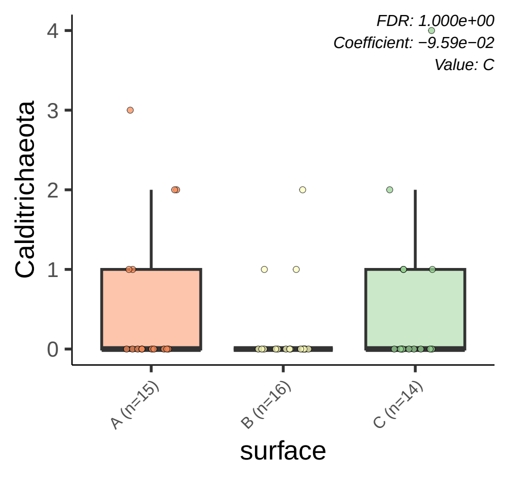
FDR: 1.000e+00
Coefficient: −9.59e−02
Value: C
Calditrichaeota
0
1
2
3
4
A (n=15)
B (n=16)
C (n=14)
surface

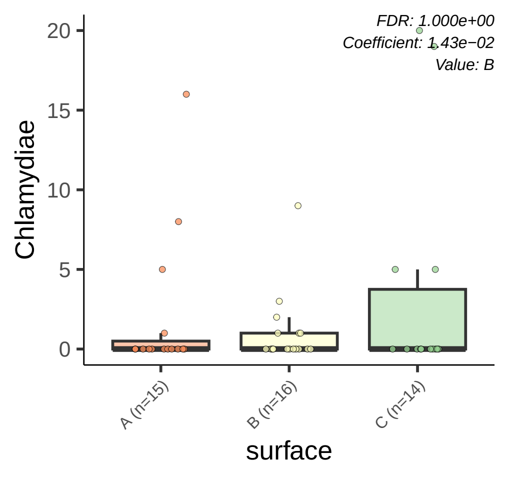
FDR: 1.000e+00
Coefficient: 1.43e−02
Value: B
Chlamydiae
20
15
10
5
0
A (n=15)
B (n=16)
C (n=14)
surface

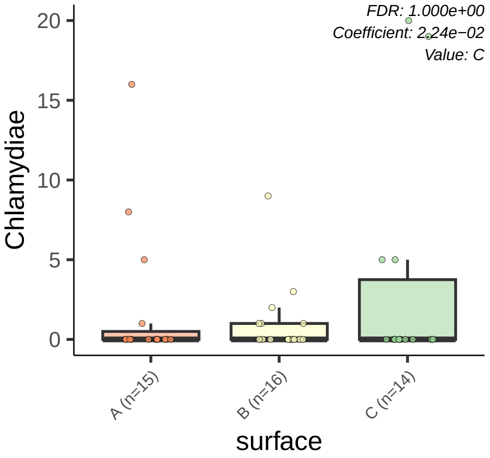

FDR: 1.000e+00
Coefficient: 2.24e−02
Value: C
Chlamydiae
20
15
10
5
0
A (n=15)
B (n=16)
C (n=14)
surface

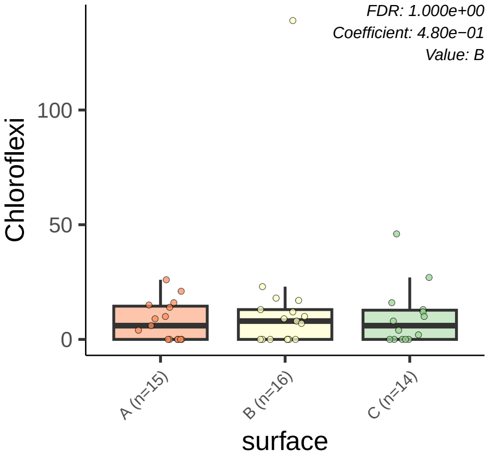

FDR: 1.000e+00
Coefficient: 4.80e−01
Value: B
Chloroflexi
100
50
0
A (n=15)
B (n=16)
C (n=14)
surface

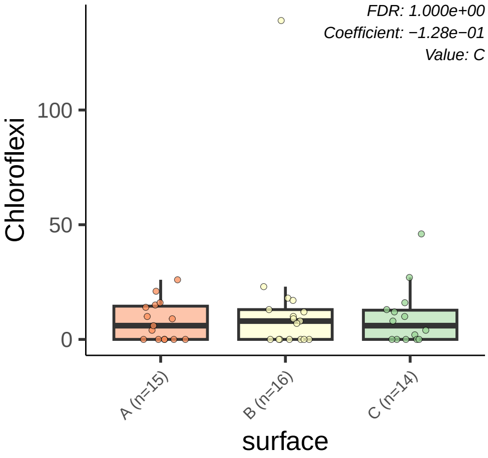
FDR: 1.000e+00
Coefficient: −1.28e−01
Value: C
Chloroflexi
100
50
0
A (n=15)
B (n=16)
C (n=14)
surface

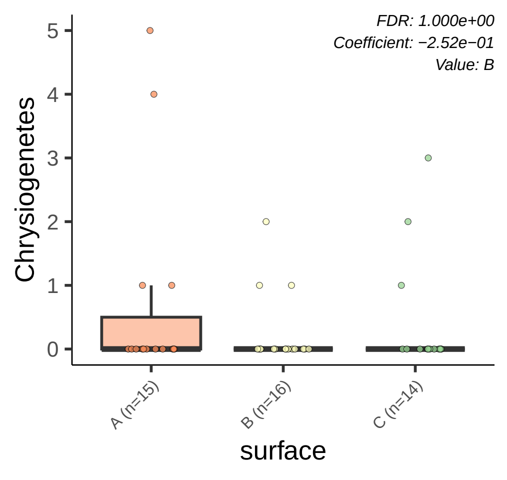
FDR: 1.000e+00
Coefficient: −2.52e−01
Value: B
Chrysiogenetes
0
1
2
3
4
5
A (n=15)
B (n=16)
C (n=14)
surface

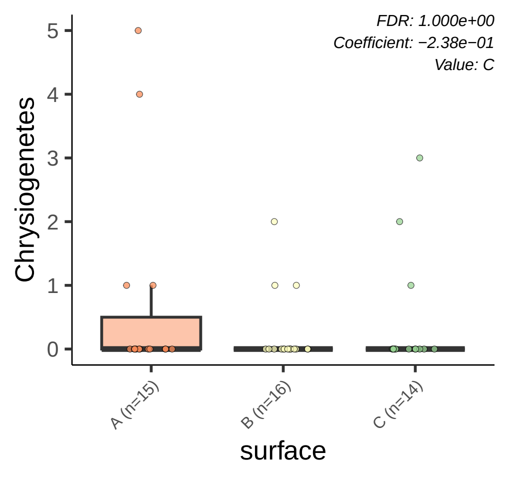

FDR: 1.000e+00
Coefficient: −2.38e−01
Value: C
Chrysiogenetes
5
4
3
2
1
0
A (n=15)
B (n=16)
C (n=14)
surface

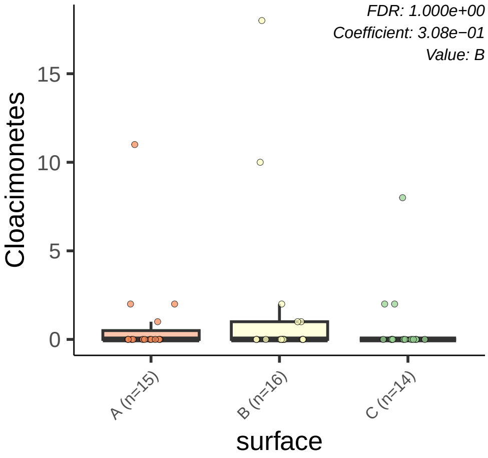
FDR: 1.000e+00
Coefficient: 3.08e−01
Value: B
Cloacimonetes
0
5
10
15
A (n=15)
B (n=16)
C (n=14)
surface

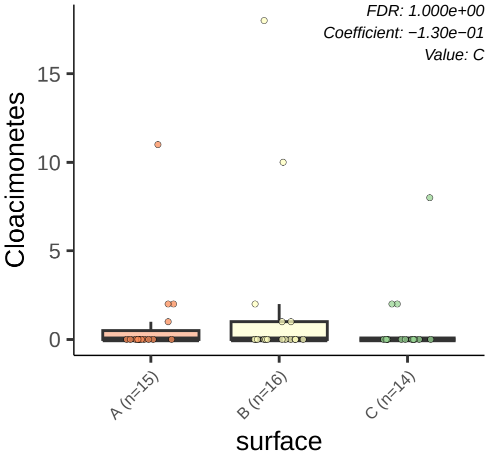

FDR: 1.000e+00
Coefficient: −1.30e−01
Value: C
Cloacimonetes
0
5
10
15
A (n=15)
B (n=16)
C (n=14)
surface

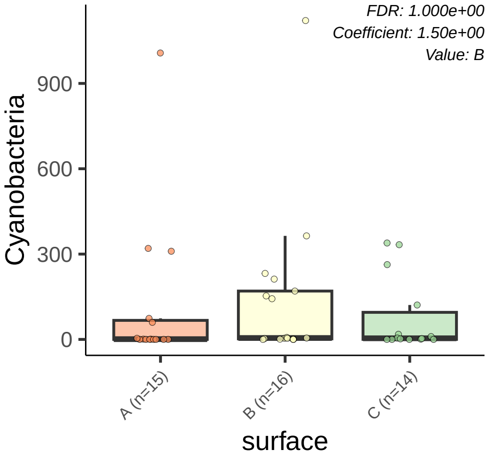
FDR: 1.000e+00
Coefficient: 1.50e+00
Value: B
Cyanobacteria
900
600
300
0
A (n=15)
B (n=16)
C (n=14)
surface

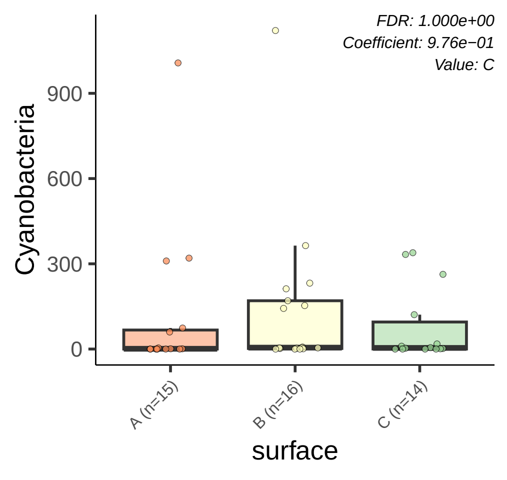
FDR: 1.000e+00
Coefficient: 9.76e−01
Value: C
Cyanobacteria
900
600
300
0
A (n=15)
B (n=16)
C (n=14)
surface

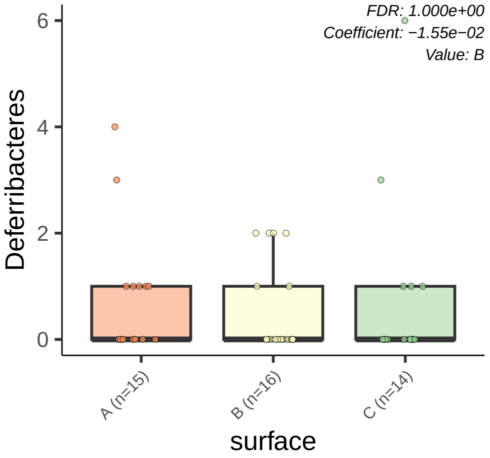

FDR: 1.000e+00
Coefficient: −1.55e−02
Value: B
Deferribacteres
0
2
4
6
A (n=15)
B (n=16)
C (n=14)
surface

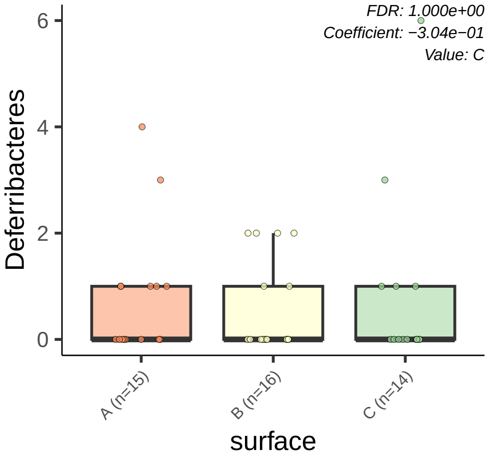
FDR: 1.000e+00
Coefficient: −3.04e−01
Value: C
Deferribacteres
6
4
2
0
A (n=15)
B (n=16)
C (n=14)
surface

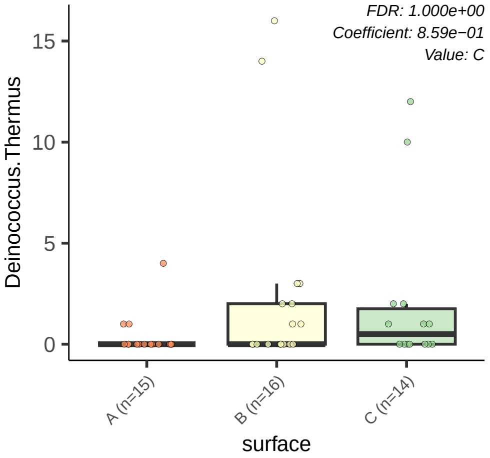

FDR: 1.000e+00
Coefficient: 8.59e−01
Value: C
Deinococcus.Thermus
15
10
5
0
A (n=15)
B (n=16)
C (n=14)
surface

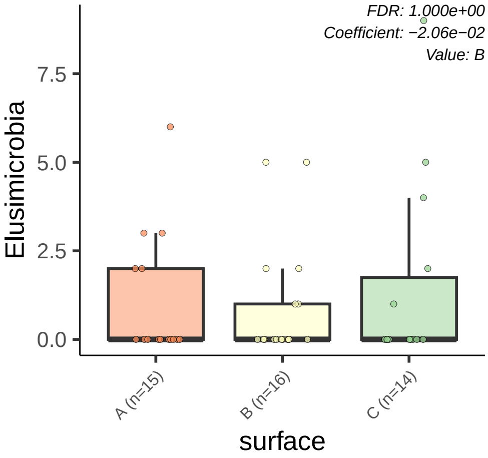

FDR: 1.000e+00
Coefficient: −2.06e−02
Value: B
Elusimicrobia
7.5
5.0
2.5
0.0
A (n=15)
B (n=16)
C (n=14)
surface

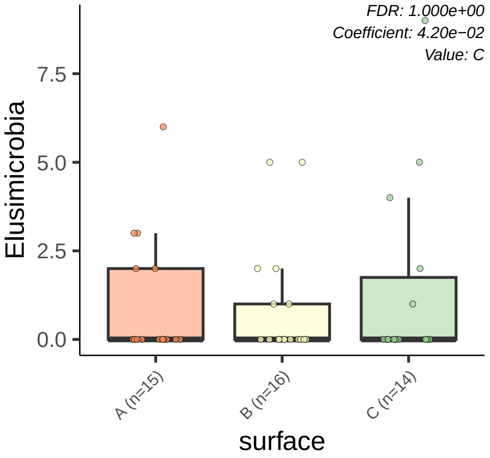
FDR: 1.000e+00
Coefficient: 4.20e−02
Value: C
Elusimicrobia
7.5
5.0
2.5
0.0
A (n=15)
B (n=16)
C (n=14)
surface

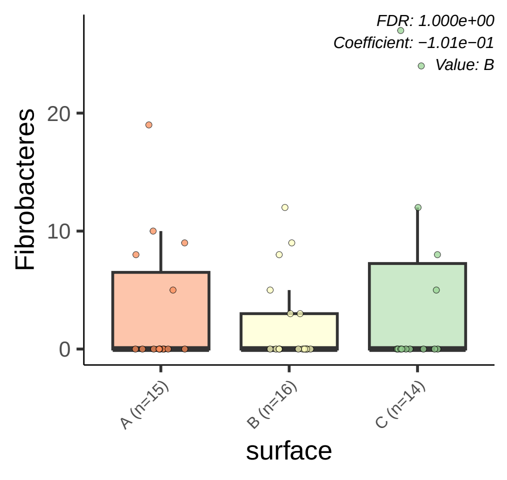

FDR: 1.000e+00
Coefficient: −1.01e−01
Value: B
Fibrobacteres
0
10
20
A (n=15)
B (n=16)
C (n=14)
surface

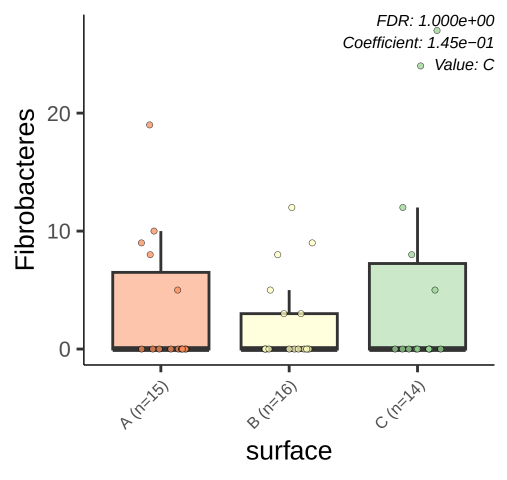

FDR: 1.000e+00
Coefficient: 1.45e−01
Value: C
Fibrobacteres
20
10
0
A (n=15)
B (n=16)
C (n=14)
surface

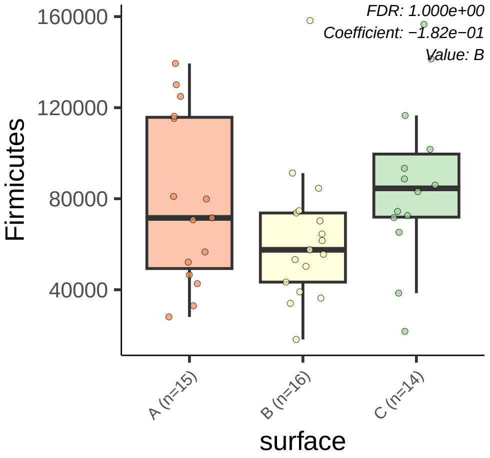

FDR: 1.000e+00
Coefficient: −1.82e−01
Value: B
160000
120000
80000
40000
Firmicutes
A (n=15)
B (n=16)
C (n=14)
surface

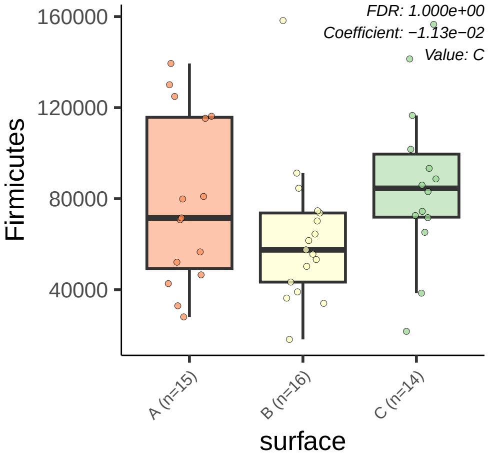

FDR: 1.000e+00
Coefficient: −1.13e−02
Value: C
Firmicutes
160000
120000
80000
40000
A (n=15)
B (n=16)
C (n=14)
surface

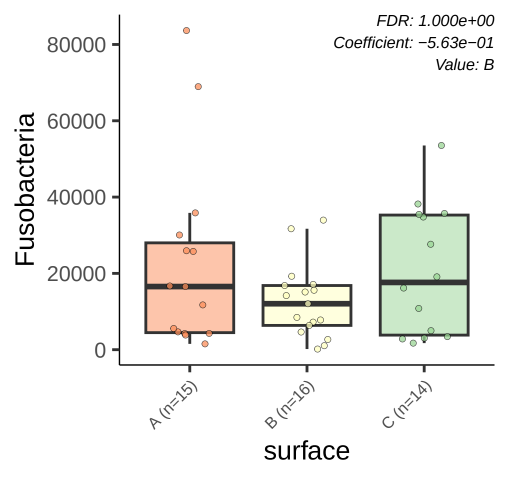
FDR: 1.000e+00
Coefficient: −5.63e−01
Value: B
Fusobacteria
80000
60000
40000
20000
0
A (n=15)
B (n=16)
C (n=14)
surface

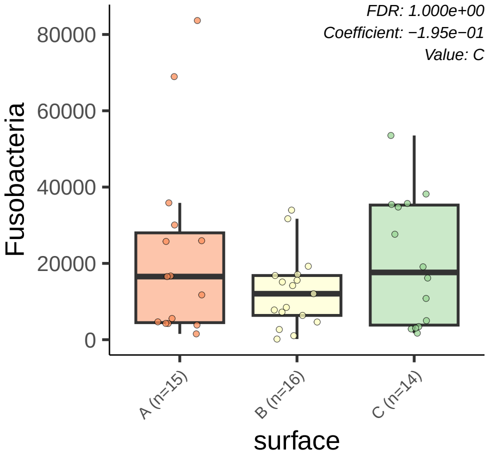

FDR: 1.000e+00
Coefficient: −1.95e−01
Value: C
Fusobacteria
80000
60000
40000
20000
0
A (n=15)
B (n=16)
C (n=14)
surface

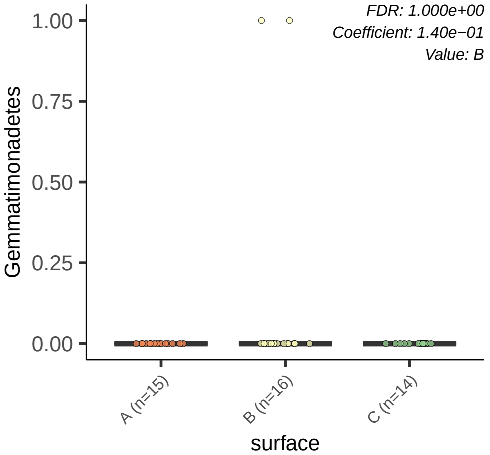

FDR: 1.000e+00
Coefficient: 1.40e−01
Value: B
Gemmatimonadetes
1.00
0.75
0.50
0.25
0.00
A (n=15)
B (n=16)
C (n=14)
surface

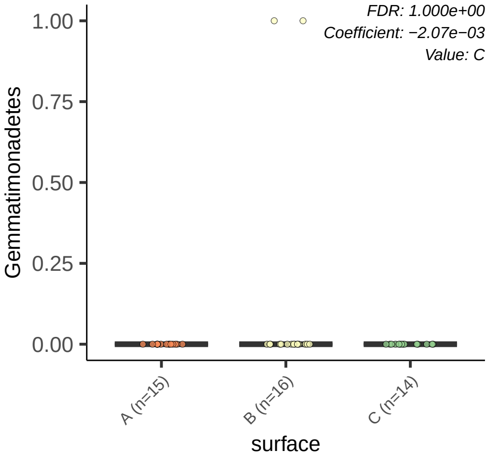

FDR: 1.000e+00
Coefficient: −2.07e−03
Value: C
Gemmatimonadetes
1.00
0.75
0.50
0.25
0.00
A (n=15)
B (n=16)
C (n=14)
surface

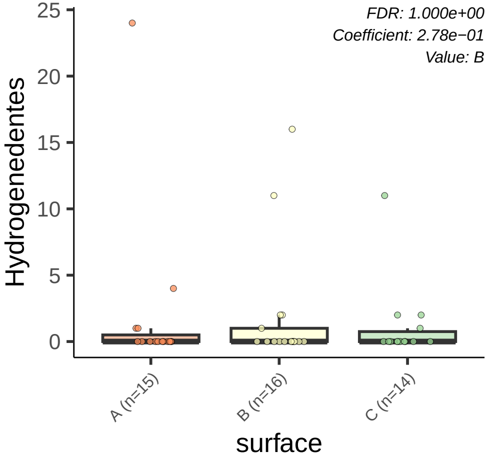
FDR: 1.000e+00
Coefficient: 2.78e−01
Value: B
Hydrogenedentes
0
5
10
15
20
25
A (n=15)
B (n=16)
C (n=14)
surface

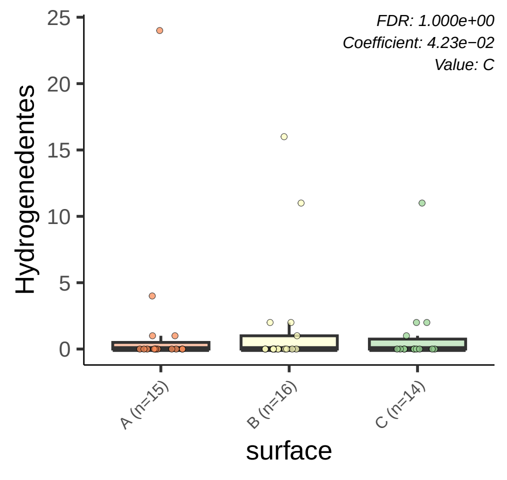
FDR: 1.000e+00
Coefficient: 4.23e−02
Value: C
Hydrogenedentes
25
20
15
10
5
0
A (n=15)
B (n=16)
C (n=14)
surface

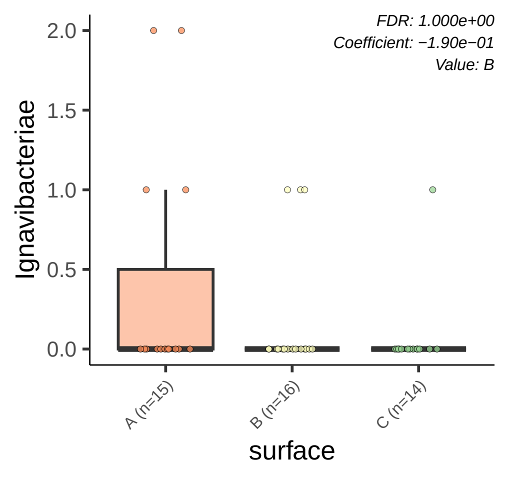
FDR: 1.000e+00
Coefficient: −1.90e−01
Value: B
Ignavibacteriae
2.0
1.5
1.0
0.5
0.0
A (n=15)
B (n=16)
C (n=14)
surface

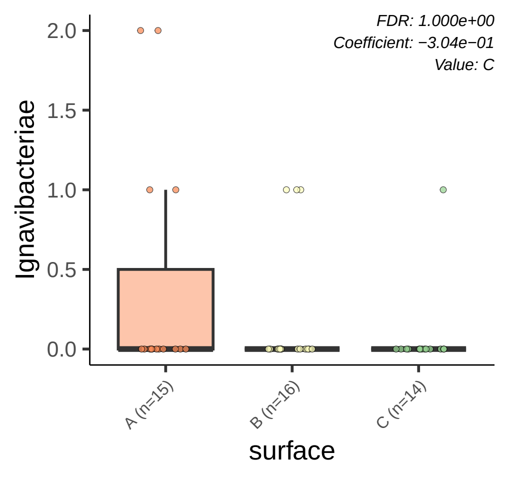

FDR: 1.000e+00
Coefficient: −3.04e−01
Value: C
Ignavibacteriae
2.0
1.5
1.0
0.5
0.0
A (n=15)
B (n=16)
C (n=14)
surface

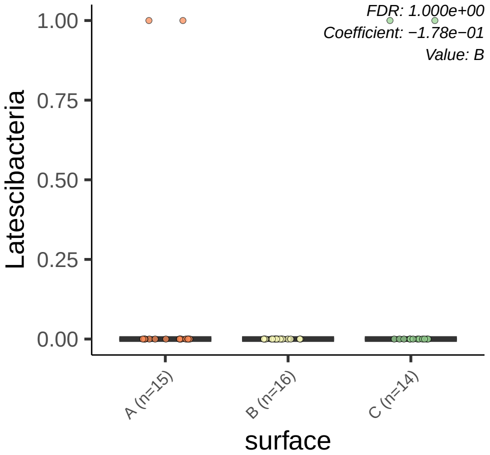
FDR: 1.000e+00
Coefficient: −1.78e−01
Value: B
Latescibacteria
1.00
0.75
0.50
0.25
0.00
A (n=15)
B (n=16)
C (n=14)
surface

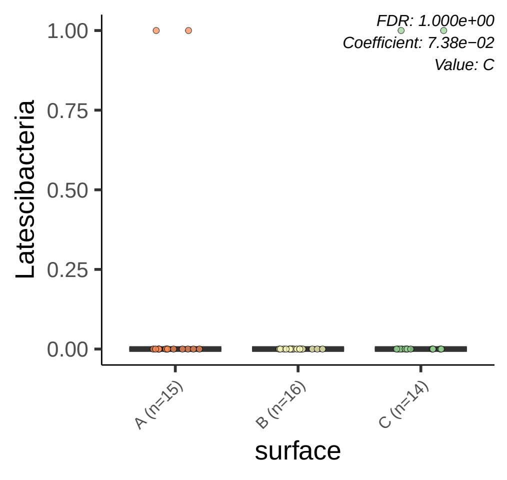
FDR: 1.000e+00
Coefficient: 7.38e−02
Value: C
Latescibacteria
1.00
0.75
0.50
0.25
0.00
A (n=15)
B (n=16)
C (n=14)
surface

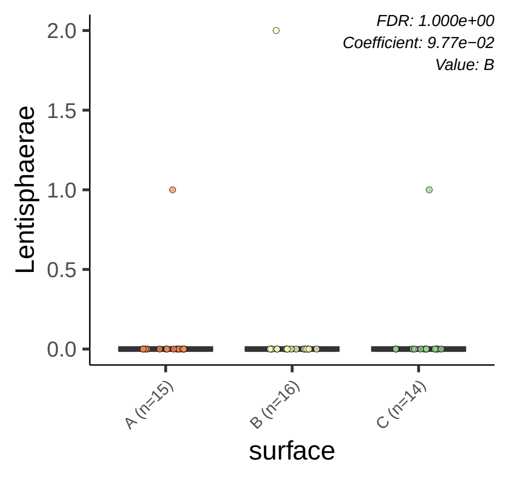
FDR: 1.000e+00
Coefficient: 9.77e−02
Value: B
Lentisphaerae
2.0
1.5
1.0
0.5
0.0
A (n=15)
B (n=16)
C (n=14)
surface

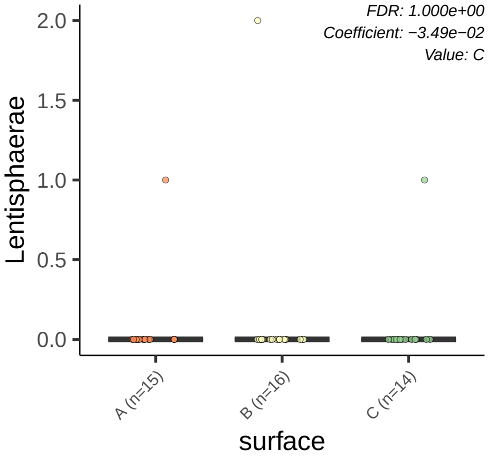
FDR: 1.000e+00
Coefficient: −3.49e−02
Value: C
Lentisphaerae
2.0
1.5
1.0
0.5
0.0
A (n=15)
B (n=16)
C (n=14)
surface

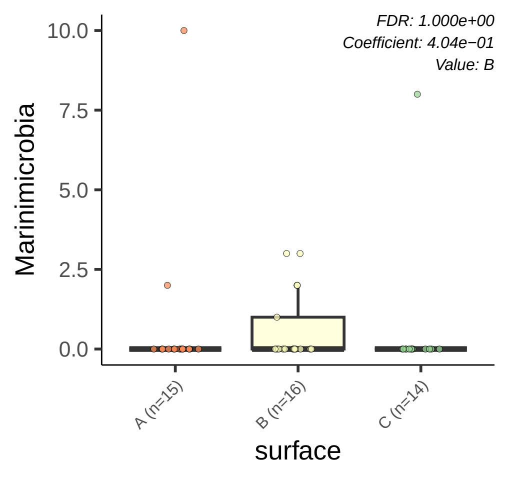
FDR: 1.000e+00
Coefficient: 4.04e−01
Value: B
Marinimicrobia
10.0
7.5
5.0
2.5
0.0
A (n=15)
B (n=16)
C (n=14)
surface

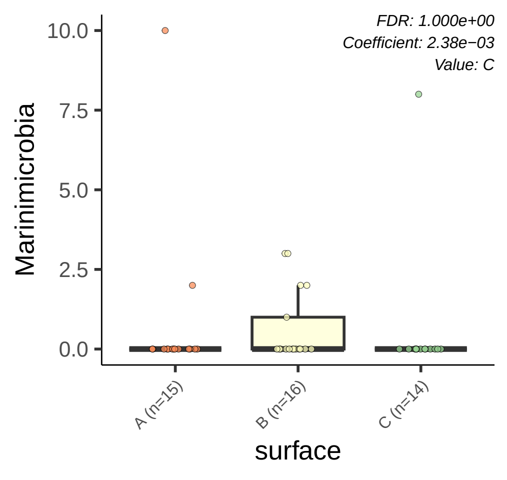
FDR: 1.000e+00
Coefficient: 2.38e−03
Value: C
Marinimicrobia
10.0
7.5
5.0
2.5
0.0
A (n=15)
B (n=16)
C (n=14)
surface

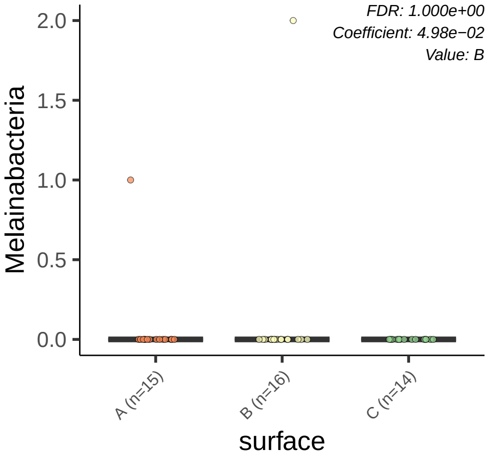
FDR: 1.000e+00
Coefficient: 4.98e−02
Value: B
Melainabacteria
2.0
1.5
1.0
0.5
0.0
A (n=15)
B (n=16)
C (n=14)
surface

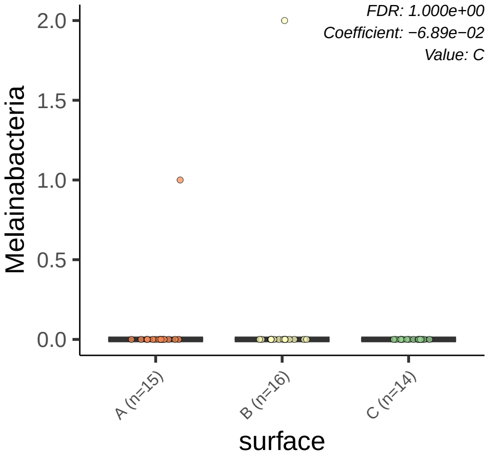
FDR: 1.000e+00
Coefficient: −6.89e−02
Value: C
Melainabacteria
2.0
1.5
1.0
0.5
0.0
A (n=15)
B (n=16)
C (n=14)
surface

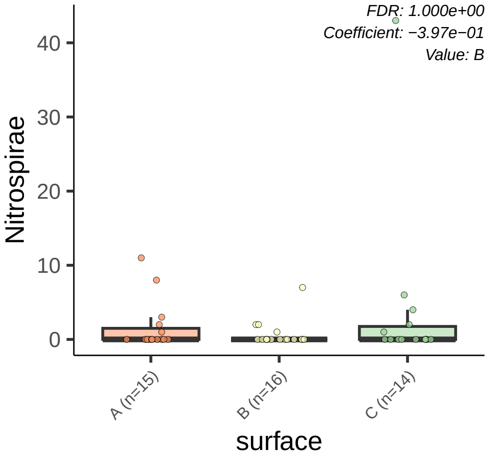

FDR: 1.000e+00
Coefficient: −3.97e−01
Value: B
Nitrospirae
40
30
20
10
0
A (n=15)
B (n=16)
C (n=14)
surface

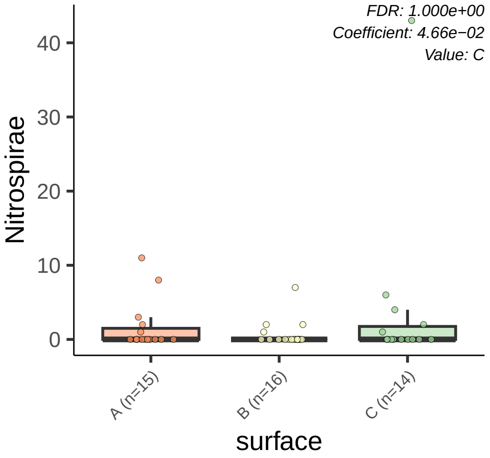
FDR: 1.000e+00
Coefficient: 4.66e−02
Value: C
Nitrospirae
40
30
20
10
0
A (n=15)
B (n=16)
C (n=14)
surface

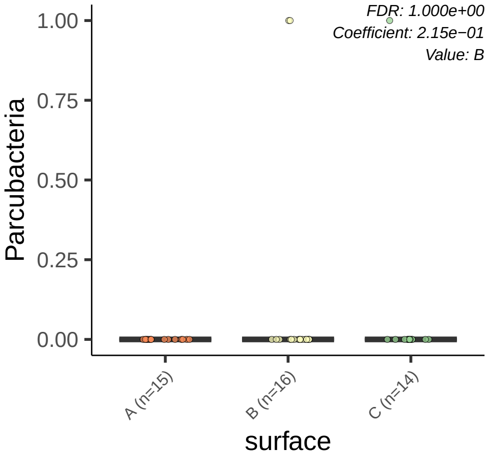

FDR: 1.000e+00
Coefficient: 2.15e−01
Value: B
Parcubacteria
1.00
0.75
0.50
0.25
0.00
A (n=15)
B (n=16)
C (n=14)
surface

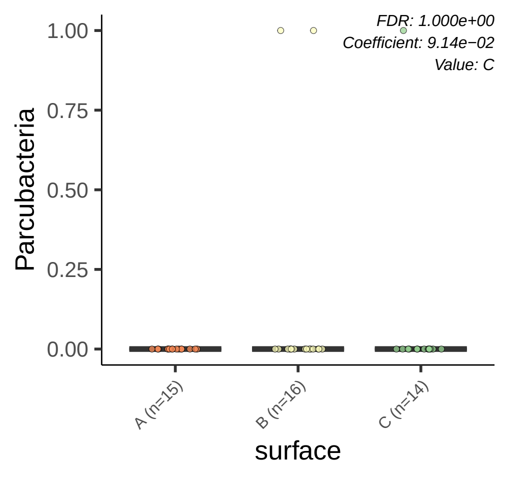

FDR: 1.000e+00
Coefficient: 9.14e−02
Value: C
Parcubacteria
1.00
0.75
0.50
0.25
0.00
A (n=15)
B (n=16)
C (n=14)
surface

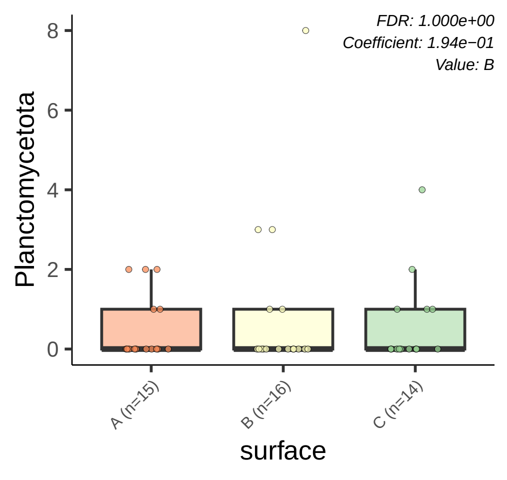
FDR: 1.000e+00
Coefficient: 1.94e−01
Value: B
Planctomycetota
0
2
4
6
8
A (n=15)
B (n=16)
C (n=14)
surface

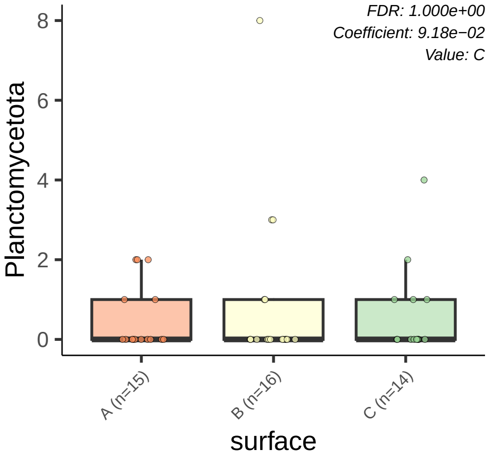

FDR: 1.000e+00
Coefficient: 9.18e−02
Value: C
Planctomycetota
0
2
4
6
8
A (n=15)
B (n=16)
C (n=14)
surface

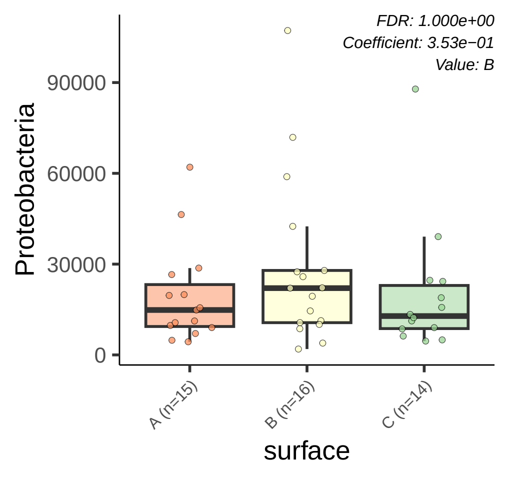
FDR: 1.000e+00
Coefficient: 3.53e−01
Value: B
Proteobacteria
90000
60000
30000
0
A (n=15)
B (n=16)
C (n=14)
surface

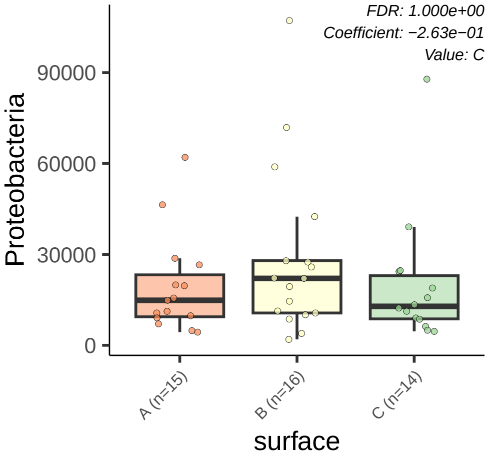

FDR: 1.000e+00
Coefficient: −2.63e−01
Value: C
Proteobacteria
90000
60000
30000
0
A (n=15)
B (n=16)
C (n=14)
surface

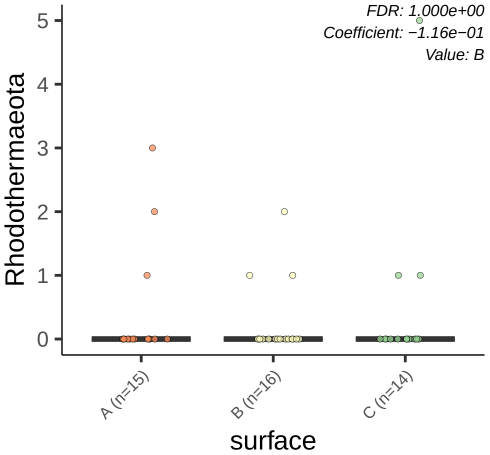

FDR: 1.000e+00
Coefficient: −1.16e−01
Value: B
Rhodothermaeota
0
1
2
3
4
5
A (n=15)
B (n=16)
C (n=14)
surface

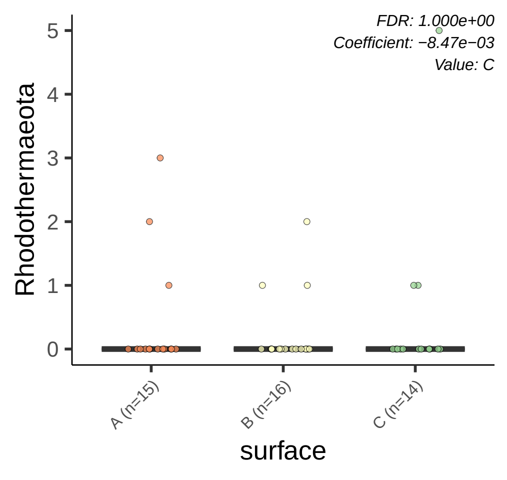
FDR: 1.000e+00
Coefficient: −8.47e−03
Value: C
Rhodothermaeota
0
1
2
3
4
5
A (n=15)
B (n=16)
C (n=14)
surface

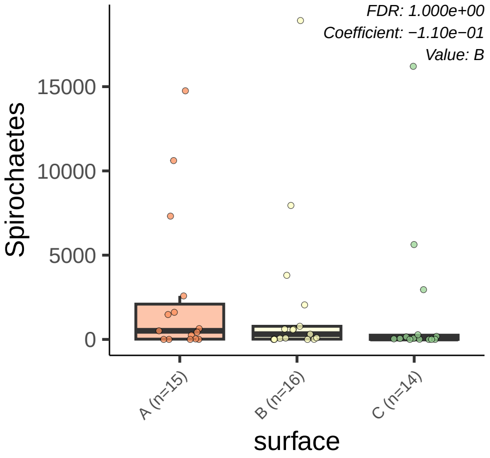

FDR: 1.000e+00
Coefficient: −1.10e−01
Value: B
Spirochaetes
15000
10000
5000
0
A (n=15)
B (n=16)
C (n=14)
surface

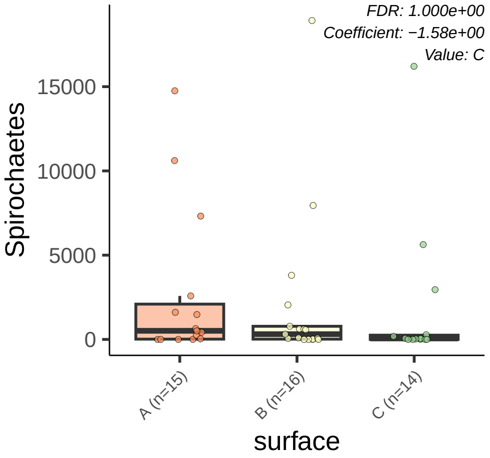

FDR: 1.000e+00
Coefficient: −1.58e+00
Value: C
Spirochaetes
15000
10000
5000
0
A (n=15)
B (n=16)
C (n=14)
surface

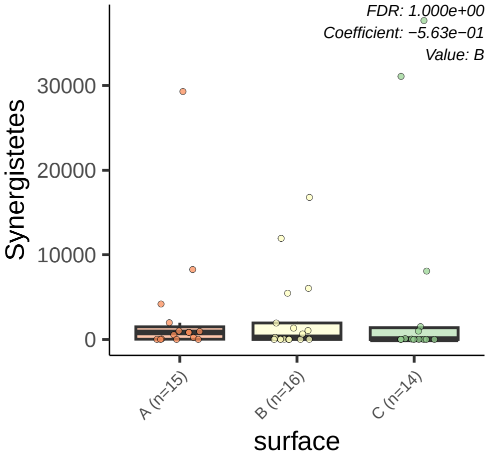

FDR: 1.000e+00
Coefficient: −5.63e−01
Value: B
Synergistetes
30000
20000
10000
0
A (n=15)
B (n=16)
C (n=14)
surface

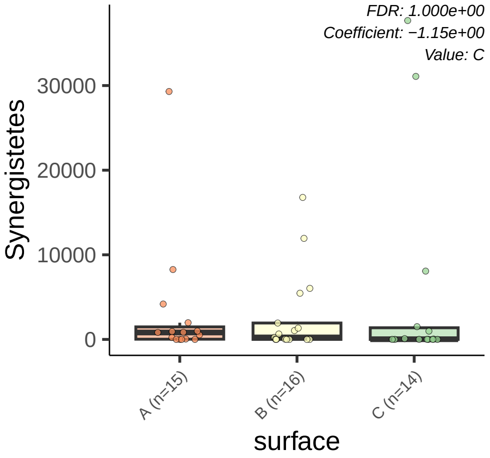

FDR: 1.000e+00
Coefficient: −1.15e+00
Value: C
Synergistetes
30000
20000
10000
0
A (n=15)
B (n=16)
C (n=14)
surface

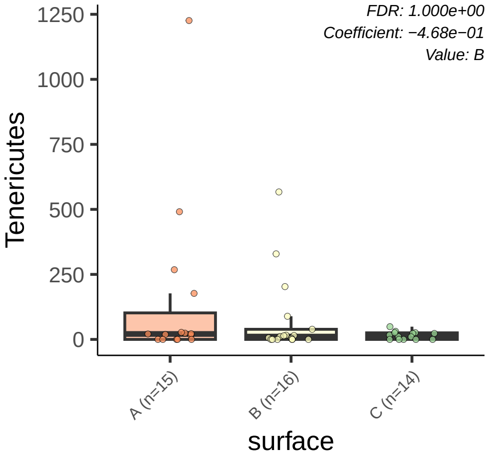
FDR: 1.000e+00
Coefficient: −4.68e−01
Value: B
Tenericutes
1250
1000
750
500
250
0
A (n=15)
B (n=16)
C (n=14)
surface

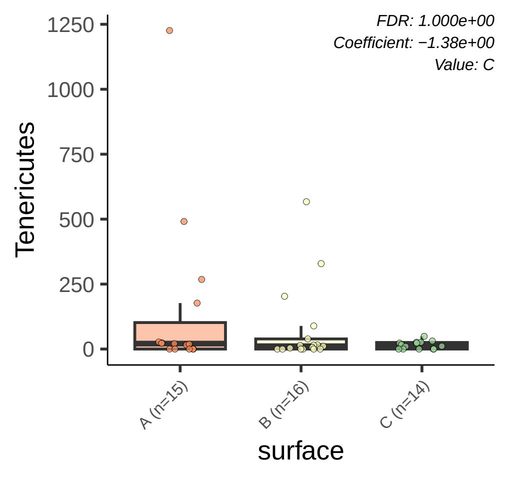

FDR: 1.000e+00
Coefficient: −1.38e+00
Value: C
Tenericutes
1250
1000
750
500
250
0
A (n=15)
B (n=16)
C (n=14)
surface

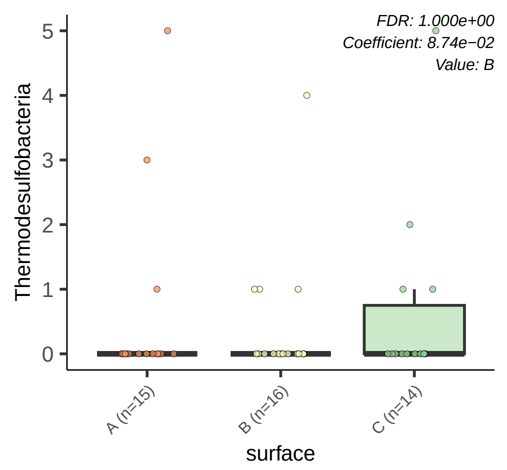
FDR: 1.000e+00
Coefficient: 8.74e−02
Value: B
Thermodesulfobacteria
0
1
2
3
4
5
A (n=15)
B (n=16)
C (n=14)
surface

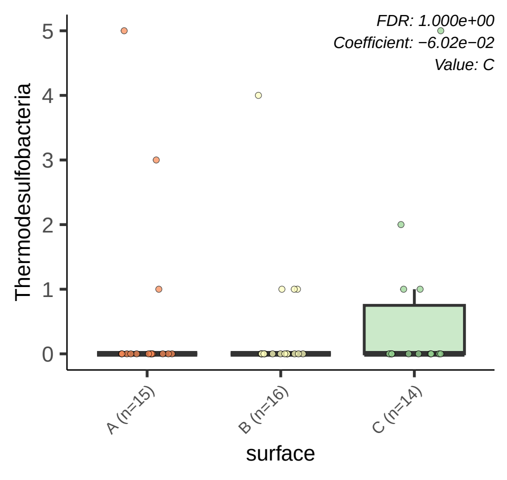
FDR: 1.000e+00
Coefficient: −6.02e−02
Value: C
Thermodesulfobacteria
0
1
2
3
4
5
A (n=15)
B (n=16)
C (n=14)
surface

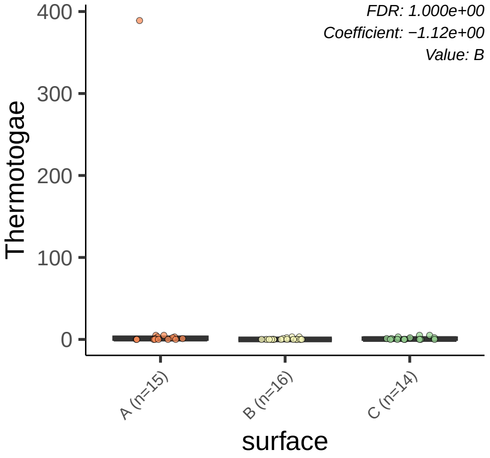
FDR: 1.000e+00
Coefficient: −1.12e+00
Value: B
Thermotogae
400
300
200
100
0
A (n=15)
B (n=16)
C (n=14)
surface

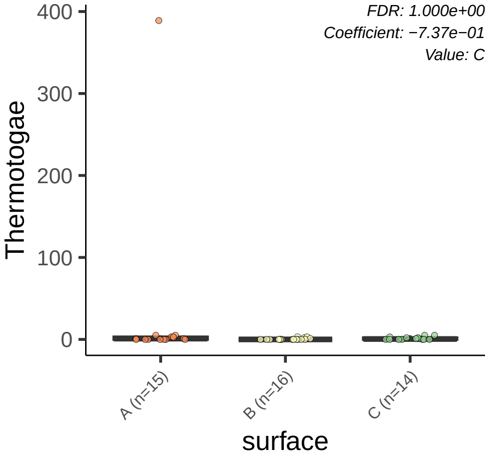
FDR: 1.000e+00
Coefficient: −7.37e−01
Value: C
Thermotogae
400
300
200
100
0
A (n=15)
B (n=16)
C (n=14)
surface

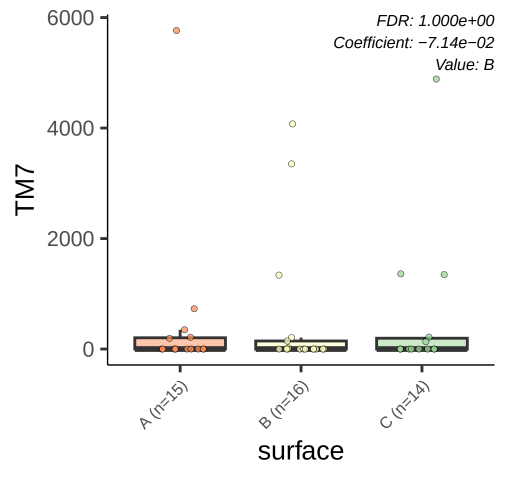
FDR: 1.000e+00
Coefficient: −7.14e−02
Value: B
6000
4000
2000
0
TM7
A (n=15)
B (n=16)
C (n=14)
surface

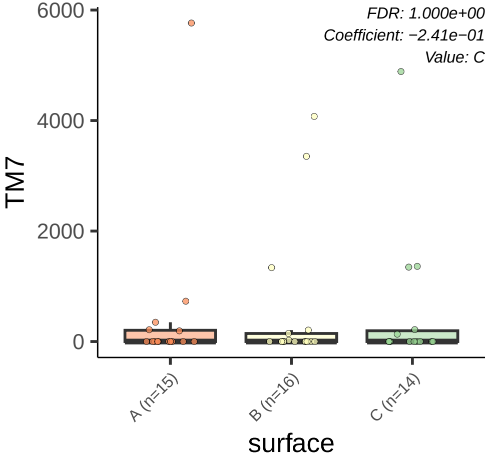

FDR: 1.000e+00
Coefficient: −2.41e−01
Value: C
TM7
6000
4000
2000
0
A (n=15)
B (n=16)
C (n=14)
surface

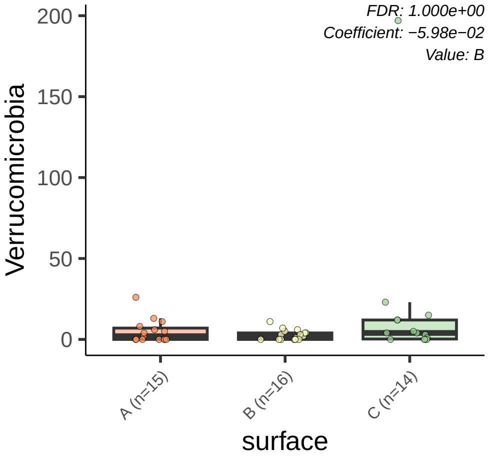

FDR: 1.000e+00
Coefficient: −5.98e−02
Value: B
Verrucomicrobia
200
150
100
50
0
A (n=15)
B (n=16)
C (n=14)
surface

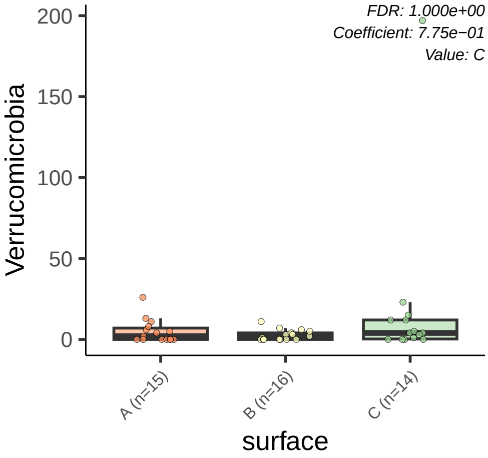
FDR: 1.000e+00
Coefficient: 7.75e−01
Value: C
Verrucomicrobia
200
150
100
50
0
A (n=15)
B (n=16)
C (n=14)
surface

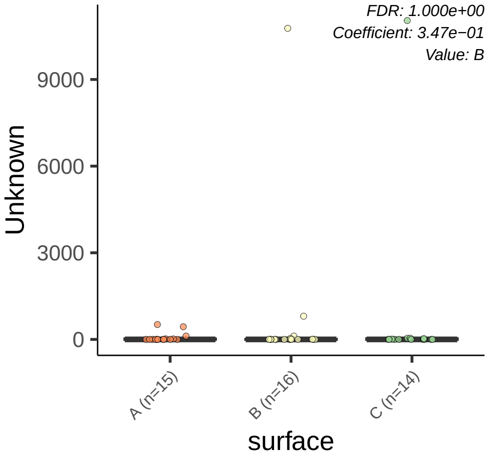

FDR: 1.000e+00
Coefficient: 3.47e−01
Value: B
Unknown
9000
6000
3000
0
A (n=15)
B (n=16)
C (n=14)
surface

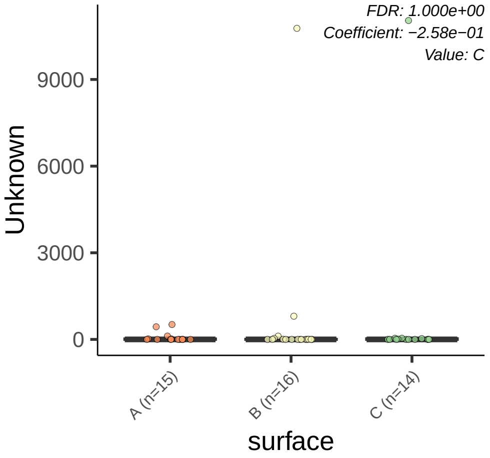
FDR: 1.000e+00
Coefficient: −2.58e−01
Value: C
Unknown
9000
6000
3000
0
A (n=15)
B (n=16)
C (n=14)
surface

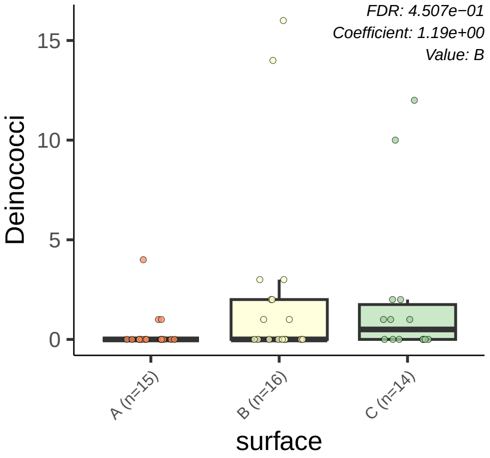
FDR: 4.507e−01
Coefficient: 1.19e+00
Value: B
Deinococci
15
10
5
0
A (n=15)
B (n=16)
C (n=14)
surface

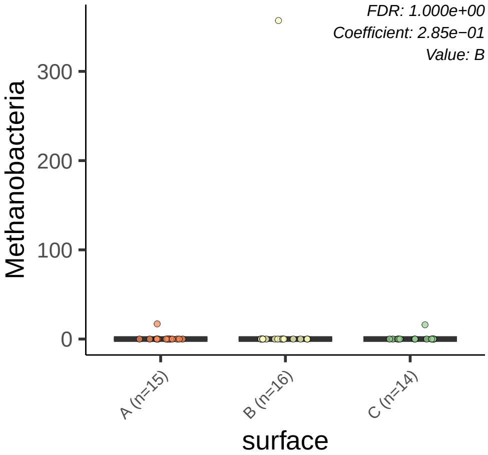

FDR: 1.000e+00
Coefficient: 2.85e−01
Value: B
Methanobacteria
300
200
100
0
A (n=15)
B (n=16)
C (n=14)
surface

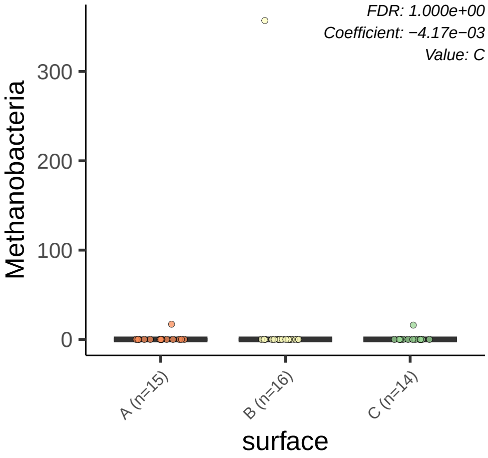

FDR: 1.000e+00
Coefficient: −4.17e−03
Value: C
Methanobacteria
300
200
100
0
A (n=15)
B (n=16)
C (n=14)
surface

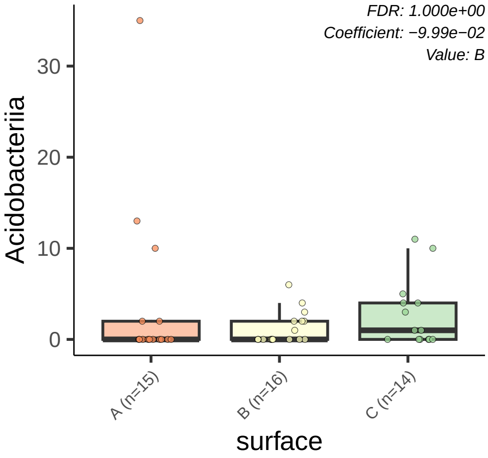

FDR: 1.000e+00
Coefficient: −9.99e−02
Value: B
Acidobacteriia
30
20
10
0
A (n=15)
B (n=16)
C (n=14)
surface

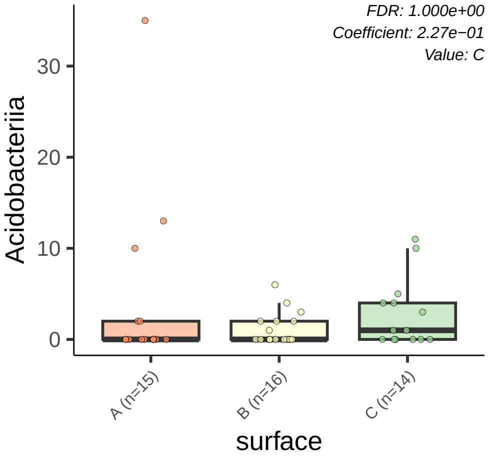
FDR: 1.000e+00
Coefficient: 2.27e−01
Value: C
Acidobacteriia
30
20
10
0
A (n=15)
B (n=16)
C (n=14)
surface

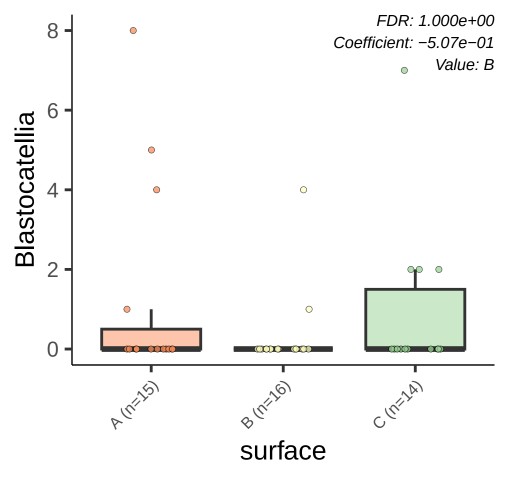
FDR: 1.000e+00
Coefficient: −5.07e−01
Value: B
Blastocatellia
0
2
4
6
8
A (n=15)
B (n=16)
C (n=14)
surface

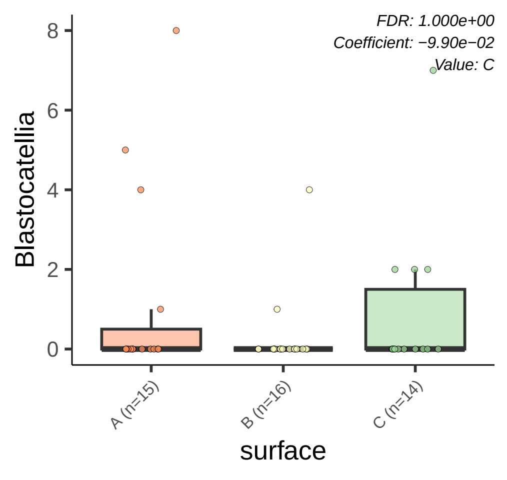

FDR: 1.000e+00
Coefficient: −9.90e−02
Value: C
Blastocatellia
8
6
4
2
0
A (n=15)
B (n=16)
C (n=14)
surface

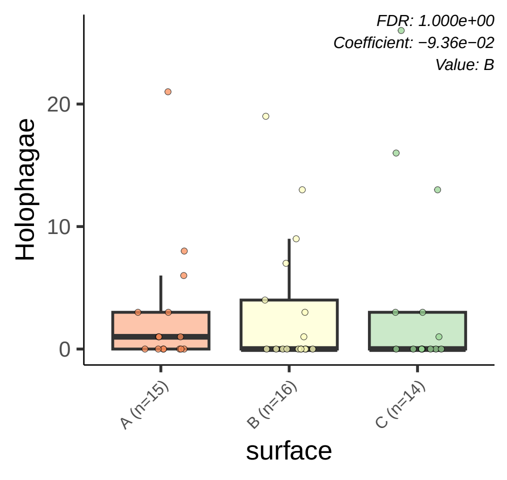
FDR: 1.000e+00
Coefficient: −9.36e−02
Value: B
Holophagae
20
10
0
A (n=15)
B (n=16)
C (n=14)
surface

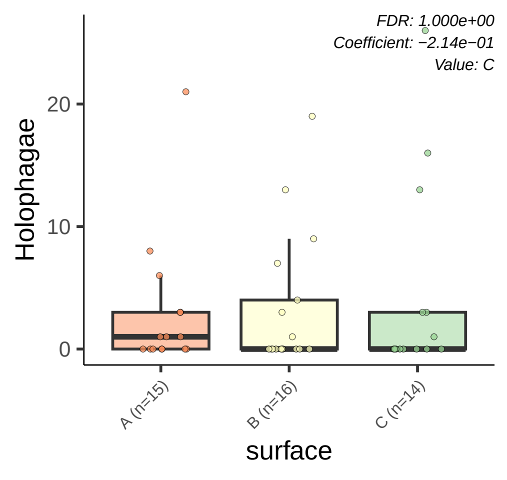
FDR: 1.000e+00
Coefficient: −2.14e−01
Value: C
Holophagae
20
10
0
A (n=15)
B (n=16)
C (n=14)
surface

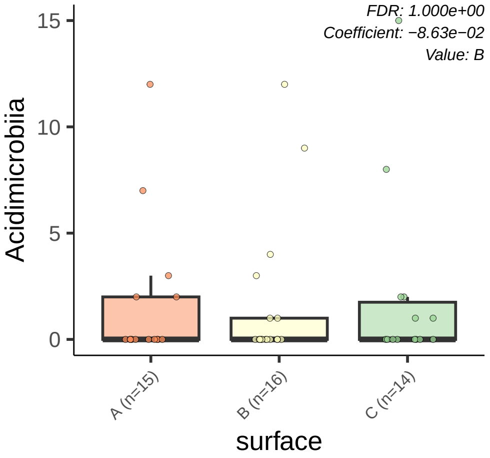
FDR: 1.000e+00
Coefficient: −8.63e−02
Value: B
Acidimicrobiia
15
10
5
0
A (n=15)
B (n=16)
C (n=14)
surface

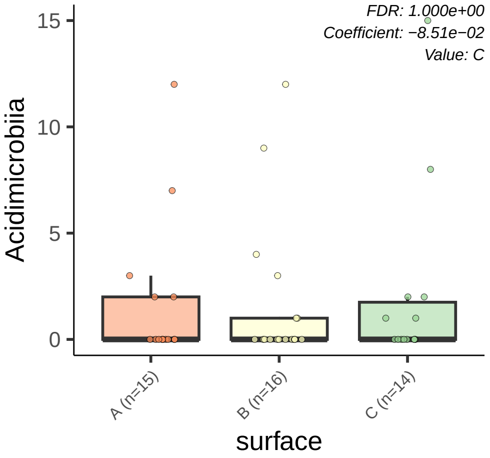
FDR: 1.000e+00
Coefficient: −8.51e−02
Value: C
Acidimicrobiia
15
10
5
0
A (n=15)
B (n=16)
C (n=14)
surface

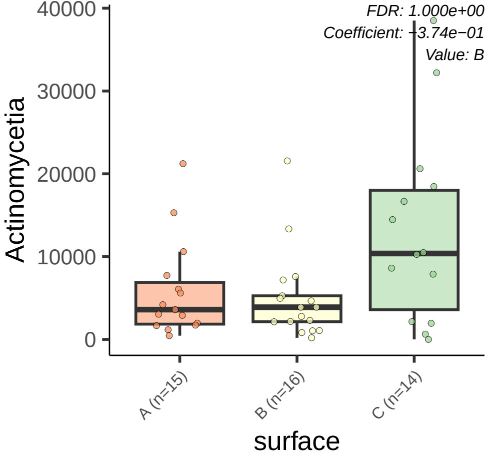

FDR: 1.000e+00
Coefficient: −3.74e−01
Value: B
Actinomycetia
40000
30000
20000
10000
0
A (n=15)
B (n=16)
C (n=14)
surface

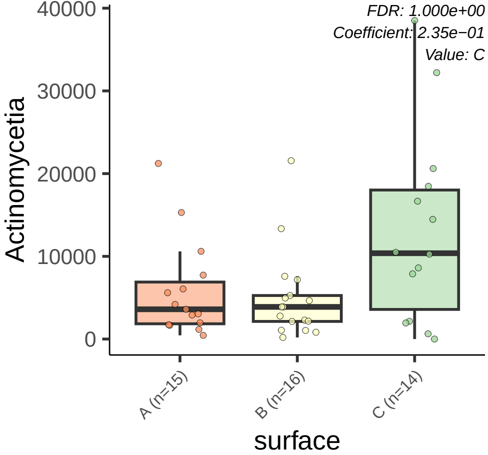

FDR: 1.000e+00
Coefficient: 2.35e−01
Value: C
Actinomycetia
40000
30000
20000
10000
0
A (n=15)
B (n=16)
C (n=14)
surface

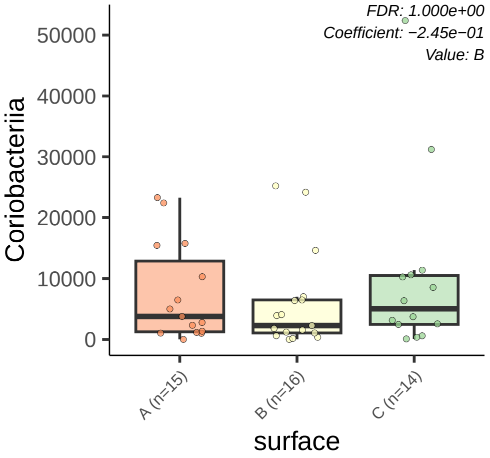
FDR: 1.000e+00
Coefficient: −2.45e−01
Value: B
Coriobacteriia
50000
40000
30000
20000
10000
0
A (n=15)
B (n=16)
C (n=14)
surface

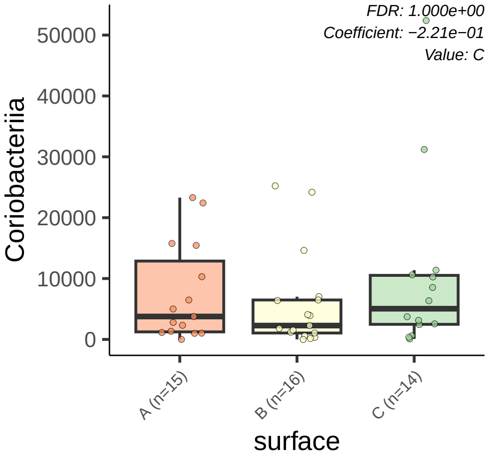

FDR: 1.000e+00
Coefficient: −2.21e−01
Value: C
Coriobacteriia
50000
40000
30000
20000
10000
0
A (n=15)
B (n=16)
C (n=14)
surface

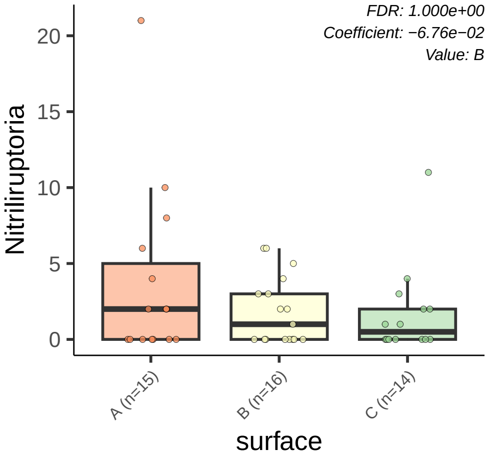
FDR: 1.000e+00
Coefficient: −6.76e−02
Value: B
Nitriliruptoria
20
15
10
5
0
A (n=15)
B (n=16)
C (n=14)
surface

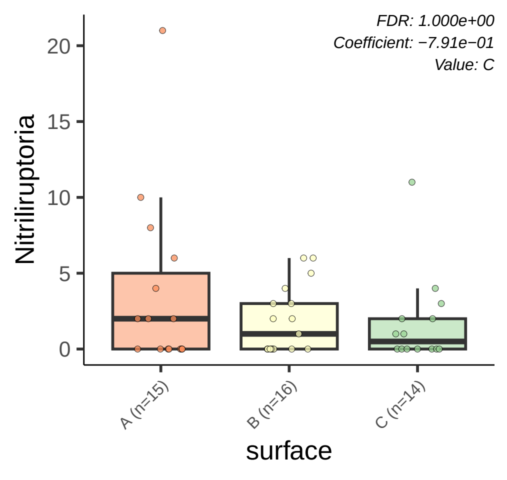
FDR: 1.000e+00
Coefficient: −7.91e−01
Value: C
Nitriliruptoria
20
15
10
5
0
A (n=15)
B (n=16)
C (n=14)
surface

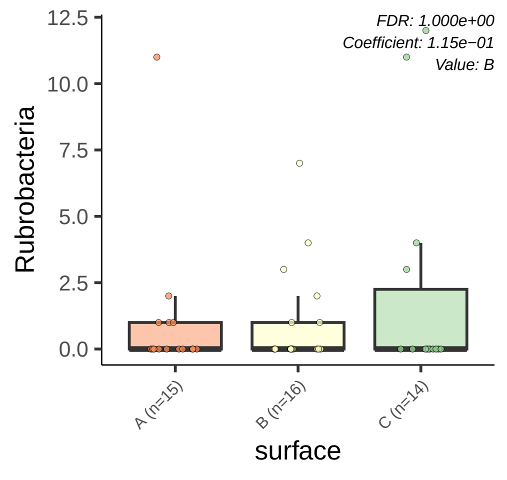
FDR: 1.000e+00
Coefficient: 1.15e−01
Value: B
Rubrobacteria
12.5
10.0
7.5
5.0
2.5
0.0
A (n=15)
B (n=16)
C (n=14)
surface

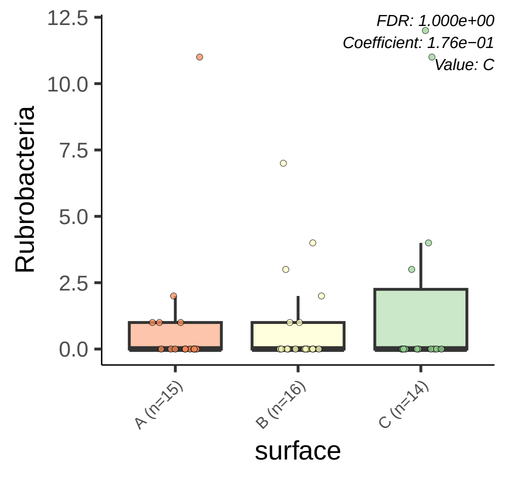

FDR: 1.000e+00
Coefficient: 1.76e−01
Value: C
Rubrobacteria
12.5
10.0
7.5
5.0
2.5
0.0
A (n=15)
B (n=16)
C (n=14)
surface

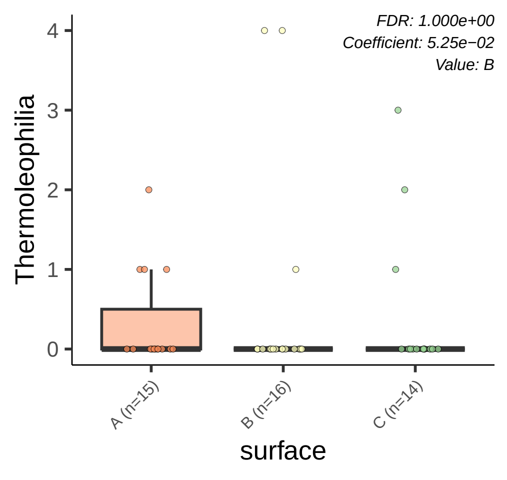

FDR: 1.000e+00
Coefficient: 5.25e−02
Value: B
Thermoleophilia
4
3
2
1
0
A (n=15)
B (n=16)
C (n=14)
surface

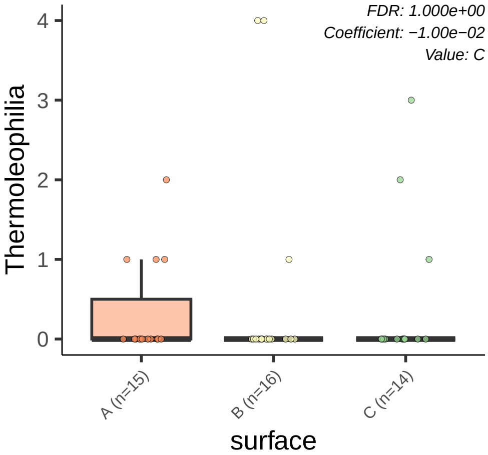

FDR: 1.000e+00
Coefficient: −1.00e−02
Value: C
Thermoleophilia
4
3
2
1
0
A (n=15)
B (n=16)
C (n=14)
surface

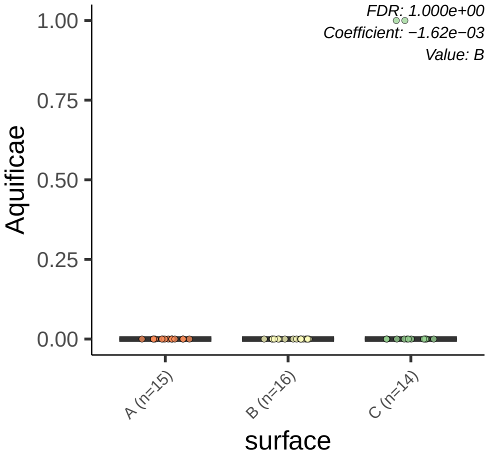
FDR: 1.000e+00
Coefficient: −1.62e−03
Value: B
1.00
0.75
0.50
0.25
0.00
Aquificae
A (n=15)
B (n=16)
C (n=14)
surface

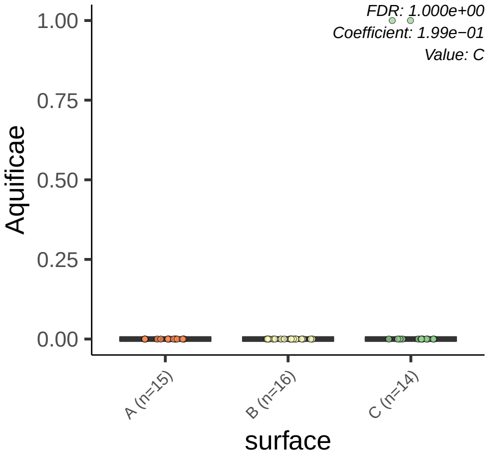

FDR: 1.000e+00
Coefficient: 1.99e−01
Value: C
1.00
0.75
0.50
0.25
0.00
Aquificae
A (n=15)
B (n=16)
C (n=14)
surface

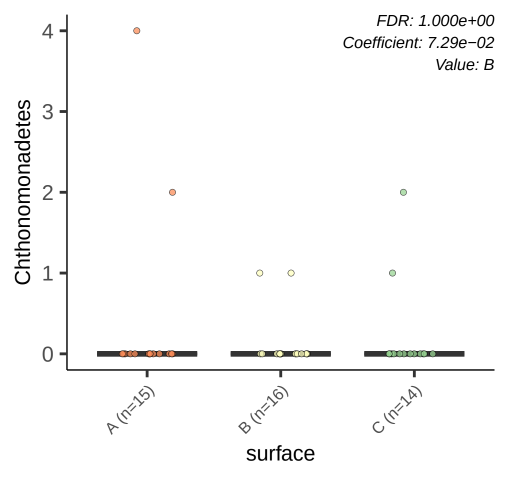
FDR: 1.000e+00
Coefficient: 7.29e−02
Value: B
Chthonomonadetes
0
1
2
3
4
A (n=15)
B (n=16)
C (n=14)
surface

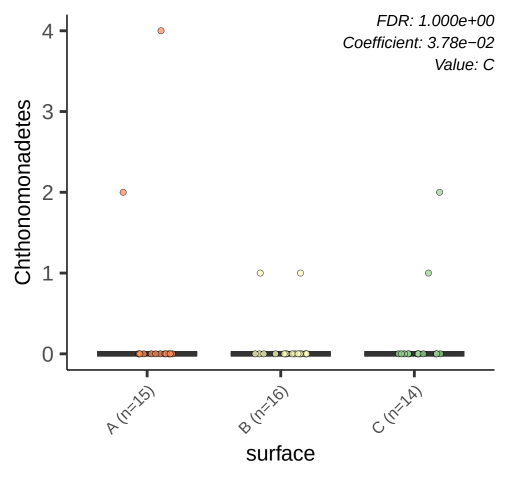
FDR: 1.000e+00
Coefficient: 3.78e−02
Value: C
Chthonomonadetes
4
3
2
1
0
A (n=15)
B (n=16)
C (n=14)
surface

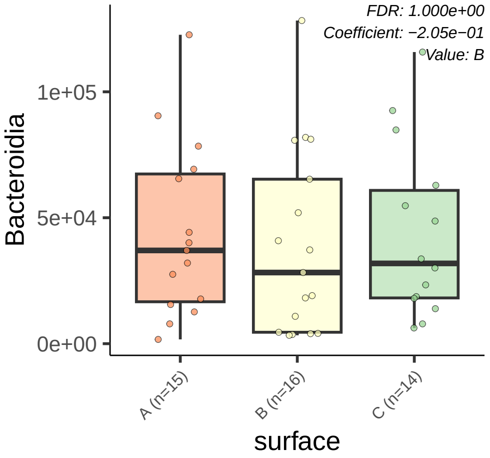

FDR: 1.000e+00
Coefficient: −2.05e−01
Value: B
1e+05
5e+04
0e+00
Bacteroidia
A (n=15)
B (n=16)
C (n=14)
surface

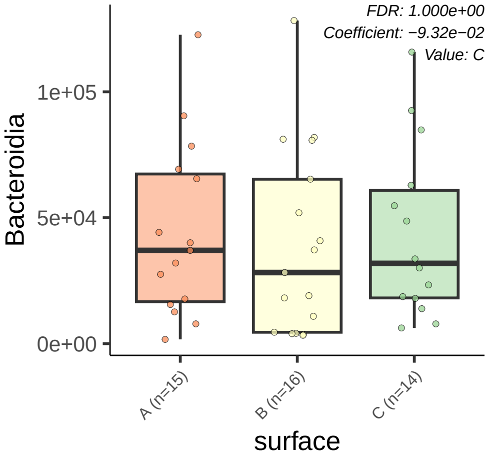
FDR: 1.000e+00
Coefficient: −9.32e−02
Value: C
Bacteroidia
1e+05
5e+04
0e+00
A (n=15)
B (n=16)
C (n=14)
surface

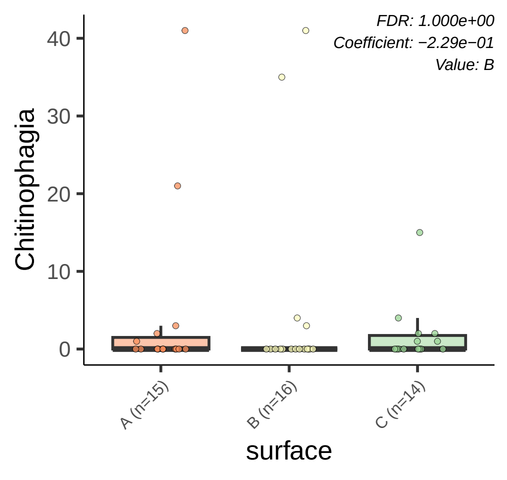

FDR: 1.000e+00
Coefficient: −2.29e−01
Value: B
Chitinophagia
40
30
20
10
0
A (n=15)
B (n=16)
C (n=14)
surface

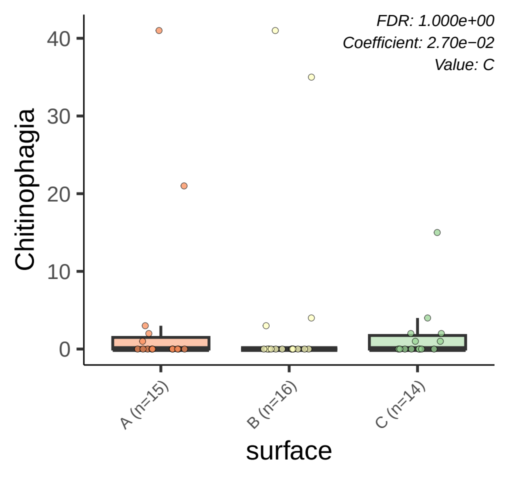

FDR: 1.000e+00
Coefficient: 2.70e−02
Value: C
Chitinophagia
0
10
20
30
40
A (n=15)
B (n=16)
C (n=14)
surface

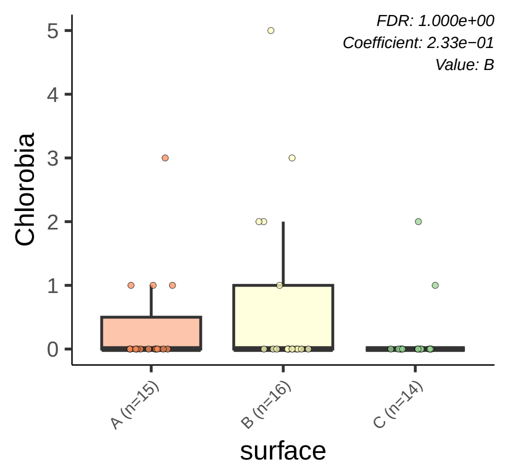
FDR: 1.000e+00
Coefficient: 2.33e−01
Value: B
Chlorobia
0
1
2
3
4
5
A (n=15)
B (n=16)
C (n=14)
surface

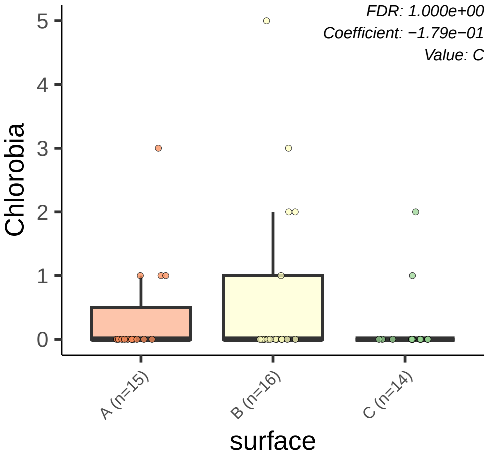
FDR: 1.000e+00
Coefficient: −1.79e−01
Value: C
Chlorobia
0
1
2
3
4
5
A (n=15)
B (n=16)
C (n=14)
surface

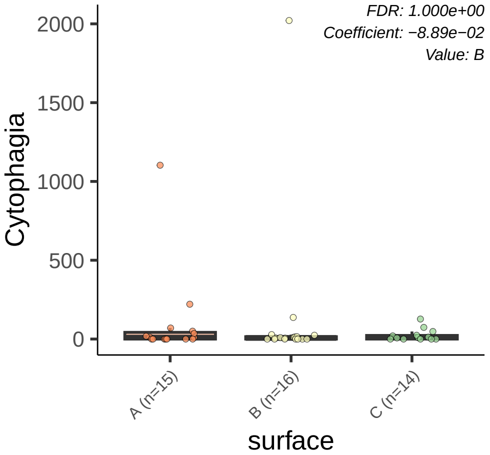
FDR: 1.000e+00
Coefficient: −8.89e−02
Value: B
Cytophagia
2000
1500
1000
500
0
A (n=15)
B (n=16)
C (n=14)
surface

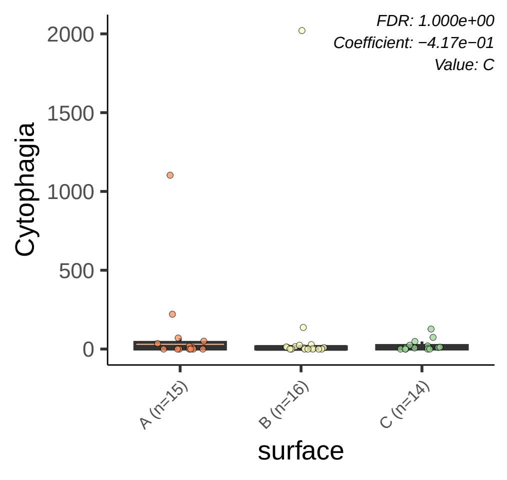
FDR: 1.000e+00
Coefficient: −4.17e−01
Value: C
Cytophagia
2000
1500
1000
500
0
A (n=15)
B (n=16)
C (n=14)
surface

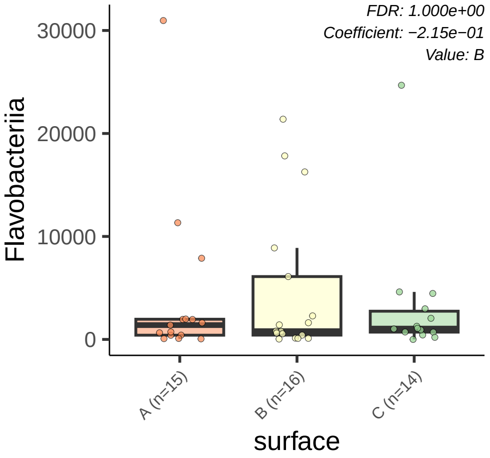

FDR: 1.000e+00
Coefficient: −2.15e−01
Value: B
Flavobacteriia
30000
20000
10000
0
A (n=15)
B (n=16)
C (n=14)
surface

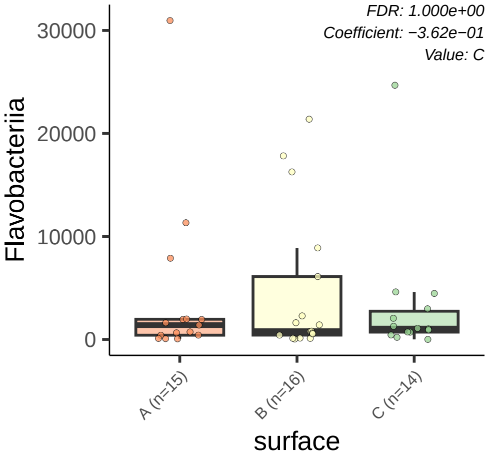

FDR: 1.000e+00
Coefficient: −3.62e−01
Value: C
Flavobacteriia
30000
20000
10000
0
A (n=15)
B (n=16)
C (n=14)
surface

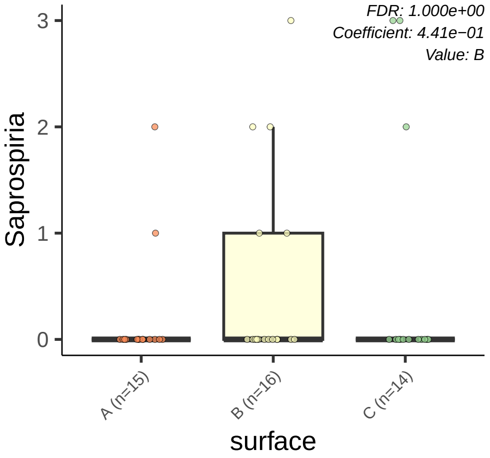
FDR: 1.000e+00
Coefficient: 4.41e−01
Value: B
Saprospiria
3
2
1
0
A (n=15)
B (n=16)
C (n=14)
surface

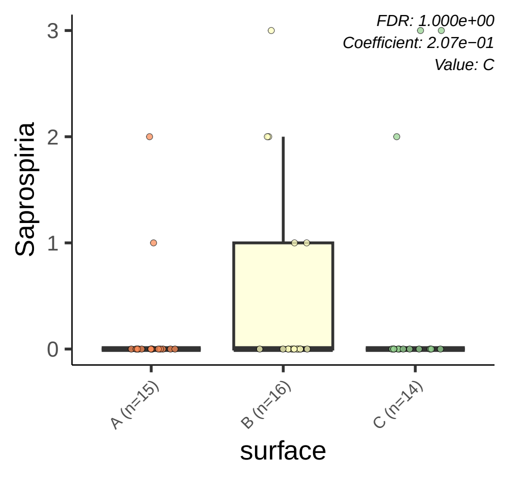
FDR: 1.000e+00
Coefficient: 2.07e−01
Value: C
Saprospiria
3
2
1
0
A (n=15)
B (n=16)
C (n=14)
surface

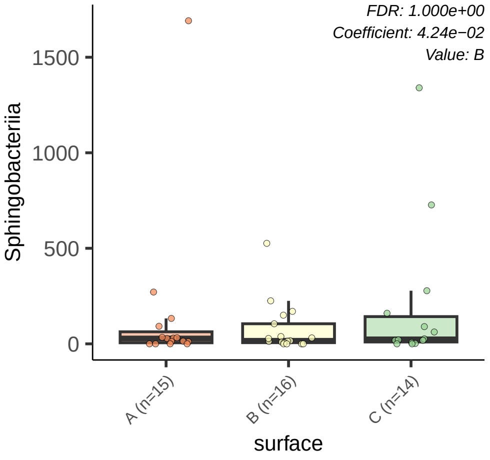
FDR: 1.000e+00
Coefficient: 4.24e−02
Value: B
Sphingobacteriia
0
500
1000
1500
A (n=15)
B (n=16)
C (n=14)
surface

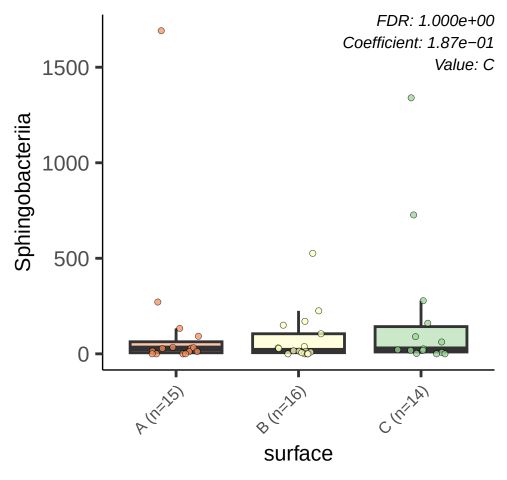
FDR: 1.000e+00
Coefficient: 1.87e−01
Value: C
Sphingobacteriia
1500
1000
500
0
A (n=15)
B (n=16)
C (n=14)
surface

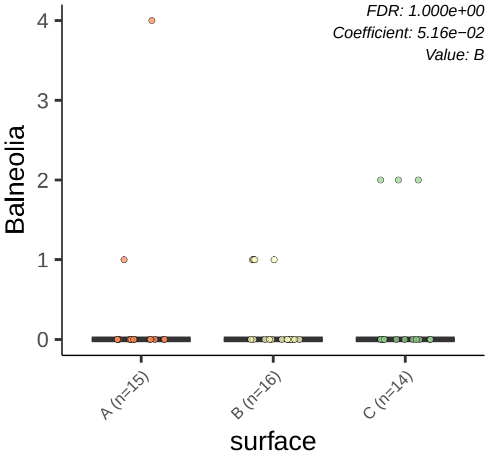

FDR: 1.000e+00
Coefficient: 5.16e−02
Value: B
Balneolia
0
1
2
3
4
A (n=15)
B (n=16)
C (n=14)
surface

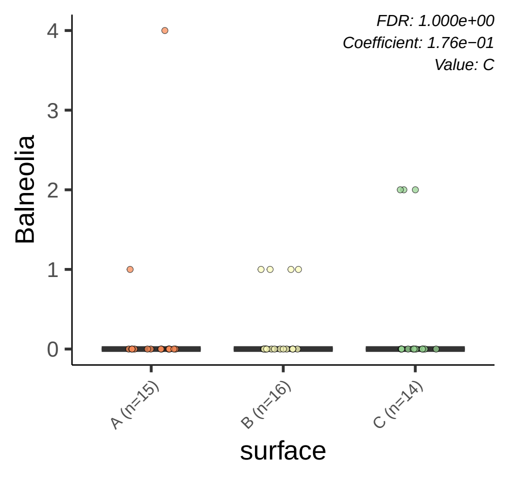

FDR: 1.000e+00
Coefficient: 1.76e−01
Value: C
Balneolia
0
1
2
3
4
A (n=15)
B (n=16)
C (n=14)
surface

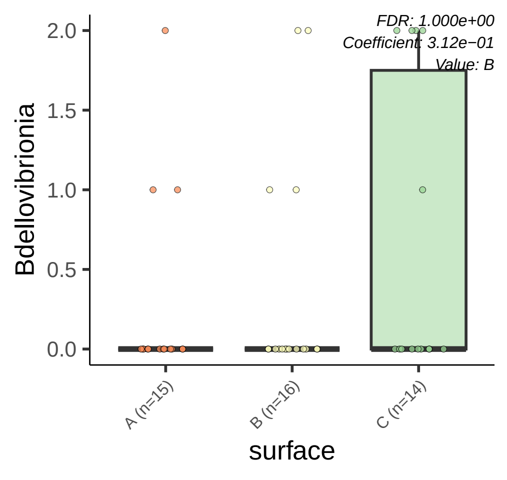

FDR: 1.000e+00
Coefficient: 3.12e−01
Value: B
Bdellovibrionia
2.0
1.5
1.0
0.5
0.0
A (n=15)
B (n=16)
C (n=14)
surface

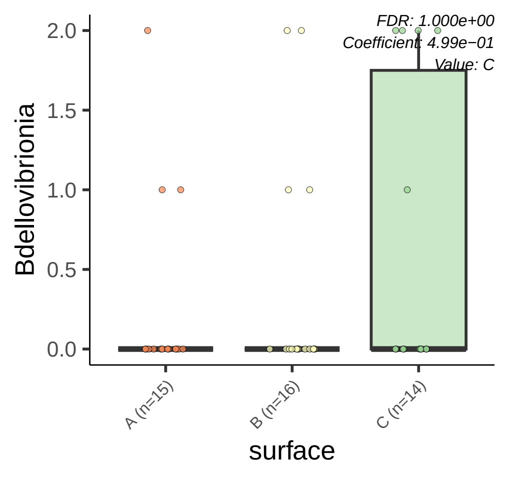

FDR: 1.000e+00
Coefficient: 4.99e−01
Value: C
Bdellovibrionia
2.0
1.5
1.0
0.5
0.0
A (n=15)
B (n=16)
C (n=14)
surface

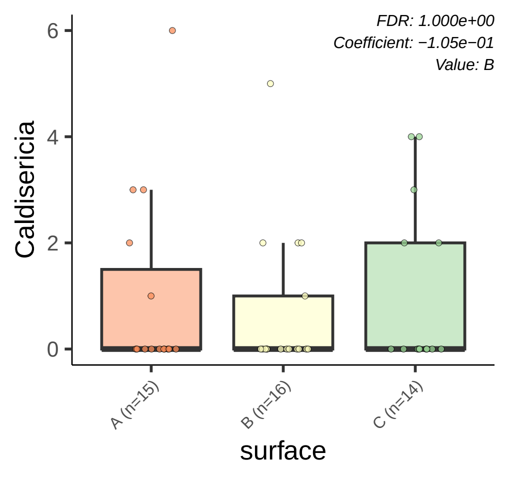

FDR: 1.000e+00
Coefficient: −1.05e−01
Value: B
Caldisericia
0
2
4
6
A (n=15)
B (n=16)
C (n=14)
surface

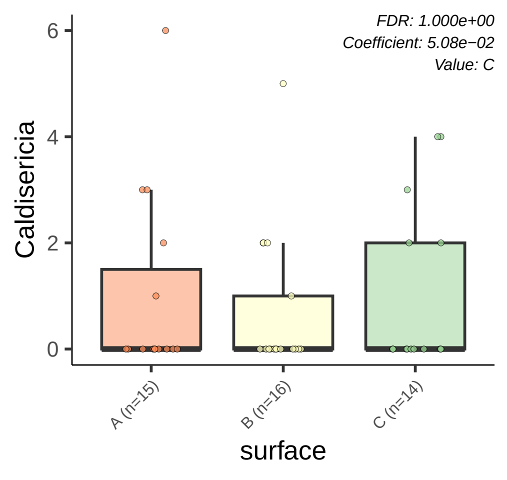
FDR: 1.000e+00
Coefficient: 5.08e−02
Value: C
Caldisericia
0
2
4
6
A (n=15)
B (n=16)
C (n=14)
surface

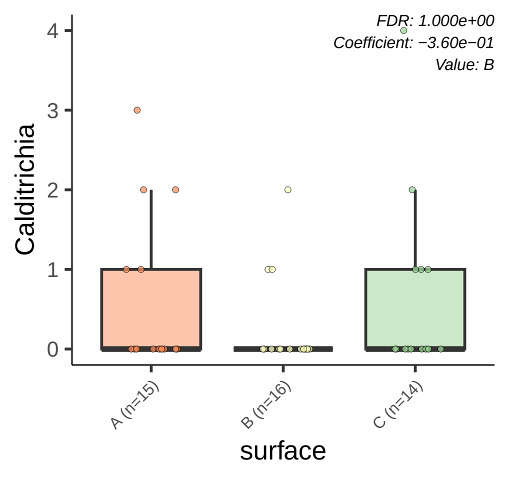
FDR: 1.000e+00
Coefficient: −3.60e−01
Value: B
Calditrichia
4
3
2
1
0
A (n=15)
B (n=16)
C (n=14)
surface

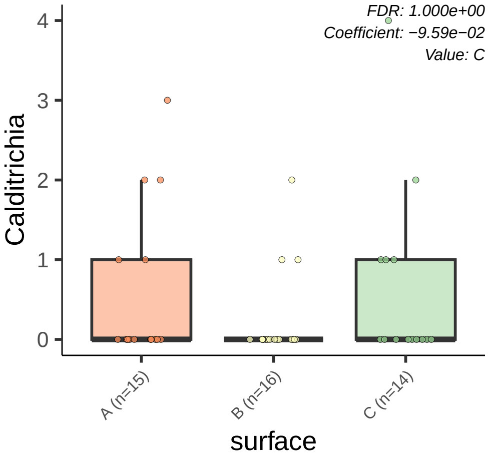
FDR: 1.000e+00
Coefficient: −9.59e−02
Value: C
Calditrichia
0
1
2
3
4
A (n=15)
B (n=16)
C (n=14)
surface

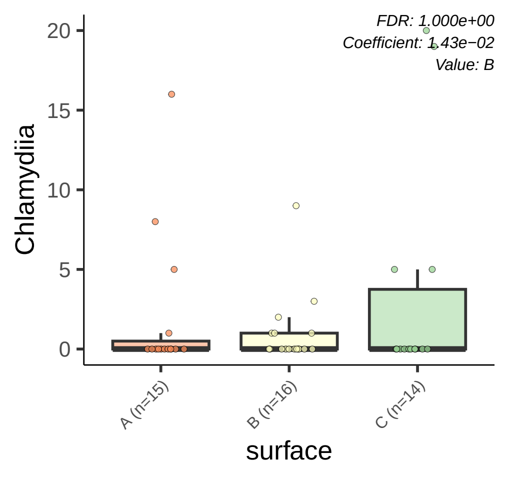
FDR: 1.000e+00
Coefficient: 1.43e−02
Value: B
Chlamydiia
20
15
10
5
0
A (n=15)
B (n=16)
C (n=14)
surface

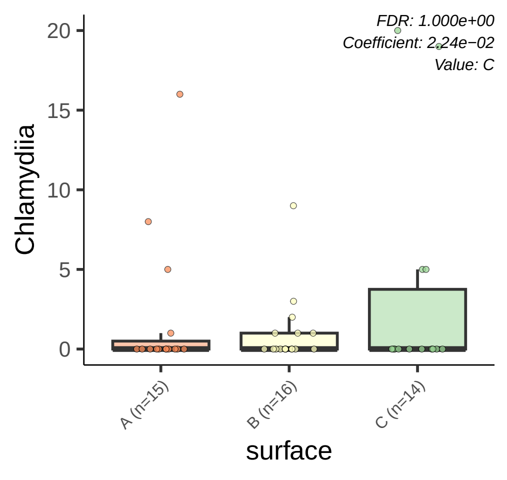
FDR: 1.000e+00
Coefficient: 2.24e−02
Value: C
Chlamydiia
0
5
10
15
20
A (n=15)
B (n=16)
C (n=14)
surface

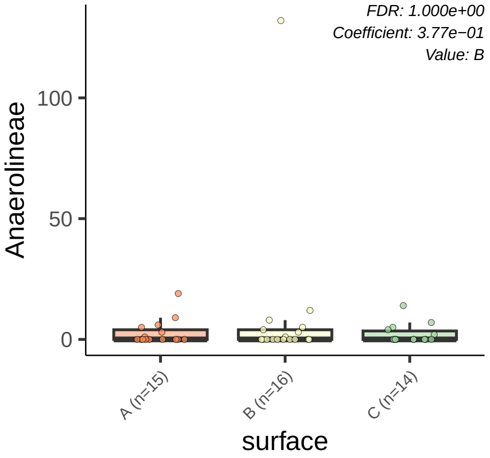
FDR: 1.000e+00
Coefficient: 3.77e−01
Value: B
Anaerolineae
100
50
0
A (n=15)
B (n=16)
C (n=14)
surface

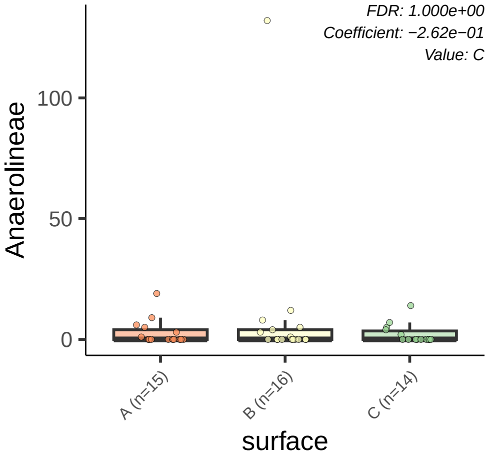

FDR: 1.000e+00
Coefficient: −2.62e−01
Value: C
Anaerolineae
100
50
0
A (n=15)
B (n=16)
C (n=14)
surface

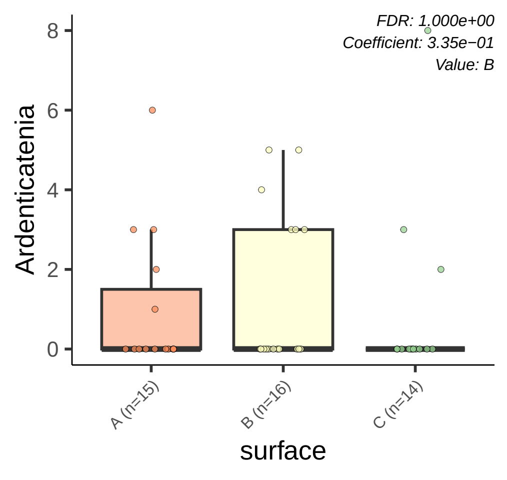

FDR: 1.000e+00
Coefficient: 3.35e−01
Value: B
Ardenticatenia
8
6
4
2
0
A (n=15)
B (n=16)
C (n=14)
surface

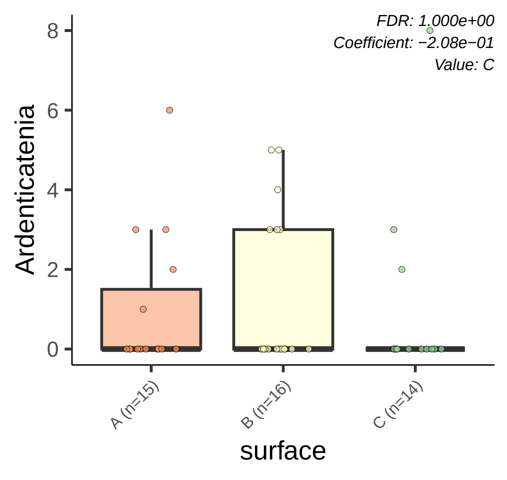
FDR: 1.000e+00
Coefficient: −2.08e−01
Value: C
Ardenticatenia
8
6
4
2
0
A (n=15)
B (n=16)
C (n=14)
surface

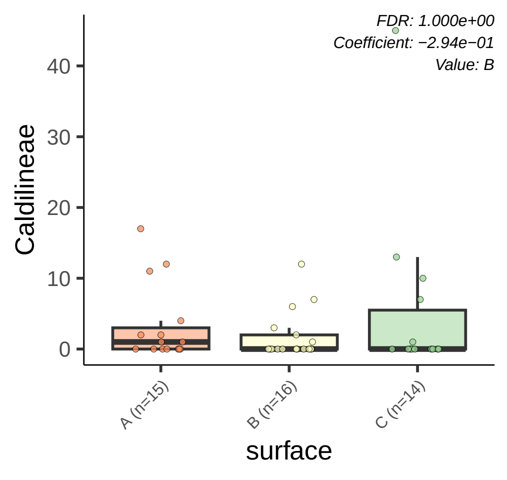

FDR: 1.000e+00
Coefficient: −2.94e−01
Value: B
Caldilineae
40
30
20
10
0
A (n=15)
B (n=16)
C (n=14)
surface

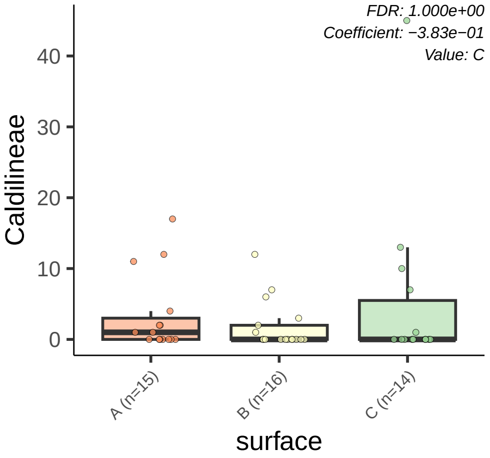
FDR: 1.000e+00
Coefficient: −3.83e−01
Value: C
Caldilineae
0
10
20
30
40
A (n=15)
B (n=16)
C (n=14)
surface

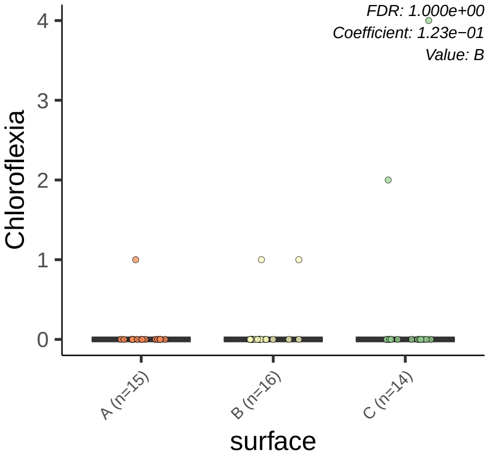
FDR: 1.000e+00
Coefficient: 1.23e−01
Value: B
Chloroflexia
0
1
2
3
4
A (n=15)
B (n=16)
C (n=14)
surface

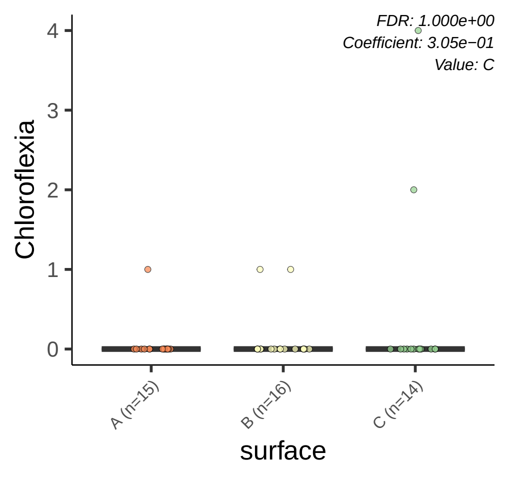

FDR: 1.000e+00
Coefficient: 3.05e−01
Value: C
Chloroflexia
0
1
2
3
4
A (n=15)
B (n=16)
C (n=14)
surface

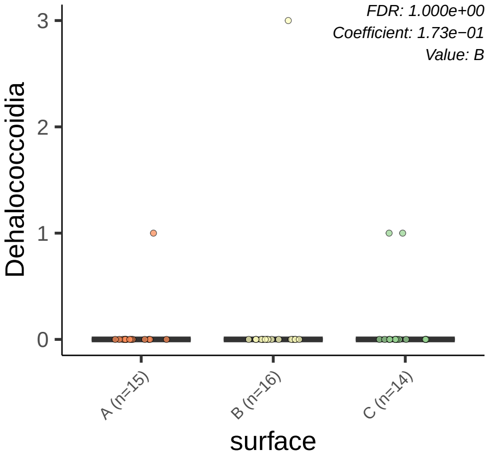
FDR: 1.000e+00
Coefficient: 1.73e−01
Value: B
Dehalococcoidia
0
1
2
3
A (n=15)
B (n=16)
C (n=14)
surface

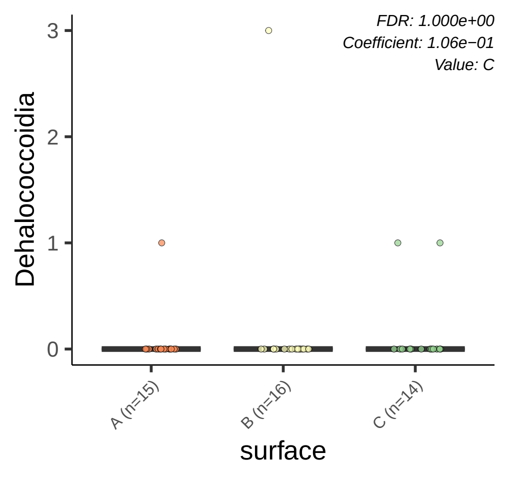
FDR: 1.000e+00
Coefficient: 1.06e−01
Value: C
Dehalococcoidia
3
2
1
0
A (n=15)
B (n=16)
C (n=14)
surface

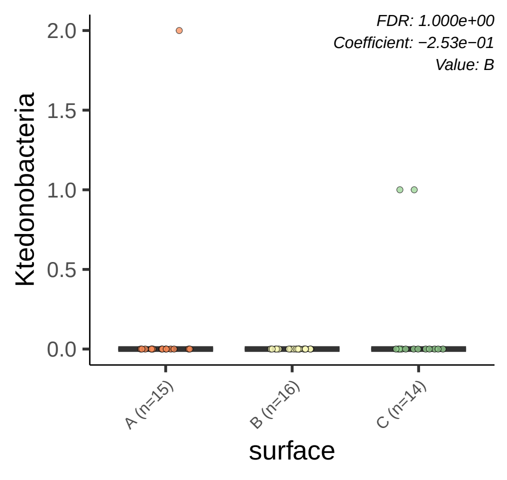
FDR: 1.000e+00
Coefficient: −2.53e−01
Value: B
Ktedonobacteria
2.0
1.5
1.0
0.5
0.0
A (n=15)
B (n=16)
C (n=14)
surface

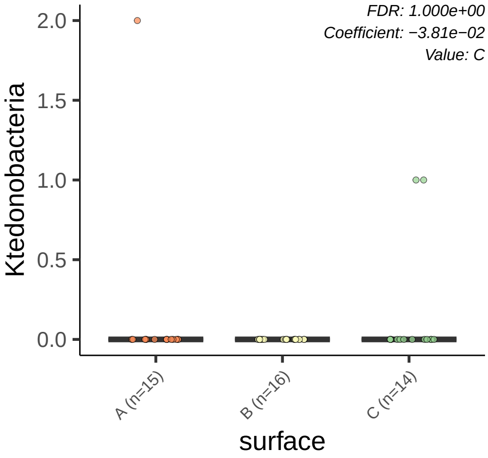
FDR: 1.000e+00
Coefficient: −3.81e−02
Value: C
Ktedonobacteria
2.0
1.5
1.0
0.5
0.0
A (n=15)
B (n=16)
C (n=14)
surface

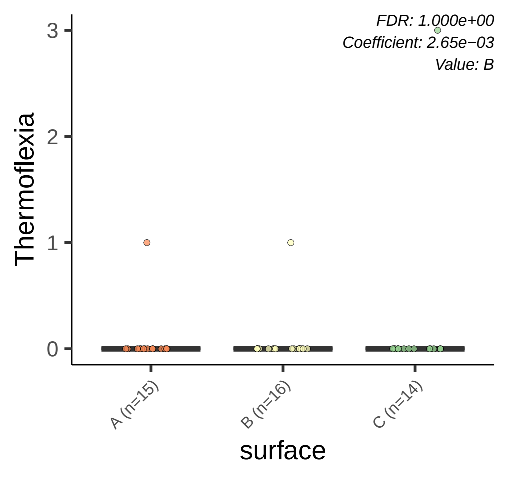
FDR: 1.000e+00
Coefficient: 2.65e−03
Value: B
Thermoflexia
3
2
1
0
A (n=15)
B (n=16)
C (n=14)
surface

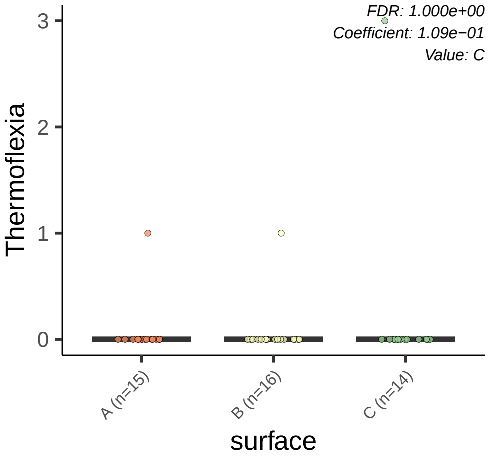
FDR: 1.000e+00
Coefficient: 1.09e−01
Value: C
Thermoflexia
0
1
2
3
A (n=15)
B (n=16)
C (n=14)
surface

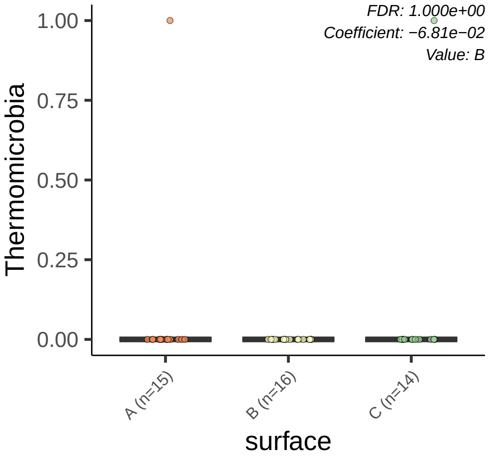

FDR: 1.000e+00
Coefficient: −6.81e−02
Value: B
Thermomicrobia
1.00
0.75
0.50
0.25
0.00
A (n=15)
B (n=16)
C (n=14)
surface

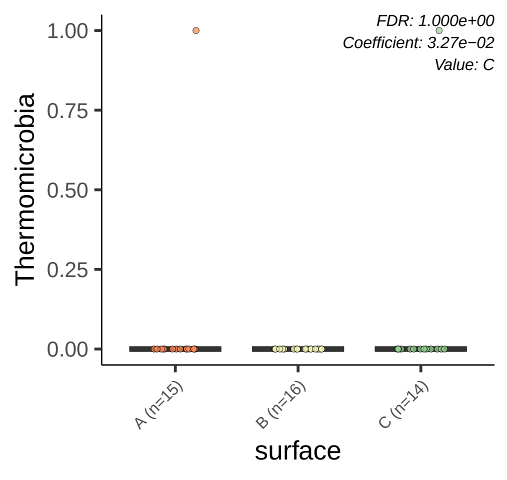
FDR: 1.000e+00
Coefficient: 3.27e−02
Value: C
Thermomicrobia
1.00
0.75
0.50
0.25
0.00
A (n=15)
B (n=16)
C (n=14)
surface

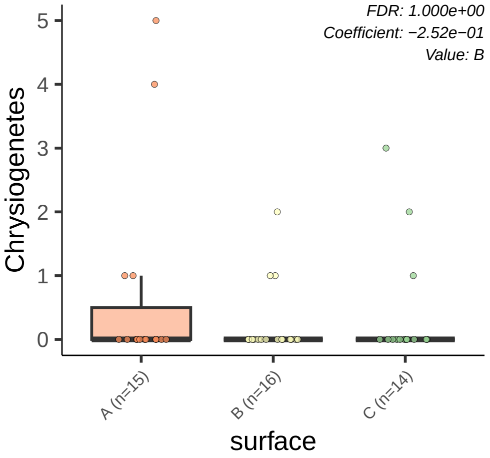

FDR: 1.000e+00
Coefficient: −2.52e−01
Value: B
Chrysiogenetes
5
4
3
2
1
0
A (n=15)
B (n=16)
C (n=14)
surface

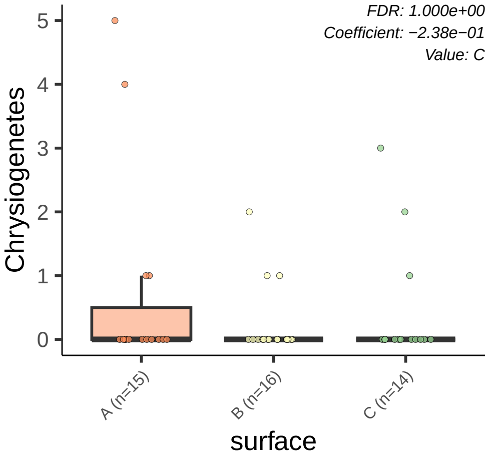
FDR: 1.000e+00
Coefficient: −2.38e−01
Value: C
Chrysiogenetes
5
4
3
2
1
0
A (n=15)
B (n=16)
C (n=14)
surface

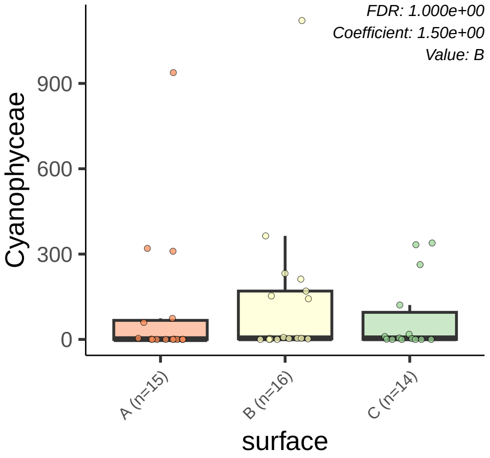

FDR: 1.000e+00
Coefficient: 1.50e+00
Value: B
Cyanophyceae
900
600
300
0
A (n=15)
B (n=16)
C (n=14)
surface

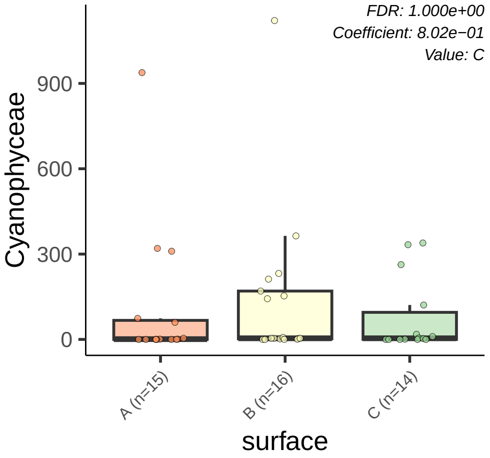

FDR: 1.000e+00
Coefficient: 8.02e−01
Value: C
Cyanophyceae
900
600
300
0
A (n=15)
B (n=16)
C (n=14)
surface

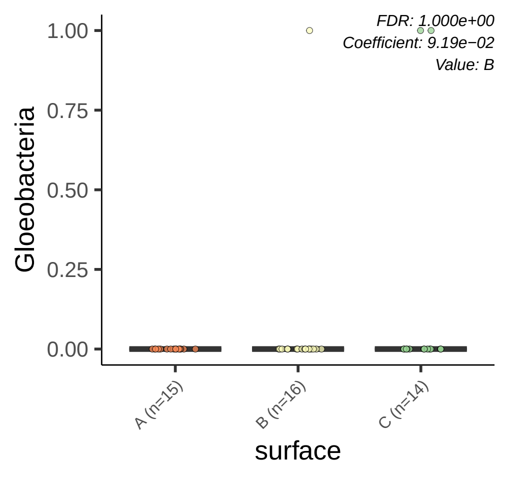
FDR: 1.000e+00
Coefficient: 9.19e−02
Value: B
Gloeobacteria
1.00
0.75
0.50
0.25
0.00
A (n=15)
B (n=16)
C (n=14)
surface

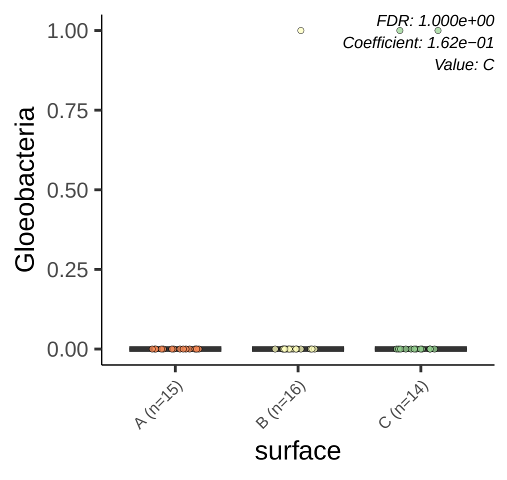
FDR: 1.000e+00
Coefficient: 1.62e−01
Value: C
Gloeobacteria
1.00
0.75
0.50
0.25
0.00
A (n=15)
B (n=16)
C (n=14)
surface

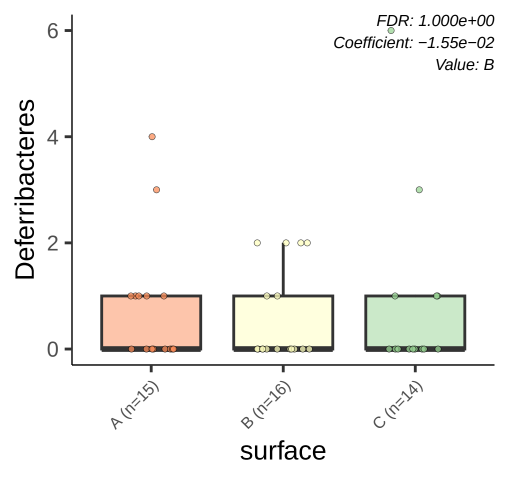

FDR: 1.000e+00
Coefficient: −1.55e−02
Value: B
Deferribacteres
0
2
4
6
A (n=15)
B (n=16)
C (n=14)
surface

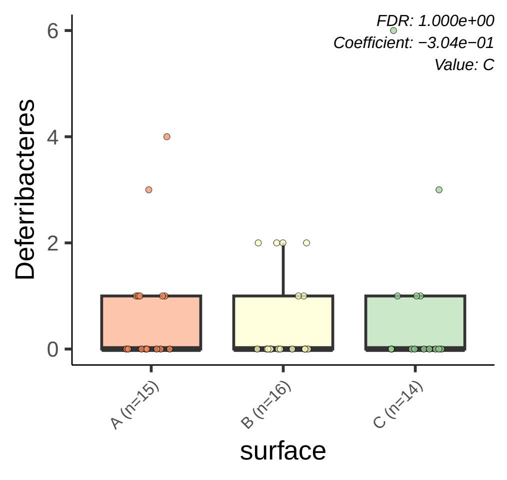
FDR: 1.000e+00
Coefficient: −3.04e−01
Value: C
Deferribacteres
0
2
4
6
A (n=15)
B (n=16)
C (n=14)
surface

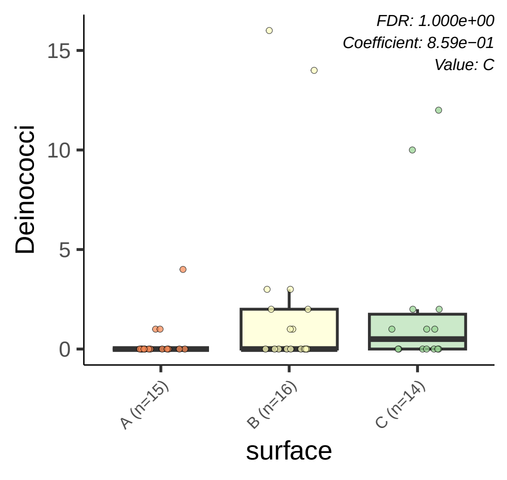

FDR: 1.000e+00
Coefficient: 8.59e−01
Value: C
Deinococci
0
5
10
15
A (n=15)
B (n=16)
C (n=14)
surface

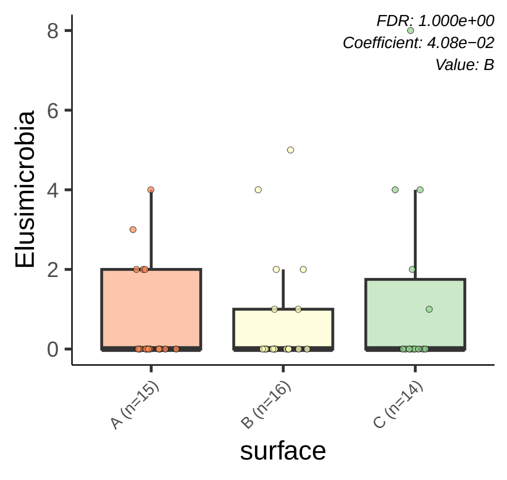
FDR: 1.000e+00
Coefficient: 4.08e−02
Value: B
Elusimicrobia
0
2
4
6
8
A (n=15)
B (n=16)
C (n=14)
surface

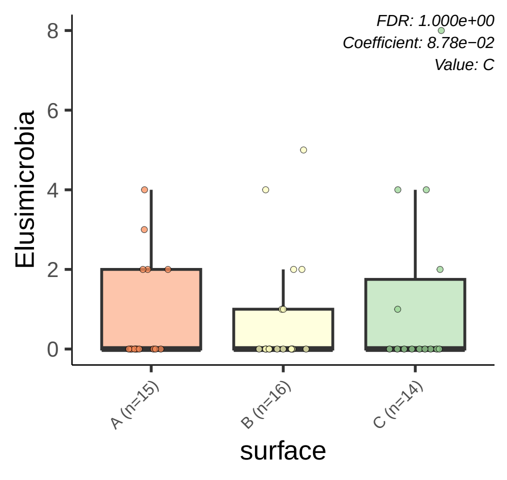

FDR: 1.000e+00
Coefficient: 8.78e−02
Value: C
Elusimicrobia
0
2
4
6
8
A (n=15)
B (n=16)
C (n=14)
surface

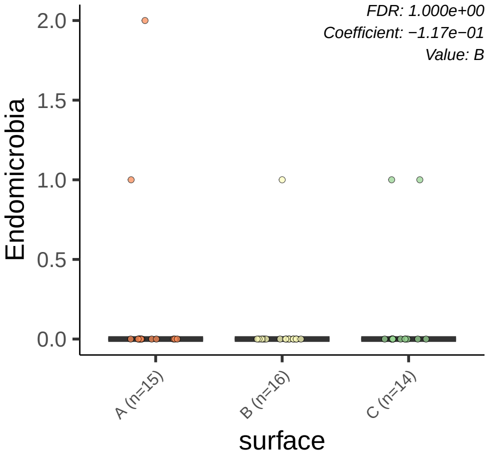
FDR: 1.000e+00
Coefficient: −1.17e−01
Value: B
Endomicrobia
2.0
1.5
1.0
0.5
0.0
A (n=15)
B (n=16)
C (n=14)
surface

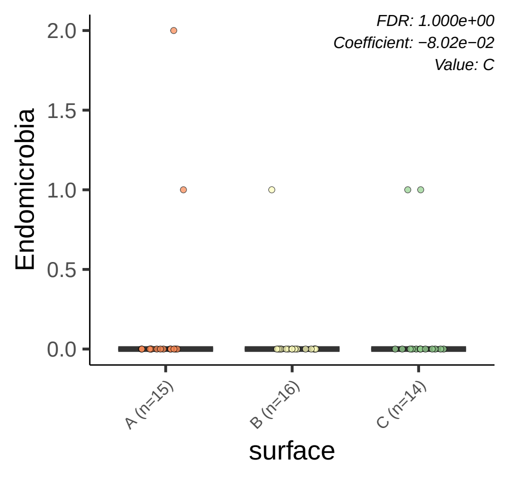
FDR: 1.000e+00
Coefficient: −8.02e−02
Value: C
Endomicrobia
2.0
1.5
1.0
0.5
0.0
A (n=15)
B (n=16)
C (n=14)
surface

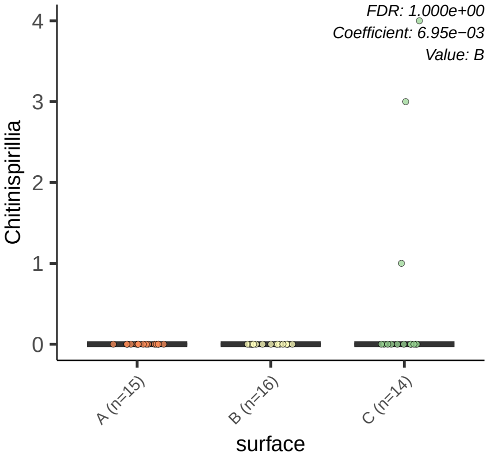
FDR: 1.000e+00
Coefficient: 6.95e−03
Value: B
Chitinispirillia
0
1
2
3
4
A (n=15)
B (n=16)
C (n=14)
surface

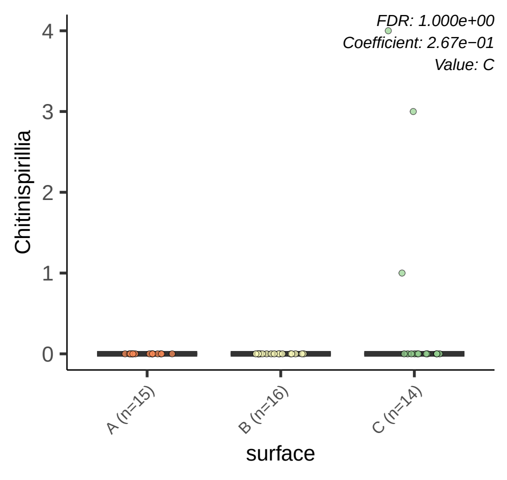

FDR: 1.000e+00
Coefficient: 2.67e−01
Value: C
Chitinispirillia
4
3
2
1
0
A (n=15)
B (n=16)
C (n=14)
surface

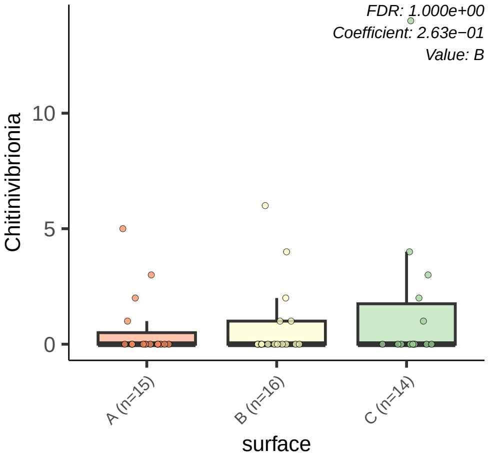

FDR: 1.000e+00
Coefficient: 2.63e−01
Value: B
Chitinivibrionia
0
5
10
A (n=15)
B (n=16)
C (n=14)
surface

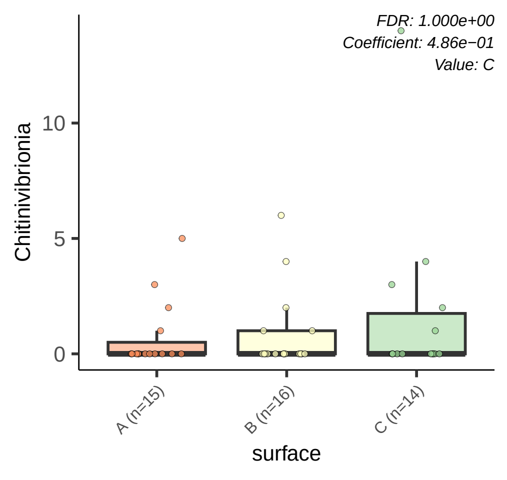

FDR: 1.000e+00
Coefficient: 4.86e−01
Value: C
Chitinivibrionia
10
5
0
A (n=15)
B (n=16)
C (n=14)
surface

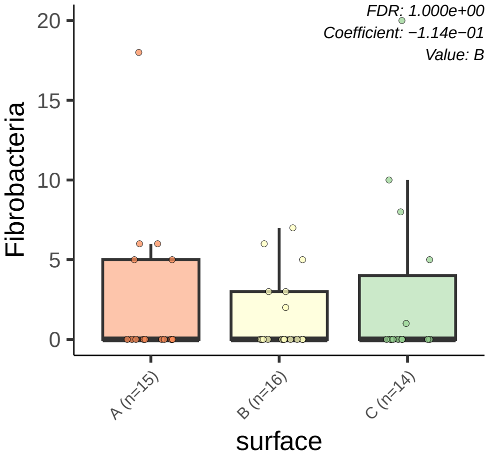

FDR: 1.000e+00
Coefficient: −1.14e−01
Value: B
Fibrobacteria
20
15
10
5
0
A (n=15)
B (n=16)
C (n=14)
surface

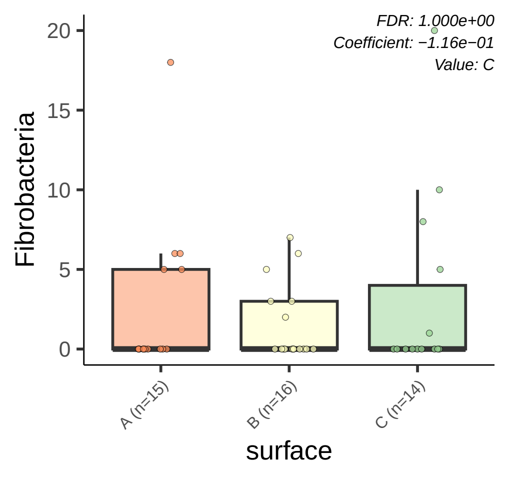
FDR: 1.000e+00
Coefficient: −1.16e−01
Value: C
Fibrobacteria
20
15
10
5
0
A (n=15)
B (n=16)
C (n=14)
surface

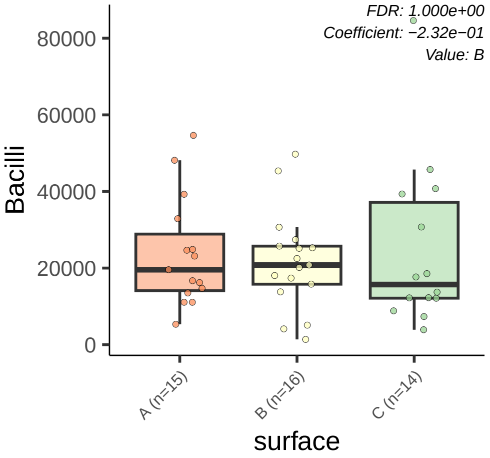

FDR: 1.000e+00
Coefficient: −2.32e−01
Value: B
Bacilli
80000
60000
40000
20000
0
A (n=15)
B (n=16)
C (n=14)
surface

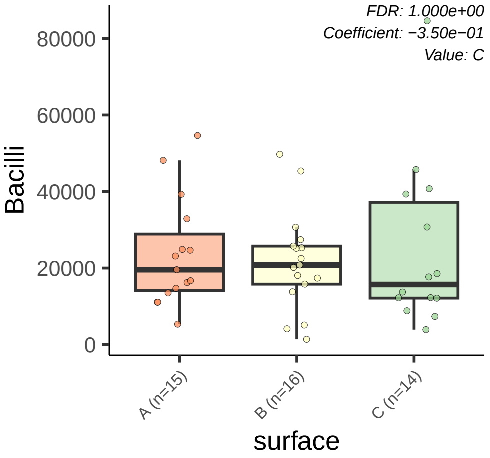

FDR: 1.000e+00
Coefficient: −3.50e−01
Value: C
Bacilli
0
20000
40000
60000
80000
A (n=15)
B (n=16)
C (n=14)
surface

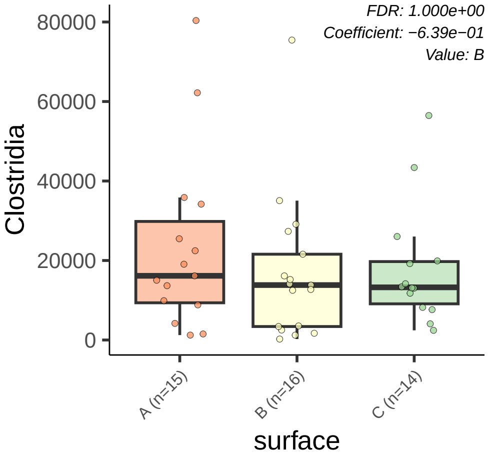

FDR: 1.000e+00
Coefficient: −6.39e−01
Value: B
80000
60000
40000
20000
0
Clostridia
A (n=15)
B (n=16)
C (n=14)
surface

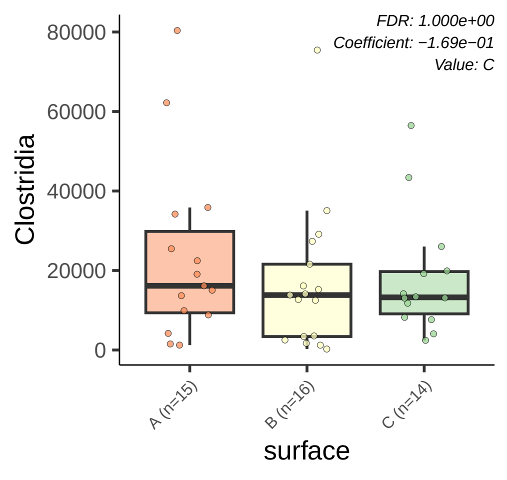

FDR: 1.000e+00
Coefficient: −1.69e−01
Value: C
Clostridia
80000
60000
40000
20000
0
A (n=15)
B (n=16)
C (n=14)
surface

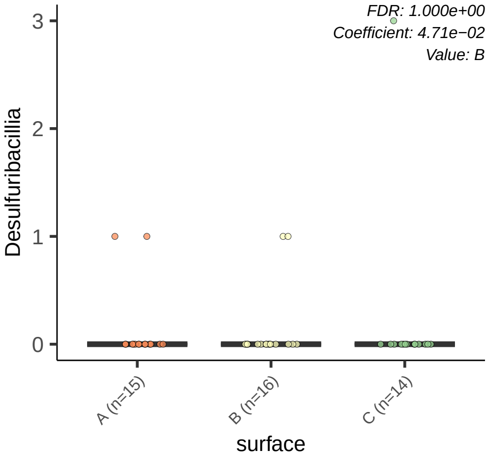

FDR: 1.000e+00
Coefficient: 4.71e−02
Value: B
Desulfuribacillia
3
2
1
0
A (n=15)
B (n=16)
C (n=14)
surface

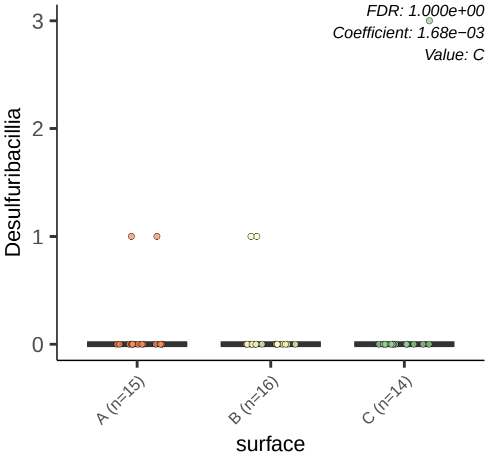
FDR: 1.000e+00
Coefficient: 1.68e−03
Value: C
Desulfuribacillia
3
2
1
0
A (n=15)
B (n=16)
C (n=14)
surface

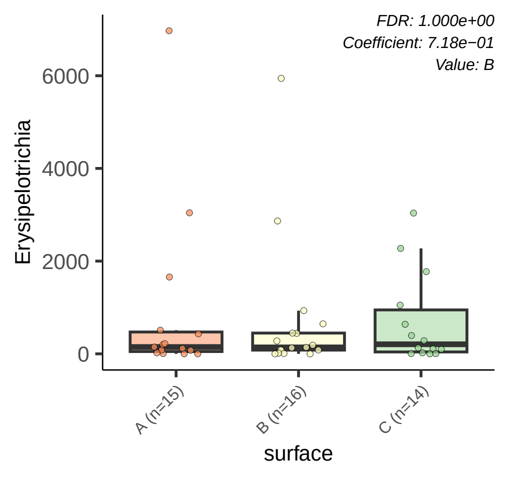

FDR: 1.000e+00
Coefficient: 7.18e−01
Value: B
Erysipelotrichia
6000
4000
2000
0
A (n=15)
B (n=16)
C (n=14)
surface

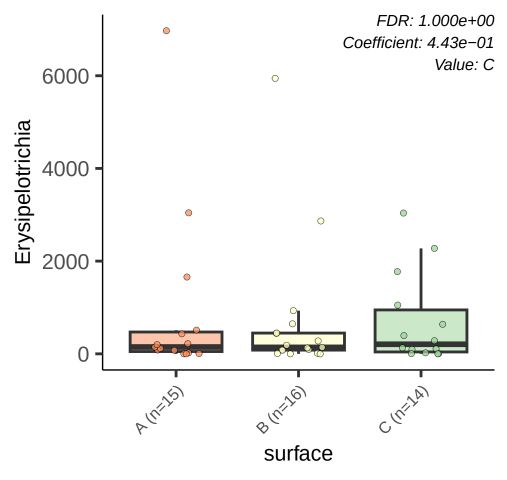
FDR: 1.000e+00
Coefficient: 4.43e−01
Value: C
Erysipelotrichia
6000
4000
2000
0
A (n=15)
B (n=16)
C (n=14)
surface

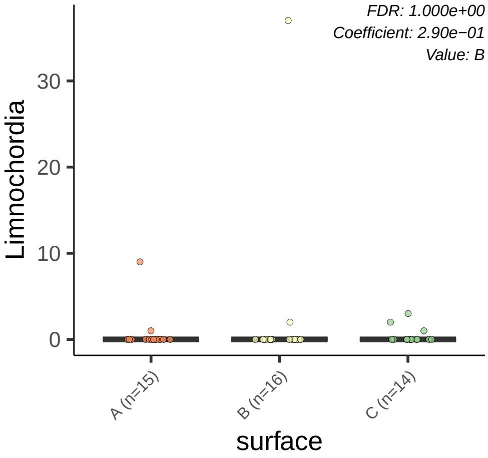
FDR: 1.000e+00
Coefficient: 2.90e−01
Value: B
Limnochordia
30
20
10
0
A (n=15)
B (n=16)
C (n=14)
surface

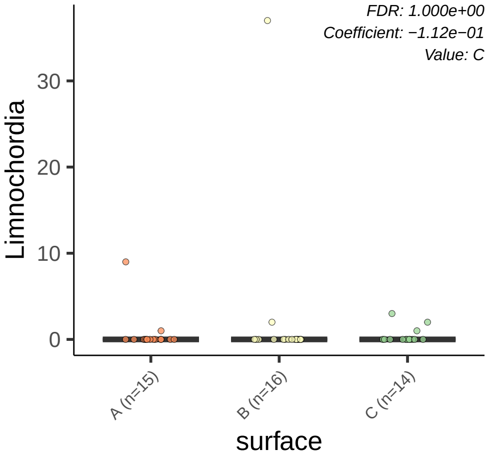

FDR: 1.000e+00
Coefficient: −1.12e−01
Value: C
Limnochordia
0
10
20
30
A (n=15)
B (n=16)
C (n=14)
surface

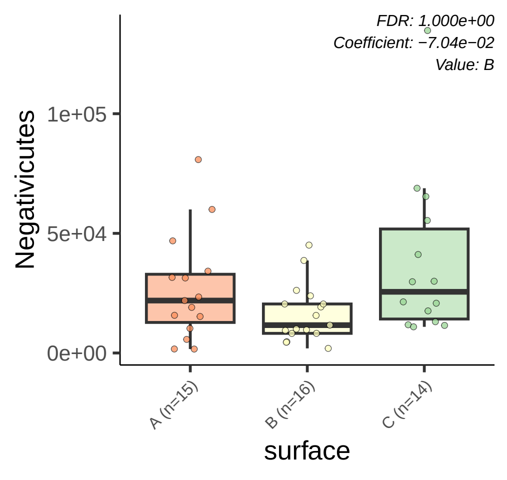
FDR: 1.000e+00
Coefficient: −7.04e−02
Value: B
Negativicutes
1e+05
5e+04
0e+00
A (n=15)
B (n=16)
C (n=14)
surface

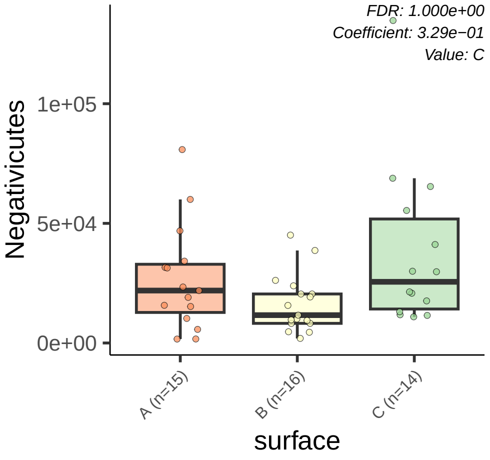

FDR: 1.000e+00
Coefficient: 3.29e−01
Value: C
Negativicutes
1e+05
5e+04
0e+00
A (n=15)
B (n=16)
C (n=14)
surface

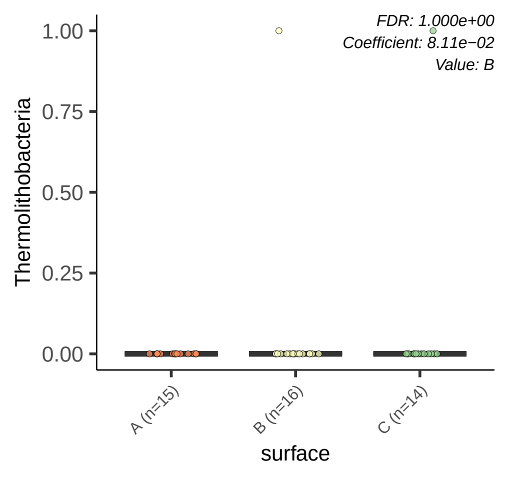

FDR: 1.000e+00
Coefficient: 8.11e−02
Value: B
Thermolithobacteria
1.00
0.75
0.50
0.25
0.00
A (n=15)
B (n=16)
C (n=14)
surface

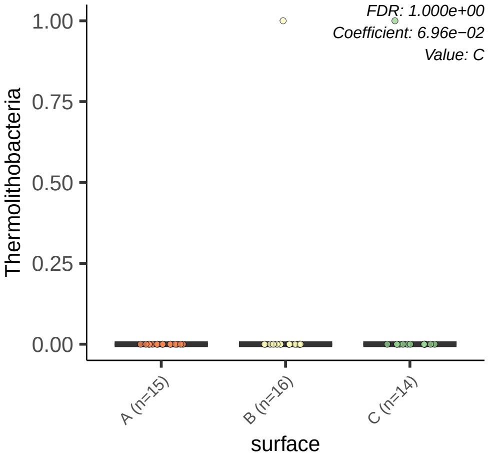
FDR: 1.000e+00
Coefficient: 6.96e−02
Value: C
Thermolithobacteria
1.00
0.75
0.50
0.25
0.00
A (n=15)
B (n=16)
C (n=14)
surface

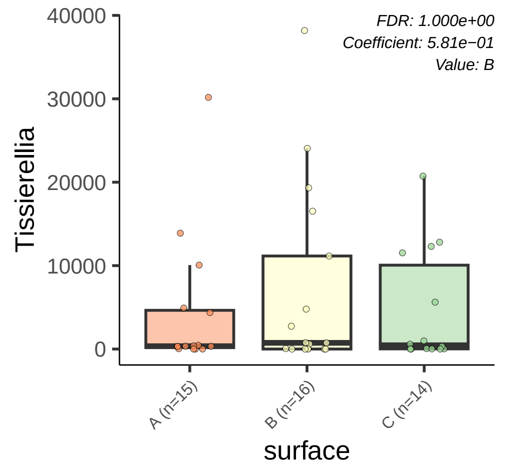
FDR: 1.000e+00
Coefficient: 5.81e−01
Value: B
Tissierellia
40000
30000
20000
10000
0
A (n=15)
B (n=16)
C (n=14)
surface

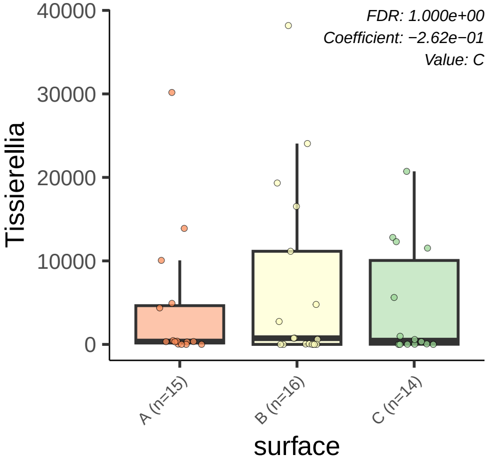

FDR: 1.000e+00
Coefficient: −2.62e−01
Value: C
Tissierellia
40000
30000
20000
10000
0
A (n=15)
B (n=16)
C (n=14)
surface

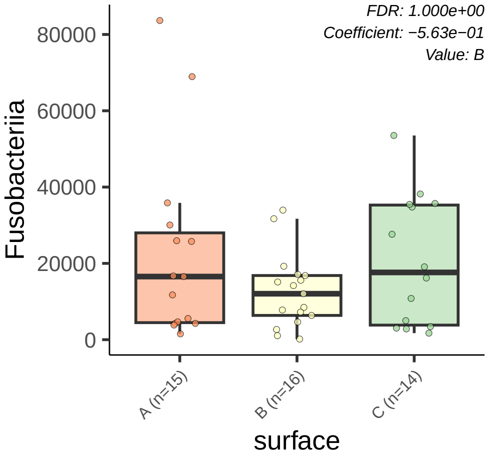

FDR: 1.000e+00
Coefficient: −5.63e−01
Value: B
Fusobacteriia
80000
60000
40000
20000
0
A (n=15)
B (n=16)
C (n=14)
surface

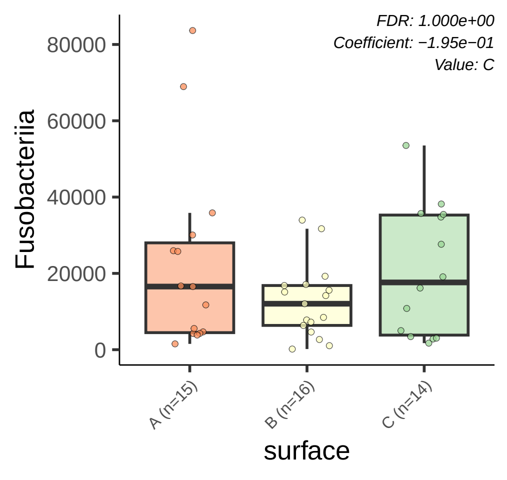

FDR: 1.000e+00
Coefficient: −1.95e−01
Value: C
Fusobacteriia
80000
60000
40000
20000
0
A (n=15)
B (n=16)
C (n=14)
surface

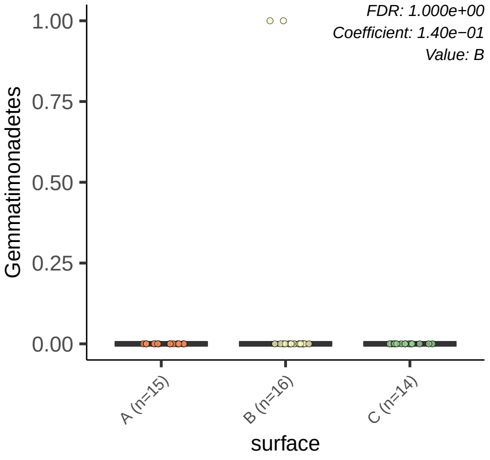
FDR: 1.000e+00
Coefficient: 1.40e−01
Value: B
Gemmatimonadetes
1.00
0.75
0.50
0.25
0.00
A (n=15)
B (n=16)
C (n=14)
surface

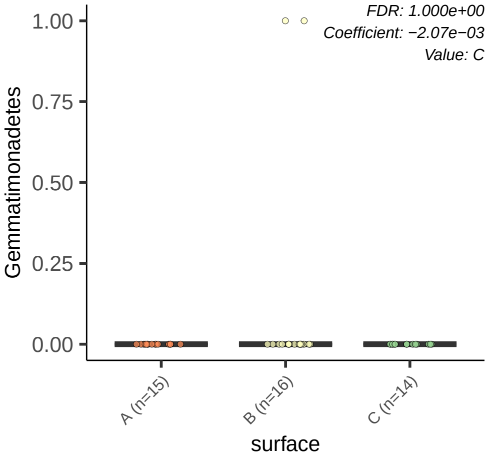

FDR: 1.000e+00
Coefficient: −2.07e−03
Value: C
Gemmatimonadetes
1.00
0.75
0.50
0.25
0.00
A (n=15)
B (n=16)
C (n=14)
surface

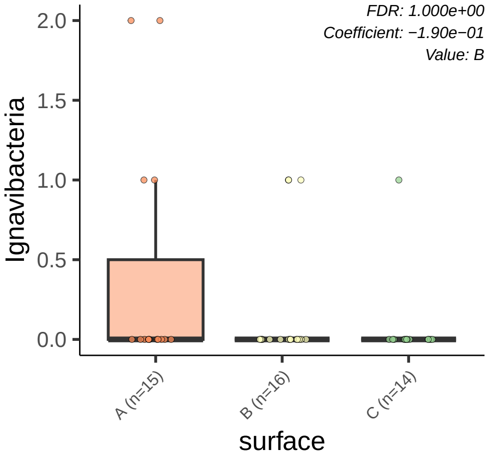
FDR: 1.000e+00
Coefficient: −1.90e−01
Value: B
Ignavibacteria
2.0
1.5
1.0
0.5
0.0
A (n=15)
B (n=16)
C (n=14)
surface

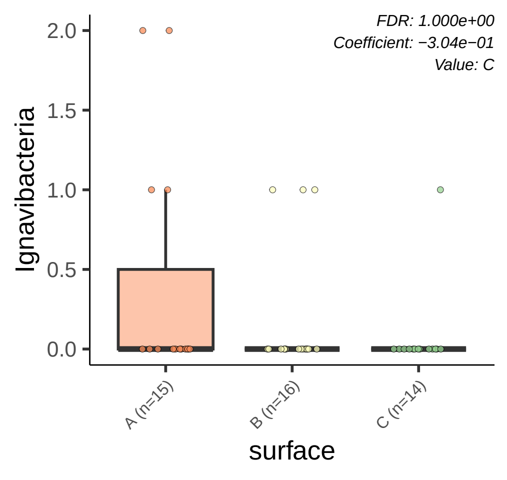

FDR: 1.000e+00
Coefficient: −3.04e−01
Value: C
Ignavibacteria
2.0
1.5
1.0
0.5
0.0
A (n=15)
B (n=16)
C (n=14)
surface

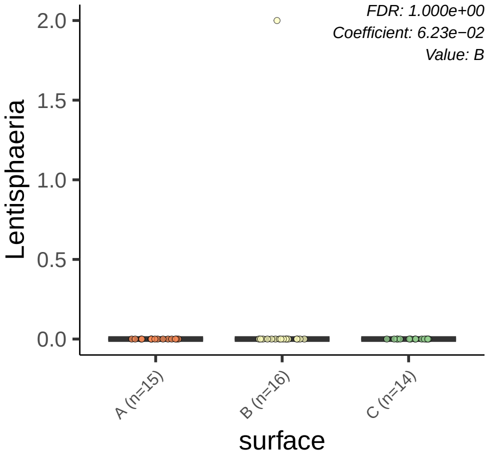
FDR: 1.000e+00
Coefficient: 6.23e−02
Value: B
Lentisphaeria
2.0
1.5
1.0
0.5
0.0
A (n=15)
B (n=16)
C (n=14)
surface

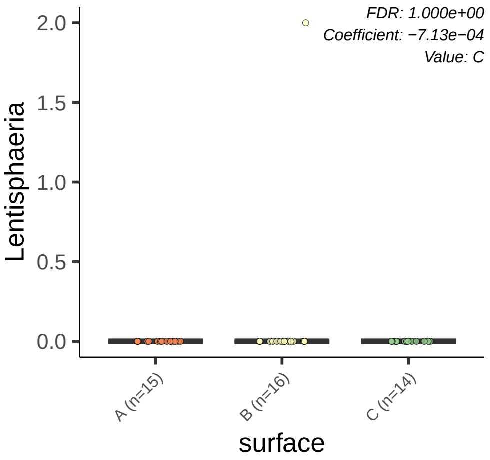

FDR: 1.000e+00
Coefficient: −7.13e−04
Value: C
Lentisphaeria
2.0
1.5
1.0
0.5
0.0
A (n=15)
B (n=16)
C (n=14)
surface

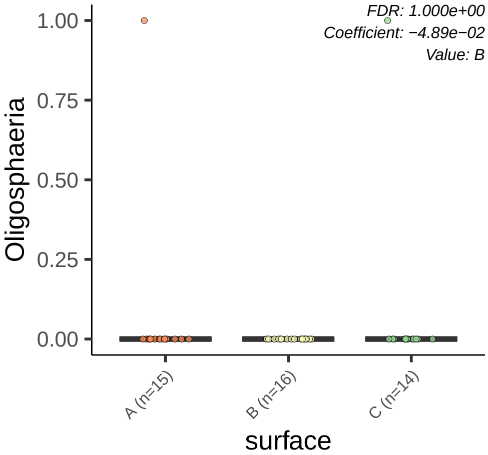

FDR: 1.000e+00
Coefficient: −4.89e−02
Value: B
Oligosphaeria
1.00
0.75
0.50
0.25
0.00
A (n=15)
B (n=16)
C (n=14)
surface

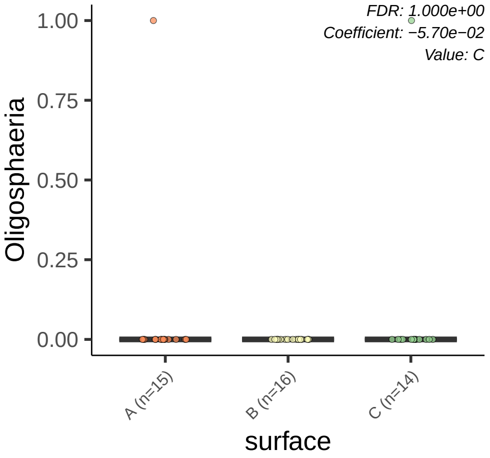

FDR: 1.000e+00
Coefficient: −5.70e−02
Value: C
Oligosphaeria
1.00
0.75
0.50
0.25
0.00
A (n=15)
B (n=16)
C (n=14)
surface

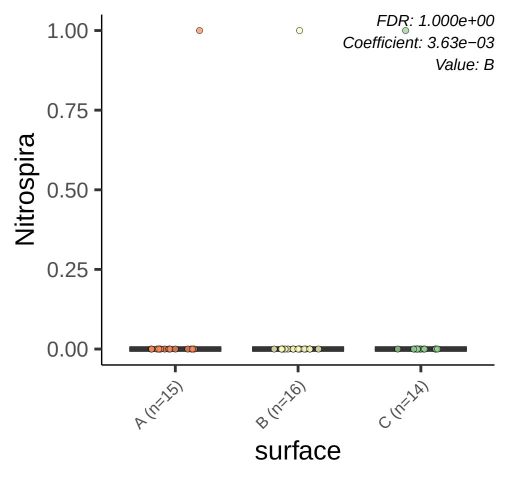

FDR: 1.000e+00
Coefficient: 3.63e−03
Value: B
Nitrospira
1.00
0.75
0.50
0.25
0.00
A (n=15)
B (n=16)
C (n=14)
surface

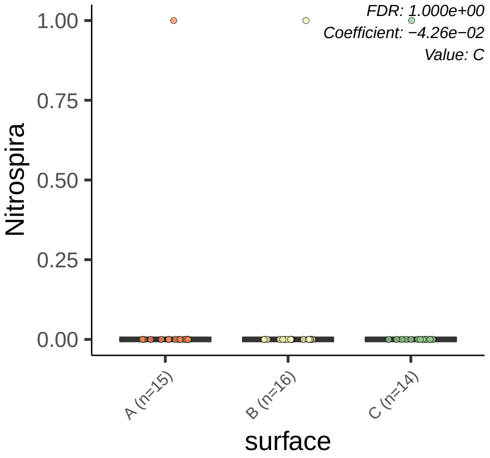

FDR: 1.000e+00
Coefficient: −4.26e−02
Value: C
Nitrospira
1.00
0.75
0.50
0.25
0.00
A (n=15)
B (n=16)
C (n=14)
surface

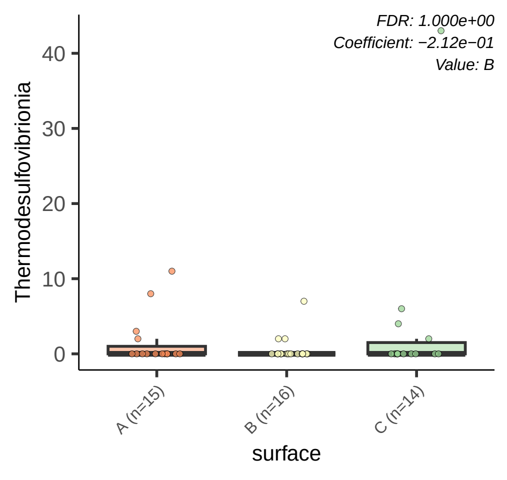
FDR: 1.000e+00
Coefficient: −2.12e−01
Value: B
Thermodesulfovibrionia
40
30
20
10
0
A (n=15)
B (n=16)
C (n=14)
surface

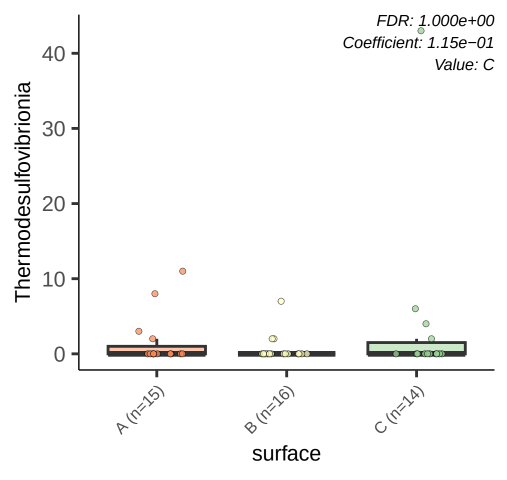
FDR: 1.000e+00
Coefficient: 1.15e−01
Value: C
Thermodesulfovibrionia
0
10
20
30
40
A (n=15)
B (n=16)
C (n=14)
surface

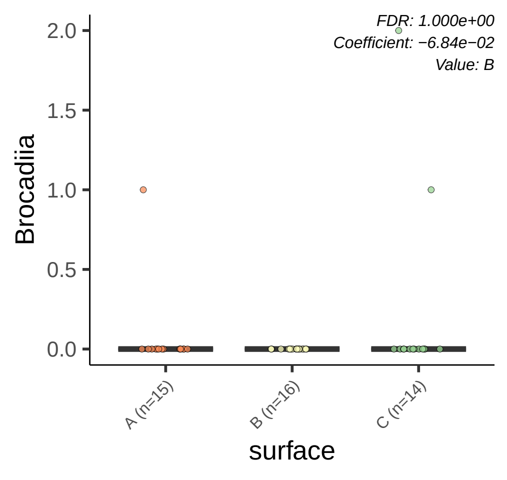
FDR: 1.000e+00
Coefficient: −6.84e−02
Value: B
Brocadiia
2.0
1.5
1.0
0.5
0.0
A (n=15)
B (n=16)
C (n=14)
surface

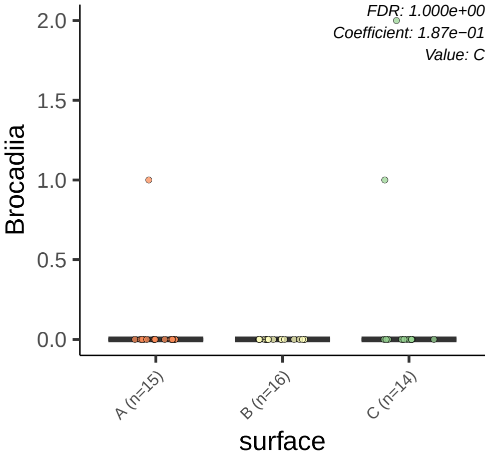

FDR: 1.000e+00
Coefficient: 1.87e−01
Value: C
Brocadiia
2.0
1.5
1.0
0.5
0.0
A (n=15)
B (n=16)
C (n=14)
surface

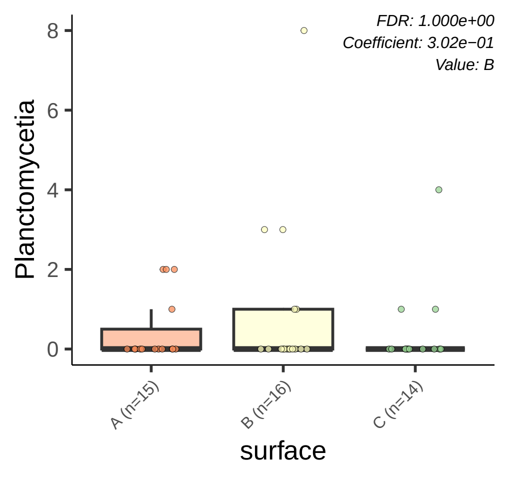

FDR: 1.000e+00
Coefficient: 3.02e−01
Value: B
Planctomycetia
0
2
4
6
8
A (n=15)
B (n=16)
C (n=14)
surface

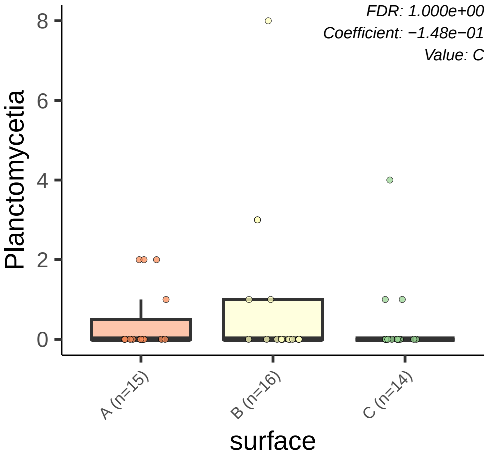
FDR: 1.000e+00
Coefficient: −1.48e−01
Value: C
Planctomycetia
0
2
4
6
8
A (n=15)
B (n=16)
C (n=14)
surface

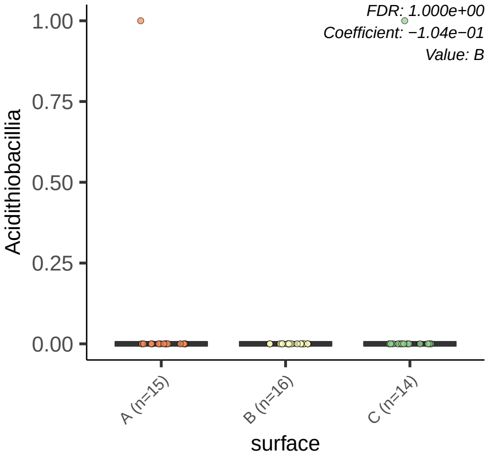
FDR: 1.000e+00
Coefficient: −1.04e−01
Value: B
Acidithiobacillia
1.00
0.75
0.50
0.25
0.00
A (n=15)
B (n=16)
C (n=14)
surface

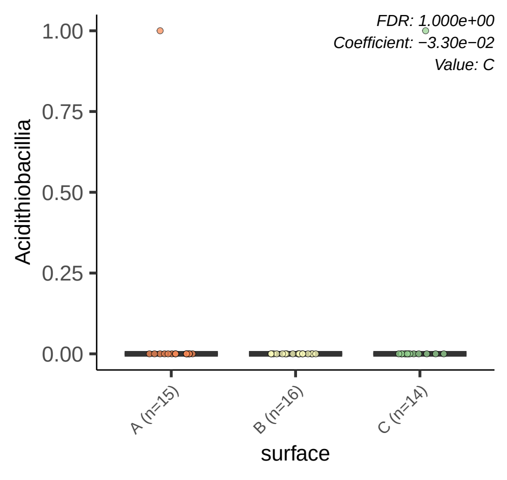
FDR: 1.000e+00
Coefficient: −3.30e−02
Value: C
Acidithiobacillia
1.00
0.75
0.50
0.25
0.00
A (n=15)
B (n=16)
C (n=14)
surface

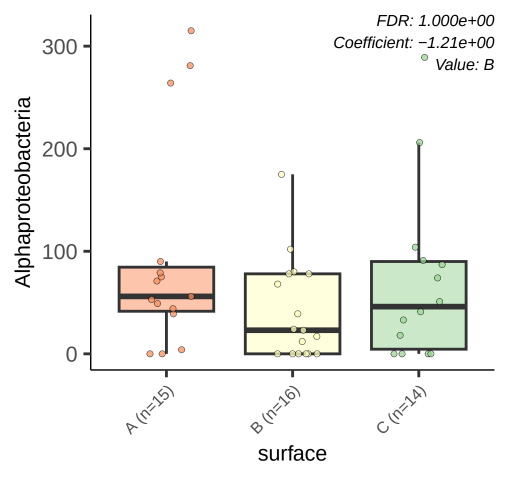

FDR: 1.000e+00
Coefficient: −1.21e+00
Value: B
Alphaproteobacteria
300
200
100
0
A (n=15)
B (n=16)
C (n=14)
surface

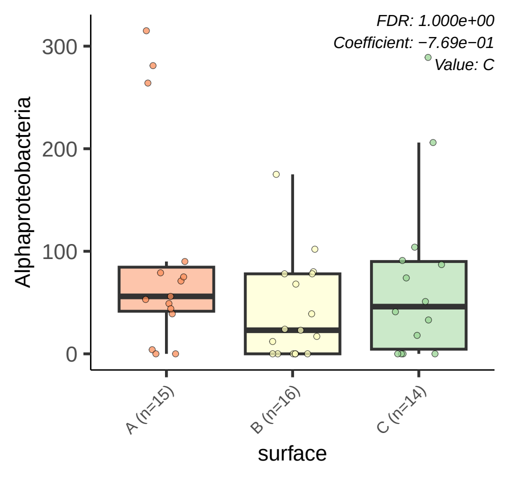
FDR: 1.000e+00
Coefficient: −7.69e−01
Value: C
Alphaproteobacteria
300
200
100
0
A (n=15)
B (n=16)
C (n=14)
surface

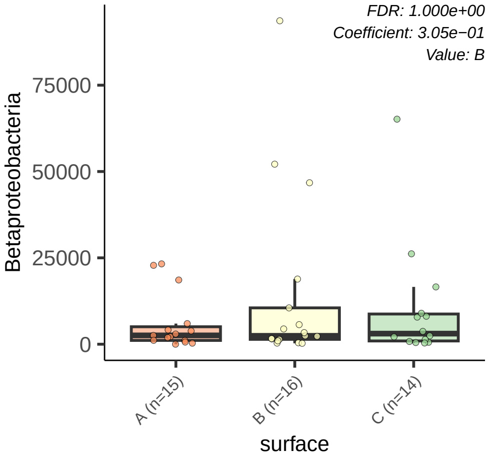
FDR: 1.000e+00
Coefficient: 3.05e−01
Value: B
Betaproteobacteria
75000
50000
25000
0
A (n=15)
B (n=16)
C (n=14)
surface

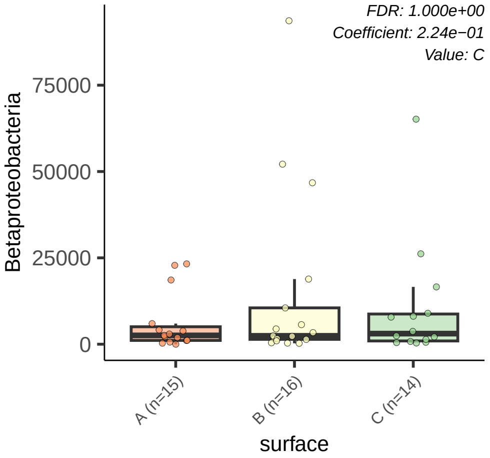
FDR: 1.000e+00
Coefficient: 2.24e−01
Value: C
Betaproteobacteria
75000
50000
25000
0
A (n=15)
B (n=16)
C (n=14)
surface

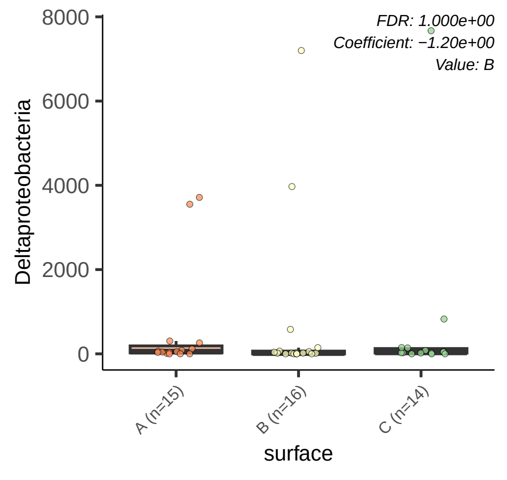

FDR: 1.000e+00
Coefficient: −1.20e+00
Value: B
Deltaproteobacteria
8000
6000
4000
2000
0
A (n=15)
B (n=16)
C (n=14)
surface

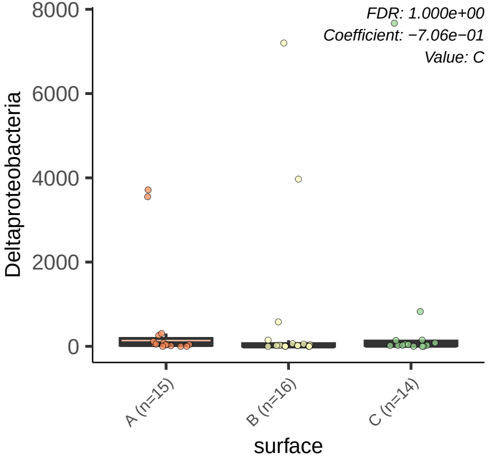

FDR: 1.000e+00
Coefficient: −7.06e−01
Value: C
8000
6000
4000
2000
0
Deltaproteobacteria
A (n=15)
B (n=16)
C (n=14)
surface

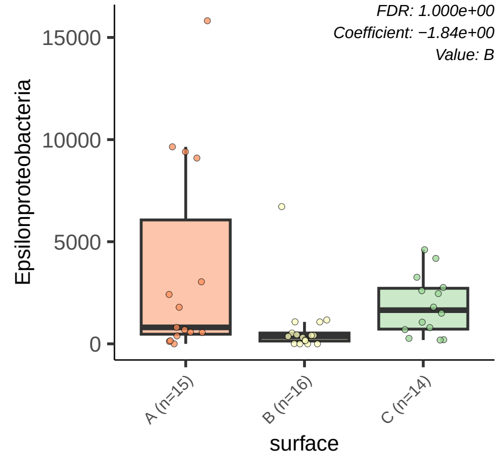

FDR: 1.000e+00
Coefficient: −1.84e+00
Value: B
15000
10000
5000
0
Epsilonproteobacteria
A (n=15)
B (n=16)
C (n=14)
surface

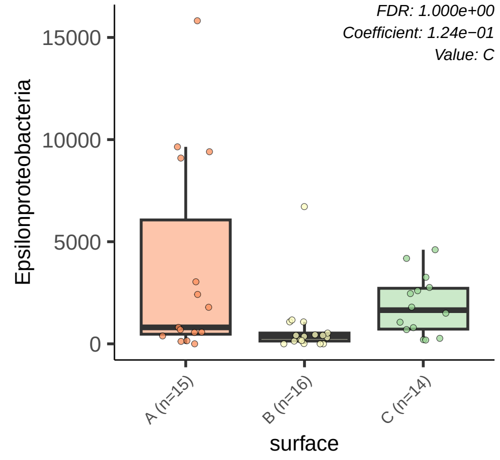
FDR: 1.000e+00
Coefficient: 1.24e−01
Value: C
15000
10000
5000
0
Epsilonproteobacteria
A (n=15)
B (n=16)
C (n=14)
surface

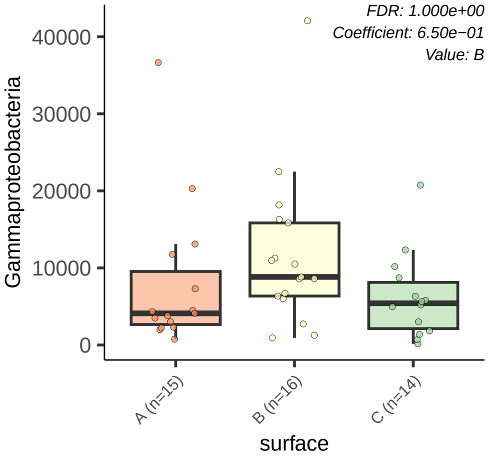
FDR: 1.000e+00
Coefficient: 6.50e−01
Value: B
Gammaproteobacteria
40000
30000
20000
10000
0
A (n=15)
B (n=16)
C (n=14)
surface

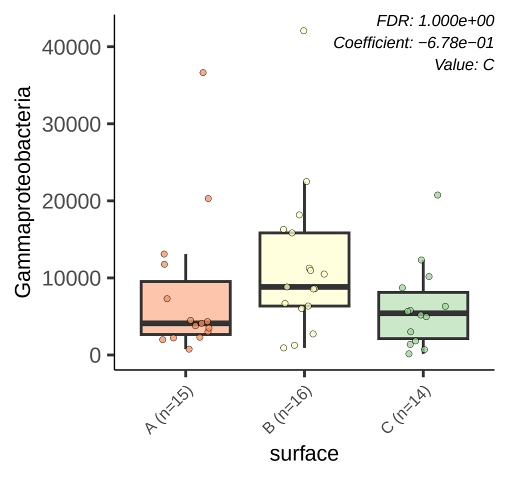

FDR: 1.000e+00
Coefficient: −6.78e−01
Value: C
Gammaproteobacteria
40000
30000
20000
10000
0
A (n=15)
B (n=16)
C (n=14)
surface

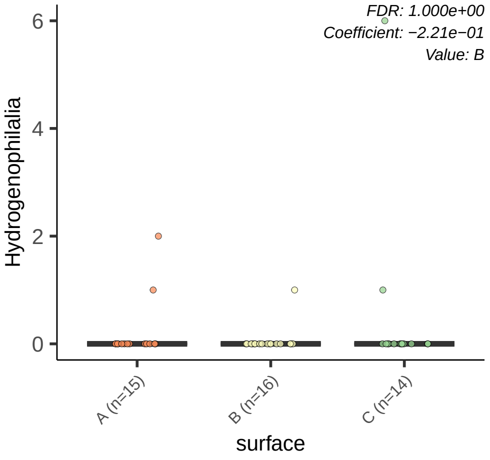
FDR: 1.000e+00
Coefficient: −2.21e−01
Value: B
Hydrogenophilalia
6
4
2
0
A (n=15)
B (n=16)
C (n=14)
surface

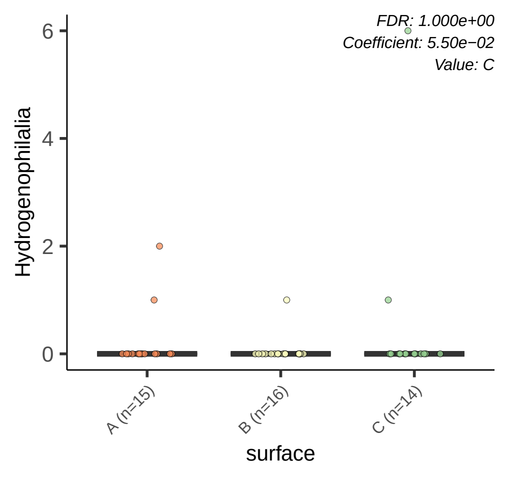

FDR: 1.000e+00
Coefficient: 5.50e−02
Value: C
Hydrogenophilalia
0
2
4
6
A (n=15)
B (n=16)
C (n=14)
surface

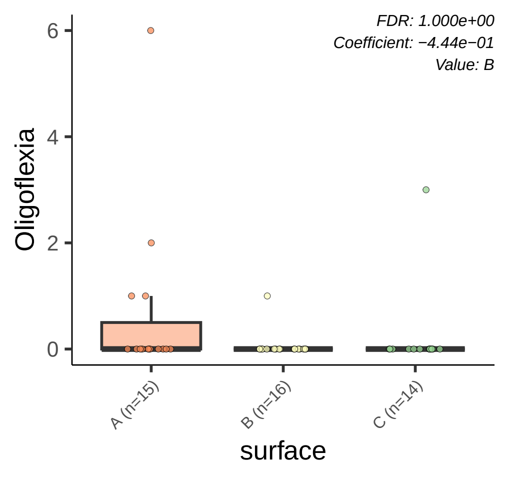

FDR: 1.000e+00
Coefficient: −4.44e−01
Value: B
Oligoflexia
6
4
2
0
A (n=15)
B (n=16)
C (n=14)
surface

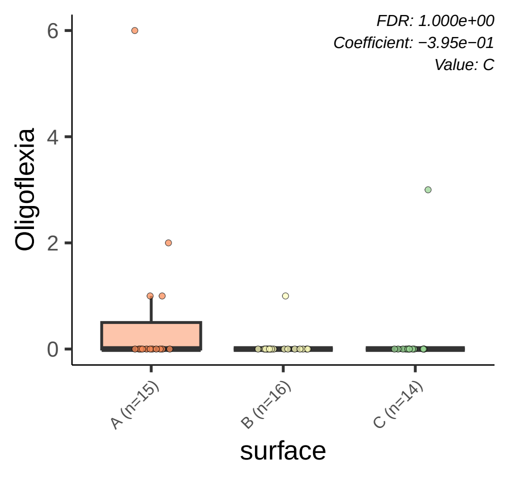
FDR: 1.000e+00
Coefficient: −3.95e−01
Value: C
Oligoflexia
0
2
4
6
A (n=15)
B (n=16)
C (n=14)
surface

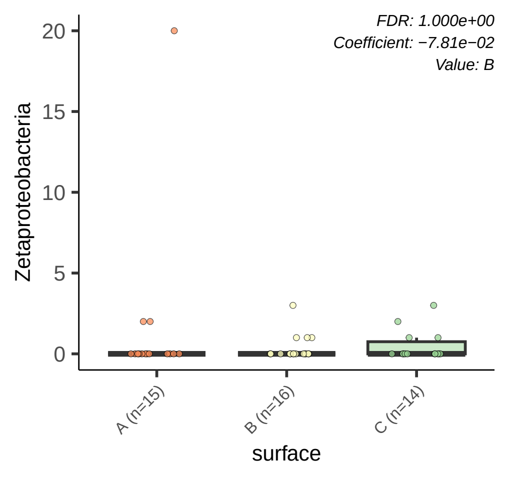

FDR: 1.000e+00
Coefficient: −7.81e−02
Value: B
Zetaproteobacteria
20
15
10
5
0
A (n=15)
B (n=16)
C (n=14)
surface

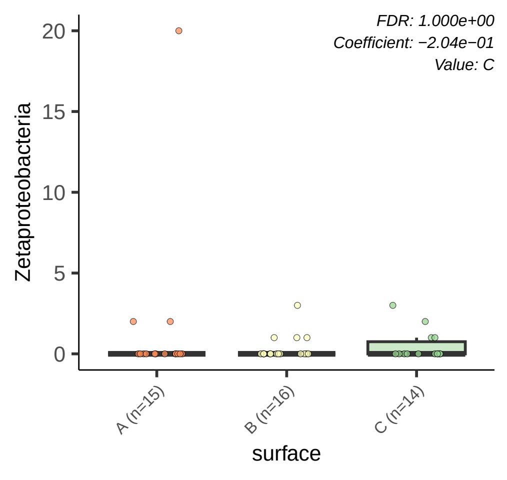

FDR: 1.000e+00
Coefficient: −2.04e−01
Value: C
Zetaproteobacteria
20
15
10
5
0
A (n=15)
B (n=16)
C (n=14)
surface

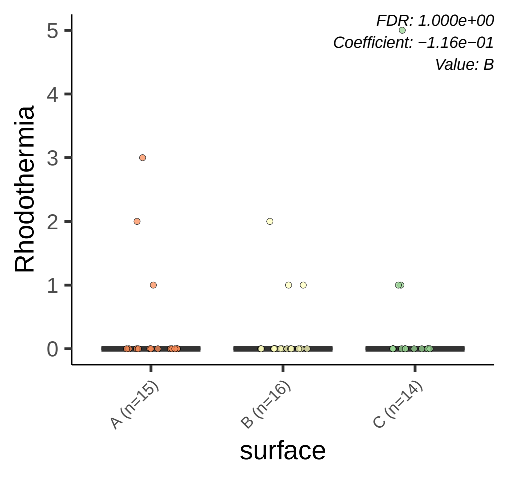
FDR: 1.000e+00
Coefficient: −1.16e−01
Value: B
Rhodothermia
0
1
2
3
4
5
A (n=15)
B (n=16)
C (n=14)
surface

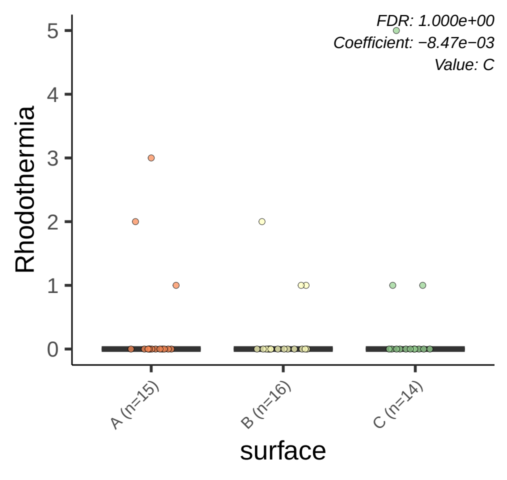

FDR: 1.000e+00
Coefficient: −8.47e−03
Value: C
Rhodothermia
0
1
2
3
4
5
A (n=15)
B (n=16)
C (n=14)
surface

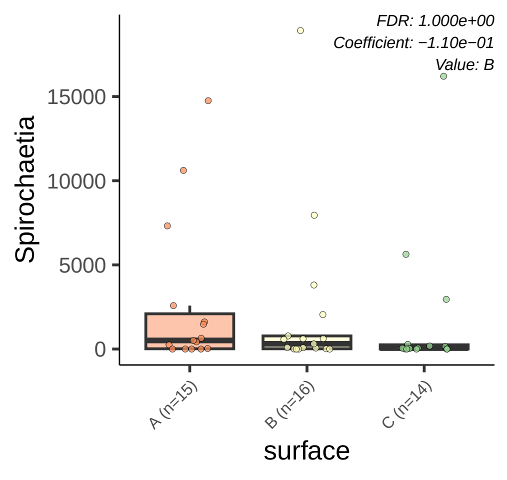
FDR: 1.000e+00
Coefficient: −1.10e−01
Value: B
Spirochaetia
15000
10000
5000
0
A (n=15)
B (n=16)
C (n=14)
surface

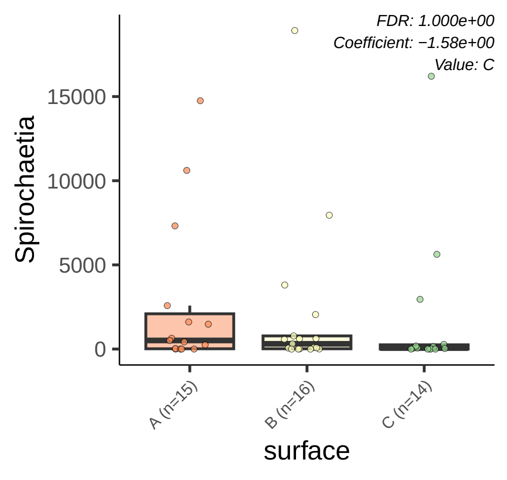

FDR: 1.000e+00
Coefficient: −1.58e+00
Value: C
Spirochaetia
15000
10000
5000
0
A (n=15)
B (n=16)
C (n=14)
surface

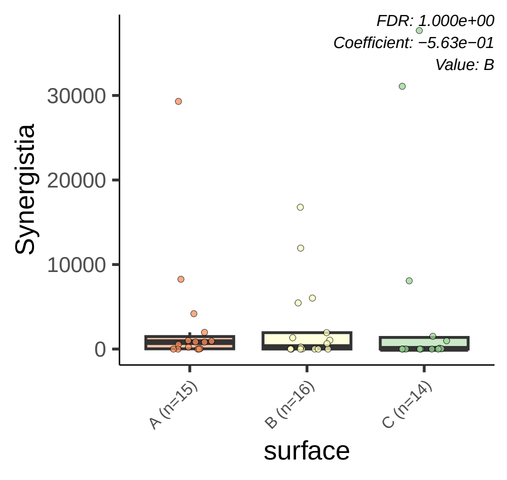

FDR: 1.000e+00
Coefficient: −5.63e−01
Value: B
Synergistia
30000
20000
10000
0
A (n=15)
B (n=16)
C (n=14)
surface

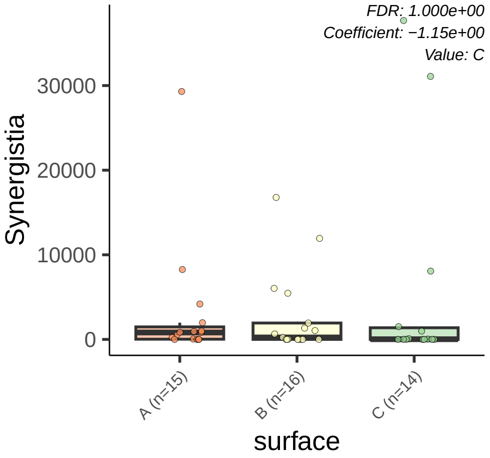

FDR: 1.000e+00
Coefficient: −1.15e+00
Value: C
Synergistia
30000
20000
10000
0
A (n=15)
B (n=16)
C (n=14)
surface

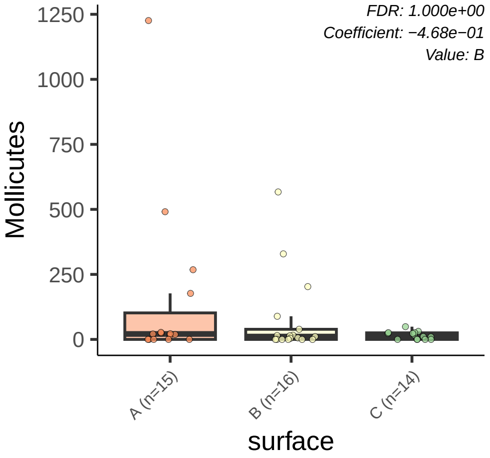
FDR: 1.000e+00
Coefficient: −4.68e−01
Value: B
Mollicutes
1250
1000
750
500
250
0
A (n=15)
B (n=16)
C (n=14)
surface

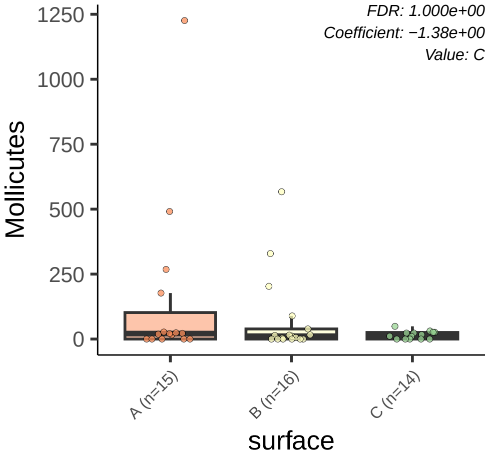

FDR: 1.000e+00
Coefficient: −1.38e+00
Value: C
Mollicutes
1250
1000
750
500
250
0
A (n=15)
B (n=16)
C (n=14)
surface

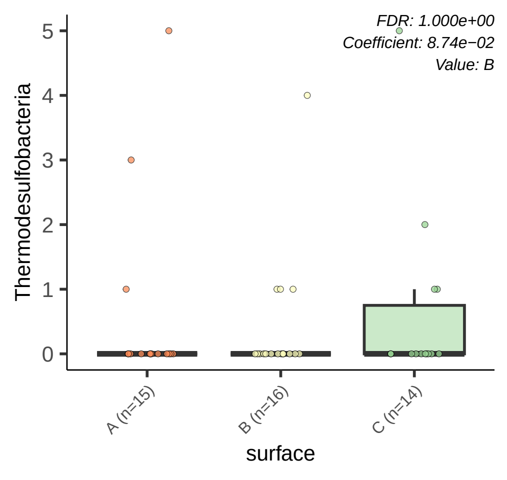

FDR: 1.000e+00
Coefficient: 8.74e−02
Value: B
Thermodesulfobacteria
0
1
2
3
4
5
A (n=15)
B (n=16)
C (n=14)
surface

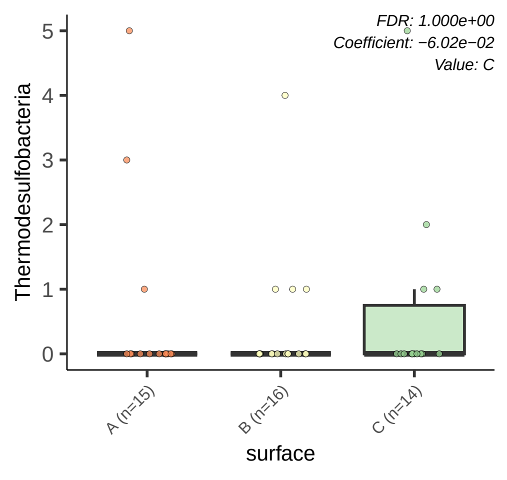
FDR: 1.000e+00
Coefficient: −6.02e−02
Value: C
Thermodesulfobacteria
0
1
2
3
4
5
A (n=15)
B (n=16)
C (n=14)
surface

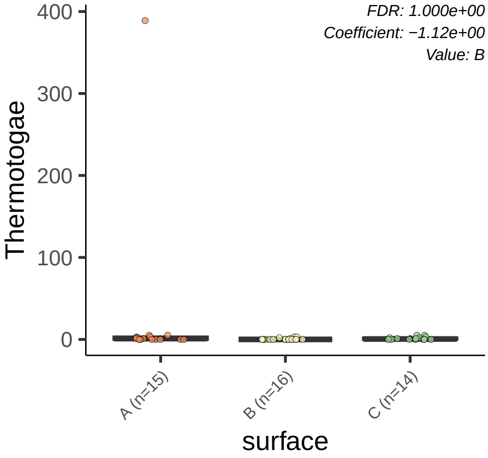

FDR: 1.000e+00
Coefficient: −1.12e+00
Value: B
Thermotogae
400
300
200
100
0
A (n=15)
B (n=16)
C (n=14)
surface

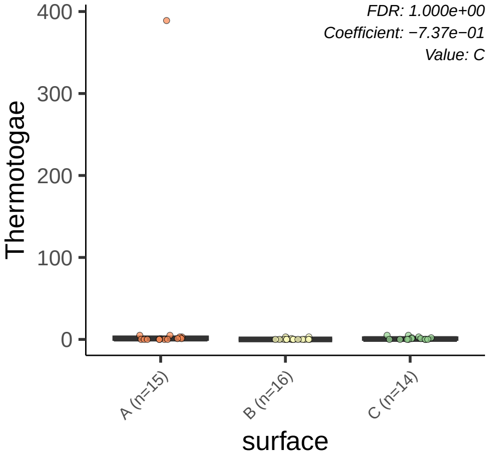
FDR: 1.000e+00
Coefficient: −7.37e−01
Value: C
Thermotogae
400
300
200
100
0
A (n=15)
B (n=16)
C (n=14)
surface

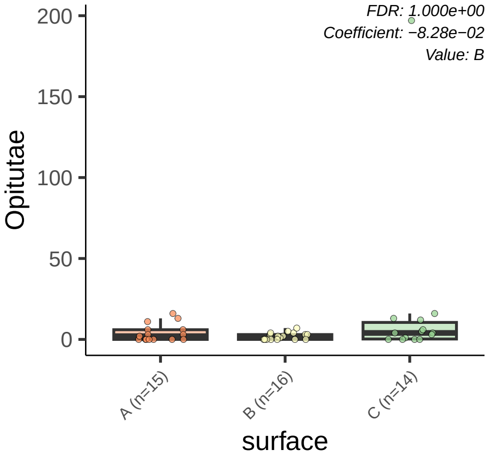

FDR: 1.000e+00
Coefficient: −8.28e−02
Value: B
Opitutae
200
150
100
50
0
A (n=15)
B (n=16)
C (n=14)
surface

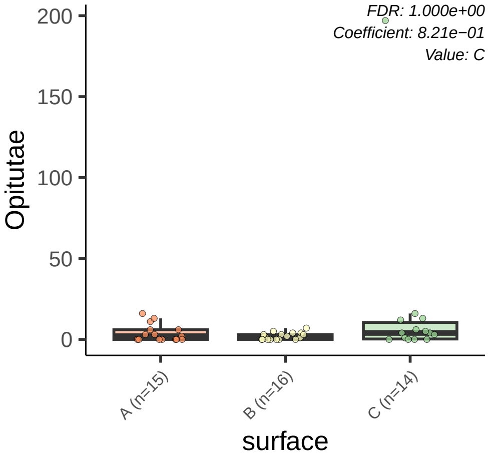
FDR: 1.000e+00
Coefficient: 8.21e−01
Value: C
Opitutae
200
150
100
50
0
A (n=15)
B (n=16)
C (n=14)
surface

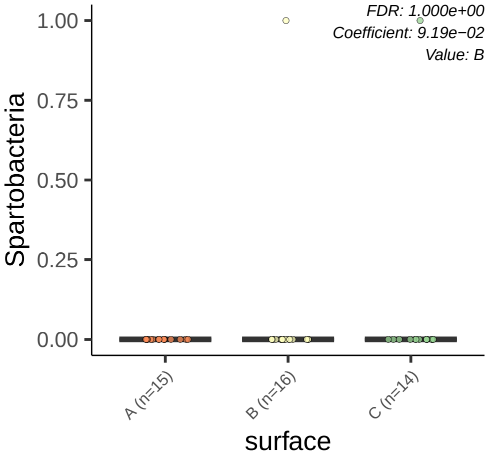
FDR: 1.000e+00
Coefficient: 9.19e−02
Value: B
Spartobacteria
1.00
0.75
0.50
0.25
0.00
A (n=15)
B (n=16)
C (n=14)
surface

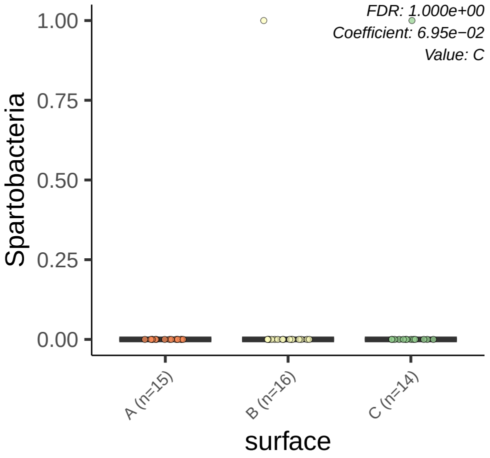
FDR: 1.000e+00
Coefficient: 6.95e−02
Value: C
Spartobacteria
1.00
0.75
0.50
0.25
0.00
A (n=15)
B (n=16)
C (n=14)
surface

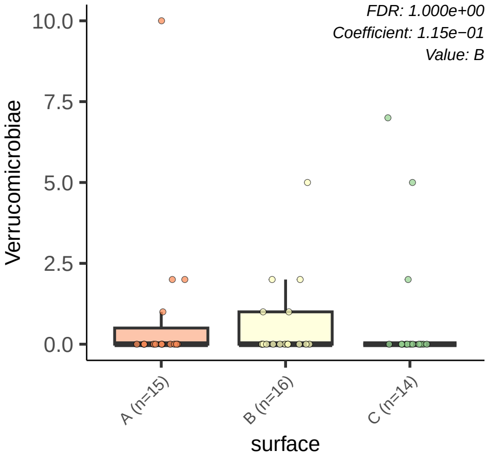
FDR: 1.000e+00
Coefficient: 1.15e−01
Value: B
Verrucomicrobiae
10.0
7.5
5.0
2.5
0.0
A (n=15)
B (n=16)
C (n=14)
surface

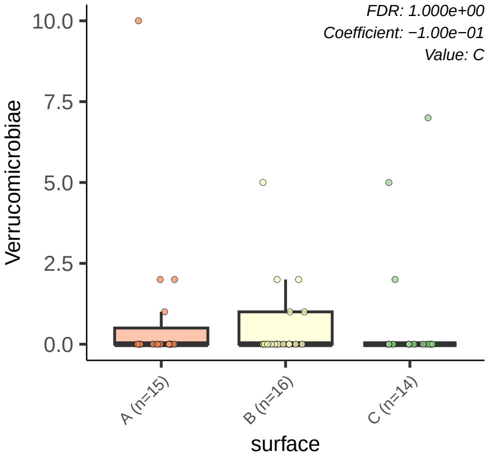
FDR: 1.000e+00
Coefficient: −1.00e−01
Value: C
Verrucomicrobiae
10.0
7.5
5.0
2.5
0.0
A (n=15)
B (n=16)
C (n=14)
surface

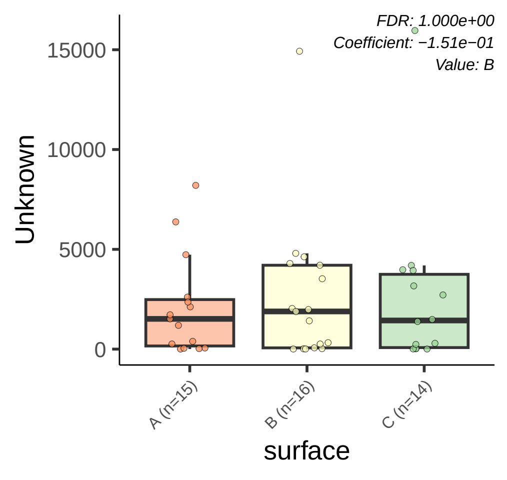

FDR: 1.000e+00
Coefficient: −1.51e−01
Value: B
15000
10000
5000
0
Unknown
A (n=15)
B (n=16)
C (n=14)
surface

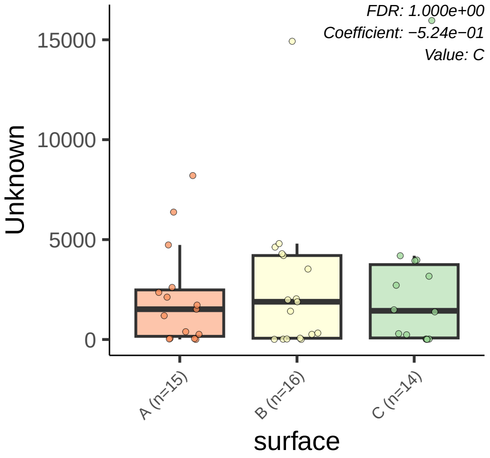

FDR: 1.000e+00
Coefficient: −5.24e−01
Value: C
15000
10000
5000
0
Unknown
A (n=15)
B (n=16)
C (n=14)
surface

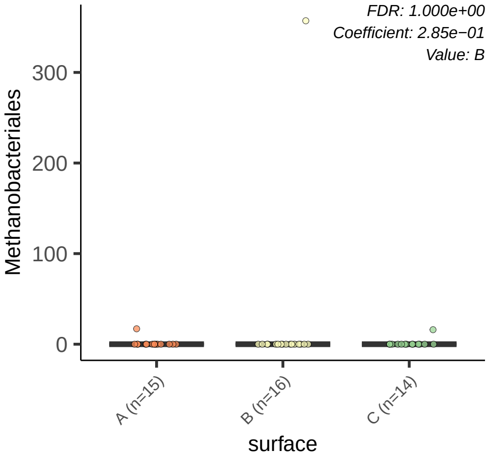
FDR: 1.000e+00
Coefficient: 2.85e−01
Value: B
Methanobacteriales
300
200
100
0
A (n=15)
B (n=16)
C (n=14)
surface

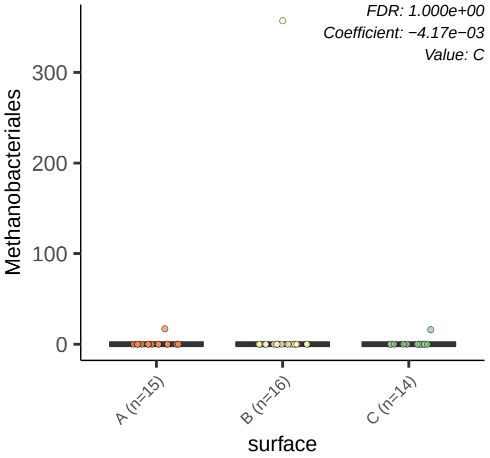
FDR: 1.000e+00
Coefficient: −4.17e−03
Value: C
Methanobacteriales
300
200
100
0
A (n=15)
B (n=16)
C (n=14)
surface

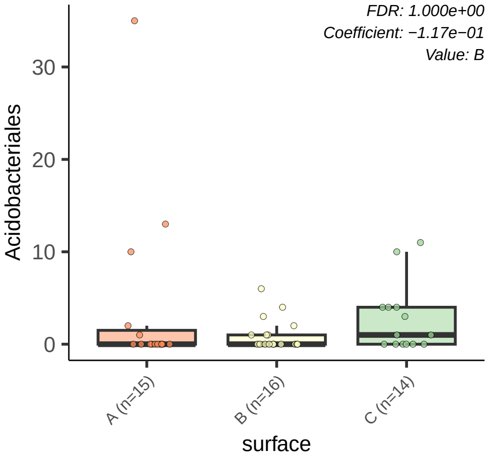

FDR: 1.000e+00
Coefficient: −1.17e−01
Value: B
Acidobacteriales
0
10
20
30
A (n=15)
B (n=16)
C (n=14)
surface

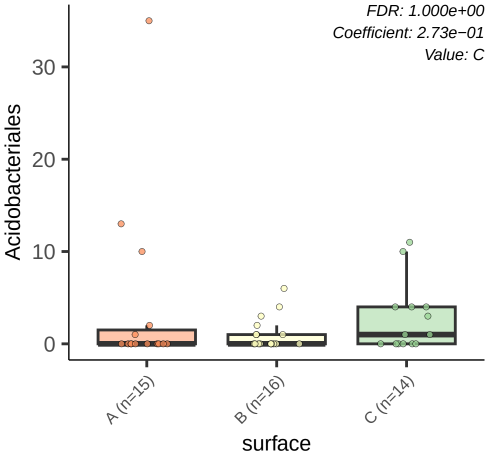
FDR: 1.000e+00
Coefficient: 2.73e−01
Value: C
Acidobacteriales
0
10
20
30
A (n=15)
B (n=16)
C (n=14)
surface

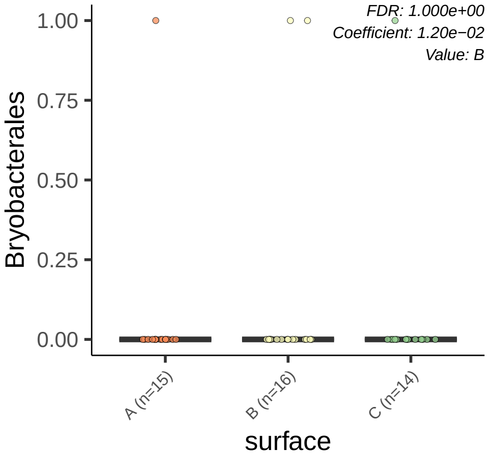

FDR: 1.000e+00
Coefficient: 1.20e−02
Value: B
Bryobacterales
1.00
0.75
0.50
0.25
0.00
A (n=15)
B (n=16)
C (n=14)
surface

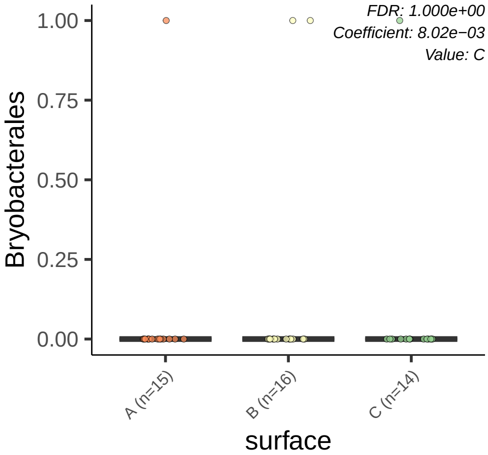
FDR: 1.000e+00
Coefficient: 8.02e−03
Value: C
Bryobacterales
1.00
0.75
0.50
0.25
0.00
A (n=15)
B (n=16)
C (n=14)
surface

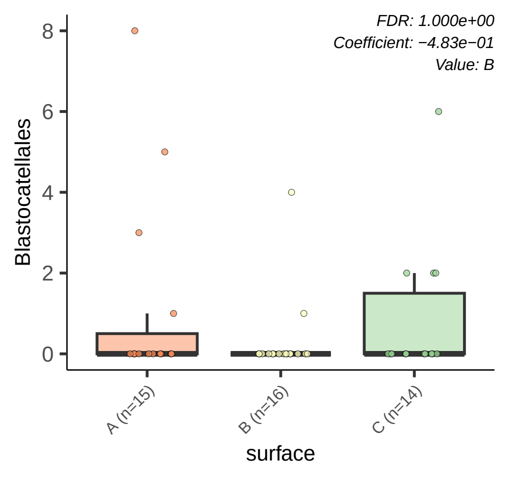

FDR: 1.000e+00
Coefficient: −4.83e−01
Value: B
Blastocatellales
0
2
4
6
8
A (n=15)
B (n=16)
C (n=14)
surface

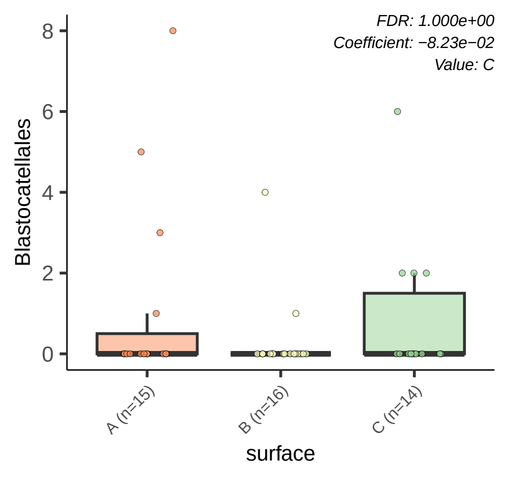

FDR: 1.000e+00
Coefficient: −8.23e−02
Value: C
Blastocatellales
8
6
4
2
0
A (n=15)
B (n=16)
C (n=14)
surface

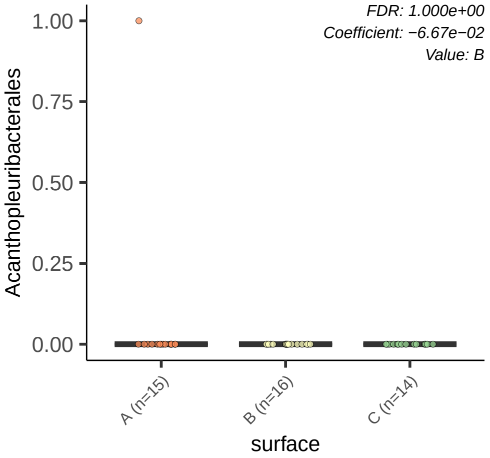
FDR: 1.000e+00
Coefficient: −6.67e−02
Value: B
Acanthopleuribacterales
1.00
0.75
0.50
0.25
0.00
A (n=15)
B (n=16)
C (n=14)
surface

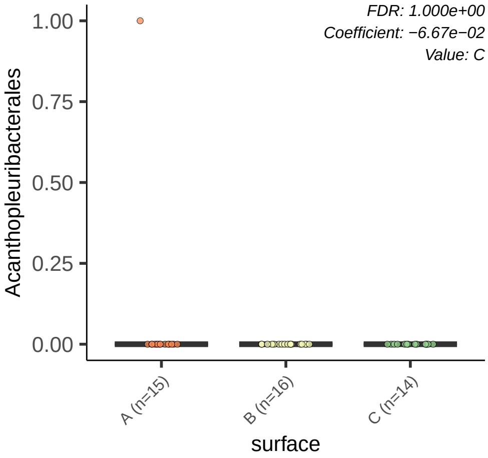

FDR: 1.000e+00
Coefficient: −6.67e−02
Value: C
Acanthopleuribacterales
1.00
0.75
0.50
0.25
0.00
A (n=15)
B (n=16)
C (n=14)
surface

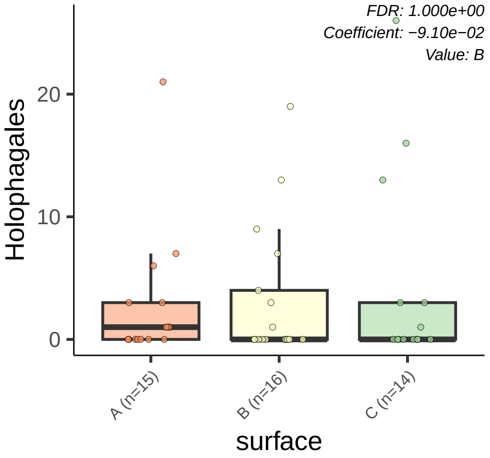

FDR: 1.000e+00
Coefficient: −9.10e−02
Value: B
Holophagales
20
10
0
A (n=15)
B (n=16)
C (n=14)
surface

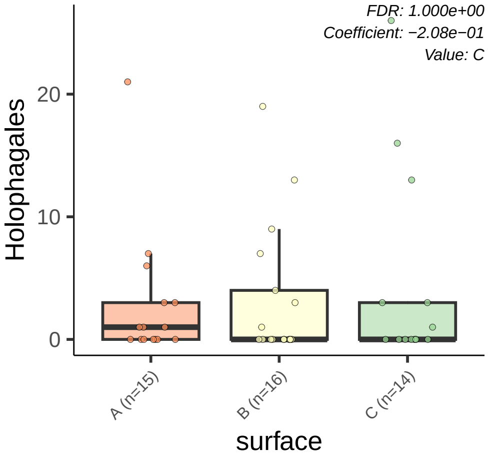
FDR: 1.000e+00
Coefficient: −2.08e−01
Value: C
Holophagales
20
10
0
A (n=15)
B (n=16)
C (n=14)
surface

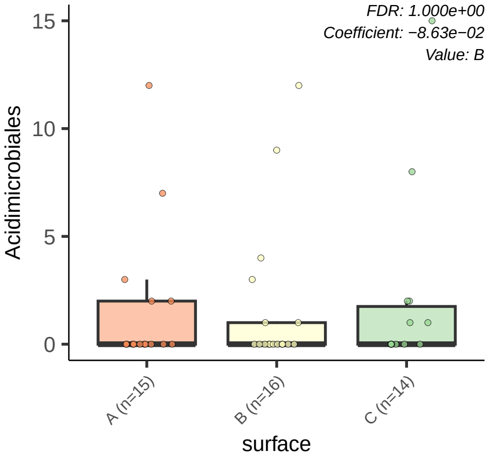
FDR: 1.000e+00
Coefficient: −8.63e−02
Value: B
Acidimicrobiales
15
10
5
0
A (n=15)
B (n=16)
C (n=14)
surface

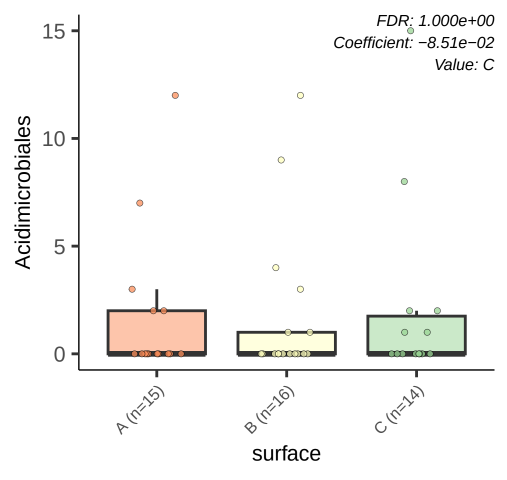
FDR: 1.000e+00
Coefficient: −8.51e−02
Value: C
Acidimicrobiales
15
10
5
0
A (n=15)
B (n=16)
C (n=14)
surface

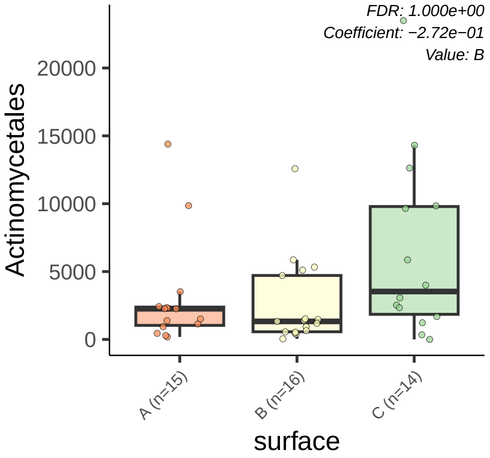
FDR: 1.000e+00
Coefficient: −2.72e−01
Value: B
Actinomycetales
20000
15000
10000
5000
0
A (n=15)
B (n=16)
C (n=14)
surface

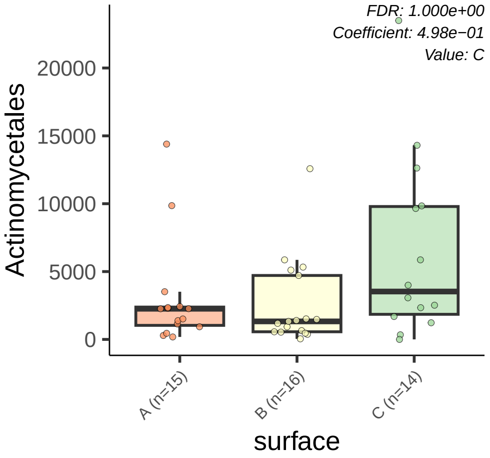
FDR: 1.000e+00
Coefficient: 4.98e−01
Value: C
Actinomycetales
20000
15000
10000
5000
0
A (n=15)
B (n=16)
C (n=14)
surface

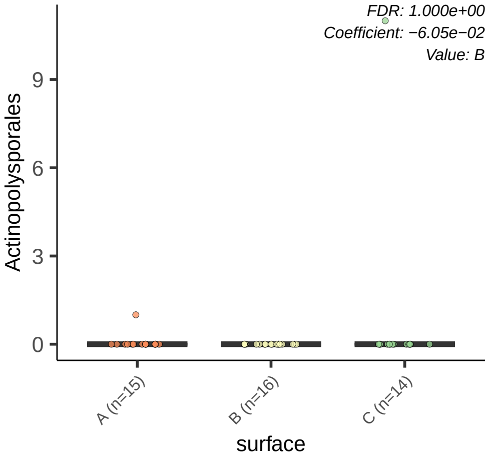

FDR: 1.000e+00
Coefficient: −6.05e−02
Value: B
Actinopolysporales
9
6
3
0
A (n=15)
B (n=16)
C (n=14)
surface

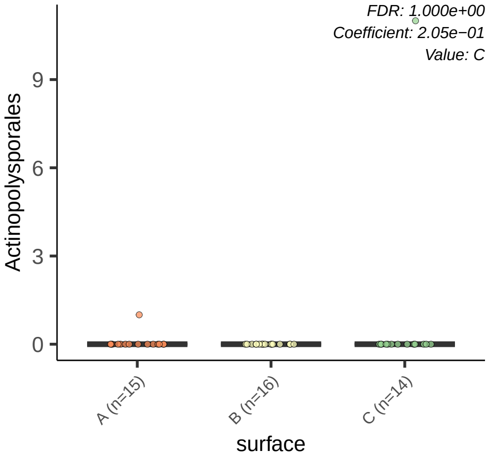
FDR: 1.000e+00
Coefficient: 2.05e−01
Value: C
Actinopolysporales
0
3
6
9
A (n=15)
B (n=16)
C (n=14)
surface

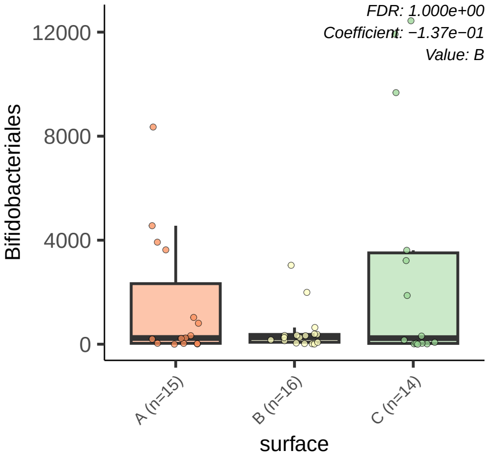

FDR: 1.000e+00
Coefficient: −1.37e−01
Value: B
Bifidobacteriales
12000
8000
4000
0
A (n=15)
B (n=16)
C (n=14)
surface

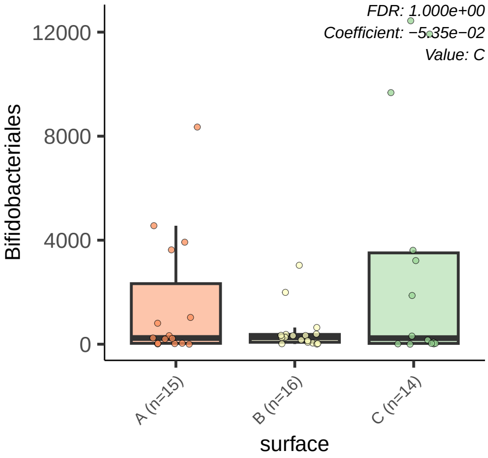

FDR: 1.000e+00
Coefficient: −5.35e−02
Value: C
Bifidobacteriales
12000
8000
4000
0
A (n=15)
B (n=16)
C (n=14)
surface

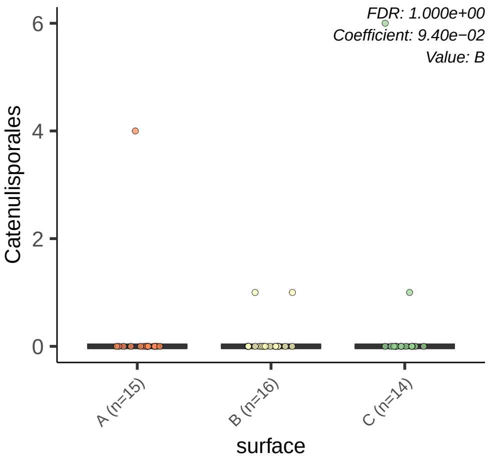

FDR: 1.000e+00
Coefficient: 9.40e−02
Value: B
Catenulisporales
6
4
2
0
A (n=15)
B (n=16)
C (n=14)
surface

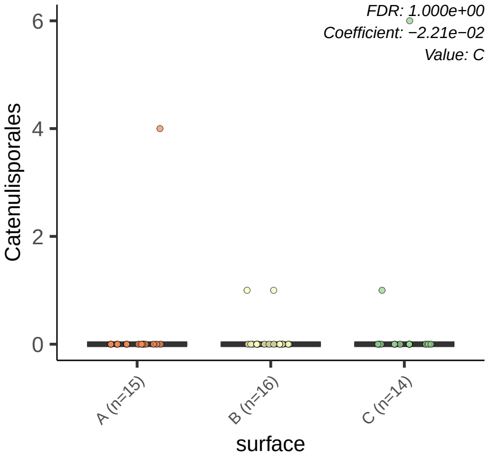
FDR: 1.000e+00
Coefficient: −2.21e−02
Value: C
Catenulisporales
6
4
2
0
A (n=15)
B (n=16)
C (n=14)
surface

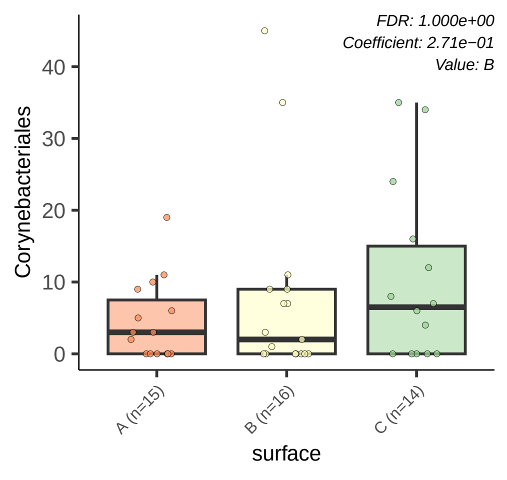
FDR: 1.000e+00
Coefficient: 2.71e−01
Value: B
Corynebacteriales
0
10
20
30
40
A (n=15)
B (n=16)
C (n=14)
surface

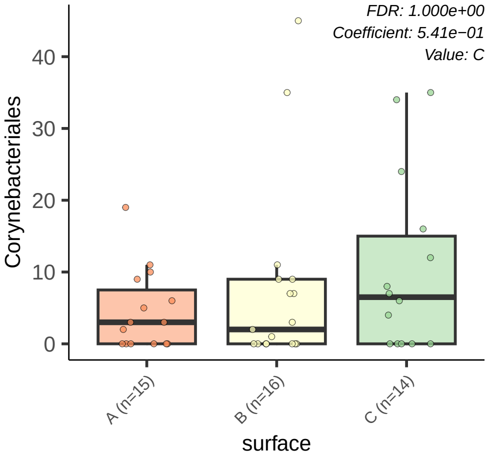

FDR: 1.000e+00
Coefficient: 5.41e−01
Value: C
Corynebacteriales
0
10
20
30
40
A (n=15)
B (n=16)
C (n=14)
surface

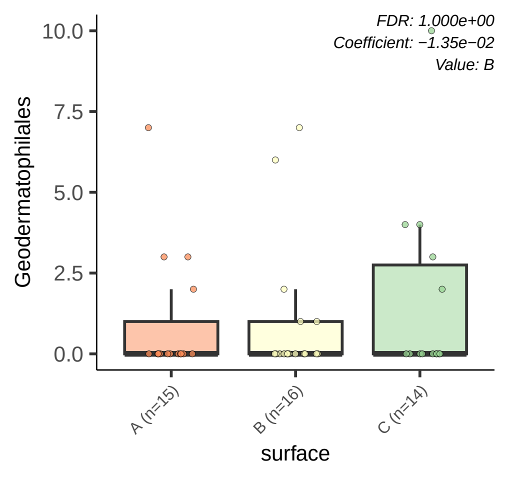
FDR: 1.000e+00
Coefficient: −1.35e−02
Value: B
Geodermatophilales
10.0
7.5
5.0
2.5
0.0
A (n=15)
B (n=16)
C (n=14)
surface

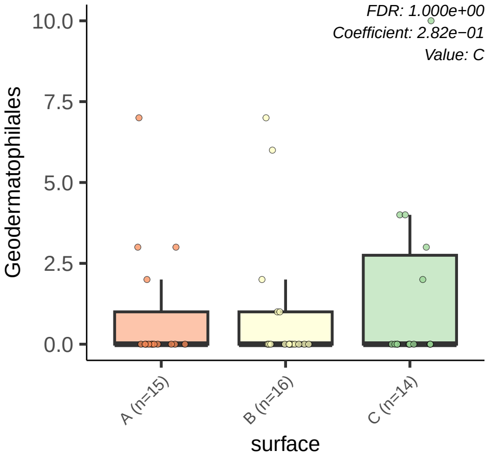
FDR: 1.000e+00
Coefficient: 2.82e−01
Value: C
Geodermatophilales
10.0
7.5
5.0
2.5
0.0
A (n=15)
B (n=16)
C (n=14)
surface

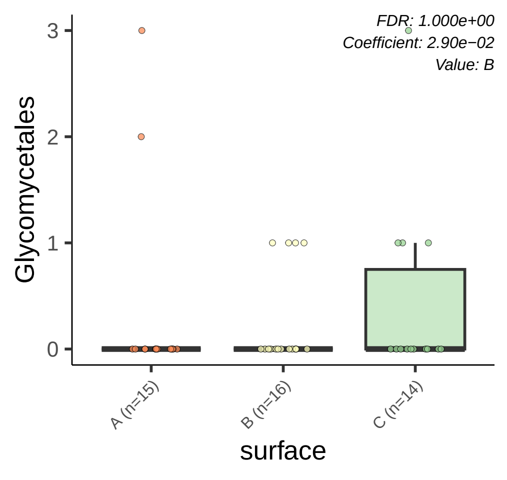

FDR: 1.000e+00
Coefficient: 2.90e−02
Value: B
Glycomycetales
0
1
2
3
A (n=15)
B (n=16)
C (n=14)
surface

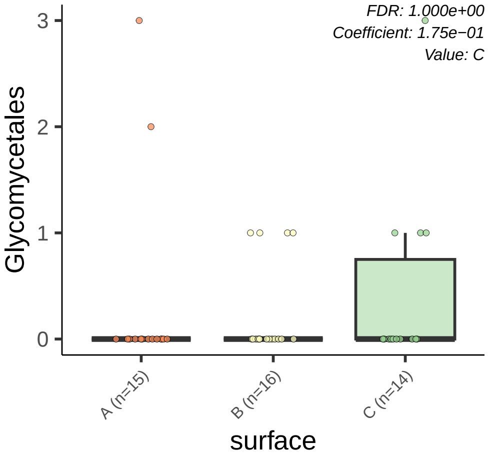

FDR: 1.000e+00
Coefficient: 1.75e−01
Value: C
Glycomycetales
3
2
1
0
A (n=15)
B (n=16)
C (n=14)
surface

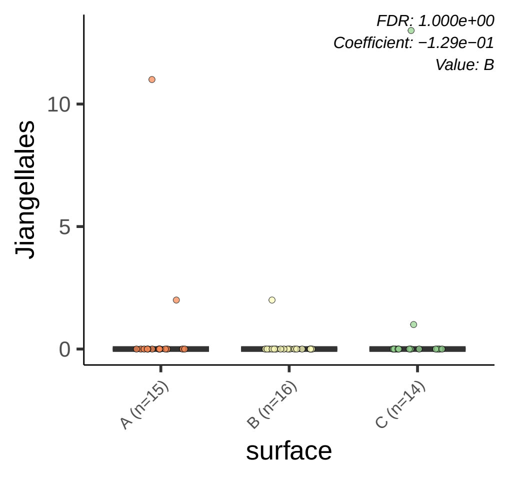
FDR: 1.000e+00
Coefficient: −1.29e−01
Value: B
Jiangellales
0
5
10
A (n=15)
B (n=16)
C (n=14)
surface

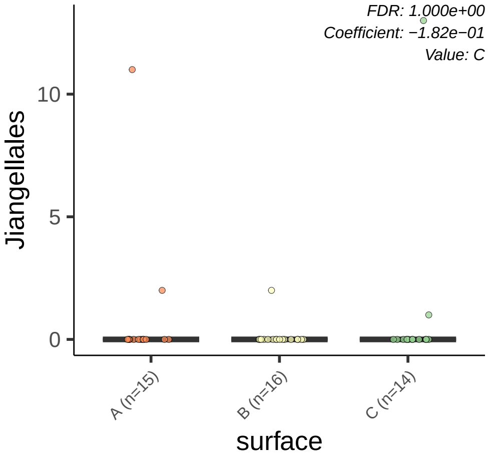
FDR: 1.000e+00
Coefficient: −1.82e−01
Value: C
Jiangellales
0
5
10
A (n=15)
B (n=16)
C (n=14)
surface

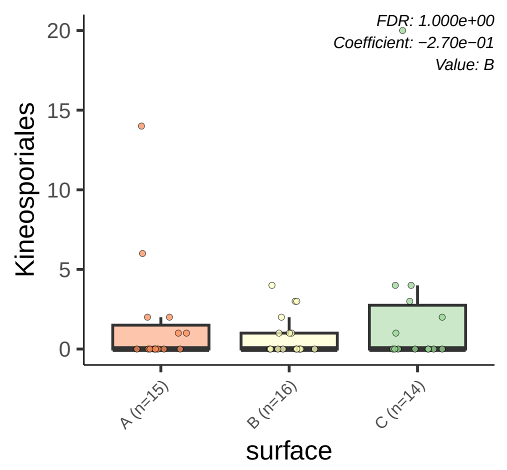
FDR: 1.000e+00
Coefficient: −2.70e−01
Value: B
Kineosporiales
20
15
10
5
0
A (n=15)
B (n=16)
C (n=14)
surface

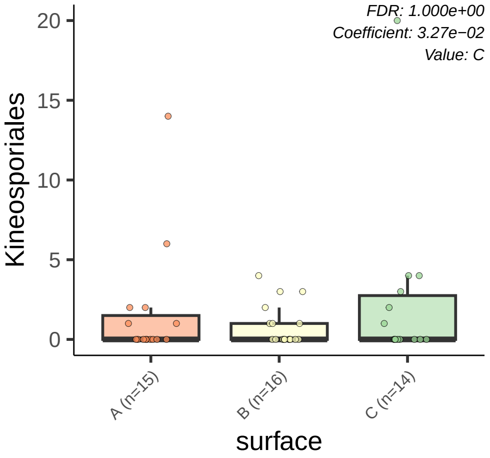

FDR: 1.000e+00
Coefficient: 3.27e−02
Value: C
Kineosporiales
20
15
10
5
0
A (n=15)
B (n=16)
C (n=14)
surface

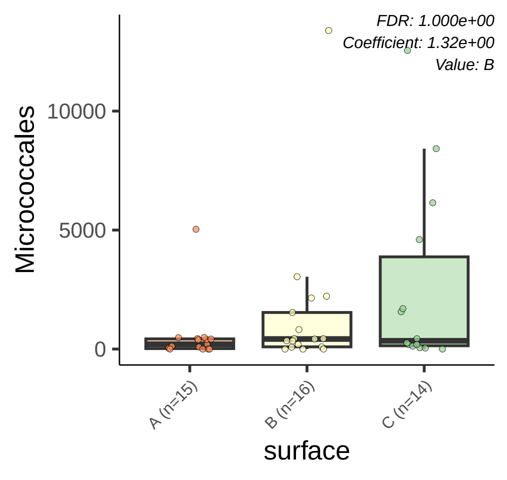

FDR: 1.000e+00
Coefficient: 1.32e+00
Value: B
Micrococcales
10000
5000
0
A (n=15)
B (n=16)
C (n=14)
surface

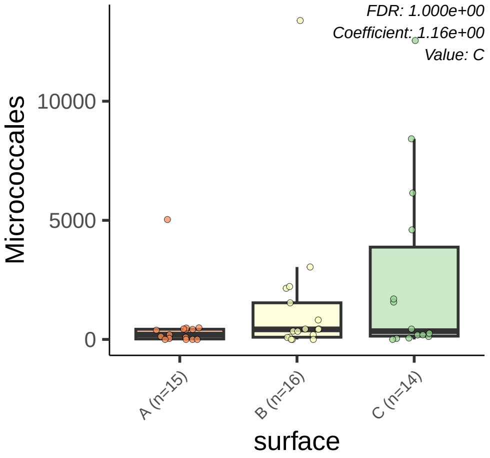
FDR: 1.000e+00
Coefficient: 1.16e+00
Value: C
Micrococcales
10000
5000
0
A (n=15)
B (n=16)
C (n=14)
surface

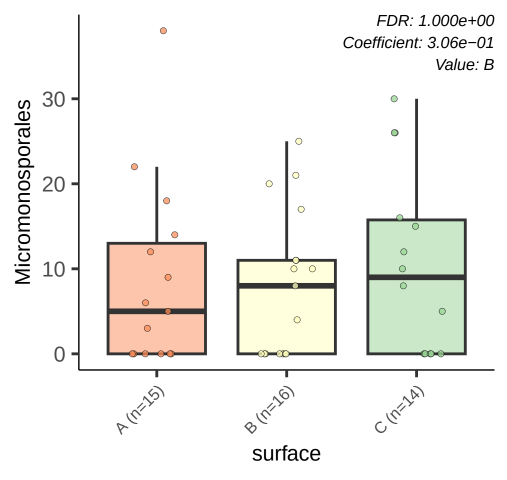
FDR: 1.000e+00
Coefficient: 3.06e−01
Value: B
Micromonosporales
0
10
20
30
A (n=15)
B (n=16)
C (n=14)
surface

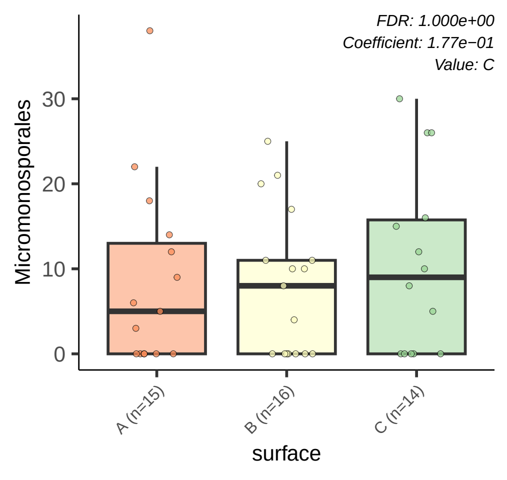
FDR: 1.000e+00
Coefficient: 1.77e−01
Value: C
Micromonosporales
30
20
10
0
A (n=15)
B (n=16)
C (n=14)
surface

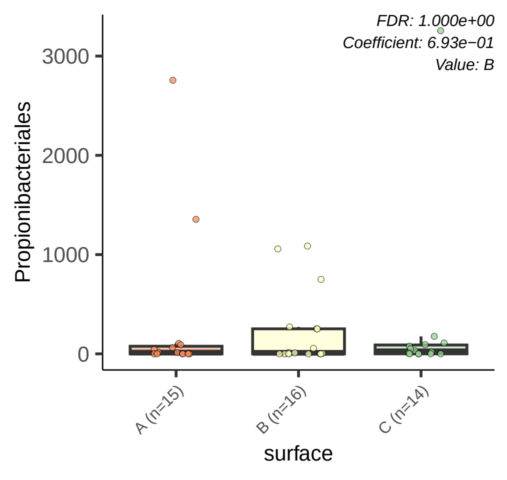

FDR: 1.000e+00
Coefficient: 6.93e−01
Value: B
Propionibacteriales
3000
2000
1000
0
A (n=15)
B (n=16)
C (n=14)
surface

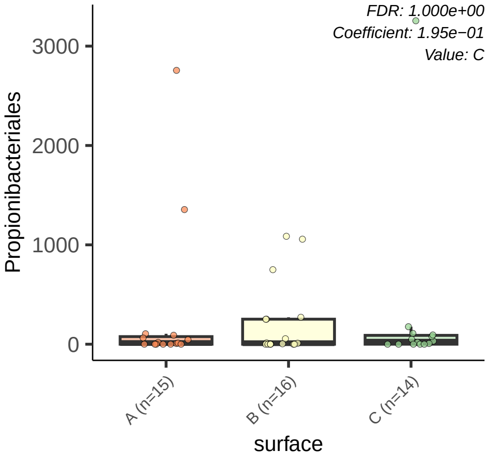

FDR: 1.000e+00
Coefficient: 1.95e−01
Value: C
Propionibacteriales
3000
2000
1000
0
A (n=15)
B (n=16)
C (n=14)
surface

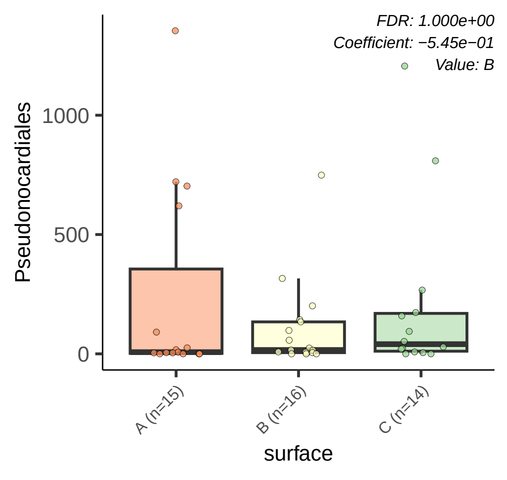

FDR: 1.000e+00
Coefficient: −5.45e−01
Value: B
Pseudonocardiales
0
500
1000
A (n=15)
B (n=16)
C (n=14)
surface

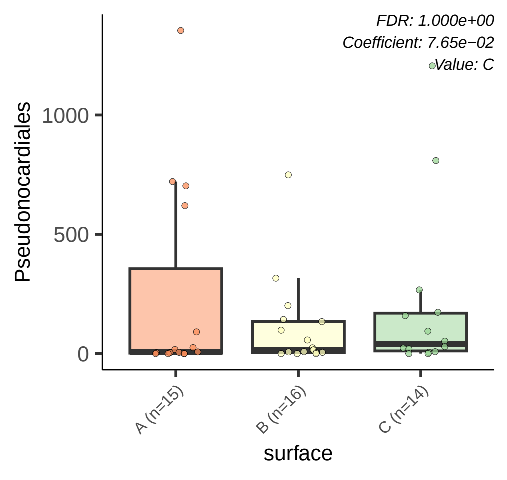
FDR: 1.000e+00
Coefficient: 7.65e−02
Value: C
Pseudonocardiales
1000
500
0
A (n=15)
B (n=16)
C (n=14)
surface

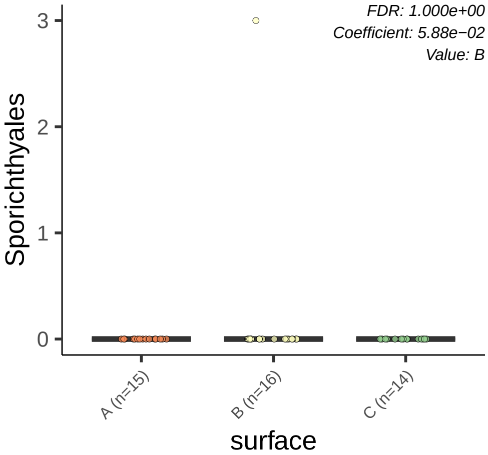
FDR: 1.000e+00
Coefficient: 5.88e−02
Value: B
Sporichthyales
3
2
1
0
A (n=15)
B (n=16)
C (n=14)
surface

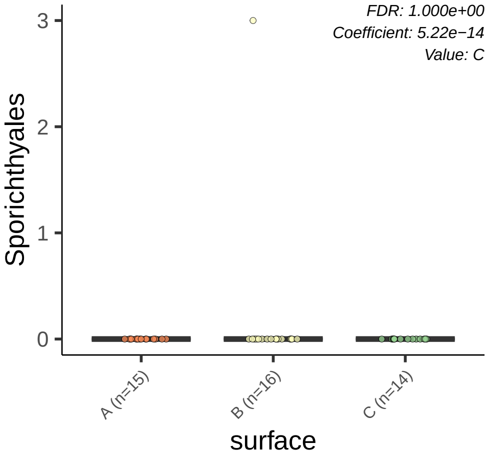
FDR: 1.000e+00
Coefficient: 5.22e−14
Value: C
Sporichthyales
0
1
2
3
A (n=15)
B (n=16)
C (n=14)
surface

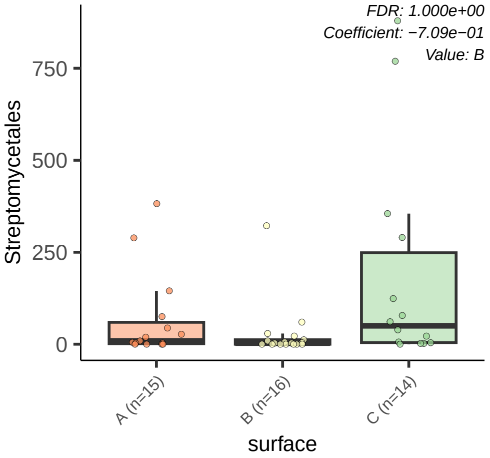
FDR: 1.000e+00
Coefficient: −7.09e−01
Value: B
Streptomycetales
750
500
250
0
A (n=15)
B (n=16)
C (n=14)
surface

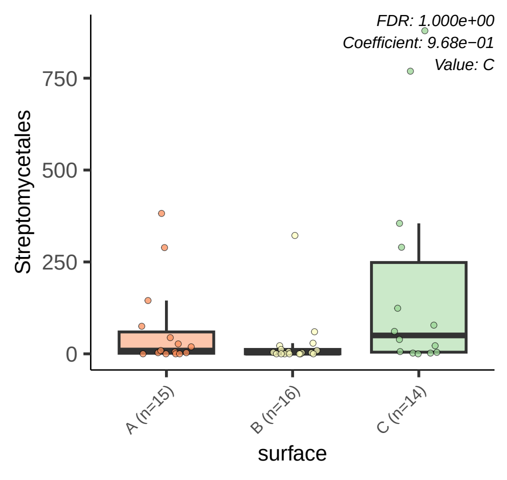
FDR: 1.000e+00
Coefficient: 9.68e−01
Value: C
Streptomycetales
750
500
250
0
A (n=15)
B (n=16)
C (n=14)
surface

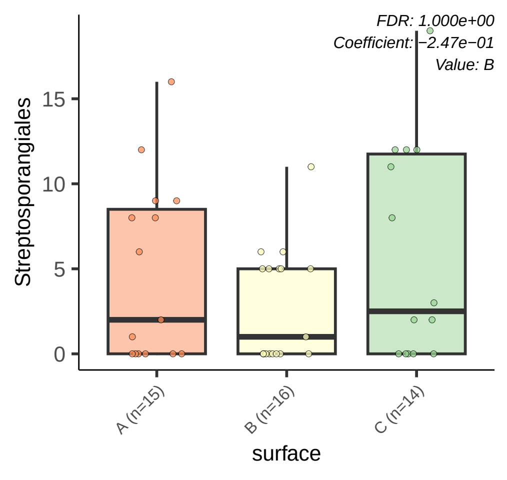

FDR: 1.000e+00
Coefficient: −2.47e−01
Value: B
Streptosporangiales
0
5
10
15
A (n=15)
B (n=16)
C (n=14)
surface

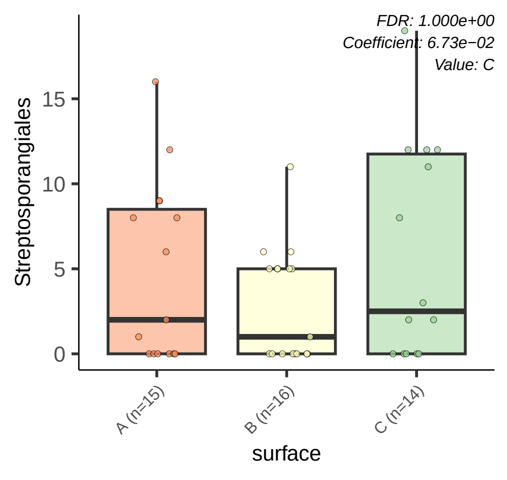
FDR: 1.000e+00
Coefficient: 6.73e−02
Value: C
Streptosporangiales
0
5
10
15
A (n=15)
B (n=16)
C (n=14)
surface

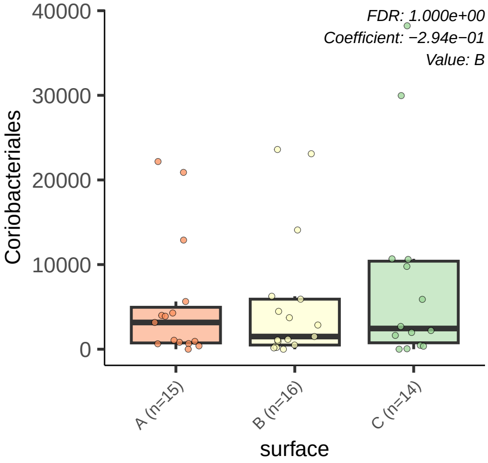
FDR: 1.000e+00
Coefficient: −2.94e−01
Value: B
Coriobacteriales
40000
30000
20000
10000
0
A (n=15)
B (n=16)
C (n=14)
surface

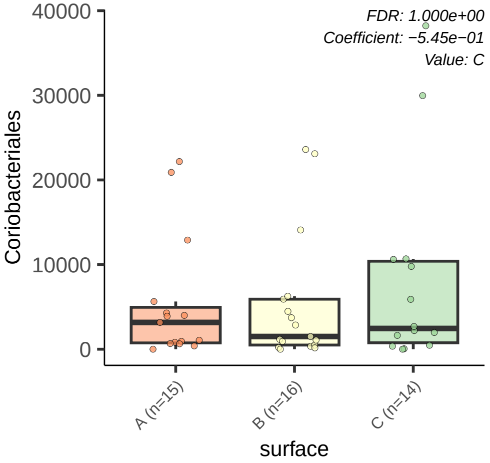

FDR: 1.000e+00
Coefficient: −5.45e−01
Value: C
40000
30000
20000
10000
0
Coriobacteriales
A (n=15)
B (n=16)
C (n=14)
surface

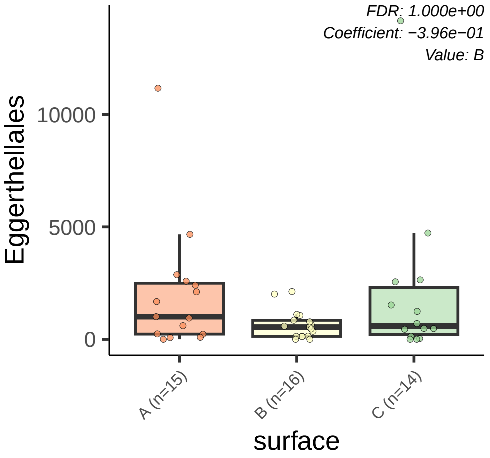

FDR: 1.000e+00
Coefficient: −3.96e−01
Value: B
Eggerthellales
10000
5000
0
A (n=15)
B (n=16)
C (n=14)
surface

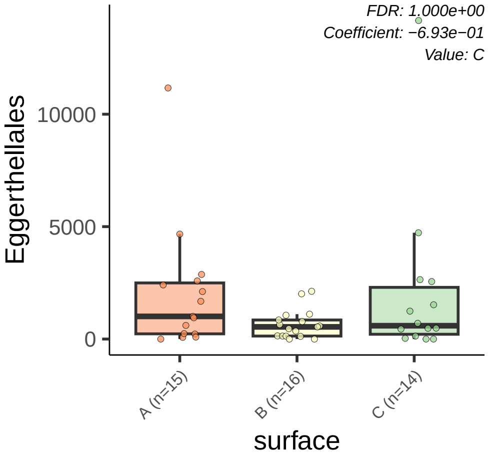
FDR: 1.000e+00
Coefficient: −6.93e−01
Value: C
Eggerthellales
10000
5000
0
A (n=15)
B (n=16)
C (n=14)
surface

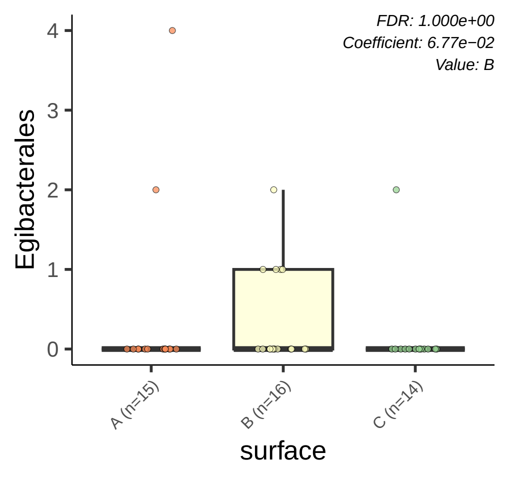

FDR: 1.000e+00
Coefficient: 6.77e−02
Value: B
Egibacterales
0
1
2
3
4
A (n=15)
B (n=16)
C (n=14)
surface

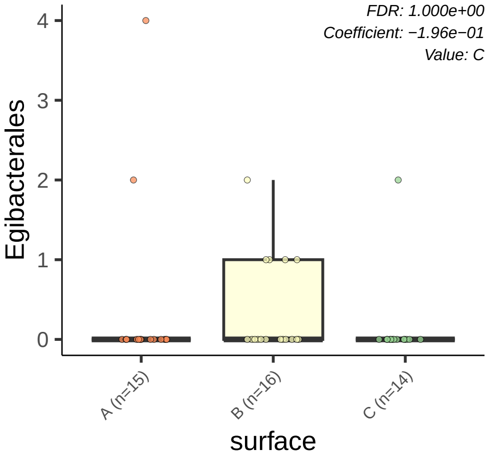
FDR: 1.000e+00
Coefficient: −1.96e−01
Value: C
Egibacterales
4
3
2
1
0
A (n=15)
B (n=16)
C (n=14)
surface

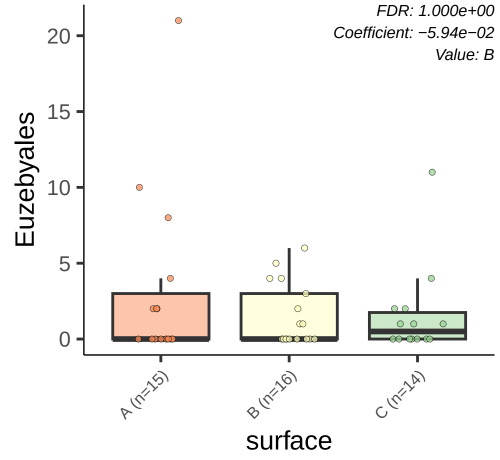
FDR: 1.000e+00
Coefficient: −5.94e−02
Value: B
Euzebyales
0
5
10
15
20
A (n=15)
B (n=16)
C (n=14)
surface

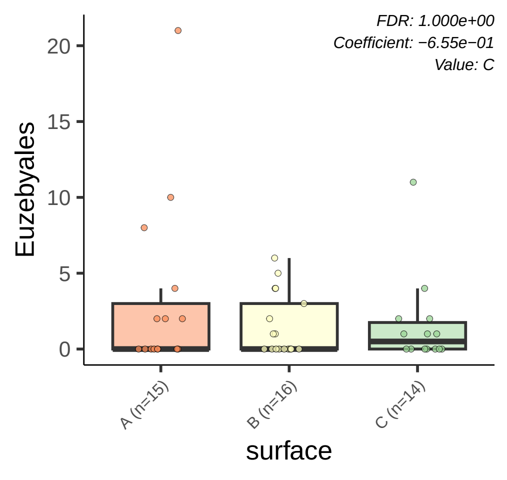
FDR: 1.000e+00
Coefficient: −6.55e−01
Value: C
Euzebyales
20
15
10
5
0
A (n=15)
B (n=16)
C (n=14)
surface

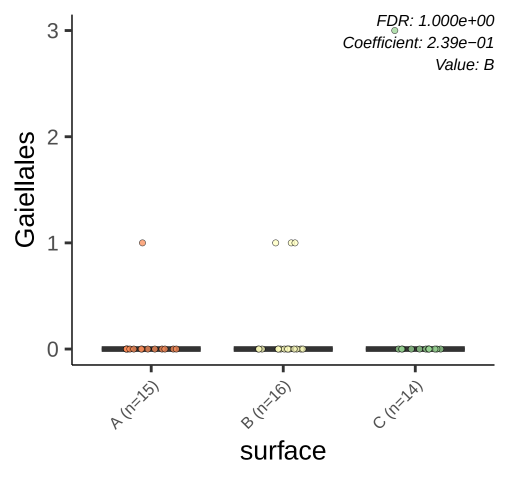

FDR: 1.000e+00
Coefficient: 2.39e−01
Value: B
Gaiellales
3
2
1
0
A (n=15)
B (n=16)
C (n=14)
surface

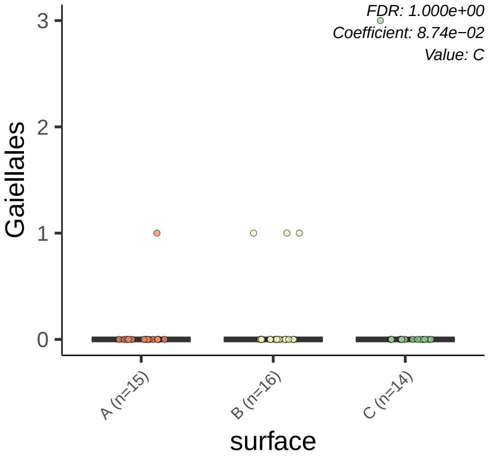

FDR: 1.000e+00
Coefficient: 8.74e−02
Value: C
Gaiellales
3
2
1
0
A (n=15)
B (n=16)
C (n=14)
surface

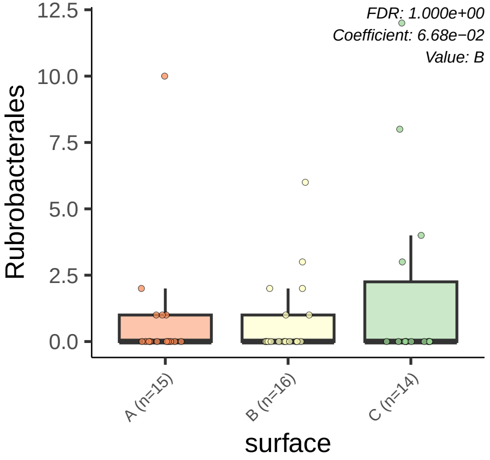

FDR: 1.000e+00
Coefficient: 6.68e−02
Value: B
Rubrobacterales
12.5
10.0
7.5
5.0
2.5
0.0
A (n=15)
B (n=16)
C (n=14)
surface

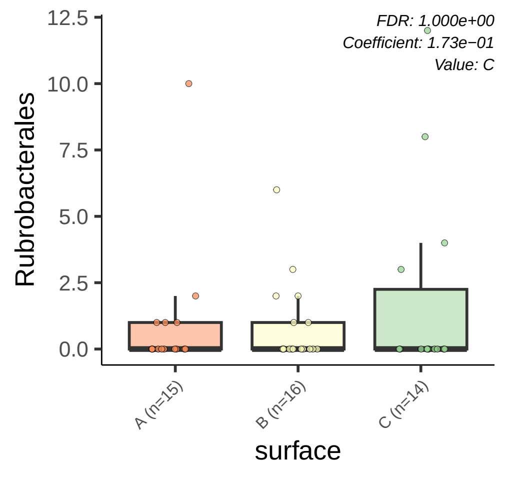

FDR: 1.000e+00
Coefficient: 1.73e−01
Value: C
Rubrobacterales
12.5
10.0
7.5
5.0
2.5
0.0
A (n=15)
B (n=16)
C (n=14)
surface

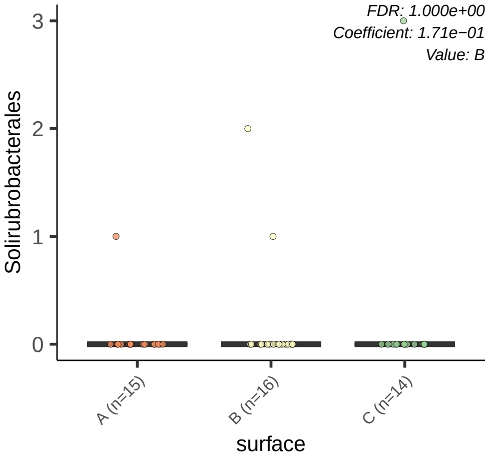
FDR: 1.000e+00
Coefficient: 1.71e−01
Value: B
Solirubrobacterales
3
2
1
0
A (n=15)
B (n=16)
C (n=14)
surface

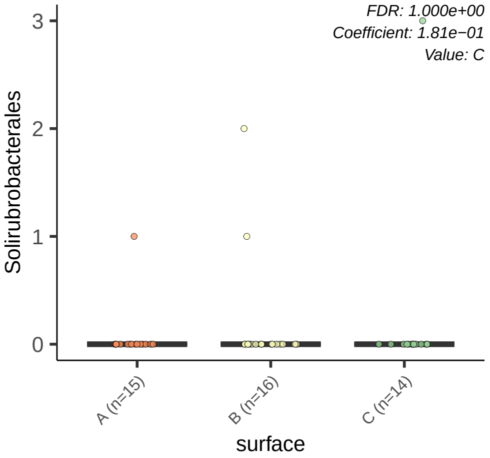

FDR: 1.000e+00
Coefficient: 1.81e−01
Value: C
Solirubrobacterales
0
1
2
3
A (n=15)
B (n=16)
C (n=14)
surface

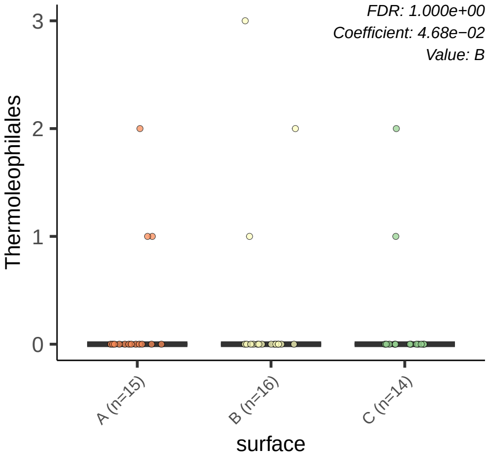

FDR: 1.000e+00
Coefficient: 4.68e−02
Value: B
Thermoleophilales
3
2
1
0
A (n=15)
B (n=16)
C (n=14)
surface

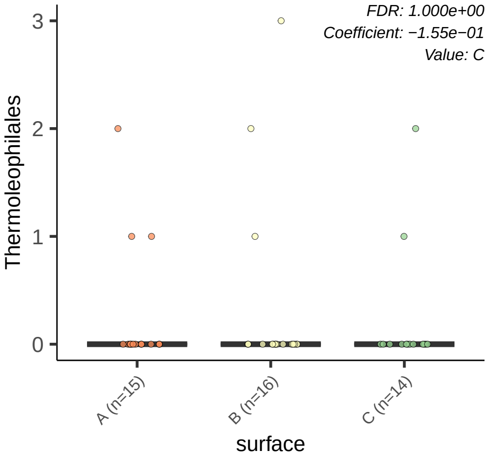

FDR: 1.000e+00
Coefficient: −1.55e−01
Value: C
Thermoleophilales
3
2
1
0
A (n=15)
B (n=16)
C (n=14)
surface

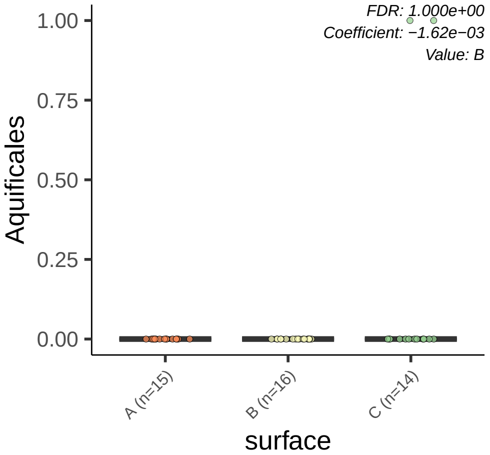

FDR: 1.000e+00
Coefficient: −1.62e−03
Value: B
Aquificales
1.00
0.75
0.50
0.25
0.00
A (n=15)
B (n=16)
C (n=14)
surface

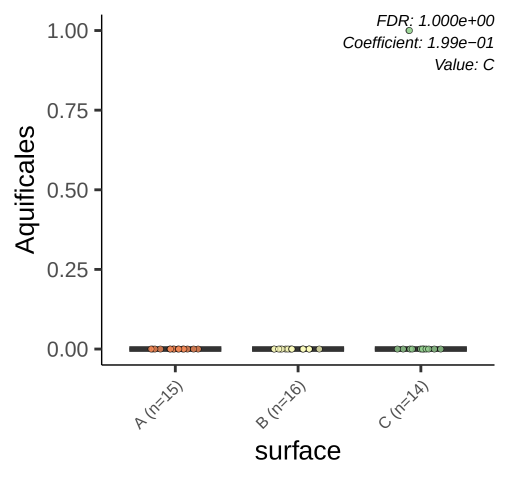

FDR: 1.000e+00
Coefficient: 1.99e−01
Value: C
Aquificales
1.00
0.75
0.50
0.25
0.00
A (n=15)
B (n=16)
C (n=14)
surface

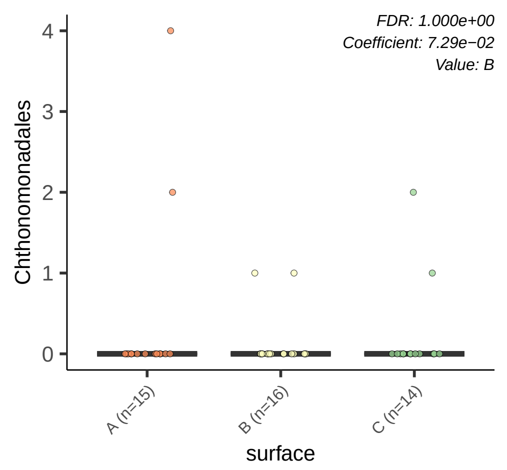
FDR: 1.000e+00
Coefficient: 7.29e−02
Value: B
Chthonomonadales
0
1
2
3
4
A (n=15)
B (n=16)
C (n=14)
surface

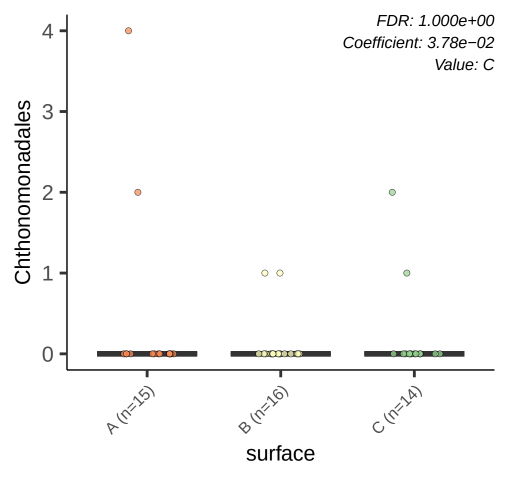

FDR: 1.000e+00
Coefficient: 3.78e−02
Value: C
Chthonomonadales
4
3
2
1
0
A (n=15)
B (n=16)
C (n=14)
surface

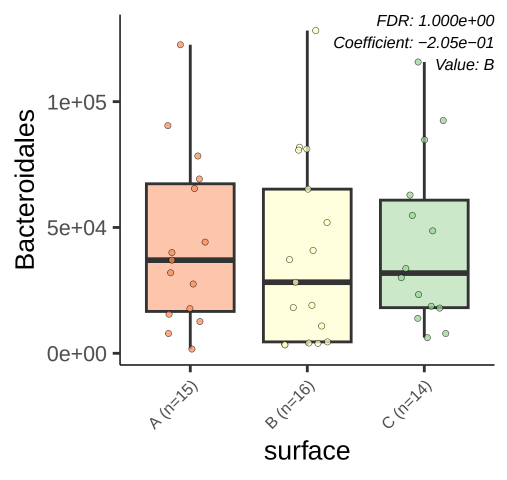
FDR: 1.000e+00
Coefficient: −2.05e−01
Value: B
Bacteroidales
1e+05
5e+04
0e+00
A (n=15)
B (n=16)
C (n=14)
surface

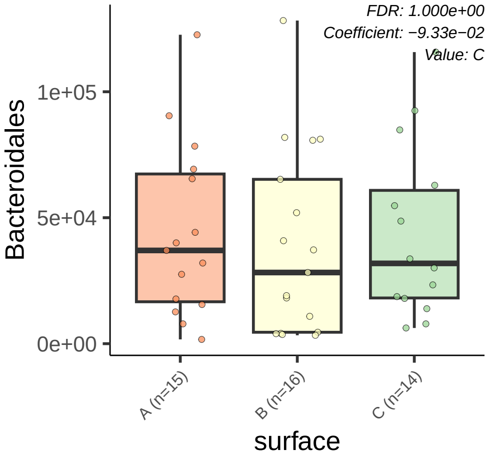
FDR: 1.000e+00
Coefficient: −9.33e−02
Value: C
Bacteroidales
1e+05
5e+04
0e+00
A (n=15)
B (n=16)
C (n=14)
surface

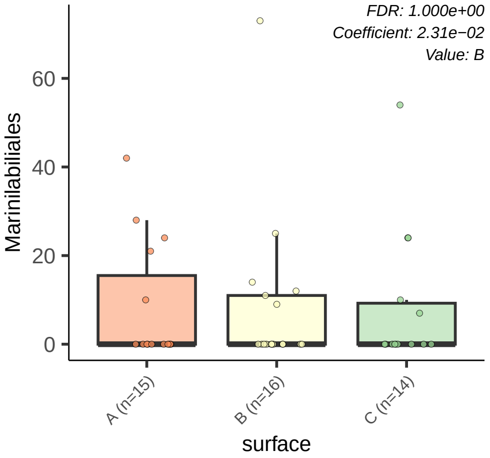

FDR: 1.000e+00
Coefficient: 2.31e−02
Value: B
Marinilabiliales
0
20
40
60
A (n=15)
B (n=16)
C (n=14)
surface

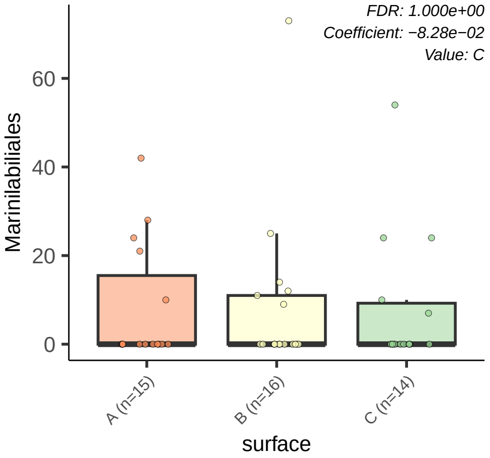
FDR: 1.000e+00
Coefficient: −8.28e−02
Value: C
Marinilabiliales
0
20
40
60
A (n=15)
B (n=16)
C (n=14)
surface

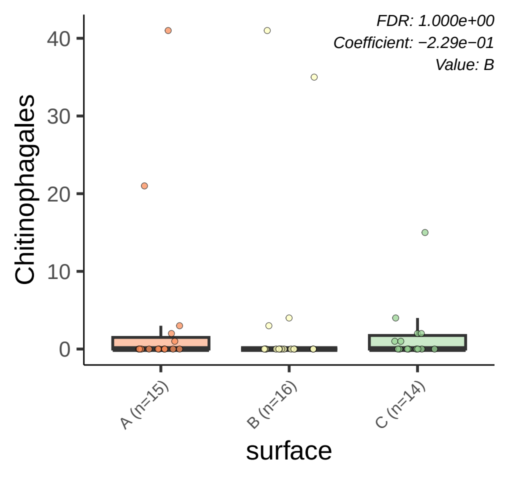

FDR: 1.000e+00
Coefficient: −2.29e−01
Value: B
Chitinophagales
0
10
20
30
40
A (n=15)
B (n=16)
C (n=14)
surface

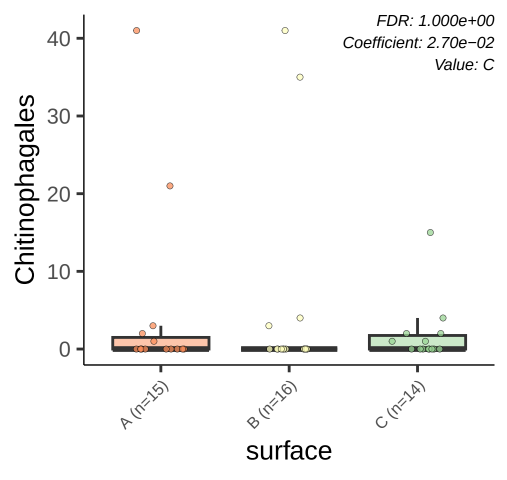
FDR: 1.000e+00
Coefficient: 2.70e−02
Value: C
Chitinophagales
40
30
20
10
0
A (n=15)
B (n=16)
C (n=14)
surface

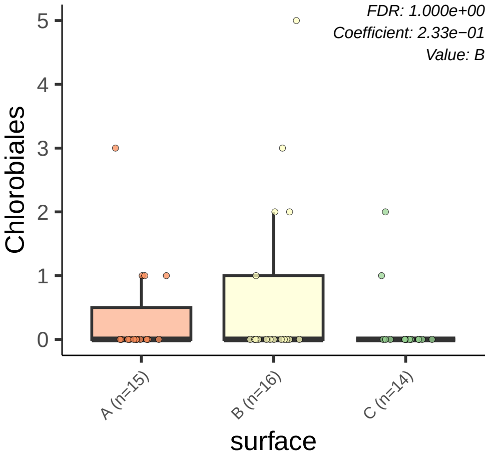
FDR: 1.000e+00
Coefficient: 2.33e−01
Value: B
Chlorobiales
0
1
2
3
4
5
A (n=15)
B (n=16)
C (n=14)
surface

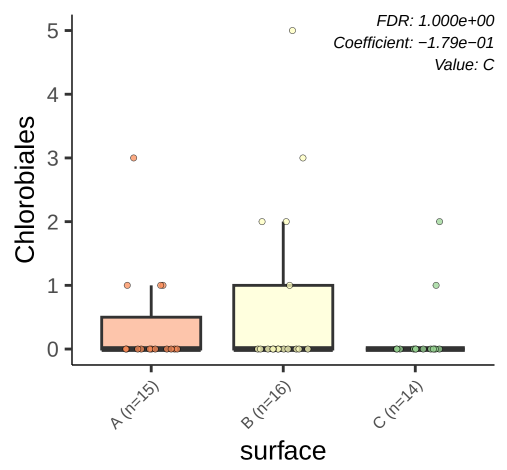

FDR: 1.000e+00
Coefficient: −1.79e−01
Value: C
Chlorobiales
0
1
2
3
4
5
A (n=15)
B (n=16)
C (n=14)
surface

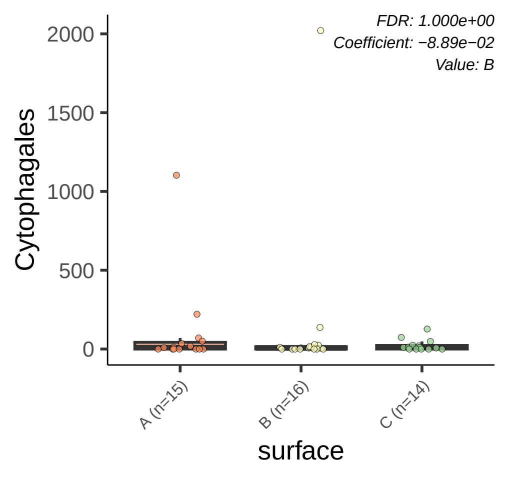

FDR: 1.000e+00
Coefficient: −8.89e−02
Value: B
Cytophagales
2000
1500
1000
500
0
A (n=15)
B (n=16)
C (n=14)
surface

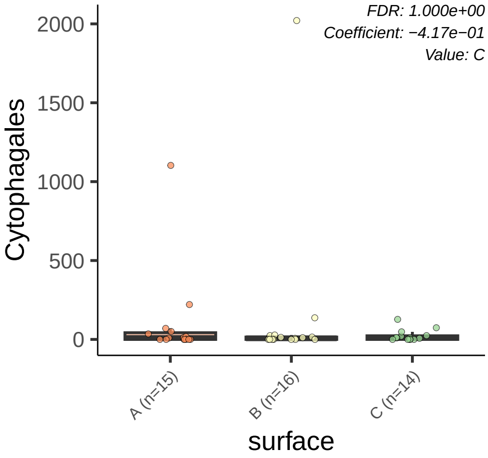
FDR: 1.000e+00
Coefficient: −4.17e−01
Value: C
Cytophagales
2000
1500
1000
500
0
A (n=15)
B (n=16)
C (n=14)
surface

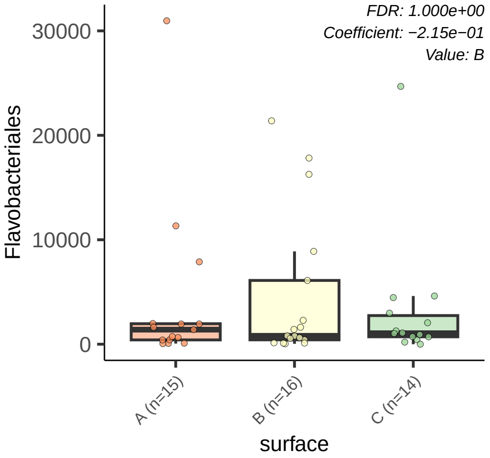

FDR: 1.000e+00
Coefficient: −2.15e−01
Value: B
Flavobacteriales
30000
20000
10000
0
A (n=15)
B (n=16)
C (n=14)
surface

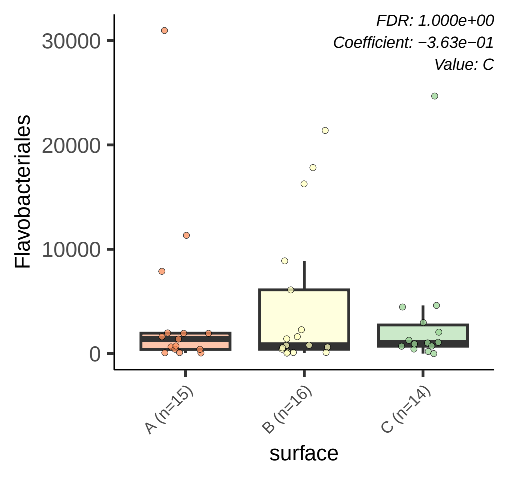
FDR: 1.000e+00
Coefficient: −3.63e−01
Value: C
Flavobacteriales
30000
20000
10000
0
A (n=15)
B (n=16)
C (n=14)
surface

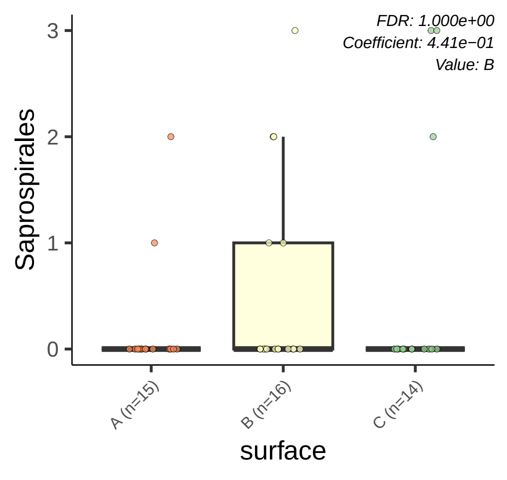

FDR: 1.000e+00
Coefficient: 4.41e−01
Value: B
Saprospirales
3
2
1
0
A (n=15)
B (n=16)
C (n=14)
surface

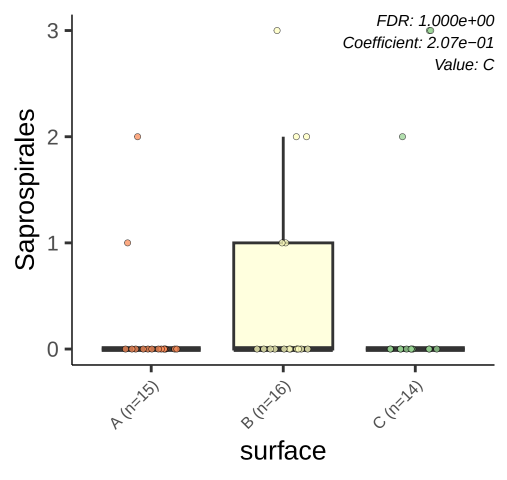
FDR: 1.000e+00
Coefficient: 2.07e−01
Value: C
Saprospirales
3
2
1
0
A (n=15)
B (n=16)
C (n=14)
surface

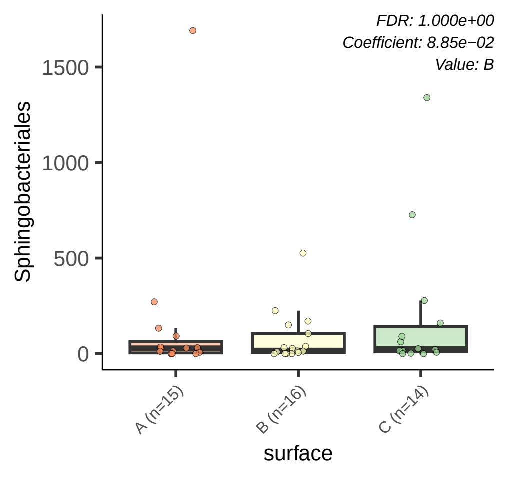

FDR: 1.000e+00
Coefficient: 8.85e−02
Value: B
Sphingobacteriales
1500
1000
500
0
A (n=15)
B (n=16)
C (n=14)
surface

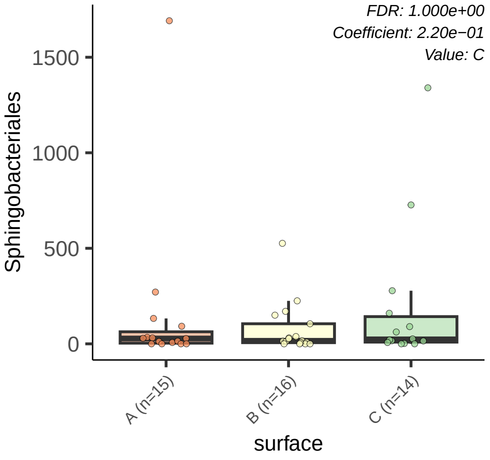

FDR: 1.000e+00
Coefficient: 2.20e−01
Value: C
Sphingobacteriales
1500
1000
500
0
A (n=15)
B (n=16)
C (n=14)
surface

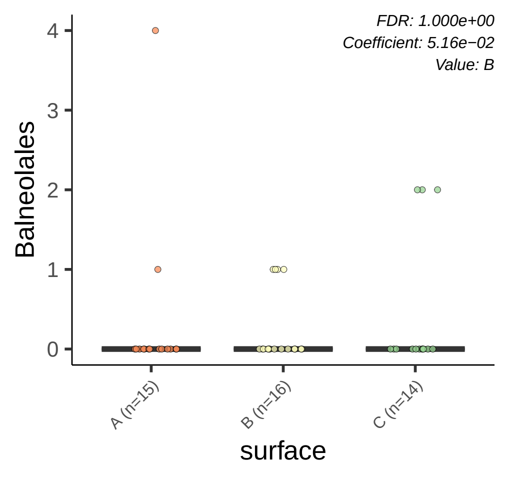

FDR: 1.000e+00
Coefficient: 5.16e−02
Value: B
Balneolales
0
1
2
3
4
A (n=15)
B (n=16)
C (n=14)
surface

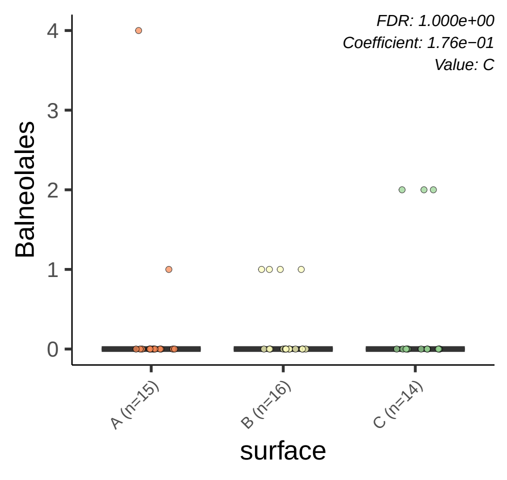
FDR: 1.000e+00
Coefficient: 1.76e−01
Value: C
Balneolales
0
1
2
3
4
A (n=15)
B (n=16)
C (n=14)
surface

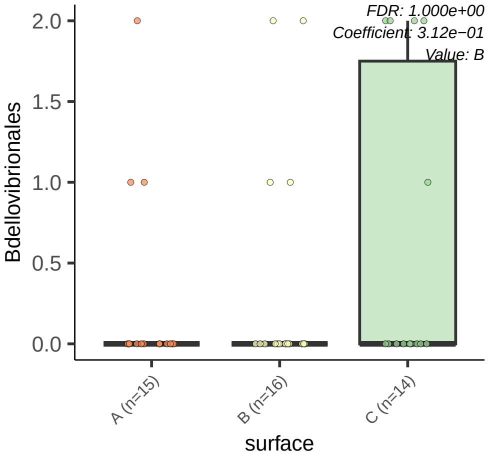
FDR: 1.000e+00
Coefficient: 3.12e−01
Value: B
Bdellovibrionales
2.0
1.5
1.0
0.5
0.0
A (n=15)
B (n=16)
C (n=14)
surface

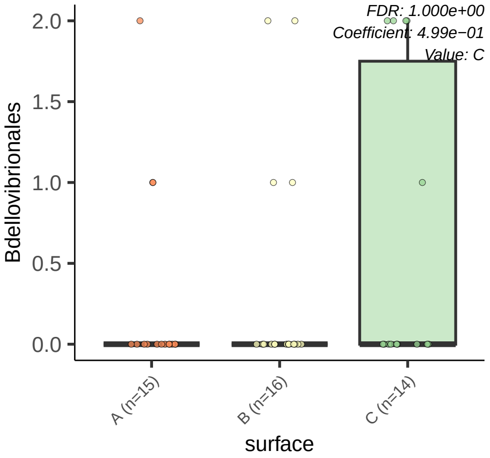
FDR: 1.000e+00
Coefficient: 4.99e−01
Value: C
2.0
1.5
1.0
0.5
0.0
Bdellovibrionales
A (n=15)
B (n=16)
C (n=14)
surface

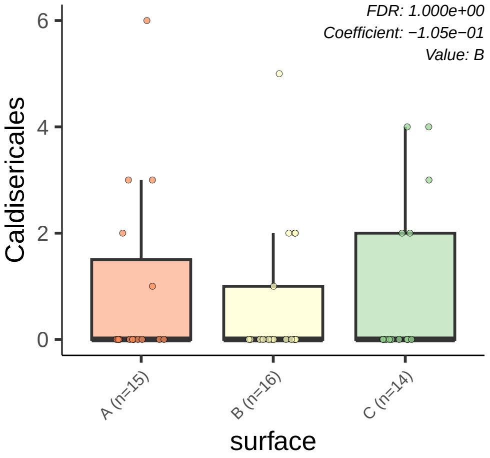
FDR: 1.000e+00
Coefficient: −1.05e−01
Value: B
Caldisericales
6
4
2
0
A (n=15)
B (n=16)
C (n=14)
surface

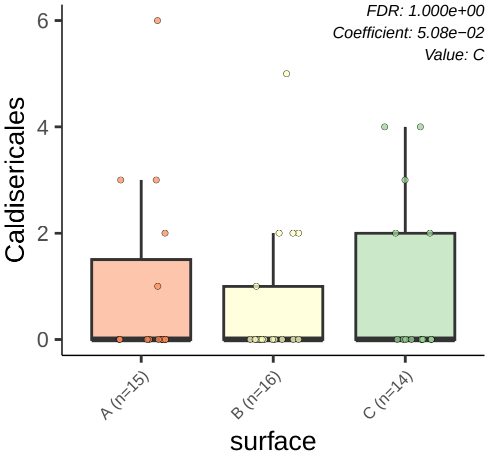

FDR: 1.000e+00
Coefficient: 5.08e−02
Value: C
Caldisericales
6
4
2
0
A (n=15)
B (n=16)
C (n=14)
surface

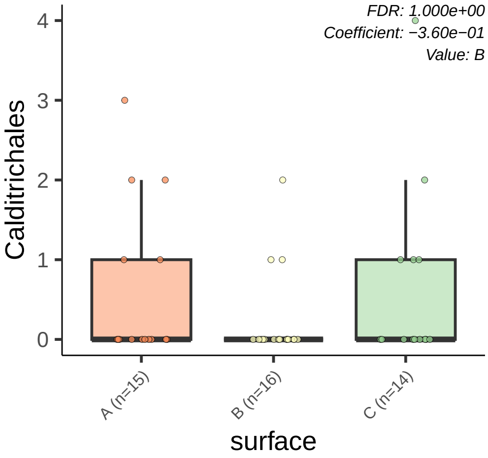
FDR: 1.000e+00
Coefficient: −3.60e−01
Value: B
Calditrichales
4
3
2
1
0
A (n=15)
B (n=16)
C (n=14)
surface

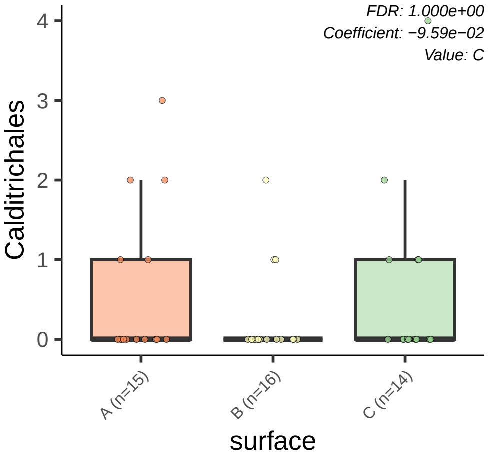
FDR: 1.000e+00
Coefficient: −9.59e−02
Value: C
Calditrichales
0
1
2
3
4
A (n=15)
B (n=16)
C (n=14)
surface

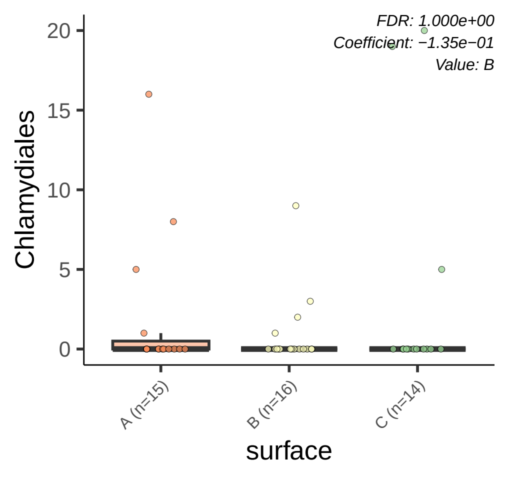
FDR: 1.000e+00
Coefficient: −1.35e−01
Value: B
Chlamydiales
20
15
10
5
0
A (n=15)
B (n=16)
C (n=14)
surface

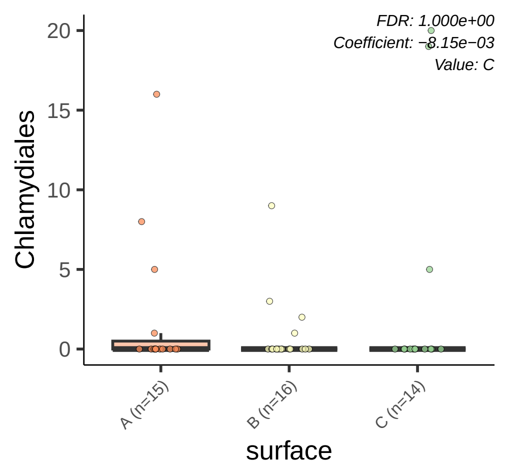

FDR: 1.000e+00
Coefficient: −8.15e−03
Value: C
Chlamydiales
0
5
10
15
20
A (n=15)
B (n=16)
C (n=14)
surface

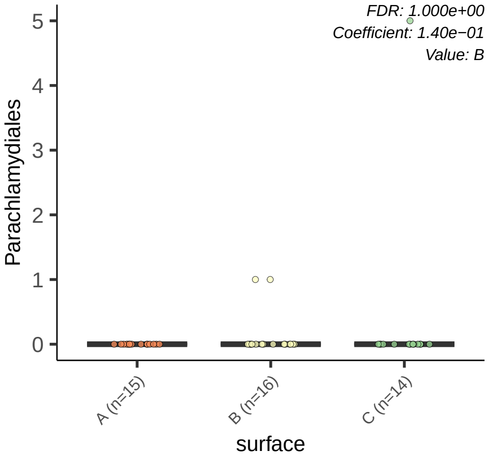
FDR: 1.000e+00
Coefficient: 1.40e−01
Value: B
Parachlamydiales
0
1
2
3
4
5
A (n=15)
B (n=16)
C (n=14)
surface

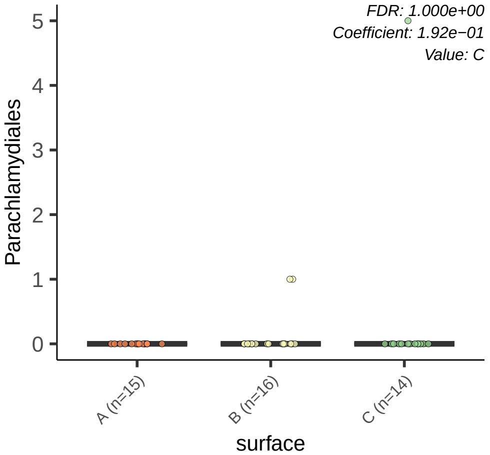

FDR: 1.000e+00
Coefficient: 1.92e−01
Value: C
Parachlamydiales
5
4
3
2
1
0
A (n=15)
B (n=16)
C (n=14)
surface

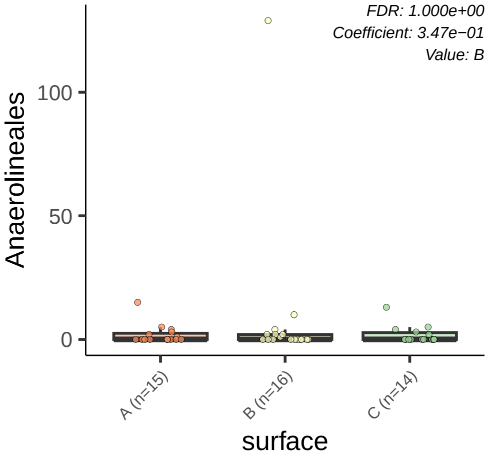
FDR: 1.000e+00
Coefficient: 3.47e−01
Value: B
Anaerolineales
100
50
0
A (n=15)
B (n=16)
C (n=14)
surface

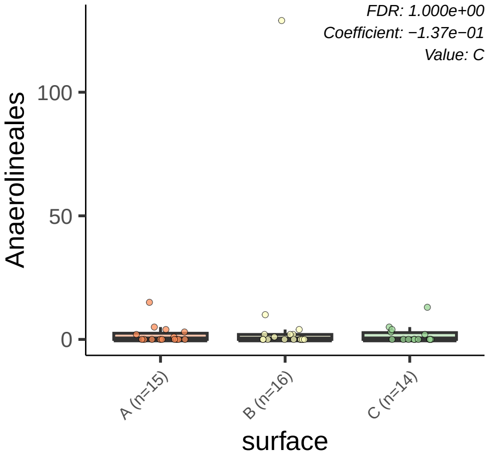
FDR: 1.000e+00
Coefficient: −1.37e−01
Value: C
Anaerolineales
100
50
0
A (n=15)
B (n=16)
C (n=14)
surface

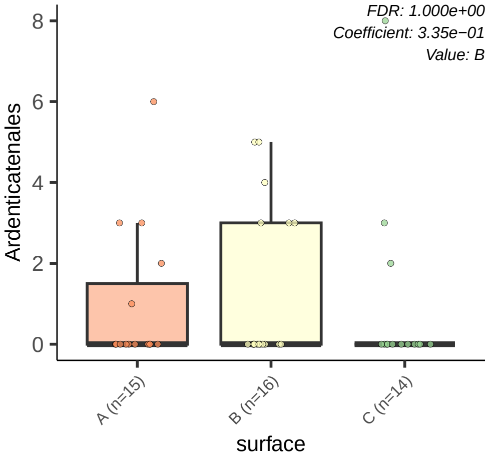

FDR: 1.000e+00
Coefficient: 3.35e−01
Value: B
Ardenticatenales
8
6
4
2
0
A (n=15)
B (n=16)
C (n=14)
surface

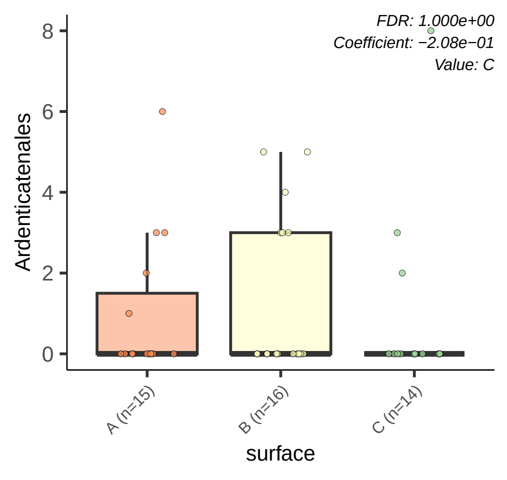
FDR: 1.000e+00
Coefficient: −2.08e−01
Value: C
Ardenticatenales
8
6
4
2
0
A (n=15)
B (n=16)
C (n=14)
surface

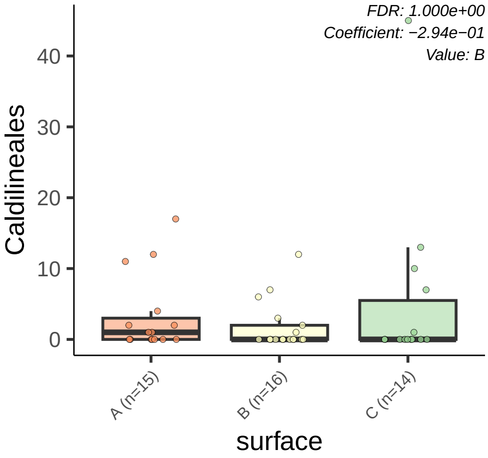
FDR: 1.000e+00
Coefficient: −2.94e−01
Value: B
Caldilineales
0
10
20
30
40
A (n=15)
B (n=16)
C (n=14)
surface

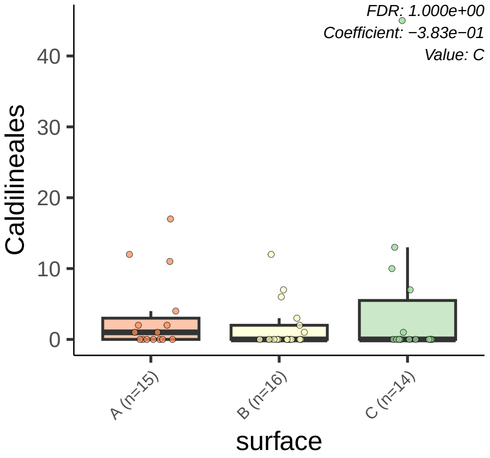

FDR: 1.000e+00
Coefficient: −3.83e−01
Value: C
Caldilineales
40
30
20
10
0
A (n=15)
B (n=16)
C (n=14)
surface

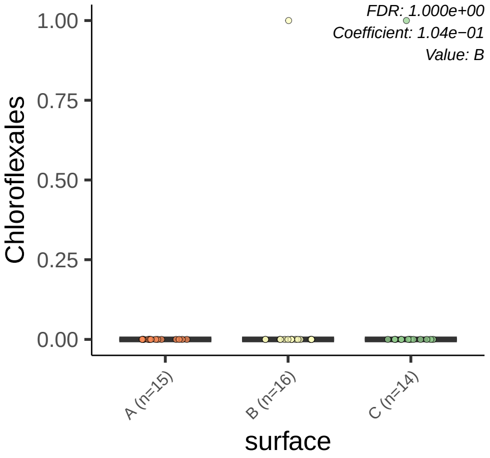
FDR: 1.000e+00
Coefficient: 1.04e−01
Value: B
Chloroflexales
1.00
0.75
0.50
0.25
0.00
A (n=15)
B (n=16)
C (n=14)
surface

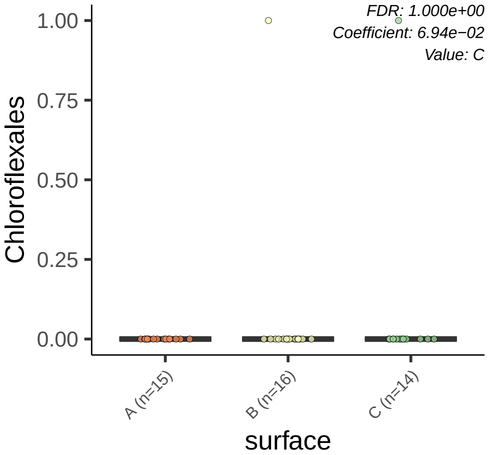
FDR: 1.000e+00
Coefficient: 6.94e−02
Value: C
Chloroflexales
1.00
0.75
0.50
0.25
0.00
A (n=15)
B (n=16)
C (n=14)
surface

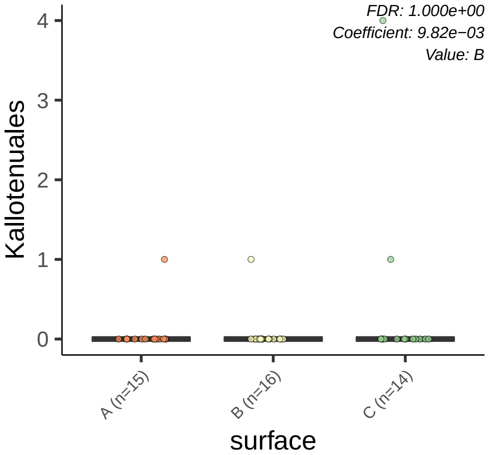
FDR: 1.000e+00
Coefficient: 9.82e−03
Value: B
Kallotenuales
4
3
2
1
0
A (n=15)
B (n=16)
C (n=14)
surface

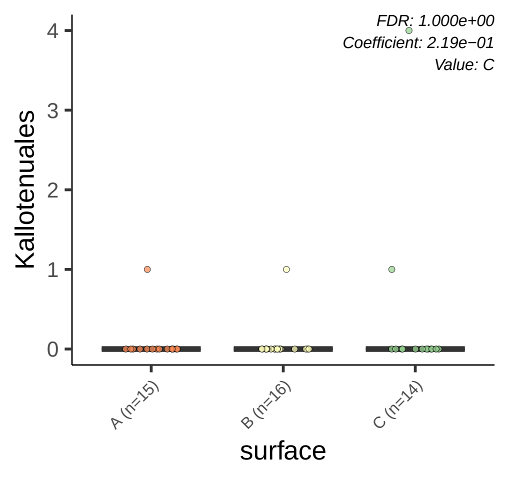
FDR: 1.000e+00
Coefficient: 2.19e−01
Value: C
Kallotenuales
4
3
2
1
0
A (n=15)
B (n=16)
C (n=14)
surface

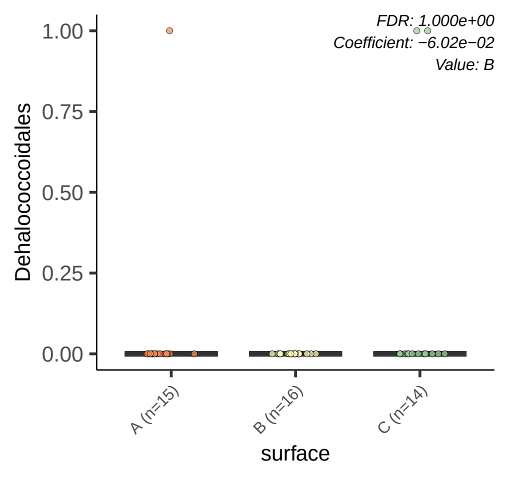
FDR: 1.000e+00
Coefficient: −6.02e−02
Value: B
Dehalococcoidales
1.00
0.75
0.50
0.25
0.00
A (n=15)
B (n=16)
C (n=14)
surface

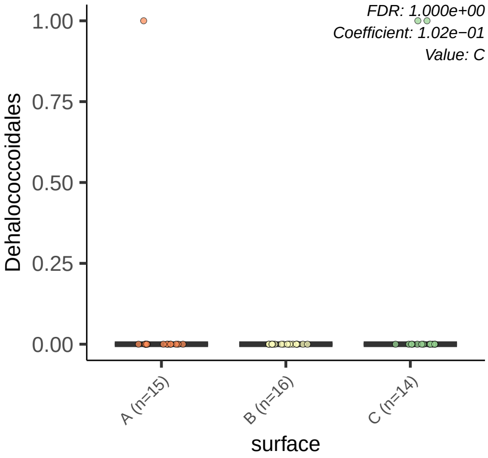
FDR: 1.000e+00
Coefficient: 1.02e−01
Value: C
Dehalococcoidales
1.00
0.75
0.50
0.25
0.00
A (n=15)
B (n=16)
C (n=14)
surface

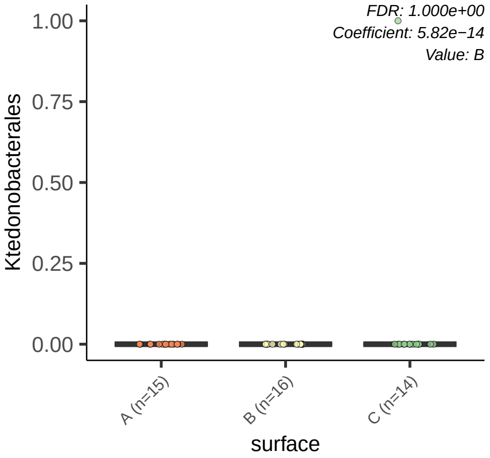
FDR: 1.000e+00
Coefficient: 5.82e−14
Value: B
1.00
0.75
0.50
0.25
0.00
Ktedonobacterales
A (n=15)
B (n=16)
C (n=14)
surface

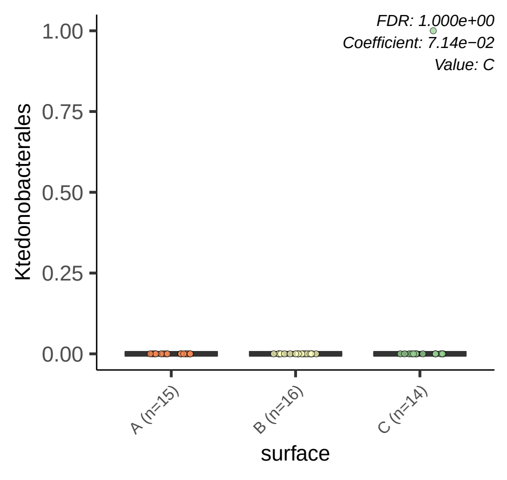
FDR: 1.000e+00
Coefficient: 7.14e−02
Value: C
Ktedonobacterales
1.00
0.75
0.50
0.25
0.00
A (n=15)
B (n=16)
C (n=14)
surface

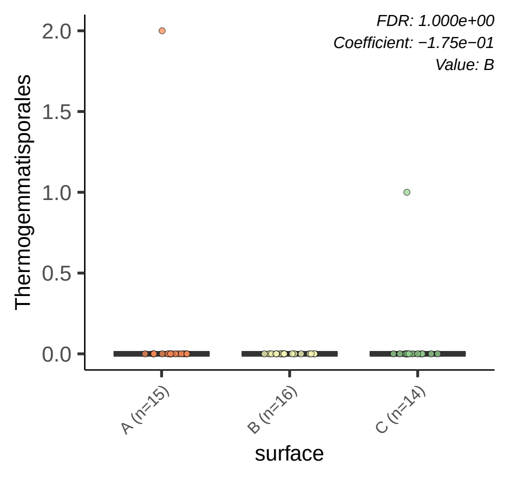
FDR: 1.000e+00
Coefficient: −1.75e−01
Value: B
Thermogemmatisporales
2.0
1.5
1.0
0.5
0.0
A (n=15)
B (n=16)
C (n=14)
surface

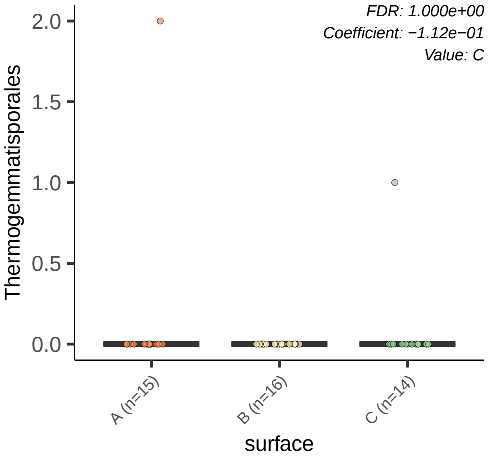

FDR: 1.000e+00
Coefficient: −1.12e−01
Value: C
Thermogemmatisporales
2.0
1.5
1.0
0.5
0.0
A (n=15)
B (n=16)
C (n=14)
surface

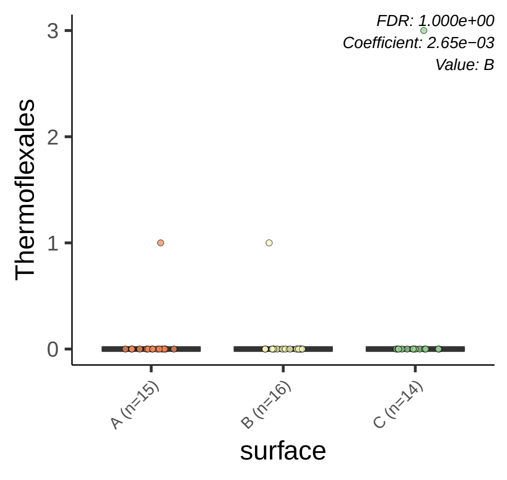

FDR: 1.000e+00
Coefficient: 2.65e−03
Value: B
Thermoflexales
3
2
1
0
A (n=15)
B (n=16)
C (n=14)
surface

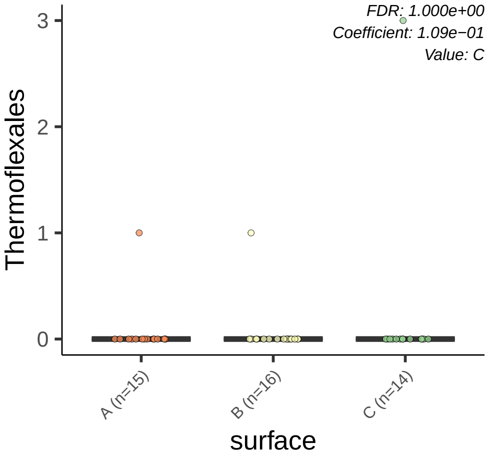
FDR: 1.000e+00
Coefficient: 1.09e−01
Value: C
Thermoflexales
3
2
1
0
A (n=15)
B (n=16)
C (n=14)
surface

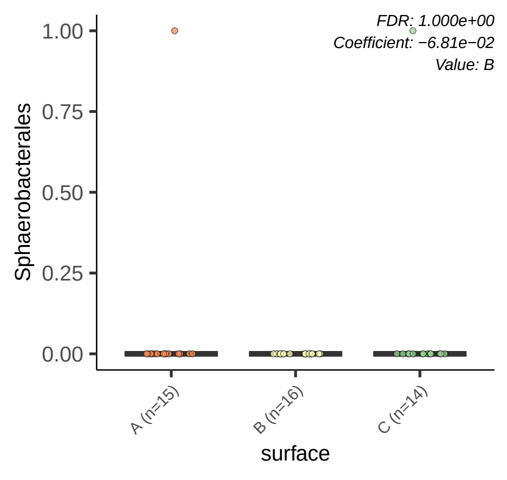

FDR: 1.000e+00
Coefficient: −6.81e−02
Value: B
Sphaerobacterales
1.00
0.75
0.50
0.25
0.00
A (n=15)
B (n=16)
C (n=14)
surface

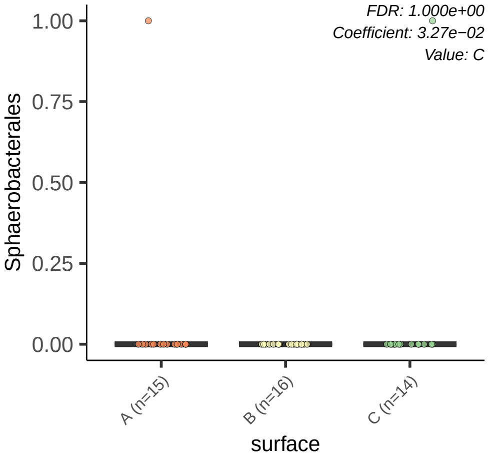

FDR: 1.000e+00
Coefficient: 3.27e−02
Value: C
Sphaerobacterales
1.00
0.75
0.50
0.25
0.00
A (n=15)
B (n=16)
C (n=14)
surface

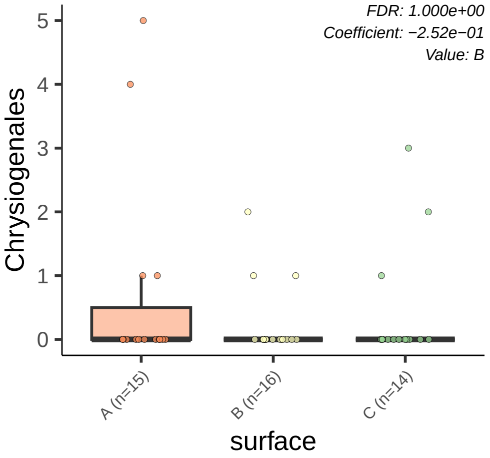

FDR: 1.000e+00
Coefficient: −2.52e−01
Value: B
Chrysiogenales
5
4
3
2
1
0
A (n=15)
B (n=16)
C (n=14)
surface

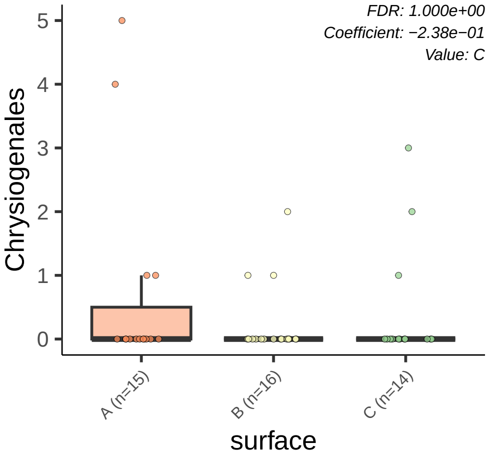
FDR: 1.000e+00
Coefficient: −2.38e−01
Value: C
Chrysiogenales
0
1
2
3
4
5
A (n=15)
B (n=16)
C (n=14)
surface

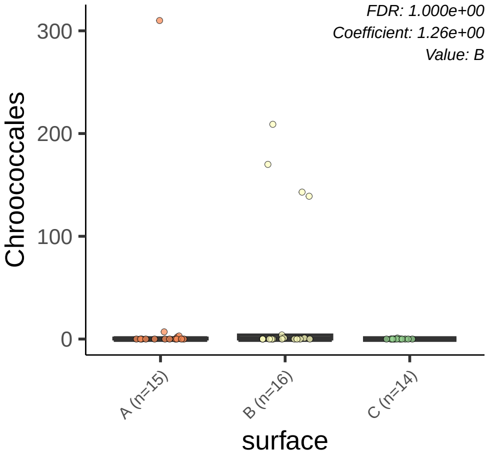

FDR: 1.000e+00
Coefficient: 1.26e+00
Value: B
Chroococcales
300
200
100
0
A (n=15)
B (n=16)
C (n=14)
surface

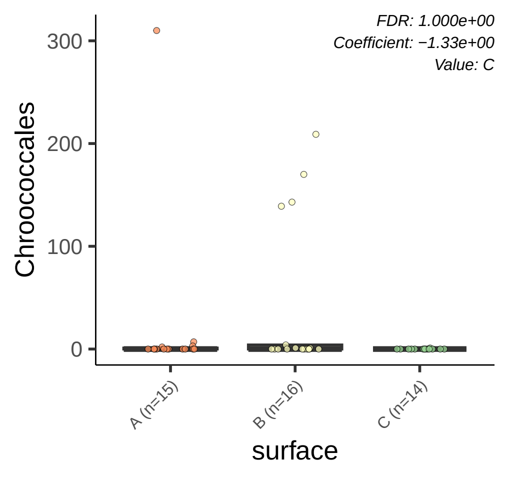

FDR: 1.000e+00
Coefficient: −1.33e+00
Value: C
Chroococcales
300
200
100
0
A (n=15)
B (n=16)
C (n=14)
surface

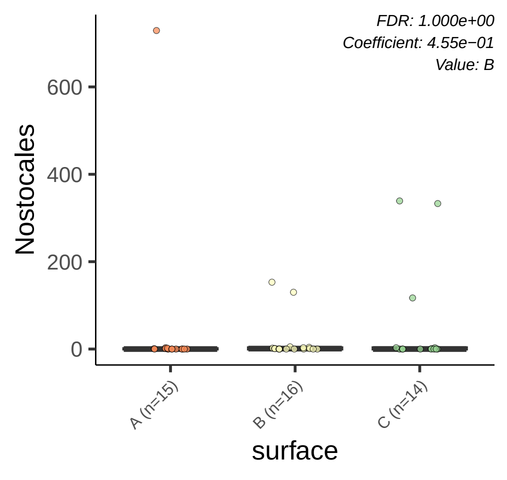
FDR: 1.000e+00
Coefficient: 4.55e−01
Value: B
Nostocales
600
400
200
0
A (n=15)
B (n=16)
C (n=14)
surface

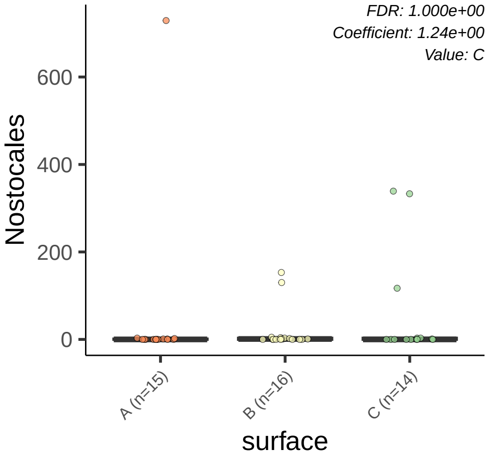
FDR: 1.000e+00
Coefficient: 1.24e+00
Value: C
Nostocales
600
400
200
0
A (n=15)
B (n=16)
C (n=14)
surface

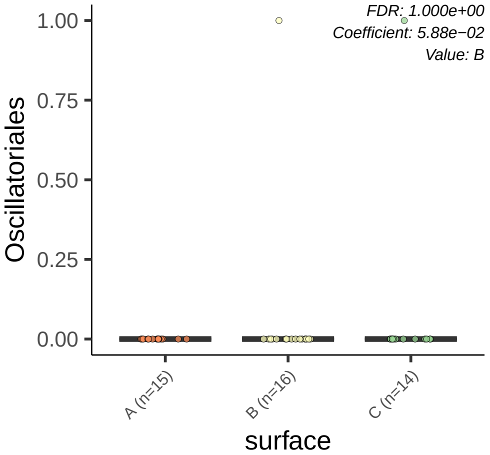

FDR: 1.000e+00
Coefficient: 5.88e−02
Value: B
Oscillatoriales
1.00
0.75
0.50
0.25
0.00
A (n=15)
B (n=16)
C (n=14)
surface

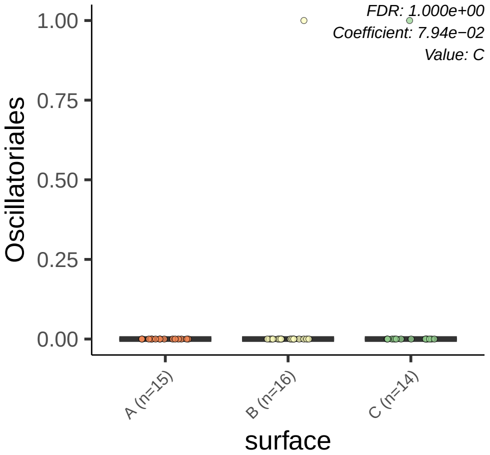

FDR: 1.000e+00
Coefficient: 7.94e−02
Value: C
Oscillatoriales
1.00
0.75
0.50
0.25
0.00
A (n=15)
B (n=16)
C (n=14)
surface

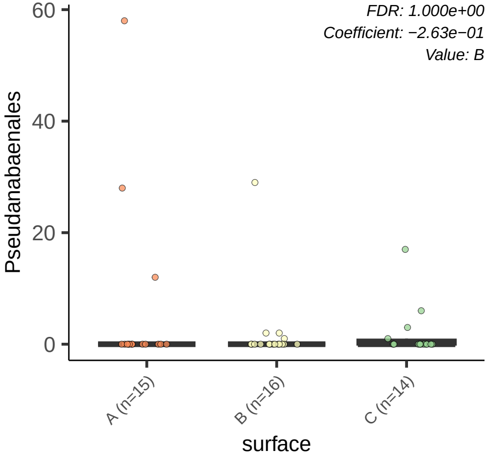
FDR: 1.000e+00
Coefficient: −2.63e−01
Value: B
Pseudanabaenales
60
40
20
0
A (n=15)
B (n=16)
C (n=14)
surface

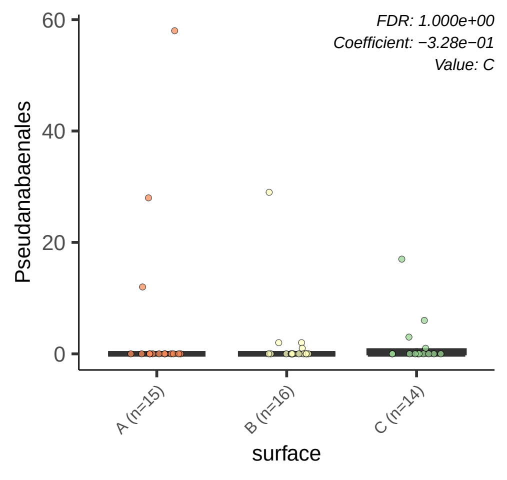

FDR: 1.000e+00
Coefficient: −3.28e−01
Value: C
Pseudanabaenales
60
40
20
0
A (n=15)
B (n=16)
C (n=14)
surface

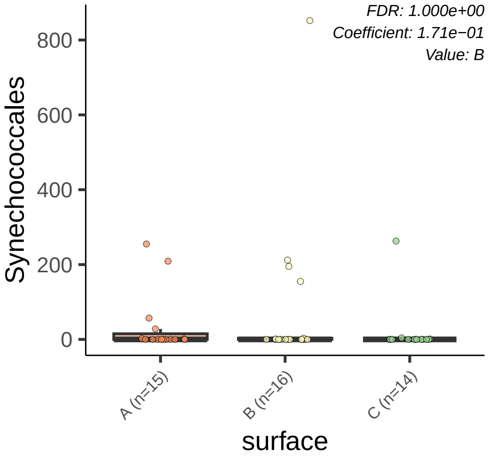

FDR: 1.000e+00
Coefficient: 1.71e−01
Value: B
Synechococcales
800
600
400
200
0
A (n=15)
B (n=16)
C (n=14)
surface

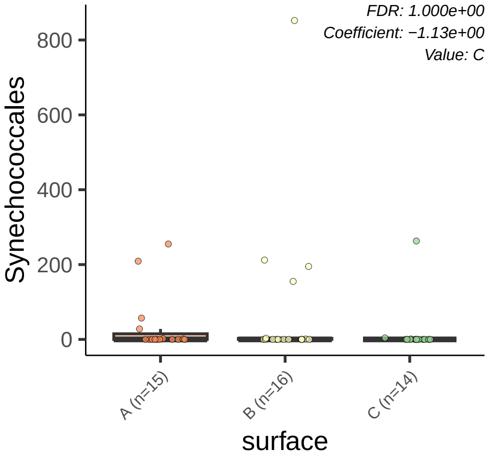
FDR: 1.000e+00
Coefficient: −1.13e+00
Value: C
Synechococcales
800
600
400
200
0
A (n=15)
B (n=16)
C (n=14)
surface

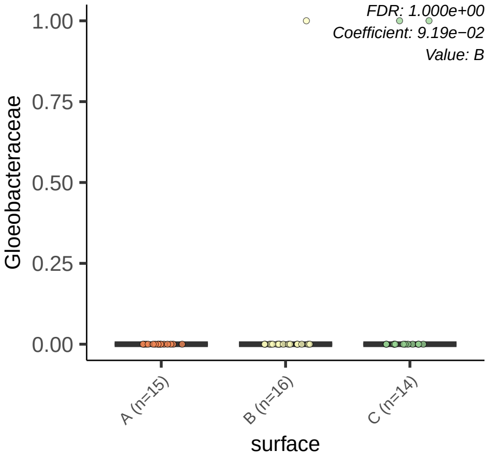
FDR: 1.000e+00
Coefficient: 9.19e−02
Value: B
Gloeobacteraceae
1.00
0.75
0.50
0.25
0.00
A (n=15)
B (n=16)
C (n=14)
surface

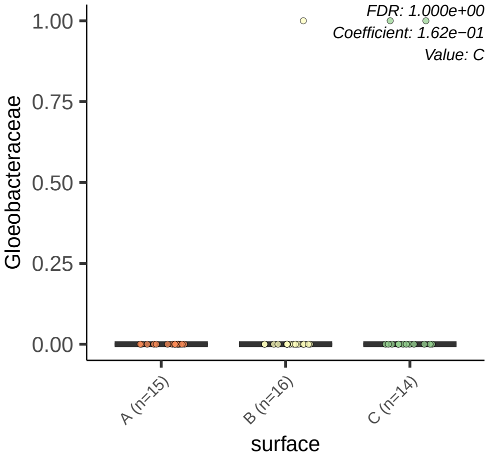
FDR: 1.000e+00
Coefficient: 1.62e−01
Value: C
Gloeobacteraceae
1.00
0.75
0.50
0.25
0.00
A (n=15)
B (n=16)
C (n=14)
surface

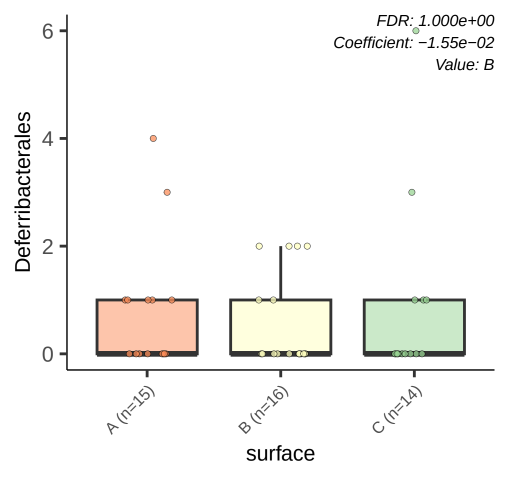
FDR: 1.000e+00
Coefficient: −1.55e−02
Value: B
Deferribacterales
0
2
4
6
A (n=15)
B (n=16)
C (n=14)
surface

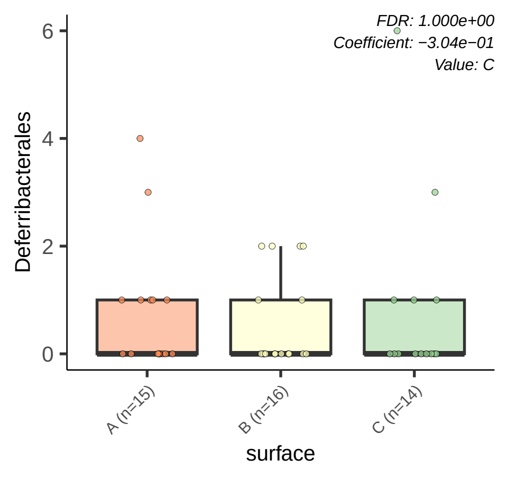
FDR: 1.000e+00
Coefficient: −3.04e−01
Value: C
Deferribacterales
0
2
4
6
A (n=15)
B (n=16)
C (n=14)
surface

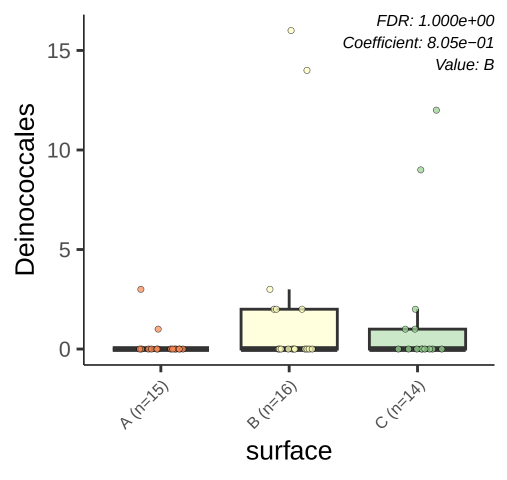

FDR: 1.000e+00
Coefficient: 8.05e−01
Value: B
Deinococcales
15
10
5
0
A (n=15)
B (n=16)
C (n=14)
surface

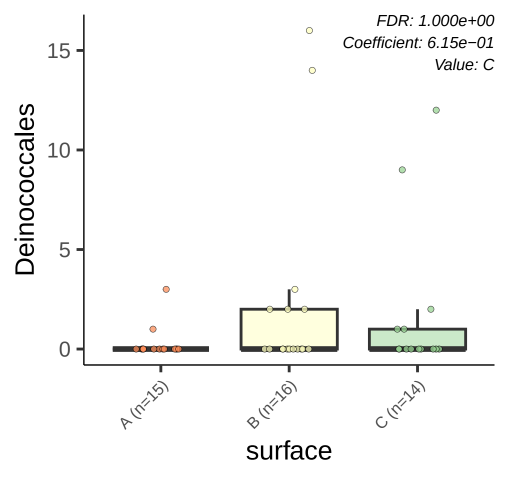
FDR: 1.000e+00
Coefficient: 6.15e−01
Value: C
Deinococcales
15
10
5
0
A (n=15)
B (n=16)
C (n=14)
surface

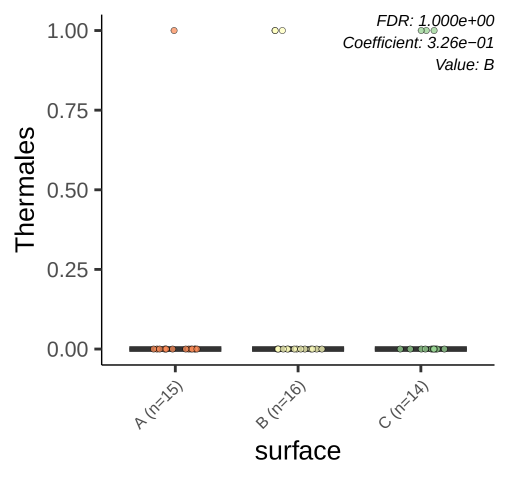

FDR: 1.000e+00
Coefficient: 3.26e−01
Value: B
Thermales
1.00
0.75
0.50
0.25
0.00
A (n=15)
B (n=16)
C (n=14)
surface

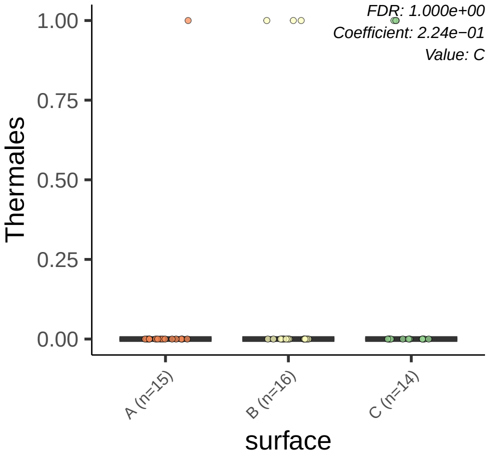
FDR: 1.000e+00
Coefficient: 2.24e−01
Value: C
Thermales
1.00
0.75
0.50
0.25
0.00
A (n=15)
B (n=16)
C (n=14)
surface

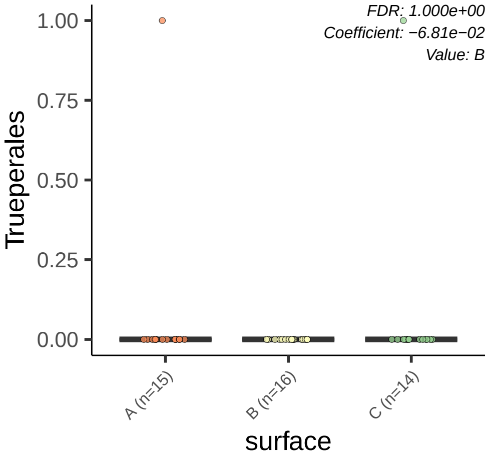
FDR: 1.000e+00
Coefficient: −6.81e−02
Value: B
Trueperales
1.00
0.75
0.50
0.25
0.00
A (n=15)
B (n=16)
C (n=14)
surface

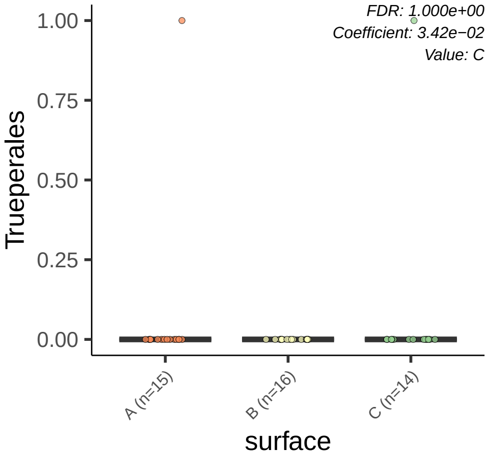
FDR: 1.000e+00
Coefficient: 3.42e−02
Value: C
Trueperales
1.00
0.75
0.50
0.25
0.00
A (n=15)
B (n=16)
C (n=14)
surface

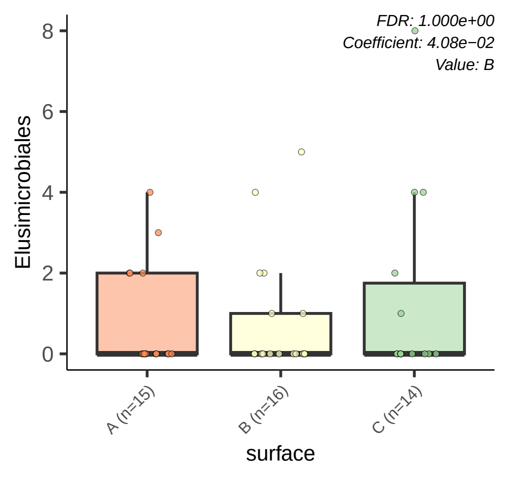
FDR: 1.000e+00
Coefficient: 4.08e−02
Value: B
Elusimicrobiales
8
6
4
2
0
A (n=15)
B (n=16)
C (n=14)
surface

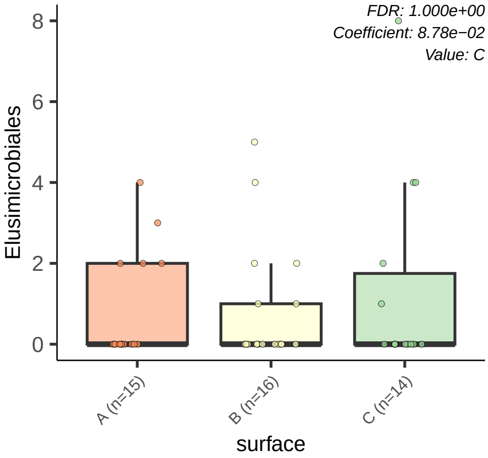

FDR: 1.000e+00
Coefficient: 8.78e−02
Value: C
Elusimicrobiales
0
2
4
6
8
A (n=15)
B (n=16)
C (n=14)
surface

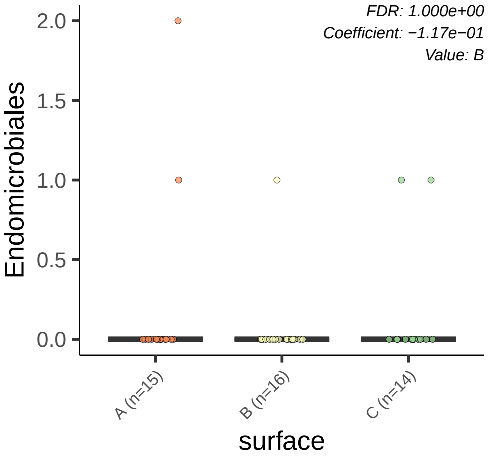
FDR: 1.000e+00
Coefficient: −1.17e−01
Value: B
Endomicrobiales
2.0
1.5
1.0
0.5
0.0
A (n=15)
B (n=16)
C (n=14)
surface

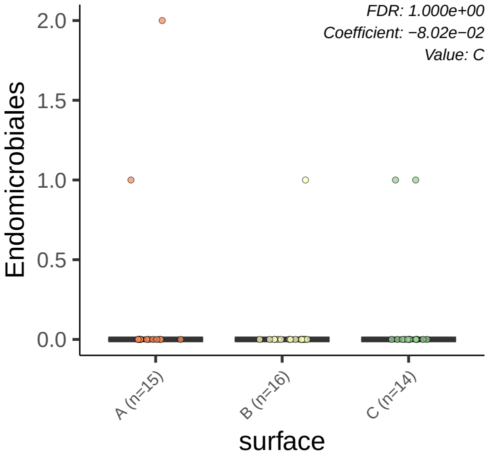
FDR: 1.000e+00
Coefficient: −8.02e−02
Value: C
Endomicrobiales
2.0
1.5
1.0
0.5
0.0
A (n=15)
B (n=16)
C (n=14)
surface

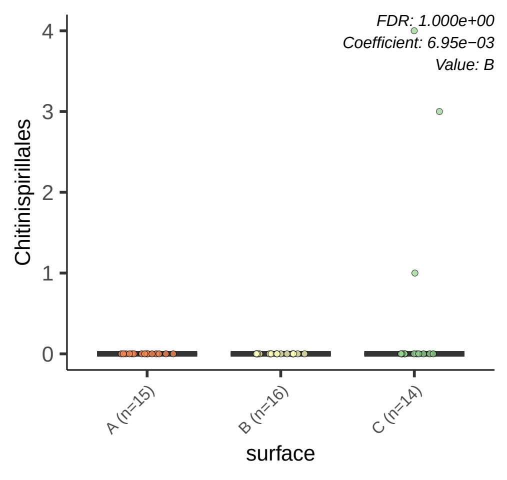

FDR: 1.000e+00
Coefficient: 6.95e−03
Value: B
Chitinispirillales
0
1
2
3
4
A (n=15)
B (n=16)
C (n=14)
surface

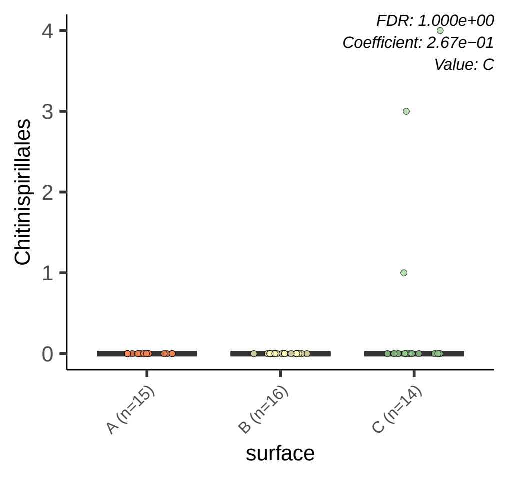

FDR: 1.000e+00
Coefficient: 2.67e−01
Value: C
Chitinispirillales
4
3
2
1
0
A (n=15)
B (n=16)
C (n=14)
surface

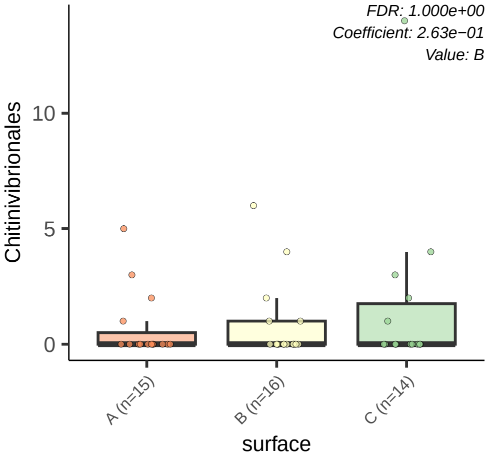
FDR: 1.000e+00
Coefficient: 2.63e−01
Value: B
Chitinivibrionales
10
5
0
A (n=15)
B (n=16)
C (n=14)
surface

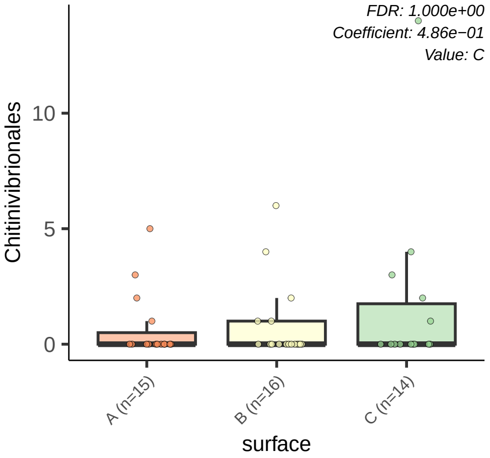
FDR: 1.000e+00
Coefficient: 4.86e−01
Value: C
Chitinivibrionales
10
5
0
A (n=15)
B (n=16)
C (n=14)
surface

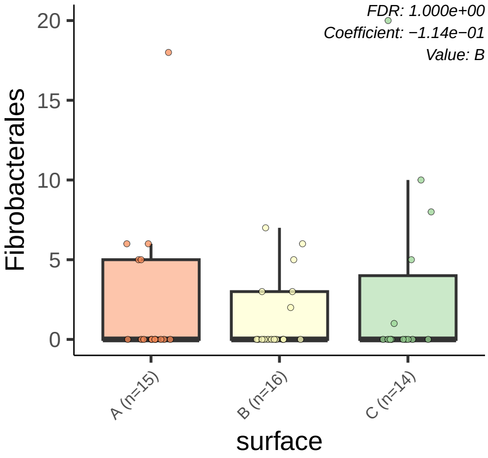
FDR: 1.000e+00
Coefficient: −1.14e−01
Value: B
Fibrobacterales
20
15
10
5
0
A (n=15)
B (n=16)
C (n=14)
surface

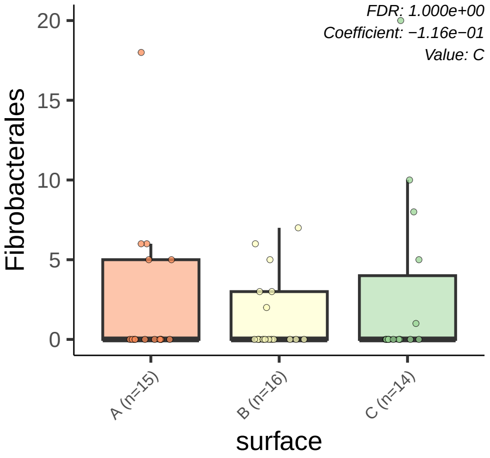
FDR: 1.000e+00
Coefficient: −1.16e−01
Value: C
Fibrobacterales
20
15
10
5
0
A (n=15)
B (n=16)
C (n=14)
surface

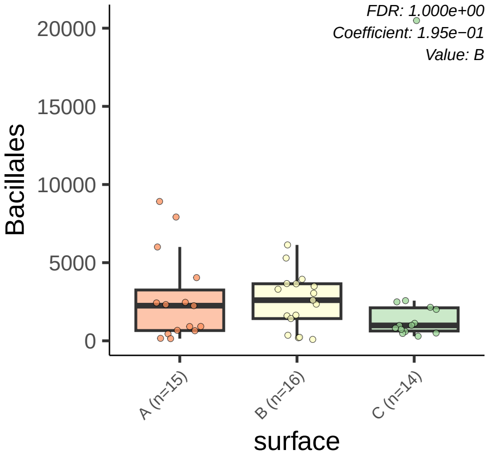

FDR: 1.000e+00
Coefficient: 1.95e−01
Value: B
Bacillales
20000
15000
10000
5000
0
A (n=15)
B (n=16)
C (n=14)
surface

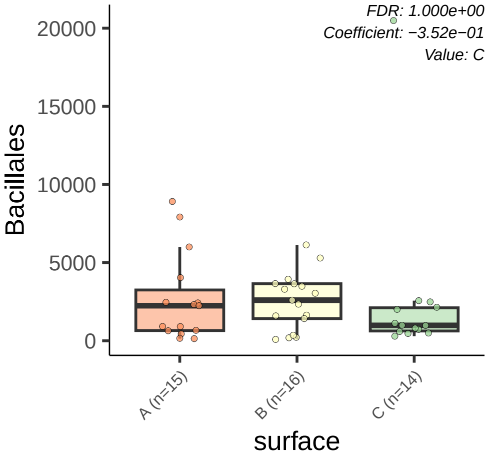
FDR: 1.000e+00
Coefficient: −3.52e−01
Value: C
20000
15000
10000
5000
0
Bacillales
A (n=15)
B (n=16)
C (n=14)
surface

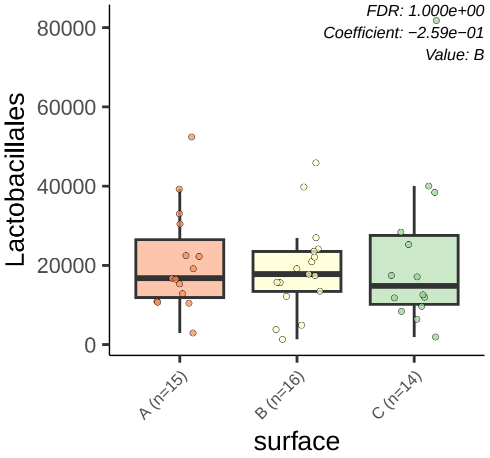

FDR: 1.000e+00
Coefficient: −2.59e−01
Value: B
80000
60000
40000
20000
0
Lactobacillales
A (n=15)
B (n=16)
C (n=14)
surface

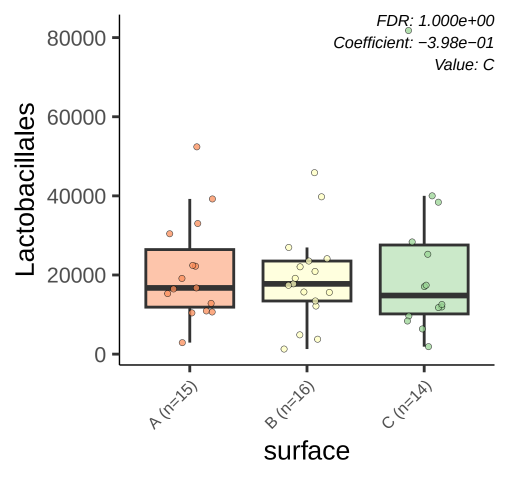

FDR: 1.000e+00
Coefficient: −3.98e−01
Value: C
Lactobacillales
80000
60000
40000
20000
0
A (n=15)
B (n=16)
C (n=14)
surface

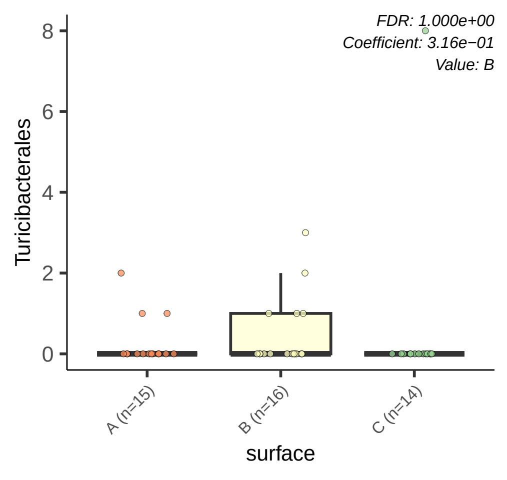
FDR: 1.000e+00
Coefficient: 3.16e−01
Value: B
Turicibacterales
8
6
4
2
0
A (n=15)
B (n=16)
C (n=14)
surface

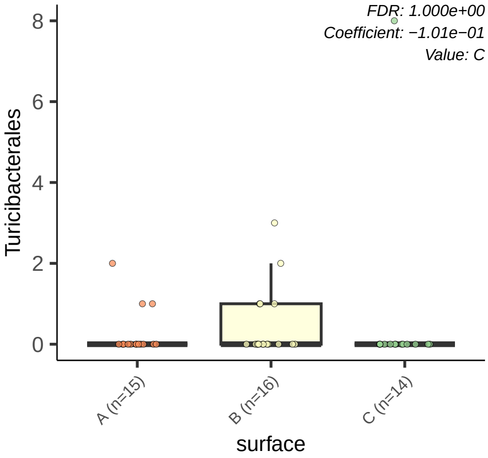

FDR: 1.000e+00
Coefficient: −1.01e−01
Value: C
Turicibacterales
8
6
4
2
0
A (n=15)
B (n=16)
C (n=14)
surface

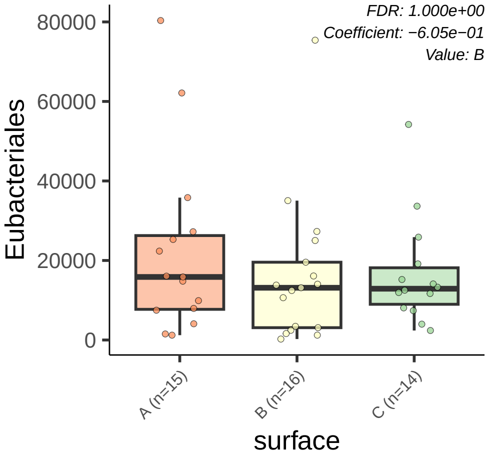
FDR: 1.000e+00
Coefficient: −6.05e−01
Value: B
Eubacteriales
80000
60000
40000
20000
0
A (n=15)
B (n=16)
C (n=14)
surface

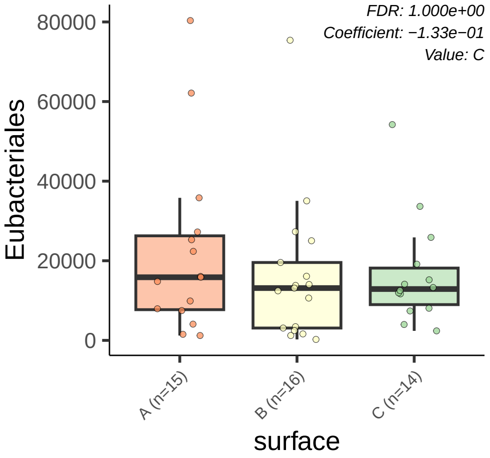
FDR: 1.000e+00
Coefficient: −1.33e−01
Value: C
Eubacteriales
80000
60000
40000
20000
0
A (n=15)
B (n=16)
C (n=14)
surface

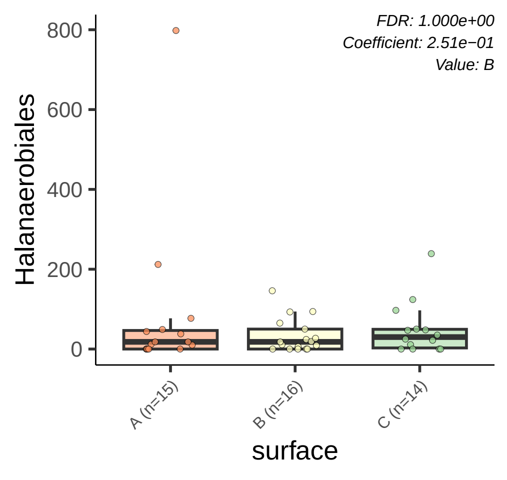
FDR: 1.000e+00
Coefficient: 2.51e−01
Value: B
Halanaerobiales
800
600
400
200
0
A (n=15)
B (n=16)
C (n=14)
surface

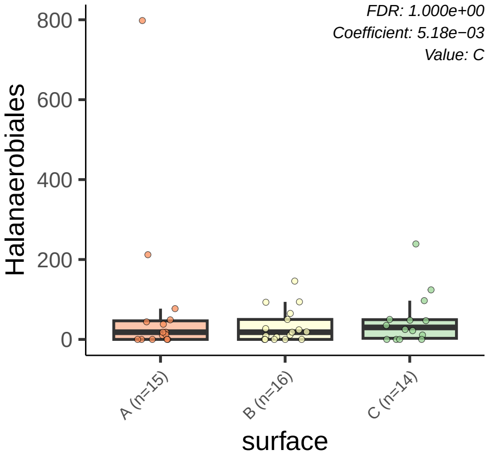

FDR: 1.000e+00
Coefficient: 5.18e−03
Value: C
Halanaerobiales
800
600
400
200
0
A (n=15)
B (n=16)
C (n=14)
surface

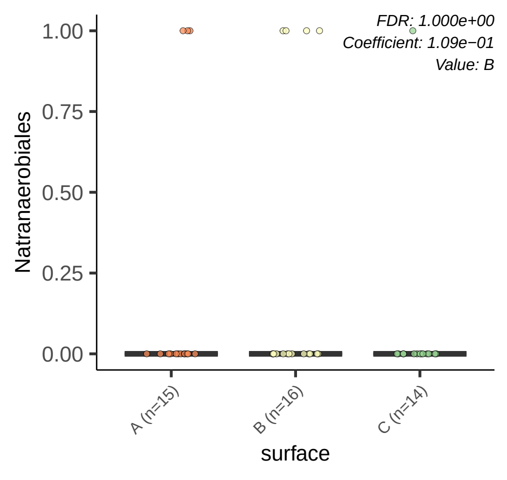
FDR: 1.000e+00
Coefficient: 1.09e−01
Value: B
Natranaerobiales
1.00
0.75
0.50
0.25
0.00
A (n=15)
B (n=16)
C (n=14)
surface

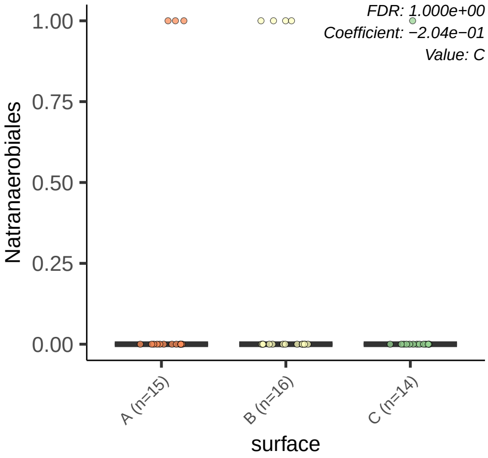
FDR: 1.000e+00
Coefficient: −2.04e−01
Value: C
Natranaerobiales
1.00
0.75
0.50
0.25
0.00
A (n=15)
B (n=16)
C (n=14)
surface

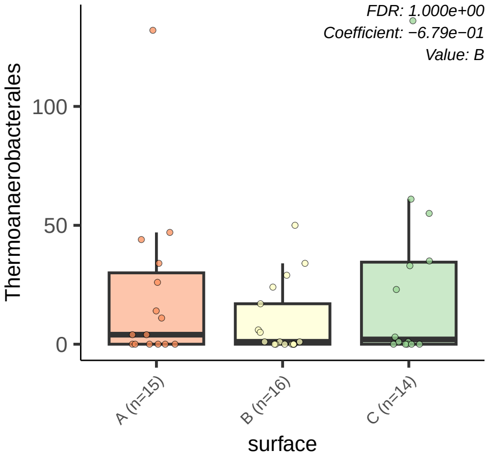
FDR: 1.000e+00
Coefficient: −6.79e−01
Value: B
Thermoanaerobacterales
100
50
0
A (n=15)
B (n=16)
C (n=14)
surface

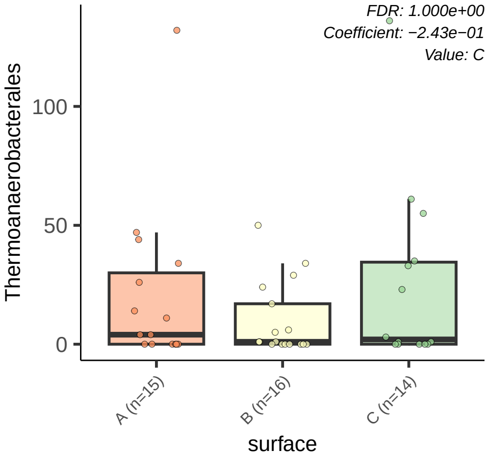
FDR: 1.000e+00
Coefficient: −2.43e−01
Value: C
Thermoanaerobacterales
100
50
0
A (n=15)
B (n=16)
C (n=14)
surface

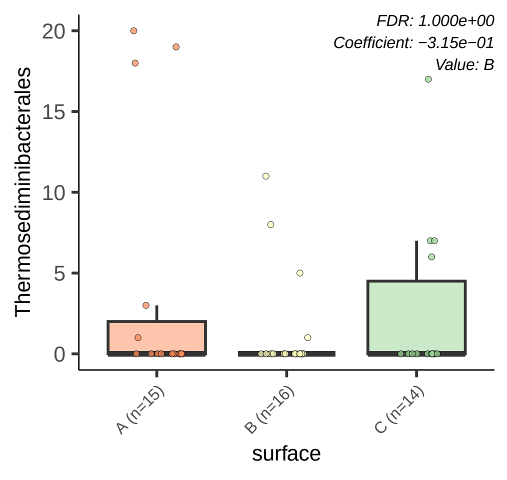
FDR: 1.000e+00
Coefficient: −3.15e−01
Value: B
Thermosediminibacterales
0
5
10
15
20
A (n=15)
B (n=16)
C (n=14)
surface

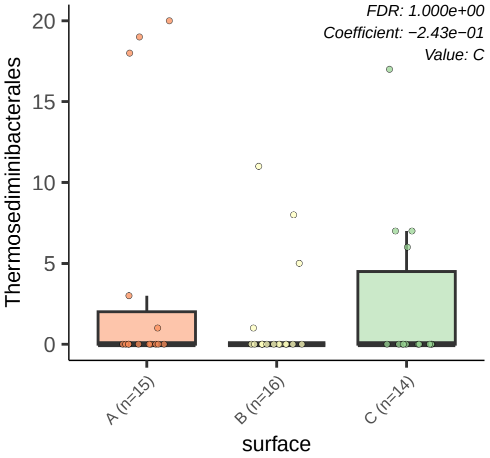

FDR: 1.000e+00
Coefficient: −2.43e−01
Value: C
Thermosediminibacterales
20
15
10
5
0
A (n=15)
B (n=16)
C (n=14)
surface

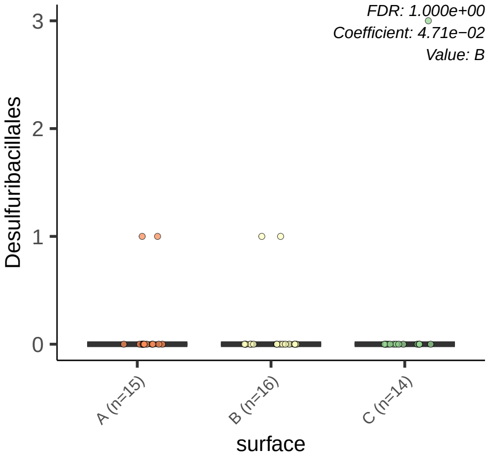
FDR: 1.000e+00
Coefficient: 4.71e−02
Value: B
3
2
1
0
Desulfuribacillales
A (n=15)
B (n=16)
C (n=14)
surface

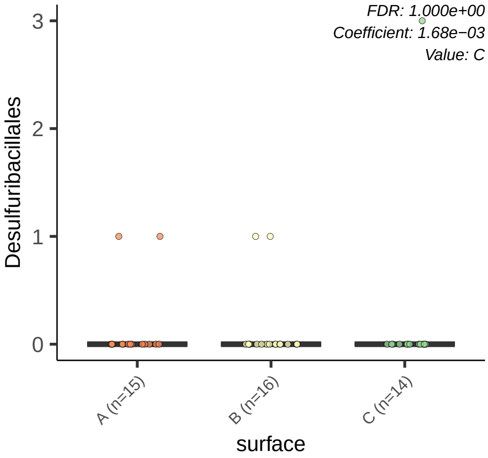

FDR: 1.000e+00
Coefficient: 1.68e−03
Value: C
Desulfuribacillales
3
2
1
0
A (n=15)
B (n=16)
C (n=14)
surface

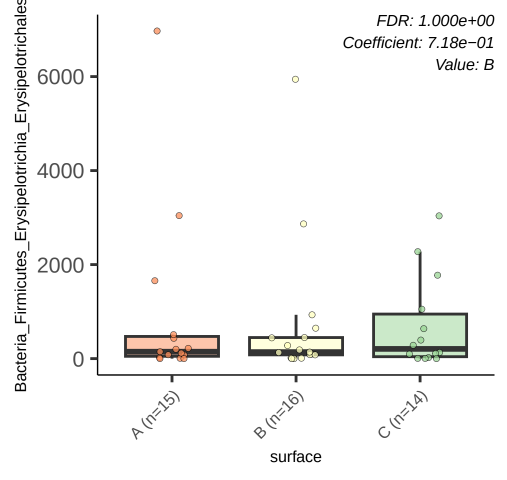
FDR: 1.000e+00
Coefficient: 7.18e−01
Value: B
Bacteria_Firmicutes_Erysipelotrichia_Erysipelotrichales
6000
4000
2000
0
A (n=15)
B (n=16)
C (n=14)
surface

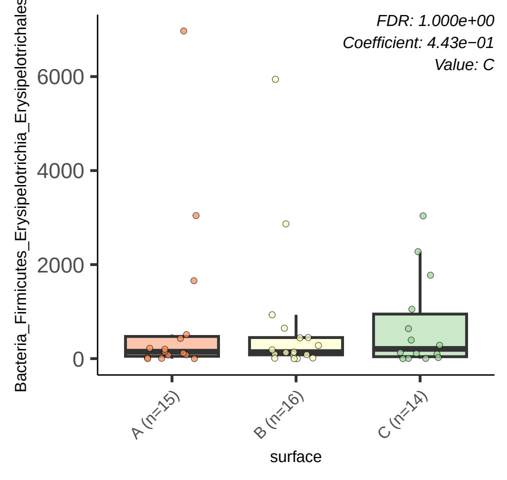

FDR: 1.000e+00
Coefficient: 4.43e−01
Value: C
Bacteria_Firmicutes_Erysipelotrichia_Erysipelotrichales
6000
4000
2000
0
A (n=15)
B (n=16)
C (n=14)
surface

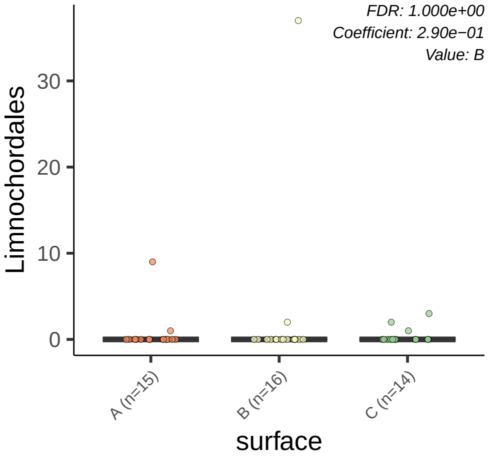

FDR: 1.000e+00
Coefficient: 2.90e−01
Value: B
Limnochordales
30
20
10
0
A (n=15)
B (n=16)
C (n=14)
surface

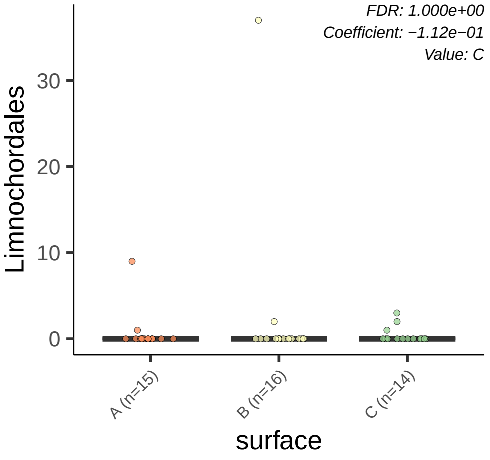
FDR: 1.000e+00
Coefficient: −1.12e−01
Value: C
Limnochordales
30
20
10
0
A (n=15)
B (n=16)
C (n=14)
surface

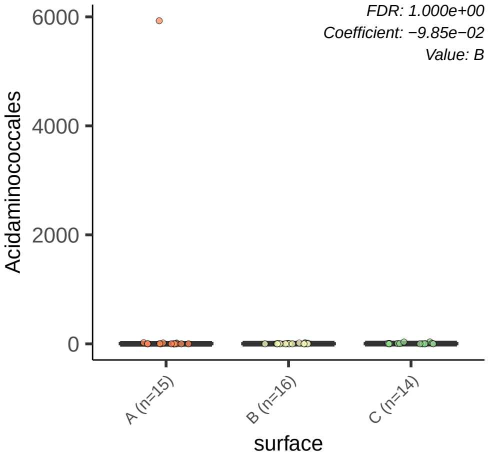
FDR: 1.000e+00
Coefficient: −9.85e−02
Value: B
Acidaminococcales
6000
4000
2000
0
A (n=15)
B (n=16)
C (n=14)
surface

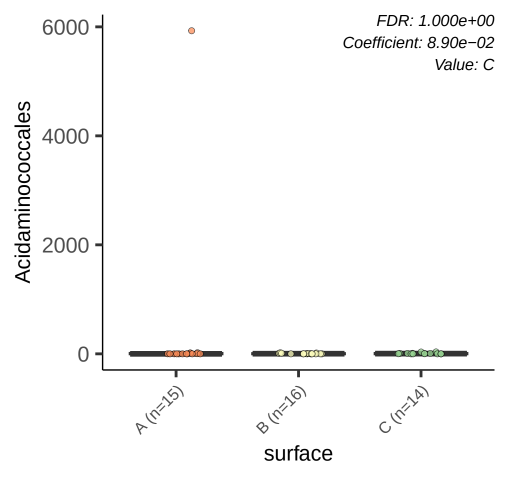

FDR: 1.000e+00
Coefficient: 8.90e−02
Value: C
6000
4000
2000
0
Acidaminococcales
A (n=15)
B (n=16)
C (n=14)
surface

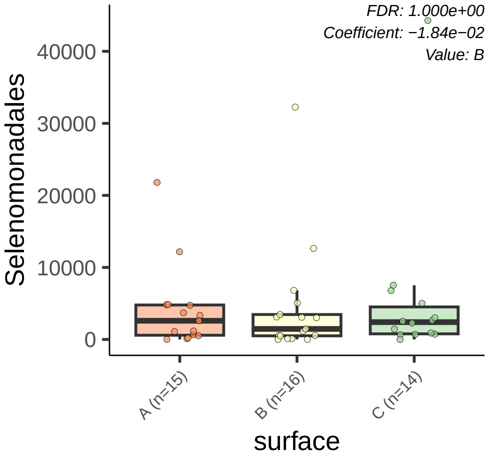

FDR: 1.000e+00
Coefficient: −1.84e−02
Value: B
Selenomonadales
40000
30000
20000
10000
0
A (n=15)
B (n=16)
C (n=14)
surface

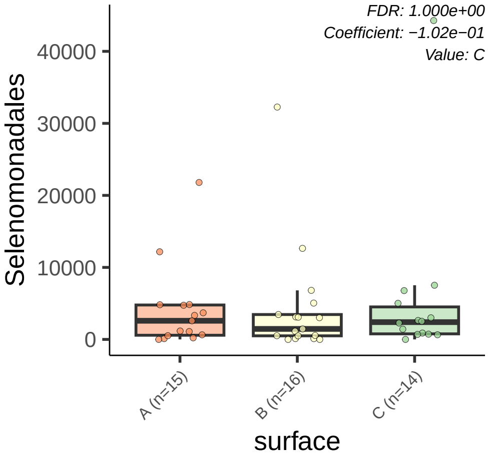
FDR: 1.000e+00
Coefficient: −1.02e−01
Value: C
Selenomonadales
40000
30000
20000
10000
0
A (n=15)
B (n=16)
C (n=14)
surface

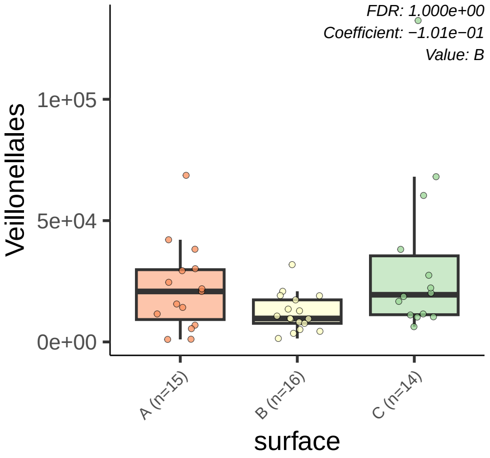

FDR: 1.000e+00
Coefficient: −1.01e−01
Value: B
Veillonellales
1e+05
5e+04
0e+00
A (n=15)
B (n=16)
C (n=14)
surface

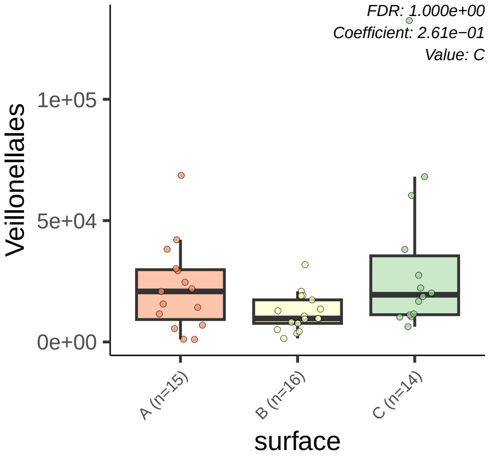

FDR: 1.000e+00
Coefficient: 2.61e−01
Value: C
Veillonellales
1e+05
5e+04
0e+00
A (n=15)
B (n=16)
C (n=14)
surface

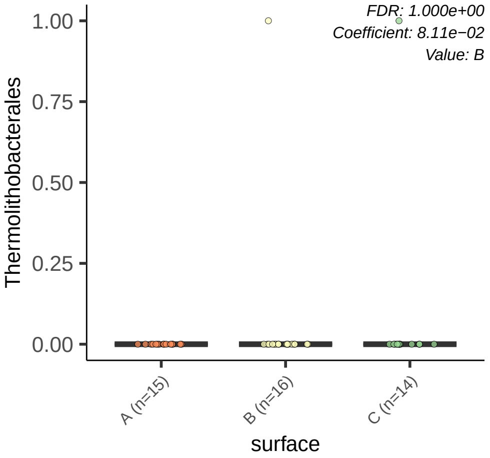

FDR: 1.000e+00
Coefficient: 8.11e−02
Value: B
Thermolithobacterales
1.00
0.75
0.50
0.25
0.00
A (n=15)
B (n=16)
C (n=14)
surface

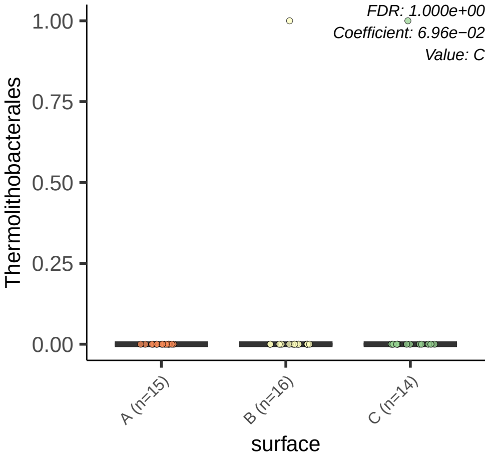

FDR: 1.000e+00
Coefficient: 6.96e−02
Value: C
Thermolithobacterales
1.00
0.75
0.50
0.25
0.00
A (n=15)
B (n=16)
C (n=14)
surface

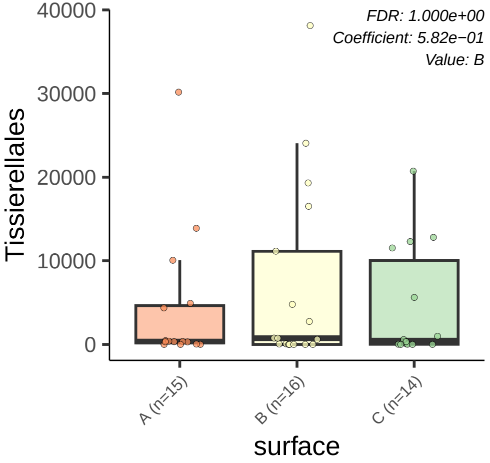
FDR: 1.000e+00
Coefficient: 5.82e−01
Value: B
Tissierellales
40000
30000
20000
10000
0
A (n=15)
B (n=16)
C (n=14)
surface

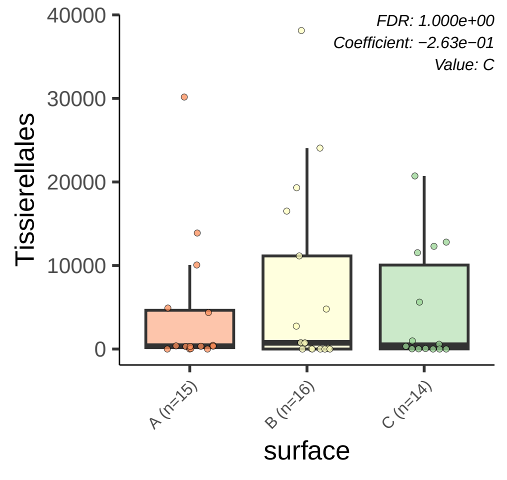

FDR: 1.000e+00
Coefficient: −2.63e−01
Value: C
40000
30000
20000
10000
0
Tissierellales
A (n=15)
B (n=16)
C (n=14)
surface

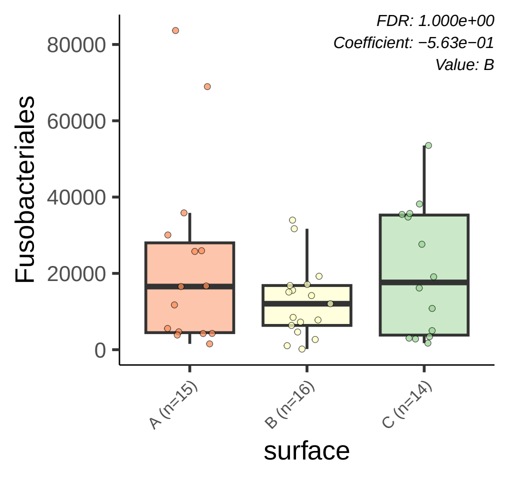

FDR: 1.000e+00
Coefficient: −5.63e−01
Value: B
Fusobacteriales
80000
60000
40000
20000
0
A (n=15)
B (n=16)
C (n=14)
surface

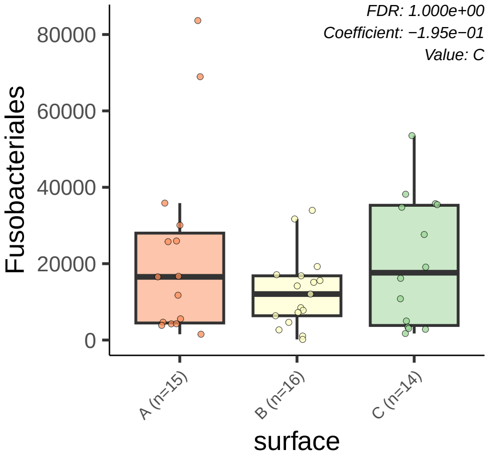

FDR: 1.000e+00
Coefficient: −1.95e−01
Value: C
Fusobacteriales
80000
60000
40000
20000
0
A (n=15)
B (n=16)
C (n=14)
surface

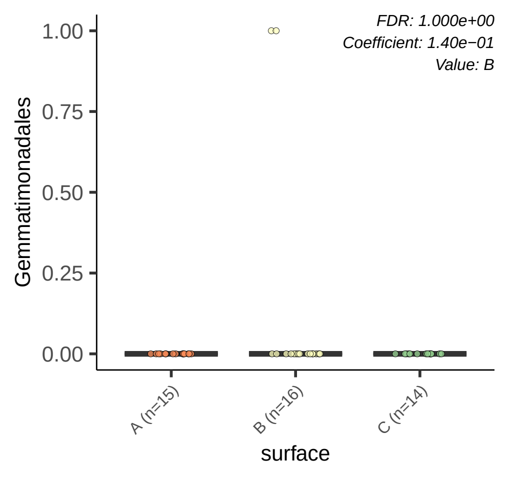
FDR: 1.000e+00
Coefficient: 1.40e−01
Value: B
Gemmatimonadales
1.00
0.75
0.50
0.25
0.00
A (n=15)
B (n=16)
C (n=14)
surface

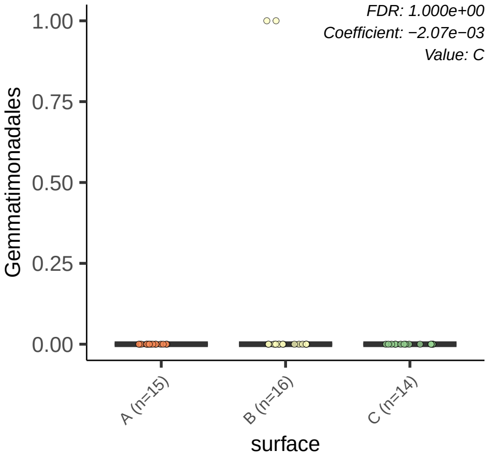

FDR: 1.000e+00
Coefficient: −2.07e−03
Value: C
Gemmatimonadales
1.00
0.75
0.50
0.25
0.00
A (n=15)
B (n=16)
C (n=14)
surface

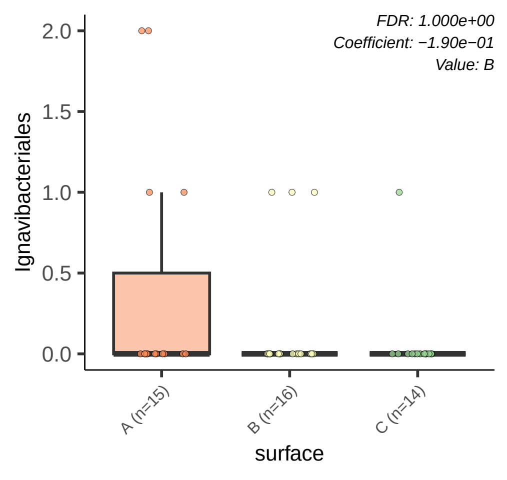
FDR: 1.000e+00
Coefficient: −1.90e−01
Value: B
Ignavibacteriales
2.0
1.5
1.0
0.5
0.0
A (n=15)
B (n=16)
C (n=14)
surface

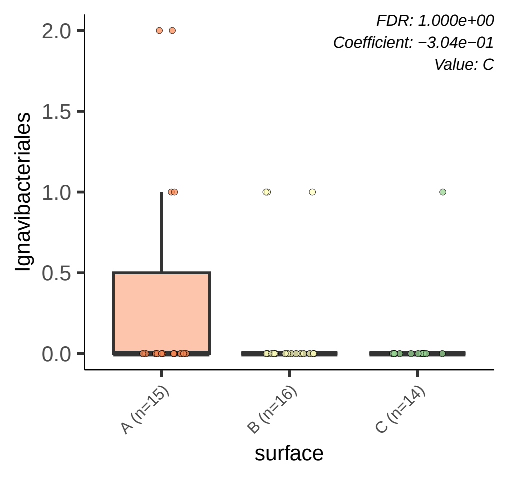
FDR: 1.000e+00
Coefficient: −3.04e−01
Value: C
Ignavibacteriales
2.0
1.5
1.0
0.5
0.0
A (n=15)
B (n=16)
C (n=14)
surface

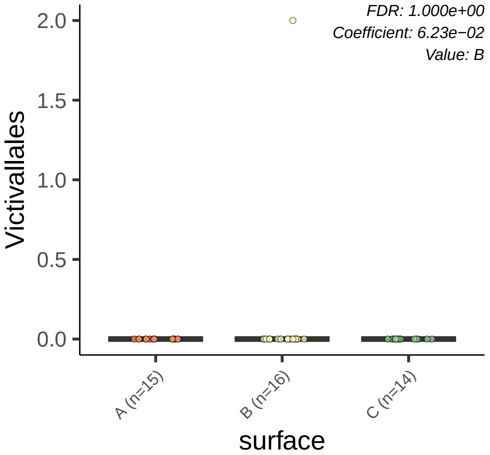

FDR: 1.000e+00
Coefficient: 6.23e−02
Value: B
Victivallales
2.0
1.5
1.0
0.5
0.0
A (n=15)
B (n=16)
C (n=14)
surface

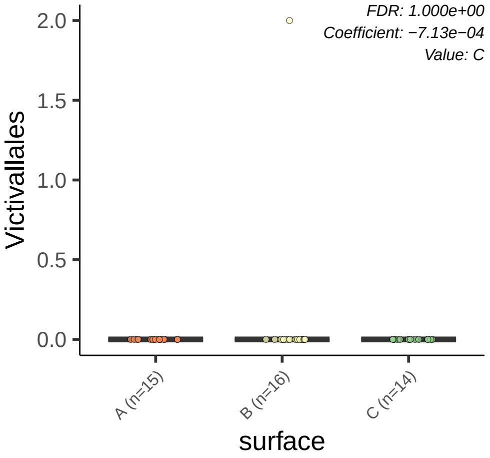
FDR: 1.000e+00
Coefficient: −7.13e−04
Value: C
Victivallales
2.0
1.5
1.0
0.5
0.0
A (n=15)
B (n=16)
C (n=14)
surface

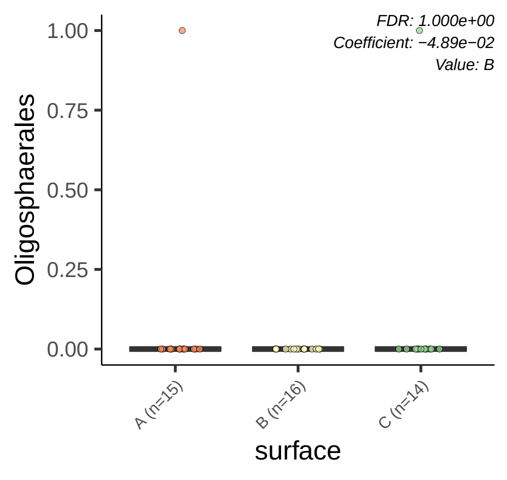

FDR: 1.000e+00
Coefficient: −4.89e−02
Value: B
Oligosphaerales
1.00
0.75
0.50
0.25
0.00
A (n=15)
B (n=16)
C (n=14)
surface

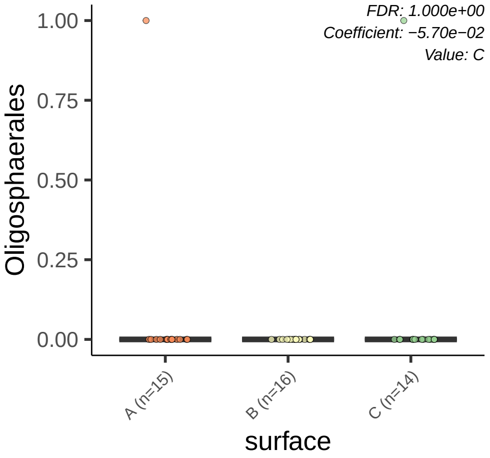
FDR: 1.000e+00
Coefficient: −5.70e−02
Value: C
Oligosphaerales
1.00
0.75
0.50
0.25
0.00
A (n=15)
B (n=16)
C (n=14)
surface

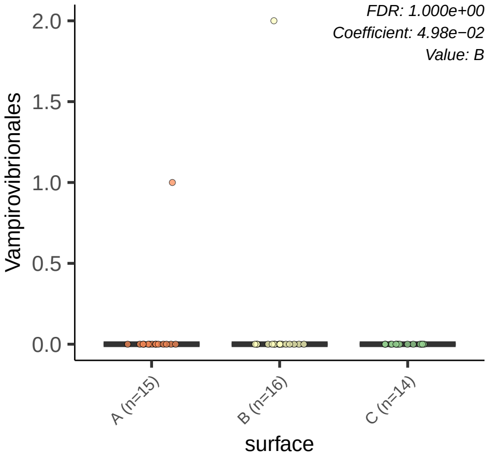

FDR: 1.000e+00
Coefficient: 4.98e−02
Value: B
Vampirovibrionales
2.0
1.5
1.0
0.5
0.0
A (n=15)
B (n=16)
C (n=14)
surface

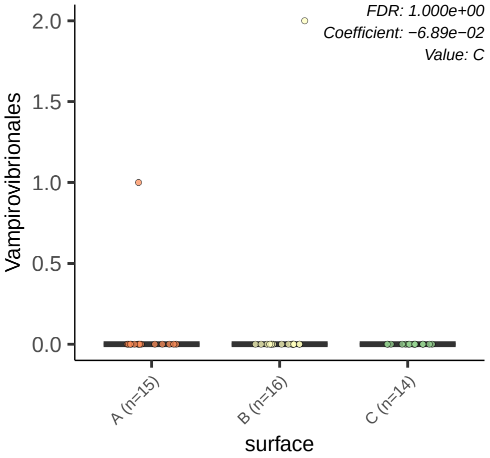
FDR: 1.000e+00
Coefficient: −6.89e−02
Value: C
Vampirovibrionales
2.0
1.5
1.0
0.5
0.0
A (n=15)
B (n=16)
C (n=14)
surface

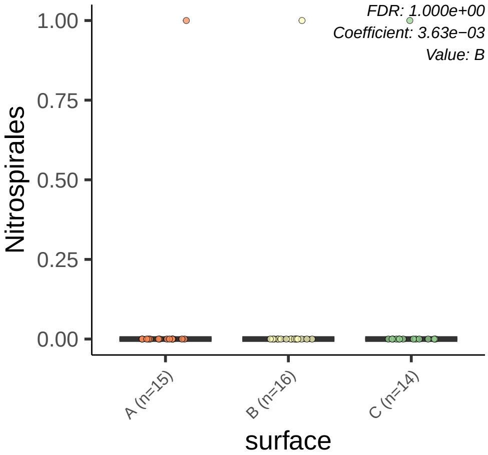
FDR: 1.000e+00
Coefficient: 3.63e−03
Value: B
Nitrospirales
1.00
0.75
0.50
0.25
0.00
A (n=15)
B (n=16)
C (n=14)
surface

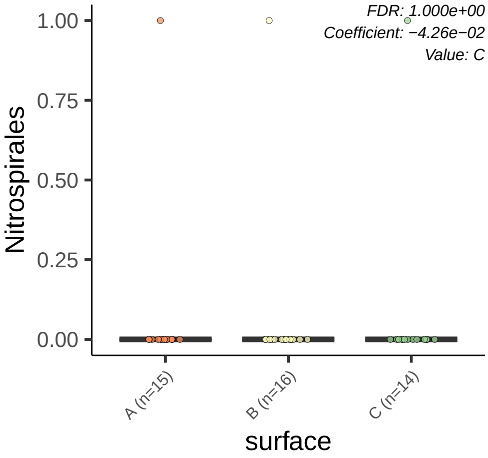

FDR: 1.000e+00
Coefficient: −4.26e−02
Value: C
Nitrospirales
1.00
0.75
0.50
0.25
0.00
A (n=15)
B (n=16)
C (n=14)
surface

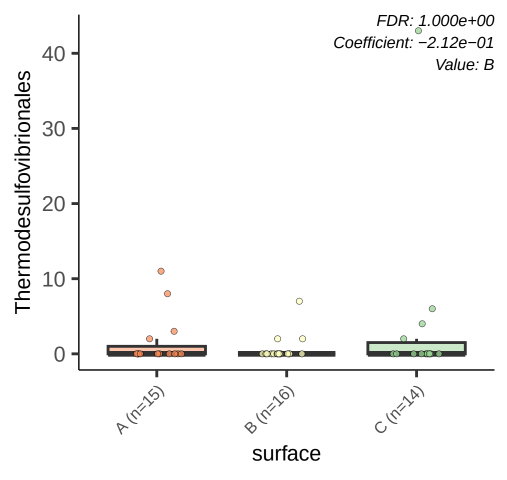
FDR: 1.000e+00
Coefficient: −2.12e−01
Value: B
Thermodesulfovibrionales
40
30
20
10
0
A (n=15)
B (n=16)
C (n=14)
surface

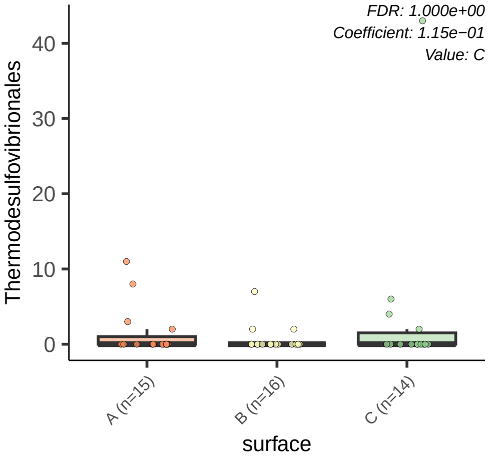
FDR: 1.000e+00
Coefficient: 1.15e−01
Value: C
Thermodesulfovibrionales
40
30
20
10
0
A (n=15)
B (n=16)
C (n=14)
surface

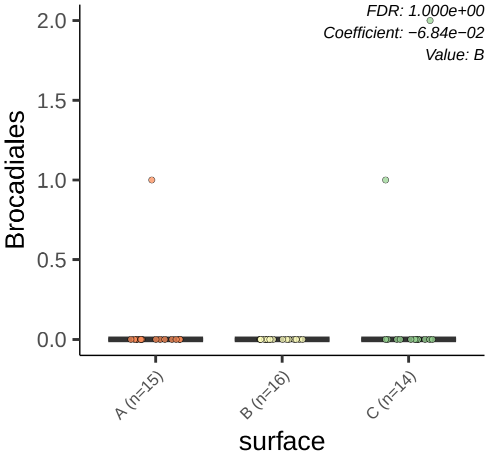
FDR: 1.000e+00
Coefficient: −6.84e−02
Value: B
Brocadiales
2.0
1.5
1.0
0.5
0.0
A (n=15)
B (n=16)
C (n=14)
surface

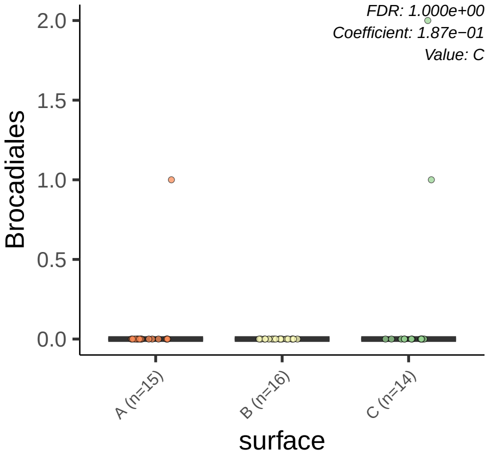

FDR: 1.000e+00
Coefficient: 1.87e−01
Value: C
Brocadiales
2.0
1.5
1.0
0.5
0.0
A (n=15)
B (n=16)
C (n=14)
surface

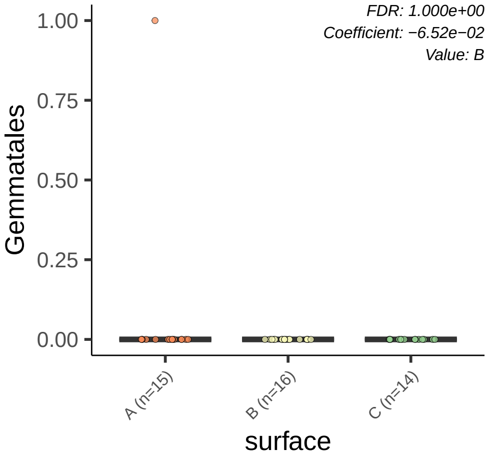

FDR: 1.000e+00
Coefficient: −6.52e−02
Value: B
Gemmatales
1.00
0.75
0.50
0.25
0.00
A (n=15)
B (n=16)
C (n=14)
surface

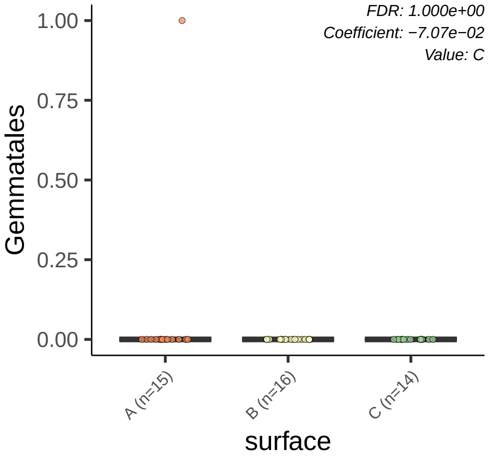
FDR: 1.000e+00
Coefficient: −7.07e−02
Value: C
Gemmatales
1.00
0.75
0.50
0.25
0.00
A (n=15)
B (n=16)
C (n=14)
surface

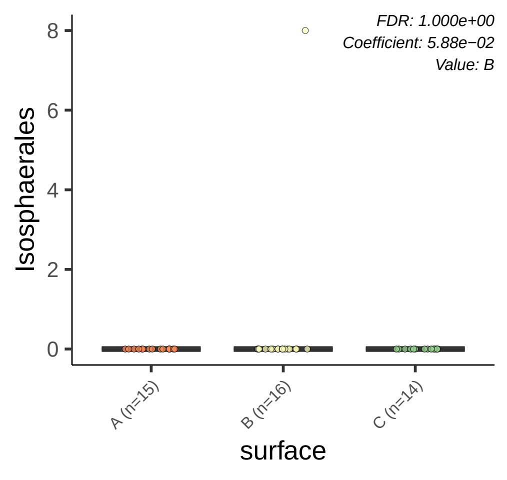

FDR: 1.000e+00
Coefficient: 5.88e−02
Value: B
Isosphaerales
8
6
4
2
0
A (n=15)
B (n=16)
C (n=14)
surface

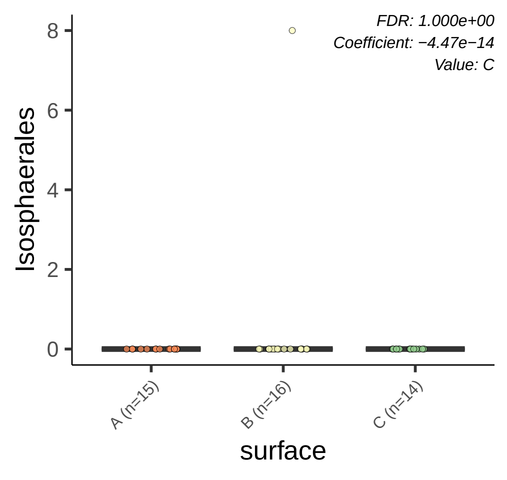
FDR: 1.000e+00
Coefficient: −4.47e−14
Value: C
Isosphaerales
8
6
4
2
0
A (n=15)
B (n=16)
C (n=14)
surface

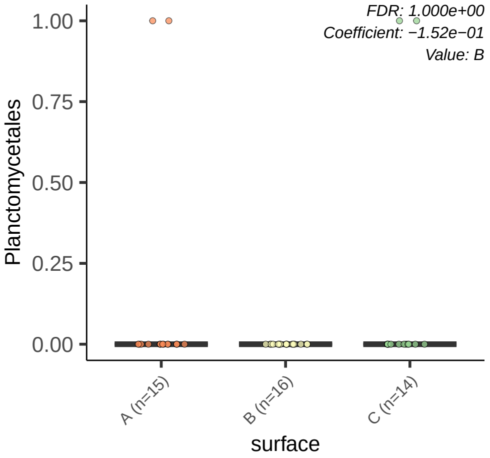
FDR: 1.000e+00
Coefficient: −1.52e−01
Value: B
Planctomycetales
1.00
0.75
0.50
0.25
0.00
A (n=15)
B (n=16)
C (n=14)
surface

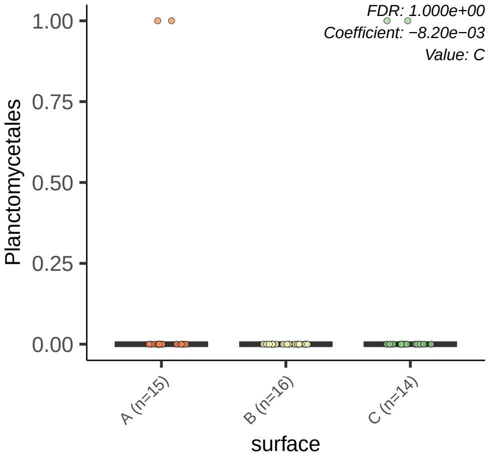

FDR: 1.000e+00
Coefficient: −8.20e−03
Value: C
Planctomycetales
1.00
0.75
0.50
0.25
0.00
A (n=15)
B (n=16)
C (n=14)
surface

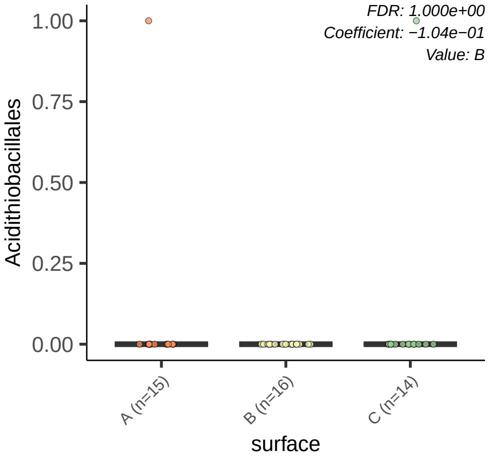
FDR: 1.000e+00
Coefficient: −1.04e−01
Value: B
Acidithiobacillales
1.00
0.75
0.50
0.25
0.00
A (n=15)
B (n=16)
C (n=14)
surface

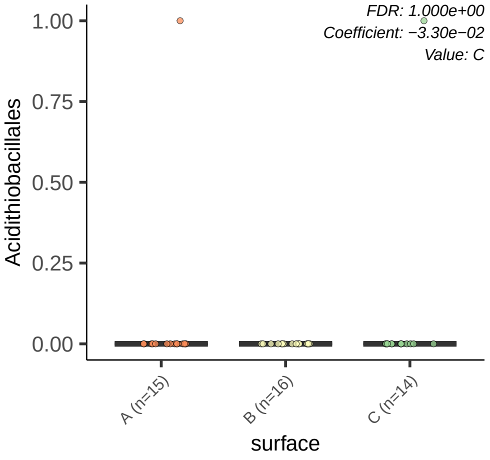
FDR: 1.000e+00
Coefficient: −3.30e−02
Value: C
Acidithiobacillales
1.00
0.75
0.50
0.25
0.00
A (n=15)
B (n=16)
C (n=14)
surface

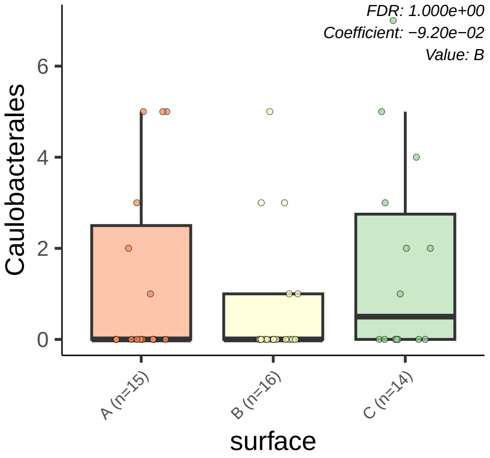
FDR: 1.000e+00
Coefficient: −9.20e−02
Value: B
Caulobacterales
0
2
4
6
A (n=15)
B (n=16)
C (n=14)
surface

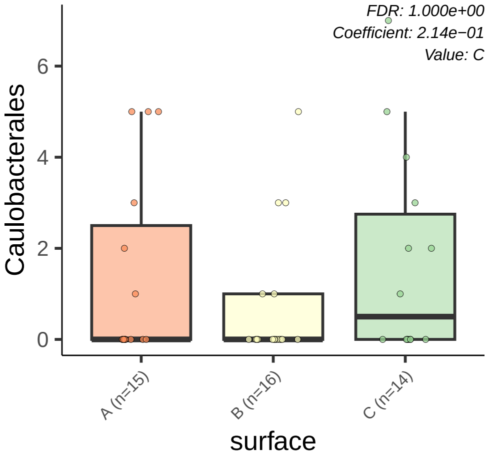
FDR: 1.000e+00
Coefficient: 2.14e−01
Value: C
Caulobacterales
0
2
4
6
A (n=15)
B (n=16)
C (n=14)
surface

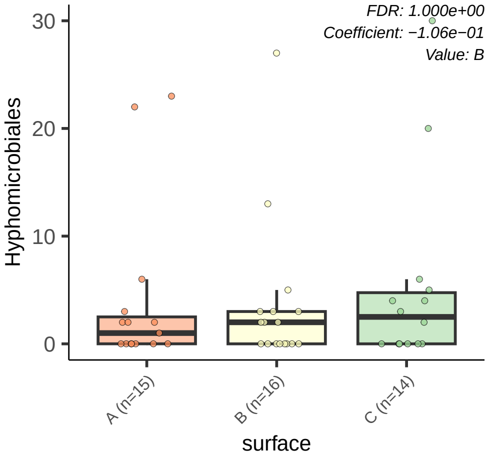
FDR: 1.000e+00
Coefficient: −1.06e−01
Value: B
Hyphomicrobiales
30
20
10
0
A (n=15)
B (n=16)
C (n=14)
surface

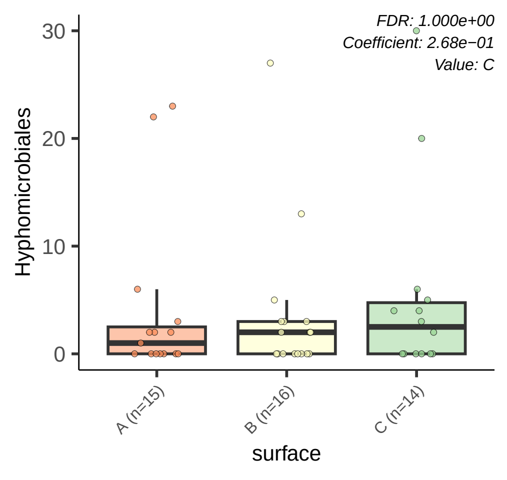

FDR: 1.000e+00
Coefficient: 2.68e−01
Value: C
Hyphomicrobiales
30
20
10
0
A (n=15)
B (n=16)
C (n=14)
surface

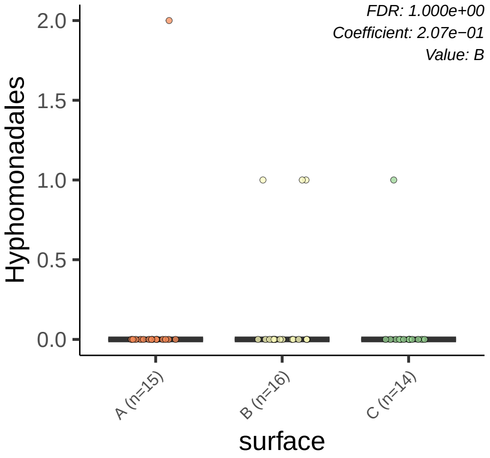

FDR: 1.000e+00
Coefficient: 2.07e−01
Value: B
Hyphomonadales
2.0
1.5
1.0
0.5
0.0
A (n=15)
B (n=16)
C (n=14)
surface

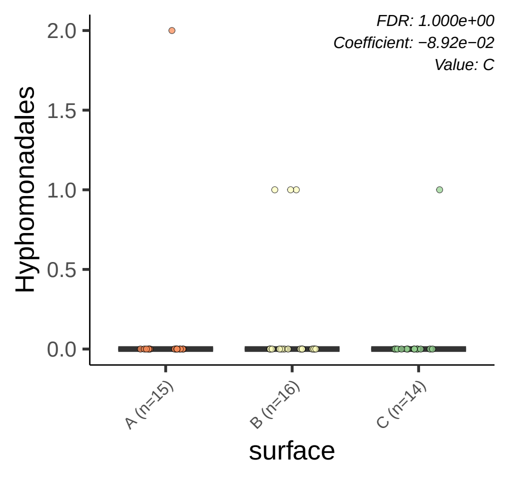
FDR: 1.000e+00
Coefficient: −8.92e−02
Value: C
Hyphomonadales
2.0
1.5
1.0
0.5
0.0
A (n=15)
B (n=16)
C (n=14)
surface

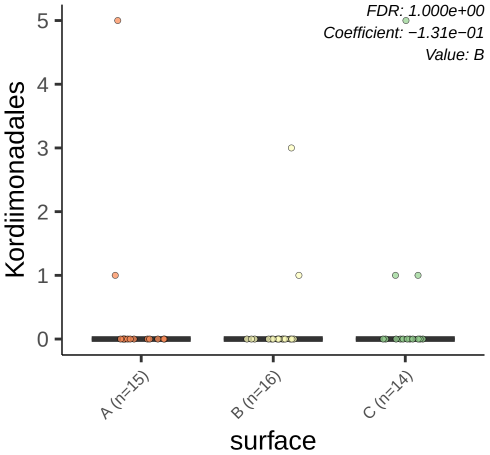
FDR: 1.000e+00
Coefficient: −1.31e−01
Value: B
Kordiimonadales
0
1
2
3
4
5
A (n=15)
B (n=16)
C (n=14)
surface

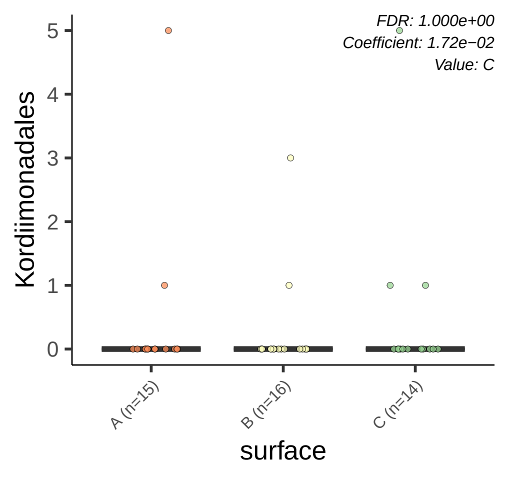
FDR: 1.000e+00
Coefficient: 1.72e−02
Value: C
Kordiimonadales
0
1
2
3
4
5
A (n=15)
B (n=16)
C (n=14)
surface

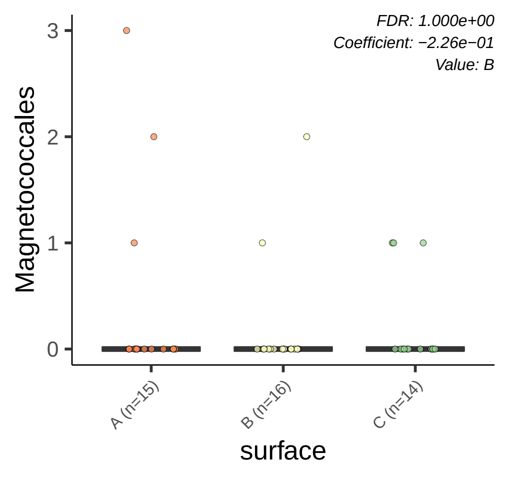
FDR: 1.000e+00
Coefficient: −2.26e−01
Value: B
Magnetococcales
3
2
1
0
A (n=15)
B (n=16)
C (n=14)
surface

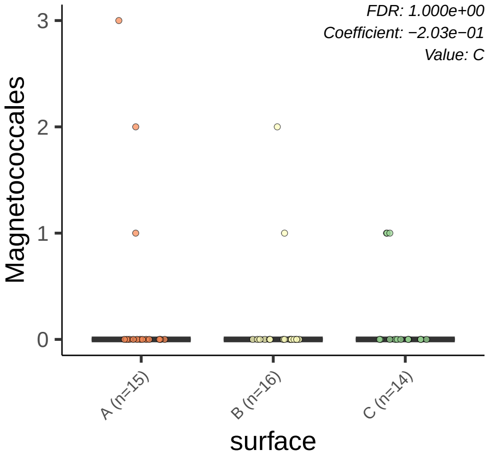
FDR: 1.000e+00
Coefficient: −2.03e−01
Value: C
Magnetococcales
3
2
1
0
A (n=15)
B (n=16)
C (n=14)
surface

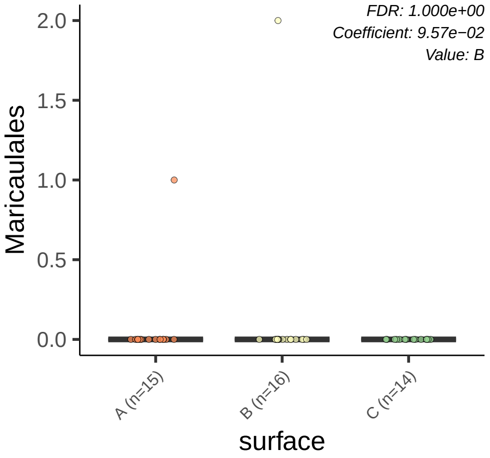

FDR: 1.000e+00
Coefficient: 9.57e−02
Value: B
Maricaulales
2.0
1.5
1.0
0.5
0.0
A (n=15)
B (n=16)
C (n=14)
surface

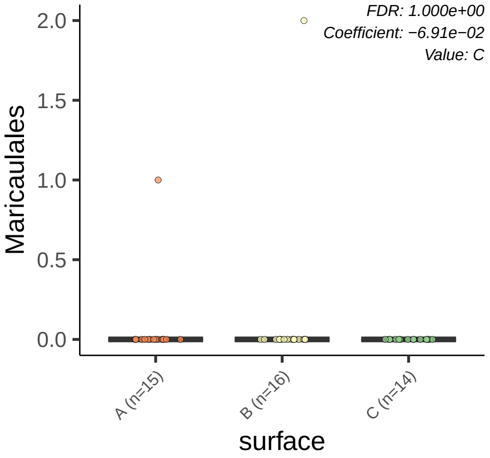

FDR: 1.000e+00
Coefficient: −6.91e−02
Value: C
Maricaulales
2.0
1.5
1.0
0.5
0.0
A (n=15)
B (n=16)
C (n=14)
surface

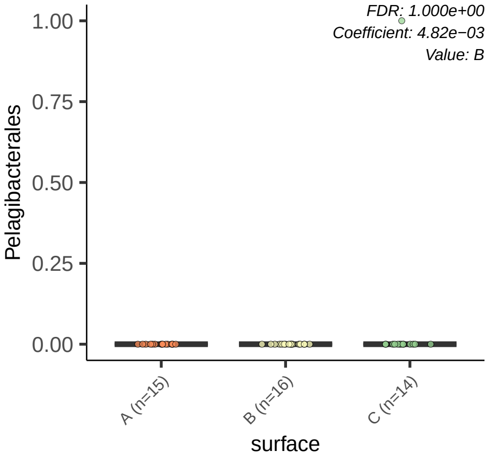

FDR: 1.000e+00
Coefficient: 4.82e−03
Value: B
1.00
0.75
0.50
0.25
0.00
Pelagibacterales
A (n=15)
B (n=16)
C (n=14)
surface

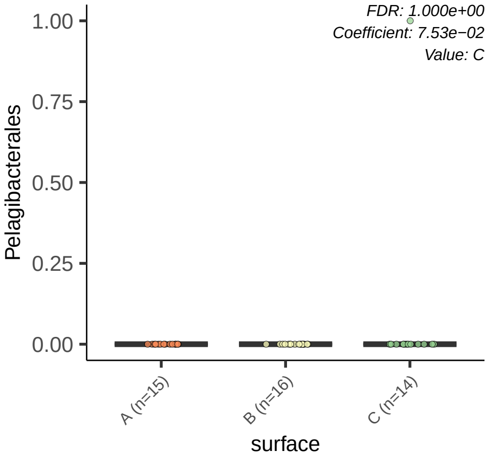
FDR: 1.000e+00
Coefficient: 7.53e−02
Value: C
Pelagibacterales
1.00
0.75
0.50
0.25
0.00
A (n=15)
B (n=16)
C (n=14)
surface

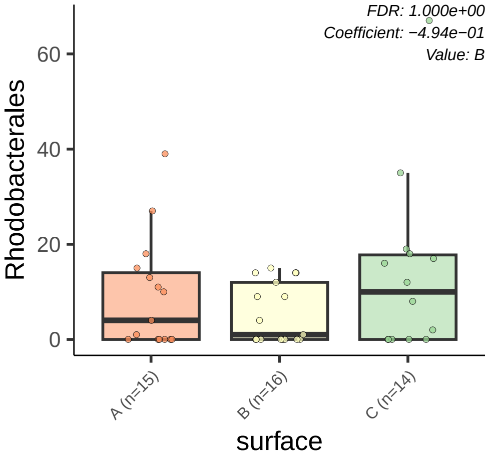

FDR: 1.000e+00
Coefficient: −4.94e−01
Value: B
Rhodobacterales
60
40
20
0
A (n=15)
B (n=16)
C (n=14)
surface

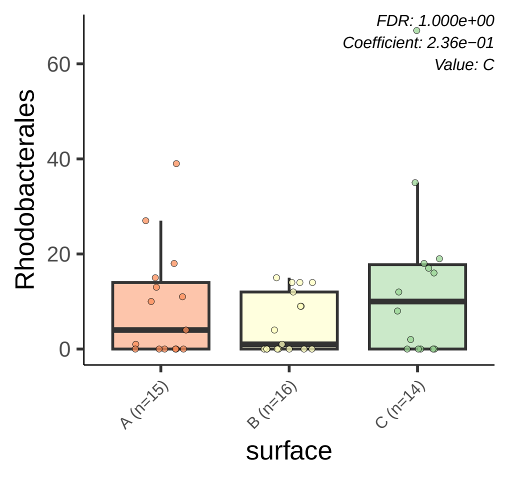

FDR: 1.000e+00
Coefficient: 2.36e−01
Value: C
Rhodobacterales
60
40
20
0
A (n=15)
B (n=16)
C (n=14)
surface

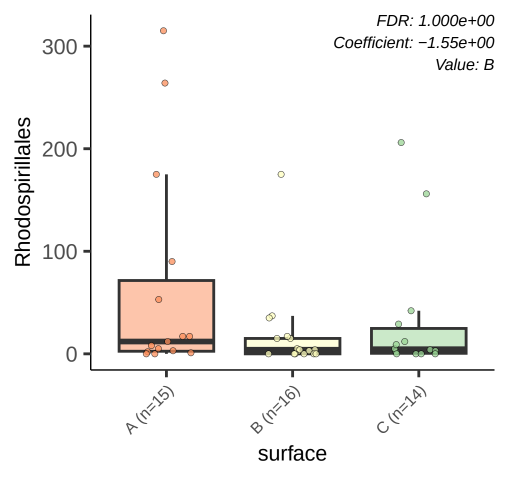

FDR: 1.000e+00
Coefficient: −1.55e+00
Value: B
Rhodospirillales
300
200
100
0
A (n=15)
B (n=16)
C (n=14)
surface

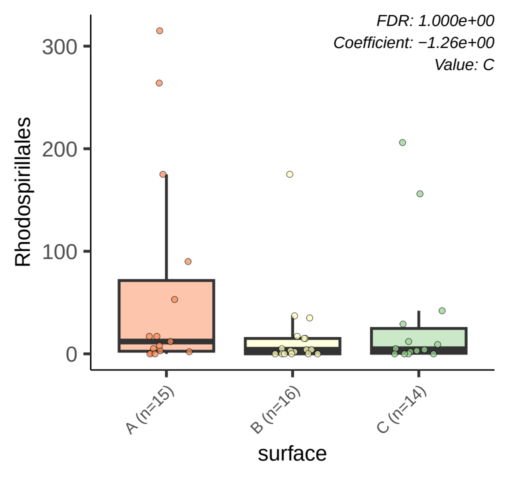
FDR: 1.000e+00
Coefficient: −1.26e+00
Value: C
Rhodospirillales
300
200
100
0
A (n=15)
B (n=16)
C (n=14)
surface

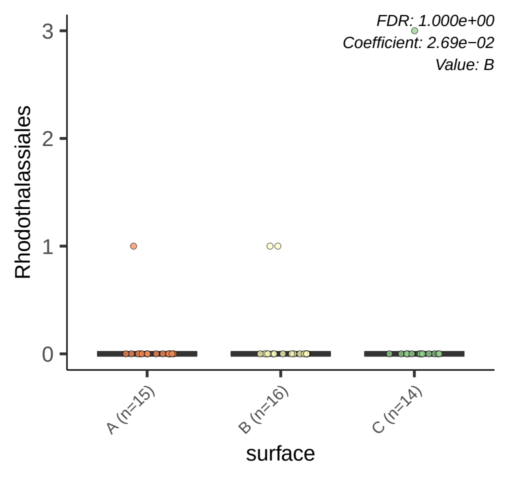
FDR: 1.000e+00
Coefficient: 2.69e−02
Value: B
Rhodothalassiales
3
2
1
0
A (n=15)
B (n=16)
C (n=14)
surface

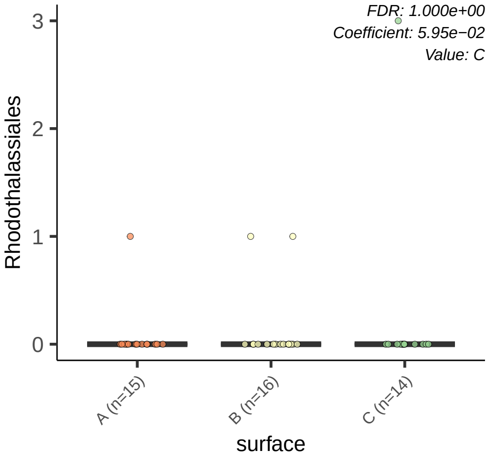
FDR: 1.000e+00
Coefficient: 5.95e−02
Value: C
Rhodothalassiales
3
2
1
0
A (n=15)
B (n=16)
C (n=14)
surface

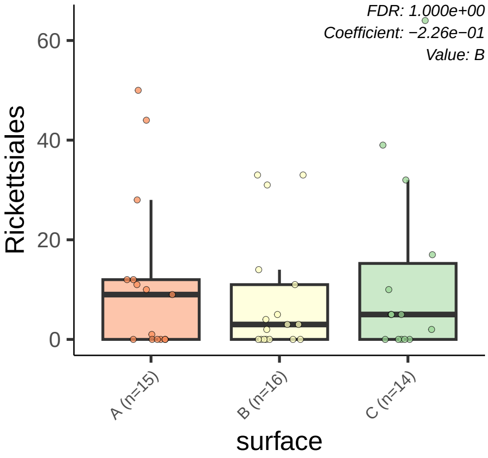

FDR: 1.000e+00
Coefficient: −2.26e−01
Value: B
Rickettsiales
60
40
20
0
A (n=15)
B (n=16)
C (n=14)
surface

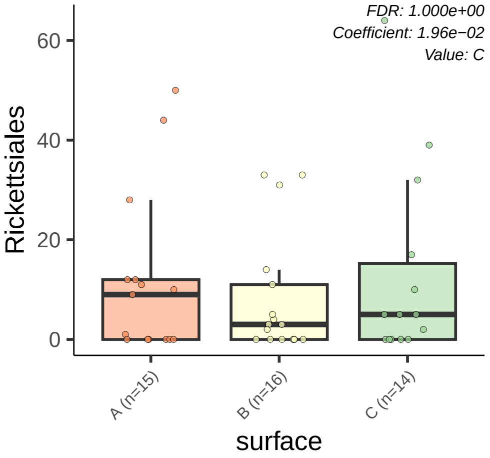

FDR: 1.000e+00
Coefficient: 1.96e−02
Value: C
Rickettsiales
0
20
40
60
A (n=15)
B (n=16)
C (n=14)
surface

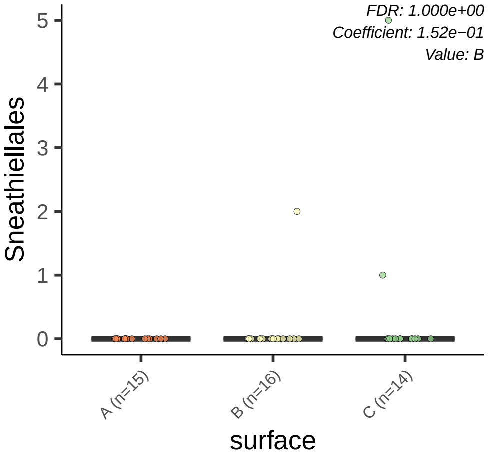
FDR: 1.000e+00
Coefficient: 1.52e−01
Value: B
Sneathiellales
5
4
3
2
1
0
A (n=15)
B (n=16)
C (n=14)
surface

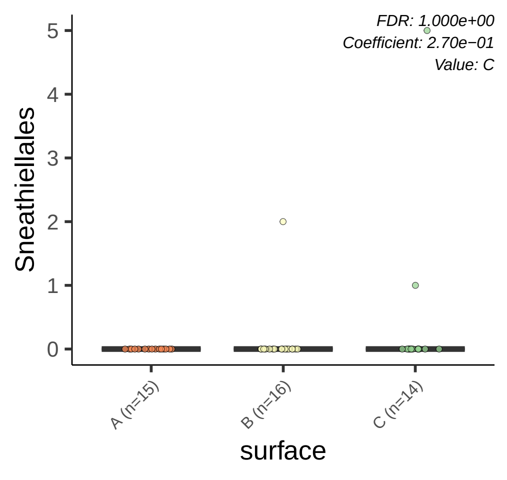
FDR: 1.000e+00
Coefficient: 2.70e−01
Value: C
Sneathiellales
0
1
2
3
4
5
A (n=15)
B (n=16)
C (n=14)
surface

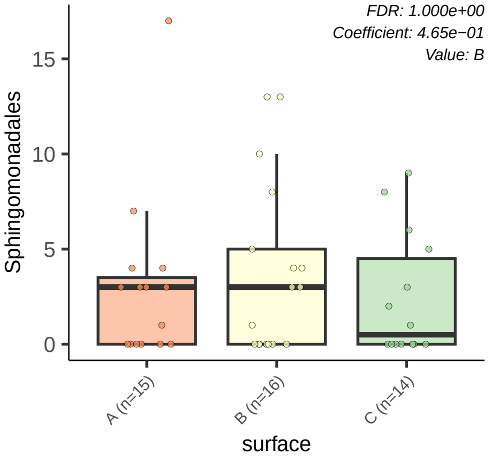
FDR: 1.000e+00
Coefficient: 4.65e−01
Value: B
Sphingomonadales
0
5
10
15
A (n=15)
B (n=16)
C (n=14)
surface

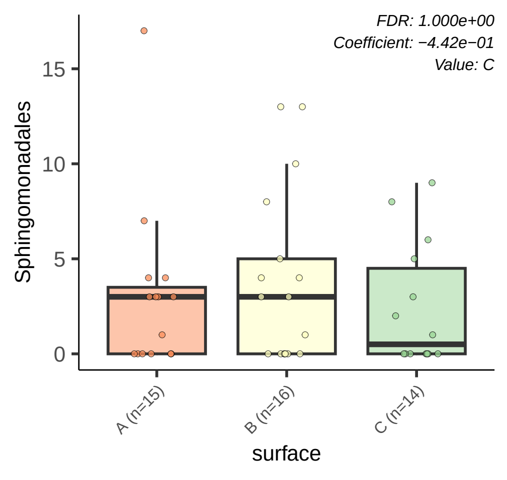
FDR: 1.000e+00
Coefficient: −4.42e−01
Value: C
Sphingomonadales
0
5
10
15
A (n=15)
B (n=16)
C (n=14)
surface

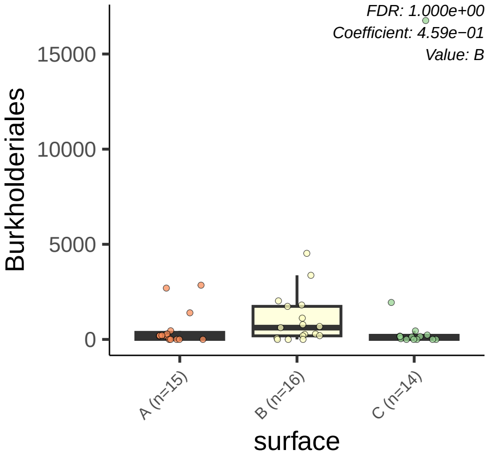

FDR: 1.000e+00
Coefficient: 4.59e−01
Value: B
Burkholderiales
15000
10000
5000
0
A (n=15)
B (n=16)
C (n=14)
surface

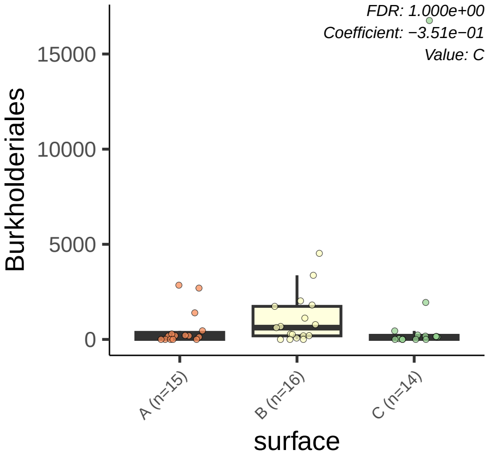
FDR: 1.000e+00
Coefficient: −3.51e−01
Value: C
Burkholderiales
15000
10000
5000
0
A (n=15)
B (n=16)
C (n=14)
surface

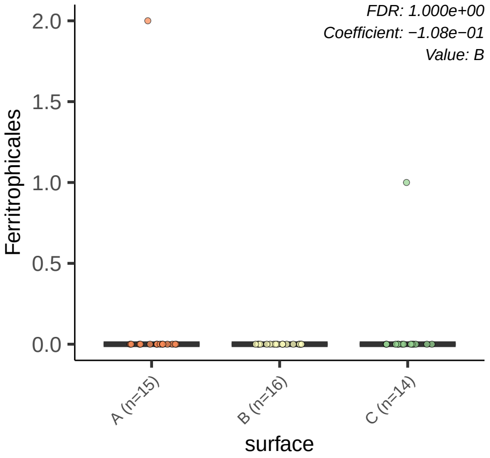

FDR: 1.000e+00
Coefficient: −1.08e−01
Value: B
Ferritrophicales
2.0
1.5
1.0
0.5
0.0
A (n=15)
B (n=16)
C (n=14)
surface

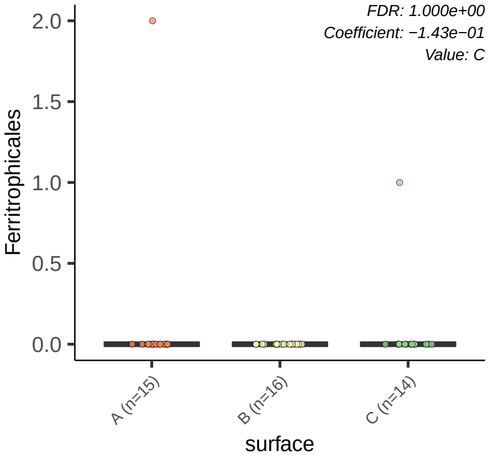

FDR: 1.000e+00
Coefficient: −1.43e−01
Value: C
Ferritrophicales
2.0
1.5
1.0
0.5
0.0
A (n=15)
B (n=16)
C (n=14)
surface

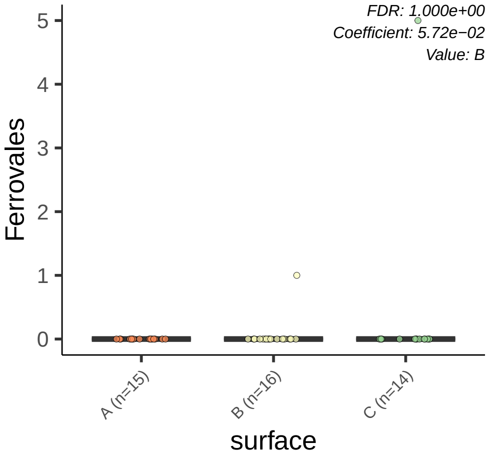
FDR: 1.000e+00
Coefficient: 5.72e−02
Value: B
Ferrovales
5
4
3
2
1
0
A (n=15)
B (n=16)
C (n=14)
surface

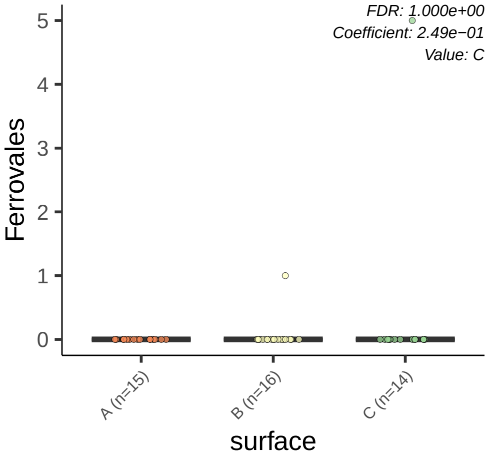
FDR: 1.000e+00
Coefficient: 2.49e−01
Value: C
Ferrovales
5
4
3
2
1
0
A (n=15)
B (n=16)
C (n=14)
surface

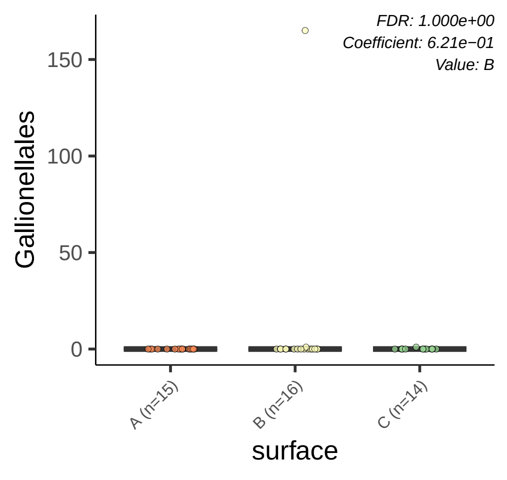
FDR: 1.000e+00
Coefficient: 6.21e−01
Value: B
Gallionellales
150
100
50
0
A (n=15)
B (n=16)
C (n=14)
surface

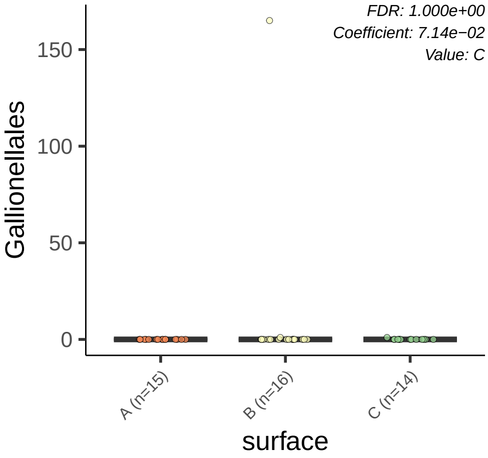
FDR: 1.000e+00
Coefficient: 7.14e−02
Value: C
Gallionellales
150
100
50
0
A (n=15)
B (n=16)
C (n=14)
surface

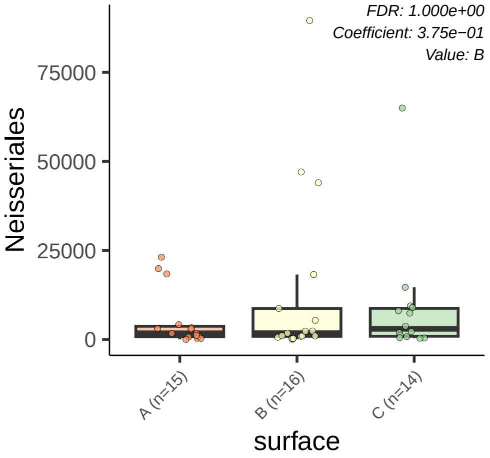

FDR: 1.000e+00
Coefficient: 3.75e−01
Value: B
Neisseriales
75000
50000
25000
0
A (n=15)
B (n=16)
C (n=14)
surface

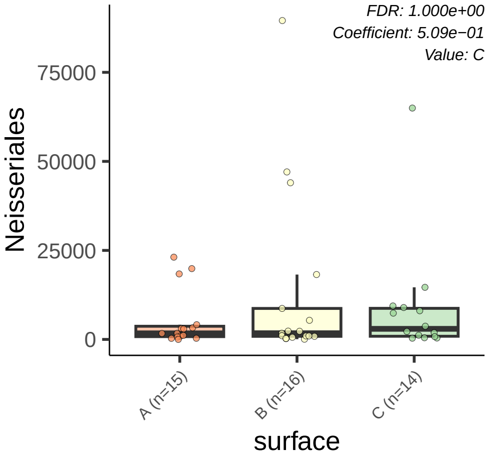

FDR: 1.000e+00
Coefficient: 5.09e−01
Value: C
Neisseriales
75000
50000
25000
0
A (n=15)
B (n=16)
C (n=14)
surface

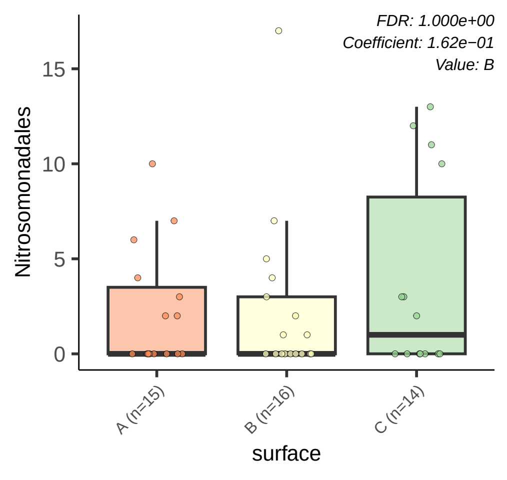

FDR: 1.000e+00
Coefficient: 1.62e−01
Value: B
Nitrosomonadales
0
5
10
15
A (n=15)
B (n=16)
C (n=14)
surface

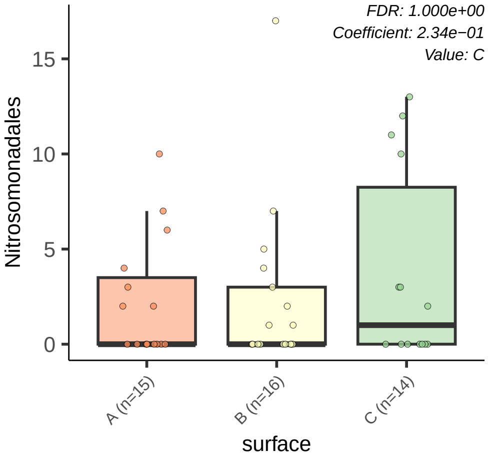
FDR: 1.000e+00
Coefficient: 2.34e−01
Value: C
Nitrosomonadales
0
5
10
15
A (n=15)
B (n=16)
C (n=14)
surface

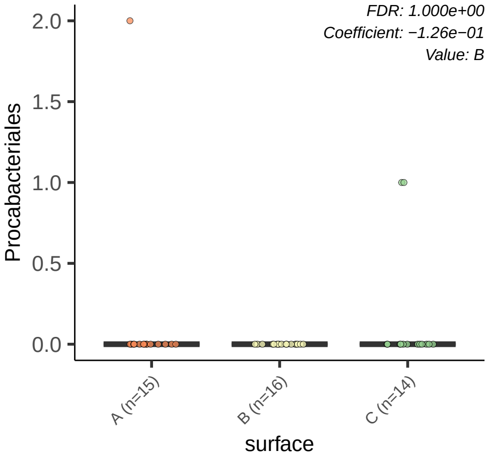
FDR: 1.000e+00
Coefficient: −1.26e−01
Value: B
Procabacteriales
2.0
1.5
1.0
0.5
0.0
A (n=15)
B (n=16)
C (n=14)
surface

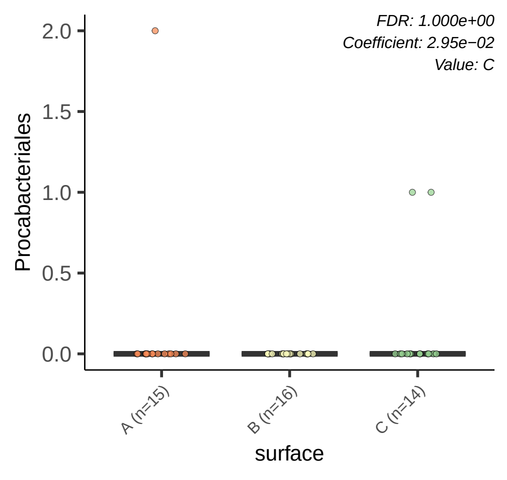

FDR: 1.000e+00
Coefficient: 2.95e−02
Value: C
Procabacteriales
2.0
1.5
1.0
0.5
0.0
A (n=15)
B (n=16)
C (n=14)
surface

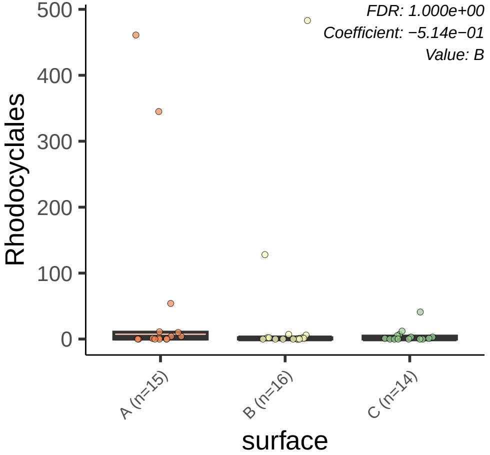
FDR: 1.000e+00
Coefficient: −5.14e−01
Value: B
Rhodocyclales
500
400
300
200
100
0
A (n=15)
B (n=16)
C (n=14)
surface

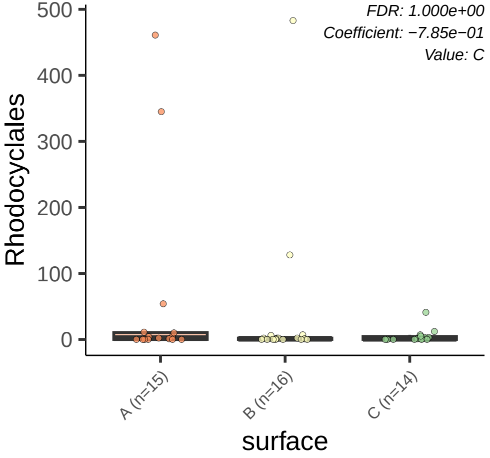

FDR: 1.000e+00
Coefficient: −7.85e−01
Value: C
Rhodocyclales
500
400
300
200
100
0
A (n=15)
B (n=16)
C (n=14)
surface

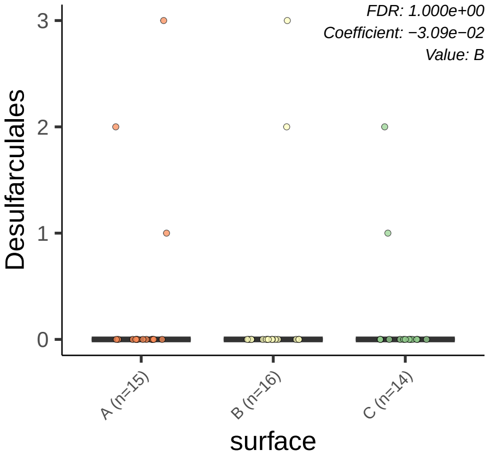
FDR: 1.000e+00
Coefficient: −3.09e−02
Value: B
Desulfarculales
3
2
1
0
A (n=15)
B (n=16)
C (n=14)
surface

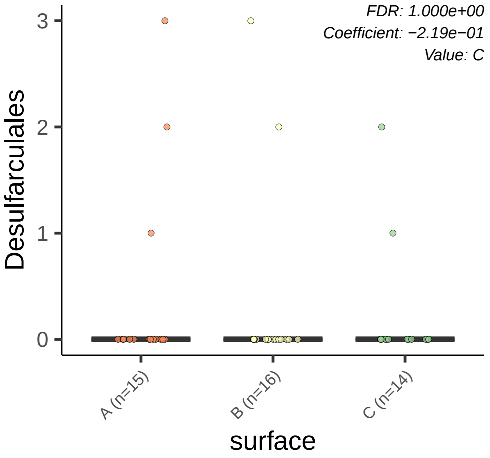
FDR: 1.000e+00
Coefficient: −2.19e−01
Value: C
Desulfarculales
3
2
1
0
A (n=15)
B (n=16)
C (n=14)
surface

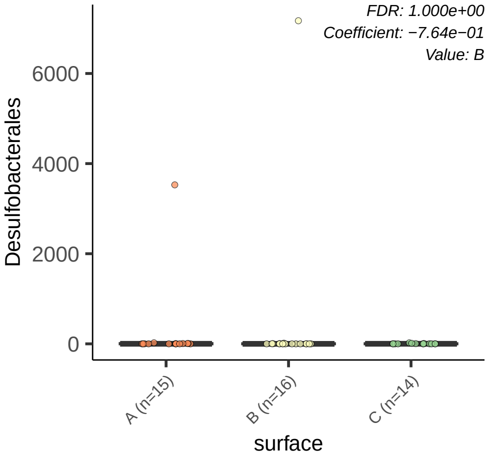
FDR: 1.000e+00
Coefficient: −7.64e−01
Value: B
Desulfobacterales
6000
4000
2000
0
A (n=15)
B (n=16)
C (n=14)
surface

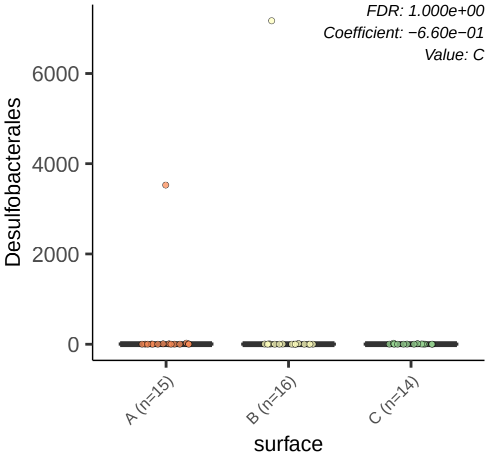

FDR: 1.000e+00
Coefficient: −6.60e−01
Value: C
Desulfobacterales
6000
4000
2000
0
A (n=15)
B (n=16)
C (n=14)
surface

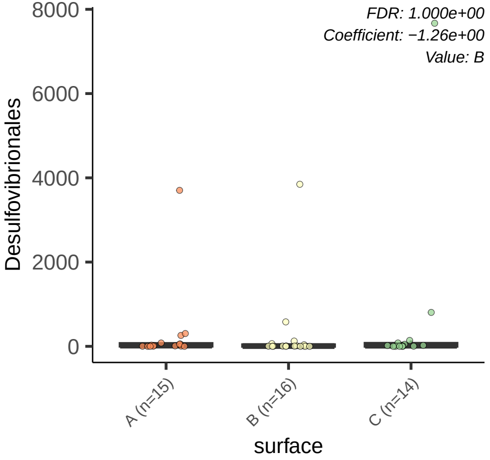
FDR: 1.000e+00
Coefficient: −1.26e+00
Value: B
8000
6000
4000
2000
0
Desulfovibrionales
A (n=15)
B (n=16)
C (n=14)
surface

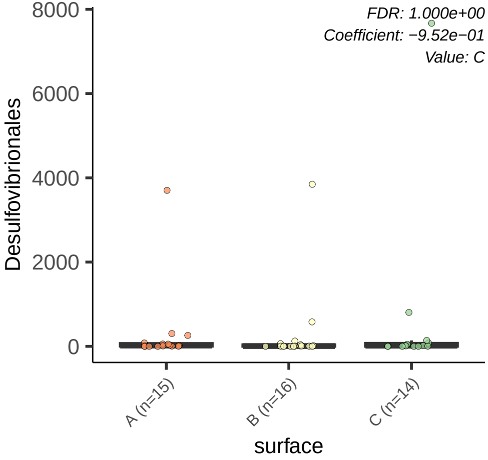
FDR: 1.000e+00
Coefficient: −9.52e−01
Value: C
Desulfovibrionales
8000
6000
4000
2000
0
A (n=15)
B (n=16)
C (n=14)
surface

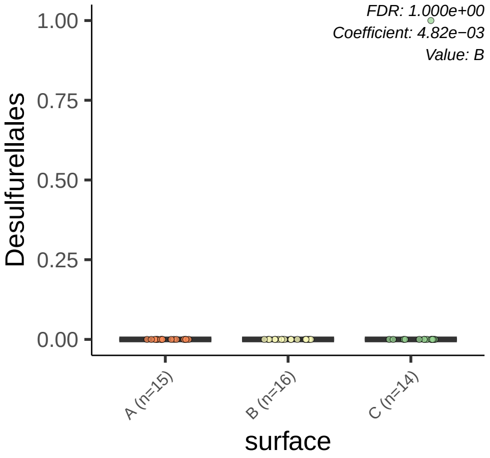

FDR: 1.000e+00
Coefficient: 4.82e−03
Value: B
Desulfurellales
1.00
0.75
0.50
0.25
0.00
A (n=15)
B (n=16)
C (n=14)
surface

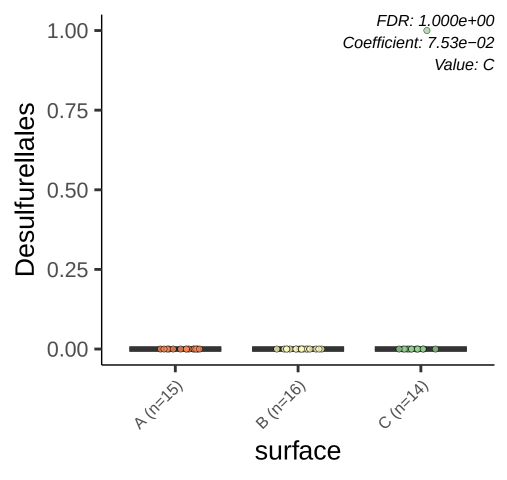
FDR: 1.000e+00
Coefficient: 7.53e−02
Value: C
Desulfurellales
1.00
0.75
0.50
0.25
0.00
A (n=15)
B (n=16)
C (n=14)
surface

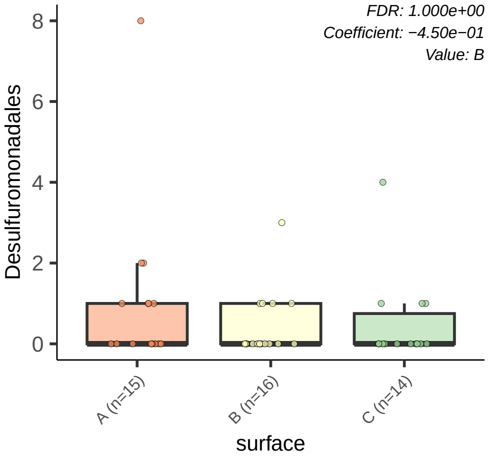

FDR: 1.000e+00
Coefficient: −4.50e−01
Value: B
Desulfuromonadales
0
2
4
6
8
A (n=15)
B (n=16)
C (n=14)
surface

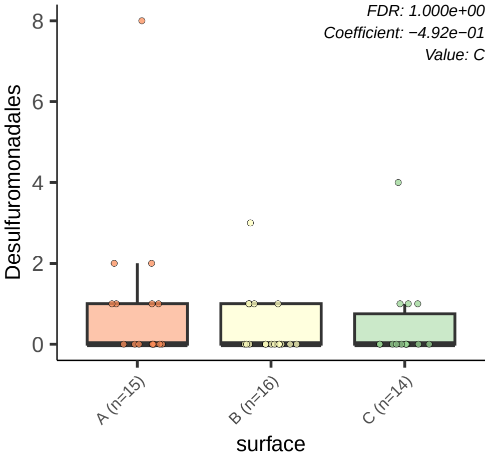
FDR: 1.000e+00
Coefficient: −4.92e−01
Value: C
Desulfuromonadales
8
6
4
2
0
A (n=15)
B (n=16)
C (n=14)
surface

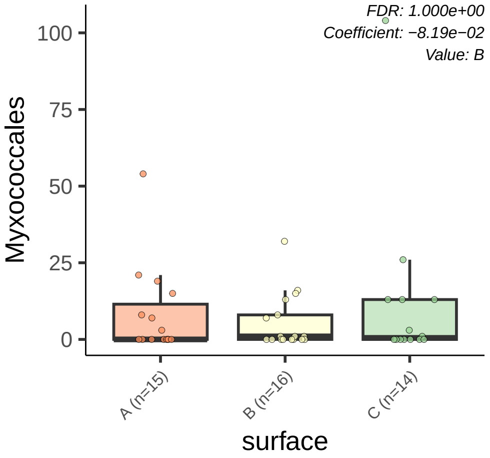

FDR: 1.000e+00
Coefficient: −8.19e−02
Value: B
Myxococcales
100
75
50
25
0
A (n=15)
B (n=16)
C (n=14)
surface

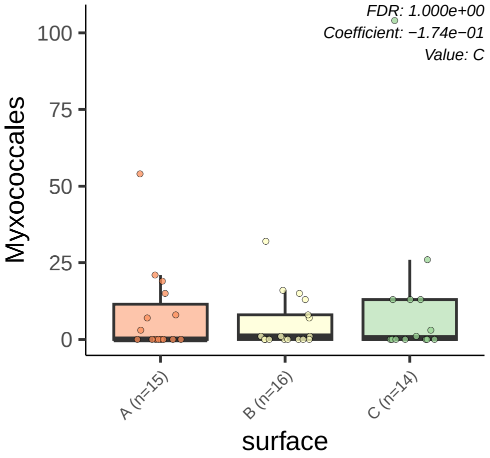

FDR: 1.000e+00
Coefficient: −1.74e−01
Value: C
Myxococcales
100
75
50
25
0
A (n=15)
B (n=16)
C (n=14)
surface

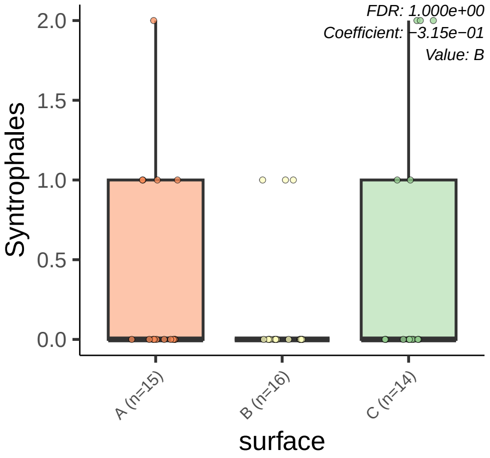

FDR: 1.000e+00
Coefficient: −3.15e−01
Value: B
Syntrophales
2.0
1.5
1.0
0.5
0.0
A (n=15)
B (n=16)
C (n=14)
surface

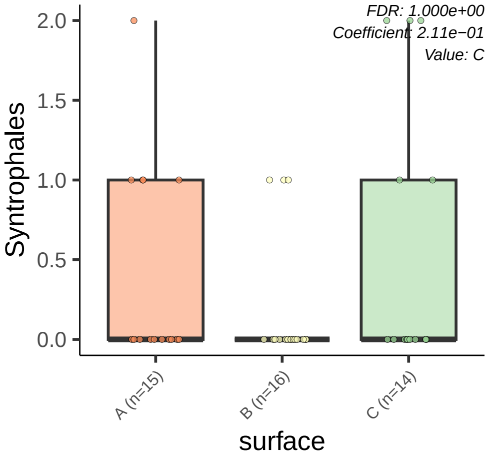
FDR: 1.000e+00
Coefficient: 2.11e−01
Value: C
Syntrophales
2.0
1.5
1.0
0.5
0.0
A (n=15)
B (n=16)
C (n=14)
surface

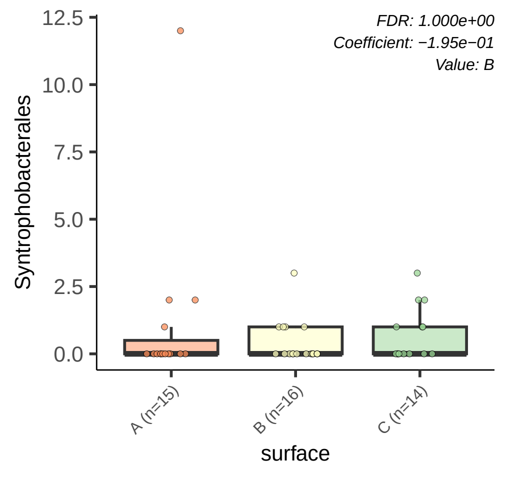
FDR: 1.000e+00
Coefficient: −1.95e−01
Value: B
Syntrophobacterales
12.5
10.0
7.5
5.0
2.5
0.0
A (n=15)
B (n=16)
C (n=14)
surface

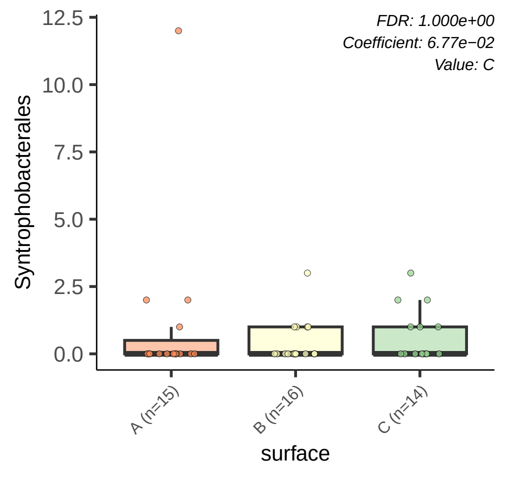
FDR: 1.000e+00
Coefficient: 6.77e−02
Value: C
Syntrophobacterales
12.5
10.0
7.5
5.0
2.5
0.0
A (n=15)
B (n=16)
C (n=14)
surface

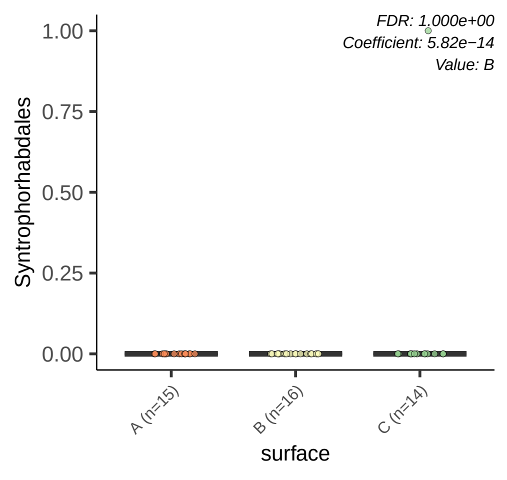
FDR: 1.000e+00
Coefficient: 5.82e−14
Value: B
Syntrophorhabdales
1.00
0.75
0.50
0.25
0.00
A (n=15)
B (n=16)
C (n=14)
surface

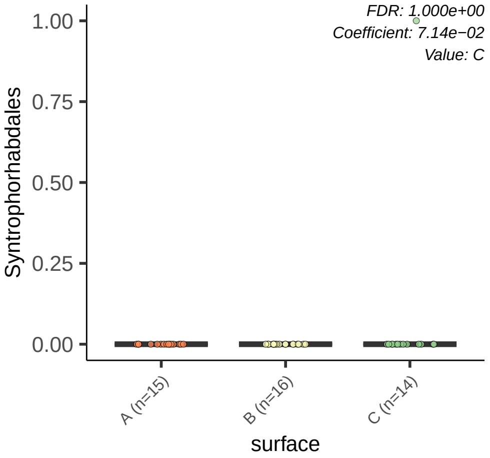
FDR: 1.000e+00
Coefficient: 7.14e−02
Value: C
Syntrophorhabdales
1.00
0.75
0.50
0.25
0.00
A (n=15)
B (n=16)
C (n=14)
surface

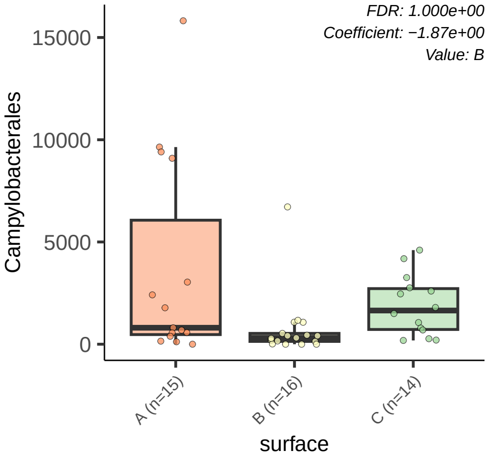

FDR: 1.000e+00
Coefficient: −1.87e+00
Value: B
Campylobacterales
15000
10000
5000
0
A (n=15)
B (n=16)
C (n=14)
surface

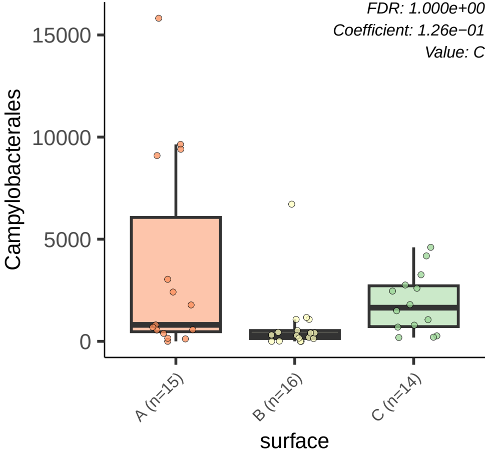
FDR: 1.000e+00
Coefficient: 1.26e−01
Value: C
Campylobacterales
15000
10000
5000
0
A (n=15)
B (n=16)
C (n=14)
surface

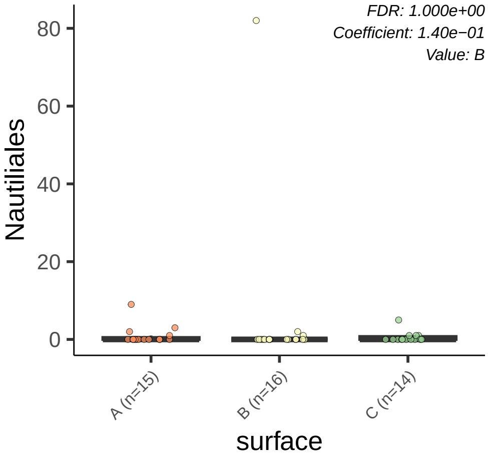
FDR: 1.000e+00
Coefficient: 1.40e−01
Value: B
Nautiliales
0
20
40
60
80
A (n=15)
B (n=16)
C (n=14)
surface

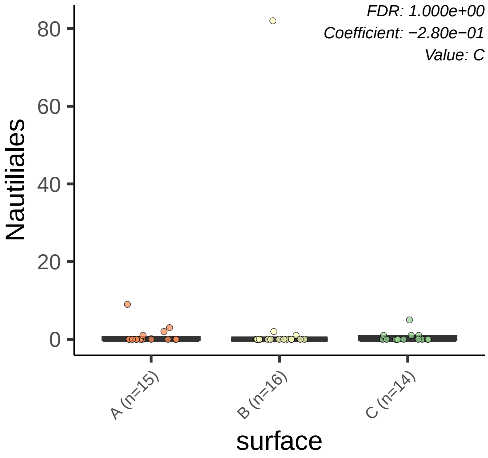
FDR: 1.000e+00
Coefficient: −2.80e−01
Value: C
Nautiliales
80
60
40
20
0
A (n=15)
B (n=16)
C (n=14)
surface

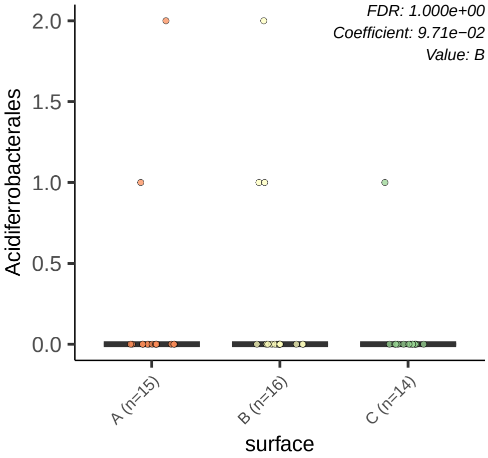
FDR: 1.000e+00
Coefficient: 9.71e−02
Value: B
Acidiferrobacterales
2.0
1.5
1.0
0.5
0.0
A (n=15)
B (n=16)
C (n=14)
surface

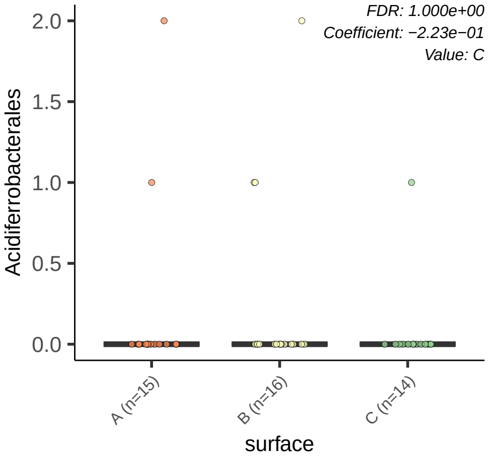

FDR: 1.000e+00
Coefficient: −2.23e−01
Value: C
Acidiferrobacterales
2.0
1.5
1.0
0.5
0.0
A (n=15)
B (n=16)
C (n=14)
surface

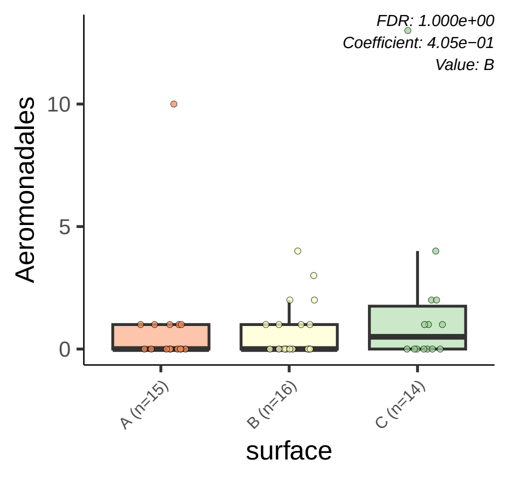
FDR: 1.000e+00
Coefficient: 4.05e−01
Value: B
Aeromonadales
0
5
10
A (n=15)
B (n=16)
C (n=14)
surface

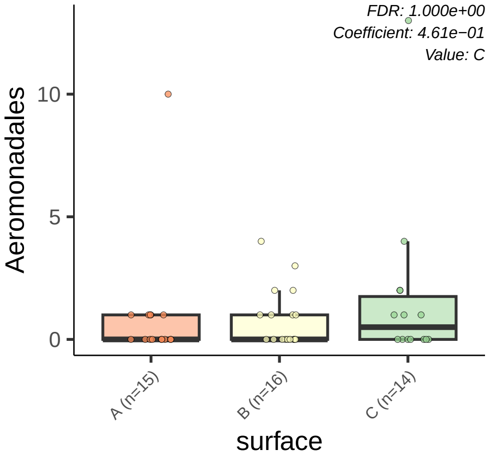
FDR: 1.000e+00
Coefficient: 4.61e−01
Value: C
Aeromonadales
10
5
0
A (n=15)
B (n=16)
C (n=14)
surface

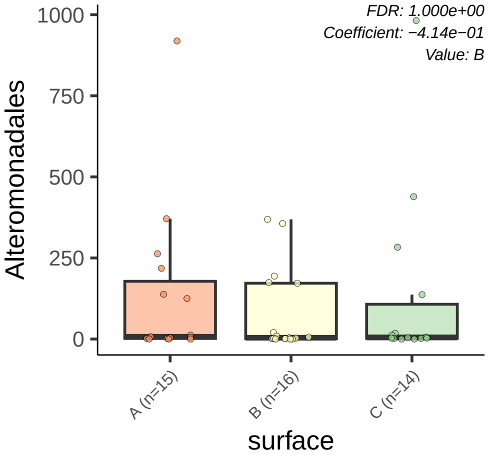
FDR: 1.000e+00
Coefficient: −4.14e−01
Value: B
Alteromonadales
1000
750
500
250
0
A (n=15)
B (n=16)
C (n=14)
surface

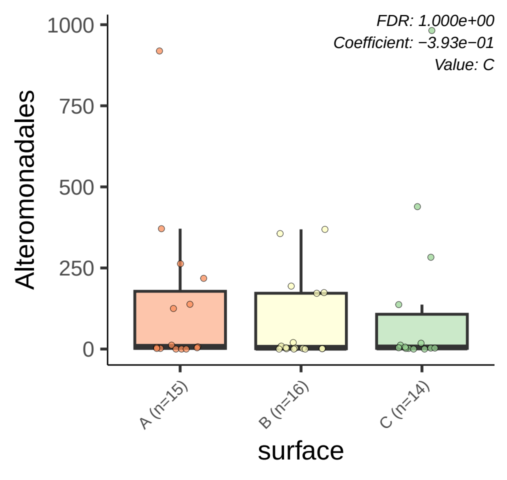
FDR: 1.000e+00
Coefficient: −3.93e−01
Value: C
Alteromonadales
1000
750
500
250
0
A (n=15)
B (n=16)
C (n=14)
surface

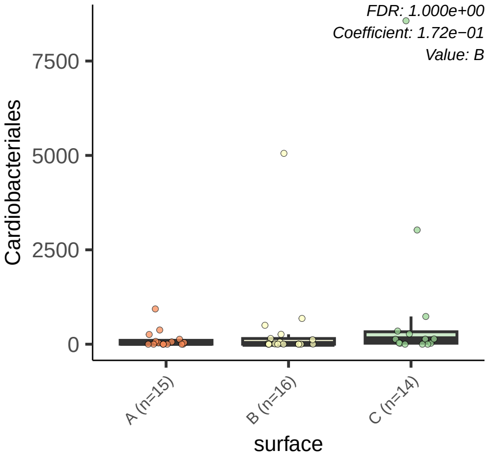

FDR: 1.000e+00
Coefficient: 1.72e−01
Value: B
Cardiobacteriales
7500
5000
2500
0
A (n=15)
B (n=16)
C (n=14)
surface

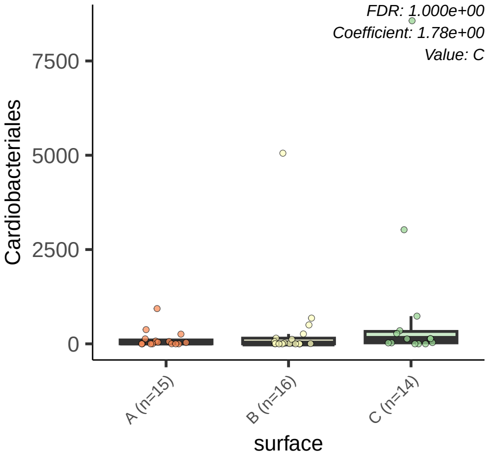
FDR: 1.000e+00
Coefficient: 1.78e+00
Value: C
Cardiobacteriales
7500
5000
2500
0
A (n=15)
B (n=16)
C (n=14)
surface

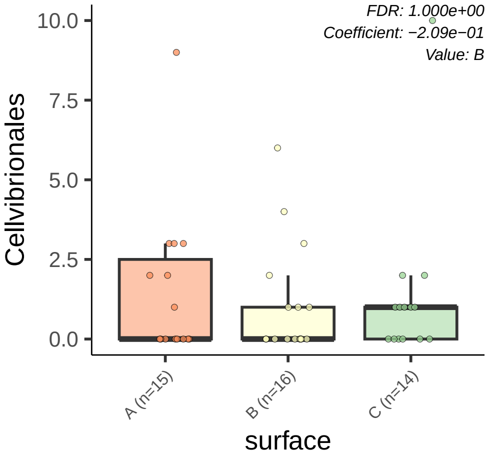

FDR: 1.000e+00
Coefficient: −2.09e−01
Value: B
Cellvibrionales
10.0
7.5
5.0
2.5
0.0
A (n=15)
B (n=16)
C (n=14)
surface

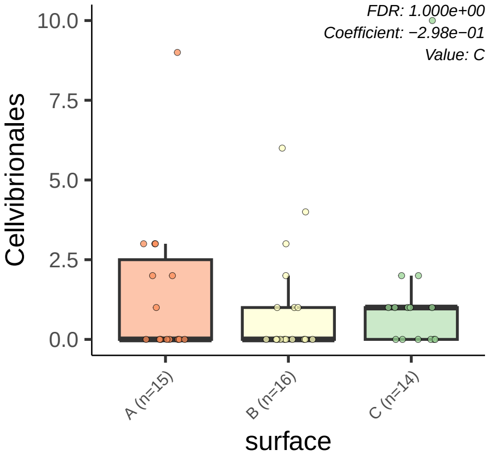
FDR: 1.000e+00
Coefficient: −2.98e−01
Value: C
10.0
7.5
5.0
2.5
0.0
Cellvibrionales
A (n=15)
B (n=16)
C (n=14)
surface

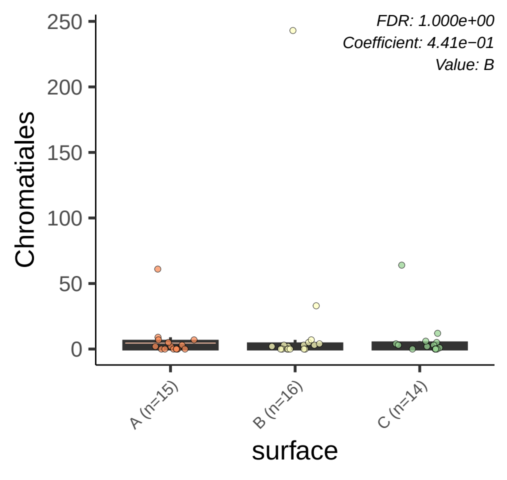
FDR: 1.000e+00
Coefficient: 4.41e−01
Value: B
Chromatiales
250
200
150
100
50
0
A (n=15)
B (n=16)
C (n=14)
surface

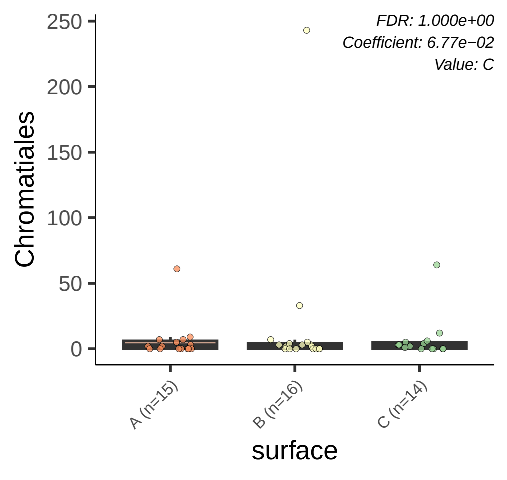

FDR: 1.000e+00
Coefficient: 6.77e−02
Value: C
Chromatiales
250
200
150
100
50
0
A (n=15)
B (n=16)
C (n=14)
surface

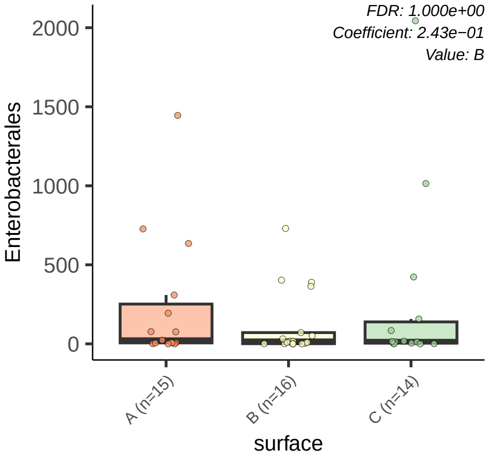
FDR: 1.000e+00
Coefficient: 2.43e−01
Value: B
Enterobacterales
2000
1500
1000
500
0
A (n=15)
B (n=16)
C (n=14)
surface

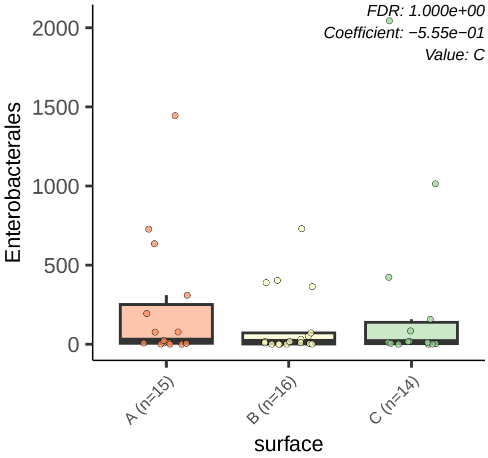

FDR: 1.000e+00
Coefficient: −5.55e−01
Value: C
2000
1500
1000
500
0
Enterobacterales
A (n=15)
B (n=16)
C (n=14)
surface

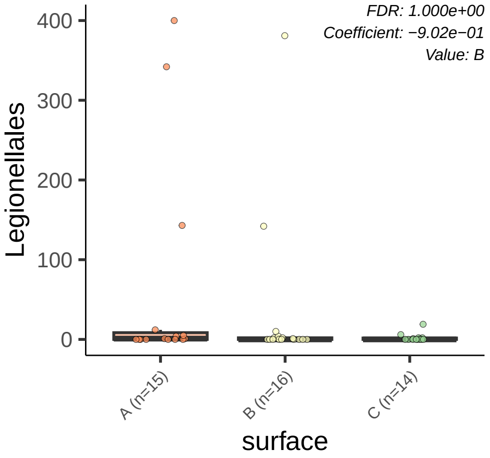

FDR: 1.000e+00
Coefficient: −9.02e−01
Value: B
Legionellales
400
300
200
100
0
A (n=15)
B (n=16)
C (n=14)
surface

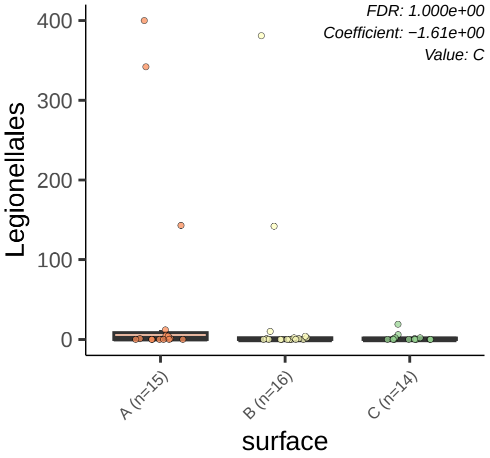

FDR: 1.000e+00
Coefficient: −1.61e+00
Value: C
Legionellales
400
300
200
100
0
A (n=15)
B (n=16)
C (n=14)
surface

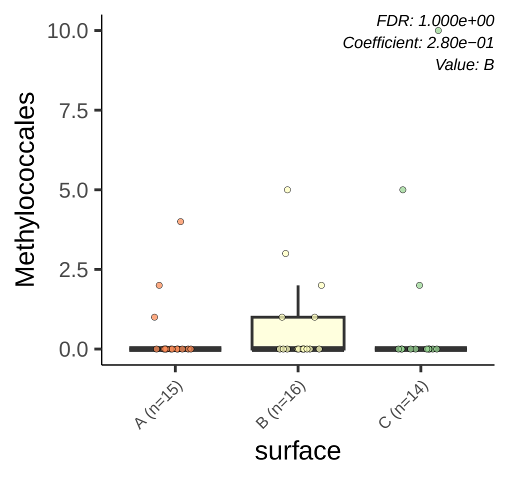

FDR: 1.000e+00
Coefficient: 2.80e−01
Value: B
Methylococcales
10.0
7.5
5.0
2.5
0.0
A (n=15)
B (n=16)
C (n=14)
surface

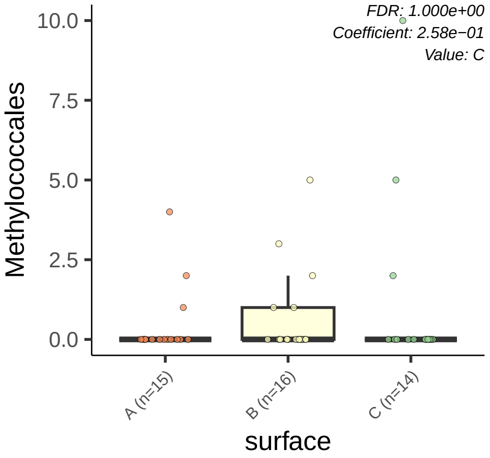

FDR: 1.000e+00
Coefficient: 2.58e−01
Value: C
Methylococcales
10.0
7.5
5.0
2.5
0.0
A (n=15)
B (n=16)
C (n=14)
surface

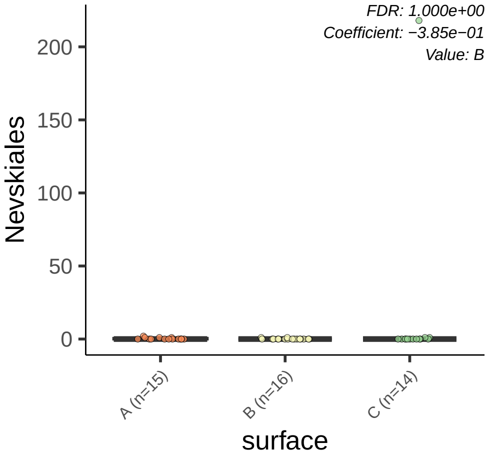
FDR: 1.000e+00
Coefficient: −3.85e−01
Value: B
Nevskiales
200
150
100
50
0
A (n=15)
B (n=16)
C (n=14)
surface

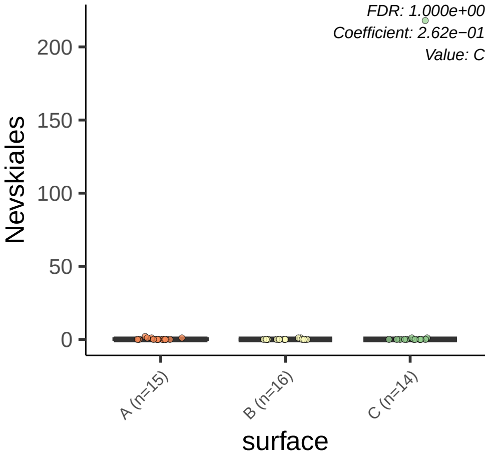
FDR: 1.000e+00
Coefficient: 2.62e−01
Value: C
Nevskiales
200
150
100
50
0
A (n=15)
B (n=16)
C (n=14)
surface

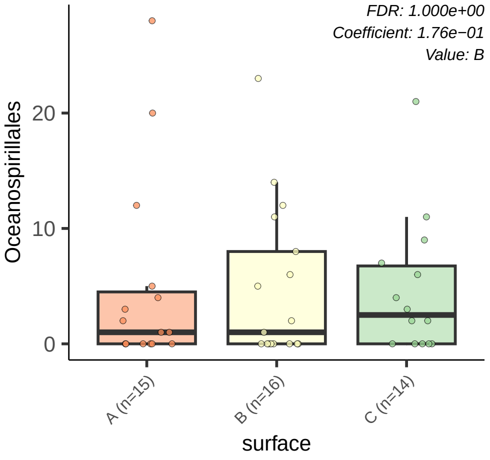
FDR: 1.000e+00
Coefficient: 1.76e−01
Value: B
Oceanospirillales
0
10
20
A (n=15)
B (n=16)
C (n=14)
surface

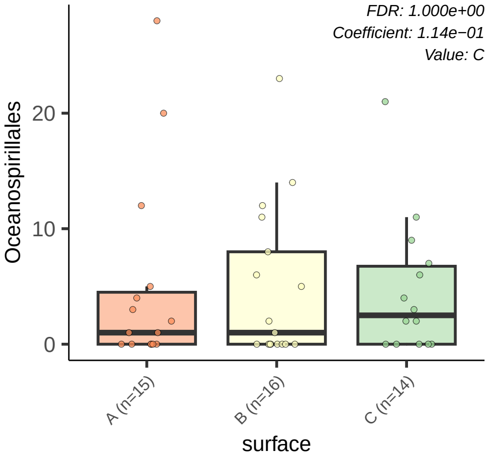

FDR: 1.000e+00
Coefficient: 1.14e−01
Value: C
Oceanospirillales
0
10
20
A (n=15)
B (n=16)
C (n=14)
surface

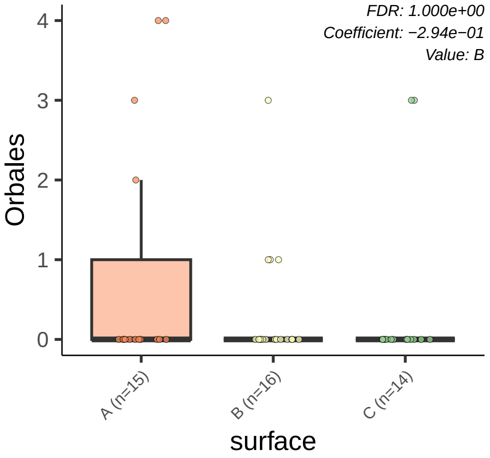
FDR: 1.000e+00
Coefficient: −2.94e−01
Value: B
Orbales
4
3
2
1
0
A (n=15)
B (n=16)
C (n=14)
surface

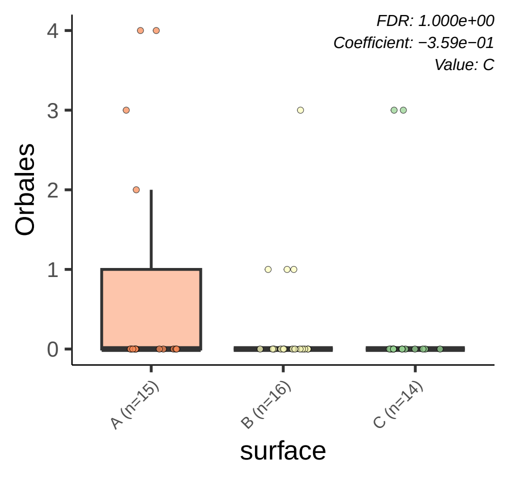

FDR: 1.000e+00
Coefficient: −3.59e−01
Value: C
Orbales
4
3
2
1
0
A (n=15)
B (n=16)
C (n=14)
surface

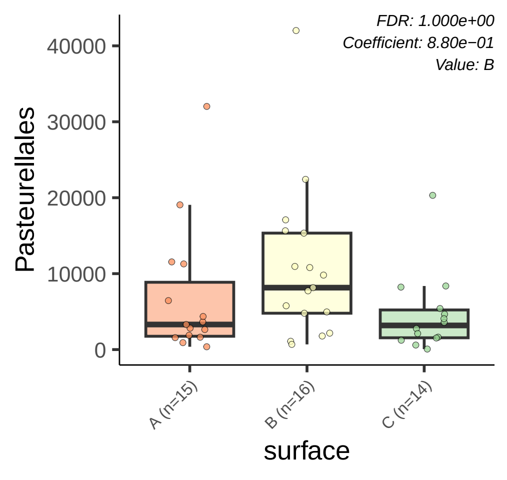

FDR: 1.000e+00
Coefficient: 8.80e−01
Value: B
Pasteurellales
40000
30000
20000
10000
0
A (n=15)
B (n=16)
C (n=14)
surface

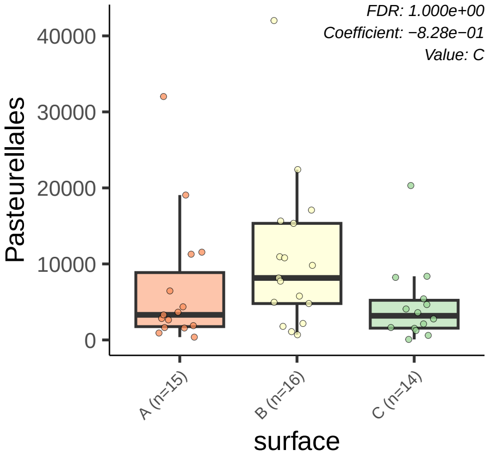
FDR: 1.000e+00
Coefficient: −8.28e−01
Value: C
Pasteurellales
40000
30000
20000
10000
0
A (n=15)
B (n=16)
C (n=14)
surface

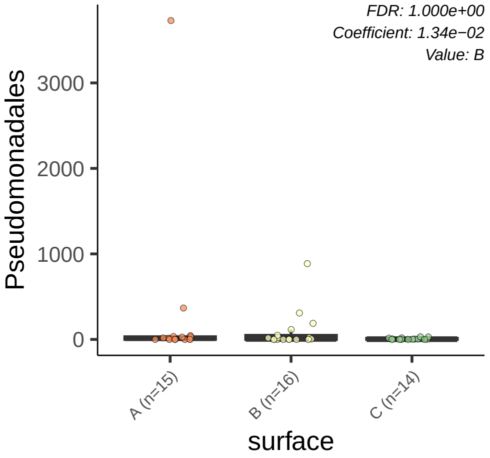
FDR: 1.000e+00
Coefficient: 1.34e−02
Value: B
Pseudomonadales
3000
2000
1000
0
A (n=15)
B (n=16)
C (n=14)
surface

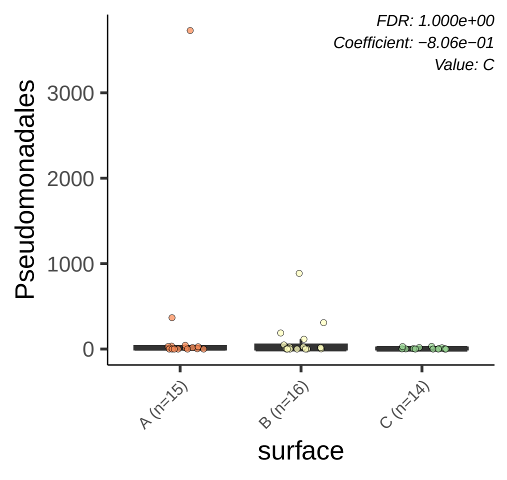

FDR: 1.000e+00
Coefficient: −8.06e−01
Value: C
Pseudomonadales
3000
2000
1000
0
A (n=15)
B (n=16)
C (n=14)
surface

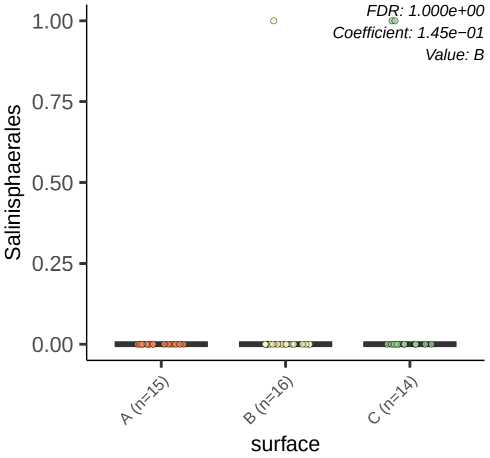

FDR: 1.000e+00
Coefficient: 1.45e−01
Value: B
Salinisphaerales
1.00
0.75
0.50
0.25
0.00
A (n=15)
B (n=16)
C (n=14)
surface

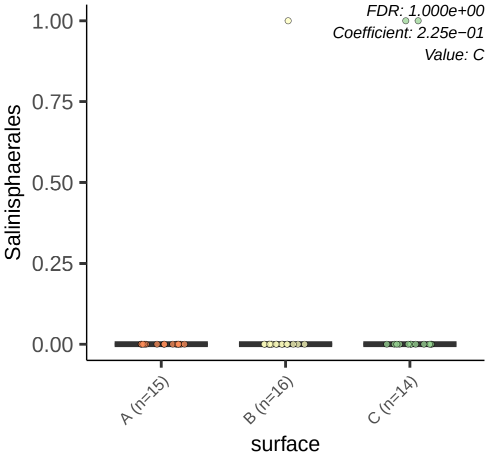
FDR: 1.000e+00
Coefficient: 2.25e−01
Value: C
Salinisphaerales
1.00
0.75
0.50
0.25
0.00
A (n=15)
B (n=16)
C (n=14)
surface

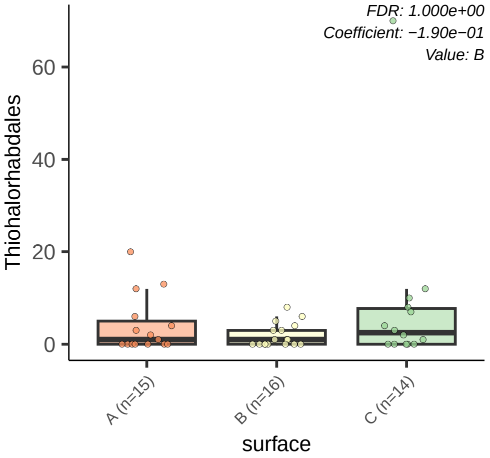

FDR: 1.000e+00
Coefficient: −1.90e−01
Value: B
Thiohalorhabdales
0
20
40
60
A (n=15)
B (n=16)
C (n=14)
surface

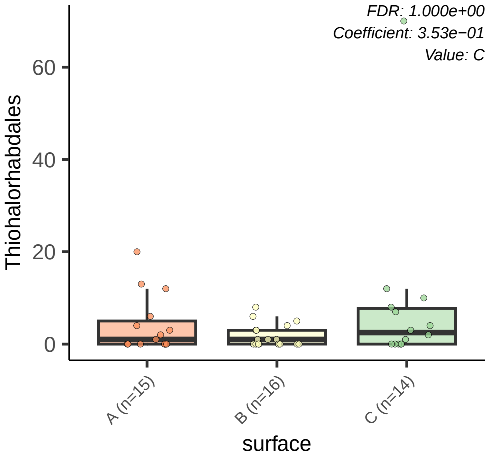

FDR: 1.000e+00
Coefficient: 3.53e−01
Value: C
Thiohalorhabdales
0
20
40
60
A (n=15)
B (n=16)
C (n=14)
surface

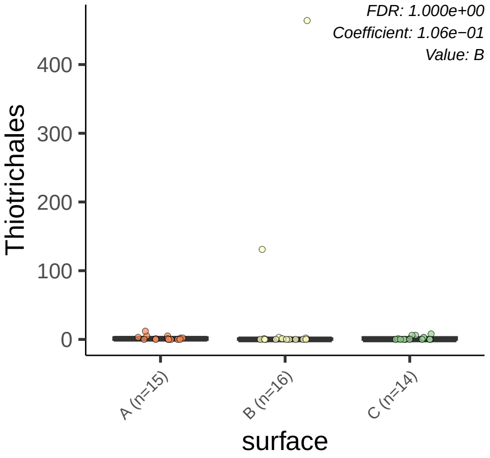
FDR: 1.000e+00
Coefficient: 1.06e−01
Value: B
Thiotrichales
400
300
200
100
0
A (n=15)
B (n=16)
C (n=14)
surface

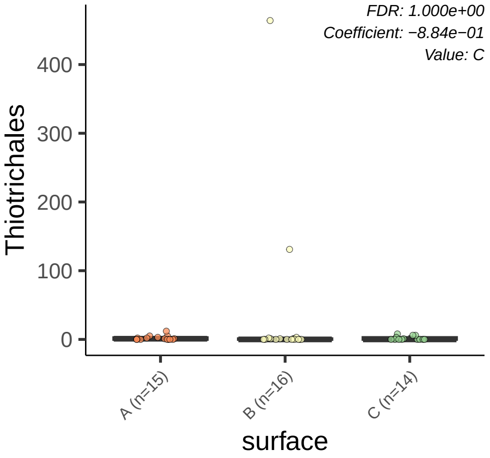
FDR: 1.000e+00
Coefficient: −8.84e−01
Value: C
Thiotrichales
400
300
200
100
0
A (n=15)
B (n=16)
C (n=14)
surface

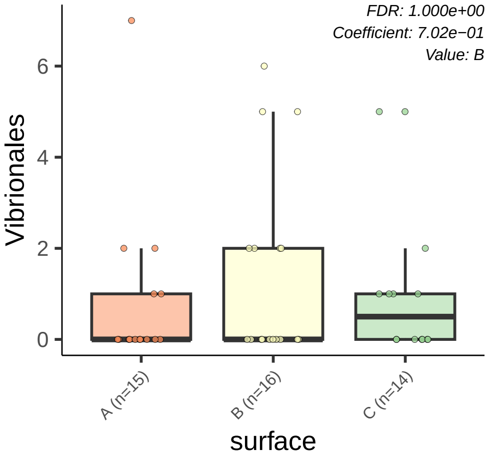

FDR: 1.000e+00
Coefficient: 7.02e−01
Value: B
Vibrionales
0
2
4
6
A (n=15)
B (n=16)
C (n=14)
surface

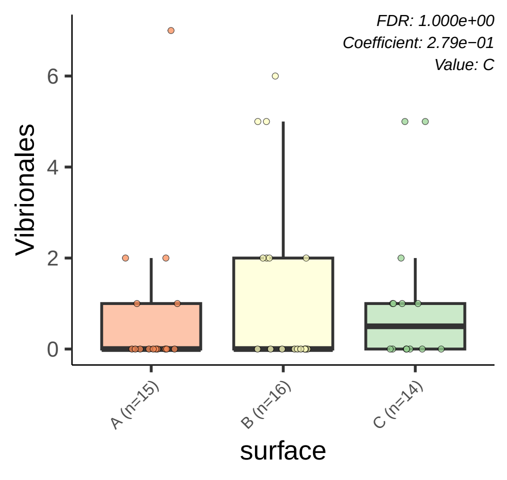
FDR: 1.000e+00
Coefficient: 2.79e−01
Value: C
Vibrionales
0
2
4
6
A (n=15)
B (n=16)
C (n=14)
surface

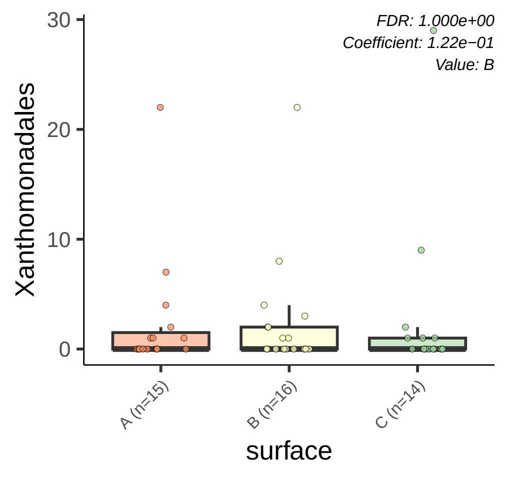

FDR: 1.000e+00
Coefficient: 1.22e−01
Value: B
Xanthomonadales
0
10
20
30
A (n=15)
B (n=16)
C (n=14)
surface

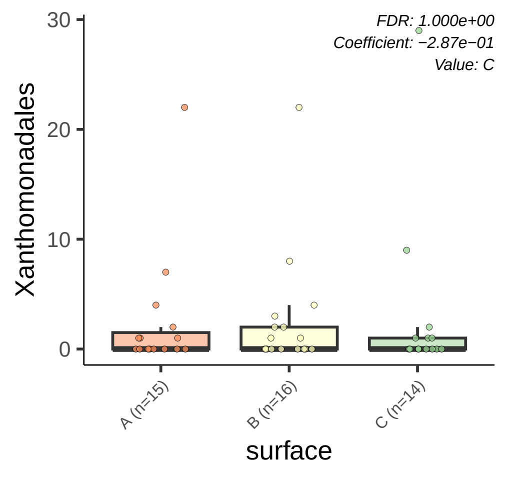
FDR: 1.000e+00
Coefficient: −2.87e−01
Value: C
Xanthomonadales
30
20
10
0
A (n=15)
B (n=16)
C (n=14)
surface

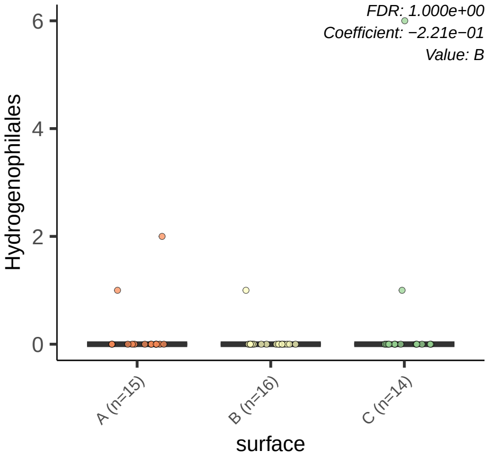
FDR: 1.000e+00
Coefficient: −2.21e−01
Value: B
Hydrogenophilales
0
2
4
6
A (n=15)
B (n=16)
C (n=14)
surface

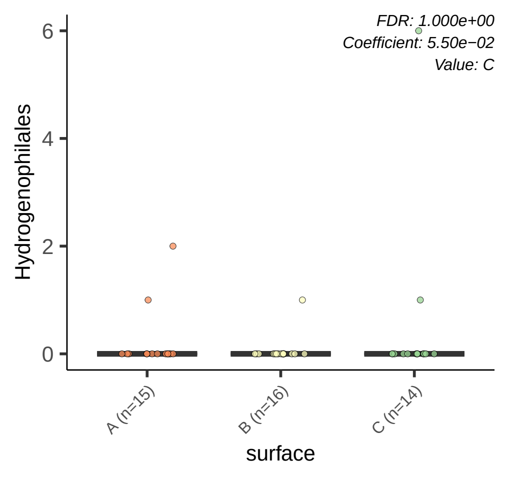

FDR: 1.000e+00
Coefficient: 5.50e−02
Value: C
Hydrogenophilales
0
2
4
6
A (n=15)
B (n=16)
C (n=14)
surface

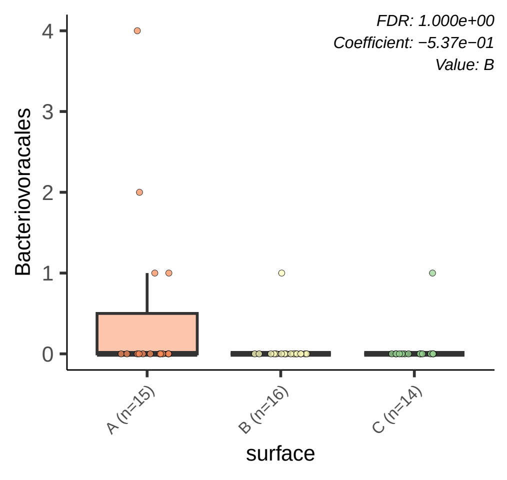

FDR: 1.000e+00
Coefficient: −5.37e−01
Value: B
Bacteriovoracales
0
1
2
3
4
A (n=15)
B (n=16)
C (n=14)
surface

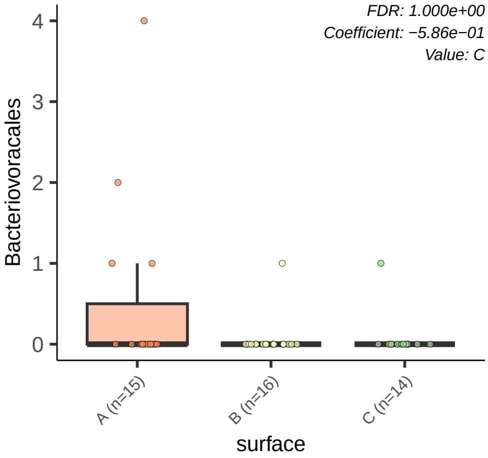
FDR: 1.000e+00
Coefficient: −5.86e−01
Value: C
Bacteriovoracales
4
3
2
1
0
A (n=15)
B (n=16)
C (n=14)
surface

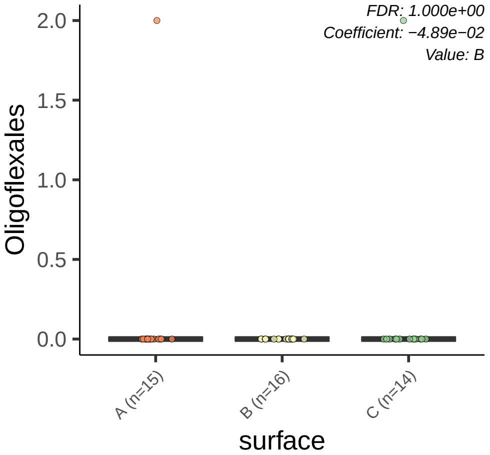

FDR: 1.000e+00
Coefficient: −4.89e−02
Value: B
Oligoflexales
2.0
1.5
1.0
0.5
0.0
A (n=15)
B (n=16)
C (n=14)
surface

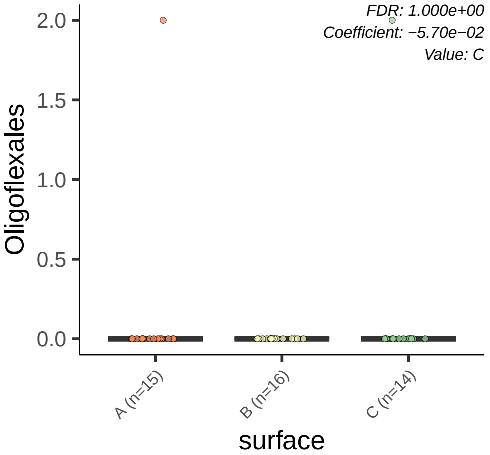

FDR: 1.000e+00
Coefficient: −5.70e−02
Value: C
Oligoflexales
2.0
1.5
1.0
0.5
0.0
A (n=15)
B (n=16)
C (n=14)
surface

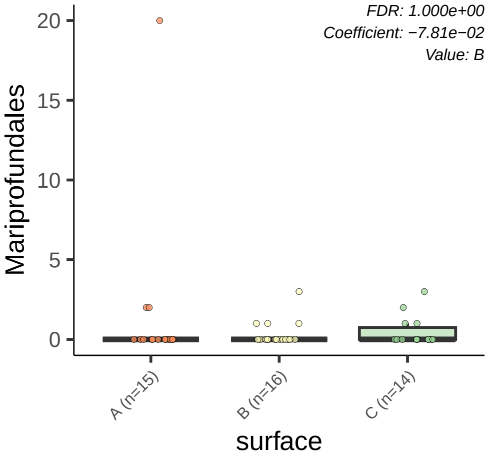
FDR: 1.000e+00
Coefficient: −7.81e−02
Value: B
Mariprofundales
20
15
10
5
0
A (n=15)
B (n=16)
C (n=14)
surface

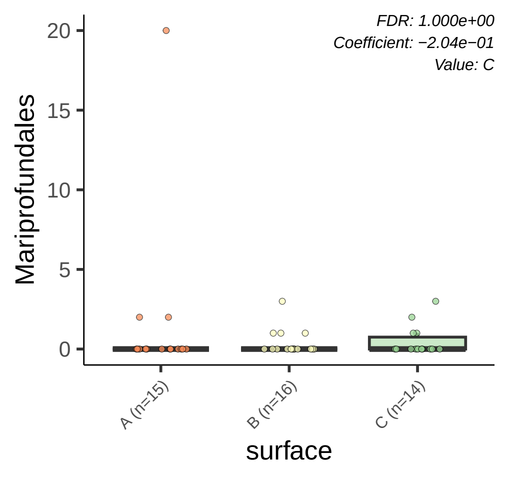
FDR: 1.000e+00
Coefficient: −2.04e−01
Value: C
Mariprofundales
20
15
10
5
0
A (n=15)
B (n=16)
C (n=14)
surface

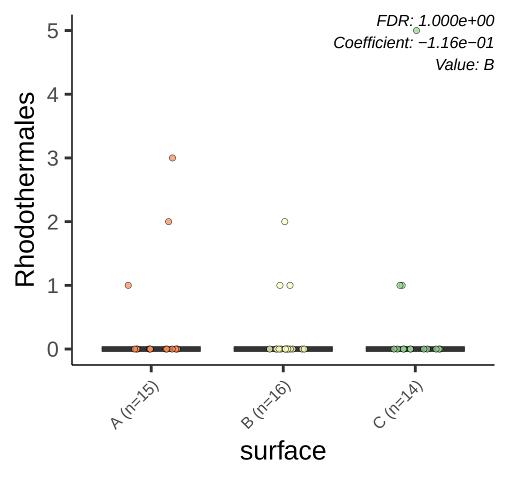
FDR: 1.000e+00
Coefficient: −1.16e−01
Value: B
Rhodothermales
5
4
3
2
1
0
A (n=15)
B (n=16)
C (n=14)
surface

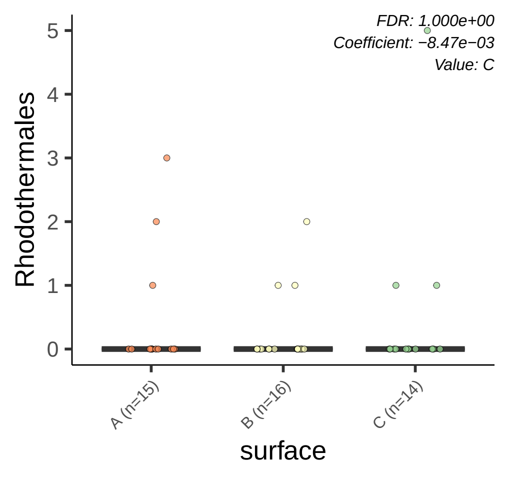

FDR: 1.000e+00
Coefficient: −8.47e−03
Value: C
Rhodothermales
0
1
2
3
4
5
A (n=15)
B (n=16)
C (n=14)
surface

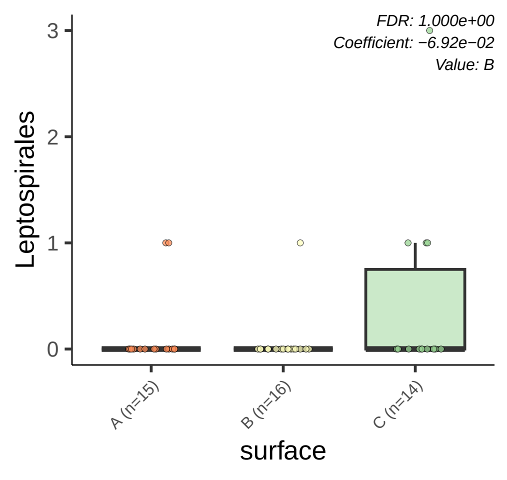

FDR: 1.000e+00
Coefficient: −6.92e−02
Value: B
Leptospirales
3
2
1
0
A (n=15)
B (n=16)
C (n=14)
surface

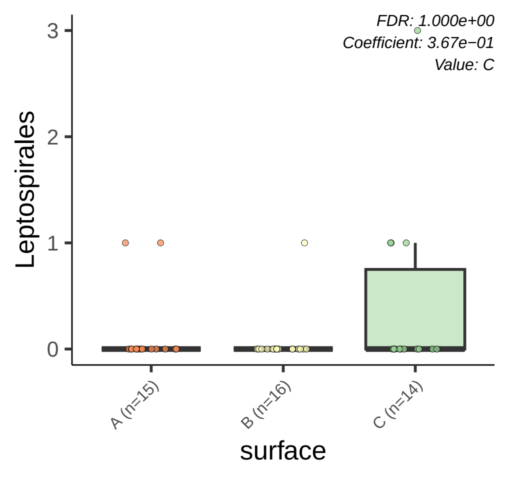
FDR: 1.000e+00
Coefficient: 3.67e−01
Value: C
Leptospirales
3
2
1
0
A (n=15)
B (n=16)
C (n=14)
surface

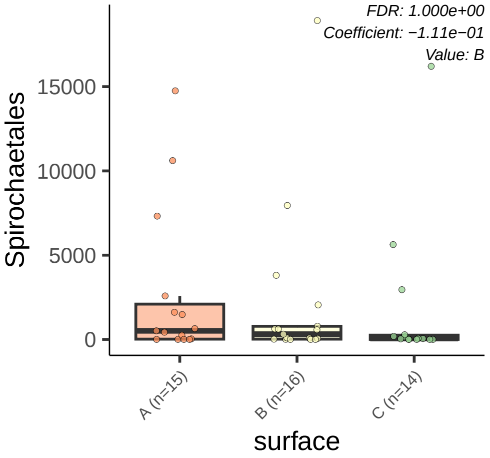

FDR: 1.000e+00
Coefficient: −1.11e−01
Value: B
Spirochaetales
15000
10000
5000
0
A (n=15)
B (n=16)
C (n=14)
surface

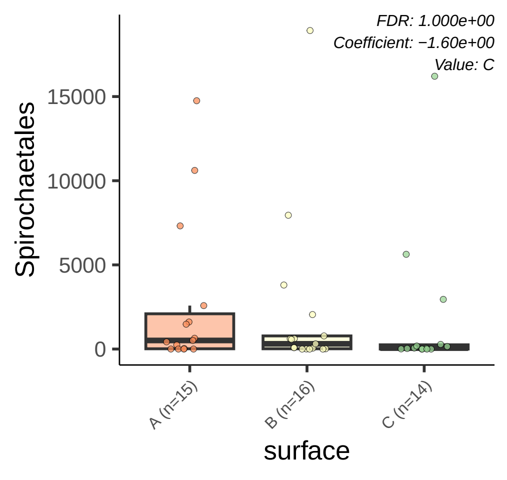
FDR: 1.000e+00
Coefficient: −1.60e+00
Value: C
Spirochaetales
15000
10000
5000
0
A (n=15)
B (n=16)
C (n=14)
surface

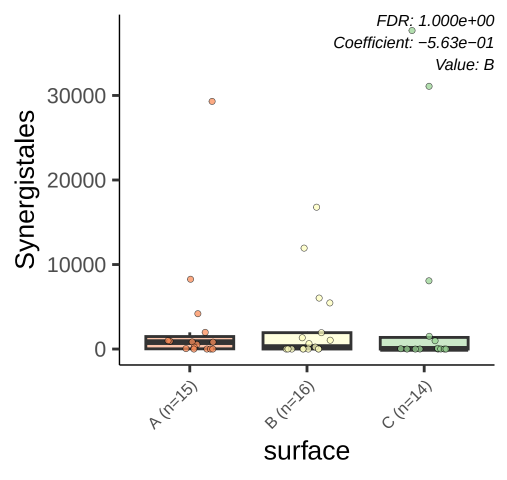
FDR: 1.000e+00
Coefficient: −5.63e−01
Value: B
Synergistales
30000
20000
10000
0
A (n=15)
B (n=16)
C (n=14)
surface

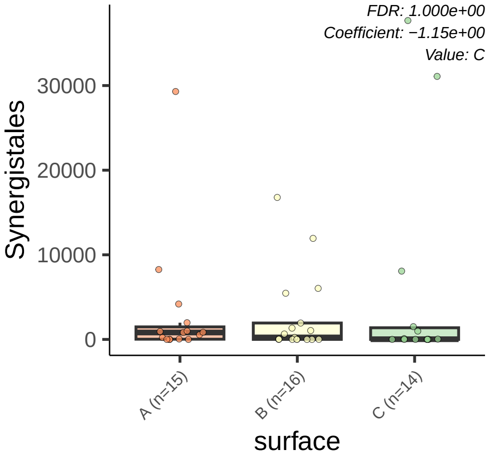

FDR: 1.000e+00
Coefficient: −1.15e+00
Value: C
Synergistales
30000
20000
10000
0
A (n=15)
B (n=16)
C (n=14)
surface

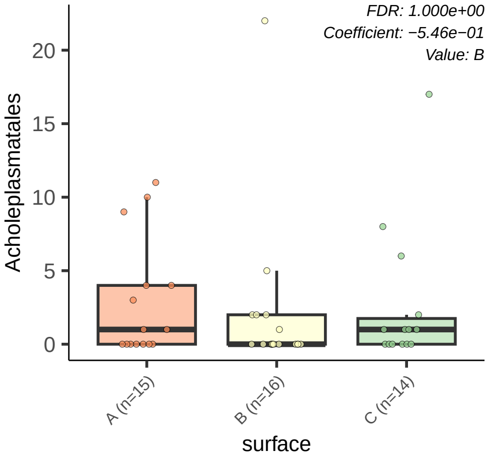

FDR: 1.000e+00
Coefficient: −5.46e−01
Value: B
Acholeplasmatales
0
5
10
15
20
A (n=15)
B (n=16)
C (n=14)
surface

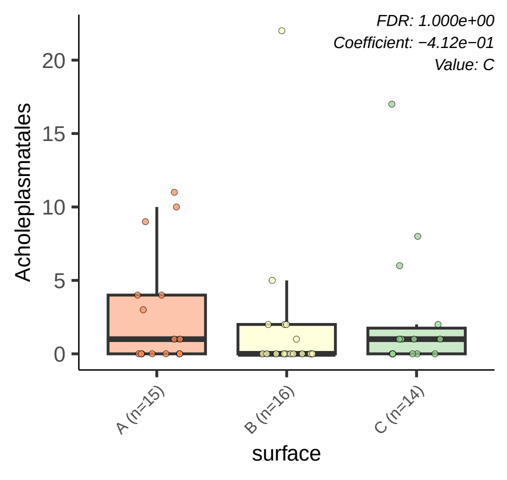
FDR: 1.000e+00
Coefficient: −4.12e−01
Value: C
Acholeplasmatales
0
5
10
15
20
A (n=15)
B (n=16)
C (n=14)
surface

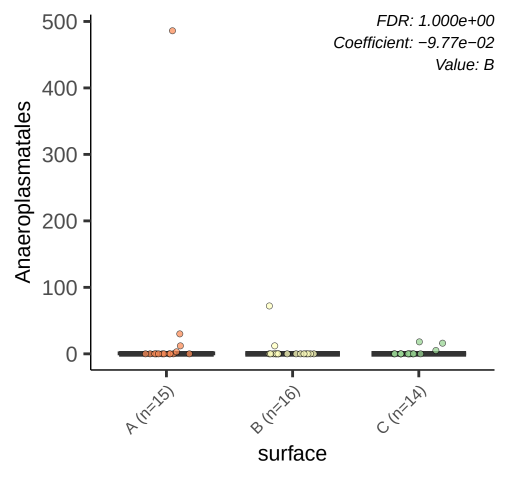
FDR: 1.000e+00
Coefficient: −9.77e−02
Value: B
Anaeroplasmatales
500
400
300
200
100
0
A (n=15)
B (n=16)
C (n=14)
surface

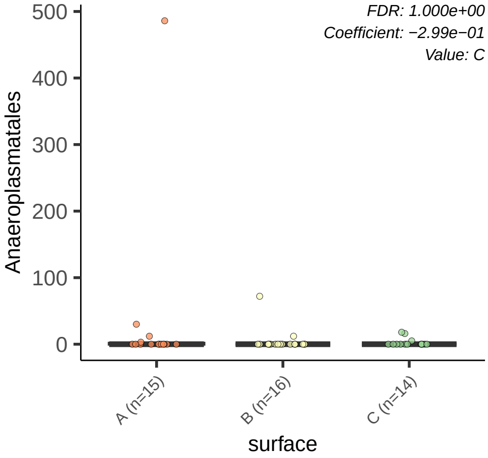

FDR: 1.000e+00
Coefficient: −2.99e−01
Value: C
Anaeroplasmatales
500
400
300
200
100
0
A (n=15)
B (n=16)
C (n=14)
surface

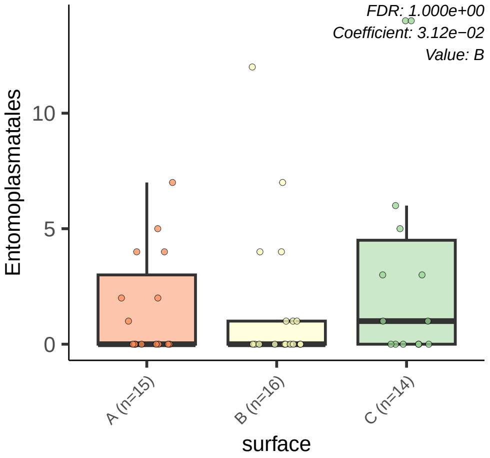
FDR: 1.000e+00
Coefficient: 3.12e−02
Value: B
Entomoplasmatales
10
5
0
A (n=15)
B (n=16)
C (n=14)
surface

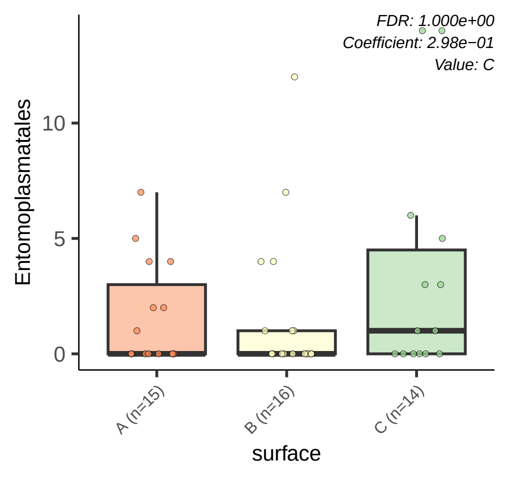
FDR: 1.000e+00
Coefficient: 2.98e−01
Value: C
Entomoplasmatales
0
5
10
A (n=15)
B (n=16)
C (n=14)
surface

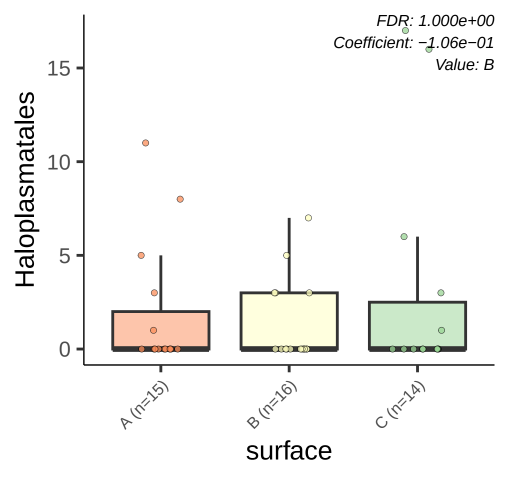
FDR: 1.000e+00
Coefficient: −1.06e−01
Value: B
Haloplasmatales
0
5
10
15
A (n=15)
B (n=16)
C (n=14)
surface

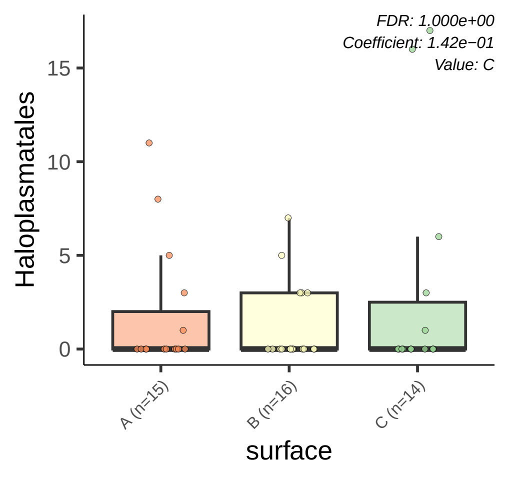
FDR: 1.000e+00
Coefficient: 1.42e−01
Value: C
Haloplasmatales
0
5
10
15
A (n=15)
B (n=16)
C (n=14)
surface

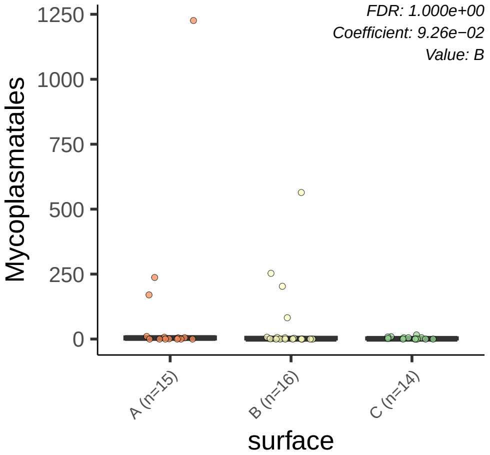

FDR: 1.000e+00
Coefficient: 9.26e−02
Value: B
Mycoplasmatales
1250
1000
750
500
250
0
A (n=15)
B (n=16)
C (n=14)
surface

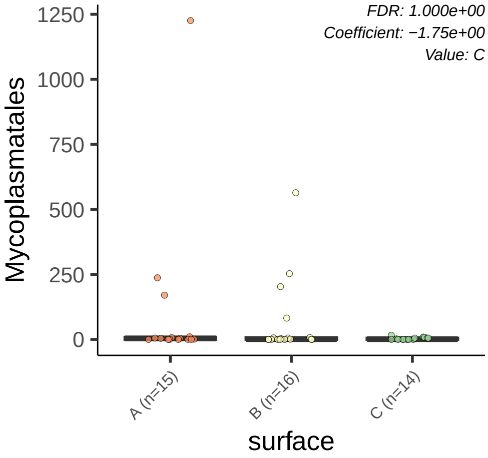

FDR: 1.000e+00
Coefficient: −1.75e+00
Value: C
Mycoplasmatales
1250
1000
750
500
250
0
A (n=15)
B (n=16)
C (n=14)
surface

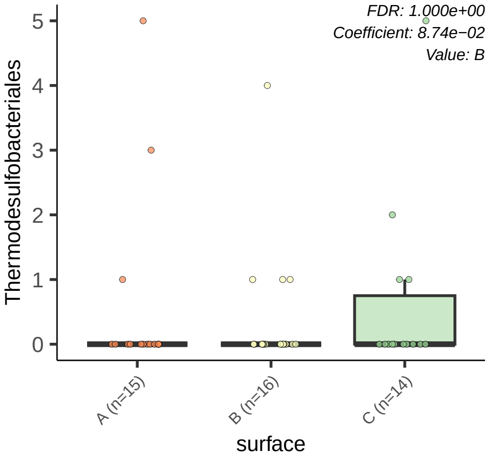
FDR: 1.000e+00
Coefficient: 8.74e−02
Value: B
Thermodesulfobacteriales
0
1
2
3
4
5
A (n=15)
B (n=16)
C (n=14)
surface

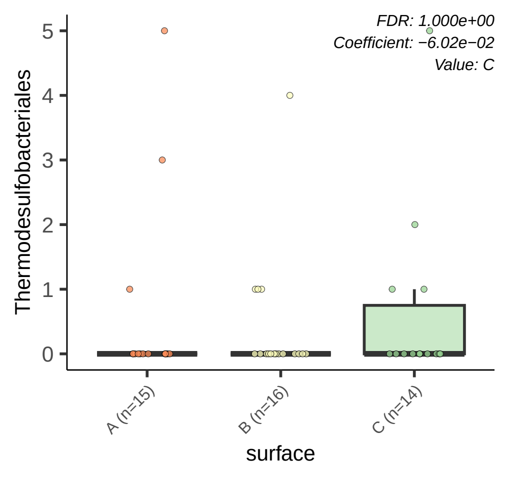

FDR: 1.000e+00
Coefficient: −6.02e−02
Value: C
Thermodesulfobacteriales
5
4
3
2
1
0
A (n=15)
B (n=16)
C (n=14)
surface

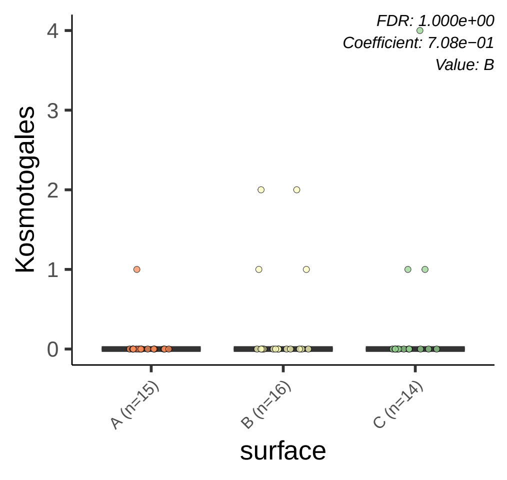

FDR: 1.000e+00
Coefficient: 7.08e−01
Value: B
Kosmotogales
4
3
2
1
0
A (n=15)
B (n=16)
C (n=14)
surface

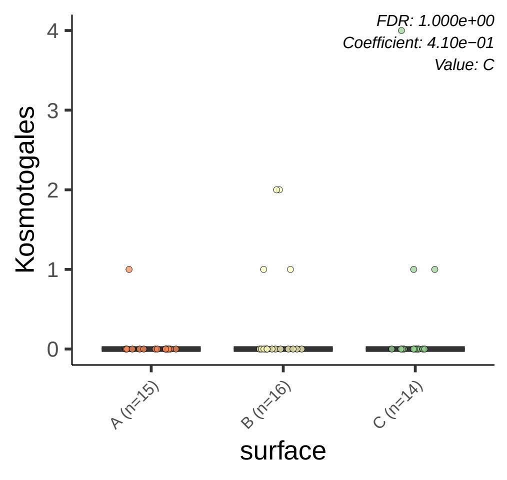
FDR: 1.000e+00
Coefficient: 4.10e−01
Value: C
Kosmotogales
0
1
2
3
4
A (n=15)
B (n=16)
C (n=14)
surface

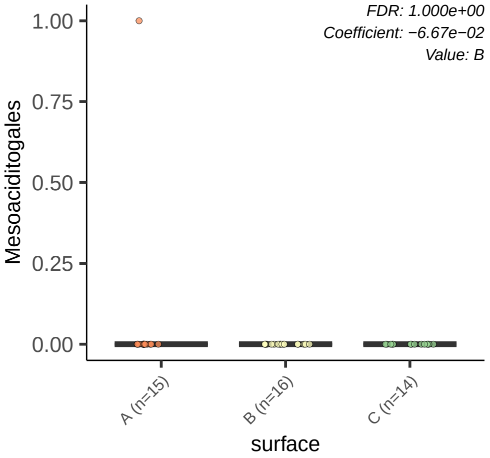
FDR: 1.000e+00
Coefficient: −6.67e−02
Value: B
Mesoaciditogales
1.00
0.75
0.50
0.25
0.00
A (n=15)
B (n=16)
C (n=14)
surface

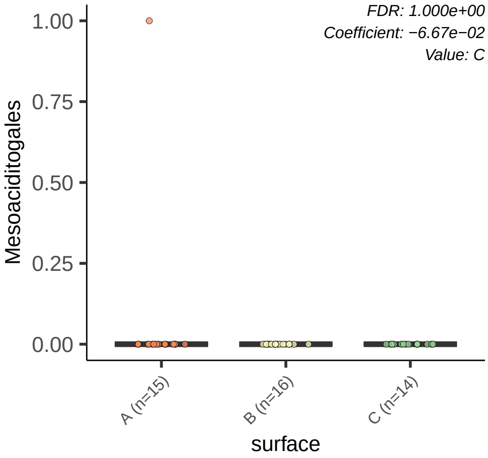
FDR: 1.000e+00
Coefficient: −6.67e−02
Value: C
Mesoaciditogales
1.00
0.75
0.50
0.25
0.00
A (n=15)
B (n=16)
C (n=14)
surface

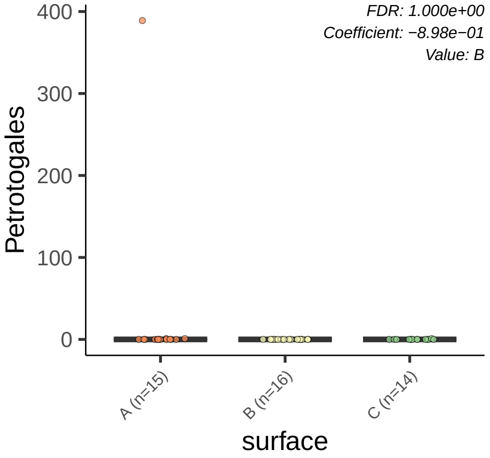
FDR: 1.000e+00
Coefficient: −8.98e−01
Value: B
Petrotogales
400
300
200
100
0
A (n=15)
B (n=16)
C (n=14)
surface

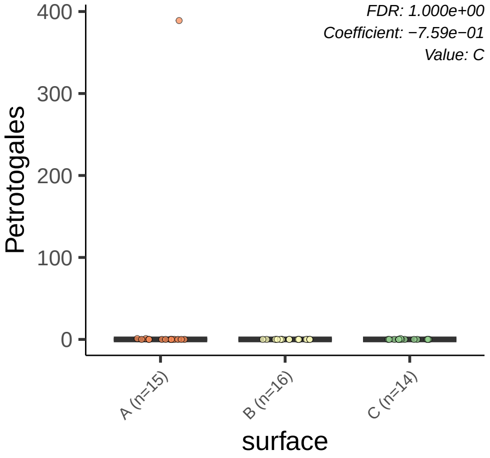

FDR: 1.000e+00
Coefficient: −7.59e−01
Value: C
Petrotogales
400
300
200
100
0
A (n=15)
B (n=16)
C (n=14)
surface

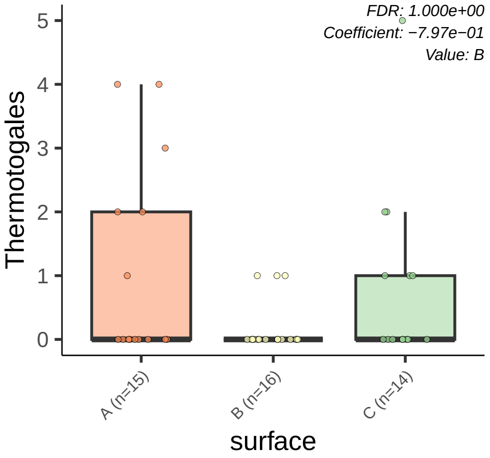
FDR: 1.000e+00
Coefficient: −7.97e−01
Value: B
Thermotogales
5
4
3
2
1
0
A (n=15)
B (n=16)
C (n=14)
surface

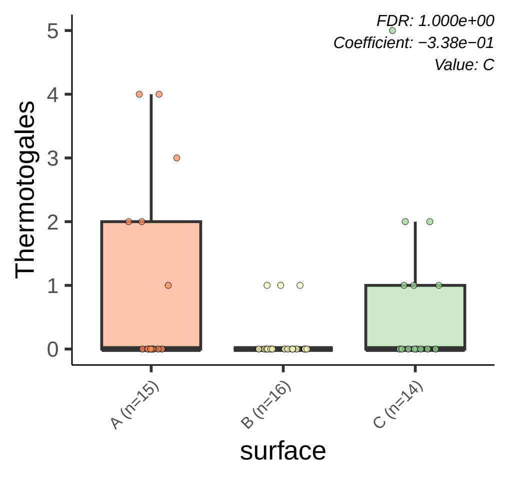
FDR: 1.000e+00
Coefficient: −3.38e−01
Value: C
Thermotogales
0
1
2
3
4
5
A (n=15)
B (n=16)
C (n=14)
surface

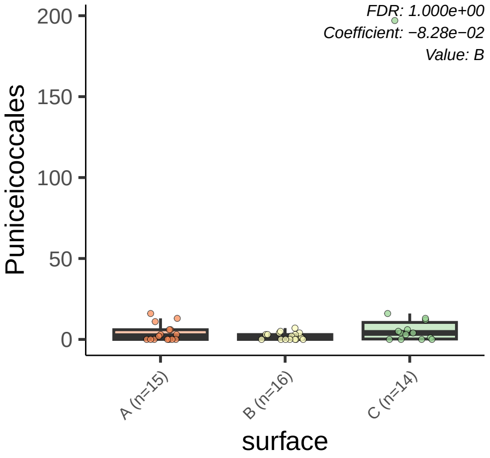
FDR: 1.000e+00
Coefficient: −8.28e−02
Value: B
Puniceicoccales
200
150
100
50
0
A (n=15)
B (n=16)
C (n=14)
surface

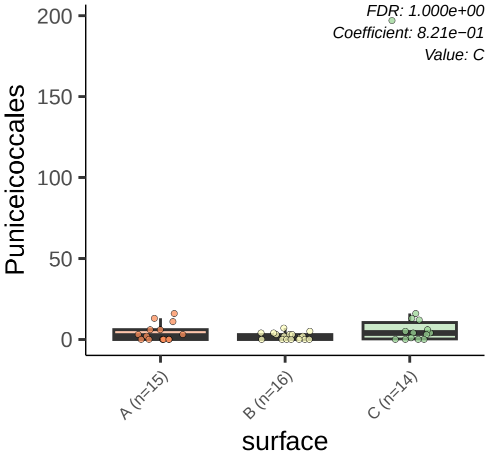

FDR: 1.000e+00
Coefficient: 8.21e−01
Value: C
Puniceicoccales
200
150
100
50
0
A (n=15)
B (n=16)
C (n=14)
surface

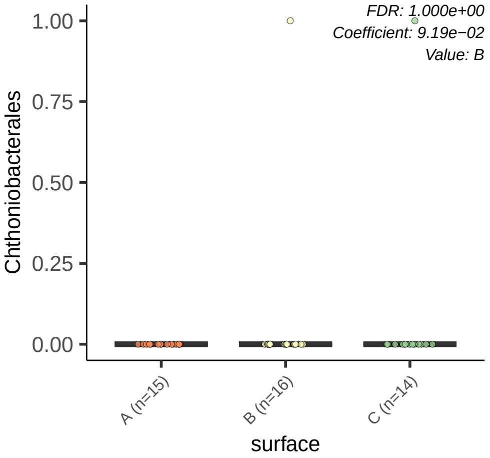
FDR: 1.000e+00
Coefficient: 9.19e−02
Value: B
1.00
0.75
0.50
0.25
0.00
Chthoniobacterales
A (n=15)
B (n=16)
C (n=14)
surface

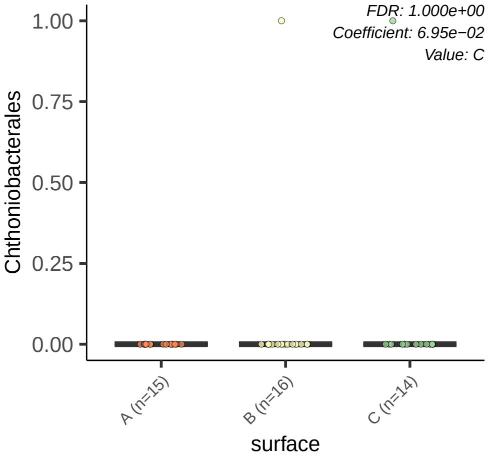
FDR: 1.000e+00
Coefficient: 6.95e−02
Value: C
Chthoniobacterales
1.00
0.75
0.50
0.25
0.00
A (n=15)
B (n=16)
C (n=14)
surface

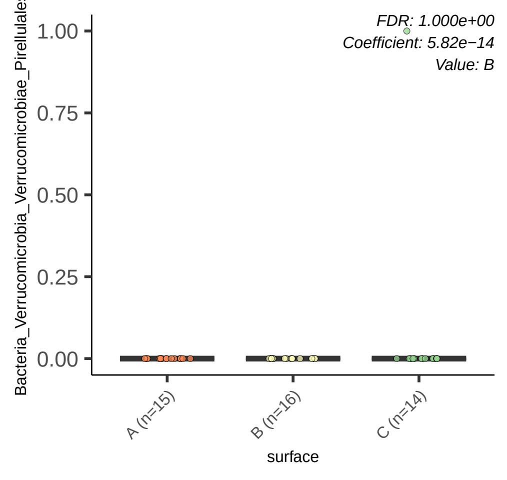

FDR: 1.000e+00
Coefficient: 5.82e−14
Value: B
Bacteria_Verrucomicrobia_Verrucomicrobiae_Pirellulales
1.00
0.75
0.50
0.25
0.00
A (n=15)
B (n=16)
C (n=14)
surface

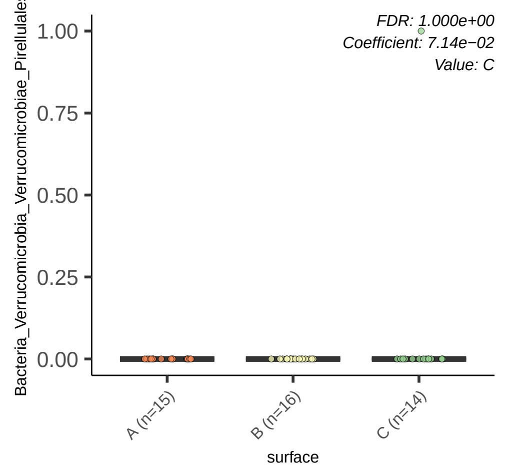

FDR: 1.000e+00
Coefficient: 7.14e−02
Value: C
Bacteria_Verrucomicrobia_Verrucomicrobiae_Pirellulales
1.00
0.75
0.50
0.25
0.00
A (n=15)
B (n=16)
C (n=14)
surface

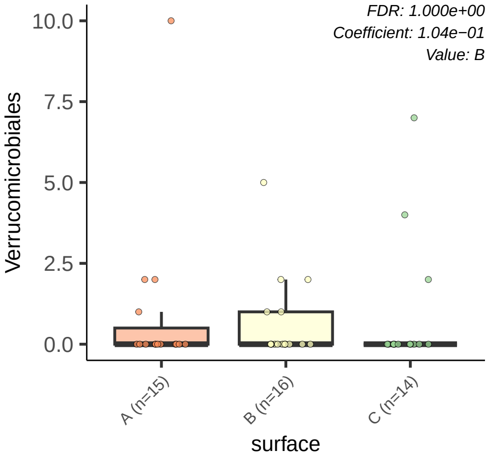

FDR: 1.000e+00
Coefficient: 1.04e−01
Value: B
Verrucomicrobiales
10.0
7.5
5.0
2.5
0.0
A (n=15)
B (n=16)
C (n=14)
surface

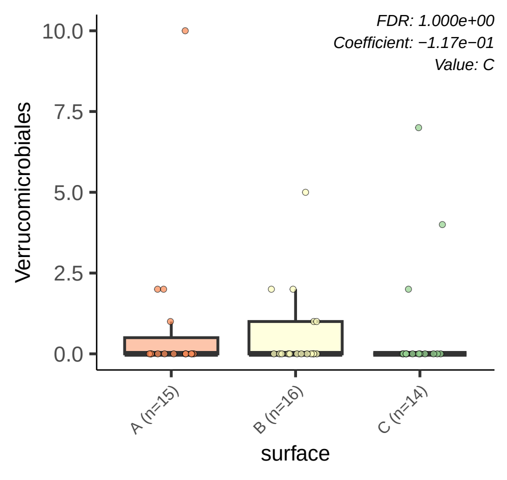

FDR: 1.000e+00
Coefficient: −1.17e−01
Value: C
Verrucomicrobiales
10.0
7.5
5.0
2.5
0.0
A (n=15)
B (n=16)
C (n=14)
surface

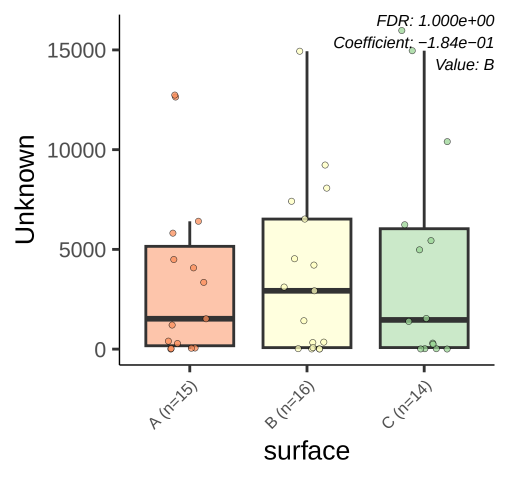

FDR: 1.000e+00
Coefficient: −1.84e−01
Value: B
Unknown
15000
10000
5000
0
A (n=15)
B (n=16)
C (n=14)
surface

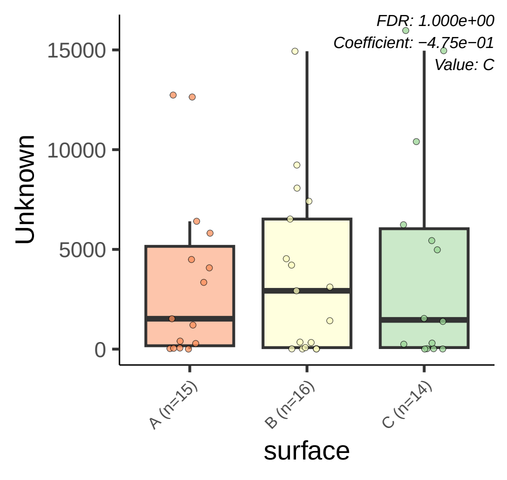

FDR: 1.000e+00
Coefficient: −4.75e−01
Value: C
Unknown
15000
10000
5000
0
A (n=15)
B (n=16)
C (n=14)
surface

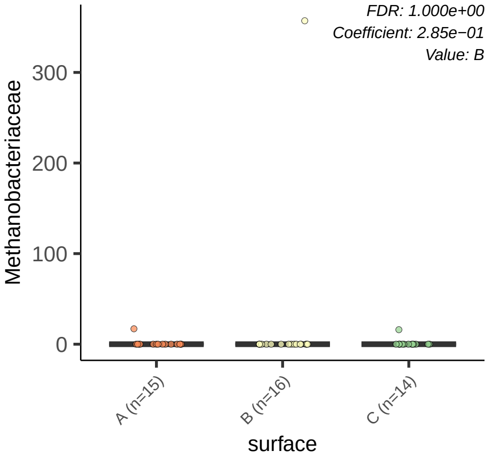

FDR: 1.000e+00
Coefficient: 2.85e−01
Value: B
Methanobacteriaceae
300
200
100
0
A (n=15)
B (n=16)
C (n=14)
surface

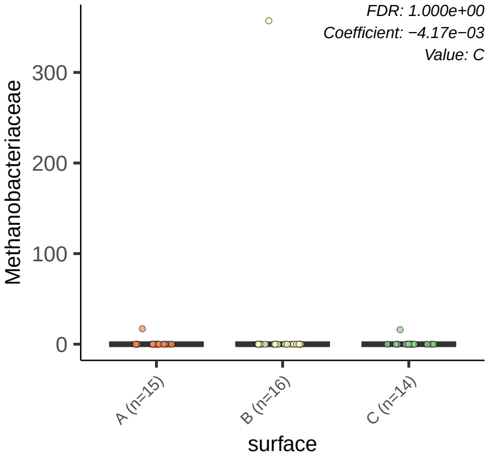
FDR: 1.000e+00
Coefficient: −4.17e−03
Value: C
Methanobacteriaceae
300
200
100
0
A (n=15)
B (n=16)
C (n=14)
surface

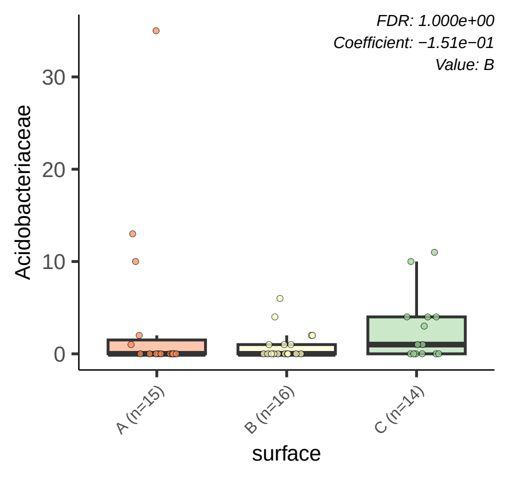
FDR: 1.000e+00
Coefficient: −1.51e−01
Value: B
Acidobacteriaceae
0
10
20
30
A (n=15)
B (n=16)
C (n=14)
surface

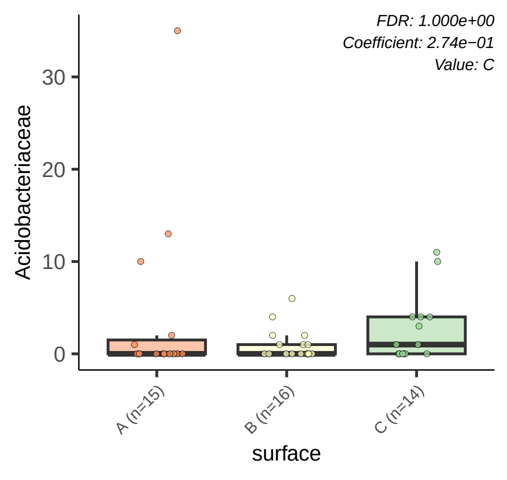

FDR: 1.000e+00
Coefficient: 2.74e−01
Value: C
Acidobacteriaceae
0
10
20
30
A (n=15)
B (n=16)
C (n=14)
surface

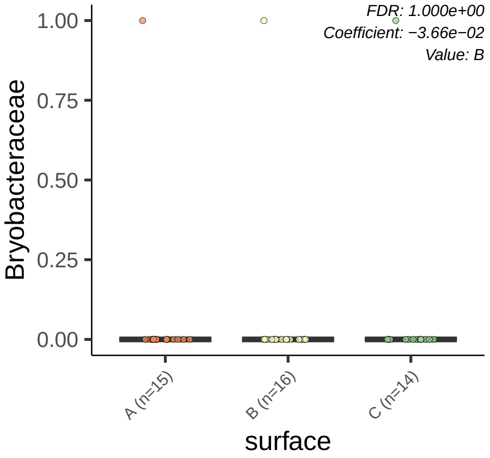
FDR: 1.000e+00
Coefficient: −3.66e−02
Value: B
Bryobacteraceae
1.00
0.75
0.50
0.25
0.00
A (n=15)
B (n=16)
C (n=14)
surface

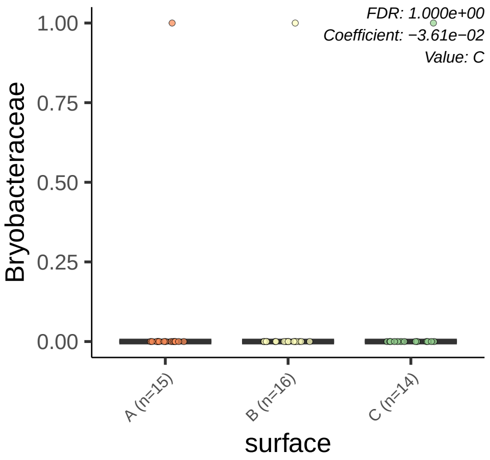
FDR: 1.000e+00
Coefficient: −3.61e−02
Value: C
Bryobacteraceae
1.00
0.75
0.50
0.25
0.00
A (n=15)
B (n=16)
C (n=14)
surface

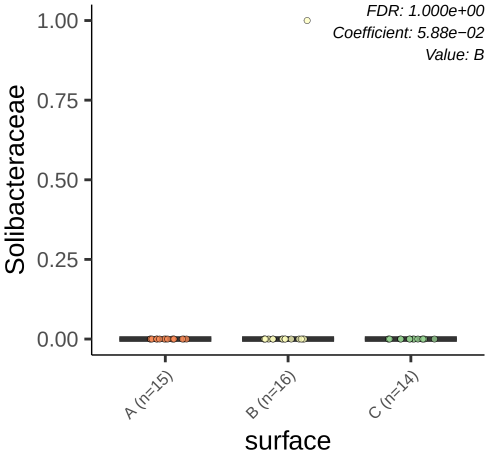

FDR: 1.000e+00
Coefficient: 5.88e−02
Value: B
Solibacteraceae
1.00
0.75
0.50
0.25
0.00
A (n=15)
B (n=16)
C (n=14)
surface

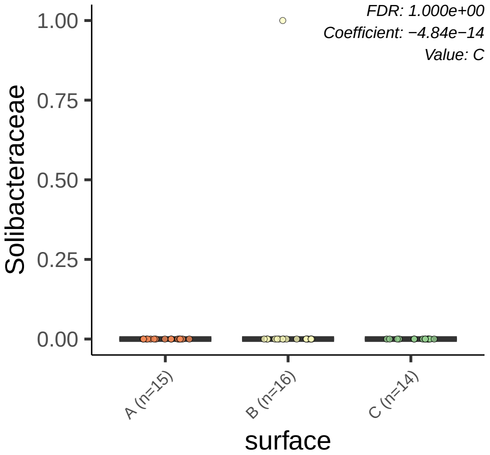
FDR: 1.000e+00
Coefficient: −4.84e−14
Value: C
Solibacteraceae
1.00
0.75
0.50
0.25
0.00
A (n=15)
B (n=16)
C (n=14)
surface

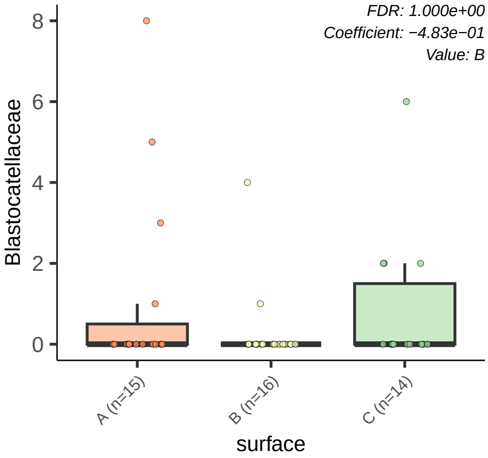

FDR: 1.000e+00
Coefficient: −4.83e−01
Value: B
Blastocatellaceae
8
6
4
2
0
A (n=15)
B (n=16)
C (n=14)
surface

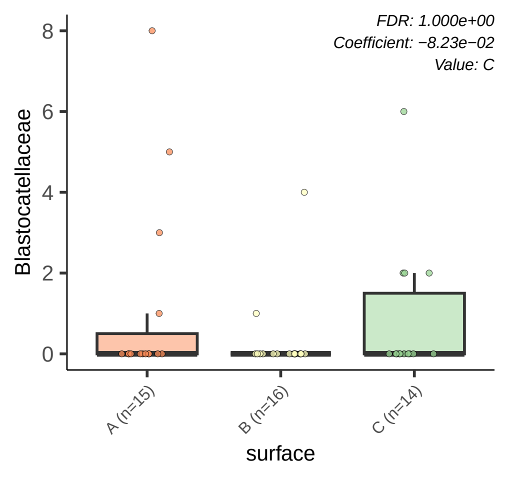

FDR: 1.000e+00
Coefficient: −8.23e−02
Value: C
Blastocatellaceae
8
6
4
2
0
A (n=15)
B (n=16)
C (n=14)
surface

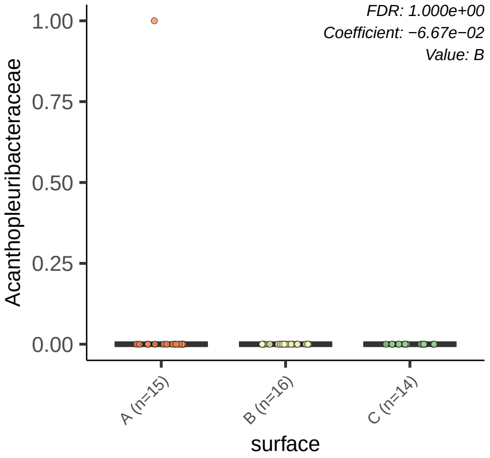
FDR: 1.000e+00
Coefficient: −6.67e−02
Value: B
Acanthopleuribacteraceae
1.00
0.75
0.50
0.25
0.00
A (n=15)
B (n=16)
C (n=14)
surface

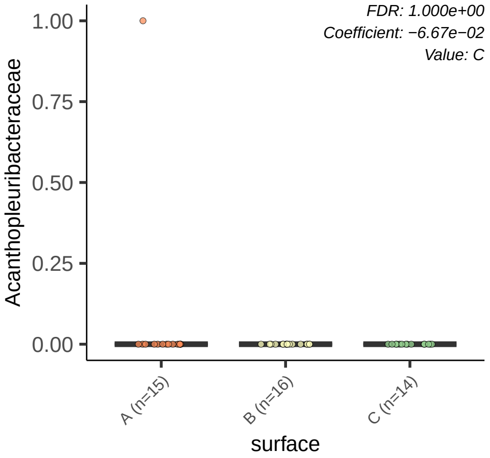
FDR: 1.000e+00
Coefficient: −6.67e−02
Value: C
Acanthopleuribacteraceae
1.00
0.75
0.50
0.25
0.00
A (n=15)
B (n=16)
C (n=14)
surface

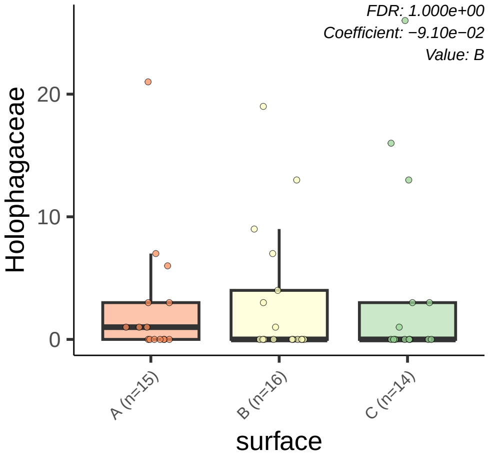

FDR: 1.000e+00
Coefficient: −9.10e−02
Value: B
Holophagaceae
20
10
0
A (n=15)
B (n=16)
C (n=14)
surface

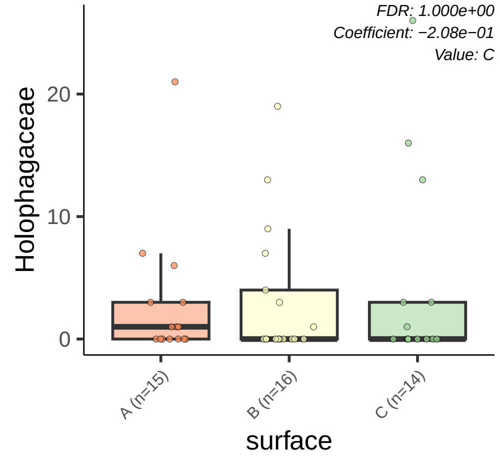
FDR: 1.000e+00
Coefficient: −2.08e−01
Value: C
Holophagaceae
0
10
20
A (n=15)
B (n=16)
C (n=14)
surface

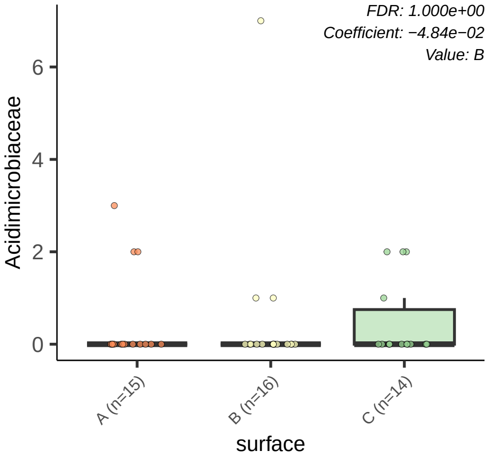

FDR: 1.000e+00
Coefficient: −4.84e−02
Value: B
Acidimicrobiaceae
0
2
4
6
A (n=15)
B (n=16)
C (n=14)
surface

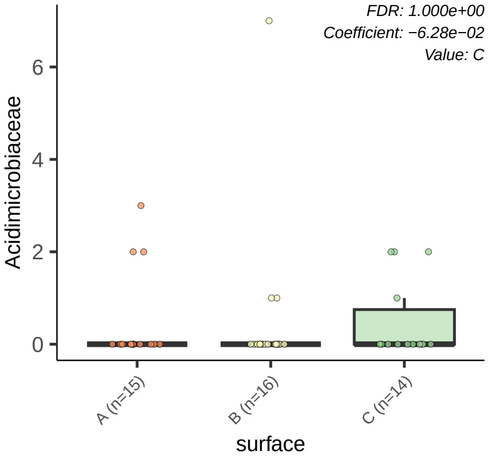

FDR: 1.000e+00
Coefficient: −6.28e−02
Value: C
Acidimicrobiaceae
0
2
4
6
A (n=15)
B (n=16)
C (n=14)
surface

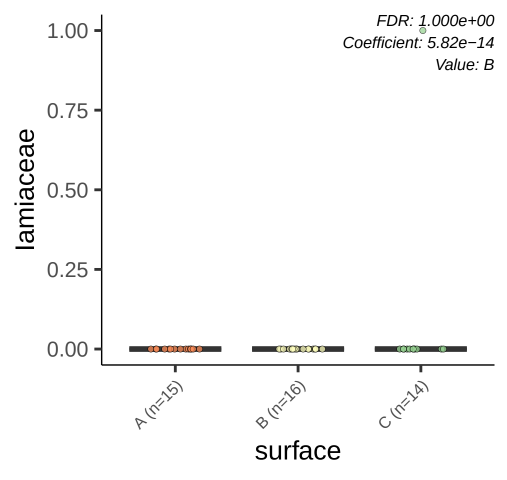
FDR: 1.000e+00
Coefficient: 5.82e−14
Value: B
lamiaceae
1.00
0.75
0.50
0.25
0.00
A (n=15)
B (n=16)
C (n=14)
surface

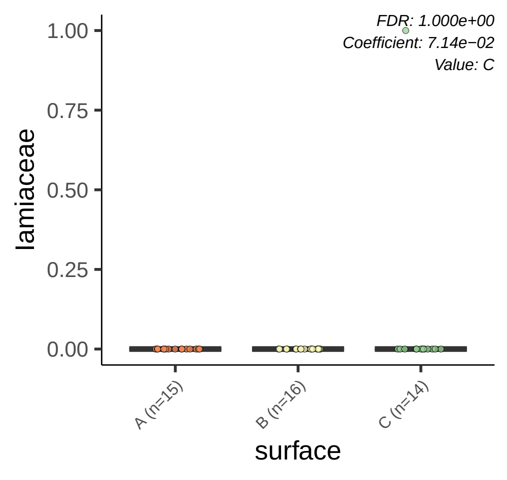
FDR: 1.000e+00
Coefficient: 7.14e−02
Value: C
lamiaceae
1.00
0.75
0.50
0.25
0.00
A (n=15)
B (n=16)
C (n=14)
surface

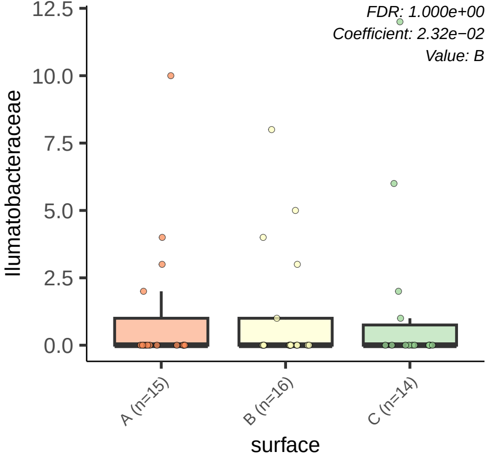

FDR: 1.000e+00
Coefficient: 2.32e−02
Value: B
Ilumatobacteraceae
12.5
10.0
7.5
5.0
2.5
0.0
A (n=15)
B (n=16)
C (n=14)
surface

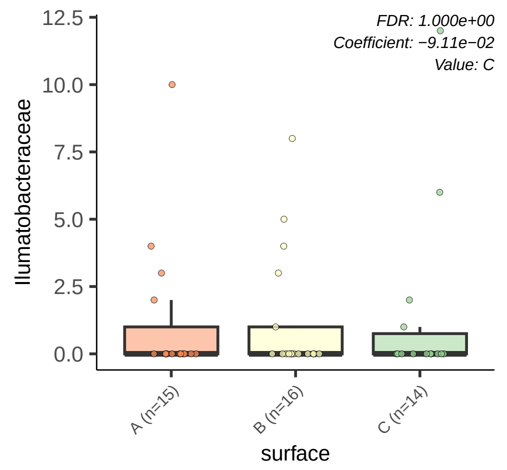

FDR: 1.000e+00
Coefficient: −9.11e−02
Value: C
Ilumatobacteraceae
12.5
10.0
7.5
5.0
2.5
0.0
A (n=15)
B (n=16)
C (n=14)
surface

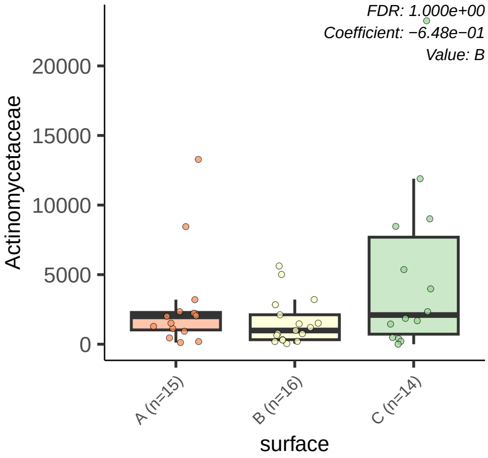
FDR: 1.000e+00
Coefficient: −6.48e−01
Value: B
Actinomycetaceae
20000
15000
10000
5000
0
A (n=15)
B (n=16)
C (n=14)
surface

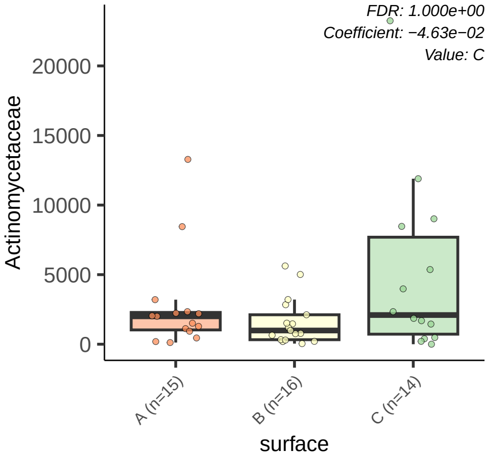
FDR: 1.000e+00
Coefficient: −4.63e−02
Value: C
Actinomycetaceae
20000
15000
10000
5000
0
A (n=15)
B (n=16)
C (n=14)
surface

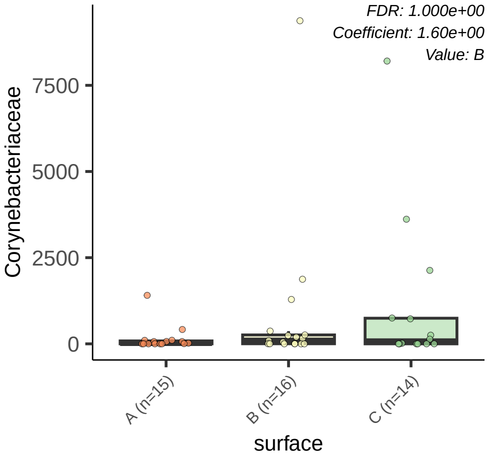

FDR: 1.000e+00
Coefficient: 1.60e+00
Value: B
Corynebacteriaceae
7500
5000
2500
0
A (n=15)
B (n=16)
C (n=14)
surface

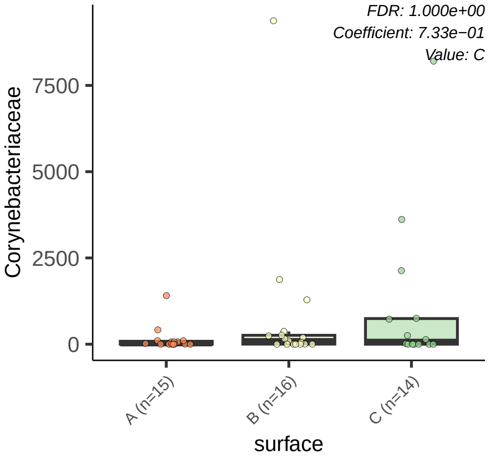
FDR: 1.000e+00
Coefficient: 7.33e−01
Value: C
Corynebacteriaceae
7500
5000
2500
0
A (n=15)
B (n=16)
C (n=14)
surface

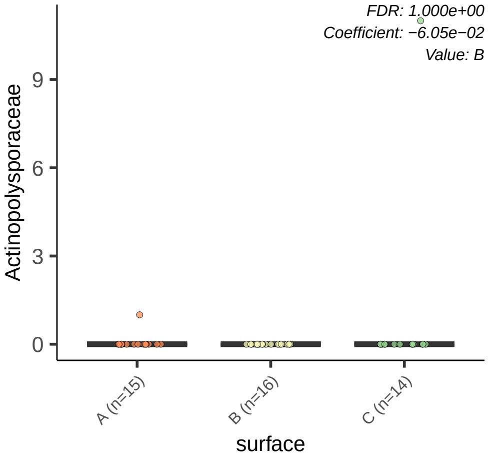
FDR: 1.000e+00
Coefficient: −6.05e−02
Value: B
Actinopolysporaceae
0
3
6
9
A (n=15)
B (n=16)
C (n=14)
surface

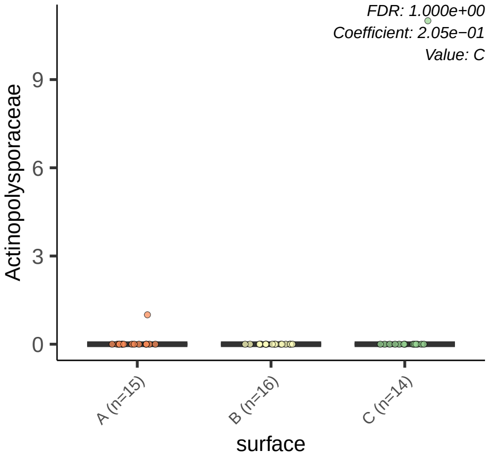
FDR: 1.000e+00
Coefficient: 2.05e−01
Value: C
Actinopolysporaceae
9
6
3
0
A (n=15)
B (n=16)
C (n=14)
surface

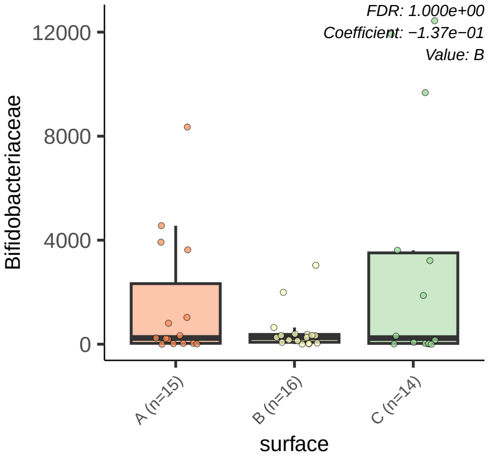

FDR: 1.000e+00
Coefficient: −1.37e−01
Value: B
Bifidobacteriaceae
12000
8000
4000
0
A (n=15)
B (n=16)
C (n=14)
surface

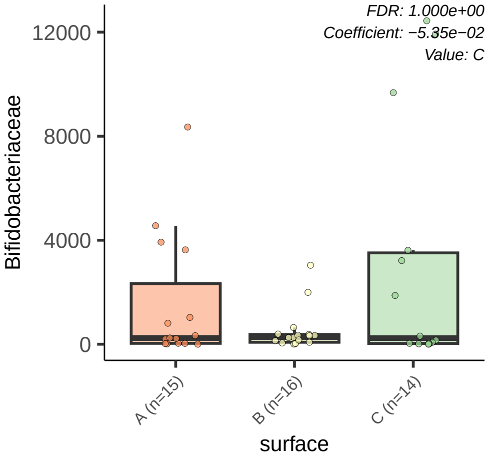
FDR: 1.000e+00
Coefficient: −5.35e−02
Value: C
Bifidobacteriaceae
12000
8000
4000
0
A (n=15)
B (n=16)
C (n=14)
surface

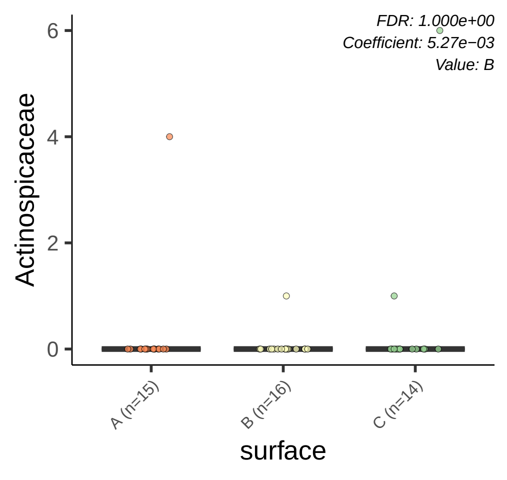
FDR: 1.000e+00
Coefficient: 5.27e−03
Value: B
Actinospicaceae
0
2
4
6
A (n=15)
B (n=16)
C (n=14)
surface

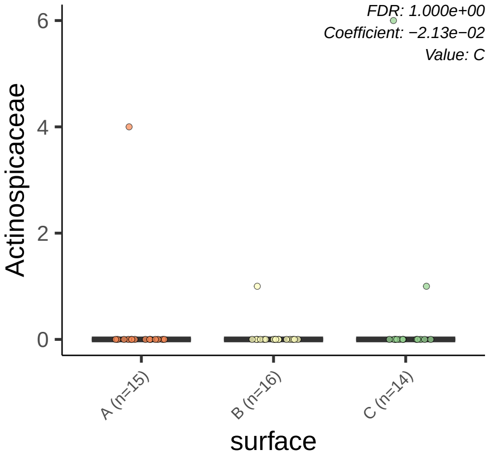

FDR: 1.000e+00
Coefficient: −2.13e−02
Value: C
Actinospicaceae
6
4
2
0
A (n=15)
B (n=16)
C (n=14)
surface

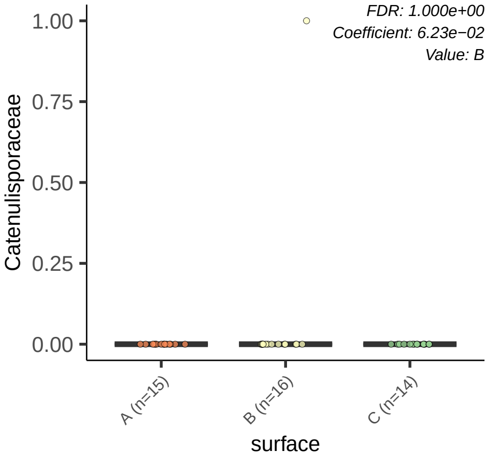
FDR: 1.000e+00
Coefficient: 6.23e−02
Value: B
Catenulisporaceae
1.00
0.75
0.50
0.25
0.00
A (n=15)
B (n=16)
C (n=14)
surface

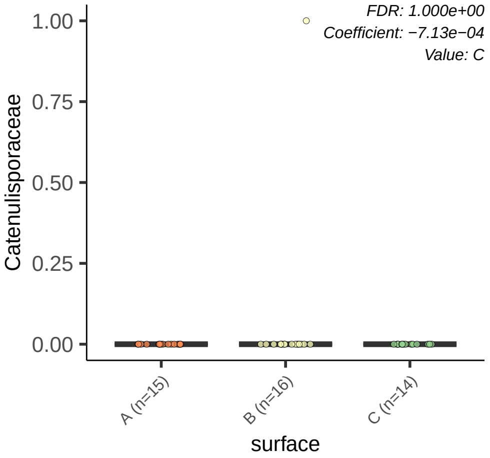
FDR: 1.000e+00
Coefficient: −7.13e−04
Value: C
Catenulisporaceae
1.00
0.75
0.50
0.25
0.00
A (n=15)
B (n=16)
C (n=14)
surface

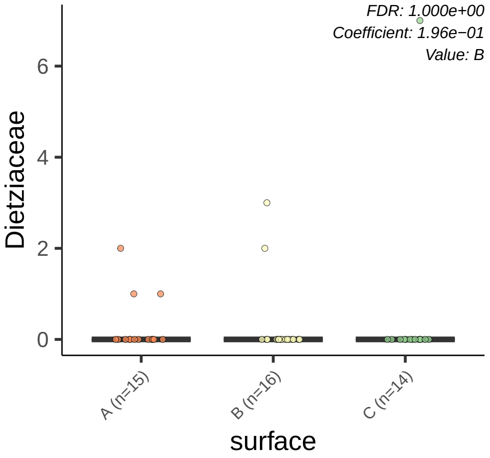

FDR: 1.000e+00
Coefficient: 1.96e−01
Value: B
Dietziaceae
0
2
4
6
A (n=15)
B (n=16)
C (n=14)
surface

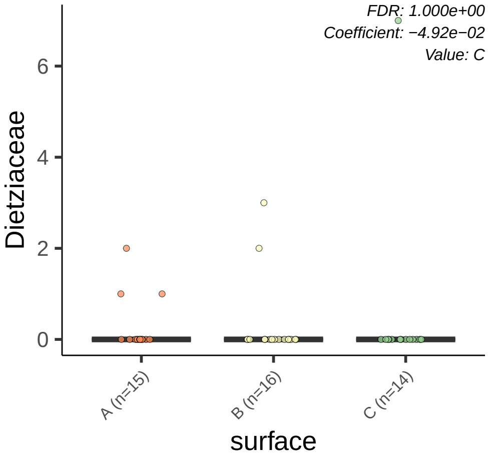
FDR: 1.000e+00
Coefficient: −4.92e−02
Value: C
Dietziaceae
0
2
4
6
A (n=15)
B (n=16)
C (n=14)
surface

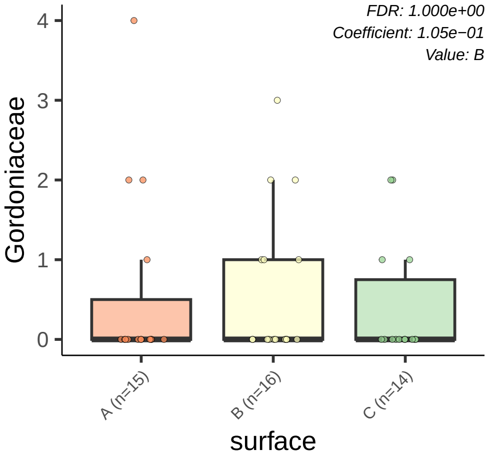
FDR: 1.000e+00
Coefficient: 1.05e−01
Value: B
Gordoniaceae
0
1
2
3
4
A (n=15)
B (n=16)
C (n=14)
surface

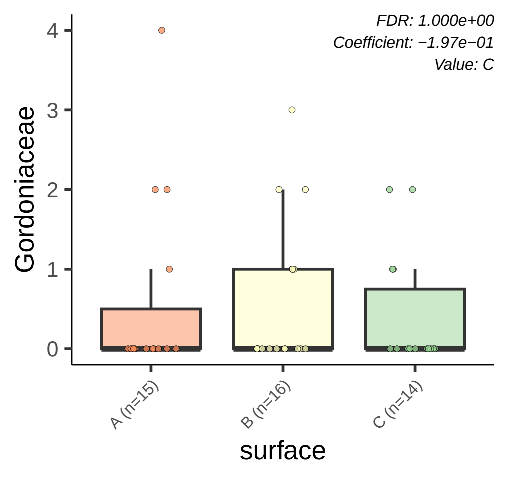

FDR: 1.000e+00
Coefficient: −1.97e−01
Value: C
Gordoniaceae
0
1
2
3
4
A (n=15)
B (n=16)
C (n=14)
surface

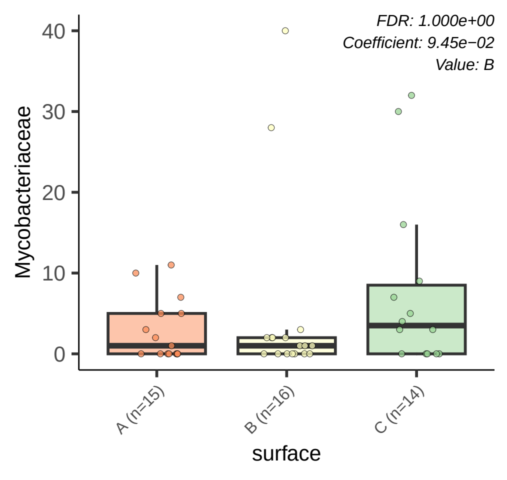

FDR: 1.000e+00
Coefficient: 9.45e−02
Value: B
Mycobacteriaceae
0
10
20
30
40
A (n=15)
B (n=16)
C (n=14)
surface

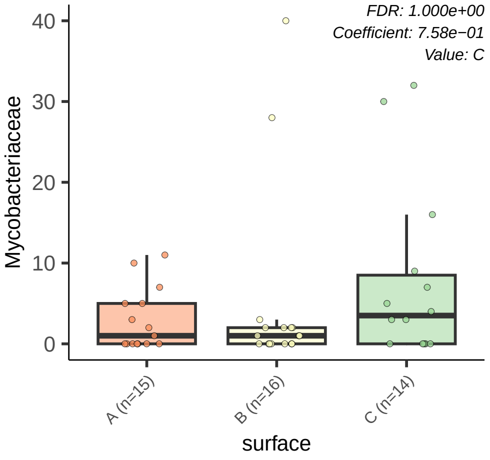

FDR: 1.000e+00
Coefficient: 7.58e−01
Value: C
Mycobacteriaceae
40
30
20
10
0
A (n=15)
B (n=16)
C (n=14)
surface

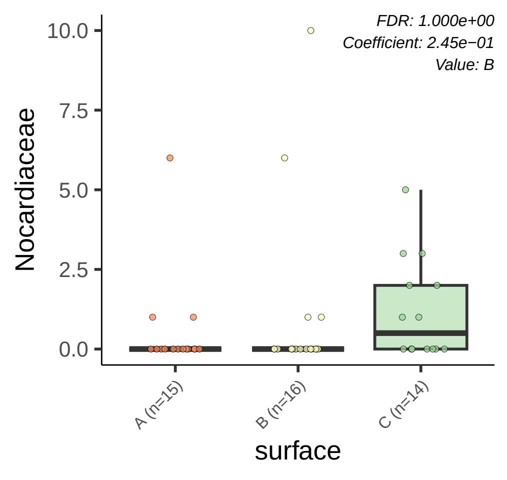

FDR: 1.000e+00
Coefficient: 2.45e−01
Value: B
Nocardiaceae
10.0
7.5
5.0
2.5
0.0
A (n=15)
B (n=16)
C (n=14)
surface

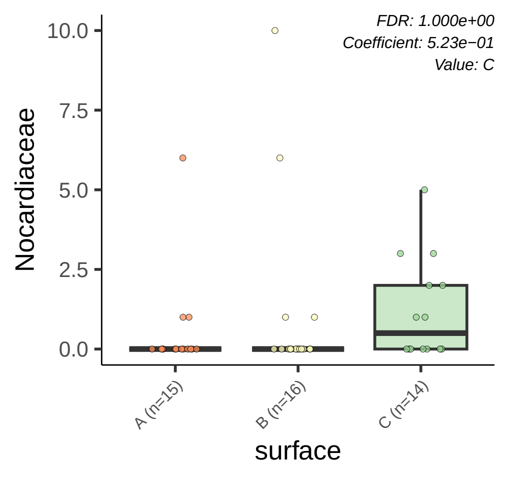

FDR: 1.000e+00
Coefficient: 5.23e−01
Value: C
Nocardiaceae
10.0
7.5
5.0
2.5
0.0
A (n=15)
B (n=16)
C (n=14)
surface

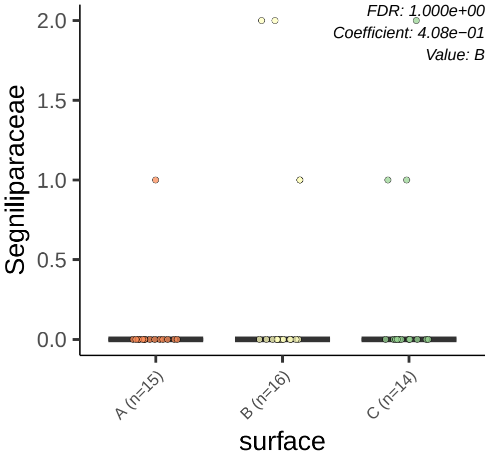
FDR: 1.000e+00
Coefficient: 4.08e−01
Value: B
Segniliparaceae
2.0
1.5
1.0
0.5
0.0
A (n=15)
B (n=16)
C (n=14)
surface

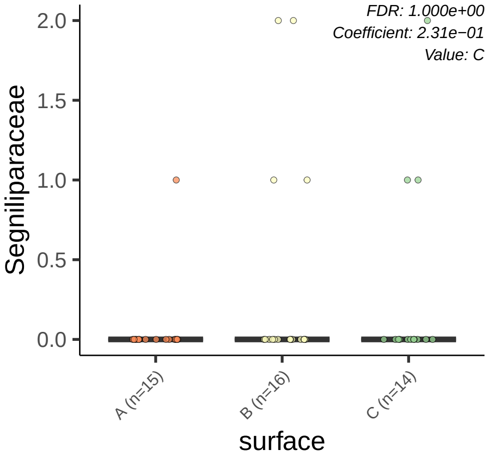
FDR: 1.000e+00
Coefficient: 2.31e−01
Value: C
Segniliparaceae
2.0
1.5
1.0
0.5
0.0
A (n=15)
B (n=16)
C (n=14)
surface

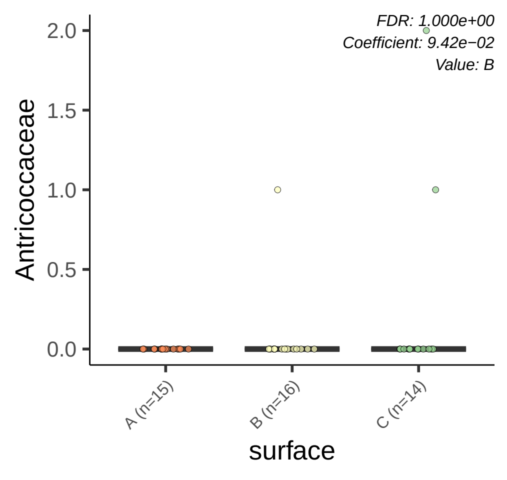
FDR: 1.000e+00
Coefficient: 9.42e−02
Value: B
Antricoccaceae
2.0
1.5
1.0
0.5
0.0
A (n=15)
B (n=16)
C (n=14)
surface

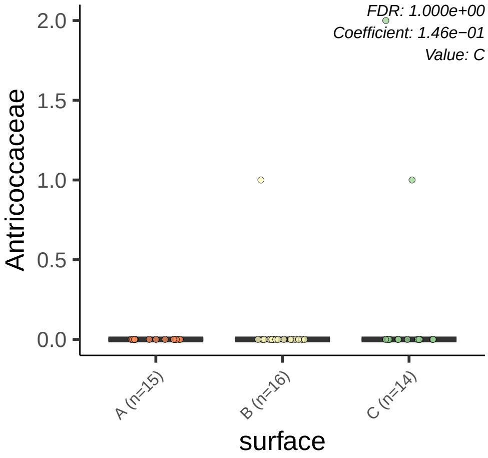

FDR: 1.000e+00
Coefficient: 1.46e−01
Value: C
Antricoccaceae
2.0
1.5
1.0
0.5
0.0
A (n=15)
B (n=16)
C (n=14)
surface

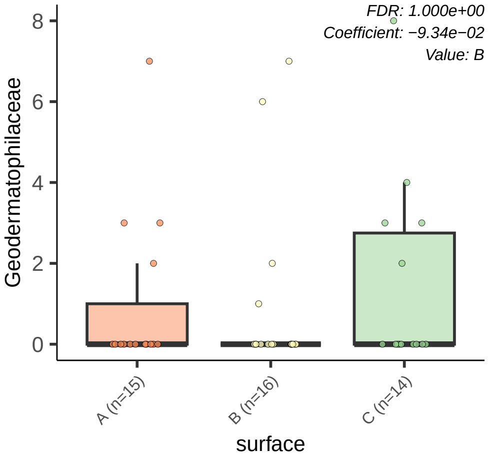
FDR: 1.000e+00
Coefficient: −9.34e−02
Value: B
Geodermatophilaceae
8
6
4
2
0
A (n=15)
B (n=16)
C (n=14)
surface

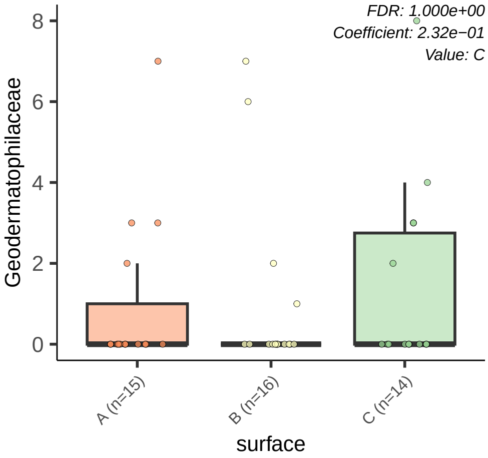

FDR: 1.000e+00
Coefficient: 2.32e−01
Value: C
Geodermatophilaceae
8
6
4
2
0
A (n=15)
B (n=16)
C (n=14)
surface

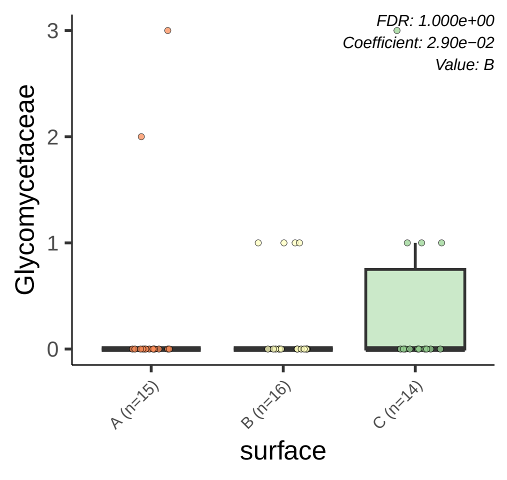

FDR: 1.000e+00
Coefficient: 2.90e−02
Value: B
Glycomycetaceae
0
1
2
3
A (n=15)
B (n=16)
C (n=14)
surface

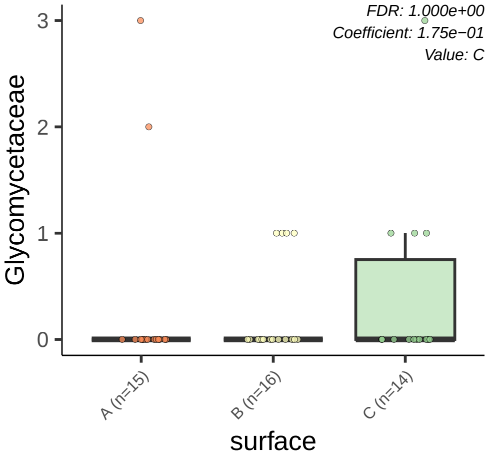
FDR: 1.000e+00
Coefficient: 1.75e−01
Value: C
Glycomycetaceae
0
1
2
3
A (n=15)
B (n=16)
C (n=14)
surface

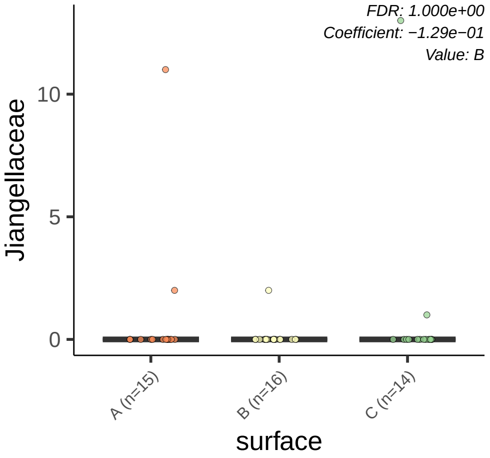
FDR: 1.000e+00
Coefficient: −1.29e−01
Value: B
Jiangellaceae
10
5
0
A (n=15)
B (n=16)
C (n=14)
surface

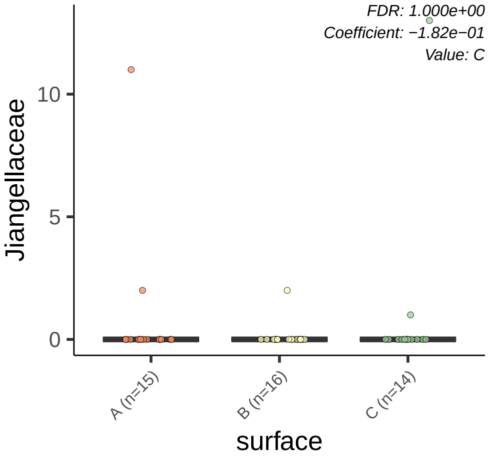
FDR: 1.000e+00
Coefficient: −1.82e−01
Value: C
Jiangellaceae
10
5
0
A (n=15)
B (n=16)
C (n=14)
surface

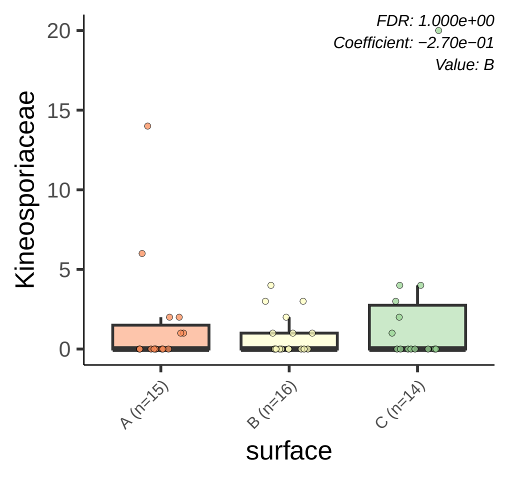
FDR: 1.000e+00
Coefficient: −2.70e−01
Value: B
Kineosporiaceae
20
15
10
5
0
A (n=15)
B (n=16)
C (n=14)
surface

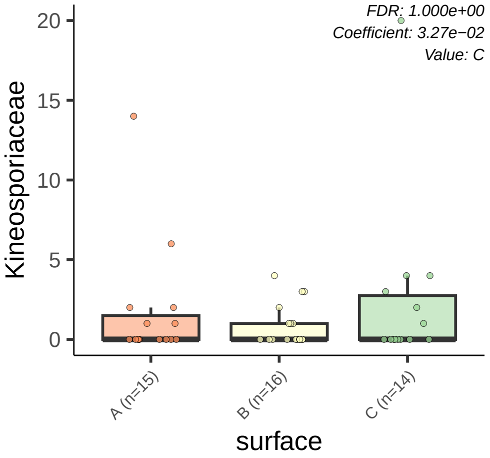

FDR: 1.000e+00
Coefficient: 3.27e−02
Value: C
Kineosporiaceae
0
5
10
15
20
A (n=15)
B (n=16)
C (n=14)
surface

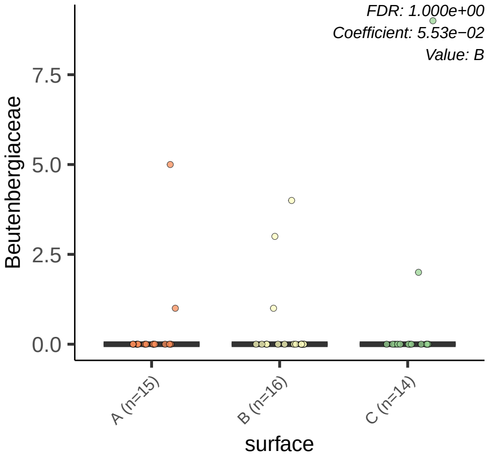

FDR: 1.000e+00
Coefficient: 5.53e−02
Value: B
Beutenbergiaceae
7.5
5.0
2.5
0.0
A (n=15)
B (n=16)
C (n=14)
surface

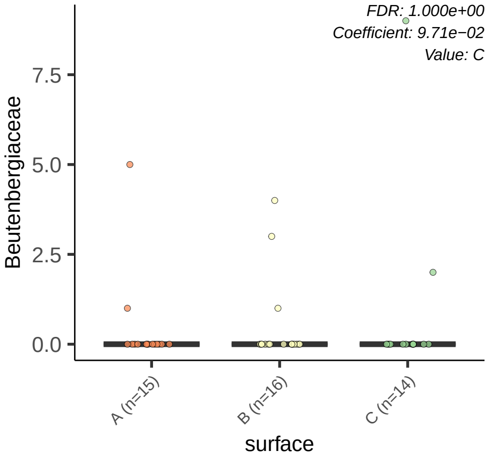
FDR: 1.000e+00
Coefficient: 9.71e−02
Value: C
Beutenbergiaceae
7.5
5.0
2.5
0.0
A (n=15)
B (n=16)
C (n=14)
surface

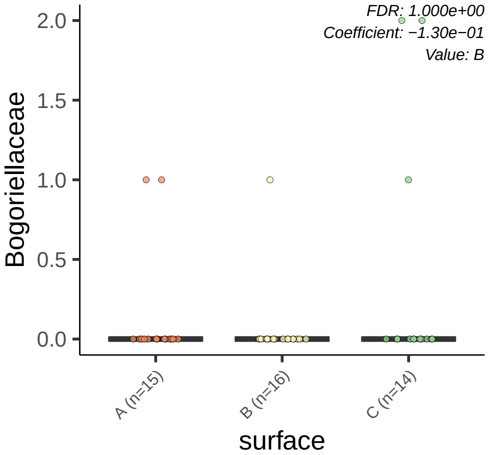
FDR: 1.000e+00
Coefficient: −1.30e−01
Value: B
Bogoriellaceae
2.0
1.5
1.0
0.5
0.0
A (n=15)
B (n=16)
C (n=14)
surface

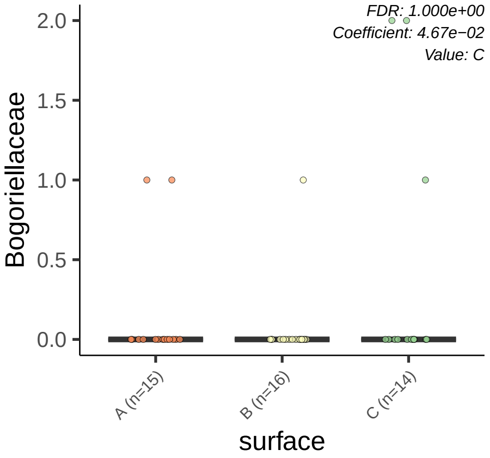

FDR: 1.000e+00
Coefficient: 4.67e−02
Value: C
Bogoriellaceae
2.0
1.5
1.0
0.5
0.0
A (n=15)
B (n=16)
C (n=14)
surface

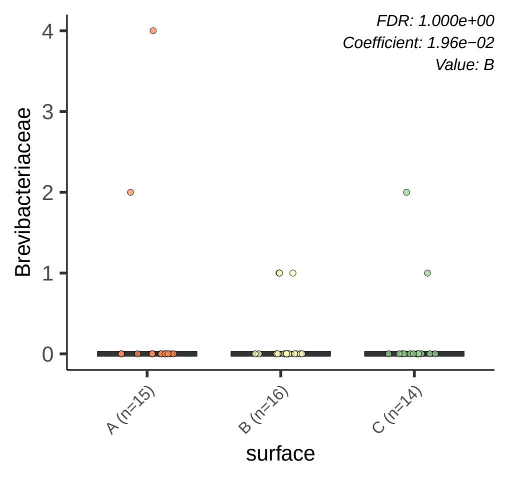
FDR: 1.000e+00
Coefficient: 1.96e−02
Value: B
Brevibacteriaceae
4
3
2
1
0
A (n=15)
B (n=16)
C (n=14)
surface

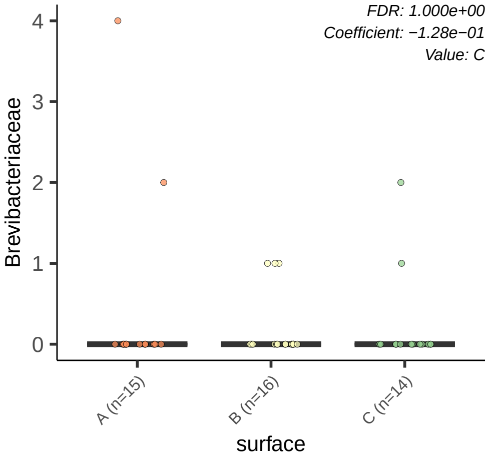
FDR: 1.000e+00
Coefficient: −1.28e−01
Value: C
Brevibacteriaceae
4
3
2
1
0
A (n=15)
B (n=16)
C (n=14)
surface

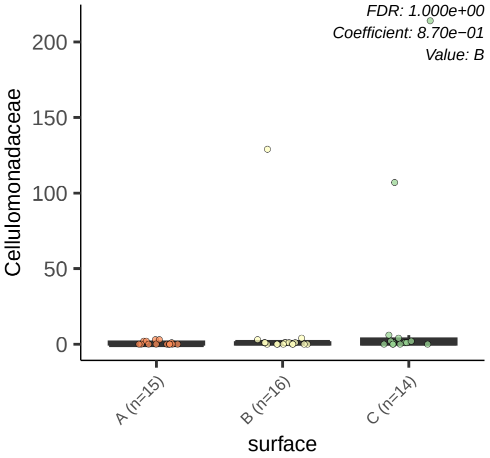
FDR: 1.000e+00
Coefficient: 8.70e−01
Value: B
Cellulomonadaceae
200
150
100
50
0
A (n=15)
B (n=16)
C (n=14)
surface

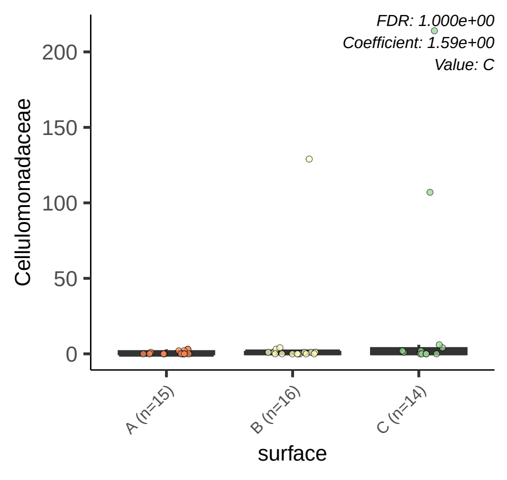
FDR: 1.000e+00
Coefficient: 1.59e+00
Value: C
Cellulomonadaceae
200
150
100
50
0
A (n=15)
B (n=16)
C (n=14)
surface

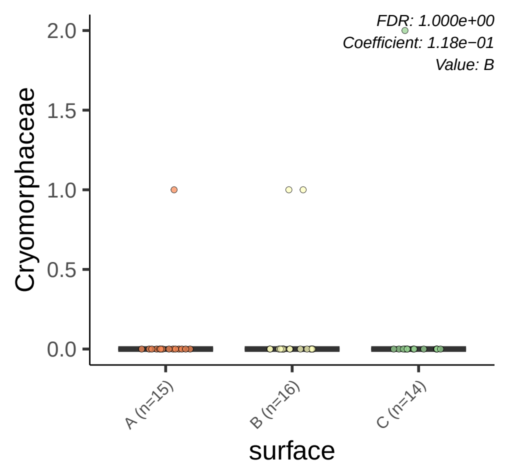

FDR: 1.000e+00
Coefficient: 1.18e−01
Value: B
Cryomorphaceae
2.0
1.5
1.0
0.5
0.0
A (n=15)
B (n=16)
C (n=14)
surface

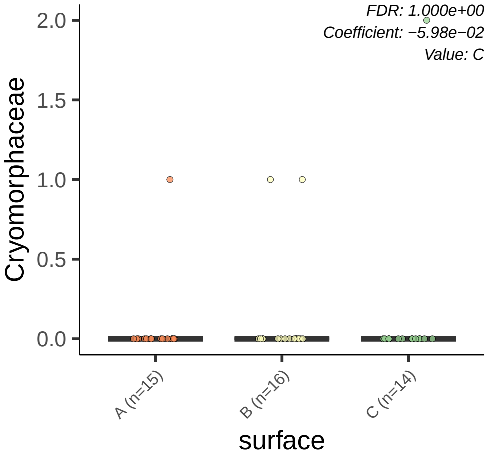
FDR: 1.000e+00
Coefficient: −5.98e−02
Value: C
Cryomorphaceae
2.0
1.5
1.0
0.5
0.0
A (n=15)
B (n=16)
C (n=14)
surface

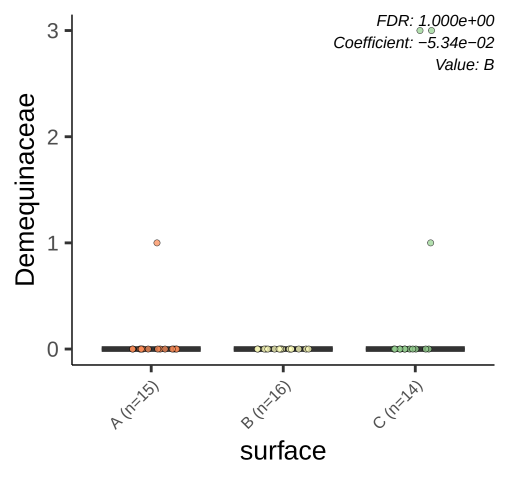

FDR: 1.000e+00
Coefficient: −5.34e−02
Value: B
Demequinaceae
3
2
1
0
A (n=15)
B (n=16)
C (n=14)
surface

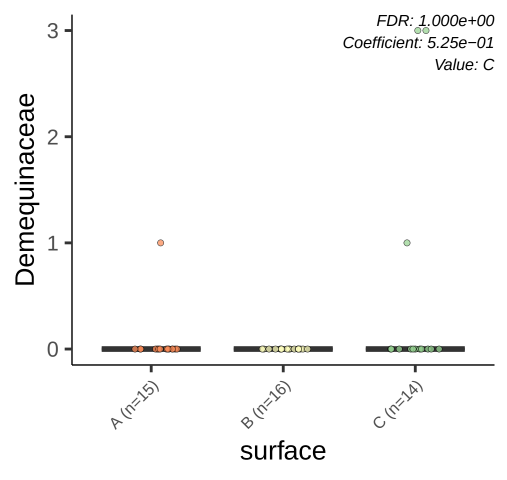

FDR: 1.000e+00
Coefficient: 5.25e−01
Value: C
Demequinaceae
3
2
1
0
A (n=15)
B (n=16)
C (n=14)
surface

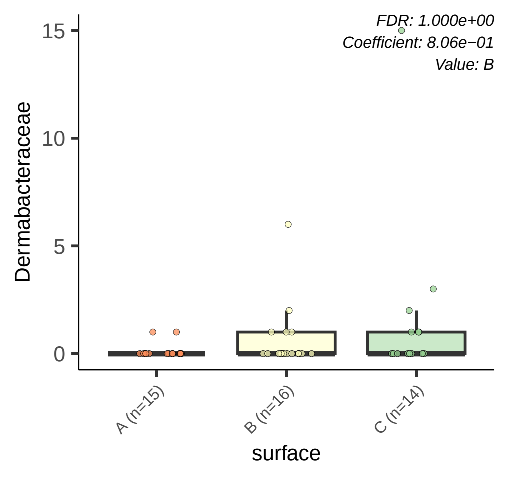

FDR: 1.000e+00
Coefficient: 8.06e−01
Value: B
Dermabacteraceae
15
10
5
0
A (n=15)
B (n=16)
C (n=14)
surface

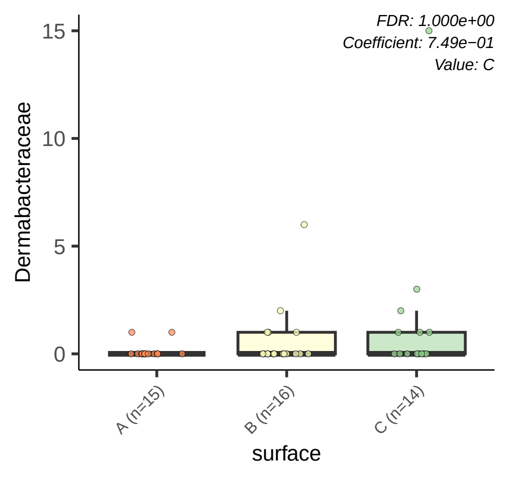

FDR: 1.000e+00
Coefficient: 7.49e−01
Value: C
Dermabacteraceae
15
10
5
0
A (n=15)
B (n=16)
C (n=14)
surface

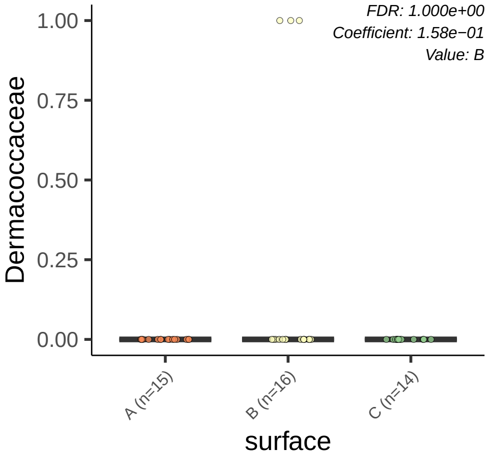
FDR: 1.000e+00
Coefficient: 1.58e−01
Value: B
Dermacoccaceae
1.00
0.75
0.50
0.25
0.00
A (n=15)
B (n=16)
C (n=14)
surface

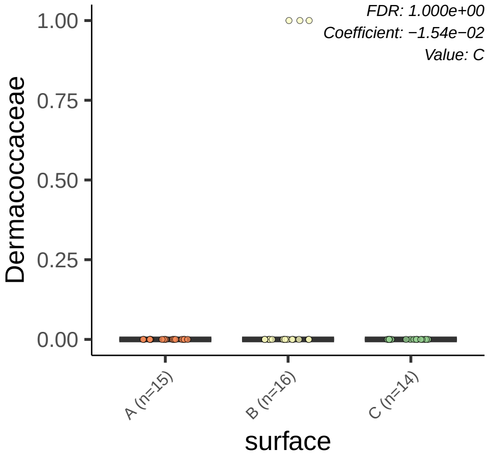
FDR: 1.000e+00
Coefficient: −1.54e−02
Value: C
Dermacoccaceae
1.00
0.75
0.50
0.25
0.00
A (n=15)
B (n=16)
C (n=14)
surface

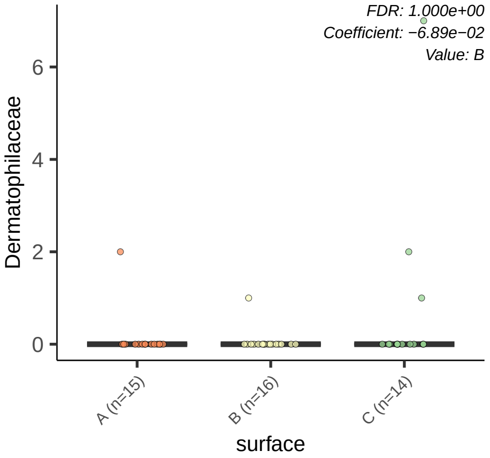

FDR: 1.000e+00
Coefficient: −6.89e−02
Value: B
Dermatophilaceae
0
2
4
6
A (n=15)
B (n=16)
C (n=14)
surface

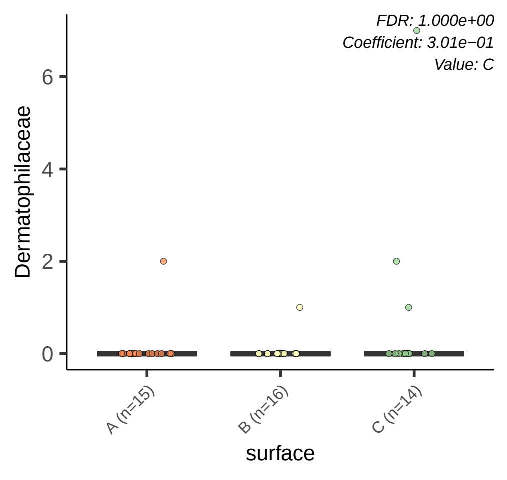
FDR: 1.000e+00
Coefficient: 3.01e−01
Value: C
Dermatophilaceae
0
2
4
6
A (n=15)
B (n=16)
C (n=14)
surface

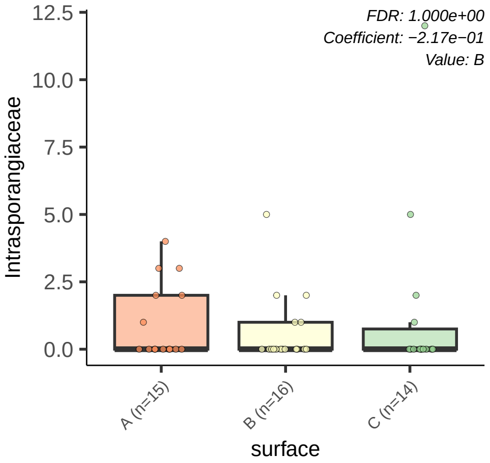
FDR: 1.000e+00
Coefficient: −2.17e−01
Value: B
Intrasporangiaceae
12.5
10.0
7.5
5.0
2.5
0.0
A (n=15)
B (n=16)
C (n=14)
surface

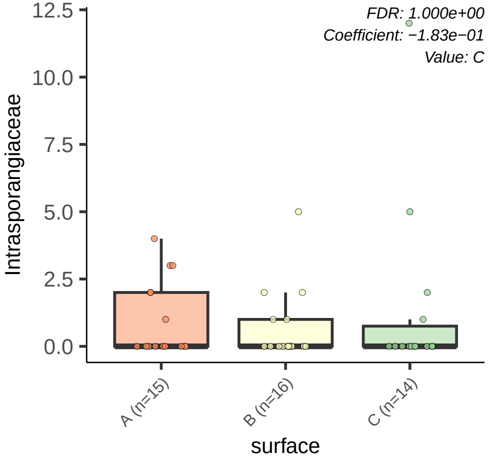
FDR: 1.000e+00
Coefficient: −1.83e−01
Value: C
Intrasporangiaceae
12.5
10.0
7.5
5.0
2.5
0.0
A (n=15)
B (n=16)
C (n=14)
surface

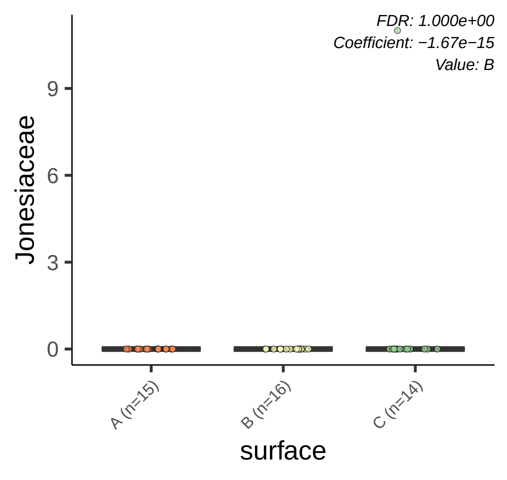
FDR: 1.000e+00
Coefficient: −1.67e−15
Value: B
Jonesiaceae
9
6
3
0
A (n=15)
B (n=16)
C (n=14)
surface

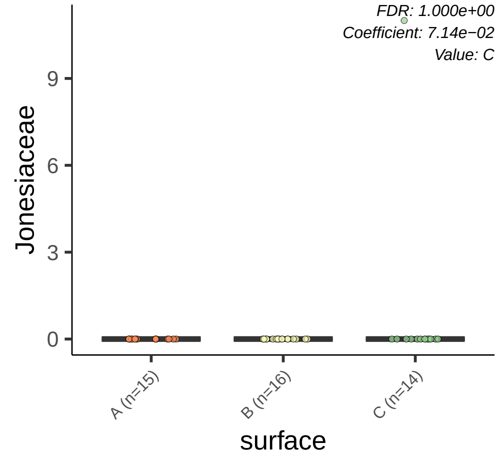
FDR: 1.000e+00
Coefficient: 7.14e−02
Value: C
Jonesiaceae
9
6
3
0
A (n=15)
B (n=16)
C (n=14)
surface

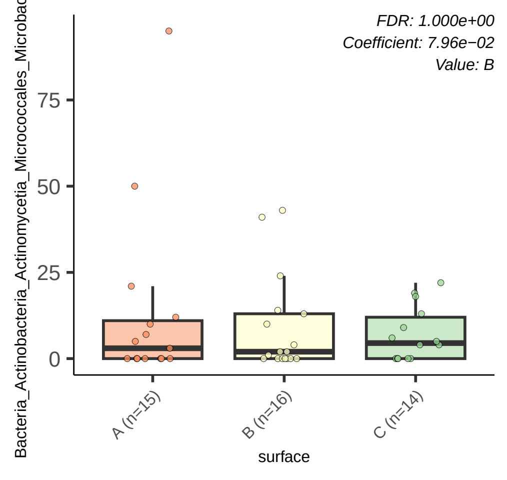

FDR: 1.000e+00
Coefficient: 7.96e−02
Value: B
Bacteria_Actinobacteria_Actinomycetia_Micrococcales_Microbac
0
25
50
75
A (n=15)
B (n=16)
C (n=14)
surface

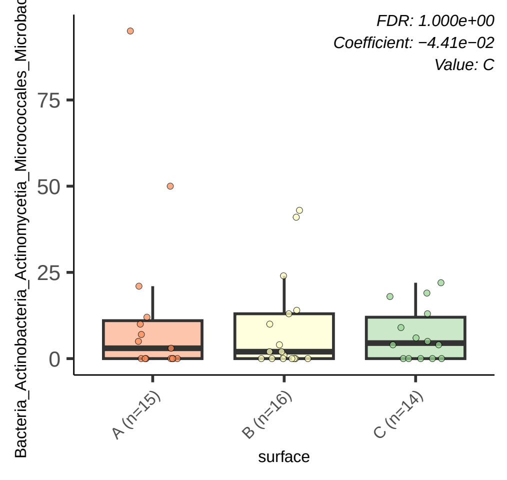
FDR: 1.000e+00
Coefficient: −4.41e−02
Value: C
Bacteria_Actinobacteria_Actinomycetia_Micrococcales_Microbac
0
25
50
75
A (n=15)
B (n=16)
C (n=14)
surface

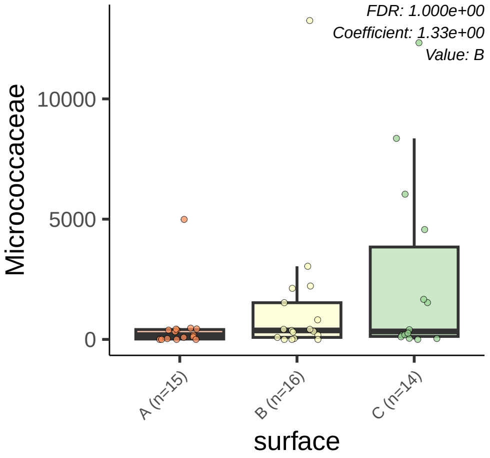

FDR: 1.000e+00
Coefficient: 1.33e+00
Value: B
Micrococcaceae
10000
5000
0
A (n=15)
B (n=16)
C (n=14)
surface

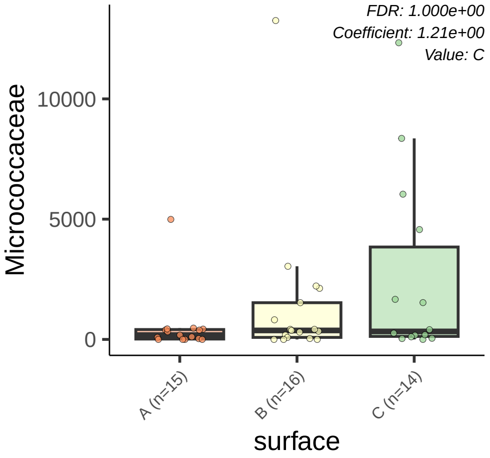

FDR: 1.000e+00
Coefficient: 1.21e+00
Value: C
Micrococcaceae
10000
5000
0
A (n=15)
B (n=16)
C (n=14)
surface

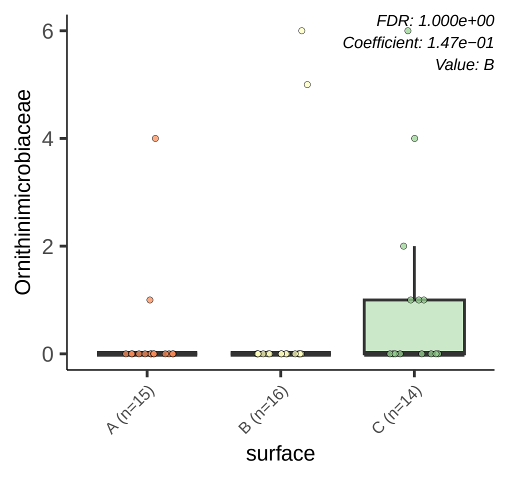
FDR: 1.000e+00
Coefficient: 1.47e−01
Value: B
Ornithinimicrobiaceae
0
2
4
6
A (n=15)
B (n=16)
C (n=14)
surface

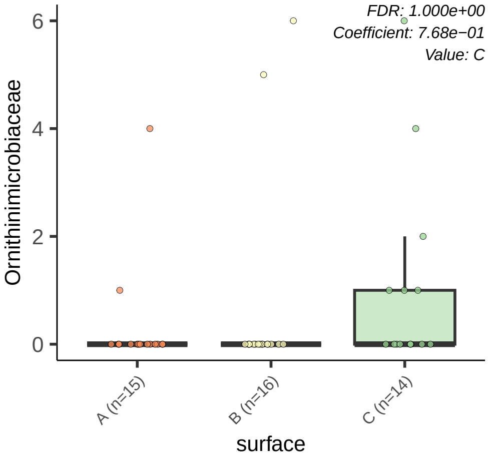

FDR: 1.000e+00
Coefficient: 7.68e−01
Value: C
Ornithinimicrobiaceae
0
2
4
6
A (n=15)
B (n=16)
C (n=14)
surface

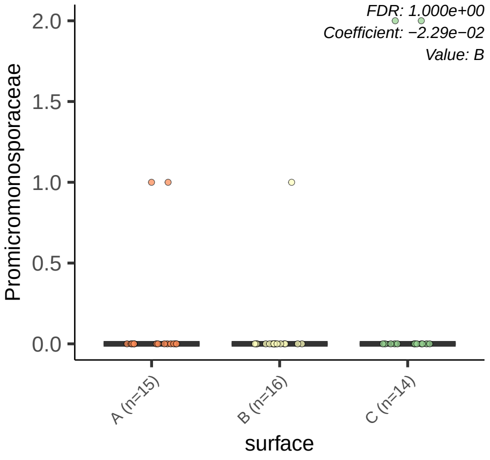

FDR: 1.000e+00
Coefficient: −2.29e−02
Value: B
Promicromonosporaceae
2.0
1.5
1.0
0.5
0.0
A (n=15)
B (n=16)
C (n=14)
surface

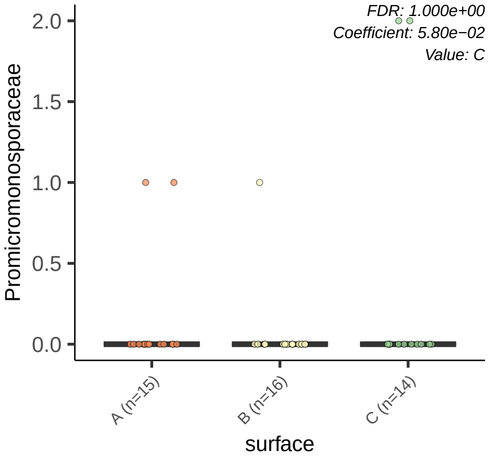

FDR: 1.000e+00
Coefficient: 5.80e−02
Value: C
Promicromonosporaceae
2.0
1.5
1.0
0.5
0.0
A (n=15)
B (n=16)
C (n=14)
surface

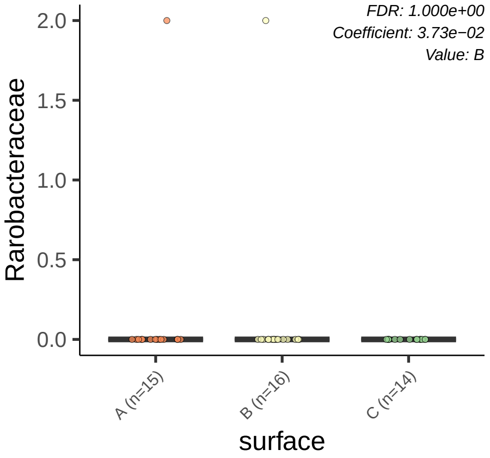

FDR: 1.000e+00
Coefficient: 3.73e−02
Value: B
Rarobacteraceae
2.0
1.5
1.0
0.5
0.0
A (n=15)
B (n=16)
C (n=14)
surface

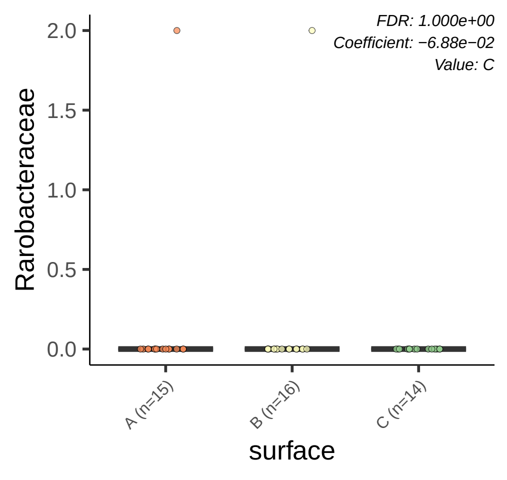

FDR: 1.000e+00
Coefficient: −6.88e−02
Value: C
Rarobacteraceae
2.0
1.5
1.0
0.5
0.0
A (n=15)
B (n=16)
C (n=14)
surface

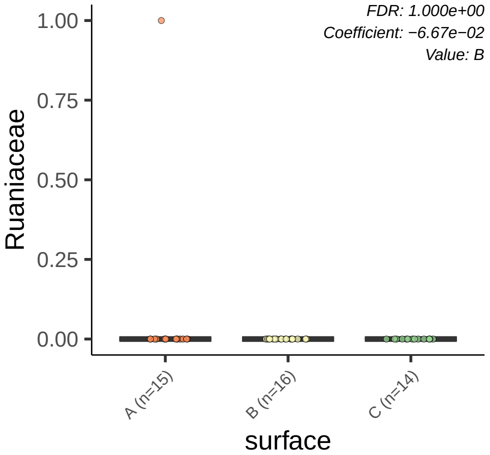
FDR: 1.000e+00
Coefficient: −6.67e−02
Value: B
Ruaniaceae
1.00
0.75
0.50
0.25
0.00
A (n=15)
B (n=16)
C (n=14)
surface

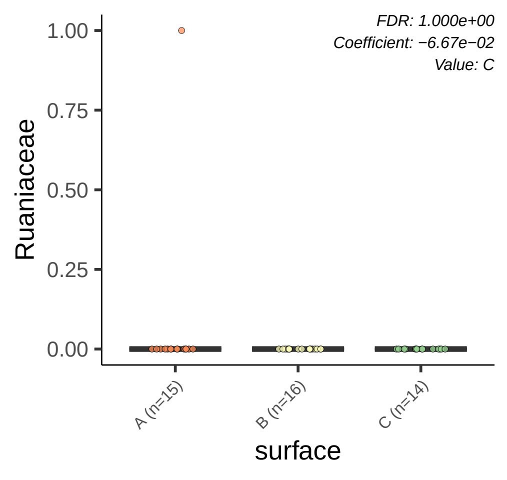
FDR: 1.000e+00
Coefficient: −6.67e−02
Value: C
Ruaniaceae
1.00
0.75
0.50
0.25
0.00
A (n=15)
B (n=16)
C (n=14)
surface

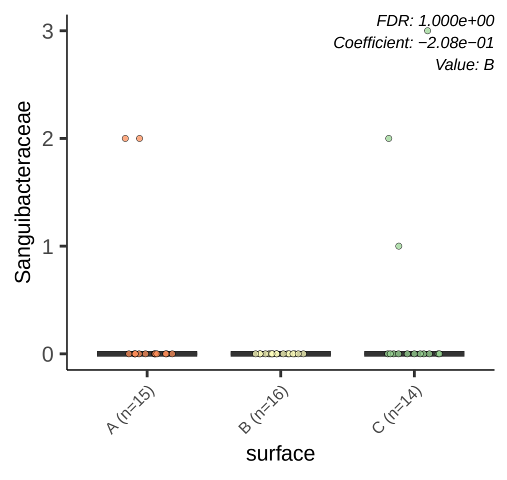

FDR: 1.000e+00
Coefficient: −2.08e−01
Value: B
Sanguibacteraceae
0
1
2
3
A (n=15)
B (n=16)
C (n=14)
surface

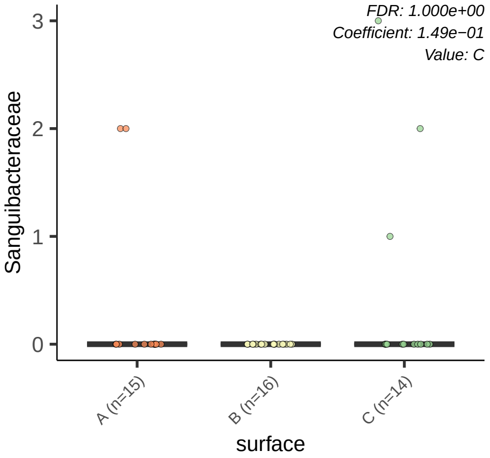

FDR: 1.000e+00
Coefficient: 1.49e−01
Value: C
Sanguibacteraceae
3
2
1
0
A (n=15)
B (n=16)
C (n=14)
surface

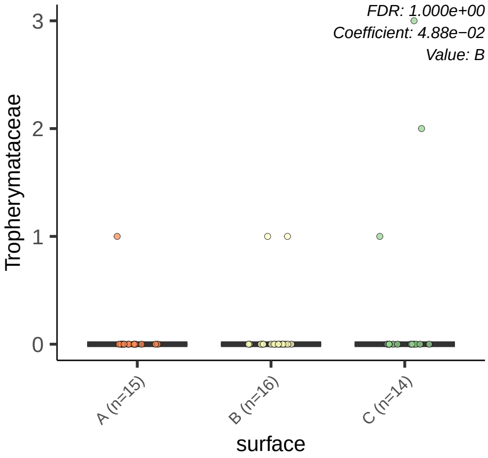
FDR: 1.000e+00
Coefficient: 4.88e−02
Value: B
Tropherymataceae
0
1
2
3
A (n=15)
B (n=16)
C (n=14)
surface

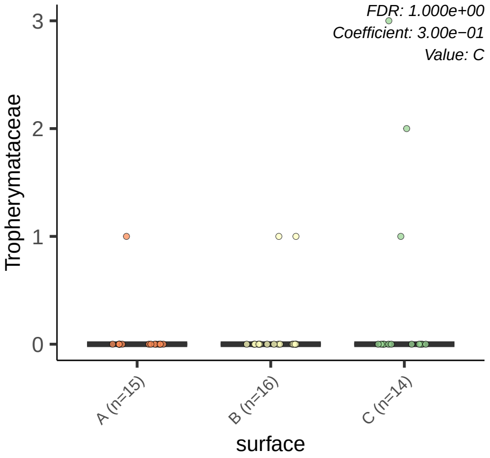
FDR: 1.000e+00
Coefficient: 3.00e−01
Value: C
Tropherymataceae
0
1
2
3
A (n=15)
B (n=16)
C (n=14)
surface

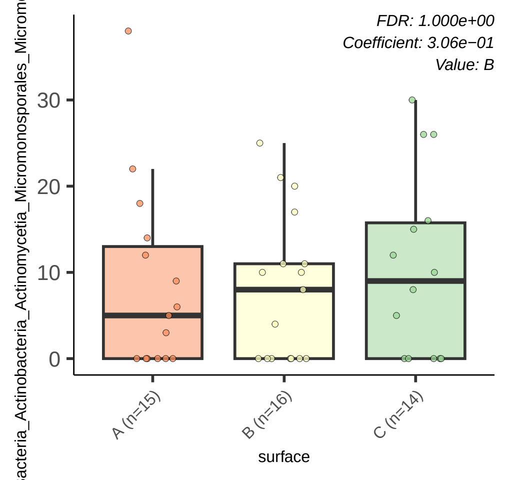

FDR: 1.000e+00
Coefficient: 3.06e−01
Value: B
30
20
10
0
A (n=15)
B (n=16)
C (n=14)
surface
bacteria_Actinobacteria_Actinomycetia_Micromonosporales_Micromo

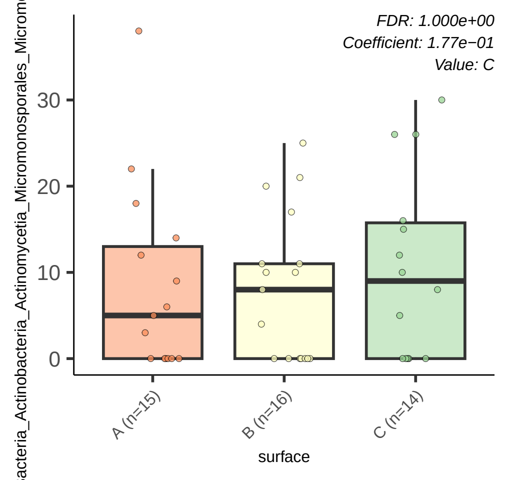

FDR: 1.000e+00
Coefficient: 1.77e−01
Value: C
30
20
10
0
A (n=15)
B (n=16)
C (n=14)
surface
bacteria_Actinobacteria_Actinomycetia_Micromonosporales_Micromo

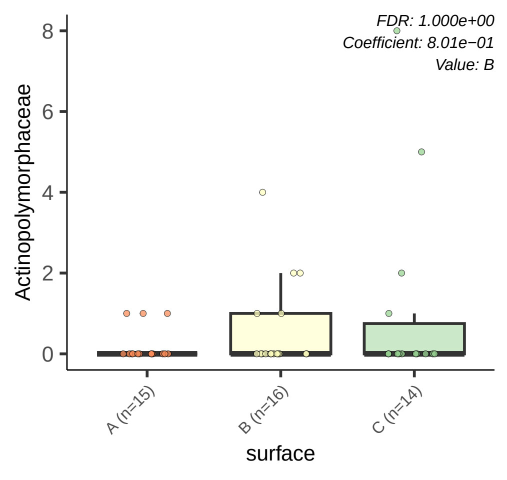

FDR: 1.000e+00
Coefficient: 8.01e−01
Value: B
Actinopolymorphaceae
0
2
4
6
8
A (n=15)
B (n=16)
C (n=14)
surface

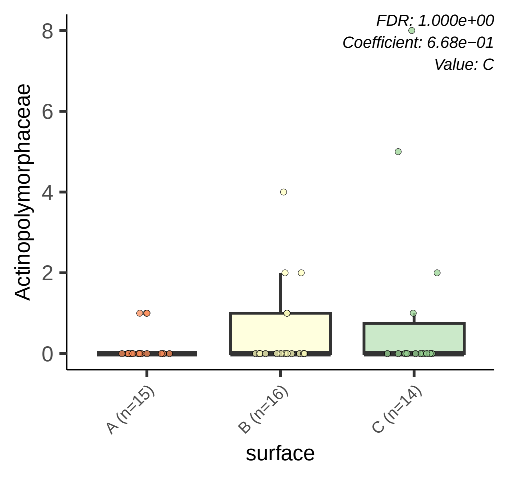
FDR: 1.000e+00
Coefficient: 6.68e−01
Value: C
Actinopolymorphaceae
8
6
4
2
0
A (n=15)
B (n=16)
C (n=14)
surface

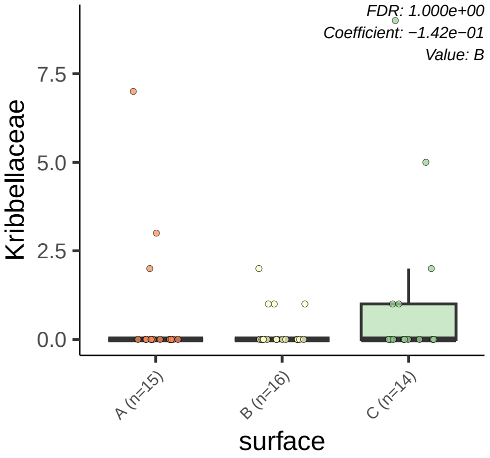
FDR: 1.000e+00
Coefficient: −1.42e−01
Value: B
Kribbellaceae
7.5
5.0
2.5
0.0
A (n=15)
B (n=16)
C (n=14)
surface

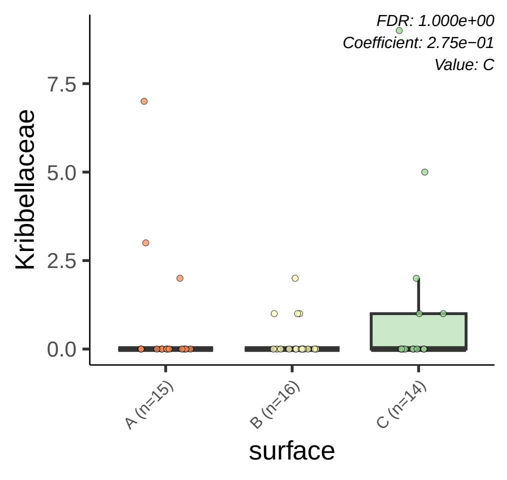
FDR: 1.000e+00
Coefficient: 2.75e−01
Value: C
Kribbellaceae
7.5
5.0
2.5
0.0
A (n=15)
B (n=16)
C (n=14)
surface

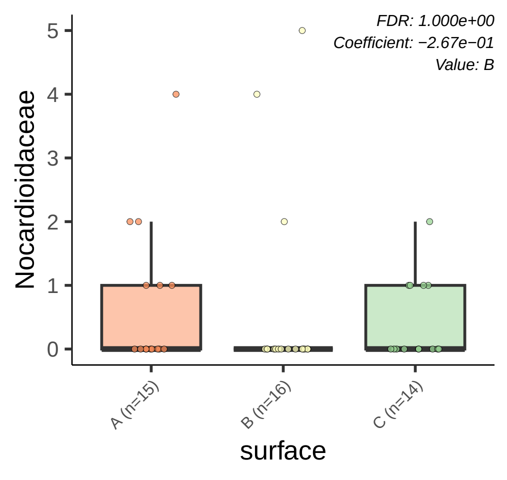

FDR: 1.000e+00
Coefficient: −2.67e−01
Value: B
Nocardioidaceae
0
1
2
3
4
5
A (n=15)
B (n=16)
C (n=14)
surface

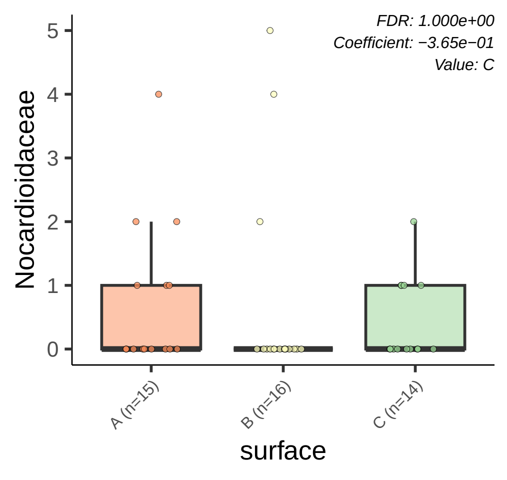
FDR: 1.000e+00
Coefficient: −3.65e−01
Value: C
Nocardioidaceae
0
1
2
3
4
5
A (n=15)
B (n=16)
C (n=14)
surface

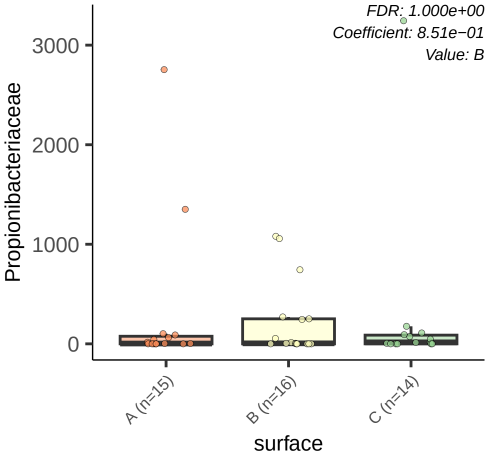
FDR: 1.000e+00
Coefficient: 8.51e−01
Value: B
3000
2000
1000
0
Propionibacteriaceae
A (n=15)
B (n=16)
C (n=14)
surface

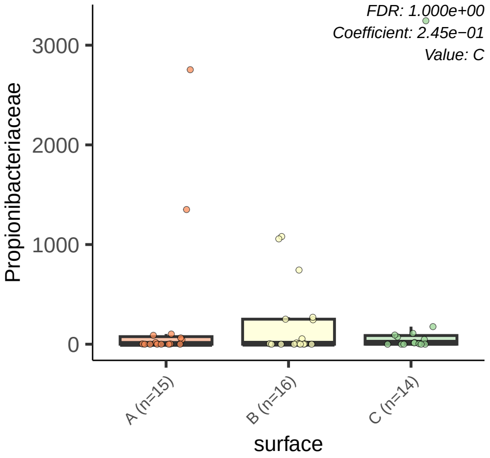

FDR: 1.000e+00
Coefficient: 2.45e−01
Value: C
Propionibacteriaceae
3000
2000
1000
0
A (n=15)
B (n=16)
C (n=14)
surface

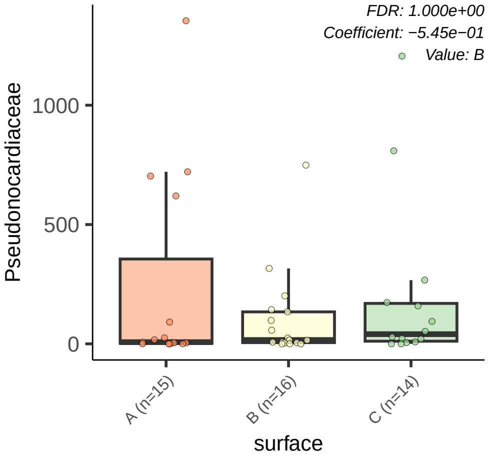

FDR: 1.000e+00
Coefficient: −5.45e−01
Value: B
Pseudonocardiaceae
1000
500
0
A (n=15)
B (n=16)
C (n=14)
surface

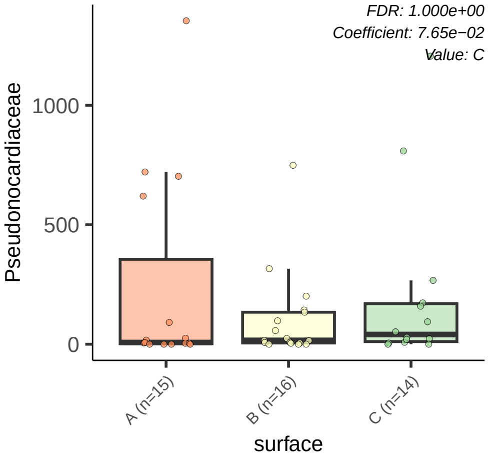
FDR: 1.000e+00
Coefficient: 7.65e−02
Value: C
Pseudonocardiaceae
1000
500
0
A (n=15)
B (n=16)
C (n=14)
surface

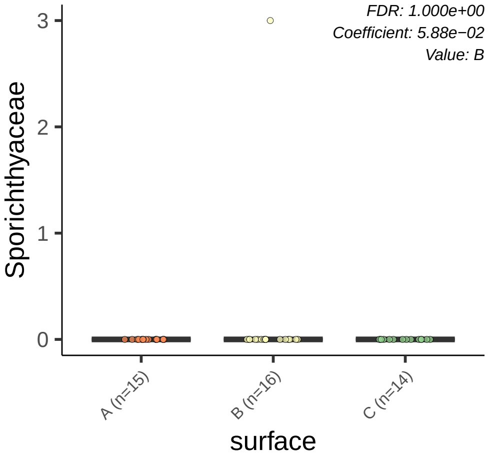
FDR: 1.000e+00
Coefficient: 5.88e−02
Value: B
Sporichthyaceae
3
2
1
0
A (n=15)
B (n=16)
C (n=14)
surface

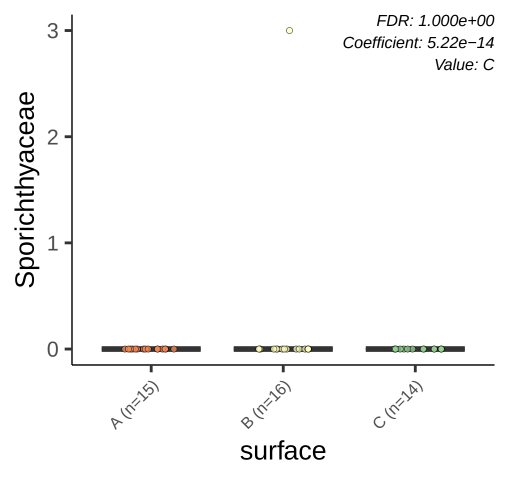

FDR: 1.000e+00
Coefficient: 5.22e−14
Value: C
Sporichthyaceae
3
2
1
0
A (n=15)
B (n=16)
C (n=14)
surface

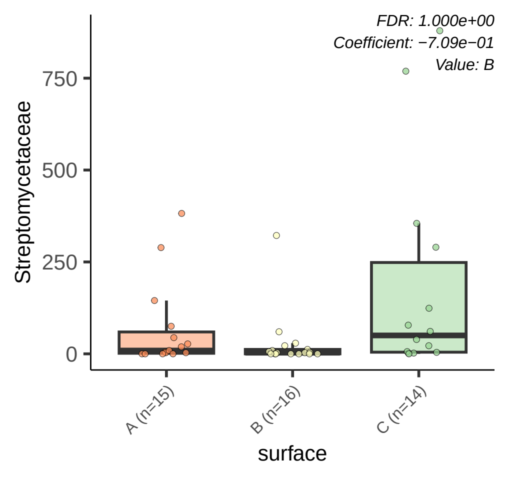

FDR: 1.000e+00
Coefficient: −7.09e−01
Value: B
Streptomycetaceae
750
500
250
0
A (n=15)
B (n=16)
C (n=14)
surface

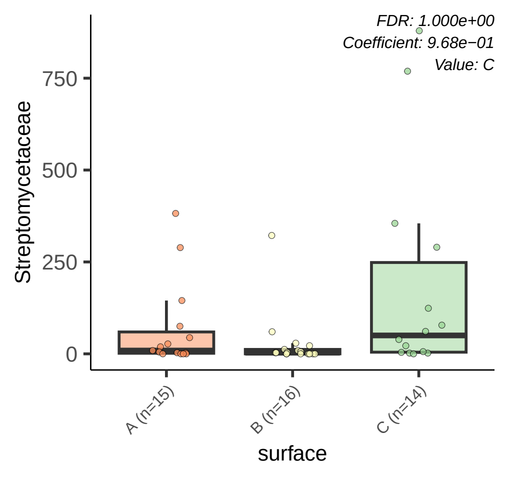

FDR: 1.000e+00
Coefficient: 9.68e−01
Value: C
Streptomycetaceae
750
500
250
0
A (n=15)
B (n=16)
C (n=14)
surface

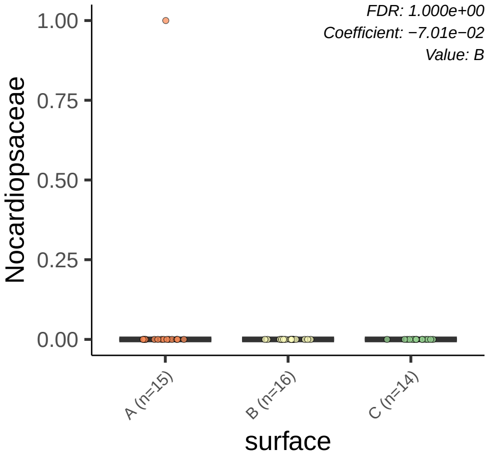

FDR: 1.000e+00
Coefficient: −7.01e−02
Value: B
Nocardiopsaceae
1.00
0.75
0.50
0.25
0.00
A (n=15)
B (n=16)
C (n=14)
surface

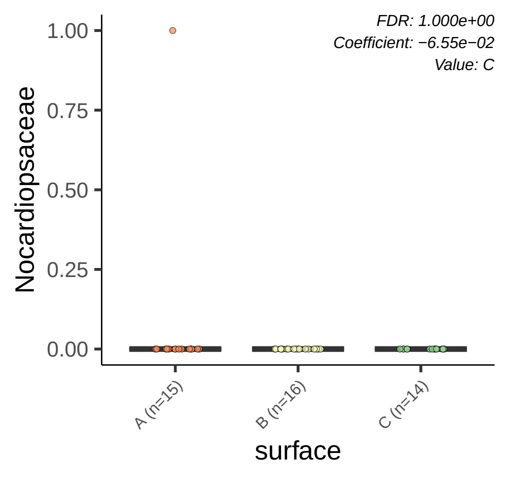
FDR: 1.000e+00
Coefficient: −6.55e−02
Value: C
Nocardiopsaceae
1.00
0.75
0.50
0.25
0.00
A (n=15)
B (n=16)
C (n=14)
surface

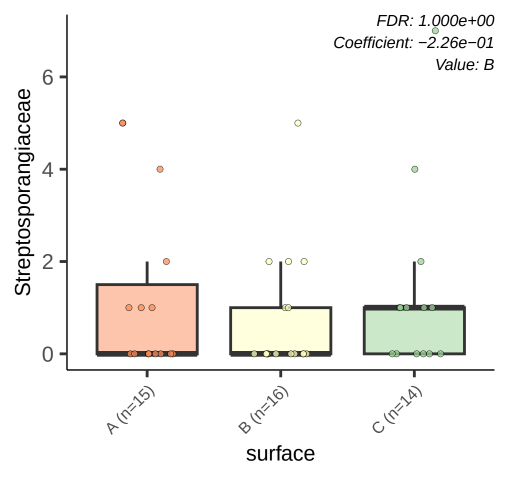
FDR: 1.000e+00
Coefficient: −2.26e−01
Value: B
Streptosporangiaceae
0
2
4
6
A (n=15)
B (n=16)
C (n=14)
surface

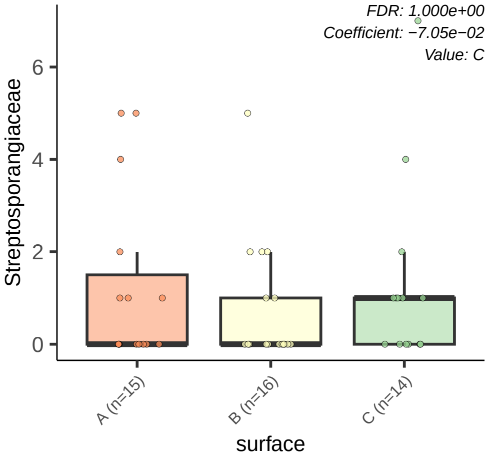
FDR: 1.000e+00
Coefficient: −7.05e−02
Value: C
Streptosporangiaceae
0
2
4
6
A (n=15)
B (n=16)
C (n=14)
surface

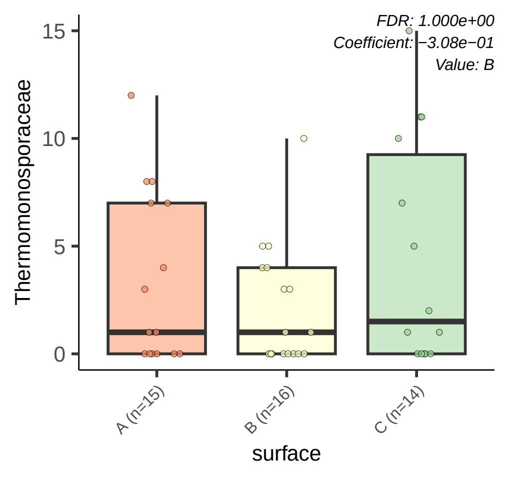

FDR: 1.000e+00
Coefficient: −3.08e−01
Value: B
Thermomonosporaceae
15
10
5
0
A (n=15)
B (n=16)
C (n=14)
surface

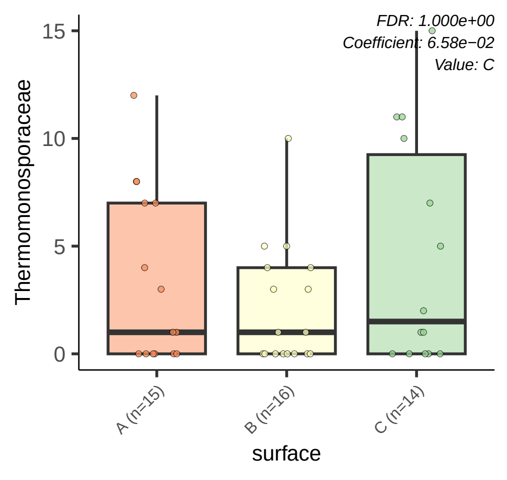
FDR: 1.000e+00
Coefficient: 6.58e−02
Value: C
Thermomonosporaceae
15
10
5
0
A (n=15)
B (n=16)
C (n=14)
surface

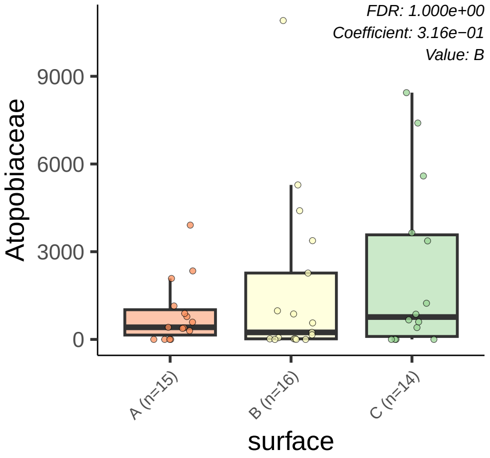
FDR: 1.000e+00
Coefficient: 3.16e−01
Value: B
Atopobiaceae
9000
6000
3000
0
A (n=15)
B (n=16)
C (n=14)
surface

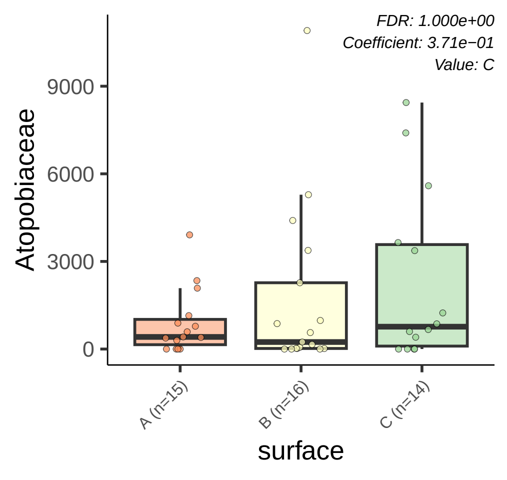

FDR: 1.000e+00
Coefficient: 3.71e−01
Value: C
Atopobiaceae
9000
6000
3000
0
A (n=15)
B (n=16)
C (n=14)
surface

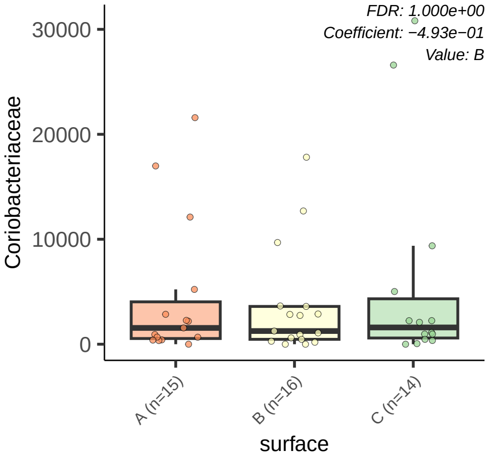

FDR: 1.000e+00
Coefficient: −4.93e−01
Value: B
30000
20000
10000
0
Coriobacteriaceae
A (n=15)
B (n=16)
C (n=14)
surface

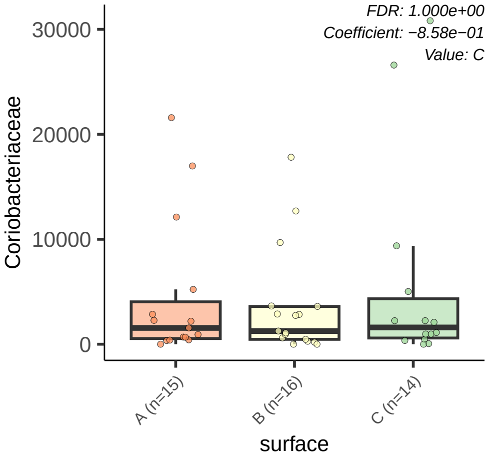
FDR: 1.000e+00
Coefficient: −8.58e−01
Value: C
30000
20000
10000
0
Coriobacteriaceae
A (n=15)
B (n=16)
C (n=14)
surface

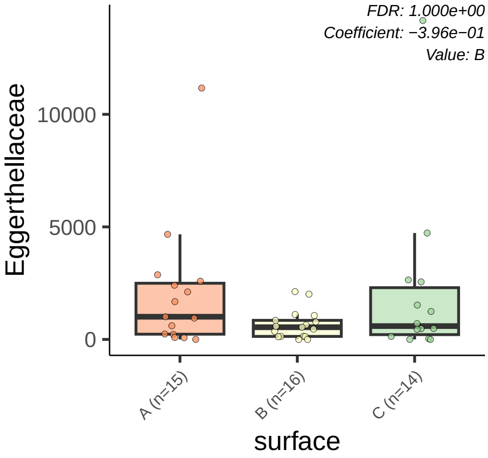
FDR: 1.000e+00
Coefficient: −3.96e−01
Value: B
Eggerthellaceae
10000
5000
0
A (n=15)
B (n=16)
C (n=14)
surface

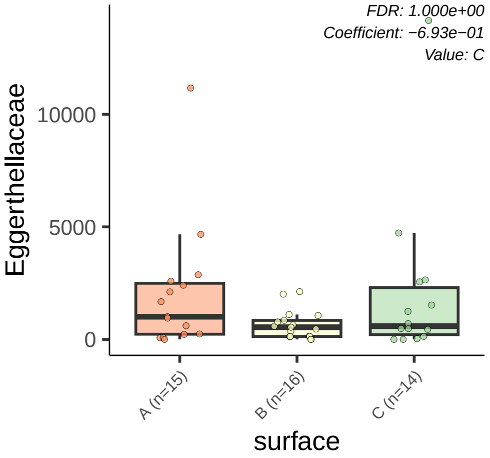
FDR: 1.000e+00
Coefficient: −6.93e−01
Value: C
Eggerthellaceae
10000
5000
0
A (n=15)
B (n=16)
C (n=14)
surface

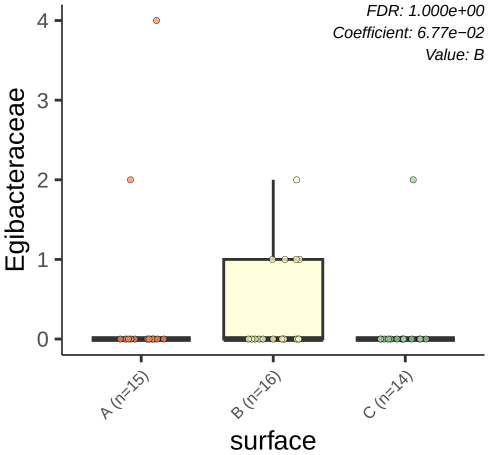

FDR: 1.000e+00
Coefficient: 6.77e−02
Value: B
Egibacteraceae
0
1
2
3
4
A (n=15)
B (n=16)
C (n=14)
surface

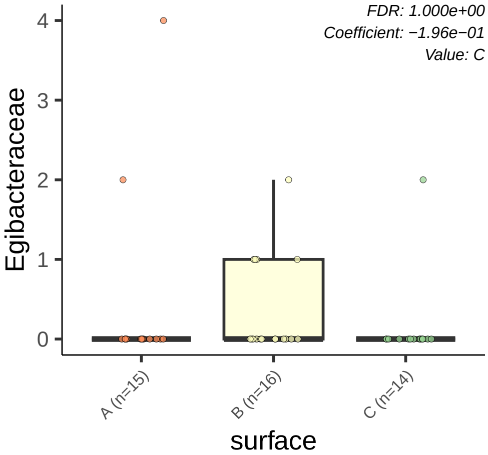

FDR: 1.000e+00
Coefficient: −1.96e−01
Value: C
Egibacteraceae
4
3
2
1
0
A (n=15)
B (n=16)
C (n=14)
surface

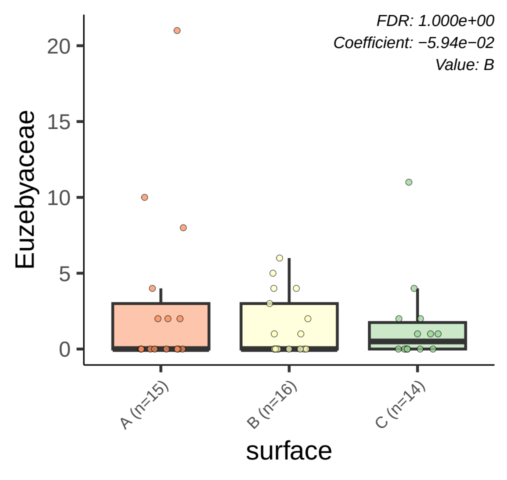
FDR: 1.000e+00
Coefficient: −5.94e−02
Value: B
Euzebyaceae
0
5
10
15
20
A (n=15)
B (n=16)
C (n=14)
surface

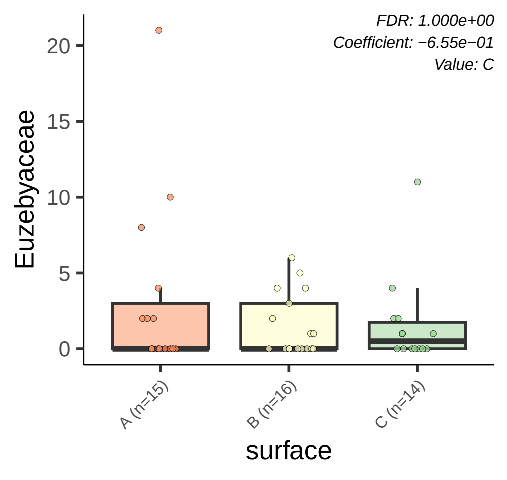
FDR: 1.000e+00
Coefficient: −6.55e−01
Value: C
Euzebyaceae
0
5
10
15
20
A (n=15)
B (n=16)
C (n=14)
surface

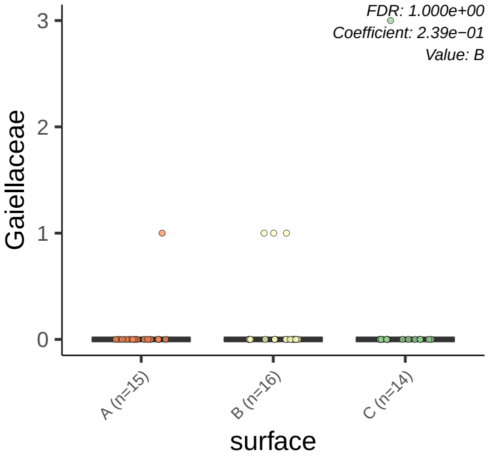

FDR: 1.000e+00
Coefficient: 2.39e−01
Value: B
Gaiellaceae
0
1
2
3
A (n=15)
B (n=16)
C (n=14)
surface

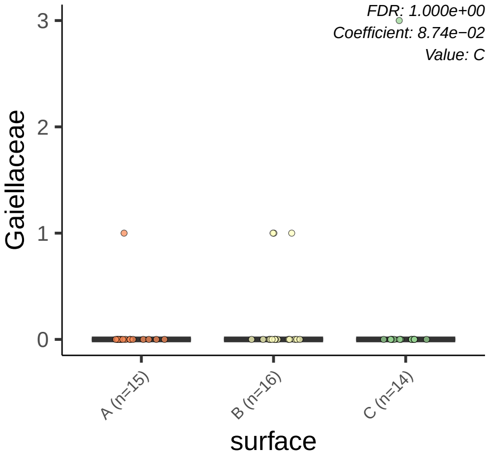
FDR: 1.000e+00
Coefficient: 8.74e−02
Value: C
Gaiellaceae
3
2
1
0
A (n=15)
B (n=16)
C (n=14)
surface

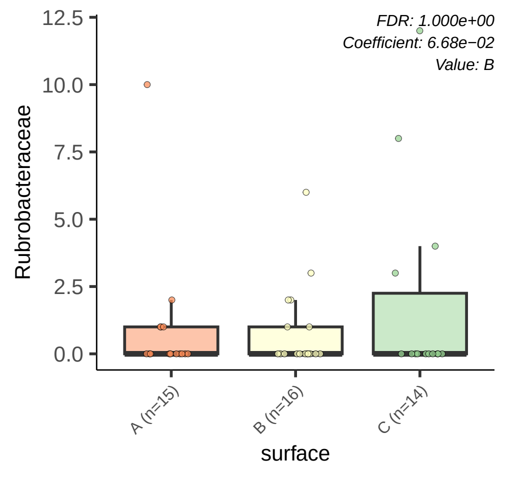

FDR: 1.000e+00
Coefficient: 6.68e−02
Value: B
Rubrobacteraceae
12.5
10.0
7.5
5.0
2.5
0.0
A (n=15)
B (n=16)
C (n=14)
surface

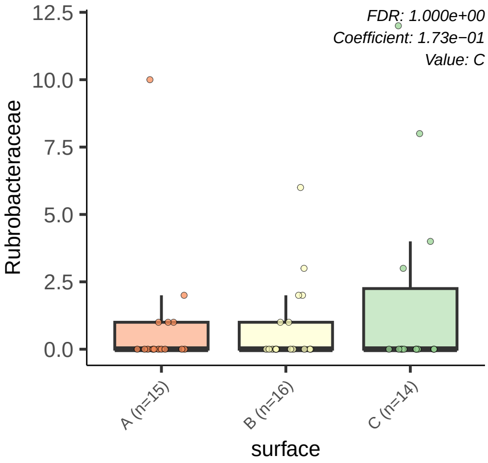

FDR: 1.000e+00
Coefficient: 1.73e−01
Value: C
Rubrobacteraceae
0.0
2.5
5.0
7.5
10.0
12.5
A (n=15)
B (n=16)
C (n=14)
surface

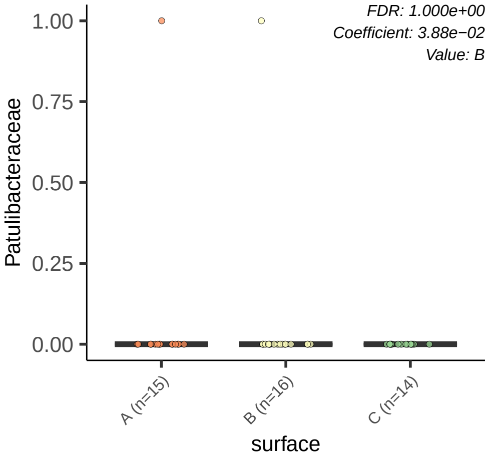

FDR: 1.000e+00
Coefficient: 3.88e−02
Value: B
Patulibacteraceae
1.00
0.75
0.50
0.25
0.00
A (n=15)
B (n=16)
C (n=14)
surface

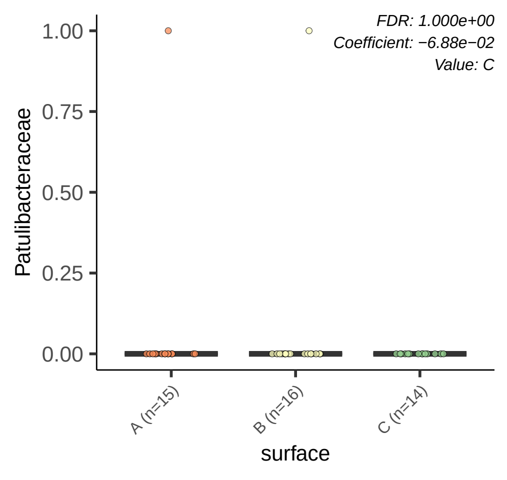
FDR: 1.000e+00
Coefficient: −6.88e−02
Value: C
Patulibacteraceae
1.00
0.75
0.50
0.25
0.00
A (n=15)
B (n=16)
C (n=14)
surface

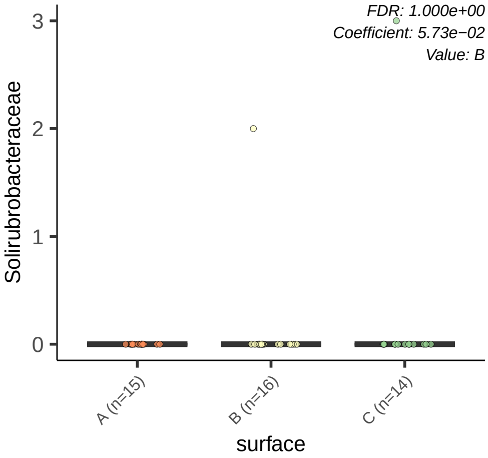

FDR: 1.000e+00
Coefficient: 5.73e−02
Value: B
Solirubrobacteraceae
3
2
1
0
A (n=15)
B (n=16)
C (n=14)
surface

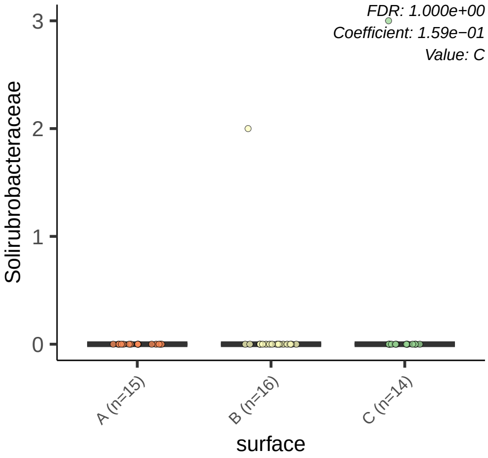
FDR: 1.000e+00
Coefficient: 1.59e−01
Value: C
Solirubrobacteraceae
0
1
2
3
A (n=15)
B (n=16)
C (n=14)
surface

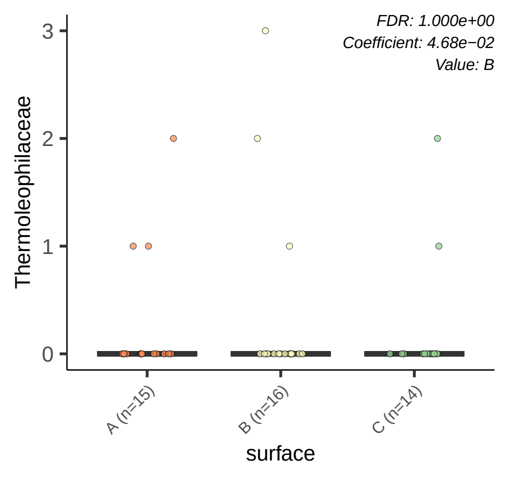
FDR: 1.000e+00
Coefficient: 4.68e−02
Value: B
Thermoleophilaceae
3
2
1
0
A (n=15)
B (n=16)
C (n=14)
surface

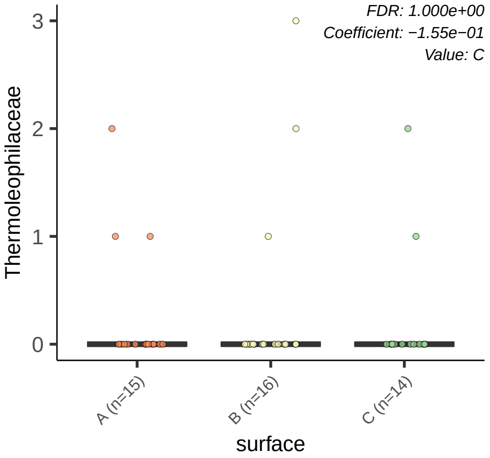
FDR: 1.000e+00
Coefficient: −1.55e−01
Value: C
Thermoleophilaceae
3
2
1
0
A (n=15)
B (n=16)
C (n=14)
surface

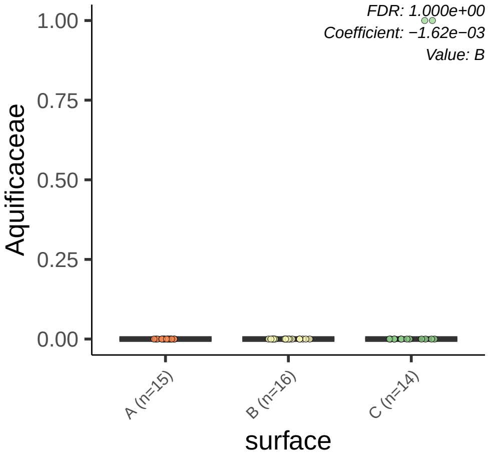

FDR: 1.000e+00
Coefficient: −1.62e−03
Value: B
Aquificaceae
1.00
0.75
0.50
0.25
0.00
A (n=15)
B (n=16)
C (n=14)
surface

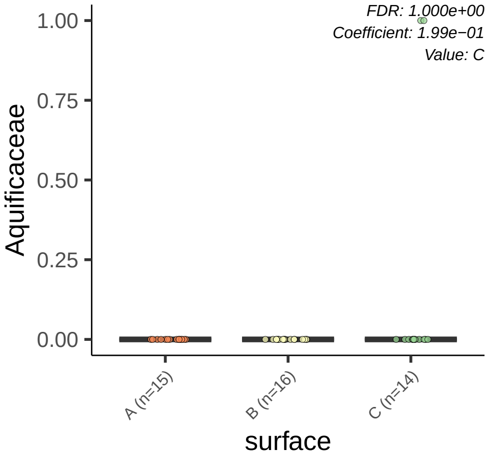
FDR: 1.000e+00
Coefficient: 1.99e−01
Value: C
Aquificaceae
1.00
0.75
0.50
0.25
0.00
A (n=15)
B (n=16)
C (n=14)
surface

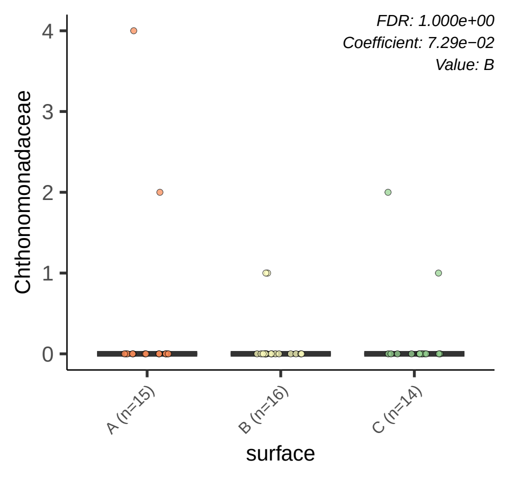
FDR: 1.000e+00
Coefficient: 7.29e−02
Value: B
Chthonomonadaceae
4
3
2
1
0
A (n=15)
B (n=16)
C (n=14)
surface

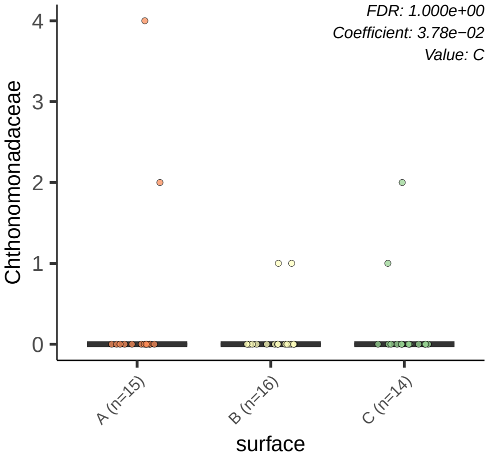

FDR: 1.000e+00
Coefficient: 3.78e−02
Value: C
Chthonomonadaceae
4
3
2
1
0
A (n=15)
B (n=16)
C (n=14)
surface

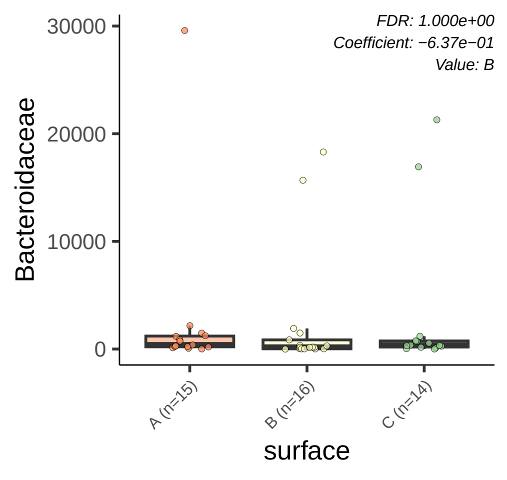

FDR: 1.000e+00
Coefficient: −6.37e−01
Value: B
Bacteroidaceae
30000
20000
10000
0
A (n=15)
B (n=16)
C (n=14)
surface

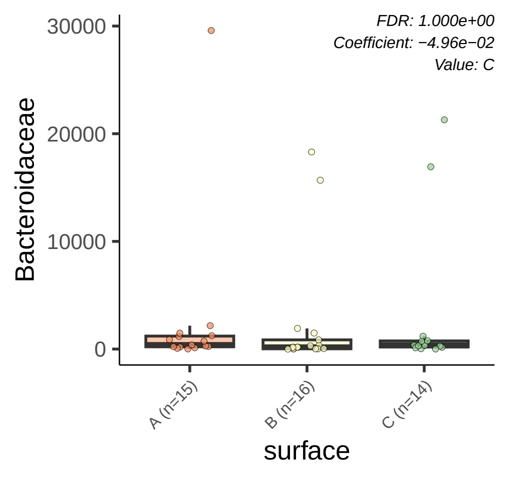
FDR: 1.000e+00
Coefficient: −4.96e−02
Value: C
Bacteroidaceae
30000
20000
10000
0
A (n=15)
B (n=16)
C (n=14)
surface

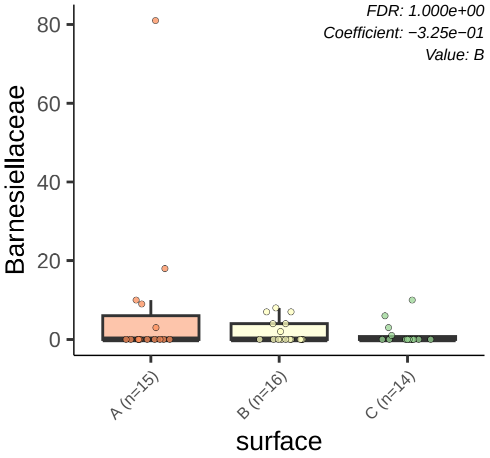

FDR: 1.000e+00
Coefficient: −3.25e−01
Value: B
Barnesiellaceae
80
60
40
20
0
A (n=15)
B (n=16)
C (n=14)
surface

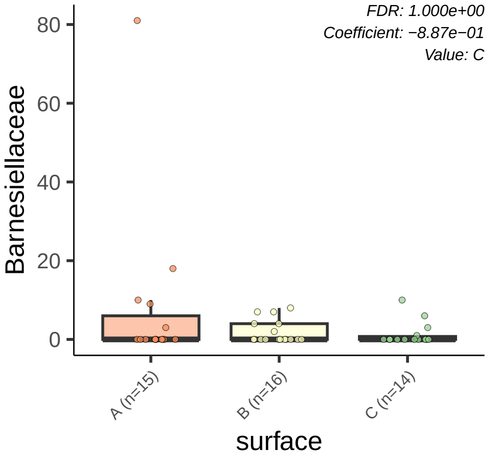

FDR: 1.000e+00
Coefficient: −8.87e−01
Value: C
Barnesiellaceae
80
60
40
20
0
A (n=15)
B (n=16)
C (n=14)
surface

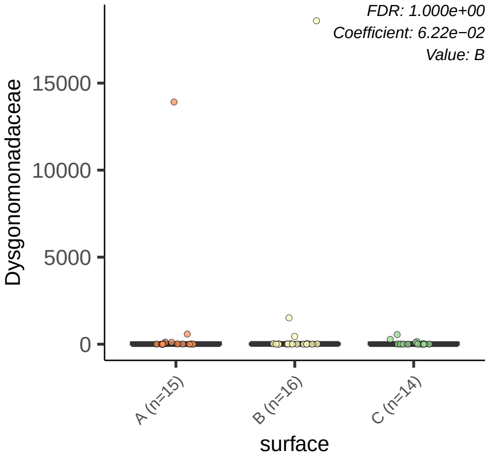
FDR: 1.000e+00
Coefficient: 6.22e−02
Value: B
Dysgonomonadaceae
15000
10000
5000
0
A (n=15)
B (n=16)
C (n=14)
surface

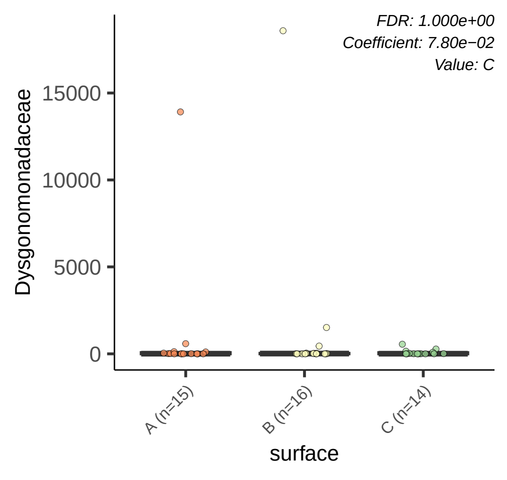

FDR: 1.000e+00
Coefficient: 7.80e−02
Value: C
Dysgonomonadaceae
15000
10000
5000
0
A (n=15)
B (n=16)
C (n=14)
surface

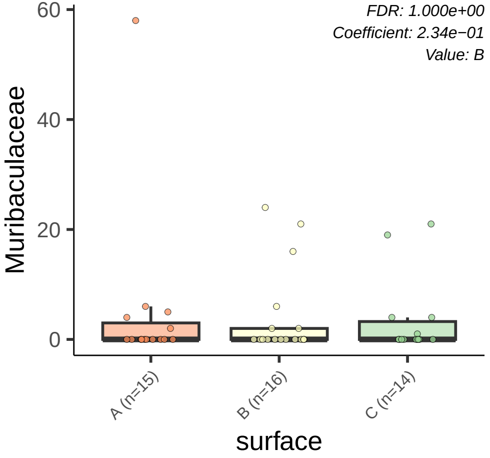
FDR: 1.000e+00
Coefficient: 2.34e−01
Value: B
Muribaculaceae
60
40
20
0
A (n=15)
B (n=16)
C (n=14)
surface

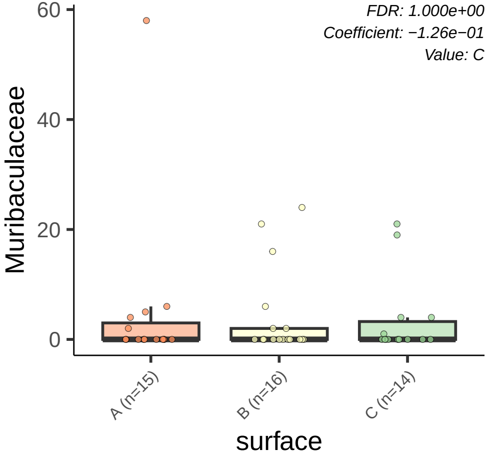
FDR: 1.000e+00
Coefficient: −1.26e−01
Value: C
Muribaculaceae
60
40
20
0
A (n=15)
B (n=16)
C (n=14)
surface

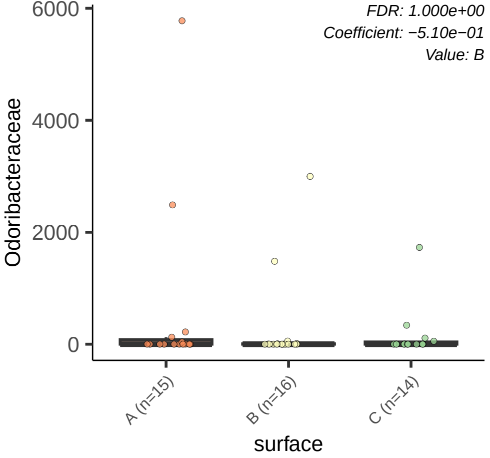

FDR: 1.000e+00
Coefficient: −5.10e−01
Value: B
Odoribacteraceae
6000
4000
2000
0
A (n=15)
B (n=16)
C (n=14)
surface

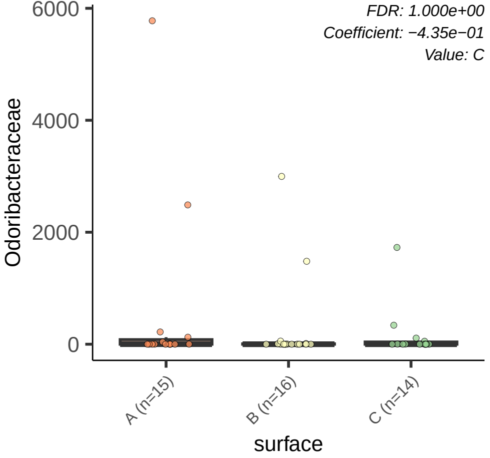
FDR: 1.000e+00
Coefficient: −4.35e−01
Value: C
Odoribacteraceae
6000
4000
2000
0
A (n=15)
B (n=16)
C (n=14)
surface

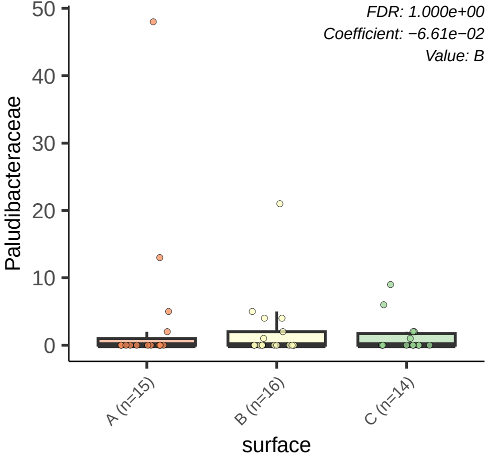
FDR: 1.000e+00
Coefficient: −6.61e−02
Value: B
Paludibacteraceae
50
40
30
20
10
0
A (n=15)
B (n=16)
C (n=14)
surface

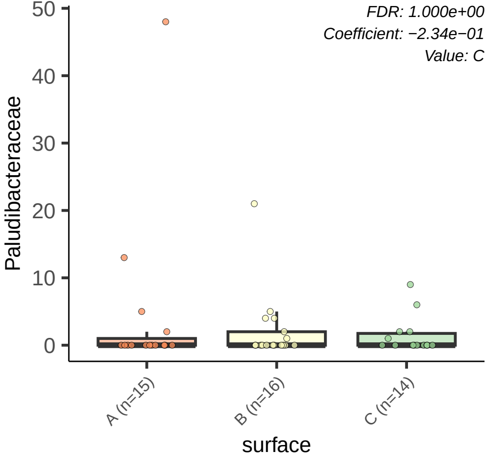
FDR: 1.000e+00
Coefficient: −2.34e−01
Value: C
Paludibacteraceae
50
40
30
20
10
0
A (n=15)
B (n=16)
C (n=14)
surface

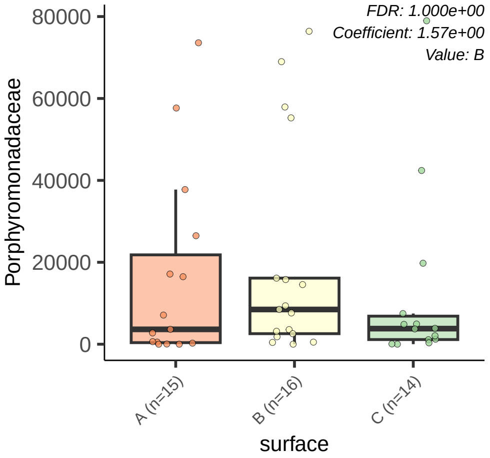
FDR: 1.000e+00
Coefficient: 1.57e+00
Value: B
Porphyromonadaceae
80000
60000
40000
20000
0
A (n=15)
B (n=16)
C (n=14)
surface

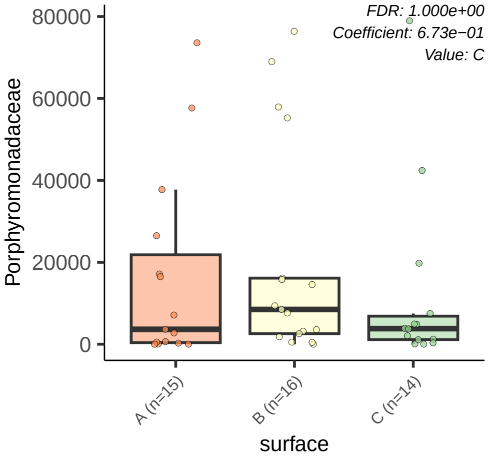
FDR: 1.000e+00
Coefficient: 6.73e−01
Value: C
Porphyromonadaceae
80000
60000
40000
20000
0
A (n=15)
B (n=16)
C (n=14)
surface

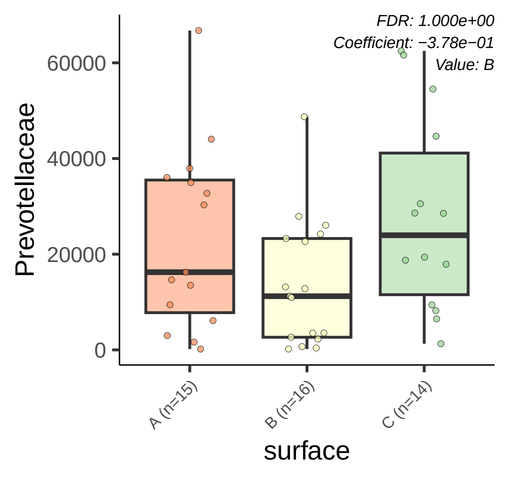
FDR: 1.000e+00
Coefficient: −3.78e−01
Value: B
Prevotellaceae
60000
40000
20000
0
A (n=15)
B (n=16)
C (n=14)
surface

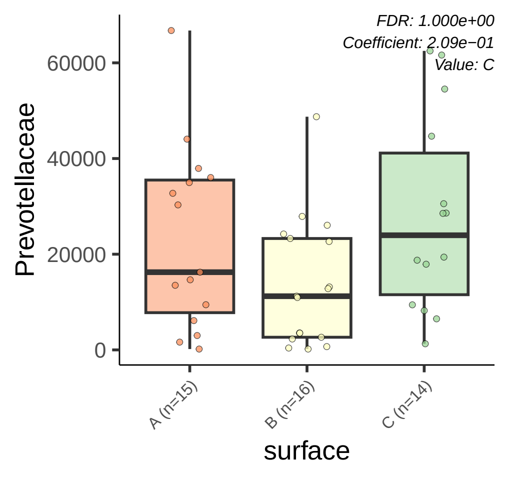

FDR: 1.000e+00
Coefficient: 2.09e−01
Value: C
Prevotellaceae
60000
40000
20000
0
A (n=15)
B (n=16)
C (n=14)
surface

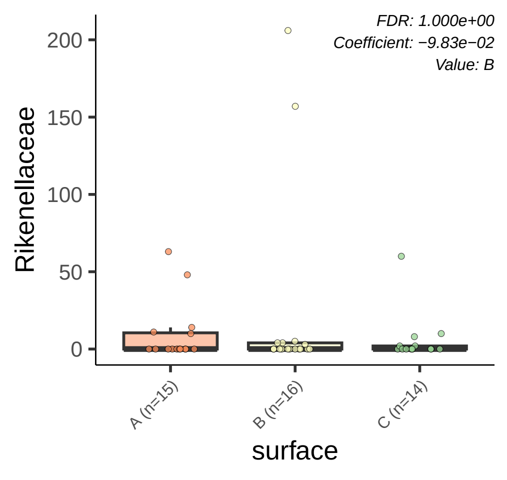
FDR: 1.000e+00
Coefficient: −9.83e−02
Value: B
Rikenellaceae
200
150
100
50
0
A (n=15)
B (n=16)
C (n=14)
surface

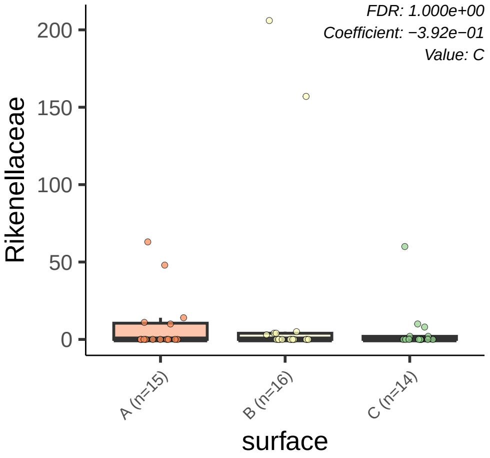

FDR: 1.000e+00
Coefficient: −3.92e−01
Value: C
Rikenellaceae
200
150
100
50
0
A (n=15)
B (n=16)
C (n=14)
surface

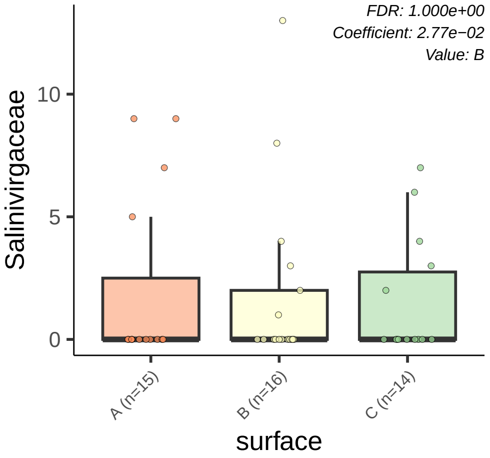
FDR: 1.000e+00
Coefficient: 2.77e−02
Value: B
Salinivirgaceae
10
5
0
A (n=15)
B (n=16)
C (n=14)
surface

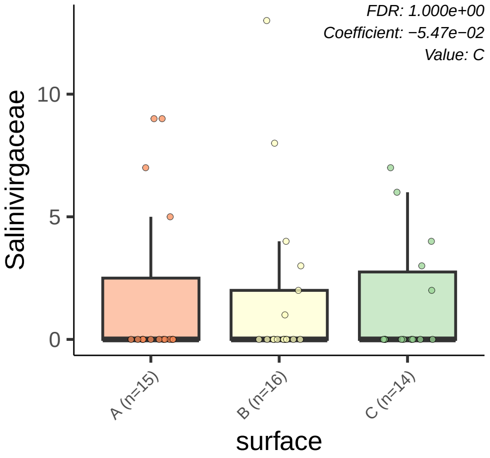
FDR: 1.000e+00
Coefficient: −5.47e−02
Value: C
Salinivirgaceae
10
5
0
A (n=15)
B (n=16)
C (n=14)
surface

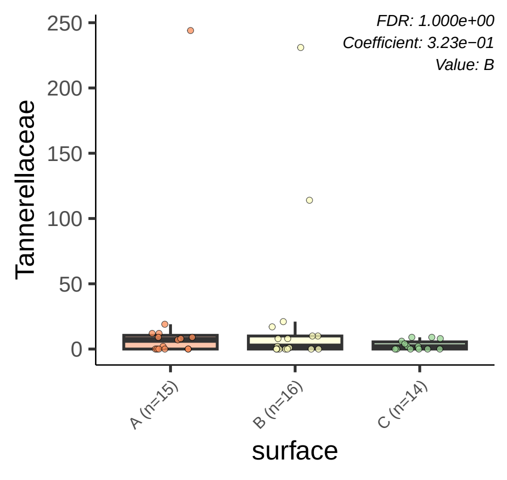
FDR: 1.000e+00
Coefficient: 3.23e−01
Value: B
Tannerellaceae
250
200
150
100
50
0
A (n=15)
B (n=16)
C (n=14)
surface

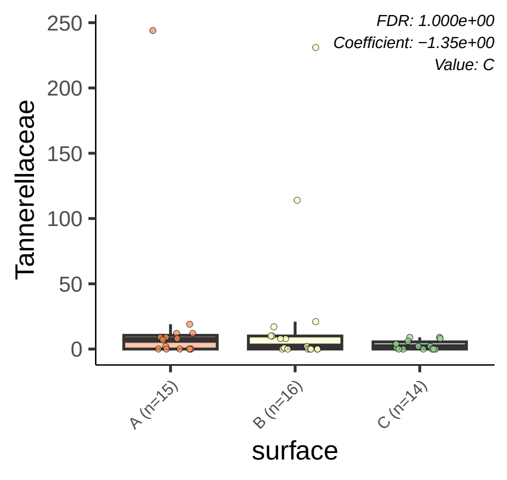

FDR: 1.000e+00
Coefficient: −1.35e+00
Value: C
Tannerellaceae
250
200
150
100
50
0
A (n=15)
B (n=16)
C (n=14)
surface

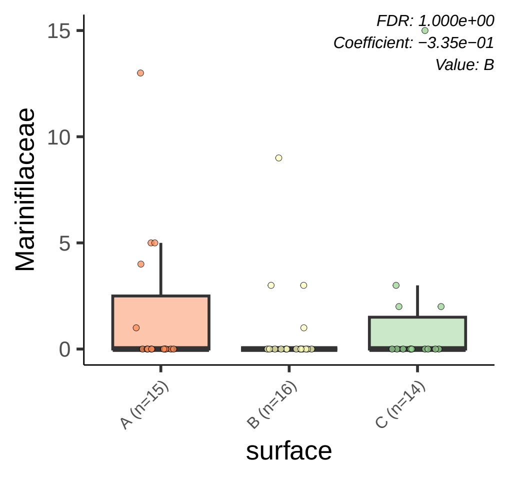
FDR: 1.000e+00
Coefficient: −3.35e−01
Value: B
Marinifilaceae
15
10
5
0
A (n=15)
B (n=16)
C (n=14)
surface

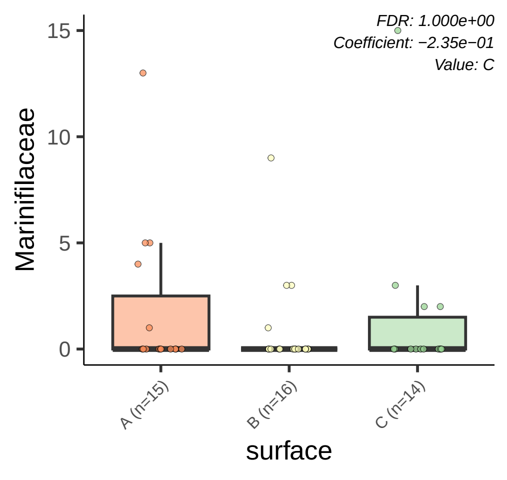

FDR: 1.000e+00
Coefficient: −2.35e−01
Value: C
Marinifilaceae
15
10
5
0
A (n=15)
B (n=16)
C (n=14)
surface

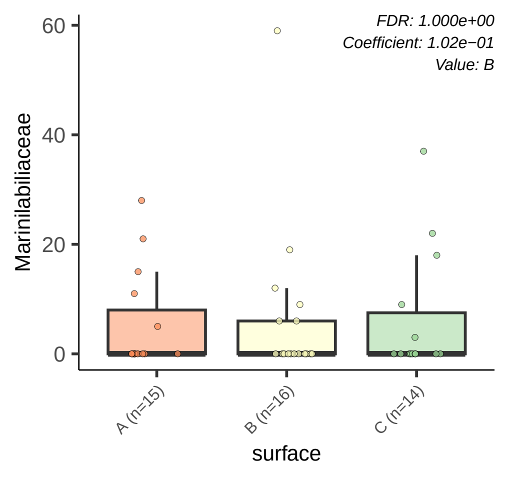
FDR: 1.000e+00
Coefficient: 1.02e−01
Value: B
Marinilabiliaceae
0
20
40
60
A (n=15)
B (n=16)
C (n=14)
surface

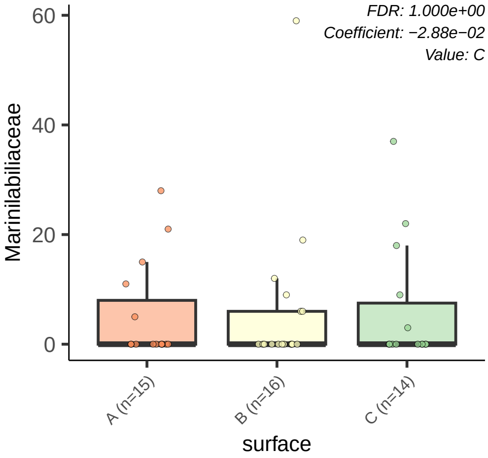
FDR: 1.000e+00
Coefficient: −2.88e−02
Value: C
Marinilabiliaceae
60
40
20
0
A (n=15)
B (n=16)
C (n=14)
surface

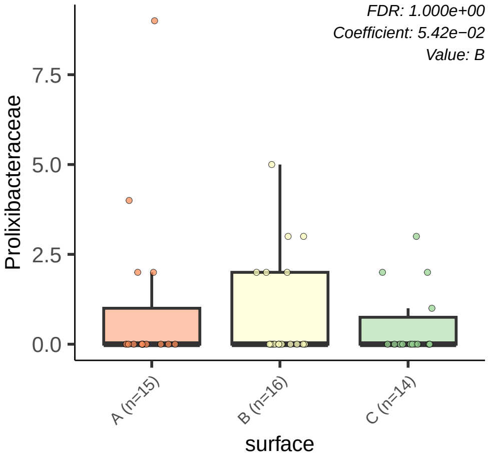

FDR: 1.000e+00
Coefficient: 5.42e−02
Value: B
Prolixibacteraceae
7.5
5.0
2.5
0.0
A (n=15)
B (n=16)
C (n=14)
surface

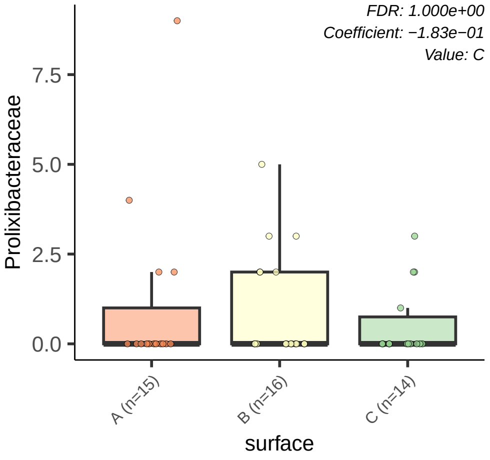
FDR: 1.000e+00
Coefficient: −1.83e−01
Value: C
Prolixibacteraceae
7.5
5.0
2.5
0.0
A (n=15)
B (n=16)
C (n=14)
surface

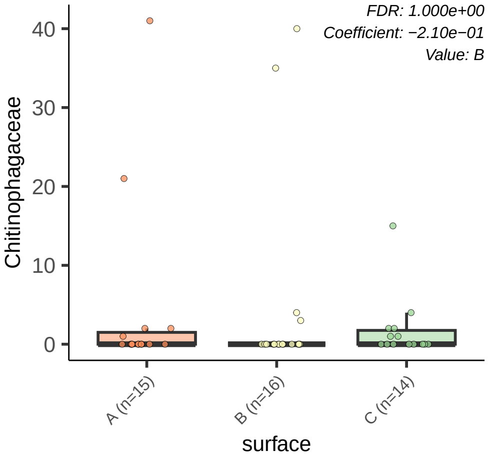
FDR: 1.000e+00
Coefficient: −2.10e−01
Value: B
Chitinophagaceae
0
10
20
30
40
A (n=15)
B (n=16)
C (n=14)
surface

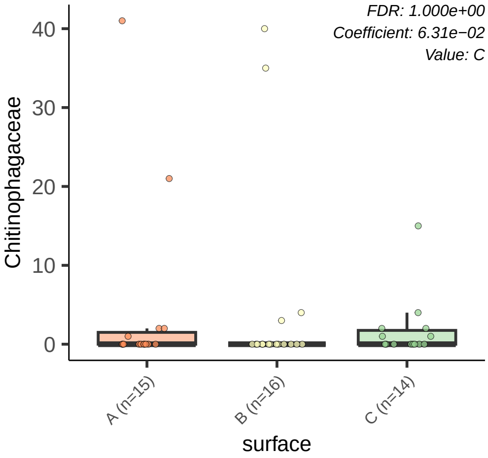

FDR: 1.000e+00
Coefficient: 6.31e−02
Value: C
Chitinophagaceae
0
10
20
30
40
A (n=15)
B (n=16)
C (n=14)
surface

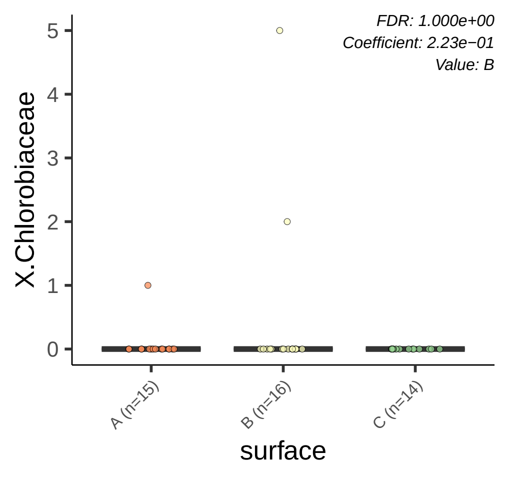
FDR: 1.000e+00
Coefficient: 2.23e−01
Value: B
X.Chlorobiaceae
0
1
2
3
4
5
A (n=15)
B (n=16)
C (n=14)
surface

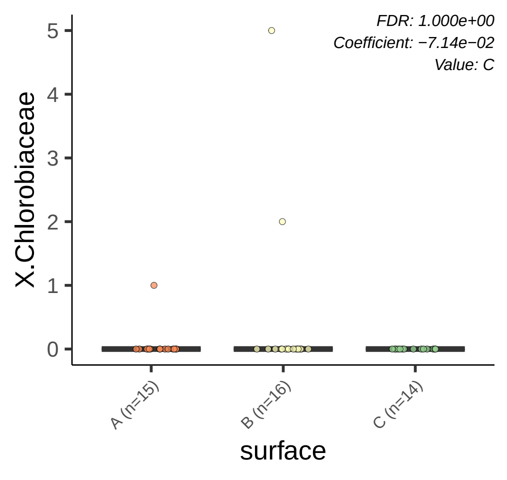

FDR: 1.000e+00
Coefficient: −7.14e−02
Value: C
X.Chlorobiaceae
0
1
2
3
4
5
A (n=15)
B (n=16)
C (n=14)
surface

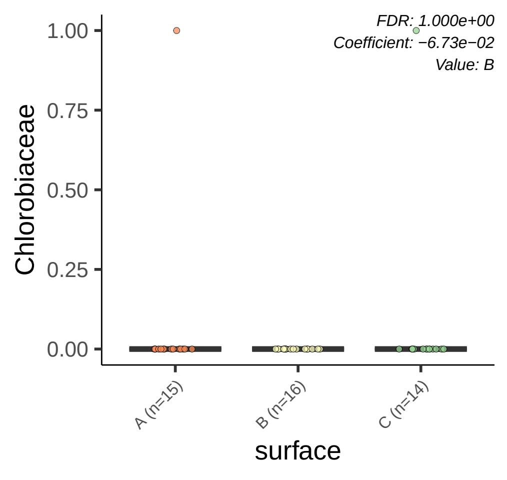

FDR: 1.000e+00
Coefficient: −6.73e−02
Value: B
Chlorobiaceae
1.00
0.75
0.50
0.25
0.00
A (n=15)
B (n=16)
C (n=14)
surface

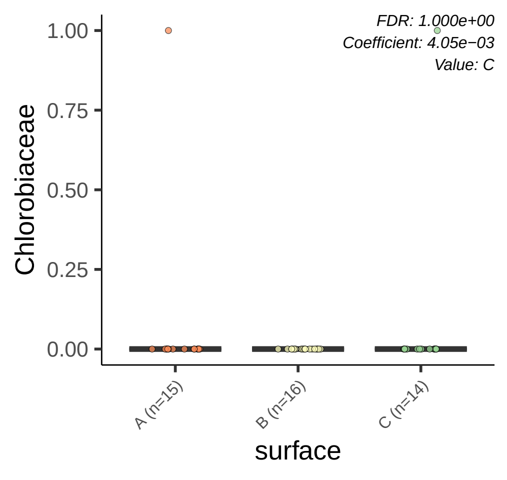
FDR: 1.000e+00
Coefficient: 4.05e−03
Value: C
Chlorobiaceae
1.00
0.75
0.50
0.25
0.00
A (n=15)
B (n=16)
C (n=14)
surface

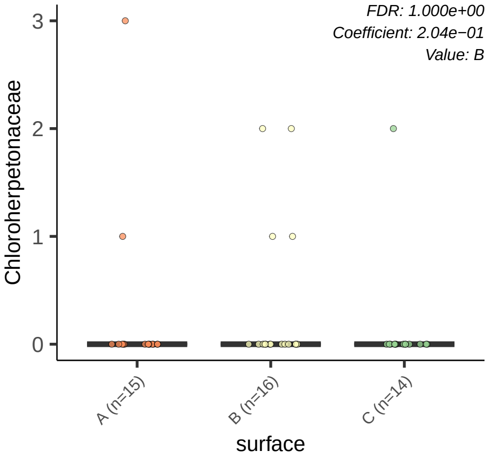
FDR: 1.000e+00
Coefficient: 2.04e−01
Value: B
Chlorohrpetonaceae
3
2
1
0
A (n=15)
B (n=16)
C (n=14)
surface

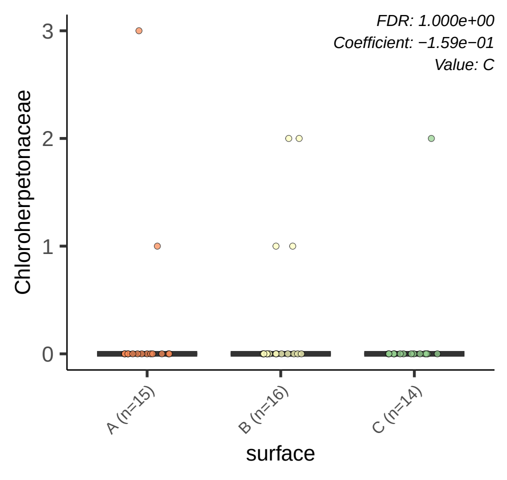
FDR: 1.000e+00
Coefficient: −1.59e−01
Value: C
Chloroherpetonaceae
0
1
2
3
A (n=15)
B (n=16)
C (n=14)
surface

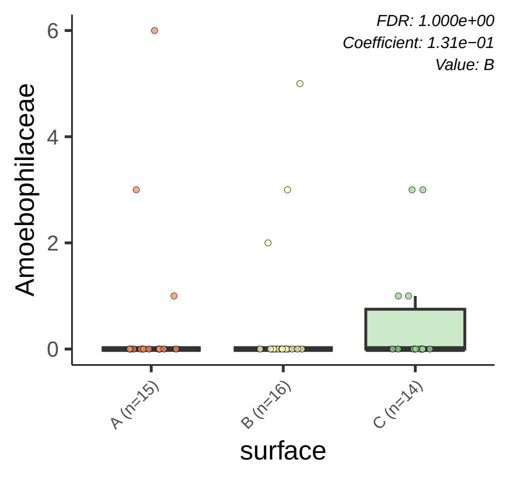

FDR: 1.000e+00
Coefficient: 1.31e−01
Value: B
Amoebophilaceae
6
4
2
0
A (n=15)
B (n=16)
C (n=14)
surface

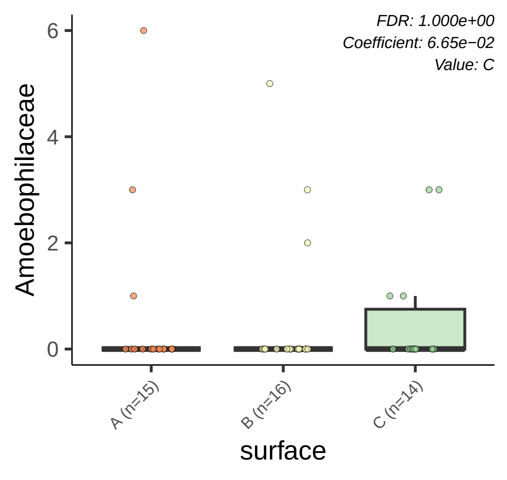
FDR: 1.000e+00
Coefficient: 6.65e−02
Value: C
Amoebophilaceae
0
2
4
6
A (n=15)
B (n=16)
C (n=14)
surface

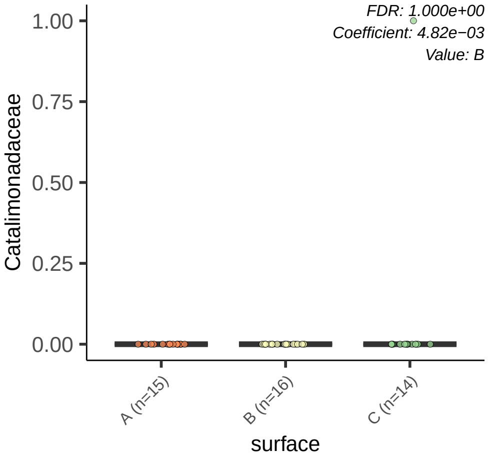
FDR: 1.000e+00
Coefficient: 4.82e−03
Value: B
1.00
0.75
0.50
0.25
0.00
Catalimonadaceae
A (n=15)
B (n=16)
C (n=14)
surface

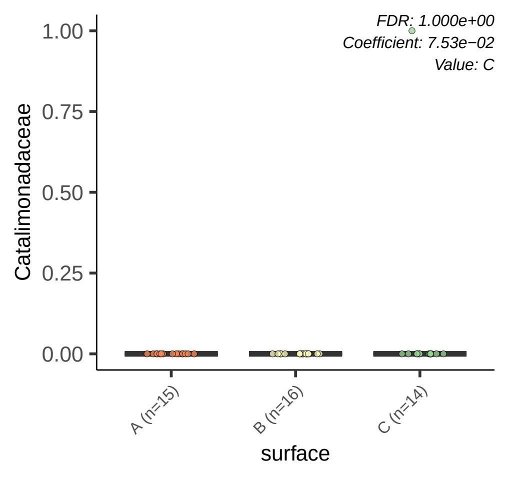
FDR: 1.000e+00
Coefficient: 7.53e−02
Value: C
Catalimonadaceae
1.00
0.75
0.50
0.25
0.00
A (n=15)
B (n=16)
C (n=14)
surface

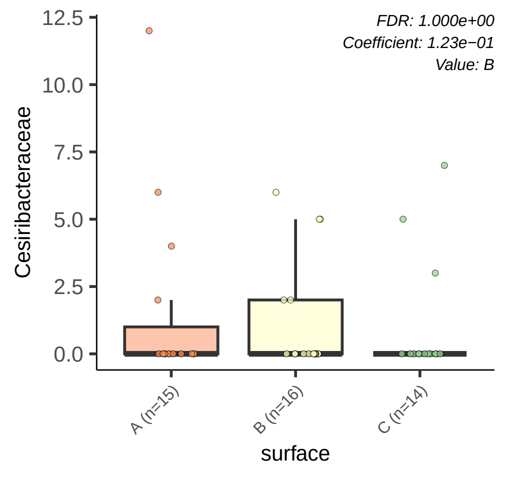
FDR: 1.000e+00
Coefficient: 1.23e−01
Value: B
Cesiribacteraceae
12.5
10.0
7.5
5.0
2.5
0.0
A (n=15)
B (n=16)
C (n=14)
surface

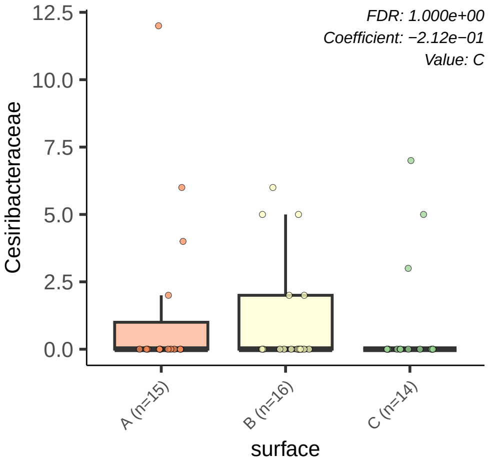
FDR: 1.000e+00
Coefficient: −2.12e−01
Value: C
Cesiribacteraceae
12.5
10.0
7.5
5.0
2.5
0.0
A (n=15)
B (n=16)
C (n=14)
surface

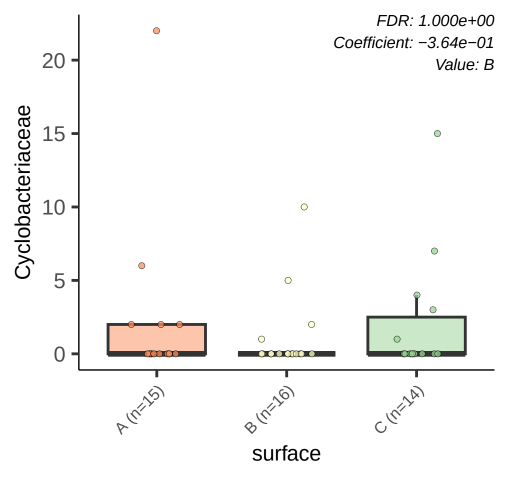

FDR: 1.000e+00
Coefficient: −3.64e−01
Value: B
Cyclobacteriaceae
0
5
10
15
20
A (n=15)
B (n=16)
C (n=14)
surface

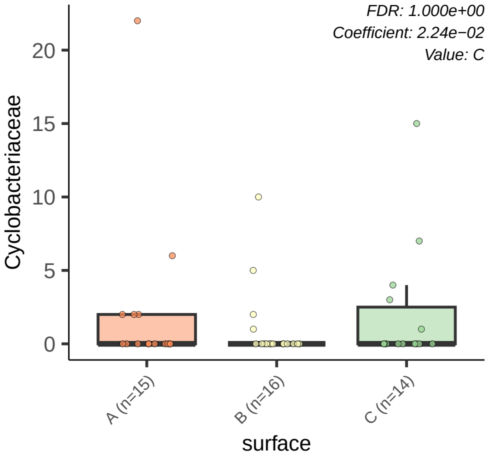
FDR: 1.000e+00
Coefficient: 2.24e−02
Value: C
Cyclobacteriaceae
0
5
10
15
20
A (n=15)
B (n=16)
C (n=14)
surface

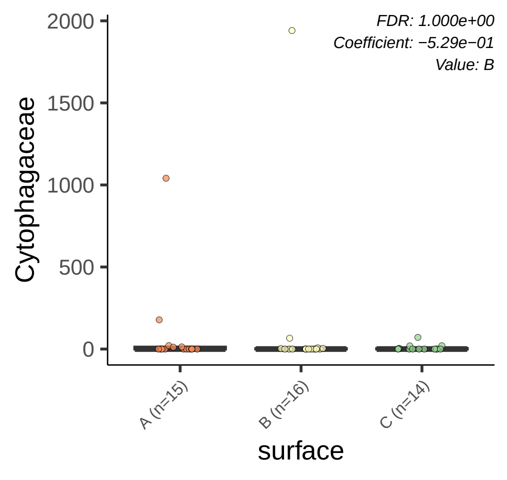
FDR: 1.000e+00
Coefficient: −5.29e−01
Value: B
Cytophagaceae
2000
1500
1000
500
0
A (n=15)
B (n=16)
C (n=14)
surface

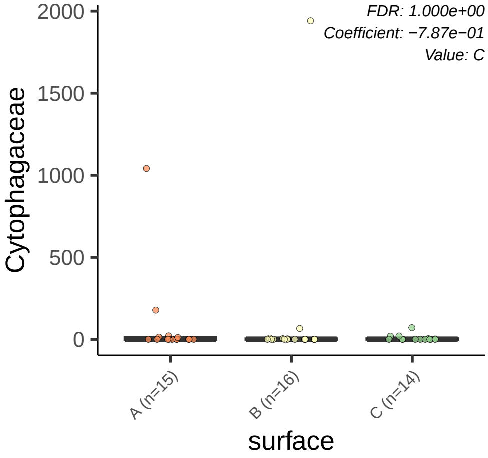
FDR: 1.000e+00
Coefficient: −7.87e−01
Value: C
Cytophagaceae
2000
1500
1000
500
0
A (n=15)
B (n=16)
C (n=14)
surface

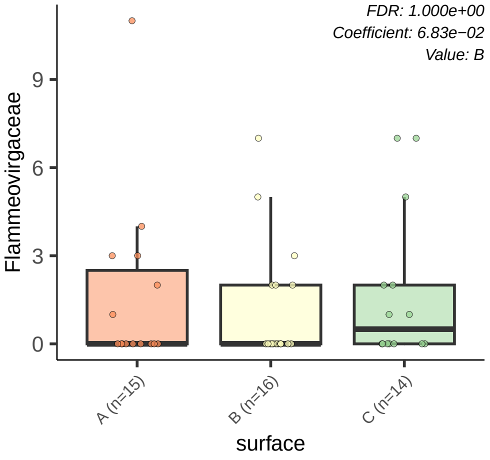

FDR: 1.000e+00
Coefficient: 6.83e−02
Value: B
Flammeovirgaceae
0
3
6
9
A (n=15)
B (n=16)
C (n=14)
surface

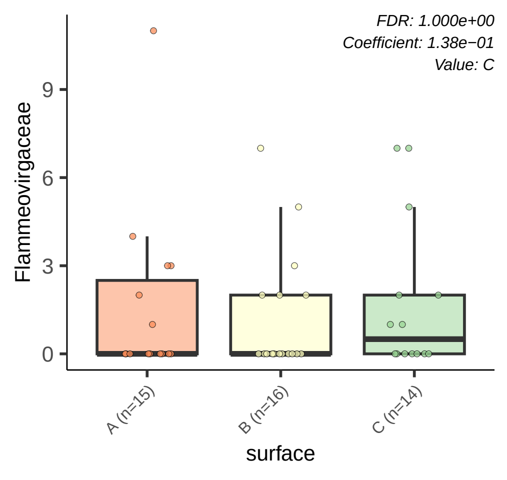
FDR: 1.000e+00
Coefficient: 1.38e−01
Value: C
Flammeovirgaceae
0
3
6
9
A (n=15)
B (n=16)
C (n=14)
surface

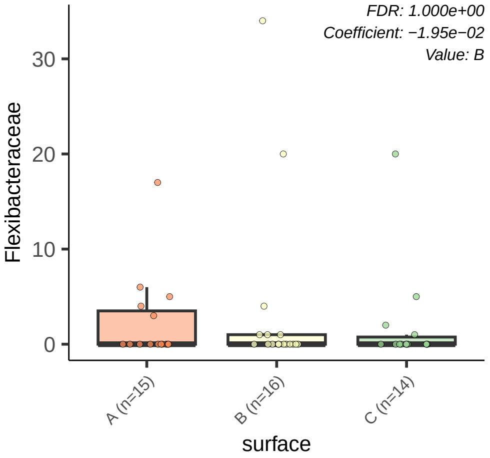
FDR: 1.000e+00
Coefficient: −1.95e−02
Value: B
Flexibacteraceae
0
10
20
30
A (n=15)
B (n=16)
C (n=14)
surface

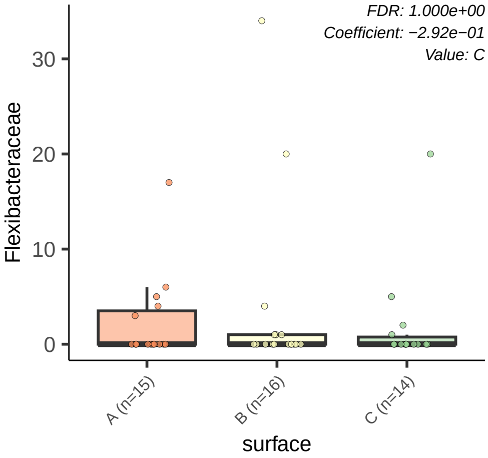

FDR: 1.000e+00
Coefficient: −2.92e−01
Value: C
Flexibacteraceae
0
10
20
30
A (n=15)
B (n=16)
C (n=14)
surface

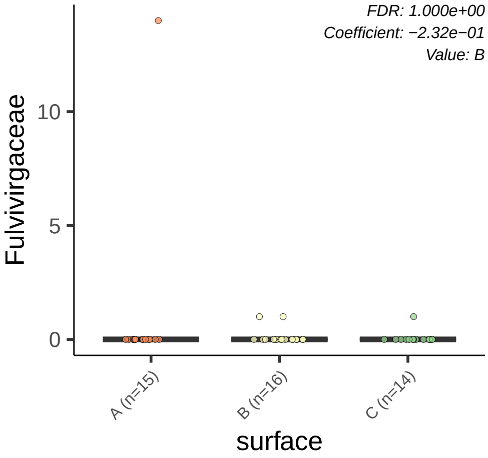
FDR: 1.000e+00
Coefficient: −2.32e−01
Value: B
Fulvivirgaceae
10
5
0
A (n=15)
B (n=16)
C (n=14)
surface

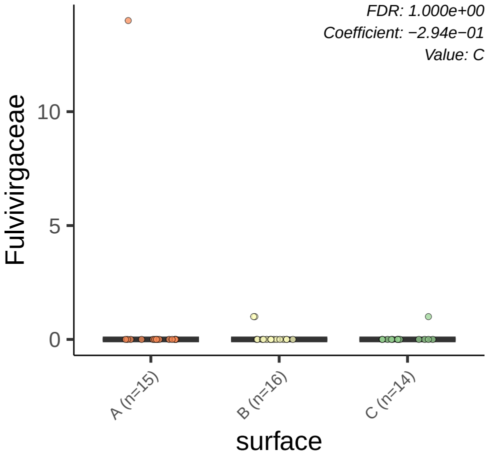
FDR: 1.000e+00
Coefficient: −2.94e−01
Value: C
Fulvivirgaceae
10
5
0
A (n=15)
B (n=16)
C (n=14)
surface

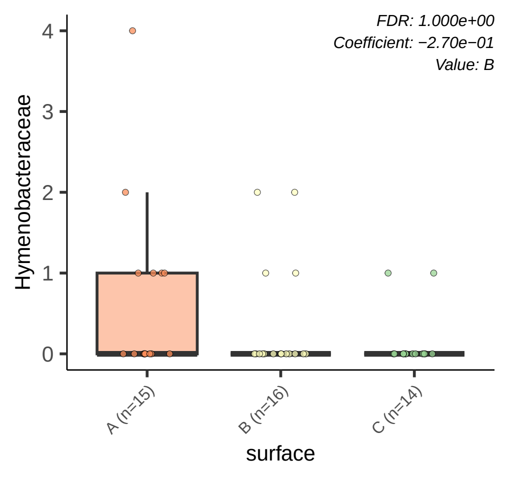
FDR: 1.000e+00
Coefficient: −2.70e−01
Value: B
Hymenobacteraceae
4
3
2
1
0
A (n=15)
B (n=16)
C (n=14)
surface

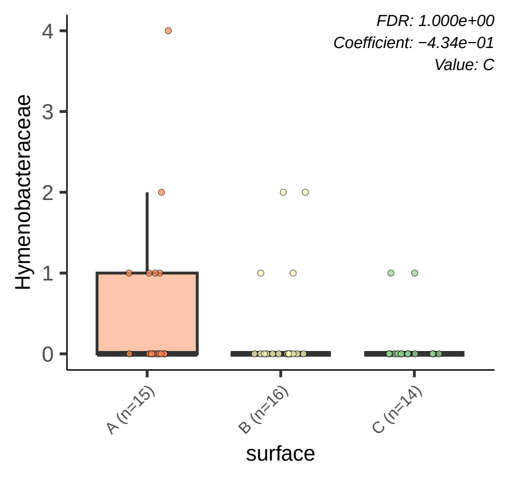
FDR: 1.000e+00
Coefficient: −4.34e−01
Value: C
Hymenobacteraceae
0
1
2
3
4
A (n=15)
B (n=16)
C (n=14)
surface

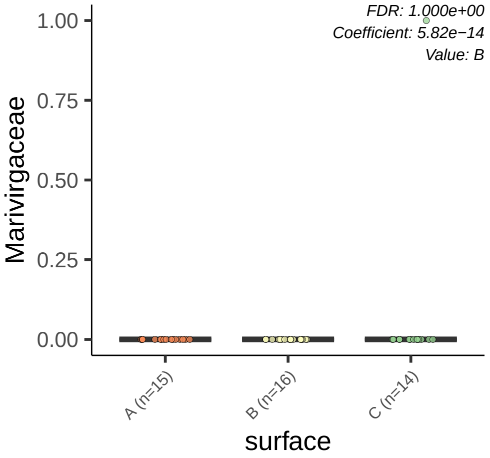

FDR: 1.000e+00
Coefficient: 5.82e−14
Value: B
Marivirgaceae
1.00
0.75
0.50
0.25
0.00
A (n=15)
B (n=16)
C (n=14)
surface

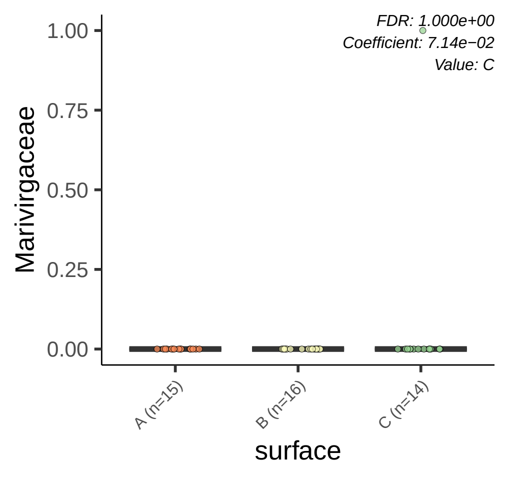
FDR: 1.000e+00
Coefficient: 7.14e−02
Value: C
Marivirgaceae
1.00
0.75
0.50
0.25
0.00
A (n=15)
B (n=16)
C (n=14)
surface

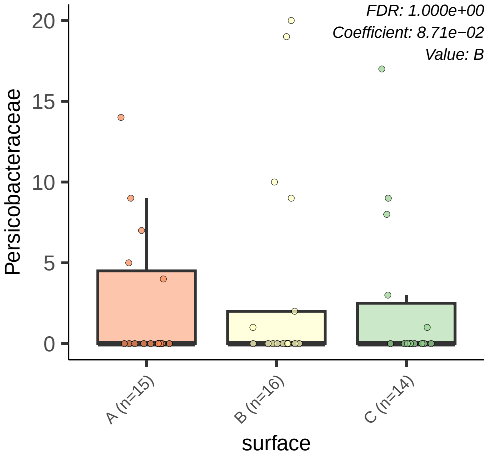
FDR: 1.000e+00
Coefficient: 8.71e−02
Value: B
Persicobacteraceae
0
5
10
15
20
A (n=15)
B (n=16)
C (n=14)
surface

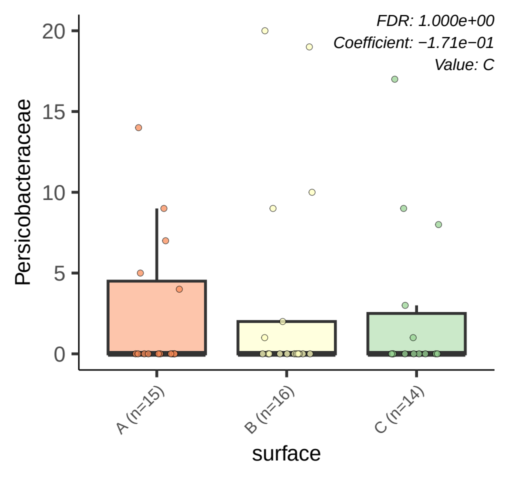

FDR: 1.000e+00
Coefficient: −1.71e−01
Value: C
Persicobacteraceae
0
5
10
15
20
A (n=15)
B (n=16)
C (n=14)
surface

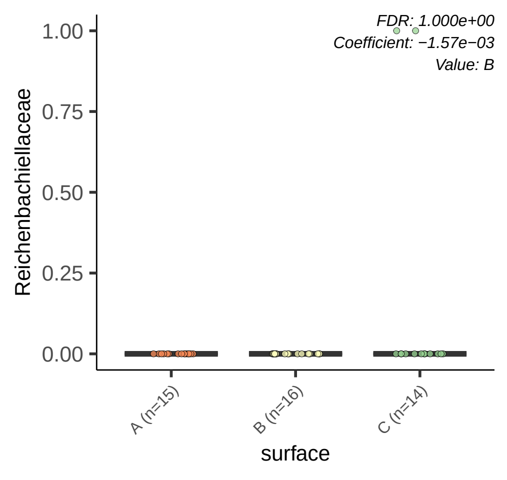
FDR: 1.000e+00
Coefficient: −1.57e−03
Value: B
Reichenbachiellaceae
1.00
0.75
0.50
0.25
0.00
A (n=15)
B (n=16)
C (n=14)
surface

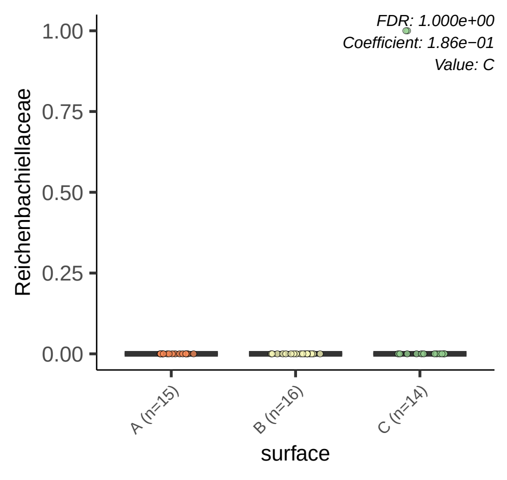

FDR: 1.000e+00
Coefficient: 1.86e−01
Value: C
1.00
0.75
0.50
0.25
0.00
Reichenbachiellaceae
A (n=15)
B (n=16)
C (n=14)
surface

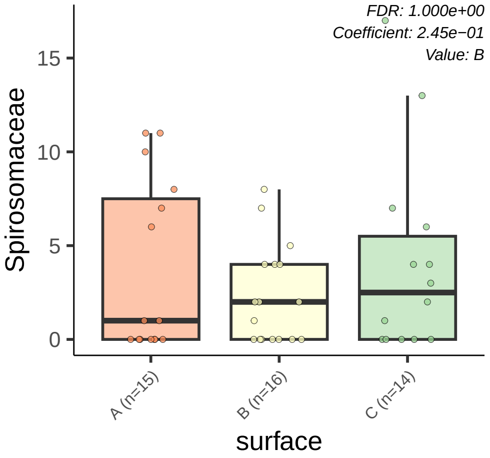
FDR: 1.000e+00
Coefficient: 2.45e−01
Value: B
Spirosomaceae
0
5
10
15
A (n=15)
B (n=16)
C (n=14)
surface

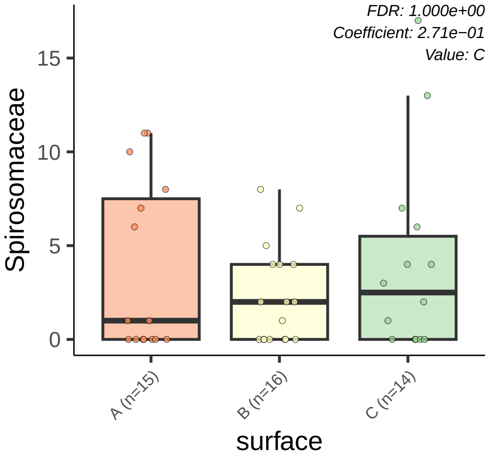

FDR: 1.000e+00
Coefficient: 2.71e−01
Value: C
Spirosomaceae
0
5
10
15
A (n=15)
B (n=16)
C (n=14)
surface

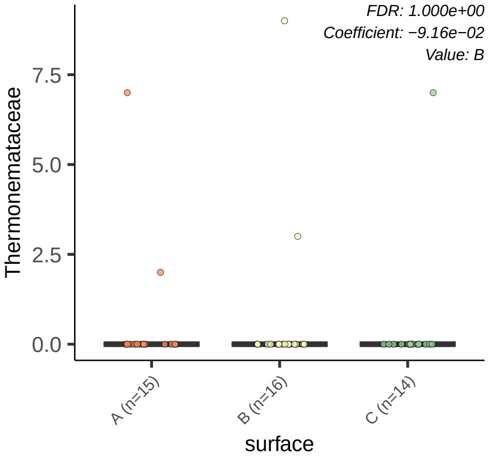
FDR: 1.000e+00
Coefficient: −9.16e−02
Value: B
Thermonemataceae
7.5
5.0
2.5
0.0
A (n=15)
B (n=16)
C (n=14)
surface

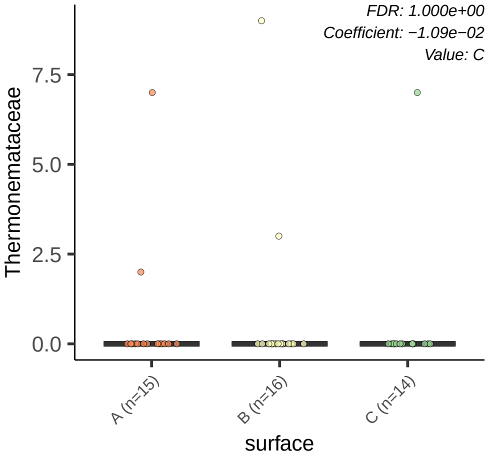
FDR: 1.000e+00
Coefficient: −1.09e−02
Value: C
Thermonemataceae
7.5
5.0
2.5
0.0
A (n=15)
B (n=16)
C (n=14)
surface

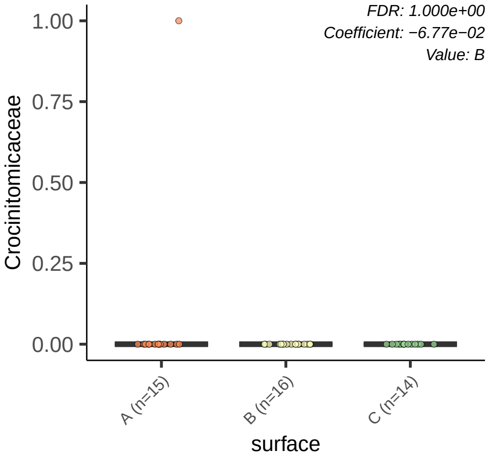
FDR: 1.000e+00
Coefficient: −6.77e−02
Value: B
Crocinitomicaceae
1.00
0.75
0.50
0.25
0.00
A (n=15)
B (n=16)
C (n=14)
surface

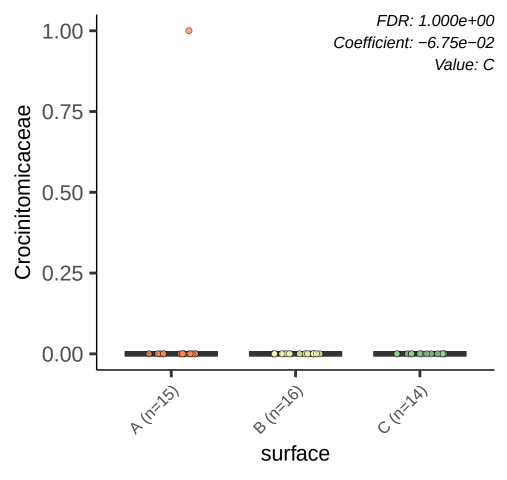
FDR: 1.000e+00
Coefficient: −6.75e−02
Value: C
Crocinitomicaceae
1.00
0.75
0.50
0.25
0.00
A (n=15)
B (n=16)
C (n=14)
surface

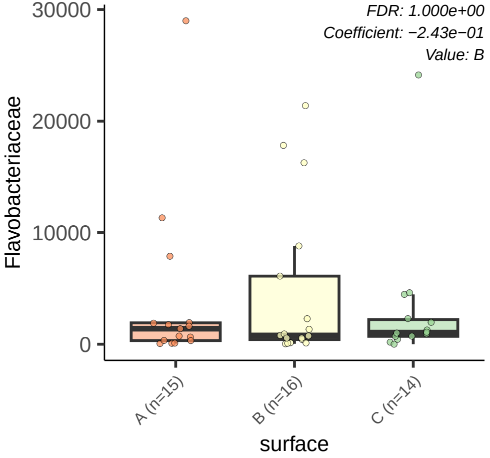

FDR: 1.000e+00
Coefficient: −2.43e−01
Value: B
30000
20000
10000
0
Flavobacteriaceae
A (n=15)
B (n=16)
C (n=14)
surface

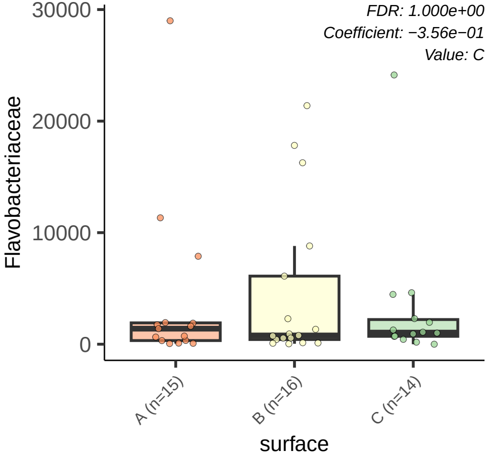

FDR: 1.000e+00
Coefficient: −3.56e−01
Value: C
Flavobacteriaceae
30000
20000
10000
0
A (n=15)
B (n=16)
C (n=14)
surface

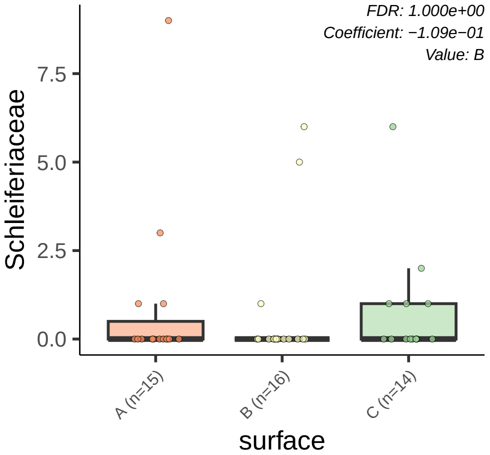
FDR: 1.000e+00
Coefficient: −1.09e−01
Value: B
Schleiferiaceae
7.5
5.0
2.5
0.0
A (n=15)
B (n=16)
C (n=14)
surface

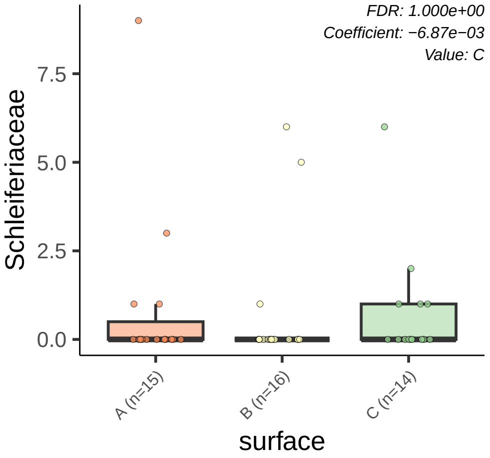

FDR: 1.000e+00
Coefficient: −6.87e−03
Value: C
Schleiferiaceae
7.5
5.0
2.5
0.0
A (n=15)
B (n=16)
C (n=14)
surface

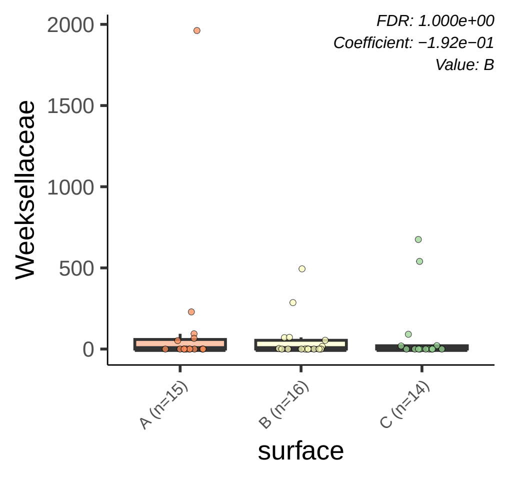

FDR: 1.000e+00
Coefficient: −1.92e−01
Value: B
Weeksellaceae
2000
1500
1000
500
0
A (n=15)
B (n=16)
C (n=14)
surface

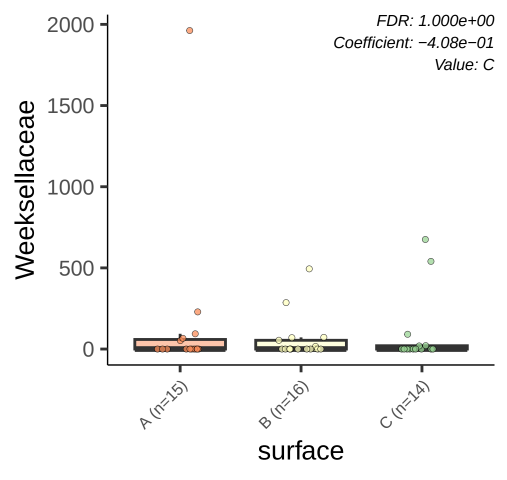
FDR: 1.000e+00
Coefficient: −4.08e−01
Value: C
2000
1500
1000
500
0
Weeksellaceae
A (n=15)
B (n=16)
C (n=14)
surface

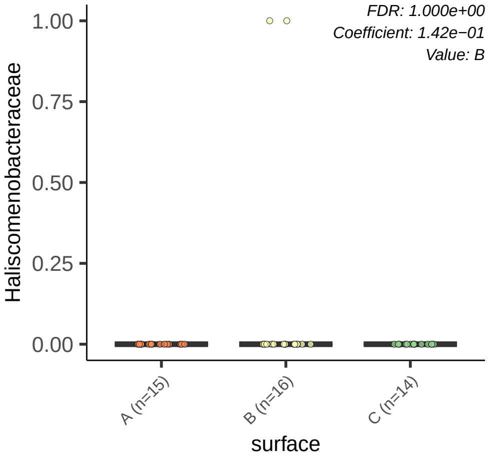
FDR: 1.000e+00
Coefficient: 1.42e−01
Value: B
Haliscomenobacteraceae
1.00
0.75
0.50
0.25
0.00
A (n=15)
B (n=16)
C (n=14)
surface

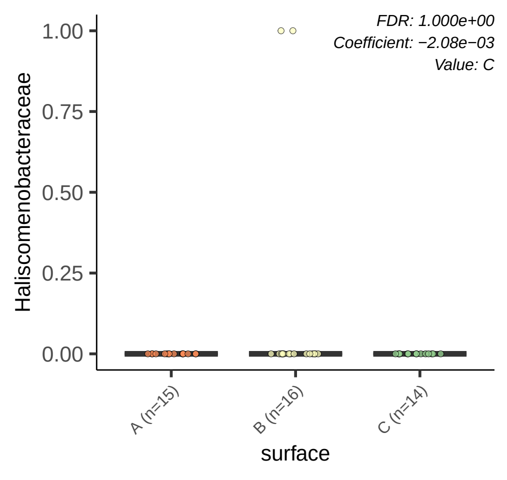
FDR: 1.000e+00
Coefficient: −2.08e−03
Value: C
Haliscomenobacteraceae
1.00
0.75
0.50
0.25
0.00
A (n=15)
B (n=16)
C (n=14)
surface

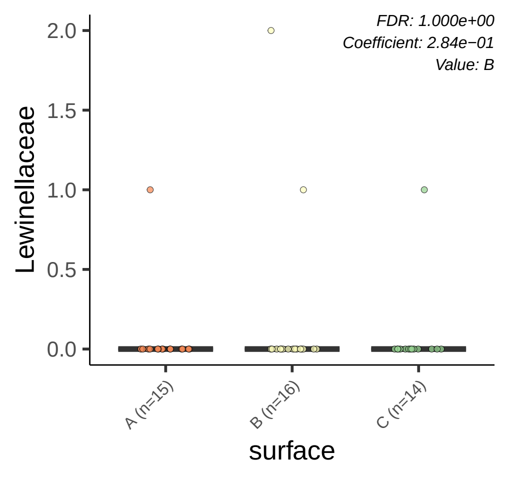
FDR: 1.000e+00
Coefficient: 2.84e−01
Value: B
Lewinellaceae
2.0
1.5
1.0
0.5
0.0
A (n=15)
B (n=16)
C (n=14)
surface

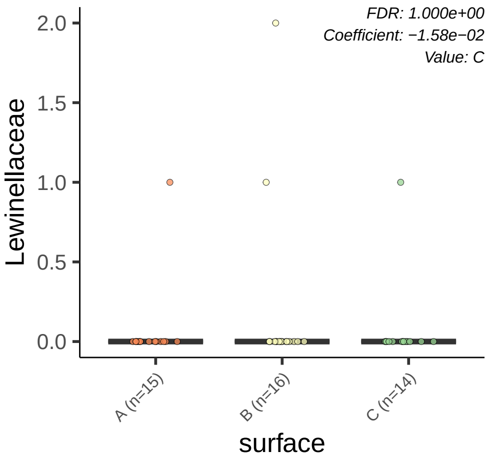

FDR: 1.000e+00
Coefficient: −1.58e−02
Value: C
Lewinellaceae
2.0
1.5
1.0
0.5
0.0
A (n=15)
B (n=16)
C (n=14)
surface

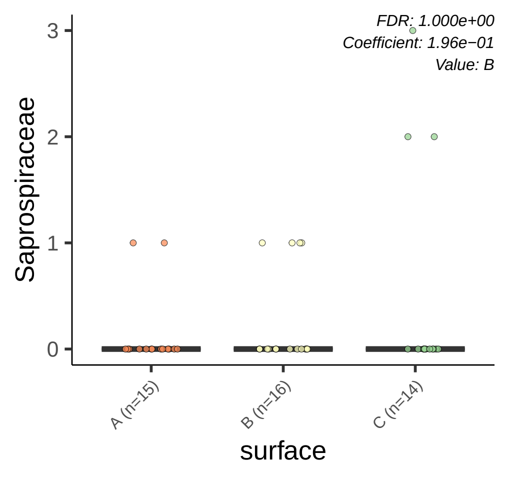
FDR: 1.000e+00
Coefficient: 1.96e−01
Value: B
Saprospiraceae
3
2
1
0
A (n=15)
B (n=16)
C (n=14)
surface

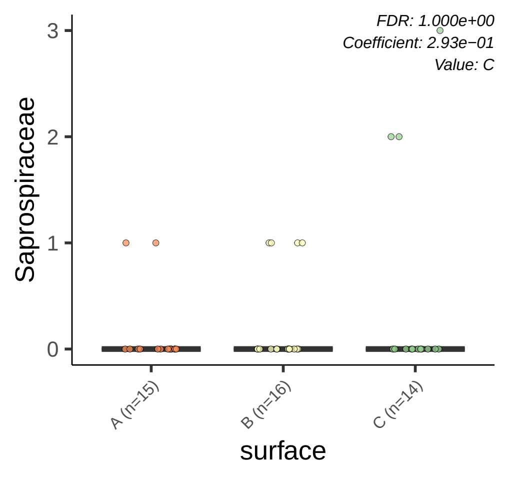
FDR: 1.000e+00
Coefficient: 2.93e−01
Value: C
Saprospiraceae
3
2
1
0
A (n=15)
B (n=16)
C (n=14)
surface

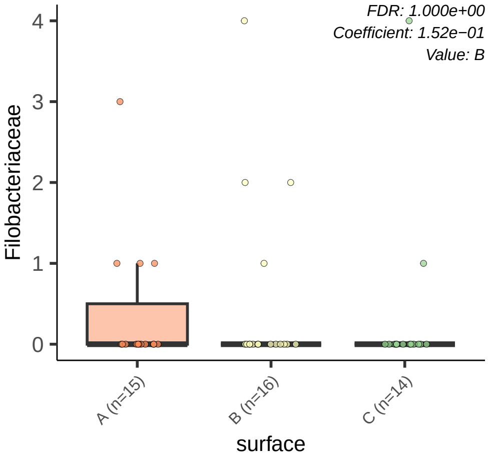

FDR: 1.000e+00
Coefficient: 1.52e−01
Value: B
Filobacteriaceae
0
1
2
3
4
A (n=15)
B (n=16)
C (n=14)
surface

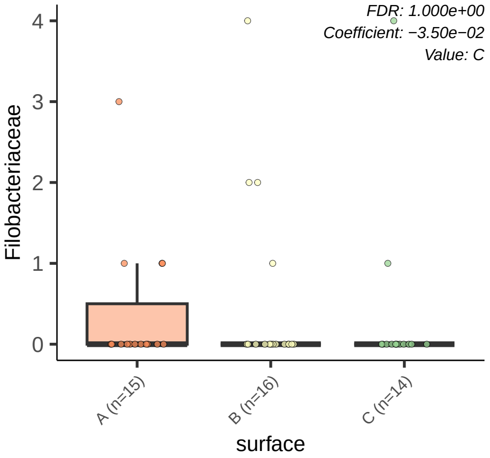
FDR: 1.000e+00
Coefficient: −3.50e−02
Value: C
Filobacteriaceae
0
1
2
3
4
A (n=15)
B (n=16)
C (n=14)
surface

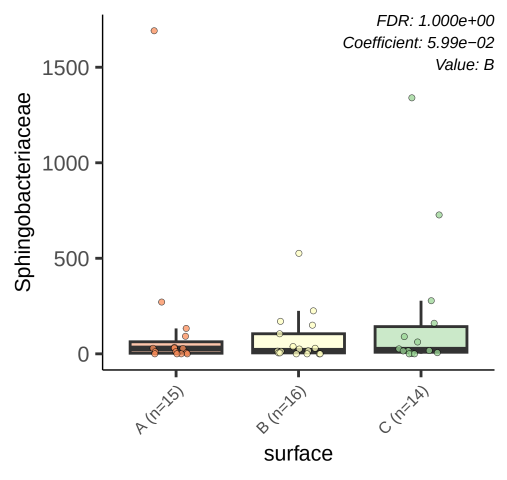
FDR: 1.000e+00
Coefficient: 5.99e−02
Value: B
Sphingobacteriaceae
1500
1000
500
0
A (n=15)
B (n=16)
C (n=14)
surface

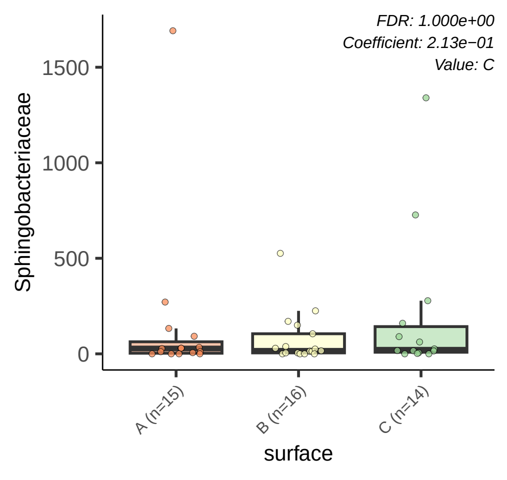
FDR: 1.000e+00
Coefficient: 2.13e−01
Value: C
Sphingobacteriaceae
1500
1000
500
0
A (n=15)
B (n=16)
C (n=14)
surface

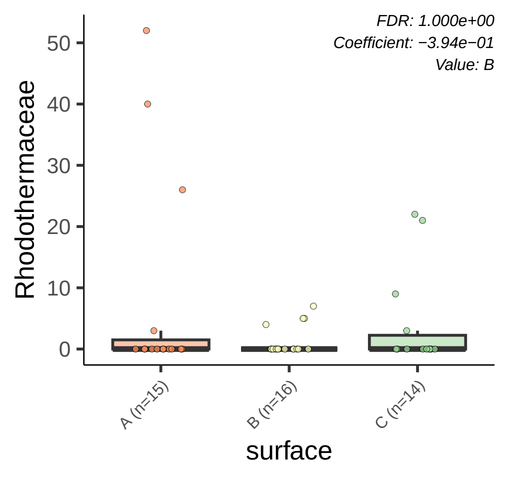

FDR: 1.000e+00
Coefficient: −3.94e−01
Value: B
Rhodothermaceae
50
40
30
20
10
0
A (n=15)
B (n=16)
C (n=14)
surface

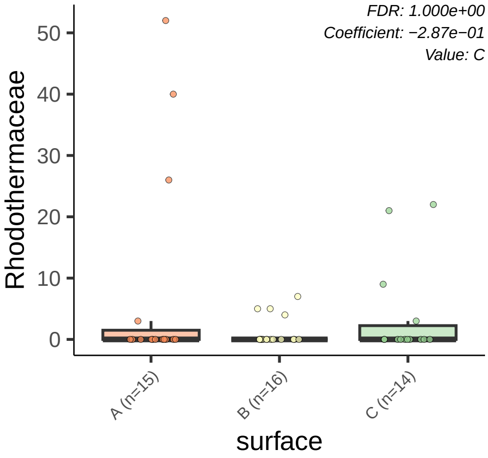
FDR: 1.000e+00
Coefficient: −2.87e−01
Value: C
Rhodothermaceae
0
10
20
30
40
50
A (n=15)
B (n=16)
C (n=14)
surface

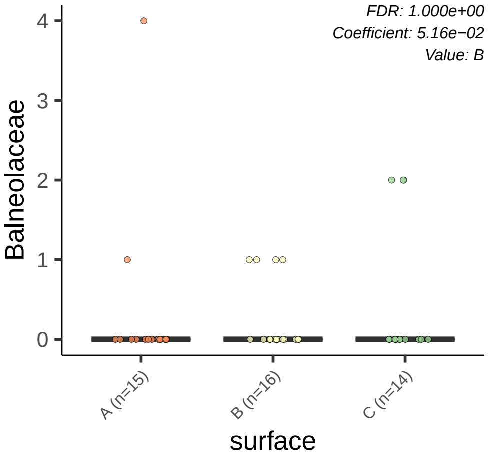

FDR: 1.000e+00
Coefficient: 5.16e−02
Value: B
Balneolaceae
0
1
2
3
4
A (n=15)
B (n=16)
C (n=14)
surface

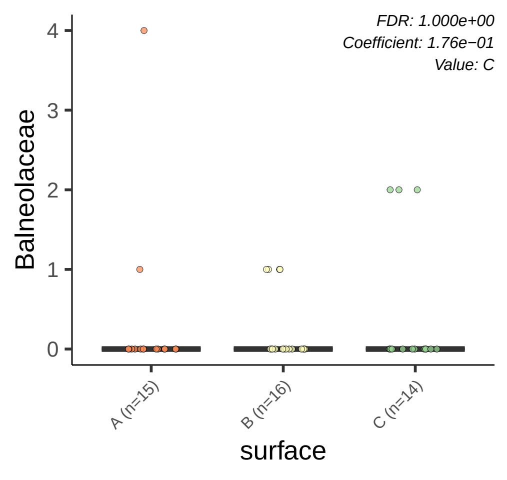
FDR: 1.000e+00
Coefficient: 1.76e−01
Value: C
Balneolaceae
4
3
2
1
0
A (n=15)
B (n=16)
C (n=14)
surface

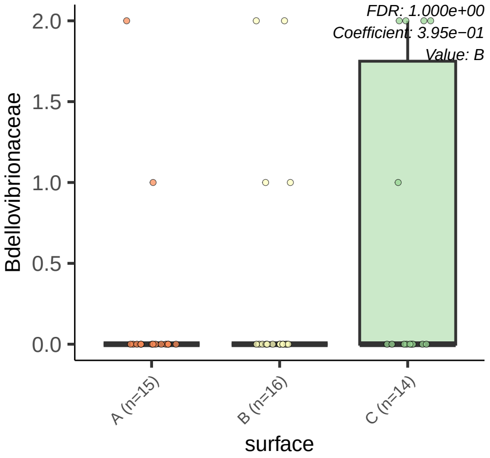

FDR: 1.000e+00
Coefficient: 3.95e−01
Value: B
2.0
1.5
1.0
0.5
0.0
Bdellovibrionaceae
A (n=15)
B (n=16)
C (n=14)
surface

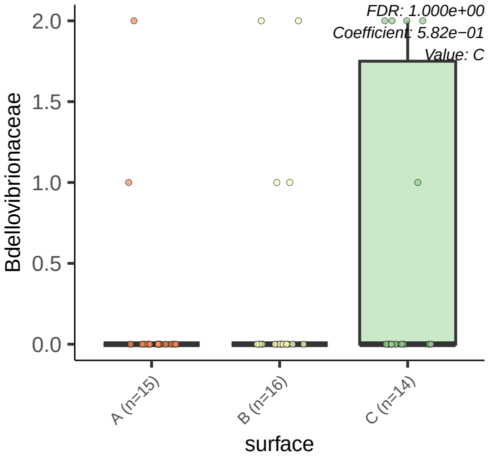
FDR: 1.000e+00
Coefficient: 5.82e−01
Value: C
Bdellovibrionaceae
2.0
1.5
1.0
0.5
0.0
A (n=15)
B (n=16)
C (n=14)
surface

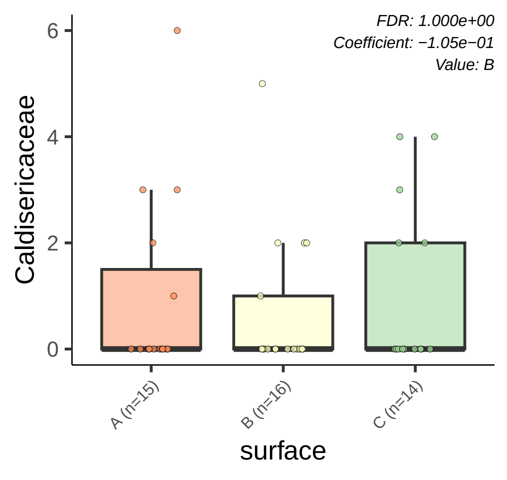
FDR: 1.000e+00
Coefficient: −1.05e−01
Value: B
Caldisericaceae
0
2
4
6
A (n=15)
B (n=16)
C (n=14)
surface

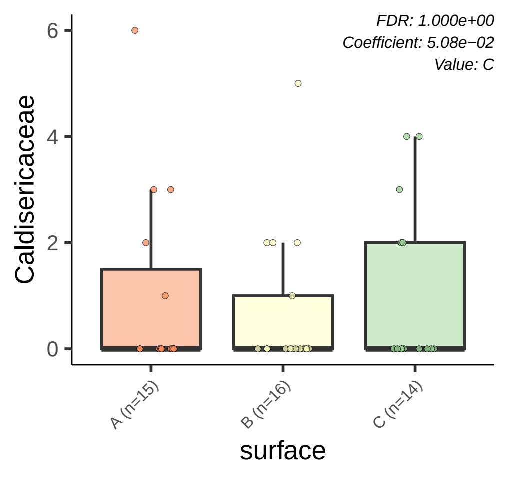

FDR: 1.000e+00
Coefficient: 5.08e−02
Value: C
Caldisericaceae
0
2
4
6
A (n=15)
B (n=16)
C (n=14)
surface

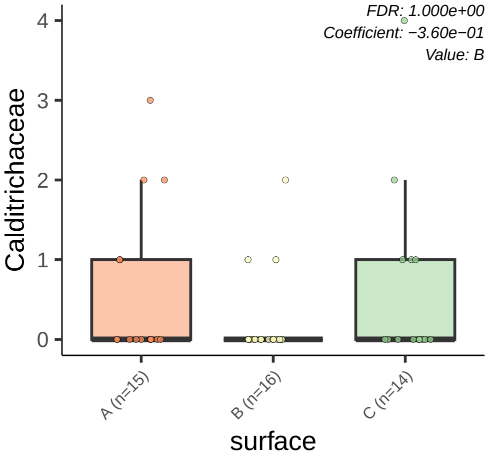
FDR: 1.000e+00
Coefficient: −3.60e−01
Value: B
Calditrichaceae
0
1
2
3
4
A (n=15)
B (n=16)
C (n=14)
surface

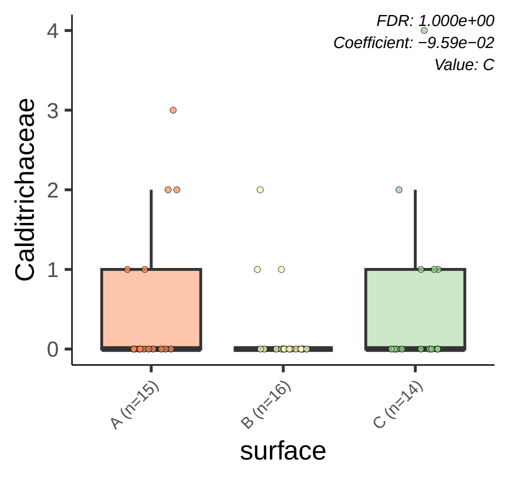
FDR: 1.000e+00
Coefficient: −9.59e−02
Value: C
Calditrichaceae
0
1
2
3
4
A (n=15)
B (n=16)
C (n=14)
surface

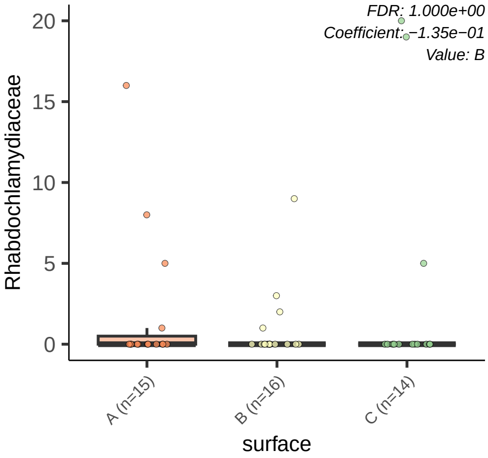
FDR: 1.000e+00
Coefficient: −1.35e−01
Value: B
Rhabdochlamydiaceae
20
15
10
5
0
A (n=15)
B (n=16)
C (n=14)
surface

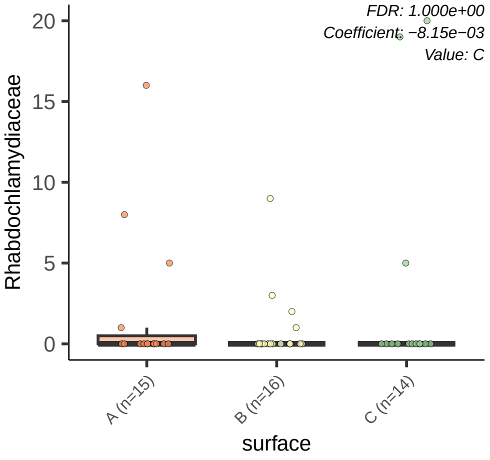
FDR: 1.000e+00
Coefficient: −8.15e−03
Value: C
Rhabdochlamydiaceae
20
15
10
5
0
A (n=15)
B (n=16)
C (n=14)
surface

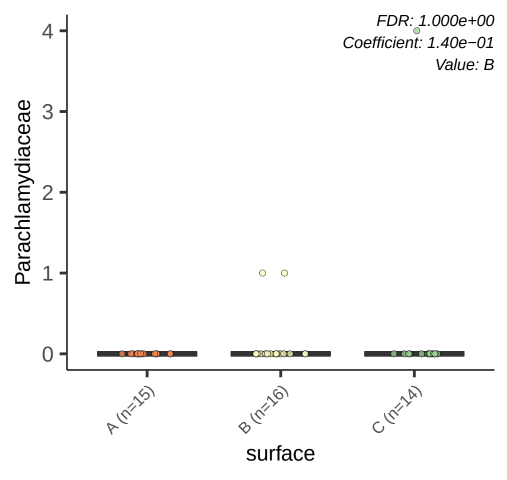

FDR: 1.000e+00
Coefficient: 1.40e−01
Value: B
Parachlamydiaceae
4
3
2
1
0
A (n=15)
B (n=16)
C (n=14)
surface

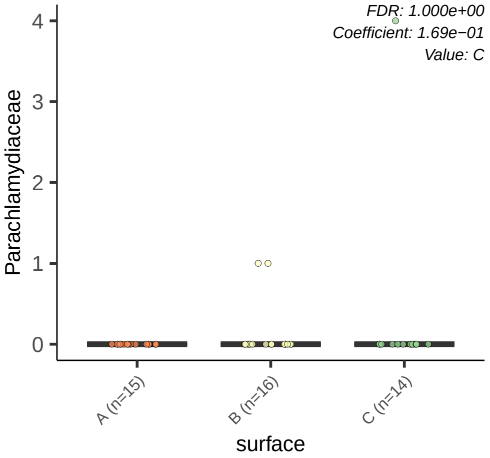
FDR: 1.000e+00
Coefficient: 1.69e−01
Value: C
Parachlamydiaceae
4
3
2
1
0
A (n=15)
B (n=16)
C (n=14)
surface

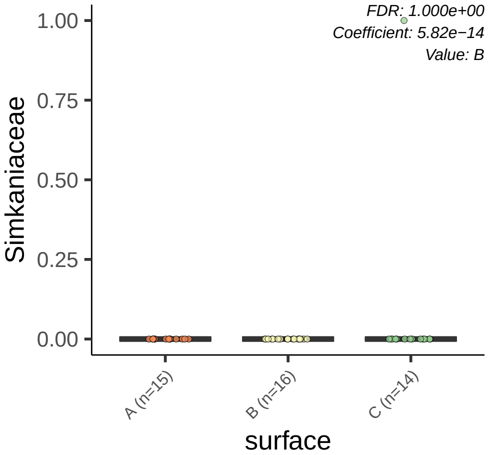

FDR: 1.000e+00
Coefficient: 5.82e−14
Value: B
Simkaniaceae
1.00
0.75
0.50
0.25
0.00
A (n=15)
B (n=16)
C (n=14)
surface

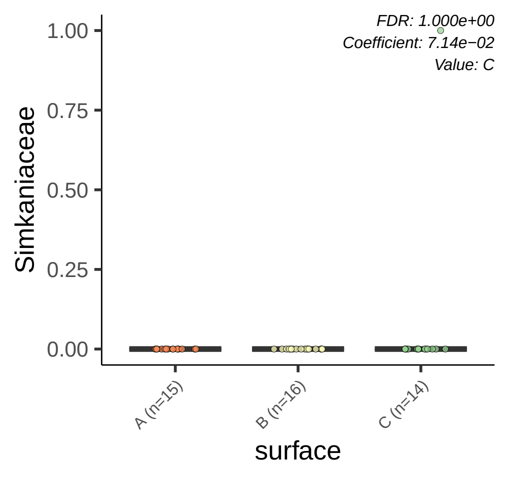
FDR: 1.000e+00
Coefficient: 7.14e−02
Value: C
Simkaniaceae
1.00
0.75
0.50
0.25
0.00
A (n=15)
B (n=16)
C (n=14)
surface

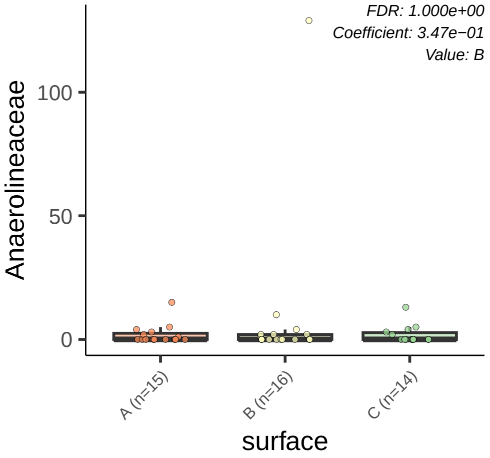

FDR: 1.000e+00
Coefficient: 3.47e−01
Value: B
Anaerolineaceae
100
50
0
A (n=15)
B (n=16)
C (n=14)
surface

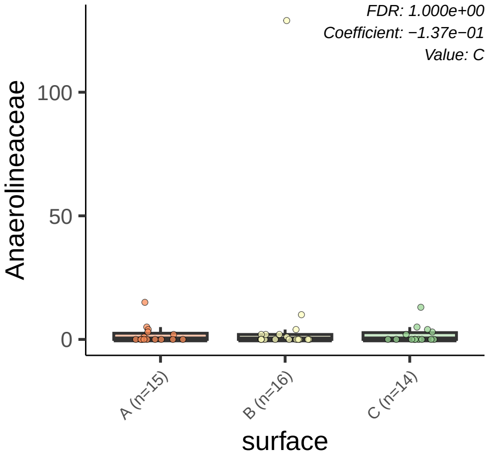

FDR: 1.000e+00
Coefficient: −1.37e−01
Value: C
Anaerolineaceae
100
50
0
A (n=15)
B (n=16)
C (n=14)
surface

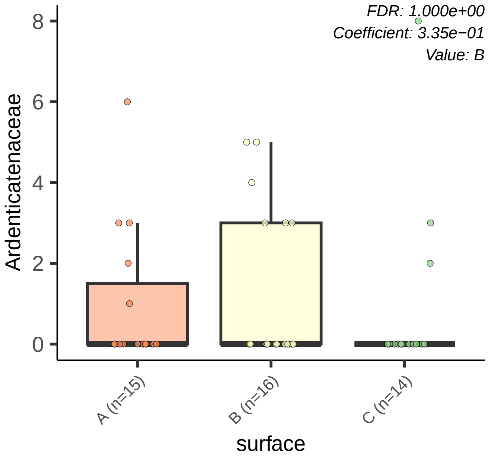

FDR: 1.000e+00
Coefficient: 3.35e−01
Value: B
Ardenticatenaceae
0
2
4
6
8
A (n=15)
B (n=16)
C (n=14)
surface

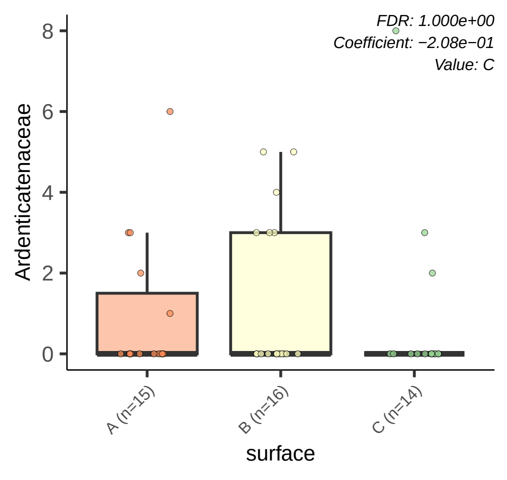
FDR: 1.000e+00
Coefficient: −2.08e−01
Value: C
Ardenticatenaceae
0
2
4
6
8
A (n=15)
B (n=16)
C (n=14)
surface

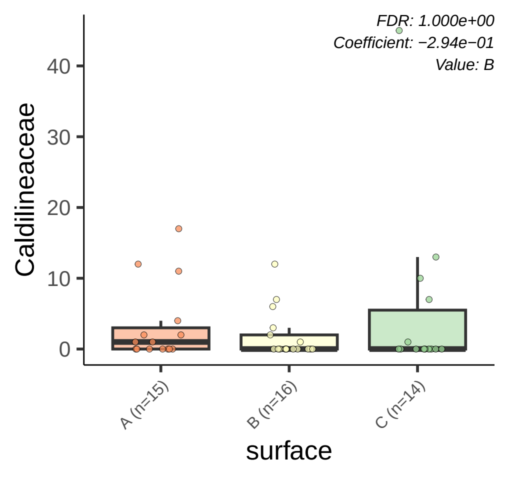
FDR: 1.000e+00
Coefficient: −2.94e−01
Value: B
Caldilineaceae
40
30
20
10
0
A (n=15)
B (n=16)
C (n=14)
surface

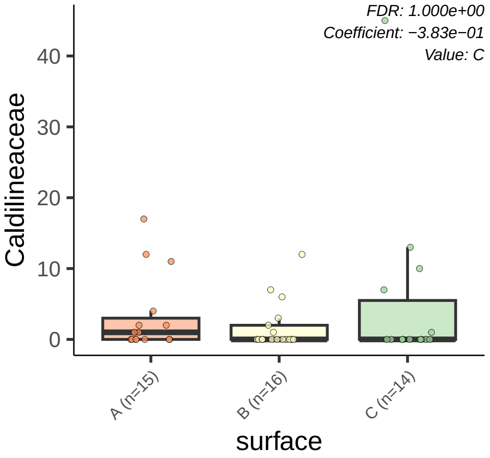
FDR: 1.000e+00
Coefficient: −3.83e−01
Value: C
Caldilineaceae
0
10
20
30
40
A (n=15)
B (n=16)
C (n=14)
surface

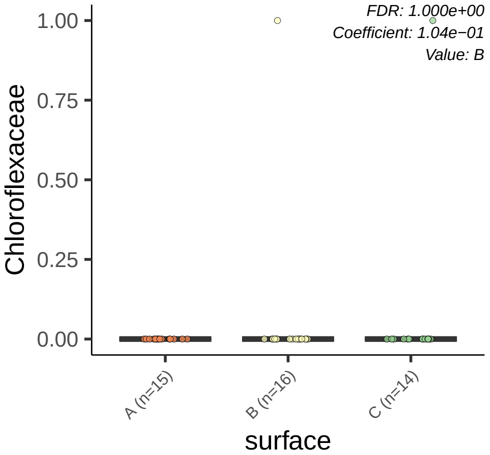

FDR: 1.000e+00
Coefficient: 1.04e−01
Value: B
Chloroflexaceae
1.00
0.75
0.50
0.25
0.00
A (n=15)
B (n=16)
C (n=14)
surface

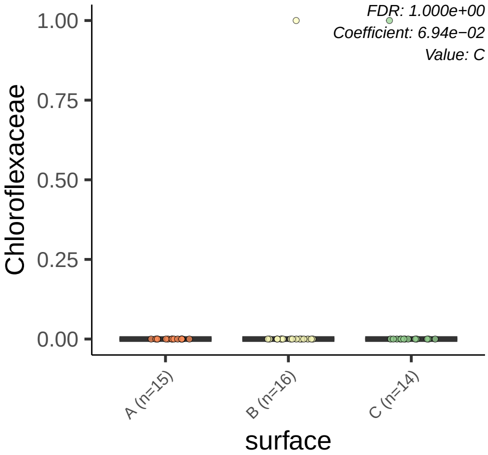
FDR: 1.000e+00
Coefficient: 6.94e−02
Value: C
Chloroflexaceae
1.00
0.75
0.50
0.25
0.00
A (n=15)
B (n=16)
C (n=14)
surface

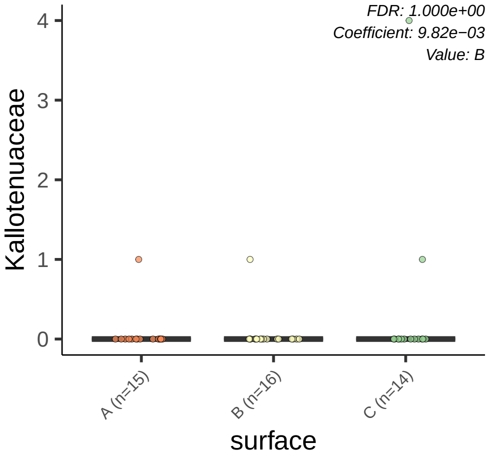

FDR: 1.000e+00
Coefficient: 9.82e−03
Value: B
Kallotenuaceae
0
1
2
3
4
A (n=15)
B (n=16)
C (n=14)
surface

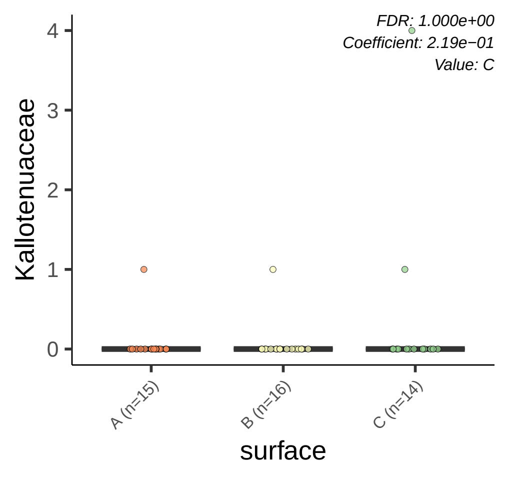
FDR: 1.000e+00
Coefficient: 2.19e−01
Value: C
Kallotenuaceae
0
1
2
3
4
A (n=15)
B (n=16)
C (n=14)
surface

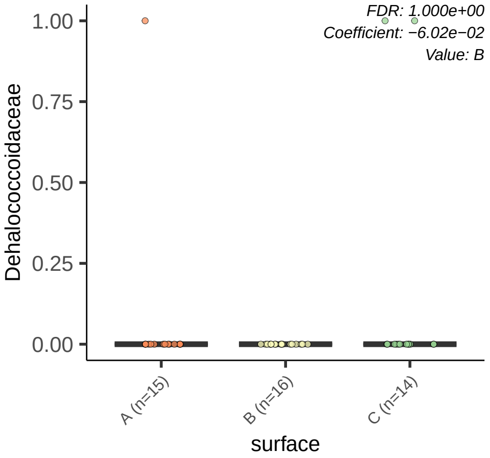
FDR: 1.000e+00
Coefficient: −6.02e−02
Value: B
Dehalococcoidaceae
1.00
0.75
0.50
0.25
0.00
A (n=15)
B (n=16)
C (n=14)
surface

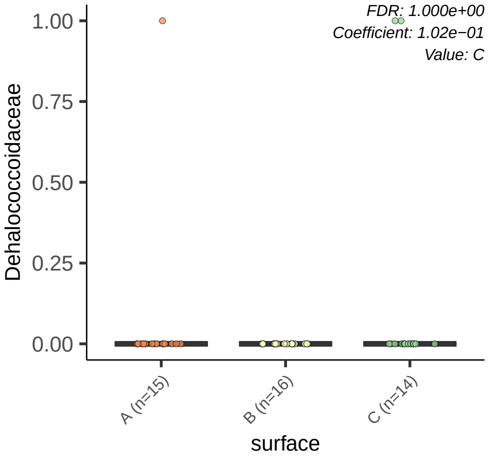

FDR: 1.000e+00
Coefficient: 1.02e−01
Value: C
Dehalococcoidaceae
1.00
0.75
0.50
0.25
0.00
A (n=15)
B (n=16)
C (n=14)
surface

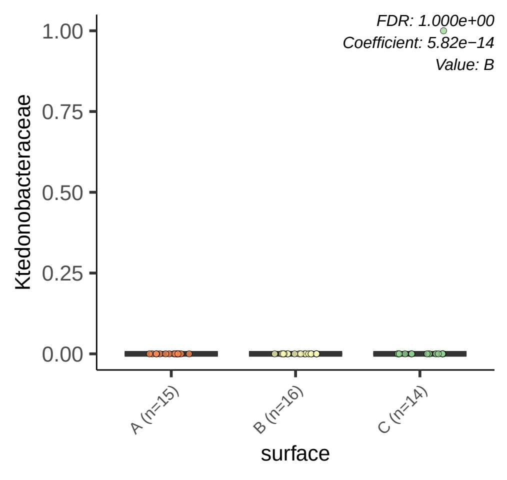
FDR: 1.000e+00
Coefficient: 5.82e−14
Value: B
Ktedonobacteraceae
1.00
0.75
0.50
0.25
0.00
A (n=15)
B (n=16)
C (n=14)
surface

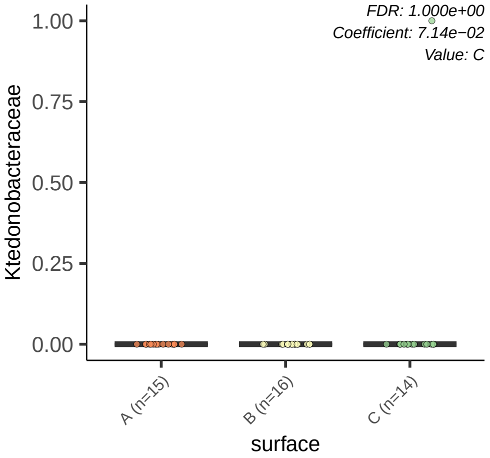
FDR: 1.000e+00
Coefficient: 7.14e−02
Value: C
Ktedonobacteraceae
1.00
0.75
0.50
0.25
0.00
A (n=15)
B (n=16)
C (n=14)
surface

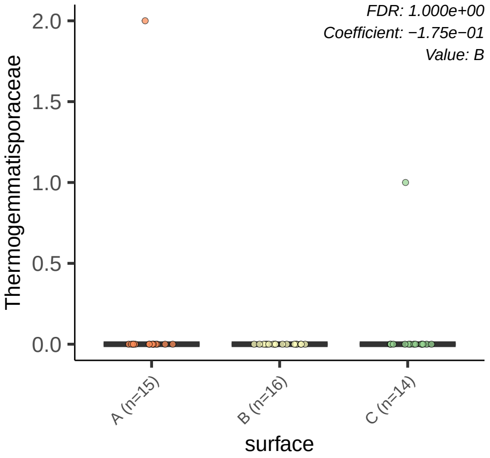

FDR: 1.000e+00
Coefficient: −1.75e−01
Value: B
Thermogemmatisporaceae
2.0
1.5
1.0
0.5
0.0
A (n=15)
B (n=16)
C (n=14)
surface

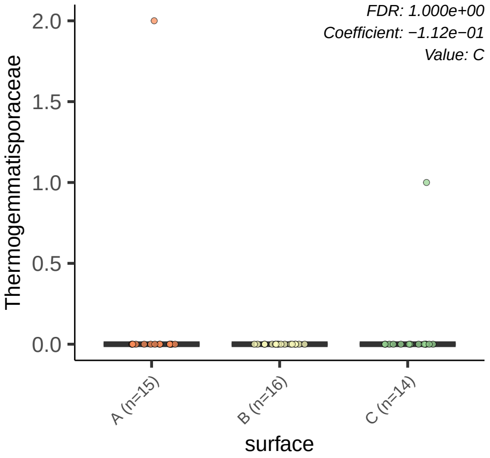
FDR: 1.000e+00
Coefficient: −1.12e−01
Value: C
Thermogemmatisporaceae
2.0
1.5
1.0
0.5
0.0
A (n=15)
B (n=16)
C (n=14)
surface

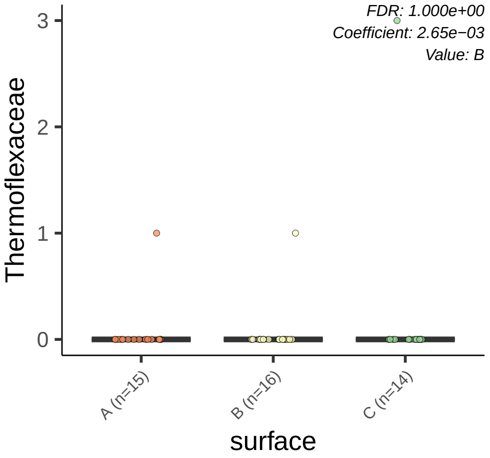
FDR: 1.000e+00
Coefficient: 2.65e−03
Value: B
Thermoflexaceae
3
2
1
0
A (n=15)
B (n=16)
C (n=14)
surface

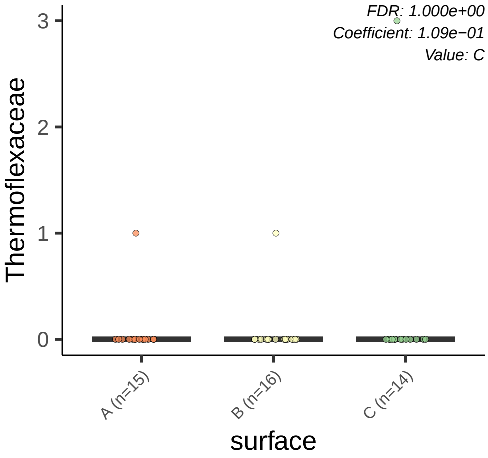

FDR: 1.000e+00
Coefficient: 1.09e−01
Value: C
Thermoflexaceae
3
2
1
0
A (n=15)
B (n=16)
C (n=14)
surface

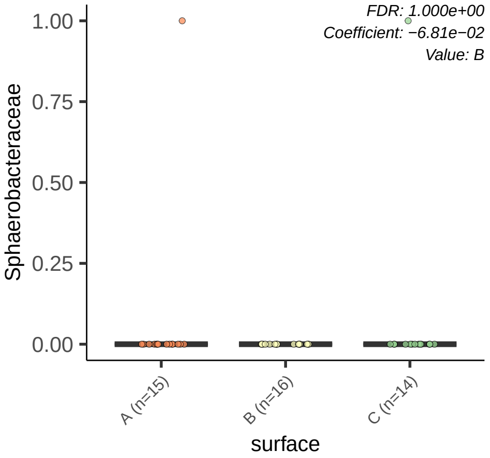
FDR: 1.000e+00
Coefficient: −6.81e−02
Value: B
Sphaerobacteraceae
1.00
0.75
0.50
0.25
0.00
A (n=15)
B (n=16)
C (n=14)
surface

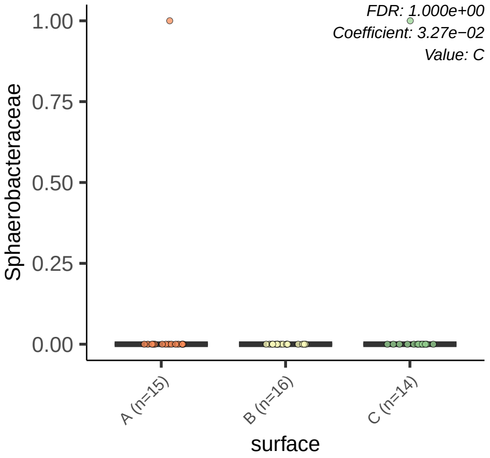
FDR: 1.000e+00
Coefficient: 3.27e−02
Value: C
Sphaerobacteraceae
1.00
0.75
0.50
0.25
0.00
A (n=15)
B (n=16)
C (n=14)
surface

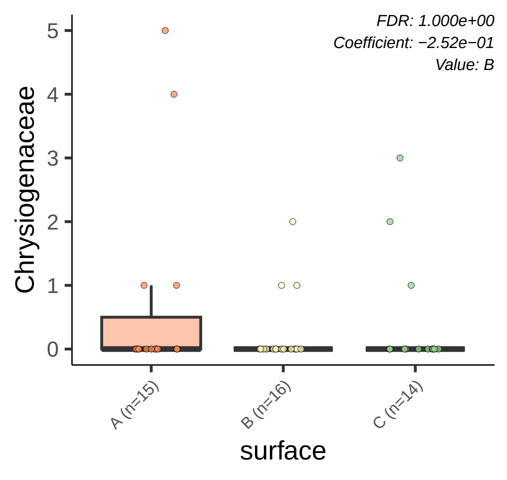
FDR: 1.000e+00
Coefficient: −2.52e−01
Value: B
Chrysiogenaceae
0
1
2
3
4
5
A (n=15)
B (n=16)
C (n=14)
surface

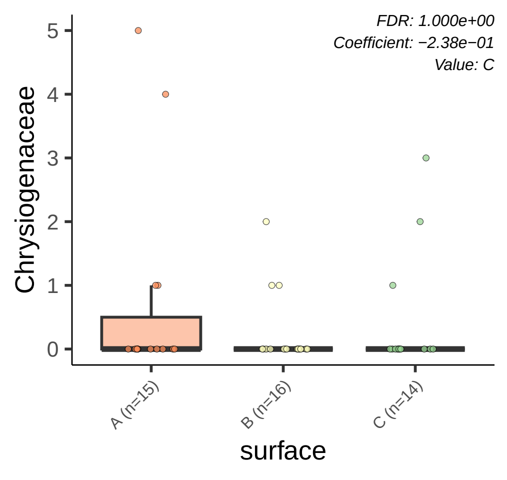
FDR: 1.000e+00
Coefficient: −2.38e−01
Value: C
Chrysiogenaceae
0
1
2
3
4
5
A (n=15)
B (n=16)
C (n=14)
surface

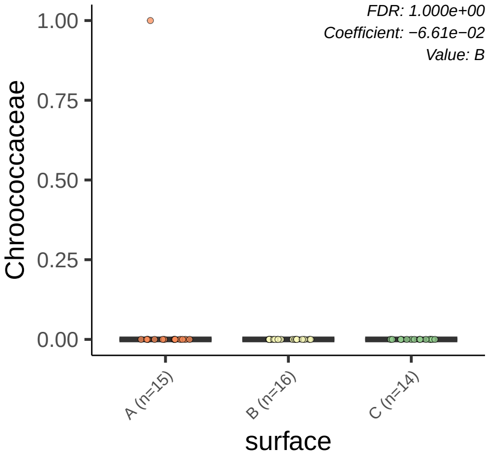
FDR: 1.000e+00
Coefficient: −6.61e−02
Value: B
Chroococcaceae
1.00
0.75
0.50
0.25
0.00
A (n=15)
B (n=16)
C (n=14)
surface

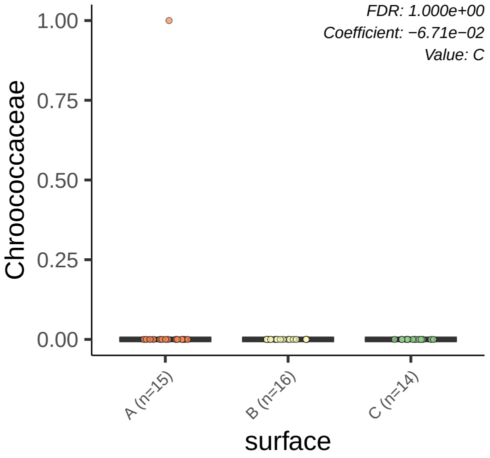

FDR: 1.000e+00
Coefficient: −6.71e−02
Value: C
Chroococcaceae
1.00
0.75
0.50
0.25
0.00
A (n=15)
B (n=16)
C (n=14)
surface

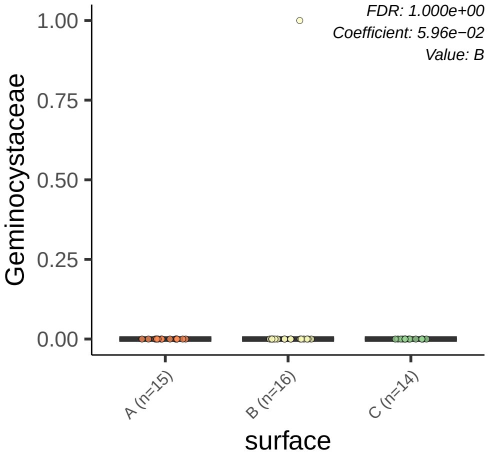

FDR: 1.000e+00
Coefficient: 5.96e−02
Value: B
Geminocystaceae
1.00
0.75
0.50
0.25
0.00
A (n=15)
B (n=16)
C (n=14)
surface

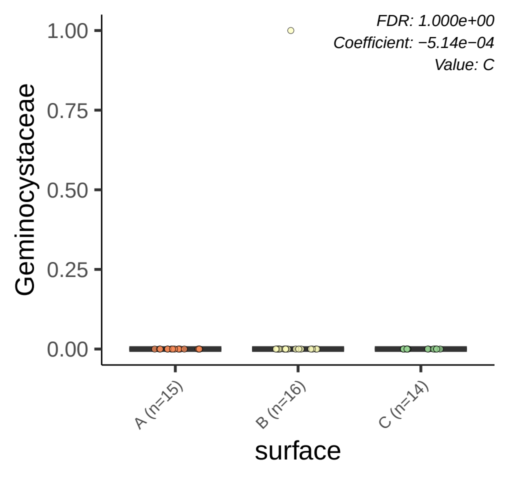

FDR: 1.000e+00
Coefficient: −5.14e−04
Value: C
Geminocystaceae
1.00
0.75
0.50
0.25
0.00
A (n=15)
B (n=16)
C (n=14)
surface

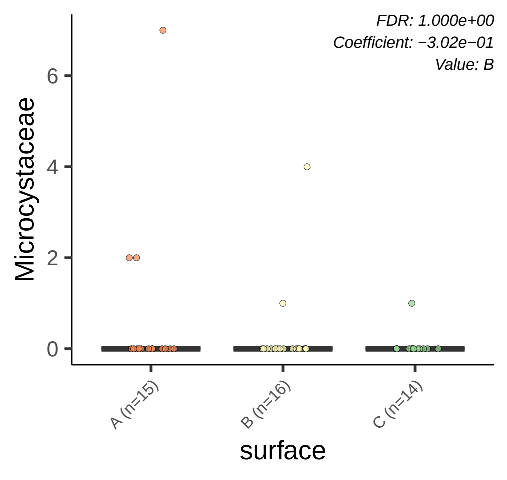
FDR: 1.000e+00
Coefficient: −3.02e−01
Value: B
Microcystaceae
0
2
4
6
A (n=15)
B (n=16)
C (n=14)
surface

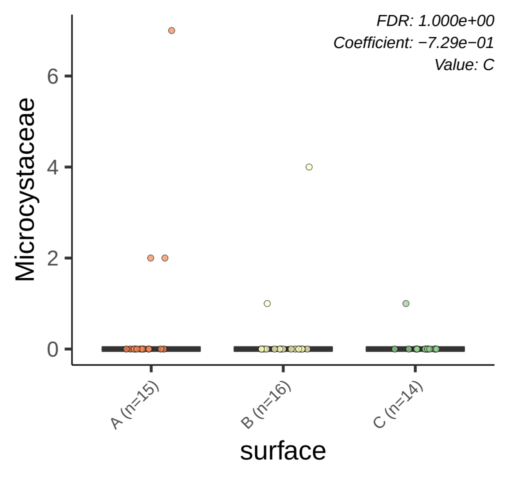
FDR: 1.000e+00
Coefficient: −7.29e−01
Value: C
Microcystaceae
0
2
4
6
A (n=15)
B (n=16)
C (n=14)
surface

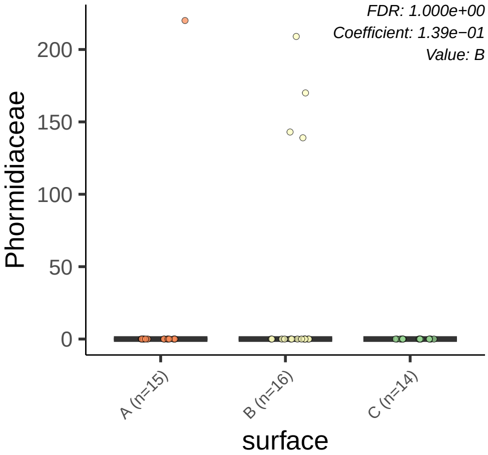
FDR: 1.000e+00
Coefficient: 1.39e−01
Value: B
Phormidiaceae
200
150
100
50
0
A (n=15)
B (n=16)
C (n=14)
surface

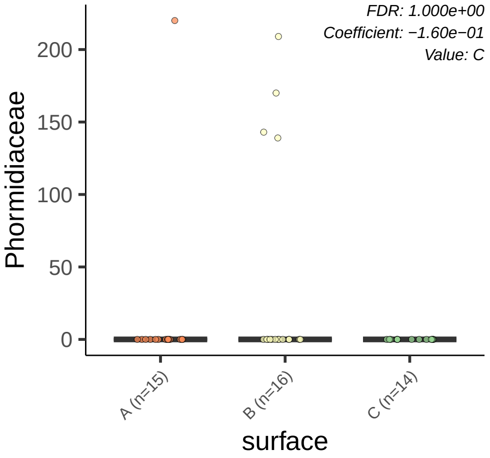

FDR: 1.000e+00
Coefficient: −1.60e−01
Value: C
Phormidiaceae
200
150
100
50
0
A (n=15)
B (n=16)
C (n=14)
surface

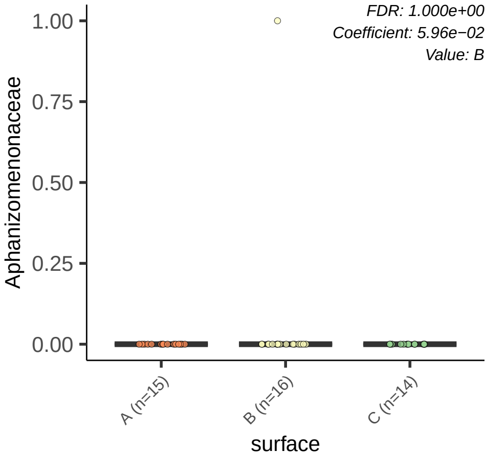

FDR: 1.000e+00
Coefficient: 5.96e−02
Value: B
Aphanizomenonaceae
1.00
0.75
0.50
0.25
0.00
A (n=15)
B (n=16)
C (n=14)
surface

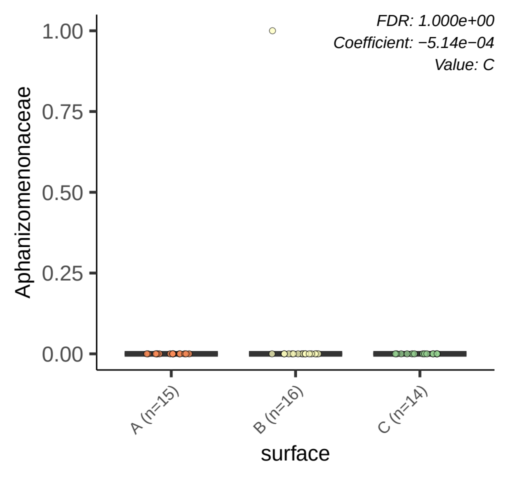
FDR: 1.000e+00
Coefficient: −5.14e−04
Value: C
Aphanizomenonaceae
1.00
0.75
0.50
0.25
0.00
A (n=15)
B (n=16)
C (n=14)
surface

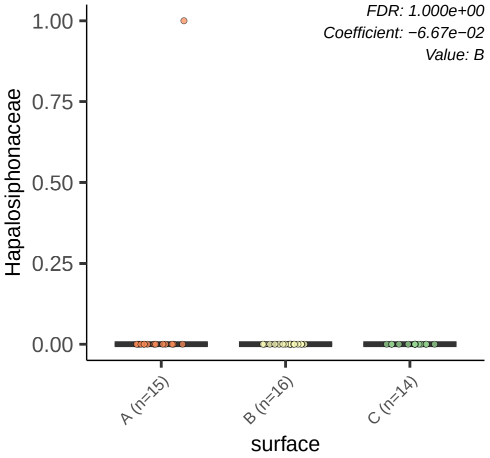
FDR: 1.000e+00
Coefficient: −6.67e−02
Value: B
Hapalosiphonaceae
1.00
0.75
0.50
0.25
0.00
A (n=15)
B (n=16)
C (n=14)
surface

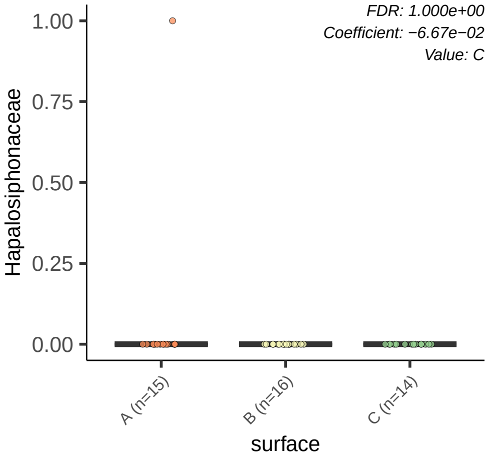
FDR: 1.000e+00
Coefficient: −6.67e−02
Value: C
Hapalosiphonaceae
1.00
0.75
0.50
0.25
0.00
A (n=15)
B (n=16)
C (n=14)
surface

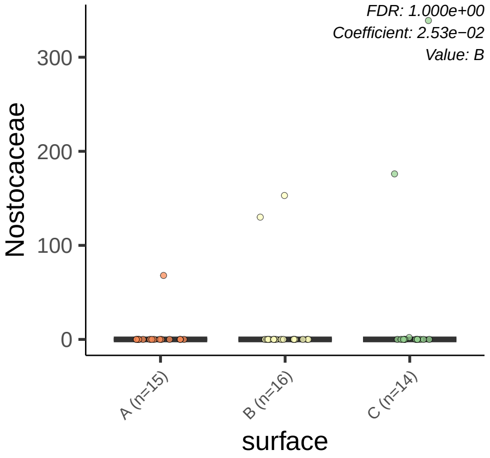
FDR: 1.000e+00
Coefficient: 2.53e−02
Value: B
Nostocaceae
300
200
100
0
A (n=15)
B (n=16)
C (n=14)
surface

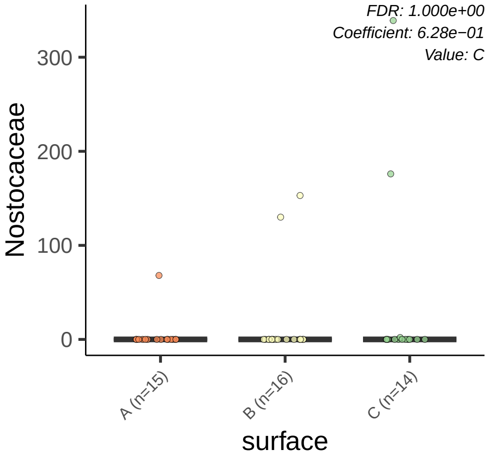
FDR: 1.000e+00
Coefficient: 6.28e−01
Value: C
Nostocaceae
300
200
100
0
A (n=15)
B (n=16)
C (n=14)
surface

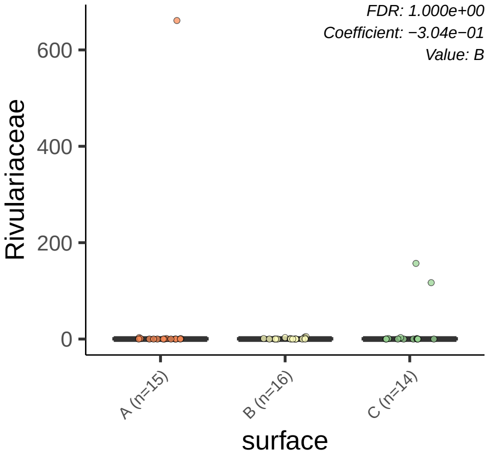
FDR: 1.000e+00
Coefficient: −3.04e−01
Value: B
Rivulariaceae
600
400
200
0
A (n=15)
B (n=16)
C (n=14)
surface

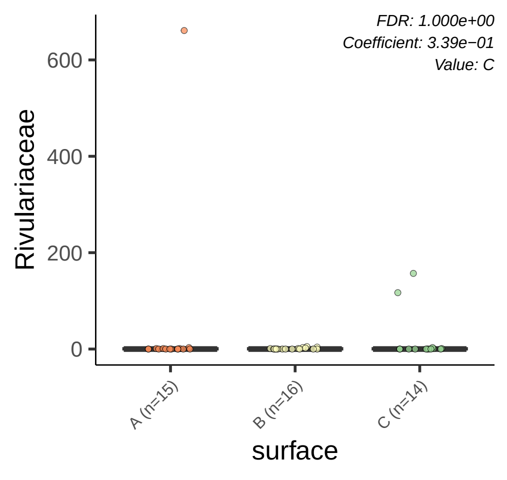

FDR: 1.000e+00
Coefficient: 3.39e−01
Value: C
Rivulariaceae
600
400
200
0
A (n=15)
B (n=16)
C (n=14)
surface

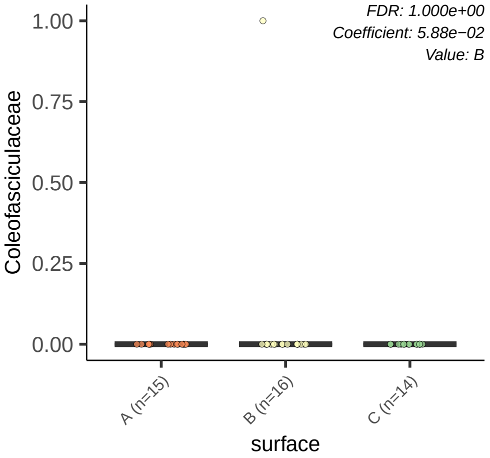
FDR: 1.000e+00
Coefficient: 5.88e−02
Value: B
Coleofasciculaceae
1.00
0.75
0.50
0.25
0.00
A (n=15)
B (n=16)
C (n=14)
surface

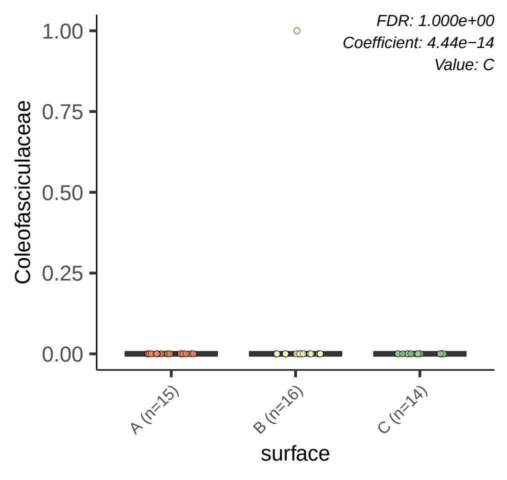
FDR: 1.000e+00
Coefficient: 4.44e−14
Value: C
Coleofasciculaceae
1.00
0.75
0.50
0.25
0.00
A (n=15)
B (n=16)
C (n=14)
surface

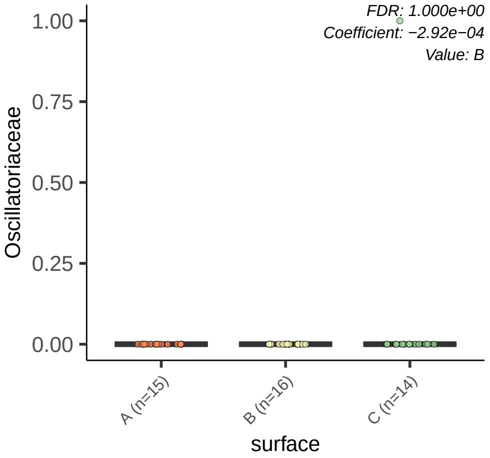
FDR: 1.000e+00
Coefficient: −2.92e−04
Value: B
1.00
0.75
0.50
0.25
0.00
Oscillatoriaceae
A (n=15)
B (n=16)
C (n=14)
surface

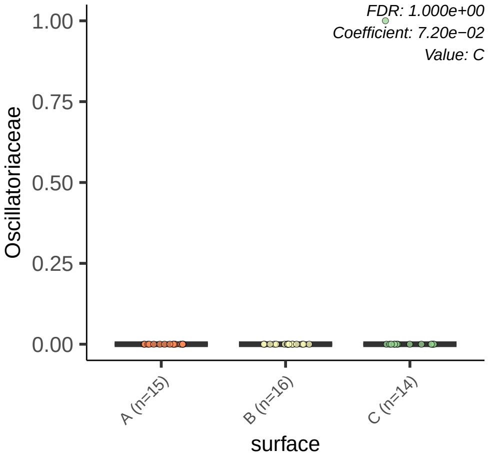
FDR: 1.000e+00
Coefficient: 7.20e−02
Value: C
Oscillatoriaceae
1.00
0.75
0.50
0.25
0.00
A (n=15)
B (n=16)
C (n=14)
surface

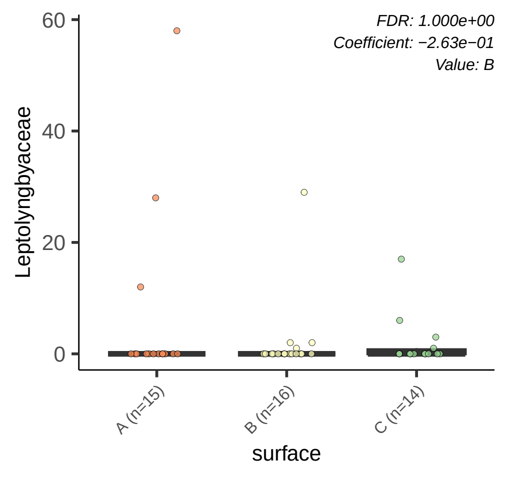
FDR: 1.000e+00
Coefficient: −2.63e−01
Value: B
Leptolyngbyaceae
60
40
20
0
A (n=15)
B (n=16)
C (n=14)
surface

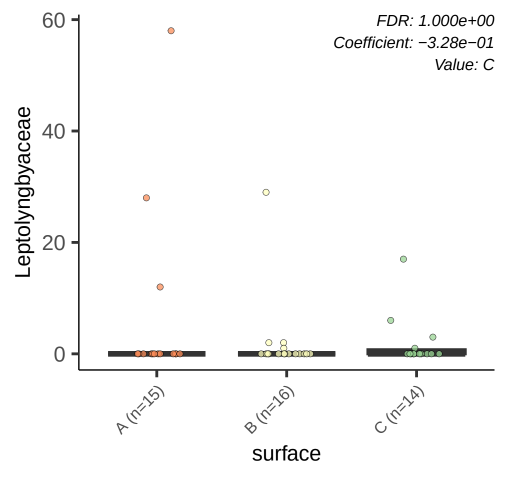

FDR: 1.000e+00
Coefficient: −3.28e−01
Value: C
Leptolyngbyaceae
60
40
20
0
A (n=15)
B (n=16)
C (n=14)
surface

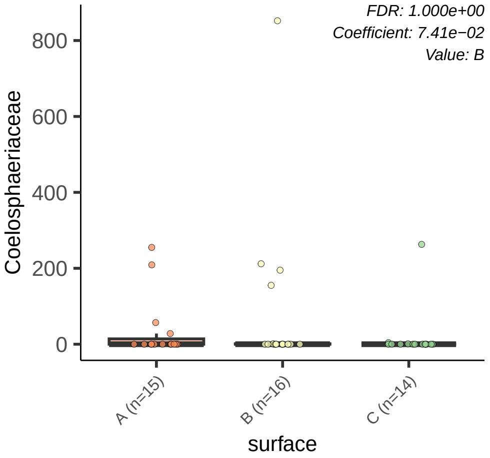

FDR: 1.000e+00
Coefficient: 7.41e−02
Value: B
Coelosphaeriaceae
800
600
400
200
0
A (n=15)
B (n=16)
C (n=14)
surface

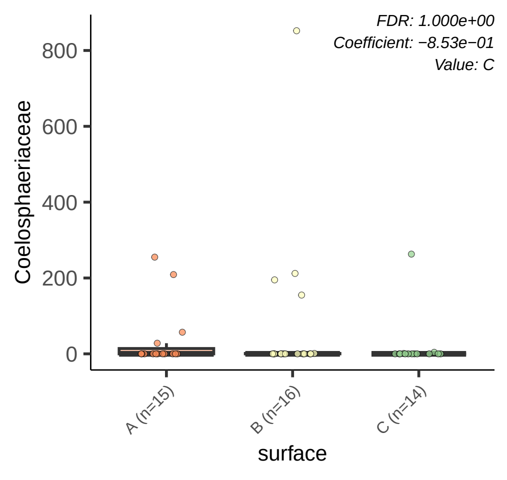
FDR: 1.000e+00
Coefficient: −8.53e−01
Value: C
Coelosphaeriaceae
800
600
400
200
0
A (n=15)
B (n=16)
C (n=14)
surface

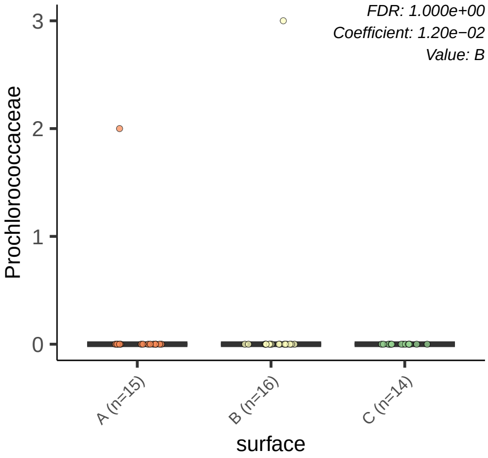
FDR: 1.000e+00
Coefficient: 1.20e−02
Value: B
Prochlorococcaceae
0
1
2
3
A (n=15)
B (n=16)
C (n=14)
surface

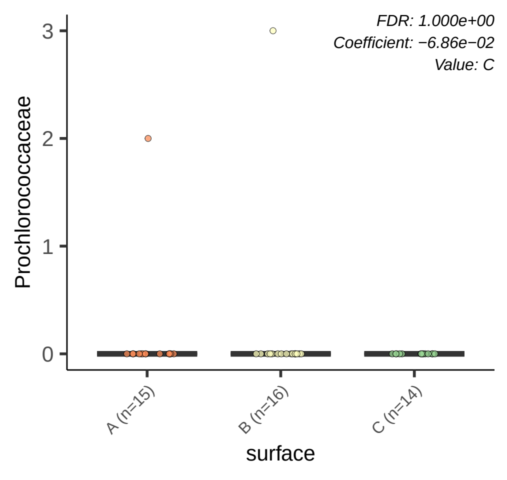

FDR: 1.000e+00
Coefficient: −6.86e−02
Value: C
Prochlorococcaceae
0
1
2
3
A (n=15)
B (n=16)
C (n=14)
surface

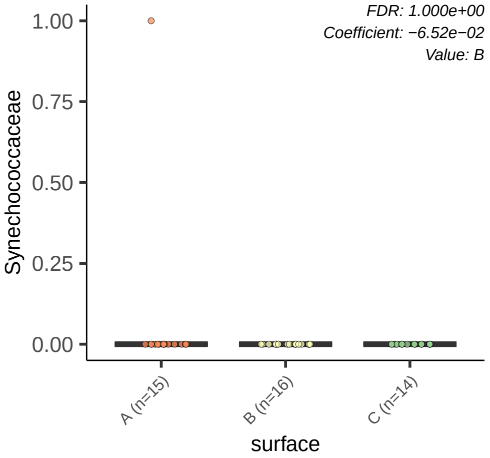

FDR: 1.000e+00
Coefficient: −6.52e−02
Value: B
Synechococcaceae
1.00
0.75
0.50
0.25
0.00
A (n=15)
B (n=16)
C (n=14)
surface

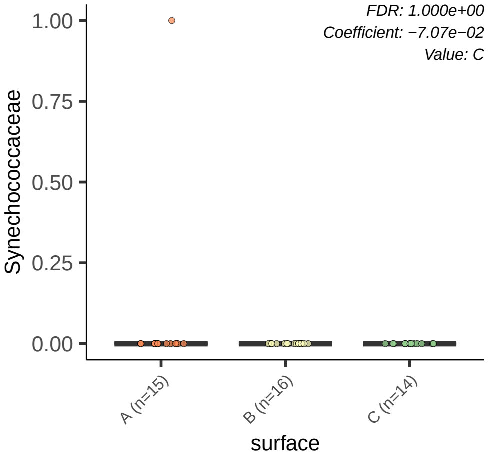

FDR: 1.000e+00
Coefficient: −7.07e−02
Value: C
Synechococcaceae
1.00
0.75
0.50
0.25
0.00
A (n=15)
B (n=16)
C (n=14)
surface

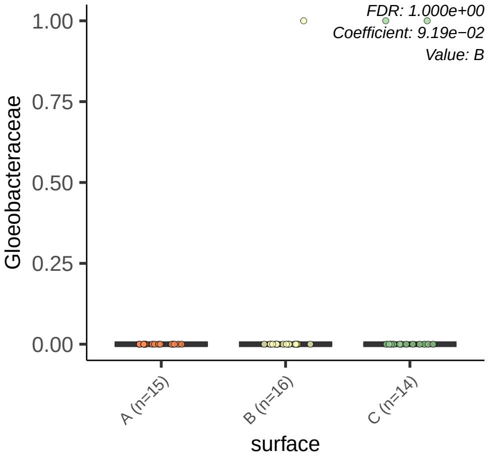

FDR: 1.000e+00
Coefficient: 9.19e−02
Value: B
Gloeobacteraceae
1.00
0.75
0.50
0.25
0.00
A (n=15)
B (n=16)
C (n=14)
surface

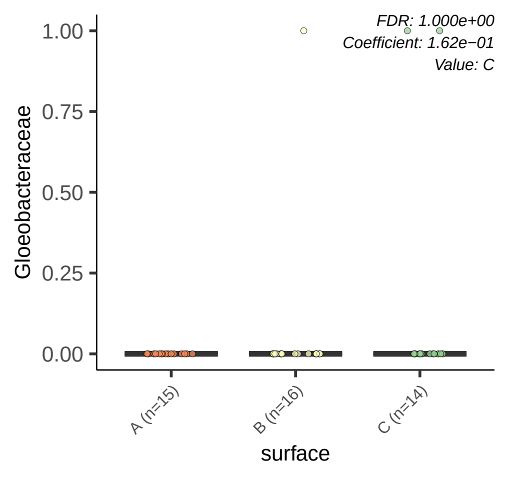
FDR: 1.000e+00
Coefficient: 1.62e−01
Value: C
Gloeobacteraceae
1.00
0.75
0.50
0.25
0.00
A (n=15)
B (n=16)
C (n=14)
surface

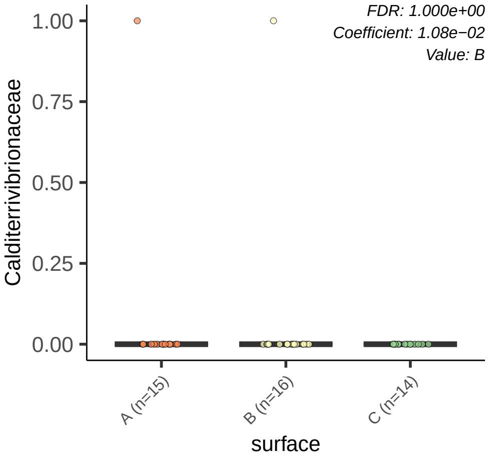
FDR: 1.000e+00
Coefficient: 1.08e−02
Value: B
Calditerrivibrionaceae
1.00
0.75
0.50
0.25
0.00
A (n=15)
B (n=16)
C (n=14)
surface

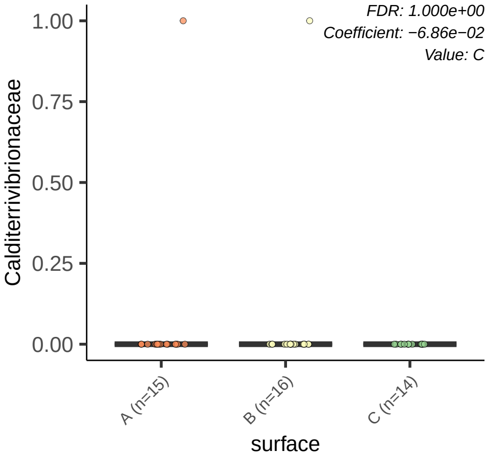
FDR: 1.000e+00
Coefficient: −6.86e−02
Value: C
Calditerrivibrionaceae
1.00
0.75
0.50
0.25
0.00
A (n=15)
B (n=16)
C (n=14)
surface

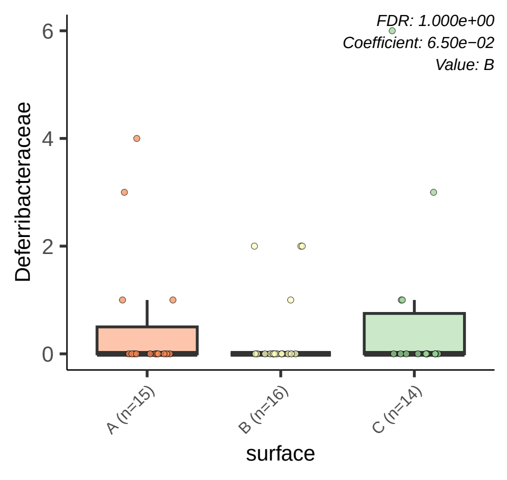

FDR: 1.000e+00
Coefficient: 6.50e−02
Value: B
Deferribacteraceae
6
4
2
0
A (n=15)
B (n=16)
C (n=14)
surface

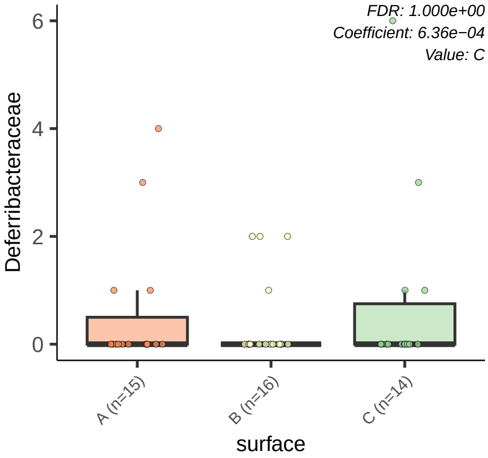

FDR: 1.000e+00
Coefficient: 6.36e−04
Value: C
Deferribacteraceae
0
2
4
6
A (n=15)
B (n=16)
C (n=14)
surface

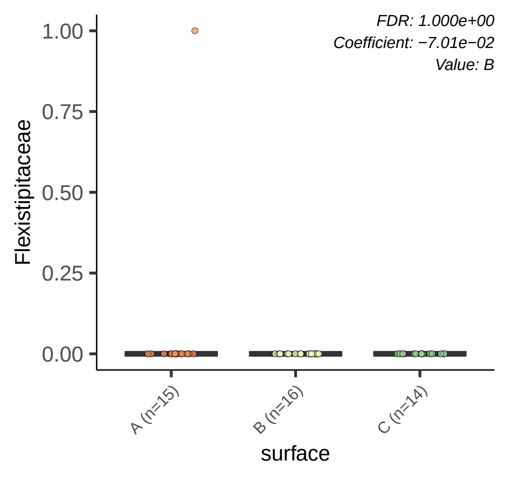
FDR: 1.000e+00
Coefficient: −7.01e−02
Value: B
Flexistipitaceae
1.00
0.75
0.50
0.25
0.00
A (n=15)
B (n=16)
C (n=14)
surface

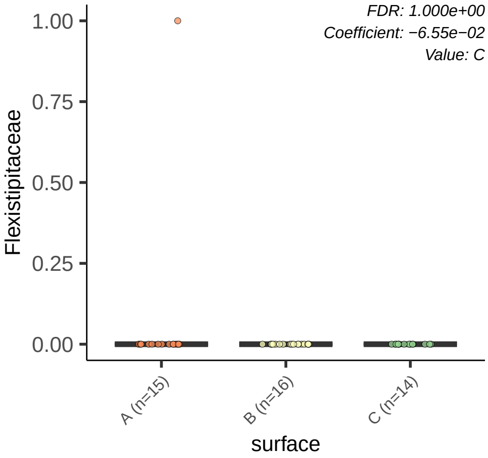

FDR: 1.000e+00
Coefficient: −6.55e−02
Value: C
1.00
0.75
0.50
0.25
0.00
Flexistipitaceae
A (n=15)
B (n=16)
C (n=14)
surface

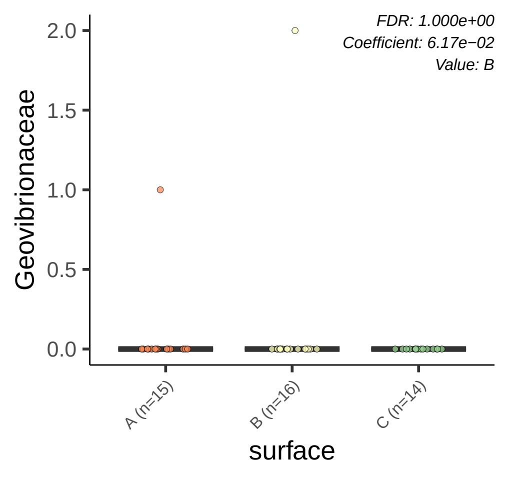
FDR: 1.000e+00
Coefficient: 6.17e−02
Value: B
Geovibrionaceae
2.0
1.5
1.0
0.5
0.0
A (n=15)
B (n=16)
C (n=14)
surface

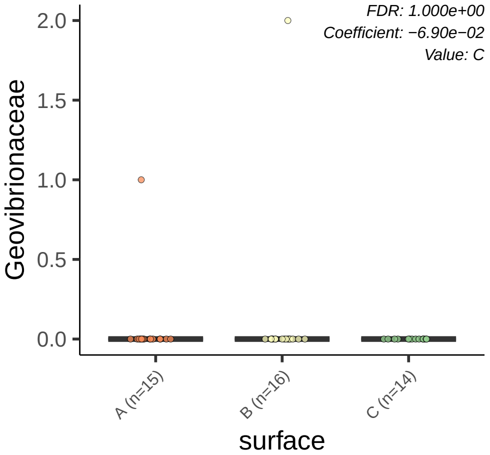
FDR: 1.000e+00
Coefficient: −6.90e−02
Value: C
Geovibrionaceae
2.0
1.5
1.0
0.5
0.0
A (n=15)
B (n=16)
C (n=14)
surface

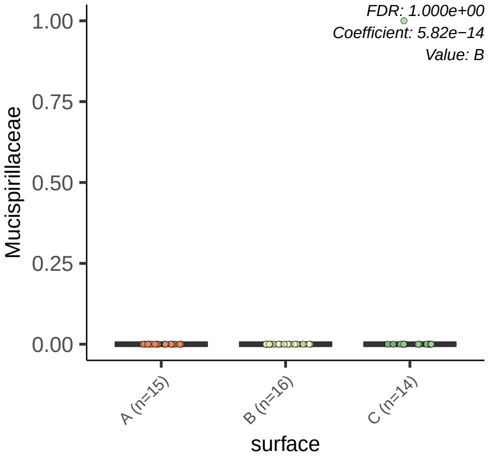
FDR: 1.000e+00
Coefficient: 5.82e−14
Value: B
1.00
0.75
0.50
0.25
0.00
Mucispirillaceae
A (n=15)
B (n=16)
C (n=14)
surface

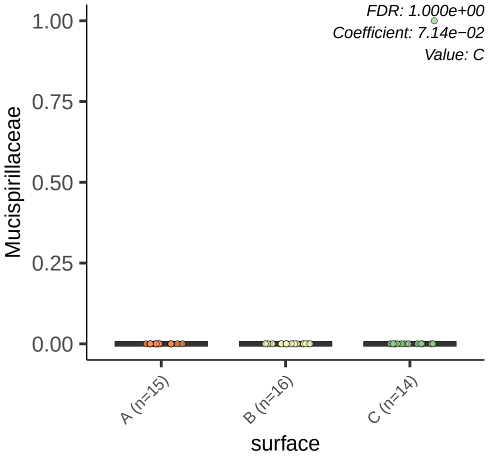
FDR: 1.000e+00
Coefficient: 7.14e−02
Value: C
Mucispirillaceae
1.00
0.75
0.50
0.25
0.00
A (n=15)
B (n=16)
C (n=14)
surface

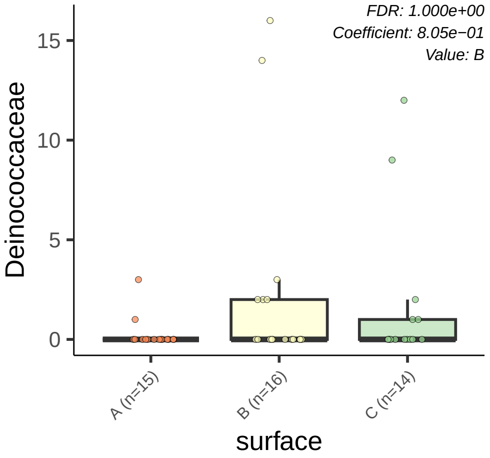

FDR: 1.000e+00
Coefficient: 8.05e−01
Value: B
Deinococcaceae
0
5
10
15
A (n=15)
B (n=16)
C (n=14)
surface

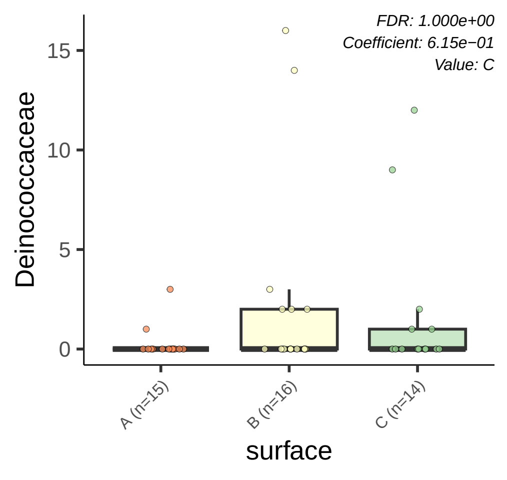
FDR: 1.000e+00
Coefficient: 6.15e−01
Value: C
Deinococcaceae
15
10
5
0
A (n=15)
B (n=16)
C (n=14)
surface

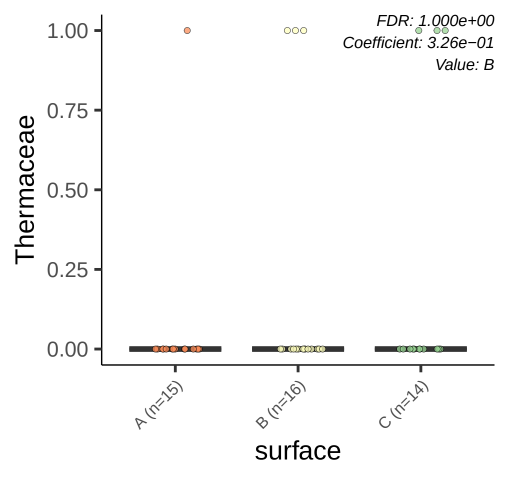
FDR: 1.000e+00
Coefficient: 3.26e−01
Value: B
Thermaceae
1.00
0.75
0.50
0.25
0.00
A (n=15)
B (n=16)
C (n=14)
surface

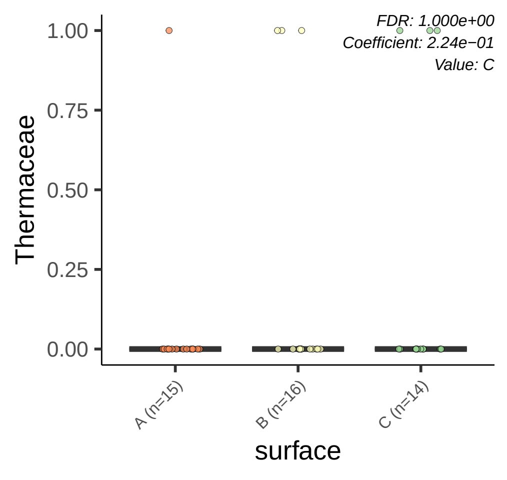

FDR: 1.000e+00
Coefficient: 2.24e−01
Value: C
Thermaceae
1.00
0.75
0.50
0.25
0.00
A (n=15)
B (n=16)
C (n=14)
surface

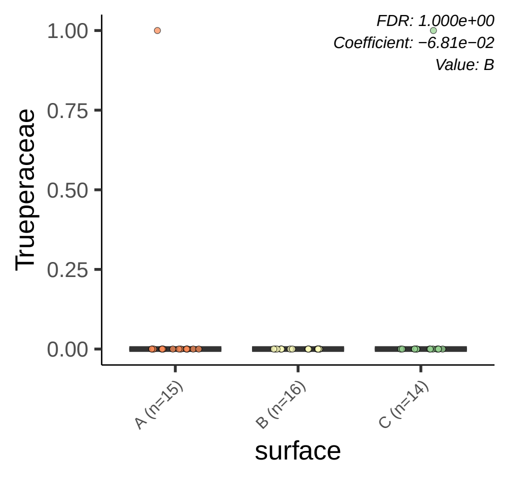

FDR: 1.000e+00
Coefficient: −6.81e−02
Value: B
Trueperaceae
1.00
0.75
0.50
0.25
0.00
A (n=15)
B (n=16)
C (n=14)
surface

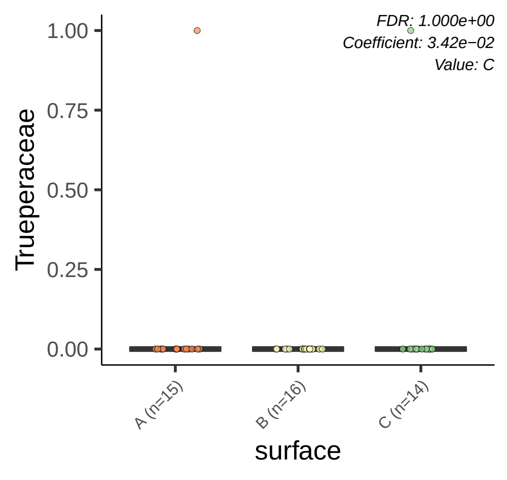
FDR: 1.000e+00
Coefficient: 3.42e−02
Value: C
Trueperaceae
1.00
0.75
0.50
0.25
0.00
A (n=15)
B (n=16)
C (n=14)
surface

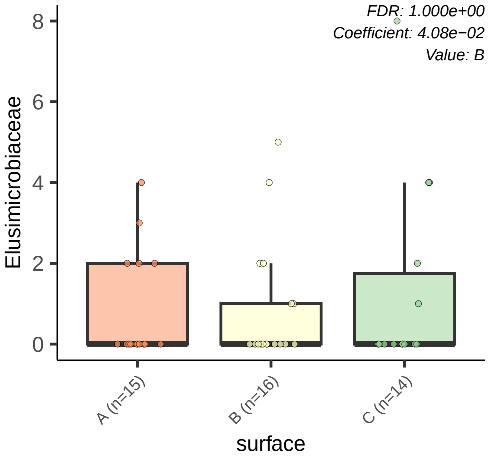

FDR: 1.000e+00
Coefficient: 4.08e−02
Value: B
Elusimicrobiaceae
0
2
4
6
8
A (n=15)
B (n=16)
C (n=14)
surface

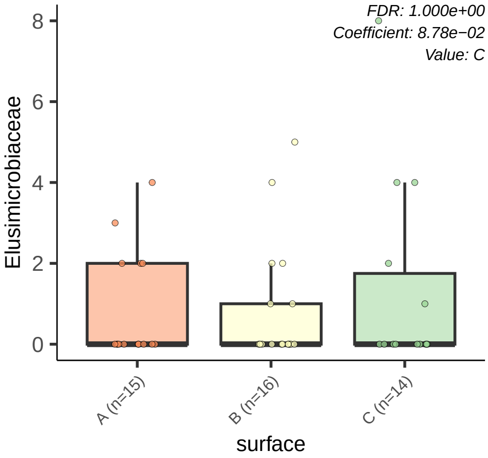
FDR: 1.000e+00
Coefficient: 8.78e−02
Value: C
Elusimicrobiaceae
8
6
4
2
0
A (n=15)
B (n=16)
C (n=14)
surface

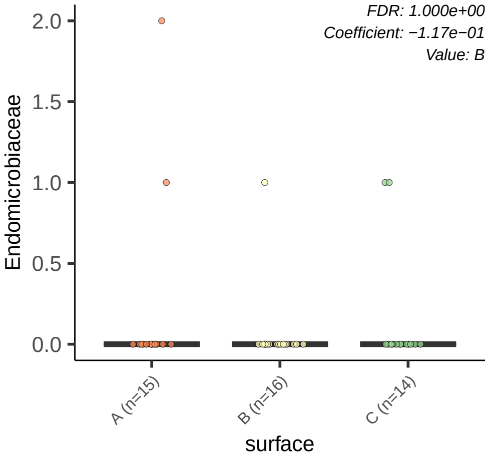
FDR: 1.000e+00
Coefficient: −1.17e−01
Value: B
Endomicrobiaceae
2.0
1.5
1.0
0.5
0.0
A (n=15)
B (n=16)
C (n=14)
surface

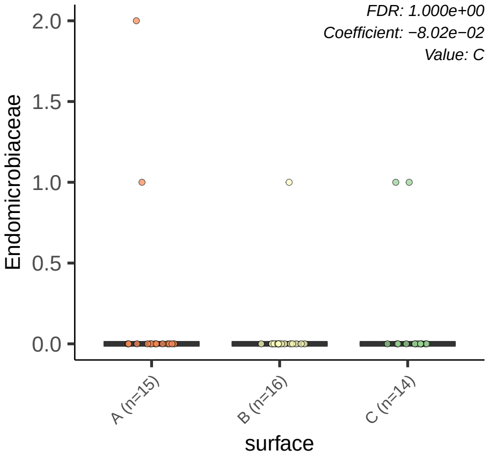
FDR: 1.000e+00
Coefficient: −8.02e−02
Value: C
Endomicrobiaceae
2.0
1.5
1.0
0.5
0.0
A (n=15)
B (n=16)
C (n=14)
surface

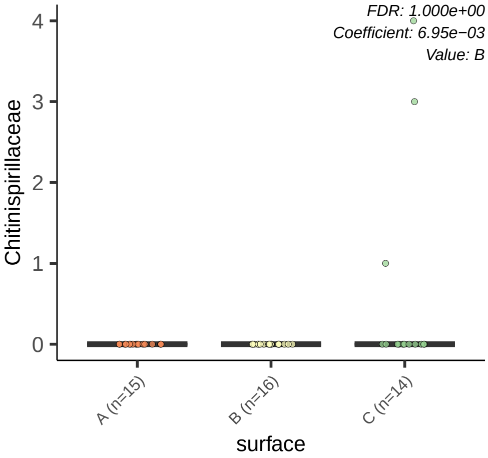
FDR: 1.000e+00
Coefficient: 6.95e−03
Value: B
Chitinispirillaceae
0
1
2
3
4
A (n=15)
B (n=16)
C (n=14)
surface

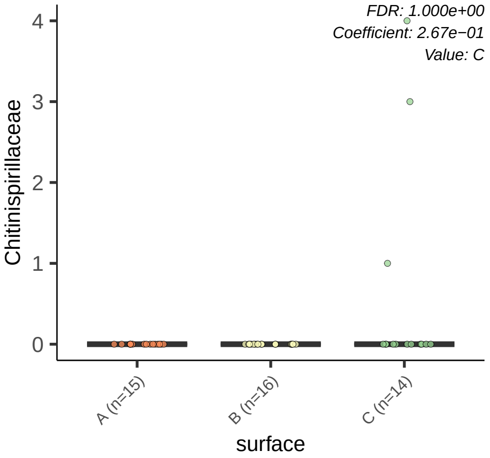
FDR: 1.000e+00
Coefficient: 2.67e−01
Value: C
Chitinispirillaceae
4
3
2
1
0
A (n=15)
B (n=16)
C (n=14)
surface

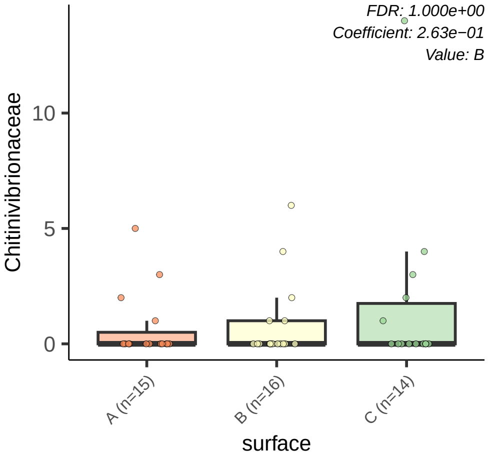

FDR: 1.000e+00
Coefficient: 2.63e−01
Value: B
Chitinivibrionaceae
0
5
10
A (n=15)
B (n=16)
C (n=14)
surface

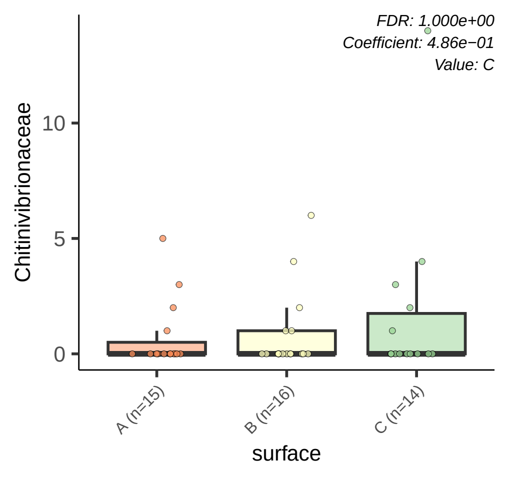
FDR: 1.000e+00
Coefficient: 4.86e−01
Value: C
Chitinivibrionaceae
10
5
0
A (n=15)
B (n=16)
C (n=14)
surface

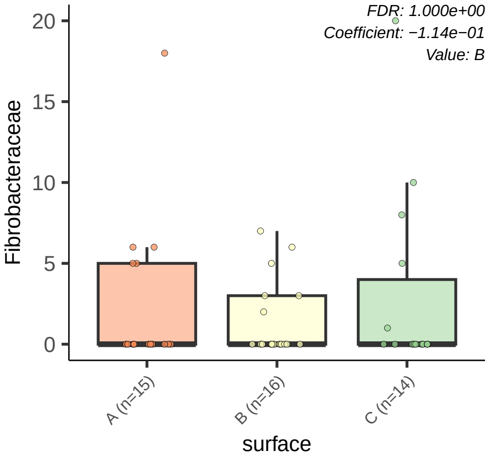
FDR: 1.000e+00
Coefficient: −1.14e−01
Value: B
Fibrobacteraceae
20
15
10
5
0
A (n=15)
B (n=16)
C (n=14)
surface

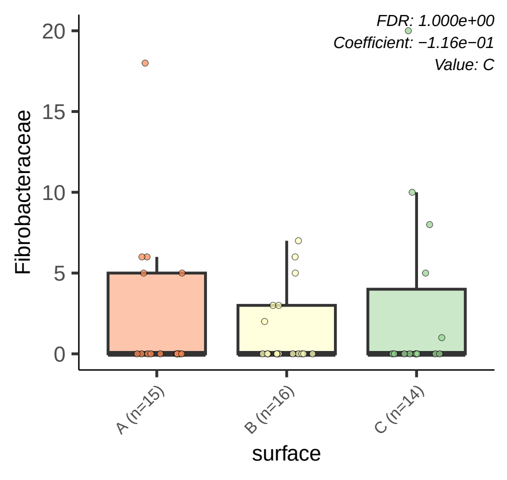

FDR: 1.000e+00
Coefficient: −1.16e−01
Value: C
Fibrobacteraceae
0
5
10
15
20
A (n=15)
B (n=16)
C (n=14)
surface

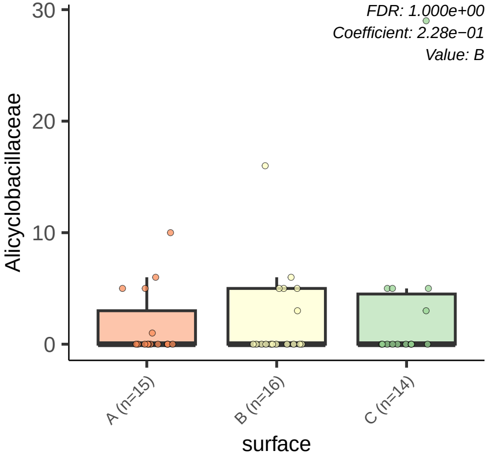
FDR: 1.000e+00
Coefficient: 2.28e−01
Value: B
Alicyclobacillaceae
0
10
20
30
A (n=15)
B (n=16)
C (n=14)
surface

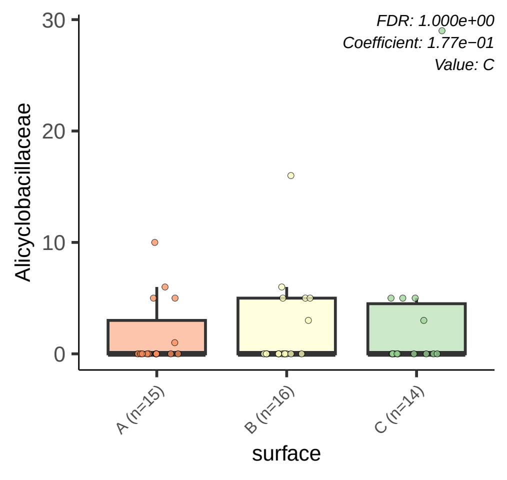
FDR: 1.000e+00
Coefficient: 1.77e−01
Value: C
Alicyclobacillaceae
30
20
10
0
A (n=15)
B (n=16)
C (n=14)
surface

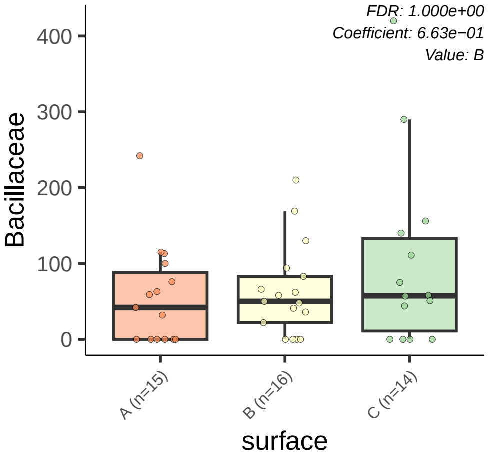
FDR: 1.000e+00
Coefficient: 6.63e−01
Value: B
Bacillaceae
400
300
200
100
0
A (n=15)
B (n=16)
C (n=14)
surface

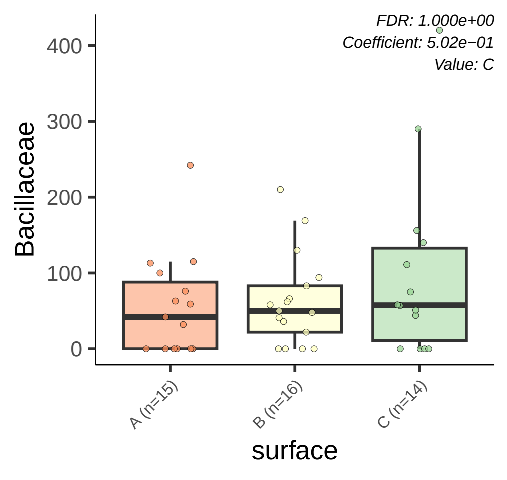
FDR: 1.000e+00
Coefficient: 5.02e−01
Value: C
Bacillaceae
400
300
200
100
0
A (n=15)
B (n=16)
C (n=14)
surface

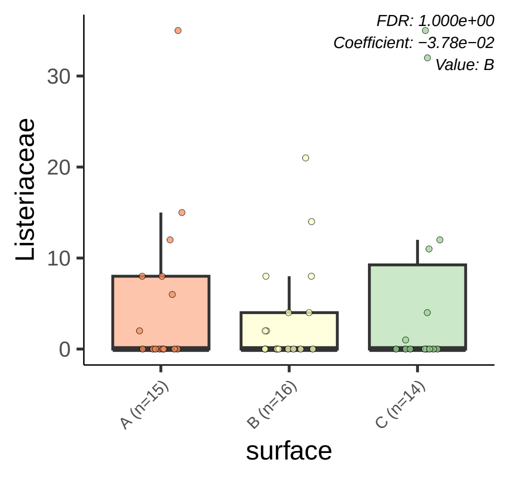
FDR: 1.000e+00
Coefficient: −3.78e−02
Value: B
Listeriaceae
0
10
20
30
A (n=15)
B (n=16)
C (n=14)
surface

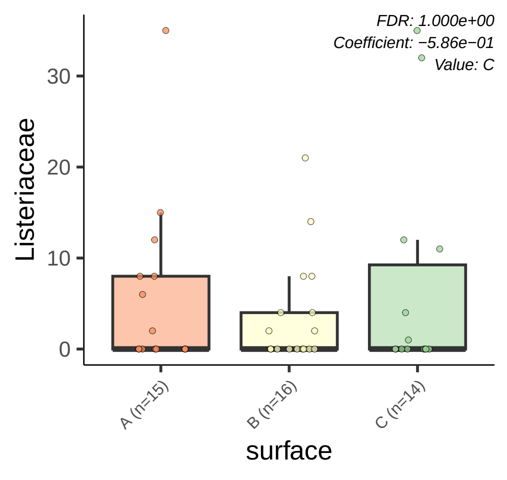

FDR: 1.000e+00
Coefficient: −5.86e−01
Value: C
Listeriaceae
0
10
20
30
A (n=15)
B (n=16)
C (n=14)
surface

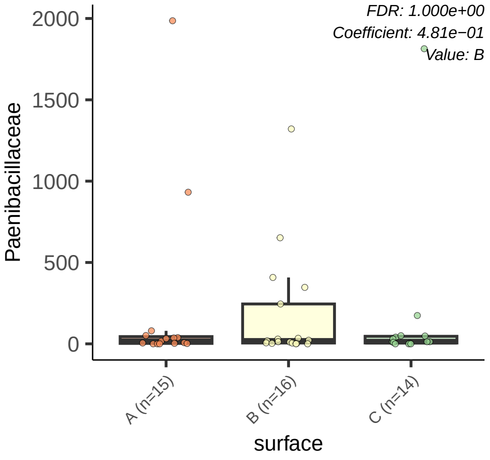

FDR: 1.000e+00
Coefficient: 4.81e−01
Value: B
Paenibacillaceae
2000
1500
1000
500
0
A (n=15)
B (n=16)
C (n=14)
surface

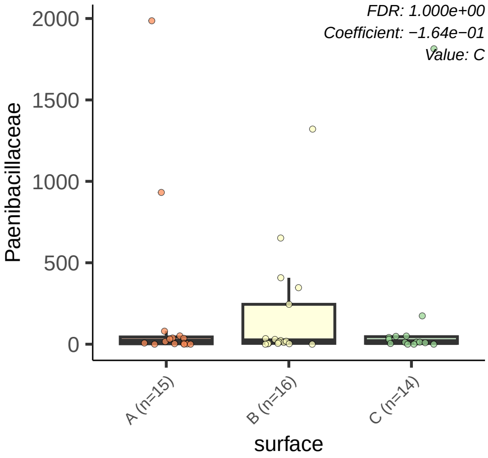
FDR: 1.000e+00
Coefficient: −1.64e−01
Value: C
Paenibacillaceae
2000
1500
1000
500
0
A (n=15)
B (n=16)
C (n=14)
surface

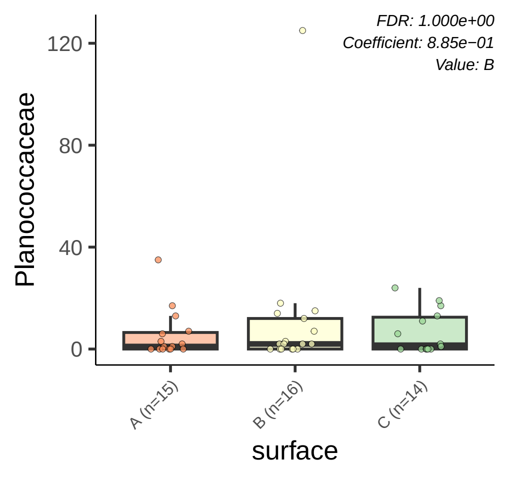

FDR: 1.000e+00
Coefficient: 8.85e−01
Value: B
Planococcaceae
120
80
40
0
A (n=15)
B (n=16)
C (n=14)
surface

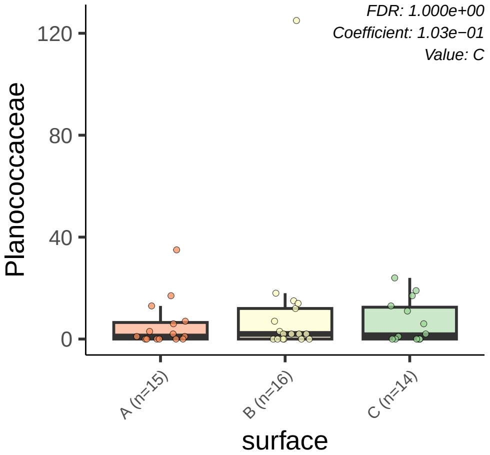

FDR: 1.000e+00
Coefficient: 1.03e−01
Value: C
Planococcaceae
0
40
80
120
A (n=15)
B (n=16)
C (n=14)
surface

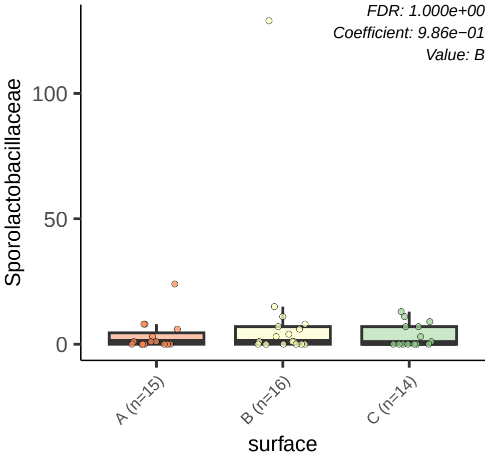

FDR: 1.000e+00
Coefficient: 9.86e−01
Value: B
Sporolactobacillaceae
100
50
0
A (n=15)
B (n=16)
C (n=14)
surface

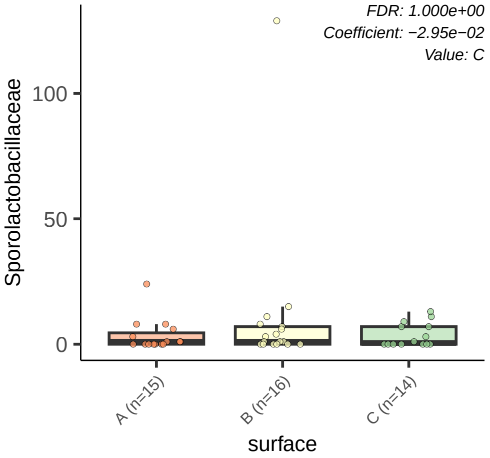

FDR: 1.000e+00
Coefficient: −2.95e−02
Value: C
Sporolactobacillaceae
100
50
0
A (n=15)
B (n=16)
C (n=14)
surface

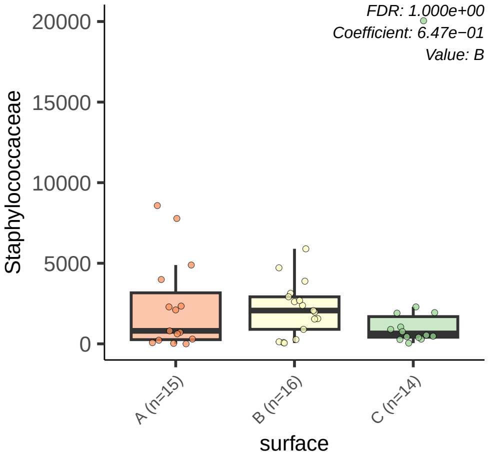
FDR: 1.000e+00
Coefficient: 6.47e−01
Value: B
Staphylococcaceae
20000
15000
10000
5000
0
A (n=15)
B (n=16)
C (n=14)
surface

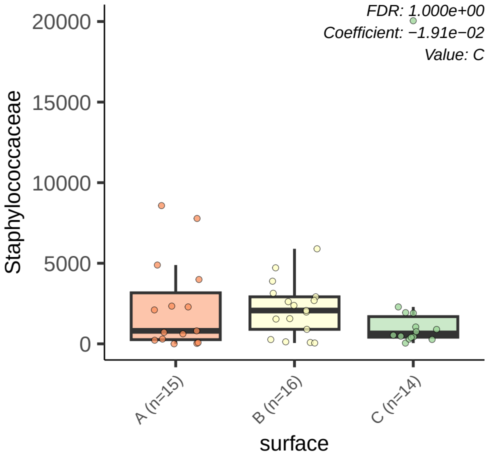
FDR: 1.000e+00
Coefficient: −1.91e−02
Value: C
Staphylococcaceae
20000
15000
10000
5000
0
A (n=15)
B (n=16)
C (n=14)
surface

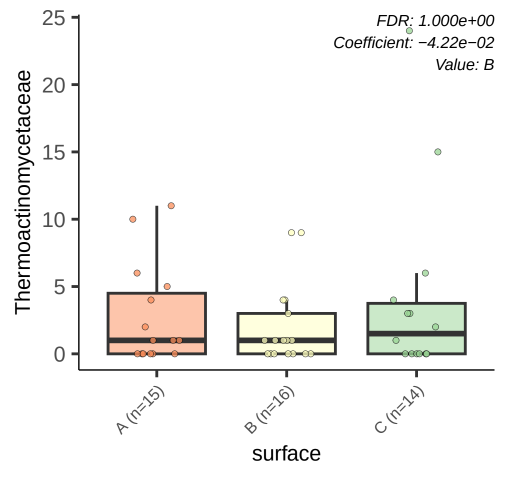
FDR: 1.000e+00
Coefficient: −4.22e−02
Value: B
Thermoactinomycetaceae
25
20
15
10
5
0
A (n=15)
B (n=16)
C (n=14)
surface

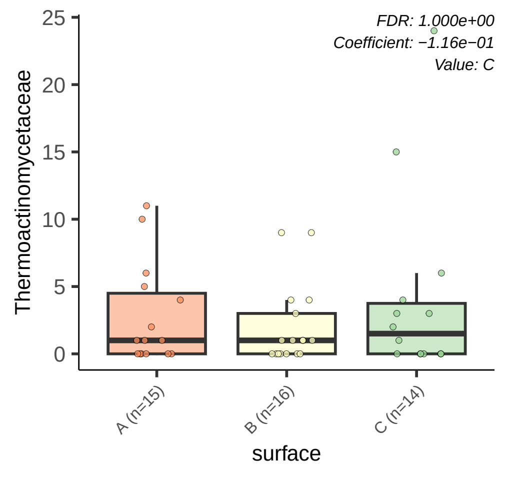
FDR: 1.000e+00
Coefficient: −1.16e−01
Value: C
Thermoactinomycetaceae
25
20
15
10
5
0
A (n=15)
B (n=16)
C (n=14)
surface

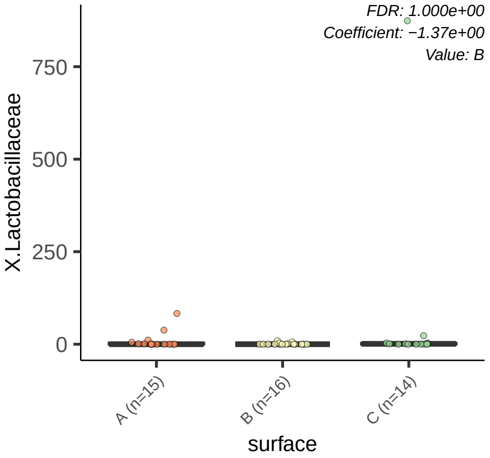
FDR: 1.000e+00
Coefficient: −1.37e+00
Value: B
X.Lactobacillaceae
750
500
250
0
A (n=15)
B (n=16)
C (n=14)
surface

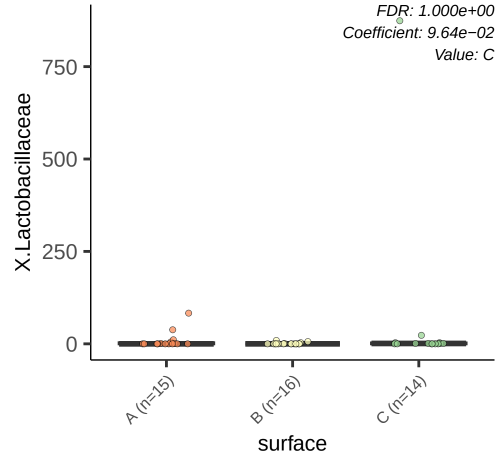

FDR: 1.000e+00
Coefficient: 9.64e−02
Value: C
X.Lactobacillaceae
750
500
250
0
A (n=15)
B (n=16)
C (n=14)
surface

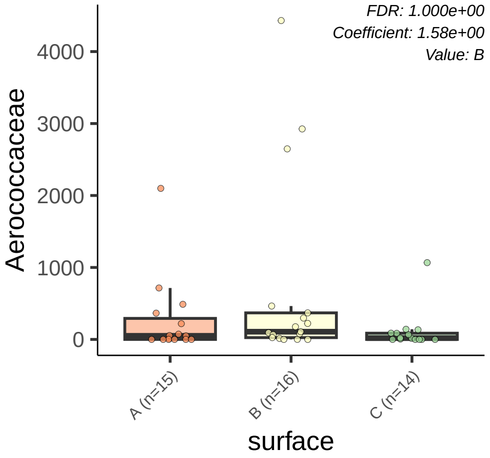
FDR: 1.000e+00
Coefficient: 1.58e+00
Value: B
Aerococcaceae
4000
3000
2000
1000
0
A (n=15)
B (n=16)
C (n=14)
surface

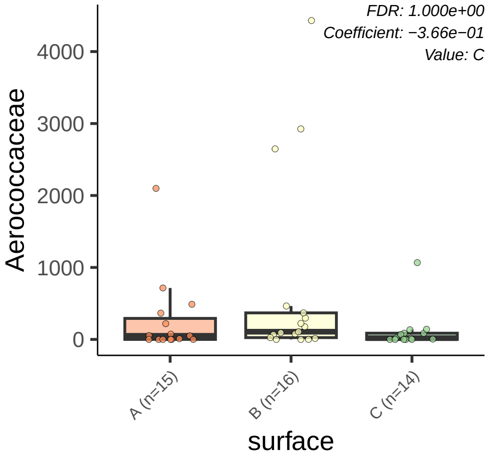
FDR: 1.000e+00
Coefficient: −3.66e−01
Value: C
Aerococcaceae
4000
3000
2000
1000
0
A (n=15)
B (n=16)
C (n=14)
surface

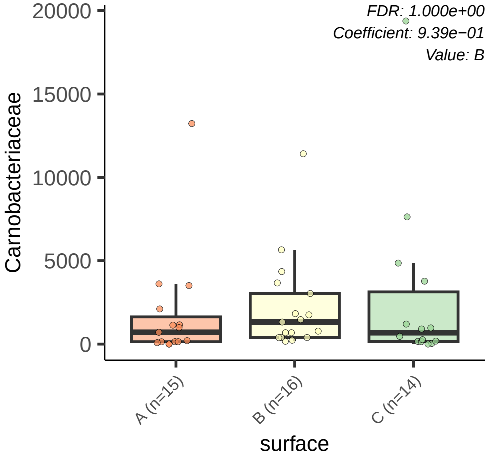

FDR: 1.000e+00
Coefficient: 9.39e−01
Value: B
Carnobacteriaceae
20000
15000
10000
5000
0
A (n=15)
B (n=16)
C (n=14)
surface

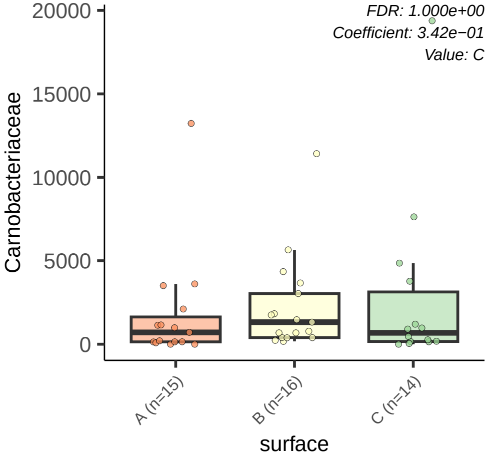

FDR: 1.000e+00
Coefficient: 3.42e−01
Value: C
Carnobacteriaceae
20000
15000
10000
5000
0
A (n=15)
B (n=16)
C (n=14)
surface

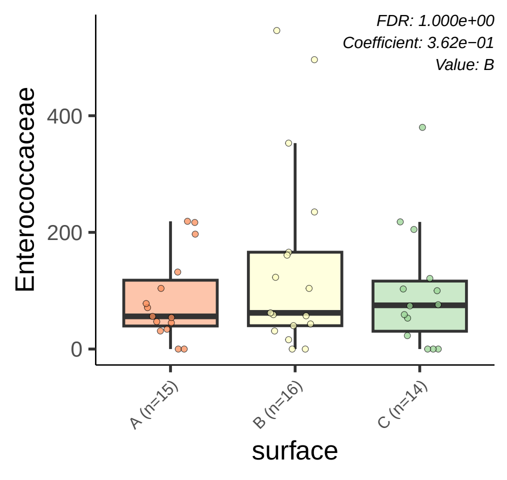
FDR: 1.000e+00
Coefficient: 3.62e−01
Value: B
Enterococcaceae
0
200
400
A (n=15)
B (n=16)
C (n=14)
surface

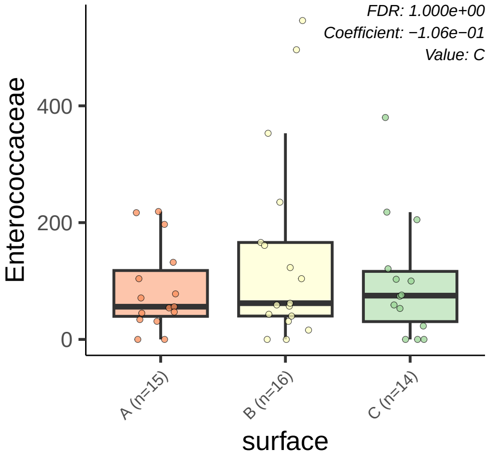

FDR: 1.000e+00
Coefficient: −1.06e−01
Value: C
Enterococcaceae
400
200
0
A (n=15)
B (n=16)
C (n=14)
surface

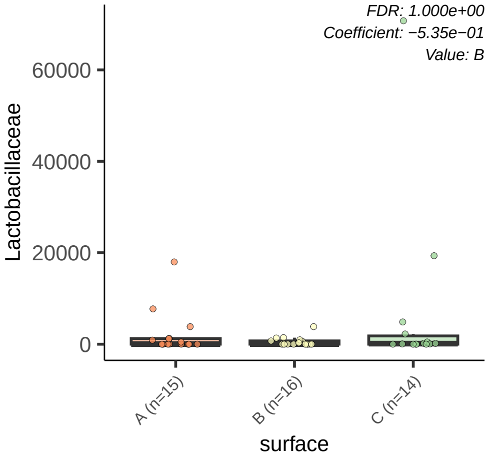

FDR: 1.000e+00
Coefficient: −5.35e−01
Value: B
Lactobacillaceae
60000
40000
20000
0
A (n=15)
B (n=16)
C (n=14)
surface

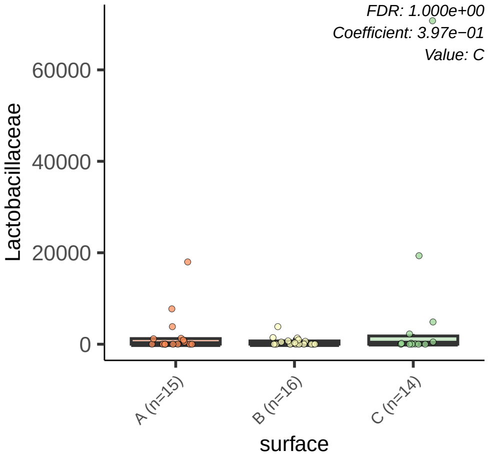
FDR: 1.000e+00
Coefficient: 3.97e−01
Value: C
Lactobacillaceae
60000
40000
20000
0
A (n=15)
B (n=16)
C (n=14)
surface

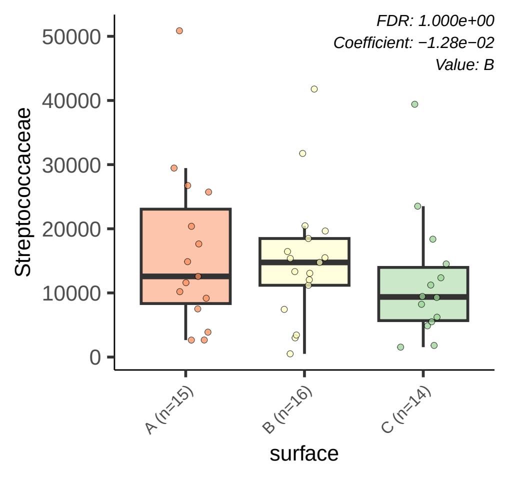

FDR: 1.000e+00
Coefficient: −1.28e−02
Value: B
Streptococcaceae
50000
40000
30000
20000
10000
0
A (n=15)
B (n=16)
C (n=14)
surface

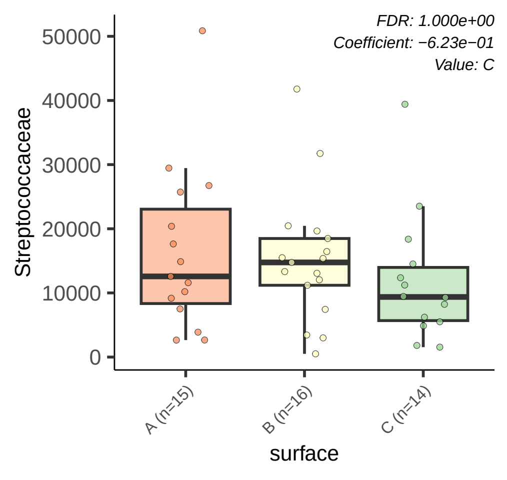
FDR: 1.000e+00
Coefficient: −6.23e−01
Value: C
Streptococcaceae
50000
40000
30000
20000
10000
0
A (n=15)
B (n=16)
C (n=14)
surface

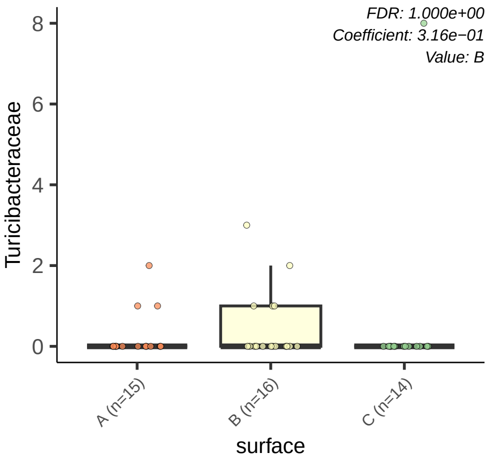
FDR: 1.000e+00
Coefficient: 3.16e−01
Value: B
Turicibacteraceae
0
2
4
6
8
A (n=15)
B (n=16)
C (n=14)
surface

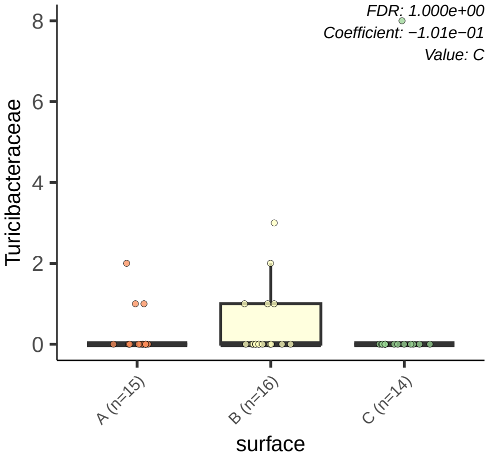

FDR: 1.000e+00
Coefficient: −1.01e−01
Value: C
Turicibacteraceae
8
6
4
2
0
A (n=15)
B (n=16)
C (n=14)
surface

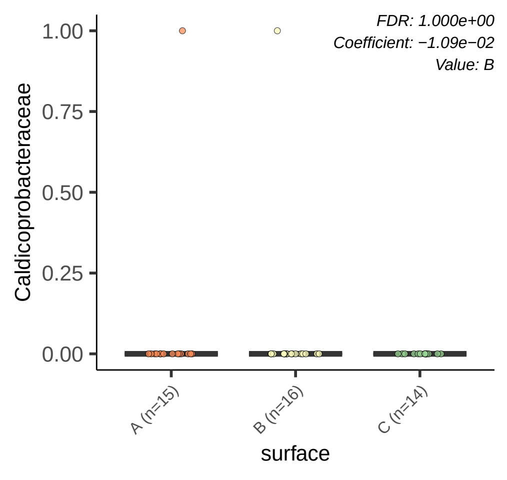
FDR: 1.000e+00
Coefficient: −1.09e−02
Value: B
Caldicoprobacteraceae
1.00
0.75
0.50
0.25
0.00
A (n=15)
B (n=16)
C (n=14)
surface

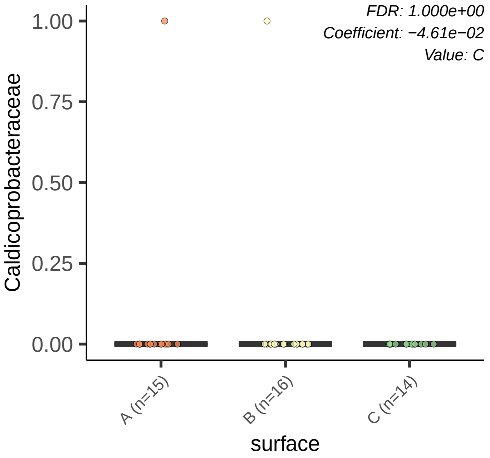
FDR: 1.000e+00
Coefficient: −4.61e−02
Value: C
Caldicoprobacteraceae
1.00
0.75
0.50
0.25
0.00
A (n=15)
B (n=16)
C (n=14)
surface

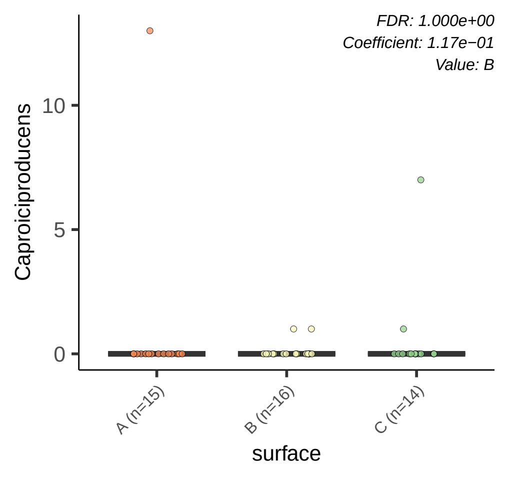

FDR: 1.000e+00
Coefficient: 1.17e−01
Value: B
Caproiciproducens
10
5
0
A (n=15)
B (n=16)
C (n=14)
surface

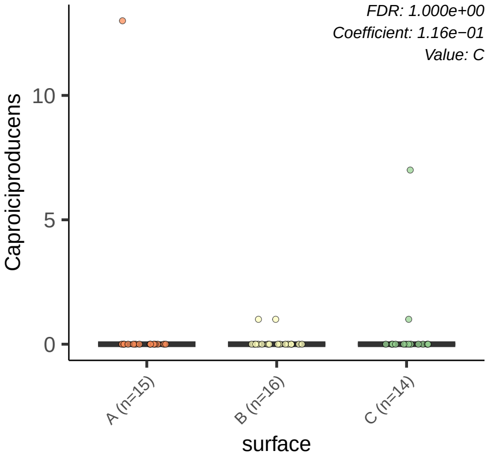
FDR: 1.000e+00
Coefficient: 1.16e−01
Value: C
Caproiciproducens
10
5
0
A (n=15)
B (n=16)
C (n=14)
surface

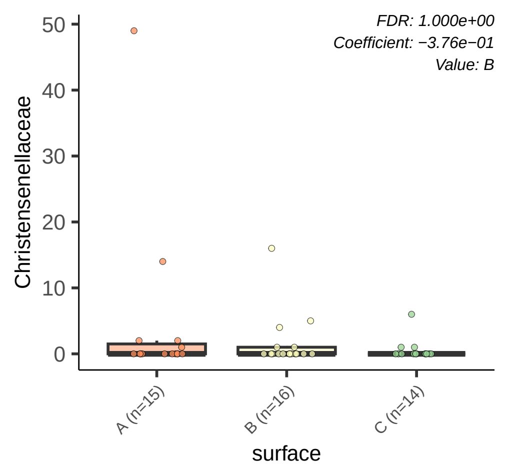

FDR: 1.000e+00
Coefficient: −3.76e−01
Value: B
Christensenellaceae
50
40
30
20
10
0
A (n=15)
B (n=16)
C (n=14)
surface

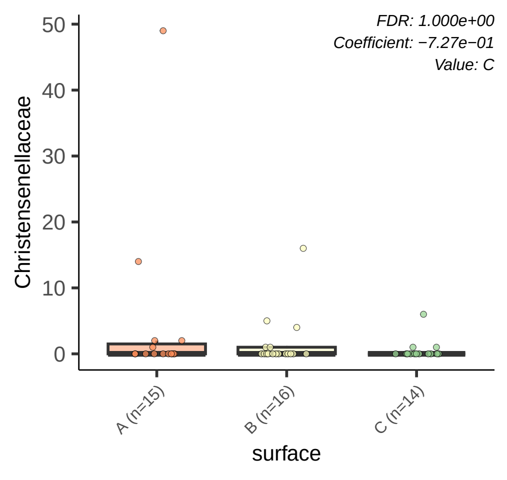
FDR: 1.000e+00
Coefficient: −7.27e−01
Value: C
Christensenellaceae
0
10
20
30
40
50
A (n=15)
B (n=16)
C (n=14)
surface

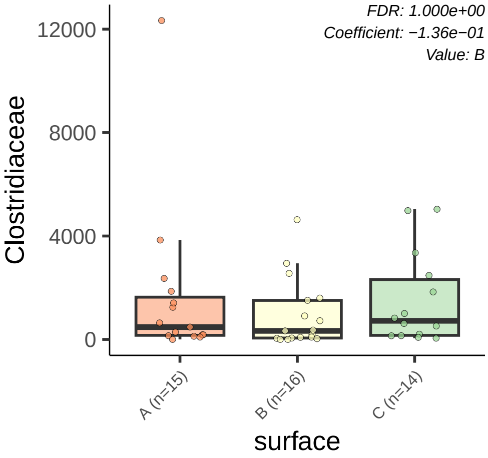

FDR: 1.000e+00
Coefficient: −1.36e−01
Value: B
Clostridiaceae
12000
8000
4000
0
A (n=15)
B (n=16)
C (n=14)
surface

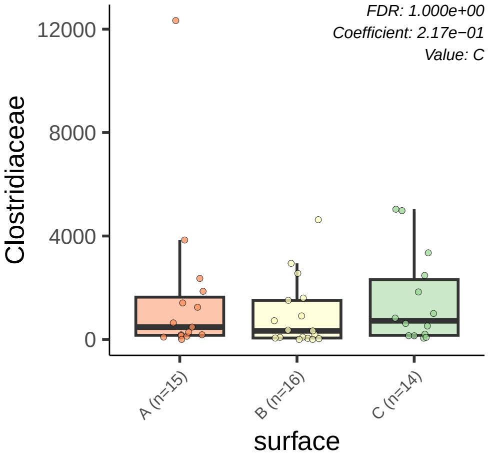
FDR: 1.000e+00
Coefficient: 2.17e−01
Value: C
12000
8000
4000
0
Clostridiaceae
A (n=15)
B (n=16)
C (n=14)
surface

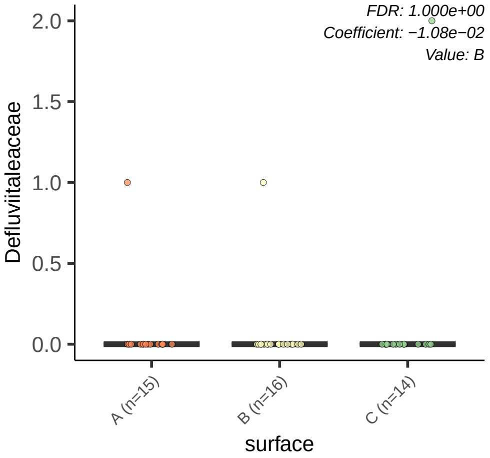
FDR: 1.000e+00
Coefficient: −1.08e−02
Value: B
Defluviitaleaceae
2.0
1.5
1.0
0.5
0.0
A (n=15)
B (n=16)
C (n=14)
surface

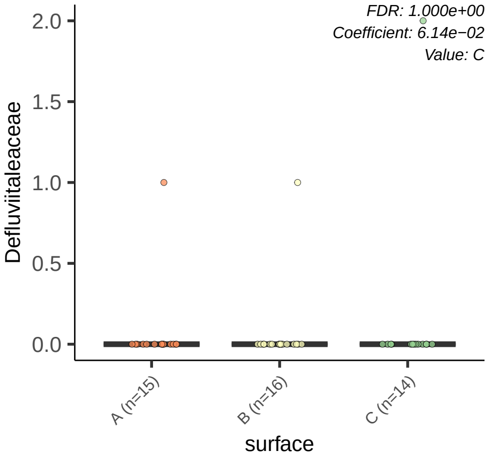

FDR: 1.000e+00
Coefficient: 6.14e−02
Value: C
Defluviitaleaceae
2.0
1.5
1.0
0.5
0.0
A (n=15)
B (n=16)
C (n=14)
surface

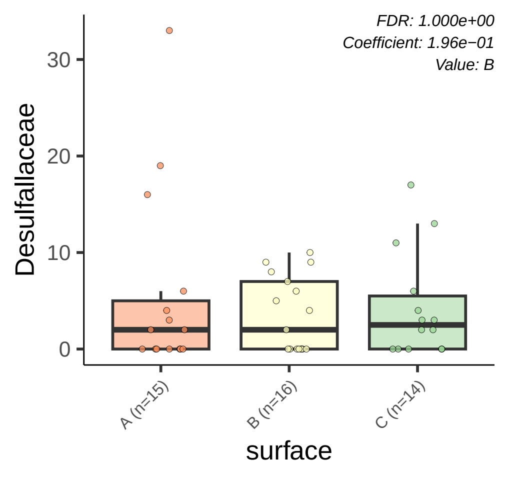

FDR: 1.000e+00
Coefficient: 1.96e−01
Value: B
Desulfallaceae
30
20
10
0
A (n=15)
B (n=16)
C (n=14)
surface

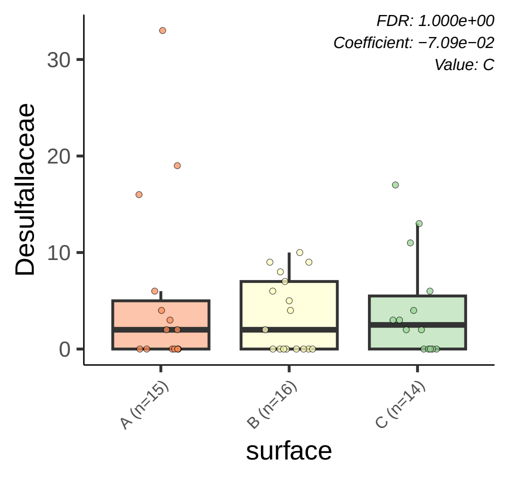
FDR: 1.000e+00
Coefficient: −7.09e−02
Value: C
Desulfallaceae
30
20
10
0
A (n=15)
B (n=16)
C (n=14)
surface

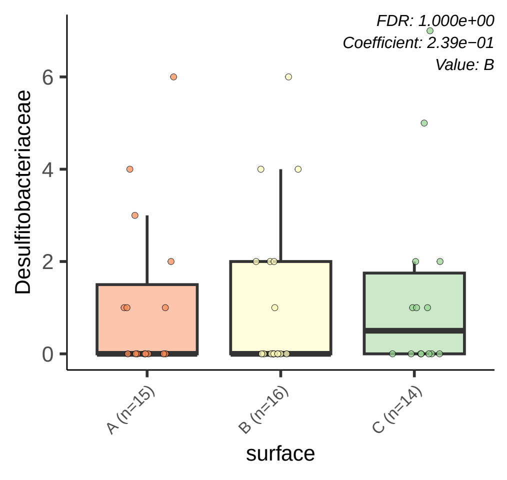

FDR: 1.000e+00
Coefficient: 2.39e−01
Value: B
Desulfitobacteriaceae
0
2
4
6
A (n=15)
B (n=16)
C (n=14)
surface

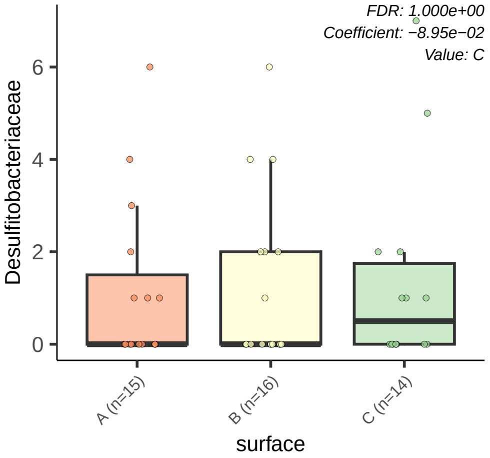

FDR: 1.000e+00
Coefficient: −8.95e−02
Value: C
Desulfitobacteriaceae
0
2
4
6
A (n=15)
B (n=16)
C (n=14)
surface

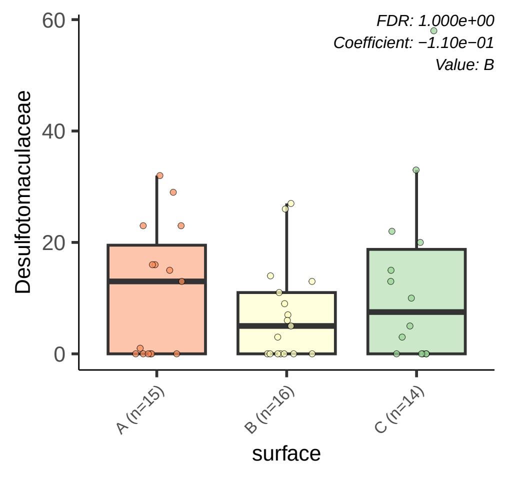
FDR: 1.000e+00
Coefficient: −1.10e−01
Value: B
Desulfotomaculaceae
0
20
40
60
A (n=15)
B (n=16)
C (n=14)
surface

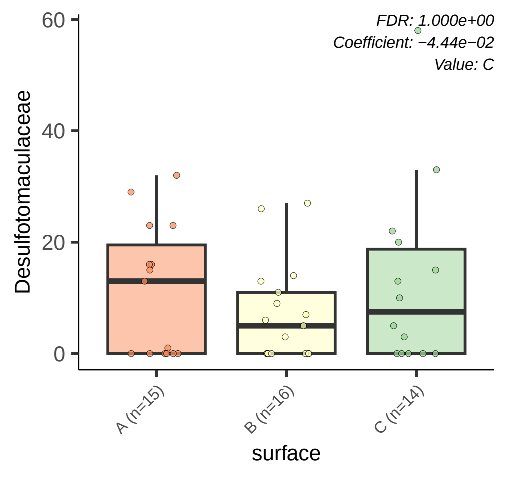
FDR: 1.000e+00
Coefficient: −4.44e−02
Value: C
Desulfotomaculaceae
0
20
40
60
A (n=15)
B (n=16)
C (n=14)
surface

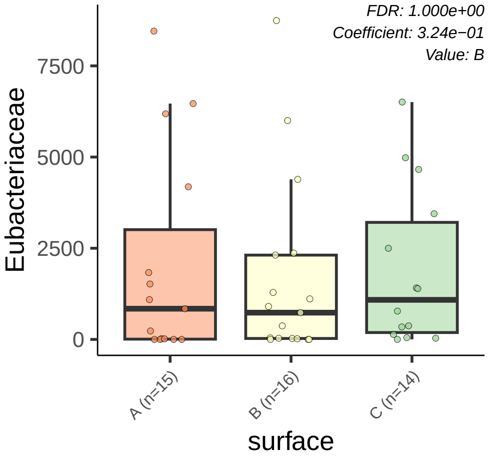

FDR: 1.000e+00
Coefficient: 3.24e−01
Value: B
Eubacteriaceae
7500
5000
2500
0
A (n=15)
B (n=16)
C (n=14)
surface

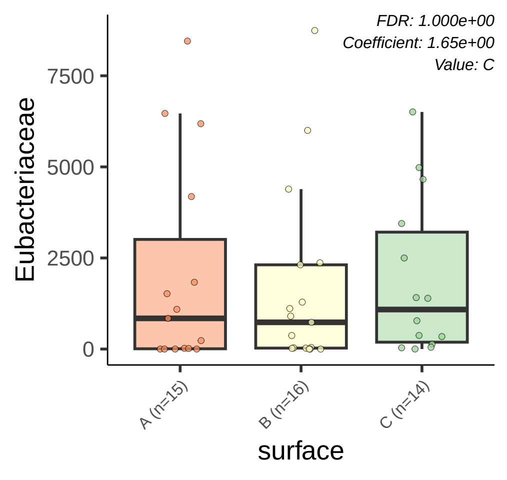

FDR: 1.000e+00
Coefficient: 1.65e+00
Value: C
Eubacteriaceae
7500
5000
2500
0
A (n=15)
B (n=16)
C (n=14)
surface

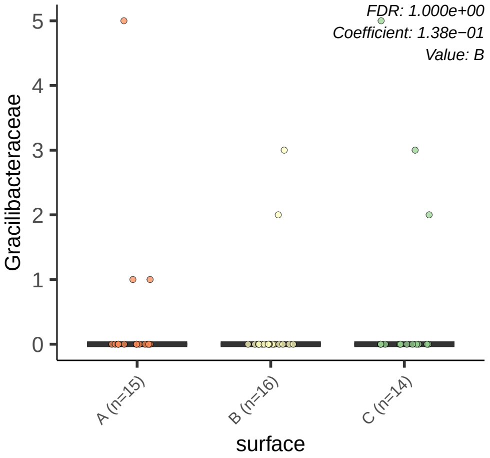
FDR: 1.000e+00
Coefficient: 1.38e−01
Value: B
Gracilibacteraceae
0
1
2
3
4
5
A (n=15)
B (n=16)
C (n=14)
surface

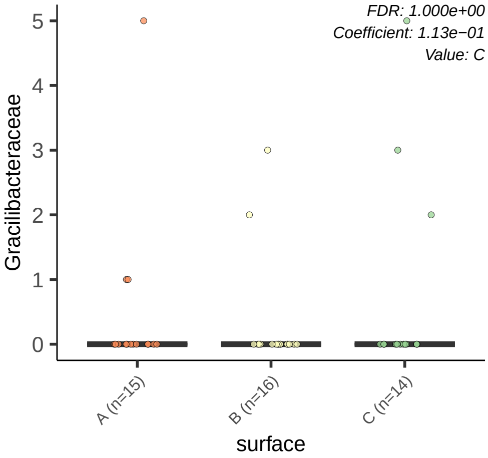

FDR: 1.000e+00
Coefficient: 1.13e−01
Value: C
Gracilibacteraceae
0
1
2
3
4
5
A (n=15)
B (n=16)
C (n=14)
surface

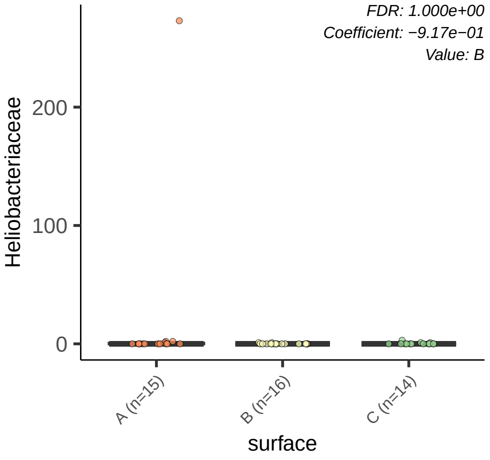

FDR: 1.000e+00
Coefficient: −9.17e−01
Value: B
Heliobacteriaceae
200
100
0
A (n=15)
B (n=16)
C (n=14)
surface

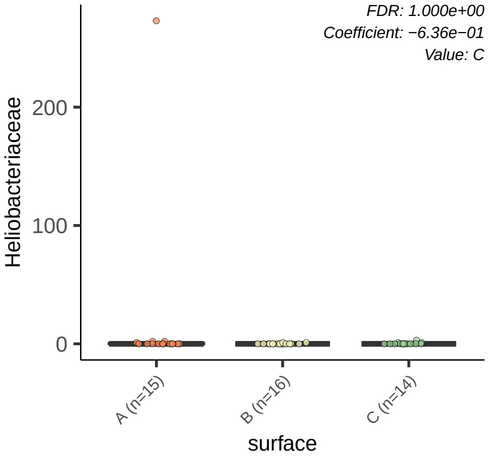

FDR: 1.000e+00
Coefficient: −6.36e−01
Value: C
Heliobacteriaceae
200
100
0
A (n=15)
B (n=16)
C (n=14)
surface

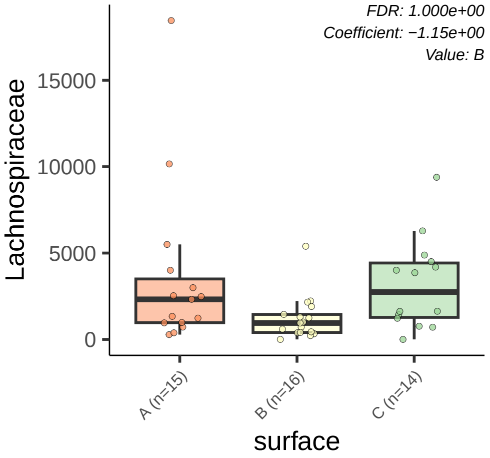
FDR: 1.000e+00
Coefficient: −1.15e+00
Value: B
Lachnospiraceae
15000
10000
5000
0
A (n=15)
B (n=16)
C (n=14)
surface

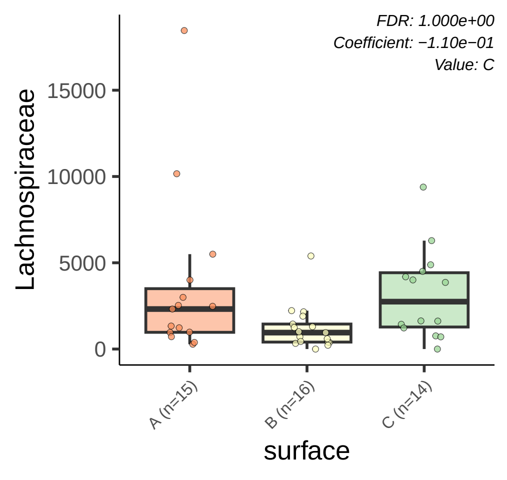

FDR: 1.000e+00
Coefficient: −1.10e−01
Value: C
Lachnospiraceae
15000
10000
5000
0
A (n=15)
B (n=16)
C (n=14)
surface

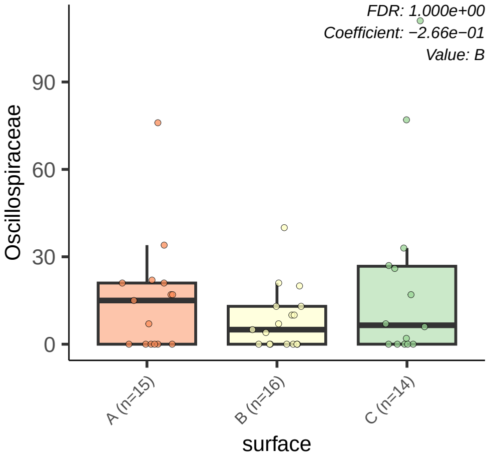

FDR: 1.000e+00
Coefficient: −2.66e−01
Value: B
Oscillospiraceae
0
30
60
90
A (n=15)
B (n=16)
C (n=14)
surface

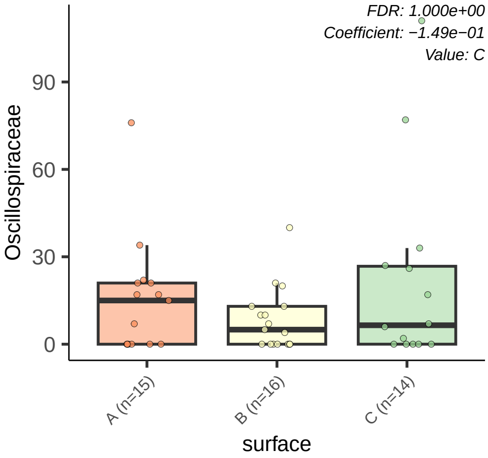
FDR: 1.000e+00
Coefficient: −1.49e−01
Value: C
Oscillospiraceae
90
60
30
0
A (n=15)
B (n=16)
C (n=14)
surface

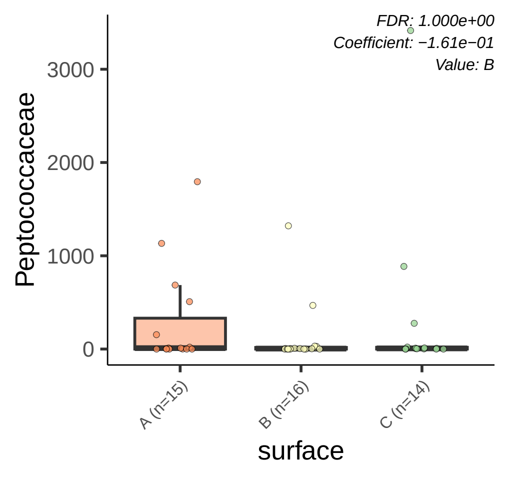

FDR: 1.000e+00
Coefficient: −1.61e−01
Value: B
Peptococcaceae
3000
2000
1000
0
A (n=15)
B (n=16)
C (n=14)
surface

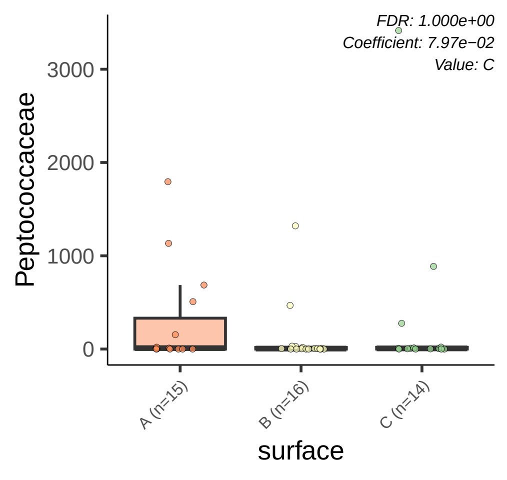

FDR: 1.000e+00
Coefficient: 7.97e−02
Value: C
Peptococcaceae
3000
2000
1000
0
A (n=15)
B (n=16)
C (n=14)
surface

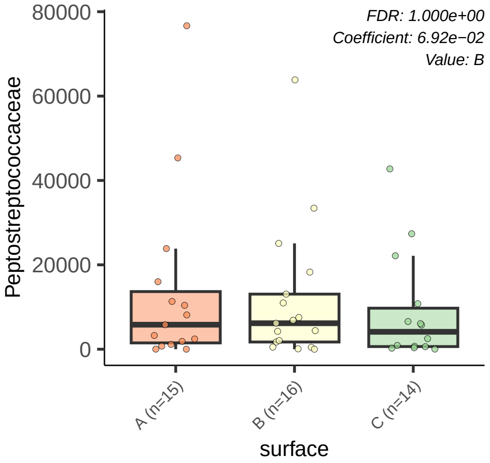

FDR: 1.000e+00
Coefficient: 6.92e−02
Value: B
80000
60000
40000
20000
0
Peptostreptococcaceae
A (n=15)
B (n=16)
C (n=14)
surface

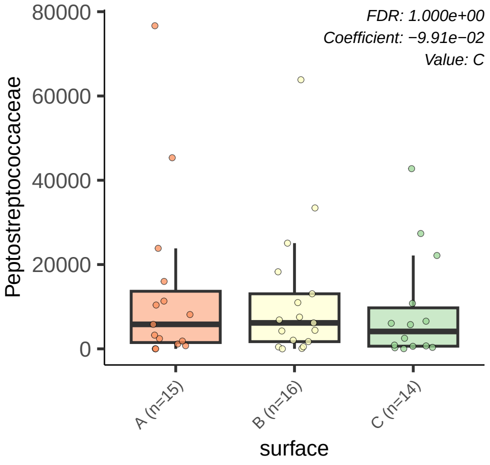
FDR: 1.000e+00
Coefficient: −9.91e−02
Value: C
Peptostreptococcaceae
80000
60000
40000
20000
0
A (n=15)
B (n=16)
C (n=14)
surface

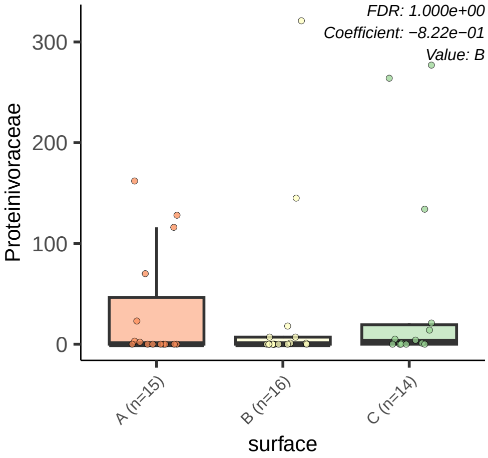

FDR: 1.000e+00
Coefficient: −8.22e−01
Value: B
Proteinivoraceae
300
200
100
0
A (n=15)
B (n=16)
C (n=14)
surface

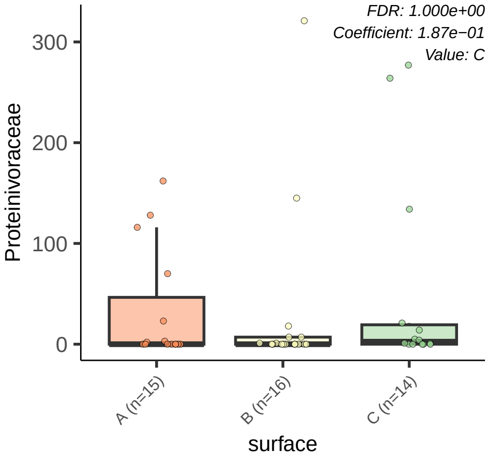

FDR: 1.000e+00
Coefficient: 1.87e−01
Value: C
Proteinivoraceae
300
200
100
0
A (n=15)
B (n=16)
C (n=14)
surface

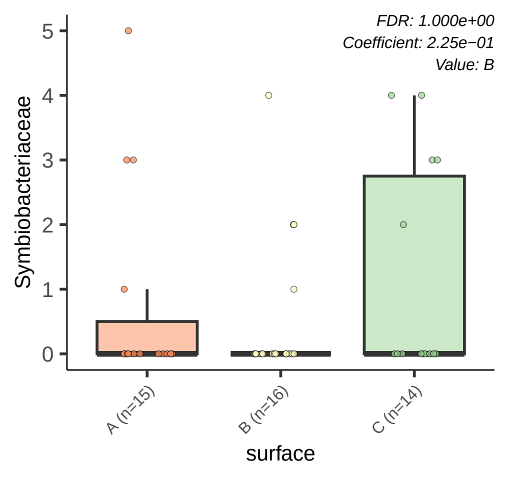
FDR: 1.000e+00
Coefficient: 2.25e−01
Value: B
Symbiobacteriaceae
0
1
2
3
4
5
A (n=15)
B (n=16)
C (n=14)
surface

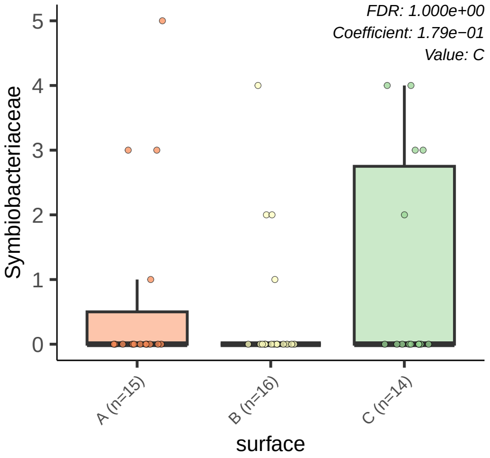
FDR: 1.000e+00
Coefficient: 1.79e−01
Value: C
Symbiobacteriaceae
0
1
2
3
4
5
A (n=15)
B (n=16)
C (n=14)
surface

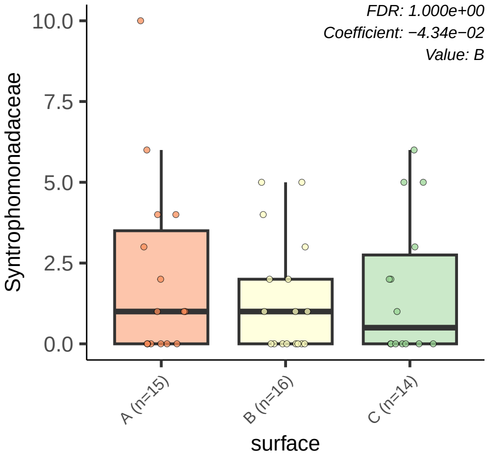

FDR: 1.000e+00
Coefficient: −4.34e−02
Value: B
Syntrophomonadaceae
10.0
7.5
5.0
2.5
0.0
A (n=15)
B (n=16)
C (n=14)
surface

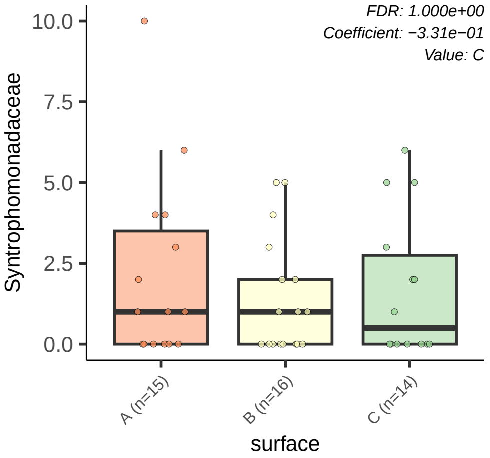
FDR: 1.000e+00
Coefficient: −3.31e−01
Value: C
Syntrophomonadaceae
10.0
7.5
5.0
2.5
0.0
A (n=15)
B (n=16)
C (n=14)
surface

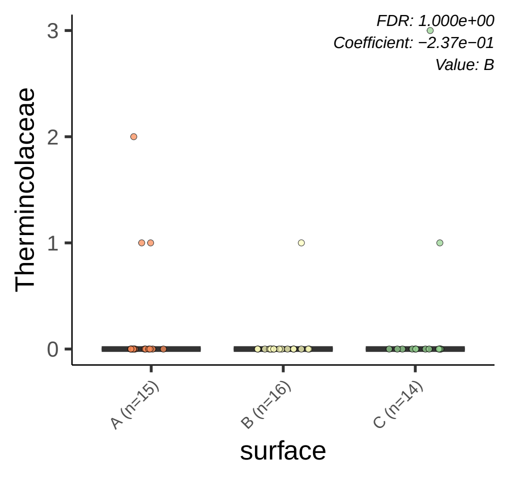

FDR: 1.000e+00
Coefficient: −2.37e−01
Value: B
Thermincolaceae
3
2
1
0
A (n=15)
B (n=16)
C (n=14)
surface

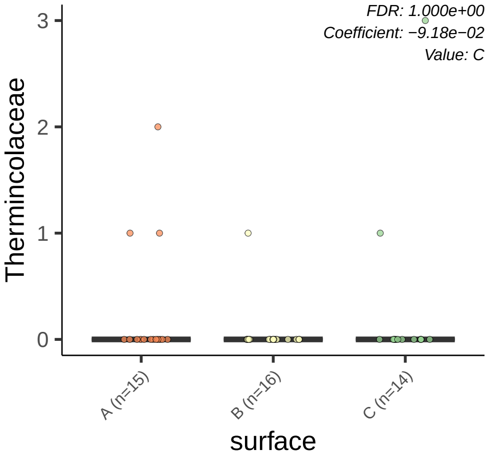
FDR: 1.000e+00
Coefficient: −9.18e−02
Value: C
Thermincolaceae
3
2
1
0
A (n=15)
B (n=16)
C (n=14)
surface

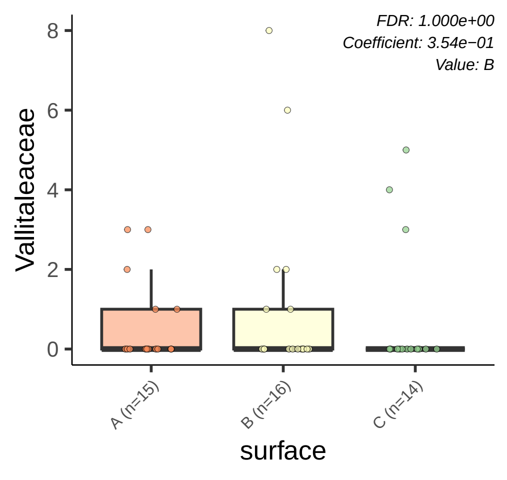

FDR: 1.000e+00
Coefficient: 3.54e−01
Value: B
Vallitaleaceae
0
2
4
6
8
A (n=15)
B (n=16)
C (n=14)
surface

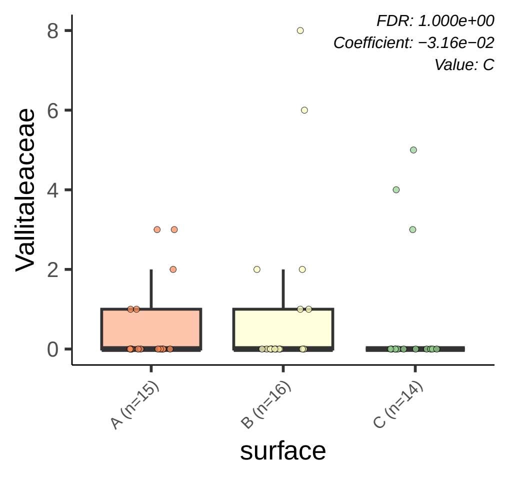

FDR: 1.000e+00
Coefficient: −3.16e−02
Value: C
Vallitaleaceae
0
2
4
6
8
A (n=15)
B (n=16)
C (n=14)
surface

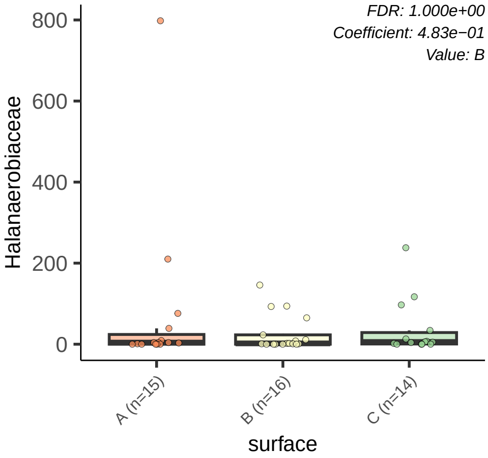
FDR: 1.000e+00
Coefficient: 4.83e−01
Value: B
Halanaerobiaceae
800
600
400
200
0
A (n=15)
B (n=16)
C (n=14)
surface

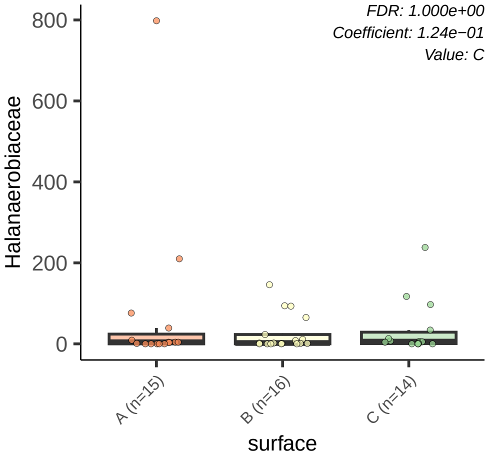

FDR: 1.000e+00
Coefficient: 1.24e−01
Value: C
Halanaerobiaceae
800
600
400
200
0
A (n=15)
B (n=16)
C (n=14)
surface

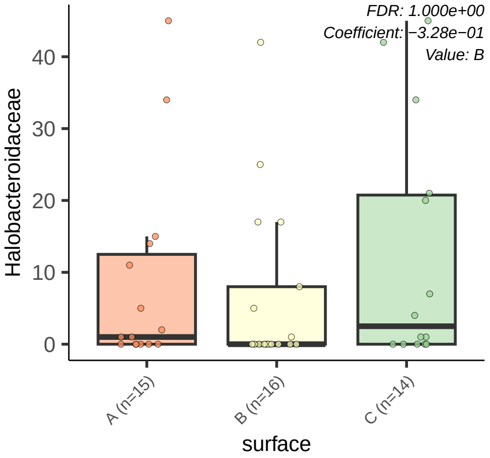
FDR: 1.000e+00
Coefficient: −3.28e−01
Value: B
Halobacteroidaceae
0
10
20
30
40
A (n=15)
B (n=16)
C (n=14)
surface

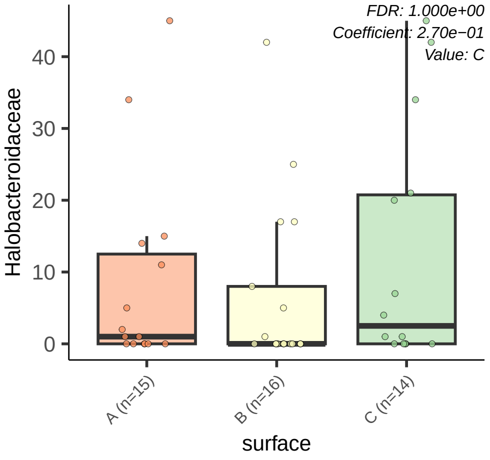

FDR: 1.000e+00
Coefficient: 2.70e−01
Value: C
Halobacteroidaceae
0
10
20
30
40
A (n=15)
B (n=16)
C (n=14)
surface

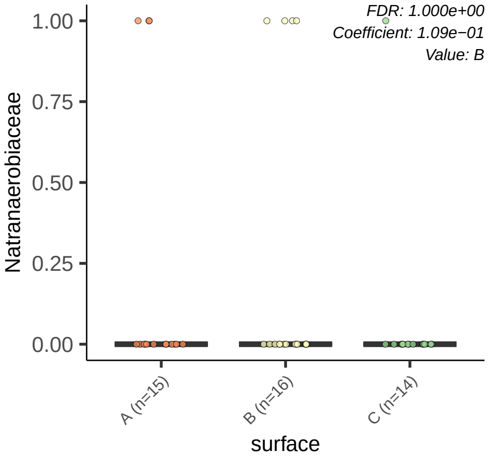

FDR: 1.000e+00
Coefficient: 1.09e−01
Value: B
1.00
0.75
0.50
0.25
0.00
Natranaerobiaceae
A (n=15)
B (n=16)
C (n=14)
surface

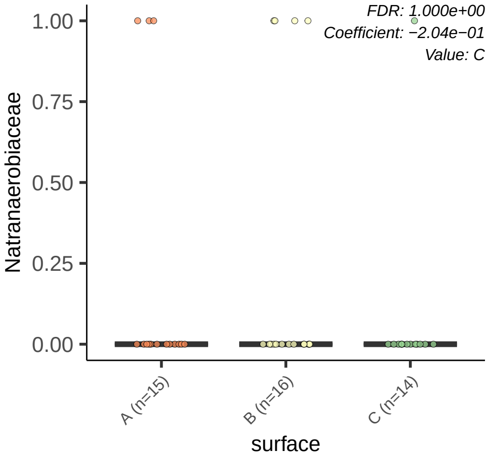
FDR: 1.000e+00
Coefficient: −2.04e−01
Value: C
1.00
0.75
0.50
0.25
0.00
Natranaerobiaceae
A (n=15)
B (n=16)
C (n=14)
surface

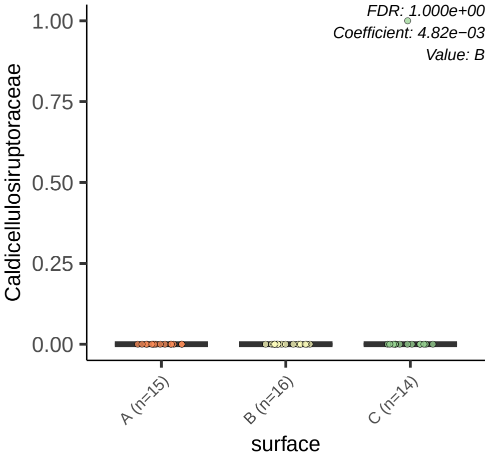

FDR: 1.000e+00
Coefficient: 4.82e−03
Value: B
Caldicellulosiruptoraceae
1.00
0.75
0.50
0.25
0.00
A (n=15)
B (n=16)
C (n=14)
surface

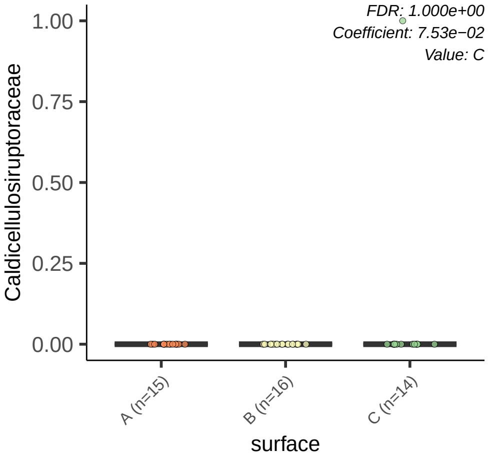
FDR: 1.000e+00
Coefficient: 7.53e−02
Value: C
Caldicellulosiruptoraceae
1.00
0.75
0.50
0.25
0.00
A (n=15)
B (n=16)
C (n=14)
surface

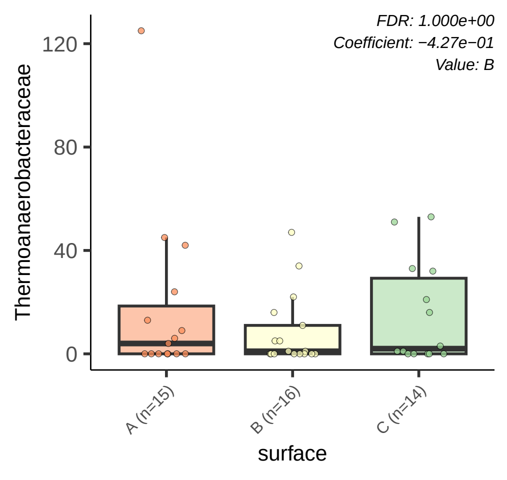

FDR: 1.000e+00
Coefficient: −4.27e−01
Value: B
Thermoanaerobacteraceae
120
80
40
0
A (n=15)
B (n=16)
C (n=14)
surface

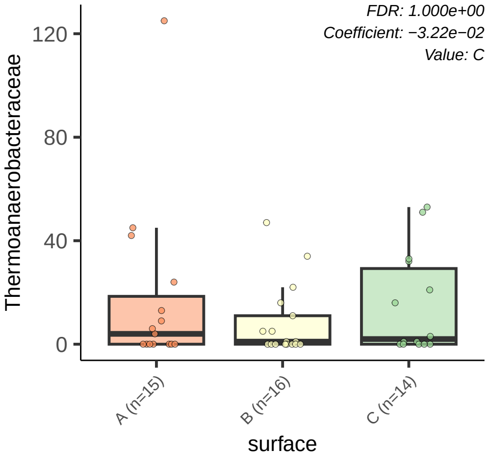
FDR: 1.000e+00
Coefficient: −3.22e−02
Value: C
Thermoanaerobacteraceae
120
80
40
0
A (n=15)
B (n=16)
C (n=14)
surface

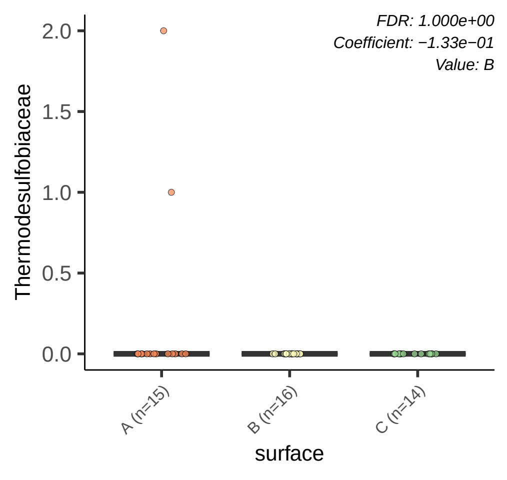

FDR: 1.000e+00
Coefficient: −1.33e−01
Value: B
Thermodesulfobiaceae
2.0
1.5
1.0
0.5
0.0
A (n=15)
B (n=16)
C (n=14)
surface

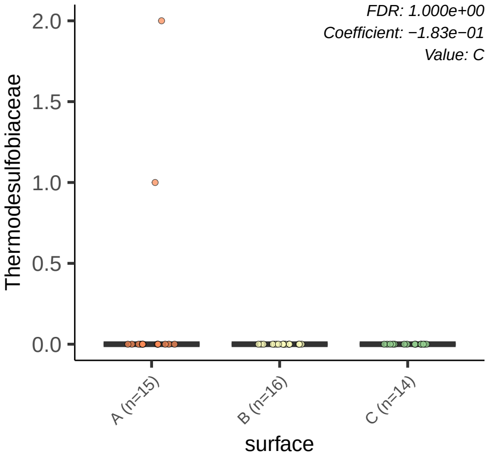

FDR: 1.000e+00
Coefficient: −1.83e−01
Value: C
Thermodesulfobiaceae
2.0
1.5
1.0
0.5
0.0
A (n=15)
B (n=16)
C (n=14)
surface

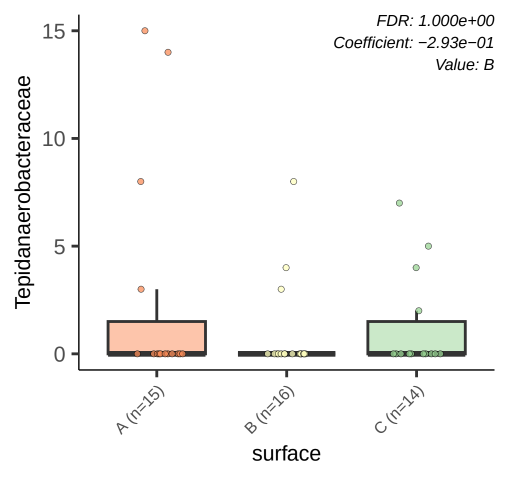
FDR: 1.000e+00
Coefficient: −2.93e−01
Value: B
Tepidanaerobacteraceae
15
10
5
0
A (n=15)
B (n=16)
C (n=14)
surface

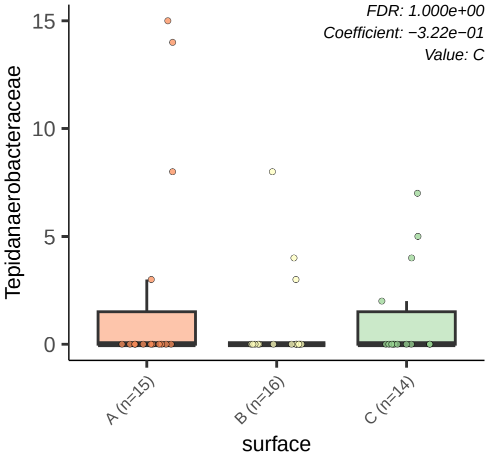

FDR: 1.000e+00
Coefficient: −3.22e−01
Value: C
15
10
5
0
Tepidanaerobacteraceae
A (n=15)
B (n=16)
C (n=14)
surface

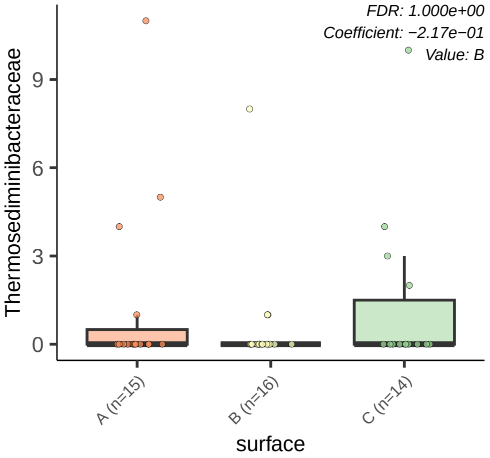

FDR: 1.000e+00
Coefficient: −2.17e−01
Value: B
Thermosediminibacteraceae
0
3
6
9
A (n=15)
B (n=16)
C (n=14)
surface

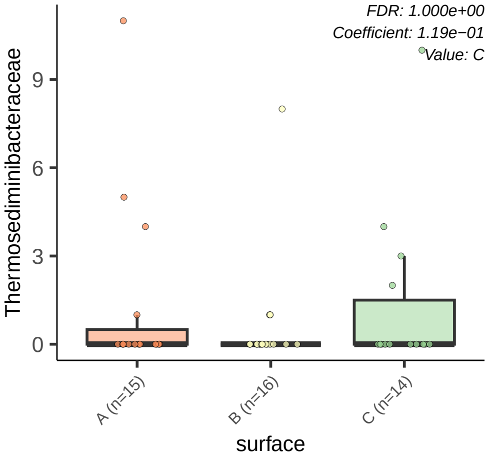

FDR: 1.000e+00
Coefficient: 1.19e−01
Value: C
Thermosediminibacteraceae
0
3
6
9
A (n=15)
B (n=16)
C (n=14)
surface

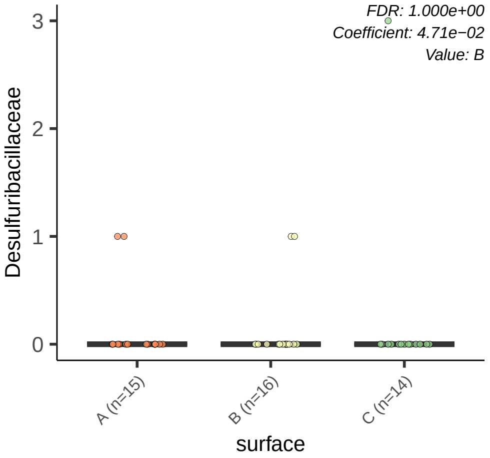

FDR: 1.000e+00
Coefficient: 4.71e−02
Value: B
Desulfuribacillaceae
3
2
1
0
A (n=15)
B (n=16)
C (n=14)
surface

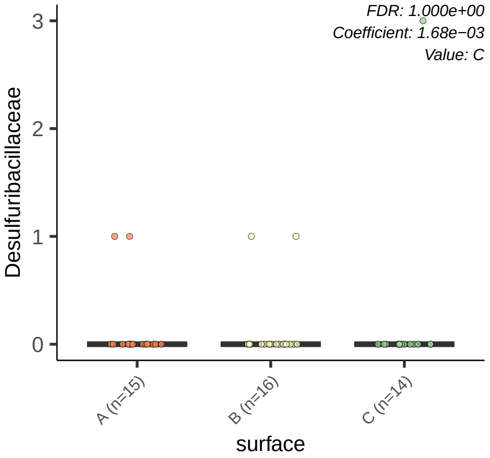
FDR: 1.000e+00
Coefficient: 1.68e−03
Value: C
Desulfuribacillaceae
3
2
1
0
A (n=15)
B (n=16)
C (n=14)
surface

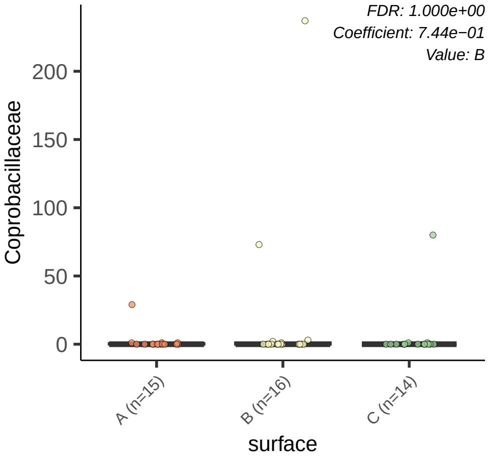
FDR: 1.000e+00
Coefficient: 7.44e−01
Value: B
Coprobacillaceae
0
50
100
150
200
A (n=15)
B (n=16)
C (n=14)
surface

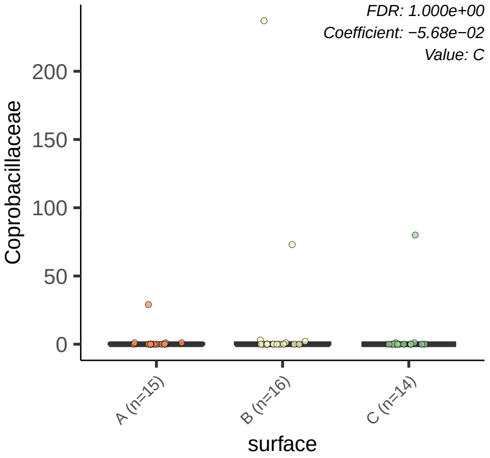
FDR: 1.000e+00
Coefficient: −5.68e−02
Value: C
Coprobacillaceae
0
50
100
150
200
A (n=15)
B (n=16)
C (n=14)
surface

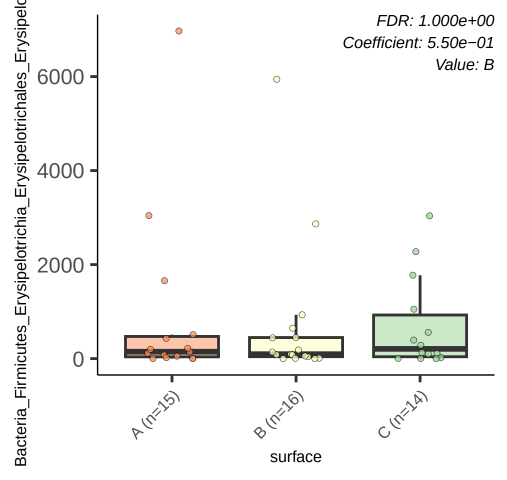

FDR: 1.000e+00
Coefficient: 5.50e−01
Value: B
Bacteria_Firmicutes_Erysipelotrichia_Erysipelotrichales_Erysipelo
6000
4000
2000
0
A (n=15)
B (n=16)
C (n=14)
surface

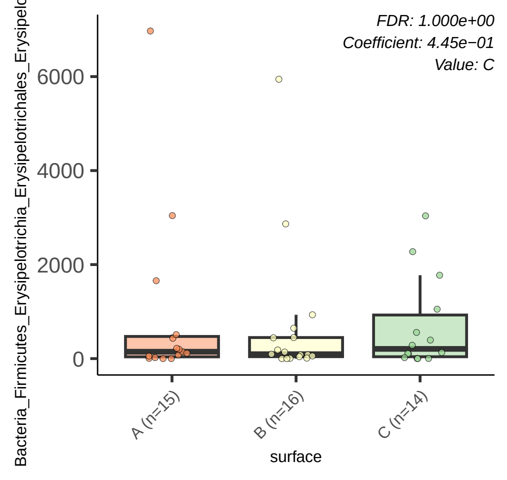

FDR: 1.000e+00
Coefficient: 4.45e−01
Value: C
Bacteria_Firmicutes_Erysipelotrichia_Erysipelotrichales_Erysipelo
6000
4000
2000
0
A (n=15)
B (n=16)
C (n=14)
surface

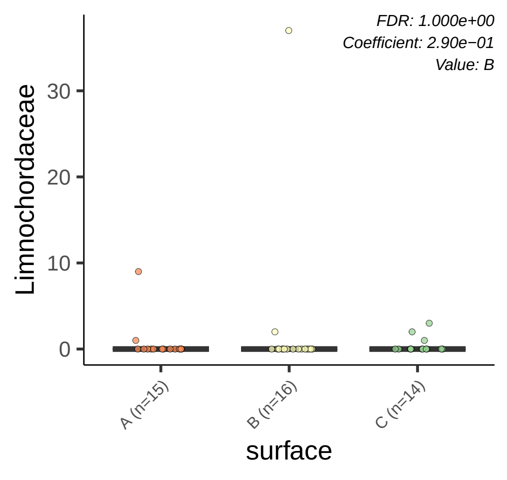
FDR: 1.000e+00
Coefficient: 2.90e−01
Value: B
Limnochordaceae
30
20
10
0
A (n=15)
B (n=16)
C (n=14)
surface

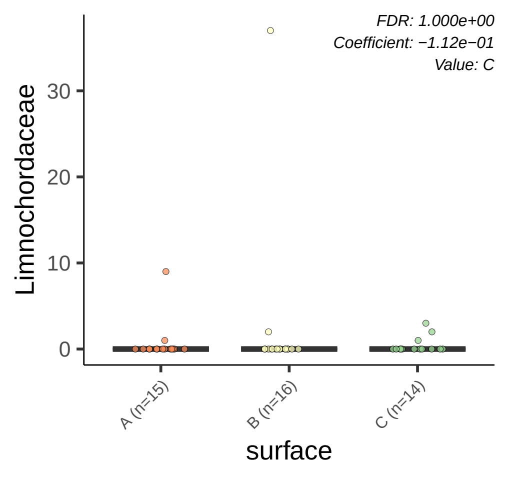
FDR: 1.000e+00
Coefficient: −1.12e−01
Value: C
Limnochordaceae
30
20
10
0
A (n=15)
B (n=16)
C (n=14)
surface

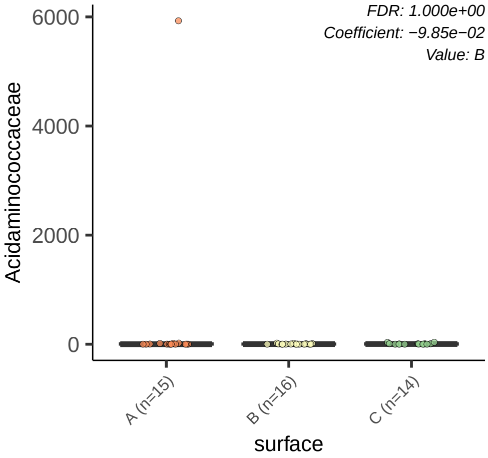

FDR: 1.000e+00
Coefficient: −9.85e−02
Value: B
Acidaminococcaceae
6000
4000
2000
0
A (n=15)
B (n=16)
C (n=14)
surface

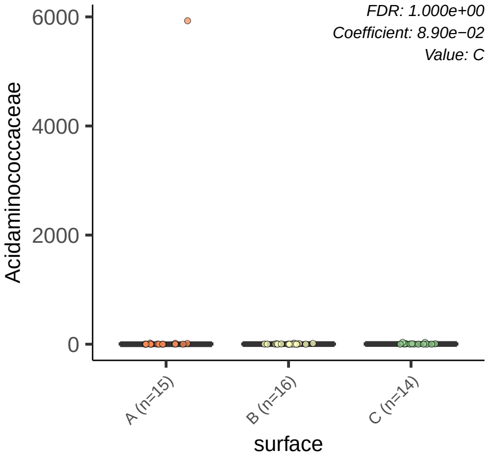
FDR: 1.000e+00
Coefficient: 8.90e−02
Value: C
6000
4000
2000
0
Acidaminococcaceae
A (n=15)
B (n=16)
C (n=14)
surface

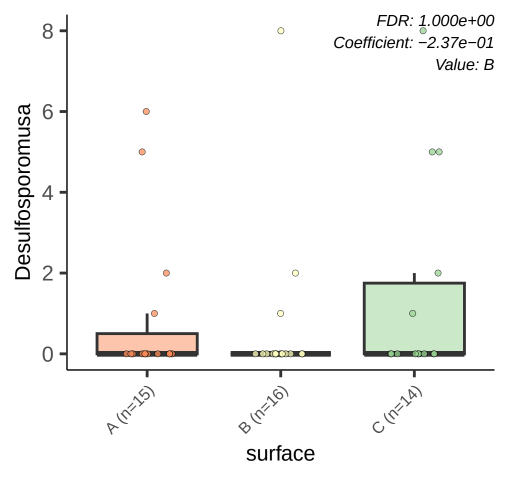

FDR: 1.000e+00
Coefficient: −2.37e−01
Value: B
Desulfosporomusa
8
6
4
2
0
A (n=15)
B (n=16)
C (n=14)
surface

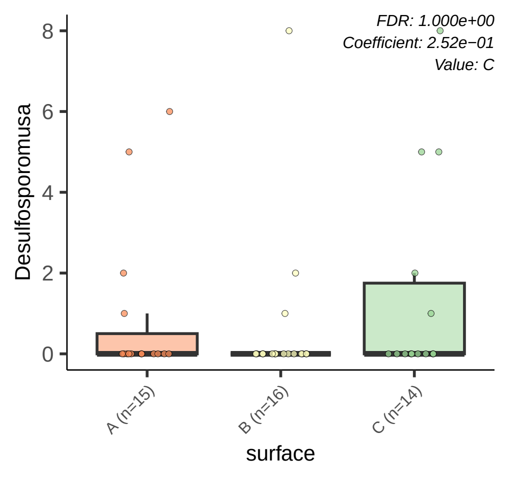

FDR: 1.000e+00
Coefficient: 2.52e−01
Value: C
Desulfosporomusa
0
2
4
6
8
A (n=15)
B (n=16)
C (n=14)
surface

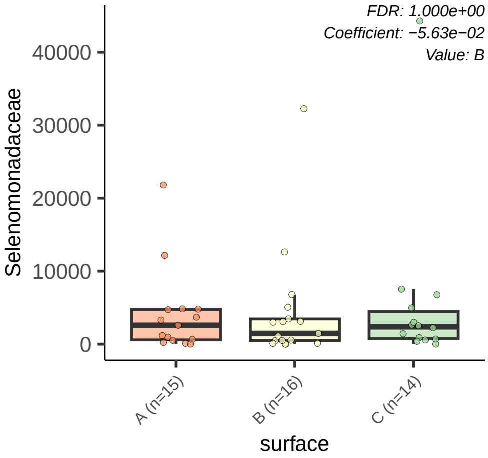

FDR: 1.000e+00
Coefficient: −5.63e−02
Value: B
Selenomonadaceae
40000
30000
20000
10000
0
A (n=15)
B (n=16)
C (n=14)
surface

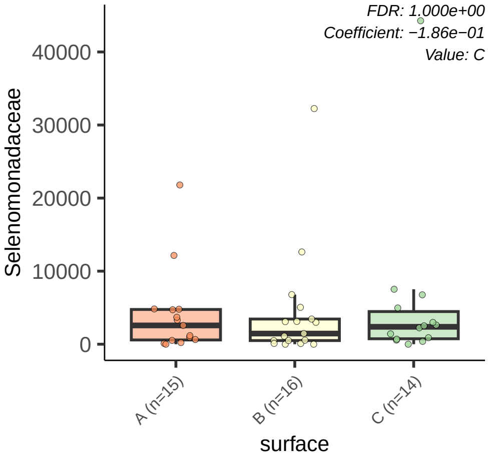

FDR: 1.000e+00
Coefficient: −1.86e−01
Value: C
Selenomonadaceae
40000
30000
20000
10000
0
A (n=15)
B (n=16)
C (n=14)
surface

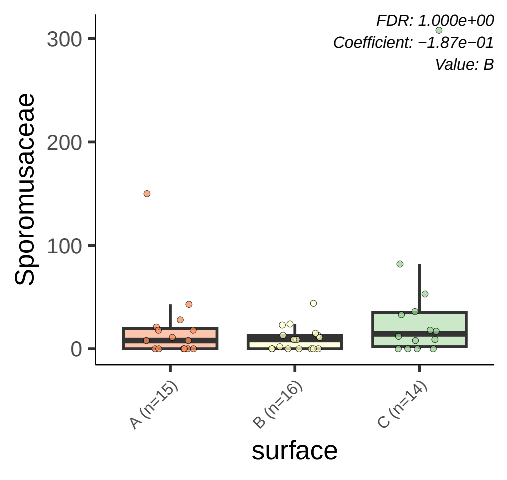
FDR: 1.000e+00
Coefficient: −1.87e−01
Value: B
Sporomusaceae
300
200
100
0
A (n=15)
B (n=16)
C (n=14)
surface

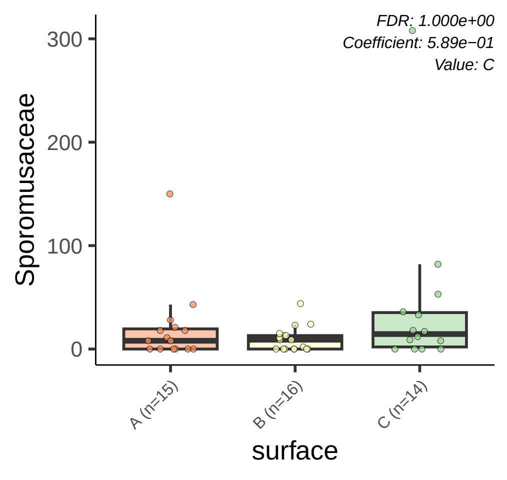
FDR: 1.000e+00
Coefficient: 5.89e−01
Value: C
Sporomusaceae
300
200
100
0
A (n=15)
B (n=16)
C (n=14)
surface

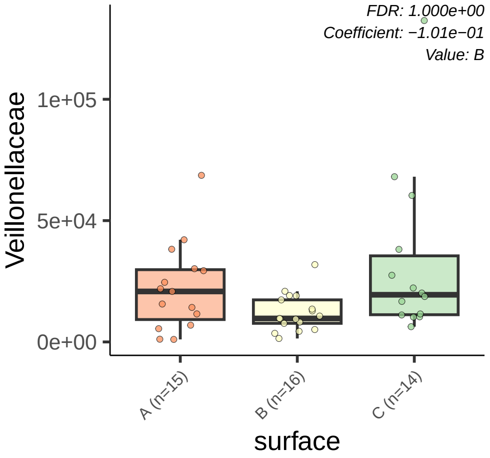

FDR: 1.000e+00
Coefficient: −1.01e−01
Value: B
Veillonellaceae
1e+05
5e+04
0e+00
A (n=15)
B (n=16)
C (n=14)
surface

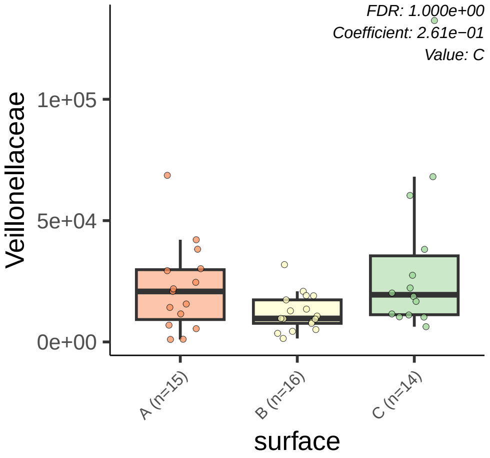
FDR: 1.000e+00
Coefficient: 2.61e−01
Value: C
Veillonellaceae
1e+05
5e+04
0e+00
A (n=15)
B (n=16)
C (n=14)
surface

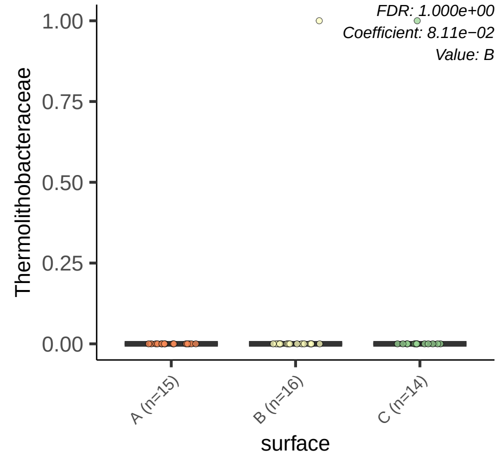

FDR: 1.000e+00
Coefficient: 8.11e−02
Value: B
Thermolithobacteraceae
1.00
0.75
0.50
0.25
0.00
A (n=15)
B (n=16)
C (n=14)
surface

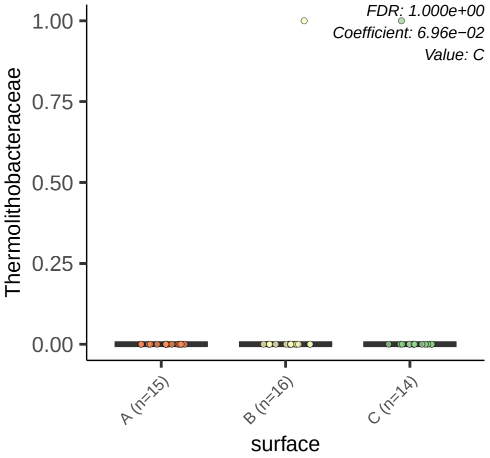
FDR: 1.000e+00
Coefficient: 6.96e−02
Value: C
Thermolithobacteraceae
1.00
0.75
0.50
0.25
0.00
A (n=15)
B (n=16)
C (n=14)
surface

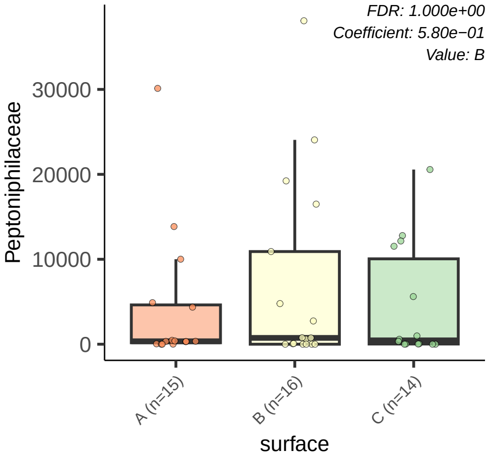

FDR: 1.000e+00
Coefficient: 5.80e−01
Value: B
Peptoniphilaceae
30000
20000
10000
0
A (n=15)
B (n=16)
C (n=14)
surface

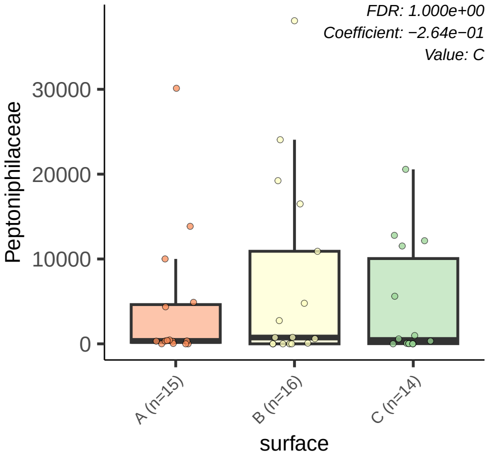

FDR: 1.000e+00
Coefficient: −2.64e−01
Value: C
Peptoniphilaceae
30000
20000
10000
0
A (n=15)
B (n=16)
C (n=14)
surface

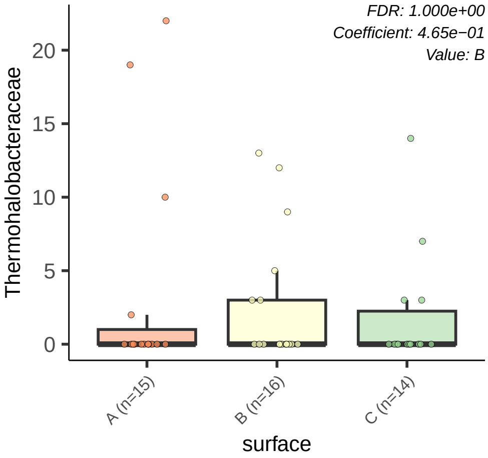

FDR: 1.000e+00
Coefficient: 4.65e−01
Value: B
Thermohalobacteraceae
0
5
10
15
20
A (n=15)
B (n=16)
C (n=14)
surface

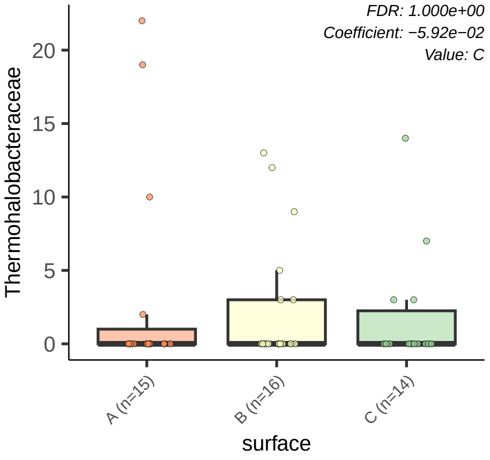
FDR: 1.000e+00
Coefficient: −5.92e−02
Value: C
Thermohalobacteraceae
0
5
10
15
20
A (n=15)
B (n=16)
C (n=14)
surface

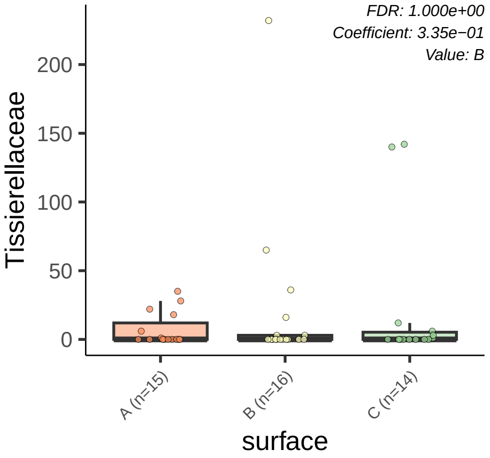

FDR: 1.000e+00
Coefficient: 3.35e−01
Value: B
Tissierellaceae
200
150
100
50
0
A (n=15)
B (n=16)
C (n=14)
surface

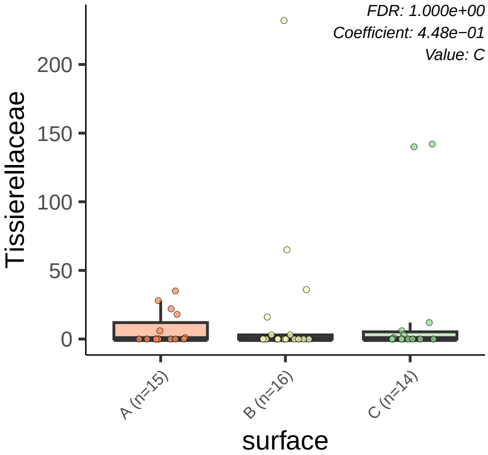

FDR: 1.000e+00
Coefficient: 4.48e−01
Value: C
Tissierellaceae
0
50
100
150
200
A (n=15)
B (n=16)
C (n=14)
surface

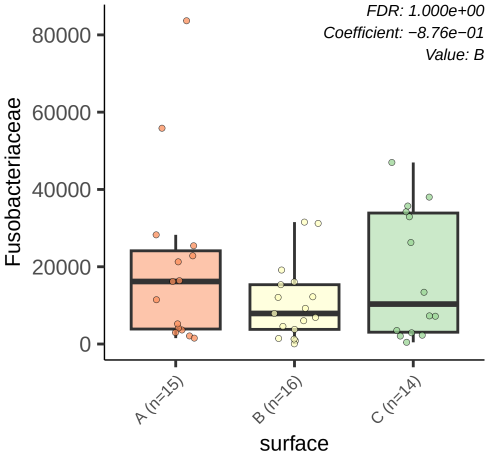

FDR: 1.000e+00
Coefficient: −8.76e−01
Value: B
Fusobacteriaceae
80000
60000
40000
20000
0
A (n=15)
B (n=16)
C (n=14)
surface

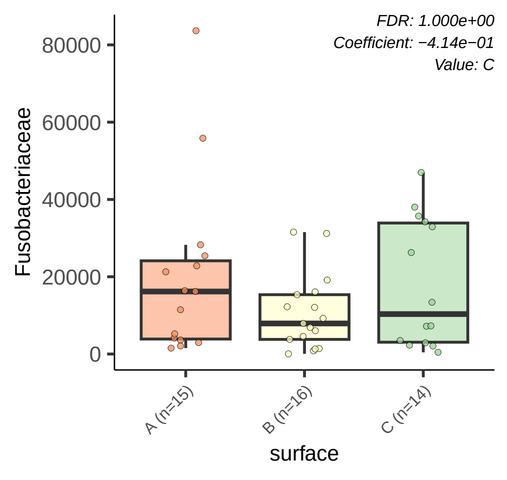

FDR: 1.000e+00
Coefficient: −4.14e−01
Value: C
Fusobacteriaceae
80000
60000
40000
20000
0
A (n=15)
B (n=16)
C (n=14)
surface

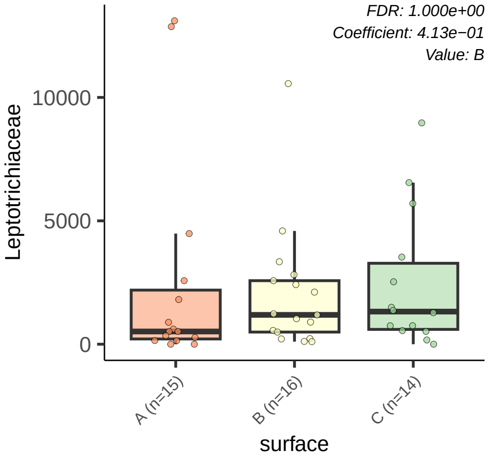
FDR: 1.000e+00
Coefficient: 4.13e−01
Value: B
Leptotrichiaceae
10000
5000
0
A (n=15)
B (n=16)
C (n=14)
surface

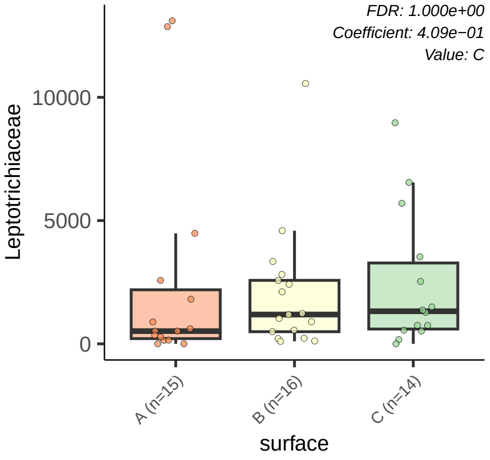
FDR: 1.000e+00
Coefficient: 4.09e−01
Value: C
Leptotrichiaceae
10000
5000
0
A (n=15)
B (n=16)
C (n=14)
surface

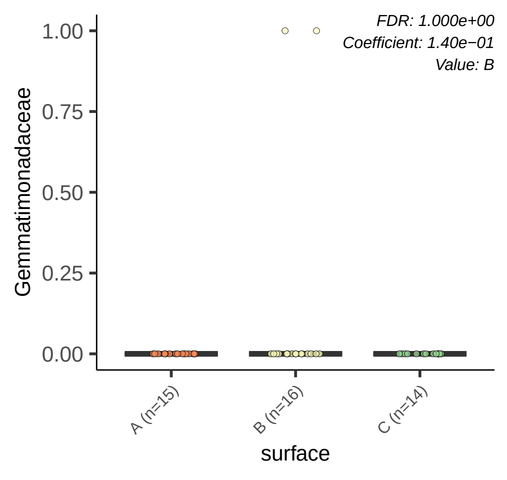

FDR: 1.000e+00
Coefficient: 1.40e−01
Value: B
Gemmatimonadaceae
1.00
0.75
0.50
0.25
0.00
A (n=15)
B (n=16)
C (n=14)
surface

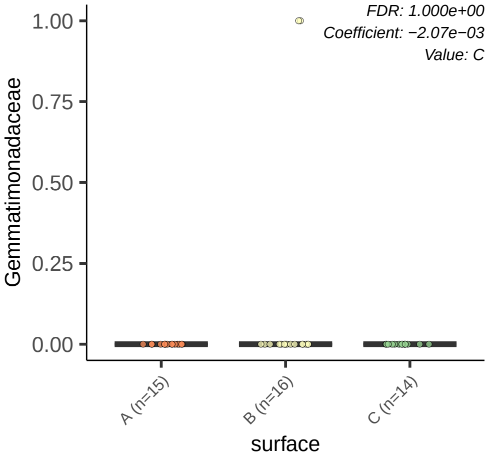
FDR: 1.000e+00
Coefficient: −2.07e−03
Value: C
Gemmatimonadaceae
1.00
0.75
0.50
0.25
0.00
A (n=15)
B (n=16)
C (n=14)
surface

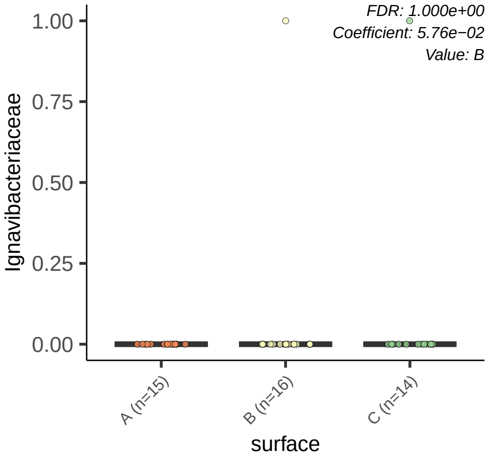

FDR: 1.000e+00
Coefficient: 5.76e−02
Value: B
1.00
0.75
0.50
0.25
0.00
Ignavibacteriaceae
A (n=15)
B (n=16)
C (n=14)
surface

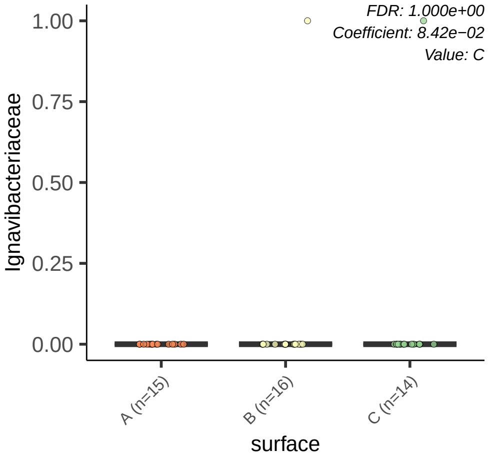
FDR: 1.000e+00
Coefficient: 8.42e−02
Value: C
1.00
0.75
0.50
0.25
0.00
Ignavibacteriaceae
A (n=15)
B (n=16)
C (n=14)
surface

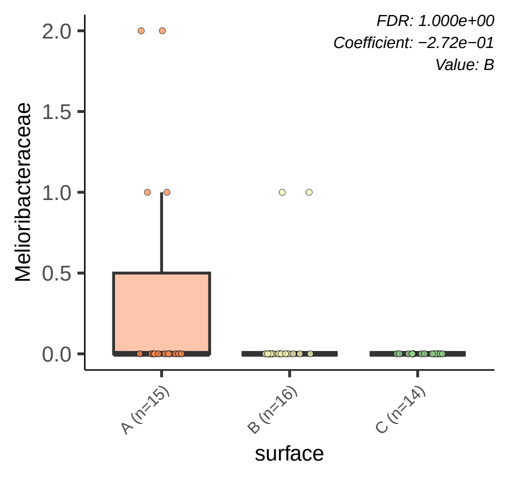
FDR: 1.000e+00
Coefficient: −2.72e−01
Value: B
Melioribacteraceae
2.0
1.5
1.0
0.5
0.0
A (n=15)
B (n=16)
C (n=14)
surface

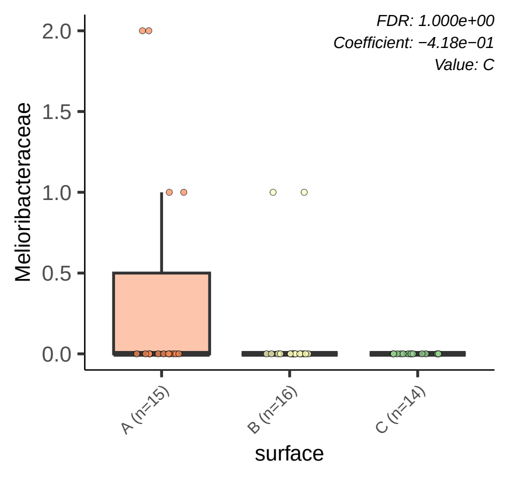

FDR: 1.000e+00
Coefficient: −4.18e−01
Value: C
Melioribacteraceae
2.0
1.5
1.0
0.5
0.0
A (n=15)
B (n=16)
C (n=14)
surface

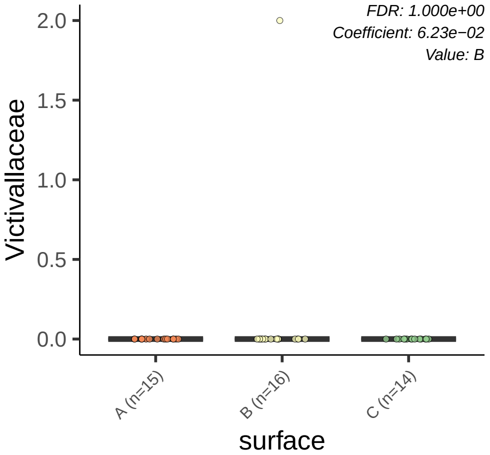
FDR: 1.000e+00
Coefficient: 6.23e−02
Value: B
Victivallaceae
2.0
1.5
1.0
0.5
0.0
A (n=15)
B (n=16)
C (n=14)
surface

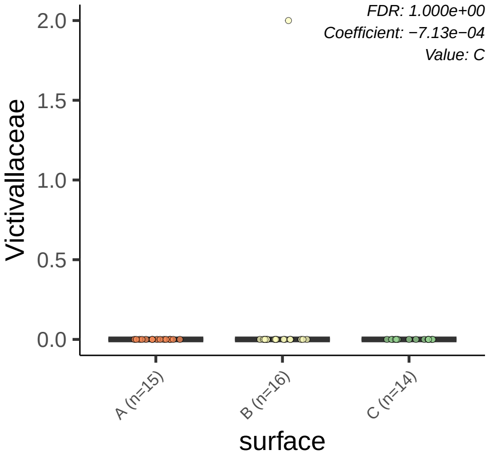
FDR: 1.000e+00
Coefficient: −7.13e−04
Value: C
Victivallaceae
2.0
1.5
1.0
0.5
0.0
A (n=15)
B (n=16)
C (n=14)
surface

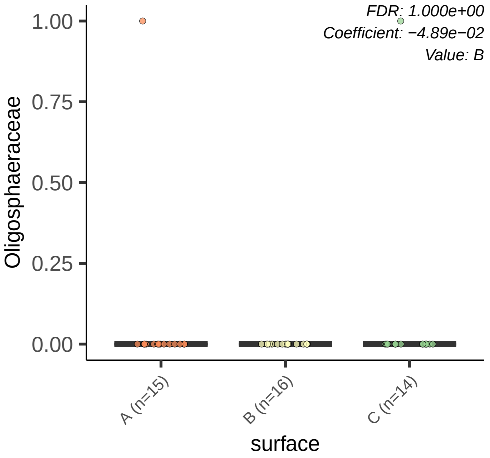
FDR: 1.000e+00
Coefficient: −4.89e−02
Value: B
Oligosphaeraceae
1.00
0.75
0.50
0.25
0.00
A (n=15)
B (n=16)
C (n=14)
surface

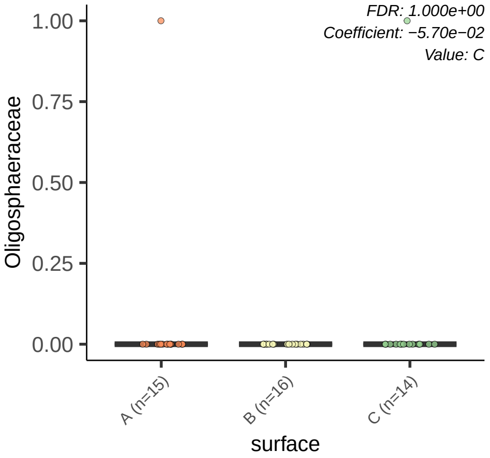

FDR: 1.000e+00
Coefficient: −5.70e−02
Value: C
Oligosphaeraceae
1.00
0.75
0.50
0.25
0.00
A (n=15)
B (n=16)
C (n=14)
surface

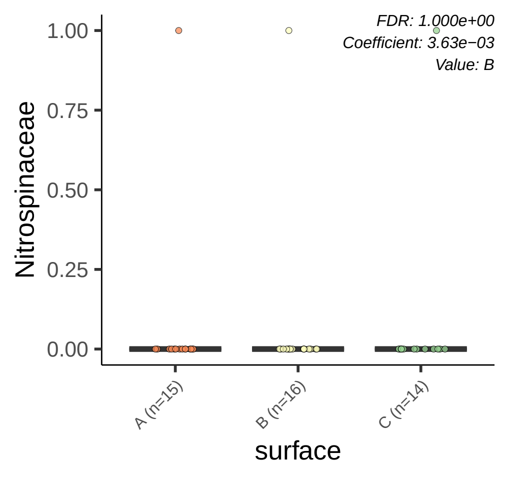
FDR: 1.000e+00
Coefficient: 3.63e−03
Value: B
Nitrospinaceae
1.00
0.75
0.50
0.25
0.00
A (n=15)
B (n=16)
C (n=14)
surface

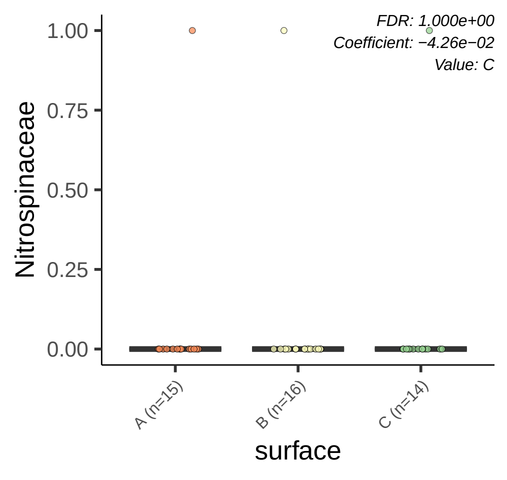
FDR: 1.000e+00
Coefficient: −4.26e−02
Value: C
Nitrospinaceae
1.00
0.75
0.50
0.25
0.00
A (n=15)
B (n=16)
C (n=14)
surface

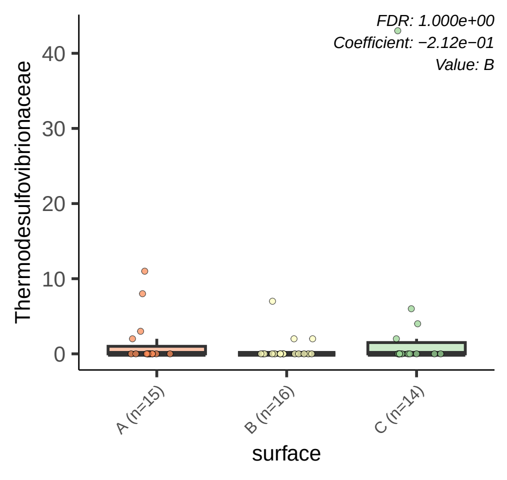
FDR: 1.000e+00
Coefficient: −2.12e−01
Value: B
Thermodesulfovibrionaceae
40
30
20
10
0
A (n=15)
B (n=16)
C (n=14)
surface

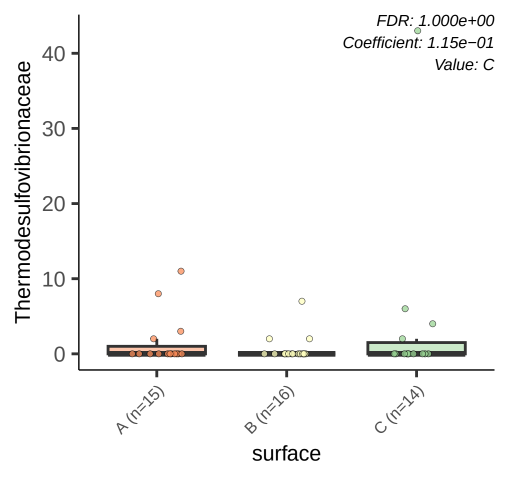
FDR: 1.000e+00
Coefficient: 1.15e−01
Value: C
Thermodesulfovibrionaceae
0
10
20
30
40
A (n=15)
B (n=16)
C (n=14)
surface

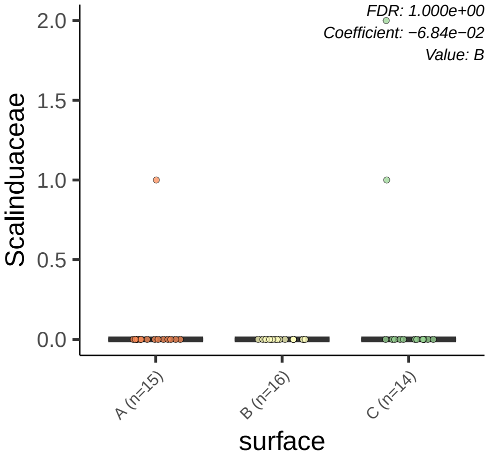

FDR: 1.000e+00
Coefficient: −6.84e−02
Value: B
Scalinduaceae
2.0
1.5
1.0
0.5
0.0
A (n=15)
B (n=16)
C (n=14)
surface

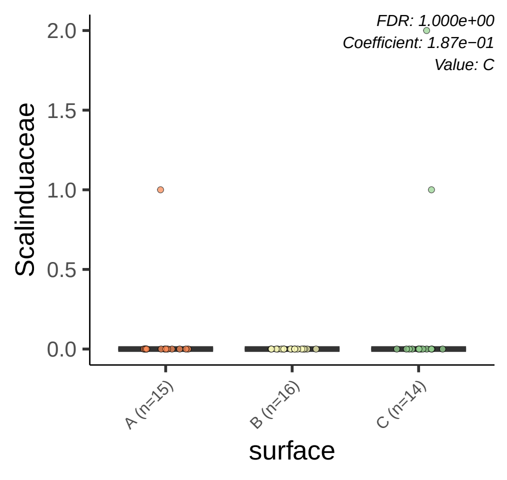

FDR: 1.000e+00
Coefficient: 1.87e−01
Value: C
Scalinduaceae
2.0
1.5
1.0
0.5
0.0
A (n=15)
B (n=16)
C (n=14)
surface

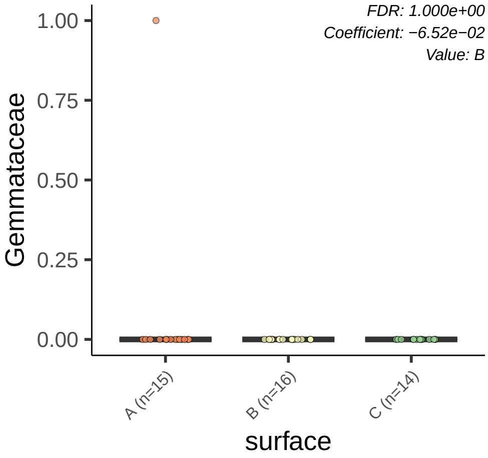
FDR: 1.000e+00
Coefficient: −6.52e−02
Value: B
Gemmataceae
1.00
0.75
0.50
0.25
0.00
A (n=15)
B (n=16)
C (n=14)
surface

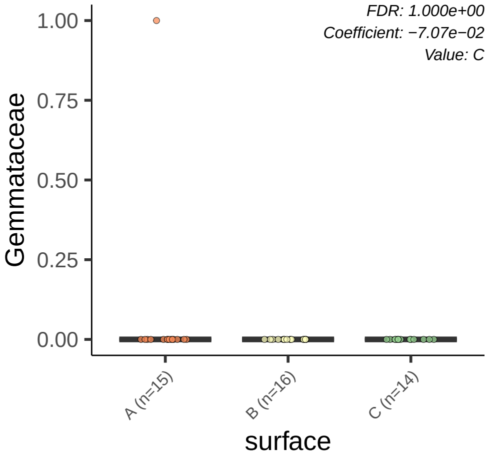
FDR: 1.000e+00
Coefficient: −7.07e−02
Value: C
Gemmataceae
1.00
0.75
0.50
0.25
0.00
A (n=15)
B (n=16)
C (n=14)
surface

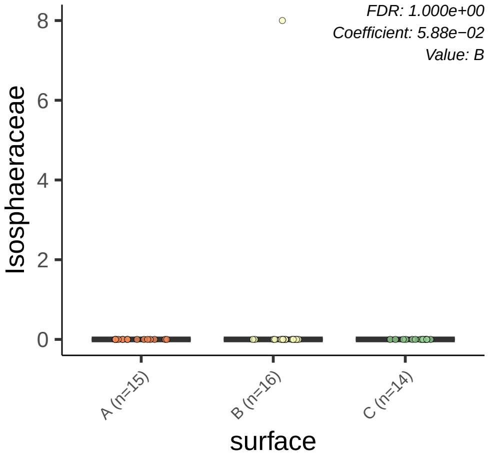
FDR: 1.000e+00
Coefficient: 5.88e−02
Value: B
Isosphaeraceae
8
6
4
2
0
A (n=15)
B (n=16)
C (n=14)
surface

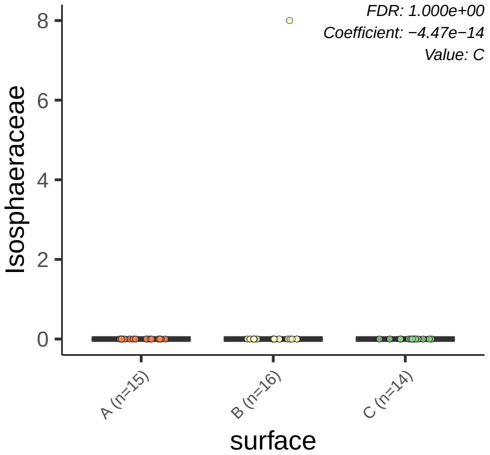

FDR: 1.000e+00
Coefficient: −4.47e−14
Value: C
Isosphaeraceae
8
6
4
2
0
A (n=15)
B (n=16)
C (n=14)
surface

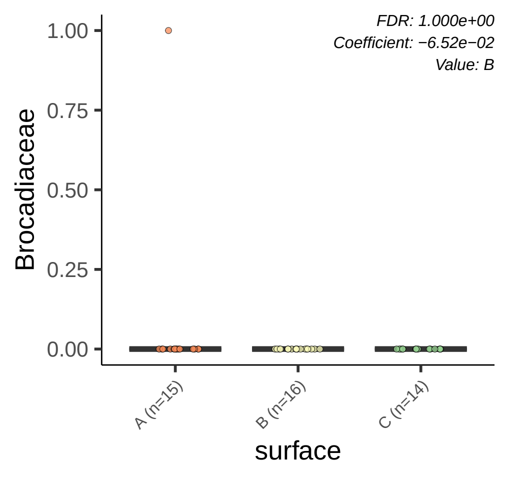
FDR: 1.000e+00
Coefficient: −6.52e−02
Value: B
1.00
0.75
0.50
0.25
0.00
Brocadiaceae
A (n=15)
B (n=16)
C (n=14)
surface

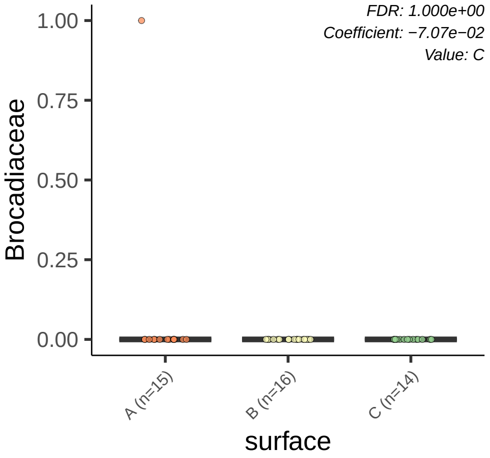
FDR: 1.000e+00
Coefficient: −7.07e−02
Value: C
Brocadiaceae
1.00
0.75
0.50
0.25
0.00
A (n=15)
B (n=16)
C (n=14)
surface

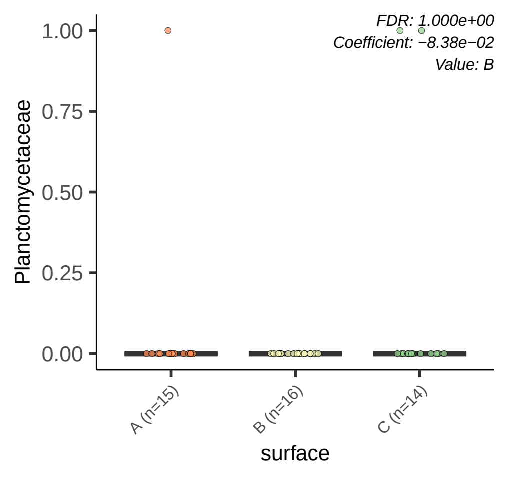

FDR: 1.000e+00
Coefficient: −8.38e−02
Value: B
Planctomycetaceae
1.00
0.75
0.50
0.25
0.00
A (n=15)
B (n=16)
C (n=14)
surface

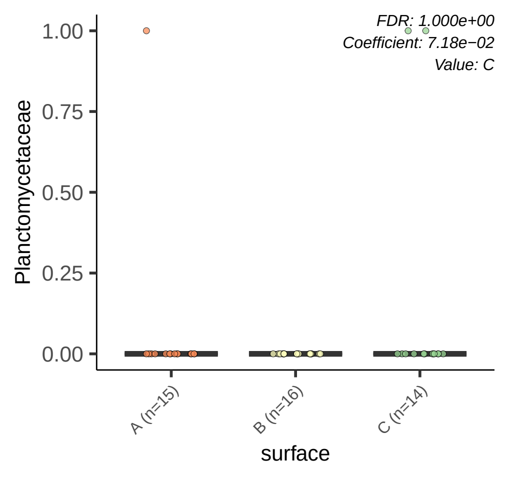
FDR: 1.000e+00
Coefficient: 7.18e−02
Value: C
Planctomycetaceae
1.00
0.75
0.50
0.25
0.00
A (n=15)
B (n=16)
C (n=14)
surface

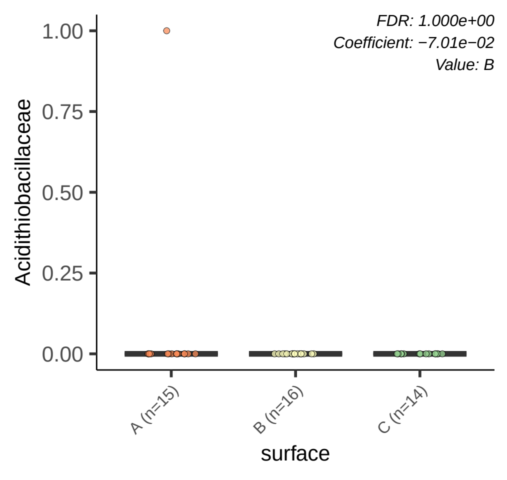
FDR: 1.000e+00
Coefficient: −7.01e−02
Value: B
Acidithiobacillaceae
1.00
0.75
0.50
0.25
0.00
A (n=15)
B (n=16)
C (n=14)
surface

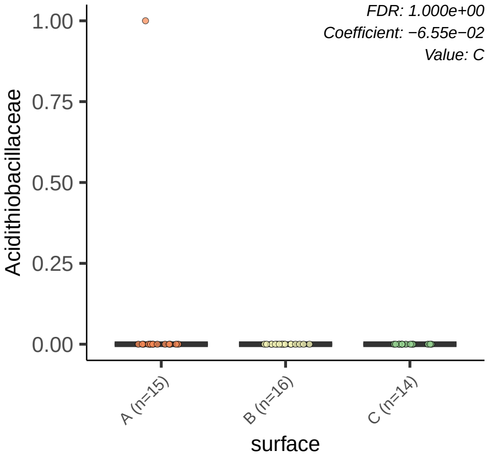
FDR: 1.000e+00
Coefficient: −6.55e−02
Value: C
1.00
0.75
0.50
0.25
0.00
Acidithiobacillaceae
A (n=15)
B (n=16)
C (n=14)
surface

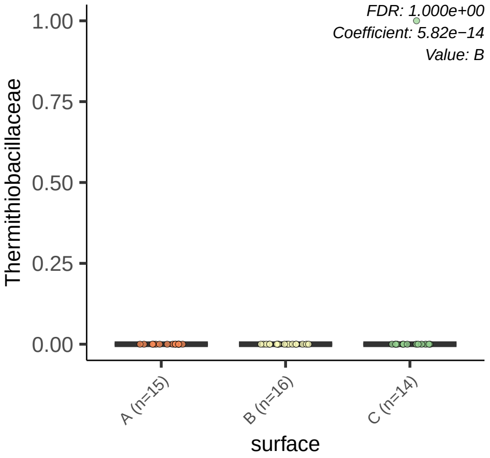

FDR: 1.000e+00
Coefficient: 5.82e−14
Value: B
1.00
0.75
0.50
0.25
0.00
Thermithiobacillaceae
A (n=15)
B (n=16)
C (n=14)
surface

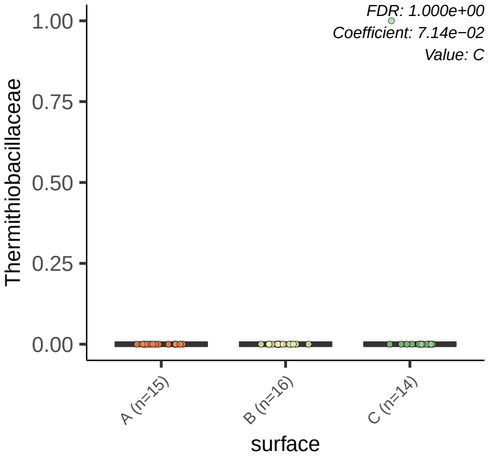

FDR: 1.000e+00
Coefficient: 7.14e−02
Value: C
1.00
0.75
0.50
0.25
0.00
Thermithiobacillaceae
A (n=15)
B (n=16)
C (n=14)
surface

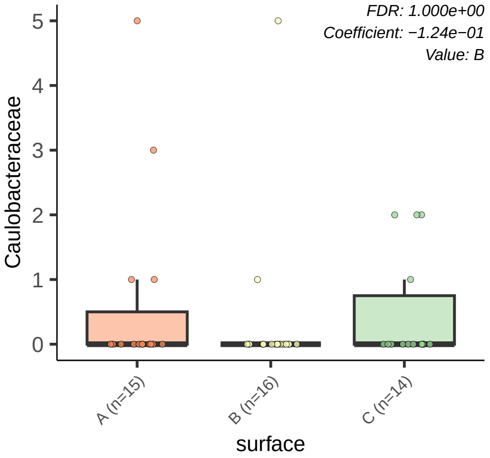
FDR: 1.000e+00
Coefficient: −1.24e−01
Value: B
Caulobacteraceae
0
1
2
3
4
5
A (n=15)
B (n=16)
C (n=14)
surface

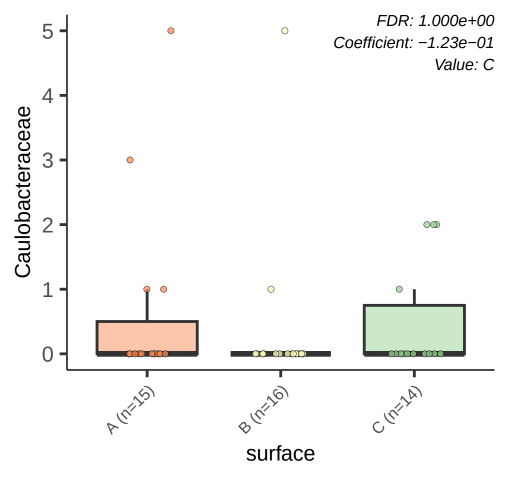

FDR: 1.000e+00
Coefficient: −1.23e−01
Value: C
Caulobacteraceae
0
1
2
3
4
5
A (n=15)
B (n=16)
C (n=14)
surface

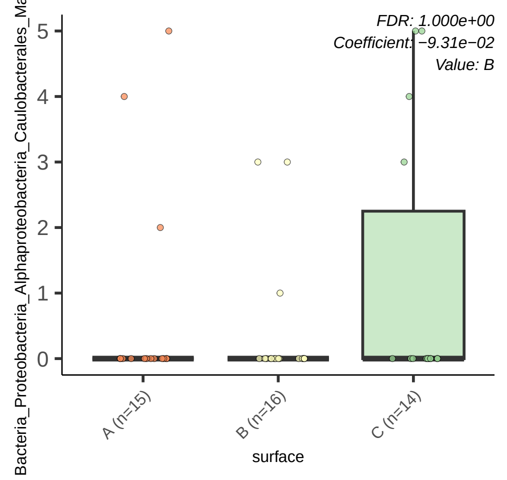

FDR: 1.000e+00
Coefficient: −9.31e−02
Value: B
Bacteria_Proteobacteria_Alphaproteobacteria_Caulobacterales_Ma
0
1
2
3
4
5
A (n=15)
B (n=16)
C (n=14)
surface

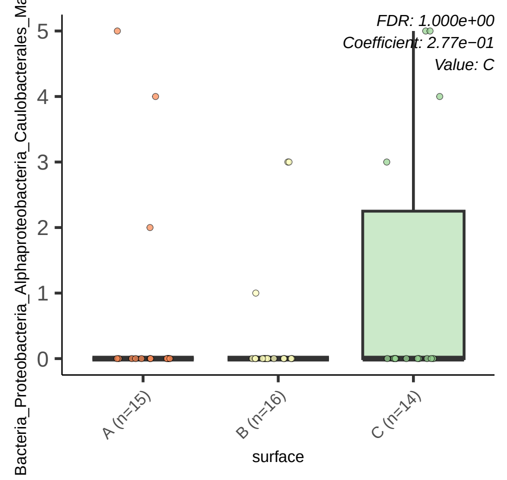
FDR: 1.000e+00
Coefficient: 2.77e−01
Value: C
Bacteria_Proteobacteria_Alphaproteobacteria_Caulobacterales_Ma
0
1
2
3
4
5
A (n=15)
B (n=16)
C (n=14)
surface

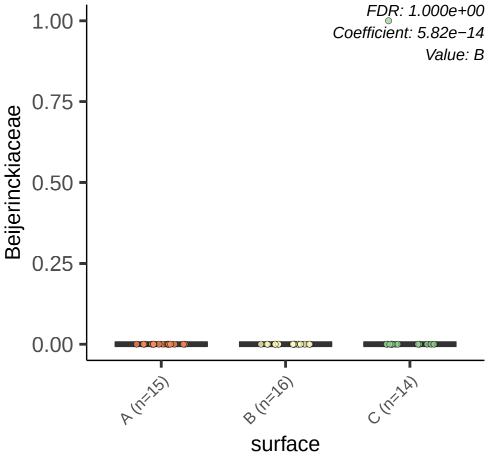

FDR: 1.000e+00
Coefficient: 5.82e−14
Value: B
Beijerinckiaceae
1.00
0.75
0.50
0.25
0.00
A (n=15)
B (n=16)
C (n=14)
surface

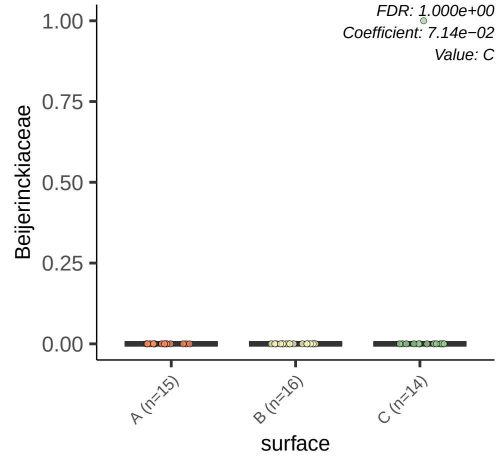
FDR: 1.000e+00
Coefficient: 7.14e−02
Value: C
Beijerinckiaceae
1.00
0.75
0.50
0.25
0.00
A (n=15)
B (n=16)
C (n=14)
surface

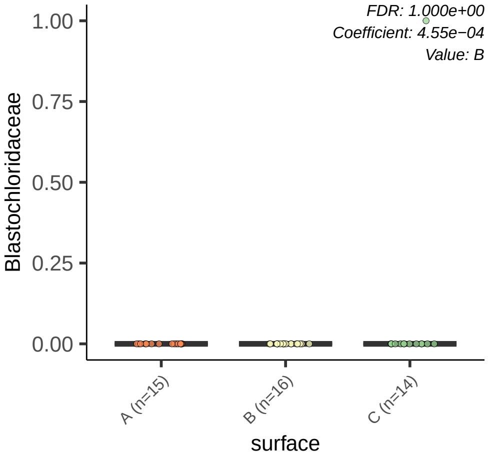
FDR: 1.000e+00
Coefficient: 4.55e−04
Value: B
Blastochloridaceae
1.00
0.75
0.50
0.25
0.00
A (n=15)
B (n=16)
C (n=14)
surface

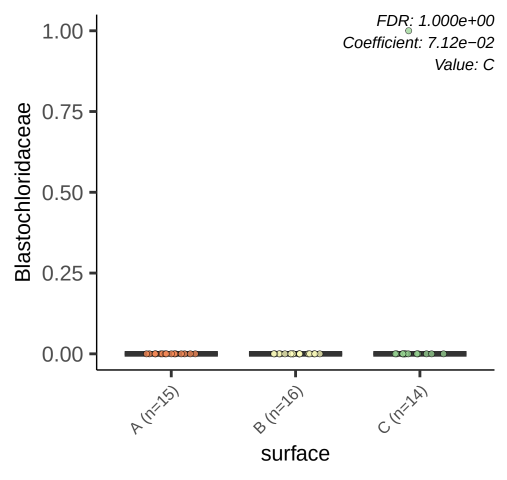
FDR: 1.000e+00
Coefficient: 7.12e−02
Value: C
Blastochloridaceae
1.00
0.75
0.50
0.25
0.00
A (n=15)
B (n=16)
C (n=14)
surface

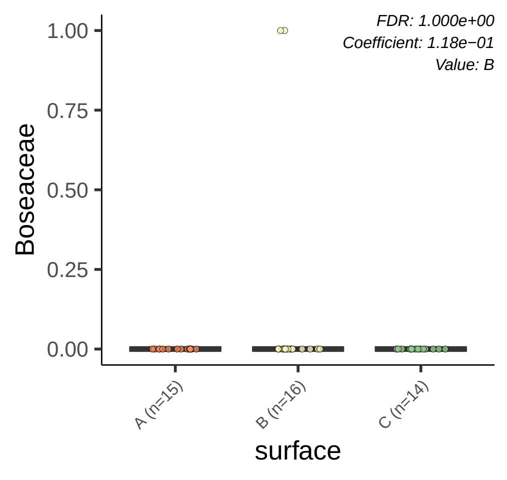

FDR: 1.000e+00
Coefficient: 1.18e−01
Value: B
1.00
0.75
0.50
0.25
0.00
Boseaceae
A (n=15)
B (n=16)
C (n=14)
surface

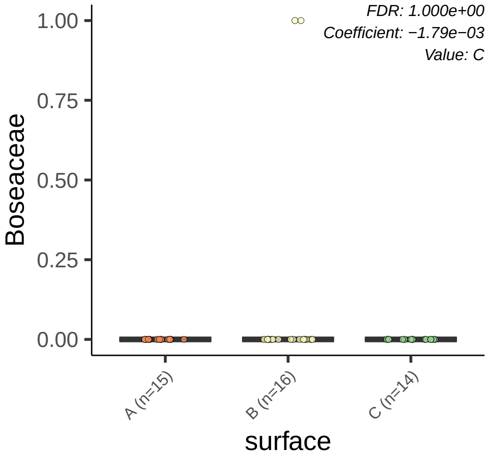
FDR: 1.000e+00
Coefficient: −1.79e−03
Value: C
Boseaceae
1.00
0.75
0.50
0.25
0.00
A (n=15)
B (n=16)
C (n=14)
surface

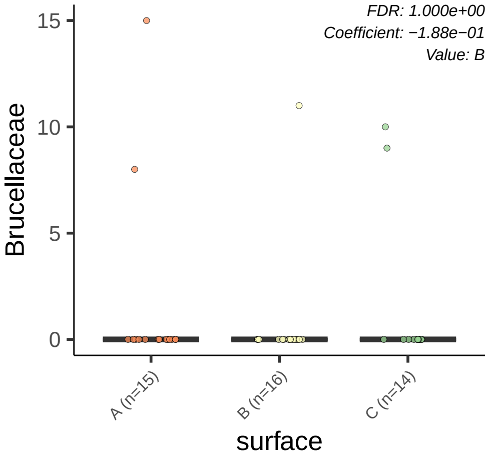
FDR: 1.000e+00
Coefficient: −1.88e−01
Value: B
Brucellaceae
15
10
5
0
A (n=15)
B (n=16)
C (n=14)
surface

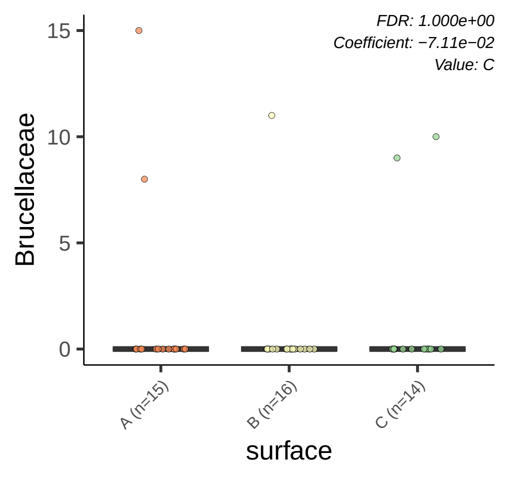
FDR: 1.000e+00
Coefficient: −7.11e−02
Value: C
Brucellaceae
15
10
5
0
A (n=15)
B (n=16)
C (n=14)
surface

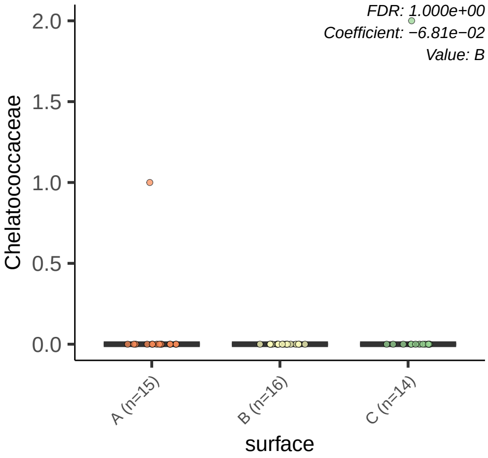

FDR: 1.000e+00
Coefficient: −6.81e−02
Value: B
Chelatococcaceae
2.0
1.5
1.0
0.5
0.0
A (n=15)
B (n=16)
C (n=14)
surface

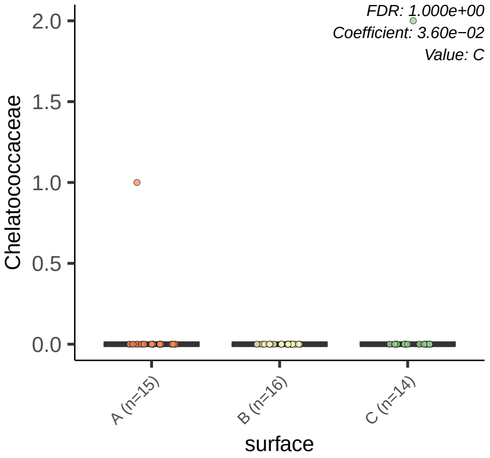
FDR: 1.000e+00
Coefficient: 3.60e−02
Value: C
Chelatococcaceae
2.0
1.5
1.0
0.5
0.0
A (n=15)
B (n=16)
C (n=14)
surface

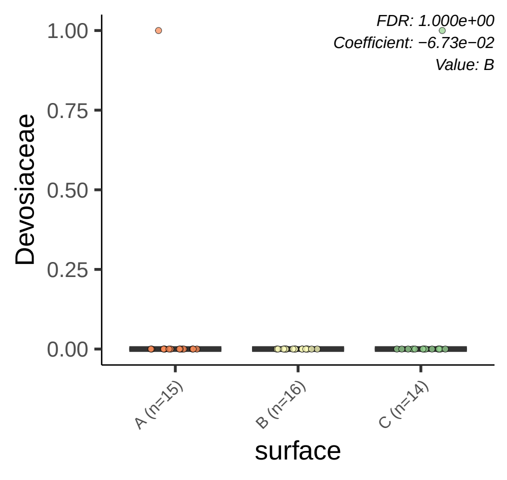
FDR: 1.000e+00
Coefficient: −6.73e−02
Value: B
Devosiaceae
1.00
0.75
0.50
0.25
0.00
A (n=15)
B (n=16)
C (n=14)
surface

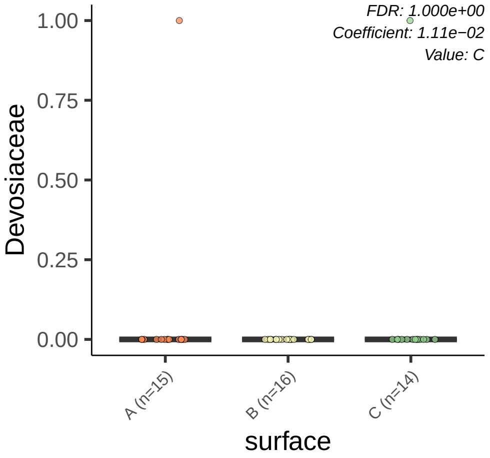
FDR: 1.000e+00
Coefficient: 1.11e−02
Value: C
Devosiaceae
1.00
0.75
0.50
0.25
0.00
A (n=15)
B (n=16)
C (n=14)
surface

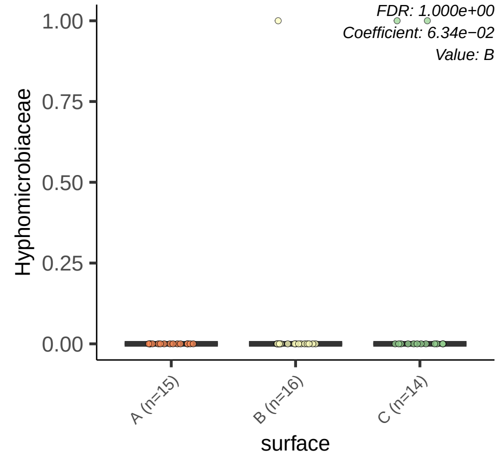
FDR: 1.000e+00
Coefficient: 6.34e−02
Value: B
Hyphomicrobiaceae
1.00
0.75
0.50
0.25
0.00
A (n=15)
B (n=16)
C (n=14)
surface

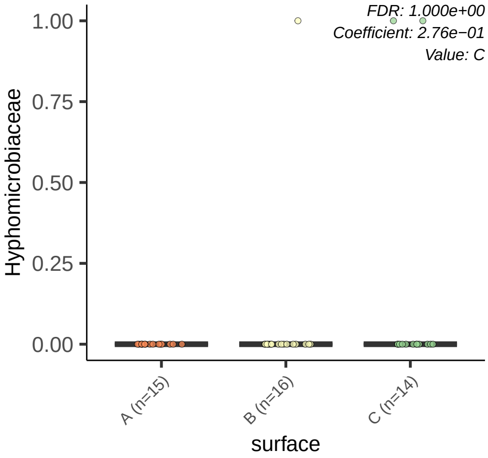

FDR: 1.000e+00
Coefficient: 2.76e−01
Value: C
Hyphomicrobiaceae
1.00
0.75
0.50
0.25
0.00
A (n=15)
B (n=16)
C (n=14)
surface

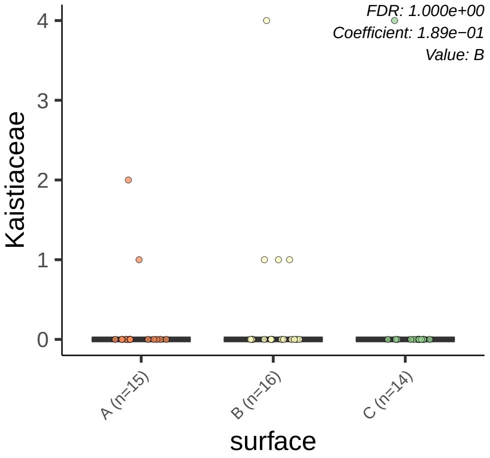
FDR: 1.000e+00
Coefficient: 1.89e−01
Value: B
Kaistiaceae
4
3
2
1
0
A (n=15)
B (n=16)
C (n=14)
surface

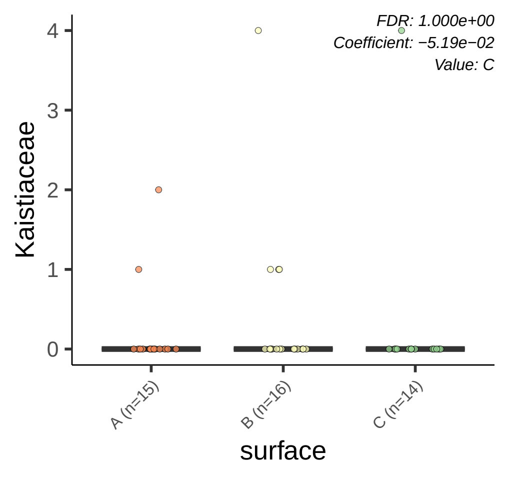
FDR: 1.000e+00
Coefficient: −5.19e−02
Value: C
Kaistiaceae
0
1
2
3
4
A (n=15)
B (n=16)
C (n=14)
surface

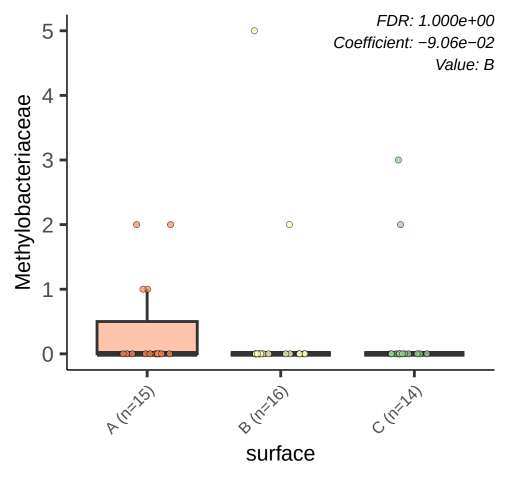

FDR: 1.000e+00
Coefficient: −9.06e−02
Value: B
Methylobacteriaceae
0
1
2
3
4
5
A (n=15)
B (n=16)
C (n=14)
surface

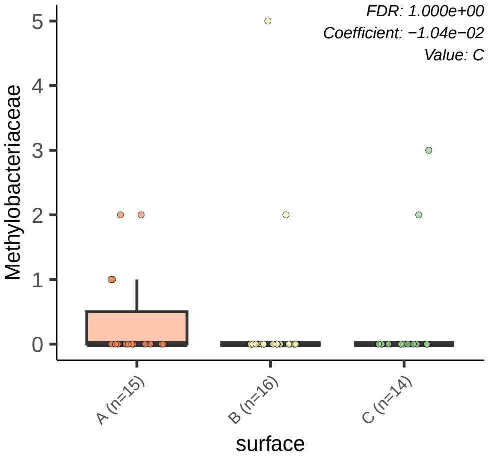

FDR: 1.000e+00
Coefficient: −1.04e−02
Value: C
Methylobacteriaceae
0
1
2
3
4
5
A (n=15)
B (n=16)
C (n=14)
surface

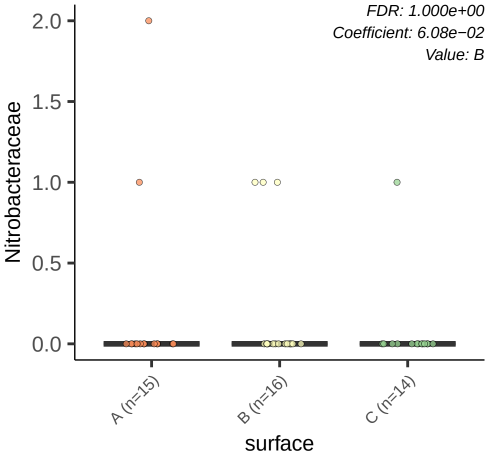
FDR: 1.000e+00
Coefficient: 6.08e−02
Value: B
Nitrobacteraceae
2.0
1.5
1.0
0.5
0.0
A (n=15)
B (n=16)
C (n=14)
surface

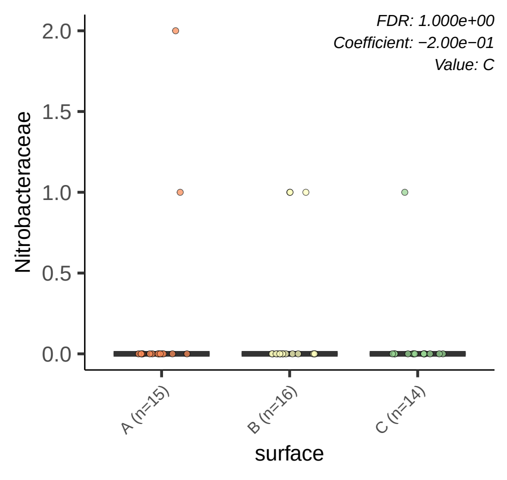

FDR: 1.000e+00
Coefficient: −2.00e−01
Value: C
Nitrobacteraceae
2.0
1.5
1.0
0.5
0.0
A (n=15)
B (n=16)
C (n=14)
surface

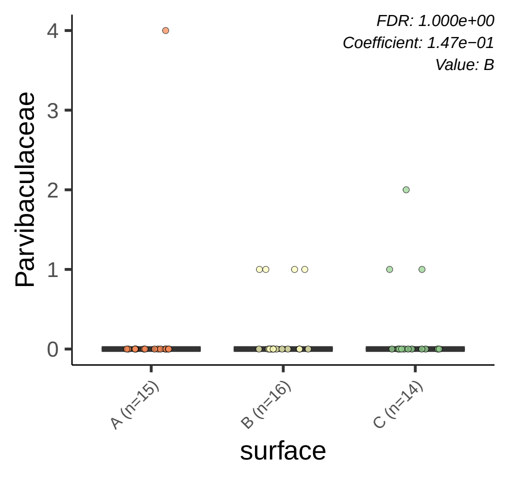

FDR: 1.000e+00
Coefficient: 1.47e−01
Value: B
Parvibaculaceae
0
1
2
3
4
A (n=15)
B (n=16)
C (n=14)
surface

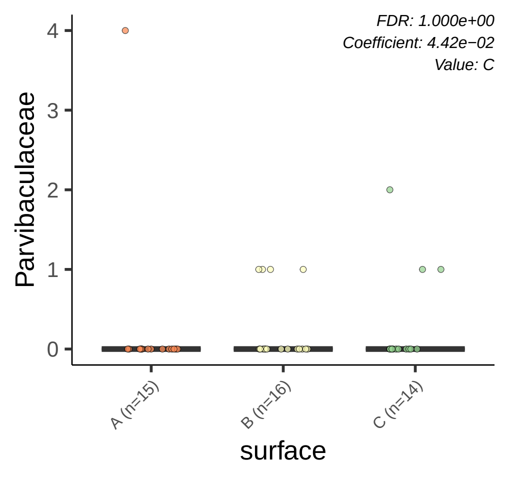
FDR: 1.000e+00
Coefficient: 4.42e−02
Value: C
Parvibaculaceae
0
1
2
3
4
A (n=15)
B (n=16)
C (n=14)
surface

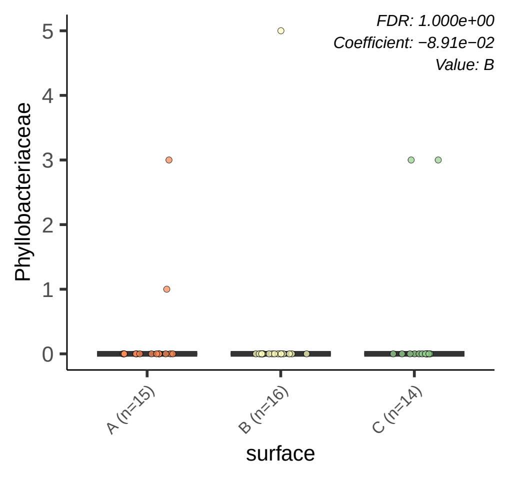
FDR: 1.000e+00
Coefficient: −8.91e−02
Value: B
Phyllobacteriaceae
0
1
2
3
4
5
A (n=15)
B (n=16)
C (n=14)
surface

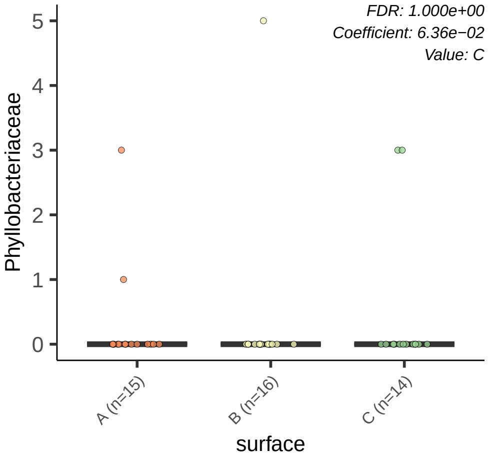
FDR: 1.000e+00
Coefficient: 6.36e−02
Value: C
Phyllobacteriaceae
0
1
2
3
4
5
A (n=15)
B (n=16)
C (n=14)
surface

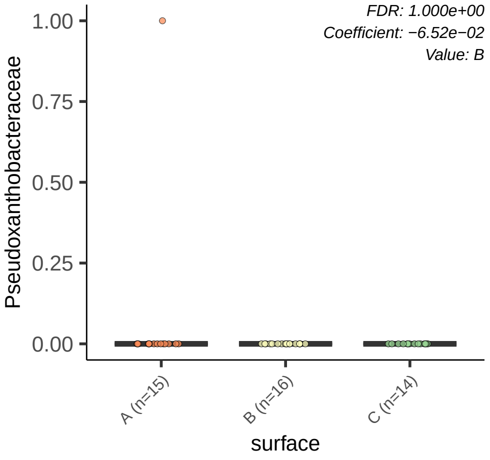
FDR: 1.000e+00
Coefficient: −6.52e−02
Value: B
Pseudoxanthobacteraceae
1.00
0.75
0.50
0.25
0.00
A (n=15)
B (n=16)
C (n=14)
surface

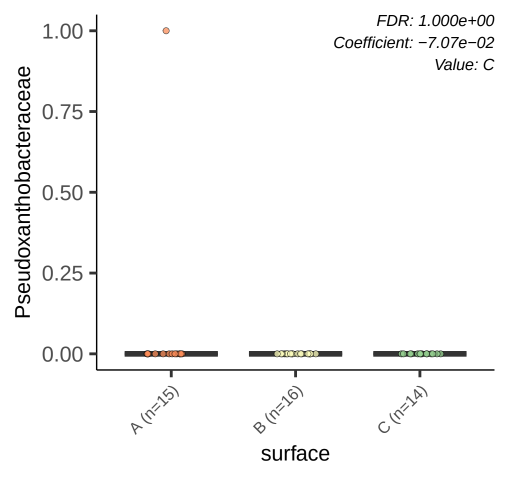
FDR: 1.000e+00
Coefficient: −7.07e−02
Value: C
Pseudoxanthobacteraceae
1.00
0.75
0.50
0.25
0.00
A (n=15)
B (n=16)
C (n=14)
surface

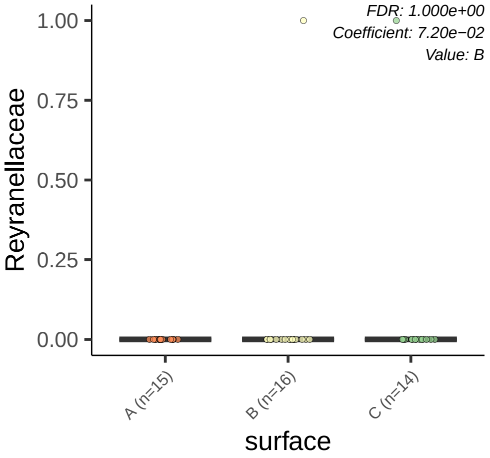
FDR: 1.000e+00
Coefficient: 7.20e−02
Value: B
Reyranellaceae
1.00
0.75
0.50
0.25
0.00
A (n=15)
B (n=16)
C (n=14)
surface

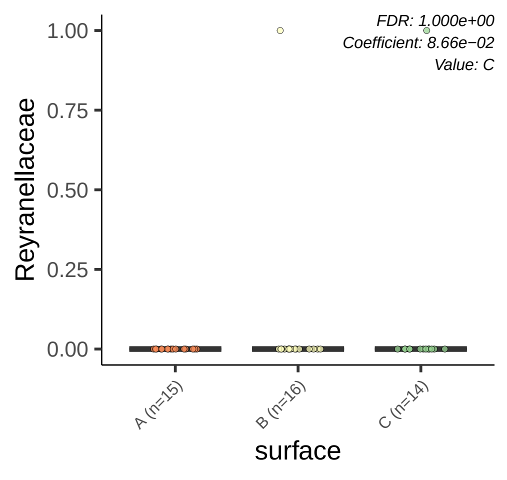

FDR: 1.000e+00
Coefficient: 8.66e−02
Value: C
Reyranellaceae
1.00
0.75
0.50
0.25
0.00
A (n=15)
B (n=16)
C (n=14)
surface

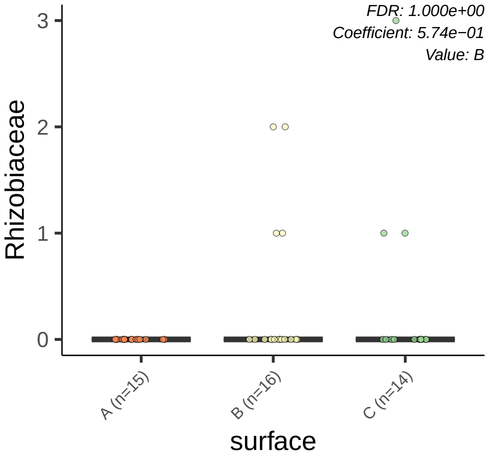

FDR: 1.000e+00
Coefficient: 5.74e−01
Value: B
Rhizobiaceae
3
2
1
0
A (n=15)
B (n=16)
C (n=14)
surface

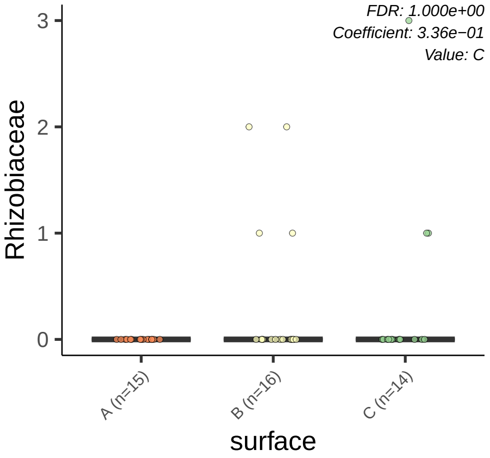
FDR: 1.000e+00
Coefficient: 3.36e−01
Value: C
Rhizobiaceae
0
1
2
3
A (n=15)
B (n=16)
C (n=14)
surface

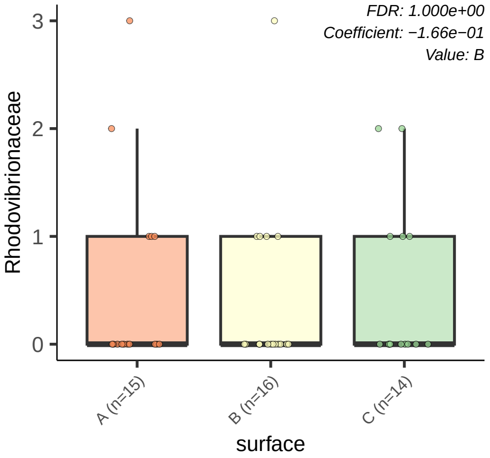

FDR: 1.000e+00
Coefficient: −1.66e−01
Value: B
Rhodovibrionaceae
3
2
1
0
A (n=15)
B (n=16)
C (n=14)
surface

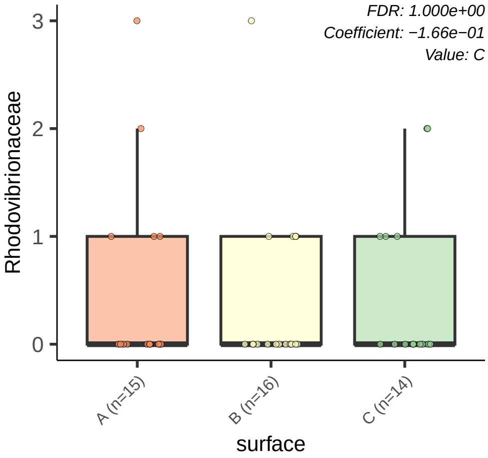
FDR: 1.000e+00
Coefficient: −1.66e−01
Value: C
Rhodovibrionaceae
3
2
1
0
A (n=15)
B (n=16)
C (n=14)
surface

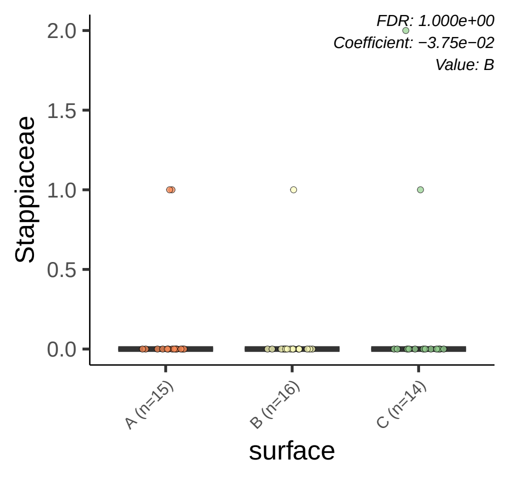

FDR: 1.000e+00
Coefficient: −3.75e−02
Value: B
Stappiaceae
2.0
1.5
1.0
0.5
0.0
A (n=15)
B (n=16)
C (n=14)
surface

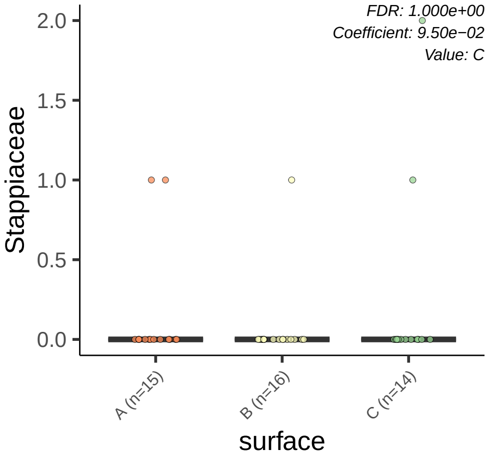

FDR: 1.000e+00
Coefficient: 9.50e−02
Value: C
Stappiaceae
2.0
1.5
1.0
0.5
0.0
A (n=15)
B (n=16)
C (n=14)
surface

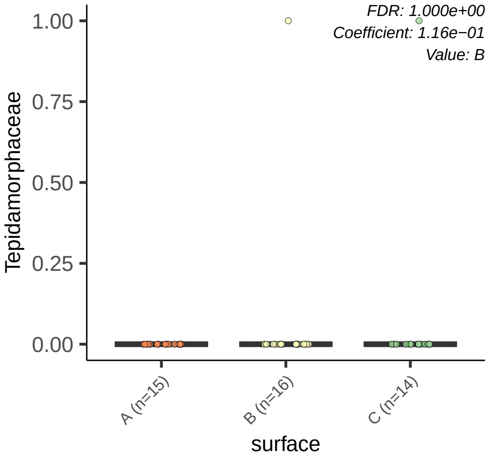
FDR: 1.000e+00
Coefficient: 1.16e−01
Value: B
Tepidamorphaceae
1.00
0.75
0.50
0.25
0.00
A (n=15)
B (n=16)
C (n=14)
surface

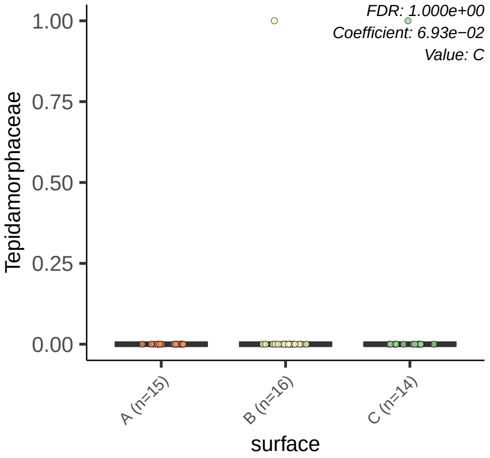
FDR: 1.000e+00
Coefficient: 6.93e−02
Value: C
Tepidamorphaceae
1.00
0.75
0.50
0.25
0.00
A (n=15)
B (n=16)
C (n=14)
surface

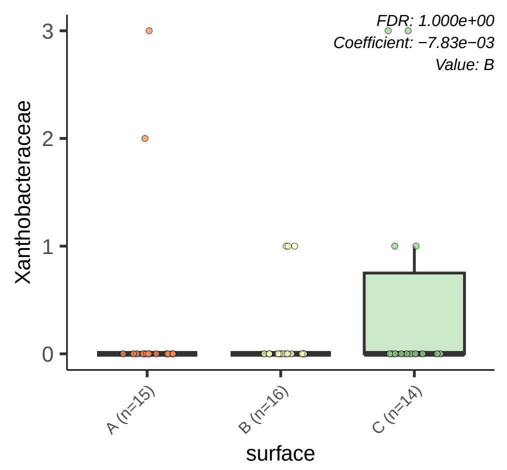

FDR: 1.000e+00
Coefficient: −7.83e−03
Value: B
Xanthobacteraceae
3
2
1
0
A (n=15)
B (n=16)
C (n=14)
surface

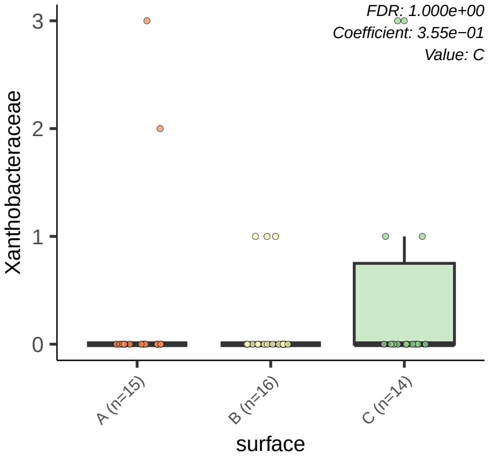
FDR: 1.000e+00
Coefficient: 3.55e−01
Value: C
Xanthobacteraceae
0
1
2
3
A (n=15)
B (n=16)
C (n=14)
surface

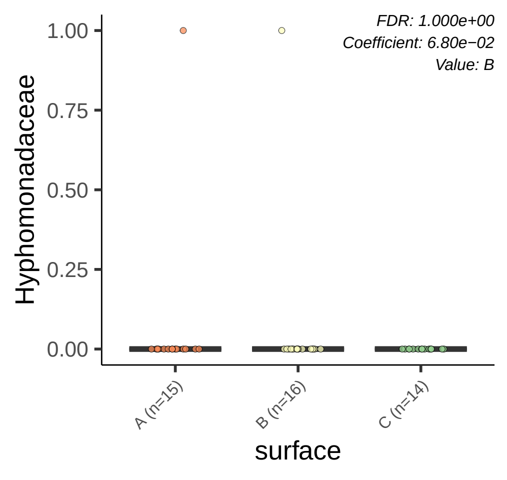

FDR: 1.000e+00
Coefficient: 6.80e−02
Value: B
Hyphomonadaceae
1.00
0.75
0.50
0.25
0.00
A (n=15)
B (n=16)
C (n=14)
surface

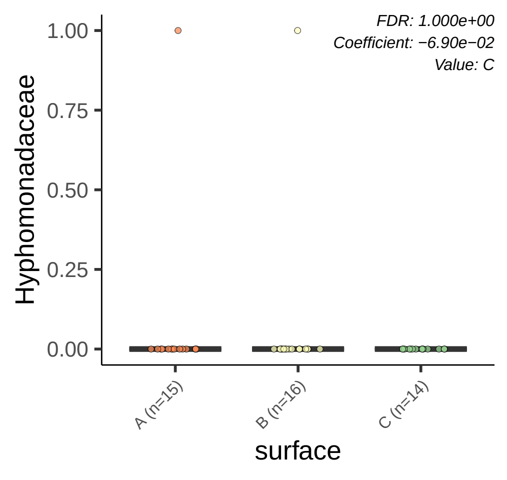

FDR: 1.000e+00
Coefficient: −6.90e−02
Value: C
Hyphomonadaceae
1.00
0.75
0.50
0.25
0.00
A (n=15)
B (n=16)
C (n=14)
surface

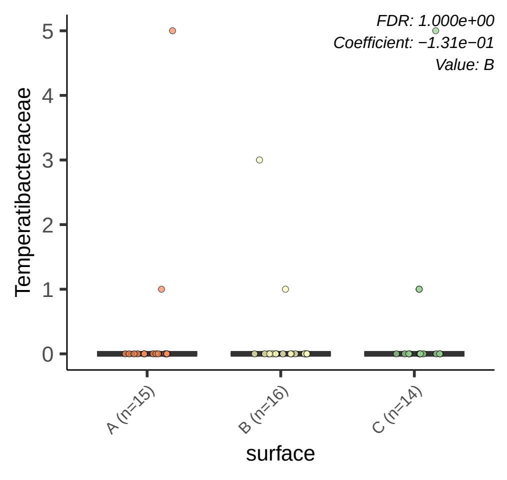
FDR: 1.000e+00
Coefficient: −1.31e−01
Value: B
Temperatibacteraceae
0
1
2
3
4
5
A (n=15)
B (n=16)
C (n=14)
surface

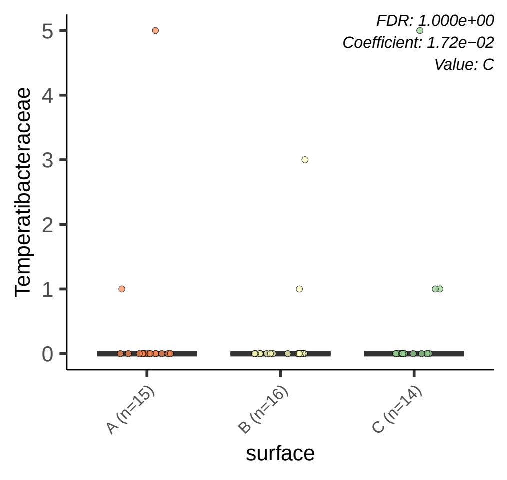
FDR: 1.000e+00
Coefficient: 1.72e−02
Value: C
Temperatibacteraceae
0
1
2
3
4
5
A (n=15)
B (n=16)
C (n=14)
surface

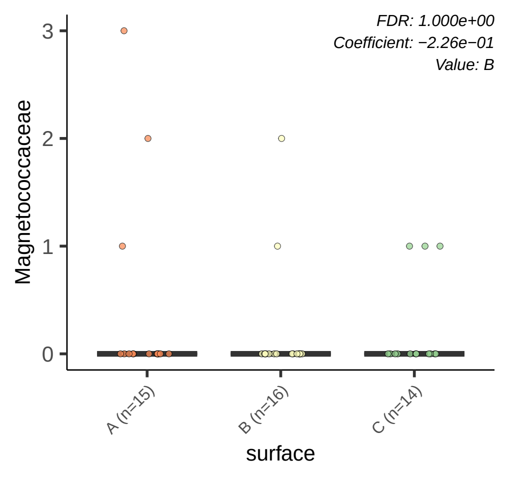

FDR: 1.000e+00
Coefficient: −2.26e−01
Value: B
Magnetococcaceae
3
2
1
0
A (n=15)
B (n=16)
C (n=14)
surface

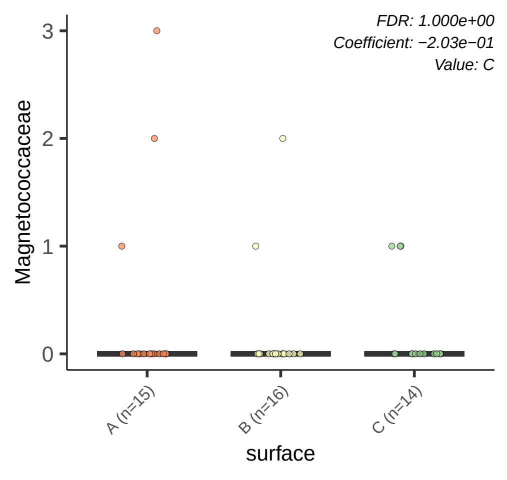
FDR: 1.000e+00
Coefficient: −2.03e−01
Value: C
Magnetococcaceae
3
2
1
0
A (n=15)
B (n=16)
C (n=14)
surface

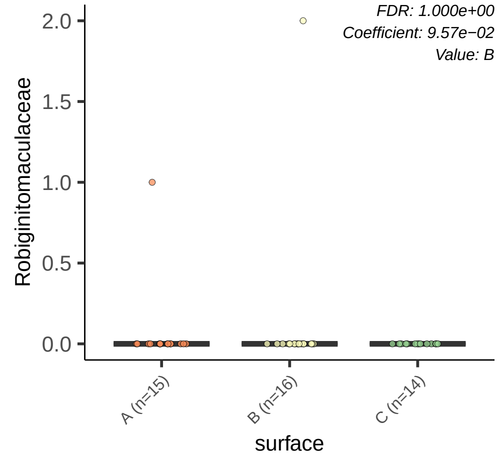
FDR: 1.000e+00
Coefficient: 9.57e−02
Value: B
Robiginitomaculaceae
2.0
1.5
1.0
0.5
0.0
A (n=15)
B (n=16)
C (n=14)
surface

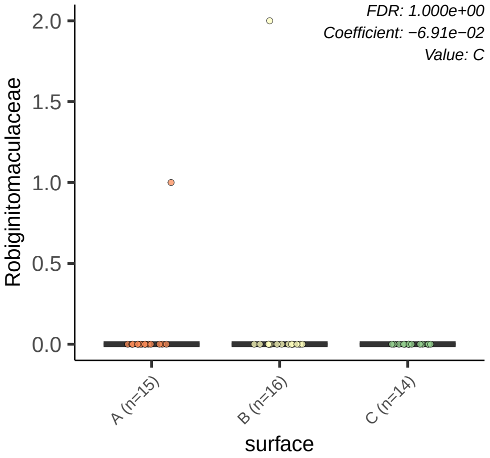
FDR: 1.000e+00
Coefficient: −6.91e−02
Value: C
Robiginitomaculaceae
2.0
1.5
1.0
0.5
0.0
A (n=15)
B (n=16)
C (n=14)
surface

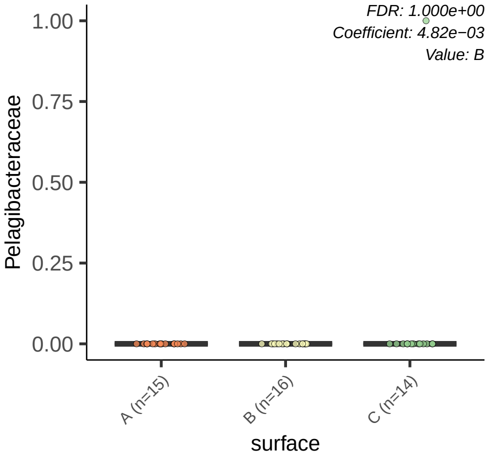

FDR: 1.000e+00
Coefficient: 4.82e−03
Value: B
1.00
0.75
0.50
0.25
0.00
Pelagibacteraceae
A (n=15)
B (n=16)
C (n=14)
surface

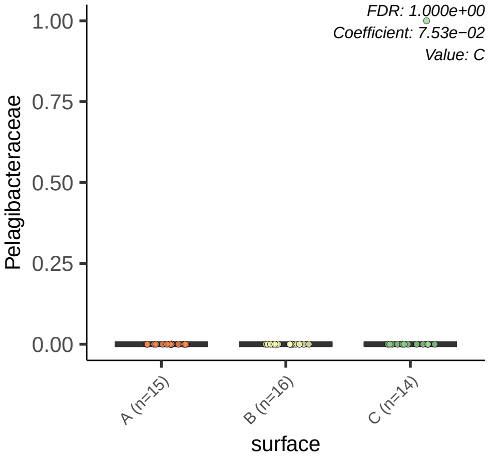
FDR: 1.000e+00
Coefficient: 7.53e−02
Value: C
1.00
0.75
0.50
0.25
0.00
Pelagibacteraceae
A (n=15)
B (n=16)
C (n=14)
surface

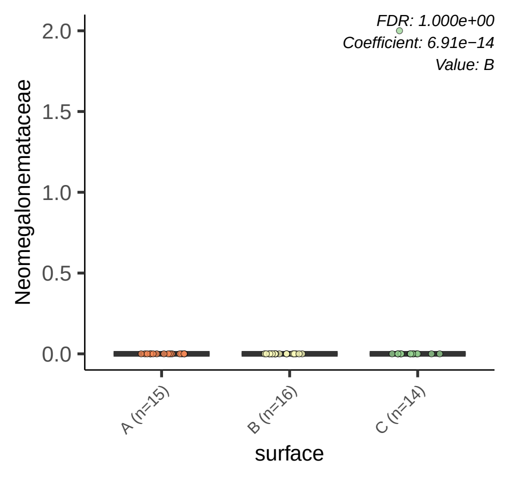
FDR: 1.000e+00
Coefficient: 6.91e−14
Value: B
Neomegalonemataceae
2.0
1.5
1.0
0.5
0.0
A (n=15)
B (n=16)
C (n=14)
surface

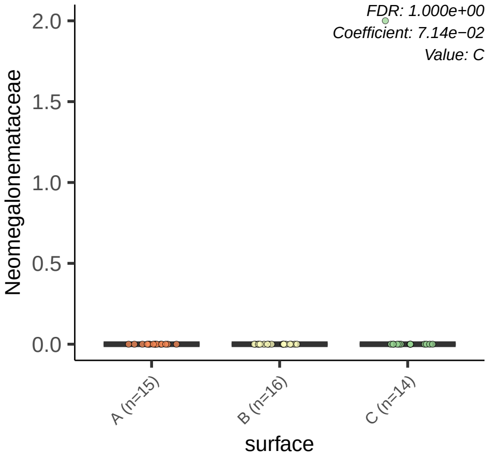
FDR: 1.000e+00
Coefficient: 7.14e−02
Value: C
Neomegalonemataceae
2.0
1.5
1.0
0.5
0.0
A (n=15)
B (n=16)
C (n=14)
surface

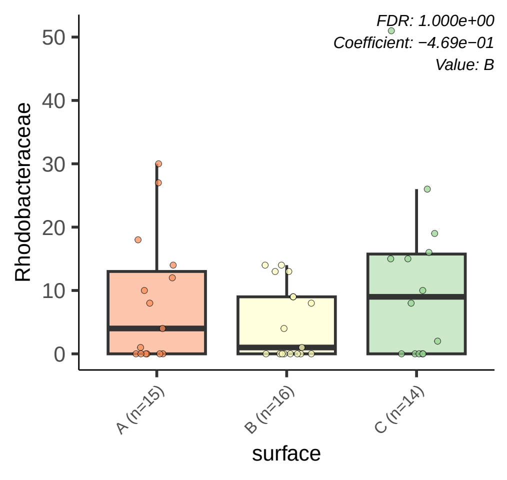
FDR: 1.000e+00
Coefficient: −4.69e−01
Value: B
Rhodobacteraceae
0
10
20
30
40
50
A (n=15)
B (n=16)
C (n=14)
surface

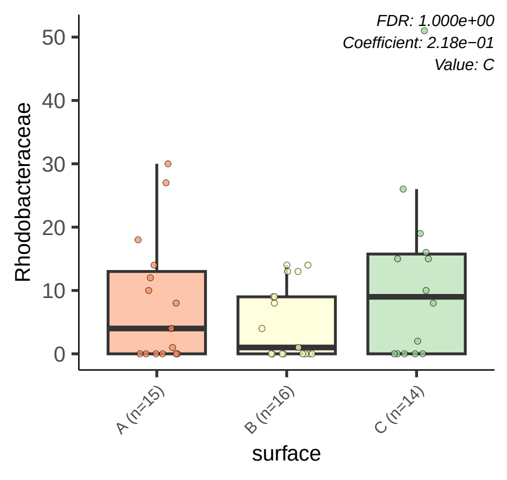
FDR: 1.000e+00
Coefficient: 2.18e−01
Value: C
Rhodobacteraceae
50
40
30
20
10
0
A (n=15)
B (n=16)
C (n=14)
surface

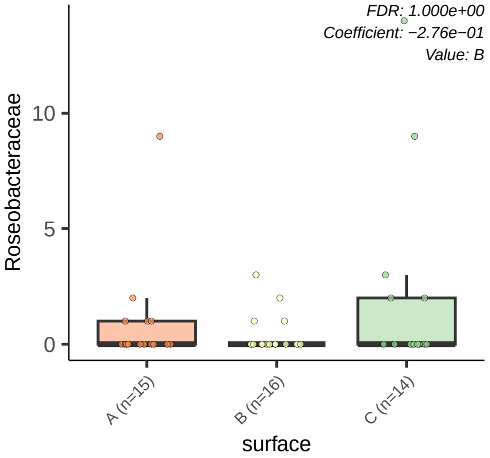
FDR: 1.000e+00
Coefficient: −2.76e−01
Value: B
Roseobacteraceae
10
5
0
A (n=15)
B (n=16)
C (n=14)
surface

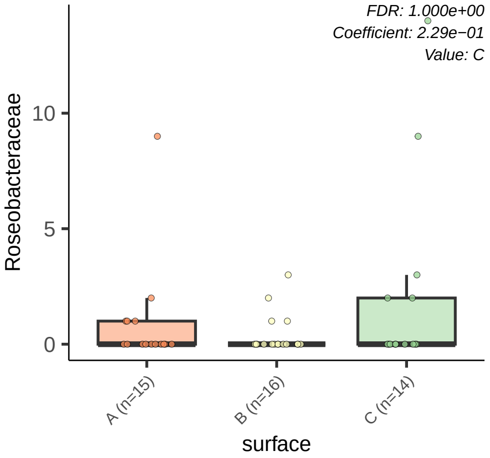
FDR: 1.000e+00
Coefficient: 2.29e−01
Value: C
Roseobacteraceae
10
5
0
A (n=15)
B (n=16)
C (n=14)
surface

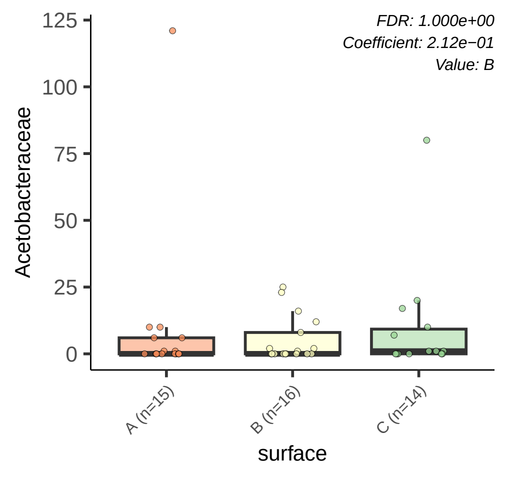

FDR: 1.000e+00
Coefficient: 2.12e−01
Value: B
Acetobacteraceae
125
100
75
50
25
0
A (n=15)
B (n=16)
C (n=14)
surface

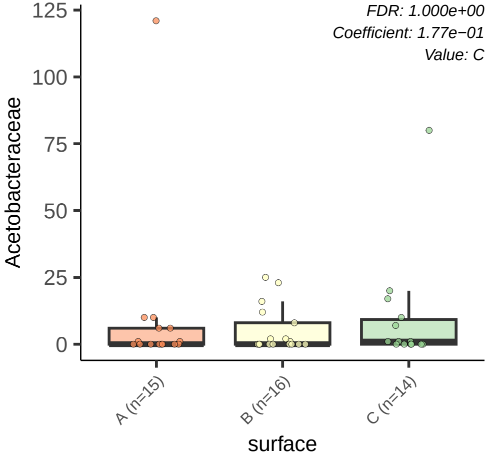
FDR: 1.000e+00
Coefficient: 1.77e−01
Value: C
Acetobacteraceae
125
100
75
50
25
0
A (n=15)
B (n=16)
C (n=14)
surface

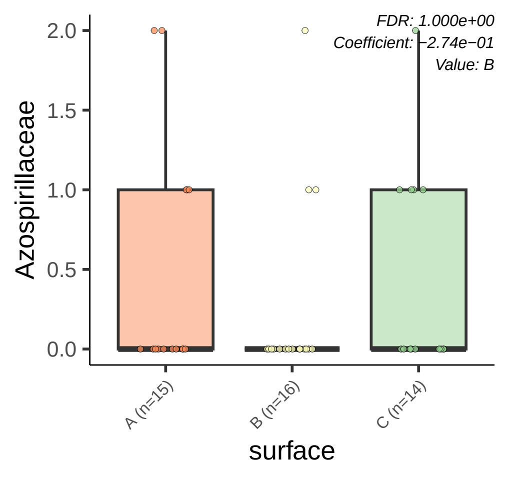

FDR: 1.000e+00
Coefficient: −2.74e−01
Value: B
Azospirillaceae
2.0
1.5
1.0
0.5
0.0
A (n=15)
B (n=16)
C (n=14)
surface

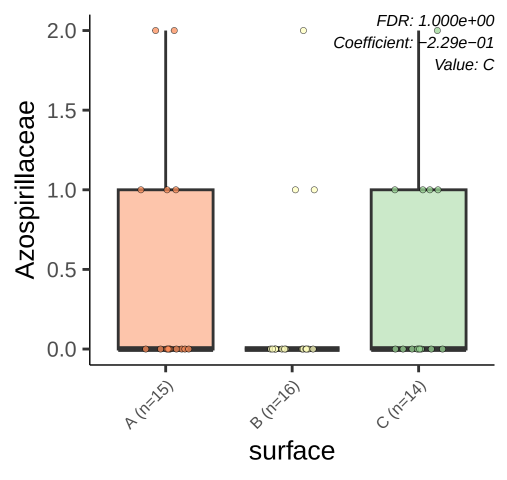
FDR: 1.000e+00
Coefficient: −2.29e−01
Value: C
Azospirillaceae
2.0
1.5
1.0
0.5
0.0
A (n=15)
B (n=16)
C (n=14)
surface

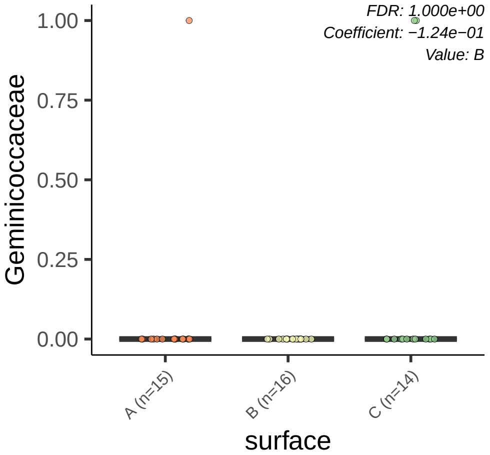
FDR: 1.000e+00
Coefficient: −1.24e−01
Value: B
Geminicoccaceae
1.00
0.75
0.50
0.25
0.00
A (n=15)
B (n=16)
C (n=14)
surface

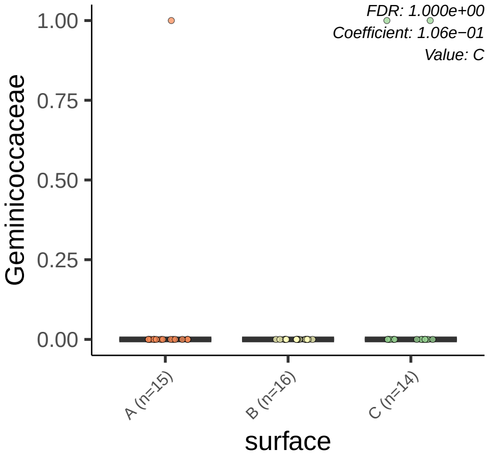

FDR: 1.000e+00
Coefficient: 1.06e−01
Value: C
Geminicoccaceae
1.00
0.75
0.50
0.25
0.00
A (n=15)
B (n=16)
C (n=14)
surface

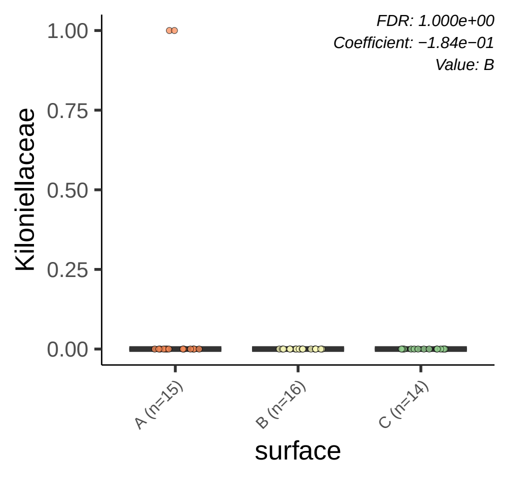
FDR: 1.000e+00
Coefficient: −1.84e−01
Value: B
Kiloniellaceae
1.00
0.75
0.50
0.25
0.00
A (n=15)
B (n=16)
C (n=14)
surface

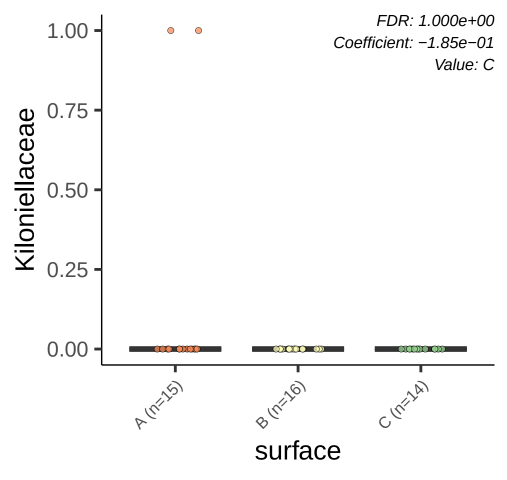
FDR: 1.000e+00
Coefficient: −1.85e−01
Value: C
Kiloniellaceae
1.00
0.75
0.50
0.25
0.00
A (n=15)
B (n=16)
C (n=14)
surface

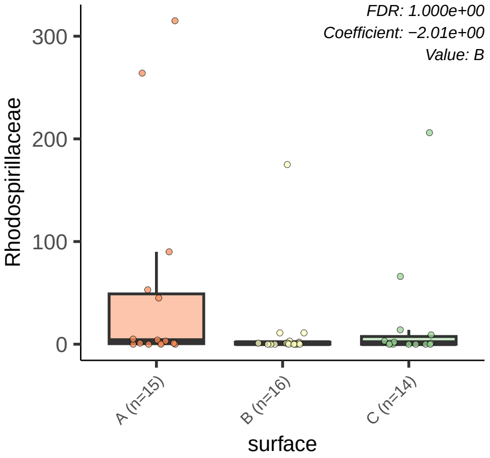
FDR: 1.000e+00
Coefficient: −2.01e+00
Value: B
Rhodospirillaceae
300
200
100
0
A (n=15)
B (n=16)
C (n=14)
surface

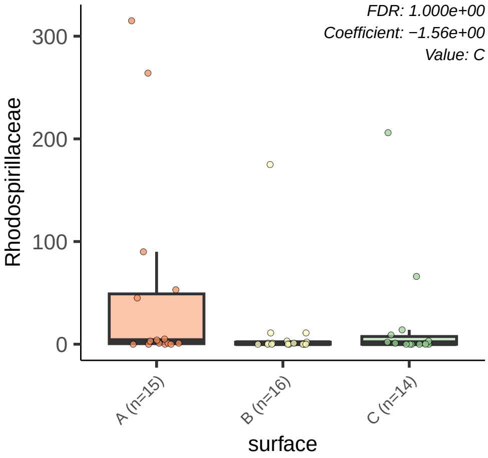
FDR: 1.000e+00
Coefficient: −1.56e+00
Value: C
Rhodospirillaceae
300
200
100
0
A (n=15)
B (n=16)
C (n=14)
surface

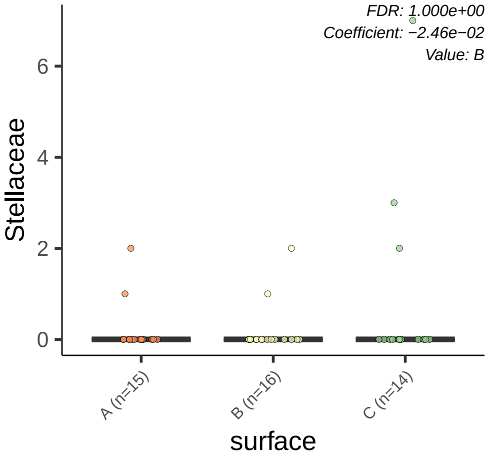
FDR: 1.000e+00
Coefficient: −2.46e−02
Value: B
Stellaceae
6
4
2
0
A (n=15)
B (n=16)
C (n=14)
surface

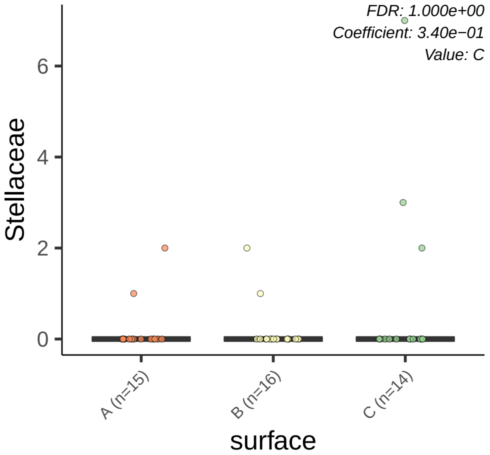

FDR: 1.000e+00
Coefficient: 3.40e−01
Value: C
Stellaceae
6
4
2
0
A (n=15)
B (n=16)
C (n=14)
surface

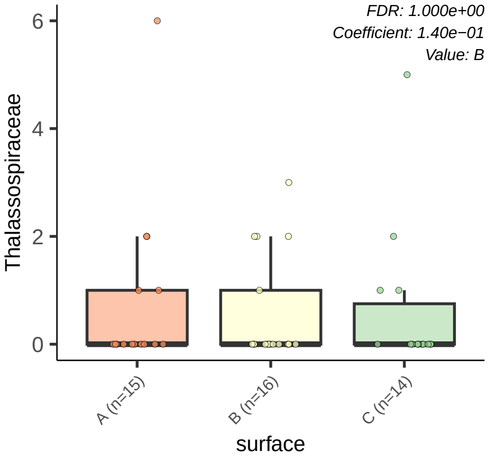
FDR: 1.000e+00
Coefficient: 1.40e−01
Value: B
Thalassospiraceae
6
4
2
0
A (n=15)
B (n=16)
C (n=14)
surface

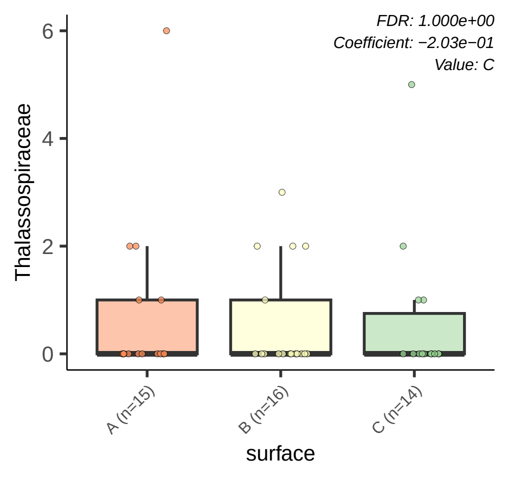
FDR: 1.000e+00
Coefficient: −2.03e−01
Value: C
Thalassospiraceae
0
2
4
6
A (n=15)
B (n=16)
C (n=14)
surface

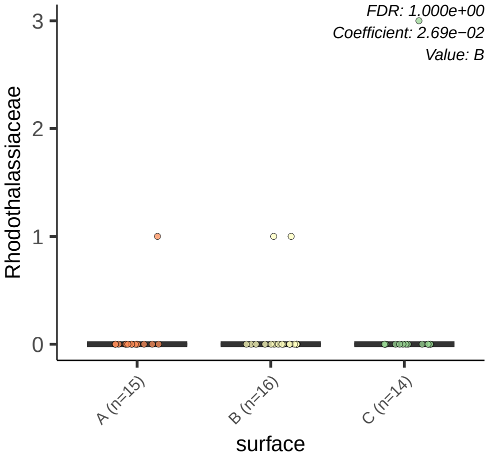
FDR: 1.000e+00
Coefficient: 2.69e−02
Value: B
Rhodothalassiaceae
3
2
1
0
A (n=15)
B (n=16)
C (n=14)
surface

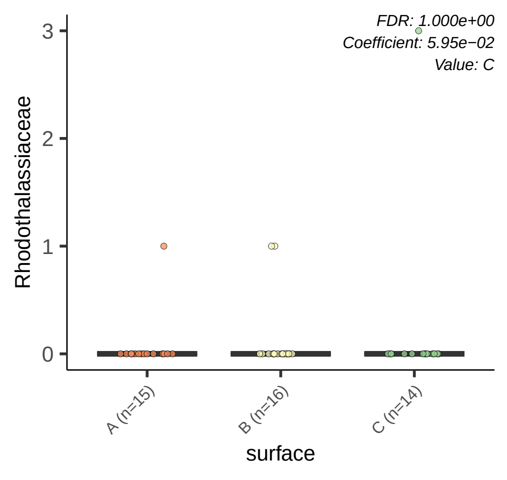

FDR: 1.000e+00
Coefficient: 5.95e−02
Value: C
Rhodothalassiaceae
3
2
1
0
A (n=15)
B (n=16)
C (n=14)
surface

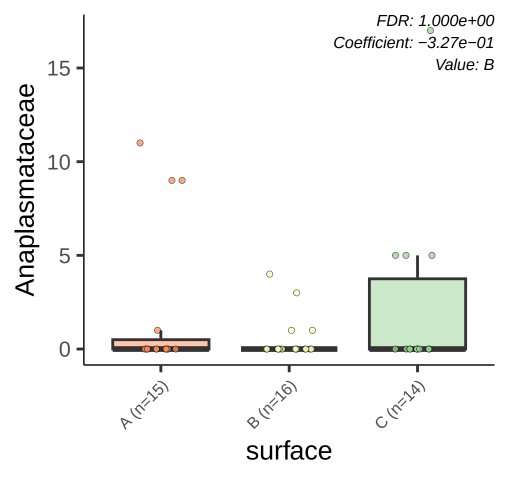
FDR: 1.000e+00
Coefficient: −3.27e−01
Value: B
Anaplasmataceae
15
10
5
0
A (n=15)
B (n=16)
C (n=14)
surface

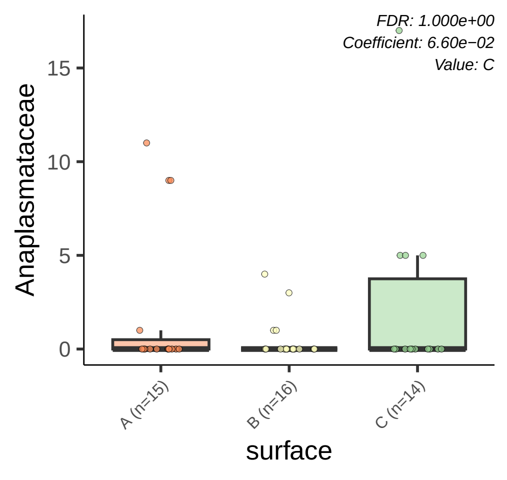
FDR: 1.000e+00
Coefficient: 6.60e−02
Value: C
Anaplasmataceae
15
10
5
0
A (n=15)
B (n=16)
C (n=14)
surface

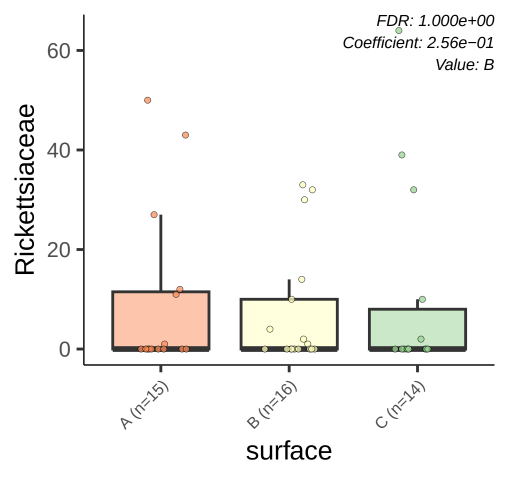

FDR: 1.000e+00
Coefficient: 2.56e−01
Value: B
Rickettsiaceae
0
20
40
60
A (n=15)
B (n=16)
C (n=14)
surface

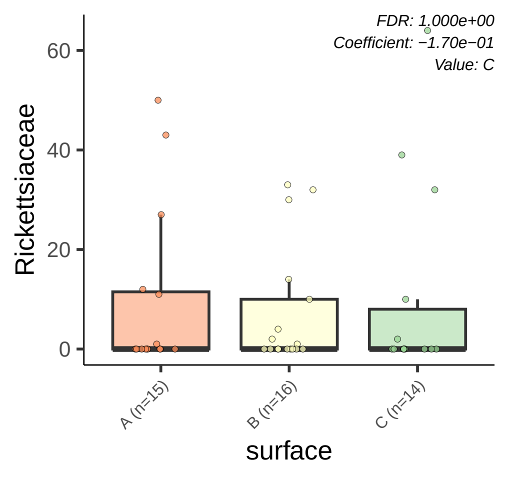
FDR: 1.000e+00
Coefficient: −1.70e−01
Value: C
Rickettsiaceae
0
20
40
60
A (n=15)
B (n=16)
C (n=14)
surface

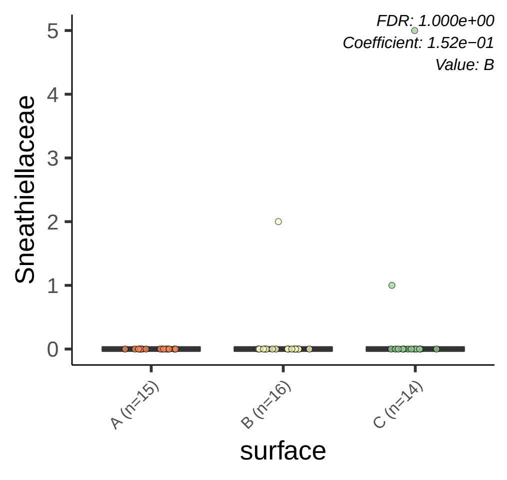
FDR: 1.000e+00
Coefficient: 1.52e−01
Value: B
Sneathiellaceae
0
1
2
3
4
5
A (n=15)
B (n=16)
C (n=14)
surface

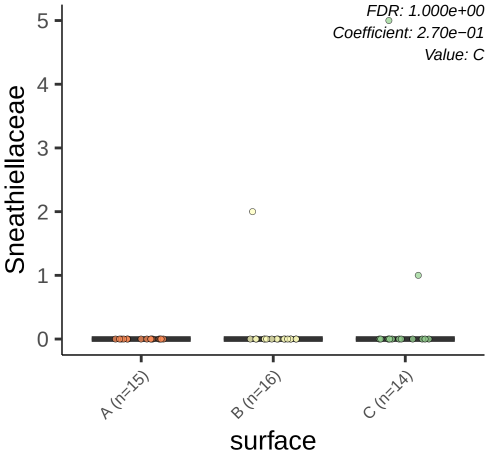
FDR: 1.000e+00
Coefficient: 2.70e−01
Value: C
Sneathiellaceae
0
1
2
3
4
5
A (n=15)
B (n=16)
C (n=14)
surface

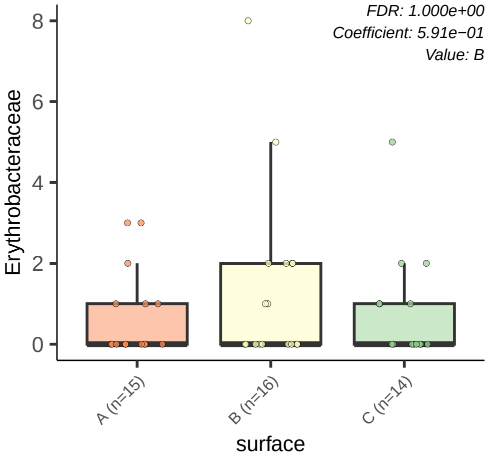
FDR: 1.000e+00
Coefficient: 5.91e−01
Value: B
Erythrobacteraceae
0
2
4
6
8
A (n=15)
B (n=16)
C (n=14)
surface

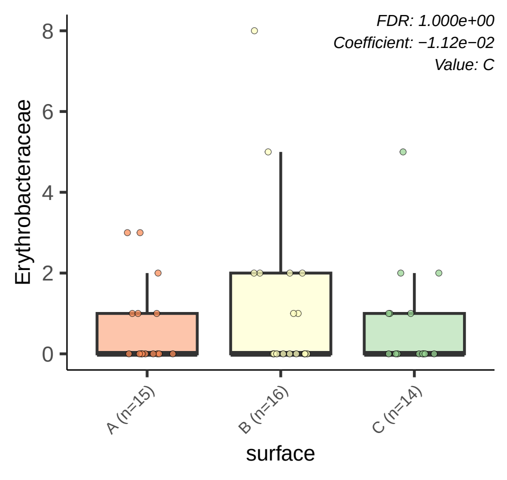

FDR: 1.000e+00
Coefficient: −1.12e−02
Value: C
Erythrobacteraceae
8
6
4
2
0
A (n=15)
B (n=16)
C (n=14)
surface

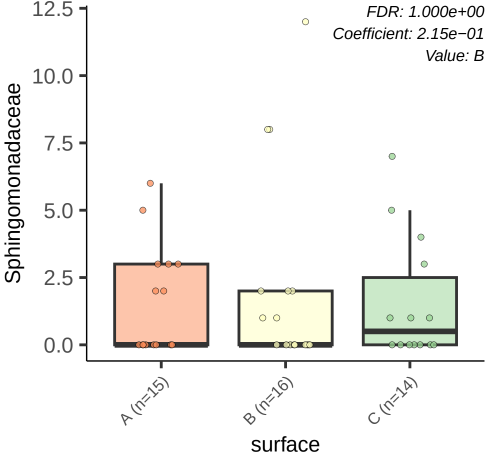
FDR: 1.000e+00
Coefficient: 2.15e−01
Value: B
Sphingomonadaceae
12.5
10.0
7.5
5.0
2.5
0.0
A (n=15)
B (n=16)
C (n=14)
surface

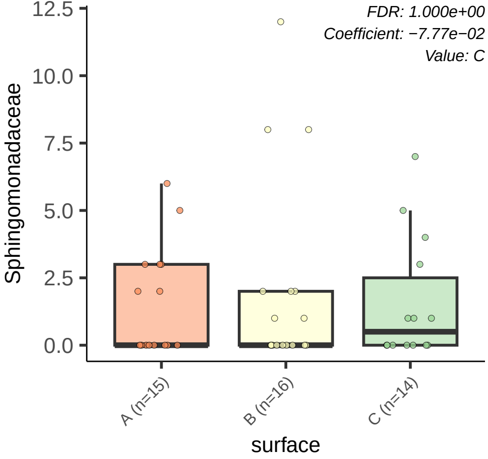

FDR: 1.000e+00
Coefficient: −7.77e−02
Value: C
Sphingomonadaceae
12.5
10.0
7.5
5.0
2.5
0.0
A (n=15)
B (n=16)
C (n=14)
surface

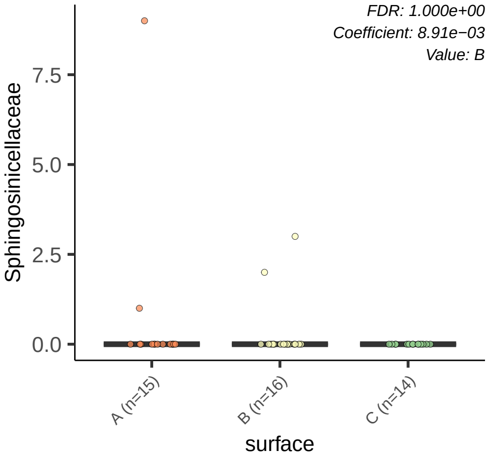
FDR: 1.000e+00
Coefficient: 8.91e−03
Value: B
Sphingosinicellaceae
7.5
5.0
2.5
0.0
A (n=15)
B (n=16)
C (n=14)
surface

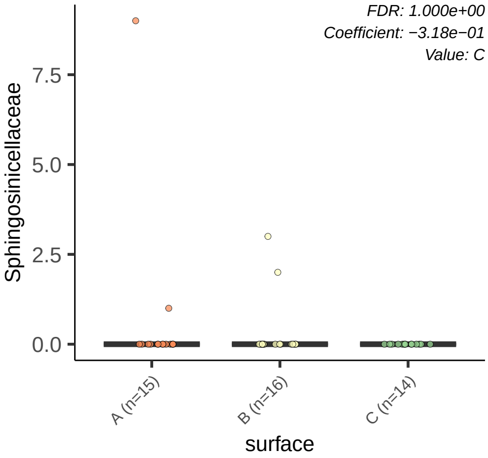

FDR: 1.000e+00
Coefficient: −3.18e−01
Value: C
Sphingosinicellaceae
7.5
5.0
2.5
0.0
A (n=15)
B (n=16)
C (n=14)
surface

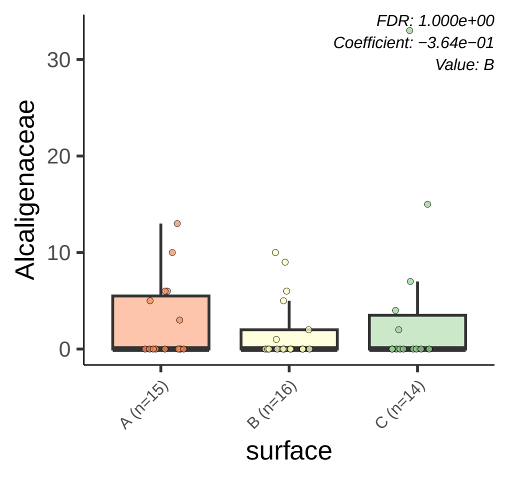
FDR: 1.000e+00
Coefficient: −3.64e−01
Value: B
Alcaligenaceae
30
20
10
0
A (n=15)
B (n=16)
C (n=14)
surface

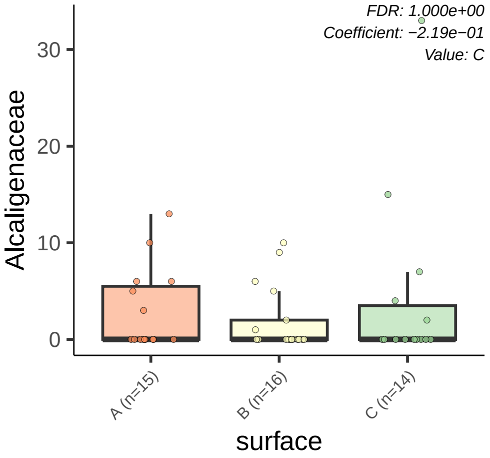
FDR: 1.000e+00
Coefficient: −2.19e−01
Value: C
Alcaligenaceae
30
20
10
0
A (n=15)
B (n=16)
C (n=14)
surface

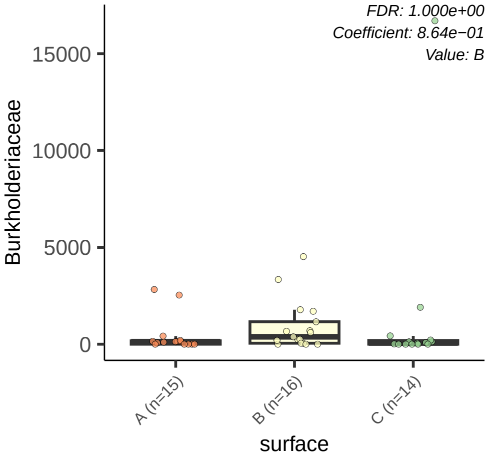

FDR: 1.000e+00
Coefficient: 8.64e−01
Value: B
Burkholderiaceae
15000
10000
5000
0
A (n=15)
B (n=16)
C (n=14)
surface

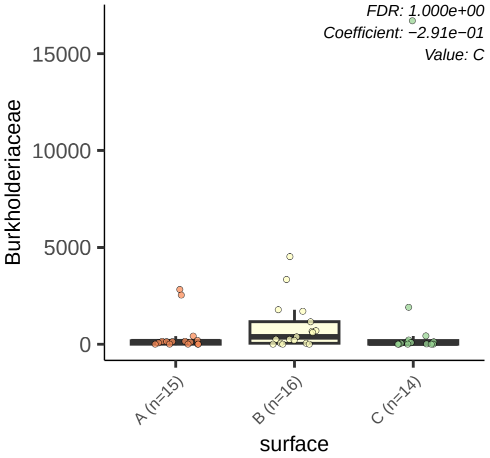
FDR: 1.000e+00
Coefficient: −2.91e−01
Value: C
15000
10000
5000
0
Burkholderiaceae
A (n=15)
B (n=16)
C (n=14)
surface

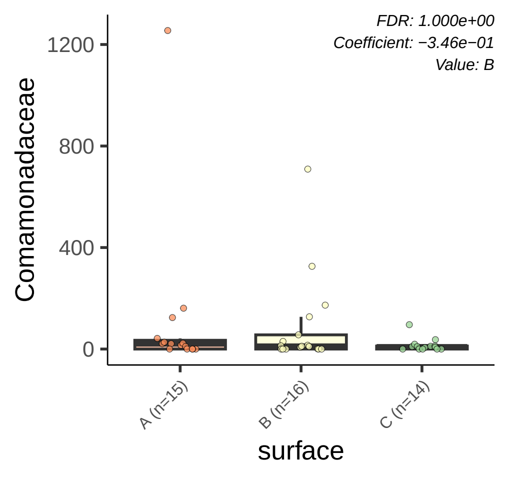
FDR: 1.000e+00
Coefficient: −3.46e−01
Value: B
1200
800
400
0
Comamonadaceae
A (n=15)
B (n=16)
C (n=14)
surface

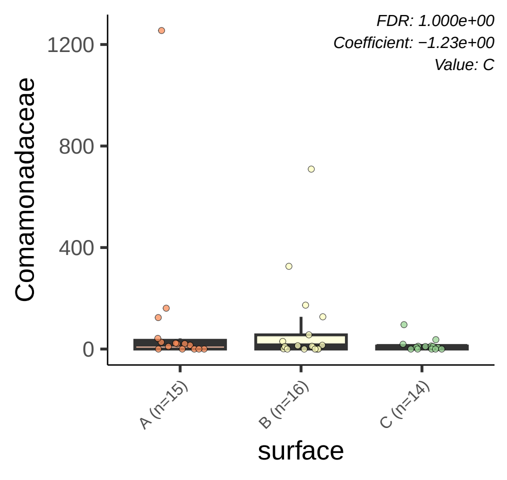
FDR: 1.000e+00
Coefficient: −1.23e+00
Value: C
Comamonadaceae
1200
800
400
0
A (n=15)
B (n=16)
C (n=14)
surface

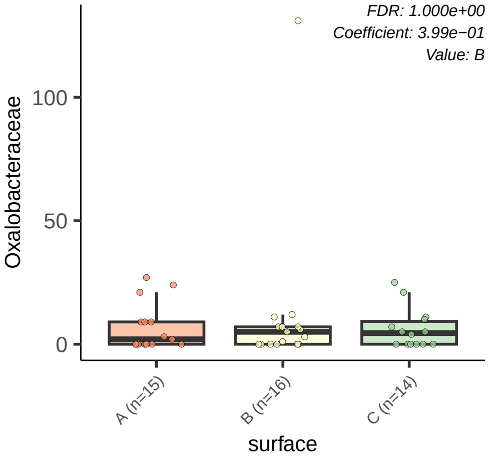

FDR: 1.000e+00
Coefficient: 3.99e−01
Value: B
Oxalobacteraceae
100
50
0
A (n=15)
B (n=16)
C (n=14)
surface

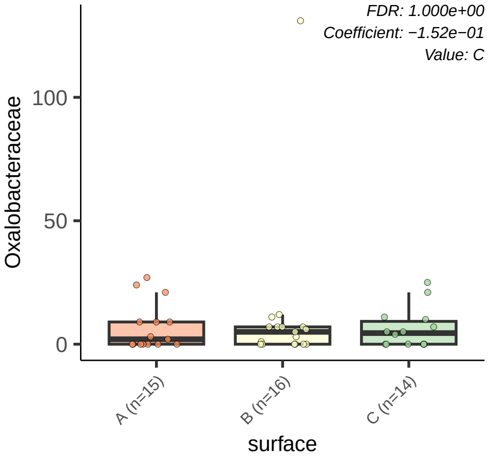

FDR: 1.000e+00
Coefficient: −1.52e−01
Value: C
Oxalobacteraceae
100
50
0
A (n=15)
B (n=16)
C (n=14)
surface

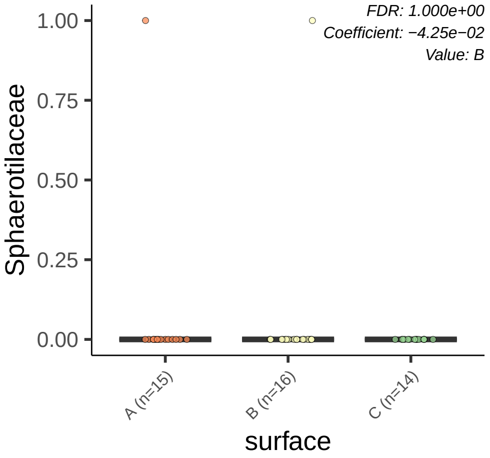
FDR: 1.000e+00
Coefficient: −4.25e−02
Value: B
Sphaerotilaceae
1.00
0.75
0.50
0.25
0.00
A (n=15)
B (n=16)
C (n=14)
surface

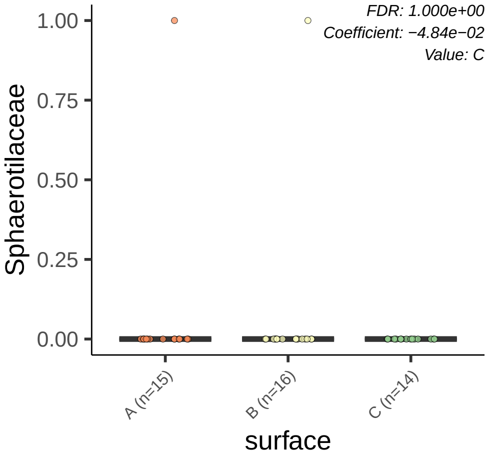
FDR: 1.000e+00
Coefficient: −4.84e−02
Value: C
1.00
0.75
0.50
0.25
0.00
Sphaerotilaceae
A (n=15)
B (n=16)
C (n=14)
surface

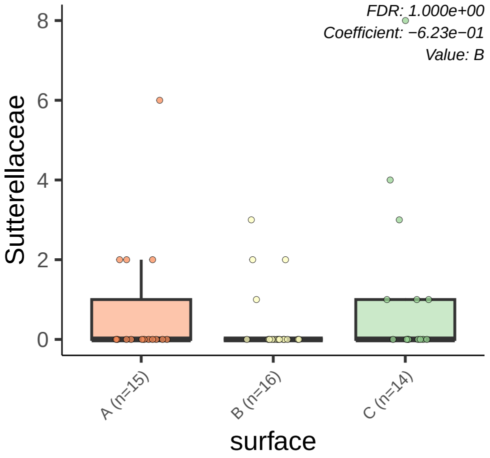

FDR: 1.000e+00
Coefficient: −6.23e−01
Value: B
Sutterellaceae
8
6
4
2
0
A (n=15)
B (n=16)
C (n=14)
surface

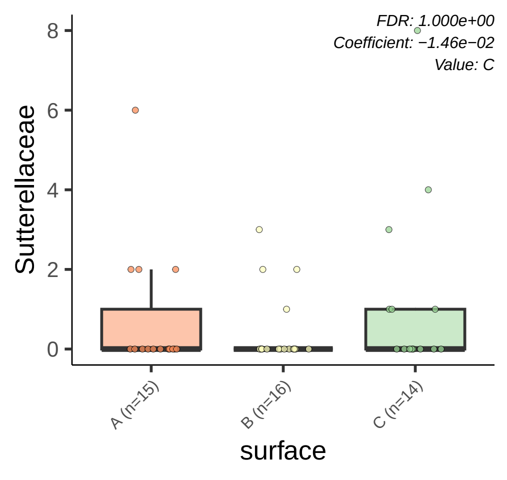
FDR: 1.000e+00
Coefficient: −1.46e−02
Value: C
Sutterellaceae
0
2
4
6
8
A (n=15)
B (n=16)
C (n=14)
surface

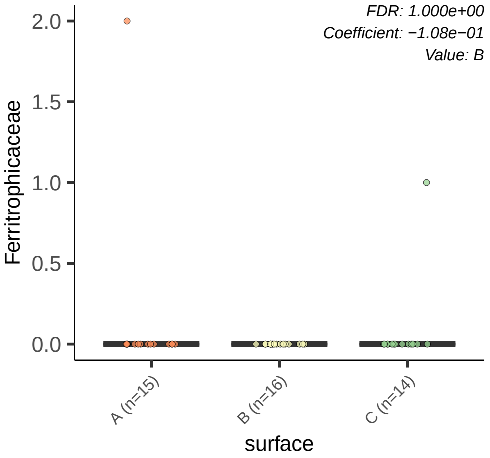
FDR: 1.000e+00
Coefficient: −1.08e−01
Value: B
Ferritrophicaceae
2.0
1.5
1.0
0.5
0.0
A (n=15)
B (n=16)
C (n=14)
surface

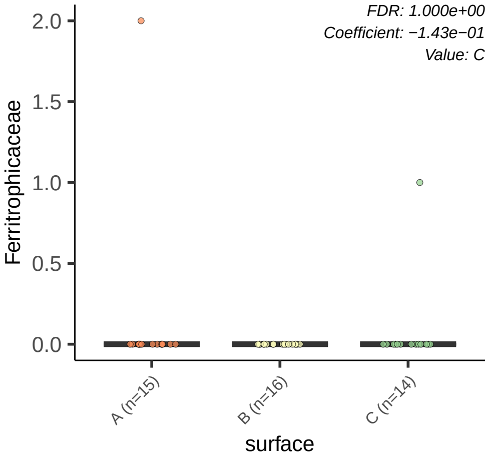

FDR: 1.000e+00
Coefficient: −1.43e−01
Value: C
Ferritrophicaceae
2.0
1.5
1.0
0.5
0.0
A (n=15)
B (n=16)
C (n=14)
surface

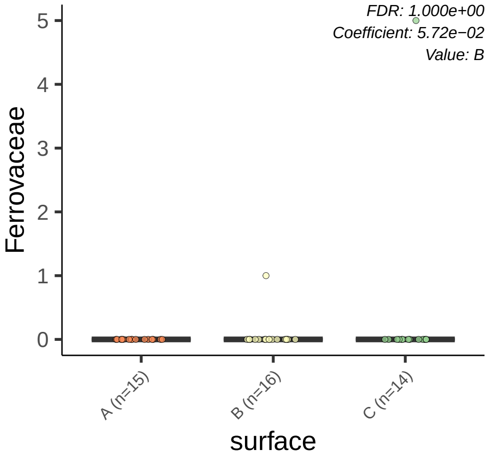

FDR: 1.000e+00
Coefficient: 5.72e−02
Value: B
Ferrovaceae
0
1
2
3
4
5
A (n=15)
B (n=16)
C (n=14)
surface

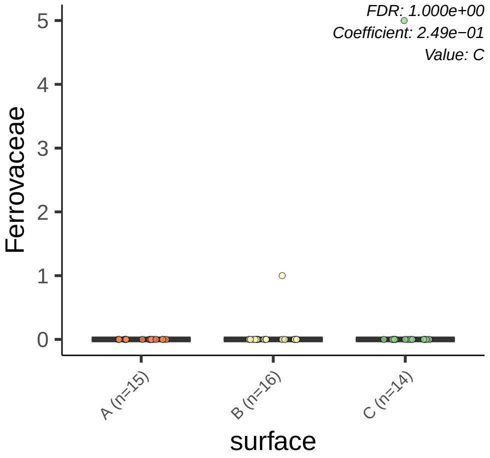
FDR: 1.000e+00
Coefficient: 2.49e−01
Value: C
Ferrovaceae
0
1
2
3
4
5
A (n=15)
B (n=16)
C (n=14)
surface

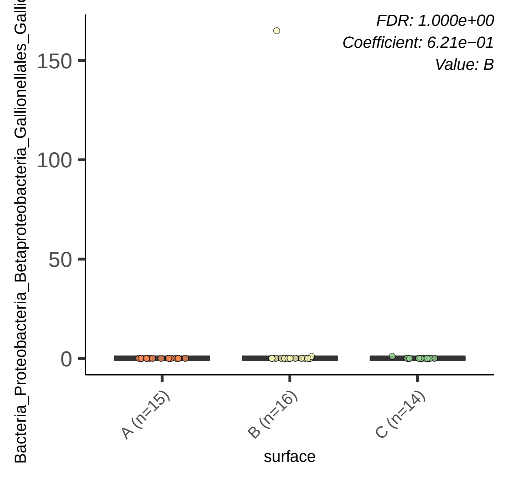
FDR: 1.000e+00
Coefficient: 6.21e−01
Value: B
Bacteria_Proteobacteria_Betaproteobacteria_Gallionellales_Gallic
150
100
50
0
A (n=15)
B (n=16)
C (n=14)
surface

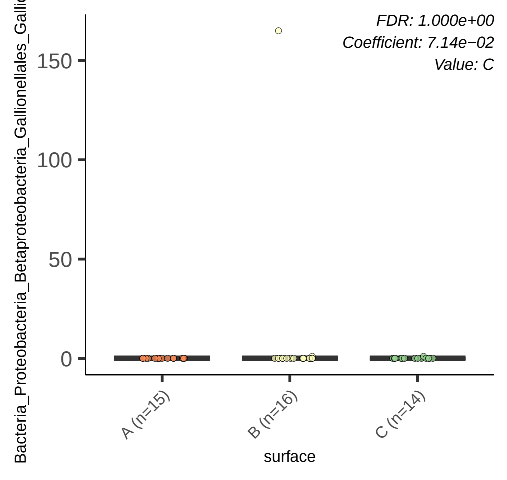

FDR: 1.000e+00
Coefficient: 7.14e−02
Value: C
Bacteria_Proteobacteria_Betaproteobacteria_Gallionellales_Gallic
150
100
50
0
A (n=15)
B (n=16)
C (n=14)
surface

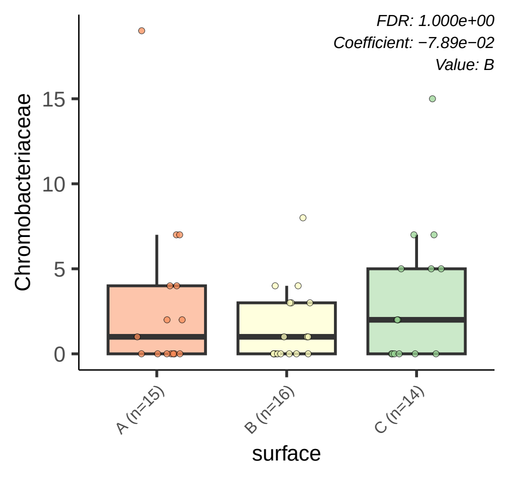

FDR: 1.000e+00
Coefficient: −7.89e−02
Value: B
Chromobacteriaceae
0
5
10
15
A (n=15)
B (n=16)
C (n=14)
surface

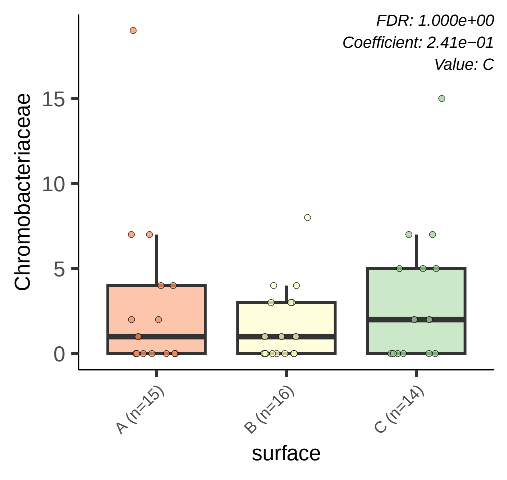
FDR: 1.000e+00
Coefficient: 2.41e−01
Value: C
Chromobacteriaceae
0
5
10
15
A (n=15)
B (n=16)
C (n=14)
surface

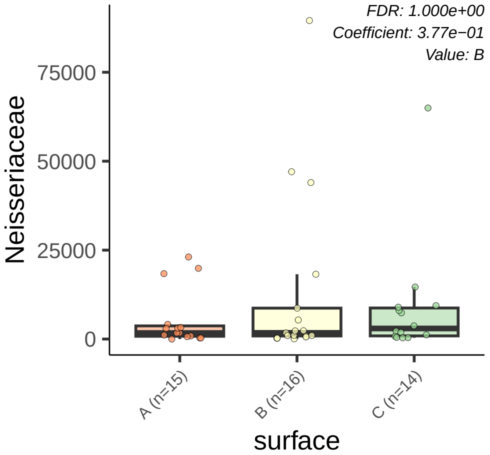

FDR: 1.000e+00
Coefficient: 3.77e−01
Value: B
Neisseriaceae
75000
50000
25000
0
A (n=15)
B (n=16)
C (n=14)
surface

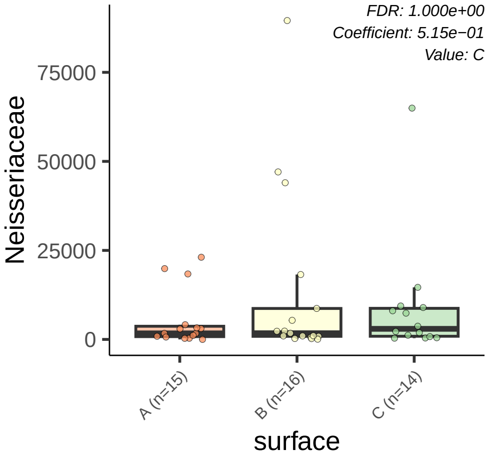
FDR: 1.000e+00
Coefficient: 5.15e−01
Value: C
Neisseriaceae
75000
50000
25000
0
A (n=15)
B (n=16)
C (n=14)
surface

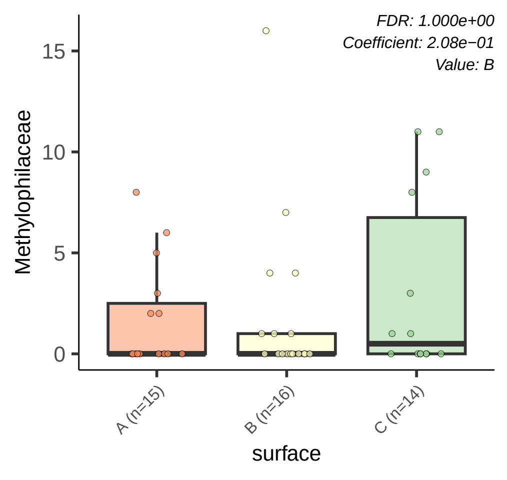
FDR: 1.000e+00
Coefficient: 2.08e−01
Value: B
Methylophilaceae
0
5
10
15
A (n=15)
B (n=16)
C (n=14)
surface

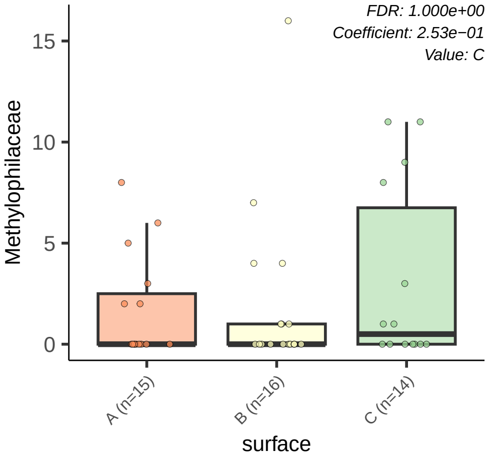

FDR: 1.000e+00
Coefficient: 2.53e−01
Value: C
Methylophilaceae
0
5
10
15
A (n=15)
B (n=16)
C (n=14)
surface

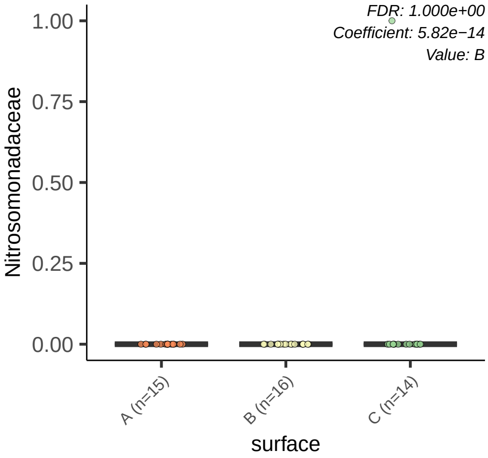

FDR: 1.000e+00
Coefficient: 5.82e−14
Value: B
Nitrosomonadaceae
1.00
0.75
0.50
0.25
0.00
A (n=15)
B (n=16)
C (n=14)
surface

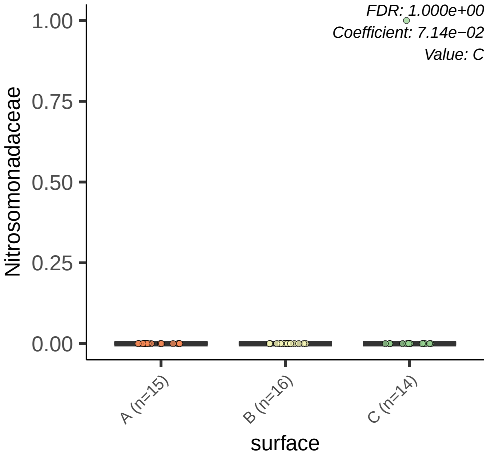

FDR: 1.000e+00
Coefficient: 7.14e−02
Value: C
1.00
0.75
0.50
0.25
0.00
Nitrosomonadaceae
A (n=15)
B (n=16)
C (n=14)
surface

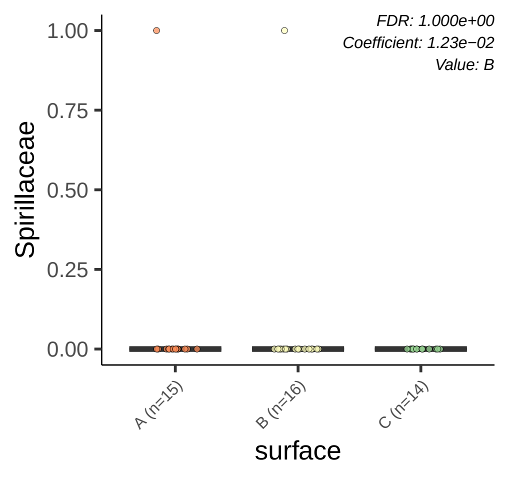
FDR: 1.000e+00
Coefficient: 1.23e−02
Value: B
Spirillaceae
1.00
0.75
0.50
0.25
0.00
A (n=15)
B (n=16)
C (n=14)
surface

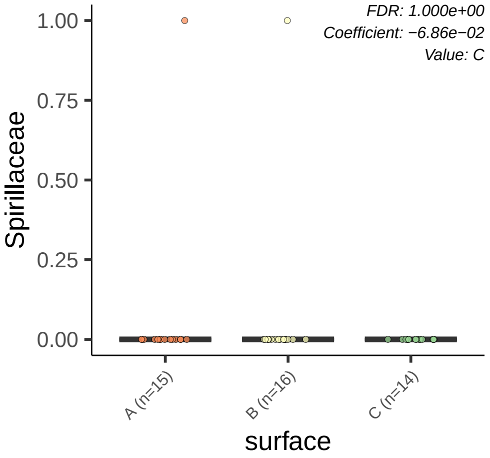

FDR: 1.000e+00
Coefficient: −6.86e−02
Value: C
Spirillaceae
1.00
0.75
0.50
0.25
0.00
A (n=15)
B (n=16)
C (n=14)
surface

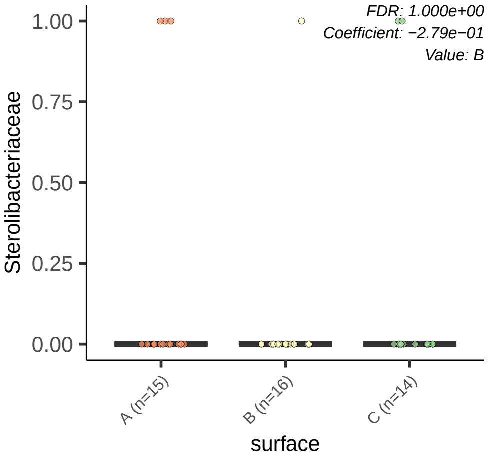

FDR: 1.000e+00
Coefficient: −2.79e−01
Value: B
Sterolibacteriaceae
1.00
0.75
0.50
0.25
0.00
A (n=15)
B (n=16)
C (n=14)
surface

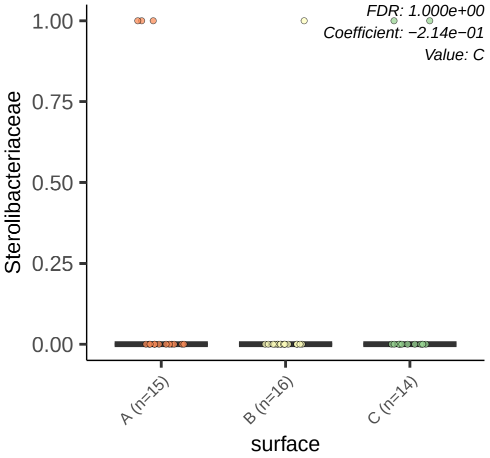
FDR: 1.000e+00
Coefficient: −2.14e−01
Value: C
Sterolibacteriaceae
1.00
0.75
0.50
0.25
0.00
A (n=15)
B (n=16)
C (n=14)
surface

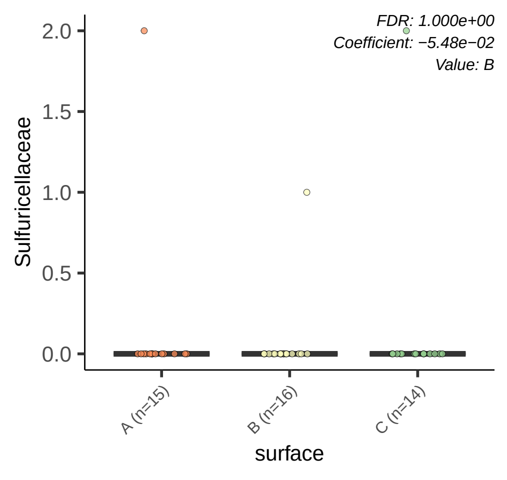
FDR: 1.000e+00
Coefficient: −5.48e−02
Value: B
Sulfuricellaceae
2.0
1.5
1.0
0.5
0.0
A (n=15)
B (n=16)
C (n=14)
surface

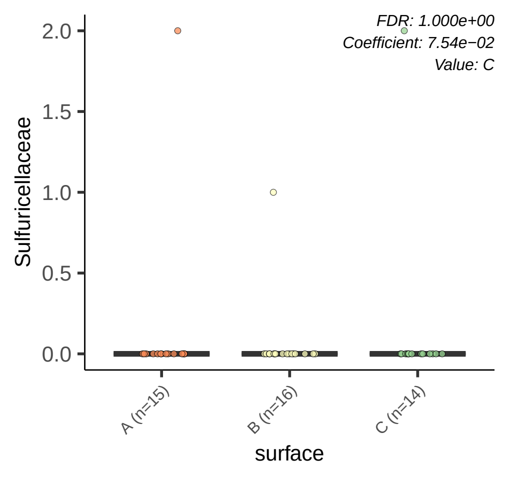

FDR: 1.000e+00
Coefficient: 7.54e−02
Value: C
Sulfuricellaceae
2.0
1.5
1.0
0.5
0.0
A (n=15)
B (n=16)
C (n=14)
surface

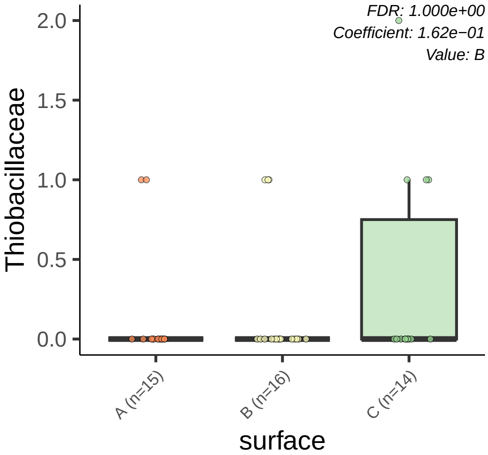

FDR: 1.000e+00
Coefficient: 1.62e−01
Value: B
Thiobacillaceae
2.0
1.5
1.0
0.5
0.0
A (n=15)
B (n=16)
C (n=14)
surface

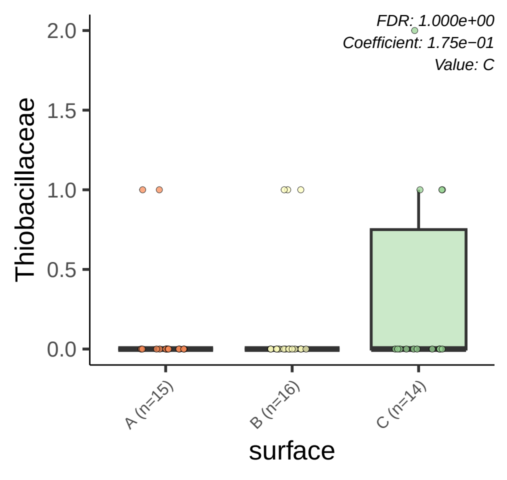

FDR: 1.000e+00
Coefficient: 1.75e−01
Value: C
Thiobacillaceae
2.0
1.5
1.0
0.5
0.0
A (n=15)
B (n=16)
C (n=14)
surface

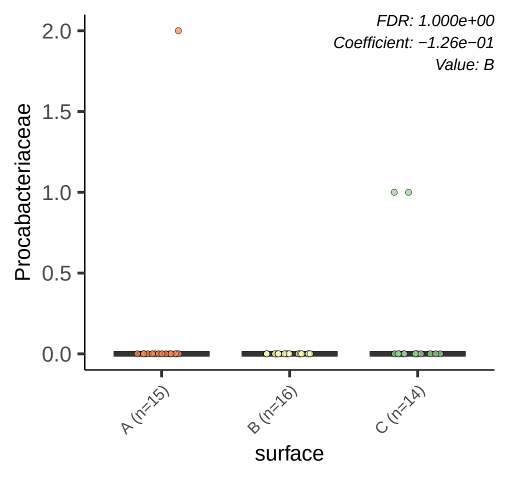
FDR: 1.000e+00
Coefficient: −1.26e−01
Value: B
Procabacteriaceae
2.0
1.5
1.0
0.5
0.0
A (n=15)
B (n=16)
C (n=14)
surface

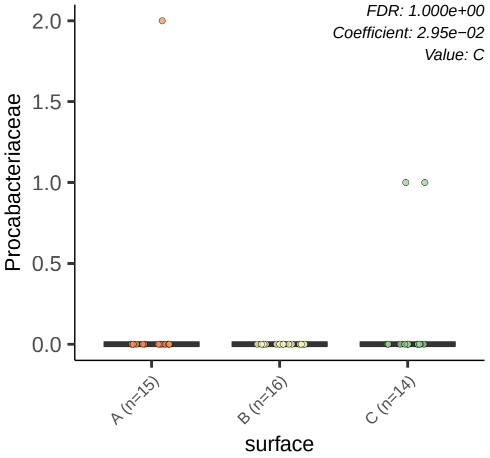

FDR: 1.000e+00
Coefficient: 2.95e−02
Value: C
Procabacteriaceae
2.0
1.5
1.0
0.5
0.0
A (n=15)
B (n=16)
C (n=14)
surface

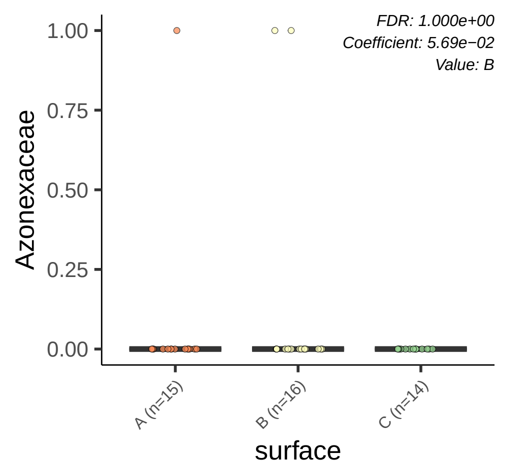

FDR: 1.000e+00
Coefficient: 5.69e−02
Value: B
Azonexaceae
1.00
0.75
0.50
0.25
0.00
A (n=15)
B (n=16)
C (n=14)
surface

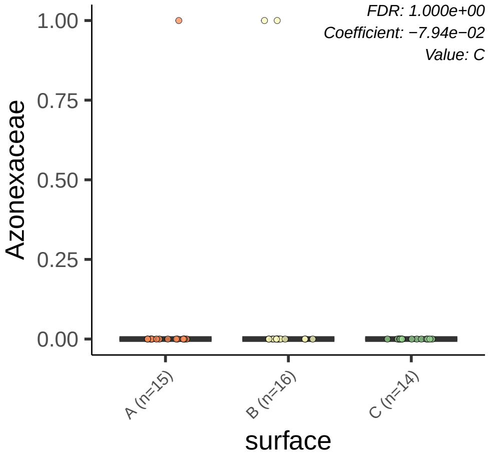

FDR: 1.000e+00
Coefficient: −7.94e−02
Value: C
Azonexaceae
1.00
0.75
0.50
0.25
0.00
A (n=15)
B (n=16)
C (n=14)
surface

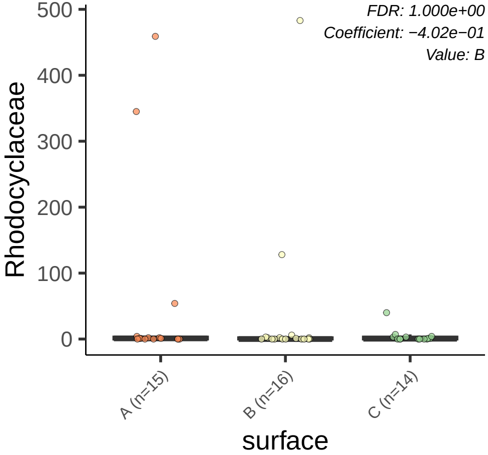
FDR: 1.000e+00
Coefficient: −4.02e−01
Value: B
Rhodocyclaceae
500
400
300
200
100
0
A (n=15)
B (n=16)
C (n=14)
surface

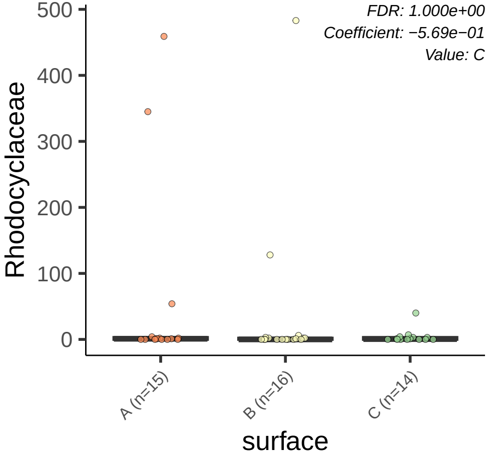

FDR: 1.000e+00
Coefficient: −5.69e−01
Value: C
Rhodocyclaceae
500
400
300
200
100
0
A (n=15)
B (n=16)
C (n=14)
surface

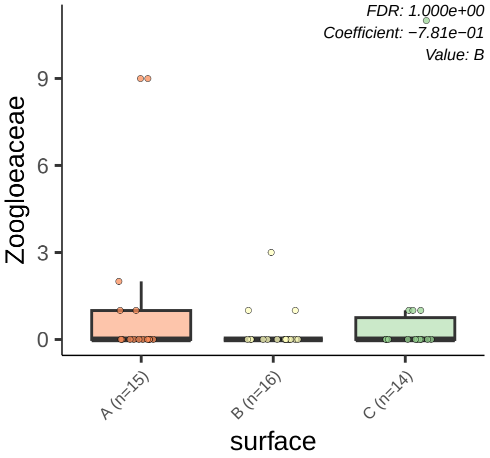
FDR: 1.000e+00
Coefficient: −7.81e−01
Value: B
Zoogloeaceae
9
6
3
0
A (n=15)
B (n=16)
C (n=14)
surface

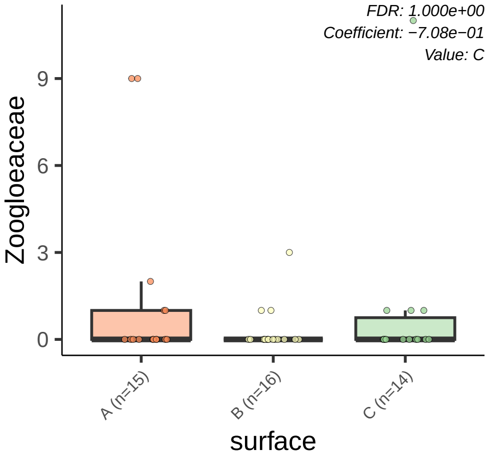

FDR: 1.000e+00
Coefficient: −7.08e−01
Value: C
Zoogloeaceae
0
3
6
9
A (n=15)
B (n=16)
C (n=14)
surface

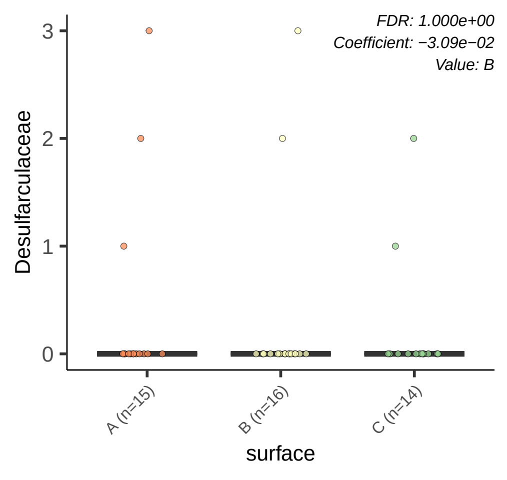

FDR: 1.000e+00
Coefficient: −3.09e−02
Value: B
Desulfarculaceae
3
2
1
0
A (n=15)
B (n=16)
C (n=14)
surface

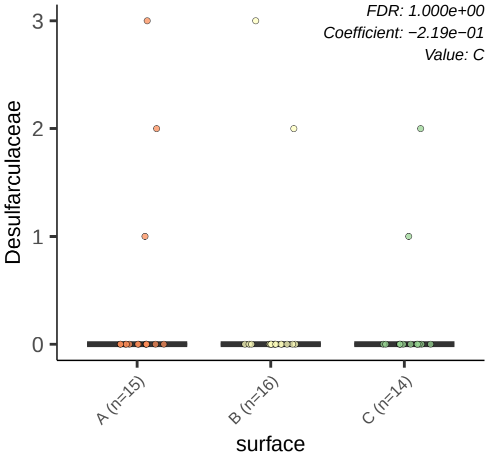
FDR: 1.000e+00
Coefficient: −2.19e−01
Value: C
Desulfarculaceae
3
2
1
0
A (n=15)
B (n=16)
C (n=14)
surface

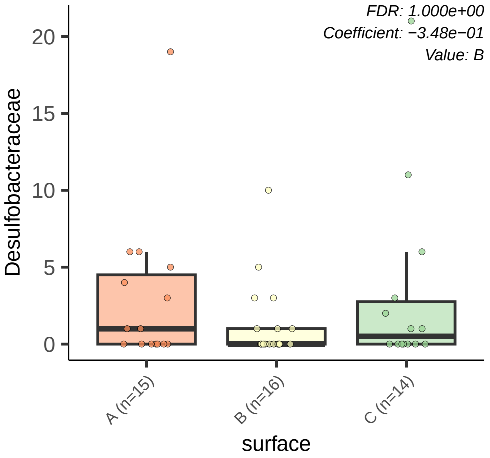
FDR: 1.000e+00
Coefficient: −3.48e−01
Value: B
Desulfobacteraceae
0
5
10
15
20
A (n=15)
B (n=16)
C (n=14)
surface

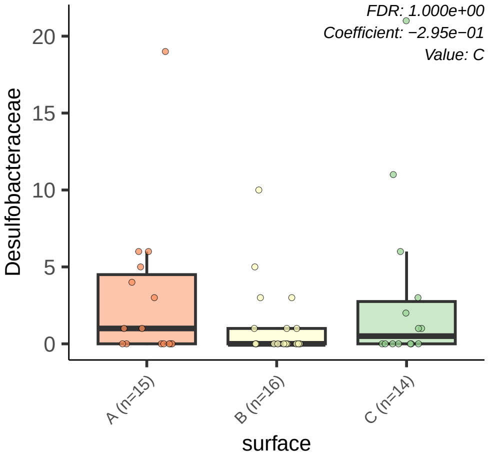

FDR: 1.000e+00
Coefficient: −2.95e−01
Value: C
Desulfobacteraceae
0
5
10
15
20
A (n=15)
B (n=16)
C (n=14)
surface

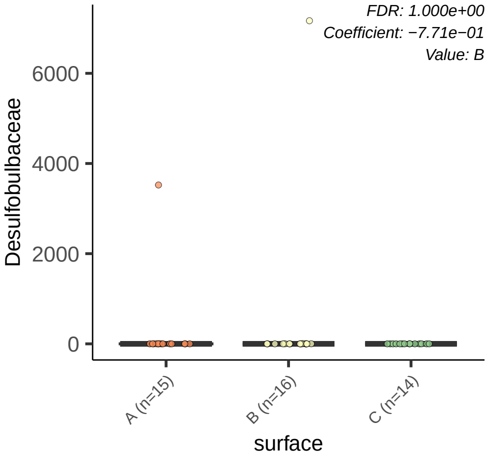
FDR: 1.000e+00
Coefficient: −7.71e−01
Value: B
Desulfobulbaceae
6000
4000
2000
0
A (n=15)
B (n=16)
C (n=14)
surface

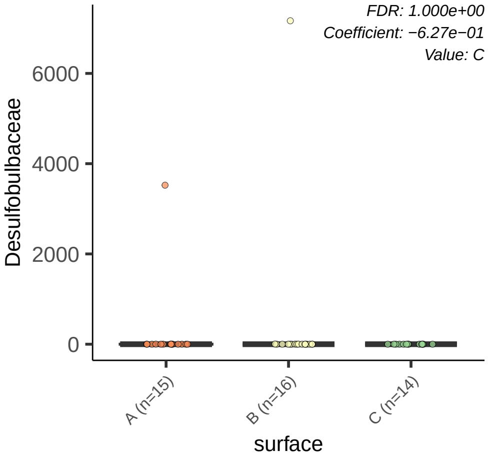
FDR: 1.000e+00
Coefficient: −6.27e−01
Value: C
Desulfobulbaceae
6000
4000
2000
0
A (n=15)
B (n=16)
C (n=14)
surface

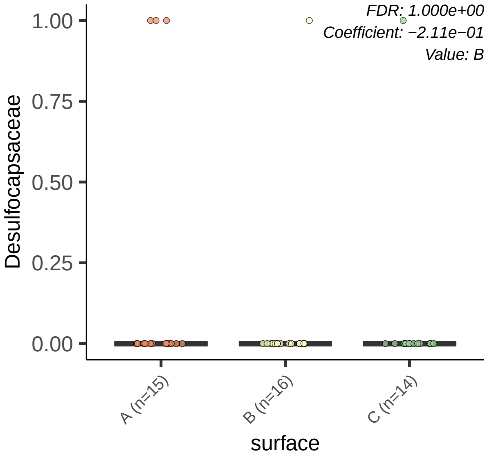
FDR: 1.000e+00
Coefficient: −2.11e−01
Value: B
Desulfocapsaceae
1.00
0.75
0.50
0.25
0.00
A (n=15)
B (n=16)
C (n=14)
surface

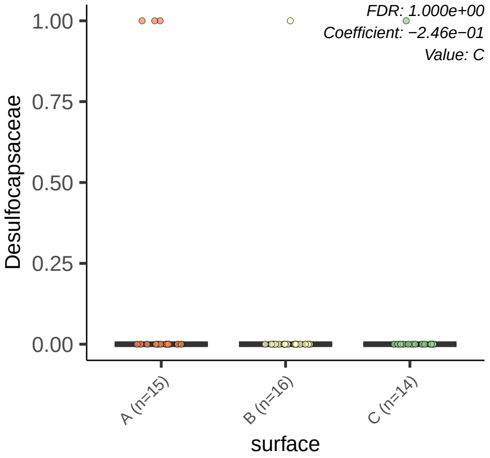
FDR: 1.000e+00
Coefficient: −2.46e−01
Value: C
Desulfocapsaceae
1.00
0.75
0.50
0.25
0.00
A (n=15)
B (n=16)
C (n=14)
surface

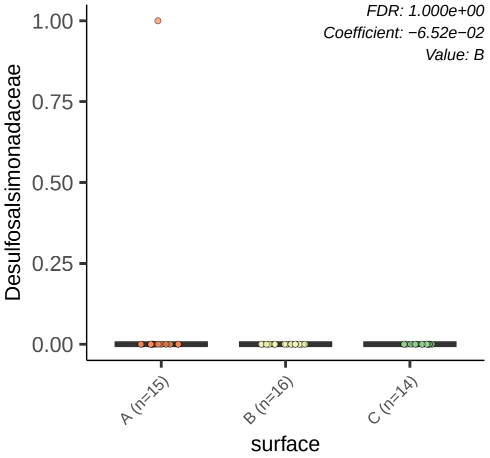
FDR: 1.000e+00
Coefficient: −6.52e−02
Value: B
Desulfosalsimonadaceae
1.00
0.75
0.50
0.25
0.00
A (n=15)
B (n=16)
C (n=14)
surface

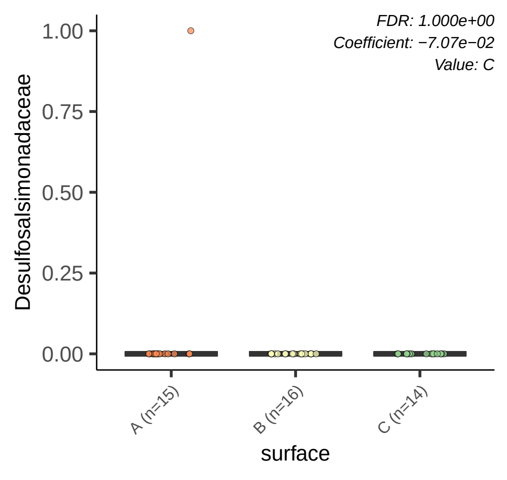

FDR: 1.000e+00
Coefficient: −7.07e−02
Value: C
Desulfosalsimonadaceae
1.00
0.75
0.50
0.25
0.00
A (n=15)
B (n=16)
C (n=14)
surface

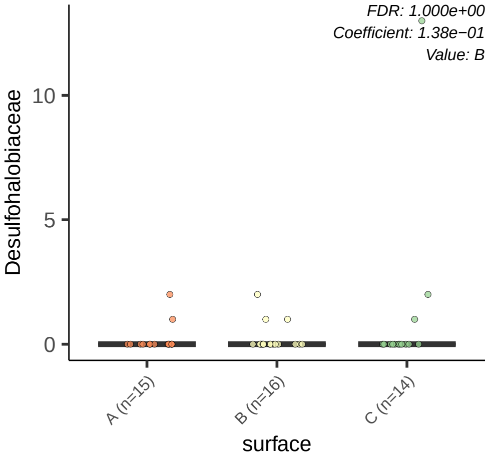

FDR: 1.000e+00
Coefficient: 1.38e−01
Value: B
Desulfohalobiaceae
10
5
0
A (n=15)
B (n=16)
C (n=14)
surface

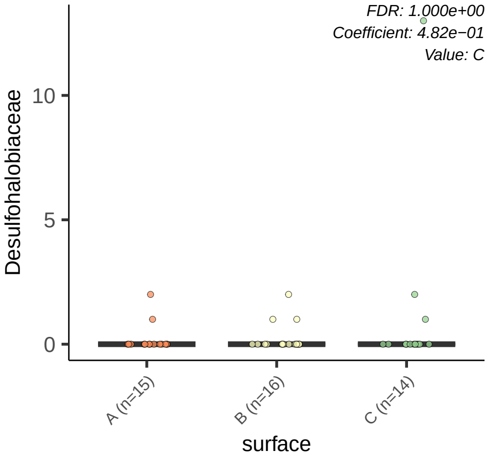

FDR: 1.000e+00
Coefficient: 4.82e−01
Value: C
Desulfohalobiaceae
10
5
0
A (n=15)
B (n=16)
C (n=14)
surface

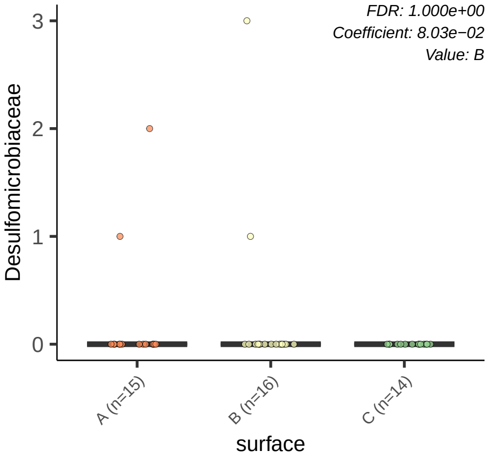

FDR: 1.000e+00
Coefficient: 8.03e−02
Value: B
Desulfomicrobiaceae
3
2
1
0
A (n=15)
B (n=16)
C (n=14)
surface

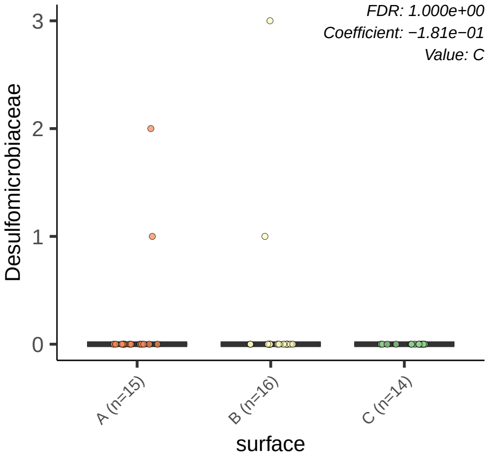

FDR: 1.000e+00
Coefficient: −1.81e−01
Value: C
Desulfomicrobiaceae
3
2
1
0
A (n=15)
B (n=16)
C (n=14)
surface

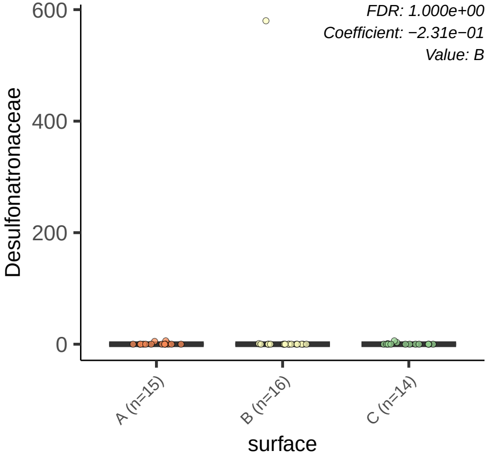
FDR: 1.000e+00
Coefficient: −2.31e−01
Value: B
Desulfonatronaceae
600
400
200
0
A (n=15)
B (n=16)
C (n=14)
surface

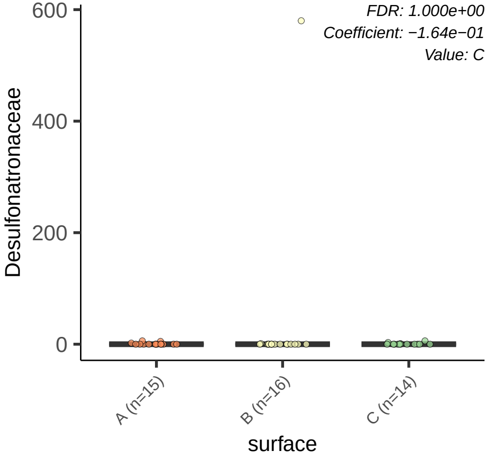
FDR: 1.000e+00
Coefficient: −1.64e−01
Value: C
Desulfonatronaceae
600
400
200
0
A (n=15)
B (n=16)
C (n=14)
surface

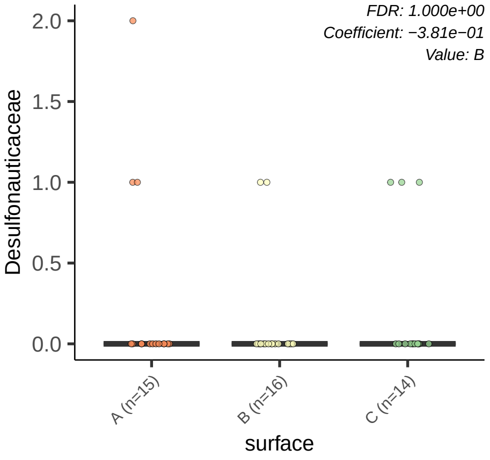
FDR: 1.000e+00
Coefficient: −3.81e−01
Value: B
Desulfonauticaceae
2.0
1.5
1.0
0.5
0.0
A (n=15)
B (n=16)
C (n=14)
surface

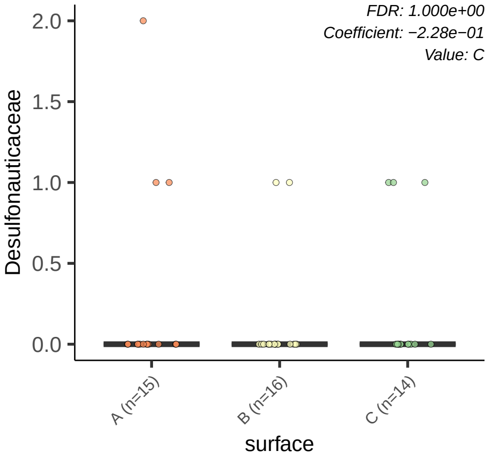
FDR: 1.000e+00
Coefficient: −2.28e−01
Value: C
2.0
1.5
1.0
0.5
0.0
Desulfonauticaceae
A (n=15)
B (n=16)
C (n=14)
surface

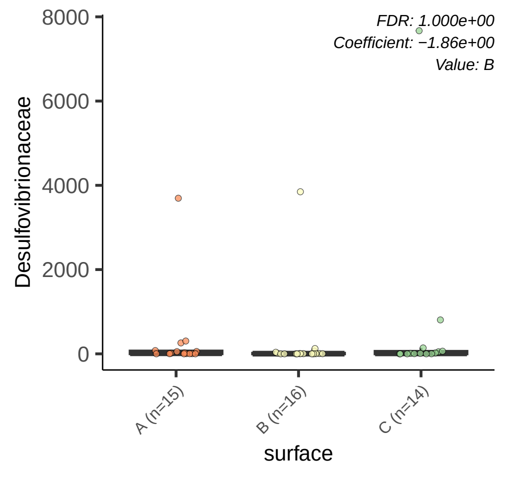
FDR: 1.000e+00
Coefficient: −1.86e+00
Value: B
Desulfovibrionaceae
8000
6000
4000
2000
0
A (n=15)
B (n=16)
C (n=14)
surface

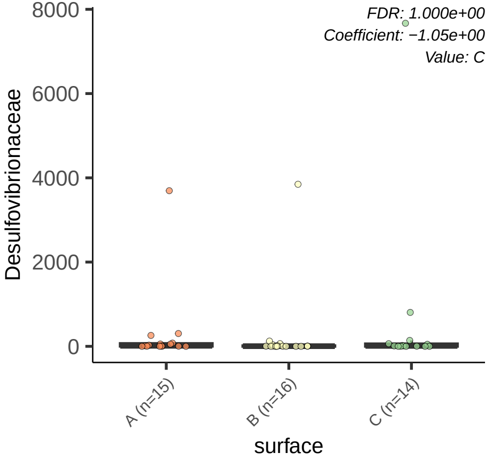

FDR: 1.000e+00
Coefficient: −1.05e+00
Value: C
8000
6000
4000
2000
0
Desulfovibrionaceae
A (n=15)
B (n=16)
C (n=14)
surface

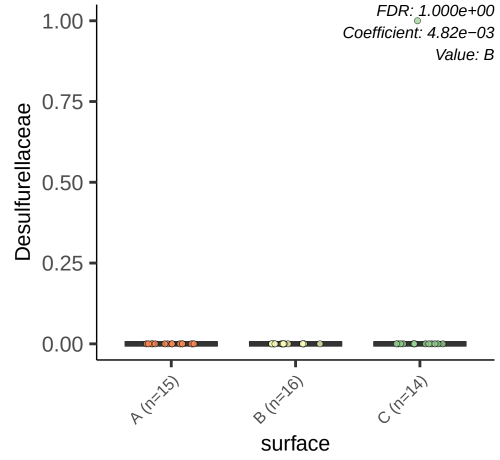

FDR: 1.000e+00
Coefficient: 4.82e−03
Value: B
1.00
0.75
0.50
0.25
0.00
Desulfurellaceae
A (n=15)
B (n=16)
C (n=14)
surface

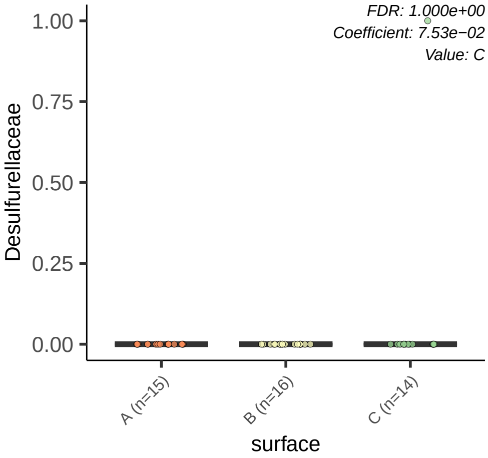
FDR: 1.000e+00
Coefficient: 7.53e−02
Value: C
Desulfurellaceae
1.00
0.75
0.50
0.25
0.00
A (n=15)
B (n=16)
C (n=14)
surface

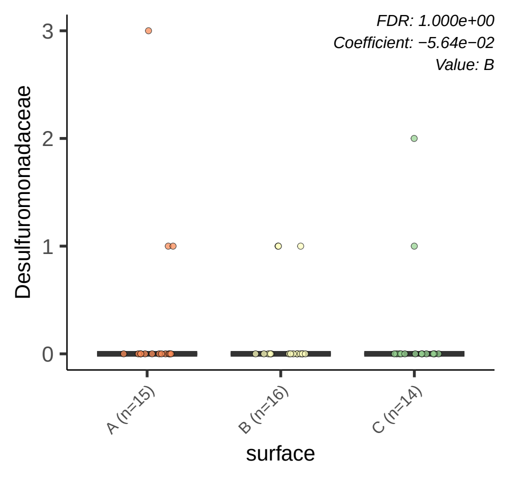
FDR: 1.000e+00
Coefficient: −5.64e−02
Value: B
Desulfuromonadaceae
0
1
2
3
A (n=15)
B (n=16)
C (n=14)
surface

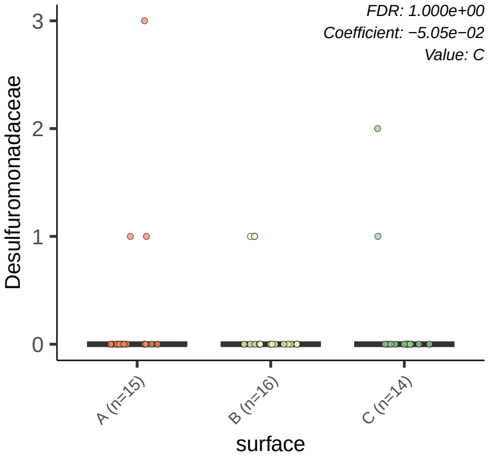

FDR: 1.000e+00
Coefficient: −5.05e−02
Value: C
Desulfuromonadaceae
3
2
1
0
A (n=15)
B (n=16)
C (n=14)
surface

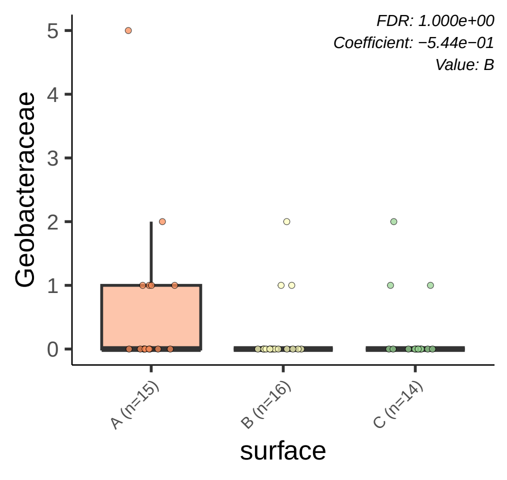

FDR: 1.000e+00
Coefficient: −5.44e−01
Value: B
Geobacteraceae
0
1
2
3
4
5
A (n=15)
B (n=16)
C (n=14)
surface

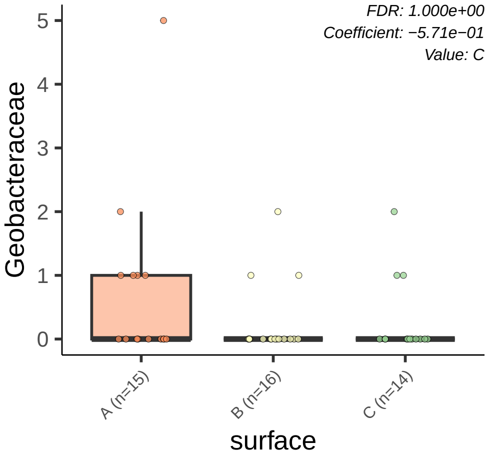
FDR: 1.000e+00
Coefficient: −5.71e−01
Value: C
Geobacteraceae
0
1
2
3
4
5
A (n=15)
B (n=16)
C (n=14)
surface

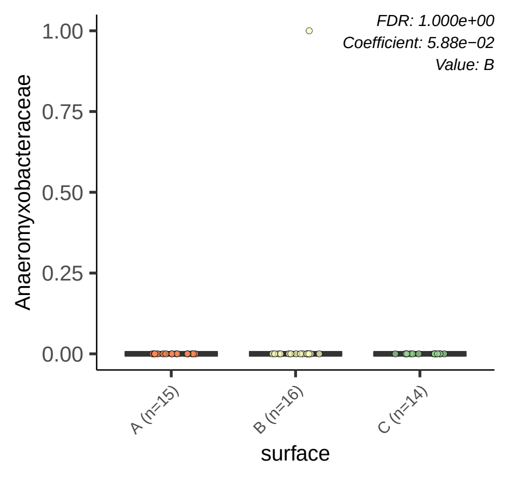
FDR: 1.000e+00
Coefficient: 5.88e−02
Value: B
1.00
0.75
0.50
0.25
0.00
Anaeromyxobacteraceae
A (n=15)
B (n=16)
C (n=14)
surface

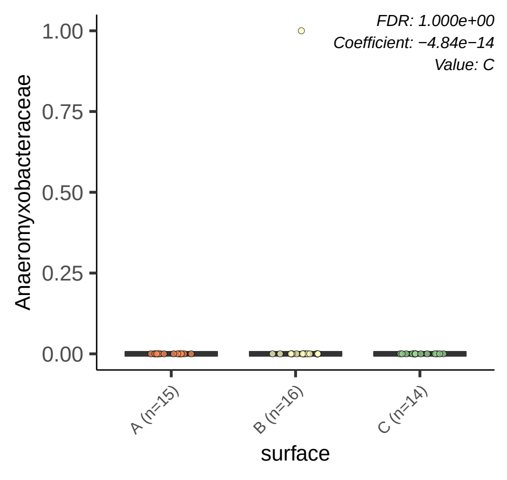

FDR: 1.000e+00
Coefficient: −4.84e−14
Value: C
Anaeromyxobacteraceae
1.00
0.75
0.50
0.25
0.00
A (n=15)
B (n=16)
C (n=14)
surface

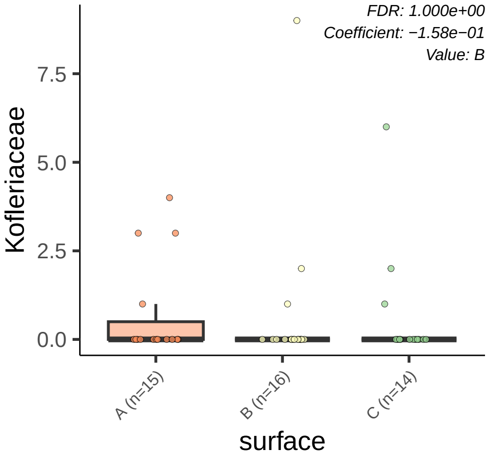

FDR: 1.000e+00
Coefficient: −1.58e−01
Value: B
Kofleriaceae
7.5
5.0
2.5
0.0
A (n=15)
B (n=16)
C (n=14)
surface

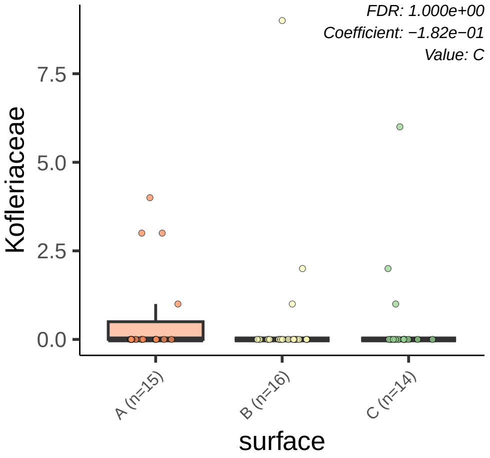

FDR: 1.000e+00
Coefficient: −1.82e−01
Value: C
Kofleriaceae
7.5
5.0
2.5
0.0
A (n=15)
B (n=16)
C (n=14)
surface

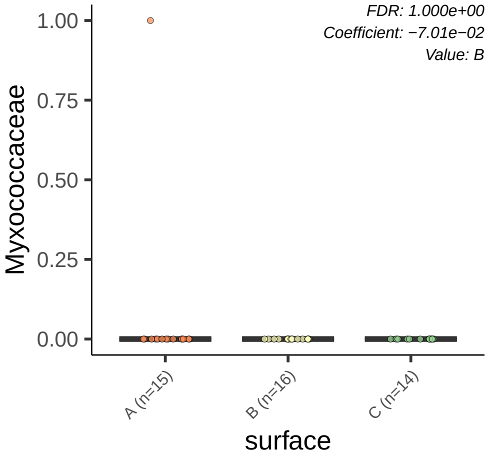
FDR: 1.000e+00
Coefficient: −7.01e−02
Value: B
1.00
0.75
0.50
0.25
0.00
Myxococcaceae
A (n=15)
B (n=16)
C (n=14)
surface

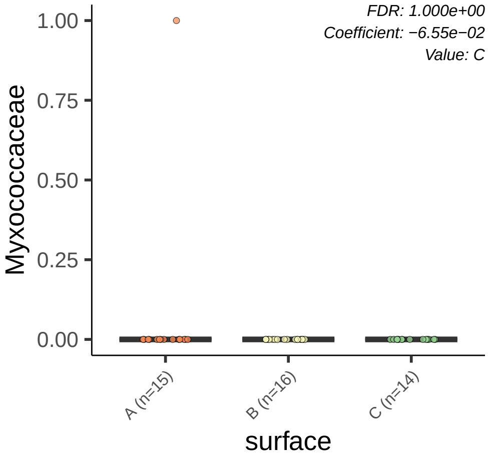
FDR: 1.000e+00
Coefficient: −6.55e−02
Value: C
Myxococcaceae
1.00
0.75
0.50
0.25
0.00
A (n=15)
B (n=16)
C (n=14)
surface

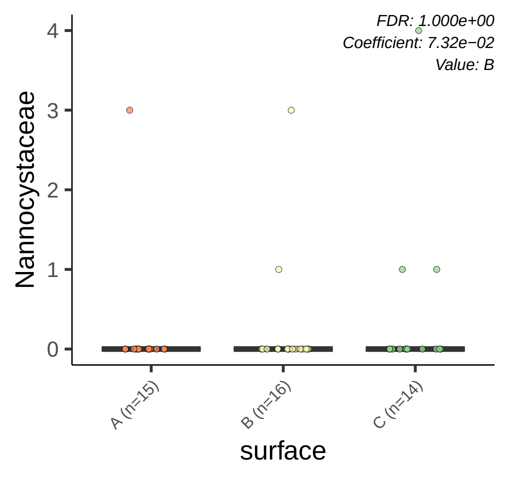

FDR: 1.000e+00
Coefficient: 7.32e−02
Value: B
Nannocystaceae
0
1
2
3
4
A (n=15)
B (n=16)
C (n=14)
surface

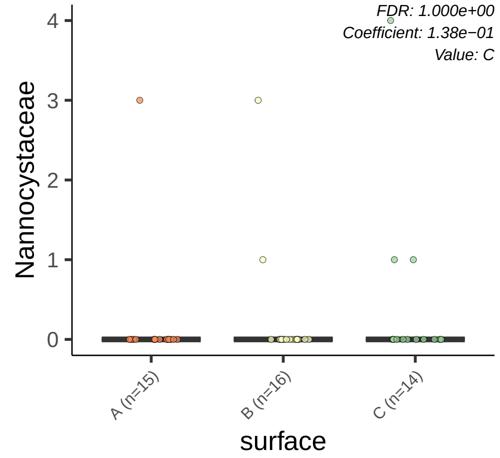
FDR: 1.000e+00
Coefficient: 1.38e−01
Value: C
Nannocystaceae
4
3
2
1
0
A (n=15)
B (n=16)
C (n=14)
surface

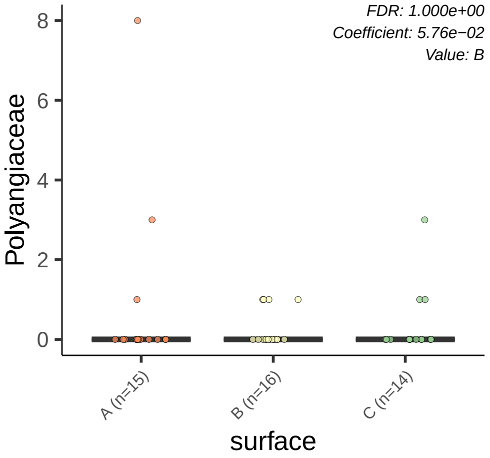

FDR: 1.000e+00
Coefficient: 5.76e−02
Value: B
Polyangiaceae
0
2
4
6
8
A (n=15)
B (n=16)
C (n=14)
surface

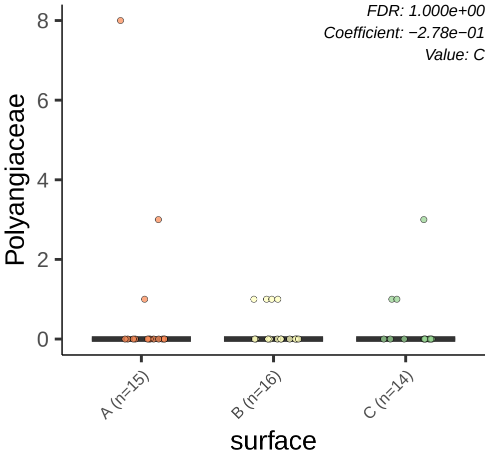

FDR: 1.000e+00
Coefficient: −2.78e−01
Value: C
Polyangiaceae
8
6
4
2
0
A (n=15)
B (n=16)
C (n=14)
surface

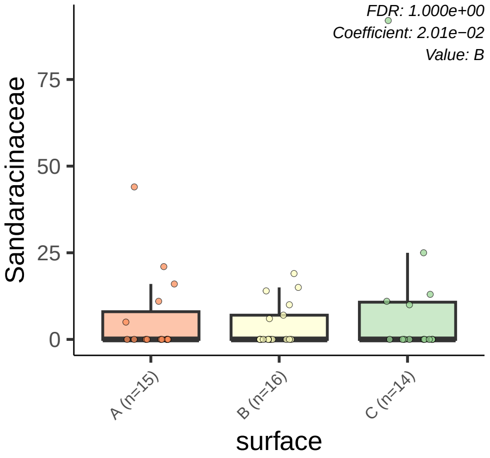

FDR: 1.000e+00
Coefficient: 2.01e−02
Value: B
Sandaracinaceae
75
50
25
0
A (n=15)
B (n=16)
C (n=14)
surface

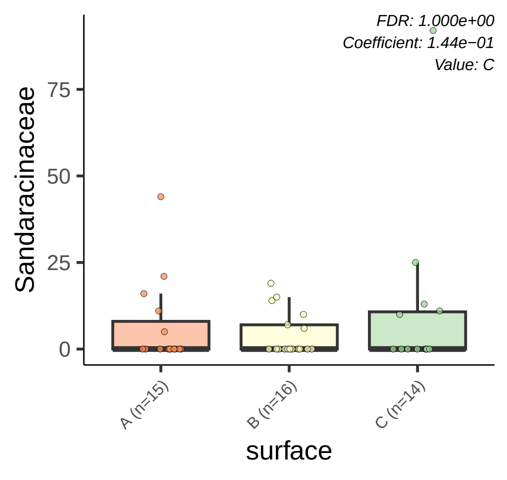

FDR: 1.000e+00
Coefficient: 1.44e−01
Value: C
Sandaracinaceae
0
25
50
75
A (n=15)
B (n=16)
C (n=14)
surface

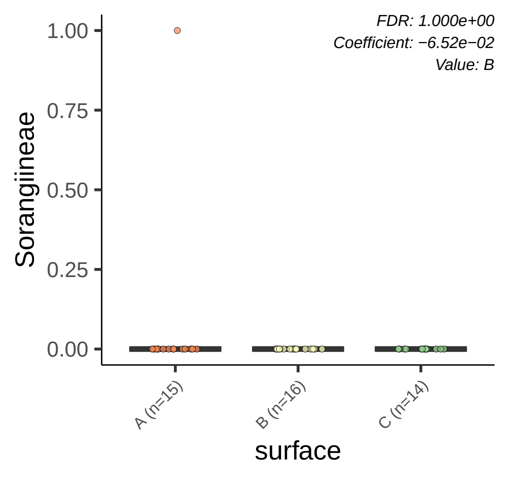
FDR: 1.000e+00
Coefficient: −6.52e−02
Value: B
1.00
0.75
0.50
0.25
0.00
Sorangineae
A (n=15)
B (n=16)
C (n=14)
surface

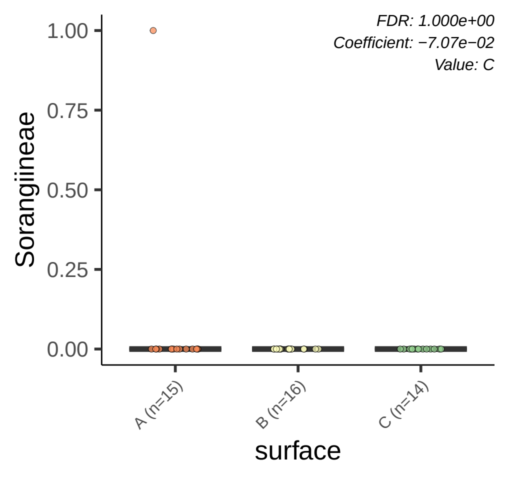
FDR: 1.000e+00
Coefficient: −7.07e−02
Value: C
Sorangiineae
1.00
0.75
0.50
0.25
0.00
A (n=15)
B (n=16)
C (n=14)
surface

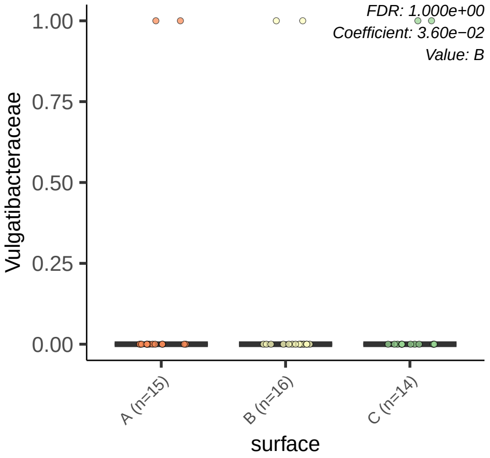

FDR: 1.000e+00
Coefficient: 3.60e−02
Value: B
1.00
0.75
0.50
0.25
0.00
Vulgatibacteraceae
A (n=15)
B (n=16)
C (n=14)
surface

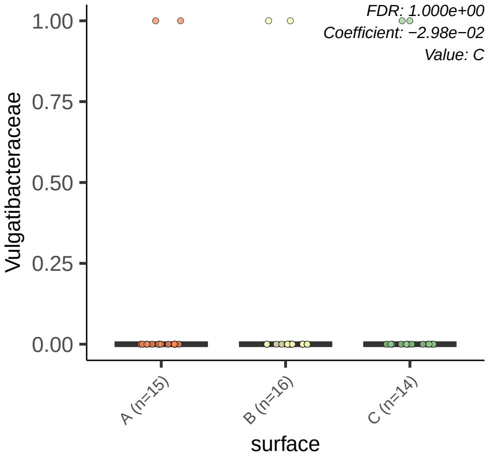
FDR: 1.000e+00
Coefficient: −2.98e−02
Value: C
Vulgatibacteraceae
1.00
0.75
0.50
0.25
0.00
A (n=15)
B (n=16)
C (n=14)
surface

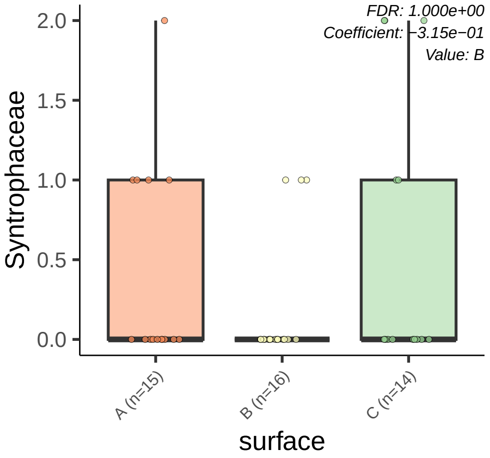

FDR: 1.000e+00
Coefficient: −3.15e−01
Value: B
Syntrophaceae
2.0
1.5
1.0
0.5
0.0
A (n=15)
B (n=16)
C (n=14)
surface

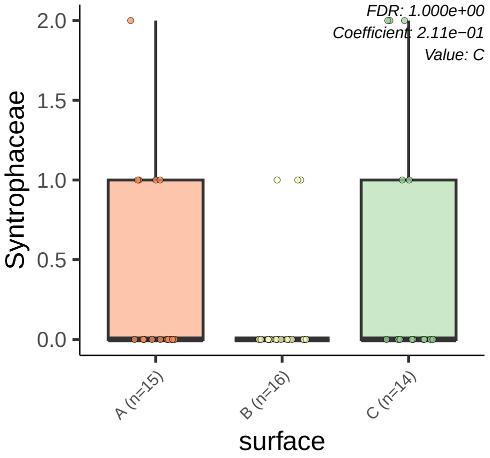

FDR: 1.000e+00
Coefficient: 2.11e−01
Value: C
Syntrophaceae
2.0
1.5
1.0
0.5
0.0
A (n=15)
B (n=16)
C (n=14)
surface

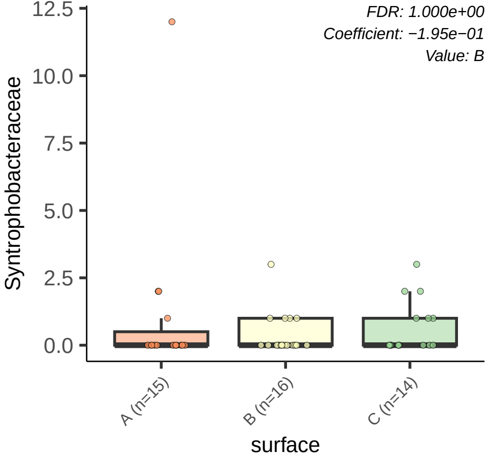
FDR: 1.000e+00
Coefficient: −1.95e−01
Value: B
Syntrophobacteraceae
12.5
10.0
7.5
5.0
2.5
0.0
A (n=15)
B (n=16)
C (n=14)
surface

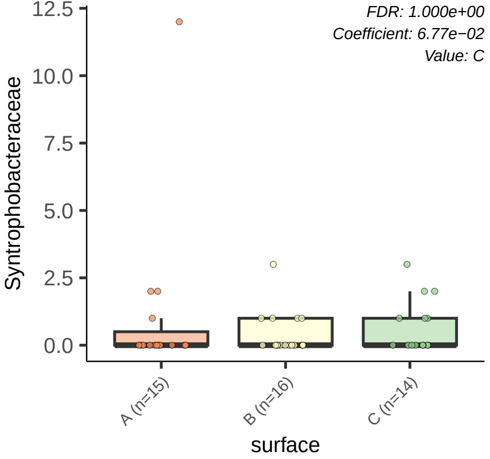

FDR: 1.000e+00
Coefficient: 6.77e−02
Value: C
Syntrophobacteraceae
12.5
10.0
7.5
5.0
2.5
0.0
A (n=15)
B (n=16)
C (n=14)
surface

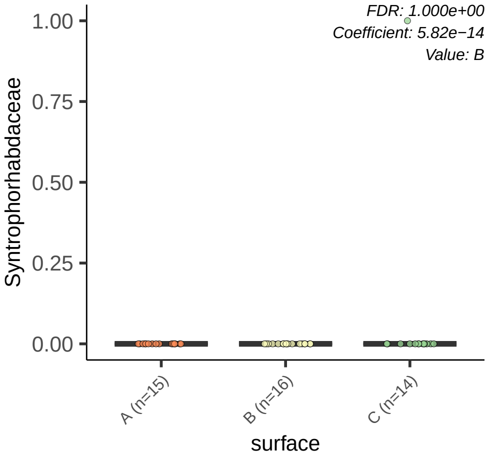
FDR: 1.000e+00
Coefficient: 5.82e−14
Value: B
Syntrophorhabdaceae
1.00
0.75
0.50
0.25
0.00
A (n=15)
B (n=16)
C (n=14)
surface

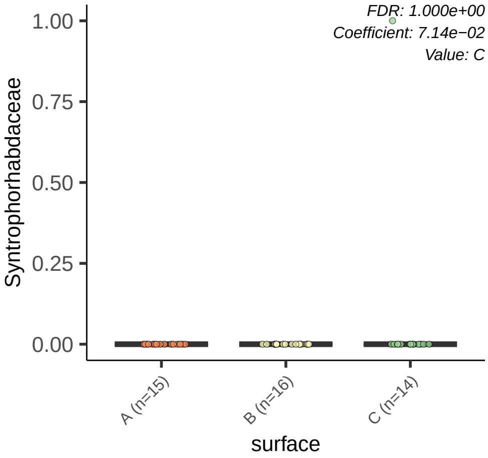

FDR: 1.000e+00
Coefficient: 7.14e−02
Value: C
Syntrophorhabdaceae
1.00
0.75
0.50
0.25
0.00
A (n=15)
B (n=16)
C (n=14)
surface

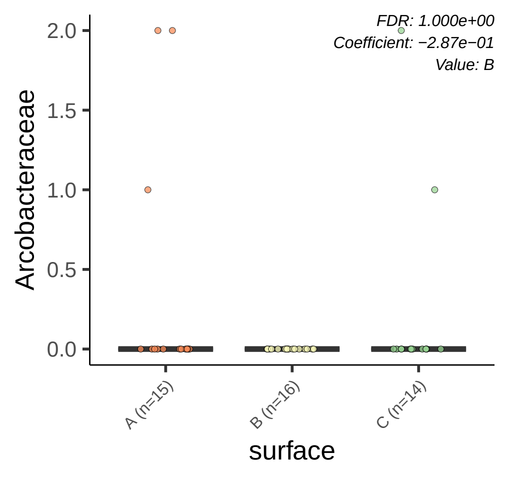
FDR: 1.000e+00
Coefficient: −2.87e−01
Value: B
Arcobacteraceae
2.0
1.5
1.0
0.5
0.0
A (n=15)
B (n=16)
C (n=14)
surface

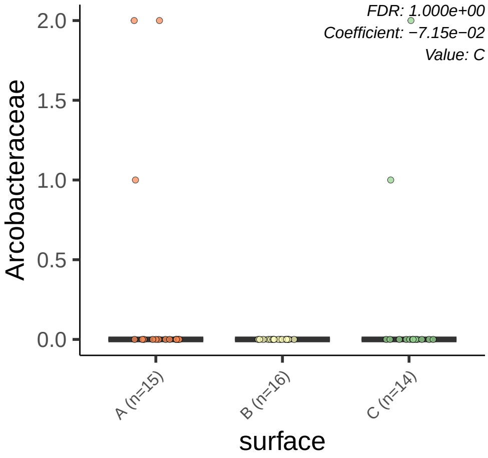

FDR: 1.000e+00
Coefficient: −7.15e−02
Value: C
Arcobacteraceae
2.0
1.5
1.0
0.5
0.0
A (n=15)
B (n=16)
C (n=14)
surface

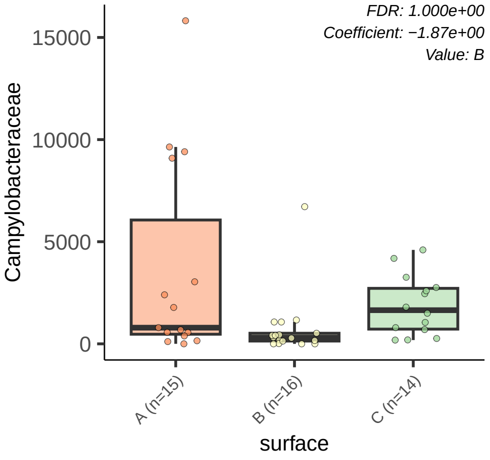

FDR: 1.000e+00
Coefficient: −1.87e+00
Value: B
Campylobacteraceae
15000
10000
5000
0
A (n=15)
B (n=16)
C (n=14)
surface

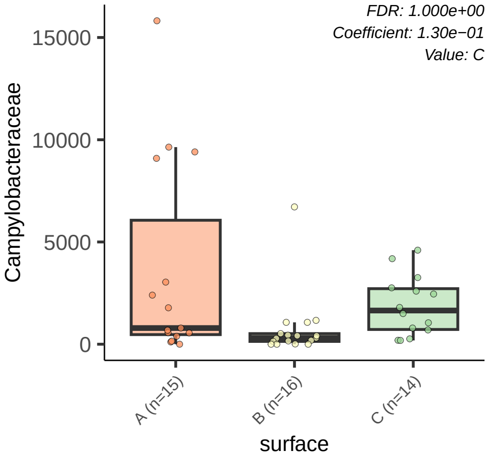

FDR: 1.000e+00
Coefficient: 1.30e−01
Value: C
Campylobacteraceae
15000
10000
5000
0
A (n=15)
B (n=16)
C (n=14)
surface

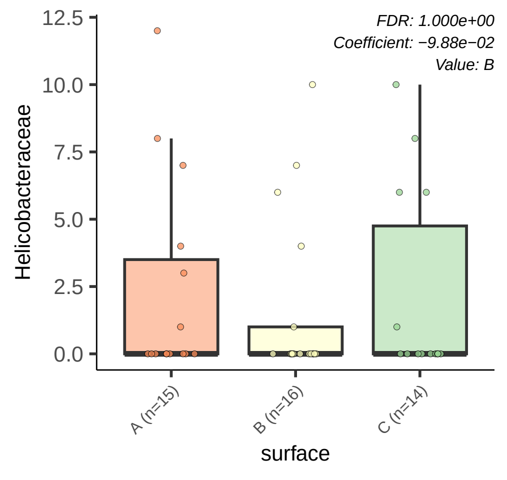

FDR: 1.000e+00
Coefficient: −9.88e−02
Value: B
Helicobacteraceae
12.5
10.0
7.5
5.0
2.5
0.0
A (n=15)
B (n=16)
C (n=14)
surface

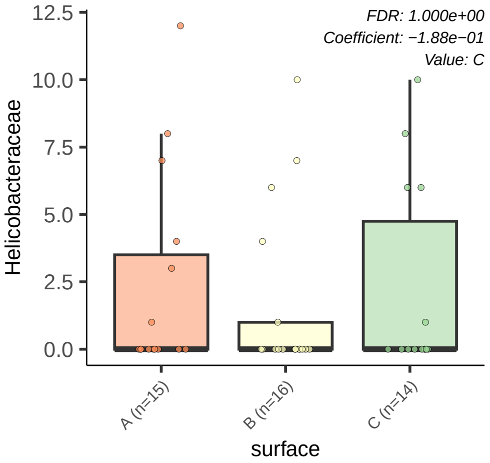

FDR: 1.000e+00
Coefficient: −1.88e−01
Value: C
Helicobacteraceae
12.5
10.0
7.5
5.0
2.5
0.0
A (n=15)
B (n=16)
C (n=14)
surface

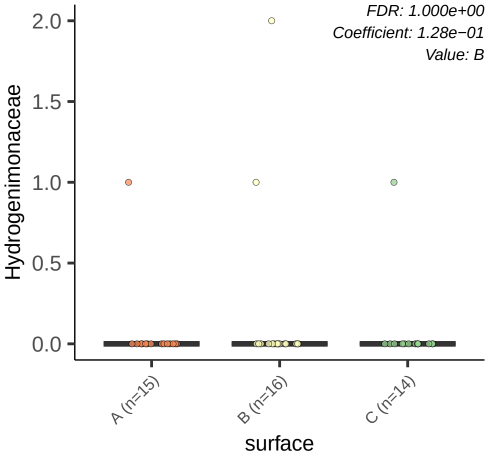
FDR: 1.000e+00
Coefficient: 1.28e−01
Value: B
Hydrogenimonaceae
2.0
1.5
1.0
0.5
0.0
A (n=15)
B (n=16)
C (n=14)
surface

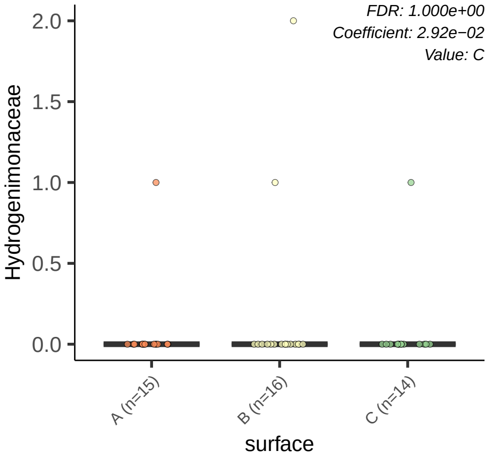

FDR: 1.000e+00
Coefficient: 2.92e−02
Value: C
Hydrogenimonaceae
2.0
1.5
1.0
0.5
0.0
A (n=15)
B (n=16)
C (n=14)
surface

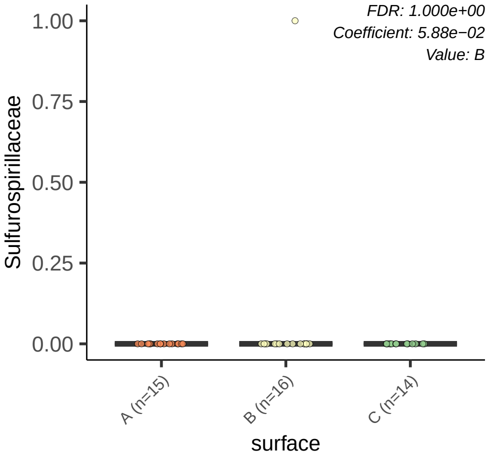

FDR: 1.000e+00
Coefficient: 5.88e−02
Value: B
Sulfurospirillaceae
1.00
0.75
0.50
0.25
0.00
A (n=15)
B (n=16)
C (n=14)
surface

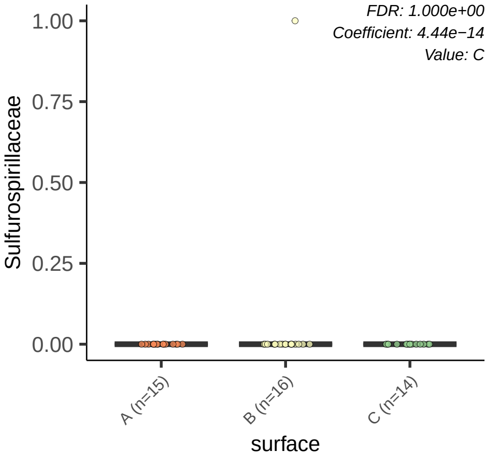

FDR: 1.000e+00
Coefficient: 4.44e−14
Value: C
Sulfurospirillaceae
1.00
0.75
0.50
0.25
0.00
A (n=15)
B (n=16)
C (n=14)
surface

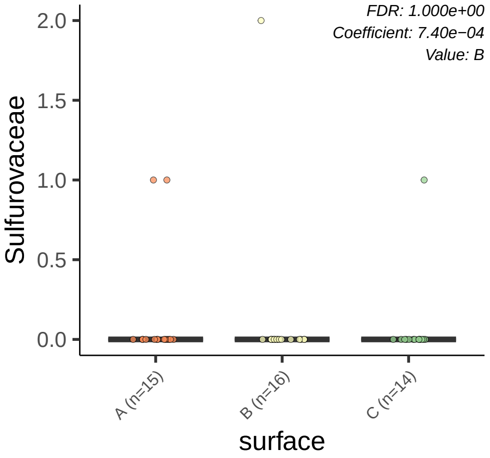
FDR: 1.000e+00
Coefficient: 7.40e−04
Value: B
Sulfurovaceae
2.0
1.5
1.0
0.5
0.0
A (n=15)
B (n=16)
C (n=14)
surface

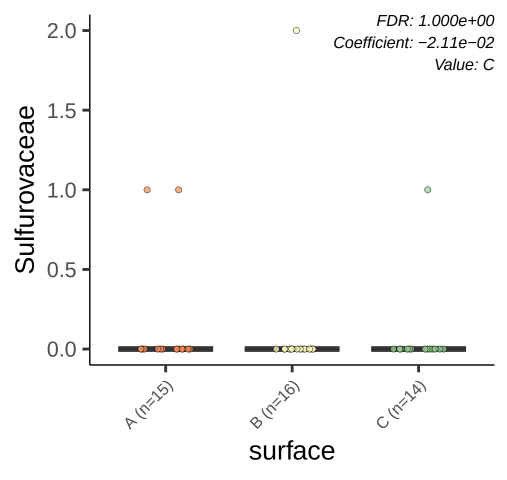
FDR: 1.000e+00
Coefficient: −2.11e−02
Value: C
Sulfurovaceae
2.0
1.5
1.0
0.5
0.0
A (n=15)
B (n=16)
C (n=14)
surface

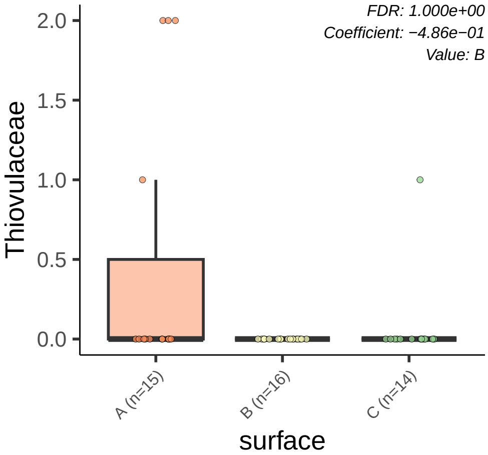
FDR: 1.000e+00
Coefficient: −4.86e−01
Value: B
Thiovulaceae
2.0
1.5
1.0
0.5
0.0
A (n=15)
B (n=16)
C (n=14)
surface

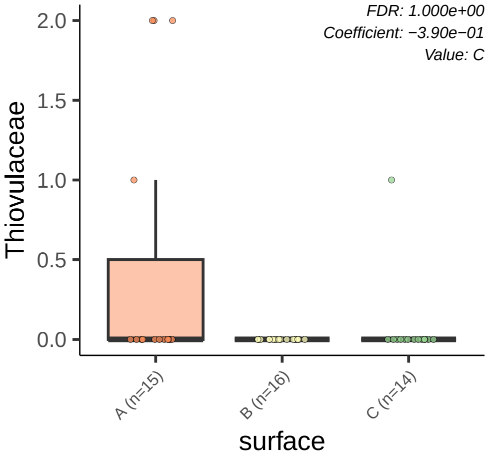

FDR: 1.000e+00
Coefficient: −3.90e−01
Value: C
Thiovulaceae
2.0
1.5
1.0
0.5
0.0
A (n=15)
B (n=16)
C (n=14)
surface

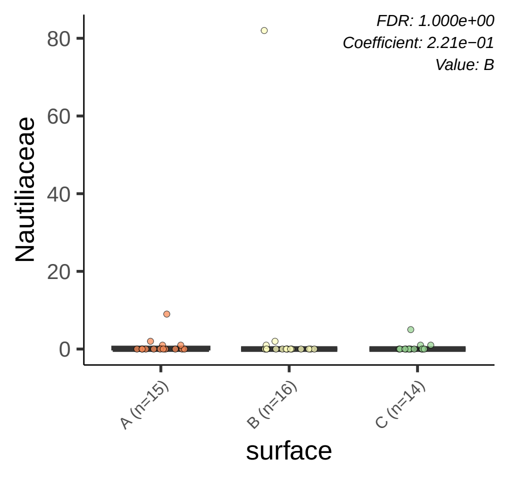
FDR: 1.000e+00
Coefficient: 2.21e−01
Value: B
Nautiliaceae
80
60
40
20
0
A (n=15)
B (n=16)
C (n=14)
surface

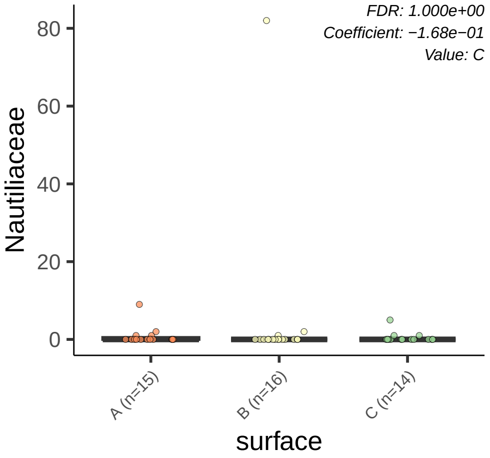
FDR: 1.000e+00
Coefficient: −1.68e−01
Value: C
Nautiliaceae
0
20
40
60
80
A (n=15)
B (n=16)
C (n=14)
surface

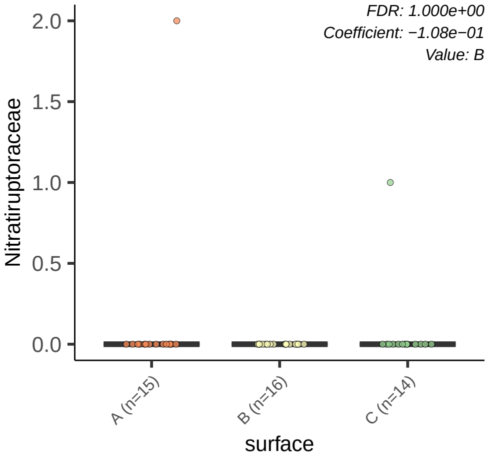
FDR: 1.000e+00
Coefficient: −1.08e−01
Value: B
Nitratiruptoraceae
2.0
1.5
1.0
0.5
0.0
A (n=15)
B (n=16)
C (n=14)
surface

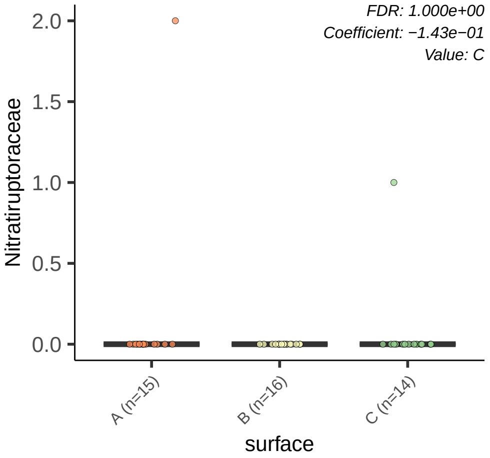

FDR: 1.000e+00
Coefficient: −1.43e−01
Value: C
Nitratiruptoraceae
2.0
1.5
1.0
0.5
0.0
A (n=15)
B (n=16)
C (n=14)
surface

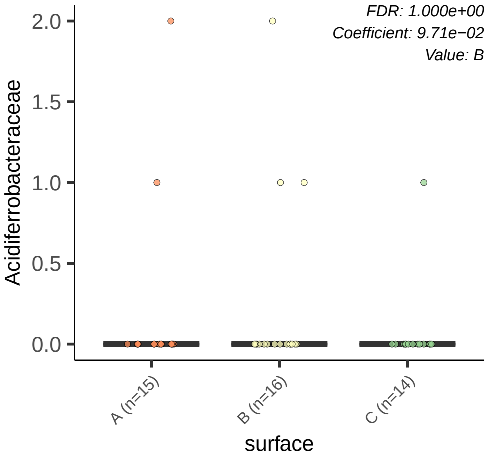
FDR: 1.000e+00
Coefficient: 9.71e−02
Value: B
Acidiferrobacteraceae
2.0
1.5
1.0
0.5
0.0
A (n=15)
B (n=16)
C (n=14)
surface

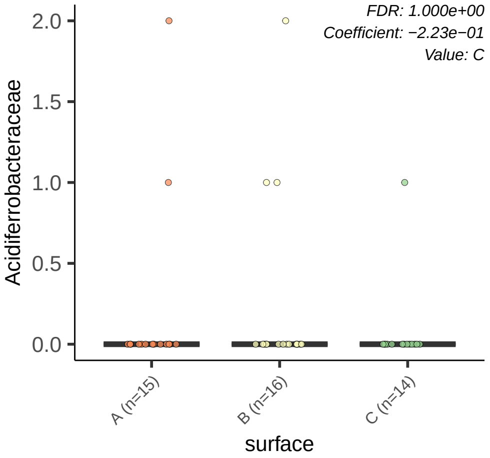
FDR: 1.000e+00
Coefficient: −2.23e−01
Value: C
Acidiferrobacteraceae
2.0
1.5
1.0
0.5
0.0
A (n=15)
B (n=16)
C (n=14)
surface

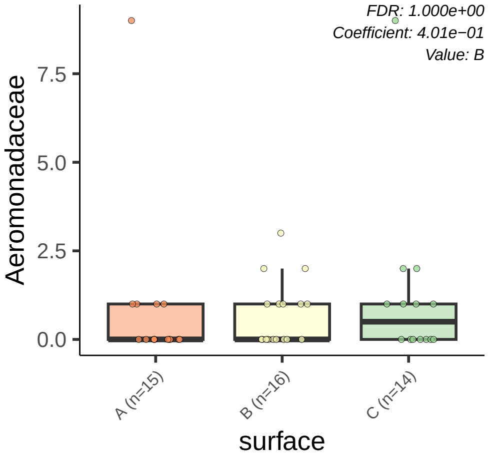

FDR: 1.000e+00
Coefficient: 4.01e−01
Value: B
Aeromonadaceae
7.5
5.0
2.5
0.0
A (n=15)
B (n=16)
C (n=14)
surface

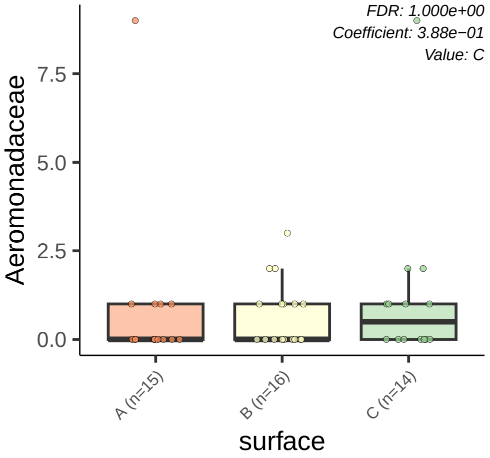
FDR: 1.000e+00
Coefficient: 3.88e−01
Value: C
Aeromonadaceae
7.5
5.0
2.5
0.0
A (n=15)
B (n=16)
C (n=14)
surface

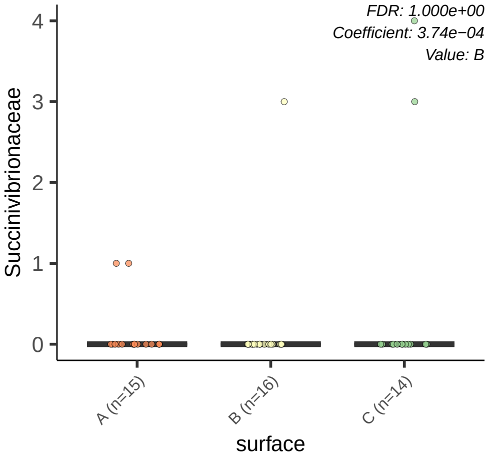
FDR: 1.000e+00
Coefficient: 3.74e−04
Value: B
Succinivibrionaceae
0
1
2
3
4
A (n=15)
B (n=16)
C (n=14)
surface

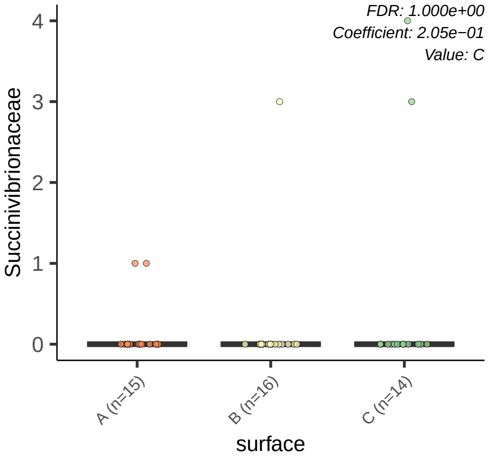
FDR: 1.000e+00
Coefficient: 2.05e−01
Value: C
Succinivibrionaceae
0
1
2
3
4
A (n=15)
B (n=16)
C (n=14)
surface

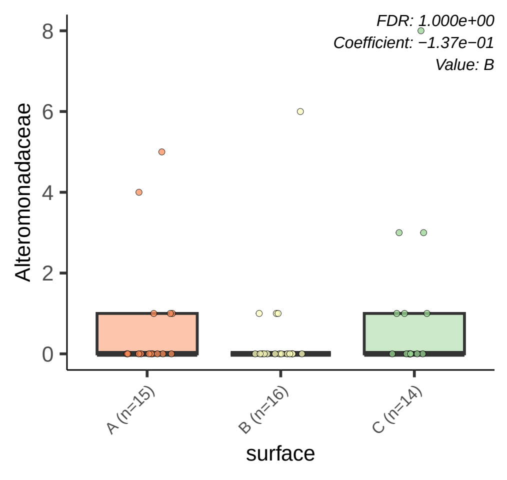
FDR: 1.000e+00
Coefficient: −1.37e−01
Value: B
Alteromonadaceae
0
2
4
6
8
A (n=15)
B (n=16)
C (n=14)
surface

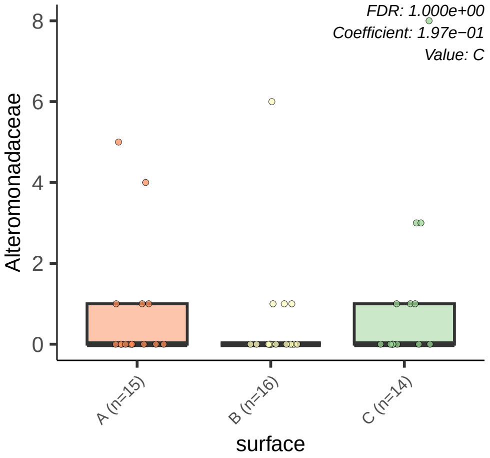

FDR: 1.000e+00
Coefficient: 1.97e−01
Value: C
Alteromonadaceae
0
2
4
6
8
A (n=15)
B (n=16)
C (n=14)
surface

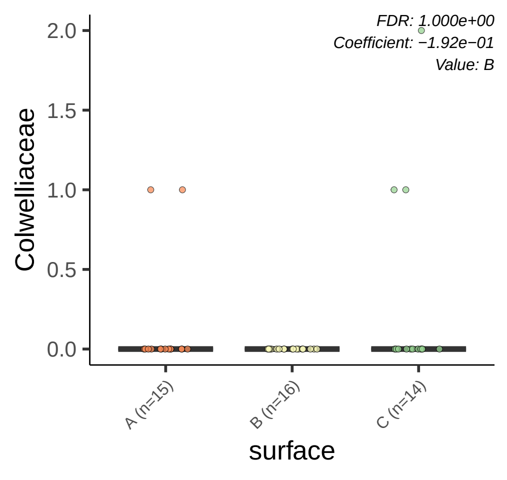

FDR: 1.000e+00
Coefficient: −1.92e−01
Value: B
Colwelliaceae
2.0
1.5
1.0
0.5
0.0
A (n=15)
B (n=16)
C (n=14)
surface

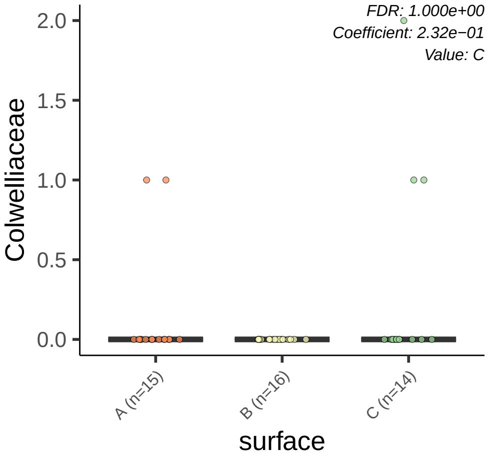

FDR: 1.000e+00
Coefficient: 2.32e−01
Value: C
Colwelliaceae
2.0
1.5
1.0
0.5
0.0
A (n=15)
B (n=16)
C (n=14)
surface

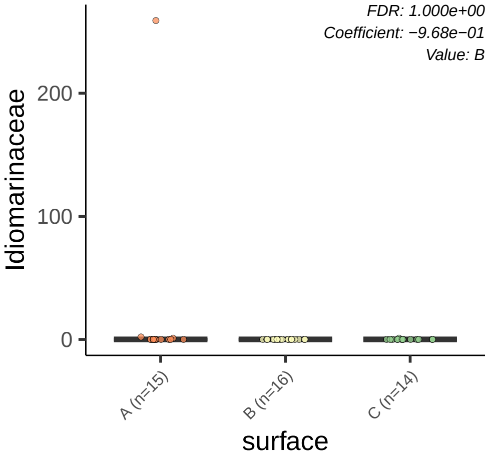

FDR: 1.000e+00
Coefficient: −9.68e−01
Value: B
Idiomarinaceae
200
100
0
A (n=15)
B (n=16)
C (n=14)
surface

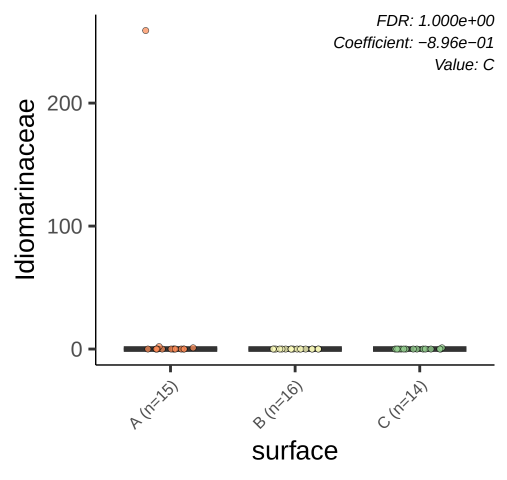

FDR: 1.000e+00
Coefficient: −8.96e−01
Value: C
Idiomarinaceae
200
100
0
A (n=15)
B (n=16)
C (n=14)
surface

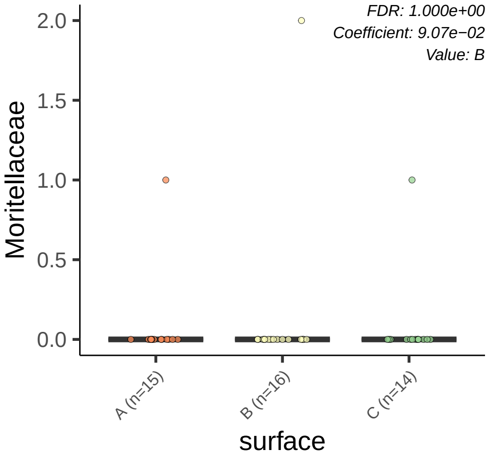

FDR: 1.000e+00
Coefficient: 9.07e−02
Value: B
Moritellaceae
2.0
1.5
1.0
0.5
0.0
A (n=15)
B (n=16)
C (n=14)
surface

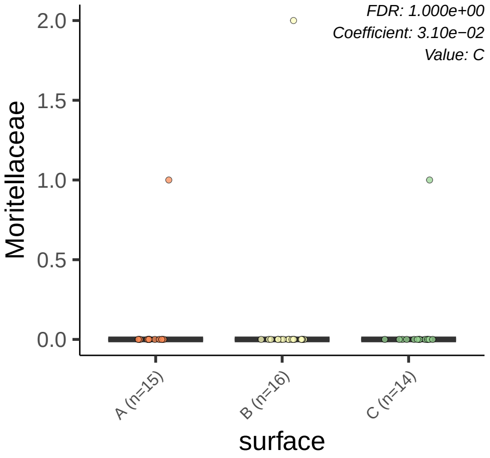
FDR: 1.000e+00
Coefficient: 3.10e−02
Value: C
Moritellaceae
2.0
1.5
1.0
0.5
0.0
A (n=15)
B (n=16)
C (n=14)
surface

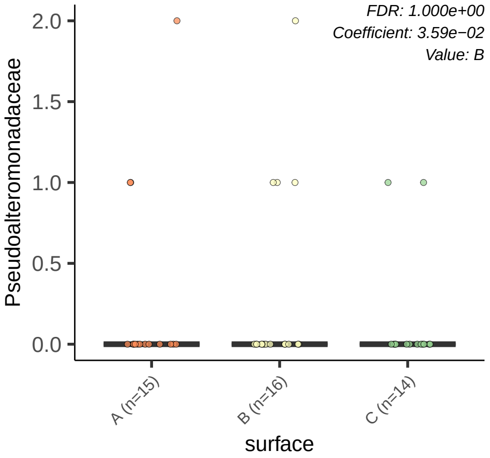

FDR: 1.000e+00
Coefficient: 3.59e−02
Value: B
Pseudoalteromonadaceae
2.0
1.5
1.0
0.5
0.0
A (n=15)
B (n=16)
C (n=14)
surface

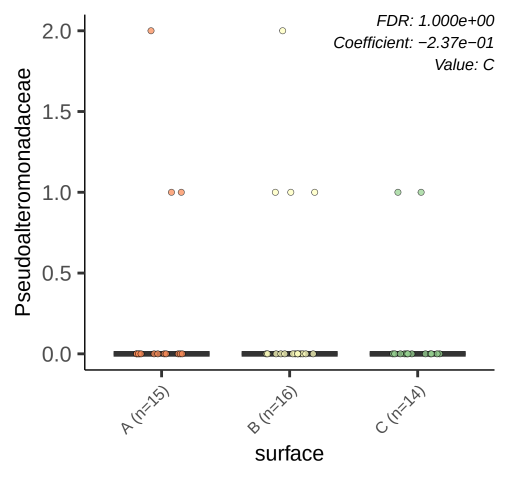
FDR: 1.000e+00
Coefficient: −2.37e−01
Value: C
Pseudoalteromonadaceae
2.0
1.5
1.0
0.5
0.0
A (n=15)
B (n=16)
C (n=14)
surface

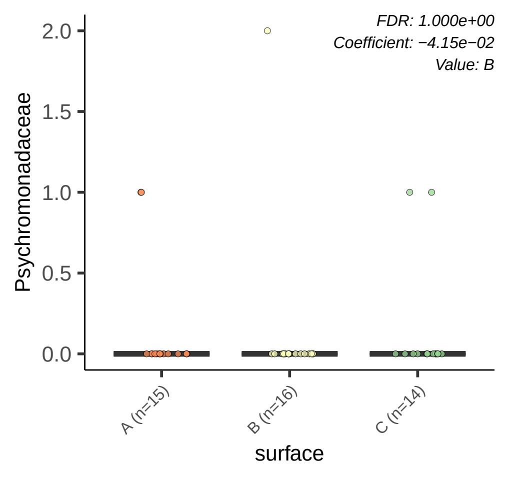
FDR: 1.000e+00
Coefficient: −4.15e−02
Value: B
Psychromonadaceae
2.0
1.5
1.0
0.5
0.0
A (n=15)
B (n=16)
C (n=14)
surface

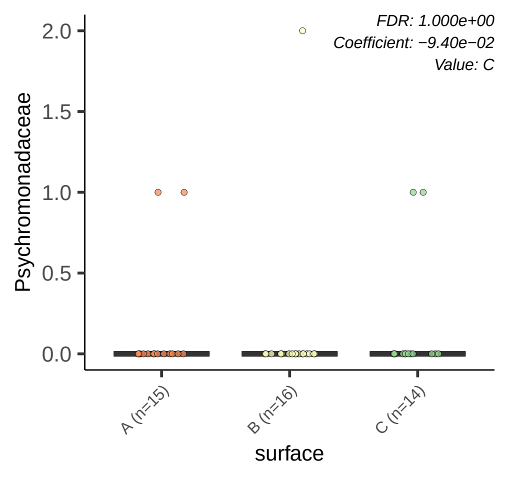

FDR: 1.000e+00
Coefficient: −9.40e−02
Value: C
Psychromonadaceae
2.0
1.5
1.0
0.5
0.0
A (n=15)
B (n=16)
C (n=14)
surface

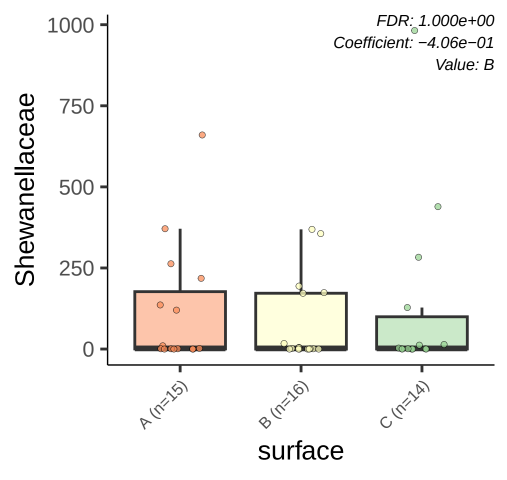
FDR: 1.000e+00
Coefficient: −4.06e−01
Value: B
Shewanellaceae
1000
750
500
250
0
A (n=15)
B (n=16)
C (n=14)
surface

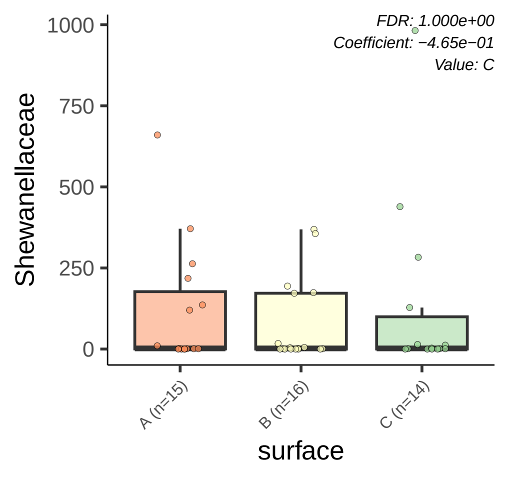

FDR: 1.000e+00
Coefficient: −4.65e−01
Value: C
Shewanellaceae
1000
750
500
250
0
A (n=15)
B (n=16)
C (n=14)
surface

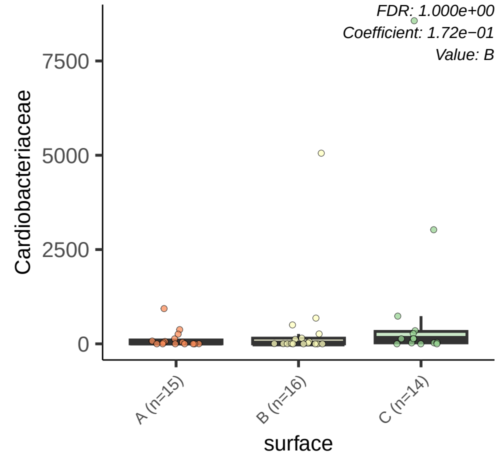
FDR: 1.000e+00
Coefficient: 1.72e−01
Value: B
Cardiobacteriaceae
7500
5000
2500
0
A (n=15)
B (n=16)
C (n=14)
surface

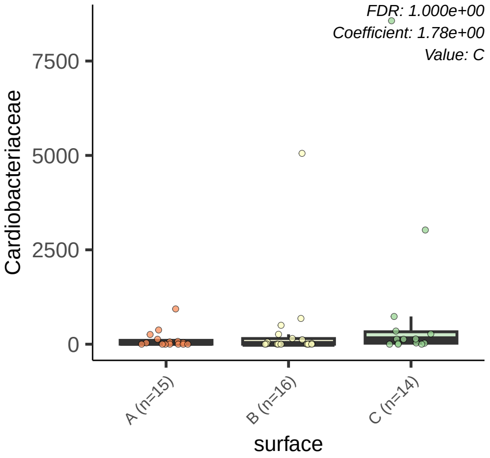
FDR: 1.000e+00
Coefficient: 1.78e+00
Value: C
Cardiobacteriaceae
7500
5000
2500
0
A (n=15)
B (n=16)
C (n=14)
surface

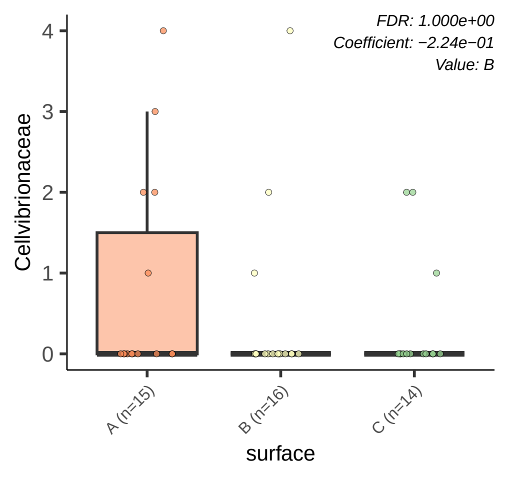
FDR: 1.000e+00
Coefficient: −2.24e−01
Value: B
Cellvibrionaceae
4
3
2
1
0
A (n=15)
B (n=16)
C (n=14)
surface

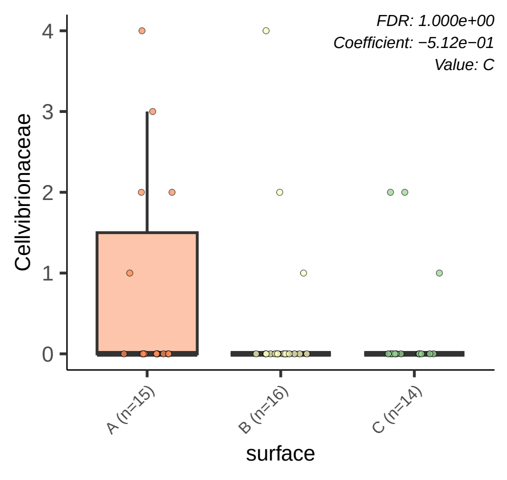
FDR: 1.000e+00
Coefficient: −5.12e−01
Value: C
Cellvibrionaceae
4
3
2
1
0
A (n=15)
B (n=16)
C (n=14)
surface

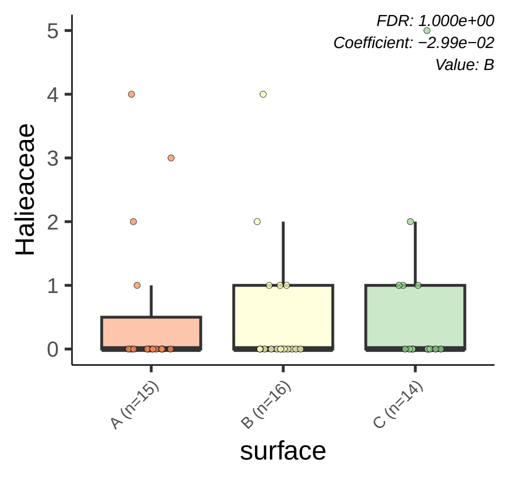

FDR: 1.000e+00
Coefficient: −2.99e−02
Value: B
Halieaceae
0
1
2
3
4
5
A (n=15)
B (n=16)
C (n=14)
surface

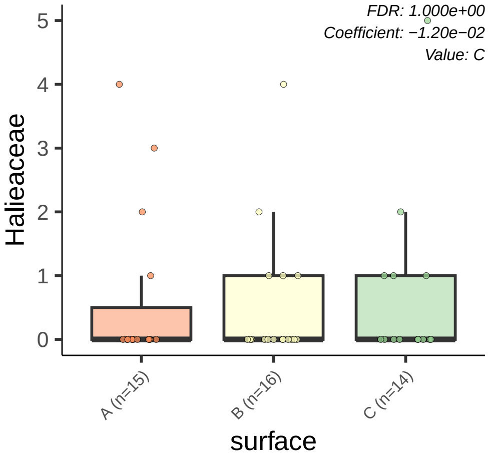

FDR: 1.000e+00
Coefficient: −1.20e−02
Value: C
Halieaceae
0
1
2
3
4
5
A (n=15)
B (n=16)
C (n=14)
surface

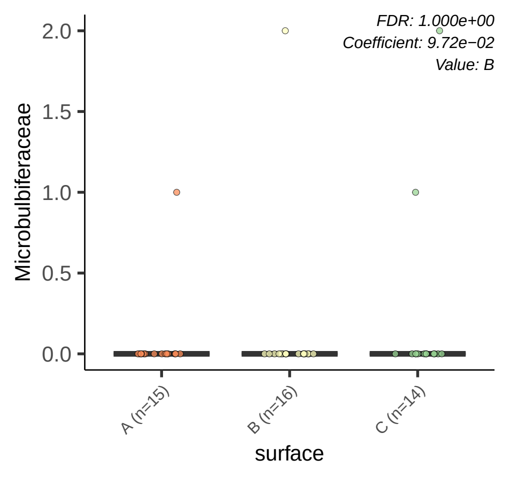
FDR: 1.000e+00
Coefficient: 9.72e−02
Value: B
Microbulbiferaceae
2.0
1.5
1.0
0.5
0.0
A (n=15)
B (n=16)
C (n=14)
surface

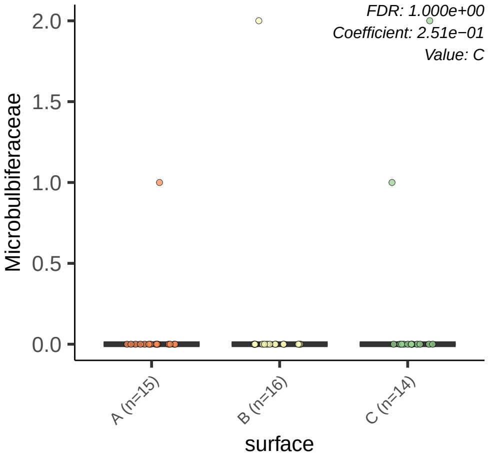
FDR: 1.000e+00
Coefficient: 2.51e−01
Value: C
Microbulbiferaceae
2.0
1.5
1.0
0.5
0.0
A (n=15)
B (n=16)
C (n=14)
surface

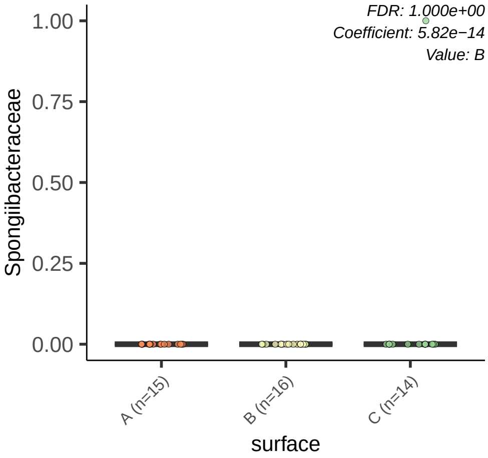

FDR: 1.000e+00
Coefficient: 5.82e−14
Value: B
Spongiibacteraceae
1.00
0.75
0.50
0.25
0.00
A (n=15)
B (n=16)
C (n=14)
surface

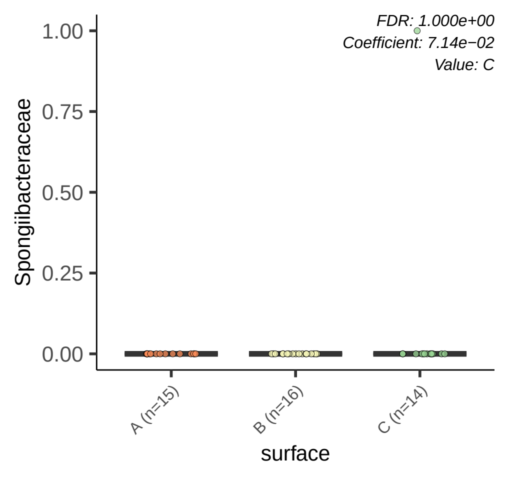
FDR: 1.000e+00
Coefficient: 7.14e−02
Value: C
Spongiibacteraceae
1.00
0.75
0.50
0.25
0.00
A (n=15)
B (n=16)
C (n=14)
surface

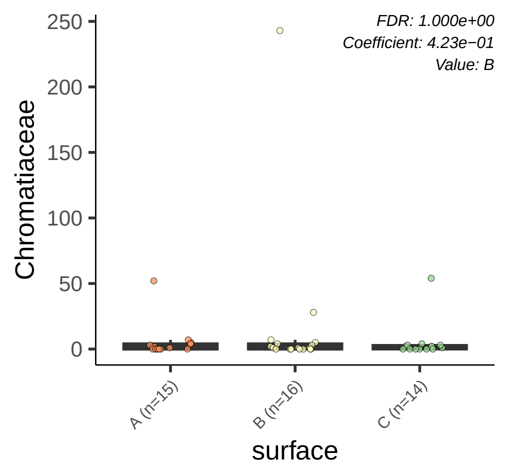
FDR: 1.000e+00
Coefficient: 4.23e−01
Value: B
Chromatiaceae
250
200
150
100
50
0
A (n=15)
B (n=16)
C (n=14)
surface

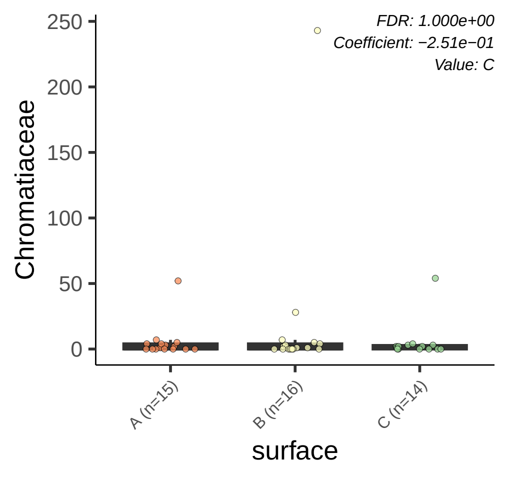

FDR: 1.000e+00
Coefficient: −2.51e−01
Value: C
Chromatiaceae
250
200
150
100
50
0
A (n=15)
B (n=16)
C (n=14)
surface

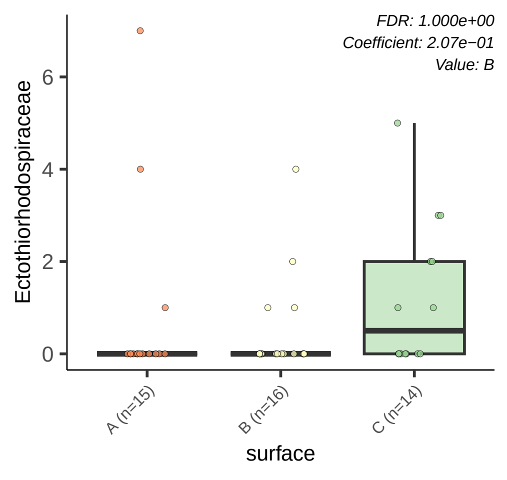

FDR: 1.000e+00
Coefficient: 2.07e−01
Value: B
Ectothiorhodospiraceae
0
2
4
6
A (n=15)
B (n=16)
C (n=14)
surface

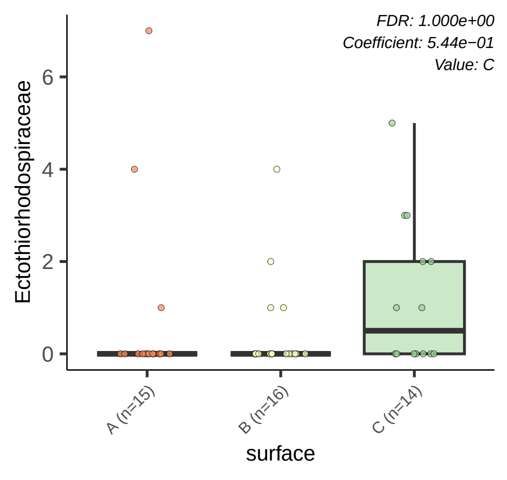

FDR: 1.000e+00
Coefficient: 5.44e−01
Value: C
Ectothiorhodospiraceae
0
2
4
6
A (n=15)
B (n=16)
C (n=14)
surface

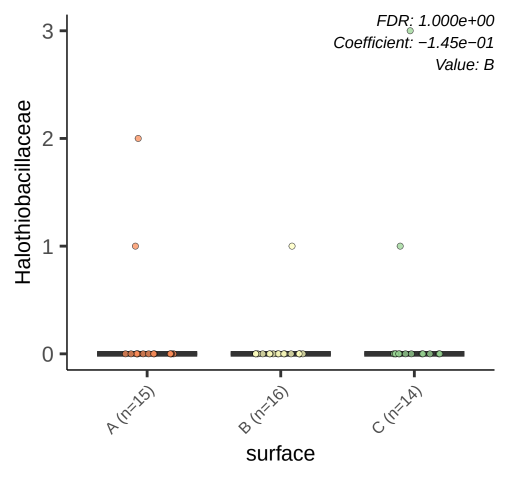

FDR: 1.000e+00
Coefficient: −1.45e−01
Value: B
Halothiobacillaceae
3
2
1
0
A (n=15)
B (n=16)
C (n=14)
surface

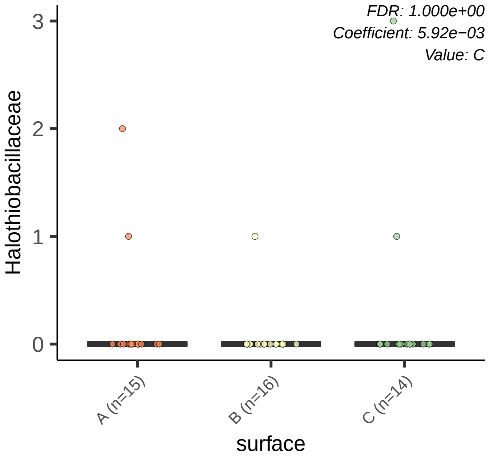

FDR: 1.000e+00
Coefficient: 5.92e−03
Value: C
Halothiobacillaceae
3
2
1
0
A (n=15)
B (n=16)
C (n=14)
surface

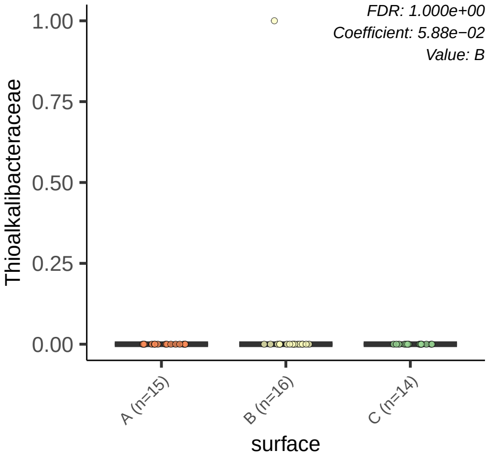

FDR: 1.000e+00
Coefficient: 5.88e−02
Value: B
Thioalkalibacteraceae
1.00
0.75
0.50
0.25
0.00
A (n=15)
B (n=16)
C (n=14)
surface

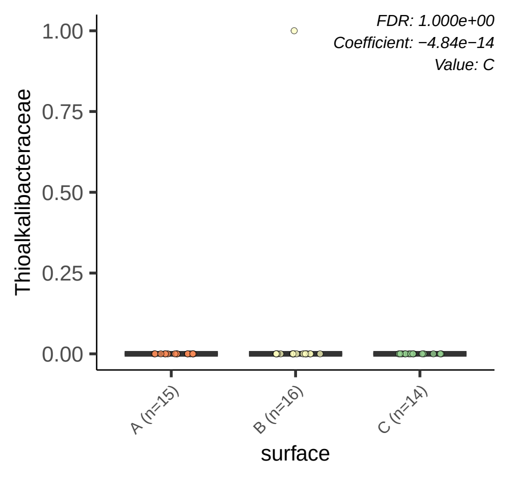
FDR: 1.000e+00
Coefficient: −4.84e−14
Value: C
Thioalkalibacteraceae
1.00
0.75
0.50
0.25
0.00
A (n=15)
B (n=16)
C (n=14)
surface

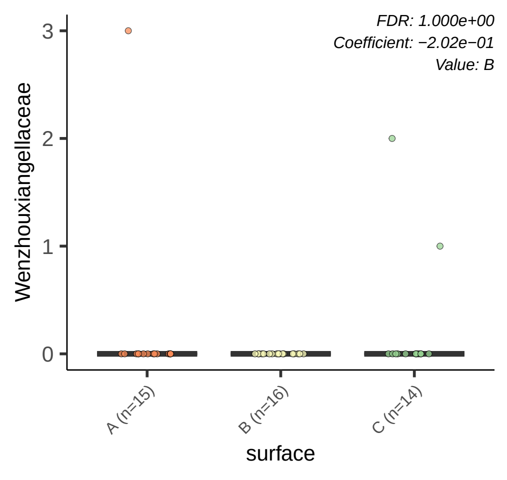
FDR: 1.000e+00
Coefficient: −2.02e−01
Value: B
Wenzhouxiangellaceae
3
2
1
0
A (n=15)
B (n=16)
C (n=14)
surface

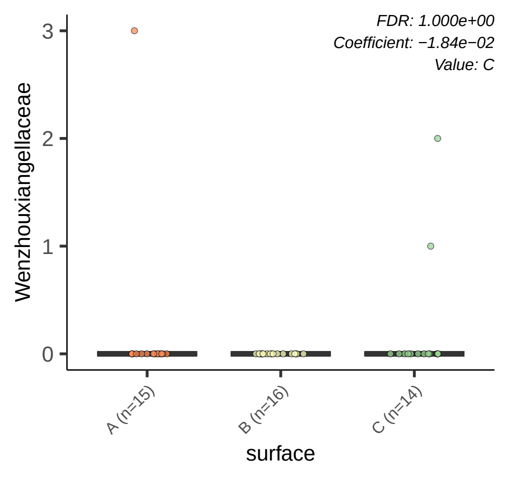

FDR: 1.000e+00
Coefficient: −1.84e−02
Value: C
Wenzhouxiangellaceae
3
2
1
0
A (n=15)
B (n=16)
C (n=14)
surface

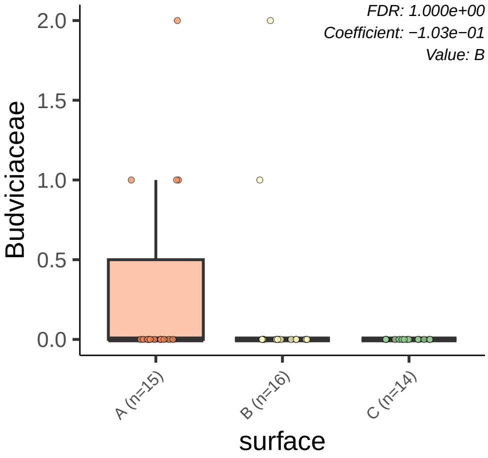
FDR: 1.000e+00
Coefficient: −1.03e−01
Value: B
Budviciaceae
2.0
1.5
1.0
0.5
0.0
A (n=15)
B (n=16)
C (n=14)
surface

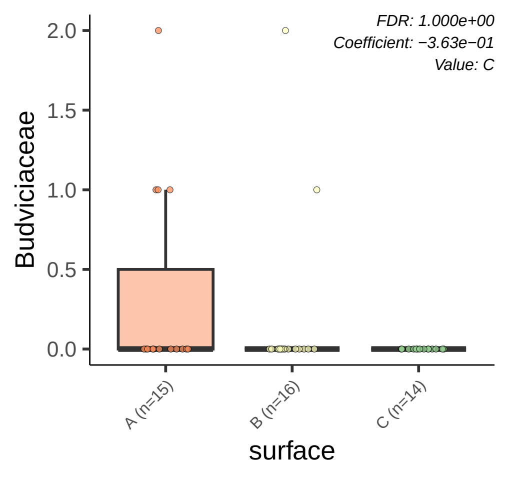

FDR: 1.000e+00
Coefficient: −3.63e−01
Value: C
Budviciaceae
2.0
1.5
1.0
0.5
0.0
A (n=15)
B (n=16)
C (n=14)
surface

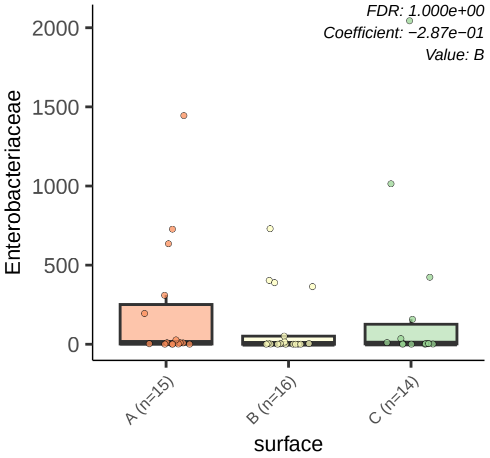
FDR: 1.000e+00
Coefficient: −2.87e−01
Value: B
Enterobacteriaceae
2000
1500
1000
500
0
A (n=15)
B (n=16)
C (n=14)
surface

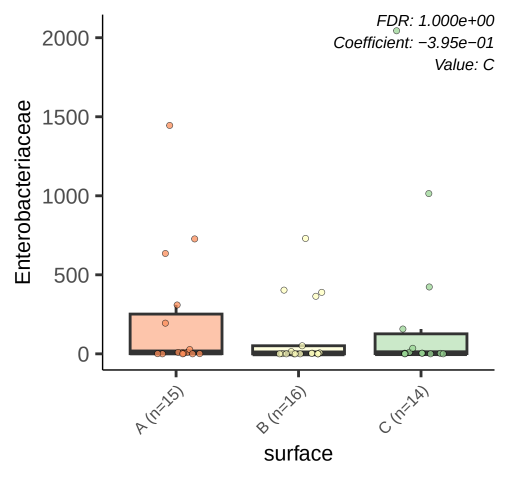

FDR: 1.000e+00
Coefficient: −3.95e−01
Value: C
Enterobacteriaceae
2000
1500
1000
500
0
A (n=15)
B (n=16)
C (n=14)
surface

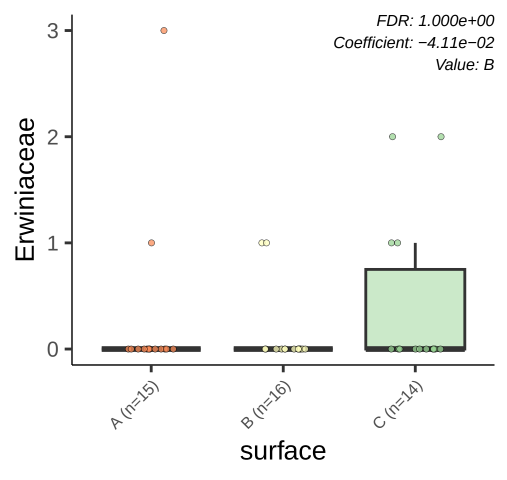

FDR: 1.000e+00
Coefficient: −4.11e−02
Value: B
Erwiniaceae
0
1
2
3
A (n=15)
B (n=16)
C (n=14)
surface

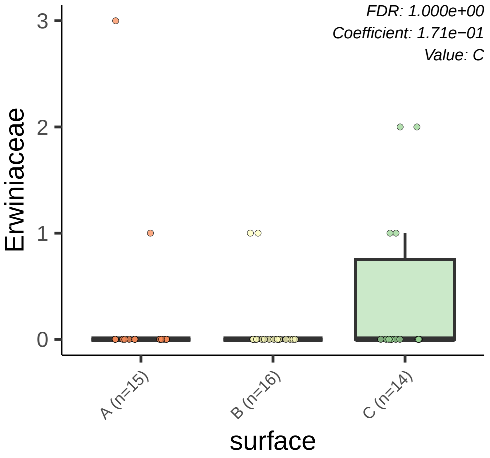
FDR: 1.000e+00
Coefficient: 1.71e−01
Value: C
Erwiniaceae
3
2
1
0
A (n=15)
B (n=16)
C (n=14)
surface

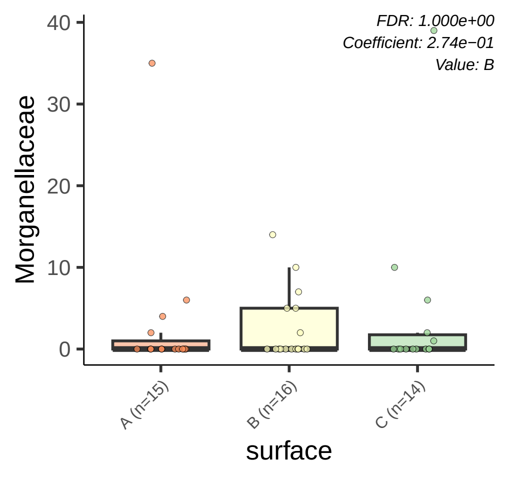
FDR: 1.000e+00
Coefficient: 2.74e−01
Value: B
Morganellaceae
0
10
20
30
40
A (n=15)
B (n=16)
C (n=14)
surface

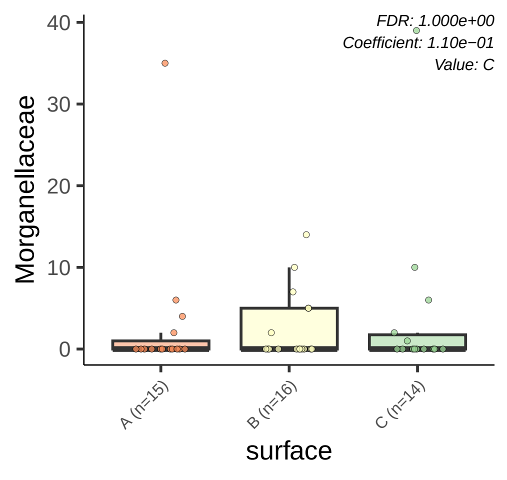
FDR: 1.000e+00
Coefficient: 1.10e−01
Value: C
Morganellaceae
40
30
20
10
0
A (n=15)
B (n=16)
C (n=14)
surface

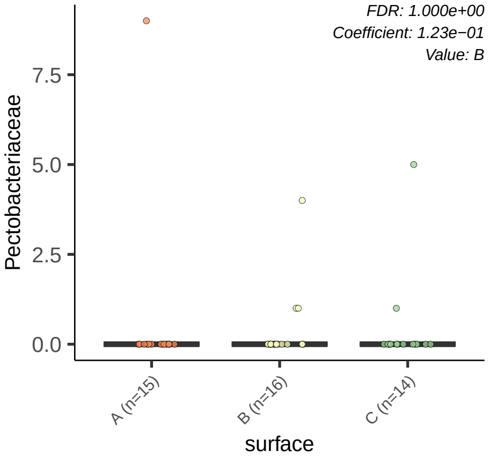
FDR: 1.000e+00
Coefficient: 1.23e−01
Value: B
Pectobacteriaceae
0.0
2.5
5.0
7.5
A (n=15)
B (n=16)
C (n=14)
surface

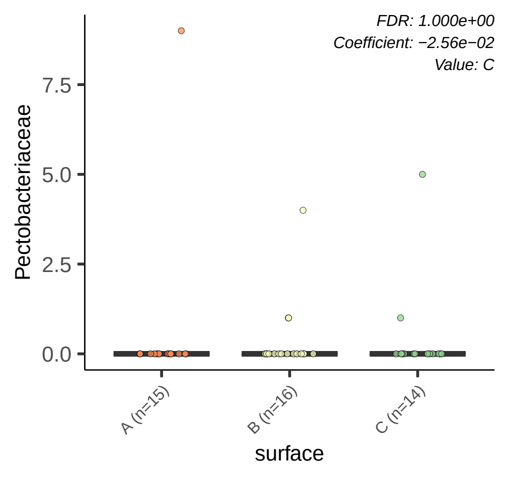
FDR: 1.000e+00
Coefficient: −2.56e−02
Value: C
Pectobacteriaceae
7.5
5.0
2.5
0.0
A (n=15)
B (n=16)
C (n=14)
surface

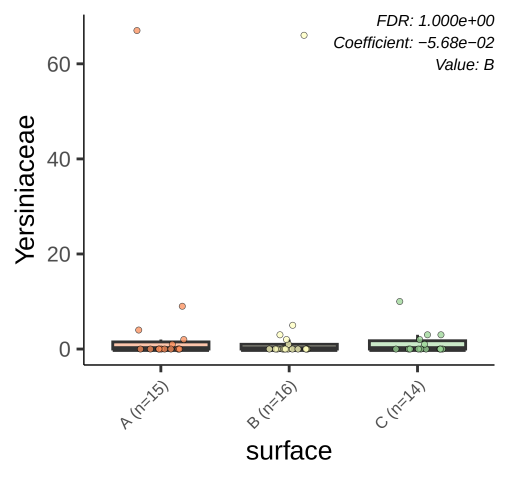

FDR: 1.000e+00
Coefficient: −5.68e−02
Value: B
Yersiniaceae
60
40
20
0
A (n=15)
B (n=16)
C (n=14)
surface

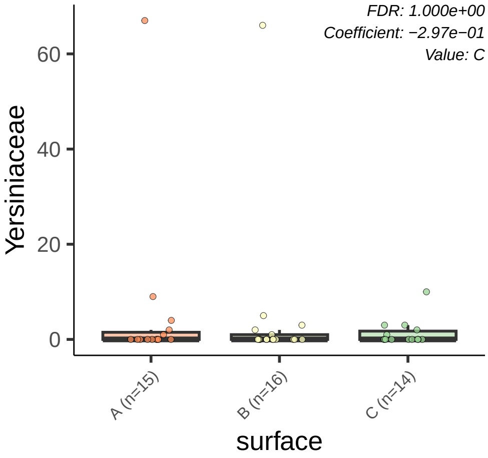

FDR: 1.000e+00
Coefficient: −2.97e−01
Value: C
Yersiniaceae
60
40
20
0
A (n=15)
B (n=16)
C (n=14)
surface

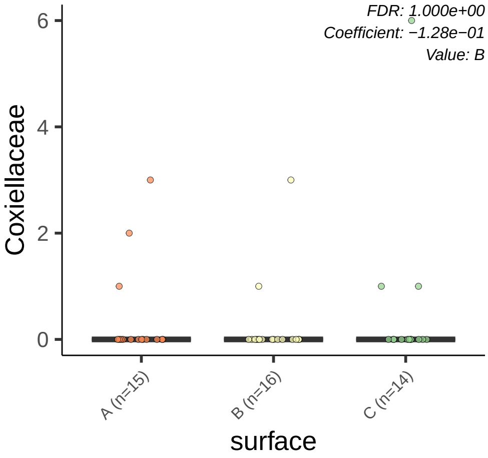
FDR: 1.000e+00
Coefficient: −1.28e−01
Value: B
Coxiellaceae
0
2
4
6
A (n=15)
B (n=16)
C (n=14)
surface

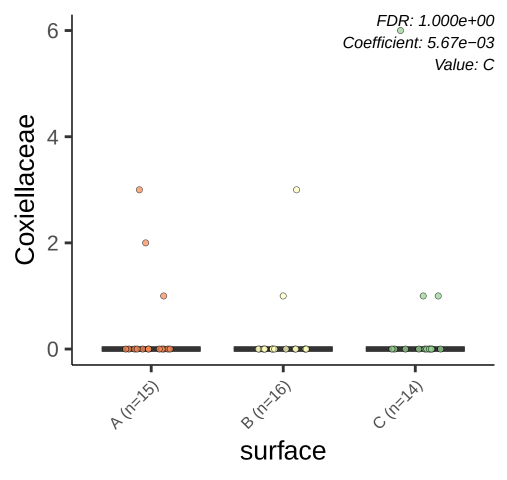
FDR: 1.000e+00
Coefficient: 5.67e−03
Value: C
Coxiellaceae
0
2
4
6
A (n=15)
B (n=16)
C (n=14)
surface

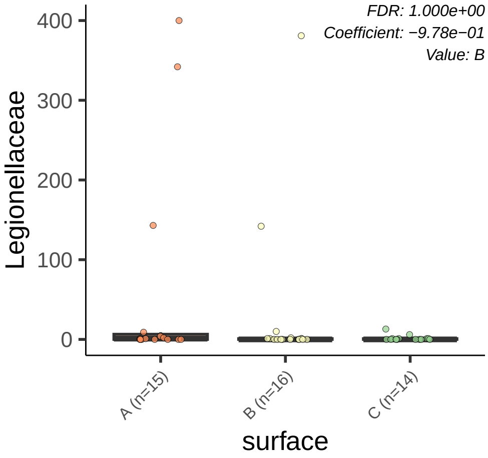
FDR: 1.000e+00
Coefficient: −9.78e−01
Value: B
Legionellaceae
400
300
200
100
0
A (n=15)
B (n=16)
C (n=14)
surface

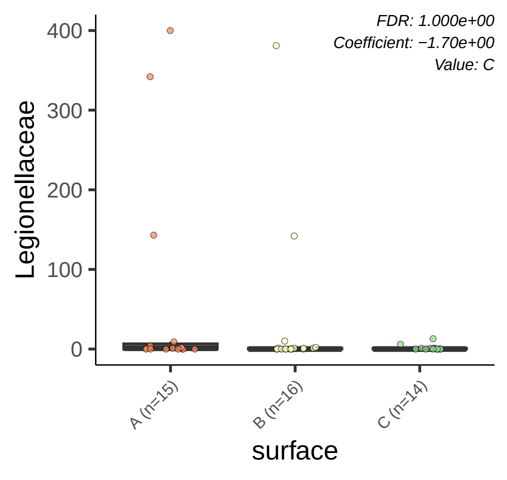
FDR: 1.000e+00
Coefficient: −1.70e+00
Value: C
Legionellaceae
400
300
200
100
0
A (n=15)
B (n=16)
C (n=14)
surface

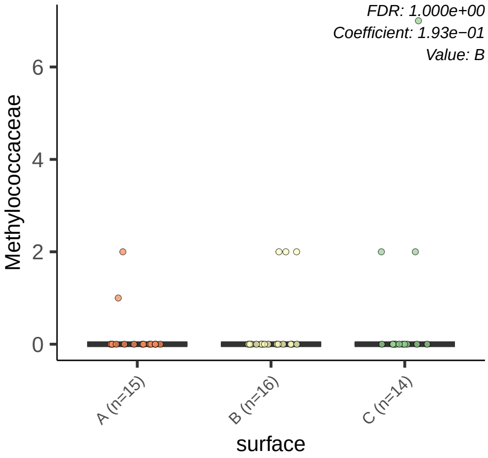

FDR: 1.000e+00
Coefficient: 1.93e−01
Value: B
Methylococcaceae
0
2
4
6
A (n=15)
B (n=16)
C (n=14)
surface

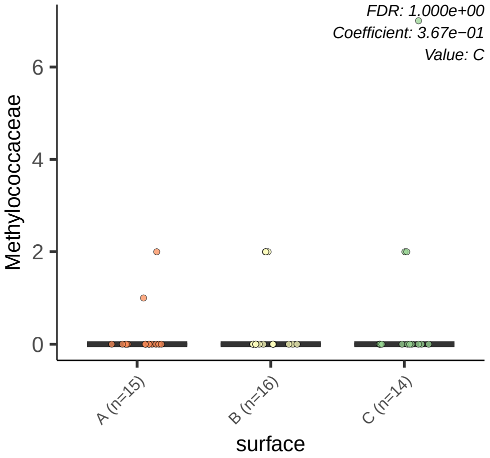

FDR: 1.000e+00
Coefficient: 3.67e−01
Value: C
Methylococcaceae
0
2
4
6
A (n=15)
B (n=16)
C (n=14)
surface

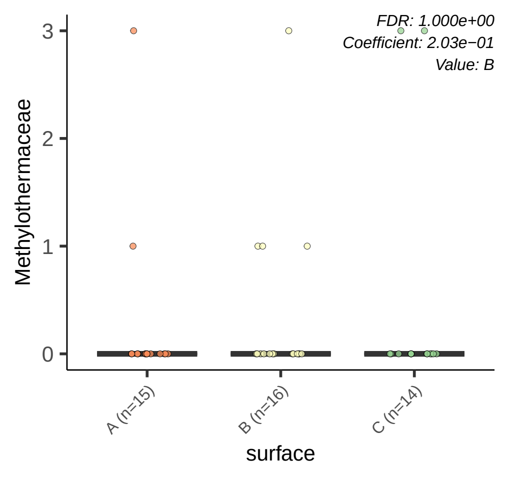
FDR: 1.000e+00
Coefficient: 2.03e−01
Value: B
Methylothermaceae
3
2
1
0
A (n=15)
B (n=16)
C (n=14)
surface

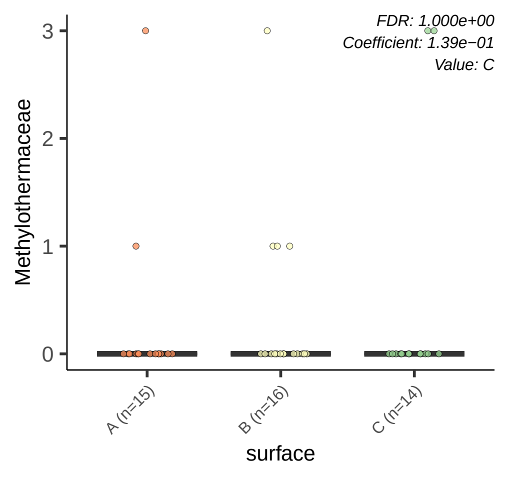

FDR: 1.000e+00
Coefficient: 1.39e−01
Value: C
Methylothermaceae
0
1
2
3
A (n=15)
B (n=16)
C (n=14)
surface

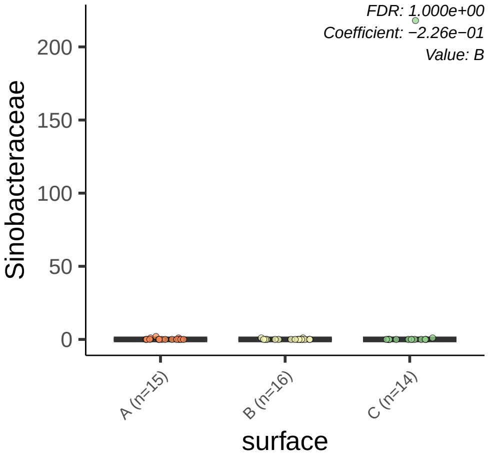
FDR: 1.000e+00
Coefficient: −2.26e−01
Value: B
Sinobacteraceae
200
150
100
50
0
A (n=15)
B (n=16)
C (n=14)
surface

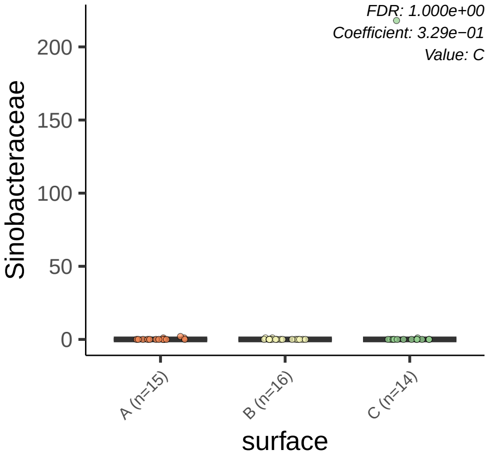
FDR: 1.000e+00
Coefficient: 3.29e−01
Value: C
Sinobacteraceae
200
150
100
50
0
A (n=15)
B (n=16)
C (n=14)
surface

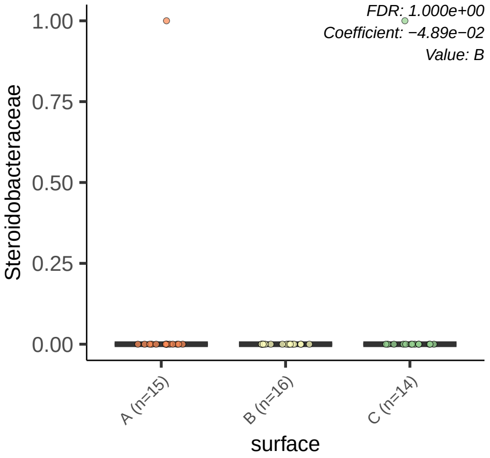

FDR: 1.000e+00
Coefficient: −4.89e−02
Value: B
Steroidobacteraceae
1.00
0.75
0.50
0.25
0.00
A (n=15)
B (n=16)
C (n=14)
surface

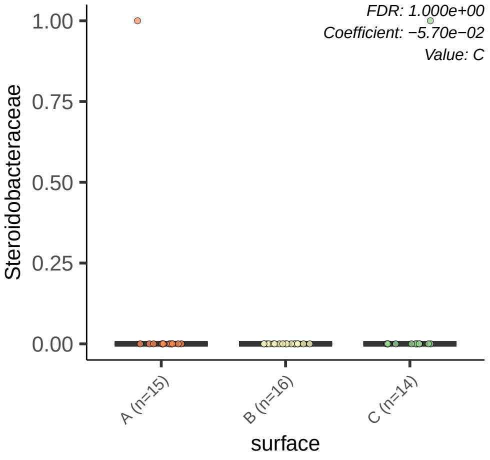
FDR: 1.000e+00
Coefficient: −5.70e−02
Value: C
Steroidobacteraceae
1.00
0.75
0.50
0.25
0.00
A (n=15)
B (n=16)
C (n=14)
surface

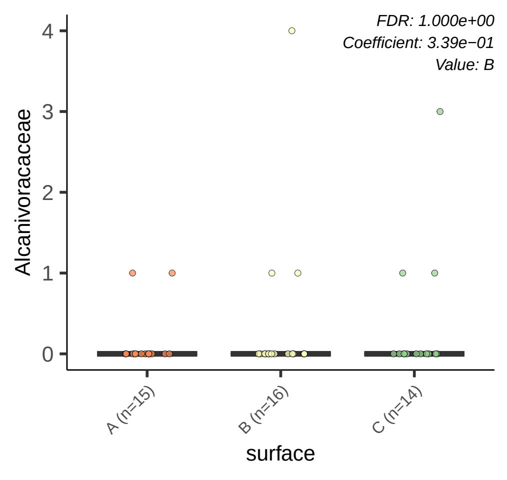
FDR: 1.000e+00
Coefficient: 3.39e−01
Value: B
Alcanivoracaceae
0
1
2
3
4
A (n=15)
B (n=16)
C (n=14)
surface

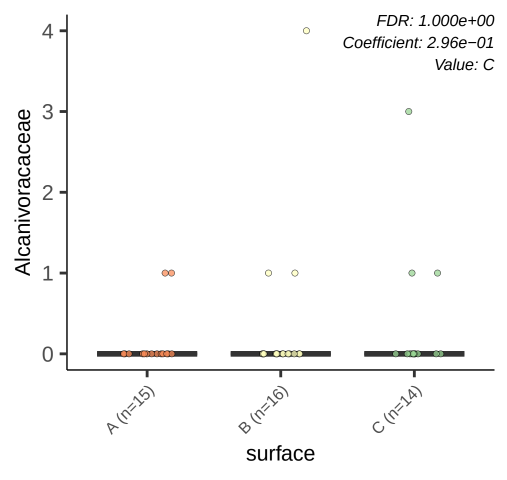
FDR: 1.000e+00
Coefficient: 2.96e−01
Value: C
Alcanivoracaceae
0
1
2
3
4
A (n=15)
B (n=16)
C (n=14)
surface

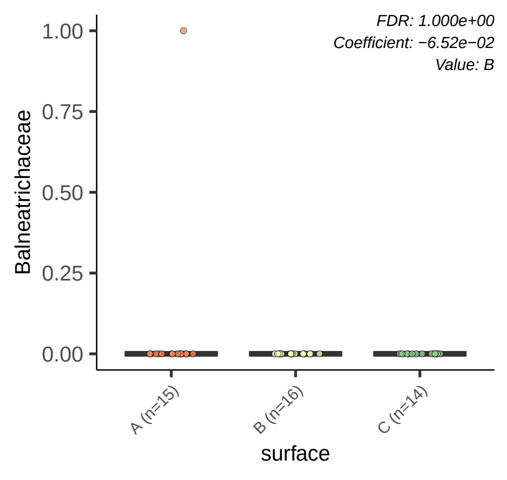
FDR: 1.000e+00
Coefficient: −6.52e−02
Value: B
Balneatrichaceae
1.00
0.75
0.50
0.25
0.00
A (n=15)
B (n=16)
C (n=14)
surface

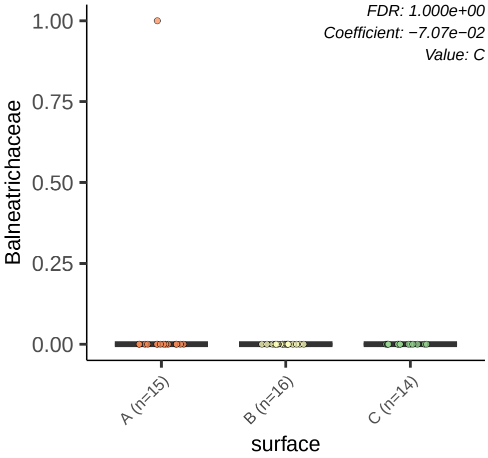
FDR: 1.000e+00
Coefficient: −7.07e−02
Value: C
Balneatrichaceae
1.00
0.75
0.50
0.25
0.00
A (n=15)
B (n=16)
C (n=14)
surface

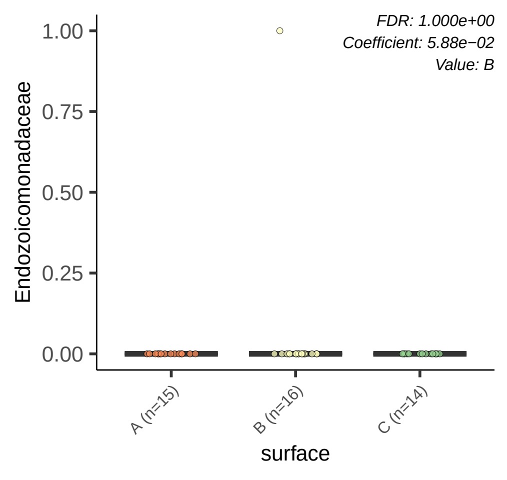
FDR: 1.000e+00
Coefficient: 5.88e−02
Value: B
Endozoicomonadaceae
1.00
0.75
0.50
0.25
0.00
A (n=15)
B (n=16)
C (n=14)
surface

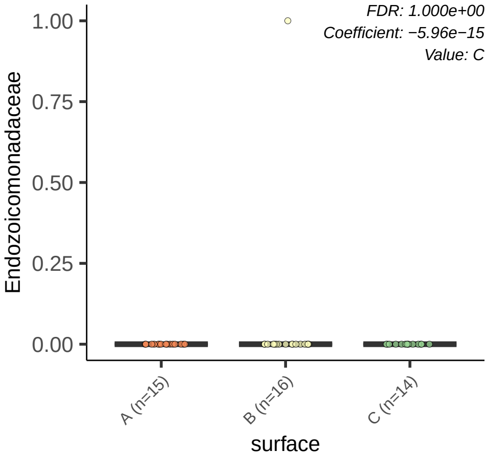

FDR: 1.000e+00
Coefficient: −5.96e−15
Value: C
Endozoicomonadaceae
1.00
0.75
0.50
0.25
0.00
A (n=15)
B (n=16)
C (n=14)
surface

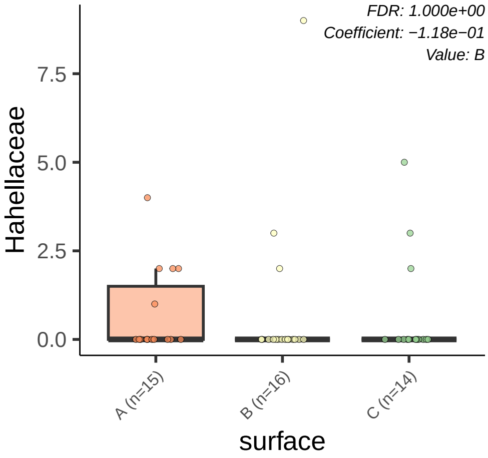

FDR: 1.000e+00
Coefficient: −1.18e−01
Value: B
Hahellaceae
7.5
5.0
2.5
0.0
A (n=15)
B (n=16)
C (n=14)
surface

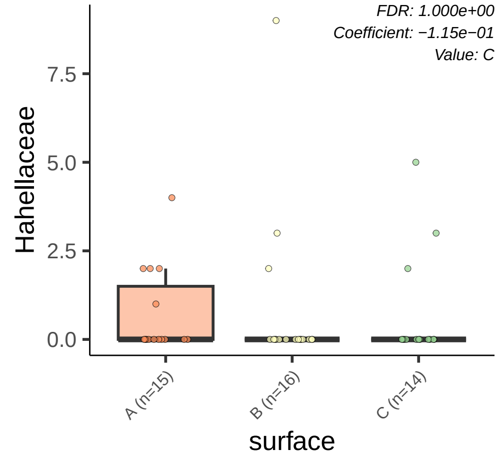

FDR: 1.000e+00
Coefficient: −1.15e−01
Value: C
Hahellaceae
7.5
5.0
2.5
0.0
A (n=15)
B (n=16)
C (n=14)
surface

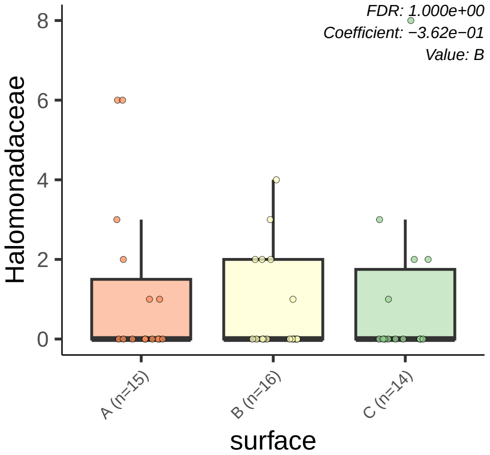

FDR: 1.000e+00
Coefficient: −3.62e−01
Value: B
Halomonadaceae
0
2
4
6
8
A (n=15)
B (n=16)
C (n=14)
surface

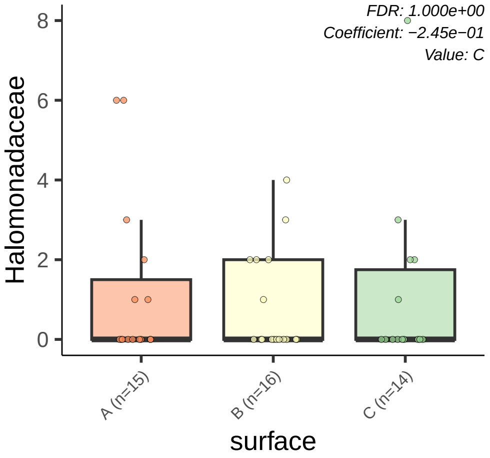

FDR: 1.000e+00
Coefficient: −2.45e−01
Value: C
Halomonadaceae
0
2
4
6
8
A (n=15)
B (n=16)
C (n=14)
surface

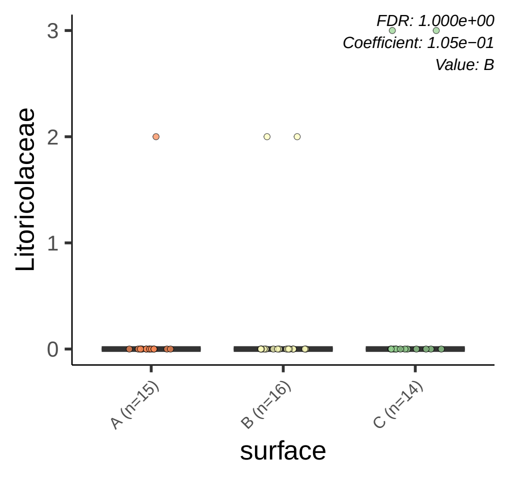

FDR: 1.000e+00
Coefficient: 1.05e−01
Value: B
Litoricolaceae
0
1
2
3
A (n=15)
B (n=16)
C (n=14)
surface

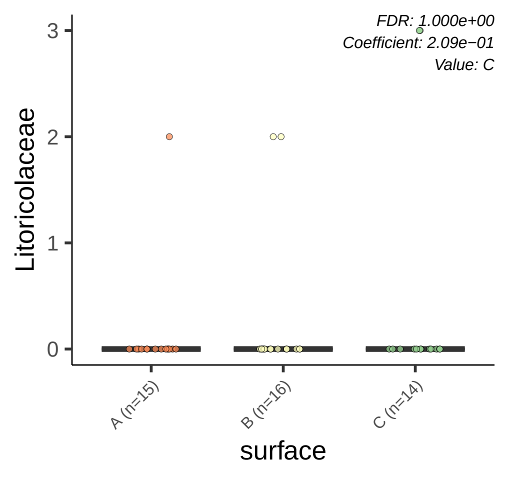

FDR: 1.000e+00
Coefficient: 2.09e−01
Value: C
Litoricolaceae
0
1
2
3
A (n=15)
B (n=16)
C (n=14)
surface

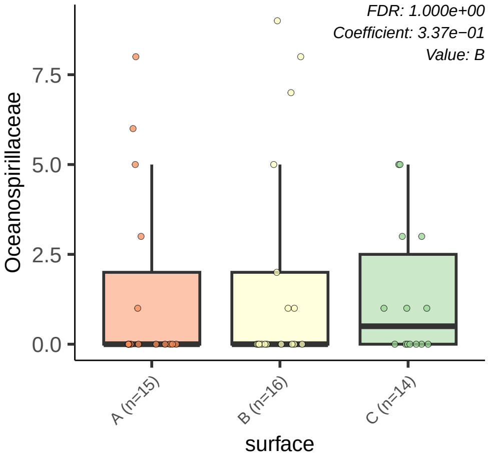

FDR: 1.000e+00
Coefficient: 3.37e−01
Value: B
Oceanospirillaceae
7.5
5.0
2.5
0.0
A (n=15)
B (n=16)
C (n=14)
surface

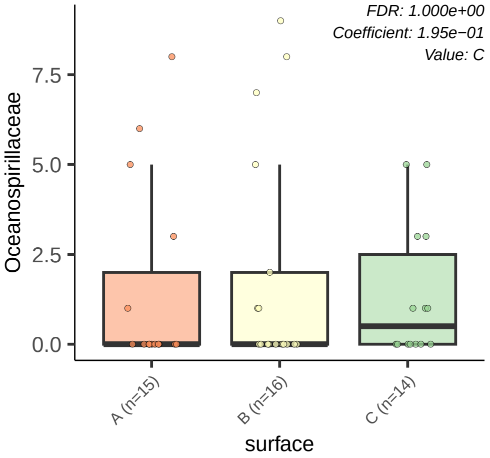
FDR: 1.000e+00
Coefficient: 1.95e−01
Value: C
Oceanospirillaceae
0.0
2.5
5.0
7.5
A (n=15)
B (n=16)
C (n=14)
surface

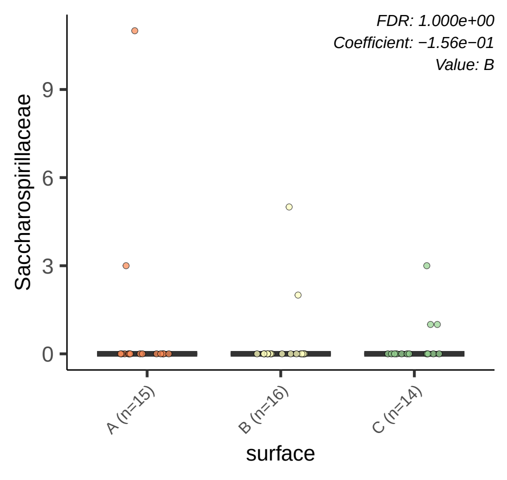
FDR: 1.000e+00
Coefficient: −1.56e−01
Value: B
Saccharospirillaceae
0
3
6
9
A (n=15)
B (n=16)
C (n=14)
surface

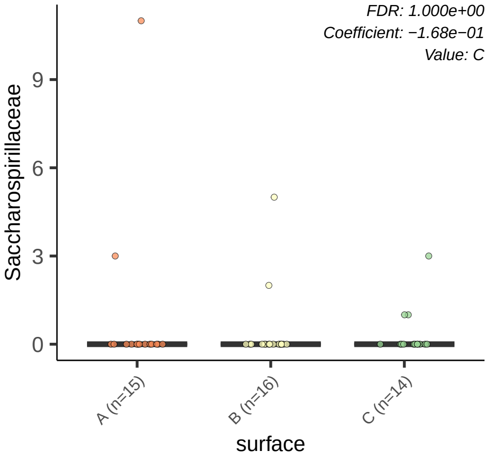
FDR: 1.000e+00
Coefficient: −1.68e−01
Value: C
Saccharospirillaceae
0
3
6
9
A (n=15)
B (n=16)
C (n=14)
surface

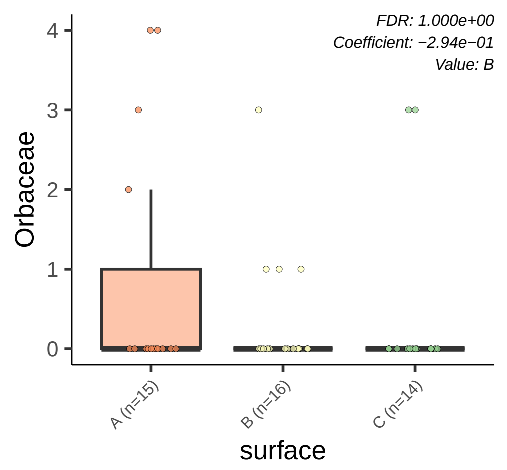
FDR: 1.000e+00
Coefficient: −2.94e−01
Value: B
Orbaceae
4
3
2
1
0
A (n=15)
B (n=16)
C (n=14)
surface

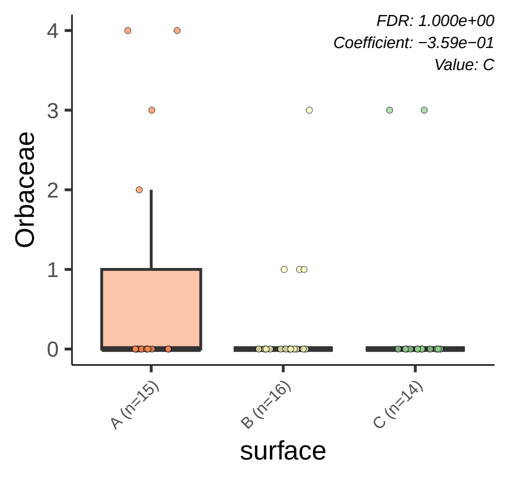

FDR: 1.000e+00
Coefficient: −3.59e−01
Value: C
Orbaceae
4
3
2
1
0
A (n=15)
B (n=16)
C (n=14)
surface

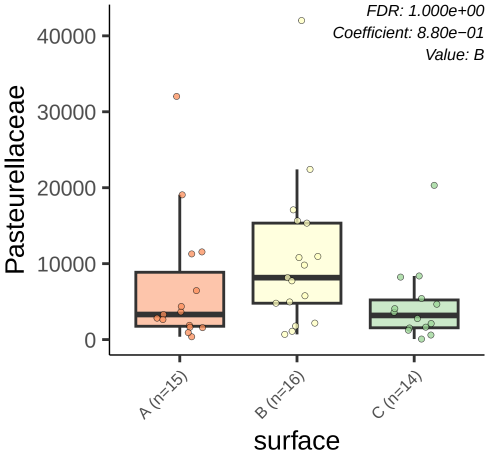
FDR: 1.000e+00
Coefficient: 8.80e−01
Value: B
Pasteurellaceae
40000
30000
20000
10000
0
A (n=15)
B (n=16)
C (n=14)
surface

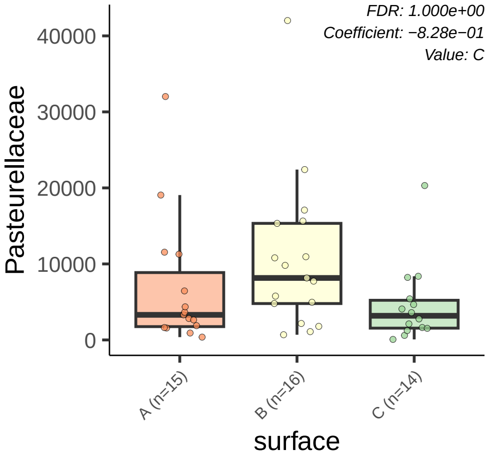

FDR: 1.000e+00
Coefficient: −8.28e−01
Value: C
Pasteurellaceae
40000
30000
20000
10000
0
A (n=15)
B (n=16)
C (n=14)
surface

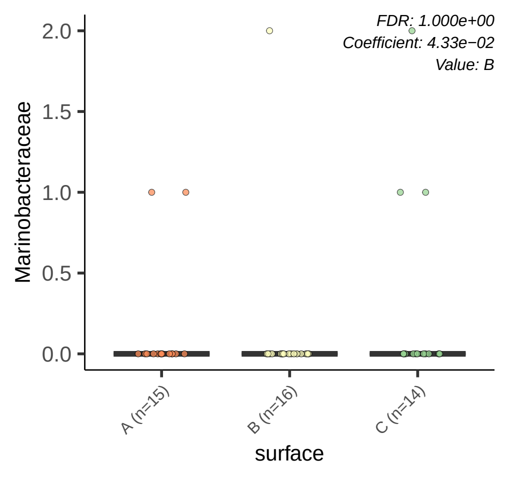
FDR: 1.000e+00
Coefficient: 4.33e−02
Value: B
Marinobacteraceae
2.0
1.5
1.0
0.5
0.0
A (n=15)
B (n=16)
C (n=14)
surface

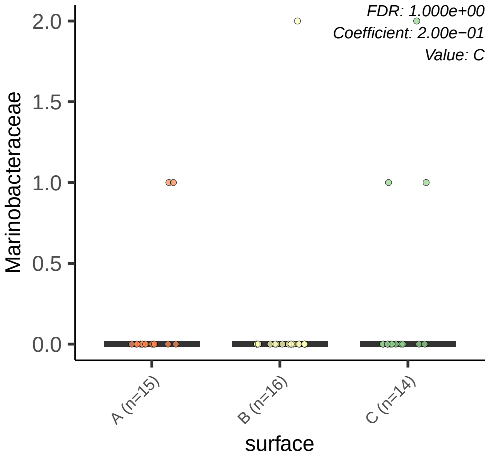

FDR: 1.000e+00
Coefficient: 2.00e−01
Value: C
2.0
1.5
1.0
0.5
0.0
Marinobacteraceae
A (n=15)
B (n=16)
C (n=14)
surface

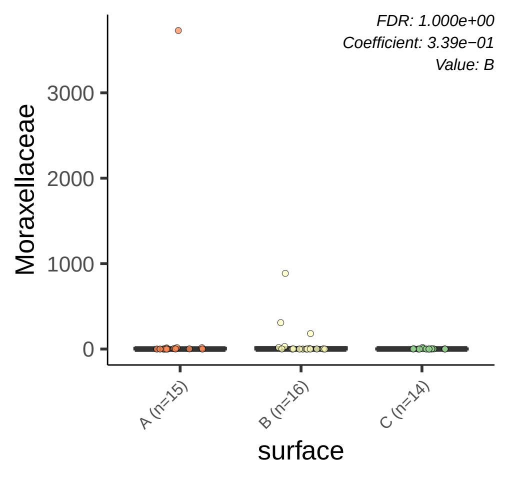
FDR: 1.000e+00
Coefficient: 3.39e−01
Value: B
Moraxellaceae
3000
2000
1000
0
A (n=15)
B (n=16)
C (n=14)
surface

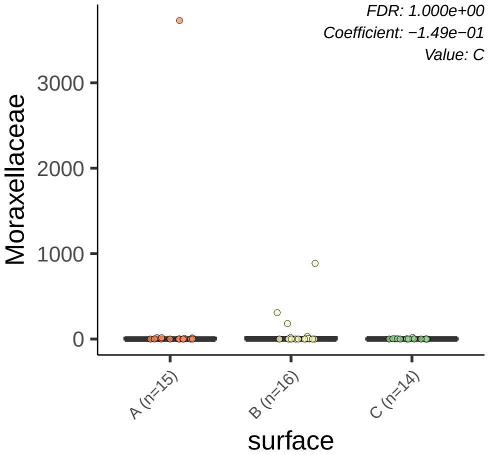
FDR: 1.000e+00
Coefficient: −1.49e−01
Value: C
Moraxellaceae
3000
2000
1000
0
A (n=15)
B (n=16)
C (n=14)
surface

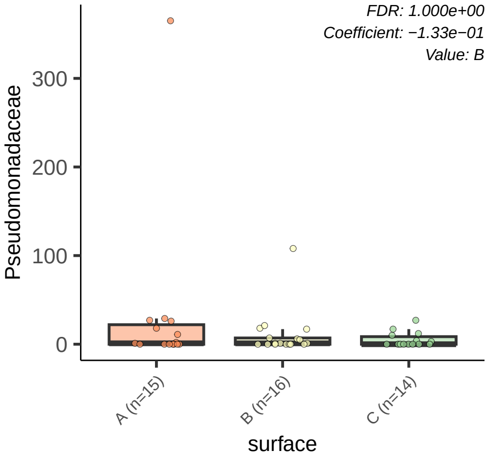

FDR: 1.000e+00
Coefficient: −1.33e−01
Value: B
Pseudomonadaceae
300
200
100
0
A (n=15)
B (n=16)
C (n=14)
surface

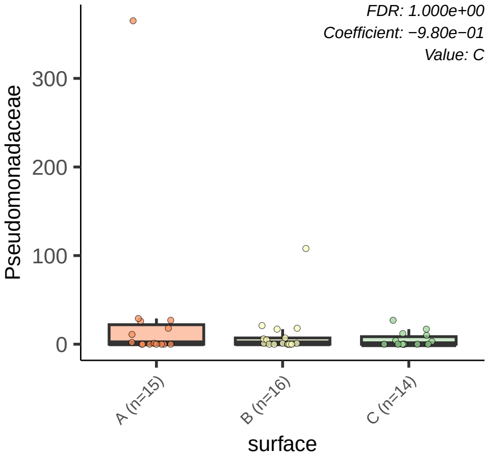
FDR: 1.000e+00
Coefficient: −9.80e−01
Value: C
Pseudomonadaceae
300
200
100
0
A (n=15)
B (n=16)
C (n=14)
surface

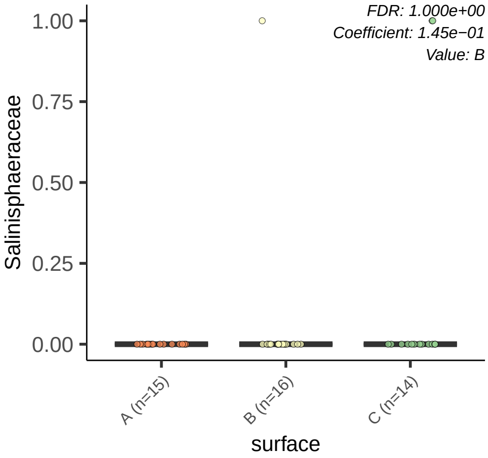
FDR: 1.000e+00
Coefficient: 1.45e−01
Value: B
Salinisphaeraceae
1.00
0.75
0.50
0.25
0.00
A (n=15)
B (n=16)
C (n=14)
surface

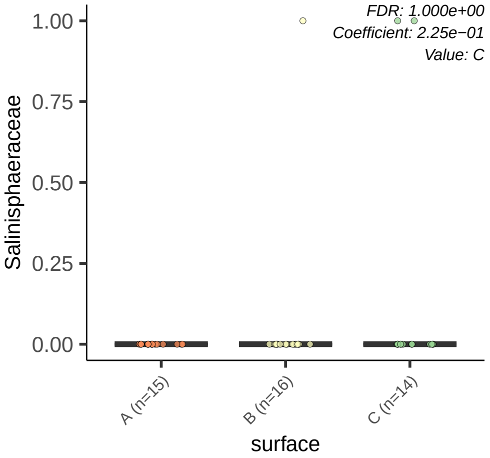

FDR: 1.000e+00
Coefficient: 2.25e−01
Value: C
Salinisphaeraceae
1.00
0.75
0.50
0.25
0.00
A (n=15)
B (n=16)
C (n=14)
surface

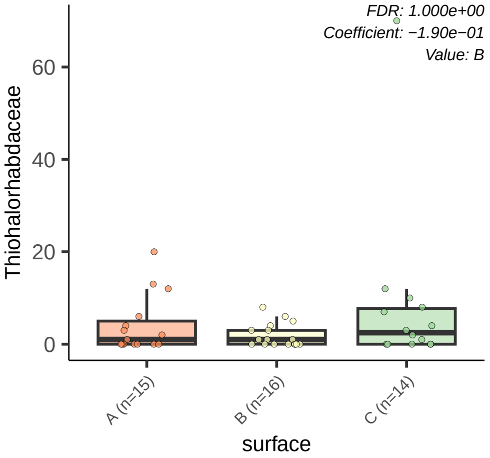
FDR: 1.000e+00
Coefficient: −1.90e−01
Value: B
Thiohalorhabdaceae
0
20
40
60
A (n=15)
B (n=16)
C (n=14)
surface

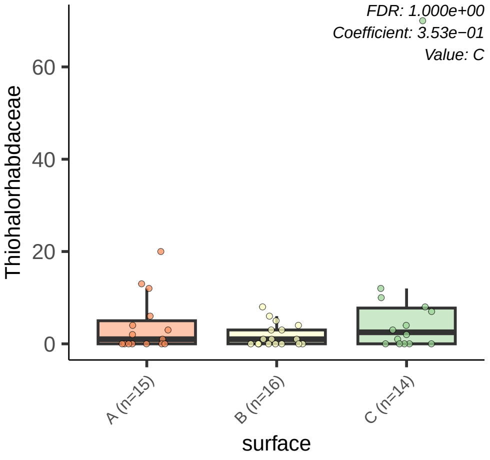
FDR: 1.000e+00
Coefficient: 3.53e−01
Value: C
Thiohalorhabdaceae
0
20
40
60
A (n=15)
B (n=16)
C (n=14)
surface

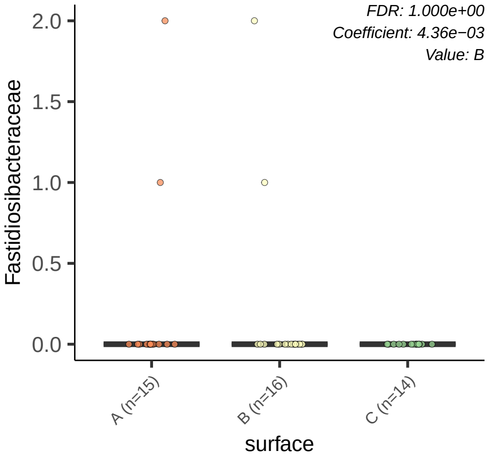

FDR: 1.000e+00
Coefficient: 4.36e−03
Value: B
Fastidiosibacteraceae
2.0
1.5
1.0
0.5
0.0
A (n=15)
B (n=16)
C (n=14)
surface

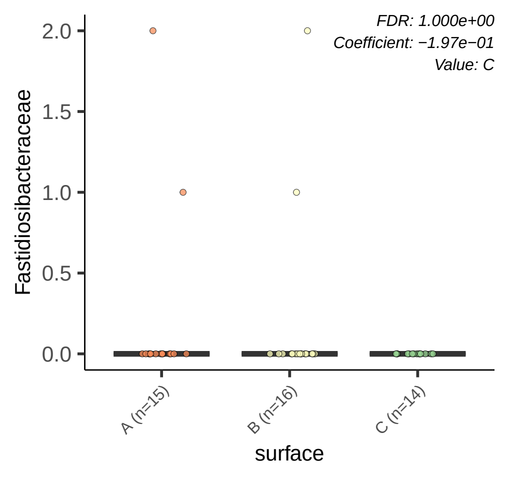
FDR: 1.000e+00
Coefficient: −1.97e−01
Value: C
Fastidiosibacteraceae
2.0
1.5
1.0
0.5
0.0
A (n=15)
B (n=16)
C (n=14)
surface

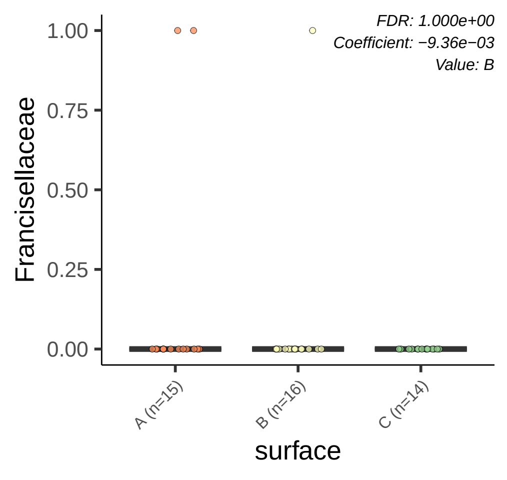

FDR: 1.000e+00
Coefficient: −9.36e−03
Value: B
Francisellaceae
1.00
0.75
0.50
0.25
0.00
A (n=15)
B (n=16)
C (n=14)
surface

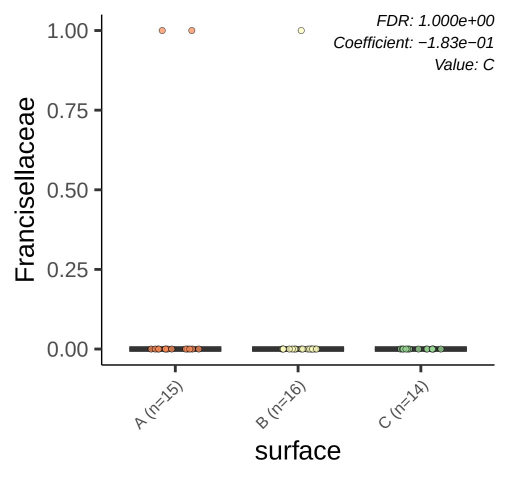

FDR: 1.000e+00
Coefficient: −1.83e−01
Value: C
Francisellaceae
1.00
0.75
0.50
0.25
0.00
A (n=15)
B (n=16)
C (n=14)
surface

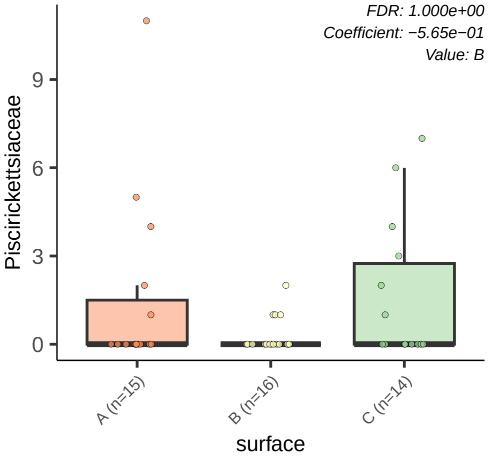

FDR: 1.000e+00
Coefficient: −5.65e−01
Value: B
Piscirickettsiaceae
0
3
6
9
A (n=15)
B (n=16)
C (n=14)
surface

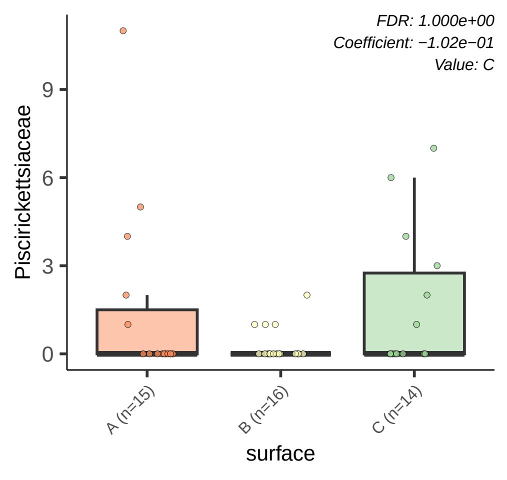
FDR: 1.000e+00
Coefficient: −1.02e−01
Value: C
Piscirickettsiaceae
0
3
6
9
A (n=15)
B (n=16)
C (n=14)
surface

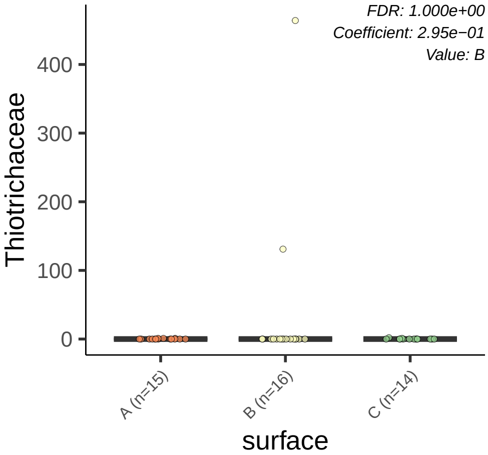
FDR: 1.000e+00
Coefficient: 2.95e−01
Value: B
Thiotrichaceae
400
300
200
100
0
A (n=15)
B (n=16)
C (n=14)
surface

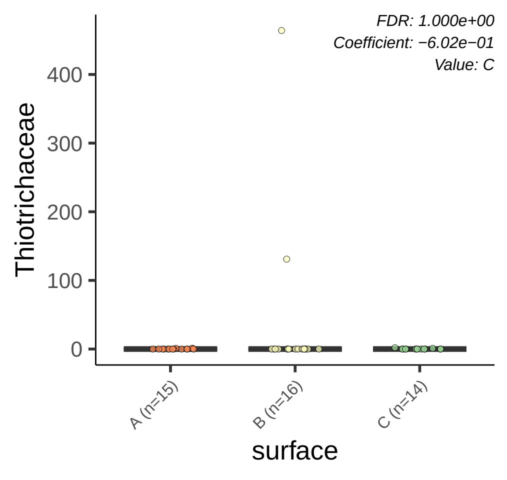

FDR: 1.000e+00
Coefficient: −6.02e−01
Value: C
Thiotrichaceae
400
300
200
100
0
A (n=15)
B (n=16)
C (n=14)
surface

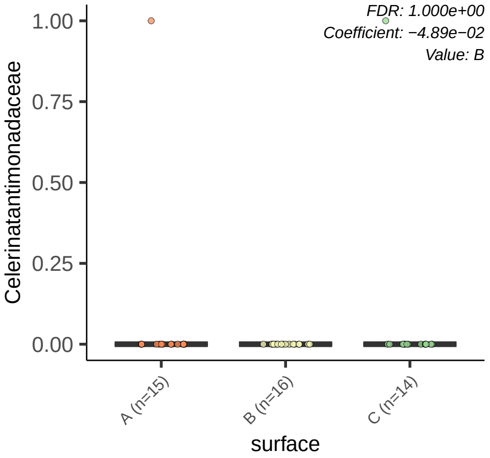

FDR: 1.000e+00
Coefficient: −4.89e−02
Value: B
1.00
0.75
0.50
0.25
0.00
Celerinatantimonadaceae
A (n=15)
B (n=16)
C (n=14)
surface

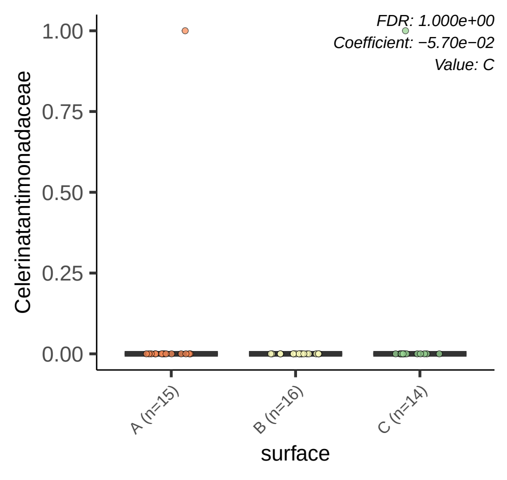
FDR: 1.000e+00
Coefficient: −5.70e−02
Value: C
Celerinatantimonadaceae
1.00
0.75
0.50
0.25
0.00
A (n=15)
B (n=16)
C (n=14)
surface

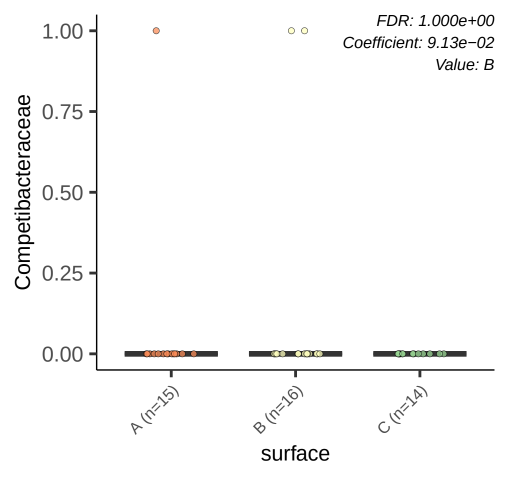
FDR: 1.000e+00
Coefficient: 9.13e−02
Value: B
Competibacteraceae
1.00
0.75
0.50
0.25
0.00
A (n=15)
B (n=16)
C (n=14)
surface

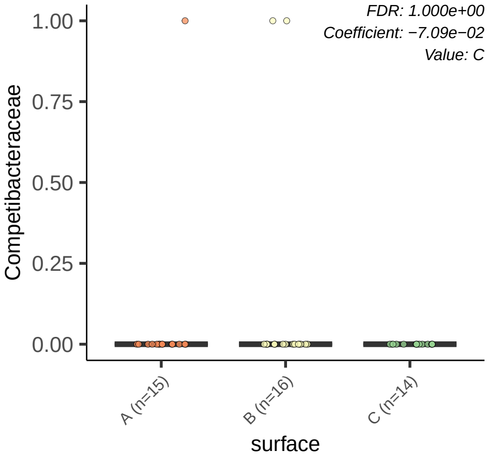
FDR: 1.000e+00
Coefficient: −7.09e−02
Value: C
Competibacteraceae
1.00
0.75
0.50
0.25
0.00
A (n=15)
B (n=16)
C (n=14)
surface

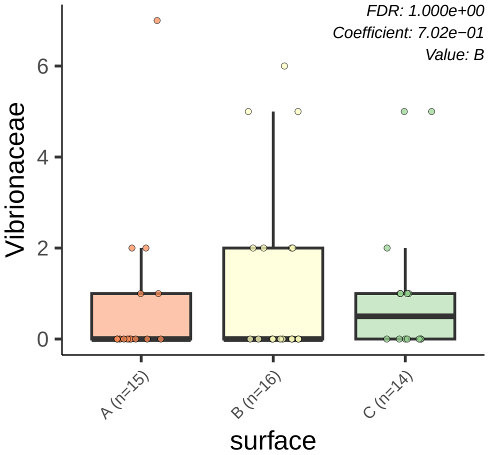

FDR: 1.000e+00
Coefficient: 7.02e−01
Value: B
Vibrionaceae
0
2
4
6
A (n=15)
B (n=16)
C (n=14)
surface

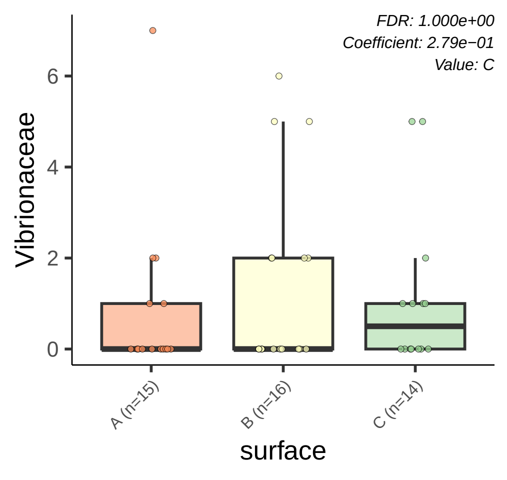

FDR: 1.000e+00
Coefficient: 2.79e−01
Value: C
Vibrionaceae
0
2
4
6
A (n=15)
B (n=16)
C (n=14)
surface

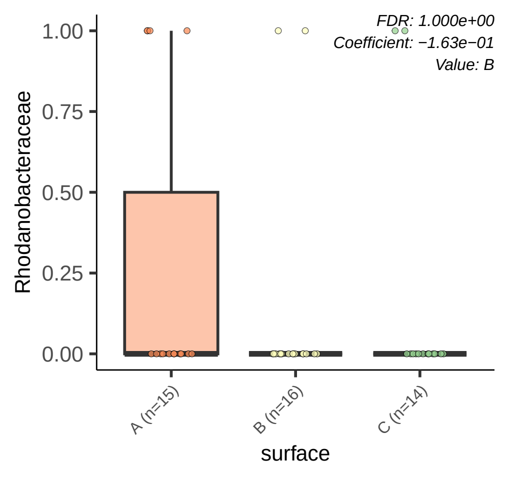
FDR: 1.000e+00
Coefficient: −1.63e−01
Value: B
1.00
0.75
0.50
0.25
0.00
Rhodanobacteraceae
A (n=15)
B (n=16)
C (n=14)
surface

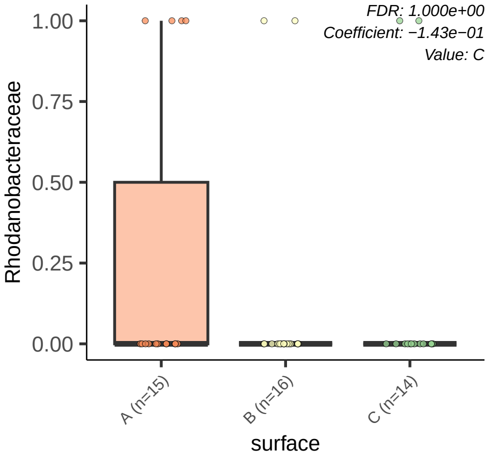

FDR: 1.000e+00
Coefficient: −1.43e−01
Value: C
Rhodanobacteraceae
1.00
0.75
0.50
0.25
0.00
A (n=15)
B (n=16)
C (n=14)
surface

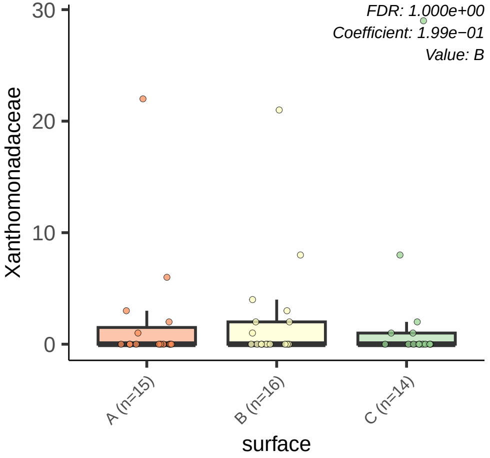
FDR: 1.000e+00
Coefficient: 1.99e−01
Value: B
Xanthomonadaceae
0
10
20
30
A (n=15)
B (n=16)
C (n=14)
surface

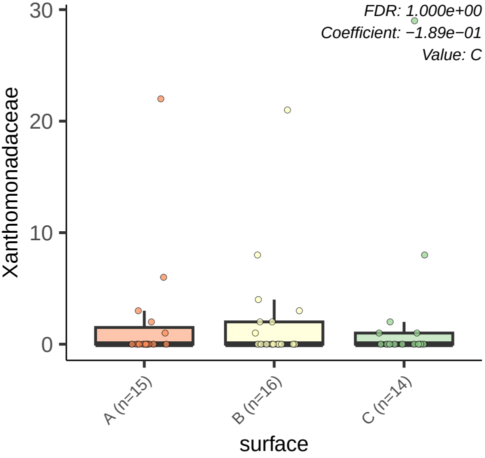

FDR: 1.000e+00
Coefficient: −1.89e−01
Value: C
Xanthomonadaceae
30
20
10
0
A (n=15)
B (n=16)
C (n=14)
surface

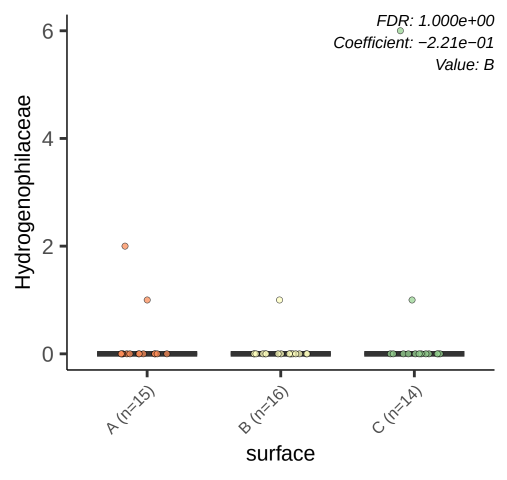
FDR: 1.000e+00
Coefficient: −2.21e−01
Value: B
Hydrogenophilaceae
6
4
2
0
A (n=15)
B (n=16)
C (n=14)
surface

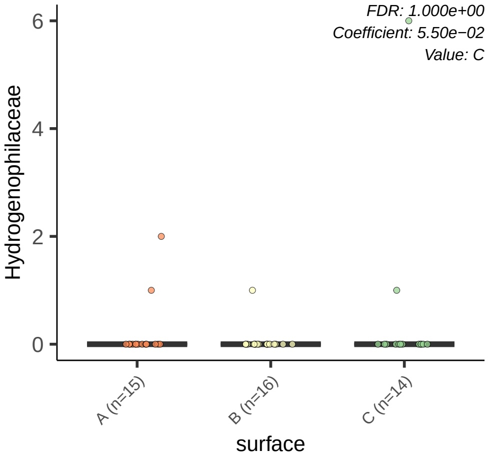

FDR: 1.000e+00
Coefficient: 5.50e−02
Value: C
Hydrogenophilaceae
0
2
4
6
A (n=15)
B (n=16)
C (n=14)
surface

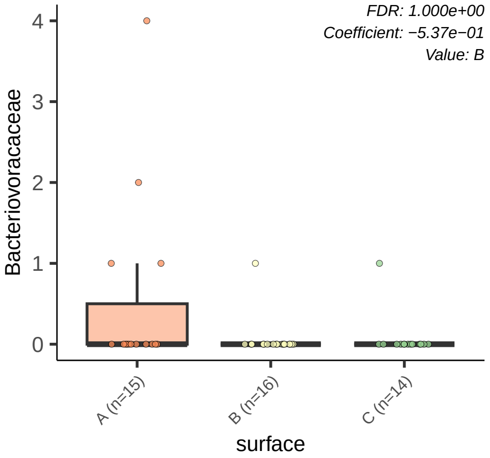
FDR: 1.000e+00
Coefficient: −5.37e−01
Value: B
Bacteriovoracaceae
4
3
2
1
0
A (n=15)
B (n=16)
C (n=14)
surface

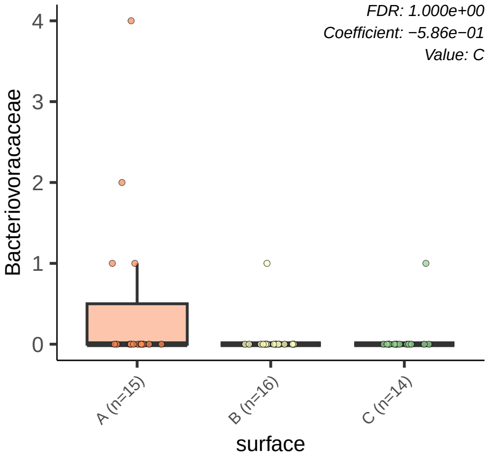
FDR: 1.000e+00
Coefficient: −5.86e−01
Value: C
Bacteriovoracaceae
0
1
2
3
4
A (n=15)
B (n=16)
C (n=14)
surface

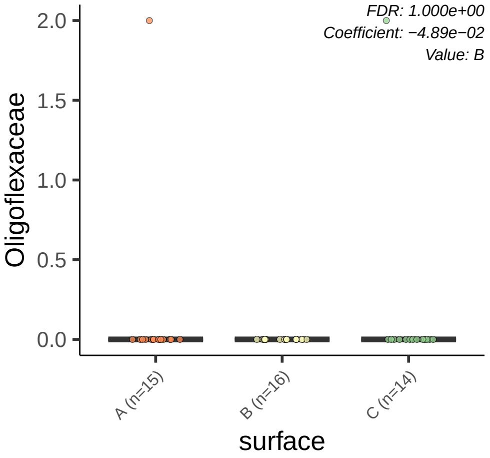

FDR: 1.000e+00
Coefficient: −4.89e−02
Value: B
Oligoflexaceae
2.0
1.5
1.0
0.5
0.0
A (n=15)
B (n=16)
C (n=14)
surface

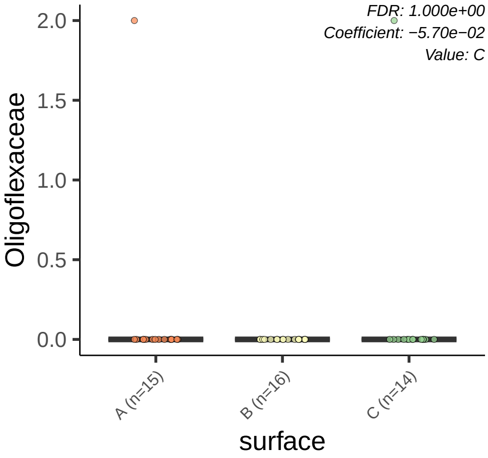

FDR: 1.000e+00
Coefficient: −5.70e−02
Value: C
Oligoflexaceae
2.0
1.5
1.0
0.5
0.0
A (n=15)
B (n=16)
C (n=14)
surface

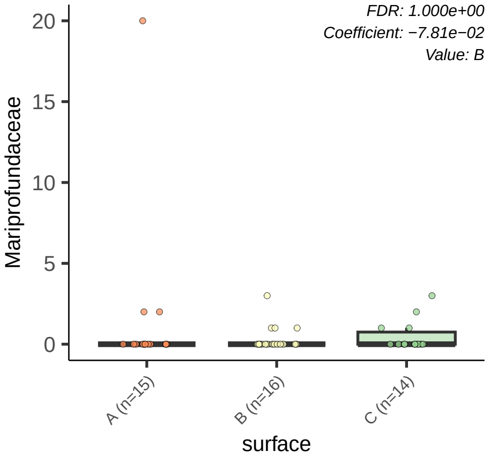

FDR: 1.000e+00
Coefficient: −7.81e−02
Value: B
Mariprofundaceae
20
15
10
5
0
A (n=15)
B (n=16)
C (n=14)
surface

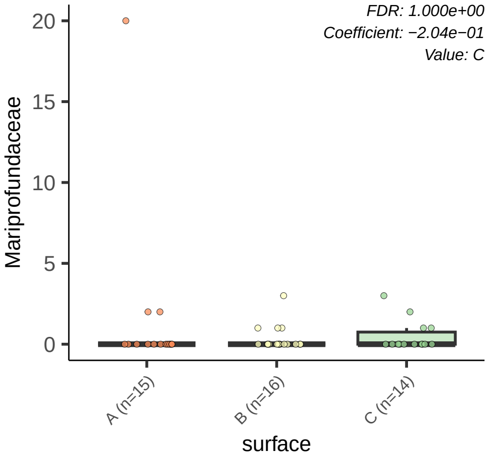

FDR: 1.000e+00
Coefficient: −2.04e−01
Value: C
Mariprofundaceae
20
15
10
5
0
A (n=15)
B (n=16)
C (n=14)
surface

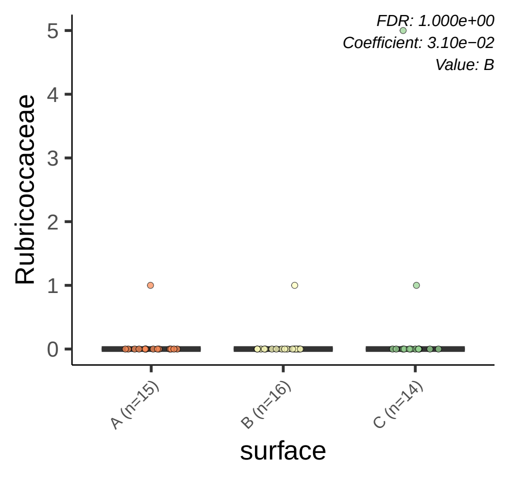
FDR: 1.000e+00
Coefficient: 3.10e−02
Value: B
Rubricoccaceae
0
1
2
3
4
5
A (n=15)
B (n=16)
C (n=14)
surface

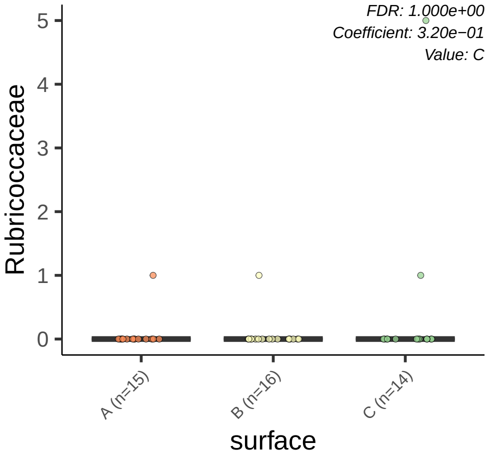

FDR: 1.000e+00
Coefficient: 3.20e−01
Value: C
Rubricoccaceae
0
1
2
3
4
5
A (n=15)
B (n=16)
C (n=14)
surface

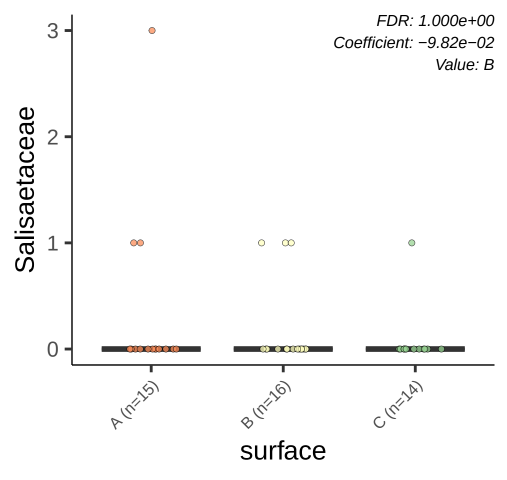
FDR: 1.000e+00
Coefficient: −9.82e−02
Value: B
Salisaetaceae
3
2
1
0
A (n=15)
B (n=16)
C (n=14)
surface

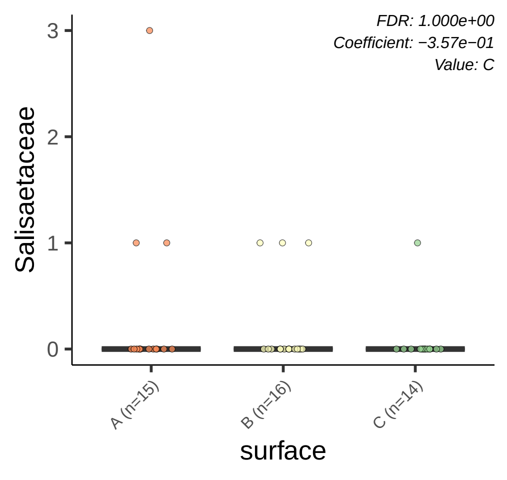

FDR: 1.000e+00
Coefficient: −3.57e−01
Value: C
Salisaetaceae
3
2
1
0
A (n=15)
B (n=16)
C (n=14)
surface

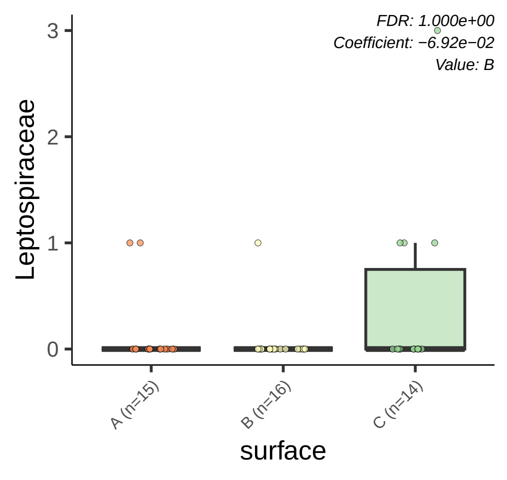

FDR: 1.000e+00
Coefficient: −6.92e−02
Value: B
Leptospiraceae
3
2
1
0
A (n=15)
B (n=16)
C (n=14)
surface

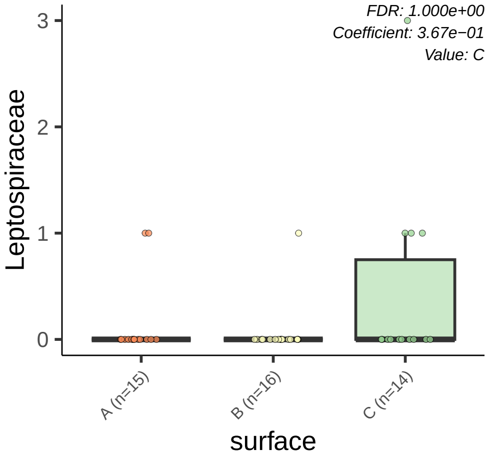

FDR: 1.000e+00
Coefficient: 3.67e−01
Value: C
Leptospiraceae
3
2
1
0
A (n=15)
B (n=16)
C (n=14)
surface

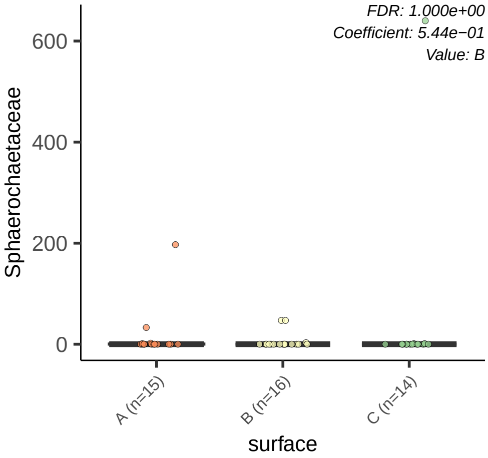
FDR: 1.000e+00
Coefficient: 5.44e−01
Value: B
Sphaerochaetaceae
600
400
200
0
A (n=15)
B (n=16)
C (n=14)
surface

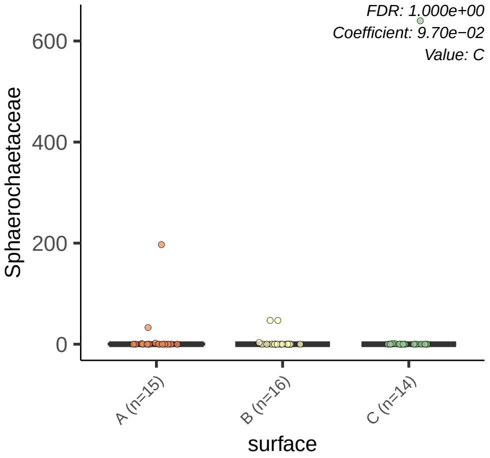
FDR: 1.000e+00
Coefficient: 9.70e−02
Value: C
Sphaerochaetaceae
600
400
200
0
A (n=15)
B (n=16)
C (n=14)
surface

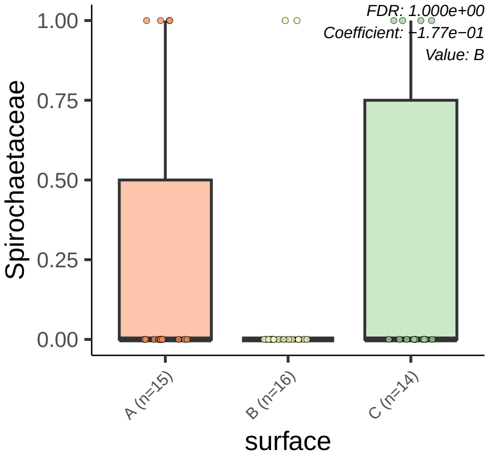

FDR: 1.000e+00
Coefficient: −1.77e−01
Value: B
Spirochaetaceae
1.00
0.75
0.50
0.25
0.00
A (n=15)
B (n=16)
C (n=14)
surface

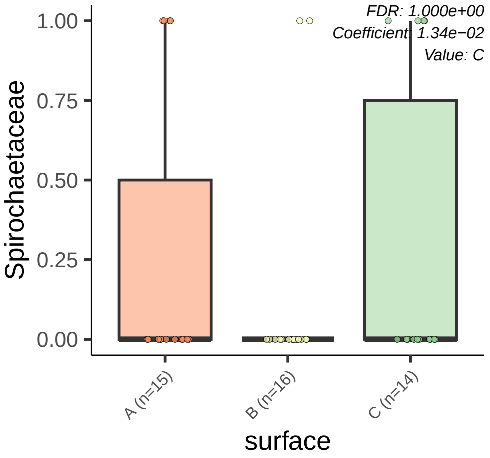

FDR: 1.000e+00
Coefficient: 1.34e−02
Value: C
Spirochaetaceae
1.00
0.75
0.50
0.25
0.00
A (n=15)
B (n=16)
C (n=14)
surface

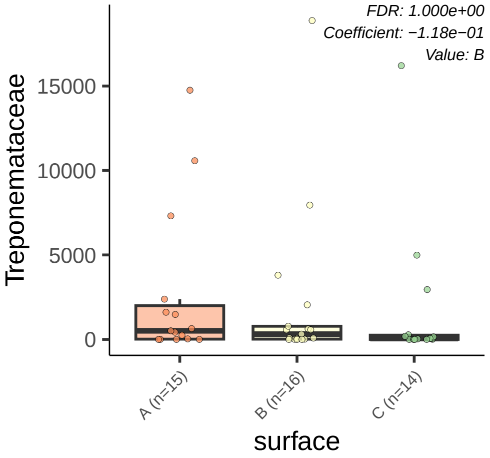
FDR: 1.000e+00
Coefficient: −1.18e−01
Value: B
Treponemataceae
15000
10000
5000
0
A (n=15)
B (n=16)
C (n=14)
surface

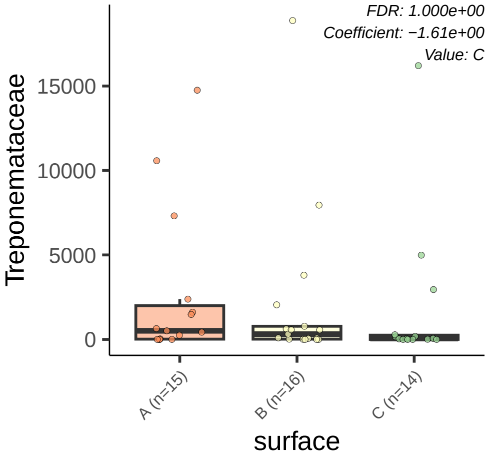
FDR: 1.000e+00
Coefficient: −1.61e+00
Value: C
Treponemataceae
15000
10000
5000
0
A (n=15)
B (n=16)
C (n=14)
surface

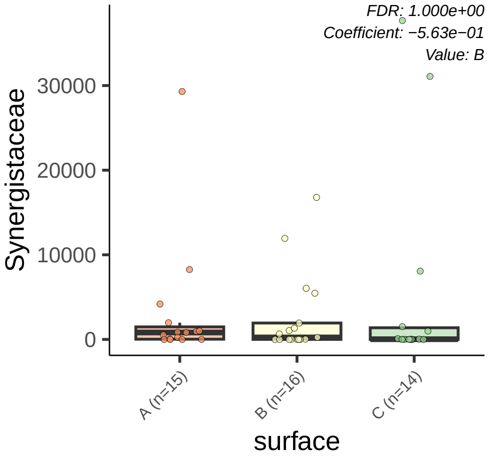

FDR: 1.000e+00
Coefficient: −5.63e−01
Value: B
Synergistaceae
30000
20000
10000
0
A (n=15)
B (n=16)
C (n=14)
surface

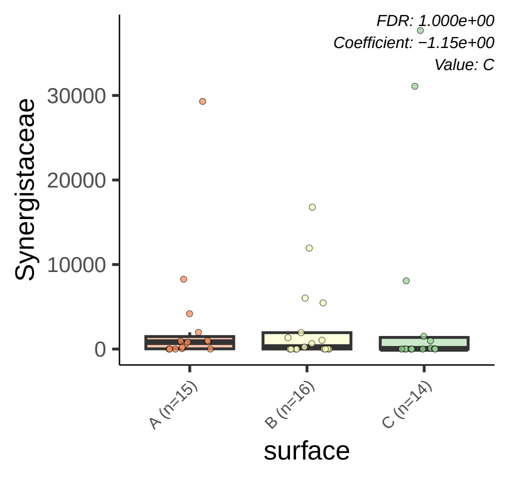
FDR: 1.000e+00
Coefficient: −1.15e+00
Value: C
Synergistaceae
30000
20000
10000
0
A (n=15)
B (n=16)
C (n=14)
surface

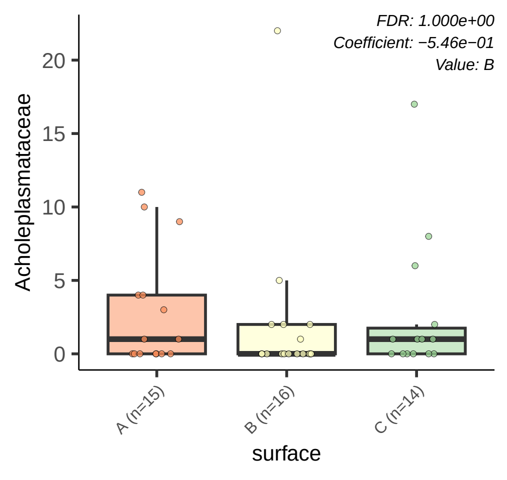

FDR: 1.000e+00
Coefficient: −5.46e−01
Value: B
Acholeplasmataceae
0
5
10
15
20
A (n=15)
B (n=16)
C (n=14)
surface

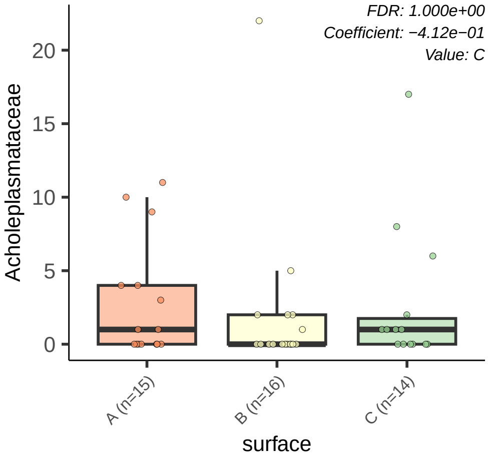

FDR: 1.000e+00
Coefficient: −4.12e−01
Value: C
Acholeplasmataceae
20
15
10
5
0
A (n=15)
B (n=16)
C (n=14)
surface

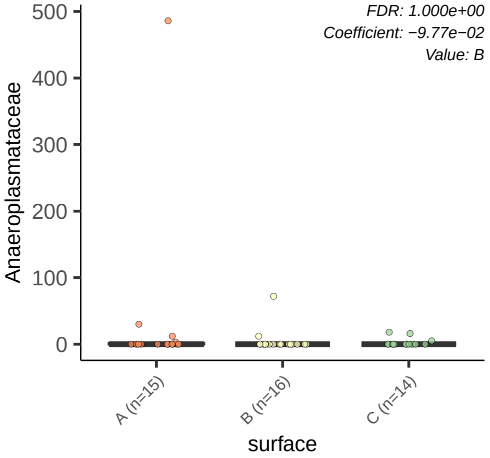

FDR: 1.000e+00
Coefficient: −9.77e−02
Value: B
Anaeroplasmataceae
500
400
300
200
100
0
A (n=15)
B (n=16)
C (n=14)
surface

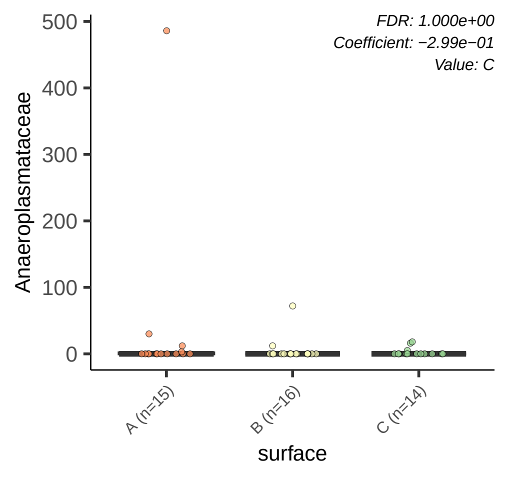
FDR: 1.000e+00
Coefficient: −2.99e−01
Value: C
Anaeroplasmataceae
0
100
200
300
400
500
A (n=15)
B (n=16)
C (n=14)
surface

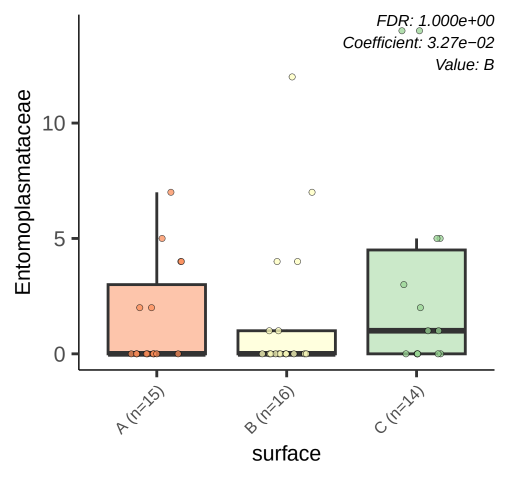
FDR: 1.000e+00
Coefficient: 3.27e−02
Value: B
Entomoplasmataceae
10
5
0
A (n=15)
B (n=16)
C (n=14)
surface

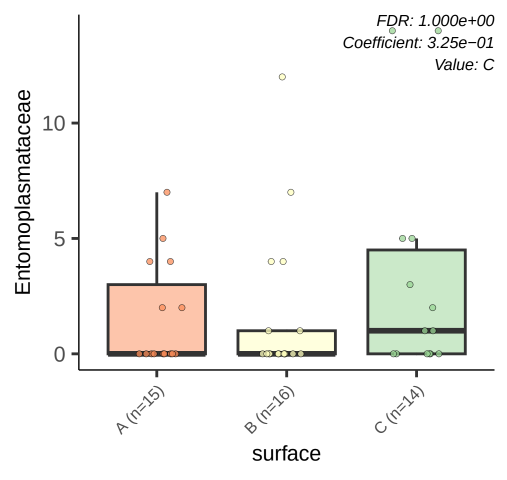

FDR: 1.000e+00
Coefficient: 3.25e−01
Value: C
Entomoplasmataceae
10
5
0
A (n=15)
B (n=16)
C (n=14)
surface

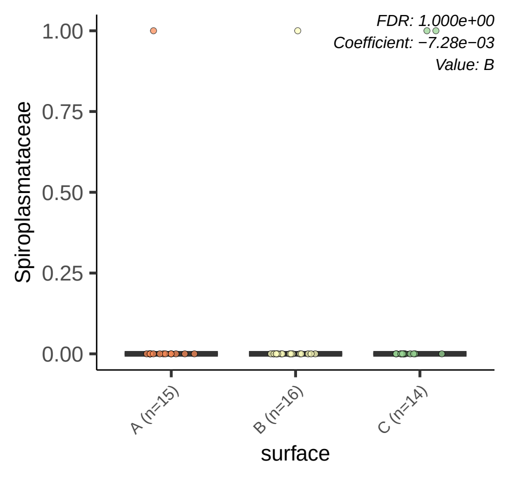
FDR: 1.000e+00
Coefficient: −7.28e−03
Value: B
Spiroplasmataceae
1.00
0.75
0.50
0.25
0.00
A (n=15)
B (n=16)
C (n=14)
surface

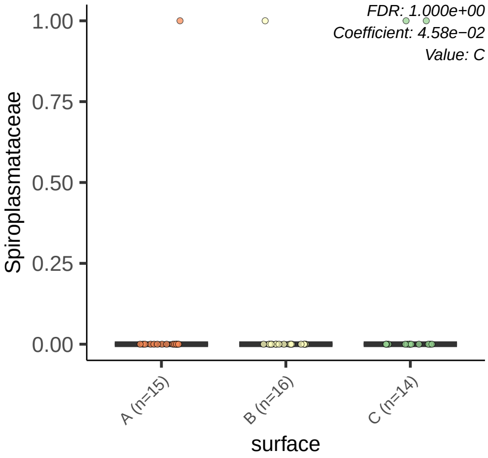

FDR: 1.000e+00
Coefficient: 4.58e−02
Value: C
Spiroplasmataceae
1.00
0.75
0.50
0.25
0.00
A (n=15)
B (n=16)
C (n=14)
surface

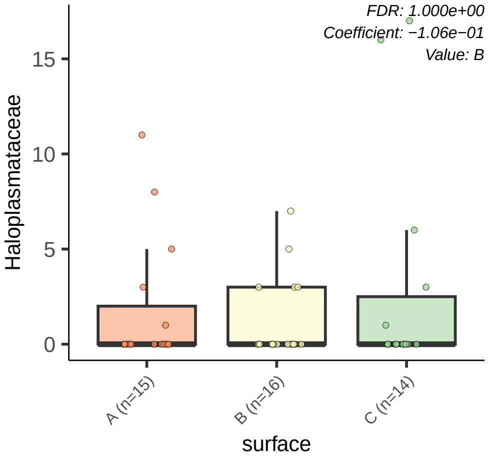

FDR: 1.000e+00
Coefficient: −1.06e−01
Value: B
Haloplasmataceae
0
5
10
15
A (n=15)
B (n=16)
C (n=14)
surface

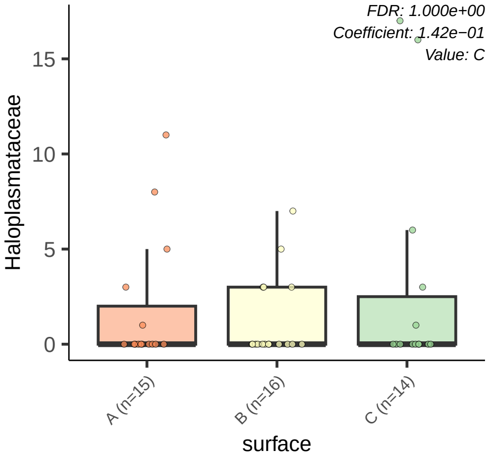

FDR: 1.000e+00
Coefficient: 1.42e−01
Value: C
Haloplasmataceae
0
5
10
15
A (n=15)
B (n=16)
C (n=14)
surface

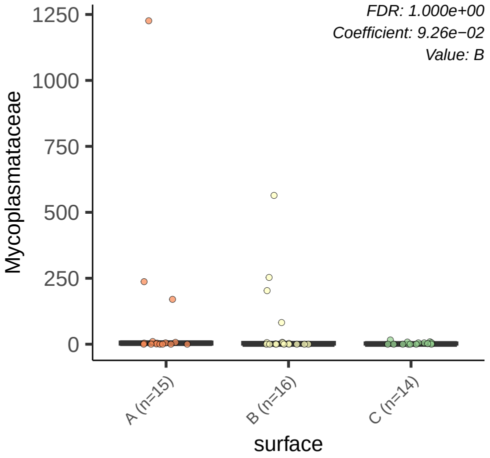
FDR: 1.000e+00
Coefficient: 9.26e−02
Value: B
Mycoplasmataceae
1250
1000
750
500
250
0
A (n=15)
B (n=16)
C (n=14)
surface

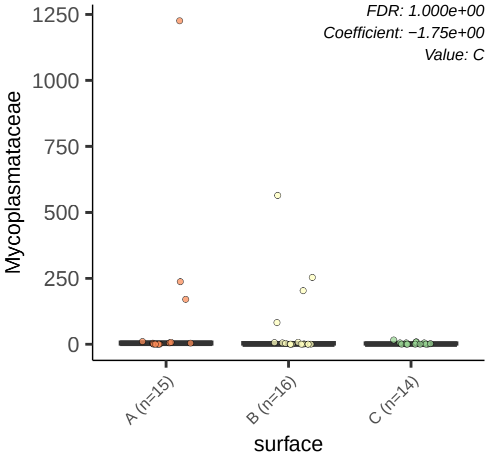
FDR: 1.000e+00
Coefficient: −1.75e+00
Value: C
Mycoplasmataceae
1250
1000
750
500
250
0
A (n=15)
B (n=16)
C (n=14)
surface

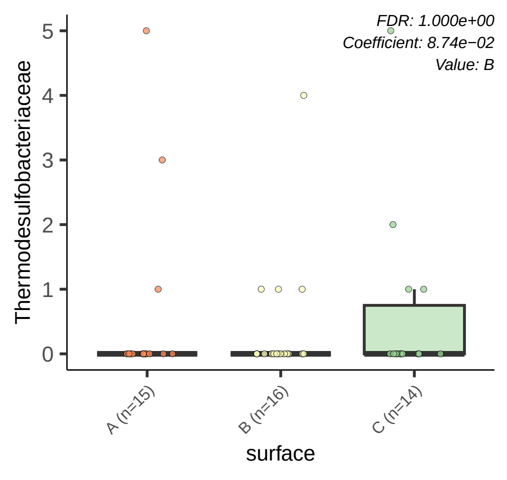
FDR: 1.000e+00
Coefficient: 8.74e−02
Value: B
Thermodesulfobacteriaceae
0
1
2
3
4
5
A (n=15)
B (n=16)
C (n=14)
surface

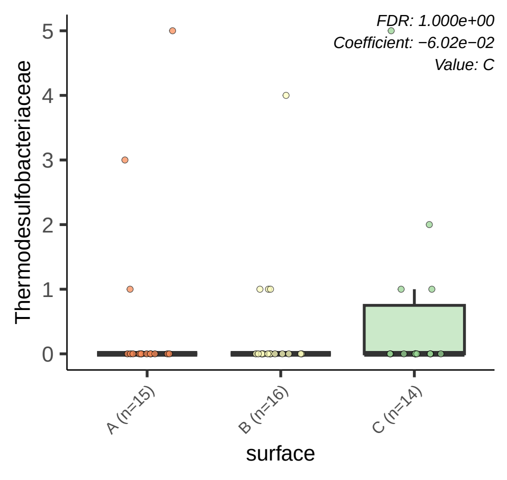

FDR: 1.000e+00
Coefficient: −6.02e−02
Value: C
Thermodesulfobacteriaceae
0
1
2
3
4
5
A (n=15)
B (n=16)
C (n=14)
surface

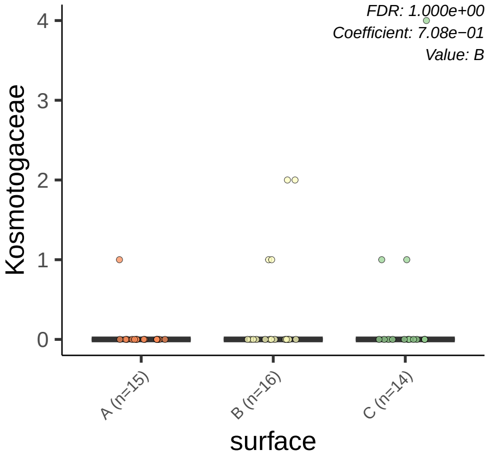
FDR: 1.000e+00
Coefficient: 7.08e−01
Value: B
Kosmotogaceae
4
3
2
1
0
A (n=15)
B (n=16)
C (n=14)
surface

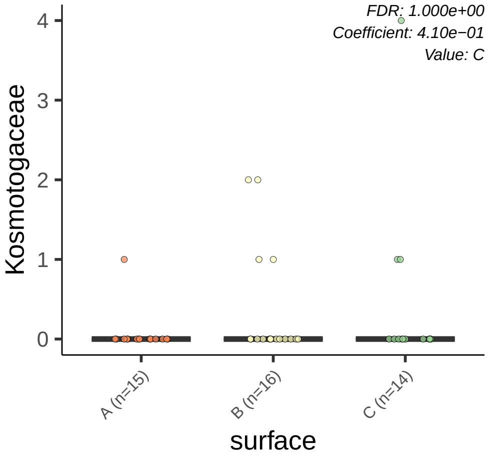
FDR: 1.000e+00
Coefficient: 4.10e−01
Value: C
Kosmotogaceae
0
1
2
3
4
A (n=15)
B (n=16)
C (n=14)
surface

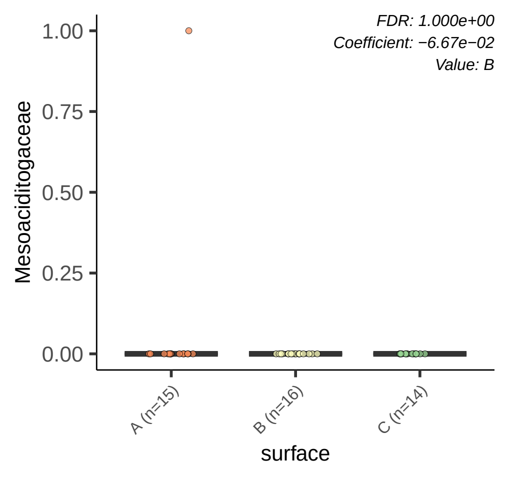
FDR: 1.000e+00
Coefficient: −6.67e−02
Value: B
Mesoaciditogaceae
1.00
0.75
0.50
0.25
0.00
A (n=15)
B (n=16)
C (n=14)
surface

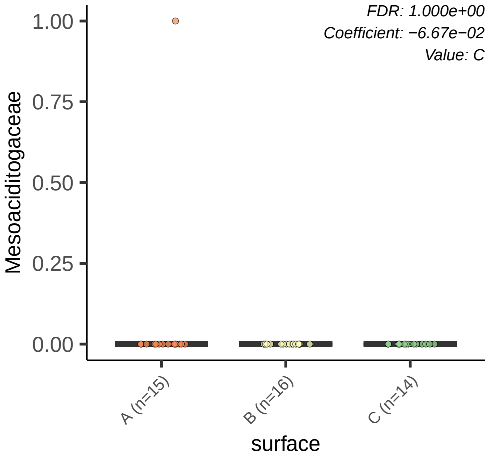

FDR: 1.000e+00
Coefficient: −6.67e−02
Value: C
Mesoaciditogaceae
1.00
0.75
0.50
0.25
0.00
A (n=15)
B (n=16)
C (n=14)
surface

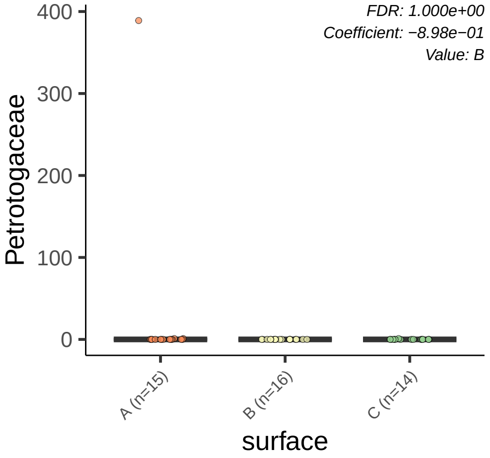

FDR: 1.000e+00
Coefficient: −8.98e−01
Value: B
Petrotogaceae
400
300
200
100
0
A (n=15)
B (n=16)
C (n=14)
surface

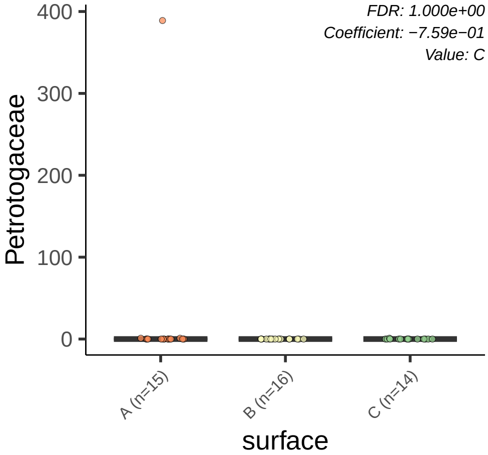
FDR: 1.000e+00
Coefficient: −7.59e−01
Value: C
Petrotogaceae
400
300
200
100
0
A (n=15)
B (n=16)
C (n=14)
surface

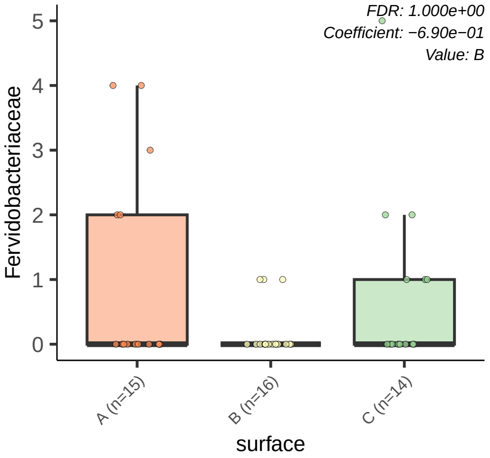

FDR: 1.000e+00
Coefficient: −6.90e−01
Value: B
Fervidobacteriaceae
5
4
3
2
1
0
A (n=15)
B (n=16)
C (n=14)
surface

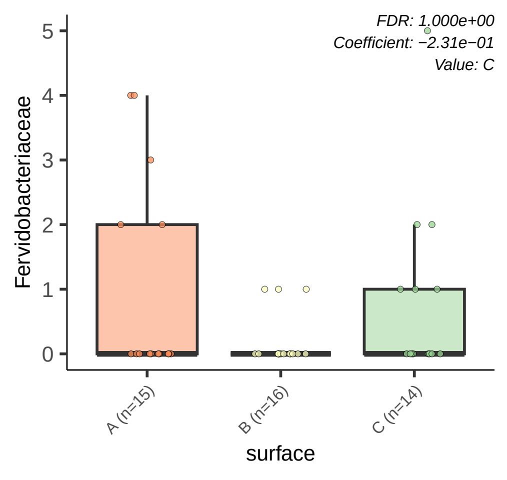
FDR: 1.000e+00
Coefficient: −2.31e−01
Value: C
Fervidobacteriaceae
0
1
2
3
4
5
A (n=15)
B (n=16)
C (n=14)
surface

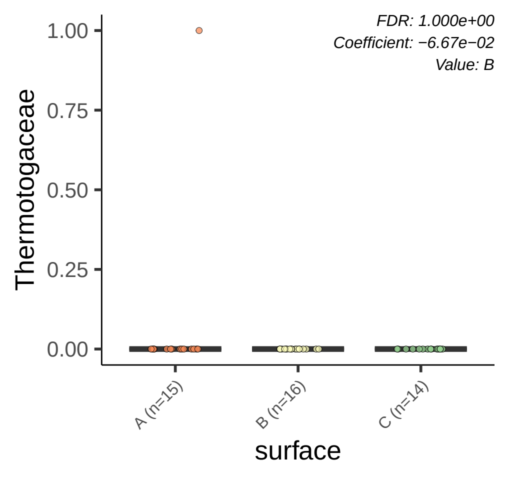
FDR: 1.000e+00
Coefficient: −6.67e−02
Value: B
Thermotogaceae
1.00
0.75
0.50
0.25
0.00
A (n=15)
B (n=16)
C (n=14)
surface

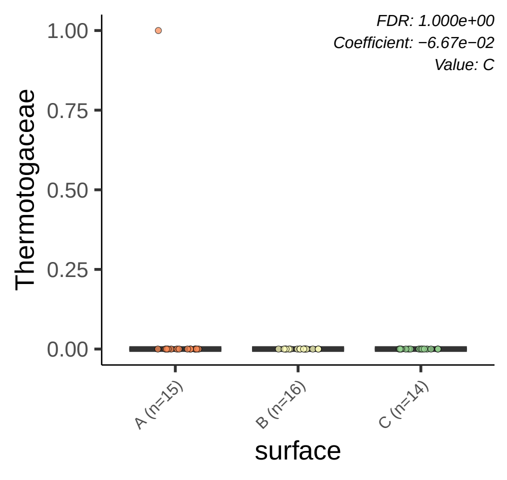
FDR: 1.000e+00
Coefficient: −6.67e−02
Value: C
Thermotogaceae
1.00
0.75
0.50
0.25
0.00
A (n=15)
B (n=16)
C (n=14)
surface

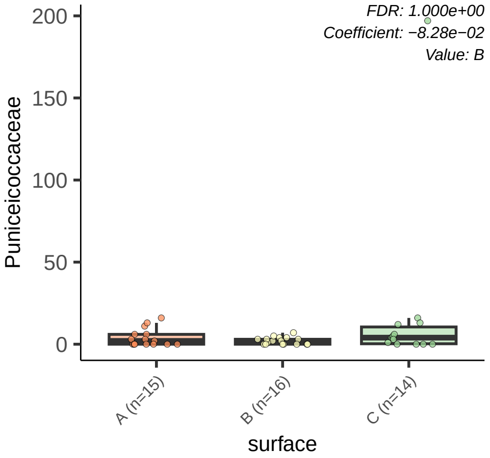
FDR: 1.000e+00
Coefficient: −8.28e−02
Value: B
Puniceicoccaceae
200
150
100
50
0
A (n=15)
B (n=16)
C (n=14)
surface

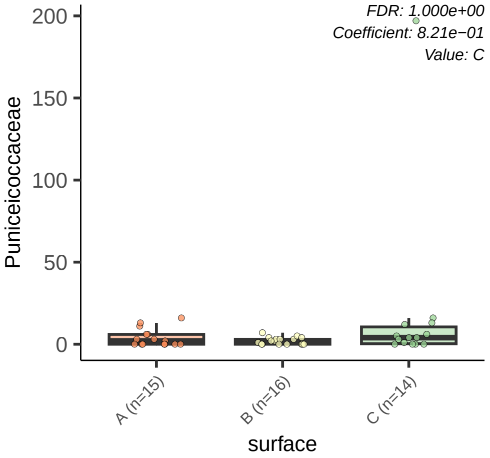
FDR: 1.000e+00
Coefficient: 8.21e−01
Value: C
Puniceicoccaceae
200
150
100
50
0
A (n=15)
B (n=16)
C (n=14)
surface

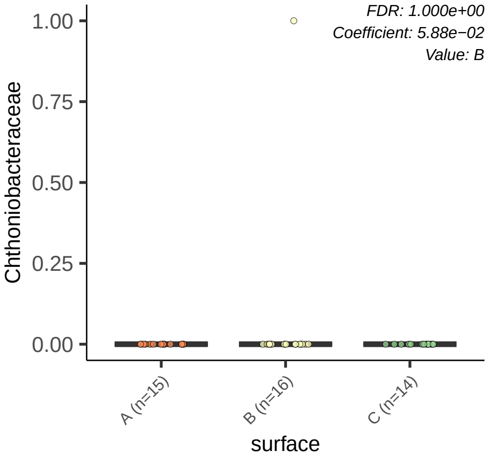

FDR: 1.000e+00
Coefficient: 5.88e−02
Value: B
Chthoniobacteraceae
1.00
0.75
0.50
0.25
0.00
A (n=15)
B (n=16)
C (n=14)
surface

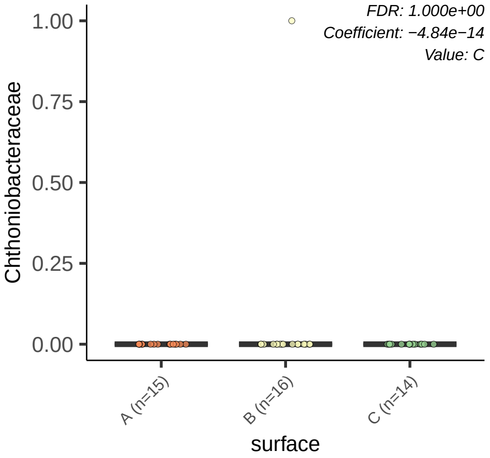
FDR: 1.000e+00
Coefficient: −4.84e−14
Value: C
Chthoniobacteraceae
1.00
0.75
0.50
0.25
0.00
A (n=15)
B (n=16)
C (n=14)
surface

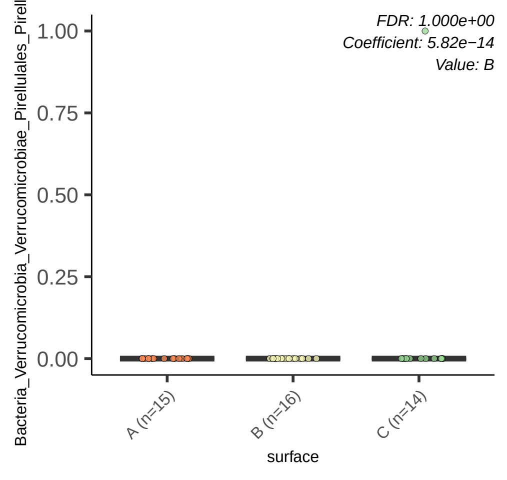
FDR: 1.000e+00
Coefficient: 5.82e−14
Value: B
Bacteria_Verrucomicrobia_Verrucomicrobiae_Pirellulales_Pirell
1.00
0.75
0.50
0.25
0.00
A (n=15)
B (n=16)
C (n=14)
surface

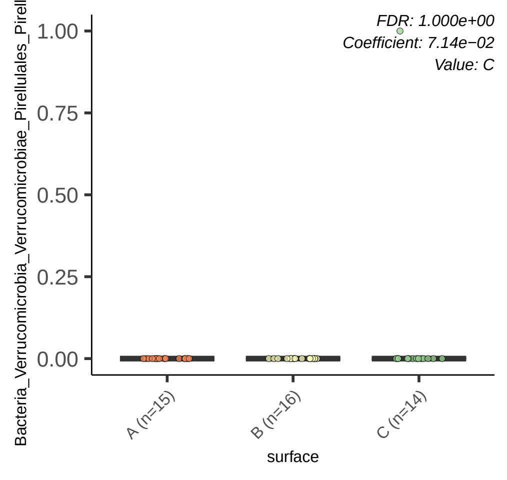

FDR: 1.000e+00
Coefficient: 7.14e−02
Value: C
Bacteria_Verrucomicrobia_Verrucomicrobiae_Pirellulales_Pirell
1.00
0.75
0.50
0.25
0.00
A (n=15)
B (n=16)
C (n=14)
surface

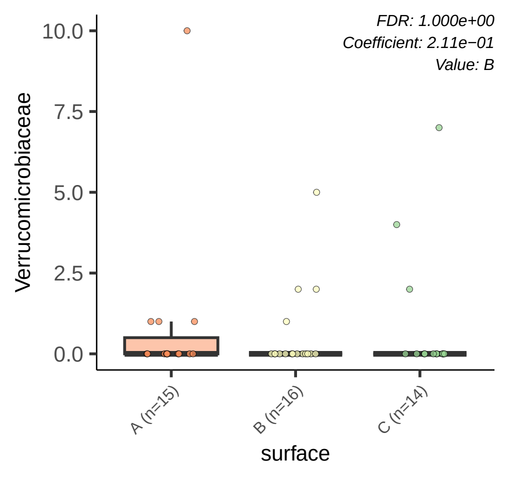
FDR: 1.000e+00
Coefficient: 2.11e−01
Value: B
Verrucomicrobiaceae
10.0
7.5
5.0
2.5
0.0
A (n=15)
B (n=16)
C (n=14)
surface

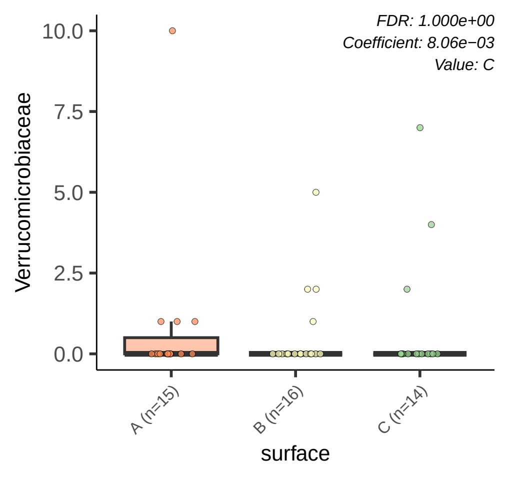
FDR: 1.000e+00
Coefficient: 8.06e−03
Value: C
Verrucomicrobiaceae
10.0
7.5
5.0
2.5
0.0
A (n=15)
B (n=16)
C (n=14)
surface

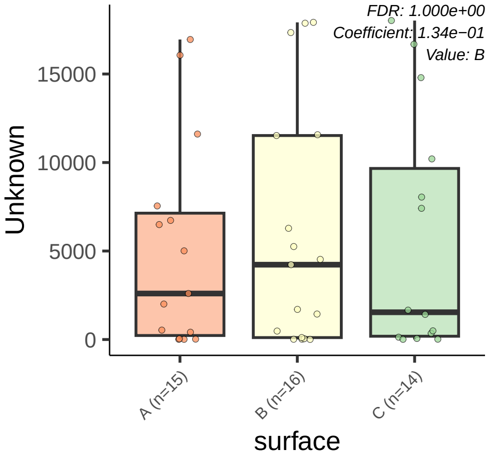
FDR: 1.000e+00
Coefficient: 1.34e−01
Value: B
15000
10000
5000
0
Unknown
A (n=15)
B (n=16)
C (n=14)
surface

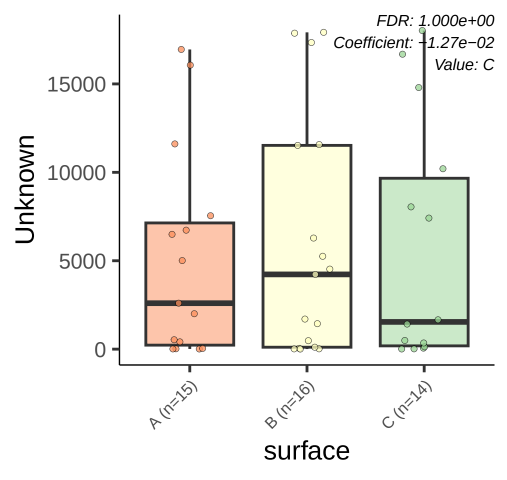
FDR: 1.000e+00
Coefficient: −1.27e−02
Value: C
Unknown
15000
10000
5000
0
A (n=15)
B (n=16)
C (n=14)
surface

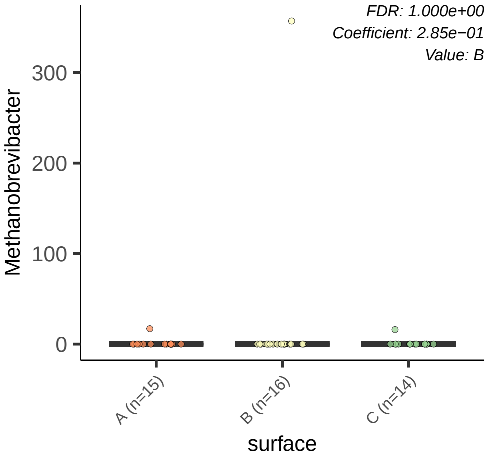

FDR: 1.000e+00
Coefficient: 2.85e−01
Value: B
Methanobrevibacter
300
200
100
0
A (n=15)
B (n=16)
C (n=14)
surface

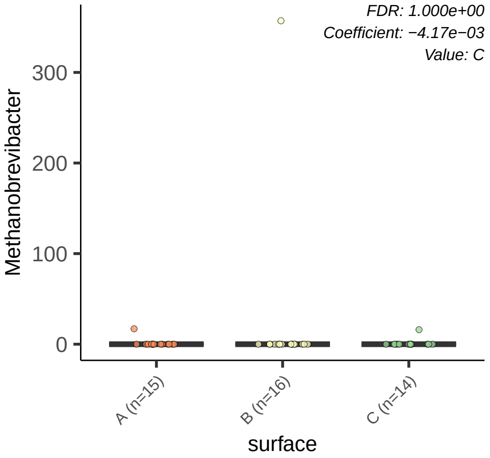
FDR: 1.000e+00
Coefficient: −4.17e−03
Value: C
Methanobrevibacter
300
200
100
0
A (n=15)
B (n=16)
C (n=14)
surface

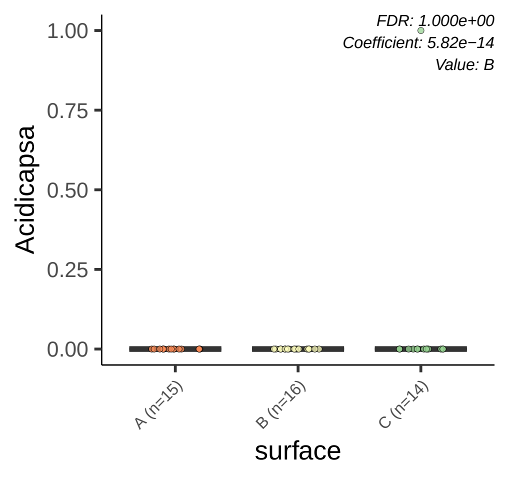

FDR: 1.000e+00
Coefficient: 5.82e−14
Value: B
Acidicapsa
1.00
0.75
0.50
0.25
0.00
A (n=15)
B (n=16)
C (n=14)
surface

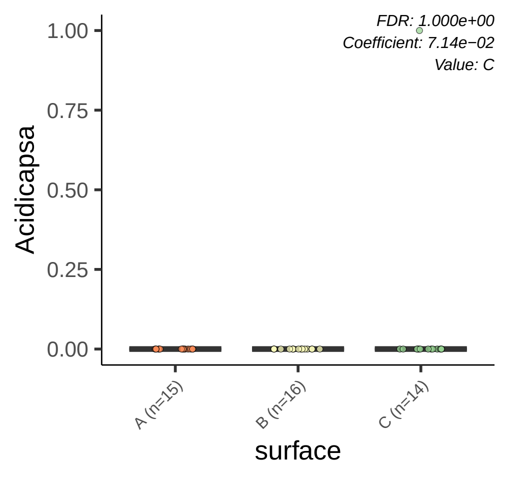
FDR: 1.000e+00
Coefficient: 7.14e−02
Value: C
1.00
0.75
0.50
0.25
0.00
Acidicapsa
A (n=15)
B (n=16)
C (n=14)
surface

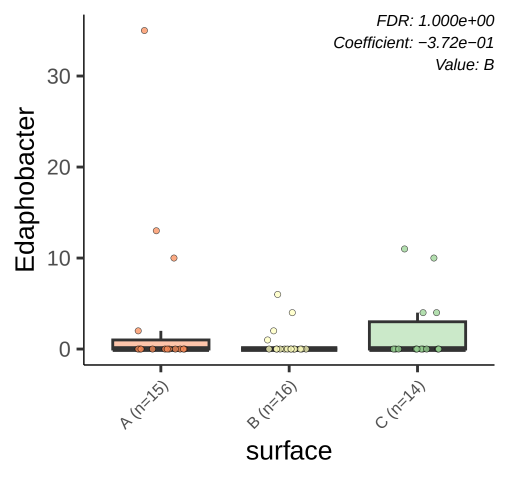
FDR: 1.000e+00
Coefficient: −3.72e−01
Value: B
Edaphobacter
0
10
20
30
A (n=15)
B (n=16)
C (n=14)
surface

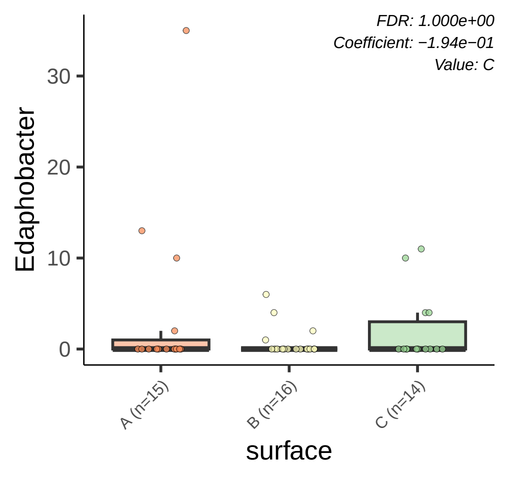

FDR: 1.000e+00
Coefficient: −1.94e−01
Value: C
Edaphobacter
30
20
10
0
A (n=15)
B (n=16)
C (n=14)
surface

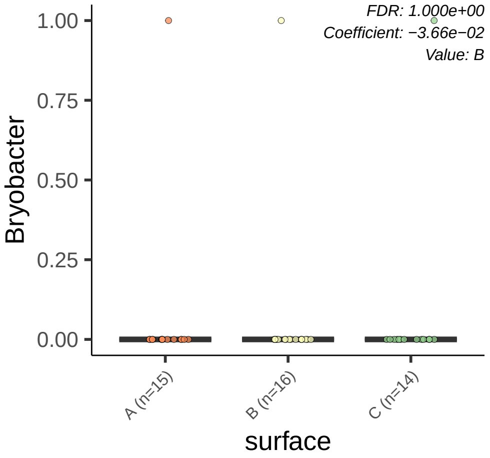

FDR: 1.000e+00
Coefficient: −3.66e−02
Value: B
Bryobacter
1.00
0.75
0.50
0.25
0.00
A (n=15)
B (n=16)
C (n=14)
surface

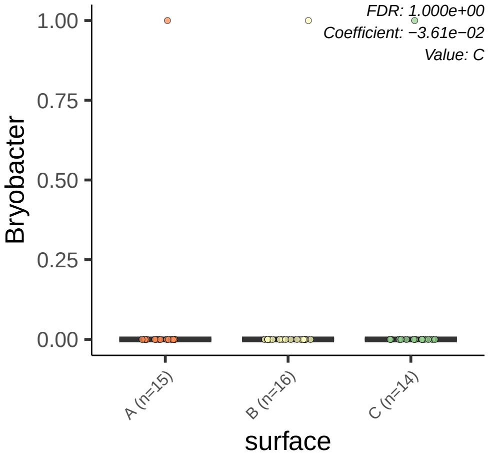
FDR: 1.000e+00
Coefficient: −3.61e−02
Value: C
Bryobacter
1.00
0.75
0.50
0.25
0.00
A (n=15)
B (n=16)
C (n=14)
surface

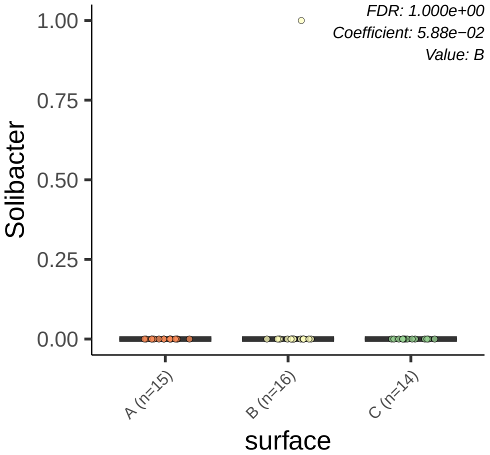
FDR: 1.000e+00
Coefficient: 5.88e−02
Value: B
Solibacter
1.00
0.75
0.50
0.25
0.00
A (n=15)
B (n=16)
C (n=14)
surface

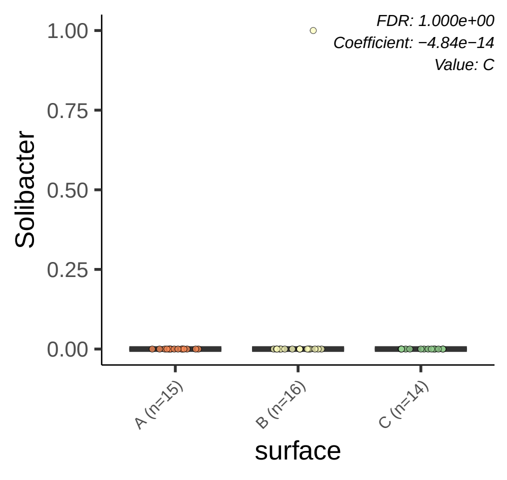
FDR: 1.000e+00
Coefficient: −4.84e−14
Value: C
Solibacter
1.00
0.75
0.50
0.25
0.00
A (n=15)
B (n=16)
C (n=14)
surface

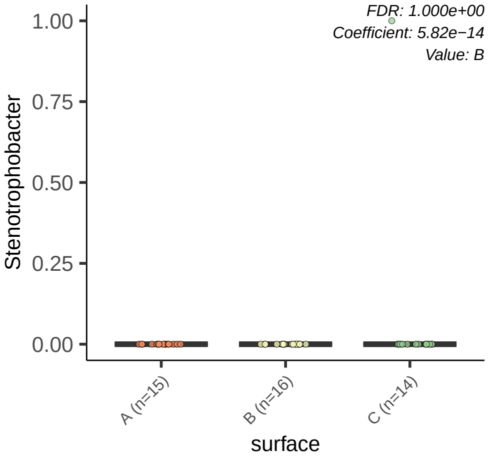

FDR: 1.000e+00
Coefficient: 5.82e−14
Value: B
Stenotrophobacter
1.00
0.75
0.50
0.25
0.00
A (n=15)
B (n=16)
C (n=14)
surface

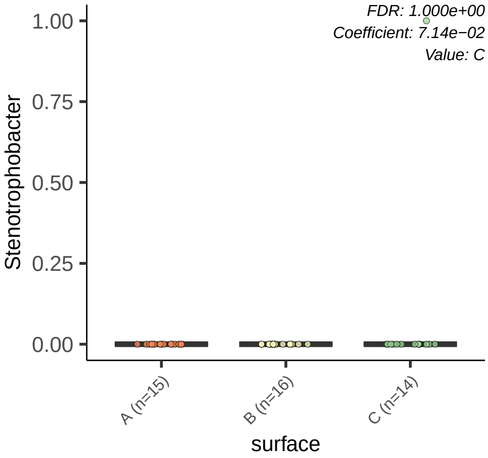
FDR: 1.000e+00
Coefficient: 7.14e−02
Value: C
Stenotrophobacter
1.00
0.75
0.50
0.25
0.00
A (n=15)
B (n=16)
C (n=14)
surface

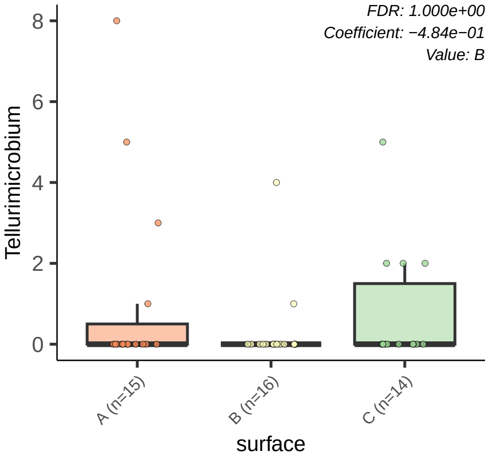

FDR: 1.000e+00
Coefficient: −4.84e−01
Value: B
Tellurimicrobium
8
6
4
2
0
A (n=15)
B (n=16)
C (n=14)
surface

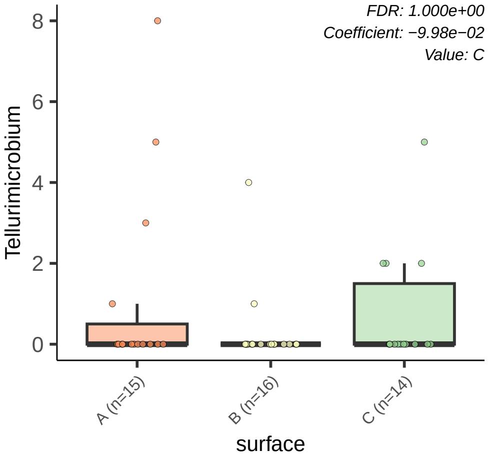
FDR: 1.000e+00
Coefficient: −9.98e−02
Value: C
Tellurimicrobium
0
2
4
6
8
A (n=15)
B (n=16)
C (n=14)
surface

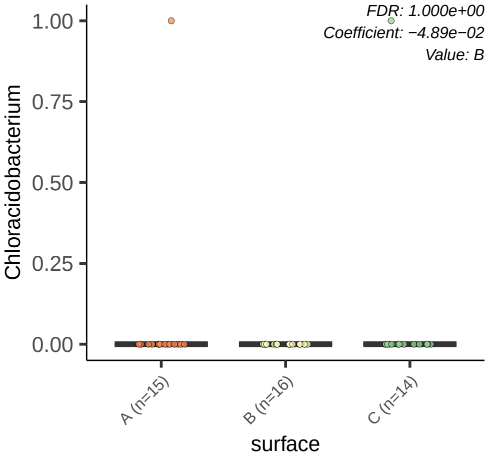
FDR: 1.000e+00
Coefficient: −4.89e−02
Value: B
Chloracidobacterium
1.00
0.75
0.50
0.25
0.00
A (n=15)
B (n=16)
C (n=14)
surface

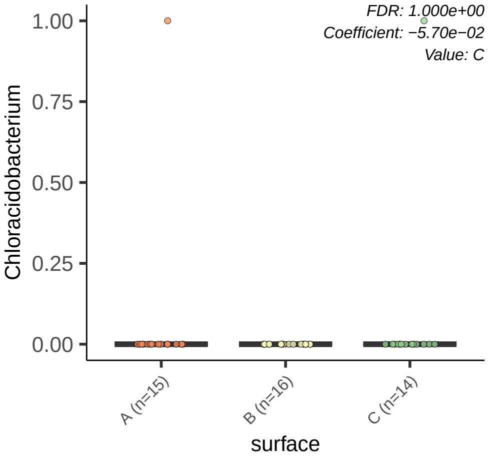
FDR: 1.000e+00
Coefficient: −5.70e−02
Value: C
Chloracidobacterium
1.00
0.75
0.50
0.25
0.00
A (n=15)
B (n=16)
C (n=14)
surface

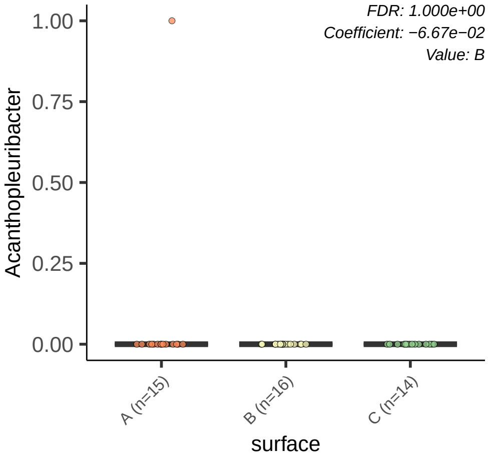
FDR: 1.000e+00
Coefficient: −6.67e−02
Value: B
Acanthopleuribacter
1.00
0.75
0.50
0.25
0.00
A (n=15)
B (n=16)
C (n=14)
surface

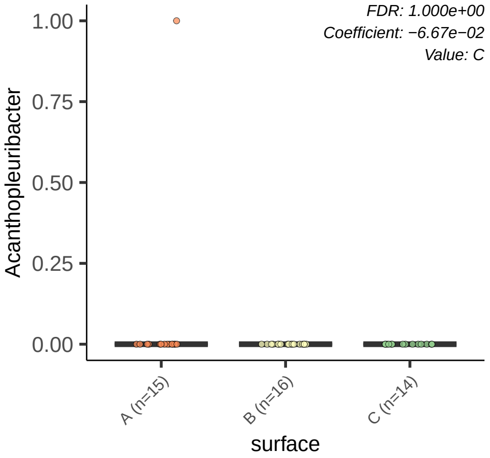
FDR: 1.000e+00
Coefficient: −6.67e−02
Value: C
Acanthopleuribacter
1.00
0.75
0.50
0.25
0.00
A (n=15)
B (n=16)
C (n=14)
surface

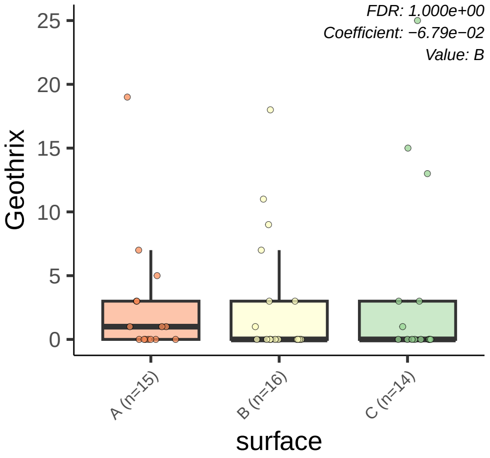
FDR: 1.000e+00
Coefficient: −6.79e−02
Value: B
Geothrix
25
20
15
10
5
0
A (n=15)
B (n=16)
C (n=14)
surface

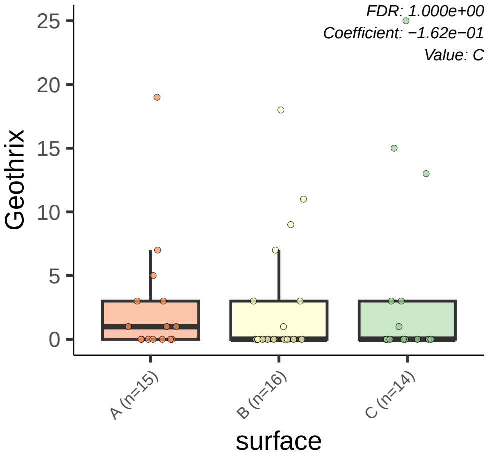
FDR: 1.000e+00
Coefficient: −1.62e−01
Value: C
Geothrix
25
20
15
10
5
0
A (n=15)
B (n=16)
C (n=14)
surface

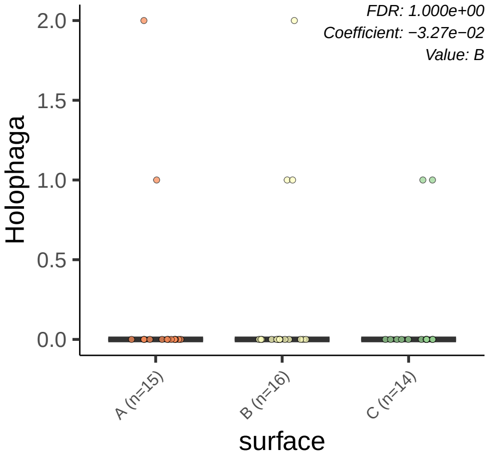

FDR: 1.000e+00
Coefficient: −3.27e−02
Value: B
Holophaga
2.0
1.5
1.0
0.5
0.0
A (n=15)
B (n=16)
C (n=14)
surface

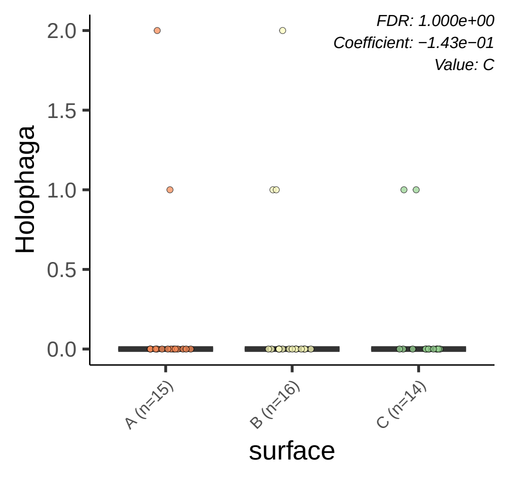

FDR: 1.000e+00
Coefficient: −1.43e−01
Value: C
Holophaga
2.0
1.5
1.0
0.5
0.0
A (n=15)
B (n=16)
C (n=14)
surface

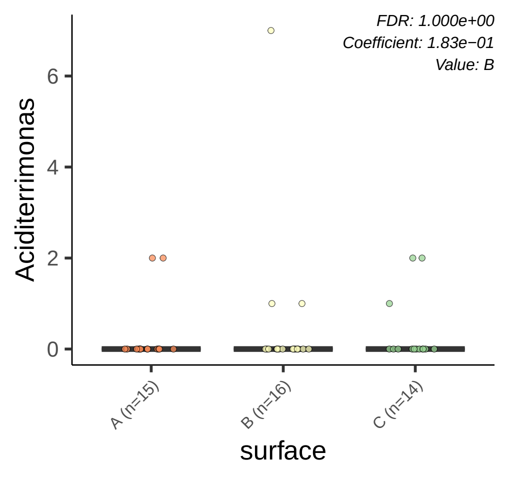
FDR: 1.000e+00
Coefficient: 1.83e−01
Value: B
Aciditerrimonas
0
2
4
6
A (n=15)
B (n=16)
C (n=14)
surface

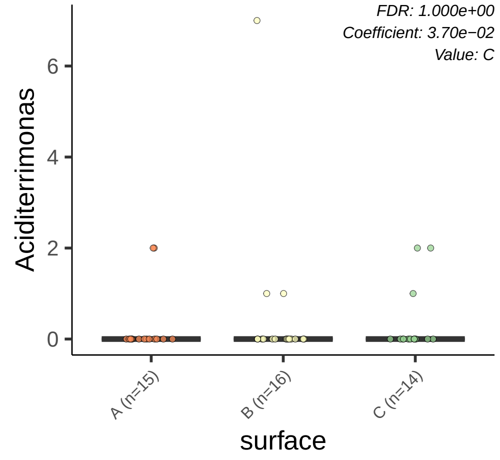
FDR: 1.000e+00
Coefficient: 3.70e−02
Value: C
Aciditerrimonas
0
2
4
6
A (n=15)
B (n=16)
C (n=14)
surface

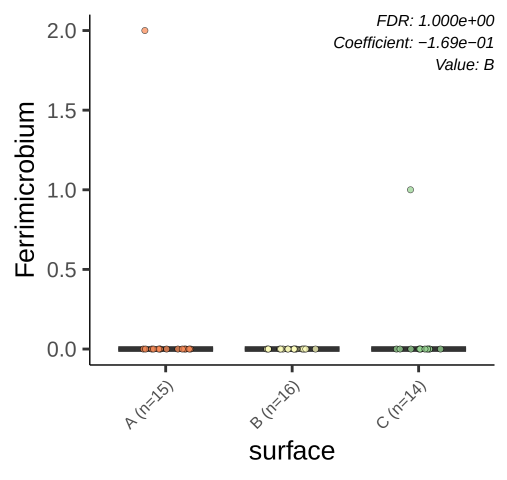
FDR: 1.000e+00
Coefficient: −1.69e−01
Value: B
Ferrimicrobium
2.0
1.5
1.0
0.5
0.0
A (n=15)
B (n=16)
C (n=14)
surface

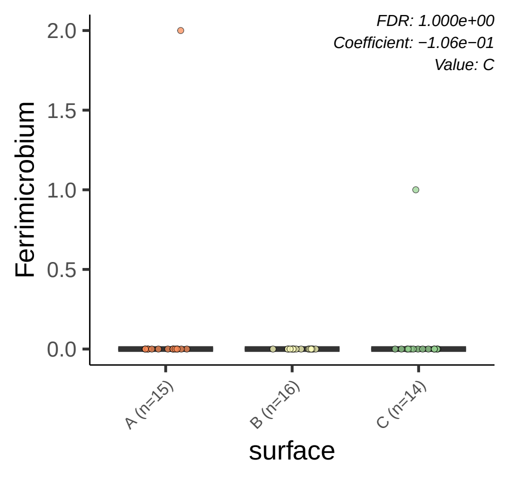
FDR: 1.000e+00
Coefficient: −1.06e−01
Value: C
Ferrimicrobium
2.0
1.5
1.0
0.5
0.0
A (n=15)
B (n=16)
C (n=14)
surface

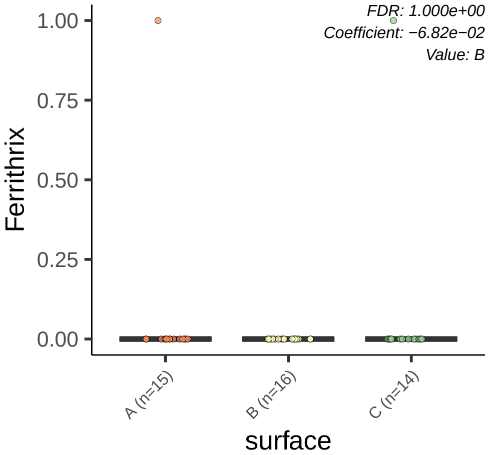
FDR: 1.000e+00
Coefficient: −6.82e−02
Value: B
1.00
0.75
0.50
0.25
0.00
Ferrithrix
A (n=15)
B (n=16)
C (n=14)
surface

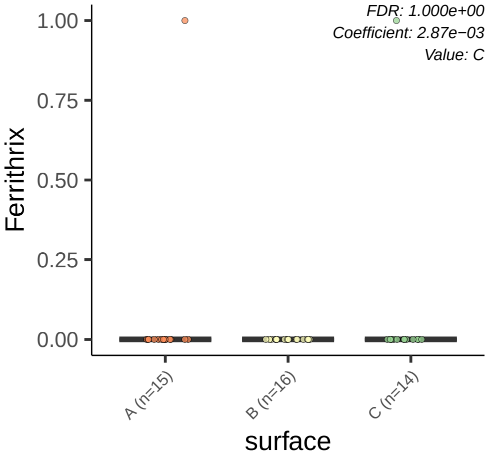

FDR: 1.000e+00
Coefficient: 2.87e−03
Value: C
Ferrithrix
1.00
0.75
0.50
0.25
0.00
A (n=15)
B (n=16)
C (n=14)
surface

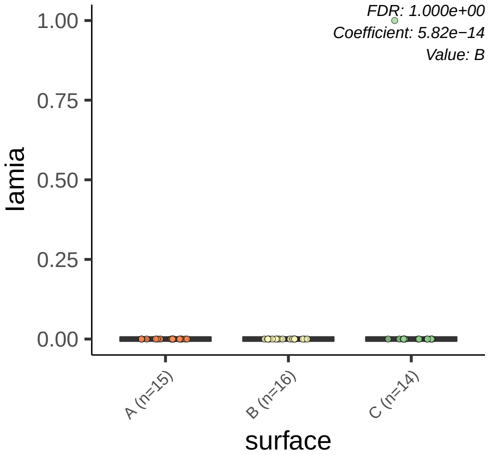

FDR: 1.000e+00
Coefficient: 5.82e−14
Value: B
1.00
0.75
0.50
0.25
0.00
lamia
A (n=15)
B (n=16)
C (n=14)
surface

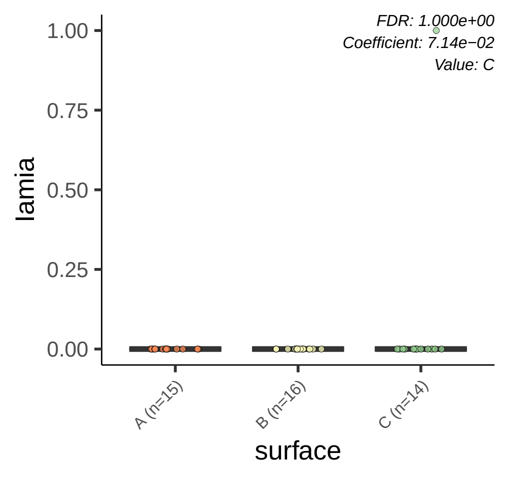

FDR: 1.000e+00
Coefficient: 7.14e−02
Value: C
lamia
1.00
0.75
0.50
0.25
0.00
A (n=15)
B (n=16)
C (n=14)
surface

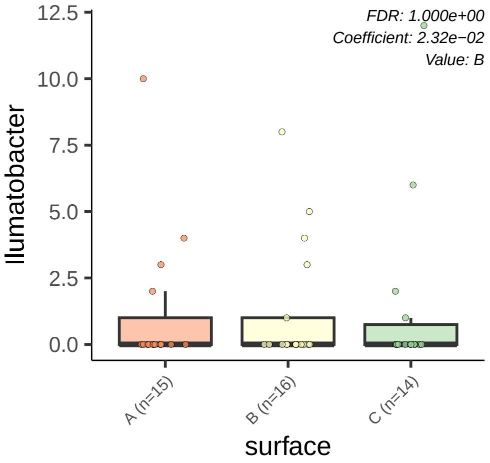

FDR: 1.000e+00
Coefficient: 2.32e−02
Value: B
Ilumatobacter
12.5
10.0
7.5
5.0
2.5
0.0
A (n=15)
B (n=16)
C (n=14)
surface

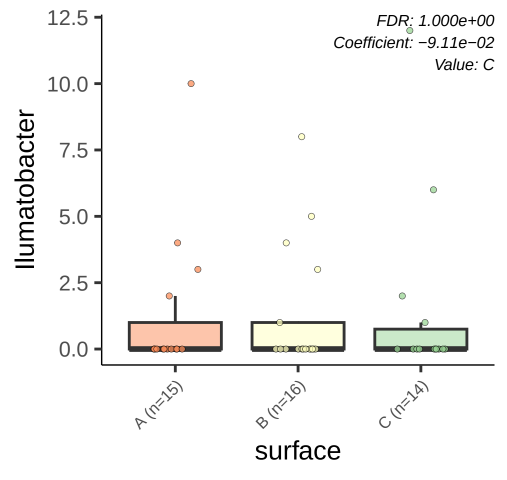

FDR: 1.000e+00
Coefficient: −9.11e−02
Value: C
Ilumatobacter
12.5
10.0
7.5
5.0
2.5
0.0
A (n=15)
B (n=16)
C (n=14)
surface

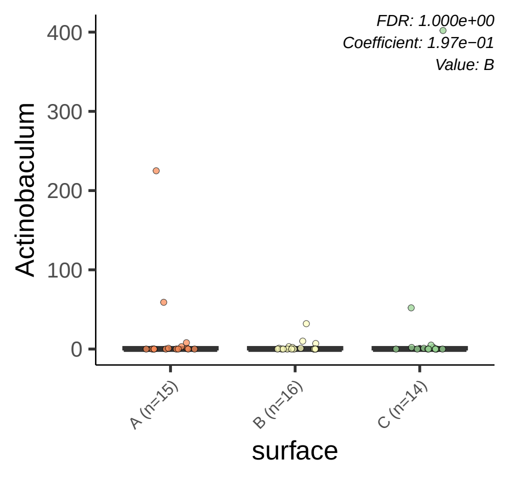

FDR: 1.000e+00
Coefficient: 1.97e−01
Value: B
Actinobaculum
400
300
200
100
0
A (n=15)
B (n=16)
C (n=14)
surface

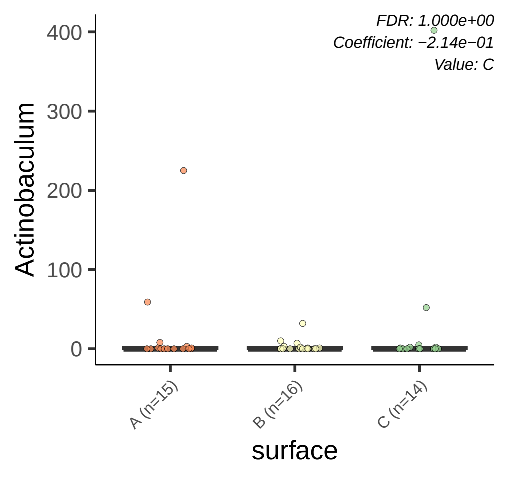
FDR: 1.000e+00
Coefficient: −2.14e−01
Value: C
Actinobaculum
400
300
200
100
0
A (n=15)
B (n=16)
C (n=14)
surface

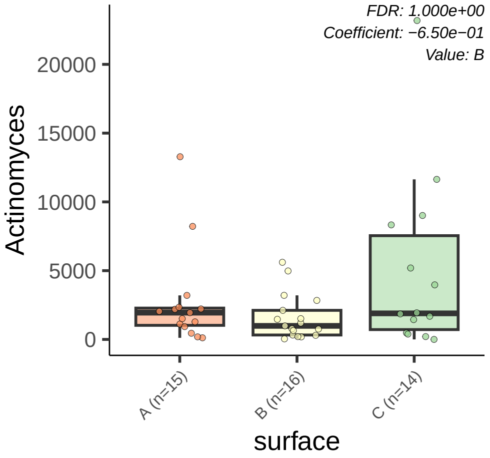

FDR: 1.000e+00
Coefficient: −6.50e−01
Value: B
Actinomyces
20000
15000
10000
5000
0
A (n=15)
B (n=16)
C (n=14)
surface

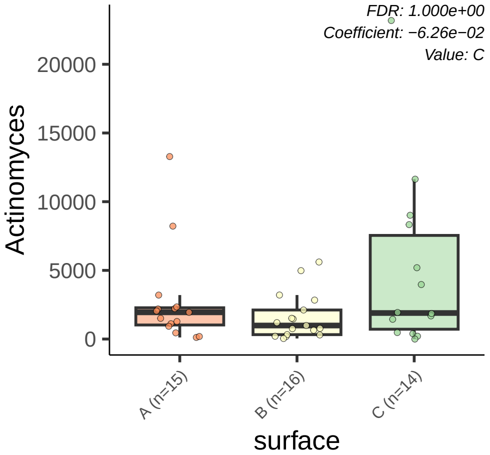
FDR: 1.000e+00
Coefficient: −6.26e−02
Value: C
Actinomyces
20000
15000
10000
5000
0
A (n=15)
B (n=16)
C (n=14)
surface

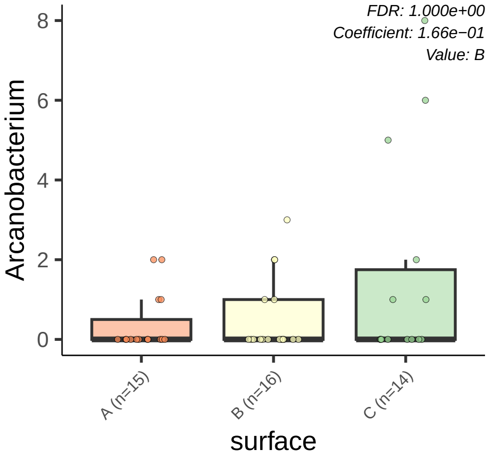

FDR: 1.000e+00
Coefficient: 1.66e−01
Value: B
Arcanobacterium
0
2
4
6
8
A (n=15)
B (n=16)
C (n=14)
surface

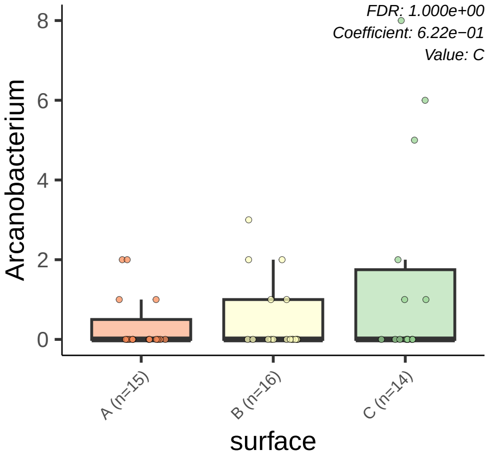
FDR: 1.000e+00
Coefficient: 6.22e−01
Value: C
Arcanobacterium
0
2
4
6
8
A (n=15)
B (n=16)
C (n=14)
surface

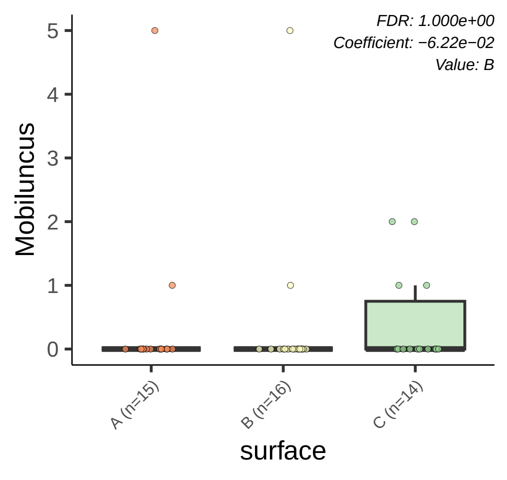
FDR: 1.000e+00
Coefficient: −6.22e−02
Value: B
Mobiluncus
0
1
2
3
4
5
A (n=15)
B (n=16)
C (n=14)
surface

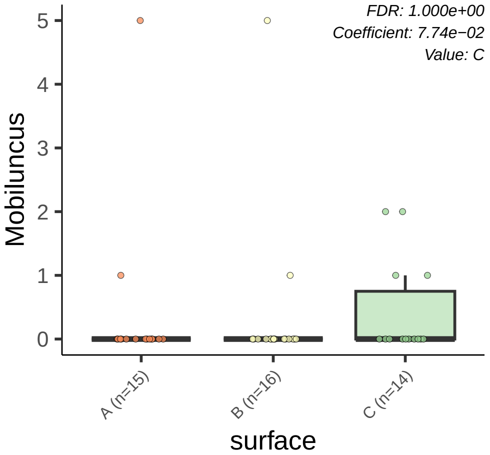
FDR: 1.000e+00
Coefficient: 7.74e−02
Value: C
Mobiluncus
5
4
3
2
1
0
A (n=15)
B (n=16)
C (n=14)
surface

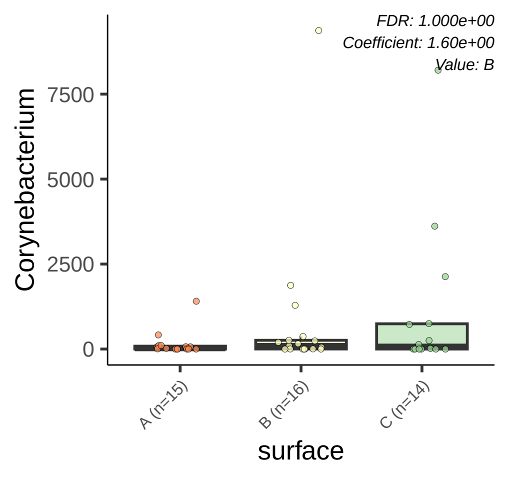
FDR: 1.000e+00
Coefficient: 1.60e+00
Value: B
Corynebacterium
7500
5000
2500
0
A (n=15)
B (n=16)
C (n=14)
surface

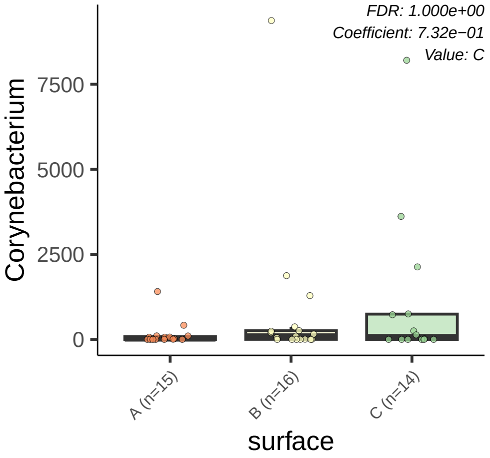

FDR: 1.000e+00
Coefficient: 7.32e−01
Value: C
Corynebacterium
7500
5000
2500
0
A (n=15)
B (n=16)
C (n=14)
surface

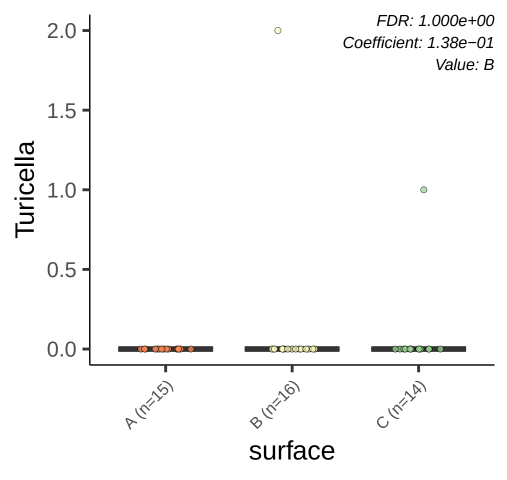
FDR: 1.000e+00
Coefficient: 1.38e−01
Value: B
Turicella
2.0
1.5
1.0
0.5
0.0
A (n=15)
B (n=16)
C (n=14)
surface

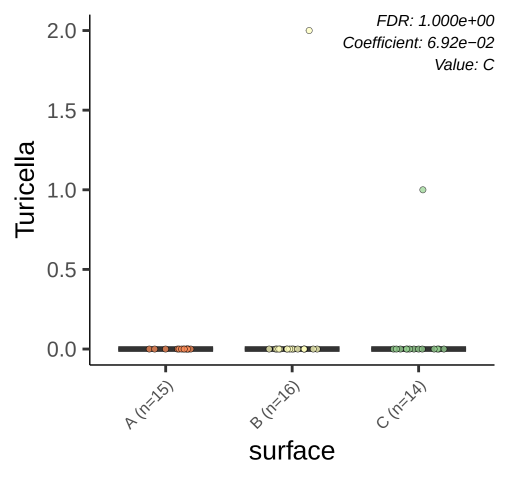
FDR: 1.000e+00
Coefficient: 6.92e−02
Value: C
Turicella
2.0
1.5
1.0
0.5
0.0
A (n=15)
B (n=16)
C (n=14)
surface

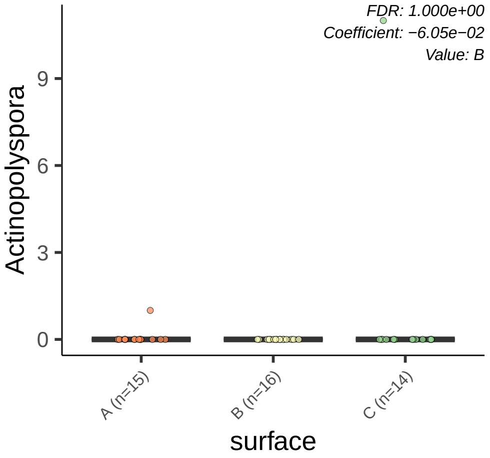
FDR: 1.000e+00
Coefficient: −6.05e−02
Value: B
Actinopolyspora
9
6
3
0
A (n=15)
B (n=16)
C (n=14)
surface

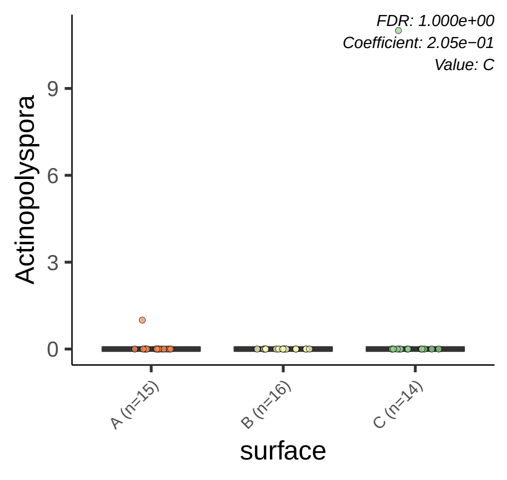
FDR: 1.000e+00
Coefficient: 2.05e−01
Value: C
Actinopolyspora
0
3
6
9
A (n=15)
B (n=16)
C (n=14)
surface

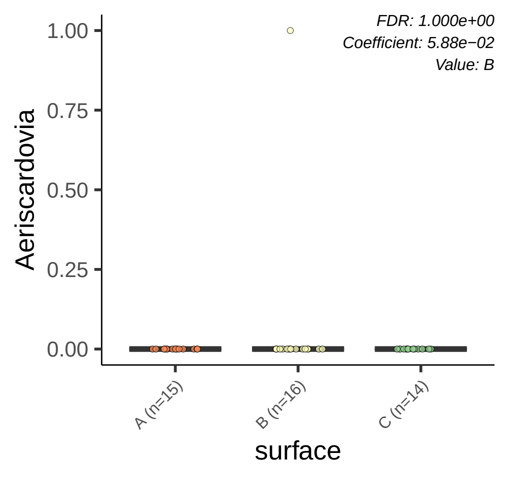

FDR: 1.000e+00
Coefficient: 5.88e−02
Value: B
Aeriscardovia
1.00
0.75
0.50
0.25
0.00
A (n=15)
B (n=16)
C (n=14)
surface

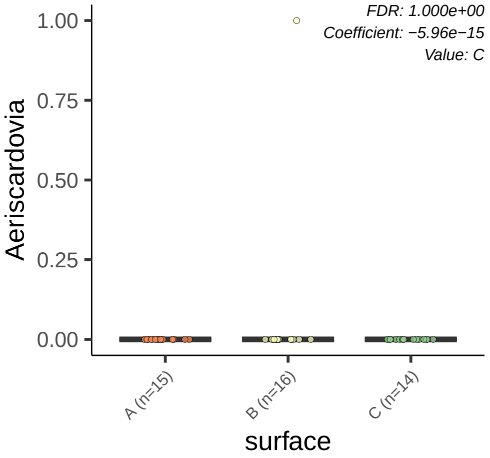
FDR: 1.000e+00
Coefficient: −5.96e−15
Value: C
Aeriscardovia
1.00
0.75
0.50
0.25
0.00
A (n=15)
B (n=16)
C (n=14)
surface

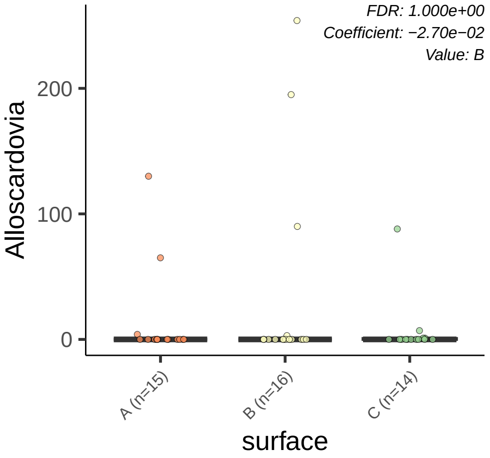

FDR: 1.000e+00
Coefficient: −2.70e−02
Value: B
Alloscardovia
200
100
0
A (n=15)
B (n=16)
C (n=14)
surface

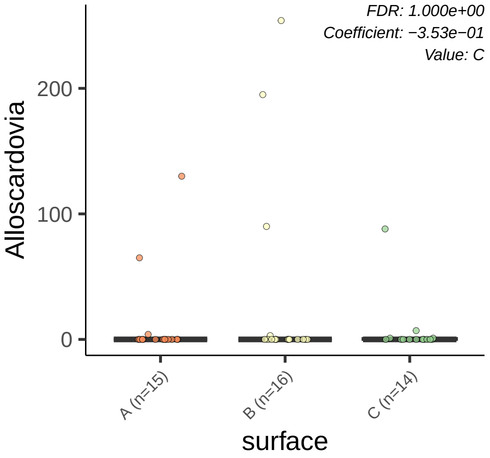
FDR: 1.000e+00
Coefficient: −3.53e−01
Value: C
Alloscardovia
200
100
0
A (n=15)
B (n=16)
C (n=14)
surface

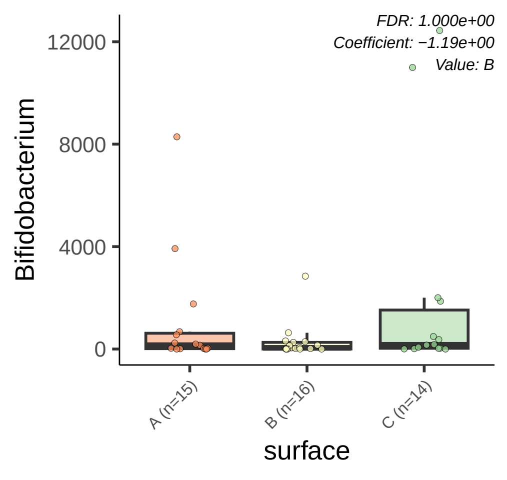

FDR: 1.000e+00
Coefficient: −1.19e+00
Value: B
Bifidobacterium
12000
8000
4000
0
A (n=15)
B (n=16)
C (n=14)
surface

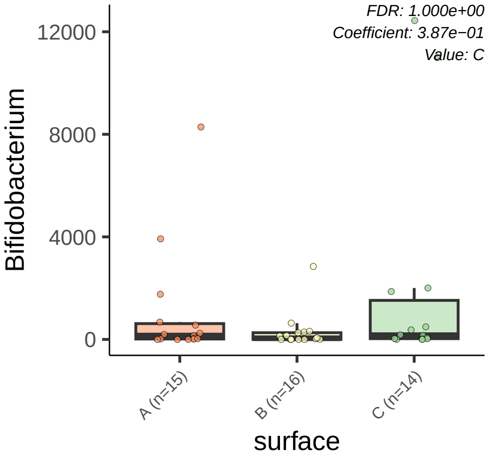
FDR: 1.000e+00
Coefficient: 3.87e−01
Value: C
12000
8000
4000
0
Bifidobacterium
A (n=15)
B (n=16)
C (n=14)
surface

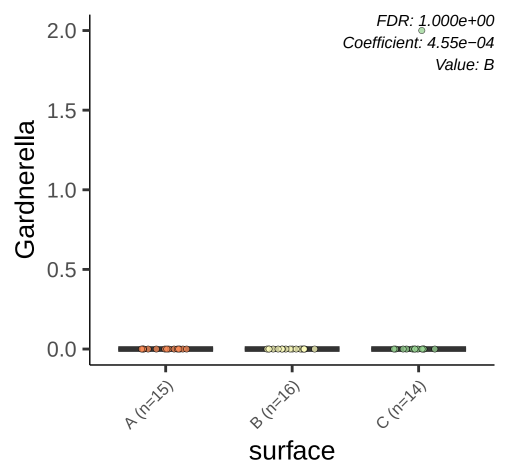
FDR: 1.000e+00
Coefficient: 4.55e−04
Value: B
Gardnerella
2.0
1.5
1.0
0.5
0.0
A (n=15)
B (n=16)
C (n=14)
surface

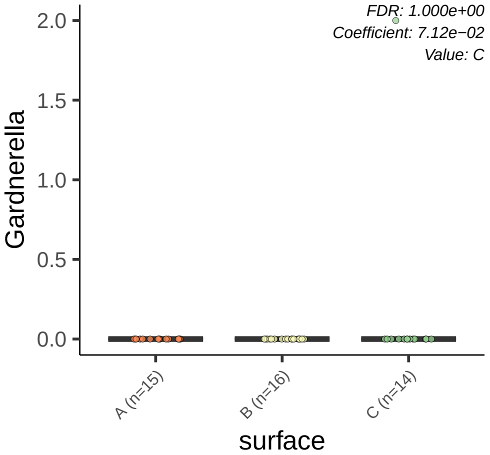
FDR: 1.000e+00
Coefficient: 7.12e−02
Value: C
2.0
1.5
1.0
0.5
0.0
Gardnerella
A (n=15)
B (n=16)
C (n=14)
surface

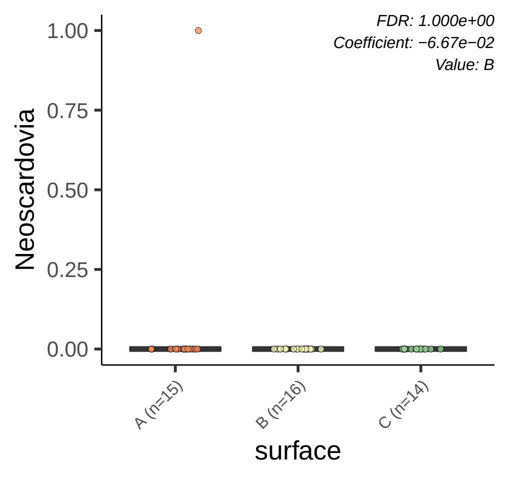
FDR: 1.000e+00
Coefficient: −6.67e−02
Value: B
Neoscardovia
1.00
0.75
0.50
0.25
0.00
A (n=15)
B (n=16)
C (n=14)
surface

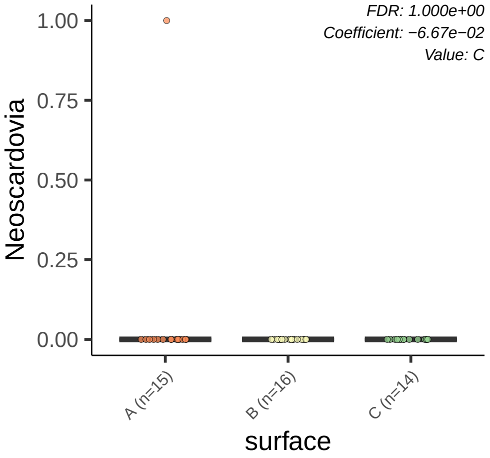
FDR: 1.000e+00
Coefficient: −6.67e−02
Value: C
Neoscardovia
1.00
0.75
0.50
0.25
0.00
A (n=15)
B (n=16)
C (n=14)
surface

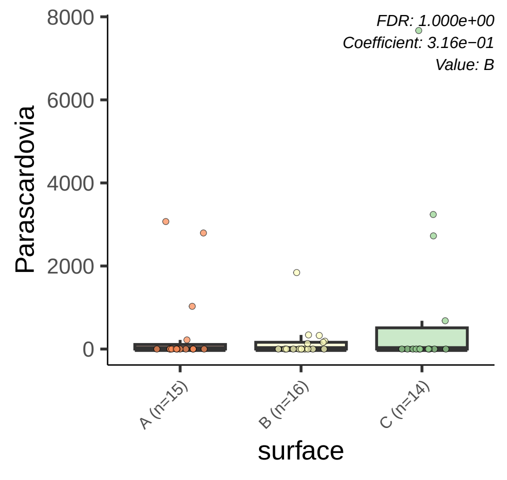

FDR: 1.000e+00
Coefficient: 3.16e−01
Value: B
Parascardovia
8000
6000
4000
2000
0
A (n=15)
B (n=16)
C (n=14)
surface

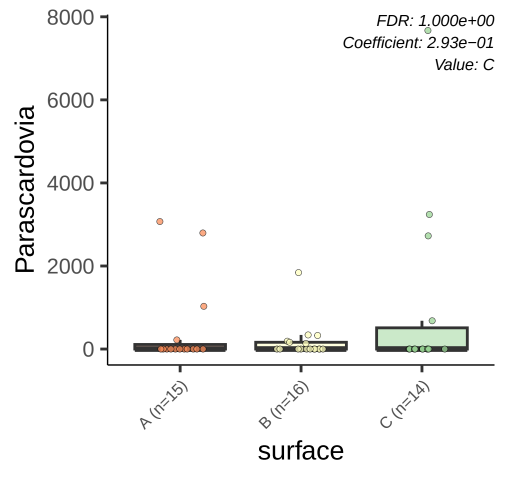

FDR: 1.000e+00
Coefficient: 2.93e−01
Value: C
Parascardovia
8000
6000
4000
2000
0
A (n=15)
B (n=16)
C (n=14)
surface

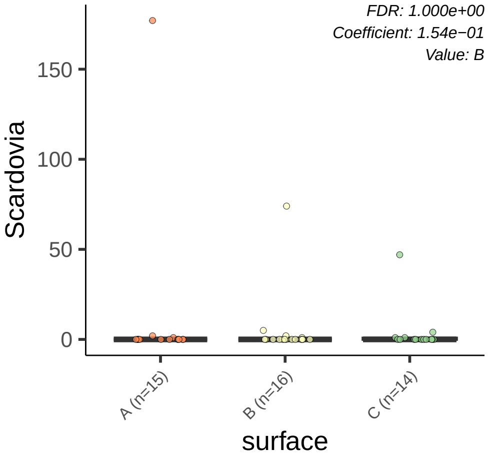

FDR: 1.000e+00
Coefficient: 1.54e−01
Value: B
Scardovia
150
100
50
0
A (n=15)
B (n=16)
C (n=14)
surface

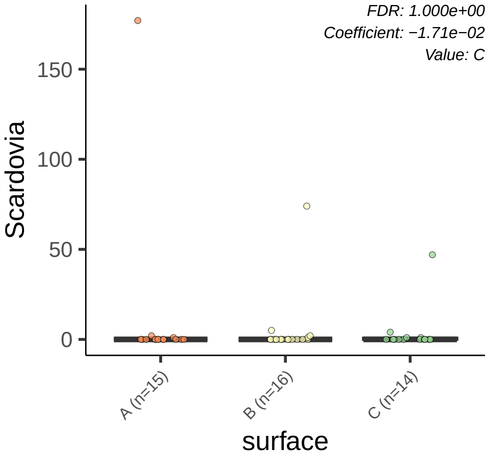

FDR: 1.000e+00
Coefficient: −1.71e−02
Value: C
Scardovia
150
100
50
0
A (n=15)
B (n=16)
C (n=14)
surface

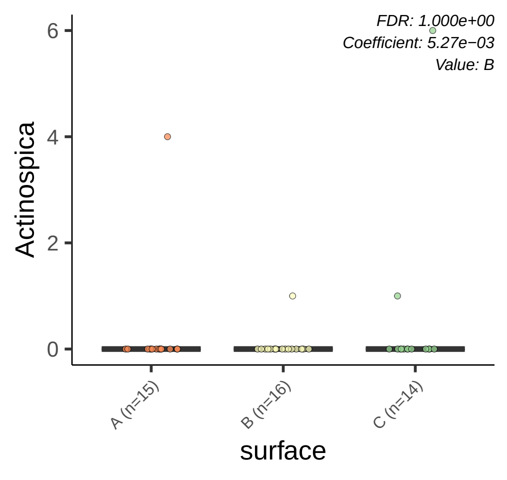
FDR: 1.000e+00
Coefficient: 5.27e−03
Value: B
Actinospica
6
4
2
0
A (n=15)
B (n=16)
C (n=14)
surface

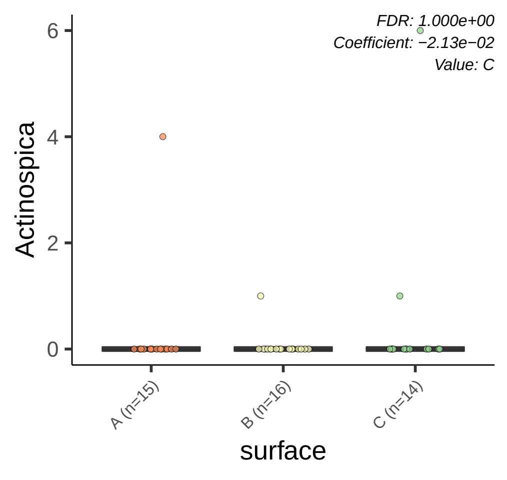

FDR: 1.000e+00
Coefficient: −2.13e−02
Value: C
Actinospica
6
4
2
0
A (n=15)
B (n=16)
C (n=14)
surface

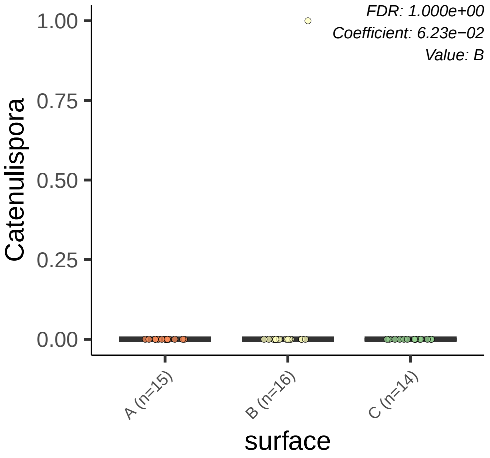
FDR: 1.000e+00
Coefficient: 6.23e−02
Value: B
Catenulispora
1.00
0.75
0.50
0.25
0.00
A (n=15)
B (n=16)
C (n=14)
surface

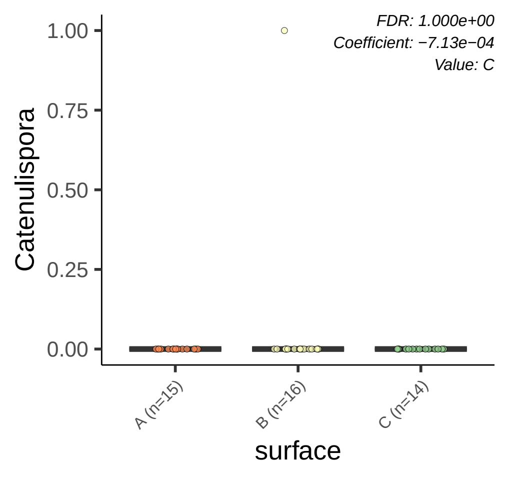
FDR: 1.000e+00
Coefficient: −7.13e−04
Value: C
Catenulispora
1.00
0.75
0.50
0.25
0.00
A (n=15)
B (n=16)
C (n=14)
surface

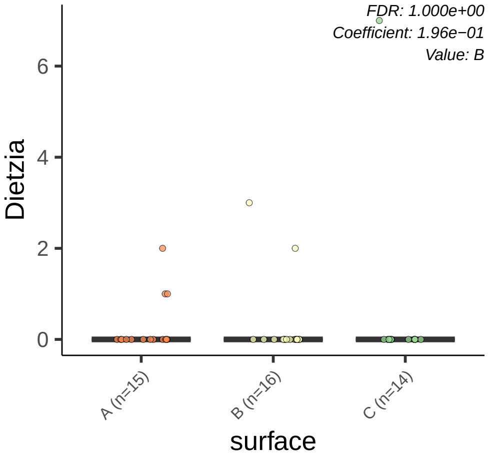

FDR: 1.000e+00
Coefficient: 1.96e−01
Value: B
Dietzia
0
2
4
6
A (n=15)
B (n=16)
C (n=14)
surface

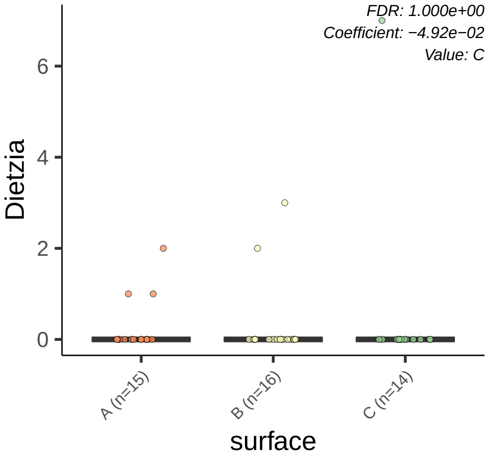

FDR: 1.000e+00
Coefficient: −4.92e−02
Value: C
Dietzia
0
2
4
6
A (n=15)
B (n=16)
C (n=14)
surface

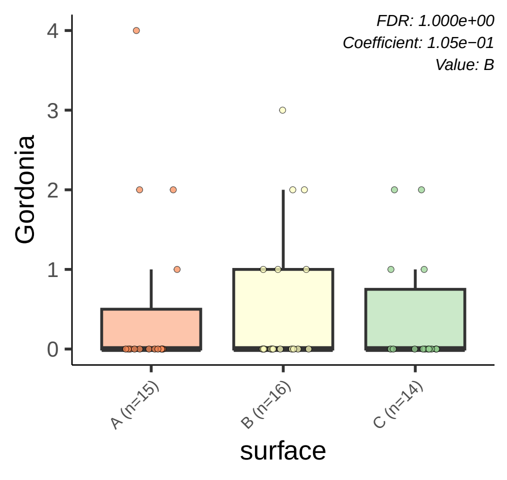
FDR: 1.000e+00
Coefficient: 1.05e−01
Value: B
Gordonia
4
3
2
1
0
A (n=15)
B (n=16)
C (n=14)
surface

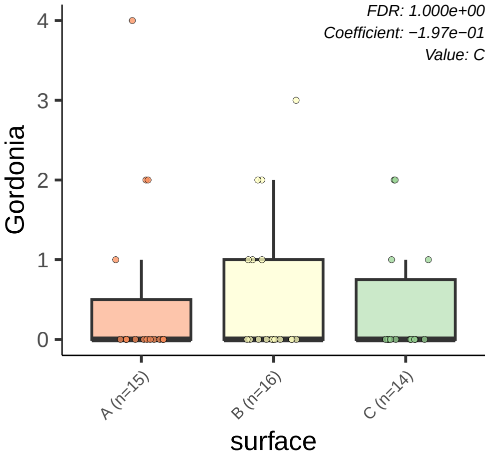
FDR: 1.000e+00
Coefficient: −1.97e−01
Value: C
Gordonia
4
3
2
1
0
A (n=15)
B (n=16)
C (n=14)
surface

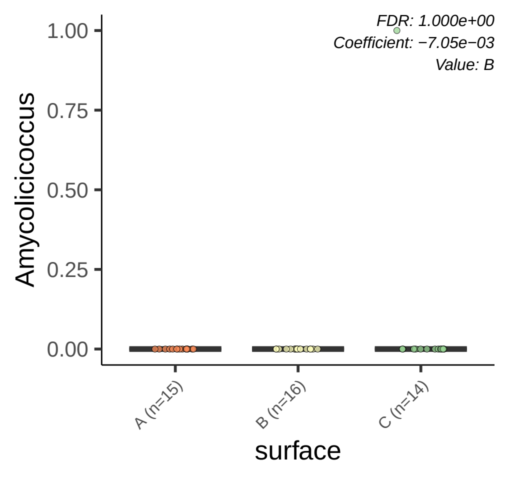
FDR: 1.000e+00
Coefficient: −7.05e−03
Value: B
Amycolicicoccus
1.00
0.75
0.50
0.25
0.00
A (n=15)
B (n=16)
C (n=14)
surface

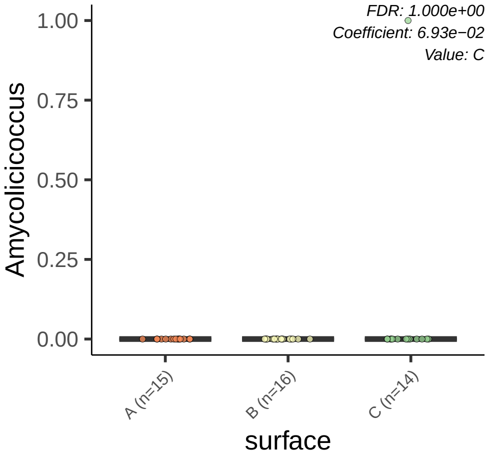
FDR: 1.000e+00
Coefficient: 6.93e−02
Value: C
1.00
0.75
0.50
0.25
0.00
Amycolicicoccus
A (n=15)
B (n=16)
C (n=14)
surface

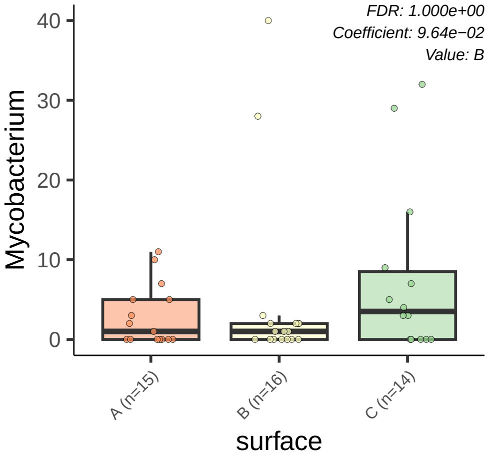
FDR: 1.000e+00
Coefficient: 9.64e−02
Value: B
Mycobacterium
0
10
20
30
40
A (n=15)
B (n=16)
C (n=14)
surface

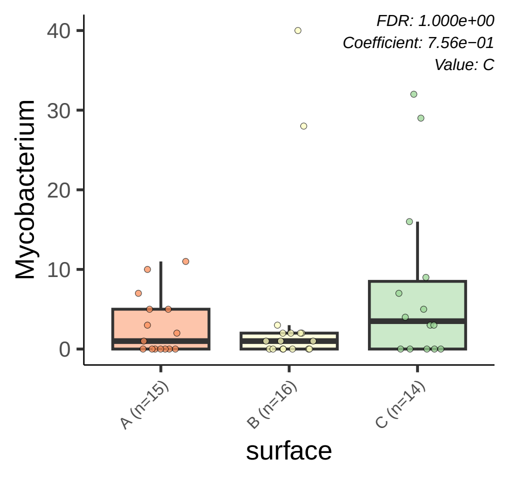

FDR: 1.000e+00
Coefficient: 7.56e−01
Value: C
Mycobacterium
40
30
20
10
0
A (n=15)
B (n=16)
C (n=14)
surface

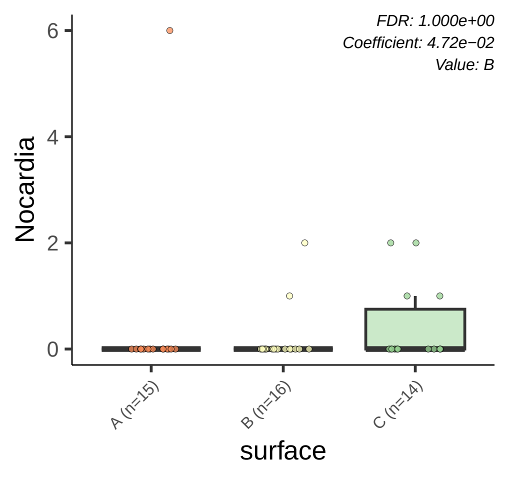
FDR: 1.000e+00
Coefficient: 4.72e−02
Value: B
Nocardia
0
2
4
6
A (n=15)
B (n=16)
C (n=14)
surface

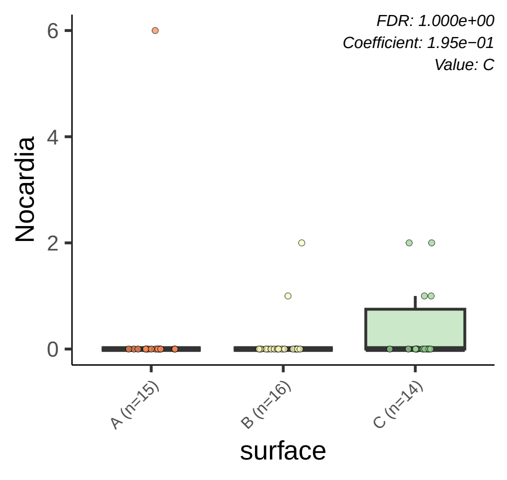
FDR: 1.000e+00
Coefficient: 1.95e−01
Value: C
Nocardia
0
2
4
6
A (n=15)
B (n=16)
C (n=14)
surface

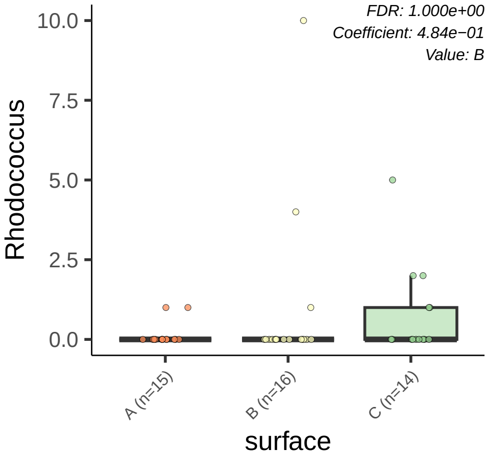
FDR: 1.000e+00
Coefficient: 4.84e−01
Value: B
Rhodococcus
10.0
7.5
5.0
2.5
0.0
A (n=15)
B (n=16)
C (n=14)
surface

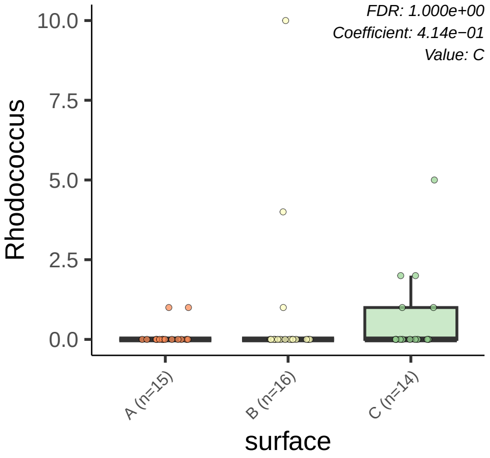

FDR: 1.000e+00
Coefficient: 4.14e−01
Value: C
Rhodococcus
10.0
7.5
5.0
2.5
0.0
A (n=15)
B (n=16)
C (n=14)
surface

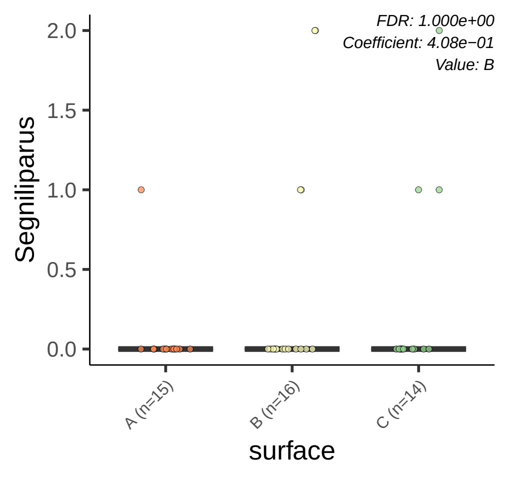
FDR: 1.000e+00
Coefficient: 4.08e−01
Value: B
Segniliparus
2.0
1.5
1.0
0.5
0.0
A (n=15)
B (n=16)
C (n=14)
surface

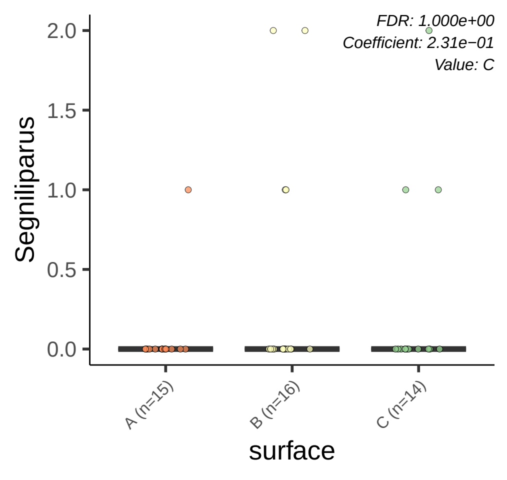

FDR: 1.000e+00
Coefficient: 2.31e−01
Value: C
Segniliparus
2.0
1.5
1.0
0.5
0.0
A (n=15)
B (n=16)
C (n=14)
surface

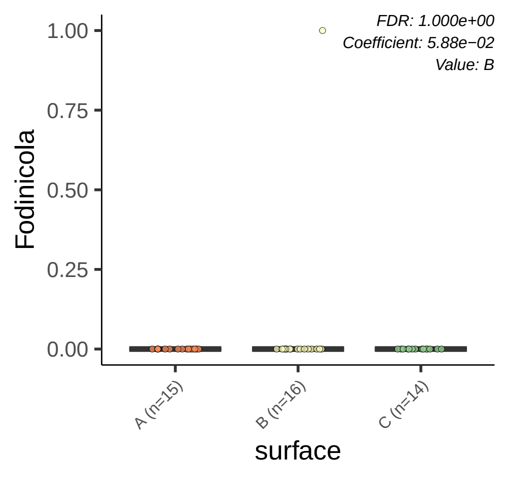
FDR: 1.000e+00
Coefficient: 5.88e−02
Value: B
1.00
0.75
0.50
0.25
0.00
Fodinicola
A (n=15)
B (n=16)
C (n=14)
surface

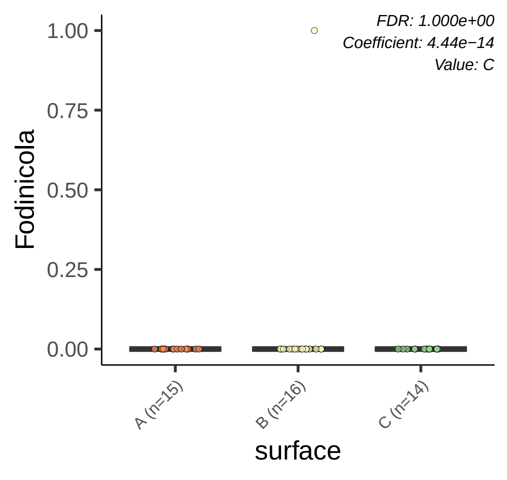

FDR: 1.000e+00
Coefficient: 4.44e−14
Value: C
1.00
0.75
0.50
0.25
0.00
Fodinicola
A (n=15)
B (n=16)
C (n=14)
surface

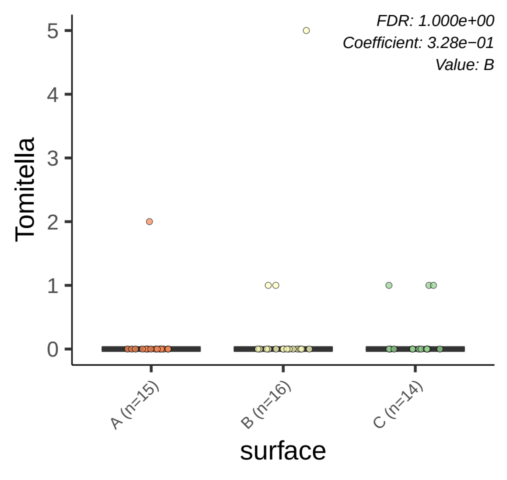

FDR: 1.000e+00
Coefficient: 3.28e−01
Value: B
Tomitella
0
1
2
3
4
5
A (n=15)
B (n=16)
C (n=14)
surface

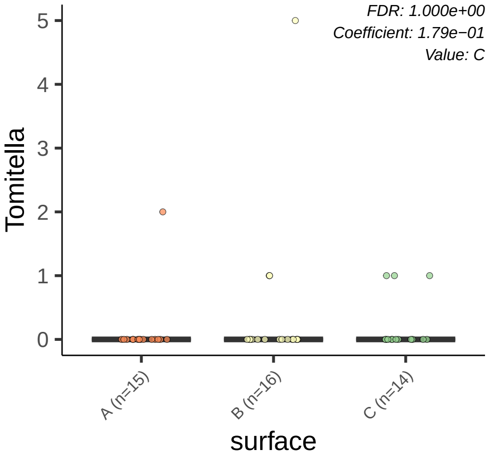

FDR: 1.000e+00
Coefficient: 1.79e−01
Value: C
Tomitella
0
1
2
3
4
5
A (n=15)
B (n=16)
C (n=14)
surface

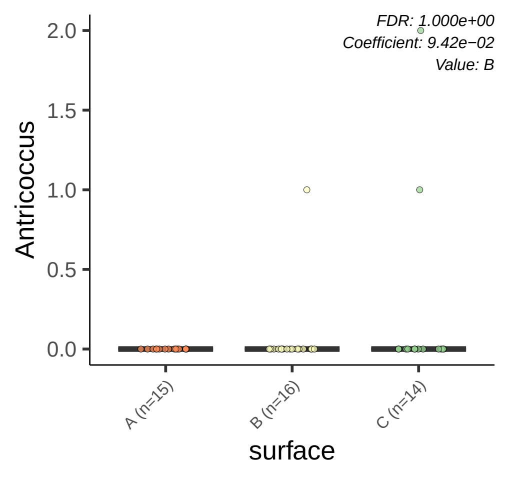

FDR: 1.000e+00
Coefficient: 9.42e−02
Value: B
Antricoccus
2.0
1.5
1.0
0.5
0.0
A (n=15)
B (n=16)
C (n=14)
surface

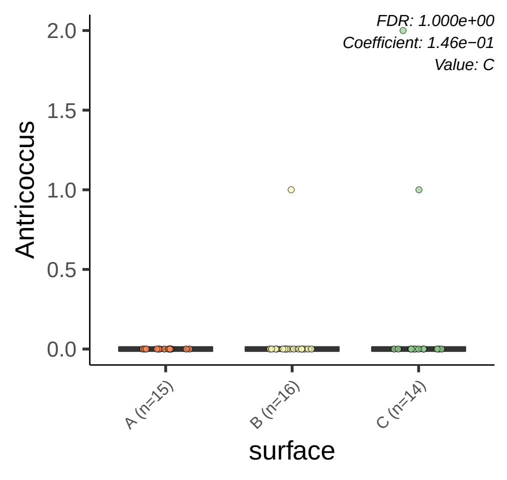

FDR: 1.000e+00
Coefficient: 1.46e−01
Value: C
Antricoccus
2.0
1.5
1.0
0.5
0.0
A (n=15)
B (n=16)
C (n=14)
surface

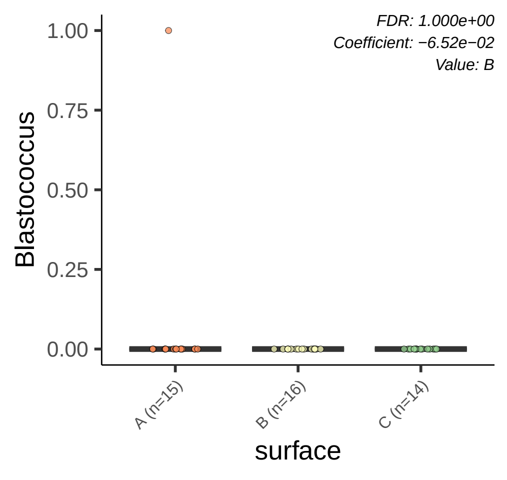

FDR: 1.000e+00
Coefficient: −6.52e−02
Value: B
Blastococcus
1.00
0.75
0.50
0.25
0.00
A (n=15)
B (n=16)
C (n=14)
surface

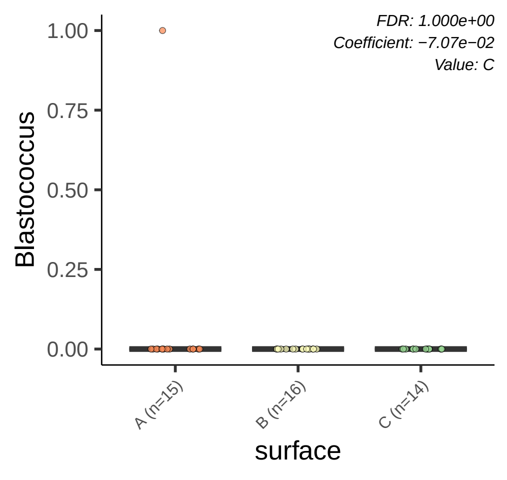

FDR: 1.000e+00
Coefficient: −7.07e−02
Value: C
Blastococcus
1.00
0.75
0.50
0.25
0.00
A (n=15)
B (n=16)
C (n=14)
surface

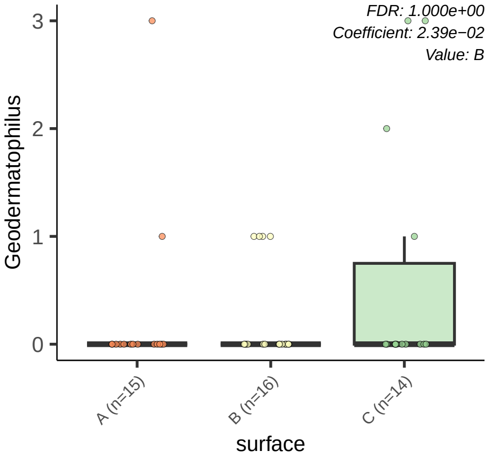
FDR: 1.000e+00
Coefficient: 2.39e−02
Value: B
Geodermatophilus
3
2
1
0
A (n=15)
B (n=16)
C (n=14)
surface

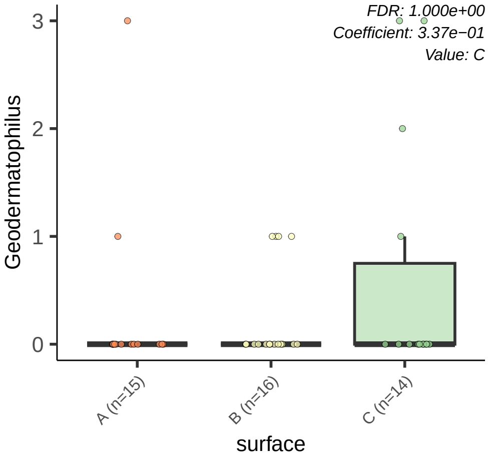

FDR: 1.000e+00
Coefficient: 3.37e−01
Value: C
Geodermatophilus
3
2
1
0
A (n=15)
B (n=16)
C (n=14)
surface

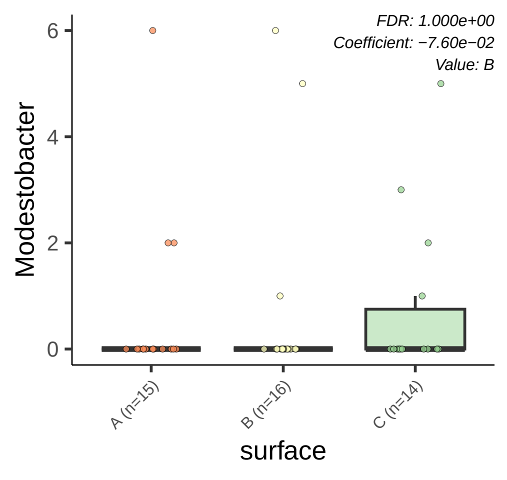

FDR: 1.000e+00
Coefficient: −7.60e−02
Value: B
Modestobacter
0
2
4
6
A (n=15)
B (n=16)
C (n=14)
surface

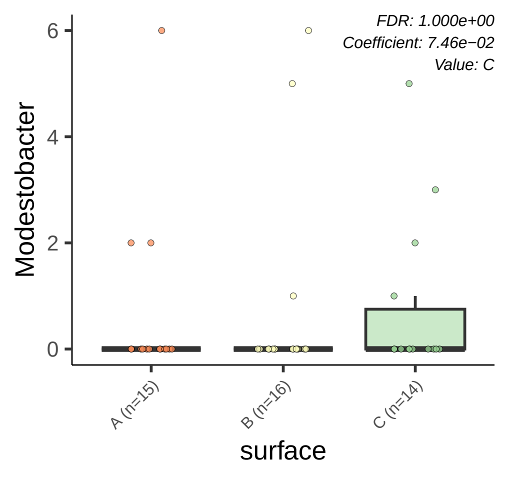
FDR: 1.000e+00
Coefficient: 7.46e−02
Value: C
Modestobacter
6
4
2
0
A (n=15)
B (n=16)
C (n=14)
surface

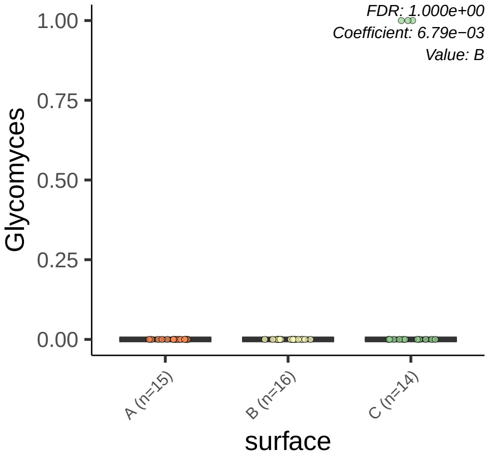
FDR: 1.000e+00
Coefficient: 6.79e−03
Value: B
Glycomyces
1.00
0.75
0.50
0.25
0.00
A (n=15)
B (n=16)
C (n=14)
surface

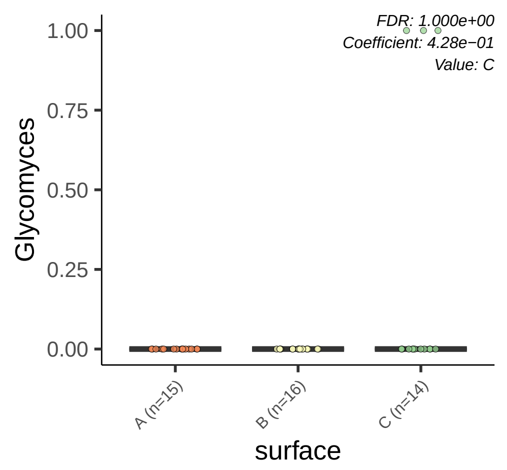

FDR: 1.000e+00
Coefficient: 4.28e−01
Value: C
Glycomyces
1.00
0.75
0.50
0.25
0.00
A (n=15)
B (n=16)
C (n=14)
surface

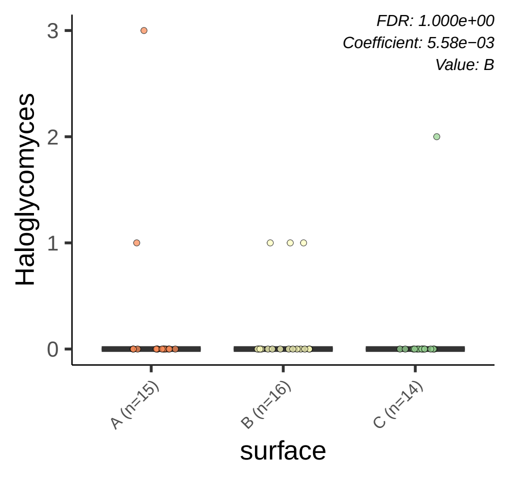
FDR: 1.000e+00
Coefficient: 5.58e−03
Value: B
Haloglycomyces
3
2
1
0
A (n=15)
B (n=16)
C (n=14)
surface

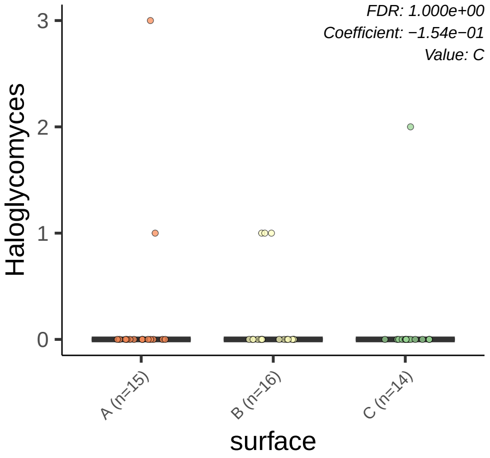

FDR: 1.000e+00
Coefficient: −1.54e−01
Value: C
Haloglycomyces
3
2
1
0
A (n=15)
B (n=16)
C (n=14)
surface

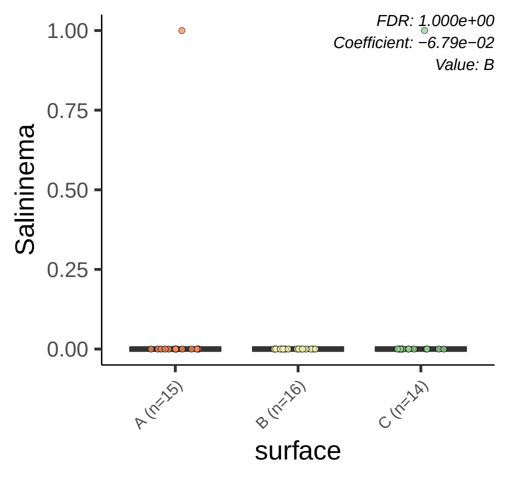
FDR: 1.000e+00
Coefficient: −6.79e−02
Value: B
Salininema
1.00
0.75
0.50
0.25
0.00
A (n=15)
B (n=16)
C (n=14)
surface

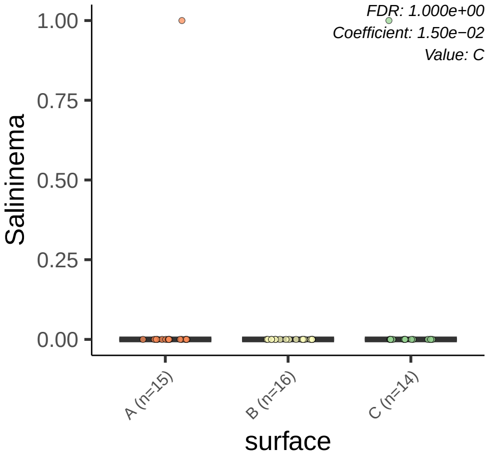
FDR: 1.000e+00
Coefficient: 1.50e−02
Value: C
Salininema
1.00
0.75
0.50
0.25
0.00
A (n=15)
B (n=16)
C (n=14)
surface

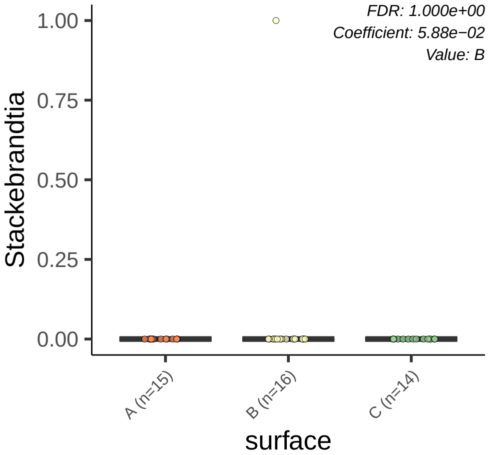
FDR: 1.000e+00
Coefficient: 5.88e−02
Value: B
Stackebrandtia
1.00
0.75
0.50
0.25
0.00
A (n=15)
B (n=16)
C (n=14)
surface

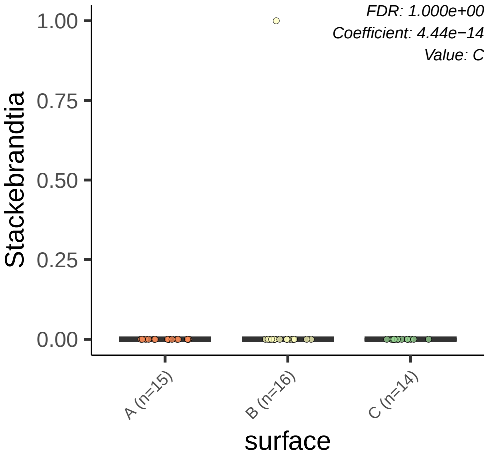
FDR: 1.000e+00
Coefficient: 4.44e−14
Value: C
Stackebrandtia
1.00
0.75
0.50
0.25
0.00
A (n=15)
B (n=16)
C (n=14)
surface

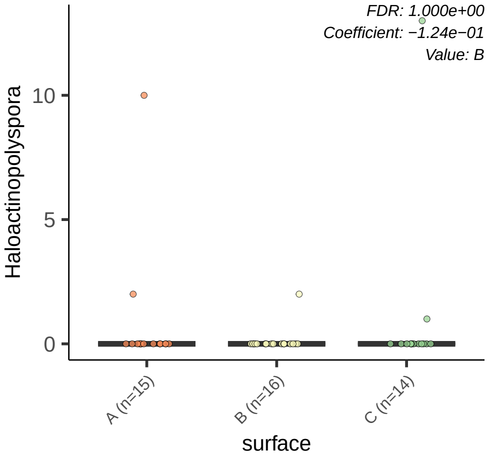

FDR: 1.000e+00
Coefficient: −1.24e−01
Value: B
Haloactinopolyspora
10
5
0
A (n=15)
B (n=16)
C (n=14)
surface

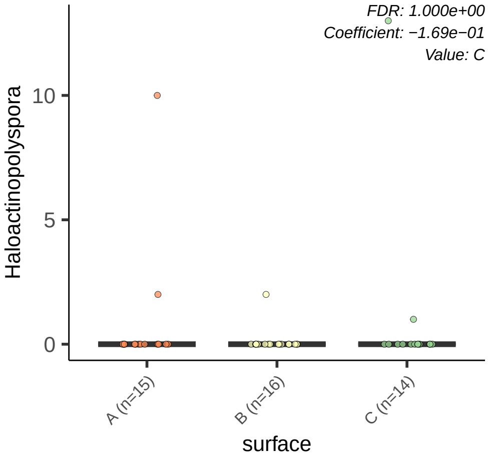
FDR: 1.000e+00
Coefficient: −1.69e−01
Value: C
Haloactinopolyspora
10
5
0
A (n=15)
B (n=16)
C (n=14)
surface

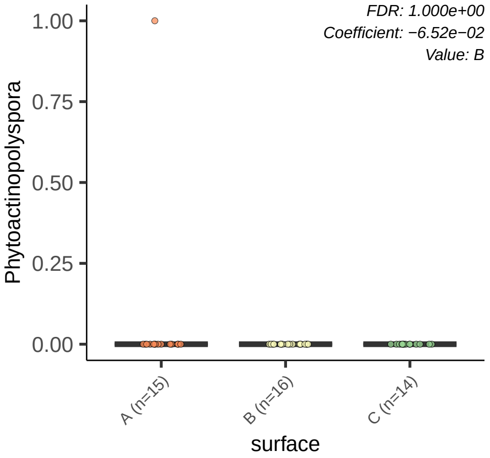
FDR: 1.000e+00
Coefficient: −6.52e−02
Value: B
Phytoactinopolyspora
1.00
0.75
0.50
0.25
0.00
A (n=15)
B (n=16)
C (n=14)
surface

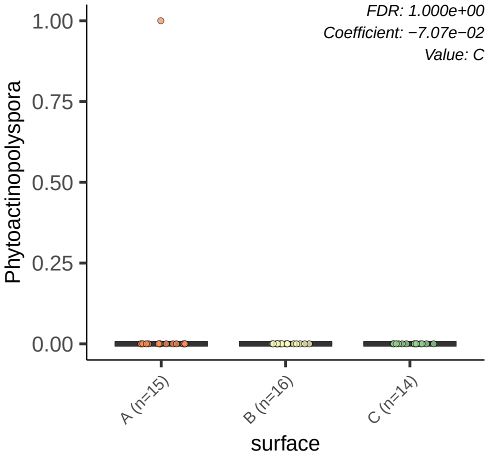
FDR: 1.000e+00
Coefficient: −7.07e−02
Value: C
Phytoactinopolyspora
1.00
0.75
0.50
0.25
0.00
A (n=15)
B (n=16)
C (n=14)
surface

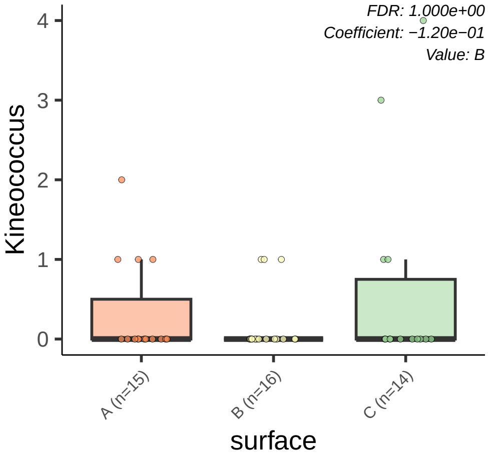
FDR: 1.000e+00
Coefficient: −1.20e−01
Value: B
Kineococcus
4
3
2
1
0
A (n=15)
B (n=16)
C (n=14)
surface

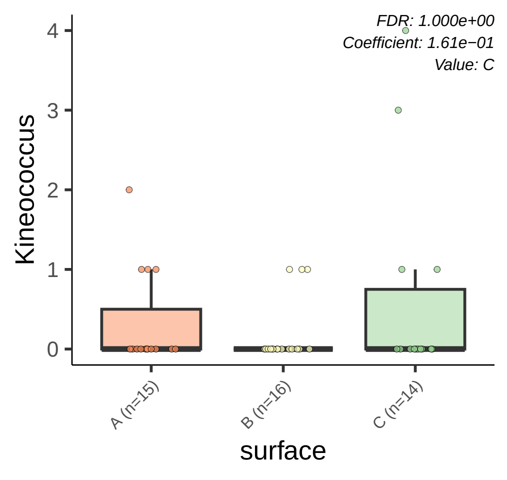

FDR: 1.000e+00
Coefficient: 1.61e−01
Value: C
Kineococcus
0
1
2
3
4
A (n=15)
B (n=16)
C (n=14)
surface

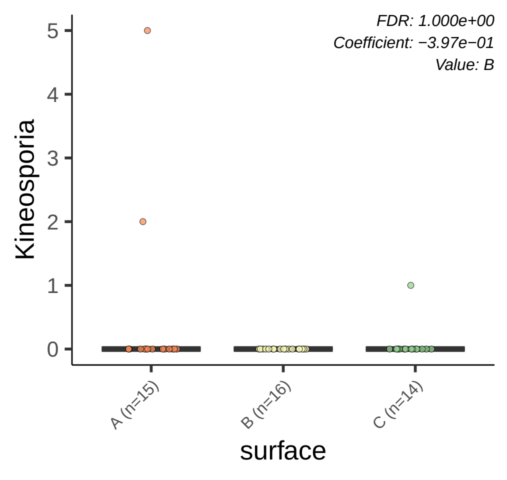
FDR: 1.000e+00
Coefficient: −3.97e−01
Value: B
Kineosporia
5
4
3
2
1
0
A (n=15)
B (n=16)
C (n=14)
surface

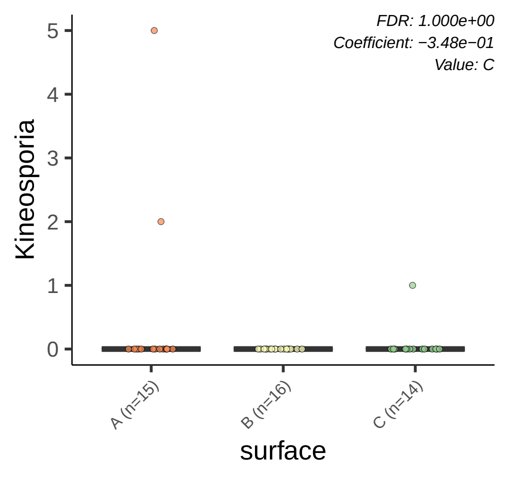
FDR: 1.000e+00
Coefficient: −3.48e−01
Value: C
Kineosporia
5
4
3
2
1
0
A (n=15)
B (n=16)
C (n=14)
surface

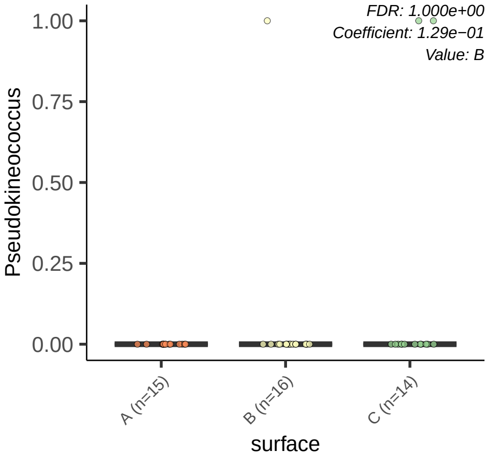
FDR: 1.000e+00
Coefficient: 1.29e−01
Value: B
Pseudokineococcus
1.00
0.75
0.50
0.25
0.00
A (n=15)
B (n=16)
C (n=14)
surface

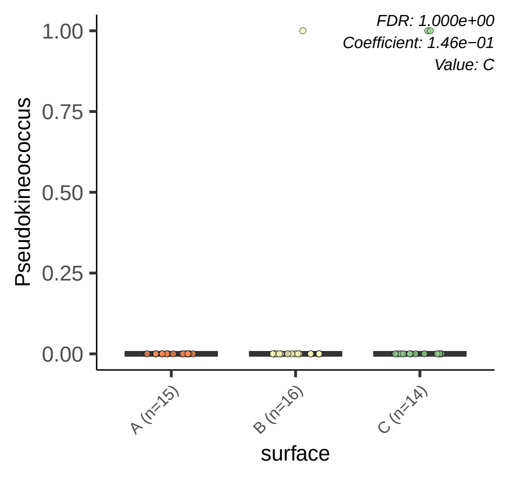

FDR: 1.000e+00
Coefficient: 1.46e−01
Value: C
Pseudokineococcus
1.00
0.75
0.50
0.25
0.00
A (n=15)
B (n=16)
C (n=14)
surface

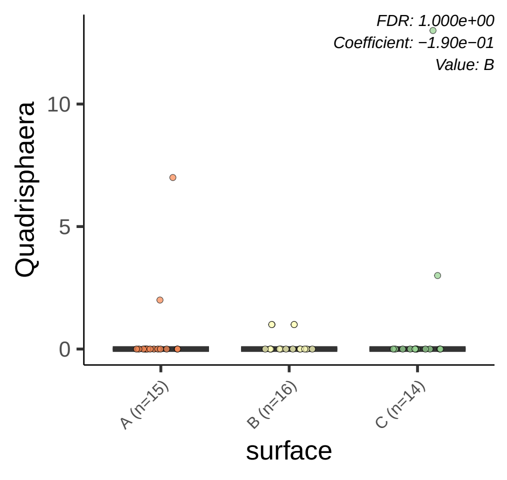
FDR: 1.000e+00
Coefficient: −1.90e−01
Value: B
Quadrisphaera
10
5
0
A (n=15)
B (n=16)
C (n=14)
surface

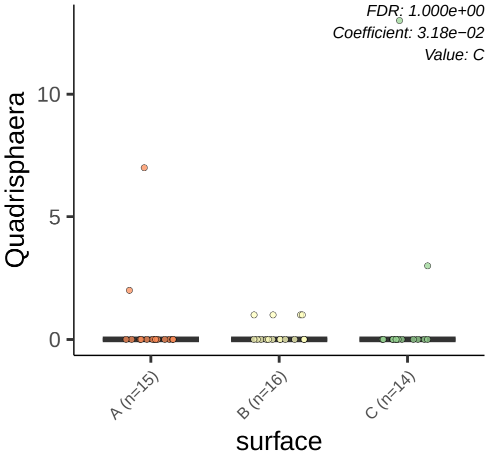
FDR: 1.000e+00
Coefficient: 3.18e−02
Value: C
Quadrisphaera
10
5
0
A (n=15)
B (n=16)
C (n=14)
surface

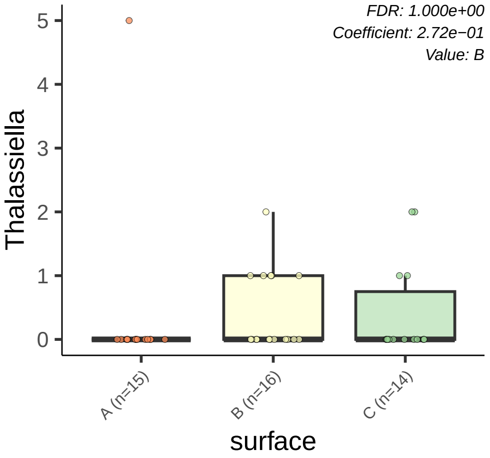

FDR: 1.000e+00
Coefficient: 2.72e−01
Value: B
Thalassiella
5
4
3
2
1
0
A (n=15)
B (n=16)
C (n=14)
surface

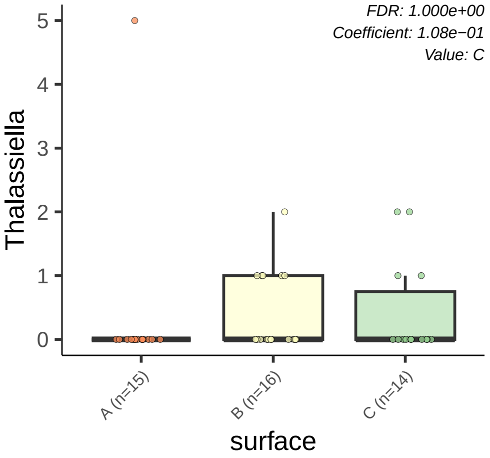

FDR: 1.000e+00
Coefficient: 1.08e−01
Value: C
Thalassiella
5
4
3
2
1
0
A (n=15)
B (n=16)
C (n=14)
surface

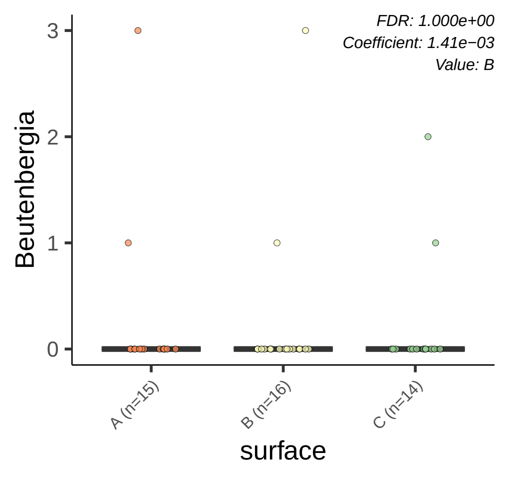

FDR: 1.000e+00
Coefficient: 1.41e−03
Value: B
Beutenbergia
3
2
1
0
A (n=15)
B (n=16)
C (n=14)
surface

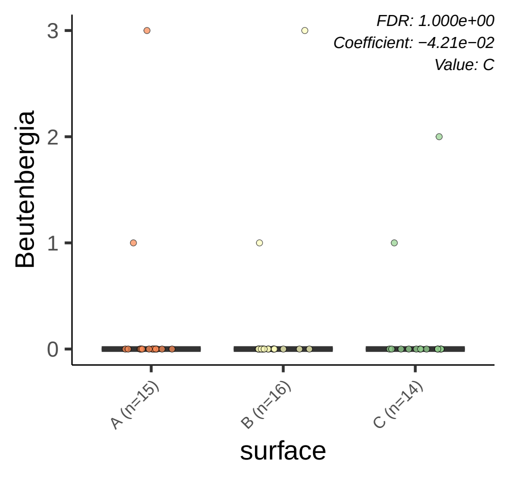
FDR: 1.000e+00
Coefficient: −4.21e−02
Value: C
Beutenbergia
3
2
1
0
A (n=15)
B (n=16)
C (n=14)
surface

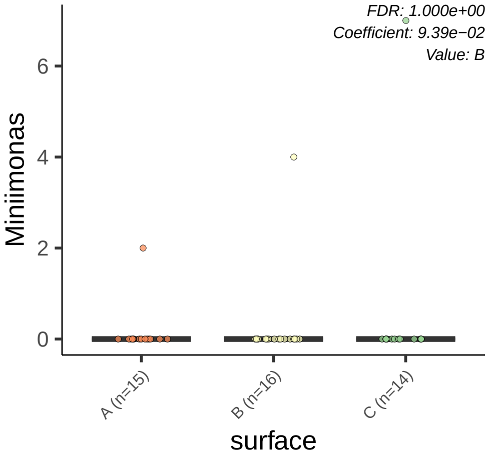

FDR: 1.000e+00
Coefficient: 9.39e−02
Value: B
Miniimonas
0
2
4
6
A (n=15)
B (n=16)
C (n=14)
surface

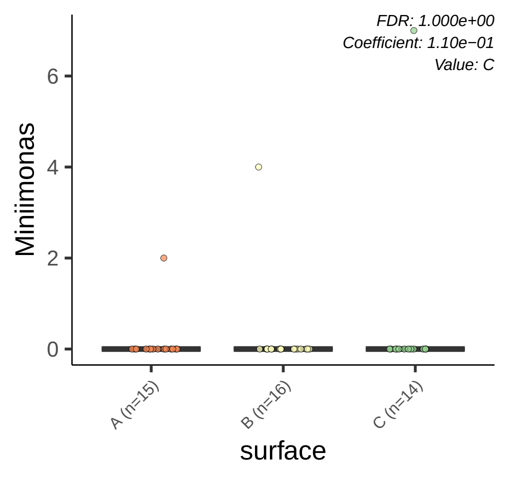
FDR: 1.000e+00
Coefficient: 1.10e−01
Value: C
Miniimonas
6
4
2
0
A (n=15)
B (n=16)
C (n=14)
surface

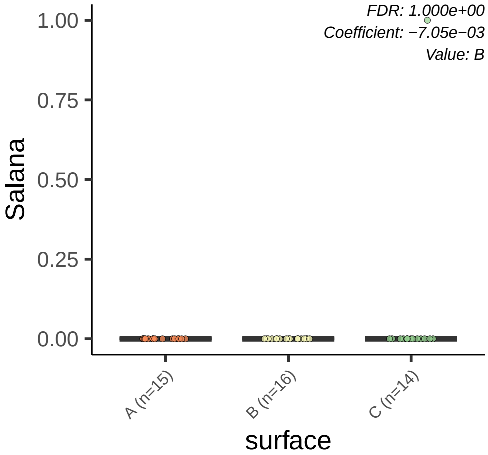

FDR: 1.000e+00
Coefficient: −7.05e−03
Value: B
Salana
1.00
0.75
0.50
0.25
0.00
A (n=15)
B (n=16)
C (n=14)
surface

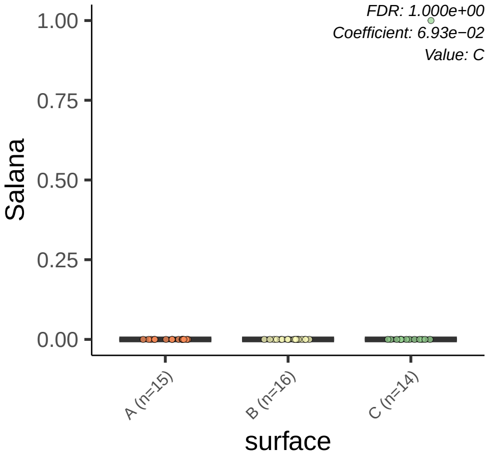

FDR: 1.000e+00
Coefficient: 6.93e−02
Value: C
Salana
1.00
0.75
0.50
0.25
0.00
A (n=15)
B (n=16)
C (n=14)
surface

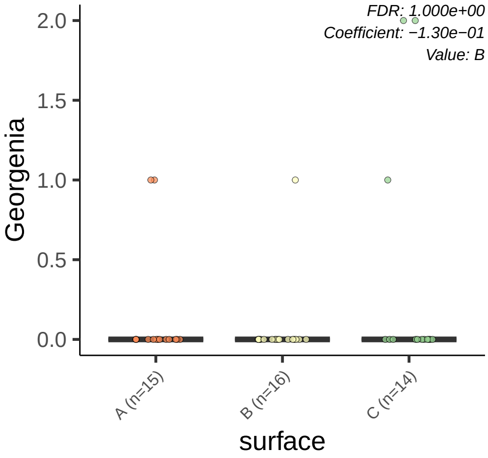
FDR: 1.000e+00
Coefficient: −1.30e−01
Value: B
Georgenia
2.0
1.5
1.0
0.5
0.0
A (n=15)
B (n=16)
C (n=14)
surface

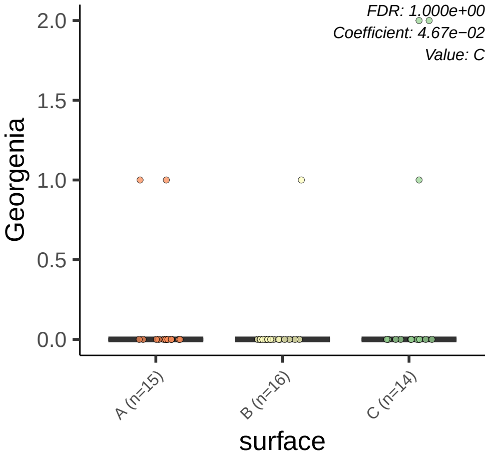

FDR: 1.000e+00
Coefficient: 4.67e−02
Value: C
Georgenia
2.0
1.5
1.0
0.5
0.0
A (n=15)
B (n=16)
C (n=14)
surface

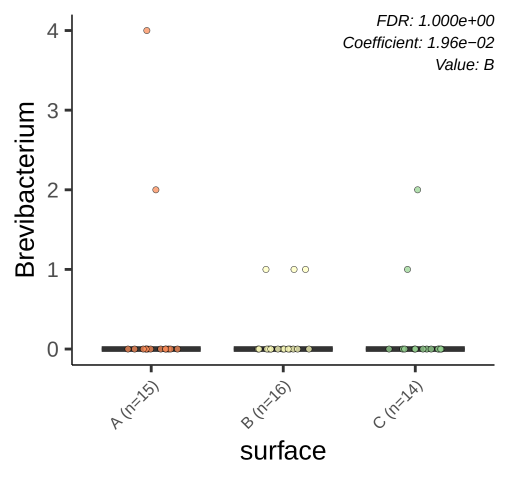

FDR: 1.000e+00
Coefficient: 1.96e−02
Value: B
Brevibacterium
0
1
2
3
4
A (n=15)
B (n=16)
C (n=14)
surface

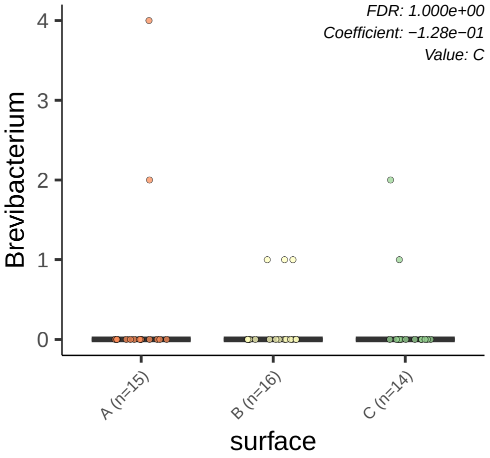

FDR: 1.000e+00
Coefficient: −1.28e−01
Value: C
Brevibacterium
4
3
2
1
0
A (n=15)
B (n=16)
C (n=14)
surface

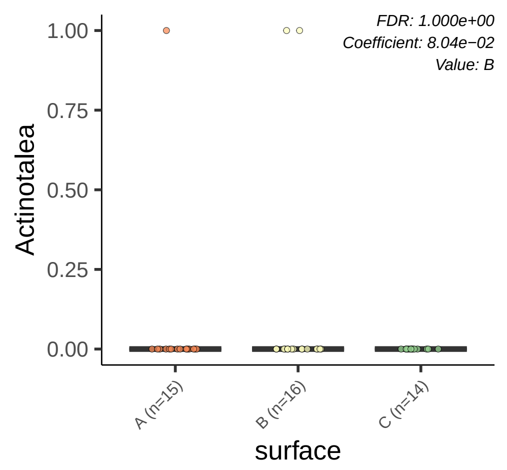
FDR: 1.000e+00
Coefficient: 8.04e−02
Value: B
Actinotalea
1.00
0.75
0.50
0.25
0.00
A (n=15)
B (n=16)
C (n=14)
surface

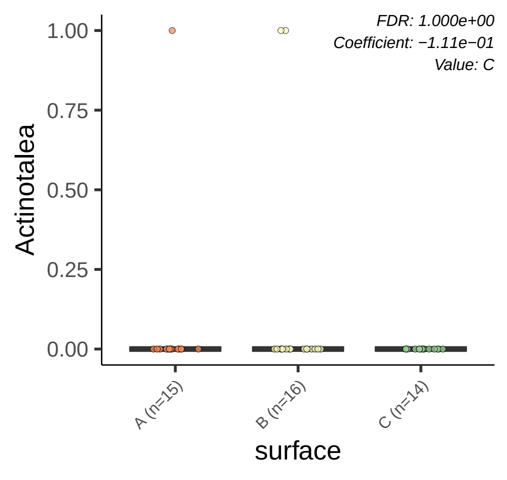
FDR: 1.000e+00
Coefficient: −1.11e−01
Value: C
Actinotalea
1.00
0.75
0.50
0.25
0.00
A (n=15)
B (n=16)
C (n=14)
surface

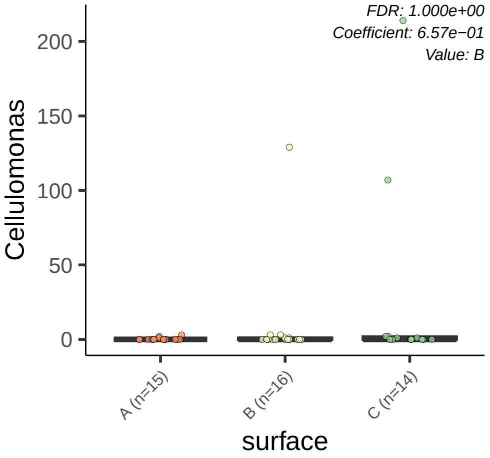
FDR: 1.000e+00
Coefficient: 6.57e−01
Value: B
Cellulomonas
200
150
100
50
0
A (n=15)
B (n=16)
C (n=14)
surface

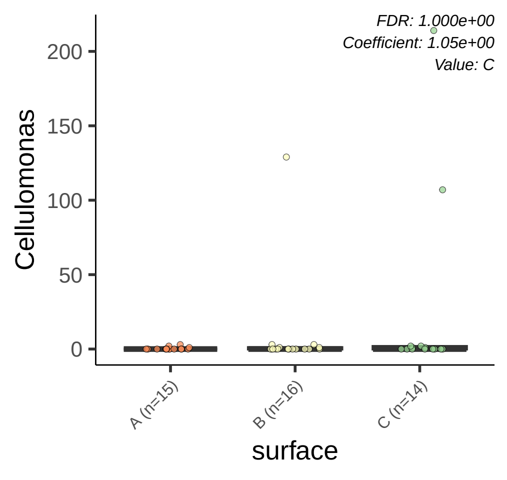

FDR: 1.000e+00
Coefficient: 1.05e+00
Value: C
Cellulomonas
200
150
100
50
0
A (n=15)
B (n=16)
C (n=14)
surface

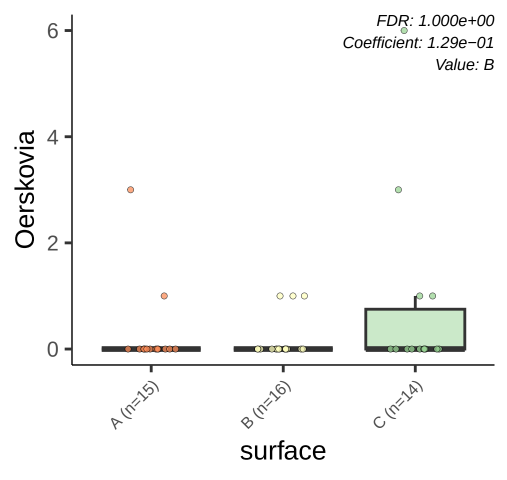
FDR: 1.000e+00
Coefficient: 1.29e−01
Value: B
Oerskovia
0
2
4
6
A (n=15)
B (n=16)
C (n=14)
surface

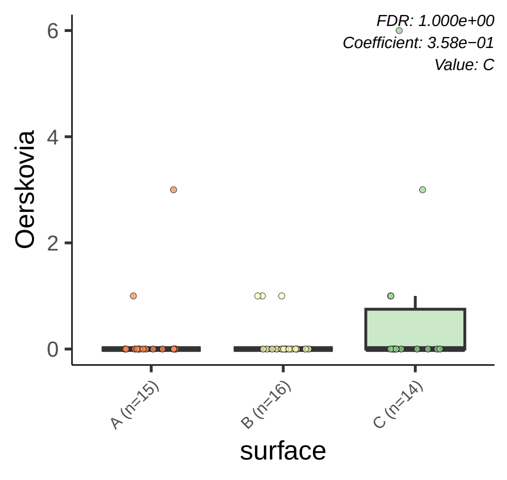

FDR: 1.000e+00
Coefficient: 3.58e−01
Value: C
Oerskovia
0
2
4
6
A (n=15)
B (n=16)
C (n=14)
surface

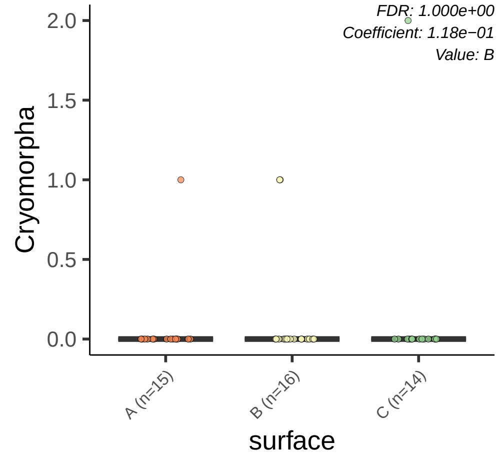
FDR: 1.000e+00
Coefficient: 1.18e−01
Value: B
Cryomorpha
2.0
1.5
1.0
0.5
0.0
A (n=15)
B (n=16)
C (n=14)
surface

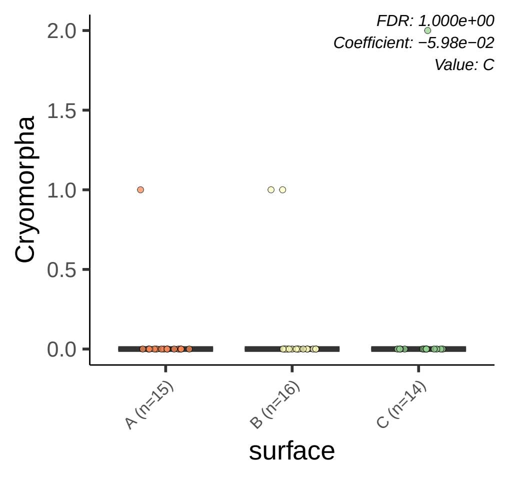

FDR: 1.000e+00
Coefficient: −5.98e−02
Value: C
Cryomorpha
2.0
1.5
1.0
0.5
0.0
A (n=15)
B (n=16)
C (n=14)
surface

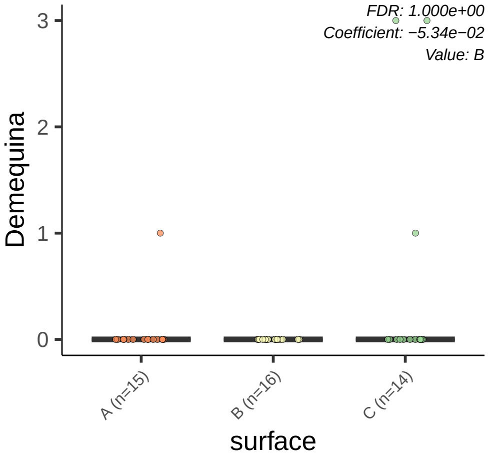
FDR: 1.000e+00
Coefficient: −5.34e−02
Value: B
Demequina
3
2
1
0
A (n=15)
B (n=16)
C (n=14)
surface

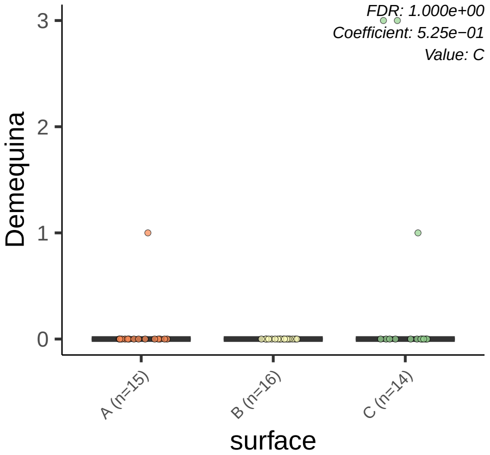
FDR: 1.000e+00
Coefficient: 5.25e−01
Value: C
Demequina
3
2
1
0
A (n=15)
B (n=16)
C (n=14)
surface

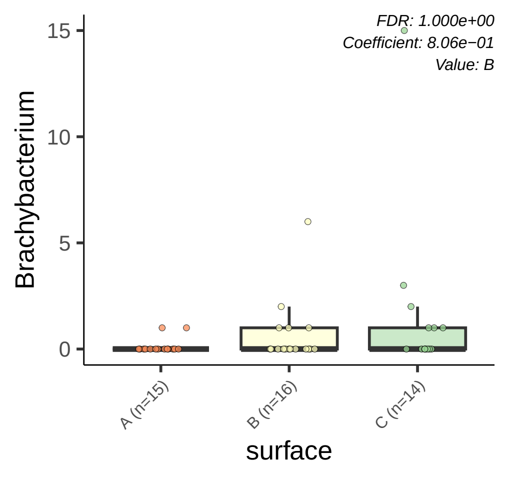

FDR: 1.000e+00
Coefficient: 8.06e−01
Value: B
Brachybacterium
15
10
5
0
A (n=15)
B (n=16)
C (n=14)
surface

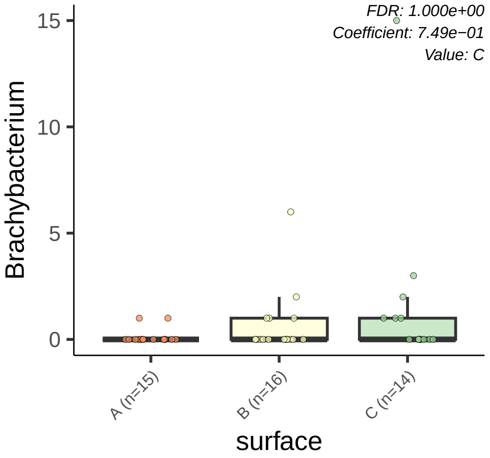
FDR: 1.000e+00
Coefficient: 7.49e−01
Value: C
Brachybacterium
15
10
5
0
A (n=15)
B (n=16)
C (n=14)
surface

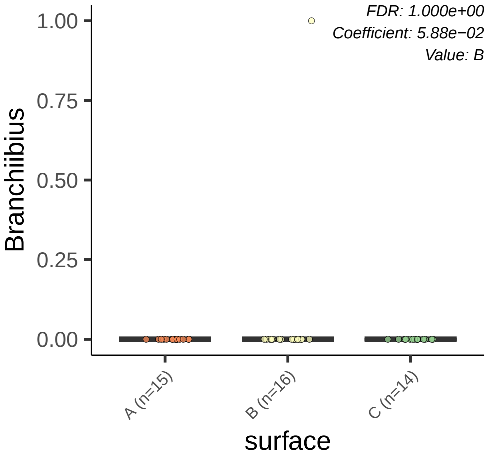
FDR: 1.000e+00
Coefficient: 5.88e−02
Value: B
Branchiibius
1.00
0.75
0.50
0.25
0.00
A (n=15)
B (n=16)
C (n=14)
surface

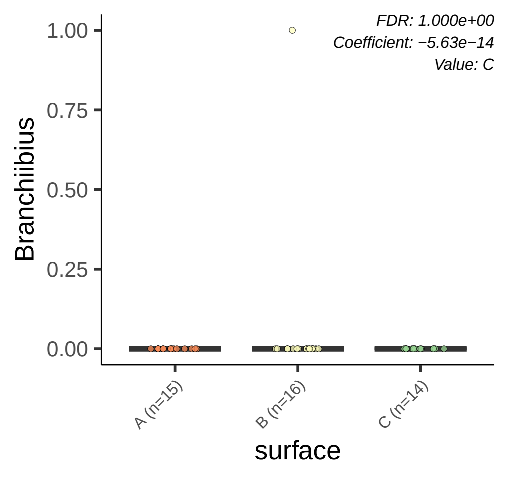
FDR: 1.000e+00
Coefficient: −5.63e−14
Value: C
Branchiibius
1.00
0.75
0.50
0.25
0.00
A (n=15)
B (n=16)
C (n=14)
surface

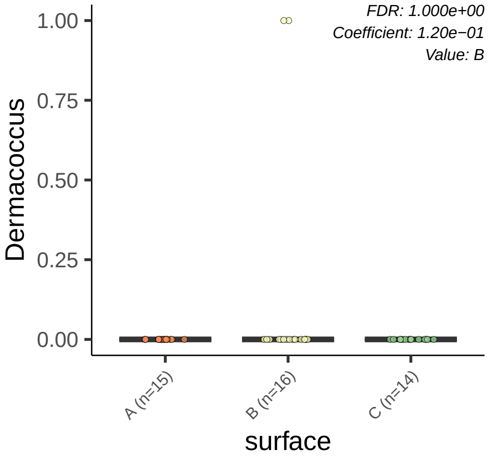
FDR: 1.000e+00
Coefficient: 1.20e−01
Value: B
Dermacoccus
1.00
0.75
0.50
0.25
0.00
A (n=15)
B (n=16)
C (n=14)
surface

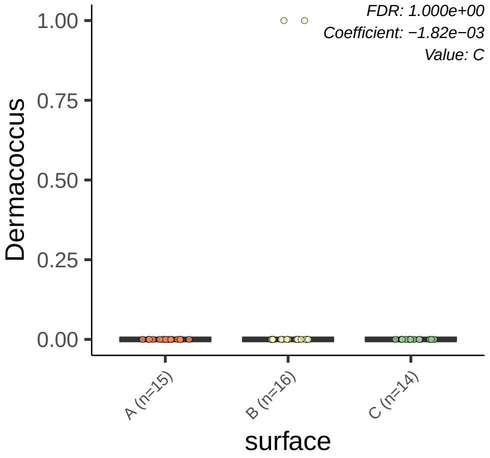
FDR: 1.000e+00
Coefficient: −1.82e−03
Value: C
Dermacoccus
1.00
0.75
0.50
0.25
0.00
A (n=15)
B (n=16)
C (n=14)
surface

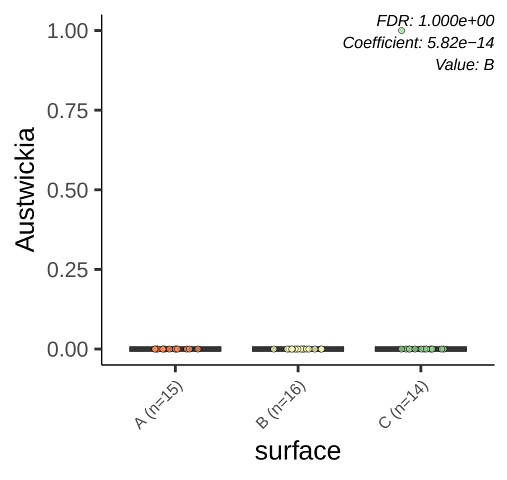

FDR: 1.000e+00
Coefficient: 5.82e−14
Value: B
Austwickia
1.00
0.75
0.50
0.25
0.00
A (n=15)
B (n=16)
C (n=14)
surface

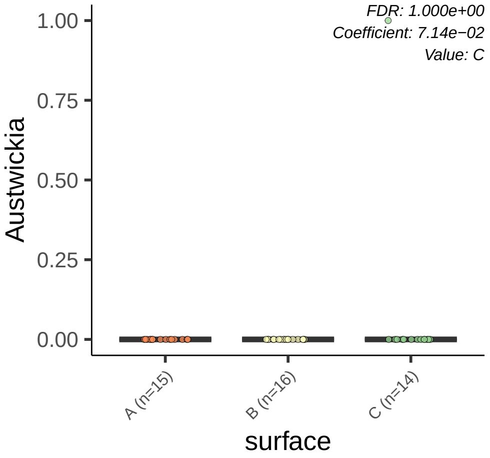
FDR: 1.000e+00
Coefficient: 7.14e−02
Value: C
Austwickia
1.00
0.75
0.50
0.25
0.00
A (n=15)
B (n=16)
C (n=14)
surface

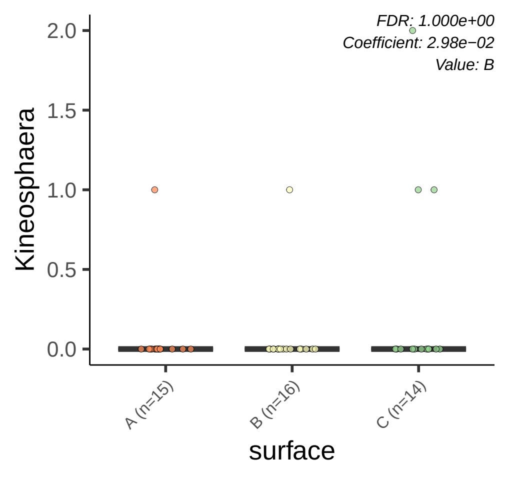
FDR: 1.000e+00
Coefficient: 2.98e−02
Value: B
Kineosphaera
2.0
1.5
1.0
0.5
0.0
A (n=15)
B (n=16)
C (n=14)
surface

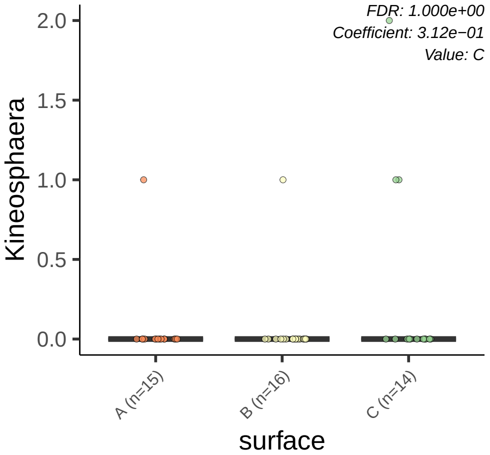

FDR: 1.000e+00
Coefficient: 3.12e−01
Value: C
Kineosphaera
2.0
1.5
1.0
0.5
0.0
A (n=15)
B (n=16)
C (n=14)
surface

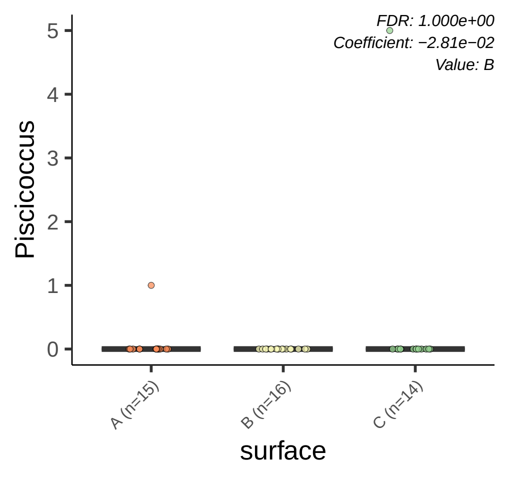
FDR: 1.000e+00
Coefficient: −2.81e−02
Value: B
Piscicoccus
0
1
2
3
4
5
A (n=15)
B (n=16)
C (n=14)
surface

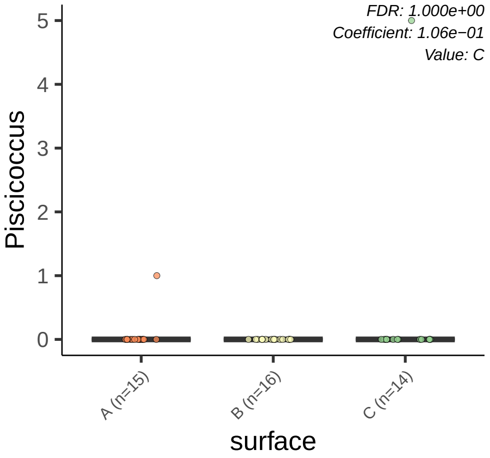
FDR: 1.000e+00
Coefficient: 1.06e−01
Value: C
Piscicoccus
0
1
2
3
4
5
A (n=15)
B (n=16)
C (n=14)
surface

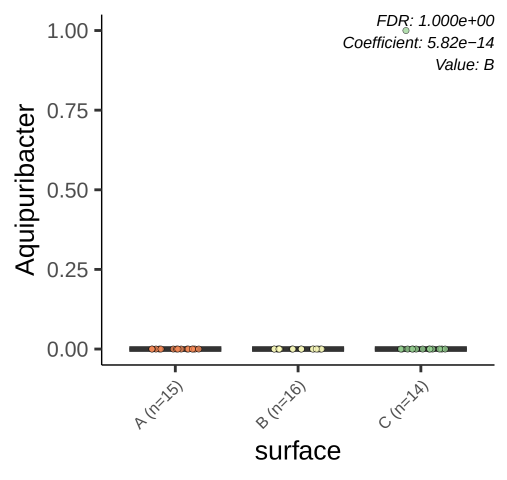

FDR: 1.000e+00
Coefficient: 5.82e−14
Value: B
Aquipuribacter
1.00
0.75
0.50
0.25
0.00
A (n=15)
B (n=16)
C (n=14)
surface

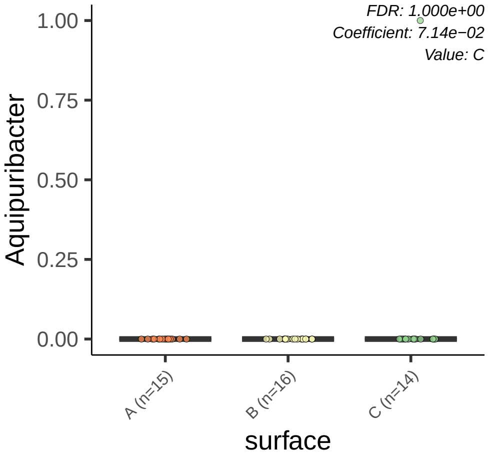

FDR: 1.000e+00
Coefficient: 7.14e−02
Value: C
Aquipuribacter
1.00
0.75
0.50
0.25
0.00
A (n=15)
B (n=16)
C (n=14)
surface

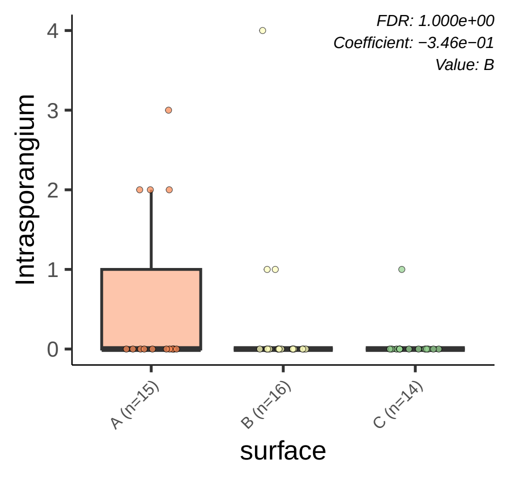
FDR: 1.000e+00
Coefficient: −3.46e−01
Value: B
Intrasporangium
0
1
2
3
4
A (n=15)
B (n=16)
C (n=14)
surface

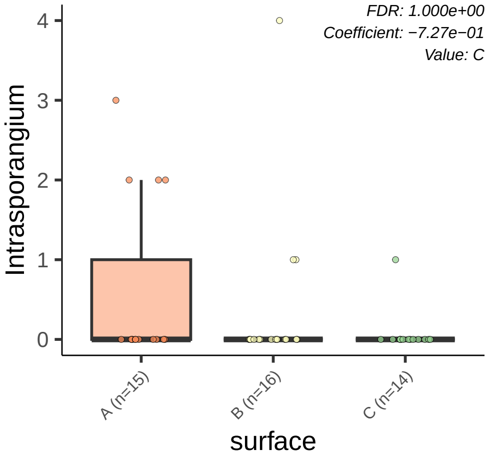

FDR: 1.000e+00
Coefficient: −7.27e−01
Value: C
Intrasporangium
0
1
2
3
4
A (n=15)
B (n=16)
C (n=14)
surface

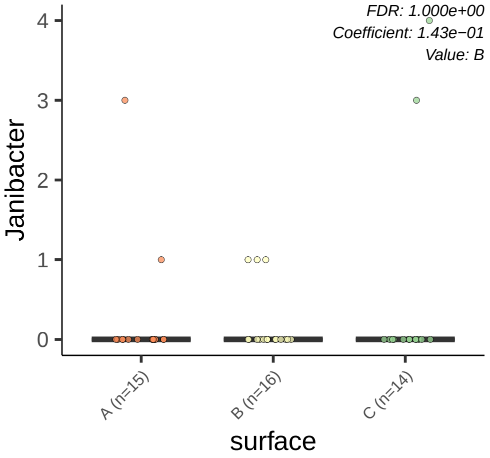

FDR: 1.000e+00
Coefficient: 1.43e−01
Value: B
Janibacter
0
1
2
3
4
A (n=15)
B (n=16)
C (n=14)
surface

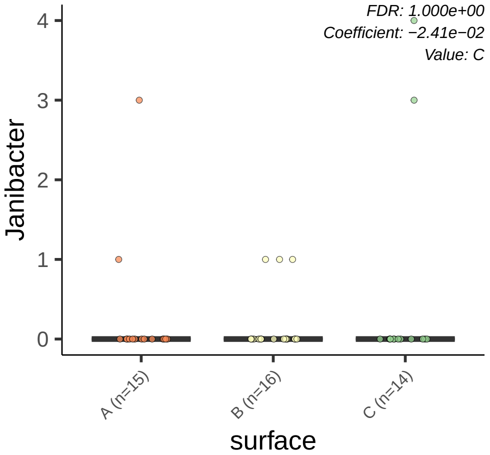
FDR: 1.000e+00
Coefficient: −2.41e−02
Value: C
Janibacter
4
3
2
1
0
A (n=15)
B (n=16)
C (n=14)
surface

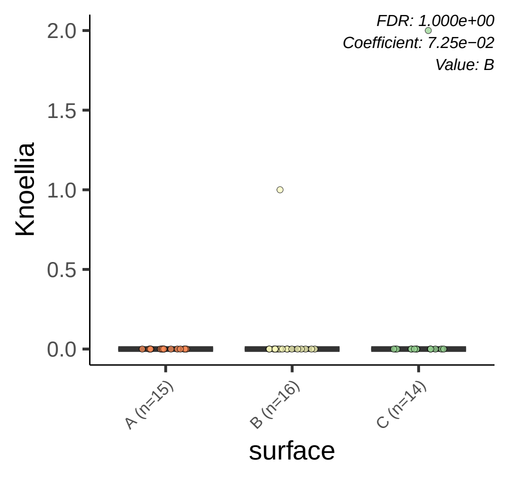
FDR: 1.000e+00
Coefficient: 7.25e−02
Value: B
Knoellia
2.0
1.5
1.0
0.5
0.0
A (n=15)
B (n=16)
C (n=14)
surface

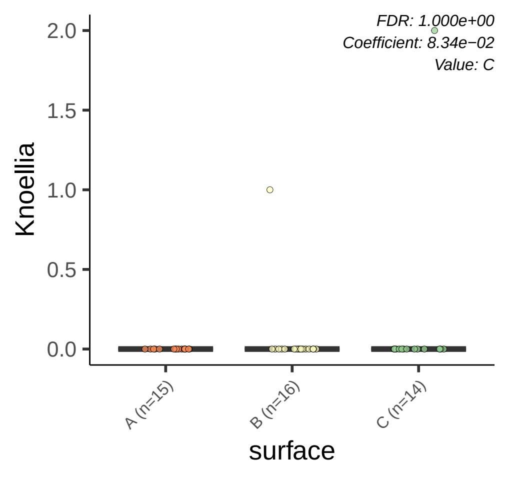
FDR: 1.000e+00
Coefficient: 8.34e−02
Value: C
Knoellia
2.0
1.5
1.0
0.5
0.0
A (n=15)
B (n=16)
C (n=14)
surface

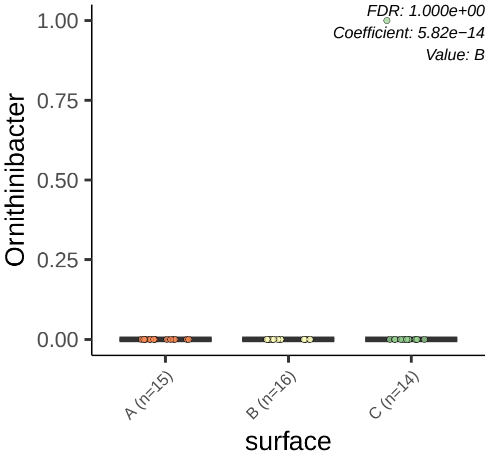
FDR: 1.000e+00
Coefficient: 5.82e−14
Value: B
Ornithinibacter
1.00
0.75
0.50
0.25
0.00
A (n=15)
B (n=16)
C (n=14)
surface

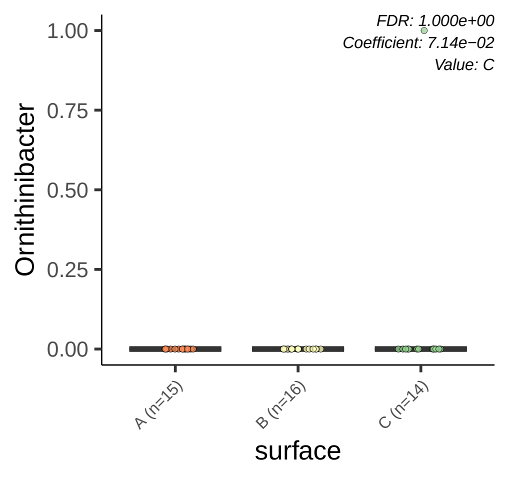
FDR: 1.000e+00
Coefficient: 7.14e−02
Value: C
Ornithinibacter
1.00
0.75
0.50
0.25
0.00
A (n=15)
B (n=16)
C (n=14)
surface

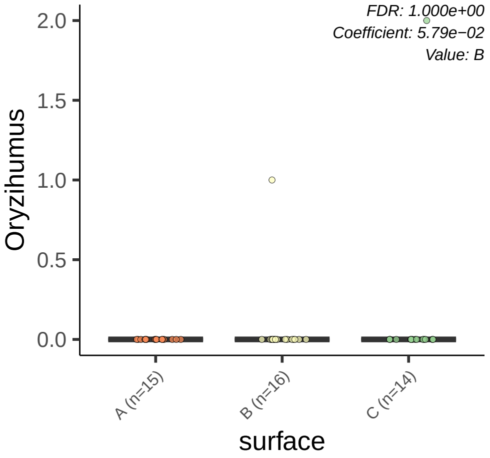
FDR: 1.000e+00
Coefficient: 5.79e−02
Value: B
Oryzihumus
2.0
1.5
1.0
0.5
0.0
A (n=15)
B (n=16)
C (n=14)
surface

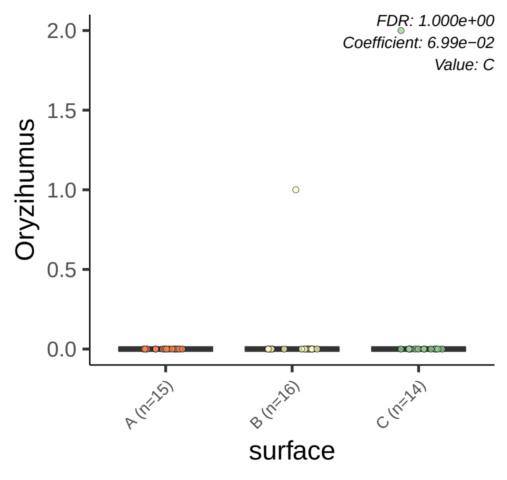

FDR: 1.000e+00
Coefficient: 6.99e−02
Value: C
Oryzihumus
2.0
1.5
1.0
0.5
0.0
A (n=15)
B (n=16)
C (n=14)
surface

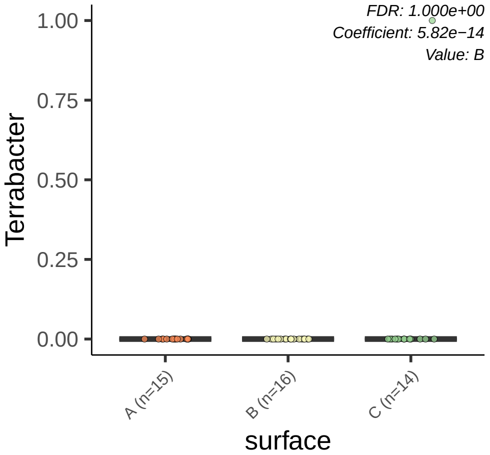
FDR: 1.000e+00
Coefficient: 5.82e−14
Value: B
Terrabacter
1.00
0.75
0.50
0.25
0.00
A (n=15)
B (n=16)
C (n=14)
surface

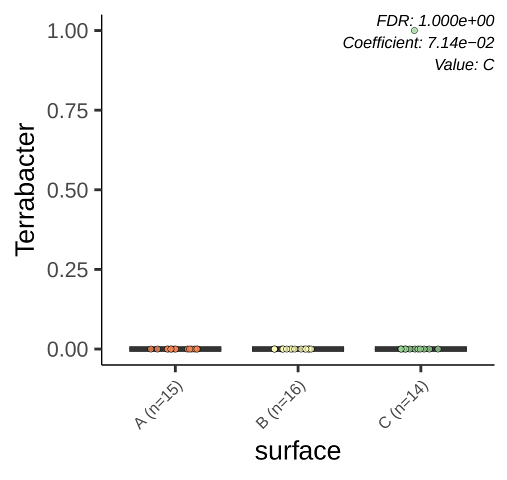

FDR: 1.000e+00
Coefficient: 7.14e−02
Value: C
Terrabacter
1.00
0.75
0.50
0.25
0.00
A (n=15)
B (n=16)
C (n=14)
surface

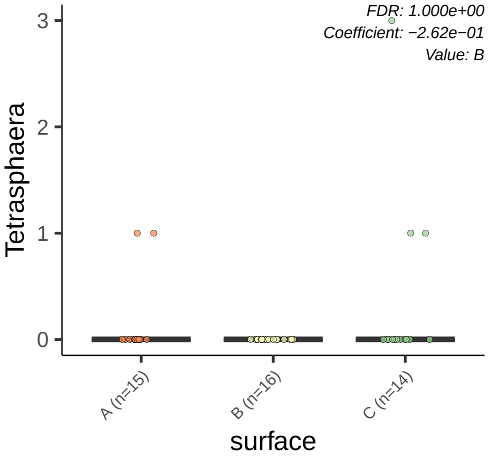
FDR: 1.000e+00
Coefficient: −2.62e−01
Value: B
Tetrasphaera
3
2
1
0
A (n=15)
B (n=16)
C (n=14)
surface

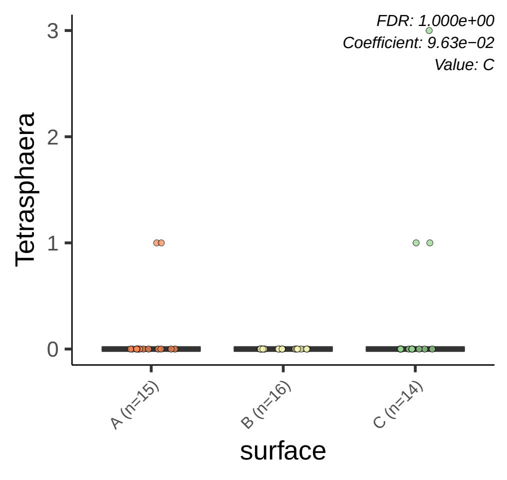
FDR: 1.000e+00
Coefficient: 9.63e−02
Value: C
Tetrasphaera
3
2
1
0
A (n=15)
B (n=16)
C (n=14)
surface

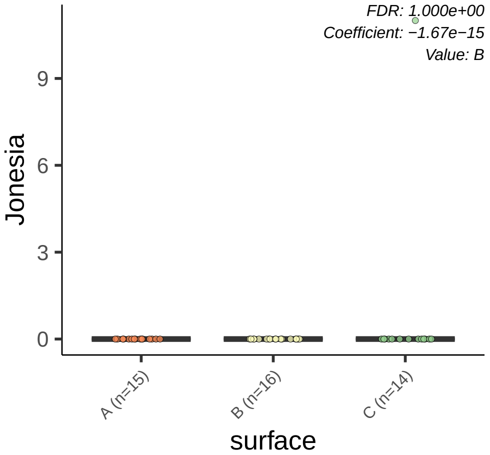
FDR: 1.000e+00
Coefficient: −1.67e−15
Value: B
Jonesia
9
6
3
0
A (n=15)
B (n=16)
C (n=14)
surface

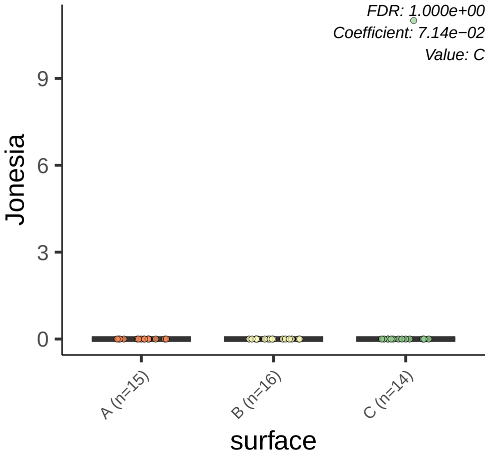

FDR: 1.000e+00
Coefficient: 7.14e−02
Value: C
Jonesia
9
6
3
0
A (n=15)
B (n=16)
C (n=14)
surface

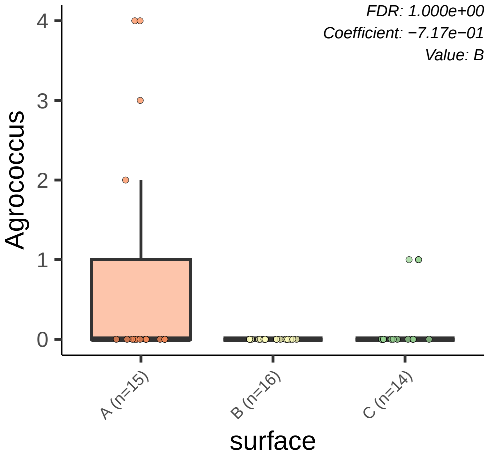
FDR: 1.000e+00
Coefficient: −7.17e−01
Value: B
Agrococcus
0
1
2
3
4
A (n=15)
B (n=16)
C (n=14)
surface

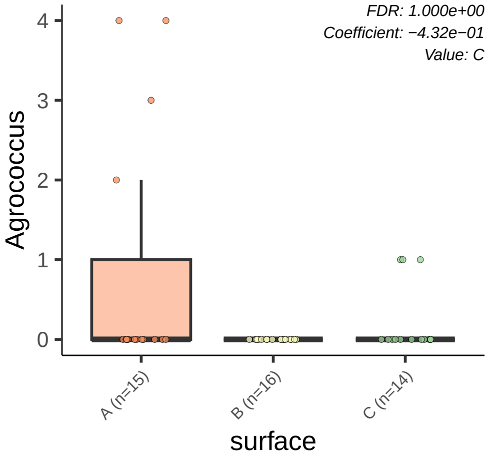
FDR: 1.000e+00
Coefficient: −4.32e−01
Value: C
Agrococcus
0
1
2
3
4
A (n=15)
B (n=16)
C (n=14)
surface

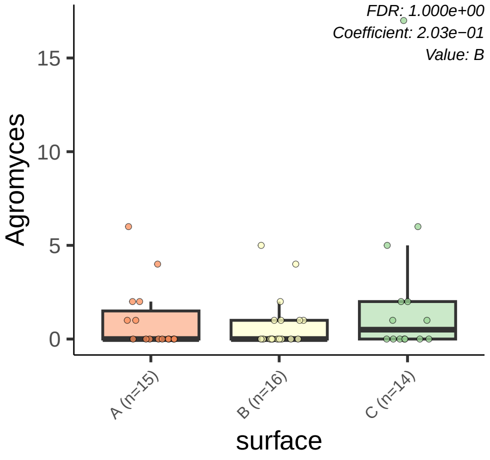

FDR: 1.000e+00
Coefficient: 2.03e−01
Value: B
Agromyces
0
5
10
15
A (n=15)
B (n=16)
C (n=14)
surface

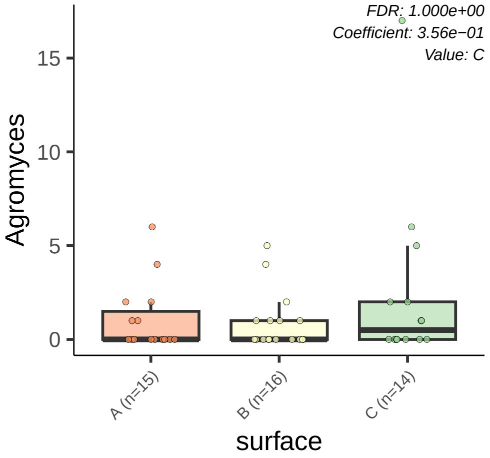

FDR: 1.000e+00
Coefficient: 3.56e−01
Value: C
Agromyces
0
5
10
15
A (n=15)
B (n=16)
C (n=14)
surface

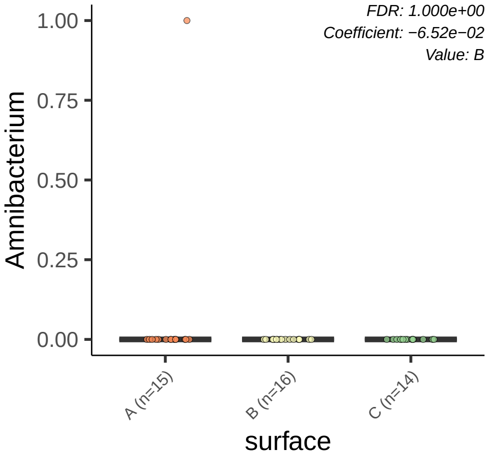
FDR: 1.000e+00
Coefficient: −6.52e−02
Value: B
Amnibacterium
1.00
0.75
0.50
0.25
0.00
A (n=15)
B (n=16)
C (n=14)
surface

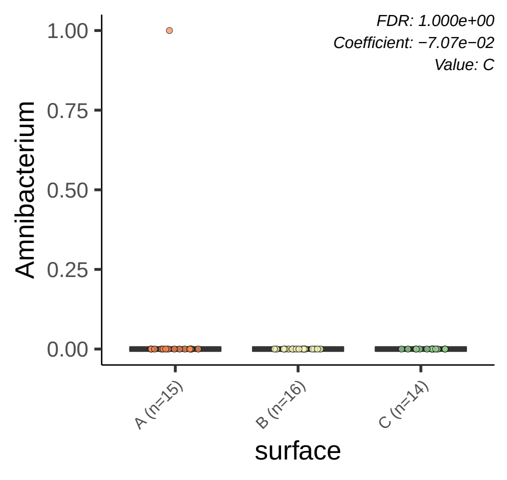
FDR: 1.000e+00
Coefficient: −7.07e−02
Value: C
Amnibacterium
1.00
0.75
0.50
0.25
0.00
A (n=15)
B (n=16)
C (n=14)
surface

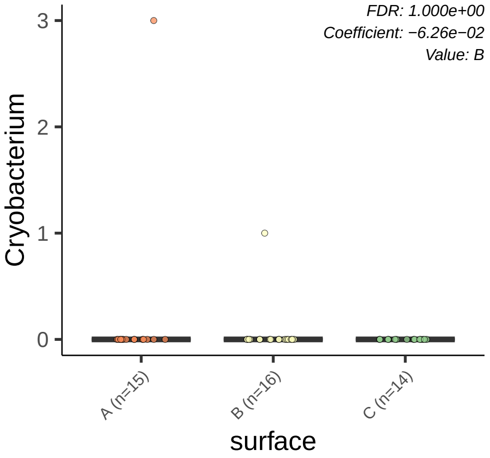
FDR: 1.000e+00
Coefficient: −6.26e−02
Value: B
Cryobacterium
3
2
1
0
A (n=15)
B (n=16)
C (n=14)
surface

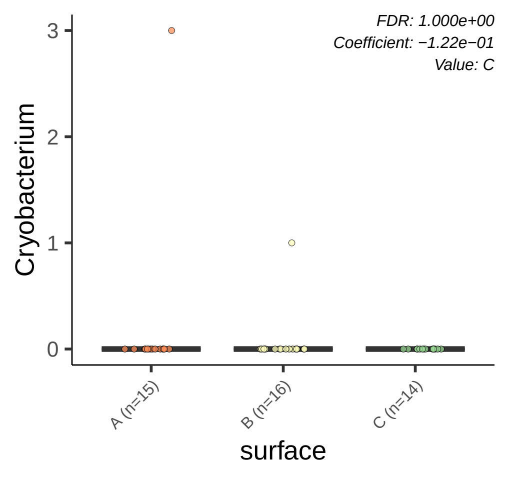

FDR: 1.000e+00
Coefficient: −1.22e−01
Value: C
Cryobacterium
3
2
1
0
A (n=15)
B (n=16)
C (n=14)
surface

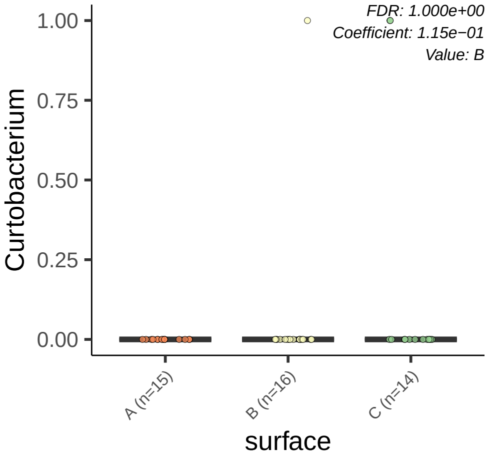
FDR: 1.000e+00
Coefficient: 1.15e−01
Value: B
Curtobacterium
1.00
0.75
0.50
0.25
0.00
A (n=15)
B (n=16)
C (n=14)
surface

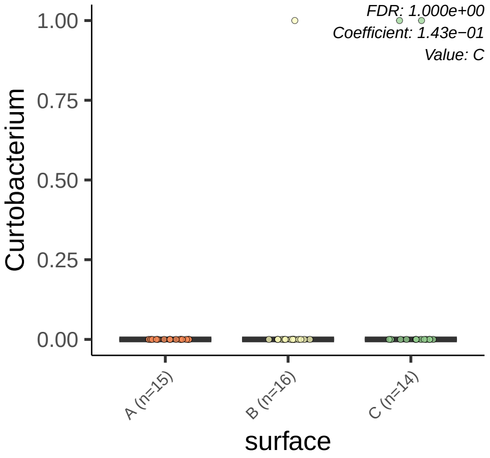

FDR: 1.000e+00
Coefficient: 1.43e−01
Value: C
Curtobacterium
1.00
0.75
0.50
0.25
0.00
A (n=15)
B (n=16)
C (n=14)
surface

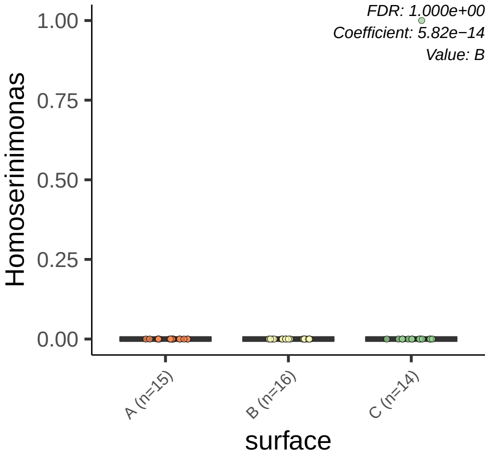

FDR: 1.000e+00
Coefficient: 5.82e−14
Value: B
Homoserinimonas
1.00
0.75
0.50
0.25
0.00
A (n=15)
B (n=16)
C (n=14)
surface

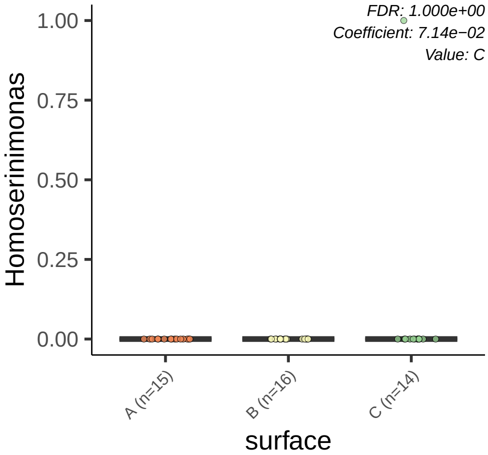

FDR: 1.000e+00
Coefficient: 7.14e−02
Value: C
Homoserinimonas
1.00
0.75
0.50
0.25
0.00
A (n=15)
B (n=16)
C (n=14)
surface

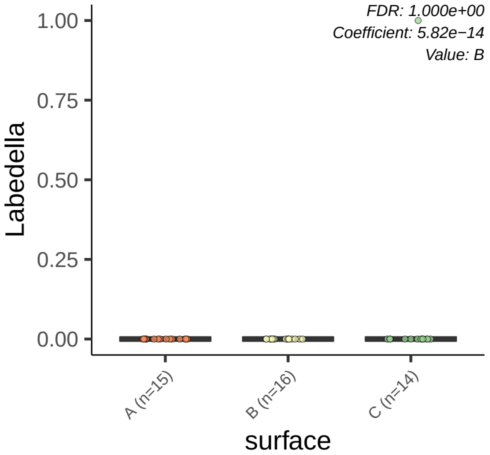
FDR: 1.000e+00
Coefficient: 5.82e−14
Value: B
1.00
0.75
0.50
0.25
0.00
Labedella
A (n=15)
B (n=16)
C (n=14)
surface

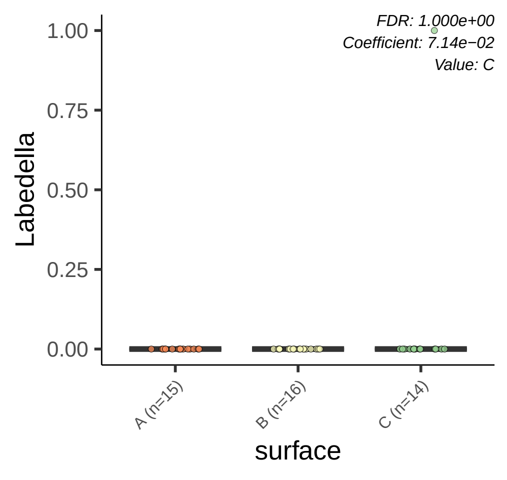
FDR: 1.000e+00
Coefficient: 7.14e−02
Value: C
Labedella
1.00
0.75
0.50
0.25
0.00
A (n=15)
B (n=16)
C (n=14)
surface

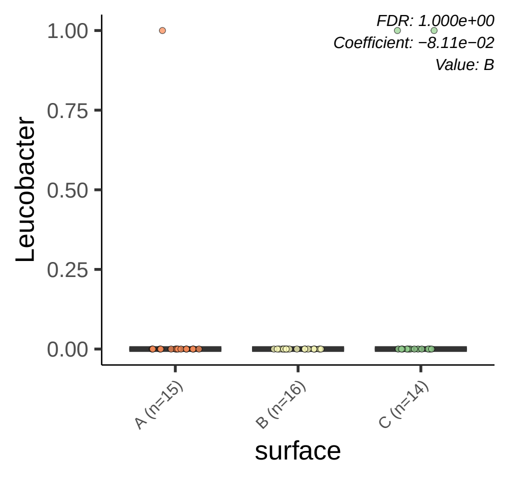
FDR: 1.000e+00
Coefficient: −8.11e−02
Value: B
Leucobacter
1.00
0.75
0.50
0.25
0.00
A (n=15)
B (n=16)
C (n=14)
surface

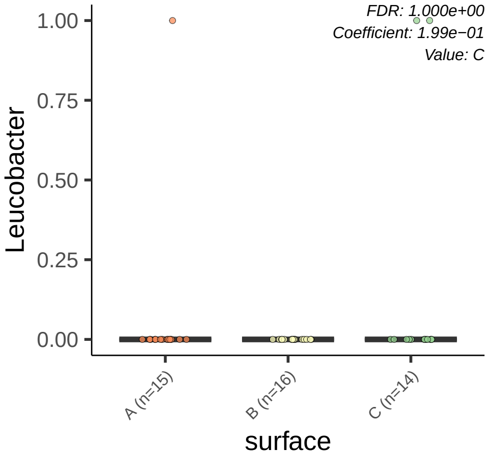

FDR: 1.000e+00
Coefficient: 1.99e−01
Value: C
Leucobacter
1.00
0.75
0.50
0.25
0.00
A (n=15)
B (n=16)
C (n=14)
surface

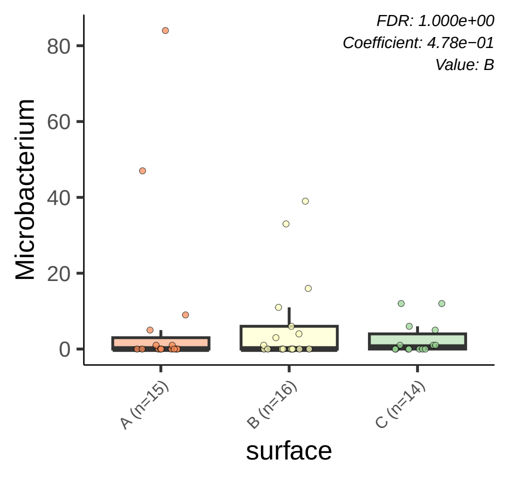
FDR: 1.000e+00
Coefficient: 4.78e−01
Value: B
Microbacterium
0
20
40
60
80
A (n=15)
B (n=16)
C (n=14)
surface

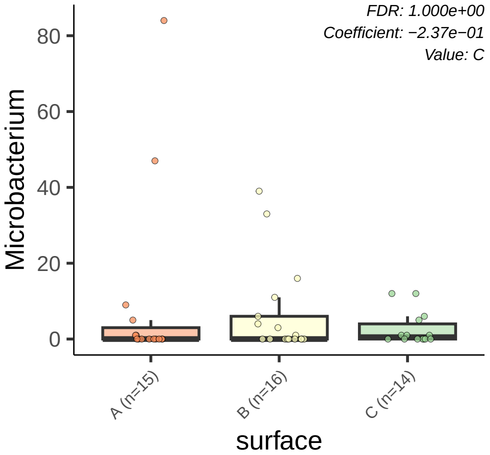
FDR: 1.000e+00
Coefficient: −2.37e−01
Value: C
Microbacterium
0
20
40
60
80
A (n=15)
B (n=16)
C (n=14)
surface

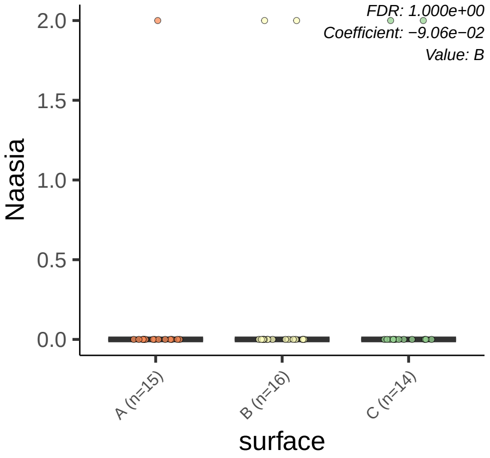

FDR: 1.000e+00
Coefficient: −9.06e−02
Value: B
2.0
1.5
1.0
0.5
0.0
Naasia
A (n=15)
B (n=16)
C (n=14)
surface

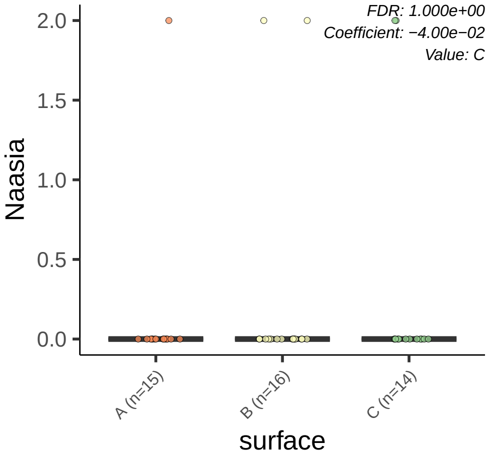

FDR: 1.000e+00
Coefficient: −4.00e−02
Value: C
2.0
1.5
1.0
0.5
0.0
Naasia
A (n=15)
B (n=16)
C (n=14)
surface

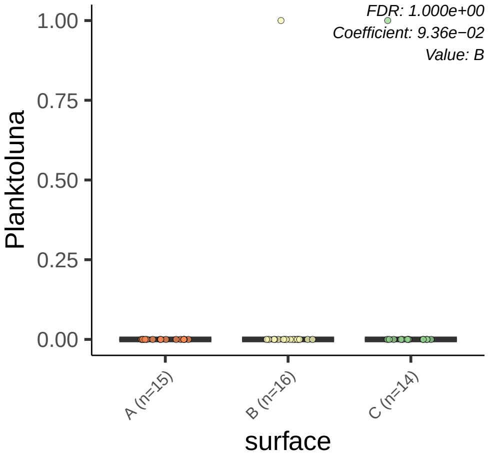
FDR: 1.000e+00
Coefficient: 9.36e−02
Value: B
Planktoluna
1.00
0.75
0.50
0.25
0.00
A (n=15)
B (n=16)
C (n=14)
surface

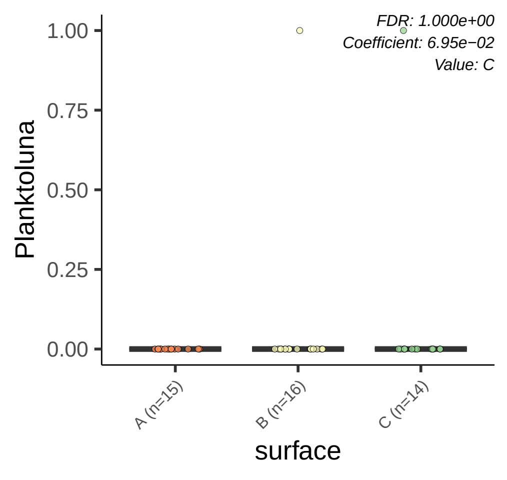
FDR: 1.000e+00
Coefficient: 6.95e−02
Value: C
Planktoluna
1.00
0.75
0.50
0.25
0.00
A (n=15)
B (n=16)
C (n=14)
surface

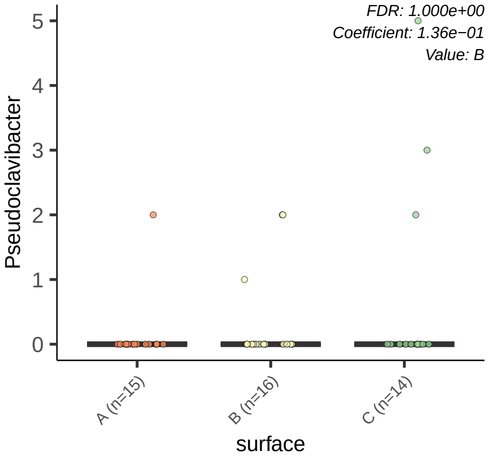

FDR: 1.000e+00
Coefficient: 1.36e−01
Value: B
Pseudoclavibacter
0
1
2
3
4
5
A (n=15)
B (n=16)
C (n=14)
surface

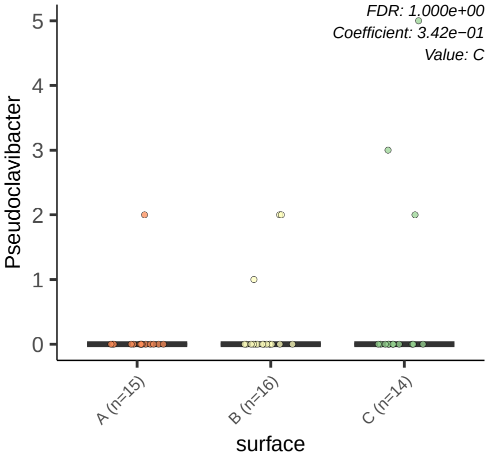
FDR: 1.000e+00
Coefficient: 3.42e−01
Value: C
Pseudoclavibacter
0
1
2
3
4
5
A (n=15)
B (n=16)
C (n=14)
surface

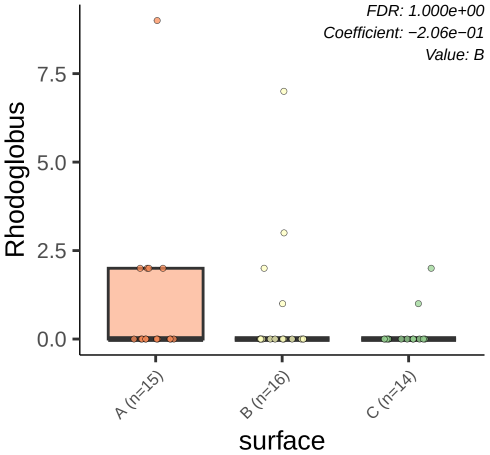
FDR: 1.000e+00
Coefficient: −2.06e−01
Value: B
Rhodoglobus
7.5
5.0
2.5
0.0
A (n=15)
B (n=16)
C (n=14)
surface

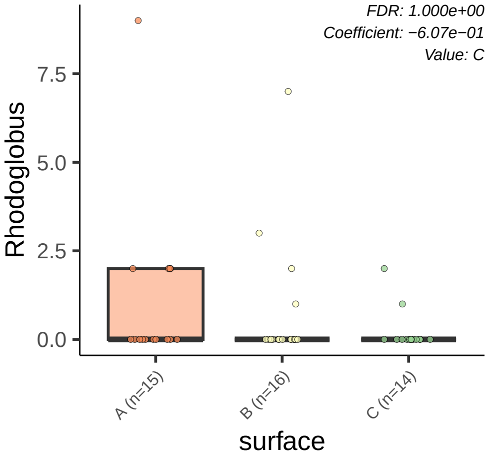
FDR: 1.000e+00
Coefficient: −6.07e−01
Value: C
Rhodoglobus
7.5
5.0
2.5
0.0
A (n=15)
B (n=16)
C (n=14)
surface

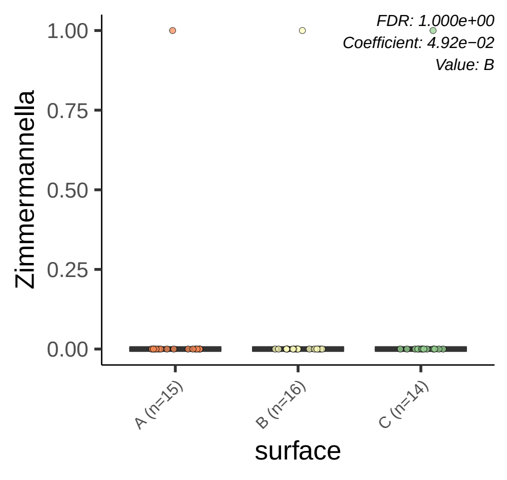
FDR: 1.000e+00
Coefficient: 4.92e−02
Value: B
Zimmermannella
1.00
0.75
0.50
0.25
0.00
A (n=15)
B (n=16)
C (n=14)
surface

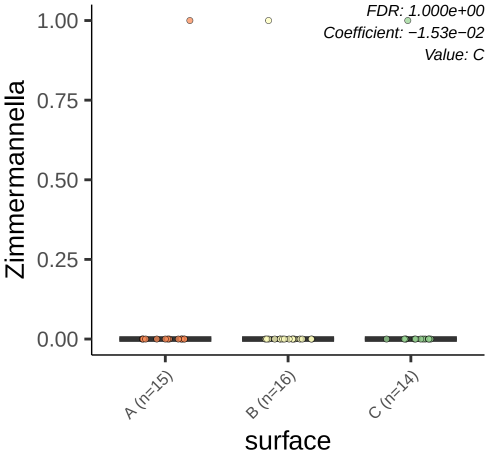
FDR: 1.000e+00
Coefficient: −1.53e−02
Value: C
Zimmermannella
1.00
0.75
0.50
0.25
0.00
A (n=15)
B (n=16)
C (n=14)
surface

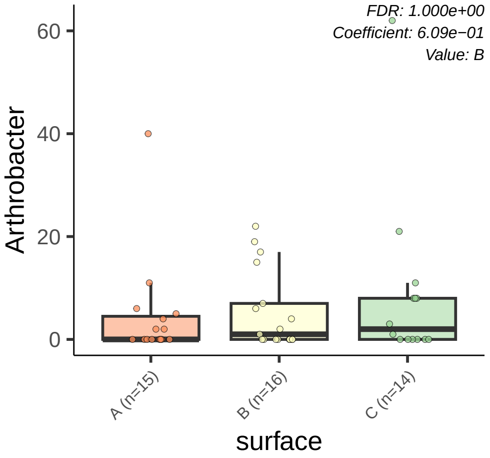
FDR: 1.000e+00
Coefficient: 6.09e−01
Value: B
Arthrobacter
0
20
40
60
A (n=15)
B (n=16)
C (n=14)
surface

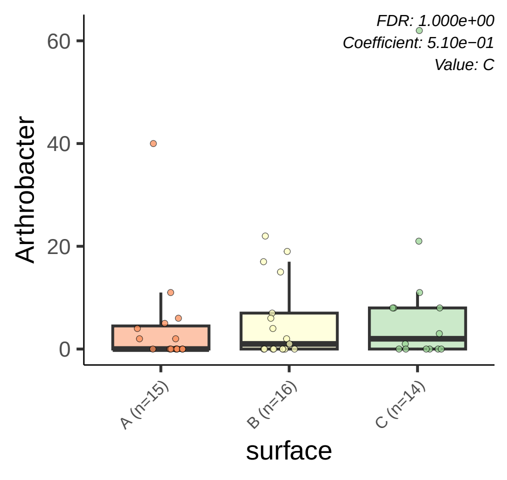

FDR: 1.000e+00
Coefficient: 5.10e−01
Value: C
Arthrobacter
60
40
20
0
A (n=15)
B (n=16)
C (n=14)
surface

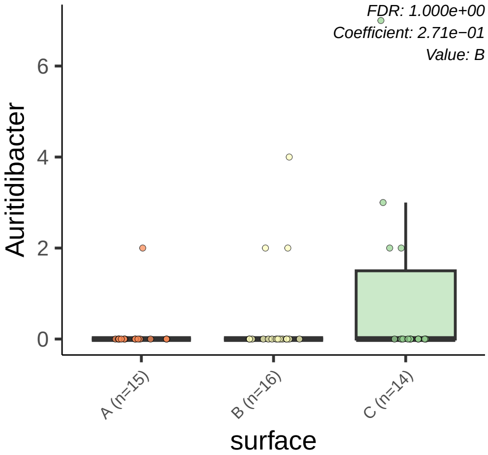

FDR: 1.000e+00
Coefficient: 2.71e−01
Value: B
Auritidibacter
0
2
4
6
A (n=15)
B (n=16)
C (n=14)
surface

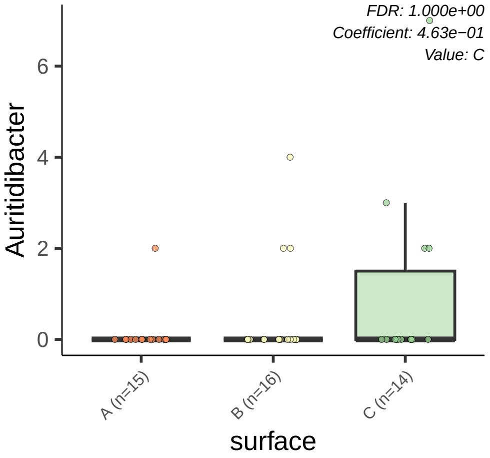
FDR: 1.000e+00
Coefficient: 4.63e−01
Value: C
Auritidibacter
0
2
4
6
A (n=15)
B (n=16)
C (n=14)
surface

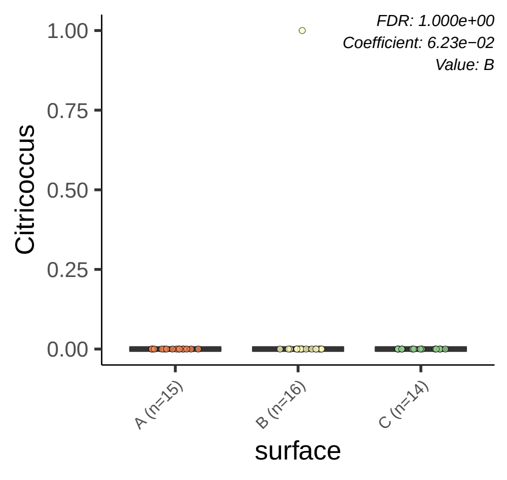
FDR: 1.000e+00
Coefficient: 6.23e−02
Value: B
1.00
0.75
0.50
0.25
0.00
Citricoccus
A (n=15)
B (n=16)
C (n=14)
surface

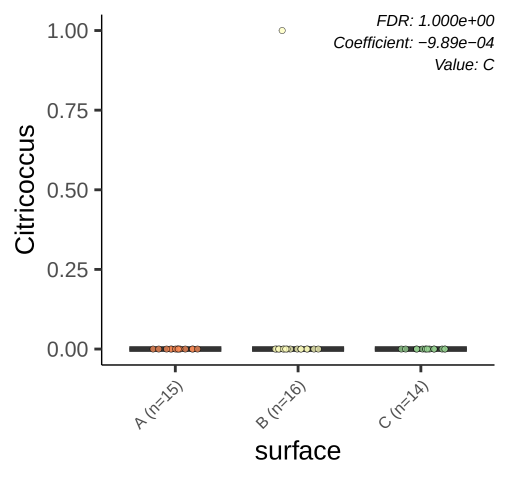
FDR: 1.000e+00
Coefficient: −9.89e−04
Value: C
Citricoccus
1.00
0.75
0.50
0.25
0.00
A (n=15)
B (n=16)
C (n=14)
surface

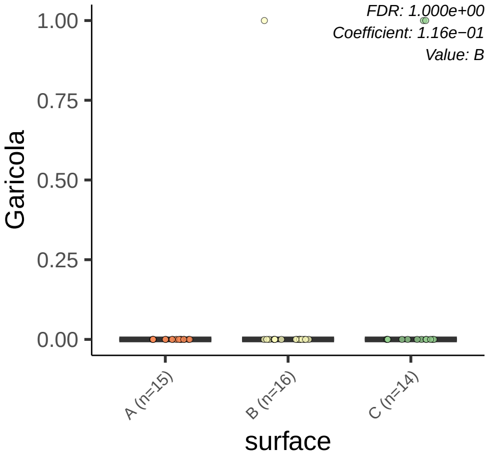
FDR: 1.000e+00
Coefficient: 1.16e−01
Value: B
1.00
0.75
0.50
0.25
0.00
Garicola
A (n=15)
B (n=16)
C (n=14)
surface

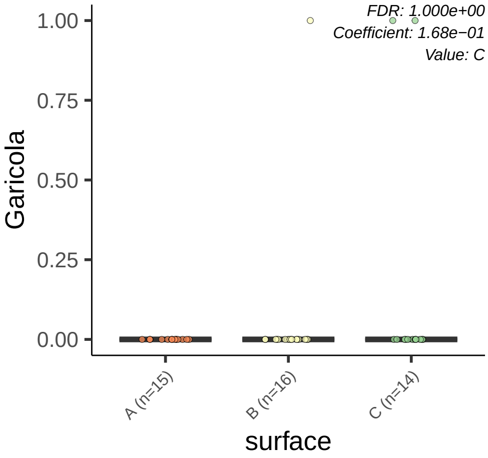

FDR: 1.000e+00
Coefficient: 1.68e−01
Value: C
Garicola
1.00
0.75
0.50
0.25
0.00
A (n=15)
B (n=16)
C (n=14)
surface

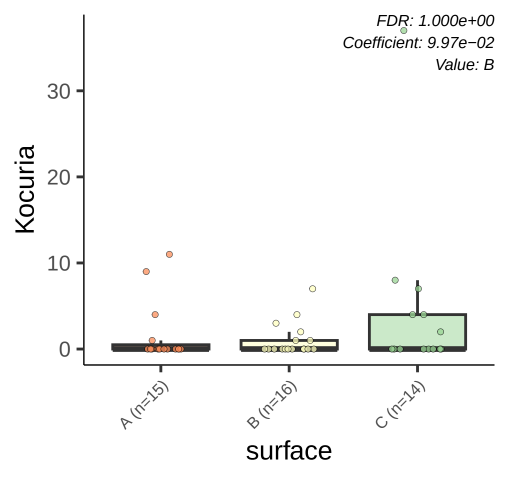
FDR: 1.000e+00
Coefficient: 9.97e−02
Value: B
Kocuria
30
20
10
0
A (n=15)
B (n=16)
C (n=14)
surface

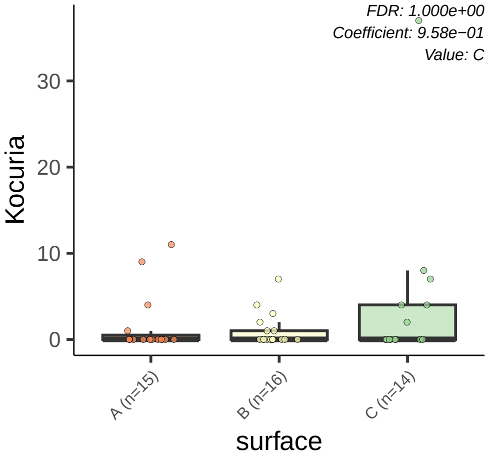

FDR: 1.000e+00
Coefficient: 9.58e−01
Value: C
Kocuria
30
20
10
0
A (n=15)
B (n=16)
C (n=14)
surface

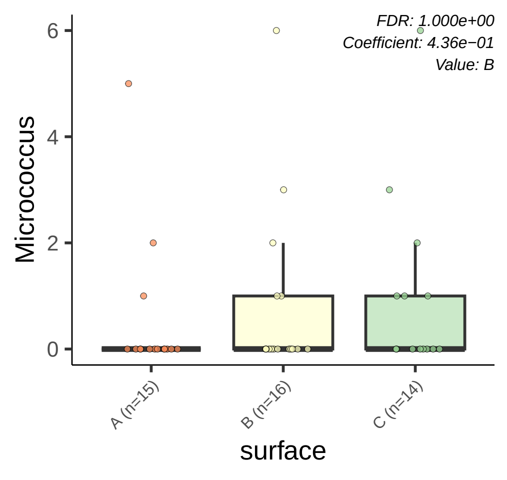

FDR: 1.000e+00
Coefficient: 4.36e−01
Value: B
Micrococcus
6
4
2
0
A (n=15)
B (n=16)
C (n=14)
surface

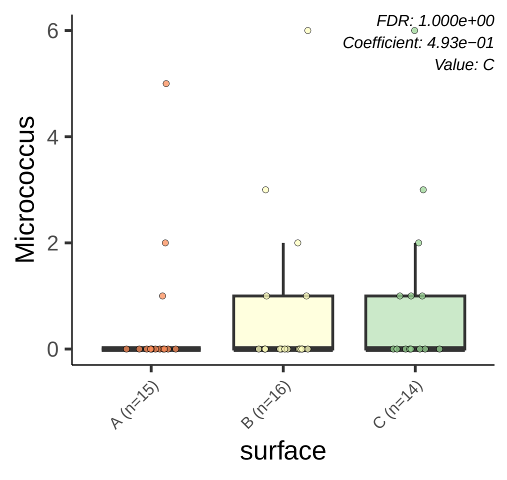
FDR: 1.000e+00
Coefficient: 4.93e−01
Value: C
Micrococcus
0
2
4
6
A (n=15)
B (n=16)
C (n=14)
surface

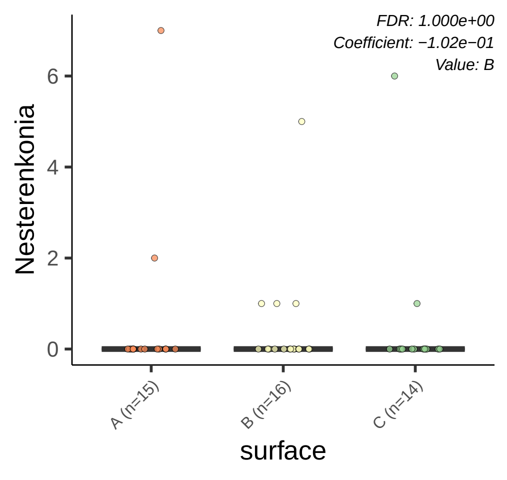

FDR: 1.000e+00
Coefficient: −1.02e−01
Value: B
Nesterenkonia
0
2
4
6
A (n=15)
B (n=16)
C (n=14)
surface

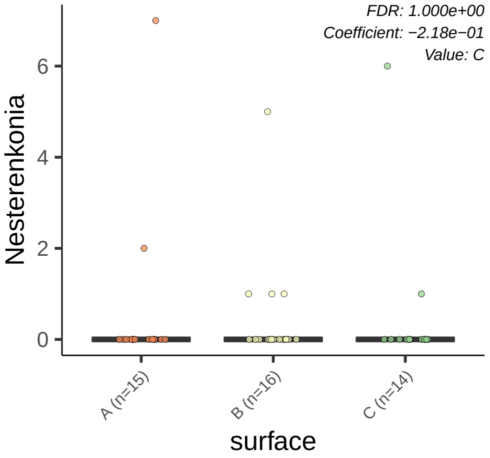
FDR: 1.000e+00
Coefficient: −2.18e−01
Value: C
Nesterenkonia
0
2
4
6
A (n=15)
B (n=16)
C (n=14)
surface

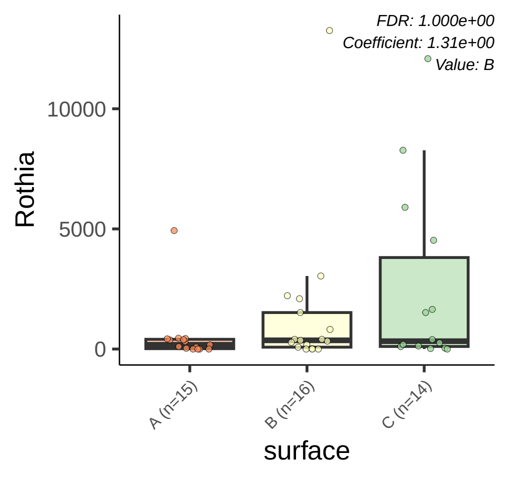

FDR: 1.000e+00
Coefficient: 1.31e+00
Value: B
Rothia
10000
5000
0
A (n=15)
B (n=16)
C (n=14)
surface

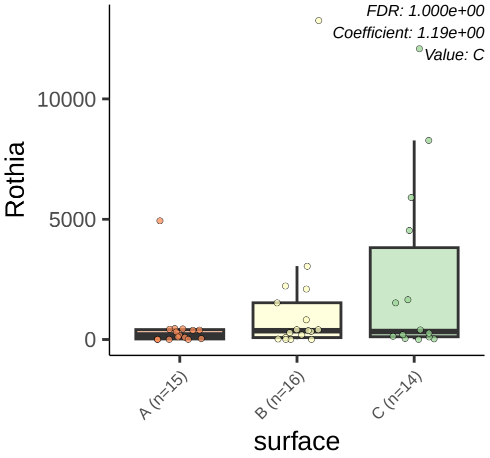
FDR: 1.000e+00
Coefficient: 1.19e+00
Value: C
Rothia
10000
5000
0
A (n=15)
B (n=16)
C (n=14)
surface

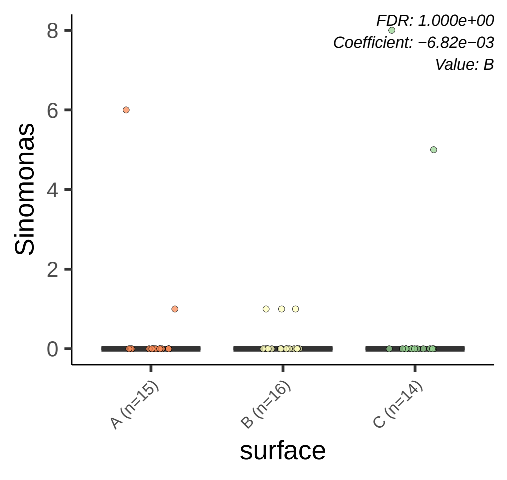
FDR: 1.000e+00
Coefficient: −6.82e−03
Value: B
Sinomonas
8
6
4
2
0
A (n=15)
B (n=16)
C (n=14)
surface

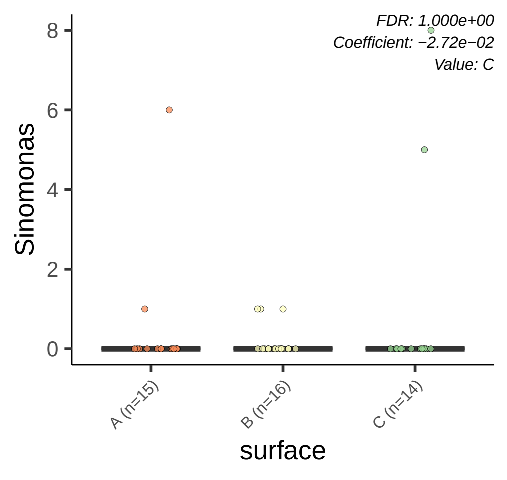
FDR: 1.000e+00
Coefficient: −2.72e−02
Value: C
Sinomonas
0
2
4
6
8
A (n=15)
B (n=16)
C (n=14)
surface

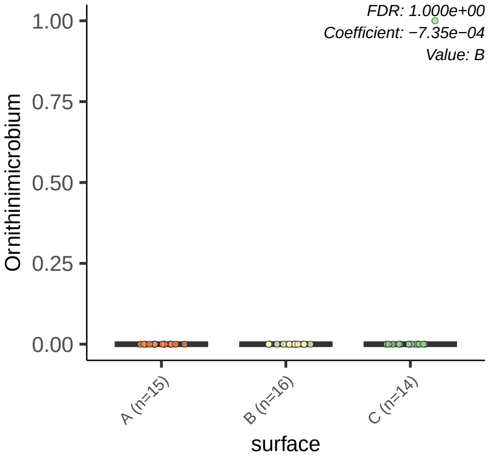
FDR: 1.000e+00
Coefficient: −7.35e−04
Value: B
Ornithinimicrobium
1.00
0.75
0.50
0.25
0.00
A (n=15)
B (n=16)
C (n=14)
surface

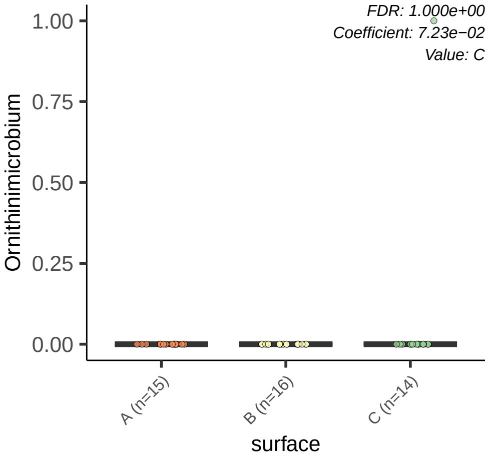

FDR: 1.000e+00
Coefficient: 7.23e−02
Value: C
Ornithinimicrobium
1.00
0.75
0.50
0.25
0.00
A (n=15)
B (n=16)
C (n=14)
surface

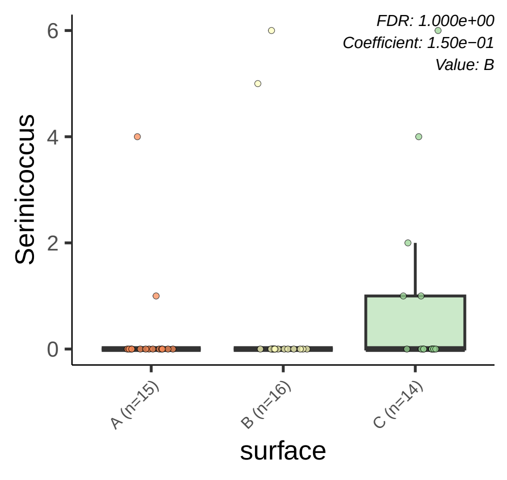

FDR: 1.000e+00
Coefficient: 1.50e−01
Value: B
Serinicoccus
0
2
4
6
A (n=15)
B (n=16)
C (n=14)
surface

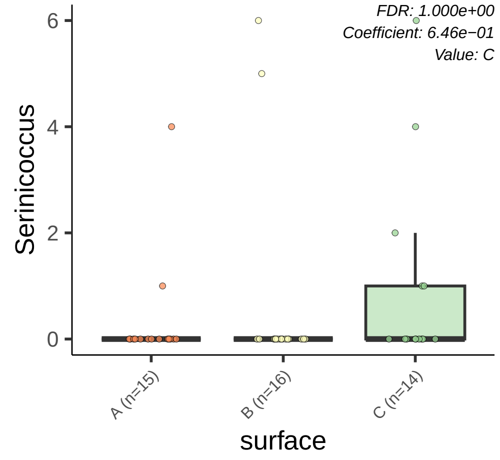

FDR: 1.000e+00
Coefficient: 6.46e−01
Value: C
Serinicoccus
0
2
4
6
A (n=15)
B (n=16)
C (n=14)
surface

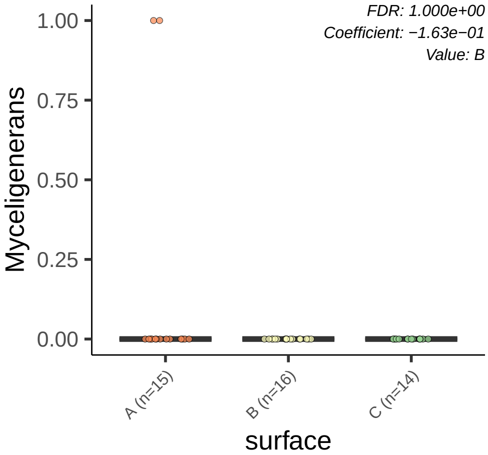

FDR: 1.000e+00
Coefficient: −1.63e−01
Value: B
Myceligenerans
1.00
0.75
0.50
0.25
0.00
A (n=15)
B (n=16)
C (n=14)
surface

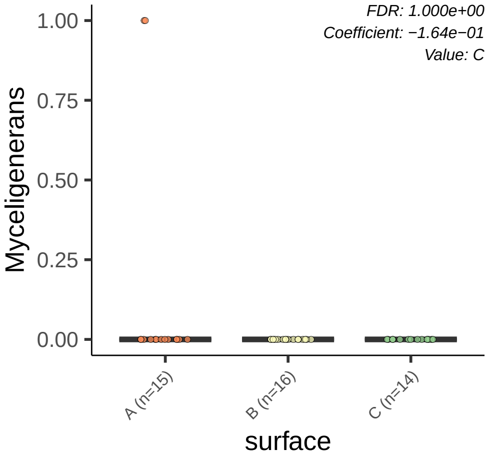
FDR: 1.000e+00
Coefficient: −1.64e−01
Value: C
Myceligenerans
1.00
0.75
0.50
0.25
0.00
A (n=15)
B (n=16)
C (n=14)
surface

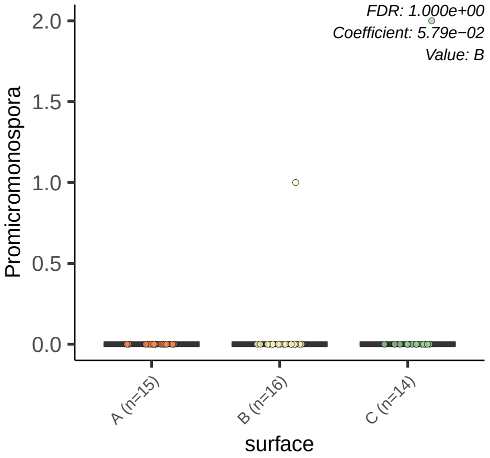

FDR: 1.000e+00
Coefficient: 5.79e−02
Value: B
Promicromonospora
2.0
1.5
1.0
0.5
0.0
A (n=15)
B (n=16)
C (n=14)
surface

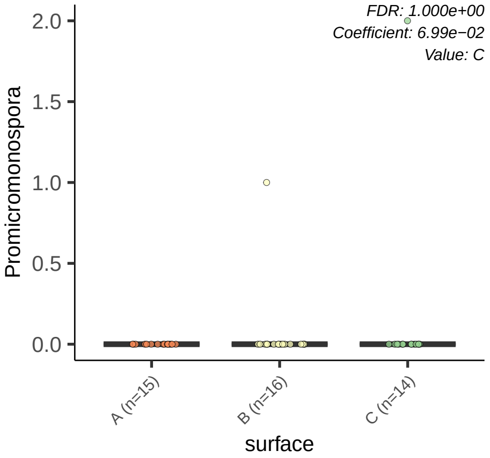

FDR: 1.000e+00
Coefficient: 6.99e−02
Value: C
Promicromonospora
2.0
1.5
1.0
0.5
0.0
A (n=15)
B (n=16)
C (n=14)
surface

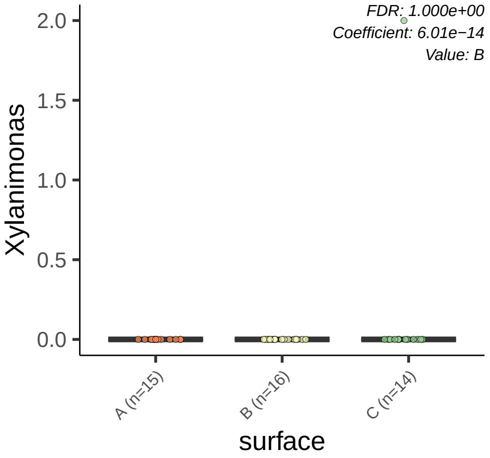
FDR: 1.000e+00
Coefficient: 6.01e−14
Value: B
Xylanimonas
2.0
1.5
1.0
0.5
0.0
A (n=15)
B (n=16)
C (n=14)
surface

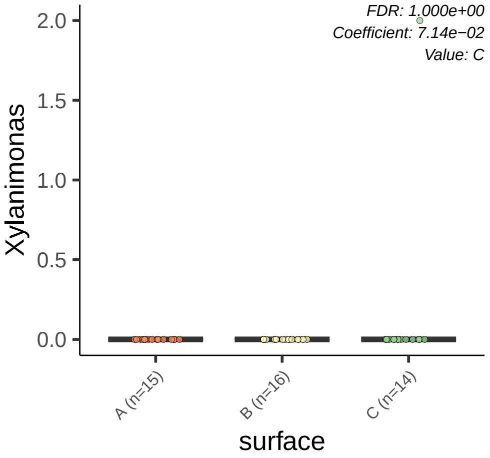
FDR: 1.000e+00
Coefficient: 7.14e−02
Value: C
Xylanimonas
2.0
1.5
1.0
0.5
0.0
A (n=15)
B (n=16)
C (n=14)
surface

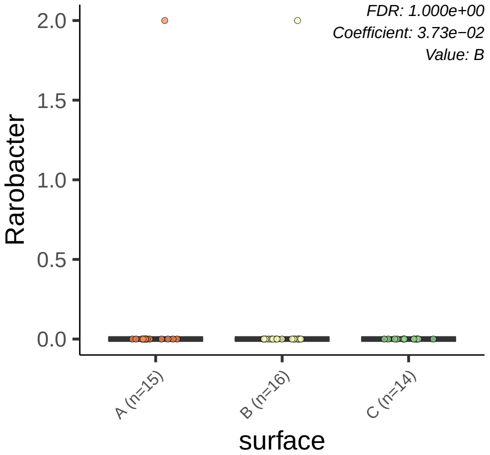
FDR: 1.000e+00
Coefficient: 3.73e−02
Value: B
Rarobacter
2.0
1.5
1.0
0.5
0.0
A (n=15)
B (n=16)
C (n=14)
surface

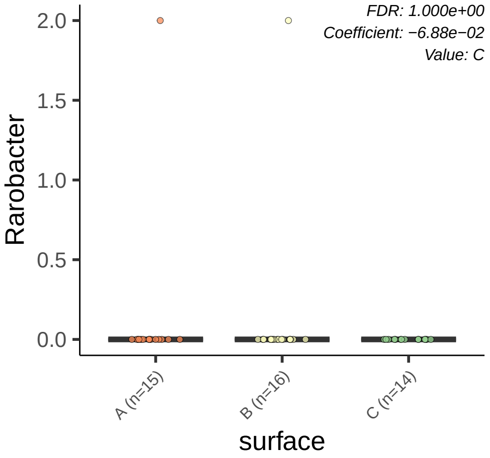

FDR: 1.000e+00
Coefficient: −6.88e−02
Value: C
Rarobacter
2.0
1.5
1.0
0.5
0.0
A (n=15)
B (n=16)
C (n=14)
surface

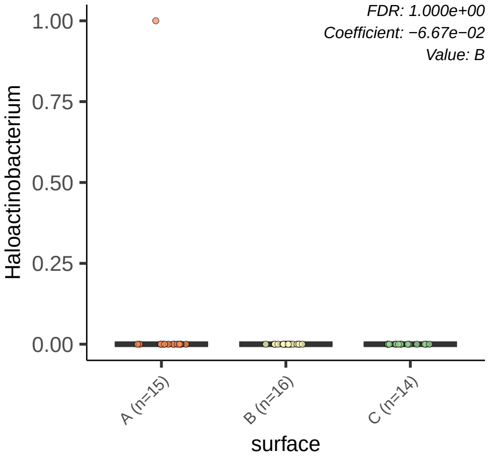

FDR: 1.000e+00
Coefficient: −6.67e−02
Value: B
Haloactinobacterium
1.00
0.75
0.50
0.25
0.00
A (n=15)
B (n=16)
C (n=14)
surface

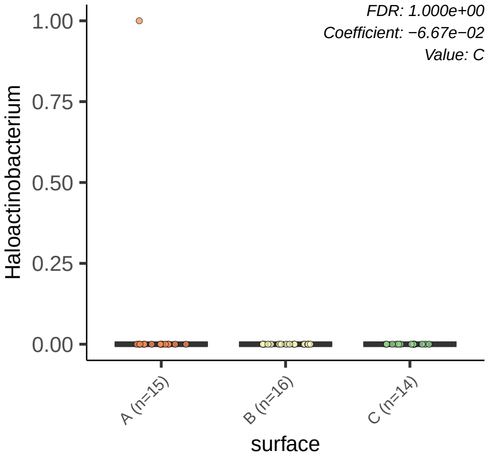
FDR: 1.000e+00
Coefficient: −6.67e−02
Value: C
Haloactinobacterium
1.00
0.75
0.50
0.25
0.00
A (n=15)
B (n=16)
C (n=14)
surface

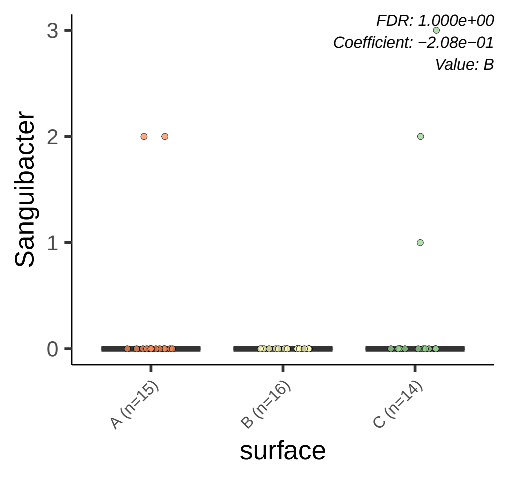
FDR: 1.000e+00
Coefficient: −2.08e−01
Value: B
Sanguibacter
3
2
1
0
A (n=15)
B (n=16)
C (n=14)
surface

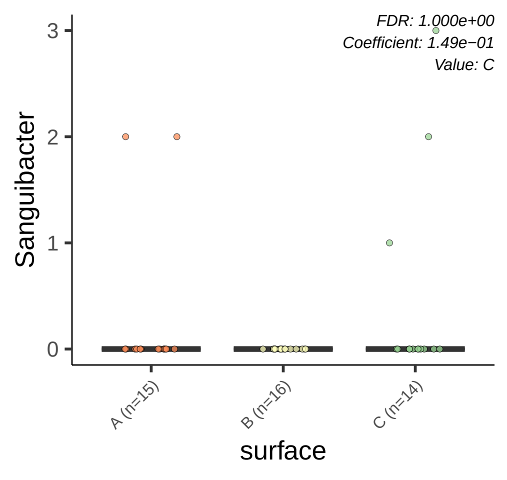
FDR: 1.000e+00
Coefficient: 1.49e−01
Value: C
Sanguibacter
3
2
1
0
A (n=15)
B (n=16)
C (n=14)
surface

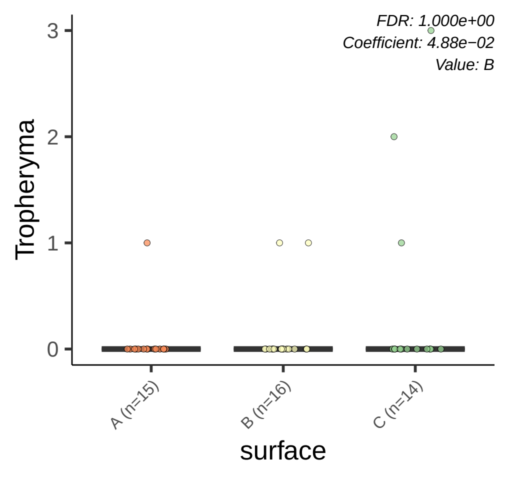
FDR: 1.000e+00
Coefficient: 4.88e−02
Value: B
Tropheryma
3
2
1
0
A (n=15)
B (n=16)
C (n=14)
surface

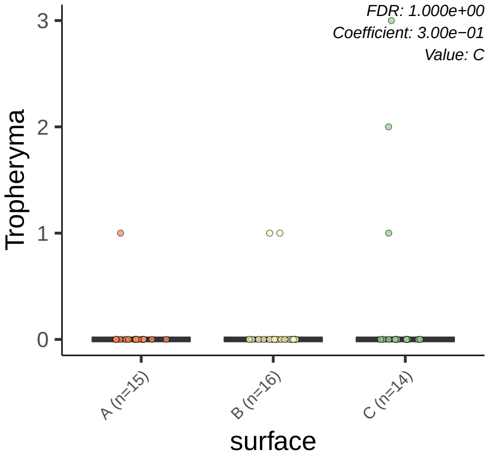
FDR: 1.000e+00
Coefficient: 3.00e−01
Value: C
Tropheryma
3
2
1
0
A (n=15)
B (n=16)
C (n=14)
surface

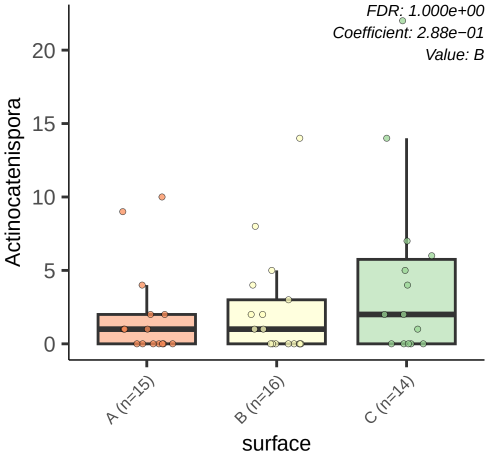
FDR: 1.000e+00
Coefficient: 2.88e−01
Value: B
Actinocatenispora
20
15
10
5
0
A (n=15)
B (n=16)
C (n=14)
surface

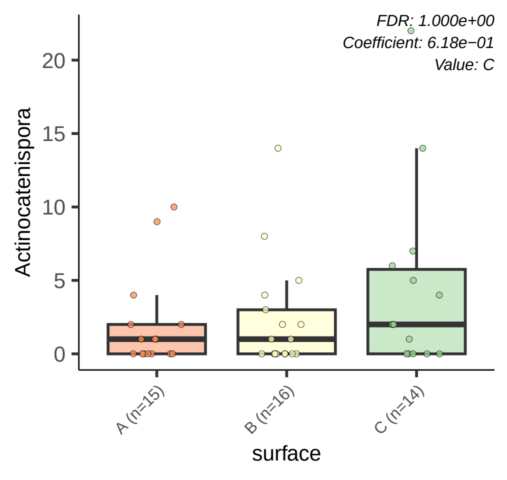
FDR: 1.000e+00
Coefficient: 6.18e−01
Value: C
Actinocatenispora
0
5
10
15
20
A (n=15)
B (n=16)
C (n=14)
surface

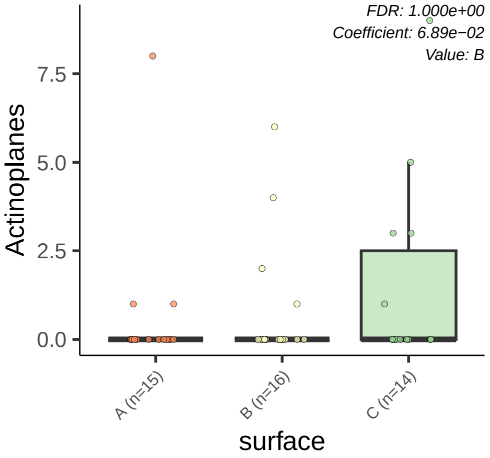

FDR: 1.000e+00
Coefficient: 6.89e−02
Value: B
Actinoplanes
7.5
5.0
2.5
0.0
A (n=15)
B (n=16)
C (n=14)
surface

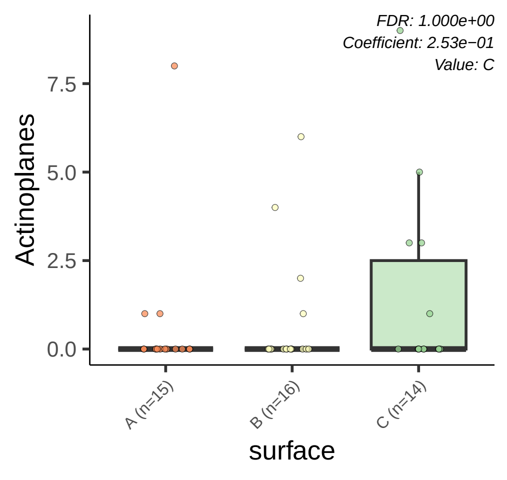
FDR: 1.000e+00
Coefficient: 2.53e−01
Value: C
Actinoplanes
7.5
5.0
2.5
0.0
A (n=15)
B (n=16)
C (n=14)
surface

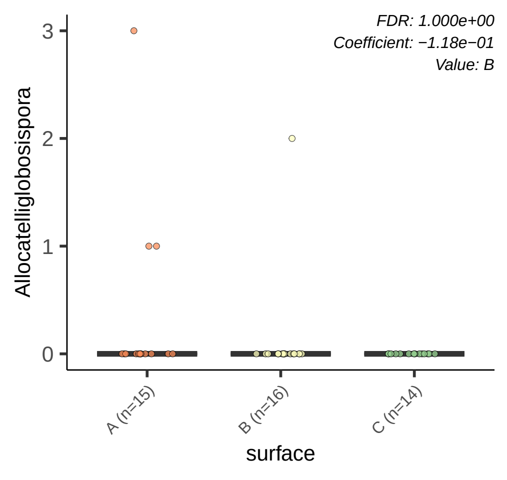

FDR: 1.000e+00
Coefficient: −1.18e−01
Value: B
Allocatelliglobosispora
3
2
1
0
A (n=15)
B (n=16)
C (n=14)
surface

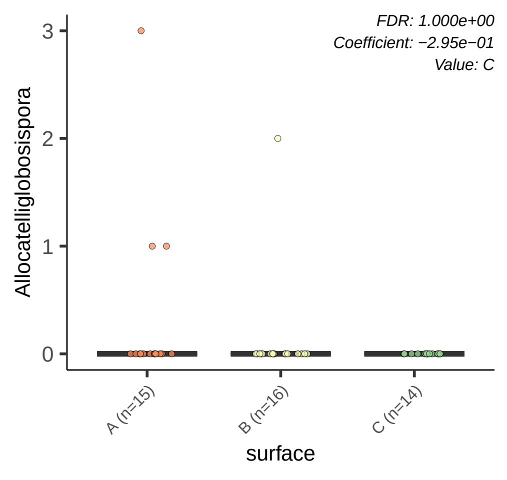

FDR: 1.000e+00
Coefficient: −2.95e−01
Value: C
Allocatelliglobosispora
3
2
1
0
A (n=15)
B (n=16)
C (n=14)
surface

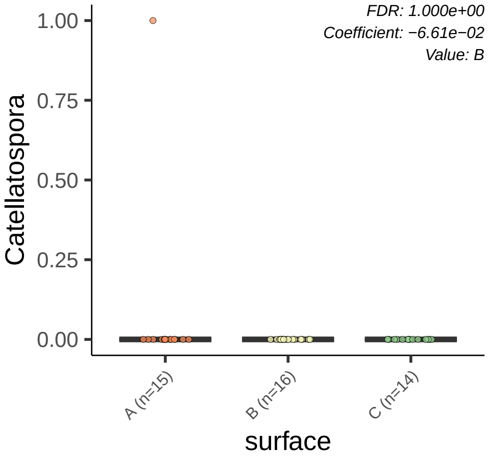
FDR: 1.000e+00
Coefficient: −6.61e−02
Value: B
Catellatospora
1.00
0.75
0.50
0.25
0.00
A (n=15)
B (n=16)
C (n=14)
surface

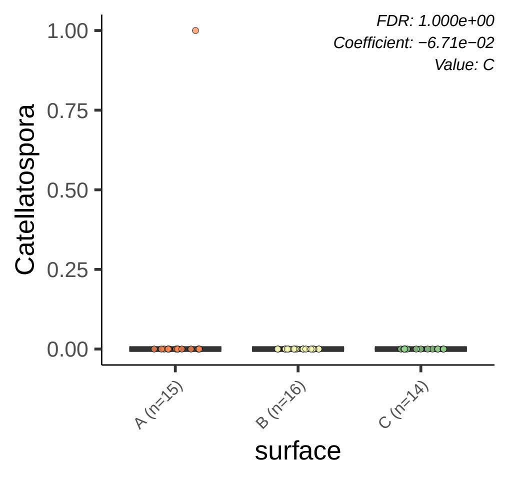
FDR: 1.000e+00
Coefficient: −6.71e−02
Value: C
Catellatospora
1.00
0.75
0.50
0.25
0.00
A (n=15)
B (n=16)
C (n=14)
surface

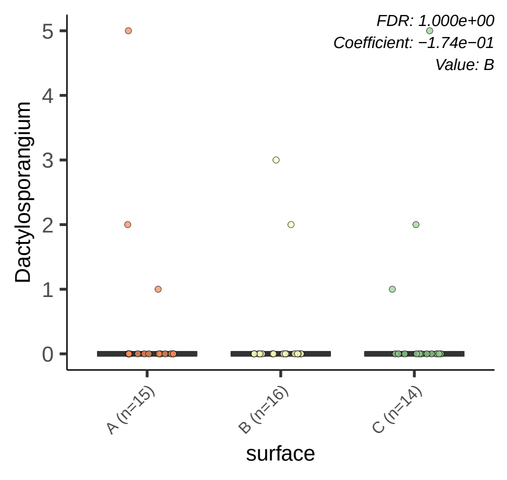
FDR: 1.000e+00
Coefficient: −1.74e−01
Value: B
Dactylosporangium
0
1
2
3
4
5
A (n=15)
B (n=16)
C (n=14)
surface

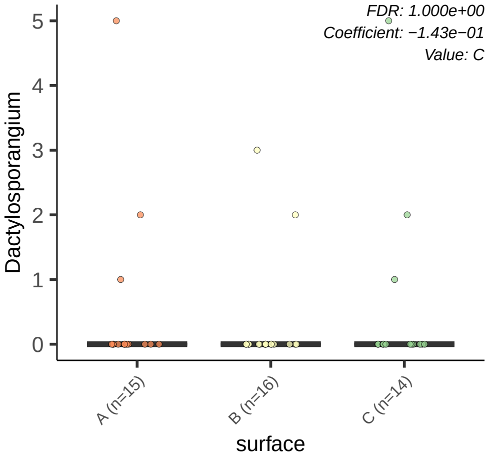

FDR: 1.000e+00
Coefficient: −1.43e−01
Value: C
Dactylosporangium
0
1
2
3
4
5
A (n=15)
B (n=16)
C (n=14)
surface

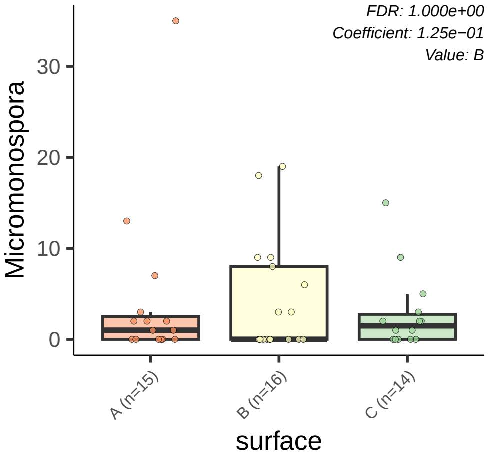

FDR: 1.000e+00
Coefficient: 1.25e−01
Value: B
Micromonospora
0
10
20
30
A (n=15)
B (n=16)
C (n=14)
surface

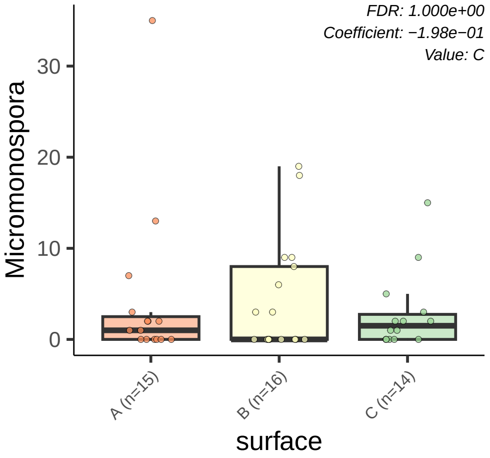
FDR: 1.000e+00
Coefficient: −1.98e−01
Value: C
Micromonospora
0
10
20
30
A (n=15)
B (n=16)
C (n=14)
surface

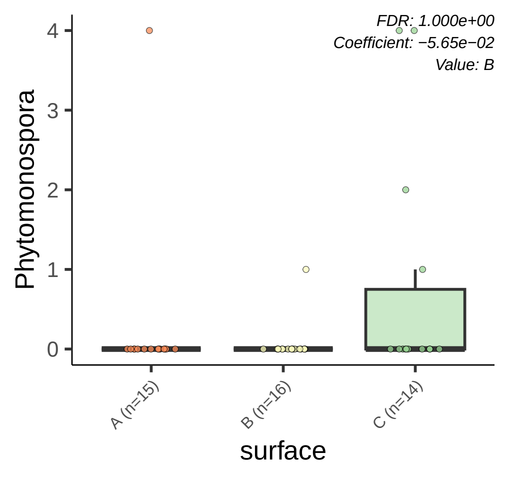
FDR: 1.000e+00
Coefficient: −5.65e−02
Value: B
Phytomonospora
4
3
2
1
0
A (n=15)
B (n=16)
C (n=14)
surface

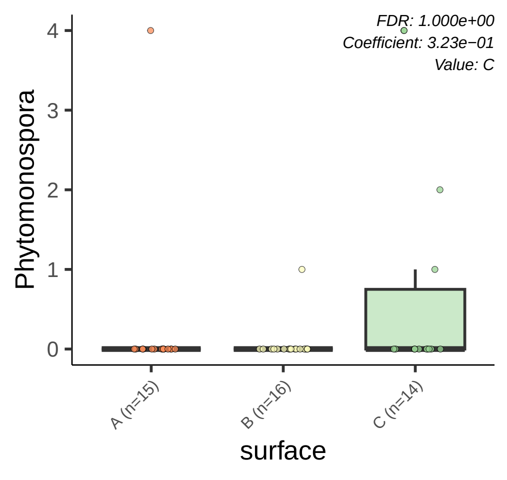
FDR: 1.000e+00
Coefficient: 3.23e−01
Value: C
Phytomonospora
4
3
2
1
0
A (n=15)
B (n=16)
C (n=14)
surface

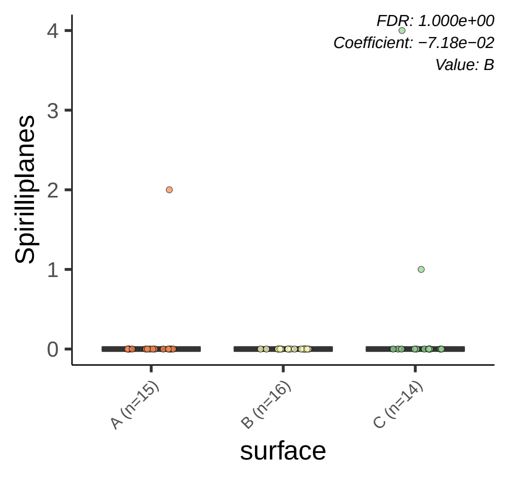

FDR: 1.000e+00
Coefficient: −7.18e−02
Value: B
Spirilliplanes
4
3
2
1
0
A (n=15)
B (n=16)
C (n=14)
surface

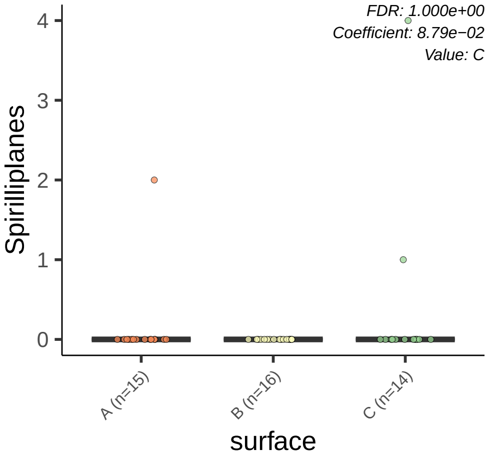
FDR: 1.000e+00
Coefficient: 8.79e−02
Value: C
Spirilliplanes
0
1
2
3
4
A (n=15)
B (n=16)
C (n=14)
surface

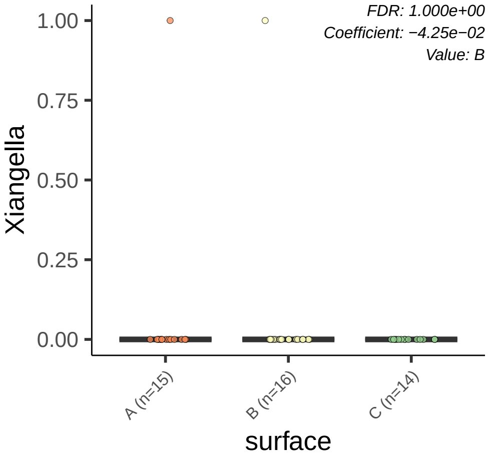
FDR: 1.000e+00
Coefficient: −4.25e−02
Value: B
Xiangella
1.00
0.75
0.50
0.25
0.00
A (n=15)
B (n=16)
C (n=14)
surface

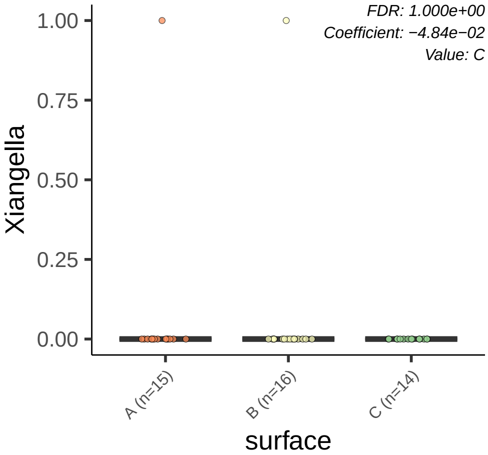

FDR: 1.000e+00
Coefficient: −4.84e−02
Value: C
Xiangella
1.00
0.75
0.50
0.25
0.00
A (n=15)
B (n=16)
C (n=14)
surface

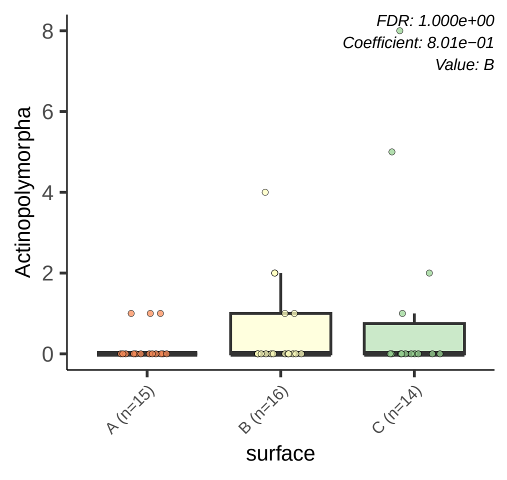
FDR: 1.000e+00
Coefficient: 8.01e−01
Value: B
Actinopolymorpha
0
2
4
6
8
A (n=15)
B (n=16)
C (n=14)
surface

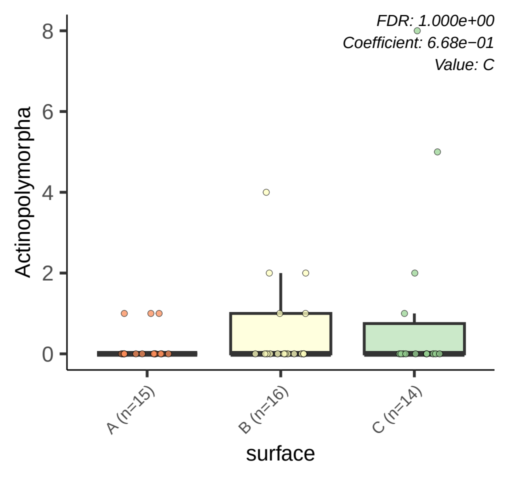
FDR: 1.000e+00
Coefficient: 6.68e−01
Value: C
Actinopolymorpha
8
6
4
2
0
A (n=15)
B (n=16)
C (n=14)
surface

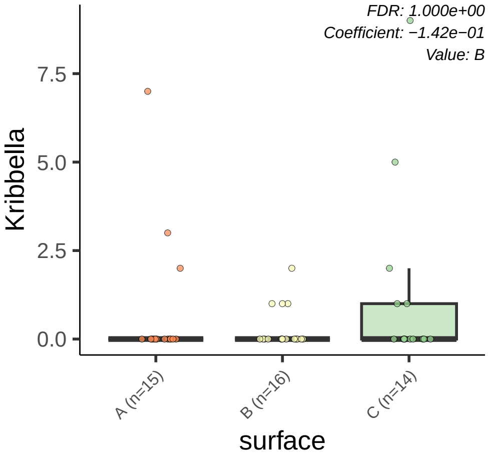
FDR: 1.000e+00
Coefficient: −1.42e−01
Value: B
Kribbella
7.5
5.0
2.5
0.0
A (n=15)
B (n=16)
C (n=14)
surface

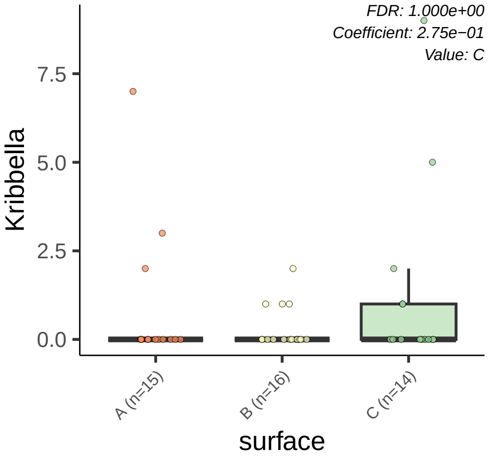

FDR: 1.000e+00
Coefficient: 2.75e−01
Value: C
Kribbella
7.5
5.0
2.5
0.0
A (n=15)
B (n=16)
C (n=14)
surface

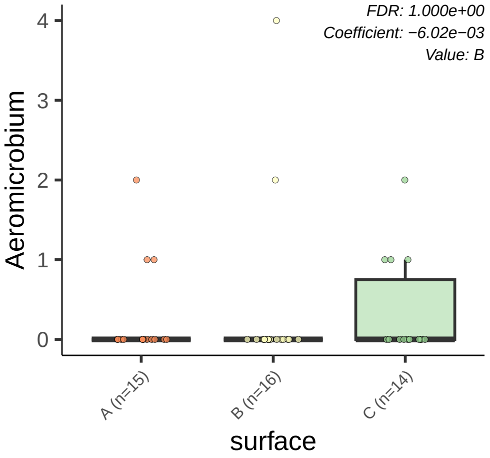

FDR: 1.000e+00
Coefficient: −6.02e−03
Value: B
Aeromicrobium
0
1
2
3
4
A (n=15)
B (n=16)
C (n=14)
surface

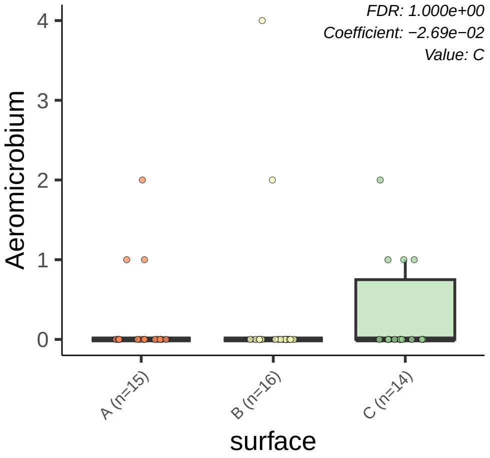

FDR: 1.000e+00
Coefficient: −2.69e−02
Value: C
Aeromicrobium
0
1
2
3
4
A (n=15)
B (n=16)
C (n=14)
surface

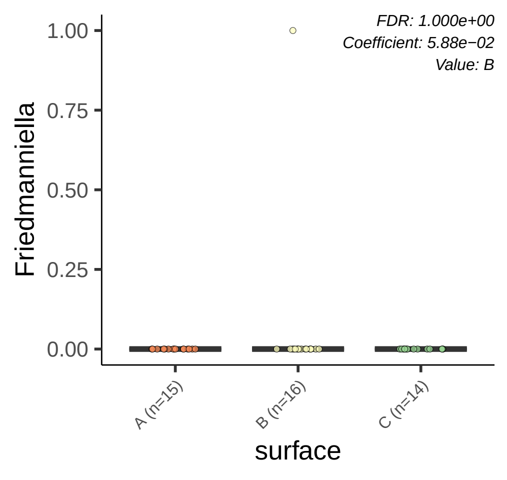

FDR: 1.000e+00
Coefficient: 5.88e−02
Value: B
Friedmanniella
1.00
0.75
0.50
0.25
0.00
A (n=15)
B (n=16)
C (n=14)
surface

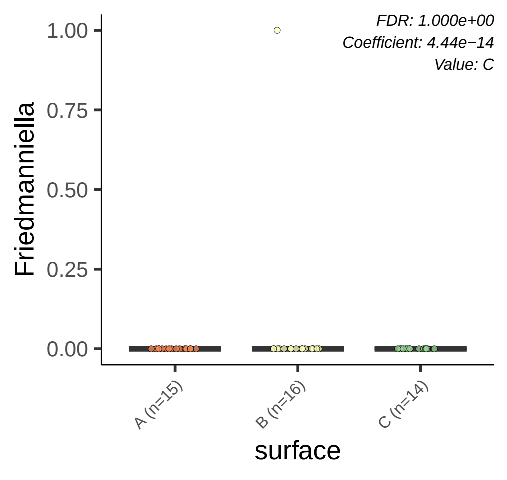
FDR: 1.000e+00
Coefficient: 4.44e−14
Value: C
Friedmanniella
1.00
0.75
0.50
0.25
0.00
A (n=15)
B (n=16)
C (n=14)
surface

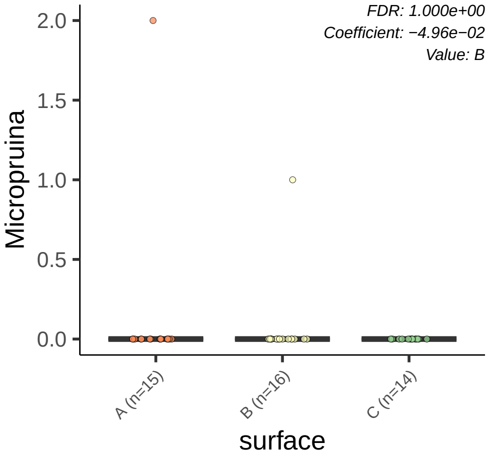

FDR: 1.000e+00
Coefficient: −4.96e−02
Value: B
Micropruina
2.0
1.5
1.0
0.5
0.0
A (n=15)
B (n=16)
C (n=14)
surface

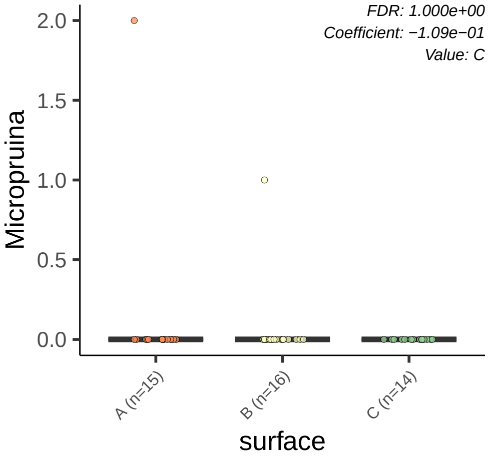

FDR: 1.000e+00
Coefficient: −1.09e−01
Value: C
Micropruina
2.0
1.5
1.0
0.5
0.0
A (n=15)
B (n=16)
C (n=14)
surface

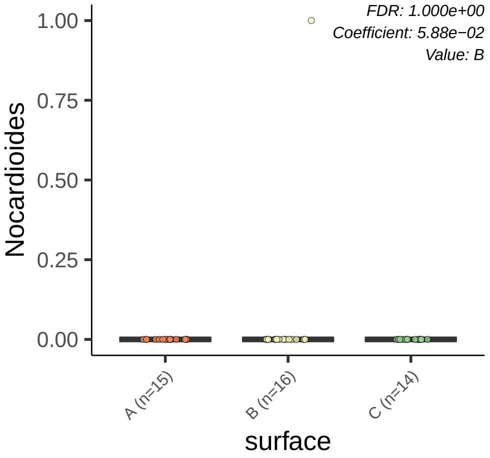
FDR: 1.000e+00
Coefficient: 5.88e−02
Value: B
Nocardioides
1.00
0.75
0.50
0.25
0.00
A (n=15)
B (n=16)
C (n=14)
surface

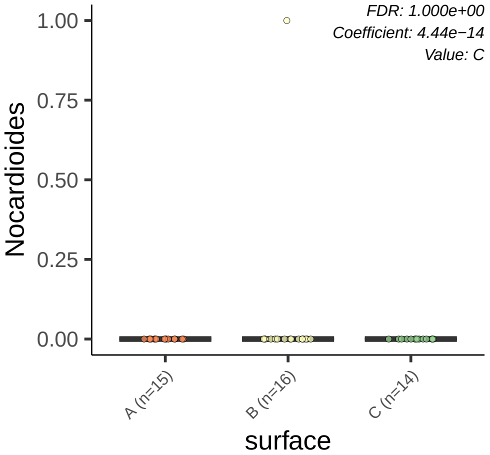
FDR: 1.000e+00
Coefficient: 4.44e−14
Value: C
Nocardioides
1.00
0.75
0.50
0.25
0.00
A (n=15)
B (n=16)
C (n=14)
surface

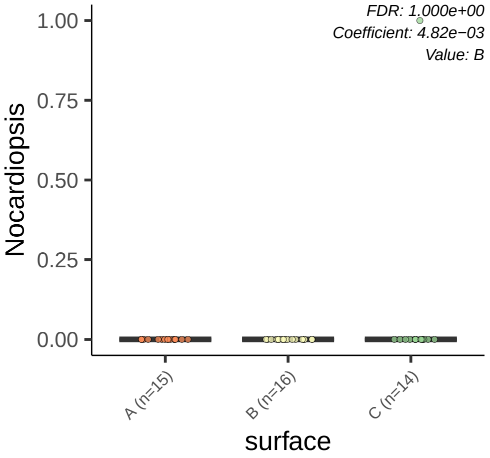
FDR: 1.000e+00
Coefficient: 4.82e−03
Value: B
Nocardiopsis
1.00
0.75
0.50
0.25
0.00
A (n=15)
B (n=16)
C (n=14)
surface

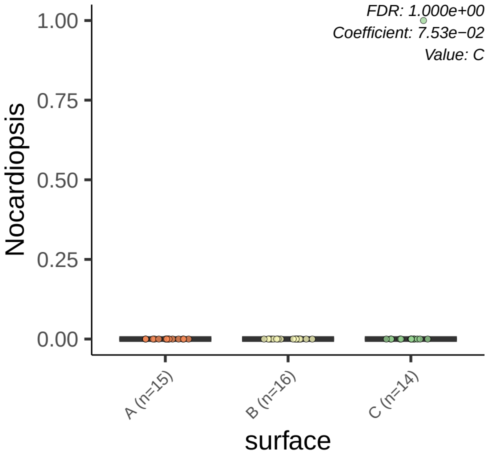
FDR: 1.000e+00
Coefficient: 7.53e−02
Value: C
Nocardiopsis
1.00
0.75
0.50
0.25
0.00
A (n=15)
B (n=16)
C (n=14)
surface

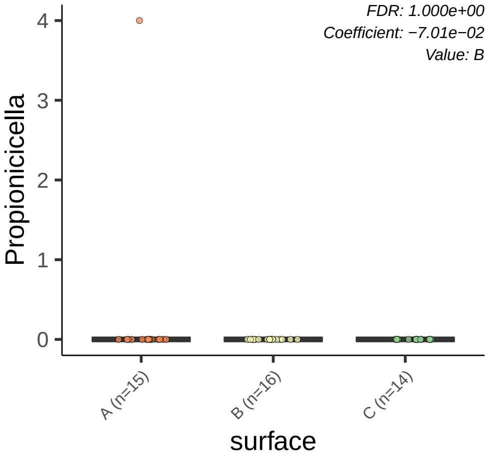

FDR: 1.000e+00
Coefficient: −7.01e−02
Value: B
Propionicicella
4
3
2
1
0
A (n=15)
B (n=16)
C (n=14)
surface

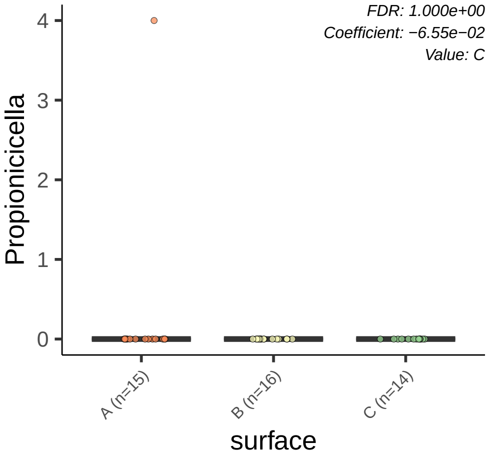
FDR: 1.000e+00
Coefficient: −6.55e−02
Value: C
Propionicicella
0
1
2
3
4
A (n=15)
B (n=16)
C (n=14)
surface

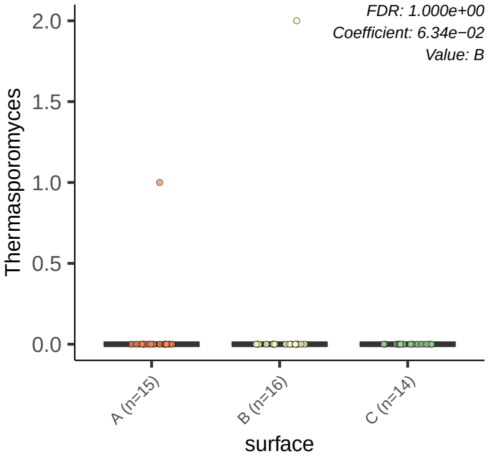
FDR: 1.000e+00
Coefficient: 6.34e−02
Value: B
Thermasporomyces
2.0
1.5
1.0
0.5
0.0
A (n=15)
B (n=16)
C (n=14)
surface

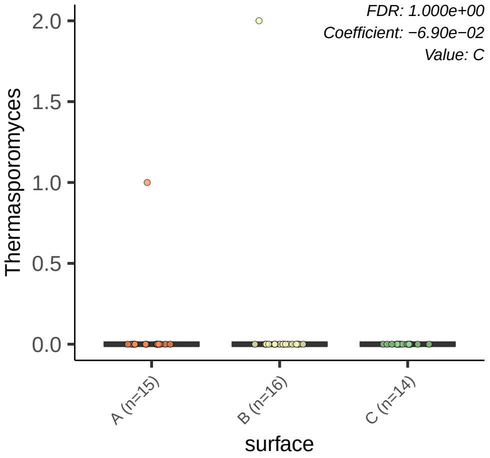
FDR: 1.000e+00
Coefficient: −6.90e−02
Value: C
Thermasporomyces
2.0
1.5
1.0
0.5
0.0
A (n=15)
B (n=16)
C (n=14)
surface

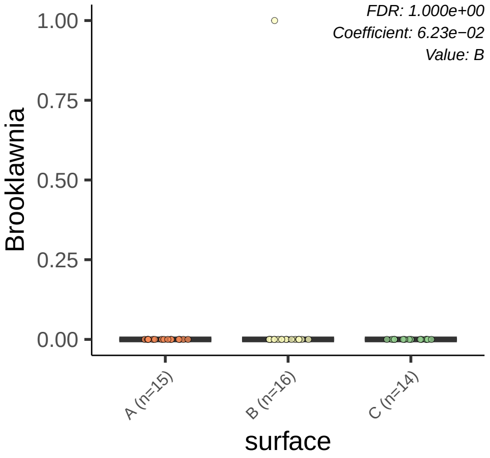
FDR: 1.000e+00
Coefficient: 6.23e−02
Value: B
Brooklawnia
1.00
0.75
0.50
0.25
0.00
A (n=15)
B (n=16)
C (n=14)
surface

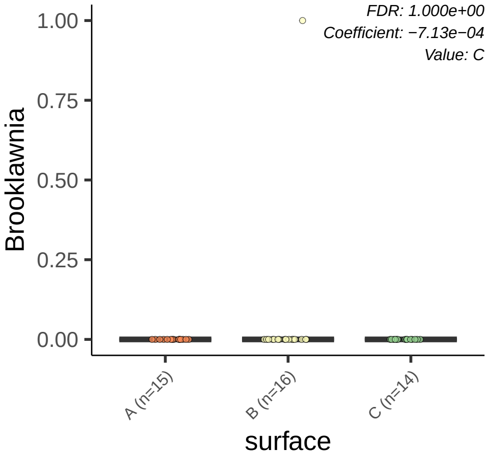

FDR: 1.000e+00
Coefficient: −7.13e−04
Value: C
Brooklawnia
1.00
0.75
0.50
0.25
0.00
A (n=15)
B (n=16)
C (n=14)
surface

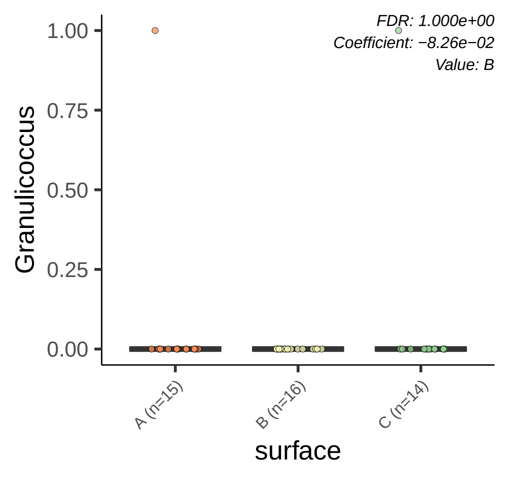
FDR: 1.000e+00
Coefficient: −8.26e−02
Value: B
Granulicoccus
1.00
0.75
0.50
0.25
0.00
A (n=15)
B (n=16)
C (n=14)
surface

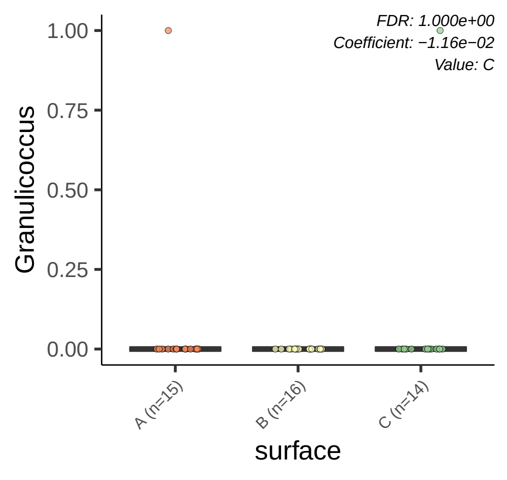
FDR: 1.000e+00
Coefficient: −1.16e−02
Value: C
Granulicoccus
1.00
0.75
0.50
0.25
0.00
A (n=15)
B (n=16)
C (n=14)
surface

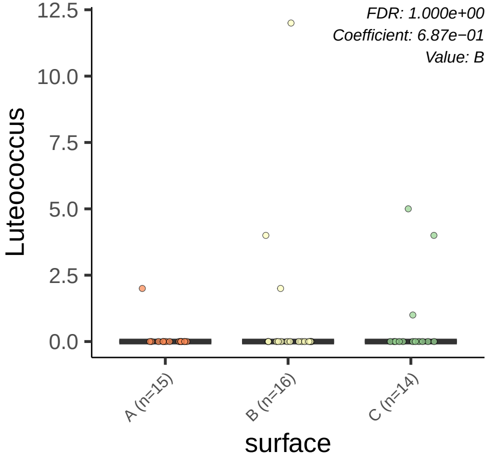
FDR: 1.000e+00
Coefficient: 6.87e−01
Value: B
Luteococcus
12.5
10.0
7.5
5.0
2.5
0.0
A (n=15)
B (n=16)
C (n=14)
surface

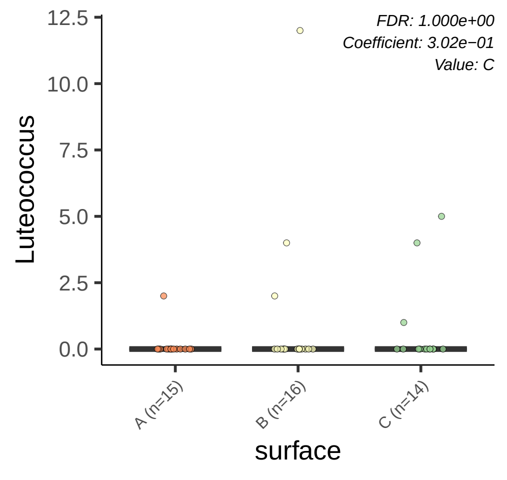
FDR: 1.000e+00
Coefficient: 3.02e−01
Value: C
Luteococcus
12.5
10.0
7.5
5.0
2.5
0.0
A (n=15)
B (n=16)
C (n=14)
surface

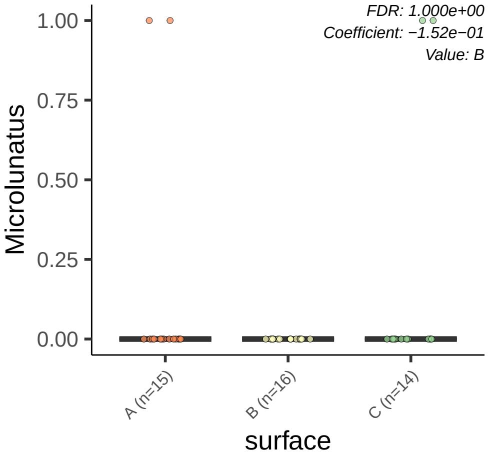
FDR: 1.000e+00
Coefficient: −1.52e−01
Value: B
Microlunatus
1.00
0.75
0.50
0.25
0.00
A (n=15)
B (n=16)
C (n=14)
surface

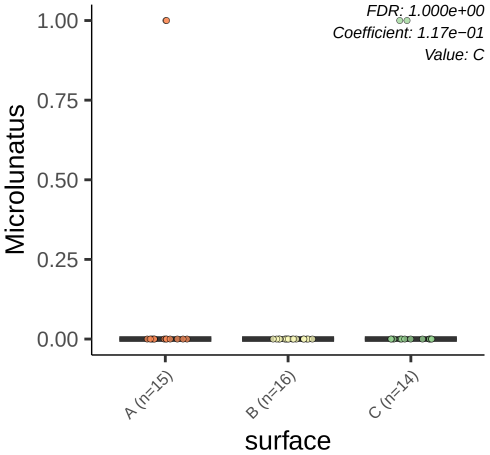

FDR: 1.000e+00
Coefficient: 1.17e−01
Value: C
1.00
0.75
0.50
0.25
0.00
Microlunatus
A (n=15)
B (n=16)
C (n=14)
surface

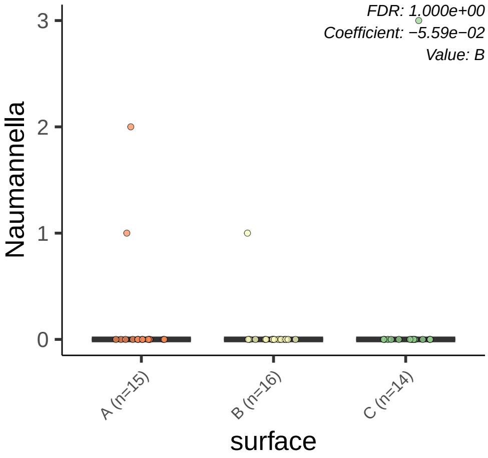
FDR: 1.000e+00
Coefficient: −5.59e−02
Value: B
Naumannella
3
2
1
0
A (n=15)
B (n=16)
C (n=14)
surface

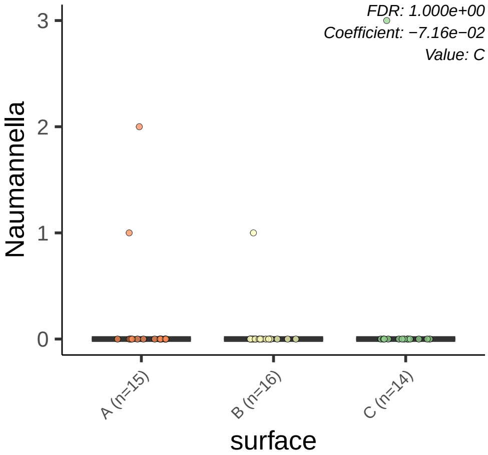

FDR: 1.000e+00
Coefficient: −7.16e−02
Value: C
Naumannella
3
2
1
0
A (n=15)
B (n=16)
C (n=14)
surface

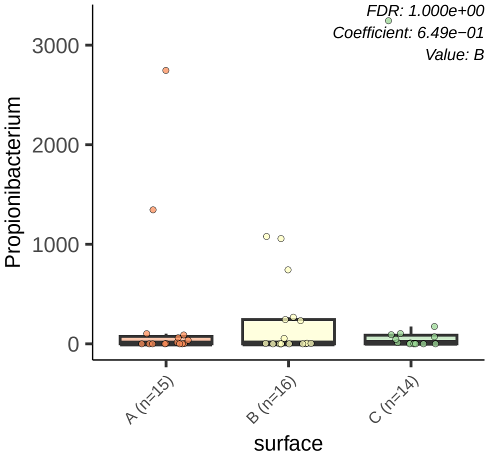
FDR: 1.000e+00
Coefficient: 6.49e−01
Value: B
Propionibacterium
3000
2000
1000
0
A (n=15)
B (n=16)
C (n=14)
surface

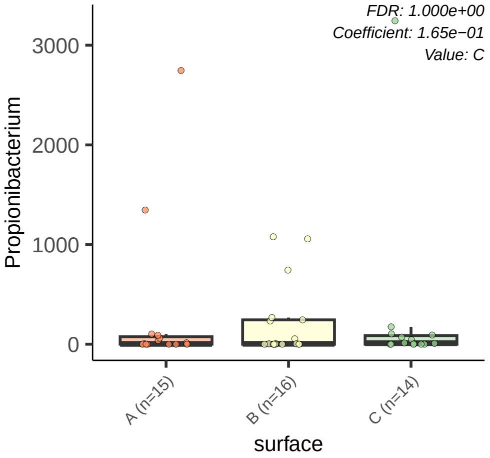

FDR: 1.000e+00
Coefficient: 1.65e−01
Value: C
Propionibacterium
3000
2000
1000
0
A (n=15)
B (n=16)
C (n=14)
surface

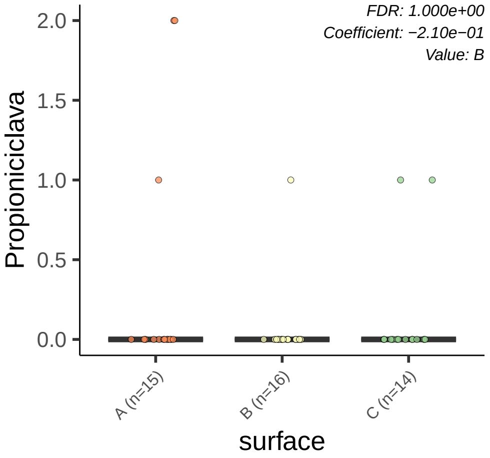
FDR: 1.000e+00
Coefficient: −2.10e−01
Value: B
Propioniciclava
2.0
1.5
1.0
0.5
0.0
A (n=15)
B (n=16)
C (n=14)
surface

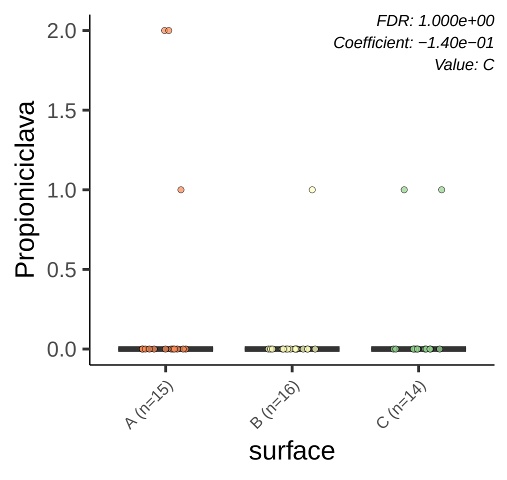

FDR: 1.000e+00
Coefficient: −1.40e−01
Value: C
Propioniciclava
2.0
1.5
1.0
0.5
0.0
A (n=15)
B (n=16)
C (n=14)
surface

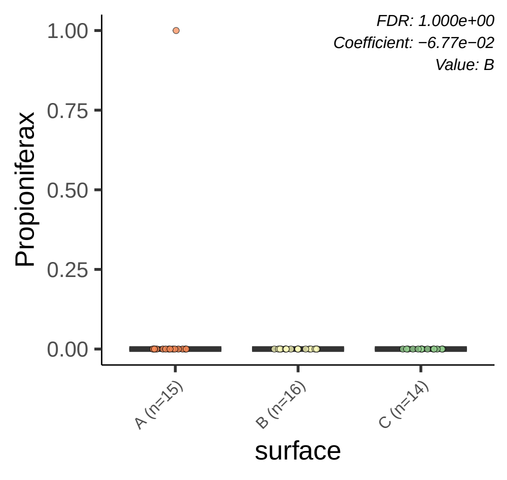
FDR: 1.000e+00
Coefficient: −6.77e−02
Value: B
Propioniferax
1.00
0.75
0.50
0.25
0.00
A (n=15)
B (n=16)
C (n=14)
surface

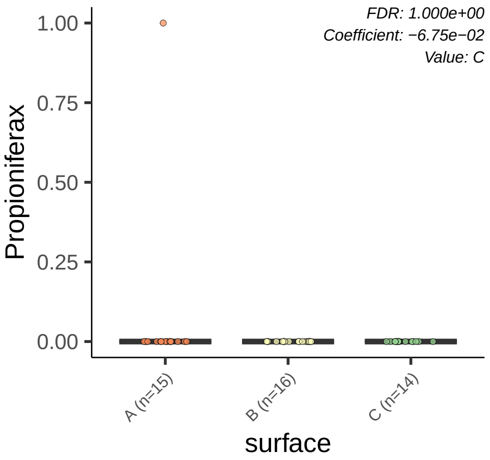
FDR: 1.000e+00
Coefficient: −6.75e−02
Value: C
Propioniferax
1.00
0.75
0.50
0.25
0.00
A (n=15)
B (n=16)
C (n=14)
surface

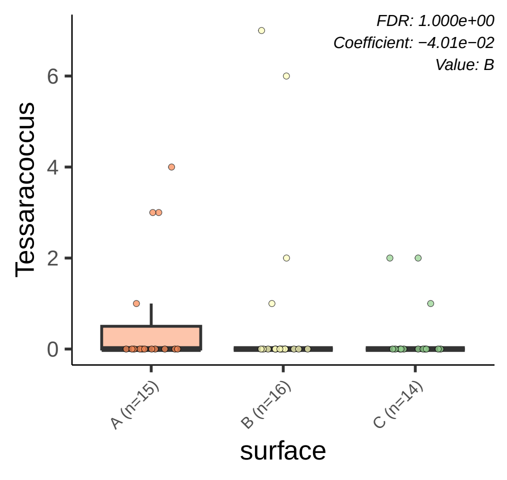
FDR: 1.000e+00
Coefficient: −4.01e−02
Value: B
Tessaracoccus
0
2
4
6
A (n=15)
B (n=16)
C (n=14)
surface

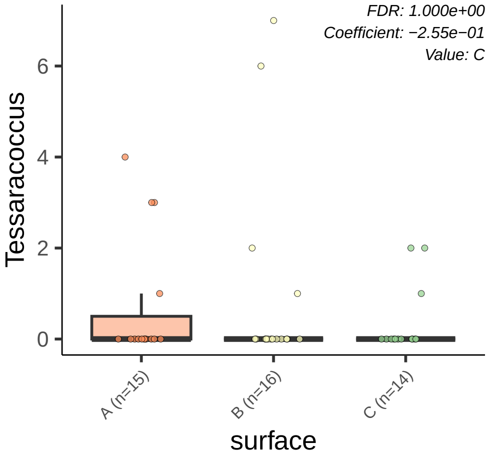
FDR: 1.000e+00
Coefficient: −2.55e−01
Value: C
Tessaracoccus
0
2
4
6
A (n=15)
B (n=16)
C (n=14)
surface

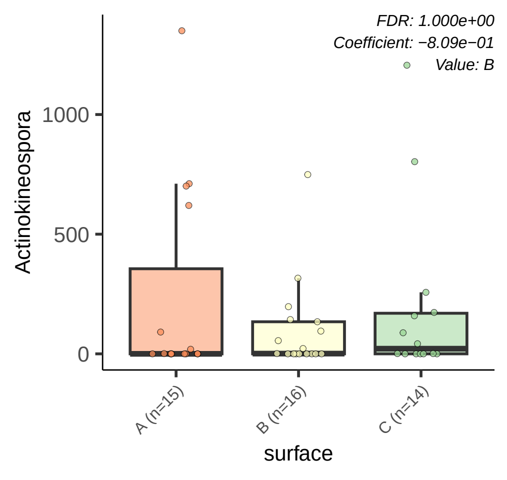
FDR: 1.000e+00
Coefficient: −8.09e−01
Value: B
Actinokineospora
1000
500
0
A (n=15)
B (n=16)
C (n=14)
surface

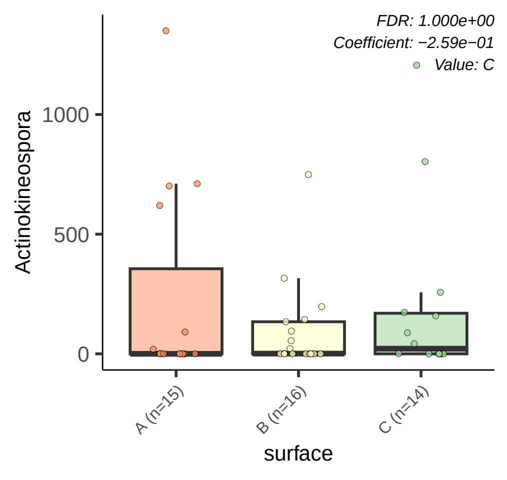

FDR: 1.000e+00
Coefficient: −2.59e−01
Value: C
Actinokineospora
1000
500
0
A (n=15)
B (n=16)
C (n=14)
surface

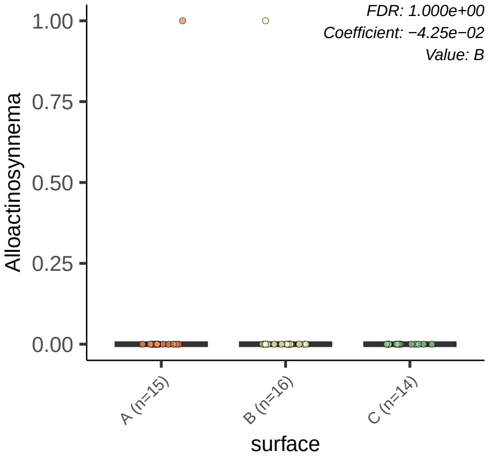
FDR: 1.000e+00
Coefficient: −4.25e−02
Value: B
Alloactinosynnema
1.00
0.75
0.50
0.25
0.00
A (n=15)
B (n=16)
C (n=14)
surface

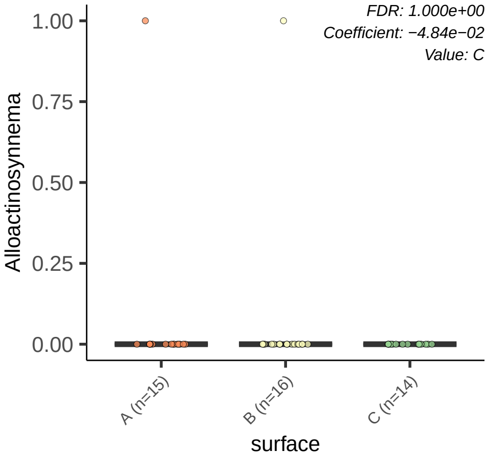
FDR: 1.000e+00
Coefficient: −4.84e−02
Value: C
Alloactinosynnema
1.00
0.75
0.50
0.25
0.00
A (n=15)
B (n=16)
C (n=14)
surface

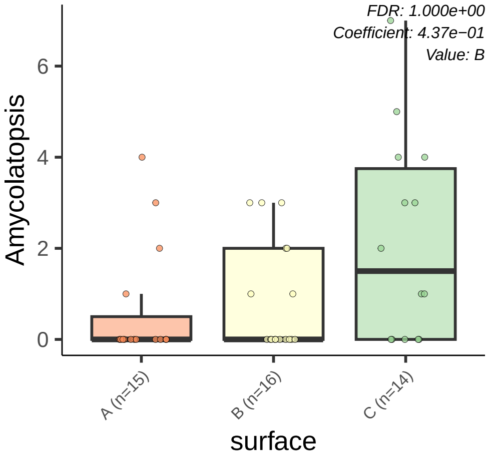
FDR: 1.000e+00
Coefficient: 4.37e−01
Value: B
Amycolatopsis
0
2
4
6
A (n=15)
B (n=16)
C (n=14)
surface

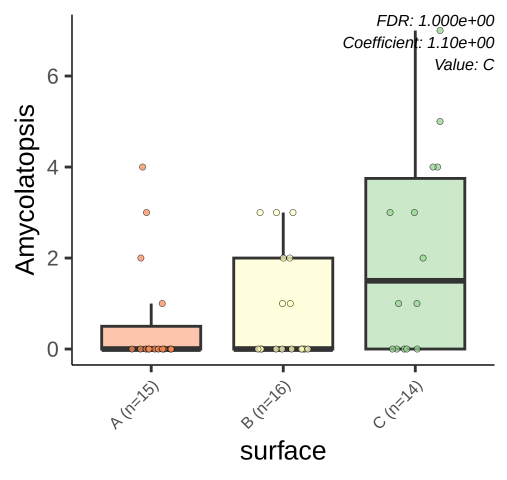

FDR: 1.000e+00
Coefficient: 1.10e+00
Value: C
Amycolatopsis
0
2
4
6
A (n=15)
B (n=16)
C (n=14)
surface

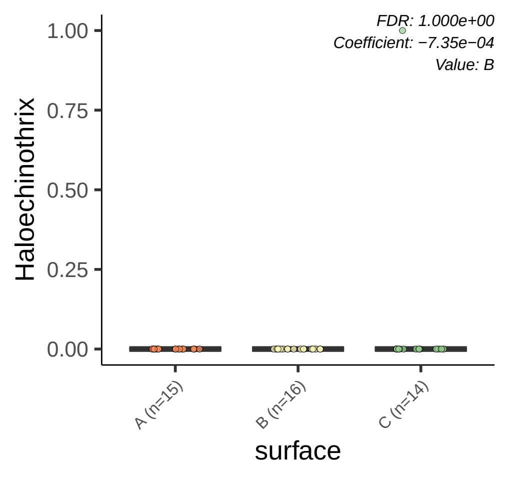

FDR: 1.000e+00
Coefficient: −7.35e−04
Value: B
Haloechinothrix
1.00
0.75
0.50
0.25
0.00
A (n=15)
B (n=16)
C (n=14)
surface

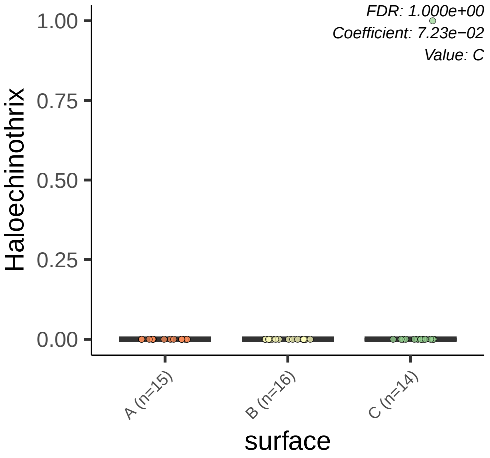

FDR: 1.000e+00
Coefficient: 7.23e−02
Value: C
Haloechinothrix
1.00
0.75
0.50
0.25
0.00
A (n=15)
B (n=16)
C (n=14)
surface

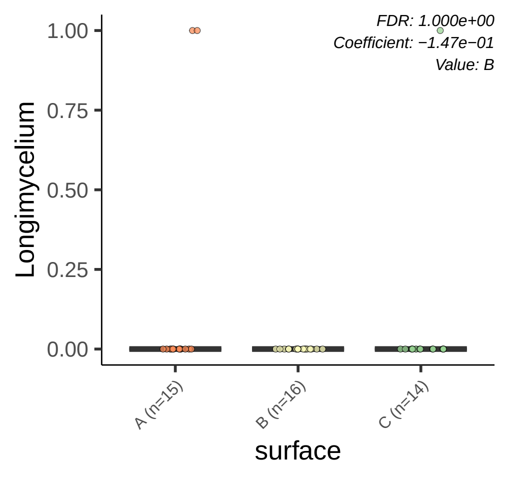
FDR: 1.000e+00
Coefficient: −1.47e−01
Value: B
Longimycelium
1.00
0.75
0.50
0.25
0.00
A (n=15)
B (n=16)
C (n=14)
surface

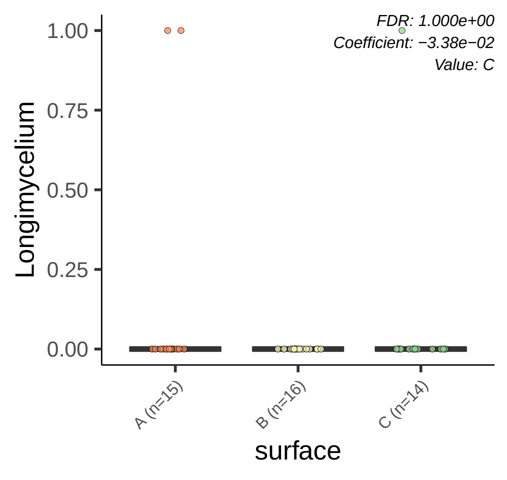

FDR: 1.000e+00
Coefficient: −3.38e−02
Value: C
Longimycelium
1.00
0.75
0.50
0.25
0.00
A (n=15)
B (n=16)
C (n=14)
surface

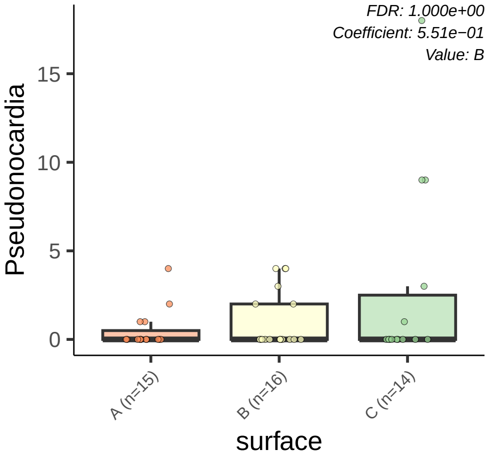
FDR: 1.000e+00
Coefficient: 5.51e−01
Value: B
Pseudonocardia
0
5
10
15
A (n=15)
B (n=16)
C (n=14)
surface

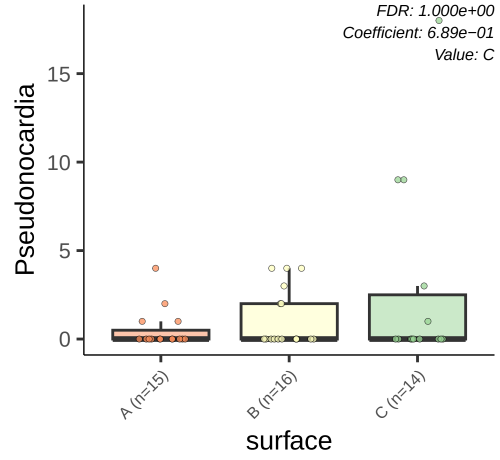
FDR: 1.000e+00
Coefficient: 6.89e−01
Value: C
Pseudonocardia
15
10
5
0
A (n=15)
B (n=16)
C (n=14)
surface

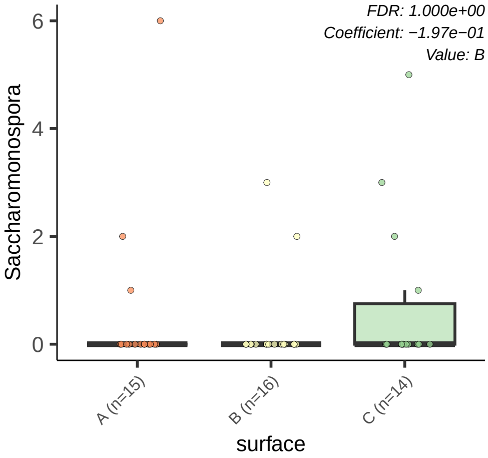

FDR: 1.000e+00
Coefficient: −1.97e−01
Value: B
Saccharomonospora
0
2
4
6
A (n=15)
B (n=16)
C (n=14)
surface

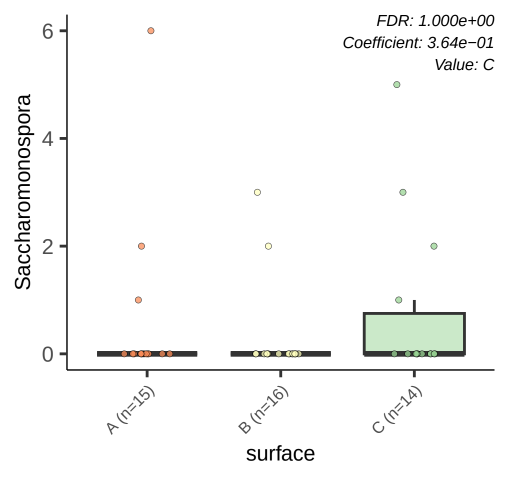

FDR: 1.000e+00
Coefficient: 3.64e−01
Value: C
Saccharomonospora
0
2
4
6
A (n=15)
B (n=16)
C (n=14)
surface

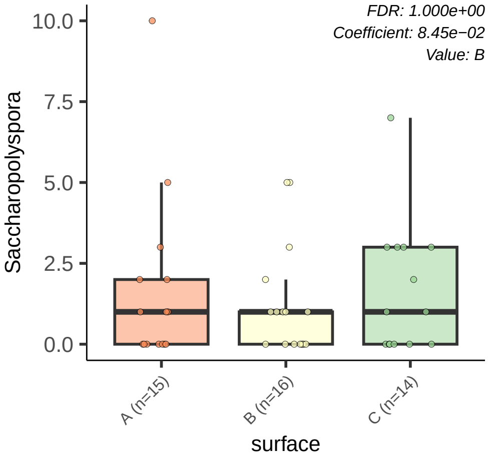

FDR: 1.000e+00
Coefficient: 8.45e−02
Value: B
Saccharopolyspora
10.0
7.5
5.0
2.5
0.0
A (n=15)
B (n=16)
C (n=14)
surface

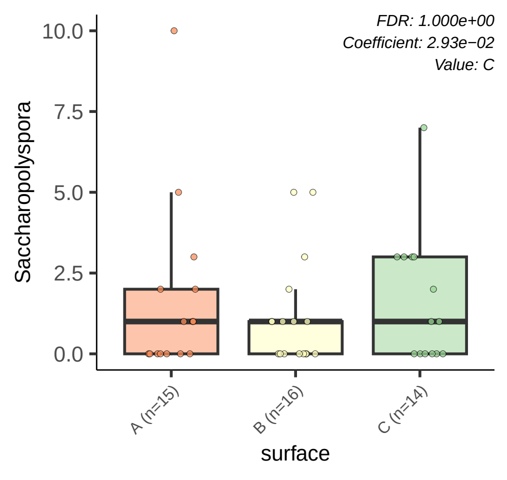
FDR: 1.000e+00
Coefficient: 2.93e−02
Value: C
Saccharopolyspora
10.0
7.5
5.0
2.5
0.0
A (n=15)
B (n=16)
C (n=14)
surface

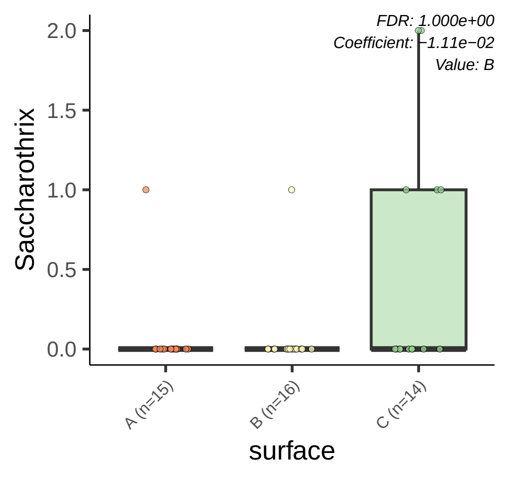
FDR: 1.000e+00
Coefficient: −1.11e−02
Value: B
Saccharothrix
2.0
1.5
1.0
0.5
0.0
A (n=15)
B (n=16)
C (n=14)
surface

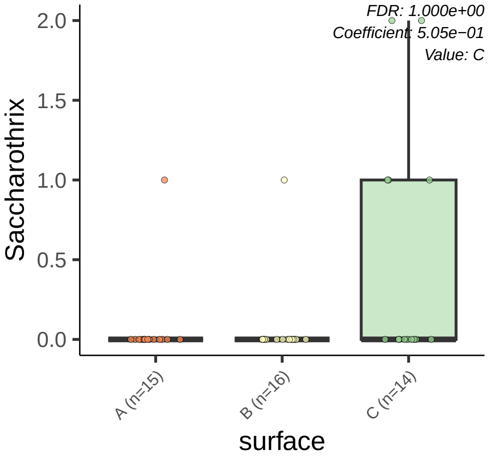

FDR: 1.000e+00
Coefficient: 5.05e−01
Value: C
Saccharothrix
2.0
1.5
1.0
0.5
0.0
A (n=15)
B (n=16)
C (n=14)
surface

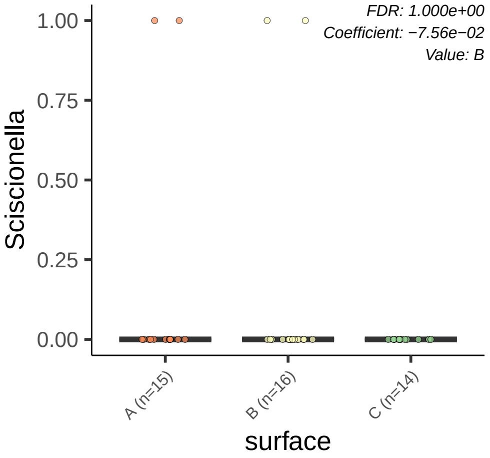

FDR: 1.000e+00
Coefficient: −7.56e−02
Value: B
Sciscionella
1.00
0.75
0.50
0.25
0.00
A (n=15)
B (n=16)
C (n=14)
surface

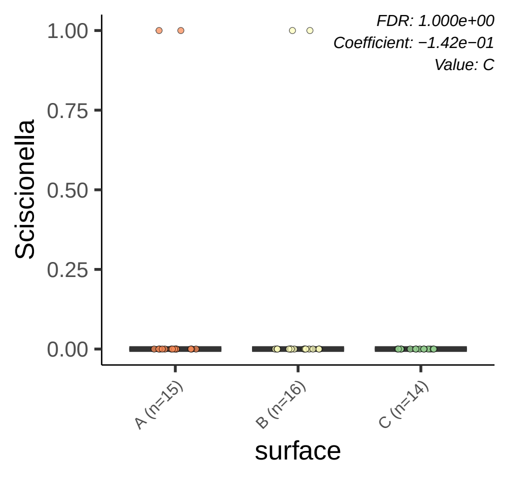
FDR: 1.000e+00
Coefficient: −1.42e−01
Value: C
Sciscionella
1.00
0.75
0.50
0.25
0.00
A (n=15)
B (n=16)
C (n=14)
surface

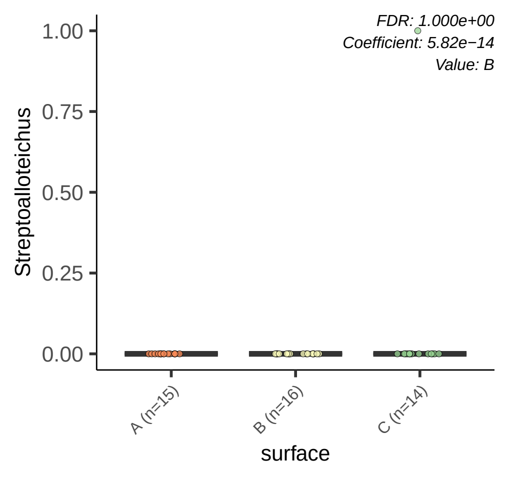
FDR: 1.000e+00
Coefficient: 5.82e−14
Value: B
Streptoalloteichus
1.00
0.75
0.50
0.25
0.00
A (n=15)
B (n=16)
C (n=14)
surface

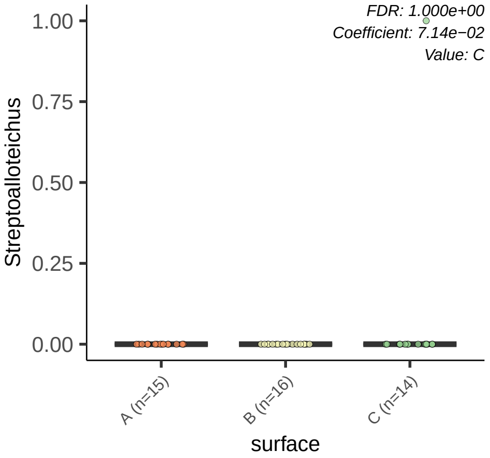

FDR: 1.000e+00
Coefficient: 7.14e−02
Value: C
Streptoalloteichus
1.00
0.75
0.50
0.25
0.00
A (n=15)
B (n=16)
C (n=14)
surface

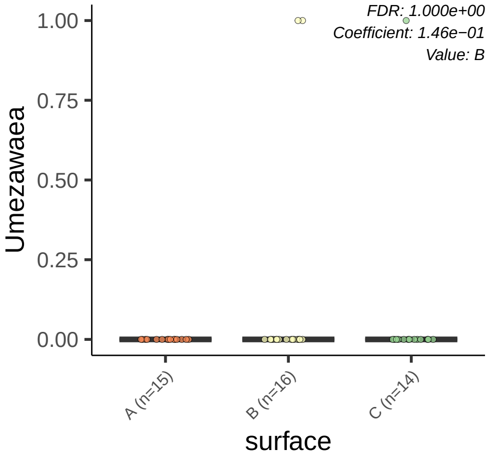
FDR: 1.000e+00
Coefficient: 1.46e−01
Value: B
Umezawaea
1.00
0.75
0.50
0.25
0.00
A (n=15)
B (n=16)
C (n=14)
surface

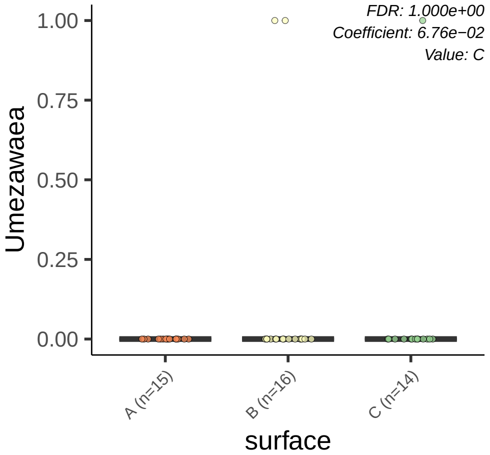
FDR: 1.000e+00
Coefficient: 6.76e−02
Value: C
Umezawaea
1.00
0.75
0.50
0.25
0.00
A (n=15)
B (n=16)
C (n=14)
surface

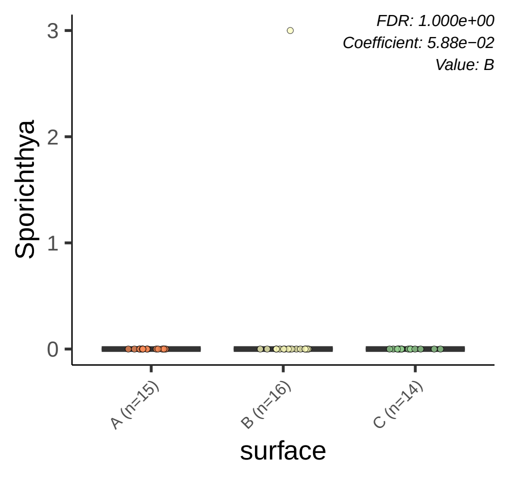

FDR: 1.000e+00
Coefficient: 5.88e−02
Value: B
Sporichthya
3
2
1
0
A (n=15)
B (n=16)
C (n=14)
surface

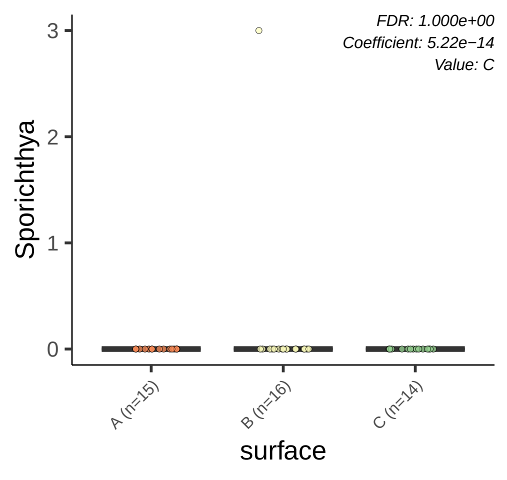

FDR: 1.000e+00
Coefficient: 5.22e−14
Value: C
Sporichthya
3
2
1
0
A (n=15)
B (n=16)
C (n=14)
surface

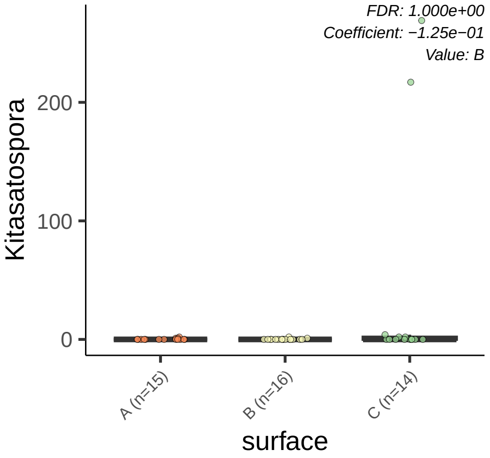
FDR: 1.000e+00
Coefficient: −1.25e−01
Value: B
Kitasatospora
200
100
0
A (n=15)
B (n=16)
C (n=14)
surface

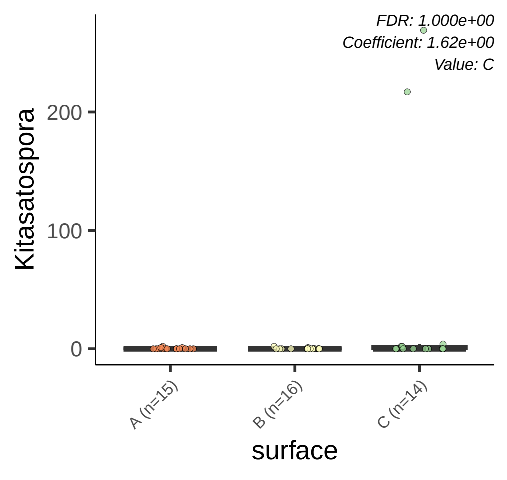
FDR: 1.000e+00
Coefficient: 1.62e+00
Value: C
Kitasatospora
200
100
0
A (n=15)
B (n=16)
C (n=14)
surface

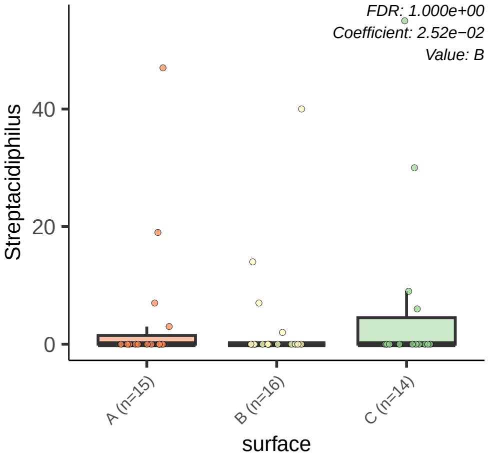

FDR: 1.000e+00
Coefficient: 2.52e−02
Value: B
Streptacidiphilus
40
20
0
A (n=15)
B (n=16)
C (n=14)
surface

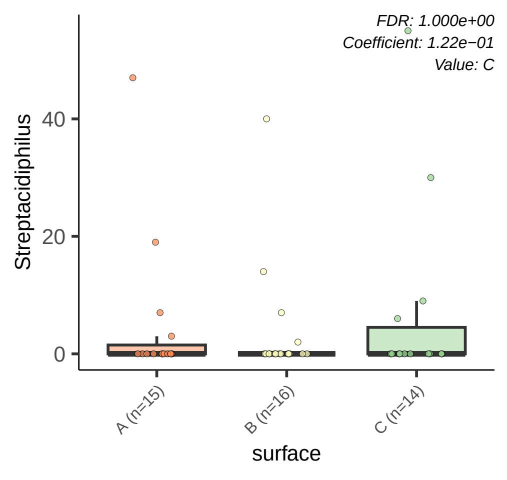
FDR: 1.000e+00
Coefficient: 1.22e−01
Value: C
Streptacidiphilus
40
20
0
A (n=15)
B (n=16)
C (n=14)
surface

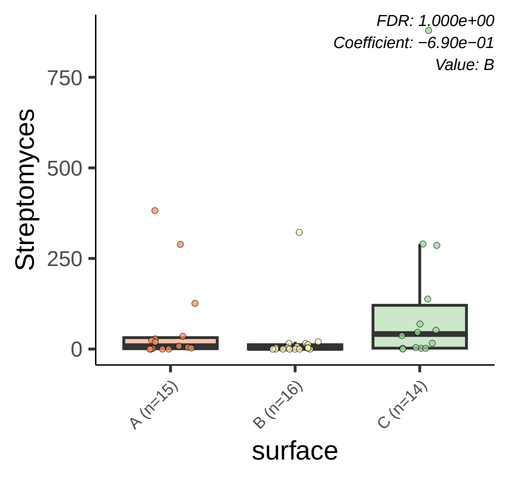

FDR: 1.000e+00
Coefficient: −6.90e−01
Value: B
Streptomyces
750
500
250
0
A (n=15)
B (n=16)
C (n=14)
surface

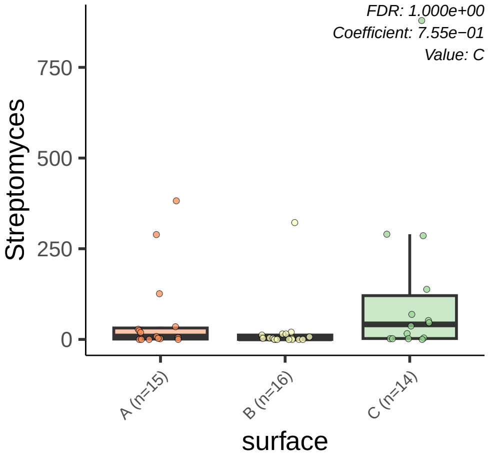
FDR: 1.000e+00
Coefficient: 7.55e−01
Value: C
Streptomyces
750
500
250
0
A (n=15)
B (n=16)
C (n=14)
surface

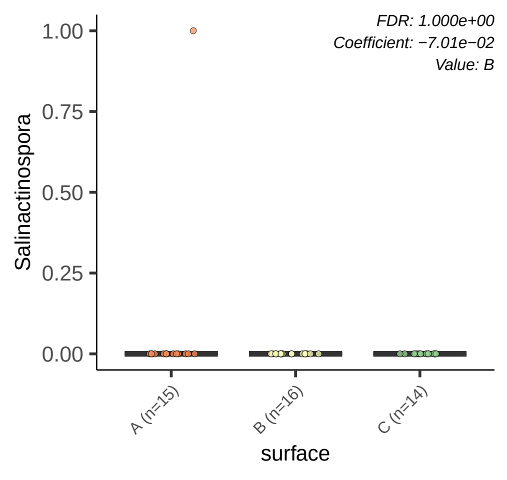
FDR: 1.000e+00
Coefficient: −7.01e−02
Value: B
Salinactinospora
1.00
0.75
0.50
0.25
0.00
A (n=15)
B (n=16)
C (n=14)
surface

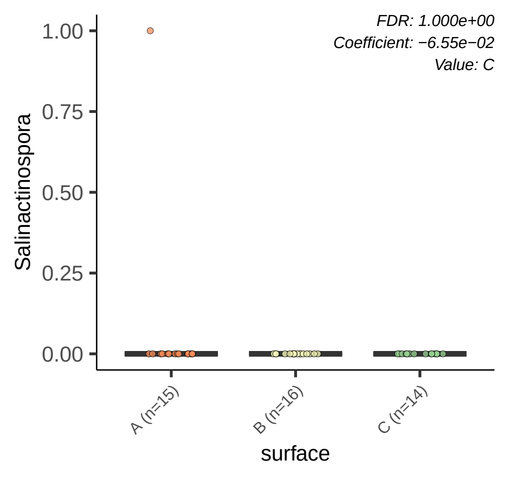

FDR: 1.000e+00
Coefficient: −6.55e−02
Value: C
Salinactinospora
1.00
0.75
0.50
0.25
0.00
A (n=15)
B (n=16)
C (n=14)
surface

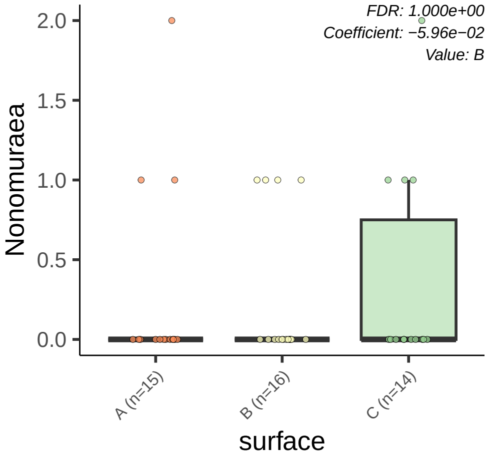

FDR: 1.000e+00
Coefficient: −5.96e−02
Value: B
2.0
1.5
1.0
0.5
0.0
Nonomuraea
A (n=15)
B (n=16)
C (n=14)
surface

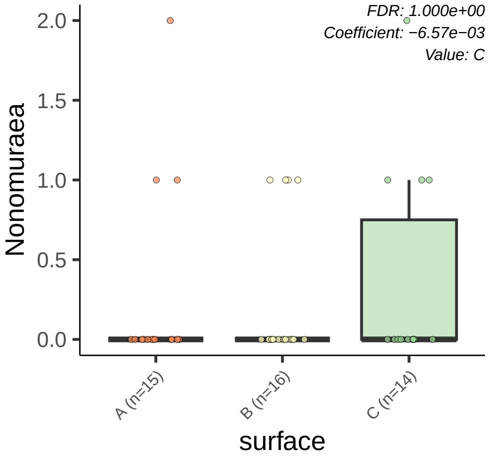
FDR: 1.000e+00
Coefficient: −6.57e−03
Value: C
Nonomuraea
2.0
1.5
1.0
0.5
0.0
A (n=15)
B (n=16)
C (n=14)
surface

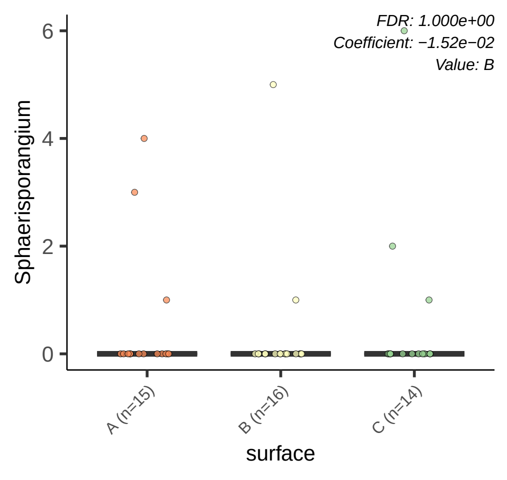
FDR: 1.000e+00
Coefficient: −1.52e−02
Value: B
Sphaerisporangium
6
4
2
0
A (n=15)
B (n=16)
C (n=14)
surface

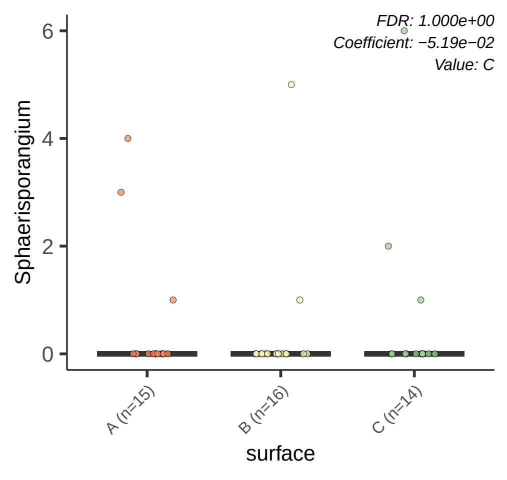

FDR: 1.000e+00
Coefficient: −5.19e−02
Value: C
Sphaerisporangium
0
2
4
6
A (n=15)
B (n=16)
C (n=14)
surface

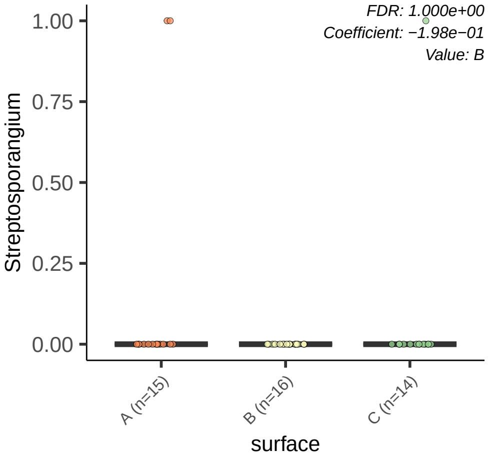

FDR: 1.000e+00
Coefficient: −1.98e−01
Value: B
Streptosporangium
1.00
0.75
0.50
0.25
0.00
A (n=15)
B (n=16)
C (n=14)
surface

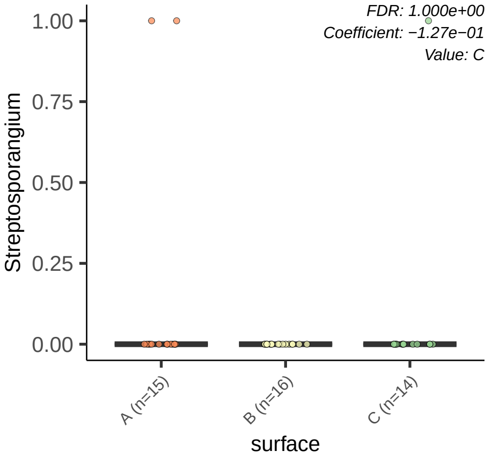
FDR: 1.000e+00
Coefficient: −1.27e−01
Value: C
Streptosporangium
1.00
0.75
0.50
0.25
0.00
A (n=15)
B (n=16)
C (n=14)
surface

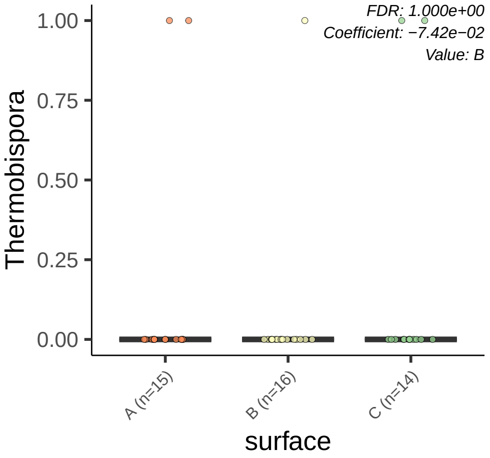

FDR: 1.000e+00
Coefficient: −7.42e−02
Value: B
Thermobispora
1.00
0.75
0.50
0.25
0.00
A (n=15)
B (n=16)
C (n=14)
surface

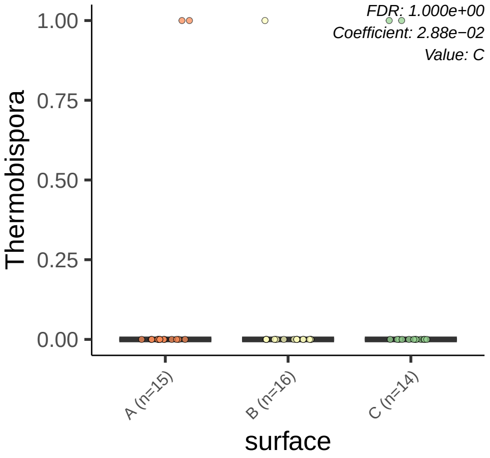
FDR: 1.000e+00
Coefficient: 2.88e−02
Value: C
Thermobispora
1.00
0.75
0.50
0.25
0.00
A (n=15)
B (n=16)
C (n=14)
surface

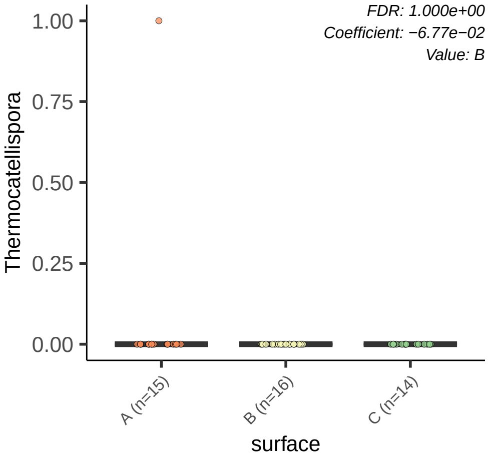
FDR: 1.000e+00
Coefficient: −6.77e−02
Value: B
Thermocatellispora
1.00
0.75
0.50
0.25
0.00
A (n=15)
B (n=16)
C (n=14)
surface

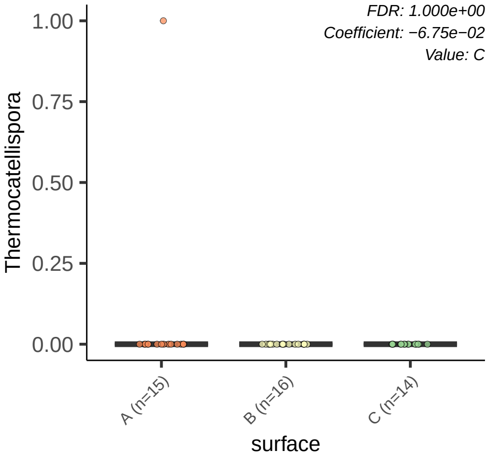
FDR: 1.000e+00
Coefficient: −6.75e−02
Value: C
Thermocatellispora
1.00
0.75
0.50
0.25
0.00
A (n=15)
B (n=16)
C (n=14)
surface

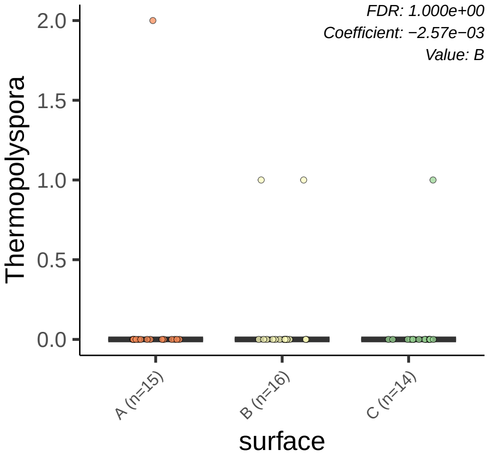

FDR: 1.000e+00
Coefficient: −2.57e−03
Value: B
Thermopolyspora
2.0
1.5
1.0
0.5
0.0
A (n=15)
B (n=16)
C (n=14)
surface

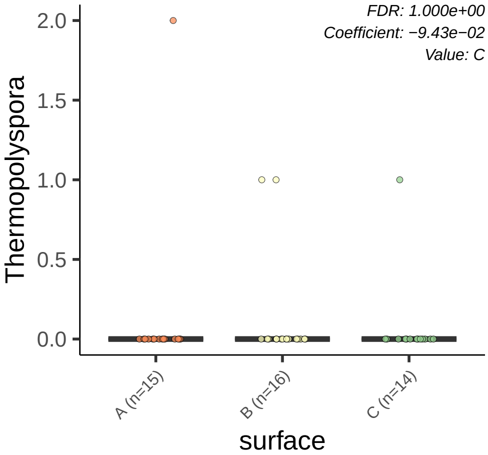
FDR: 1.000e+00
Coefficient: −9.43e−02
Value: C
Thermopolyspora
2.0
1.5
1.0
0.5
0.0
A (n=15)
B (n=16)
C (n=14)
surface

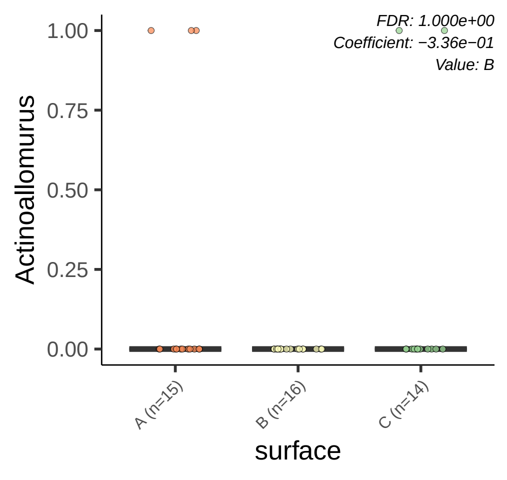
FDR: 1.000e+00
Coefficient: −3.36e−01
Value: B
Actinoallomurus
1.00
0.75
0.50
0.25
0.00
A (n=15)
B (n=16)
C (n=14)
surface

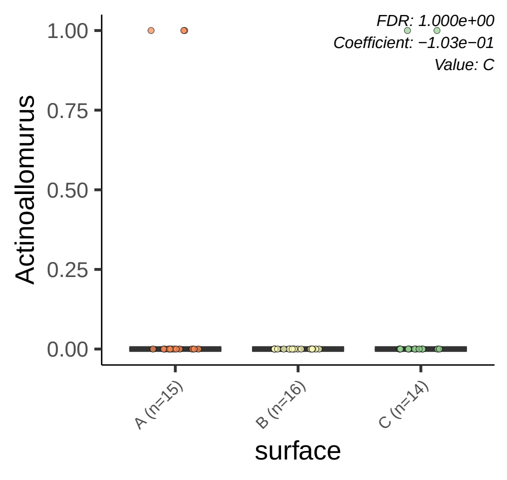

FDR: 1.000e+00
Coefficient: −1.03e−01
Value: C
Actinoallomurus
1.00
0.75
0.50
0.25
0.00
A (n=15)
B (n=16)
C (n=14)
surface

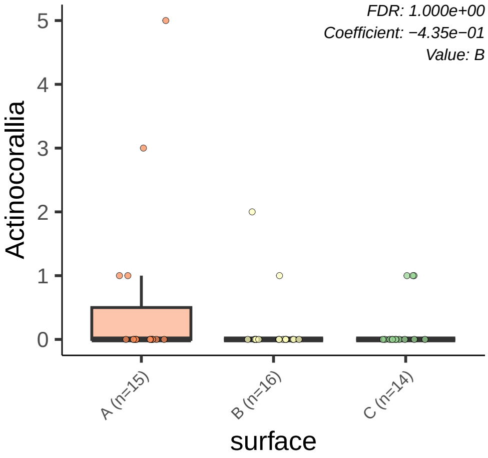

FDR: 1.000e+00
Coefficient: −4.35e−01
Value: B
Actinocorallia
5
4
3
2
1
0
A (n=15)
B (n=16)
C (n=14)
surface

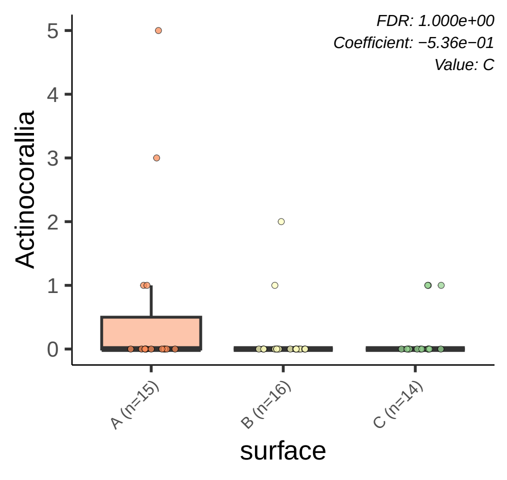
FDR: 1.000e+00
Coefficient: −5.36e−01
Value: C
Actinocorallia
0
1
2
3
4
5
A (n=15)
B (n=16)
C (n=14)
surface

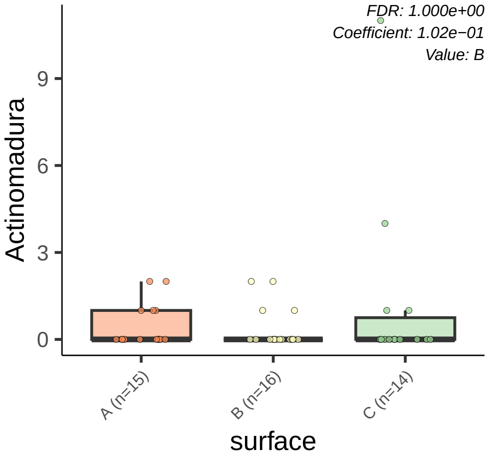
FDR: 1.000e+00
Coefficient: 1.02e−01
Value: B
Actinomadura
0
3
6
9
A (n=15)
B (n=16)
C (n=14)
surface

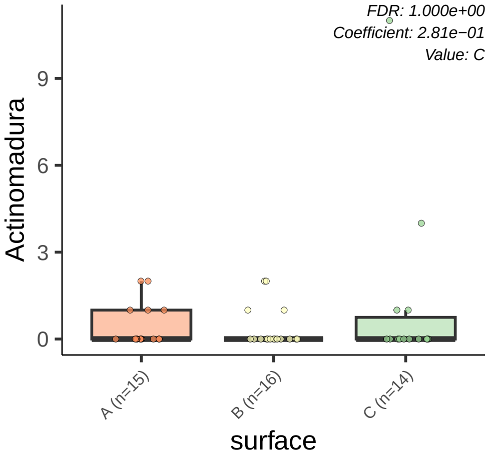
FDR: 1.000e+00
Coefficient: 2.81e−01
Value: C
Actinomadura
9
6
3
0
A (n=15)
B (n=16)
C (n=14)
surface

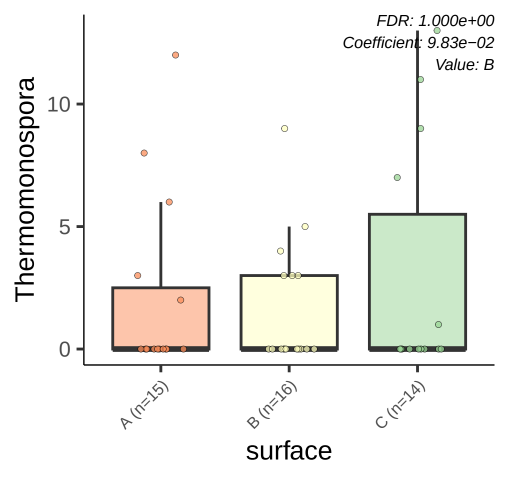

FDR: 1.000e+00
Coefficient: 9.83e−02
Value: B
Thermomonospora
0
5
10
A (n=15)
B (n=16)
C (n=14)
surface

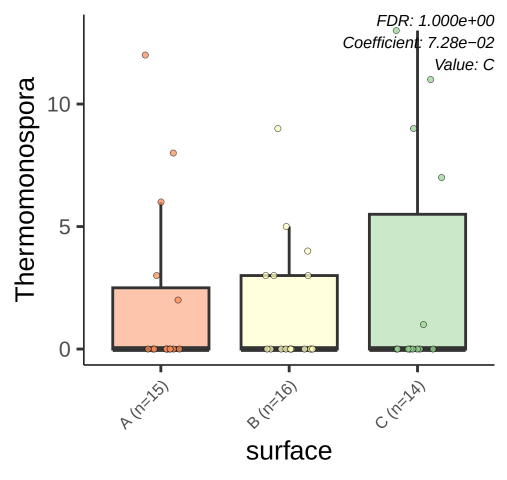

FDR: 1.000e+00
Coefficient: 7.28e−02
Value: C
Thermomonospora
0
5
10
A (n=15)
B (n=16)
C (n=14)
surface

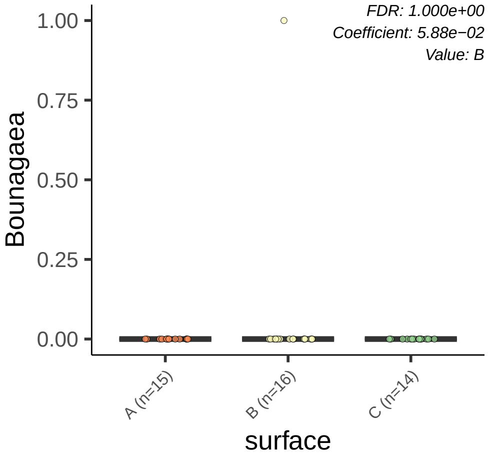
FDR: 1.000e+00
Coefficient: 5.88e−02
Value: B
Bounagaea
1.00
0.75
0.50
0.25
0.00
A (n=15)
B (n=16)
C (n=14)
surface

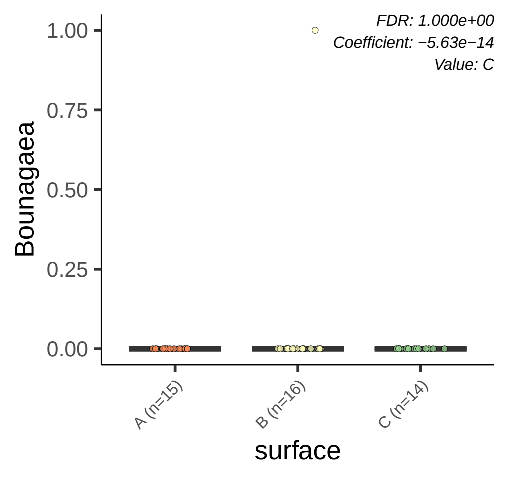
FDR: 1.000e+00
Coefficient: −5.63e−14
Value: C
Bounagaea
1.00
0.75
0.50
0.25
0.00
A (n=15)
B (n=16)
C (n=14)
surface

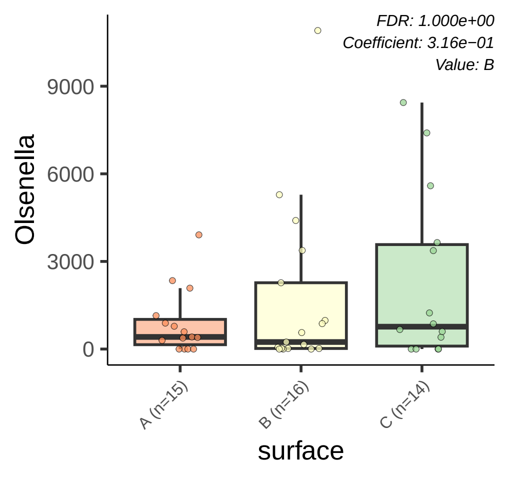
FDR: 1.000e+00
Coefficient: 3.16e−01
Value: B
Olsenella
9000
6000
3000
0
A (n=15)
B (n=16)
C (n=14)
surface

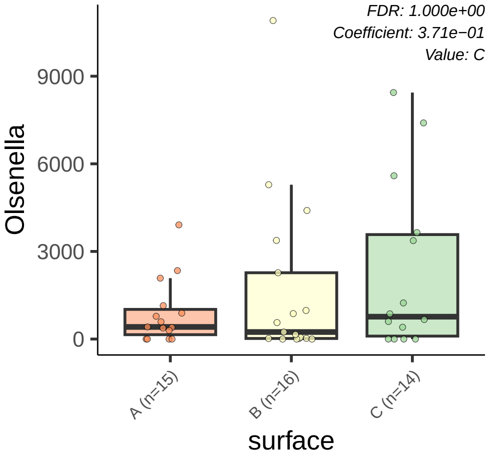
FDR: 1.000e+00
Coefficient: 3.71e−01
Value: C
Olsenella
9000
6000
3000
0
A (n=15)
B (n=16)
C (n=14)
surface

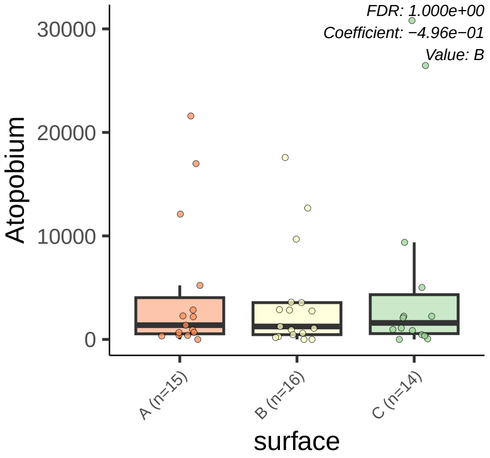

FDR: 1.000e+00
Coefficient: −4.96e−01
Value: B
Atopobium
30000
20000
10000
0
A (n=15)
B (n=16)
C (n=14)
surface

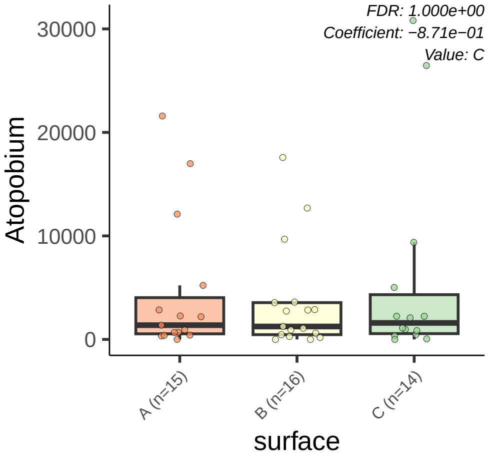

FDR: 1.000e+00
Coefficient: −8.71e−01
Value: C
30000
20000
10000
0
Atopobium
A (n=15)
B (n=16)
C (n=14)
surface

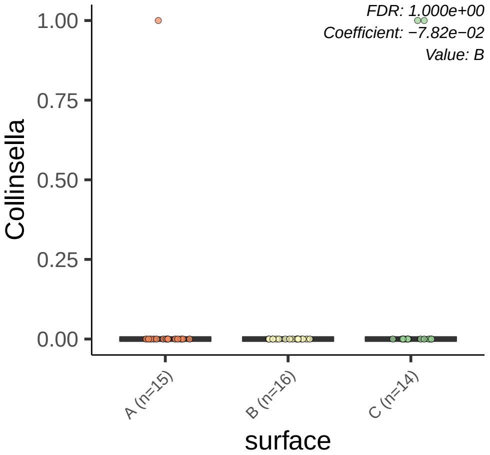

FDR: 1.000e+00
Coefficient: −7.82e−02
Value: B
Collinsella
1.00
0.75
0.50
0.25
0.00
A (n=15)
B (n=16)
C (n=14)
surface

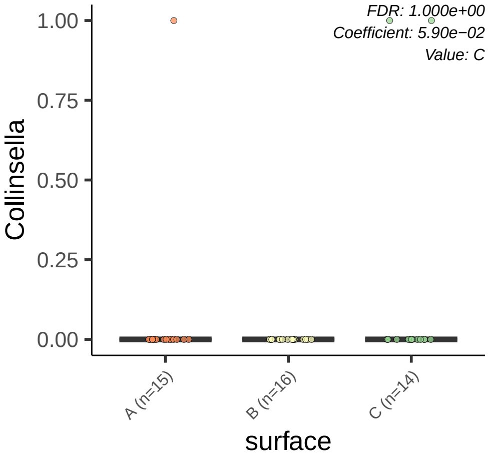

FDR: 1.000e+00
Coefficient: 5.90e−02
Value: C
Collinsella
1.00
0.75
0.50
0.25
0.00
A (n=15)
B (n=16)
C (n=14)
surface

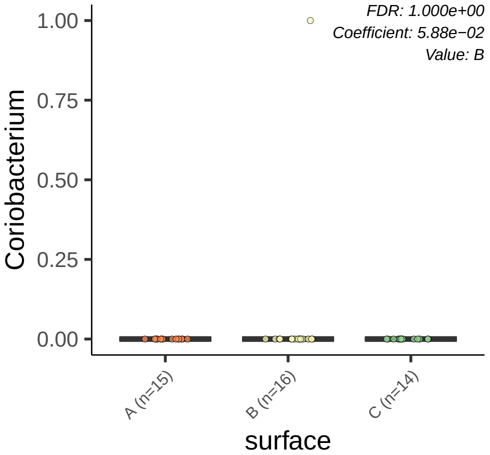

FDR: 1.000e+00
Coefficient: 5.88e−02
Value: B
Coriobacterium
1.00
0.75
0.50
0.25
0.00
A (n=15)
B (n=16)
C (n=14)
surface

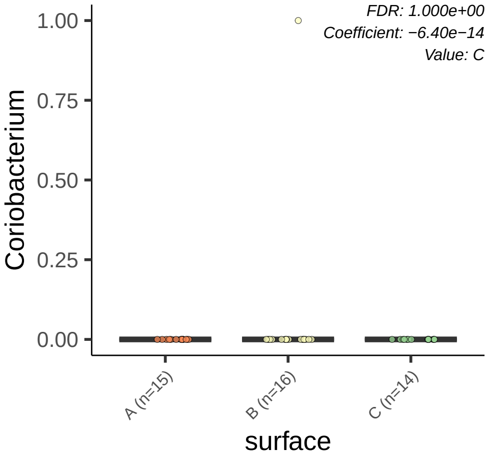

FDR: 1.000e+00
Coefficient: −6.40e−14
Value: C
Coriobacterium
1.00
0.75
0.50
0.25
0.00
A (n=15)
B (n=16)
C (n=14)
surface

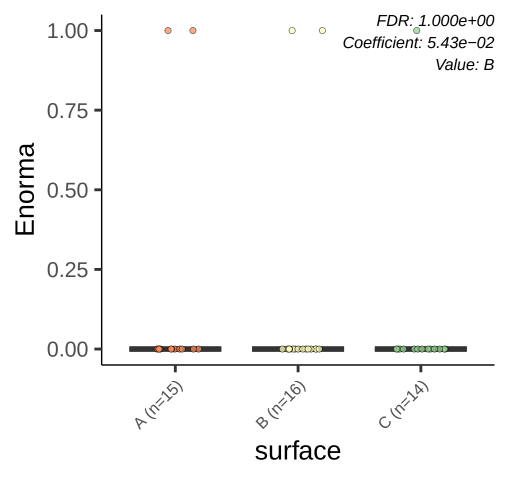

FDR: 1.000e+00
Coefficient: 5.43e−02
Value: B
1.00
0.75
0.50
0.25
0.00
Enorma
A (n=15)
B (n=16)
C (n=14)
surface

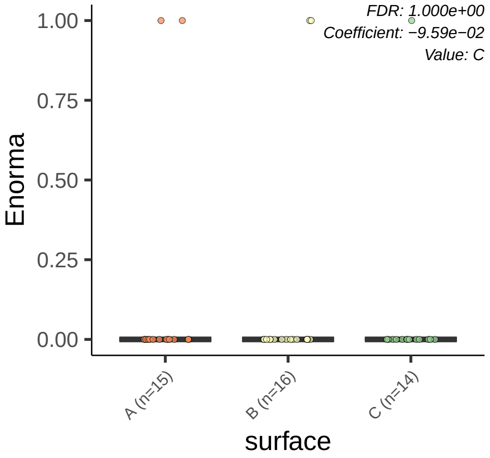

FDR: 1.000e+00
Coefficient: −9.59e−02
Value: C
Enorma
1.00
0.75
0.50
0.25
0.00
A (n=15)
B (n=16)
C (n=14)
surface

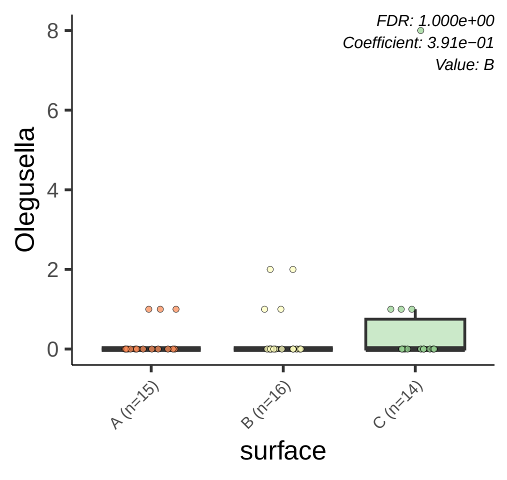
FDR: 1.000e+00
Coefficient: 3.91e−01
Value: B
Olegusella
8
6
4
2
0
A (n=15)
B (n=16)
C (n=14)
surface

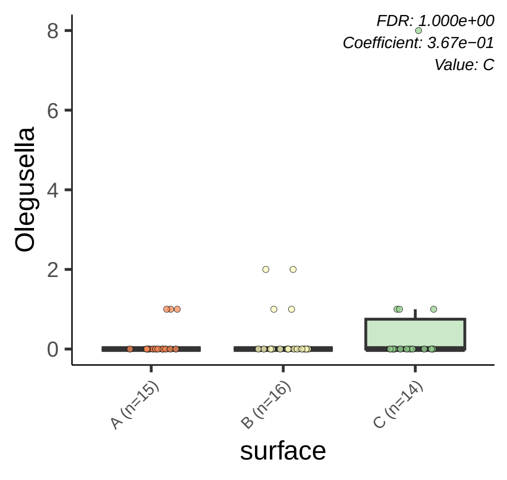
FDR: 1.000e+00
Coefficient: 3.67e−01
Value: C
Olegusella
8
6
4
2
0
A (n=15)
B (n=16)
C (n=14)
surface

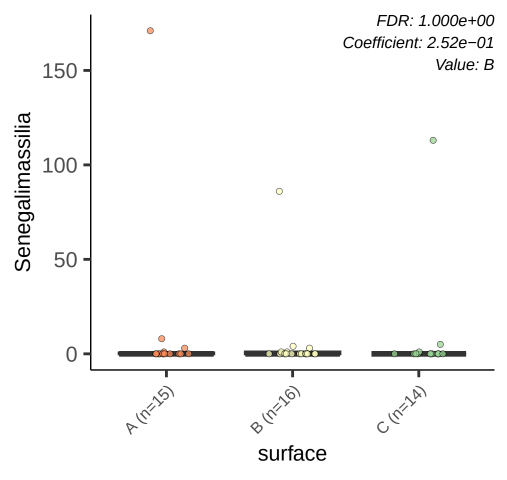
FDR: 1.000e+00
Coefficient: 2.52e−01
Value: B
Senegalimassilia
150
100
50
0
A (n=15)
B (n=16)
C (n=14)
surface

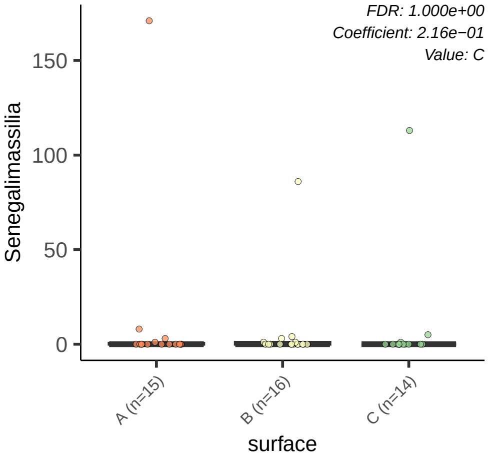

FDR: 1.000e+00
Coefficient: 2.16e−01
Value: C
Senegalimassilia
150
100
50
0
A (n=15)
B (n=16)
C (n=14)
surface

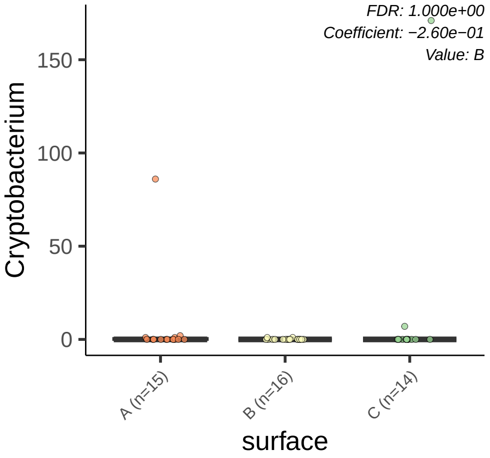
FDR: 1.000e+00
Coefficient: −2.60e−01
Value: B
Cryptobacterium
150
100
50
0
A (n=15)
B (n=16)
C (n=14)
surface

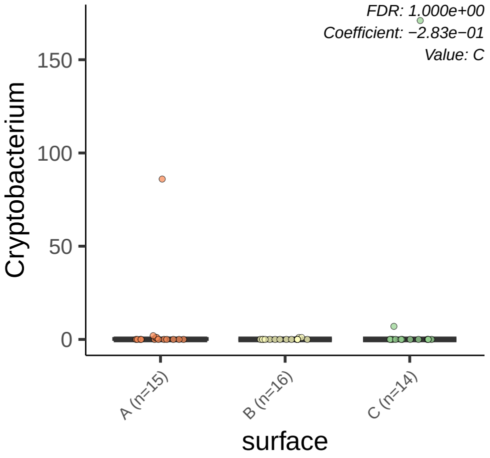
FDR: 1.000e+00
Coefficient: −2.83e−01
Value: C
Cryptobacterium
150
100
50
0
A (n=15)
B (n=16)
C (n=14)
surface

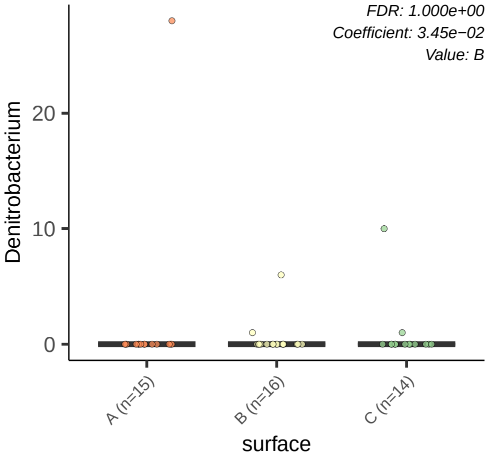

FDR: 1.000e+00
Coefficient: 3.45e−02
Value: B
Denitrobacterium
20
10
0
A (n=15)
B (n=16)
C (n=14)
surface

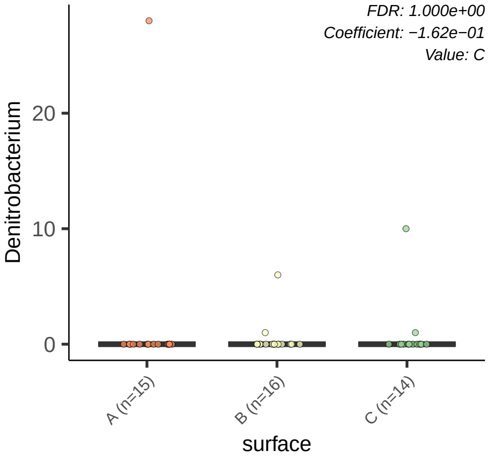
FDR: 1.000e+00
Coefficient: −1.62e−01
Value: C
Denitrobacterium
20
10
0
A (n=15)
B (n=16)
C (n=14)
surface

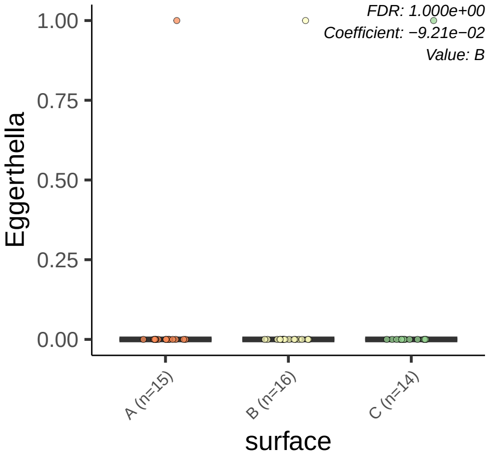
FDR: 1.000e+00
Coefficient: −9.21e−02
Value: B
1.00
0.75
0.50
0.25
0.00
Eggerthella
A (n=15)
B (n=16)
C (n=14)
surface

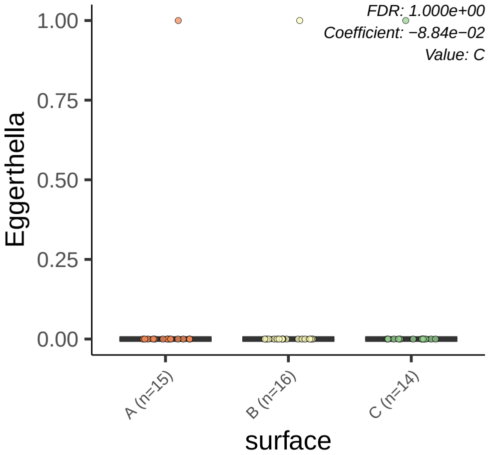
FDR: 1.000e+00
Coefficient: −8.84e−02
Value: C
1.00
0.75
0.50
0.25
0.00
Eggerthella
A (n=15)
B (n=16)
C (n=14)
surface

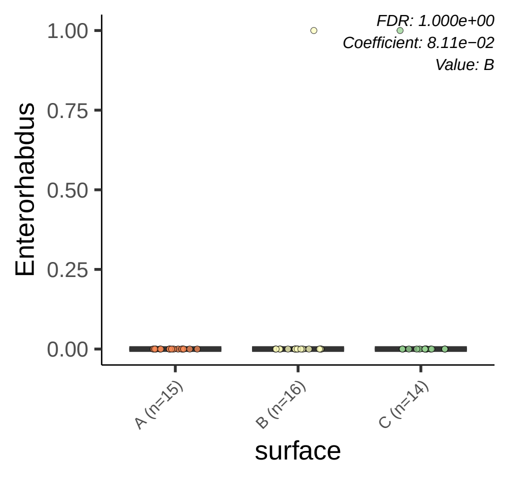
FDR: 1.000e+00
Coefficient: 8.11e−02
Value: B
Enterorhabdus
1.00
0.75
0.50
0.25
0.00
A (n=15)
B (n=16)
C (n=14)
surface

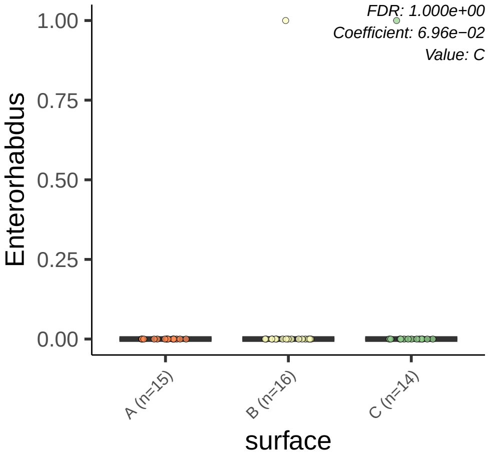
FDR: 1.000e+00
Coefficient: 6.96e−02
Value: C
Enterorhabdus
1.00
0.75
0.50
0.25
0.00
A (n=15)
B (n=16)
C (n=14)
surface

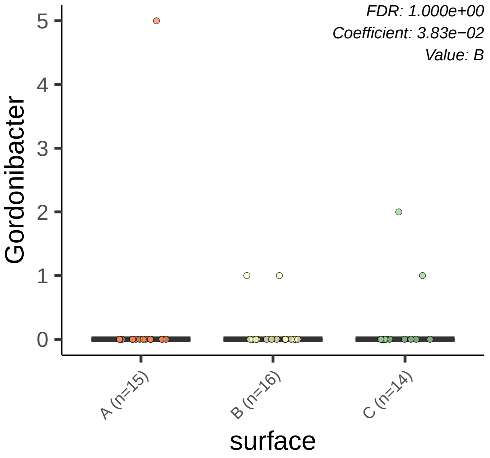
FDR: 1.000e+00
Coefficient: 3.83e−02
Value: B
Gordonibacter
0
1
2
3
4
5
A (n=15)
B (n=16)
C (n=14)
surface

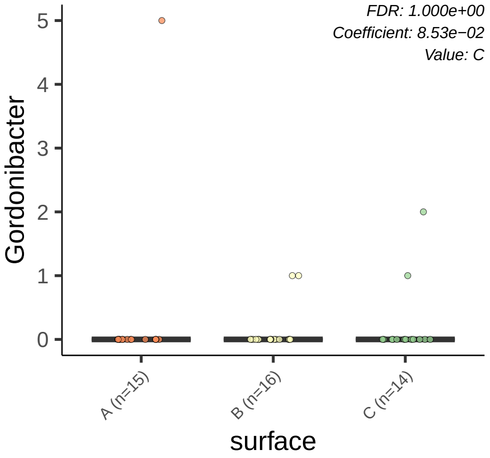

FDR: 1.000e+00
Coefficient: 8.53e−02
Value: C
Gordonibacter
5
4
3
2
1
0
A (n=15)
B (n=16)
C (n=14)
surface

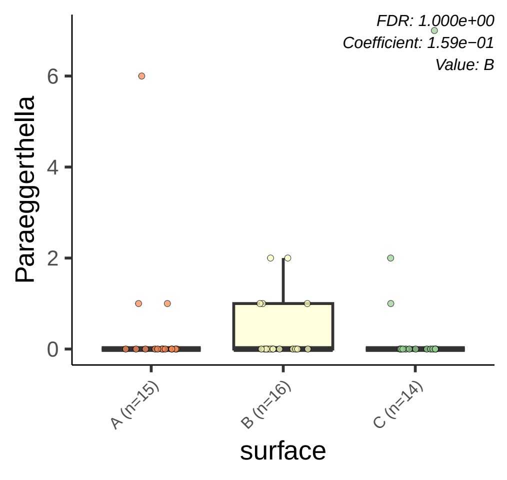

FDR: 1.000e+00
Coefficient: 1.59e−01
Value: B
Paraeggerthella
0
2
4
6
A (n=15)
B (n=16)
C (n=14)
surface

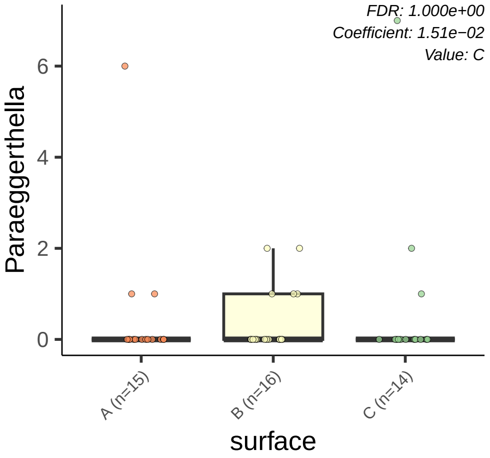
FDR: 1.000e+00
Coefficient: 1.51e−02
Value: C
Paraeggerthella
0
2
4
6
A (n=15)
B (n=16)
C (n=14)
surface

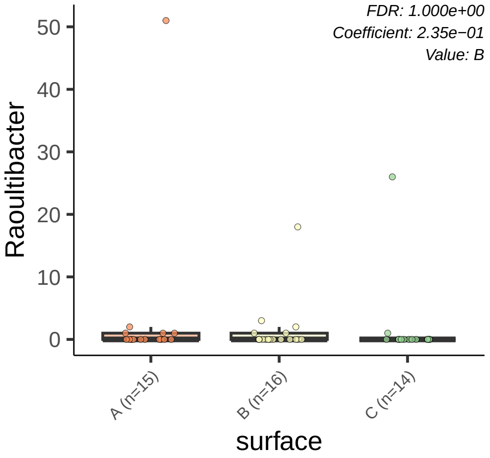
FDR: 1.000e+00
Coefficient: 2.35e−01
Value: B
Raoultibacter
50
40
30
20
10
0
A (n=15)
B (n=16)
C (n=14)
surface

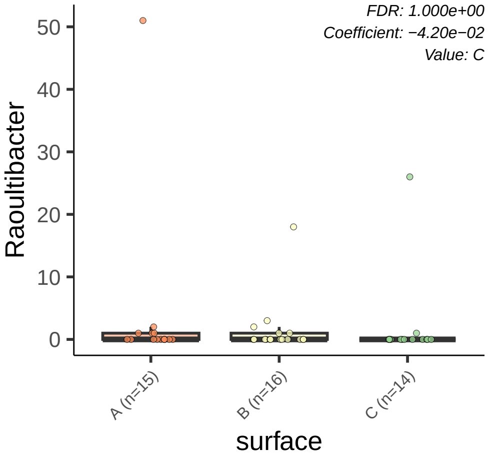
FDR: 1.000e+00
Coefficient: −4.20e−02
Value: C
Raoultibacter
50
40
30
20
10
0
A (n=15)
B (n=16)
C (n=14)
surface

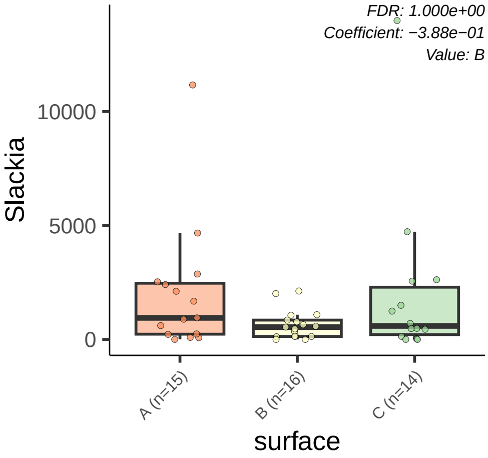
FDR: 1.000e+00
Coefficient: −3.88e−01
Value: B
Slackia
10000
5000
0
A (n=15)
B (n=16)
C (n=14)
surface

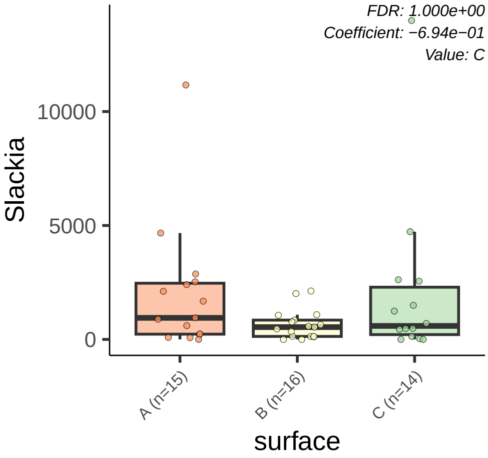
FDR: 1.000e+00
Coefficient: −6.94e−01
Value: C
10000
5000
0
Slackia
A (n=15)
B (n=16)
C (n=14)
surface

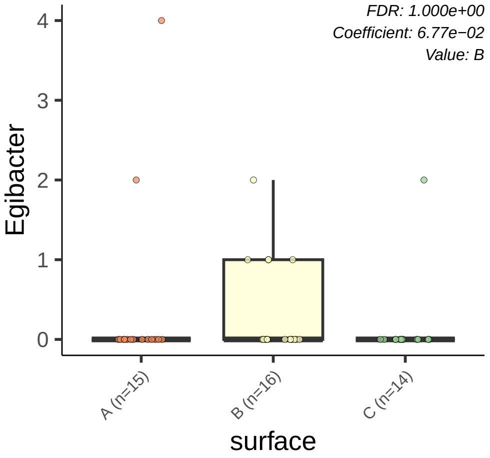
FDR: 1.000e+00
Coefficient: 6.77e−02
Value: B
Egibacter
0
1
2
3
4
A (n=15)
B (n=16)
C (n=14)
surface

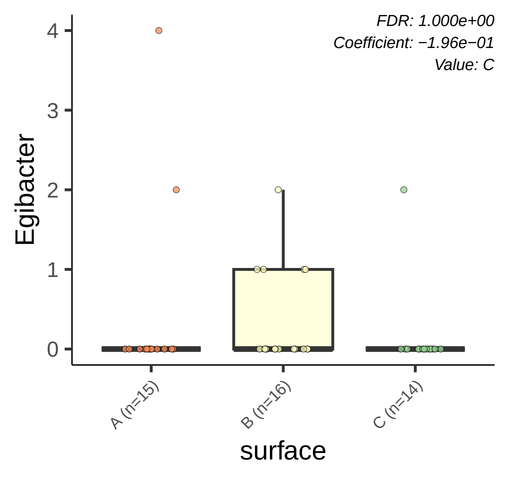
FDR: 1.000e+00
Coefficient: −1.96e−01
Value: C
Egibacter
0
1
2
3
4
A (n=15)
B (n=16)
C (n=14)
surface

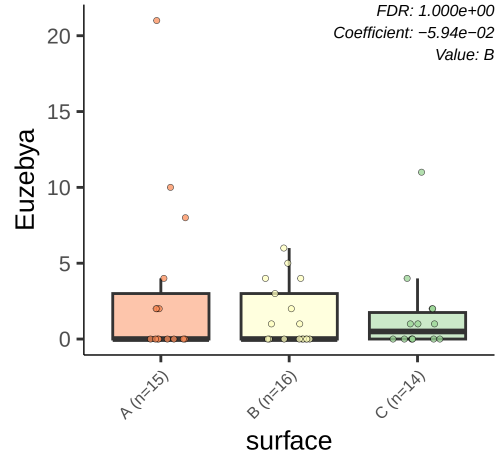

FDR: 1.000e+00
Coefficient: −5.94e−02
Value: B
Euzebya
20
15
10
5
0
A (n=15)
B (n=16)
C (n=14)
surface

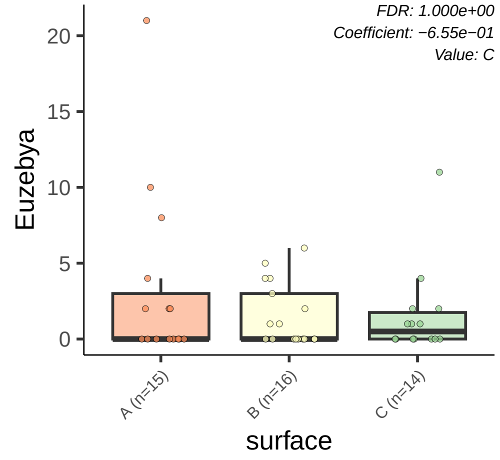

FDR: 1.000e+00
Coefficient: −6.55e−01
Value: C
Euzebya
20
15
10
5
0
A (n=15)
B (n=16)
C (n=14)
surface

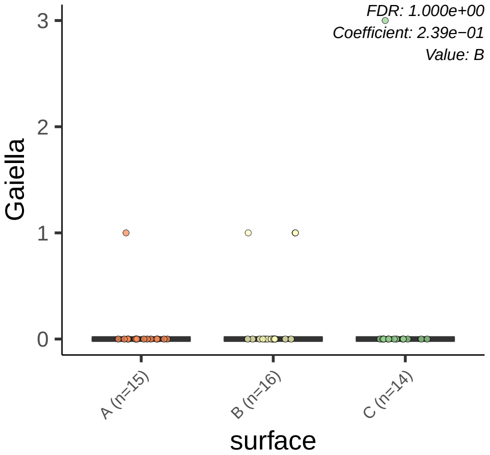

FDR: 1.000e+00
Coefficient: 2.39e−01
Value: B
Gaiella
3
2
1
0
A (n=15)
B (n=16)
C (n=14)
surface

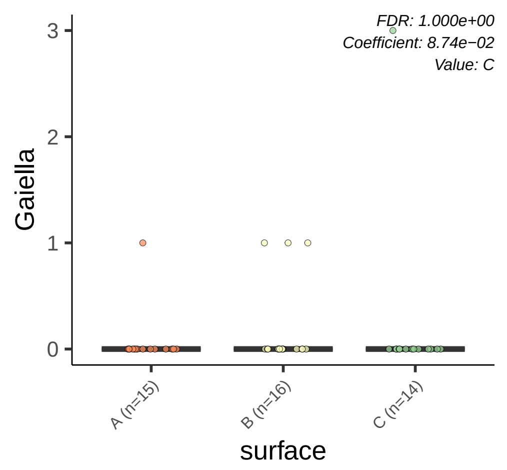
FDR: 1.000e+00
Coefficient: 8.74e−02
Value: C
Gaiella
3
2
1
0
A (n=15)
B (n=16)
C (n=14)
surface

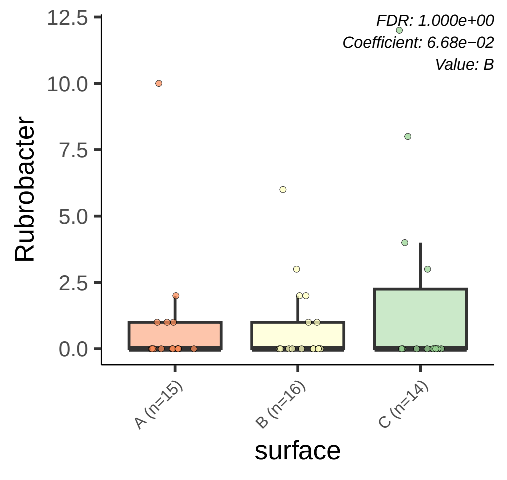
FDR: 1.000e+00
Coefficient: 6.68e−02
Value: B
Rubrobacter
12.5
10.0
7.5
5.0
2.5
0.0
A (n=15)
B (n=16)
C (n=14)
surface

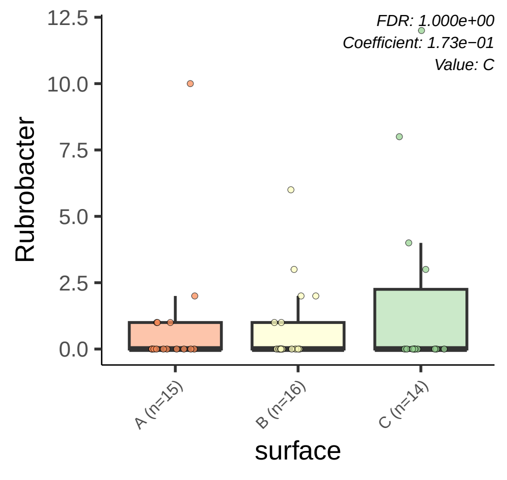
FDR: 1.000e+00
Coefficient: 1.73e−01
Value: C
Rubrobacter
12.5
10.0
7.5
5.0
2.5
0.0
A (n=15)
B (n=16)
C (n=14)
surface

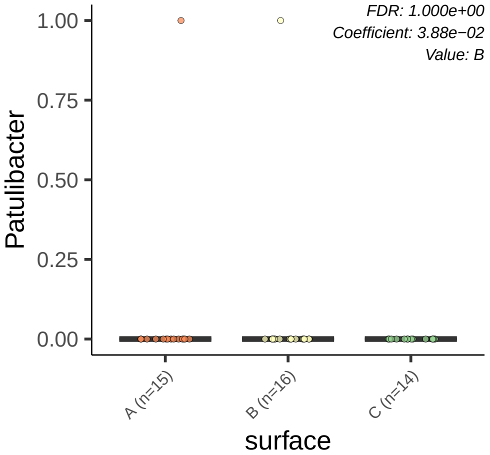

FDR: 1.000e+00
Coefficient: 3.88e−02
Value: B
Patulibacter
1.00
0.75
0.50
0.25
0.00
A (n=15)
B (n=16)
C (n=14)
surface

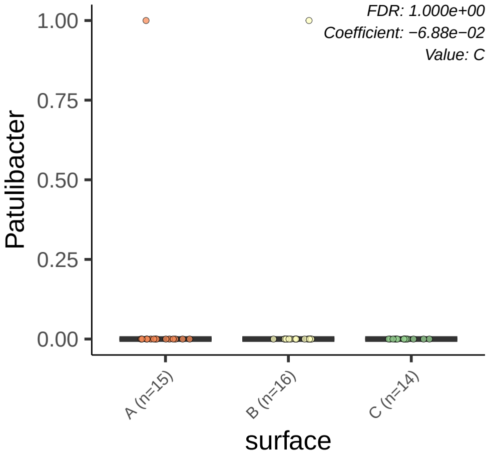

FDR: 1.000e+00
Coefficient: −6.88e−02
Value: C
1.00
0.75
0.50
0.25
0.00
Patulibacter
A (n=15)
B (n=16)
C (n=14)
surface

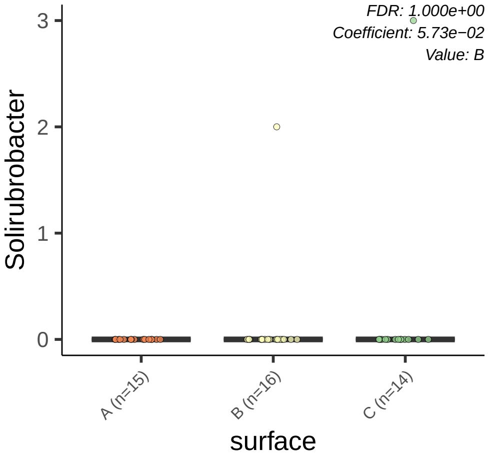

FDR: 1.000e+00
Coefficient: 5.73e−02
Value: B
Solirubrobacter
3
2
1
0
A (n=15)
B (n=16)
C (n=14)
surface

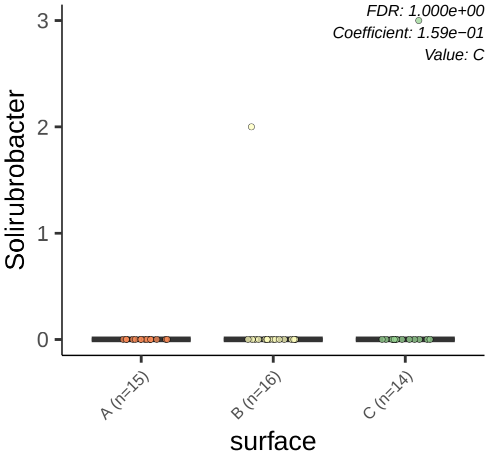

FDR: 1.000e+00
Coefficient: 1.59e−01
Value: C
Solirubrobacter
0
1
2
3
A (n=15)
B (n=16)
C (n=14)
surface

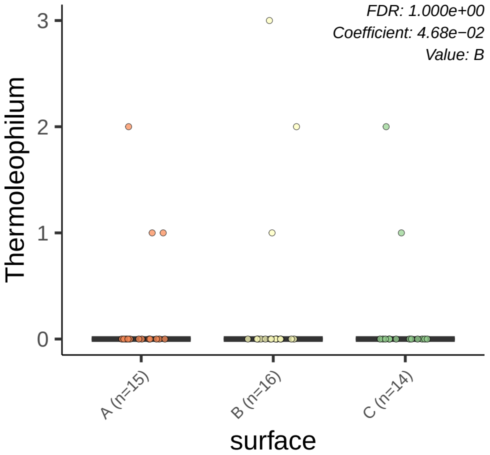
FDR: 1.000e+00
Coefficient: 4.68e−02
Value: B
Thermoleophilum
3
2
1
0
A (n=15)
B (n=16)
C (n=14)
surface

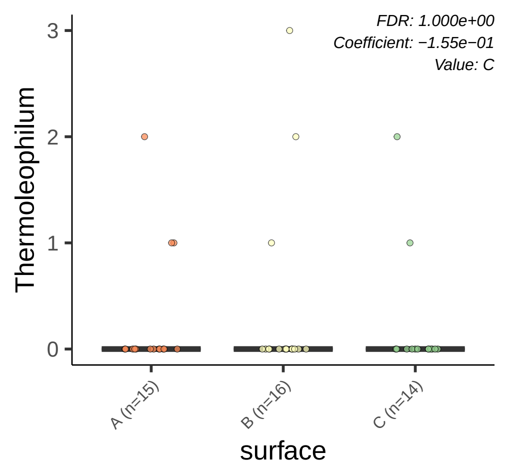
FDR: 1.000e+00
Coefficient: −1.55e−01
Value: C
Thermoleophilum
3
2
1
0
A (n=15)
B (n=16)
C (n=14)
surface

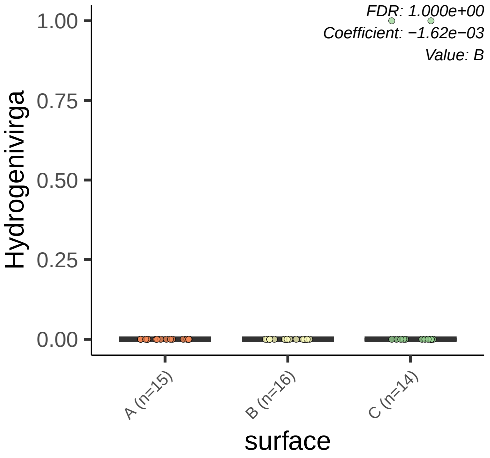
FDR: 1.000e+00
Coefficient: −1.62e−03
Value: B
Hydrogenivirga
1.00
0.75
0.50
0.25
0.00
A (n=15)
B (n=16)
C (n=14)
surface

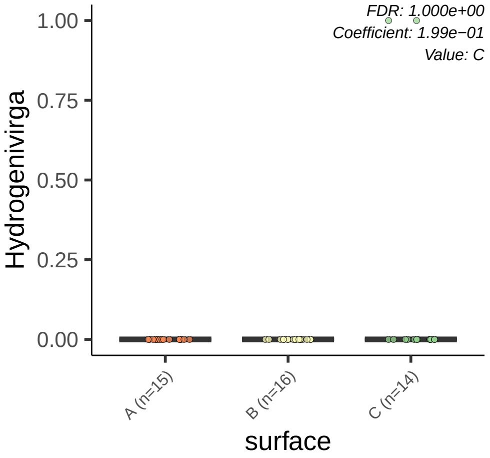

FDR: 1.000e+00
Coefficient: 1.99e−01
Value: C
Hydrogenivirga
1.00
0.75
0.50
0.25
0.00
A (n=15)
B (n=16)
C (n=14)
surface

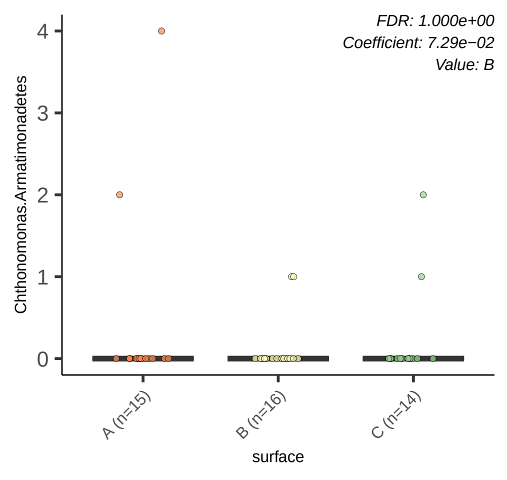

FDR: 1.000e+00
Coefficient: 7.29e−02
Value: B
Chthonomonas.Armatimonadetes
4
3
2
1
0
A (n=15)
B (n=16)
C (n=14)
surface

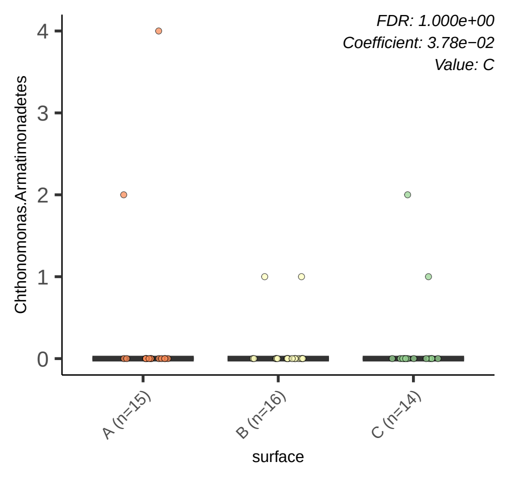
FDR: 1.000e+00
Coefficient: 3.78e−02
Value: C
Chthonomonas.Armatimonadetes
4
3
2
1
0
A (n=15)
B (n=16)
C (n=14)
surface

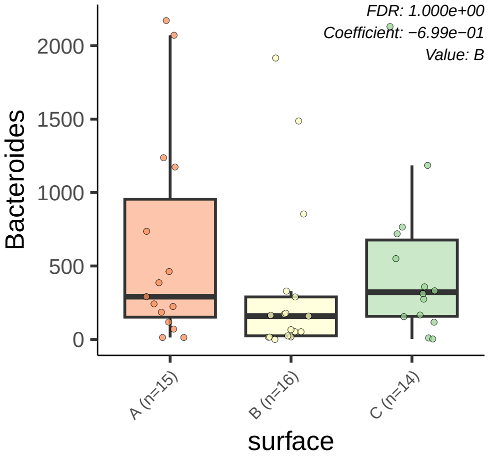
FDR: 1.000e+00
Coefficient: −6.99e−01
Value: B
Bacteroides
2000
1500
1000
500
0
A (n=15)
B (n=16)
C (n=14)
surface

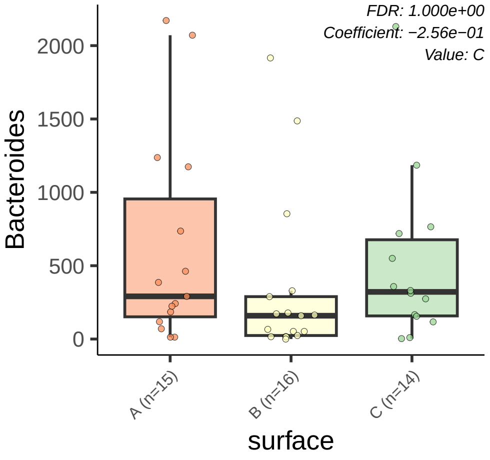
FDR: 1.000e+00
Coefficient: −2.56e−01
Value: C
Bacteroides
2000
1500
1000
500
0
A (n=15)
B (n=16)
C (n=14)
surface

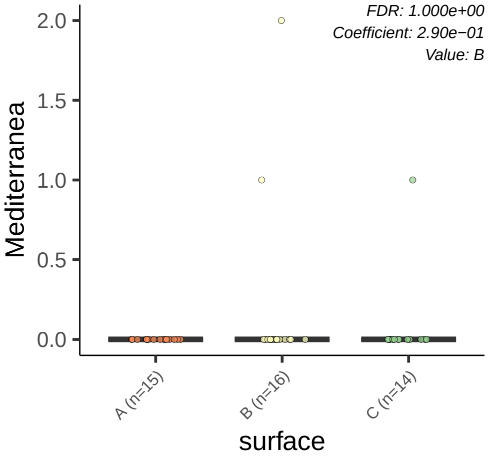

FDR: 1.000e+00
Coefficient: 2.90e−01
Value: B
Mediterranea
2.0
1.5
1.0
0.5
0.0
A (n=15)
B (n=16)
C (n=14)
surface

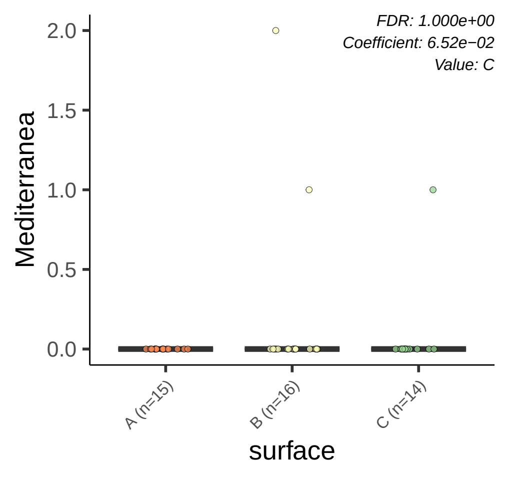
FDR: 1.000e+00
Coefficient: 6.52e−02
Value: C
Mediterranea
2.0
1.5
1.0
0.5
0.0
A (n=15)
B (n=16)
C (n=14)
surface

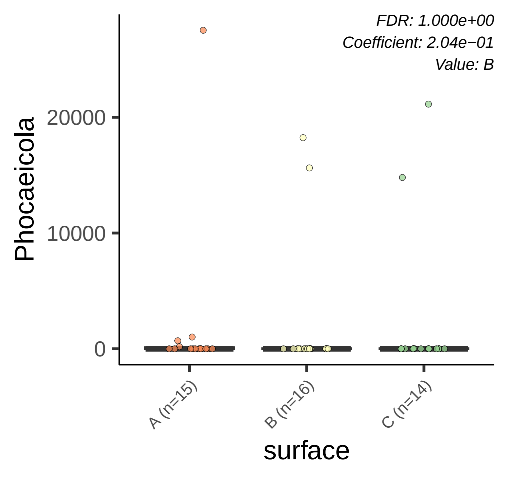

FDR: 1.000e+00
Coefficient: 2.04e−01
Value: B
Phocaeicola
20000
10000
0
A (n=15)
B (n=16)
C (n=14)
surface

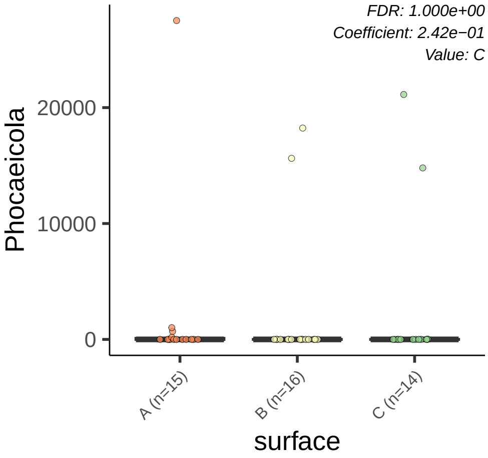
FDR: 1.000e+00
Coefficient: 2.42e−01
Value: C
Phocaeicola
20000
10000
0
A (n=15)
B (n=16)
C (n=14)
surface

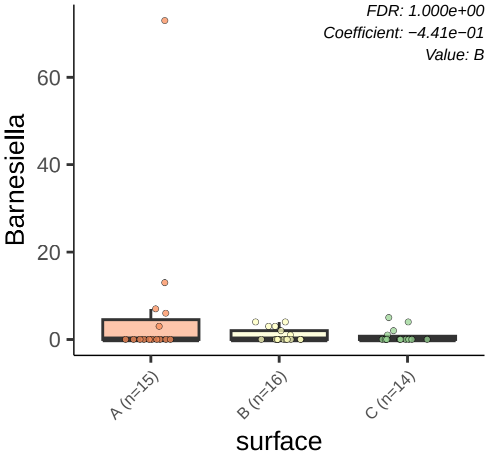
FDR: 1.000e+00
Coefficient: −4.41e−01
Value: B
Barnesiella
0
20
40
60
A (n=15)
B (n=16)
C (n=14)
surface

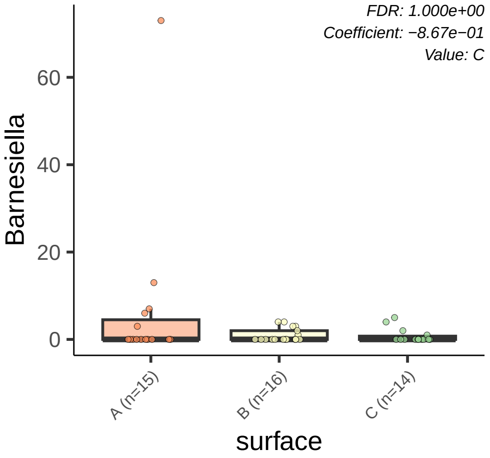
FDR: 1.000e+00
Coefficient: −8.67e−01
Value: C
Barnesiella
60
40
20
0
A (n=15)
B (n=16)
C (n=14)
surface

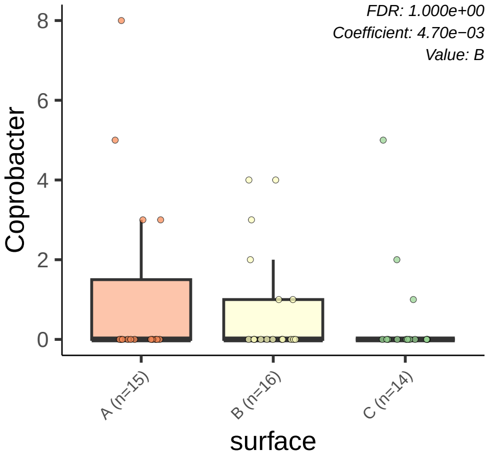
FDR: 1.000e+00
Coefficient: 4.70e−03
Value: B
Coprobacter
0
2
4
6
8
A (n=15)
B (n=16)
C (n=14)
surface

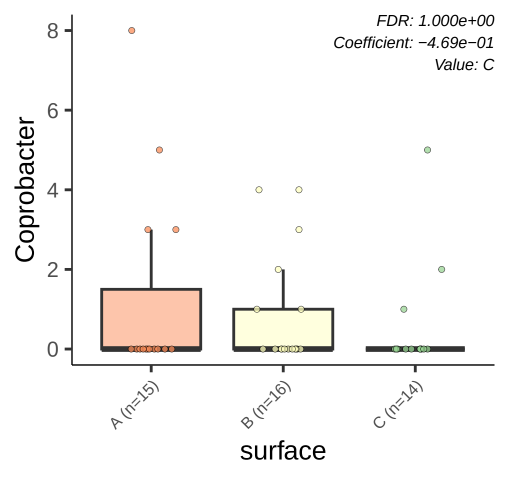

FDR: 1.000e+00
Coefficient: −4.69e−01
Value: C
Coprobacter
0
2
4
6
8
A (n=15)
B (n=16)
C (n=14)
surface

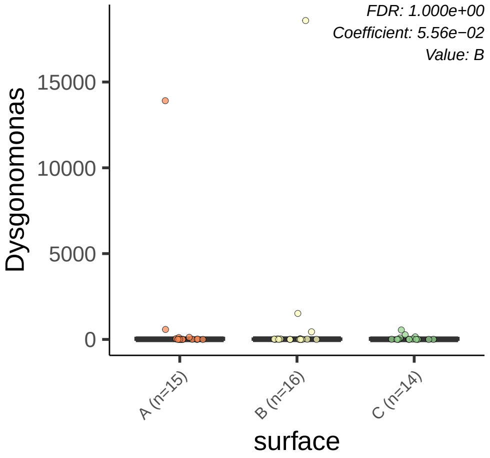
FDR: 1.000e+00
Coefficient: 5.56e−02
Value: B
Dysgonomonas
15000
10000
5000
0
A (n=15)
B (n=16)
C (n=14)
surface

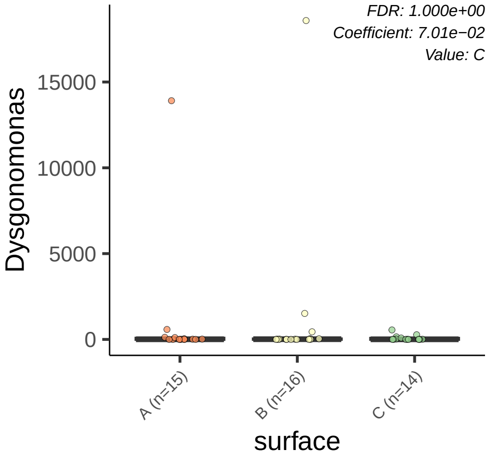
FDR: 1.000e+00
Coefficient: 7.01e−02
Value: C
Dysgonomonas
15000
10000
5000
0
A (n=15)
B (n=16)
C (n=14)
surface

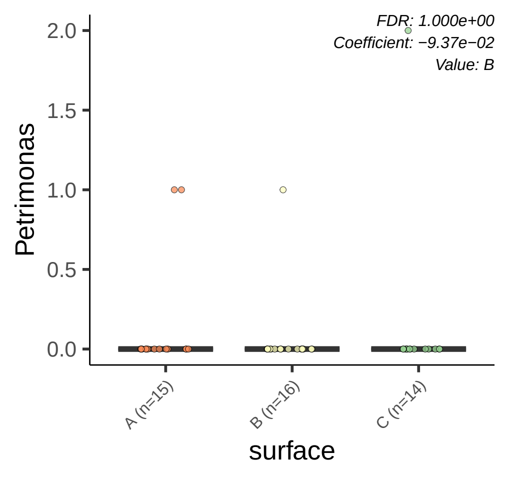

FDR: 1.000e+00
Coefficient: −9.37e−02
Value: B
Petrimonas
2.0
1.5
1.0
0.5
0.0
A (n=15)
B (n=16)
C (n=14)
surface

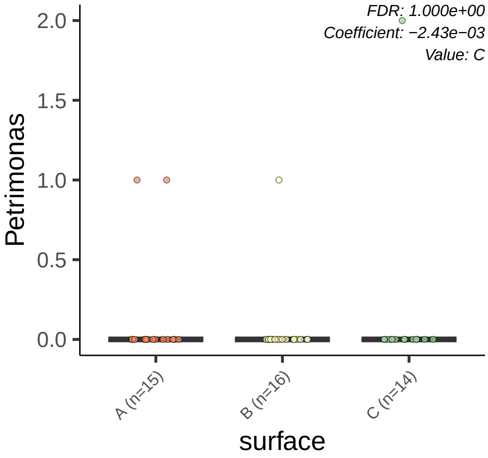

FDR: 1.000e+00
Coefficient: −2.43e−03
Value: C
Petrimonas
2.0
1.5
1.0
0.5
0.0
A (n=15)
B (n=16)
C (n=14)
surface

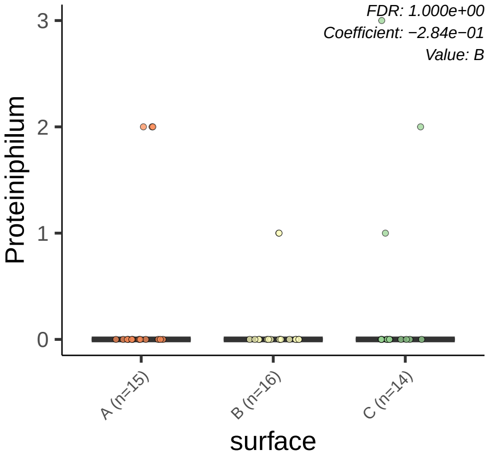
FDR: 1.000e+00
Coefficient: −2.84e−01
Value: B
Proteiniphilum
3
2
1
0
A (n=15)
B (n=16)
C (n=14)
surface

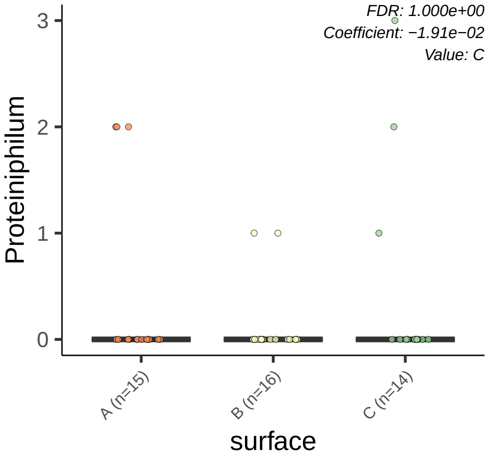
FDR: 1.000e+00
Coefficient: −1.91e−02
Value: C
Proteiniphilum
3
2
1
0
A (n=15)
B (n=16)
C (n=14)
surface

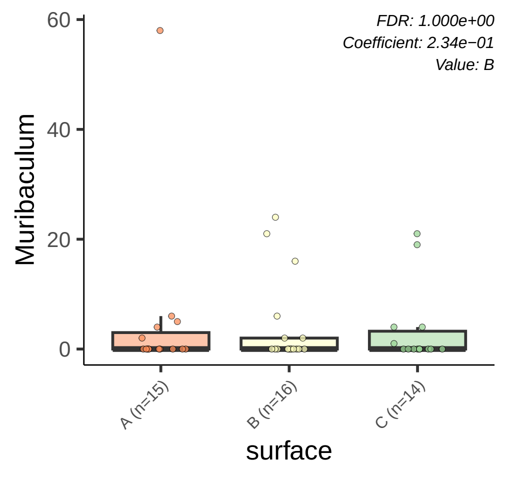
FDR: 1.000e+00
Coefficient: 2.34e−01
Value: B
Muribaculum
60
40
20
0
A (n=15)
B (n=16)
C (n=14)
surface

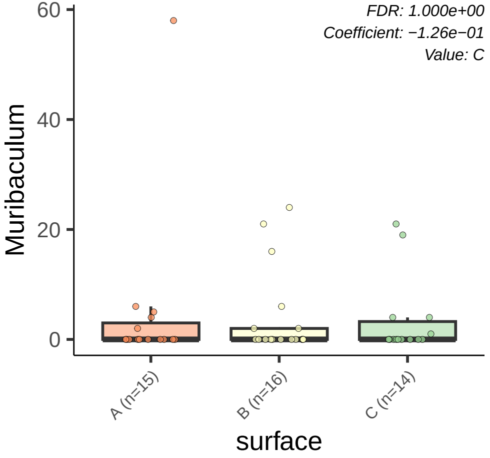

FDR: 1.000e+00
Coefficient: −1.26e−01
Value: C
Muribaculum
60
40
20
0
A (n=15)
B (n=16)
C (n=14)
surface

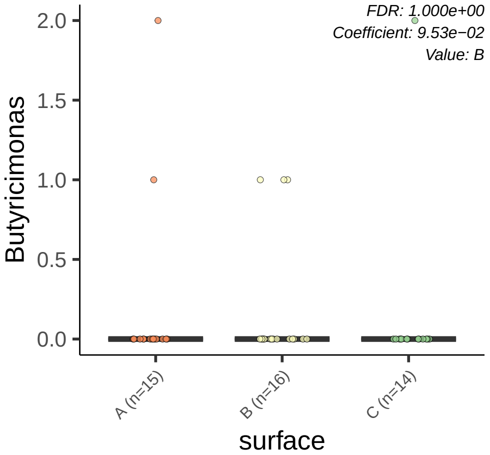

FDR: 1.000e+00
Coefficient: 9.53e−02
Value: B
Butyricimonas
2.0
1.5
1.0
0.5
0.0
A (n=15)
B (n=16)
C (n=14)
surface

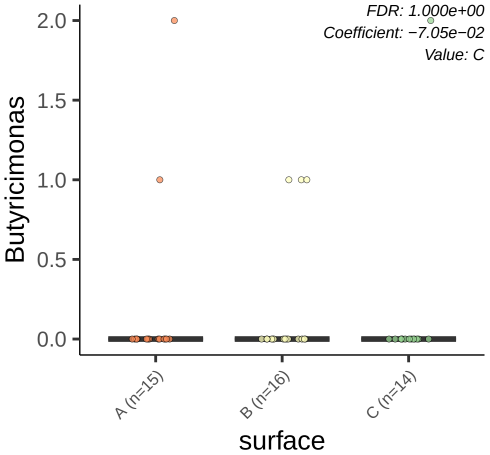
FDR: 1.000e+00
Coefficient: −7.05e−02
Value: C
Butyricimonas
2.0
1.5
1.0
0.5
0.0
A (n=15)
B (n=16)
C (n=14)
surface

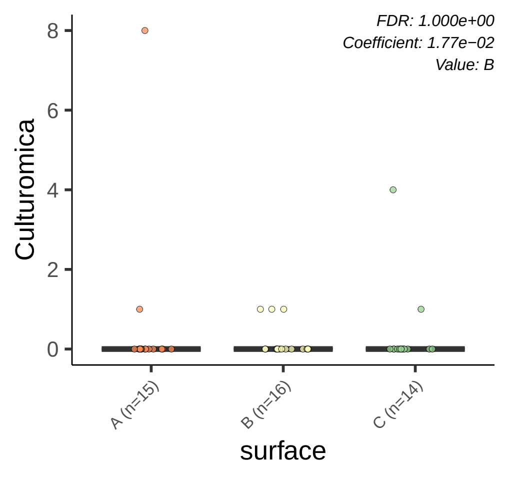
FDR: 1.000e+00
Coefficient: 1.77e−02
Value: B
Culturomica
0
2
4
6
8
A (n=15)
B (n=16)
C (n=14)
surface

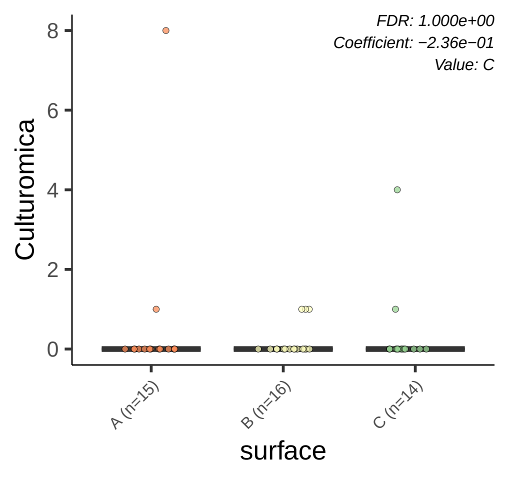

FDR: 1.000e+00
Coefficient: −2.36e−01
Value: C
Culturomica
0
2
4
6
8
A (n=15)
B (n=16)
C (n=14)
surface

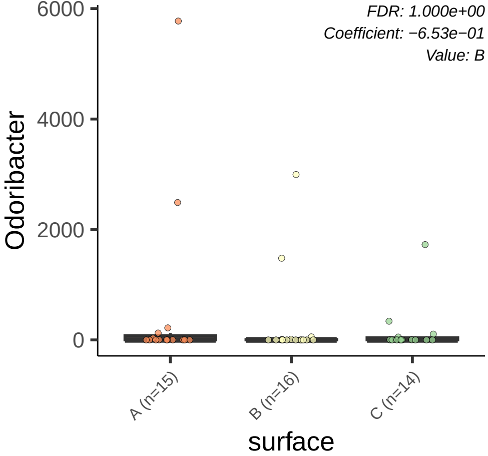

FDR: 1.000e+00
Coefficient: −6.53e−01
Value: B
Odoribacter
6000
4000
2000
0
A (n=15)
B (n=16)
C (n=14)
surface

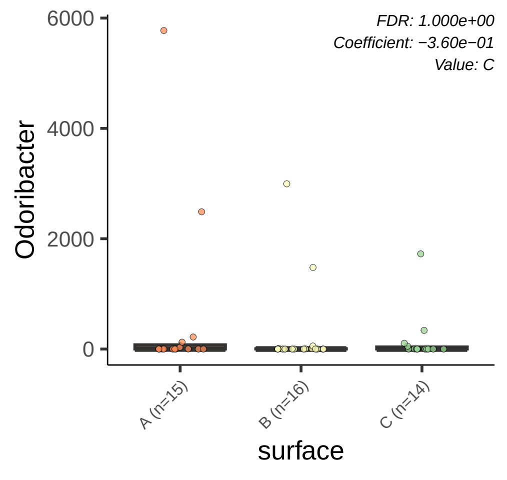
FDR: 1.000e+00
Coefficient: −3.60e−01
Value: C
6000
4000
2000
0
Odoribacter
A (n=15)
B (n=16)
C (n=14)
surface

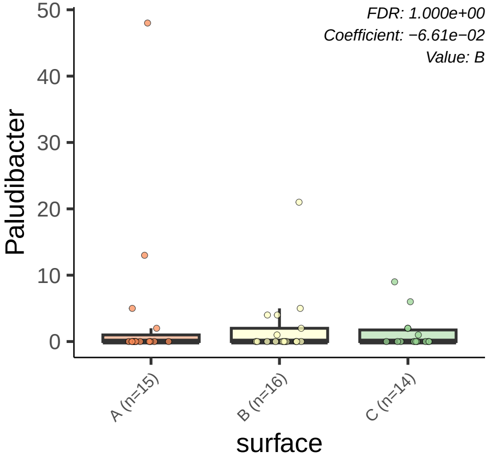
FDR: 1.000e+00
Coefficient: −6.61e−02
Value: B
Paludibacter
50
40
30
20
10
0
A (n=15)
B (n=16)
C (n=14)
surface

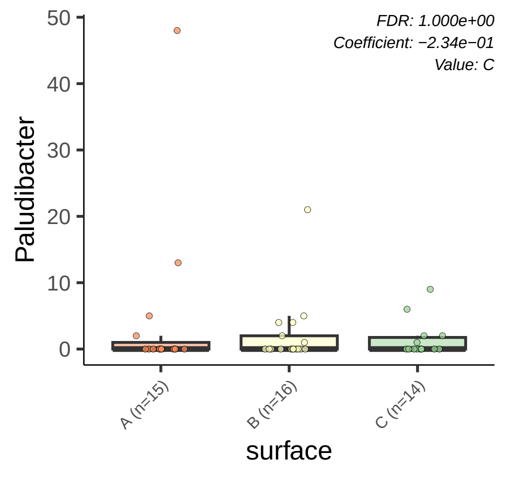

FDR: 1.000e+00
Coefficient: −2.34e−01
Value: C
Paludibacter
50
40
30
20
10
0
A (n=15)
B (n=16)
C (n=14)
surface

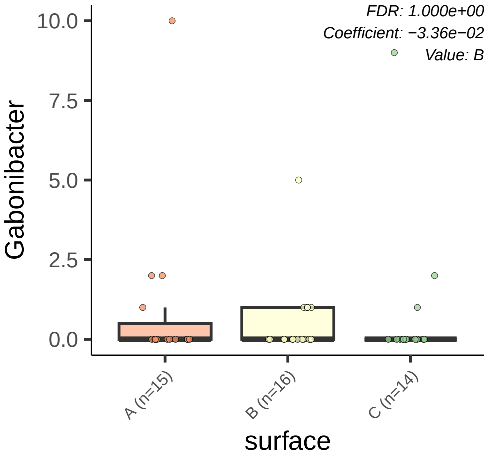

FDR: 1.000e+00
Coefficient: −3.36e−02
Value: B
Gabonibacter
10.0
7.5
5.0
2.5
0.0
A (n=15)
B (n=16)
C (n=14)
surface

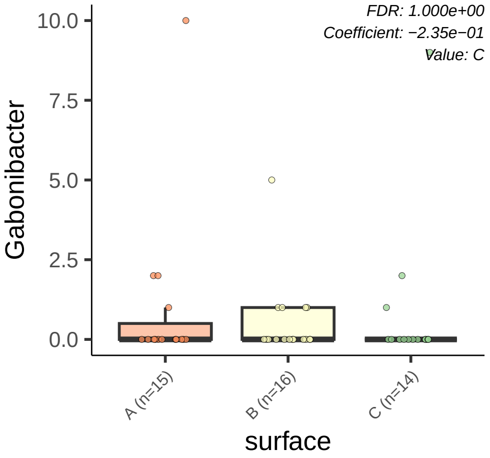
FDR: 1.000e+00
Coefficient: −2.35e−01
Value: C
Gabonibacter
10.0
7.5
5.0
2.5
0.0
A (n=15)
B (n=16)
C (n=14)
surface

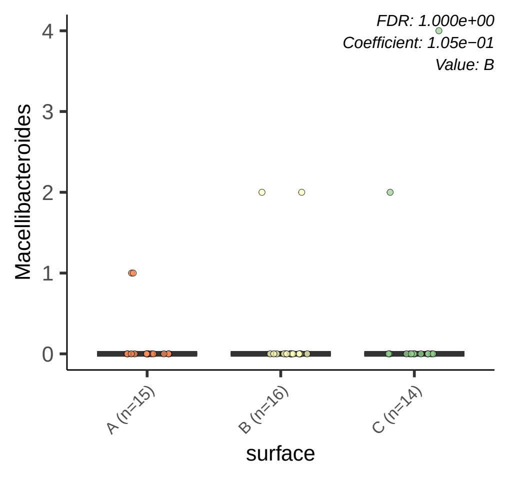
FDR: 1.000e+00
Coefficient: 1.05e−01
Value: B
Macellibacteroides
0
1
2
3
4
A (n=15)
B (n=16)
C (n=14)
surface

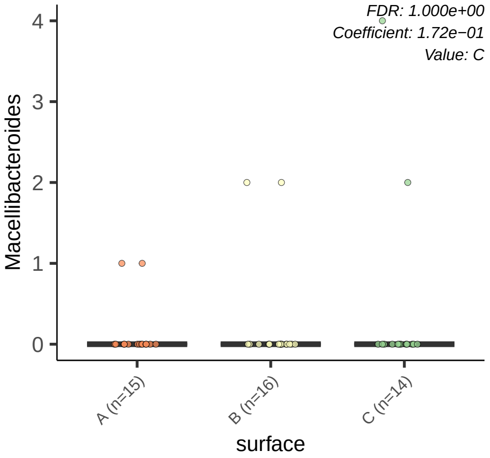
FDR: 1.000e+00
Coefficient: 1.72e−01
Value: C
Macellibacteroides
0
1
2
3
4
A (n=15)
B (n=16)
C (n=14)
surface

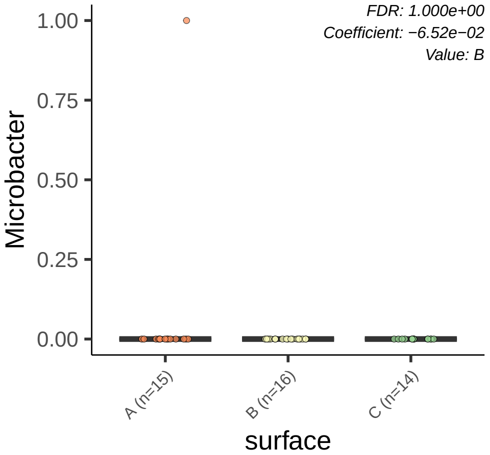

FDR: 1.000e+00
Coefficient: −6.52e−02
Value: B
Microbacter
1.00
0.75
0.50
0.25
0.00
A (n=15)
B (n=16)
C (n=14)
surface

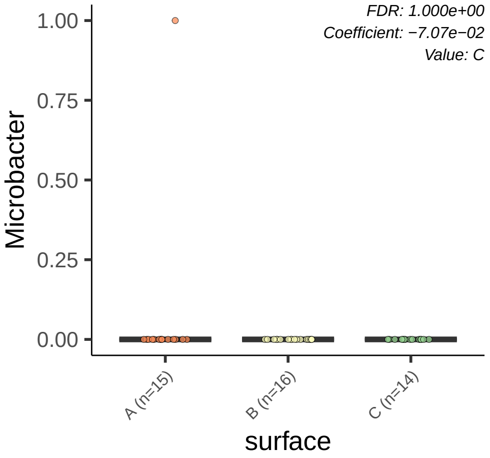
FDR: 1.000e+00
Coefficient: −7.07e−02
Value: C
Microbacter
1.00
0.75
0.50
0.25
0.00
A (n=15)
B (n=16)
C (n=14)
surface

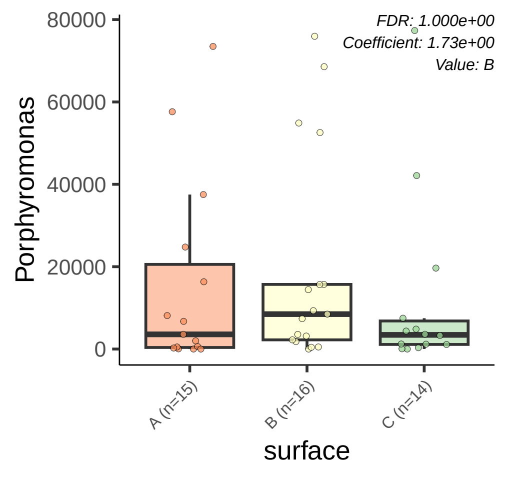

FDR: 1.000e+00
Coefficient: 1.73e+00
Value: B
Porphyromonas
80000
60000
40000
20000
0
A (n=15)
B (n=16)
C (n=14)
surface

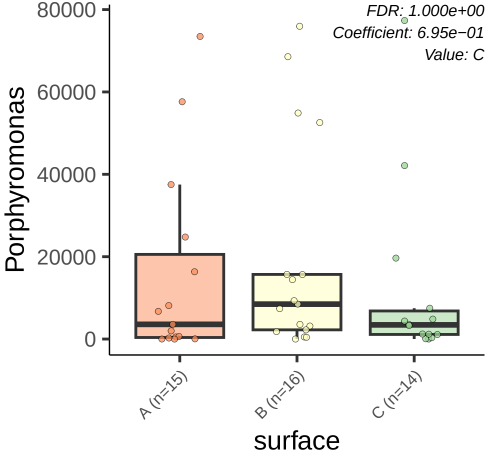

FDR: 1.000e+00
Coefficient: 6.95e−01
Value: C
80000
60000
40000
20000
0
Porphyromonas
A (n=15)
B (n=16)
C (n=14)
surface

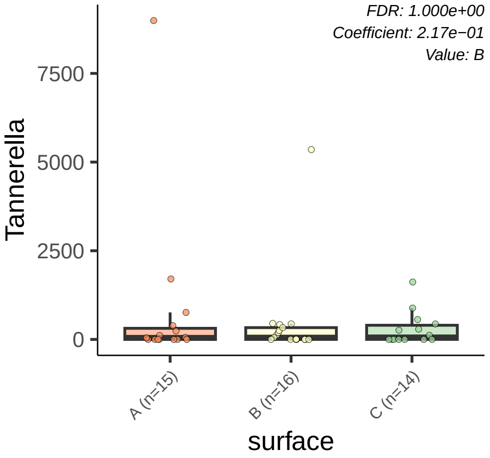
FDR: 1.000e+00
Coefficient: 2.17e−01
Value: B
Tannerella
7500
5000
2500
0
A (n=15)
B (n=16)
C (n=14)
surface

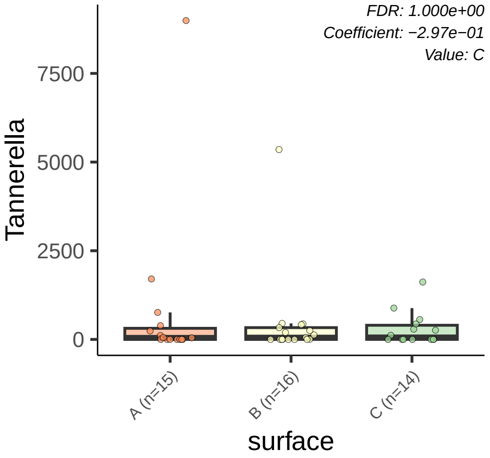
FDR: 1.000e+00
Coefficient: −2.97e−01
Value: C
Tannerella
7500
5000
2500
0
A (n=15)
B (n=16)
C (n=14)
surface

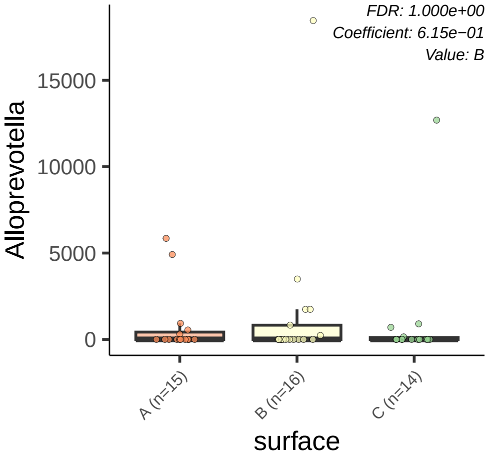
FDR: 1.000e+00
Coefficient: 6.15e−01
Value: B
Alloprevotella
15000
10000
5000
0
A (n=15)
B (n=16)
C (n=14)
surface

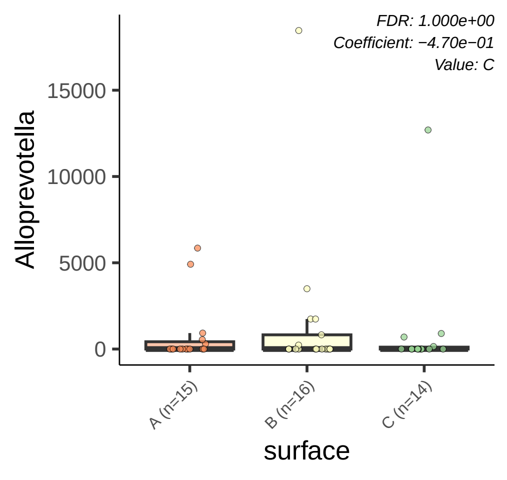

FDR: 1.000e+00
Coefficient: −4.70e−01
Value: C
Alloprevotella
15000
10000
5000
0
A (n=15)
B (n=16)
C (n=14)
surface

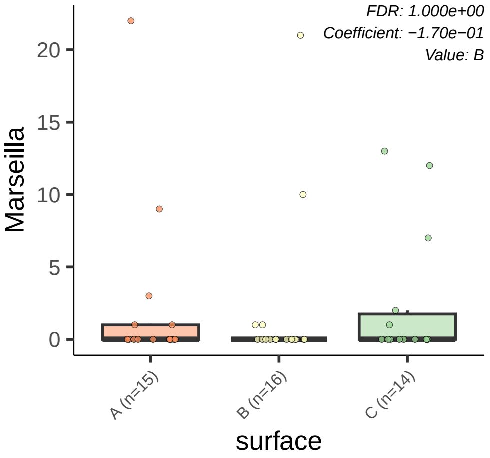

FDR: 1.000e+00
Coefficient: −1.70e−01
Value: B
Marseilla
20
15
10
5
0
A (n=15)
B (n=16)
C (n=14)
surface

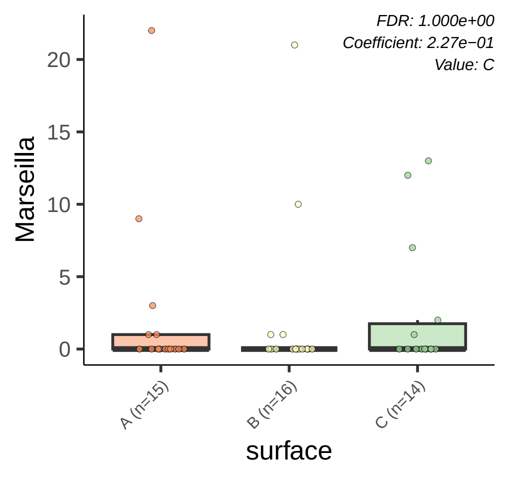
FDR: 1.000e+00
Coefficient: 2.27e−01
Value: C
Marseilla
0
5
10
15
20
A (n=15)
B (n=16)
C (n=14)
surface

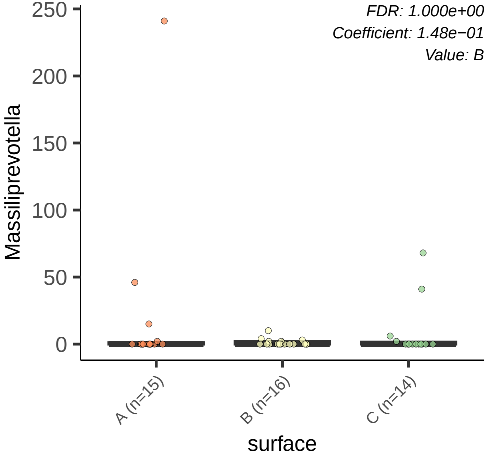
FDR: 1.000e+00
Coefficient: 1.48e−01
Value: B
Massiliprevotella
250
200
150
100
50
0
A (n=15)
B (n=16)
C (n=14)
surface

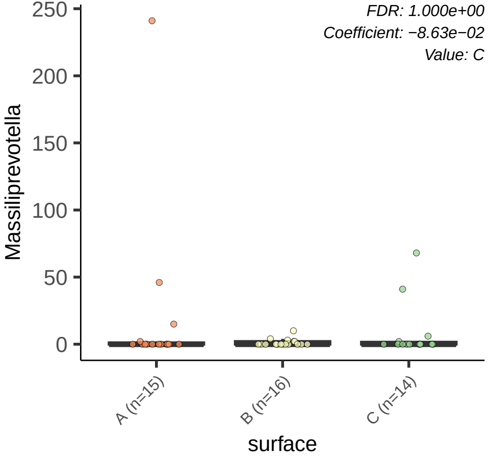

FDR: 1.000e+00
Coefficient: −8.63e−02
Value: C
Massiliprevotella
250
200
150
100
50
0
A (n=15)
B (n=16)
C (n=14)
surface

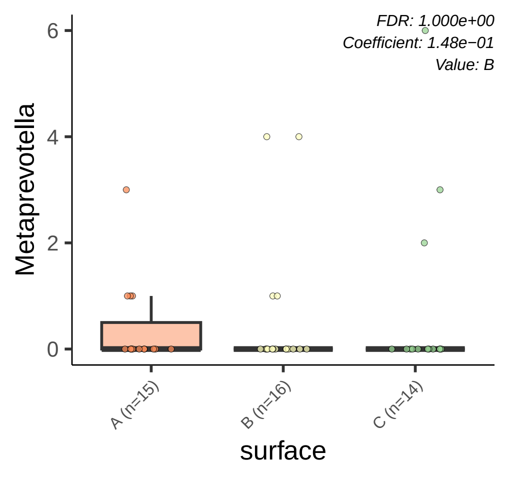

FDR: 1.000e+00
Coefficient: 1.48e−01
Value: B
Metaprevotella
0
2
4
6
A (n=15)
B (n=16)
C (n=14)
surface

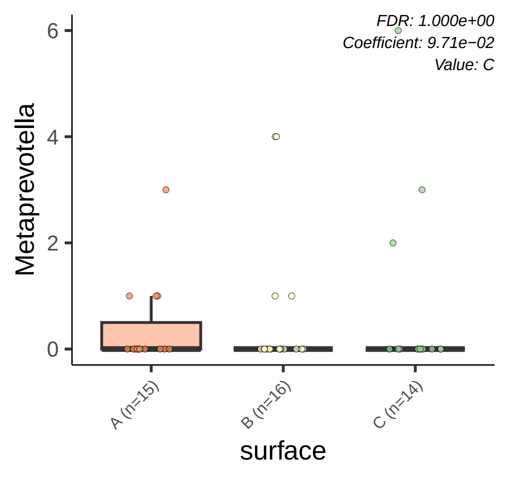

FDR: 1.000e+00
Coefficient: 9.71e−02
Value: C
Metaprevotella
0
2
4
6
A (n=15)
B (n=16)
C (n=14)
surface

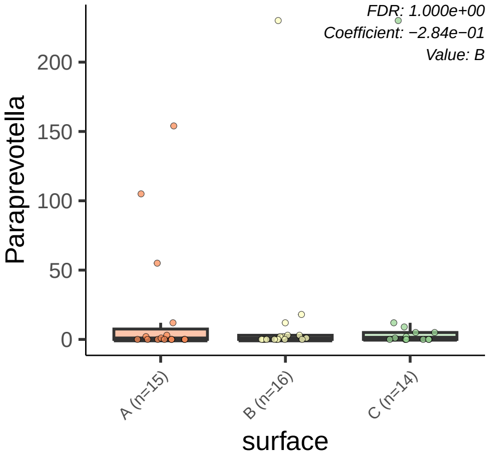
FDR: 1.000e+00
Coefficient: −2.84e−01
Value: B
Paraprevotella
200
150
100
50
0
A (n=15)
B (n=16)
C (n=14)
surface

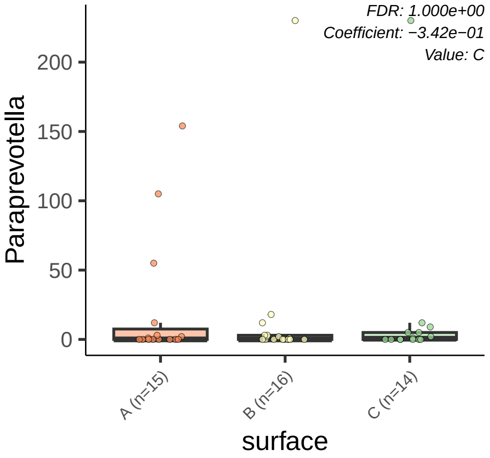
FDR: 1.000e+00
Coefficient: −3.42e−01
Value: C
Paraprevotella
0
50
100
150
200
A (n=15)
B (n=16)
C (n=14)
surface

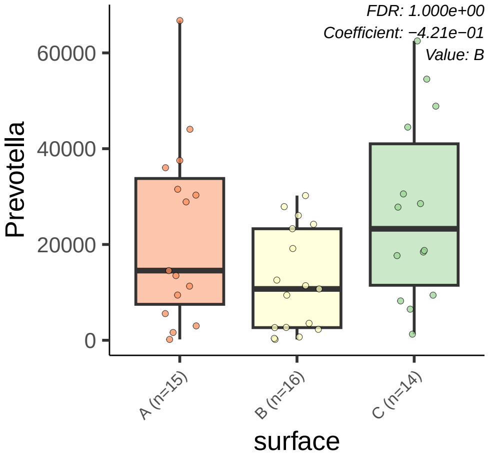

FDR: 1.000e+00
Coefficient: −4.21e−01
Value: B
Prevotella
60000
40000
20000
0
A (n=15)
B (n=16)
C (n=14)
surface

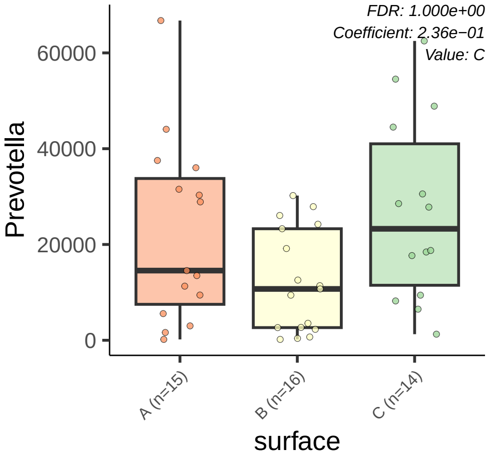

FDR: 1.000e+00
Coefficient: 2.36e−01
Value: C
Prevotella
60000
40000
20000
0
A (n=15)
B (n=16)
C (n=14)
surface

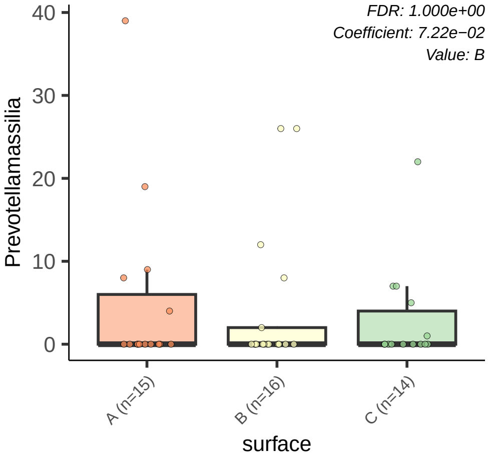

FDR: 1.000e+00
Coefficient: 7.22e−02
Value: B
Prevotellamassilia
40
30
20
10
0
A (n=15)
B (n=16)
C (n=14)
surface

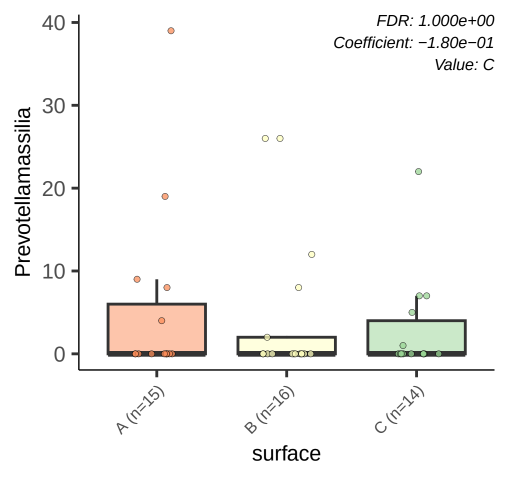
FDR: 1.000e+00
Coefficient: −1.80e−01
Value: C
Prevotellamassilia
0
10
20
30
40
A (n=15)
B (n=16)
C (n=14)
surface

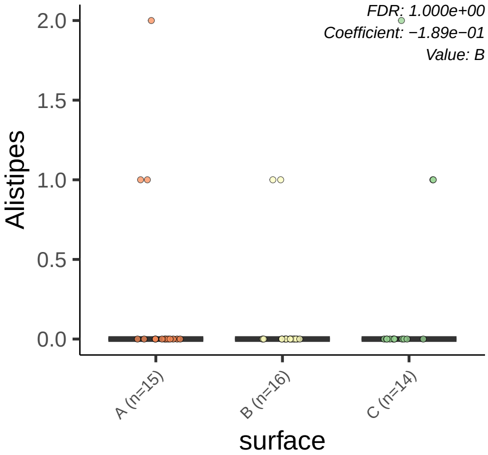
FDR: 1.000e+00
Coefficient: −1.89e−01
Value: B
Alistipes
2.0
1.5
1.0
0.5
0.0
A (n=15)
B (n=16)
C (n=14)
surface

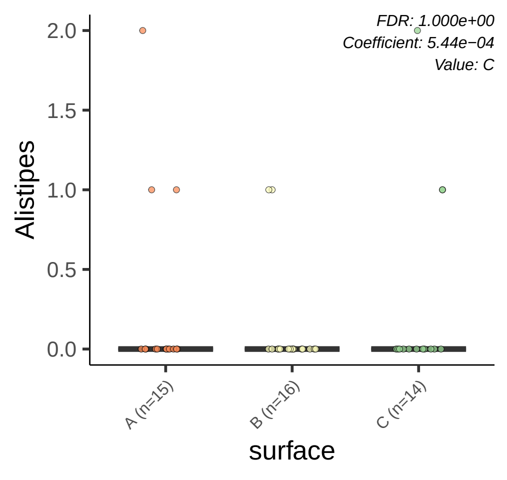
FDR: 1.000e+00
Coefficient: 5.44e−04
Value: C
Alistipes
2.0
1.5
1.0
0.5
0.0
A (n=15)
B (n=16)
C (n=14)
surface

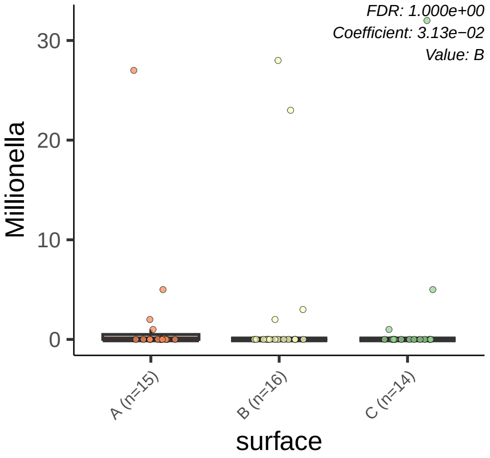

FDR: 1.000e+00
Coefficient: 3.13e−02
Value: B
Millionella
30
20
10
0
A (n=15)
B (n=16)
C (n=14)
surface

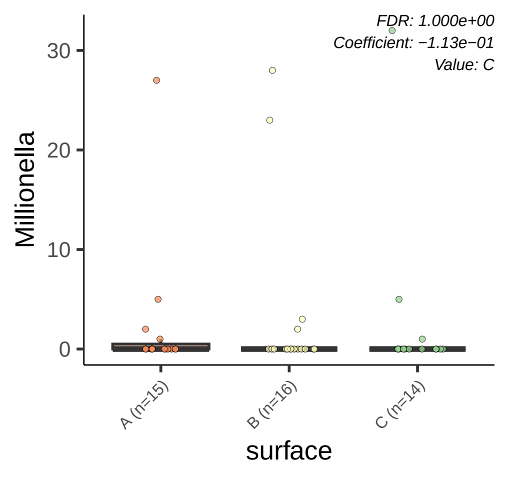

FDR: 1.000e+00
Coefficient: −1.13e−01
Value: C
Millionella
30
20
10
0
A (n=15)
B (n=16)
C (n=14)
surface

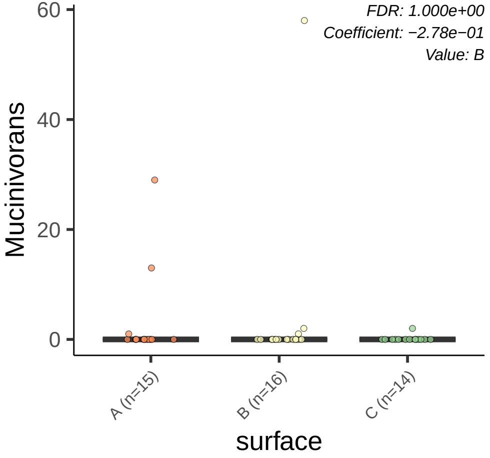

FDR: 1.000e+00
Coefficient: −2.78e−01
Value: B
Mucinivorans
60
40
20
0
A (n=15)
B (n=16)
C (n=14)
surface

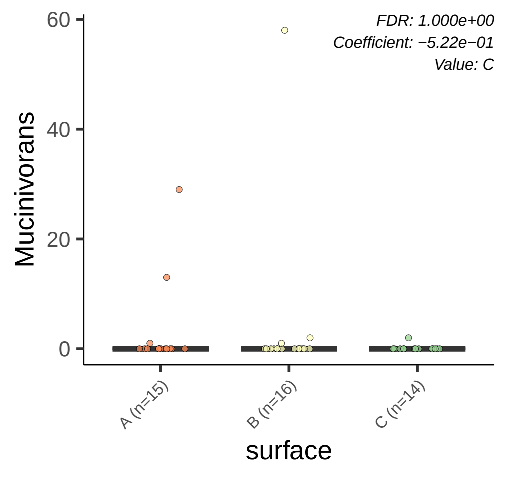
FDR: 1.000e+00
Coefficient: −5.22e−01
Value: C
Mucinivorans
0
20
40
60
A (n=15)
B (n=16)
C (n=14)
surface

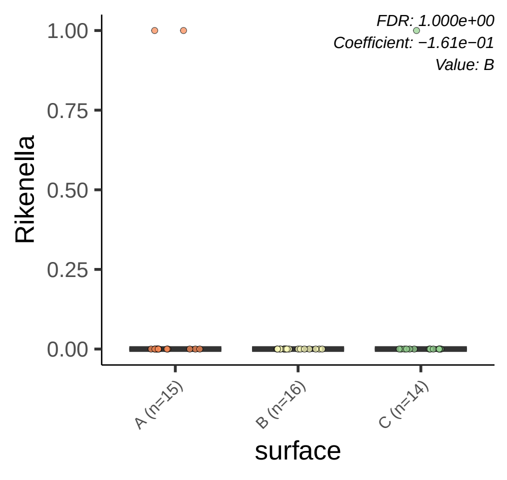

FDR: 1.000e+00
Coefficient: −1.61e−01
Value: B
Rikenella
1.00
0.75
0.50
0.25
0.00
A (n=15)
B (n=16)
C (n=14)
surface

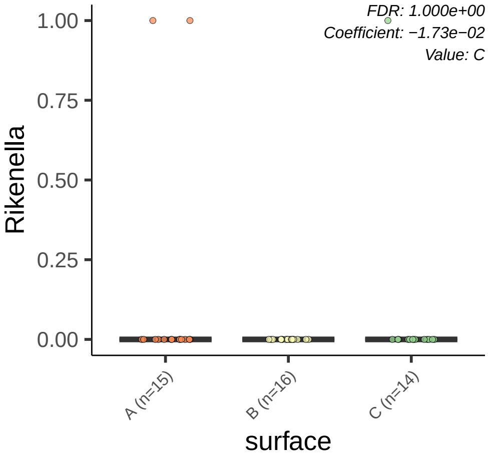

FDR: 1.000e+00
Coefficient: −1.73e−02
Value: C
1.00
0.75
0.50
0.25
0.00
Rikenella
A (n=15)
B (n=16)
C (n=14)
surface

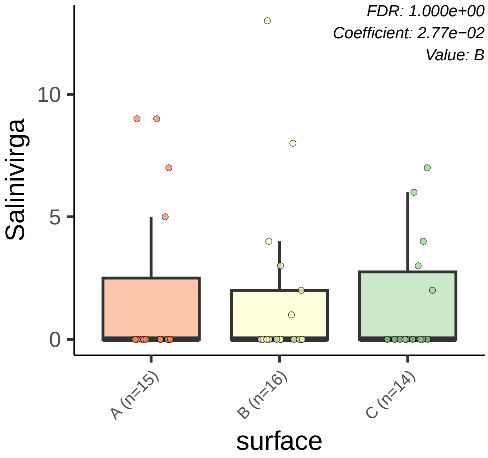
FDR: 1.000e+00
Coefficient: 2.77e−02
Value: B
Salinivirga
0
5
10
A (n=15)
B (n=16)
C (n=14)
surface

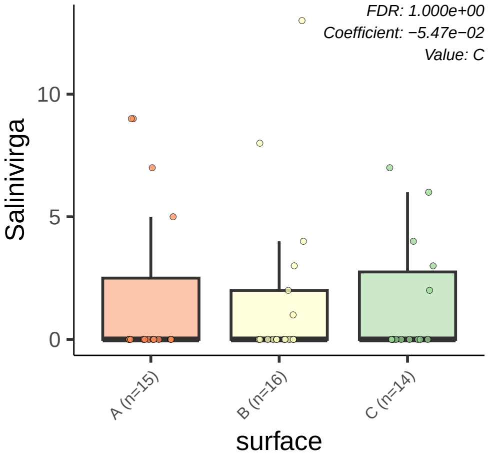

FDR: 1.000e+00
Coefficient: −5.47e−02
Value: C
Salinivirga
10
5
0
A (n=15)
B (n=16)
C (n=14)
surface

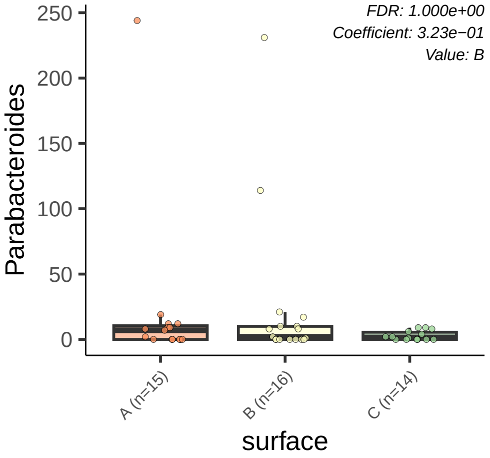

FDR: 1.000e+00
Coefficient: 3.23e−01
Value: B
Parabacteroides
250
200
150
100
50
0
A (n=15)
B (n=16)
C (n=14)
surface

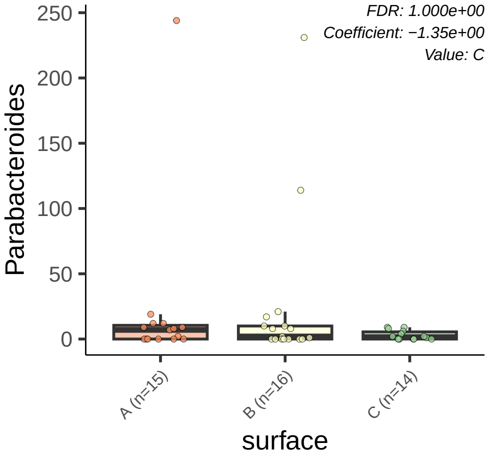

FDR: 1.000e+00
Coefficient: −1.35e+00
Value: C
Parabacteroides
250
200
150
100
50
0
A (n=15)
B (n=16)
C (n=14)
surface

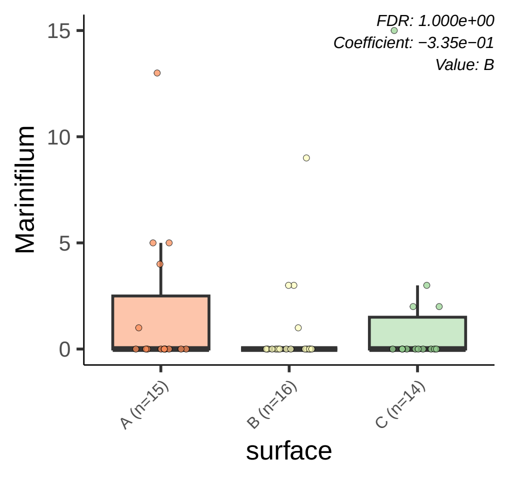
FDR: 1.000e+00
Coefficient: −3.35e−01
Value: B
Marinifilum
15
10
5
0
A (n=15)
B (n=16)
C (n=14)
surface

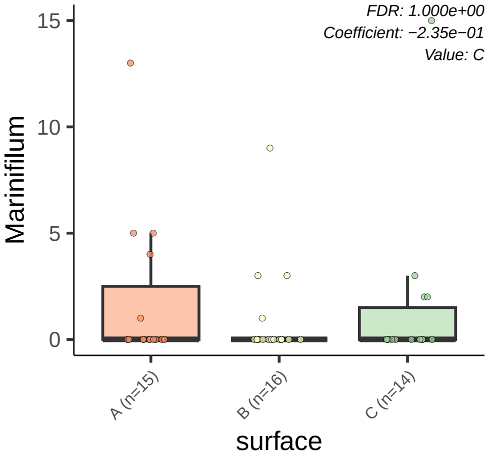
FDR: 1.000e+00
Coefficient: −2.35e−01
Value: C
Marinifilum
15
10
5
0
A (n=15)
B (n=16)
C (n=14)
surface

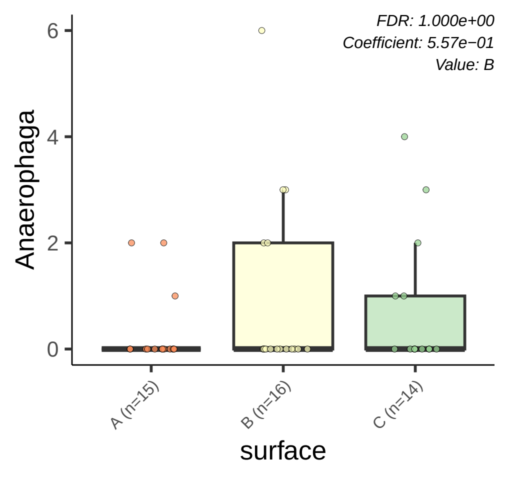

FDR: 1.000e+00
Coefficient: 5.57e−01
Value: B
Anaerophaga
6
4
2
0
A (n=15)
B (n=16)
C (n=14)
surface

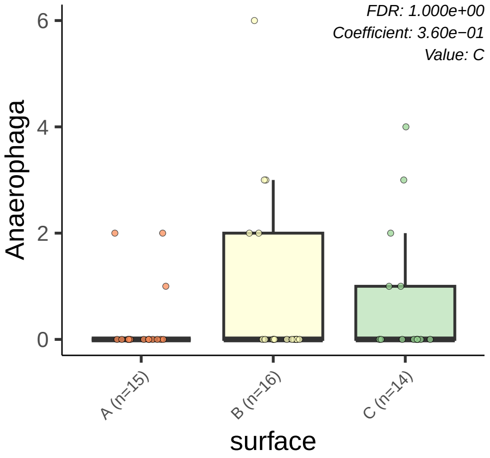
FDR: 1.000e+00
Coefficient: 3.60e−01
Value: C
Anaerophaga
0
2
4
6
A (n=15)
B (n=16)
C (n=14)
surface

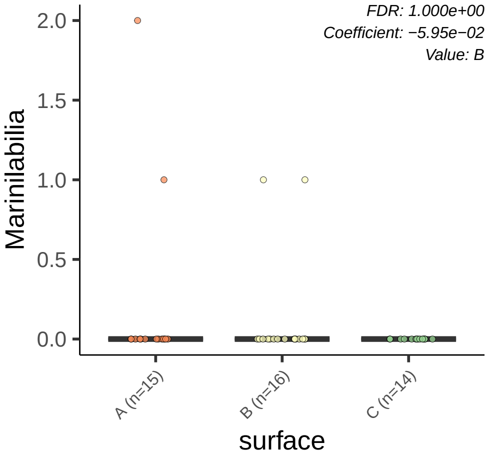
FDR: 1.000e+00
Coefficient: −5.95e−02
Value: B
Marinilabilia
2.0
1.5
1.0
0.5
0.0
A (n=15)
B (n=16)
C (n=14)
surface

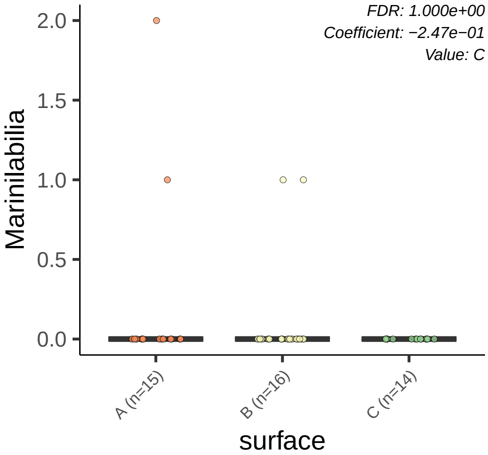

FDR: 1.000e+00
Coefficient: −2.47e−01
Value: C
Marinilabilia
2.0
1.5
1.0
0.5
0.0
A (n=15)
B (n=16)
C (n=14)
surface

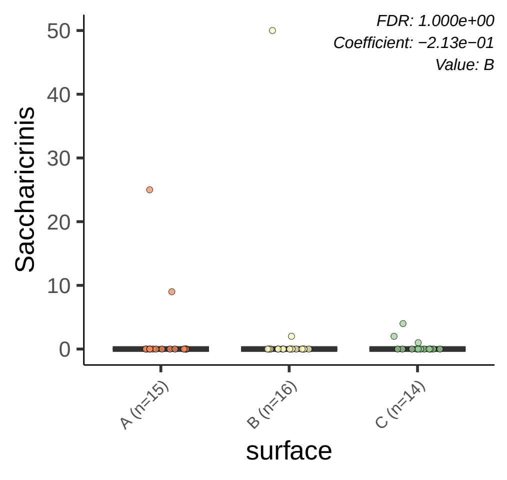

FDR: 1.000e+00
Coefficient: −2.13e−01
Value: B
Saccharicrinis
50
40
30
20
10
0
A (n=15)
B (n=16)
C (n=14)
surface

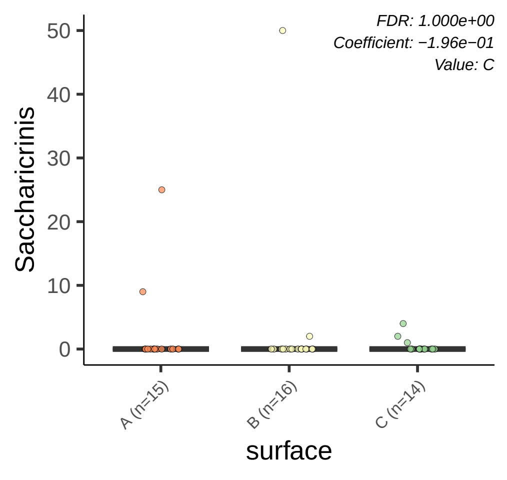

FDR: 1.000e+00
Coefficient: −1.96e−01
Value: C
Saccharicrinis
50
40
30
20
10
0
A (n=15)
B (n=16)
C (n=14)
surface

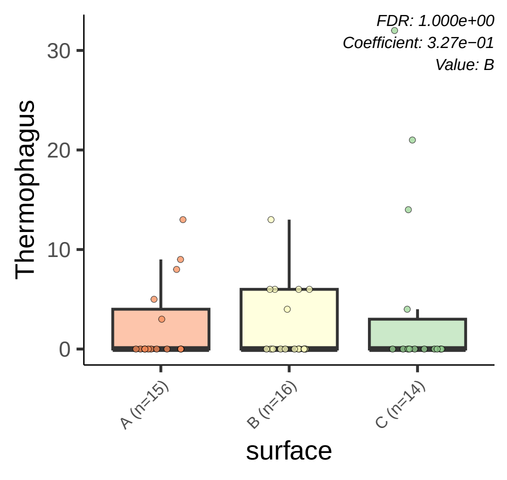
FDR: 1.000e+00
Coefficient: 3.27e−01
Value: B
Thermophagus
30
20
10
0
A (n=15)
B (n=16)
C (n=14)
surface

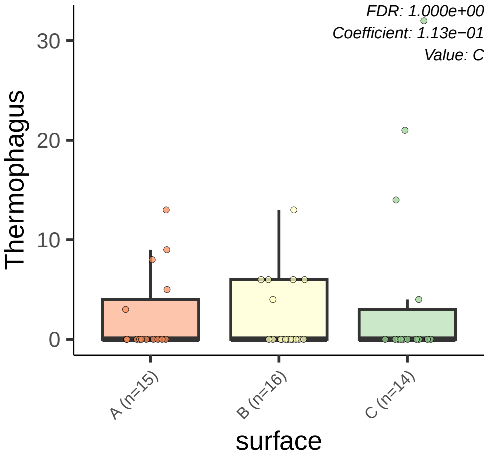
FDR: 1.000e+00
Coefficient: 1.13e−01
Value: C
Thermophagus
0
10
20
30
A (n=15)
B (n=16)
C (n=14)
surface

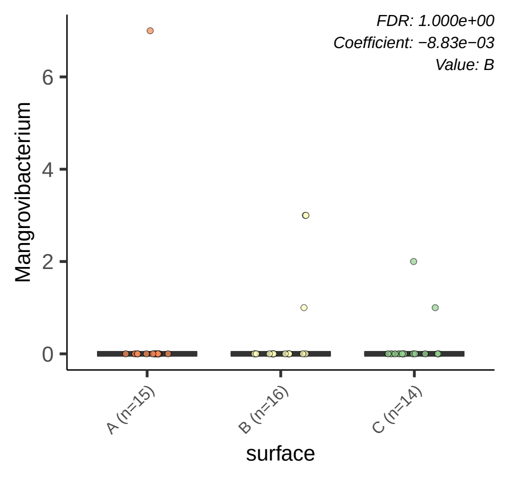

FDR: 1.000e+00
Coefficient: −8.83e−03
Value: B
Mangrovibacterium
0
2
4
6
A (n=15)
B (n=16)
C (n=14)
surface

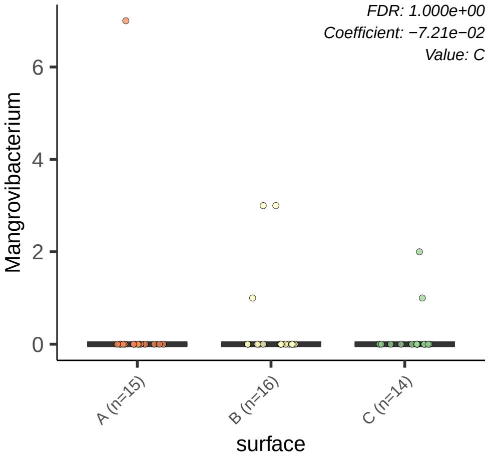

FDR: 1.000e+00
Coefficient: −7.21e−02
Value: C
Mangrovibacterium
0
2
4
6
A (n=15)
B (n=16)
C (n=14)
surface

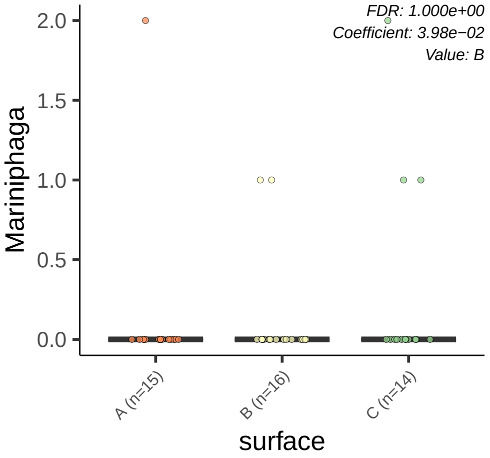
FDR: 1.000e+00
Coefficient: 3.98e−02
Value: B
2.0
1.5
1.0
0.5
0.0
Mariniphaga
A (n=15)
B (n=16)
C (n=14)
surface

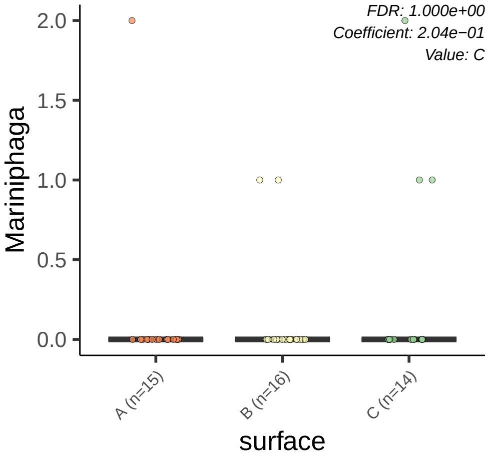

FDR: 1.000e+00
Coefficient: 2.04e−01
Value: C
Mariniphaga
2.0
1.5
1.0
0.5
0.0
A (n=15)
B (n=16)
C (n=14)
surface

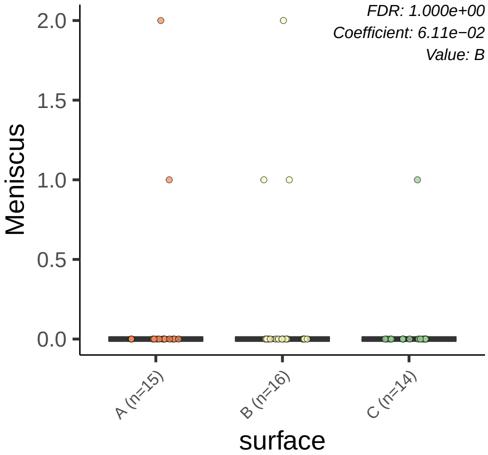

FDR: 1.000e+00
Coefficient: 6.11e−02
Value: B
Meniscus
2.0
1.5
1.0
0.5
0.0
A (n=15)
B (n=16)
C (n=14)
surface

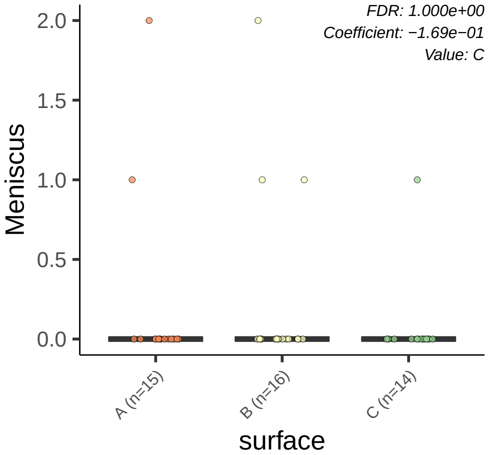

FDR: 1.000e+00
Coefficient: −1.69e−01
Value: C
Meniscus
2.0
1.5
1.0
0.5
0.0
A (n=15)
B (n=16)
C (n=14)
surface

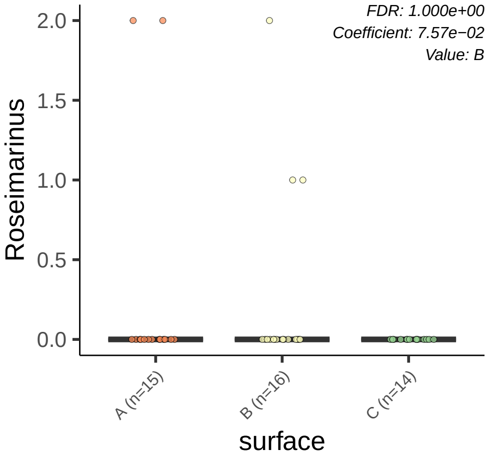

FDR: 1.000e+00
Coefficient: 7.57e−02
Value: B
Roseimarinus
2.0
1.5
1.0
0.5
0.0
A (n=15)
B (n=16)
C (n=14)
surface

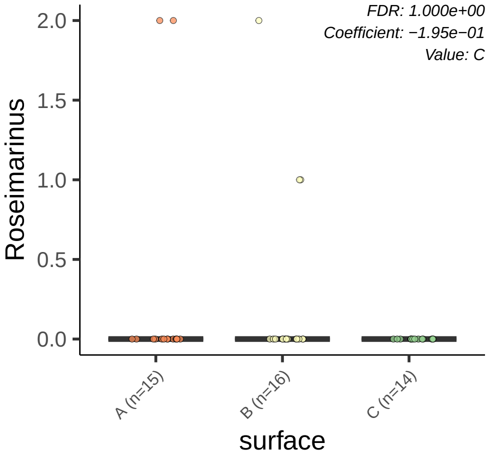

FDR: 1.000e+00
Coefficient: −1.95e−01
Value: C
Roseimarinus
2.0
1.5
1.0
0.5
0.0
A (n=15)
B (n=16)
C (n=14)
surface

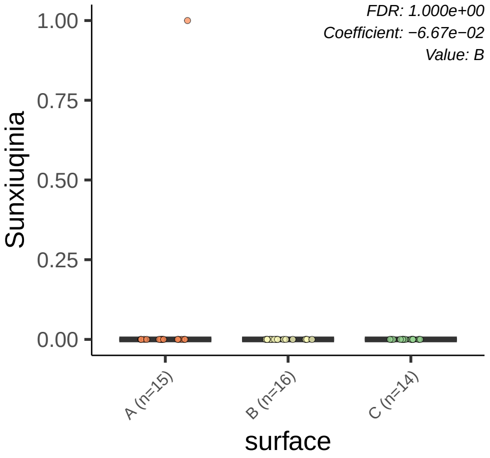

FDR: 1.000e+00
Coefficient: −6.67e−02
Value: B
Sunxiuqinia
1.00
0.75
0.50
0.25
0.00
A (n=15)
B (n=16)
C (n=14)
surface

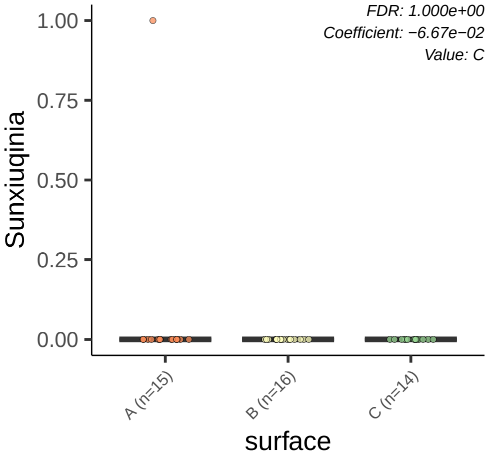

FDR: 1.000e+00
Coefficient: −6.67e−02
Value: C
Sunxiuqinia
1.00
0.75
0.50
0.25
0.00
A (n=15)
B (n=16)
C (n=14)
surface

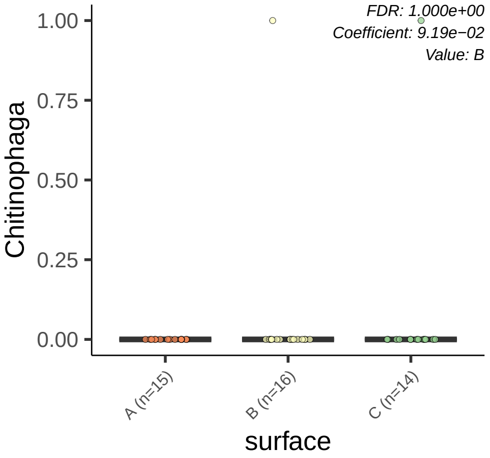

FDR: 1.000e+00
Coefficient: 9.19e−02
Value: B
Chitinophaga
1.00
0.75
0.50
0.25
0.00
A (n=15)
B (n=16)
C (n=14)
surface

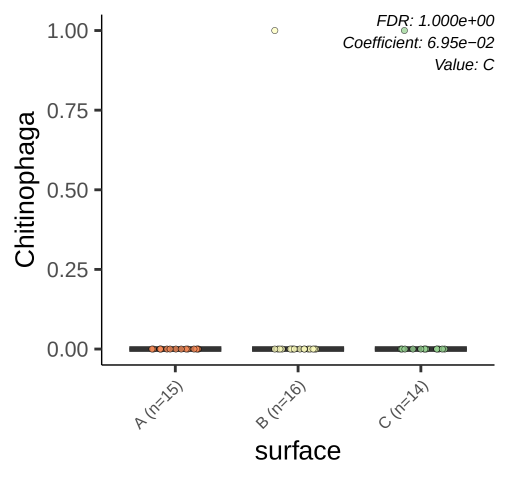

FDR: 1.000e+00
Coefficient: 6.95e−02
Value: C
Chitinophaga
1.00
0.75
0.50
0.25
0.00
A (n=15)
B (n=16)
C (n=14)
surface

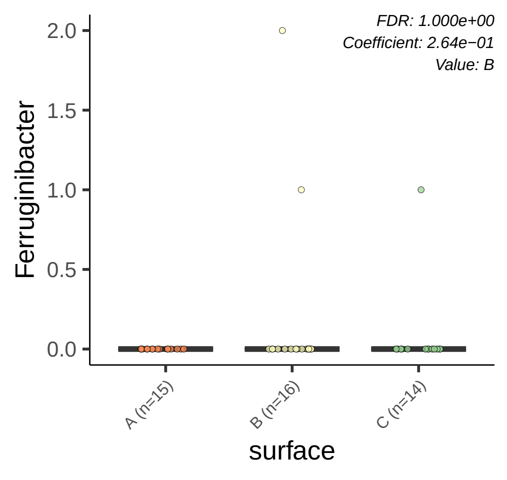
FDR: 1.000e+00
Coefficient: 2.64e−01
Value: B
Ferruginibacter
2.0
1.5
1.0
0.5
0.0
A (n=15)
B (n=16)
C (n=14)
surface

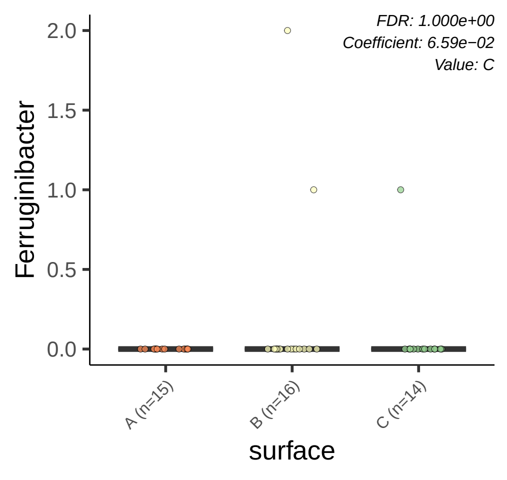
FDR: 1.000e+00
Coefficient: 6.59e−02
Value: C
Ferruginibacter
2.0
1.5
1.0
0.5
0.0
A (n=15)
B (n=16)
C (n=14)
surface

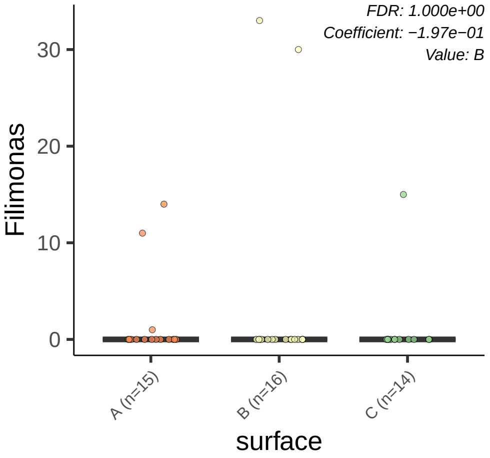

FDR: 1.000e+00
Coefficient: −1.97e−01
Value: B
Filimonas
0
10
20
30
A (n=15)
B (n=16)
C (n=14)
surface

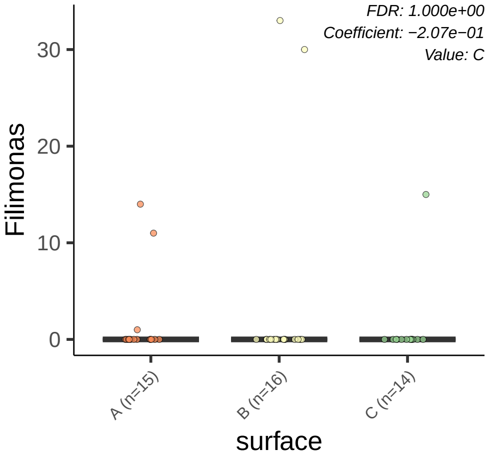

FDR: 1.000e+00
Coefficient: −2.07e−01
Value: C
Filimonas
0
10
20
30
A (n=15)
B (n=16)
C (n=14)
surface

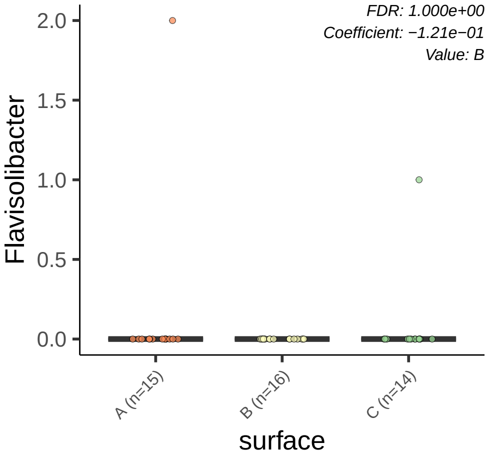
FDR: 1.000e+00
Coefficient: −1.21e−01
Value: B
Flavisolibacter
2.0
1.5
1.0
0.5
0.0
A (n=15)
B (n=16)
C (n=14)
surface

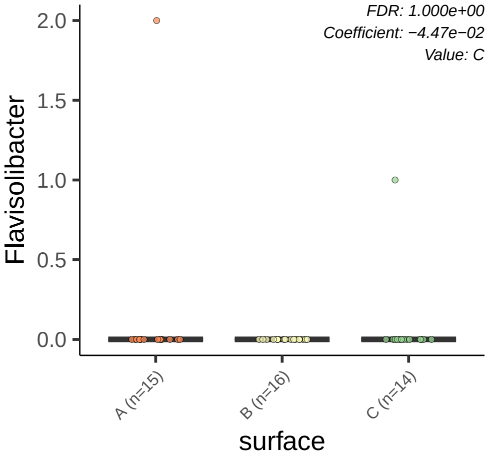
FDR: 1.000e+00
Coefficient: −4.47e−02
Value: C
Flavisolibacter
2.0
1.5
1.0
0.5
0.0
A (n=15)
B (n=16)
C (n=14)
surface

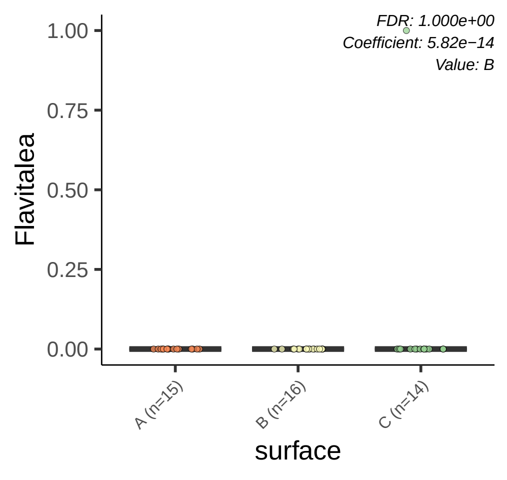

FDR: 1.000e+00
Coefficient: 5.82e−14
Value: B
1.00
0.75
0.50
0.25
0.00
Flavitalea
A (n=15)
B (n=16)
C (n=14)
surface

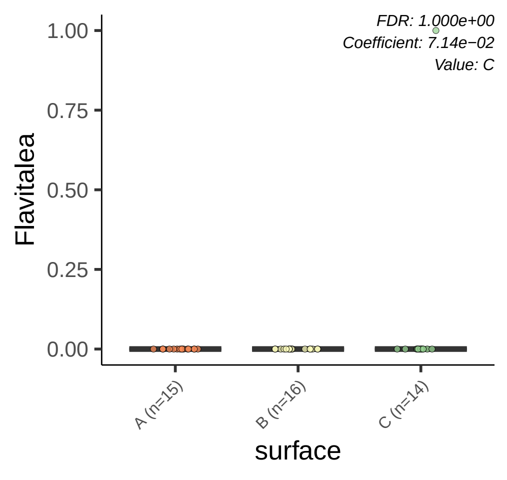

FDR: 1.000e+00
Coefficient: 7.14e−02
Value: C
1.00
0.75
0.50
0.25
0.00
Flavitalea
A (n=15)
B (n=16)
C (n=14)
surface

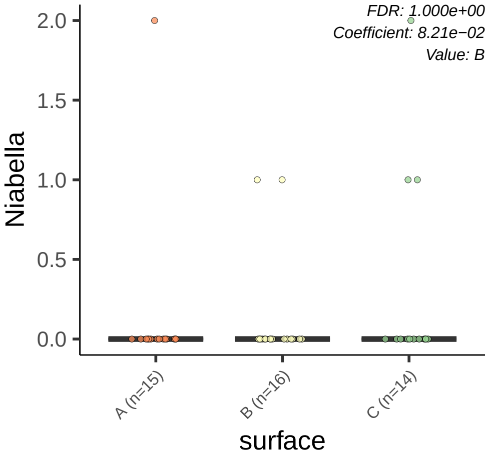
FDR: 1.000e+00
Coefficient: 8.21e−02
Value: B
Niabella
2.0
1.5
1.0
0.5
0.0
A (n=15)
B (n=16)
C (n=14)
surface

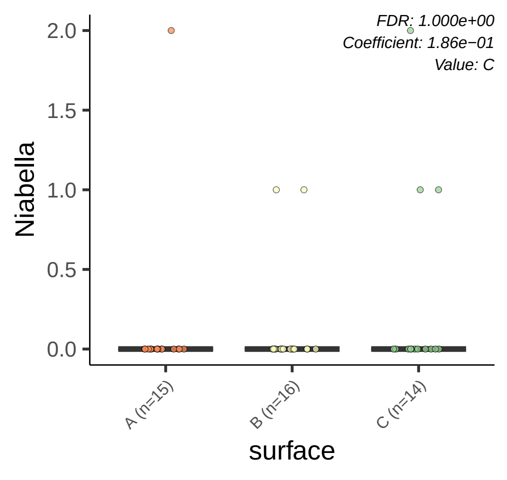
FDR: 1.000e+00
Coefficient: 1.86e−01
Value: C
Niabella
2.0
1.5
1.0
0.5
0.0
A (n=15)
B (n=16)
C (n=14)
surface

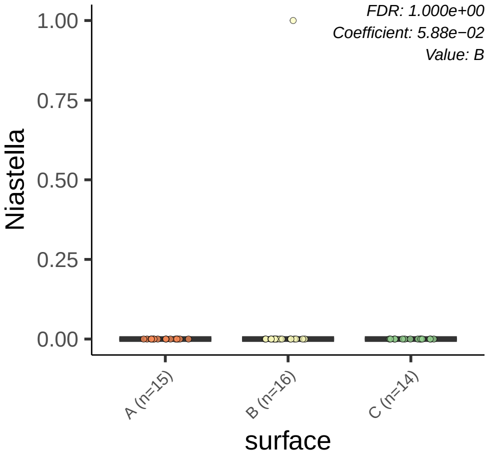
FDR: 1.000e+00
Coefficient: 5.88e−02
Value: B
Niastella
1.00
0.75
0.50
0.25
0.00
A (n=15)
B (n=16)
C (n=14)
surface

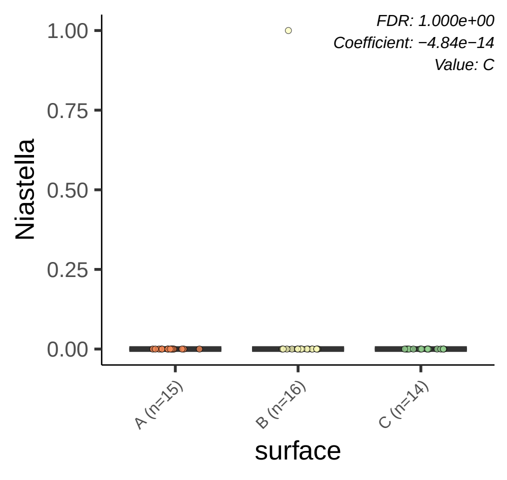
FDR: 1.000e+00
Coefficient: −4.84e−14
Value: C
Niastella
1.00
0.75
0.50
0.25
0.00
A (n=15)
B (n=16)
C (n=14)
surface

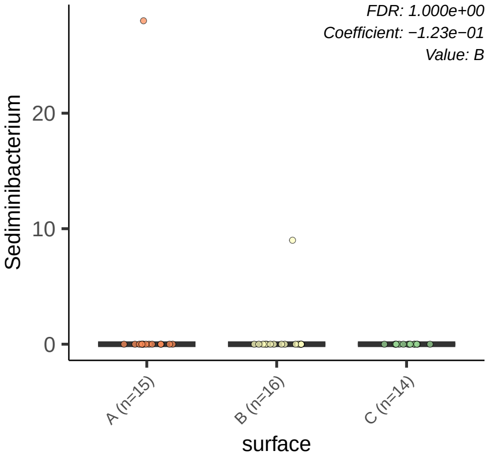
FDR: 1.000e+00
Coefficient: −1.23e−01
Value: B
Sediminibacterium
20
10
0
A (n=15)
B (n=16)
C (n=14)
surface

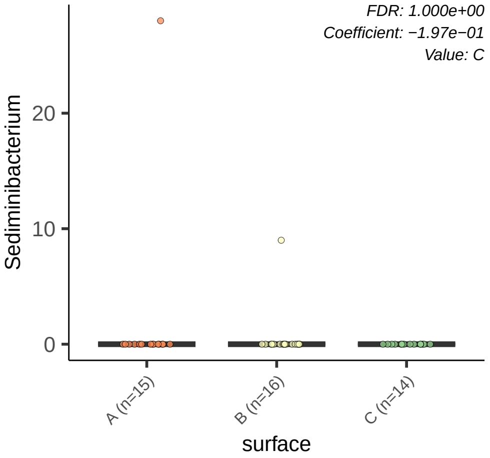
FDR: 1.000e+00
Coefficient: −1.97e−01
Value: C
Sediminibacterium
20
10
0
A (n=15)
B (n=16)
C (n=14)
surface

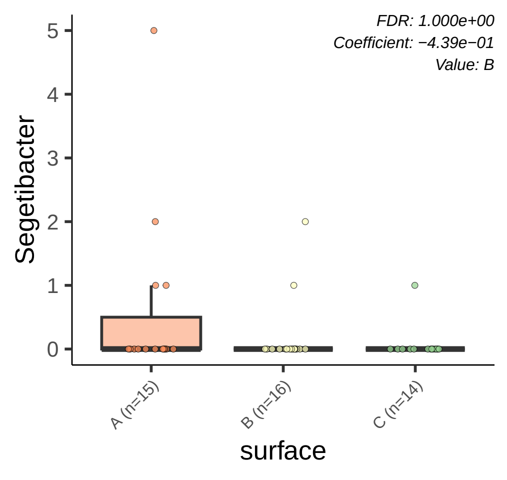
FDR: 1.000e+00
Coefficient: −4.39e−01
Value: B
Segetibacter
0
1
2
3
4
5
A (n=15)
B (n=16)
C (n=14)
surface

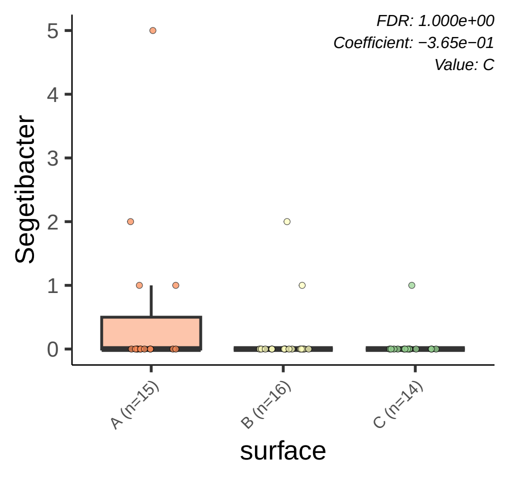
FDR: 1.000e+00
Coefficient: −3.65e−01
Value: C
Segetibacter
5
4
3
2
1
0
A (n=15)
B (n=16)
C (n=14)
surface

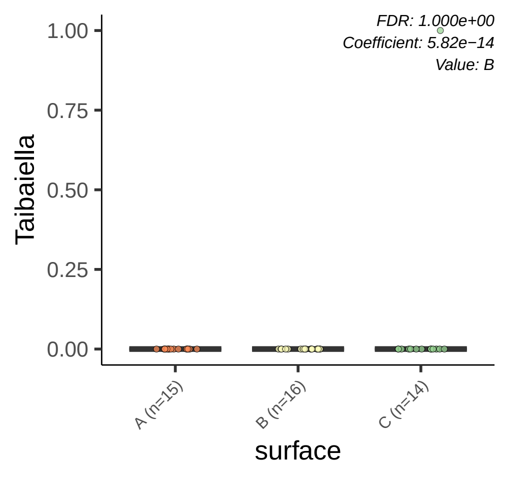
FDR: 1.000e+00
Coefficient: 5.82e−14
Value: B
Taibaiella
1.00
0.75
0.50
0.25
0.00
A (n=15)
B (n=16)
C (n=14)
surface

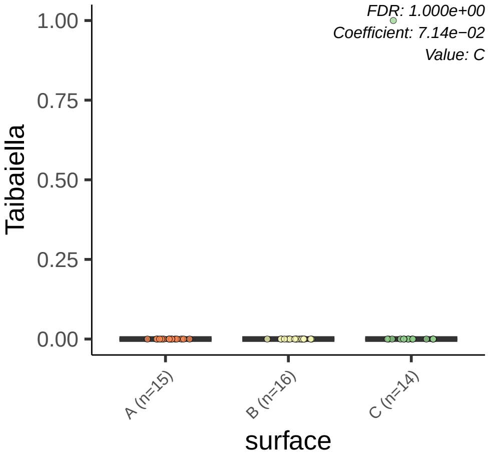
FDR: 1.000e+00
Coefficient: 7.14e−02
Value: C
Taibaiella
1.00
0.75
0.50
0.25
0.00
A (n=15)
B (n=16)
C (n=14)
surface

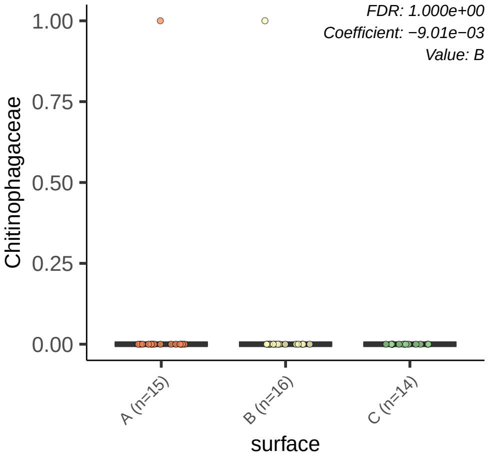
FDR: 1.000e+00
Coefficient: −9.01e−03
Value: B
Chitinophagaceae
1.00
0.75
0.50
0.25
0.00
A (n=15)
B (n=16)
C (n=14)
surface

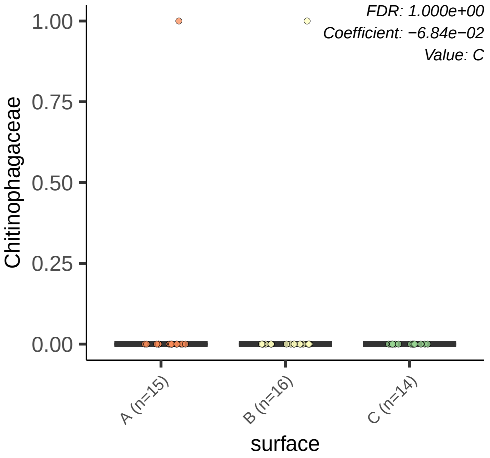
FDR: 1.000e+00
Coefficient: −6.84e−02
Value: C
Chitinophagaceae
1.00
0.75
0.50
0.25
0.00
A (n=15)
B (n=16)
C (n=14)
surface

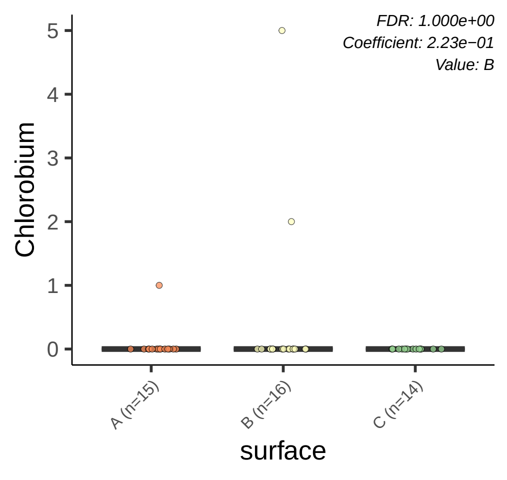
FDR: 1.000e+00
Coefficient: 2.23e−01
Value: B
Chlorobium
0
1
2
3
4
5
A (n=15)
B (n=16)
C (n=14)
surface

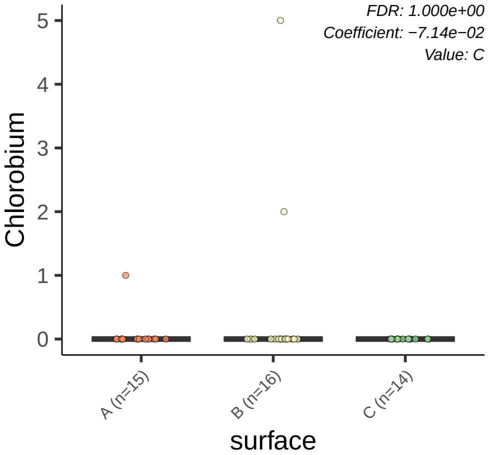
FDR: 1.000e+00
Coefficient: −7.14e−02
Value: C
Chlorobium
0
1
2
3
4
5
A (n=15)
B (n=16)
C (n=14)
surface

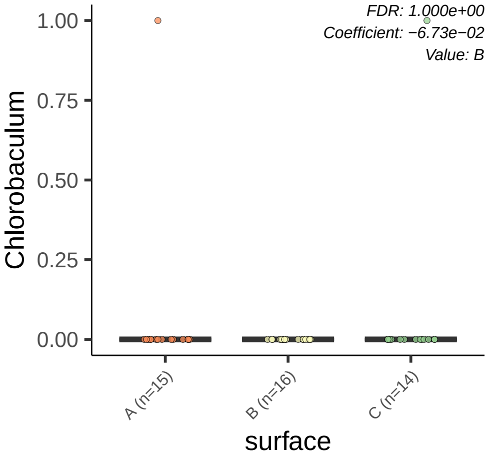
FDR: 1.000e+00
Coefficient: −6.73e−02
Value: B
Chlorobaculum
1.00
0.75
0.50
0.25
0.00
A (n=15)
B (n=16)
C (n=14)
surface

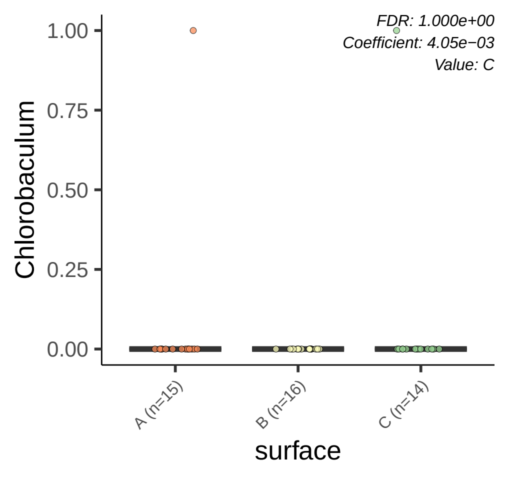

FDR: 1.000e+00
Coefficient: 4.05e−03
Value: C
Chlorobaculum
1.00
0.75
0.50
0.25
0.00
A (n=15)
B (n=16)
C (n=14)
surface

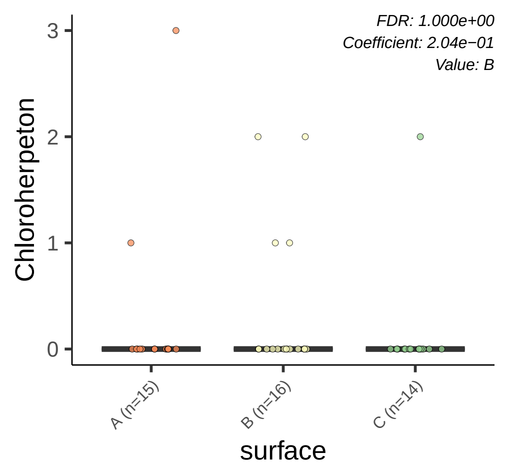
FDR: 1.000e+00
Coefficient: 2.04e−01
Value: B
Chloroherpeton
3
2
1
0
A (n=15)
B (n=16)
C (n=14)
surface

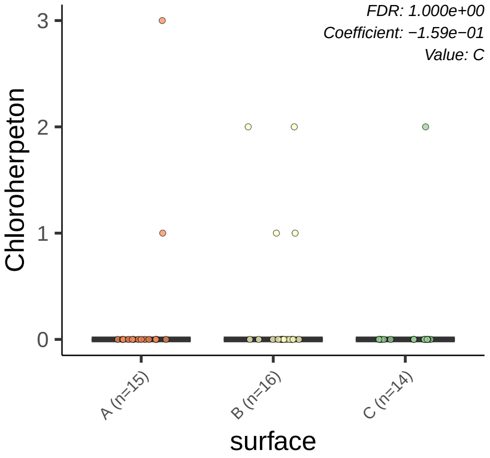

FDR: 1.000e+00
Coefficient: −1.59e−01
Value: C
Chloroherpeton
3
2
1
0
A (n=15)
B (n=16)
C (n=14)
surface

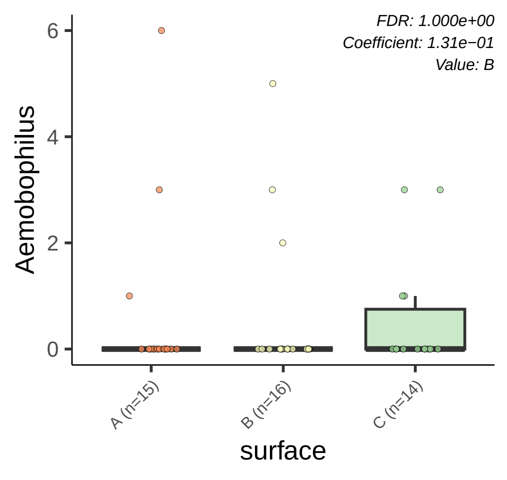
FDR: 1.000e+00
Coefficient: 1.31e−01
Value: B
Aemobophilus
0
2
4
6
A (n=15)
B (n=16)
C (n=14)
surface

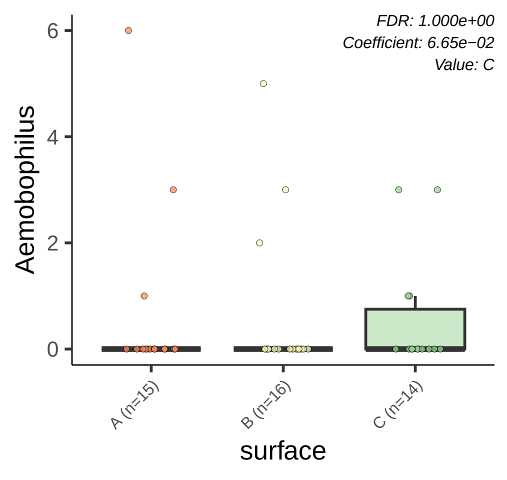
FDR: 1.000e+00
Coefficient: 6.65e−02
Value: C
Aemobophilus
6
4
2
0
A (n=15)
B (n=16)
C (n=14)
surface

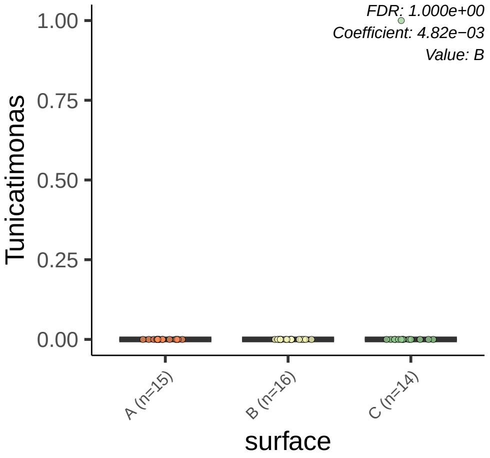
FDR: 1.000e+00
Coefficient: 4.82e−03
Value: B
Tunicatimonas
1.00
0.75
0.50
0.25
0.00
A (n=15)
B (n=16)
C (n=14)
surface

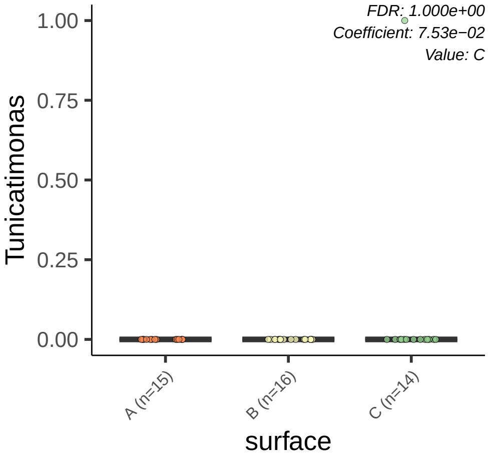

FDR: 1.000e+00
Coefficient: 7.53e−02
Value: C
Tunicatimonas
1.00
0.75
0.50
0.25
0.00
A (n=15)
B (n=16)
C (n=14)
surface

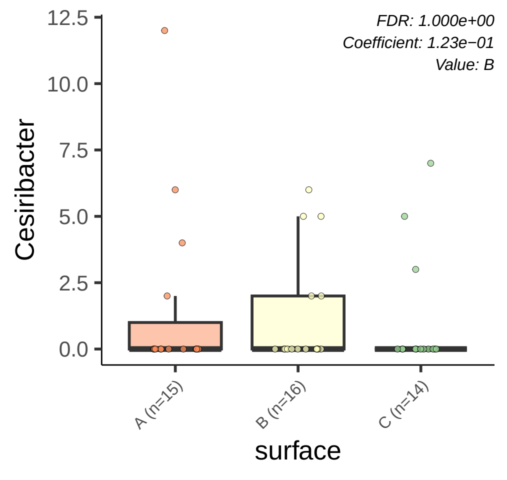

FDR: 1.000e+00
Coefficient: 1.23e−01
Value: B
Cesiribacter
12.5
10.0
7.5
5.0
2.5
0.0
A (n=15)
B (n=16)
C (n=14)
surface

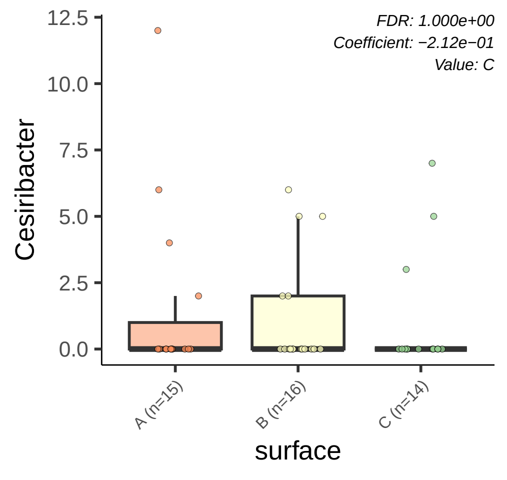

FDR: 1.000e+00
Coefficient: −2.12e−01
Value: C
Cesiribacter
12.5
10.0
7.5
5.0
2.5
0.0
A (n=15)
B (n=16)
C (n=14)
surface

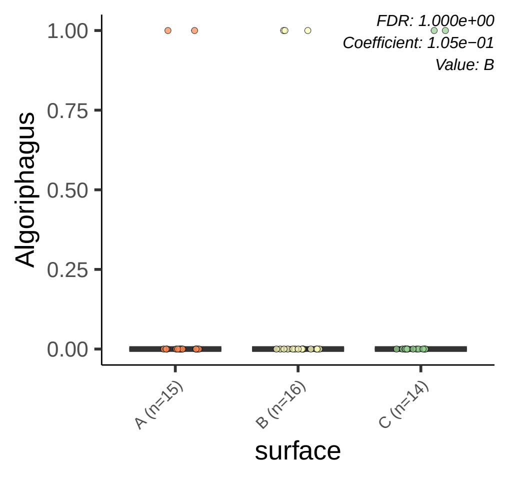
FDR: 1.000e+00
Coefficient: 1.05e−01
Value: B
Algoriphagus
1.00
0.75
0.50
0.25
0.00
A (n=15)
B (n=16)
C (n=14)
surface

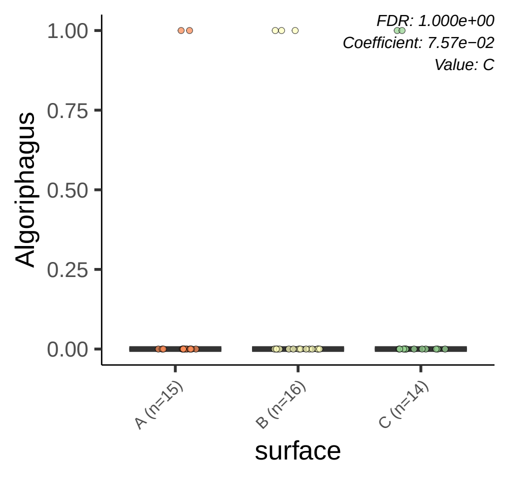
FDR: 1.000e+00
Coefficient: 7.57e−02
Value: C
Algoriphagus
1.00
0.75
0.50
0.25
0.00
A (n=15)
B (n=16)
C (n=14)
surface

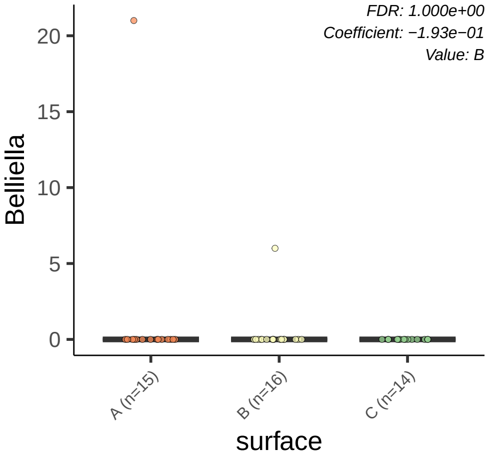
FDR: 1.000e+00
Coefficient: −1.93e−01
Value: B
Belliella
20
15
10
5
0
A (n=15)
B (n=16)
C (n=14)
surface

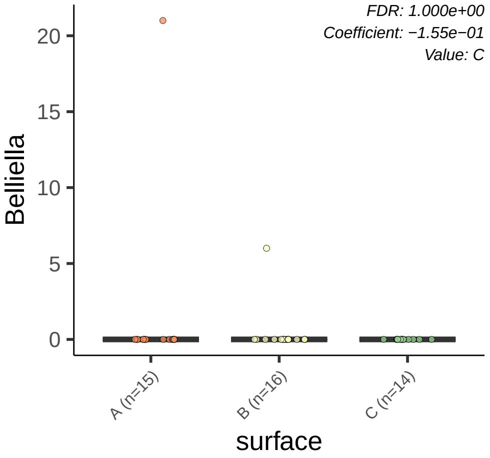
FDR: 1.000e+00
Coefficient: −1.55e−01
Value: C
Belliella
20
15
10
5
0
A (n=15)
B (n=16)
C (n=14)
surface

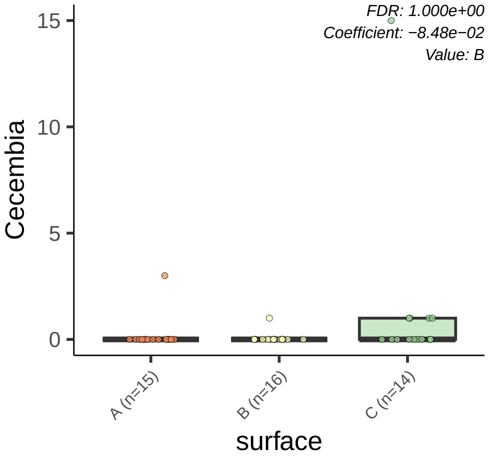
FDR: 1.000e+00
Coefficient: −8.48e−02
Value: B
Cecembia
15
10
5
0
A (n=15)
B (n=16)
C (n=14)
surface

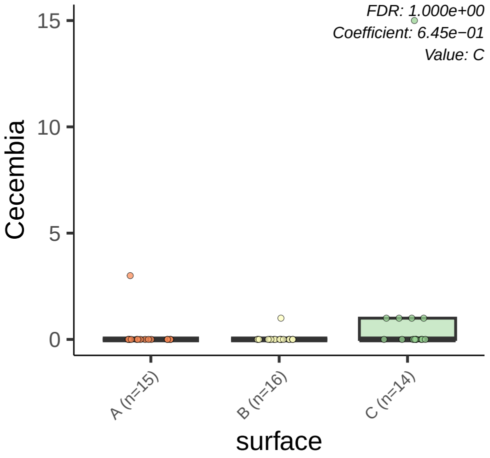
FDR: 1.000e+00
Coefficient: 6.45e−01
Value: C
Cecembia
15
10
5
0
A (n=15)
B (n=16)
C (n=14)
surface

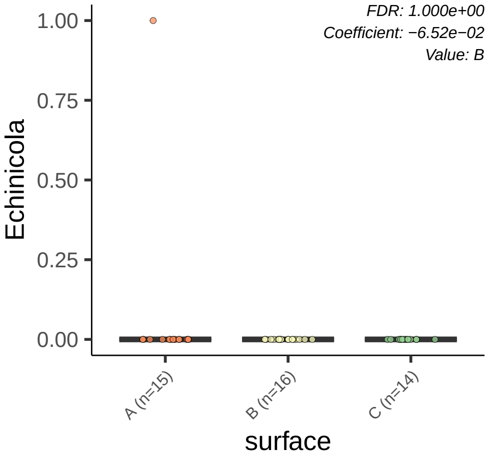
FDR: 1.000e+00
Coefficient: −6.52e−02
Value: B
Echinicola
1.00
0.75
0.50
0.25
0.00
A (n=15)
B (n=16)
C (n=14)
surface

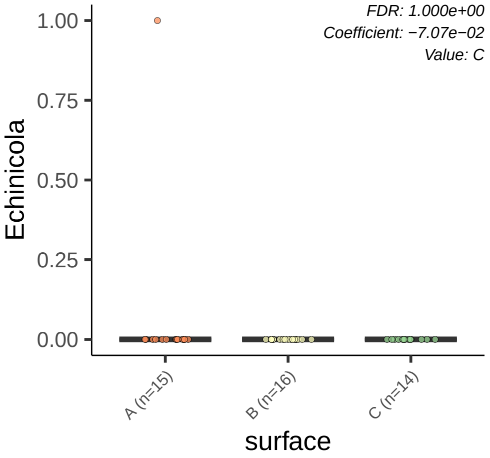
FDR: 1.000e+00
Coefficient: −7.07e−02
Value: C
Echinicola
1.00
0.75
0.50
0.25
0.00
A (n=15)
B (n=16)
C (n=14)
surface

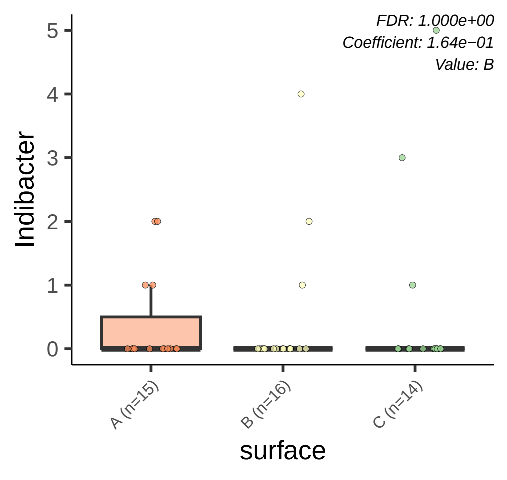
FDR: 1.000e+00
Coefficient: 1.64e−01
Value: B
Indibacter
0
1
2
3
4
5
A (n=15)
B (n=16)
C (n=14)
surface

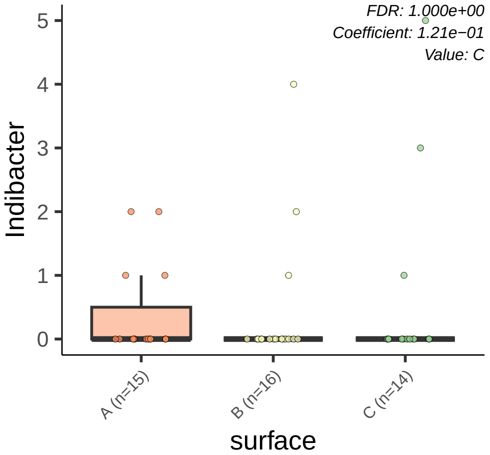

FDR: 1.000e+00
Coefficient: 1.21e−01
Value: C
Indibacter
0
1
2
3
4
5
A (n=15)
B (n=16)
C (n=14)
surface

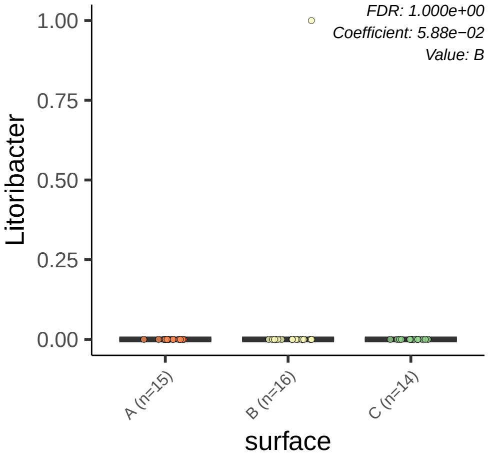
FDR: 1.000e+00
Coefficient: 5.88e−02
Value: B
Litoribacter
1.00
0.75
0.50
0.25
0.00
A (n=15)
B (n=16)
C (n=14)
surface

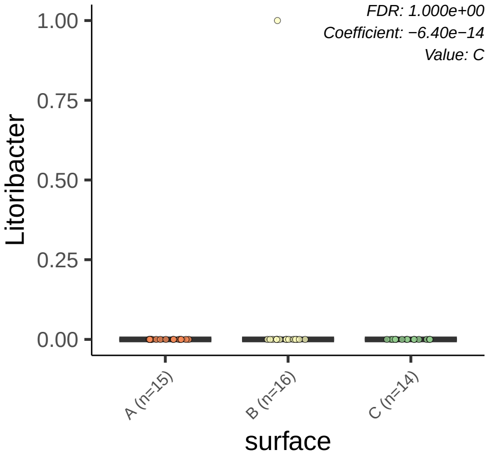
FDR: 1.000e+00
Coefficient: −6.40e−14
Value: C
Litoribacter
1.00
0.75
0.50
0.25
0.00
A (n=15)
B (n=16)
C (n=14)
surface

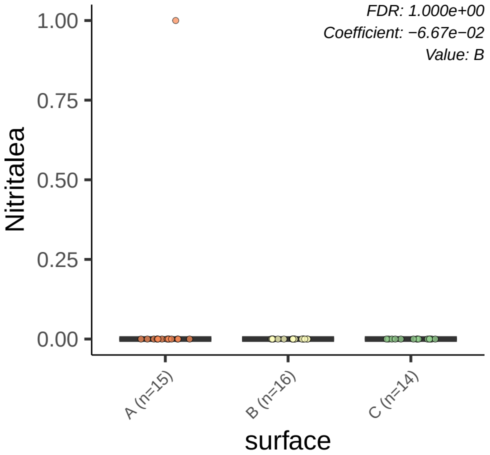
FDR: 1.000e+00
Coefficient: −6.67e−02
Value: B
Nitritalea
1.00
0.75
0.50
0.25
0.00
A (n=15)
B (n=16)
C (n=14)
surface

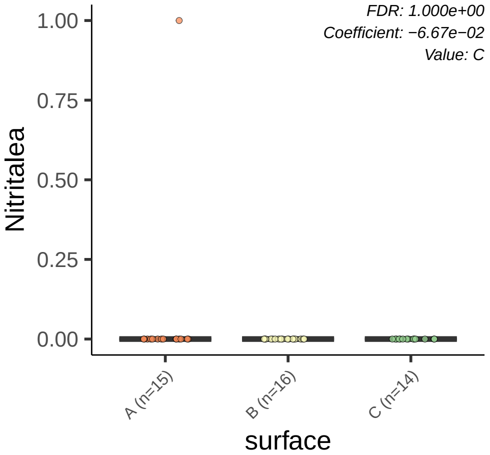
FDR: 1.000e+00
Coefficient: −6.67e−02
Value: C
Nitritalea
1.00
0.75
0.50
0.25
0.00
A (n=15)
B (n=16)
C (n=14)
surface

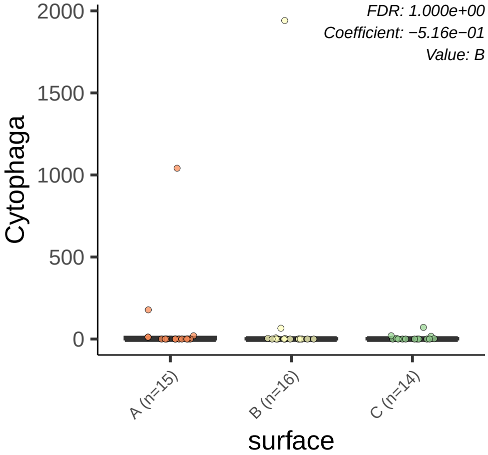
FDR: 1.000e+00
Coefficient: −5.16e−01
Value: B
Cytophaga
2000
1500
1000
500
0
A (n=15)
B (n=16)
C (n=14)
surface

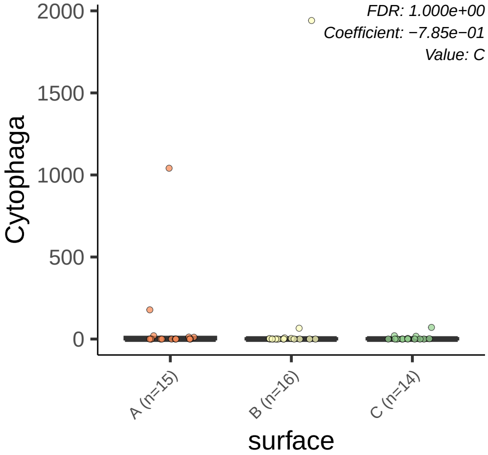

FDR: 1.000e+00
Coefficient: −7.85e−01
Value: C
Cytophaga
2000
1500
1000
500
0
A (n=15)
B (n=16)
C (n=14)
surface

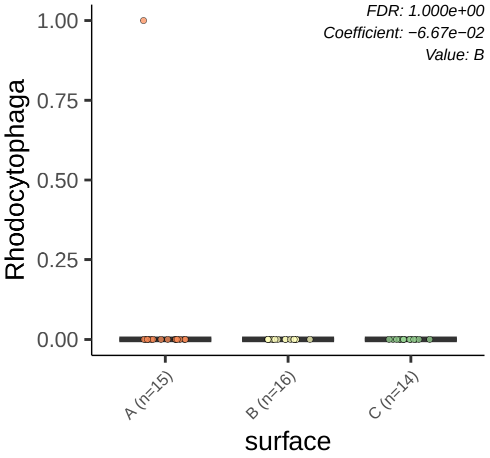
FDR: 1.000e+00
Coefficient: −6.67e−02
Value: B
Rhodocytophaga
1.00
0.75
0.50
0.25
0.00
A (n=15)
B (n=16)
C (n=14)
surface

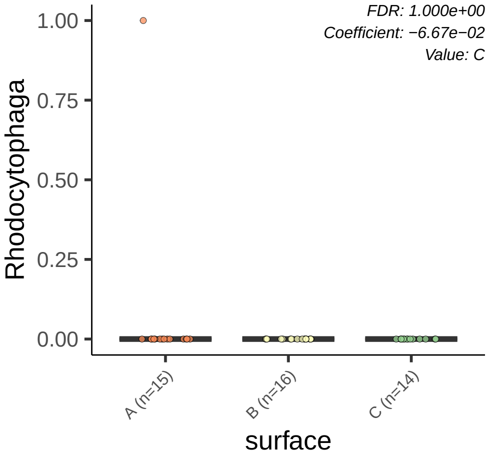

FDR: 1.000e+00
Coefficient: −6.67e−02
Value: C
Rhodocytophaga
1.00
0.75
0.50
0.25
0.00
A (n=15)
B (n=16)
C (n=14)
surface

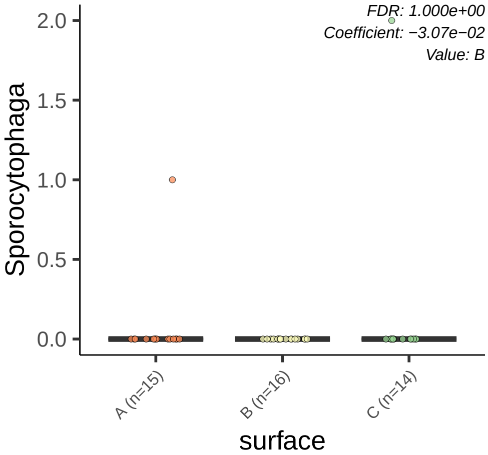
FDR: 1.000e+00
Coefficient: −3.07e−02
Value: B
Sporocytophaga
2.0
1.5
1.0
0.5
0.0
A (n=15)
B (n=16)
C (n=14)
surface

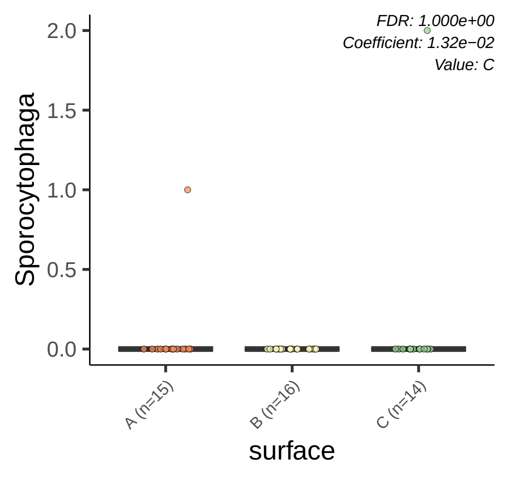

FDR: 1.000e+00
Coefficient: 1.32e−02
Value: C
Sporocytophaga
2.0
1.5
1.0
0.5
0.0
A (n=15)
B (n=16)
C (n=14)
surface

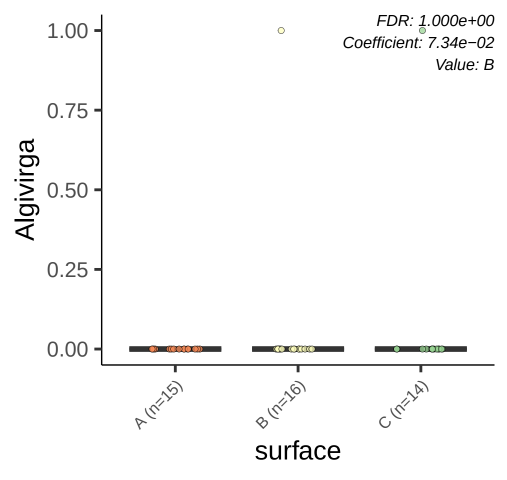

FDR: 1.000e+00
Coefficient: 7.34e−02
Value: B
1.00
0.75
0.50
0.25
0.00
Algivirga
A (n=15)
B (n=16)
C (n=14)
surface

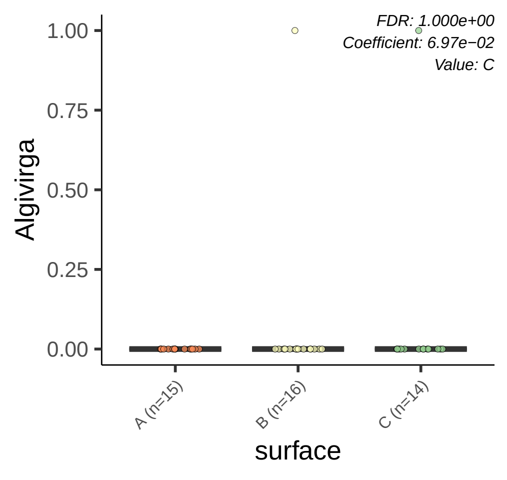
FDR: 1.000e+00
Coefficient: 6.97e−02
Value: C
Algivirga
1.00
0.75
0.50
0.25
0.00
A (n=15)
B (n=16)
C (n=14)
surface

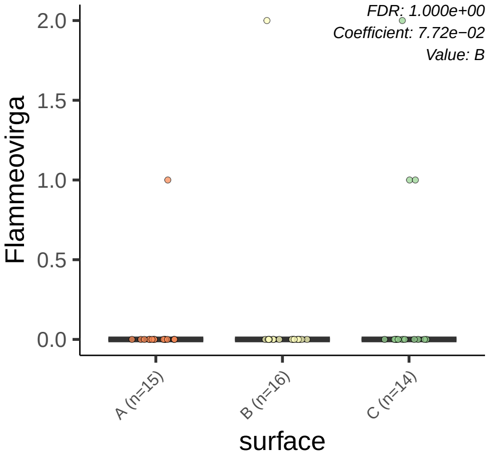
FDR: 1.000e+00
Coefficient: 7.72e−02
Value: B
Flammeovirga
2.0
1.5
1.0
0.5
0.0
A (n=15)
B (n=16)
C (n=14)
surface

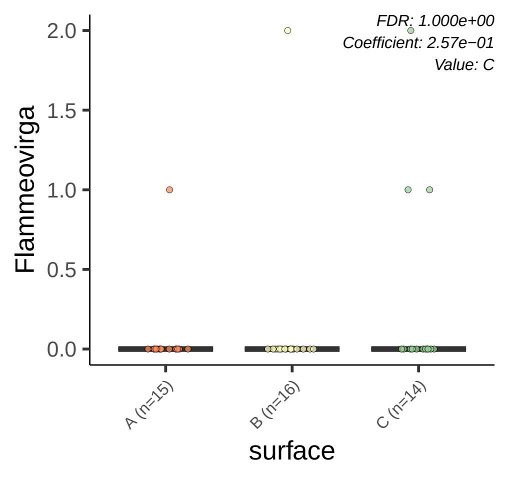
FDR: 1.000e+00
Coefficient: 2.57e−01
Value: C
Flammeovirga
2.0
1.5
1.0
0.5
0.0
A (n=15)
B (n=16)
C (n=14)
surface

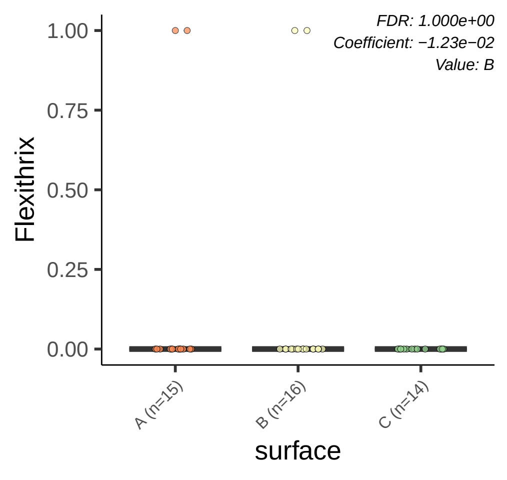
FDR: 1.000e+00
Coefficient: −1.23e−02
Value: B
Flexithrix
1.00
0.75
0.50
0.25
0.00
A (n=15)
B (n=16)
C (n=14)
surface

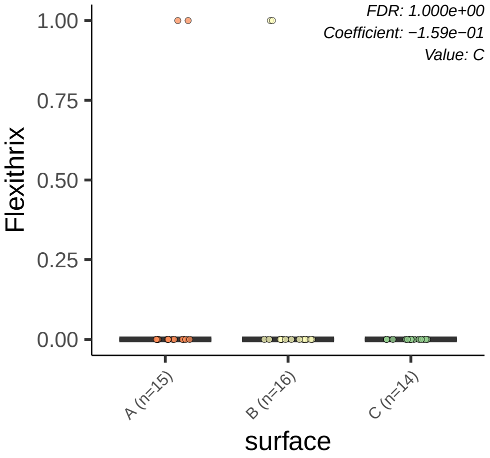
FDR: 1.000e+00
Coefficient: −1.59e−01
Value: C
Flexithrix
1.00
0.75
0.50
0.25
0.00
A (n=15)
B (n=16)
C (n=14)
surface

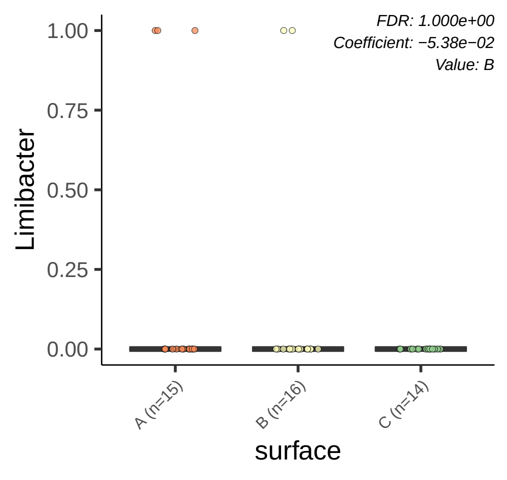

FDR: 1.000e+00
Coefficient: −5.38e−02
Value: B
Limibacter
1.00
0.75
0.50
0.25
0.00
A (n=15)
B (n=16)
C (n=14)
surface

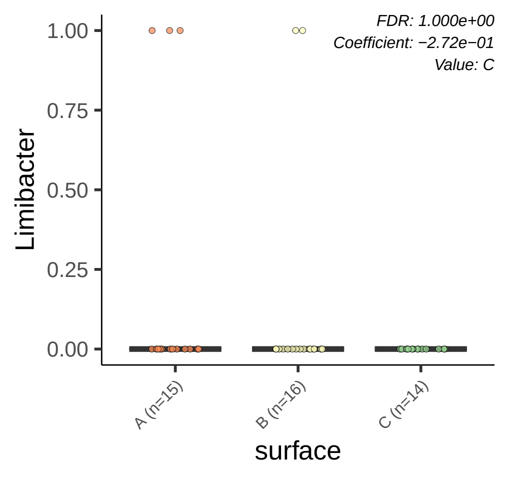
FDR: 1.000e+00
Coefficient: −2.72e−01
Value: C
Limibacter
1.00
0.75
0.50
0.25
0.00
A (n=15)
B (n=16)
C (n=14)
surface

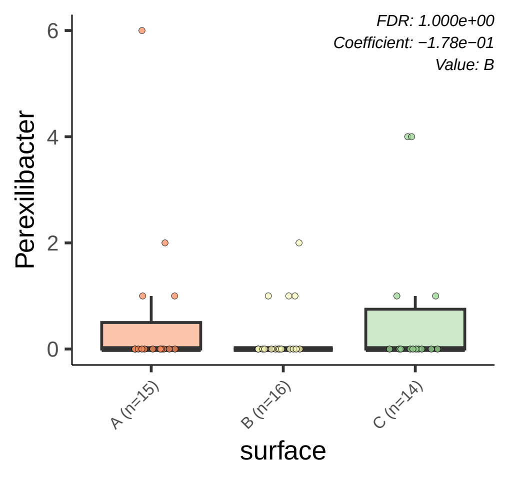
FDR: 1.000e+00
Coefficient: −1.78e−01
Value: B
Perexilibacter
6
4
2
0
A (n=15)
B (n=16)
C (n=14)
surface

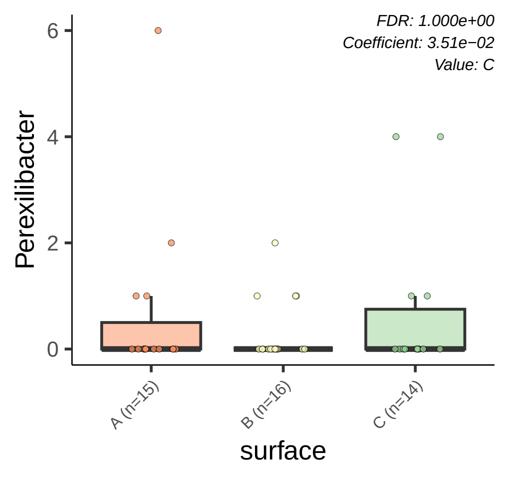
FDR: 1.000e+00
Coefficient: 3.51e−02
Value: C
Perexilibacter
6
4
2
0
A (n=15)
B (n=16)
C (n=14)
surface

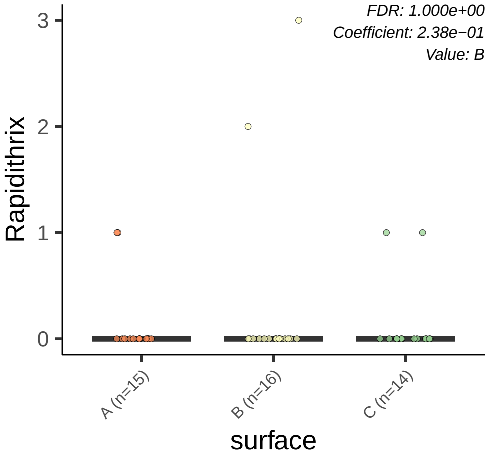

FDR: 1.000e+00
Coefficient: 2.38e−01
Value: B
Rapidithrix
3
2
1
0
A (n=15)
B (n=16)
C (n=14)
surface

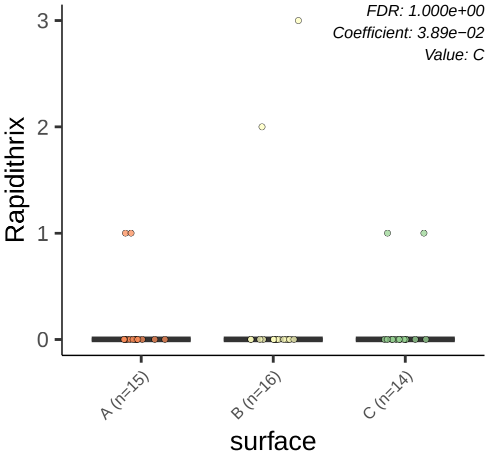
FDR: 1.000e+00
Coefficient: 3.89e−02
Value: C
Rapidithrix
3
2
1
0
A (n=15)
B (n=16)
C (n=14)
surface

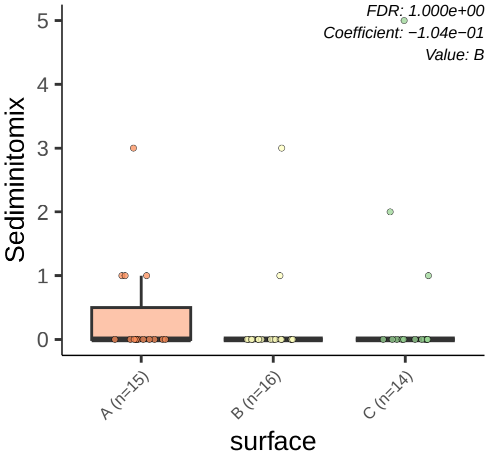

FDR: 1.000e+00
Coefficient: −1.04e−01
Value: B
Sedimintomix
0
1
2
3
4
5
A (n=15)
B (n=16)
C (n=14)
surface

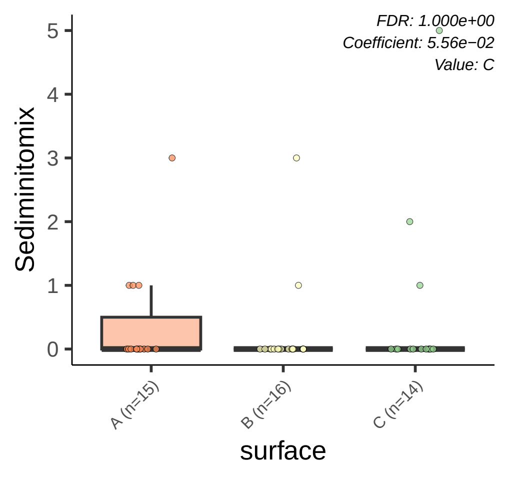

FDR: 1.000e+00
Coefficient: 5.56e−02
Value: C
Sediminitomix
0
1
2
3
4
5
A (n=15)
B (n=16)
C (n=14)
surface

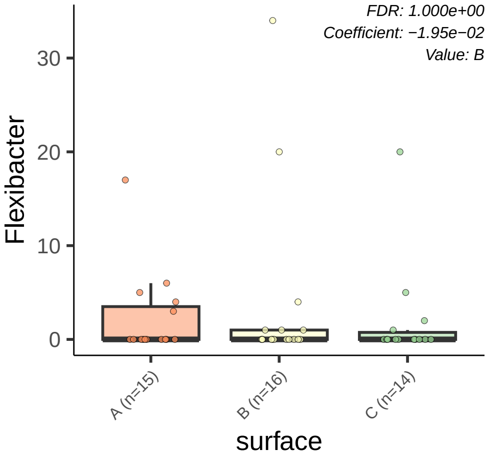
FDR: 1.000e+00
Coefficient: −1.95e−02
Value: B
Flexibacter
30
20
10
0
A (n=15)
B (n=16)
C (n=14)
surface

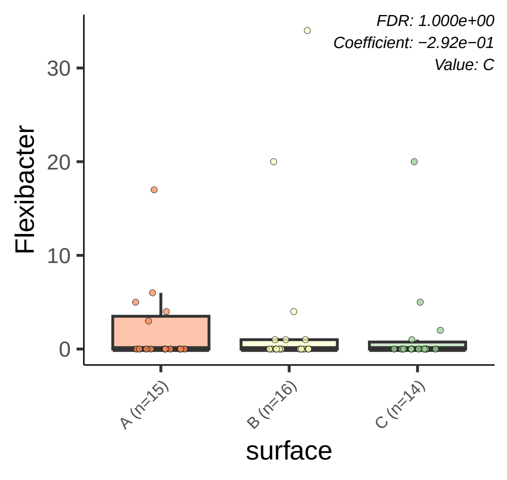
FDR: 1.000e+00
Coefficient: −2.92e−01
Value: C
Flexibacter
30
20
10
0
A (n=15)
B (n=16)
C (n=14)
surface

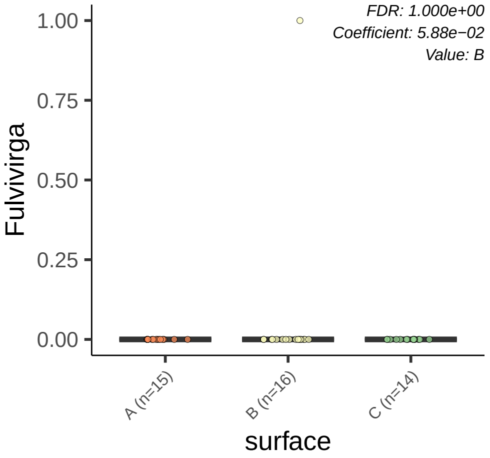

FDR: 1.000e+00
Coefficient: 5.88e−02
Value: B
1.00
0.75
0.50
0.25
0.00
Fulvivirga
A (n=15)
B (n=16)
C (n=14)
surface

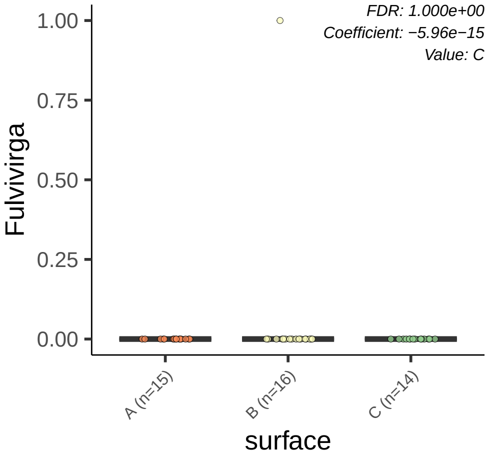

FDR: 1.000e+00
Coefficient: −5.96e−15
Value: C
Fulvivirga
1.00
0.75
0.50
0.25
0.00
A (n=15)
B (n=16)
C (n=14)
surface

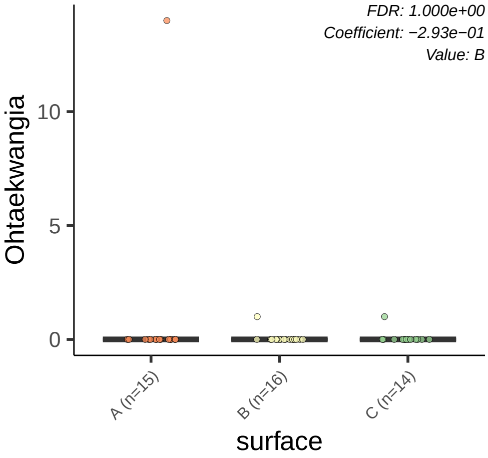

FDR: 1.000e+00
Coefficient: −2.93e−01
Value: B
Ohtaekwangia
10
5
0
A (n=15)
B (n=16)
C (n=14)
surface

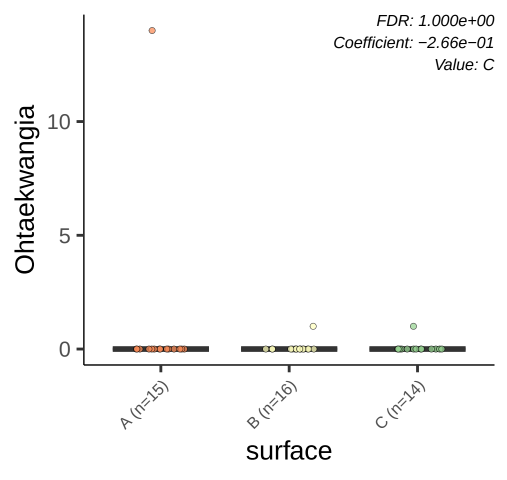

FDR: 1.000e+00
Coefficient: −2.66e−01
Value: C
Ohtaekwangia
10
5
0
A (n=15)
B (n=16)
C (n=14)
surface

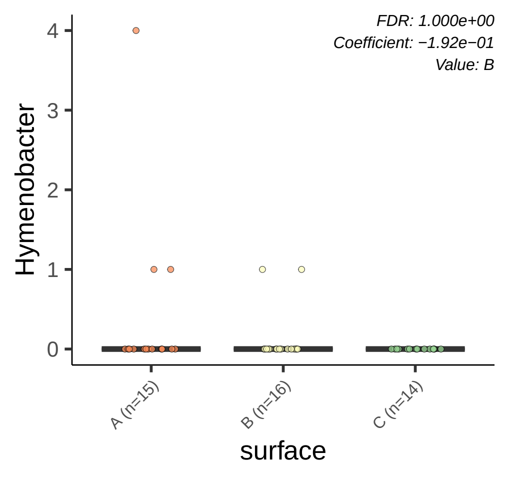
FDR: 1.000e+00
Coefficient: −1.92e−01
Value: B
Hymenobacter
4
3
2
1
0
A (n=15)
B (n=16)
C (n=14)
surface

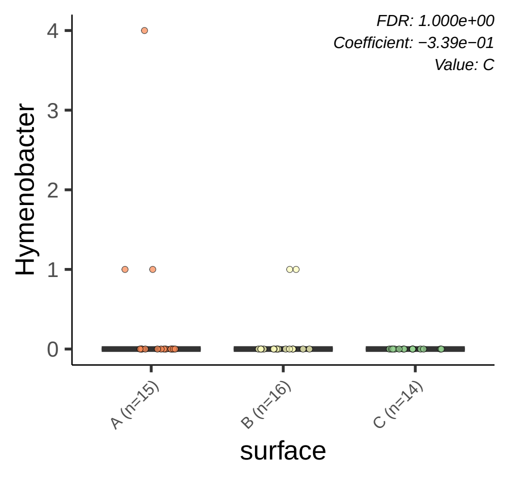

FDR: 1.000e+00
Coefficient: −3.39e−01
Value: C
Hymenobacter
0
1
2
3
4
A (n=15)
B (n=16)
C (n=14)
surface

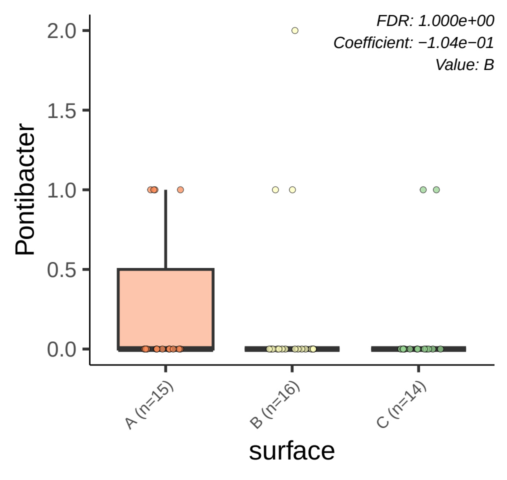
FDR: 1.000e+00
Coefficient: −1.04e−01
Value: B
Pontibacter
2.0
1.5
1.0
0.5
0.0
A (n=15)
B (n=16)
C (n=14)
surface

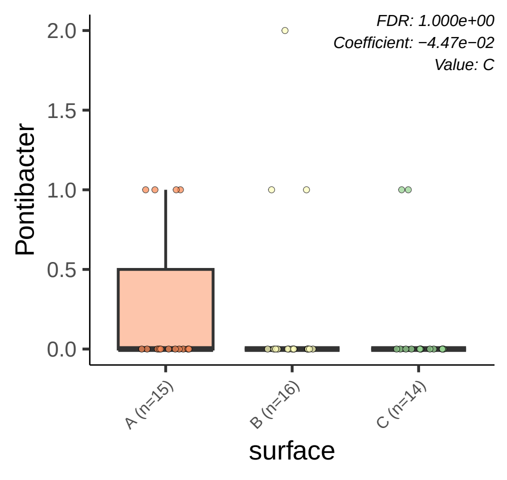

FDR: 1.000e+00
Coefficient: −4.47e−02
Value: C
Pontibacter
2.0
1.5
1.0
0.5
0.0
A (n=15)
B (n=16)
C (n=14)
surface

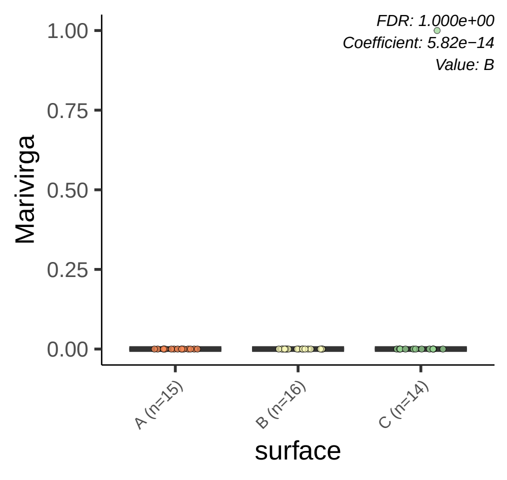
FDR: 1.000e+00
Coefficient: 5.82e−14
Value: B
Marivirga
1.00
0.75
0.50
0.25
0.00
A (n=15)
B (n=16)
C (n=14)
surface

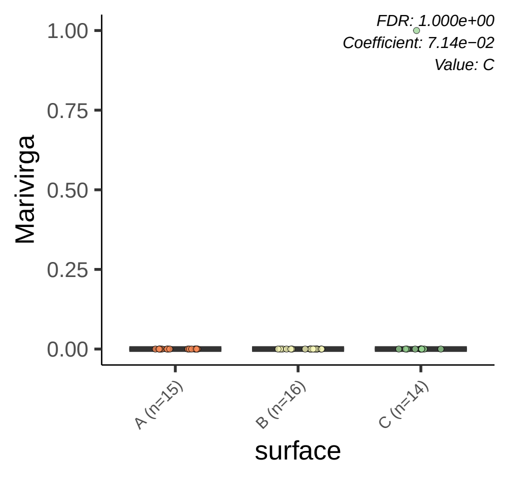
FDR: 1.000e+00
Coefficient: 7.14e−02
Value: C
1.00
0.75
0.50
0.25
0.00
Marivirga
A (n=15)
B (n=16)
C (n=14)
surface

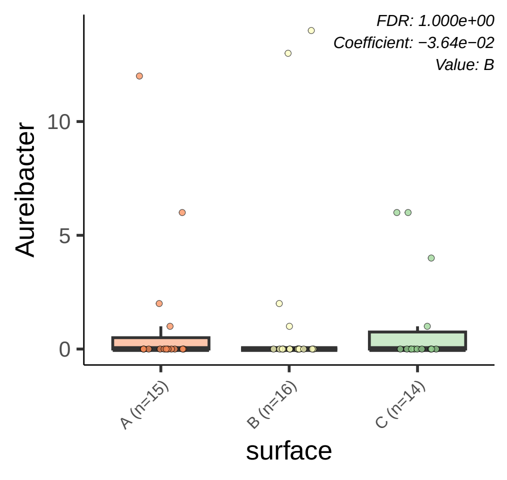
FDR: 1.000e+00
Coefficient: −3.64e−02
Value: B
Aureibacter
10
5
0
A (n=15)
B (n=16)
C (n=14)
surface

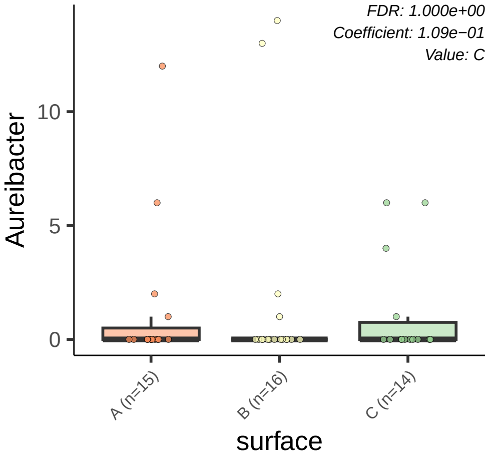
FDR: 1.000e+00
Coefficient: 1.09e−01
Value: C
Aureibacter
10
5
0
A (n=15)
B (n=16)
C (n=14)
surface

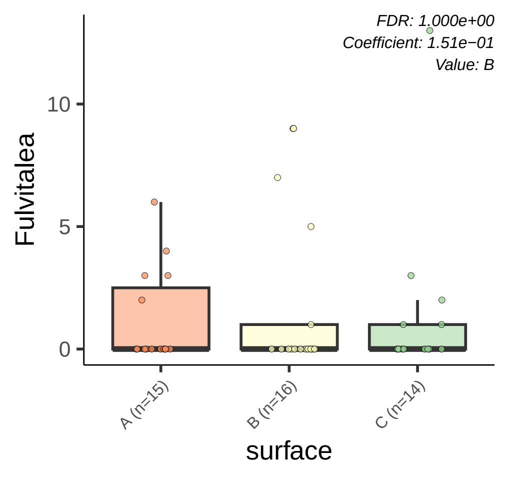

FDR: 1.000e+00
Coefficient: 1.51e−01
Value: B
Fulvitalea
0
5
10
A (n=15)
B (n=16)
C (n=14)
surface

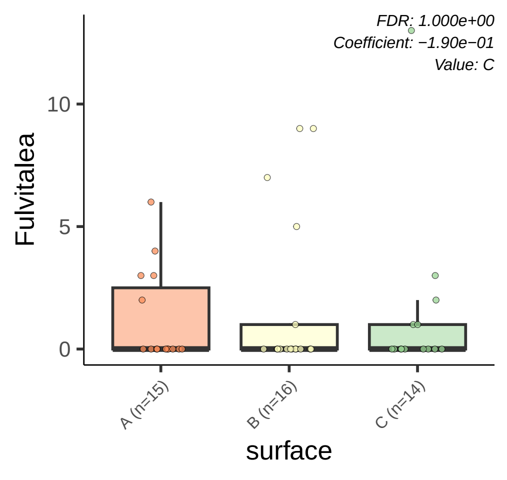
FDR: 1.000e+00
Coefficient: −1.90e−01
Value: C
Fulvitalea
10
5
0
A (n=15)
B (n=16)
C (n=14)
surface

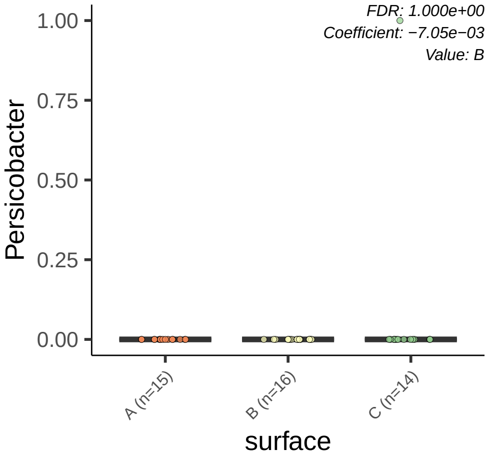
FDR: 1.000e+00
Coefficient: −7.05e−03
Value: B
Persicobacter
1.00
0.75
0.50
0.25
0.00
A (n=15)
B (n=16)
C (n=14)
surface

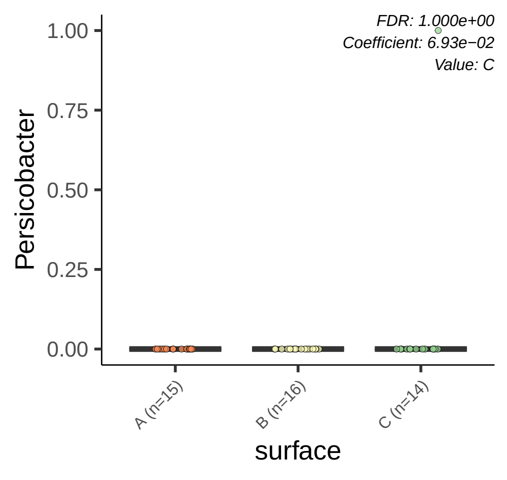

FDR: 1.000e+00
Coefficient: 6.93e−02
Value: C
Persicobacter
1.00
0.75
0.50
0.25
0.00
A (n=15)
B (n=16)
C (n=14)
surface

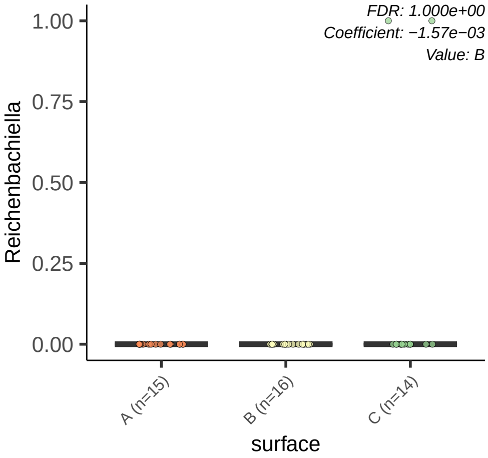
FDR: 1.000e+00
Coefficient: −1.57e−03
Value: B
1.00
0.75
0.50
0.25
0.00
Reichenbachiella
A (n=15)
B (n=16)
C (n=14)
surface

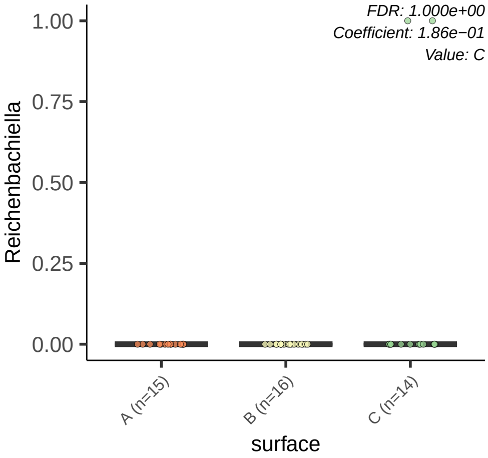
FDR: 1.000e+00
Coefficient: 1.86e−01
Value: C
Reichenbachiella
1.00
0.75
0.50
0.25
0.00
A (n=15)
B (n=16)
C (n=14)
surface

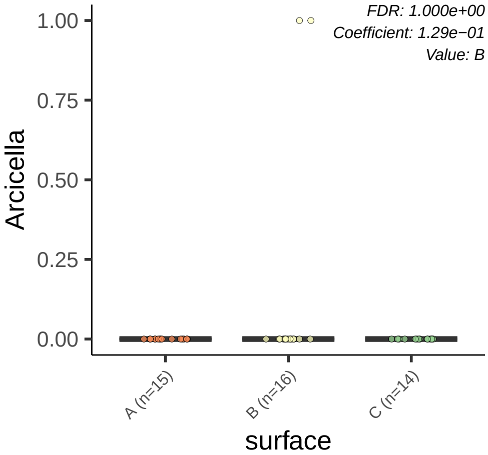

FDR: 1.000e+00
Coefficient: 1.29e−01
Value: B
1.00
0.75
0.50
0.25
0.00
Arcicella
A (n=15)
B (n=16)
C (n=14)
surface

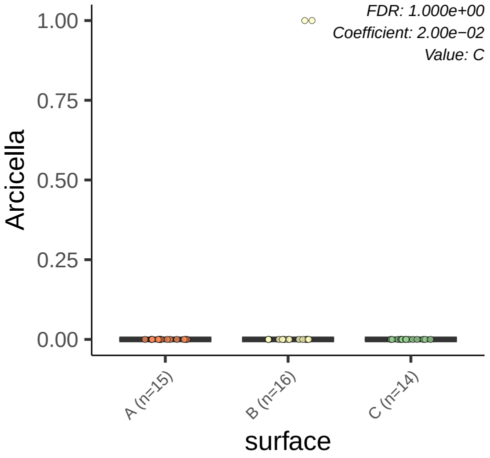

FDR: 1.000e+00
Coefficient: 2.00e−02
Value: C
Arcicella
1.00
0.75
0.50
0.25
0.00
A (n=15)
B (n=16)
C (n=14)
surface

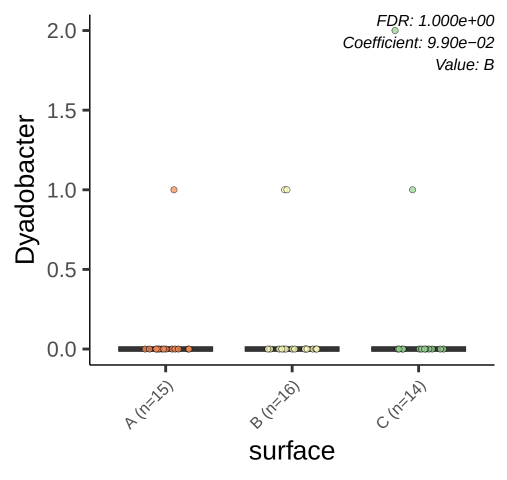
FDR: 1.000e+00
Coefficient: 9.90e−02
Value: B
Dyadobacter
2.0
1.5
1.0
0.5
0.0
A (n=15)
B (n=16)
C (n=14)
surface

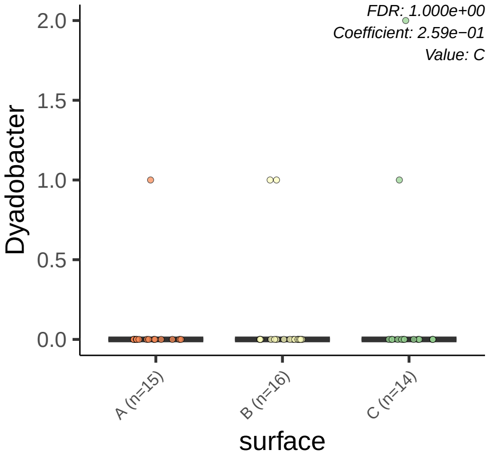

FDR: 1.000e+00
Coefficient: 2.59e−01
Value: C
Dyadobacter
2.0
1.5
1.0
0.5
0.0
A (n=15)
B (n=16)
C (n=14)
surface

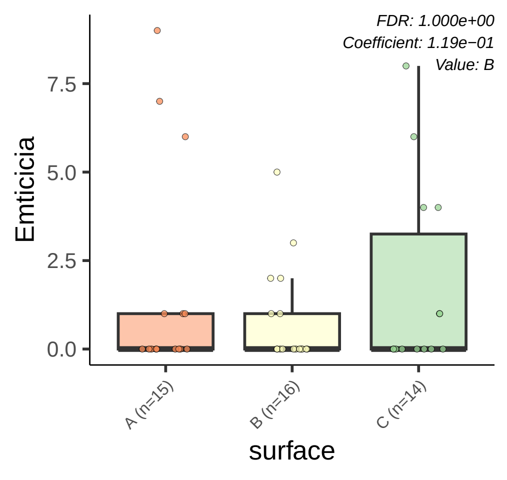
FDR: 1.000e+00
Coefficient: 1.19e−01
Value: B
Emticicia
7.5
5.0
2.5
0.0
A (n=15)
B (n=16)
C (n=14)
surface

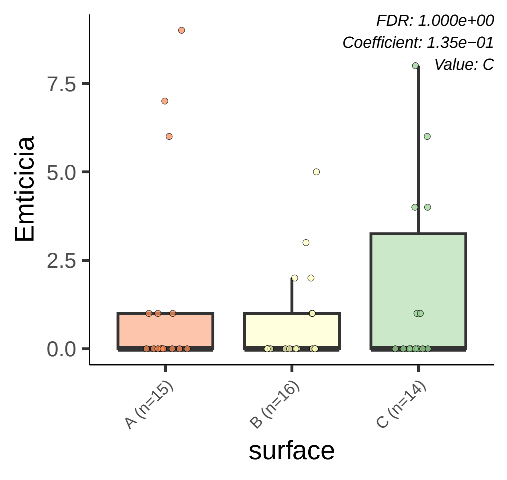
FDR: 1.000e+00
Coefficient: 1.35e−01
Value: C
Emticicia
7.5
5.0
2.5
0.0
A (n=15)
B (n=16)
C (n=14)
surface

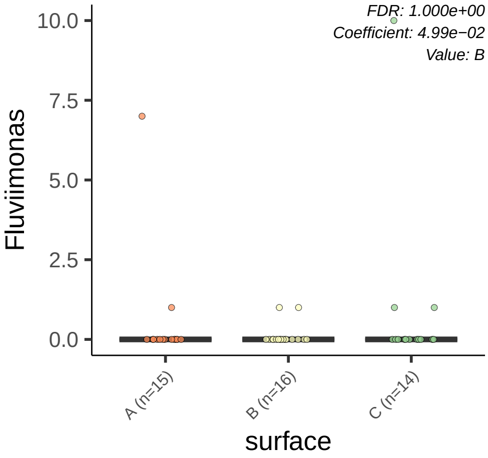

FDR: 1.000e+00
Coefficient: 4.99e−02
Value: B
Fluviimonas
10.0
7.5
5.0
2.5
0.0
A (n=15)
B (n=16)
C (n=14)
surface

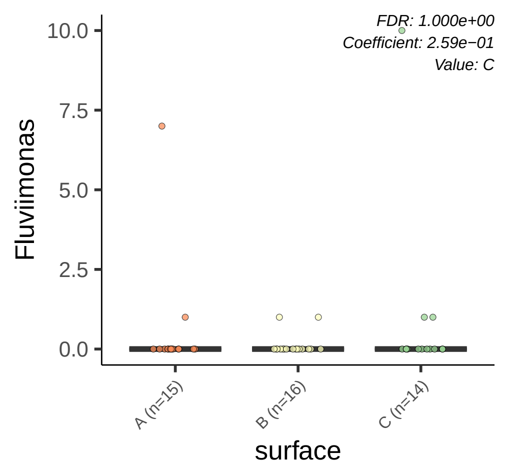
FDR: 1.000e+00
Coefficient: 2.59e−01
Value: C
Fluviimonas
10.0
7.5
5.0
2.5
0.0
A (n=15)
B (n=16)
C (n=14)
surface

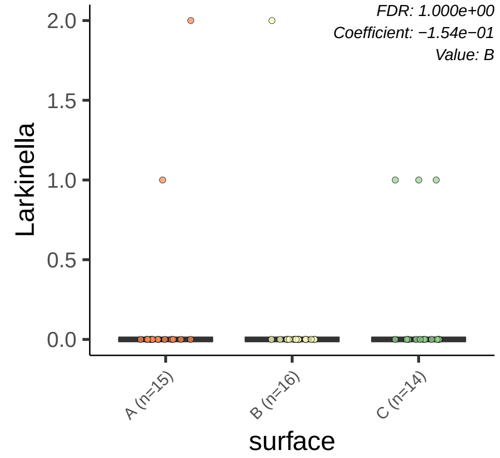

FDR: 1.000e+00
Coefficient: −1.54e−01
Value: B
Larkinella
2.0
1.5
1.0
0.5
0.0
A (n=15)
B (n=16)
C (n=14)
surface

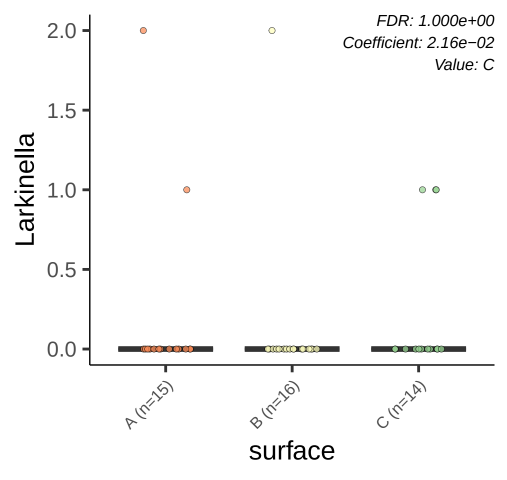
FDR: 1.000e+00
Coefficient: 2.16e−02
Value: C
Larkinella
2.0
1.5
1.0
0.5
0.0
A (n=15)
B (n=16)
C (n=14)
surface

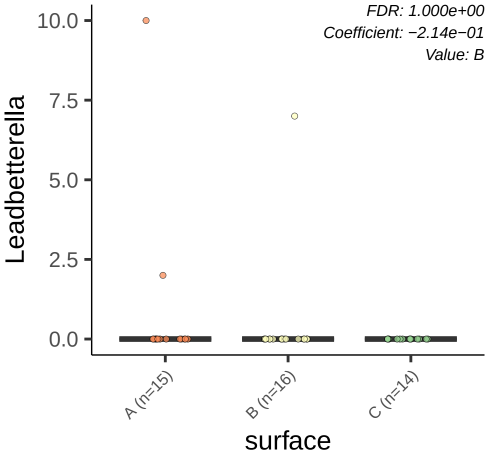
FDR: 1.000e+00
Coefficient: −2.14e−01
Value: B
Leadbetterella
10.0
7.5
5.0
2.5
0.0
A (n=15)
B (n=16)
C (n=14)
surface

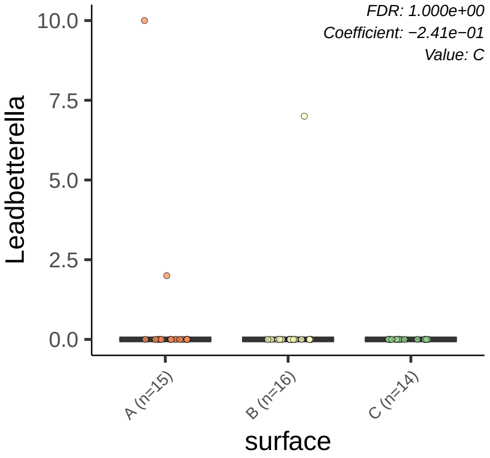
FDR: 1.000e+00
Coefficient: −2.41e−01
Value: C
Leadbetterella
10.0
7.5
5.0
2.5
0.0
A (n=15)
B (n=16)
C (n=14)
surface

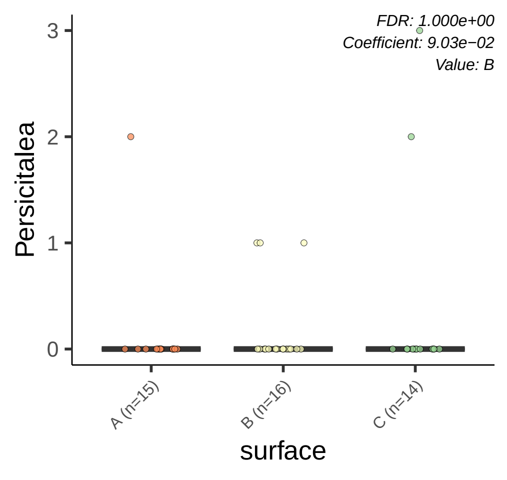
FDR: 1.000e+00
Coefficient: 9.03e−02
Value: B
Persicitalea
3
2
1
0
A (n=15)
B (n=16)
C (n=14)
surface

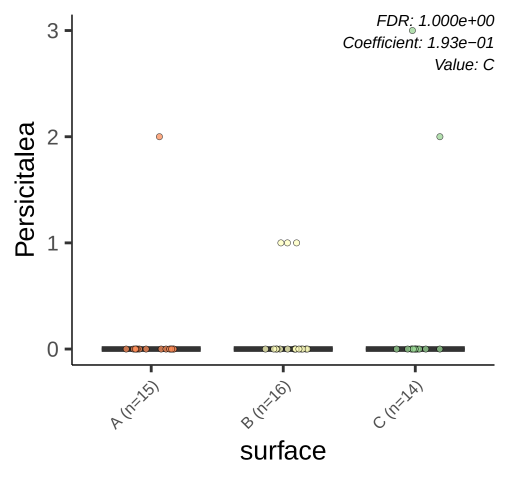

FDR: 1.000e+00
Coefficient: 1.93e−01
Value: C
Persicitalea
3
2
1
0
A (n=15)
B (n=16)
C (n=14)
surface

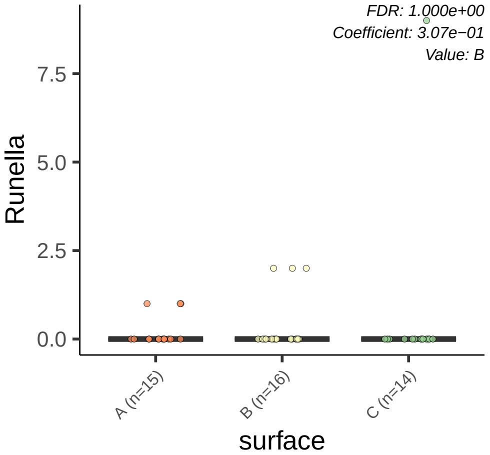

FDR: 1.000e+00
Coefficient: 3.07e−01
Value: B
Runella
7.5
5.0
2.5
0.0
A (n=15)
B (n=16)
C (n=14)
surface

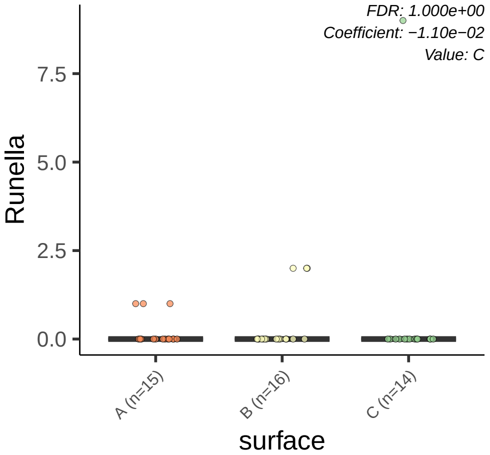

FDR: 1.000e+00
Coefficient: −1.10e−02
Value: C
Runella
7.5
5.0
2.5
0.0
A (n=15)
B (n=16)
C (n=14)
surface

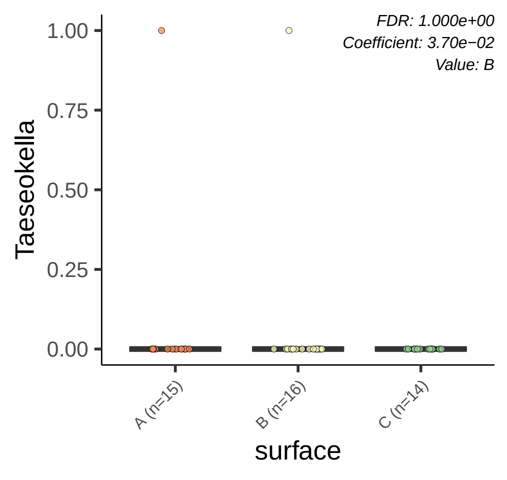

FDR: 1.000e+00
Coefficient: 3.70e−02
Value: B
1.00
0.75
0.50
0.25
0.00
Taeseokella
A (n=15)
B (n=16)
C (n=14)
surface

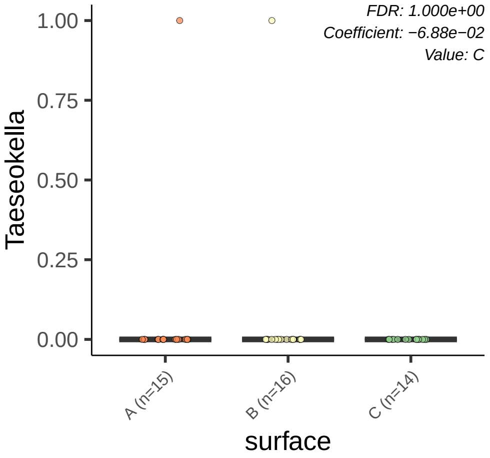

FDR: 1.000e+00
Coefficient: −6.88e−02
Value: C
Taeseokella
1.00
0.75
0.50
0.25
0.00
A (n=15)
B (n=16)
C (n=14)
surface

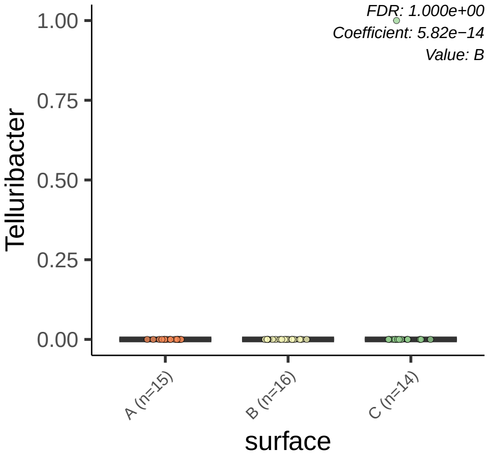
FDR: 1.000e+00
Coefficient: 5.82e−14
Value: B
1.00
0.75
0.50
0.25
0.00
Telluribacter
A (n=15)
B (n=16)
C (n=14)
surface

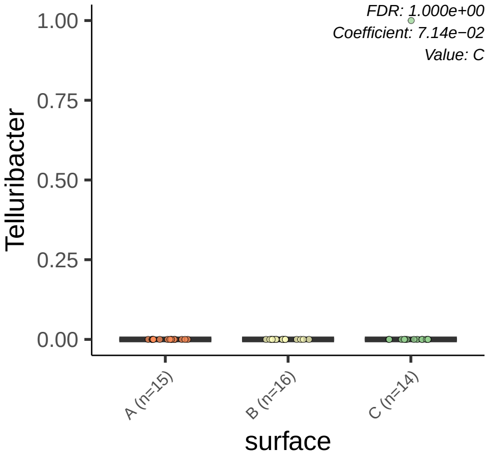

FDR: 1.000e+00
Coefficient: 7.14e−02
Value: C
Telluribacter
1.00
0.75
0.50
0.25
0.00
A (n=15)
B (n=16)
C (n=14)
surface

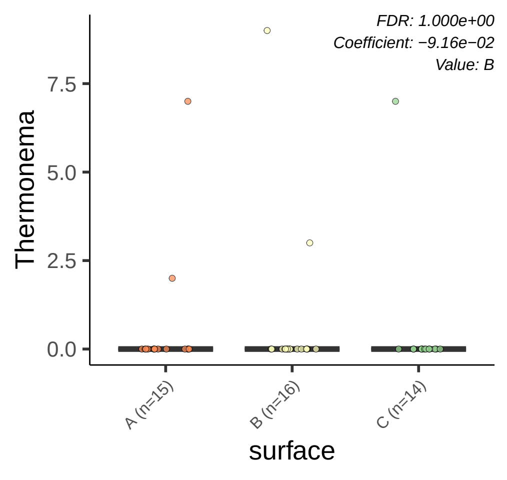

FDR: 1.000e+00
Coefficient: −9.16e−02
Value: B
Thermonema
7.5
5.0
2.5
0.0
A (n=15)
B (n=16)
C (n=14)
surface

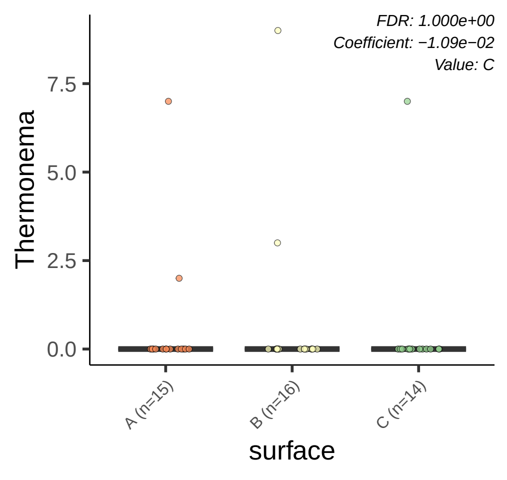
FDR: 1.000e+00
Coefficient: −1.09e−02
Value: C
Thermonema
7.5
5.0
2.5
0.0
A (n=15)
B (n=16)
C (n=14)
surface

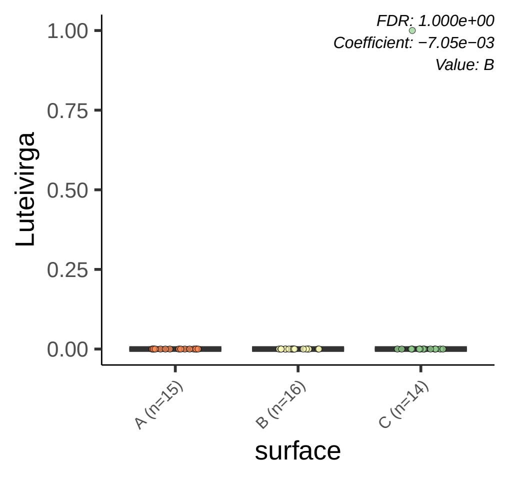

FDR: 1.000e+00
Coefficient: −7.05e−03
Value: B
Luteivirga
1.00
0.75
0.50
0.25
0.00
A (n=15)
B (n=16)
C (n=14)
surface

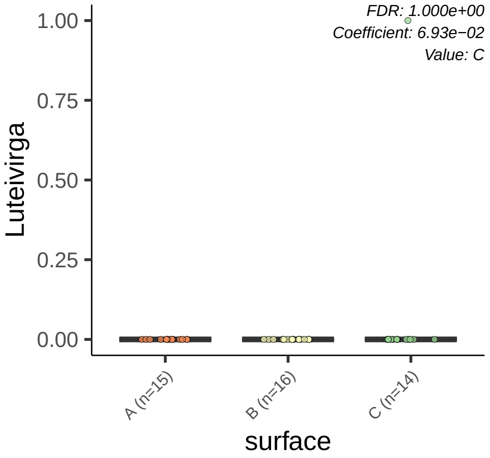
FDR: 1.000e+00
Coefficient: 6.93e−02
Value: C
1.00
0.75
0.50
0.25
0.00
Luteivirga
A (n=15)
B (n=16)
C (n=14)
surface

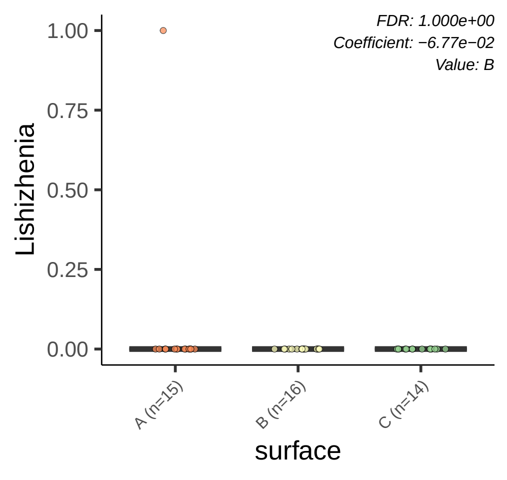
FDR: 1.000e+00
Coefficient: −6.77e−02
Value: B
Lishizhenia
1.00
0.75
0.50
0.25
0.00
A (n=15)
B (n=16)
C (n=14)
surface

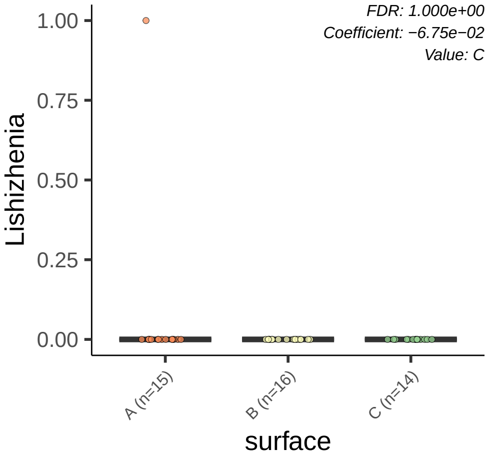
FDR: 1.000e+00
Coefficient: −6.75e−02
Value: C
Lishizhenia
1.00
0.75
0.50
0.25
0.00
A (n=15)
B (n=16)
C (n=14)
surface

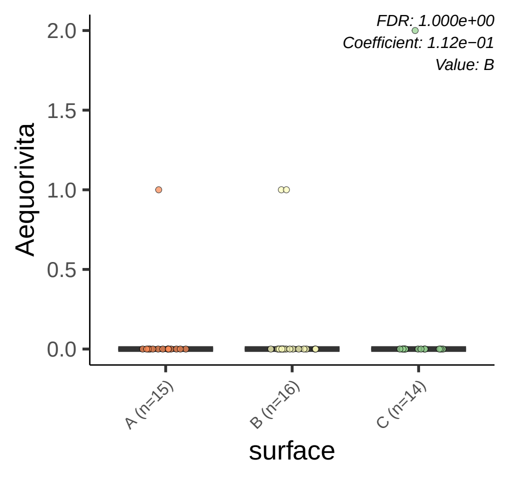
FDR: 1.000e+00
Coefficient: 1.12e−01
Value: B
Aequorivita
2.0
1.5
1.0
0.5
0.0
A (n=15)
B (n=16)
C (n=14)
surface

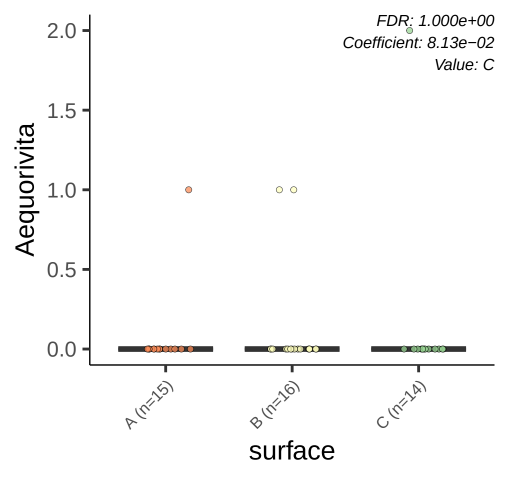
FDR: 1.000e+00
Coefficient: 8.13e−02
Value: C
Aequorivita
2.0
1.5
1.0
0.5
0.0
A (n=15)
B (n=16)
C (n=14)
surface

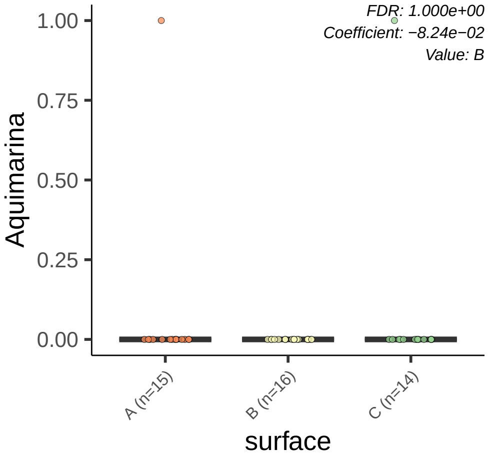

FDR: 1.000e+00
Coefficient: −8.24e−02
Value: B
Aquimarina
1.00
0.75
0.50
0.25
0.00
A (n=15)
B (n=16)
C (n=14)
surface

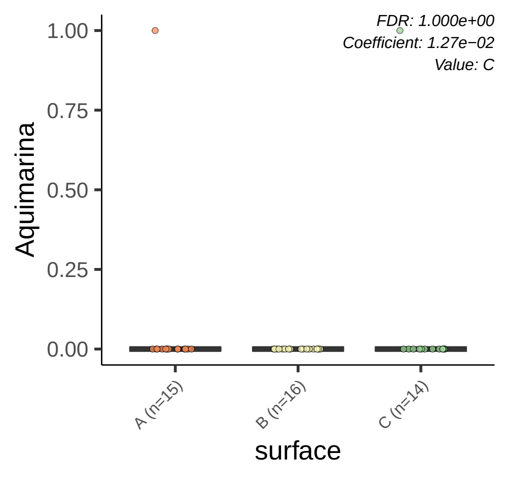

FDR: 1.000e+00
Coefficient: 1.27e−02
Value: C
Aquimarina
1.00
0.75
0.50
0.25
0.00
A (n=15)
B (n=16)
C (n=14)
surface

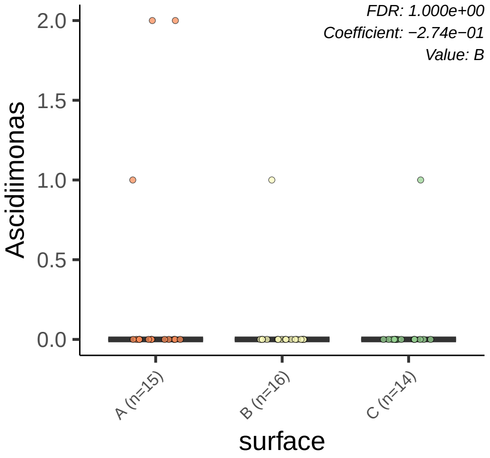

FDR: 1.000e+00
Coefficient: −2.74e−01
Value: B
Ascidimonas
2.0
1.5
1.0
0.5
0.0
A (n=15)
B (n=16)
C (n=14)
surface

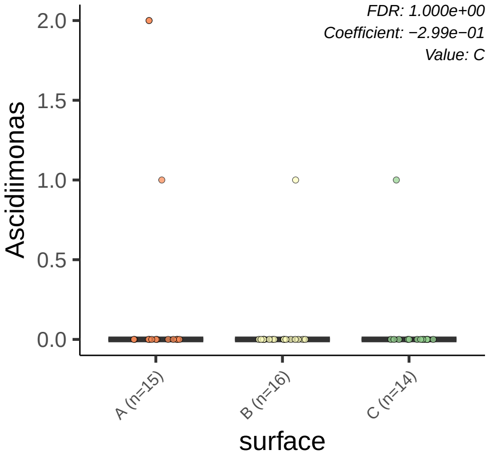

FDR: 1.000e+00
Coefficient: −2.99e−01
Value: C
Ascidimonas
2.0
1.5
1.0
0.5
0.0
A (n=15)
B (n=16)
C (n=14)
surface

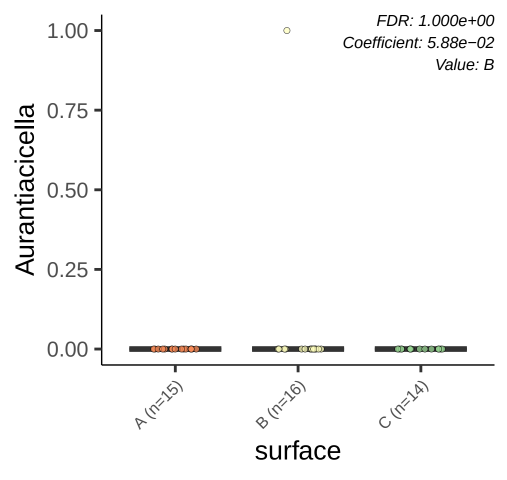
FDR: 1.000e+00
Coefficient: 5.88e−02
Value: B
Aurantiacicella
1.00
0.75
0.50
0.25
0.00
A (n=15)
B (n=16)
C (n=14)
surface

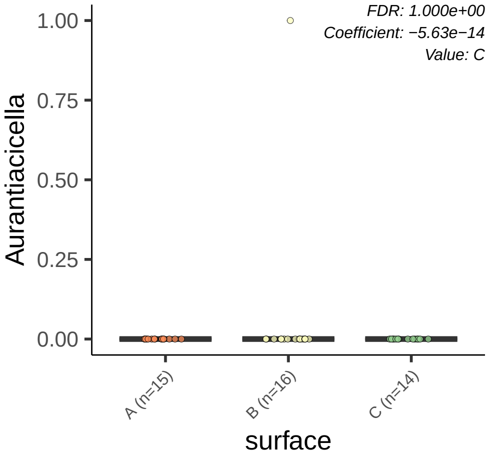

FDR: 1.000e+00
Coefficient: −5.63e−14
Value: C
Aurantiacicella
1.00
0.75
0.50
0.25
0.00
A (n=15)
B (n=16)
C (n=14)
surface

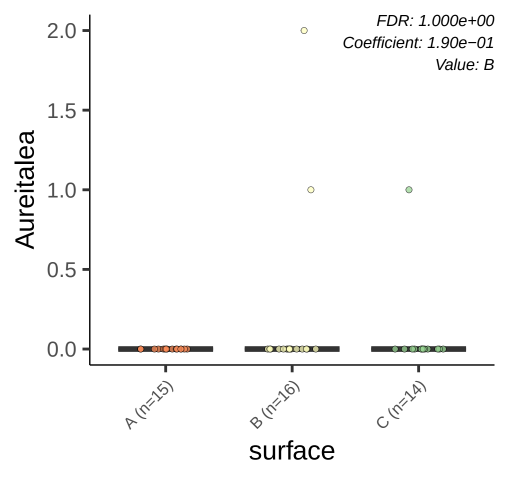

FDR: 1.000e+00
Coefficient: 1.90e−01
Value: B
2.0
1.5
1.0
0.5
0.0
Aureitalea
A (n=15)
B (n=16)
C (n=14)
surface

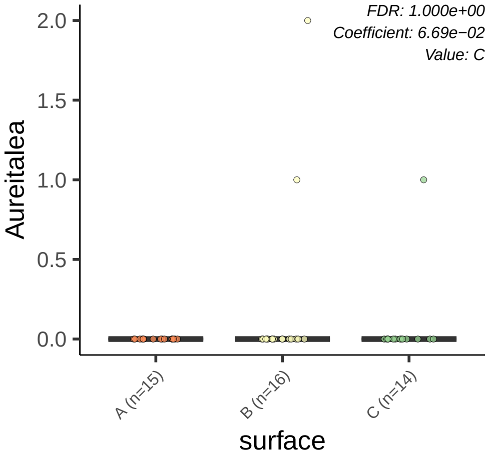
FDR: 1.000e+00
Coefficient: 6.69e−02
Value: C
Aureitalea
2.0
1.5
1.0
0.5
0.0
A (n=15)
B (n=16)
C (n=14)
surface

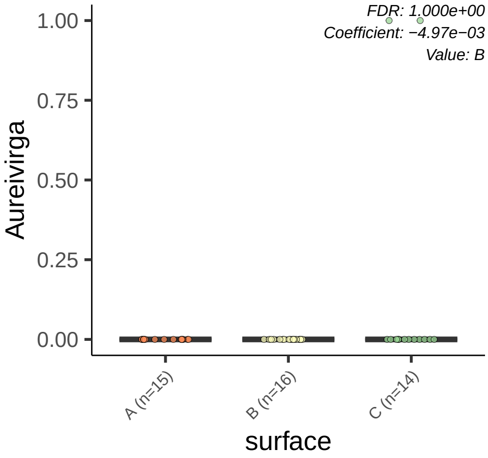

FDR: 1.000e+00
Coefficient: −4.97e−03
Value: B
Aureivirga
1.00
0.75
0.50
0.25
0.00
A (n=15)
B (n=16)
C (n=14)
surface

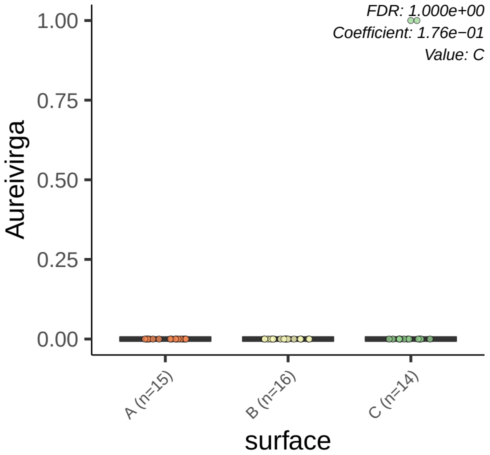
FDR: 1.000e+00
Coefficient: 1.76e−01
Value: C
Aureivirga
1.00
0.75
0.50
0.25
0.00
A (n=15)
B (n=16)
C (n=14)
surface

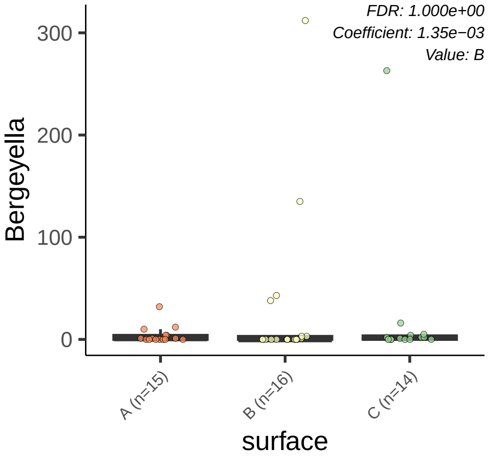

FDR: 1.000e+00
Coefficient: 1.35e−03
Value: B
Bergeyella
300
200
100
0
A (n=15)
B (n=16)
C (n=14)
surface

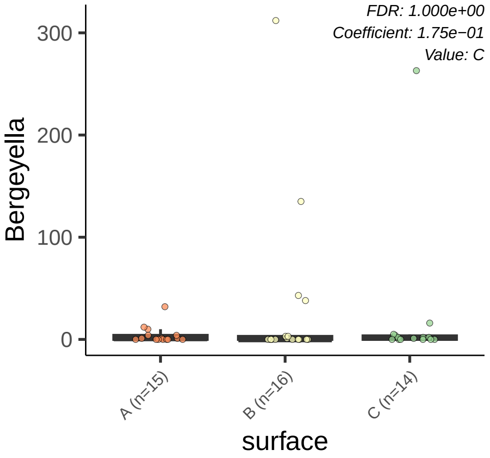

FDR: 1.000e+00
Coefficient: 1.75e−01
Value: C
Bergeyella
0
100
200
300
A (n=15)
B (n=16)
C (n=14)
surface

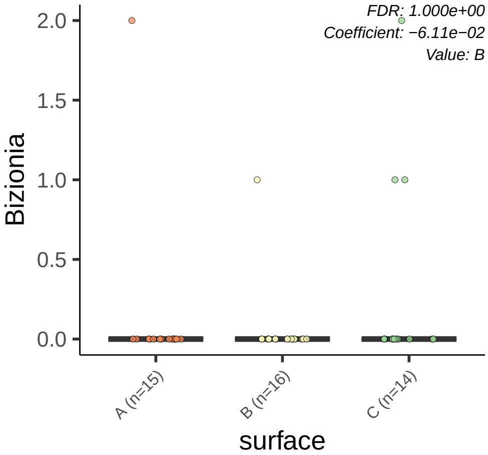

FDR: 1.000e+00
Coefficient: −6.11e−02
Value: B
2.0
1.5
1.0
0.5
0.0
Bizionia
A (n=15)
B (n=16)
C (n=14)
surface

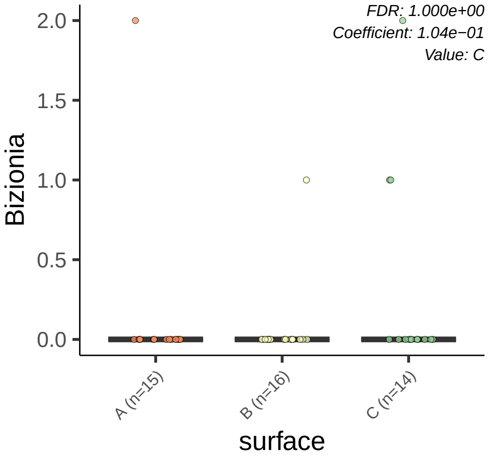
FDR: 1.000e+00
Coefficient: 1.04e−01
Value: C
Bizionia
2.0
1.5
1.0
0.5
0.0
A (n=15)
B (n=16)
C (n=14)
surface

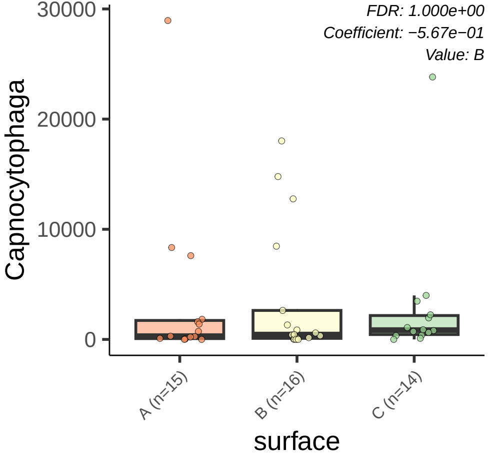
FDR: 1.000e+00
Coefficient: −5.67e−01
Value: B
Capnocytophaga
30000
20000
10000
0
A (n=15)
B (n=16)
C (n=14)
surface

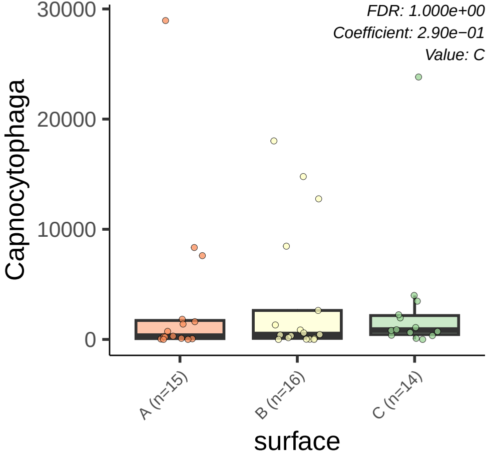
FDR: 1.000e+00
Coefficient: 2.90e−01
Value: C
Capnocytophaga
30000
20000
10000
0
A (n=15)
B (n=16)
C (n=14)
surface

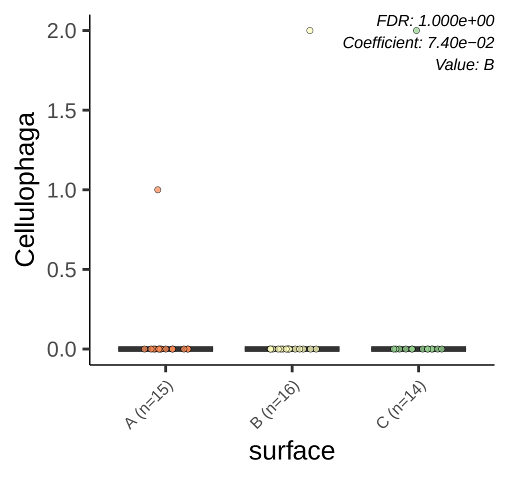
FDR: 1.000e+00
Coefficient: 7.40e−02
Value: B
Cellulophaga
2.0
1.5
1.0
0.5
0.0
A (n=15)
B (n=16)
C (n=14)
surface

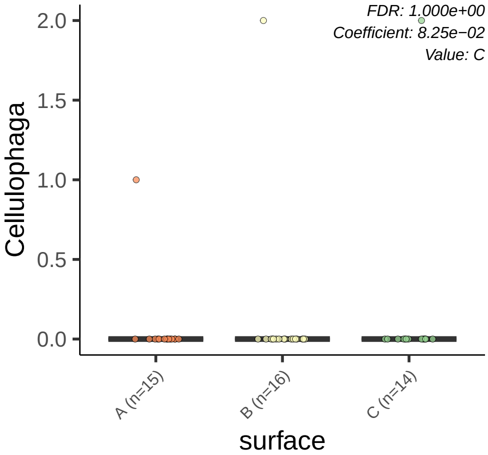
FDR: 1.000e+00
Coefficient: 8.25e−02
Value: C
2.0
1.5
1.0
0.5
0.0
Cellulophaga
A (n=15)
B (n=16)
C (n=14)
surface

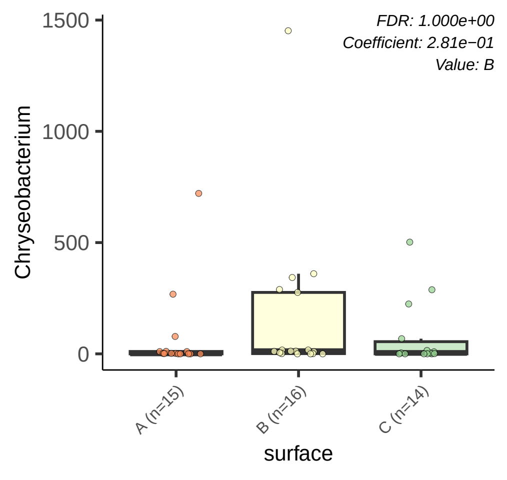
FDR: 1.000e+00
Coefficient: 2.81e−01
Value: B
1500
1000
500
0
Chryseobacterium
A (n=15)
B (n=16)
C (n=14)
surface

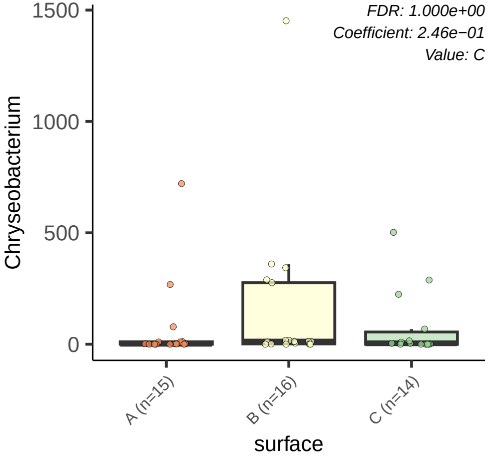
FDR: 1.000e+00
Coefficient: 2.46e−01
Value: C
Chryseobacterium
1500
1000
500
0
A (n=15)
B (n=16)
C (n=14)
surface

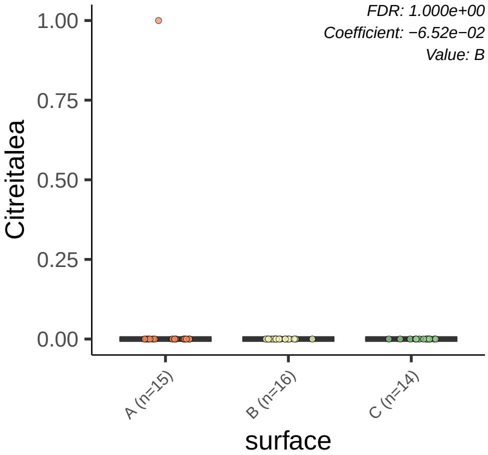
FDR: 1.000e+00
Coefficient: −6.52e−02
Value: B
Citreitalea
1.00
0.75
0.50
0.25
0.00
A (n=15)
B (n=16)
C (n=14)
surface

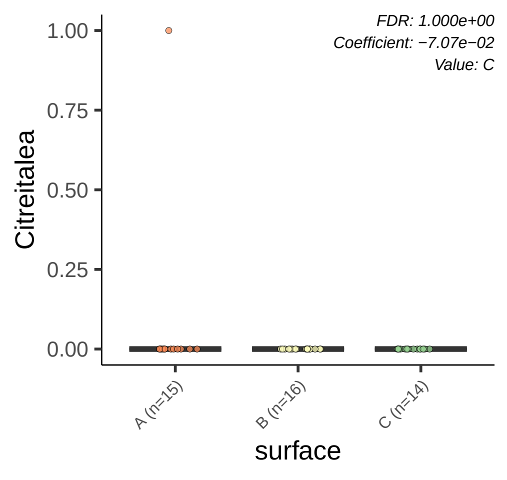
FDR: 1.000e+00
Coefficient: −7.07e−02
Value: C
Citreitalea
1.00
0.75
0.50
0.25
0.00
A (n=15)
B (n=16)
C (n=14)
surface

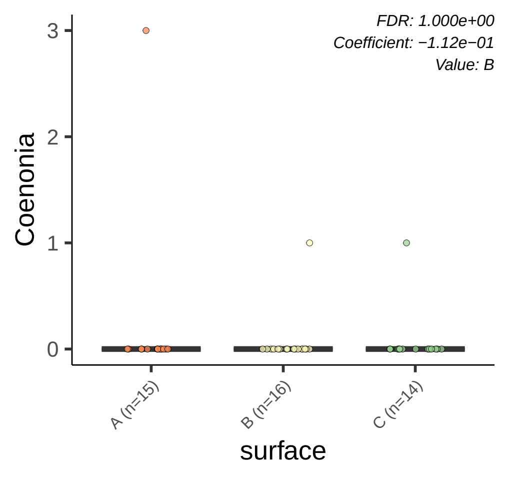

FDR: 1.000e+00
Coefficient: −1.12e−01
Value: B
Coenonia
0
1
2
3
A (n=15)
B (n=16)
C (n=14)
surface

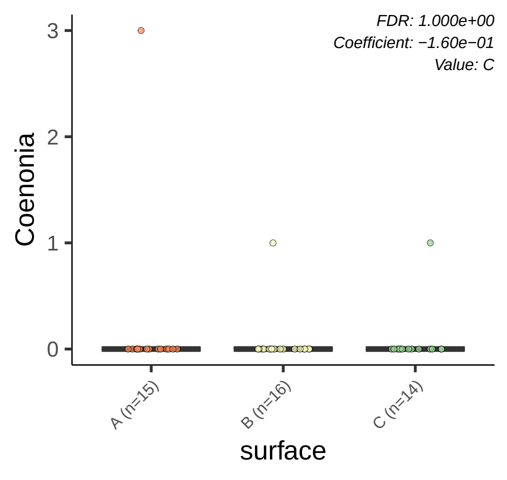
FDR: 1.000e+00
Coefficient: −1.60e−01
Value: C
Coenonia
3
2
1
0
A (n=15)
B (n=16)
C (n=14)
surface

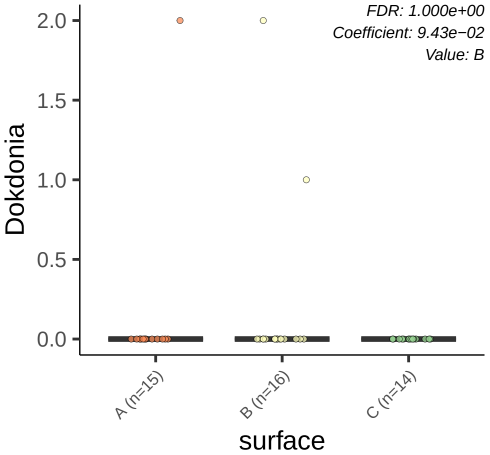

FDR: 1.000e+00
Coefficient: 9.43e−02
Value: B
Dokdonia
2.0
1.5
1.0
0.5
0.0
A (n=15)
B (n=16)
C (n=14)
surface

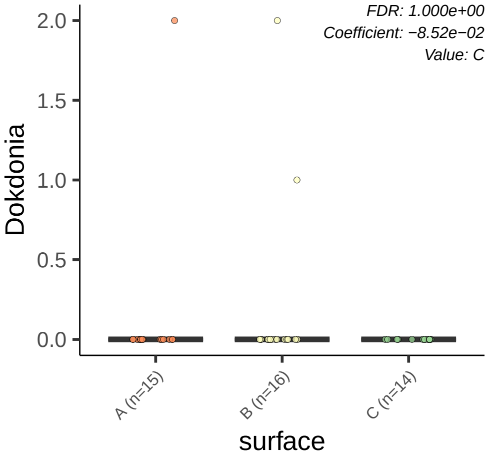
FDR: 1.000e+00
Coefficient: −8.52e−02
Value: C
Dokdonia
2.0
1.5
1.0
0.5
0.0
A (n=15)
B (n=16)
C (n=14)
surface

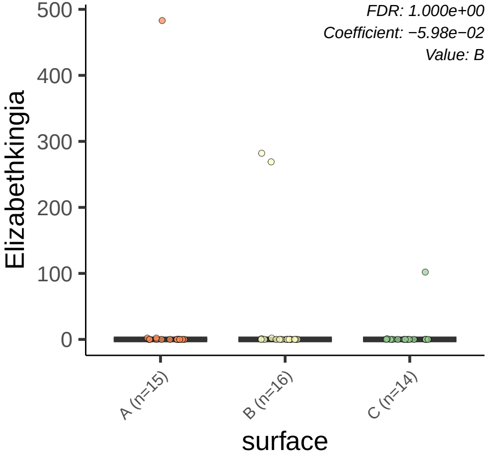
FDR: 1.000e+00
Coefficient: −5.98e−02
Value: B
Elizabethkingia
500
400
300
200
100
0
A (n=15)
B (n=16)
C (n=14)
surface

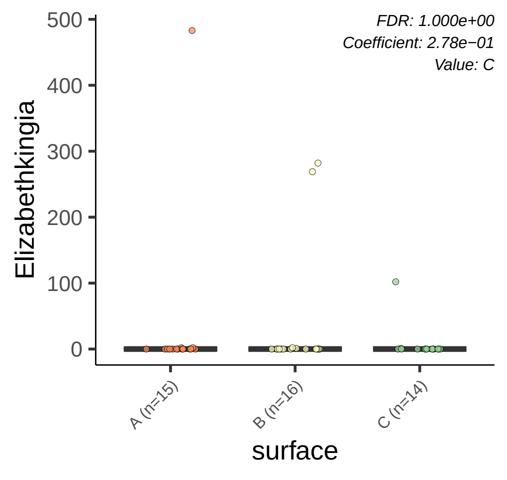
FDR: 1.000e+00
Coefficient: 2.78e−01
Value: C
Elizabethkingia
500
400
300
200
100
0
A (n=15)
B (n=16)
C (n=14)
surface

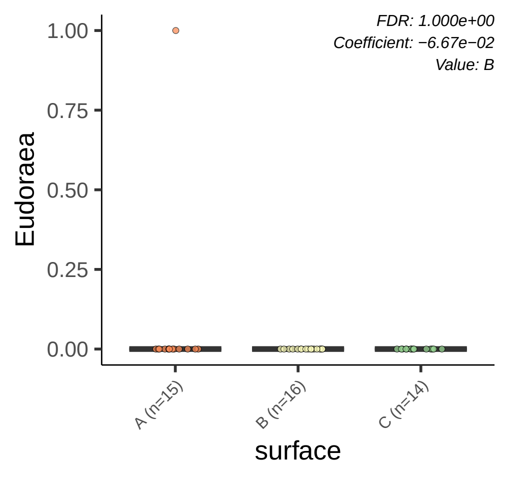
FDR: 1.000e+00
Coefficient: −6.67e−02
Value: B
Eudoraea
1.00
0.75
0.50
0.25
0.00
A (n=15)
B (n=16)
C (n=14)
surface

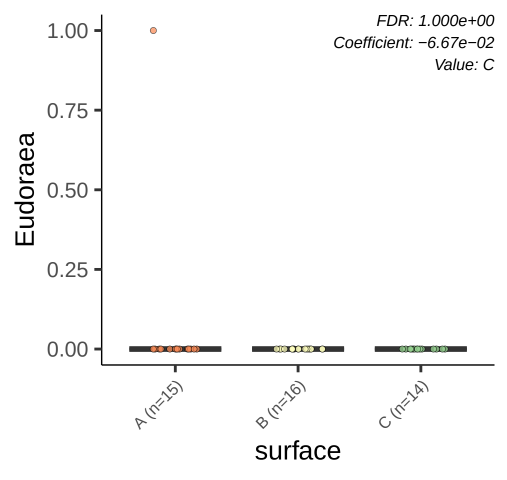
FDR: 1.000e+00
Coefficient: −6.67e−02
Value: C
1.00
0.75
0.50
0.25
0.00
Eudoraea
A (n=15)
B (n=16)
C (n=14)
surface

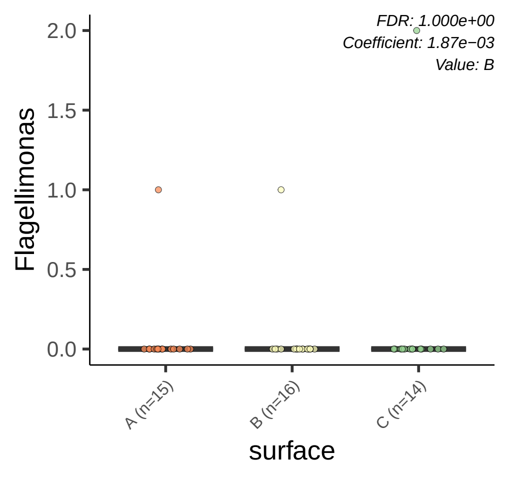

FDR: 1.000e+00
Coefficient: 1.87e−03
Value: B
Flagellimonas
2.0
1.5
1.0
0.5
0.0
A (n=15)
B (n=16)
C (n=14)
surface

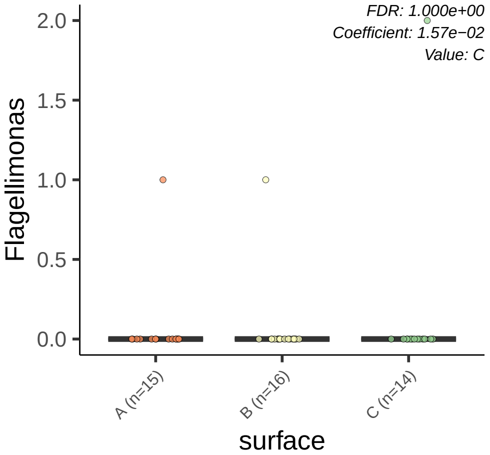

FDR: 1.000e+00
Coefficient: 1.57e−02
Value: C
Flagellimonas
2.0
1.5
1.0
0.5
0.0
A (n=15)
B (n=16)
C (n=14)
surface

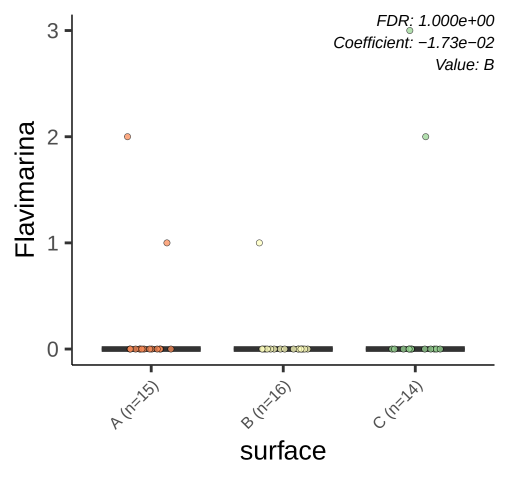
FDR: 1.000e+00
Coefficient: −1.73e−02
Value: B
Flavimarina
3
2
1
0
A (n=15)
B (n=16)
C (n=14)
surface

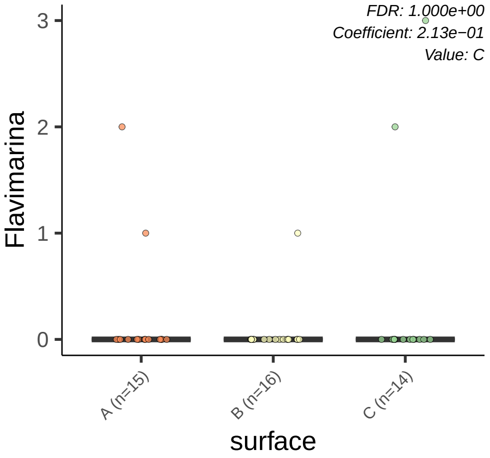
FDR: 1.000e+00
Coefficient: 2.13e−01
Value: C
Flavimarina
3
2
1
0
A (n=15)
B (n=16)
C (n=14)
surface

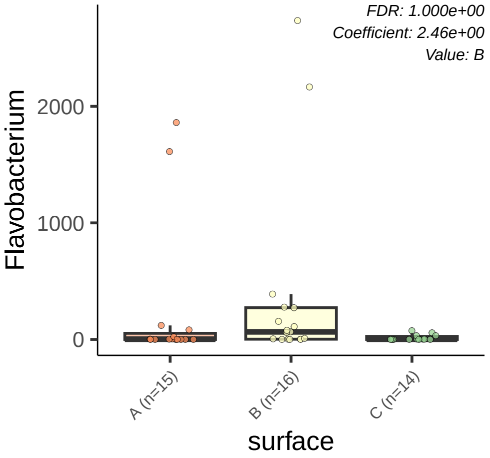

FDR: 1.000e+00
Coefficient: 2.46e+00
Value: B
Flavobacterium
2000
1000
0
A (n=15)
B (n=16)
C (n=14)
surface

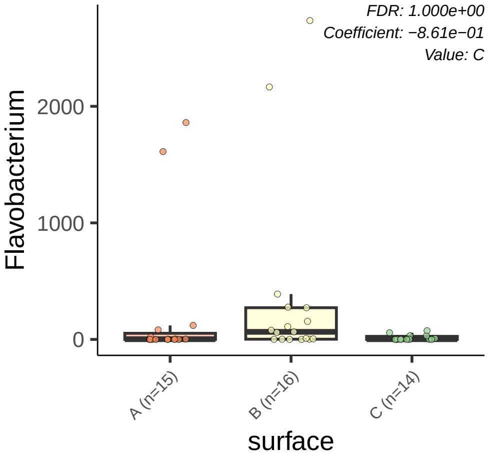

FDR: 1.000e+00
Coefficient: −8.61e−01
Value: C
Flavobacterium
2000
1000
0
A (n=15)
B (n=16)
C (n=14)
surface

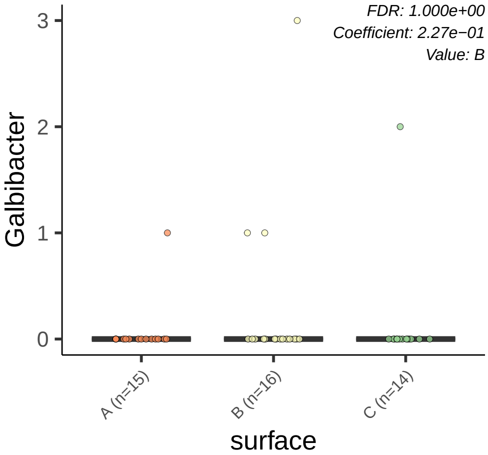
FDR: 1.000e+00
Coefficient: 2.27e−01
Value: B
Galbibacter
0
1
2
3
A (n=15)
B (n=16)
C (n=14)
surface

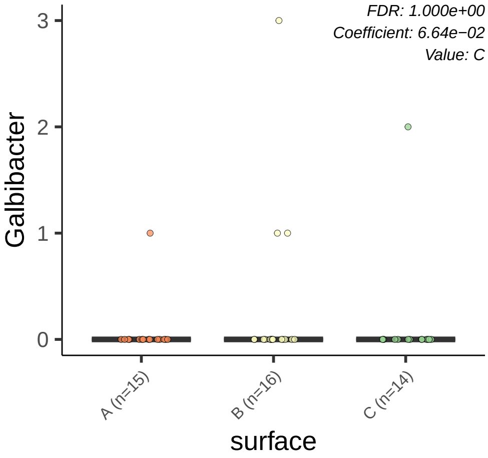

FDR: 1.000e+00
Coefficient: 6.64e−02
Value: C
3
2
1
0
Galbibacter
A (n=15)
B (n=16)
C (n=14)
surface

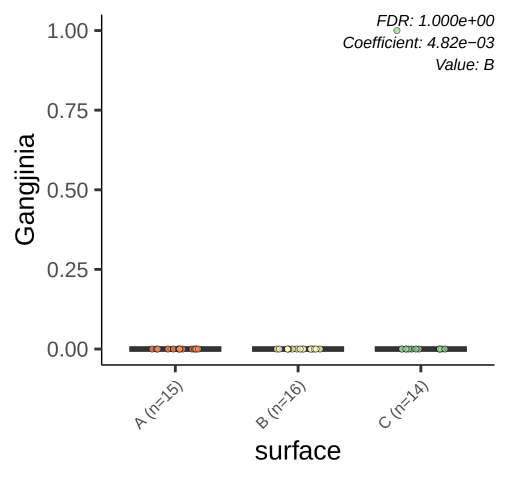
FDR: 1.000e+00
Coefficient: 4.82e−03
Value: B
Gangjinia
1.00
0.75
0.50
0.25
0.00
A (n=15)
B (n=16)
C (n=14)
surface

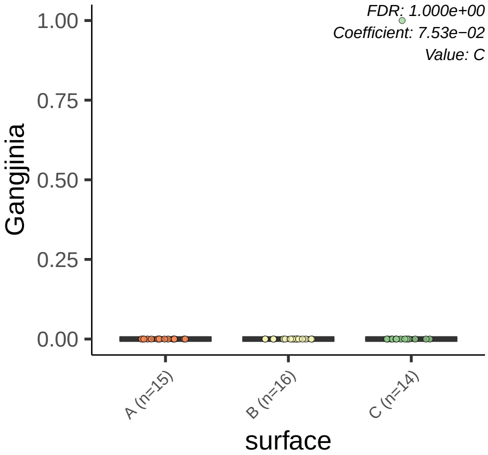
FDR: 1.000e+00
Coefficient: 7.53e−02
Value: C
Gangjinia
1.00
0.75
0.50
0.25
0.00
A (n=15)
B (n=16)
C (n=14)
surface

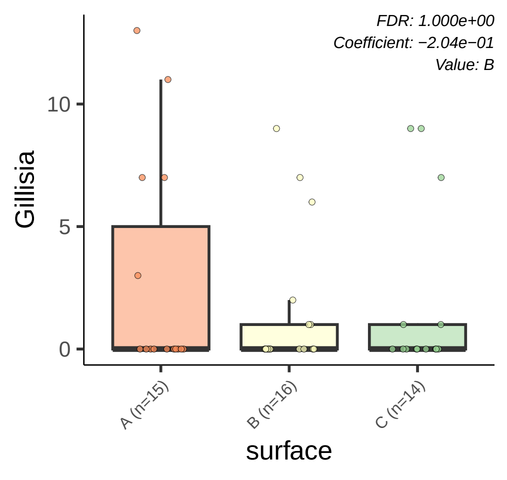

FDR: 1.000e+00
Coefficient: −2.04e−01
Value: B
Gillisia
10
5
0
A (n=15)
B (n=16)
C (n=14)
surface

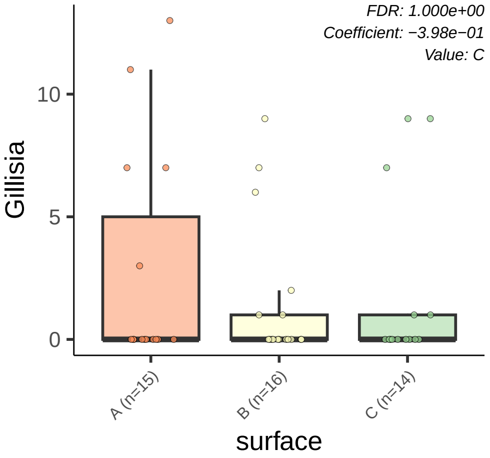
FDR: 1.000e+00
Coefficient: −3.98e−01
Value: C
Gillisia
10
5
0
A (n=15)
B (n=16)
C (n=14)
surface

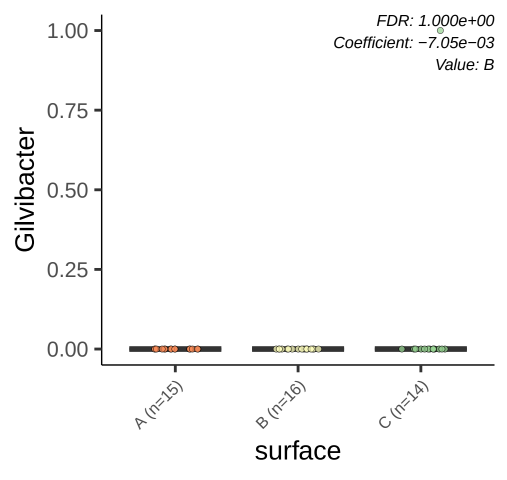
FDR: 1.000e+00
Coefficient: −7.05e−03
Value: B
1.00
0.75
0.50
0.25
0.00
Gilvibacter
A (n=15)
B (n=16)
C (n=14)
surface

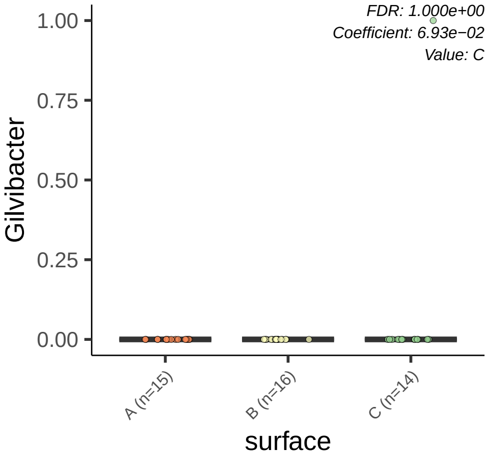

FDR: 1.000e+00
Coefficient: 6.93e−02
Value: C
1.00
0.75
0.50
0.25
0.00
Gilvibacter
A (n=15)
B (n=16)
C (n=14)
surface

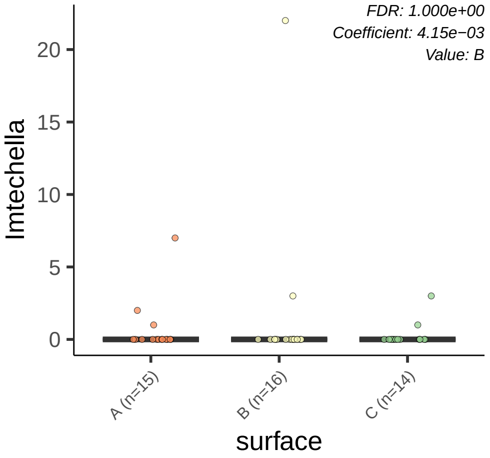

FDR: 1.000e+00
Coefficient: 4.15e−03
Value: B
Imtechella
0
5
10
15
20
A (n=15)
B (n=16)
C (n=14)
surface

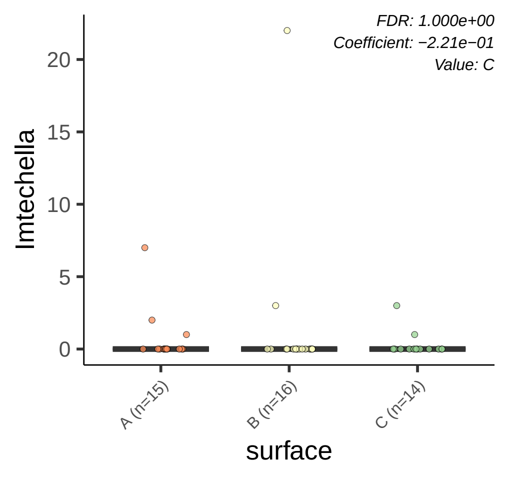

FDR: 1.000e+00
Coefficient: −2.21e−01
Value: C
Imtechella
20
15
10
5
0
A (n=15)
B (n=16)
C (n=14)
surface

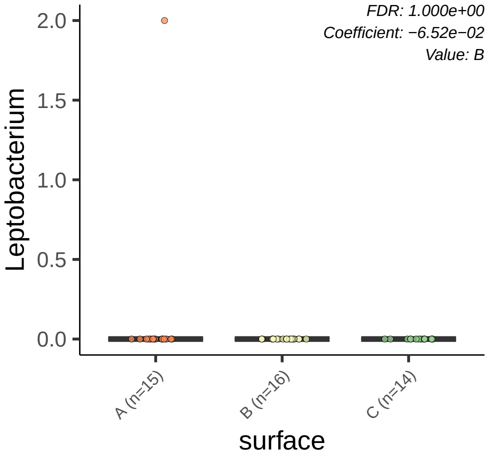
FDR: 1.000e+00
Coefficient: −6.52e−02
Value: B
Leptobacterium
2.0
1.5
1.0
0.5
0.0
A (n=15)
B (n=16)
C (n=14)
surface

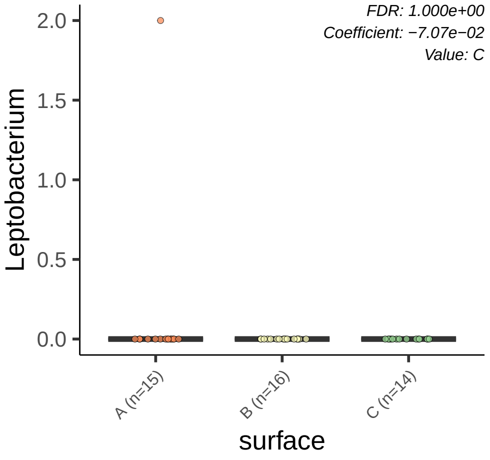
FDR: 1.000e+00
Coefficient: −7.07e−02
Value: C
Leptobacterium
2.0
1.5
1.0
0.5
0.0
A (n=15)
B (n=16)
C (n=14)
surface

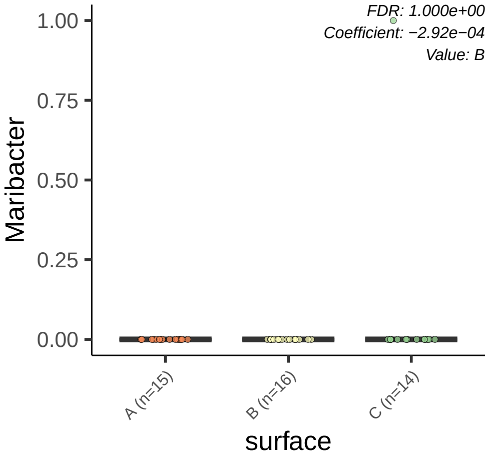
FDR: 1.000e+00
Coefficient: −2.92e−04
Value: B
1.00
0.75
0.50
0.25
0.00
Maribacter
A (n=15)
B (n=16)
C (n=14)
surface

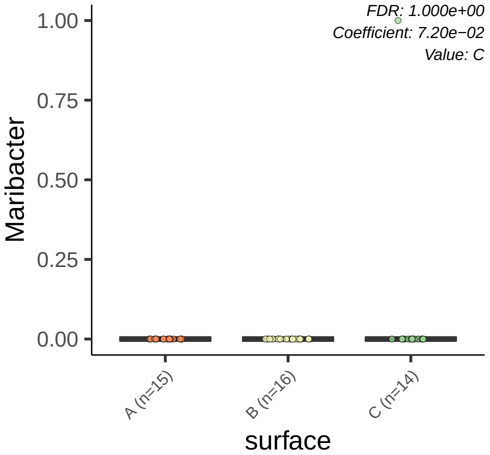
FDR: 1.000e+00
Coefficient: 7.20e−02
Value: C
1.00
0.75
0.50
0.25
0.00
Maribacter
A (n=15)
B (n=16)
C (n=14)
surface

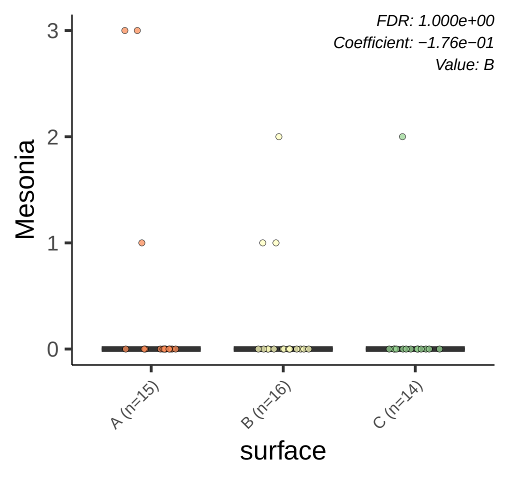
FDR: 1.000e+00
Coefficient: −1.76e−01
Value: B
Mesonia
3
2
1
0
A (n=15)
B (n=16)
C (n=14)
surface

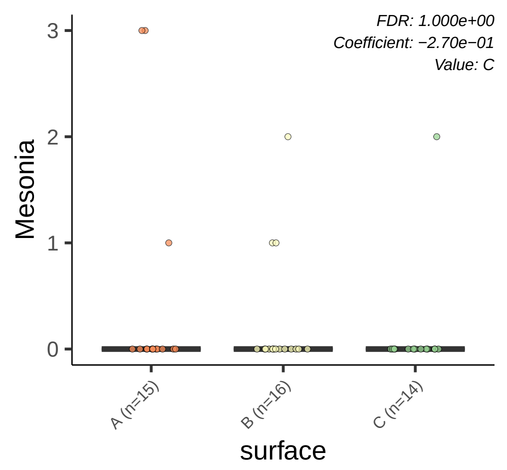
FDR: 1.000e+00
Coefficient: −2.70e−01
Value: C
Mesonia
3
2
1
0
A (n=15)
B (n=16)
C (n=14)
surface

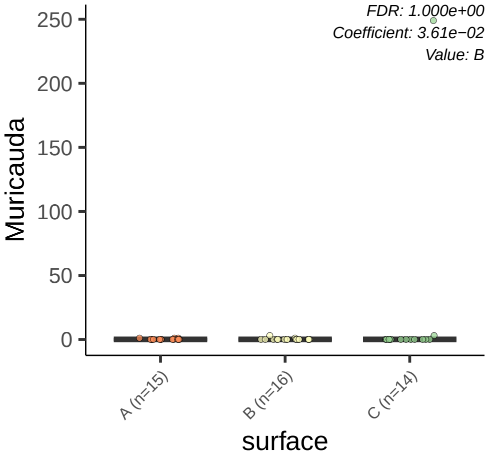
FDR: 1.000e+00
Coefficient: 3.61e−02
Value: B
Muricauda
250
200
150
100
50
0
A (n=15)
B (n=16)
C (n=14)
surface

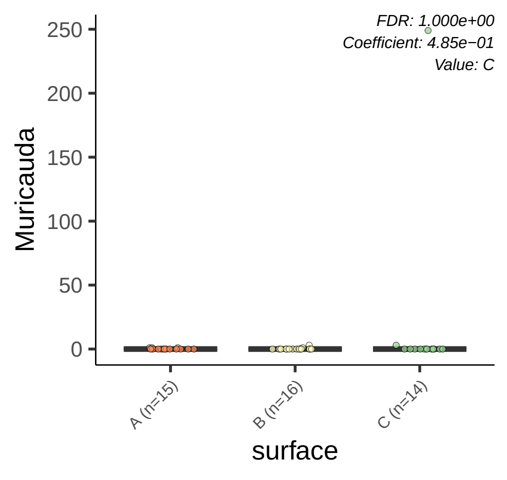
FDR: 1.000e+00
Coefficient: 4.85e−01
Value: C
250
200
150
100
50
0
Muricauda
A (n=15)
B (n=16)
C (n=14)
surface

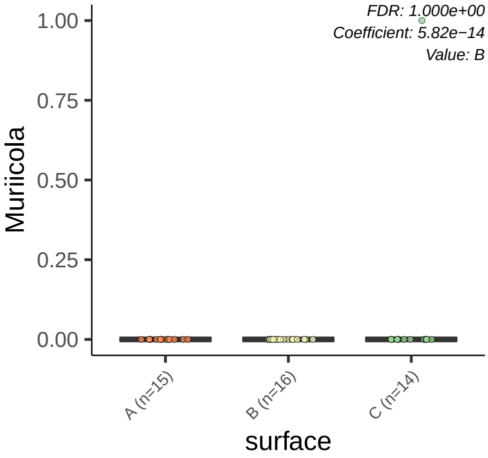
FDR: 1.000e+00
Coefficient: 5.82e−14
Value: B
1.00
0.75
0.50
0.25
0.00
Muriicola
A (n=15)
B (n=16)
C (n=14)
surface

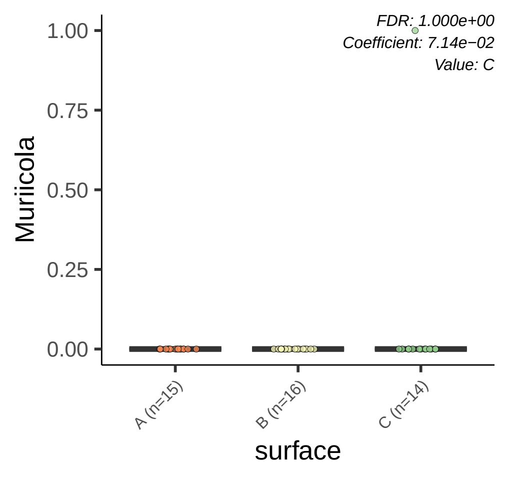
FDR: 1.000e+00
Coefficient: 7.14e−02
Value: C
Muriicola
1.00
0.75
0.50
0.25
0.00
A (n=15)
B (n=16)
C (n=14)
surface

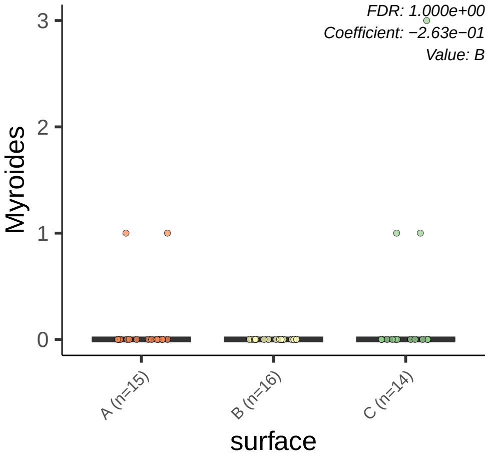

FDR: 1.000e+00
Coefficient: −2.63e−01
Value: B
Myroides
3
2
1
0
A (n=15)
B (n=16)
C (n=14)
surface

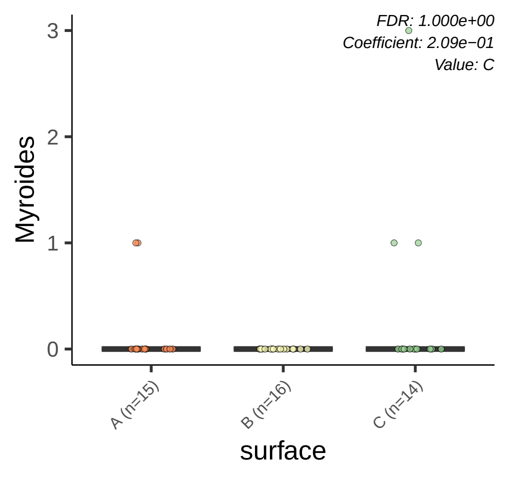

FDR: 1.000e+00
Coefficient: 2.09e−01
Value: C
Myroides
3
2
1
0
A (n=15)
B (n=16)
C (n=14)
surface

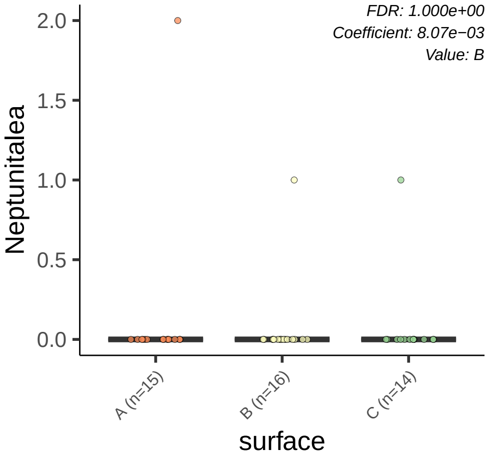

FDR: 1.000e+00
Coefficient: 8.07e−03
Value: B
Neptunitalea
2.0
1.5
1.0
0.5
0.0
A (n=15)
B (n=16)
C (n=14)
surface

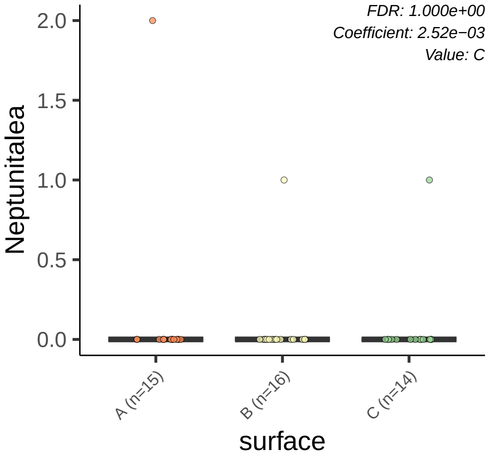
FDR: 1.000e+00
Coefficient: 2.52e−03
Value: C
Neptunitalea
2.0
1.5
1.0
0.5
0.0
A (n=15)
B (n=16)
C (n=14)
surface

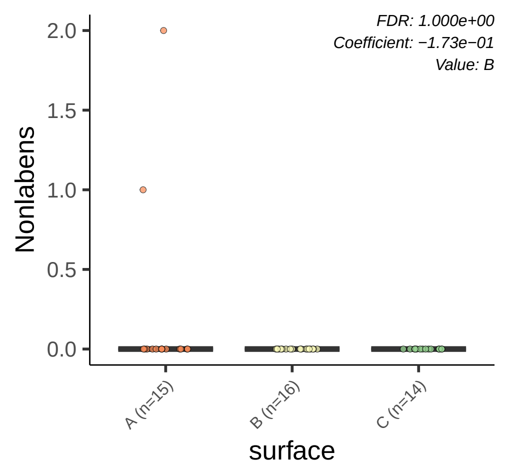

FDR: 1.000e+00
Coefficient: −1.73e−01
Value: B
Nonlabens
2.0
1.5
1.0
0.5
0.0
A (n=15)
B (n=16)
C (n=14)
surface

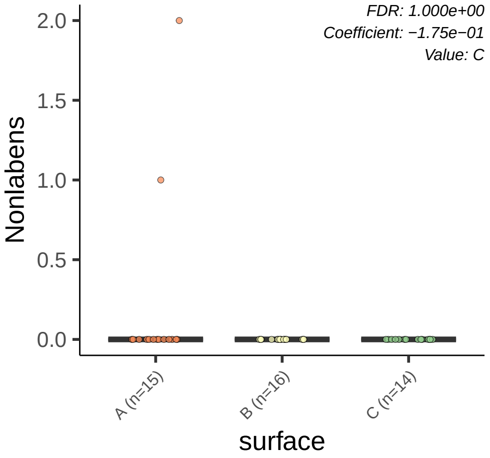

FDR: 1.000e+00
Coefficient: −1.75e−01
Value: C
Nonlabens
2.0
1.5
1.0
0.5
0.0
A (n=15)
B (n=16)
C (n=14)
surface

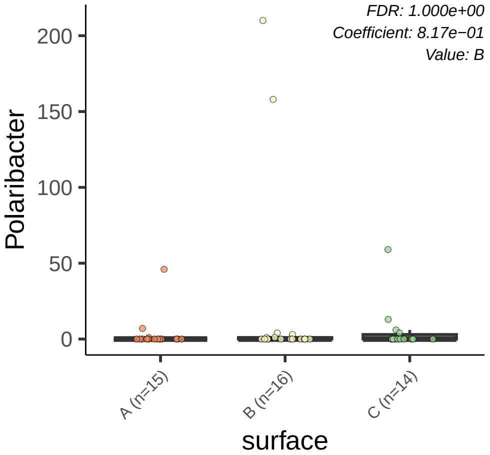

FDR: 1.000e+00
Coefficient: 8.17e−01
Value: B
Polaribacter
200
150
100
50
0
A (n=15)
B (n=16)
C (n=14)
surface

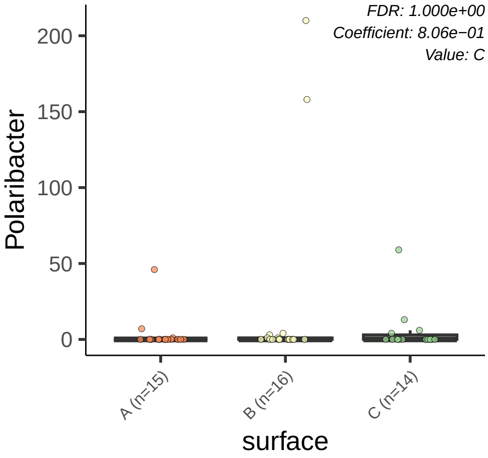
FDR: 1.000e+00
Coefficient: 8.06e−01
Value: C
Polaribacter
200
150
100
50
0
A (n=15)
B (n=16)
C (n=14)
surface

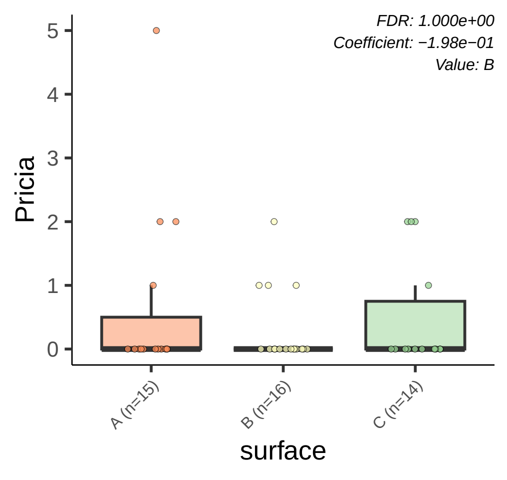

FDR: 1.000e+00
Coefficient: −1.98e−01
Value: B
Pricia
5
4
3
2
1
0
A (n=15)
B (n=16)
C (n=14)
surface

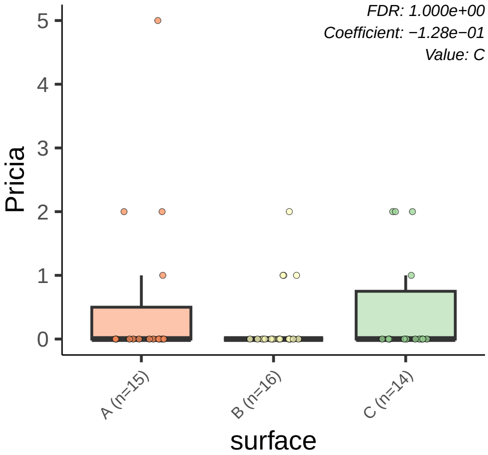

FDR: 1.000e+00
Coefficient: −1.28e−01
Value: C
Pricia
5
4
3
2
1
0
A (n=15)
B (n=16)
C (n=14)
surface

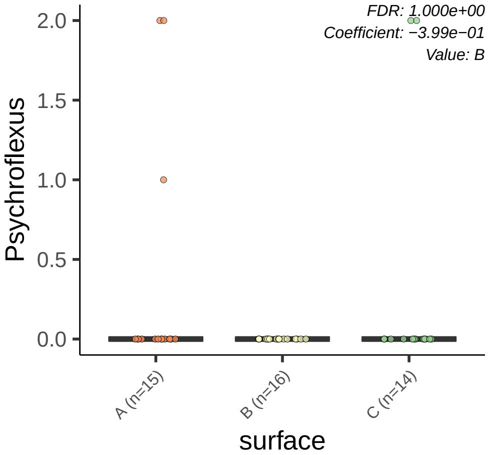

FDR: 1.000e+00
Coefficient: −3.99e−01
Value: B
Psychroflexus
2.0
1.5
1.0
0.5
0.0
A (n=15)
B (n=16)
C (n=14)
surface

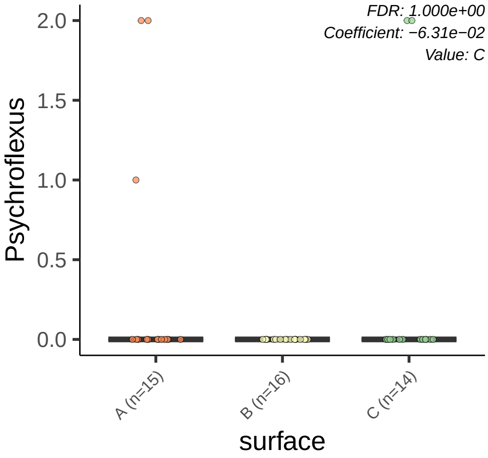

FDR: 1.000e+00
Coefficient: −6.31e−02
Value: C
Psychroflexus
2.0
1.5
1.0
0.5
0.0
A (n=15)
B (n=16)
C (n=14)
surface

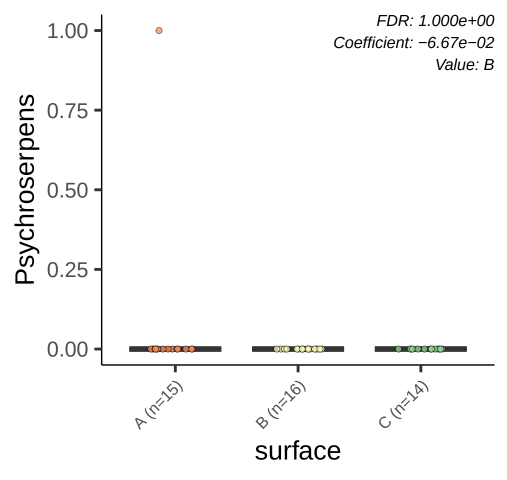
FDR: 1.000e+00
Coefficient: −6.67e−02
Value: B
Psychroserpens
1.00
0.75
0.50
0.25
0.00
A (n=15)
B (n=16)
C (n=14)
surface

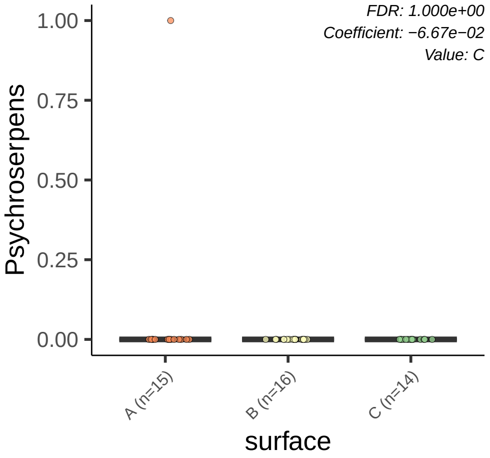
FDR: 1.000e+00
Coefficient: −6.67e−02
Value: C
Psychroserpens
1.00
0.75
0.50
0.25
0.00
A (n=15)
B (n=16)
C (n=14)
surface

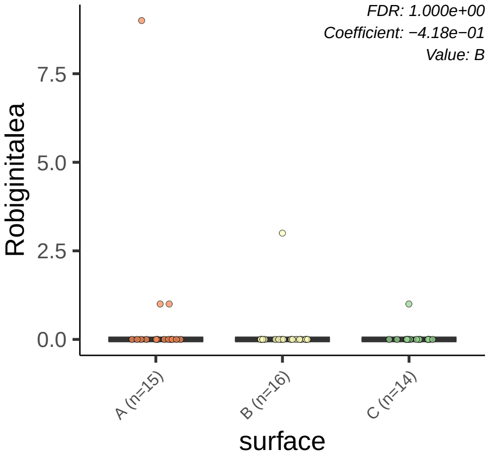
FDR: 1.000e+00
Coefficient: −4.18e−01
Value: B
Robiginitalea
7.5
5.0
2.5
0.0
A (n=15)
B (n=16)
C (n=14)
surface

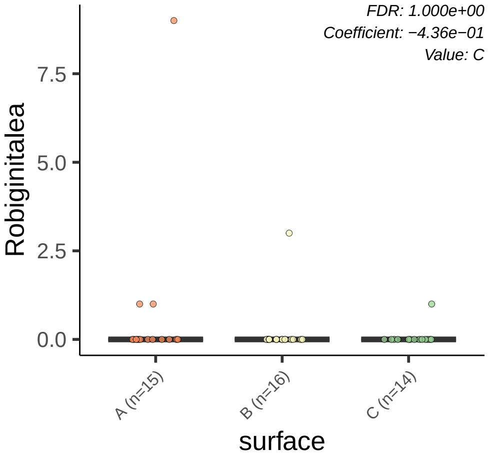

FDR: 1.000e+00
Coefficient: −4.36e−01
Value: C
Robiginitalea
7.5
5.0
2.5
0.0
A (n=15)
B (n=16)
C (n=14)
surface

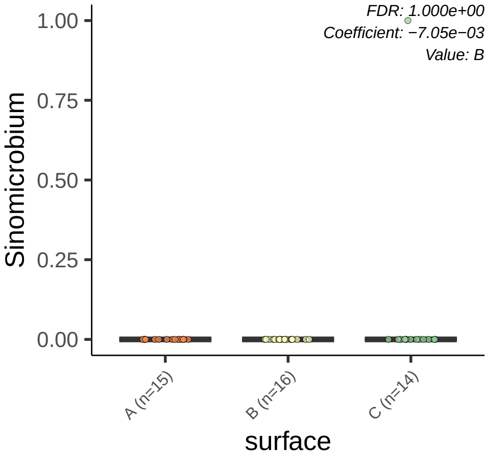

FDR: 1.000e+00
Coefficient: −7.05e−03
Value: B
1.00
0.75
0.50
0.25
0.00
Sinomicrobium
A (n=15)
B (n=16)
C (n=14)
surface

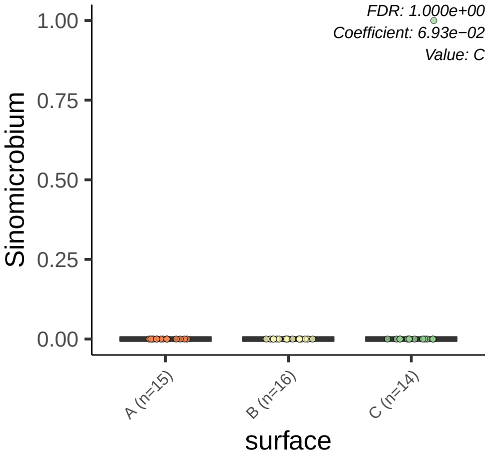
FDR: 1.000e+00
Coefficient: 6.93e−02
Value: C
Sinomicrobium
1.00
0.75
0.50
0.25
0.00
A (n=15)
B (n=16)
C (n=14)
surface

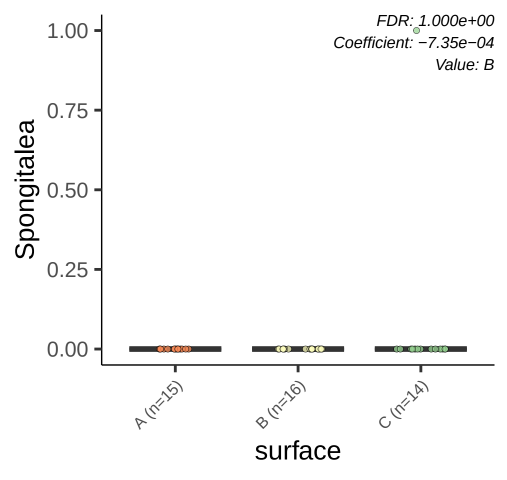

FDR: 1.000e+00
Coefficient: −7.35e−04
Value: B
1.00
0.75
0.50
0.25
0.00
Spongitalea
A (n=15)
B (n=16)
C (n=14)
surface

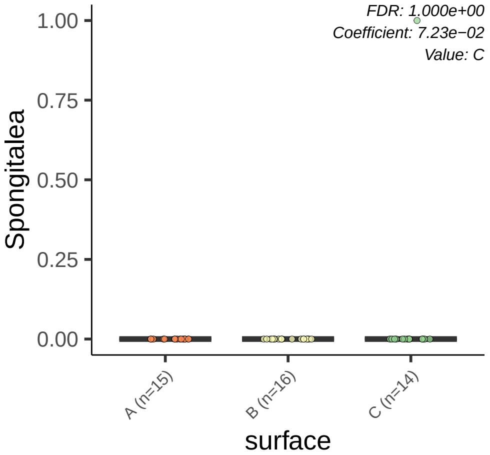

FDR: 1.000e+00
Coefficient: 7.23e−02
Value: C
Spongitalea
1.00
0.75
0.50
0.25
0.00
A (n=15)
B (n=16)
C (n=14)
surface

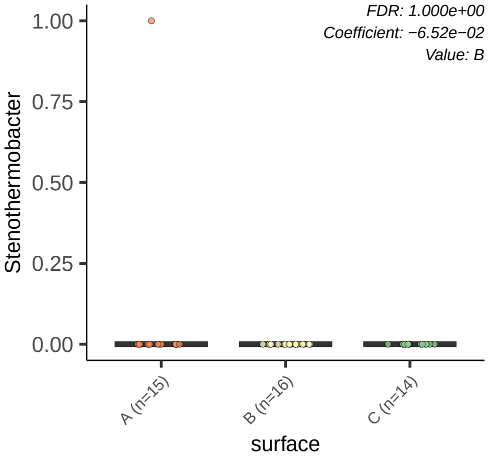
FDR: 1.000e+00
Coefficient: −6.52e−02
Value: B
Stenothermobacter
1.00
0.75
0.50
0.25
0.00
A (n=15)
B (n=16)
C (n=14)
surface

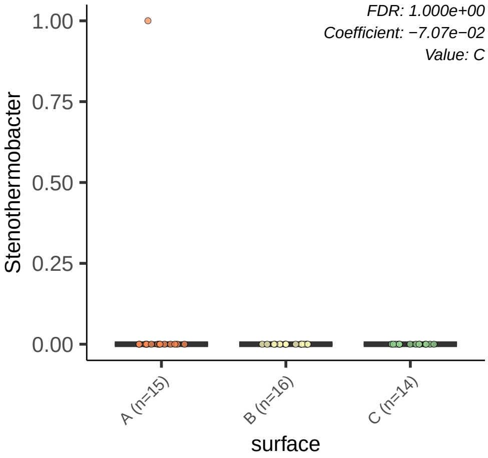

FDR: 1.000e+00
Coefficient: −7.07e−02
Value: C
Stenothermobacter
1.00
0.75
0.50
0.25
0.00
A (n=15)
B (n=16)
C (n=14)
surface

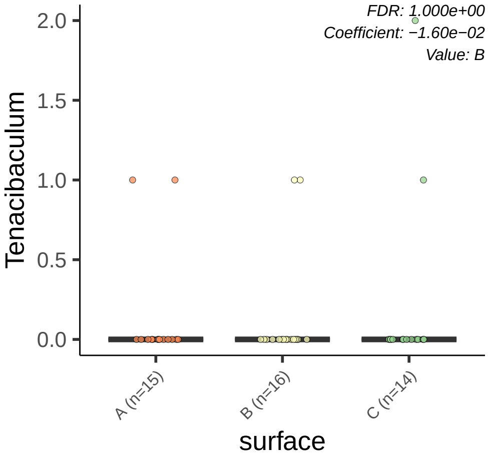

FDR: 1.000e+00
Coefficient: −1.60e−02
Value: B
Tenacibaculum
2.0
1.5
1.0
0.5
0.0
A (n=15)
B (n=16)
C (n=14)
surface

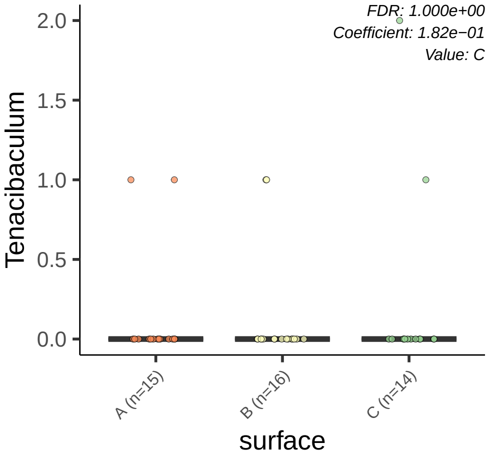

FDR: 1.000e+00
Coefficient: 1.82e−01
Value: C
Tenacibaculum
2.0
1.5
1.0
0.5
0.0
A (n=15)
B (n=16)
C (n=14)
surface

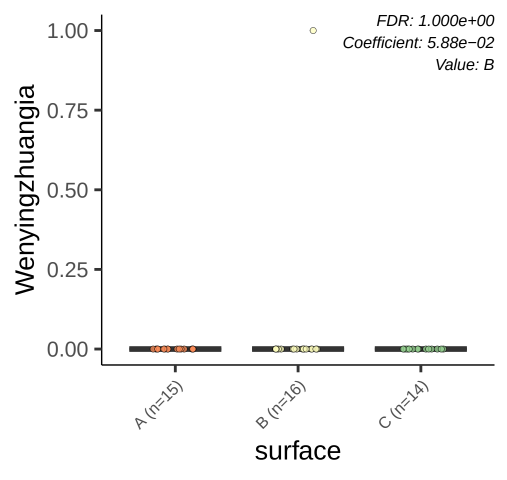

FDR: 1.000e+00
Coefficient: 5.88e−02
Value: B
Wenyingzhuangia
1.00
0.75
0.50
0.25
0.00
A (n=15)
B (n=16)
C (n=14)
surface

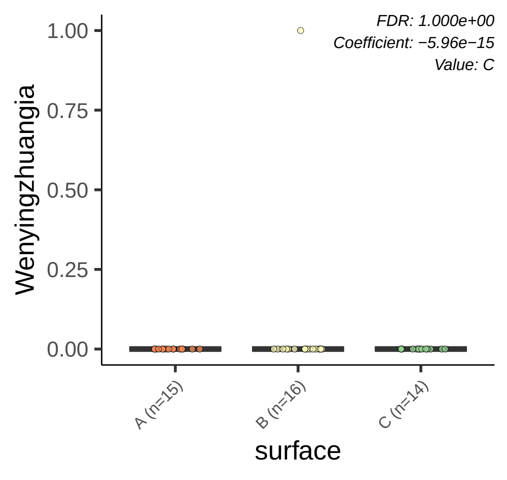
FDR: 1.000e+00
Coefficient: −5.96e−15
Value: C
Wenyingzhuangia
1.00
0.75
0.50
0.25
0.00
A (n=15)
B (n=16)
C (n=14)
surface

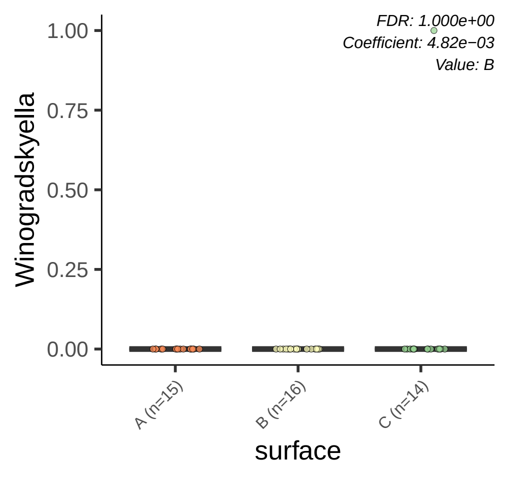

FDR: 1.000e+00
Coefficient: 4.82e−03
Value: B
1.00
0.75
0.50
0.25
0.00
Winogradskyella
A (n=15)
B (n=16)
C (n=14)
surface

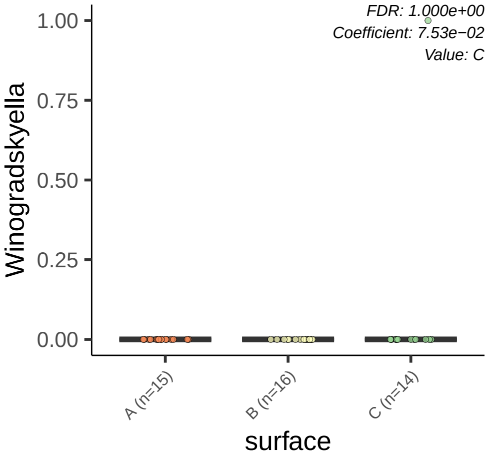

FDR: 1.000e+00
Coefficient: 7.53e−02
Value: C
Winogradskyella
1.00
0.75
0.50
0.25
0.00
A (n=15)
B (n=16)
C (n=14)
surface

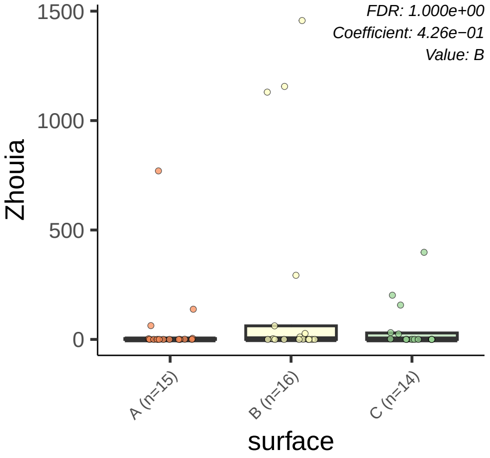

FDR: 1.000e+00
Coefficient: 4.26e−01
Value: B
Zhouia
1500
1000
500
0
A (n=15)
B (n=16)
C (n=14)
surface

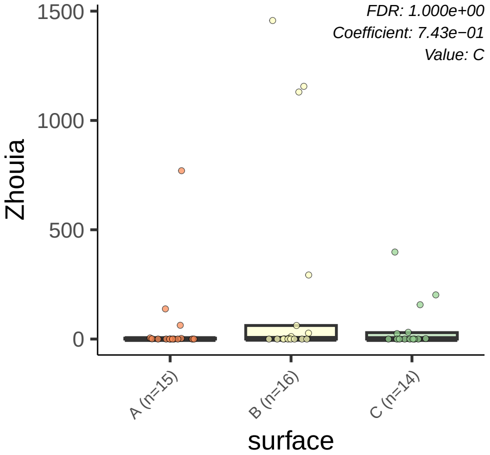

FDR: 1.000e+00
Coefficient: 7.43e−01
Value: C
Zhouia
1500
1000
500
0
A (n=15)
B (n=16)
C (n=14)
surface

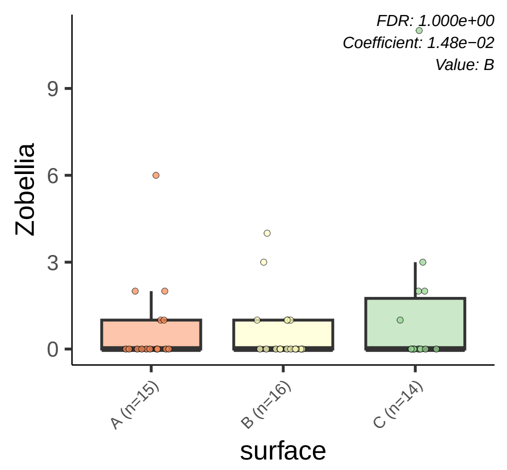
FDR: 1.000e+00
Coefficient: 1.48e−02
Value: B
Zobellia
9
6
3
0
A (n=15)
B (n=16)
C (n=14)
surface

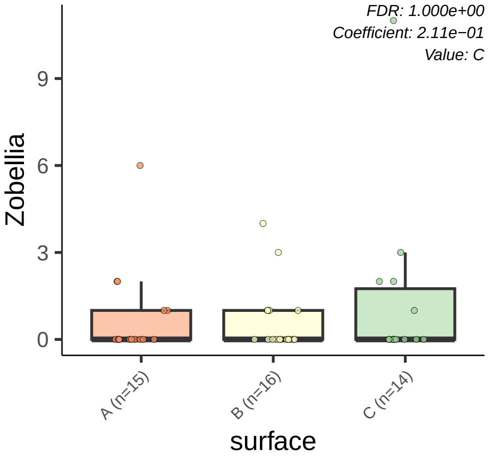

FDR: 1.000e+00
Coefficient: 2.11e−01
Value: C
Zobellia
0
3
6
9
A (n=15)
B (n=16)
C (n=14)
surface

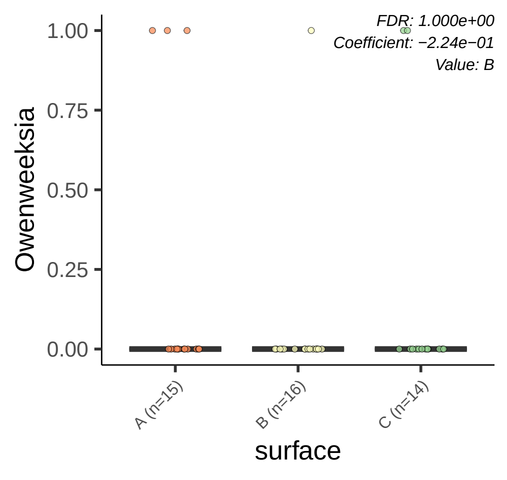
FDR: 1.000e+00
Coefficient: −2.24e−01
Value: B
Owenweeksia
1.00
0.75
0.50
0.25
0.00
A (n=15)
B (n=16)
C (n=14)
surface

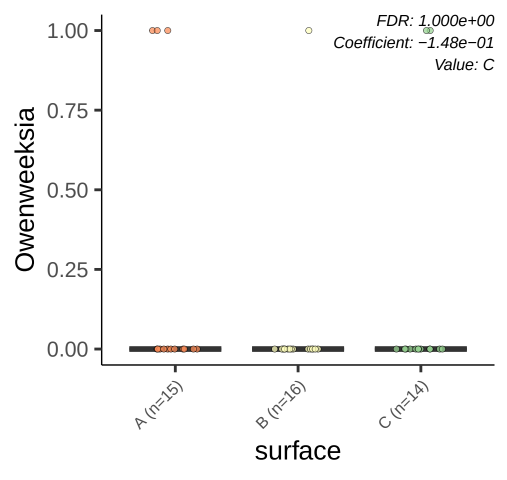
FDR: 1.000e+00
Coefficient: −1.48e−01
Value: C
Owenweeksia
1.00
0.75
0.50
0.25
0.00
A (n=15)
B (n=16)
C (n=14)
surface

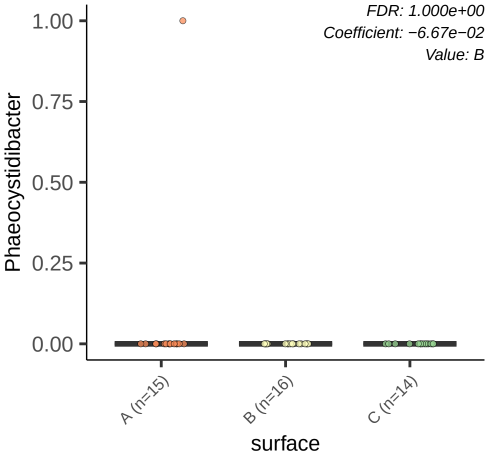
FDR: 1.000e+00
Coefficient: −6.67e−02
Value: B
Phaeocystidibacter
1.00
0.75
0.50
0.25
0.00
A (n=15)
B (n=16)
C (n=14)
surface

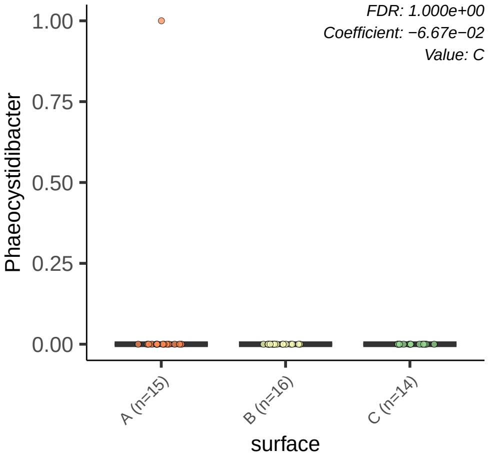

FDR: 1.000e+00
Coefficient: −6.67e−02
Value: C
Phaeocystidibacter
1.00
0.75
0.50
0.25
0.00
A (n=15)
B (n=16)
C (n=14)
surface

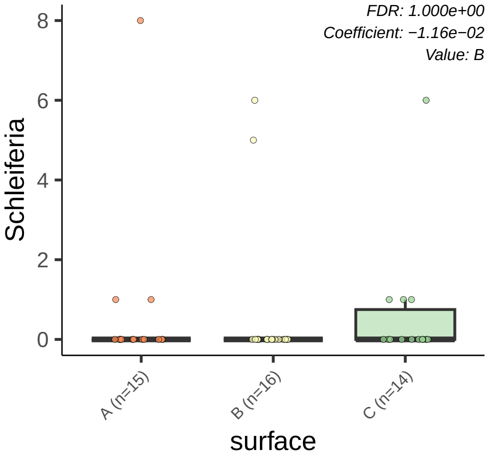
FDR: 1.000e+00
Coefficient: −1.16e−02
Value: B
Schleiferia
8
6
4
2
0
A (n=15)
B (n=16)
C (n=14)
surface

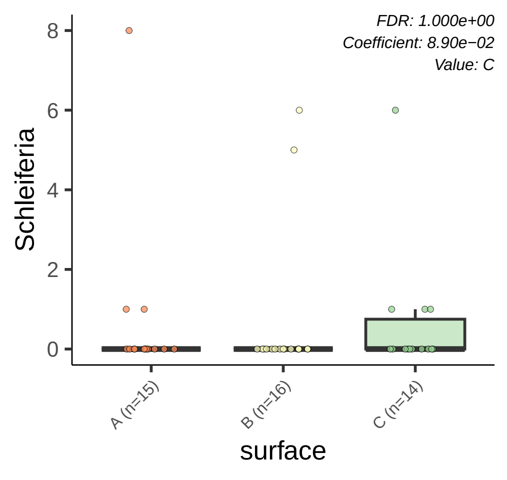
FDR: 1.000e+00
Coefficient: 8.90e−02
Value: C
Schleiferia
8
6
4
2
0
A (n=15)
B (n=16)
C (n=14)
surface

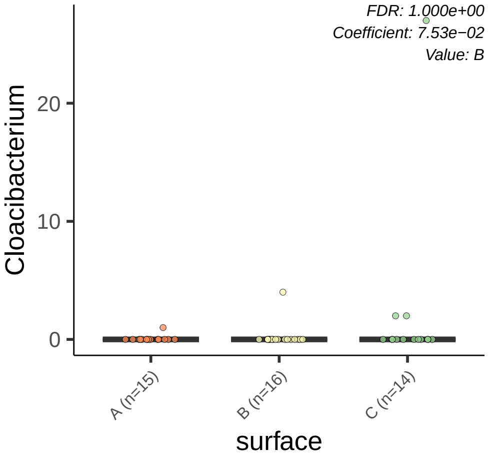

FDR: 1.000e+00
Coefficient: 7.53e−02
Value: B
Cloacibacterium
20
10
0
A (n=15)
B (n=16)
C (n=14)
surface

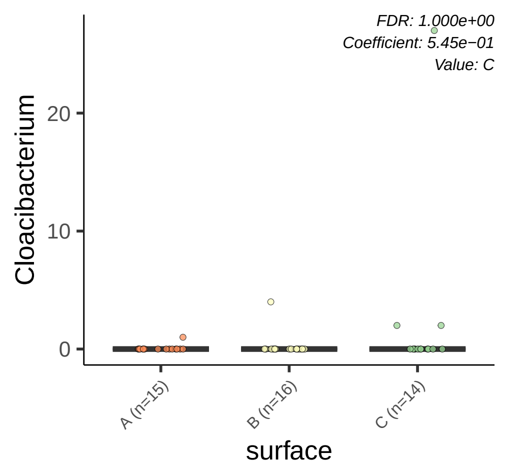

FDR: 1.000e+00
Coefficient: 5.45e−01
Value: C
Cloacibacterium
20
10
0
A (n=15)
B (n=16)
C (n=14)
surface

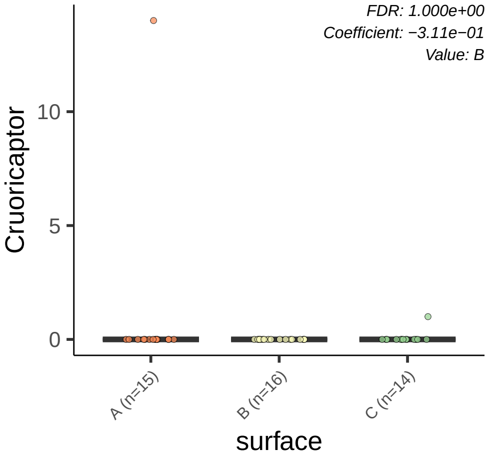
FDR: 1.000e+00
Coefficient: −3.11e−01
Value: B
Cruoricaptor
10
5
0
A (n=15)
B (n=16)
C (n=14)
surface

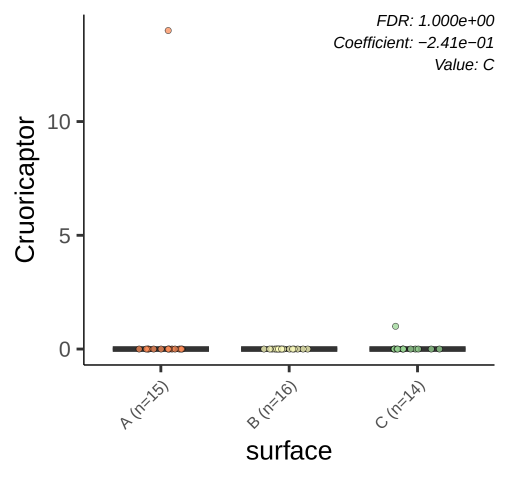
FDR: 1.000e+00
Coefficient: −2.41e−01
Value: C
Cruoricaptor
10
5
0
A (n=15)
B (n=16)
C (n=14)
surface

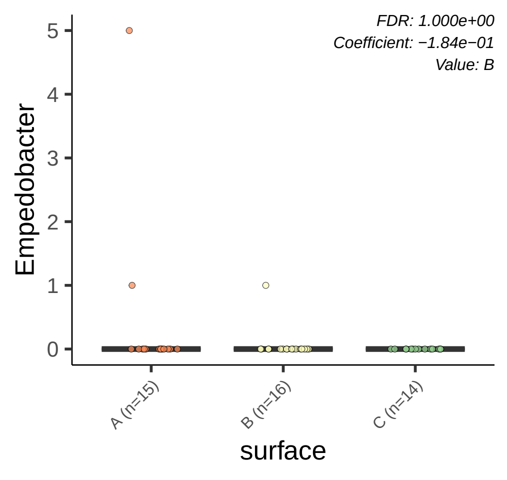

FDR: 1.000e+00
Coefficient: −1.84e−01
Value: B
Empedobacter
5
4
3
2
1
0
A (n=15)
B (n=16)
C (n=14)
surface

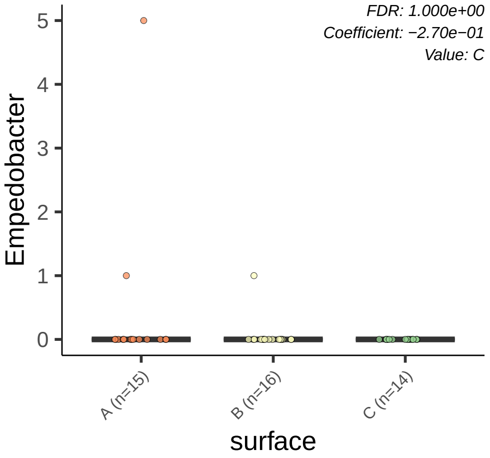

FDR: 1.000e+00
Coefficient: −2.70e−01
Value: C
Empedobacter
0
1
2
3
4
5
A (n=15)
B (n=16)
C (n=14)
surface

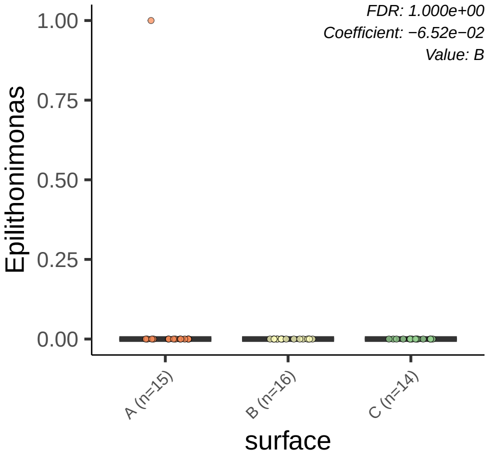

FDR: 1.000e+00
Coefficient: −6.52e−02
Value: B
Epilithonimonas
1.00
0.75
0.50
0.25
0.00
A (n=15)
B (n=16)
C (n=14)
surface

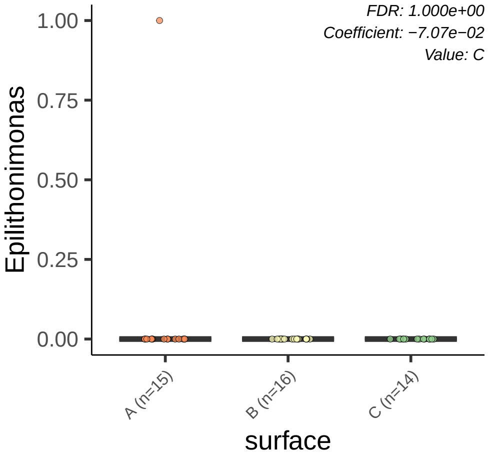

FDR: 1.000e+00
Coefficient: −7.07e−02
Value: C
Epilithonimonas
1.00
0.75
0.50
0.25
0.00
A (n=15)
B (n=16)
C (n=14)
surface

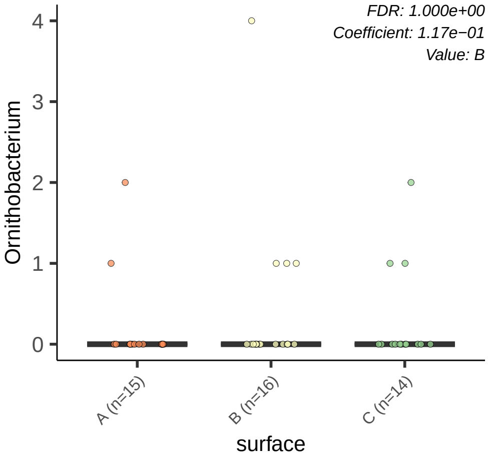

FDR: 1.000e+00
Coefficient: 1.17e−01
Value: B
Ornithobacterium
4
3
2
1
0
A (n=15)
B (n=16)
C (n=14)
surface

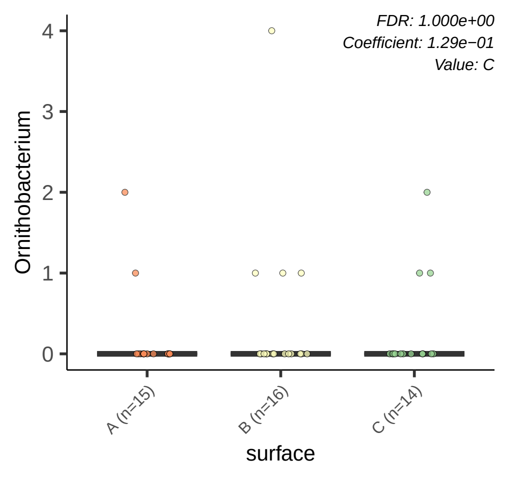
FDR: 1.000e+00
Coefficient: 1.29e−01
Value: C
Ornithobacterium
0
1
2
3
4
A (n=15)
B (n=16)
C (n=14)
surface

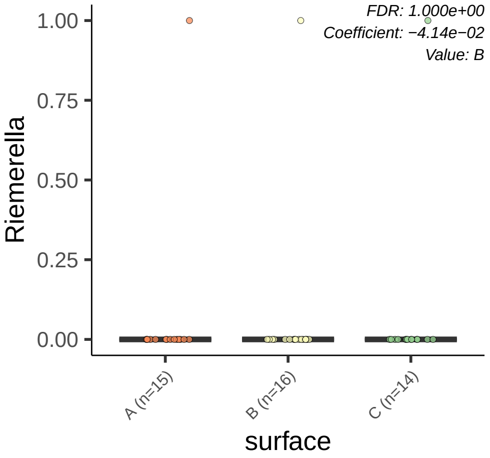
FDR: 1.000e+00
Coefficient: −4.14e−02
Value: B
Riemerella
1.00
0.75
0.50
0.25
0.00
A (n=15)
B (n=16)
C (n=14)
surface

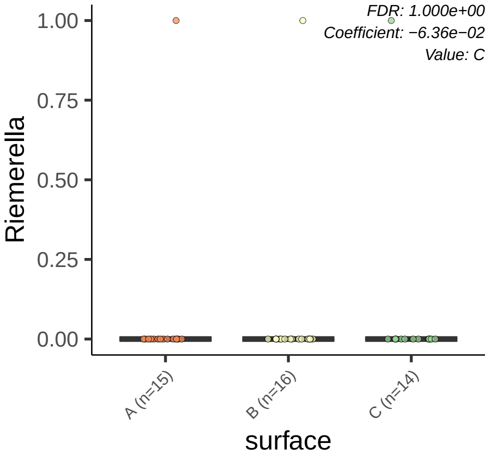

FDR: 1.000e+00
Coefficient: −6.36e−02
Value: C
Riemerella
1.00
0.75
0.50
0.25
0.00
A (n=15)
B (n=16)
C (n=14)
surface

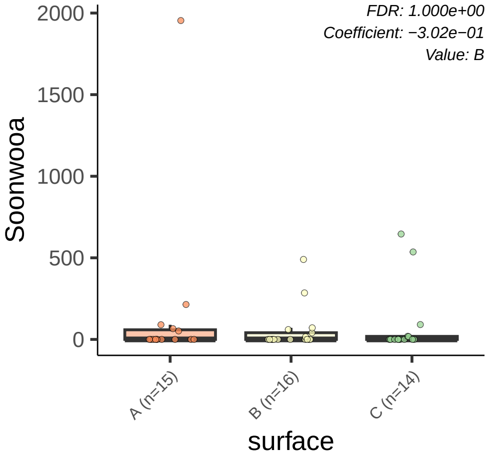

FDR: 1.000e+00
Coefficient: −3.02e−01
Value: B
2000
1500
1000
500
0
Soonwooa
A (n=15)
B (n=16)
C (n=14)
surface

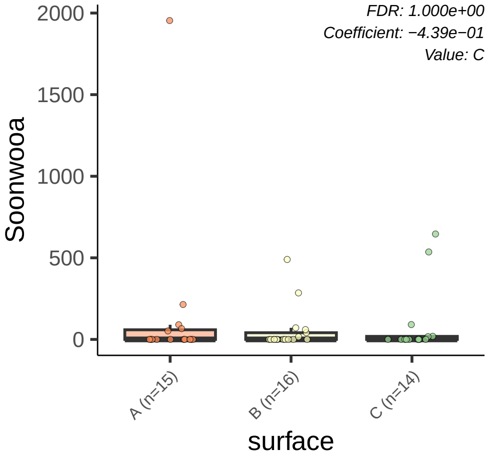
FDR: 1.000e+00
Coefficient: −4.39e−01
Value: C
Soonwooa
2000
1500
1000
500
0
A (n=15)
B (n=16)
C (n=14)
surface

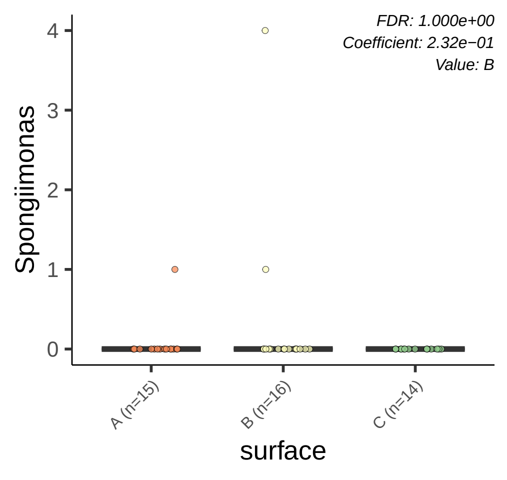

FDR: 1.000e+00
Coefficient: 2.32e−01
Value: B
Spongiimonas
0
1
2
3
4
A (n=15)
B (n=16)
C (n=14)
surface

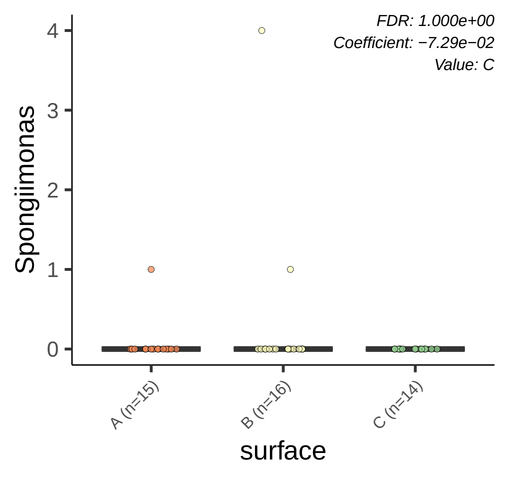
FDR: 1.000e+00
Coefficient: −7.29e−02
Value: C
Spongiimonas
0
1
2
3
4
A (n=15)
B (n=16)
C (n=14)
surface

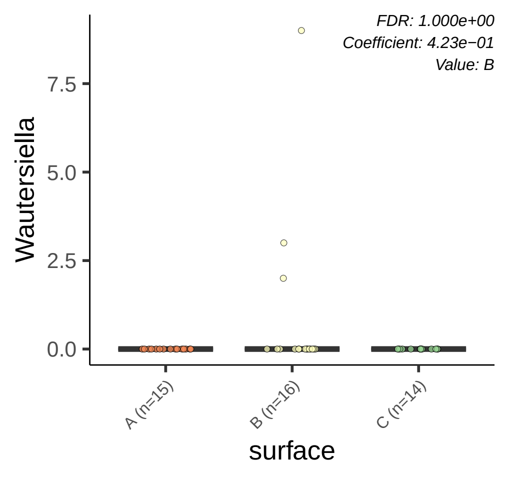
FDR: 1.000e+00
Coefficient: 4.23e−01
Value: B
Wautersiella
7.5
5.0
2.5
0.0
A (n=15)
B (n=16)
C (n=14)
surface

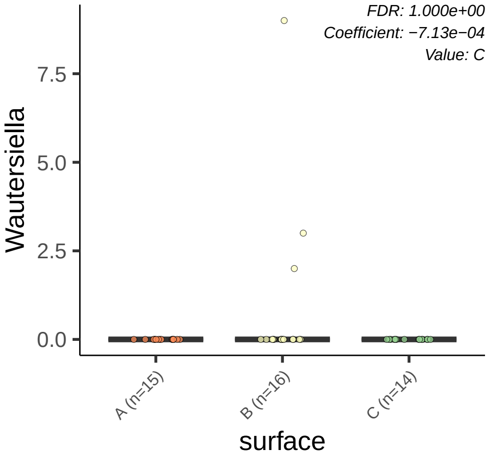

FDR: 1.000e+00
Coefficient: −7.13e−04
Value: C
Wautersiella
7.5
5.0
2.5
0.0
A (n=15)
B (n=16)
C (n=14)
surface

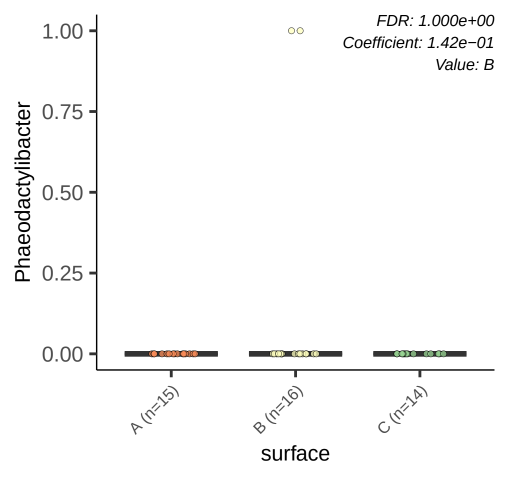
FDR: 1.000e+00
Coefficient: 1.42e−01
Value: B
Phaeodactylibacter
1.00
0.75
0.50
0.25
0.00
A (n=15)
B (n=16)
C (n=14)
surface

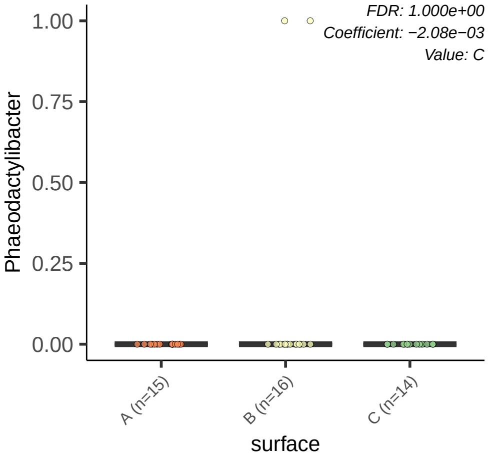

FDR: 1.000e+00
Coefficient: −2.08e−03
Value: C
Phaeodactylibacter
1.00
0.75
0.50
0.25
0.00
A (n=15)
B (n=16)
C (n=14)
surface

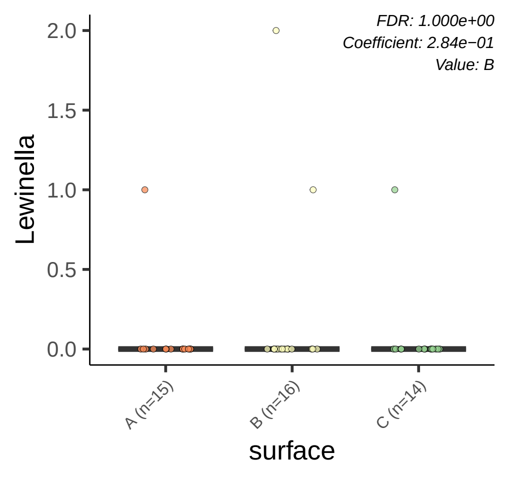
FDR: 1.000e+00
Coefficient: 2.84e−01
Value: B
Lewinella
2.0
1.5
1.0
0.5
0.0
A (n=15)
B (n=16)
C (n=14)
surface

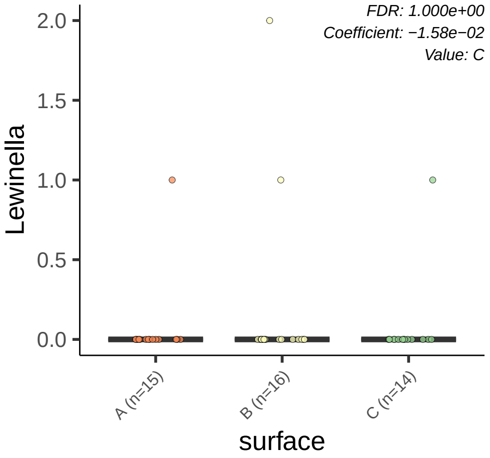
FDR: 1.000e+00
Coefficient: −1.58e−02
Value: C
Lewinella
2.0
1.5
1.0
0.5
0.0
A (n=15)
B (n=16)
C (n=14)
surface

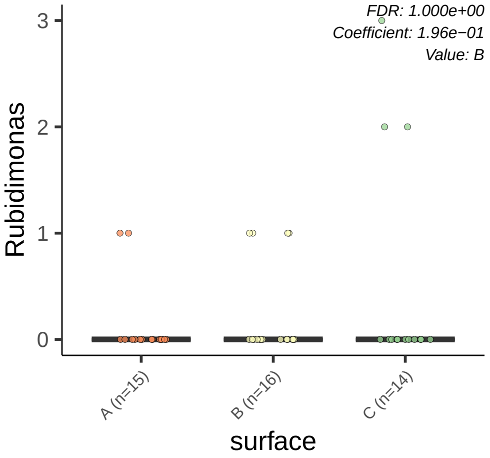
FDR: 1.000e+00
Coefficient: 1.96e−01
Value: B
Rubidimonas
3
2
1
0
A (n=15)
B (n=16)
C (n=14)
surface

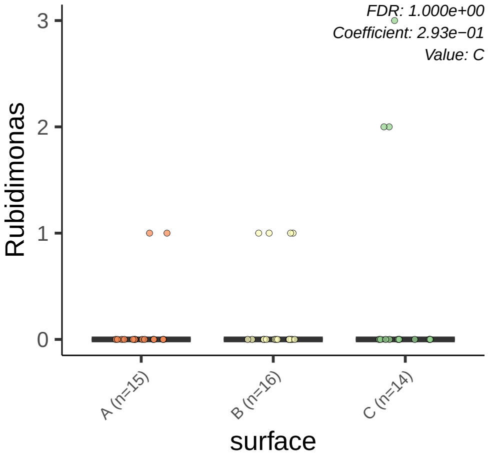
FDR: 1.000e+00
Coefficient: 2.93e−01
Value: C
3
2
1
0
Rubidimonas
A (n=15)
B (n=16)
C (n=14)
surface

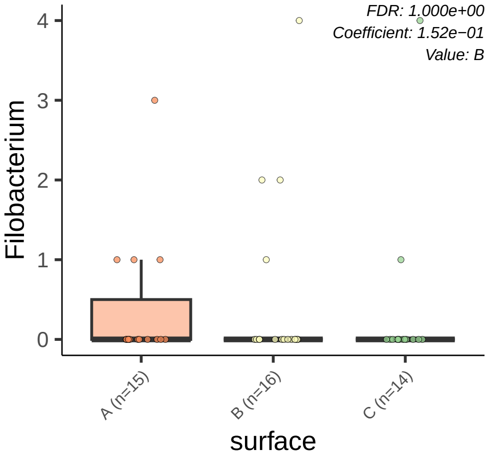
FDR: 1.000e+00
Coefficient: 1.52e−01
Value: B
Filobacterium
0
1
2
3
4
A (n=15)
B (n=16)
C (n=14)
surface

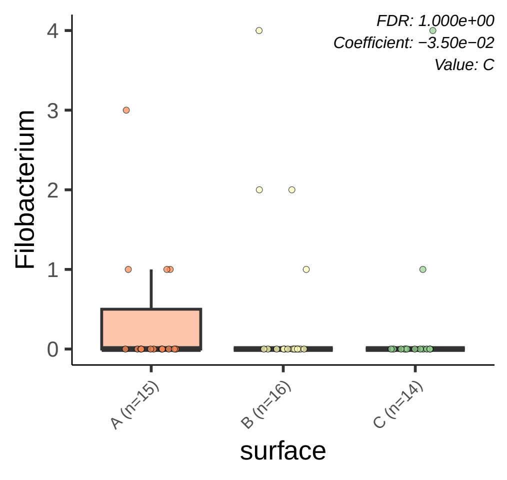

FDR: 1.000e+00
Coefficient: −3.50e−02
Value: C
Filobacterium
0
1
2
3
4
A (n=15)
B (n=16)
C (n=14)
surface

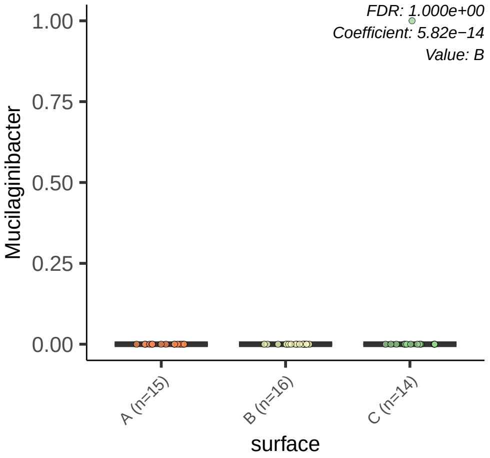
FDR: 1.000e+00
Coefficient: 5.82e−14
Value: B
Mucilaginibacter
1.00
0.75
0.50
0.25
0.00
A (n=15)
B (n=16)
C (n=14)
surface

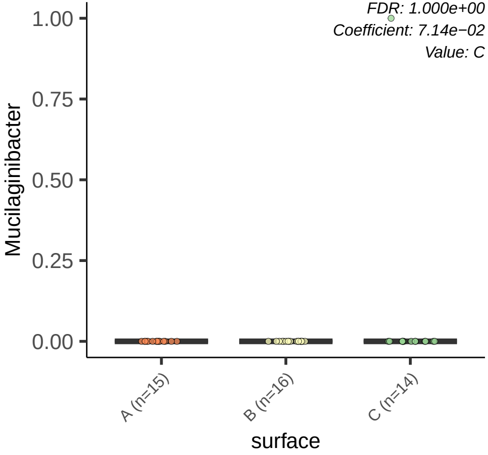

FDR: 1.000e+00
Coefficient: 7.14e−02
Value: C
Mucilaginibacter
1.00
0.75
0.50
0.25
0.00
A (n=15)
B (n=16)
C (n=14)
surface

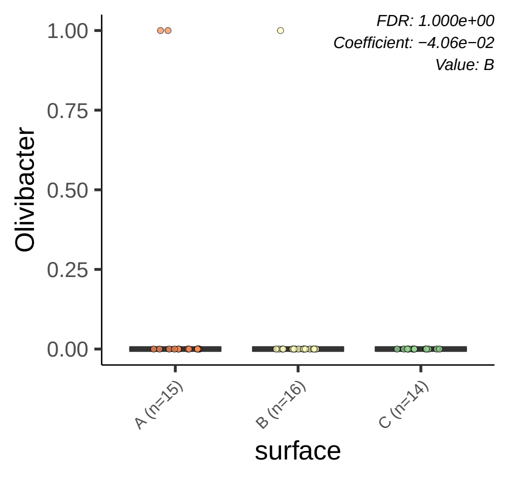

FDR: 1.000e+00
Coefficient: −4.06e−02
Value: B
Olivibacter
1.00
0.75
0.50
0.25
0.00
A (n=15)
B (n=16)
C (n=14)
surface

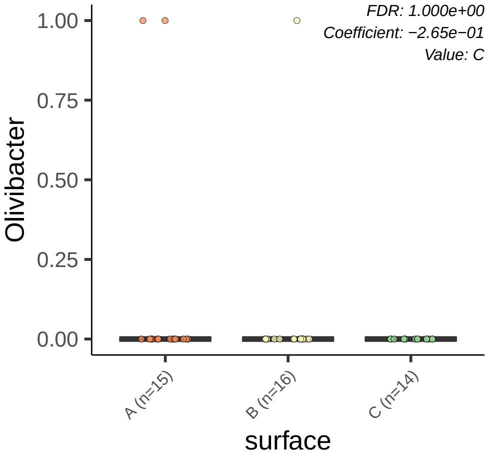

FDR: 1.000e+00
Coefficient: −2.65e−01
Value: C
Olivibacter
1.00
0.75
0.50
0.25
0.00
A (n=15)
B (n=16)
C (n=14)
surface

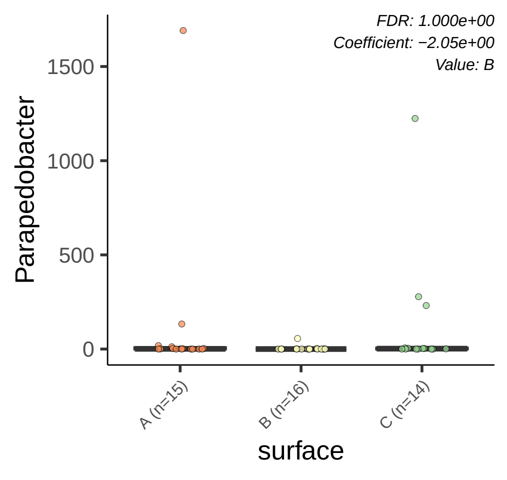
FDR: 1.000e+00
Coefficient: −2.05e+00
Value: B
Parapedobacter
1500
1000
500
0
A (n=15)
B (n=16)
C (n=14)
surface

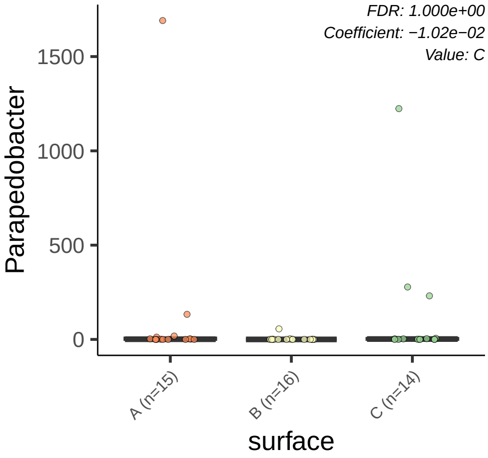
FDR: 1.000e+00
Coefficient: −1.02e−02
Value: C
Parapedobacter
1500
1000
500
0
A (n=15)
B (n=16)
C (n=14)
surface

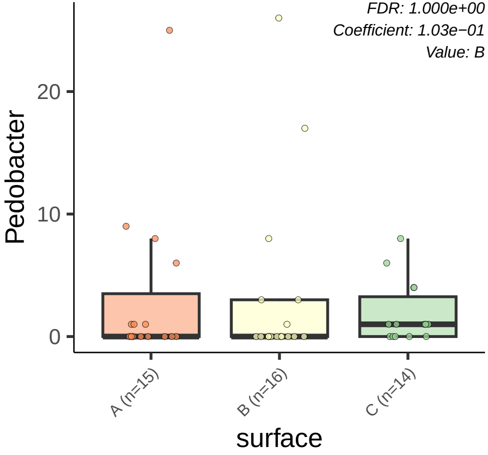

FDR: 1.000e+00
Coefficient: 1.03e−01
Value: B
Pedobacter
20
10
0
A (n=15)
B (n=16)
C (n=14)
surface

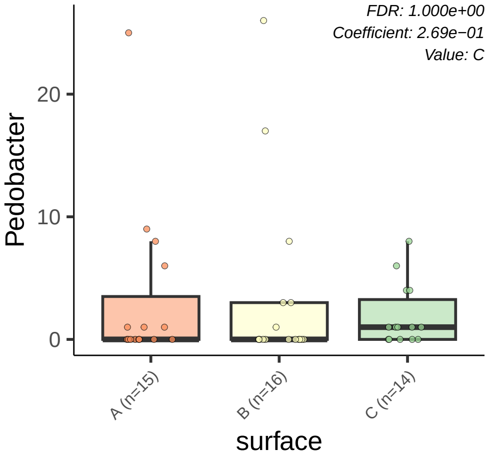

FDR: 1.000e+00
Coefficient: 2.69e−01
Value: C
Pedobacter
0
10
20
A (n=15)
B (n=16)
C (n=14)
surface

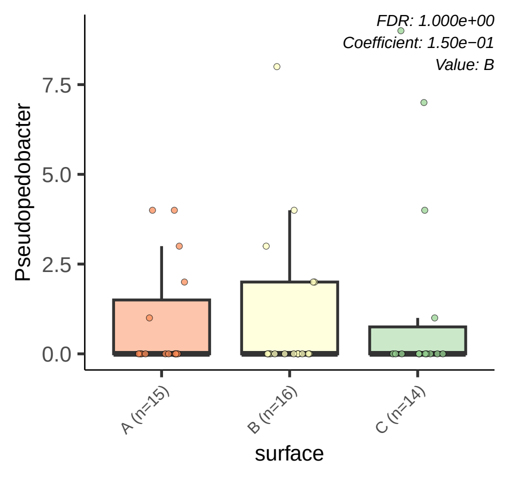

FDR: 1.000e+00
Coefficient: 1.50e−01
Value: B
Pseudopedobacter
7.5
5.0
2.5
0.0
A (n=15)
B (n=16)
C (n=14)
surface

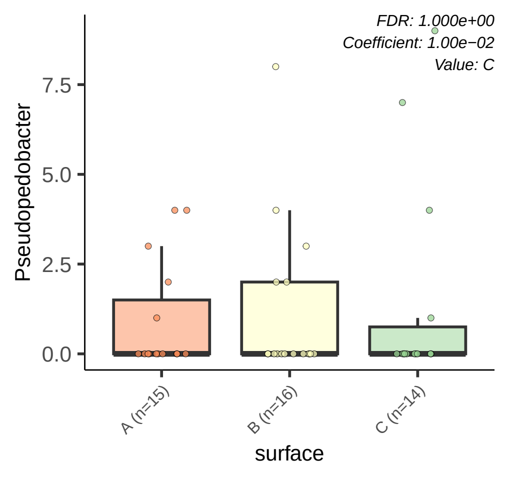

FDR: 1.000e+00
Coefficient: 1.00e−02
Value: C
Pseudopedobacter
7.5
5.0
2.5
0.0
A (n=15)
B (n=16)
C (n=14)
surface

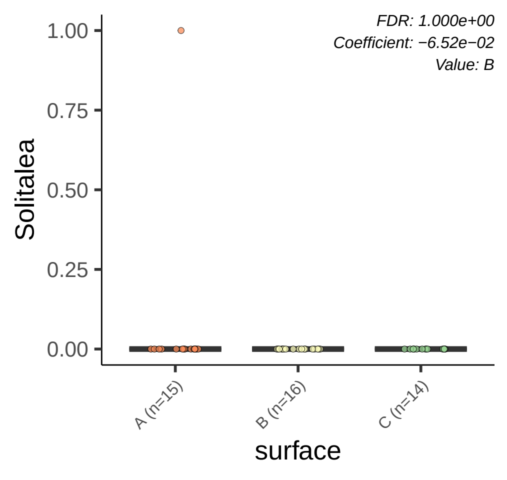
FDR: 1.000e+00
Coefficient: −6.52e−02
Value: B
1.00
0.75
0.50
0.25
0.00
Solitalea
A (n=15)
B (n=16)
C (n=14)
surface

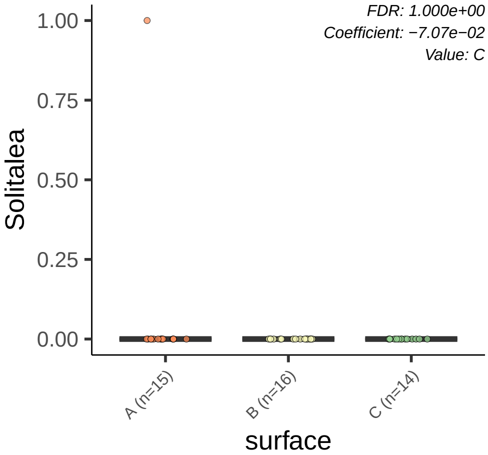

FDR: 1.000e+00
Coefficient: −7.07e−02
Value: C
1.00
0.75
0.50
0.25
0.00
Solitalea
A (n=15)
B (n=16)
C (n=14)
surface

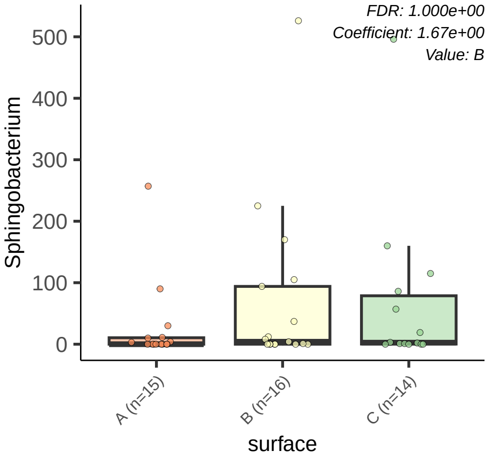

FDR: 1.000e+00
Coefficient: 1.67e+00
Value: B
Sphingobacterium
500
400
300
200
100
0
A (n=15)
B (n=16)
C (n=14)
surface

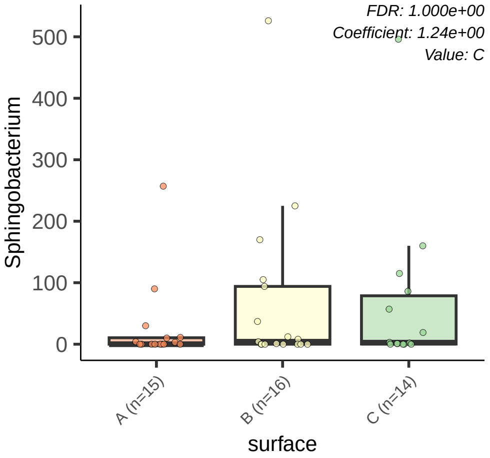
FDR: 1.000e+00
Coefficient: 1.24e+00
Value: C
Sphingobacterium
0
100
200
300
400
500
A (n=15)
B (n=16)
C (n=14)
surface

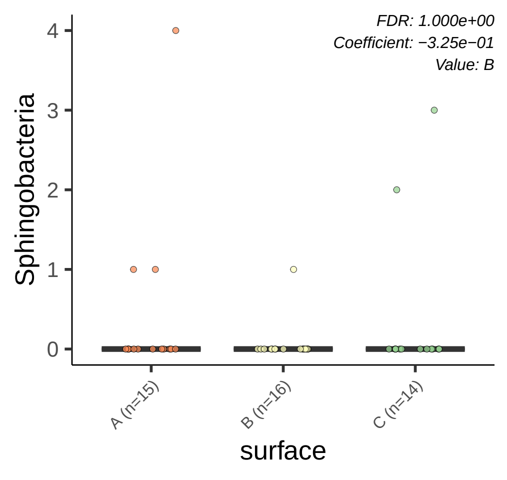
FDR: 1.000e+00
Coefficient: −3.25e−01
Value: B
Sphingobacteria
0
1
2
3
4
A (n=15)
B (n=16)
C (n=14)
surface

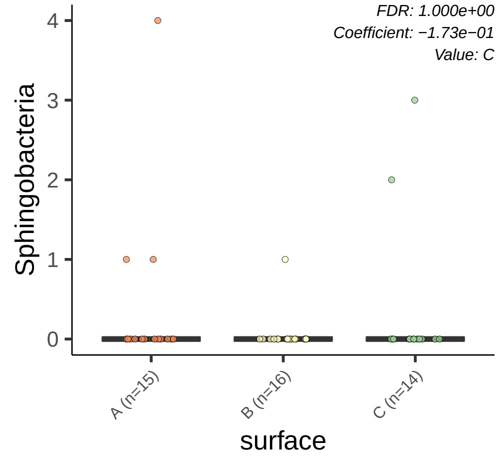
FDR: 1.000e+00
Coefficient: −1.73e−01
Value: C
Sphingobacteria
0
1
2
3
4
A (n=15)
B (n=16)
C (n=14)
surface

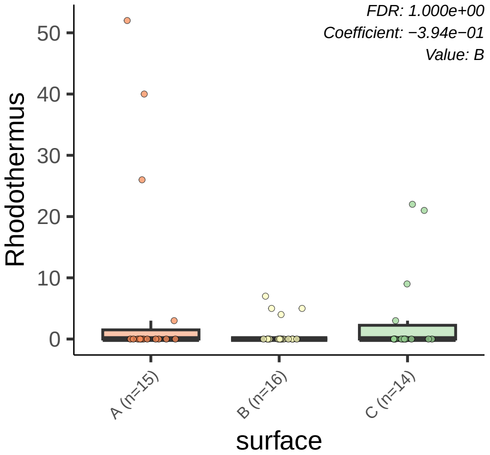

FDR: 1.000e+00
Coefficient: −3.94e−01
Value: B
Rhodothermus
0
10
20
30
40
50
A (n=15)
B (n=16)
C (n=14)
surface

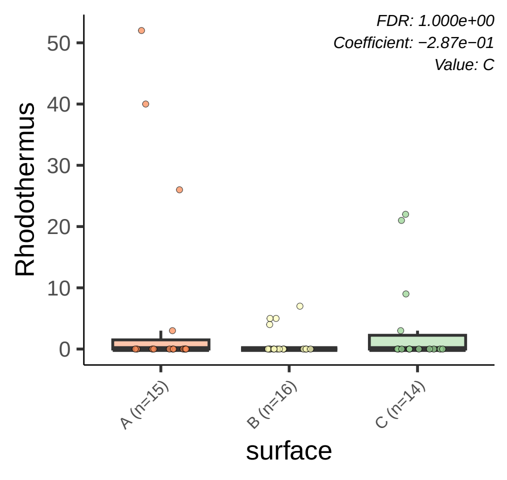
FDR: 1.000e+00
Coefficient: −2.87e−01
Value: C
Rhodothermus
50
40
30
20
10
0
A (n=15)
B (n=16)
C (n=14)
surface

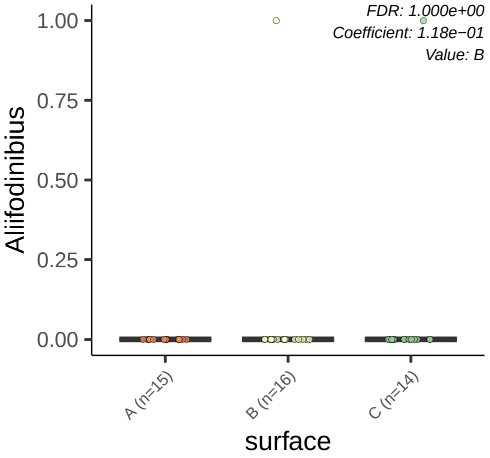
FDR: 1.000e+00
Coefficient: 1.18e−01
Value: B
Aliifodinibius
1.00
0.75
0.50
0.25
0.00
A (n=15)
B (n=16)
C (n=14)
surface

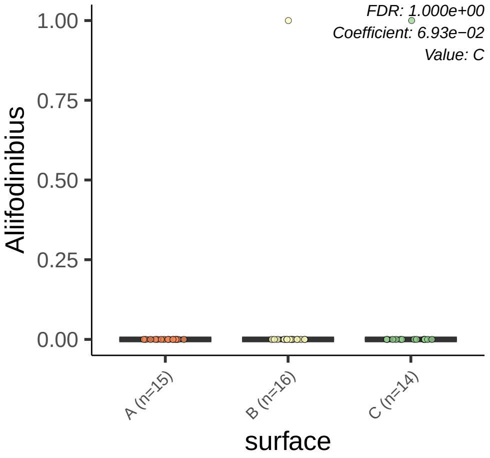

FDR: 1.000e+00
Coefficient: 6.93e−02
Value: C
Aliifodinibius
1.00
0.75
0.50
0.25
0.00
A (n=15)
B (n=16)
C (n=14)
surface

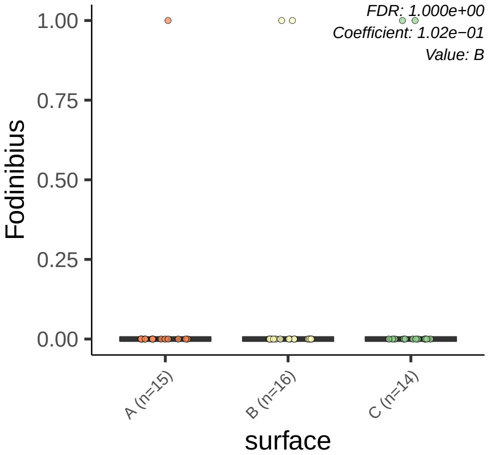
FDR: 1.000e+00
Coefficient: 1.02e−01
Value: B
Fodinibius
1.00
0.75
0.50
0.25
0.00
A (n=15)
B (n=16)
C (n=14)
surface

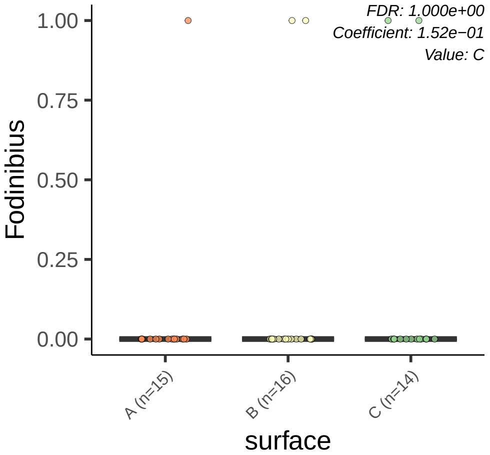

FDR: 1.000e+00
Coefficient: 1.52e−01
Value: C
1.00
0.75
0.50
0.25
0.00
Fodinibius
A (n=15)
B (n=16)
C (n=14)
surface

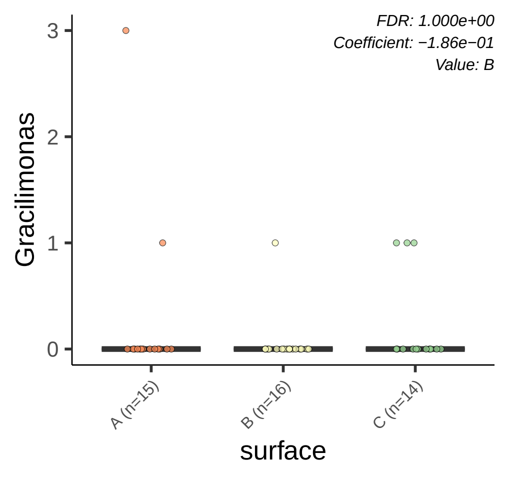

FDR: 1.000e+00
Coefficient: −1.86e−01
Value: B
Gracilimonas
3
2
1
0
A (n=15)
B (n=16)
C (n=14)
surface

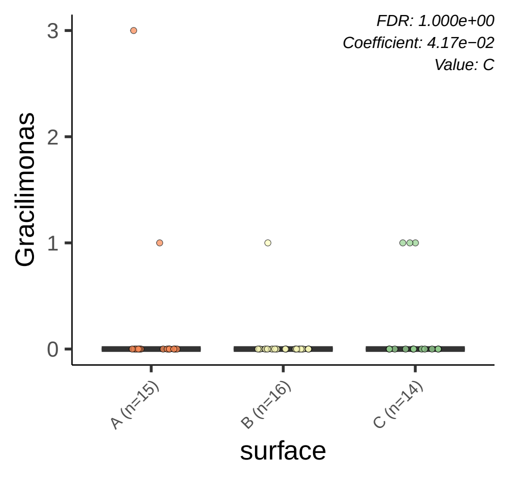

FDR: 1.000e+00
Coefficient: 4.17e−02
Value: C
Gracilimonas
3
2
1
0
A (n=15)
B (n=16)
C (n=14)
surface

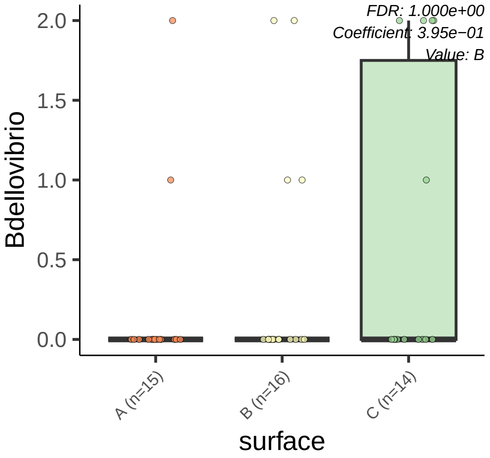
FDR: 1.000e+00
Coefficient: 3.95e−01
Value: B
Bdellovibrio
2.0
1.5
1.0
0.5
0.0
A (n=15)
B (n=16)
C (n=14)
surface

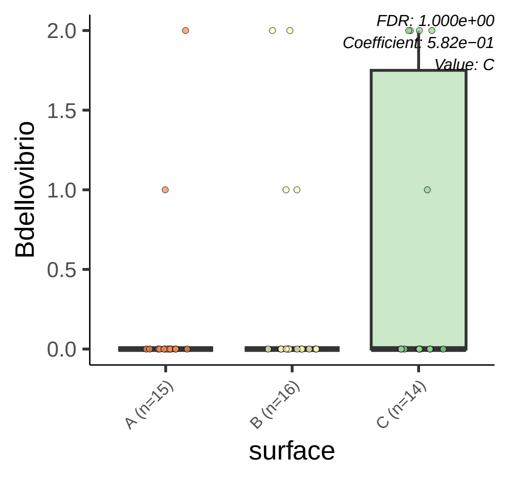
FDR: 1.000e+00
Coefficient: 5.82e−01
Value: C
Bdellovibrio
2.0
1.5
1.0
0.5
0.0
A (n=15)
B (n=16)
C (n=14)
surface

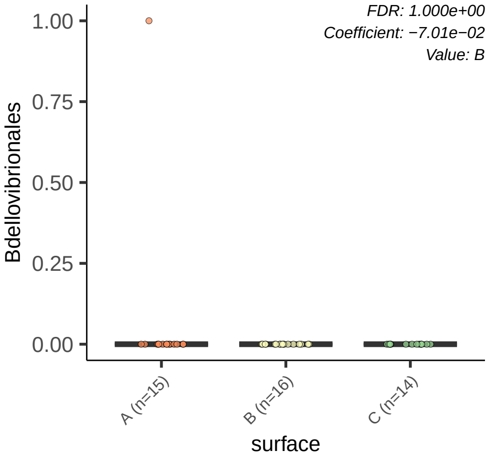
FDR: 1.000e+00
Coefficient: −7.01e−02
Value: B
Bdellovibrionales
1.00
0.75
0.50
0.25
0.00
A (n=15)
B (n=16)
C (n=14)
surface

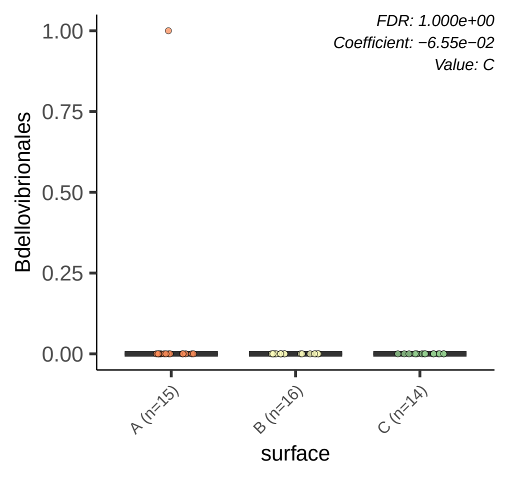

FDR: 1.000e+00
Coefficient: −6.55e−02
Value: C
Bdellovibrionales
1.00
0.75
0.50
0.25
0.00
A (n=15)
B (n=16)
C (n=14)
surface

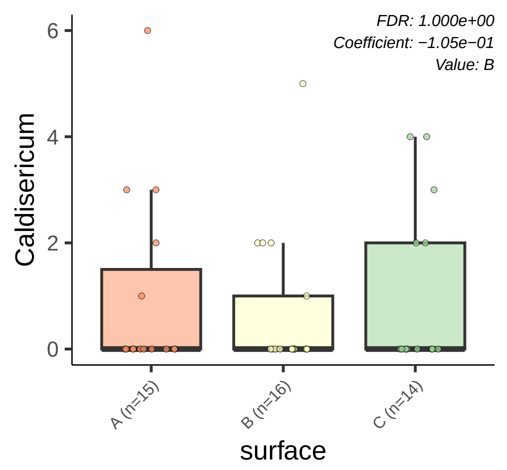

FDR: 1.000e+00
Coefficient: −1.05e−01
Value: B
Caldisericum
6
4
2
0
A (n=15)
B (n=16)
C (n=14)
surface

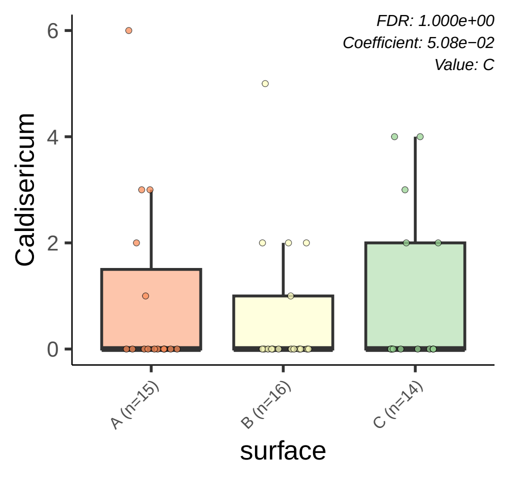

FDR: 1.000e+00
Coefficient: 5.08e−02
Value: C
Caldisericum
0
2
4
6
A (n=15)
B (n=16)
C (n=14)
surface

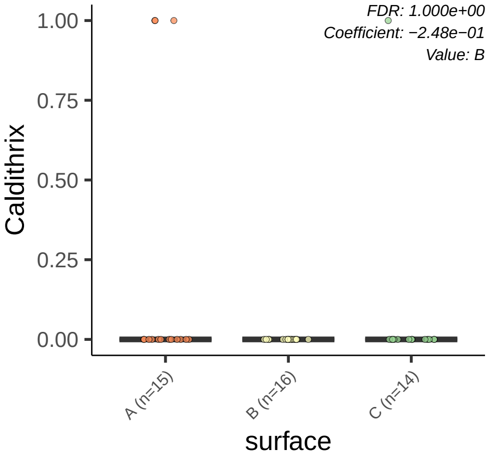
FDR: 1.000e+00
Coefficient: −2.48e−01
Value: B
Caldithrix
1.00
0.75
0.50
0.25
0.00
A (n=15)
B (n=16)
C (n=14)
surface

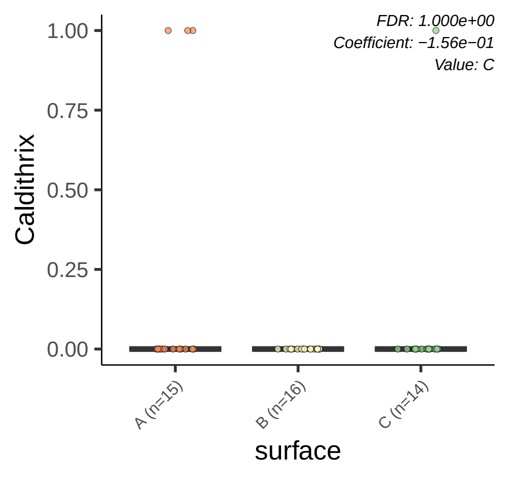
FDR: 1.000e+00
Coefficient: −1.56e−01
Value: C
Caldithrix
1.00
0.75
0.50
0.25
0.00
A (n=15)
B (n=16)
C (n=14)
surface

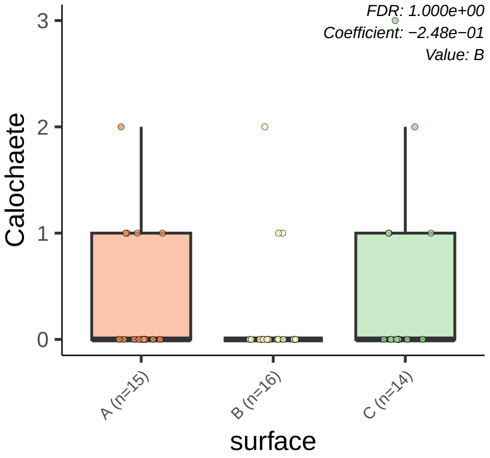

FDR: 1.000e+00
Coefficient: −2.48e−01
Value: B
Calochaete
3
2
1
0
A (n=15)
B (n=16)
C (n=14)
surface

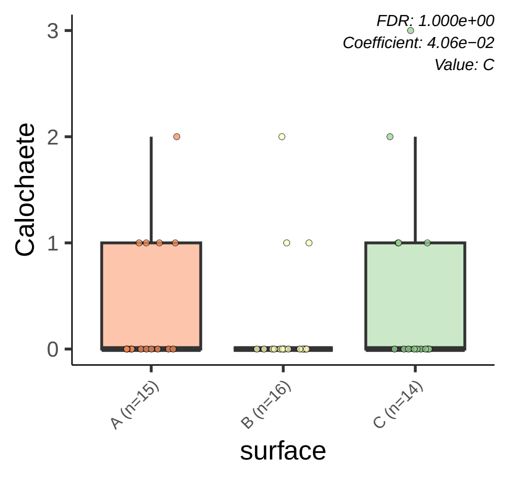
FDR: 1.000e+00
Coefficient: 4.06e−02
Value: C
Calochaete
3
2
1
0
A (n=15)
B (n=16)
C (n=14)
surface

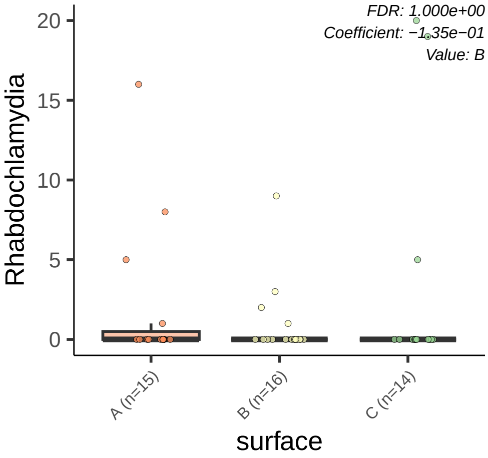

FDR: 1.000e+00
Coefficient: −1.35e−01
Value: B
Rhabdochlamydia
0
5
10
15
20
A (n=15)
B (n=16)
C (n=14)
surface

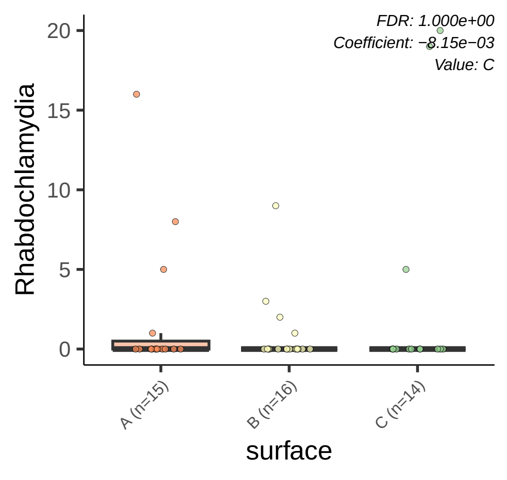
FDR: 1.000e+00
Coefficient: −8.15e−03
Value: C
Rhabdochlamydia
20
15
10
5
0
A (n=15)
B (n=16)
C (n=14)
surface

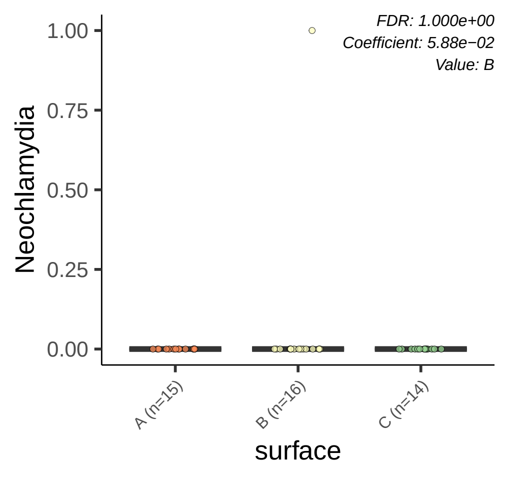

FDR: 1.000e+00
Coefficient: 5.88e−02
Value: B
Neochlamydia
1.00
0.75
0.50
0.25
0.00
A (n=15)
B (n=16)
C (n=14)
surface

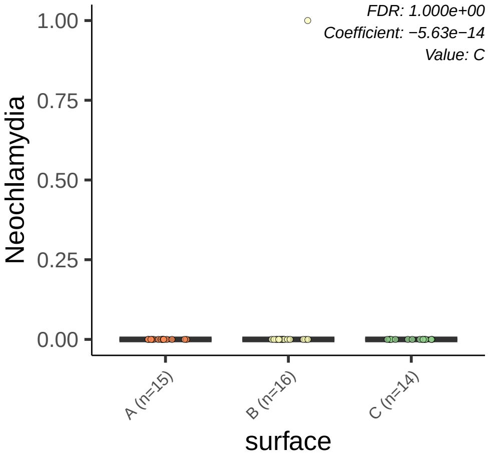
FDR: 1.000e+00
Coefficient: −5.63e−14
Value: C
Neochlamydia
1.00
0.75
0.50
0.25
0.00
A (n=15)
B (n=16)
C (n=14)
surface

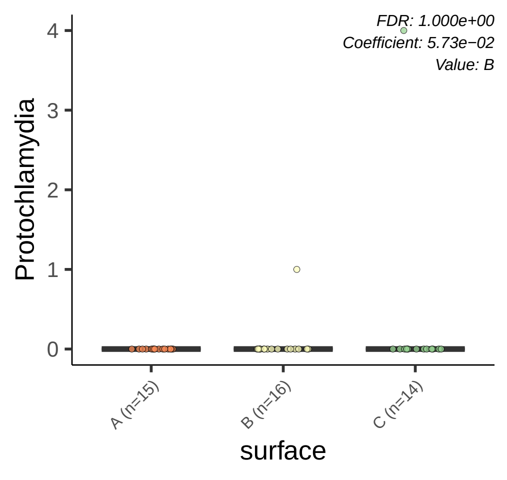

FDR: 1.000e+00
Coefficient: 5.73e−02
Value: B
Protochlamydia
0
1
2
3
4
A (n=15)
B (n=16)
C (n=14)
surface

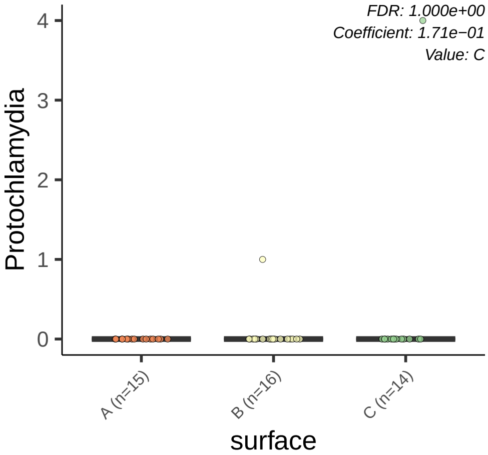
FDR: 1.000e+00
Coefficient: 1.71e−01
Value: C
Protochlamydia
0
1
2
3
4
A (n=15)
B (n=16)
C (n=14)
surface

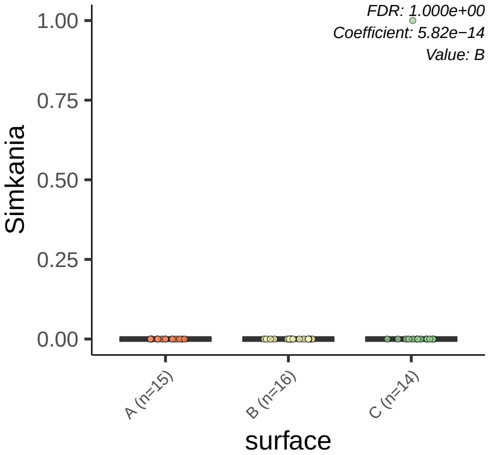
FDR: 1.000e+00
Coefficient: 5.82e−14
Value: B
1.00
0.75
0.50
0.25
0.00
Simkania
A (n=15)
B (n=16)
C (n=14)
surface

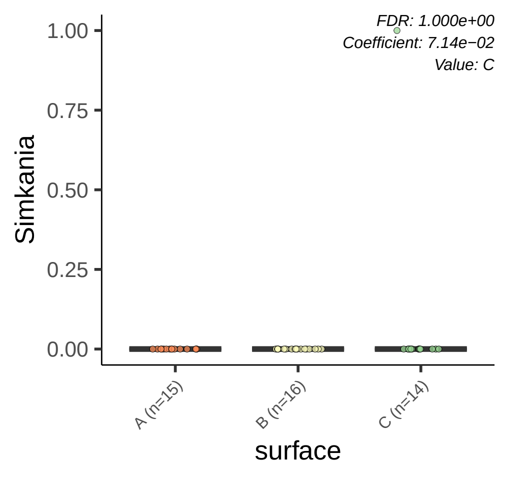

FDR: 1.000e+00
Coefficient: 7.14e−02
Value: C
Simkania
1.00
0.75
0.50
0.25
0.00
A (n=15)
B (n=16)
C (n=14)
surface

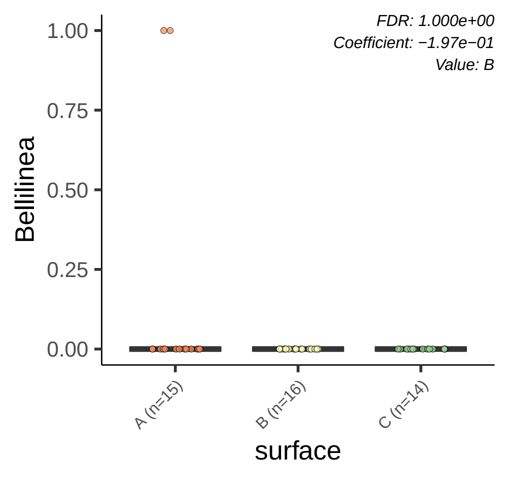
FDR: 1.000e+00
Coefficient: −1.97e−01
Value: B
Bellilinea
1.00
0.75
0.50
0.25
0.00
A (n=15)
B (n=16)
C (n=14)
surface

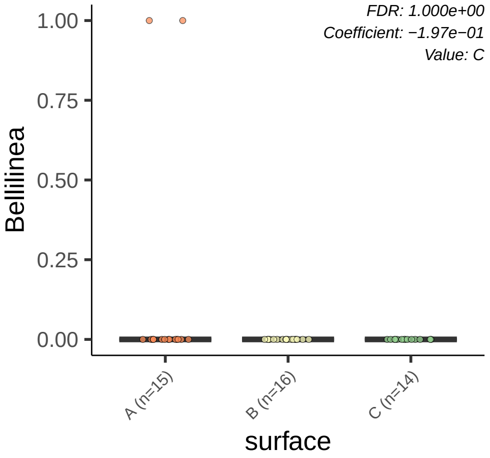

FDR: 1.000e+00
Coefficient: −1.97e−01
Value: C
Bellilinea
1.00
0.75
0.50
0.25
0.00
A (n=15)
B (n=16)
C (n=14)
surface

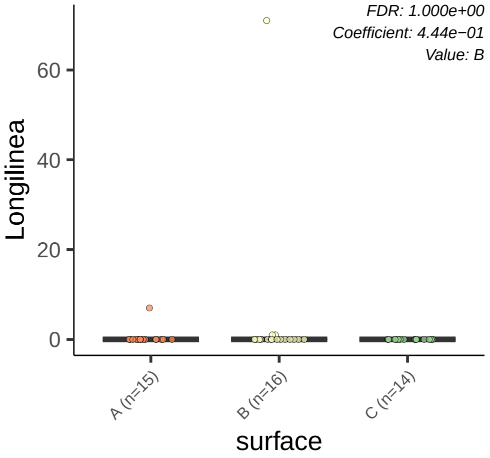
FDR: 1.000e+00
Coefficient: 4.44e−01
Value: B
Longilinea
0
20
40
60
A (n=15)
B (n=16)
C (n=14)
surface

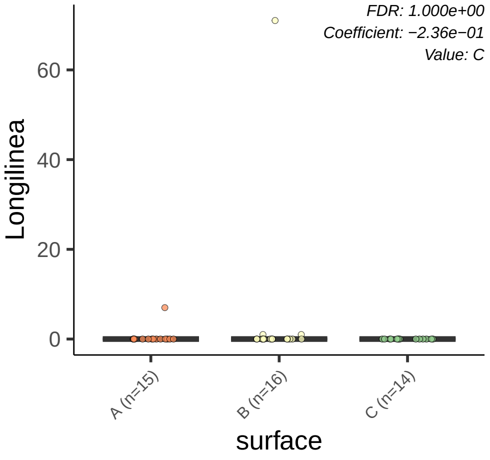
FDR: 1.000e+00
Coefficient: −2.36e−01
Value: C
Longilinea
60
40
20
0
A (n=15)
B (n=16)
C (n=14)
surface

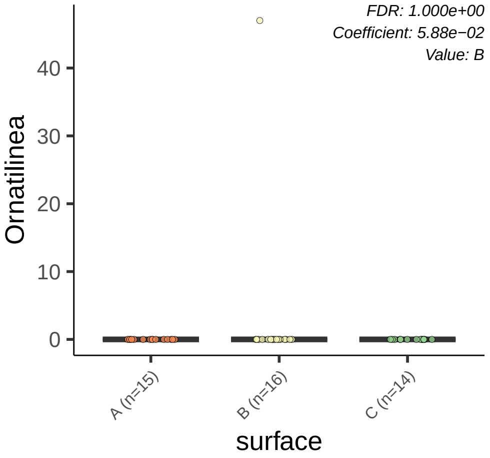
FDR: 1.000e+00
Coefficient: 5.88e−02
Value: B
Ornatilinea
0
10
20
30
40
A (n=15)
B (n=16)
C (n=14)
surface

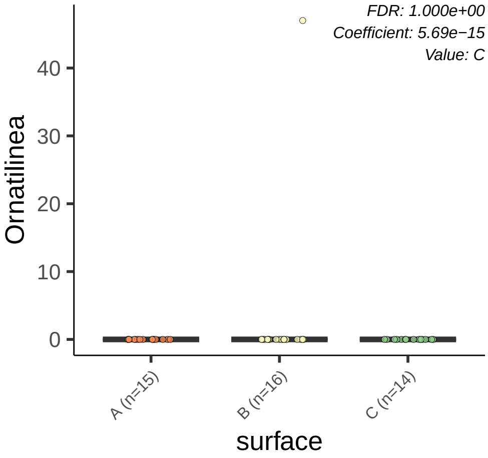

FDR: 1.000e+00
Coefficient: 5.69e−15
Value: C
Ornatilinea
40
30
20
10
0
A (n=15)
B (n=16)
C (n=14)
surface

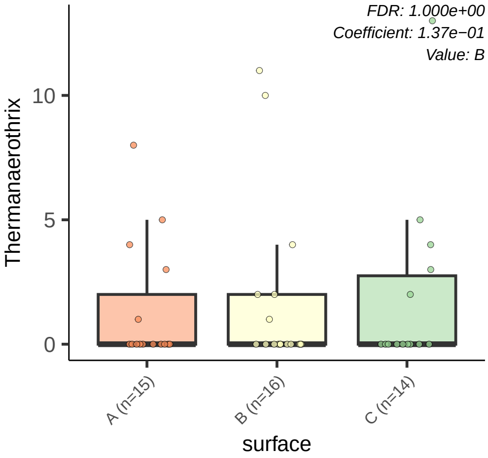
FDR: 1.000e+00
Coefficient: 1.37e−01
Value: B
Thermanaerothrix
10
5
0
A (n=15)
B (n=16)
C (n=14)
surface

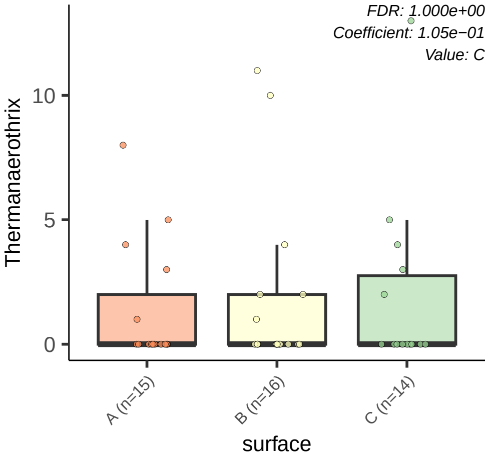

FDR: 1.000e+00
Coefficient: 1.05e−01
Value: C
Thermanaerothrix
10
5
0
A (n=15)
B (n=16)
C (n=14)
surface

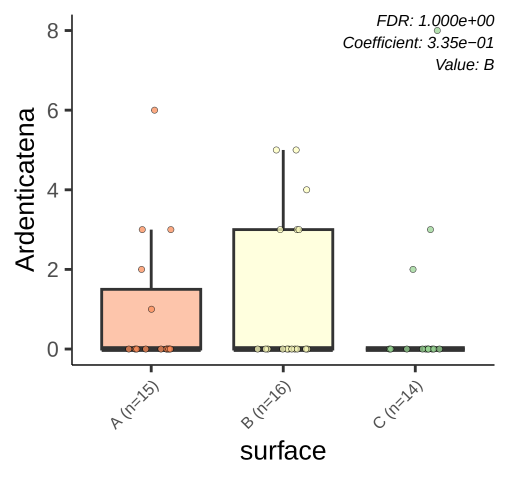

FDR: 1.000e+00
Coefficient: 3.35e−01
Value: B
8
6
4
2
0
Ardenticatena
A (n=15)
B (n=16)
C (n=14)
surface

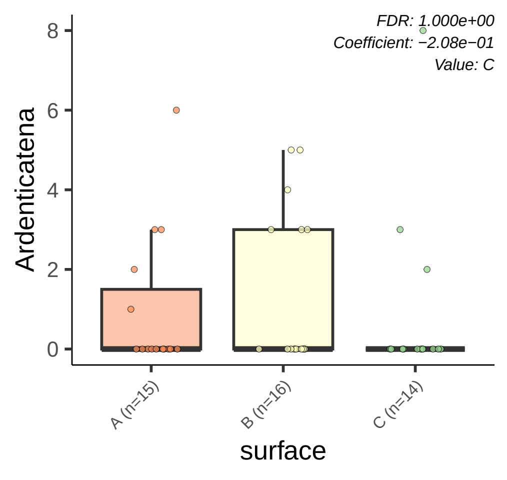
FDR: 1.000e+00
Coefficient: −2.08e−01
Value: C
Ardenticatena
0
2
4
6
8
A (n=15)
B (n=16)
C (n=14)
surface

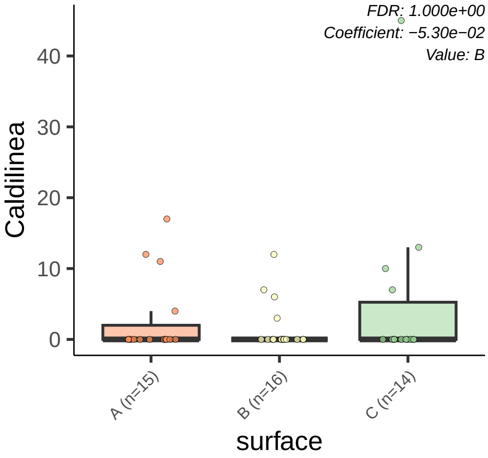
FDR: 1.000e+00
Coefficient: −5.30e−02
Value: B
Caldilinea
40
30
20
10
0
A (n=15)
B (n=16)
C (n=14)
surface

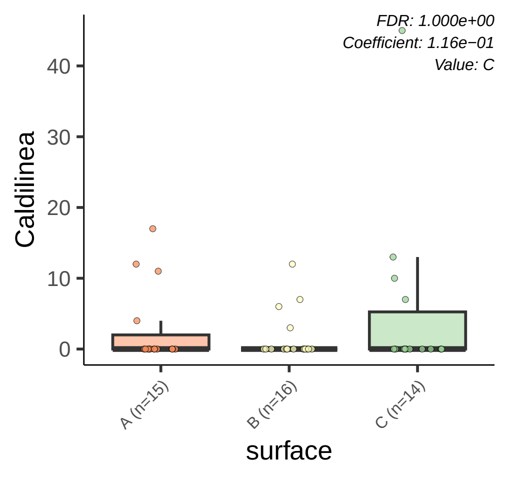
FDR: 1.000e+00
Coefficient: 1.16e−01
Value: C
Caldilinea
40
30
20
10
0
A (n=15)
B (n=16)
C (n=14)
surface

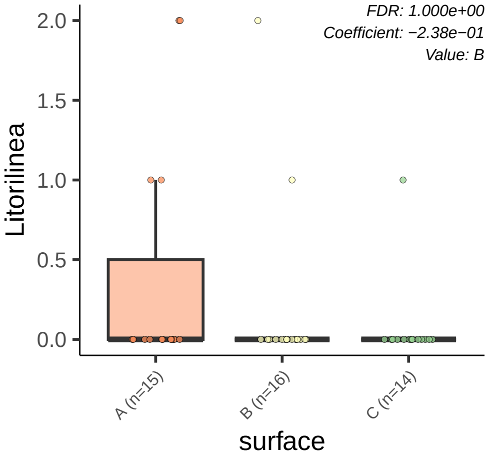
FDR: 1.000e+00
Coefficient: −2.38e−01
Value: B
Litorilinea
2.0
1.5
1.0
0.5
0.0
A (n=15)
B (n=16)
C (n=14)
surface

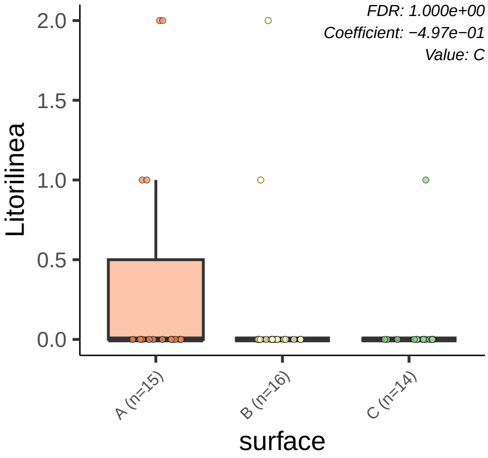

FDR: 1.000e+00
Coefficient: −4.97e−01
Value: C
Litorilinea
2.0
1.5
1.0
0.5
0.0
A (n=15)
B (n=16)
C (n=14)
surface

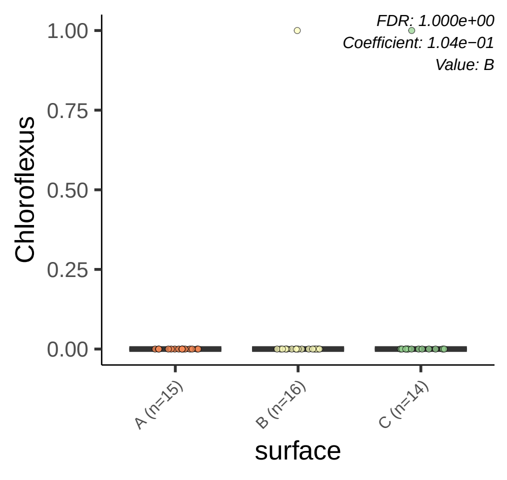

FDR: 1.000e+00
Coefficient: 1.04e−01
Value: B
1.00
0.75
0.50
0.25
0.00
Chloroflexus
A (n=15)
B (n=16)
C (n=14)
surface

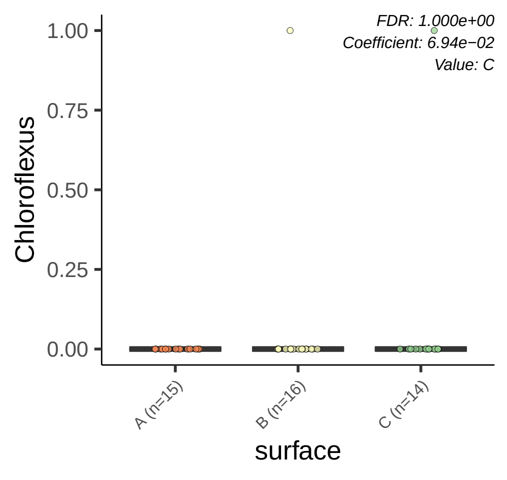
FDR: 1.000e+00
Coefficient: 6.94e−02
Value: C
1.00
0.75
0.50
0.25
0.00
Chloroflexus
A (n=15)
B (n=16)
C (n=14)
surface

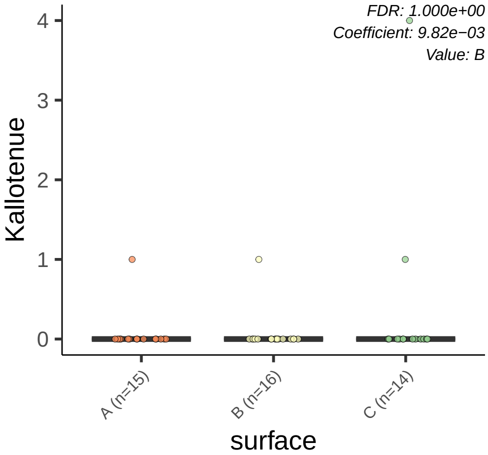
FDR: 1.000e+00
Coefficient: 9.82e−03
Value: B
Kallotenue
0
1
2
3
4
A (n=15)
B (n=16)
C (n=14)
surface

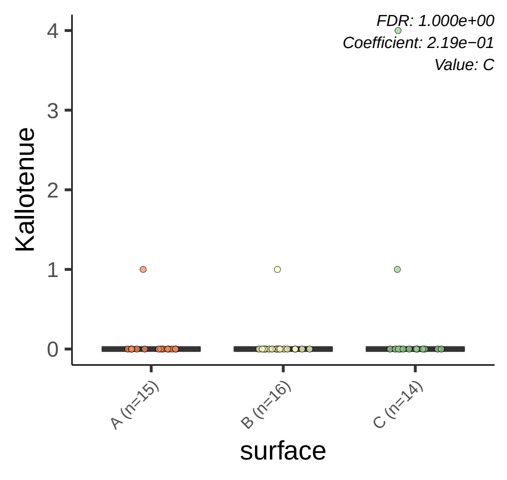
FDR: 1.000e+00
Coefficient: 2.19e−01
Value: C
Kallotenue
4
3
2
1
0
A (n=15)
B (n=16)
C (n=14)
surface

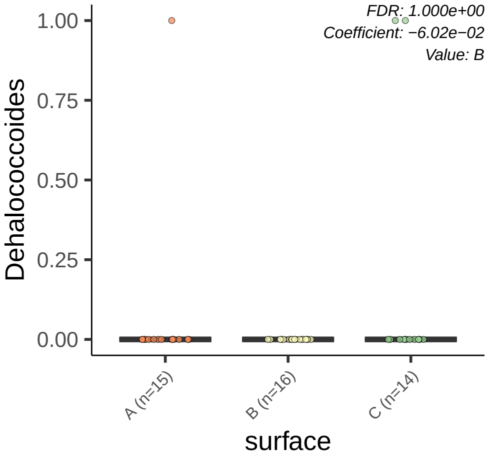

FDR: 1.000e+00
Coefficient: −6.02e−02
Value: B
Dehalococcoides
1.00
0.75
0.50
0.25
0.00
A (n=15)
B (n=16)
C (n=14)
surface

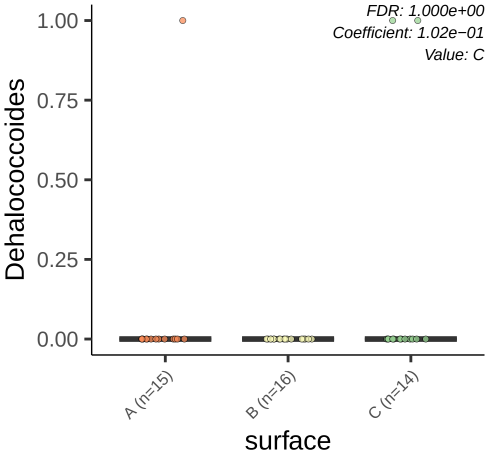
FDR: 1.000e+00
Coefficient: 1.02e−01
Value: C
1.00
0.75
0.50
0.25
0.00
Dehalococcoides
A (n=15)
B (n=16)
C (n=14)
surface

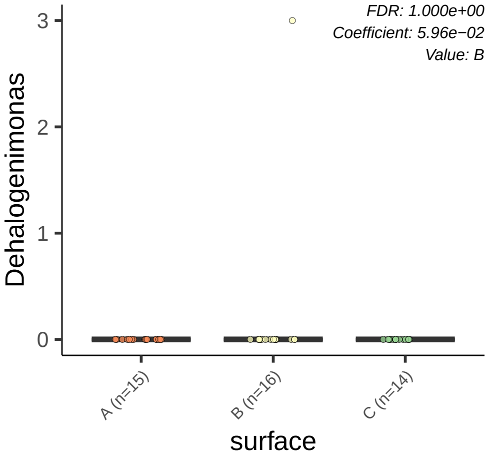
FDR: 1.000e+00
Coefficient: 5.96e−02
Value: B
Dehalogenimonas
3
2
1
0
A (n=15)
B (n=16)
C (n=14)
surface

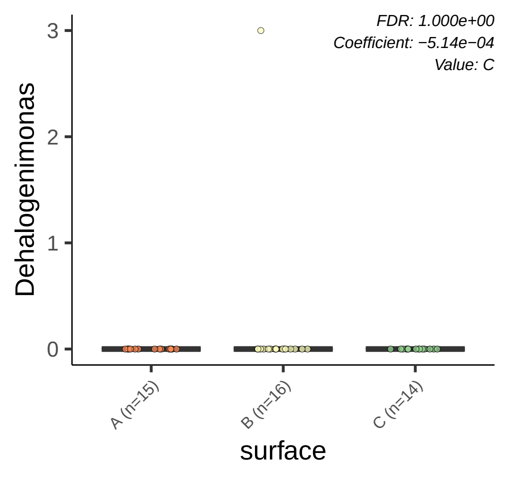
FDR: 1.000e+00
Coefficient: −5.14e−04
Value: C
Dehalogenimonas
0
1
2
3
A (n=15)
B (n=16)
C (n=14)
surface

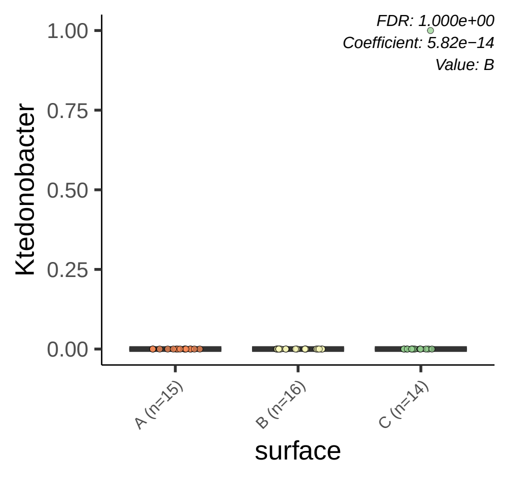

FDR: 1.000e+00
Coefficient: 5.82e−14
Value: B
Ktedonobacter
1.00
0.75
0.50
0.25
0.00
A (n=15)
B (n=16)
C (n=14)
surface

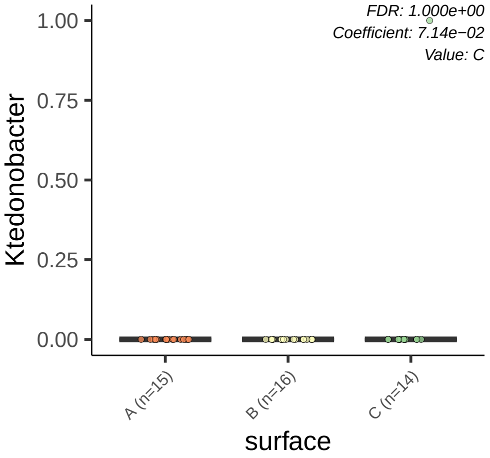
FDR: 1.000e+00
Coefficient: 7.14e−02
Value: C
Ktedonobacter
1.00
0.75
0.50
0.25
0.00
A (n=15)
B (n=16)
C (n=14)
surface

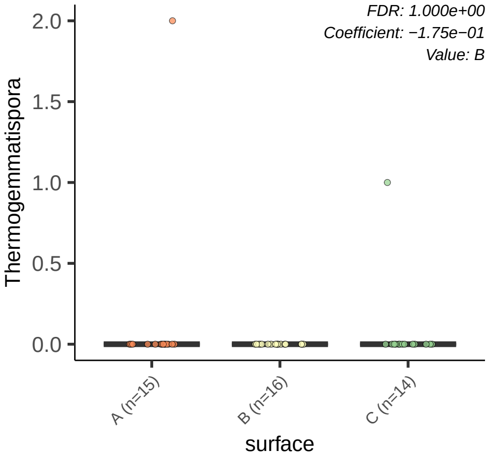
FDR: 1.000e+00
Coefficient: −1.75e−01
Value: B
Thermogemmatispora
2.0
1.5
1.0
0.5
0.0
A (n=15)
B (n=16)
C (n=14)
surface

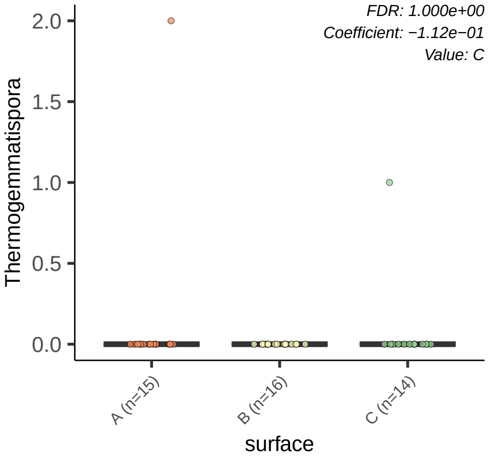
FDR: 1.000e+00
Coefficient: −1.12e−01
Value: C
Thermogemmatispora
2.0
1.5
1.0
0.5
0.0
A (n=15)
B (n=16)
C (n=14)
surface

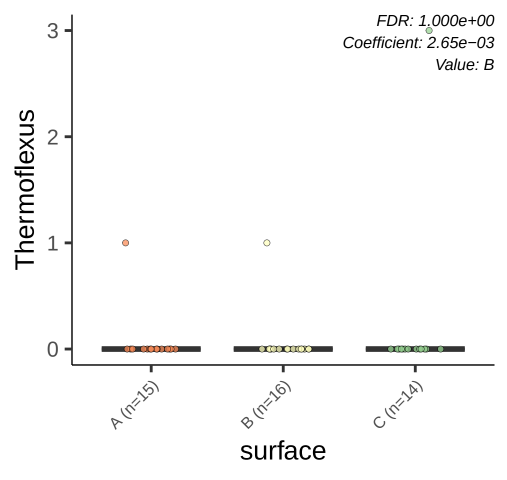

FDR: 1.000e+00
Coefficient: 2.65e−03
Value: B
Thermoflexus
3
2
1
0
A (n=15)
B (n=16)
C (n=14)
surface

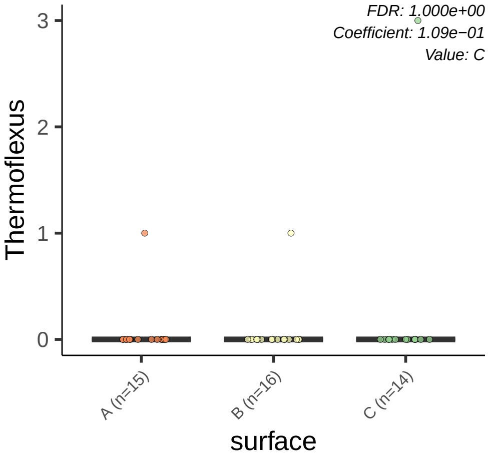
FDR: 1.000e+00
Coefficient: 1.09e−01
Value: C
Thermoflexus
3
2
1
0
A (n=15)
B (n=16)
C (n=14)
surface

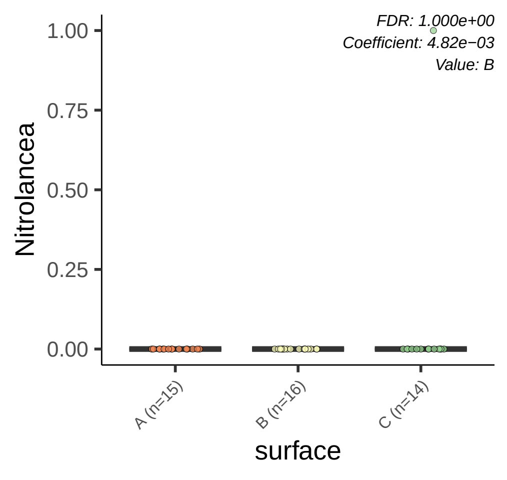
FDR: 1.000e+00
Coefficient: 4.82e−03
Value: B
Nitrolancea
1.00
0.75
0.50
0.25
0.00
A (n=15)
B (n=16)
C (n=14)
surface

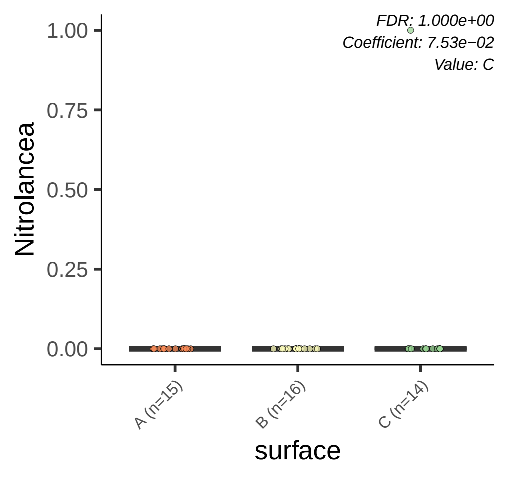

FDR: 1.000e+00
Coefficient: 7.53e−02
Value: C
Nitrolancea
1.00
0.75
0.50
0.25
0.00
A (n=15)
B (n=16)
C (n=14)
surface

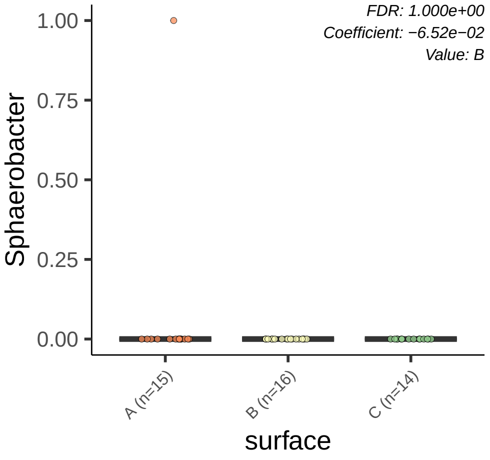

FDR: 1.000e+00
Coefficient: −6.52e−02
Value: B
Sphaerobacter
1.00
0.75
0.50
0.25
0.00
A (n=15)
B (n=16)
C (n=14)
surface

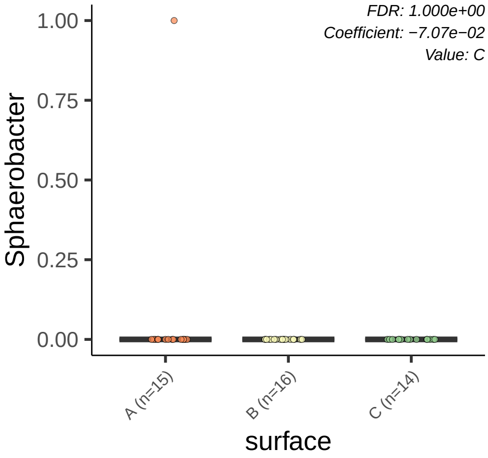

FDR: 1.000e+00
Coefficient: −7.07e−02
Value: C
Sphaerobacter
1.00
0.75
0.50
0.25
0.00
A (n=15)
B (n=16)
C (n=14)
surface

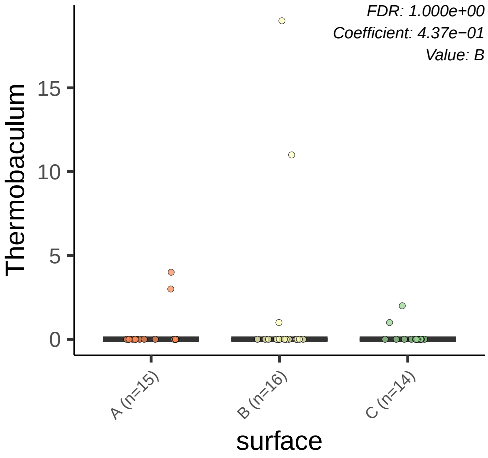

FDR: 1.000e+00
Coefficient: 4.37e−01
Value: B
Thermobaculum
0
5
10
15
A (n=15)
B (n=16)
C (n=14)
surface

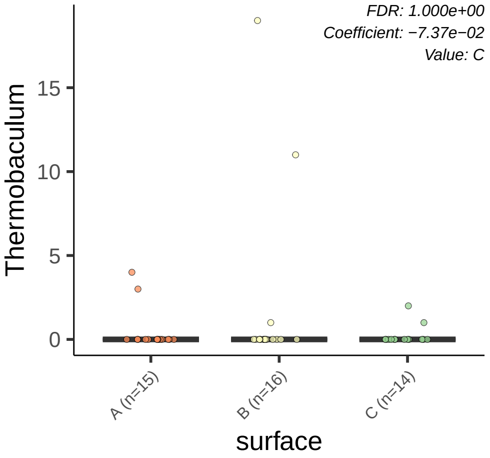
FDR: 1.000e+00
Coefficient: −7.37e−02
Value: C
Thermobaculum
15
10
5
0
A (n=15)
B (n=16)
C (n=14)
surface

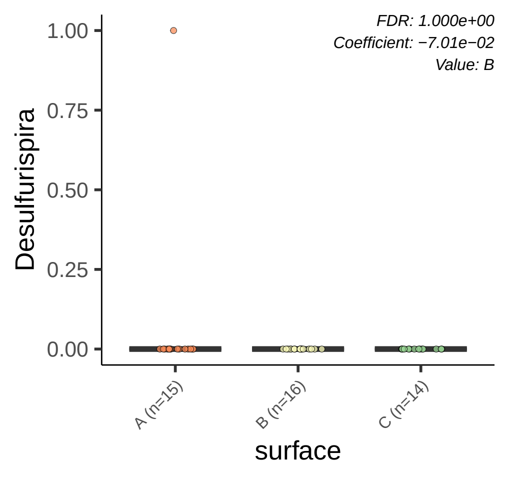

FDR: 1.000e+00
Coefficient: −7.01e−02
Value: B
Desulfurispira
1.00
0.75
0.50
0.25
0.00
A (n=15)
B (n=16)
C (n=14)
surface

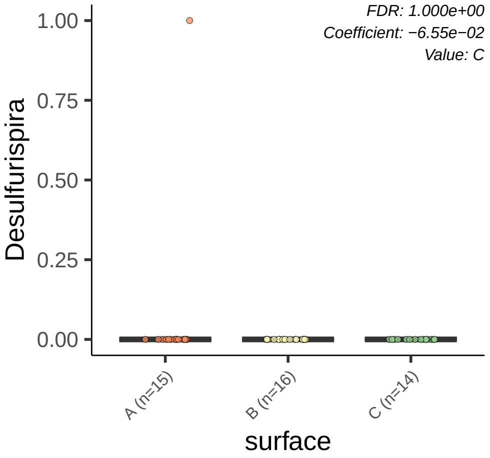
FDR: 1.000e+00
Coefficient: −6.55e−02
Value: C
Desulfurispira
1.00
0.75
0.50
0.25
0.00
A (n=15)
B (n=16)
C (n=14)
surface

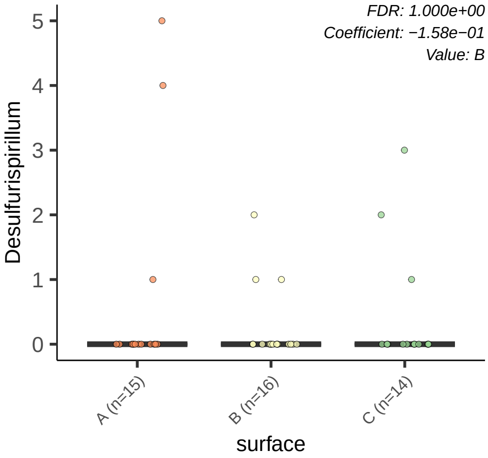

FDR: 1.000e+00
Coefficient: −1.58e−01
Value: B
Desulfurispirillum
0
1
2
3
4
5
A (n=15)
B (n=16)
C (n=14)
surface

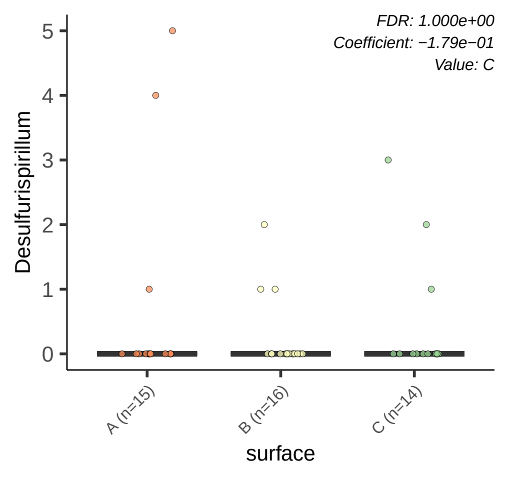
FDR: 1.000e+00
Coefficient: −1.79e−01
Value: C
Desulfurispirillum
0
1
2
3
4
5
A (n=15)
B (n=16)
C (n=14)
surface

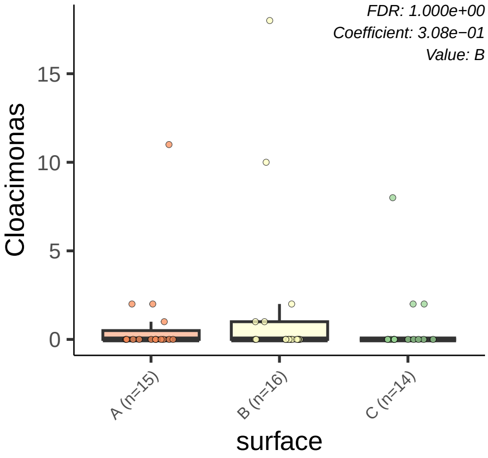

FDR: 1.000e+00
Coefficient: 3.08e−01
Value: B
Cloacimonas
15
10
5
0
A (n=15)
B (n=16)
C (n=14)
surface

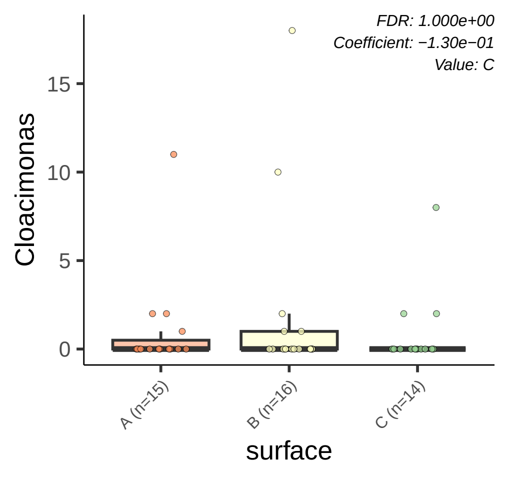
FDR: 1.000e+00
Coefficient: −1.30e−01
Value: C
Cloacimonas
0
5
10
15
A (n=15)
B (n=16)
C (n=14)
surface

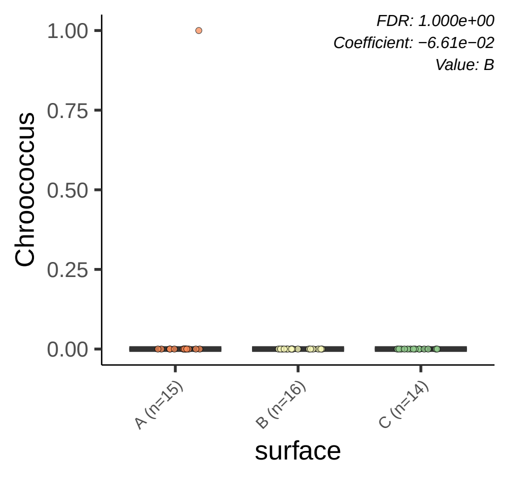

FDR: 1.000e+00
Coefficient: −6.61e−02
Value: B
Chroococcus
1.00
0.75
0.50
0.25
0.00
A (n=15)
B (n=16)
C (n=14)
surface

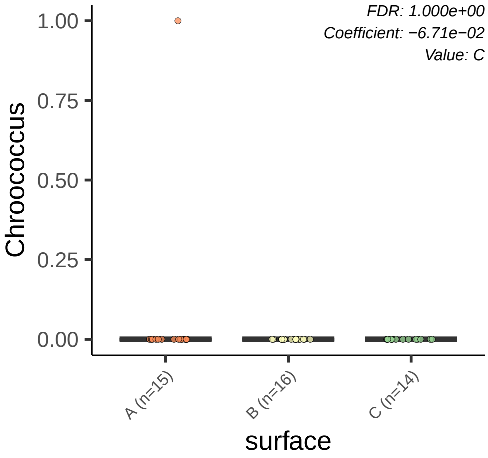

FDR: 1.000e+00
Coefficient: −6.71e−02
Value: C
Chroococcus
1.00
0.75
0.50
0.25
0.00
A (n=15)
B (n=16)
C (n=14)
surface

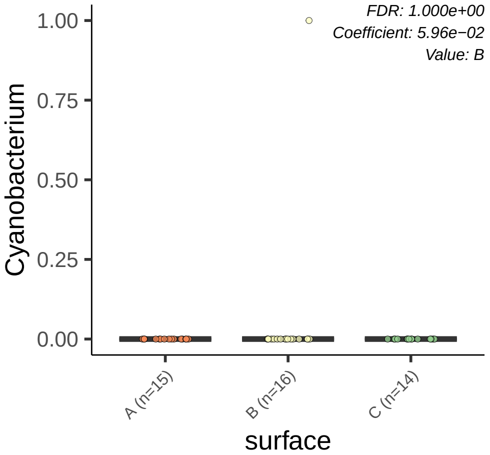
FDR: 1.000e+00
Coefficient: 5.96e−02
Value: B
1.00
0.75
0.50
0.25
0.00
Cyanobacterium
A (n=15)
B (n=16)
C (n=14)
surface

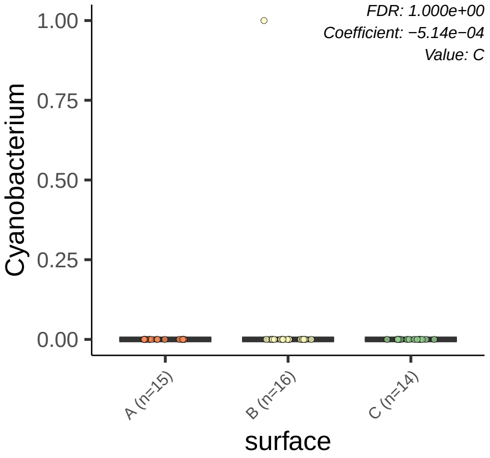
FDR: 1.000e+00
Coefficient: −5.14e−04
Value: C
Cyanobacterium
1.00
0.75
0.50
0.25
0.00
A (n=15)
B (n=16)
C (n=14)
surface

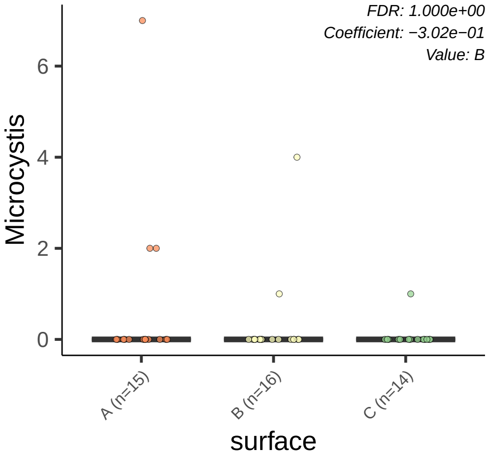
FDR: 1.000e+00
Coefficient: −3.02e−01
Value: B
Microcystis
0
2
4
6
A (n=15)
B (n=16)
C (n=14)
surface

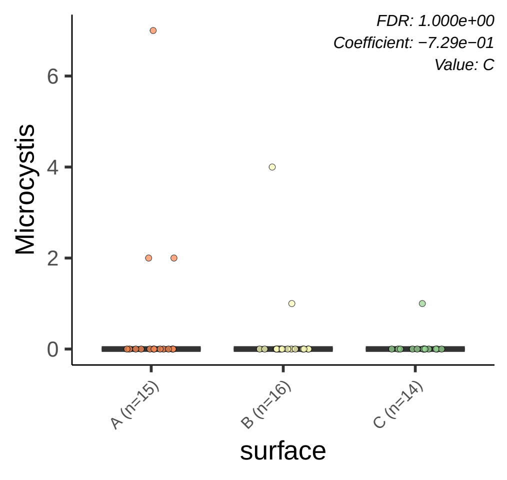

FDR: 1.000e+00
Coefficient: −7.29e−01
Value: C
Microcystis
0
2
4
6
A (n=15)
B (n=16)
C (n=14)
surface

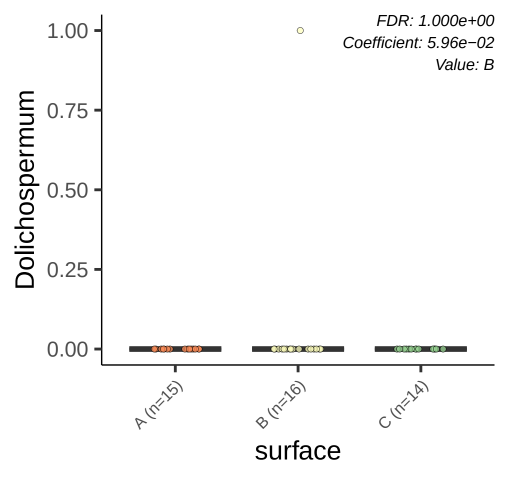
FDR: 1.000e+00
Coefficient: 5.96e−02
Value: B
Dolichospermum
1.00
0.75
0.50
0.25
0.00
A (n=15)
B (n=16)
C (n=14)
surface

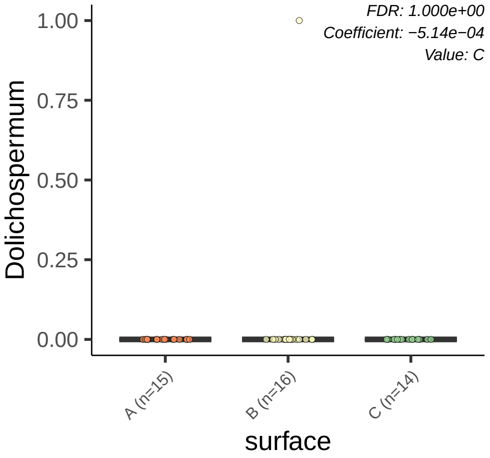
FDR: 1.000e+00
Coefficient: −5.14e−04
Value: C
Dolichospermum
1.00
0.75
0.50
0.25
0.00
A (n=15)
B (n=16)
C (n=14)
surface

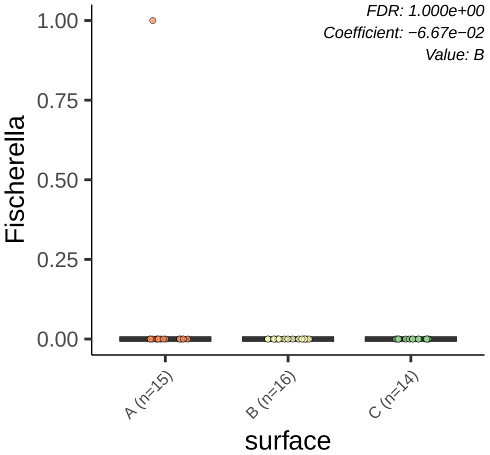
FDR: 1.000e+00
Coefficient: −6.67e−02
Value: B
Fischerella
1.00
0.75
0.50
0.25
0.00
A (n=15)
B (n=16)
C (n=14)
surface

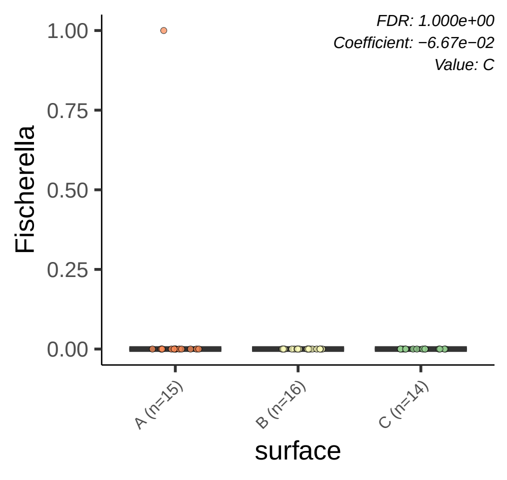
FDR: 1.000e+00
Coefficient: −6.67e−02
Value: C
Fischerella
1.00
0.75
0.50
0.25
0.00
A (n=15)
B (n=16)
C (n=14)
surface

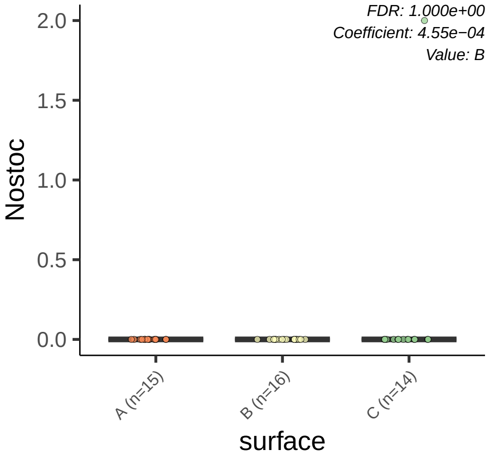
FDR: 1.000e+00
Coefficient: 4.55e−04
Value: B
Nostoc
2.0
1.5
1.0
0.5
0.0
A (n=15)
B (n=16)
C (n=14)
surface

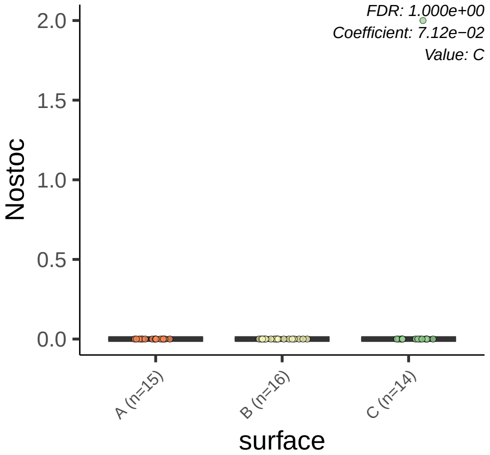

FDR: 1.000e+00
Coefficient: 7.12e−02
Value: C
2.0
1.5
1.0
0.5
0.0
Nostoc
A (n=15)
B (n=16)
C (n=14)
surface

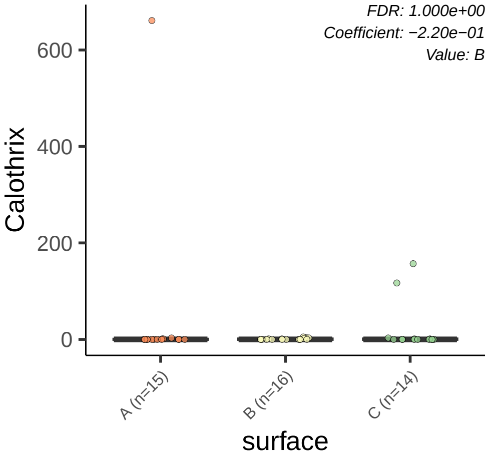
FDR: 1.000e+00
Coefficient: −2.20e−01
Value: B
Calothrix
600
400
200
0
A (n=15)
B (n=16)
C (n=14)
surface

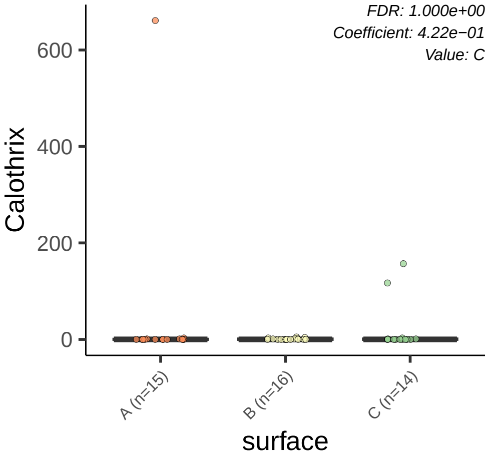
FDR: 1.000e+00
Coefficient: 4.22e−01
Value: C
Calothrix
600
400
200
0
A (n=15)
B (n=16)
C (n=14)
surface

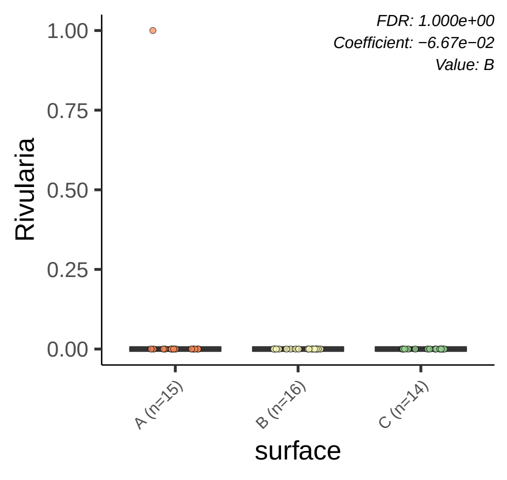
FDR: 1.000e+00
Coefficient: −6.67e−02
Value: B
Rivularia
1.00
0.75
0.50
0.25
0.00
A (n=15)
B (n=16)
C (n=14)
surface

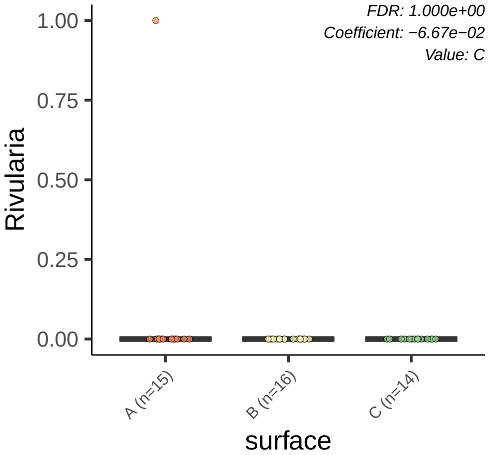
FDR: 1.000e+00
Coefficient: −6.67e−02
Value: C
1.00
0.75
0.50
0.25
0.00
Rivularia
A (n=15)
B (n=16)
C (n=14)
surface

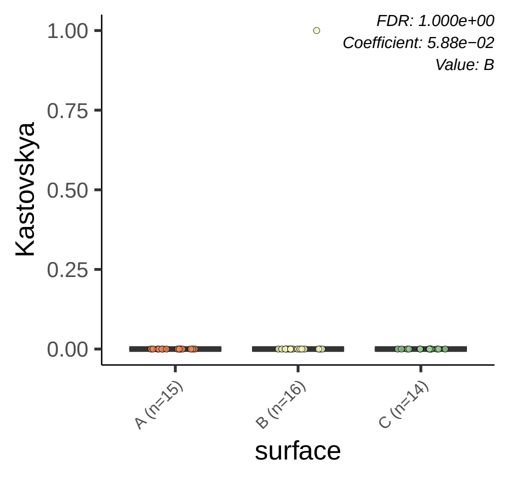
FDR: 1.000e+00
Coefficient: 5.88e−02
Value: B
Kastovskya
1.00
0.75
0.50
0.25
0.00
A (n=15)
B (n=16)
C (n=14)
surface

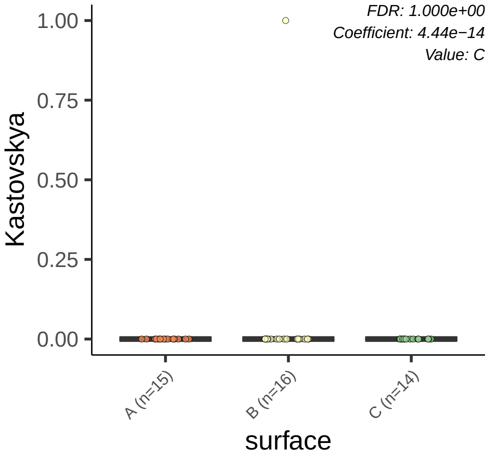
FDR: 1.000e+00
Coefficient: 4.44e−14
Value: C
Kastovskya
1.00
0.75
0.50
0.25
0.00
A (n=15)
B (n=16)
C (n=14)
surface

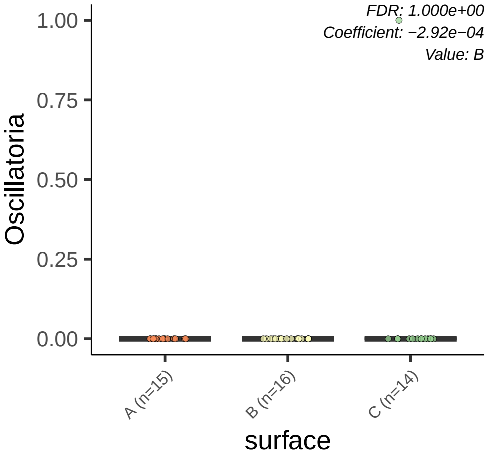
FDR: 1.000e+00
Coefficient: −2.92e−04
Value: B
Oscillatoria
1.00
0.75
0.50
0.25
0.00
A (n=15)
B (n=16)
C (n=14)
surface

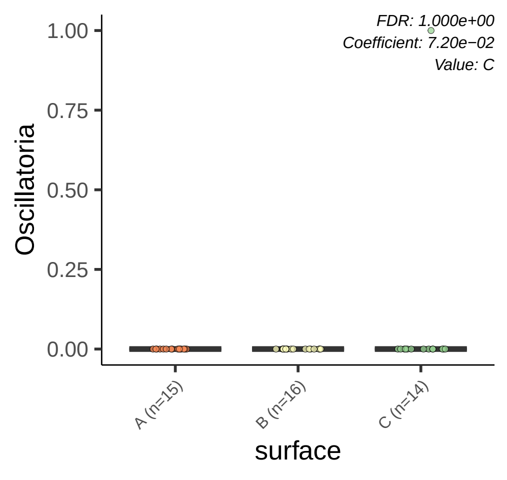
FDR: 1.000e+00
Coefficient: 7.20e−02
Value: C
Oscillatoria
1.00
0.75
0.50
0.25
0.00
A (n=15)
B (n=16)
C (n=14)
surface

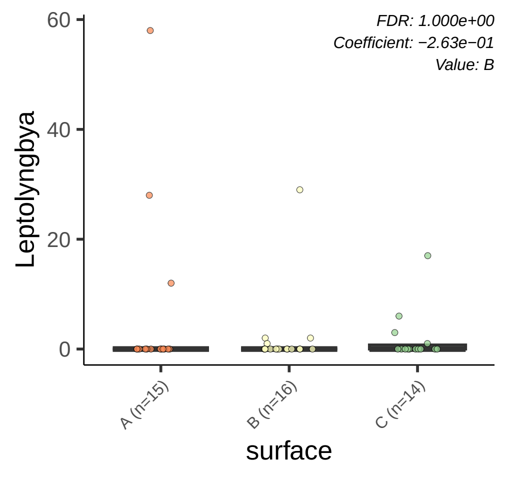

FDR: 1.000e+00
Coefficient: −2.63e−01
Value: B
Leptolyngbya
60
40
20
0
A (n=15)
B (n=16)
C (n=14)
surface

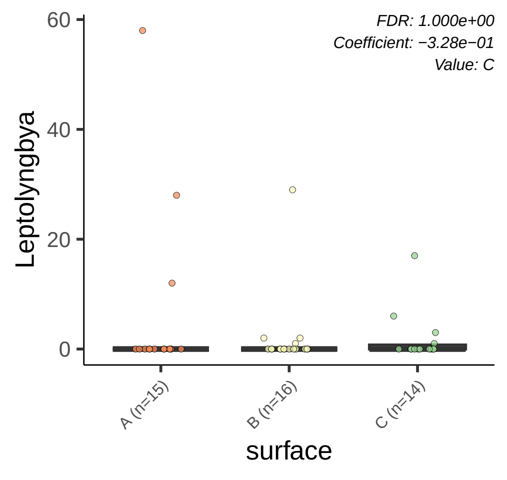
FDR: 1.000e+00
Coefficient: −3.28e−01
Value: C
Leptolyngbya
60
40
20
0
A (n=15)
B (n=16)
C (n=14)
surface

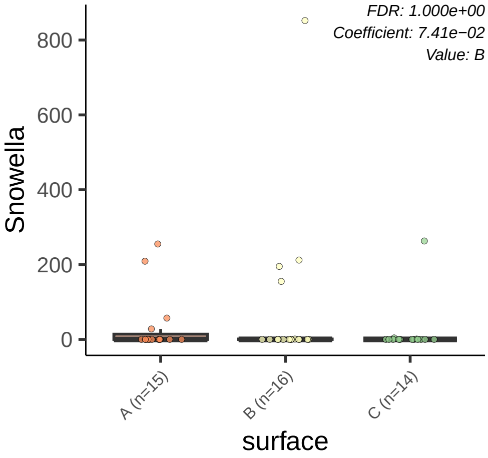

FDR: 1.000e+00
Coefficient: 7.41e−02
Value: B
Snowella
800
600
400
200
0
A (n=15)
B (n=16)
C (n=14)
surface

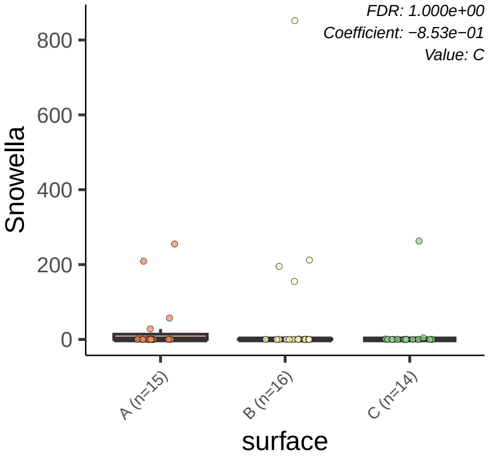

FDR: 1.000e+00
Coefficient: −8.53e−01
Value: C
Snowella
800
600
400
200
0
A (n=15)
B (n=16)
C (n=14)
surface

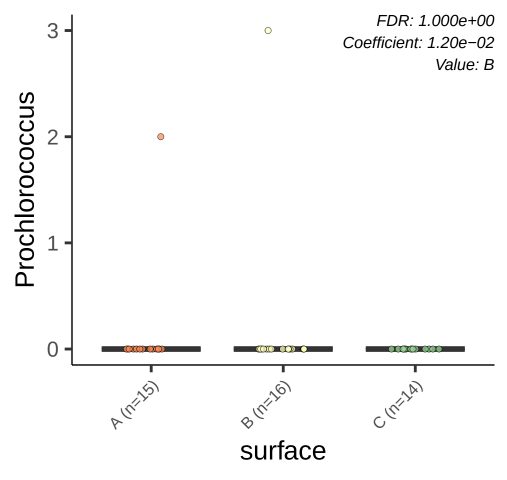
FDR: 1.000e+00
Coefficient: 1.20e−02
Value: B
Prochlorococcus
3
2
1
0
A (n=15)
B (n=16)
C (n=14)
surface

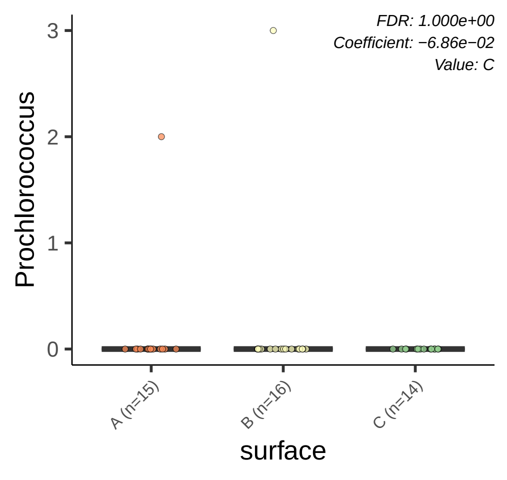
FDR: 1.000e+00
Coefficient: −6.86e−02
Value: C
Prochlorococcus
0
1
2
3
A (n=15)
B (n=16)
C (n=14)
surface

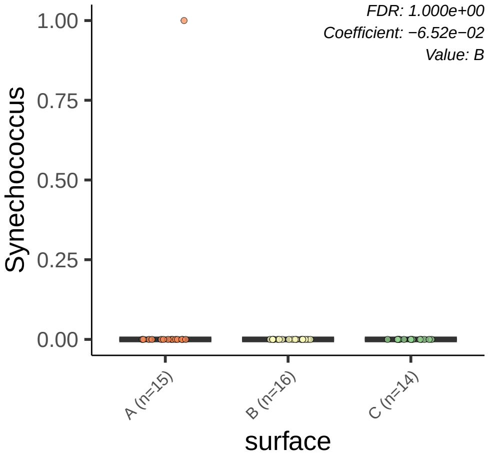
FDR: 1.000e+00
Coefficient: −6.52e−02
Value: B
Synechococcus
1.00
0.75
0.50
0.25
0.00
A (n=15)
B (n=16)
C (n=14)
surface

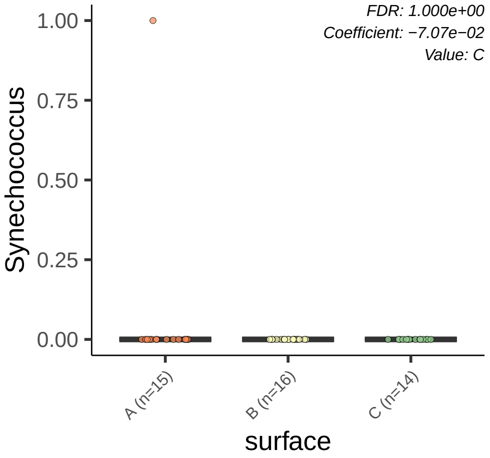
FDR: 1.000e+00
Coefficient: −7.07e−02
Value: C
Synechococcus
1.00
0.75
0.50
0.25
0.00
A (n=15)
B (n=16)
C (n=14)
surface

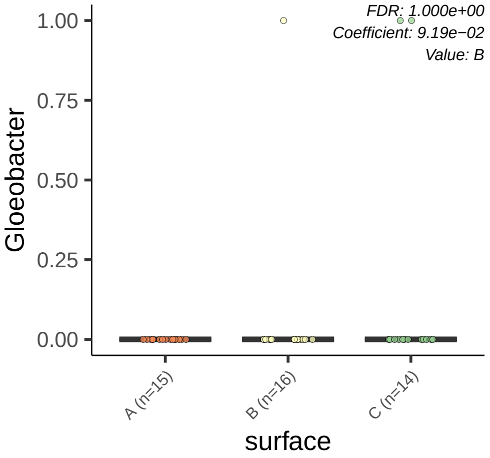
FDR: 1.000e+00
Coefficient: 9.19e−02
Value: B
Gloeobacter
1.00
0.75
0.50
0.25
0.00
A (n=15)
B (n=16)
C (n=14)
surface

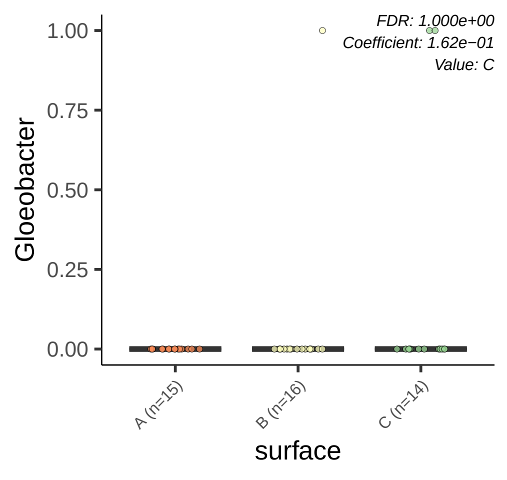
FDR: 1.000e+00
Coefficient: 1.62e−01
Value: C
Gloeobacter
1.00
0.75
0.50
0.25
0.00
A (n=15)
B (n=16)
C (n=14)
surface

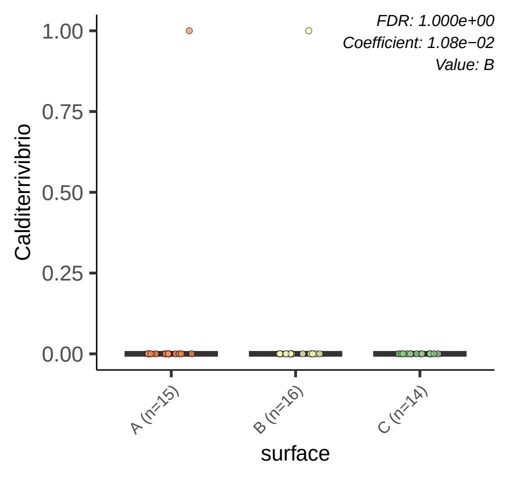

FDR: 1.000e+00
Coefficient: 1.08e−02
Value: B
Calditerrivibrio
1.00
0.75
0.50
0.25
0.00
A (n=15)
B (n=16)
C (n=14)
surface

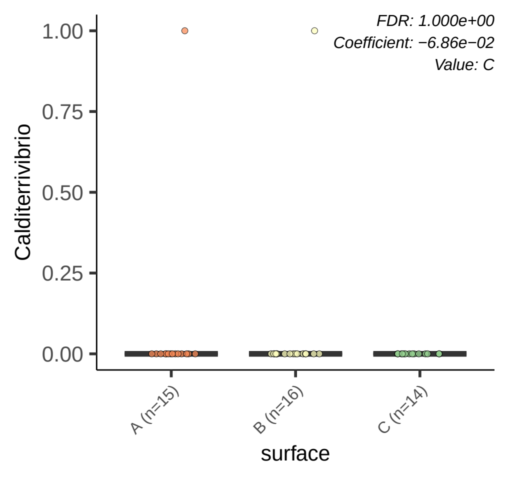
FDR: 1.000e+00
Coefficient: −6.86e−02
Value: C
1.00
0.75
0.50
0.25
0.00
Calditerrivibrio
A (n=15)
B (n=16)
C (n=14)
surface

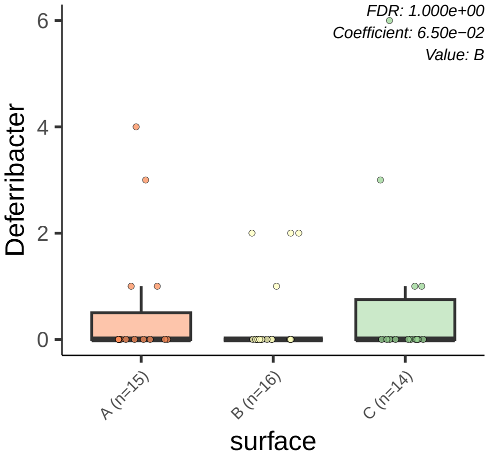

FDR: 1.000e+00
Coefficient: 6.50e−02
Value: B
Deferribacter
0
2
4
6
A (n=15)
B (n=16)
C (n=14)
surface

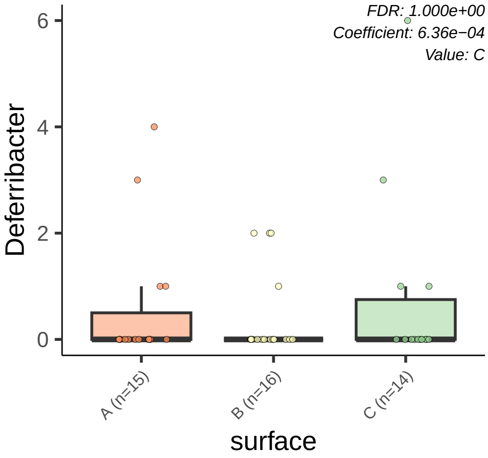

FDR: 1.000e+00
Coefficient: 6.36e−04
Value: C
Deferribacter
0
2
4
6
A (n=15)
B (n=16)
C (n=14)
surface

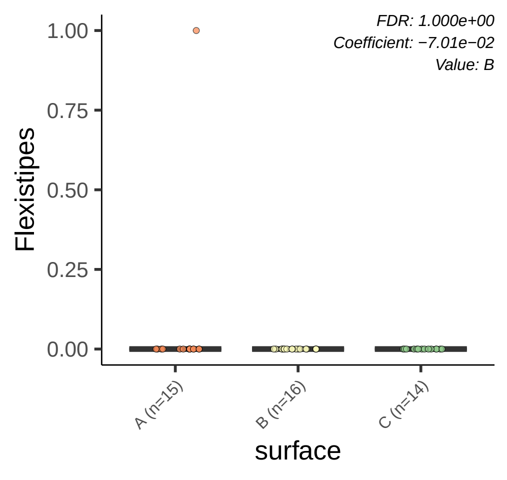
FDR: 1.000e+00
Coefficient: −7.01e−02
Value: B
Flexistipes
1.00
0.75
0.50
0.25
0.00
A (n=15)
B (n=16)
C (n=14)
surface

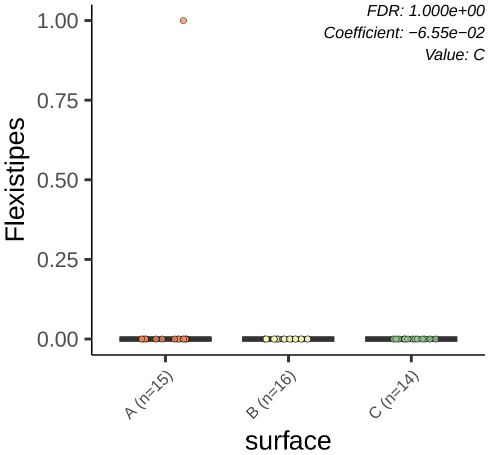
FDR: 1.000e+00
Coefficient: −6.55e−02
Value: C
Flexistipes
1.00
0.75
0.50
0.25
0.00
A (n=15)
B (n=16)
C (n=14)
surface

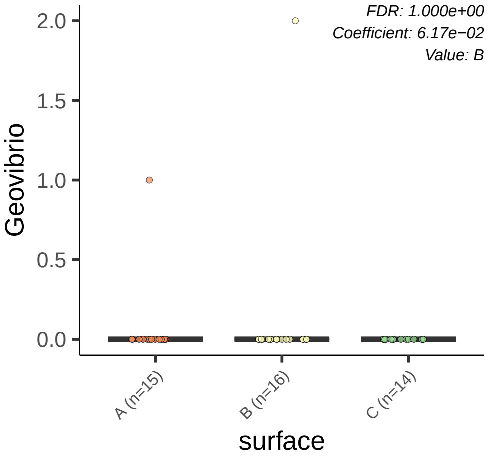
FDR: 1.000e+00
Coefficient: 6.17e−02
Value: B
Geovibrio
2.0
1.5
1.0
0.5
0.0
A (n=15)
B (n=16)
C (n=14)
surface

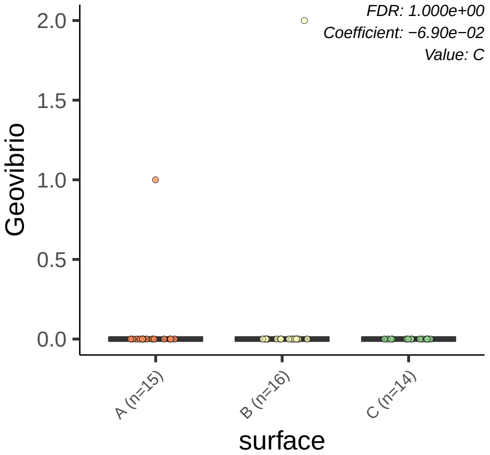
FDR: 1.000e+00
Coefficient: −6.90e−02
Value: C
Geovibrio
2.0
1.5
1.0
0.5
0.0
A (n=15)
B (n=16)
C (n=14)
surface

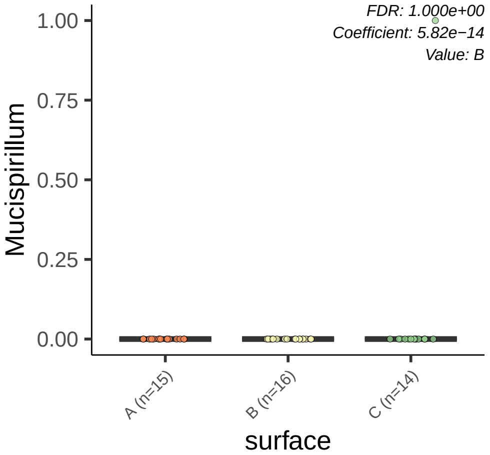

FDR: 1.000e+00
Coefficient: 5.82e−14
Value: B
Mucispirillum
1.00
0.75
0.50
0.25
0.00
A (n=15)
B (n=16)
C (n=14)
surface

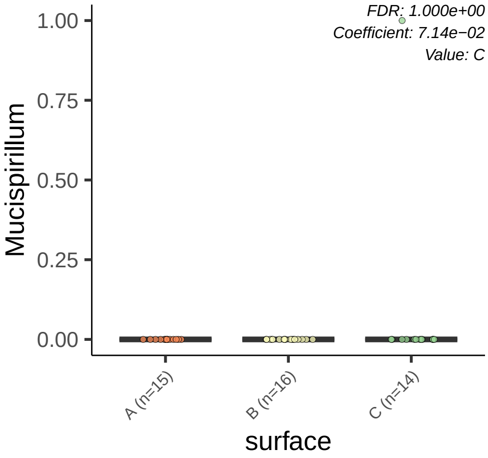

FDR: 1.000e+00
Coefficient: 7.14e−02
Value: C
Mucispirillum
1.00
0.75
0.50
0.25
0.00
A (n=15)
B (n=16)
C (n=14)
surface

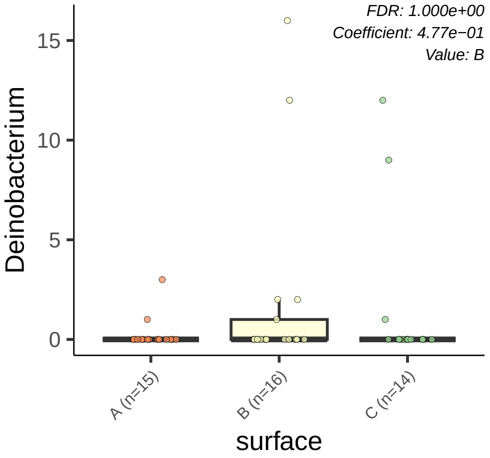
FDR: 1.000e+00
Coefficient: 4.77e−01
Value: B
Deinobacterium
15
10
5
0
A (n=15)
B (n=16)
C (n=14)
surface

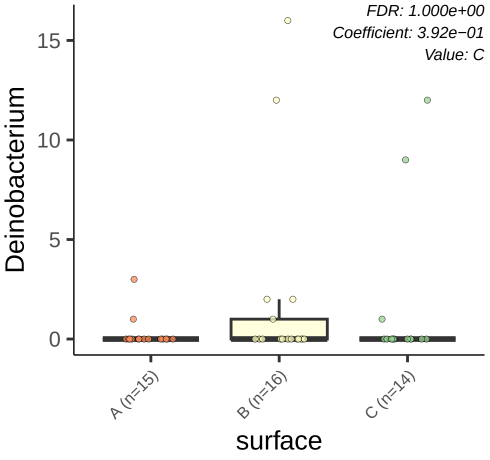
FDR: 1.000e+00
Coefficient: 3.92e−01
Value: C
Deinobacterium
15
10
5
0
A (n=15)
B (n=16)
C (n=14)
surface

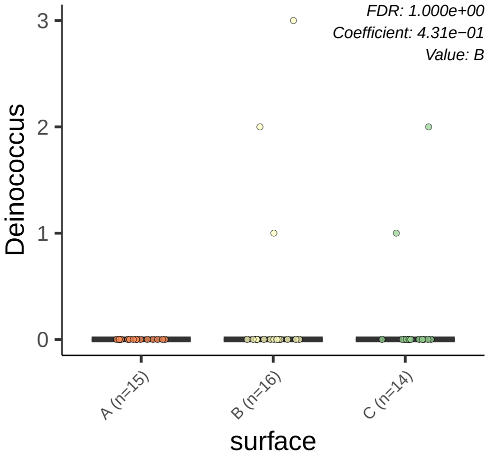

FDR: 1.000e+00
Coefficient: 4.31e−01
Value: B
Deinococcus
3
2
1
0
A (n=15)
B (n=16)
C (n=14)
surface

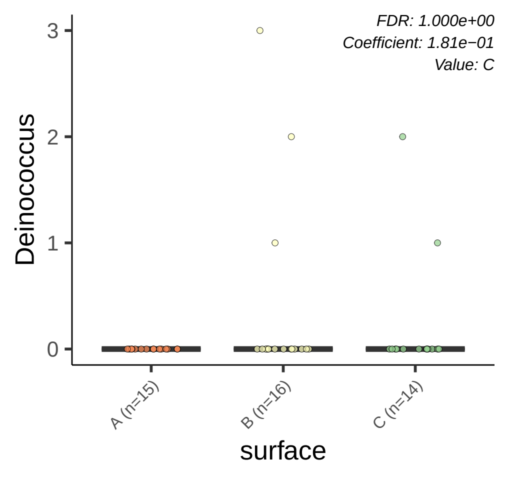

FDR: 1.000e+00
Coefficient: 1.81e−01
Value: C
Deinococcus
3
2
1
0
A (n=15)
B (n=16)
C (n=14)
surface

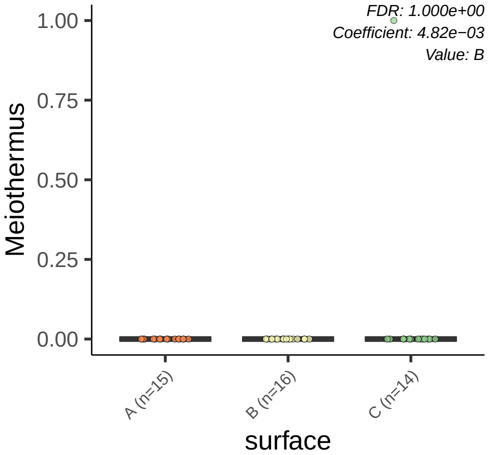

FDR: 1.000e+00
Coefficient: 4.82e−03
Value: B
Meiothermus
1.00
0.75
0.50
0.25
0.00
A (n=15)
B (n=16)
C (n=14)
surface

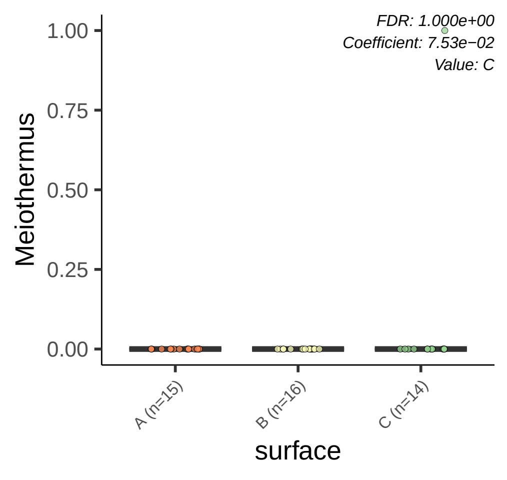
FDR: 1.000e+00
Coefficient: 7.53e−02
Value: C
Meiothermus
1.00
0.75
0.50
0.25
0.00
A (n=15)
B (n=16)
C (n=14)
surface

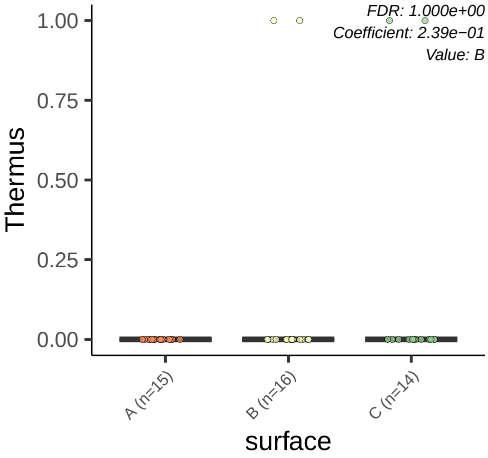
FDR: 1.000e+00
Coefficient: 2.39e−01
Value: B
Thermus
1.00
0.75
0.50
0.25
0.00
A (n=15)
B (n=16)
C (n=14)
surface

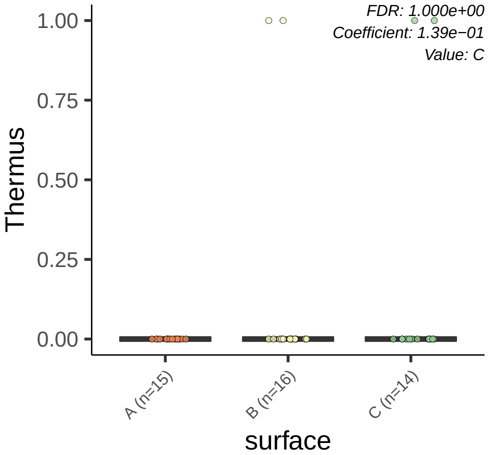

FDR: 1.000e+00
Coefficient: 1.39e−01
Value: C
Thermus
1.00
0.75
0.50
0.25
0.00
A (n=15)
B (n=16)
C (n=14)
surface

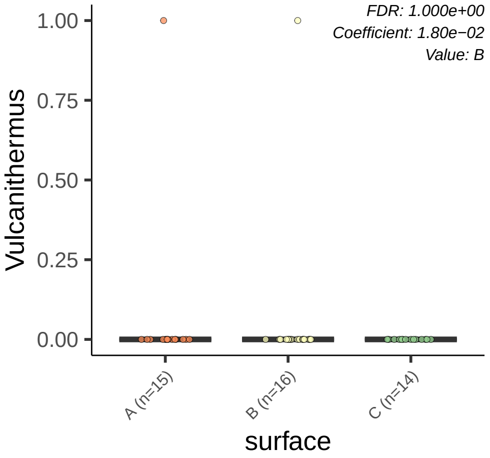
FDR: 1.000e+00
Coefficient: 1.80e−02
Value: B
Vulcanithermus
1.00
0.75
0.50
0.25
0.00
A (n=15)
B (n=16)
C (n=14)
surface

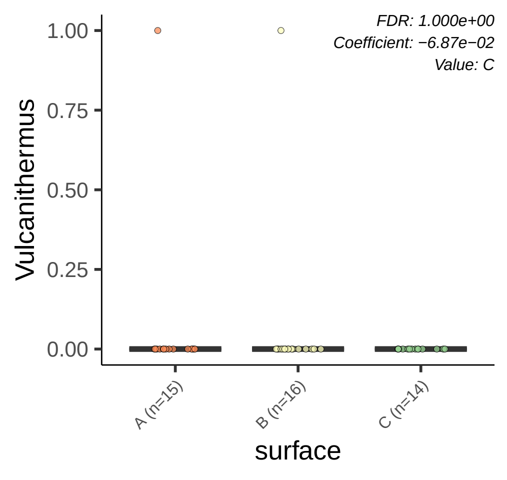

FDR: 1.000e+00
Coefficient: −6.87e−02
Value: C
Vulcanithermus
1.00
0.75
0.50
0.25
0.00
A (n=15)
B (n=16)
C (n=14)
surface

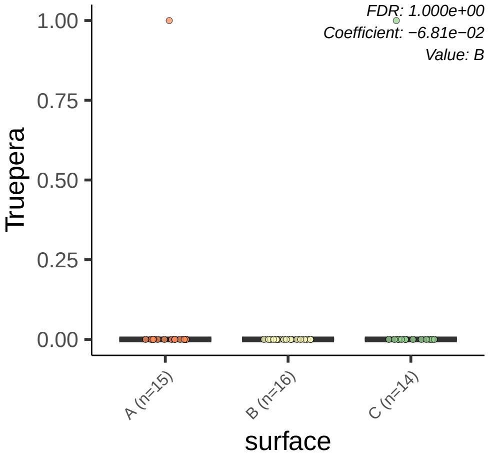

FDR: 1.000e+00
Coefficient: −6.81e−02
Value: B
Truepera
1.00
0.75
0.50
0.25
0.00
A (n=15)
B (n=16)
C (n=14)
surface

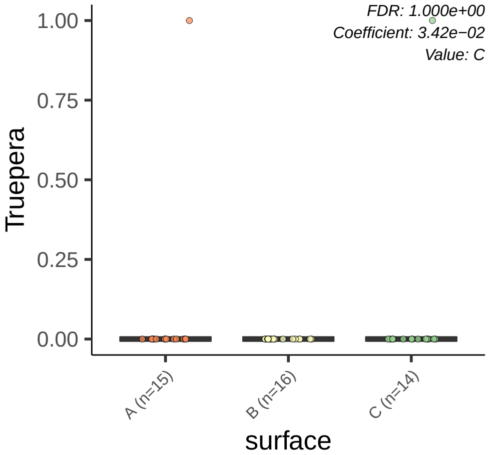
FDR: 1.000e+00
Coefficient: 3.42e−02
Value: C
1.00
0.75
0.50
0.25
0.00
Truepera
A (n=15)
B (n=16)
C (n=14)
surface

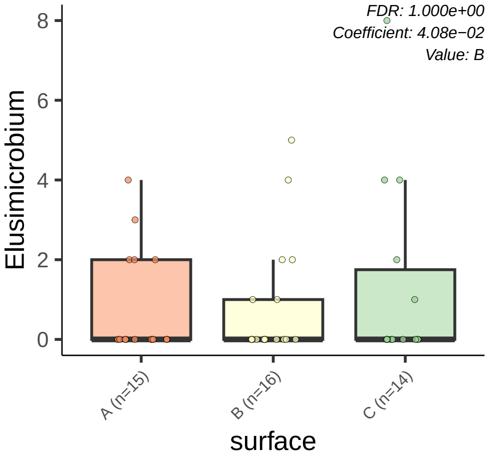

FDR: 1.000e+00
Coefficient: 4.08e−02
Value: B
Elusimicrobium
0
2
4
6
8
A (n=15)
B (n=16)
C (n=14)
surface

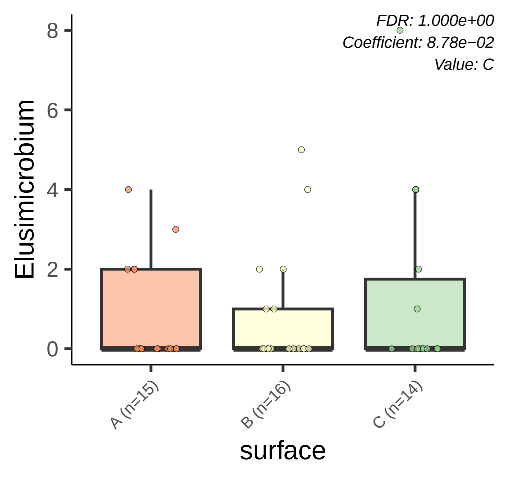
FDR: 1.000e+00
Coefficient: 8.78e−02
Value: C
Elusimicrobium
0
2
4
6
8
A (n=15)
B (n=16)
C (n=14)
surface

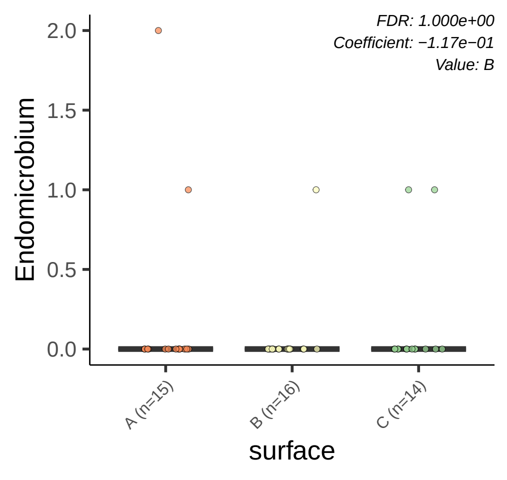
FDR: 1.000e+00
Coefficient: −1.17e−01
Value: B
Endomicrobium
2.0
1.5
1.0
0.5
0.0
A (n=15)
B (n=16)
C (n=14)
surface

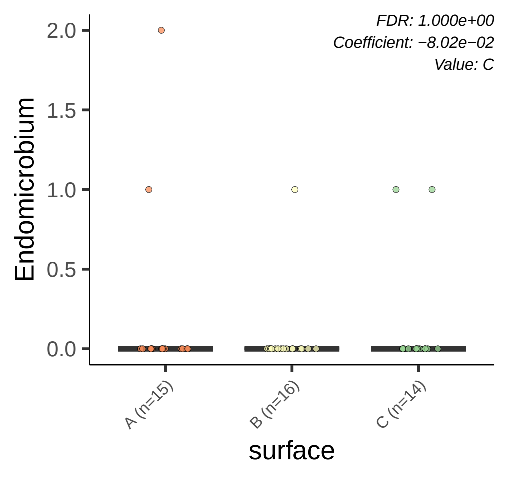
FDR: 1.000e+00
Coefficient: −8.02e−02
Value: C
Endomicrobium
2.0
1.5
1.0
0.5
0.0
A (n=15)
B (n=16)
C (n=14)
surface

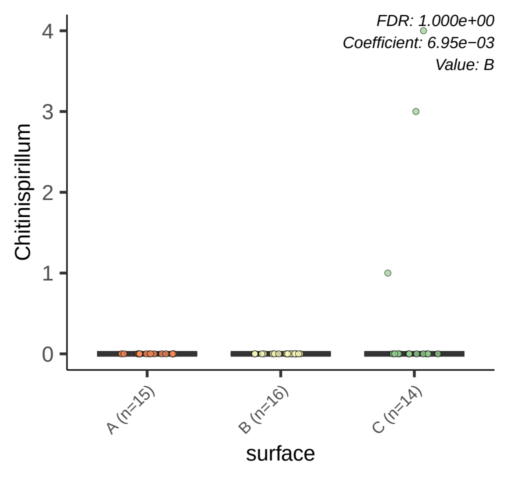

FDR: 1.000e+00
Coefficient: 6.95e−03
Value: B
Chitinispirillum
0
1
2
3
4
A (n=15)
B (n=16)
C (n=14)
surface

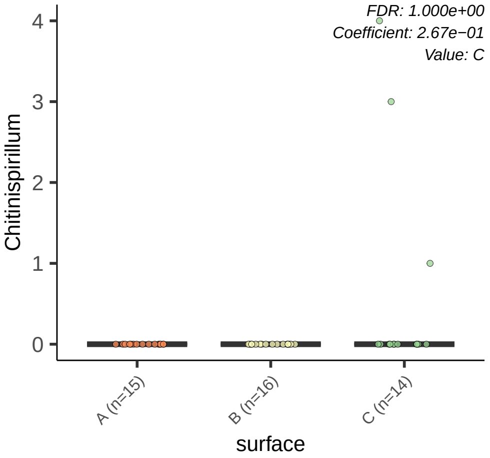

FDR: 1.000e+00
Coefficient: 2.67e−01
Value: C
Chitinispirillum
0
1
2
3
4
A (n=15)
B (n=16)
C (n=14)
surface

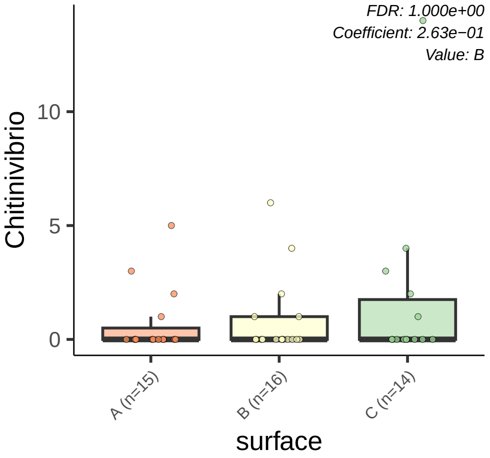

FDR: 1.000e+00
Coefficient: 2.63e−01
Value: B
Chitinivibrio
10
5
0
A (n=15)
B (n=16)
C (n=14)
surface

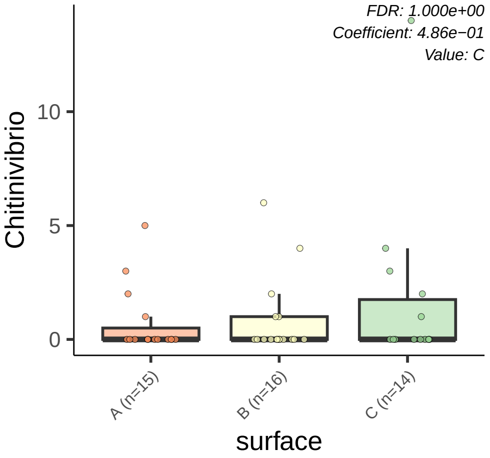

FDR: 1.000e+00
Coefficient: 4.86e−01
Value: C
Chitinivibrio
10
5
0
A (n=15)
B (n=16)
C (n=14)
surface

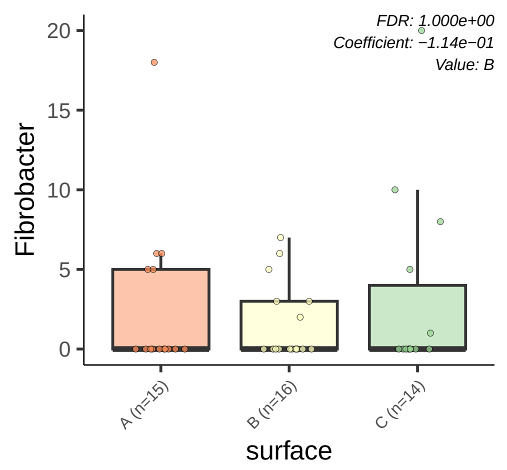
FDR: 1.000e+00
Coefficient: −1.14e−01
Value: B
Fibrobacter
20
15
10
5
0
A (n=15)
B (n=16)
C (n=14)
surface

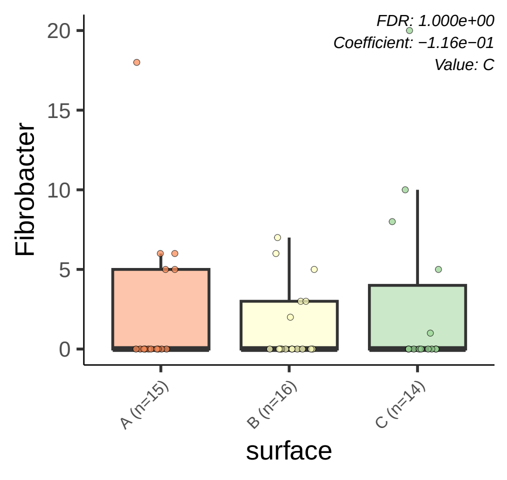
FDR: 1.000e+00
Coefficient: −1.16e−01
Value: C
Fibrobacter
20
15
10
5
0
A (n=15)
B (n=16)
C (n=14)
surface

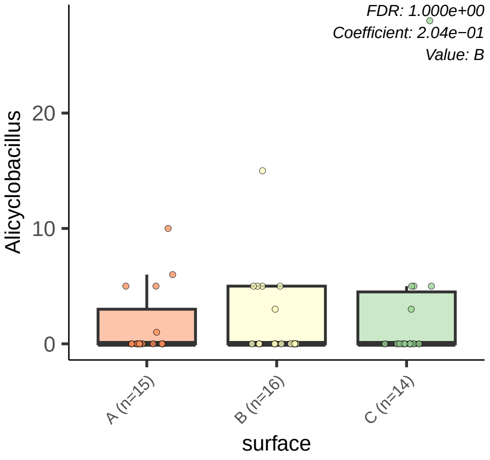
FDR: 1.000e+00
Coefficient: 2.04e−01
Value: B
Alicyclobacillus
0
10
20
A (n=15)
B (n=16)
C (n=14)
surface

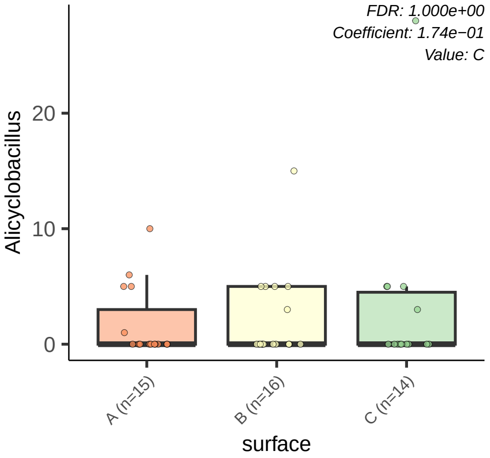

FDR: 1.000e+00
Coefficient: 1.74e−01
Value: C
Alicyclobacillus
0
10
20
A (n=15)
B (n=16)
C (n=14)
surface

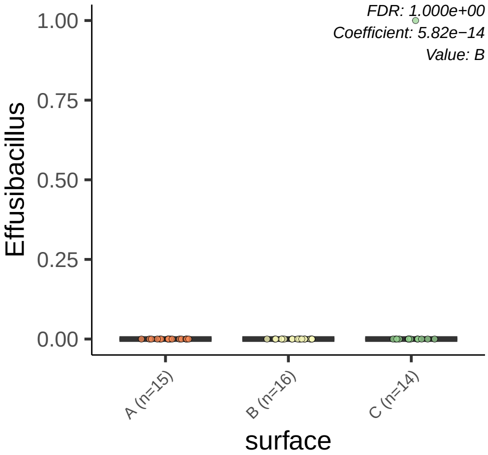
FDR: 1.000e+00
Coefficient: 5.82e−14
Value: B
1.00
0.75
0.50
0.25
0.00
Effusibacillus
A (n=15)
B (n=16)
C (n=14)
surface

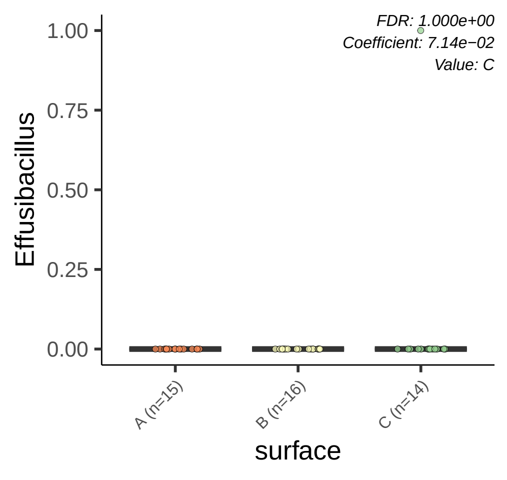

FDR: 1.000e+00
Coefficient: 7.14e−02
Value: C
Effusibacillus
1.00
0.75
0.50
0.25
0.00
A (n=15)
B (n=16)
C (n=14)
surface

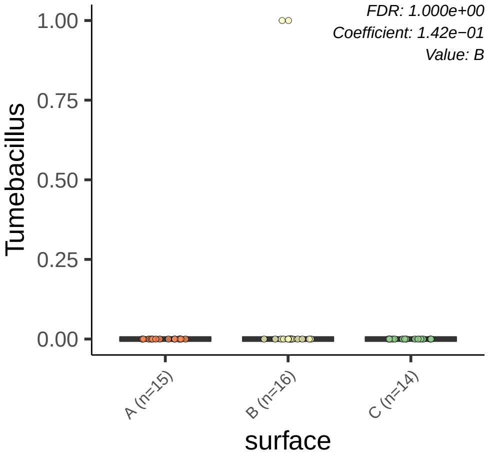
FDR: 1.000e+00
Coefficient: 1.42e−01
Value: B
Tumebacillus
1.00
0.75
0.50
0.25
0.00
A (n=15)
B (n=16)
C (n=14)
surface

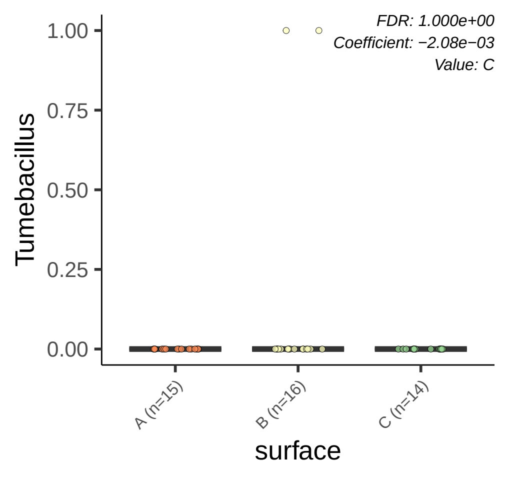
FDR: 1.000e+00
Coefficient: −2.08e−03
Value: C
Tumebacillus
1.00
0.75
0.50
0.25
0.00
A (n=15)
B (n=16)
C (n=14)
surface

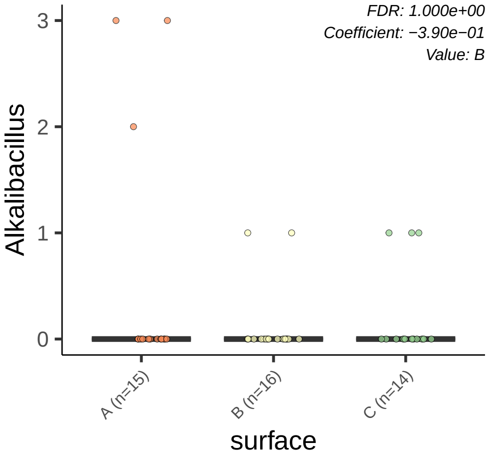

FDR: 1.000e+00
Coefficient: −3.90e−01
Value: B
Alkalibacillus
0
1
2
3
A (n=15)
B (n=16)
C (n=14)
surface

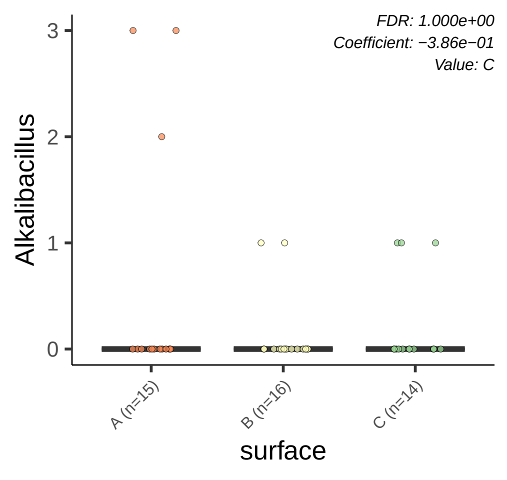

FDR: 1.000e+00
Coefficient: −3.86e−01
Value: C
Alkalibacillus
3
2
1
0
A (n=15)
B (n=16)
C (n=14)
surface

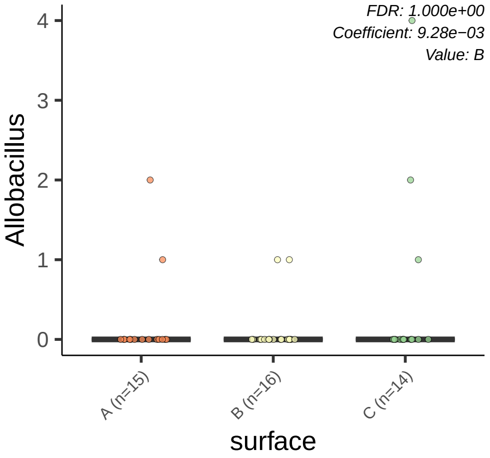

FDR: 1.000e+00
Coefficient: 9.28e−03
Value: B
Allobacillus
0
1
2
3
4
A (n=15)
B (n=16)
C (n=14)
surface

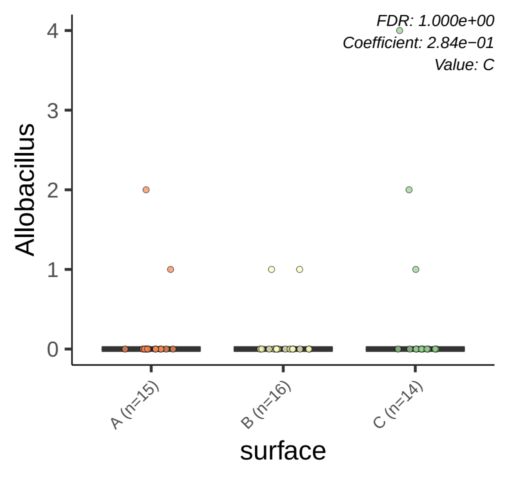
FDR: 1.000e+00
Coefficient: 2.84e−01
Value: C
Allobacillus
4
3
2
1
0
A (n=15)
B (n=16)
C (n=14)
surface

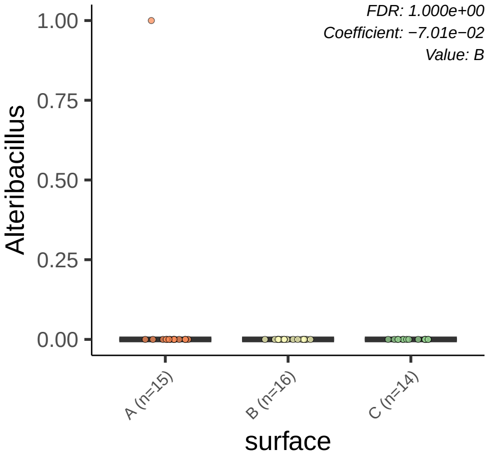

FDR: 1.000e+00
Coefficient: −7.01e−02
Value: B
Alteribacillus
1.00
0.75
0.50
0.25
0.00
A (n=15)
B (n=16)
C (n=14)
surface

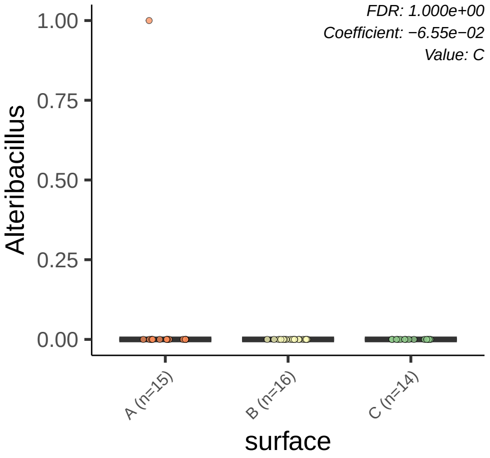

FDR: 1.000e+00
Coefficient: −6.55e−02
Value: C
Alteribacillus
1.00
0.75
0.50
0.25
0.00
A (n=15)
B (n=16)
C (n=14)
surface

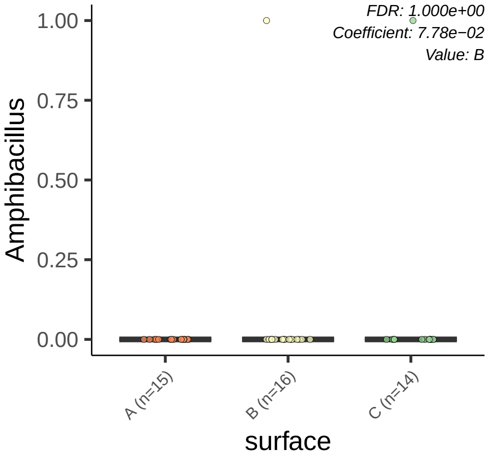

FDR: 1.000e+00
Coefficient: 7.78e−02
Value: B
Amphibacillus
1.00
0.75
0.50
0.25
0.00
A (n=15)
B (n=16)
C (n=14)
surface

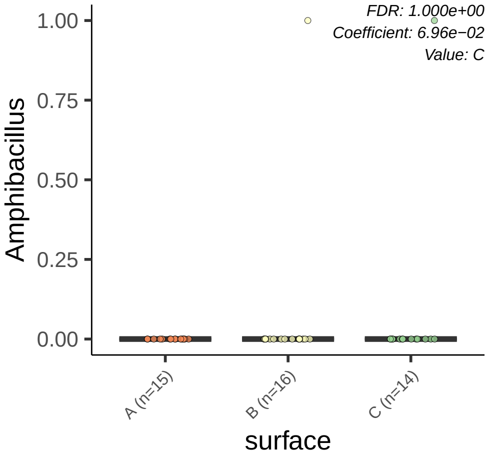
FDR: 1.000e+00
Coefficient: 6.96e−02
Value: C
Amphibacillus
1.00
0.75
0.50
0.25
0.00
A (n=15)
B (n=16)
C (n=14)
surface

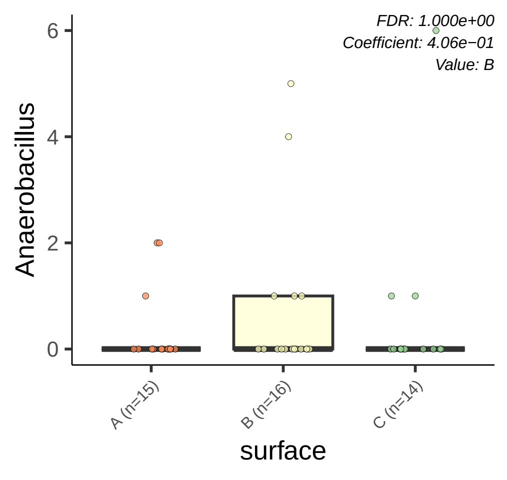

FDR: 1.000e+00
Coefficient: 4.06e−01
Value: B
Anaerobacillus
0
2
4
6
A (n=15)
B (n=16)
C (n=14)
surface

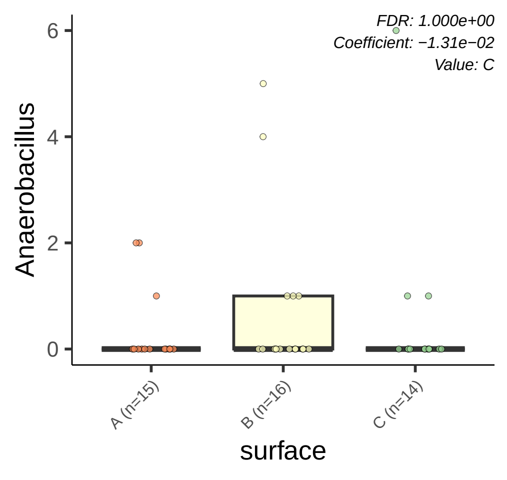
FDR: 1.000e+00
Coefficient: −1.31e−02
Value: C
Anaerobacillus
0
2
4
6
A (n=15)
B (n=16)
C (n=14)
surface

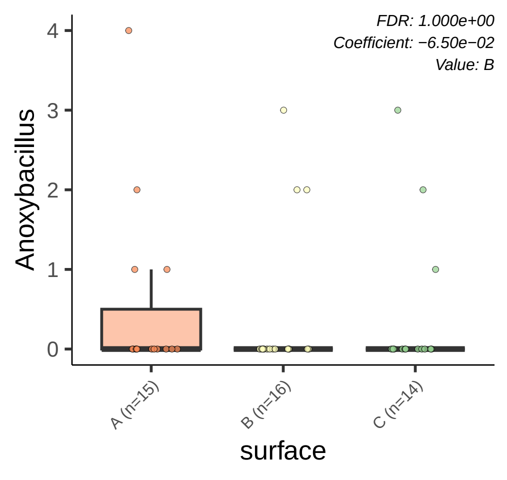

FDR: 1.000e+00
Coefficient: −6.50e−02
Value: B
Anoxybacillus
0
1
2
3
4
A (n=15)
B (n=16)
C (n=14)
surface

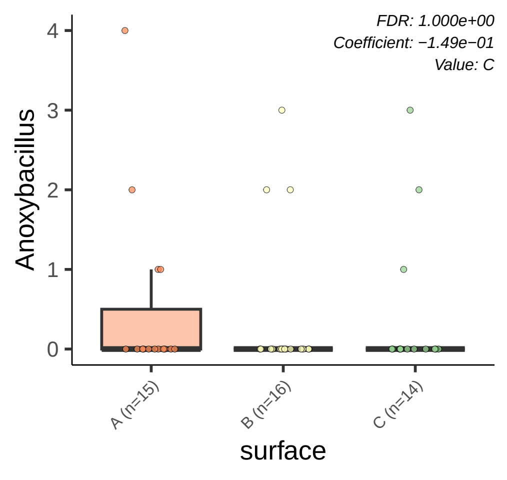
FDR: 1.000e+00
Coefficient: −1.49e−01
Value: C
Anoxybacillus
4
3
2
1
0
A (n=15)
B (n=16)
C (n=14)
surface

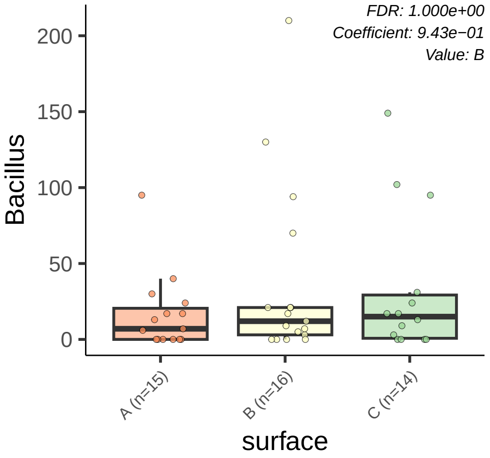
FDR: 1.000e+00
Coefficient: 9.43e−01
Value: B
Bacillus
200
150
100
50
0
A (n=15)
B (n=16)
C (n=14)
surface

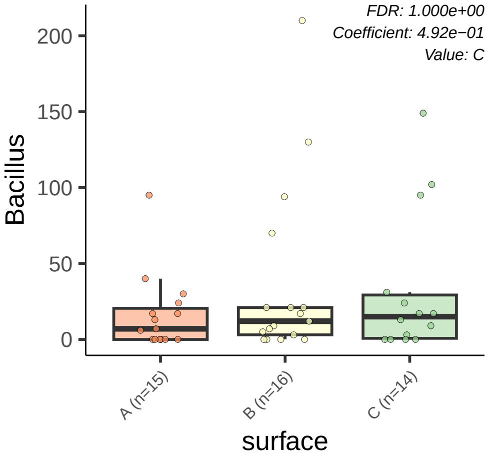
FDR: 1.000e+00
Coefficient: 4.92e−01
Value: C
Bacillus
200
150
100
50
0
A (n=15)
B (n=16)
C (n=14)
surface

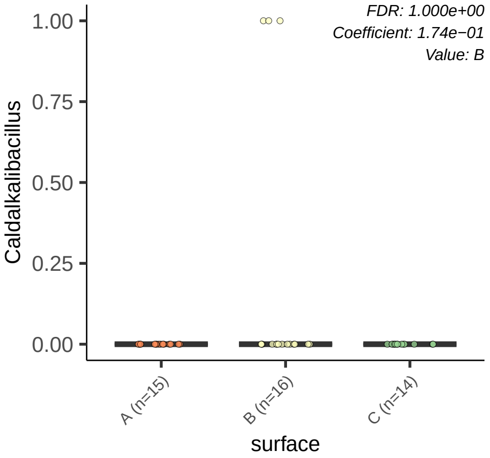
FDR: 1.000e+00
Coefficient: 1.74e−01
Value: B
Caldalkalibacillus
1.00
0.75
0.50
0.25
0.00
A (n=15)
B (n=16)
C (n=14)
surface

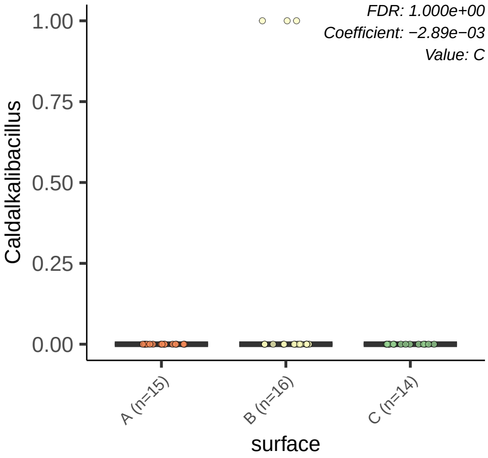

FDR: 1.000e+00
Coefficient: −2.89e−03
Value: C
1.00
0.75
0.50
0.25
0.00
Caldalkalibacillus
A (n=15)
B (n=16)
C (n=14)
surface

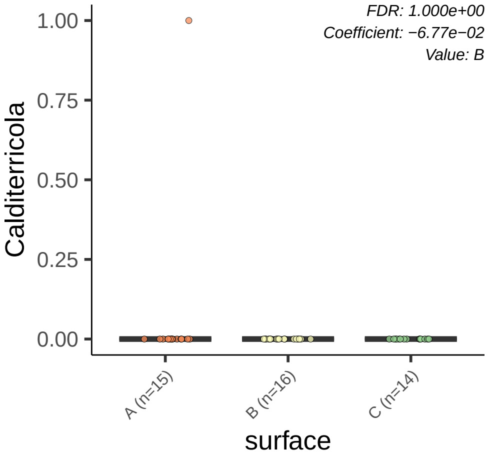
FDR: 1.000e+00
Coefficient: −6.77e−02
Value: B
Calditerricola
1.00
0.75
0.50
0.25
0.00
A (n=15)
B (n=16)
C (n=14)
surface

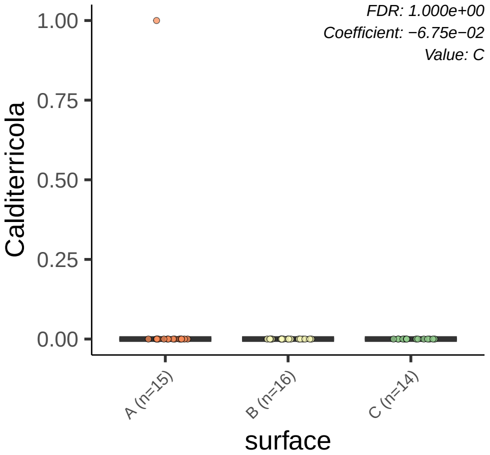

FDR: 1.000e+00
Coefficient: −6.75e−02
Value: C
Calditerricola
1.00
0.75
0.50
0.25
0.00
A (n=15)
B (n=16)
C (n=14)
surface

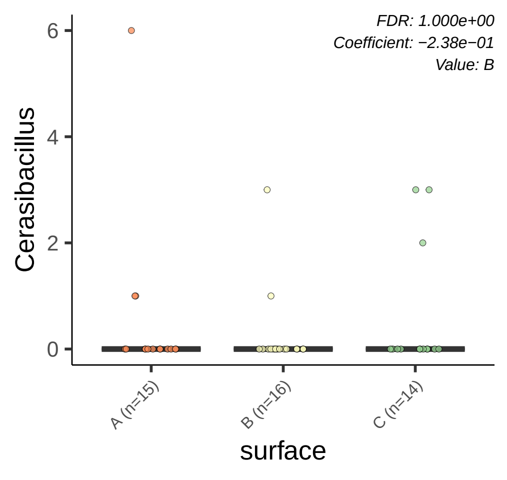

FDR: 1.000e+00
Coefficient: −2.38e−01
Value: B
Cerasibacillus
0
2
4
6
A (n=15)
B (n=16)
C (n=14)
surface

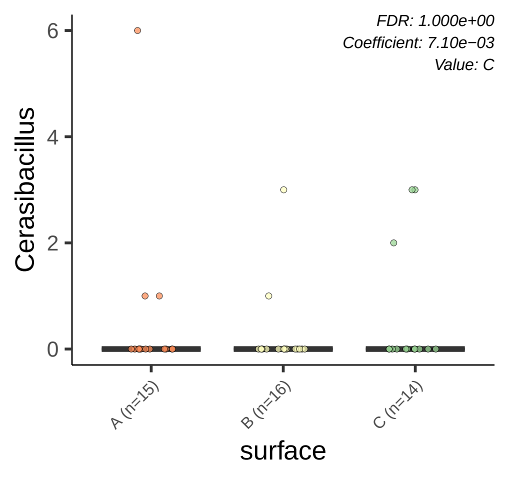

FDR: 1.000e+00
Coefficient: 7.10e−03
Value: C
Cerasibacillus
0
2
4
6
A (n=15)
B (n=16)
C (n=14)
surface

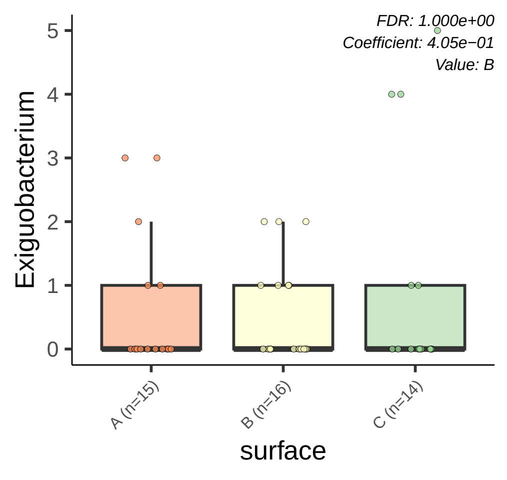

FDR: 1.000e+00
Coefficient: 4.05e−01
Value: B
Exiguobacterium
0
1
2
3
4
5
A (n=15)
B (n=16)
C (n=14)
surface

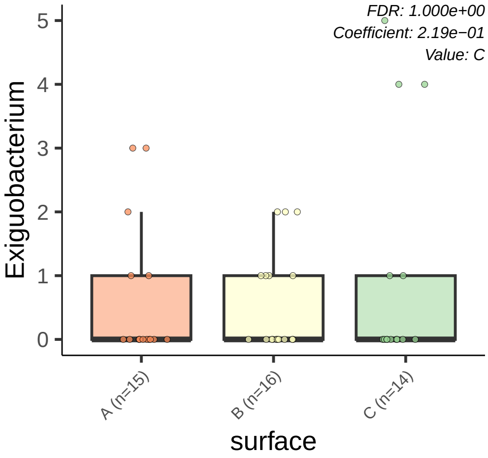
FDR: 1.000e+00
Coefficient: 2.19e−01
Value: C
Exiguobacterium
0
1
2
3
4
5
A (n=15)
B (n=16)
C (n=14)
surface

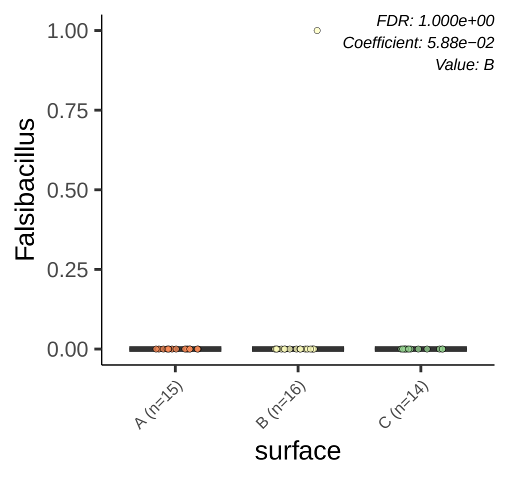
FDR: 1.000e+00
Coefficient: 5.88e−02
Value: B
Falsibacillus
1.00
0.75
0.50
0.25
0.00
A (n=15)
B (n=16)
C (n=14)
surface

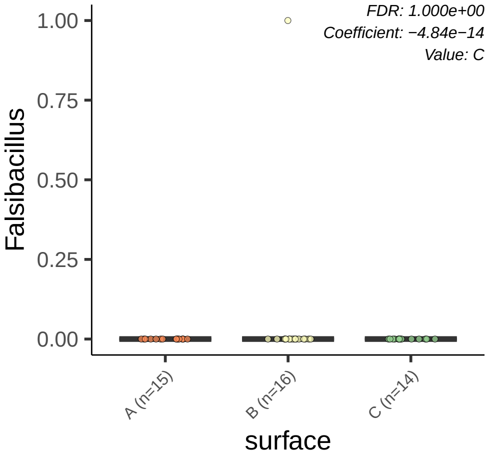
FDR: 1.000e+00
Coefficient: −4.84e−14
Value: C
Falsibacillus
1.00
0.75
0.50
0.25
0.00
A (n=15)
B (n=16)
C (n=14)
surface

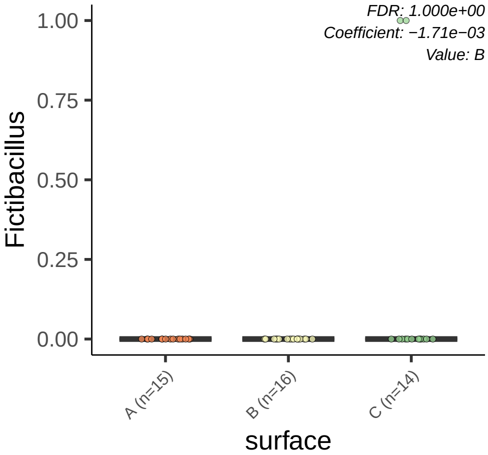
FDR: 1.000e+00
Coefficient: −1.71e−03
Value: B
Fictibacillus
1.00
0.75
0.50
0.25
0.00
A (n=15)
B (n=16)
C (n=14)
surface

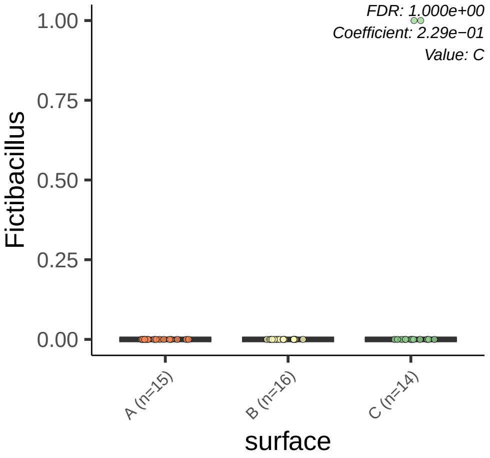
FDR: 1.000e+00
Coefficient: 2.29e−01
Value: C
1.00
0.75
0.50
0.25
0.00
Fictibacillus
A (n=15)
B (n=16)
C (n=14)
surface

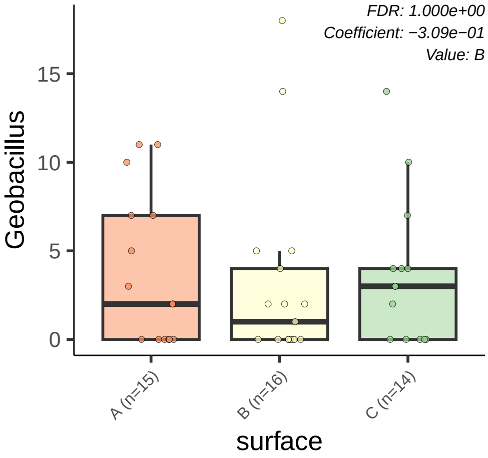

FDR: 1.000e+00
Coefficient: −3.09e−01
Value: B
Geobacillus
15
10
5
0
A (n=15)
B (n=16)
C (n=14)
surface

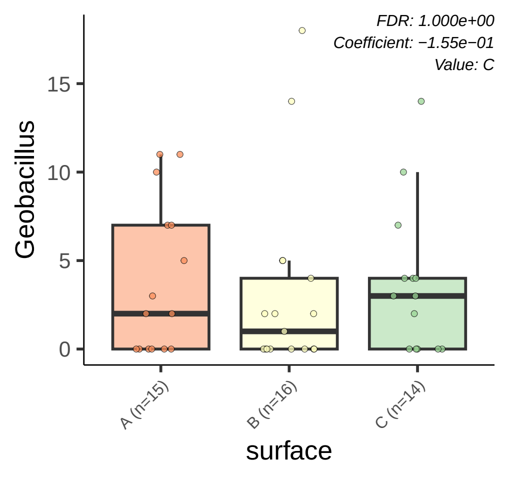
FDR: 1.000e+00
Coefficient: −1.55e−01
Value: C
Geobacillus
15
10
5
0
A (n=15)
B (n=16)
C (n=14)
surface

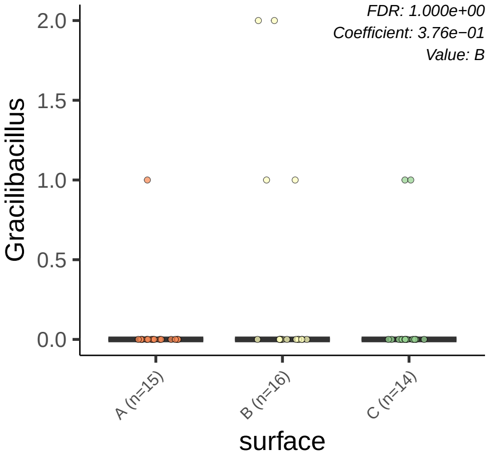

FDR: 1.000e+00
Coefficient: 3.76e−01
Value: B
Gracilibacillus
2.0
1.5
1.0
0.5
0.0
A (n=15)
B (n=16)
C (n=14)
surface

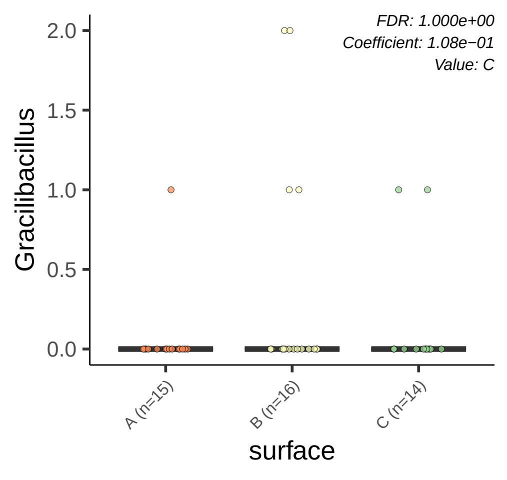
FDR: 1.000e+00
Coefficient: 1.08e−01
Value: C
Gracilibacillus
2.0
1.5
1.0
0.5
0.0
A (n=15)
B (n=16)
C (n=14)
surface

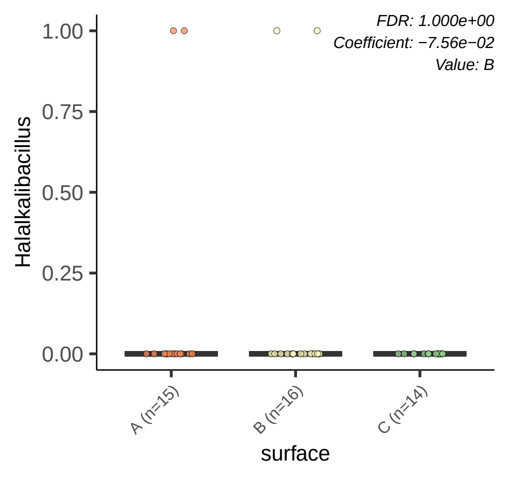
FDR: 1.000e+00
Coefficient: −7.56e−02
Value: B
Halalkalibacillus
1.00
0.75
0.50
0.25
0.00
A (n=15)
B (n=16)
C (n=14)
surface

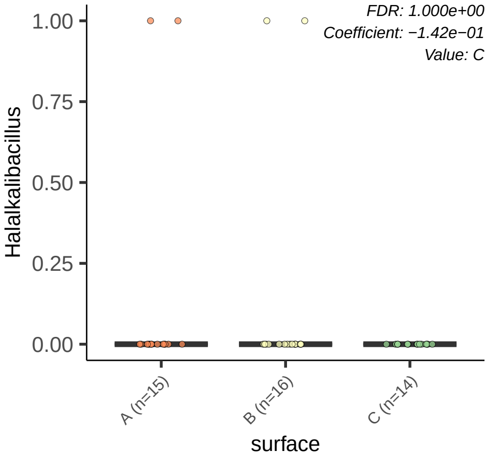

FDR: 1.000e+00
Coefficient: −1.42e−01
Value: C
Halalkalibacillus
1.00
0.75
0.50
0.25
0.00
A (n=15)
B (n=16)
C (n=14)
surface

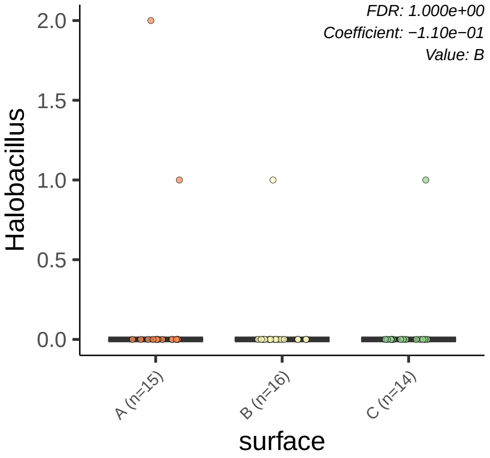
FDR: 1.000e+00
Coefficient: −1.10e−01
Value: B
Halobacillus
2.0
1.5
1.0
0.5
0.0
A (n=15)
B (n=16)
C (n=14)
surface

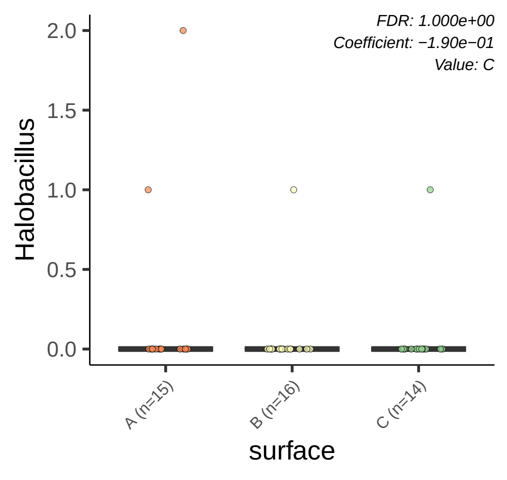
FDR: 1.000e+00
Coefficient: −1.90e−01
Value: C
Halobacillus
2.0
1.5
1.0
0.5
0.0
A (n=15)
B (n=16)
C (n=14)
surface

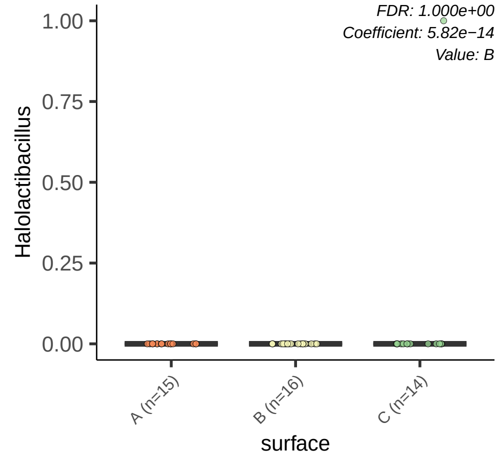

FDR: 1.000e+00
Coefficient: 5.82e−14
Value: B
Halolactibacillus
1.00
0.75
0.50
0.25
0.00
A (n=15)
B (n=16)
C (n=14)
surface

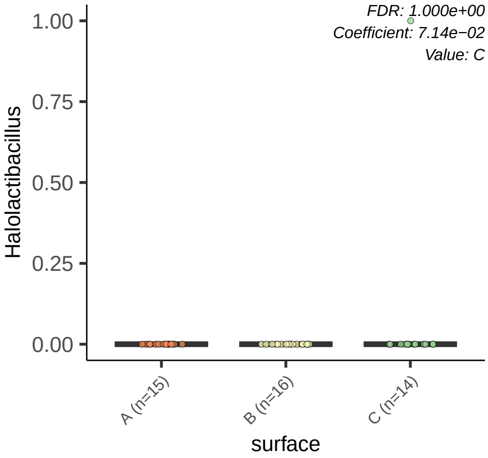

FDR: 1.000e+00
Coefficient: 7.14e−02
Value: C
1.00
0.75
0.50
0.25
0.00
Halolactibacillus
A (n=15)
B (n=16)
C (n=14)
surface

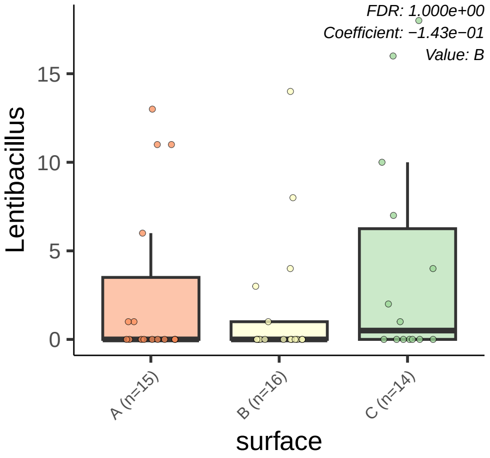

FDR: 1.000e+00
Coefficient: −1.43e−01
Value: B
Lentibacillus
15
10
5
0
A (n=15)
B (n=16)
C (n=14)
surface

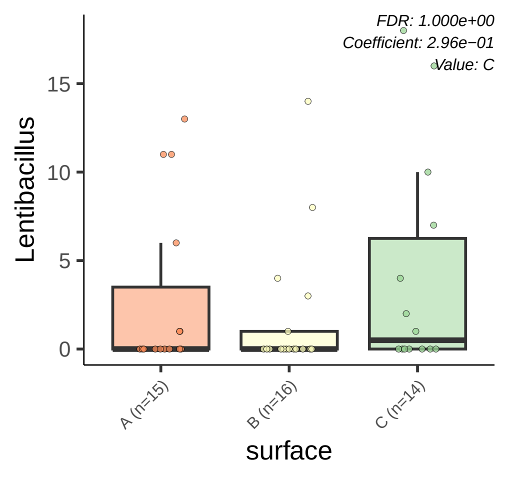
FDR: 1.000e+00
Coefficient: 2.96e−01
Value: C
Lentibacillus
0
5
10
15
A (n=15)
B (n=16)
C (n=14)
surface

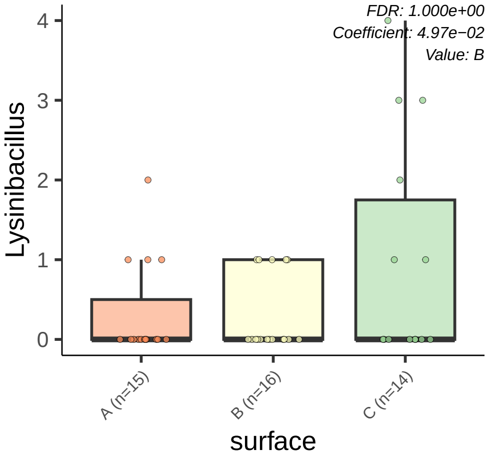

FDR: 1.000e+00
Coefficient: 4.97e−02
Value: B
Lysinibacillus
4
3
2
1
0
A (n=15)
B (n=16)
C (n=14)
surface

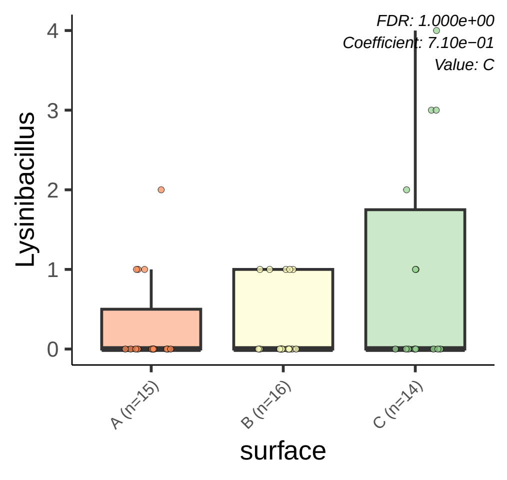

FDR: 1.000e+00
Coefficient: 7.10e−01
Value: C
Lysinibacillus
0
1
2
3
4
A (n=15)
B (n=16)
C (n=14)
surface

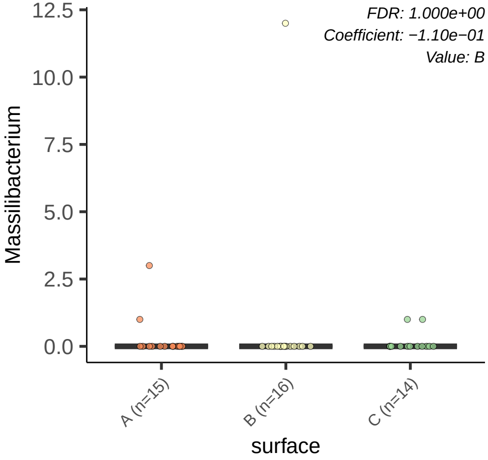

FDR: 1.000e+00
Coefficient: −1.10e−01
Value: B
Massilibacterium
12.5
10.0
7.5
5.0
2.5
0.0
A (n=15)
B (n=16)
C (n=14)
surface

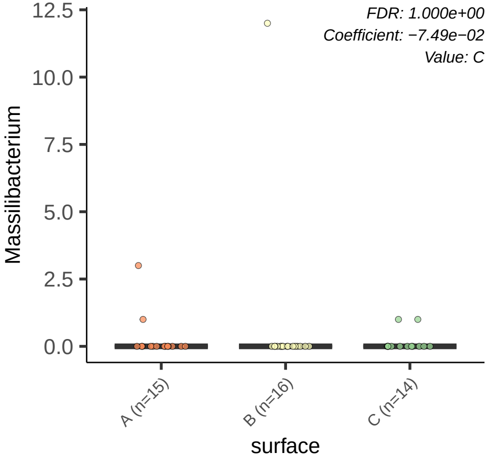

FDR: 1.000e+00
Coefficient: −7.49e−02
Value: C
Massilibacterium
12.5
10.0
7.5
5.0
2.5
0.0
A (n=15)
B (n=16)
C (n=14)
surface

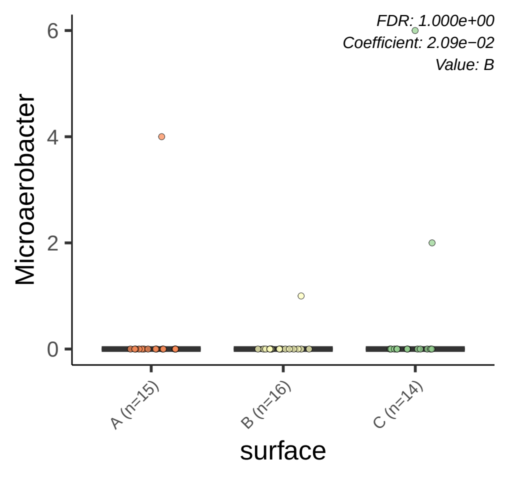
FDR: 1.000e+00
Coefficient: 2.09e−02
Value: B
Microaerobacter
6
4
2
0
A (n=15)
B (n=16)
C (n=14)
surface

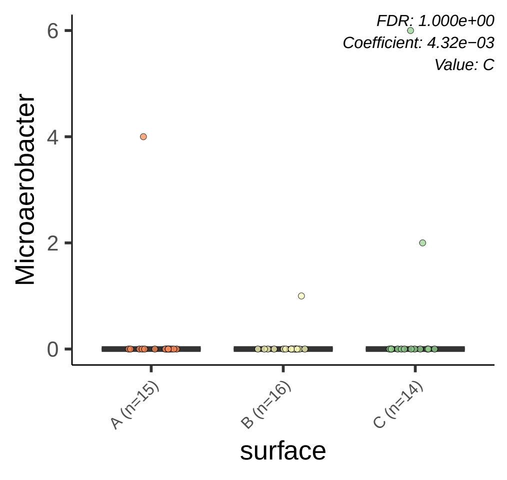

FDR: 1.000e+00
Coefficient: 4.32e−03
Value: C
Microaerobacter
0
2
4
6
A (n=15)
B (n=16)
C (n=14)
surface

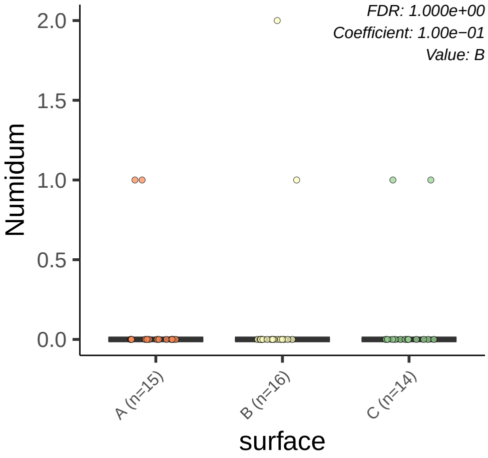
FDR: 1.000e+00
Coefficient: 1.00e−01
Value: B
Numidum
2.0
1.5
1.0
0.5
0.0
A (n=15)
B (n=16)
C (n=14)
surface

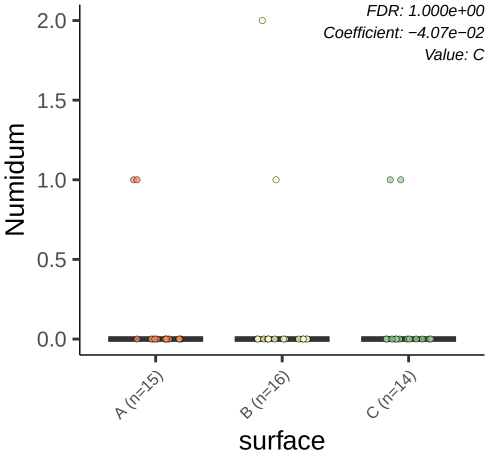

FDR: 1.000e+00
Coefficient: −4.07e−02
Value: C
2.0
1.5
1.0
0.5
0.0
Numidum
A (n=15)
B (n=16)
C (n=14)
surface

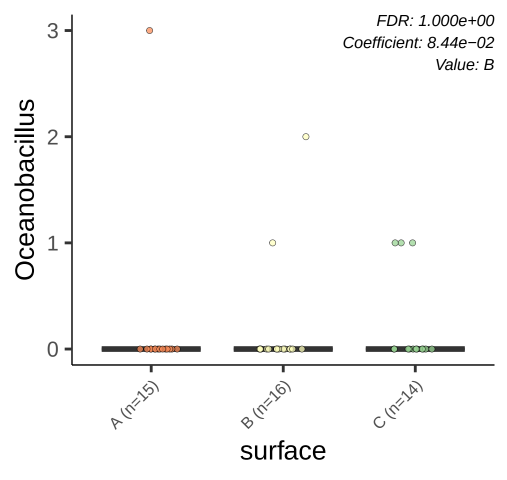
FDR: 1.000e+00
Coefficient: 8.44e−02
Value: B
Oceanobacillus
3
2
1
0
A (n=15)
B (n=16)
C (n=14)
surface

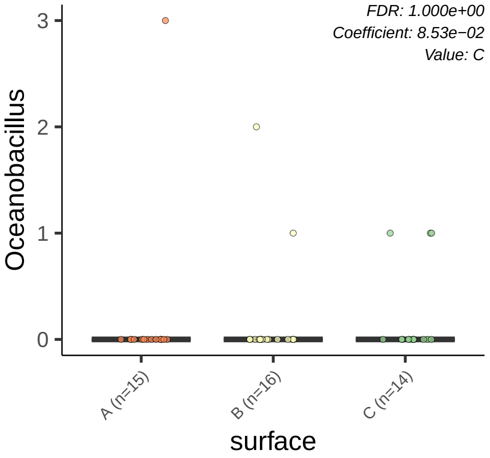

FDR: 1.000e+00
Coefficient: 8.53e−02
Value: C
Oceanobacillus
3
2
1
0
A (n=15)
B (n=16)
C (n=14)
surface

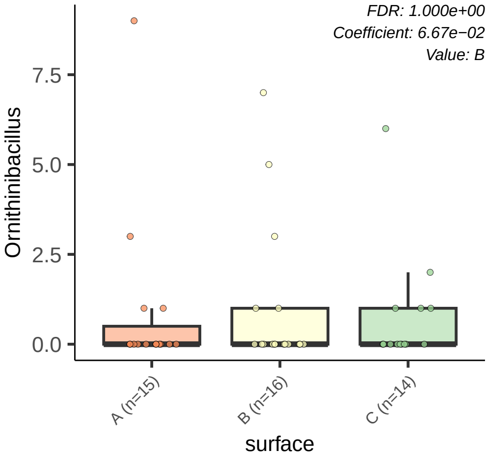
FDR: 1.000e+00
Coefficient: 6.67e−02
Value: B
Ornithinibacillus
7.5
5.0
2.5
0.0
A (n=15)
B (n=16)
C (n=14)
surface

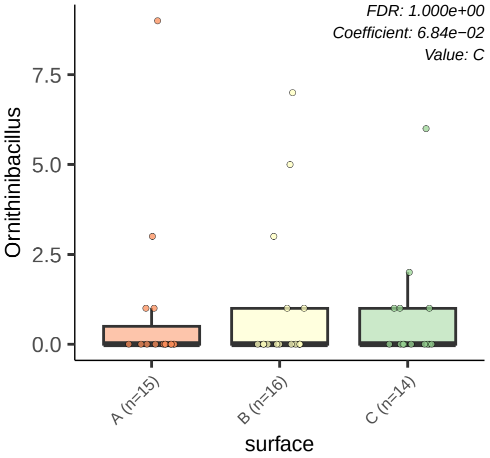

FDR: 1.000e+00
Coefficient: 6.84e−02
Value: C
Ornithinibacillus
7.5
5.0
2.5
0.0
A (n=15)
B (n=16)
C (n=14)
surface

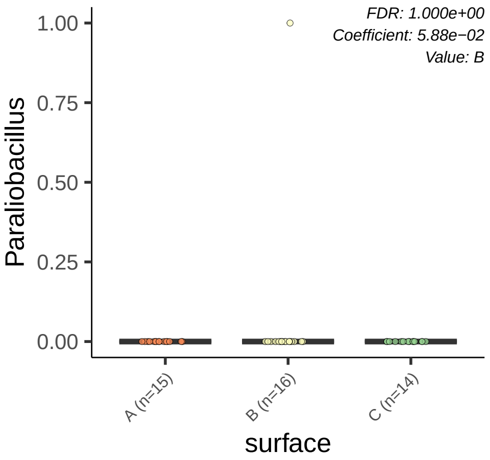
FDR: 1.000e+00
Coefficient: 5.88e−02
Value: B
Paraliobacillus
1.00
0.75
0.50
0.25
0.00
A (n=15)
B (n=16)
C (n=14)
surface

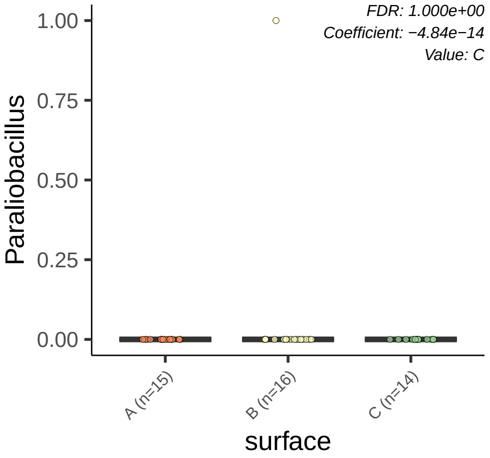

FDR: 1.000e+00
Coefficient: −4.84e−14
Value: C
1.00
0.75
0.50
0.25
0.00
Paraliobacillus
A (n=15)
B (n=16)
C (n=14)
surface

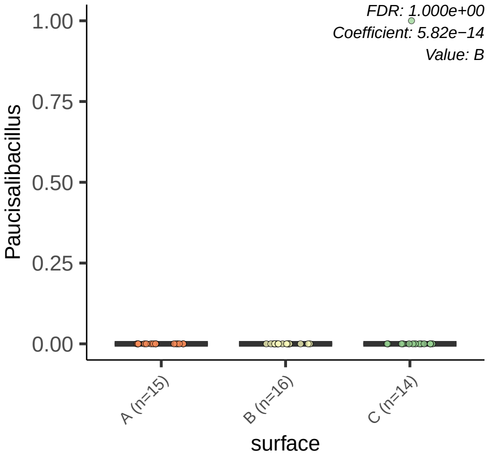

FDR: 1.000e+00
Coefficient: 5.82e−14
Value: B
Paucisalibacillus
1.00
0.75
0.50
0.25
0.00
A (n=15)
B (n=16)
C (n=14)
surface

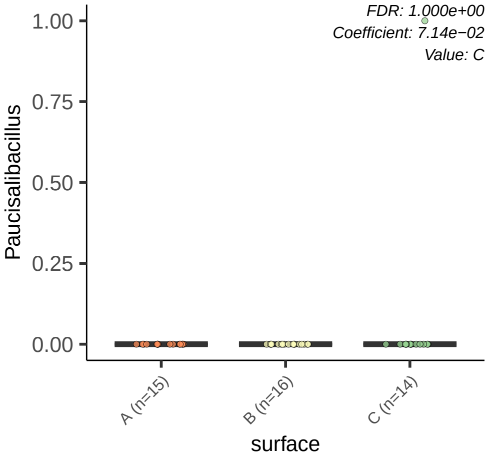
FDR: 1.000e+00
Coefficient: 7.14e−02
Value: C
Paucisalibacillus
1.00
0.75
0.50
0.25
0.00
A (n=15)
B (n=16)
C (n=14)
surface

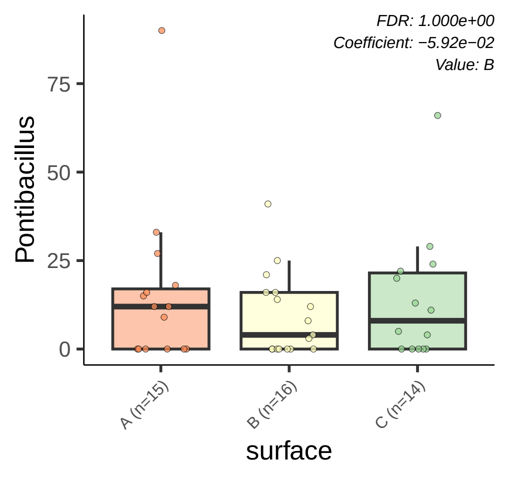
FDR: 1.000e+00
Coefficient: −5.92e−02
Value: B
Pontibacillus
75
50
25
0
A (n=15)
B (n=16)
C (n=14)
surface

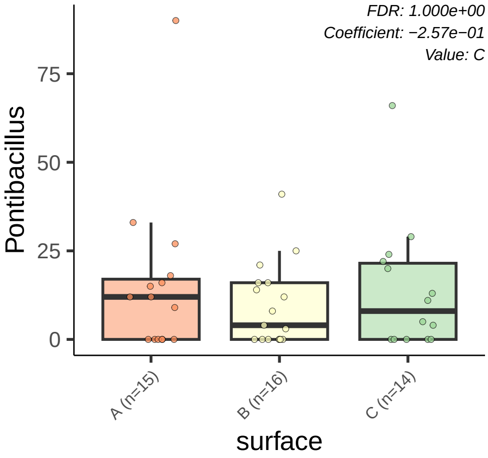
FDR: 1.000e+00
Coefficient: −2.57e−01
Value: C
Pontibacillus
75
50
25
0
A (n=15)
B (n=16)
C (n=14)
surface

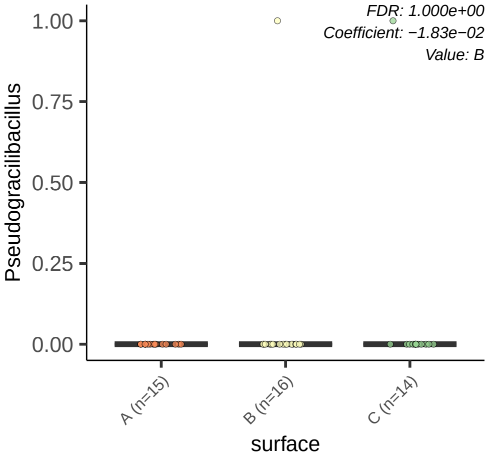
FDR: 1.000e+00
Coefficient: −1.83e−02
Value: B
Pseudogracilibacillus
1.00
0.75
0.50
0.25
0.00
A (n=15)
B (n=16)
C (n=14)
surface

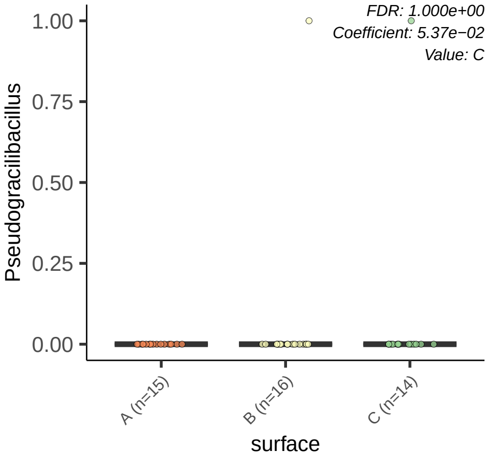

FDR: 1.000e+00
Coefficient: 5.37e−02
Value: C
Pseudogracilibacillus
1.00
0.75
0.50
0.25
0.00
A (n=15)
B (n=16)
C (n=14)
surface

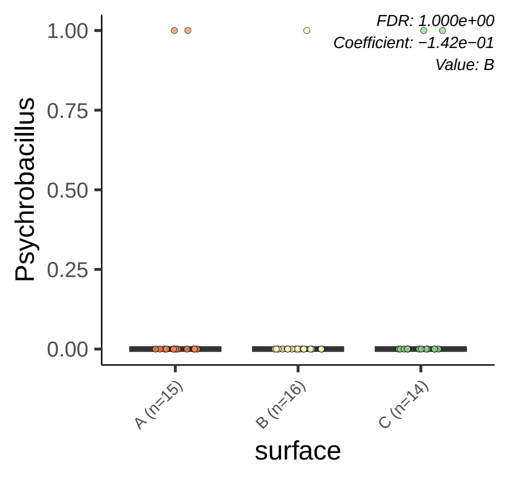
FDR: 1.000e+00
Coefficient: −1.42e−01
Value: B
Psychrobacillus
1.00
0.75
0.50
0.25
0.00
A (n=15)
B (n=16)
C (n=14)
surface

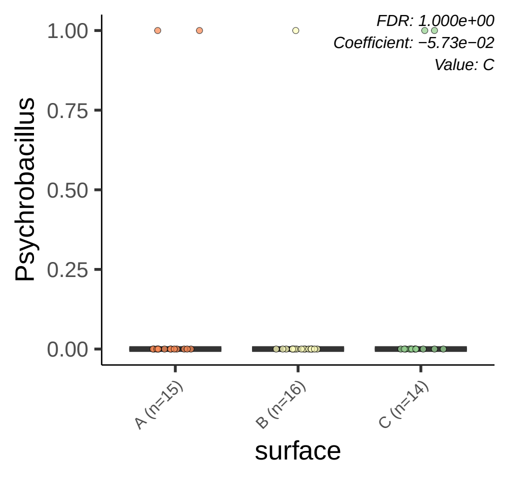
FDR: 1.000e+00
Coefficient: −5.73e−02
Value: C
Psychrobacillus
1.00
0.75
0.50
0.25
0.00
A (n=15)
B (n=16)
C (n=14)
surface

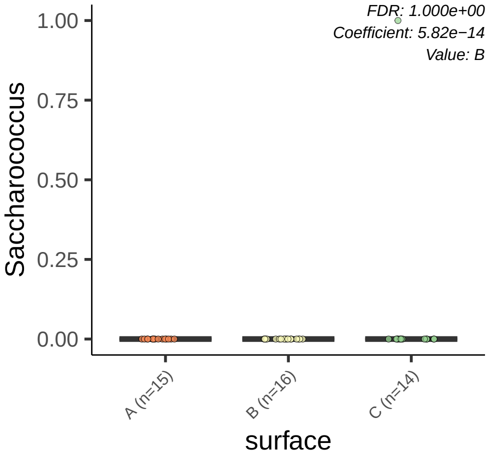
FDR: 1.000e+00
Coefficient: 5.82e−14
Value: B
Saccharococcus
1.00
0.75
0.50
0.25
0.00
A (n=15)
B (n=16)
C (n=14)
surface

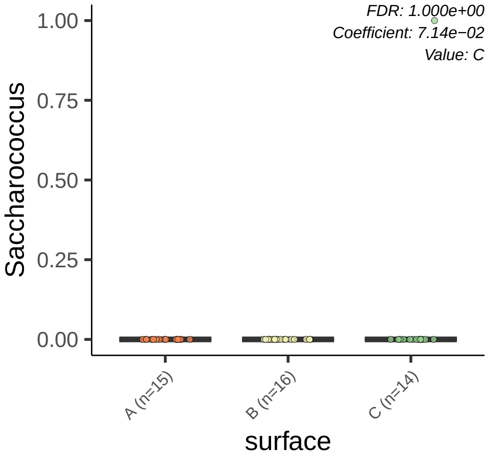
FDR: 1.000e+00
Coefficient: 7.14e−02
Value: C
Saccharococcus
1.00
0.75
0.50
0.25
0.00
A (n=15)
B (n=16)
C (n=14)
surface

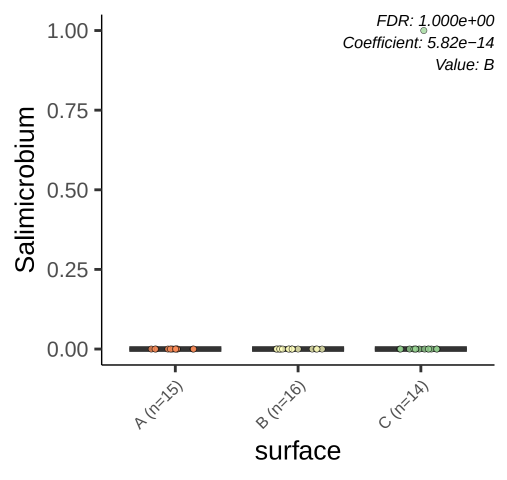
FDR: 1.000e+00
Coefficient: 5.82e−14
Value: B
Salimicrobium
1.00
0.75
0.50
0.25
0.00
A (n=15)
B (n=16)
C (n=14)
surface

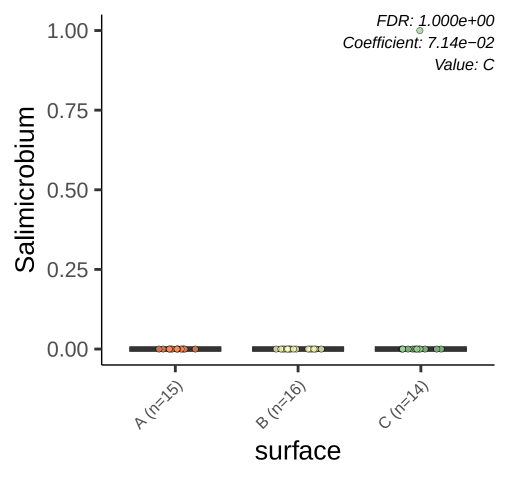

FDR: 1.000e+00
Coefficient: 7.14e−02
Value: C
1.00
0.75
0.50
0.25
0.00
Salimicrobium
A (n=15)
B (n=16)
C (n=14)
surface

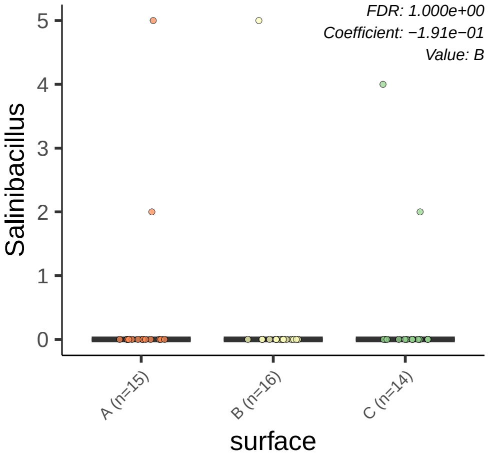
FDR: 1.000e+00
Coefficient: −1.91e−01
Value: B
Salinibacillus
5
4
3
2
1
0
A (n=15)
B (n=16)
C (n=14)
surface

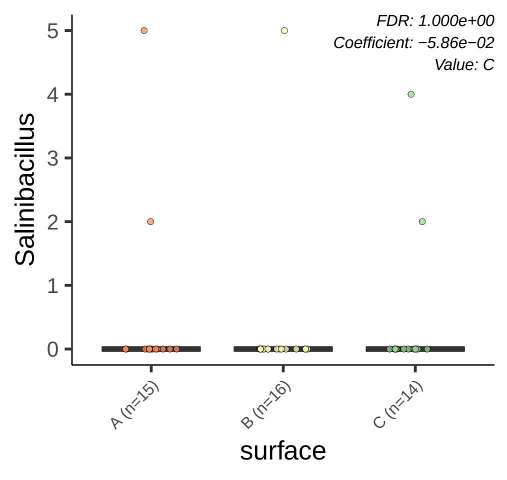
FDR: 1.000e+00
Coefficient: −5.86e−02
Value: C
Salinibacillus
5
4
3
2
1
0
A (n=15)
B (n=16)
C (n=14)
surface

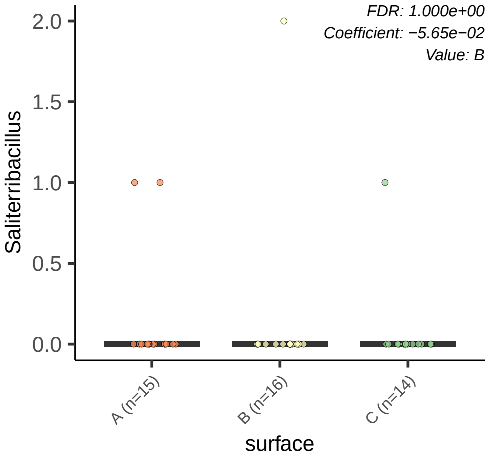
FDR: 1.000e+00
Coefficient: −5.65e−02
Value: B
Saliterribacillus
2.0
1.5
1.0
0.5
0.0
A (n=15)
B (n=16)
C (n=14)
surface

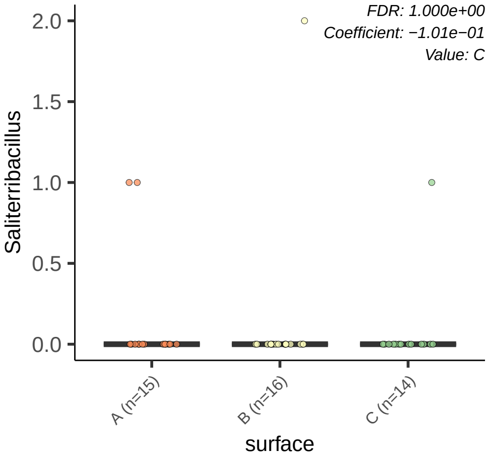

FDR: 1.000e+00
Coefficient: −1.01e−01
Value: C
Saliterribacillus
2.0
1.5
1.0
0.5
0.0
A (n=15)
B (n=16)
C (n=14)
surface

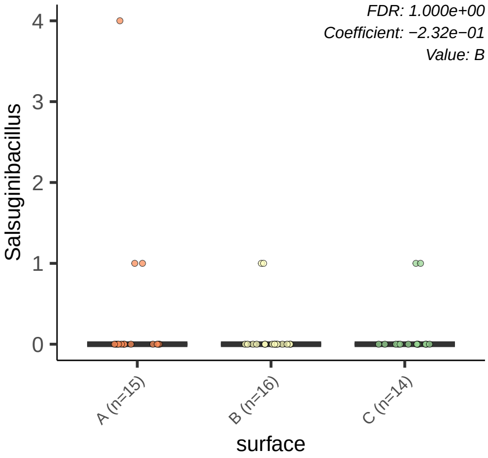

FDR: 1.000e+00
Coefficient: −2.32e−01
Value: B
Salsuginibacillus
0
1
2
3
4
A (n=15)
B (n=16)
C (n=14)
surface

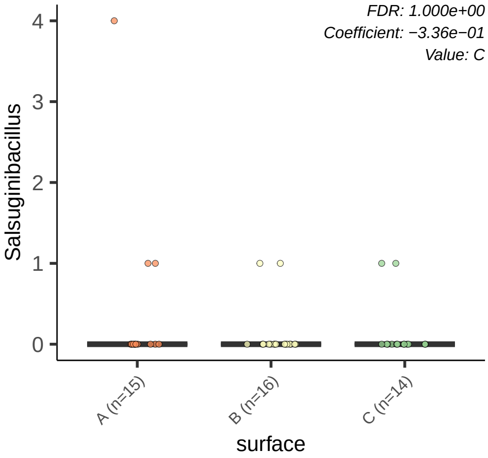
FDR: 1.000e+00
Coefficient: −3.36e−01
Value: C
Salsuginibacillus
4
3
2
1
0
A (n=15)
B (n=16)
C (n=14)
surface

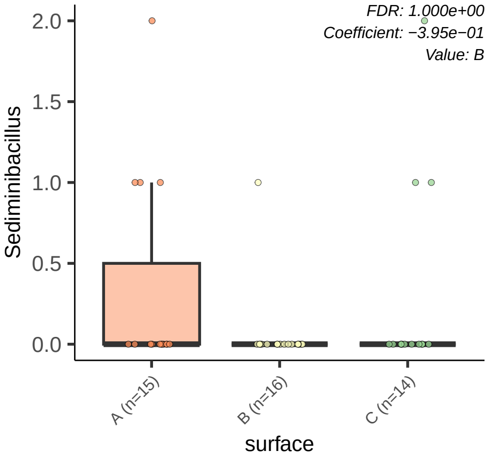
FDR: 1.000e+00
Coefficient: −3.95e−01
Value: B
Sediminibacillus
2.0
1.5
1.0
0.5
0.0
A (n=15)
B (n=16)
C (n=14)
surface

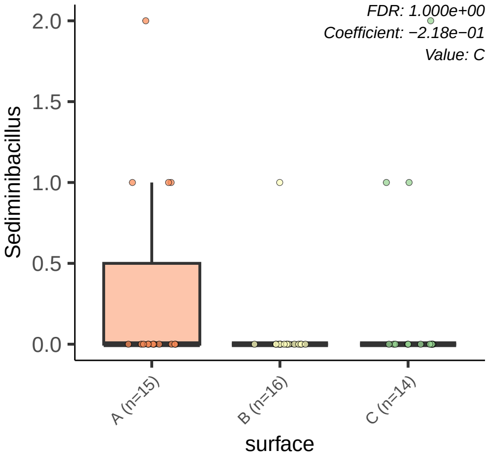

FDR: 1.000e+00
Coefficient: −2.18e−01
Value: C
Sediminibacillus
2.0
1.5
1.0
0.5
0.0
A (n=15)
B (n=16)
C (n=14)
surface

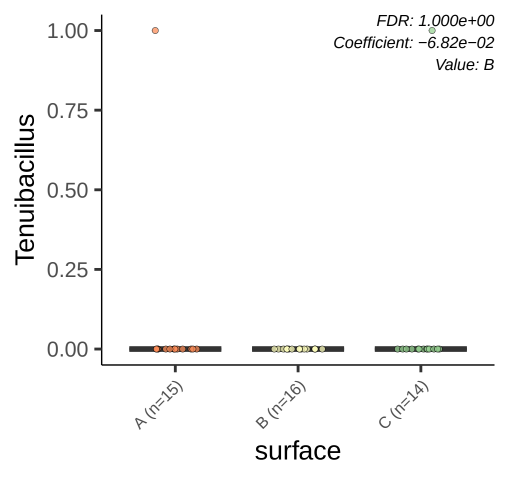
FDR: 1.000e+00
Coefficient: −6.82e−02
Value: B
Tenuibacillus
1.00
0.75
0.50
0.25
0.00
A (n=15)
B (n=16)
C (n=14)
surface

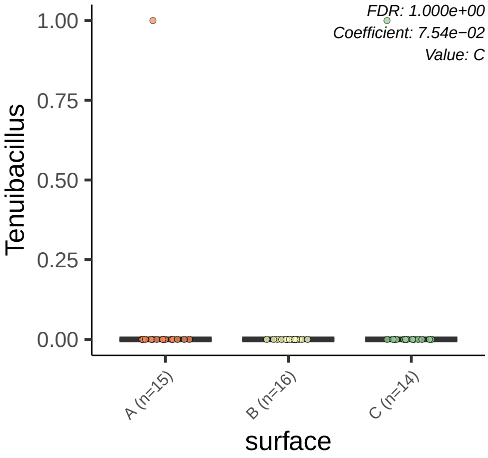
FDR: 1.000e+00
Coefficient: 7.54e−02
Value: C
Tenuibacillus
1.00
0.75
0.50
0.25
0.00
A (n=15)
B (n=16)
C (n=14)
surface

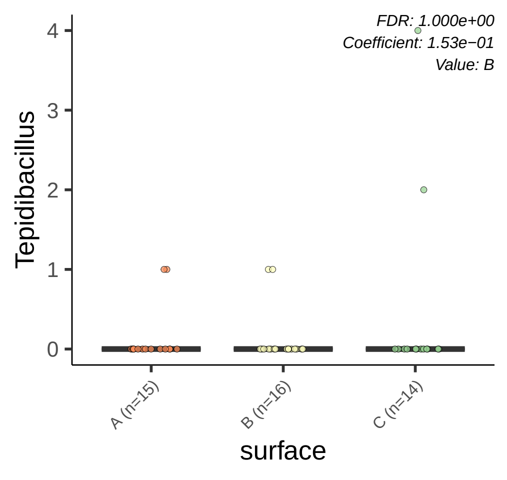
FDR: 1.000e+00
Coefficient: 1.53e−01
Value: B
Tepidibacillus
0
1
2
3
4
A (n=15)
B (n=16)
C (n=14)
surface

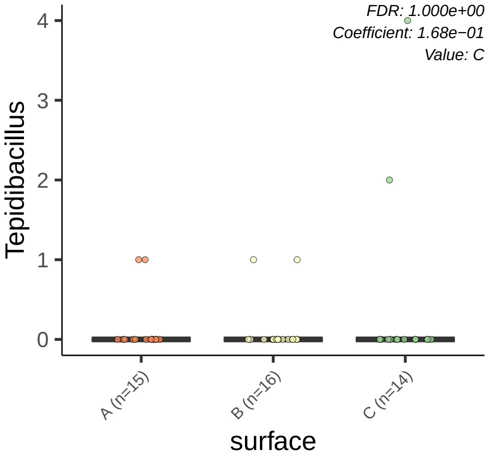
FDR: 1.000e+00
Coefficient: 1.68e−01
Value: C
Tepidibacillus
0
1
2
3
4
A (n=15)
B (n=16)
C (n=14)
surface

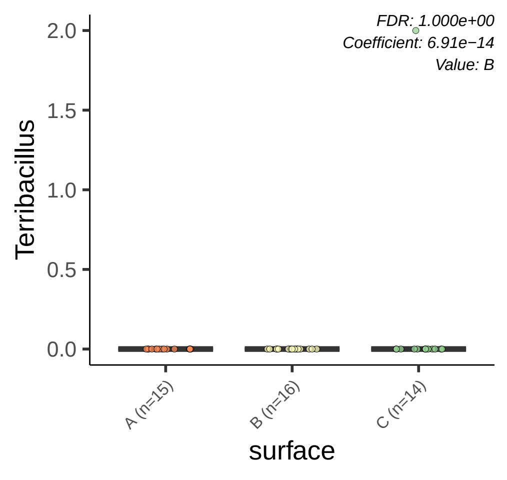
FDR: 1.000e+00
Coefficient: 6.91e−14
Value: B
Terribacillus
2.0
1.5
1.0
0.5
0.0
A (n=15)
B (n=16)
C (n=14)
surface

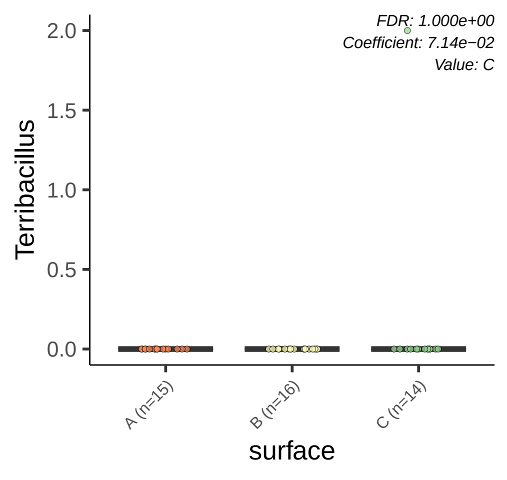
FDR: 1.000e+00
Coefficient: 7.14e−02
Value: C
Terribacillus
2.0
1.5
1.0
0.5
0.0
A (n=15)
B (n=16)
C (n=14)
surface

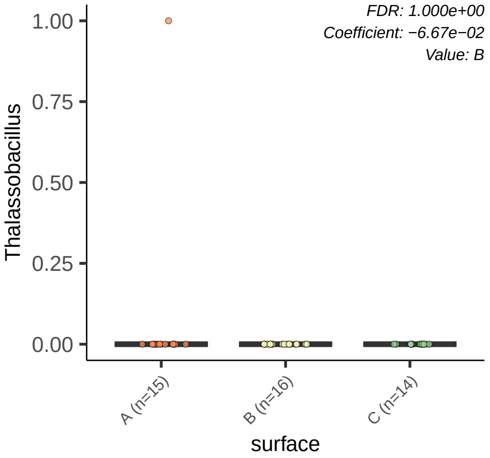
FDR: 1.000e+00
Coefficient: −6.67e−02
Value: B
Thalassobacillus
1.00
0.75
0.50
0.25
0.00
A (n=15)
B (n=16)
C (n=14)
surface

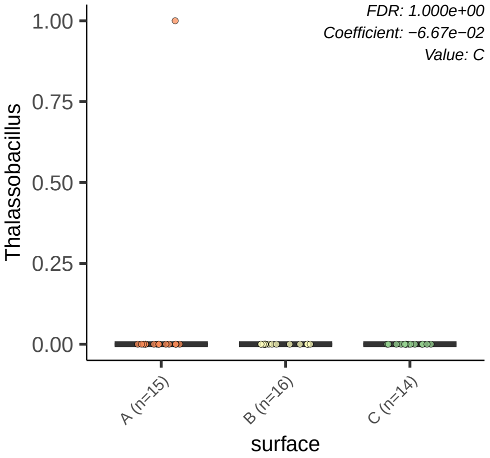
FDR: 1.000e+00
Coefficient: −6.67e−02
Value: C
Thalassobacillus
1.00
0.75
0.50
0.25
0.00
A (n=15)
B (n=16)
C (n=14)
surface

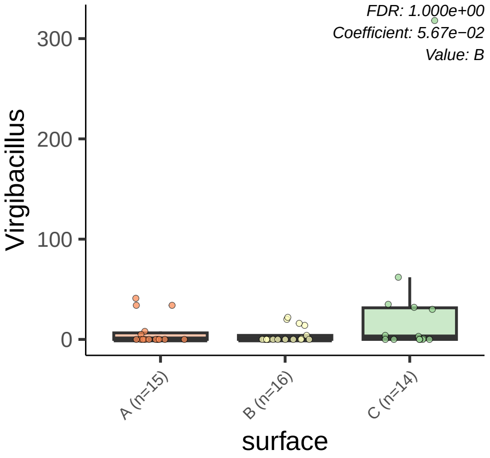
FDR: 1.000e+00
Coefficient: 5.67e−02
Value: B
Virgibacillus
300
200
100
0
A (n=15)
B (n=16)
C (n=14)
surface

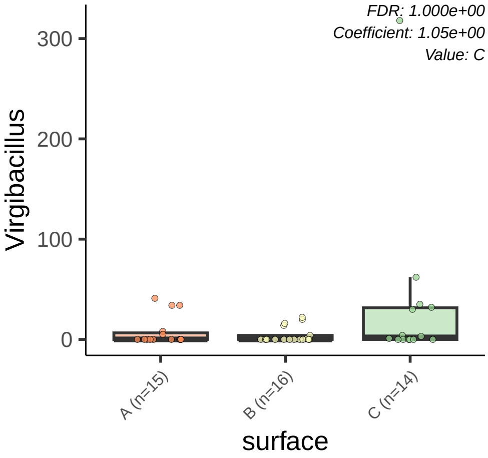

FDR: 1.000e+00
Coefficient: 1.05e+00
Value: C
Virgibacillus
300
200
100
0
A (n=15)
B (n=16)
C (n=14)
surface

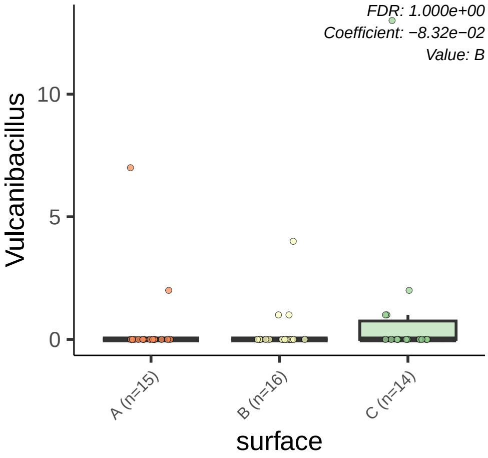
FDR: 1.000e+00
Coefficient: −8.32e−02
Value: B
Vulcanibacillus
10
5
0
A (n=15)
B (n=16)
C (n=14)
surface

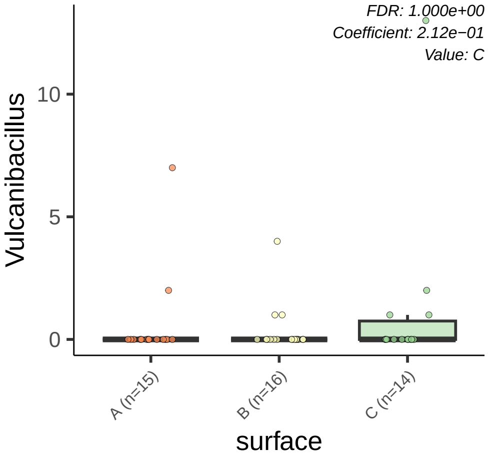
FDR: 1.000e+00
Coefficient: 2.12e−01
Value: C
Vulcanibacillus
10
5
0
A (n=15)
B (n=16)
C (n=14)
surface

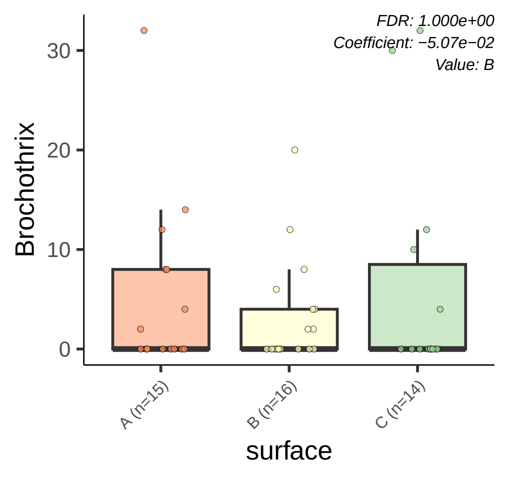
FDR: 1.000e+00
Coefficient: −5.07e−02
Value: B
Brochothrix
30
20
10
0
A (n=15)
B (n=16)
C (n=14)
surface

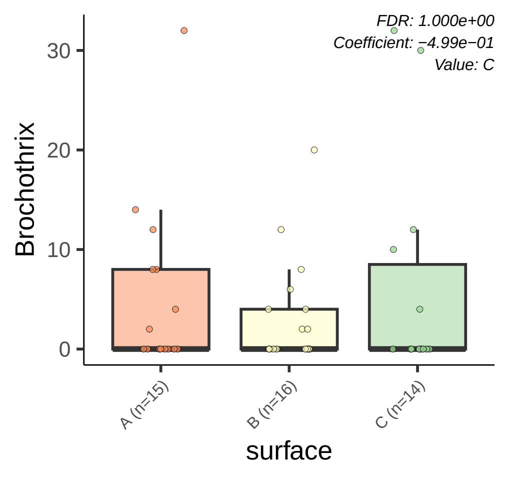

FDR: 1.000e+00
Coefficient: −4.99e−01
Value: C
Brochothrix
30
20
10
0
A (n=15)
B (n=16)
C (n=14)
surface

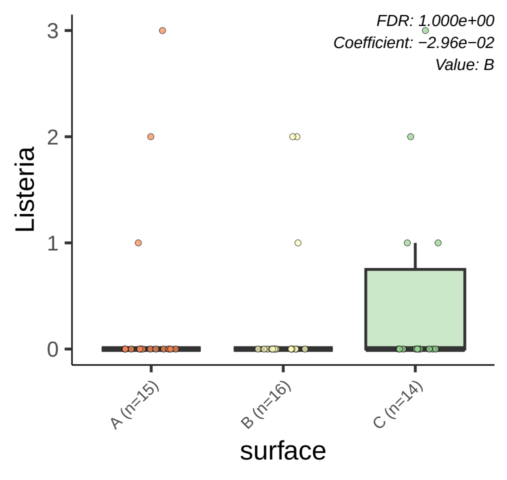
FDR: 1.000e+00
Coefficient: −2.96e−02
Value: B
Listeria
0
1
2
3
A (n=15)
B (n=16)
C (n=14)
surface

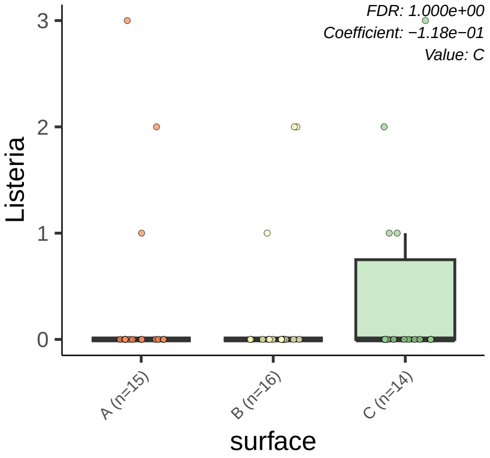
FDR: 1.000e+00
Coefficient: −1.18e−01
Value: C
Listeria
3
2
1
0
A (n=15)
B (n=16)
C (n=14)
surface

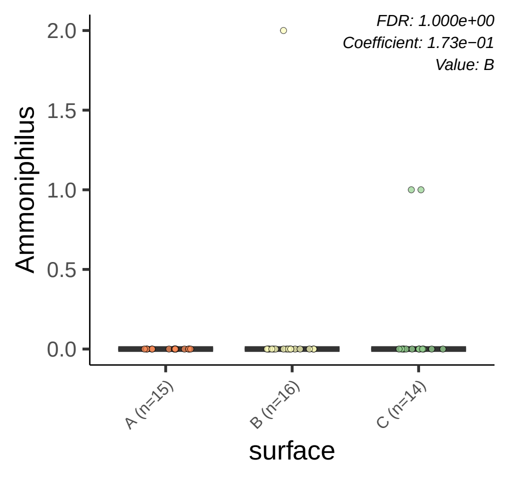
FDR: 1.000e+00
Coefficient: 1.73e−01
Value: B
Ammoniphilus
2.0
1.5
1.0
0.5
0.0
A (n=15)
B (n=16)
C (n=14)
surface

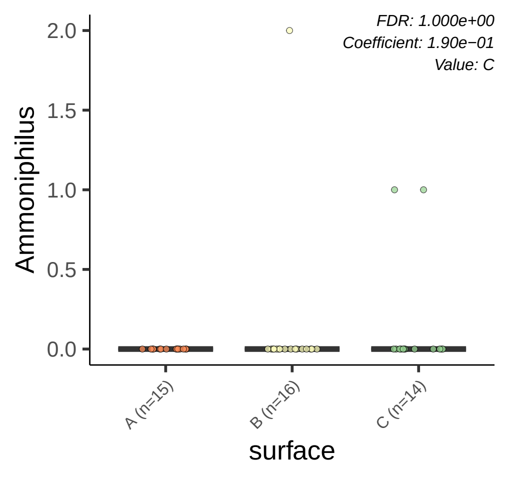
FDR: 1.000e+00
Coefficient: 1.90e−01
Value: C
Ammoniphilus
2.0
1.5
1.0
0.5
0.0
A (n=15)
B (n=16)
C (n=14)
surface

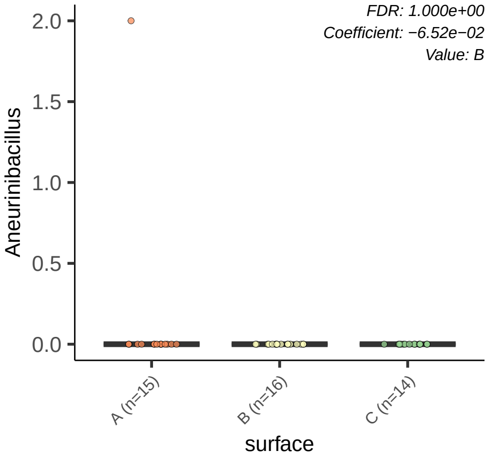

FDR: 1.000e+00
Coefficient: −6.52e−02
Value: B
Aneurinibacillus
2.0
1.5
1.0
0.5
0.0
A (n=15)
B (n=16)
C (n=14)
surface

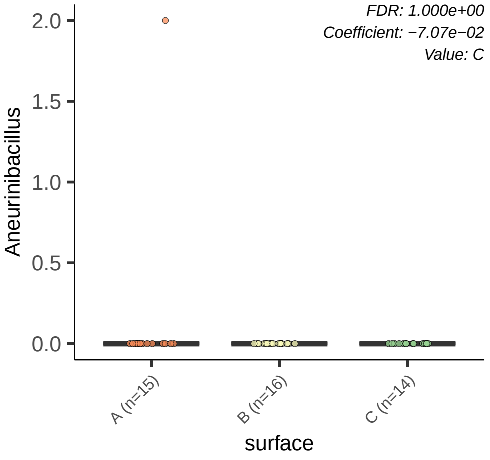
FDR: 1.000e+00
Coefficient: −7.07e−02
Value: C
Aneurinibacillus
2.0
1.5
1.0
0.5
0.0
A (n=15)
B (n=16)
C (n=14)
surface

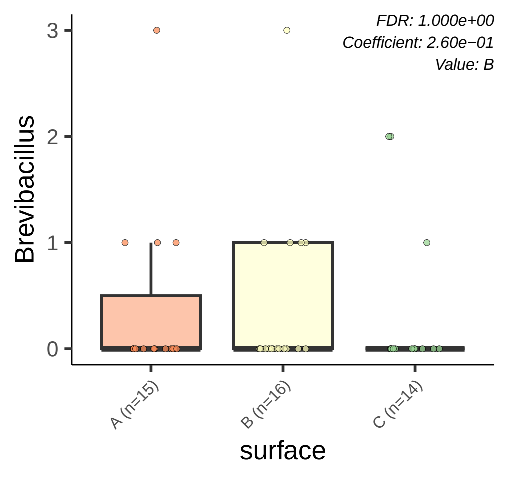
FDR: 1.000e+00
Coefficient: 2.60e−01
Value: B
Brevibacillus
0
1
2
3
A (n=15)
B (n=16)
C (n=14)
surface

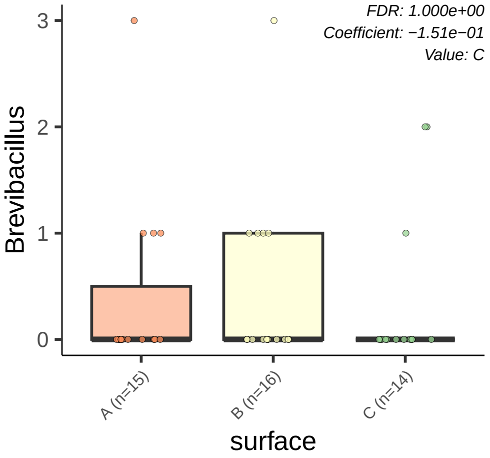
FDR: 1.000e+00
Coefficient: −1.51e−01
Value: C
Brevibacillus
3
2
1
0
A (n=15)
B (n=16)
C (n=14)
surface

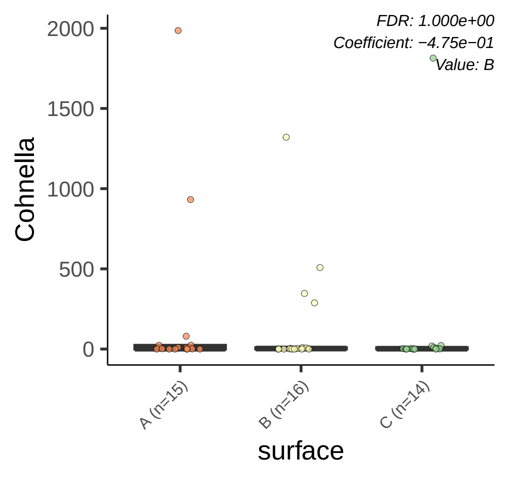
FDR: 1.000e+00
Coefficient: −4.75e−01
Value: B
Cohnella
2000
1500
1000
500
0
A (n=15)
B (n=16)
C (n=14)
surface

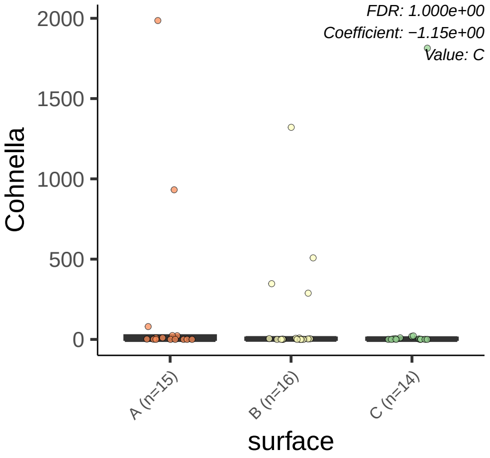
FDR: 1.000e+00
Coefficient: −1.15e+00
Value: C
Cohnella
2000
1500
1000
500
0
A (n=15)
B (n=16)
C (n=14)
surface

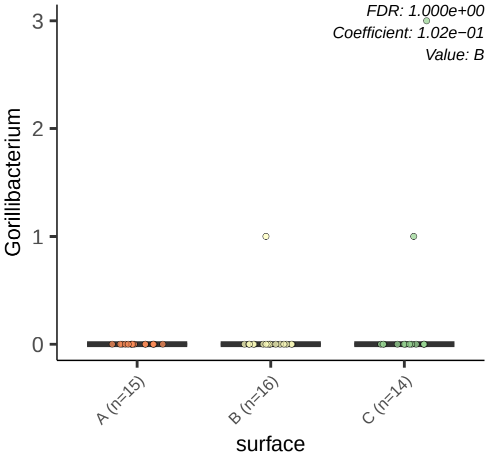
FDR: 1.000e+00
Coefficient: 1.02e−01
Value: B
Gorillibacterium
3
2
1
0
A (n=15)
B (n=16)
C (n=14)
surface

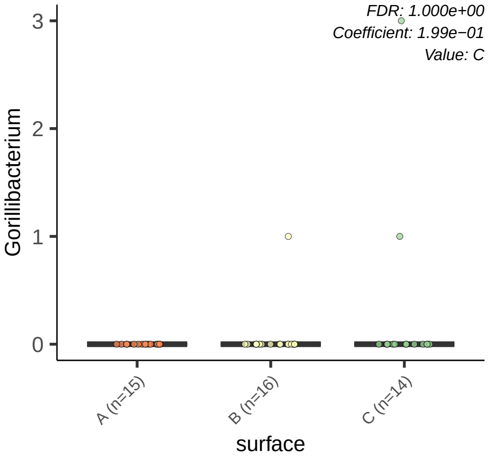
FDR: 1.000e+00
Coefficient: 1.99e−01
Value: C
3
2
1
0
Gorillibacterium
A (n=15)
B (n=16)
C (n=14)
surface

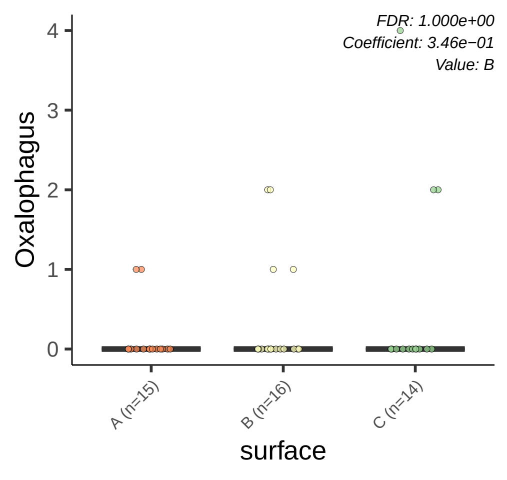
FDR: 1.000e+00
Coefficient: 3.46e−01
Value: B
Oxalophagus
4
3
2
1
0
A (n=15)
B (n=16)
C (n=14)
surface

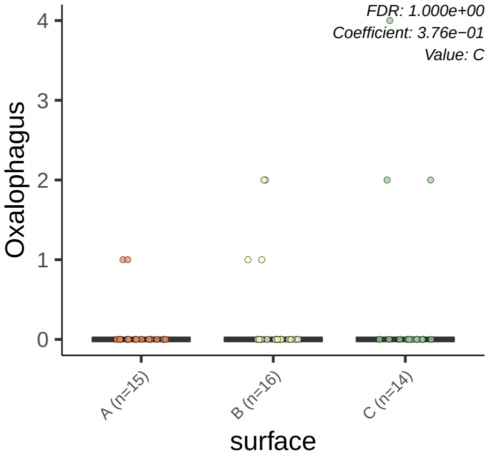

FDR: 1.000e+00
Coefficient: 3.76e−01
Value: C
Oxalophagus
0
1
2
3
4
A (n=15)
B (n=16)
C (n=14)
surface

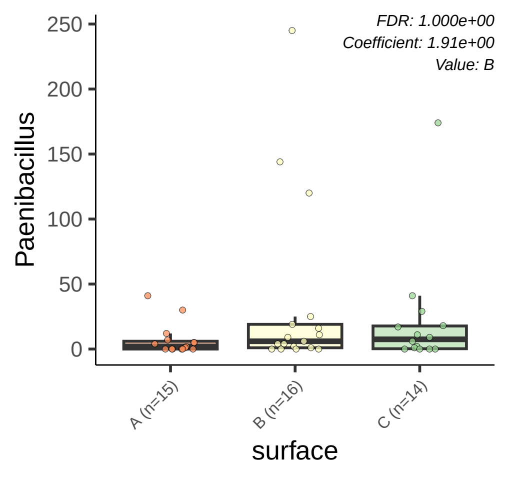

FDR: 1.000e+00
Coefficient: 1.91e+00
Value: B
Paenibacillus
250
200
150
100
50
0
A (n=15)
B (n=16)
C (n=14)
surface

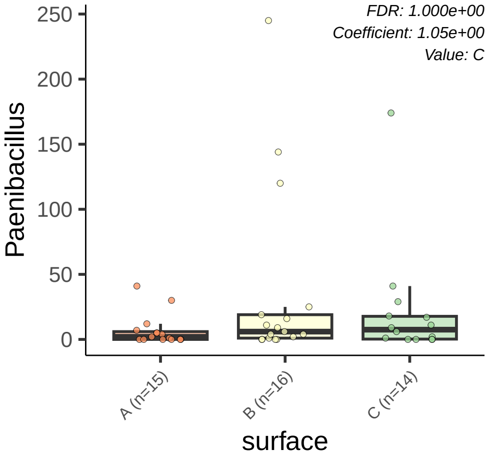

FDR: 1.000e+00
Coefficient: 1.05e+00
Value: C
Paenibacillus
250
200
150
100
50
0
A (n=15)
B (n=16)
C (n=14)
surface

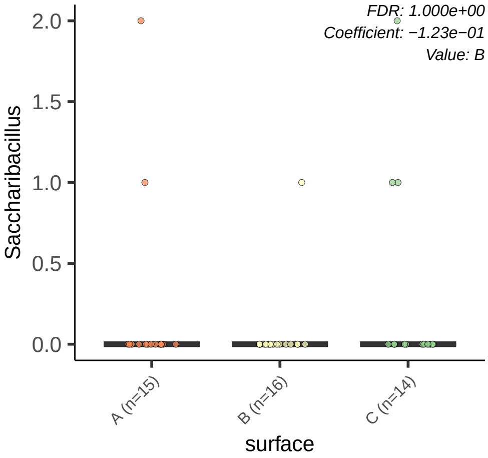

FDR: 1.000e+00
Coefficient: −1.23e−01
Value: B
Saccharibacillus
2.0
1.5
1.0
0.5
0.0
A (n=15)
B (n=16)
C (n=14)
surface

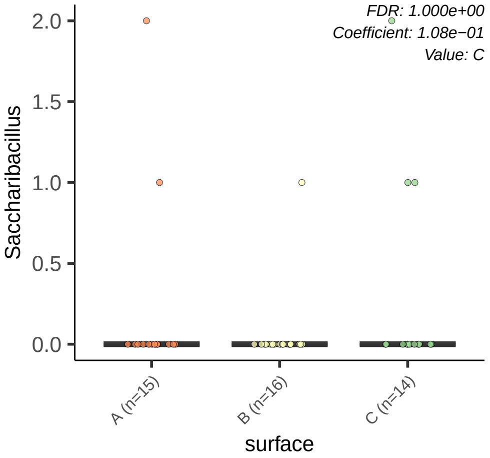
FDR: 1.000e+00
Coefficient: 1.08e−01
Value: C
Saccharibacillus
2.0
1.5
1.0
0.5
0.0
A (n=15)
B (n=16)
C (n=14)
surface

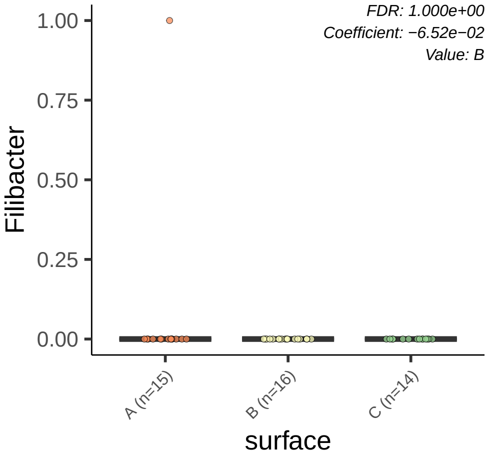

FDR: 1.000e+00
Coefficient: −6.52e−02
Value: B
Filibacter
1.00
0.75
0.50
0.25
0.00
A (n=15)
B (n=16)
C (n=14)
surface

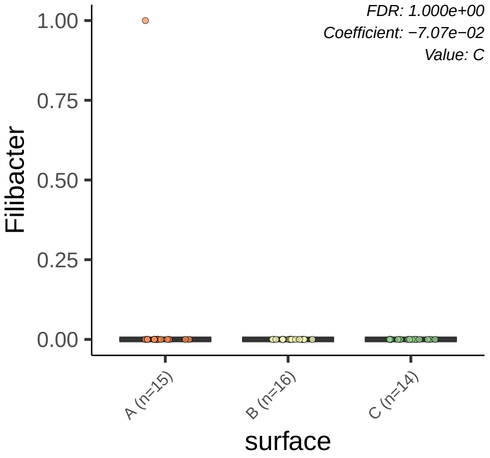
FDR: 1.000e+00
Coefficient: −7.07e−02
Value: C
Filibacter
1.00
0.75
0.50
0.25
0.00
A (n=15)
B (n=16)
C (n=14)
surface

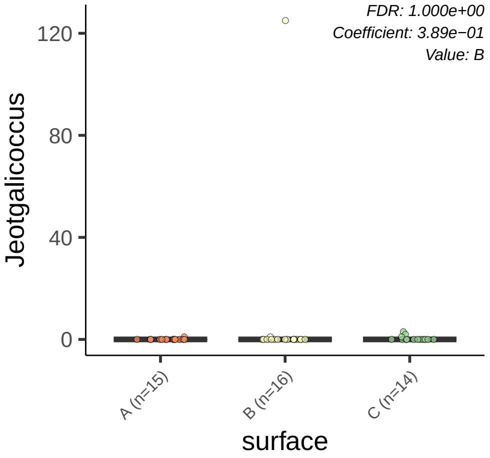

FDR: 1.000e+00
Coefficient: 3.89e−01
Value: B
Jeotgalicoccus
120
80
40
0
A (n=15)
B (n=16)
C (n=14)
surface

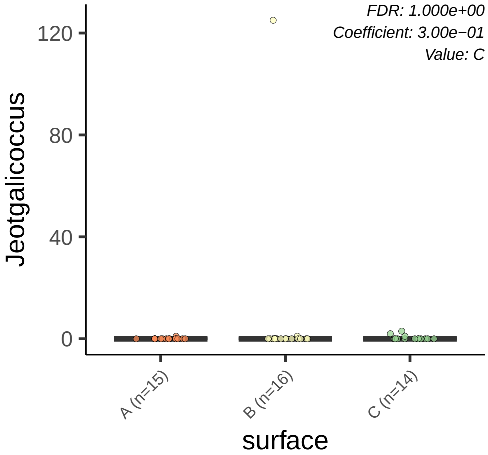

FDR: 1.000e+00
Coefficient: 3.00e−01
Value: C
Jeotgalicoccus
120
80
40
0
A (n=15)
B (n=16)
C (n=14)
surface

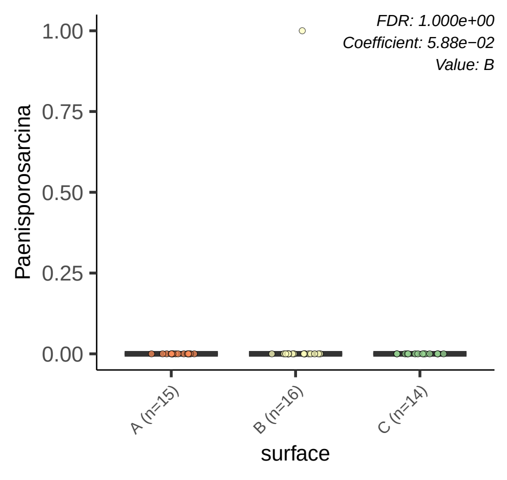

FDR: 1.000e+00
Coefficient: 5.88e−02
Value: B
Paenisporosarcina
1.00
0.75
0.50
0.25
0.00
A (n=15)
B (n=16)
C (n=14)
surface

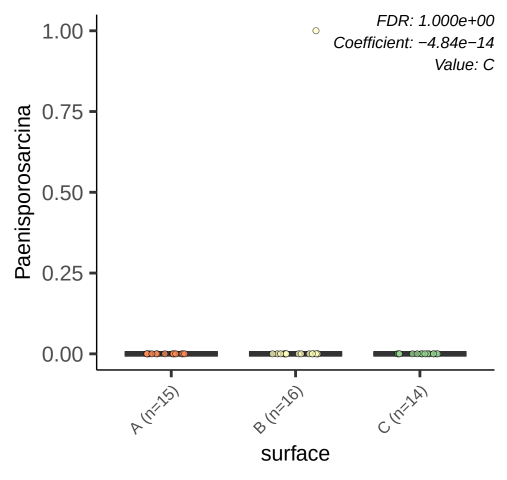
FDR: 1.000e+00
Coefficient: −4.84e−14
Value: C
Paenisporosarcina
1.00
0.75
0.50
0.25
0.00
A (n=15)
B (n=16)
C (n=14)
surface

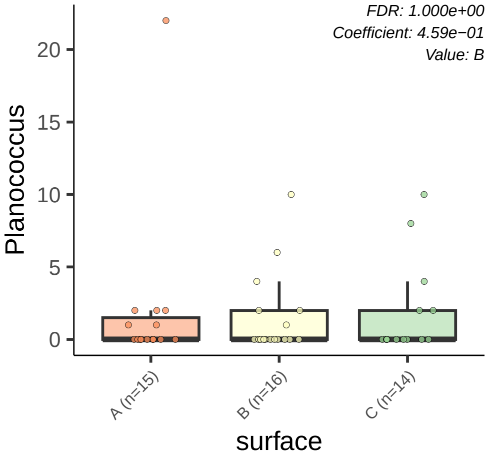
FDR: 1.000e+00
Coefficient: 4.59e−01
Value: B
Planococcus
0
5
10
15
20
A (n=15)
B (n=16)
C (n=14)
surface

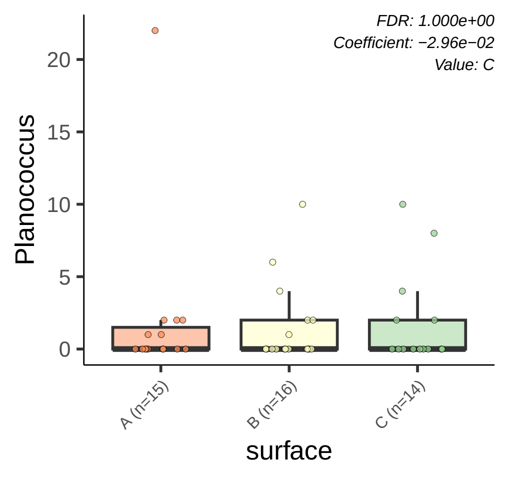
FDR: 1.000e+00
Coefficient: −2.96e−02
Value: C
Planococcus
0
5
10
15
20
A (n=15)
B (n=16)
C (n=14)
surface

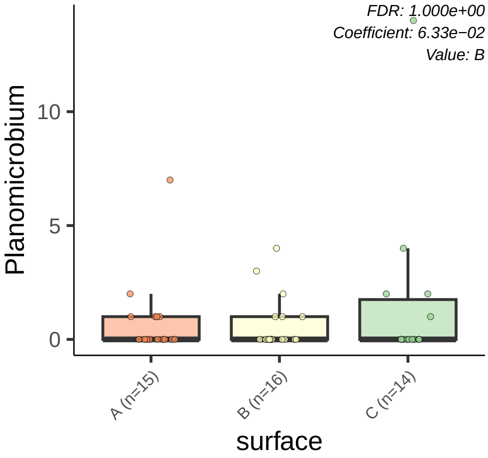
FDR: 1.000e+00
Coefficient: 6.33e−02
Value: B
Planomicrobium
0
5
10
A (n=15)
B (n=16)
C (n=14)
surface

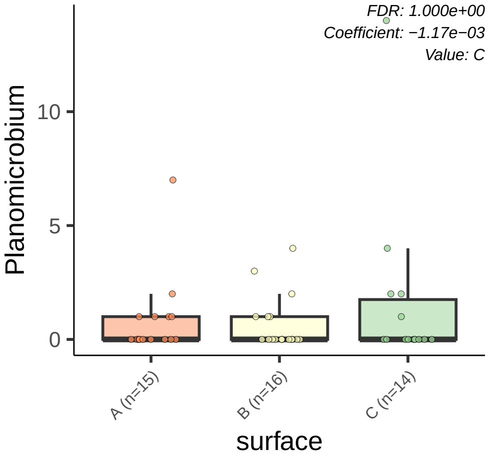

FDR: 1.000e+00
Coefficient: −1.17e−03
Value: C
Planomicrobium
10
5
0
A (n=15)
B (n=16)
C (n=14)
surface

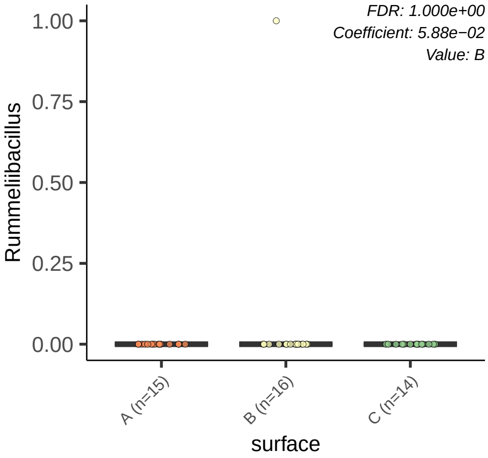
FDR: 1.000e+00
Coefficient: 5.88e−02
Value: B
Rummeliibacillus
1.00
0.75
0.50
0.25
0.00
A (n=15)
B (n=16)
C (n=14)
surface

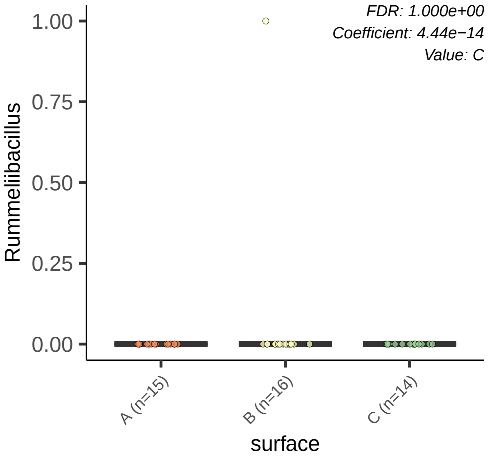

FDR: 1.000e+00
Coefficient: 4.44e−14
Value: C
Rummeliibacillus
1.00
0.75
0.50
0.25
0.00
A (n=15)
B (n=16)
C (n=14)
surface

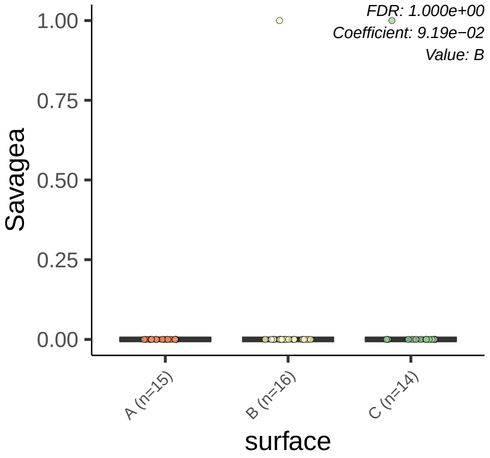
FDR: 1.000e+00
Coefficient: 9.19e−02
Value: B
Savagea
1.00
0.75
0.50
0.25
0.00
A (n=15)
B (n=16)
C (n=14)
surface

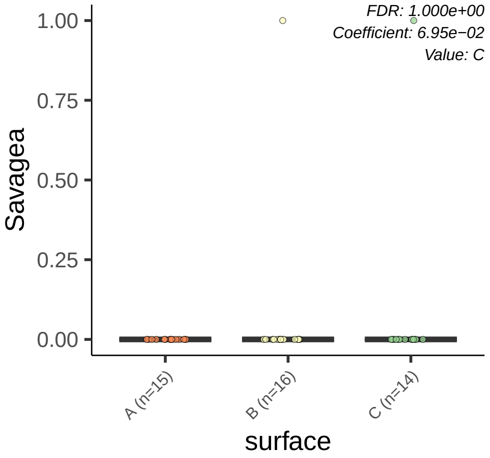

FDR: 1.000e+00
Coefficient: 6.95e−02
Value: C
Savagea
1.00
0.75
0.50
0.25
0.00
A (n=15)
B (n=16)
C (n=14)
surface

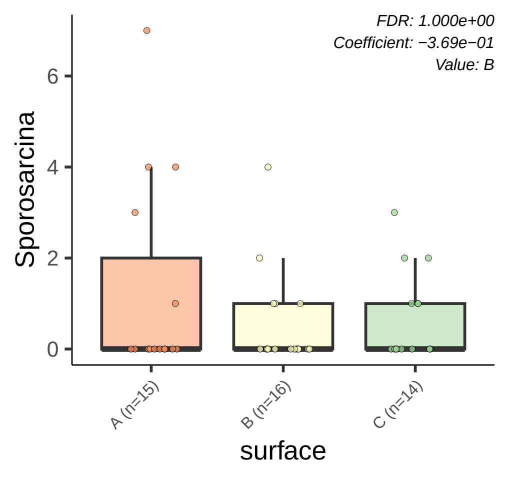

FDR: 1.000e+00
Coefficient: −3.69e−01
Value: B
Sporosarcina
0
2
4
6
A (n=15)
B (n=16)
C (n=14)
surface

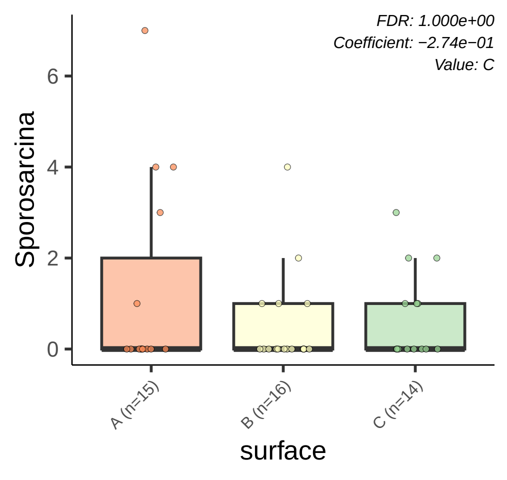
FDR: 1.000e+00
Coefficient: −2.74e−01
Value: C
Sporosarcina
0
2
4
6
A (n=15)
B (n=16)
C (n=14)
surface

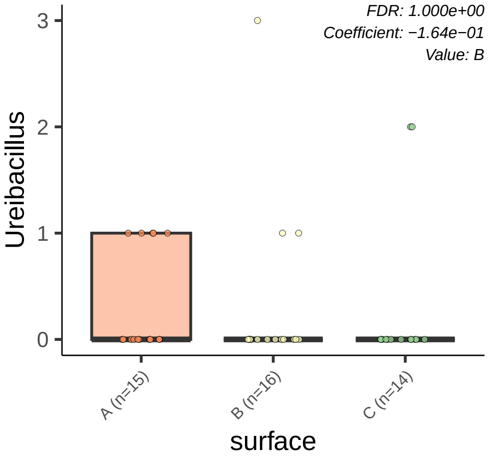

FDR: 1.000e+00
Coefficient: −1.64e−01
Value: B
Ureibacillus
3
2
1
0
A (n=15)
B (n=16)
C (n=14)
surface

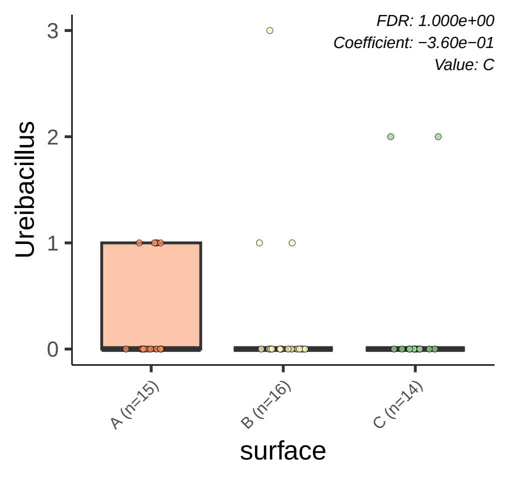
FDR: 1.000e+00
Coefficient: −3.60e−01
Value: C
Ureibacillus
0
1
2
3
A (n=15)
B (n=16)
C (n=14)
surface

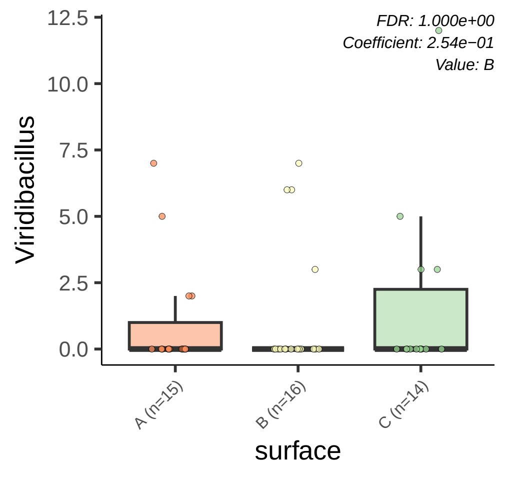
FDR: 1.000e+00
Coefficient: 2.54e−01
Value: B
Viridibacillus
12.5
10.0
7.5
5.0
2.5
0.0
A (n=15)
B (n=16)
C (n=14)
surface

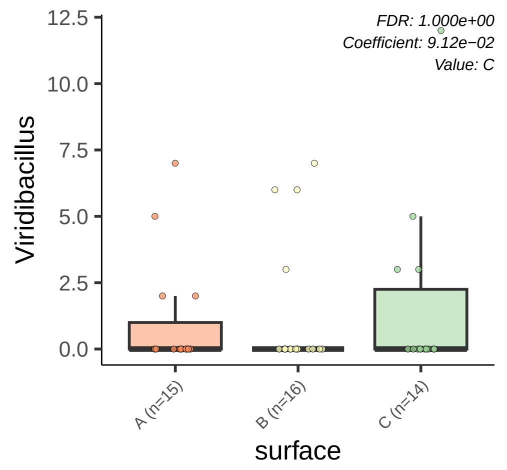
FDR: 1.000e+00
Coefficient: 9.12e−02
Value: C
Viridibacillus
12.5
10.0
7.5
5.0
2.5
0.0
A (n=15)
B (n=16)
C (n=14)
surface

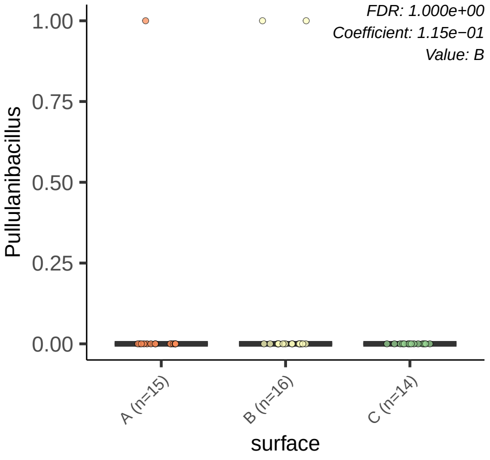

FDR: 1.000e+00
Coefficient: 1.15e−01
Value: B
Pullulanibacillus
1.00
0.75
0.50
0.25
0.00
A (n=15)
B (n=16)
C (n=14)
surface

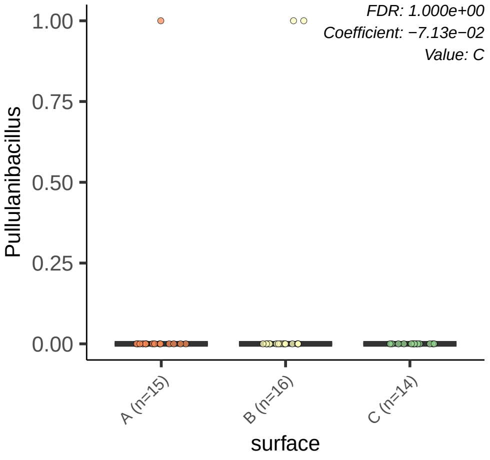
FDR: 1.000e+00
Coefficient: −7.13e−02
Value: C
Pullulanibacillus
1.00
0.75
0.50
0.25
0.00
A (n=15)
B (n=16)
C (n=14)
surface

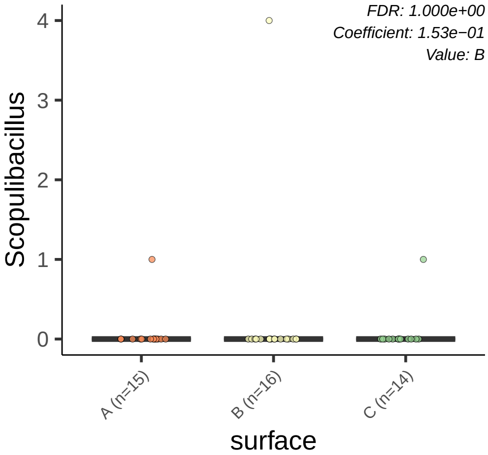

FDR: 1.000e+00
Coefficient: 1.53e−01
Value: B
Scopulibacillus
4
3
2
1
0
A (n=15)
B (n=16)
C (n=14)
surface

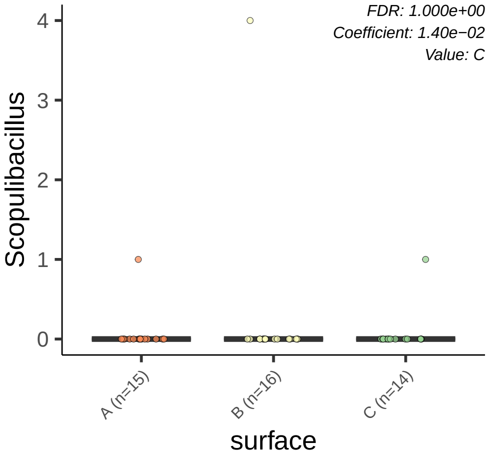

FDR: 1.000e+00
Coefficient: 1.40e−02
Value: C
Scopulibacillus
0
1
2
3
4
A (n=15)
B (n=16)
C (n=14)
surface

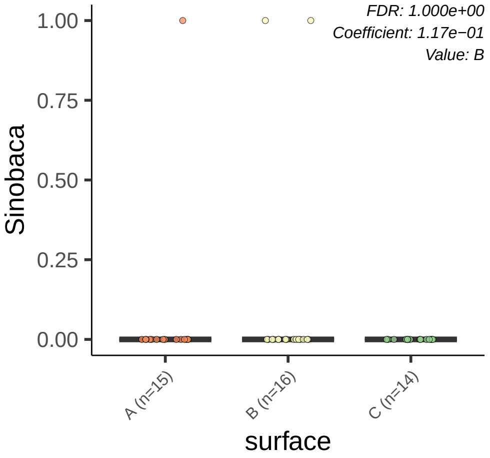
FDR: 1.000e+00
Coefficient: 1.17e−01
Value: B
Sinobaca
1.00
0.75
0.50
0.25
0.00
A (n=15)
B (n=16)
C (n=14)
surface

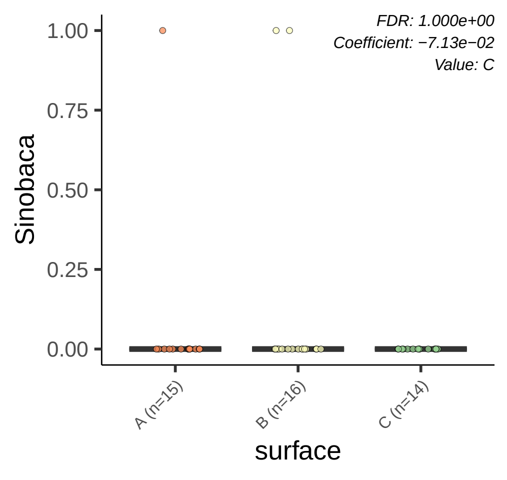
FDR: 1.000e+00
Coefficient: −7.13e−02
Value: C
Sinobaca
1.00
0.75
0.50
0.25
0.00
A (n=15)
B (n=16)
C (n=14)
surface

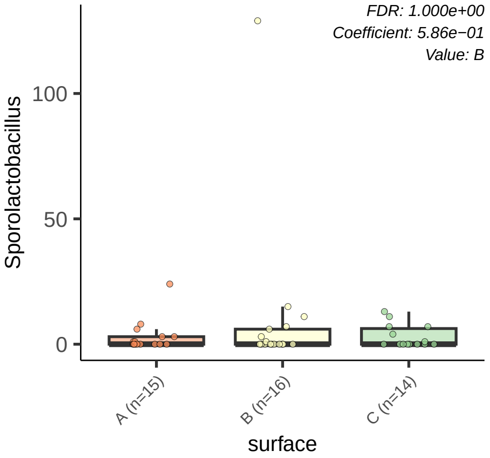

FDR: 1.000e+00
Coefficient: 5.86e−01
Value: B
Sporolactobacillus
100
50
0
A (n=15)
B (n=16)
C (n=14)
surface

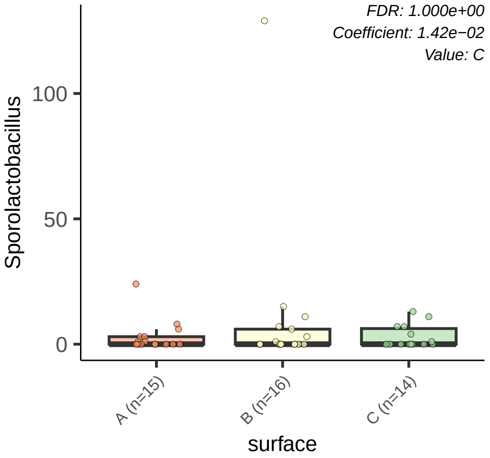

FDR: 1.000e+00
Coefficient: 1.42e−02
Value: C
Sporolactobacillus
100
50
0
A (n=15)
B (n=16)
C (n=14)
surface

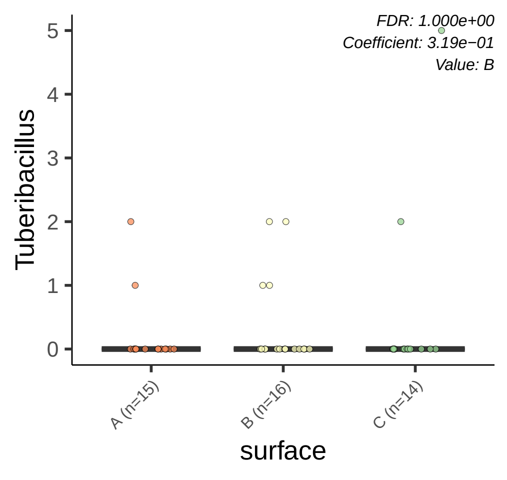

FDR: 1.000e+00
Coefficient: 3.19e−01
Value: B
Tuberibacillus
0
1
2
3
4
5
A (n=15)
B (n=16)
C (n=14)
surface

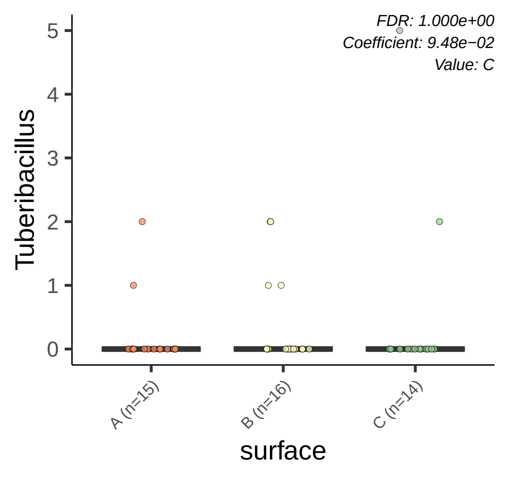
FDR: 1.000e+00
Coefficient: 9.48e−02
Value: C
Tuberibacillus
0
1
2
3
4
5
A (n=15)
B (n=16)
C (n=14)
surface

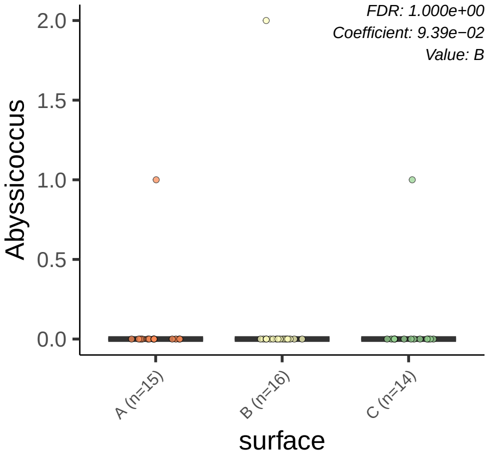

FDR: 1.000e+00
Coefficient: 9.39e−02
Value: B
Abyssicoccus
2.0
1.5
1.0
0.5
0.0
A (n=15)
B (n=16)
C (n=14)
surface

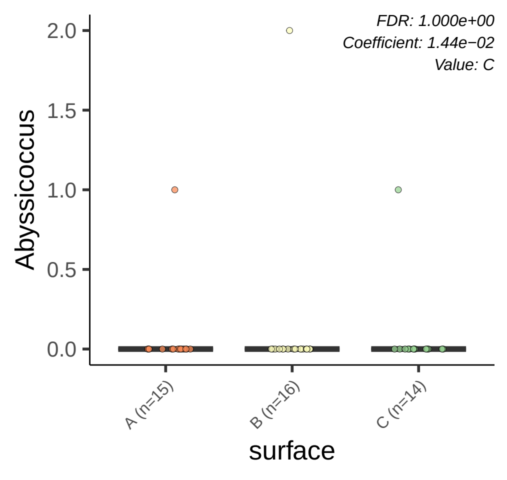

FDR: 1.000e+00
Coefficient: 1.44e−02
Value: C
Abyssicoccus
2.0
1.5
1.0
0.5
0.0
A (n=15)
B (n=16)
C (n=14)
surface

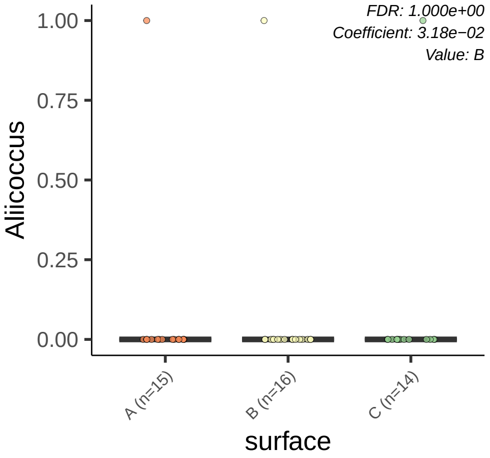

FDR: 1.000e+00
Coefficient: 3.18e−02
Value: B
Aliicoccus
1.00
0.75
0.50
0.25
0.00
A (n=15)
B (n=16)
C (n=14)
surface

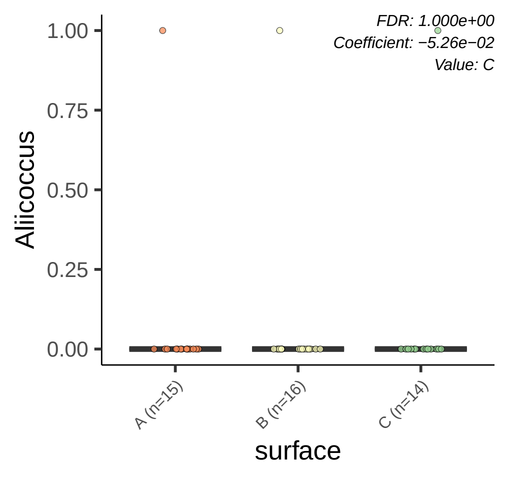

FDR: 1.000e+00
Coefficient: −5.26e−02
Value: C
1.00
0.75
0.50
0.25
0.00
Aliicoccus
A (n=15)
B (n=16)
C (n=14)
surface

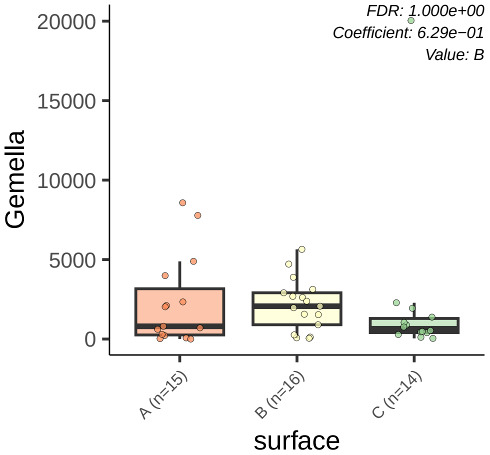

FDR: 1.000e+00
Coefficient: 6.29e−01
Value: B
20000
15000
10000
5000
0
Gemella
A (n=15)
B (n=16)
C (n=14)
surface

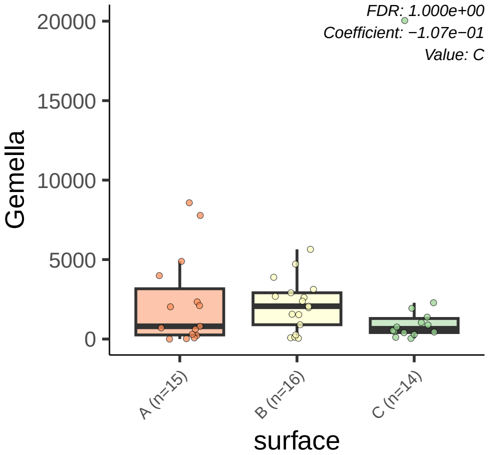

FDR: 1.000e+00
Coefficient: −1.07e−01
Value: C
Gemella
20000
15000
10000
5000
0
A (n=15)
B (n=16)
C (n=14)
surface

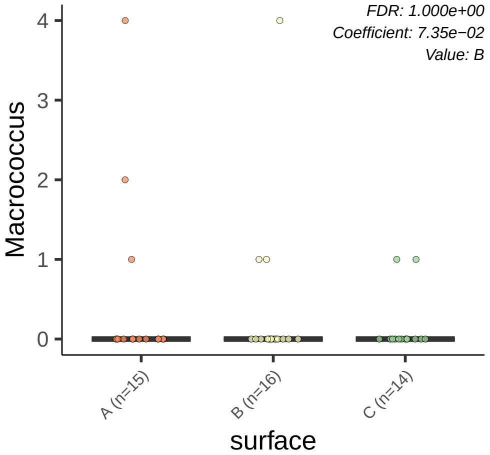

FDR: 1.000e+00
Coefficient: 7.35e−02
Value: B
Macrococcus
0
1
2
3
4
A (n=15)
B (n=16)
C (n=14)
surface

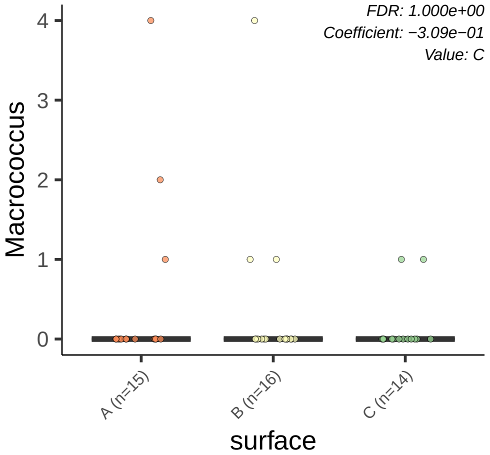
FDR: 1.000e+00
Coefficient: −3.09e−01
Value: C
Macrococcus
0
1
2
3
4
A (n=15)
B (n=16)
C (n=14)
surface

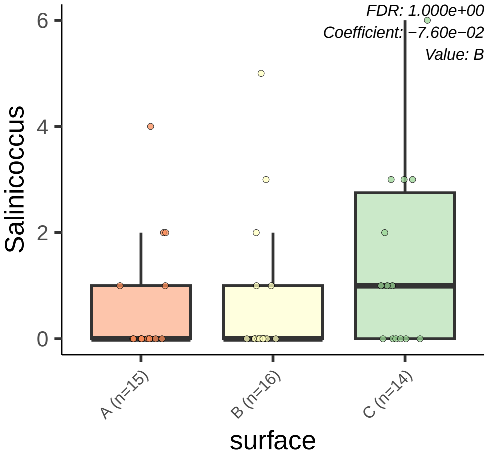

FDR: 1.000e+00
Coefficient: −7.60e−02
Value: B
Salinicoccus
0
2
4
6
A (n=15)
B (n=16)
C (n=14)
surface

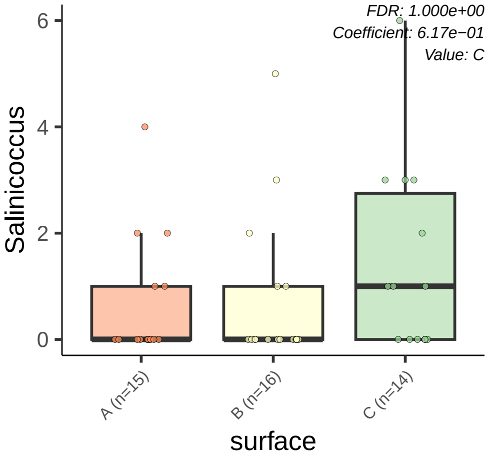
FDR: 1.000e+00
Coefficient: 6.17e−01
Value: C
Salinicoccus
0
2
4
6
A (n=15)
B (n=16)
C (n=14)
surface

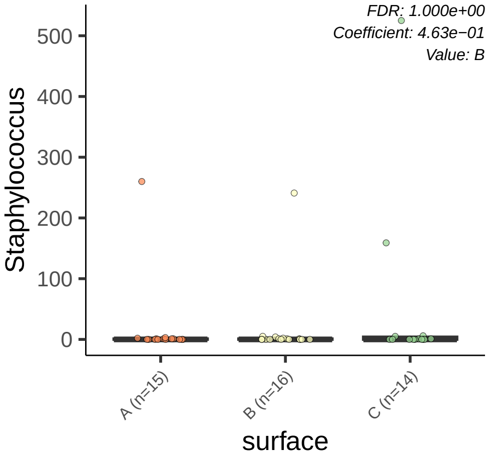
FDR: 1.000e+00
Coefficient: 4.63e−01
Value: B
Staphylococcus
500
400
300
200
100
0
A (n=15)
B (n=16)
C (n=14)
surface

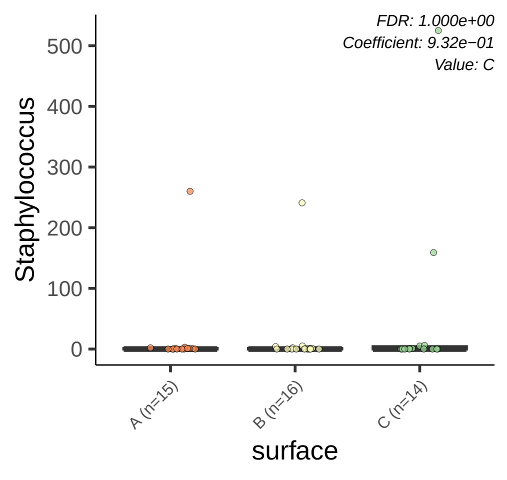
FDR: 1.000e+00
Coefficient: 9.32e−01
Value: C
Staphylococcus
500
400
300
200
100
0
A (n=15)
B (n=16)
C (n=14)
surface

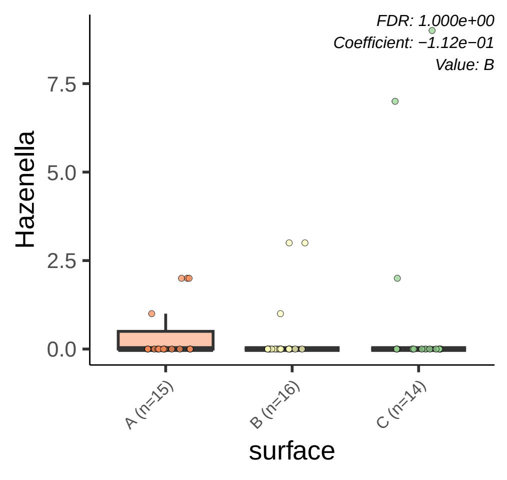
FDR: 1.000e+00
Coefficient: −1.12e−01
Value: B
Hazenella
7.5
5.0
2.5
0.0
A (n=15)
B (n=16)
C (n=14)
surface

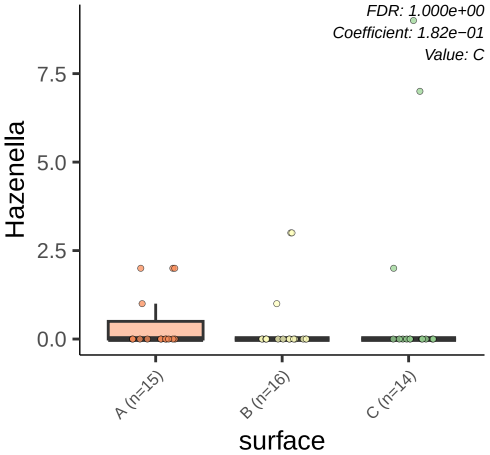

FDR: 1.000e+00
Coefficient: 1.82e−01
Value: C
Hazenella
7.5
5.0
2.5
0.0
A (n=15)
B (n=16)
C (n=14)
surface

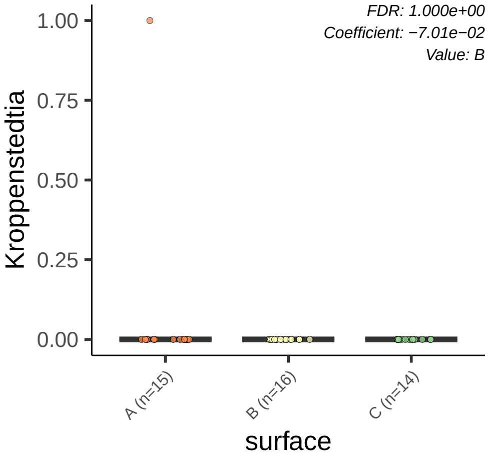
FDR: 1.000e+00
Coefficient: −7.01e−02
Value: B
Kroppenstedtia
1.00
0.75
0.50
0.25
0.00
A (n=15)
B (n=16)
C (n=14)
surface

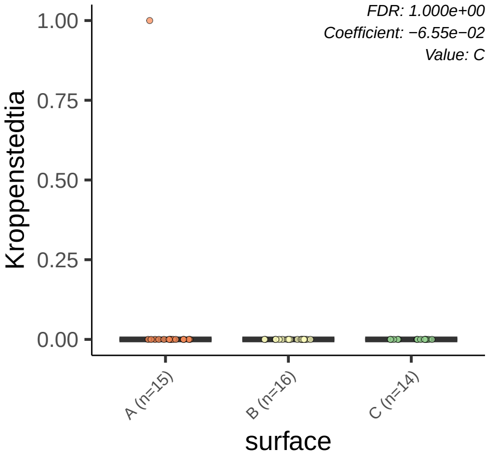

FDR: 1.000e+00
Coefficient: −6.55e−02
Value: C
Kroppenstedtia
1.00
0.75
0.50
0.25
0.00
A (n=15)
B (n=16)
C (n=14)
surface

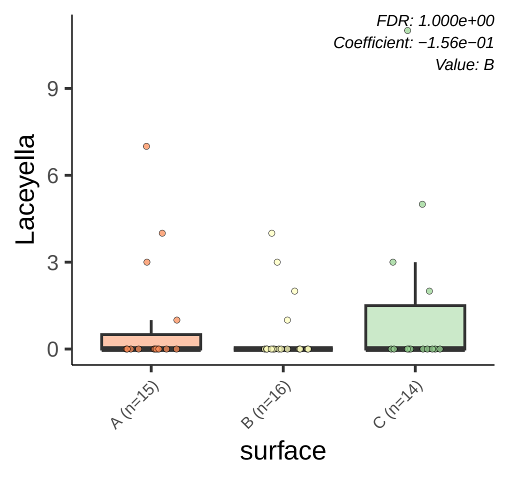

FDR: 1.000e+00
Coefficient: −1.56e−01
Value: B
Laceyella
9
6
3
0
A (n=15)
B (n=16)
C (n=14)
surface

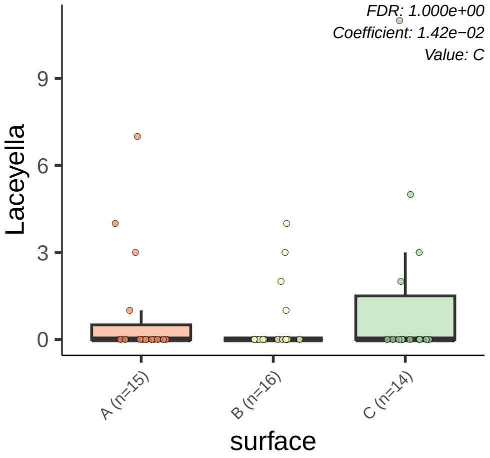

FDR: 1.000e+00
Coefficient: 1.42e−02
Value: C
Laceyella
9
6
3
0
A (n=15)
B (n=16)
C (n=14)
surface

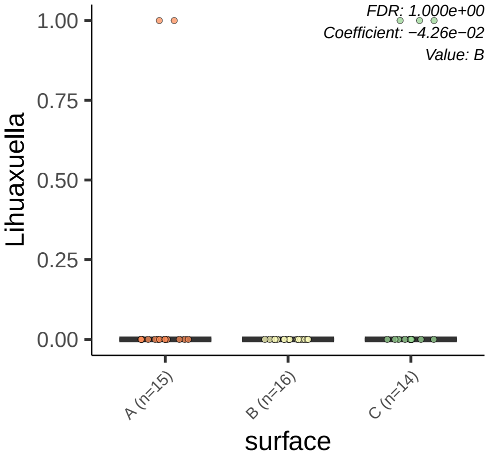
FDR: 1.000e+00
Coefficient: −4.26e−02
Value: B
Lihuaxuella
1.00
0.75
0.50
0.25
0.00
A (n=15)
B (n=16)
C (n=14)
surface

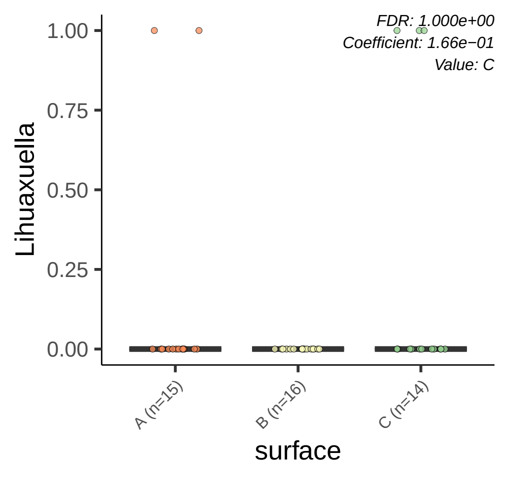
FDR: 1.000e+00
Coefficient: 1.66e−01
Value: C
1.00
0.75
0.50
0.25
0.00
Lihuaxuella
A (n=15)
B (n=16)
C (n=14)
surface

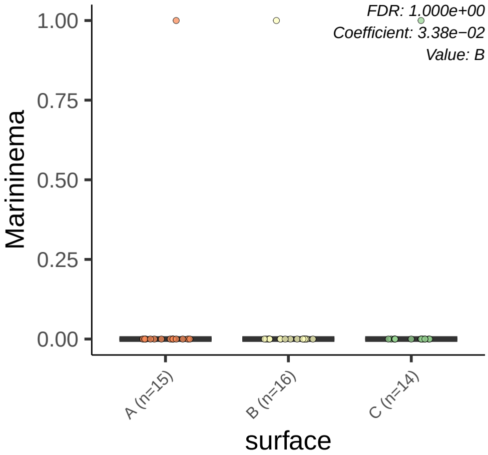

FDR: 1.000e+00
Coefficient: 3.38e−02
Value: B
Marininema
1.00
0.75
0.50
0.25
0.00
A (n=15)
B (n=16)
C (n=14)
surface

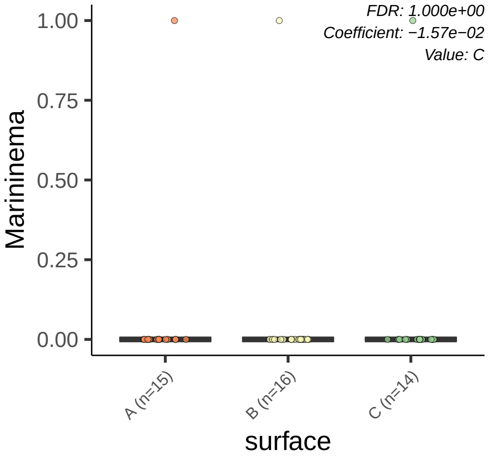
FDR: 1.000e+00
Coefficient: −1.57e−02
Value: C
Marininema
1.00
0.75
0.50
0.25
0.00
A (n=15)
B (n=16)
C (n=14)
surface

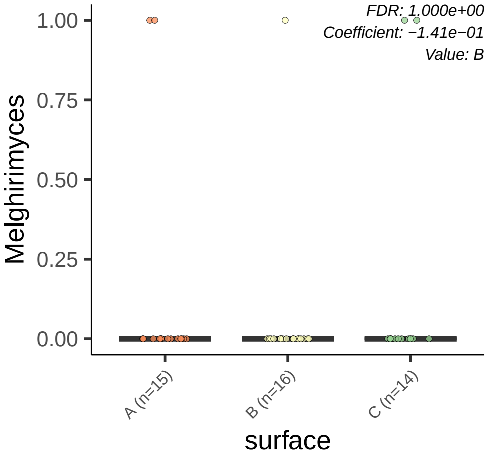
FDR: 1.000e+00
Coefficient: −1.41e−01
Value: B
Melghirimyces
1.00
0.75
0.50
0.25
0.00
A (n=15)
B (n=16)
C (n=14)
surface

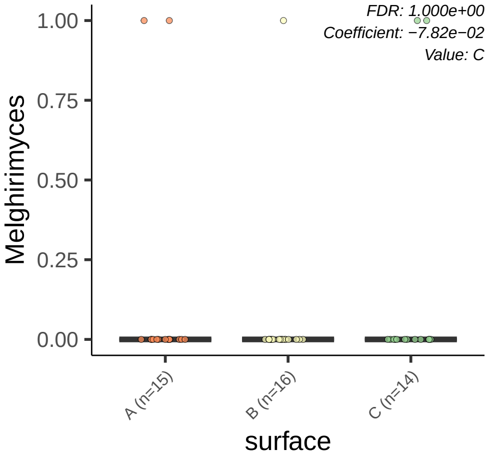
FDR: 1.000e+00
Coefficient: −7.82e−02
Value: C
Melghirimyces
1.00
0.75
0.50
0.25
0.00
A (n=15)
B (n=16)
C (n=14)
surface

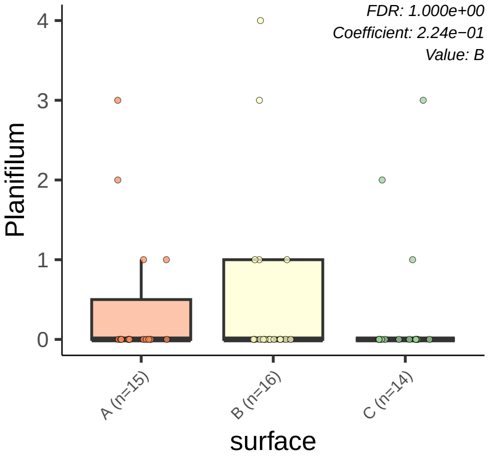
FDR: 1.000e+00
Coefficient: 2.24e−01
Value: B
Planifilum
0
1
2
3
4
A (n=15)
B (n=16)
C (n=14)
surface

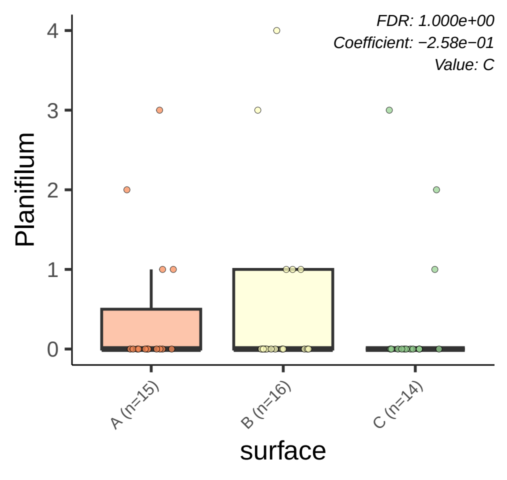
FDR: 1.000e+00
Coefficient: −2.58e−01
Value: C
Planifilum
0
1
2
3
4
A (n=15)
B (n=16)
C (n=14)
surface

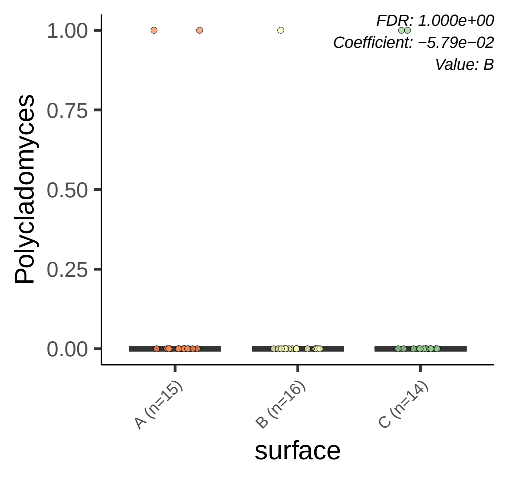
FDR: 1.000e+00
Coefficient: −5.79e−02
Value: B
Polycladomyces
1.00
0.75
0.50
0.25
0.00
A (n=15)
B (n=16)
C (n=14)
surface

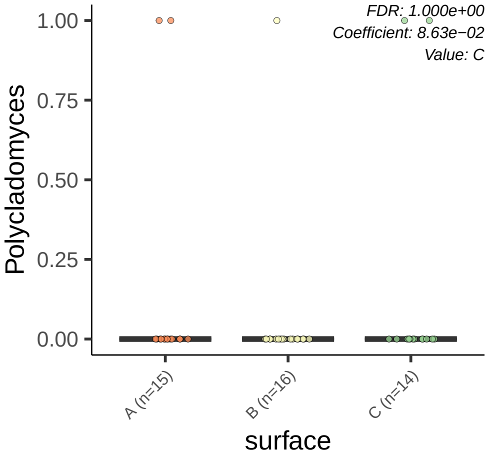

FDR: 1.000e+00
Coefficient: 8.63e−02
Value: C
Polycladomyces
1.00
0.75
0.50
0.25
0.00
A (n=15)
B (n=16)
C (n=14)
surface

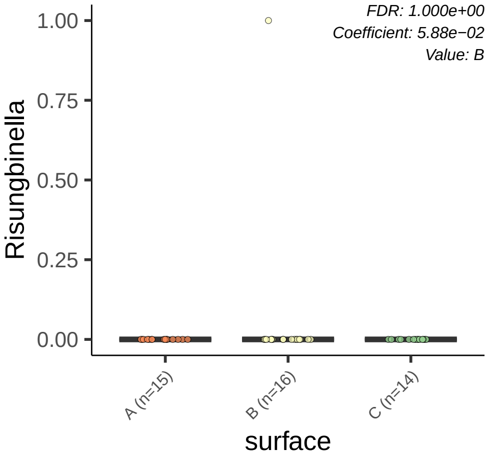

FDR: 1.000e+00
Coefficient: 5.88e−02
Value: B
Risungbinella
1.00
0.75
0.50
0.25
0.00
A (n=15)
B (n=16)
C (n=14)
surface

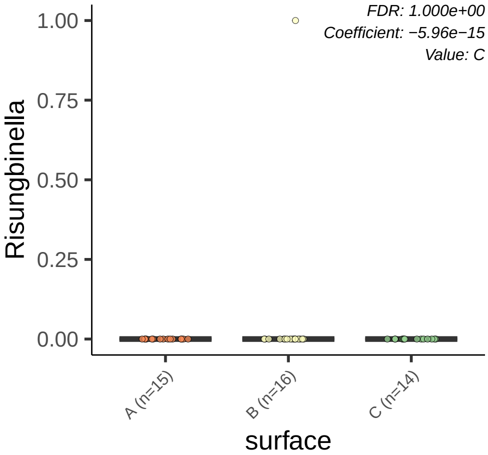
FDR: 1.000e+00
Coefficient: −5.96e−15
Value: C
Risungbinella
1.00
0.75
0.50
0.25
0.00
A (n=15)
B (n=16)
C (n=14)
surface

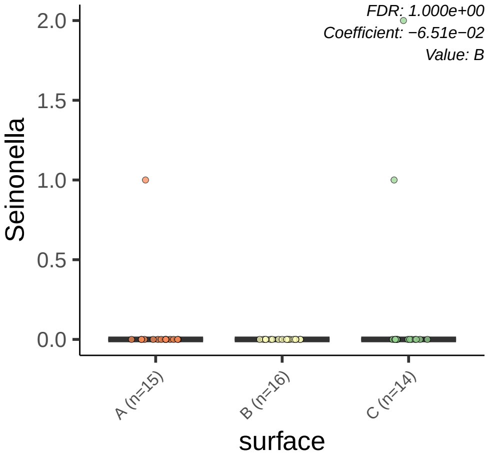

FDR: 1.000e+00
Coefficient: −6.51e−02
Value: B
Seinonella
2.0
1.5
1.0
0.5
0.0
A (n=15)
B (n=16)
C (n=14)
surface

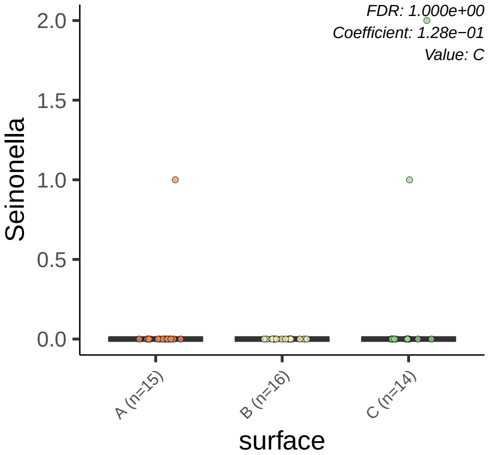
FDR: 1.000e+00
Coefficient: 1.28e−01
Value: C
Seinonella
2.0
1.5
1.0
0.5
0.0
A (n=15)
B (n=16)
C (n=14)
surface

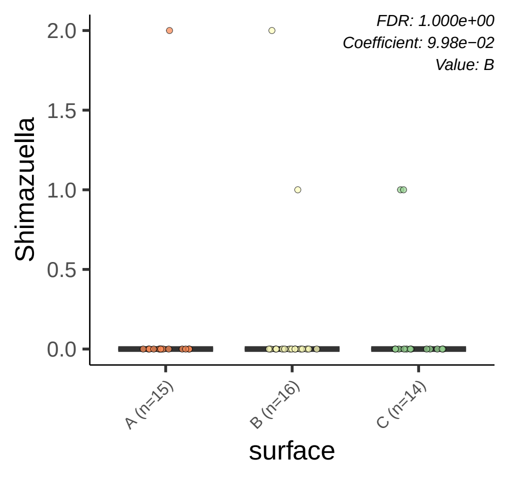
FDR: 1.000e+00
Coefficient: 9.98e−02
Value: B
Shimazuella
2.0
1.5
1.0
0.5
0.0
A (n=15)
B (n=16)
C (n=14)
surface

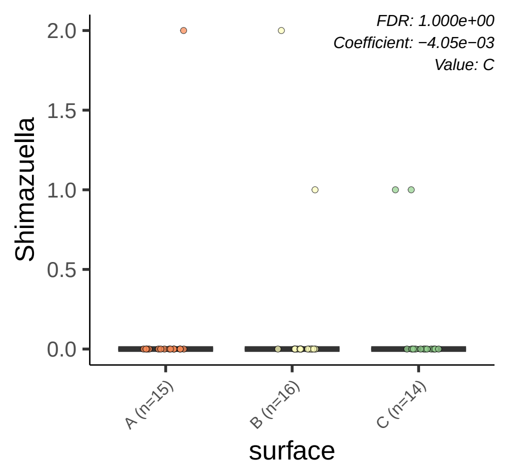

FDR: 1.000e+00
Coefficient: −4.05e−03
Value: C
Shimazuella
2.0
1.5
1.0
0.5
0.0
A (n=15)
B (n=16)
C (n=14)
surface

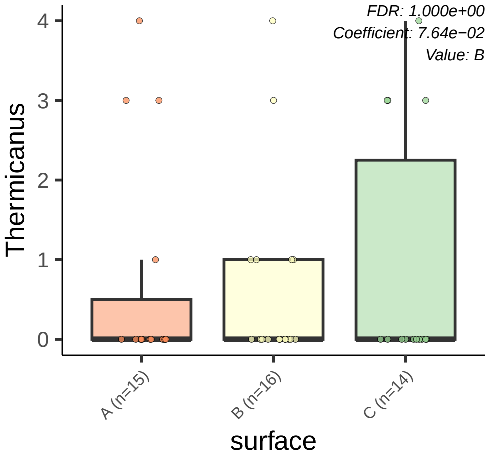
FDR: 1.000e+00
Coefficient: 7.64e−02
Value: B
Thermicanus
0
1
2
3
4
A (n=15)
B (n=16)
C (n=14)
surface

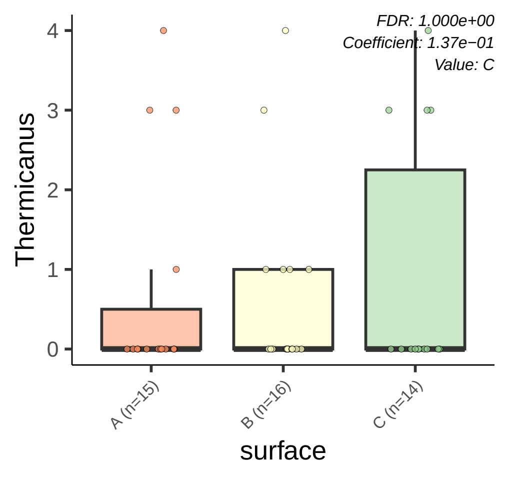
FDR: 1.000e+00
Coefficient: 1.37e−01
Value: C
Thermicanus
0
1
2
3
4
A (n=15)
B (n=16)
C (n=14)
surface

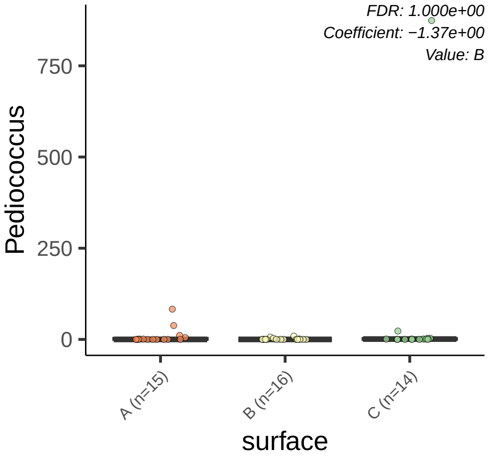

FDR: 1.000e+00
Coefficient: −1.37e+00
Value: B
Pediococcus
750
500
250
0
A (n=15)
B (n=16)
C (n=14)
surface

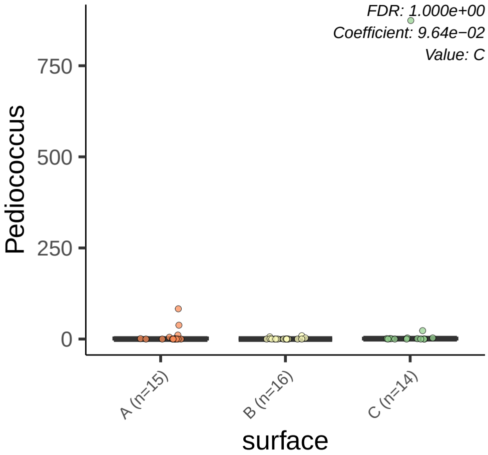

FDR: 1.000e+00
Coefficient: 9.64e−02
Value: C
Pediococcus
750
500
250
0
A (n=15)
B (n=16)
C (n=14)
surface

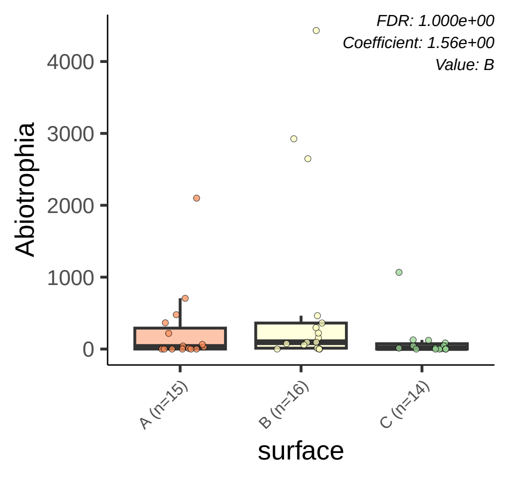
FDR: 1.000e+00
Coefficient: 1.56e+00
Value: B
Abiotrophia
4000
3000
2000
1000
0
A (n=15)
B (n=16)
C (n=14)
surface

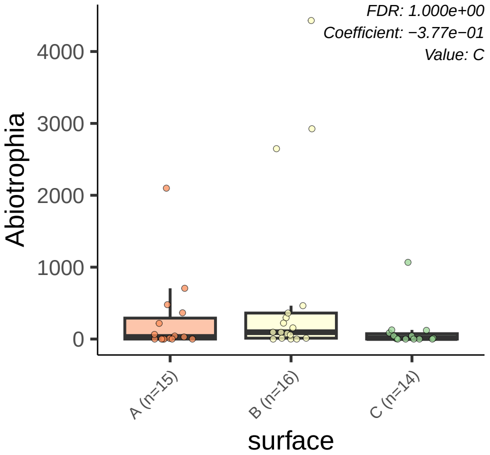
FDR: 1.000e+00
Coefficient: −3.77e−01
Value: C
Abiotrophia
4000
3000
2000
1000
0
A (n=15)
B (n=16)
C (n=14)
surface

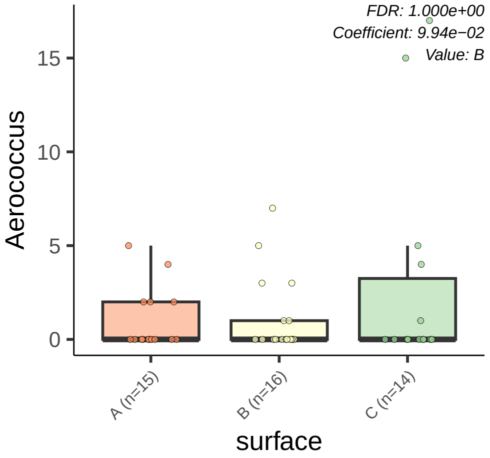

FDR: 1.000e+00
Coefficient: 9.94e−02
Value: B
Aerococcus
15
10
5
0
A (n=15)
B (n=16)
C (n=14)
surface

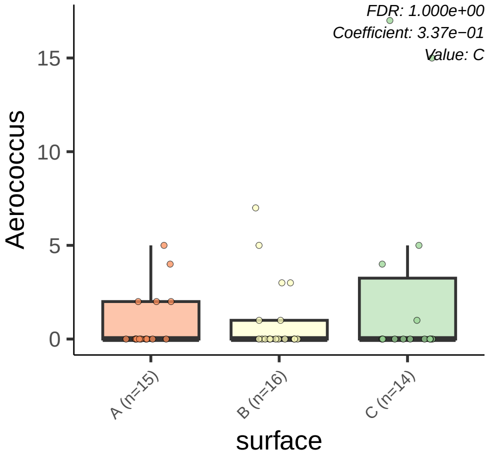
FDR: 1.000e+00
Coefficient: 3.37e−01
Value: C
Aerococcus
15
10
5
0
A (n=15)
B (n=16)
C (n=14)
surface

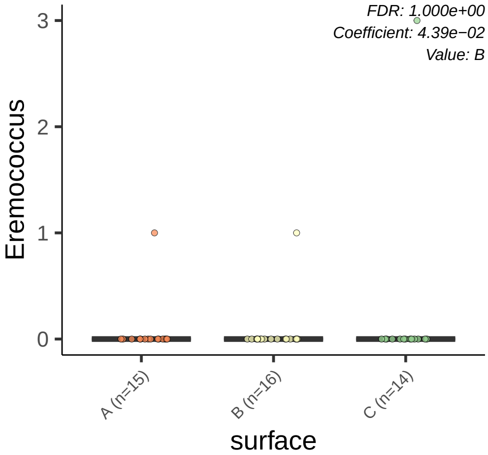
FDR: 1.000e+00
Coefficient: 4.39e−02
Value: B
Eremococcus
3
2
1
0
A (n=15)
B (n=16)
C (n=14)
surface

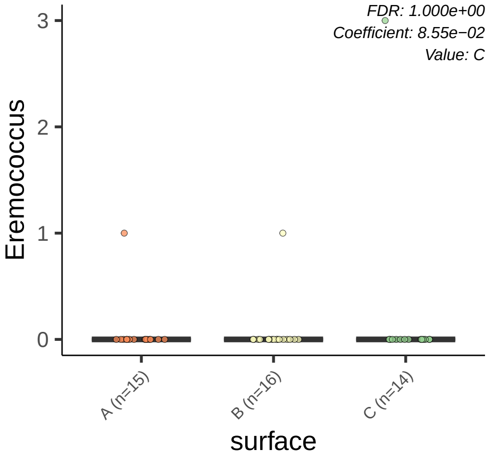
FDR: 1.000e+00
Coefficient: 8.55e−02
Value: C
Eremococcus
3
2
1
0
A (n=15)
B (n=16)
C (n=14)
surface

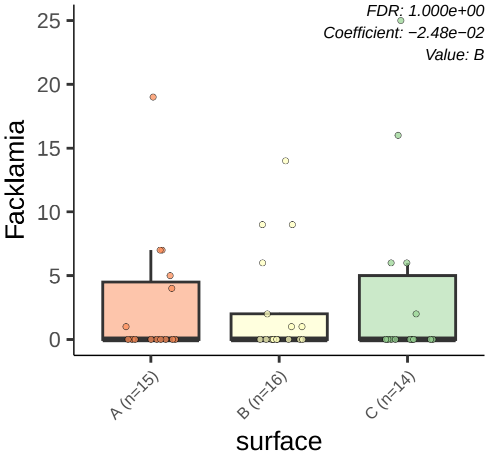

FDR: 1.000e+00
Coefficient: −2.48e−02
Value: B
Facklamia
25
20
15
10
5
0
A (n=15)
B (n=16)
C (n=14)
surface

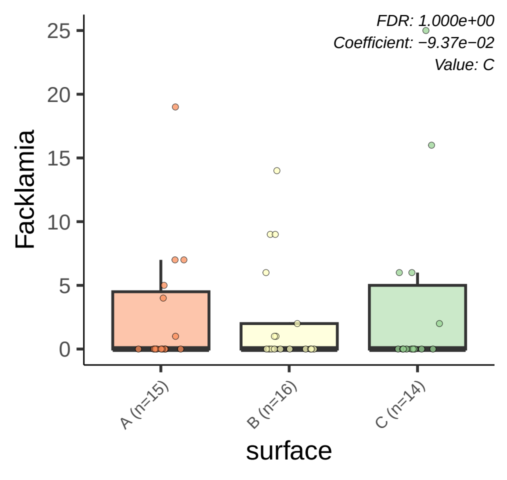
FDR: 1.000e+00
Coefficient: −9.37e−02
Value: C
Facklamia
25
20
15
10
5
0
A (n=15)
B (n=16)
C (n=14)
surface

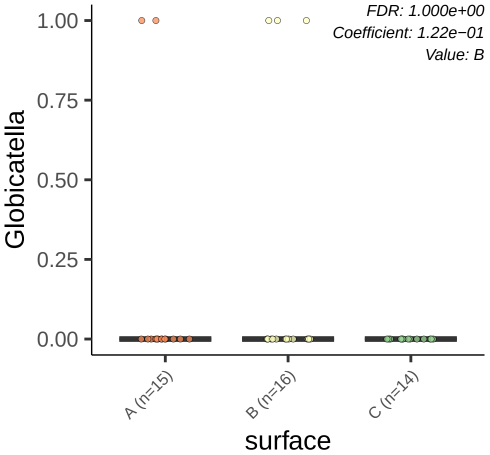
FDR: 1.000e+00
Coefficient: 1.22e−01
Value: B
Globicatella
1.00
0.75
0.50
0.25
0.00
A (n=15)
B (n=16)
C (n=14)
surface

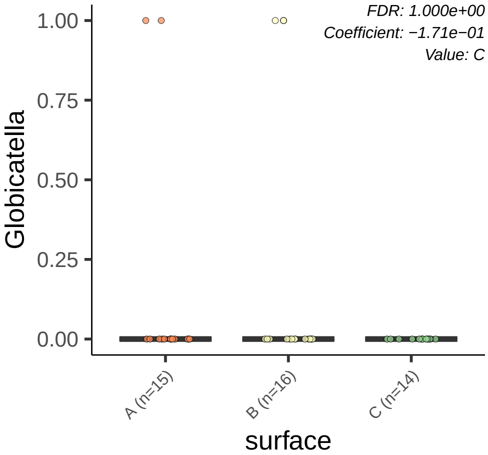
FDR: 1.000e+00
Coefficient: −1.71e−01
Value: C
Globicatella
1.00
0.75
0.50
0.25
0.00
A (n=15)
B (n=16)
C (n=14)
surface

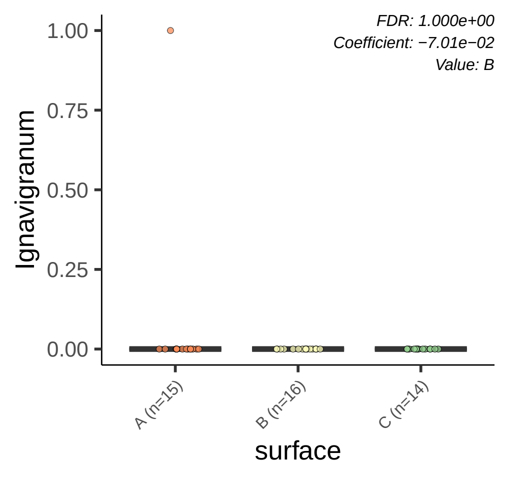
FDR: 1.000e+00
Coefficient: −7.01e−02
Value: B
Ignavigranum
1.00
0.75
0.50
0.25
0.00
A (n=15)
B (n=16)
C (n=14)
surface

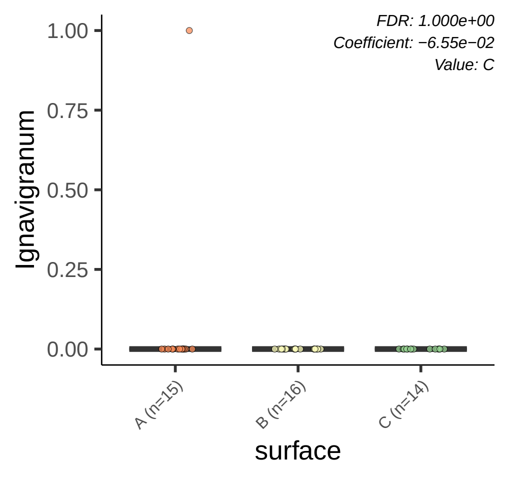
FDR: 1.000e+00
Coefficient: −6.55e−02
Value: C
Ignavigranum
1.00
0.75
0.50
0.25
0.00
A (n=15)
B (n=16)
C (n=14)
surface

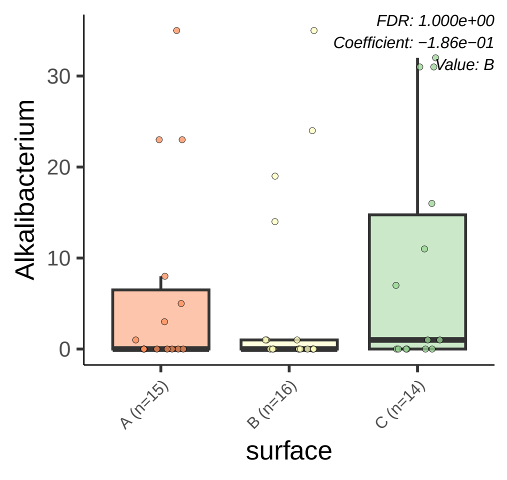

FDR: 1.000e+00
Coefficient: −1.86e−01
Value: B
Alkalibacterium
30
20
10
0
A (n=15)
B (n=16)
C (n=14)
surface

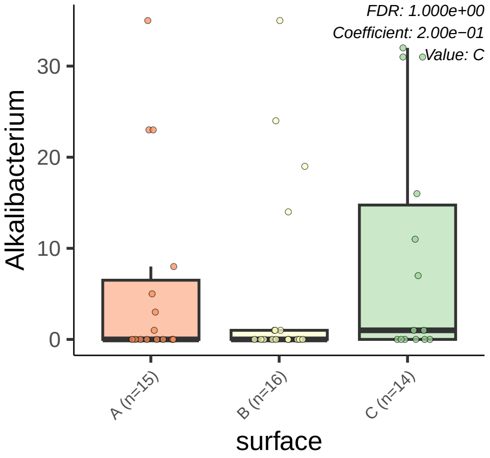

FDR: 1.000e+00
Coefficient: 2.00e−01
Value: C
Alkalibacterium
0
10
20
30
A (n=15)
B (n=16)
C (n=14)
surface

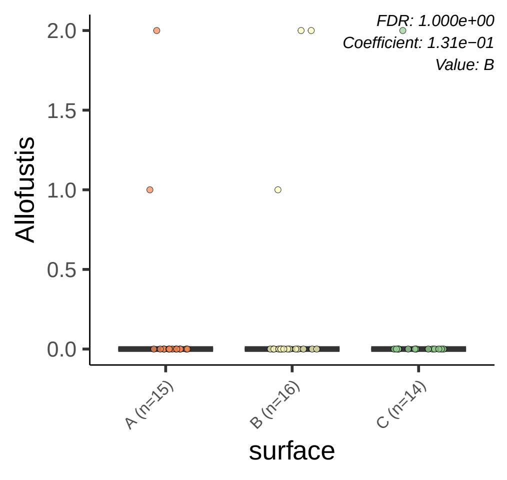
FDR: 1.000e+00
Coefficient: 1.31e−01
Value: B
Allofustis
2.0
1.5
1.0
0.5
0.0
A (n=15)
B (n=16)
C (n=14)
surface

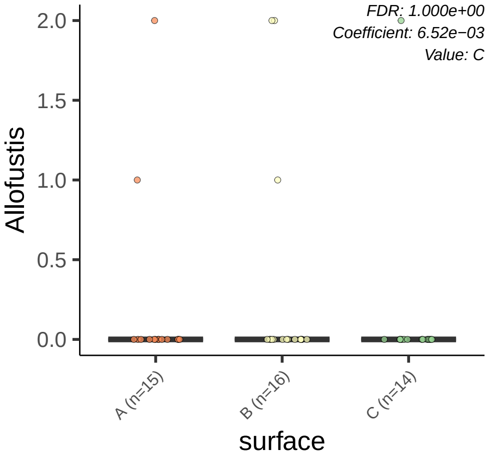
FDR: 1.000e+00
Coefficient: 6.52e−03
Value: C
Allofustis
2.0
1.5
1.0
0.5
0.0
A (n=15)
B (n=16)
C (n=14)
surface

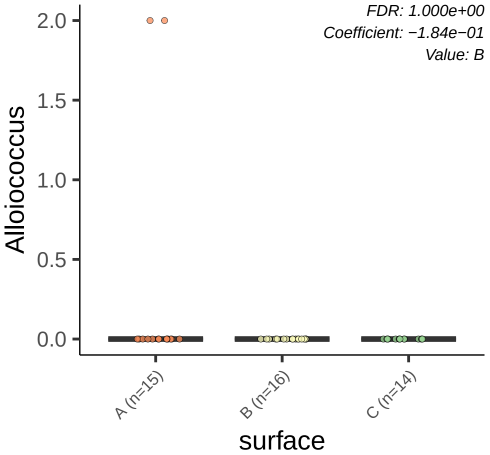
FDR: 1.000e+00
Coefficient: −1.84e−01
Value: B
Alloiococcus
2.0
1.5
1.0
0.5
0.0
A (n=15)
B (n=16)
C (n=14)
surface

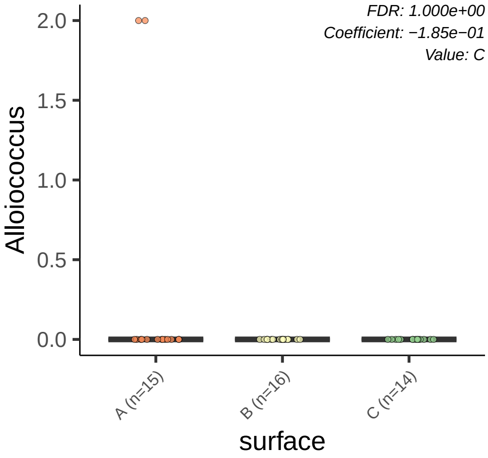
FDR: 1.000e+00
Coefficient: −1.85e−01
Value: C
2.0
1.5
1.0
0.5
0.0
Alloiococcus
A (n=15)
B (n=16)
C (n=14)
surface

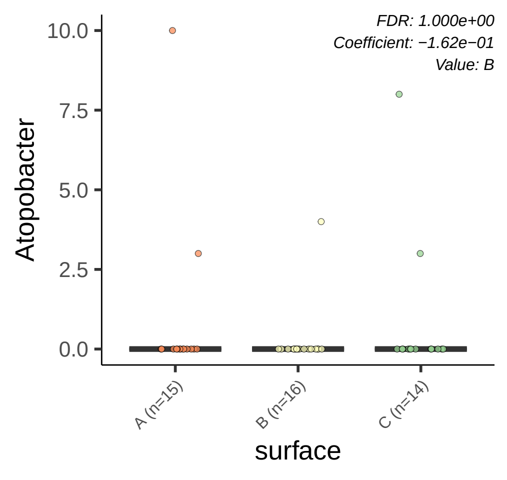
FDR: 1.000e+00
Coefficient: −1.62e−01
Value: B
Atopobacter
10.0
7.5
5.0
2.5
0.0
A (n=15)
B (n=16)
C (n=14)
surface

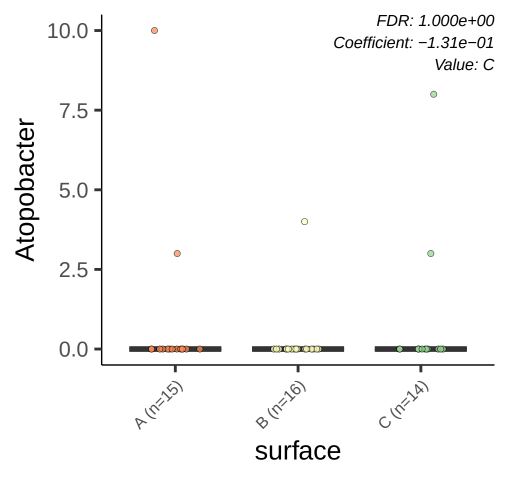
FDR: 1.000e+00
Coefficient: −1.31e−01
Value: C
Atopobacter
10.0
7.5
5.0
2.5
0.0
A (n=15)
B (n=16)
C (n=14)
surface

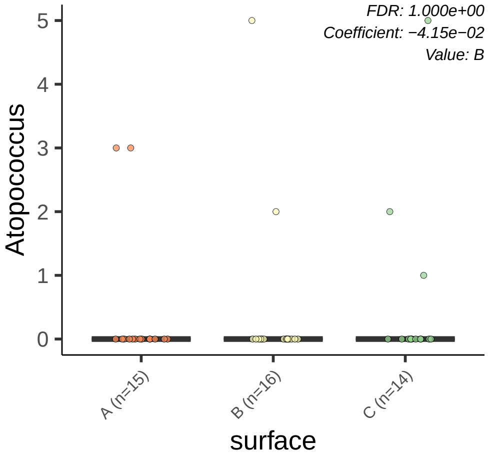

FDR: 1.000e+00
Coefficient: −4.15e−02
Value: B
Atopococcus
5
4
3
2
1
0
A (n=15)
B (n=16)
C (n=14)
surface

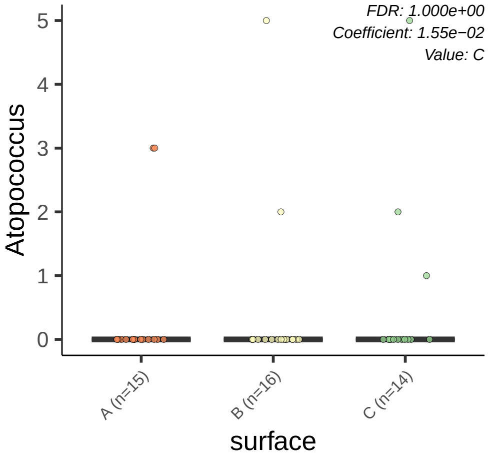
FDR: 1.000e+00
Coefficient: 1.55e−02
Value: C
Atopococcus
0
1
2
3
4
5
A (n=15)
B (n=16)
C (n=14)
surface

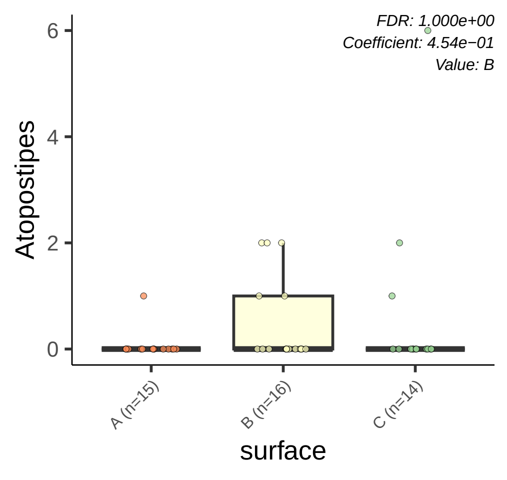

FDR: 1.000e+00
Coefficient: 4.54e−01
Value: B
Atopostipes
0
2
4
6
A (n=15)
B (n=16)
C (n=14)
surface

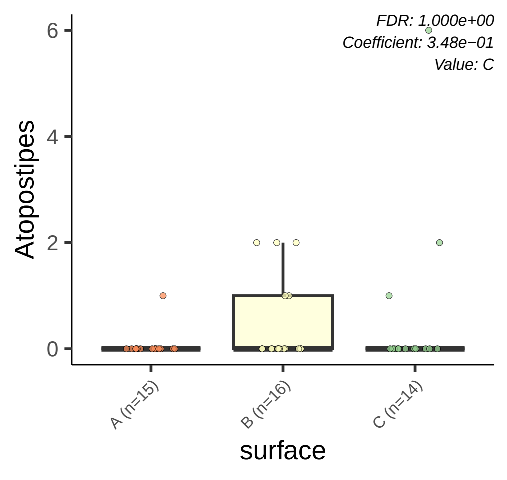
FDR: 1.000e+00
Coefficient: 3.48e−01
Value: C
6
4
2
0
Atopostipes
A (n=15)
B (n=16)
C (n=14)
surface

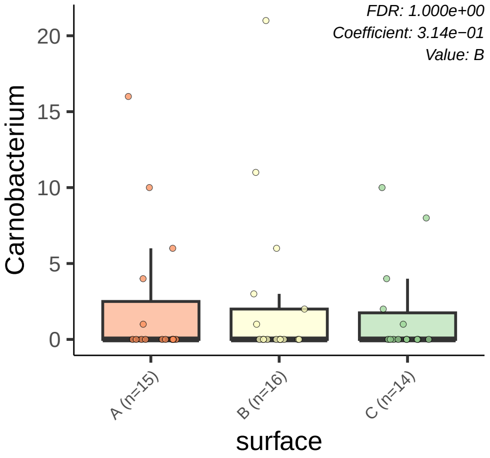
FDR: 1.000e+00
Coefficient: 3.14e−01
Value: B
Carnobacterium
0
5
10
15
20
A (n=15)
B (n=16)
C (n=14)
surface

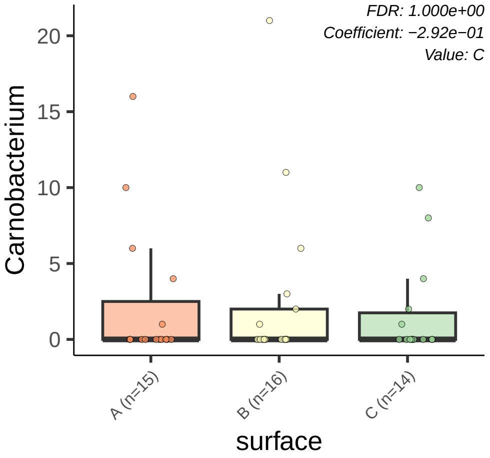
FDR: 1.000e+00
Coefficient: −2.92e−01
Value: C
Carnobacterium
20
15
10
5
0
A (n=15)
B (n=16)
C (n=14)
surface

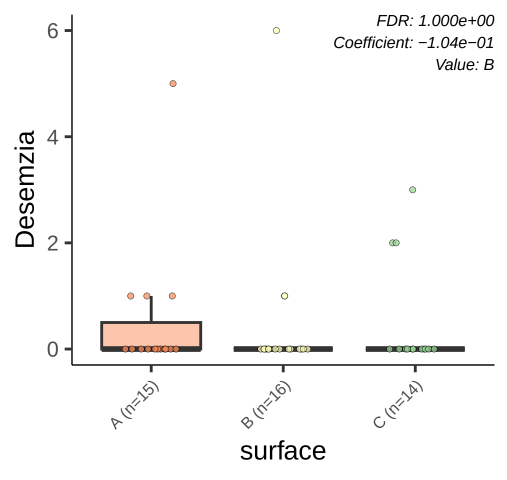
FDR: 1.000e+00
Coefficient: −1.04e−01
Value: B
Desemzia
6
4
2
0
A (n=15)
B (n=16)
C (n=14)
surface

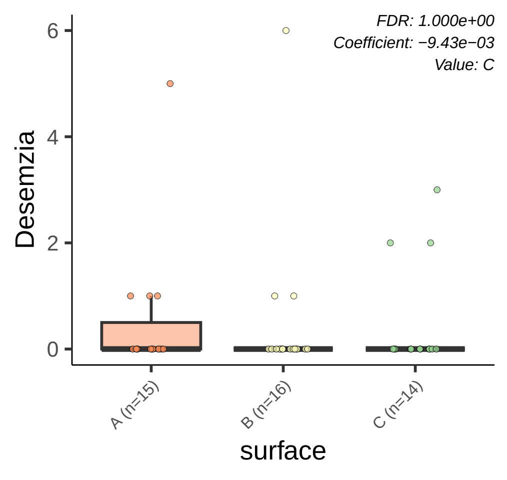
FDR: 1.000e+00
Coefficient: −9.43e−03
Value: C
Desemzia
6
4
2
0
A (n=15)
B (n=16)
C (n=14)
surface

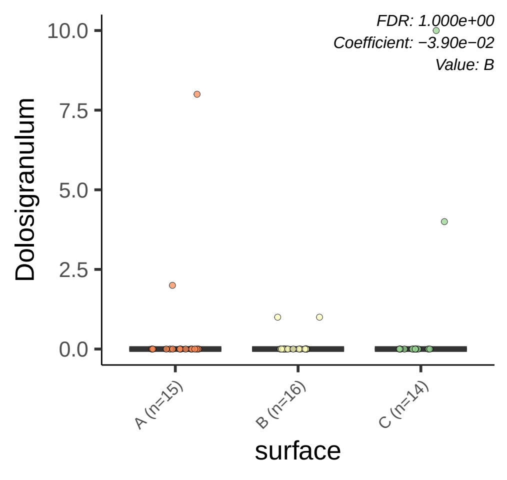
FDR: 1.000e+00
Coefficient: −3.90e−02
Value: B
Dolosigranulum
10.0
7.5
5.0
2.5
0.0
A (n=15)
B (n=16)
C (n=14)
surface

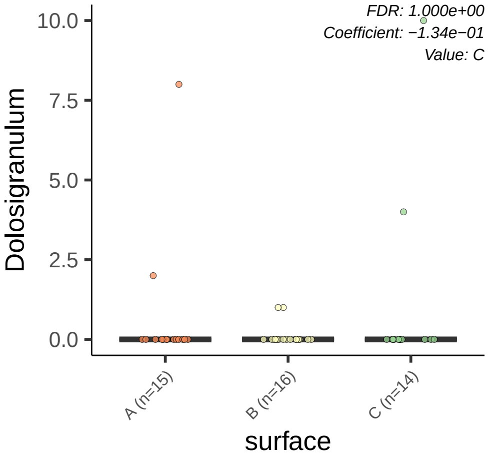

FDR: 1.000e+00
Coefficient: −1.34e−01
Value: C
Dolosigranulum
10.0
7.5
5.0
2.5
0.0
A (n=15)
B (n=16)
C (n=14)
surface

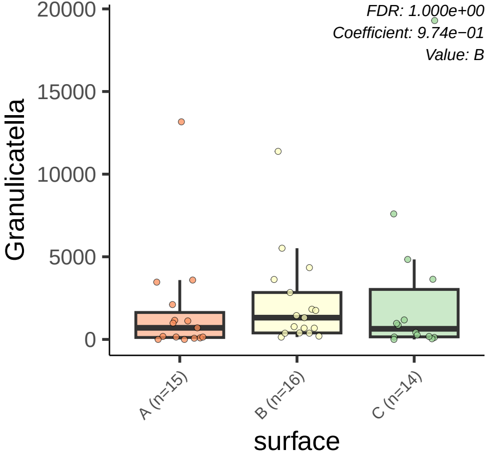

FDR: 1.000e+00
Coefficient: 9.74e−01
Value: B
20000
15000
10000
5000
0
Granulicatella
A (n=15)
B (n=16)
C (n=14)
surface

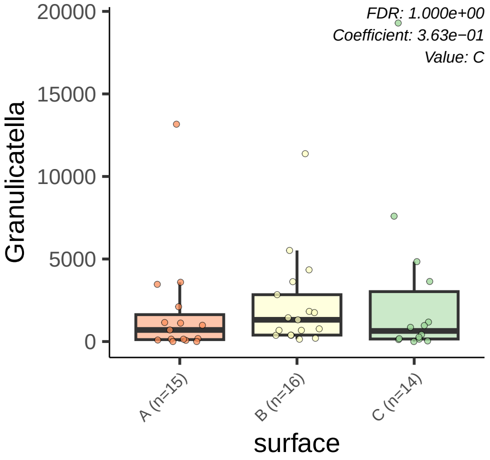
FDR: 1.000e+00
Coefficient: 3.63e−01
Value: C
Granulicatella
20000
15000
10000
5000
0
A (n=15)
B (n=16)
C (n=14)
surface

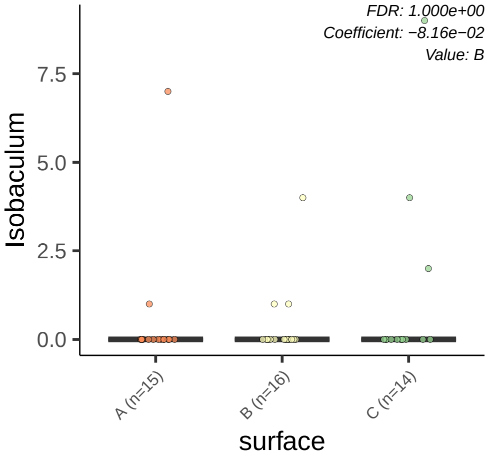

FDR: 1.000e+00
Coefficient: −8.16e−02
Value: B
7.5
5.0
2.5
0.0
Isobaculum
A (n=15)
B (n=16)
C (n=14)
surface

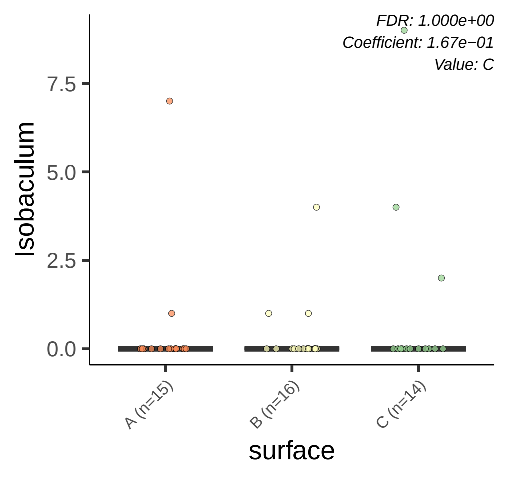
FDR: 1.000e+00
Coefficient: 1.67e−01
Value: C
Isobaculum
7.5
5.0
2.5
0.0
A (n=15)
B (n=16)
C (n=14)
surface

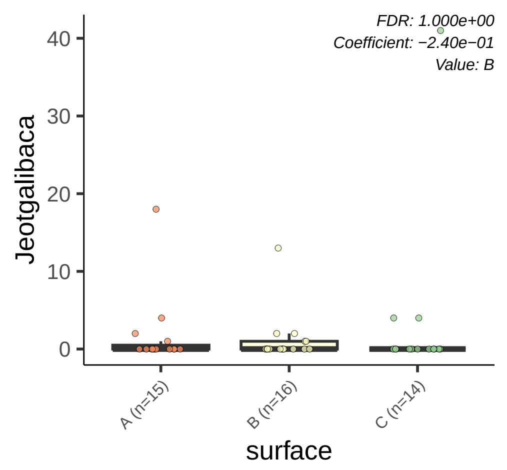
FDR: 1.000e+00
Coefficient: −2.40e−01
Value: B
Jeotgalibaca
0
10
20
30
40
A (n=15)
B (n=16)
C (n=14)
surface

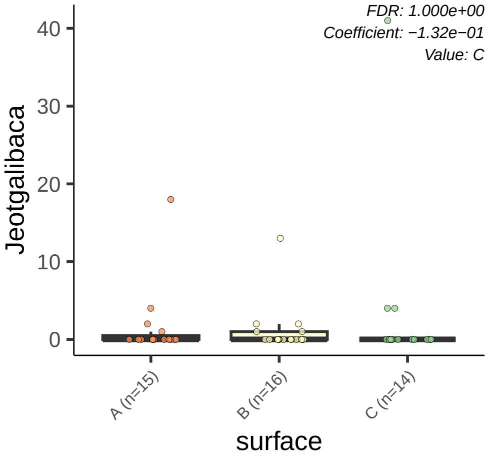

FDR: 1.000e+00
Coefficient: −1.32e−01
Value: C
Jeotgalibaca
40
30
20
10
0
A (n=15)
B (n=16)
C (n=14)
surface

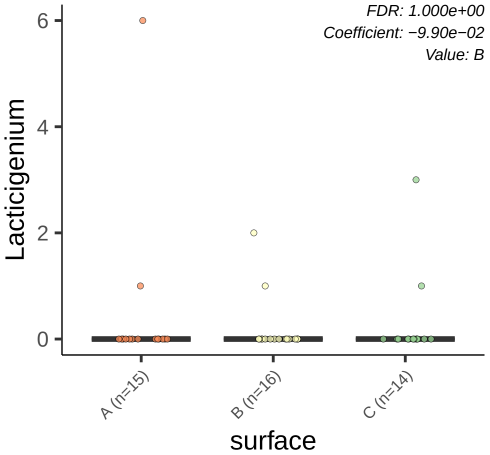

FDR: 1.000e+00
Coefficient: −9.90e−02
Value: B
Lacticigenium
6
4
2
0
A (n=15)
B (n=16)
C (n=14)
surface

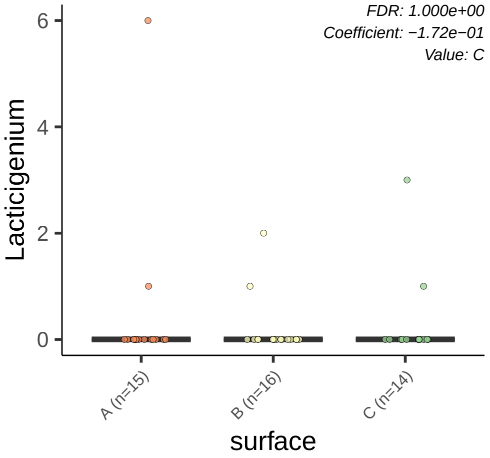
FDR: 1.000e+00
Coefficient: −1.72e−01
Value: C
Lacticigenium
0
2
4
6
A (n=15)
B (n=16)
C (n=14)
surface

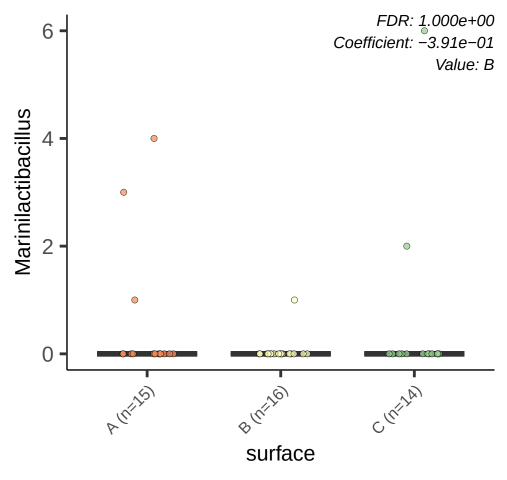

FDR: 1.000e+00
Coefficient: −3.91e−01
Value: B
Marinilactibacillus
6
4
2
0
A (n=15)
B (n=16)
C (n=14)
surface

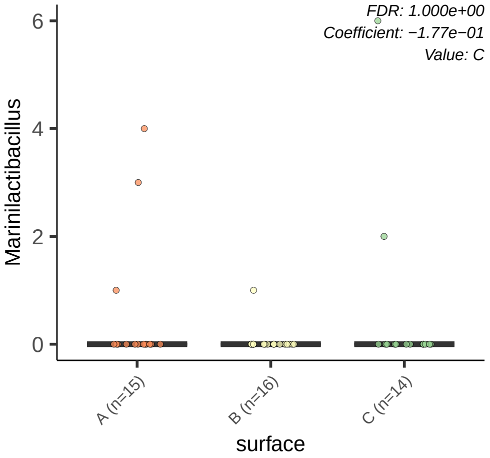
FDR: 1.000e+00
Coefficient: −1.77e−01
Value: C
Marinilactibacillus
6
4
2
0
A (n=15)
B (n=16)
C (n=14)
surface

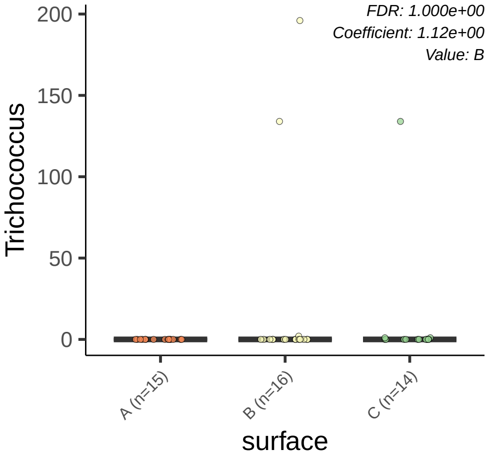
FDR: 1.000e+00
Coefficient: 1.12e+00
Value: B
Trichococcus
200
150
100
50
0
A (n=15)
B (n=16)
C (n=14)
surface

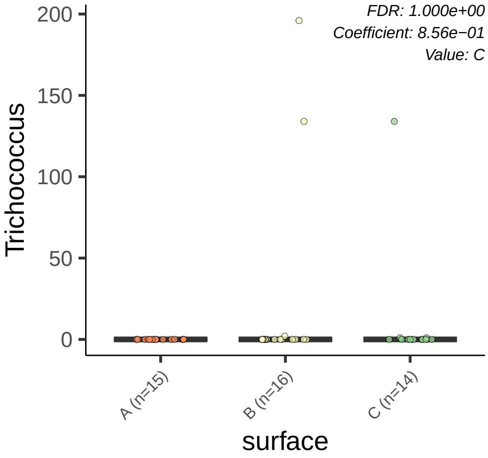

FDR: 1.000e+00
Coefficient: 8.56e−01
Value: C
Trichococcus
200
150
100
50
0
A (n=15)
B (n=16)
C (n=14)
surface

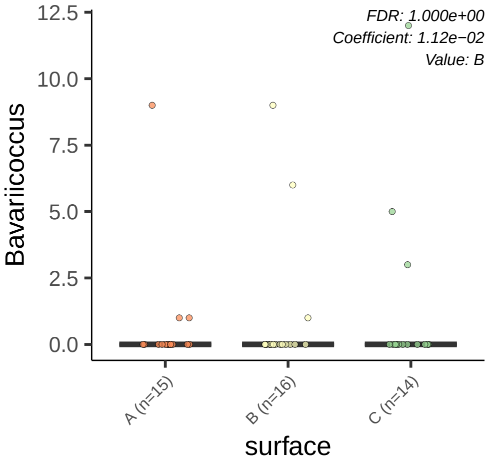

FDR: 1.000e+00
Coefficient: 1.12e−02
Value: B
Bavariicoccus
12.5
10.0
7.5
5.0
2.5
0.0
A (n=15)
B (n=16)
C (n=14)
surface

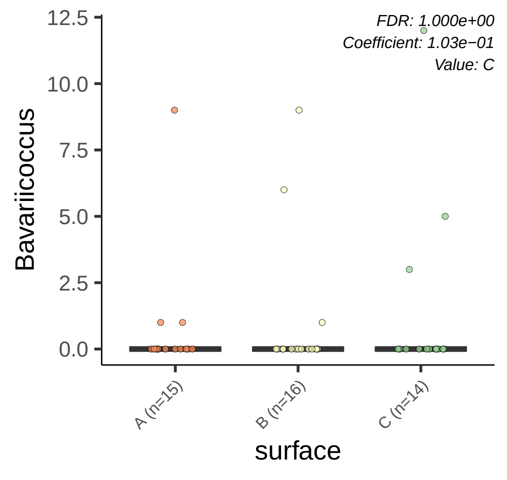

FDR: 1.000e+00
Coefficient: 1.03e−01
Value: C
Bavariicoccus
12.5
10.0
7.5
5.0
2.5
0.0
A (n=15)
B (n=16)
C (n=14)
surface

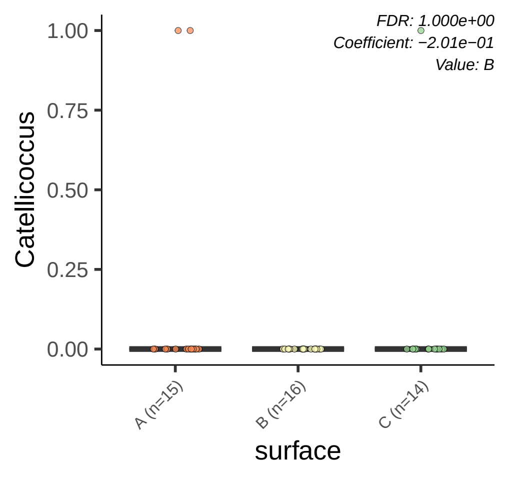
FDR: 1.000e+00
Coefficient: −2.01e−01
Value: B
Catellicoccus
1.00
0.75
0.50
0.25
0.00
A (n=15)
B (n=16)
C (n=14)
surface

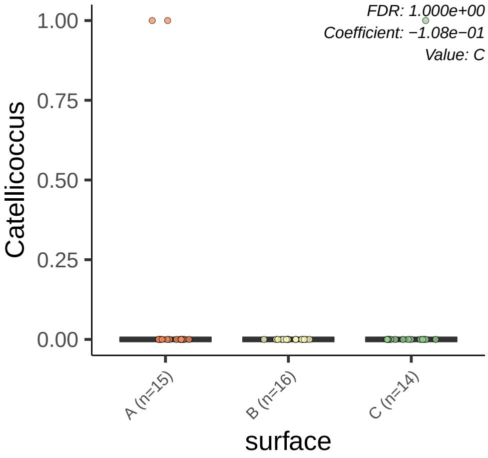

FDR: 1.000e+00
Coefficient: −1.08e−01
Value: C
Catellicoccus
1.00
0.75
0.50
0.25
0.00
A (n=15)
B (n=16)
C (n=14)
surface

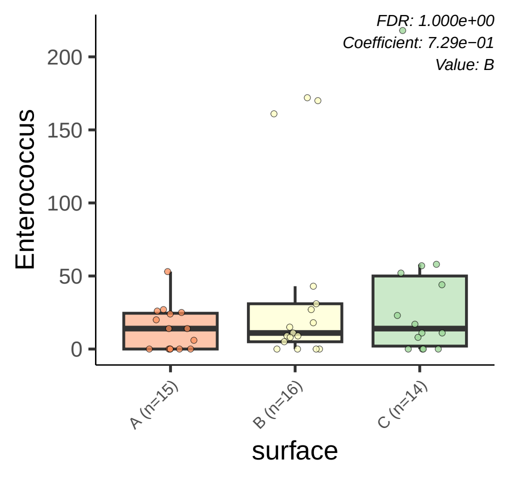
FDR: 1.000e+00
Coefficient: 7.29e−01
Value: B
Enterococcus
200
150
100
50
0
A (n=15)
B (n=16)
C (n=14)
surface

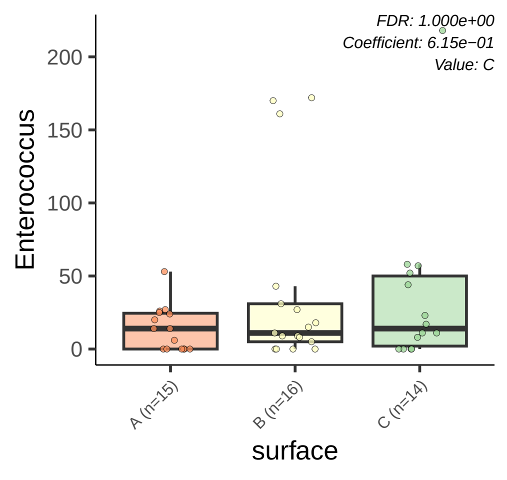
FDR: 1.000e+00
Coefficient: 6.15e−01
Value: C
Enterococcus
200
150
100
50
0
A (n=15)
B (n=16)
C (n=14)
surface

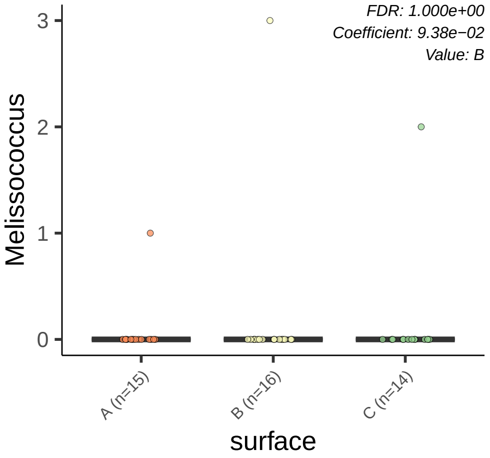

FDR: 1.000e+00
Coefficient: 9.38e−02
Value: B
Melissococcus
3
2
1
0
A (n=15)
B (n=16)
C (n=14)
surface

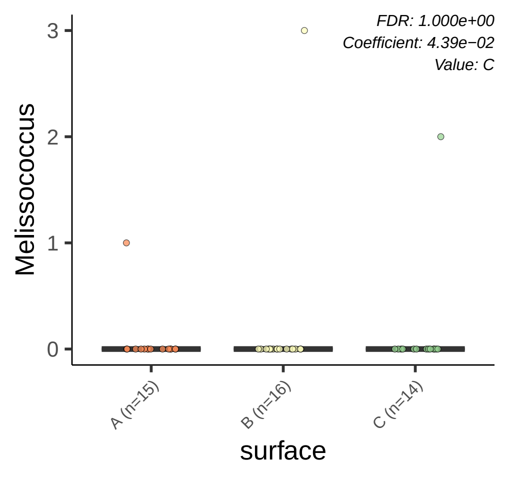
FDR: 1.000e+00
Coefficient: 4.39e−02
Value: C
Melissococcus
0
1
2
3
A (n=15)
B (n=16)
C (n=14)
surface

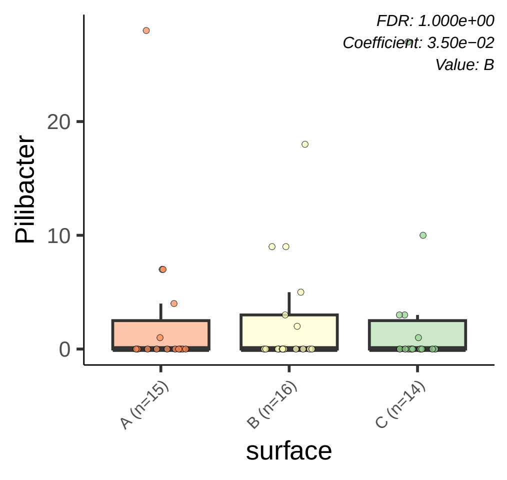
FDR: 1.000e+00
Coefficient: 3.50e−02
Value: B
Pilibacter
20
10
0
A (n=15)
B (n=16)
C (n=14)
surface

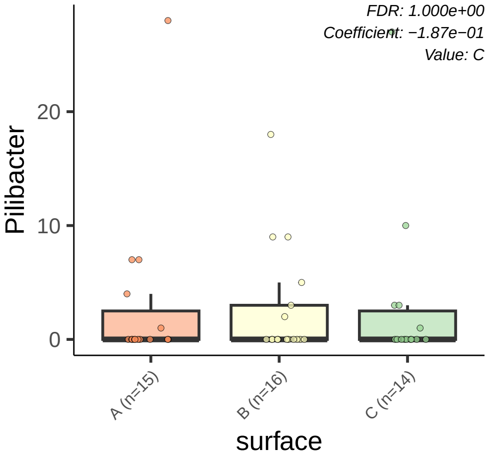
FDR: 1.000e+00
Coefficient: −1.87e−01
Value: C
Pilibacter
0
10
20
A (n=15)
B (n=16)
C (n=14)
surface

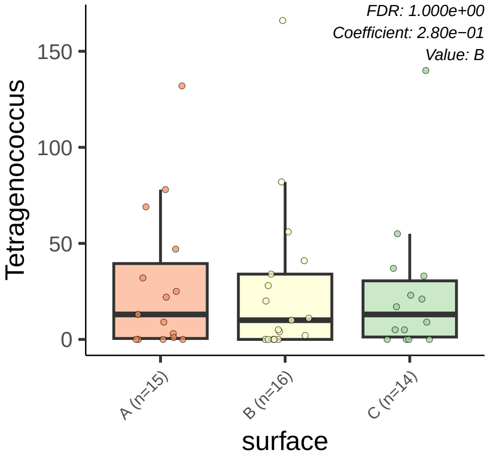
FDR: 1.000e+00
Coefficient: 2.80e−01
Value: B
Tetragenococcus
150
100
50
0
A (n=15)
B (n=16)
C (n=14)
surface

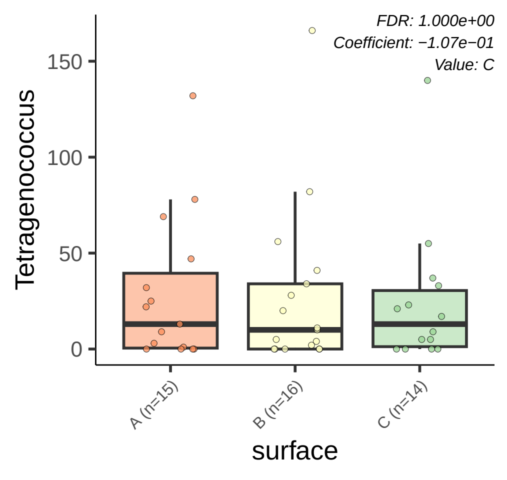

FDR: 1.000e+00
Coefficient: −1.07e−01
Value: C
Tetragenococcus
150
100
50
0
A (n=15)
B (n=16)
C (n=14)
surface

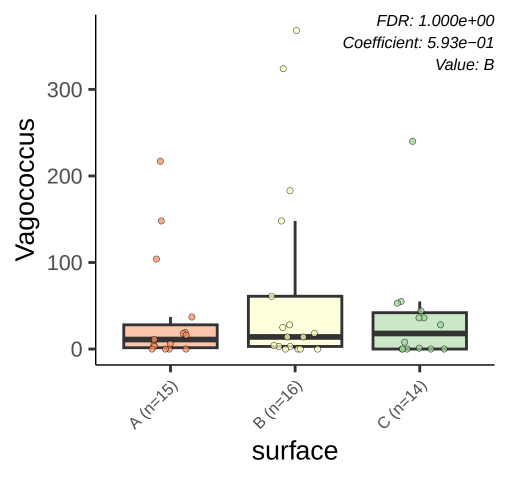
FDR: 1.000e+00
Coefficient: 5.93e−01
Value: B
Vagococcus
300
200
100
0
A (n=15)
B (n=16)
C (n=14)
surface

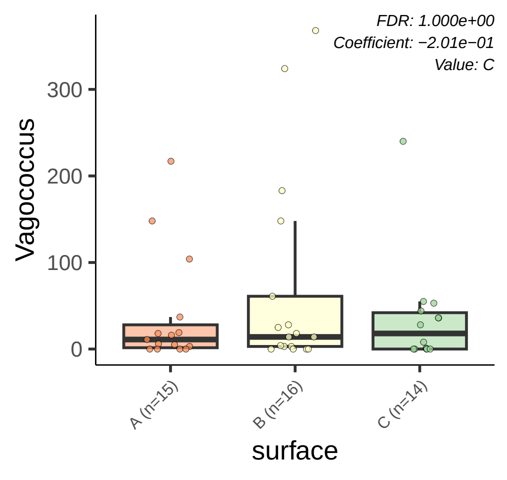

FDR: 1.000e+00
Coefficient: −2.01e−01
Value: C
Vagococcus
300
200
100
0
A (n=15)
B (n=16)
C (n=14)
surface

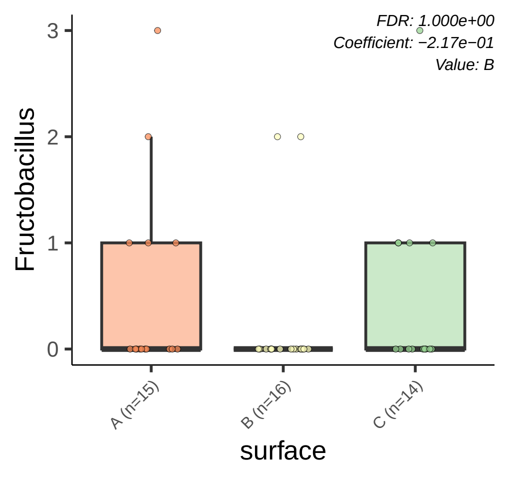

FDR: 1.000e+00
Coefficient: −2.17e−01
Value: B
Fructobacillus
0
1
2
3
A (n=15)
B (n=16)
C (n=14)
surface

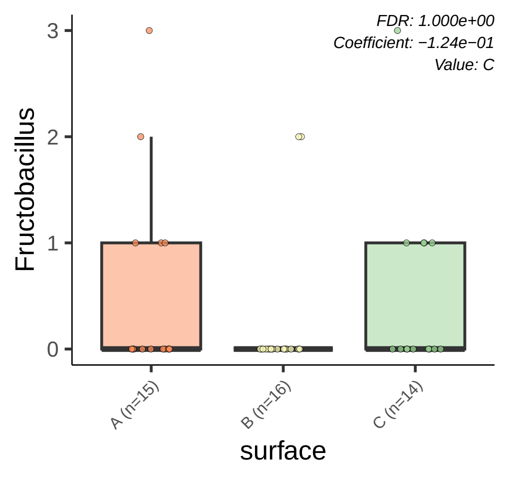
FDR: 1.000e+00
Coefficient: −1.24e−01
Value: C
Fructobacillus
3
2
1
0
A (n=15)
B (n=16)
C (n=14)
surface

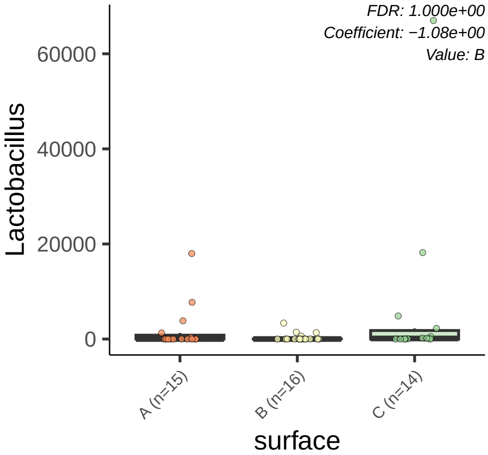

FDR: 1.000e+00
Coefficient: −1.08e+00
Value: B
Lactobacillus
60000
40000
20000
0
A (n=15)
B (n=16)
C (n=14)
surface

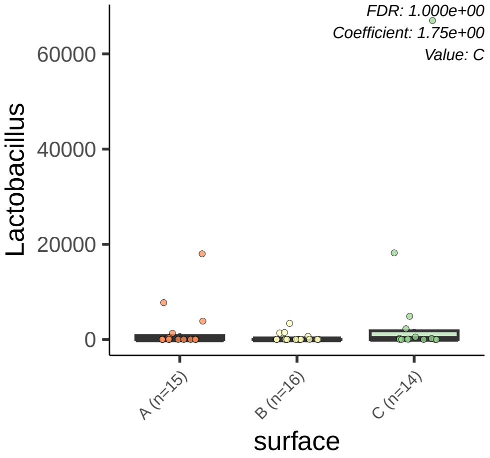
FDR: 1.000e+00
Coefficient: 1.75e+00
Value: C
Lactobacillus
60000
40000
20000
0
A (n=15)
B (n=16)
C (n=14)
surface

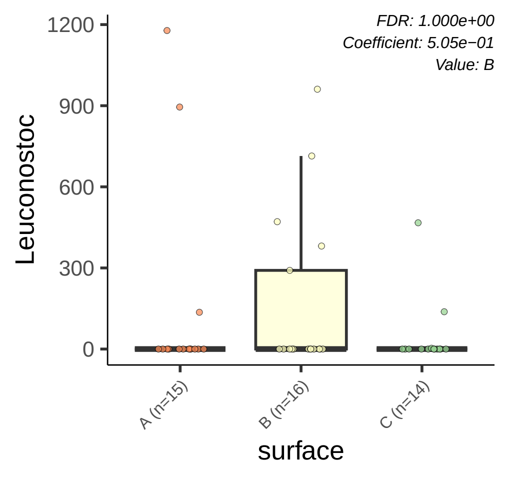
FDR: 1.000e+00
Coefficient: 5.05e−01
Value: B
1200
900
600
300
0
Leuconostoc
A (n=15)
B (n=16)
C (n=14)
surface

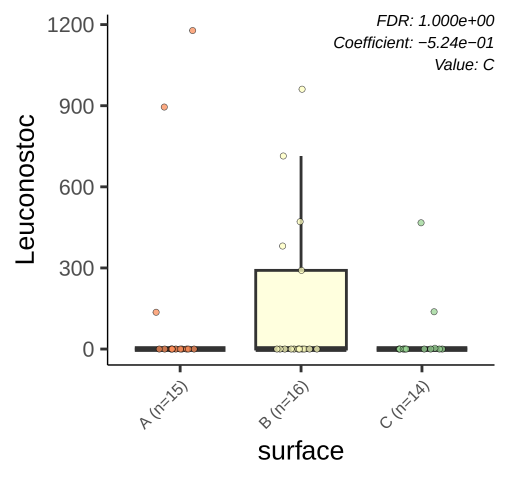

FDR: 1.000e+00
Coefficient: −5.24e−01
Value: C
Leuconostoc
1200
900
600
300
0
A (n=15)
B (n=16)
C (n=14)
surface

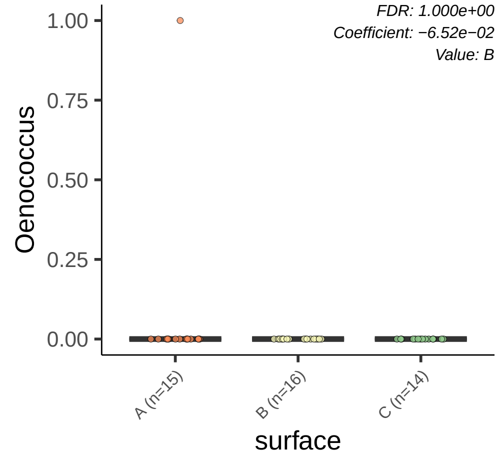
FDR: 1.000e+00
Coefficient: −6.52e−02
Value: B
1.00
0.75
0.50
0.25
0.00
Oenococcus
A (n=15)
B (n=16)
C (n=14)
surface

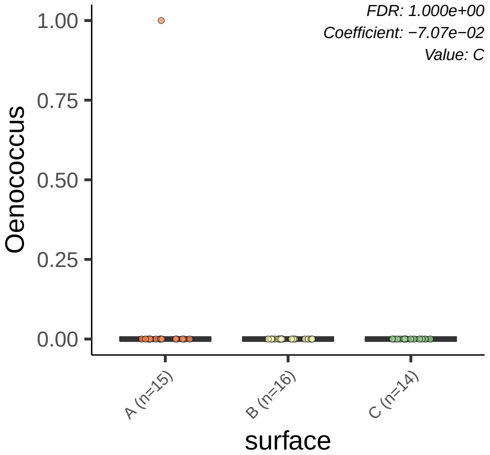
FDR: 1.000e+00
Coefficient: −7.07e−02
Value: C
Oenococcus
1.00
0.75
0.50
0.25
0.00
A (n=15)
B (n=16)
C (n=14)
surface

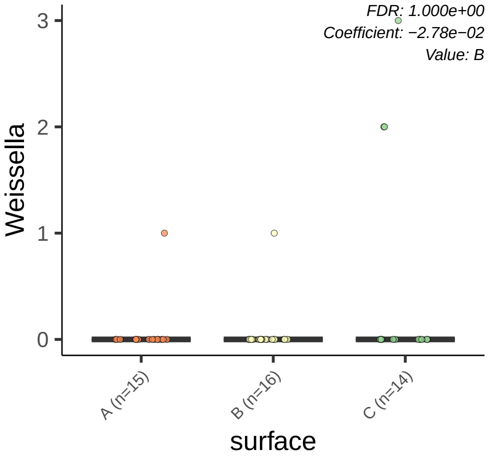
FDR: 1.000e+00
Coefficient: −2.78e−02
Value: B
Weissella
3
2
1
0
A (n=15)
B (n=16)
C (n=14)
surface

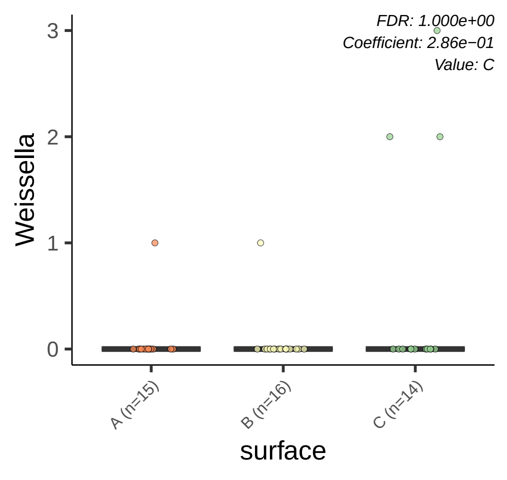
FDR: 1.000e+00
Coefficient: 2.86e−01
Value: C
Weissella
0
1
2
3
A (n=15)
B (n=16)
C (n=14)
surface

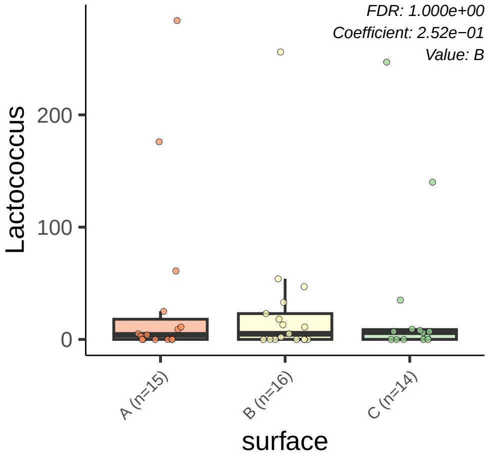

FDR: 1.000e+00
Coefficient: 2.52e−01
Value: B
Lactococcus
200
100
0
A (n=15)
B (n=16)
C (n=14)
surface

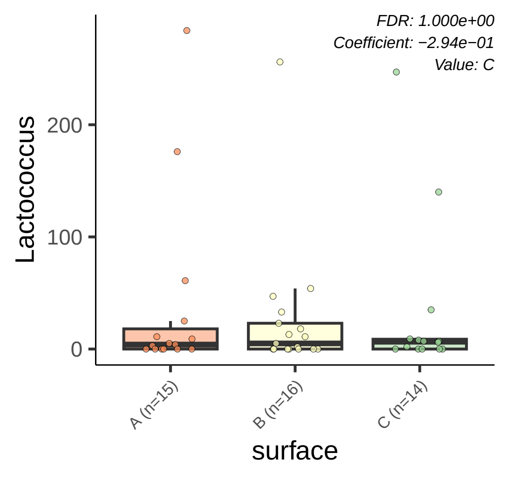
FDR: 1.000e+00
Coefficient: −2.94e−01
Value: C
Lactococcus
200
100
0
A (n=15)
B (n=16)
C (n=14)
surface

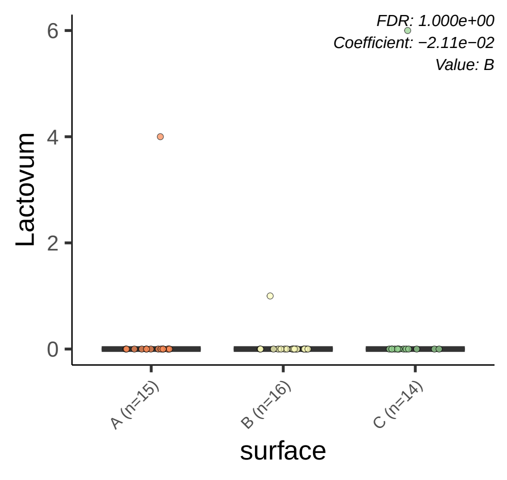
FDR: 1.000e+00
Coefficient: −2.11e−02
Value: B
Lactovum
6
4
2
0
A (n=15)
B (n=16)
C (n=14)
surface

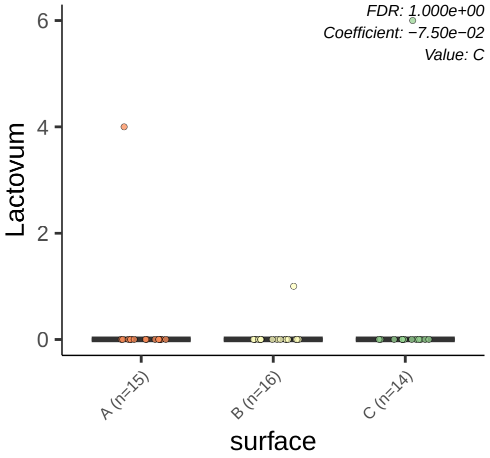
FDR: 1.000e+00
Coefficient: −7.50e−02
Value: C
Lactovum
6
4
2
0
A (n=15)
B (n=16)
C (n=14)
surface

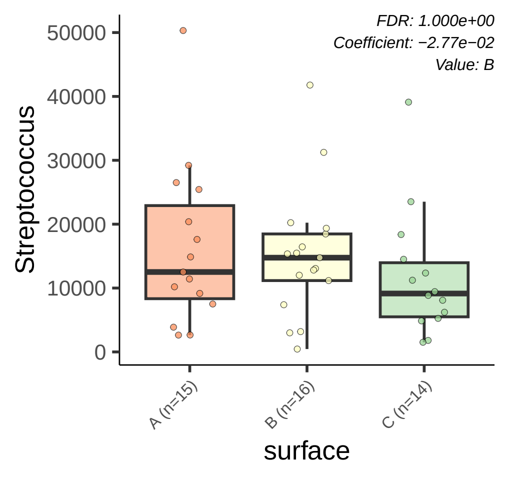
FDR: 1.000e+00
Coefficient: −2.77e−02
Value: B
Streptococcus
50000
40000
30000
20000
10000
0
A (n=15)
B (n=16)
C (n=14)
surface

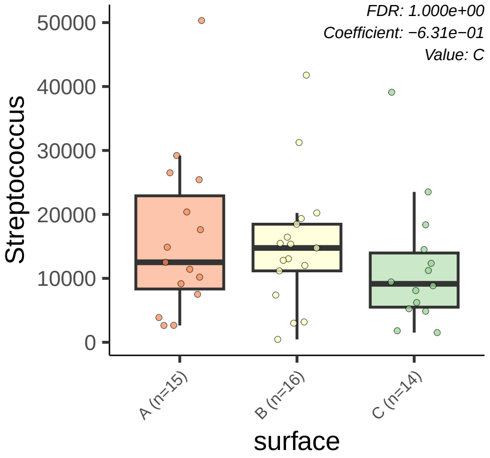
FDR: 1.000e+00
Coefficient: −6.31e−01
Value: C
Streptococcus
50000
40000
30000
20000
10000
0
A (n=15)
B (n=16)
C (n=14)
surface

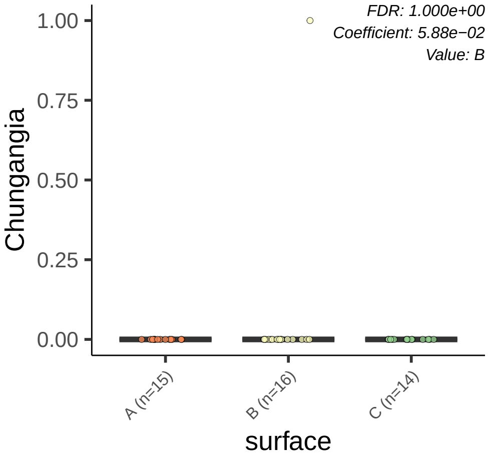
FDR: 1.000e+00
Coefficient: 5.88e−02
Value: B
Chungangia
1.00
0.75
0.50
0.25
0.00
A (n=15)
B (n=16)
C (n=14)
surface

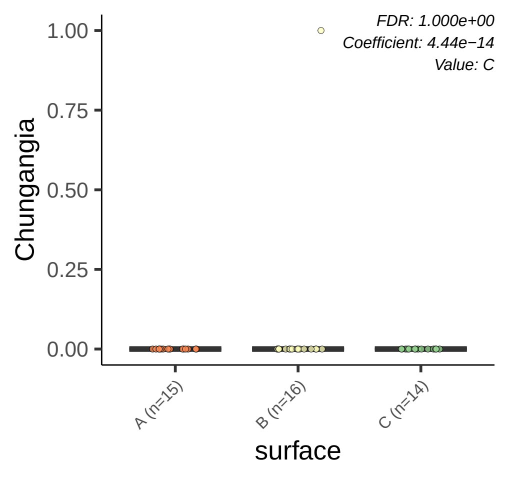

FDR: 1.000e+00
Coefficient: 4.44e−14
Value: C
1.00
0.75
0.50
0.25
0.00
Chungangia
A (n=15)
B (n=16)
C (n=14)
surface

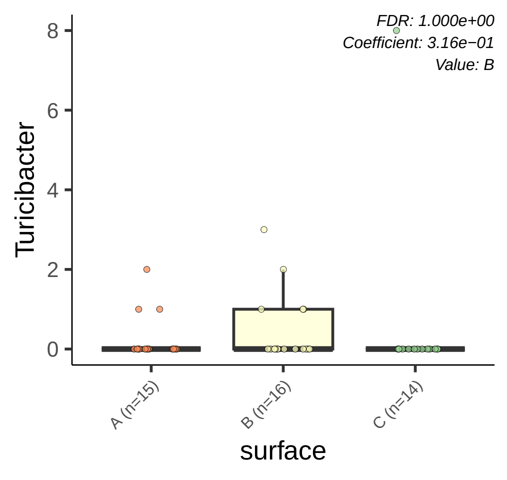
FDR: 1.000e+00
Coefficient: 3.16e−01
Value: B
Turicibacter
0
2
4
6
8
A (n=15)
B (n=16)
C (n=14)
surface

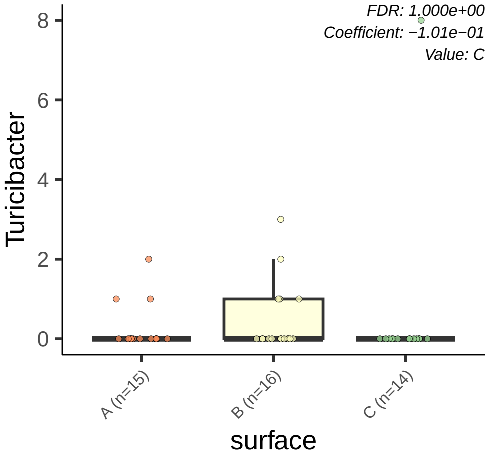
FDR: 1.000e+00
Coefficient: −1.01e−01
Value: C
Turicibacter
0
2
4
6
8
A (n=15)
B (n=16)
C (n=14)
surface

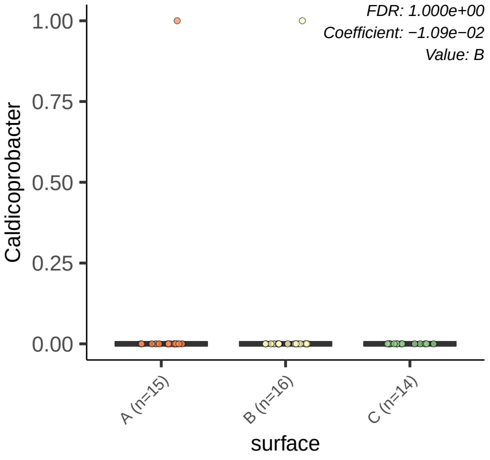

FDR: 1.000e+00
Coefficient: −1.09e−02
Value: B
Caldicoprobacter
1.00
0.75
0.50
0.25
0.00
A (n=15)
B (n=16)
C (n=14)
surface

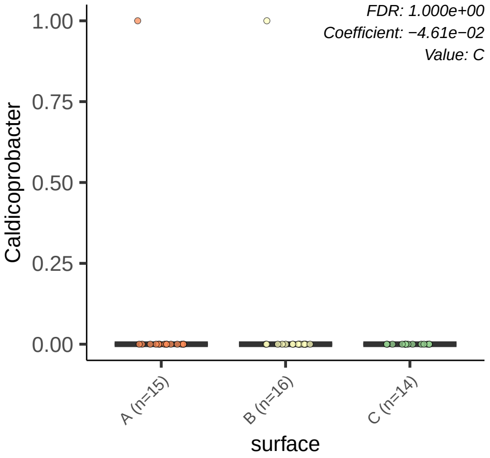
FDR: 1.000e+00
Coefficient: −4.61e−02
Value: C
Caldicoprobacter
1.00
0.75
0.50
0.25
0.00
A (n=15)
B (n=16)
C (n=14)
surface

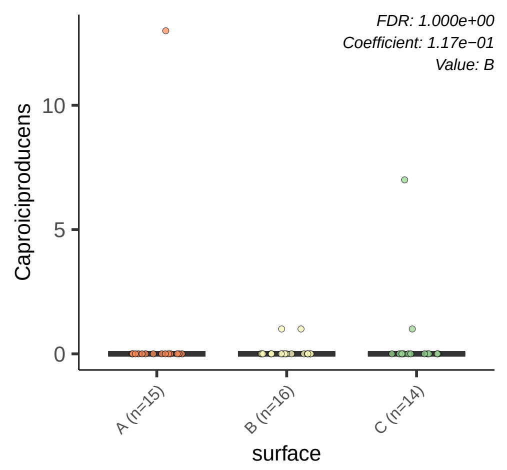

FDR: 1.000e+00
Coefficient: 1.17e−01
Value: B
Caproiciproducens
10
5
0
A (n=15)
B (n=16)
C (n=14)
surface

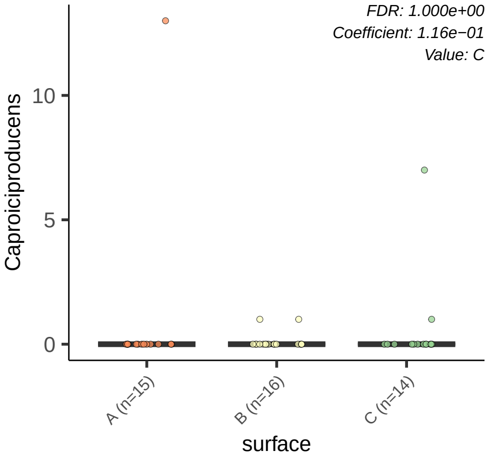
FDR: 1.000e+00
Coefficient: 1.16e−01
Value: C
Caproiciproducens
10
5
0
A (n=15)
B (n=16)
C (n=14)
surface

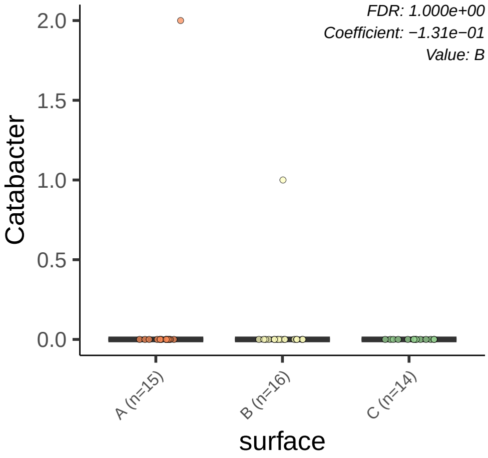

FDR: 1.000e+00
Coefficient: −1.31e−01
Value: B
Catabacter
2.0
1.5
1.0
0.5
0.0
A (n=15)
B (n=16)
C (n=14)
surface

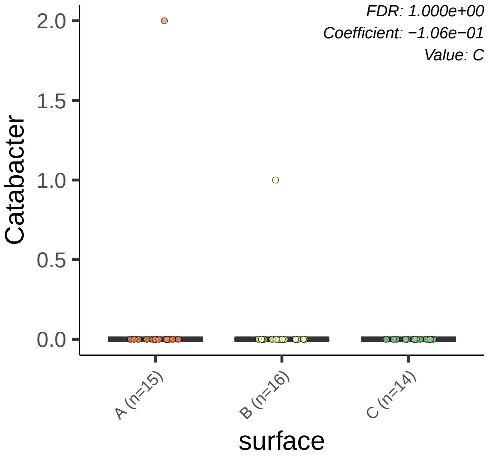
FDR: 1.000e+00
Coefficient: −1.06e−01
Value: C
Catabacter
2.0
1.5
1.0
0.5
0.0
A (n=15)
B (n=16)
C (n=14)
surface

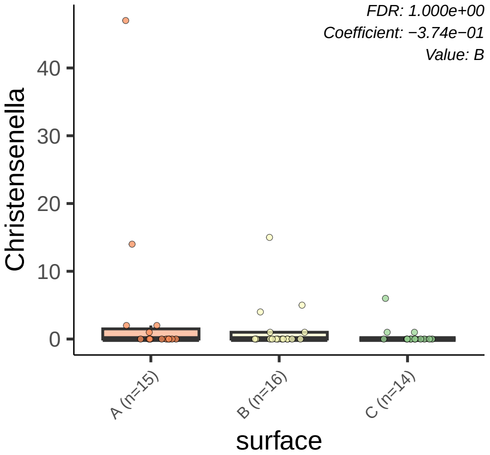

FDR: 1.000e+00
Coefficient: −3.74e−01
Value: B
Christensenella
0
10
20
30
40
A (n=15)
B (n=16)
C (n=14)
surface

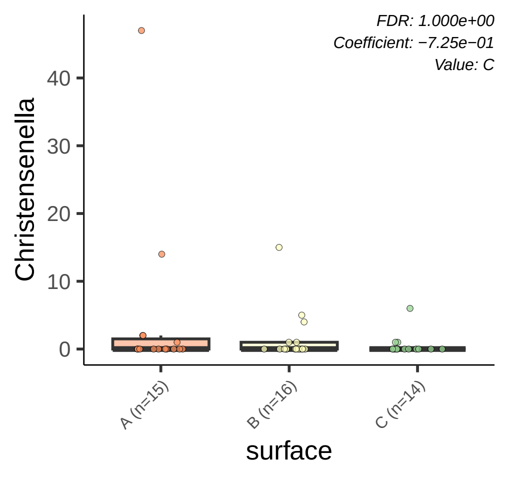

FDR: 1.000e+00
Coefficient: −7.25e−01
Value: C
Christensenella
40
30
20
10
0
A (n=15)
B (n=16)
C (n=14)
surface

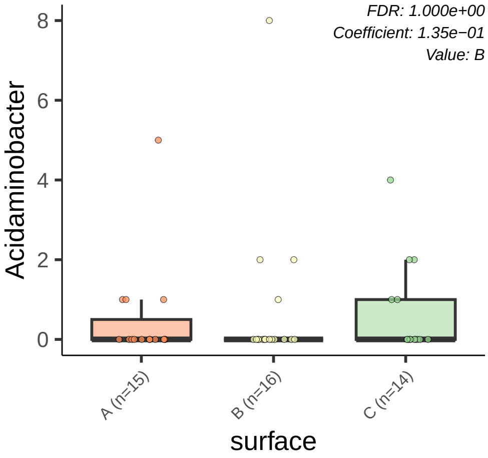

FDR: 1.000e+00
Coefficient: 1.35e−01
Value: B
8
6
4
2
0
Acidaminobacter
A (n=15)
B (n=16)
C (n=14)
surface

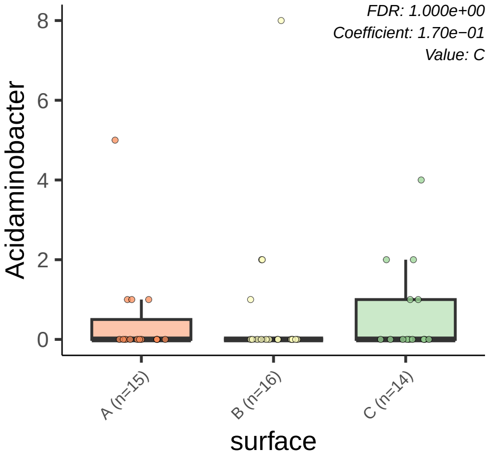

FDR: 1.000e+00
Coefficient: 1.70e−01
Value: C
Acidaminobacter
0
2
4
6
8
A (n=15)
B (n=16)
C (n=14)
surface

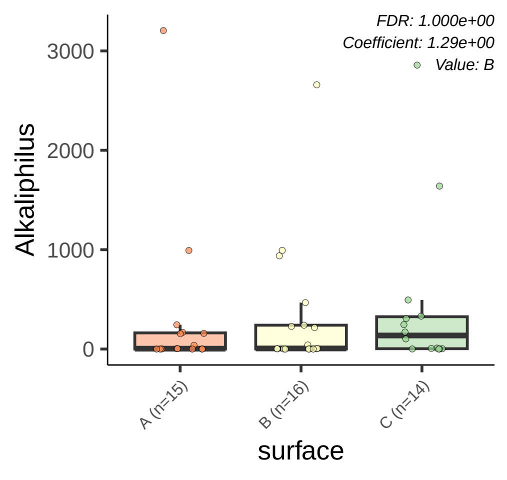
FDR: 1.000e+00
Coefficient: 1.29e+00
Value: B
Alkaliphilus
3000
2000
1000
0
A (n=15)
B (n=16)
C (n=14)
surface

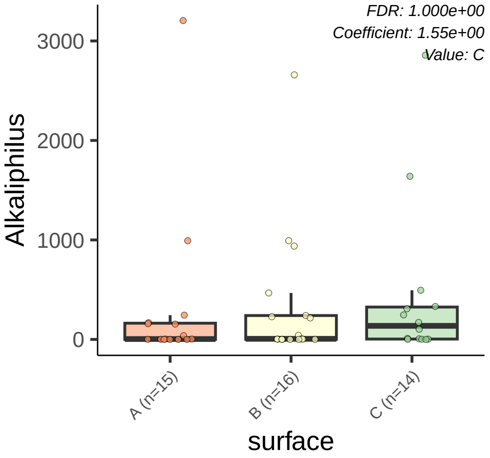
FDR: 1.000e+00
Coefficient: 1.55e+00
Value: C
Alkaliphilus
3000
2000
1000
0
A (n=15)
B (n=16)
C (n=14)
surface

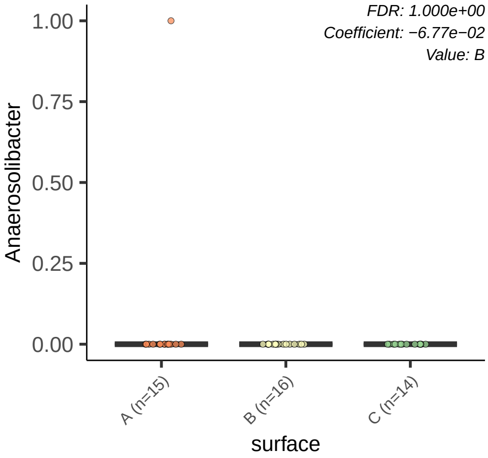

FDR: 1.000e+00
Coefficient: −6.77e−02
Value: B
Anaerosolibacter
1.00
0.75
0.50
0.25
0.00
A (n=15)
B (n=16)
C (n=14)
surface

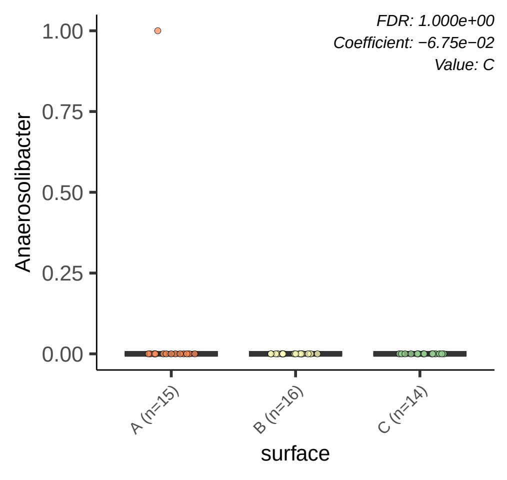
FDR: 1.000e+00
Coefficient: −6.75e−02
Value: C
Anaerosolibacter
1.00
0.75
0.50
0.25
0.00
A (n=15)
B (n=16)
C (n=14)
surface

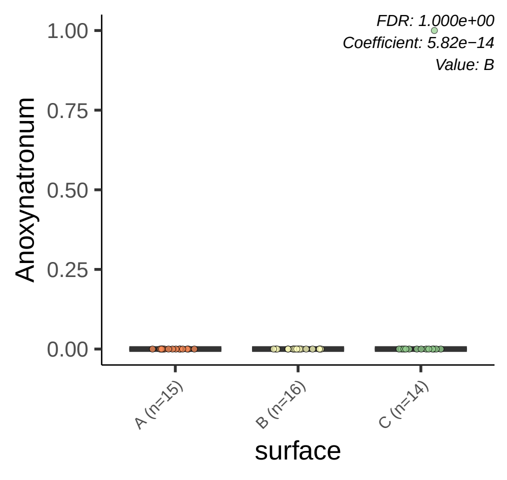
FDR: 1.000e+00
Coefficient: 5.82e−14
Value: B
Anoxynatronum
1.00
0.75
0.50
0.25
0.00
A (n=15)
B (n=16)
C (n=14)
surface

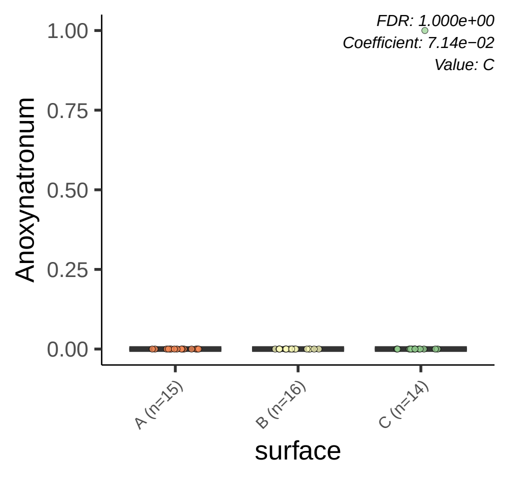
FDR: 1.000e+00
Coefficient: 7.14e−02
Value: C
Anoxynatronum
1.00
0.75
0.50
0.25
0.00
A (n=15)
B (n=16)
C (n=14)
surface

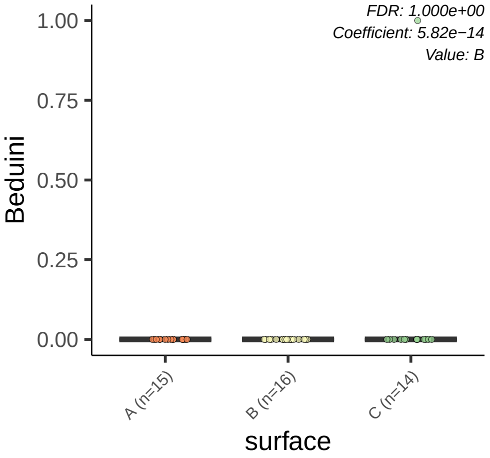
FDR: 1.000e+00
Coefficient: 5.82e−14
Value: B
Beduini
1.00
0.75
0.50
0.25
0.00
A (n=15)
B (n=16)
C (n=14)
surface

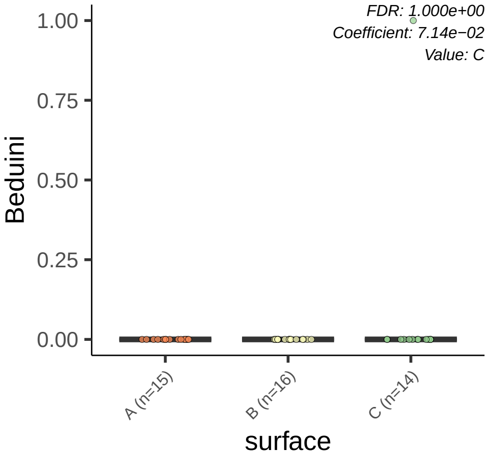
FDR: 1.000e+00
Coefficient: 7.14e−02
Value: C
Beduini
1.00
0.75
0.50
0.25
0.00
A (n=15)
B (n=16)
C (n=14)
surface

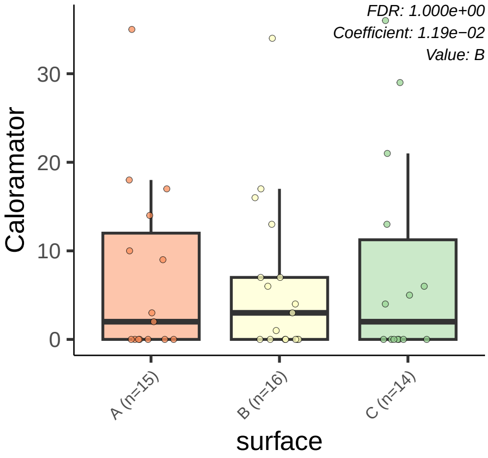
FDR: 1.000e+00
Coefficient: 1.19e−02
Value: B
Caloramator
0
10
20
30
A (n=15)
B (n=16)
C (n=14)
surface

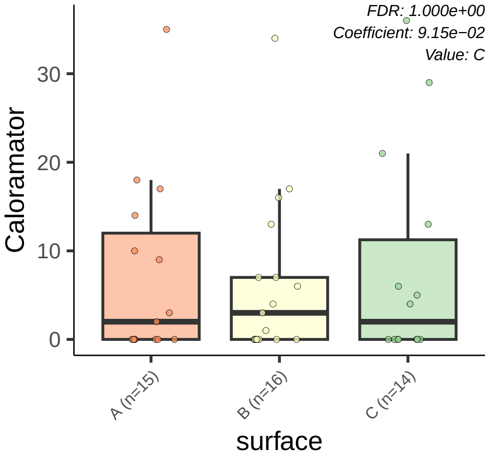

FDR: 1.000e+00
Coefficient: 9.15e−02
Value: C
Caloramator
0
10
20
30
A (n=15)
B (n=16)
C (n=14)
surface

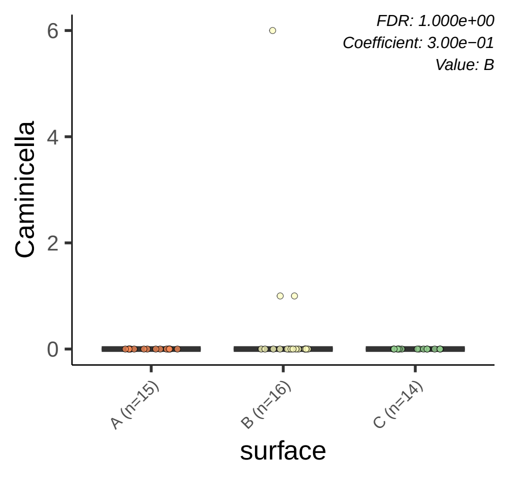
FDR: 1.000e+00
Coefficient: 3.00e−01
Value: B
Caminicella
6
4
2
0
A (n=15)
B (n=16)
C (n=14)
surface

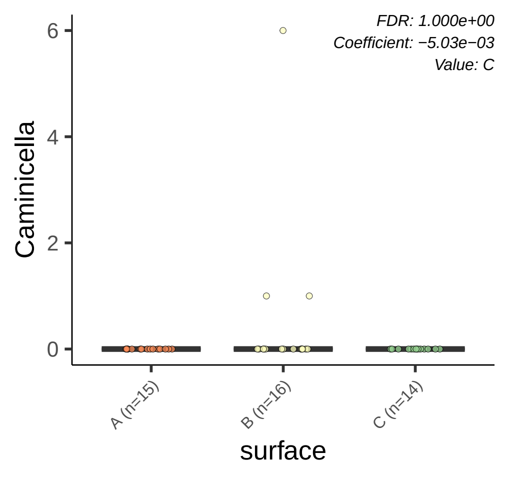

FDR: 1.000e+00
Coefficient: −5.03e−03
Value: C
Caminicella
6
4
2
0
A (n=15)
B (n=16)
C (n=14)
surface

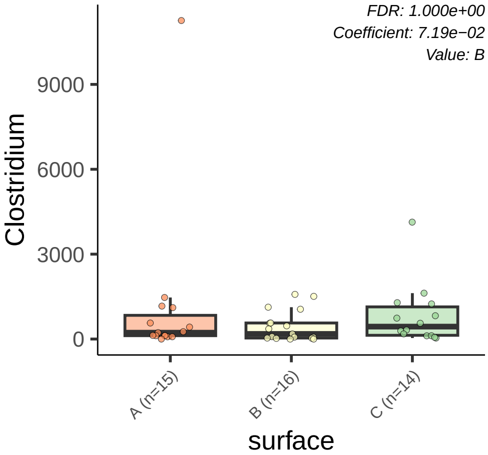
FDR: 1.000e+00
Coefficient: 7.19e−02
Value: B
9000
6000
3000
0
Clostridium
A (n=15)
B (n=16)
C (n=14)
surface

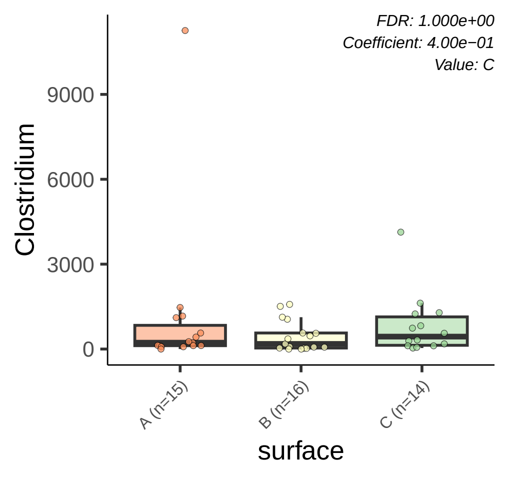
FDR: 1.000e+00
Coefficient: 4.00e−01
Value: C
9000
6000
3000
0
Clostridium
A (n=15)
B (n=16)
C (n=14)
surface

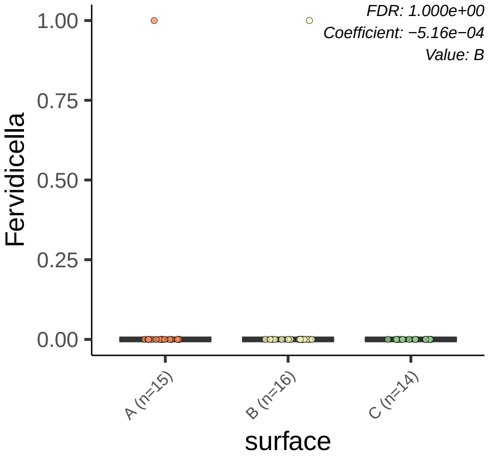
FDR: 1.000e+00
Coefficient: −5.16e−04
Value: B
1.00
0.75
0.50
0.25
0.00
Fervidicella
A (n=15)
B (n=16)
C (n=14)
surface

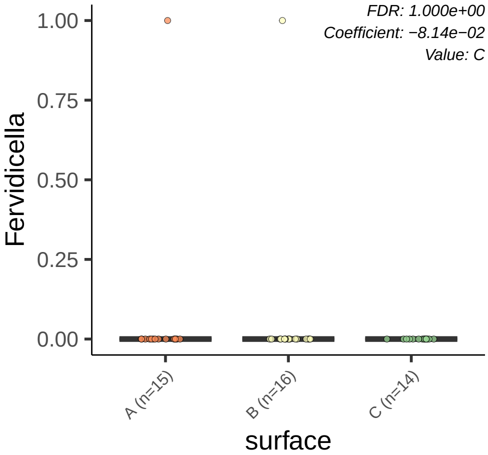

FDR: 1.000e+00
Coefficient: −8.14e−02
Value: C
Fervidicella
1.00
0.75
0.50
0.25
0.00
A (n=15)
B (n=16)
C (n=14)
surface

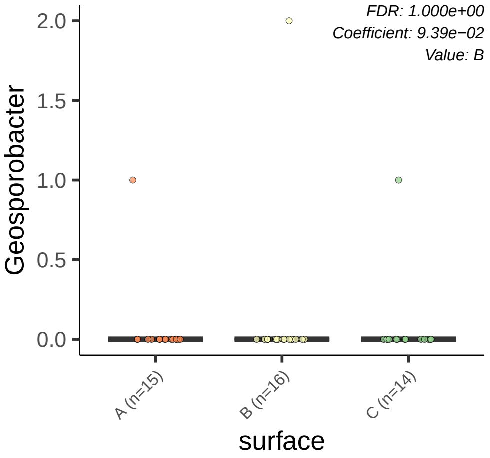

FDR: 1.000e+00
Coefficient: 9.39e−02
Value: B
Geosporobacter
2.0
1.5
1.0
0.5
0.0
A (n=15)
B (n=16)
C (n=14)
surface

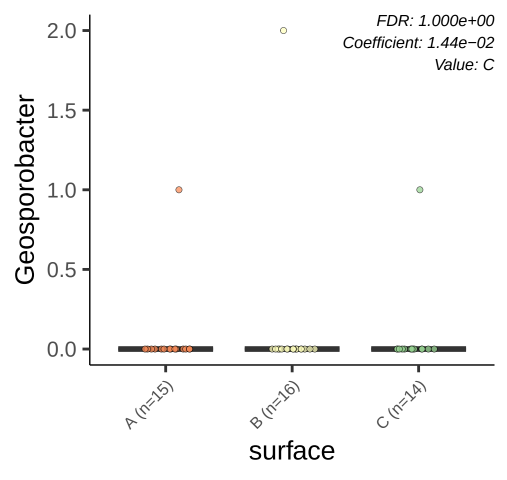
FDR: 1.000e+00
Coefficient: 1.44e−02
Value: C
Geosporobacter
2.0
1.5
1.0
0.5
0.0
A (n=15)
B (n=16)
C (n=14)
surface

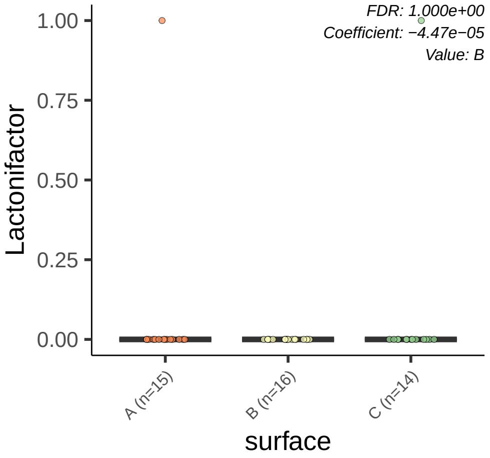
FDR: 1.000e+00
Coefficient: −4.47e−05
Value: B
Lactonifactor
1.00
0.75
0.50
0.25
0.00
A (n=15)
B (n=16)
C (n=14)
surface

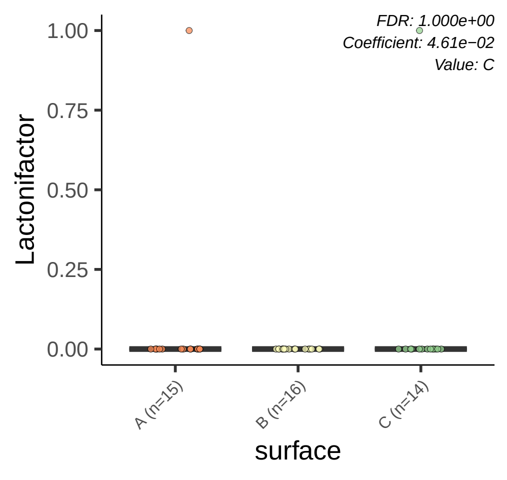
FDR: 1.000e+00
Coefficient: 4.61e−02
Value: C
Lactonifactor
1.00
0.75
0.50
0.25
0.00
A (n=15)
B (n=16)
C (n=14)
surface

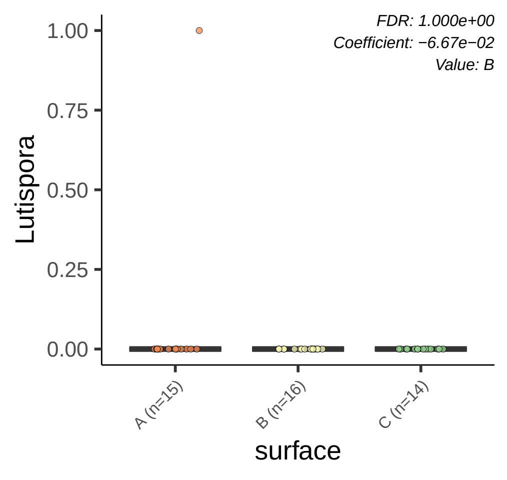
FDR: 1.000e+00
Coefficient: −6.67e−02
Value: B
Lutispora
1.00
0.75
0.50
0.25
0.00
A (n=15)
B (n=16)
C (n=14)
surface

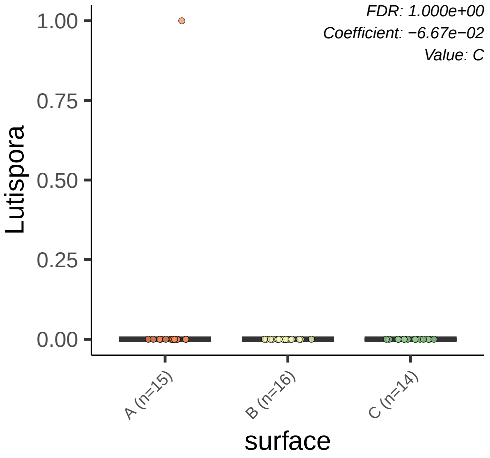
FDR: 1.000e+00
Coefficient: −6.67e−02
Value: C
Lutispora
1.00
0.75
0.50
0.25
0.00
A (n=15)
B (n=16)
C (n=14)
surface

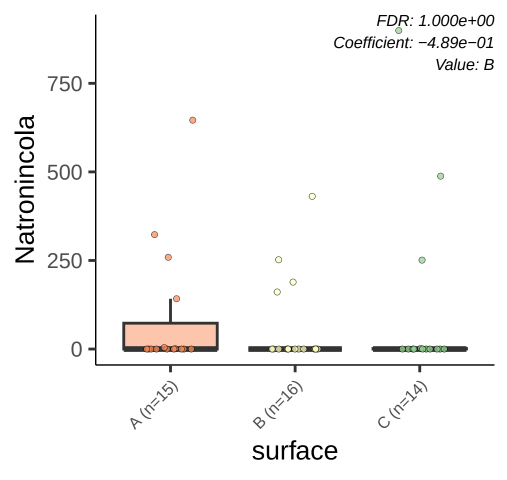
FDR: 1.000e+00
Coefficient: −4.89e−01
Value: B
Natronincola
750
500
250
0
A (n=15)
B (n=16)
C (n=14)
surface

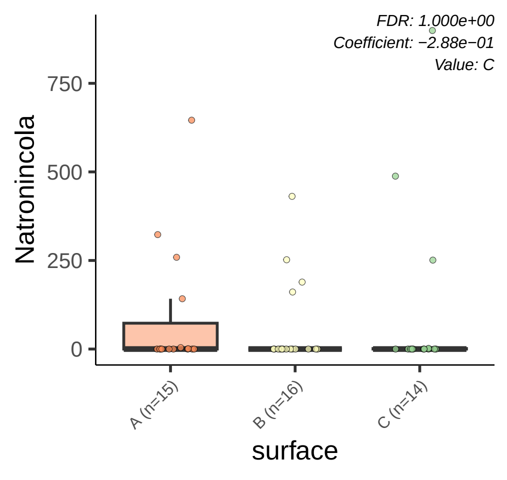
FDR: 1.000e+00
Coefficient: −2.88e−01
Value: C
Natronincola
750
500
250
0
A (n=15)
B (n=16)
C (n=14)
surface

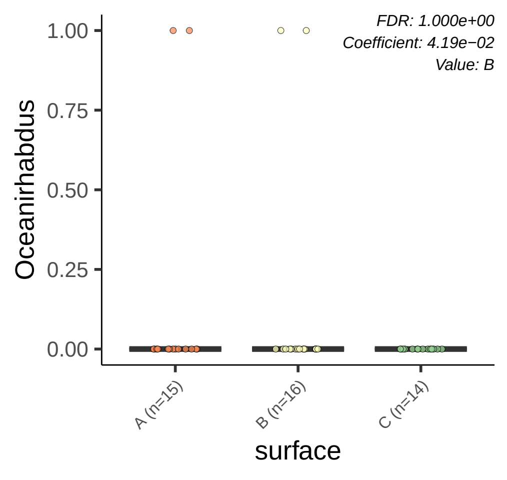

FDR: 1.000e+00
Coefficient: 4.19e−02
Value: B
Oceanirhabdus
1.00
0.75
0.50
0.25
0.00
A (n=15)
B (n=16)
C (n=14)
surface

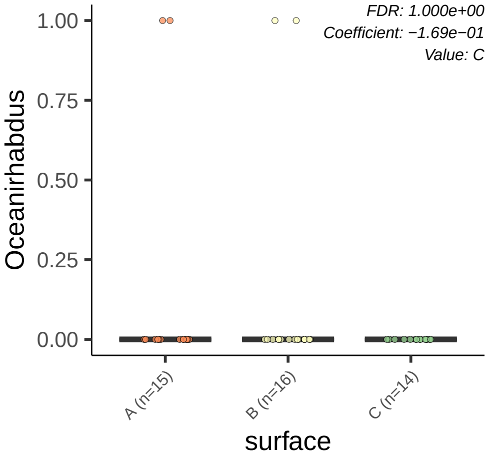
FDR: 1.000e+00
Coefficient: −1.69e−01
Value: C
Oceanirhabdus
1.00
0.75
0.50
0.25
0.00
A (n=15)
B (n=16)
C (n=14)
surface

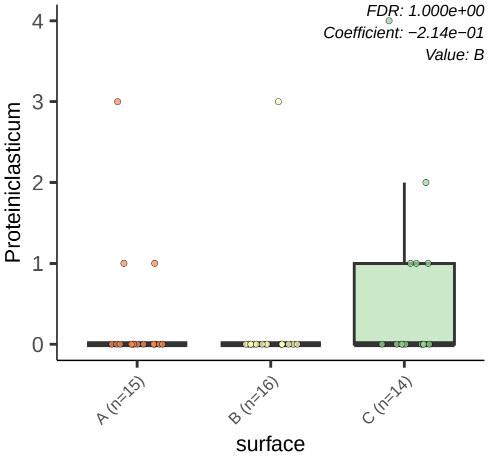

FDR: 1.000e+00
Coefficient: −2.14e−01
Value: B
Proteiniclasticum
4
3
2
1
0
A (n=15)
B (n=16)
C (n=14)
surface

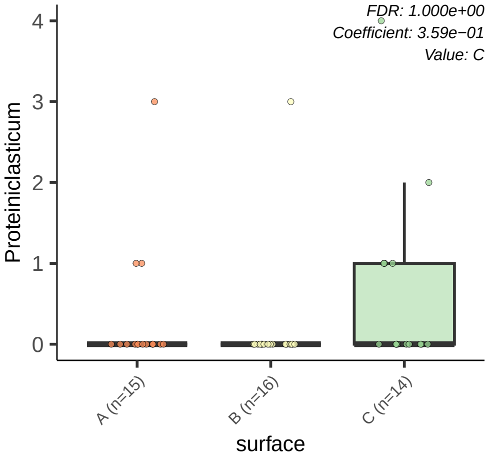

FDR: 1.000e+00
Coefficient: 3.59e−01
Value: C
Proteiniclasticum
0
1
2
3
4
A (n=15)
B (n=16)
C (n=14)
surface

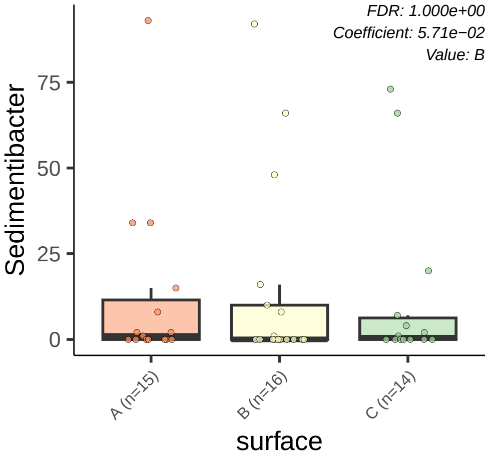

FDR: 1.000e+00
Coefficient: 5.71e−02
Value: B
Sedimentibacter
75
50
25
0
A (n=15)
B (n=16)
C (n=14)
surface

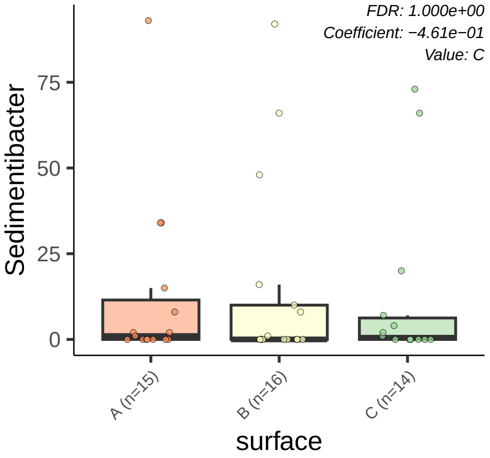

FDR: 1.000e+00
Coefficient: −4.61e−01
Value: C
Sedimentibacter
75
50
25
0
A (n=15)
B (n=16)
C (n=14)
surface

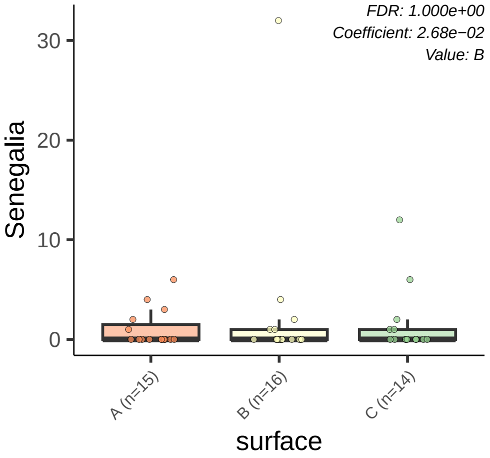
FDR: 1.000e+00
Coefficient: 2.68e−02
Value: B
Senegalia
30
20
10
0
A (n=15)
B (n=16)
C (n=14)
surface

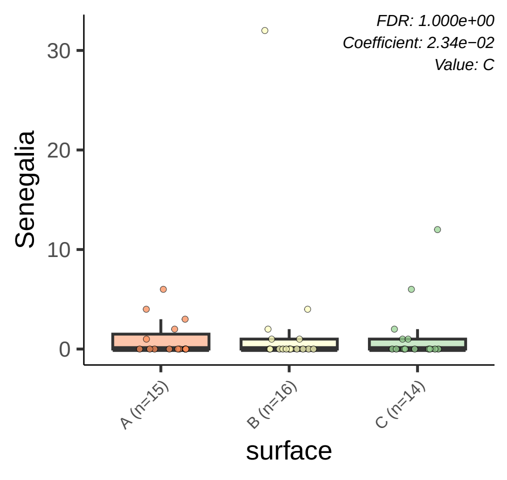

FDR: 1.000e+00
Coefficient: 2.34e−02
Value: C
Senegalia
30
20
10
0
A (n=15)
B (n=16)
C (n=14)
surface

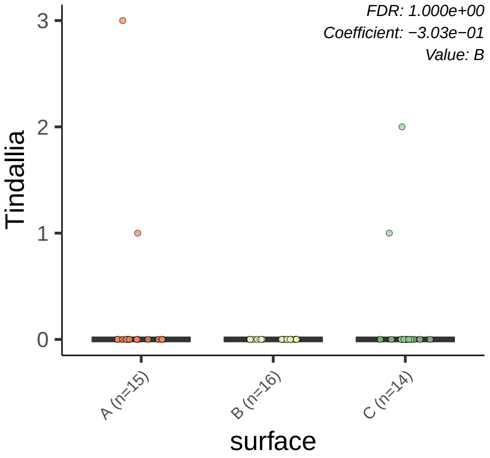
FDR: 1.000e+00
Coefficient: −3.03e−01
Value: B
Tindallia
3
2
1
0
A (n=15)
B (n=16)
C (n=14)
surface

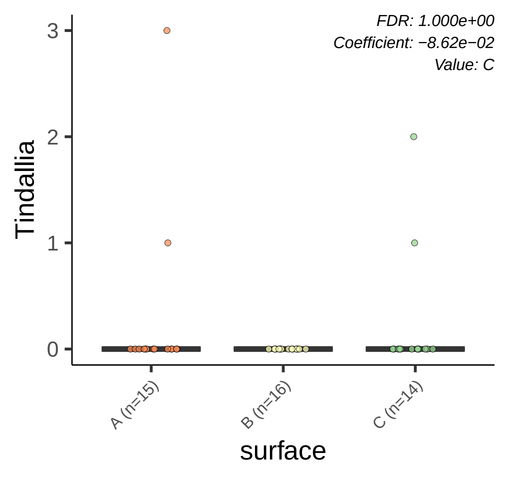

FDR: 1.000e+00
Coefficient: −8.62e−02
Value: C
Tindallia
3
2
1
0
A (n=15)
B (n=16)
C (n=14)
surface

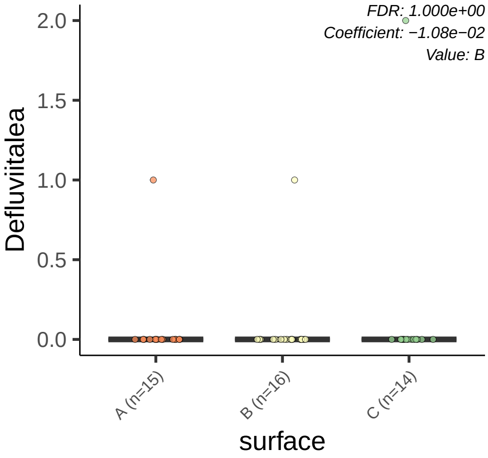

FDR: 1.000e+00
Coefficient: −1.08e−02
Value: B
Defluviitalea
2.0
1.5
1.0
0.5
0.0
A (n=15)
B (n=16)
C (n=14)
surface

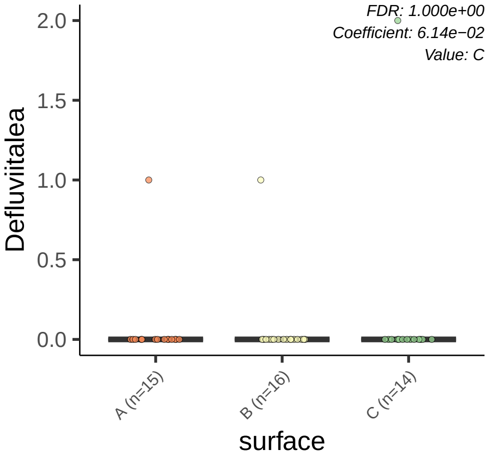
FDR: 1.000e+00
Coefficient: 6.14e−02
Value: C
Defluviitalea
2.0
1.5
1.0
0.5
0.0
A (n=15)
B (n=16)
C (n=14)
surface

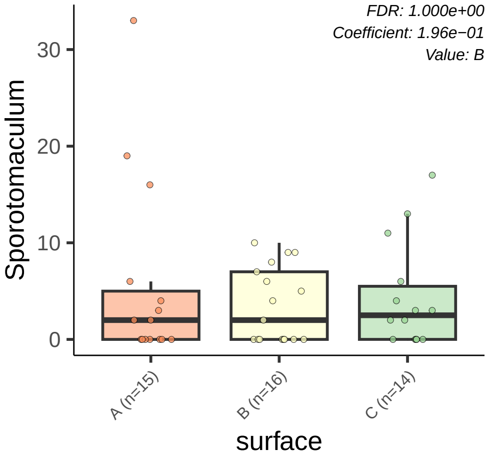

FDR: 1.000e+00
Coefficient: 1.96e−01
Value: B
Sporotomaculum
30
20
10
0
A (n=15)
B (n=16)
C (n=14)
surface

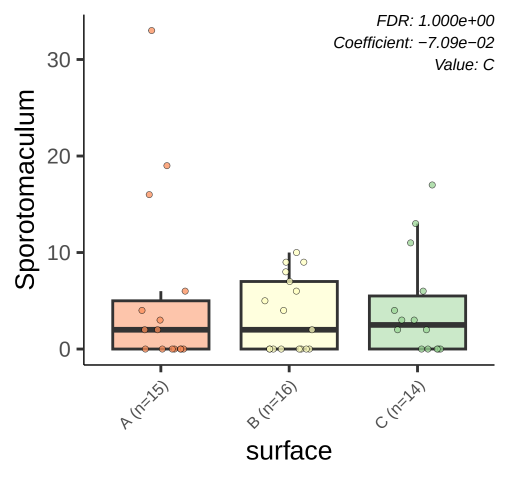
FDR: 1.000e+00
Coefficient: −7.09e−02
Value: C
Sporotomaculum
30
20
10
0
A (n=15)
B (n=16)
C (n=14)
surface

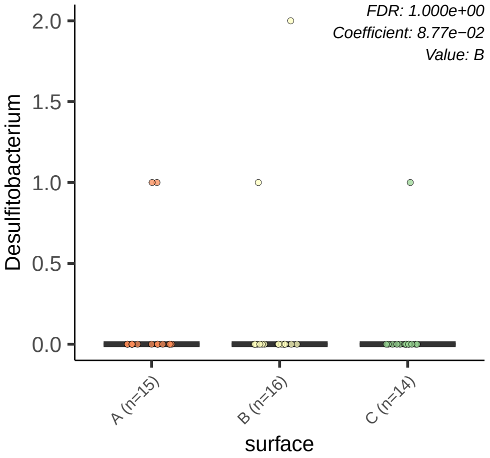

FDR: 1.000e+00
Coefficient: 8.77e−02
Value: B
Desulfitobacterium
2.0
1.5
1.0
0.5
0.0
A (n=15)
B (n=16)
C (n=14)
surface

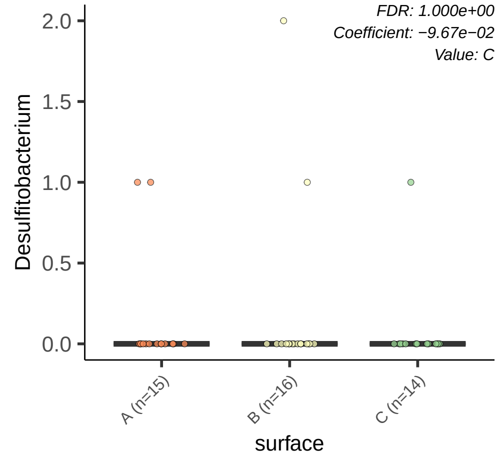

FDR: 1.000e+00
Coefficient: −9.67e−02
Value: C
Desulfitobacterium
2.0
1.5
1.0
0.5
0.0
A (n=15)
B (n=16)
C (n=14)
surface

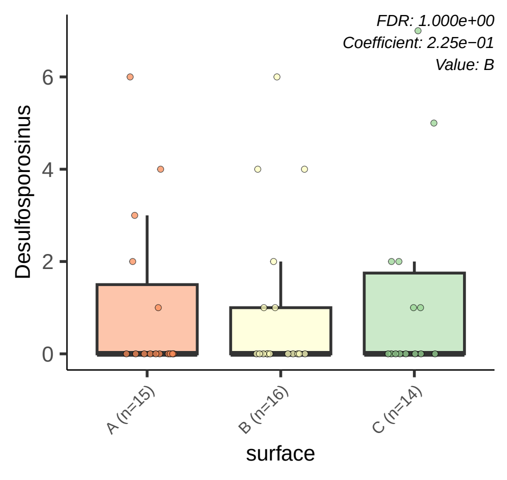

FDR: 1.000e+00
Coefficient: 2.25e−01
Value: B
Desulfosporosinus
0
2
4
6
A (n=15)
B (n=16)
C (n=14)
surface

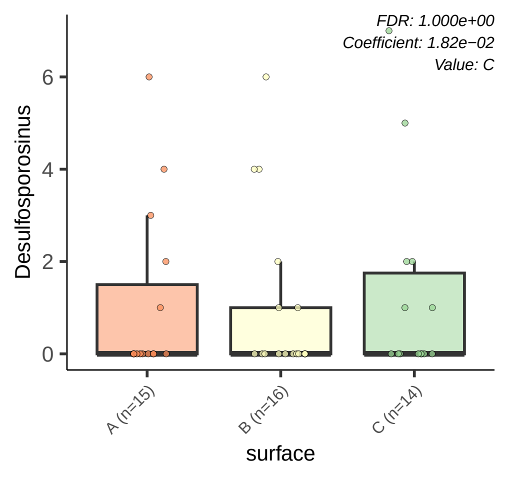
FDR: 1.000e+00
Coefficient: 1.82e−02
Value: C
Desulfosporosinus
0
2
4
6
A (n=15)
B (n=16)
C (n=14)
surface

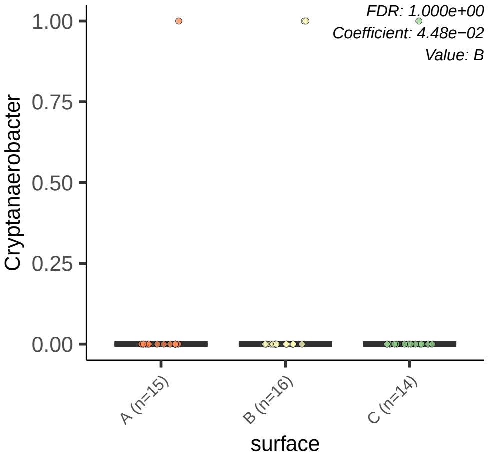
FDR: 1.000e+00
Coefficient: 4.48e−02
Value: B
Cryptanaerobacter
1.00
0.75
0.50
0.25
0.00
A (n=15)
B (n=16)
C (n=14)
surface

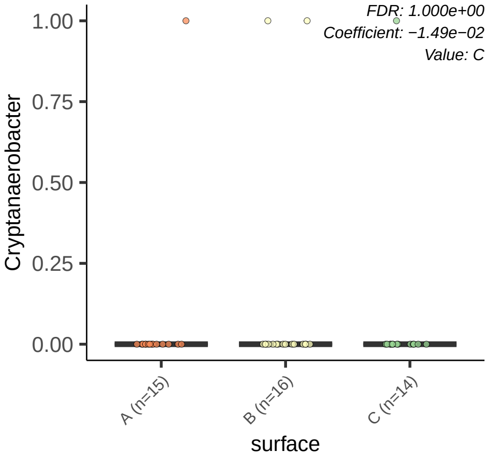

FDR: 1.000e+00
Coefficient: −1.49e−02
Value: C
1.00
0.75
0.50
0.25
0.00
Cryptanaerobacter
A (n=15)
B (n=16)
C (n=14)
surface

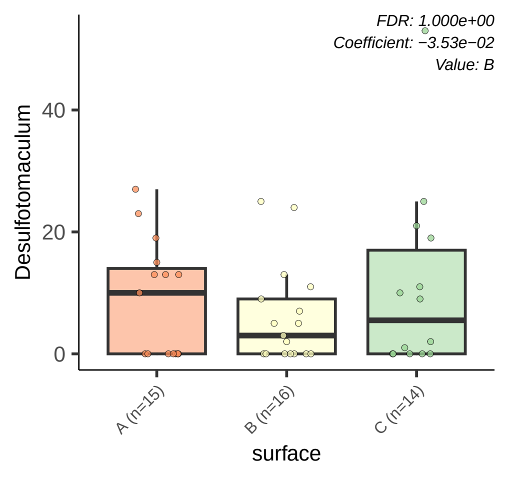
FDR: 1.000e+00
Coefficient: −3.53e−02
Value: B
Desulfotomaculum
0
20
40
A (n=15)
B (n=16)
C (n=14)
surface

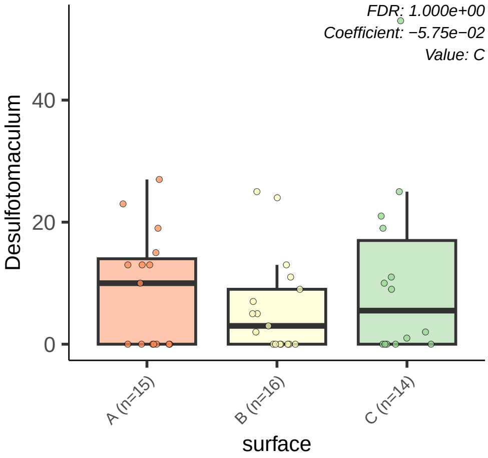

FDR: 1.000e+00
Coefficient: −5.75e−02
Value: C
Desulfotomaculum
0
20
40
A (n=15)
B (n=16)
C (n=14)
surface

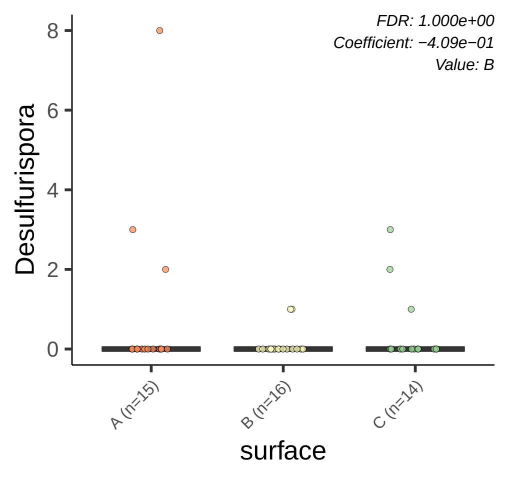
FDR: 1.000e+00
Coefficient: −4.09e−01
Value: B
Desulfurispora
8
6
4
2
0
A (n=15)
B (n=16)
C (n=14)
surface

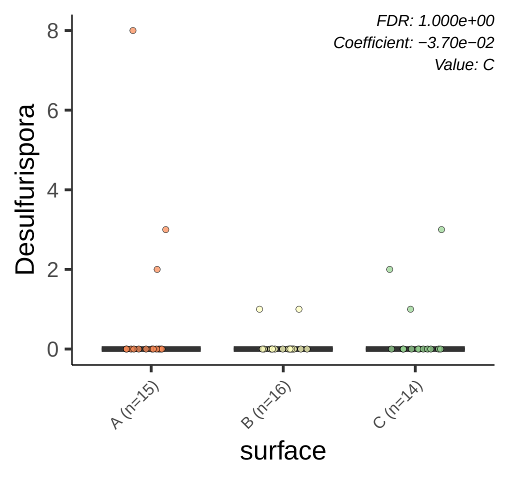
FDR: 1.000e+00
Coefficient: −3.70e−02
Value: C
Desulfurispora
8
6
4
2
0
A (n=15)
B (n=16)
C (n=14)
surface

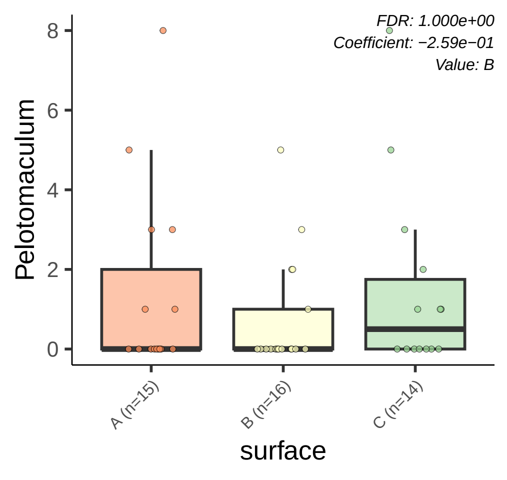
FDR: 1.000e+00
Coefficient: −2.59e−01
Value: B
Pelotomaculum
0
2
4
6
8
A (n=15)
B (n=16)
C (n=14)
surface

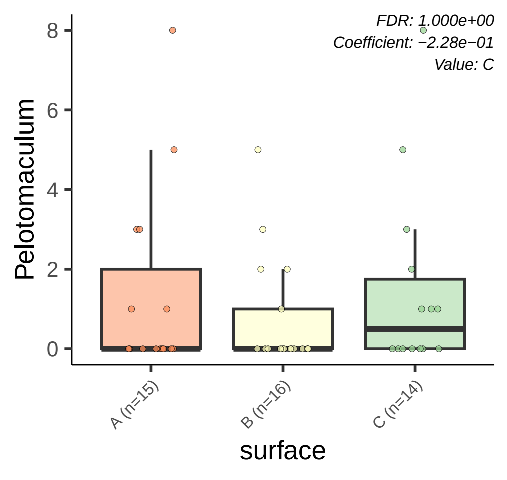
FDR: 1.000e+00
Coefficient: −2.28e−01
Value: C
Pelotomaculum
0
2
4
6
8
A (n=15)
B (n=16)
C (n=14)
surface

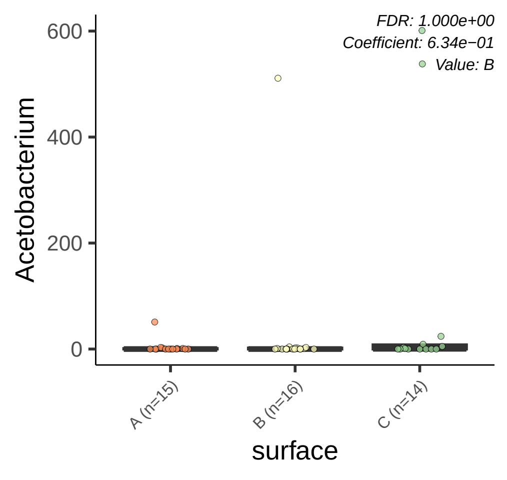

FDR: 1.000e+00
Coefficient: 6.34e−01
Value: B
Acetobacterium
600
400
200
0
A (n=15)
B (n=16)
C (n=14)
surface

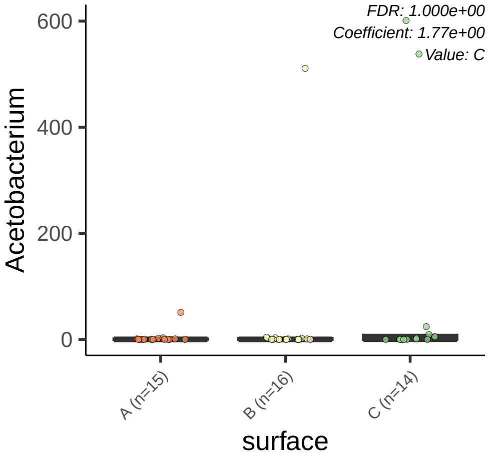

FDR: 1.000e+00
Coefficient: 1.77e+00
Value: C
Acetobacterium
600
400
200
0
A (n=15)
B (n=16)
C (n=14)
surface

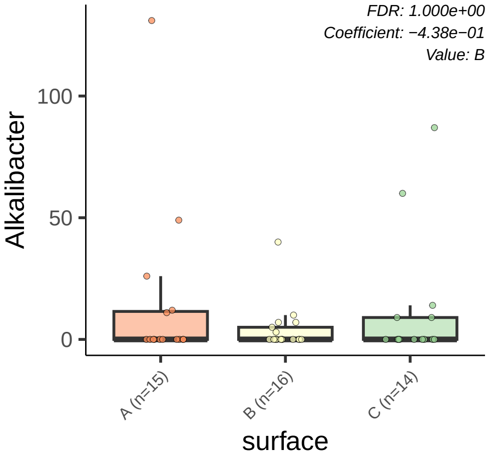
FDR: 1.000e+00
Coefficient: −4.38e−01
Value: B
Alkalibacter
100
50
0
A (n=15)
B (n=16)
C (n=14)
surface

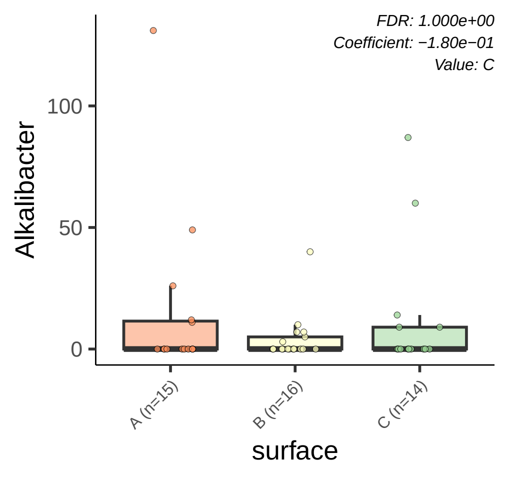

FDR: 1.000e+00
Coefficient: −1.80e−01
Value: C
Alkalibacter
100
50
0
A (n=15)
B (n=16)
C (n=14)
surface

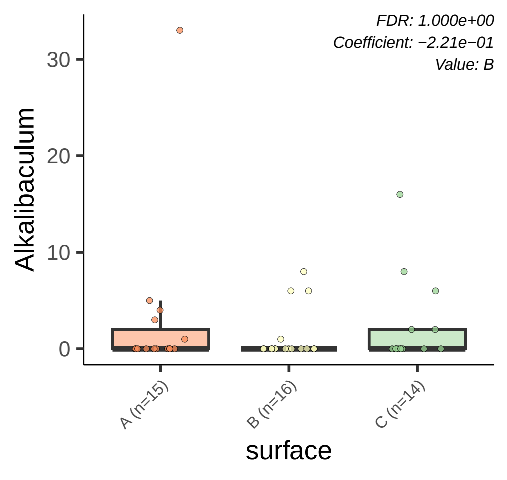
FDR: 1.000e+00
Coefficient: −2.21e−01
Value: B
Alkalibaculum
30
20
10
0
A (n=15)
B (n=16)
C (n=14)
surface

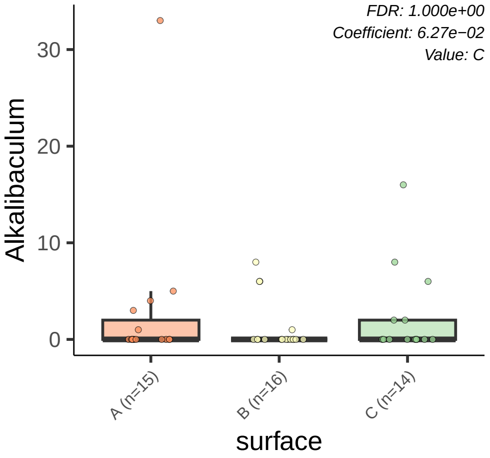
FDR: 1.000e+00
Coefficient: 6.27e−02
Value: C
Alkalibaculum
30
20
10
0
A (n=15)
B (n=16)
C (n=14)
surface

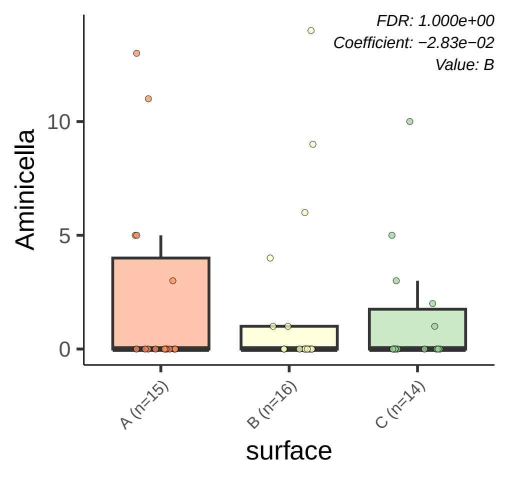

FDR: 1.000e+00
Coefficient: −2.83e−02
Value: B
Aminicella
10
5
0
A (n=15)
B (n=16)
C (n=14)
surface

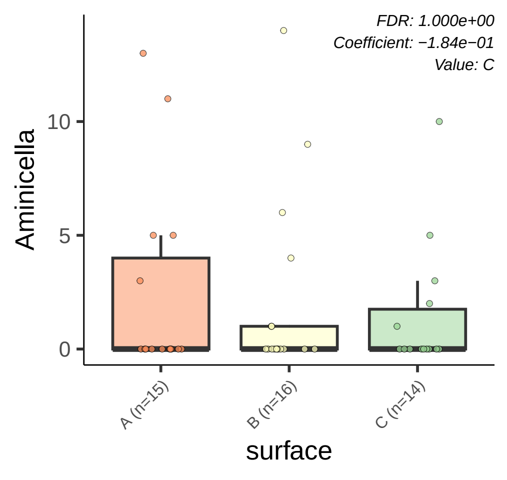
FDR: 1.000e+00
Coefficient: −1.84e−01
Value: C
Aminicella
0
5
10
A (n=15)
B (n=16)
C (n=14)
surface

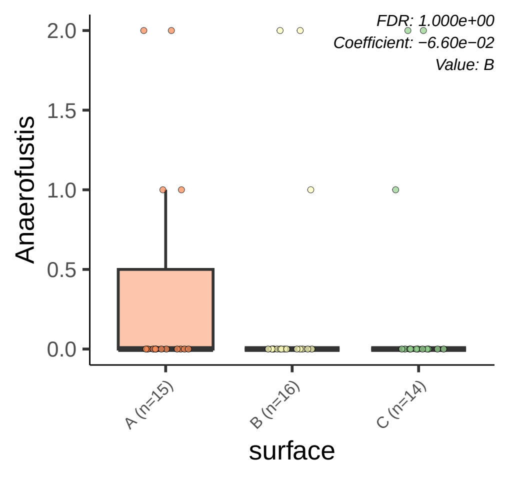

FDR: 1.000e+00
Coefficient: −6.60e−02
Value: B
Anaerofustis
2.0
1.5
1.0
0.5
0.0
A (n=15)
B (n=16)
C (n=14)
surface

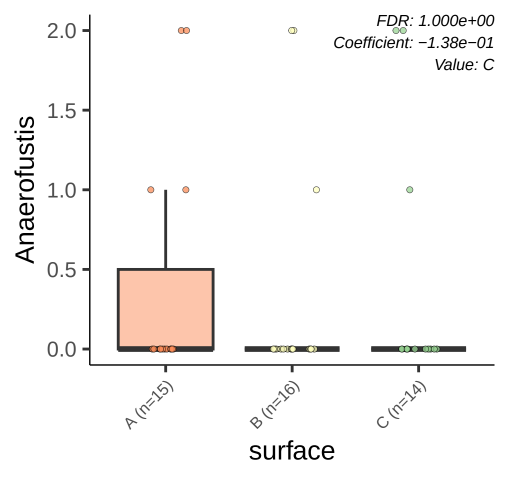
FDR: 1.000e+00
Coefficient: −1.38e−01
Value: C
2.0
1.5
1.0
0.5
0.0
Anaerofustis
A (n=15)
B (n=16)
C (n=14)
surface

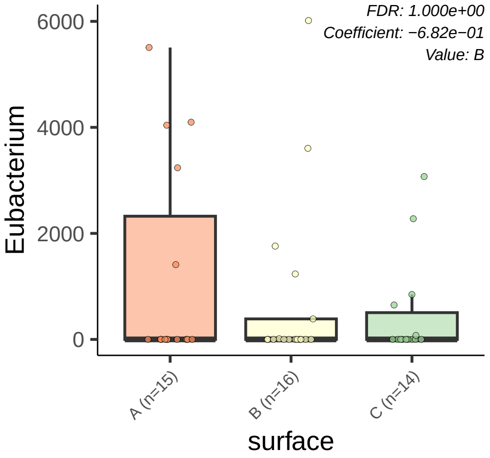

FDR: 1.000e+00
Coefficient: −6.82e−01
Value: B
Eubacterium
6000
4000
2000
0
A (n=15)
B (n=16)
C (n=14)
surface

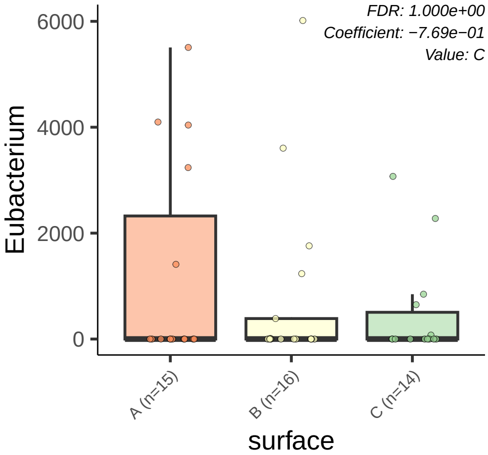

FDR: 1.000e+00
Coefficient: −7.69e−01
Value: C
6000
4000
2000
0
Eubacterium
A (n=15)
B (n=16)
C (n=14)
surface

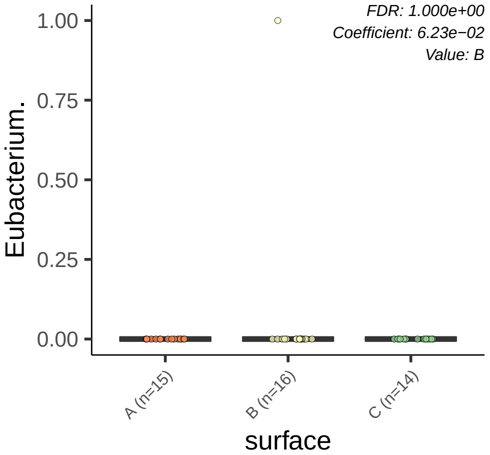

FDR: 1.000e+00
Coefficient: 6.23e−02
Value: B
Eubacterium.
1.00
0.75
0.50
0.25
0.00
A (n=15)
B (n=16)
C (n=14)
surface

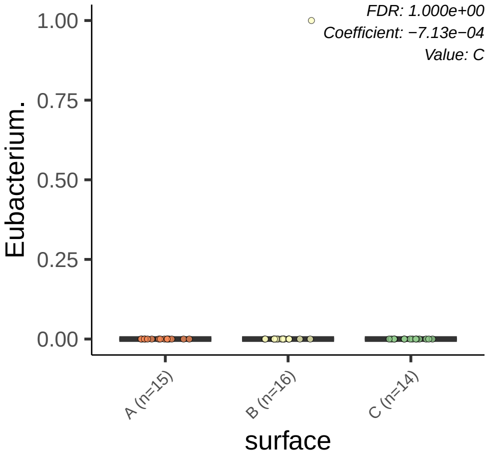
FDR: 1.000e+00
Coefficient: −7.13e−04
Value: C
Eubacterium.
1.00
0.75
0.50
0.25
0.00
A (n=15)
B (n=16)
C (n=14)
surface

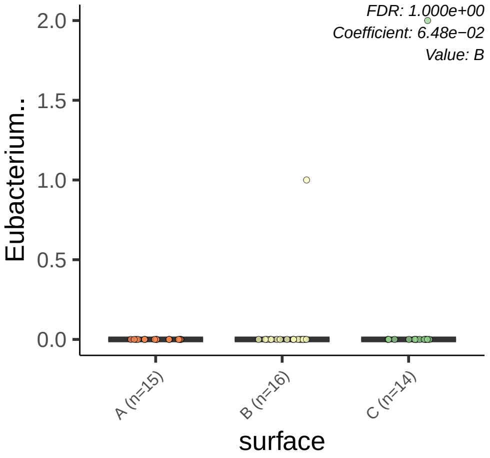

FDR: 1.000e+00
Coefficient: 6.48e−02
Value: B
Eubacterium..
2.0
1.5
1.0
0.5
0.0
A (n=15)
B (n=16)
C (n=14)
surface

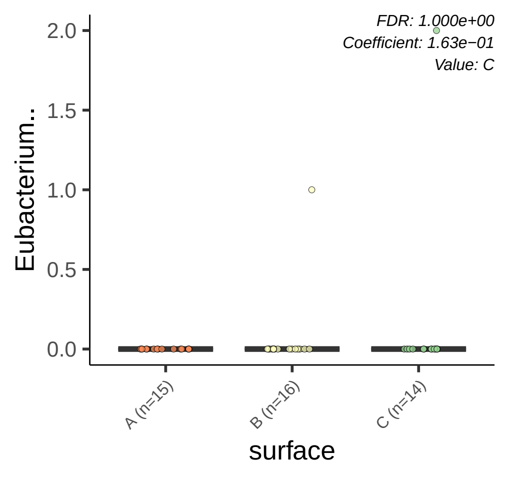
FDR: 1.000e+00
Coefficient: 1.63e−01
Value: C
Eubacterium..
2.0
1.5
1.0
0.5
0.0
A (n=15)
B (n=16)
C (n=14)
surface

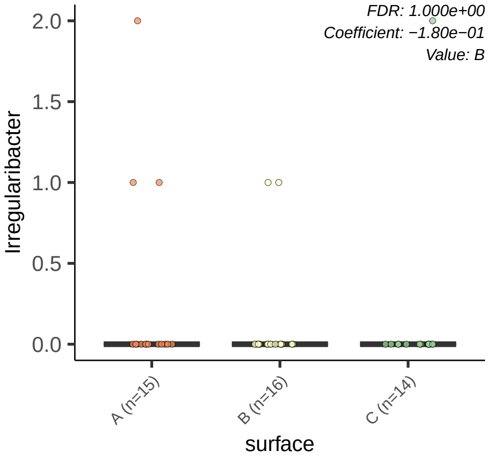
FDR: 1.000e+00
Coefficient: −1.80e−01
Value: B
Irregularibacter
2.0
1.5
1.0
0.5
0.0
A (n=15)
B (n=16)
C (n=14)
surface

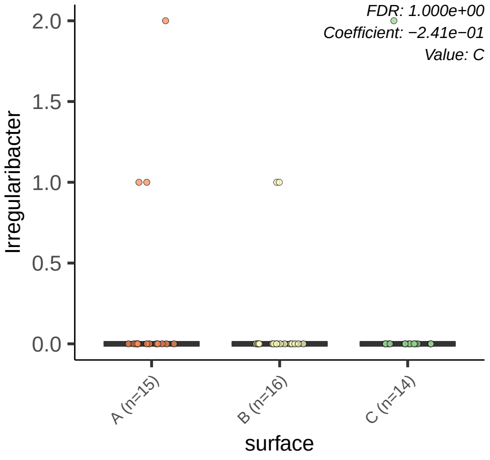
FDR: 1.000e+00
Coefficient: −2.41e−01
Value: C
Irregularibacter
2.0
1.5
1.0
0.5
0.0
A (n=15)
B (n=16)
C (n=14)
surface

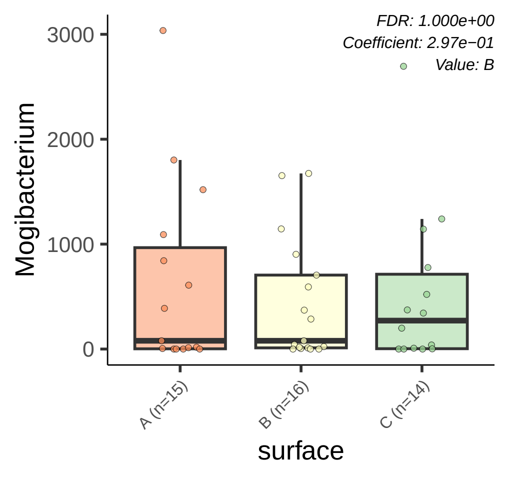
FDR: 1.000e+00
Coefficient: 2.97e−01
Value: B
Mogibacterium
3000
2000
1000
0
A (n=15)
B (n=16)
C (n=14)
surface

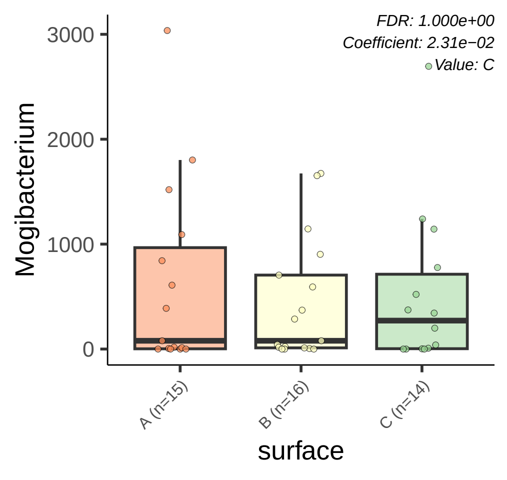

FDR: 1.000e+00
Coefficient: 2.31e−02
Value: C
Mogibacterium
3000
2000
1000
0
A (n=15)
B (n=16)
C (n=14)
surface

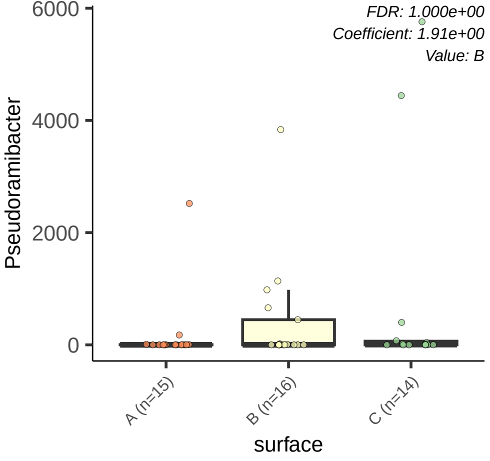

FDR: 1.000e+00
Coefficient: 1.91e+00
Value: B
6000
4000
2000
0
Pseudoramibacter
A (n=15)
B (n=16)
C (n=14)
surface

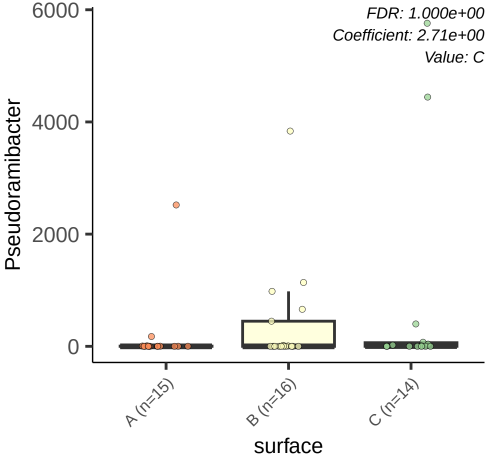
FDR: 1.000e+00
Coefficient: 2.71e+00
Value: C
Pseudoramibacter
6000
4000
2000
0
A (n=15)
B (n=16)
C (n=14)
surface

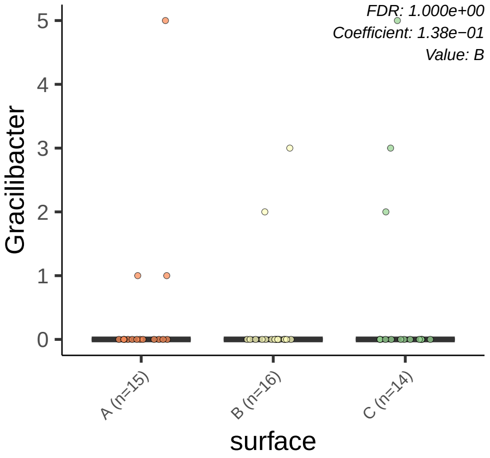
FDR: 1.000e+00
Coefficient: 1.38e−01
Value: B
Gracilibacter
0
1
2
3
4
5
A (n=15)
B (n=16)
C (n=14)
surface

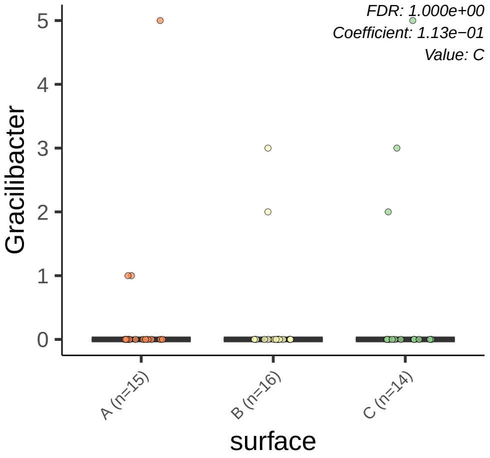
FDR: 1.000e+00
Coefficient: 1.13e−01
Value: C
Gracilibacter
0
1
2
3
4
5
A (n=15)
B (n=16)
C (n=14)
surface

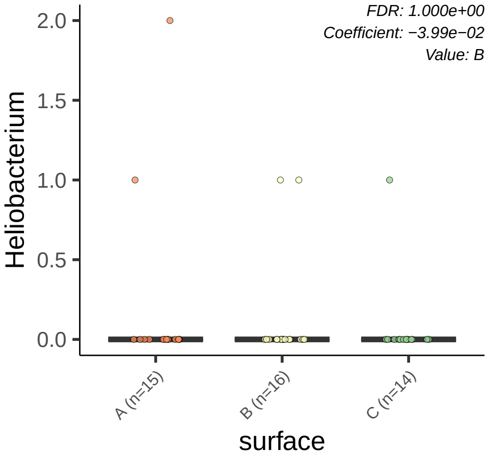
FDR: 1.000e+00
Coefficient: −3.99e−02
Value: B
Heliobacterium
2.0
1.5
1.0
0.5
0.0
A (n=15)
B (n=16)
C (n=14)
surface

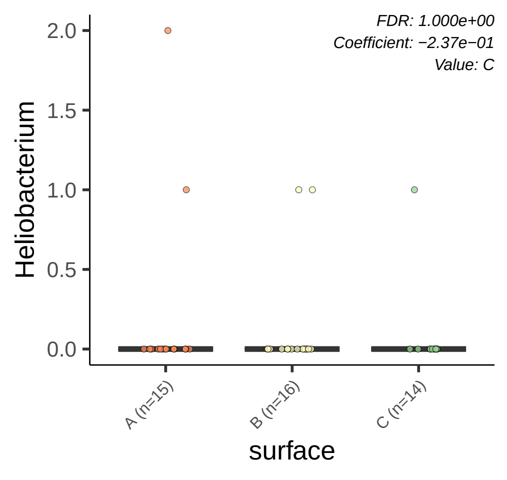
FDR: 1.000e+00
Coefficient: −2.37e−01
Value: C
Heliobacterium
2.0
1.5
1.0
0.5
0.0
A (n=15)
B (n=16)
C (n=14)
surface

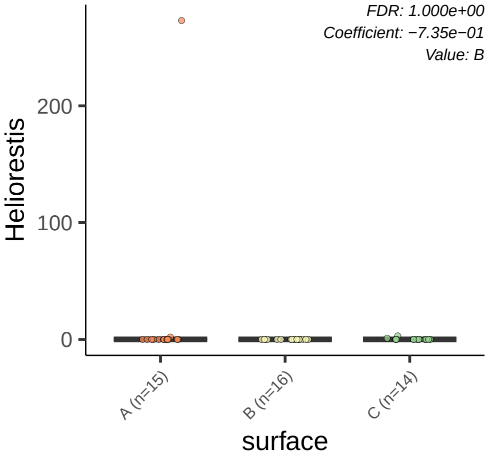

FDR: 1.000e+00
Coefficient: −7.35e−01
Value: B
Heliorestis
200
100
0
A (n=15)
B (n=16)
C (n=14)
surface

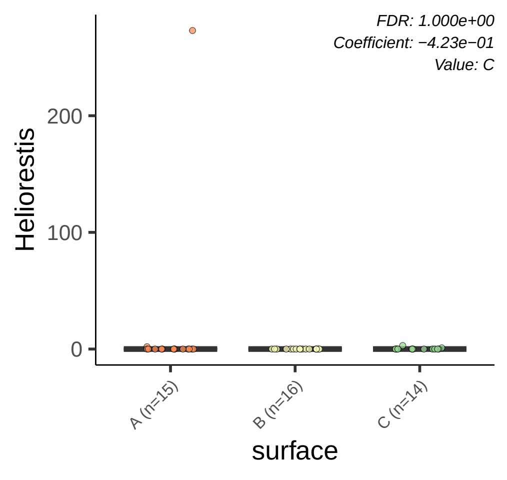

FDR: 1.000e+00
Coefficient: −4.23e−01
Value: C
Heliorestis
200
100
0
A (n=15)
B (n=16)
C (n=14)
surface

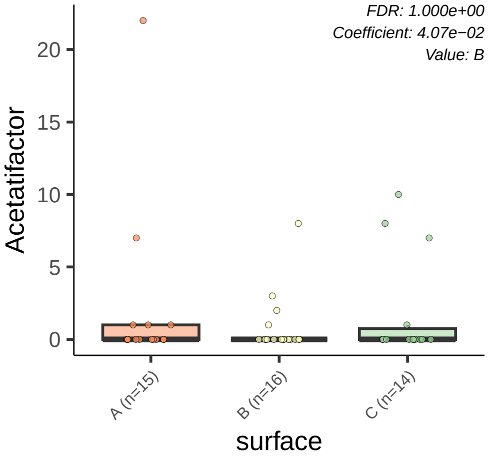

FDR: 1.000e+00
Coefficient: 4.07e−02
Value: B
Acetatifactor
20
15
10
5
0
A (n=15)
B (n=16)
C (n=14)
surface

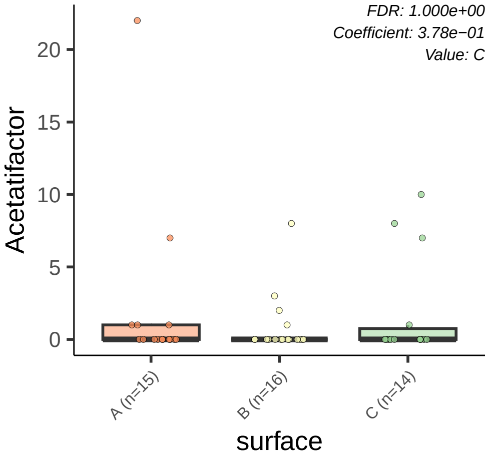

FDR: 1.000e+00
Coefficient: 3.78e−01
Value: C
Acetatifactor
0
5
10
15
20
A (n=15)
B (n=16)
C (n=14)
surface

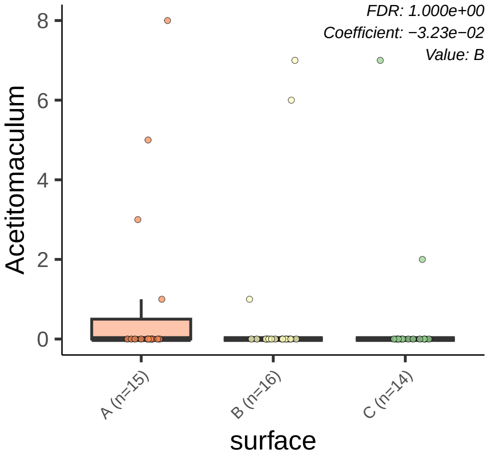
FDR: 1.000e+00
Coefficient: −3.23e−02
Value: B
Acetitomaculum
8
6
4
2
0
A (n=15)
B (n=16)
C (n=14)
surface

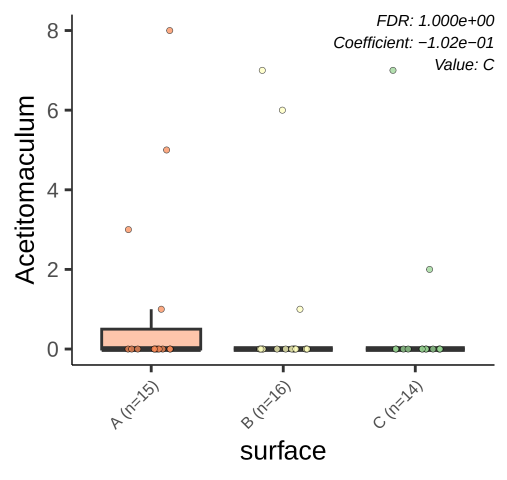

FDR: 1.000e+00
Coefficient: −1.02e−01
Value: C
Acetitomaculum
8
6
4
2
0
A (n=15)
B (n=16)
C (n=14)
surface

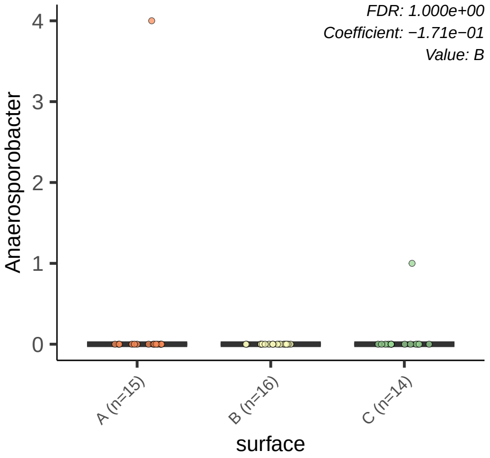
FDR: 1.000e+00
Coefficient: −1.71e−01
Value: B
Anaerosporobacter
0
1
2
3
4
A (n=15)
B (n=16)
C (n=14)
surface

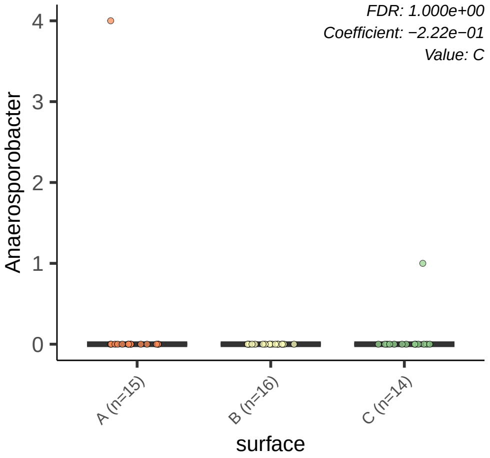
FDR: 1.000e+00
Coefficient: −2.22e−01
Value: C
Anaerosporobacter
4
3
2
1
0
A (n=15)
B (n=16)
C (n=14)
surface

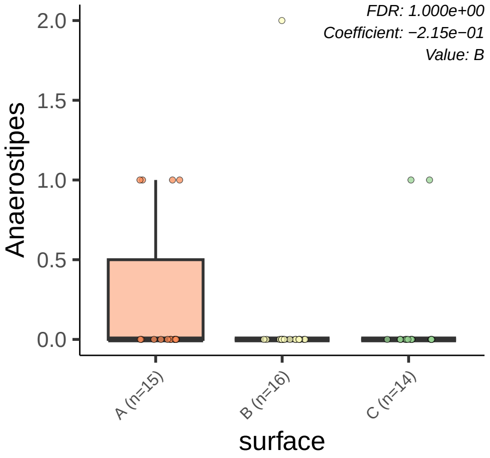
FDR: 1.000e+00
Coefficient: −2.15e−01
Value: B
Anaerostipes
2.0
1.5
1.0
0.5
0.0
A (n=15)
B (n=16)
C (n=14)
surface

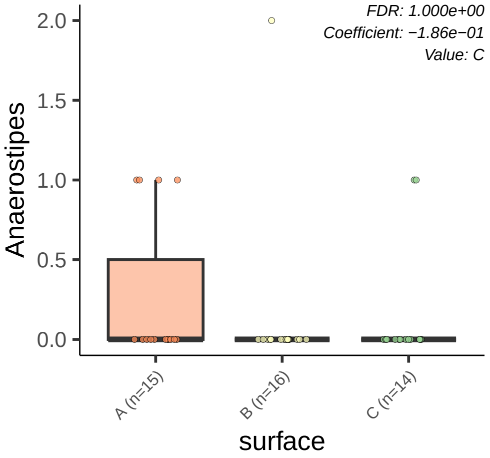

FDR: 1.000e+00
Coefficient: −1.86e−01
Value: C
Anaerostipes
2.0
1.5
1.0
0.5
0.0
A (n=15)
B (n=16)
C (n=14)
surface

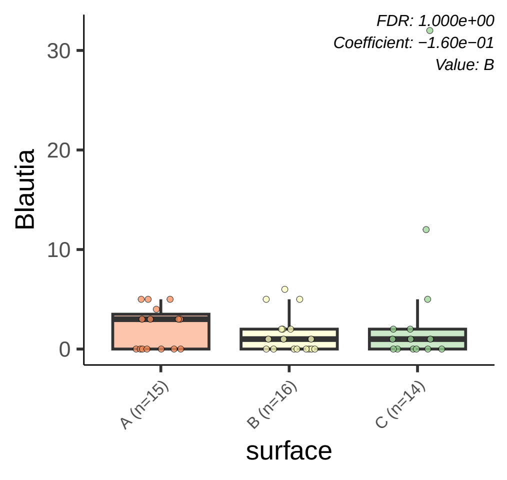

FDR: 1.000e+00
Coefficient: −1.60e−01
Value: B
Blautia
0
10
20
30
A (n=15)
B (n=16)
C (n=14)
surface

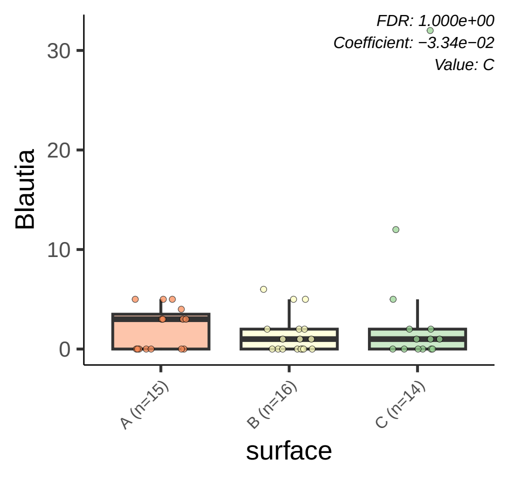
FDR: 1.000e+00
Coefficient: −3.34e−02
Value: C
Blautia
30
20
10
0
A (n=15)
B (n=16)
C (n=14)
surface

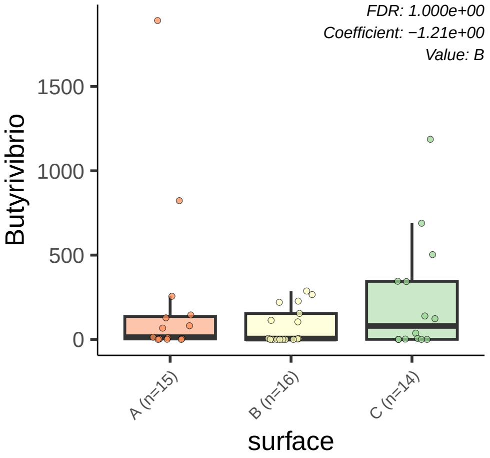
FDR: 1.000e+00
Coefficient: −1.21e+00
Value: B
Butyrivibrio
1500
1000
500
0
A (n=15)
B (n=16)
C (n=14)
surface

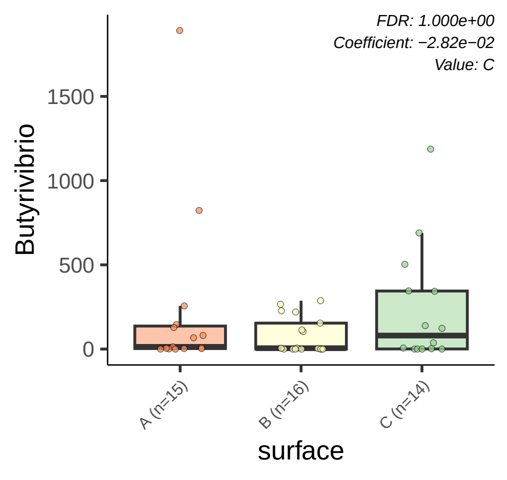
FDR: 1.000e+00
Coefficient: −2.82e−02
Value: C
Butyrivibrio
1500
1000
500
0
A (n=15)
B (n=16)
C (n=14)
surface

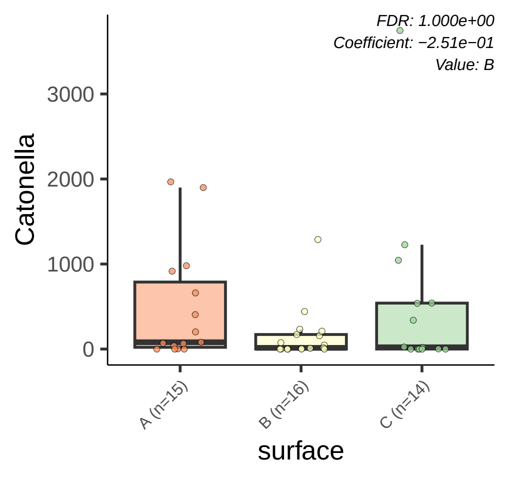
FDR: 1.000e+00
Coefficient: −2.51e−01
Value: B
Catonella
3000
2000
1000
0
A (n=15)
B (n=16)
C (n=14)
surface

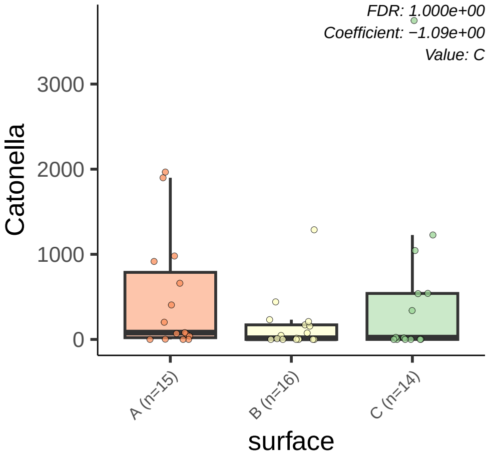
FDR: 1.000e+00
Coefficient: −1.09e+00
Value: C
3000
2000
1000
0
Catonella
A (n=15)
B (n=16)
C (n=14)
surface

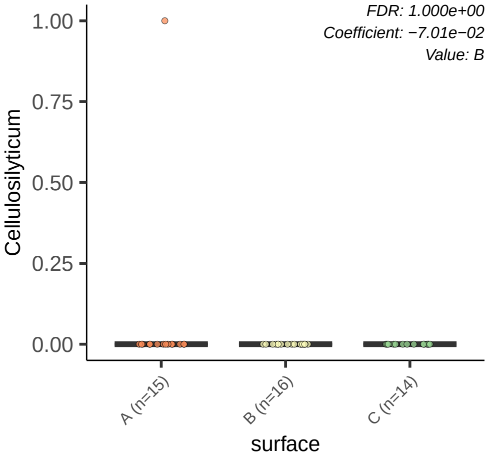

FDR: 1.000e+00
Coefficient: −7.01e−02
Value: B
Cellulosilyticum
1.00
0.75
0.50
0.25
0.00
A (n=15)
B (n=16)
C (n=14)
surface

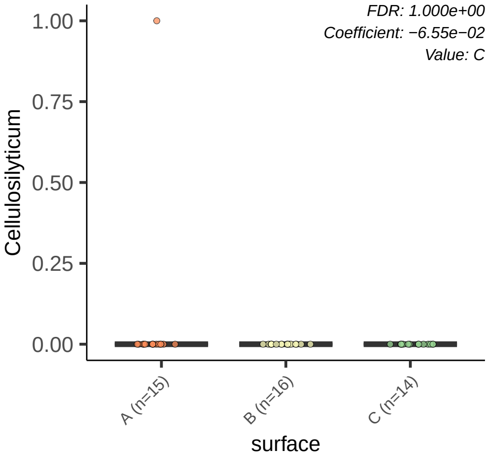

FDR: 1.000e+00
Coefficient: −6.55e−02
Value: C
Cellulosilyticum
1.00
0.75
0.50
0.25
0.00
A (n=15)
B (n=16)
C (n=14)
surface

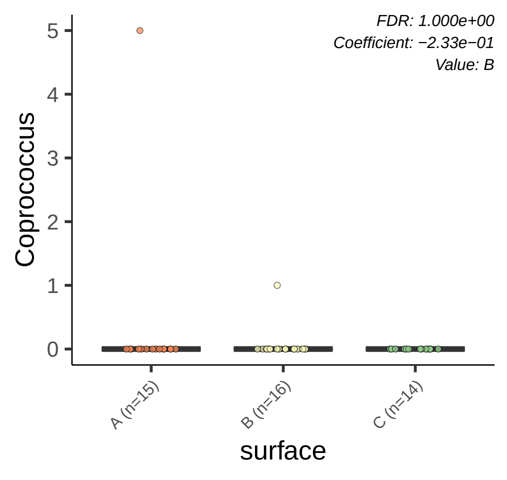
FDR: 1.000e+00
Coefficient: −2.33e−01
Value: B
Coprococcus
0
1
2
3
4
5
A (n=15)
B (n=16)
C (n=14)
surface

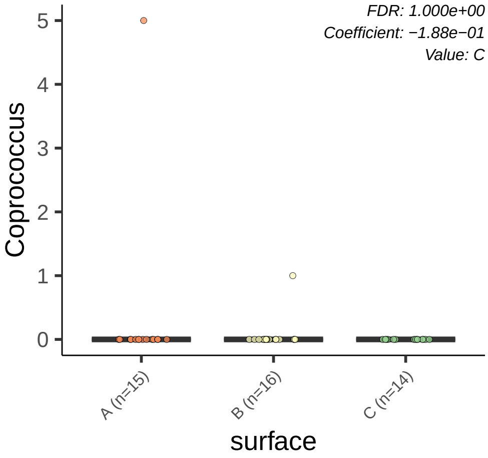
FDR: 1.000e+00
Coefficient: −1.88e−01
Value: C
Coprococcus
0
1
2
3
4
5
A (n=15)
B (n=16)
C (n=14)
surface

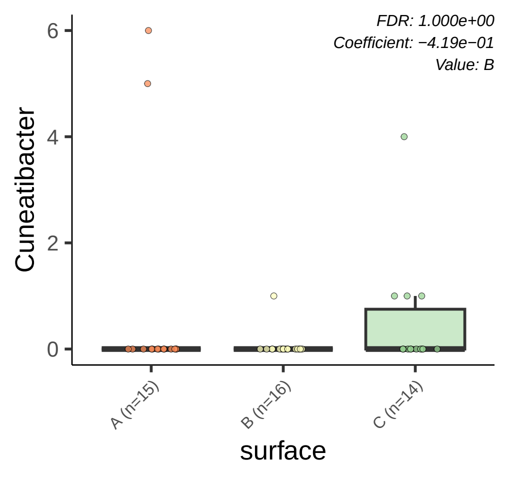

FDR: 1.000e+00
Coefficient: −4.19e−01
Value: B
Cuneatibacter
6
4
2
0
A (n=15)
B (n=16)
C (n=14)
surface

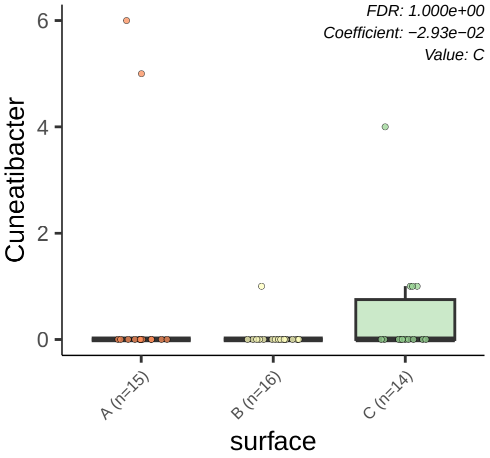
FDR: 1.000e+00
Coefficient: −2.93e−02
Value: C
Cuneatibacter
0
2
4
6
A (n=15)
B (n=16)
C (n=14)
surface

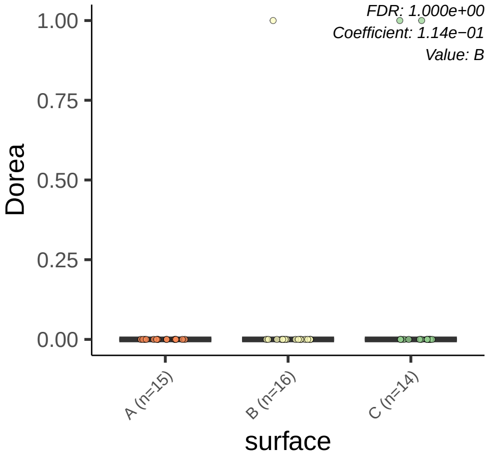

FDR: 1.000e+00
Coefficient: 1.14e−01
Value: B
1.00
0.75
0.50
0.25
0.00
Dorea
A (n=15)
B (n=16)
C (n=14)
surface

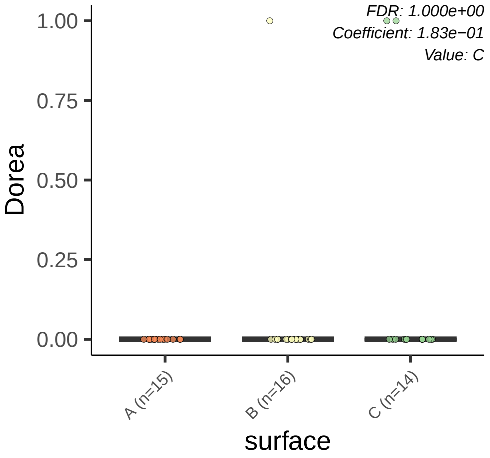
FDR: 1.000e+00
Coefficient: 1.83e−01
Value: C
Dorea
1.00
0.75
0.50
0.25
0.00
A (n=15)
B (n=16)
C (n=14)
surface

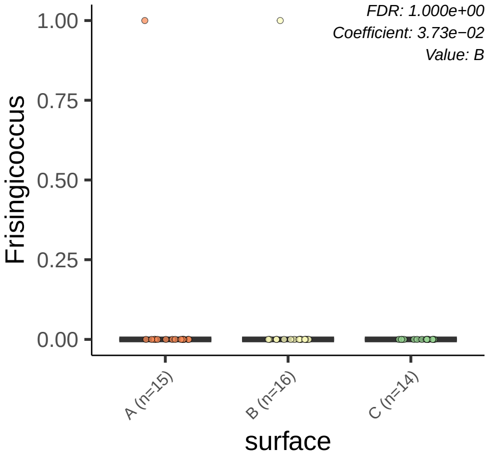

FDR: 1.000e+00
Coefficient: 3.73e−02
Value: B
Frisingicoccus
1.00
0.75
0.50
0.25
0.00
A (n=15)
B (n=16)
C (n=14)
surface

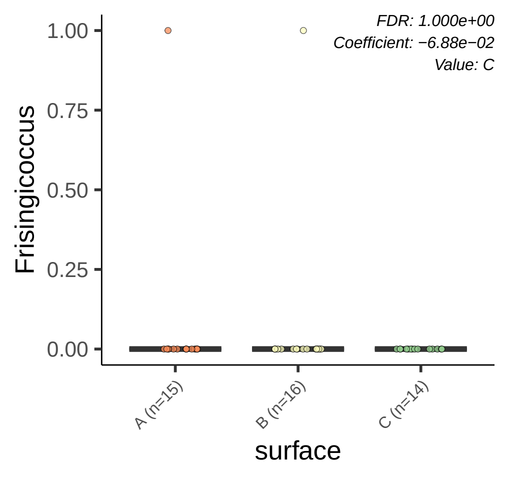

FDR: 1.000e+00
Coefficient: −6.88e−02
Value: C
Frisingicoccus
1.00
0.75
0.50
0.25
0.00
A (n=15)
B (n=16)
C (n=14)
surface

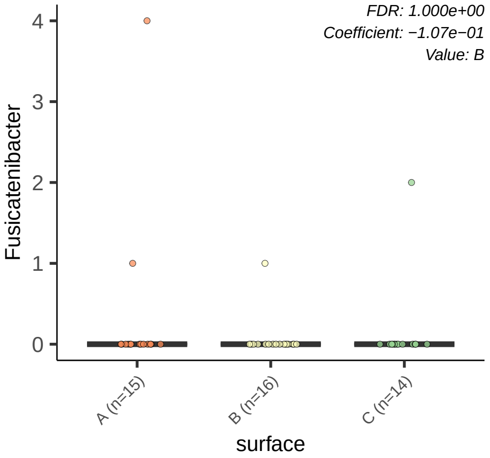
FDR: 1.000e+00
Coefficient: −1.07e−01
Value: B
Fusicatenibacter
4
3
2
1
0
A (n=15)
B (n=16)
C (n=14)
surface

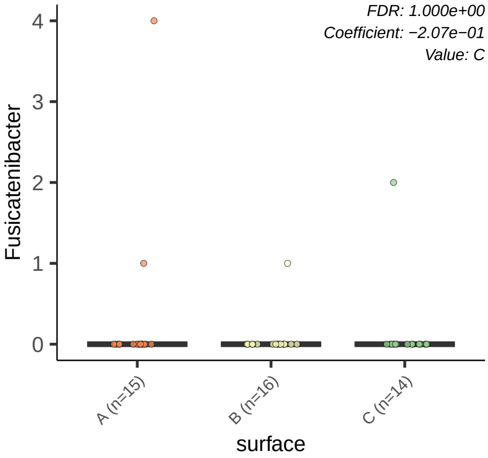

FDR: 1.000e+00
Coefficient: −2.07e−01
Value: C
Fusicatenibacter
0
1
2
3
4
A (n=15)
B (n=16)
C (n=14)
surface

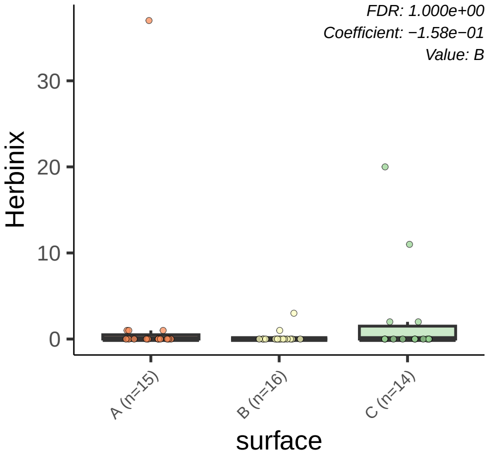

FDR: 1.000e+00
Coefficient: −1.58e−01
Value: B
Herbinix
30
20
10
0
A (n=15)
B (n=16)
C (n=14)
surface

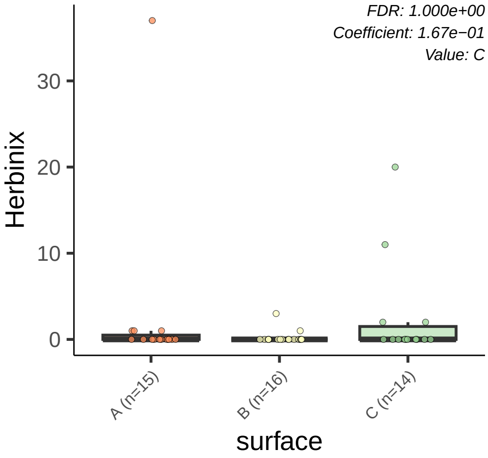
FDR: 1.000e+00
Coefficient: 1.67e−01
Value: C
Herbinix
0
10
20
30
A (n=15)
B (n=16)
C (n=14)
surface

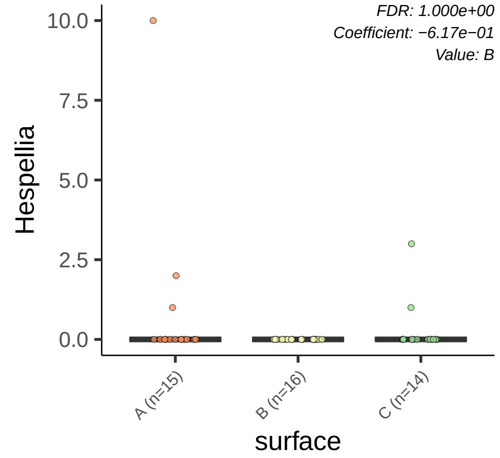

FDR: 1.000e+00
Coefficient: −6.17e−01
Value: B
Hespellia
10.0
7.5
5.0
2.5
0.0
A (n=15)
B (n=16)
C (n=14)
surface

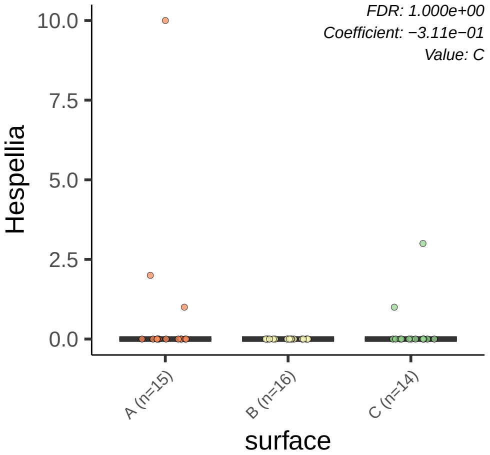

FDR: 1.000e+00
Coefficient: −3.11e−01
Value: C
Hespellia
10.0
7.5
5.0
2.5
0.0
A (n=15)
B (n=16)
C (n=14)
surface

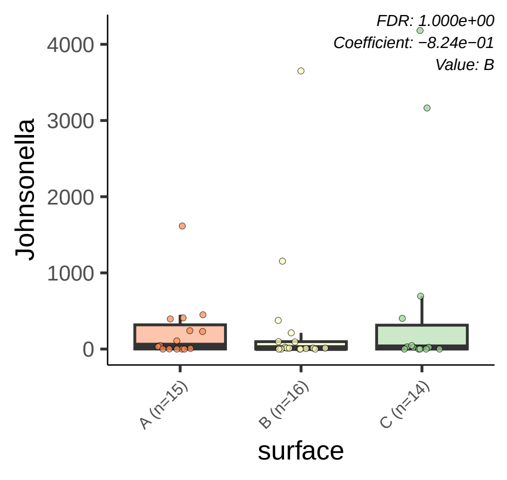

FDR: 1.000e+00
Coefficient: −8.24e−01
Value: B
4000
3000
2000
1000
0
Johnsonella
A (n=15)
B (n=16)
C (n=14)
surface

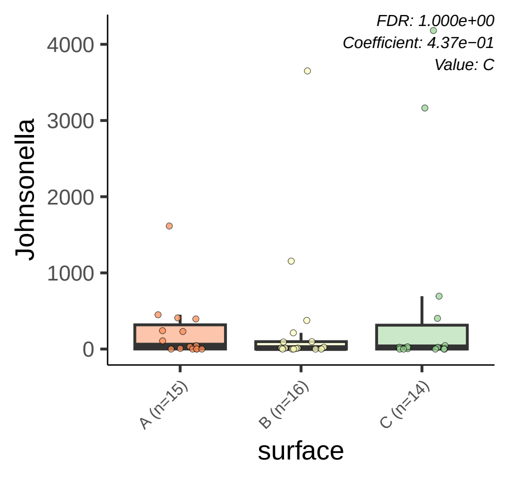
FDR: 1.000e+00
Coefficient: 4.37e−01
Value: C
Johnsonella
4000
3000
2000
1000
0
A (n=15)
B (n=16)
C (n=14)
surface

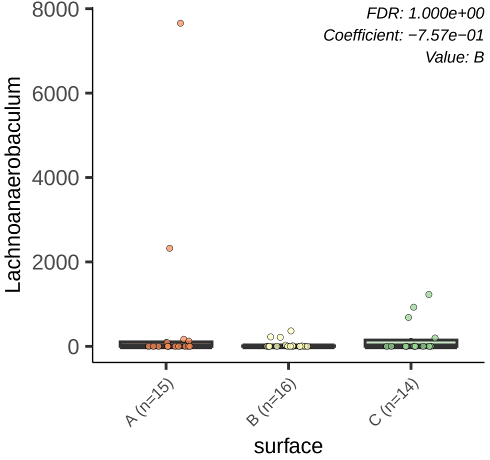

FDR: 1.000e+00
Coefficient: −7.57e−01
Value: B
8000
6000
4000
2000
0
Lachnoanaerobaculum
A (n=15)
B (n=16)
C (n=14)
surface

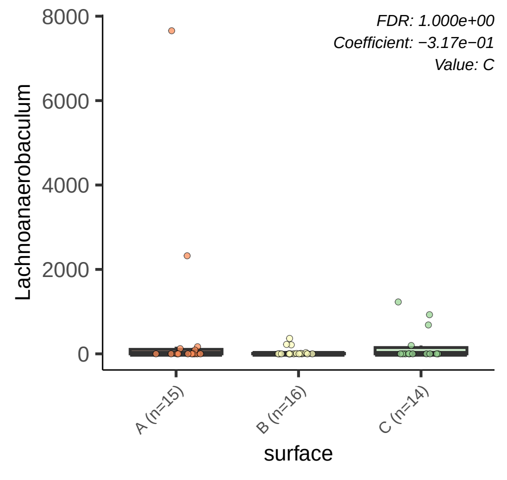
FDR: 1.000e+00
Coefficient: −3.17e−01
Value: C
Lachnoanaerobaculum
8000
6000
4000
2000
0
A (n=15)
B (n=16)
C (n=14)
surface

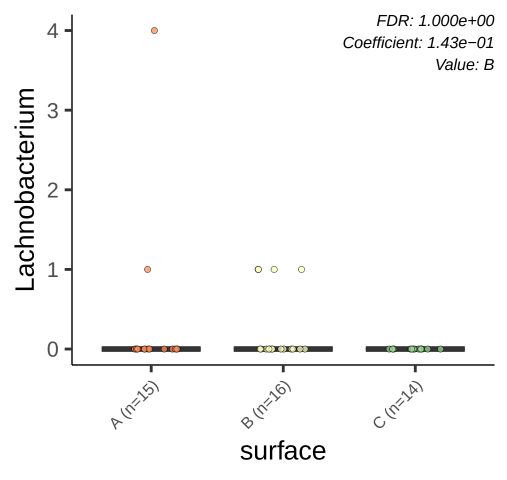
FDR: 1.000e+00
Coefficient: 1.43e−01
Value: B
Lachnobacterium
0
1
2
3
4
A (n=15)
B (n=16)
C (n=14)
surface

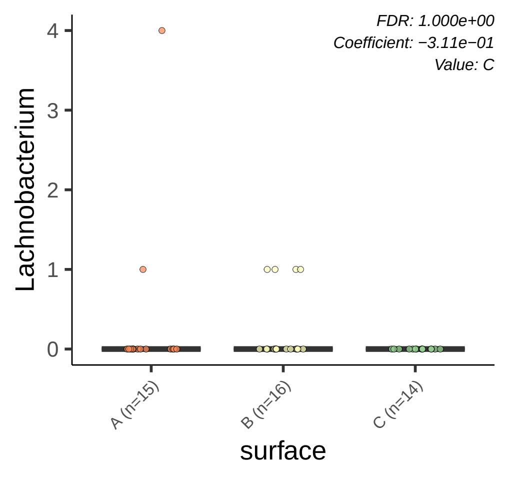
FDR: 1.000e+00
Coefficient: −3.11e−01
Value: C
Lachnobacterium
4
3
2
1
0
A (n=15)
B (n=16)
C (n=14)
surface

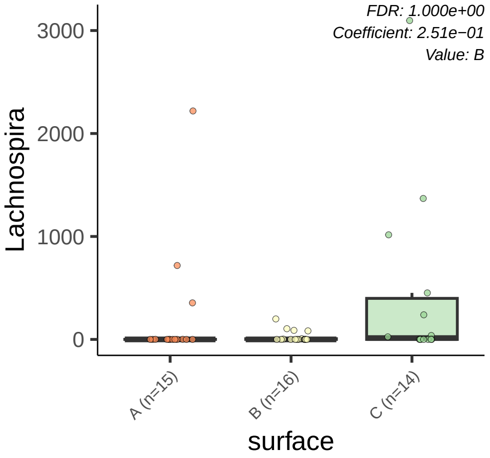

FDR: 1.000e+00
Coefficient: 2.51e−01
Value: B
3000
2000
1000
0
Lachnospira
A (n=15)
B (n=16)
C (n=14)
surface

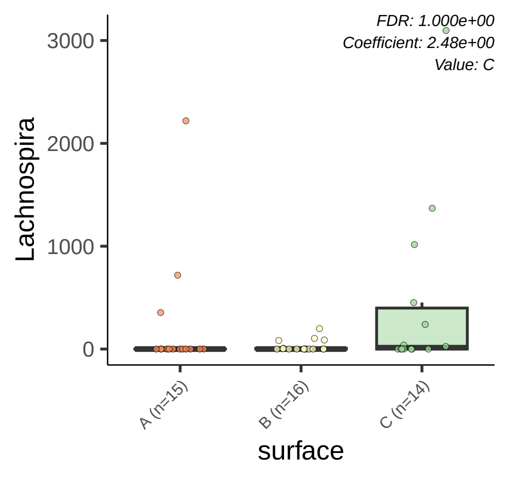
FDR: 1.000e+00
Coefficient: 2.48e+00
Value: C
Lachnospira
3000
2000
1000
0
A (n=15)
B (n=16)
C (n=14)
surface

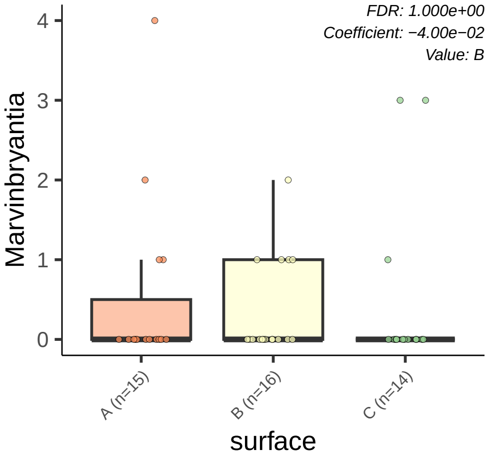
FDR: 1.000e+00
Coefficient: −4.00e−02
Value: B
Marvinbryantia
0
1
2
3
4
A (n=15)
B (n=16)
C (n=14)
surface

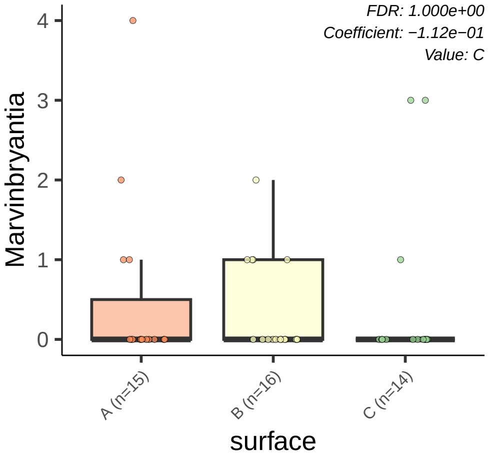

FDR: 1.000e+00
Coefficient: −1.12e−01
Value: C
Marvinbryantia
4
3
2
1
0
A (n=15)
B (n=16)
C (n=14)
surface

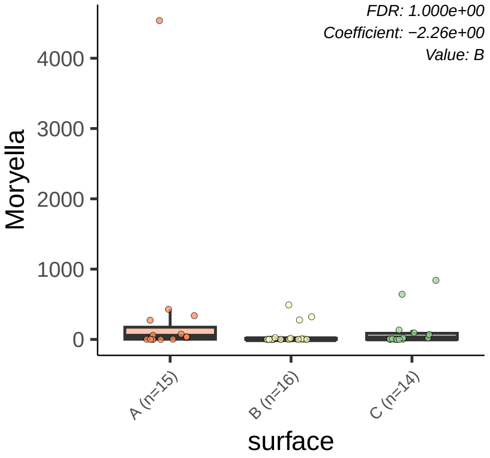
FDR: 1.000e+00
Coefficient: −2.26e+00
Value: B
4000
3000
2000
1000
0
Moryella
A (n=15)
B (n=16)
C (n=14)
surface

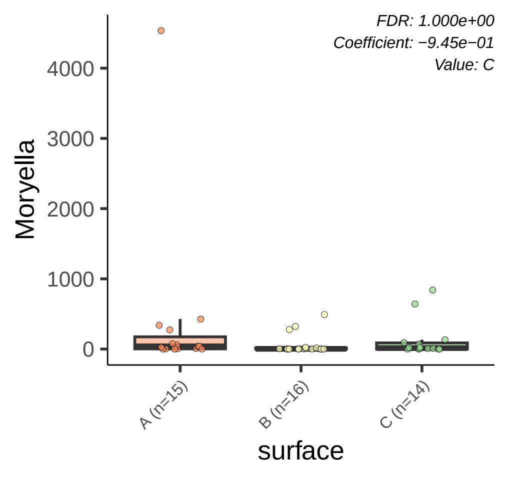
FDR: 1.000e+00
Coefficient: −9.45e−01
Value: C
Moryella
4000
3000
2000
1000
0
A (n=15)
B (n=16)
C (n=14)
surface

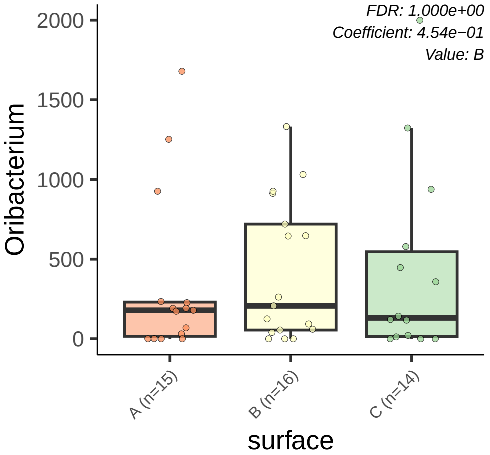

FDR: 1.000e+00
Coefficient: 4.54e−01
Value: B
Oribacterium
2000
1500
1000
500
0
A (n=15)
B (n=16)
C (n=14)
surface

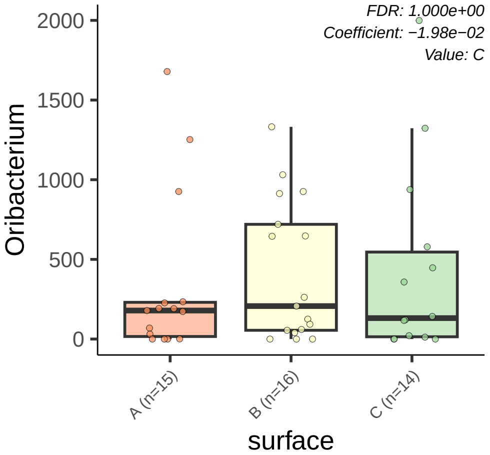
FDR: 1.000e+00
Coefficient: −1.98e−02
Value: C
Oribacterium
2000
1500
1000
500
0
A (n=15)
B (n=16)
C (n=14)
surface

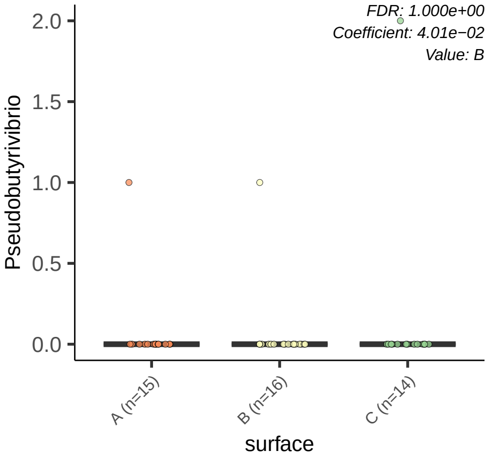
FDR: 1.000e+00
Coefficient: 4.01e−02
Value: B
Pseudobutyrivibrio
2.0
1.5
1.0
0.5
0.0
A (n=15)
B (n=16)
C (n=14)
surface

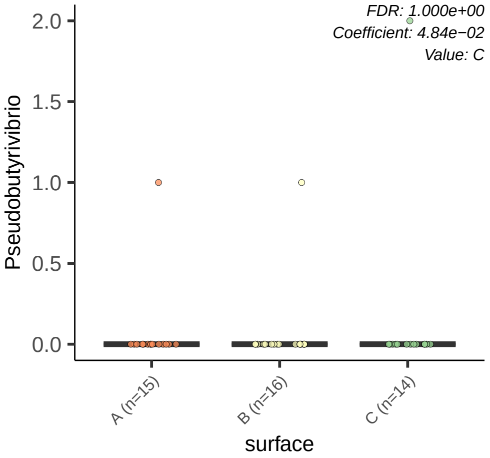
FDR: 1.000e+00
Coefficient: 4.84e−02
Value: C
Pseudobutyrivibrio
2.0
1.5
1.0
0.5
0.0
A (n=15)
B (n=16)
C (n=14)
surface

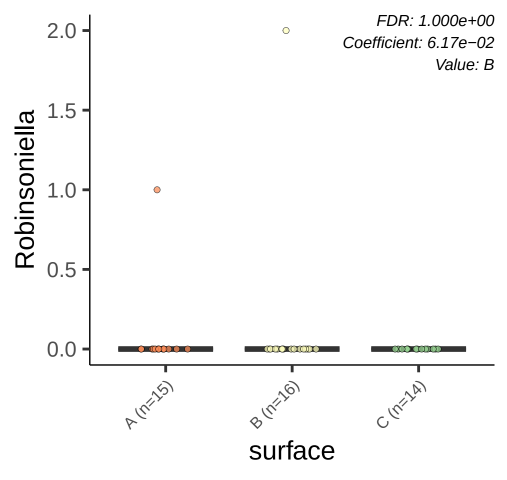
FDR: 1.000e+00
Coefficient: 6.17e−02
Value: B
Robinsoniella
2.0
1.5
1.0
0.5
0.0
A (n=15)
B (n=16)
C (n=14)
surface

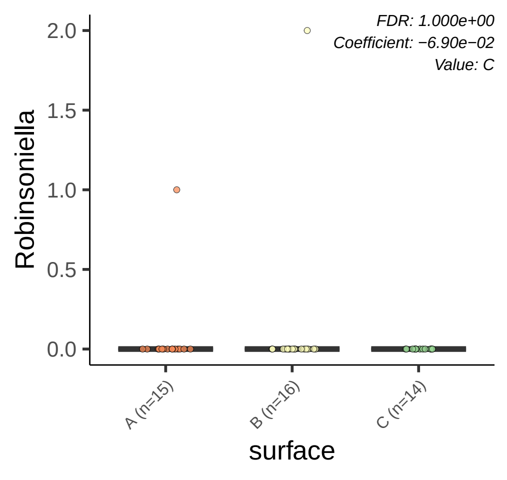

FDR: 1.000e+00
Coefficient: −6.90e−02
Value: C
Robinsoniella
2.0
1.5
1.0
0.5
0.0
A (n=15)
B (n=16)
C (n=14)
surface

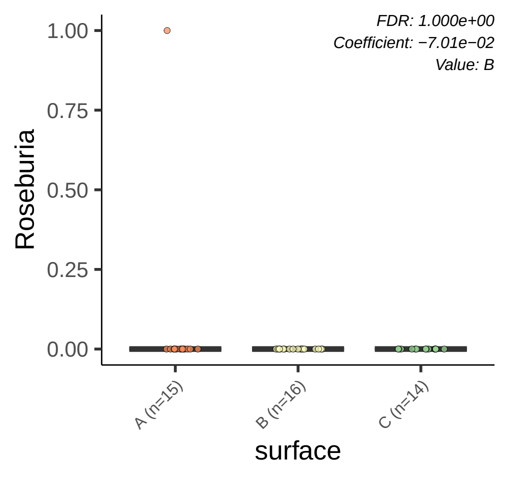

FDR: 1.000e+00
Coefficient: −7.01e−02
Value: B
Roseburia
1.00
0.75
0.50
0.25
0.00
A (n=15)
B (n=16)
C (n=14)
surface

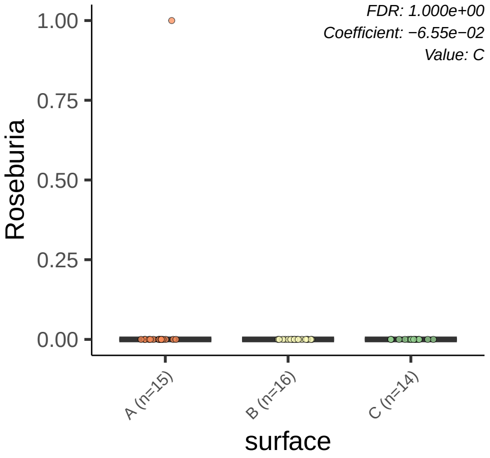
FDR: 1.000e+00
Coefficient: −6.55e−02
Value: C
Roseburia
1.00
0.75
0.50
0.25
0.00
A (n=15)
B (n=16)
C (n=14)
surface

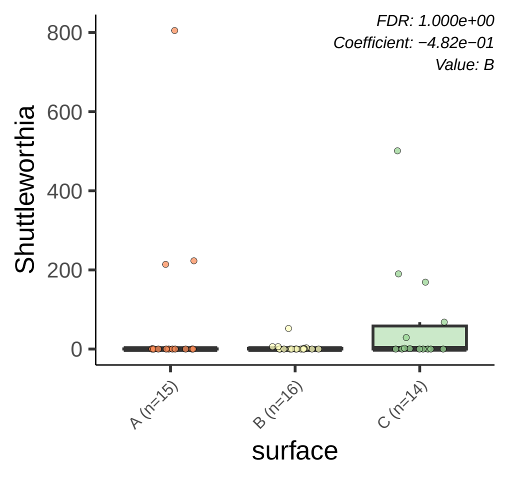

FDR: 1.000e+00
Coefficient: −4.82e−01
Value: B
Shuttleworthia
800
600
400
200
0
A (n=15)
B (n=16)
C (n=14)
surface

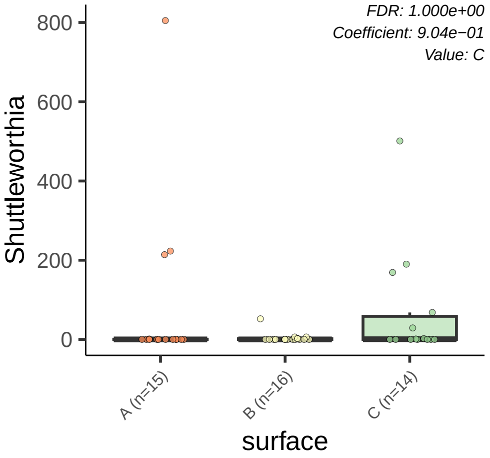
FDR: 1.000e+00
Coefficient: 9.04e−01
Value: C
Shuttleworthia
800
600
400
200
0
A (n=15)
B (n=16)
C (n=14)
surface

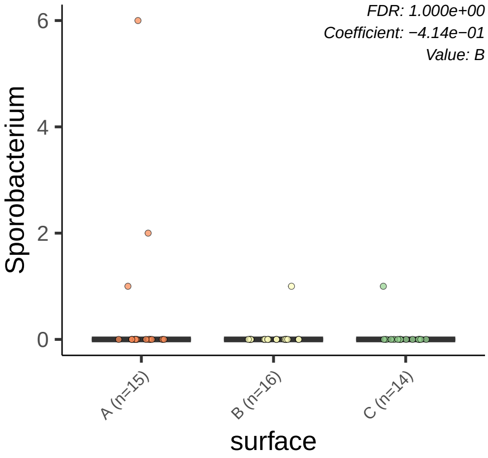
FDR: 1.000e+00
Coefficient: −4.14e−01
Value: B
Sporobacterium
6
4
2
0
A (n=15)
B (n=16)
C (n=14)
surface

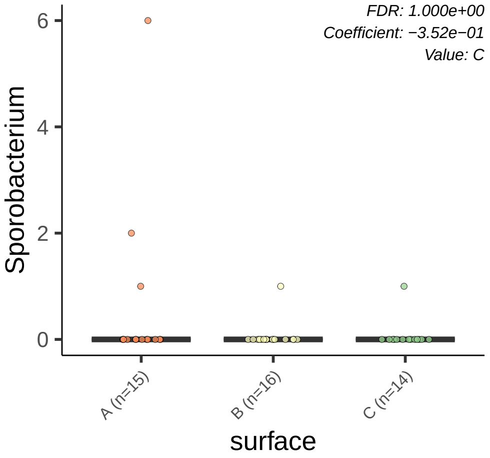
FDR: 1.000e+00
Coefficient: −3.52e−01
Value: C
Sporobacterium
0
2
4
6
A (n=15)
B (n=16)
C (n=14)
surface

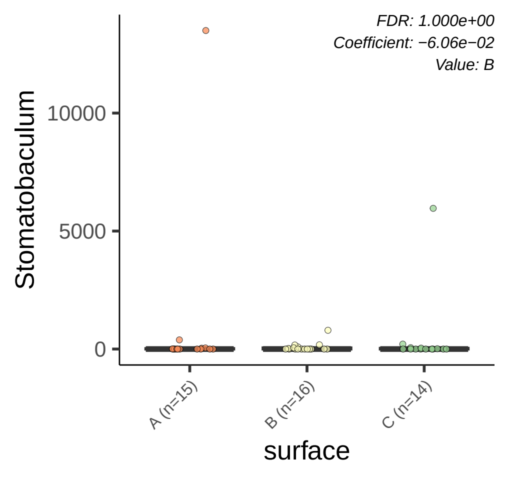
FDR: 1.000e+00
Coefficient: −6.06e−02
Value: B
Stomatobaculum
10000
5000
0
A (n=15)
B (n=16)
C (n=14)
surface

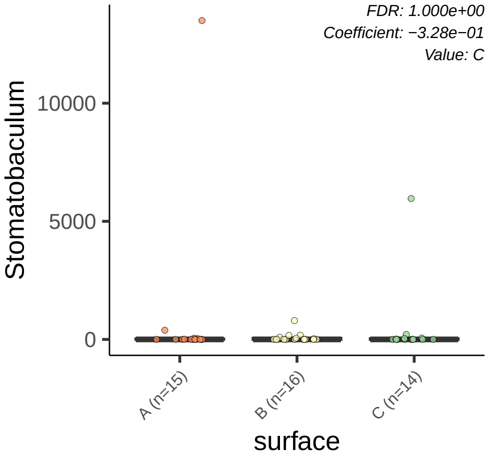

FDR: 1.000e+00
Coefficient: −3.28e−01
Value: C
Stomatobaculum
10000
5000
0
A (n=15)
B (n=16)
C (n=14)
surface

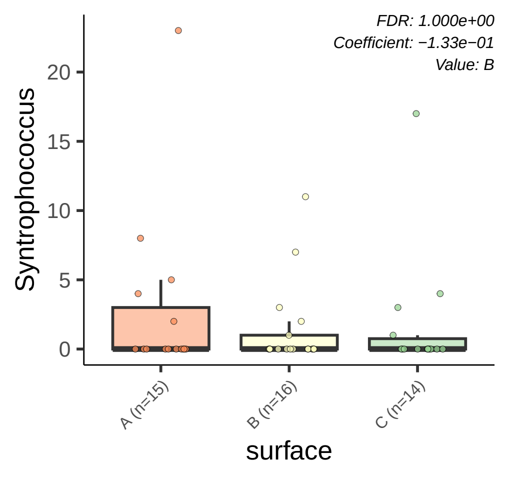

FDR: 1.000e+00
Coefficient: −1.33e−01
Value: B
Syntrophococcus
20
15
10
5
0
A (n=15)
B (n=16)
C (n=14)
surface

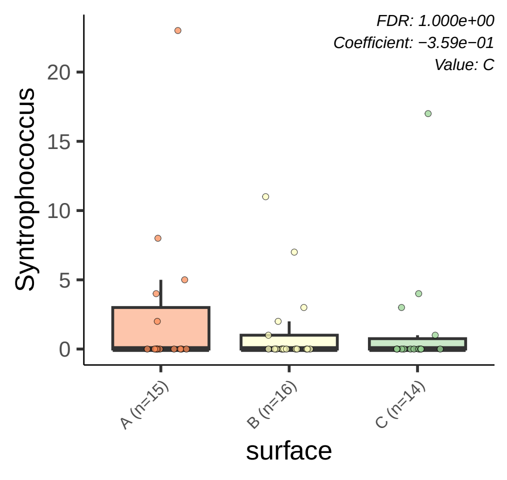
FDR: 1.000e+00
Coefficient: −3.59e−01
Value: C
Syntrophococcus
20
15
10
5
0
A (n=15)
B (n=16)
C (n=14)
surface

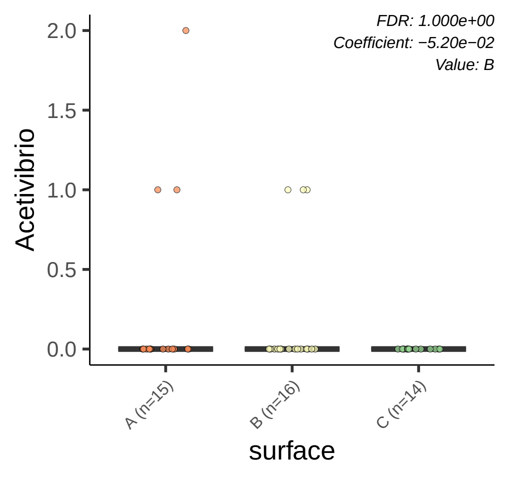

FDR: 1.000e+00
Coefficient: −5.20e−02
Value: B
Acetivibrio
2.0
1.5
1.0
0.5
0.0
A (n=15)
B (n=16)
C (n=14)
surface

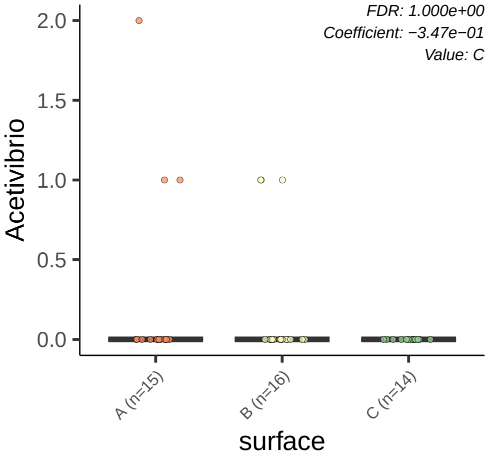

FDR: 1.000e+00
Coefficient: −3.47e−01
Value: C
Acetivibrio
2.0
1.5
1.0
0.5
0.0
A (n=15)
B (n=16)
C (n=14)
surface

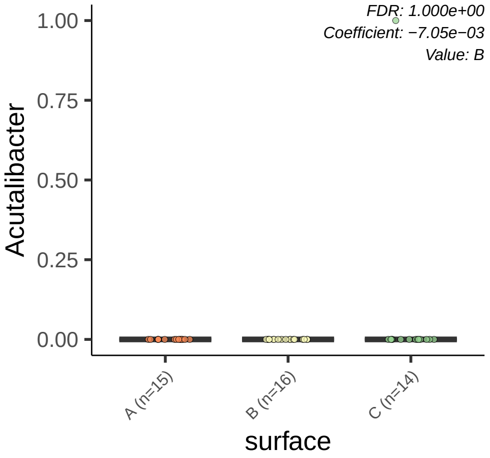
FDR: 1.000e+00
Coefficient: −7.05e−03
Value: B
1.00
0.75
0.50
0.25
0.00
Acutalibacter
A (n=15)
B (n=16)
C (n=14)
surface

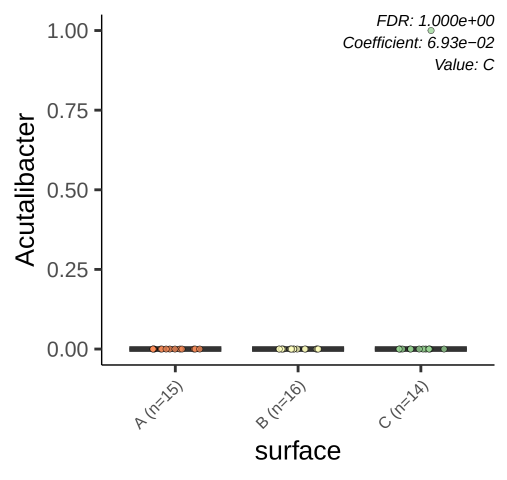
FDR: 1.000e+00
Coefficient: 6.93e−02
Value: C
Acutalibacter
1.00
0.75
0.50
0.25
0.00
A (n=15)
B (n=16)
C (n=14)
surface

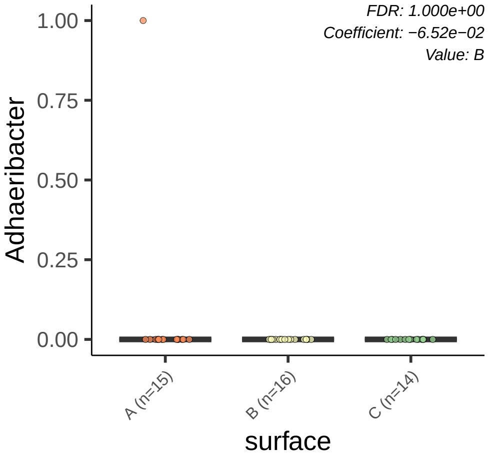

FDR: 1.000e+00
Coefficient: −6.52e−02
Value: B
Adhaeribacter
1.00
0.75
0.50
0.25
0.00
A (n=15)
B (n=16)
C (n=14)
surface

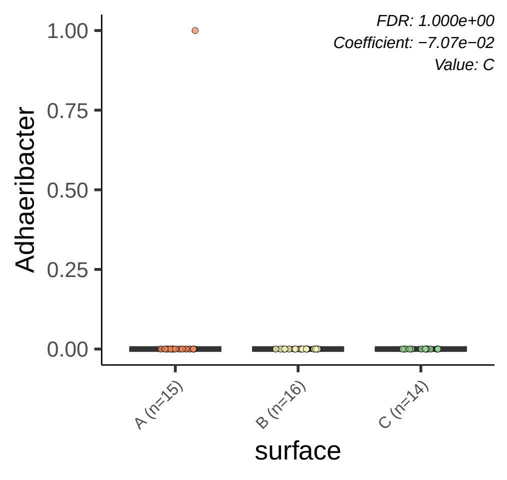
FDR: 1.000e+00
Coefficient: −7.07e−02
Value: C
1.00
0.75
0.50
0.25
0.00
Adhaeribacter
A (n=15)
B (n=16)
C (n=14)
surface

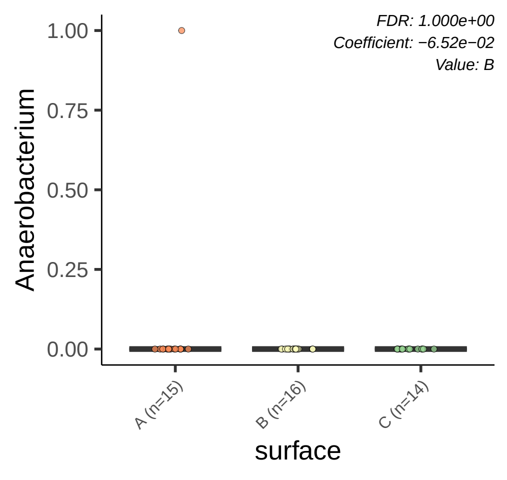

FDR: 1.000e+00
Coefficient: −6.52e−02
Value: B
Anaerobacterium
1.00
0.75
0.50
0.25
0.00
A (n=15)
B (n=16)
C (n=14)
surface

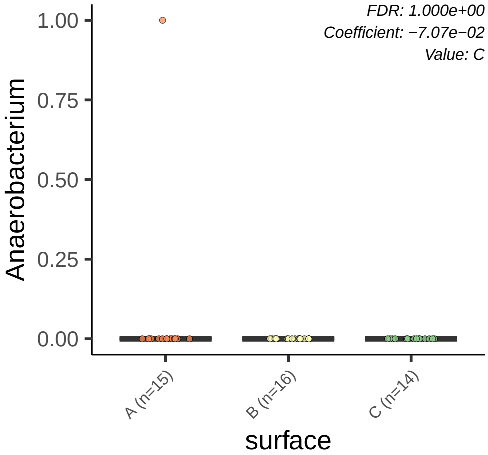
FDR: 1.000e+00
Coefficient: −7.07e−02
Value: C
Anaerobacterium
1.00
0.75
0.50
0.25
0.00
A (n=15)
B (n=16)
C (n=14)
surface

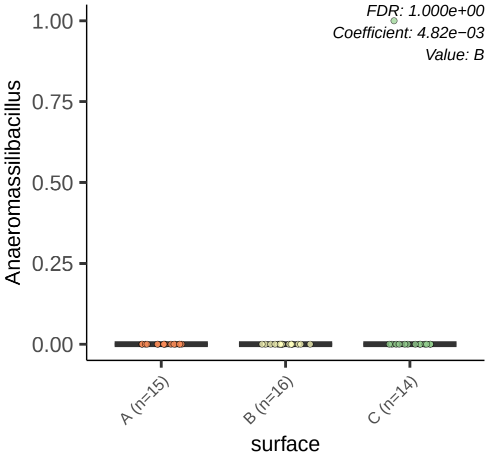
FDR: 1.000e+00
Coefficient: 4.82e−03
Value: B
1.00
0.75
0.50
0.25
0.00
Anaeromassilibacillus
A (n=15)
B (n=16)
C (n=14)
surface

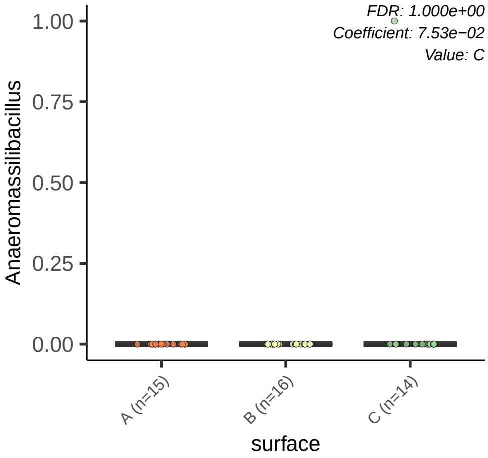
FDR: 1.000e+00
Coefficient: 7.53e−02
Value: C
Anaeromassilibacillus
1.00
0.75
0.50
0.25
0.00
A (n=15)
B (n=16)
C (n=14)
surface

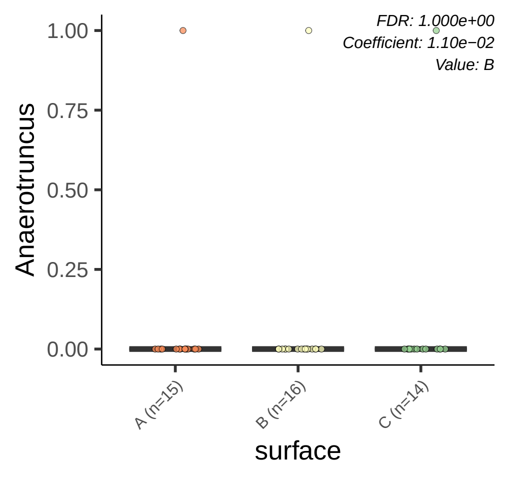

FDR: 1.000e+00
Coefficient: 1.10e−02
Value: B
Anaerotruncus
1.00
0.75
0.50
0.25
0.00
A (n=15)
B (n=16)
C (n=14)
surface

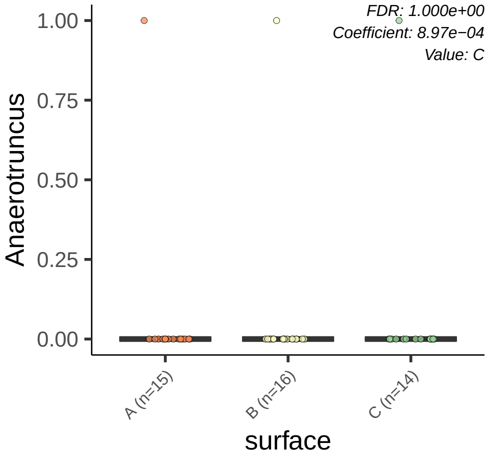

FDR: 1.000e+00
Coefficient: 8.97e−04
Value: C
Anaerotruncus
1.00
0.75
0.50
0.25
0.00
A (n=15)
B (n=16)
C (n=14)
surface

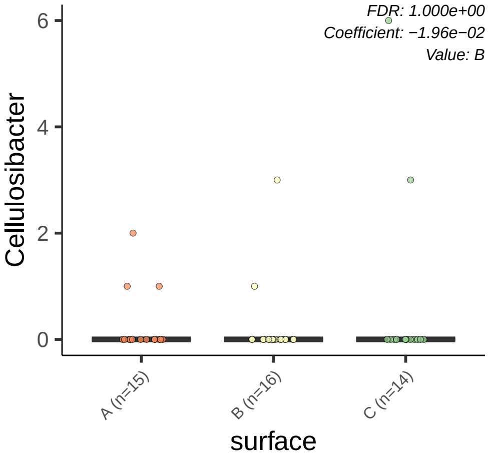

FDR: 1.000e+00
Coefficient: −1.96e−02
Value: B
Cellulosibacter
0
2
4
6
A (n=15)
B (n=16)
C (n=14)
surface

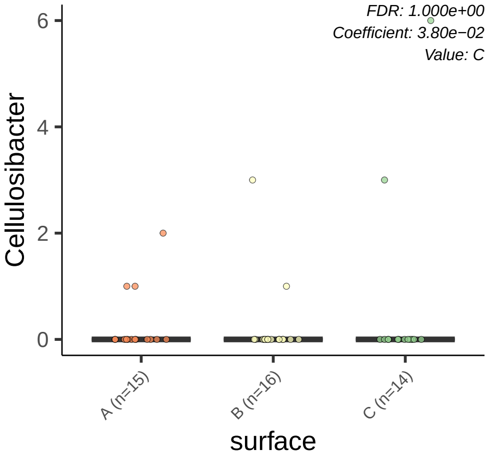
FDR: 1.000e+00
Coefficient: 3.80e−02
Value: C
Cellulosibacter
6
4
2
0
A (n=15)
B (n=16)
C (n=14)
surface

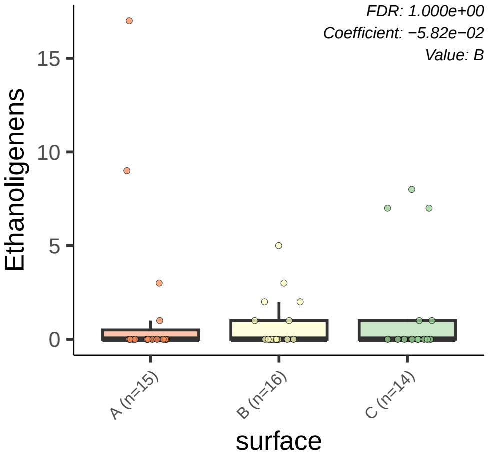
FDR: 1.000e+00
Coefficient: −5.82e−02
Value: B
Ethanoligenens
0
5
10
15
A (n=15)
B (n=16)
C (n=14)
surface

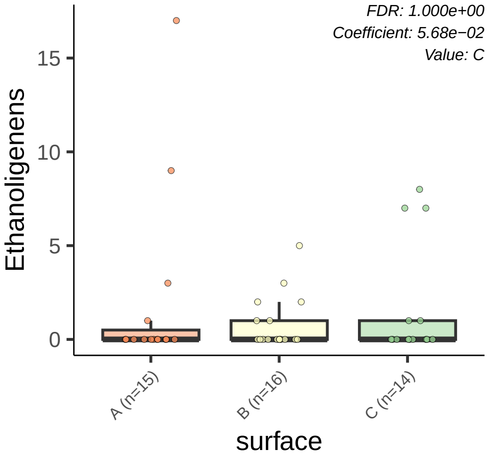

FDR: 1.000e+00
Coefficient: 5.68e−02
Value: C
Ethanoligenens
15
10
5
0
A (n=15)
B (n=16)
C (n=14)
surface

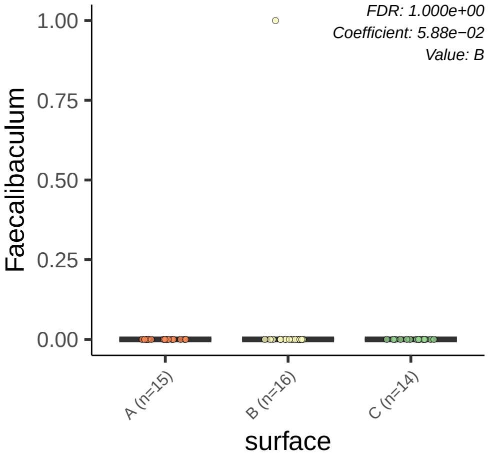

FDR: 1.000e+00
Coefficient: 5.88e−02
Value: B
Faecalibaculum
1.00
0.75
0.50
0.25
0.00
A (n=15)
B (n=16)
C (n=14)
surface

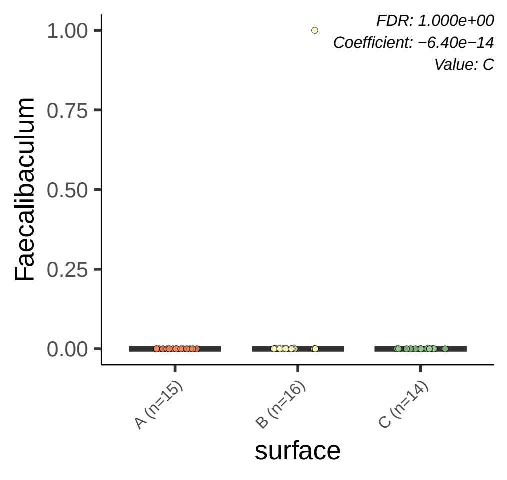

FDR: 1.000e+00
Coefficient: −6.40e−14
Value: C
1.00
0.75
0.50
0.25
0.00
Faecalibaculum
A (n=15)
B (n=16)
C (n=14)
surface

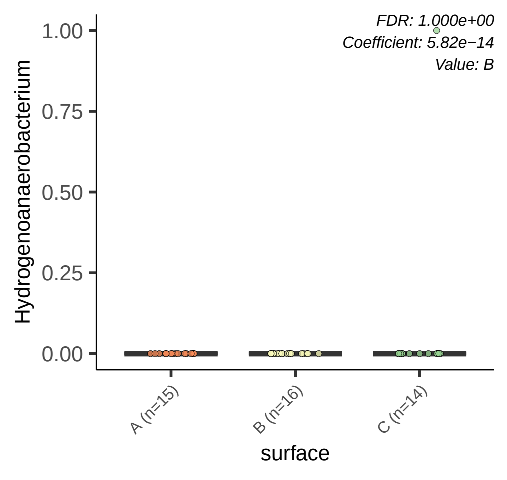
FDR: 1.000e+00
Coefficient: 5.82e−14
Value: B
Hydrogenoanaerobacterium
1.00
0.75
0.50
0.25
0.00
A (n=15)
B (n=16)
C (n=14)
surface

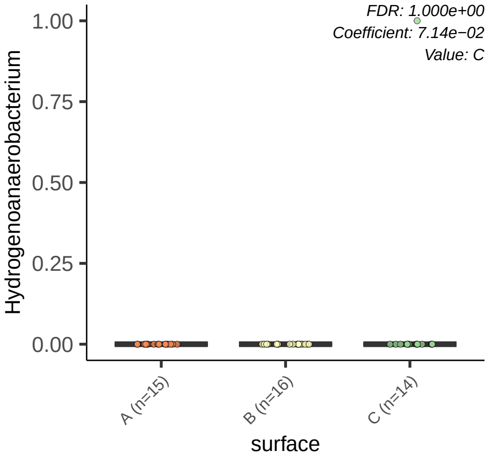
FDR: 1.000e+00
Coefficient: 7.14e−02
Value: C
Hydrogenoanaerobacterium
1.00
0.75
0.50
0.25
0.00
A (n=15)
B (n=16)
C (n=14)
surface

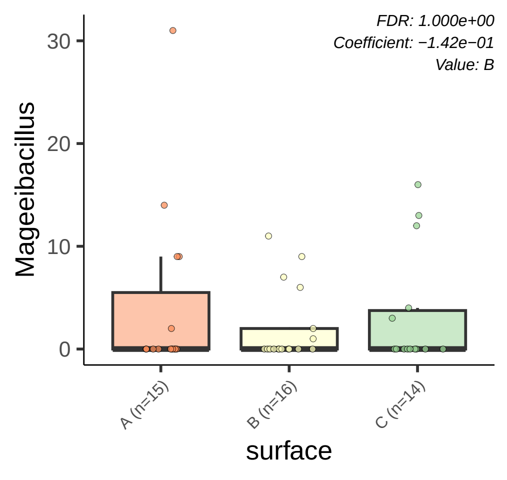

FDR: 1.000e+00
Coefficient: −1.42e−01
Value: B
Mageeibacillus
30
20
10
0
A (n=15)
B (n=16)
C (n=14)
surface

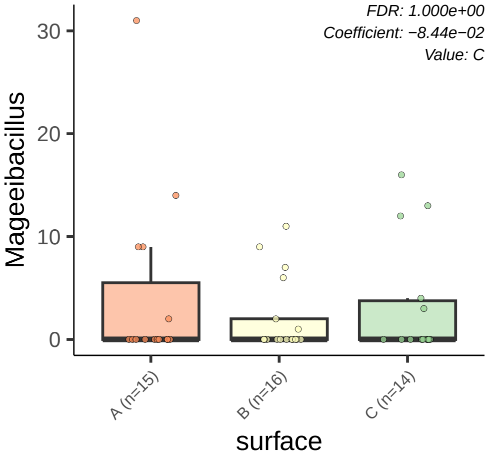
FDR: 1.000e+00
Coefficient: −8.44e−02
Value: C
Mageeibacillus
30
20
10
0
A (n=15)
B (n=16)
C (n=14)
surface

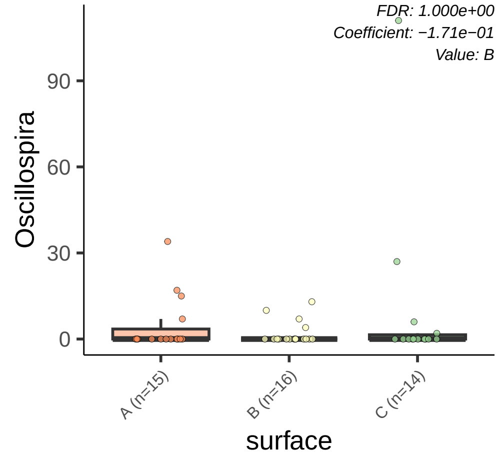
FDR: 1.000e+00
Coefficient: −1.71e−01
Value: B
Oscillospira
90
60
30
0
A (n=15)
B (n=16)
C (n=14)
surface

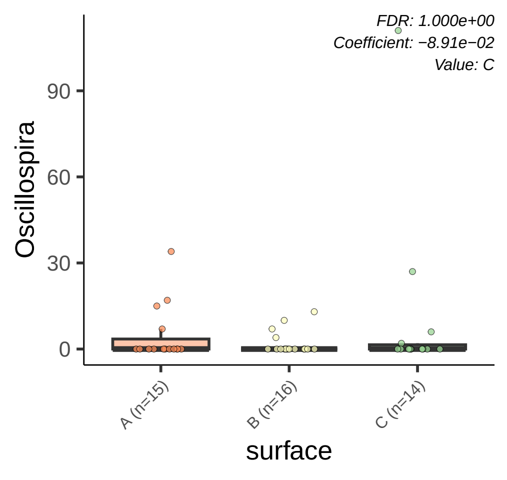

FDR: 1.000e+00
Coefficient: −8.91e−02
Value: C
Oscillospira
90
60
30
0
A (n=15)
B (n=16)
C (n=14)
surface

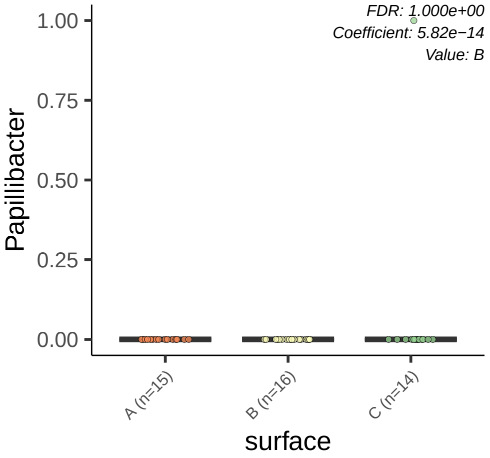

FDR: 1.000e+00
Coefficient: 5.82e−14
Value: B
Papillibacter
1.00
0.75
0.50
0.25
0.00
A (n=15)
B (n=16)
C (n=14)
surface

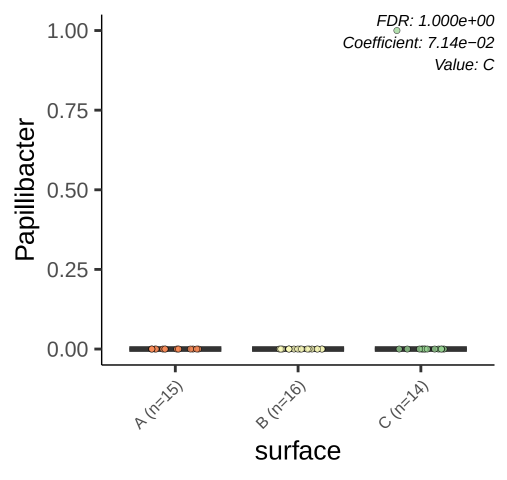
FDR: 1.000e+00
Coefficient: 7.14e−02
Value: C
1.00
0.75
0.50
0.25
0.00
Papillibacter
A (n=15)
B (n=16)
C (n=14)
surface

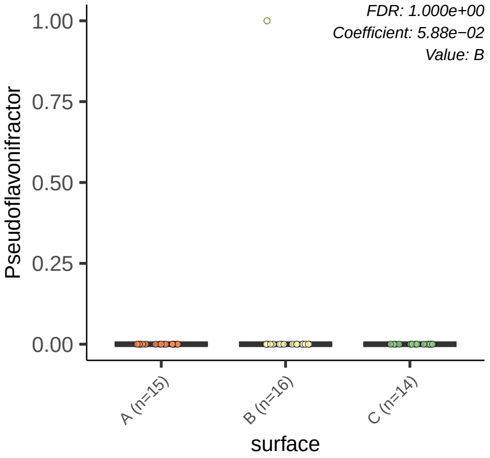

FDR: 1.000e+00
Coefficient: 5.88e−02
Value: B
Pseudoflavonifractor
1.00
0.75
0.50
0.25
0.00
A (n=15)
B (n=16)
C (n=14)
surface

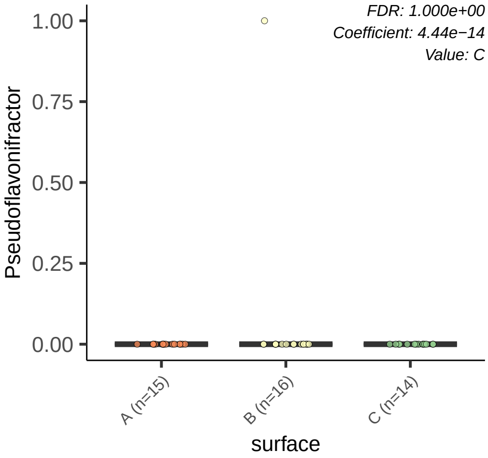
FDR: 1.000e+00
Coefficient: 4.44e−14
Value: C
Pseudoflavonifractor
1.00
0.75
0.50
0.25
0.00
A (n=15)
B (n=16)
C (n=14)
surface

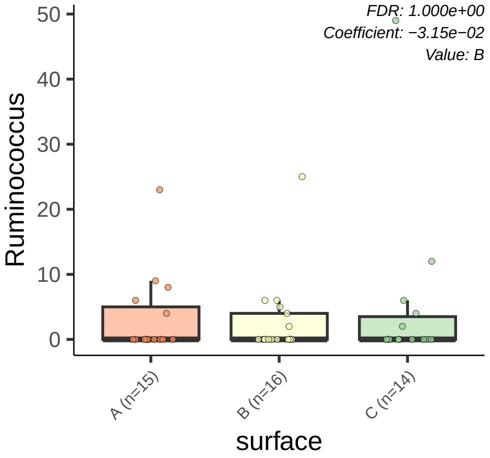
FDR: 1.000e+00
Coefficient: −3.15e−02
Value: B
Ruminococcus
0
10
20
30
40
50
A (n=15)
B (n=16)
C (n=14)
surface

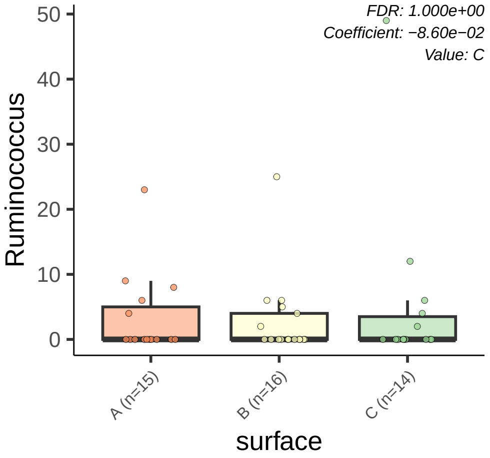

FDR: 1.000e+00
Coefficient: −8.60e−02
Value: C
Ruminococcus
0
10
20
30
40
50
A (n=15)
B (n=16)
C (n=14)
surface

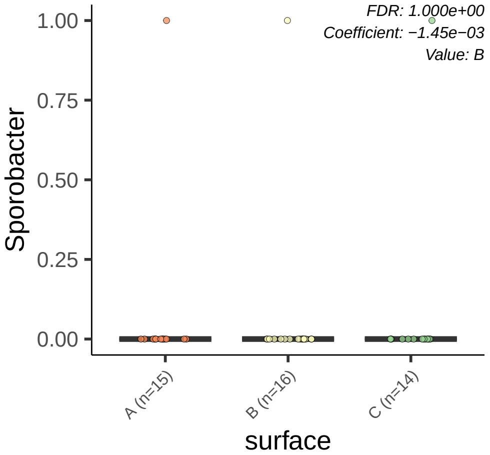

FDR: 1.000e+00
Coefficient: −1.45e−03
Value: B
Sporobacter
1.00
0.75
0.50
0.25
0.00
A (n=15)
B (n=16)
C (n=14)
surface

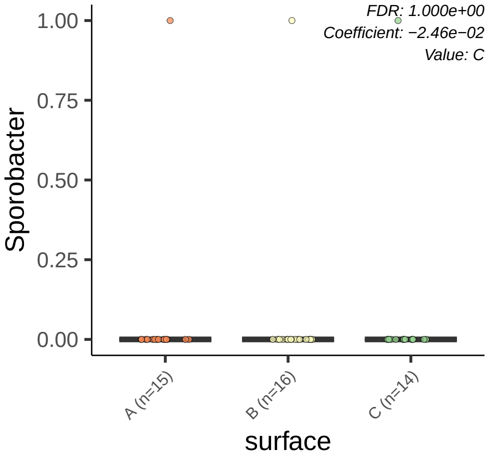

FDR: 1.000e+00
Coefficient: −2.46e−02
Value: C
Sporobacter
1.00
0.75
0.50
0.25
0.00
A (n=15)
B (n=16)
C (n=14)
surface

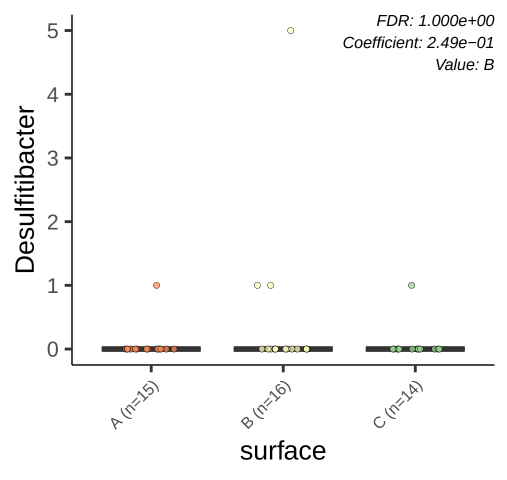
FDR: 1.000e+00
Coefficient: 2.49e−01
Value: B
Desulfitibacter
0
1
2
3
4
5
A (n=15)
B (n=16)
C (n=14)
surface

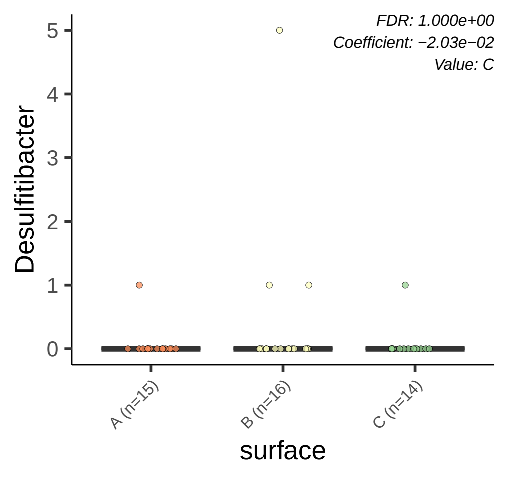
FDR: 1.000e+00
Coefficient: −2.03e−02
Value: C
Desulfitibacter
5
4
3
2
1
0
A (n=15)
B (n=16)
C (n=14)
surface

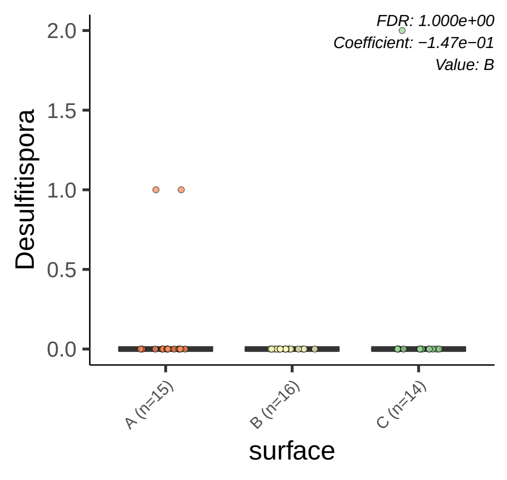

FDR: 1.000e+00
Coefficient: −1.47e−01
Value: B
Desulfitispora
2.0
1.5
1.0
0.5
0.0
A (n=15)
B (n=16)
C (n=14)
surface

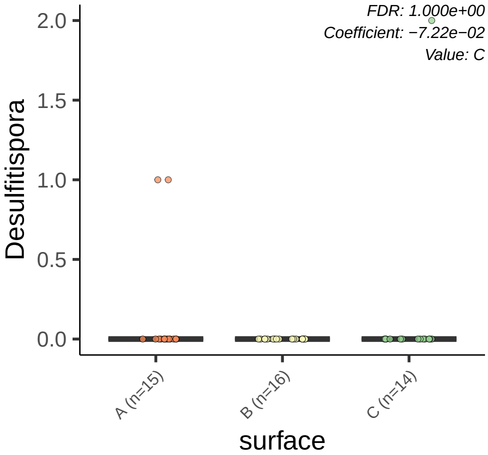
FDR: 1.000e+00
Coefficient: −7.22e−02
Value: C
Desulfitispora
2.0
1.5
1.0
0.5
0.0
A (n=15)
B (n=16)
C (n=14)
surface

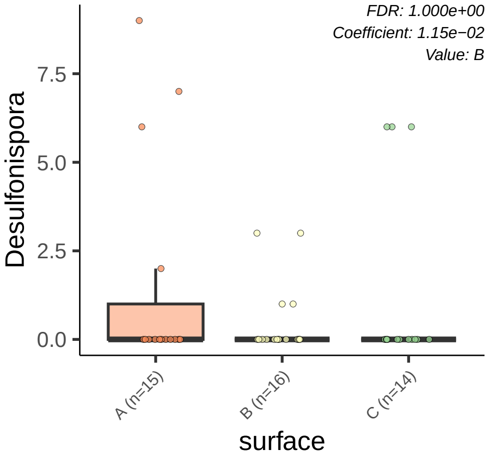
FDR: 1.000e+00
Coefficient: 1.15e−02
Value: B
Desulfonispora
7.5
5.0
2.5
0.0
A (n=15)
B (n=16)
C (n=14)
surface

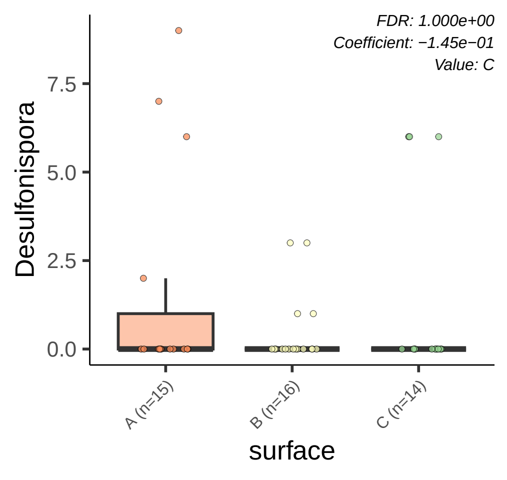

FDR: 1.000e+00
Coefficient: −1.45e−01
Value: C
Desulfonispora
7.5
5.0
2.5
0.0
A (n=15)
B (n=16)
C (n=14)
surface

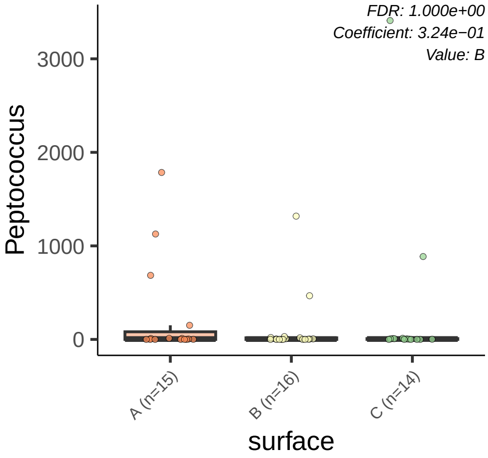

FDR: 1.000e+00
Coefficient: 3.24e−01
Value: B
3000
2000
1000
0
Peptococcus
A (n=15)
B (n=16)
C (n=14)
surface

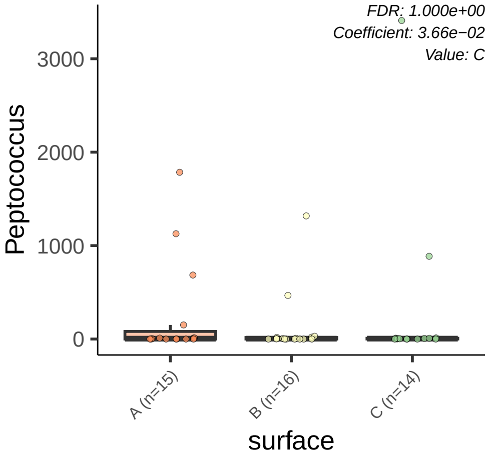

FDR: 1.000e+00
Coefficient: 3.66e−02
Value: C
Peptococcus
3000
2000
1000
0
A (n=15)
B (n=16)
C (n=14)
surface

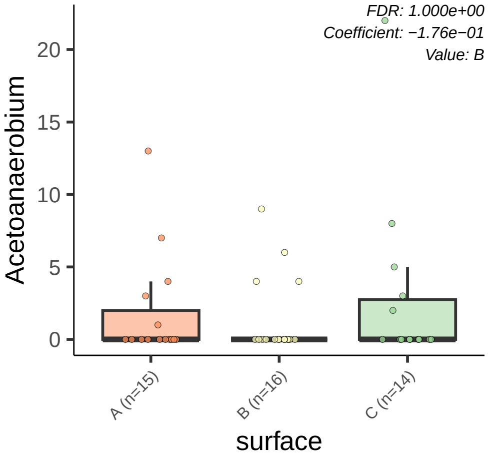
FDR: 1.000e+00
Coefficient: −1.76e−01
Value: B
Acetoanaerobium
0
5
10
15
20
A (n=15)
B (n=16)
C (n=14)
surface

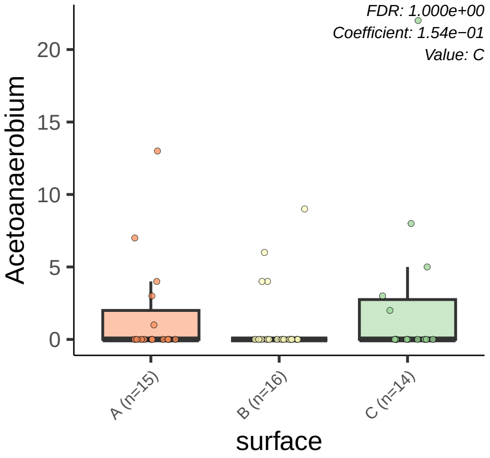
FDR: 1.000e+00
Coefficient: 1.54e−01
Value: C
Acetoanaerobium
0
5
10
15
20
A (n=15)
B (n=16)
C (n=14)
surface

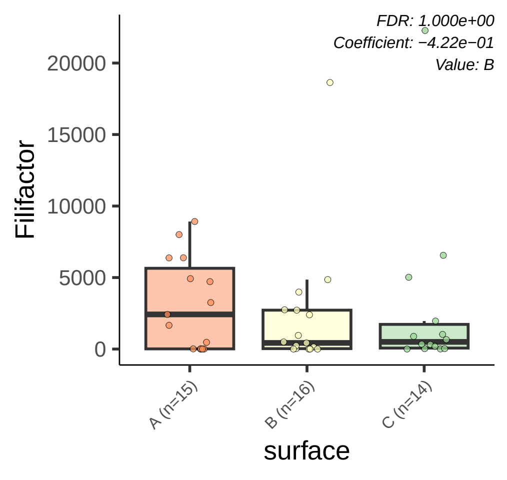
FDR: 1.000e+00
Coefficient: −4.22e−01
Value: B
Filifactor
20000
15000
10000
5000
0
A (n=15)
B (n=16)
C (n=14)
surface

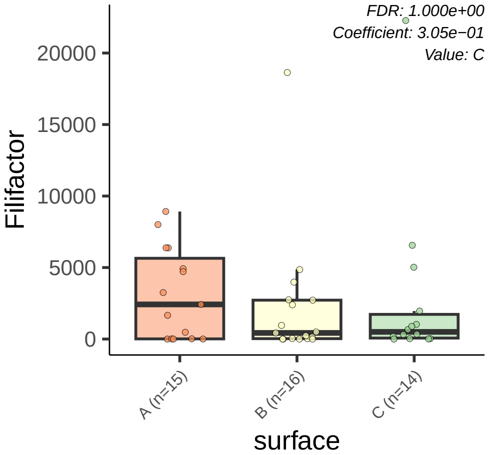

FDR: 1.000e+00
Coefficient: 3.05e−01
Value: C
Filifactor
20000
15000
10000
5000
0
A (n=15)
B (n=16)
C (n=14)
surface

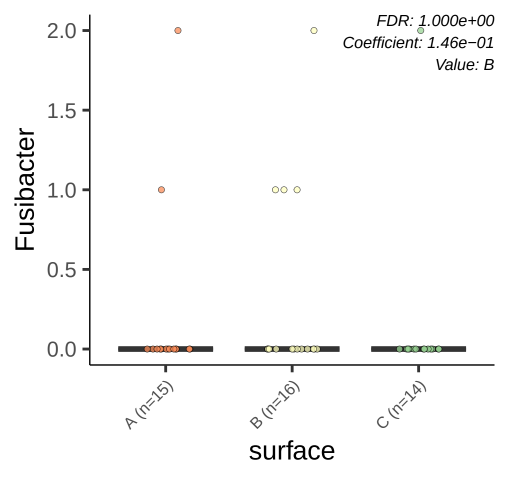

FDR: 1.000e+00
Coefficient: 1.46e−01
Value: B
Fusibacter
2.0
1.5
1.0
0.5
0.0
A (n=15)
B (n=16)
C (n=14)
surface

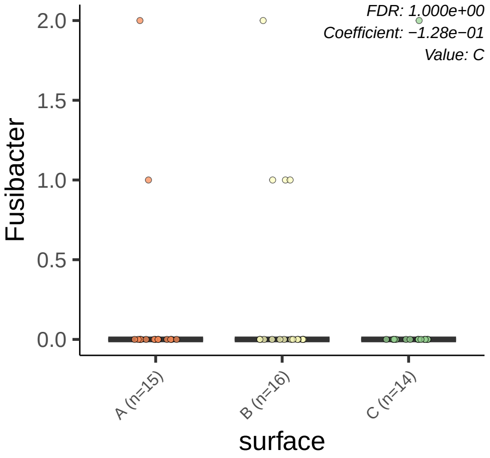
FDR: 1.000e+00
Coefficient: −1.28e−01
Value: C
Fusibacter
2.0
1.5
1.0
0.5
0.0
A (n=15)
B (n=16)
C (n=14)
surface

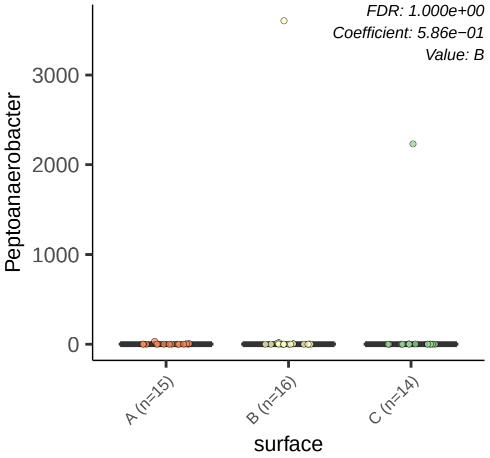

FDR: 1.000e+00
Coefficient: 5.86e−01
Value: B
Peptoanaerobacter
3000
2000
1000
0
A (n=15)
B (n=16)
C (n=14)
surface

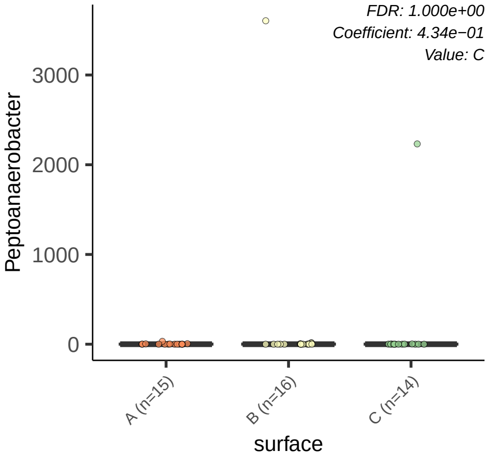
FDR: 1.000e+00
Coefficient: 4.34e−01
Value: C
Peptoanaerobacter
3000
2000
1000
0
A (n=15)
B (n=16)
C (n=14)
surface

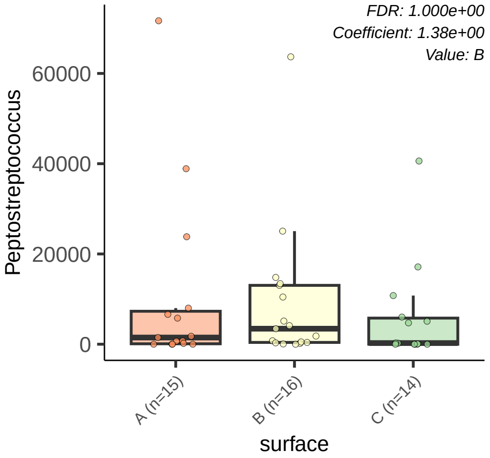
FDR: 1.000e+00
Coefficient: 1.38e+00
Value: B
Peptostreptococcus
60000
40000
20000
0
A (n=15)
B (n=16)
C (n=14)
surface

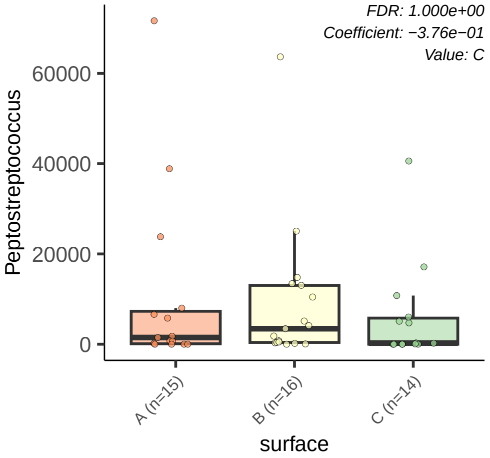

FDR: 1.000e+00
Coefficient: −3.76e−01
Value: C
Peptostreptococcus
60000
40000
20000
0
A (n=15)
B (n=16)
C (n=14)
surface

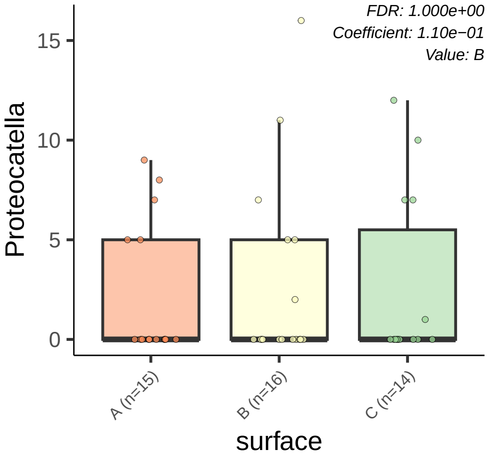

FDR: 1.000e+00
Coefficient: 1.10e−01
Value: B
Proteocatella
15
10
5
0
A (n=15)
B (n=16)
C (n=14)
surface

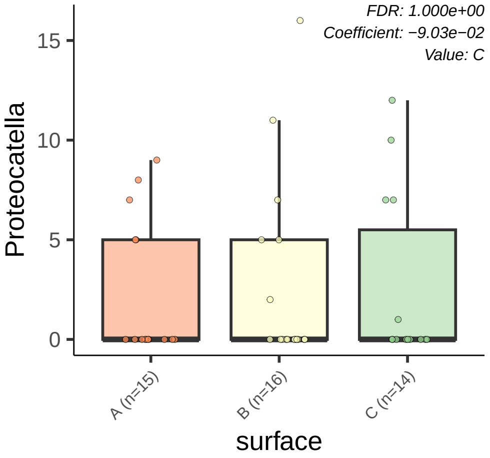

FDR: 1.000e+00
Coefficient: −9.03e−02
Value: C
Proteocatella
0
5
10
15
A (n=15)
B (n=16)
C (n=14)
surface

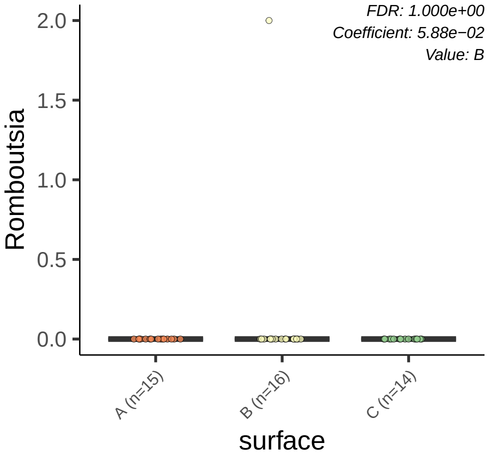
FDR: 1.000e+00
Coefficient: 5.88e−02
Value: B
Romboutsia
2.0
1.5
1.0
0.5
0.0
A (n=15)
B (n=16)
C (n=14)
surface

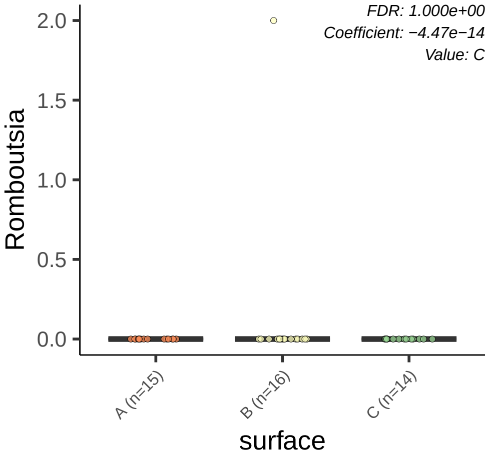
FDR: 1.000e+00
Coefficient: −4.47e−14
Value: C
Rombousia
2.0
1.5
1.0
0.5
0.0
A (n=15)
B (n=16)
C (n=14)
surface

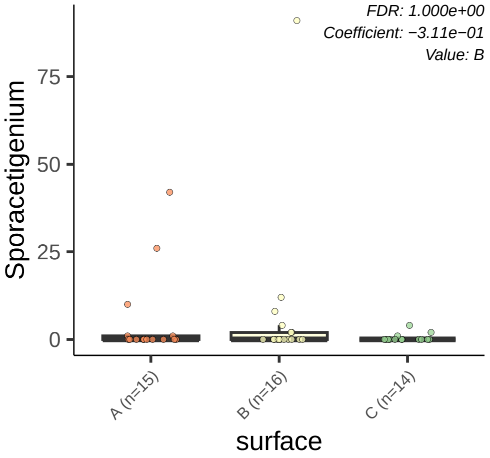

FDR: 1.000e+00
Coefficient: −3.11e−01
Value: B
Sporacetigenium
0
25
50
75
A (n=15)
B (n=16)
C (n=14)
surface

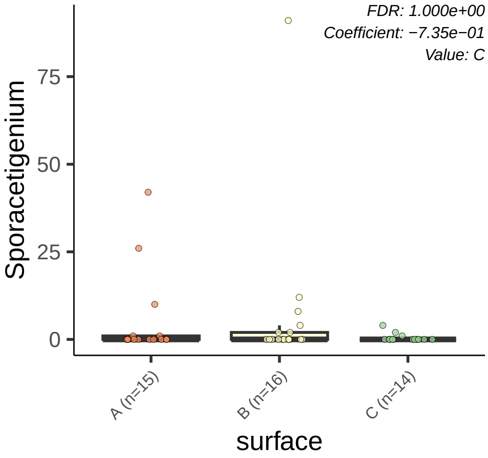
FDR: 1.000e+00
Coefficient: −7.35e−01
Value: C
Sporacetigenium
0
25
50
75
A (n=15)
B (n=16)
C (n=14)
surface

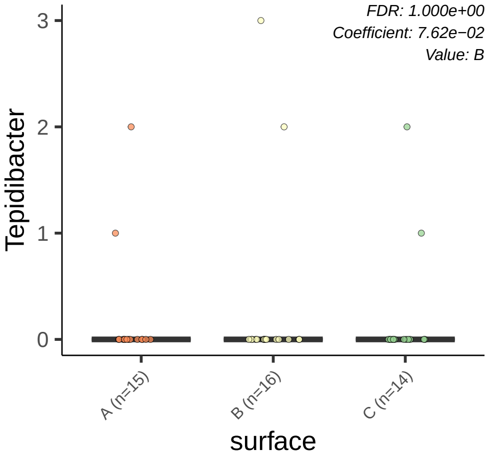

FDR: 1.000e+00
Coefficient: 7.62e−02
Value: B
Tepidibacter
3
2
1
0
A (n=15)
B (n=16)
C (n=14)
surface

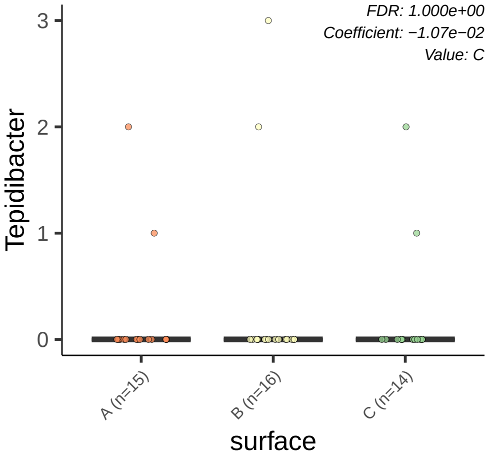
FDR: 1.000e+00
Coefficient: −1.07e−02
Value: C
Tepidibacter
3
2
1
0
A (n=15)
B (n=16)
C (n=14)
surface

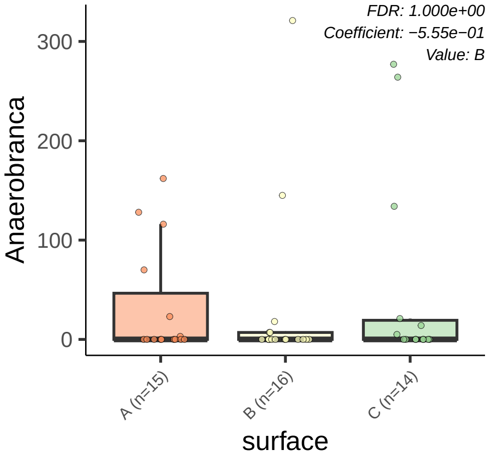

FDR: 1.000e+00
Coefficient: −5.55e−01
Value: B
Anaerobranca
300
200
100
0
A (n=15)
B (n=16)
C (n=14)
surface

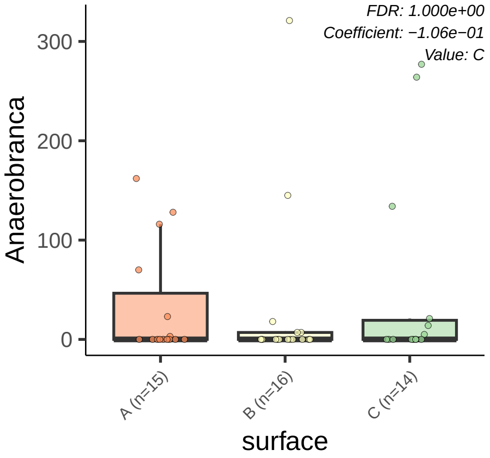

FDR: 1.000e+00
Coefficient: −1.06e−01
Value: C
Anaerobranca
300
200
100
0
A (n=15)
B (n=16)
C (n=14)
surface

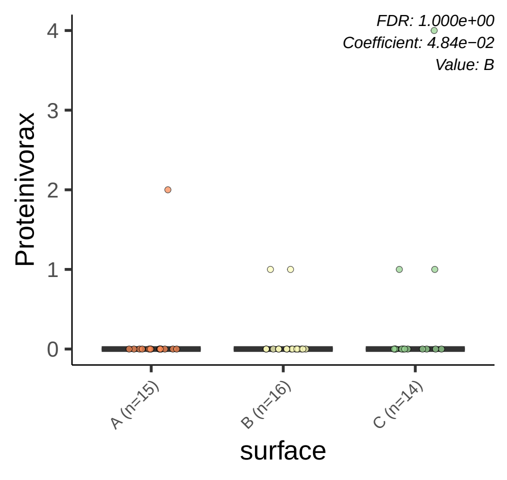
FDR: 1.000e+00
Coefficient: 4.84e−02
Value: B
Proteinivorax
0
1
2
3
4
A (n=15)
B (n=16)
C (n=14)
surface

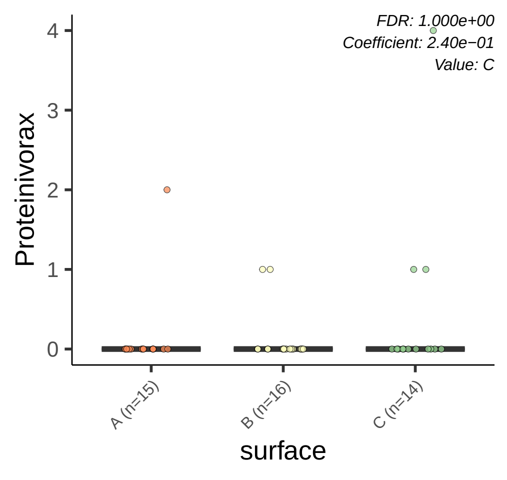

FDR: 1.000e+00
Coefficient: 2.40e−01
Value: C
Proteinivorax
4
3
2
1
0
A (n=15)
B (n=16)
C (n=14)
surface

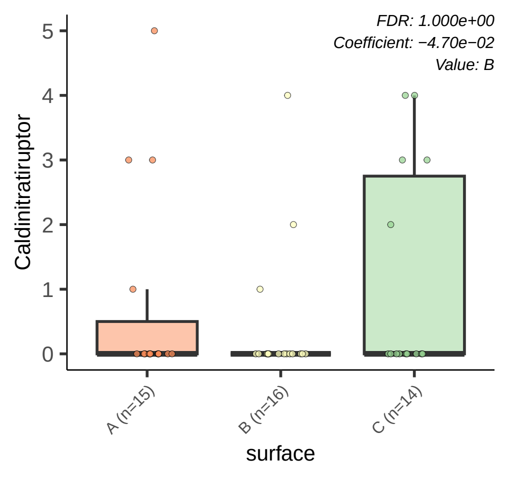

FDR: 1.000e+00
Coefficient: −4.70e−02
Value: B
Caldinitratiruptor
0
1
2
3
4
5
A (n=15)
B (n=16)
C (n=14)
surface

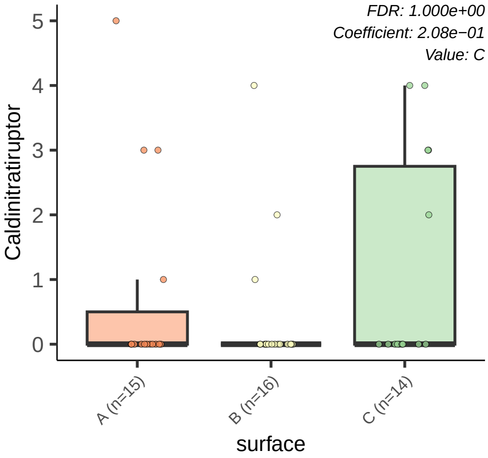
FDR: 1.000e+00
Coefficient: 2.08e−01
Value: C
Caldinitratiruptor
0
1
2
3
4
5
A (n=15)
B (n=16)
C (n=14)
surface

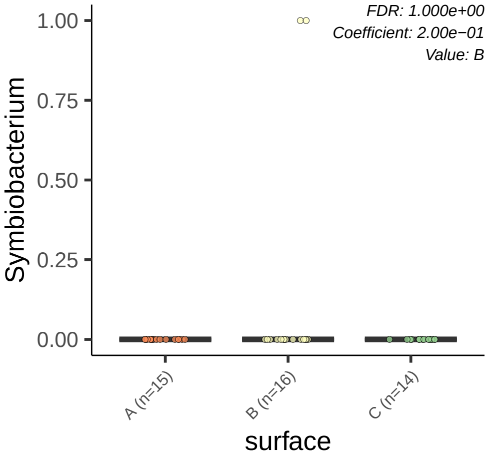

FDR: 1.000e+00
Coefficient: 2.00e−01
Value: B
Symbiobacterium
1.00
0.75
0.50
0.25
0.00
A (n=15)
B (n=16)
C (n=14)
surface

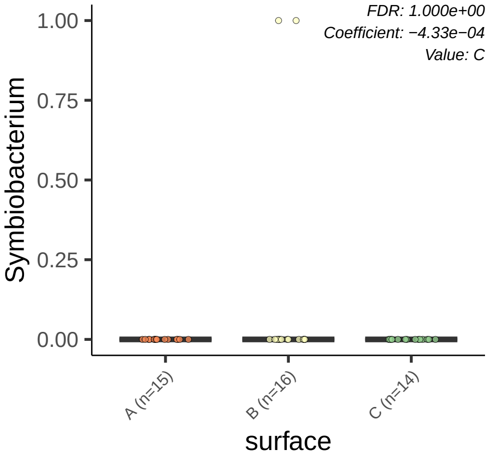
FDR: 1.000e+00
Coefficient: −4.33e−04
Value: C
Symbiobacterium
1.00
0.75
0.50
0.25
0.00
A (n=15)
B (n=16)
C (n=14)
surface

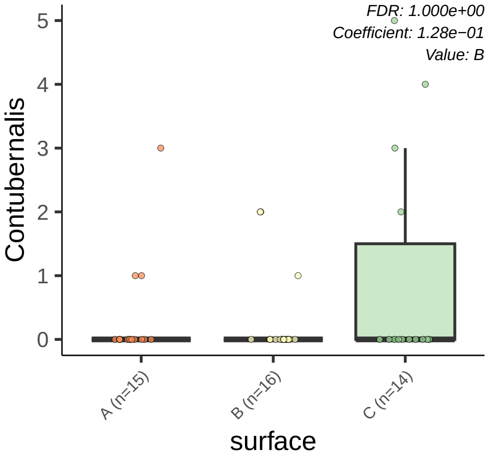

FDR: 1.000e+00
Coefficient: 1.28e−01
Value: B
Contubernalis
0
1
2
3
4
5
A (n=15)
B (n=16)
C (n=14)
surface

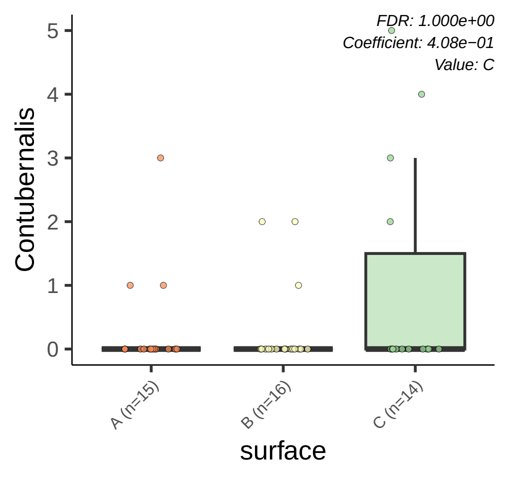

FDR: 1.000e+00
Coefficient: 4.08e−01
Value: C
Contubernalis
0
1
2
3
4
5
A (n=15)
B (n=16)
C (n=14)
surface

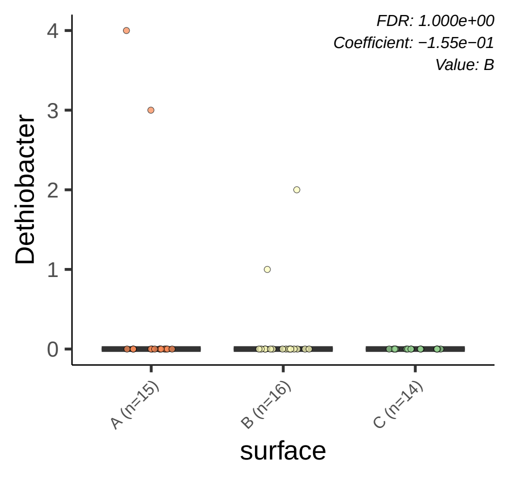
FDR: 1.000e+00
Coefficient: −1.55e−01
Value: B
Dethiobacter
4
3
2
1
0
A (n=15)
B (n=16)
C (n=14)
surface

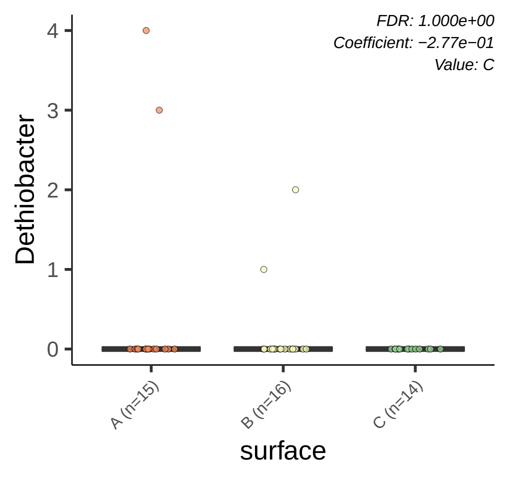
FDR: 1.000e+00
Coefficient: −2.77e−01
Value: C
Dethiobacter
4
3
2
1
0
A (n=15)
B (n=16)
C (n=14)
surface

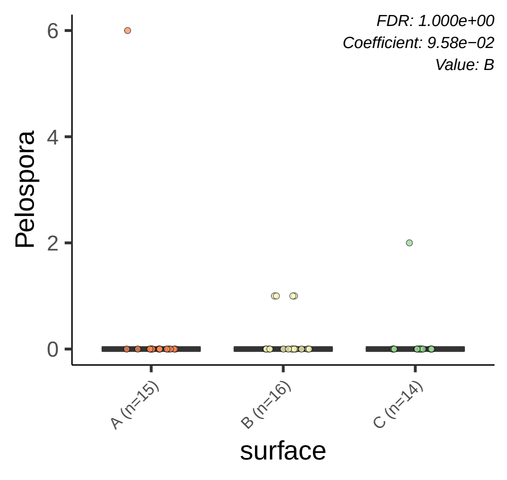
FDR: 1.000e+00
Coefficient: 9.58e−02
Value: B
Pelospora
6
4
2
0
A (n=15)
B (n=16)
C (n=14)
surface

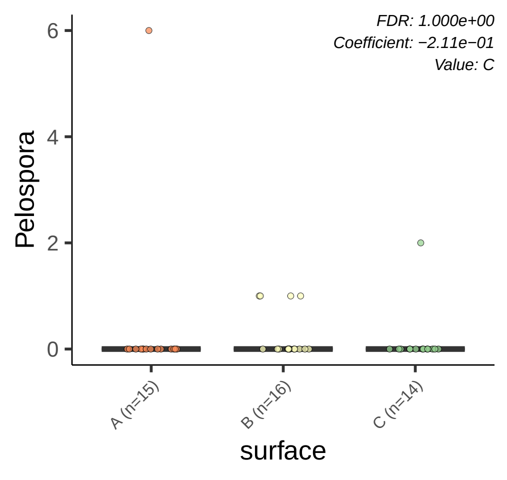
FDR: 1.000e+00
Coefficient: −2.11e−01
Value: C
Pelospora
6
4
2
0
A (n=15)
B (n=16)
C (n=14)
surface

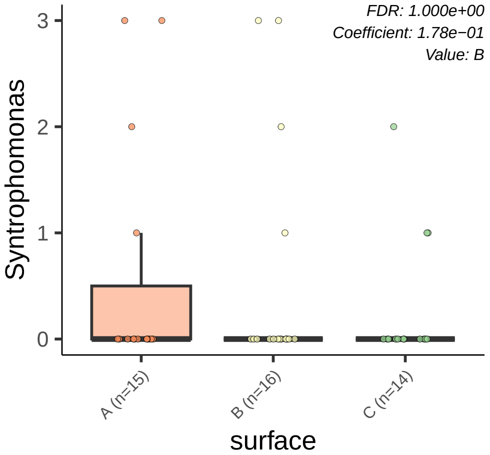

FDR: 1.000e+00
Coefficient: 1.78e−01
Value: B
Syntrophomonas
3
2
1
0
A (n=15)
B (n=16)
C (n=14)
surface

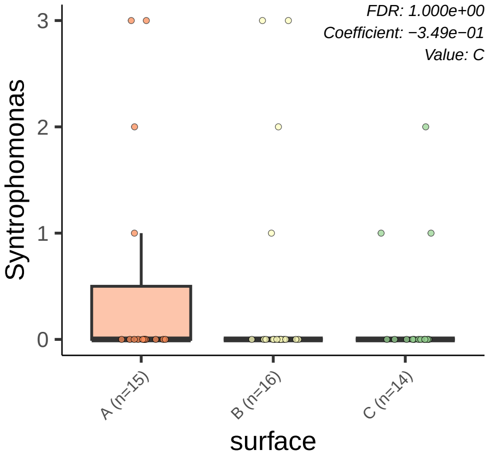

FDR: 1.000e+00
Coefficient: −3.49e−01
Value: C
Syntrophomonas
3
2
1
0
A (n=15)
B (n=16)
C (n=14)
surface

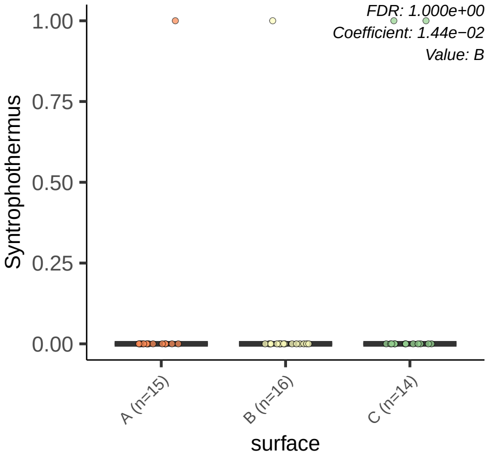
FDR: 1.000e+00
Coefficient: 1.44e−02
Value: B
Syntrophothermus
1.00
0.75
0.50
0.25
0.00
A (n=15)
B (n=16)
C (n=14)
surface

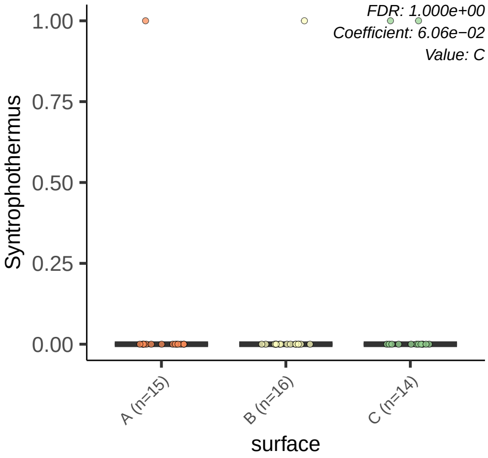

FDR: 1.000e+00
Coefficient: 6.06e−02
Value: C
Syntrophothermus
1.00
0.75
0.50
0.25
0.00
A (n=15)
B (n=16)
C (n=14)
surface

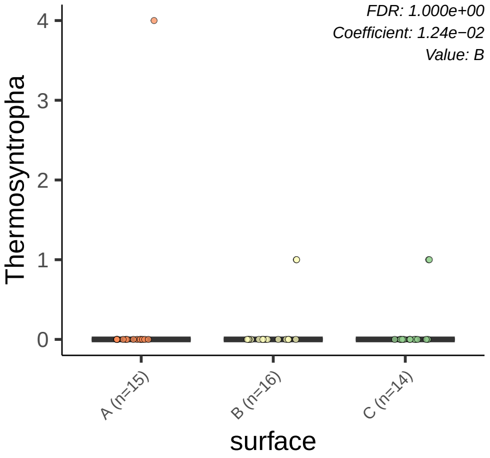

FDR: 1.000e+00
Coefficient: 1.24e−02
Value: B
Thermosyntropha
4
3
2
1
0
A (n=15)
B (n=16)
C (n=14)
surface

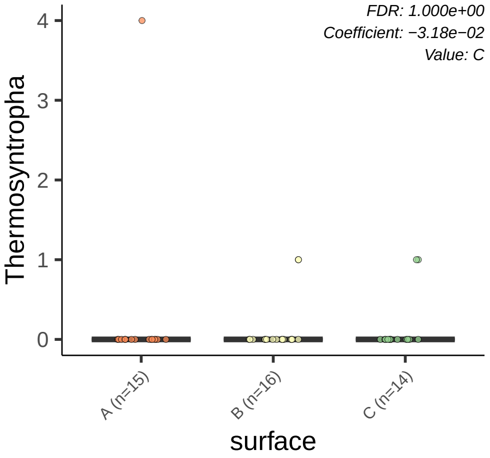

FDR: 1.000e+00
Coefficient: −3.18e−02
Value: C
Thermosyntropha
4
3
2
1
0
A (n=15)
B (n=16)
C (n=14)
surface

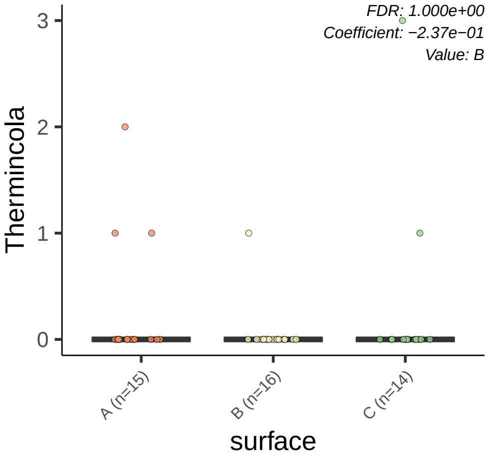
FDR: 1.000e+00
Coefficient: −2.37e−01
Value: B
Thermincola
3
2
1
0
A (n=15)
B (n=16)
C (n=14)
surface

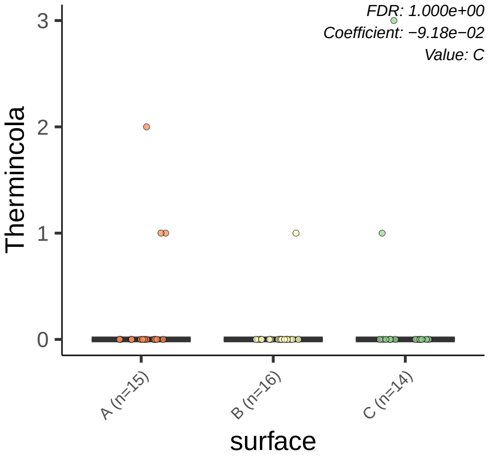
FDR: 1.000e+00
Coefficient: −9.18e−02
Value: C
Thermincola
3
2
1
0
A (n=15)
B (n=16)
C (n=14)
surface

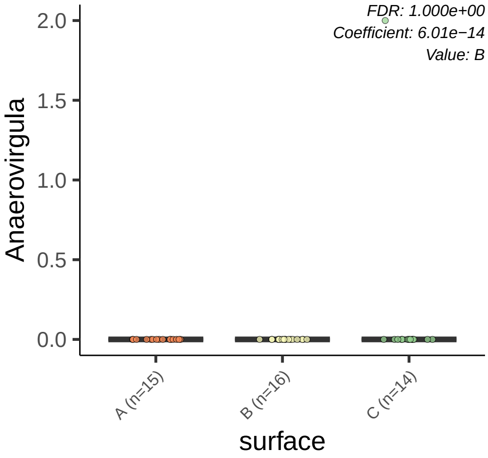
FDR: 1.000e+00
Coefficient: 6.01e−14
Value: B
Anaerovirgula
2.0
1.5
1.0
0.5
0.0
A (n=15)
B (n=16)
C (n=14)
surface

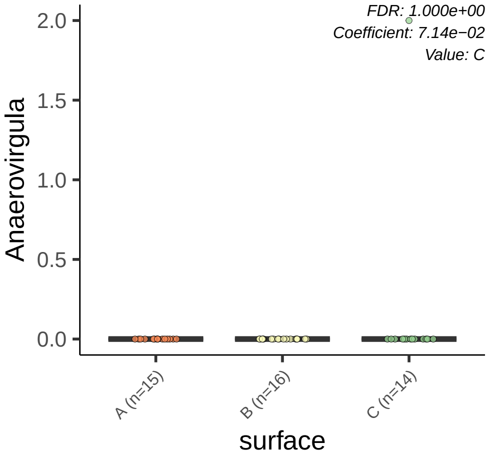

FDR: 1.000e+00
Coefficient: 7.14e−02
Value: C
Anaerovirgula
2.0
1.5
1.0
0.5
0.0
A (n=15)
B (n=16)
C (n=14)
surface

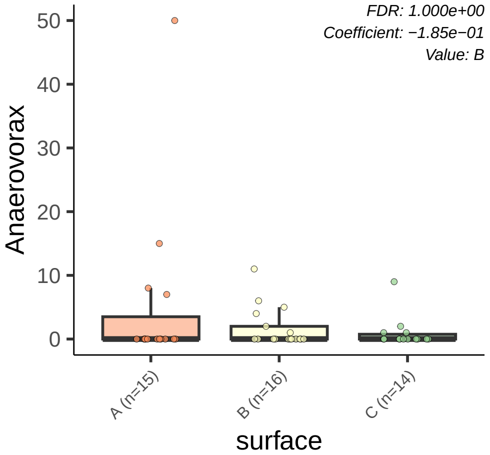

FDR: 1.000e+00
Coefficient: −1.85e−01
Value: B
Anaerovorax
0
10
20
30
40
50
A (n=15)
B (n=16)
C (n=14)
surface

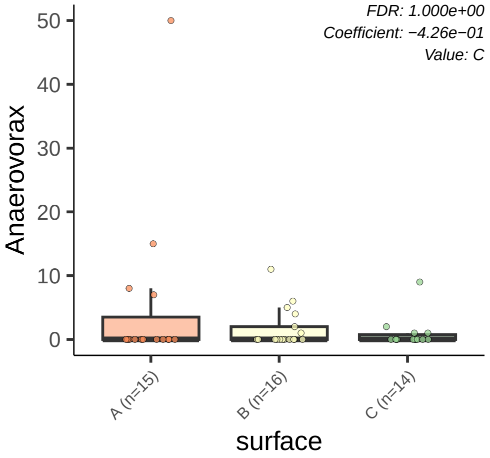
FDR: 1.000e+00
Coefficient: −4.26e−01
Value: C
Anaerovorax
50
40
30
20
10
0
A (n=15)
B (n=16)
C (n=14)
surface

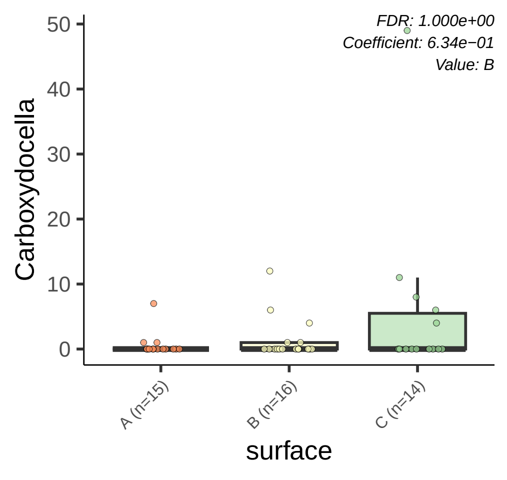
FDR: 1.000e+00
Coefficient: 6.34e−01
Value: B
Carboxydocella
50
40
30
20
10
0
A (n=15)
B (n=16)
C (n=14)
surface

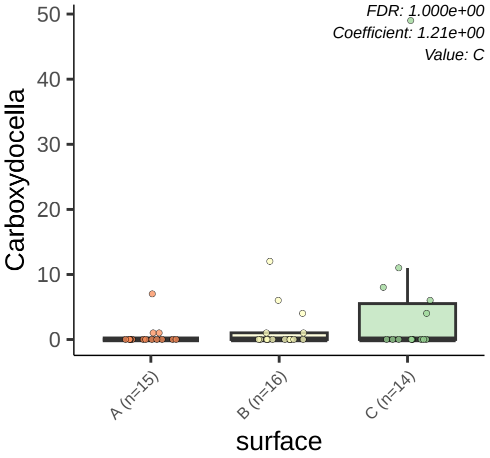

FDR: 1.000e+00
Coefficient: 1.21e+00
Value: C
Carboxydocella
50
40
30
20
10
0
A (n=15)
B (n=16)
C (n=14)
surface

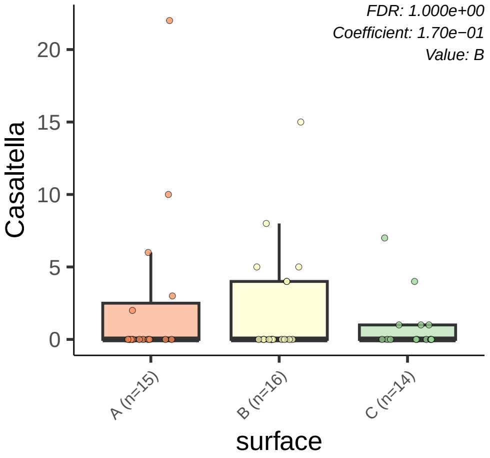
FDR: 1.000e+00
Coefficient: 1.70e−01
Value: B
Casaltella
20
15
10
5
0
A (n=15)
B (n=16)
C (n=14)
surface

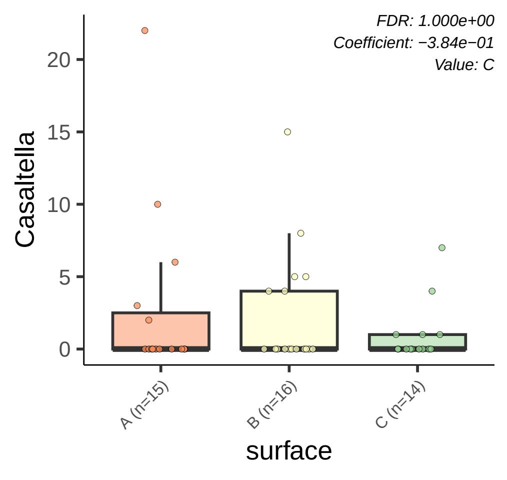

FDR: 1.000e+00
Coefficient: −3.84e−01
Value: C
Casaltella
0
5
10
15
20
A (n=15)
B (n=16)
C (n=14)
surface

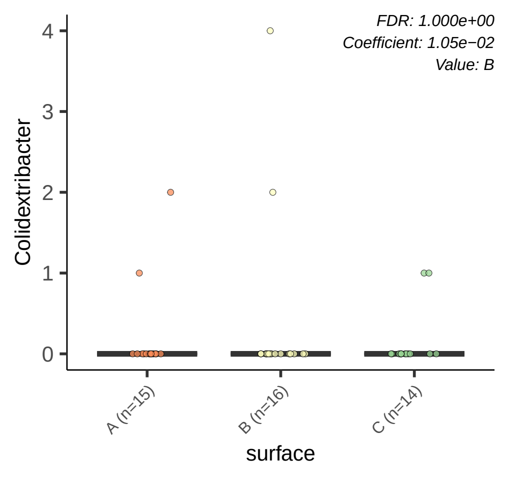

FDR: 1.000e+00
Coefficient: 1.05e−02
Value: B
Colidextribacter
0
1
2
3
4
A (n=15)
B (n=16)
C (n=14)
surface

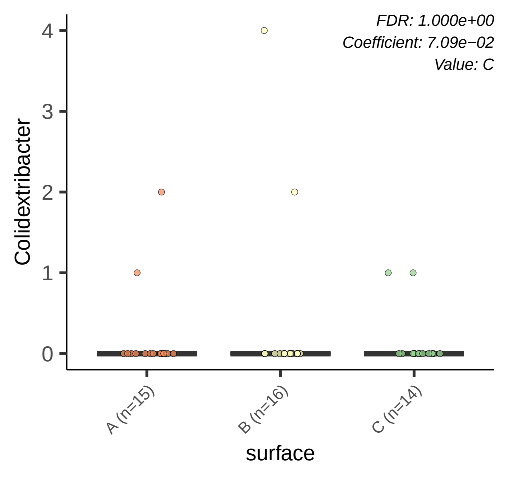
FDR: 1.000e+00
Coefficient: 7.09e−02
Value: C
Colidextribacter
0
1
2
3
4
A (n=15)
B (n=16)
C (n=14)
surface

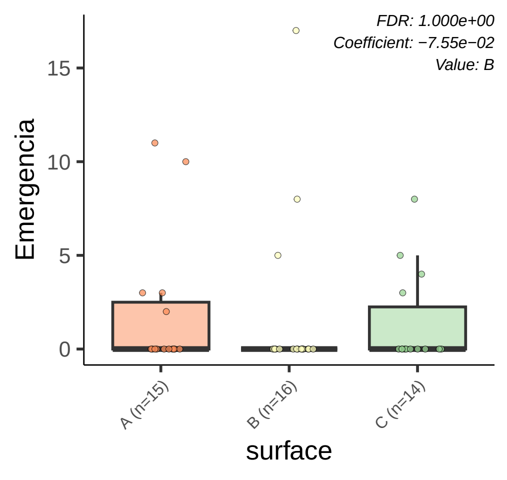
FDR: 1.000e+00
Coefficient: −7.55e−02
Value: B
Emergencia
0
5
10
15
A (n=15)
B (n=16)
C (n=14)
surface

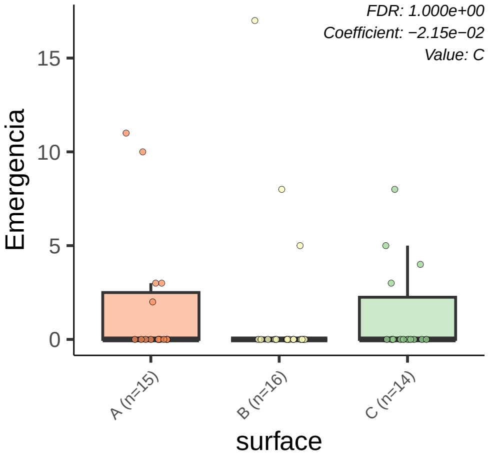
FDR: 1.000e+00
Coefficient: −2.15e−02
Value: C
Emergencia
0
5
10
15
A (n=15)
B (n=16)
C (n=14)
surface

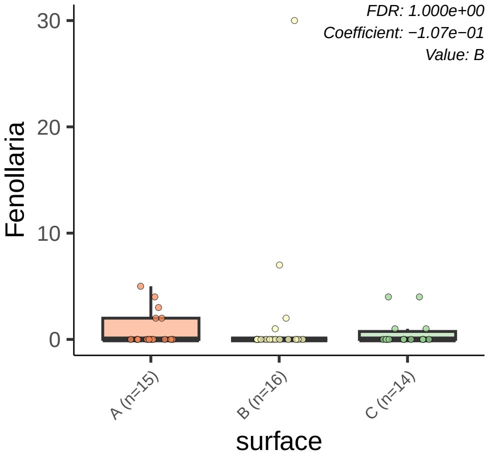
FDR: 1.000e+00
Coefficient: −1.07e−01
Value: B
Fenollaria
30
20
10
0
A (n=15)
B (n=16)
C (n=14)
surface

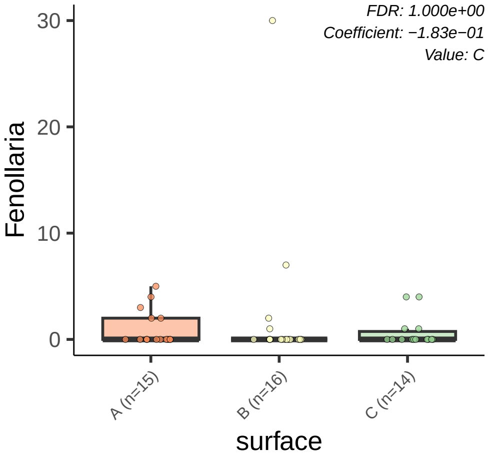

FDR: 1.000e+00
Coefficient: −1.83e−01
Value: C
Fenollaria
30
20
10
0
A (n=15)
B (n=16)
C (n=14)
surface

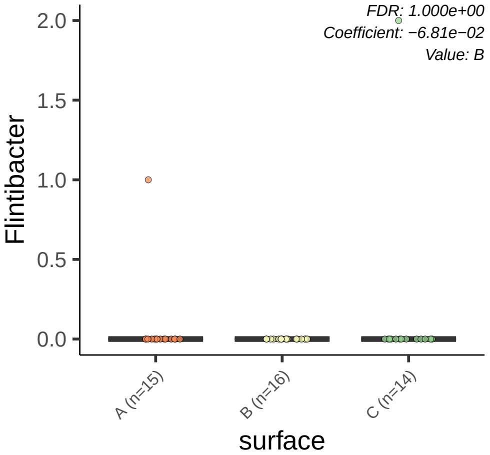
FDR: 1.000e+00
Coefficient: −6.81e−02
Value: B
Flintibacter
2.0
1.5
1.0
0.5
0.0
A (n=15)
B (n=16)
C (n=14)
surface

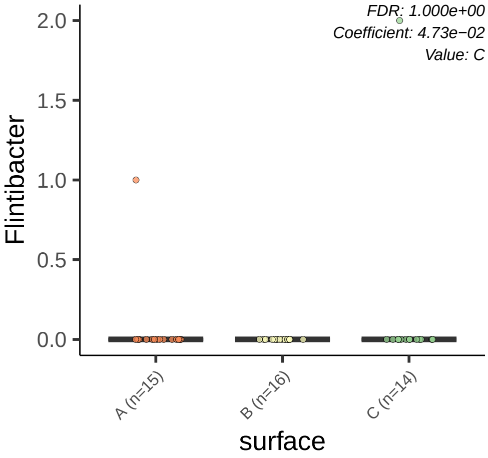
FDR: 1.000e+00
Coefficient: 4.73e−02
Value: C
Flintibacter
2.0
1.5
1.0
0.5
0.0
A (n=15)
B (n=16)
C (n=14)
surface

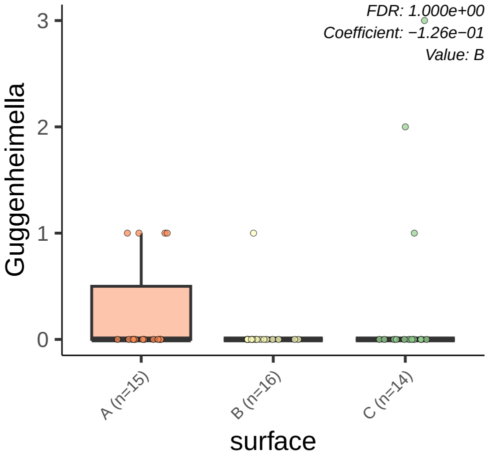
FDR: 1.000e+00
Coefficient: −1.26e−01
Value: B
Guggenheimella
0
1
2
3
A (n=15)
B (n=16)
C (n=14)
surface

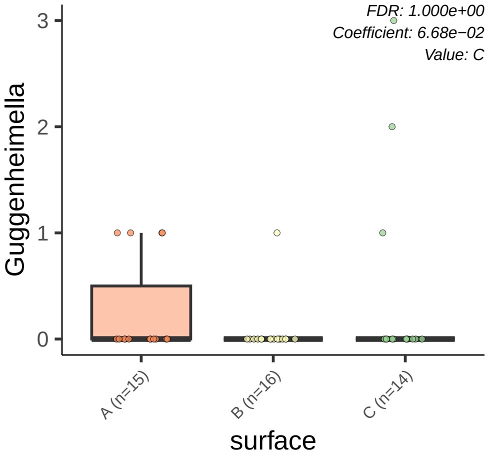

FDR: 1.000e+00
Coefficient: 6.68e−02
Value: C
Guggenheimella
3
2
1
0
A (n=15)
B (n=16)
C (n=14)
surface

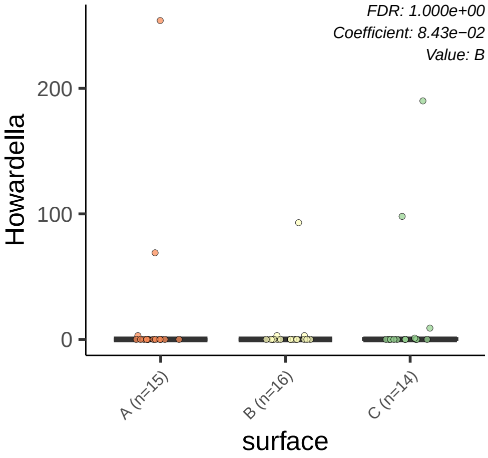

FDR: 1.000e+00
Coefficient: 8.43e−02
Value: B
Howardella
200
100
0
A (n=15)
B (n=16)
C (n=14)
surface

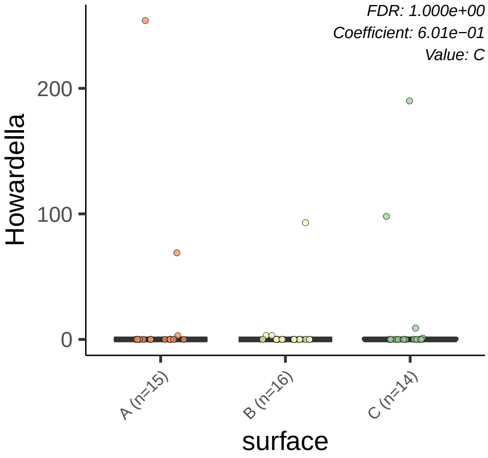
FDR: 1.000e+00
Coefficient: 6.01e−01
Value: C
Howardella
200
100
0
A (n=15)
B (n=16)
C (n=14)
surface

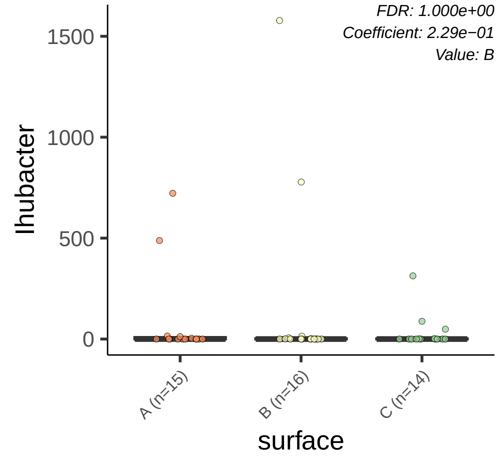
FDR: 1.000e+00
Coefficient: 2.29e−01
Value: B
Ihubacter
1500
1000
500
0
A (n=15)
B (n=16)
C (n=14)
surface

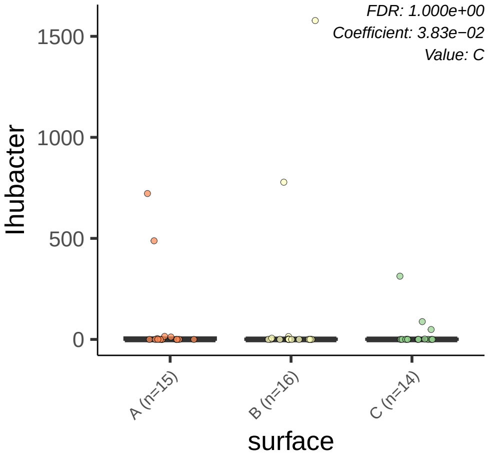

FDR: 1.000e+00
Coefficient: 3.83e−02
Value: C
Ihubacter
1500
1000
500
0
A (n=15)
B (n=16)
C (n=14)
surface

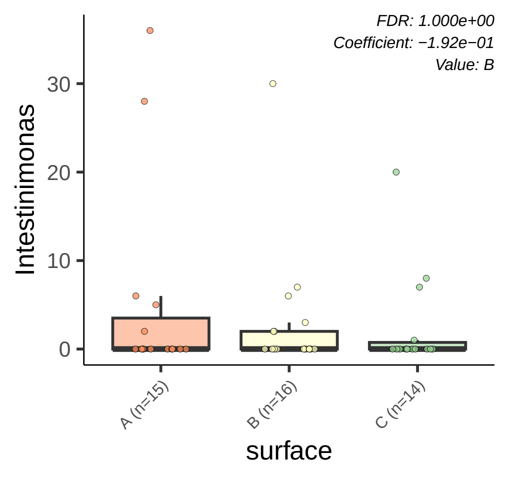
FDR: 1.000e+00
Coefficient: −1.92e−01
Value: B
Intestinimonas
30
20
10
0
A (n=15)
B (n=16)
C (n=14)
surface

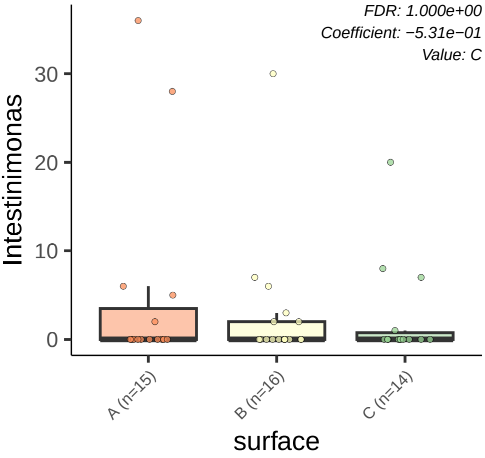
FDR: 1.000e+00
Coefficient: −5.31e−01
Value: C
Intestinimonas
30
20
10
0
A (n=15)
B (n=16)
C (n=14)
surface

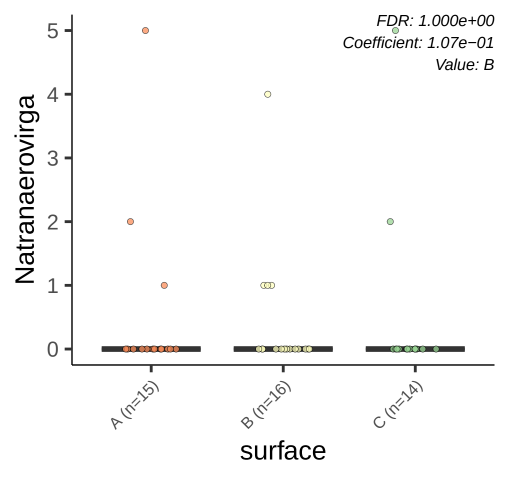
FDR: 1.000e+00
Coefficient: 1.07e−01
Value: B
Natranaerovirga
5
4
3
2
1
0
A (n=15)
B (n=16)
C (n=14)
surface

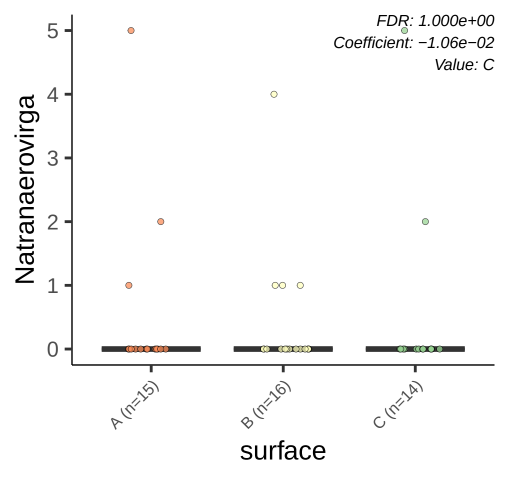

FDR: 1.000e+00
Coefficient: −1.06e−02
Value: C
Natranaerovirga
0
1
2
3
4
5
A (n=15)
B (n=16)
C (n=14)
surface

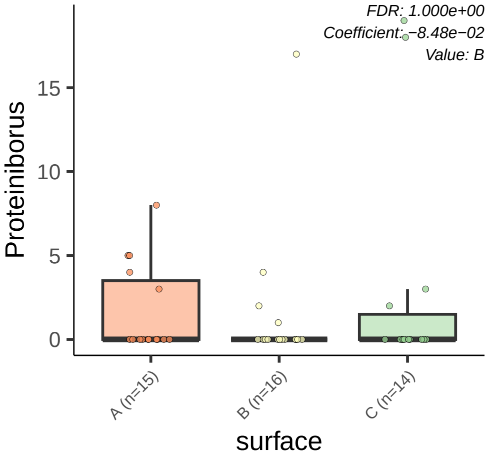
FDR: 1.000e+00
Coefficient: −8.48e−02
Value: B
Proteiniborus
0
5
10
15
A (n=15)
B (n=16)
C (n=14)
surface

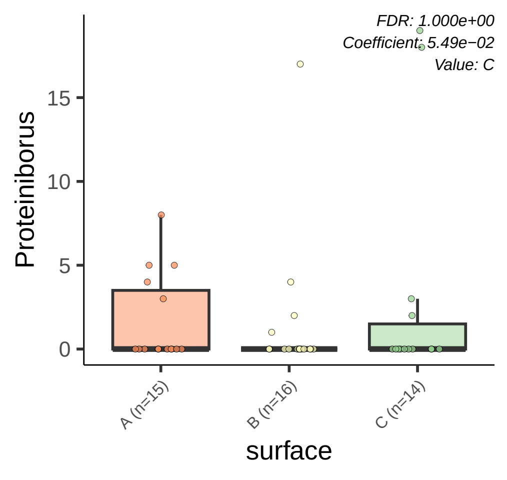

FDR: 1.000e+00
Coefficient: 5.49e−02
Value: C
Proteiniborus
0
5
10
15
A (n=15)
B (n=16)
C (n=14)
surface

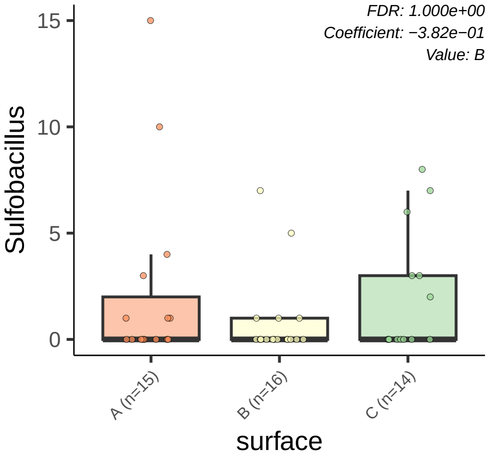

FDR: 1.000e+00
Coefficient: −3.82e−01
Value: B
Sulfobacillus
15
10
5
0
A (n=15)
B (n=16)
C (n=14)
surface

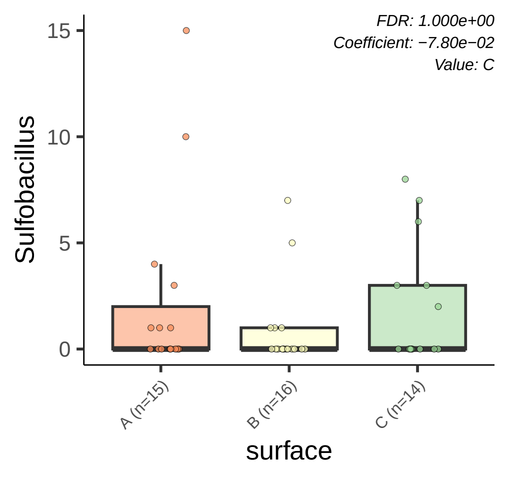

FDR: 1.000e+00
Coefficient: −7.80e−02
Value: C
Sulfobacillus
15
10
5
0
A (n=15)
B (n=16)
C (n=14)
surface

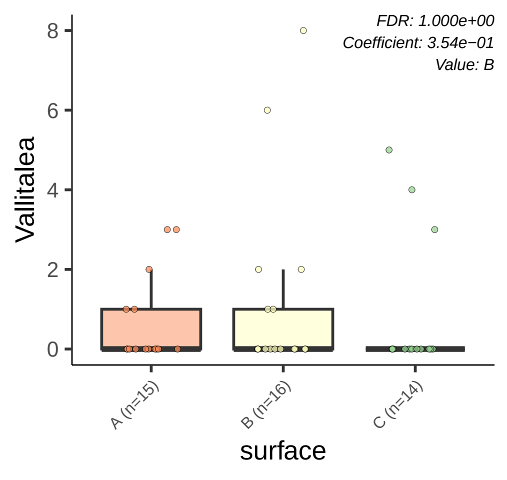
FDR: 1.000e+00
Coefficient: 3.54e−01
Value: B
Vallitalea
0
2
4
6
8
A (n=15)
B (n=16)
C (n=14)
surface

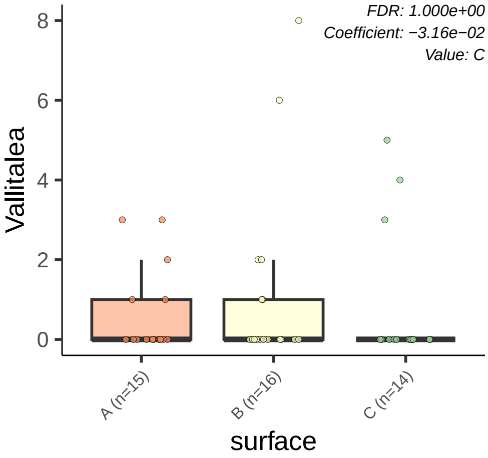

FDR: 1.000e+00
Coefficient: −3.16e−02
Value: C
Vallitalea
0
2
4
6
8
A (n=15)
B (n=16)
C (n=14)
surface

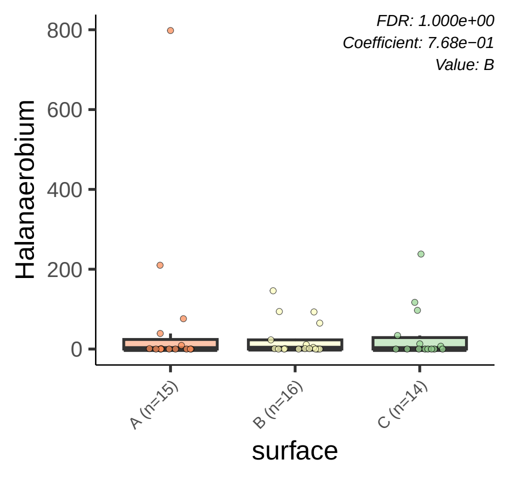

FDR: 1.000e+00
Coefficient: 7.68e−01
Value: B
Halanaerobium
800
600
400
200
0
A (n=15)
B (n=16)
C (n=14)
surface

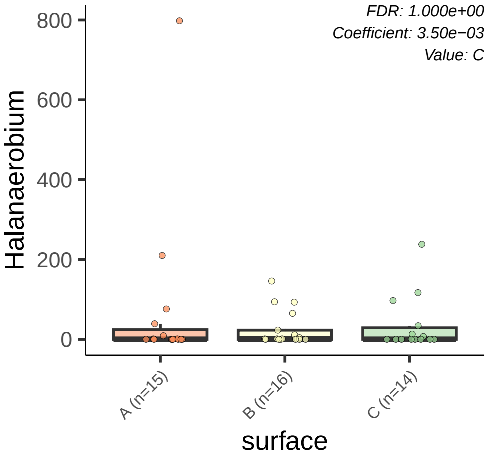
FDR: 1.000e+00
Coefficient: 3.50e−03
Value: C
Halanaerobium
800
600
400
200
0
A (n=15)
B (n=16)
C (n=14)
surface

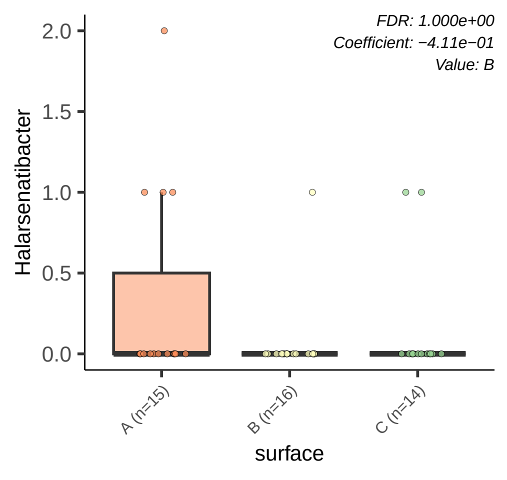

FDR: 1.000e+00
Coefficient: −4.11e−01
Value: B
Halarsenatibacter
2.0
1.5
1.0
0.5
0.0
A (n=15)
B (n=16)
C (n=14)
surface

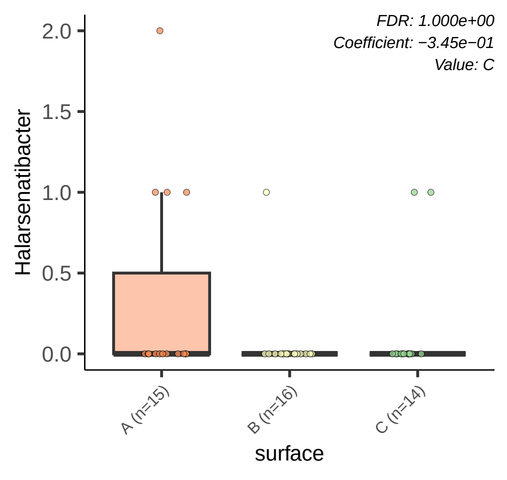
FDR: 1.000e+00
Coefficient: −3.45e−01
Value: C
Halarsenatibacter
2.0
1.5
1.0
0.5
0.0
A (n=15)
B (n=16)
C (n=14)
surface

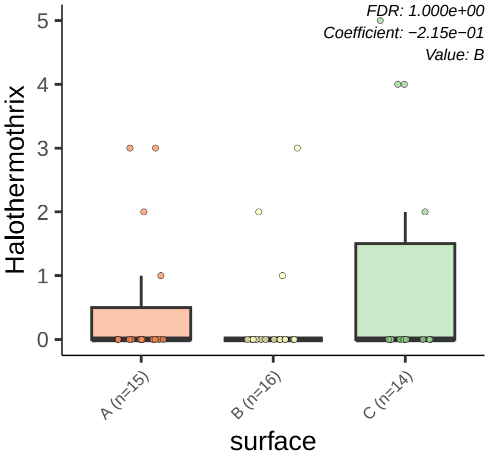
FDR: 1.000e+00
Coefficient: −2.15e−01
Value: B
Halothermothrix
0
1
2
3
4
5
A (n=15)
B (n=16)
C (n=14)
surface

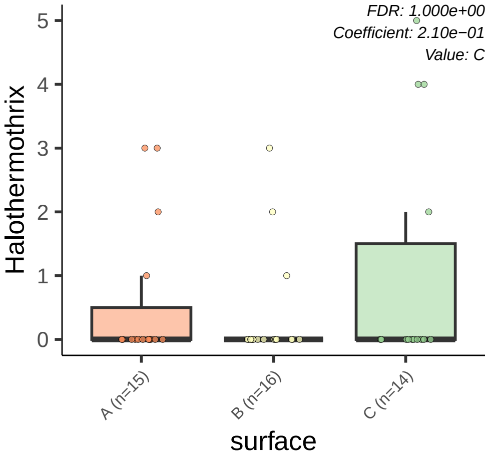
FDR: 1.000e+00
Coefficient: 2.10e−01
Value: C
Halothermothrix
0
1
2
3
4
5
A (n=15)
B (n=16)
C (n=14)
surface

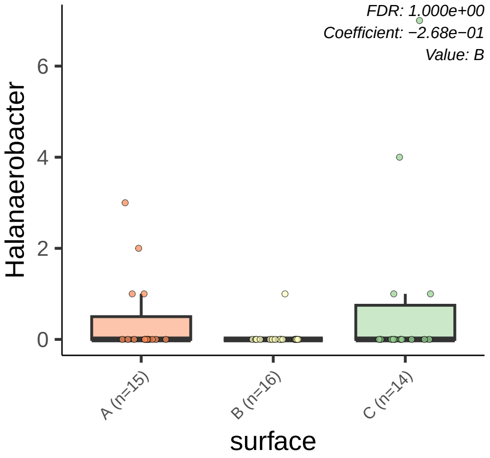
FDR: 1.000e+00
Coefficient: −2.68e−01
Value: B
Halanaerobacter
0
2
4
6
A (n=15)
B (n=16)
C (n=14)
surface

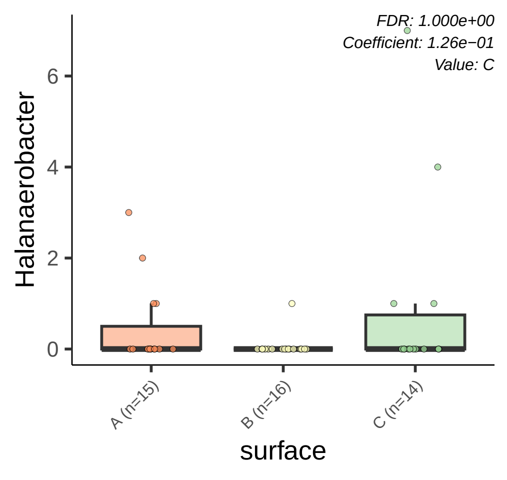

FDR: 1.000e+00
Coefficient: 1.26e−01
Value: C
Halanaerobacter
0
2
4
6
A (n=15)
B (n=16)
C (n=14)
surface

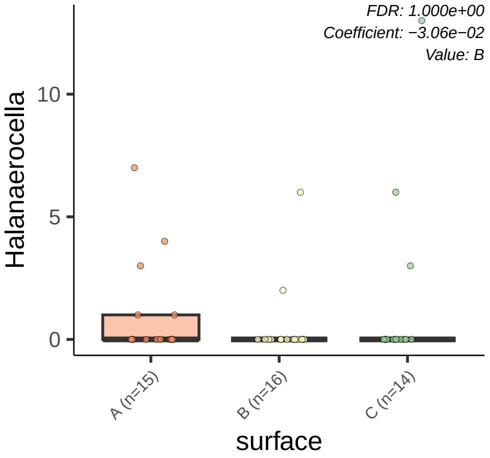

FDR: 1.000e+00
Coefficient: −3.06e−02
Value: B
Halanaerocella
10
5
0
A (n=15)
B (n=16)
C (n=14)
surface

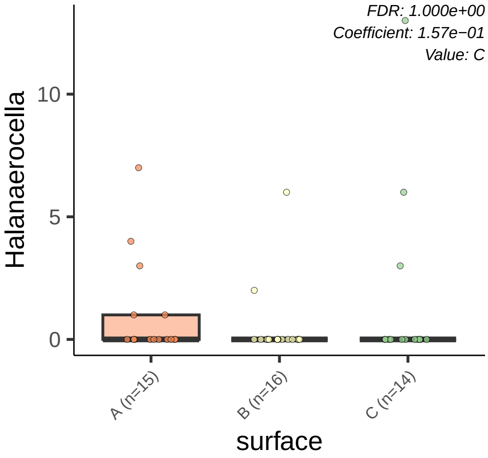
FDR: 1.000e+00
Coefficient: 1.57e−01
Value: C
Halanaerocella
10
5
0
A (n=15)
B (n=16)
C (n=14)
surface

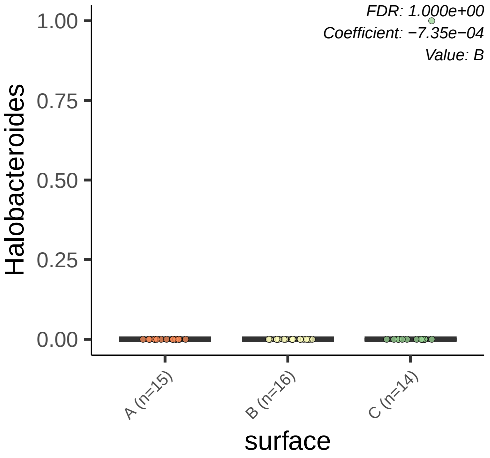
FDR: 1.000e+00
Coefficient: −7.35e−04
Value: B
Halobacteroides
1.00
0.75
0.50
0.25
0.00
A (n=15)
B (n=16)
C (n=14)
surface

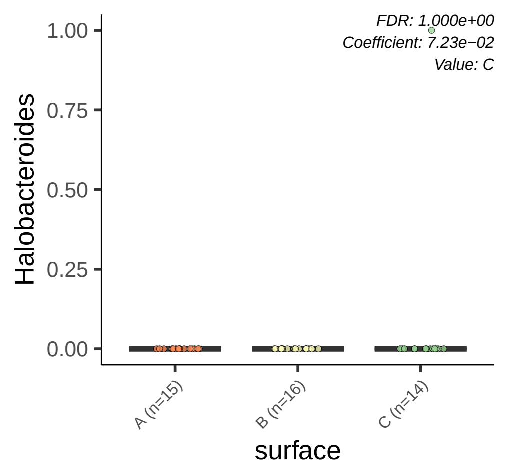
FDR: 1.000e+00
Coefficient: 7.23e−02
Value: C
Halobacteroides
1.00
0.75
0.50
0.25
0.00
A (n=15)
B (n=16)
C (n=14)
surface

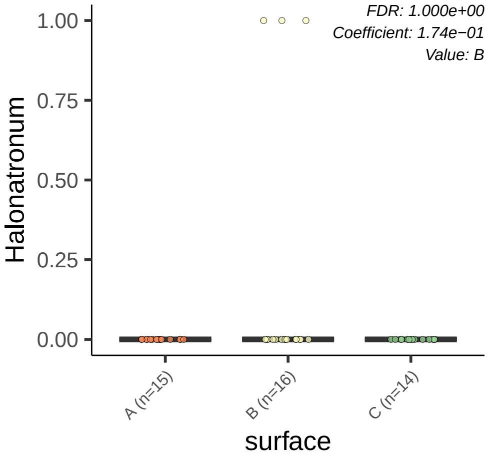
FDR: 1.000e+00
Coefficient: 1.74e−01
Value: B
Halonatronum
1.00
0.75
0.50
0.25
0.00
A (n=15)
B (n=16)
C (n=14)
surface

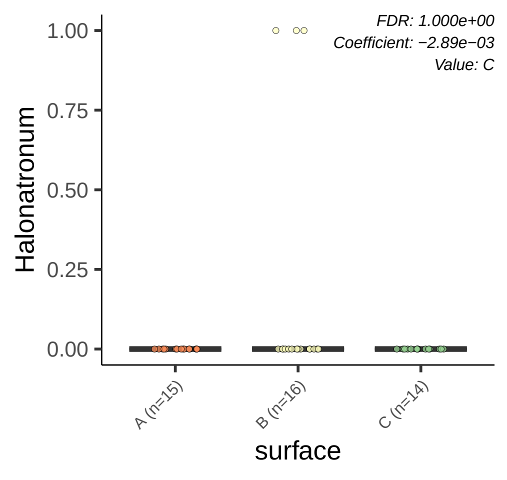

FDR: 1.000e+00
Coefficient: −2.89e−03
Value: C
Halonatronum
1.00
0.75
0.50
0.25
0.00
A (n=15)
B (n=16)
C (n=14)
surface

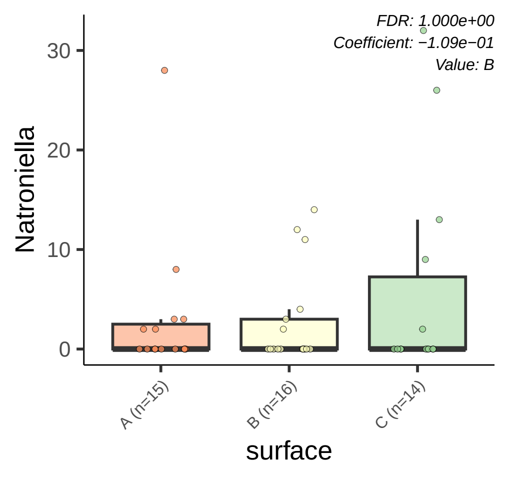

FDR: 1.000e+00
Coefficient: −1.09e−01
Value: B
Natroniella
30
20
10
0
A (n=15)
B (n=16)
C (n=14)
surface

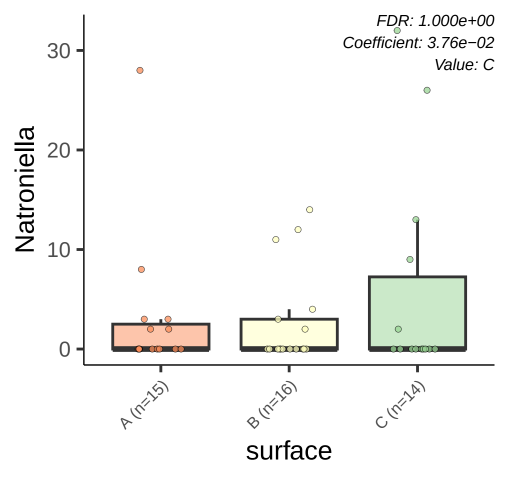
FDR: 1.000e+00
Coefficient: 3.76e−02
Value: C
Natroniella
30
20
10
0
A (n=15)
B (n=16)
C (n=14)
surface

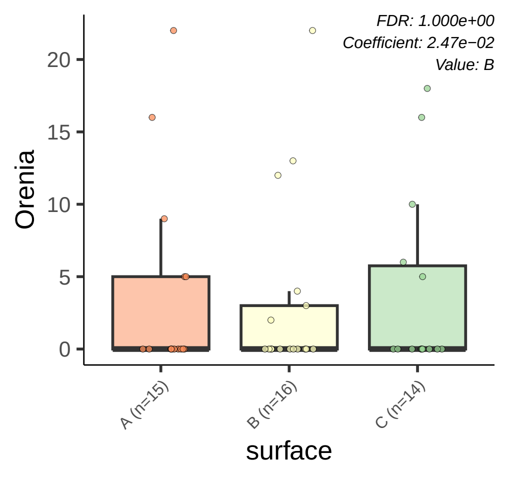

FDR: 1.000e+00
Coefficient: 2.47e−02
Value: B
Orenia
20
15
10
5
0
A (n=15)
B (n=16)
C (n=14)
surface

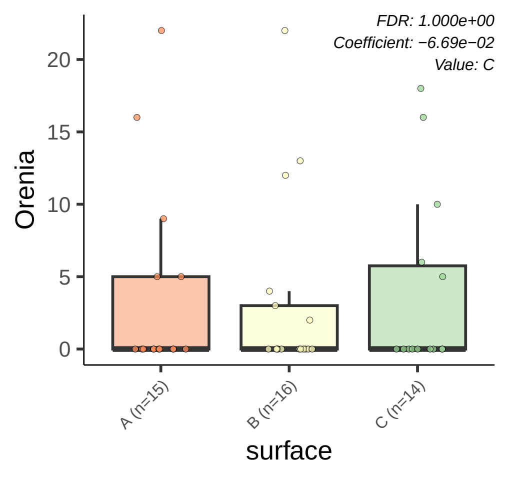
FDR: 1.000e+00
Coefficient: −6.69e−02
Value: C
Orenia
20
15
10
5
0
A (n=15)
B (n=16)
C (n=14)
surface

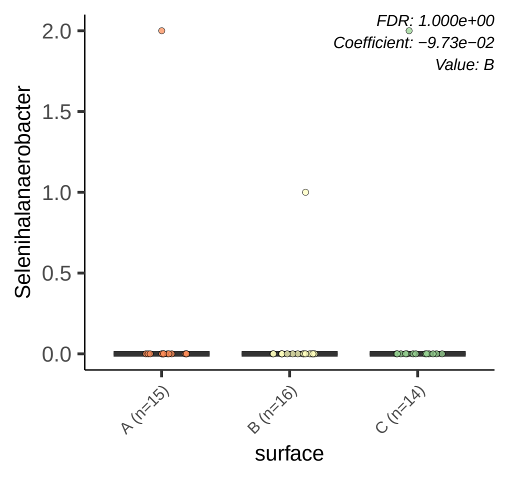

FDR: 1.000e+00
Coefficient: −9.73e−02
Value: B
Selenihalanaerobacter
2.0
1.5
1.0
0.5
0.0
A (n=15)
B (n=16)
C (n=14)
surface

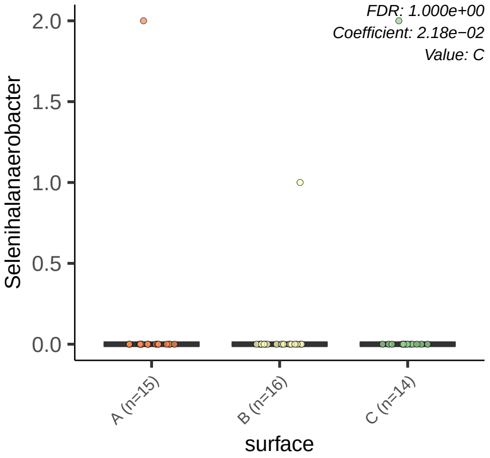

FDR: 1.000e+00
Coefficient: 2.18e−02
Value: C
Selenihalanaerobacter
2.0
1.5
1.0
0.5
0.0
A (n=15)
B (n=16)
C (n=14)
surface

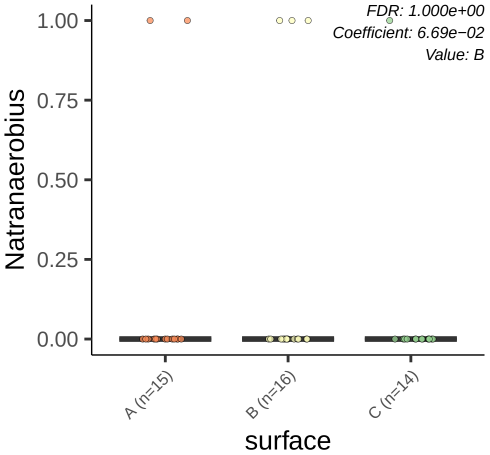
FDR: 1.000e+00
Coefficient: 6.69e−02
Value: B
Natranaerobius
1.00
0.75
0.50
0.25
0.00
A (n=15)
B (n=16)
C (n=14)
surface

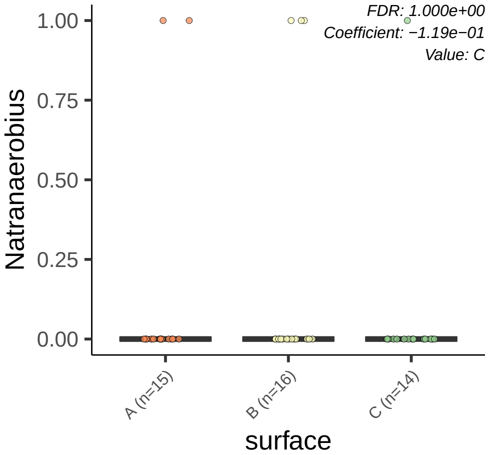

FDR: 1.000e+00
Coefficient: −1.19e−01
Value: C
Natranaerobius
1.00
0.75
0.50
0.25
0.00
A (n=15)
B (n=16)
C (n=14)
surface

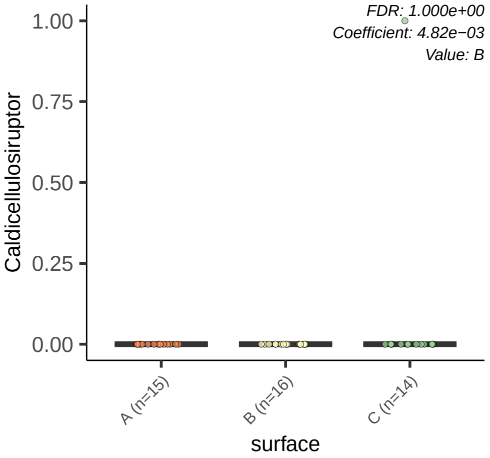

FDR: 1.000e+00
Coefficient: 4.82e−03
Value: B
Caldicellulosiruptor
1.00
0.75
0.50
0.25
0.00
A (n=15)
B (n=16)
C (n=14)
surface

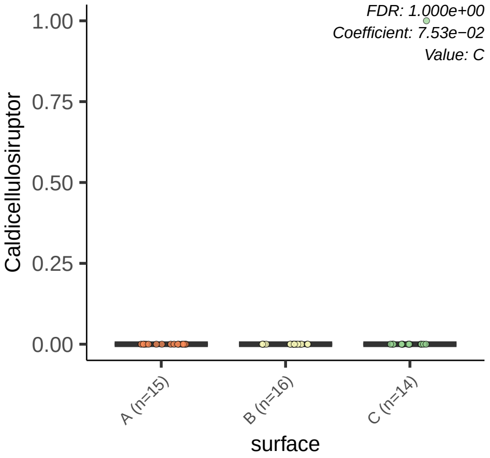

FDR: 1.000e+00
Coefficient: 7.53e−02
Value: C
Caldicellulosiruptor
1.00
0.75
0.50
0.25
0.00
A (n=15)
B (n=16)
C (n=14)
surface

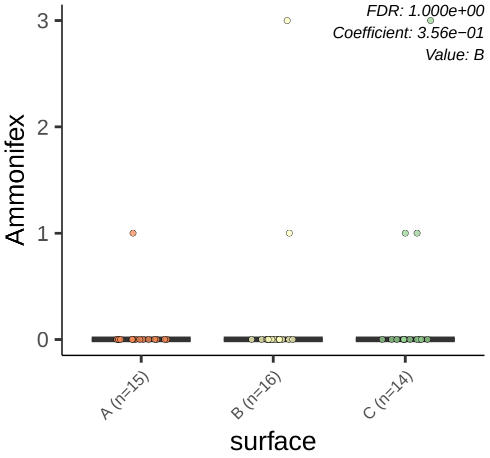

FDR: 1.000e+00
Coefficient: 3.56e−01
Value: B
Ammonifex
0
1
2
3
A (n=15)
B (n=16)
C (n=14)
surface

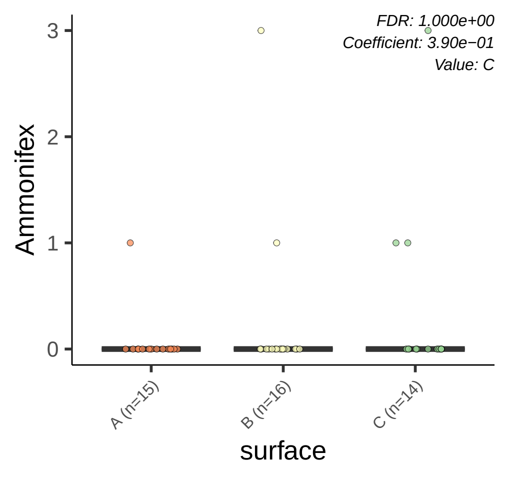

FDR: 1.000e+00
Coefficient: 3.90e−01
Value: C
Ammonifex
0
1
2
3
A (n=15)
B (n=16)
C (n=14)
surface

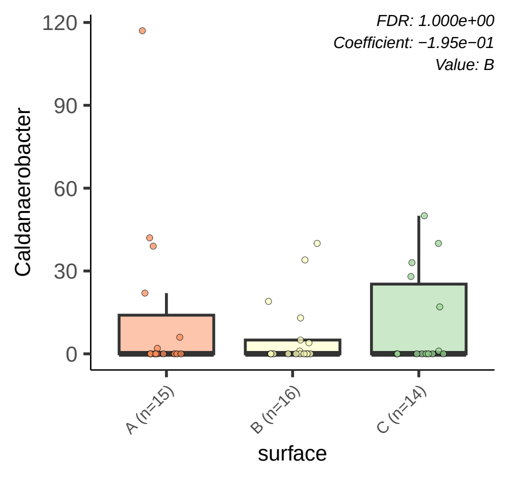
FDR: 1.000e+00
Coefficient: −1.95e−01
Value: B
120
90
60
30
0
Caldanaerobacter
A (n=15)
B (n=16)
C (n=14)
surface

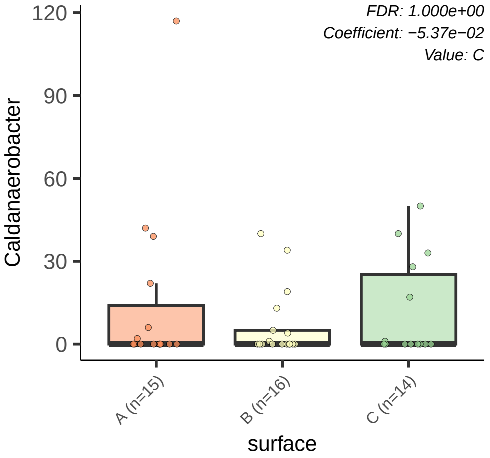

FDR: 1.000e+00
Coefficient: −5.37e−02
Value: C
Caldanaerobacter
120
90
60
30
0
A (n=15)
B (n=16)
C (n=14)
surface

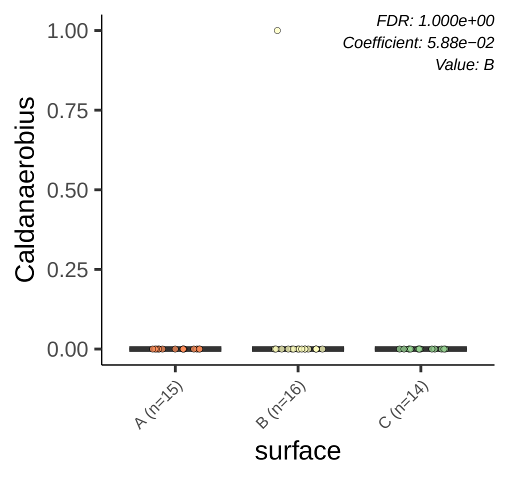
FDR: 1.000e+00
Coefficient: 5.88e−02
Value: B
Caldanaerobius
1.00
0.75
0.50
0.25
0.00
A (n=15)
B (n=16)
C (n=14)
surface

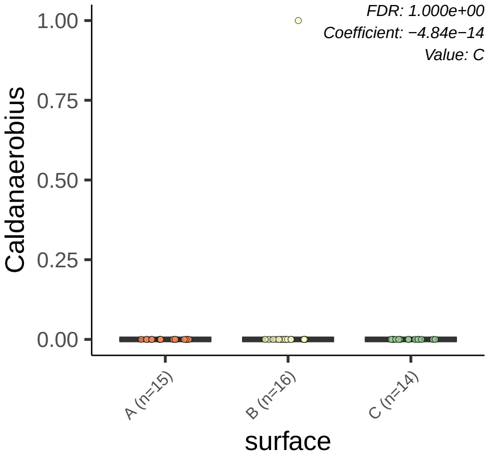
FDR: 1.000e+00
Coefficient: −4.84e−14
Value: C
Caldanaerobius
1.00
0.75
0.50
0.25
0.00
A (n=15)
B (n=16)
C (n=14)
surface

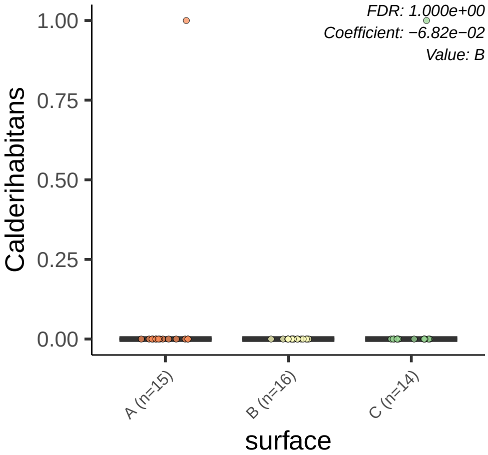
FDR: 1.000e+00
Coefficient: −6.82e−02
Value: B
Calderihabitans
1.00
0.75
0.50
0.25
0.00
A (n=15)
B (n=16)
C (n=14)
surface

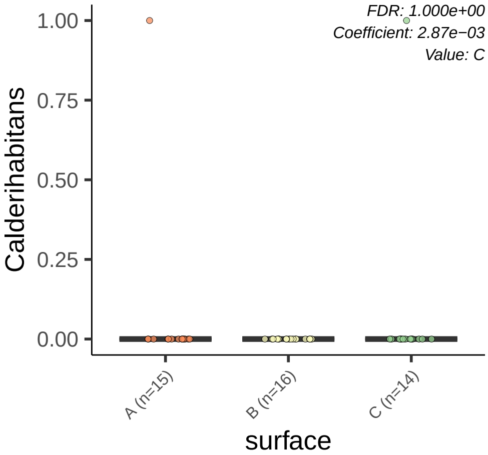

FDR: 1.000e+00
Coefficient: 2.87e−03
Value: C
Calderihabitans
1.00
0.75
0.50
0.25
0.00
A (n=15)
B (n=16)
C (n=14)
surface

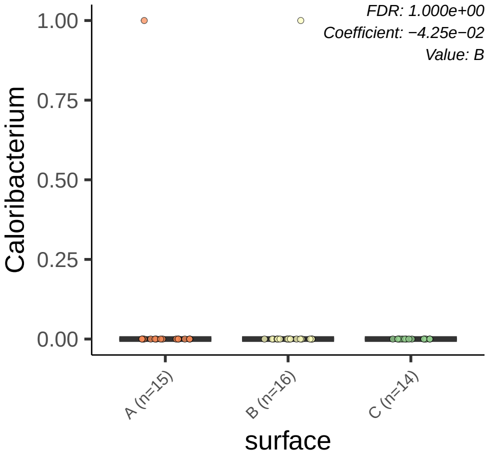
FDR: 1.000e+00
Coefficient: −4.25e−02
Value: B
Caloribacterium
1.00
0.75
0.50
0.25
0.00
A (n=15)
B (n=16)
C (n=14)
surface

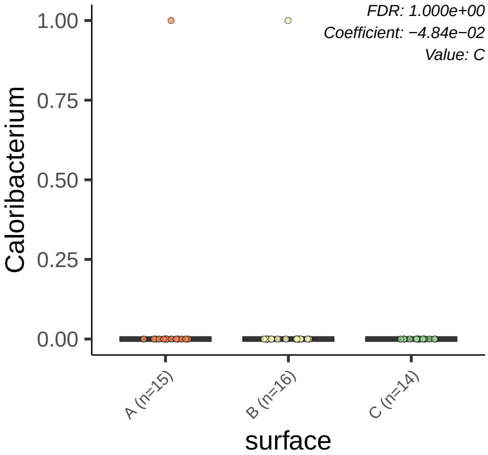

FDR: 1.000e+00
Coefficient: −4.84e−02
Value: C
Caloribacterium
1.00
0.75
0.50
0.25
0.00
A (n=15)
B (n=16)
C (n=14)
surface

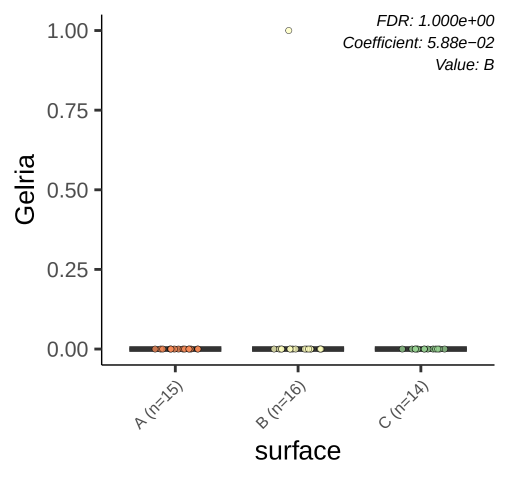

FDR: 1.000e+00
Coefficient: 5.88e−02
Value: B
Gelria
1.00
0.75
0.50
0.25
0.00
A (n=15)
B (n=16)
C (n=14)
surface

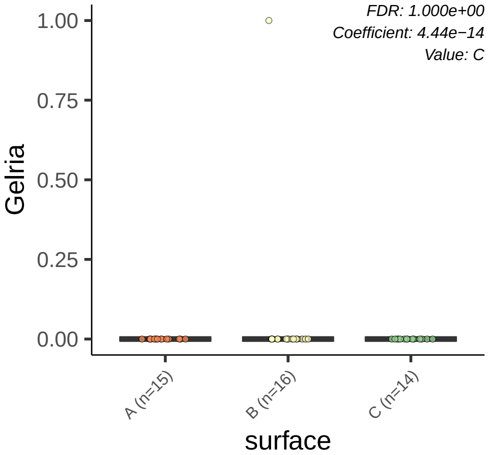
FDR: 1.000e+00
Coefficient: 4.44e−14
Value: C
Gelria
1.00
0.75
0.50
0.25
0.00
A (n=15)
B (n=16)
C (n=14)
surface

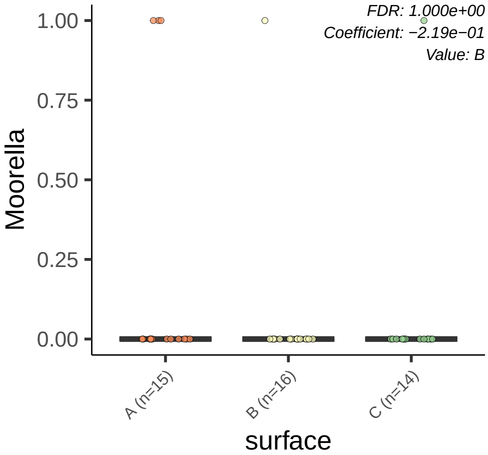
FDR: 1.000e+00
Coefficient: −2.19e−01
Value: B
Moorella
1.00
0.75
0.50
0.25
0.00
A (n=15)
B (n=16)
C (n=14)
surface

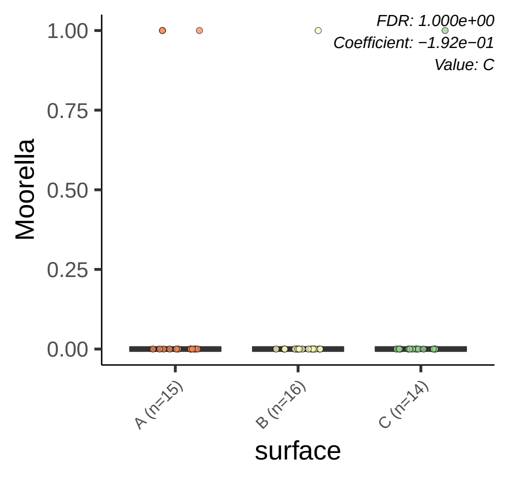
FDR: 1.000e+00
Coefficient: −1.92e−01
Value: C
Moorella
1.00
0.75
0.50
0.25
0.00
A (n=15)
B (n=16)
C (n=14)
surface

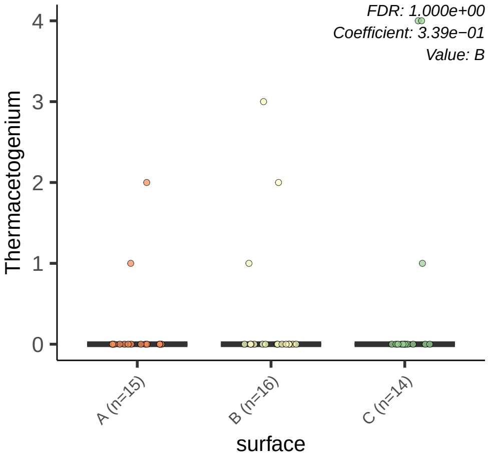

FDR: 1.000e+00
Coefficient: 3.39e−01
Value: B
Thermacetogenium
0
1
2
3
4
A (n=15)
B (n=16)
C (n=14)
surface

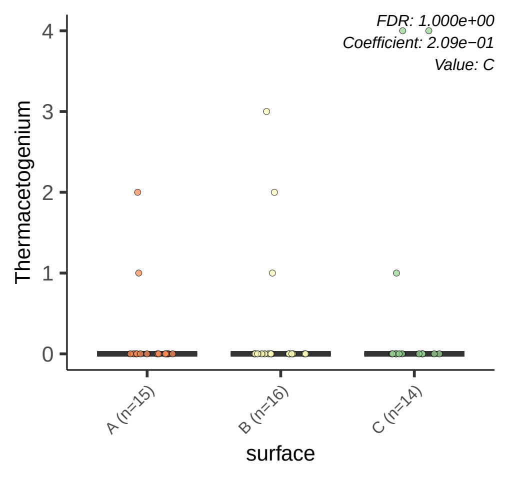

FDR: 1.000e+00
Coefficient: 2.09e−01
Value: C
Thermacetogenium
4
3
2
1
0
A (n=15)
B (n=16)
C (n=14)
surface

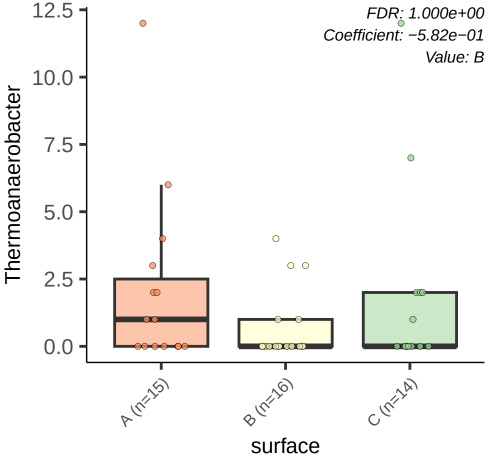
FDR: 1.000e+00
Coefficient: −5.82e−01
Value: B
Thermoanaerobacter
12.5
10.0
7.5
5.0
2.5
0.0
A (n=15)
B (n=16)
C (n=14)
surface

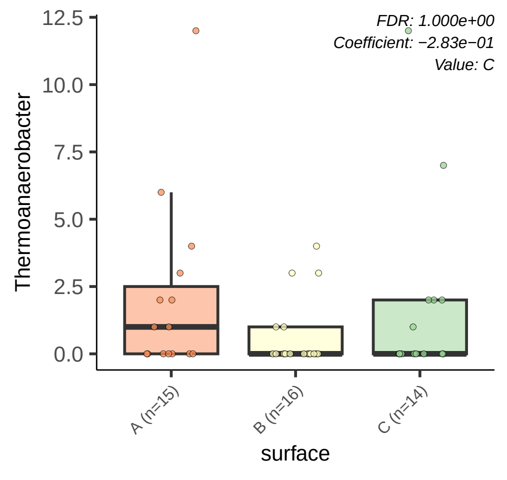

FDR: 1.000e+00
Coefficient: −2.83e−01
Value: C
Thermoanaerobacter
12.5
10.0
7.5
5.0
2.5
0.0
A (n=15)
B (n=16)
C (n=14)
surface

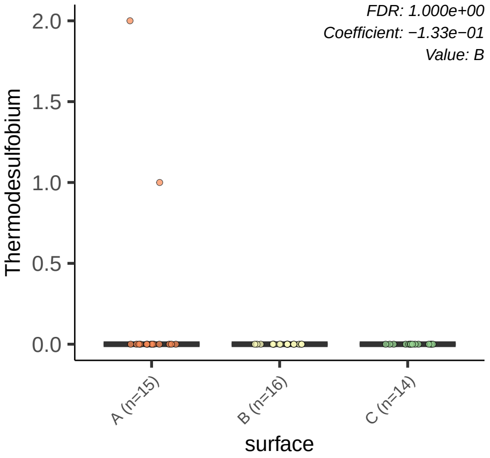
FDR: 1.000e+00
Coefficient: −1.33e−01
Value: B
Thermodesulfobium
2.0
1.5
1.0
0.5
0.0
A (n=15)
B (n=16)
C (n=14)
surface

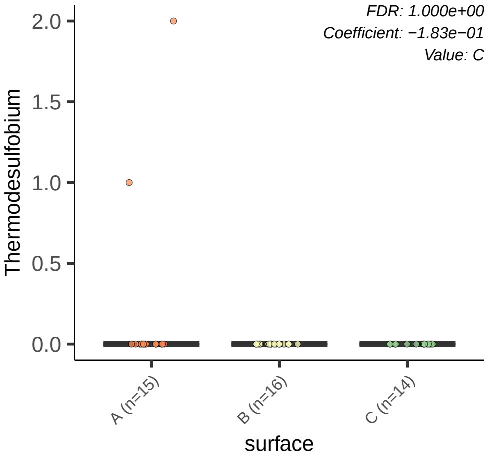
FDR: 1.000e+00
Coefficient: −1.83e−01
Value: C
Thermodesulfobium
2.0
1.5
1.0
0.5
0.0
A (n=15)
B (n=16)
C (n=14)
surface

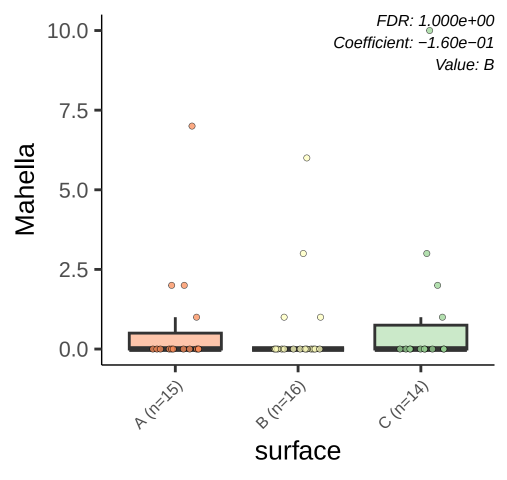
FDR: 1.000e+00
Coefficient: −1.60e−01
Value: B
Mahella
10.0
7.5
5.0
2.5
0.0
A (n=15)
B (n=16)
C (n=14)
surface

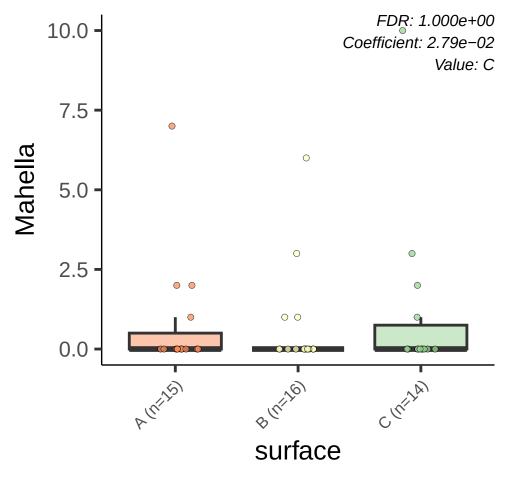
FDR: 1.000e+00
Coefficient: 2.79e−02
Value: C
Mahella
10.0
7.5
5.0
2.5
0.0
A (n=15)
B (n=16)
C (n=14)
surface

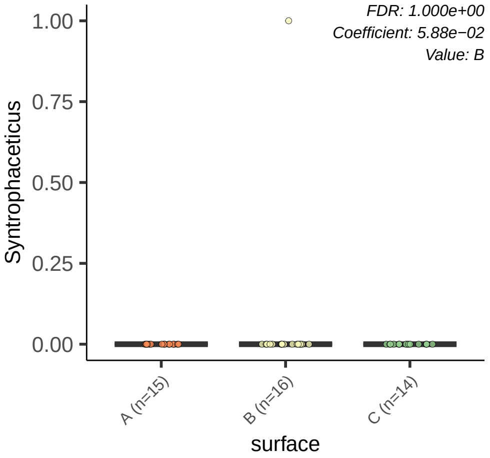

FDR: 1.000e+00
Coefficient: 5.88e−02
Value: B
Syntrophaceticus
1.00
0.75
0.50
0.25
0.00
A (n=15)
B (n=16)
C (n=14)
surface

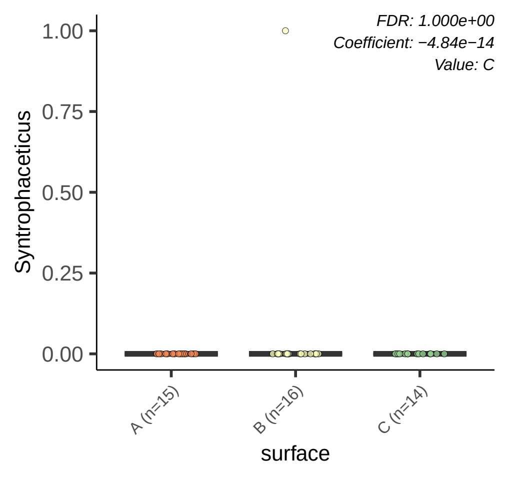
FDR: 1.000e+00
Coefficient: −4.84e−14
Value: C
Syntrophaceticus
1.00
0.75
0.50
0.25
0.00
A (n=15)
B (n=16)
C (n=14)
surface

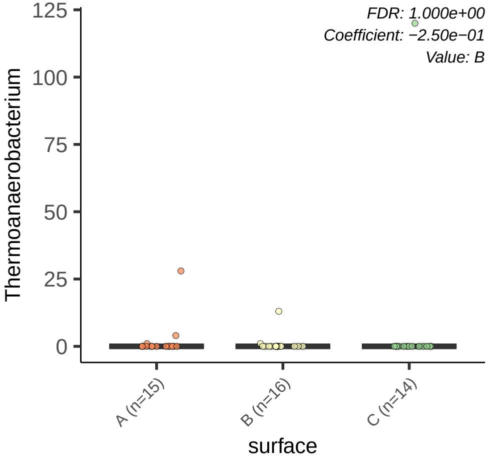

FDR: 1.000e+00
Coefficient: −2.50e−01
Value: B
Thermoanaerobacterium
125
100
75
50
25
0
A (n=15)
B (n=16)
C (n=14)
surface

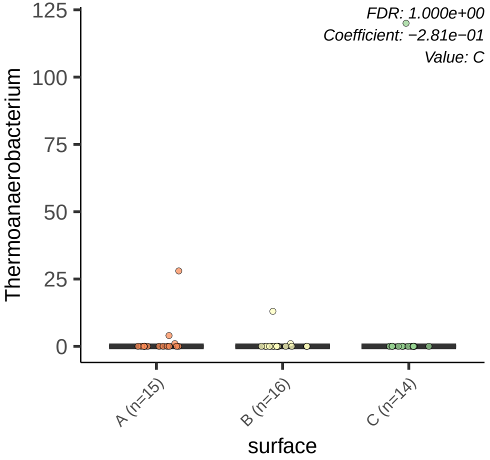
FDR: 1.000e+00
Coefficient: −2.81e−01
Value: C
Thermoanaerobacterium
125
100
75
50
25
0
A (n=15)
B (n=16)
C (n=14)
surface

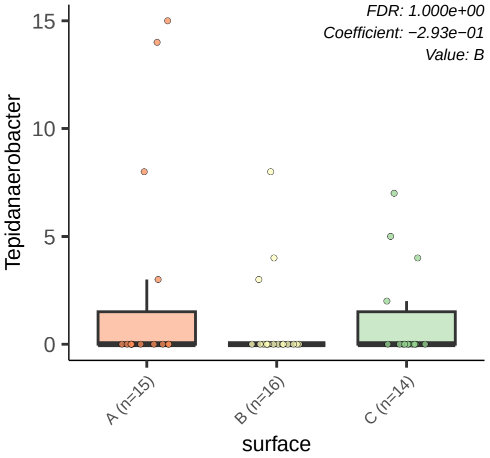

FDR: 1.000e+00
Coefficient: −2.93e−01
Value: B
Tepidanaerobacter
15
10
5
0
A (n=15)
B (n=16)
C (n=14)
surface

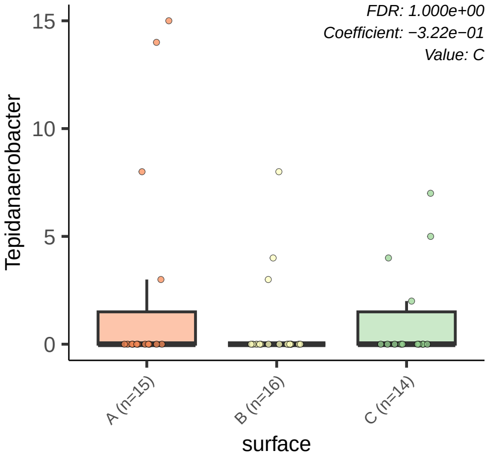
FDR: 1.000e+00
Coefficient: −3.22e−01
Value: C
Tepidanaerobacter
15
10
5
0
A (n=15)
B (n=16)
C (n=14)
surface

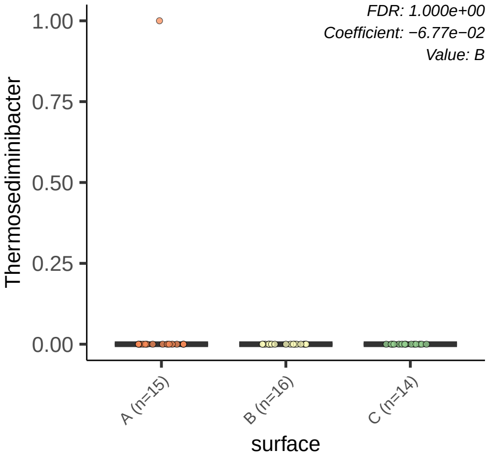

FDR: 1.000e+00
Coefficient: −6.77e−02
Value: B
Thermosediminibacter
1.00
0.75
0.50
0.25
0.00
A (n=15)
B (n=16)
C (n=14)
surface

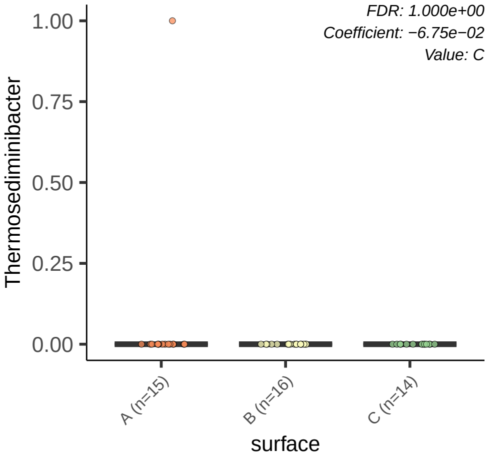
FDR: 1.000e+00
Coefficient: −6.75e−02
Value: C
Thermosediminibacter
1.00
0.75
0.50
0.25
0.00
A (n=15)
B (n=16)
C (n=14)
surface

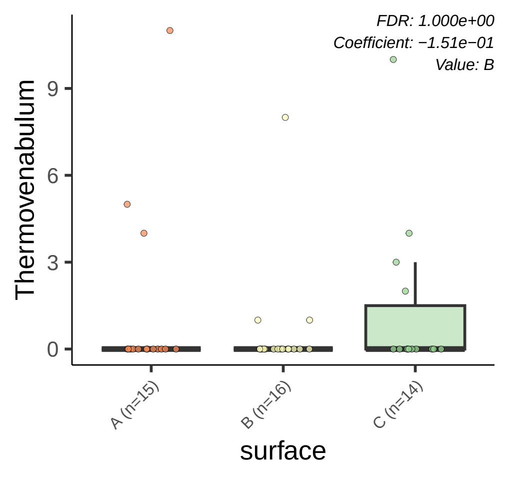

FDR: 1.000e+00
Coefficient: −1.51e−01
Value: B
Thermovenabulum
0
3
6
9
A (n=15)
B (n=16)
C (n=14)
surface

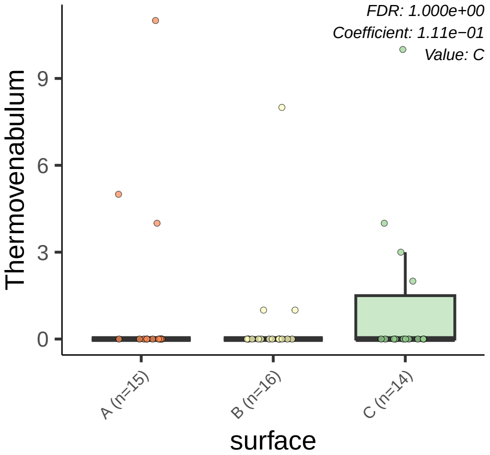
FDR: 1.000e+00
Coefficient: 1.11e−01
Value: C
Thermovenabulum
0
3
6
9
A (n=15)
B (n=16)
C (n=14)
surface

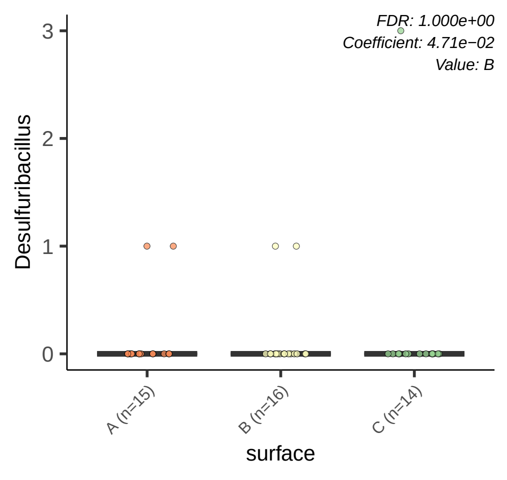
FDR: 1.000e+00
Coefficient: 4.71e−02
Value: B
Desulfuribacillus
3
2
1
0
A (n=15)
B (n=16)
C (n=14)
surface

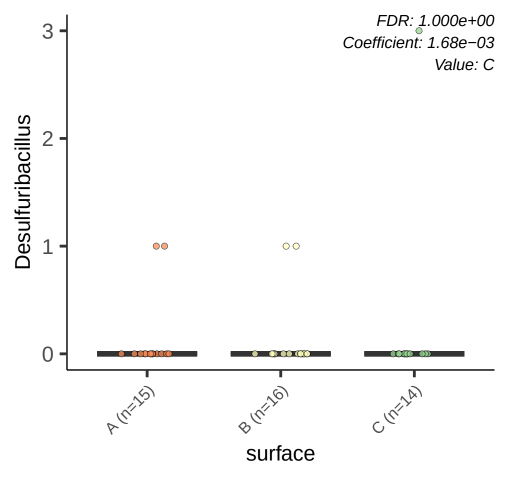
FDR: 1.000e+00
Coefficient: 1.68e−03
Value: C
Desulfuribacillus
3
2
1
0
A (n=15)
B (n=16)
C (n=14)
surface

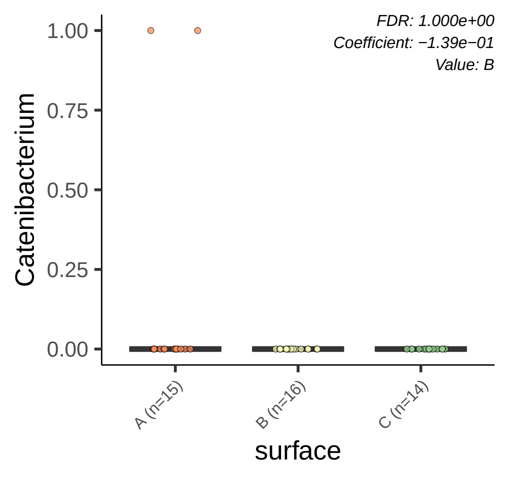
FDR: 1.000e+00
Coefficient: −1.39e−01
Value: B
Catenibacterium
1.00
0.75
0.50
0.25
0.00
A (n=15)
B (n=16)
C (n=14)
surface

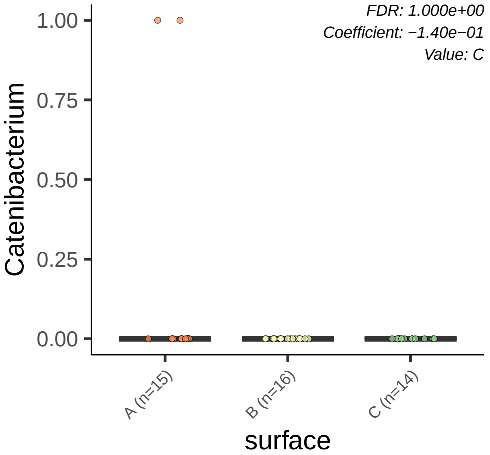
FDR: 1.000e+00
Coefficient: −1.40e−01
Value: C
Catenibacterium
1.00
0.75
0.50
0.25
0.00
A (n=15)
B (n=16)
C (n=14)
surface

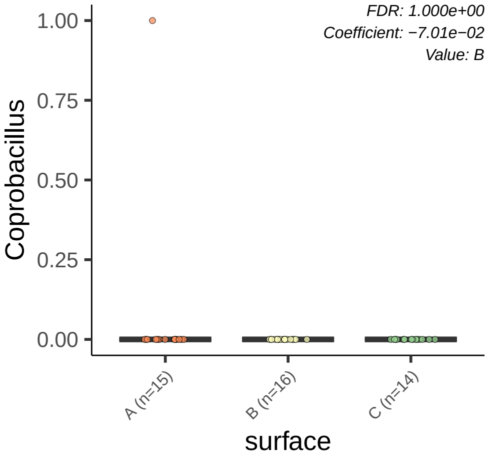
FDR: 1.000e+00
Coefficient: −7.01e−02
Value: B
Coprobacillus
1.00
0.75
0.50
0.25
0.00
A (n=15)
B (n=16)
C (n=14)
surface

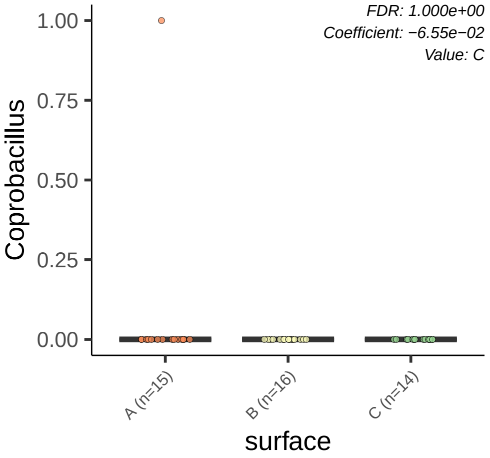
FDR: 1.000e+00
Coefficient: −6.55e−02
Value: C
Coprobacillus
1.00
0.75
0.50
0.25
0.00
A (n=15)
B (n=16)
C (n=14)
surface

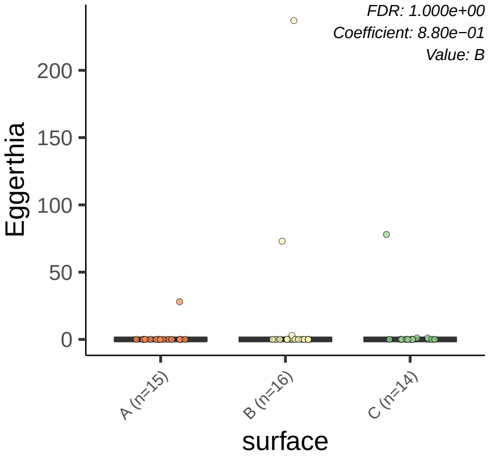
FDR: 1.000e+00
Coefficient: 8.80e−01
Value: B
Eggerthia
0
50
100
150
200
A (n=15)
B (n=16)
C (n=14)
surface

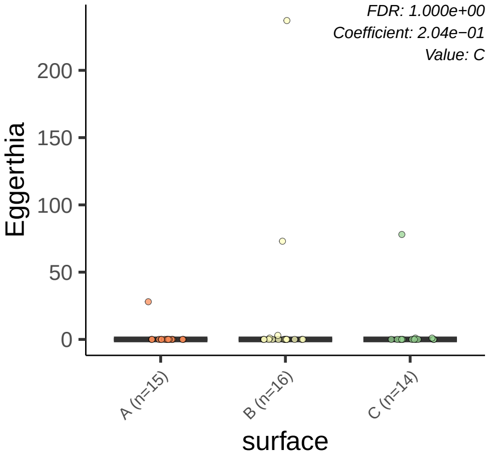

FDR: 1.000e+00
Coefficient: 2.04e−01
Value: C
Eggerthia
200
150
100
50
0
A (n=15)
B (n=16)
C (n=14)
surface

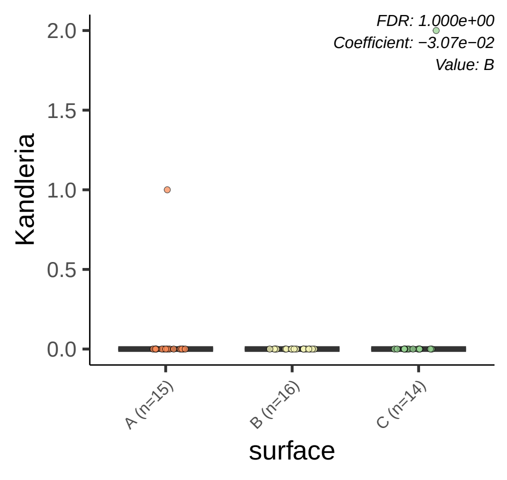
FDR: 1.000e+00
Coefficient: −3.07e−02
Value: B
Kandleria
2.0
1.5
1.0
0.5
0.0
A (n=15)
B (n=16)
C (n=14)
surface

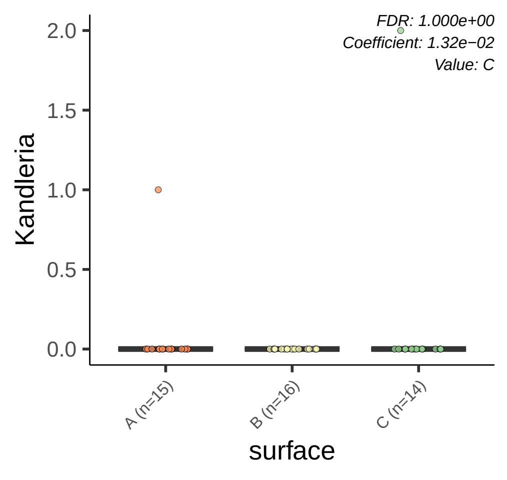

FDR: 1.000e+00
Coefficient: 1.32e−02
Value: C
Kandleria
2.0
1.5
1.0
0.5
0.0
A (n=15)
B (n=16)
C (n=14)
surface

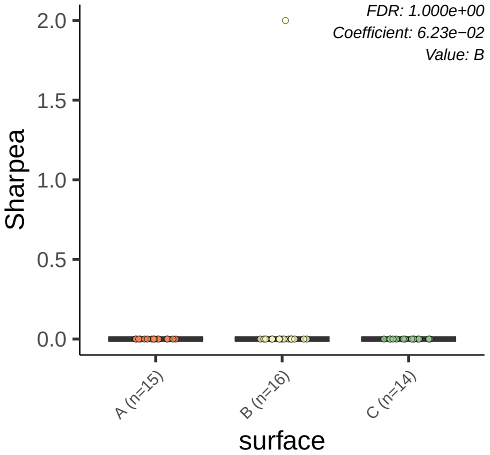
FDR: 1.000e+00
Coefficient: 6.23e−02
Value: B
Sharpea
2.0
1.5
1.0
0.5
0.0
A (n=15)
B (n=16)
C (n=14)
surface

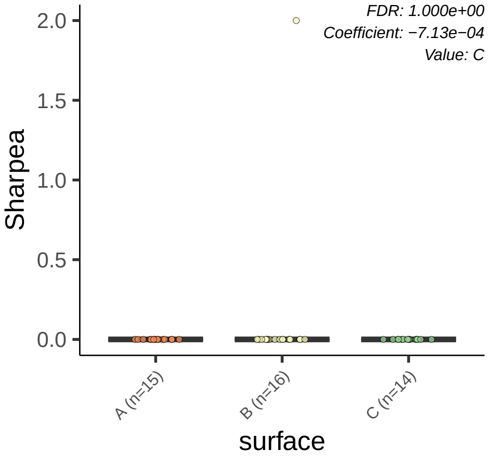

FDR: 1.000e+00
Coefficient: −7.13e−04
Value: C
Sharpea
2.0
1.5
1.0
0.5
0.0
A (n=15)
B (n=16)
C (n=14)
surface

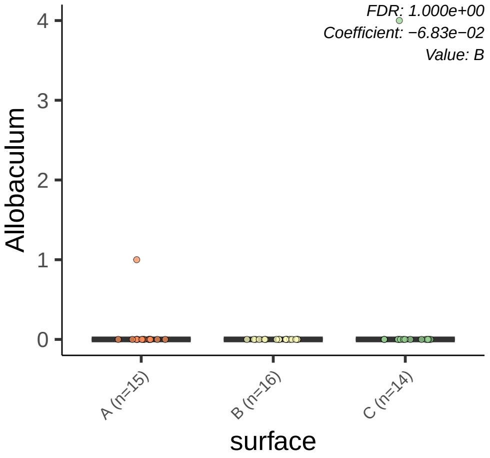

FDR: 1.000e+00
Coefficient: −6.83e−02
Value: B
Allobaculum
4
3
2
1
0
A (n=15)
B (n=16)
C (n=14)
surface

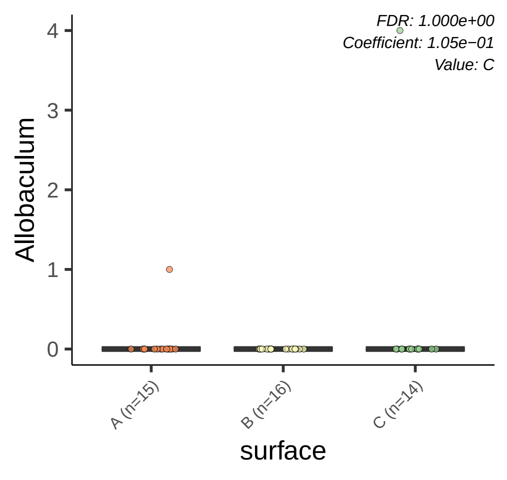

FDR: 1.000e+00
Coefficient: 1.05e−01
Value: C
Allobaculum
4
3
2
1
0
A (n=15)
B (n=16)
C (n=14)
surface

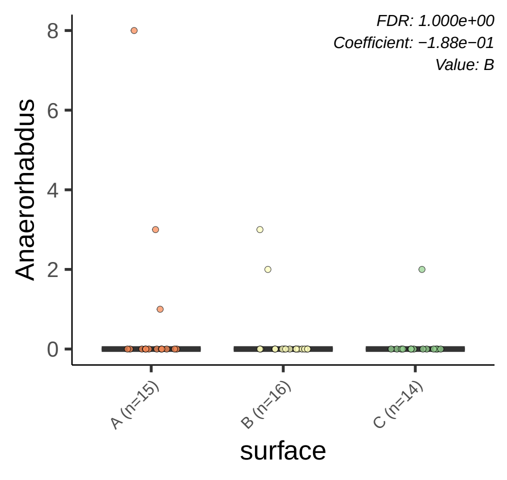
FDR: 1.000e+00
Coefficient: −1.88e−01
Value: B
Anaerorhabdus
8
6
4
2
0
A (n=15)
B (n=16)
C (n=14)
surface

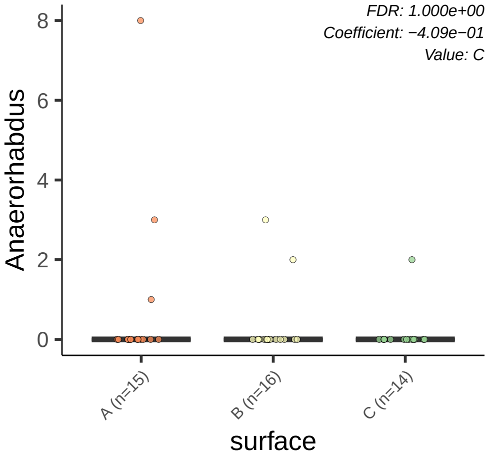
FDR: 1.000e+00
Coefficient: −4.09e−01
Value: C
Anaerorhabdus
8
6
4
2
0
A (n=15)
B (n=16)
C (n=14)
surface

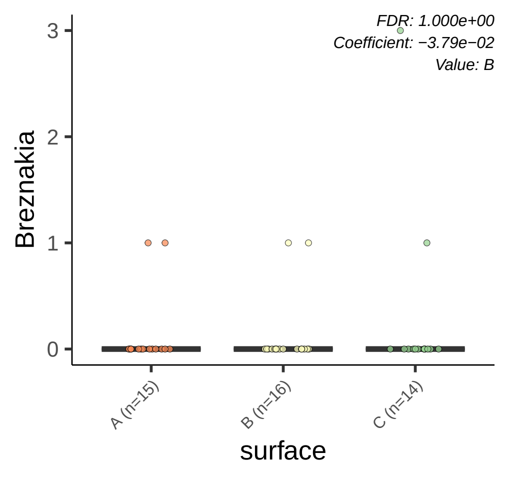
FDR: 1.000e+00
Coefficient: −3.79e−02
Value: B
Breznakia
0
1
2
3
A (n=15)
B (n=16)
C (n=14)
surface

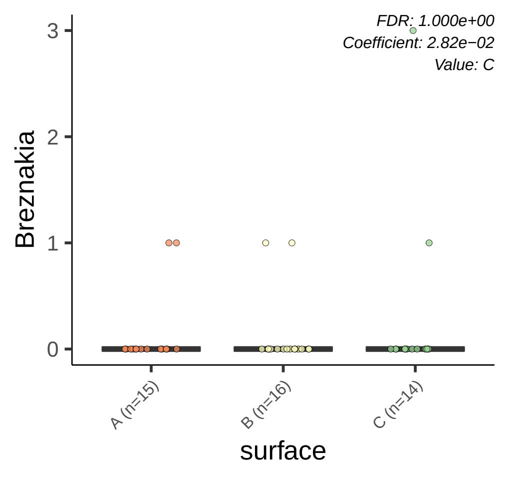

FDR: 1.000e+00
Coefficient: 2.82e−02
Value: C
Breznakia
3
2
1
0
A (n=15)
B (n=16)
C (n=14)
surface

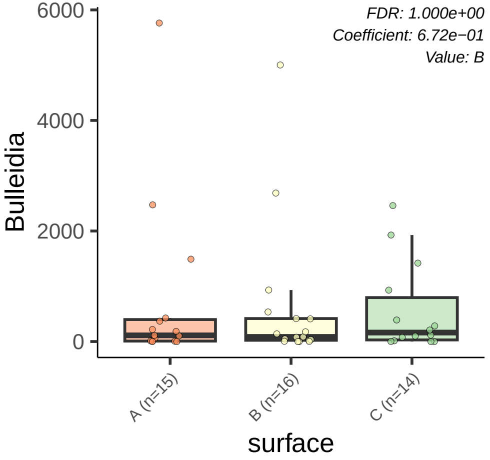

FDR: 1.000e+00
Coefficient: 6.72e−01
Value: B
6000
4000
2000
0
Bulleidia
A (n=15)
B (n=16)
C (n=14)
surface

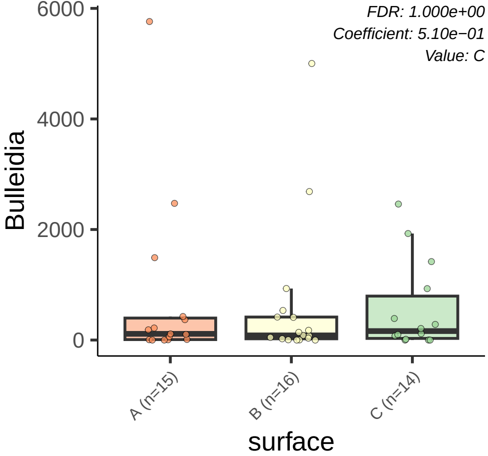

FDR: 1.000e+00
Coefficient: 5.10e−01
Value: C
6000
4000
2000
0
Bulleidia
A (n=15)
B (n=16)
C (n=14)
surface

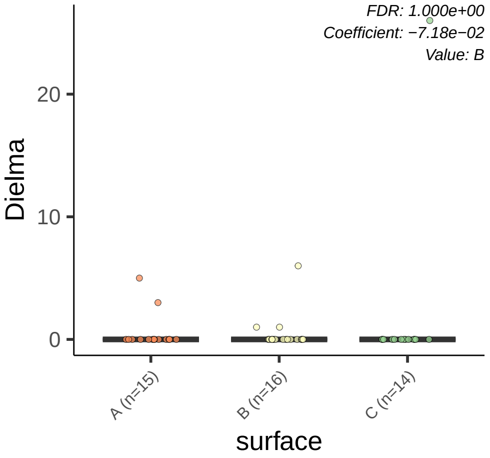
FDR: 1.000e+00
Coefficient: −7.18e−02
Value: B
Dielma
20
10
0
A (n=15)
B (n=16)
C (n=14)
surface

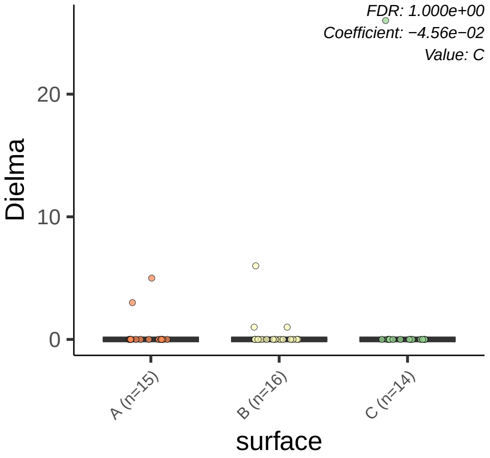
FDR: 1.000e+00
Coefficient: −4.56e−02
Value: C
Dielma
20
10
0
A (n=15)
B (n=16)
C (n=14)
surface

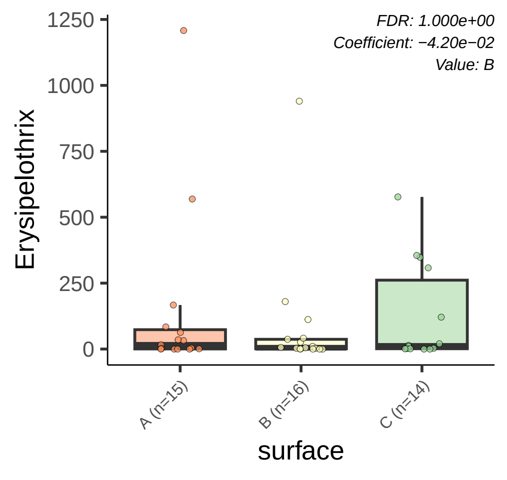
FDR: 1.000e+00
Coefficient: −4.20e−02
Value: B
Erysipelothrix
1250
1000
750
500
250
0
A (n=15)
B (n=16)
C (n=14)
surface

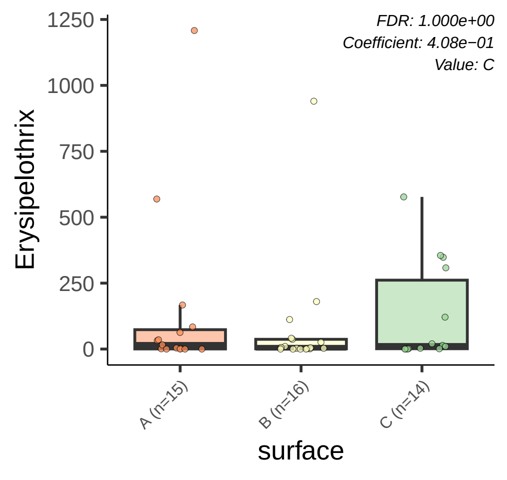

FDR: 1.000e+00
Coefficient: 4.08e−01
Value: C
Erysipelothrix
1250
1000
750
500
250
0
A (n=15)
B (n=16)
C (n=14)
surface

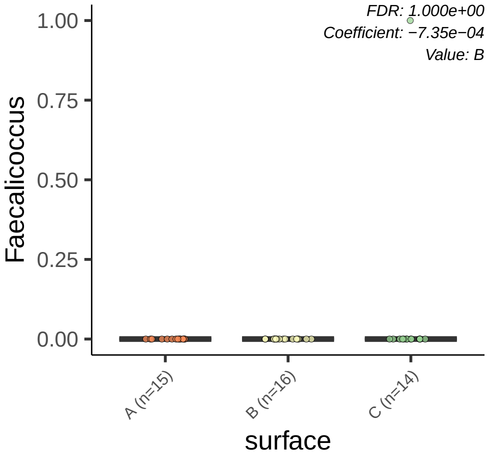

FDR: 1.000e+00
Coefficient: −7.35e−04
Value: B
Faecalicoccus
1.00
0.75
0.50
0.25
0.00
A (n=15)
B (n=16)
C (n=14)
surface

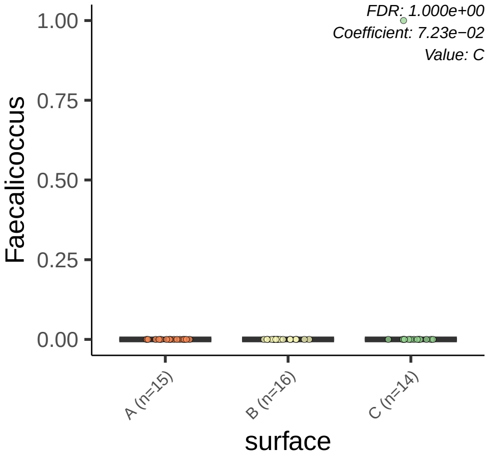
FDR: 1.000e+00
Coefficient: 7.23e−02
Value: C
Faecalicoccus
1.00
0.75
0.50
0.25
0.00
A (n=15)
B (n=16)
C (n=14)
surface

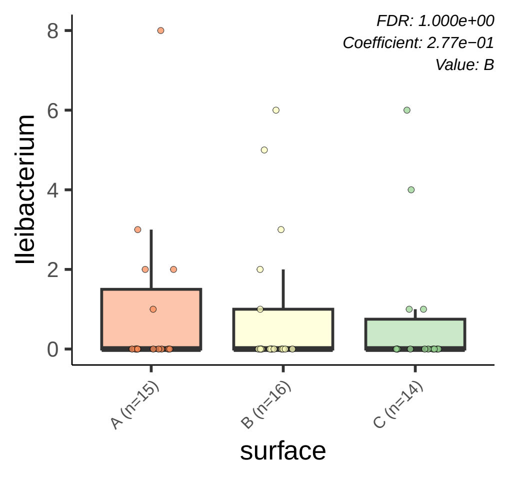
FDR: 1.000e+00
Coefficient: 2.77e−01
Value: B
Ileibacterium
0
2
4
6
8
A (n=15)
B (n=16)
C (n=14)
surface

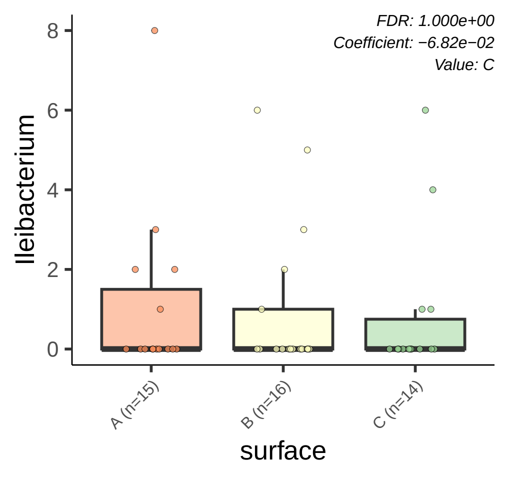

FDR: 1.000e+00
Coefficient: −6.82e−02
Value: C
Ileibacterium
8
6
4
2
0
A (n=15)
B (n=16)
C (n=14)
surface

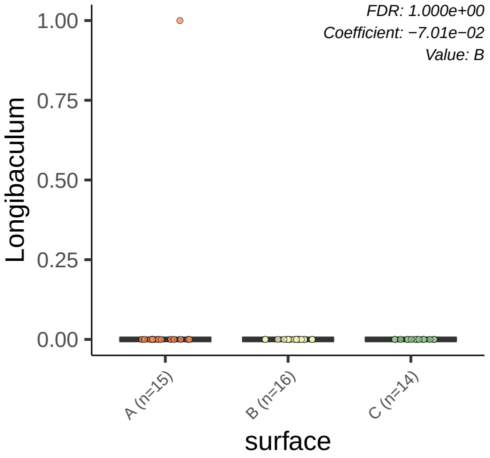
FDR: 1.000e+00
Coefficient: −7.01e−02
Value: B
Longibaculum
1.00
0.75
0.50
0.25
0.00
A (n=15)
B (n=16)
C (n=14)
surface

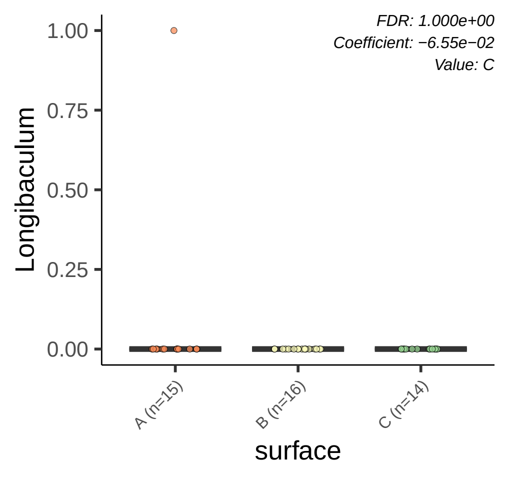
FDR: 1.000e+00
Coefficient: −6.55e−02
Value: C
Longibaculum
1.00
0.75
0.50
0.25
0.00
A (n=15)
B (n=16)
C (n=14)
surface

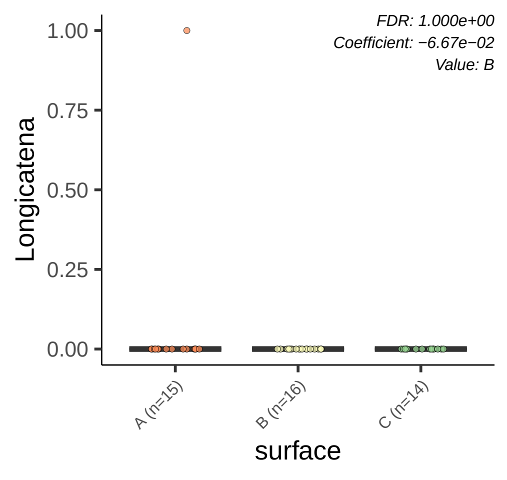

FDR: 1.000e+00
Coefficient: −6.67e−02
Value: B
1.00
0.75
0.50
0.25
0.00
Longicatena
A (n=15)
B (n=16)
C (n=14)
surface

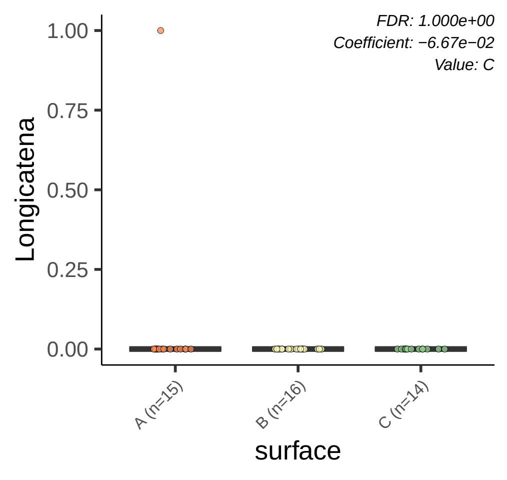
FDR: 1.000e+00
Coefficient: −6.67e−02
Value: C
Longicatena
1.00
0.75
0.50
0.25
0.00
A (n=15)
B (n=16)
C (n=14)
surface

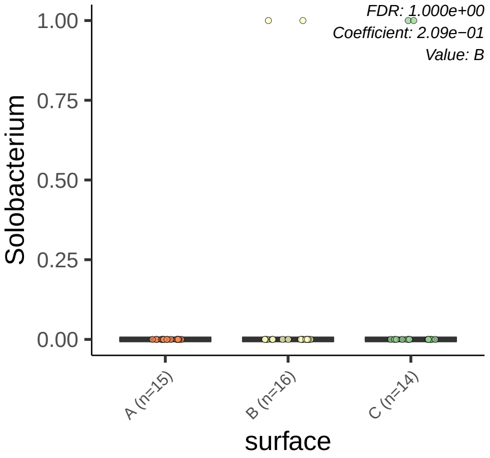
FDR: 1.000e+00
Coefficient: 2.09e−01
Value: B
Solobacterium
1.00
0.75
0.50
0.25
0.00
A (n=15)
B (n=16)
C (n=14)
surface

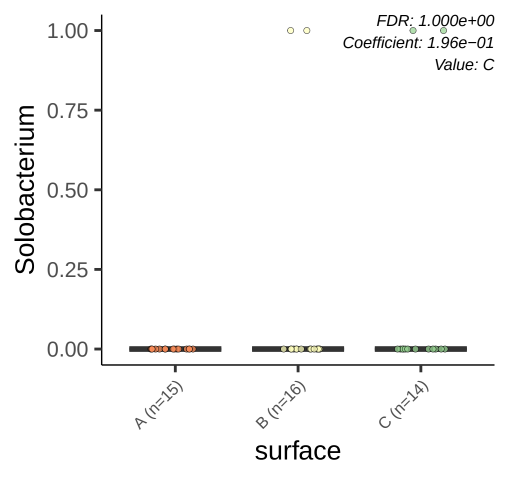
FDR: 1.000e+00
Coefficient: 1.96e−01
Value: C
Solobacterium
1.00
0.75
0.50
0.25
0.00
A (n=15)
B (n=16)
C (n=14)
surface

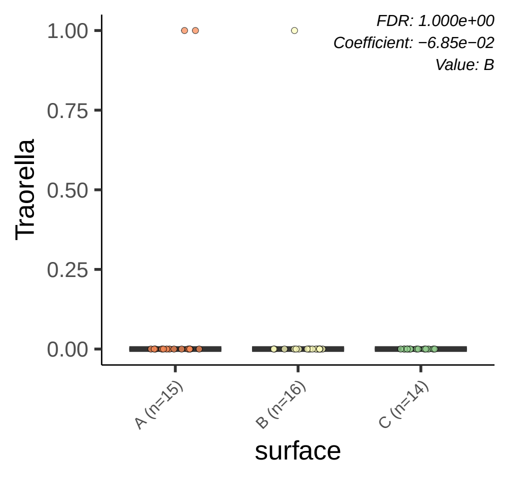
FDR: 1.000e+00
Coefficient: −6.85e−02
Value: B
Traorella
1.00
0.75
0.50
0.25
0.00
A (n=15)
B (n=16)
C (n=14)
surface

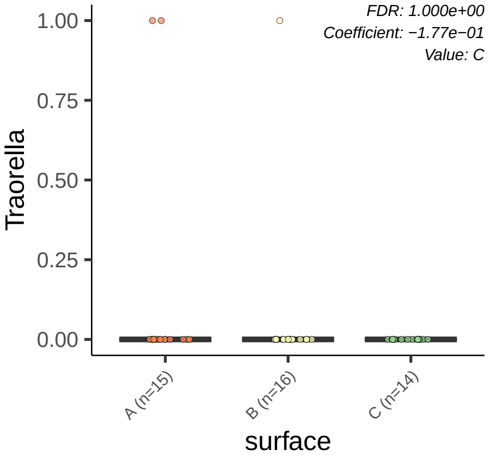
FDR: 1.000e+00
Coefficient: −1.77e−01
Value: C
Traorella
1.00
0.75
0.50
0.25
0.00
A (n=15)
B (n=16)
C (n=14)
surface

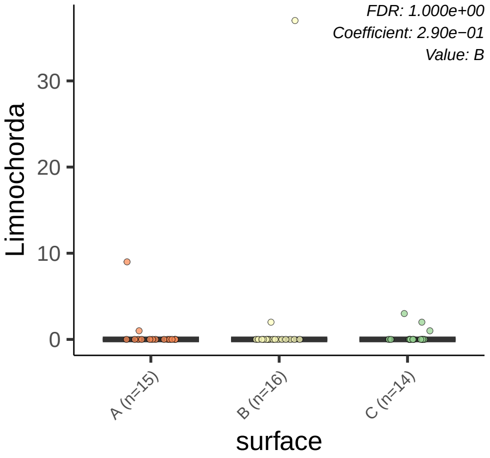

FDR: 1.000e+00
Coefficient: 2.90e−01
Value: B
Limnochorda
30
20
10
0
A (n=15)
B (n=16)
C (n=14)
surface

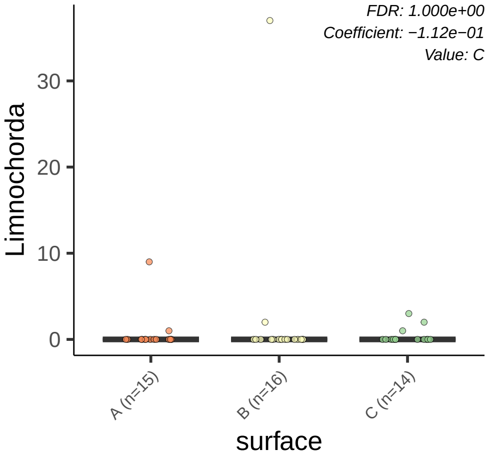

FDR: 1.000e+00
Coefficient: −1.12e−01
Value: C
Limnochorda
30
20
10
0
A (n=15)
B (n=16)
C (n=14)
surface

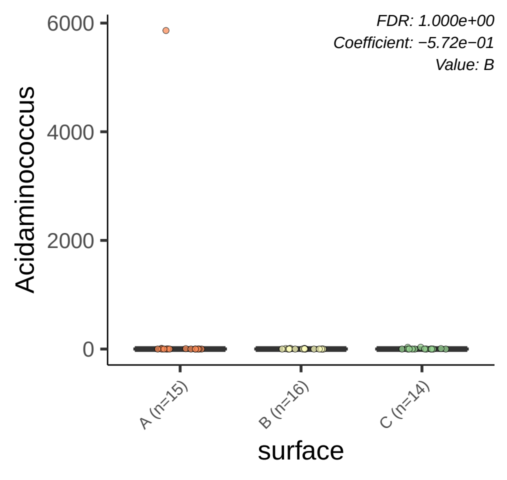
FDR: 1.000e+00
Coefficient: −5.72e−01
Value: B
Acidaminococcus
6000
4000
2000
0
A (n=15)
B (n=16)
C (n=14)
surface

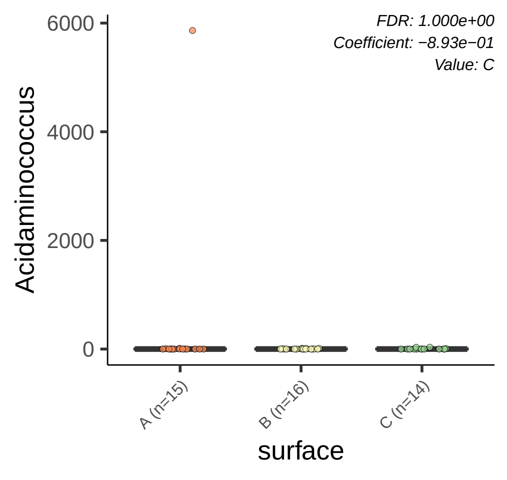
FDR: 1.000e+00
Coefficient: −8.93e−01
Value: C
Acidaminococcus
6000
4000
2000
0
A (n=15)
B (n=16)
C (n=14)
surface

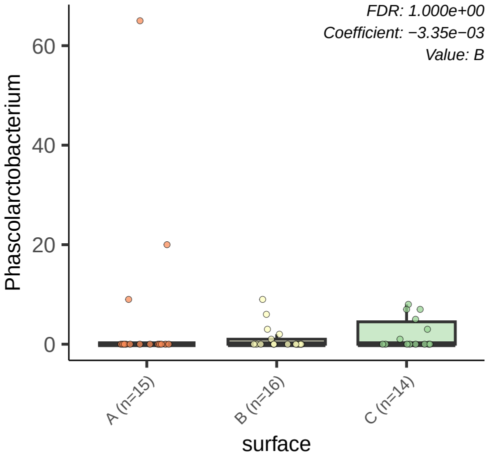

FDR: 1.000e+00
Coefficient: −3.35e−03
Value: B
Phascolarctobacterium
60
40
20
0
A (n=15)
B (n=16)
C (n=14)
surface

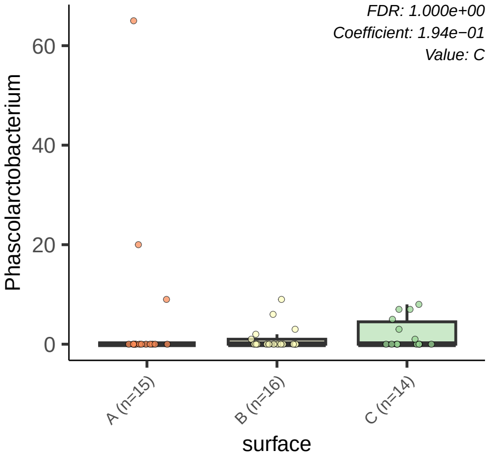

FDR: 1.000e+00
Coefficient: 1.94e−01
Value: C
Phascolarctobacterium
0
20
40
60
A (n=15)
B (n=16)
C (n=14)
surface

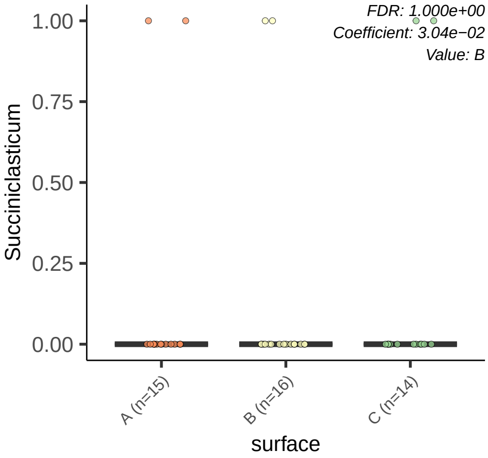

FDR: 1.000e+00
Coefficient: 3.04e−02
Value: B
Succiniclasticum
1.00
0.75
0.50
0.25
0.00
A (n=15)
B (n=16)
C (n=14)
surface

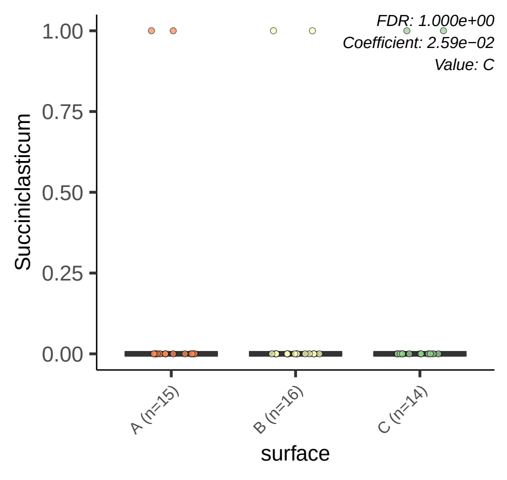
FDR: 1.000e+00
Coefficient: 2.59e−02
Value: C
Succiniclasticum
1.00
0.75
0.50
0.25
0.00
A (n=15)
B (n=16)
C (n=14)
surface

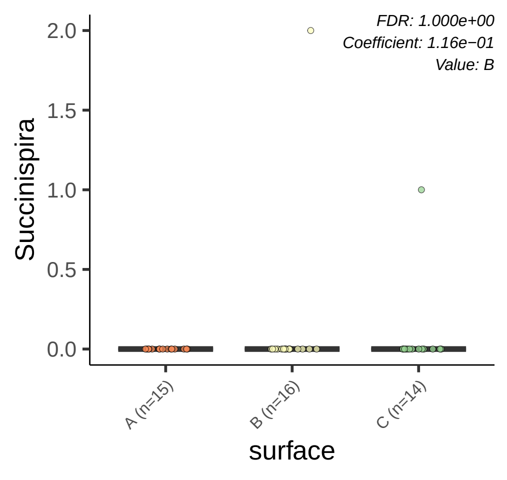
FDR: 1.000e+00
Coefficient: 1.16e−01
Value: B
Succinispira
2.0
1.5
1.0
0.5
0.0
A (n=15)
B (n=16)
C (n=14)
surface

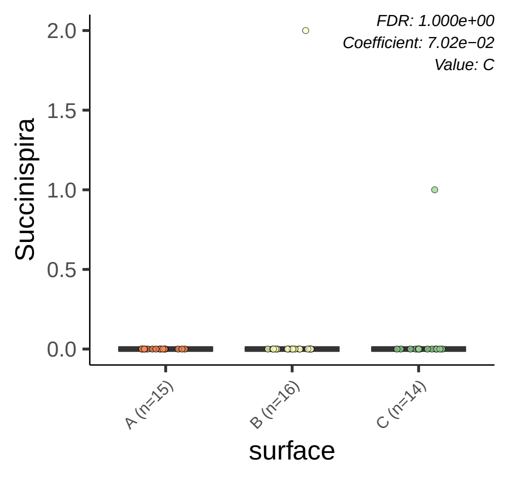
FDR: 1.000e+00
Coefficient: 7.02e−02
Value: C
Succinispira
2.0
1.5
1.0
0.5
0.0
A (n=15)
B (n=16)
C (n=14)
surface

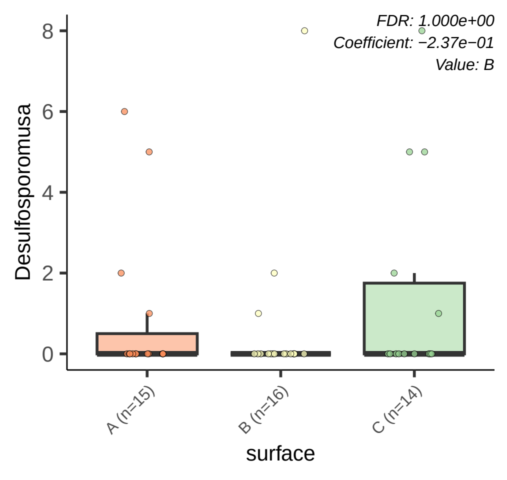
FDR: 1.000e+00
Coefficient: −2.37e−01
Value: B
Desulfosporomusa
0
2
4
6
8
A (n=15)
B (n=16)
C (n=14)
surface

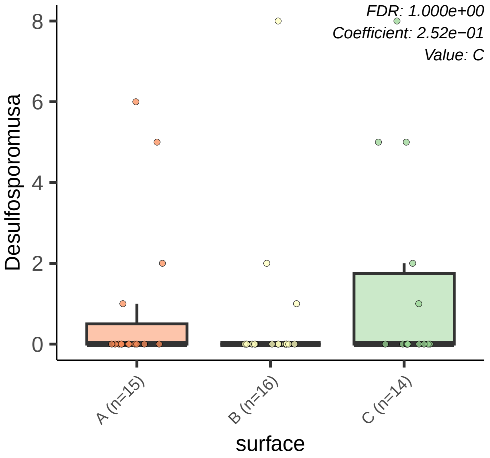
FDR: 1.000e+00
Coefficient: 2.52e−01
Value: C
Desulfosporomusa
0
2
4
6
8
A (n=15)
B (n=16)
C (n=14)
surface

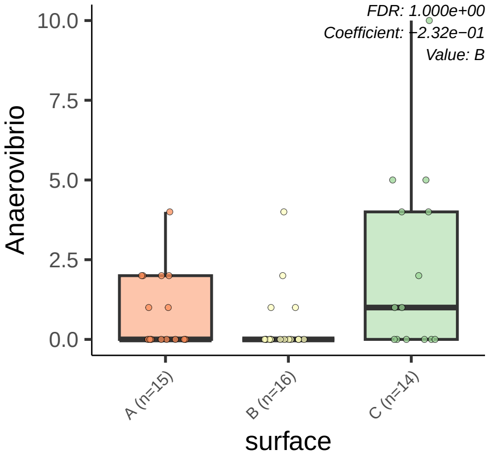

FDR: 1.000e+00
Coefficient: −2.32e−01
Value: B
Anaerovibrio
10.0
7.5
5.0
2.5
0.0
A (n=15)
B (n=16)
C (n=14)
surface

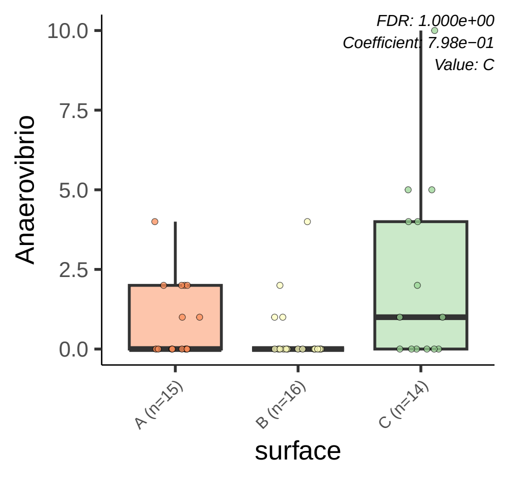

FDR: 1.000e+00
Coefficient: 7.98e−01
Value: C
Anaerovibrio
10.0
7.5
5.0
2.5
0.0
A (n=15)
B (n=16)
C (n=14)
surface

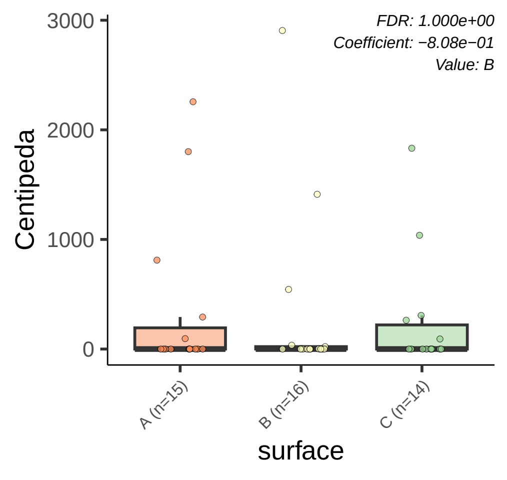

FDR: 1.000e+00
Coefficient: −8.08e−01
Value: B
Centipeda
3000
2000
1000
0
A (n=15)
B (n=16)
C (n=14)
surface

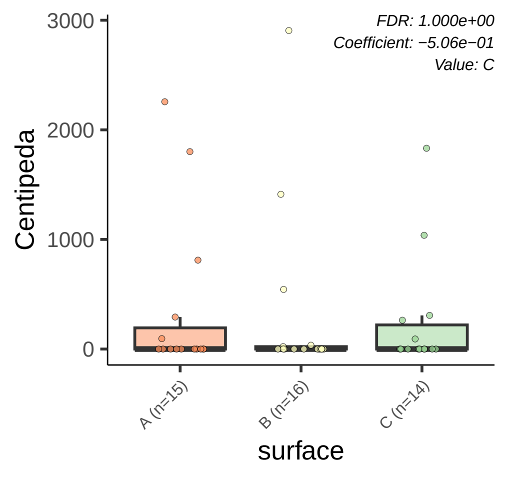
FDR: 1.000e+00
Coefficient: −5.06e−01
Value: C
Centipeda
3000
2000
1000
0
A (n=15)
B (n=16)
C (n=14)
surface

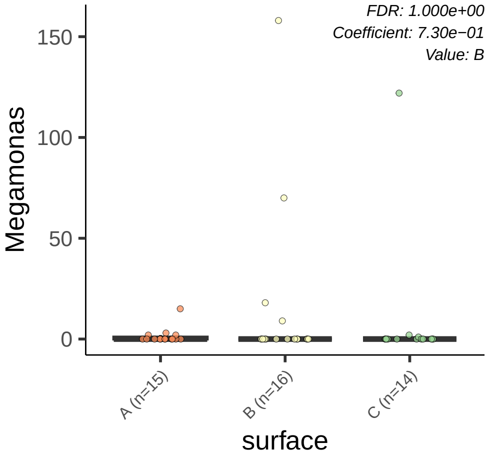
FDR: 1.000e+00
Coefficient: 7.30e−01
Value: B
Megamonas
150
100
50
0
A (n=15)
B (n=16)
C (n=14)
surface

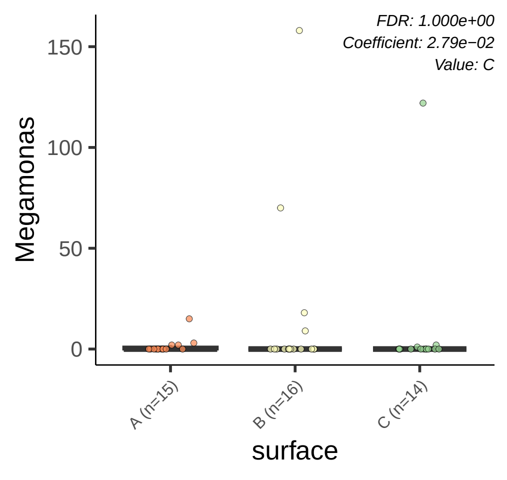

FDR: 1.000e+00
Coefficient: 2.79e−02
Value: C
Megamonas
150
100
50
0
A (n=15)
B (n=16)
C (n=14)
surface

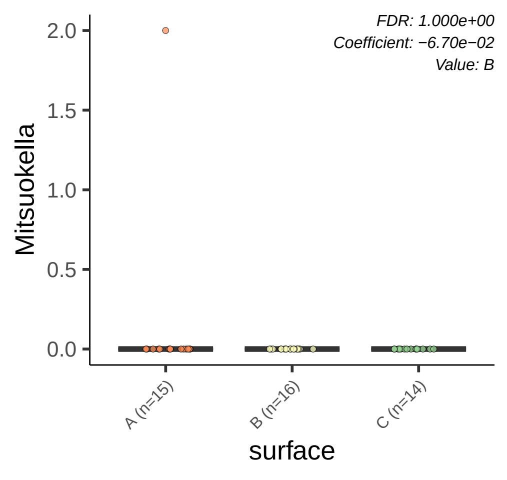
FDR: 1.000e+00
Coefficient: −6.70e−02
Value: B
Mitsuokella
2.0
1.5
1.0
0.5
0.0
A (n=15)
B (n=16)
C (n=14)
surface

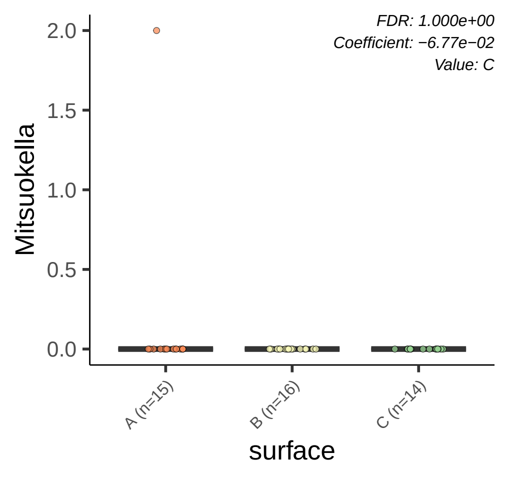
FDR: 1.000e+00
Coefficient: −6.77e−02
Value: C
2.0
1.5
1.0
0.5
0.0
Mitsuokella
A (n=15)
B (n=16)
C (n=14)
surface

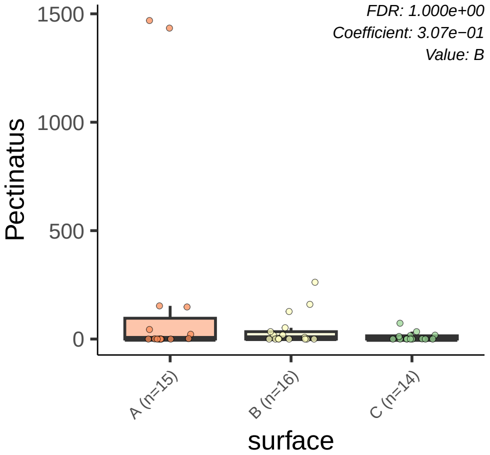
FDR: 1.000e+00
Coefficient: 3.07e−01
Value: B
Pectinatus
1500
1000
500
0
A (n=15)
B (n=16)
C (n=14)
surface

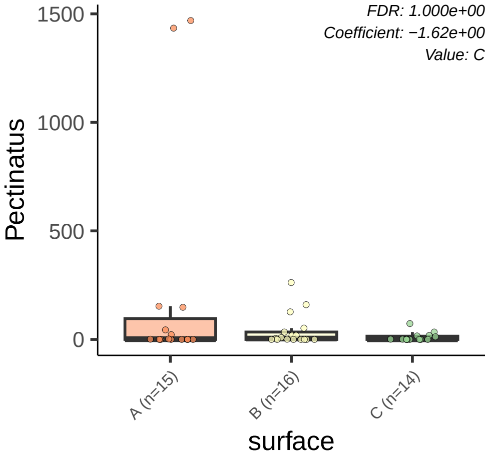
FDR: 1.000e+00
Coefficient: −1.62e+00
Value: C
Pectinatus
1500
1000
500
0
A (n=15)
B (n=16)
C (n=14)
surface

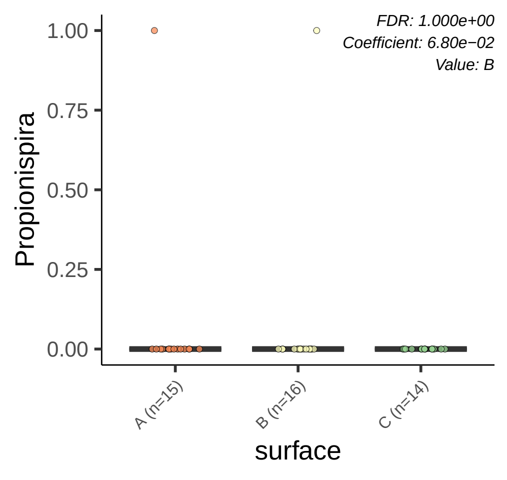

FDR: 1.000e+00
Coefficient: 6.80e−02
Value: B
Propionispira
1.00
0.75
0.50
0.25
0.00
A (n=15)
B (n=16)
C (n=14)
surface

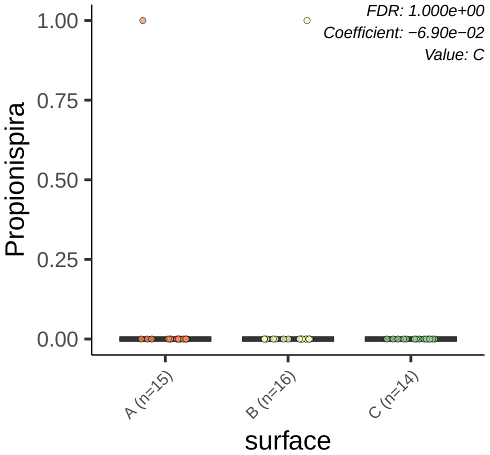
FDR: 1.000e+00
Coefficient: −6.90e−02
Value: C
Propionispira
1.00
0.75
0.50
0.25
0.00
A (n=15)
B (n=16)
C (n=14)
surface

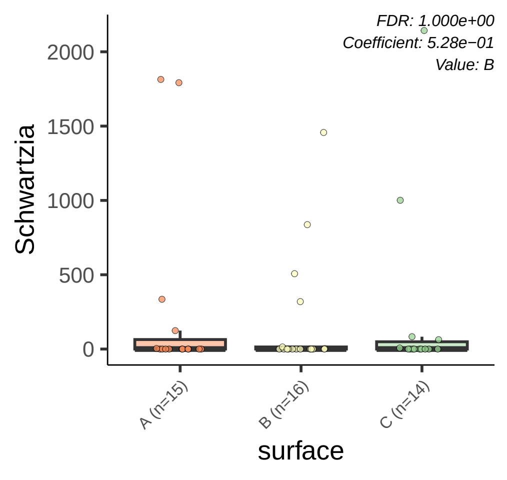

FDR: 1.000e+00
Coefficient: 5.28e−01
Value: B
Schwartzia
2000
1500
1000
500
0
A (n=15)
B (n=16)
C (n=14)
surface

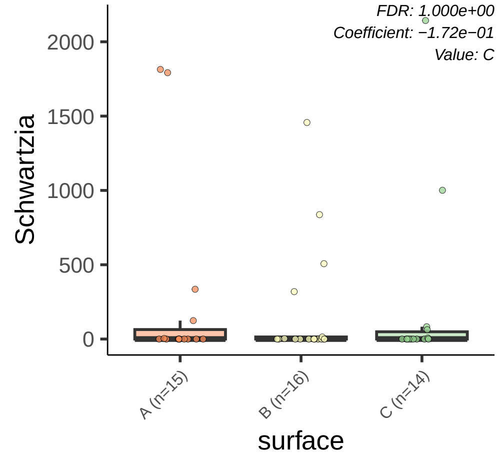

FDR: 1.000e+00
Coefficient: −1.72e−01
Value: C
Schwartzia
2000
1500
1000
500
0
A (n=15)
B (n=16)
C (n=14)
surface

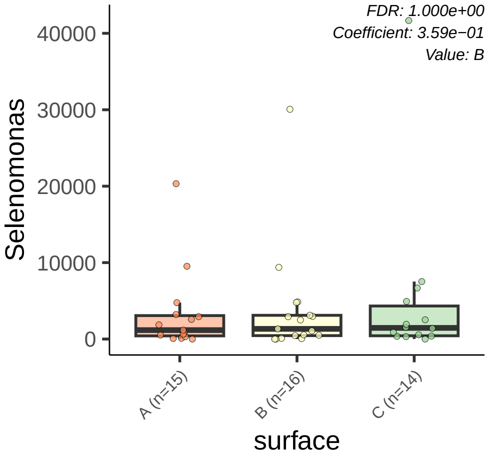
FDR: 1.000e+00
Coefficient: 3.59e−01
Value: B
Selenomonas
40000
30000
20000
10000
0
A (n=15)
B (n=16)
C (n=14)
surface

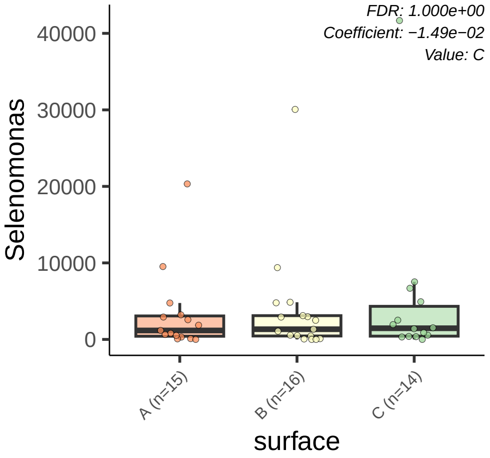

FDR: 1.000e+00
Coefficient: −1.49e−02
Value: C
Selenomonas
40000
30000
20000
10000
0
A (n=15)
B (n=16)
C (n=14)
surface

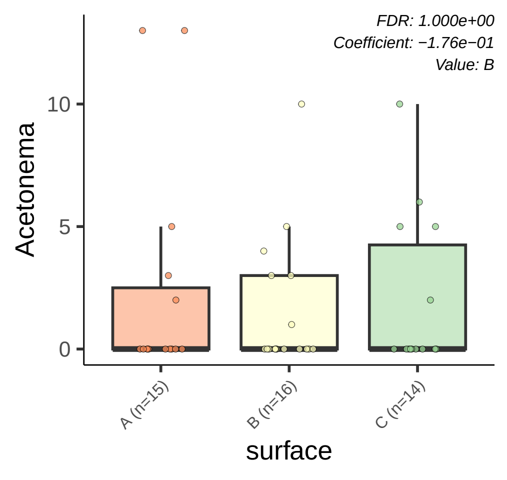

FDR: 1.000e+00
Coefficient: −1.76e−01
Value: B
Acetonema
10
5
0
A (n=15)
B (n=16)
C (n=14)
surface

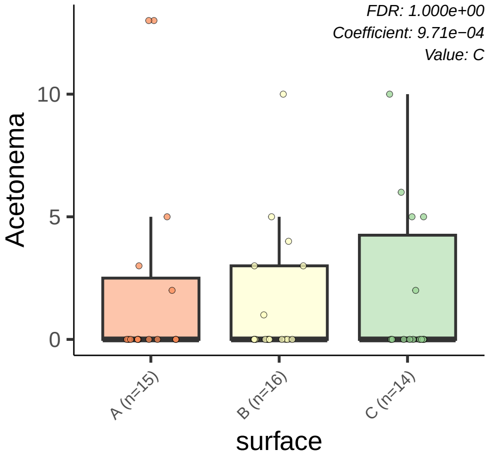

FDR: 1.000e+00
Coefficient: 9.71e−04
Value: C
Acetonema
10
5
0
A (n=15)
B (n=16)
C (n=14)
surface

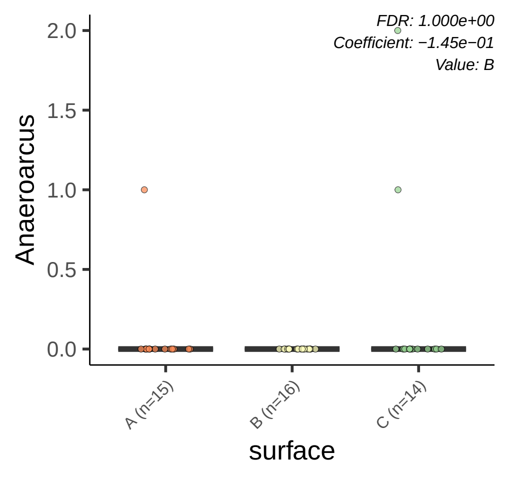
FDR: 1.000e+00
Coefficient: −1.45e−01
Value: B
Anaeroarcus
2.0
1.5
1.0
0.5
0.0
A (n=15)
B (n=16)
C (n=14)
surface

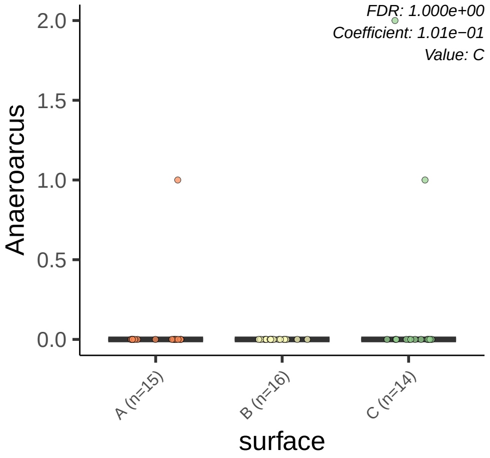
FDR: 1.000e+00
Coefficient: 1.01e−01
Value: C
Anaeroarcus
2.0
1.5
1.0
0.5
0.0
A (n=15)
B (n=16)
C (n=14)
surface

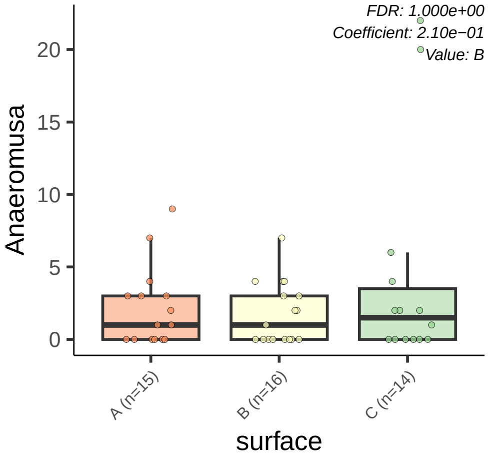
FDR: 1.000e+00
Coefficient: 2.10e−01
Value: B
Anaeromusa
0
5
10
15
20
A (n=15)
B (n=16)
C (n=14)
surface

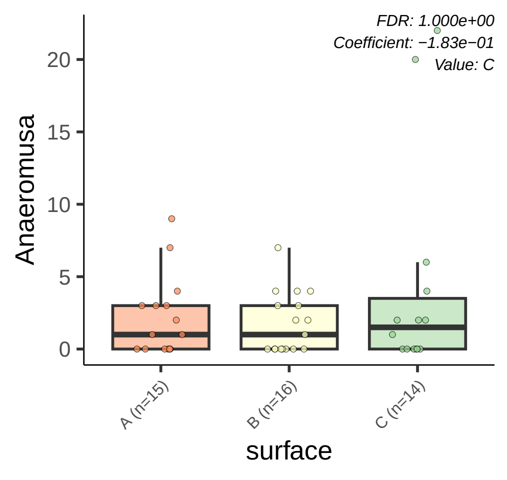
FDR: 1.000e+00
Coefficient: −1.83e−01
Value: C
Anaeromusa
20
15
10
5
0
A (n=15)
B (n=16)
C (n=14)
surface

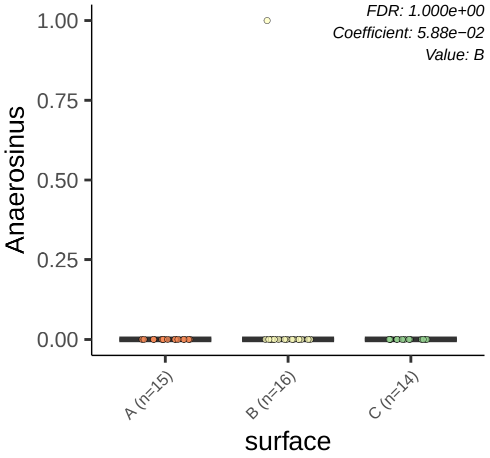
FDR: 1.000e+00
Coefficient: 5.88e−02
Value: B
Anaerosinus
1.00
0.75
0.50
0.25
0.00
A (n=15)
B (n=16)
C (n=14)
surface

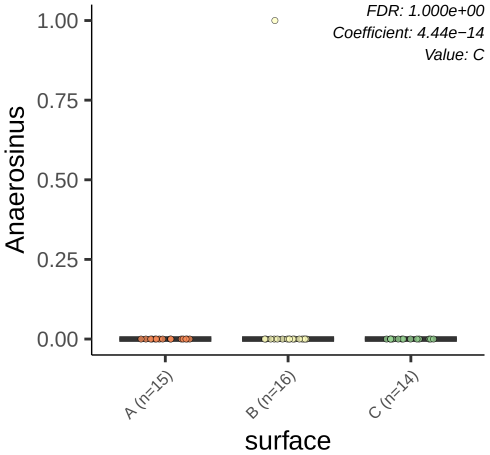

FDR: 1.000e+00
Coefficient: 4.44e−14
Value: C
Anaerosinus
1.00
0.75
0.50
0.25
0.00
A (n=15)
B (n=16)
C (n=14)
surface

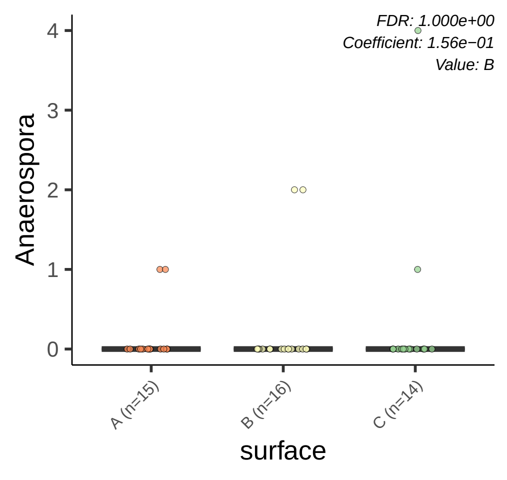
FDR: 1.000e+00
Coefficient: 1.56e−01
Value: B
Anaerospora
4
3
2
1
0
A (n=15)
B (n=16)
C (n=14)
surface

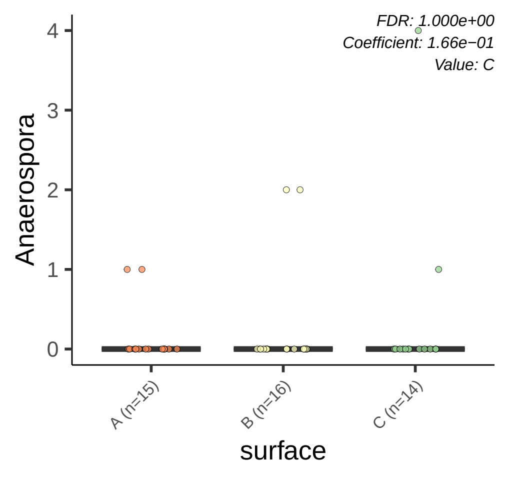
FDR: 1.000e+00
Coefficient: 1.66e−01
Value: C
Anaerospora
0
1
2
3
4
A (n=15)
B (n=16)
C (n=14)
surface

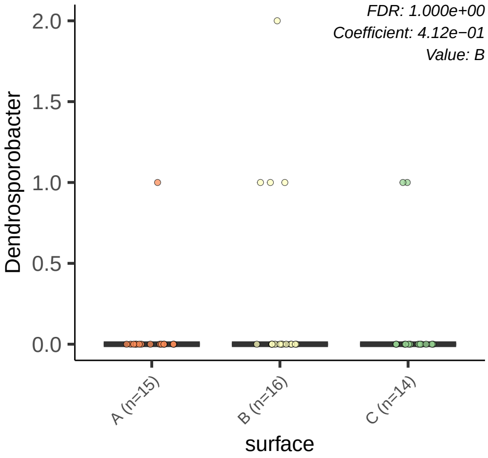
FDR: 1.000e+00
Coefficient: 4.12e−01
Value: B
Dendrosporobacter
2.0
1.5
1.0
0.5
0.0
A (n=15)
B (n=16)
C (n=14)
surface

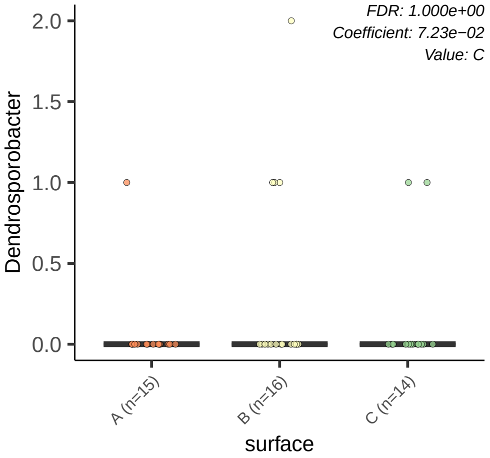
FDR: 1.000e+00
Coefficient: 7.23e−02
Value: C
Dendrosporobacter
2.0
1.5
1.0
0.5
0.0
A (n=15)
B (n=16)
C (n=14)
surface

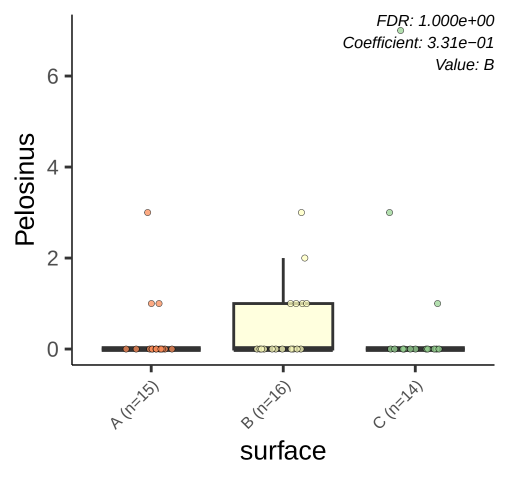
FDR: 1.000e+00
Coefficient: 3.31e−01
Value: B
Pelosinus
0
2
4
6
A (n=15)
B (n=16)
C (n=14)
surface

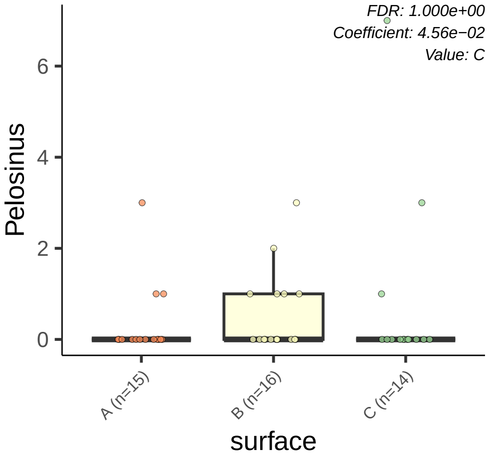
FDR: 1.000e+00
Coefficient: 4.56e−02
Value: C
Pelosinus
0
2
4
6
A (n=15)
B (n=16)
C (n=14)
surface

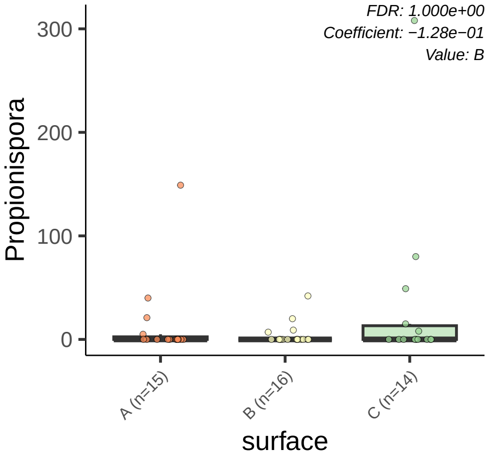

FDR: 1.000e+00
Coefficient: −1.28e−01
Value: B
Propionispora
300
200
100
0
A (n=15)
B (n=16)
C (n=14)
surface

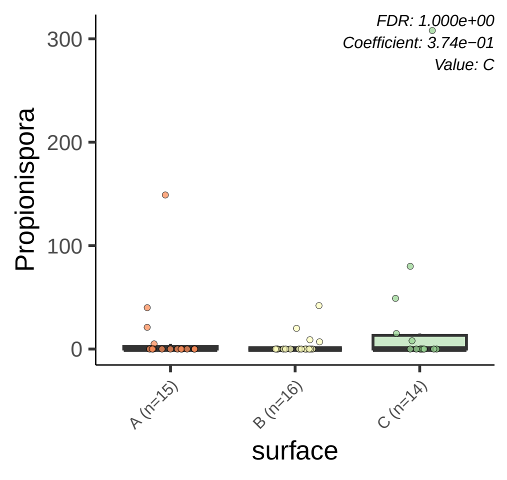

FDR: 1.000e+00
Coefficient: 3.74e−01
Value: C
Propionispora
300
200
100
0
A (n=15)
B (n=16)
C (n=14)
surface

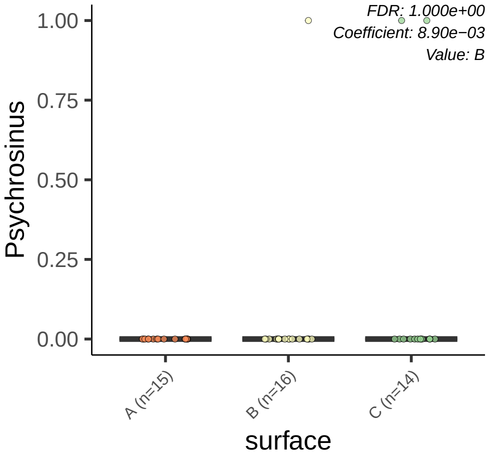

FDR: 1.000e+00
Coefficient: 8.90e−03
Value: B
Psychrosinus
1.00
0.75
0.50
0.25
0.00
A (n=15)
B (n=16)
C (n=14)
surface

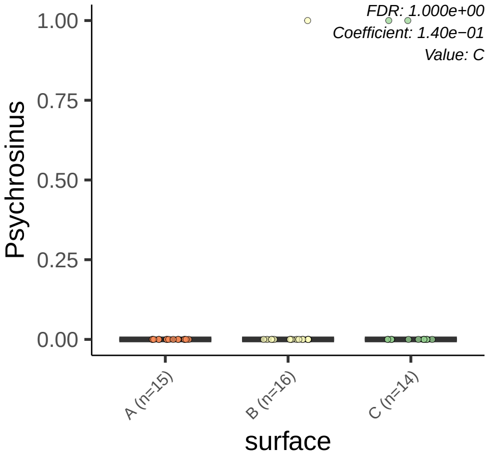

FDR: 1.000e+00
Coefficient: 1.40e−01
Value: C
1.00
0.75
0.50
0.25
0.00
Psychrosinus
A (n=15)
B (n=16)
C (n=14)
surface

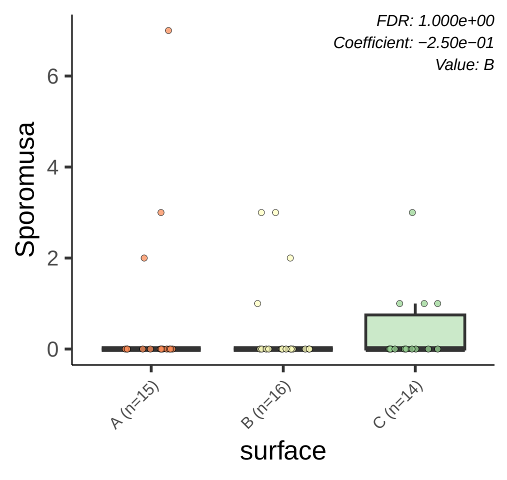

FDR: 1.000e+00
Coefficient: −2.50e−01
Value: B
Sporomusa
0
2
4
6
A (n=15)
B (n=16)
C (n=14)
surface

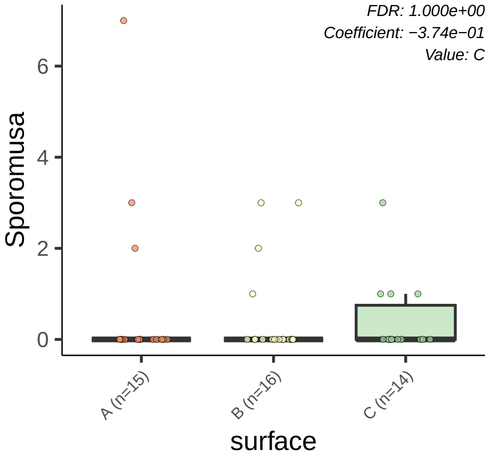

FDR: 1.000e+00
Coefficient: −3.74e−01
Value: C
Sporomusa
0
2
4
6
A (n=15)
B (n=16)
C (n=14)
surface

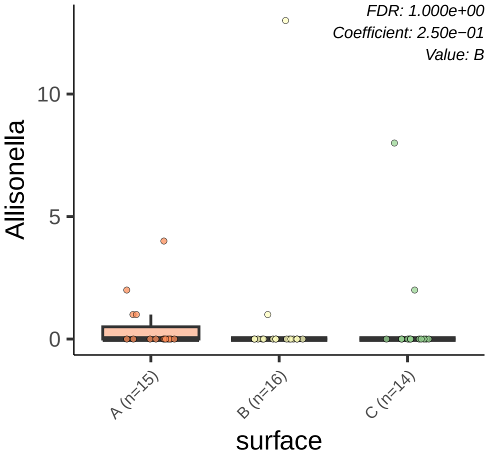
FDR: 1.000e+00
Coefficient: 2.50e−01
Value: B
Allisonella
10
5
0
A (n=15)
B (n=16)
C (n=14)
surface

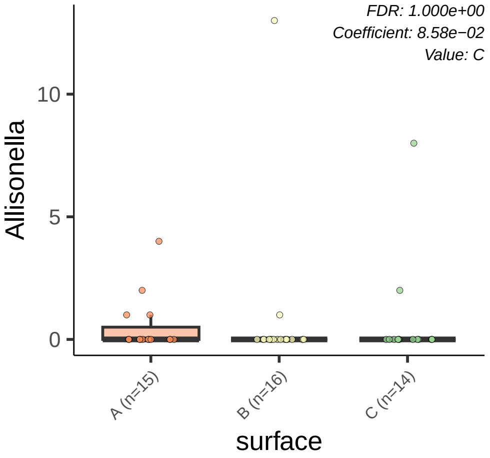
FDR: 1.000e+00
Coefficient: 8.58e−02
Value: C
Allisonella
10
5
0
A (n=15)
B (n=16)
C (n=14)
surface

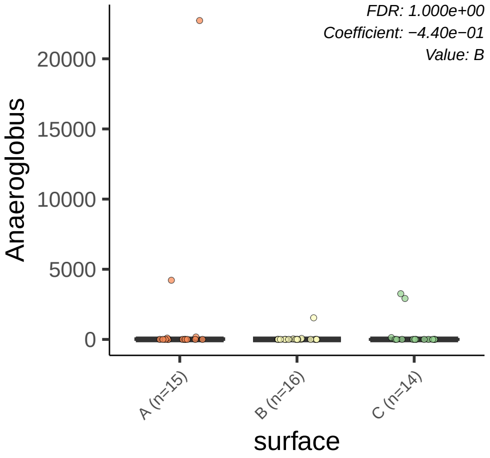
FDR: 1.000e+00
Coefficient: −4.40e−01
Value: B
Anaeroglobus
20000
15000
10000
5000
0
A (n=15)
B (n=16)
C (n=14)
surface

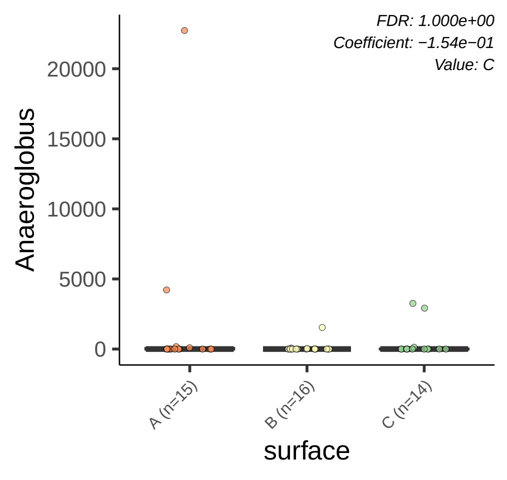

FDR: 1.000e+00
Coefficient: −1.54e−01
Value: C
Anaeroglobus
20000
15000
10000
5000
0
A (n=15)
B (n=16)
C (n=14)
surface

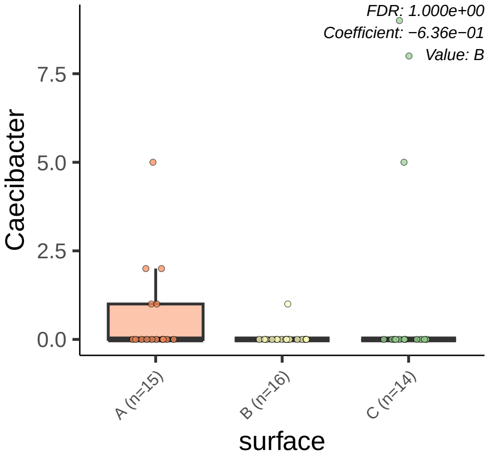
FDR: 1.000e+00
Coefficient: −6.36e−01
Value: B
Caecibacter
7.5
5.0
2.5
0.0
A (n=15)
B (n=16)
C (n=14)
surface

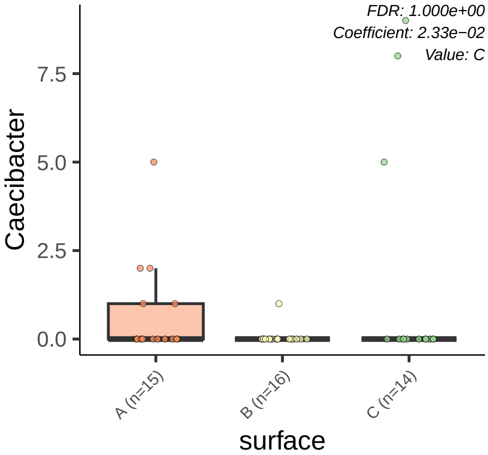
FDR: 1.000e+00
Coefficient: 2.33e−02
Value: C
Caecibacter
7.5
5.0
2.5
0.0
A (n=15)
B (n=16)
C (n=14)
surface

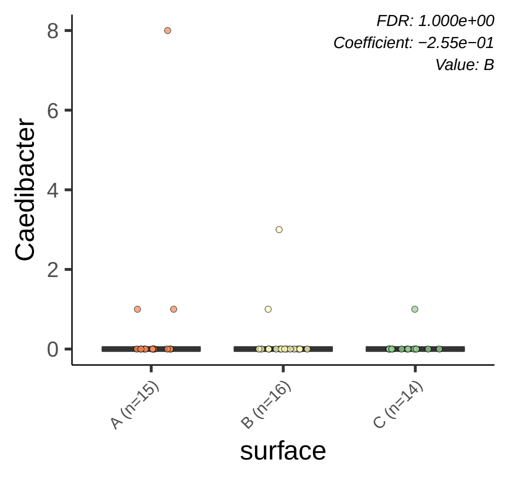
FDR: 1.000e+00
Coefficient: −2.55e−01
Value: B
Caedibacter
8
6
4
2
0
A (n=15)
B (n=16)
C (n=14)
surface

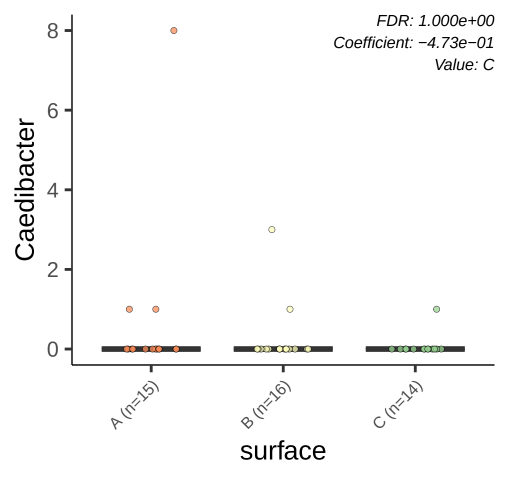

FDR: 1.000e+00
Coefficient: −4.73e−01
Value: C
Caedibacter
0
2
4
6
8
A (n=15)
B (n=16)
C (n=14)
surface

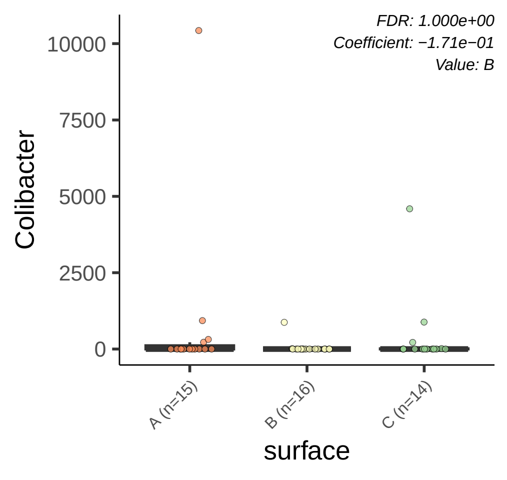
FDR: 1.000e+00
Coefficient: −1.71e−01
Value: B
Colibacter
10000
7500
5000
2500
0
A (n=15)
B (n=16)
C (n=14)
surface

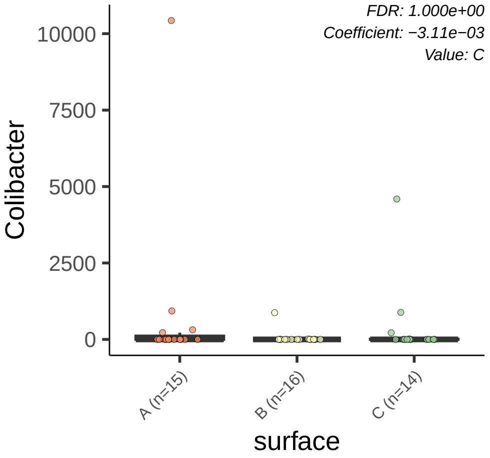
FDR: 1.000e+00
Coefficient: −3.11e−03
Value: C
Colibacter
10000
7500
5000
2500
0
A (n=15)
B (n=16)
C (n=14)
surface

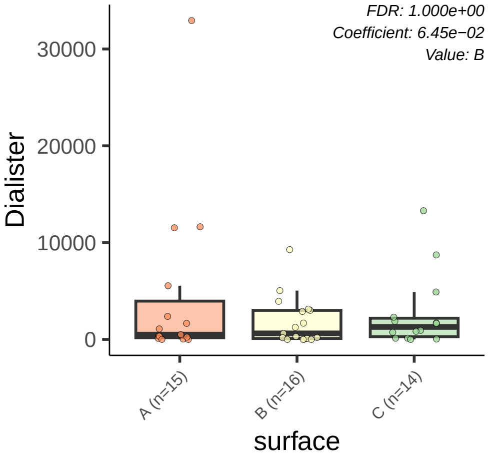

FDR: 1.000e+00
Coefficient: 6.45e−02
Value: B
Dialister
30000
20000
10000
0
A (n=15)
B (n=16)
C (n=14)
surface

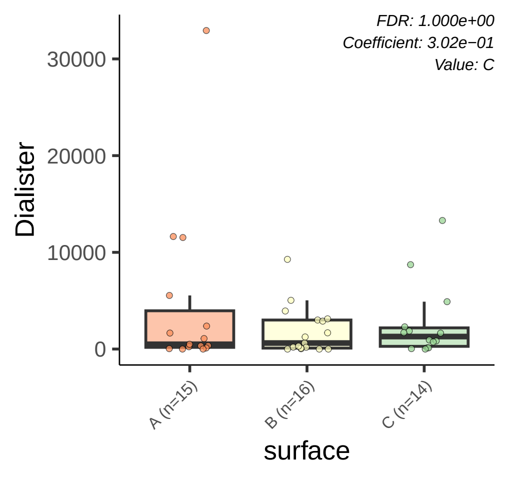
FDR: 1.000e+00
Coefficient: 3.02e−01
Value: C
Dialister
30000
20000
10000
0
A (n=15)
B (n=16)
C (n=14)
surface

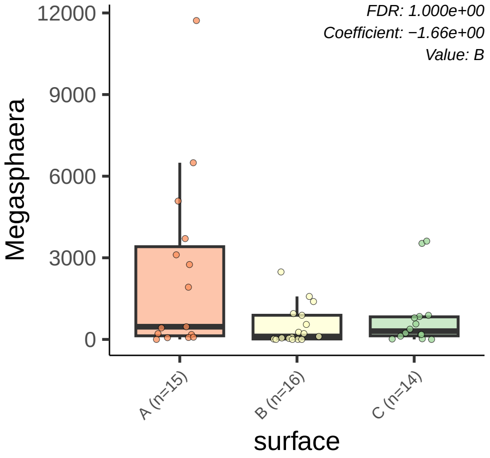

FDR: 1.000e+00
Coefficient: −1.66e+00
Value: B
Megasphaera
12000
9000
6000
3000
0
A (n=15)
B (n=16)
C (n=14)
surface

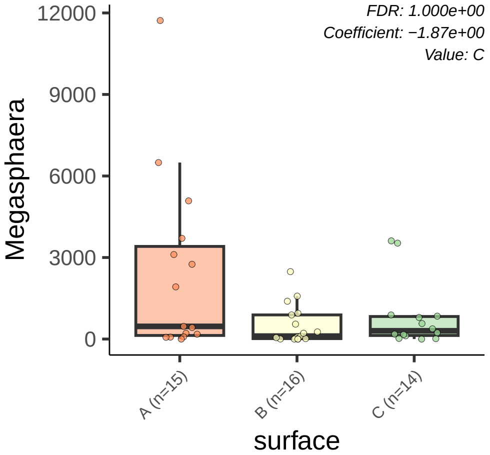

FDR: 1.000e+00
Coefficient: −1.87e+00
Value: C
Megasphaera
12000
9000
6000
3000
0
A (n=15)
B (n=16)
C (n=14)
surface

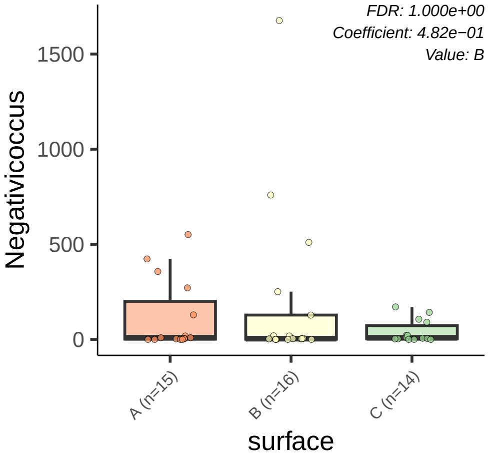
FDR: 1.000e+00
Coefficient: 4.82e−01
Value: B
Negativicoccus
1500
1000
500
0
A (n=15)
B (n=16)
C (n=14)
surface

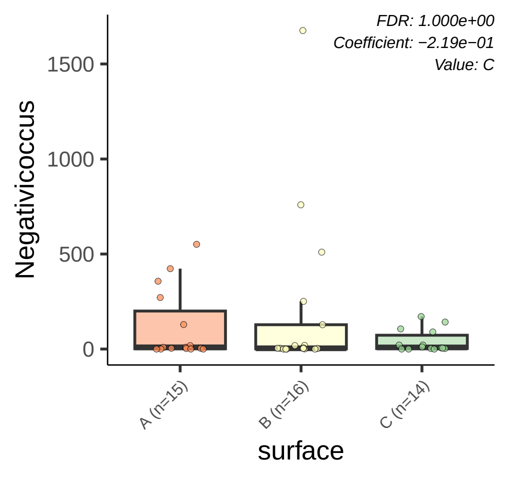

FDR: 1.000e+00
Coefficient: −2.19e−01
Value: C
Negativicoccus
1500
1000
500
0
A (n=15)
B (n=16)
C (n=14)
surface

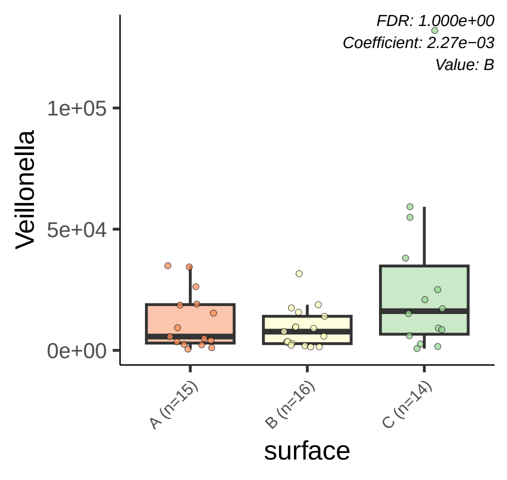
FDR: 1.000e+00
Coefficient: 2.27e−03
Value: B
Veillonella
1e+05
5e+04
0e+00
A (n=15)
B (n=16)
C (n=14)
surface

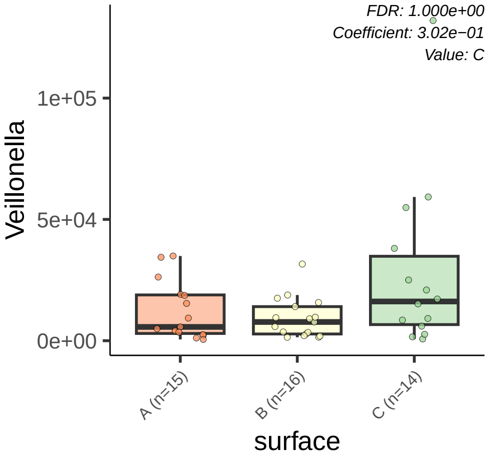

FDR: 1.000e+00
Coefficient: 3.02e−01
Value: C
Veillonella
1e+05
5e+04
0e+00
A (n=15)
B (n=16)
C (n=14)
surface

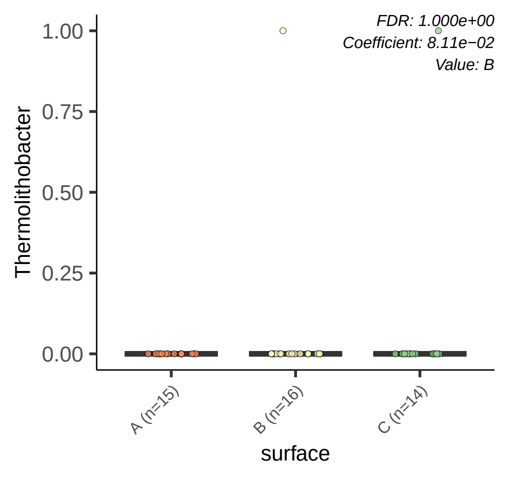

FDR: 1.000e+00
Coefficient: 8.11e−02
Value: B
Thermolithobacter
1.00
0.75
0.50
0.25
0.00
A (n=15)
B (n=16)
C (n=14)
surface

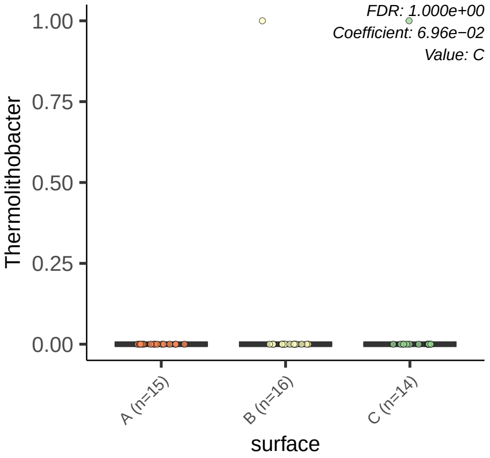

FDR: 1.000e+00
Coefficient: 6.96e−02
Value: C
Thermolithobacter
1.00
0.75
0.50
0.25
0.00
A (n=15)
B (n=16)
C (n=14)
surface

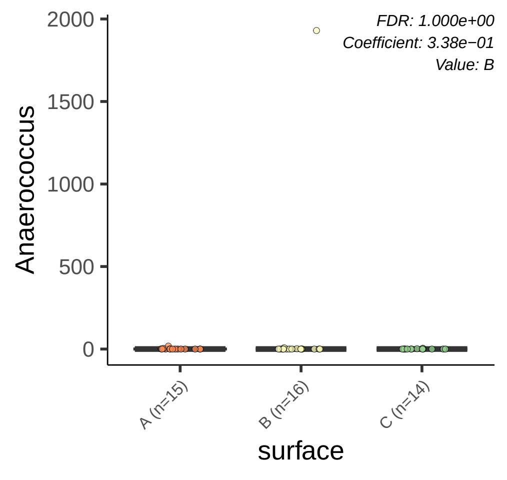

FDR: 1.000e+00
Coefficient: 3.38e−01
Value: B
Anaerococcus
2000
1500
1000
500
0
A (n=15)
B (n=16)
C (n=14)
surface

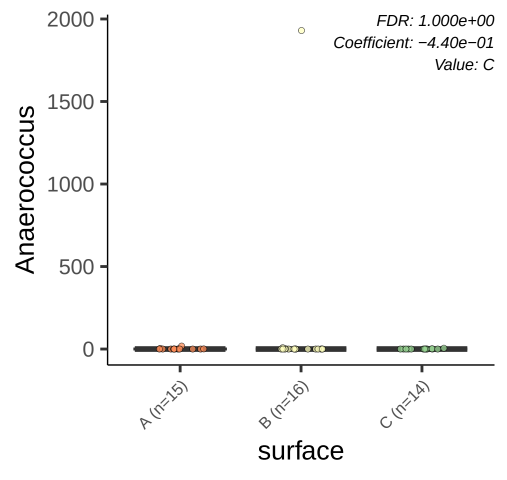
FDR: 1.000e+00
Coefficient: −4.40e−01
Value: C
2000
1500
1000
500
0
Anaerococcus
A (n=15)
B (n=16)
C (n=14)
surface

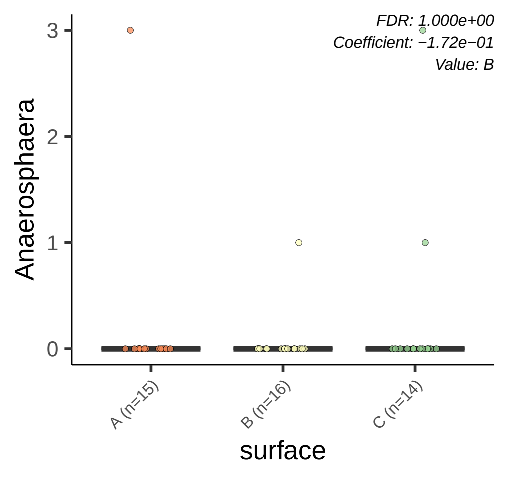
FDR: 1.000e+00
Coefficient: −1.72e−01
Value: B
Anaerosphaera
3
2
1
0
A (n=15)
B (n=16)
C (n=14)
surface

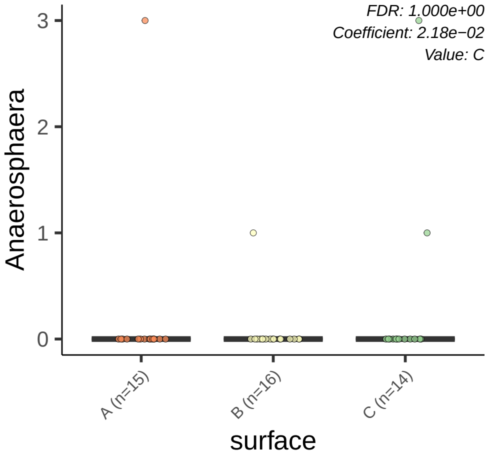

FDR: 1.000e+00
Coefficient: 2.18e−02
Value: C
Anaerosphaera
3
2
1
0
A (n=15)
B (n=16)
C (n=14)
surface

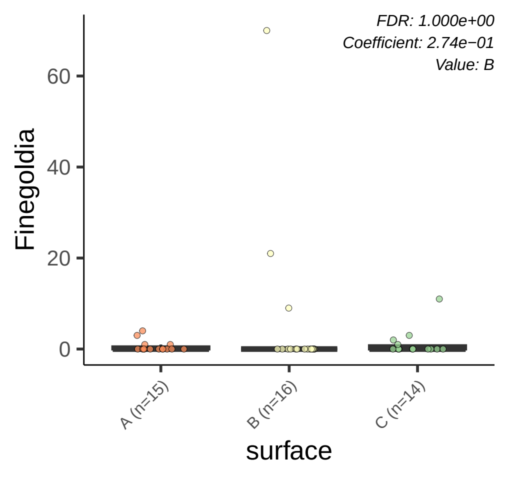

FDR: 1.000e+00
Coefficient: 2.74e−01
Value: B
Finegoldia
60
40
20
0
A (n=15)
B (n=16)
C (n=14)
surface

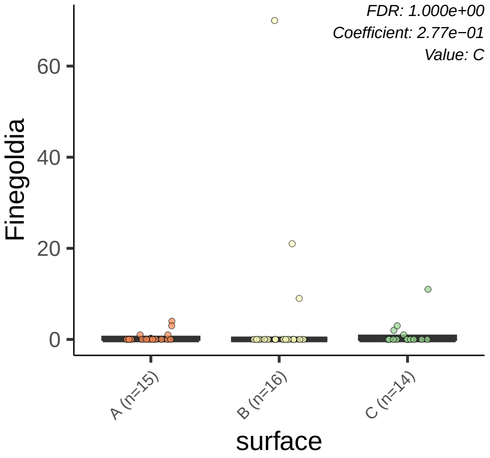
FDR: 1.000e+00
Coefficient: 2.77e−01
Value: C
Finegoldia
0
20
40
60
A (n=15)
B (n=16)
C (n=14)
surface

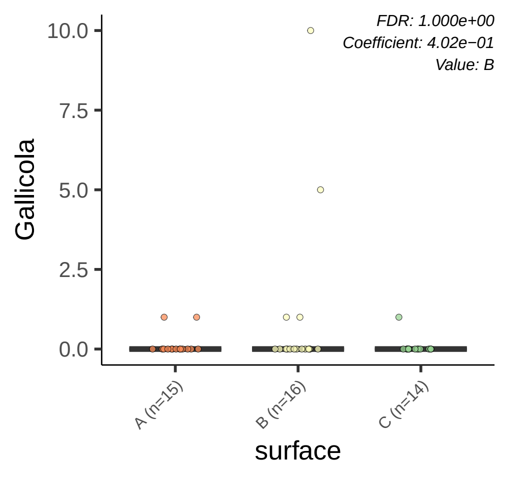
FDR: 1.000e+00
Coefficient: 4.02e−01
Value: B
Gallicola
10.0
7.5
5.0
2.5
0.0
A (n=15)
B (n=16)
C (n=14)
surface

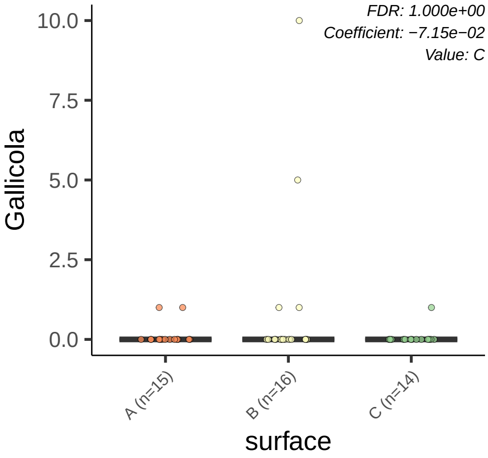

FDR: 1.000e+00
Coefficient: −7.15e−02
Value: C
Gallicola
10.0
7.5
5.0
2.5
0.0
A (n=15)
B (n=16)
C (n=14)
surface

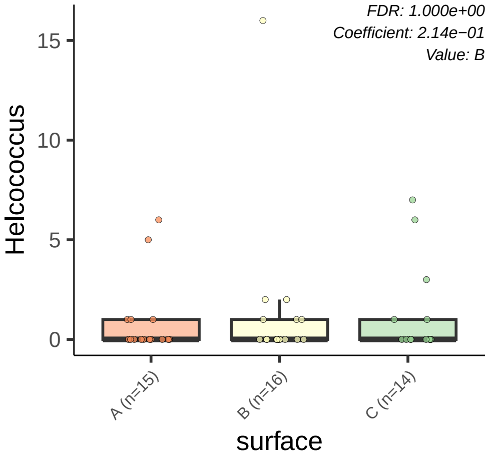
FDR: 1.000e+00
Coefficient: 2.14e−01
Value: B
Helcococcus
15
10
5
0
A (n=15)
B (n=16)
C (n=14)
surface

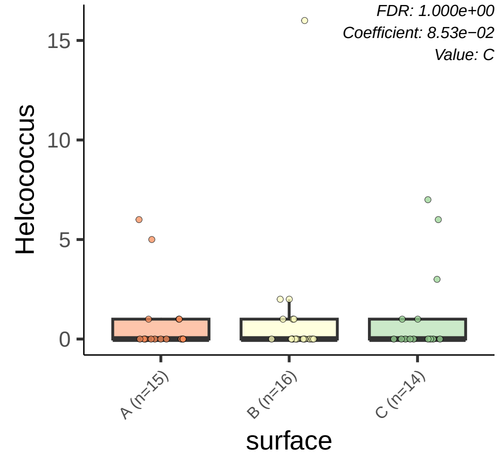

FDR: 1.000e+00
Coefficient: 8.53e−02
Value: C
Helcococcus
15
10
5
0
A (n=15)
B (n=16)
C (n=14)
surface

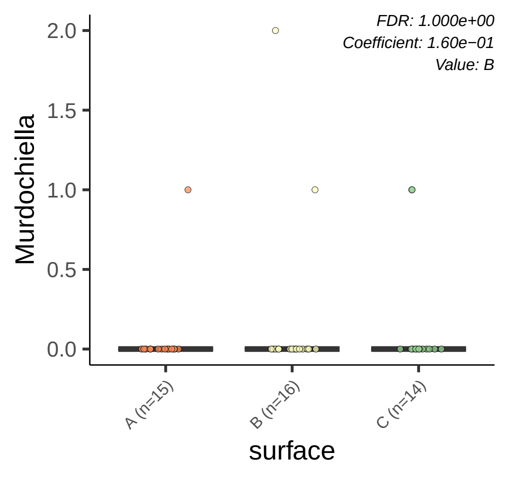
FDR: 1.000e+00
Coefficient: 1.60e−01
Value: B
Murdochiella
2.0
1.5
1.0
0.5
0.0
A (n=15)
B (n=16)
C (n=14)
surface

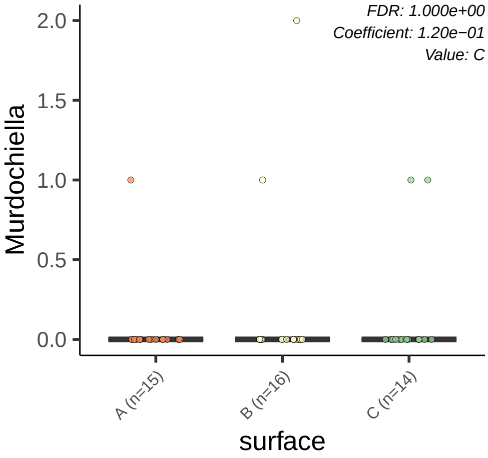
FDR: 1.000e+00
Coefficient: 1.20e−01
Value: C
Murdochiella
2.0
1.5
1.0
0.5
0.0
A (n=15)
B (n=16)
C (n=14)
surface

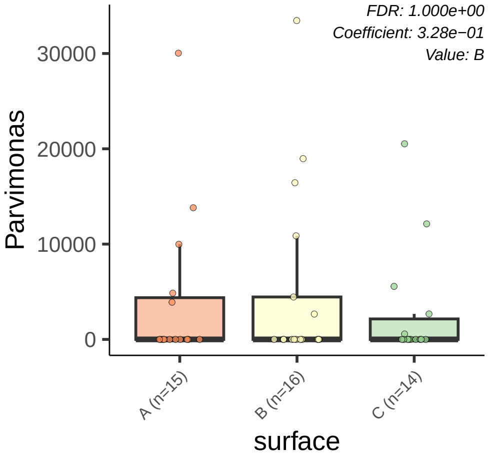

FDR: 1.000e+00
Coefficient: 3.28e−01
Value: B
Parvimonas
30000
20000
10000
0
A (n=15)
B (n=16)
C (n=14)
surface

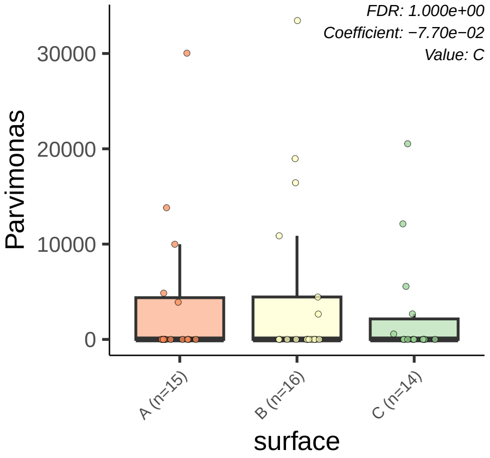
FDR: 1.000e+00
Coefficient: −7.70e−02
Value: C
Parvimonas
30000
20000
10000
0
A (n=15)
B (n=16)
C (n=14)
surface

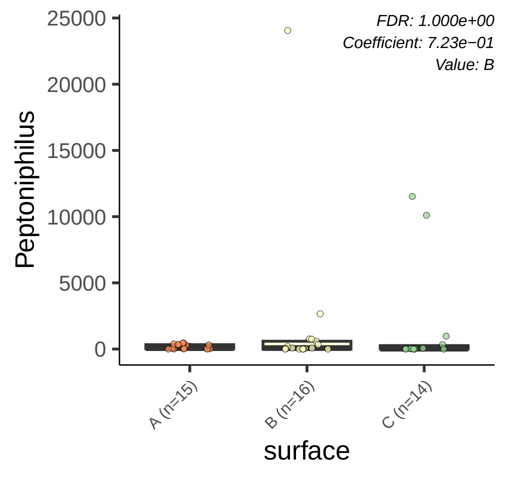
FDR: 1.000e+00
Coefficient: 7.23e−01
Value: B
25000
20000
15000
10000
5000
0
Peptoniphilus
A (n=15)
B (n=16)
C (n=14)
surface

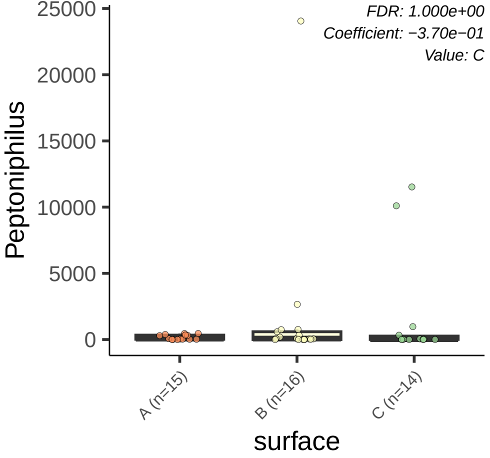

FDR: 1.000e+00
Coefficient: −3.70e−01
Value: C
Peptoniphilus
25000
20000
15000
10000
5000
0
A (n=15)
B (n=16)
C (n=14)
surface

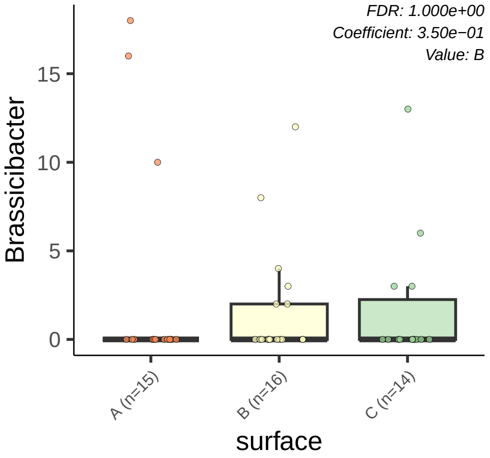

FDR: 1.000e+00
Coefficient: 3.50e−01
Value: B
Brassicibacter
0
5
10
15
A (n=15)
B (n=16)
C (n=14)
surface

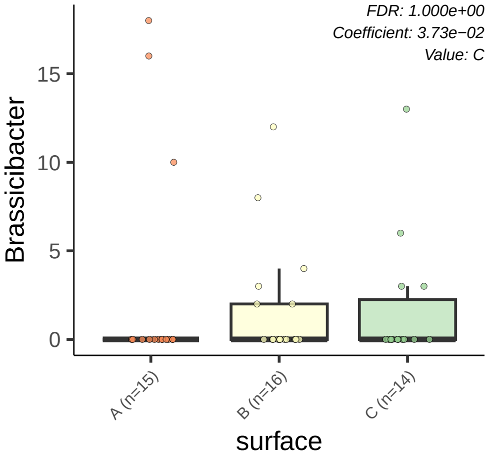

FDR: 1.000e+00
Coefficient: 3.73e−02
Value: C
Brassicibacter
0
5
10
15
A (n=15)
B (n=16)
C (n=14)
surface

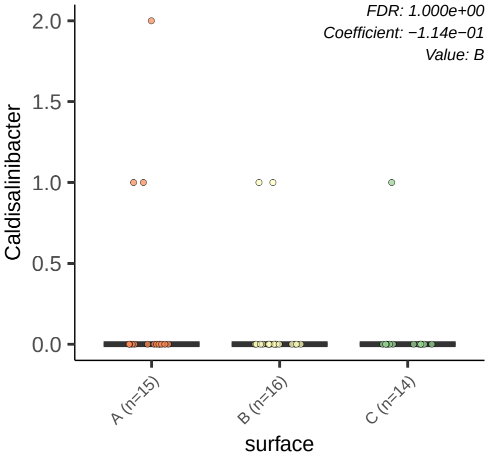
FDR: 1.000e+00
Coefficient: −1.14e−01
Value: B
Caldisalinibacter
2.0
1.5
1.0
0.5
0.0
A (n=15)
B (n=16)
C (n=14)
surface

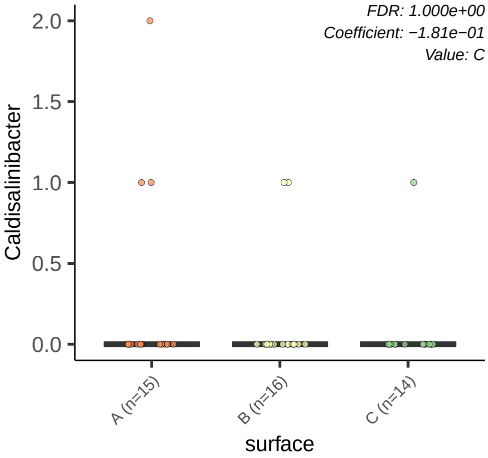

FDR: 1.000e+00
Coefficient: −1.81e−01
Value: C
Caldisalinibacter
2.0
1.5
1.0
0.5
0.0
A (n=15)
B (n=16)
C (n=14)
surface

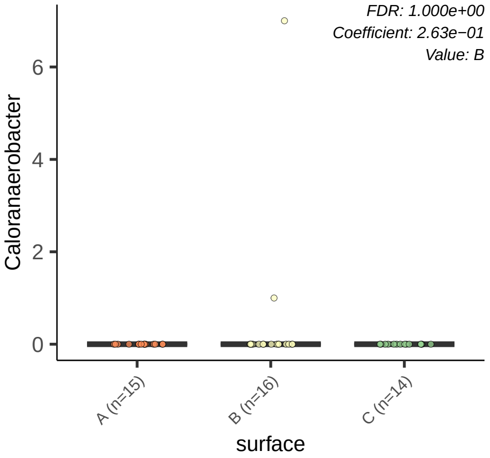
FDR: 1.000e+00
Coefficient: 2.63e−01
Value: B
Caloranaerobacter
0
2
4
6
A (n=15)
B (n=16)
C (n=14)
surface

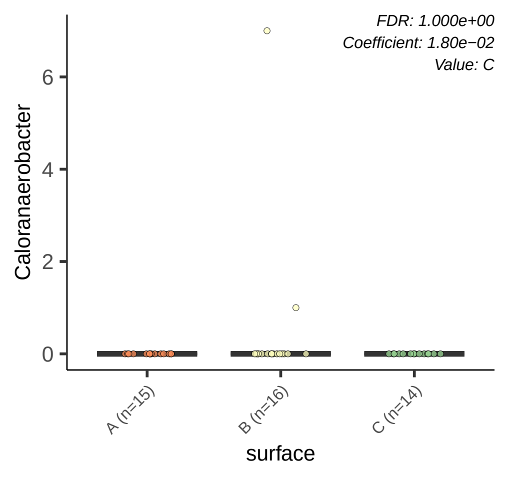
FDR: 1.000e+00
Coefficient: 1.80e−02
Value: C
Caloranaerobacter
0
2
4
6
A (n=15)
B (n=16)
C (n=14)
surface

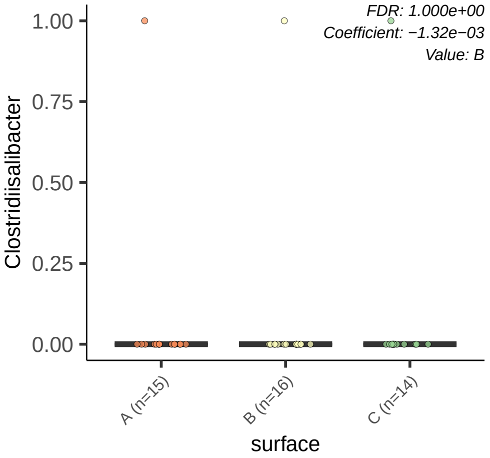

FDR: 1.000e+00
Coefficient: −1.32e−03
Value: B
Clostridiisalibacter
1.00
0.75
0.50
0.25
0.00
A (n=15)
B (n=16)
C (n=14)
surface

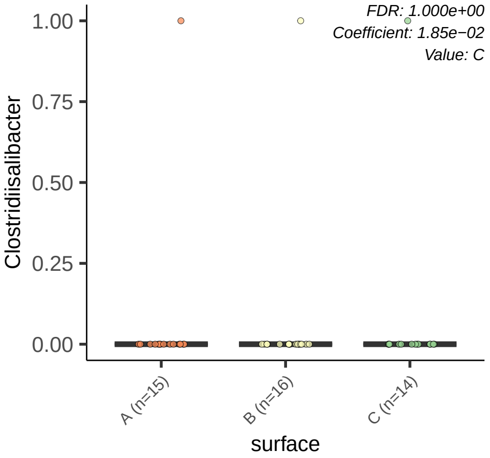
FDR: 1.000e+00
Coefficient: 1.85e−02
Value: C
1.00
0.75
0.50
0.25
0.00
Clostridiisalibacter
A (n=15)
B (n=16)
C (n=14)
surface

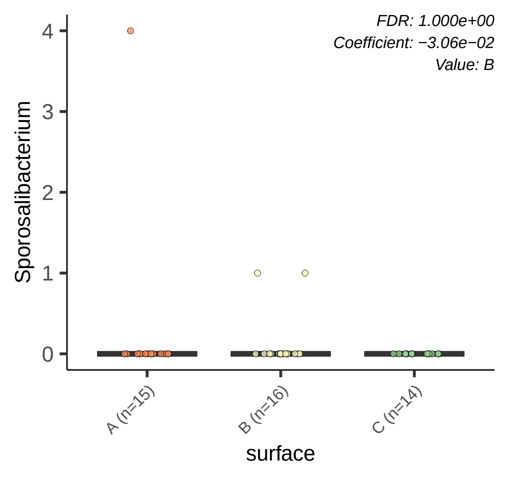
FDR: 1.000e+00
Coefficient: −3.06e−02
Value: B
Sporosalibacterium
4
3
2
1
0
A (n=15)
B (n=16)
C (n=14)
surface

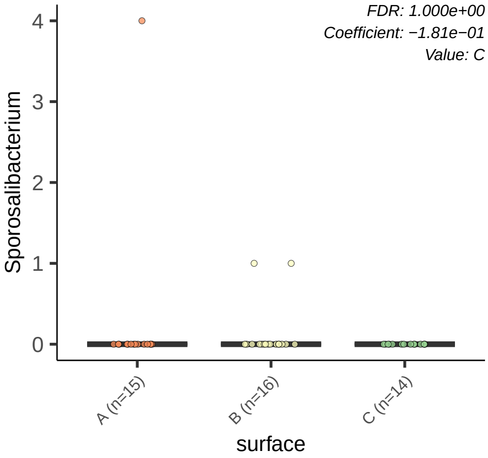
FDR: 1.000e+00
Coefficient: −1.81e−01
Value: C
Sporosalibacterium
4
3
2
1
0
A (n=15)
B (n=16)
C (n=14)
surface

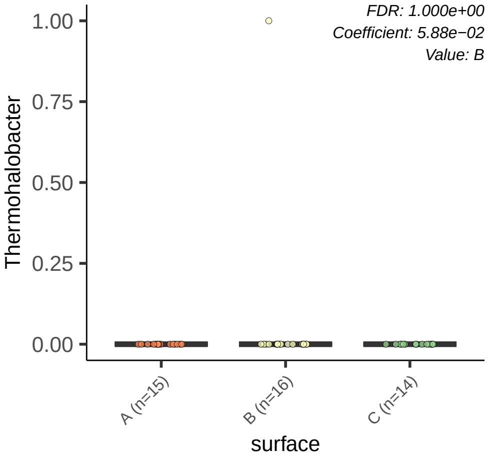
FDR: 1.000e+00
Coefficient: 5.88e−02
Value: B
Thermohalobacter
1.00
0.75
0.50
0.25
0.00
A (n=15)
B (n=16)
C (n=14)
surface

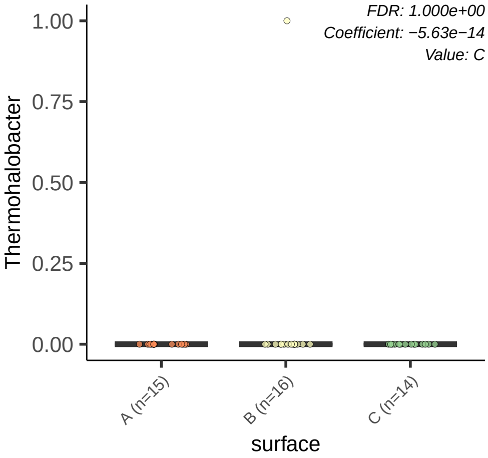
FDR: 1.000e+00
Coefficient: −5.63e−14
Value: C
Thermohalobacter
1.00
0.75
0.50
0.25
0.00
A (n=15)
B (n=16)
C (n=14)
surface

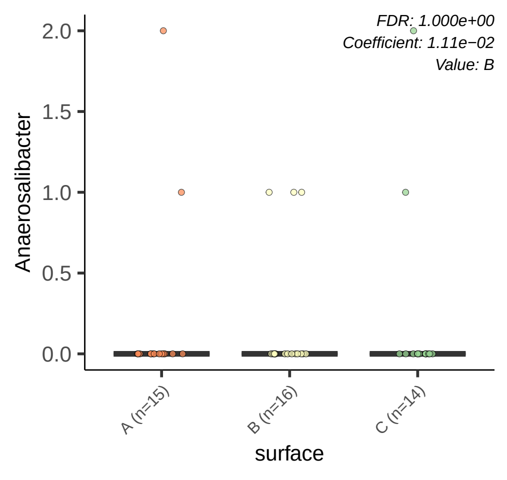
FDR: 1.000e+00
Coefficient: 1.11e−02
Value: B
Anaerosalibacter
2.0
1.5
1.0
0.5
0.0
A (n=15)
B (n=16)
C (n=14)
surface

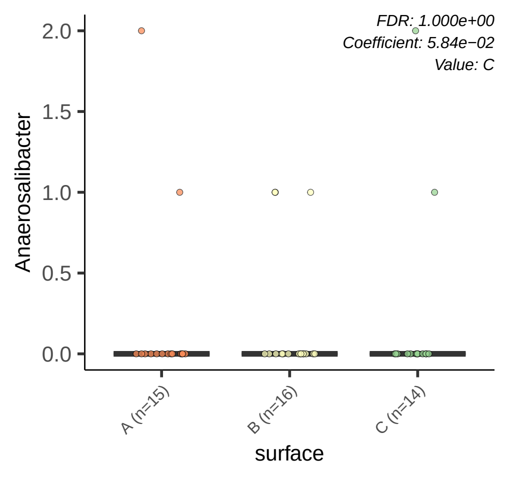

FDR: 1.000e+00
Coefficient: 5.84e−02
Value: C
Anaerosalibacter
2.0
1.5
1.0
0.5
0.0
A (n=15)
B (n=16)
C (n=14)
surface

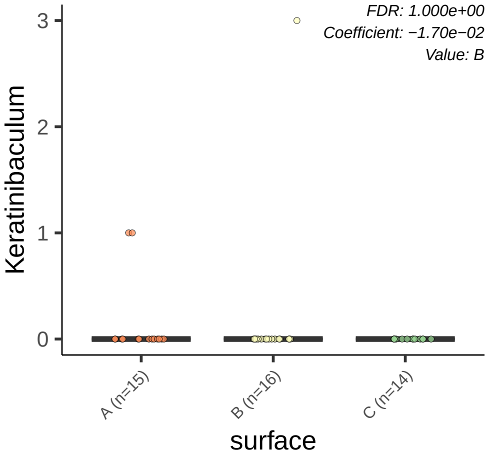
FDR: 1.000e+00
Coefficient: −1.70e−02
Value: B
Keratinibaculum
0
1
2
3
A (n=15)
B (n=16)
C (n=14)
surface

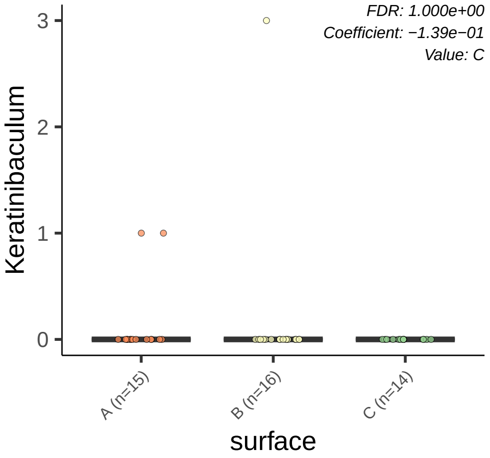
FDR: 1.000e+00
Coefficient: −1.39e−01
Value: C
Keratinibaculum
3
2
1
0
A (n=15)
B (n=16)
C (n=14)
surface

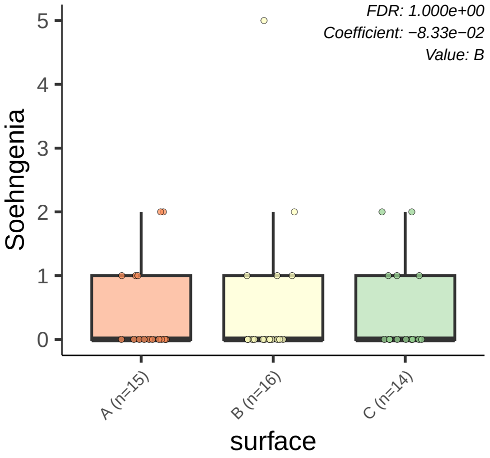

FDR: 1.000e+00
Coefficient: −8.33e−02
Value: B
Soehngenia
5
4
3
2
1
0
A (n=15)
B (n=16)
C (n=14)
surface

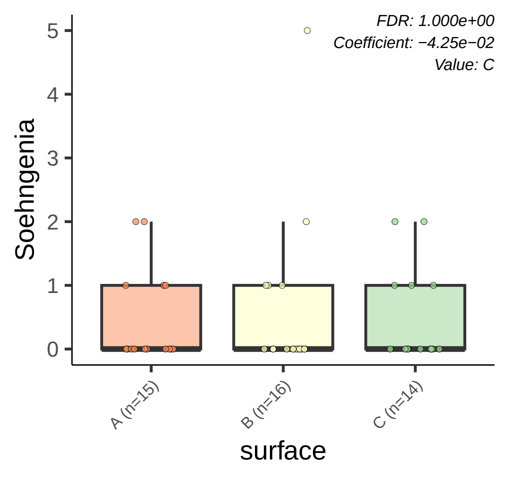
FDR: 1.000e+00
Coefficient: −4.25e−02
Value: C
Soehngenia
5
4
3
2
1
0
A (n=15)
B (n=16)
C (n=14)
surface

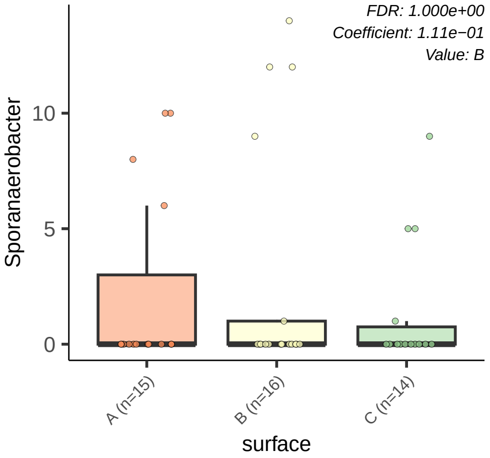

FDR: 1.000e+00
Coefficient: 1.11e−01
Value: B
Sporanaerobacter
10
5
0
A (n=15)
B (n=16)
C (n=14)
surface

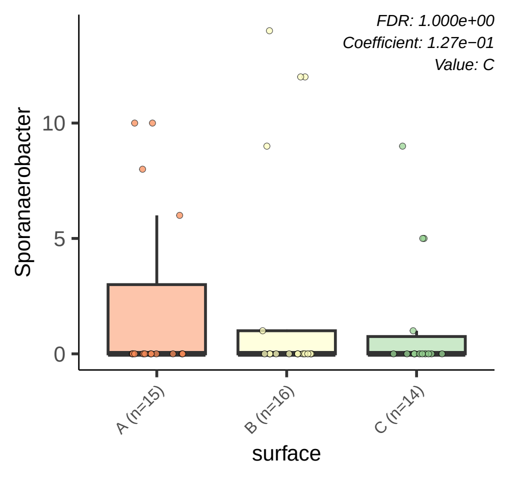
FDR: 1.000e+00
Coefficient: 1.27e−01
Value: C
Sporanaerobacter
10
5
0
A (n=15)
B (n=16)
C (n=14)
surface

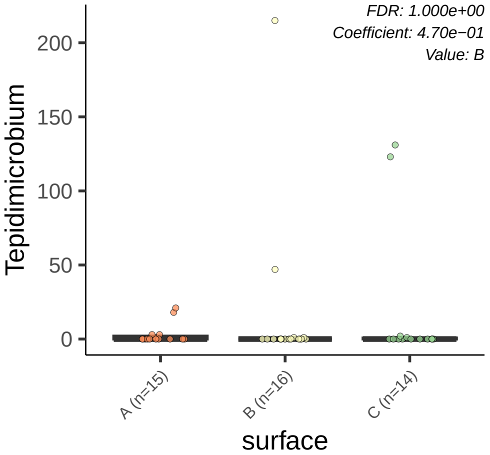
FDR: 1.000e+00
Coefficient: 4.70e−01
Value: B
Tepidimicrobium
200
150
100
50
0
A (n=15)
B (n=16)
C (n=14)
surface

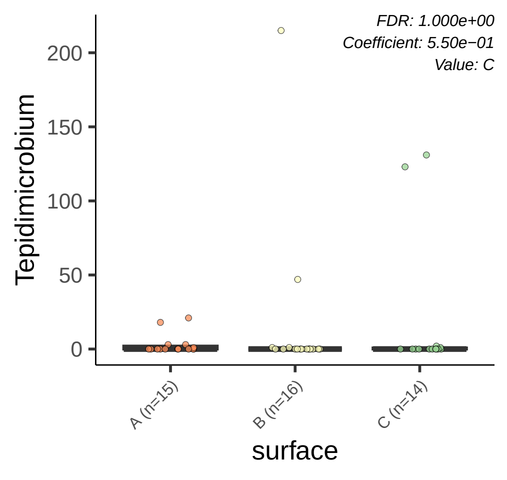
FDR: 1.000e+00
Coefficient: 5.50e−01
Value: C
Tepidimicrobium
200
150
100
50
0
A (n=15)
B (n=16)
C (n=14)
surface

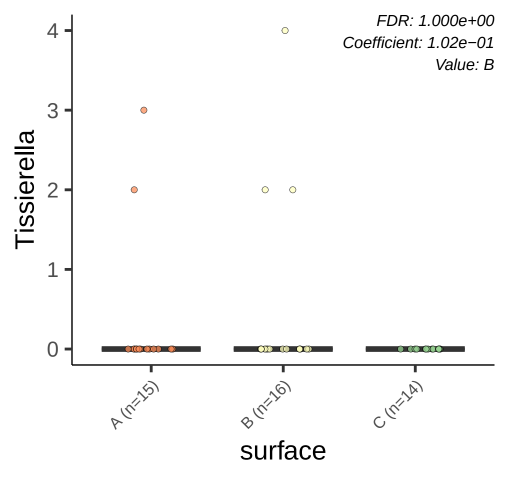
FDR: 1.000e+00
Coefficient: 1.02e−01
Value: B
Tissierella
4
3
2
1
0
A (n=15)
B (n=16)
C (n=14)
surface

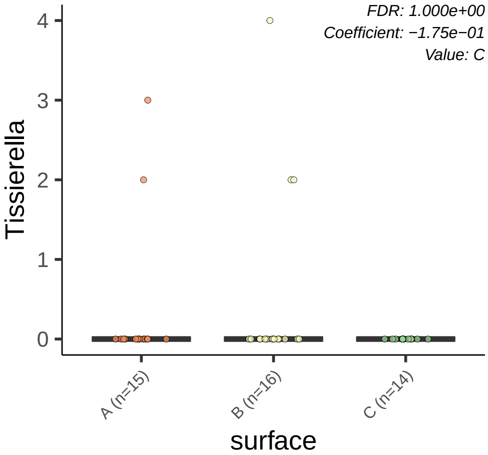
FDR: 1.000e+00
Coefficient: −1.75e−01
Value: C
Tissierella
4
3
2
1
0
A (n=15)
B (n=16)
C (n=14)
surface

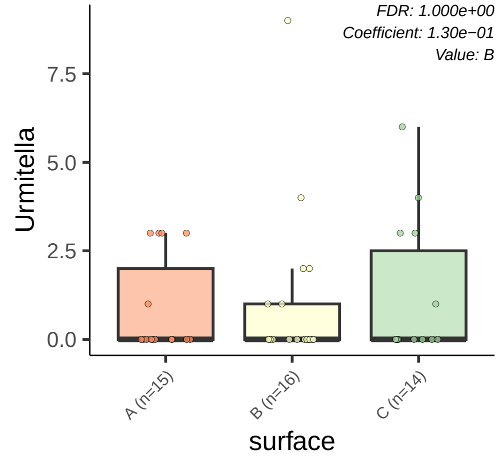

FDR: 1.000e+00
Coefficient: 1.30e−01
Value: B
Urmitella
7.5
5.0
2.5
0.0
A (n=15)
B (n=16)
C (n=14)
surface

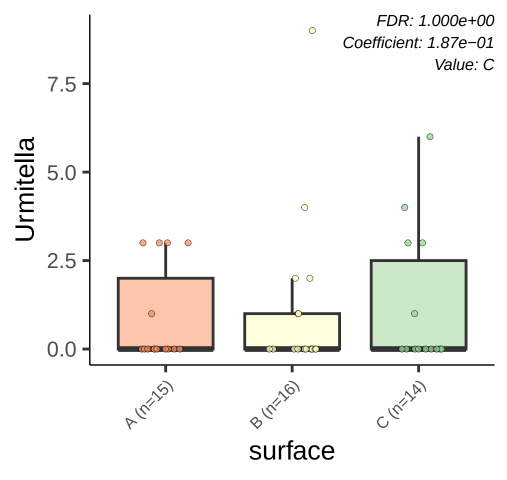
FDR: 1.000e+00
Coefficient: 1.87e−01
Value: C
Urmitella
7.5
5.0
2.5
0.0
A (n=15)
B (n=16)
C (n=14)
surface

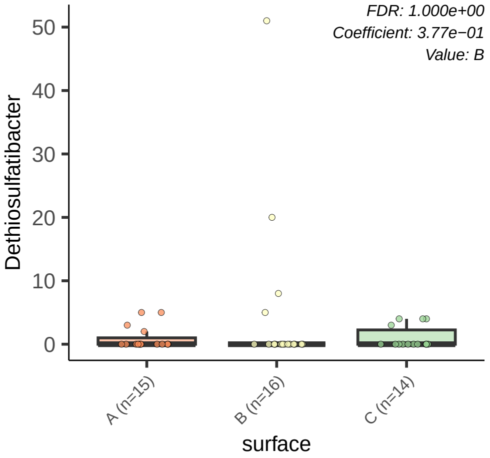

FDR: 1.000e+00
Coefficient: 3.77e−01
Value: B
Dethiosulfatibacter
0
10
20
30
40
50
A (n=15)
B (n=16)
C (n=14)
surface

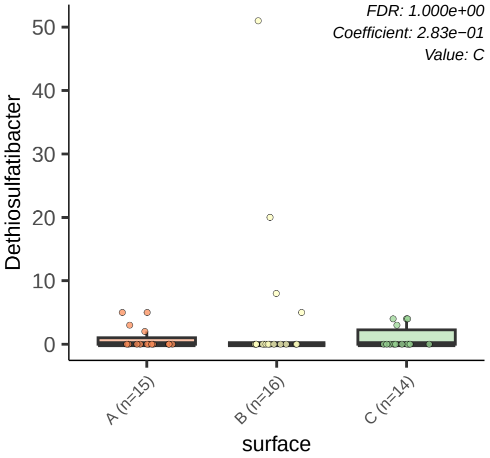
FDR: 1.000e+00
Coefficient: 2.83e−01
Value: C
Dethiosulfatibacter
50
40
30
20
10
0
A (n=15)
B (n=16)
C (n=14)
surface

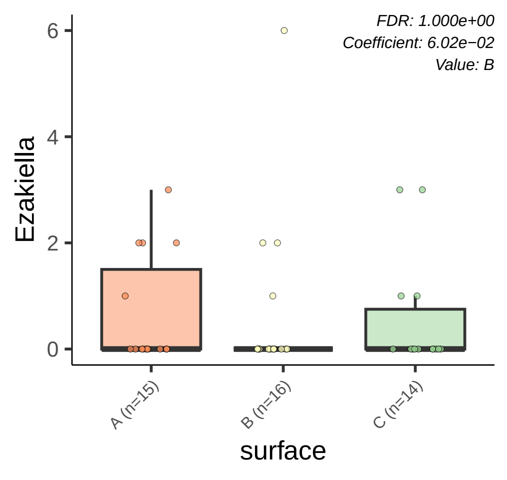
FDR: 1.000e+00
Coefficient: 6.02e−02
Value: B
6
4
2
0
Ezakiella
A (n=15)
B (n=16)
C (n=14)
surface

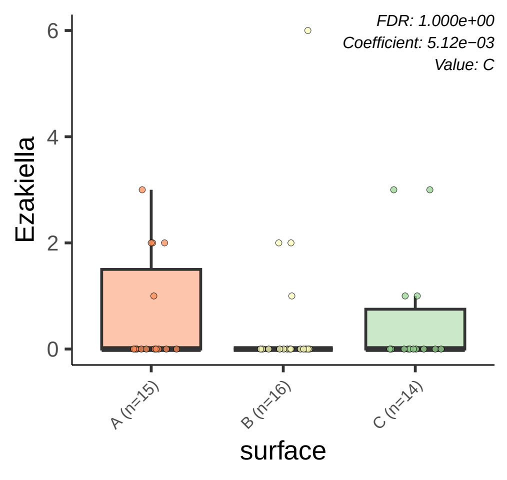

FDR: 1.000e+00
Coefficient: 5.12e−03
Value: C
Ezakiella
0
2
4
6
A (n=15)
B (n=16)
C (n=14)
surface

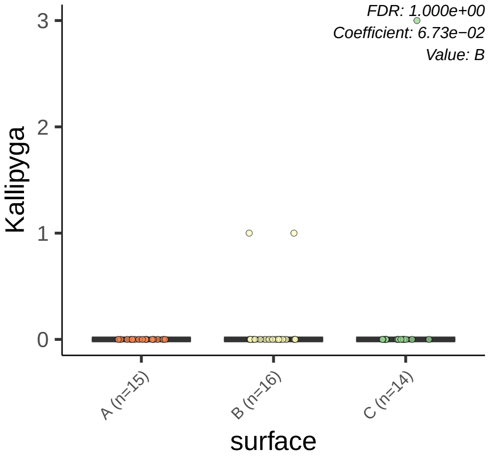
FDR: 1.000e+00
Coefficient: 6.73e−02
Value: B
Kallipyga
3
2
1
0
A (n=15)
B (n=16)
C (n=14)
surface

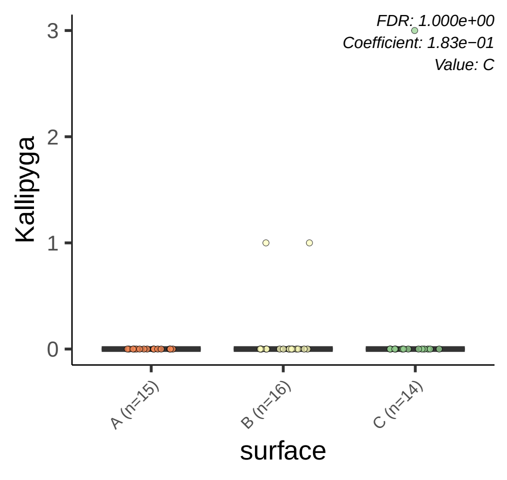
FDR: 1.000e+00
Coefficient: 1.83e−01
Value: C
Kallipyga
3
2
1
0
A (n=15)
B (n=16)
C (n=14)
surface

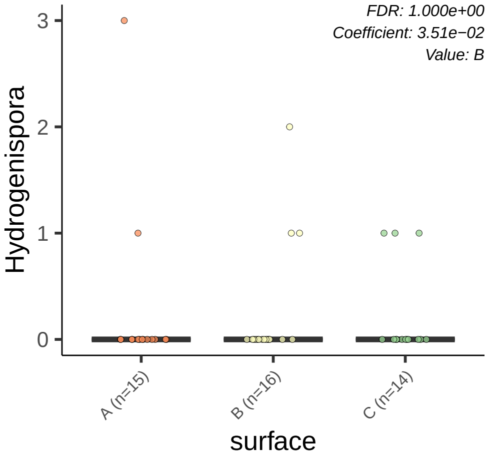
FDR: 1.000e+00
Coefficient: 3.51e−02
Value: B
Hydrogenispora
3
2
1
0
A (n=15)
B (n=16)
C (n=14)
surface

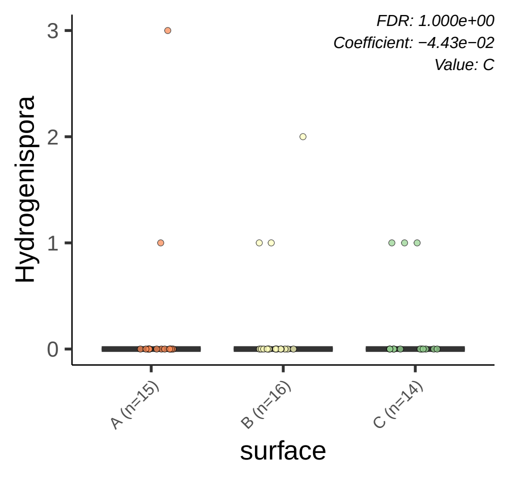

FDR: 1.000e+00
Coefficient: −4.43e−02
Value: C
Hydrogenispora
3
2
1
0
A (n=15)
B (n=16)
C (n=14)
surface

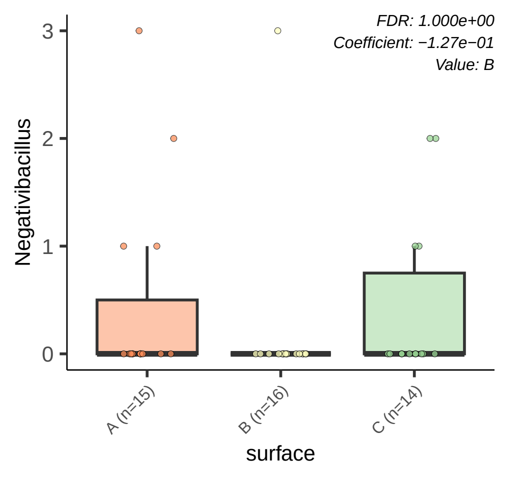

FDR: 1.000e+00
Coefficient: −1.27e−01
Value: B
Negativibacillus
3
2
1
0
A (n=15)
B (n=16)
C (n=14)
surface

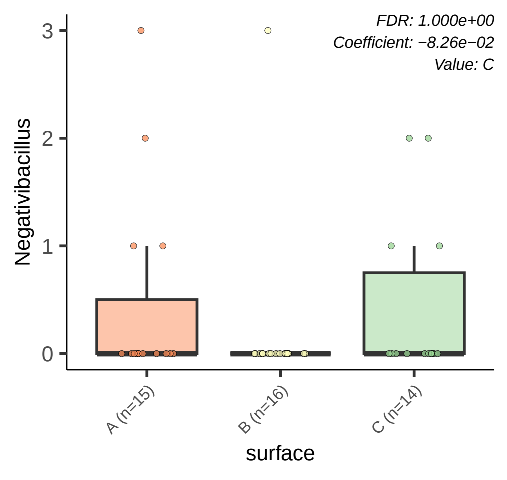
FDR: 1.000e+00
Coefficient: −8.26e−02
Value: C
Negativibacillus
3
2
1
0
A (n=15)
B (n=16)
C (n=14)
surface

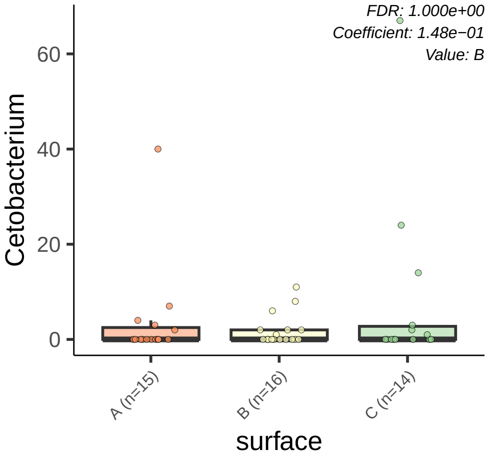

FDR: 1.000e+00
Coefficient: 1.48e−01
Value: B
Cetobacterium
60
40
20
0
A (n=15)
B (n=16)
C (n=14)
surface

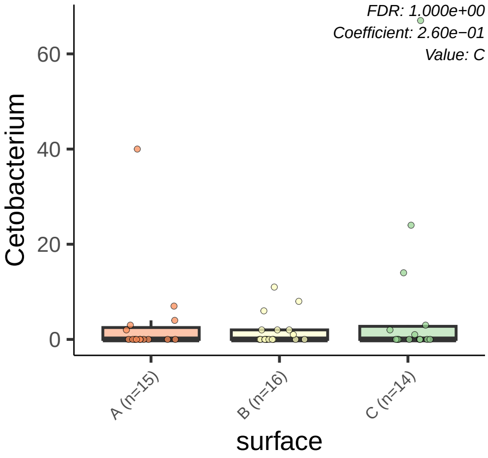
FDR: 1.000e+00
Coefficient: 2.60e−01
Value: C
Cetobacterium
60
40
20
0
A (n=15)
B (n=16)
C (n=14)
surface

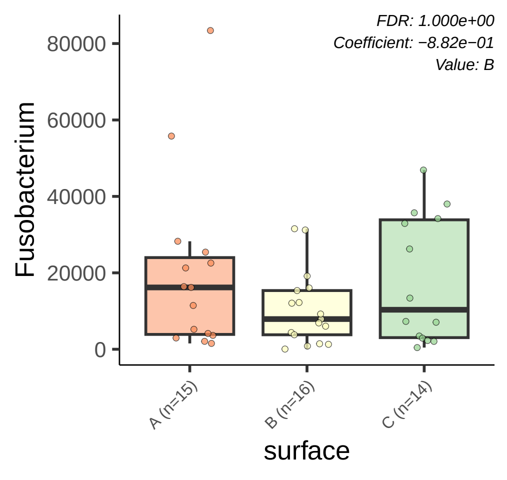
FDR: 1.000e+00
Coefficient: −8.82e−01
Value: B
Fusobacterium
80000
60000
40000
20000
0
A (n=15)
B (n=16)
C (n=14)
surface

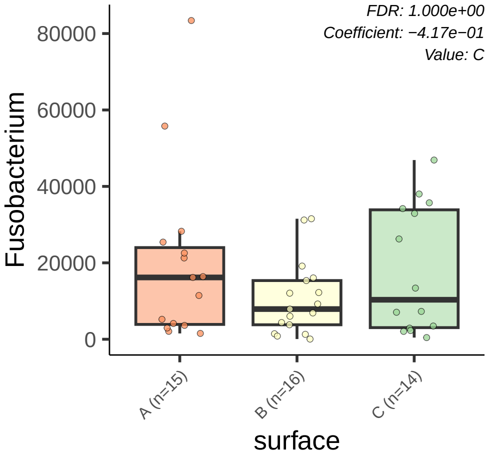
FDR: 1.000e+00
Coefficient: −4.17e−01
Value: C
Fusobacterium
80000
60000
40000
20000
0
A (n=15)
B (n=16)
C (n=14)
surface

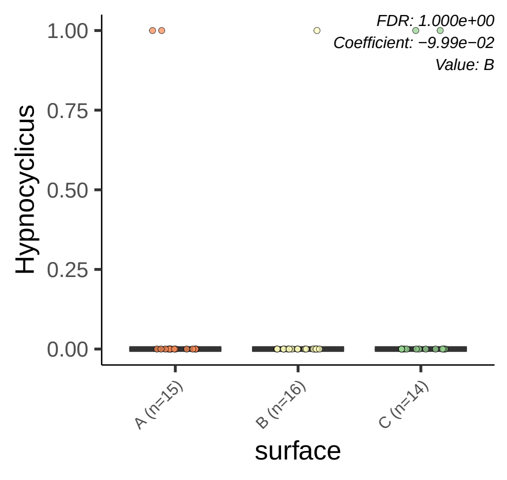

FDR: 1.000e+00
Coefficient: −9.99e−02
Value: B
Hypnocyclicus
1.00
0.75
0.50
0.25
0.00
A (n=15)
B (n=16)
C (n=14)
surface

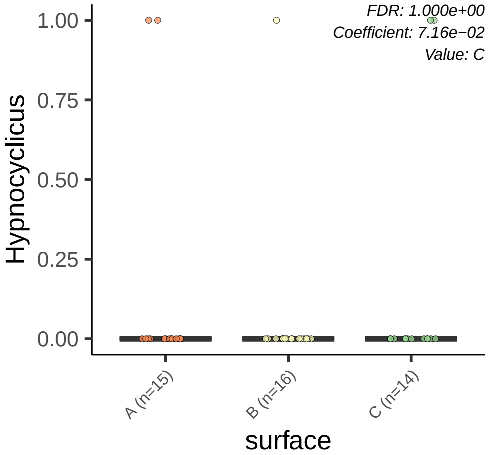

FDR: 1.000e+00
Coefficient: 7.16e−02
Value: C
Hypnocyclicus
1.00
0.75
0.50
0.25
0.00
A (n=15)
B (n=16)
C (n=14)
surface

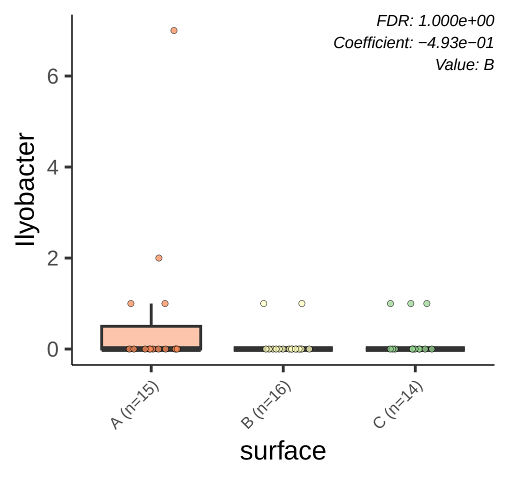
FDR: 1.000e+00
Coefficient: −4.93e−01
Value: B
Ilyobacter
0
2
4
6
A (n=15)
B (n=16)
C (n=14)
surface

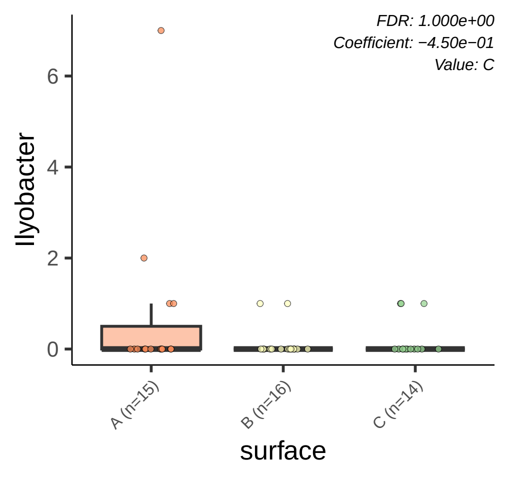
FDR: 1.000e+00
Coefficient: −4.50e−01
Value: C
Ilyobacter
0
2
4
6
A (n=15)
B (n=16)
C (n=14)
surface

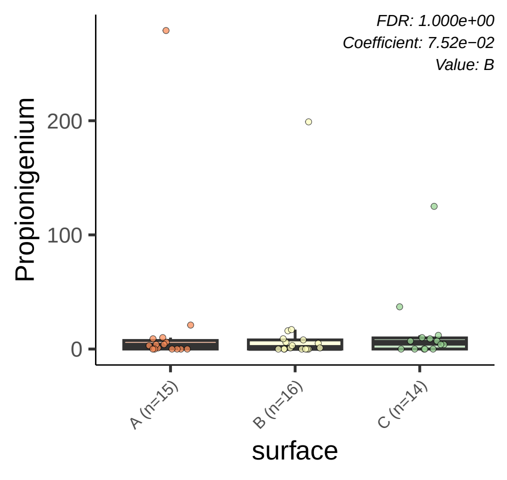

FDR: 1.000e+00
Coefficient: 7.52e−02
Value: B
Propionigenium
200
100
0
A (n=15)
B (n=16)
C (n=14)
surface

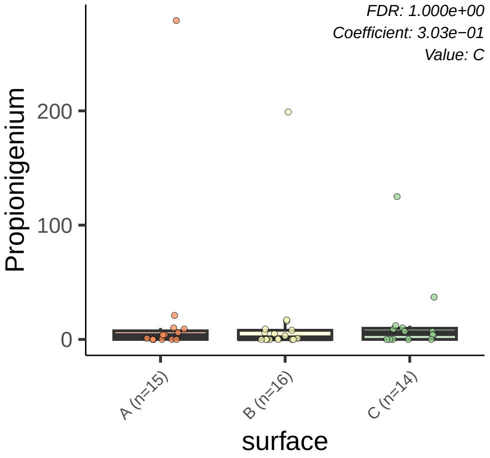
FDR: 1.000e+00
Coefficient: 3.03e−01
Value: C
Propionigenium
200
100
0
A (n=15)
B (n=16)
C (n=14)
surface

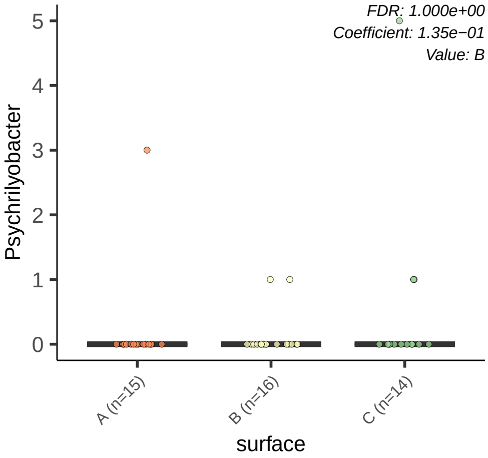

FDR: 1.000e+00
Coefficient: 1.35e−01
Value: B
Psychrilyobacter
0
1
2
3
4
5
A (n=15)
B (n=16)
C (n=14)
surface

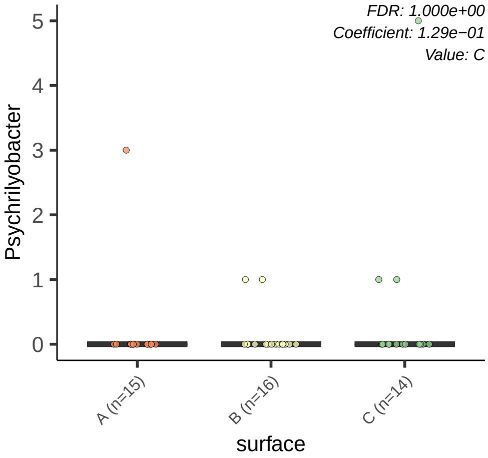

FDR: 1.000e+00
Coefficient: 1.29e−01
Value: C
Psychrilyobacter
0
1
2
3
4
5
A (n=15)
B (n=16)
C (n=14)
surface

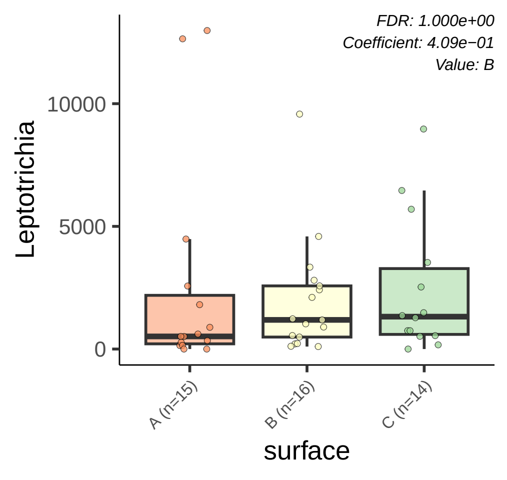
FDR: 1.000e+00
Coefficient: 4.09e−01
Value: B
Leptotrichia
10000
5000
0
A (n=15)
B (n=16)
C (n=14)
surface

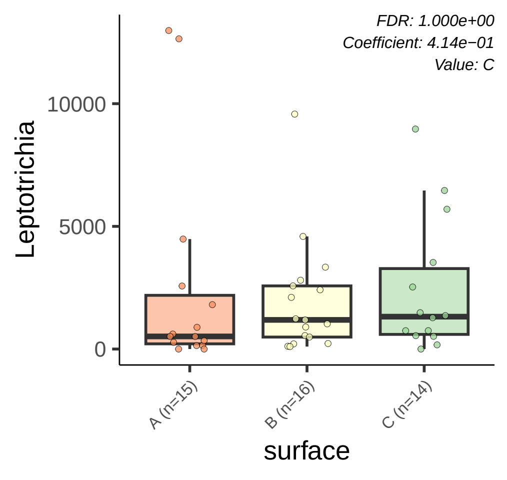
FDR: 1.000e+00
Coefficient: 4.14e−01
Value: C
Leptotrichia
10000
5000
0
A (n=15)
B (n=16)
C (n=14)
surface

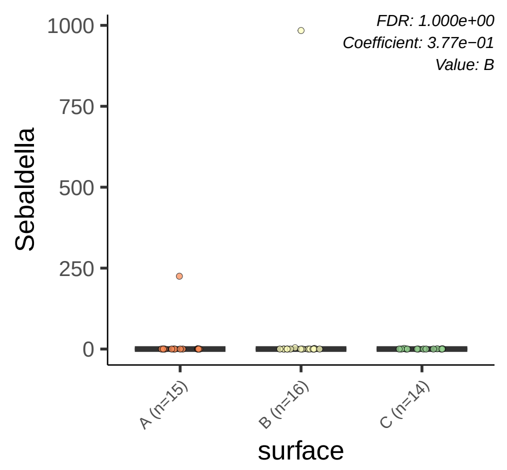

FDR: 1.000e+00
Coefficient: 3.77e−01
Value: B
1000
750
500
250
0
Sebaldella
A (n=15)
B (n=16)
C (n=14)
surface

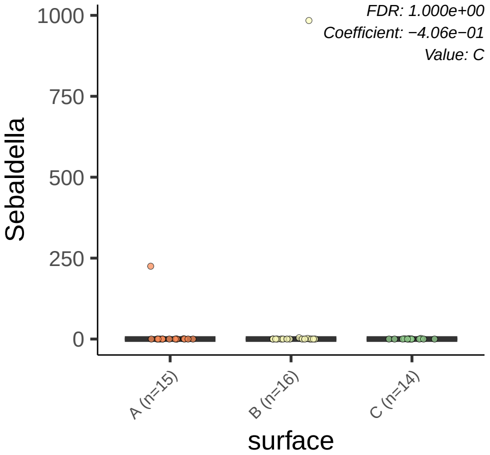
FDR: 1.000e+00
Coefficient: −4.06e−01
Value: C
1000
750
500
250
0
Sebaldella
A (n=15)
B (n=16)
C (n=14)
surface

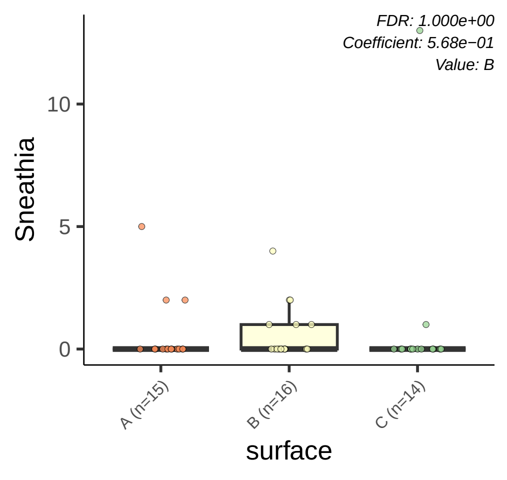
FDR: 1.000e+00
Coefficient: 5.68e−01
Value: B
Sneathia
10
5
0
A (n=15)
B (n=16)
C (n=14)
surface

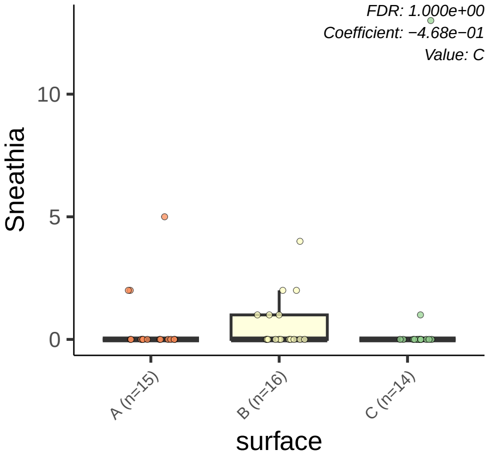
FDR: 1.000e+00
Coefficient: −4.68e−01
Value: C
Sneathia
10
5
0
A (n=15)
B (n=16)
C (n=14)
surface

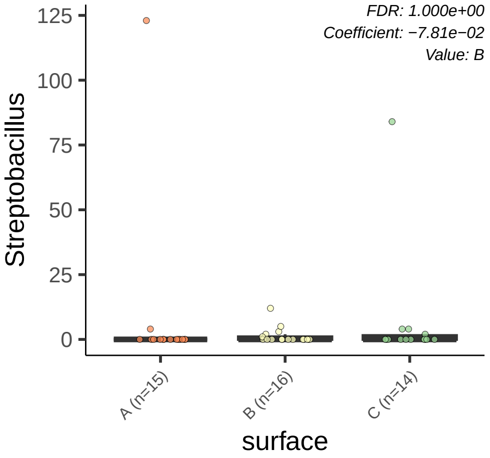
FDR: 1.000e+00
Coefficient: −7.81e−02
Value: B
Streptobacillus
125
100
75
50
25
0
A (n=15)
B (n=16)
C (n=14)
surface

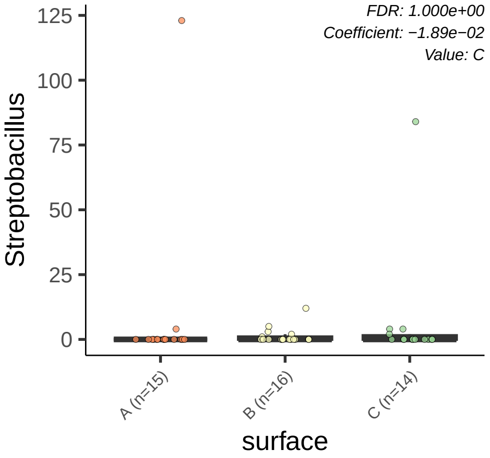
FDR: 1.000e+00
Coefficient: −1.89e−02
Value: C
Streptobacillus
125
100
75
50
25
0
A (n=15)
B (n=16)
C (n=14)
surface

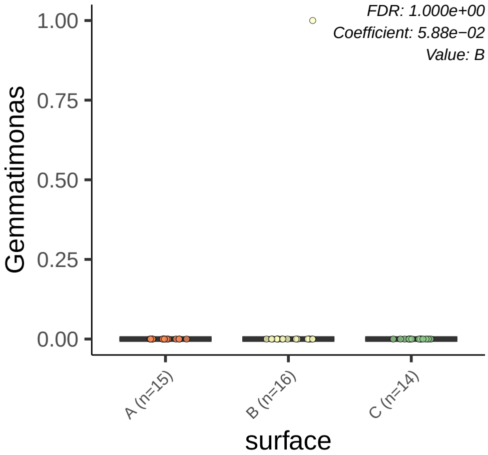
FDR: 1.000e+00
Coefficient: 5.88e−02
Value: B
Gemmatimonas
1.00
0.75
0.50
0.25
0.00
A (n=15)
B (n=16)
C (n=14)
surface

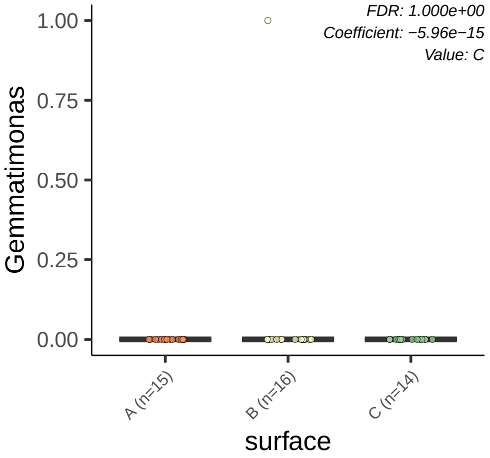
FDR: 1.000e+00
Coefficient: −5.96e−15
Value: C
Gemmatimonas
1.00
0.75
0.50
0.25
0.00
A (n=15)
B (n=16)
C (n=14)
surface

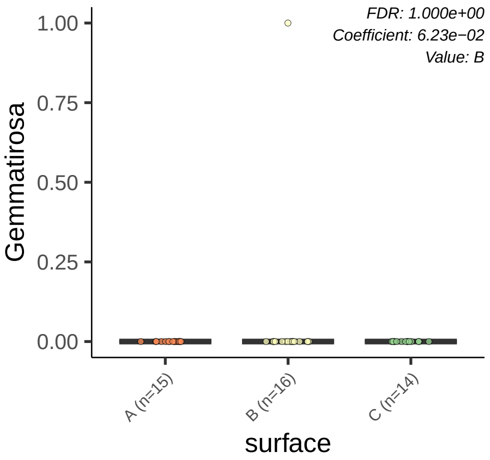
FDR: 1.000e+00
Coefficient: 6.23e−02
Value: B
Gemmatirosa
1.00
0.75
0.50
0.25
0.00
A (n=15)
B (n=16)
C (n=14)
surface

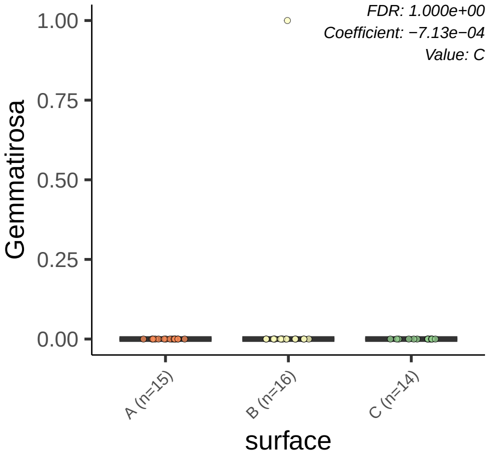

FDR: 1.000e+00
Coefficient: −7.13e−04
Value: C
Gemmatirosa
1.00
0.75
0.50
0.25
0.00
A (n=15)
B (n=16)
C (n=14)
surface

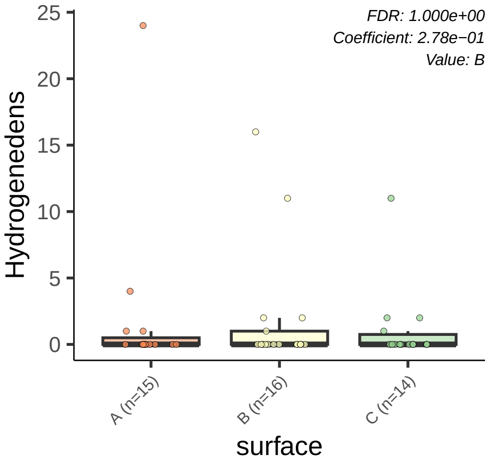
FDR: 1.000e+00
Coefficient: 2.78e−01
Value: B
Hydrogenedens
0
5
10
15
20
25
A (n=15)
B (n=16)
C (n=14)
surface

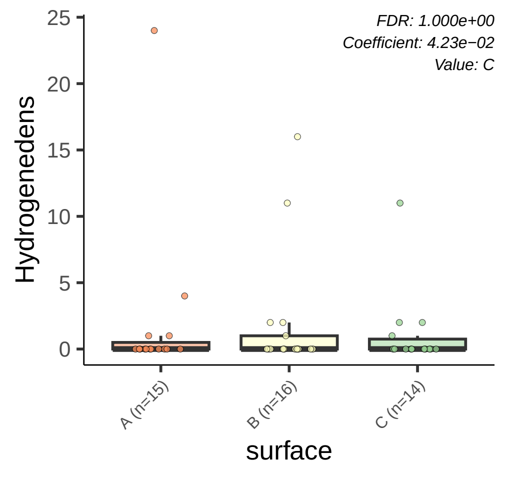

FDR: 1.000e+00
Coefficient: 4.23e−02
Value: C
Hydrogenedens
25
20
15
10
5
0
A (n=15)
B (n=16)
C (n=14)
surface

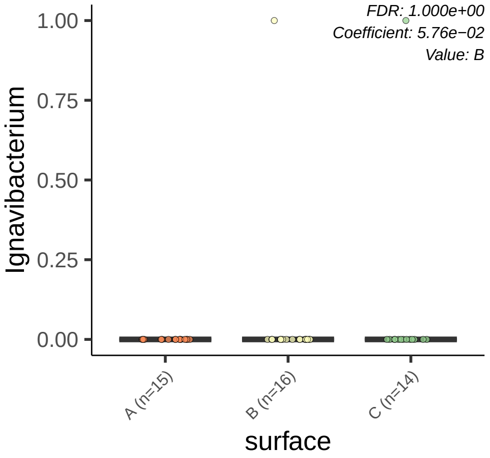

FDR: 1.000e+00
Coefficient: 5.76e−02
Value: B
Ignavibacterium
1.00
0.75
0.50
0.25
0.00
A (n=15)
B (n=16)
C (n=14)
surface

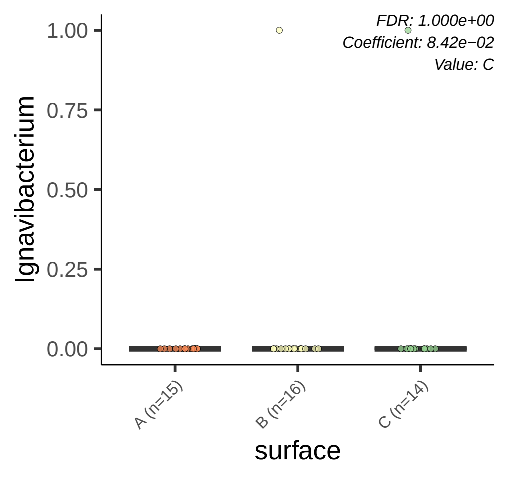
FDR: 1.000e+00
Coefficient: 8.42e−02
Value: C
Ignavibacterium
1.00
0.75
0.50
0.25
0.00
A (n=15)
B (n=16)
C (n=14)
surface

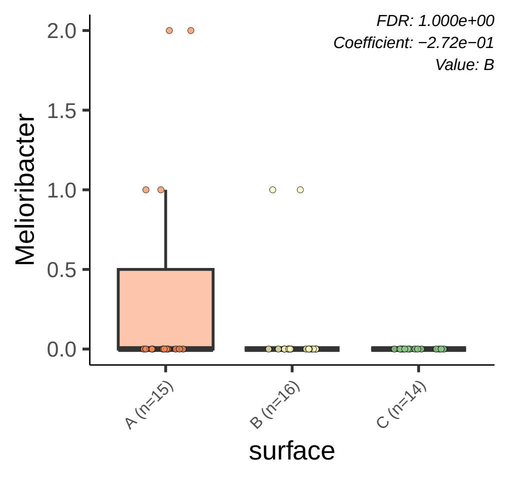
FDR: 1.000e+00
Coefficient: −2.72e−01
Value: B
Melioribacter
2.0
1.5
1.0
0.5
0.0
A (n=15)
B (n=16)
C (n=14)
surface

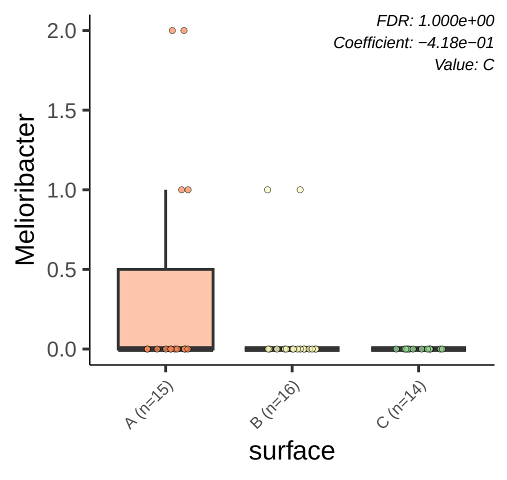
FDR: 1.000e+00
Coefficient: −4.18e−01
Value: C
Melioribacter
2.0
1.5
1.0
0.5
0.0
A (n=15)
B (n=16)
C (n=14)
surface

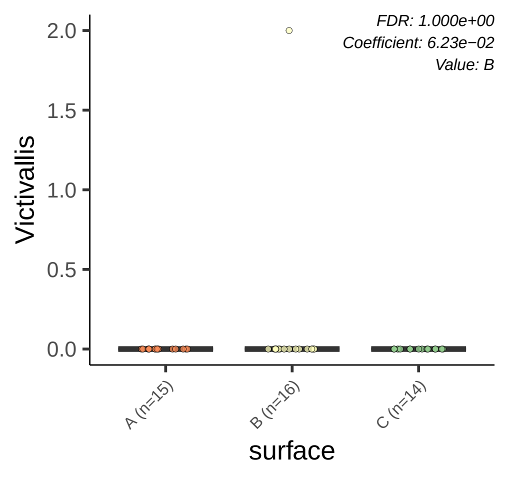
FDR: 1.000e+00
Coefficient: 6.23e−02
Value: B
Victivallis
2.0
1.5
1.0
0.5
0.0
A (n=15)
B (n=16)
C (n=14)
surface

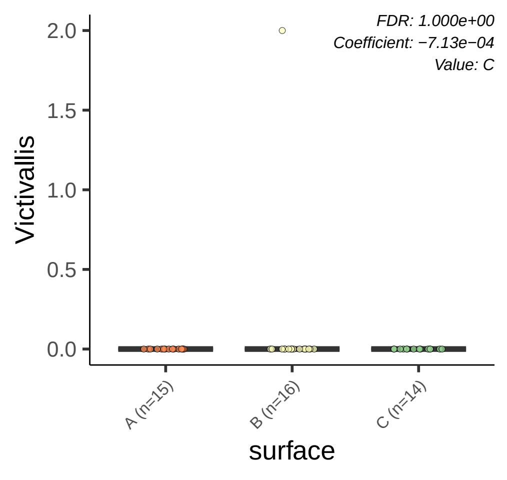

FDR: 1.000e+00
Coefficient: −7.13e−04
Value: C
Victivallis
2.0
1.5
1.0
0.5
0.0
A (n=15)
B (n=16)
C (n=14)
surface

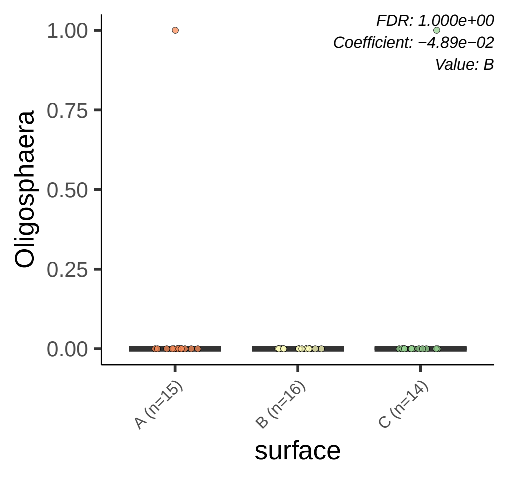
FDR: 1.000e+00
Coefficient: −4.89e−02
Value: B
Oligosphaera
1.00
0.75
0.50
0.25
0.00
A (n=15)
B (n=16)
C (n=14)
surface

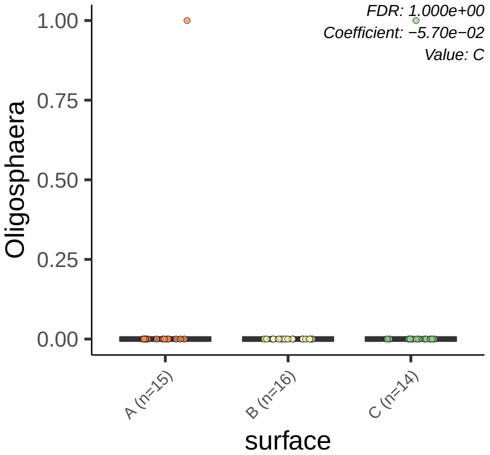
FDR: 1.000e+00
Coefficient: −5.70e−02
Value: C
Oligosphaera
1.00
0.75
0.50
0.25
0.00
A (n=15)
B (n=16)
C (n=14)
surface

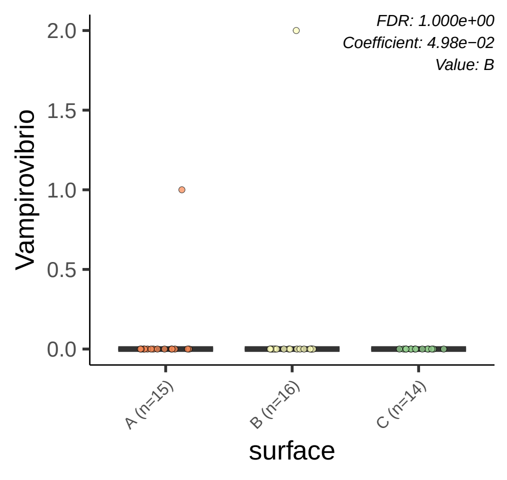
FDR: 1.000e+00
Coefficient: 4.98e−02
Value: B
Vampirovibrio
2.0
1.5
1.0
0.5
0.0
A (n=15)
B (n=16)
C (n=14)
surface

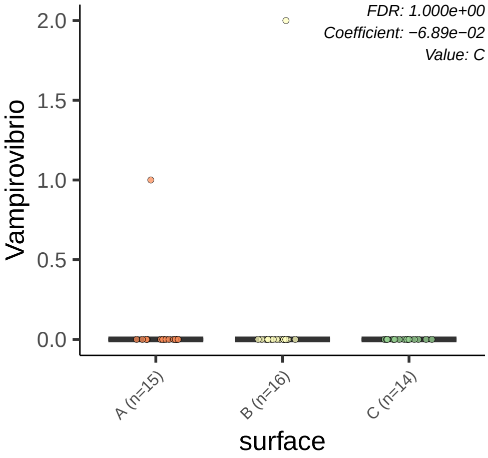

FDR: 1.000e+00
Coefficient: −6.89e−02
Value: C
Vampirovibrio
2.0
1.5
1.0
0.5
0.0
A (n=15)
B (n=16)
C (n=14)
surface

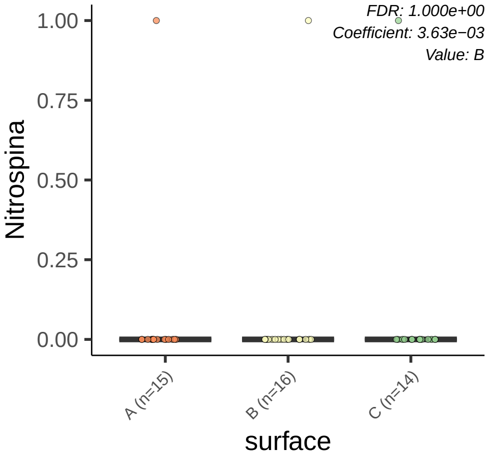
FDR: 1.000e+00
Coefficient: 3.63e−03
Value: B
Nitrospina
1.00
0.75
0.50
0.25
0.00
A (n=15)
B (n=16)
C (n=14)
surface

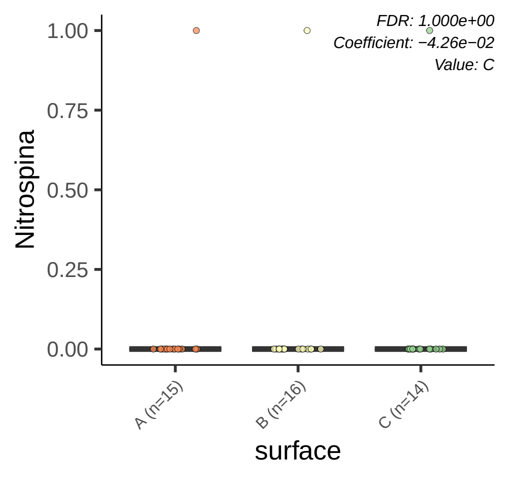
FDR: 1.000e+00
Coefficient: −4.26e−02
Value: C
Nitrospina
1.00
0.75
0.50
0.25
0.00
A (n=15)
B (n=16)
C (n=14)
surface

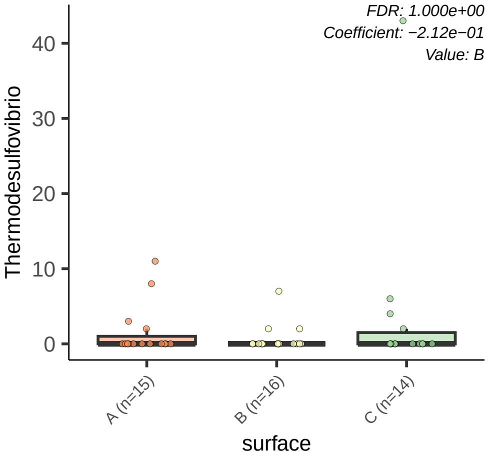

FDR: 1.000e+00
Coefficient: −2.12e−01
Value: B
Thermodesulfovibrio
0
10
20
30
40
A (n=15)
B (n=16)
C (n=14)
surface

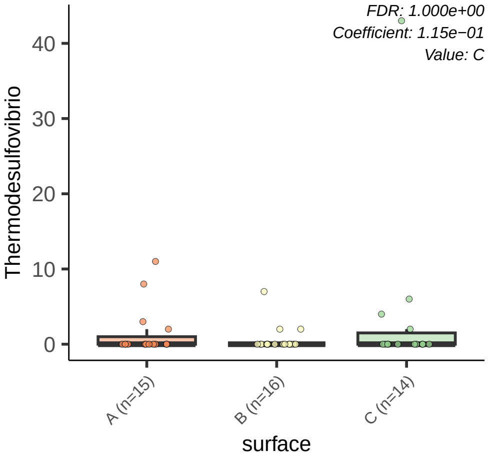

FDR: 1.000e+00
Coefficient: 1.15e−01
Value: C
Thermodesulfovibrio
0
10
20
30
40
A (n=15)
B (n=16)
C (n=14)
surface

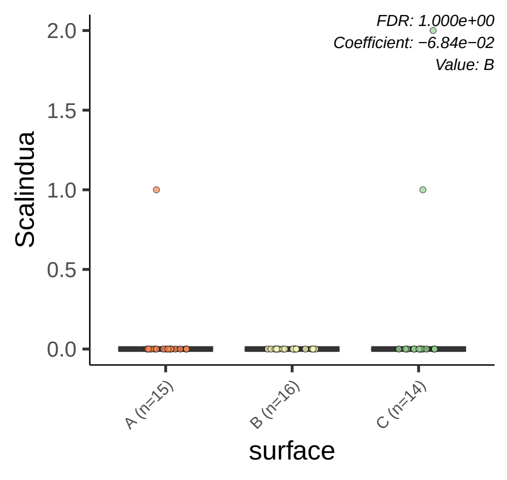
FDR: 1.000e+00
Coefficient: −6.84e−02
Value: B
2.0
1.5
1.0
0.5
0.0
Scalindua
A (n=15)
B (n=16)
C (n=14)
surface

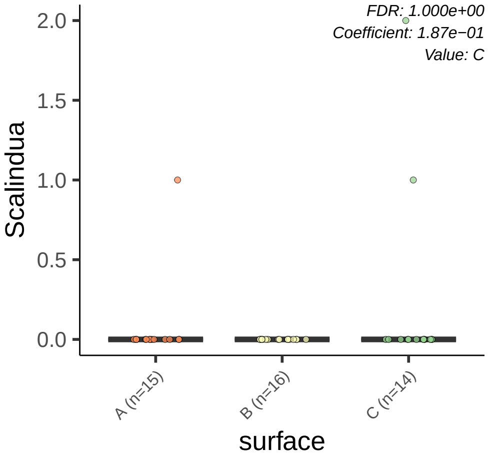

FDR: 1.000e+00
Coefficient: 1.87e−01
Value: C
Scalindua
2.0
1.5
1.0
0.5
0.0
A (n=15)
B (n=16)
C (n=14)
surface

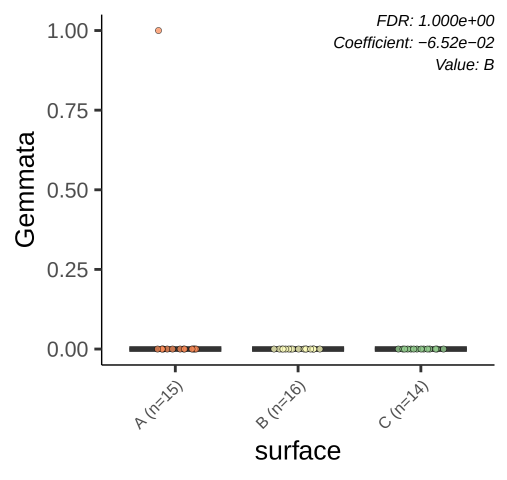
FDR: 1.000e+00
Coefficient: −6.52e−02
Value: B
Gemmata
1.00
0.75
0.50
0.25
0.00
A (n=15)
B (n=16)
C (n=14)
surface

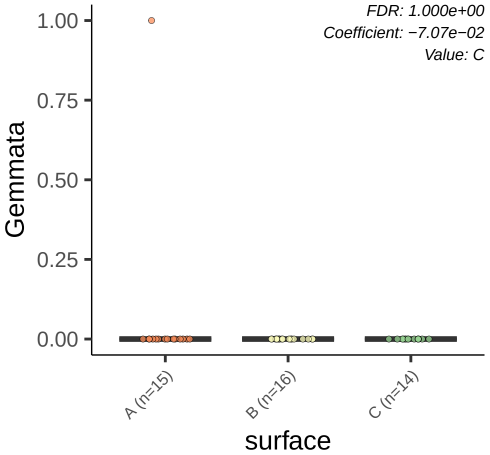
FDR: 1.000e+00
Coefficient: −7.07e−02
Value: C
Gemmata
1.00
0.75
0.50
0.25
0.00
A (n=15)
B (n=16)
C (n=14)
surface

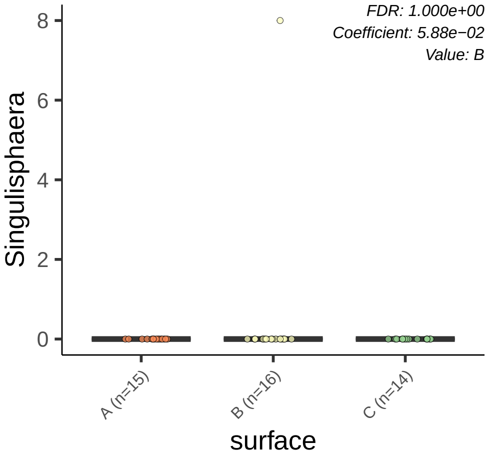

FDR: 1.000e+00
Coefficient: 5.88e−02
Value: B
Singulisphaera
8
6
4
2
0
A (n=15)
B (n=16)
C (n=14)
surface

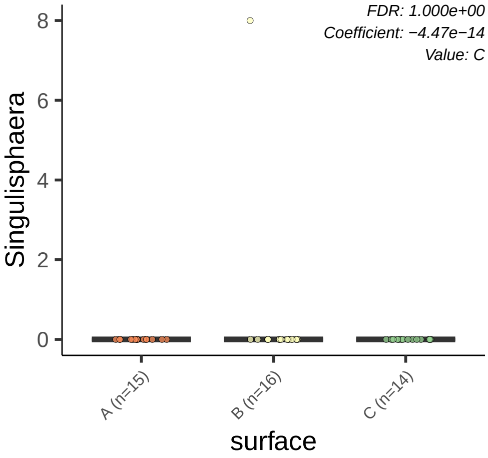

FDR: 1.000e+00
Coefficient: −4.47e−14
Value: C
Singulisphaera
8
6
4
2
0
A (n=15)
B (n=16)
C (n=14)
surface

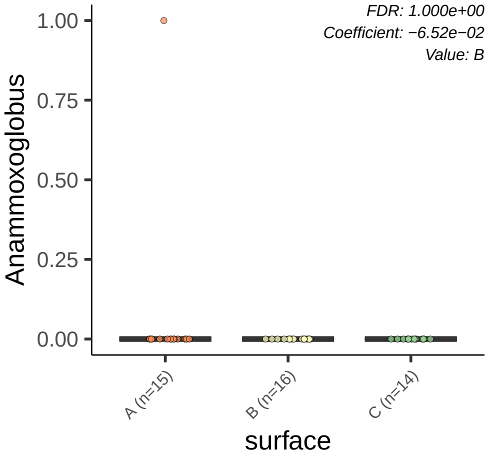
FDR: 1.000e+00
Coefficient: −6.52e−02
Value: B
Anammoxoglobus
1.00
0.75
0.50
0.25
0.00
A (n=15)
B (n=16)
C (n=14)
surface

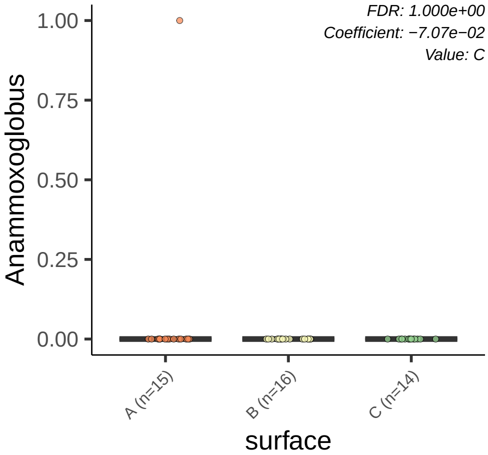

FDR: 1.000e+00
Coefficient: −7.07e−02
Value: C
Anammoxoglobus
1.00
0.75
0.50
0.25
0.00
A (n=15)
B (n=16)
C (n=14)
surface

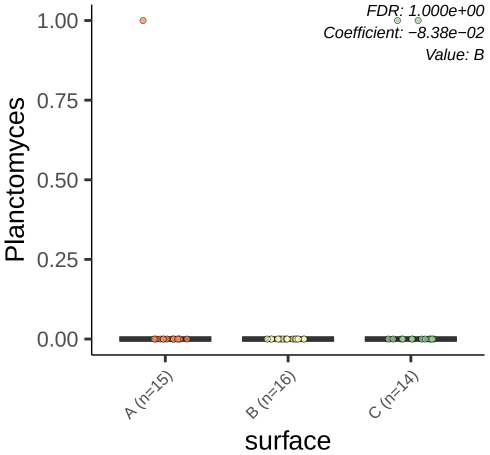

FDR: 1.000e+00
Coefficient: −8.38e−02
Value: B
1.00
0.75
0.50
0.25
0.00
Planctomyces
A (n=15)
B (n=16)
C (n=14)
surface

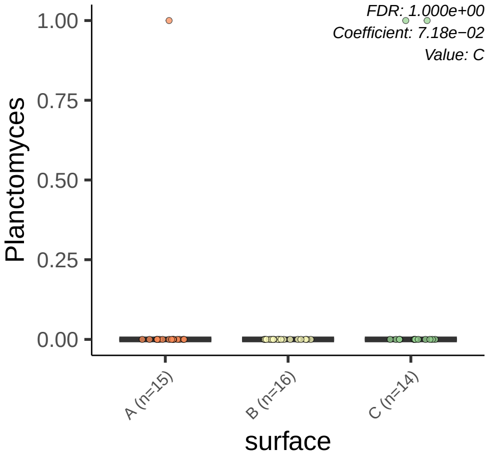
FDR: 1.000e+00
Coefficient: 7.18e−02
Value: C
Planctomyces
1.00
0.75
0.50
0.25
0.00
A (n=15)
B (n=16)
C (n=14)
surface

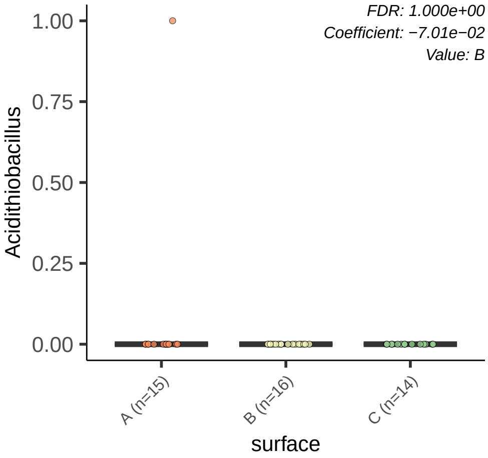

FDR: 1.000e+00
Coefficient: −7.01e−02
Value: B
Acidithiobacillus
1.00
0.75
0.50
0.25
0.00
A (n=15)
B (n=16)
C (n=14)
surface

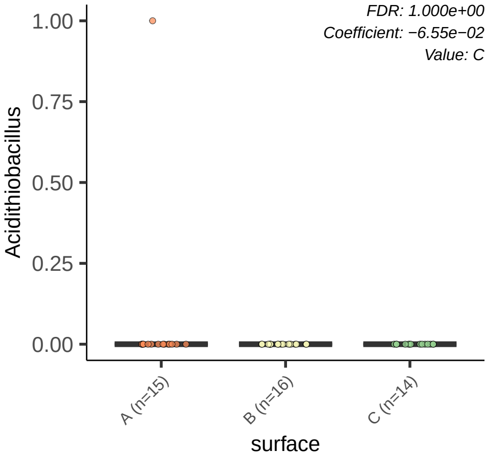

FDR: 1.000e+00
Coefficient: −6.55e−02
Value: C
Acidithiobacillus
1.00
0.75
0.50
0.25
0.00
A (n=15)
B (n=16)
C (n=14)
surface

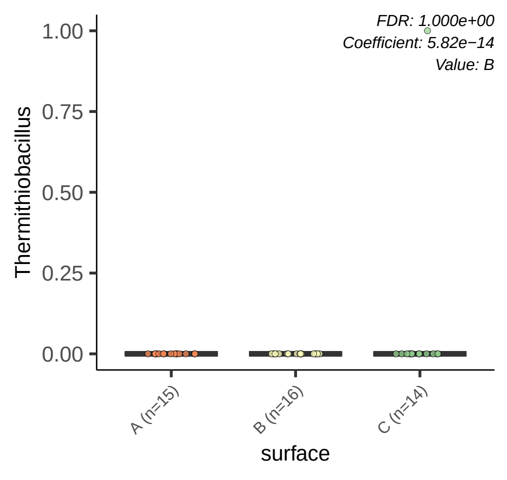
FDR: 1.000e+00
Coefficient: 5.82e−14
Value: B
Thermithiobacillus
1.00
0.75
0.50
0.25
0.00
A (n=15)
B (n=16)
C (n=14)
surface

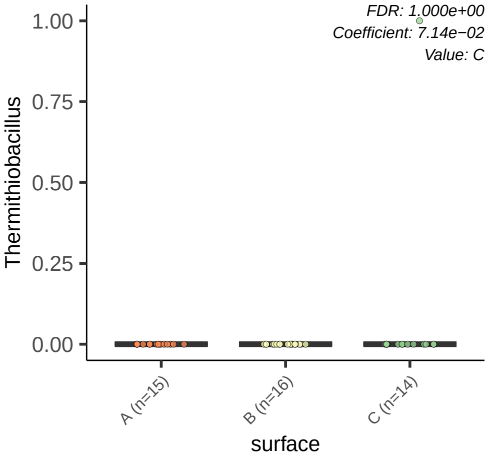
FDR: 1.000e+00
Coefficient: 7.14e−02
Value: C
Thermithiobacillus
1.00
0.75
0.50
0.25
0.00
A (n=15)
B (n=16)
C (n=14)
surface

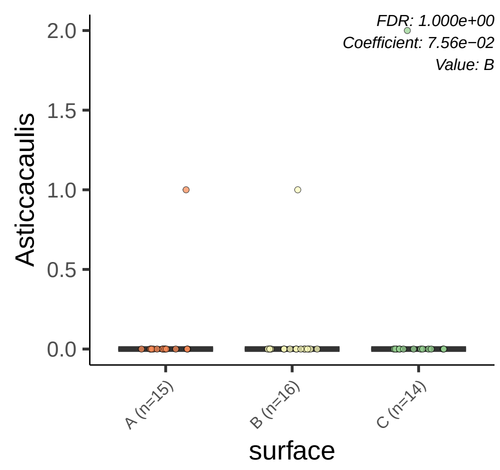

FDR: 1.000e+00
Coefficient: 7.56e−02
Value: B
Asticcacaulis
2.0
1.5
1.0
0.5
0.0
A (n=15)
B (n=16)
C (n=14)
surface

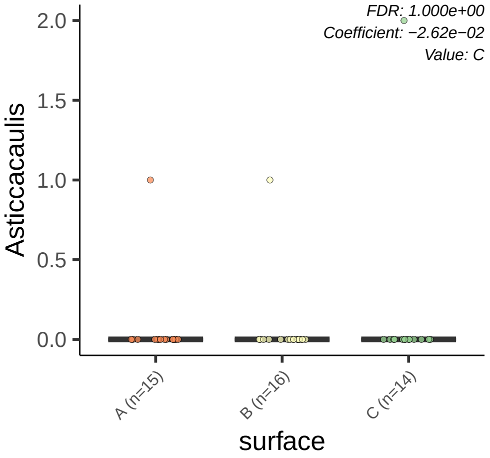
FDR: 1.000e+00
Coefficient: −2.62e−02
Value: C
Asticcacaulis
2.0
1.5
1.0
0.5
0.0
A (n=15)
B (n=16)
C (n=14)
surface

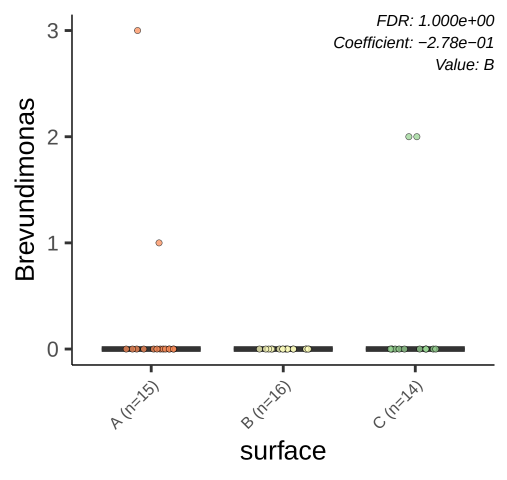

FDR: 1.000e+00
Coefficient: −2.78e−01
Value: B
Brevundimonas
3
2
1
0
A (n=15)
B (n=16)
C (n=14)
surface

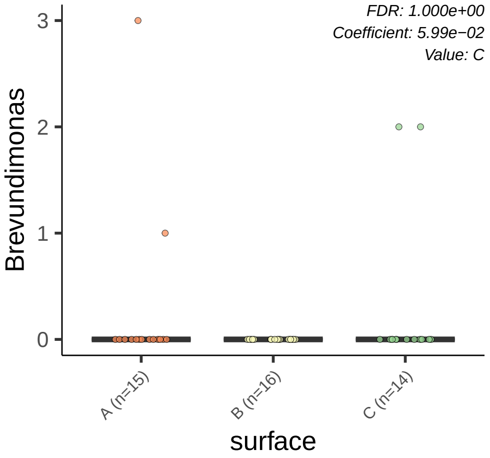
FDR: 1.000e+00
Coefficient: 5.99e−02
Value: C
Brevundimonas
3
2
1
0
A (n=15)
B (n=16)
C (n=14)
surface

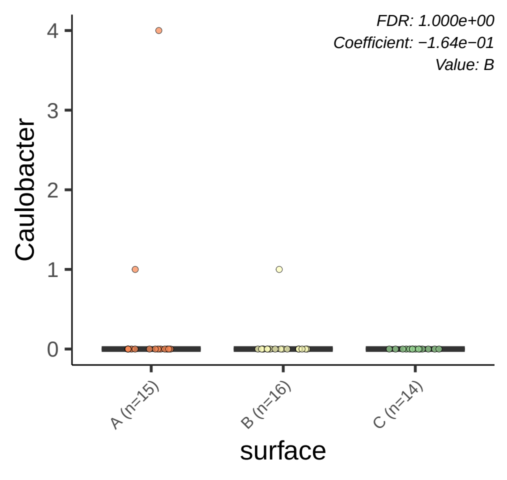
FDR: 1.000e+00
Coefficient: −1.64e−01
Value: B
Caulobacter
4
3
2
1
0
A (n=15)
B (n=16)
C (n=14)
surface

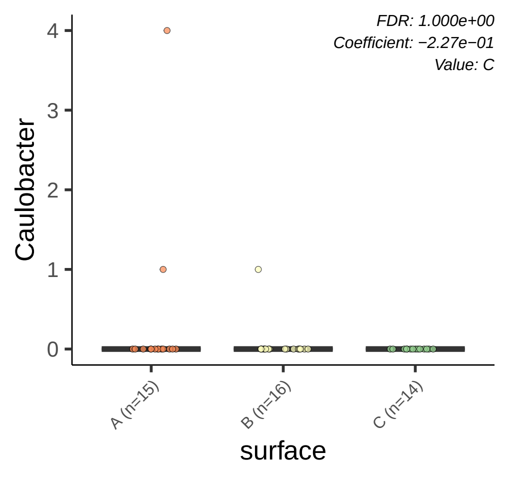
FDR: 1.000e+00
Coefficient: −2.27e−01
Value: C
Caulobacter
0
1
2
3
4
A (n=15)
B (n=16)
C (n=14)
surface

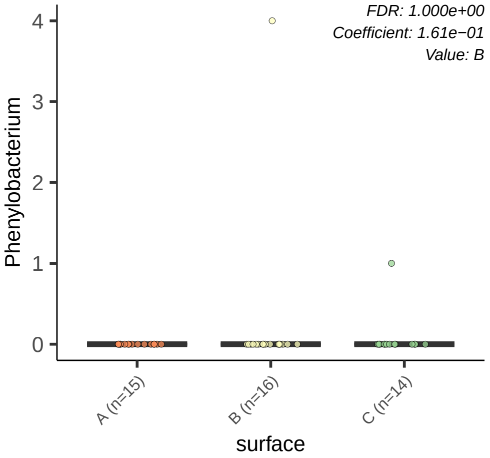
FDR: 1.000e+00
Coefficient: 1.61e−01
Value: B
Phenylobacterium
0
1
2
3
4
A (n=15)
B (n=16)
C (n=14)
surface

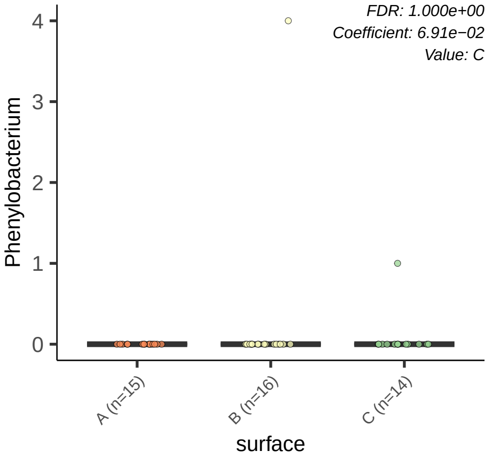

FDR: 1.000e+00
Coefficient: 6.91e−02
Value: C
Phenylobacterium
0
1
2
3
4
A (n=15)
B (n=16)
C (n=14)
surface

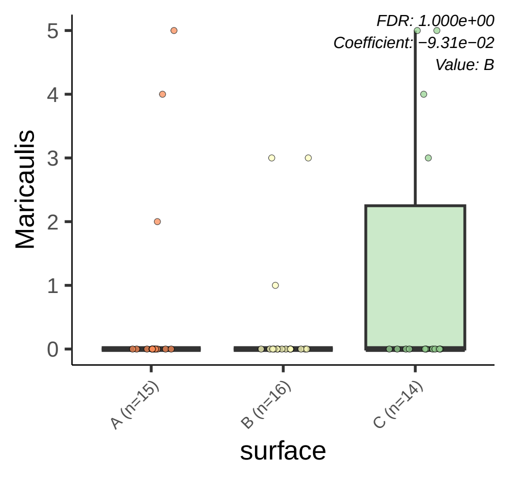

FDR: 1.000e+00
Coefficient: −9.31e−02
Value: B
Maricaulis
0
1
2
3
4
5
A (n=15)
B (n=16)
C (n=14)
surface

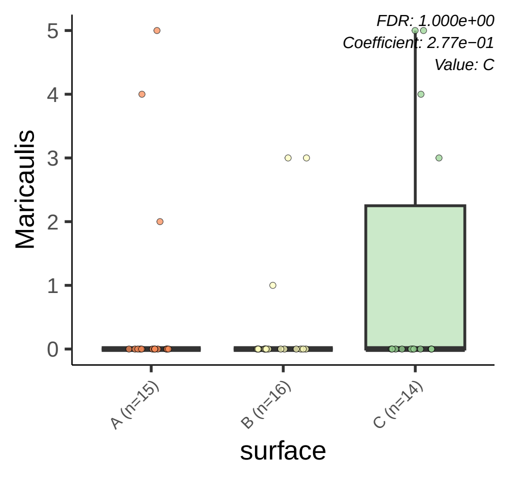
FDR: 1.000e+00
Coefficient: 2.77e−01
Value: C
Maricaulis
0
1
2
3
4
5
A (n=15)
B (n=16)
C (n=14)
surface

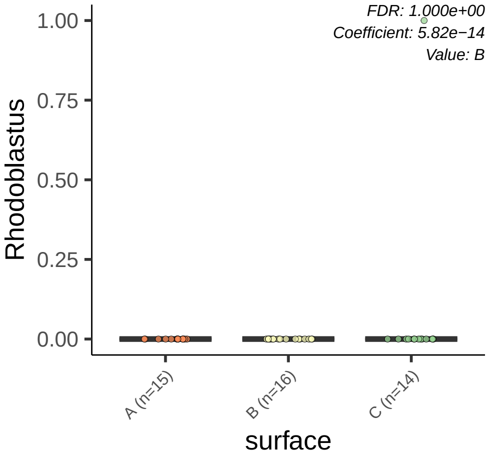

FDR: 1.000e+00
Coefficient: 5.82e−14
Value: B
Rhodoblastus
1.00
0.75
0.50
0.25
0.00
A (n=15)
B (n=16)
C (n=14)
surface

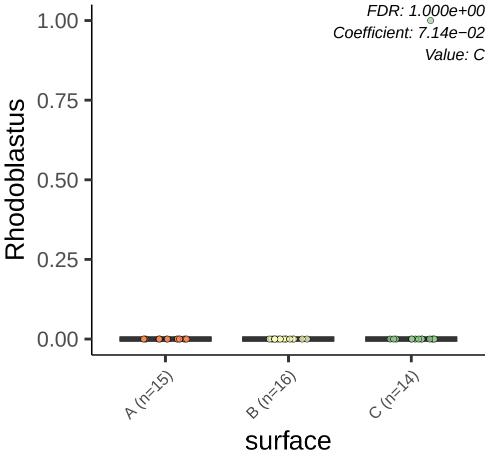

FDR: 1.000e+00
Coefficient: 7.14e−02
Value: C
Rhodoblastus
1.00
0.75
0.50
0.25
0.00
A (n=15)
B (n=16)
C (n=14)
surface

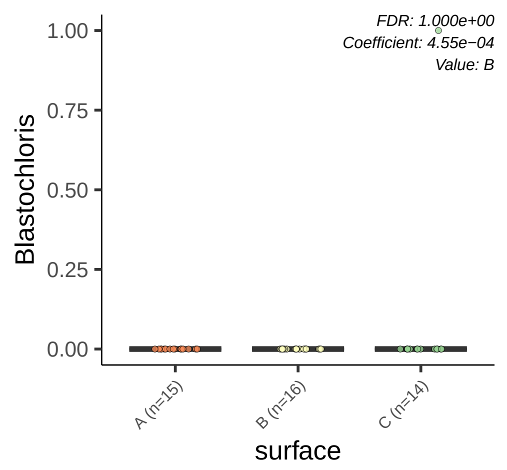
FDR: 1.000e+00
Coefficient: 4.55e−04
Value: B
1.00
0.75
0.50
0.25
0.00
Blastochloris
A (n=15)
B (n=16)
C (n=14)
surface

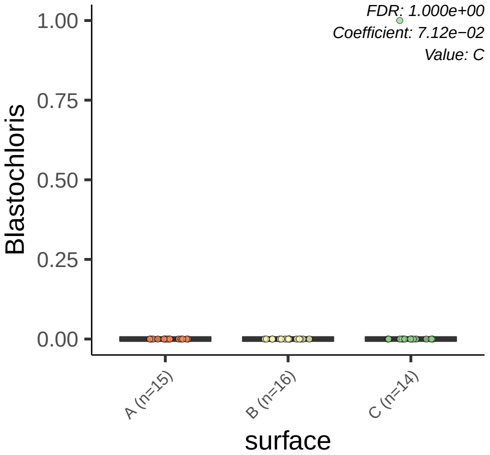
FDR: 1.000e+00
Coefficient: 7.12e−02
Value: C
1.00
0.75
0.50
0.25
0.00
Blastochloris
A (n=15)
B (n=16)
C (n=14)
surface

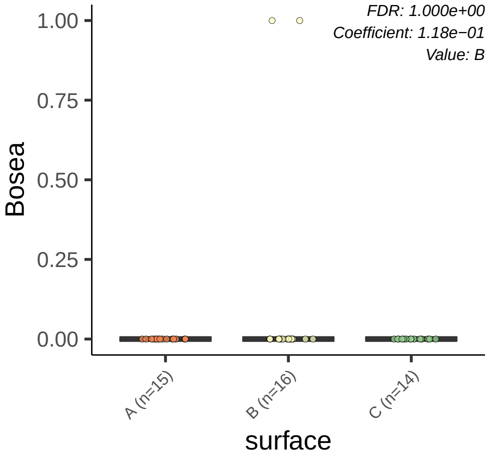

FDR: 1.000e+00
Coefficient: 1.18e−01
Value: B
Bosea
1.00
0.75
0.50
0.25
0.00
A (n=15)
B (n=16)
C (n=14)
surface

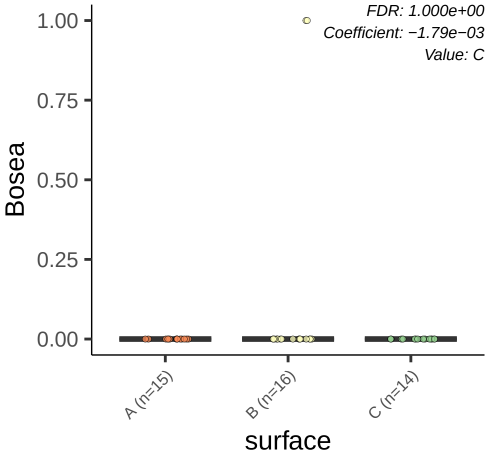
FDR: 1.000e+00
Coefficient: −1.79e−03
Value: C
Bosea
1.00
0.75
0.50
0.25
0.00
A (n=15)
B (n=16)
C (n=14)
surface

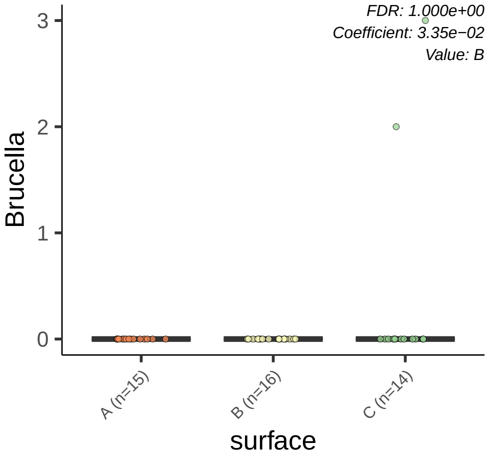
FDR: 1.000e+00
Coefficient: 3.35e−02
Value: B
Brucella
3
2
1
0
A (n=15)
B (n=16)
C (n=14)
surface

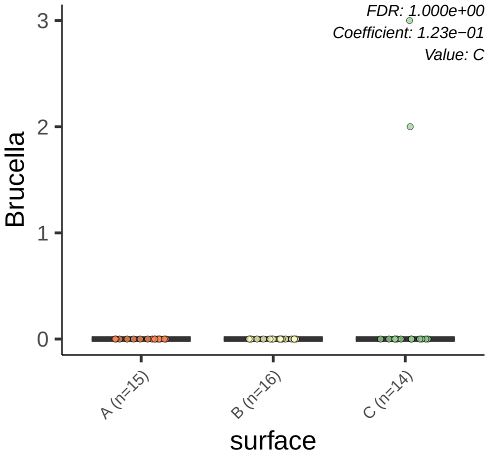
FDR: 1.000e+00
Coefficient: 1.23e−01
Value: C
Brucella
3
2
1
0
A (n=15)
B (n=16)
C (n=14)
surface

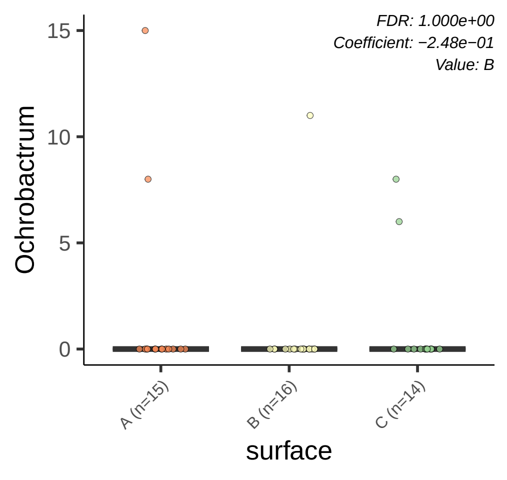

FDR: 1.000e+00
Coefficient: −2.48e−01
Value: B
Ochrobactrum
15
10
5
0
A (n=15)
B (n=16)
C (n=14)
surface

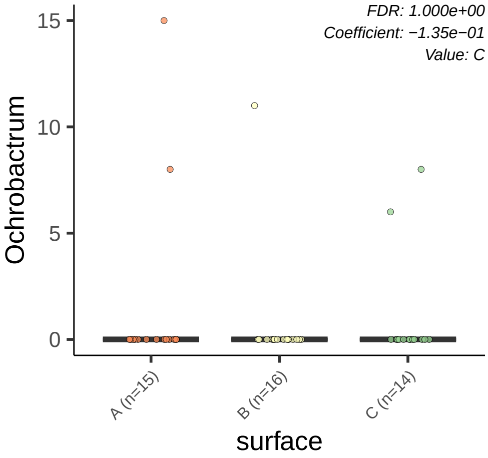

FDR: 1.000e+00
Coefficient: −1.35e−01
Value: C
Ochrobactrum
0
5
10
15
A (n=15)
B (n=16)
C (n=14)
surface

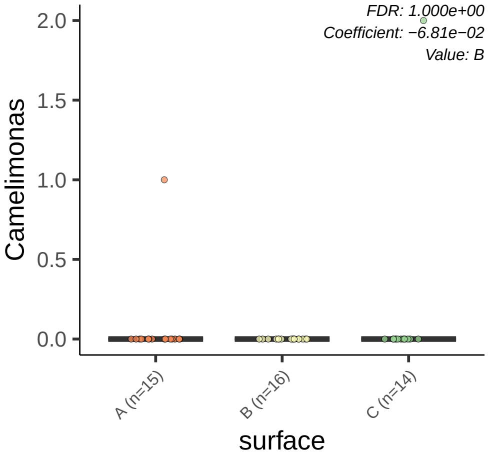
FDR: 1.000e+00
Coefficient: −6.81e−02
Value: B
Camelimonas
2.0
1.5
1.0
0.5
0.0
A (n=15)
B (n=16)
C (n=14)
surface

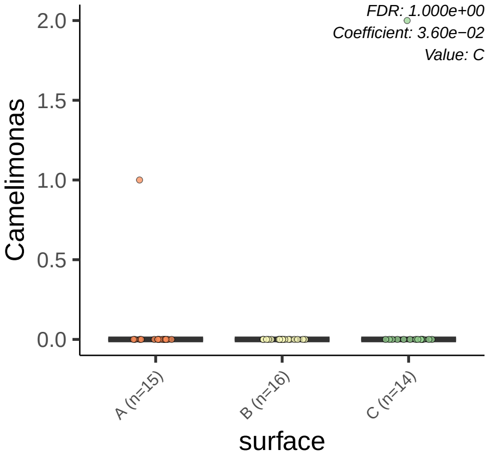
FDR: 1.000e+00
Coefficient: 3.60e−02
Value: C
Camelimonas
2.0
1.5
1.0
0.5
0.0
A (n=15)
B (n=16)
C (n=14)
surface

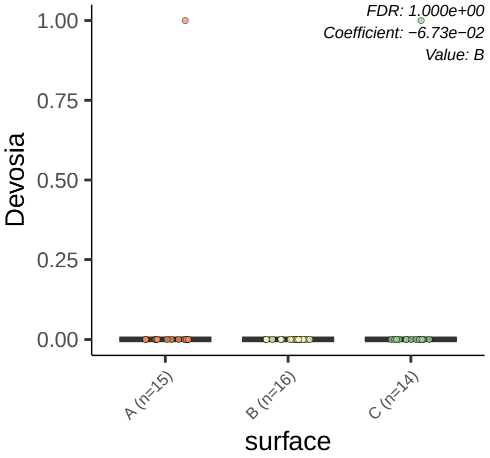

FDR: 1.000e+00
Coefficient: −6.73e−02
Value: B
Devosia
1.00
0.75
0.50
0.25
0.00
A (n=15)
B (n=16)
C (n=14)
surface

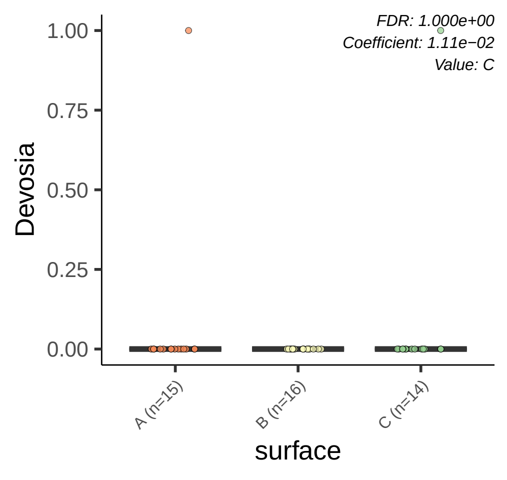
FDR: 1.000e+00
Coefficient: 1.11e−02
Value: C
Devosia
1.00
0.75
0.50
0.25
0.00
A (n=15)
B (n=16)
C (n=14)
surface

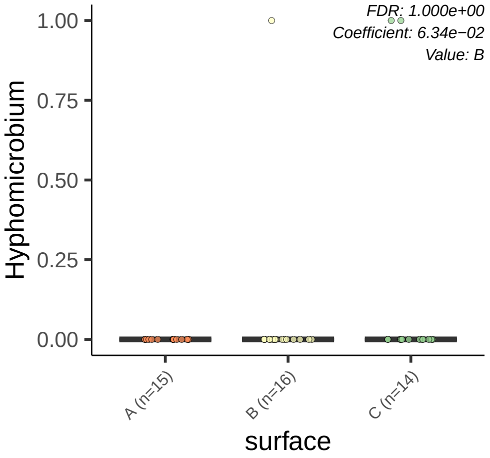

FDR: 1.000e+00
Coefficient: 6.34e−02
Value: B
Hyphomicrobium
1.00
0.75
0.50
0.25
0.00
A (n=15)
B (n=16)
C (n=14)
surface

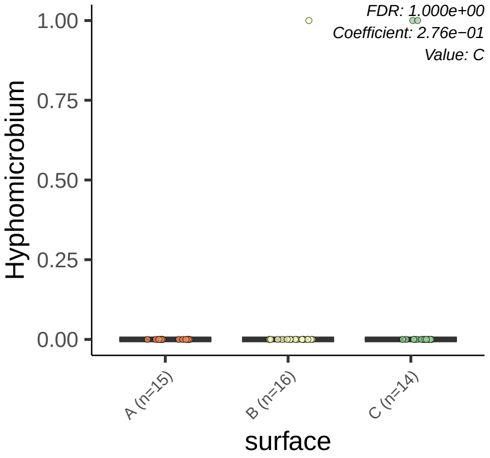

FDR: 1.000e+00
Coefficient: 2.76e−01
Value: C
Hyphomicrobium
1.00
0.75
0.50
0.25
0.00
A (n=15)
B (n=16)
C (n=14)
surface

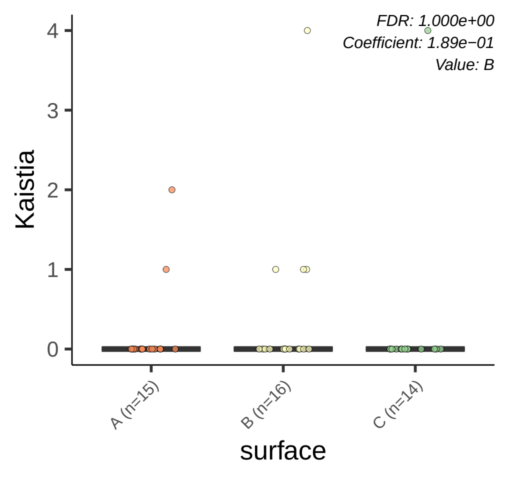
FDR: 1.000e+00
Coefficient: 1.89e−01
Value: B
Kaistia
0
1
2
3
4
A (n=15)
B (n=16)
C (n=14)
surface

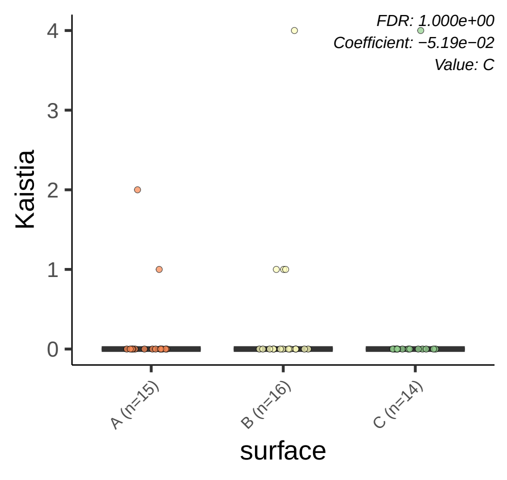

FDR: 1.000e+00
Coefficient: −5.19e−02
Value: C
Kaistia
4
3
2
1
0
A (n=15)
B (n=16)
C (n=14)
surface

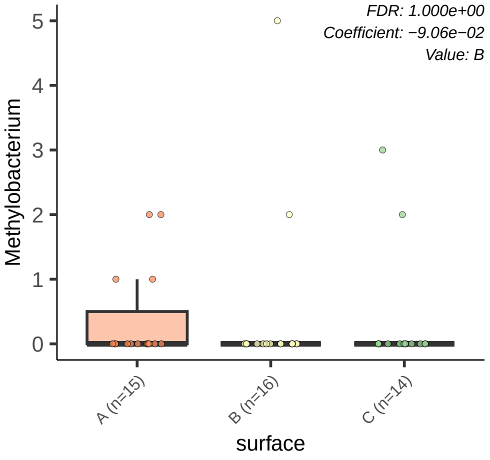
FDR: 1.000e+00
Coefficient: −9.06e−02
Value: B
Methylobacterium
0
1
2
3
4
5
A (n=15)
B (n=16)
C (n=14)
surface

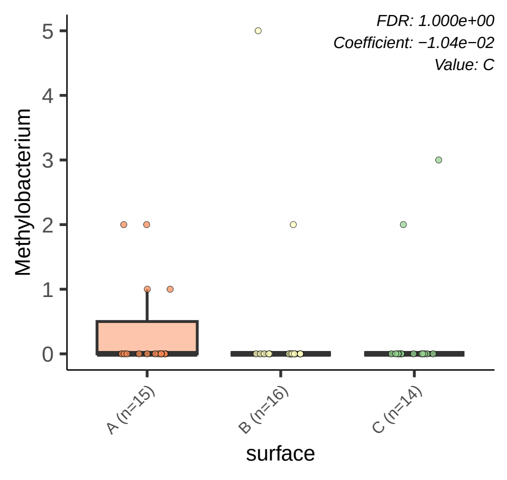
FDR: 1.000e+00
Coefficient: −1.04e−02
Value: C
Methylobacterium
0
1
2
3
4
5
A (n=15)
B (n=16)
C (n=14)
surface

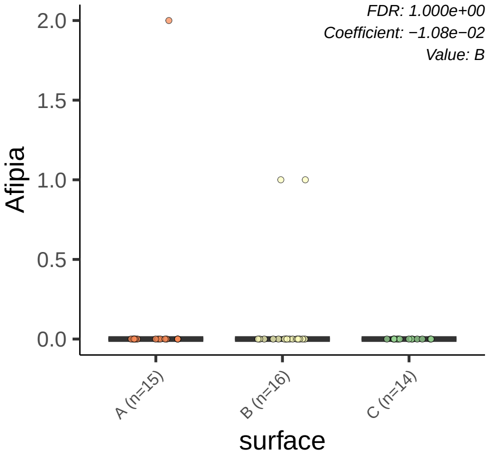

FDR: 1.000e+00
Coefficient: −1.08e−02
Value: B
Afipia
2.0
1.5
1.0
0.5
0.0
A (n=15)
B (n=16)
C (n=14)
surface

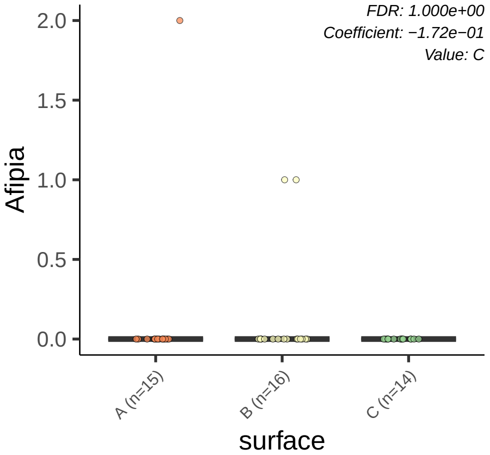

FDR: 1.000e+00
Coefficient: −1.72e−01
Value: C
Afipia
2.0
1.5
1.0
0.5
0.0
A (n=15)
B (n=16)
C (n=14)
surface

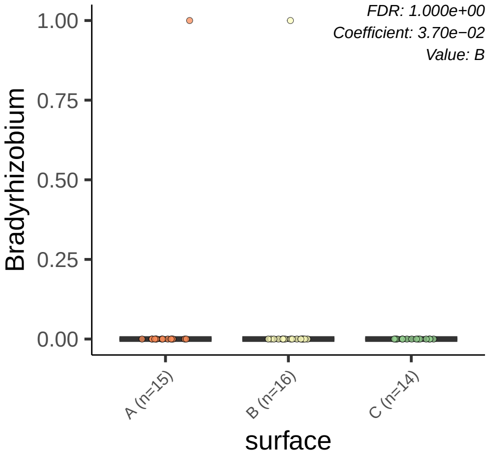

FDR: 1.000e+00
Coefficient: 3.70e−02
Value: B
Bradyrhizobium
1.00
0.75
0.50
0.25
0.00
A (n=15)
B (n=16)
C (n=14)
surface

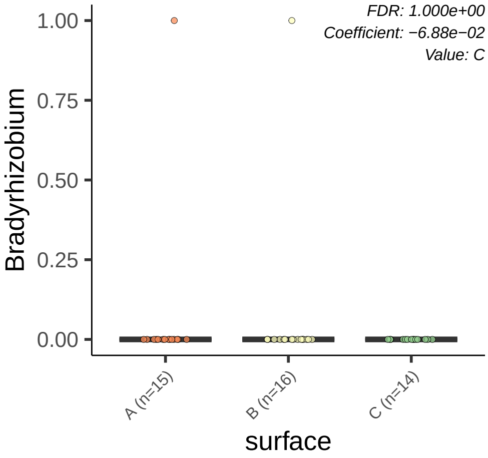

FDR: 1.000e+00
Coefficient: −6.88e−02
Value: C
1.00
0.75
0.50
0.25
0.00
Bradyrhizobium
A (n=15)
B (n=16)
C (n=14)
surface

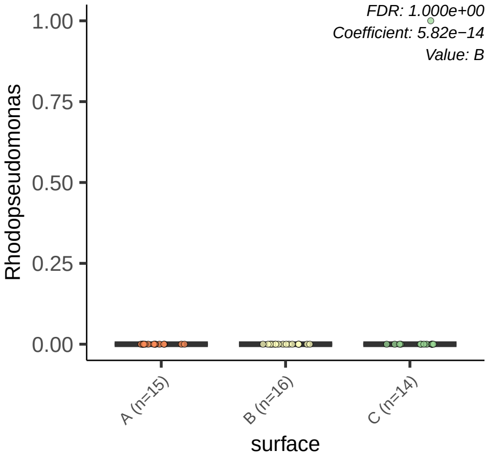
FDR: 1.000e+00
Coefficient: 5.82e−14
Value: B
Rhodopseudomonas
1.00
0.75
0.50
0.25
0.00
A (n=15)
B (n=16)
C (n=14)
surface

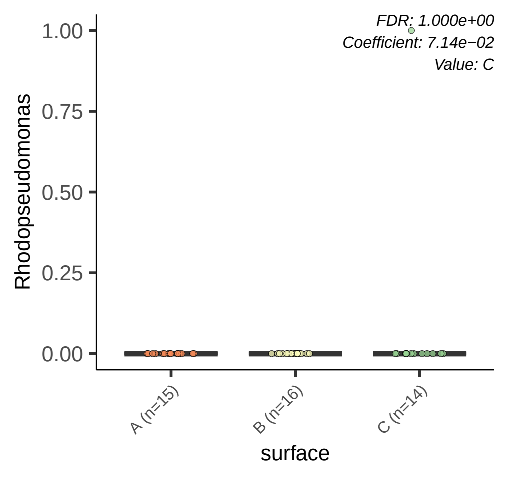

FDR: 1.000e+00
Coefficient: 7.14e−02
Value: C
1.00
0.75
0.50
0.25
0.00
Rhodopseudomonas
A (n=15)
B (n=16)
C (n=14)
surface

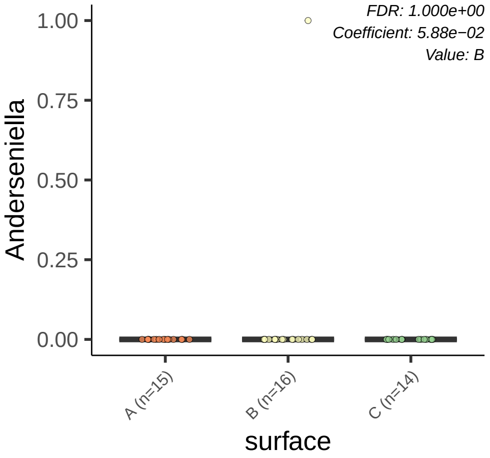
FDR: 1.000e+00
Coefficient: 5.88e−02
Value: B
Anderseniella
1.00
0.75
0.50
0.25
0.00
A (n=15)
B (n=16)
C (n=14)
surface

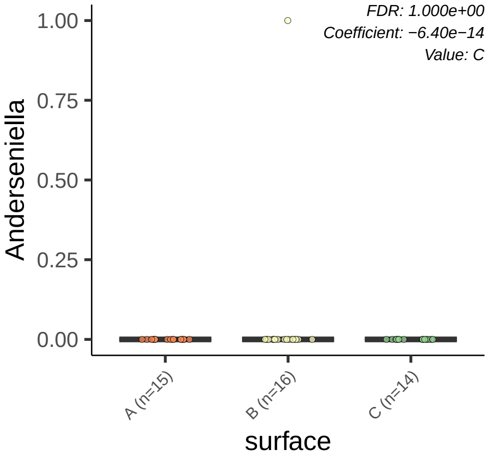

FDR: 1.000e+00
Coefficient: −6.40e−14
Value: C
Anderseniella
1.00
0.75
0.50
0.25
0.00
A (n=15)
B (n=16)
C (n=14)
surface

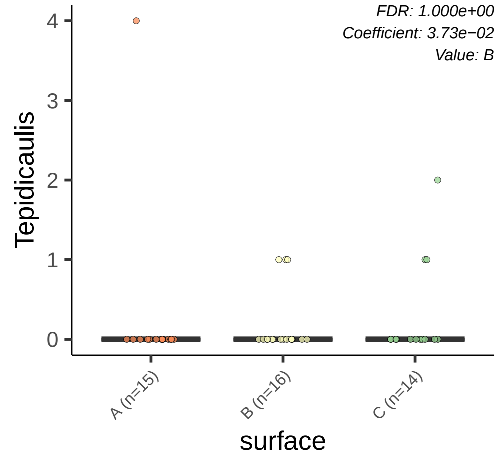
FDR: 1.000e+00
Coefficient: 3.73e−02
Value: B
Tepidicaulis
4
3
2
1
0
A (n=15)
B (n=16)
C (n=14)
surface

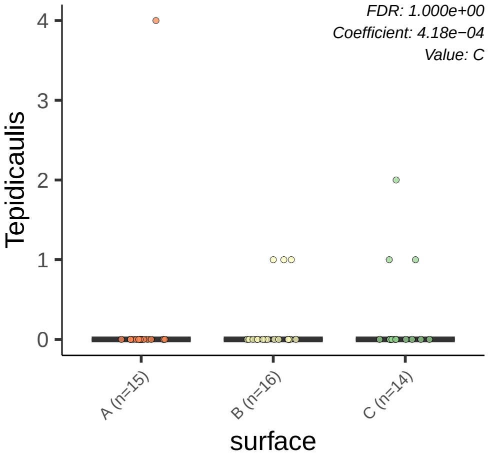
FDR: 1.000e+00
Coefficient: 4.18e−04
Value: C
Tepidicaulis
0
1
2
3
4
A (n=15)
B (n=16)
C (n=14)
surface

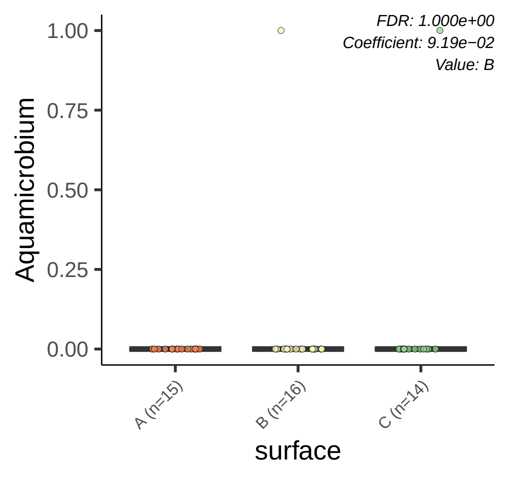
FDR: 1.000e+00
Coefficient: 9.19e−02
Value: B
Aquamicrobium
1.00
0.75
0.50
0.25
0.00
A (n=15)
B (n=16)
C (n=14)
surface

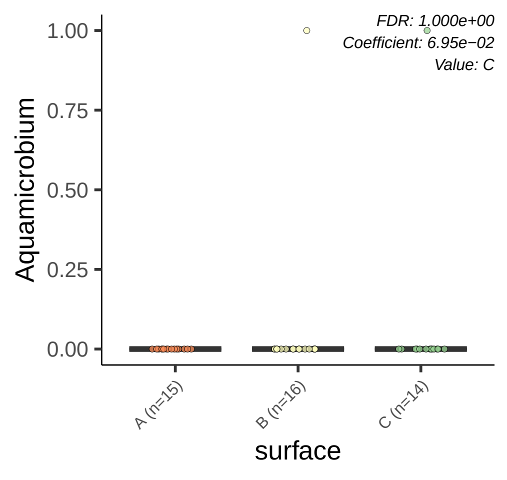
FDR: 1.000e+00
Coefficient: 6.95e−02
Value: C
Aquamicrobium
1.00
0.75
0.50
0.25
0.00
A (n=15)
B (n=16)
C (n=14)
surface

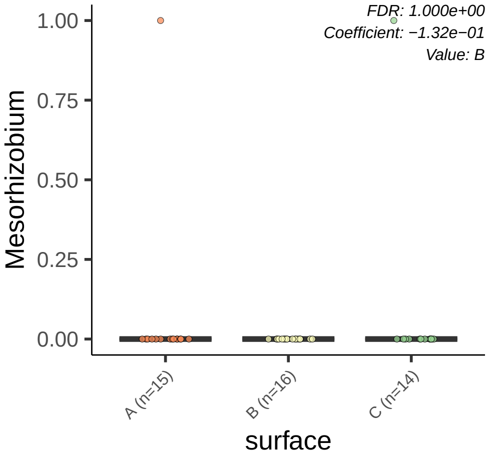

FDR: 1.000e+00
Coefficient: −1.32e−01
Value: B
Mesorhizobium
1.00
0.75
0.50
0.25
0.00
A (n=15)
B (n=16)
C (n=14)
surface

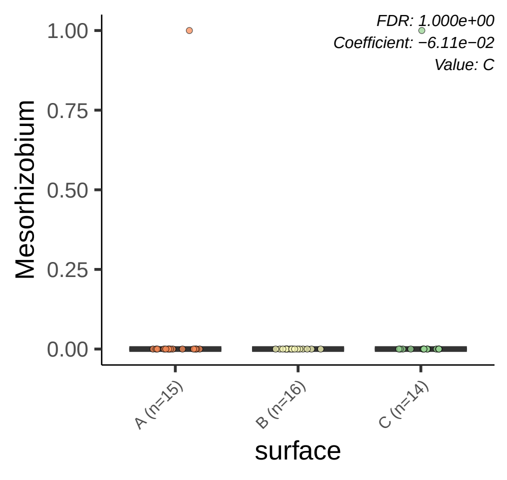
FDR: 1.000e+00
Coefficient: −6.11e−02
Value: C
Mesorhizobium
1.00
0.75
0.50
0.25
0.00
A (n=15)
B (n=16)
C (n=14)
surface

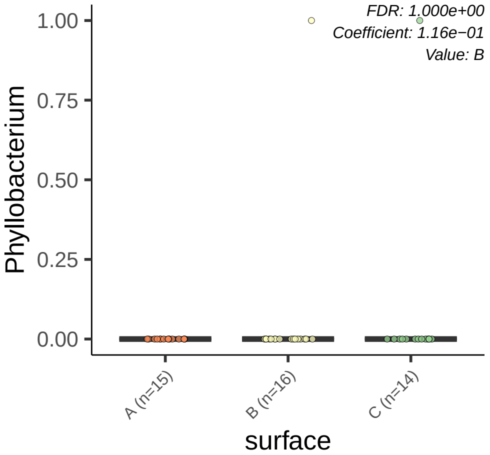

FDR: 1.000e+00
Coefficient: 1.16e−01
Value: B
Phyllobacterium
1.00
0.75
0.50
0.25
0.00
A (n=15)
B (n=16)
C (n=14)
surface

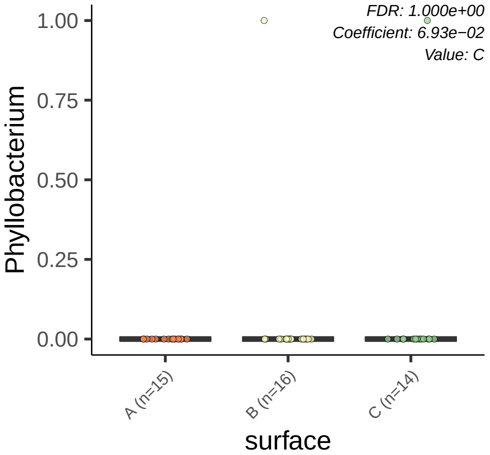

FDR: 1.000e+00
Coefficient: 6.93e−02
Value: C
Phyllobacterium
1.00
0.75
0.50
0.25
0.00
A (n=15)
B (n=16)
C (n=14)
surface

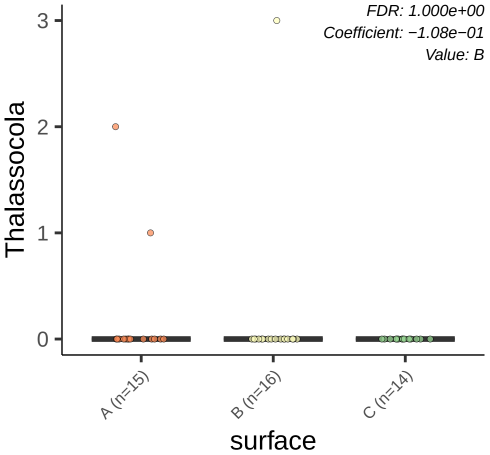
FDR: 1.000e+00
Coefficient: −1.08e−01
Value: B
Thalassocola
3
2
1
0
A (n=15)
B (n=16)
C (n=14)
surface

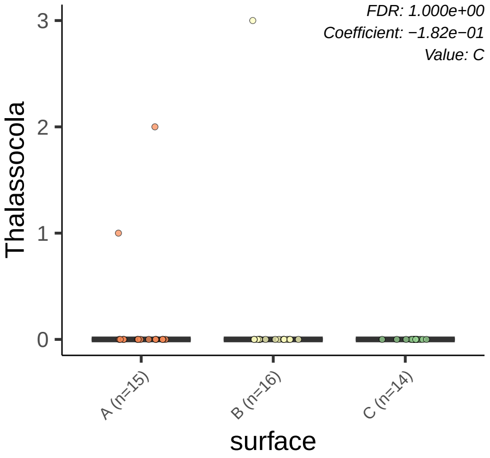

FDR: 1.000e+00
Coefficient: −1.82e−01
Value: C
Thalassocola
0
1
2
3
A (n=15)
B (n=16)
C (n=14)
surface

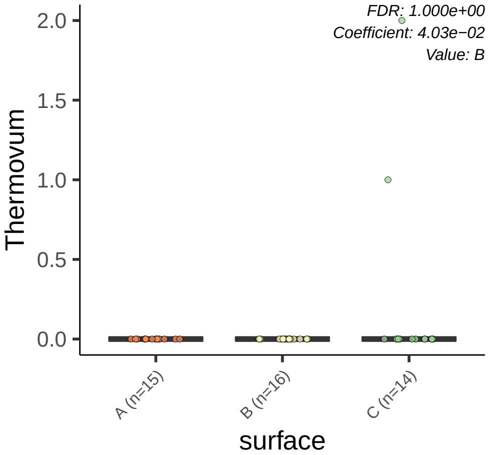

FDR: 1.000e+00
Coefficient: 4.03e−02
Value: B
Thermovum
2.0
1.5
1.0
0.5
0.0
A (n=15)
B (n=16)
C (n=14)
surface

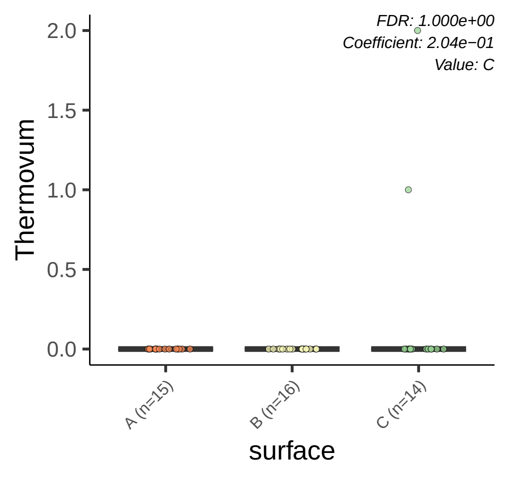
FDR: 1.000e+00
Coefficient: 2.04e−01
Value: C
Thermovum
2.0
1.5
1.0
0.5
0.0
A (n=15)
B (n=16)
C (n=14)
surface

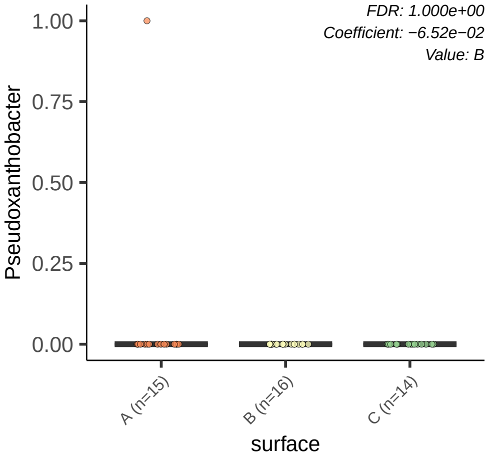

FDR: 1.000e+00
Coefficient: −6.52e−02
Value: B
Pseudoxanthobacter
1.00
0.75
0.50
0.25
0.00
A (n=15)
B (n=16)
C (n=14)
surface

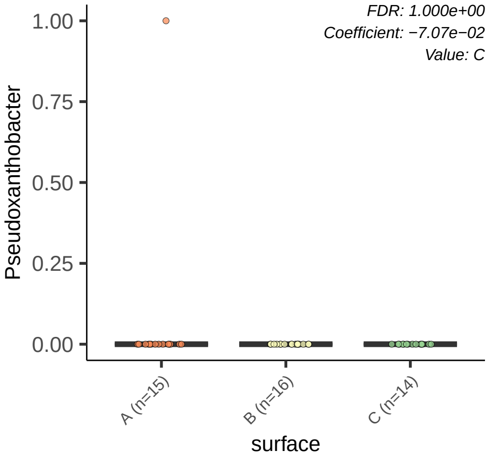

FDR: 1.000e+00
Coefficient: −7.07e−02
Value: C
Pseudoxanthobacter
1.00
0.75
0.50
0.25
0.00
A (n=15)
B (n=16)
C (n=14)
surface

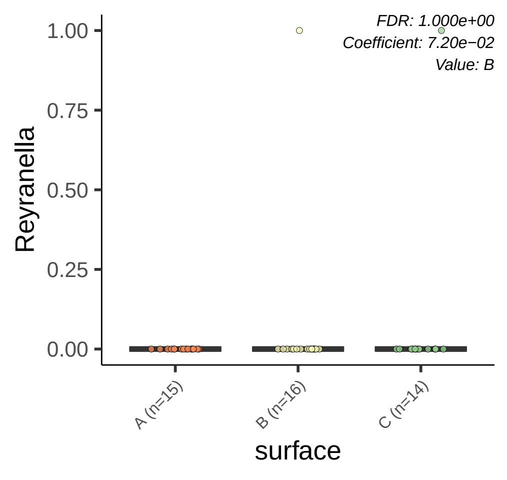
FDR: 1.000e+00
Coefficient: 7.20e−02
Value: B
Reyranella
1.00
0.75
0.50
0.25
0.00
A (n=15)
B (n=16)
C (n=14)
surface

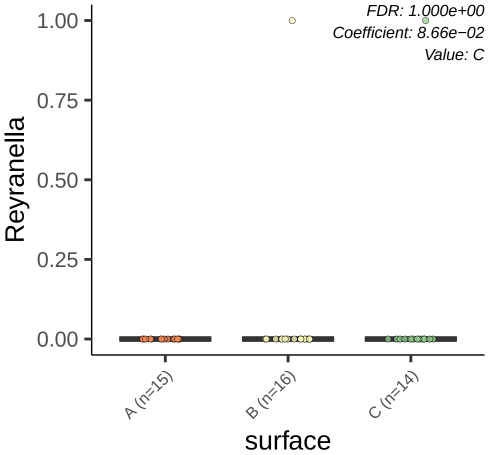

FDR: 1.000e+00
Coefficient: 8.66e−02
Value: C
Reyranella
1.00
0.75
0.50
0.25
0.00
A (n=15)
B (n=16)
C (n=14)
surface

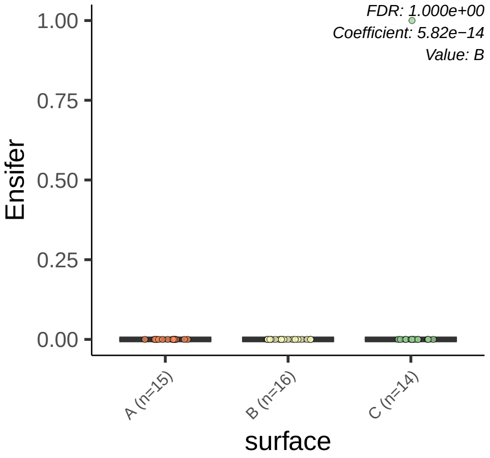
FDR: 1.000e+00
Coefficient: 5.82e−14
Value: B
1.00
0.75
0.50
0.25
0.00
Ensifer
A (n=15)
B (n=16)
C (n=14)
surface

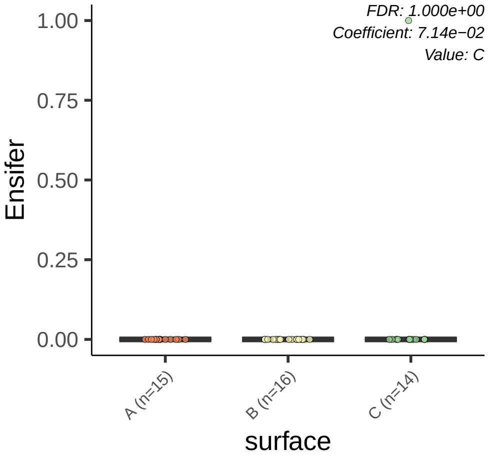

FDR: 1.000e+00
Coefficient: 7.14e−02
Value: C
1.00
0.75
0.50
0.25
0.00
Ensifer
A (n=15)
B (n=16)
C (n=14)
surface

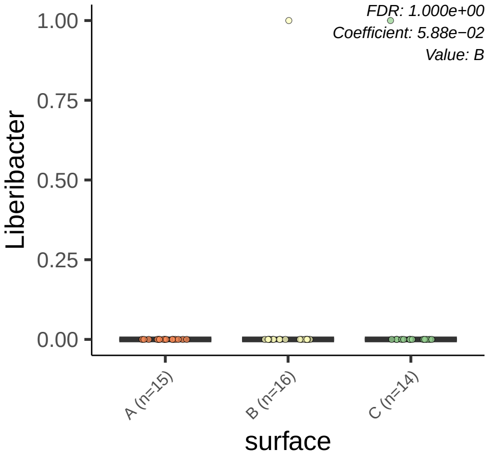
FDR: 1.000e+00
Coefficient: 5.88e−02
Value: B
Liberibacter
1.00
0.75
0.50
0.25
0.00
A (n=15)
B (n=16)
C (n=14)
surface

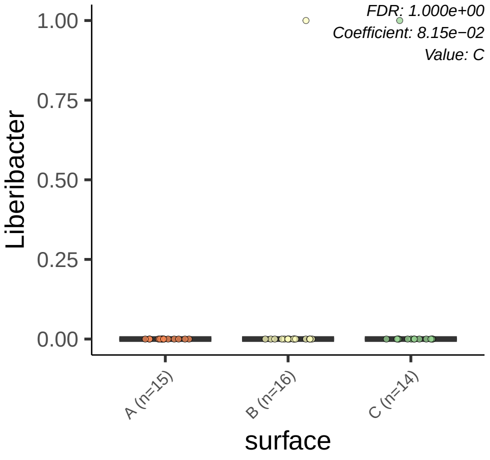
FDR: 1.000e+00
Coefficient: 8.15e−02
Value: C
Liberibacter
1.00
0.75
0.50
0.25
0.00
A (n=15)
B (n=16)
C (n=14)
surface

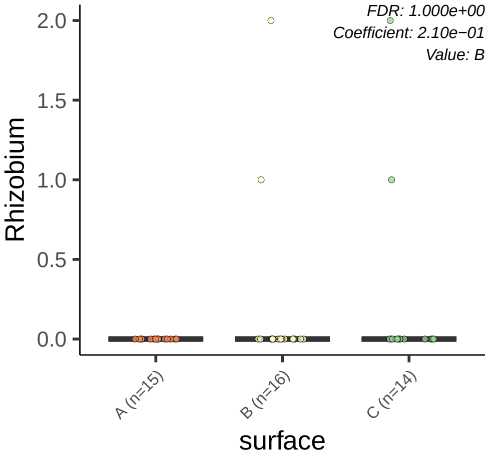

FDR: 1.000e+00
Coefficient: 2.10e−01
Value: B
Rhizobium
2.0
1.5
1.0
0.5
0.0
A (n=15)
B (n=16)
C (n=14)
surface

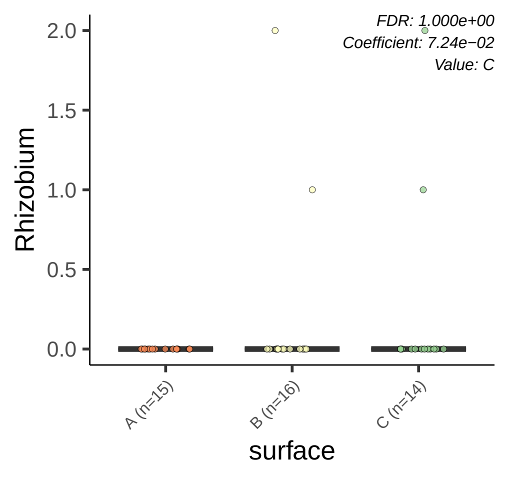

FDR: 1.000e+00
Coefficient: 7.24e−02
Value: C
Rhizobium
2.0
1.5
1.0
0.5
0.0
A (n=15)
B (n=16)
C (n=14)
surface

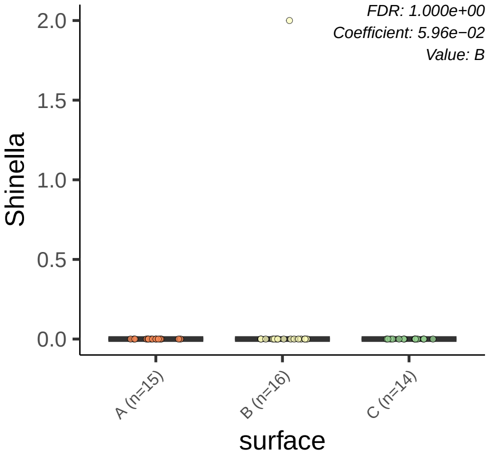

FDR: 1.000e+00
Coefficient: 5.96e−02
Value: B
Shinella
2.0
1.5
1.0
0.5
0.0
A (n=15)
B (n=16)
C (n=14)
surface

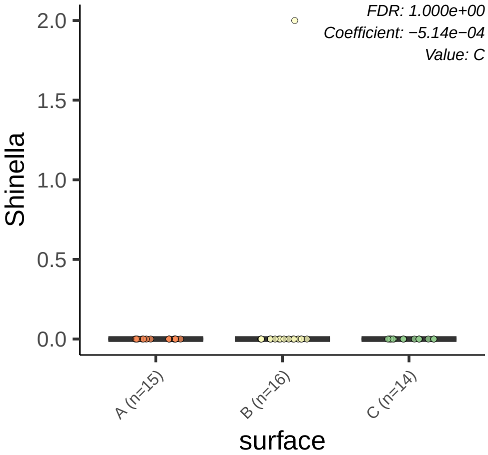

FDR: 1.000e+00
Coefficient: −5.14e−04
Value: C
Shinella
2.0
1.5
1.0
0.5
0.0
A (n=15)
B (n=16)
C (n=14)
surface

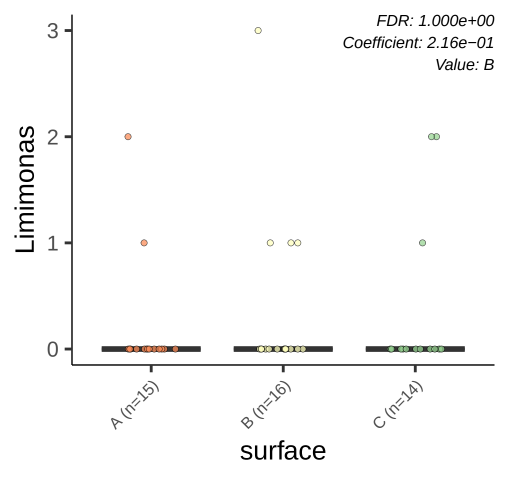
FDR: 1.000e+00
Coefficient: 2.16e−01
Value: B
Limimonas
3
2
1
0
A (n=15)
B (n=16)
C (n=14)
surface

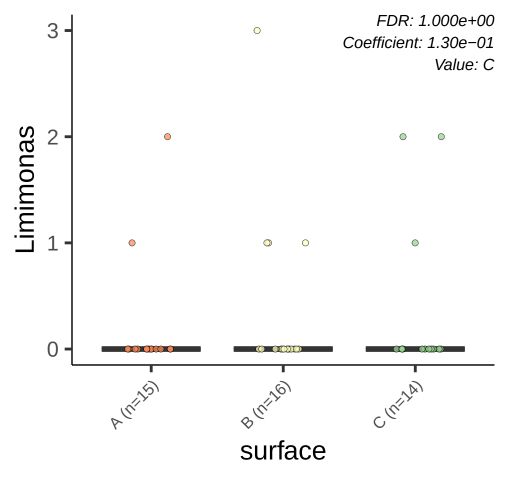
FDR: 1.000e+00
Coefficient: 1.30e−01
Value: C
Limimonas
3
2
1
0
A (n=15)
B (n=16)
C (n=14)
surface

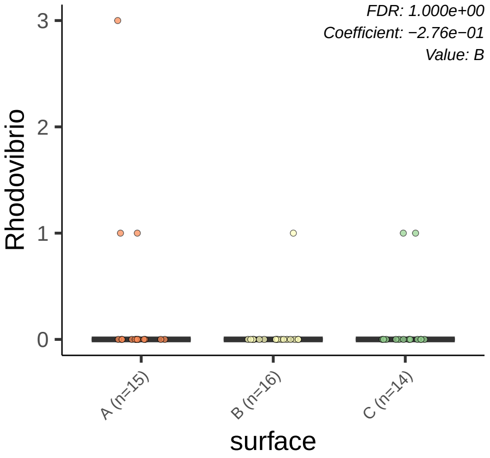
FDR: 1.000e+00
Coefficient: −2.76e−01
Value: B
Rhodovibrio
3
2
1
0
A (n=15)
B (n=16)
C (n=14)
surface

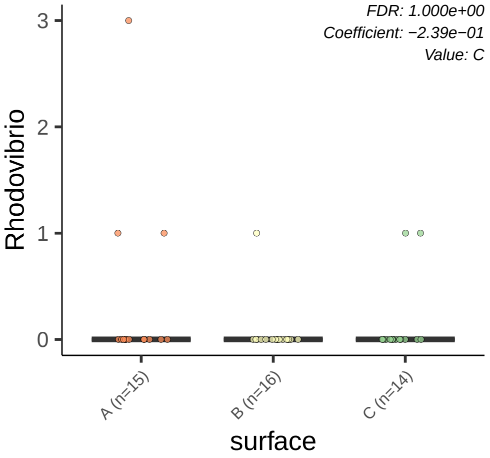
FDR: 1.000e+00
Coefficient: −2.39e−01
Value: C
Rhodovibrio
3
2
1
0
A (n=15)
B (n=16)
C (n=14)
surface

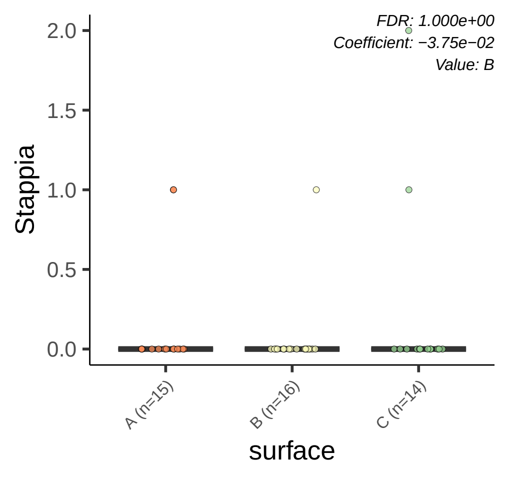

FDR: 1.000e+00
Coefficient: −3.75e−02
Value: B
Stappia
2.0
1.5
1.0
0.5
0.0
A (n=15)
B (n=16)
C (n=14)
surface

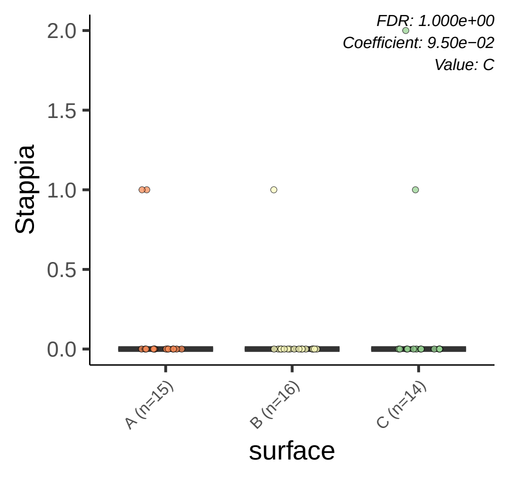

FDR: 1.000e+00
Coefficient: 9.50e−02
Value: C
Stappia
2.0
1.5
1.0
0.5
0.0
A (n=15)
B (n=16)
C (n=14)
surface

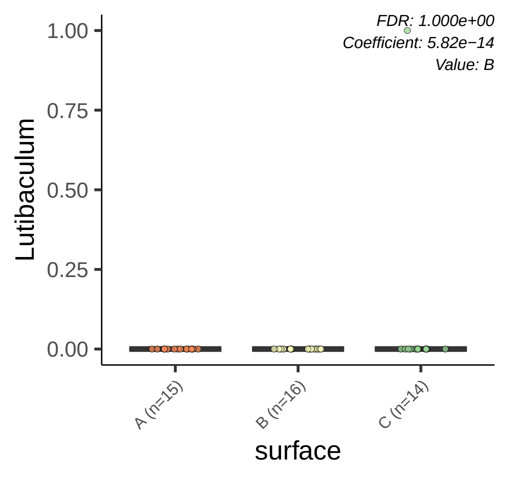
FDR: 1.000e+00
Coefficient: 5.82e−14
Value: B
Lutibaculum
1.00
0.75
0.50
0.25
0.00
A (n=15)
B (n=16)
C (n=14)
surface

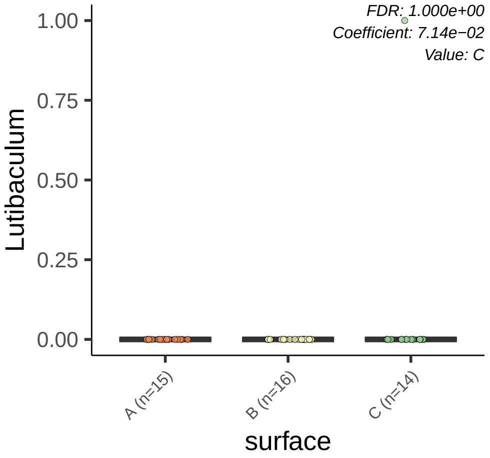
FDR: 1.000e+00
Coefficient: 7.14e−02
Value: C
Lutibaculum
1.00
0.75
0.50
0.25
0.00
A (n=15)
B (n=16)
C (n=14)
surface

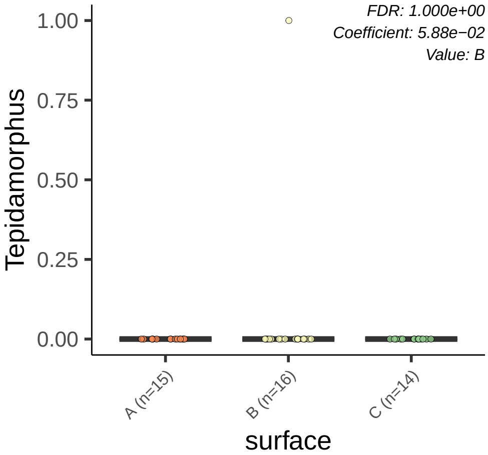
FDR: 1.000e+00
Coefficient: 5.88e−02
Value: B
Tepidamorphus
1.00
0.75
0.50
0.25
0.00
A (n=15)
B (n=16)
C (n=14)
surface

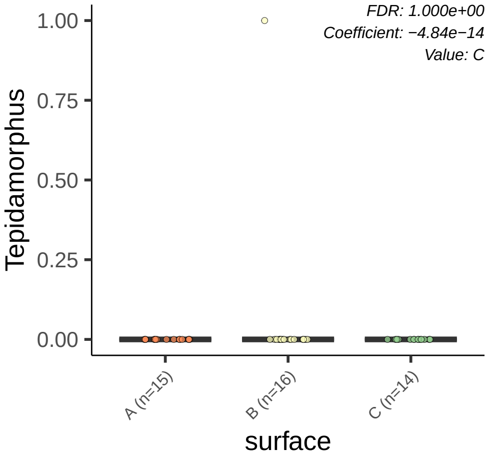

FDR: 1.000e+00
Coefficient: −4.84e−14
Value: C
Tepidamorphus
1.00
0.75
0.50
0.25
0.00
A (n=15)
B (n=16)
C (n=14)
surface

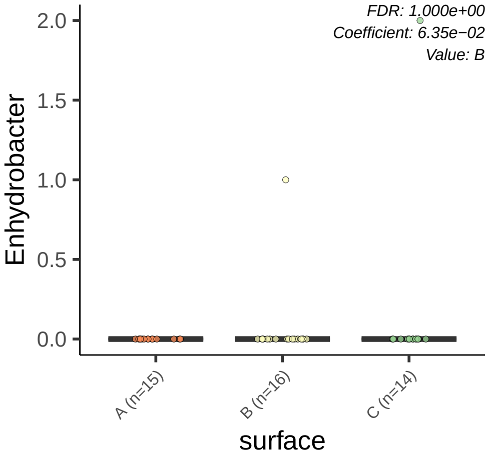

FDR: 1.000e+00
Coefficient: 6.35e−02
Value: B
Enhydrobacter
2.0
1.5
1.0
0.5
0.0
A (n=15)
B (n=16)
C (n=14)
surface

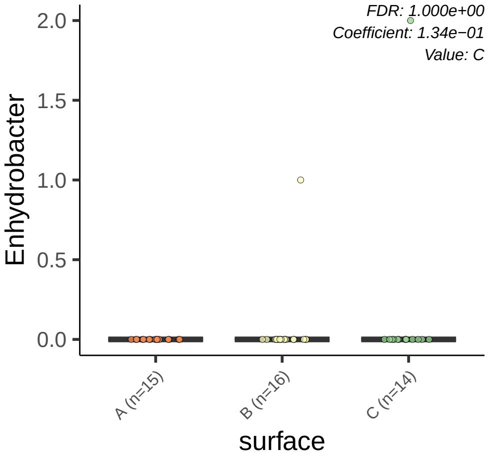
FDR: 1.000e+00
Coefficient: 1.34e−01
Value: C
Enhydrobacter
2.0
1.5
1.0
0.5
0.0
A (n=15)
B (n=16)
C (n=14)
surface

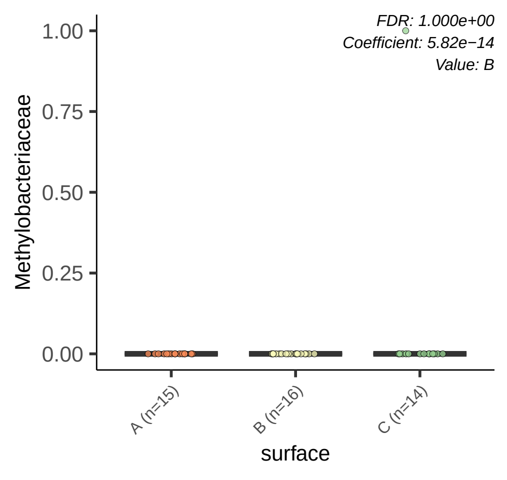
FDR: 1.000e+00
Coefficient: 5.82e−14
Value: B
Methylobacteriaceae
1.00
0.75
0.50
0.25
0.00
A (n=15)
B (n=16)
C (n=14)
surface

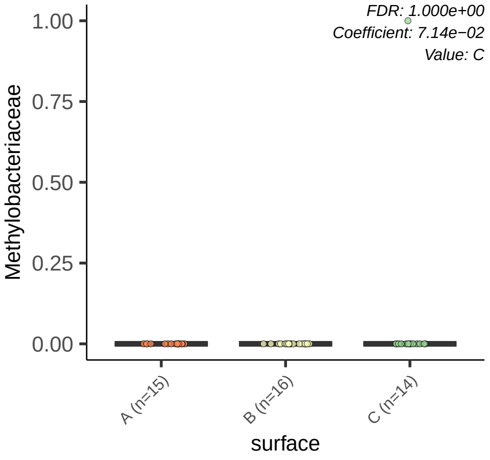
FDR: 1.000e+00
Coefficient: 7.14e−02
Value: C
Methylobacteriaceae
1.00
0.75
0.50
0.25
0.00
A (n=15)
B (n=16)
C (n=14)
surface

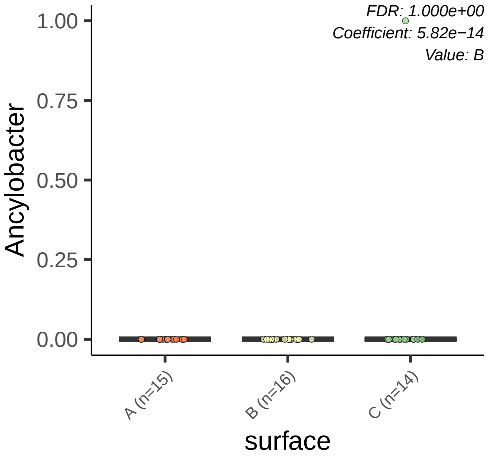
FDR: 1.000e+00
Coefficient: 5.82e−14
Value: B
Ancylobacter
1.00
0.75
0.50
0.25
0.00
A (n=15)
B (n=16)
C (n=14)
surface

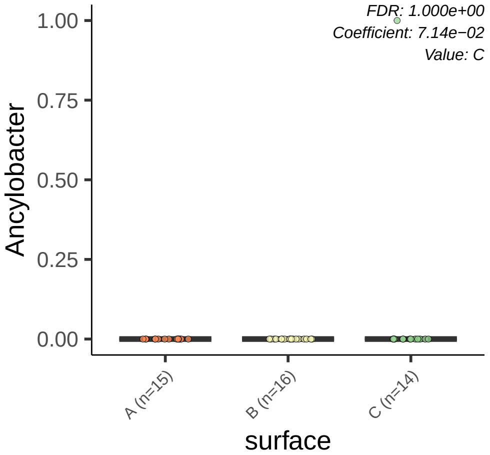
FDR: 1.000e+00
Coefficient: 7.14e−02
Value: C
Ancylobacter
1.00
0.75
0.50
0.25
0.00
A (n=15)
B (n=16)
C (n=14)
surface

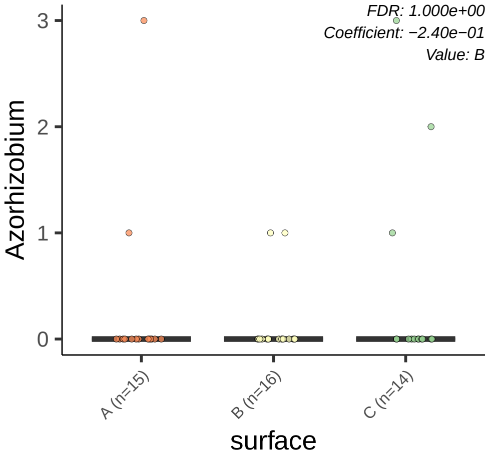

FDR: 1.000e+00
Coefficient: −2.40e−01
Value: B
Azorhizobium
3
2
1
0
A (n=15)
B (n=16)
C (n=14)
surface

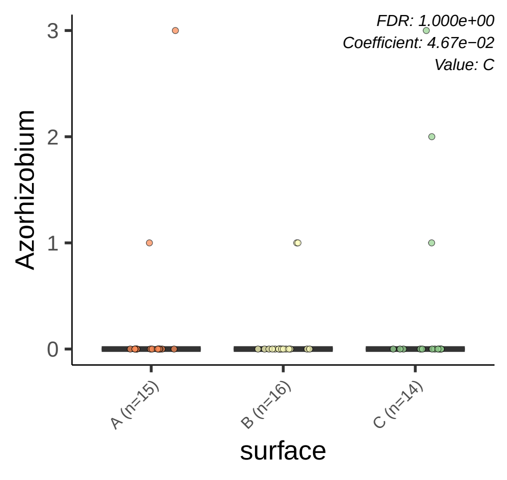
FDR: 1.000e+00
Coefficient: 4.67e−02
Value: C
Azorhizobium
0
1
2
3
A (n=15)
B (n=16)
C (n=14)
surface

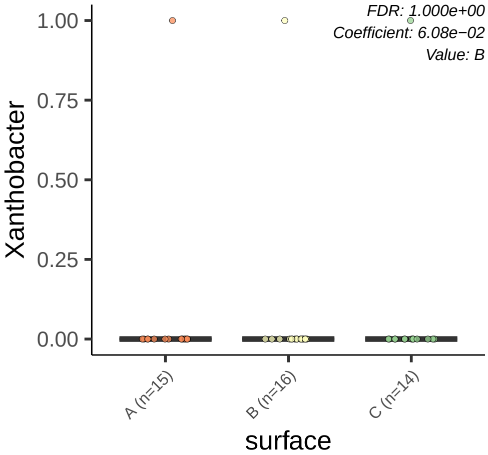

FDR: 1.000e+00
Coefficient: 6.08e−02
Value: B
1.00
0.75
0.50
0.25
0.00
Xanthobacter
A (n=15)
B (n=16)
C (n=14)
surface

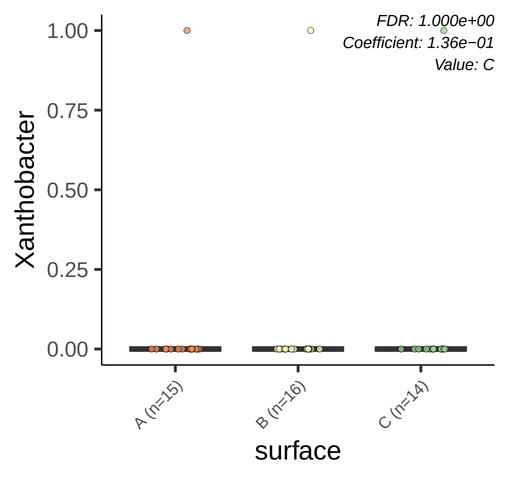

FDR: 1.000e+00
Coefficient: 1.36e−01
Value: C
Xanthobacter
1.00
0.75
0.50
0.25
0.00
A (n=15)
B (n=16)
C (n=14)
surface

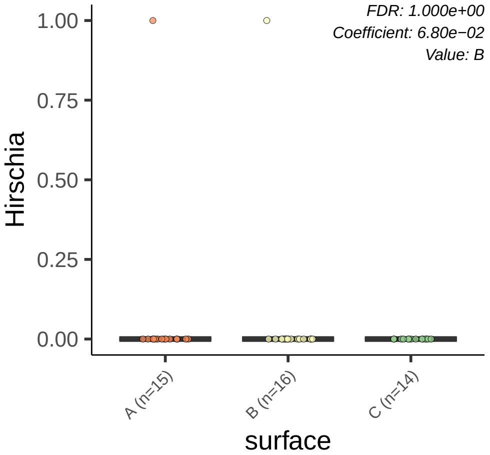
FDR: 1.000e+00
Coefficient: 6.80e−02
Value: B
Hirschia
1.00
0.75
0.50
0.25
0.00
A (n=15)
B (n=16)
C (n=14)
surface

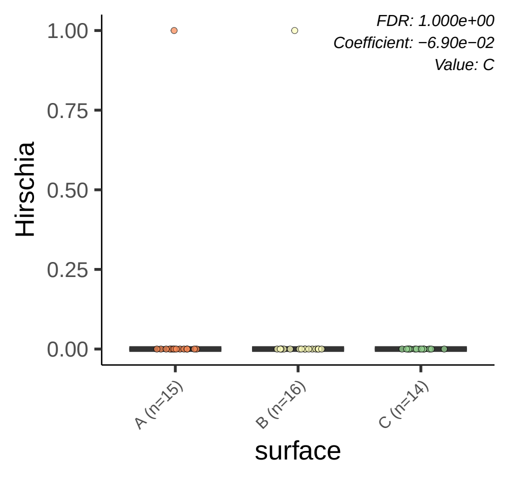
FDR: 1.000e+00
Coefficient: −6.90e−02
Value: C
Hirschia
1.00
0.75
0.50
0.25
0.00
A (n=15)
B (n=16)
C (n=14)
surface

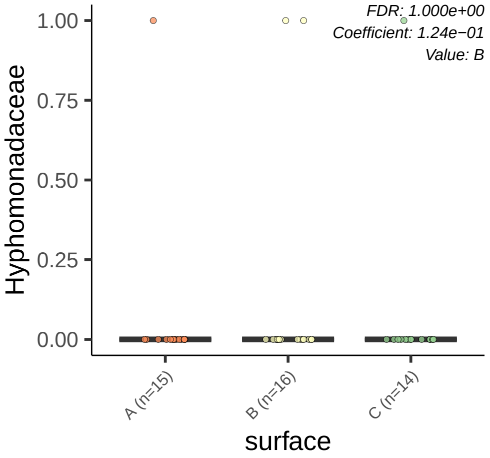
FDR: 1.000e+00
Coefficient: 1.24e−01
Value: B
Hyphomonadaceae
1.00
0.75
0.50
0.25
0.00
A (n=15)
B (n=16)
C (n=14)
surface

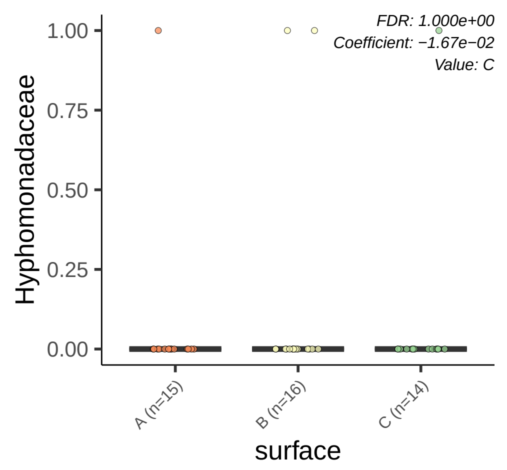

FDR: 1.000e+00
Coefficient: −1.67e−02
Value: C
Hyphomonadaceae
1.00
0.75
0.50
0.25
0.00
A (n=15)
B (n=16)
C (n=14)
surface

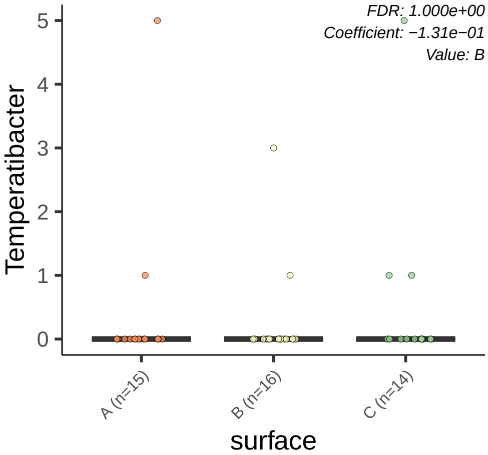

FDR: 1.000e+00
Coefficient: −1.31e−01
Value: B
Temperatibacter
5
4
3
2
1
0
A (n=15)
B (n=16)
C (n=14)
surface

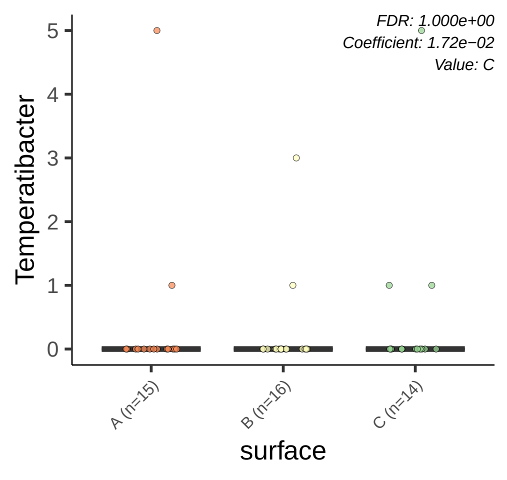
FDR: 1.000e+00
Coefficient: 1.72e−02
Value: C
Temperatibacter
0
1
2
3
4
5
A (n=15)
B (n=16)
C (n=14)
surface

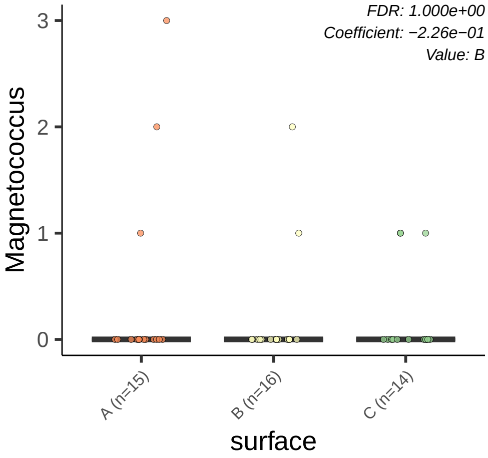

FDR: 1.000e+00
Coefficient: −2.26e−01
Value: B
Magnetococcus
3
2
1
0
A (n=15)
B (n=16)
C (n=14)
surface

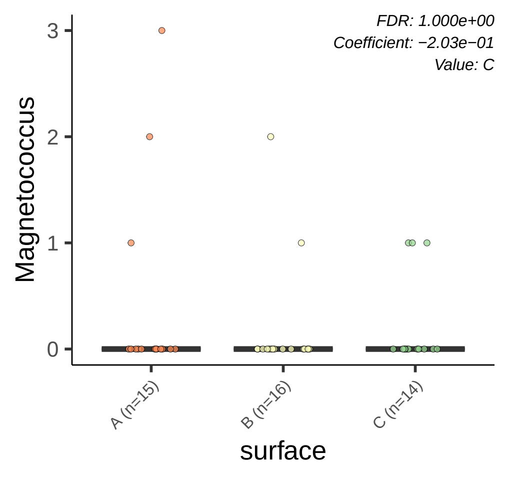
FDR: 1.000e+00
Coefficient: −2.03e−01
Value: C
Magnetococcus
0
1
2
3
A (n=15)
B (n=16)
C (n=14)
surface

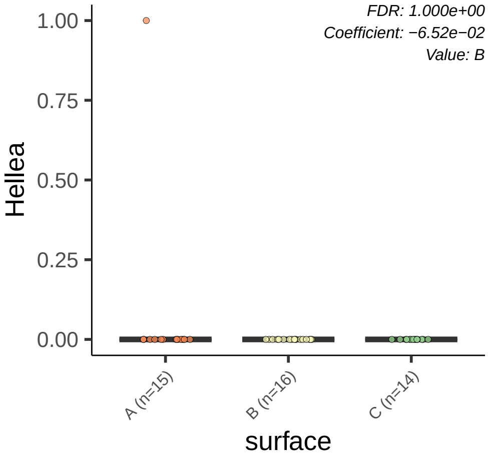
FDR: 1.000e+00
Coefficient: −6.52e−02
Value: B
Hellea
1.00
0.75
0.50
0.25
0.00
A (n=15)
B (n=16)
C (n=14)
surface

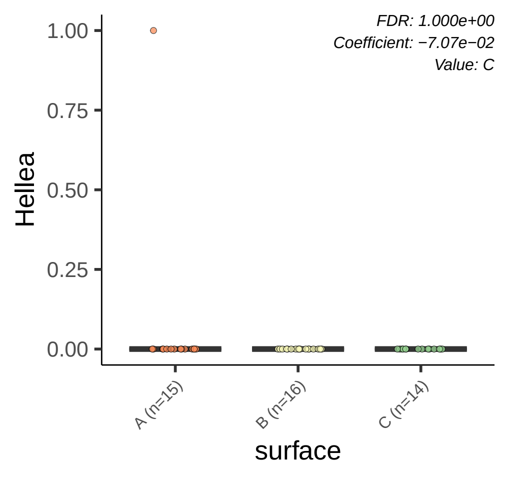

FDR: 1.000e+00
Coefficient: −7.07e−02
Value: C
Hellea
1.00
0.75
0.50
0.25
0.00
A (n=15)
B (n=16)
C (n=14)
surface

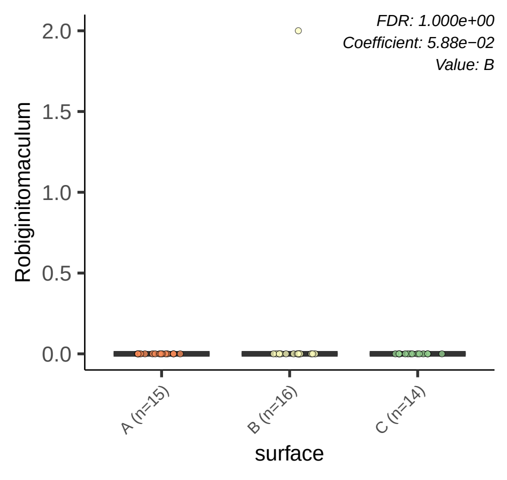
FDR: 1.000e+00
Coefficient: 5.88e−02
Value: B
Robiginitomaculum
2.0
1.5
1.0
0.5
0.0
A (n=15)
B (n=16)
C (n=14)
surface

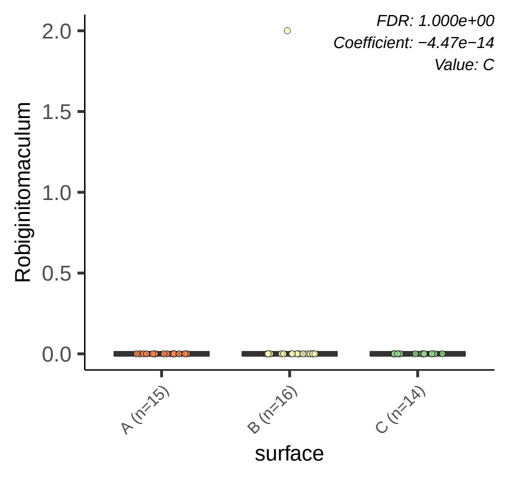
FDR: 1.000e+00
Coefficient: −4.47e−14
Value: C
Robiginitomaculum
2.0
1.5
1.0
0.5
0.0
A (n=15)
B (n=16)
C (n=14)
surface

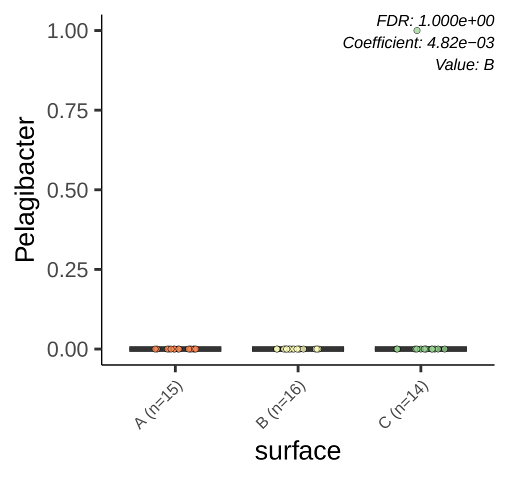
FDR: 1.000e+00
Coefficient: 4.82e−03
Value: B
Pelagibacter
1.00
0.75
0.50
0.25
0.00
A (n=15)
B (n=16)
C (n=14)
surface

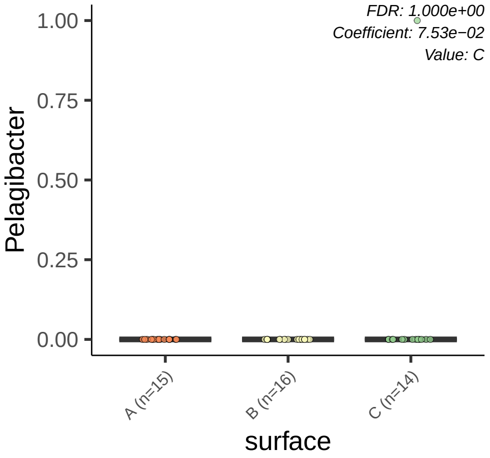

FDR: 1.000e+00
Coefficient: 7.53e−02
Value: C
Pelagibacter
1.00
0.75
0.50
0.25
0.00
A (n=15)
B (n=16)
C (n=14)
surface

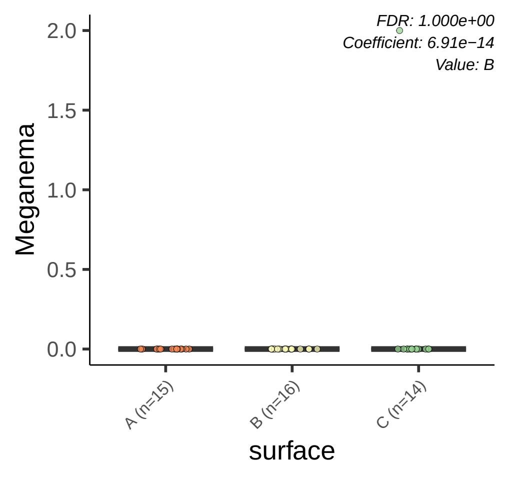
FDR: 1.000e+00
Coefficient: 6.91e−14
Value: B
Meganema
2.0
1.5
1.0
0.5
0.0
A (n=15)
B (n=16)
C (n=14)
surface

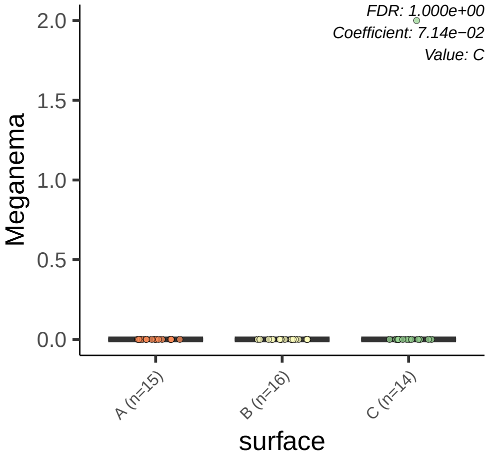
FDR: 1.000e+00
Coefficient: 7.14e−02
Value: C
Meganema
2.0
1.5
1.0
0.5
0.0
A (n=15)
B (n=16)
C (n=14)
surface

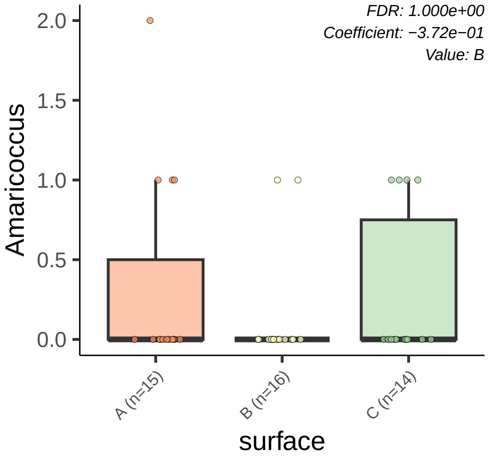
FDR: 1.000e+00
Coefficient: −3.72e−01
Value: B
Amaricoccus
2.0
1.5
1.0
0.5
0.0
A (n=15)
B (n=16)
C (n=14)
surface

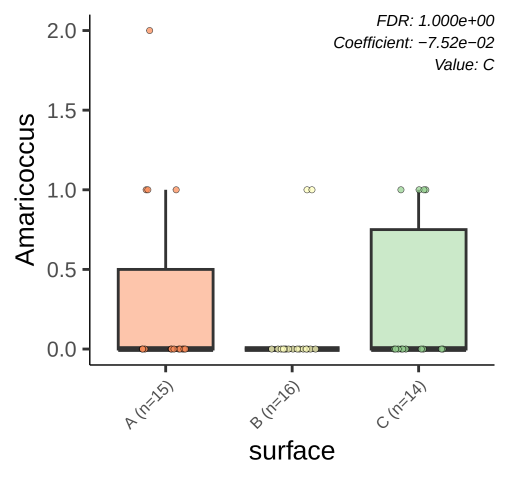
FDR: 1.000e+00
Coefficient: −7.52e−02
Value: C
Amaricoccus
2.0
1.5
1.0
0.5
0.0
A (n=15)
B (n=16)
C (n=14)
surface

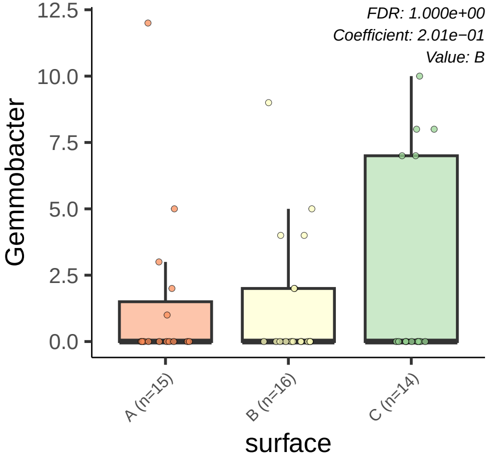
FDR: 1.000e+00
Coefficient: 2.01e−01
Value: B
Gemmobacter
12.5
10.0
7.5
5.0
2.5
0.0
A (n=15)
B (n=16)
C (n=14)
surface

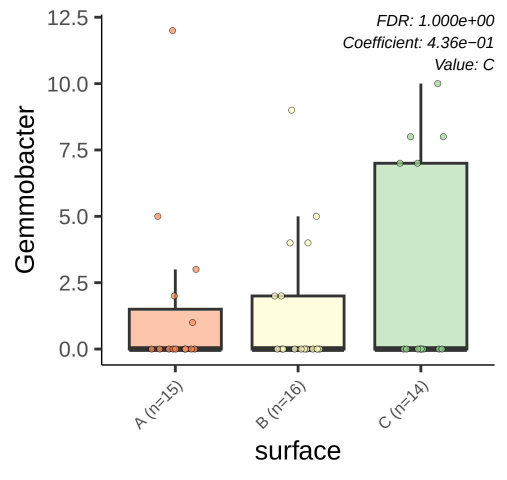
FDR: 1.000e+00
Coefficient: 4.36e−01
Value: C
Gemmobacter
12.5
10.0
7.5
5.0
2.5
0.0
A (n=15)
B (n=16)
C (n=14)
surface

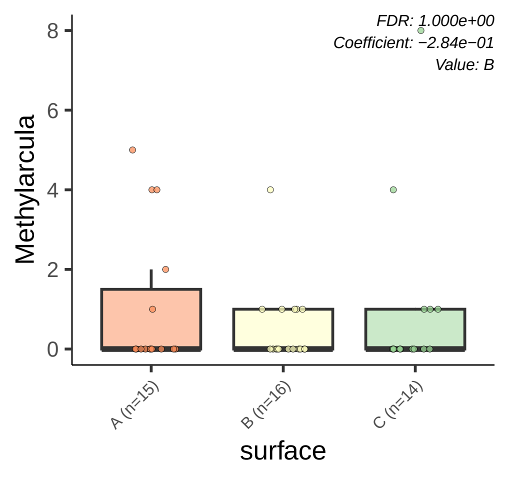

FDR: 1.000e+00
Coefficient: −2.84e−01
Value: B
Methylarcula
8
6
4
2
0
A (n=15)
B (n=16)
C (n=14)
surface

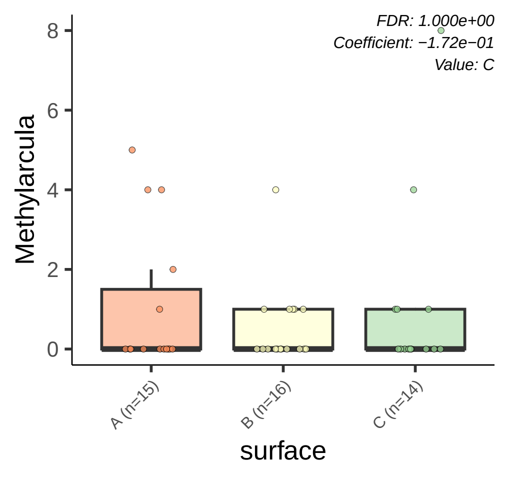

FDR: 1.000e+00
Coefficient: −1.72e−01
Value: C
Methylarcula
8
6
4
2
0
A (n=15)
B (n=16)
C (n=14)
surface

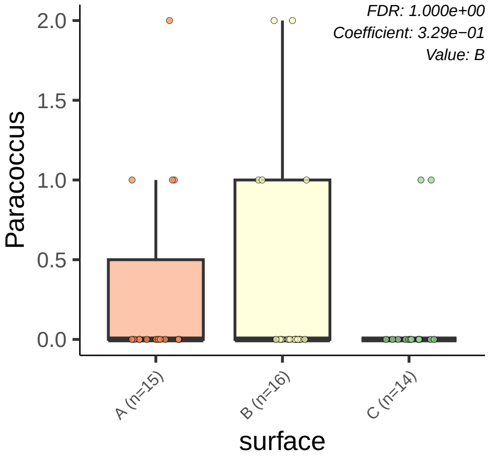
FDR: 1.000e+00
Coefficient: 3.29e−01
Value: B
Paracoccus
2.0
1.5
1.0
0.5
0.0
A (n=15)
B (n=16)
C (n=14)
surface

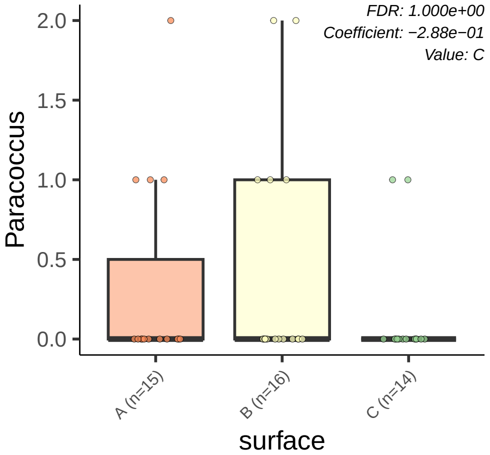

FDR: 1.000e+00
Coefficient: −2.88e−01
Value: C
Paracoccus
2.0
1.5
1.0
0.5
0.0
A (n=15)
B (n=16)
C (n=14)
surface

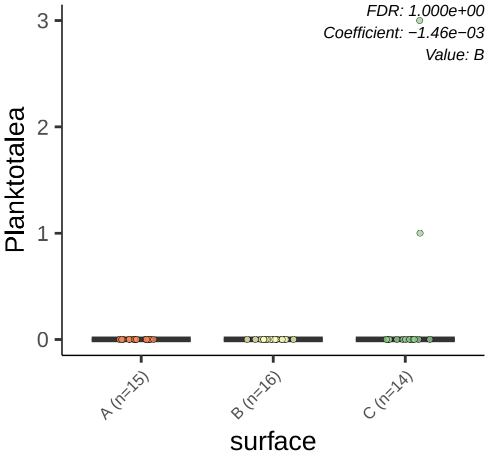

FDR: 1.000e+00
Coefficient: −1.46e−03
Value: B
Planktotalea
3
2
1
0
A (n=15)
B (n=16)
C (n=14)
surface

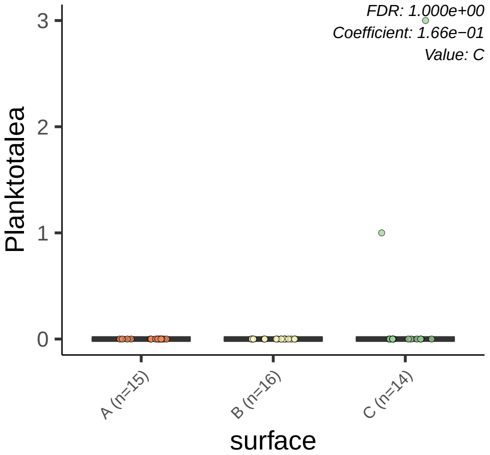
FDR: 1.000e+00
Coefficient: 1.66e−01
Value: C
Planktotalea
3
2
1
0
A (n=15)
B (n=16)
C (n=14)
surface

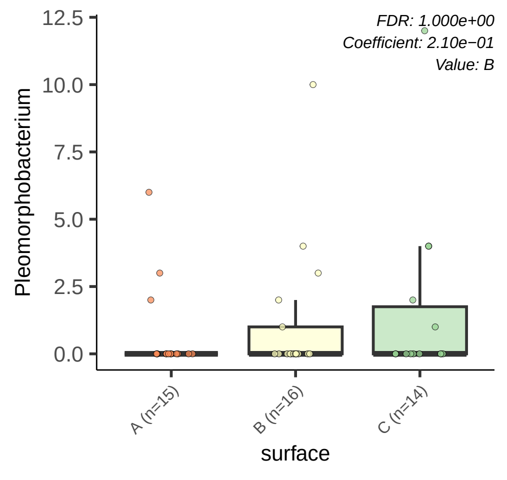

FDR: 1.000e+00
Coefficient: 2.10e−01
Value: B
Pleomorphobacterium
12.5
10.0
7.5
5.0
2.5
0.0
A (n=15)
B (n=16)
C (n=14)
surface

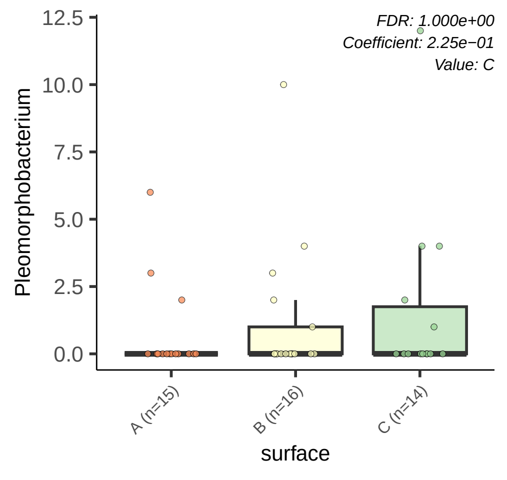
FDR: 1.000e+00
Coefficient: 2.25e−01
Value: C
Pleomorphobacterium
12.5
10.0
7.5
5.0
2.5
0.0
A (n=15)
B (n=16)
C (n=14)
surface

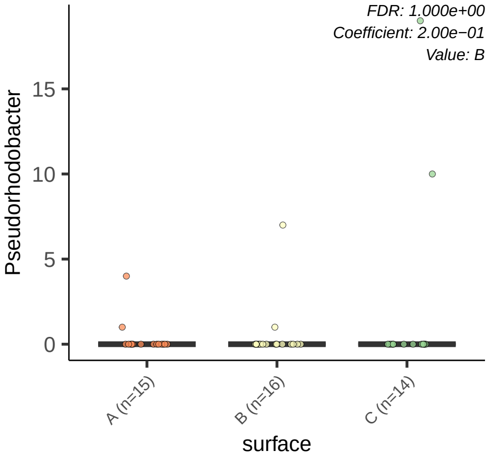

FDR: 1.000e+00
Coefficient: 2.00e−01
Value: B
Pseudorhodobacter
15
10
5
0
A (n=15)
B (n=16)
C (n=14)
surface

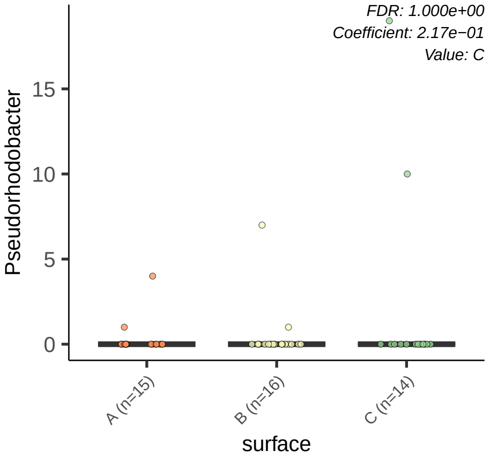
FDR: 1.000e+00
Coefficient: 2.17e−01
Value: C
Pseudorhodobacter
15
10
5
0
A (n=15)
B (n=16)
C (n=14)
surface

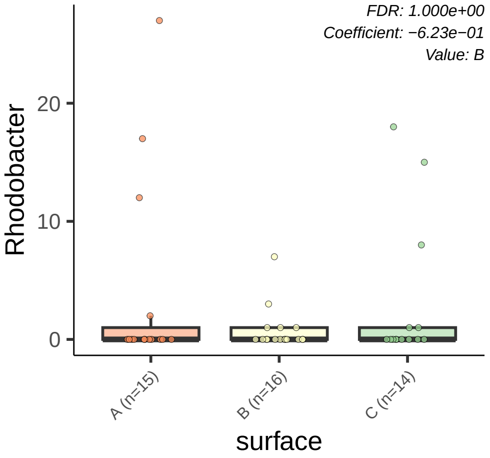
FDR: 1.000e+00
Coefficient: −6.23e−01
Value: B
Rhodobacter
20
10
0
A (n=15)
B (n=16)
C (n=14)
surface

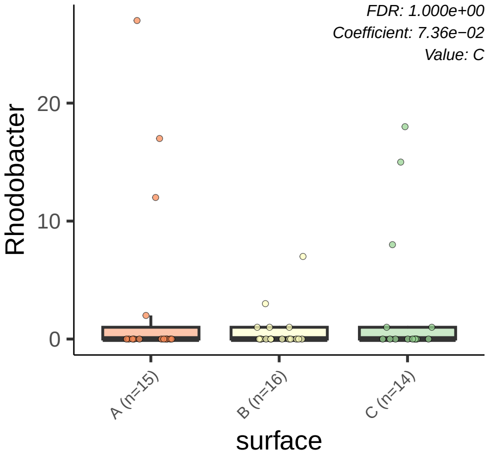
FDR: 1.000e+00
Coefficient: 7.36e−02
Value: C
Rhodobacter
0
10
20
A (n=15)
B (n=16)
C (n=14)
surface

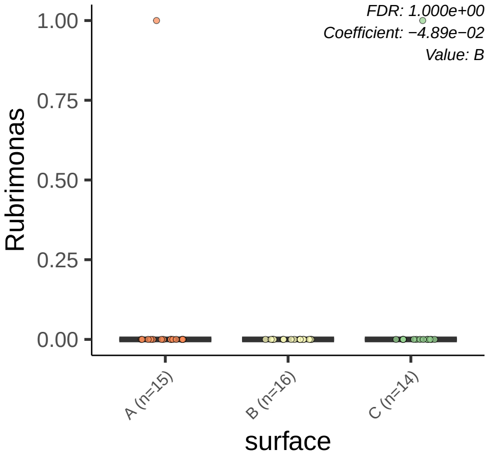
FDR: 1.000e+00
Coefficient: −4.89e−02
Value: B
Rubrimonas
1.00
0.75
0.50
0.25
0.00
A (n=15)
B (n=16)
C (n=14)
surface

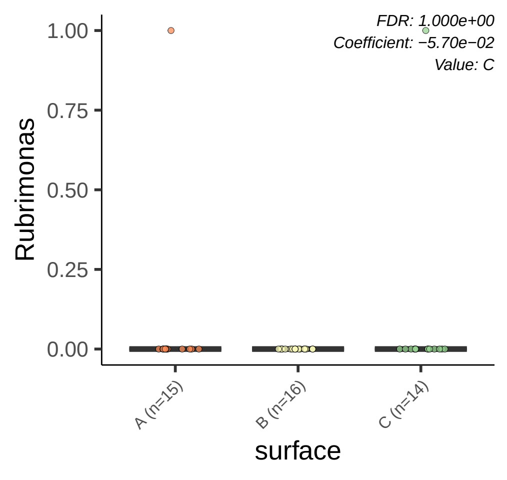

FDR: 1.000e+00
Coefficient: −5.70e−02
Value: C
Rubrimonas
1.00
0.75
0.50
0.25
0.00
A (n=15)
B (n=16)
C (n=14)
surface

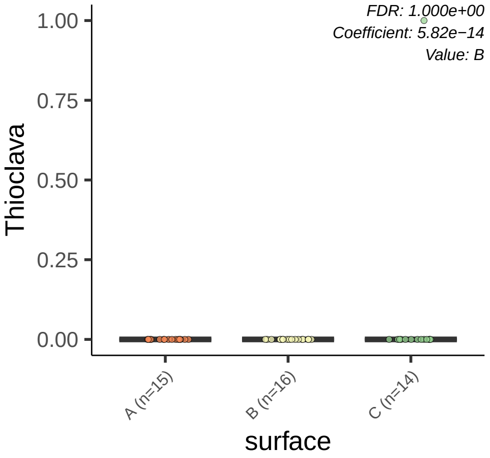
FDR: 1.000e+00
Coefficient: 5.82e−14
Value: B
1.00
0.75
0.50
0.25
0.00
Thioclava
A (n=15)
B (n=16)
C (n=14)
surface

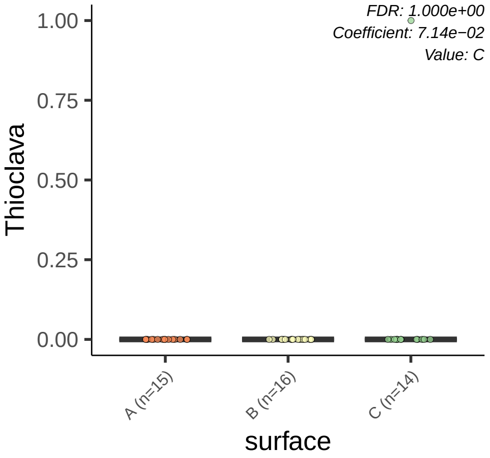
FDR: 1.000e+00
Coefficient: 7.14e−02
Value: C
1.00
0.75
0.50
0.25
0.00
Thioclava
A (n=15)
B (n=16)
C (n=14)
surface

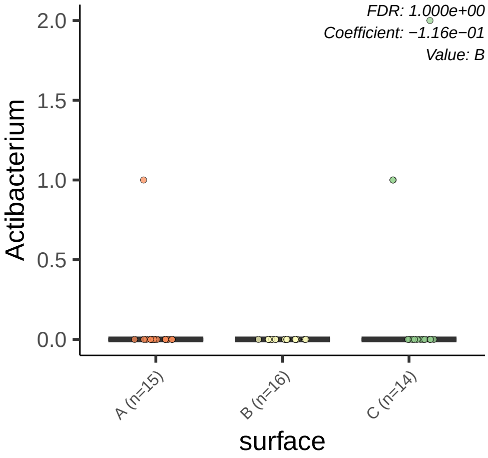

FDR: 1.000e+00
Coefficient: −1.16e−01
Value: B
Actibacterium
2.0
1.5
1.0
0.5
0.0
A (n=15)
B (n=16)
C (n=14)
surface

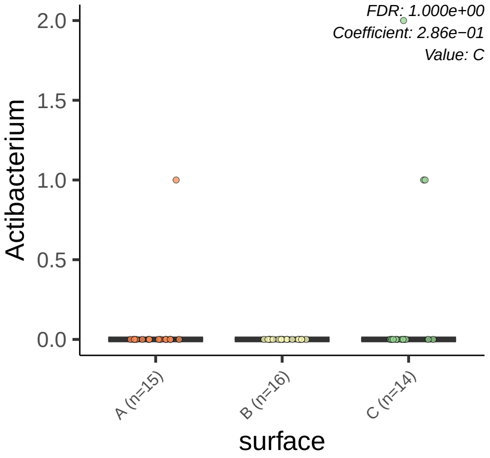
FDR: 1.000e+00
Coefficient: 2.86e−01
Value: C
Actibacterium
2.0
1.5
1.0
0.5
0.0
A (n=15)
B (n=16)
C (n=14)
surface

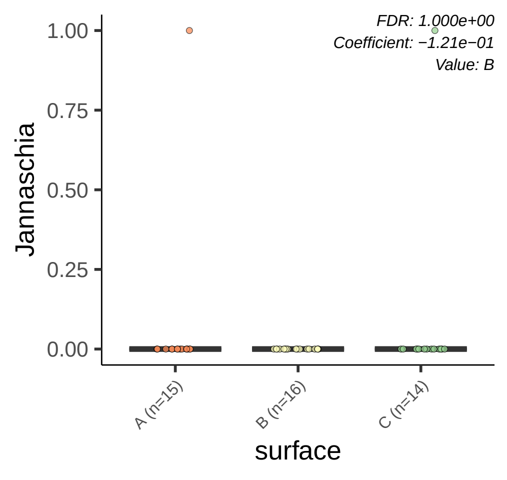
FDR: 1.000e+00
Coefficient: −1.21e−01
Value: B
Jannaschia
1.00
0.75
0.50
0.25
0.00
A (n=15)
B (n=16)
C (n=14)
surface

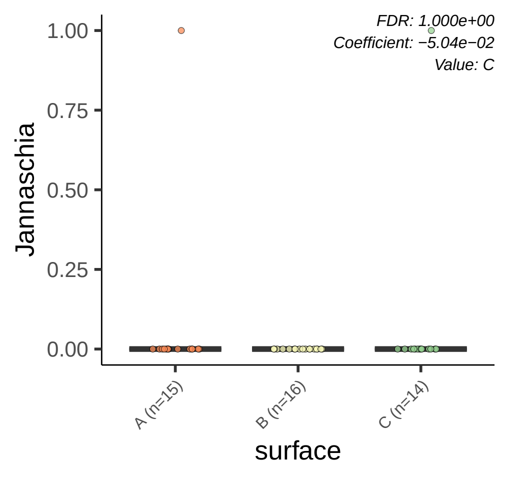
FDR: 1.000e+00
Coefficient: −5.04e−02
Value: C
Jannaschia
1.00
0.75
0.50
0.25
0.00
A (n=15)
B (n=16)
C (n=14)
surface

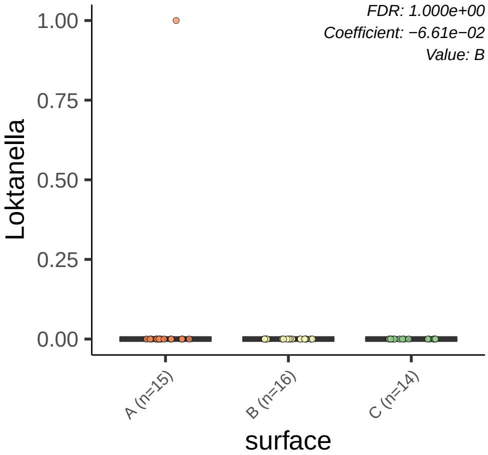
FDR: 1.000e+00
Coefficient: −6.61e−02
Value: B
Loktanella
1.00
0.75
0.50
0.25
0.00
A (n=15)
B (n=16)
C (n=14)
surface

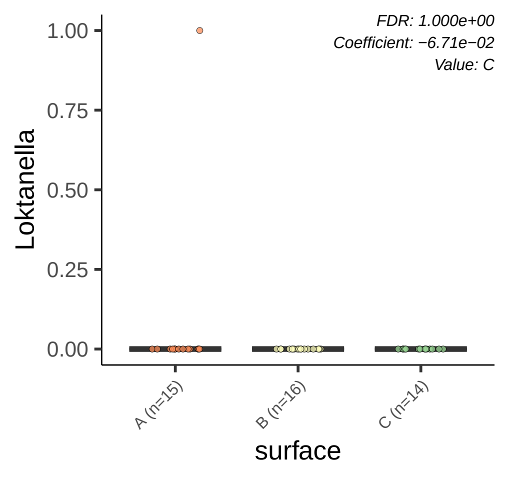

FDR: 1.000e+00
Coefficient: −6.71e−02
Value: C
Loktanella
1.00
0.75
0.50
0.25
0.00
A (n=15)
B (n=16)
C (n=14)
surface

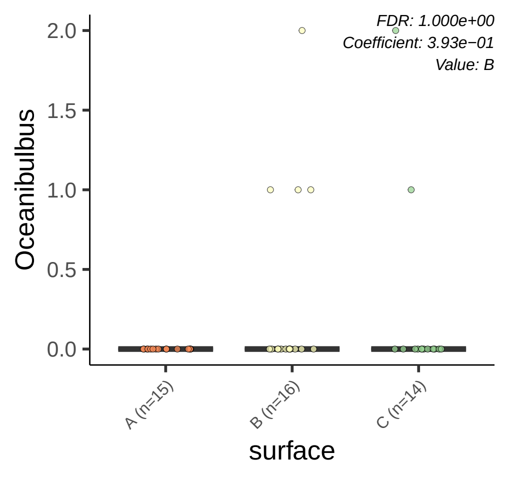

FDR: 1.000e+00
Coefficient: 3.93e−01
Value: B
Oceanibulbus
2.0
1.5
1.0
0.5
0.0
A (n=15)
B (n=16)
C (n=14)
surface

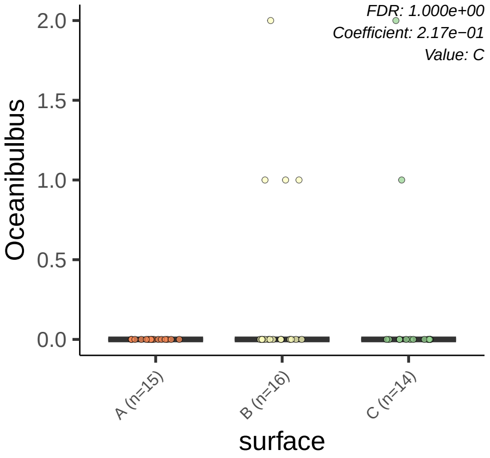
FDR: 1.000e+00
Coefficient: 2.17e−01
Value: C
Oceanibulbus
2.0
1.5
1.0
0.5
0.0
A (n=15)
B (n=16)
C (n=14)
surface

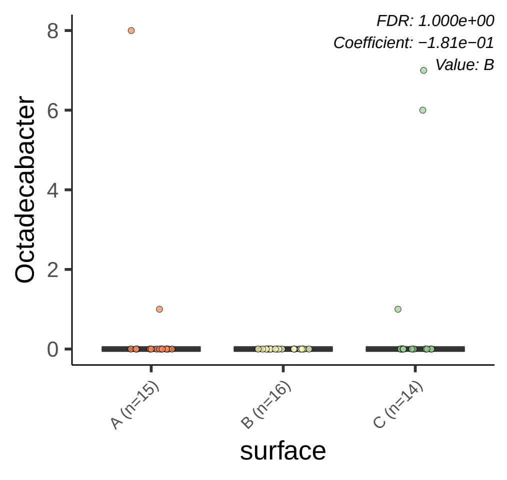
FDR: 1.000e+00
Coefficient: −1.81e−01
Value: B
Octadecabacter
0
2
4
6
8
A (n=15)
B (n=16)
C (n=14)
surface

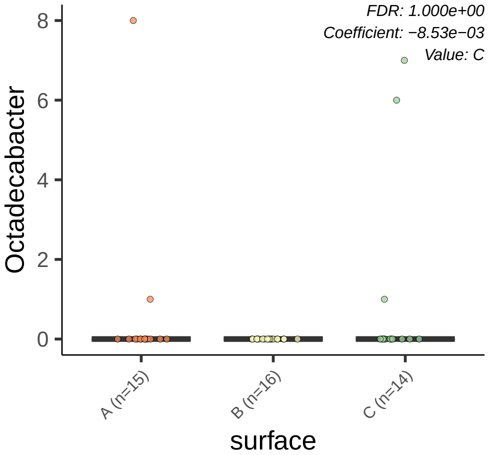
FDR: 1.000e+00
Coefficient: −8.53e−03
Value: C
Octadecabacter
8
6
4
2
0
A (n=15)
B (n=16)
C (n=14)
surface

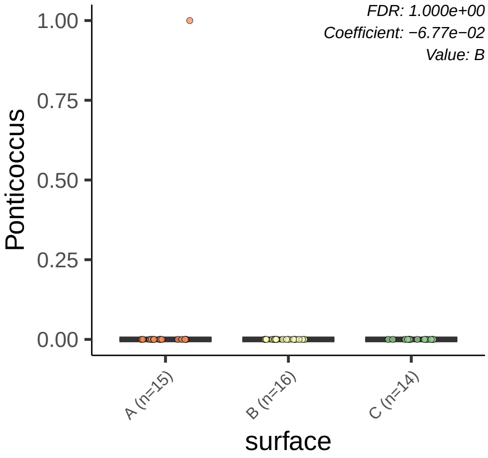
FDR: 1.000e+00
Coefficient: −6.77e−02
Value: B
Ponticoccus
1.00
0.75
0.50
0.25
0.00
A (n=15)
B (n=16)
C (n=14)
surface

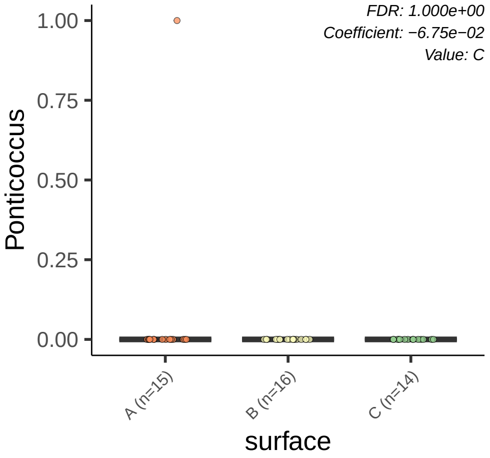

FDR: 1.000e+00
Coefficient: −6.75e−02
Value: C
Ponticoccus
1.00
0.75
0.50
0.25
0.00
A (n=15)
B (n=16)
C (n=14)
surface

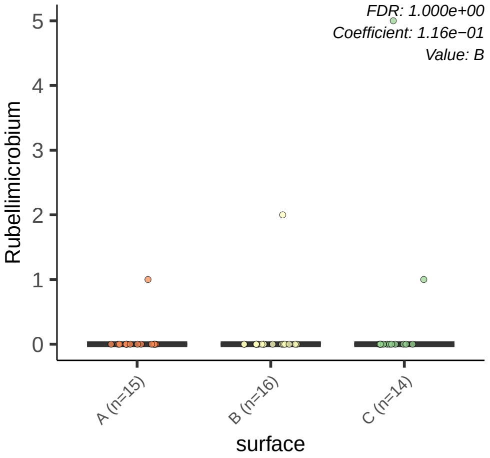

FDR: 1.000e+00
Coefficient: 1.16e−01
Value: B
Rubellimicrobium
0
1
2
3
4
5
A (n=15)
B (n=16)
C (n=14)
surface

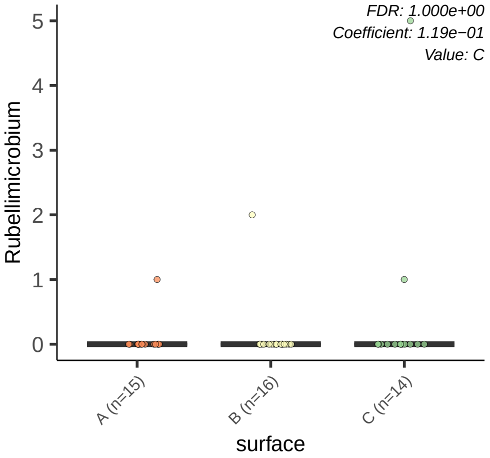

FDR: 1.000e+00
Coefficient: 1.19e−01
Value: C
Rubellimicrobium
0
1
2
3
4
5
A (n=15)
B (n=16)
C (n=14)
surface

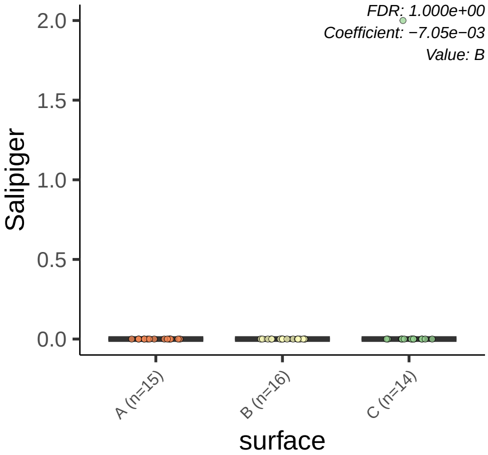
FDR: 1.000e+00
Coefficient: −7.05e−03
Value: B
Salipiger
2.0
1.5
1.0
0.5
0.0
A (n=15)
B (n=16)
C (n=14)
surface

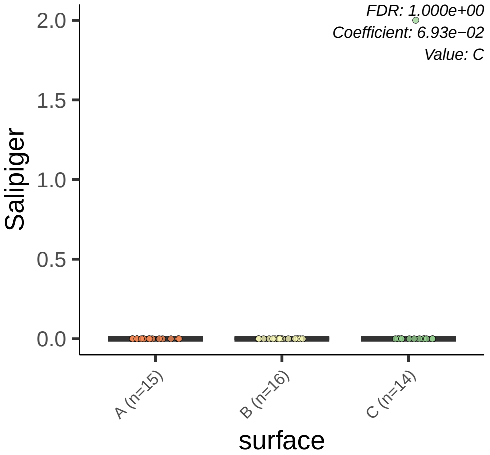
FDR: 1.000e+00
Coefficient: 6.93e−02
Value: C
Salipiger
2.0
1.5
1.0
0.5
0.0
A (n=15)
B (n=16)
C (n=14)
surface

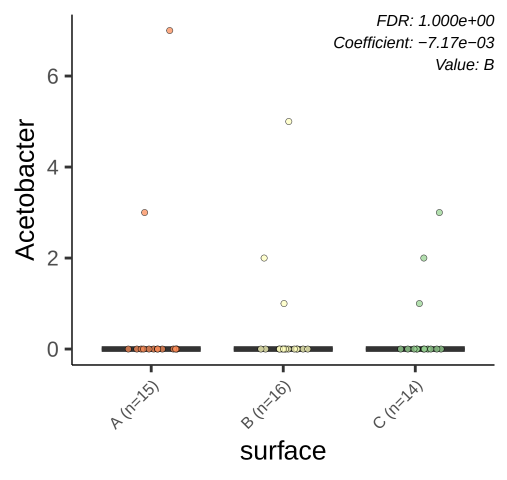

FDR: 1.000e+00
Coefficient: −7.17e−03
Value: B
Acetobacter
6
4
2
0
A (n=15)
B (n=16)
C (n=14)
surface

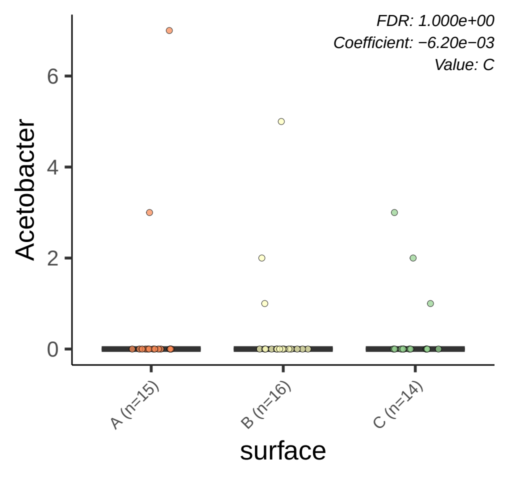
FDR: 1.000e+00
Coefficient: −6.20e−03
Value: C
Acetobacter
0
2
4
6
A (n=15)
B (n=16)
C (n=14)
surface

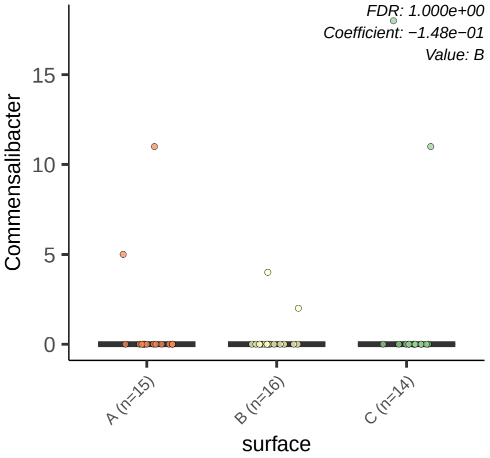
FDR: 1.000e+00
Coefficient: −1.48e−01
Value: B
Commensalibacter
15
10
5
0
A (n=15)
B (n=16)
C (n=14)
surface

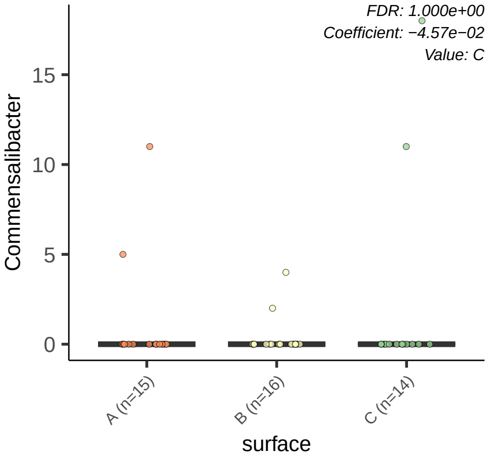
FDR: 1.000e+00
Coefficient: −4.57e−02
Value: C
Commensalibacter
15
10
5
0
A (n=15)
B (n=16)
C (n=14)
surface

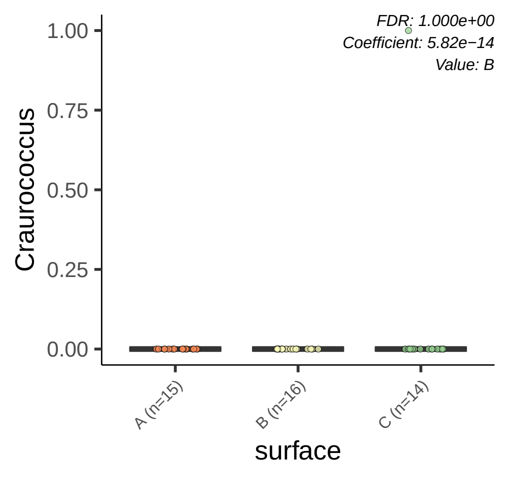

FDR: 1.000e+00
Coefficient: 5.82e−14
Value: B
1.00
0.75
0.50
0.25
0.00
Craurococcus
A (n=15)
B (n=16)
C (n=14)
surface

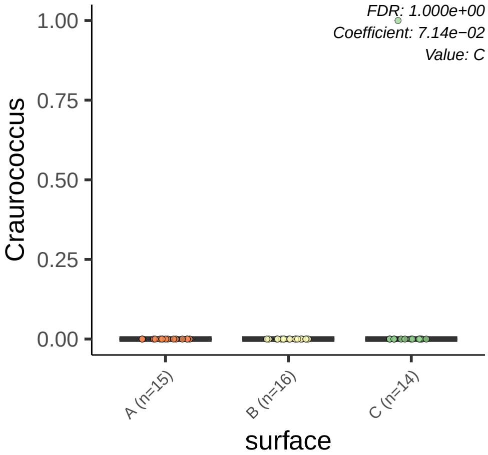
FDR: 1.000e+00
Coefficient: 7.14e−02
Value: C
1.00
0.75
0.50
0.25
0.00
Craurococcus
A (n=15)
B (n=16)
C (n=14)
surface

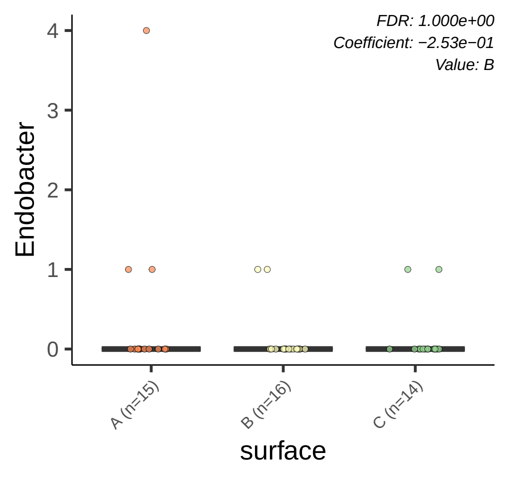
FDR: 1.000e+00
Coefficient: −2.53e−01
Value: B
Endobacter
0
1
2
3
4
A (n=15)
B (n=16)
C (n=14)
surface

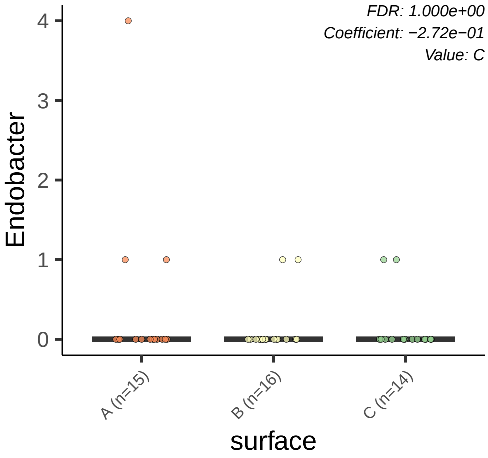

FDR: 1.000e+00
Coefficient: −2.72e−01
Value: C
Endobacter
4
3
2
1
0
A (n=15)
B (n=16)
C (n=14)
surface

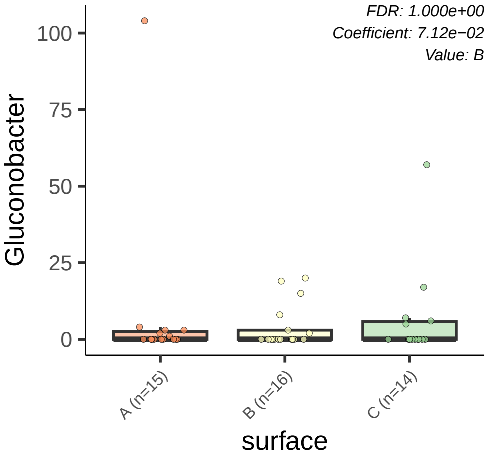
FDR: 1.000e+00
Coefficient: 7.12e−02
Value: B
Gluconobacter
100
75
50
25
0
A (n=15)
B (n=16)
C (n=14)
surface

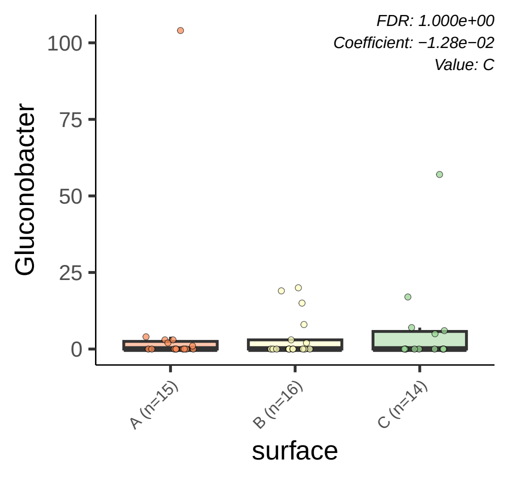

FDR: 1.000e+00
Coefficient: −1.28e−02
Value: C
Gluconobacter
100
75
50
25
0
A (n=15)
B (n=16)
C (n=14)
surface

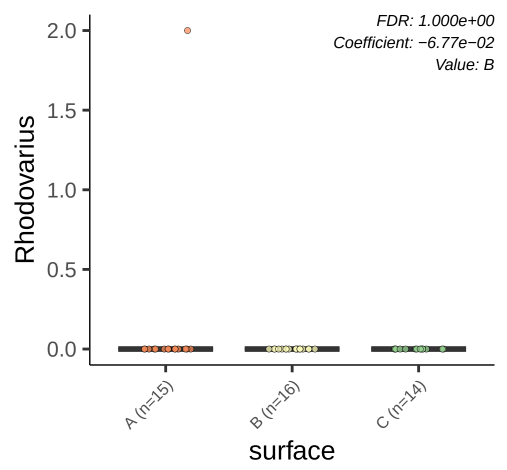

FDR: 1.000e+00
Coefficient: −6.77e−02
Value: B
Rhodovarius
2.0
1.5
1.0
0.5
0.0
A (n=15)
B (n=16)
C (n=14)
surface

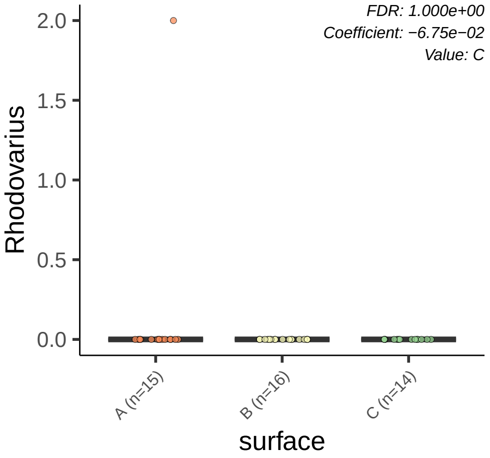
FDR: 1.000e+00
Coefficient: −6.75e−02
Value: C
Rhodovarius
2.0
1.5
1.0
0.5
0.0
A (n=15)
B (n=16)
C (n=14)
surface

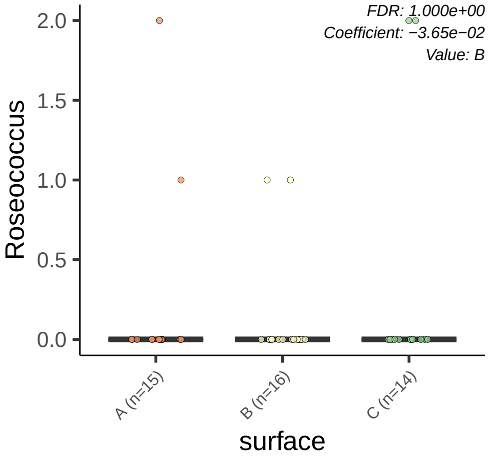
FDR: 1.000e+00
Coefficient: −3.65e−02
Value: B
Roseococcus
2.0
1.5
1.0
0.5
0.0
A (n=15)
B (n=16)
C (n=14)
surface

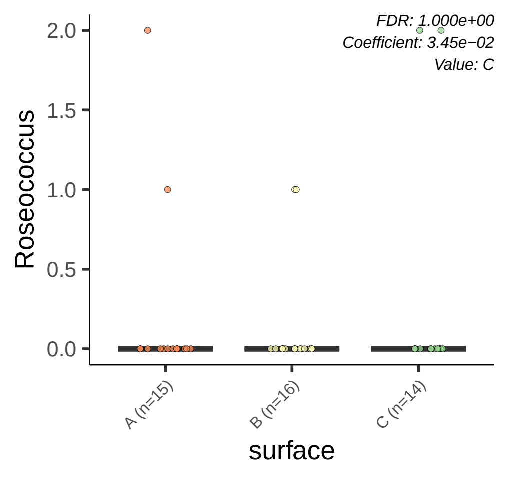

FDR: 1.000e+00
Coefficient: 3.45e−02
Value: C
Roseococcus
2.0
1.5
1.0
0.5
0.0
A (n=15)
B (n=16)
C (n=14)
surface

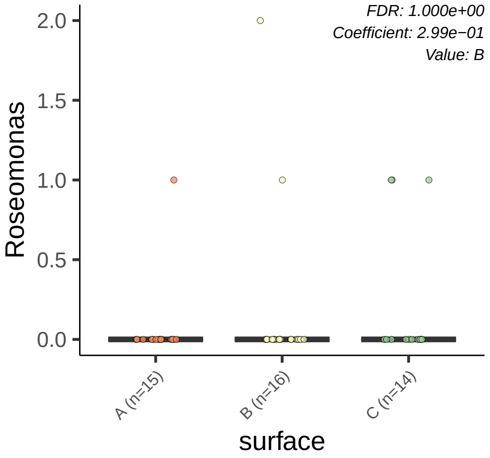

FDR: 1.000e+00
Coefficient: 2.99e−01
Value: B
Roseomonas
2.0
1.5
1.0
0.5
0.0
A (n=15)
B (n=16)
C (n=14)
surface

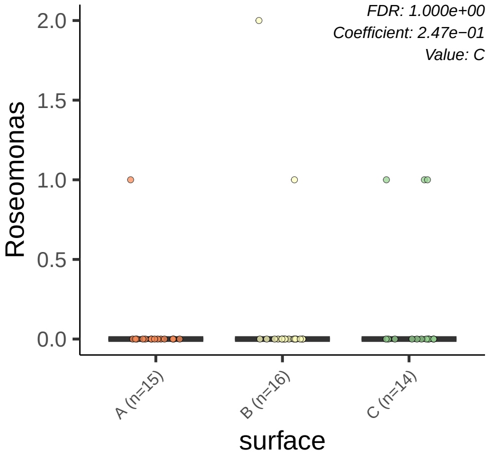

FDR: 1.000e+00
Coefficient: 2.47e−01
Value: C
Roseomonas
2.0
1.5
1.0
0.5
0.0
A (n=15)
B (n=16)
C (n=14)
surface

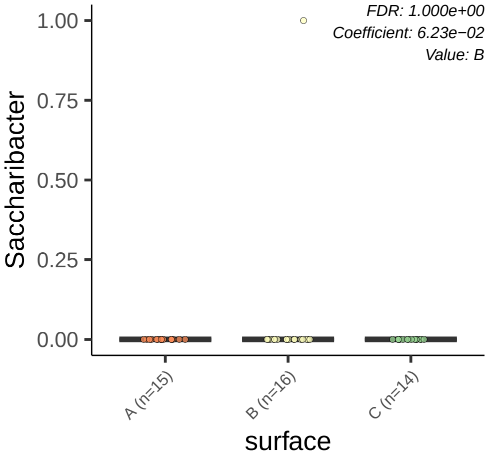

FDR: 1.000e+00
Coefficient: 6.23e−02
Value: B
Saccharibacter
1.00
0.75
0.50
0.25
0.00
A (n=15)
B (n=16)
C (n=14)
surface

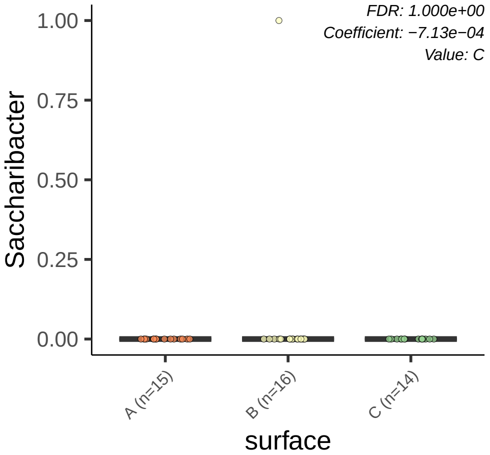
FDR: 1.000e+00
Coefficient: −7.13e−04
Value: C
Saccharibacter
1.00
0.75
0.50
0.25
0.00
A (n=15)
B (n=16)
C (n=14)
surface

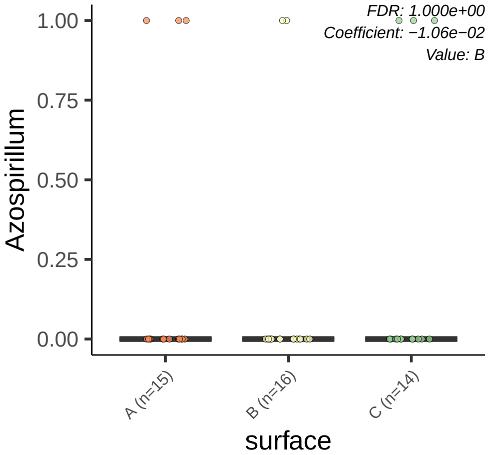

FDR: 1.000e+00
Coefficient: −1.06e−02
Value: B
Azospirillum
1.00
0.75
0.50
0.25
0.00
A (n=15)
B (n=16)
C (n=14)
surface

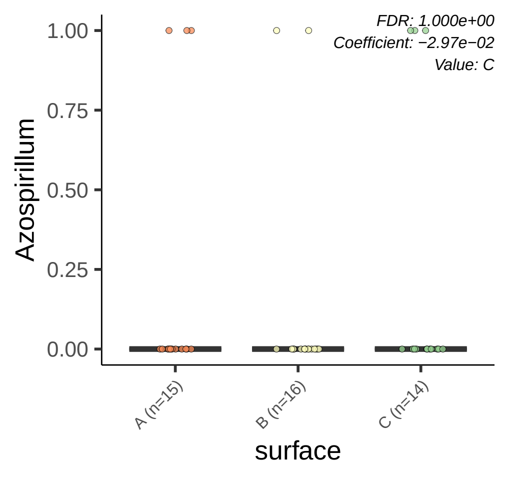
FDR: 1.000e+00
Coefficient: −2.97e−02
Value: C
Azospirillum
1.00
0.75
0.50
0.25
0.00
A (n=15)
B (n=16)
C (n=14)
surface

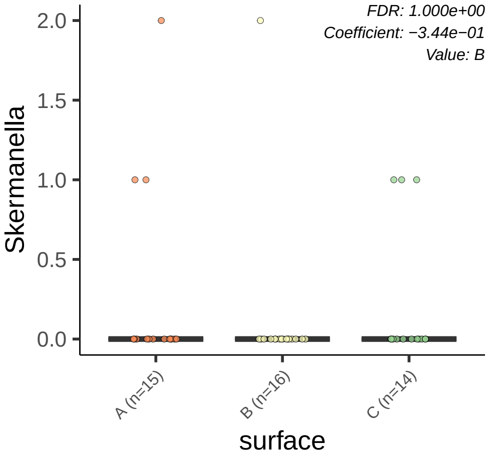
FDR: 1.000e+00
Coefficient: −3.44e−01
Value: B
Skermanella
2.0
1.5
1.0
0.5
0.0
A (n=15)
B (n=16)
C (n=14)
surface

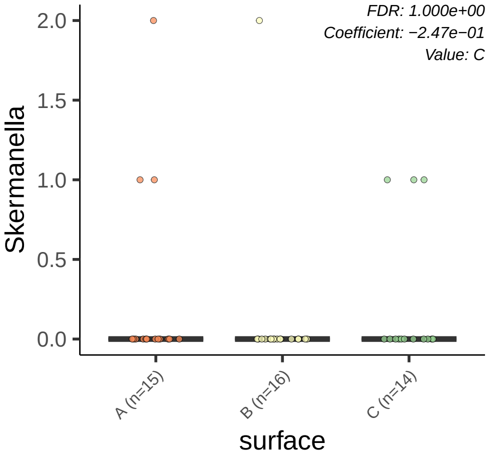

FDR: 1.000e+00
Coefficient: −2.47e−01
Value: C
Skermanella
2.0
1.5
1.0
0.5
0.0
A (n=15)
B (n=16)
C (n=14)
surface

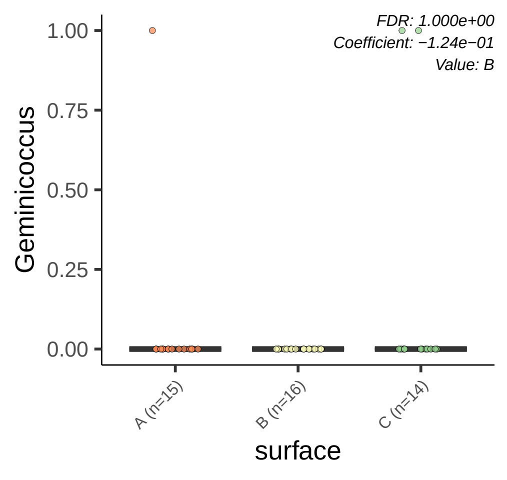

FDR: 1.000e+00
Coefficient: −1.24e−01
Value: B
Geminicoccus
1.00
0.75
0.50
0.25
0.00
A (n=15)
B (n=16)
C (n=14)
surface

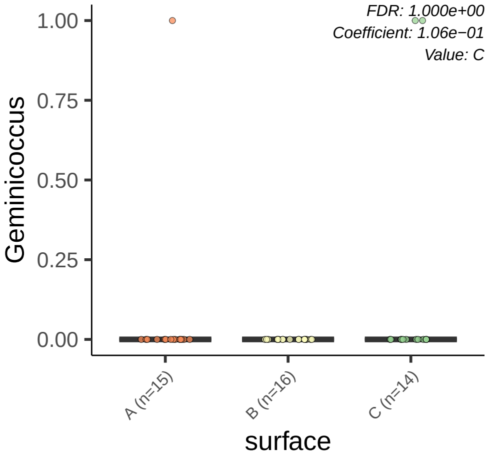

FDR: 1.000e+00
Coefficient: 1.06e−01
Value: C
Geminicoccus
1.00
0.75
0.50
0.25
0.00
A (n=15)
B (n=16)
C (n=14)
surface

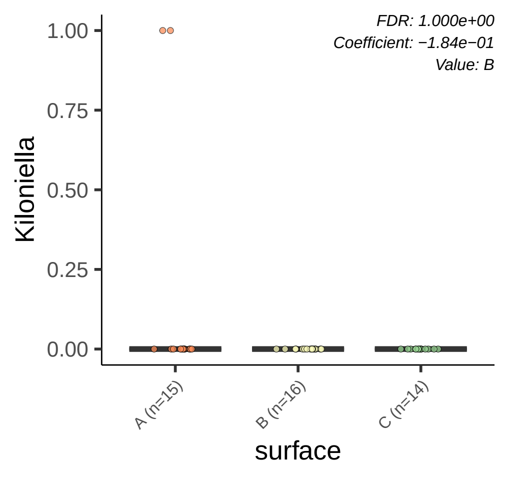
FDR: 1.000e+00
Coefficient: −1.84e−01
Value: B
Kiloniella
1.00
0.75
0.50
0.25
0.00
A (n=15)
B (n=16)
C (n=14)
surface

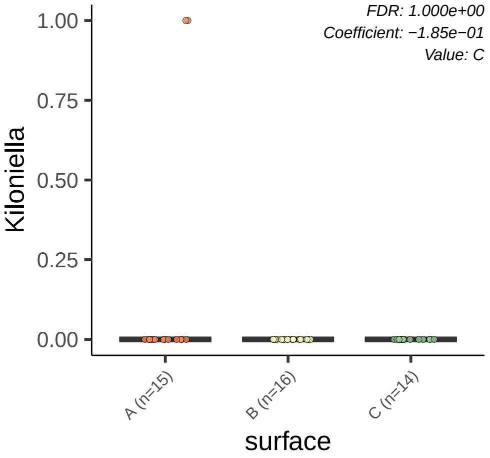
FDR: 1.000e+00
Coefficient: −1.85e−01
Value: C
Kiloniella
1.00
0.75
0.50
0.25
0.00
A (n=15)
B (n=16)
C (n=14)
surface

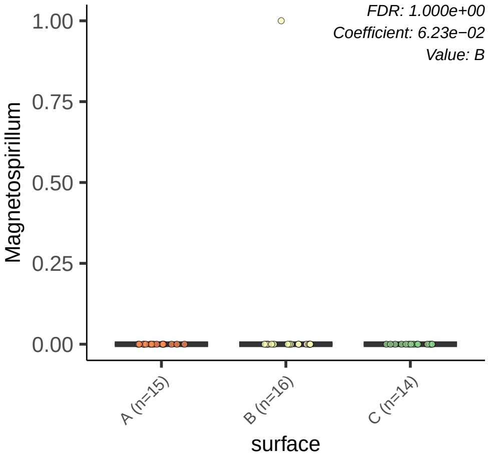

FDR: 1.000e+00
Coefficient: 6.23e−02
Value: B
Magnetospirillum
1.00
0.75
0.50
0.25
0.00
A (n=15)
B (n=16)
C (n=14)
surface

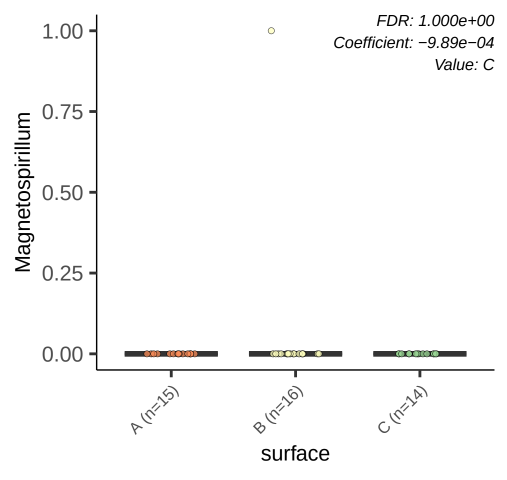
FDR: 1.000e+00
Coefficient: −9.89e−04
Value: C
Magnetospirillum
1.00
0.75
0.50
0.25
0.00
A (n=15)
B (n=16)
C (n=14)
surface

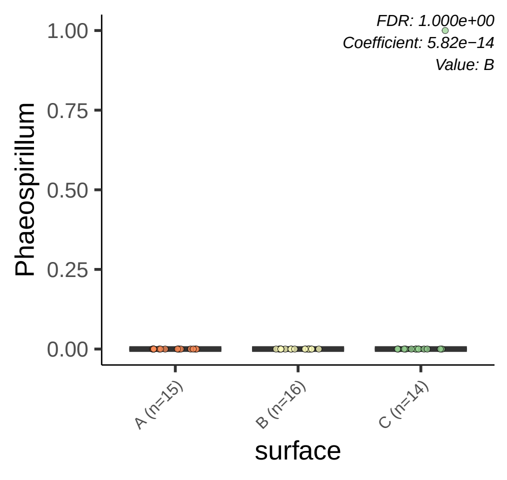
FDR: 1.000e+00
Coefficient: 5.82e−14
Value: B
Phaeospirillum
1.00
0.75
0.50
0.25
0.00
A (n=15)
B (n=16)
C (n=14)
surface

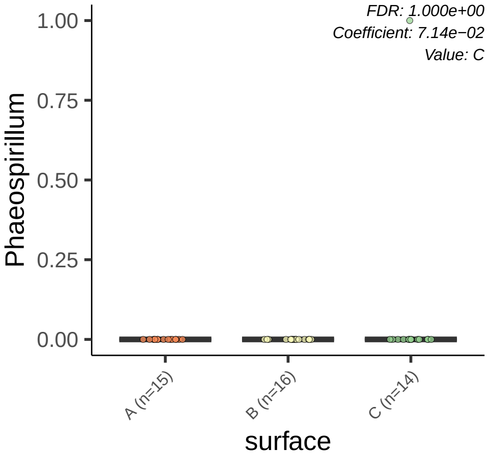
FDR: 1.000e+00
Coefficient: 7.14e−02
Value: C
Phaeospirillum
1.00
0.75
0.50
0.25
0.00
A (n=15)
B (n=16)
C (n=14)
surface

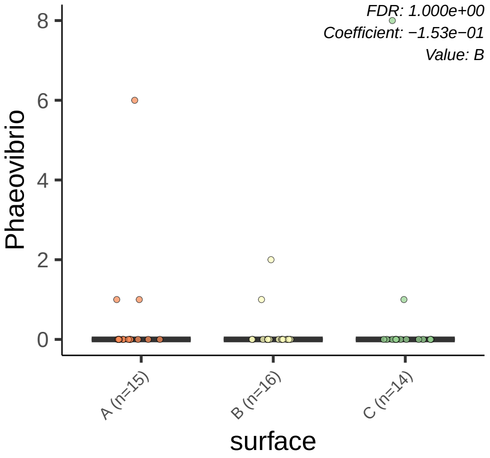

FDR: 1.000e+00
Coefficient: −1.53e−01
Value: B
Phaeovibrio
0
2
4
6
8
A (n=15)
B (n=16)
C (n=14)
surface

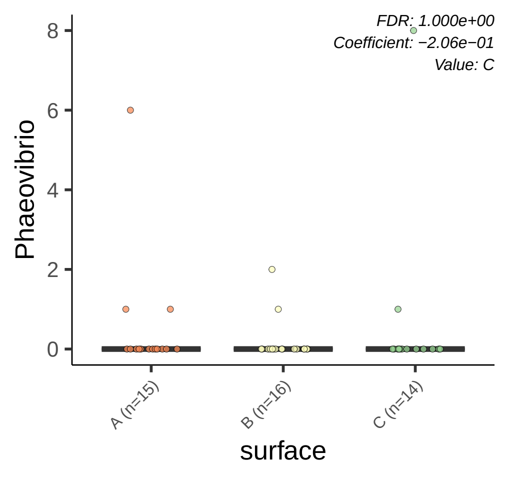
FDR: 1.000e+00
Coefficient: −2.06e−01
Value: C
Phaeovibrio
0
2
4
6
8
A (n=15)
B (n=16)
C (n=14)
surface

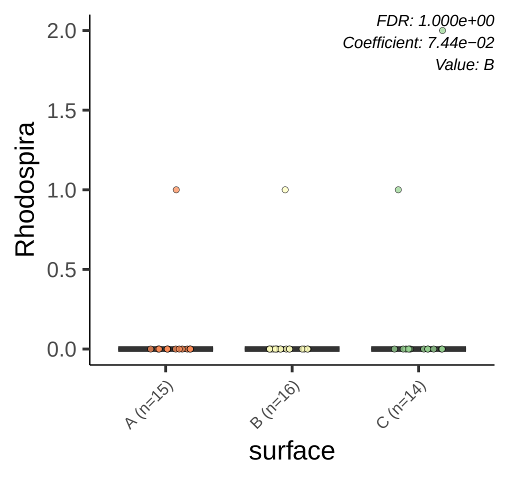

FDR: 1.000e+00
Coefficient: 7.44e−02
Value: B
Rhodospira
2.0
1.5
1.0
0.5
0.0
A (n=15)
B (n=16)
C (n=14)
surface

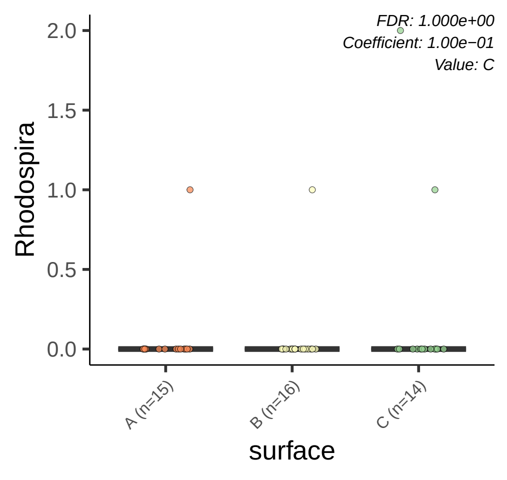
FDR: 1.000e+00
Coefficient: 1.00e−01
Value: C
Rhodospira
2.0
1.5
1.0
0.5
0.0
A (n=15)
B (n=16)
C (n=14)
surface

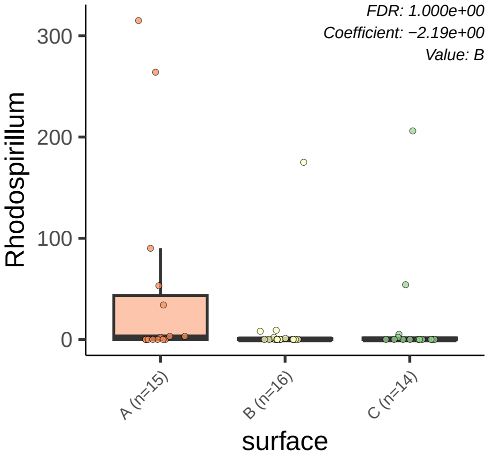

FDR: 1.000e+00
Coefficient: −2.19e+00
Value: B
Rhodospirillum
300
200
100
0
A (n=15)
B (n=16)
C (n=14)
surface

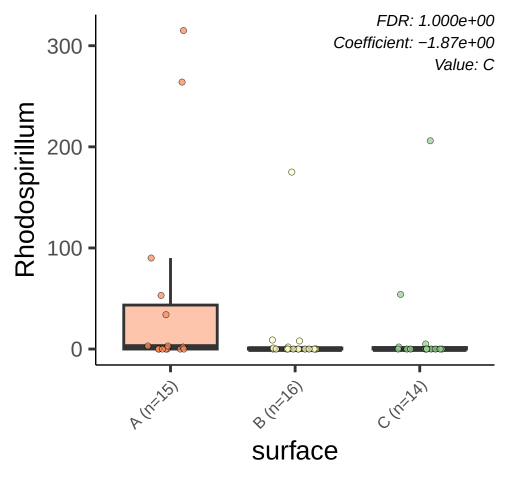
FDR: 1.000e+00
Coefficient: −1.87e+00
Value: C
Rhodospirillum
300
200
100
0
A (n=15)
B (n=16)
C (n=14)
surface

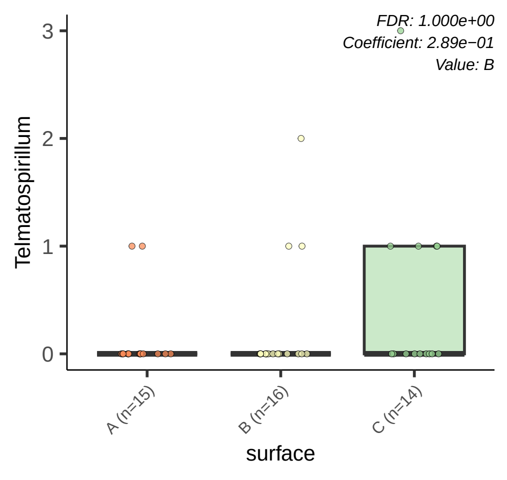
FDR: 1.000e+00
Coefficient: 2.89e−01
Value: B
Telmatospirillum
0
1
2
3
A (n=15)
B (n=16)
C (n=14)
surface

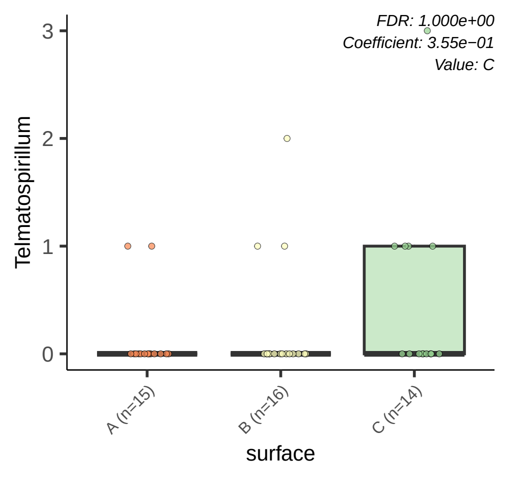
FDR: 1.000e+00
Coefficient: 3.55e−01
Value: C
Telmatospirillum
0
1
2
3
A (n=15)
B (n=16)
C (n=14)
surface

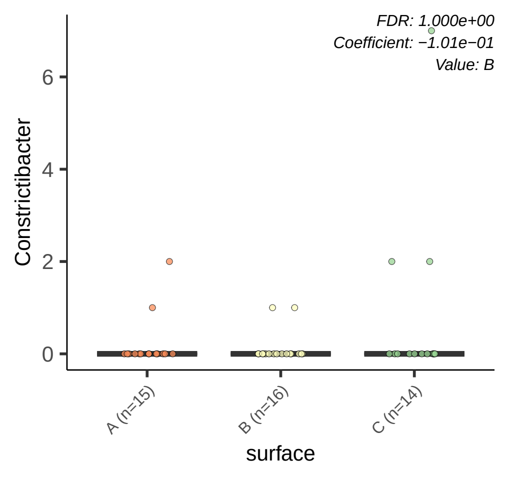
FDR: 1.000e+00
Coefficient: −1.01e−01
Value: B
Constrictibacter
0
2
4
6
A (n=15)
B (n=16)
C (n=14)
surface

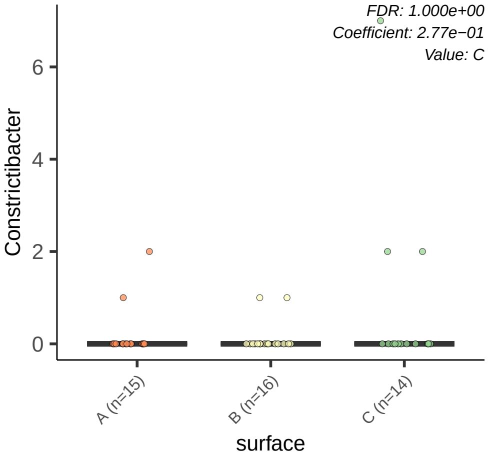
FDR: 1.000e+00
Coefficient: 2.77e−01
Value: C
Constrictibacter
0
2
4
6
A (n=15)
B (n=16)
C (n=14)
surface

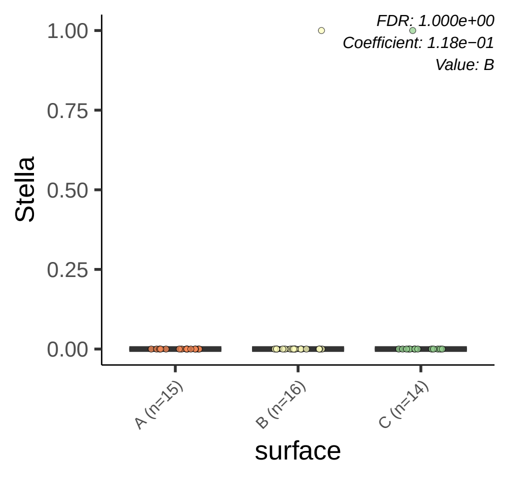
FDR: 1.000e+00
Coefficient: 1.18e−01
Value: B
Stella
1.00
0.75
0.50
0.25
0.00
A (n=15)
B (n=16)
C (n=14)
surface

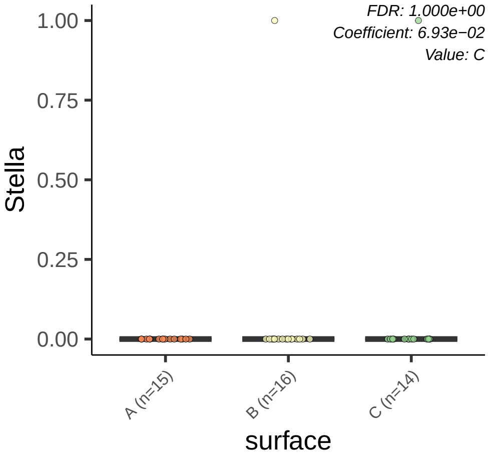
FDR: 1.000e+00
Coefficient: 6.93e−02
Value: C
Stella
1.00
0.75
0.50
0.25
0.00
A (n=15)
B (n=16)
C (n=14)
surface

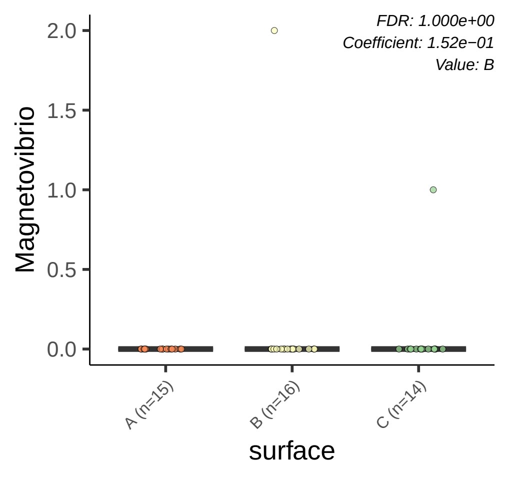
FDR: 1.000e+00
Coefficient: 1.52e−01
Value: B
Magnetovibrio
2.0
1.5
1.0
0.5
0.0
A (n=15)
B (n=16)
C (n=14)
surface

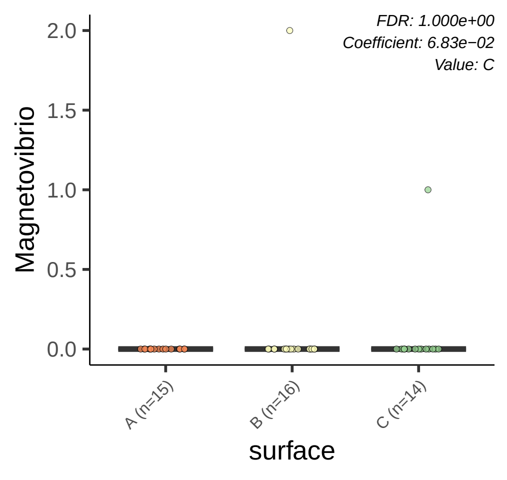

FDR: 1.000e+00
Coefficient: 6.83e−02
Value: C
Magnetovibrio
2.0
1.5
1.0
0.5
0.0
A (n=15)
B (n=16)
C (n=14)
surface

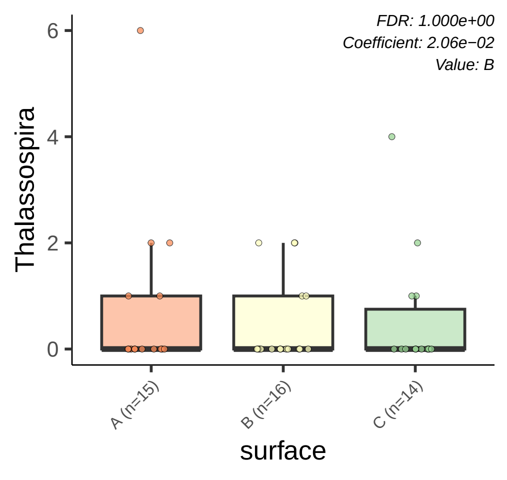

FDR: 1.000e+00
Coefficient: 2.06e−02
Value: B
Thalassospira
6
4
2
0
A (n=15)
B (n=16)
C (n=14)
surface

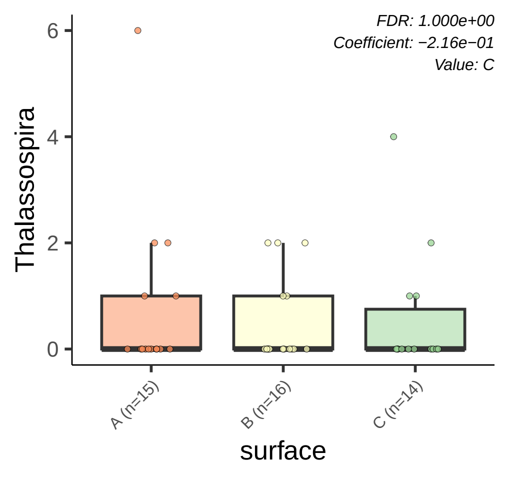

FDR: 1.000e+00
Coefficient: −2.16e−01
Value: C
Thalassospira
6
4
2
0
A (n=15)
B (n=16)
C (n=14)
surface

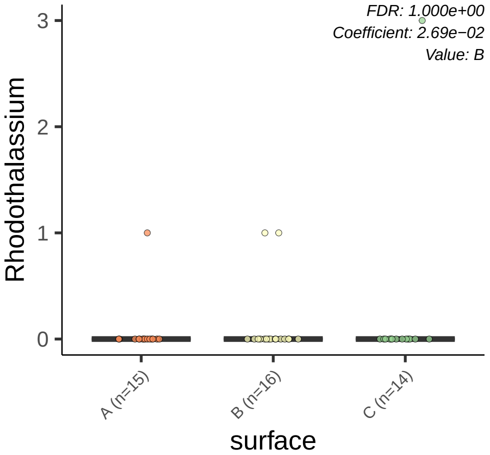
FDR: 1.000e+00
Coefficient: 2.69e−02
Value: B
Rhodothalassium
3
2
1
0
A (n=15)
B (n=16)
C (n=14)
surface

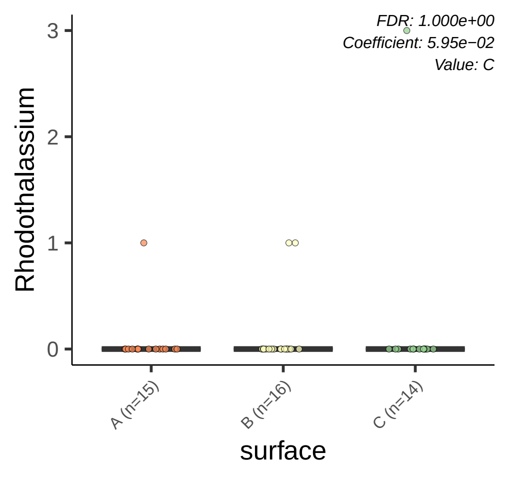
FDR: 1.000e+00
Coefficient: 5.95e−02
Value: C
Rhodothalassium
3
2
1
0
A (n=15)
B (n=16)
C (n=14)
surface

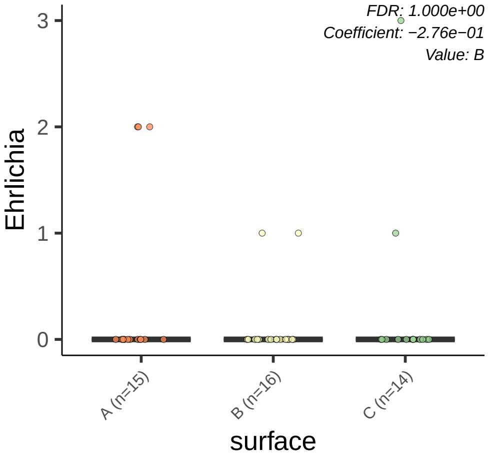

FDR: 1.000e+00
Coefficient: −2.76e−01
Value: B
Ehrlichia
3
2
1
0
A (n=15)
B (n=16)
C (n=14)
surface

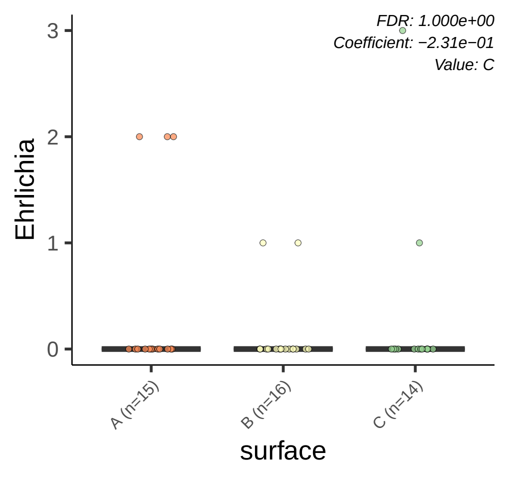
FDR: 1.000e+00
Coefficient: −2.31e−01
Value: C
Ehrlichia
3
2
1
0
A (n=15)
B (n=16)
C (n=14)
surface

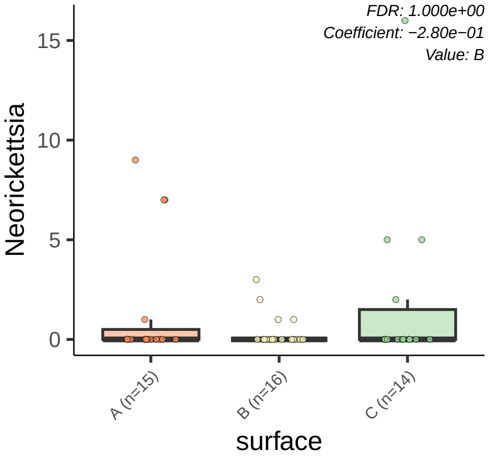
FDR: 1.000e+00
Coefficient: −2.80e−01
Value: B
Neorickettsia
15
10
5
0
A (n=15)
B (n=16)
C (n=14)
surface

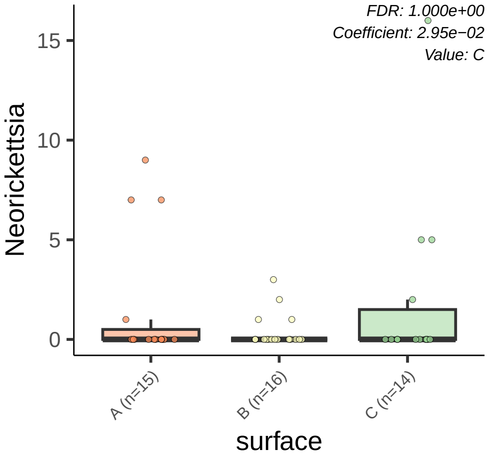

FDR: 1.000e+00
Coefficient: 2.95e−02
Value: C
Neorickettsia
15
10
5
0
A (n=15)
B (n=16)
C (n=14)
surface

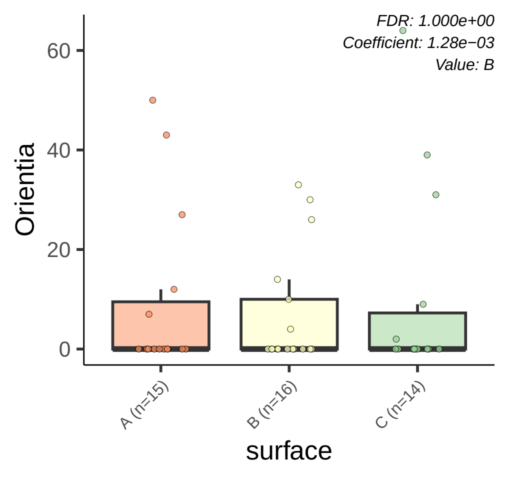
FDR: 1.000e+00
Coefficient: 1.28e−03
Value: B
Orientia
60
40
20
0
A (n=15)
B (n=16)
C (n=14)
surface

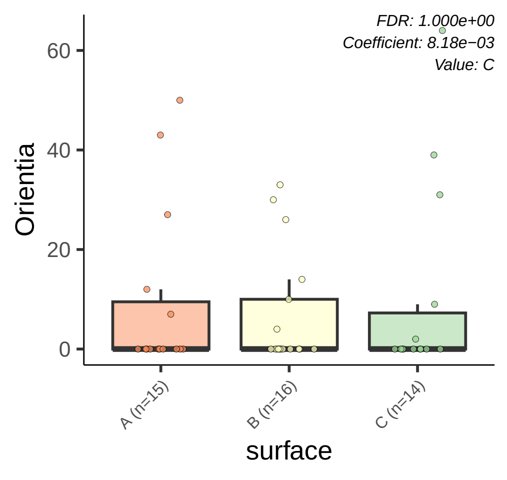
FDR: 1.000e+00
Coefficient: 8.18e−03
Value: C
Orientia
0
20
40
60
A (n=15)
B (n=16)
C (n=14)
surface

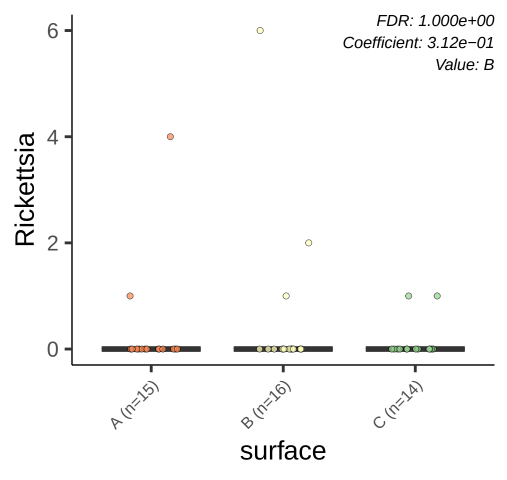

FDR: 1.000e+00
Coefficient: 3.12e−01
Value: B
Rickettsia
0
2
4
6
A (n=15)
B (n=16)
C (n=14)
surface

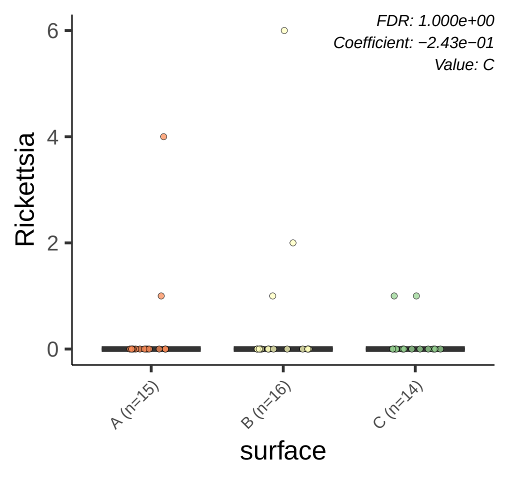
FDR: 1.000e+00
Coefficient: −2.43e−01
Value: C
Rickettsia
0
2
4
6
A (n=15)
B (n=16)
C (n=14)
surface

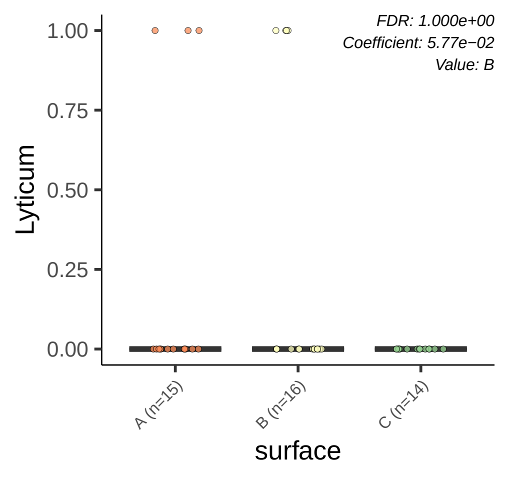

FDR: 1.000e+00
Coefficient: 5.77e−02
Value: B
1.00
0.75
0.50
0.25
0.00
Lyticum
A (n=15)
B (n=16)
C (n=14)
surface

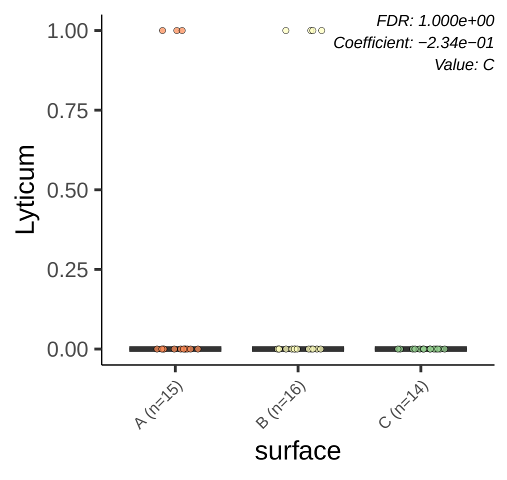
FDR: 1.000e+00
Coefficient: −2.34e−01
Value: C
1.00
0.75
0.50
0.25
0.00
Lyticum
A (n=15)
B (n=16)
C (n=14)
surface

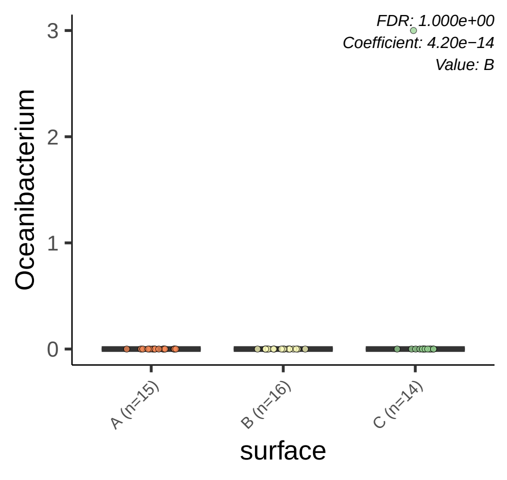
FDR: 1.000e+00
Coefficient: 4.20e−14
Value: B
Oceanibacterium
3
2
1
0
A (n=15)
B (n=16)
C (n=14)
surface

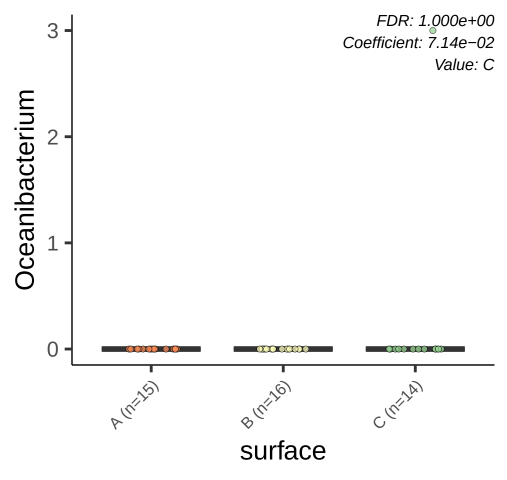
FDR: 1.000e+00
Coefficient: 7.14e−02
Value: C
Oceanibacterium
3
2
1
0
A (n=15)
B (n=16)
C (n=14)
surface

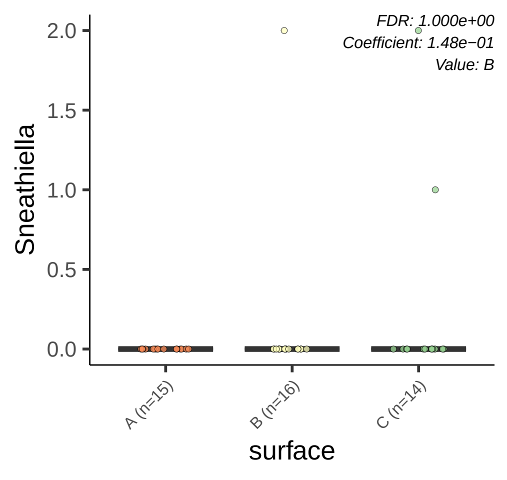

FDR: 1.000e+00
Coefficient: 1.48e−01
Value: B
Sneathiella
2.0
1.5
1.0
0.5
0.0
A (n=15)
B (n=16)
C (n=14)
surface

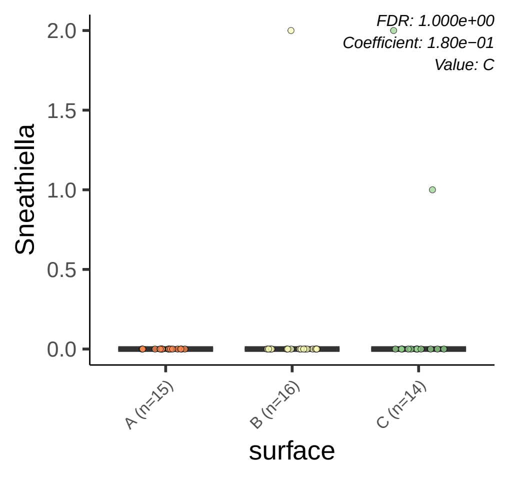
FDR: 1.000e+00
Coefficient: 1.80e−01
Value: C
Sneathiella
2.0
1.5
1.0
0.5
0.0
A (n=15)
B (n=16)
C (n=14)
surface

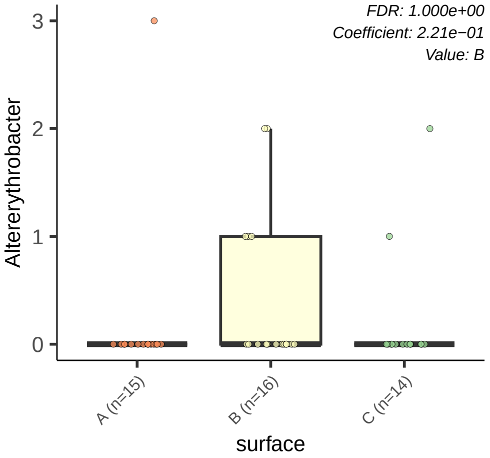

FDR: 1.000e+00
Coefficient: 2.21e−01
Value: B
Altererythrobacter
3
2
1
0
A (n=15)
B (n=16)
C (n=14)
surface

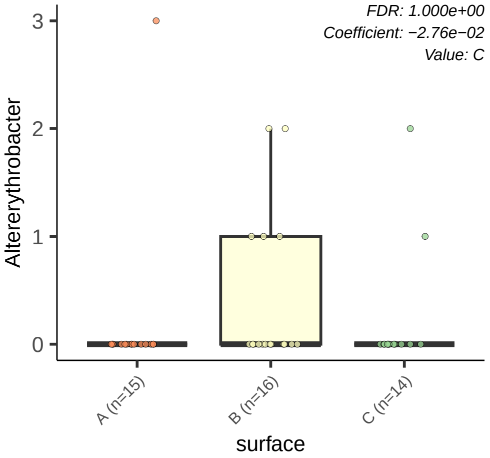
FDR: 1.000e+00
Coefficient: −2.76e−02
Value: C
Altererythrobacter
3
2
1
0
A (n=15)
B (n=16)
C (n=14)
surface

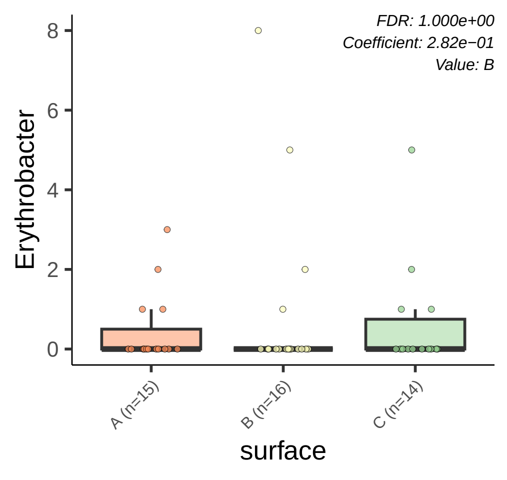

FDR: 1.000e+00
Coefficient: 2.82e−01
Value: B
Erythrobacter
8
6
4
2
0
A (n=15)
B (n=16)
C (n=14)
surface

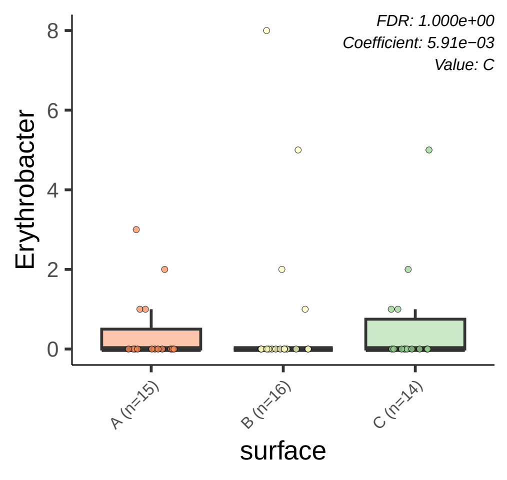
FDR: 1.000e+00
Coefficient: 5.91e−03
Value: C
Erythrobacter
0
2
4
6
8
A (n=15)
B (n=16)
C (n=14)
surface

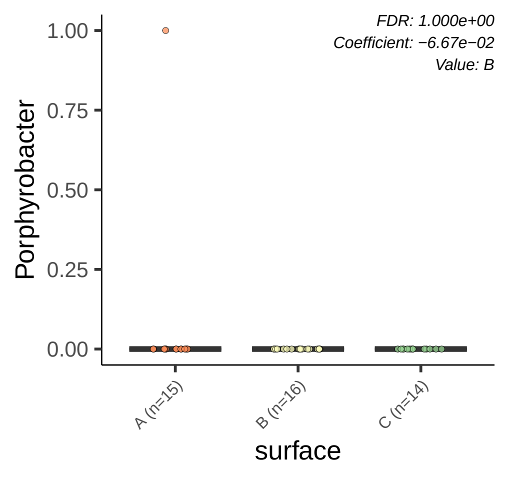
FDR: 1.000e+00
Coefficient: −6.67e−02
Value: B
Porphyrobacter
1.00
0.75
0.50
0.25
0.00
A (n=15)
B (n=16)
C (n=14)
surface

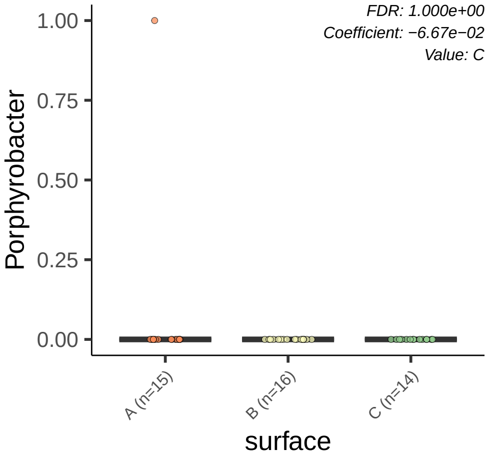
FDR: 1.000e+00
Coefficient: −6.67e−02
Value: C
Porphyrobacter
1.00
0.75
0.50
0.25
0.00
A (n=15)
B (n=16)
C (n=14)
surface

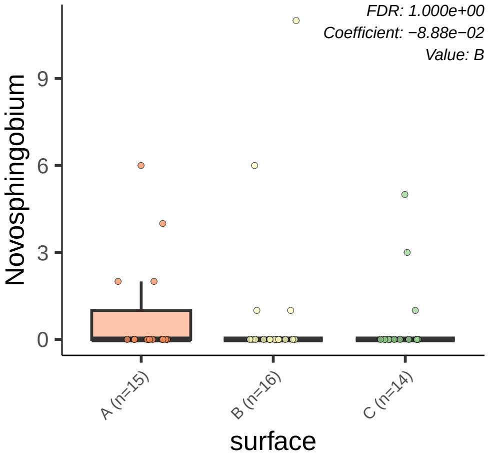

FDR: 1.000e+00
Coefficient: −8.88e−02
Value: B
Novosphingobium
0
3
6
9
A (n=15)
B (n=16)
C (n=14)
surface

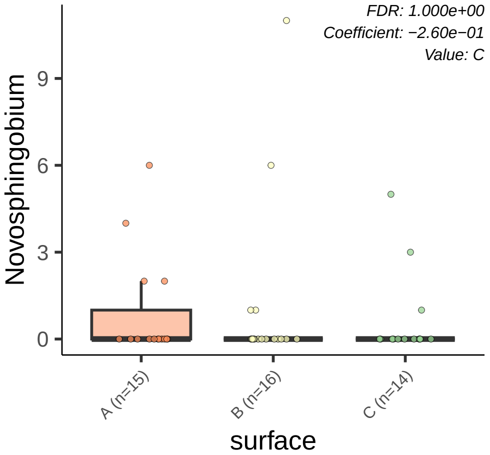

FDR: 1.000e+00
Coefficient: −2.60e−01
Value: C
Novosphingobium
0
3
6
9
A (n=15)
B (n=16)
C (n=14)
surface

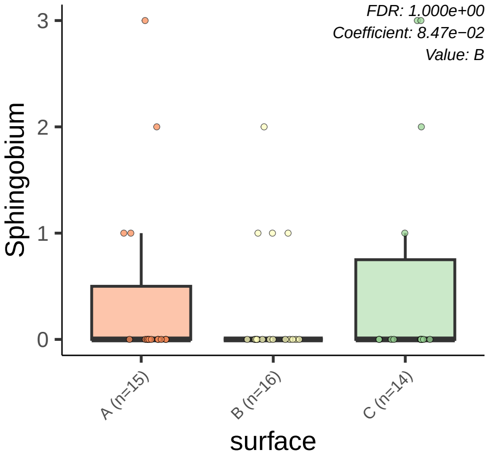

FDR: 1.000e+00
Coefficient: 8.47e−02
Value: B
Sphingobium
0
1
2
3
A (n=15)
B (n=16)
C (n=14)
surface

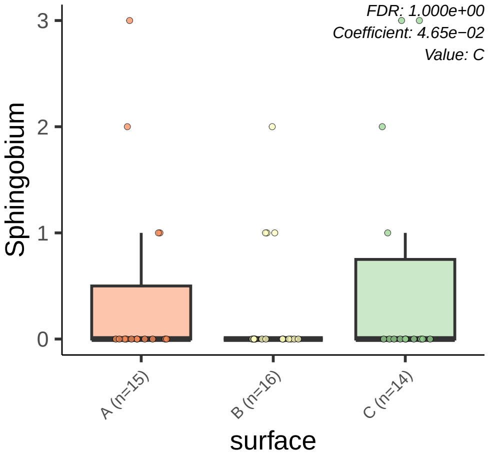
FDR: 1.000e+00
Coefficient: 4.65e−02
Value: C
Sphingobium
0
1
2
3
A (n=15)
B (n=16)
C (n=14)
surface

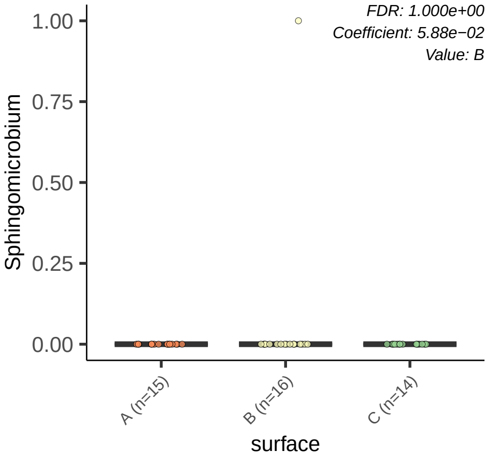
FDR: 1.000e+00
Coefficient: 5.88e−02
Value: B
Sphingomicrobium
1.00
0.75
0.50
0.25
0.00
A (n=15)
B (n=16)
C (n=14)
surface

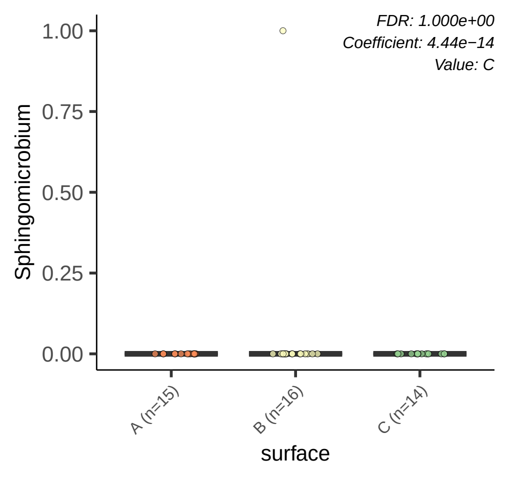
FDR: 1.000e+00
Coefficient: 4.44e−14
Value: C
Sphingomicrobium
1.00
0.75
0.50
0.25
0.00
A (n=15)
B (n=16)
C (n=14)
surface

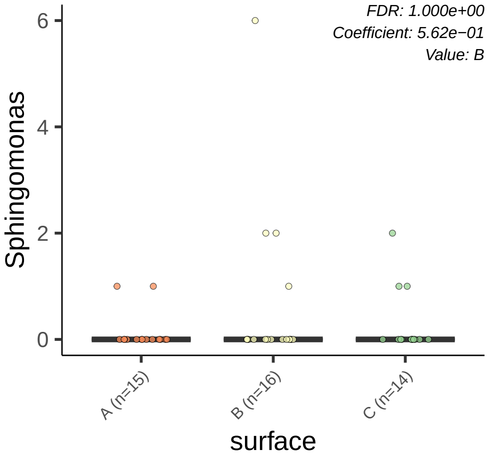
FDR: 1.000e+00
Coefficient: 5.62e−01
Value: B
Sphingomonas
0
2
4
6
A (n=15)
B (n=16)
C (n=14)
surface

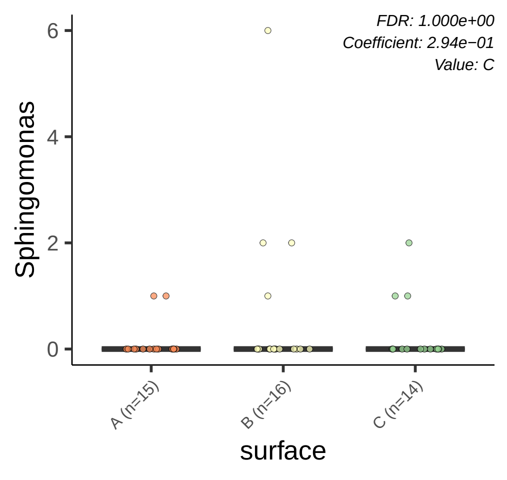
FDR: 1.000e+00
Coefficient: 2.94e−01
Value: C
Sphingomonas
0
2
4
6
A (n=15)
B (n=16)
C (n=14)
surface

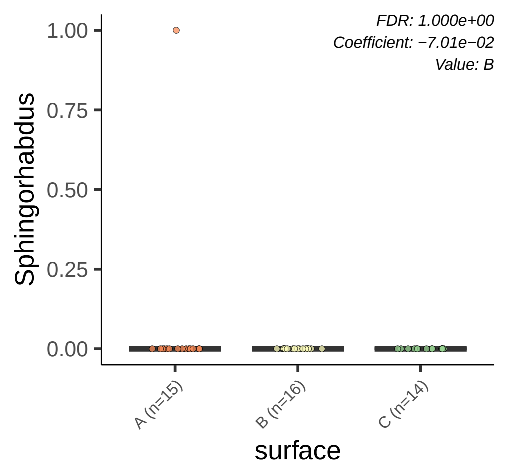
FDR: 1.000e+00
Coefficient: −7.01e−02
Value: B
Sphingorhabdus
1.00
0.75
0.50
0.25
0.00
A (n=15)
B (n=16)
C (n=14)
surface

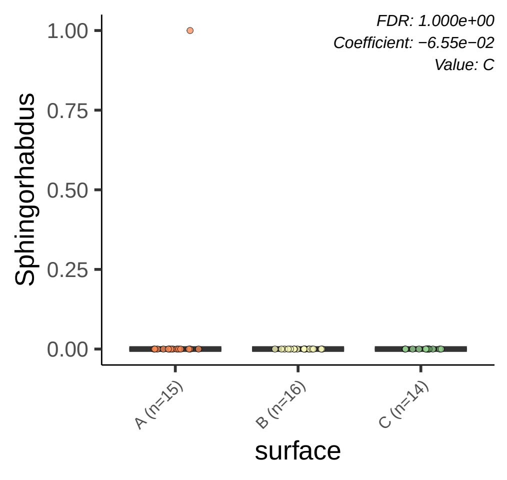
FDR: 1.000e+00
Coefficient: −6.55e−02
Value: C
Sphingorhabdus
1.00
0.75
0.50
0.25
0.00
A (n=15)
B (n=16)
C (n=14)
surface

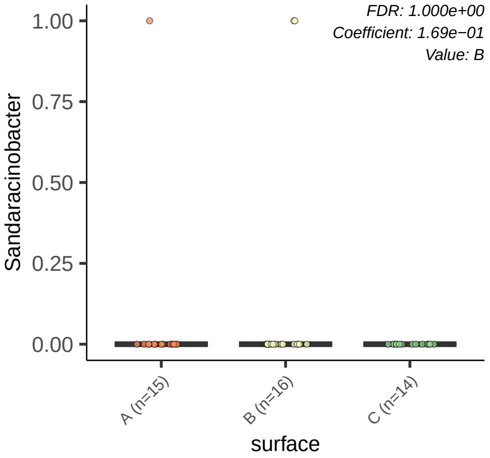

FDR: 1.000e+00
Coefficient: 1.69e−01
Value: B
Sandaracinobacter
1.00
0.75
0.50
0.25
0.00
A (n=15)
B (n=16)
C (n=14)
surface

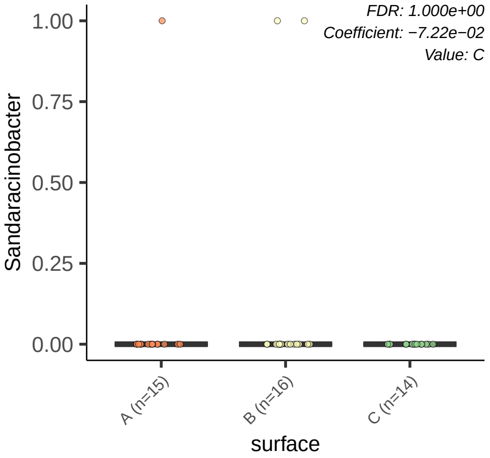
FDR: 1.000e+00
Coefficient: −7.22e−02
Value: C
Sandaracinobacter
1.00
0.75
0.50
0.25
0.00
A (n=15)
B (n=16)
C (n=14)
surface

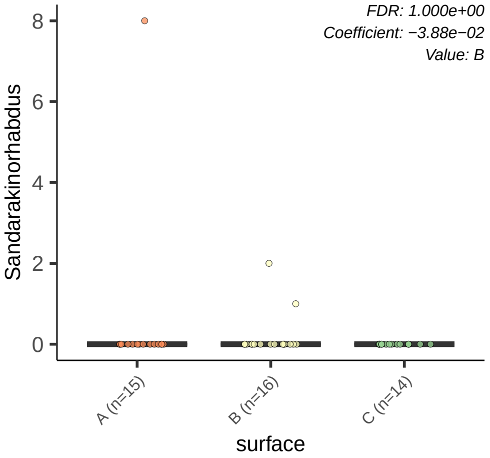
FDR: 1.000e+00
Coefficient: −3.88e−02
Value: B
Sandarakinorhabdus
8
6
4
2
0
A (n=15)
B (n=16)
C (n=14)
surface

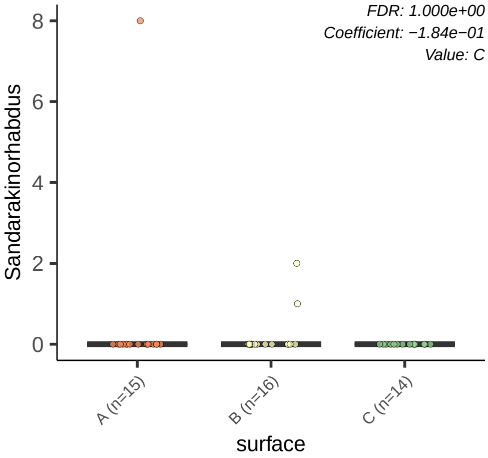

FDR: 1.000e+00
Coefficient: −1.84e−01
Value: C
Sandarakinorhabdus
8
6
4
2
0
A (n=15)
B (n=16)
C (n=14)
surface

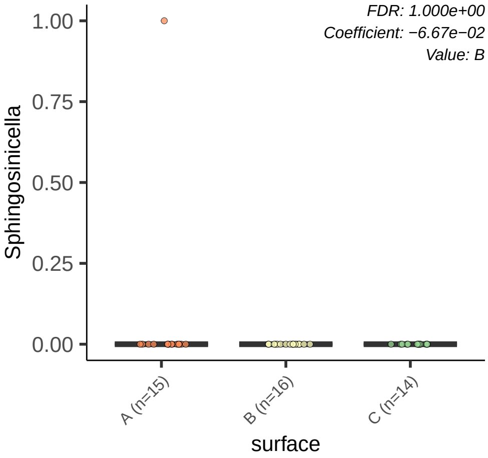

FDR: 1.000e+00
Coefficient: −6.67e−02
Value: B
Sphingosinicella
1.00
0.75
0.50
0.25
0.00
A (n=15)
B (n=16)
C (n=14)
surface

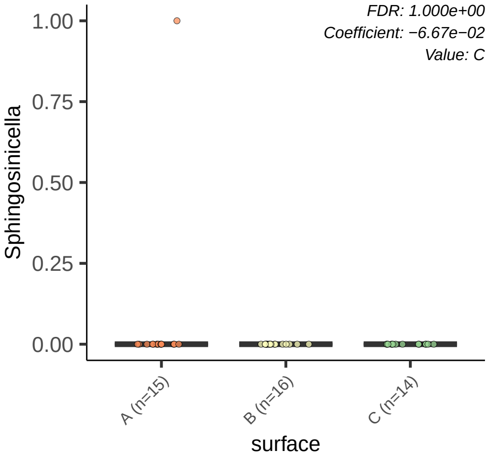

FDR: 1.000e+00
Coefficient: −6.67e−02
Value: C
Sphingosinicella
1.00
0.75
0.50
0.25
0.00
A (n=15)
B (n=16)
C (n=14)
surface

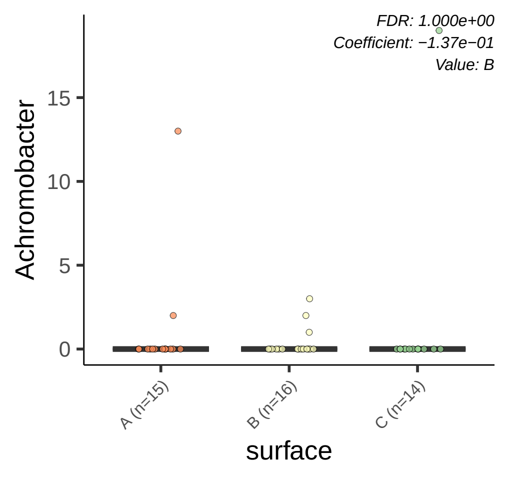

FDR: 1.000e+00
Coefficient: −1.37e−01
Value: B
Achromobacter
15
10
5
0
A (n=15)
B (n=16)
C (n=14)
surface

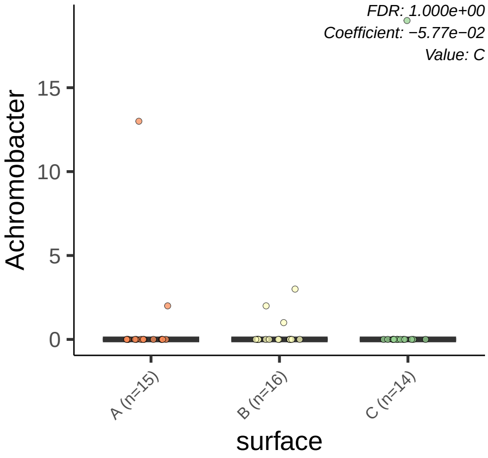

FDR: 1.000e+00
Coefficient: −5.77e−02
Value: C
Achromobacter
15
10
5
0
A (n=15)
B (n=16)
C (n=14)
surface

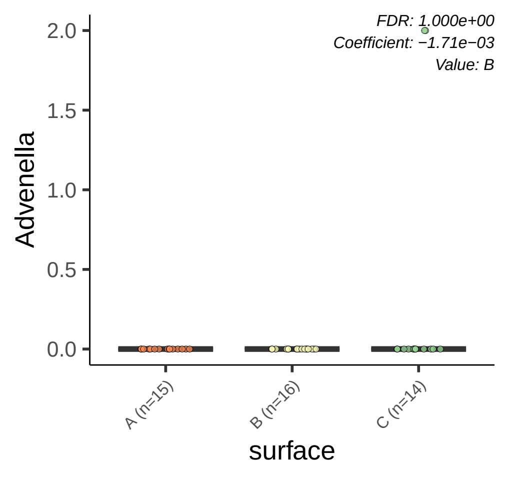

FDR: 1.000e+00
Coefficient: −1.71e−03
Value: B
Advenella
2.0
1.5
1.0
0.5
0.0
A (n=15)
B (n=16)
C (n=14)
surface

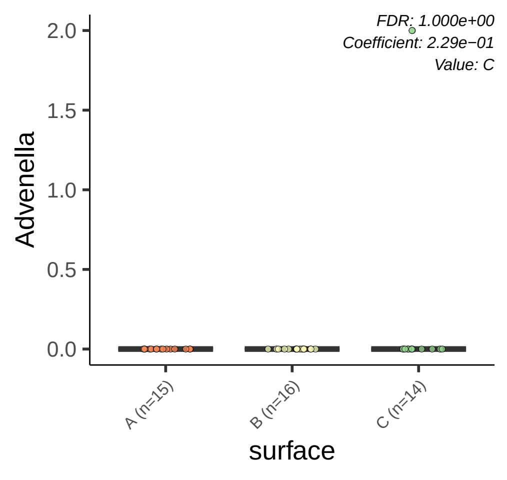
FDR: 1.000e+00
Coefficient: 2.29e−01
Value: C
2.0
1.5
1.0
0.5
0.0
Advenella
A (n=15)
B (n=16)
C (n=14)
surface

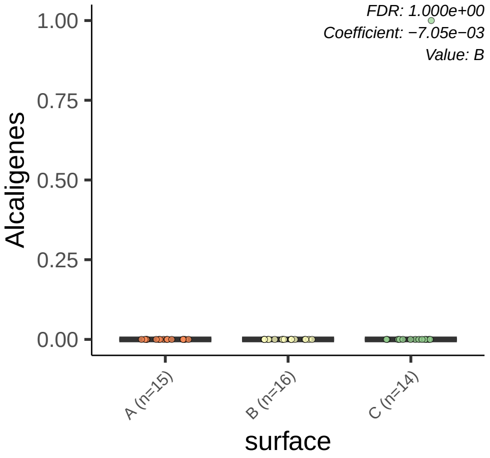
FDR: 1.000e+00
Coefficient: −7.05e−03
Value: B
Alcaligenes
1.00
0.75
0.50
0.25
0.00
A (n=15)
B (n=16)
C (n=14)
surface

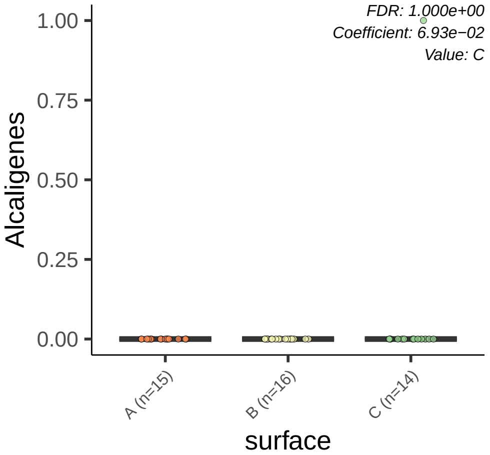
FDR: 1.000e+00
Coefficient: 6.93e−02
Value: C
Alcaligenes
1.00
0.75
0.50
0.25
0.00
A (n=15)
B (n=16)
C (n=14)
surface

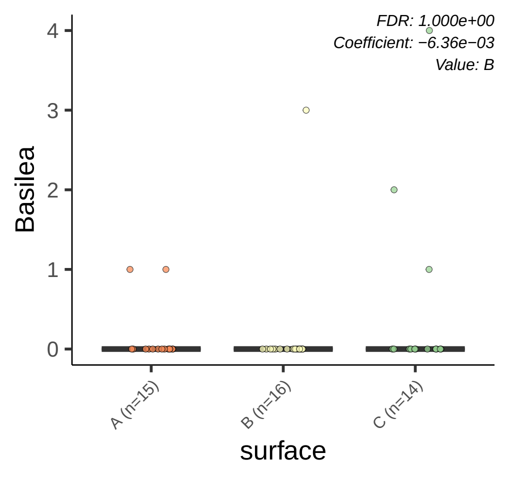

FDR: 1.000e+00
Coefficient: −6.36e−03
Value: B
Basilea
4
3
2
1
0
A (n=15)
B (n=16)
C (n=14)
surface

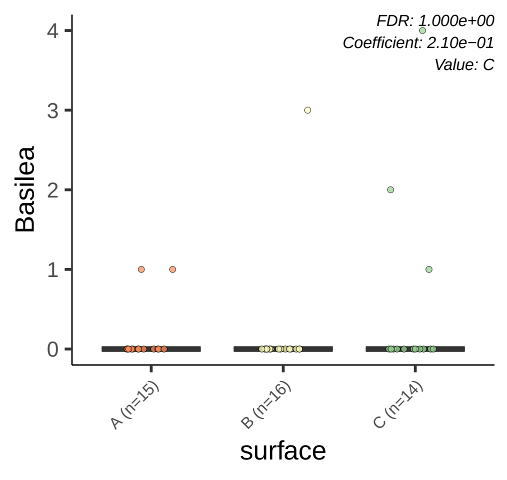
FDR: 1.000e+00
Coefficient: 2.10e−01
Value: C
Basilea
0
1
2
3
4
A (n=15)
B (n=16)
C (n=14)
surface

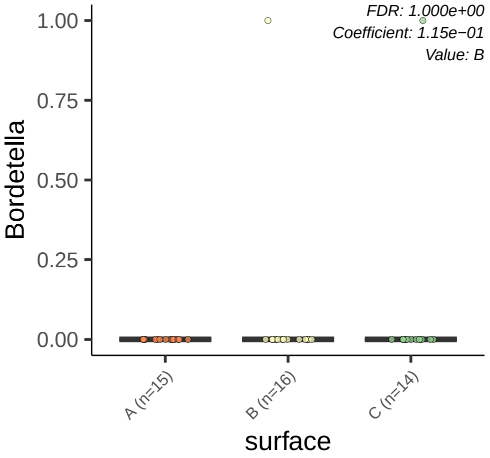
FDR: 1.000e+00
Coefficient: 1.15e−01
Value: B
Bordetella
1.00
0.75
0.50
0.25
0.00
A (n=15)
B (n=16)
C (n=14)
surface

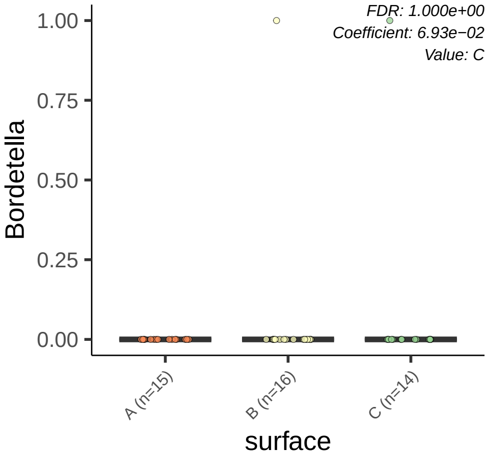
FDR: 1.000e+00
Coefficient: 6.93e−02
Value: C
1.00
0.75
0.50
0.25
0.00
Bordetella
A (n=15)
B (n=16)
C (n=14)
surface

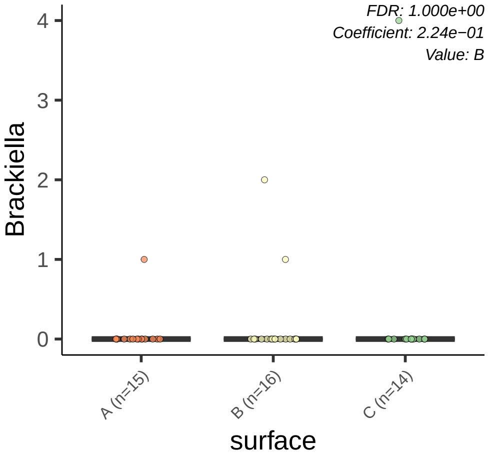

FDR: 1.000e+00
Coefficient: 2.24e−01
Value: B
Brackiella
0
1
2
3
4
A (n=15)
B (n=16)
C (n=14)
surface

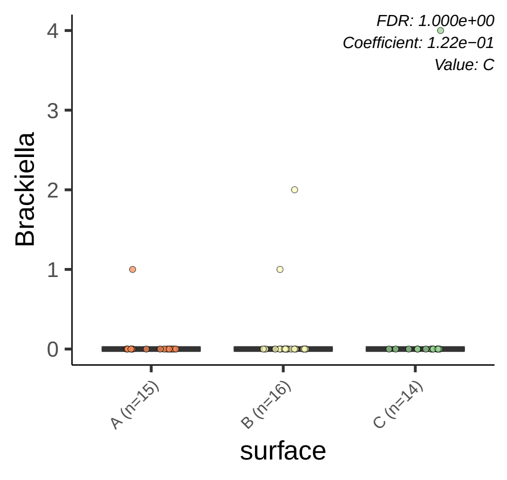

FDR: 1.000e+00
Coefficient: 1.22e−01
Value: C
Brackiella
0
1
2
3
4
A (n=15)
B (n=16)
C (n=14)
surface

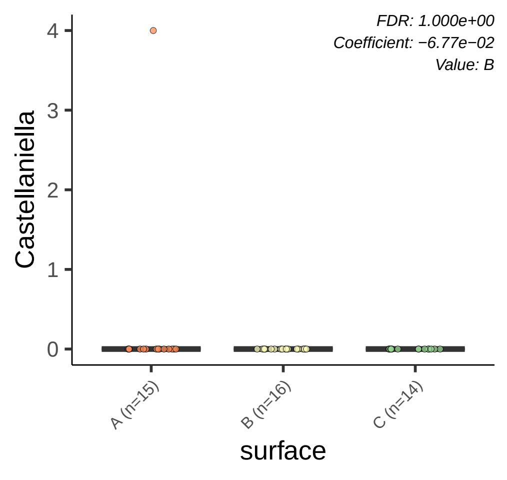
FDR: 1.000e+00
Coefficient: −6.77e−02
Value: B
Castellaniella
0
1
2
3
4
A (n=15)
B (n=16)
C (n=14)
surface

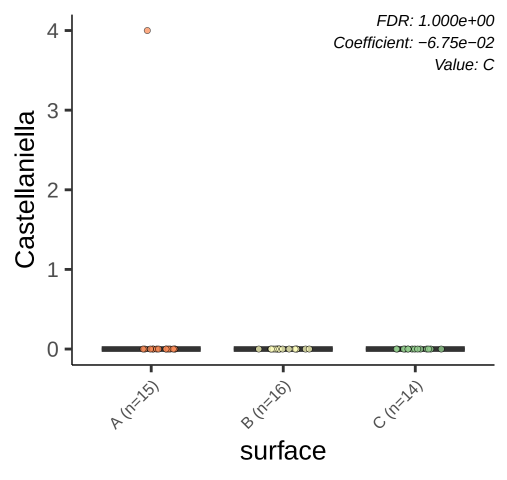
FDR: 1.000e+00
Coefficient: −6.75e−02
Value: C
Castellaniella
4
3
2
1
0
A (n=15)
B (n=16)
C (n=14)
surface

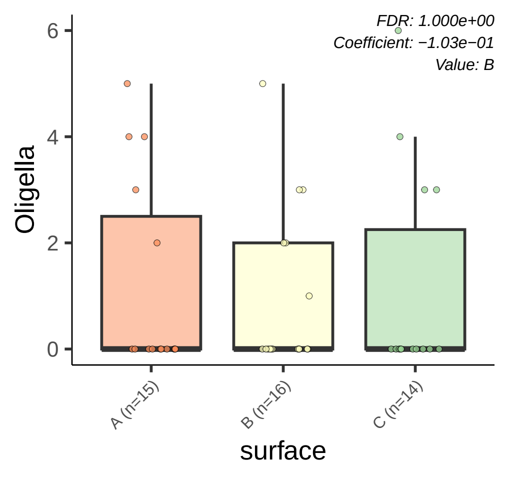

FDR: 1.000e+00
Coefficient: −1.03e−01
Value: B
Oligella
0
2
4
6
A (n=15)
B (n=16)
C (n=14)
surface

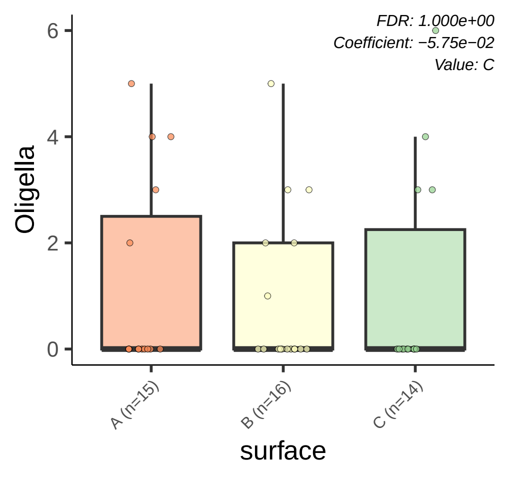

FDR: 1.000e+00
Coefficient: −5.75e−02
Value: C
Oligella
0
2
4
6
A (n=15)
B (n=16)
C (n=14)
surface

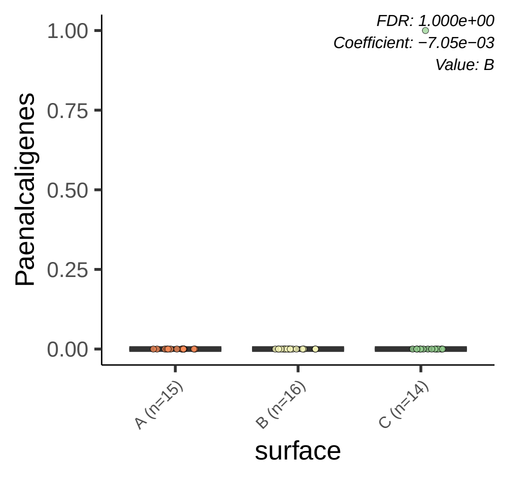
FDR: 1.000e+00
Coefficient: −7.05e−03
Value: B
Paenalcaligenes
1.00
0.75
0.50
0.25
0.00
A (n=15)
B (n=16)
C (n=14)
surface

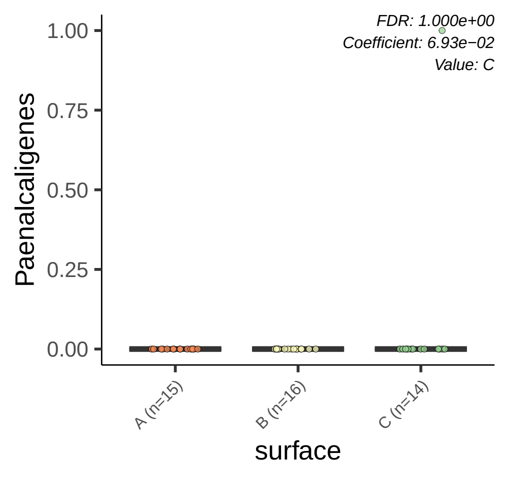
FDR: 1.000e+00
Coefficient: 6.93e−02
Value: C
Paenalcaligenes
1.00
0.75
0.50
0.25
0.00
A (n=15)
B (n=16)
C (n=14)
surface

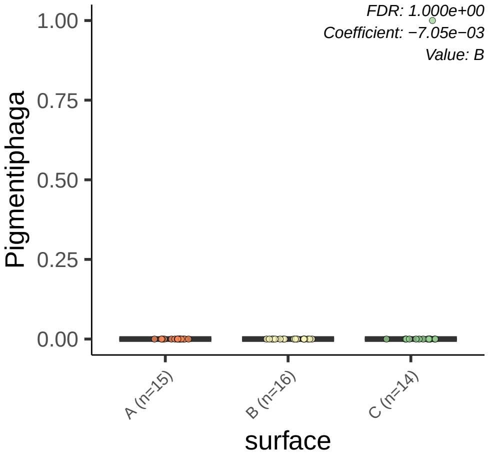

FDR: 1.000e+00
Coefficient: −7.05e−03
Value: B
Pigmentiphaga
1.00
0.75
0.50
0.25
0.00
A (n=15)
B (n=16)
C (n=14)
surface

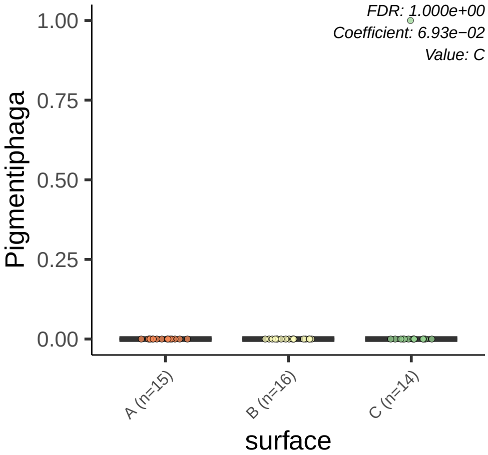
FDR: 1.000e+00
Coefficient: 6.93e−02
Value: C
1.00
0.75
0.50
0.25
0.00
Pigmentiphaga
A (n=15)
B (n=16)
C (n=14)
surface

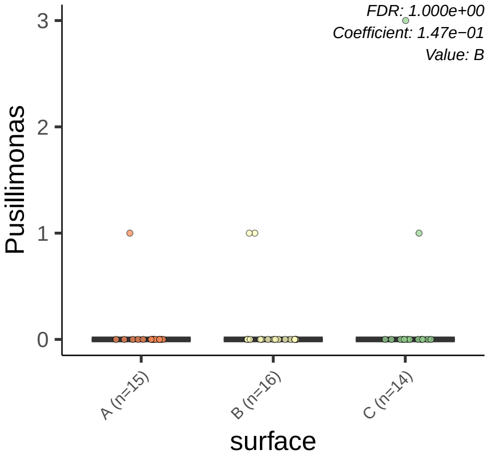
FDR: 1.000e+00
Coefficient: 1.47e−01
Value: B
Pusillimonas
3
2
1
0
A (n=15)
B (n=16)
C (n=14)
surface

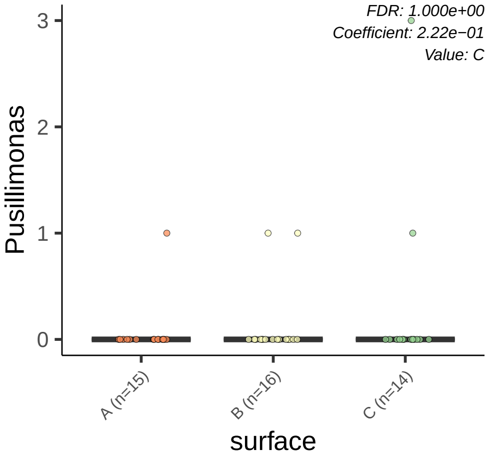
FDR: 1.000e+00
Coefficient: 2.22e−01
Value: C
Pusillimonas
3
2
1
0
A (n=15)
B (n=16)
C (n=14)
surface

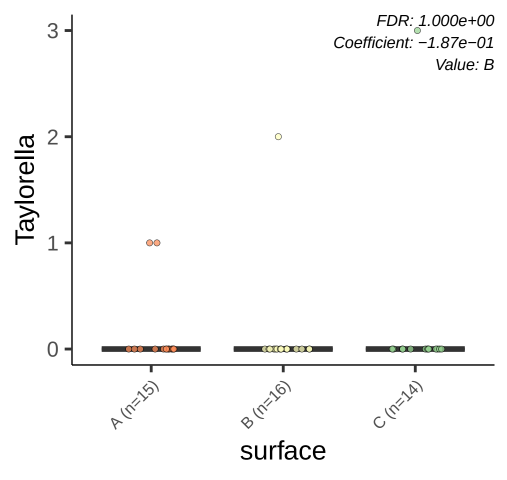
FDR: 1.000e+00
Coefficient: −1.87e−01
Value: B
Taylorella
3
2
1
0
A (n=15)
B (n=16)
C (n=14)
surface

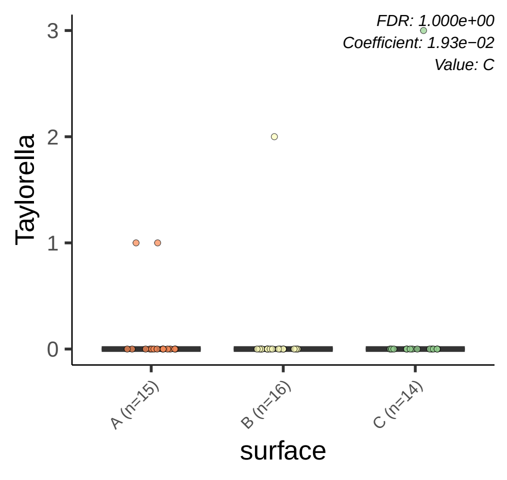

FDR: 1.000e+00
Coefficient: 1.93e−02
Value: C
Taylorella
3
2
1
0
A (n=15)
B (n=16)
C (n=14)
surface

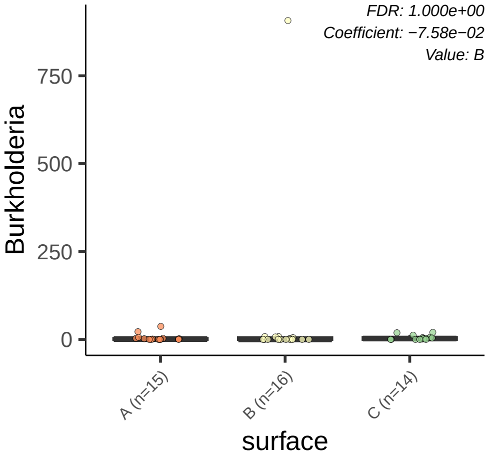

FDR: 1.000e+00
Coefficient: −7.58e−02
Value: B
Burkholderia
750
500
250
0
A (n=15)
B (n=16)
C (n=14)
surface

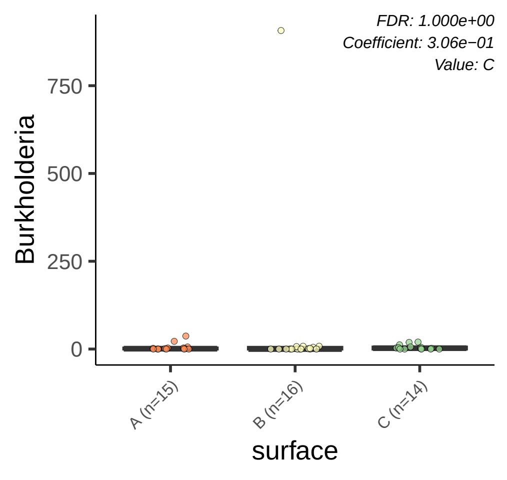
FDR: 1.000e+00
Coefficient: 3.06e−01
Value: C
Burkholderia
750
500
250
0
A (n=15)
B (n=16)
C (n=14)
surface

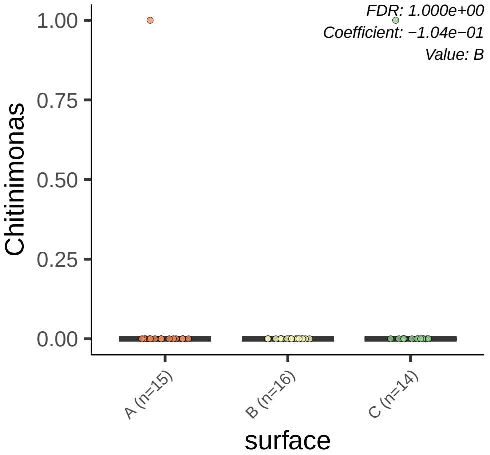

FDR: 1.000e+00
Coefficient: −1.04e−01
Value: B
Chitinimonas
1.00
0.75
0.50
0.25
0.00
A (n=15)
B (n=16)
C (n=14)
surface

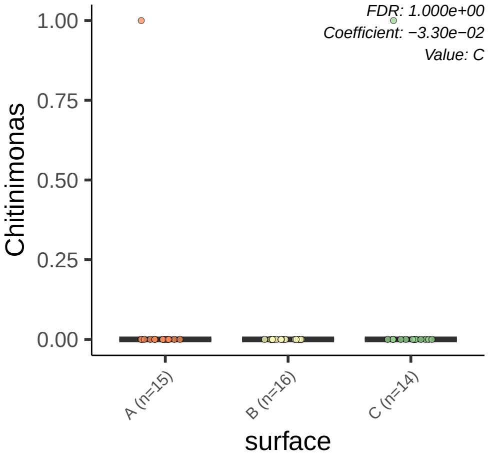

FDR: 1.000e+00
Coefficient: −3.30e−02
Value: C
Chitinimonas
1.00
0.75
0.50
0.25
0.00
A (n=15)
B (n=16)
C (n=14)
surface

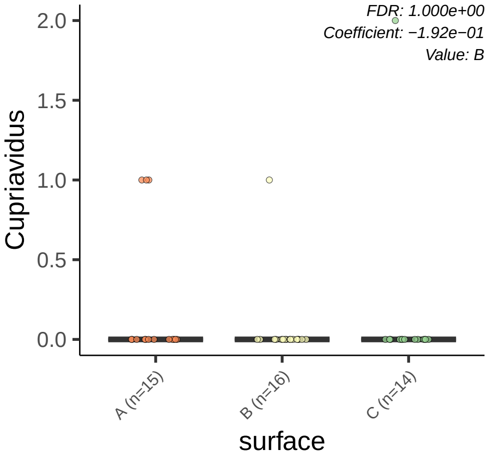
FDR: 1.000e+00
Coefficient: −1.92e−01
Value: B
2.0
1.5
1.0
0.5
0.0
Cupriavidus
A (n=15)
B (n=16)
C (n=14)
surface

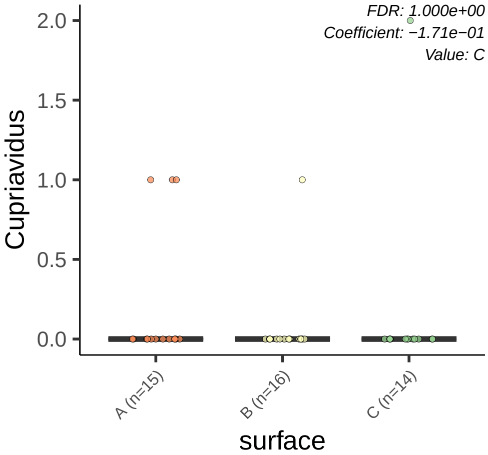

FDR: 1.000e+00
Coefficient: −1.71e−01
Value: C
Cupriavidus
2.0
1.5
1.0
0.5
0.0
A (n=15)
B (n=16)
C (n=14)
surface

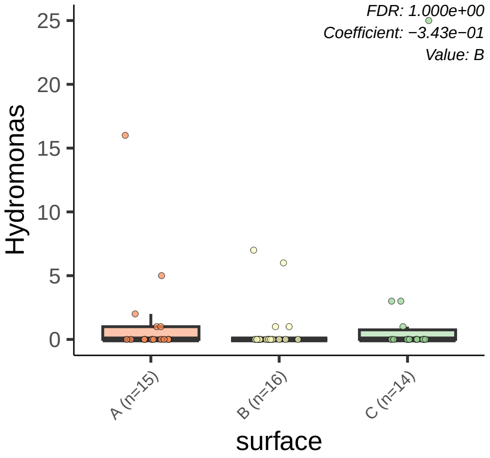

FDR: 1.000e+00
Coefficient: −3.43e−01
Value: B
Hydromonas
25
20
15
10
5
0
A (n=15)
B (n=16)
C (n=14)
surface

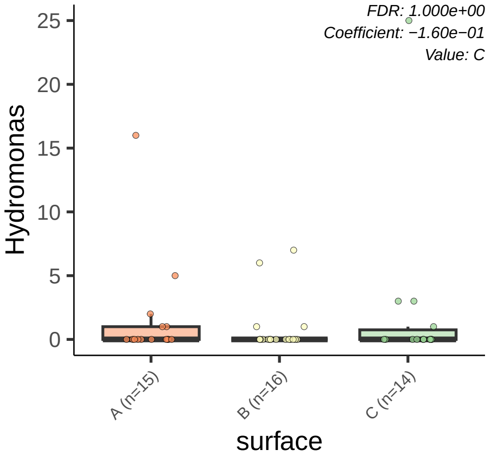

FDR: 1.000e+00
Coefficient: −1.60e−01
Value: C
Hydromonas
25
20
15
10
5
0
A (n=15)
B (n=16)
C (n=14)
surface

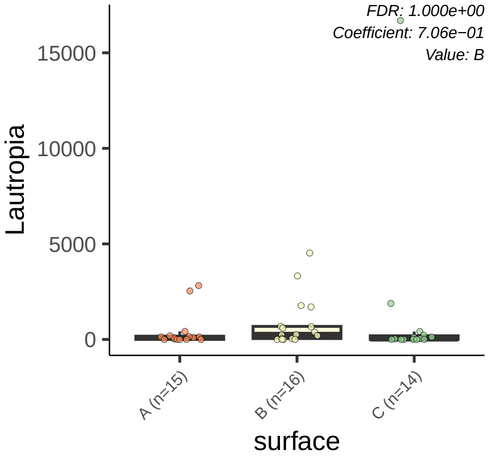
FDR: 1.000e+00
Coefficient: 7.06e−01
Value: B
Lautropia
15000
10000
5000
0
A (n=15)
B (n=16)
C (n=14)
surface

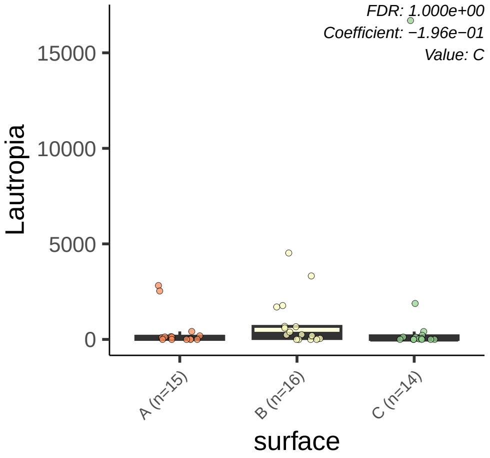
FDR: 1.000e+00
Coefficient: −1.96e−01
Value: C
Lautropia
15000
10000
5000
0
A (n=15)
B (n=16)
C (n=14)
surface

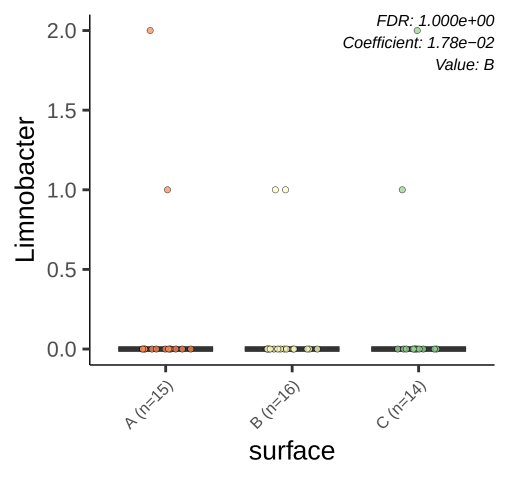
FDR: 1.000e+00
Coefficient: 1.78e−02
Value: B
Limnobacter
2.0
1.5
1.0
0.5
0.0
A (n=15)
B (n=16)
C (n=14)
surface

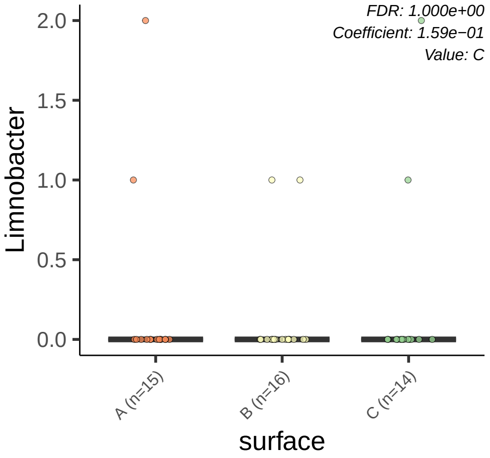
FDR: 1.000e+00
Coefficient: 1.59e−01
Value: C
Limnobacter
2.0
1.5
1.0
0.5
0.0
A (n=15)
B (n=16)
C (n=14)
surface

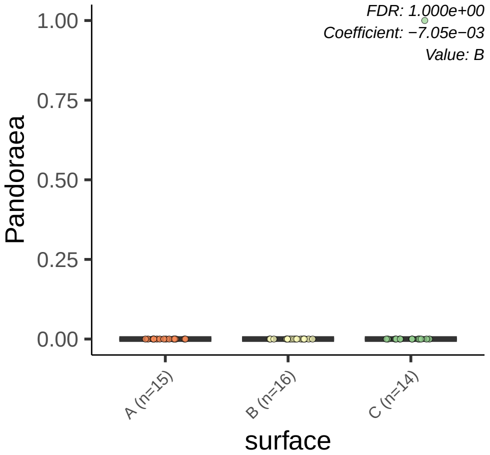
FDR: 1.000e+00
Coefficient: −7.05e−03
Value: B
Pandoraea
1.00
0.75
0.50
0.25
0.00
A (n=15)
B (n=16)
C (n=14)
surface

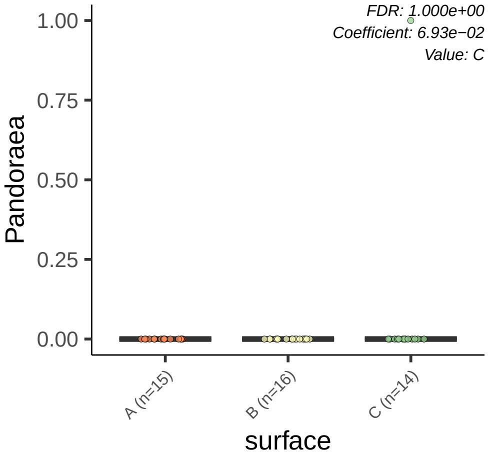

FDR: 1.000e+00
Coefficient: 6.93e−02
Value: C
1.00
0.75
0.50
0.25
0.00
Pandoraea
A (n=15)
B (n=16)
C (n=14)
surface

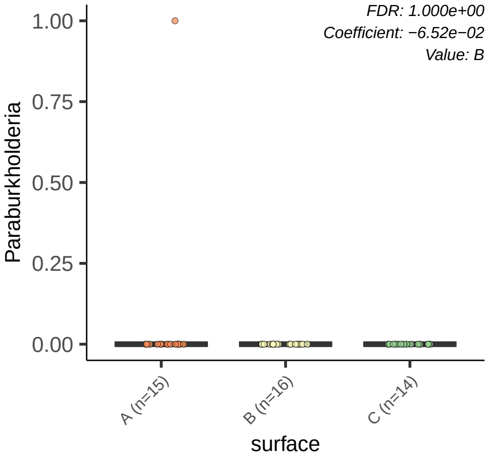

FDR: 1.000e+00
Coefficient: −6.52e−02
Value: B
Paraburkholderia
1.00
0.75
0.50
0.25
0.00
A (n=15)
B (n=16)
C (n=14)
surface

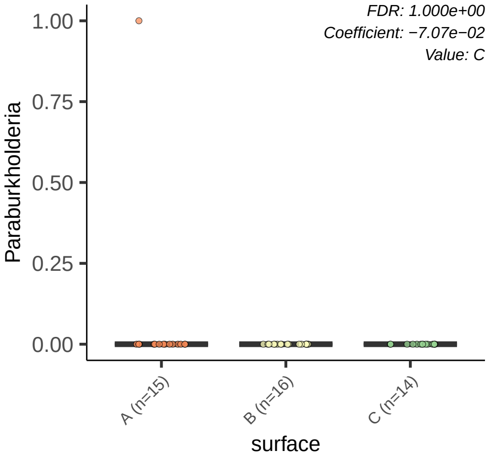
FDR: 1.000e+00
Coefficient: −7.07e−02
Value: C
Paraburkholderia
1.00
0.75
0.50
0.25
0.00
A (n=15)
B (n=16)
C (n=14)
surface

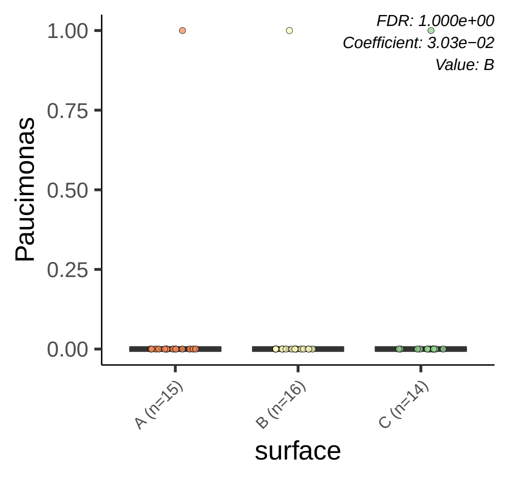

FDR: 1.000e+00
Coefficient: 3.03e−02
Value: B
Paucimonas
1.00
0.75
0.50
0.25
0.00
A (n=15)
B (n=16)
C (n=14)
surface

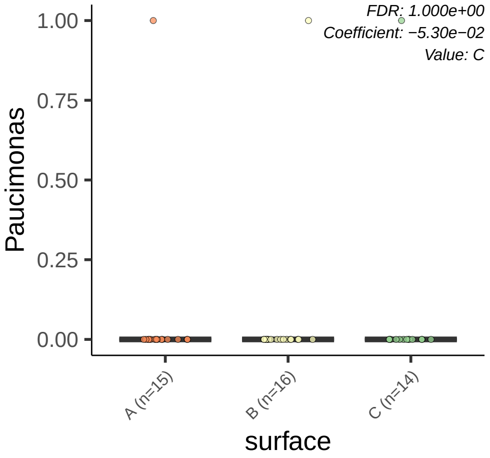

FDR: 1.000e+00
Coefficient: −5.30e−02
Value: C
Paucimonas
1.00
0.75
0.50
0.25
0.00
A (n=15)
B (n=16)
C (n=14)
surface

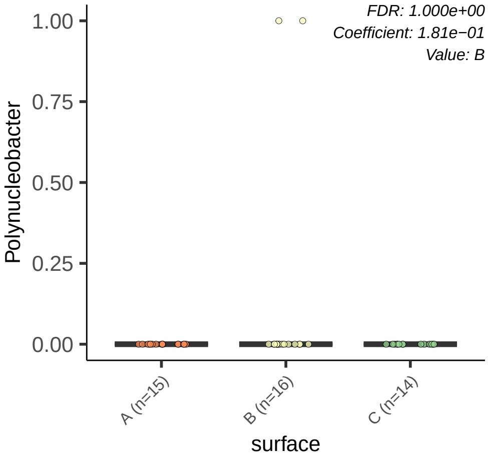
FDR: 1.000e+00
Coefficient: 1.81e−01
Value: B
Polynucleobacter
1.00
0.75
0.50
0.25
0.00
A (n=15)
B (n=16)
C (n=14)
surface

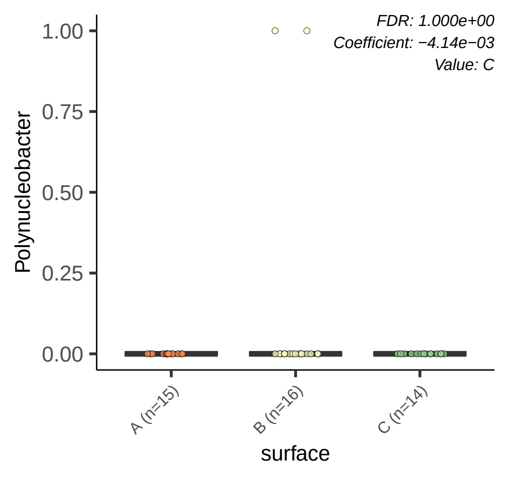

FDR: 1.000e+00
Coefficient: −4.14e−03
Value: C
Polynucleobacter
1.00
0.75
0.50
0.25
0.00
A (n=15)
B (n=16)
C (n=14)
surface

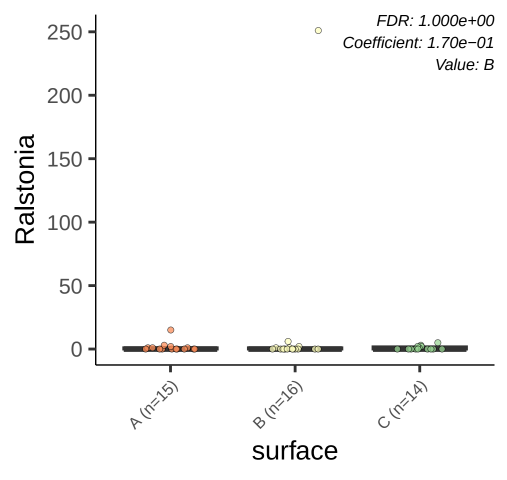
FDR: 1.000e+00
Coefficient: 1.70e−01
Value: B
Ralstonia
250
200
150
100
50
0
A (n=15)
B (n=16)
C (n=14)
surface

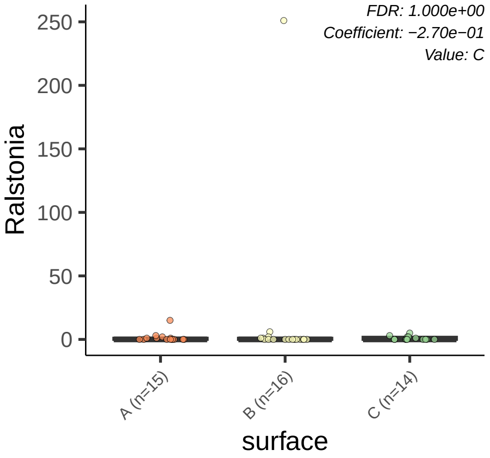

FDR: 1.000e+00
Coefficient: −2.70e−01
Value: C
Ralstonia
250
200
150
100
50
0
A (n=15)
B (n=16)
C (n=14)
surface

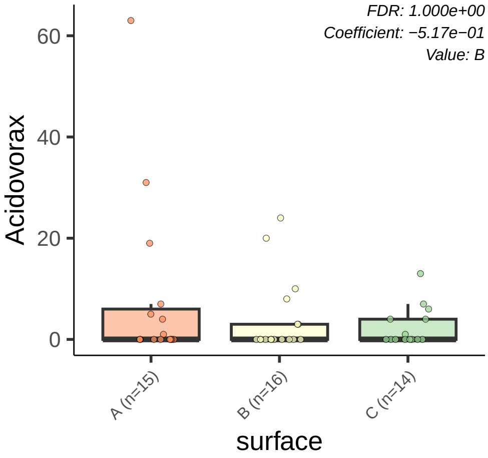
FDR: 1.000e+00
Coefficient: −5.17e−01
Value: B
Acidovorax
0
20
40
60
A (n=15)
B (n=16)
C (n=14)
surface

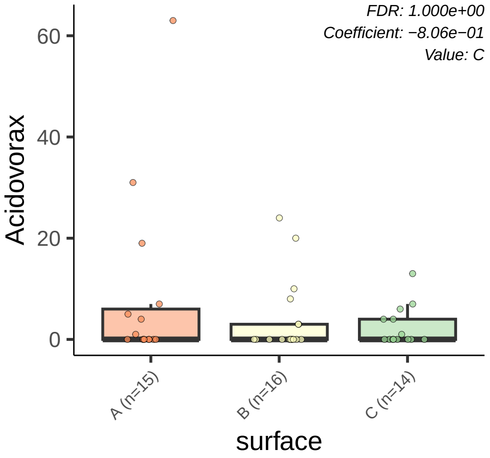
FDR: 1.000e+00
Coefficient: −8.06e−01
Value: C
Acidovorax
60
40
20
0
A (n=15)
B (n=16)
C (n=14)
surface

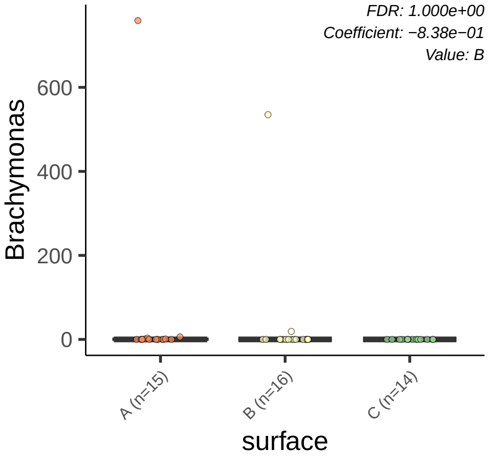

FDR: 1.000e+00
Coefficient: −8.38e−01
Value: B
Brachymonas
600
400
200
0
A (n=15)
B (n=16)
C (n=14)
surface

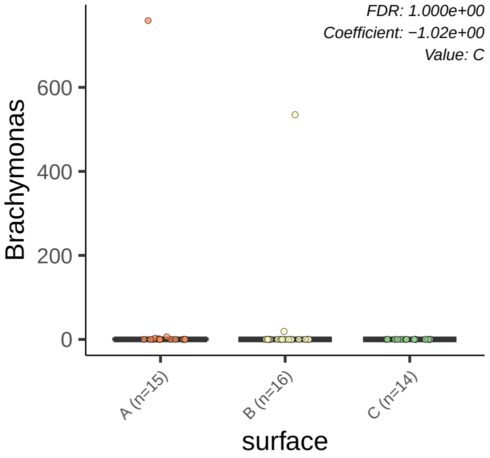
FDR: 1.000e+00
Coefficient: −1.02e+00
Value: C
Brachymonas
600
400
200
0
A (n=15)
B (n=16)
C (n=14)
surface

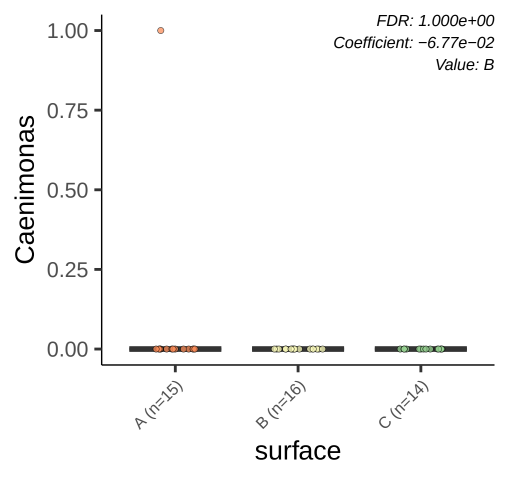

FDR: 1.000e+00
Coefficient: −6.77e−02
Value: B
1.00
0.75
0.50
0.25
0.00
Caenimonas
A (n=15)
B (n=16)
C (n=14)
surface

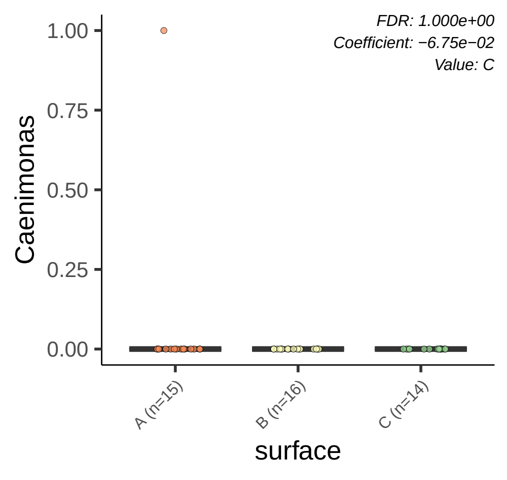
FDR: 1.000e+00
Coefficient: −6.75e−02
Value: C
Caenimonas
1.00
0.75
0.50
0.25
0.00
A (n=15)
B (n=16)
C (n=14)
surface

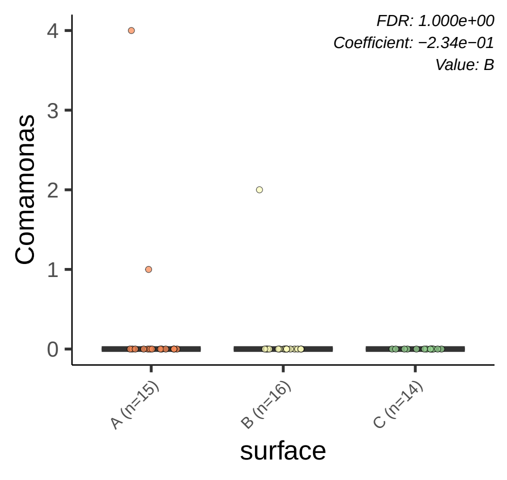
FDR: 1.000e+00
Coefficient: −2.34e−01
Value: B
Comamonas
4
3
2
1
0
A (n=15)
B (n=16)
C (n=14)
surface

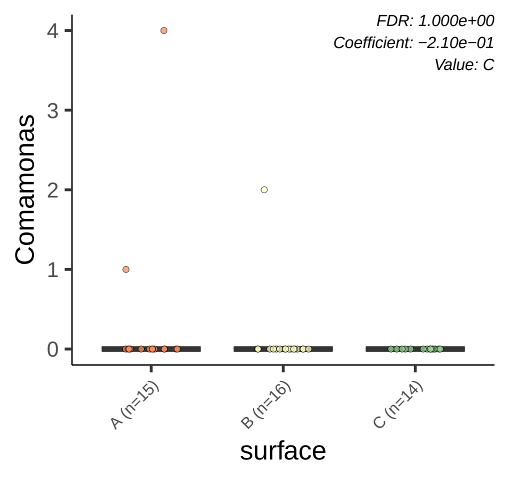
FDR: 1.000e+00
Coefficient: −2.10e−01
Value: C
Comamonas
0
1
2
3
4
A (n=15)
B (n=16)
C (n=14)
surface

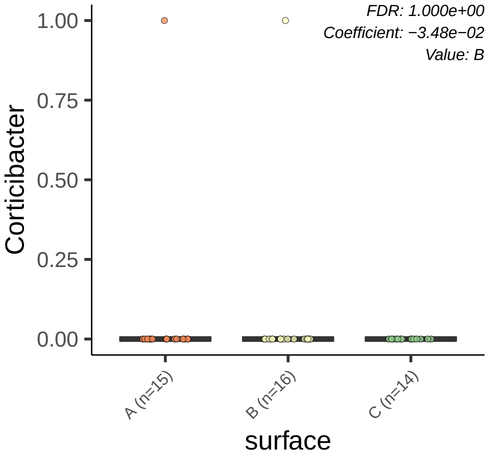

FDR: 1.000e+00
Coefficient: −3.48e−02
Value: B
1.00
0.75
0.50
0.25
0.00
Corticibacter
A (n=15)
B (n=16)
C (n=14)
surface

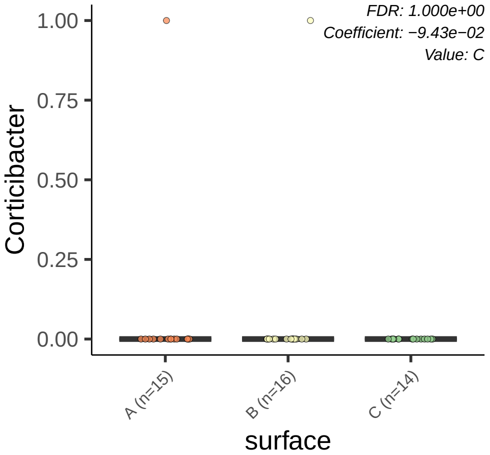
FDR: 1.000e+00
Coefficient: −9.43e−02
Value: C
Corticibacter
1.00
0.75
0.50
0.25
0.00
A (n=15)
B (n=16)
C (n=14)
surface

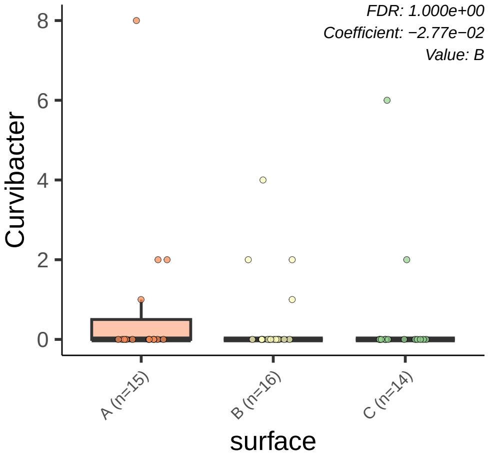

FDR: 1.000e+00
Coefficient: −2.77e−02
Value: B
Curvibacter
8
6
4
2
0
A (n=15)
B (n=16)
C (n=14)
surface

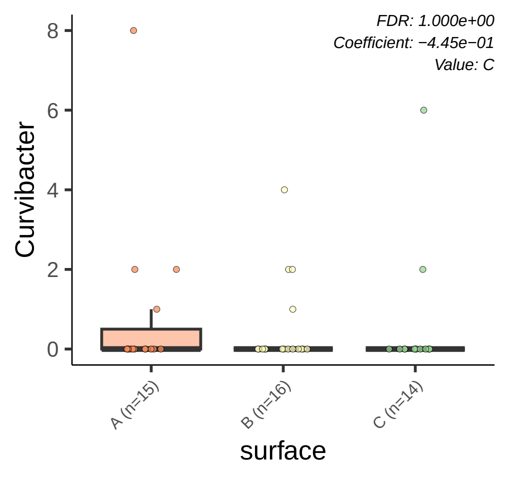

FDR: 1.000e+00
Coefficient: −4.45e−01
Value: C
Curvibacter
0
2
4
6
8
A (n=15)
B (n=16)
C (n=14)
surface

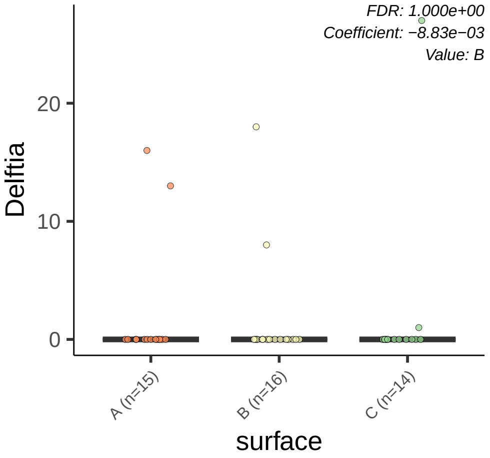
FDR: 1.000e+00
Coefficient: −8.83e−03
Value: B
Delftia
20
10
0
A (n=15)
B (n=16)
C (n=14)
surface

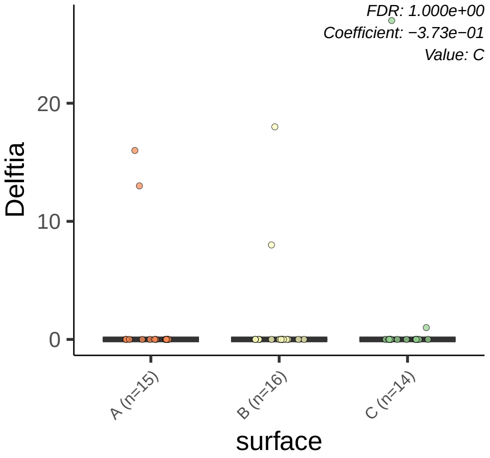

FDR: 1.000e+00
Coefficient: −3.73e−01
Value: C
Delftia
20
10
0
A (n=15)
B (n=16)
C (n=14)
surface

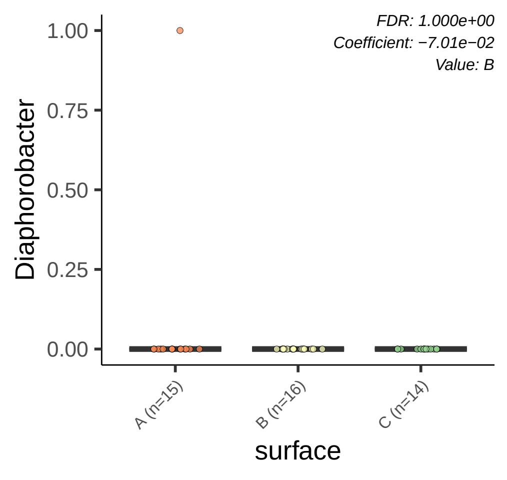
FDR: 1.000e+00
Coefficient: −7.01e−02
Value: B
Diaphorobacter
1.00
0.75
0.50
0.25
0.00
A (n=15)
B (n=16)
C (n=14)
surface

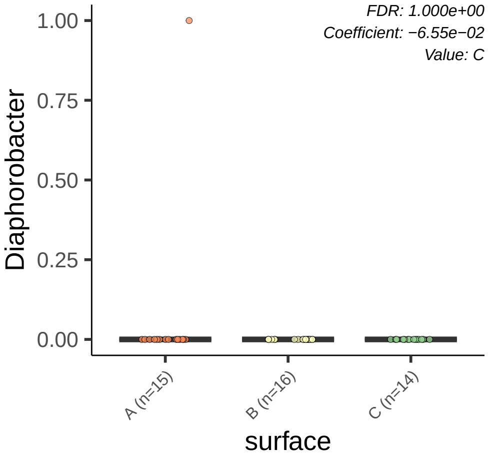
FDR: 1.000e+00
Coefficient: −6.55e−02
Value: C
Diaphorobacter
1.00
0.75
0.50
0.25
0.00
A (n=15)
B (n=16)
C (n=14)
surface

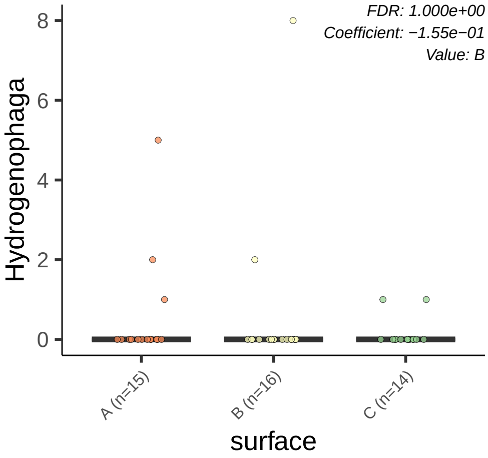

FDR: 1.000e+00
Coefficient: −1.55e−01
Value: B
Hydrogenophaga
8
6
4
2
0
A (n=15)
B (n=16)
C (n=14)
surface

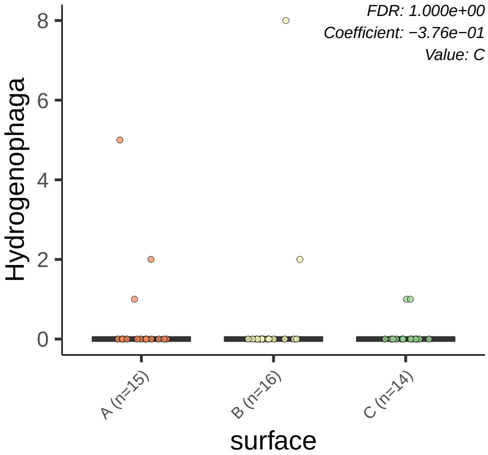
FDR: 1.000e+00
Coefficient: −3.76e−01
Value: C
Hydrogenophaga
0
2
4
6
8
A (n=15)
B (n=16)
C (n=14)
surface

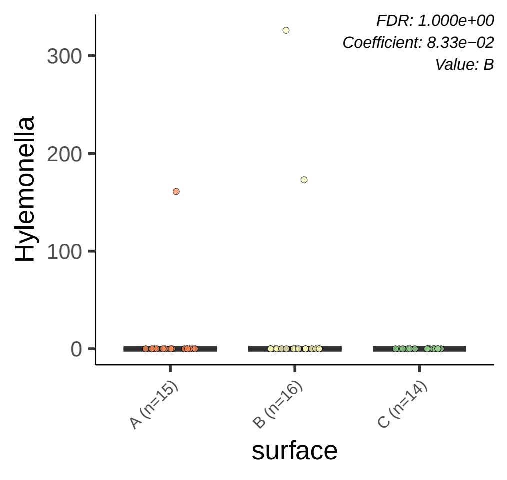

FDR: 1.000e+00
Coefficient: 8.33e−02
Value: B
Hylemonella
300
200
100
0
A (n=15)
B (n=16)
C (n=14)
surface

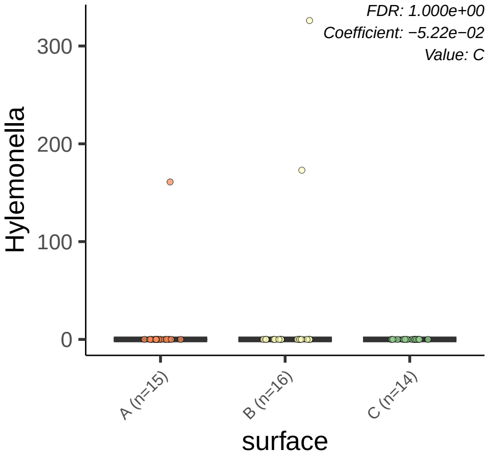

FDR: 1.000e+00
Coefficient: −5.22e−02
Value: C
Hylemonella
300
200
100
0
A (n=15)
B (n=16)
C (n=14)
surface

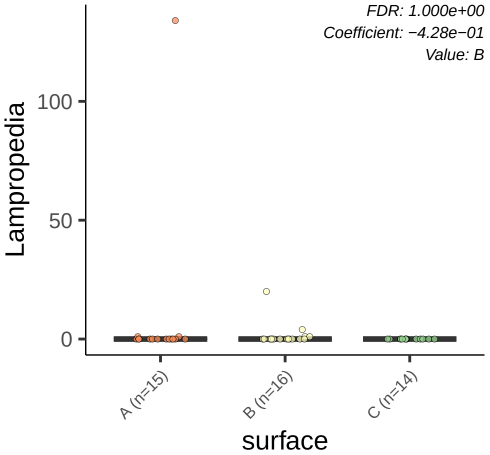

FDR: 1.000e+00
Coefficient: −4.28e−01
Value: B
Lampropedia
100
50
0
A (n=15)
B (n=16)
C (n=14)
surface

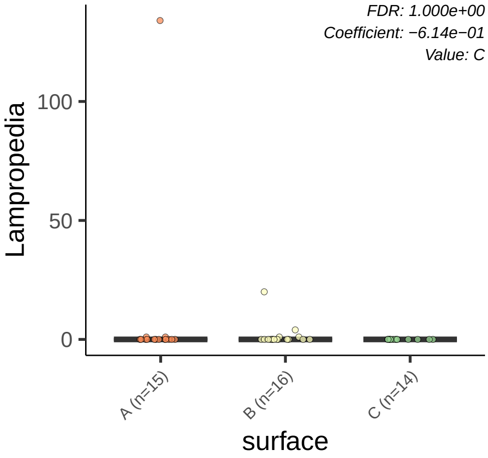

FDR: 1.000e+00
Coefficient: −6.14e−01
Value: C
Lampropedia
100
50
0
A (n=15)
B (n=16)
C (n=14)
surface

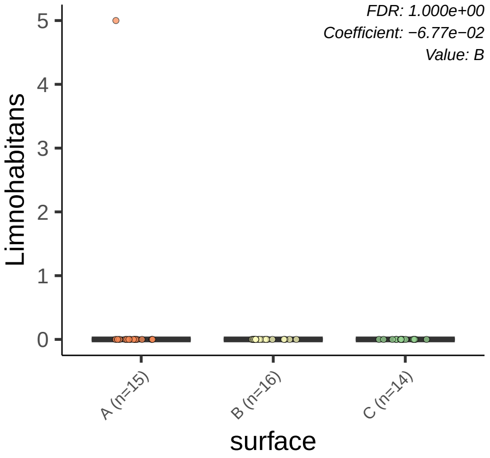
FDR: 1.000e+00
Coefficient: −6.77e−02
Value: B
Limnohabitans
5
4
3
2
1
0
A (n=15)
B (n=16)
C (n=14)
surface

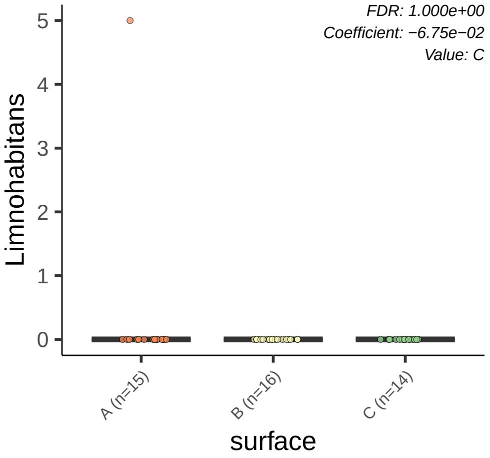

FDR: 1.000e+00
Coefficient: −6.75e−02
Value: C
Limnohabitans
5
4
3
2
1
0
A (n=15)
B (n=16)
C (n=14)
surface

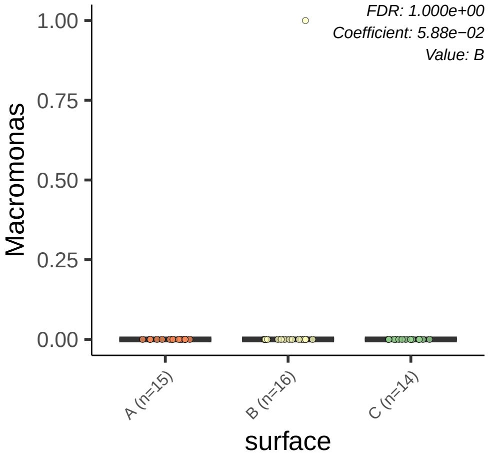
FDR: 1.000e+00
Coefficient: 5.88e−02
Value: B
Macromonas
1.00
0.75
0.50
0.25
0.00
A (n=15)
B (n=16)
C (n=14)
surface

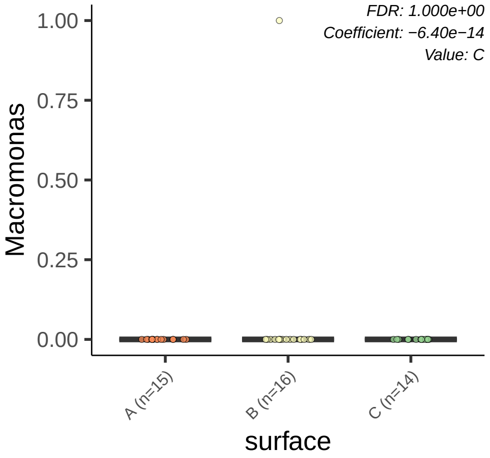

FDR: 1.000e+00
Coefficient: −6.40e−14
Value: C
Macromonas
1.00
0.75
0.50
0.25
0.00
A (n=15)
B (n=16)
C (n=14)
surface

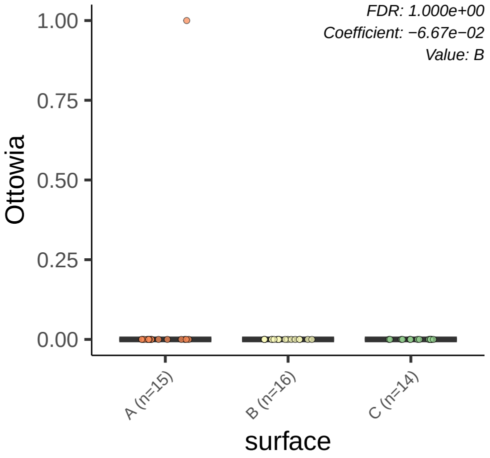
FDR: 1.000e+00
Coefficient: −6.67e−02
Value: B
Ottowia
1.00
0.75
0.50
0.25
0.00
A (n=15)
B (n=16)
C (n=14)
surface

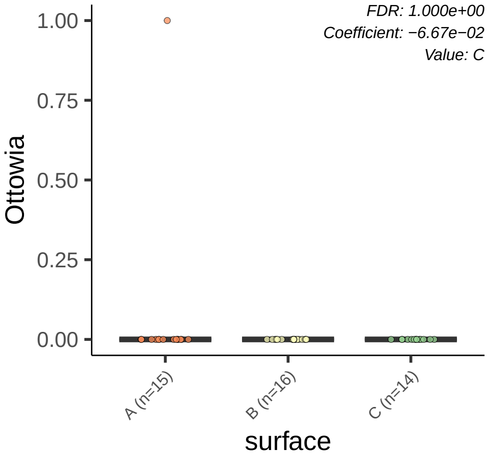

FDR: 1.000e+00
Coefficient: −6.67e−02
Value: C
Ottowia
1.00
0.75
0.50
0.25
0.00
A (n=15)
B (n=16)
C (n=14)
surface

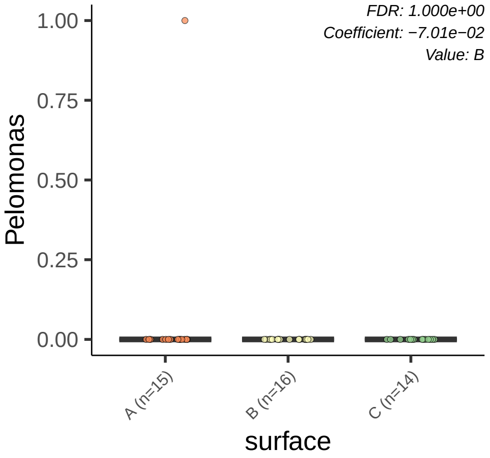

FDR: 1.000e+00
Coefficient: −7.01e−02
Value: B
1.00
0.75
0.50
0.25
0.00
Pelomonas
A (n=15)
B (n=16)
C (n=14)
surface

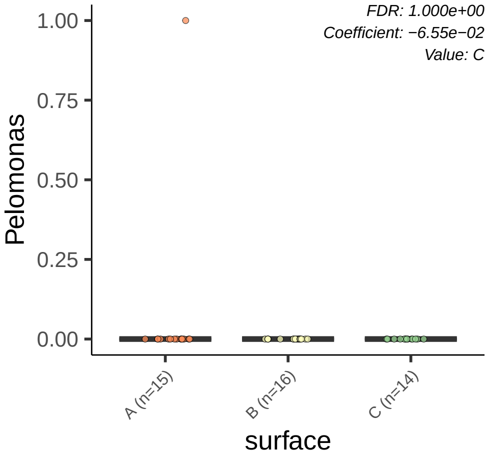
FDR: 1.000e+00
Coefficient: −6.55e−02
Value: C
Pelomonas
1.00
0.75
0.50
0.25
0.00
A (n=15)
B (n=16)
C (n=14)
surface

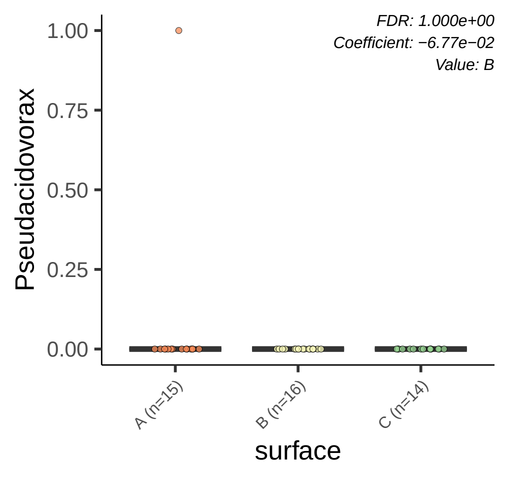
FDR: 1.000e+00
Coefficient: −6.77e−02
Value: B
Pseudacidovorax
1.00
0.75
0.50
0.25
0.00
A (n=15)
B (n=16)
C (n=14)
surface

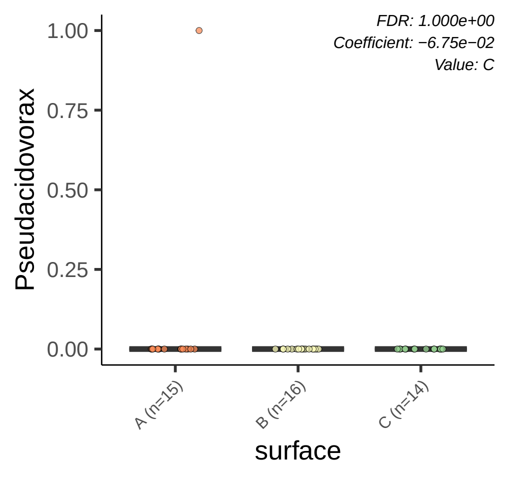
FDR: 1.000e+00
Coefficient: −6.75e−02
Value: C
Pseudacidovorax
1.00
0.75
0.50
0.25
0.00
A (n=15)
B (n=16)
C (n=14)
surface

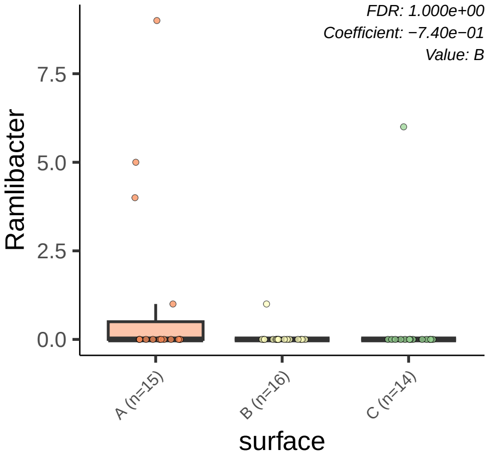
FDR: 1.000e+00
Coefficient: −7.40e−01
Value: B
Ramlibacter
7.5
5.0
2.5
0.0
A (n=15)
B (n=16)
C (n=14)
surface

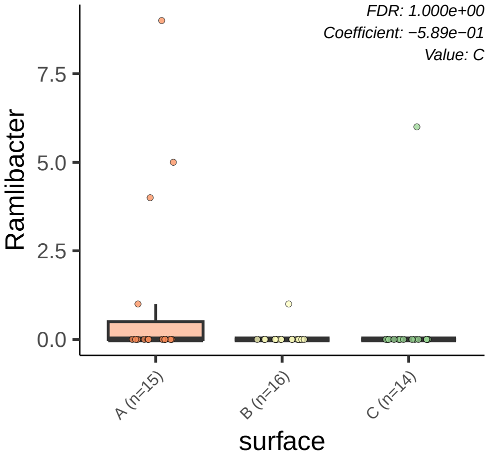

FDR: 1.000e+00
Coefficient: −5.89e−01
Value: C
Ramlibacter
7.5
5.0
2.5
0.0
A (n=15)
B (n=16)
C (n=14)
surface

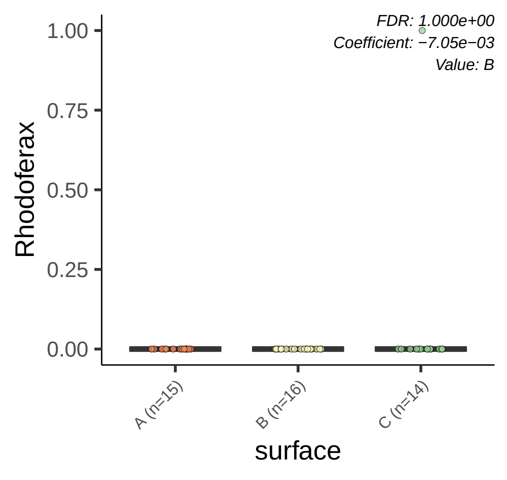

FDR: 1.000e+00
Coefficient: −7.05e−03
Value: B
Rhodoferax
1.00
0.75
0.50
0.25
0.00
A (n=15)
B (n=16)
C (n=14)
surface

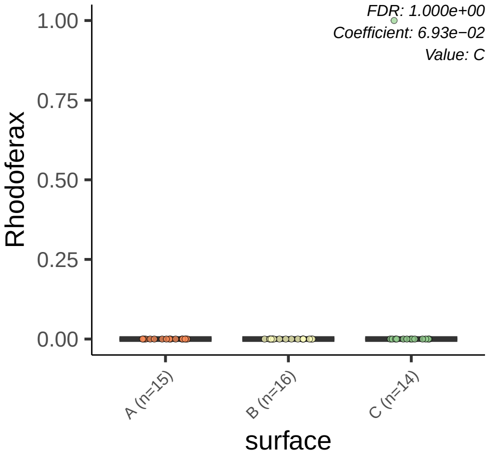
FDR: 1.000e+00
Coefficient: 6.93e−02
Value: C
Rhodoferax
1.00
0.75
0.50
0.25
0.00
A (n=15)
B (n=16)
C (n=14)
surface

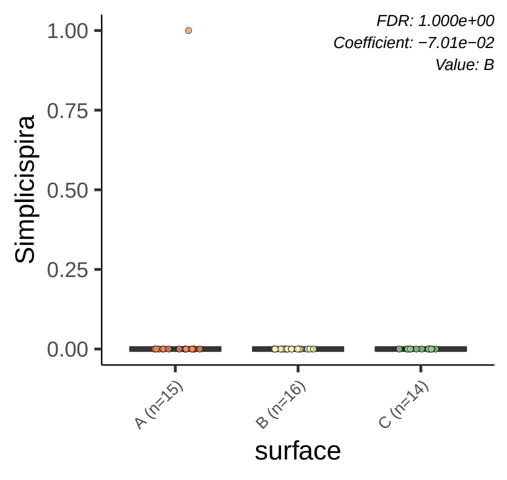
FDR: 1.000e+00
Coefficient: −7.01e−02
Value: B
Simplicispira
1.00
0.75
0.50
0.25
0.00
A (n=15)
B (n=16)
C (n=14)
surface

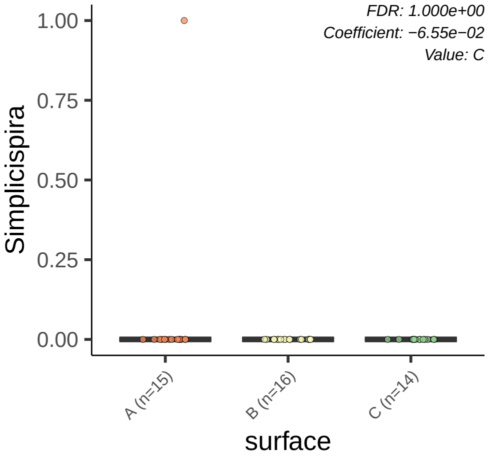
FDR: 1.000e+00
Coefficient: −6.55e−02
Value: C
Simplicispira
1.00
0.75
0.50
0.25
0.00
A (n=15)
B (n=16)
C (n=14)
surface

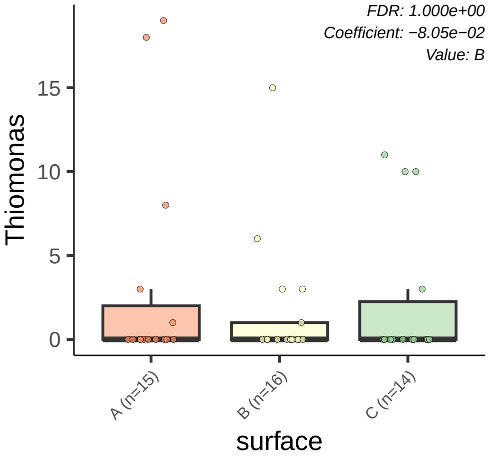

FDR: 1.000e+00
Coefficient: −8.05e−02
Value: B
Thiomonas
0
5
10
15
A (n=15)
B (n=16)
C (n=14)
surface

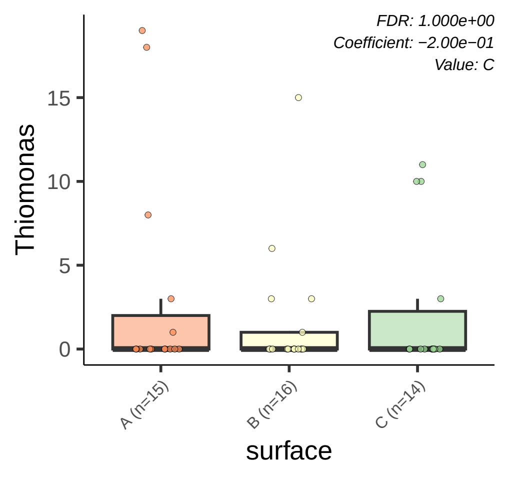

FDR: 1.000e+00
Coefficient: −2.00e−01
Value: C
Thiomonas
0
5
10
15
A (n=15)
B (n=16)
C (n=14)
surface

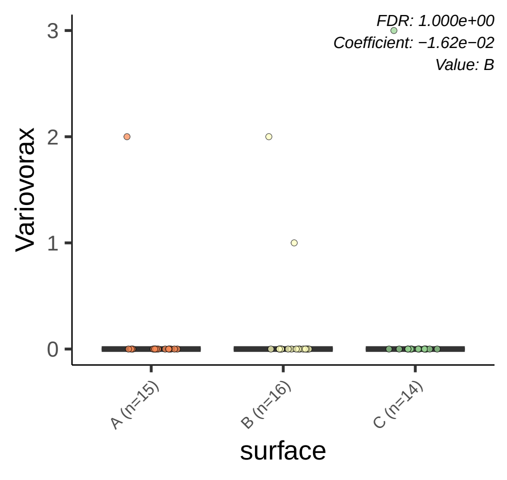
FDR: 1.000e+00
Coefficient: −1.62e−02
Value: B
Variovorax
3
2
1
0
A (n=15)
B (n=16)
C (n=14)
surface

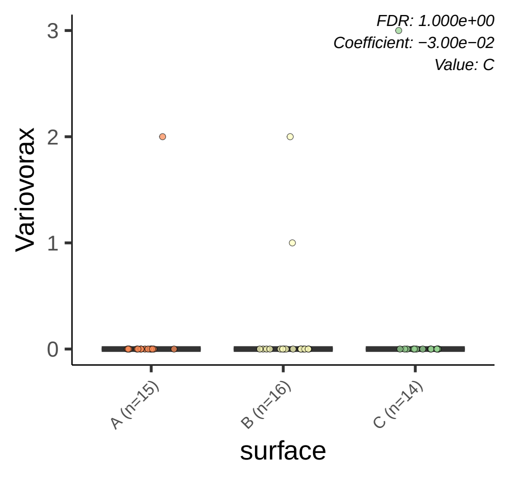

FDR: 1.000e+00
Coefficient: −3.00e−02
Value: C
Variovorax
3
2
1
0
A (n=15)
B (n=16)
C (n=14)
surface

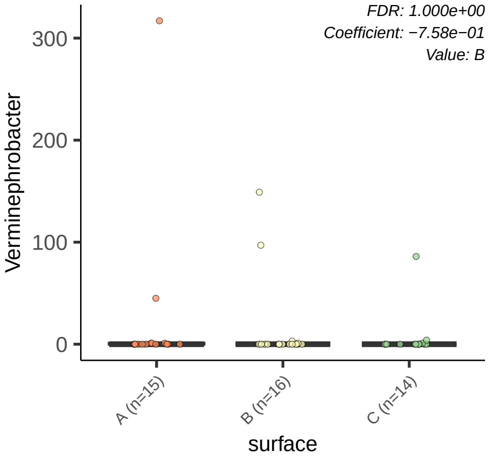
FDR: 1.000e+00
Coefficient: −7.58e−01
Value: B
Verminephrobacter
300
200
100
0
A (n=15)
B (n=16)
C (n=14)
surface

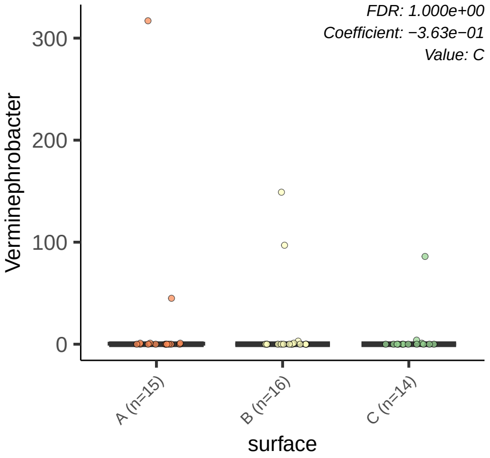
FDR: 1.000e+00
Coefficient: −3.63e−01
Value: C
300
200
100
0
Verminephrobacter
A (n=15)
B (n=16)
C (n=14)
surface

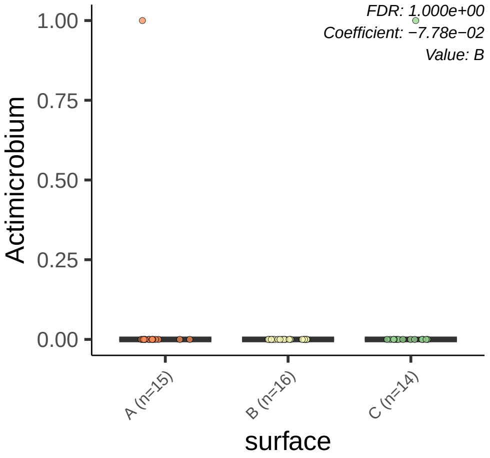
FDR: 1.000e+00
Coefficient: −7.78e−02
Value: B
Actimicrobium
1.00
0.75
0.50
0.25
0.00
A (n=15)
B (n=16)
C (n=14)
surface

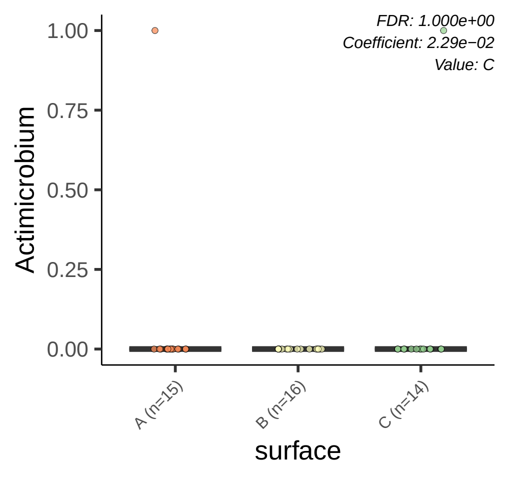

FDR: 1.000e+00
Coefficient: 2.29e−02
Value: C
Actimicrobium
1.00
0.75
0.50
0.25
0.00
A (n=15)
B (n=16)
C (n=14)
surface

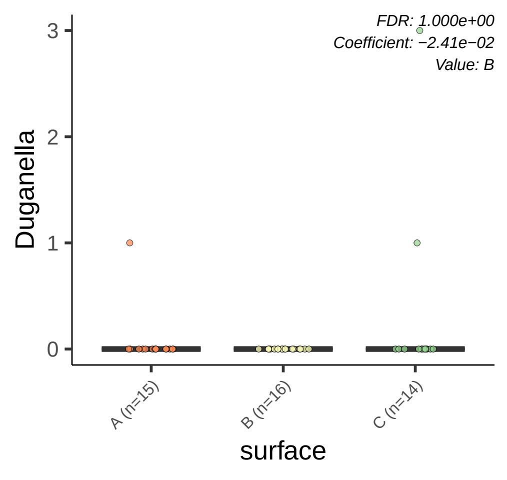
FDR: 1.000e+00
Coefficient: −2.41e−02
Value: B
Duganella
3
2
1
0
A (n=15)
B (n=16)
C (n=14)
surface

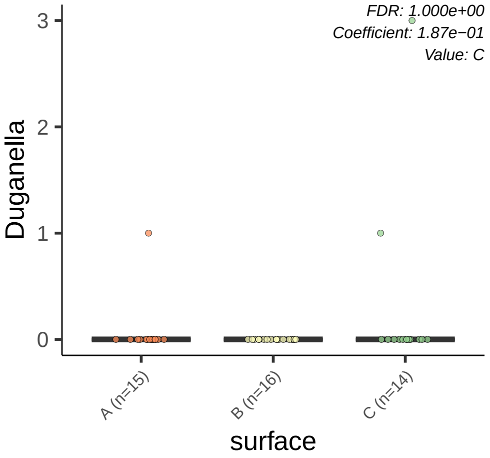

FDR: 1.000e+00
Coefficient: 1.87e−01
Value: C
Duganella
3
2
1
0
A (n=15)
B (n=16)
C (n=14)
surface

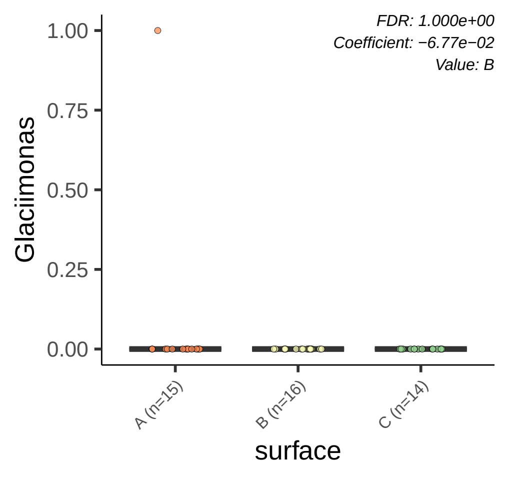
FDR: 1.000e+00
Coefficient: −6.77e−02
Value: B
Glaciimonas
1.00
0.75
0.50
0.25
0.00
A (n=15)
B (n=16)
C (n=14)
surface

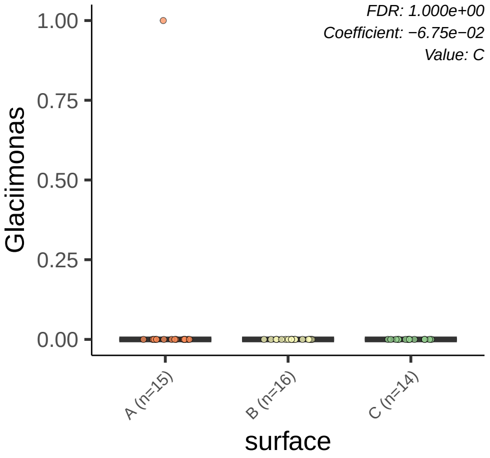
FDR: 1.000e+00
Coefficient: −6.75e−02
Value: C
Glaciimonas
1.00
0.75
0.50
0.25
0.00
A (n=15)
B (n=16)
C (n=14)
surface

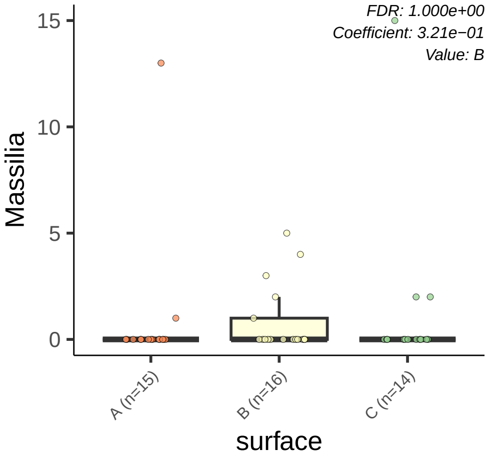
FDR: 1.000e+00
Coefficient: 3.21e−01
Value: B
Massilia
15
10
5
0
A (n=15)
B (n=16)
C (n=14)
surface

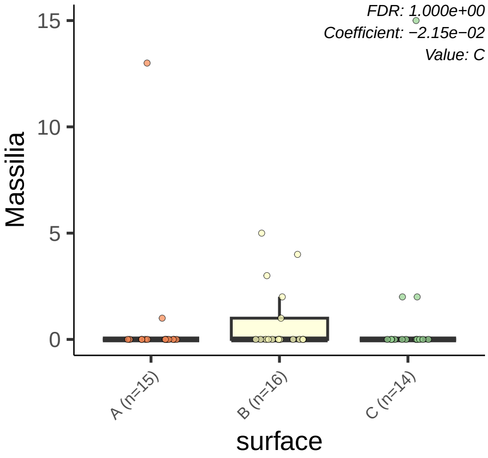

FDR: 1.000e+00
Coefficient: −2.15e−02
Value: C
Massilia
15
10
5
0
A (n=15)
B (n=16)
C (n=14)
surface

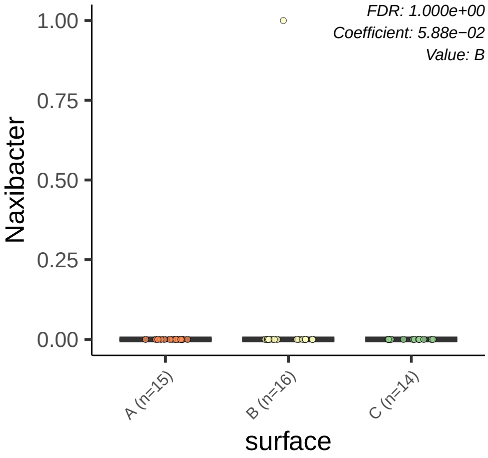
FDR: 1.000e+00
Coefficient: 5.88e−02
Value: B
Naxibacter
1.00
0.75
0.50
0.25
0.00
A (n=15)
B (n=16)
C (n=14)
surface

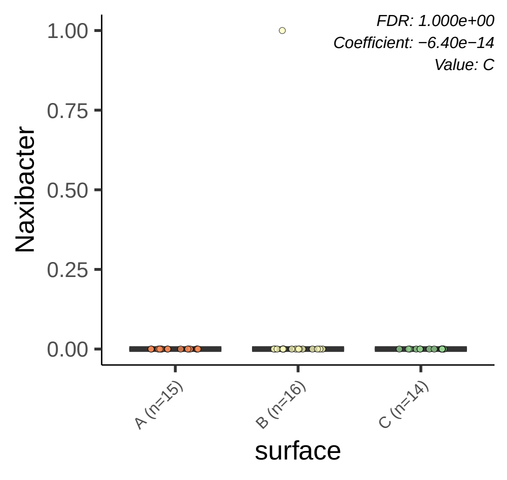

FDR: 1.000e+00
Coefficient: −6.40e−14
Value: C
Naxibacter
1.00
0.75
0.50
0.25
0.00
A (n=15)
B (n=16)
C (n=14)
surface

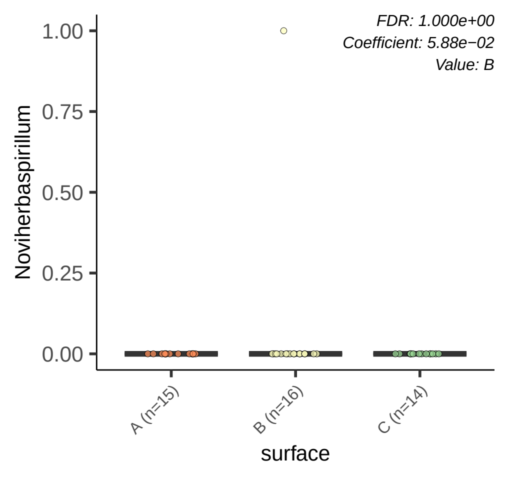

FDR: 1.000e+00
Coefficient: 5.88e−02
Value: B
1.00
0.75
0.50
0.25
0.00
Noviherbaspirillum
A (n=15)
B (n=16)
C (n=14)
surface

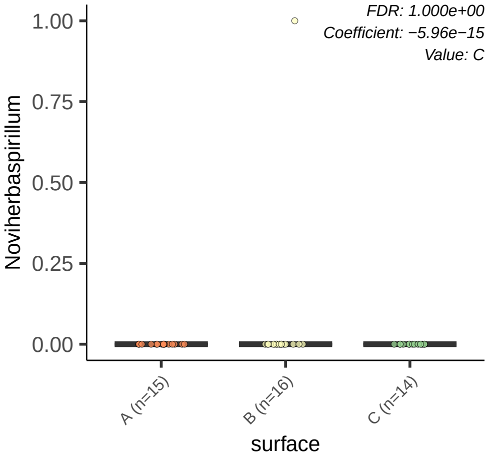
FDR: 1.000e+00
Coefficient: −5.96e−15
Value: C
Noviherbaspirillum
1.00
0.75
0.50
0.25
0.00
A (n=15)
B (n=16)
C (n=14)
surface

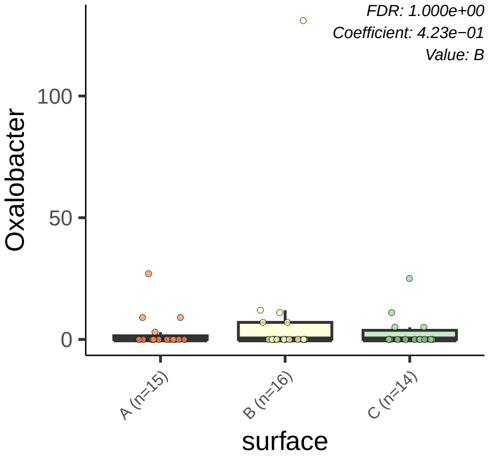

FDR: 1.000e+00
Coefficient: 4.23e−01
Value: B
Oxalobacter
100
50
0
A (n=15)
B (n=16)
C (n=14)
surface

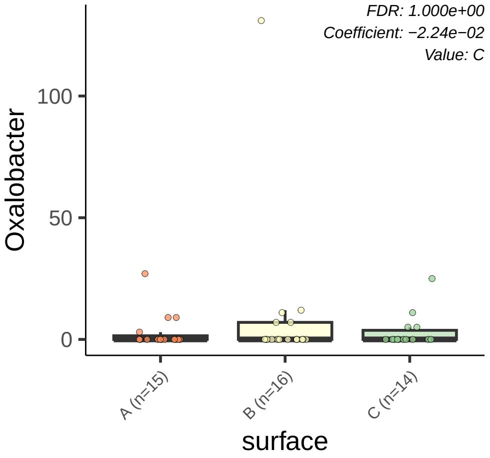
FDR: 1.000e+00
Coefficient: −2.24e−02
Value: C
Oxalobacter
100
50
0
A (n=15)
B (n=16)
C (n=14)
surface

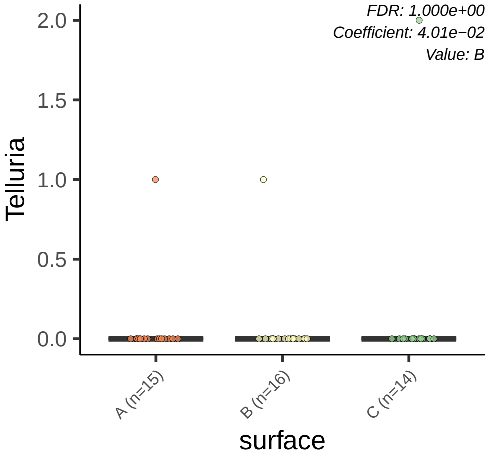
FDR: 1.000e+00
Coefficient: 4.01e−02
Value: B
Telluria
2.0
1.5
1.0
0.5
0.0
A (n=15)
B (n=16)
C (n=14)
surface

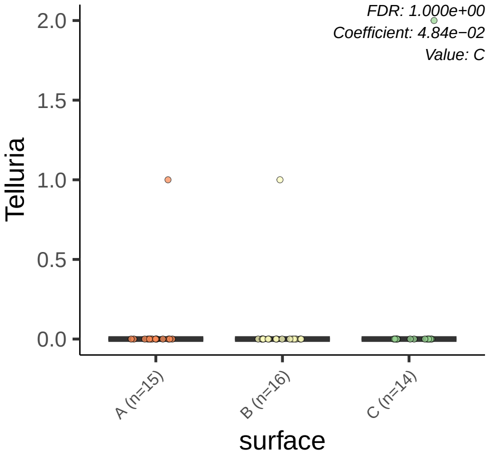
FDR: 1.000e+00
Coefficient: 4.84e−02
Value: C
Telluria
2.0
1.5
1.0
0.5
0.0
A (n=15)
B (n=16)
C (n=14)
surface

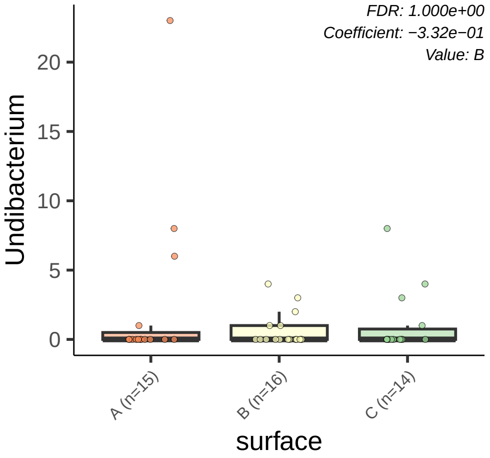

FDR: 1.000e+00
Coefficient: −3.32e−01
Value: B
Undibacterium
0
5
10
15
20
A (n=15)
B (n=16)
C (n=14)
surface

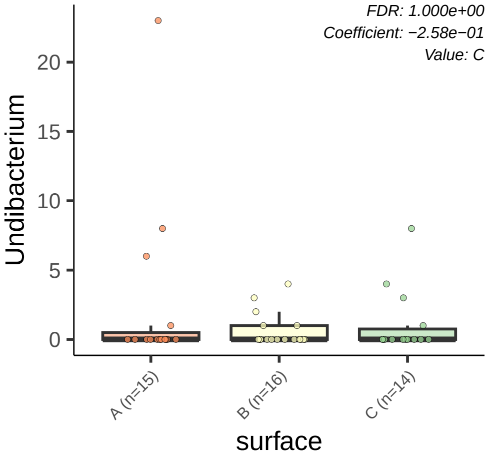
FDR: 1.000e+00
Coefficient: −2.58e−01
Value: C
Undibacterium
0
5
10
15
20
A (n=15)
B (n=16)
C (n=14)
surface

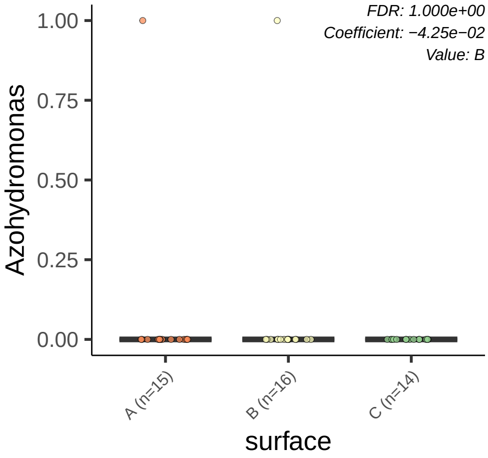

FDR: 1.000e+00
Coefficient: −4.25e−02
Value: B
Azohydromonas
1.00
0.75
0.50
0.25
0.00
A (n=15)
B (n=16)
C (n=14)
surface

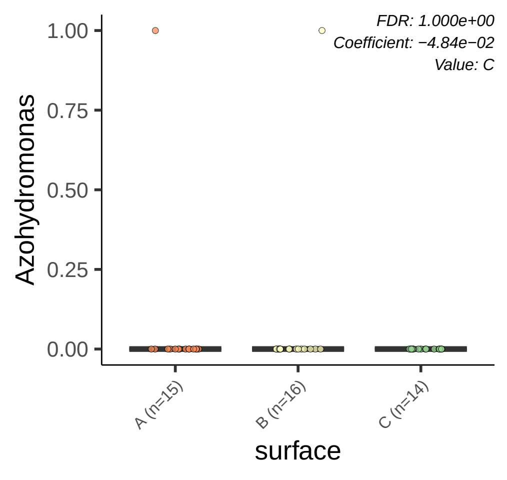

FDR: 1.000e+00
Coefficient: −4.84e−02
Value: C
Azohydromonas
1.00
0.75
0.50
0.25
0.00
A (n=15)
B (n=16)
C (n=14)
surface

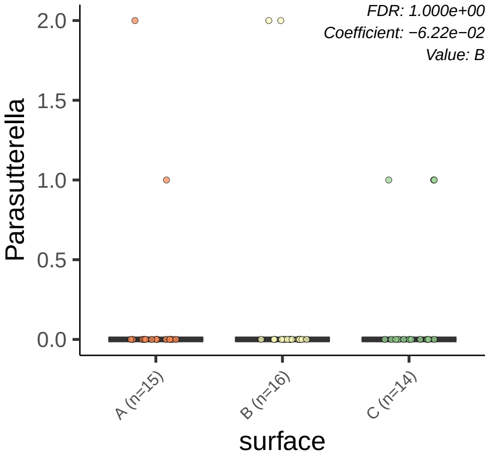

FDR: 1.000e+00
Coefficient: −6.22e−02
Value: B
Parasutterella
2.0
1.5
1.0
0.5
0.0
A (n=15)
B (n=16)
C (n=14)
surface

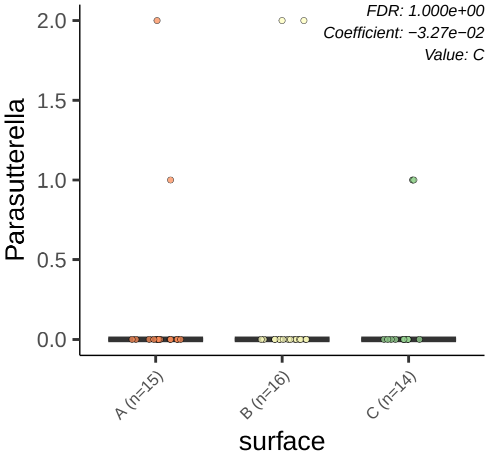
FDR: 1.000e+00
Coefficient: −3.27e−02
Value: C
Parasutterella
2.0
1.5
1.0
0.5
0.0
A (n=15)
B (n=16)
C (n=14)
surface

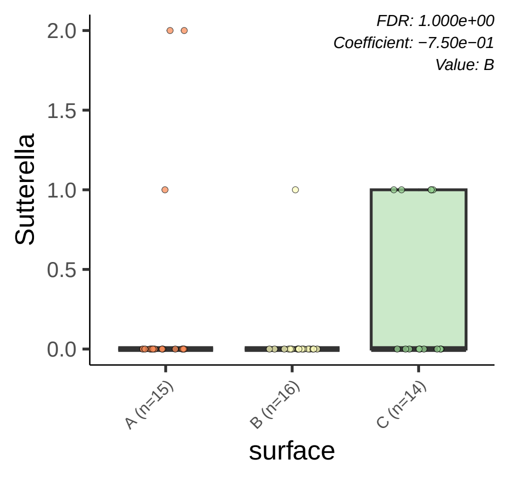
FDR: 1.000e+00
Coefficient: −7.50e−01
Value: B
Sutterella
2.0
1.5
1.0
0.5
0.0
A (n=15)
B (n=16)
C (n=14)
surface

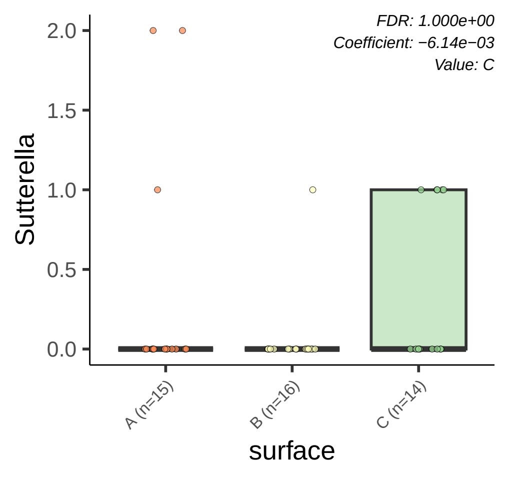
FDR: 1.000e+00
Coefficient: −6.14e−03
Value: C
Sutterella
2.0
1.5
1.0
0.5
0.0
A (n=15)
B (n=16)
C (n=14)
surface

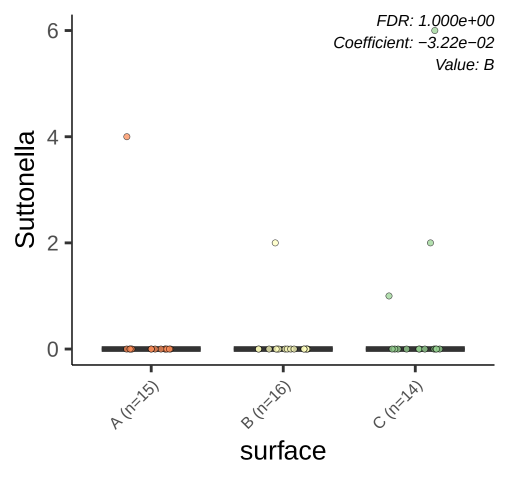
FDR: 1.000e+00
Coefficient: −3.22e−02
Value: B
Suttonella
0
2
4
6
A (n=15)
B (n=16)
C (n=14)
surface

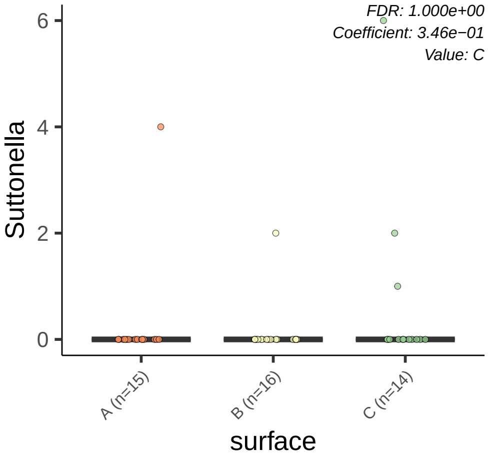
FDR: 1.000e+00
Coefficient: 3.46e−01
Value: C
Suttonella
6
4
2
0
A (n=15)
B (n=16)
C (n=14)
surface

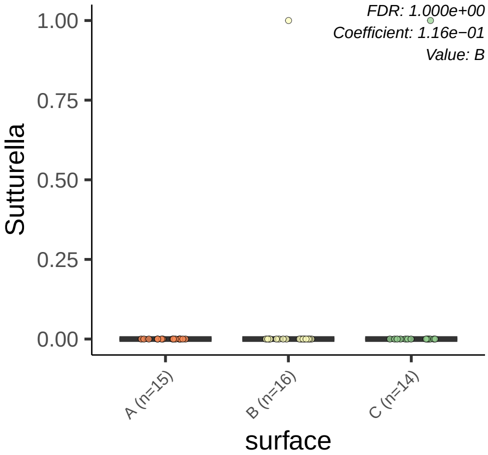
FDR: 1.000e+00
Coefficient: 1.16e−01
Value: B
Sutturella
1.00
0.75
0.50
0.25
0.00
A (n=15)
B (n=16)
C (n=14)
surface

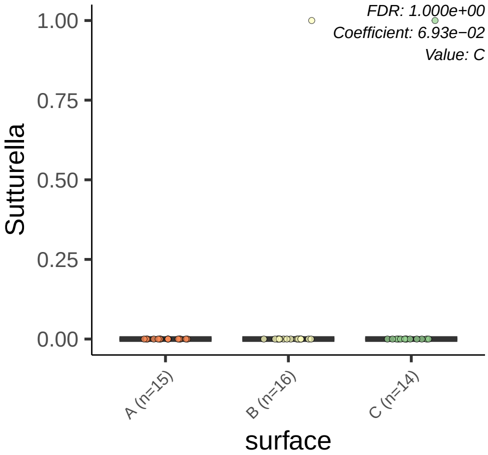
FDR: 1.000e+00
Coefficient: 6.93e−02
Value: C
Sutturella
1.00
0.75
0.50
0.25
0.00
A (n=15)
B (n=16)
C (n=14)
surface

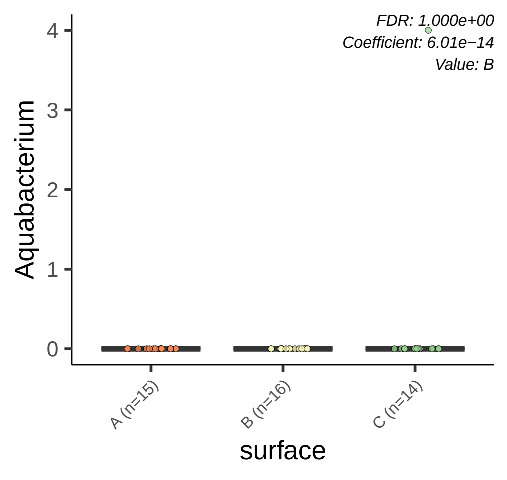
FDR: 1.000e+00
Coefficient: 6.01e−14
Value: B
Aquabacterium
0
1
2
3
4
A (n=15)
B (n=16)
C (n=14)
surface

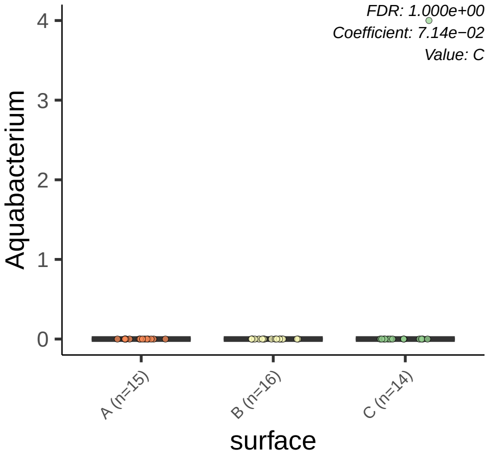

FDR: 1.000e+00
Coefficient: 7.14e−02
Value: C
Aquabacterium
4
3
2
1
0
A (n=15)
B (n=16)
C (n=14)
surface

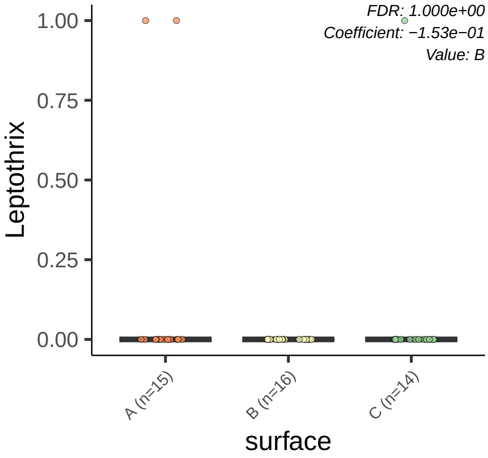
FDR: 1.000e+00
Coefficient: −1.53e−01
Value: B
Leptothrix
1.00
0.75
0.50
0.25
0.00
A (n=15)
B (n=16)
C (n=14)
surface

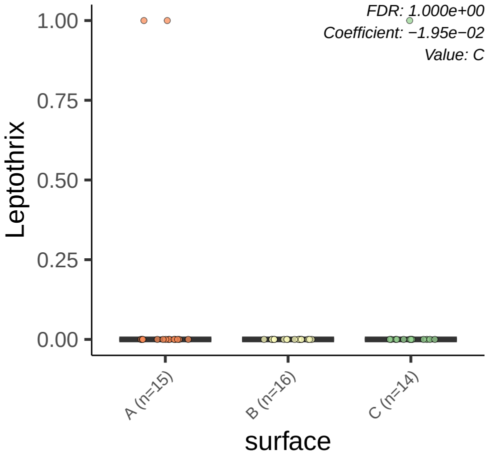
FDR: 1.000e+00
Coefficient: −1.95e−02
Value: C
Leptothrix
1.00
0.75
0.50
0.25
0.00
A (n=15)
B (n=16)
C (n=14)
surface

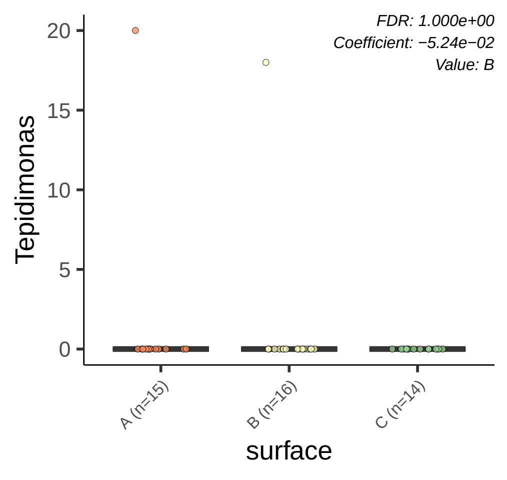

FDR: 1.000e+00
Coefficient: −5.24e−02
Value: B
Tepidimonas
20
15
10
5
0
A (n=15)
B (n=16)
C (n=14)
surface

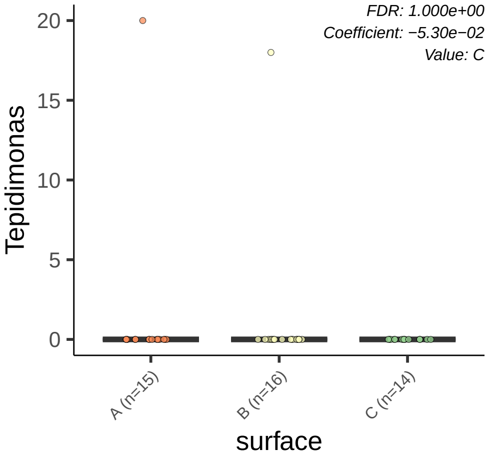
FDR: 1.000e+00
Coefficient: −5.30e−02
Value: C
Tepidimonas
20
15
10
5
0
A (n=15)
B (n=16)
C (n=14)
surface

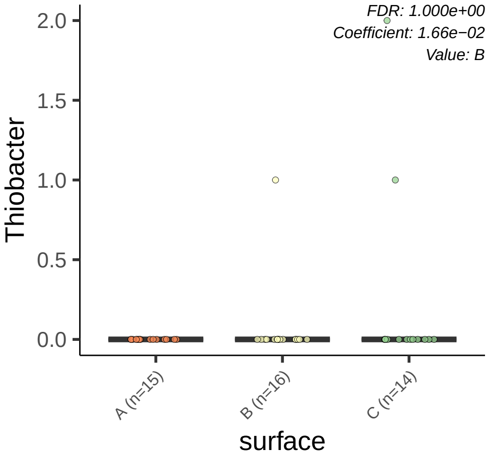

FDR: 1.000e+00
Coefficient: 1.66e−02
Value: B
Thiobacter
2.0
1.5
1.0
0.5
0.0
A (n=15)
B (n=16)
C (n=14)
surface

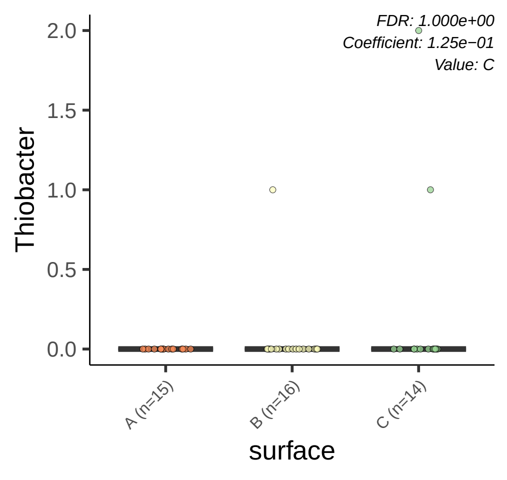
FDR: 1.000e+00
Coefficient: 1.25e−01
Value: C
Thiobacter
2.0
1.5
1.0
0.5
0.0
A (n=15)
B (n=16)
C (n=14)
surface

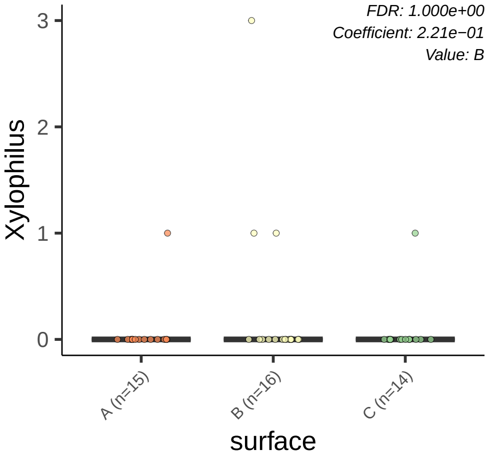
FDR: 1.000e+00
Coefficient: 2.21e−01
Value: B
Xylophilus
3
2
1
0
A (n=15)
B (n=16)
C (n=14)
surface

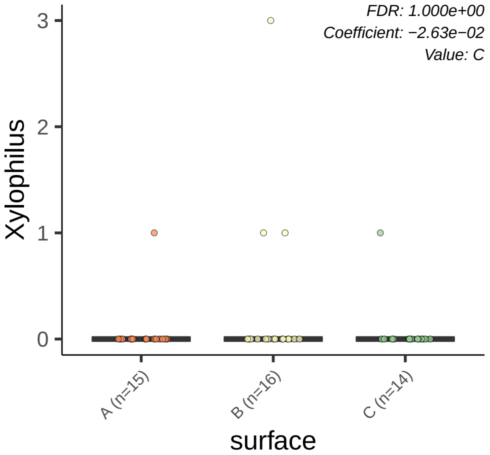
FDR: 1.000e+00
Coefficient: −2.63e−02
Value: C
Xylophilus
3
2
1
0
A (n=15)
B (n=16)
C (n=14)
surface

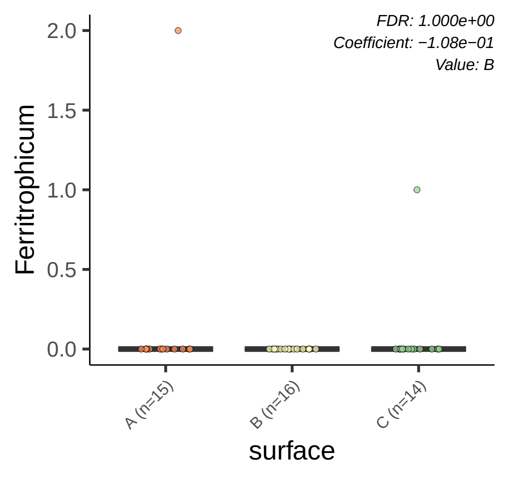
FDR: 1.000e+00
Coefficient: −1.08e−01
Value: B
Ferritrophicum
2.0
1.5
1.0
0.5
0.0
A (n=15)
B (n=16)
C (n=14)
surface

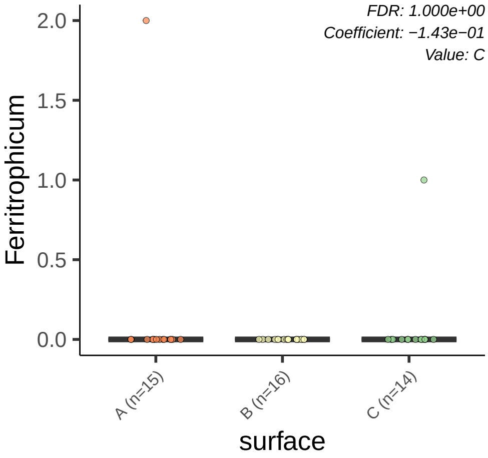

FDR: 1.000e+00
Coefficient: −1.43e−01
Value: C
Ferritrophicum
2.0
1.5
1.0
0.5
0.0
A (n=15)
B (n=16)
C (n=14)
surface

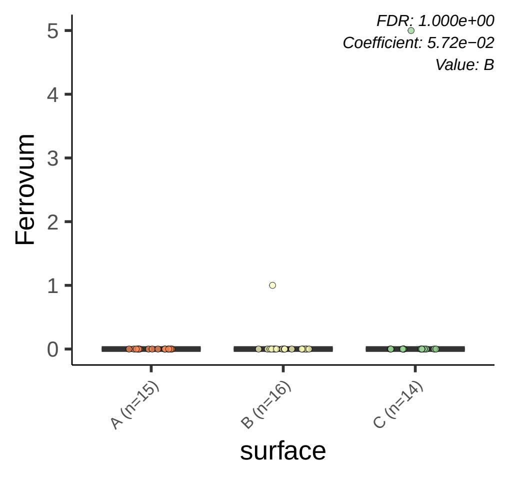
FDR: 1.000e+00
Coefficient: 5.72e−02
Value: B
Ferrovum
5
4
3
2
1
0
A (n=15)
B (n=16)
C (n=14)
surface

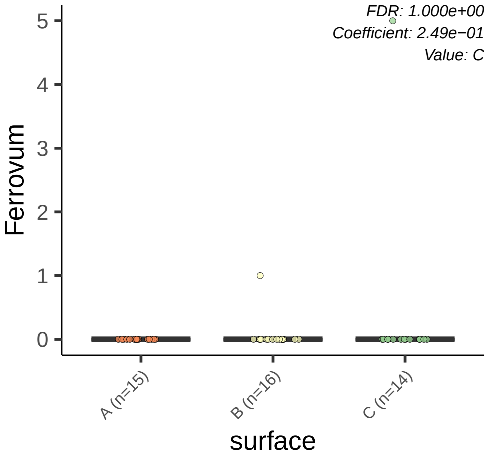
FDR: 1.000e+00
Coefficient: 2.49e−01
Value: C
Ferrovum
5
4
3
2
1
0
A (n=15)
B (n=16)
C (n=14)
surface

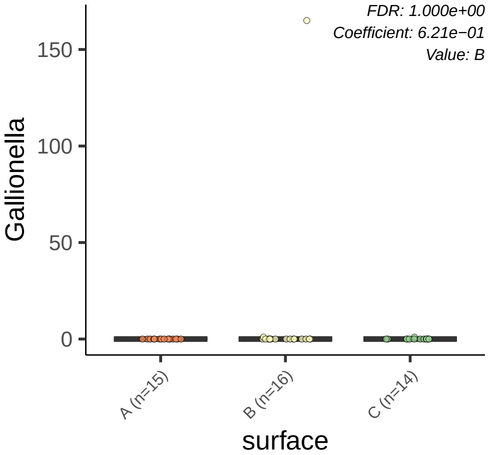

FDR: 1.000e+00
Coefficient: 6.21e−01
Value: B
Gallionella
150
100
50
0
A (n=15)
B (n=16)
C (n=14)
surface

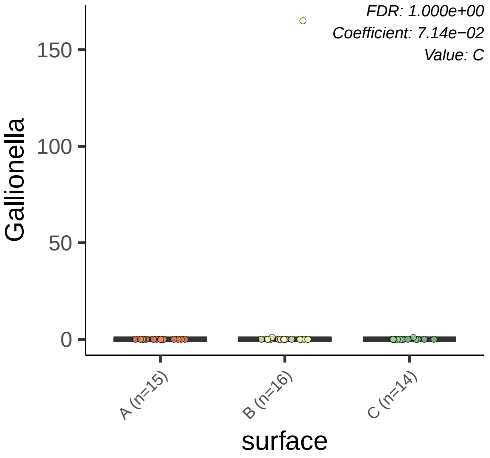
FDR: 1.000e+00
Coefficient: 7.14e−02
Value: C
Gallionella
150
100
50
0
A (n=15)
B (n=16)
C (n=14)
surface

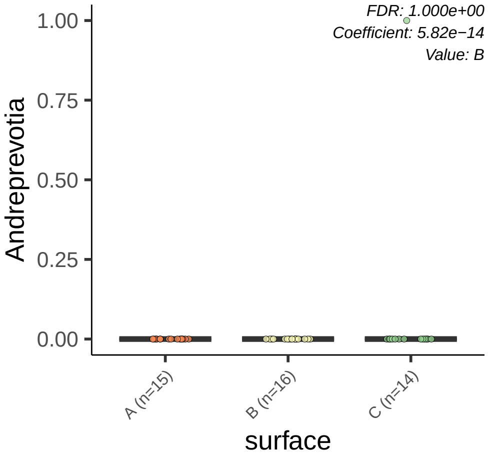
FDR: 1.000e+00
Coefficient: 5.82e−14
Value: B
Andreprevotia
1.00
0.75
0.50
0.25
0.00
A (n=15)
B (n=16)
C (n=14)
surface

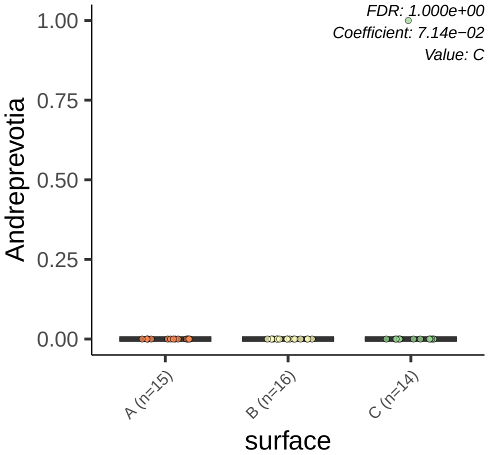
FDR: 1.000e+00
Coefficient: 7.14e−02
Value: C
1.00
0.75
0.50
0.25
0.00
Andreprevotia
A (n=15)
B (n=16)
C (n=14)
surface

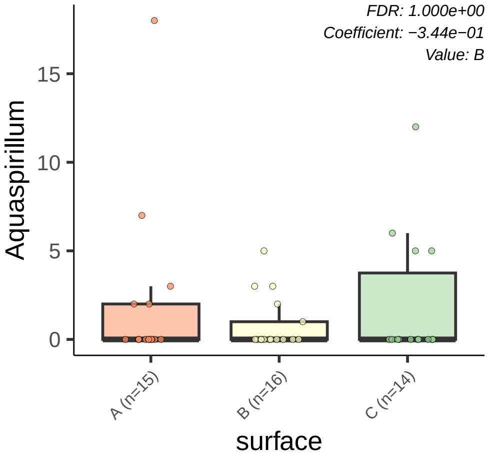
FDR: 1.000e+00
Coefficient: −3.44e−01
Value: B
Aquaspirillum
15
10
5
0
A (n=15)
B (n=16)
C (n=14)
surface

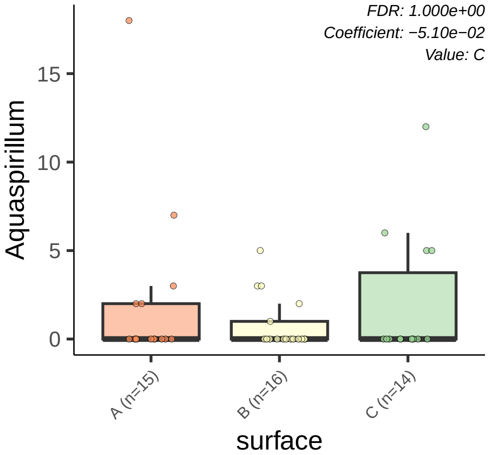

FDR: 1.000e+00
Coefficient: −5.10e−02
Value: C
Aquaspirillum
15
10
5
0
A (n=15)
B (n=16)
C (n=14)
surface

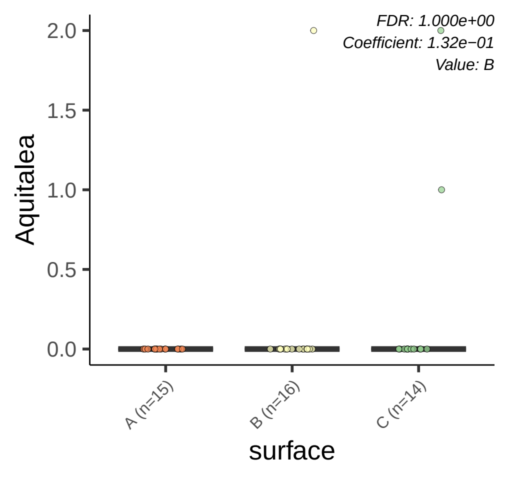

FDR: 1.000e+00
Coefficient: 1.32e−01
Value: B
Aquitalea
2.0
1.5
1.0
0.5
0.0
A (n=15)
B (n=16)
C (n=14)
surface

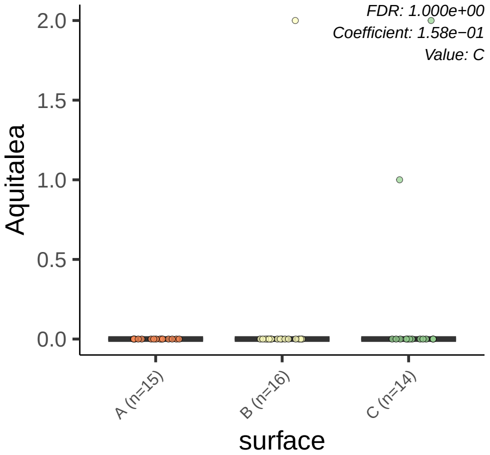
FDR: 1.000e+00
Coefficient: 1.58e−01
Value: C
Aquitalea
2.0
1.5
1.0
0.5
0.0
A (n=15)
B (n=16)
C (n=14)
surface

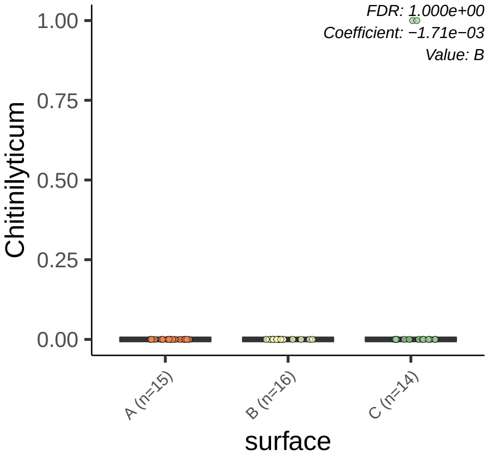
FDR: 1.000e+00
Coefficient: −1.71e−03
Value: B
1.00
0.75
0.50
0.25
0.00
Chitinilyticum
A (n=15)
B (n=16)
C (n=14)
surface

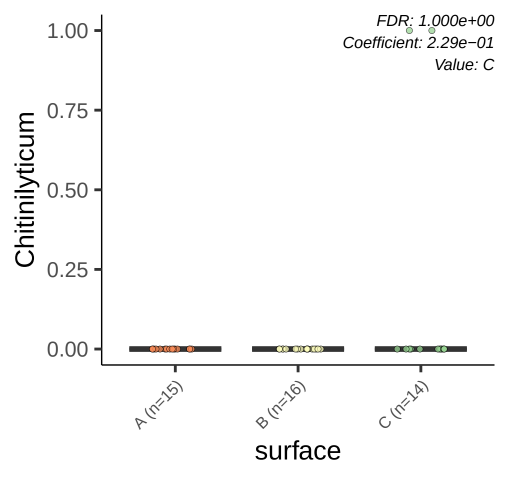
FDR: 1.000e+00
Coefficient: 2.29e−01
Value: C
1.00
0.75
0.50
0.25
0.00
Chitinilyticum
A (n=15)
B (n=16)
C (n=14)
surface

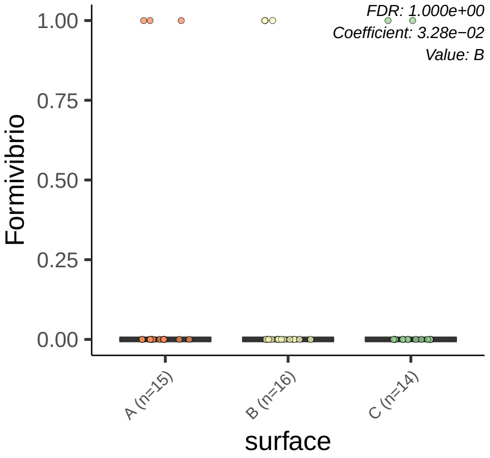
FDR: 1.000e+00
Coefficient: 3.28e−02
Value: B
Formivibrio
1.00
0.75
0.50
0.25
0.00
A (n=15)
B (n=16)
C (n=14)
surface

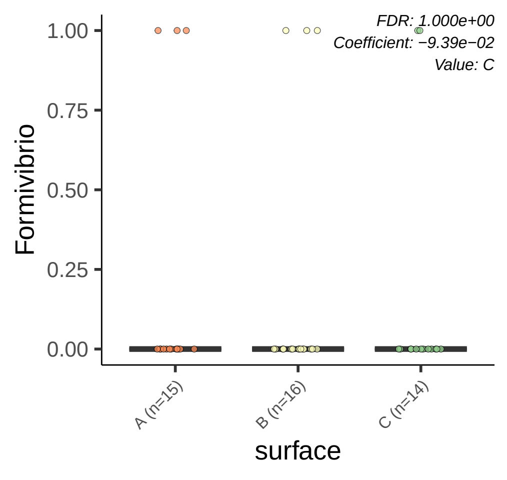
FDR: 1.000e+00
Coefficient: −9.39e−02
Value: C
1.00
0.75
0.50
0.25
0.00
Formivibrio
A (n=15)
B (n=16)
C (n=14)
surface

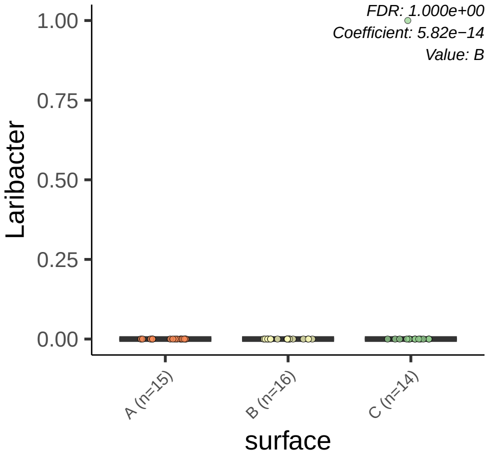
FDR: 1.000e+00
Coefficient: 5.82e−14
Value: B
1.00
0.75
0.50
0.25
0.00
Laribacter
A (n=15)
B (n=16)
C (n=14)
surface

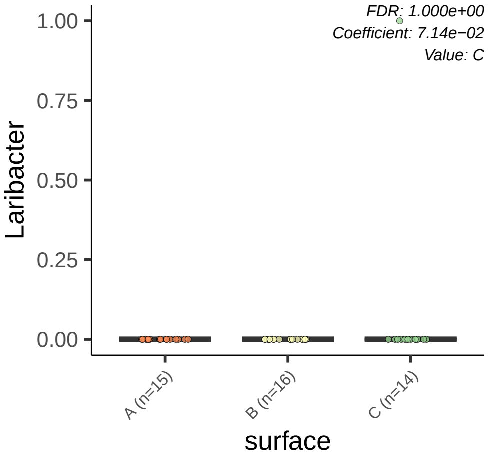
FDR: 1.000e+00
Coefficient: 7.14e−02
Value: C
Laribacter
1.00
0.75
0.50
0.25
0.00
A (n=15)
B (n=16)
C (n=14)
surface

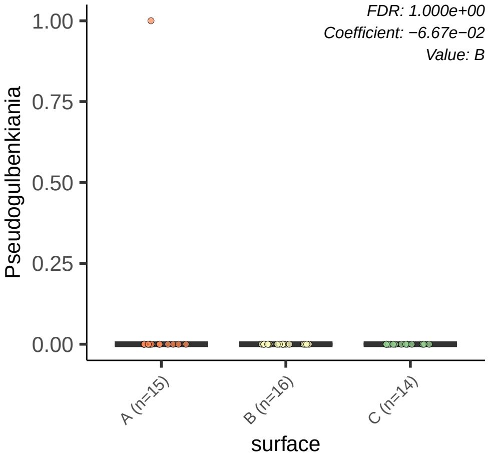

FDR: 1.000e+00
Coefficient: −6.67e−02
Value: B
Pseudogulbenkiania
1.00
0.75
0.50
0.25
0.00
A (n=15)
B (n=16)
C (n=14)
surface

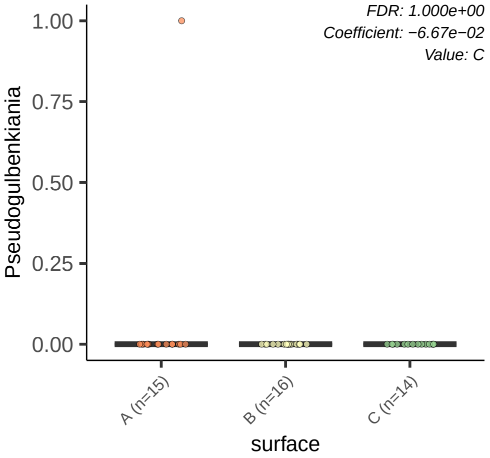
FDR: 1.000e+00
Coefficient: −6.67e−02
Value: C
Pseudogulbenkiania
1.00
0.75
0.50
0.25
0.00
A (n=15)
B (n=16)
C (n=14)
surface

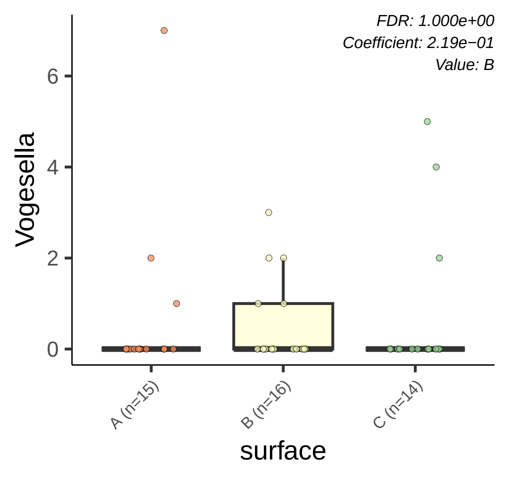
FDR: 1.000e+00
Coefficient: 2.19e−01
Value: B
Vogesella
0
2
4
6
A (n=15)
B (n=16)
C (n=14)
surface

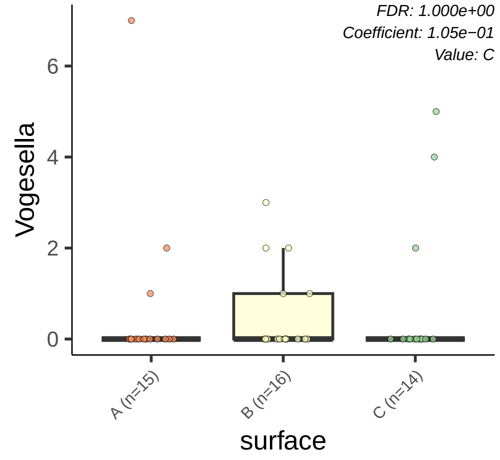
FDR: 1.000e+00
Coefficient: 1.05e−01
Value: C
Vogesella
0
2
4
6
A (n=15)
B (n=16)
C (n=14)
surface

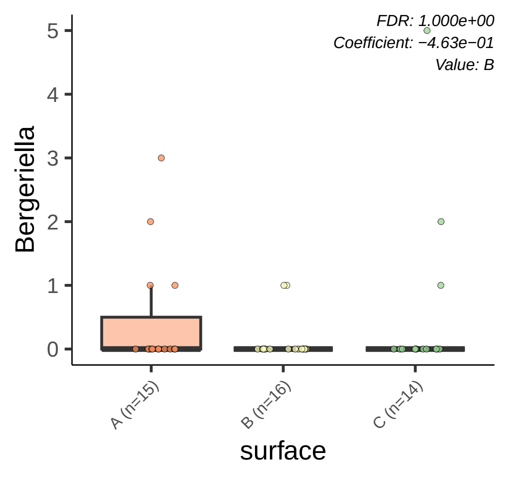

FDR: 1.000e+00
Coefficient: −4.63e−01
Value: B
Bergeriella
0
1
2
3
4
5
A (n=15)
B (n=16)
C (n=14)
surface

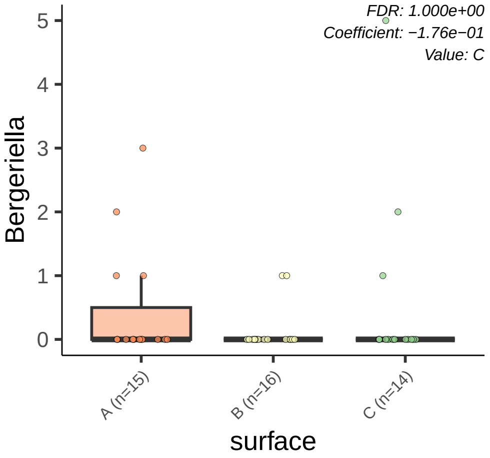
FDR: 1.000e+00
Coefficient: −1.76e−01
Value: C
Bergeriella
0
1
2
3
4
5
A (n=15)
B (n=16)
C (n=14)
surface

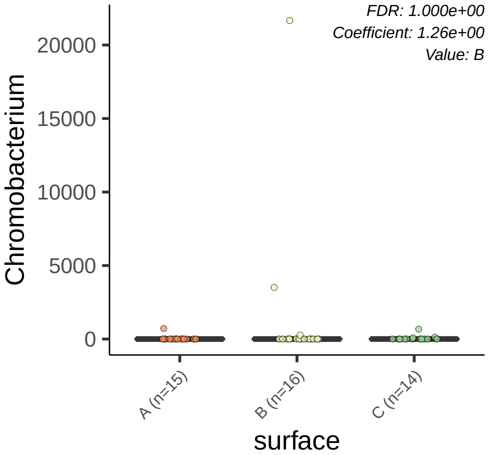

FDR: 1.000e+00
Coefficient: 1.26e+00
Value: B
Chromobacterium
20000
15000
10000
5000
0
A (n=15)
B (n=16)
C (n=14)
surface

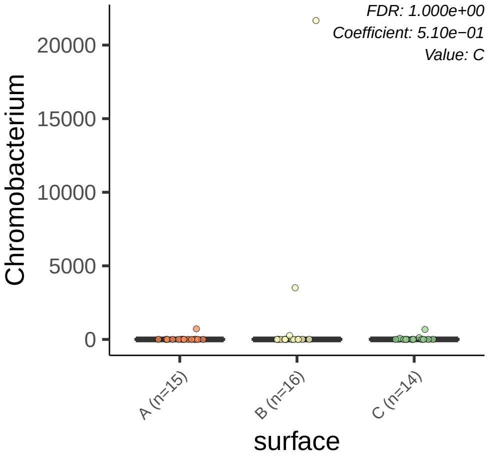
FDR: 1.000e+00
Coefficient: 5.10e−01
Value: C
Chromobacterium
20000
15000
10000
5000
0
A (n=15)
B (n=16)
C (n=14)
surface

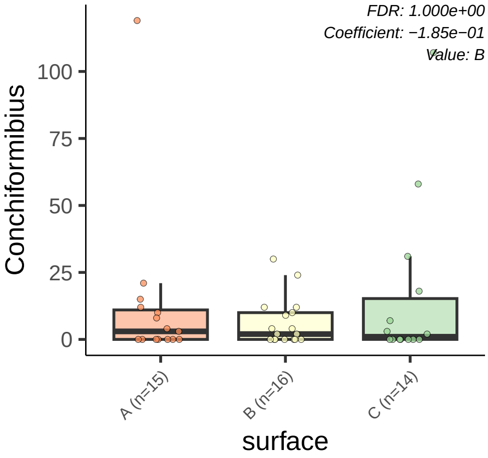

FDR: 1.000e+00
Coefficient: −1.85e−01
Value: B
Conchiformibius
100
75
50
25
0
A (n=15)
B (n=16)
C (n=14)
surface

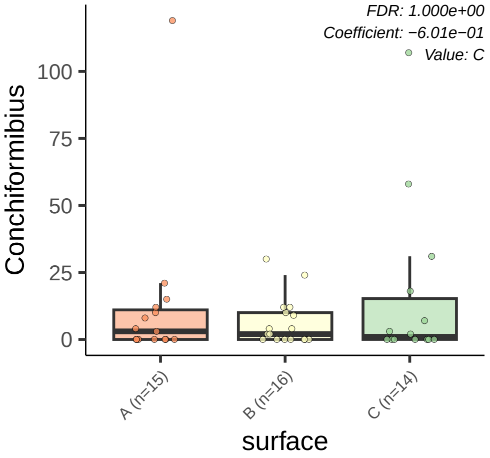
FDR: 1.000e+00
Coefficient: −6.01e−01
Value: C
Conchiformibius
100
75
50
25
0
A (n=15)
B (n=16)
C (n=14)
surface

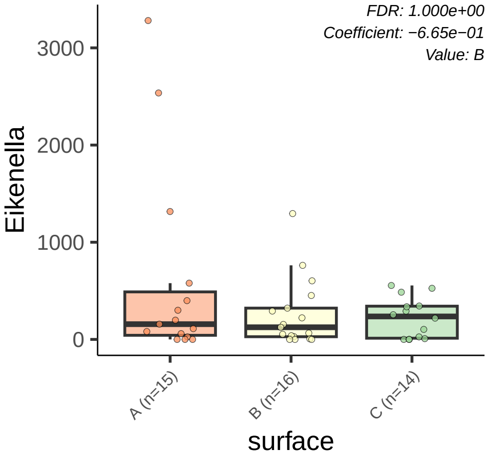

FDR: 1.000e+00
Coefficient: −6.65e−01
Value: B
Eikenella
3000
2000
1000
0
A (n=15)
B (n=16)
C (n=14)
surface

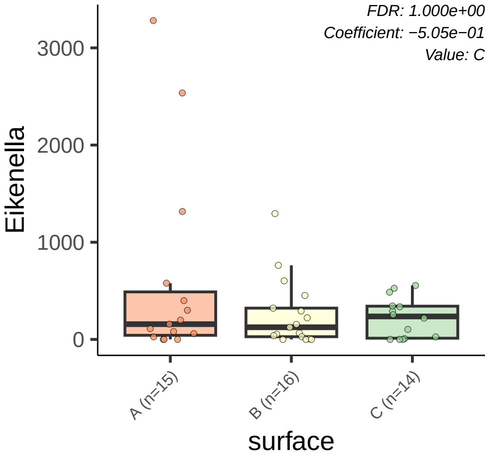

FDR: 1.000e+00
Coefficient: −5.05e−01
Value: C
3000
2000
1000
0
Eikenella
A (n=15)
B (n=16)
C (n=14)
surface

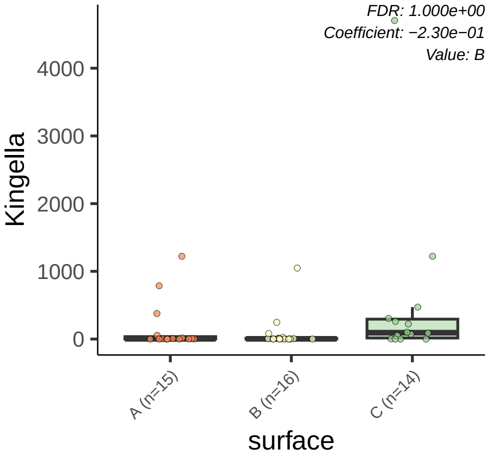
FDR: 1.000e+00
Coefficient: −2.30e−01
Value: B
Kingella
4000
3000
2000
1000
0
A (n=15)
B (n=16)
C (n=14)
surface

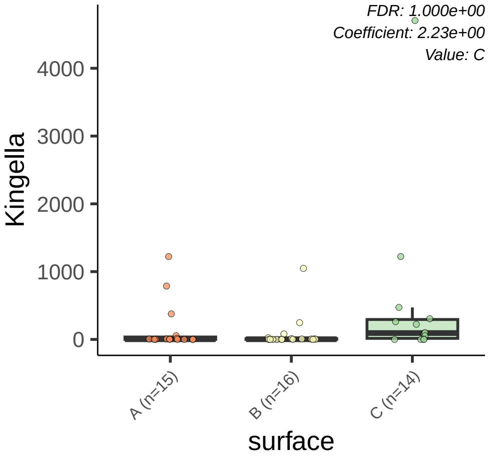
FDR: 1.000e+00
Coefficient: 2.23e+00
Value: C
Kingella
4000
3000
2000
1000
0
A (n=15)
B (n=16)
C (n=14)
surface

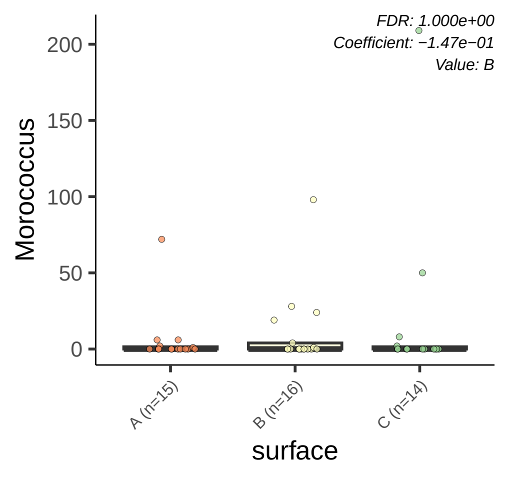

FDR: 1.000e+00
Coefficient: −1.47e−01
Value: B
Morococcus
200
150
100
50
0
A (n=15)
B (n=16)
C (n=14)
surface

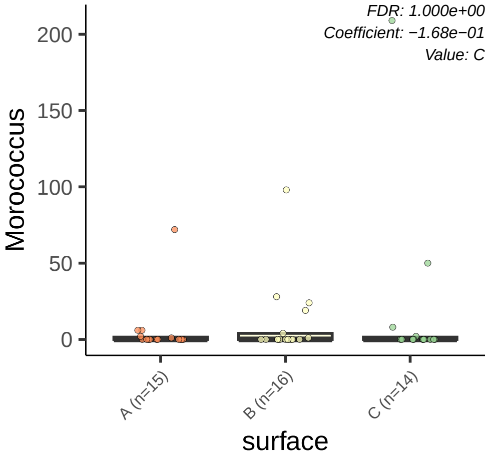

FDR: 1.000e+00
Coefficient: −1.68e−01
Value: C
Morococcus
0
50
100
150
200
A (n=15)
B (n=16)
C (n=14)
surface

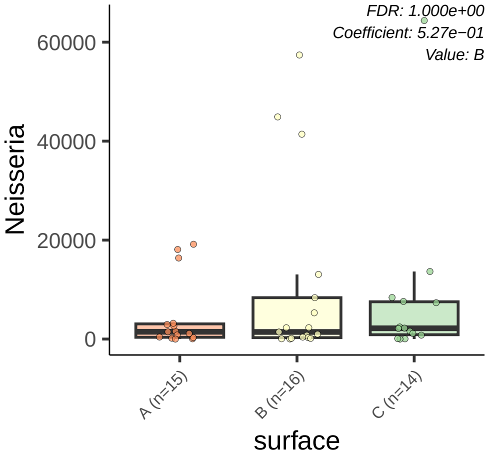

FDR: 1.000e+00
Coefficient: 5.27e−01
Value: B
60000
40000
20000
0
Neisseria
A (n=15)
B (n=16)
C (n=14)
surface

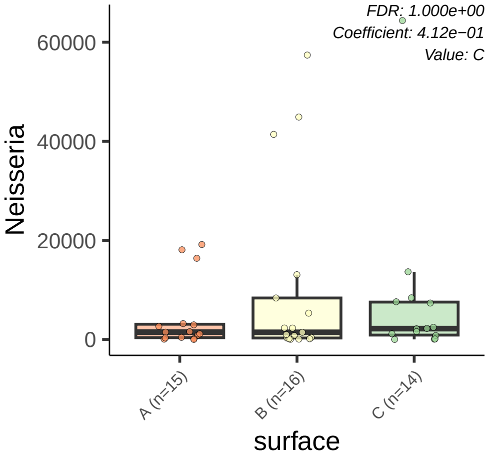
FDR: 1.000e+00
Coefficient: 4.12e−01
Value: C
60000
40000
20000
0
Neisseria
A (n=15)
B (n=16)
C (n=14)
surface

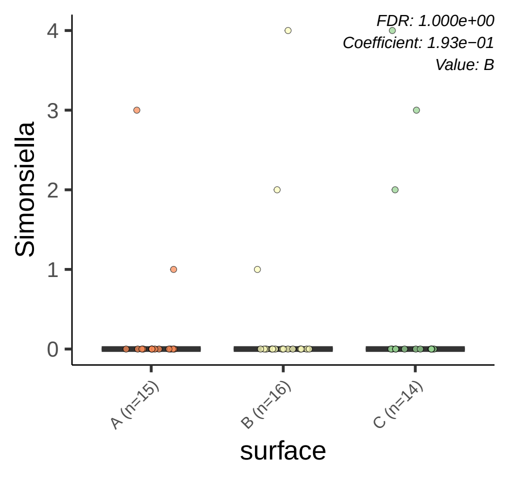

FDR: 1.000e+00
Coefficient: 1.93e−01
Value: B
Simonsiella
4
3
2
1
0
A (n=15)
B (n=16)
C (n=14)
surface

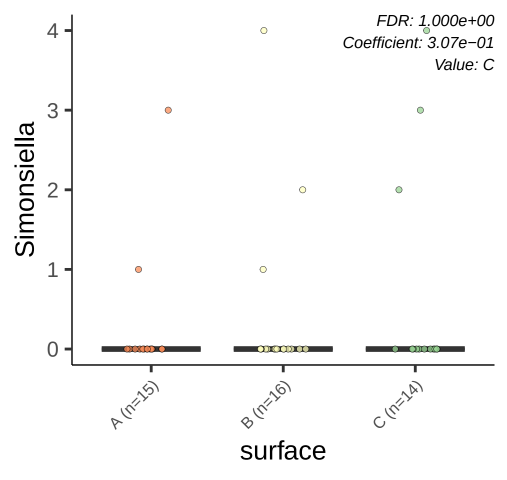

FDR: 1.000e+00
Coefficient: 3.07e−01
Value: C
Simonsiella
0
1
2
3
4
A (n=15)
B (n=16)
C (n=14)
surface

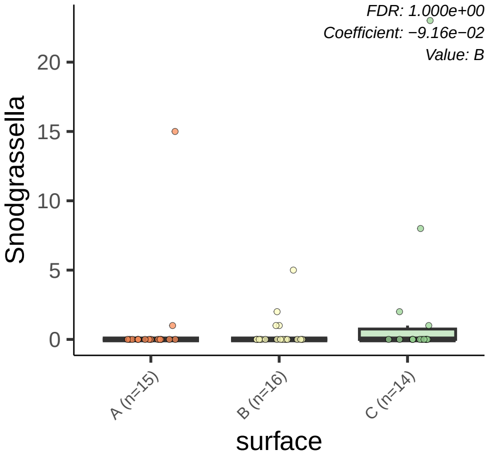
FDR: 1.000e+00
Coefficient: −9.16e−02
Value: B
Snodgrassella
20
15
10
5
0
A (n=15)
B (n=16)
C (n=14)
surface

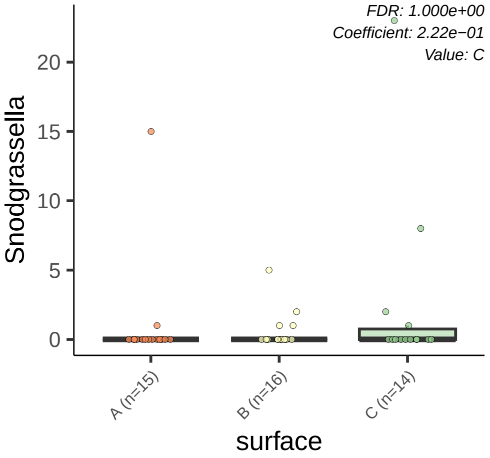

FDR: 1.000e+00
Coefficient: 2.22e−01
Value: C
Snodgrassella
20
15
10
5
0
A (n=15)
B (n=16)
C (n=14)
surface

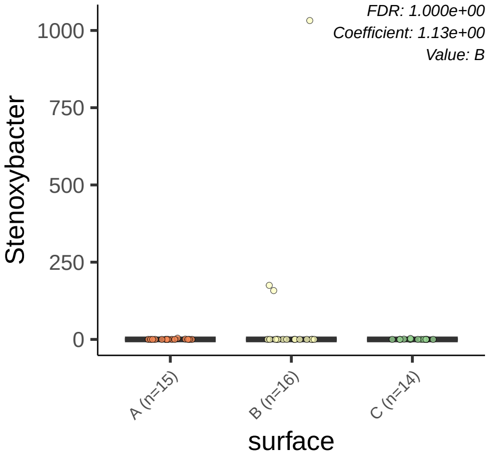

FDR: 1.000e+00
Coefficient: 1.13e+00
Value: B
Stenoxybacter
1000
750
500
250
0
A (n=15)
B (n=16)
C (n=14)
surface

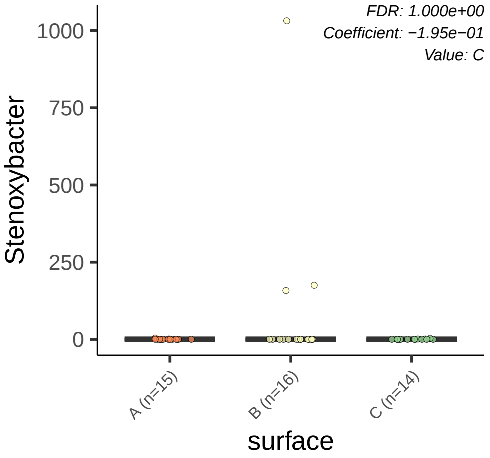

FDR: 1.000e+00
Coefficient: −1.95e−01
Value: C
Stenoxybacter
1000
750
500
250
0
A (n=15)
B (n=16)
C (n=14)
surface

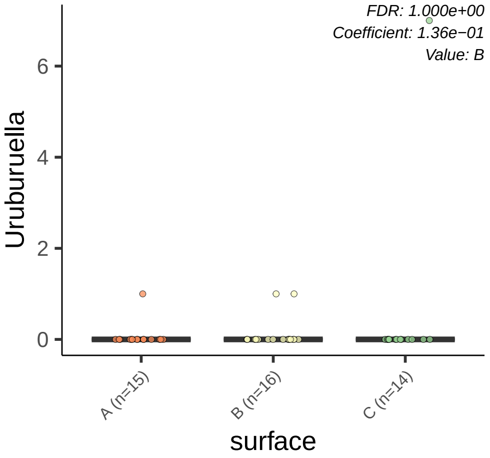
FDR: 1.000e+00
Coefficient: 1.36e−01
Value: B
Uruburuella
6
4
2
0
A (n=15)
B (n=16)
C (n=14)
surface

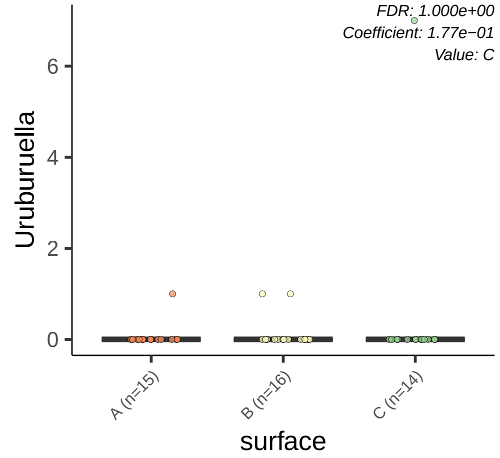

FDR: 1.000e+00
Coefficient: 1.77e−01
Value: C
Uruburuella
6
4
2
0
A (n=15)
B (n=16)
C (n=14)
surface

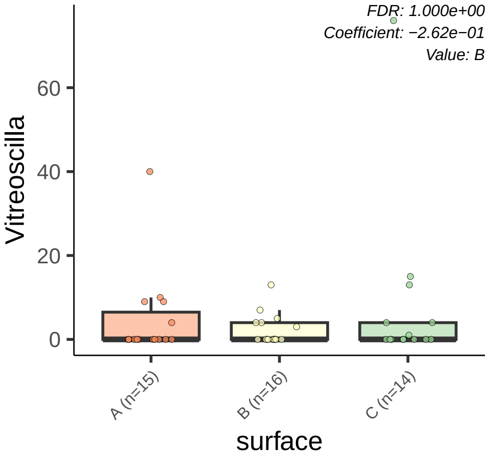
FDR: 1.000e+00
Coefficient: −2.62e−01
Value: B
Vitreoscilla
60
40
20
0
A (n=15)
B (n=16)
C (n=14)
surface

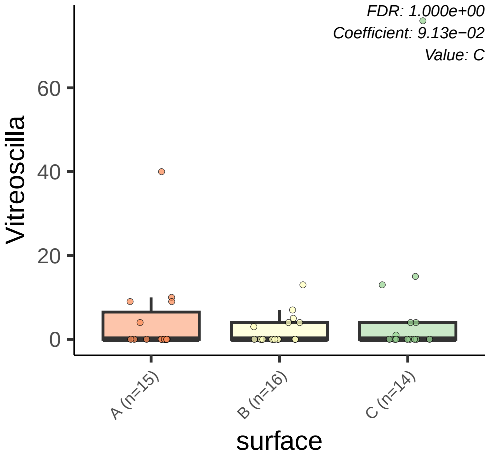

FDR: 1.000e+00
Coefficient: 9.13e−02
Value: C
Vitreoscilla
60
40
20
0
A (n=15)
B (n=16)
C (n=14)
surface

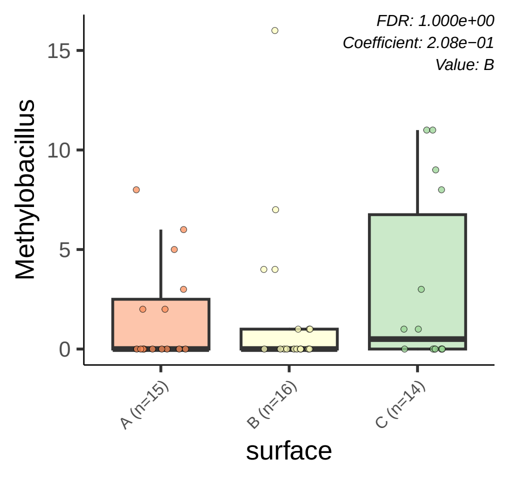
FDR: 1.000e+00
Coefficient: 2.08e−01
Value: B
Methylobacillus
0
5
10
15
A (n=15)
B (n=16)
C (n=14)
surface

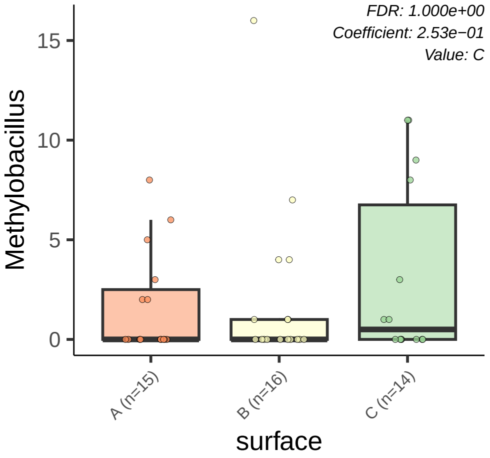

FDR: 1.000e+00
Coefficient: 2.53e−01
Value: C
Methylobacillus
15
10
5
0
A (n=15)
B (n=16)
C (n=14)
surface

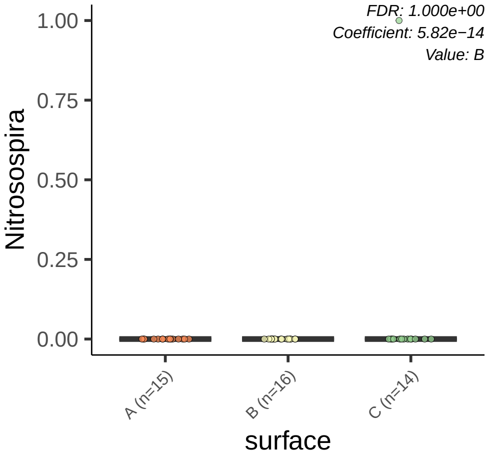
FDR: 1.000e+00
Coefficient: 5.82e−14
Value: B
1.00
0.75
0.50
0.25
0.00
Nitrosospira
A (n=15)
B (n=16)
C (n=14)
surface

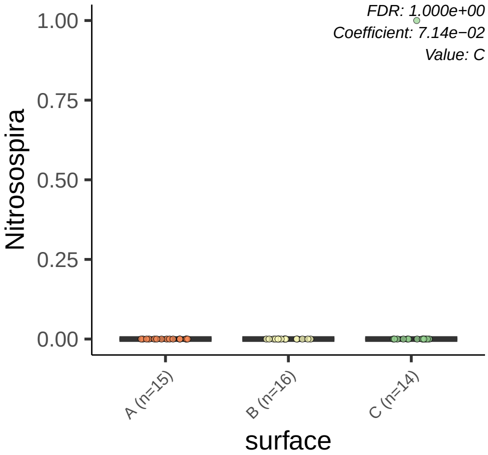
FDR: 1.000e+00
Coefficient: 7.14e−02
Value: C
Nitrosospira
1.00
0.75
0.50
0.25
0.00
A (n=15)
B (n=16)
C (n=14)
surface

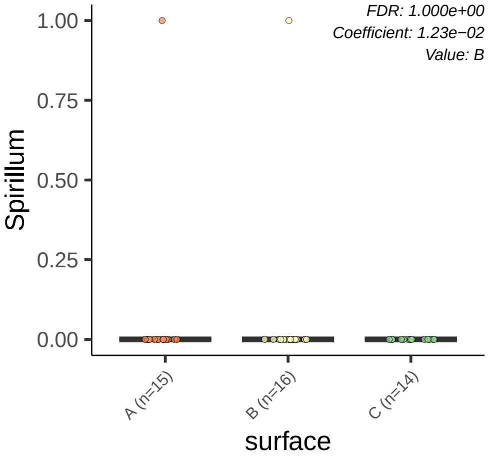
FDR: 1.000e+00
Coefficient: 1.23e−02
Value: B
Spirillum
1.00
0.75
0.50
0.25
0.00
A (n=15)
B (n=16)
C (n=14)
surface

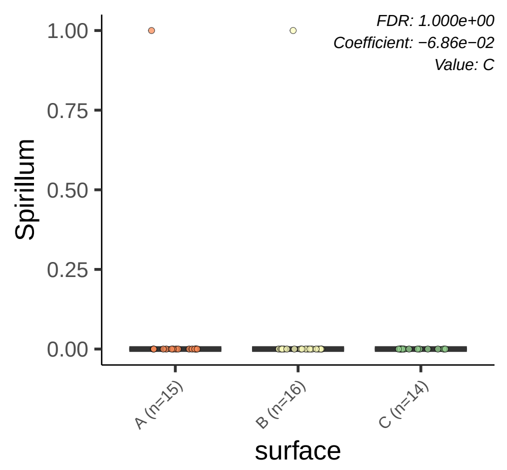
FDR: 1.000e+00
Coefficient: −6.86e−02
Value: C
1.00
0.75
0.50
0.25
0.00
Spirillum
A (n=15)
B (n=16)
C (n=14)
surface

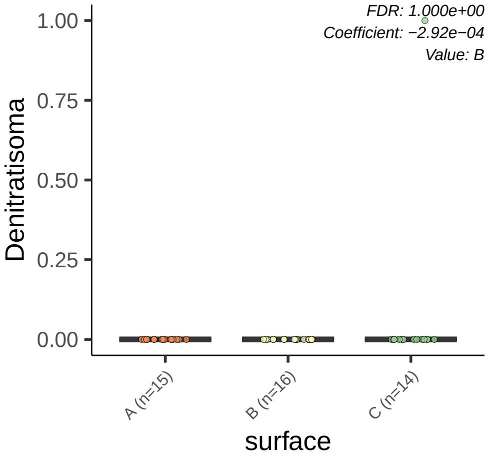
FDR: 1.000e+00
Coefficient: −2.92e−04
Value: B
Denitratisoma
1.00
0.75
0.50
0.25
0.00
A (n=15)
B (n=16)
C (n=14)
surface

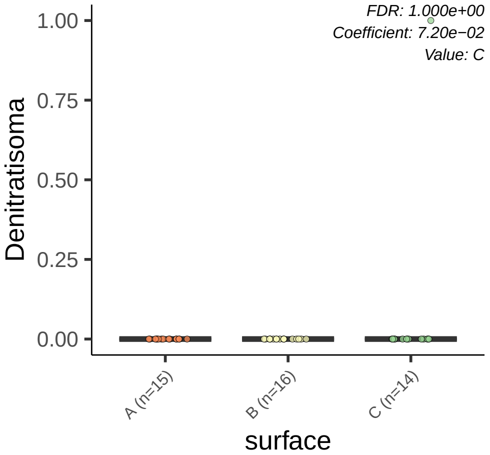
FDR: 1.000e+00
Coefficient: 7.20e−02
Value: C
Denitratisoma
1.00
0.75
0.50
0.25
0.00
A (n=15)
B (n=16)
C (n=14)
surface

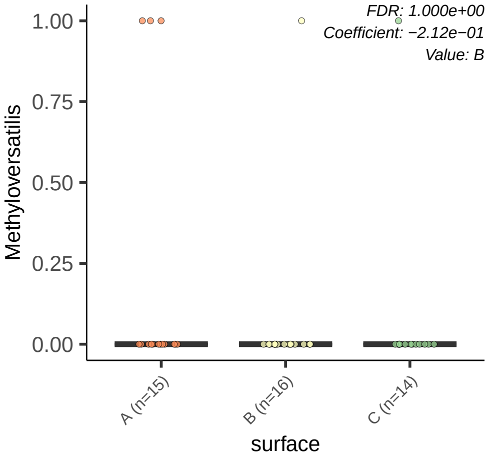

FDR: 1.000e+00
Coefficient: −2.12e−01
Value: B
Methyloversatilis
1.00
0.75
0.50
0.25
0.00
A (n=15)
B (n=16)
C (n=14)
surface

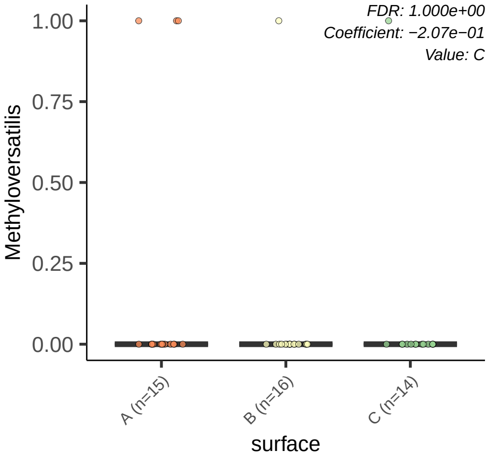
FDR: 1.000e+00
Coefficient: −2.07e−01
Value: C
1.00
0.75
0.50
0.25
0.00
Methyloversatilis
A (n=15)
B (n=16)
C (n=14)
surface

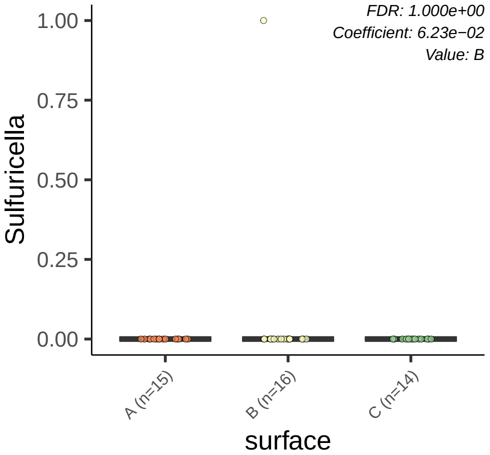

FDR: 1.000e+00
Coefficient: 6.23e−02
Value: B
Sulfuricella
1.00
0.75
0.50
0.25
0.00
A (n=15)
B (n=16)
C (n=14)
surface

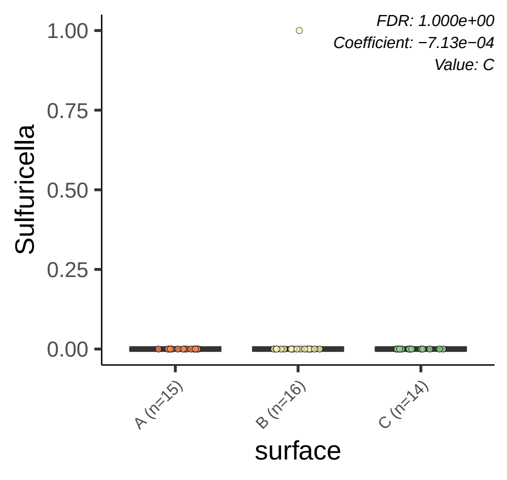

FDR: 1.000e+00
Coefficient: −7.13e−04
Value: C
Sulfuricella
1.00
0.75
0.50
0.25
0.00
A (n=15)
B (n=16)
C (n=14)
surface

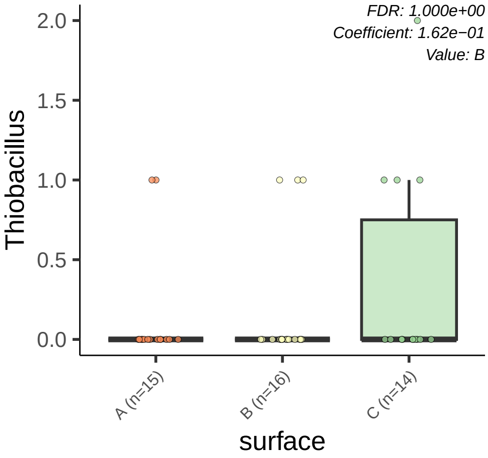

FDR: 1.000e+00
Coefficient: 1.62e−01
Value: B
Thiobacillus
2.0
1.5
1.0
0.5
0.0
A (n=15)
B (n=16)
C (n=14)
surface

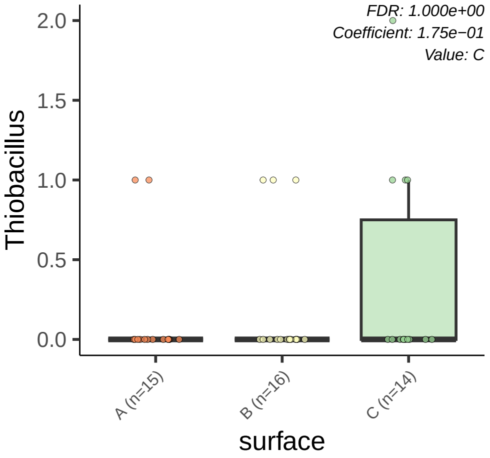

FDR: 1.000e+00
Coefficient: 1.75e−01
Value: C
Thiobacillus
2.0
1.5
1.0
0.5
0.0
A (n=15)
B (n=16)
C (n=14)
surface

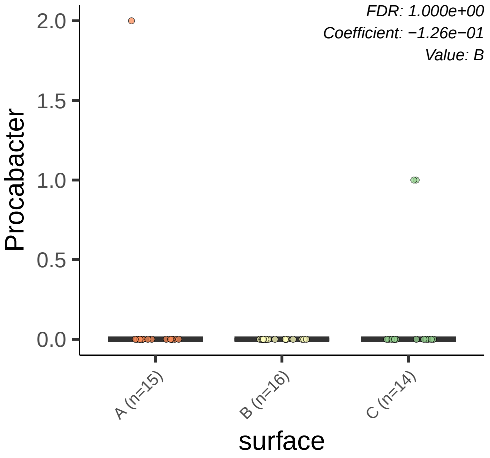
FDR: 1.000e+00
Coefficient: −1.26e−01
Value: B
Procabacter
2.0
1.5
1.0
0.5
0.0
A (n=15)
B (n=16)
C (n=14)
surface

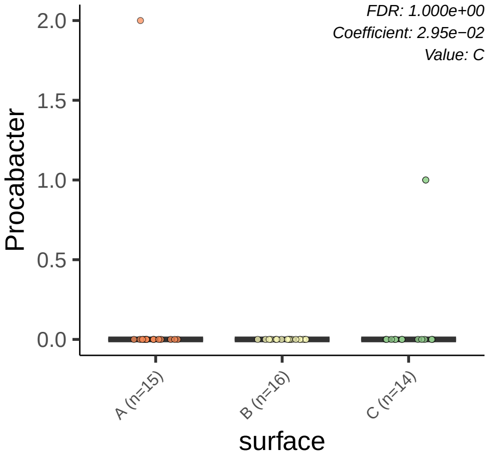

FDR: 1.000e+00
Coefficient: 2.95e−02
Value: C
Procabacter
2.0
1.5
1.0
0.5
0.0
A (n=15)
B (n=16)
C (n=14)
surface

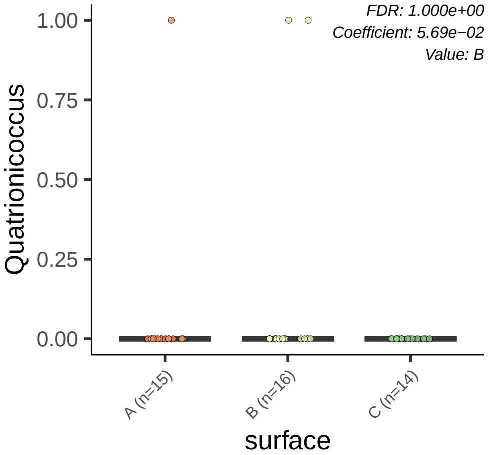

FDR: 1.000e+00
Coefficient: 5.69e−02
Value: B
Quatrionicoccus
1.00
0.75
0.50
0.25
0.00
A (n=15)
B (n=16)
C (n=14)
surface

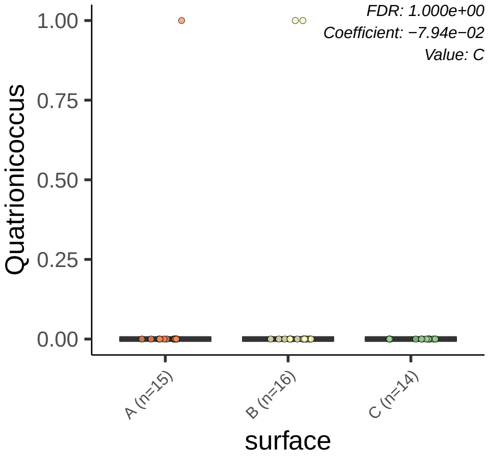
FDR: 1.000e+00
Coefficient: −7.94e−02
Value: C
Quatrionicoccus
1.00
0.75
0.50
0.25
0.00
A (n=15)
B (n=16)
C (n=14)
surface

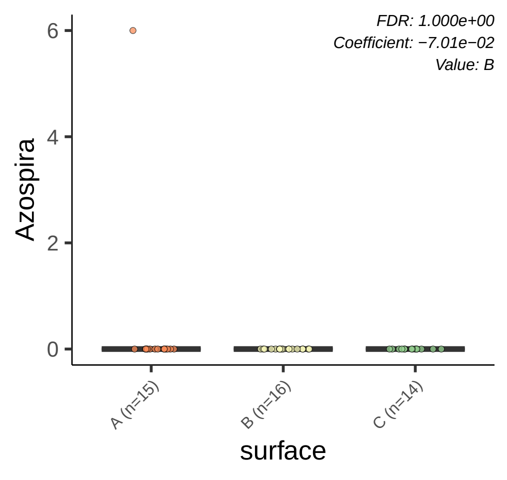

FDR: 1.000e+00
Coefficient: −7.01e−02
Value: B
Azospira
0
2
4
6
A (n=15)
B (n=16)
C (n=14)
surface

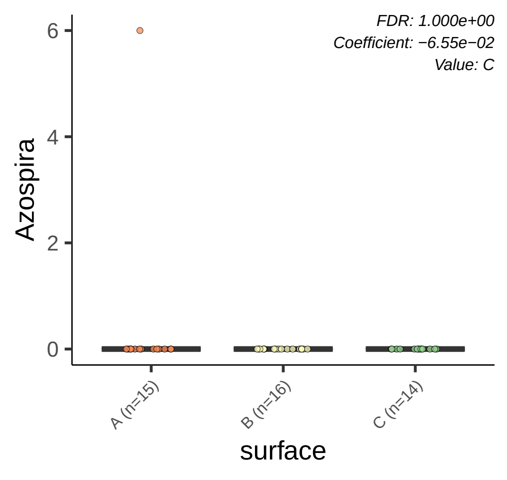
FDR: 1.000e+00
Coefficient: −6.55e−02
Value: C
Azospira
6
4
2
0
A (n=15)
B (n=16)
C (n=14)
surface

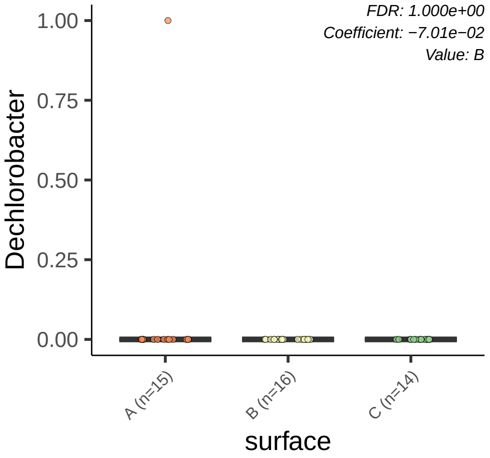
FDR: 1.000e+00
Coefficient: −7.01e−02
Value: B
Dechlorobacter
1.00
0.75
0.50
0.25
0.00
A (n=15)
B (n=16)
C (n=14)
surface

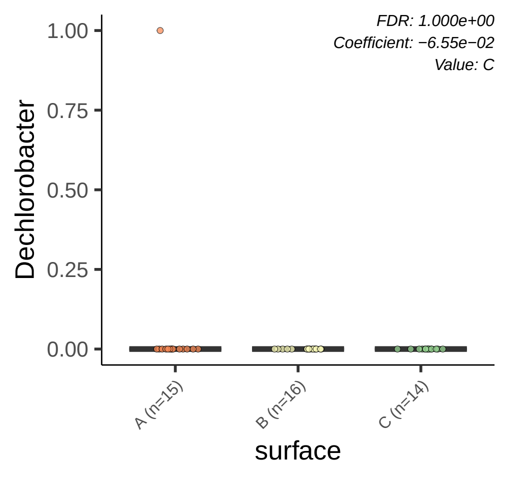

FDR: 1.000e+00
Coefficient: −6.55e−02
Value: C
Dechlorobacter
1.00
0.75
0.50
0.25
0.00
A (n=15)
B (n=16)
C (n=14)
surface

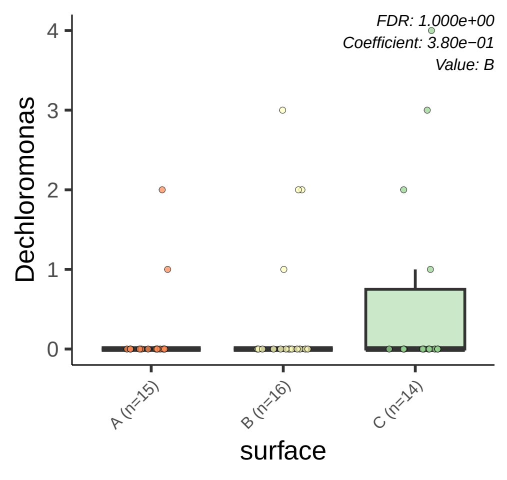

FDR: 1.000e+00
Coefficient: 3.80e−01
Value: B
Dechloromonas
0
1
2
3
4
A (n=15)
B (n=16)
C (n=14)
surface

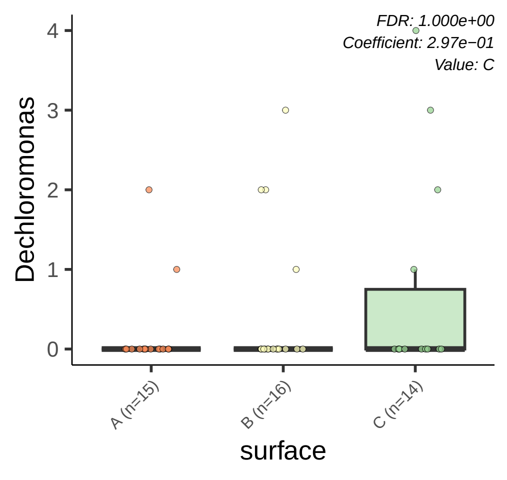

FDR: 1.000e+00
Coefficient: 2.97e−01
Value: C
Dechloromonas
0
1
2
3
4
A (n=15)
B (n=16)
C (n=14)
surface

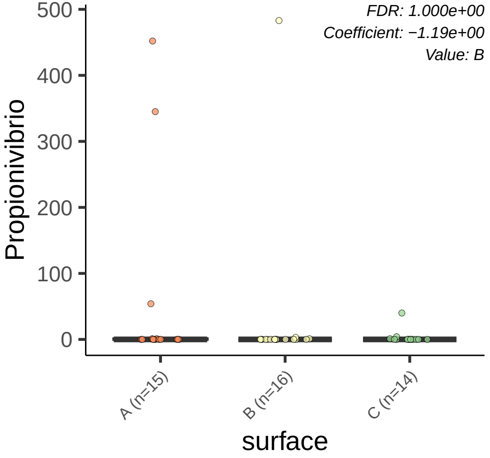
FDR: 1.000e+00
Coefficient: −1.19e+00
Value: B
Propionivibrio
500
400
300
200
100
0
A (n=15)
B (n=16)
C (n=14)
surface

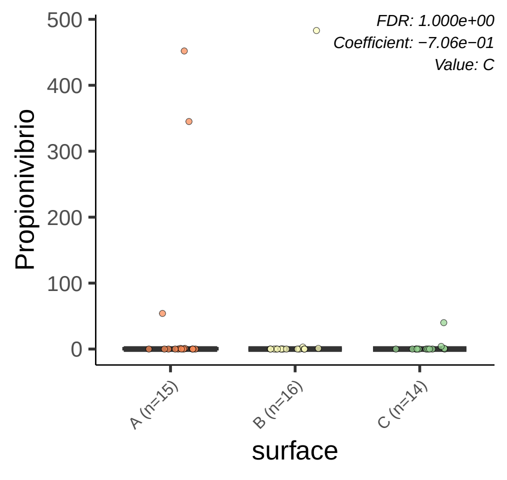
FDR: 1.000e+00
Coefficient: −7.06e−01
Value: C
Propionivibrio
500
400
300
200
100
0
A (n=15)
B (n=16)
C (n=14)
surface

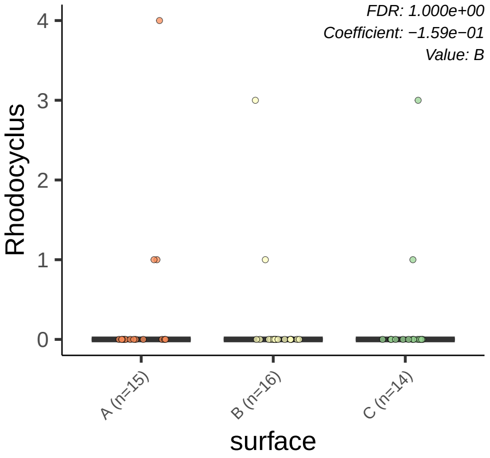
FDR: 1.000e+00
Coefficient: −1.59e−01
Value: B
Rhodocyclus
4
3
2
1
0
A (n=15)
B (n=16)
C (n=14)
surface

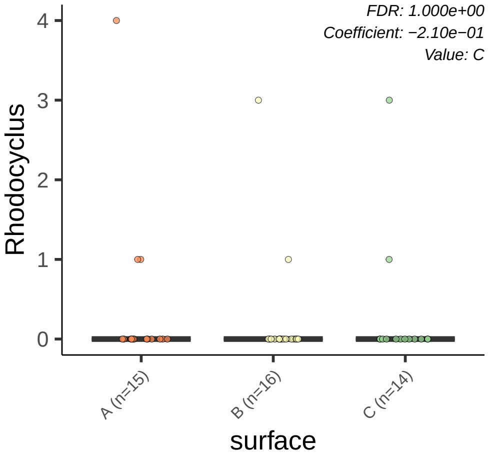

FDR: 1.000e+00
Coefficient: −2.10e−01
Value: C
Rhodocyclus
4
3
2
1
0
A (n=15)
B (n=16)
C (n=14)
surface

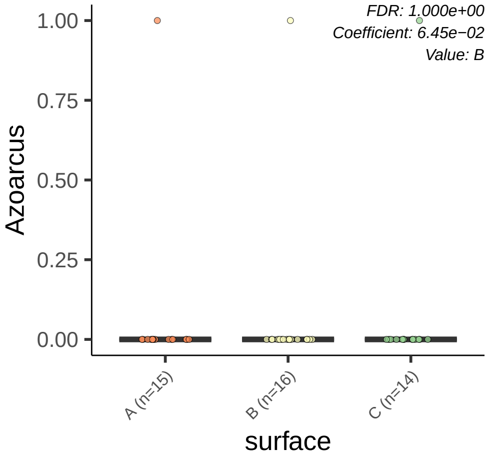
FDR: 1.000e+00
Coefficient: 6.45e−02
Value: B
Azoarcus
1.00
0.75
0.50
0.25
0.00
A (n=15)
B (n=16)
C (n=14)
surface

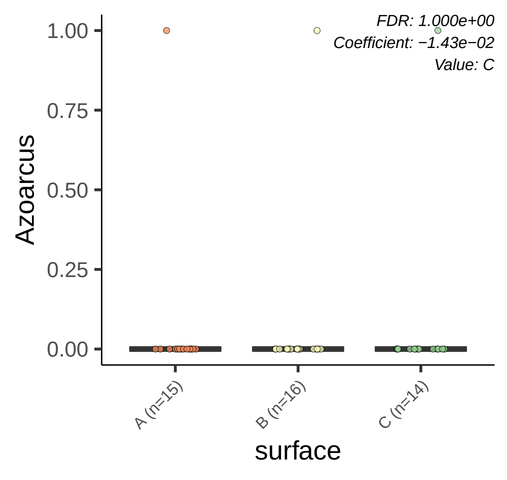
FDR: 1.000e+00
Coefficient: −1.43e−02
Value: C
Azoarcus
1.00
0.75
0.50
0.25
0.00
A (n=15)
B (n=16)
C (n=14)
surface

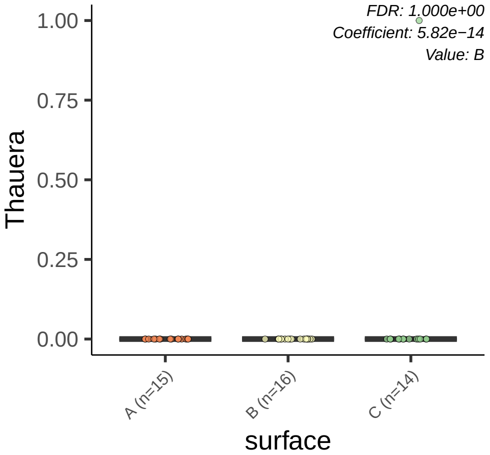
FDR: 1.000e+00
Coefficient: 5.82e−14
Value: B
Thauera
1.00
0.75
0.50
0.25
0.00
A (n=15)
B (n=16)
C (n=14)
surface

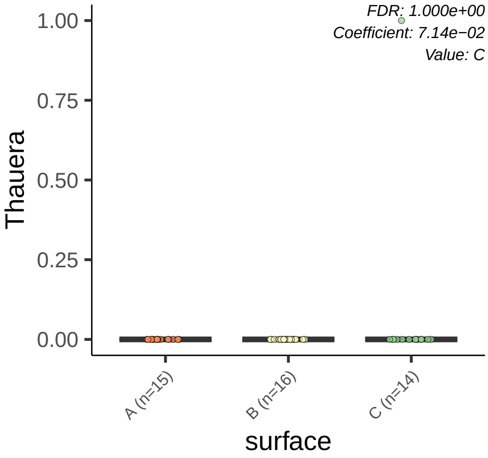

FDR: 1.000e+00
Coefficient: 7.14e−02
Value: C
Thauera
1.00
0.75
0.50
0.25
0.00
A (n=15)
B (n=16)
C (n=14)
surface

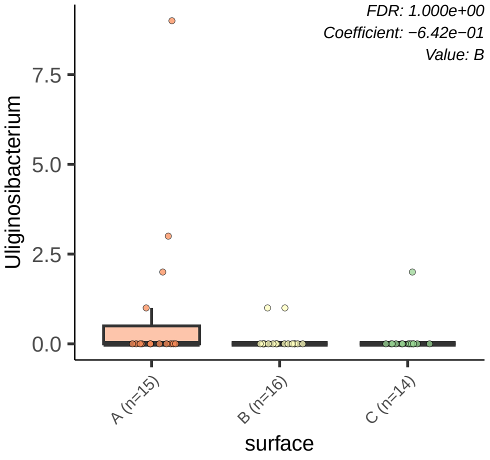
FDR: 1.000e+00
Coefficient: −6.42e−01
Value: B
Uliginosibacterium
7.5
5.0
2.5
0.0
A (n=15)
B (n=16)
C (n=14)
surface

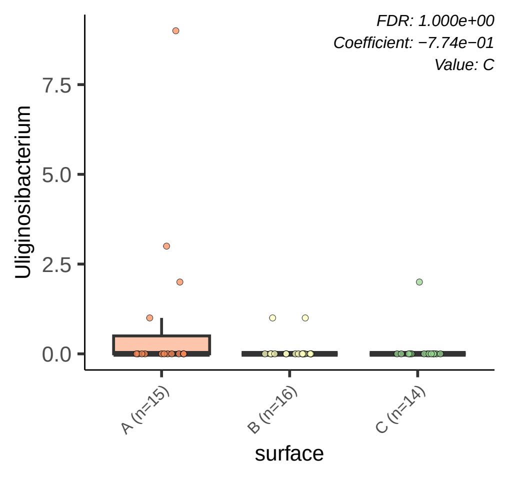

FDR: 1.000e+00
Coefficient: −7.74e−01
Value: C
Uliginosibacterium
7.5
5.0
2.5
0.0
A (n=15)
B (n=16)
C (n=14)
surface

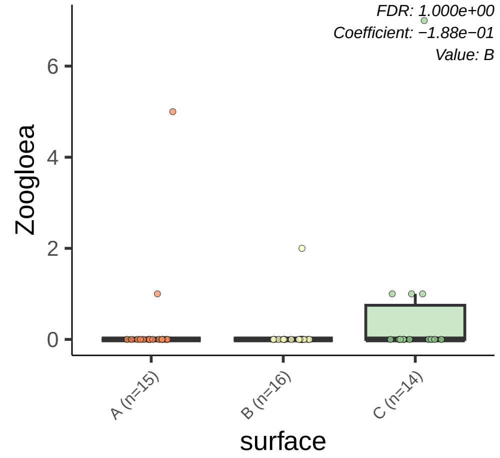

FDR: 1.000e+00
Coefficient: −1.88e−01
Value: B
Zoogloea
0
2
4
6
A (n=15)
B (n=16)
C (n=14)
surface

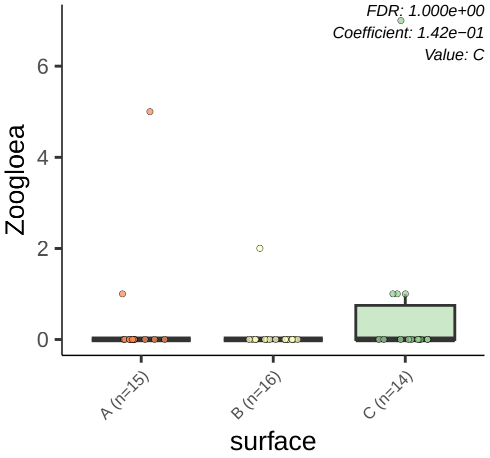
FDR: 1.000e+00
Coefficient: 1.42e−01
Value: C
Zoogloea
0
2
4
6
A (n=15)
B (n=16)
C (n=14)
surface

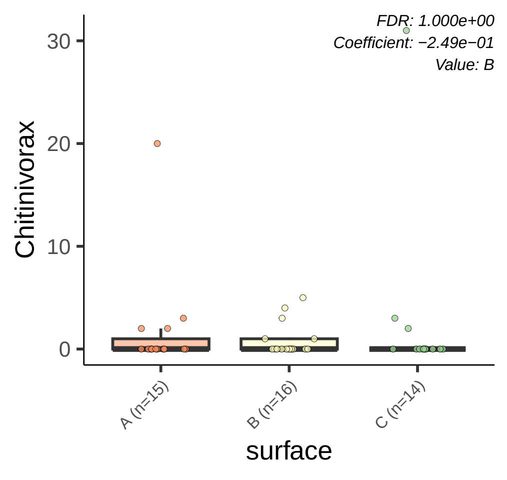

FDR: 1.000e+00
Coefficient: −2.49e−01
Value: B
Chitinivorax
30
20
10
0
A (n=15)
B (n=16)
C (n=14)
surface

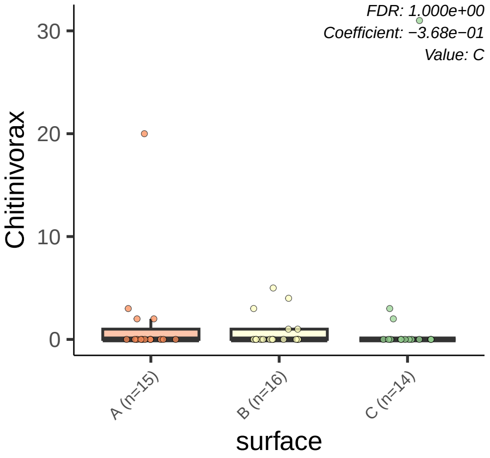
FDR: 1.000e+00
Coefficient: −3.68e−01
Value: C
Chitinivorax
30
20
10
0
A (n=15)
B (n=16)
C (n=14)
surface

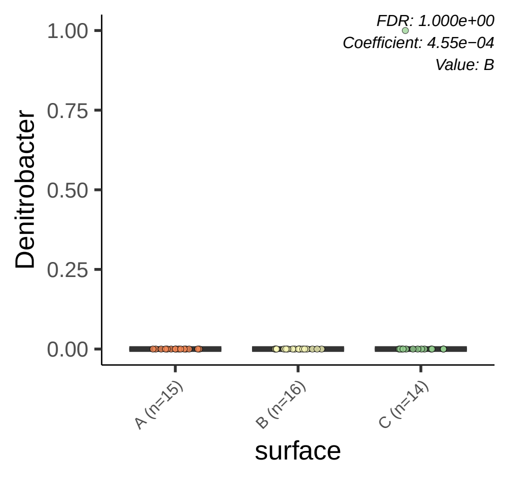
FDR: 1.000e+00
Coefficient: 4.55e−04
Value: B
Denitrobacter
1.00
0.75
0.50
0.25
0.00
A (n=15)
B (n=16)
C (n=14)
surface

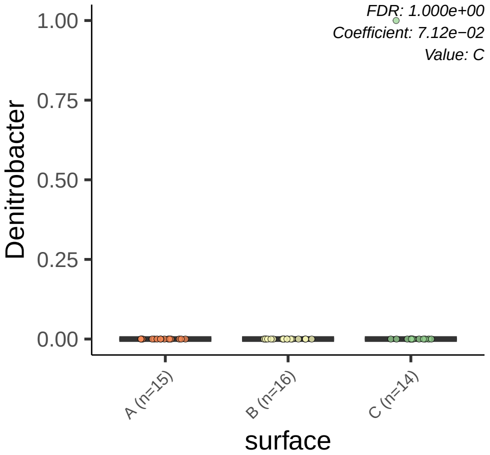

FDR: 1.000e+00
Coefficient: 7.12e−02
Value: C
Denitrobacter
1.00
0.75
0.50
0.25
0.00
A (n=15)
B (n=16)
C (n=14)
surface

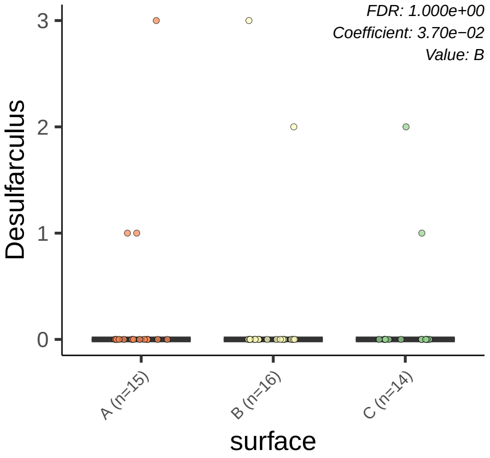
FDR: 1.000e+00
Coefficient: 3.70e−02
Value: B
Desulfarculus
3
2
1
0
A (n=15)
B (n=16)
C (n=14)
surface

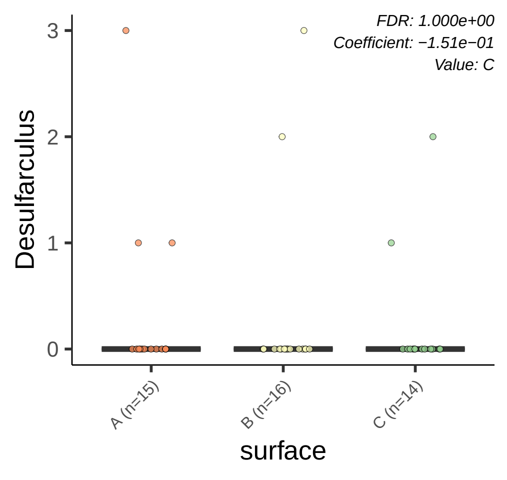
FDR: 1.000e+00
Coefficient: −1.51e−01
Value: C
Desulfarculus
3
2
1
0
A (n=15)
B (n=16)
C (n=14)
surface

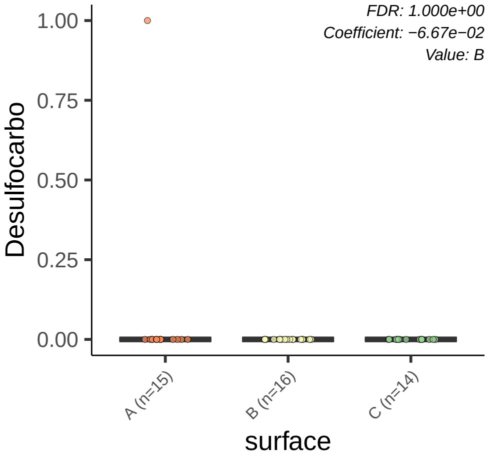
FDR: 1.000e+00
Coefficient: −6.67e−02
Value: B
Desulfocarbo
1.00
0.75
0.50
0.25
0.00
A (n=15)
B (n=16)
C (n=14)
surface

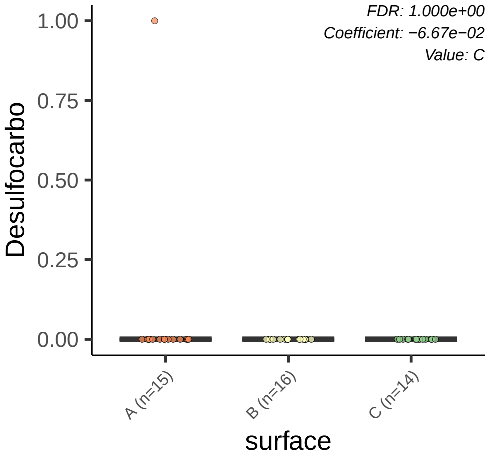
FDR: 1.000e+00
Coefficient: −6.67e−02
Value: C
Desulfocarbo
1.00
0.75
0.50
0.25
0.00
A (n=15)
B (n=16)
C (n=14)
surface

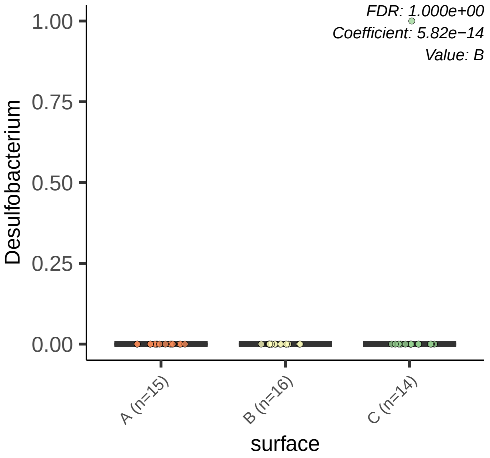

FDR: 1.000e+00
Coefficient: 5.82e−14
Value: B
1.00
0.75
0.50
0.25
0.00
Desulfobacterium
A (n=15)
B (n=16)
C (n=14)
surface

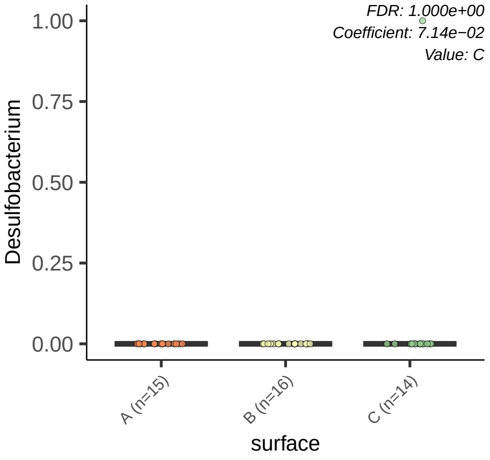
FDR: 1.000e+00
Coefficient: 7.14e−02
Value: C
Desulfobacterium
1.00
0.75
0.50
0.25
0.00
A (n=15)
B (n=16)
C (n=14)
surface

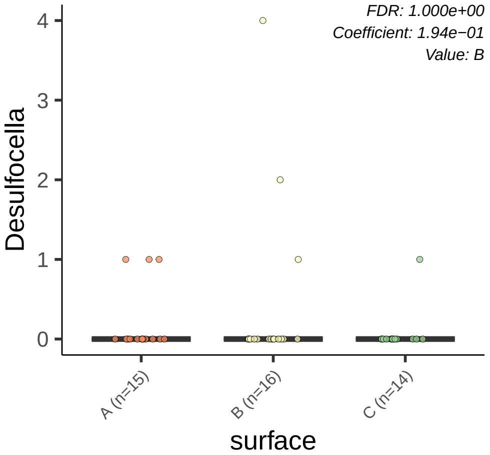

FDR: 1.000e+00
Coefficient: 1.94e−01
Value: B
Desulfocella
4
3
2
1
0
A (n=15)
B (n=16)
C (n=14)
surface

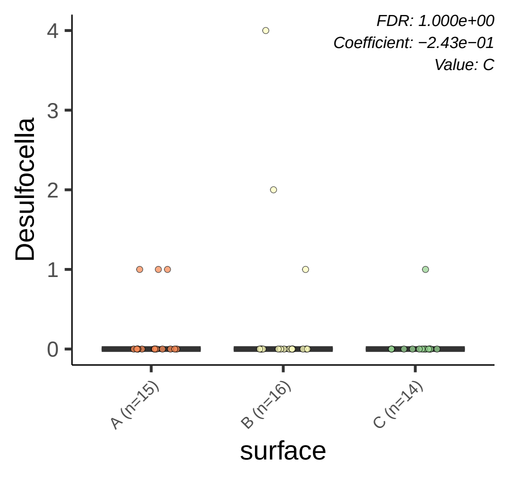
FDR: 1.000e+00
Coefficient: −2.43e−01
Value: C
Desulfocella
4
3
2
1
0
A (n=15)
B (n=16)
C (n=14)
surface

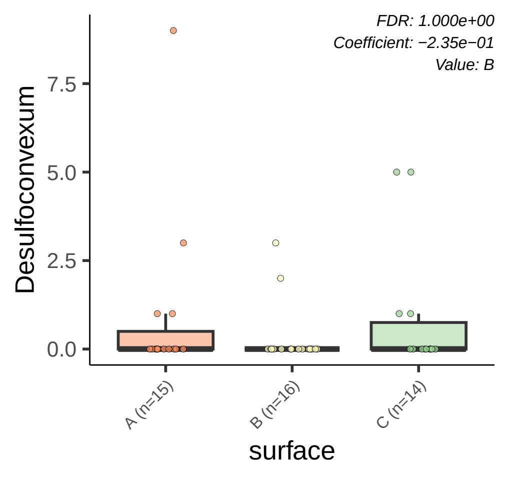
FDR: 1.000e+00
Coefficient: −2.35e−01
Value: B
Desulfoconvexum
7.5
5.0
2.5
0.0
A (n=15)
B (n=16)
C (n=14)
surface

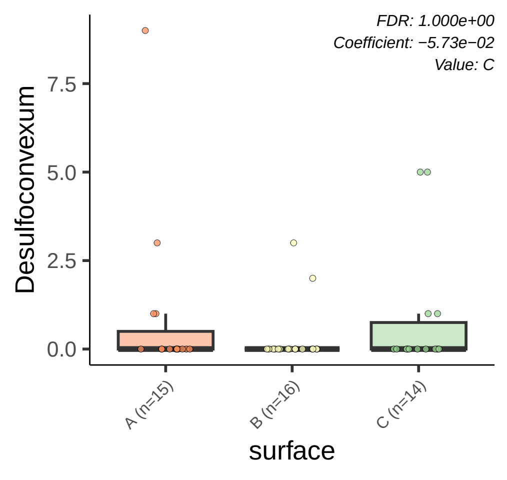
FDR: 1.000e+00
Coefficient: −5.73e−02
Value: C
Desulfoconvexum
7.5
5.0
2.5
0.0
A (n=15)
B (n=16)
C (n=14)
surface

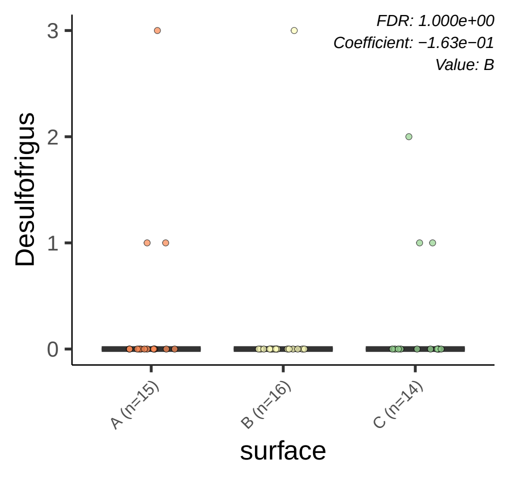
FDR: 1.000e+00
Coefficient: −1.63e−01
Value: B
Desulfofrigus
3
2
1
0
A (n=15)
B (n=16)
C (n=14)
surface

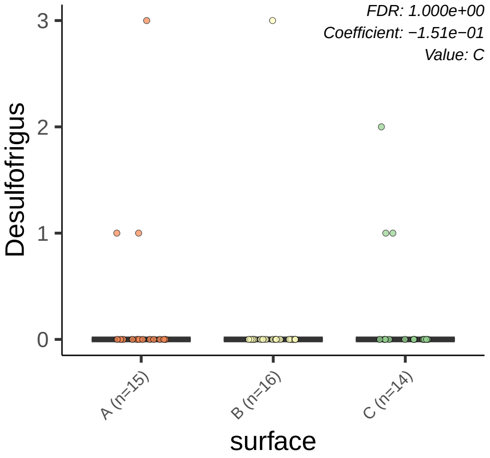
FDR: 1.000e+00
Coefficient: −1.51e−01
Value: C
Desulfofrigus
3
2
1
0
A (n=15)
B (n=16)
C (n=14)
surface

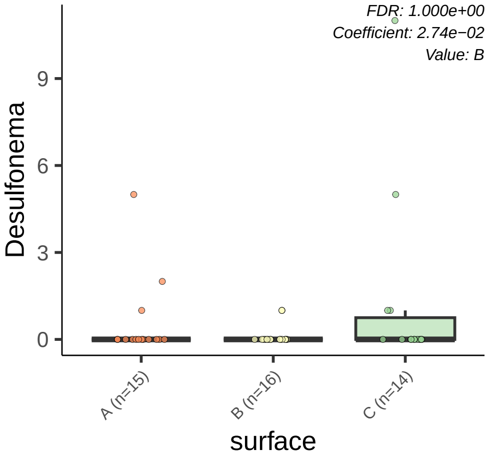
FDR: 1.000e+00
Coefficient: 2.74e−02
Value: B
Desulfonema
9
6
3
0
A (n=15)
B (n=16)
C (n=14)
surface

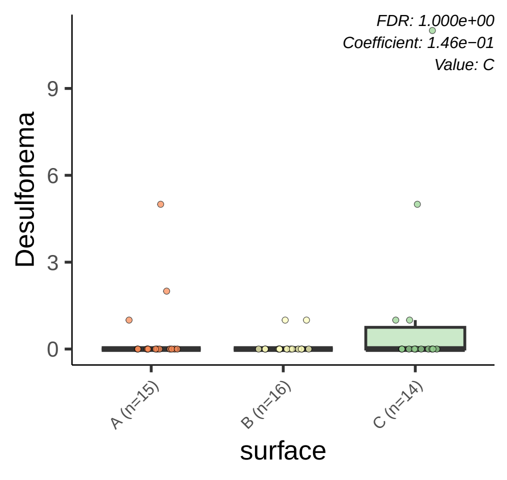
FDR: 1.000e+00
Coefficient: 1.46e−01
Value: C
Desulfonema
0
3
6
9
A (n=15)
B (n=16)
C (n=14)
surface

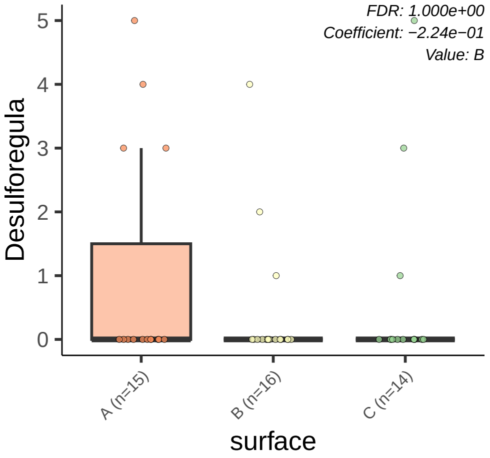
FDR: 1.000e+00
Coefficient: −2.24e−01
Value: B
Desulforegula
0
1
2
3
4
5
A (n=15)
B (n=16)
C (n=14)
surface

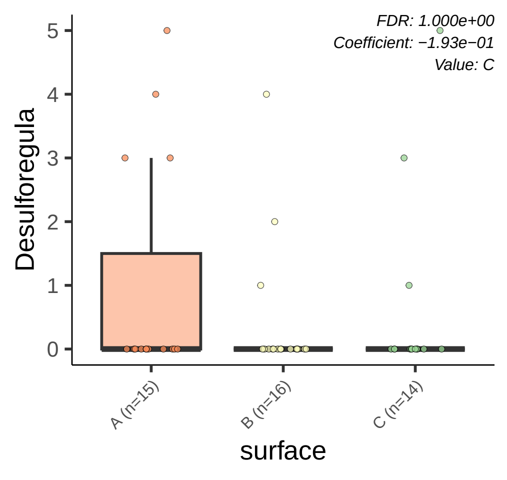

FDR: 1.000e+00
Coefficient: −1.93e−01
Value: C
Desulforegula
0
1
2
3
4
5
A (n=15)
B (n=16)
C (n=14)
surface

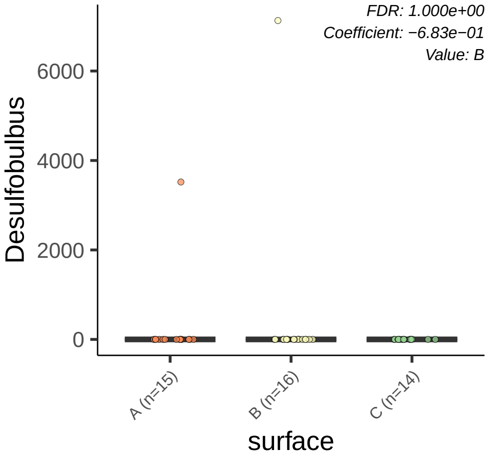

FDR: 1.000e+00
Coefficient: −6.83e−01
Value: B
Desulfobulbus
6000
4000
2000
0
A (n=15)
B (n=16)
C (n=14)
surface

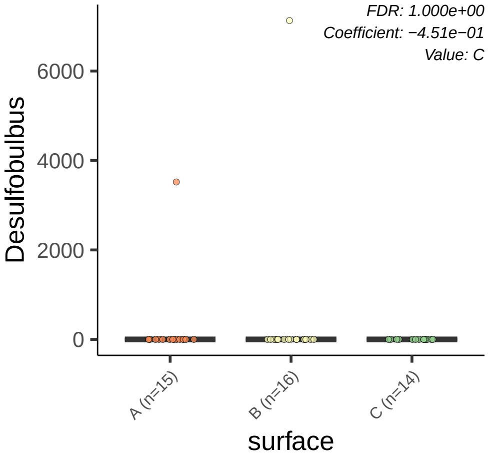
FDR: 1.000e+00
Coefficient: −4.51e−01
Value: C
Desulfobulbus
6000
4000
2000
0
A (n=15)
B (n=16)
C (n=14)
surface

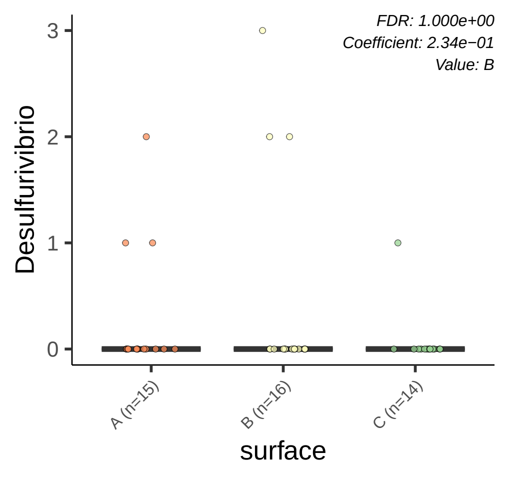
FDR: 1.000e+00
Coefficient: 2.34e−01
Value: B
Desulfurivibrio
3
2
1
0
A (n=15)
B (n=16)
C (n=14)
surface

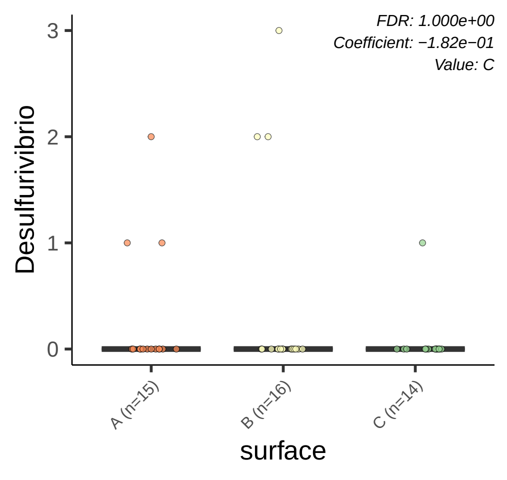
FDR: 1.000e+00
Coefficient: −1.82e−01
Value: C
Desulfurivibrio
3
2
1
0
A (n=15)
B (n=16)
C (n=14)
surface

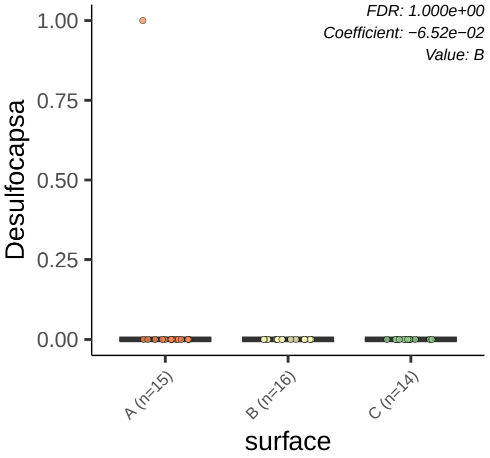
FDR: 1.000e+00
Coefficient: −6.52e−02
Value: B
Desulfocapsa
1.00
0.75
0.50
0.25
0.00
A (n=15)
B (n=16)
C (n=14)
surface

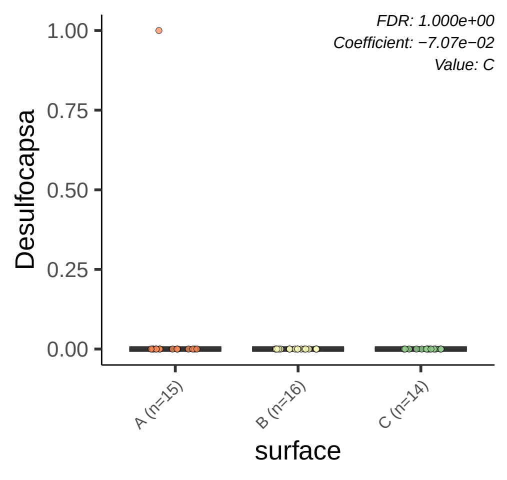
FDR: 1.000e+00
Coefficient: −7.07e−02
Value: C
Desulfocapsa
1.00
0.75
0.50
0.25
0.00
A (n=15)
B (n=16)
C (n=14)
surface

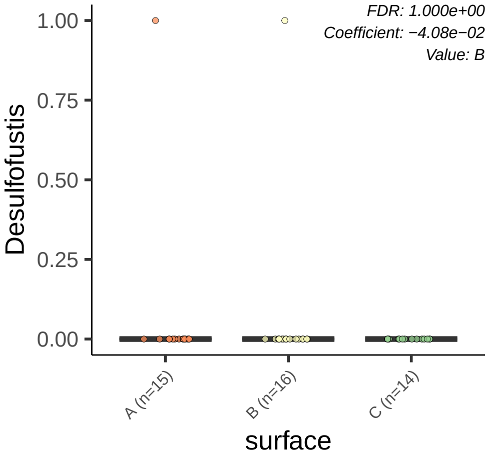
FDR: 1.000e+00
Coefficient: −4.08e−02
Value: B
Desulfofustis
1.00
0.75
0.50
0.25
0.00
A (n=15)
B (n=16)
C (n=14)
surface

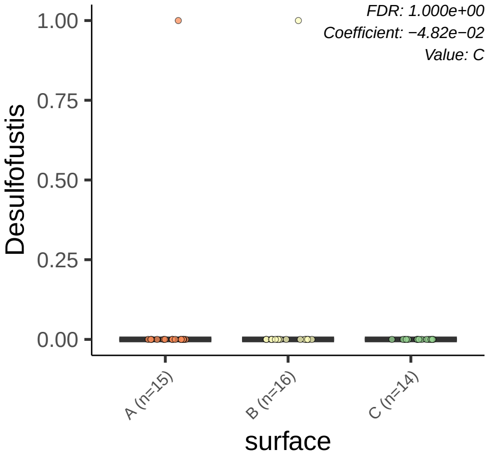
FDR: 1.000e+00
Coefficient: −4.82e−02
Value: C
Desulfofustis
1.00
0.75
0.50
0.25
0.00
A (n=15)
B (n=16)
C (n=14)
surface

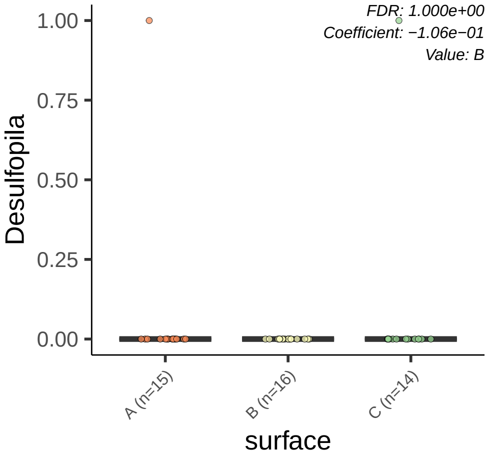
FDR: 1.000e+00
Coefficient: −1.06e−01
Value: B
Desulfopila
1.00
0.75
0.50
0.25
0.00
A (n=15)
B (n=16)
C (n=14)
surface

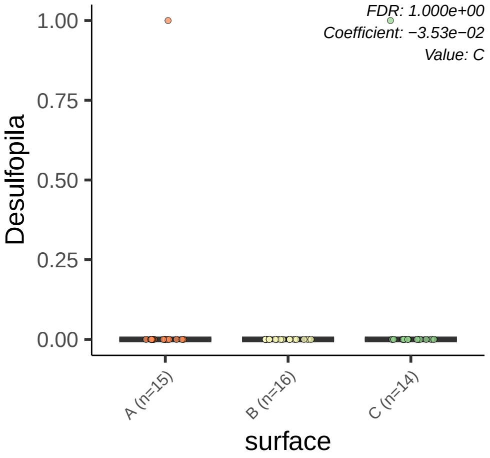
FDR: 1.000e+00
Coefficient: −3.53e−02
Value: C
Desulfopila
1.00
0.75
0.50
0.25
0.00
A (n=15)
B (n=16)
C (n=14)
surface

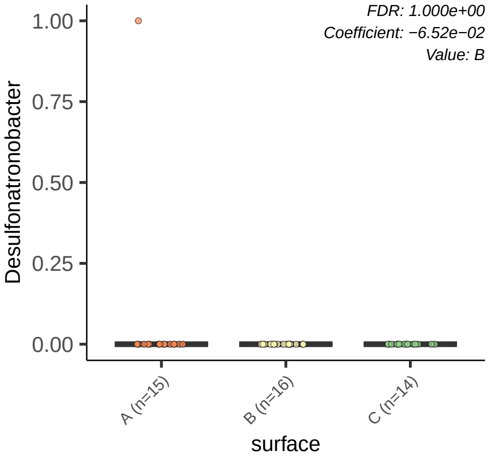

FDR: 1.000e+00
Coefficient: −6.52e−02
Value: B
Desulfonatronobacter
1.00
0.75
0.50
0.25
0.00
A (n=15)
B (n=16)
C (n=14)
surface

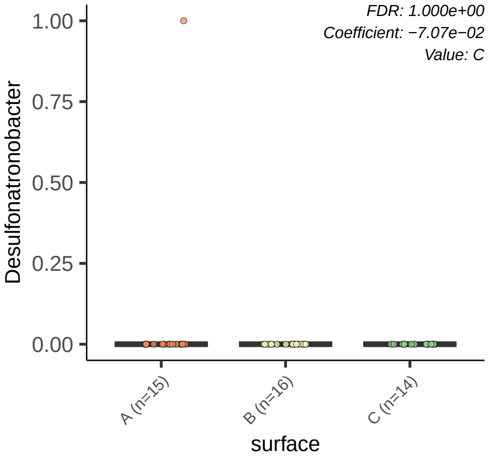

FDR: 1.000e+00
Coefficient: −7.07e−02
Value: C
Desulfonatronobacter
1.00
0.75
0.50
0.25
0.00
A (n=15)
B (n=16)
C (n=14)
surface

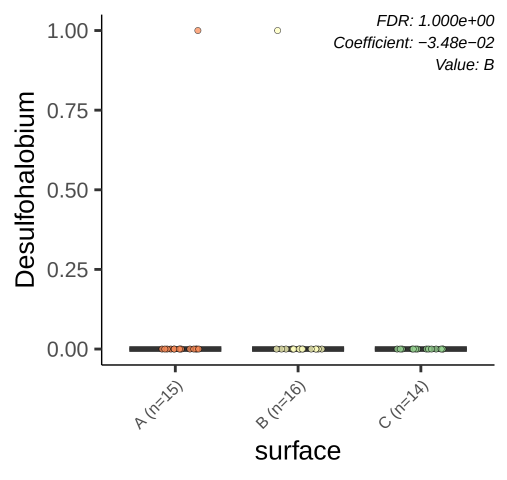
FDR: 1.000e+00
Coefficient: −3.48e−02
Value: B
Desulfohalobium
1.00
0.75
0.50
0.25
0.00
A (n=15)
B (n=16)
C (n=14)
surface

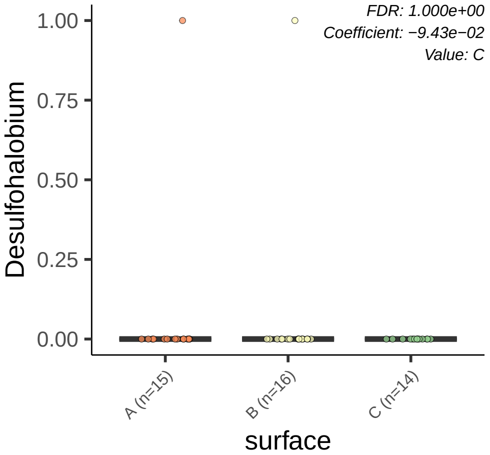

FDR: 1.000e+00
Coefficient: −9.43e−02
Value: C
Desulfohalobium
1.00
0.75
0.50
0.25
0.00
A (n=15)
B (n=16)
C (n=14)
surface

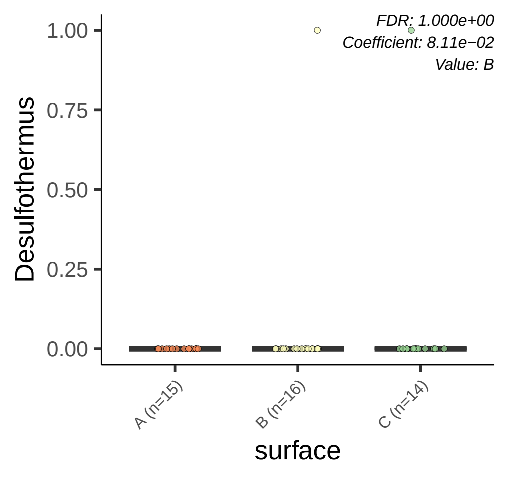

FDR: 1.000e+00
Coefficient: 8.11e−02
Value: B
Desulfothermus
1.00
0.75
0.50
0.25
0.00
A (n=15)
B (n=16)
C (n=14)
surface

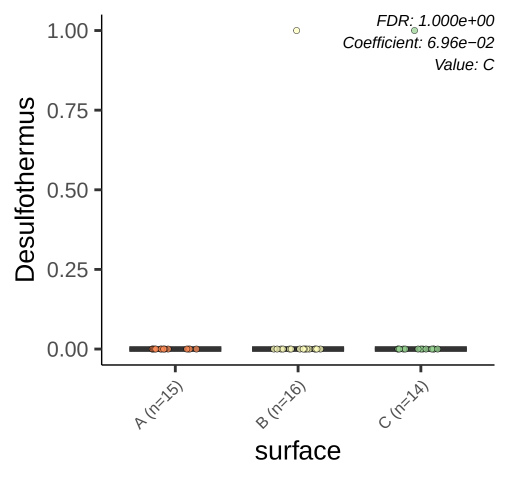
FDR: 1.000e+00
Coefficient: 6.96e−02
Value: C
Desulfothermus
1.00
0.75
0.50
0.25
0.00
A (n=15)
B (n=16)
C (n=14)
surface

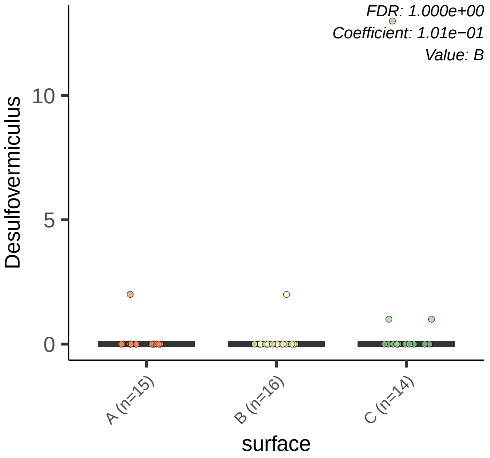
FDR: 1.000e+00
Coefficient: 1.01e−01
Value: B
Desulfovermiculus
10
5
0
A (n=15)
B (n=16)
C (n=14)
surface

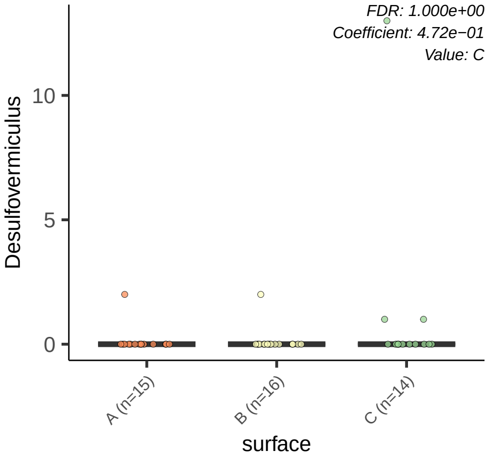

FDR: 1.000e+00
Coefficient: 4.72e−01
Value: C
Desulfovermiculus
0
5
10
A (n=15)
B (n=16)
C (n=14)
surface

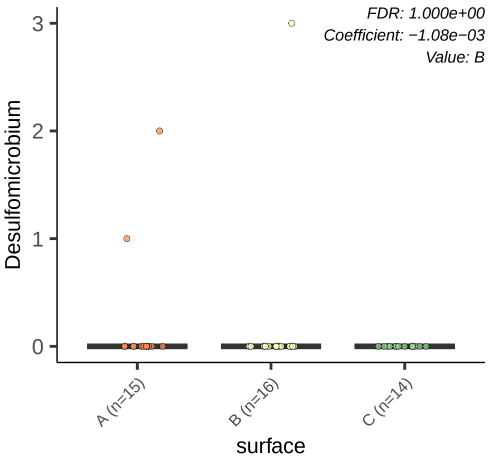
FDR: 1.000e+00
Coefficient: −1.08e−03
Value: B
Desulfomicrobium
3
2
1
0
A (n=15)
B (n=16)
C (n=14)
surface

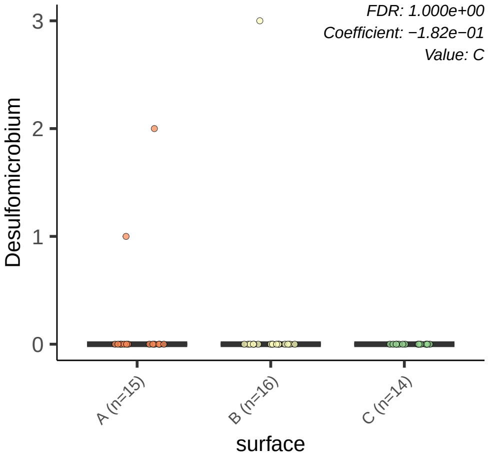

FDR: 1.000e+00
Coefficient: −1.82e−01
Value: C
Desulfomicrobium
3
2
1
0
A (n=15)
B (n=16)
C (n=14)
surface

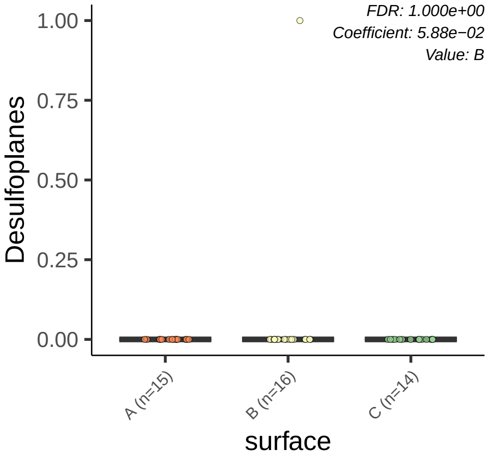

FDR: 1.000e+00
Coefficient: 5.88e−02
Value: B
Desulfoplanes
1.00
0.75
0.50
0.25
0.00
A (n=15)
B (n=16)
C (n=14)
surface

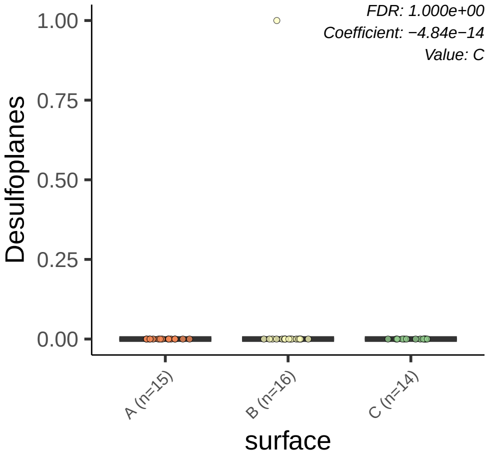

FDR: 1.000e+00
Coefficient: −4.84e−14
Value: C
Desulfoplanes
1.00
0.75
0.50
0.25
0.00
A (n=15)
B (n=16)
C (n=14)
surface

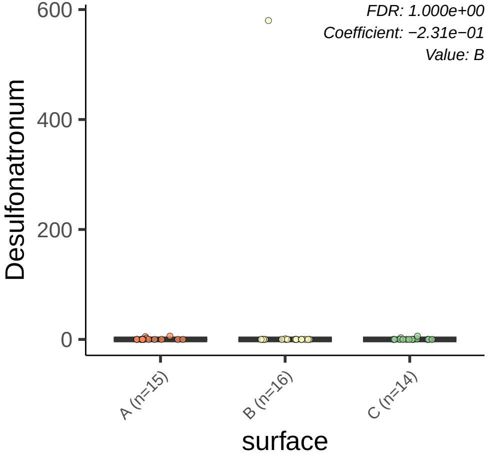

FDR: 1.000e+00
Coefficient: −2.31e−01
Value: B
600
400
200
0
Desulfonatronum
A (n=15)
B (n=16)
C (n=14)
surface

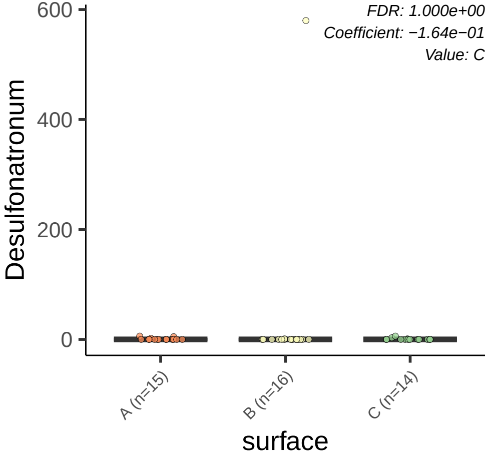

FDR: 1.000e+00
Coefficient: −1.64e−01
Value: C
Desulfonatronum
600
400
200
0
A (n=15)
B (n=16)
C (n=14)
surface

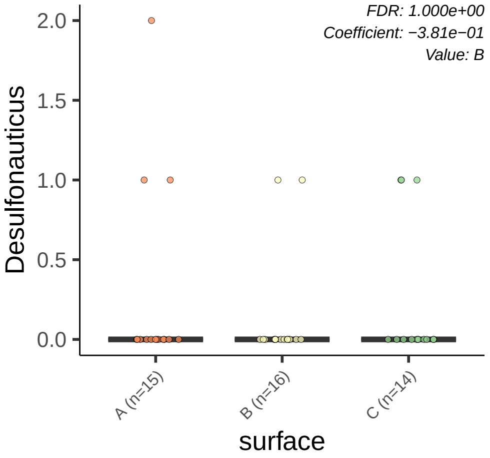
FDR: 1.000e+00
Coefficient: −3.81e−01
Value: B
Desulfonauticus
2.0
1.5
1.0
0.5
0.0
A (n=15)
B (n=16)
C (n=14)
surface

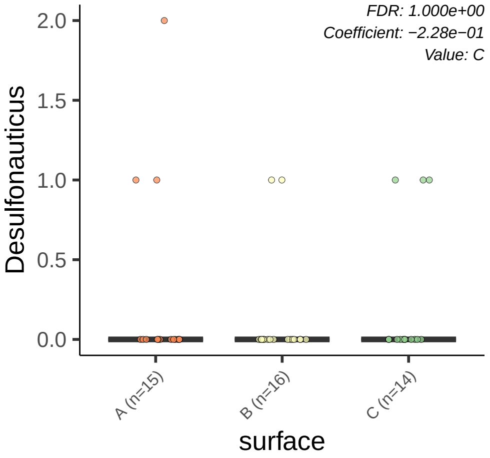

FDR: 1.000e+00
Coefficient: −2.28e−01
Value: C
2.0
1.5
1.0
0.5
0.0
Desulfonauticus
A (n=15)
B (n=16)
C (n=14)
surface

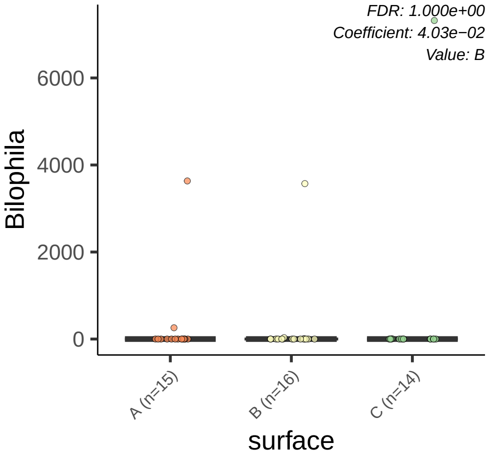

FDR: 1.000e+00
Coefficient: 4.03e−02
Value: B
6000
4000
2000
0
Bilophila
A (n=15)
B (n=16)
C (n=14)
surface

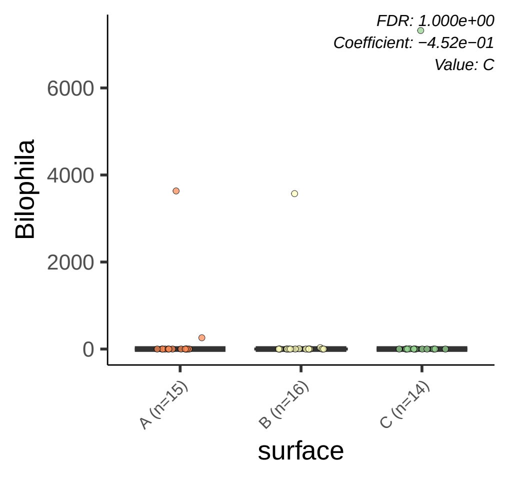

FDR: 1.000e+00
Coefficient: −4.52e−01
Value: C
6000
4000
2000
0
Bilophila
A (n=15)
B (n=16)
C (n=14)
surface

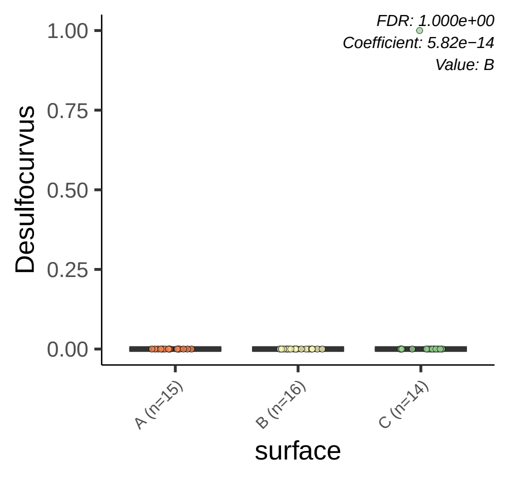
FDR: 1.000e+00
Coefficient: 5.82e−14
Value: B
Desulfocurvus
1.00
0.75
0.50
0.25
0.00
A (n=15)
B (n=16)
C (n=14)
surface

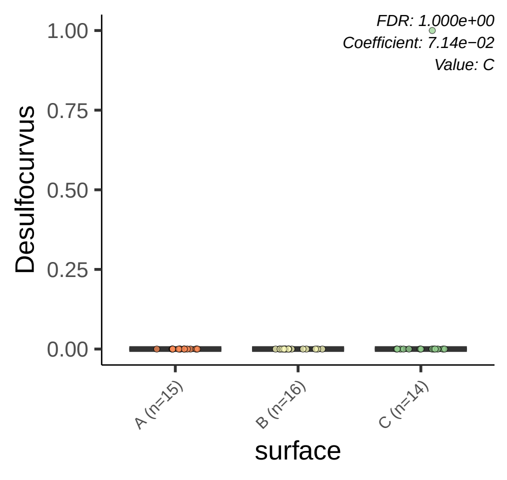
FDR: 1.000e+00
Coefficient: 7.14e−02
Value: C
1.00
0.75
0.50
0.25
0.00
Desulfocurvus
A (n=15)
B (n=16)
C (n=14)
surface

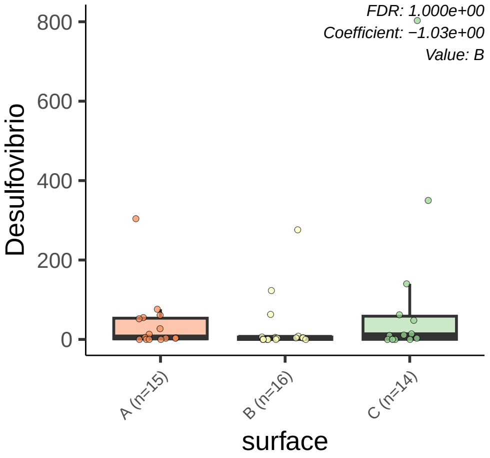
FDR: 1.000e+00
Coefficient: −1.03e+00
Value: B
Desulfovibrio
800
600
400
200
0
A (n=15)
B (n=16)
C (n=14)
surface

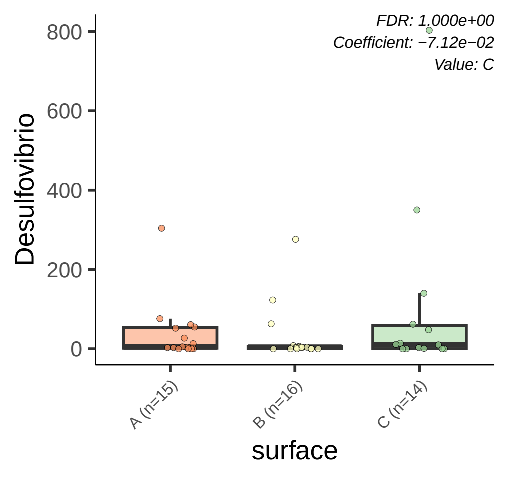
FDR: 1.000e+00
Coefficient: −7.12e−02
Value: C
Desulfovibrio
800
600
400
200
0
A (n=15)
B (n=16)
C (n=14)
surface

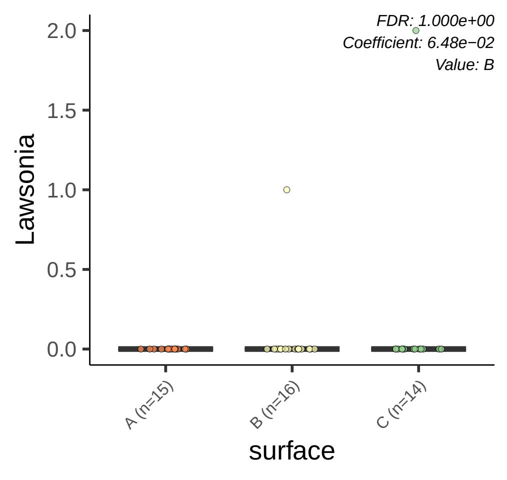
FDR: 1.000e+00
Coefficient: 6.48e−02
Value: B
Lawsonia
2.0
1.5
1.0
0.5
0.0
A (n=15)
B (n=16)
C (n=14)
surface

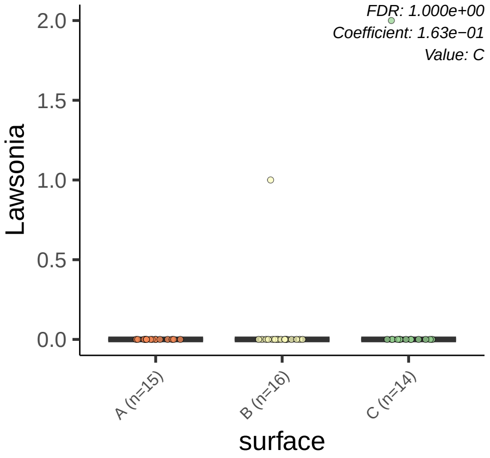

FDR: 1.000e+00
Coefficient: 1.63e−01
Value: C
Lawsonia
2.0
1.5
1.0
0.5
0.0
A (n=15)
B (n=16)
C (n=14)
surface

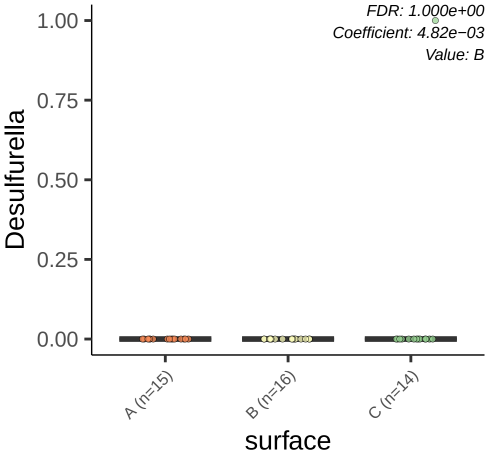

FDR: 1.000e+00
Coefficient: 4.82e−03
Value: B
Desulfurella
1.00
0.75
0.50
0.25
0.00
A (n=15)
B (n=16)
C (n=14)
surface

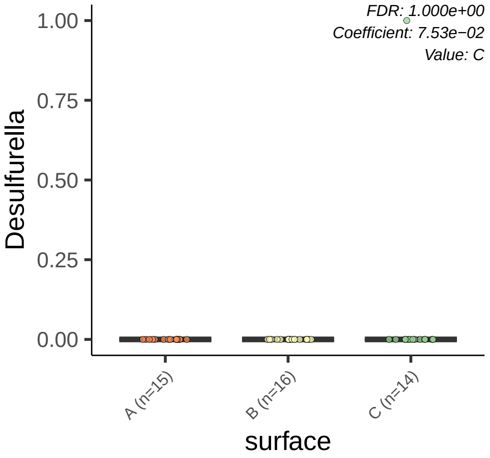
FDR: 1.000e+00
Coefficient: 7.53e−02
Value: C
Desulfurella
1.00
0.75
0.50
0.25
0.00
A (n=15)
B (n=16)
C (n=14)
surface

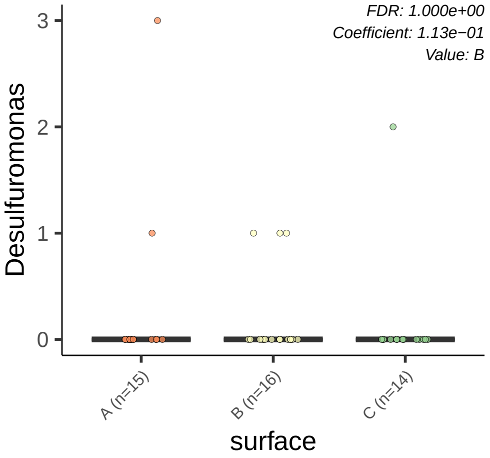
FDR: 1.000e+00
Coefficient: 1.13e−01
Value: B
Desulfuromonas
3
2
1
0
A (n=15)
B (n=16)
C (n=14)
surface

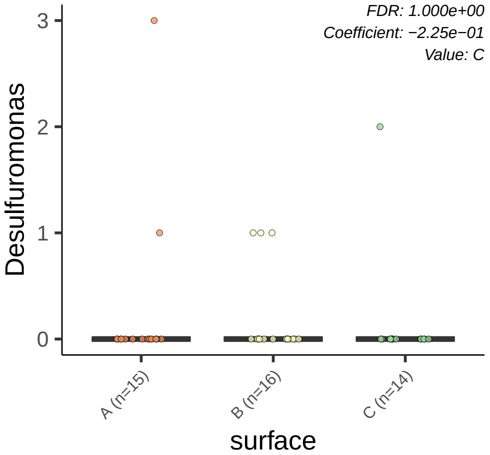
FDR: 1.000e+00
Coefficient: −2.25e−01
Value: C
Desulfuromonas
3
2
1
0
A (n=15)
B (n=16)
C (n=14)
surface

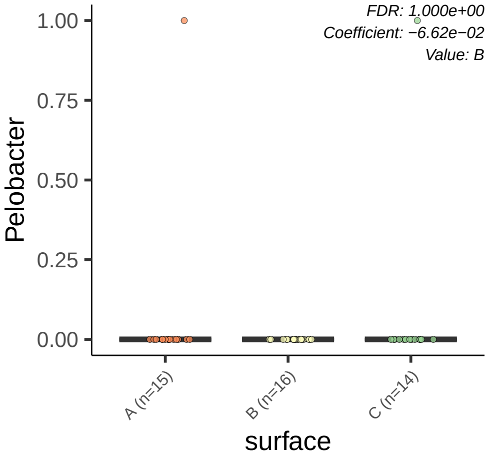
FDR: 1.000e+00
Coefficient: −6.62e−02
Value: B
Pelobacter
1.00
0.75
0.50
0.25
0.00
A (n=15)
B (n=16)
C (n=14)
surface

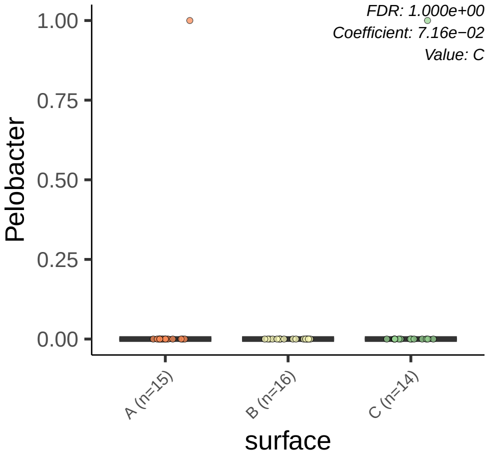
FDR: 1.000e+00
Coefficient: 7.16e−02
Value: C
Pelobacter
1.00
0.75
0.50
0.25
0.00
A (n=15)
B (n=16)
C (n=14)
surface

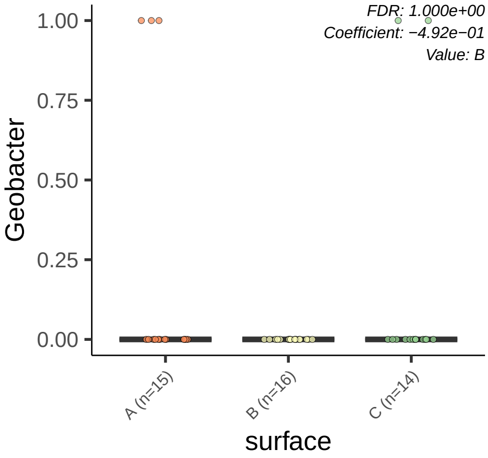
FDR: 1.000e+00
Coefficient: −4.92e−01
Value: B
1.00
0.75
0.50
0.25
0.00
Geobacter
A (n=15)
B (n=16)
C (n=14)
surface

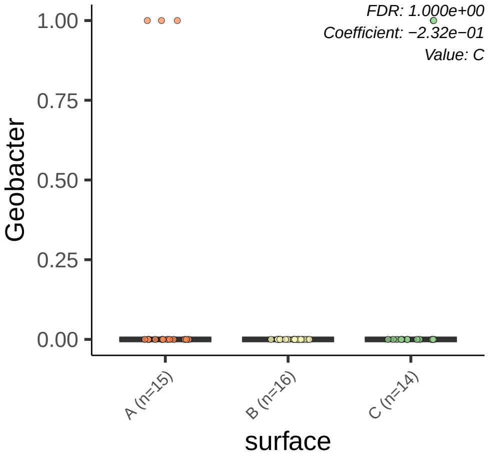
FDR: 1.000e+00
Coefficient: −2.32e−01
Value: C
Geobacter
1.00
0.75
0.50
0.25
0.00
A (n=15)
B (n=16)
C (n=14)
surface

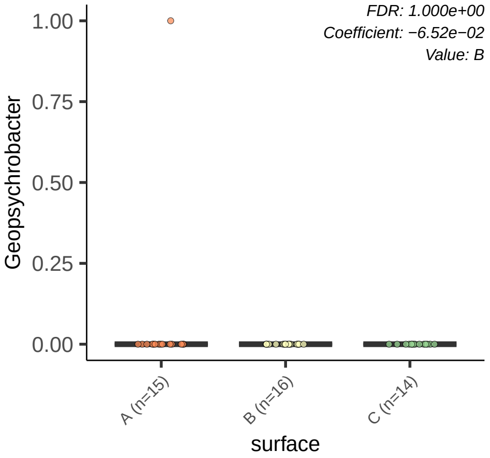
FDR: 1.000e+00
Coefficient: −6.52e−02
Value: B
Geopsychrobacter
1.00
0.75
0.50
0.25
0.00
A (n=15)
B (n=16)
C (n=14)
surface

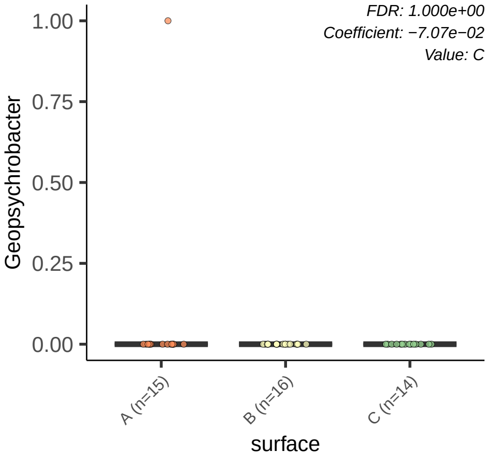
FDR: 1.000e+00
Coefficient: −7.07e−02
Value: C
Geopsychrobacter
1.00
0.75
0.50
0.25
0.00
A (n=15)
B (n=16)
C (n=14)
surface

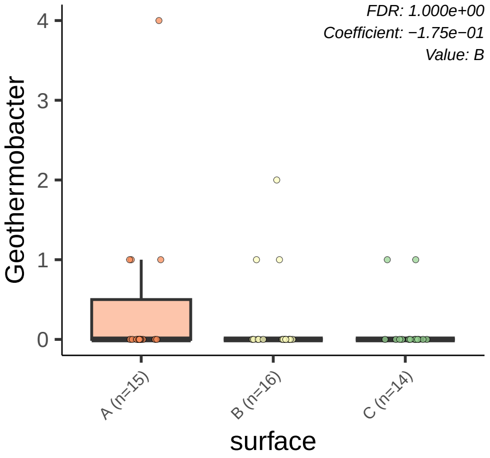
FDR: 1.000e+00
Coefficient: −1.75e−01
Value: B
Geothermobacter
4
3
2
1
0
A (n=15)
B (n=16)
C (n=14)
surface

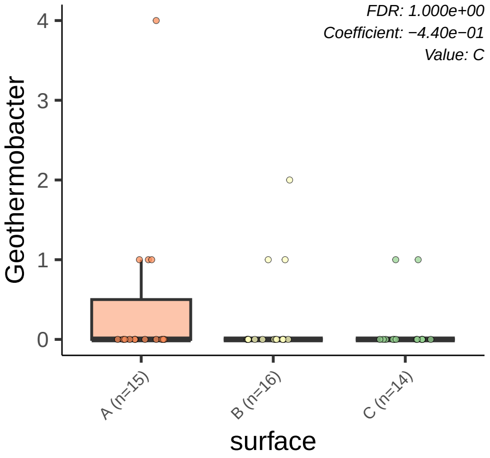
FDR: 1.000e+00
Coefficient: −4.40e−01
Value: C
Geothermobacter
0
1
2
3
4
A (n=15)
B (n=16)
C (n=14)
surface

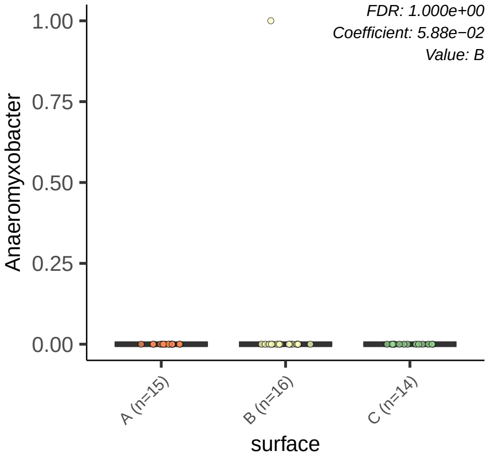
FDR: 1.000e+00
Coefficient: 5.88e−02
Value: B
Anaeromyxobacter
1.00
0.75
0.50
0.25
0.00
A (n=15)
B (n=16)
C (n=14)
surface

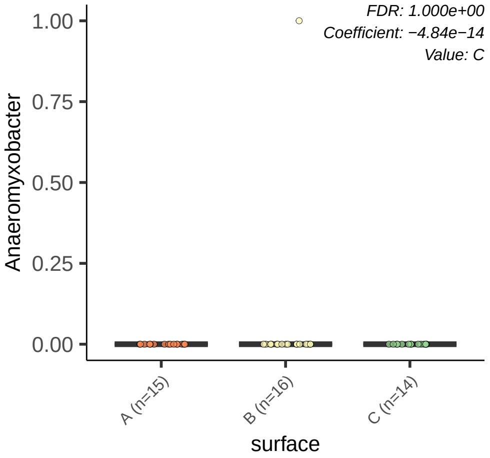
FDR: 1.000e+00
Coefficient: −4.84e−14
Value: C
Anaeromyxobacter
1.00
0.75
0.50
0.25
0.00
A (n=15)
B (n=16)
C (n=14)
surface

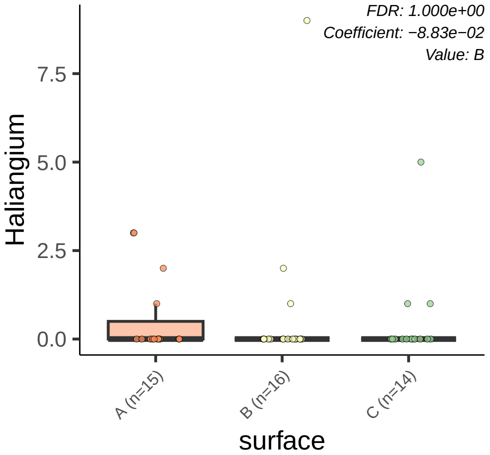

FDR: 1.000e+00
Coefficient: −8.83e−02
Value: B
Haliangium
7.5
5.0
2.5
0.0
A (n=15)
B (n=16)
C (n=14)
surface

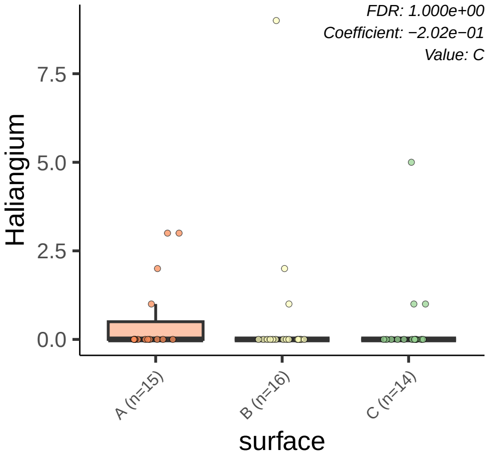
FDR: 1.000e+00
Coefficient: −2.02e−01
Value: C
Haliangium
0.0
2.5
5.0
7.5
A (n=15)
B (n=16)
C (n=14)
surface

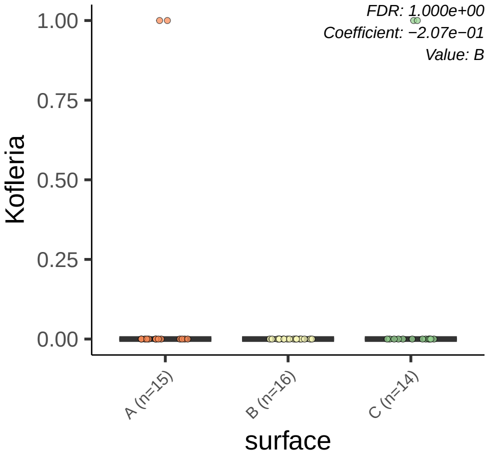
FDR: 1.000e+00
Coefficient: −2.07e−01
Value: B
Kofleria
1.00
0.75
0.50
0.25
0.00
A (n=15)
B (n=16)
C (n=14)
surface

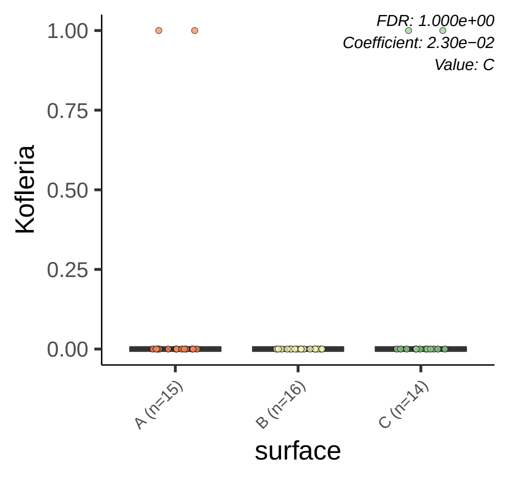

FDR: 1.000e+00
Coefficient: 2.30e−02
Value: C
Kofleria
1.00
0.75
0.50
0.25
0.00
A (n=15)
B (n=16)
C (n=14)
surface

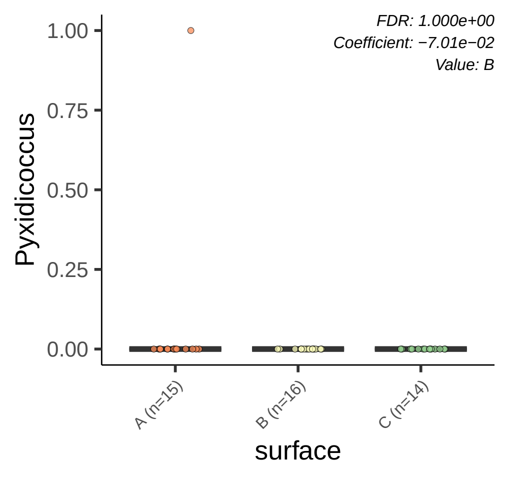
FDR: 1.000e+00
Coefficient: −7.01e−02
Value: B
Pyxidicoccus
1.00
0.75
0.50
0.25
0.00
A (n=15)
B (n=16)
C (n=14)
surface

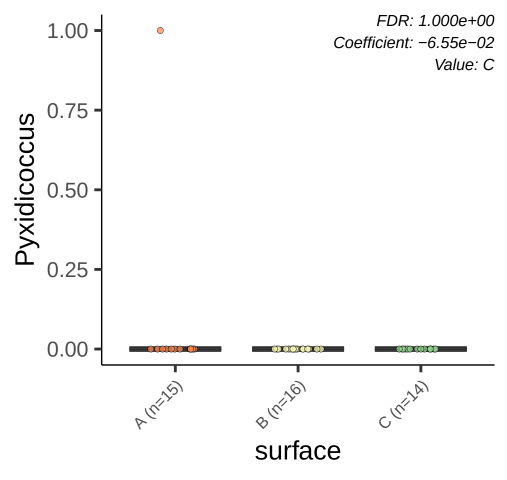
FDR: 1.000e+00
Coefficient: −6.55e−02
Value: C
Pyxidicoccus
1.00
0.75
0.50
0.25
0.00
A (n=15)
B (n=16)
C (n=14)
surface

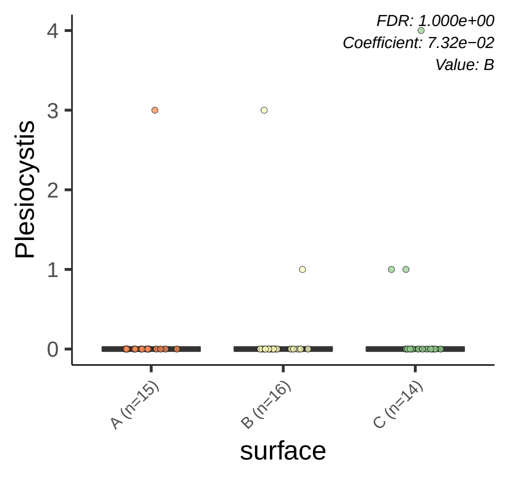
FDR: 1.000e+00
Coefficient: 7.32e−02
Value: B
Plesiocystis
0
1
2
3
4
A (n=15)
B (n=16)
C (n=14)
surface

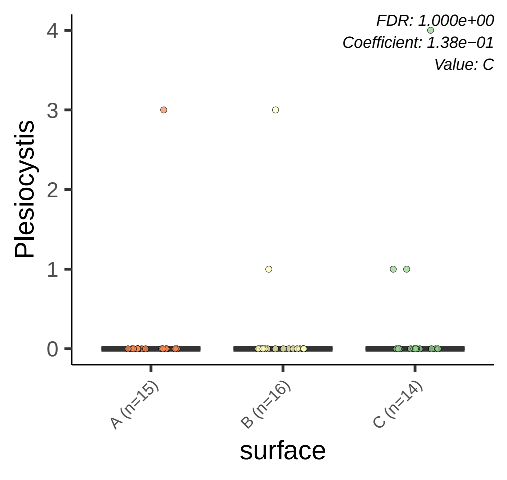
FDR: 1.000e+00
Coefficient: 1.38e−01
Value: C
Plesiocystis
4
3
2
1
0
A (n=15)
B (n=16)
C (n=14)
surface

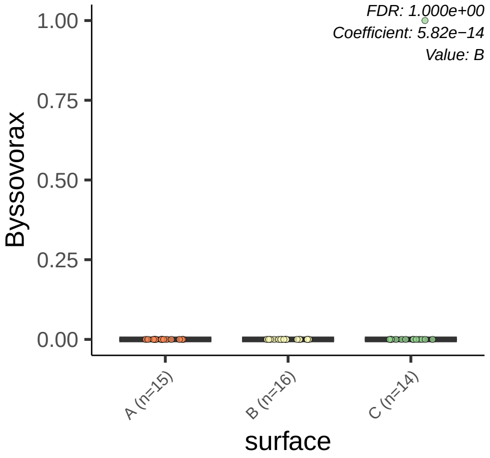

FDR: 1.000e+00
Coefficient: 5.82e−14
Value: B
1.00
0.75
0.50
0.25
0.00
Byssovorax
A (n=15)
B (n=16)
C (n=14)
surface

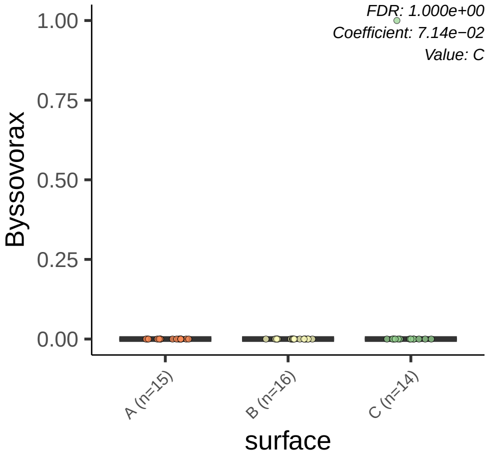
FDR: 1.000e+00
Coefficient: 7.14e−02
Value: C
Byssovorax
1.00
0.75
0.50
0.25
0.00
A (n=15)
B (n=16)
C (n=14)
surface

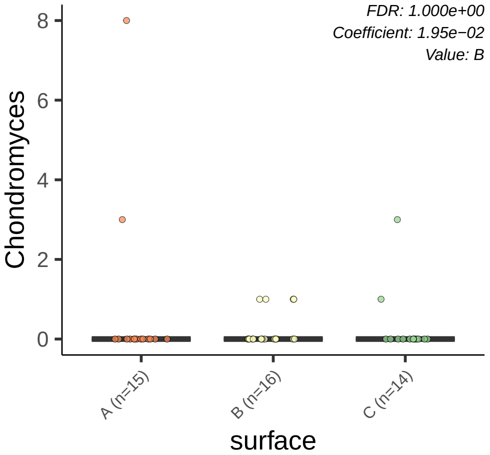
FDR: 1.000e+00
Coefficient: 1.95e−02
Value: B
Chondromyces
0
2
4
6
8
A (n=15)
B (n=16)
C (n=14)
surface

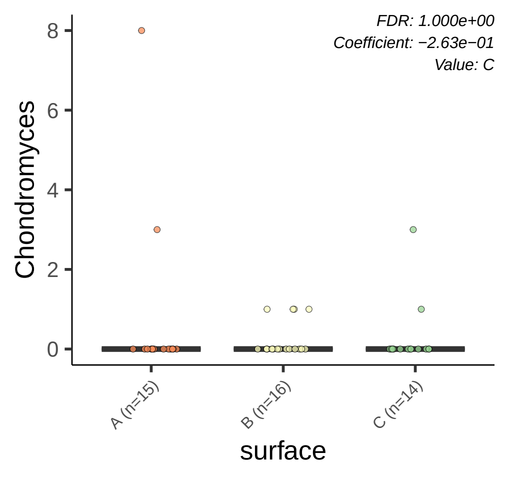

FDR: 1.000e+00
Coefficient: −2.63e−01
Value: C
Chondromyces
8
6
4
2
0
A (n=15)
B (n=16)
C (n=14)
surface

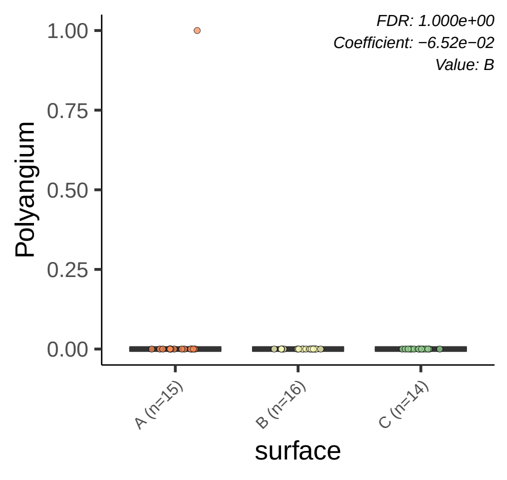
FDR: 1.000e+00
Coefficient: −6.52e−02
Value: B
Polyangium
1.00
0.75
0.50
0.25
0.00
A (n=15)
B (n=16)
C (n=14)
surface

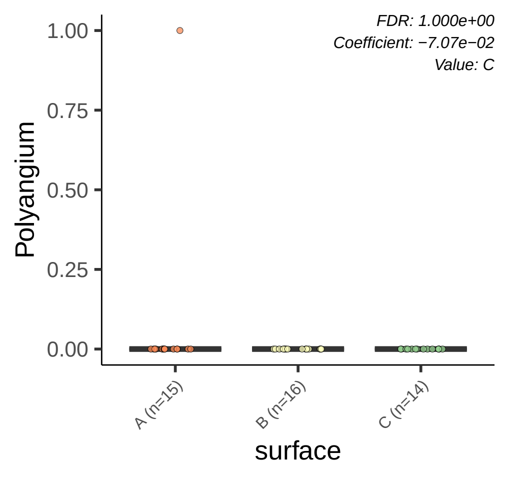
FDR: 1.000e+00
Coefficient: −7.07e−02
Value: C
Polyangium
1.00
0.75
0.50
0.25
0.00
A (n=15)
B (n=16)
C (n=14)
surface

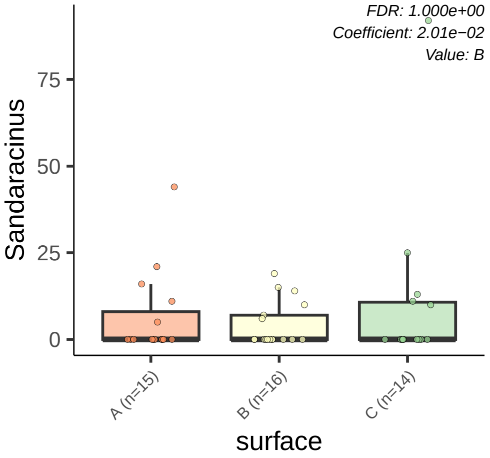
FDR: 1.000e+00
Coefficient: 2.01e−02
Value: B
Sandaracinus
75
50
25
0
A (n=15)
B (n=16)
C (n=14)
surface

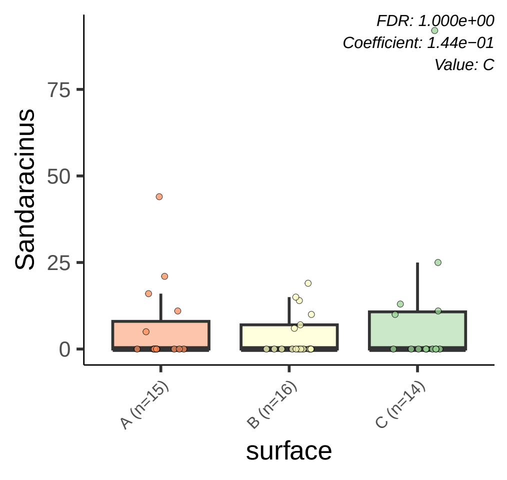
FDR: 1.000e+00
Coefficient: 1.44e−01
Value: C
Sandaracinus
75
50
25
0
A (n=15)
B (n=16)
C (n=14)
surface

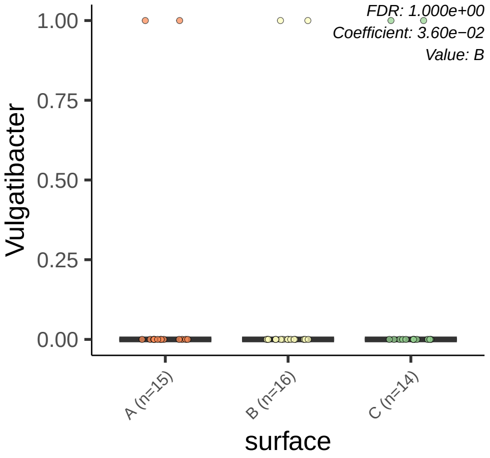
FDR: 1.000e+00
Coefficient: 3.60e−02
Value: B
Vulgatibacter
1.00
0.75
0.50
0.25
0.00
A (n=15)
B (n=16)
C (n=14)
surface

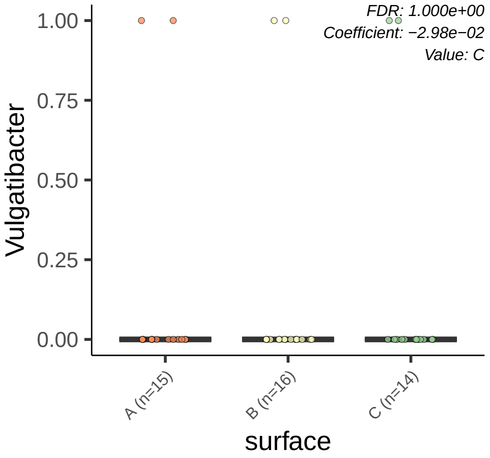
FDR: 1.000e+00
Coefficient: −2.98e−02
Value: C
1.00
0.75
0.50
0.25
0.00
Vulgatibacter
A (n=15)
B (n=16)
C (n=14)
surface

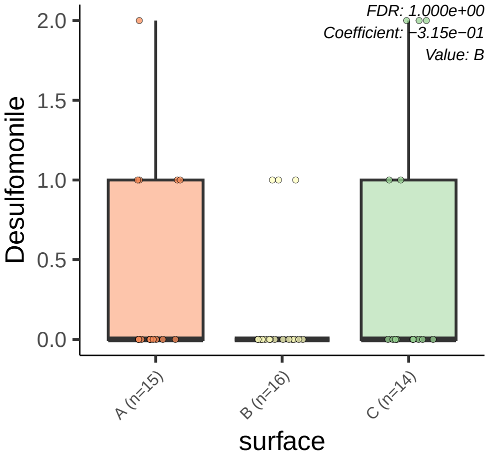
FDR: 1.000e+00
Coefficient: −3.15e−01
Value: B
Desulfomonile
2.0
1.5
1.0
0.5
0.0
A (n=15)
B (n=16)
C (n=14)
surface

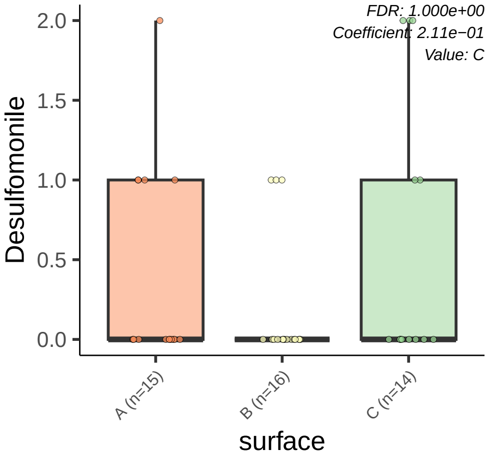
FDR: 1.000e+00
Coefficient: 2.11e−01
Value: C
Desulfomonile
2.0
1.5
1.0
0.5
0.0
A (n=15)
B (n=16)
C (n=14)
surface

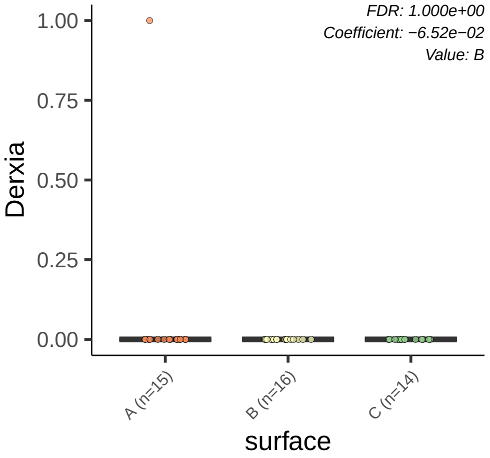

FDR: 1.000e+00
Coefficient: −6.52e−02
Value: B
Derxia
1.00
0.75
0.50
0.25
0.00
A (n=15)
B (n=16)
C (n=14)
surface

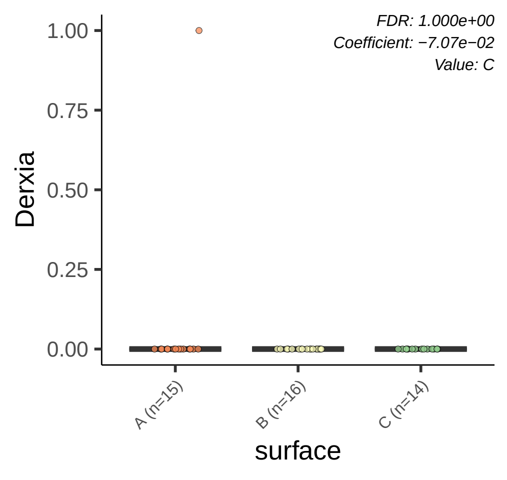
FDR: 1.000e+00
Coefficient: −7.07e−02
Value: C
Derxia
1.00
0.75
0.50
0.25
0.00
A (n=15)
B (n=16)
C (n=14)
surface

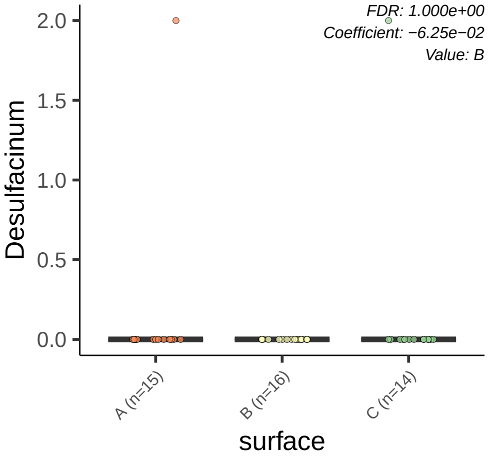
FDR: 1.000e+00
Coefficient: −6.25e−02
Value: B
Desulfacinum
2.0
1.5
1.0
0.5
0.0
A (n=15)
B (n=16)
C (n=14)
surface

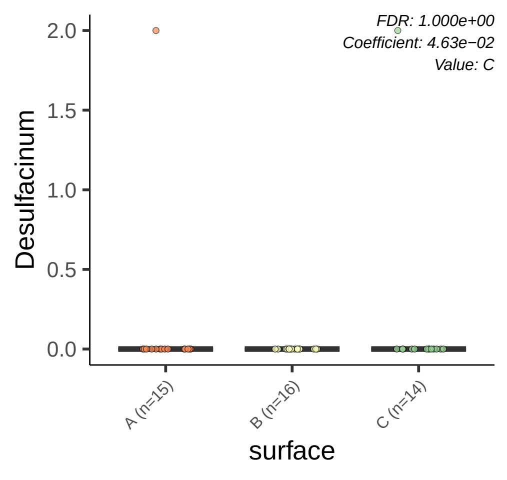
FDR: 1.000e+00
Coefficient: 4.63e−02
Value: C
Desulfacinum
2.0
1.5
1.0
0.5
0.0
A (n=15)
B (n=16)
C (n=14)
surface

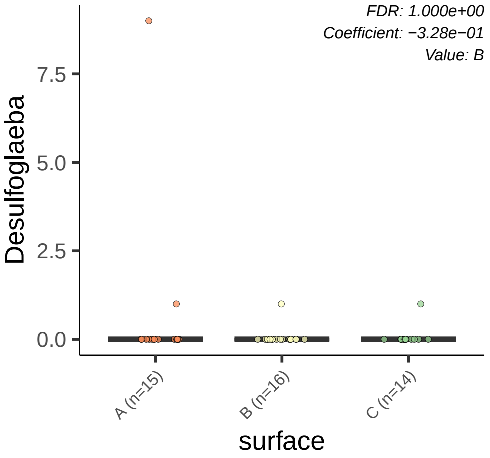
FDR: 1.000e+00
Coefficient: −3.28e−01
Value: B
Desulfoglaeba
7.5
5.0
2.5
0.0
A (n=15)
B (n=16)
C (n=14)
surface

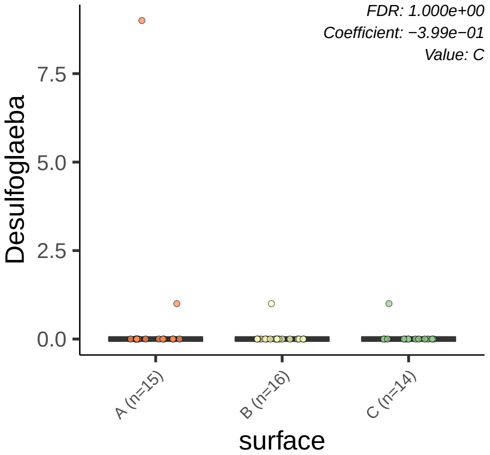
FDR: 1.000e+00
Coefficient: −3.99e−01
Value: C
Desulfoglaeba
7.5
5.0
2.5
0.0
A (n=15)
B (n=16)
C (n=14)
surface

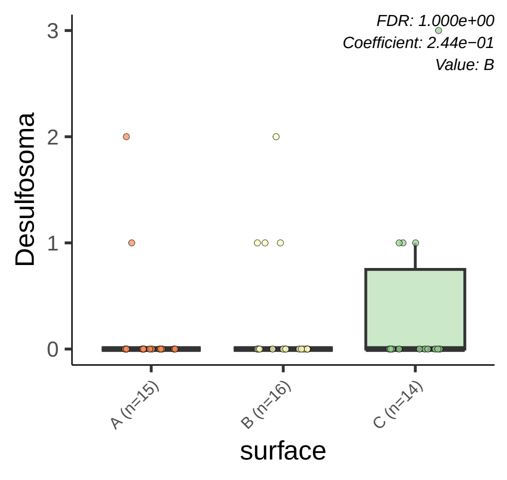
FDR: 1.000e+00
Coefficient: 2.44e−01
Value: B
Desulfosoma
3
2
1
0
A (n=15)
B (n=16)
C (n=14)
surface

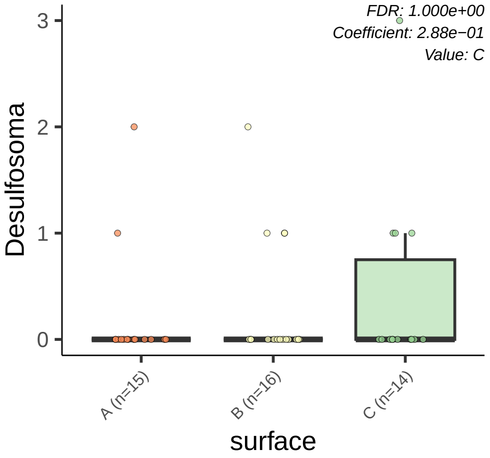

FDR: 1.000e+00
Coefficient: 2.88e−01
Value: C
Desulfosoma
3
2
1
0
A (n=15)
B (n=16)
C (n=14)
surface

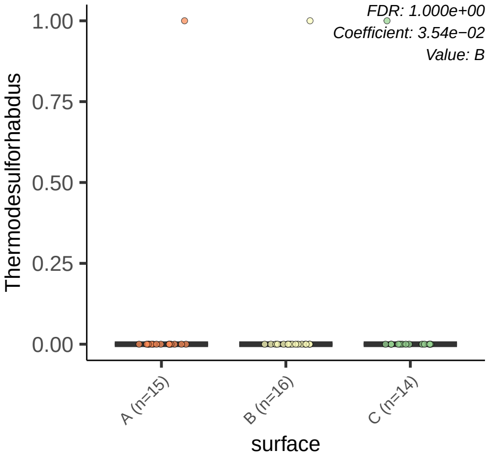
FDR: 1.000e+00
Coefficient: 3.54e−02
Value: B
Thermodesulforhabdus
1.00
0.75
0.50
0.25
0.00
A (n=15)
B (n=16)
C (n=14)
surface

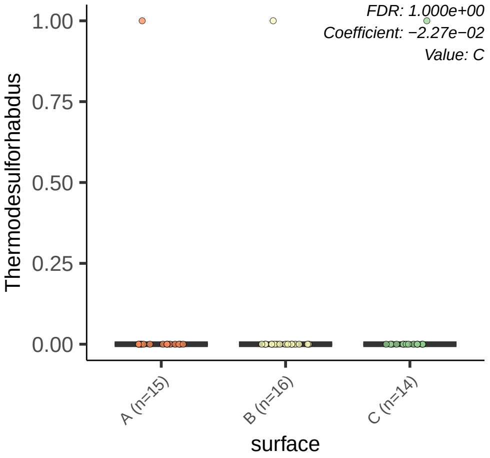
FDR: 1.000e+00
Coefficient: −2.27e−02
Value: C
Thermodesulforhabdus
1.00
0.75
0.50
0.25
0.00
A (n=15)
B (n=16)
C (n=14)
surface

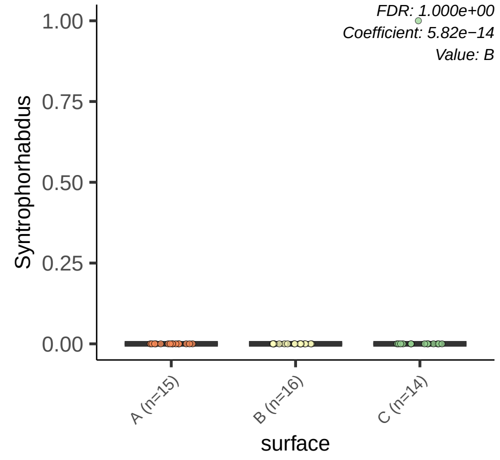

FDR: 1.000e+00
Coefficient: 5.82e−14
Value: B
Syntrophorhabdus
1.00
0.75
0.50
0.25
0.00
A (n=15)
B (n=16)
C (n=14)
surface

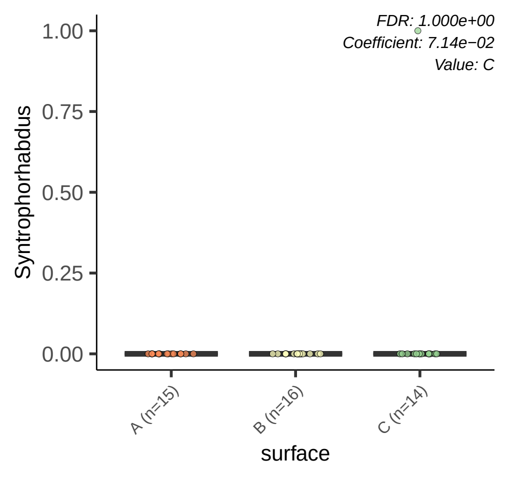

FDR: 1.000e+00
Coefficient: 7.14e−02
Value: C
Syntrophorhabdus
1.00
0.75
0.50
0.25
0.00
A (n=15)
B (n=16)
C (n=14)
surface

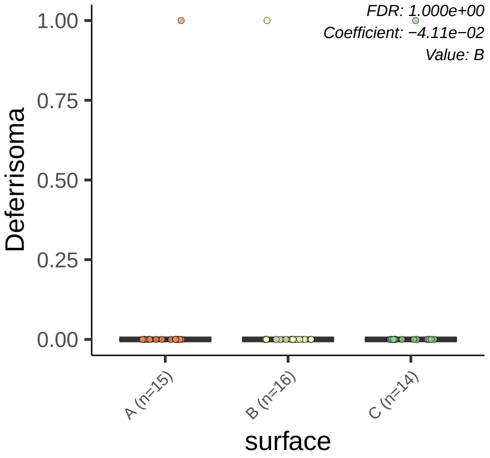
FDR: 1.000e+00
Coefficient: −4.11e−02
Value: B
Deferrisoma
1.00
0.75
0.50
0.25
0.00
A (n=15)
B (n=16)
C (n=14)
surface

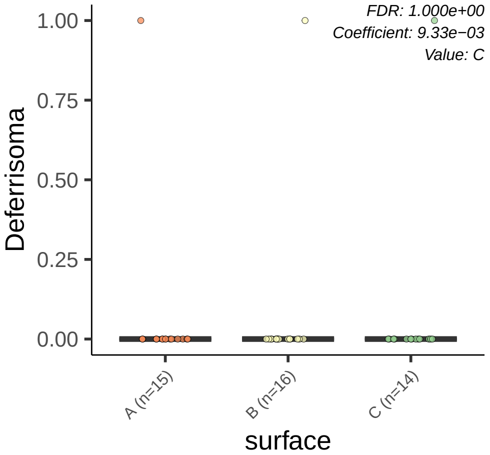
FDR: 1.000e+00
Coefficient: 9.33e−03
Value: C
Deferrisoma
1.00
0.75
0.50
0.25
0.00
A (n=15)
B (n=16)
C (n=14)
surface

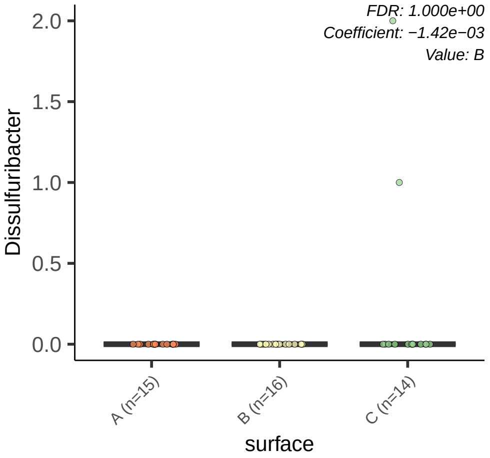

FDR: 1.000e+00
Coefficient: −1.42e−03
Value: B
Dissulfuribacter
2.0
1.5
1.0
0.5
0.0
A (n=15)
B (n=16)
C (n=14)
surface

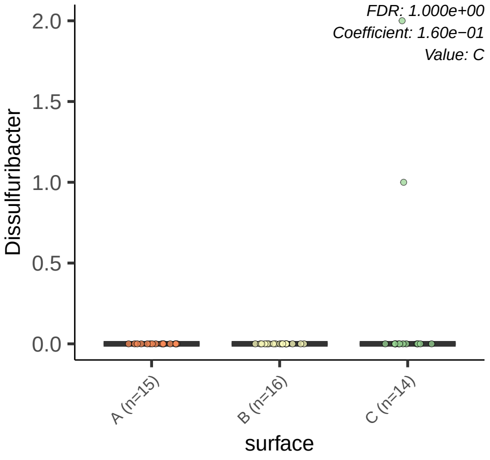
FDR: 1.000e+00
Coefficient: 1.60e−01
Value: C
Dissulfuribacter
2.0
1.5
1.0
0.5
0.0
A (n=15)
B (n=16)
C (n=14)
surface

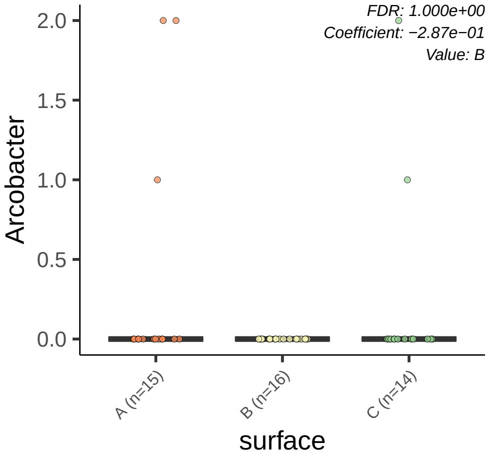
FDR: 1.000e+00
Coefficient: −2.87e−01
Value: B
Arcobacter
2.0
1.5
1.0
0.5
0.0
A (n=15)
B (n=16)
C (n=14)
surface

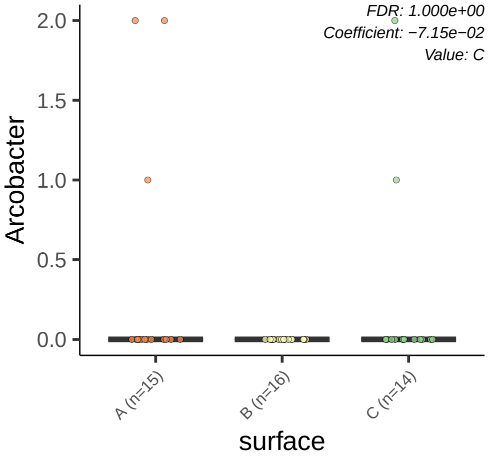

FDR: 1.000e+00
Coefficient: −7.15e−02
Value: C
Arcobacter
2.0
1.5
1.0
0.5
0.0
A (n=15)
B (n=16)
C (n=14)
surface

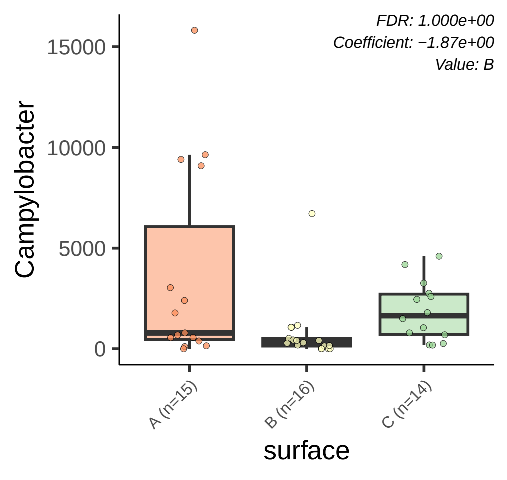

FDR: 1.000e+00
Coefficient: −1.87e+00
Value: B
Campylobacter
15000
10000
5000
0
A (n=15)
B (n=16)
C (n=14)
surface

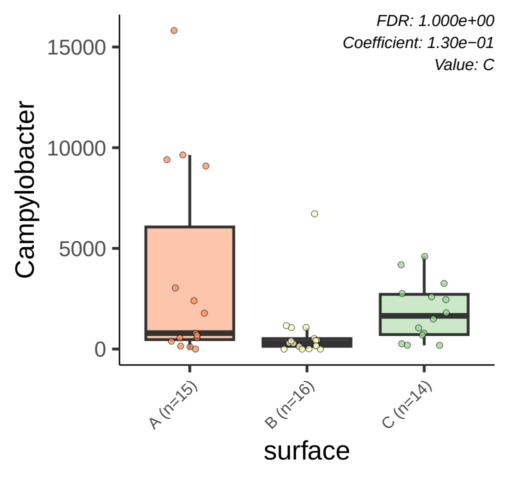

FDR: 1.000e+00
Coefficient: 1.30e−01
Value: C
Campylobacter
15000
10000
5000
0
A (n=15)
B (n=16)
C (n=14)
surface

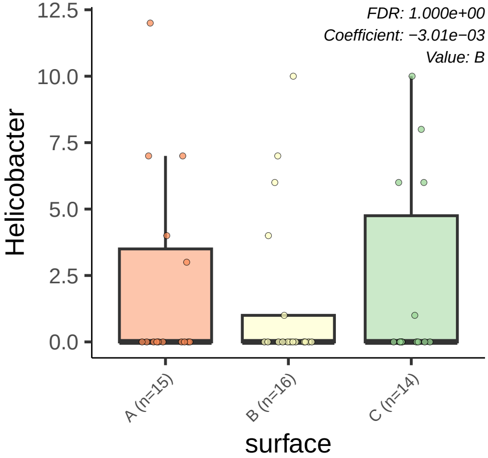
FDR: 1.000e+00
Coefficient: −3.01e−03
Value: B
Helicobacter
12.5
10.0
7.5
5.0
2.5
0.0
A (n=15)
B (n=16)
C (n=14)
surface

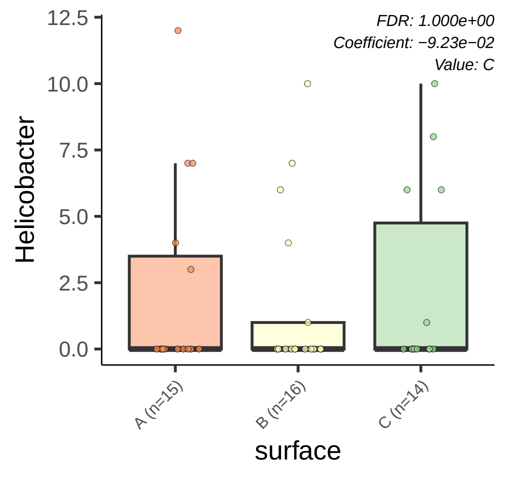
FDR: 1.000e+00
Coefficient: −9.23e−02
Value: C
Helicobacter
12.5
10.0
7.5
5.0
2.5
0.0
A (n=15)
B (n=16)
C (n=14)
surface

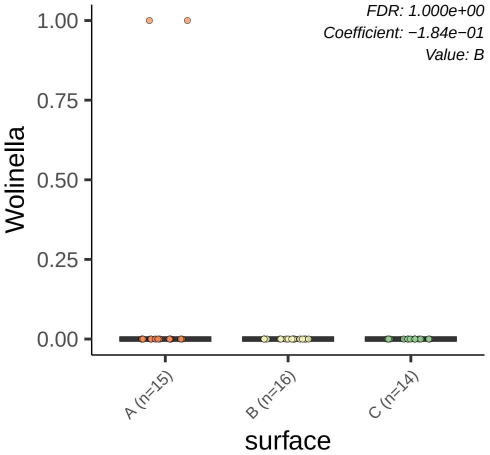
FDR: 1.000e+00
Coefficient: −1.84e−01
Value: B
1.00
0.75
0.50
0.25
0.00
Wolinella
A (n=15)
B (n=16)
C (n=14)
surface

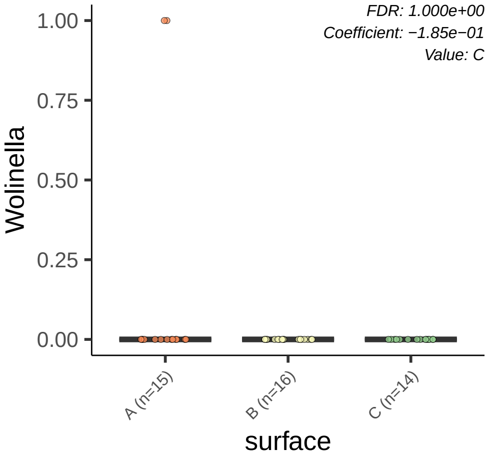

FDR: 1.000e+00
Coefficient: −1.85e−01
Value: C
Wolinella
1.00
0.75
0.50
0.25
0.00
A (n=15)
B (n=16)
C (n=14)
surface

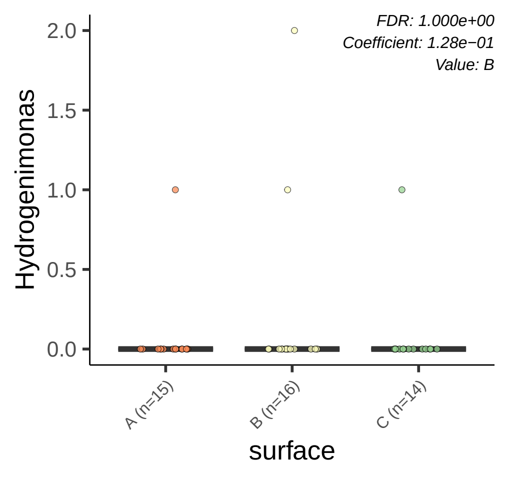
FDR: 1.000e+00
Coefficient: 1.28e−01
Value: B
Hydrogenimonas
2.0
1.5
1.0
0.5
0.0
A (n=15)
B (n=16)
C (n=14)
surface

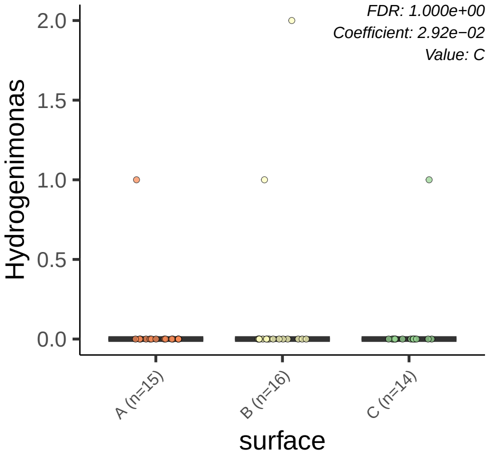
FDR: 1.000e+00
Coefficient: 2.92e−02
Value: C
Hydrogenimonas
2.0
1.5
1.0
0.5
0.0
A (n=15)
B (n=16)
C (n=14)
surface

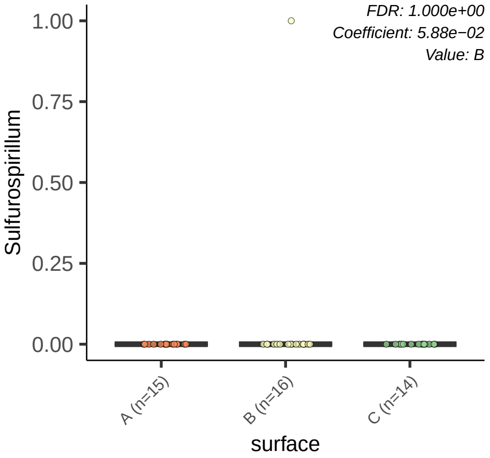

FDR: 1.000e+00
Coefficient: 5.88e−02
Value: B
1.00
0.75
0.50
0.25
0.00
Sulfurospirillum
A (n=15)
B (n=16)
C (n=14)
surface

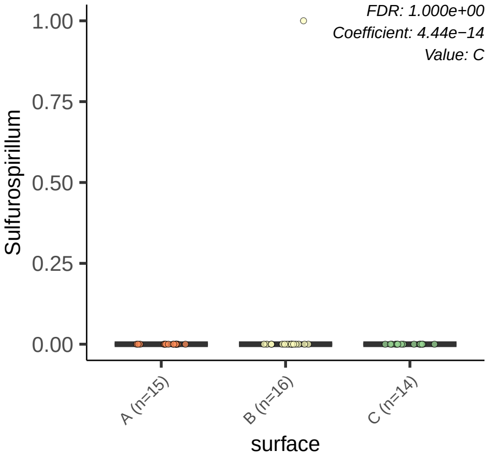
FDR: 1.000e+00
Coefficient: 4.44e−14
Value: C
Sulfurospirillum
1.00
0.75
0.50
0.25
0.00
A (n=15)
B (n=16)
C (n=14)
surface

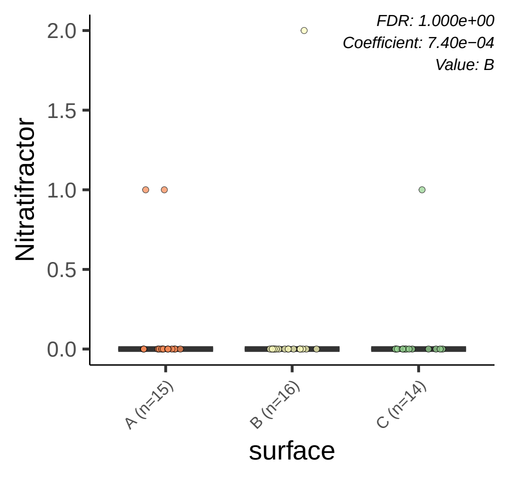

FDR: 1.000e+00
Coefficient: 7.40e−04
Value: B
Nitratifractor
2.0
1.5
1.0
0.5
0.0
A (n=15)
B (n=16)
C (n=14)
surface

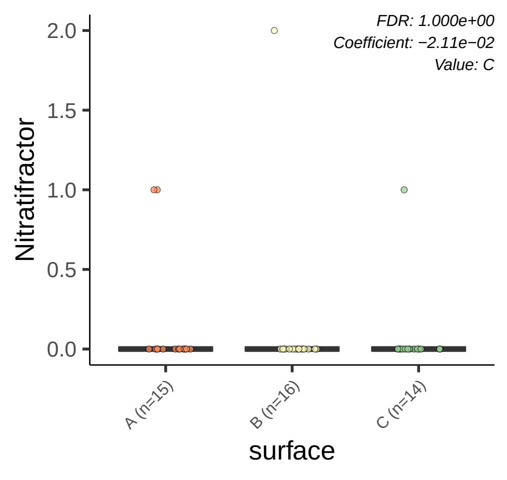
FDR: 1.000e+00
Coefficient: −2.11e−02
Value: C
Nitratifractor
2.0
1.5
1.0
0.5
0.0
A (n=15)
B (n=16)
C (n=14)
surface

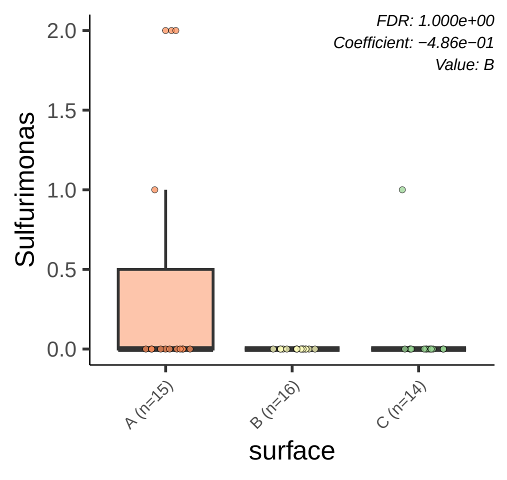

FDR: 1.000e+00
Coefficient: −4.86e−01
Value: B
Sulfurimonas
2.0
1.5
1.0
0.5
0.0
A (n=15)
B (n=16)
C (n=14)
surface

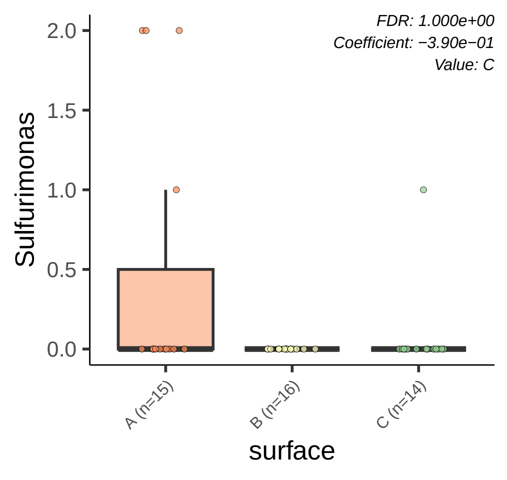
FDR: 1.000e+00
Coefficient: −3.90e−01
Value: C
Sulfurimonas
2.0
1.5
1.0
0.5
0.0
A (n=15)
B (n=16)
C (n=14)
surface

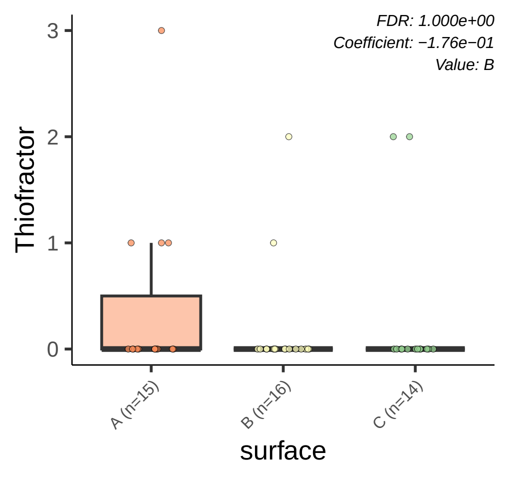
FDR: 1.000e+00
Coefficient: −1.76e−01
Value: B
Thiofractor
0
1
2
3
A (n=15)
B (n=16)
C (n=14)
surface

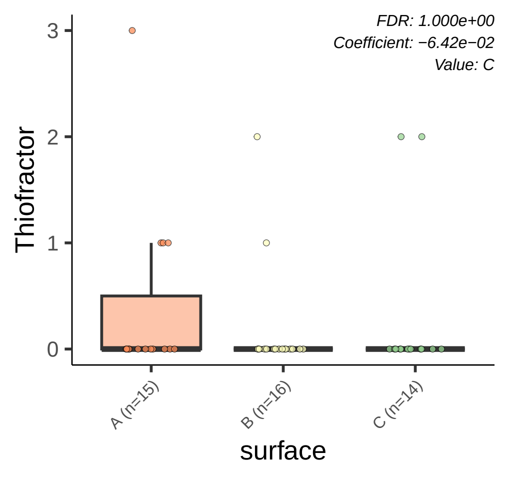

FDR: 1.000e+00
Coefficient: −6.42e−02
Value: C
Thiofractor
3
2
1
0
A (n=15)
B (n=16)
C (n=14)
surface

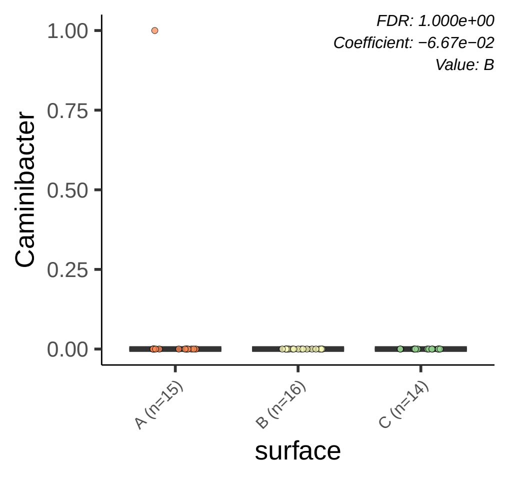

FDR: 1.000e+00
Coefficient: −6.67e−02
Value: B
1.00
0.75
0.50
0.25
0.00
Caminibacter
A (n=15)
B (n=16)
C (n=14)
surface

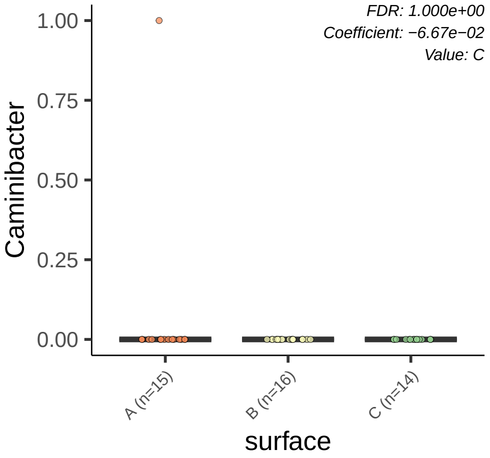
FDR: 1.000e+00
Coefficient: −6.67e−02
Value: C
Caminibacter
1.00
0.75
0.50
0.25
0.00
A (n=15)
B (n=16)
C (n=14)
surface

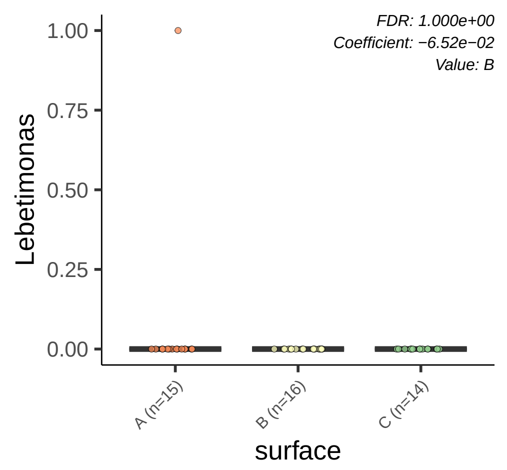
FDR: 1.000e+00
Coefficient: −6.52e−02
Value: B
Lebetimonas
1.00
0.75
0.50
0.25
0.00
A (n=15)
B (n=16)
C (n=14)
surface

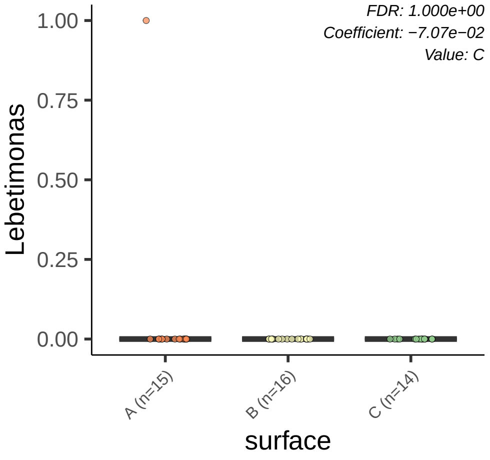
FDR: 1.000e+00
Coefficient: −7.07e−02
Value: C
Lebetimonas
1.00
0.75
0.50
0.25
0.00
A (n=15)
B (n=16)
C (n=14)
surface

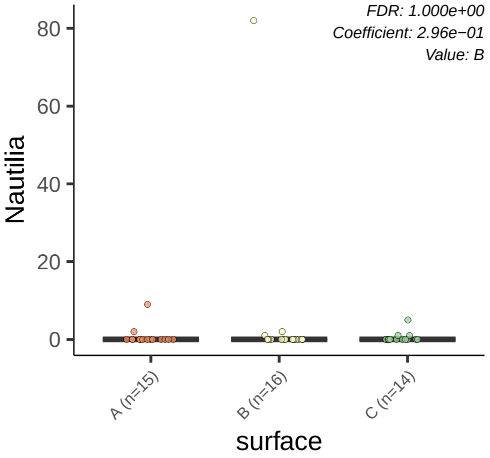

FDR: 1.000e+00
Coefficient: 2.96e−01
Value: B
Nautilia
0
20
40
60
80
A (n=15)
B (n=16)
C (n=14)
surface

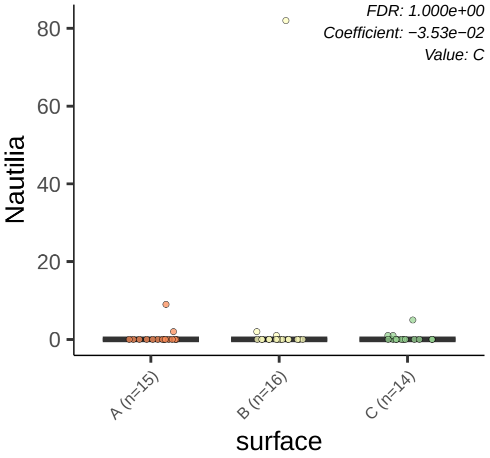

FDR: 1.000e+00
Coefficient: −3.53e−02
Value: C
Nautilia
80
60
40
20
0
A (n=15)
B (n=16)
C (n=14)
surface

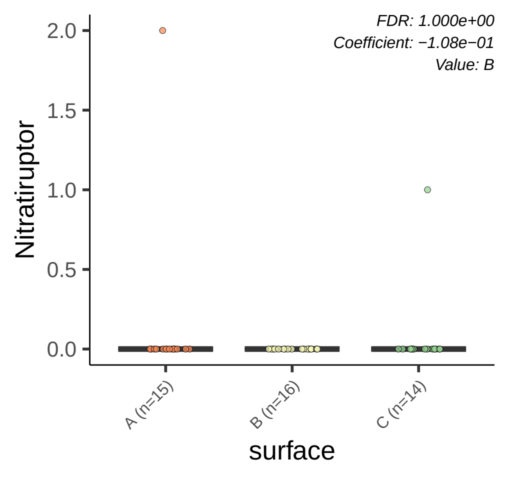
FDR: 1.000e+00
Coefficient: −1.08e−01
Value: B
Nitratiruptor
2.0
1.5
1.0
0.5
0.0
A (n=15)
B (n=16)
C (n=14)
surface

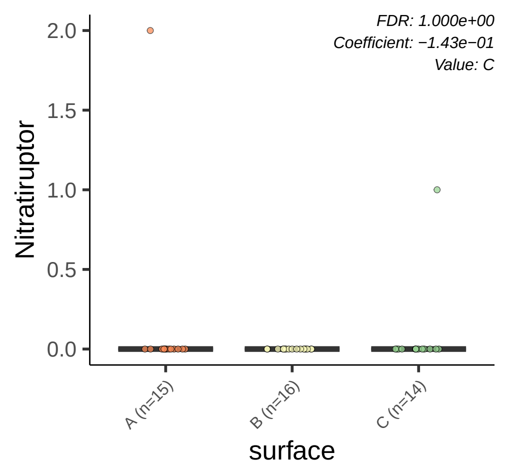
FDR: 1.000e+00
Coefficient: −1.43e−01
Value: C
Nitratiruptor
2.0
1.5
1.0
0.5
0.0
A (n=15)
B (n=16)
C (n=14)
surface

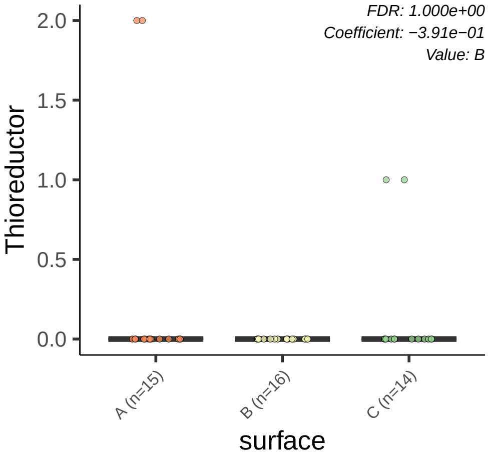

FDR: 1.000e+00
Coefficient: −3.91e−01
Value: B
Thioreductor
2.0
1.5
1.0
0.5
0.0
A (n=15)
B (n=16)
C (n=14)
surface

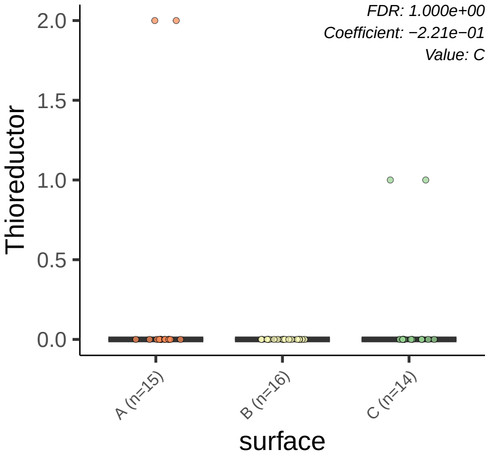
FDR: 1.000e+00
Coefficient: −2.21e−01
Value: C
Thioreductor
2.0
1.5
1.0
0.5
0.0
A (n=15)
B (n=16)
C (n=14)
surface

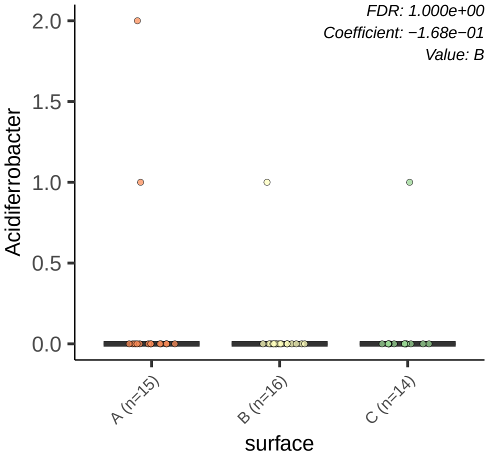
FDR: 1.000e+00
Coefficient: −1.68e−01
Value: B
Acidiferrobacter
2.0
1.5
1.0
0.5
0.0
A (n=15)
B (n=16)
C (n=14)
surface

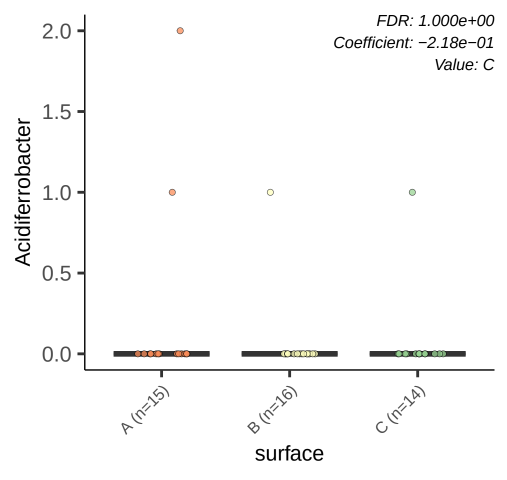
FDR: 1.000e+00
Coefficient: −2.18e−01
Value: C
Acidiferrobacter
2.0
1.5
1.0
0.5
0.0
A (n=15)
B (n=16)
C (n=14)
surface

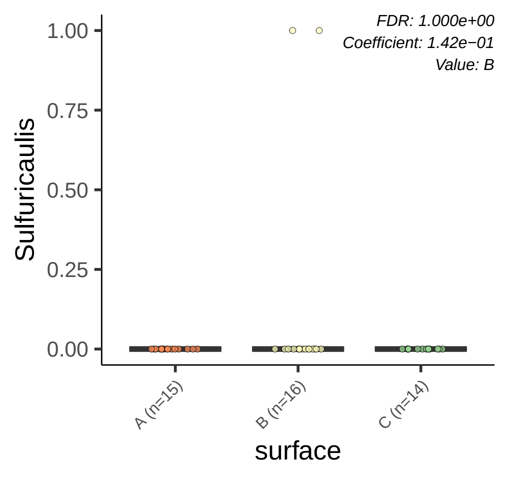
FDR: 1.000e+00
Coefficient: 1.42e−01
Value: B
Sulfuricaulis
1.00
0.75
0.50
0.25
0.00
A (n=15)
B (n=16)
C (n=14)
surface

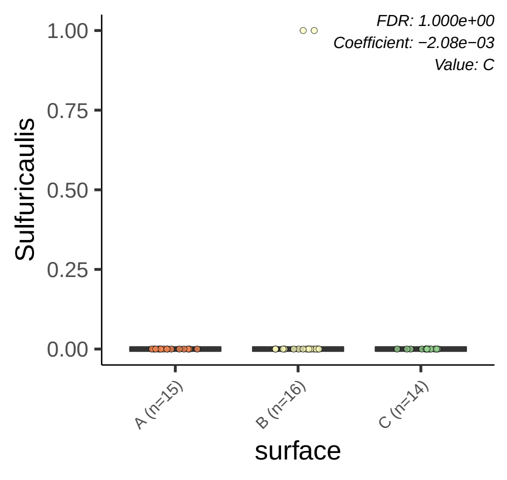
FDR: 1.000e+00
Coefficient: −2.08e−03
Value: C
Sulfuricaulis
1.00
0.75
0.50
0.25
0.00
A (n=15)
B (n=16)
C (n=14)
surface

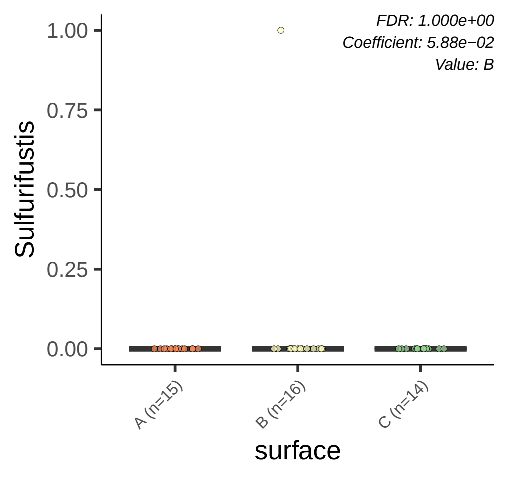
FDR: 1.000e+00
Coefficient: 5.88e−02
Value: B
Sulfurifustis
1.00
0.75
0.50
0.25
0.00
A (n=15)
B (n=16)
C (n=14)
surface

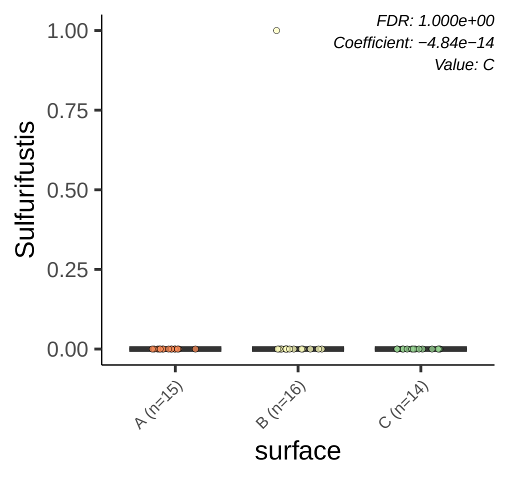

FDR: 1.000e+00
Coefficient: −4.84e−14
Value: C
1.00
0.75
0.50
0.25
0.00
Sulfurifustis
A (n=15)
B (n=16)
C (n=14)
surface

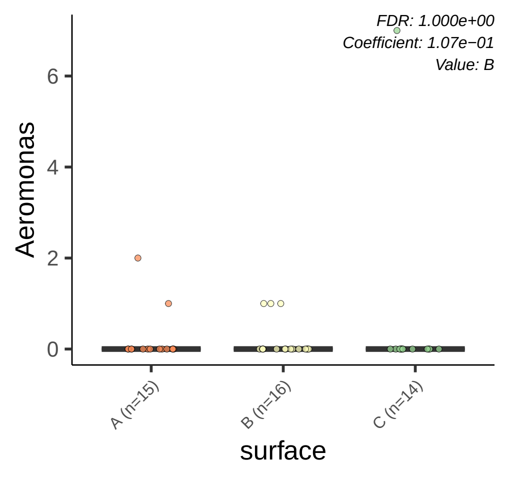
FDR: 1.000e+00
Coefficient: 1.07e−01
Value: B
Aeromonas
0
2
4
6
A (n=15)
B (n=16)
C (n=14)
surface

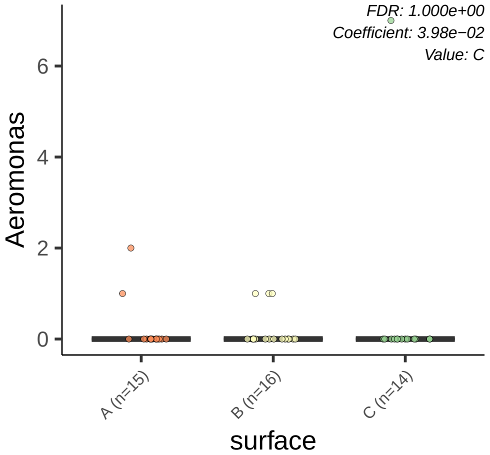
FDR: 1.000e+00
Coefficient: 3.98e−02
Value: C
Aeromonas
0
2
4
6
A (n=15)
B (n=16)
C (n=14)
surface

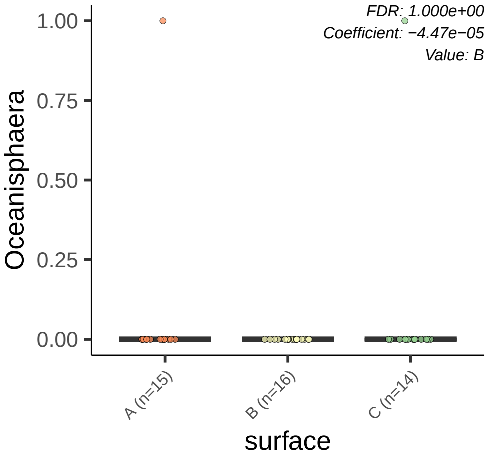

FDR: 1.000e+00
Coefficient: −4.47e−05
Value: B
Oceanisphaera
1.00
0.75
0.50
0.25
0.00
A (n=15)
B (n=16)
C (n=14)
surface

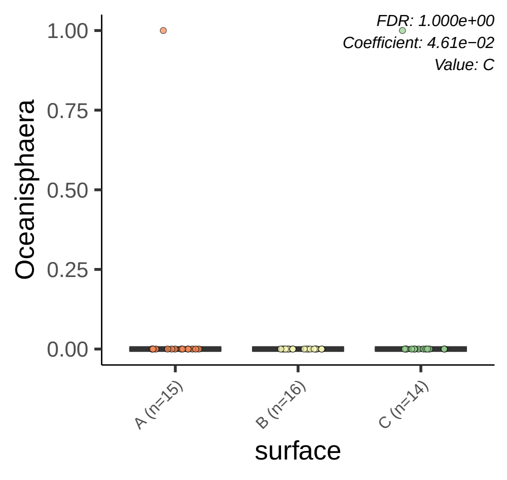
FDR: 1.000e+00
Coefficient: 4.61e−02
Value: C
Oceanisphaera
1.00
0.75
0.50
0.25
0.00
A (n=15)
B (n=16)
C (n=14)
surface

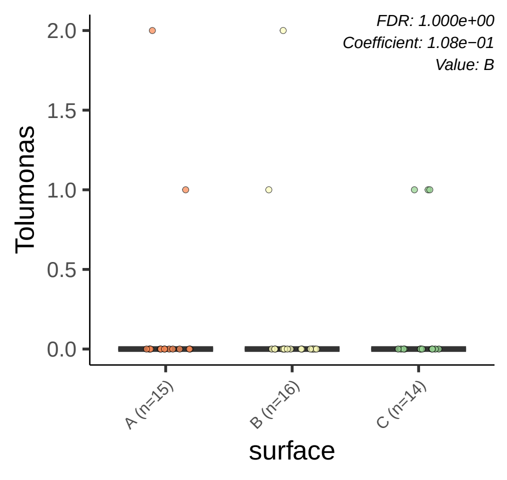
FDR: 1.000e+00
Coefficient: 1.08e−01
Value: B
Tolumonas
2.0
1.5
1.0
0.5
0.0
A (n=15)
B (n=16)
C (n=14)
surface

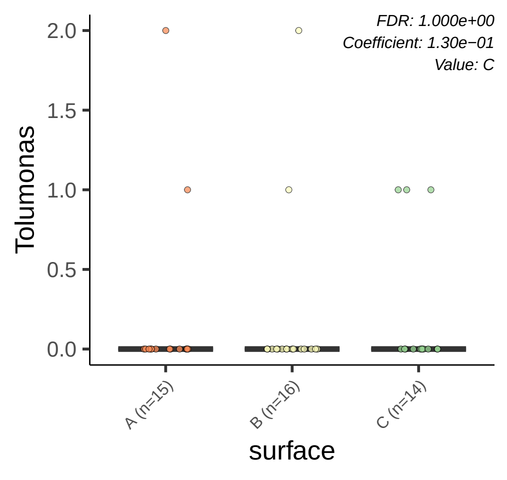
FDR: 1.000e+00
Coefficient: 1.30e−01
Value: C
Tolumonas
2.0
1.5
1.0
0.5
0.0
A (n=15)
B (n=16)
C (n=14)
surface

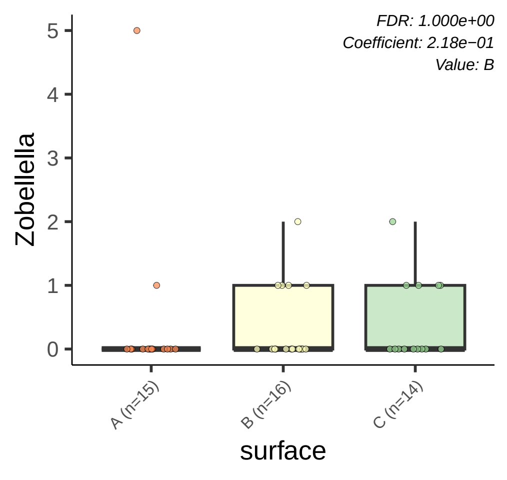

FDR: 1.000e+00
Coefficient: 2.18e−01
Value: B
Zobellella
0
1
2
3
4
5
A (n=15)
B (n=16)
C (n=14)
surface

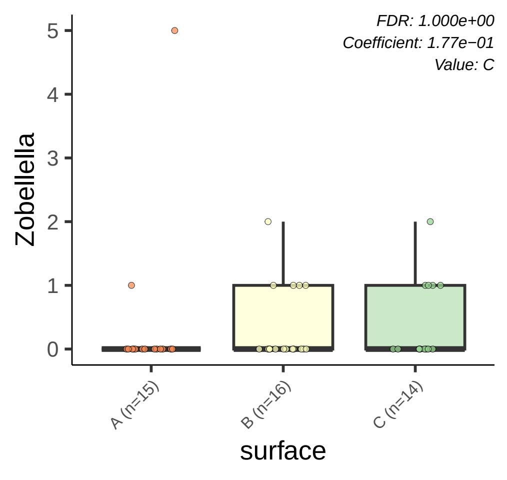
FDR: 1.000e+00
Coefficient: 1.77e−01
Value: C
Zobellella
5
4
3
2
1
0
A (n=15)
B (n=16)
C (n=14)
surface

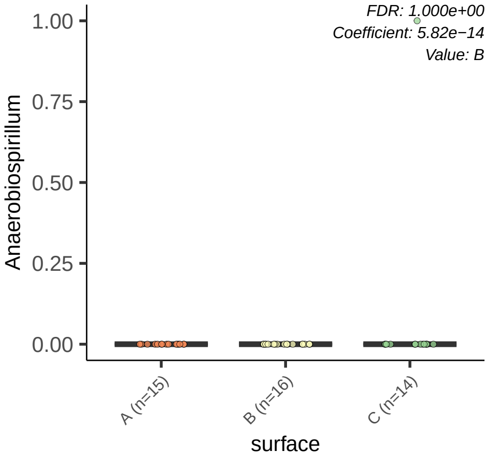
FDR: 1.000e+00
Coefficient: 5.82e−14
Value: B
Anaerobiospirillum
1.00
0.75
0.50
0.25
0.00
A (n=15)
B (n=16)
C (n=14)
surface

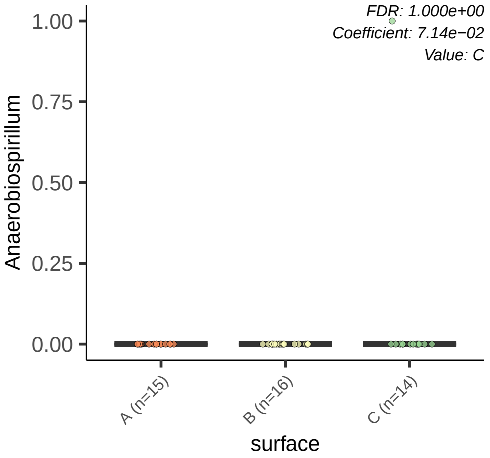

FDR: 1.000e+00
Coefficient: 7.14e−02
Value: C
1.00
0.75
0.50
0.25
0.00
Anaerobiospirillum
A (n=15)
B (n=16)
C (n=14)
surface

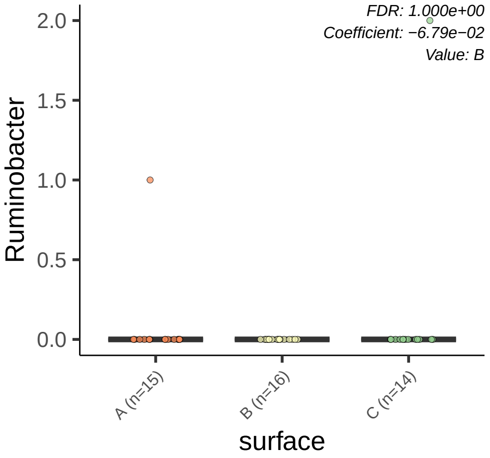
FDR: 1.000e+00
Coefficient: −6.79e−02
Value: B
Ruminobacter
2.0
1.5
1.0
0.5
0.0
A (n=15)
B (n=16)
C (n=14)
surface

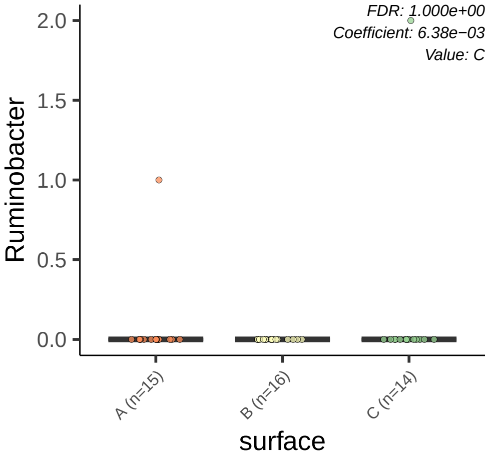
FDR: 1.000e+00
Coefficient: 6.38e−03
Value: C
Ruminobacter
2.0
1.5
1.0
0.5
0.0
A (n=15)
B (n=16)
C (n=14)
surface

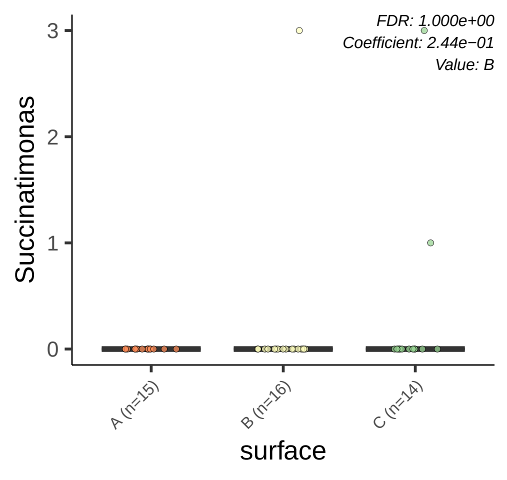

FDR: 1.000e+00
Coefficient: 2.44e−01
Value: B
Succinatimonas
3
2
1
0
A (n=15)
B (n=16)
C (n=14)
surface

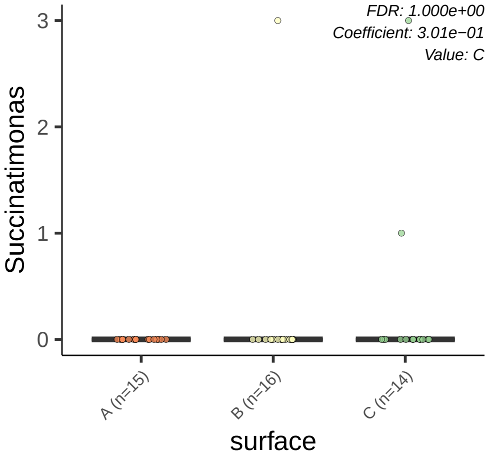
FDR: 1.000e+00
Coefficient: 3.01e−01
Value: C
Succinatimonas
3
2
1
0
A (n=15)
B (n=16)
C (n=14)
surface

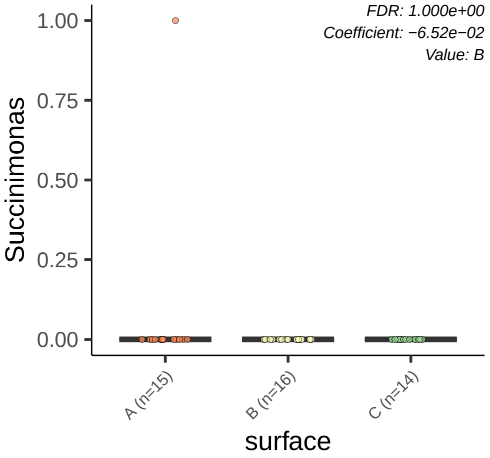

FDR: 1.000e+00
Coefficient: −6.52e−02
Value: B
Succinimonas
1.00
0.75
0.50
0.25
0.00
A (n=15)
B (n=16)
C (n=14)
surface

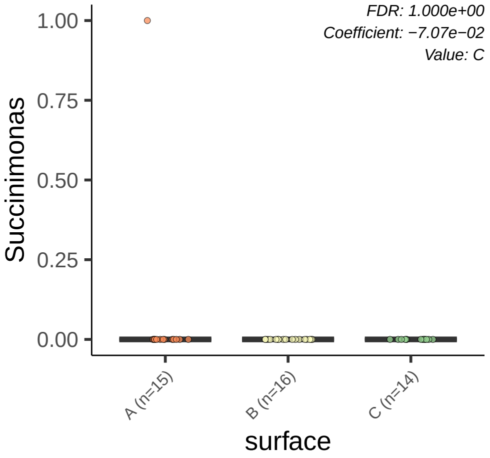

FDR: 1.000e+00
Coefficient: −7.07e−02
Value: C
Succinimonas
1.00
0.75
0.50
0.25
0.00
A (n=15)
B (n=16)
C (n=14)
surface

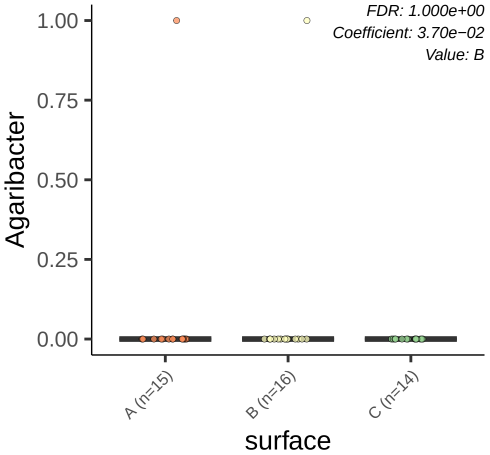
FDR: 1.000e+00
Coefficient: 3.70e−02
Value: B
Agaribacter
1.00
0.75
0.50
0.25
0.00
A (n=15)
B (n=16)
C (n=14)
surface

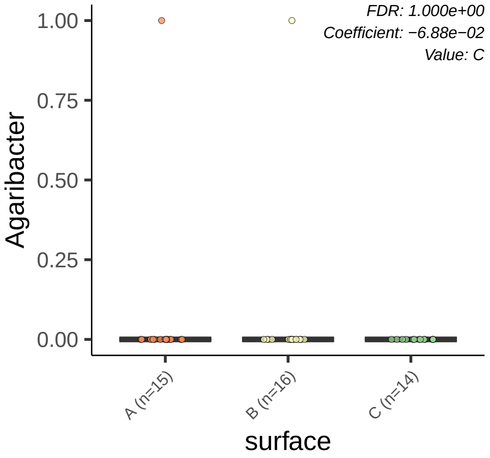
FDR: 1.000e+00
Coefficient: −6.88e−02
Value: C
Agaribacter
1.00
0.75
0.50
0.25
0.00
A (n=15)
B (n=16)
C (n=14)
surface

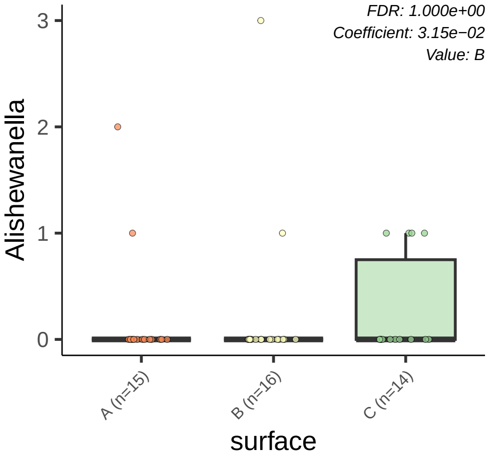

FDR: 1.000e+00
Coefficient: 3.15e−02
Value: B
Alishewanella
0
1
2
3
A (n=15)
B (n=16)
C (n=14)
surface

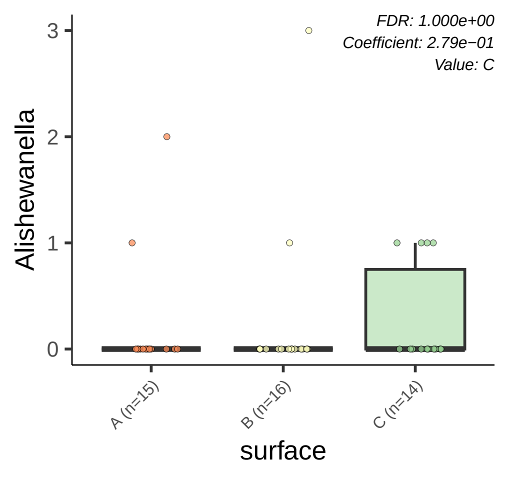
FDR: 1.000e+00
Coefficient: 2.79e−01
Value: C
Alishewanella
3
2
1
0
A (n=15)
B (n=16)
C (n=14)
surface

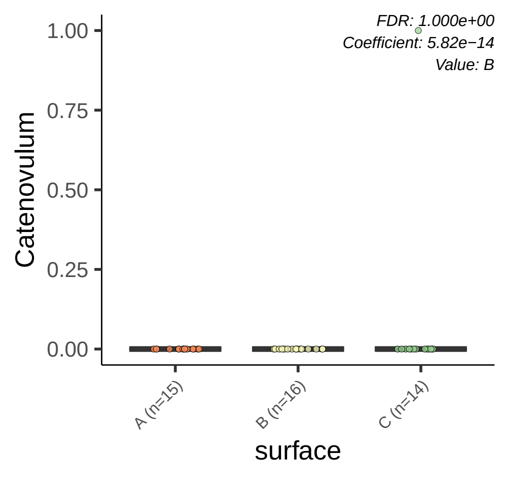
FDR: 1.000e+00
Coefficient: 5.82e−14
Value: B
1.00
0.75
0.50
0.25
0.00
Catenovulum
A (n=15)
B (n=16)
C (n=14)
surface

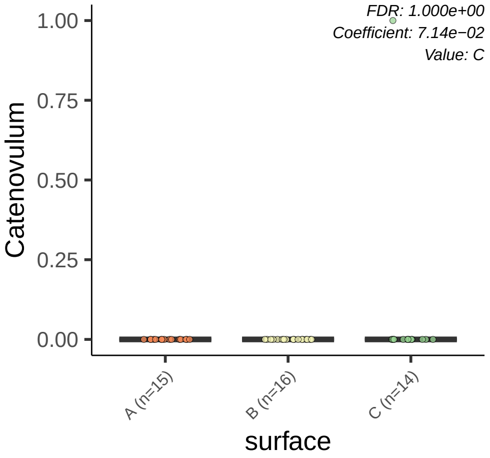
FDR: 1.000e+00
Coefficient: 7.14e−02
Value: C
Catenovulum
1.00
0.75
0.50
0.25
0.00
A (n=15)
B (n=16)
C (n=14)
surface

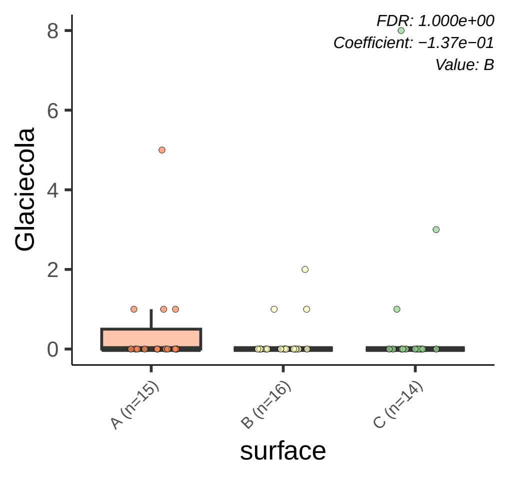

FDR: 1.000e+00
Coefficient: −1.37e−01
Value: B
Glaciecola
8
6
4
2
0
A (n=15)
B (n=16)
C (n=14)
surface

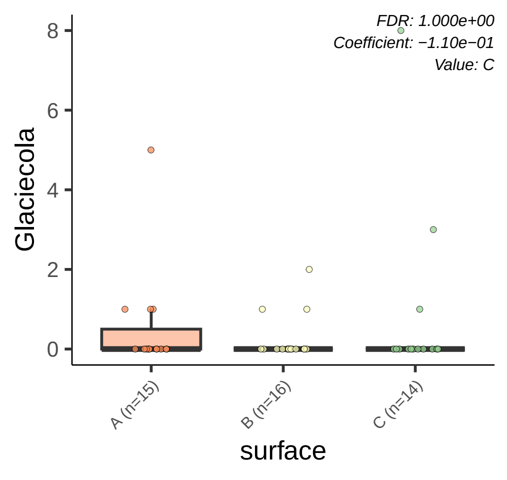

FDR: 1.000e+00
Coefficient: −1.10e−01
Value: C
Glaciecola
0
2
4
6
8
A (n=15)
B (n=16)
C (n=14)
surface

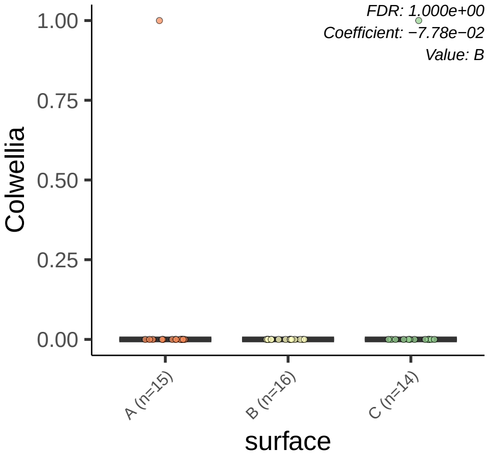
FDR: 1.000e+00
Coefficient: −7.78e−02
Value: B
1.00
0.75
0.50
0.25
0.00
Colwellia
A (n=15)
B (n=16)
C (n=14)
surface

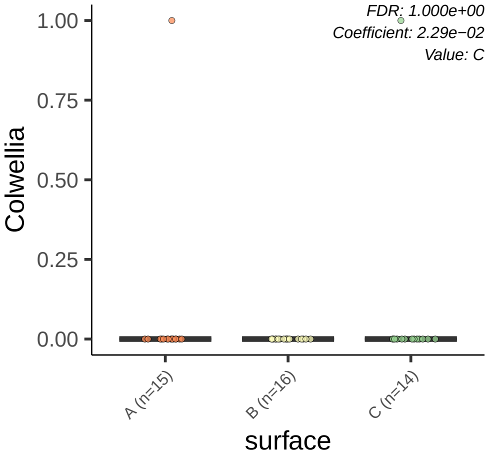
FDR: 1.000e+00
Coefficient: 2.29e−02
Value: C
Colwellia
1.00
0.75
0.50
0.25
0.00
A (n=15)
B (n=16)
C (n=14)
surface

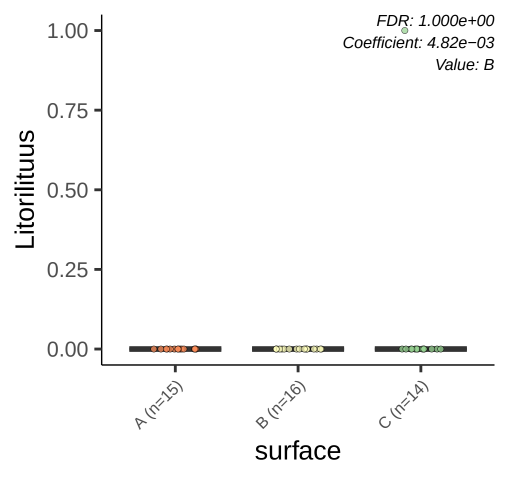

FDR: 1.000e+00
Coefficient: 4.82e−03
Value: B
1.00
0.75
0.50
0.25
0.00
Litorilituus
A (n=15)
B (n=16)
C (n=14)
surface

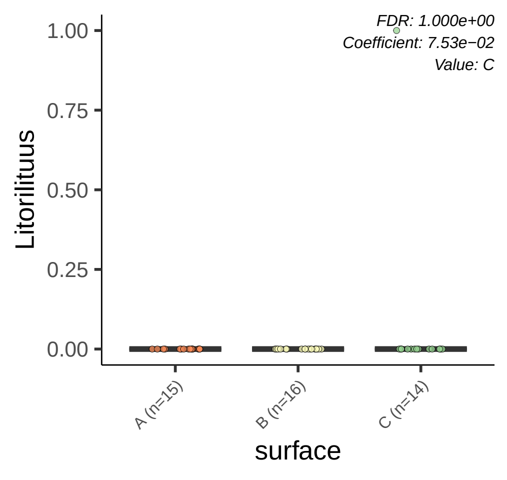

FDR: 1.000e+00
Coefficient: 7.53e−02
Value: C
1.00
0.75
0.50
0.25
0.00
Litorilituus
A (n=15)
B (n=16)
C (n=14)
surface

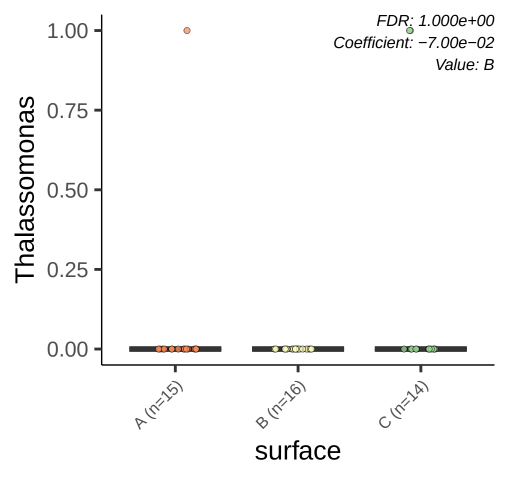

FDR: 1.000e+00
Coefficient: −7.00e−02
Value: B
Thalassomonas
1.00
0.75
0.50
0.25
0.00
A (n=15)
B (n=16)
C (n=14)
surface

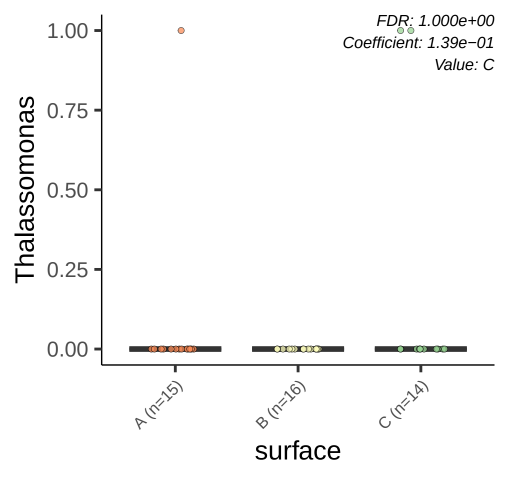
FDR: 1.000e+00
Coefficient: 1.39e−01
Value: C
Thalassomonas
1.00
0.75
0.50
0.25
0.00
A (n=15)
B (n=16)
C (n=14)
surface

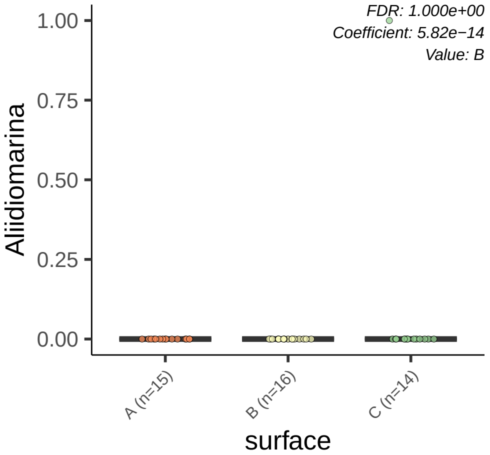

FDR: 1.000e+00
Coefficient: 5.82e−14
Value: B
Aliidiomarina
1.00
0.75
0.50
0.25
0.00
A (n=15)
B (n=16)
C (n=14)
surface

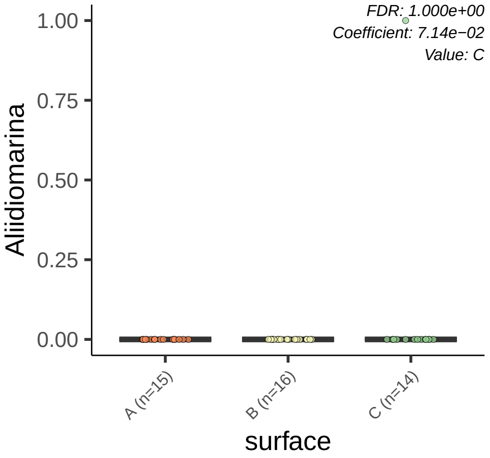

FDR: 1.000e+00
Coefficient: 7.14e−02
Value: C
Aliidiomarina
1.00
0.75
0.50
0.25
0.00
A (n=15)
B (n=16)
C (n=14)
surface

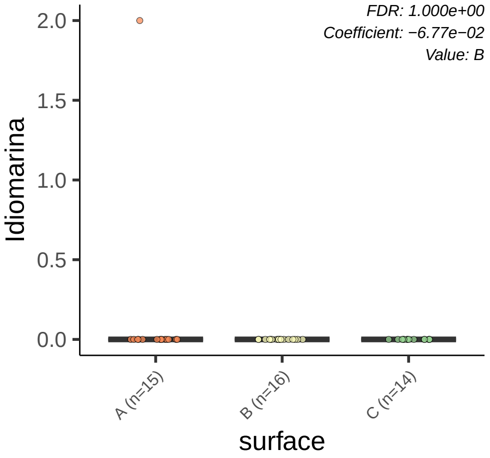
FDR: 1.000e+00
Coefficient: −6.77e−02
Value: B
Idiomarina
2.0
1.5
1.0
0.5
0.0
A (n=15)
B (n=16)
C (n=14)
surface

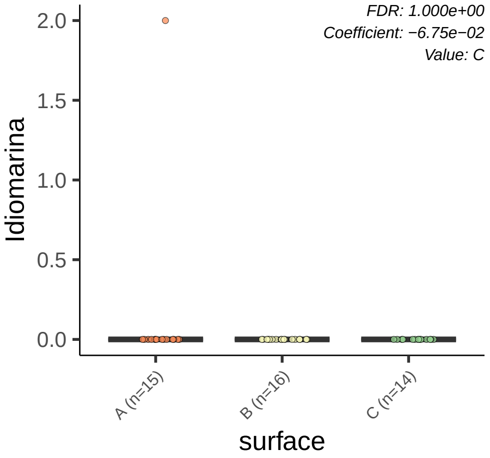
FDR: 1.000e+00
Coefficient: −6.75e−02
Value: C
Idiomarina
2.0
1.5
1.0
0.5
0.0
A (n=15)
B (n=16)
C (n=14)
surface

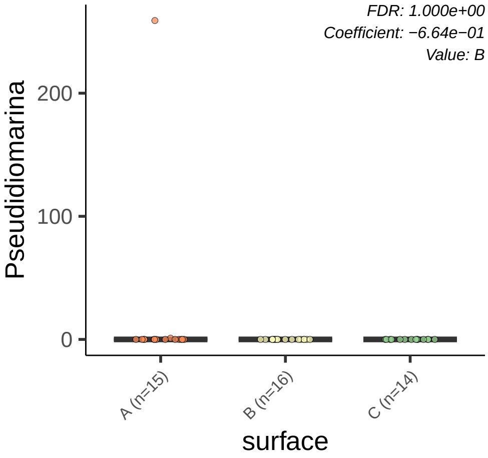

FDR: 1.000e+00
Coefficient: −6.64e−01
Value: B
Pseudidiomarina
200
100
0
A (n=15)
B (n=16)
C (n=14)
surface

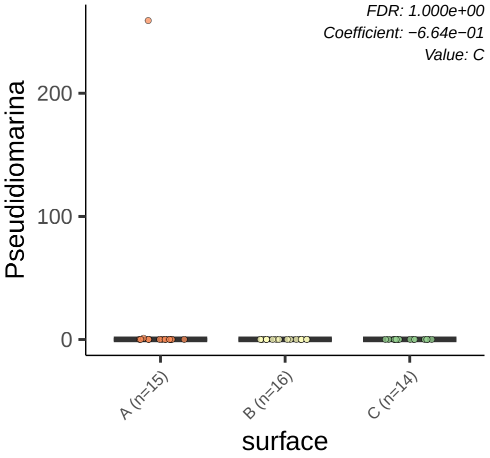
FDR: 1.000e+00
Coefficient: −6.64e−01
Value: C
Pseudidiomarina
200
100
0
A (n=15)
B (n=16)
C (n=14)
surface

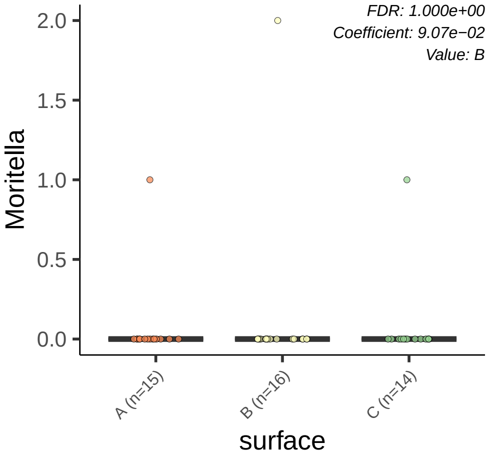

FDR: 1.000e+00
Coefficient: 9.07e−02
Value: B
Moritella
2.0
1.5
1.0
0.5
0.0
A (n=15)
B (n=16)
C (n=14)
surface

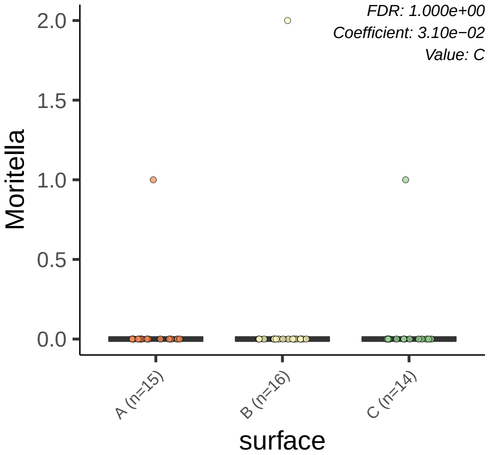
FDR: 1.000e+00
Coefficient: 3.10e−02
Value: C
Moritella
2.0
1.5
1.0
0.5
0.0
A (n=15)
B (n=16)
C (n=14)
surface

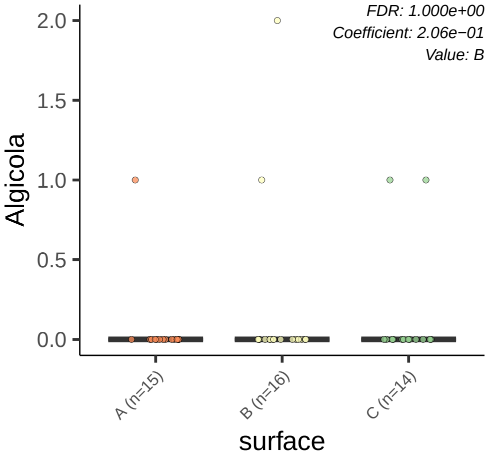
FDR: 1.000e+00
Coefficient: 2.06e−01
Value: B
Algicola
2.0
1.5
1.0
0.5
0.0
A (n=15)
B (n=16)
C (n=14)
surface

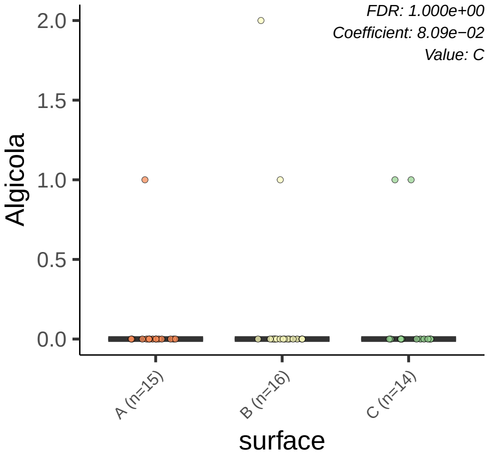

FDR: 1.000e+00
Coefficient: 8.09e−02
Value: C
Algicola
2.0
1.5
1.0
0.5
0.0
A (n=15)
B (n=16)
C (n=14)
surface

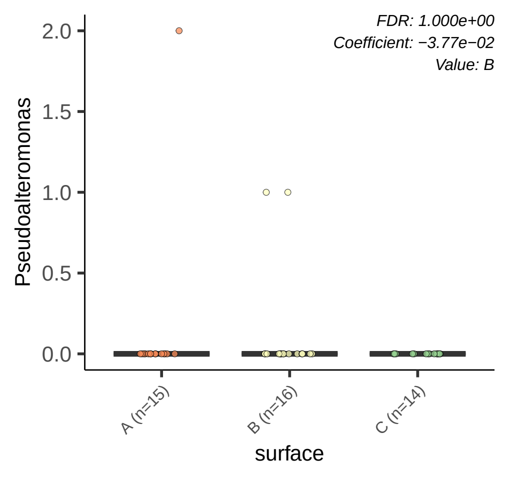
FDR: 1.000e+00
Coefficient: −3.77e−02
Value: B
Pseudoalteromonas
2.0
1.5
1.0
0.5
0.0
A (n=15)
B (n=16)
C (n=14)
surface

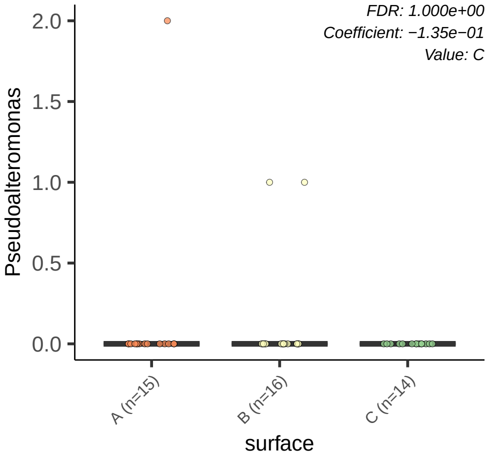
FDR: 1.000e+00
Coefficient: −1.35e−01
Value: C
Pseudoalteromonas
2.0
1.5
1.0
0.5
0.0
A (n=15)
B (n=16)
C (n=14)
surface

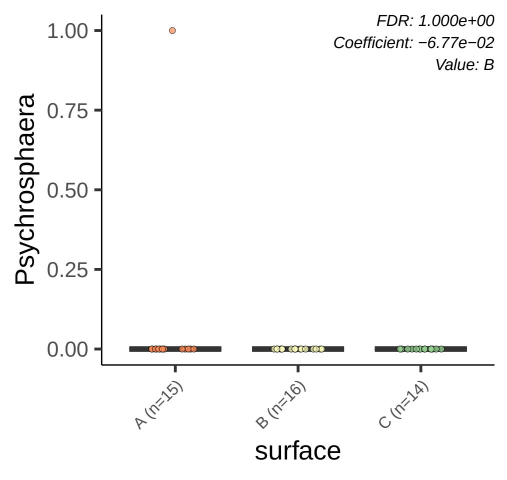
FDR: 1.000e+00
Coefficient: −6.77e−02
Value: B
Psychrosphaera
1.00
0.75
0.50
0.25
0.00
A (n=15)
B (n=16)
C (n=14)
surface

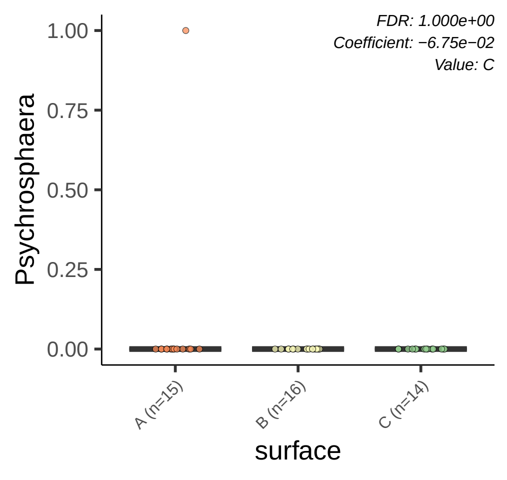
FDR: 1.000e+00
Coefficient: −6.75e−02
Value: C
Psychrosphaera
1.00
0.75
0.50
0.25
0.00
A (n=15)
B (n=16)
C (n=14)
surface

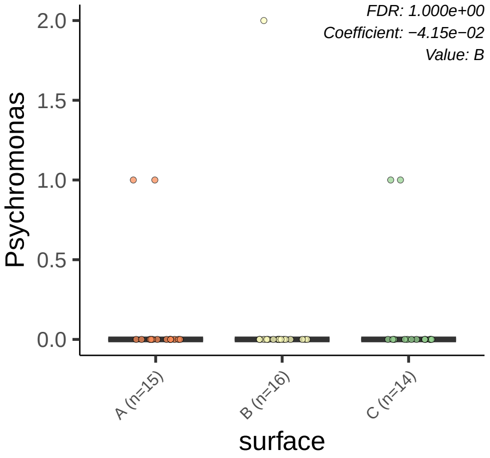
FDR: 1.000e+00
Coefficient: −4.15e−02
Value: B
Psychromonas
2.0
1.5
1.0
0.5
0.0
A (n=15)
B (n=16)
C (n=14)
surface

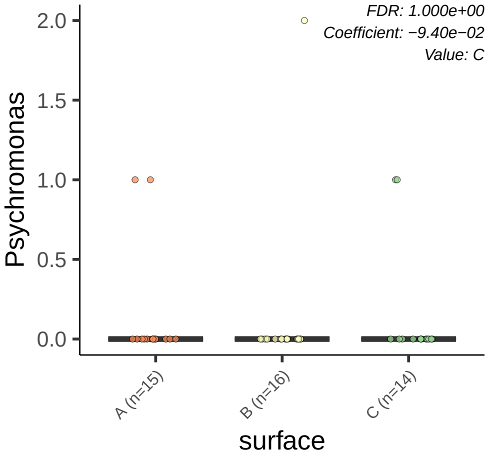

FDR: 1.000e+00
Coefficient: −9.40e−02
Value: C
Psychromonas
2.0
1.5
1.0
0.5
0.0
A (n=15)
B (n=16)
C (n=14)
surface

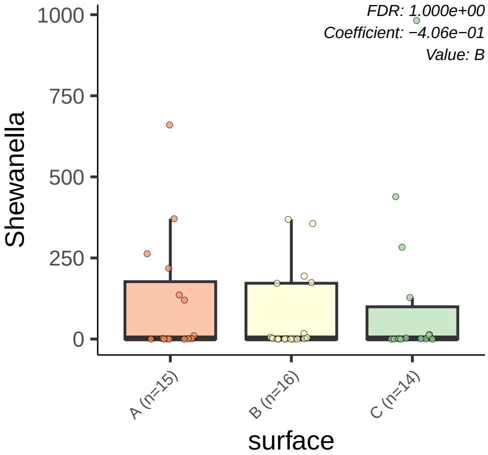

FDR: 1.000e+00
Coefficient: −4.06e−01
Value: B
Shewanella
1000
750
500
250
0
A (n=15)
B (n=16)
C (n=14)
surface

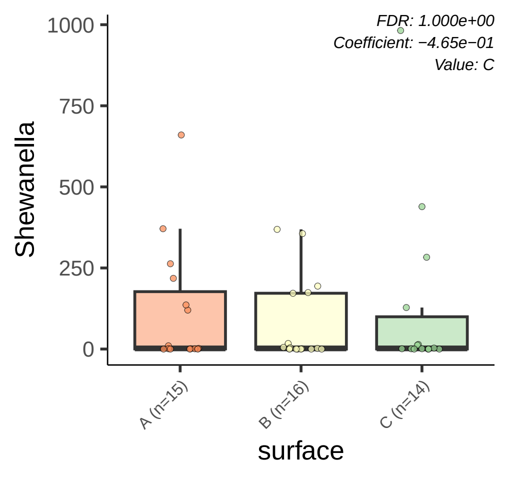
FDR: 1.000e+00
Coefficient: −4.65e−01
Value: C
Shewanella
1000
750
500
250
0
A (n=15)
B (n=16)
C (n=14)
surface

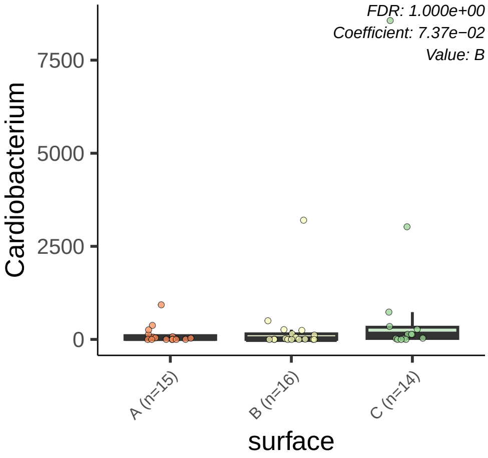
FDR: 1.000e+00
Coefficient: 7.37e−02
Value: B
Cardiobacterium
7500
5000
2500
0
A (n=15)
B (n=16)
C (n=14)
surface

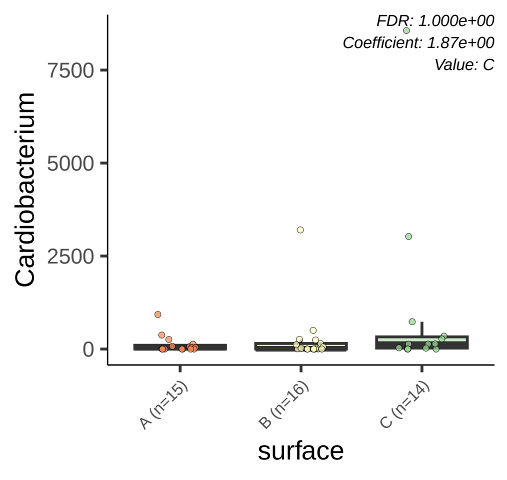

FDR: 1.000e+00
Coefficient: 1.87e+00
Value: C
Cardiobacterium
7500
5000
2500
0
A (n=15)
B (n=16)
C (n=14)
surface

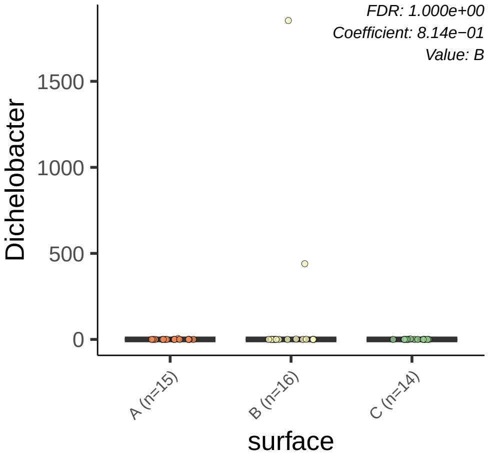
FDR: 1.000e+00
Coefficient: 8.14e−01
Value: B
Dichelobacter
1500
1000
500
0
A (n=15)
B (n=16)
C (n=14)
surface

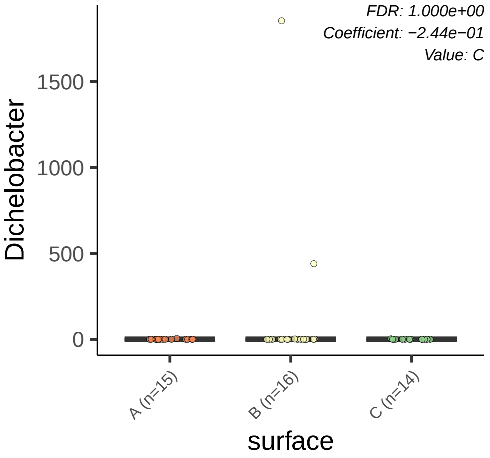
FDR: 1.000e+00
Coefficient: −2.44e−01
Value: C
Dichelobacter
1500
1000
500
0
A (n=15)
B (n=16)
C (n=14)
surface

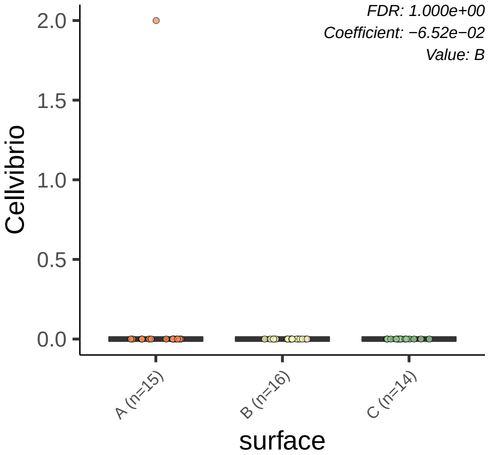
FDR: 1.000e+00
Coefficient: −6.52e−02
Value: B
Cellvibrio
2.0
1.5
1.0
0.5
0.0
A (n=15)
B (n=16)
C (n=14)
surface

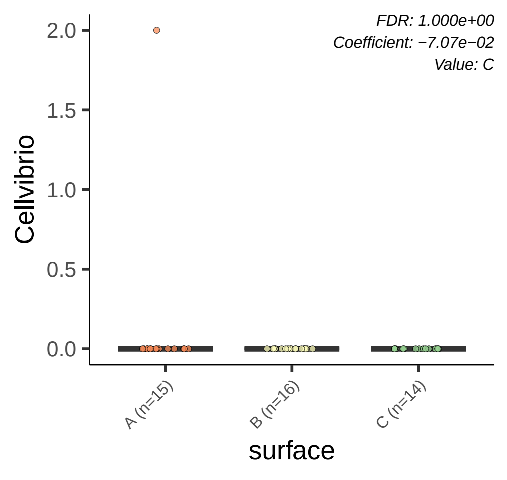
FDR: 1.000e+00
Coefficient: −7.07e−02
Value: C
Cellvibrio
2.0
1.5
1.0
0.5
0.0
A (n=15)
B (n=16)
C (n=14)
surface

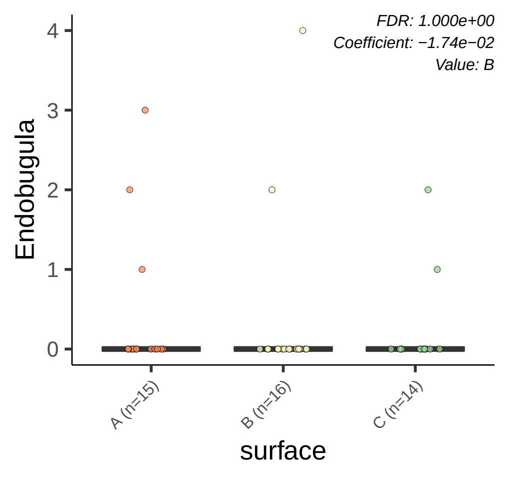

FDR: 1.000e+00
Coefficient: −1.74e−02
Value: B
Endobugula
0
1
2
3
4
A (n=15)
B (n=16)
C (n=14)
surface

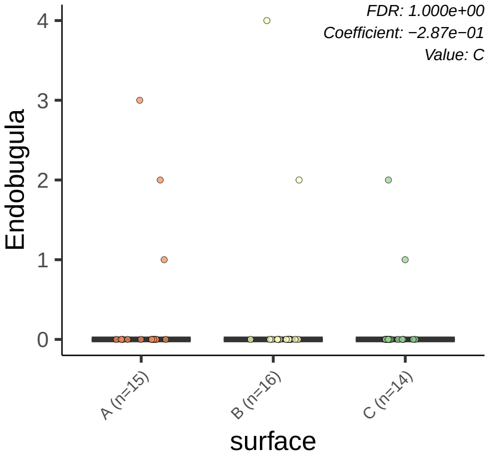

FDR: 1.000e+00
Coefficient: −2.87e−01
Value: C
Endobugula
0
1
2
3
4
A (n=15)
B (n=16)
C (n=14)
surface

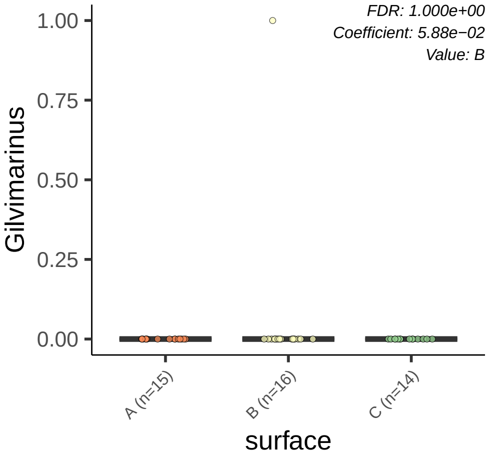
FDR: 1.000e+00
Coefficient: 5.88e−02
Value: B
Gilvimarinus
1.00
0.75
0.50
0.25
0.00
A (n=15)
B (n=16)
C (n=14)
surface

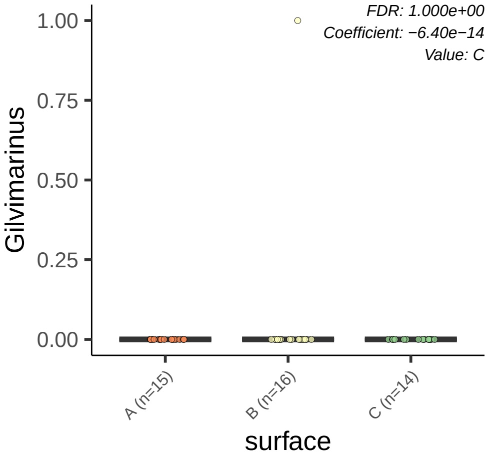
FDR: 1.000e+00
Coefficient: −6.40e−14
Value: C
Gilvimarinus
1.00
0.75
0.50
0.25
0.00
A (n=15)
B (n=16)
C (n=14)
surface

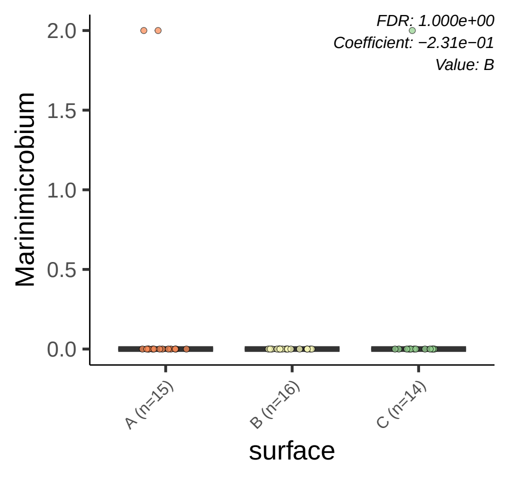

FDR: 1.000e+00
Coefficient: −2.31e−01
Value: B
Marinimicrobium
2.0
1.5
1.0
0.5
0.0
A (n=15)
B (n=16)
C (n=14)
surface

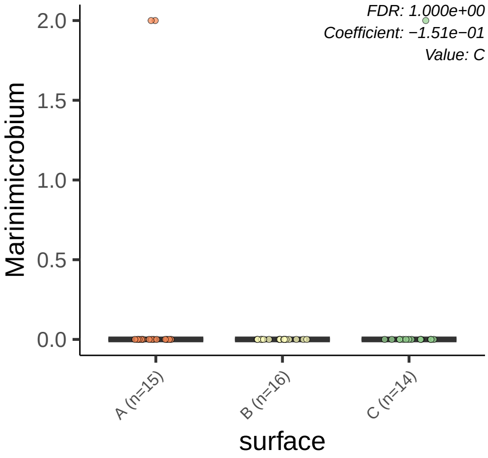

FDR: 1.000e+00
Coefficient: −1.51e−01
Value: C
Marinimicrobium
2.0
1.5
1.0
0.5
0.0
A (n=15)
B (n=16)
C (n=14)
surface

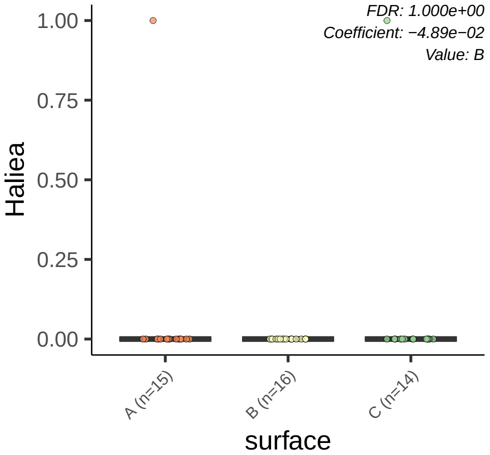
FDR: 1.000e+00
Coefficient: −4.89e−02
Value: B
Haliea
1.00
0.75
0.50
0.25
0.00
A (n=15)
B (n=16)
C (n=14)
surface

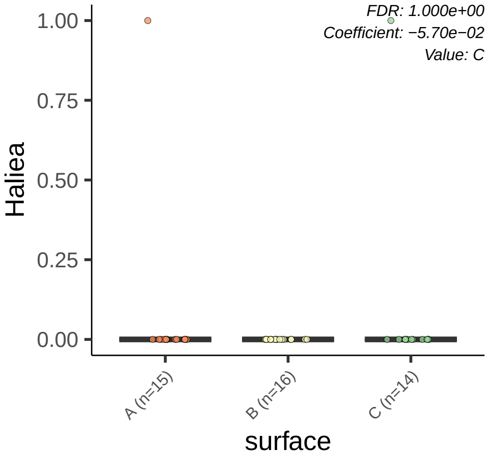
FDR: 1.000e+00
Coefficient: −5.70e−02
Value: C
Haliea
1.00
0.75
0.50
0.25
0.00
A (n=15)
B (n=16)
C (n=14)
surface

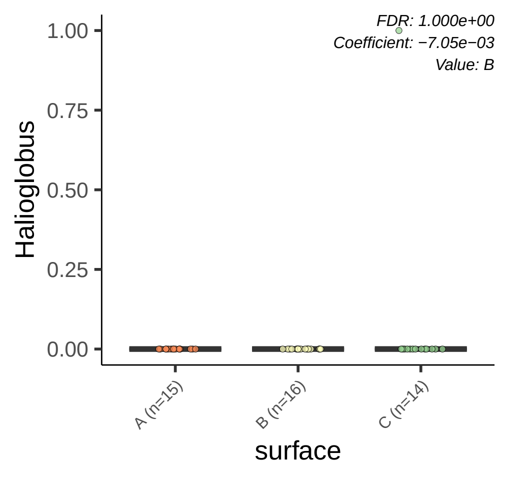
FDR: 1.000e+00
Coefficient: −7.05e−03
Value: B
Halioglobus
1.00
0.75
0.50
0.25
0.00
A (n=15)
B (n=16)
C (n=14)
surface

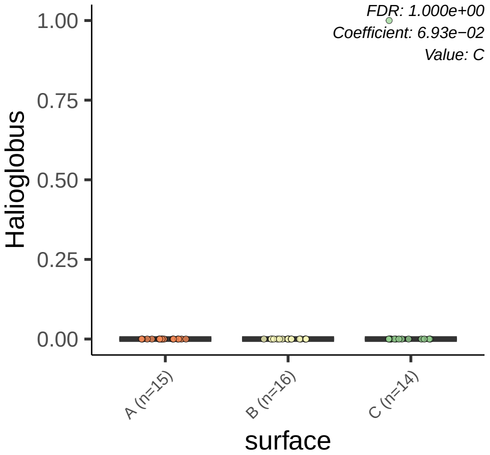
FDR: 1.000e+00
Coefficient: 6.93e−02
Value: C
1.00
0.75
0.50
0.25
0.00
Halioglobus
A (n=15)
B (n=16)
C (n=14)
surface

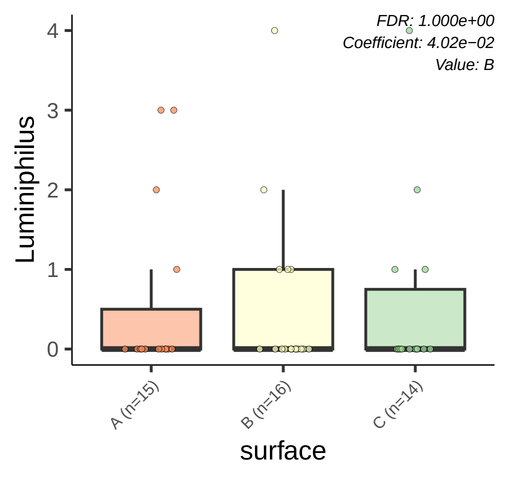
FDR: 1.000e+00
Coefficient: 4.02e−02
Value: B
Luminiphilus
0
1
2
3
4
A (n=15)
B (n=16)
C (n=14)
surface

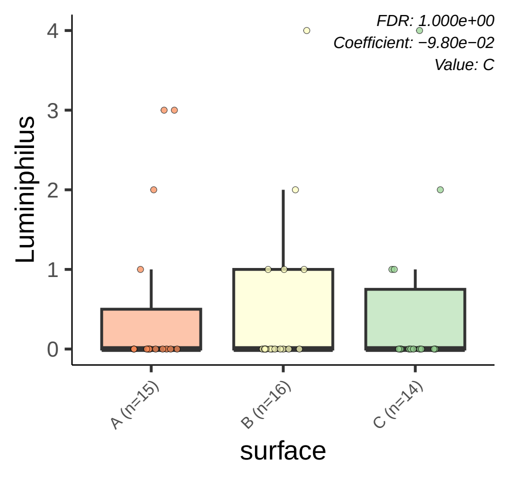

FDR: 1.000e+00
Coefficient: −9.80e−02
Value: C
Luminiphilus
0
1
2
3
4
A (n=15)
B (n=16)
C (n=14)
surface

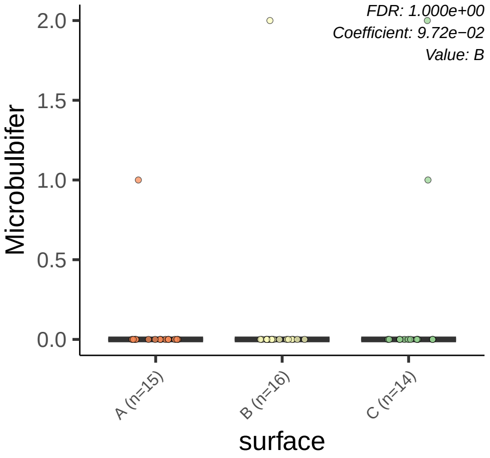

FDR: 1.000e+00
Coefficient: 9.72e−02
Value: B
Microbulbifer
2.0
1.5
1.0
0.5
0.0
A (n=15)
B (n=16)
C (n=14)
surface

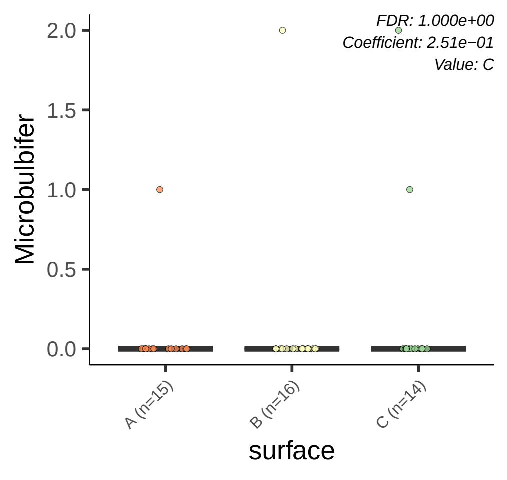
FDR: 1.000e+00
Coefficient: 2.51e−01
Value: C
Microbulbifer
2.0
1.5
1.0
0.5
0.0
A (n=15)
B (n=16)
C (n=14)
surface

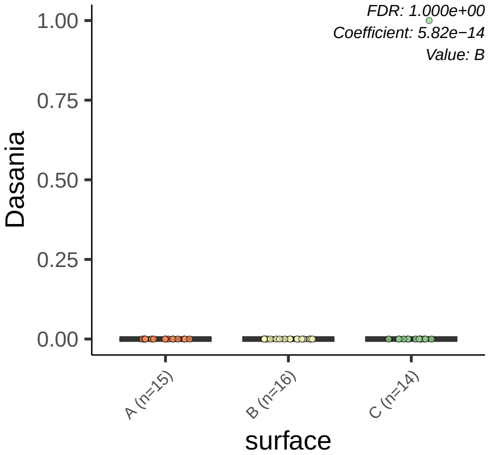
FDR: 1.000e+00
Coefficient: 5.82e−14
Value: B
1.00
0.75
0.50
0.25
0.00
Dasania
A (n=15)
B (n=16)
C (n=14)
surface

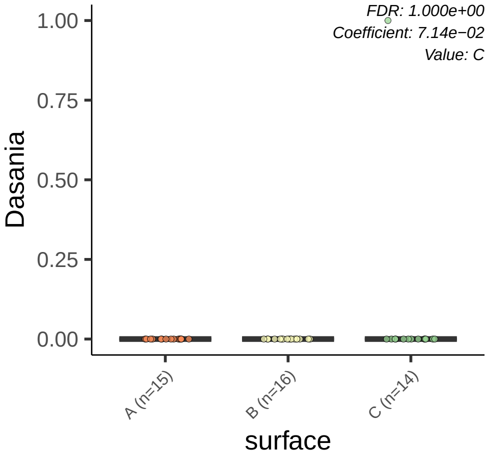
FDR: 1.000e+00
Coefficient: 7.14e−02
Value: C
Dasania
1.00
0.75
0.50
0.25
0.00
A (n=15)
B (n=16)
C (n=14)
surface

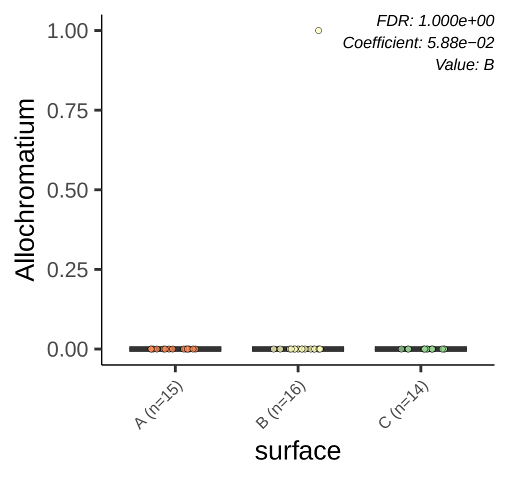
FDR: 1.000e+00
Coefficient: 5.88e−02
Value: B
Allochromatium
1.00
0.75
0.50
0.25
0.00
A (n=15)
B (n=16)
C (n=14)
surface

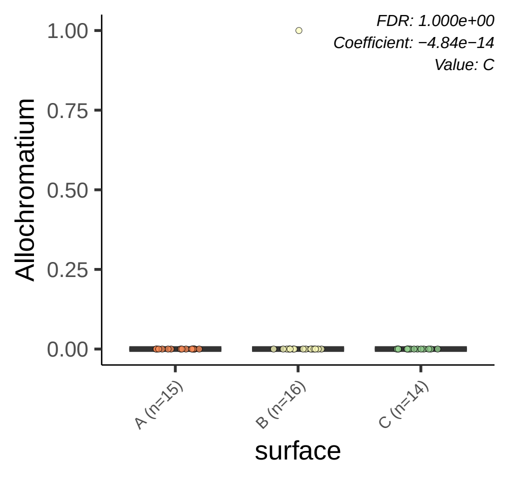

FDR: 1.000e+00
Coefficient: −4.84e−14
Value: C
Allochromatium
1.00
0.75
0.50
0.25
0.00
A (n=15)
B (n=16)
C (n=14)
surface

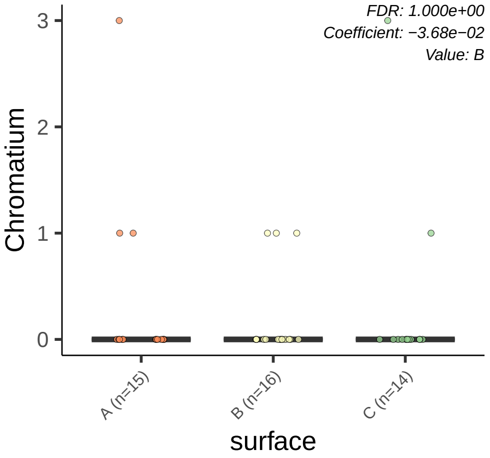
FDR: 1.000e+00
Coefficient: −3.68e−02
Value: B
Chromatium
3
2
1
0
A (n=15)
B (n=16)
C (n=14)
surface

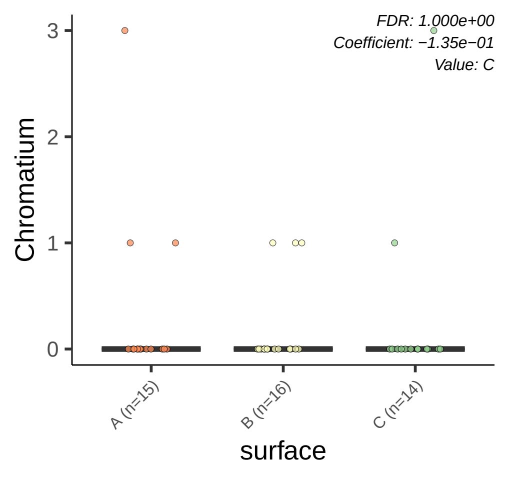

FDR: 1.000e+00
Coefficient: −1.35e−01
Value: C
Chromatium
3
2
1
0
A (n=15)
B (n=16)
C (n=14)
surface

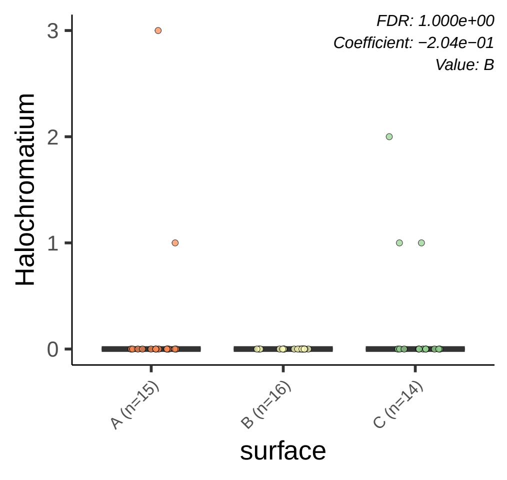
FDR: 1.000e+00
Coefficient: −2.04e−01
Value: B
Halochromatium
3
2
1
0
A (n=15)
B (n=16)
C (n=14)
surface

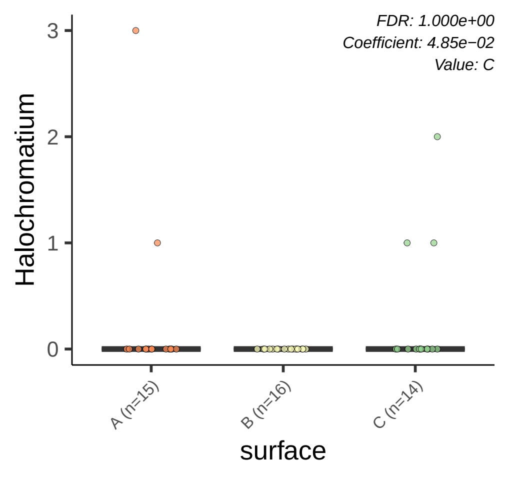

FDR: 1.000e+00
Coefficient: 4.85e−02
Value: C
Halochromatium
3
2
1
0
A (n=15)
B (n=16)
C (n=14)
surface

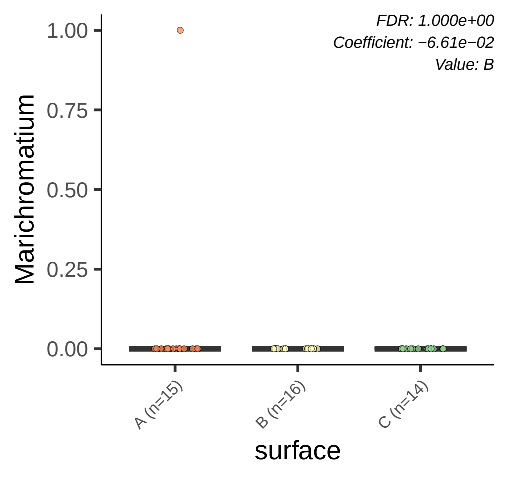

FDR: 1.000e+00
Coefficient: −6.61e−02
Value: B
Marichromatium
1.00
0.75
0.50
0.25
0.00
A (n=15)
B (n=16)
C (n=14)
surface

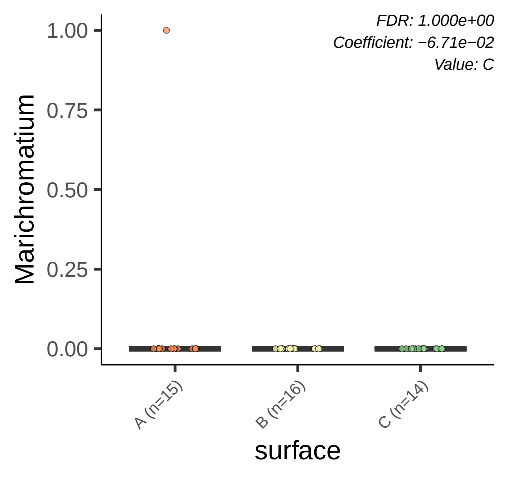

FDR: 1.000e+00
Coefficient: −6.71e−02
Value: C
Marichromatium
1.00
0.75
0.50
0.25
0.00
A (n=15)
B (n=16)
C (n=14)
surface

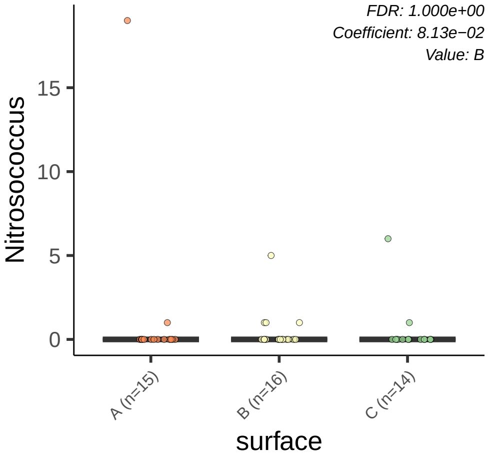
FDR: 1.000e+00
Coefficient: 8.13e−02
Value: B
Nitrosococcus
0
5
10
15
A (n=15)
B (n=16)
C (n=14)
surface

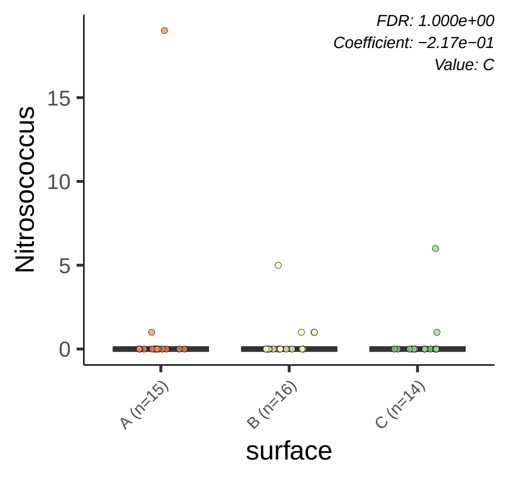
FDR: 1.000e+00
Coefficient: −2.17e−01
Value: C
Nitrosococcus
15
10
5
0
A (n=15)
B (n=16)
C (n=14)
surface

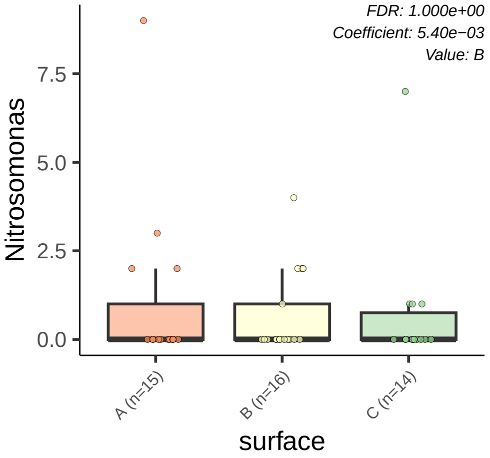
FDR: 1.000e+00
Coefficient: 5.40e−03
Value: B
Nitrosomonas
7.5
5.0
2.5
0.0
A (n=15)
B (n=16)
C (n=14)
surface

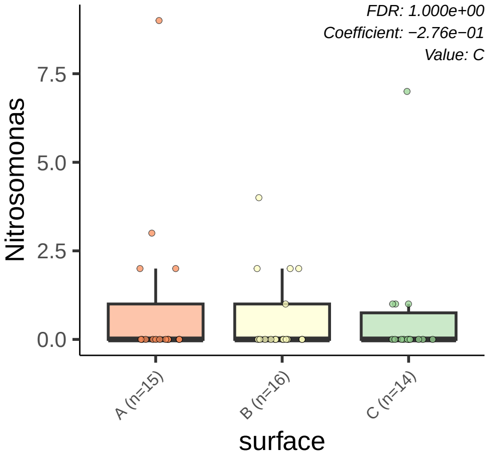
FDR: 1.000e+00
Coefficient: −2.76e−01
Value: C
Nitrosomonas
7.5
5.0
2.5
0.0
A (n=15)
B (n=16)
C (n=14)
surface

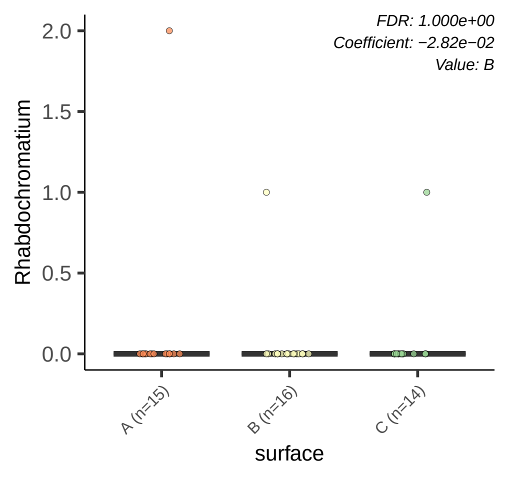

FDR: 1.000e+00
Coefficient: −2.82e−02
Value: B
Rhabdochromatium
2.0
1.5
1.0
0.5
0.0
A (n=15)
B (n=16)
C (n=14)
surface

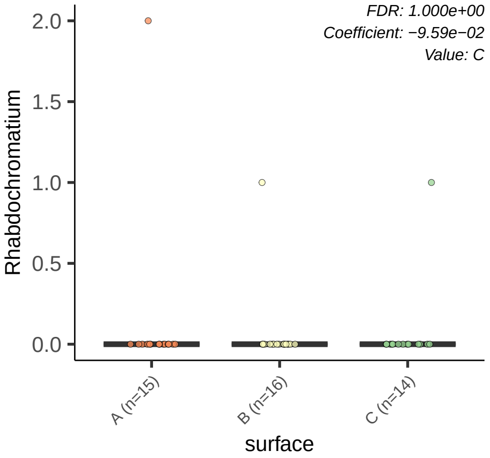

FDR: 1.000e+00
Coefficient: −9.59e−02
Value: C
Rhabdochromatium
2.0
1.5
1.0
0.5
0.0
A (n=15)
B (n=16)
C (n=14)
surface

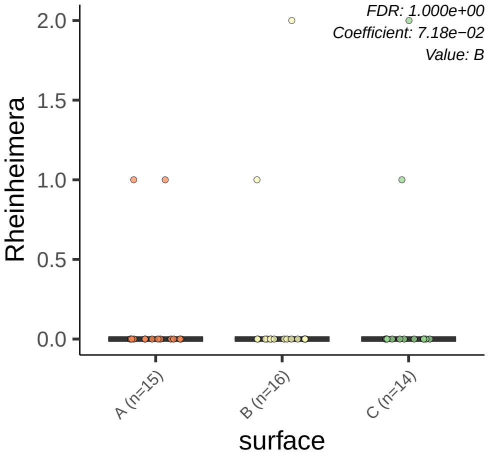
FDR: 1.000e+00
Coefficient: 7.18e−02
Value: B
Rheinheimera
2.0
1.5
1.0
0.5
0.0
A (n=15)
B (n=16)
C (n=14)
surface

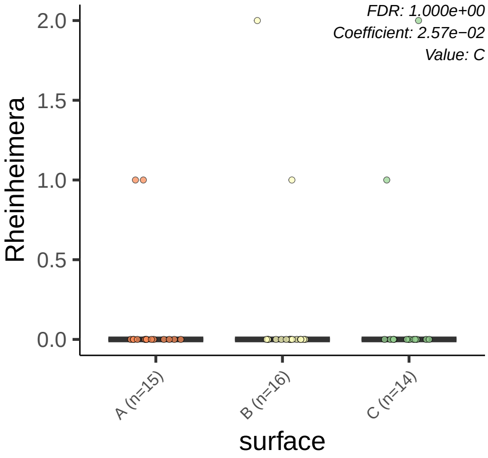
FDR: 1.000e+00
Coefficient: 2.57e−02
Value: C
Rheinheimera
2.0
1.5
1.0
0.5
0.0
A (n=15)
B (n=16)
C (n=14)
surface

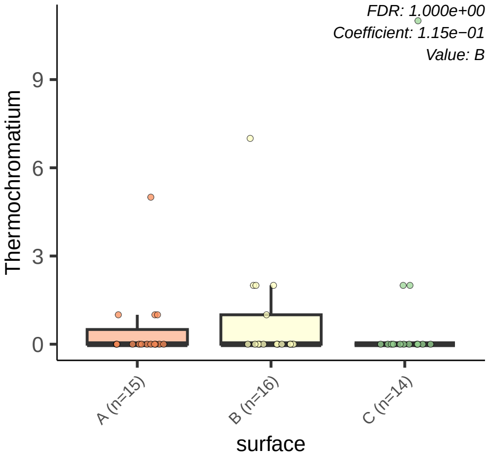
FDR: 1.000e+00
Coefficient: 1.15e−01
Value: B
Thermochromatium
0
3
6
9
A (n=15)
B (n=16)
C (n=14)
surface

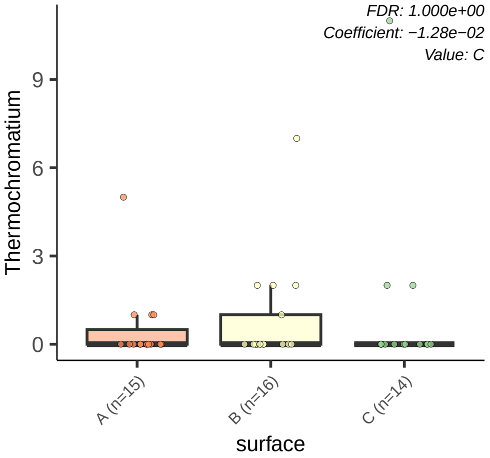

FDR: 1.000e+00
Coefficient: −1.28e−02
Value: C
Thermochromatium
0
3
6
9
A (n=15)
B (n=16)
C (n=14)
surface

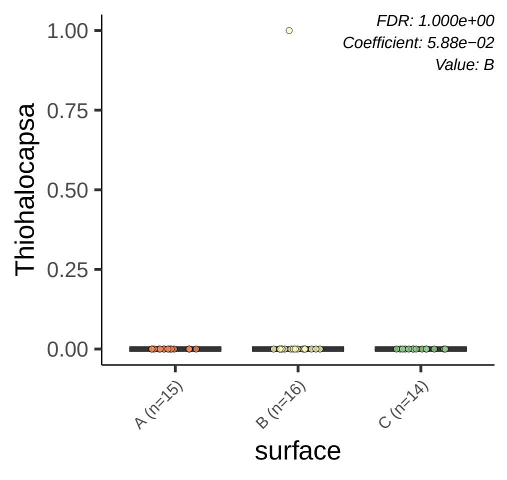

FDR: 1.000e+00
Coefficient: 5.88e−02
Value: B
Thiohalocapsa
1.00
0.75
0.50
0.25
0.00
A (n=15)
B (n=16)
C (n=14)
surface

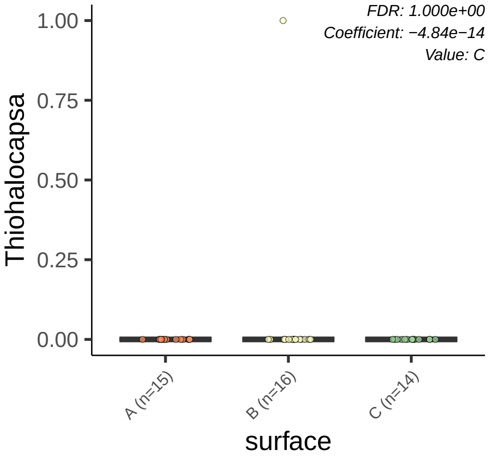
FDR: 1.000e+00
Coefficient: −4.84e−14
Value: C
Thiohalocapsa
1.00
0.75
0.50
0.25
0.00
A (n=15)
B (n=16)
C (n=14)
surface

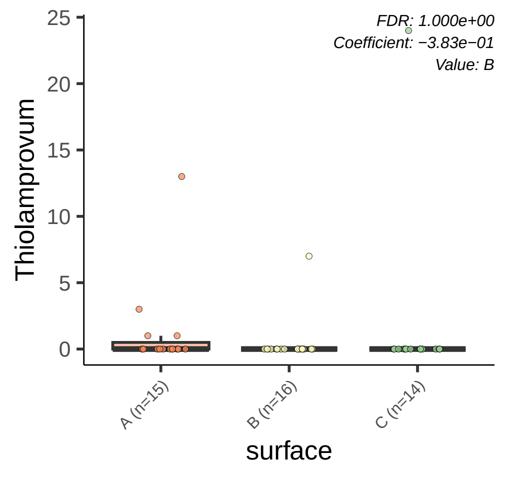
FDR: 1.000e+00
Coefficient: −3.83e−01
Value: B
Thiolamprovum
25
20
15
10
5
0
A (n=15)
B (n=16)
C (n=14)
surface

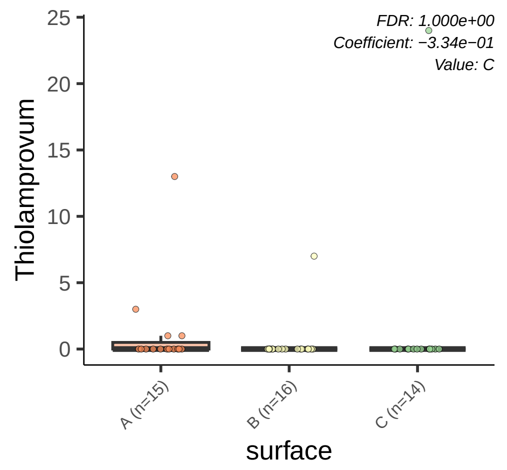
FDR: 1.000e+00
Coefficient: −3.34e−01
Value: C
Thiolamprovum
25
20
15
10
5
0
A (n=15)
B (n=16)
C (n=14)
surface

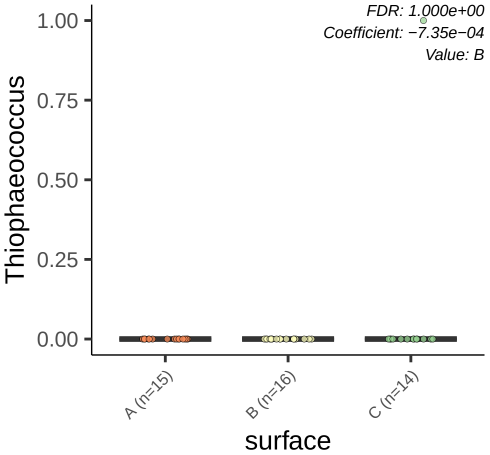

FDR: 1.000e+00
Coefficient: −7.35e−04
Value: B
1.00
0.75
0.50
0.25
0.00
Thiophaeococcus
A (n=15)
B (n=16)
C (n=14)
surface

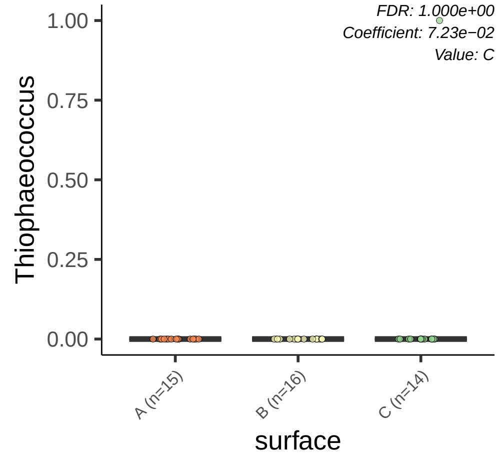

FDR: 1.000e+00
Coefficient: 7.23e−02
Value: C
Thiophaeococcus
1.00
0.75
0.50
0.25
0.00
A (n=15)
B (n=16)
C (n=14)
surface

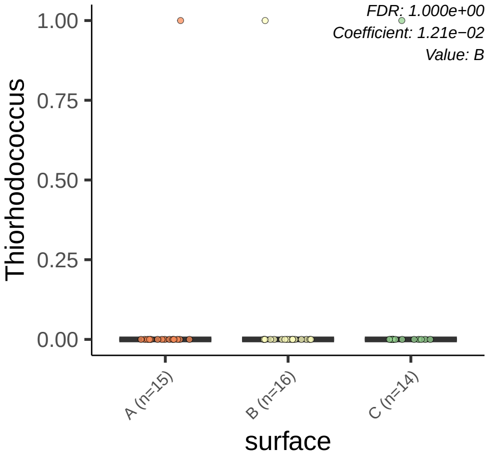
FDR: 1.000e+00
Coefficient: 1.21e−02
Value: B
Thiorhodococcus
1.00
0.75
0.50
0.25
0.00
A (n=15)
B (n=16)
C (n=14)
surface

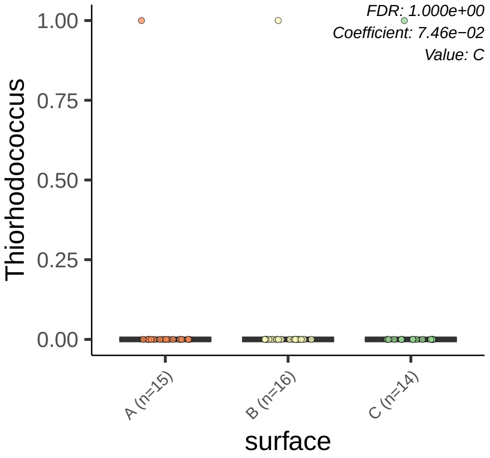

FDR: 1.000e+00
Coefficient: 7.46e−02
Value: C
Thiorhodococcus
1.00
0.75
0.50
0.25
0.00
A (n=15)
B (n=16)
C (n=14)
surface

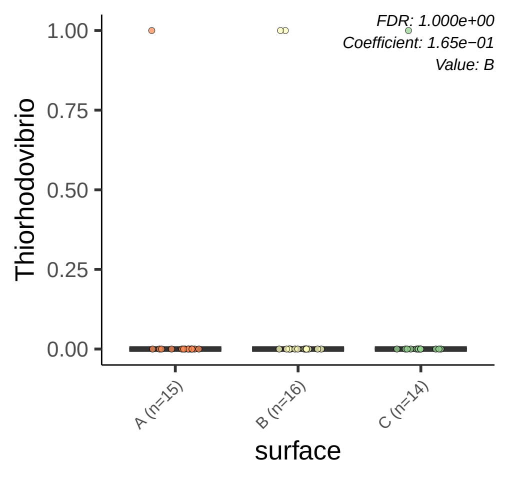

FDR: 1.000e+00
Coefficient: 1.65e−01
Value: B
Thiorhodovibrio
1.00
0.75
0.50
0.25
0.00
A (n=15)
B (n=16)
C (n=14)
surface

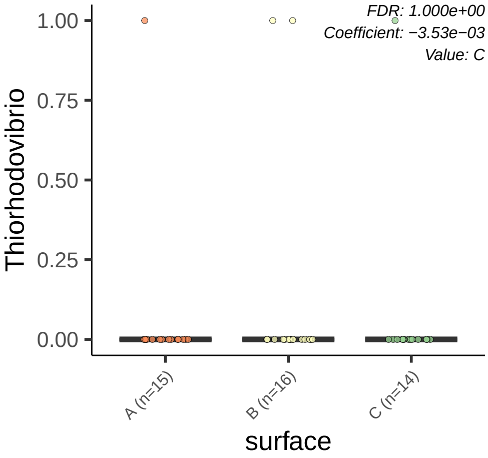

FDR: 1.000e+00
Coefficient: −3.53e−03
Value: C
Thiorhodovibrio
1.00
0.75
0.50
0.25
0.00
A (n=15)
B (n=16)
C (n=14)
surface

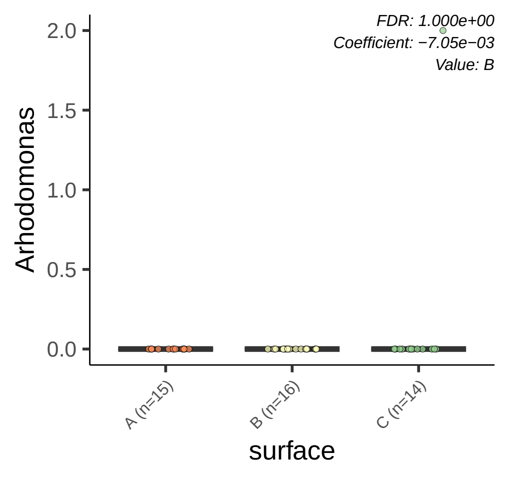
FDR: 1.000e+00
Coefficient: −7.05e−03
Value: B
Arhodomonas
2.0
1.5
1.0
0.5
0.0
A (n=15)
B (n=16)
C (n=14)
surface

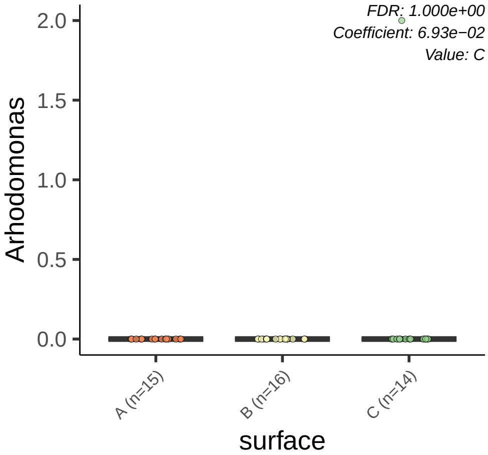

FDR: 1.000e+00
Coefficient: 6.93e−02
Value: C
Arhodomonas
2.0
1.5
1.0
0.5
0.0
A (n=15)
B (n=16)
C (n=14)
surface

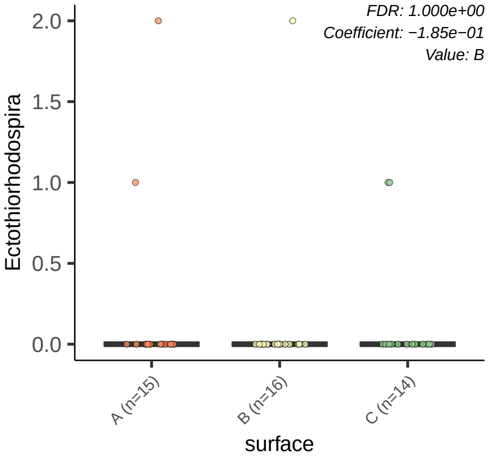
FDR: 1.000e+00
Coefficient: −1.85e−01
Value: B
Ectothiorhodospira
2.0
1.5
1.0
0.5
0.0
A (n=15)
B (n=16)
C (n=14)
surface

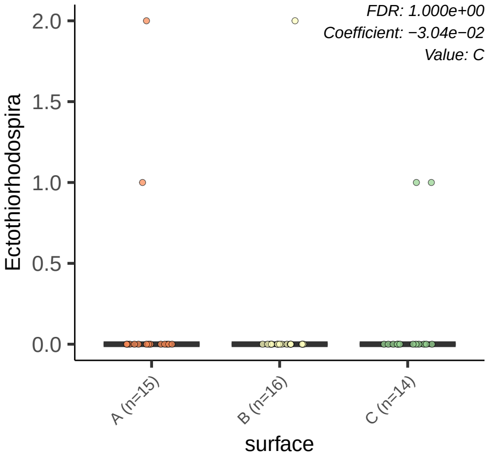
FDR: 1.000e+00
Coefficient: −3.04e−02
Value: C
Ectothiorhodospira
2.0
1.5
1.0
0.5
0.0
A (n=15)
B (n=16)
C (n=14)
surface

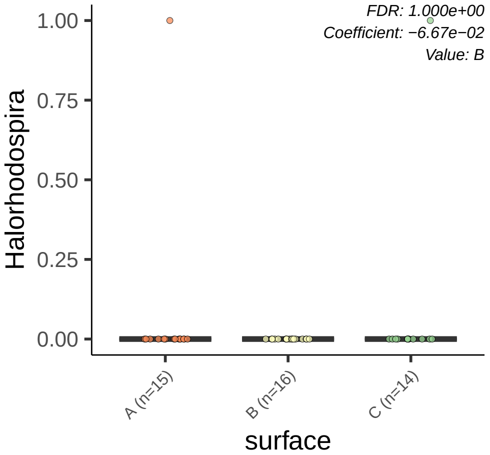
FDR: 1.000e+00
Coefficient: −6.67e−02
Value: B
Halorhodospira
1.00
0.75
0.50
0.25
0.00
A (n=15)
B (n=16)
C (n=14)
surface

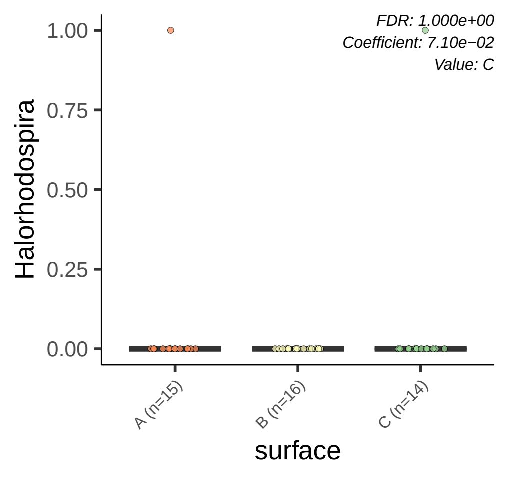
FDR: 1.000e+00
Coefficient: 7.10e−02
Value: C
Halorhodospira
1.00
0.75
0.50
0.25
0.00
A (n=15)
B (n=16)
C (n=14)
surface

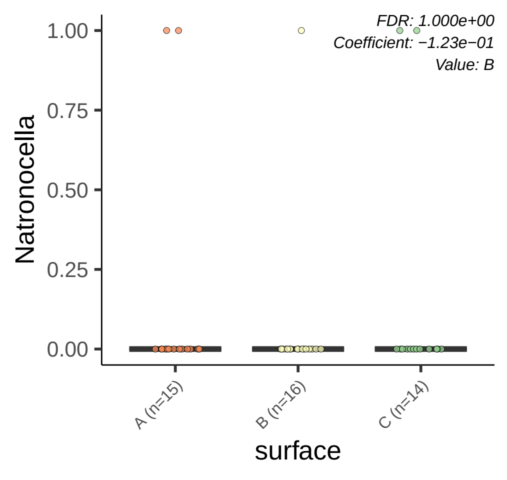

FDR: 1.000e+00
Coefficient: −1.23e−01
Value: B
Natronocella
1.00
0.75
0.50
0.25
0.00
A (n=15)
B (n=16)
C (n=14)
surface

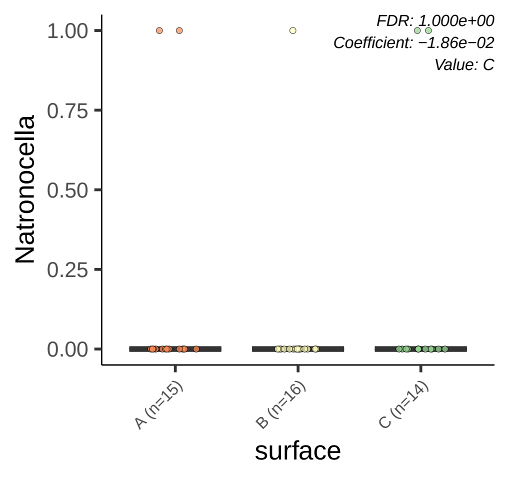
FDR: 1.000e+00
Coefficient: −1.86e−02
Value: C
Natronocella
1.00
0.75
0.50
0.25
0.00
A (n=15)
B (n=16)
C (n=14)
surface

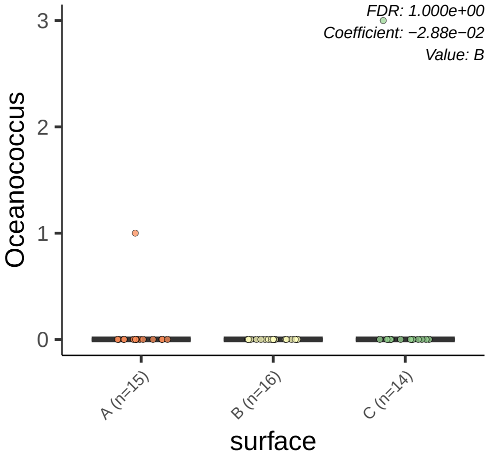
FDR: 1.000e+00
Coefficient: −2.88e−02
Value: B
Oceanococcus
3
2
1
0
A (n=15)
B (n=16)
C (n=14)
surface

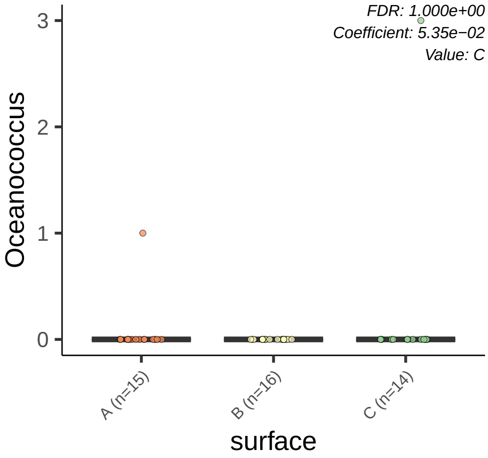
FDR: 1.000e+00
Coefficient: 5.35e−02
Value: C
Oceanococcus
3
2
1
0
A (n=15)
B (n=16)
C (n=14)
surface

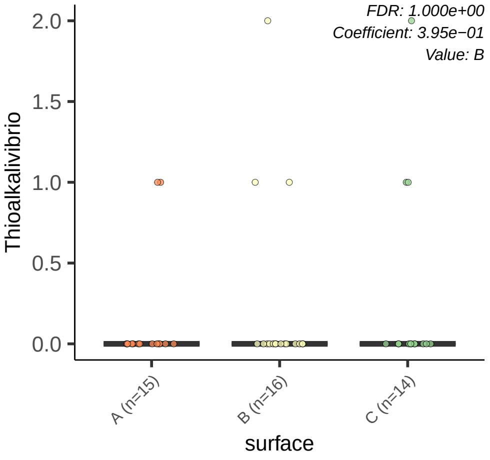

FDR: 1.000e+00
Coefficient: 3.95e−01
Value: B
Thioalkalivibrio
2.0
1.5
1.0
0.5
0.0
A (n=15)
B (n=16)
C (n=14)
surface

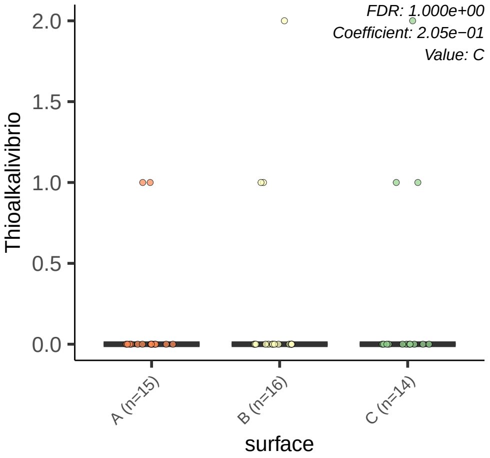
FDR: 1.000e+00
Coefficient: 2.05e−01
Value: C
Thioalkalivibrio
2.0
1.5
1.0
0.5
0.0
A (n=15)
B (n=16)
C (n=14)
surface

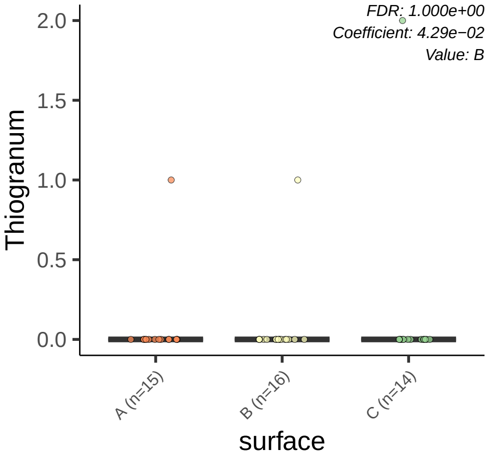
FDR: 1.000e+00
Coefficient: 4.29e−02
Value: B
Thiogranum
2.0
1.5
1.0
0.5
0.0
A (n=15)
B (n=16)
C (n=14)
surface

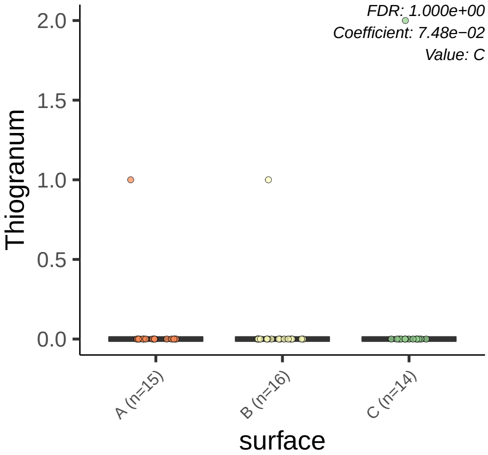

FDR: 1.000e+00
Coefficient: 7.48e−02
Value: C
Thiogranum
2.0
1.5
1.0
0.5
0.0
A (n=15)
B (n=16)
C (n=14)
surface

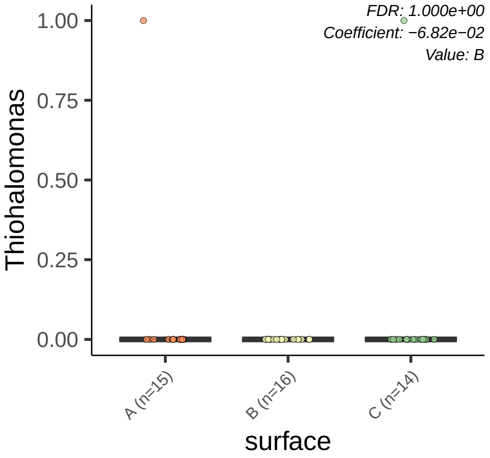
FDR: 1.000e+00
Coefficient: −6.82e−02
Value: B
Thiohalomonas
1.00
0.75
0.50
0.25
0.00
A (n=15)
B (n=16)
C (n=14)
surface

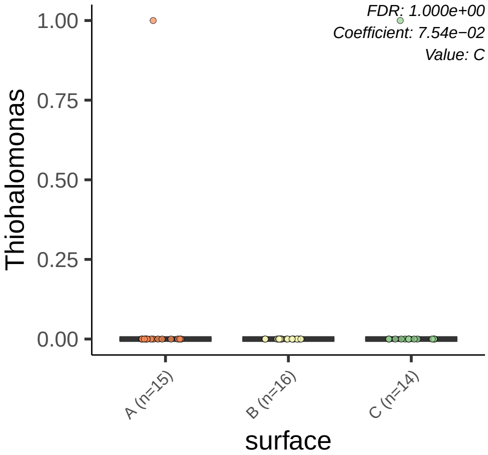

FDR: 1.000e+00
Coefficient: 7.54e−02
Value: C
Thiohalomonas
1.00
0.75
0.50
0.25
0.00
A (n=15)
B (n=16)
C (n=14)
surface

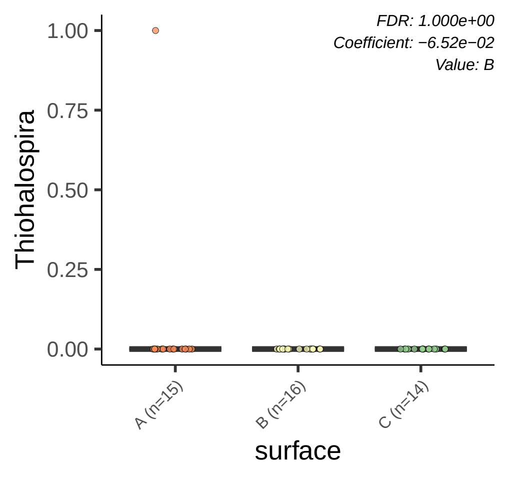
FDR: 1.000e+00
Coefficient: −6.52e−02
Value: B
Thiohalospira
1.00
0.75
0.50
0.25
0.00
A (n=15)
B (n=16)
C (n=14)
surface

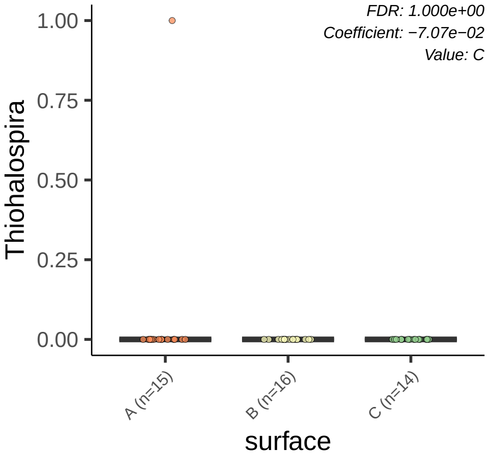
FDR: 1.000e+00
Coefficient: −7.07e−02
Value: C
Thiohalospira
1.00
0.75
0.50
0.25
0.00
A (n=15)
B (n=16)
C (n=14)
surface

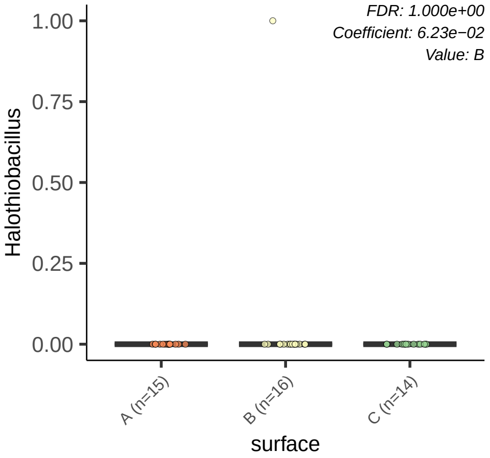

FDR: 1.000e+00
Coefficient: 6.23e−02
Value: B
Halothiobacillus
1.00
0.75
0.50
0.25
0.00
A (n=15)
B (n=16)
C (n=14)
surface

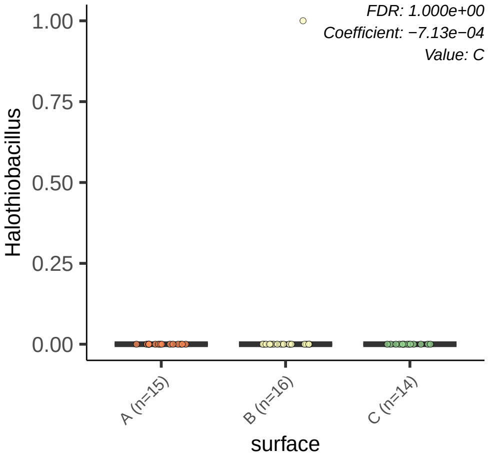
FDR: 1.000e+00
Coefficient: −7.13e−04
Value: C
Halothiobacillus
1.00
0.75
0.50
0.25
0.00
A (n=15)
B (n=16)
C (n=14)
surface

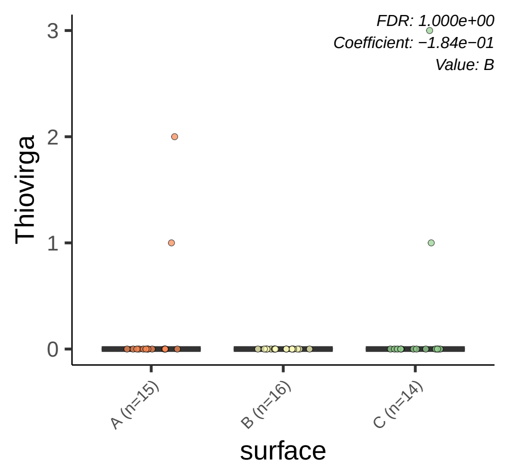
FDR: 1.000e+00
Coefficient: −1.84e−01
Value: B
Thiovirga
3
2
1
0
A (n=15)
B (n=16)
C (n=14)
surface

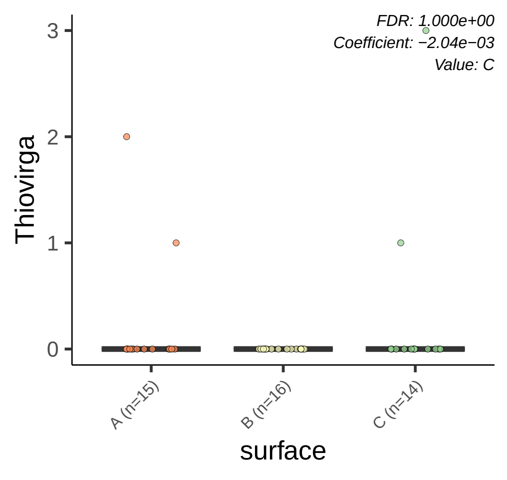

FDR: 1.000e+00
Coefficient: −2.04e−03
Value: C
Thiovirga
0
1
2
3
A (n=15)
B (n=16)
C (n=14)
surface

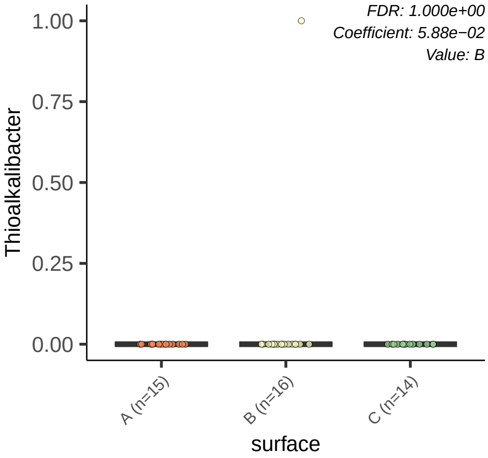
FDR: 1.000e+00
Coefficient: 5.88e−02
Value: B
Thioalkalibacter
1.00
0.75
0.50
0.25
0.00
A (n=15)
B (n=16)
C (n=14)
surface

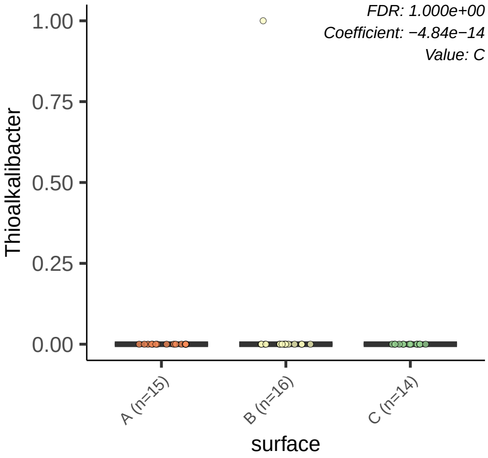

FDR: 1.000e+00
Coefficient: −4.84e−14
Value: C
Thioalkalibacter
1.00
0.75
0.50
0.25
0.00
A (n=15)
B (n=16)
C (n=14)
surface

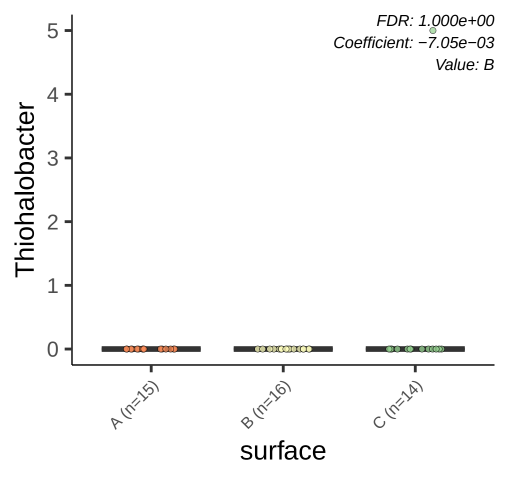
FDR: 1.000e+00
Coefficient: −7.05e−03
Value: B
Thiohalobacter
5
4
3
2
1
0
A (n=15)
B (n=16)
C (n=14)
surface

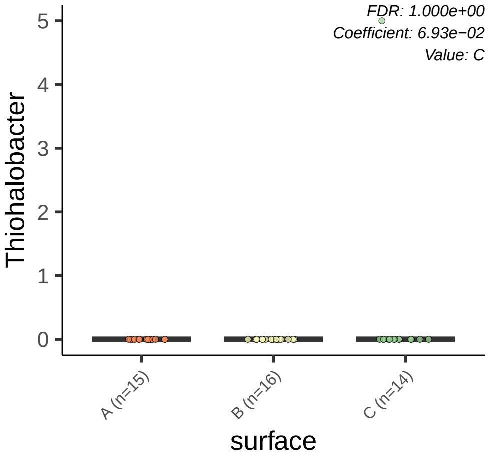
FDR: 1.000e+00
Coefficient: 6.93e−02
Value: C
Thiohalobacter
5
4
3
2
1
0
A (n=15)
B (n=16)
C (n=14)
surface

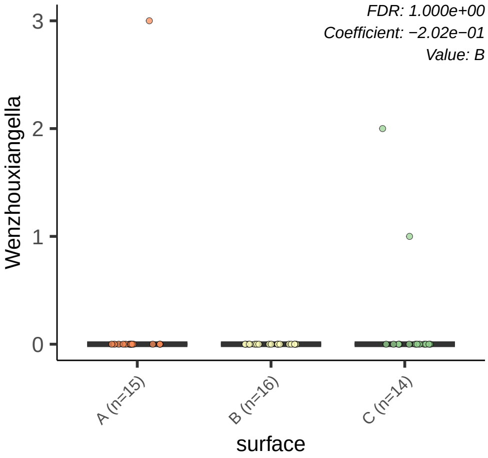
FDR: 1.000e+00
Coefficient: −2.02e−01
Value: B
Wenzhouxiangella
3
2
1
0
A (n=15)
B (n=16)
C (n=14)
surface

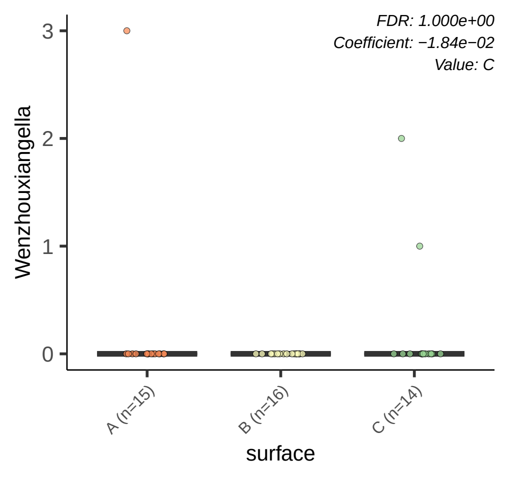

FDR: 1.000e+00
Coefficient: −1.84e−02
Value: C
Wenzhouxiangella
3
2
1
0
A (n=15)
B (n=16)
C (n=14)
surface

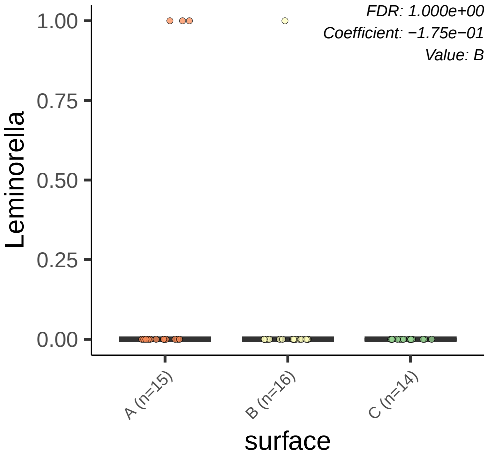
FDR: 1.000e+00
Coefficient: −1.75e−01
Value: B
Leminorella
1.00
0.75
0.50
0.25
0.00
A (n=15)
B (n=16)
C (n=14)
surface

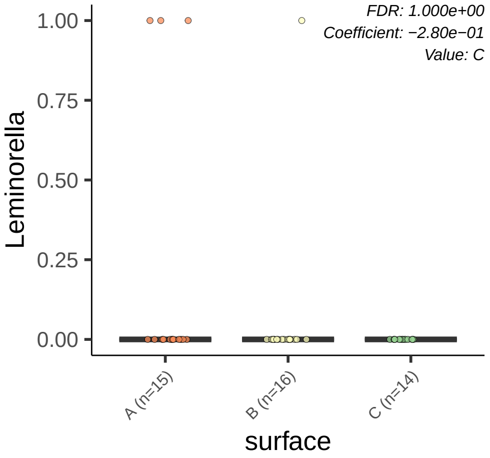

FDR: 1.000e+00
Coefficient: −2.80e−01
Value: C
Leminorella
1.00
0.75
0.50
0.25
0.00
A (n=15)
B (n=16)
C (n=14)
surface

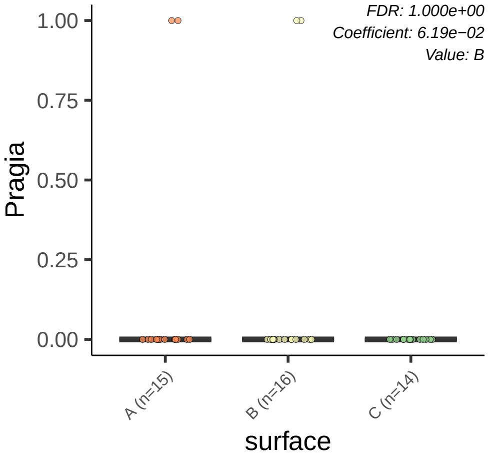

FDR: 1.000e+00
Coefficient: 6.19e−02
Value: B
Pragia
1.00
0.75
0.50
0.25
0.00
A (n=15)
B (n=16)
C (n=14)
surface

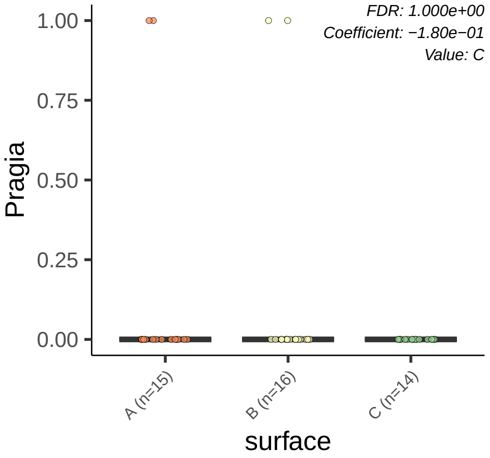
FDR: 1.000e+00
Coefficient: −1.80e−01
Value: C
Pragia
1.00
0.75
0.50
0.25
0.00
A (n=15)
B (n=16)
C (n=14)
surface

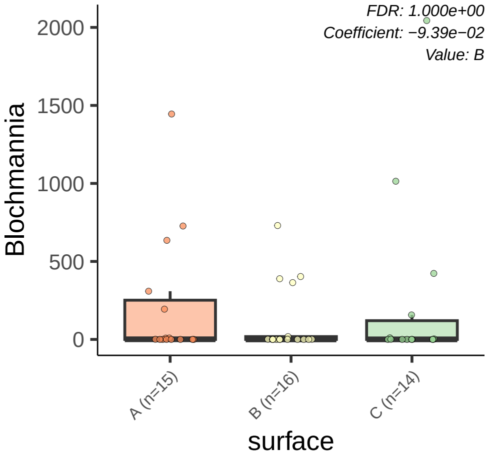
FDR: 1.000e+00
Coefficient: −9.39e−02
Value: B
Blochmannia
2000
1500
1000
500
0
A (n=15)
B (n=16)
C (n=14)
surface

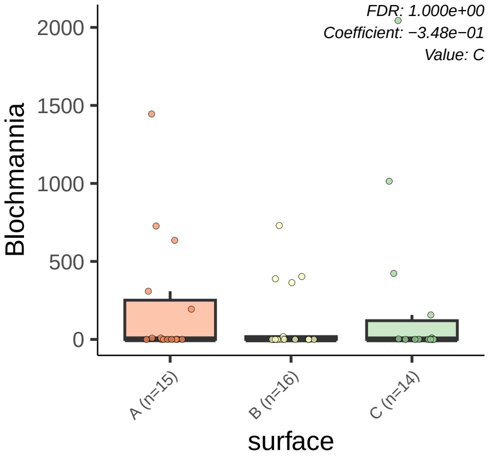

FDR: 1.000e+00
Coefficient: −3.48e−01
Value: C
2000
1500
1000
500
0
Blochmannia
A (n=15)
B (n=16)
C (n=14)
surface

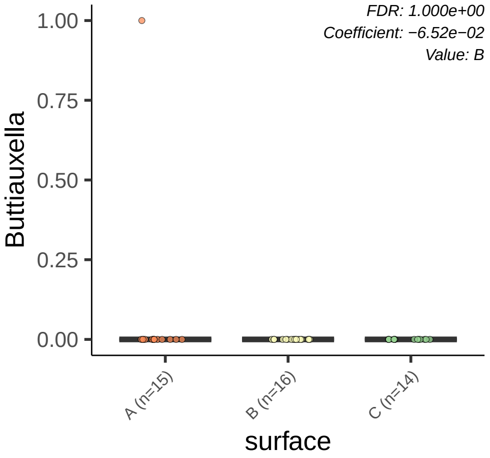
FDR: 1.000e+00
Coefficient: −6.52e−02
Value: B
1.00
0.75
0.50
0.25
0.00
Buttiauxella
A (n=15)
B (n=16)
C (n=14)
surface

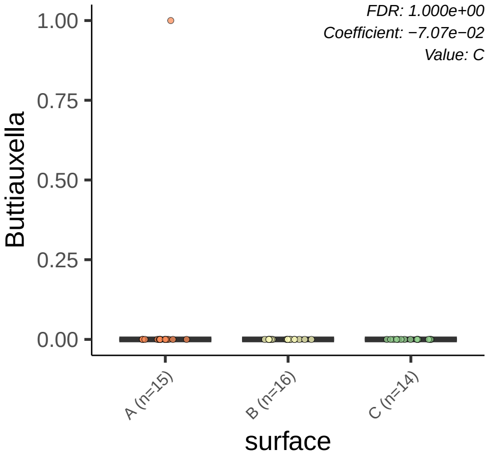

FDR: 1.000e+00
Coefficient: −7.07e−02
Value: C
Buttiauxella
1.00
0.75
0.50
0.25
0.00
A (n=15)
B (n=16)
C (n=14)
surface

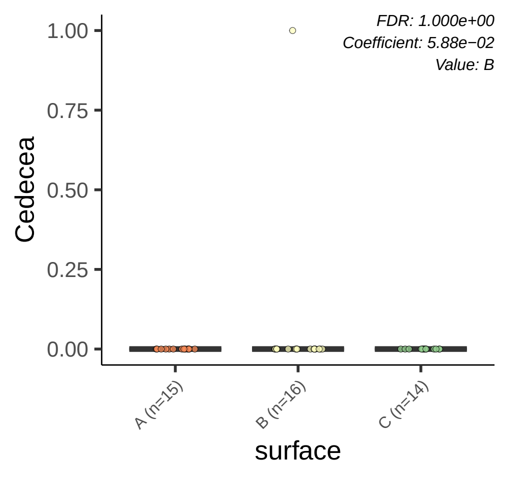
FDR: 1.000e+00
Coefficient: 5.88e−02
Value: B
Cedecea
1.00
0.75
0.50
0.25
0.00
A (n=15)
B (n=16)
C (n=14)
surface

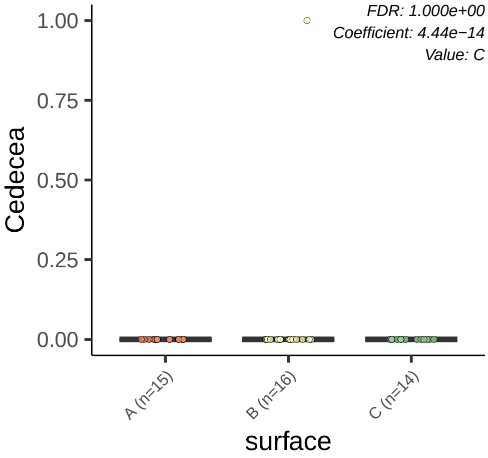

FDR: 1.000e+00
Coefficient: 4.44e−14
Value: C
1.00
0.75
0.50
0.25
0.00
Cedecea
A (n=15)
B (n=16)
C (n=14)
surface

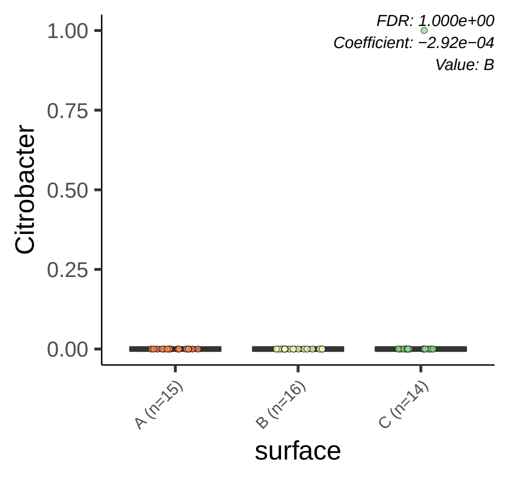
FDR: 1.000e+00
Coefficient: −2.92e−04
Value: B
1.00
0.75
0.50
0.25
0.00
Citrobacter
A (n=15)
B (n=16)
C (n=14)
surface

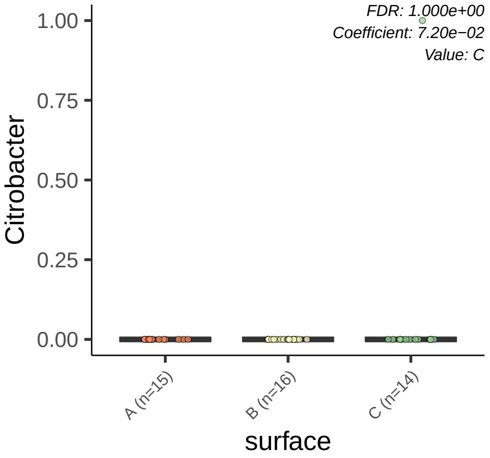

FDR: 1.000e+00
Coefficient: 7.20e−02
Value: C
Citrobacter
1.00
0.75
0.50
0.25
0.00
A (n=15)
B (n=16)
C (n=14)
surface

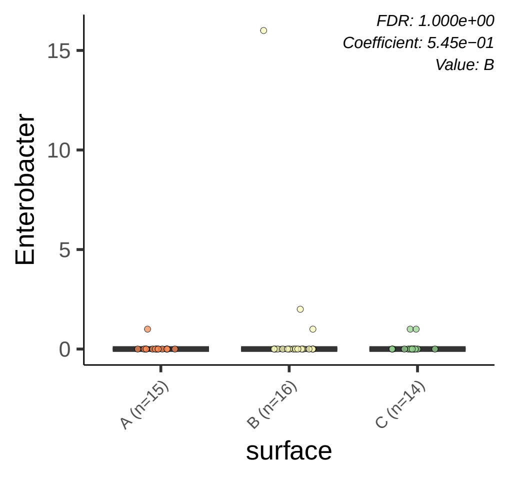
FDR: 1.000e+00
Coefficient: 5.45e−01
Value: B
Enterobacter
15
10
5
0
A (n=15)
B (n=16)
C (n=14)
surface

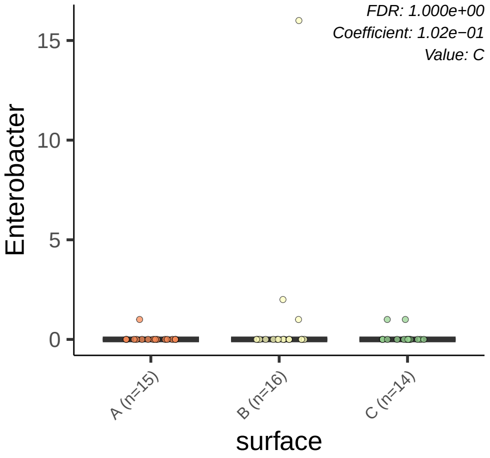
FDR: 1.000e+00
Coefficient: 1.02e−01
Value: C
Enterobacter
15
10
5
0
A (n=15)
B (n=16)
C (n=14)
surface

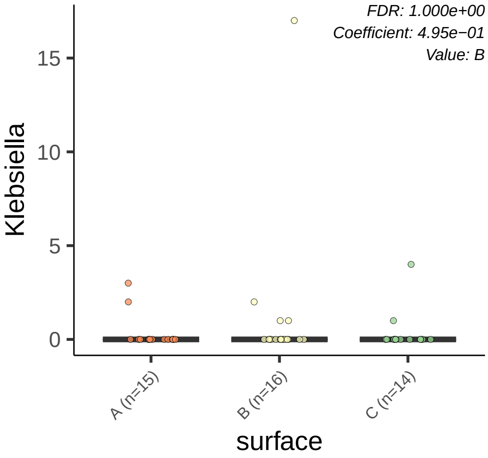
FDR: 1.000e+00
Coefficient: 4.95e−01
Value: B
Klebsiella
15
10
5
0
A (n=15)
B (n=16)
C (n=14)
surface

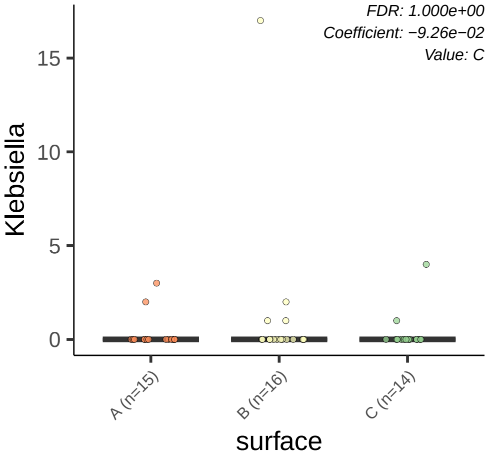
FDR: 1.000e+00
Coefficient: −9.26e−02
Value: C
Klebsiella
15
10
5
0
A (n=15)
B (n=16)
C (n=14)
surface

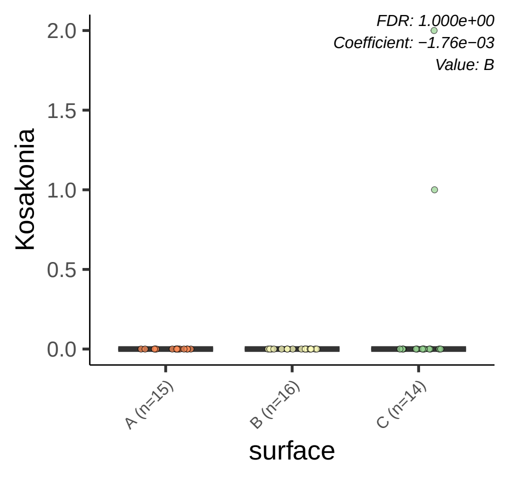
FDR: 1.000e+00
Coefficient: −1.76e−03
Value: B
Kosakonia
2.0
1.5
1.0
0.5
0.0
A (n=15)
B (n=16)
C (n=14)
surface

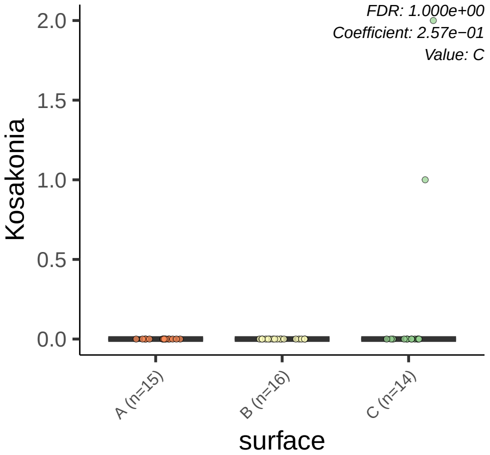
FDR: 1.000e+00
Coefficient: 2.57e−01
Value: C
Kosakonia
2.0
1.5
1.0
0.5
0.0
A (n=15)
B (n=16)
C (n=14)
surface

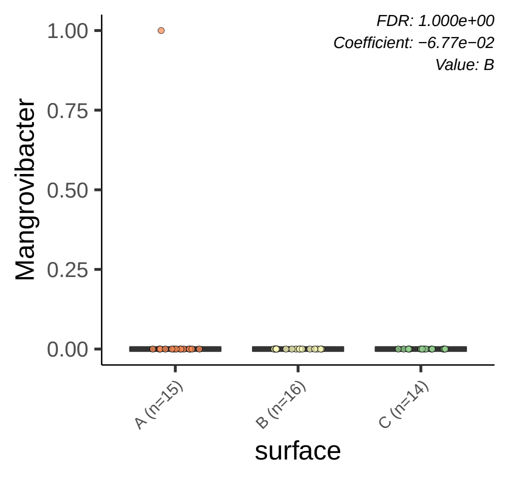
FDR: 1.000e+00
Coefficient: −6.77e−02
Value: B
Mangrovibacter
1.00
0.75
0.50
0.25
0.00
A (n=15)
B (n=16)
C (n=14)
surface

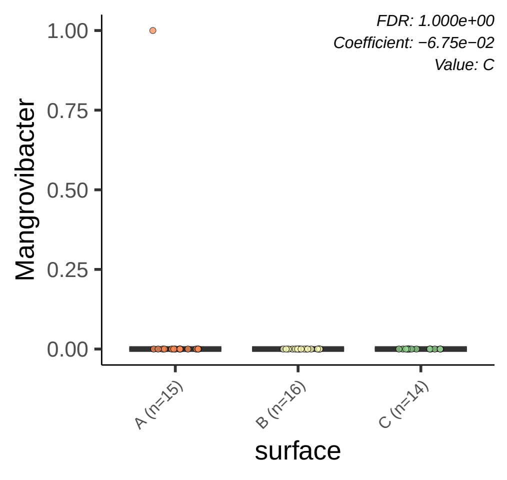
FDR: 1.000e+00
Coefficient: −6.75e−02
Value: C
Mangrovibacter
1.00
0.75
0.50
0.25
0.00
A (n=15)
B (n=16)
C (n=14)
surface

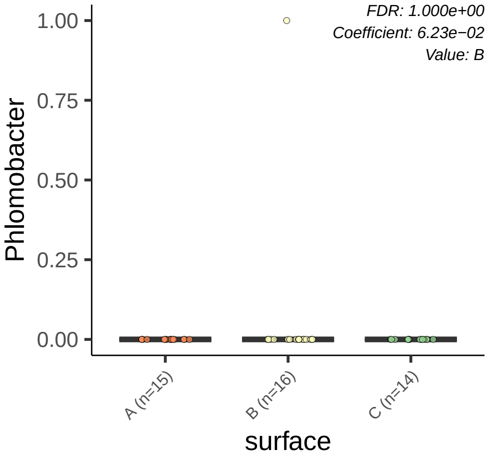
FDR: 1.000e+00
Coefficient: 6.23e−02
Value: B
Phlomobacter
1.00
0.75
0.50
0.25
0.00
A (n=15)
B (n=16)
C (n=14)
surface

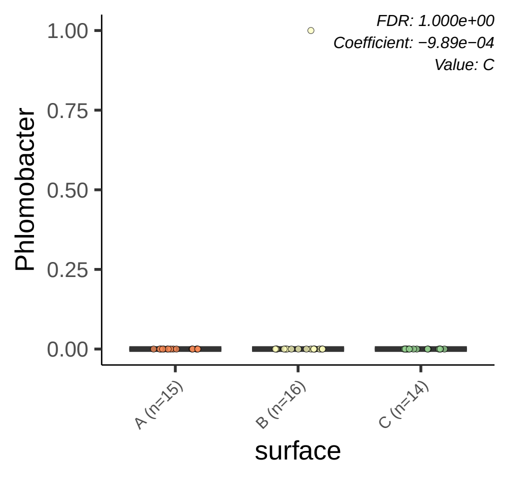
FDR: 1.000e+00
Coefficient: −9.89e−04
Value: C
Phlomobacter
1.00
0.75
0.50
0.25
0.00
A (n=15)
B (n=16)
C (n=14)
surface

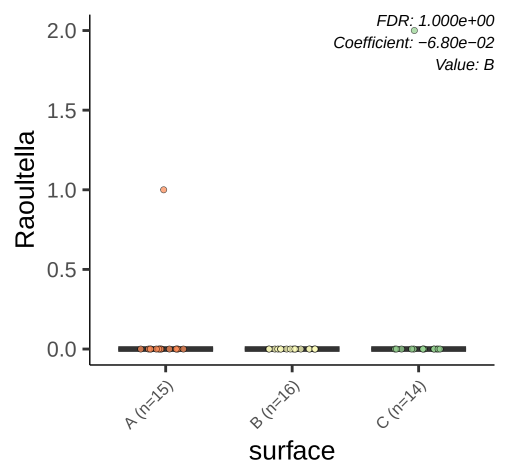

FDR: 1.000e+00
Coefficient: −6.80e−02
Value: B
Raoultella
2.0
1.5
1.0
0.5
0.0
A (n=15)
B (n=16)
C (n=14)
surface

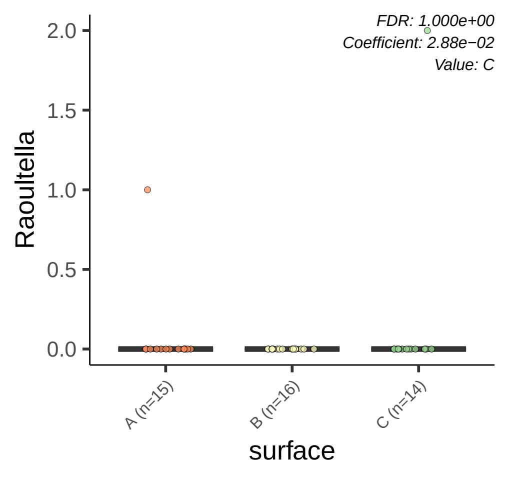

FDR: 1.000e+00
Coefficient: 2.88e−02
Value: C
Raoultella
2.0
1.5
1.0
0.5
0.0
A (n=15)
B (n=16)
C (n=14)
surface

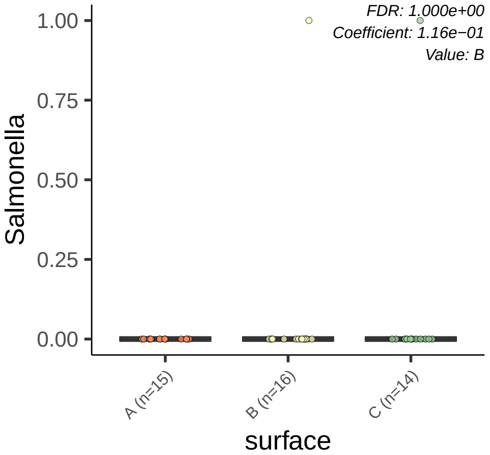
FDR: 1.000e+00
Coefficient: 1.16e−01
Value: B
1.00
0.75
0.50
0.25
0.00
Salmonella
A (n=15)
B (n=16)
C (n=14)
surface

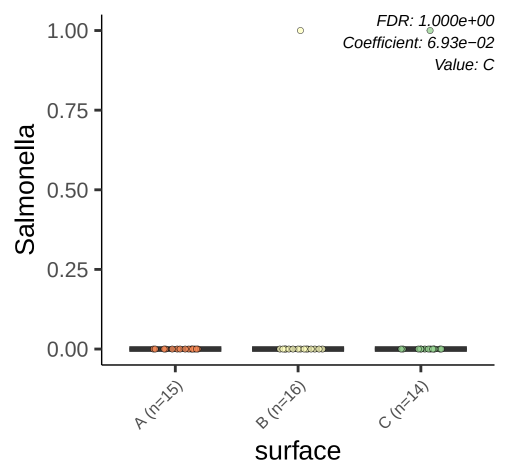
FDR: 1.000e+00
Coefficient: 6.93e−02
Value: C
Salmonella
1.00
0.75
0.50
0.25
0.00
A (n=15)
B (n=16)
C (n=14)
surface

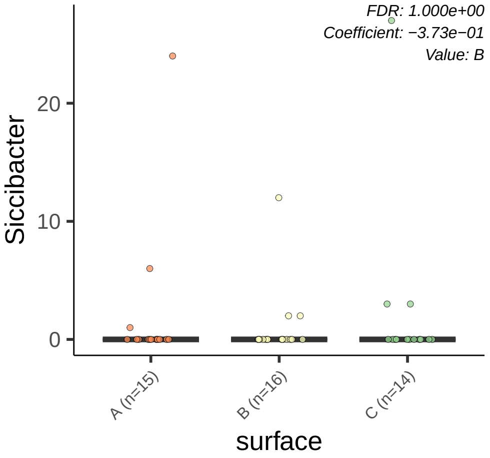

FDR: 1.000e+00
Coefficient: −3.73e−01
Value: B
Siccibacter
20
10
0
A (n=15)
B (n=16)
C (n=14)
surface

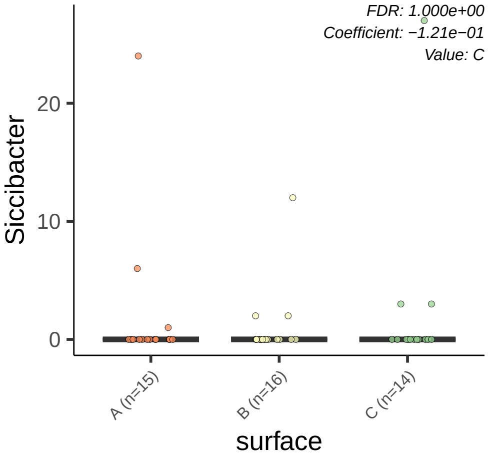

FDR: 1.000e+00
Coefficient: −1.21e−01
Value: C
Siccibacter
20
10
0
A (n=15)
B (n=16)
C (n=14)
surface

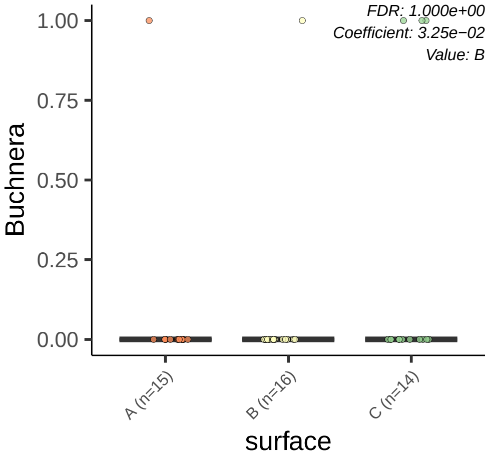
FDR: 1.000e+00
Coefficient: 3.25e−02
Value: B
Buchnera
1.00
0.75
0.50
0.25
0.00
A (n=15)
B (n=16)
C (n=14)
surface

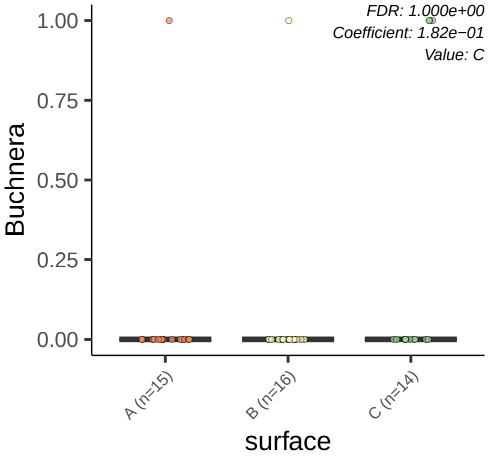
FDR: 1.000e+00
Coefficient: 1.82e−01
Value: C
1.00
0.75
0.50
0.25
0.00
Buchnera
A (n=15)
B (n=16)
C (n=14)
surface

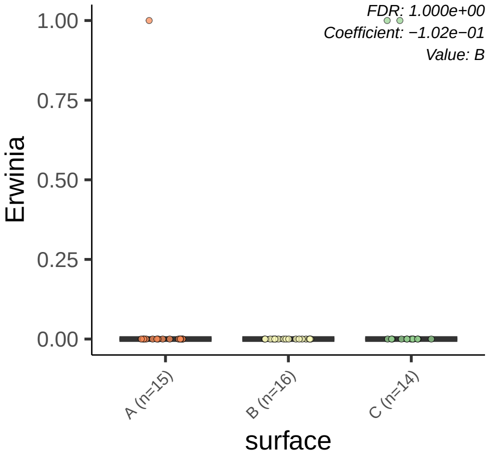

FDR: 1.000e+00
Coefficient: −1.02e−01
Value: B
Erwinia
1.00
0.75
0.50
0.25
0.00
A (n=15)
B (n=16)
C (n=14)
surface

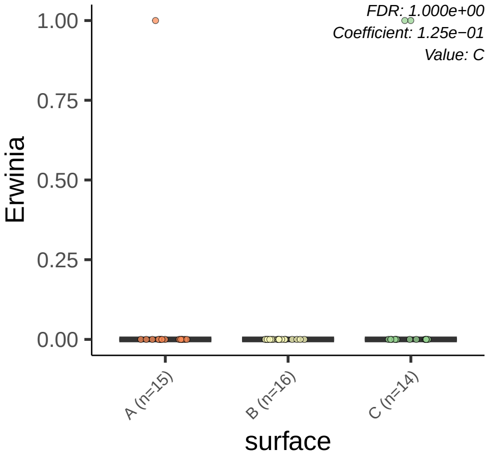
FDR: 1.000e+00
Coefficient: 1.25e−01
Value: C
1.00
0.75
0.50
0.25
0.00
Erwinia
A (n=15)
B (n=16)
C (n=14)
surface

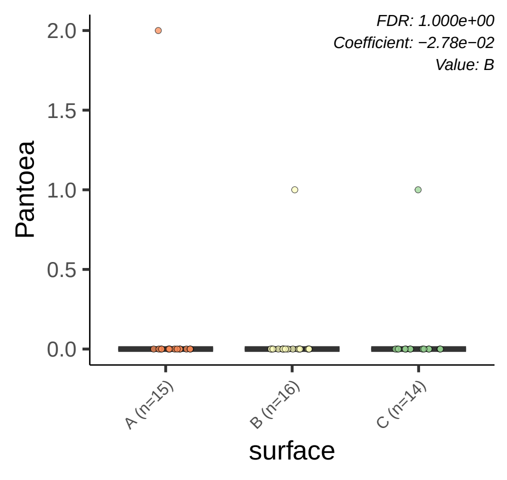

FDR: 1.000e+00
Coefficient: −2.78e−02
Value: B
Pantoea
2.0
1.5
1.0
0.5
0.0
A (n=15)
B (n=16)
C (n=14)
surface

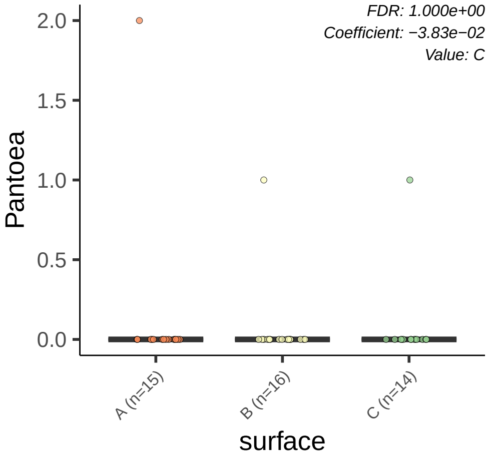

FDR: 1.000e+00
Coefficient: −3.83e−02
Value: C
Pantoea
2.0
1.5
1.0
0.5
0.0
A (n=15)
B (n=16)
C (n=14)
surface

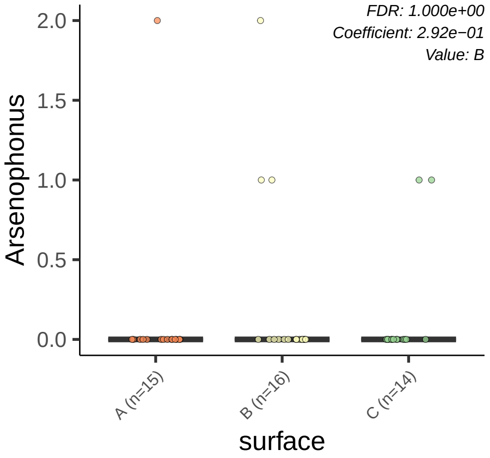
FDR: 1.000e+00
Coefficient: 2.92e−01
Value: B
Arsenophonus
2.0
1.5
1.0
0.5
0.0
A (n=15)
B (n=16)
C (n=14)
surface

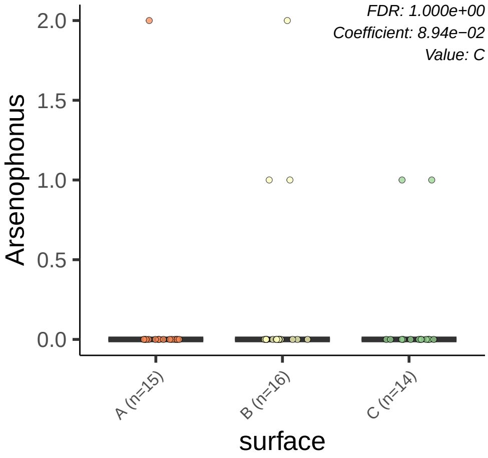
FDR: 1.000e+00
Coefficient: 8.94e−02
Value: C
Arsenophonus
2.0
1.5
1.0
0.5
0.0
A (n=15)
B (n=16)
C (n=14)
surface

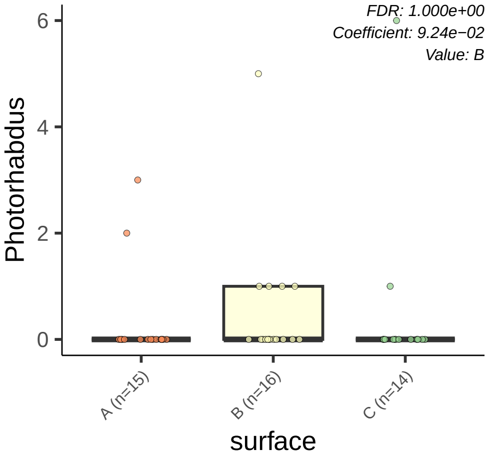

FDR: 1.000e+00
Coefficient: 9.24e−02
Value: B
Photorhabdus
0
2
4
6
A (n=15)
B (n=16)
C (n=14)
surface

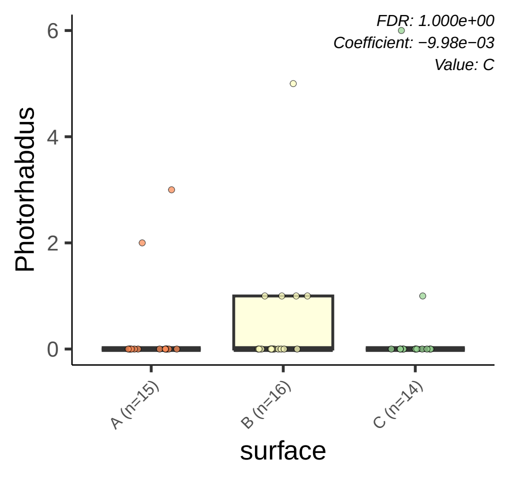
FDR: 1.000e+00
Coefficient: −9.98e−03
Value: C
Photorhabdus
0
2
4
6
A (n=15)
B (n=16)
C (n=14)
surface

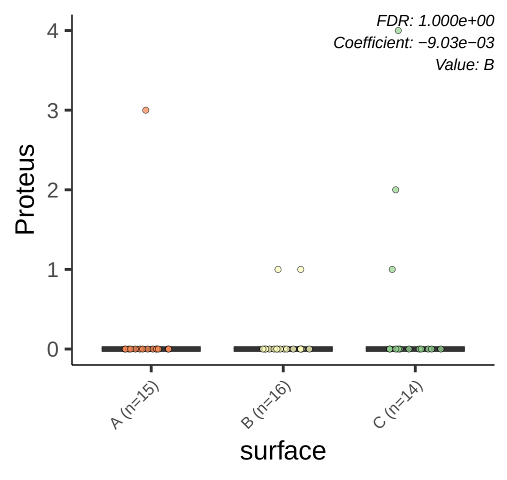
FDR: 1.000e+00
Coefficient: −9.03e−03
Value: B
Proteus
4
3
2
1
0
A (n=15)
B (n=16)
C (n=14)
surface

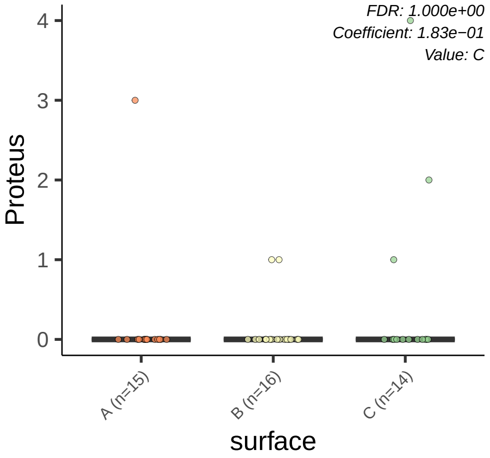

FDR: 1.000e+00
Coefficient: 1.83e−01
Value: C
Proteus
4
3
2
1
0
A (n=15)
B (n=16)
C (n=14)
surface

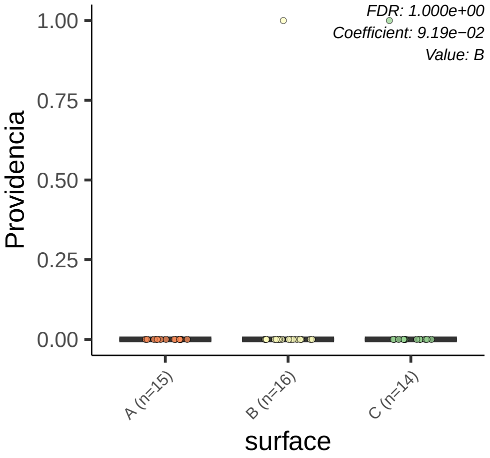
FDR: 1.000e+00
Coefficient: 9.19e−02
Value: B
Providencia
1.00
0.75
0.50
0.25
0.00
A (n=15)
B (n=16)
C (n=14)
surface

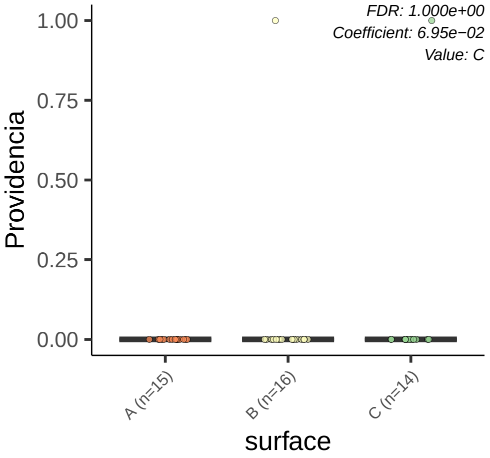
FDR: 1.000e+00
Coefficient: 6.95e−02
Value: C
1.00
0.75
0.50
0.25
0.00
Providencia
A (n=15)
B (n=16)
C (n=14)
surface

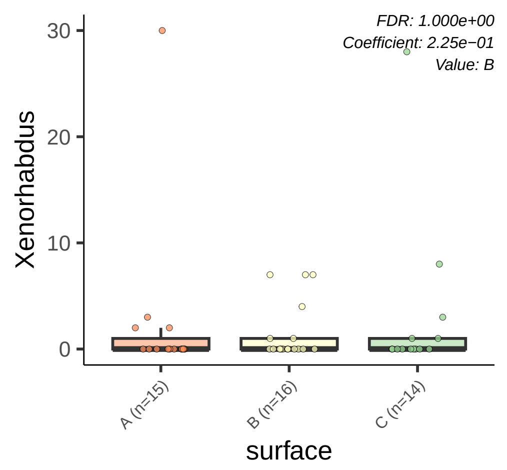

FDR: 1.000e+00
Coefficient: 2.25e−01
Value: B
Xenorhabdus
30
20
10
0
A (n=15)
B (n=16)
C (n=14)
surface

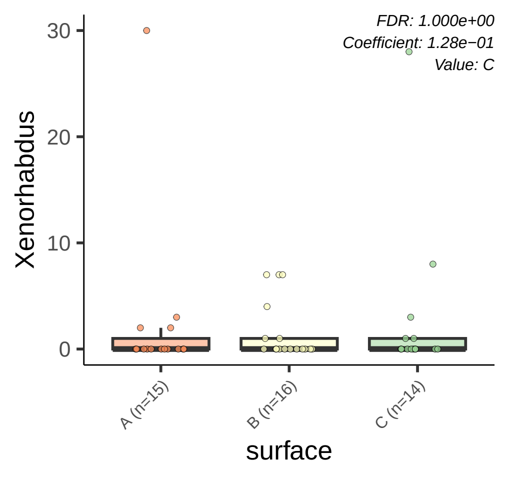
FDR: 1.000e+00
Coefficient: 1.28e−01
Value: C
30
20
10
0
Xenorhabdus
A (n=15)
B (n=16)
C (n=14)
surface

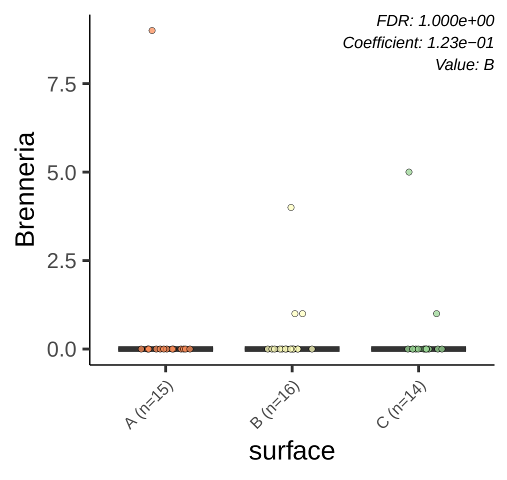

FDR: 1.000e+00
Coefficient: 1.23e−01
Value: B
Brenneria
7.5
5.0
2.5
0.0
A (n=15)
B (n=16)
C (n=14)
surface

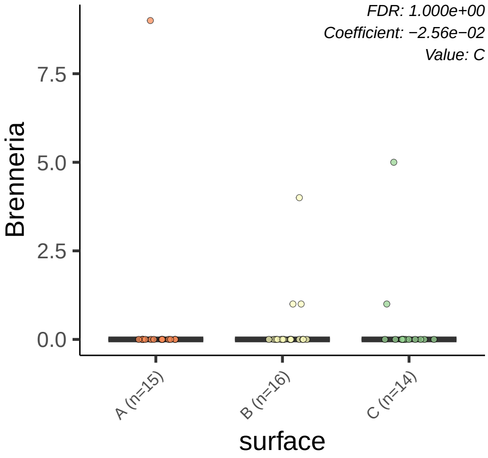

FDR: 1.000e+00
Coefficient: −2.56e−02
Value: C
Brenneria
7.5
5.0
2.5
0.0
A (n=15)
B (n=16)
C (n=14)
surface

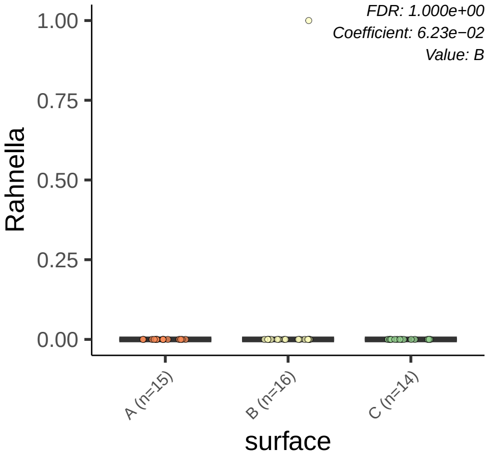
FDR: 1.000e+00
Coefficient: 6.23e−02
Value: B
Rahnella
1.00
0.75
0.50
0.25
0.00
A (n=15)
B (n=16)
C (n=14)
surface

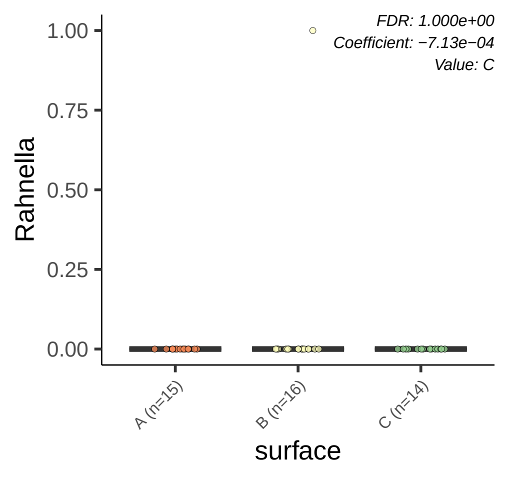
FDR: 1.000e+00
Coefficient: −7.13e−04
Value: C
1.00
0.75
0.50
0.25
0.00
Rahnella
A (n=15)
B (n=16)
C (n=14)
surface

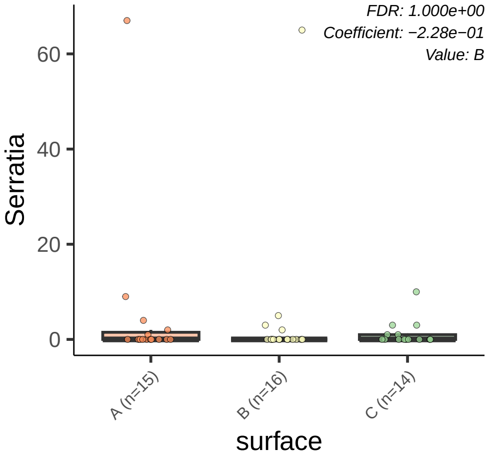
FDR: 1.000e+00
Coefficient: −2.28e−01
Value: B
Serratia
60
40
20
0
A (n=15)
B (n=16)
C (n=14)
surface

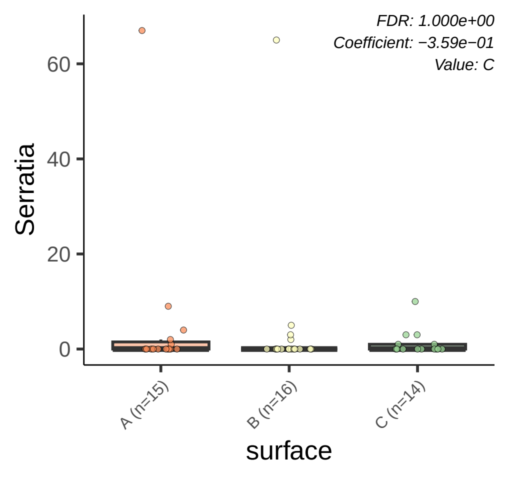

FDR: 1.000e+00
Coefficient: −3.59e−01
Value: C
Serratia
60
40
20
0
A (n=15)
B (n=16)
C (n=14)
surface

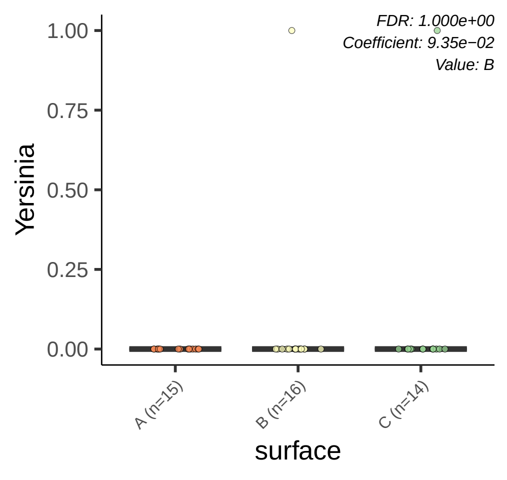
FDR: 1.000e+00
Coefficient: 9.35e−02
Value: B
Yersinia
1.00
0.75
0.50
0.25
0.00
A (n=15)
B (n=16)
C (n=14)
surface

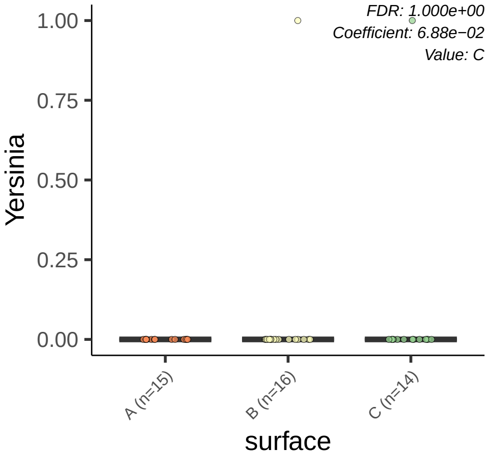
FDR: 1.000e+00
Coefficient: 6.88e−02
Value: C
Yersinia
1.00
0.75
0.50
0.25
0.00
A (n=15)
B (n=16)
C (n=14)
surface

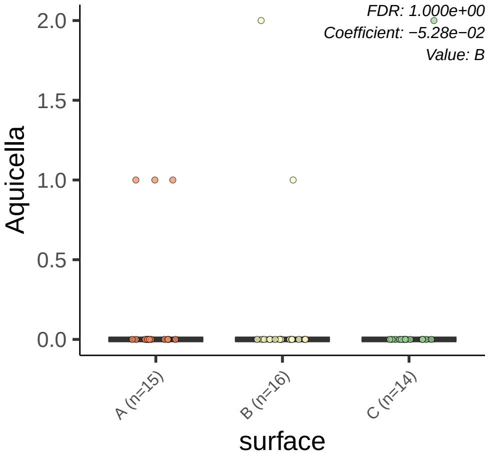
FDR: 1.000e+00
Coefficient: −5.28e−02
Value: B
Aquicella
2.0
1.5
1.0
0.5
0.0
A (n=15)
B (n=16)
C (n=14)
surface

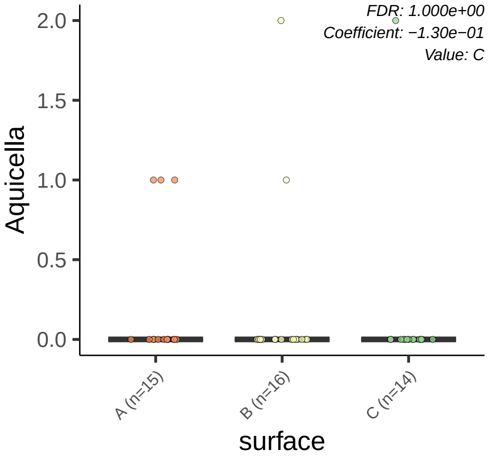
FDR: 1.000e+00
Coefficient: −1.30e−01
Value: C
Aquicella
2.0
1.5
1.0
0.5
0.0
A (n=15)
B (n=16)
C (n=14)
surface

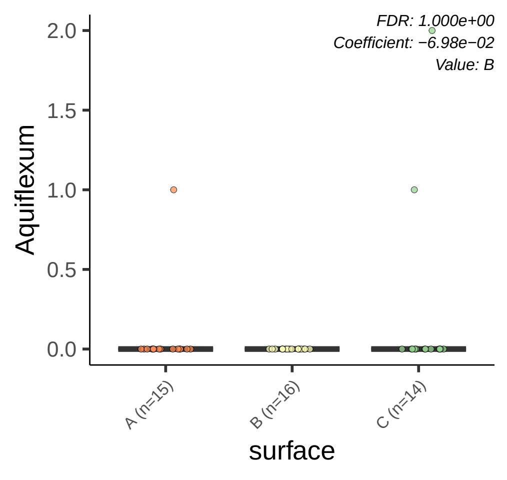
FDR: 1.000e+00
Coefficient: −6.98e−02
Value: B
Aquiflexum
2.0
1.5
1.0
0.5
0.0
A (n=15)
B (n=16)
C (n=14)
surface

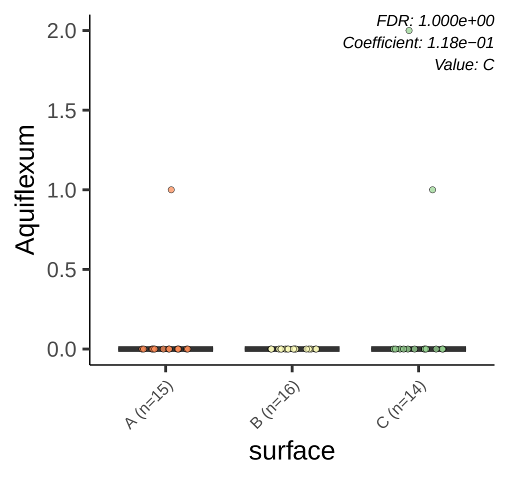
FDR: 1.000e+00
Coefficient: 1.18e−01
Value: C
Aquiflexum
2.0
1.5
1.0
0.5
0.0
A (n=15)
B (n=16)
C (n=14)
surface

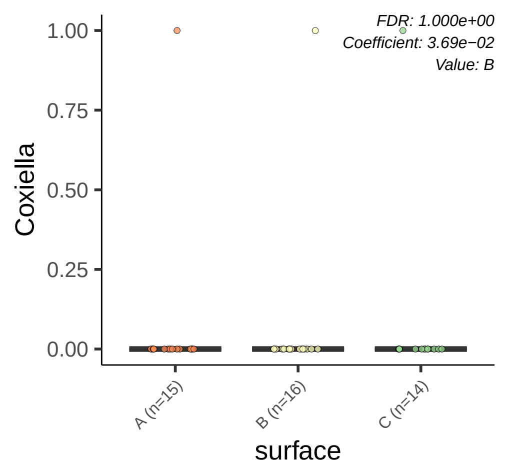
FDR: 1.000e+00
Coefficient: 3.69e−02
Value: B
1.00
0.75
0.50
0.25
0.00
Coxiella
A (n=15)
B (n=16)
C (n=14)
surface

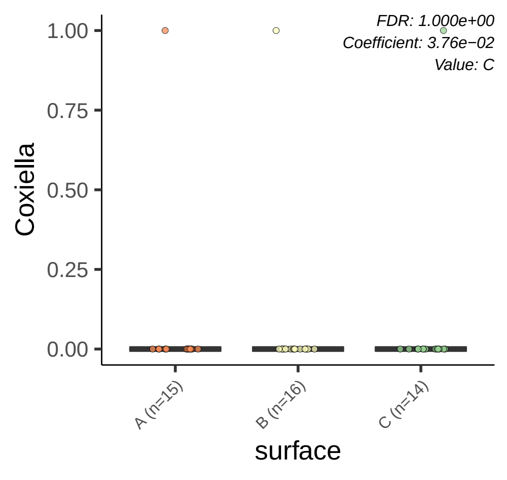
FDR: 1.000e+00
Coefficient: 3.76e−02
Value: C
Coxiella
1.00
0.75
0.50
0.25
0.00
A (n=15)
B (n=16)
C (n=14)
surface

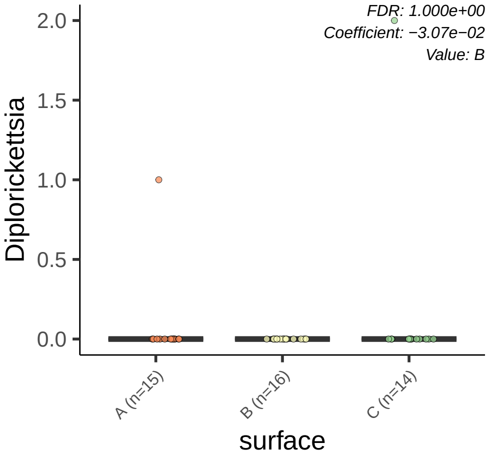
FDR: 1.000e+00
Coefficient: −3.07e−02
Value: B
Diplorickettsia
2.0
1.5
1.0
0.5
0.0
A (n=15)
B (n=16)
C (n=14)
surface

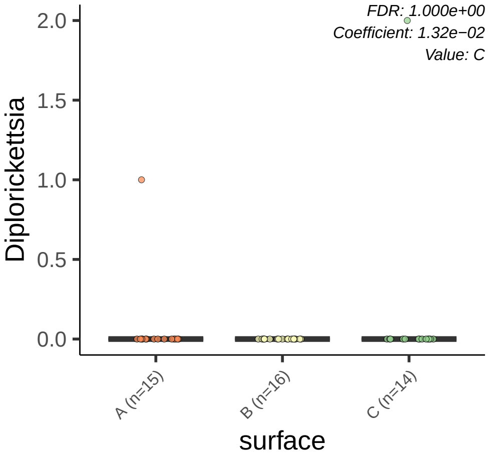
FDR: 1.000e+00
Coefficient: 1.32e−02
Value: C
Diplorickettsia
2.0
1.5
1.0
0.5
0.0
A (n=15)
B (n=16)
C (n=14)
surface

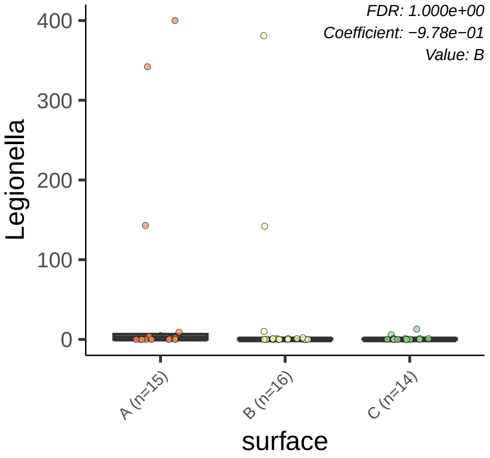

FDR: 1.000e+00
Coefficient: −9.78e−01
Value: B
Legionella
400
300
200
100
0
A (n=15)
B (n=16)
C (n=14)
surface

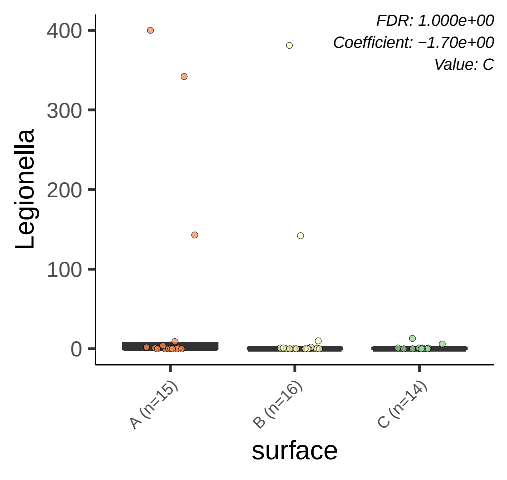
FDR: 1.000e+00
Coefficient: −1.70e+00
Value: C
Legionella
400
300
200
100
0
A (n=15)
B (n=16)
C (n=14)
surface

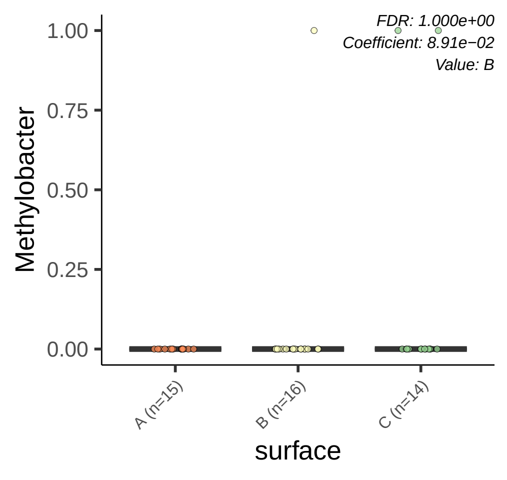
FDR: 1.000e+00
Coefficient: 8.91e−02
Value: B
Methylobacter
1.00
0.75
0.50
0.25
0.00
A (n=15)
B (n=16)
C (n=14)
surface

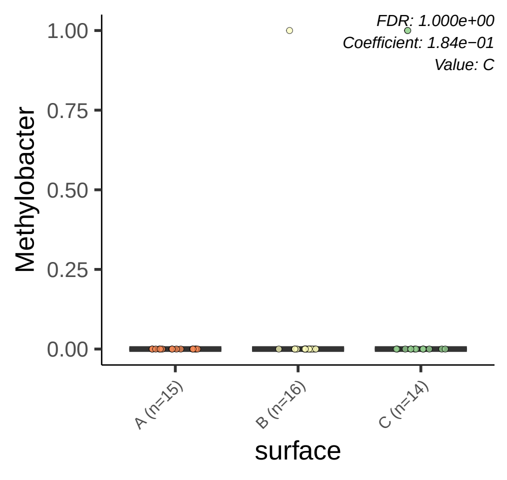
FDR: 1.000e+00
Coefficient: 1.84e−01
Value: C
Methylobacter
1.00
0.75
0.50
0.25
0.00
A (n=15)
B (n=16)
C (n=14)
surface

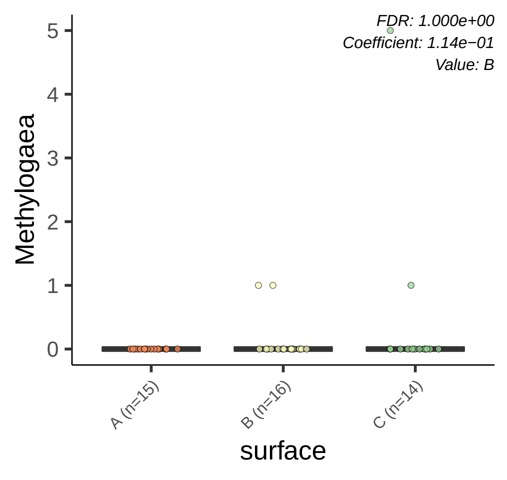
FDR: 1.000e+00
Coefficient: 1.14e−01
Value: B
Methylogaea
0
1
2
3
4
5
A (n=15)
B (n=16)
C (n=14)
surface

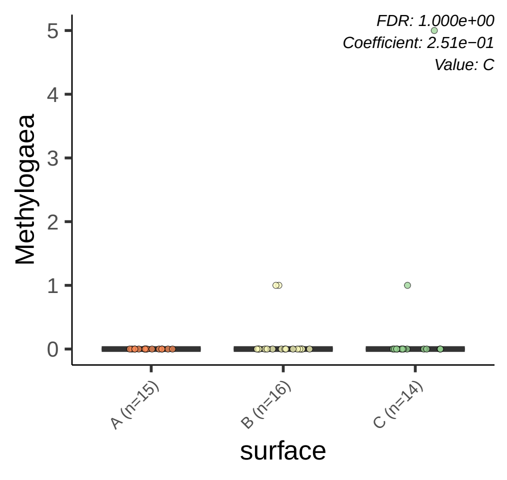
FDR: 1.000e+00
Coefficient: 2.51e−01
Value: C
Methylogaea
5
4
3
2
1
0
A (n=15)
B (n=16)
C (n=14)
surface

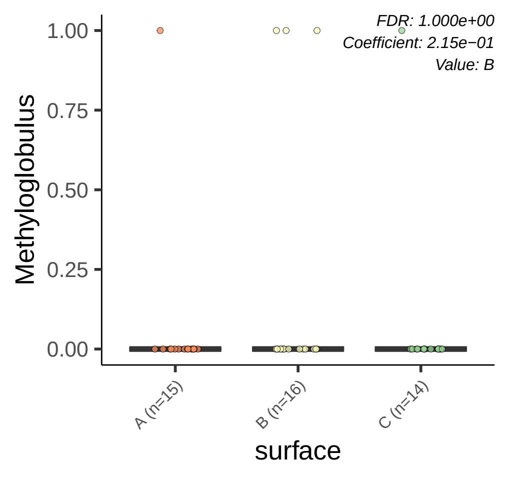
FDR: 1.000e+00
Coefficient: 2.15e−01
Value: B
Methyloglobulus
1.00
0.75
0.50
0.25
0.00
A (n=15)
B (n=16)
C (n=14)
surface

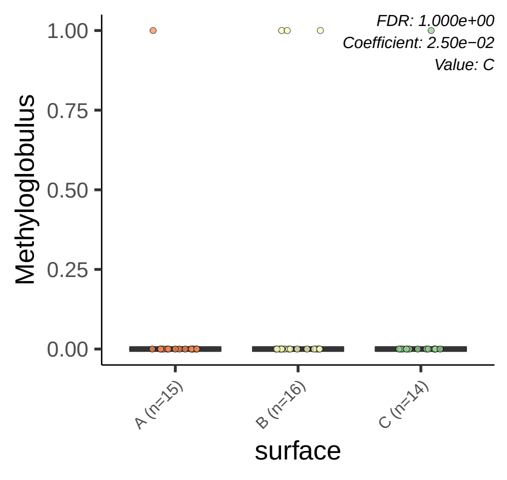

FDR: 1.000e+00
Coefficient: 2.50e−02
Value: C
Methyloglobulus
1.00
0.75
0.50
0.25
0.00
A (n=15)
B (n=16)
C (n=14)
surface

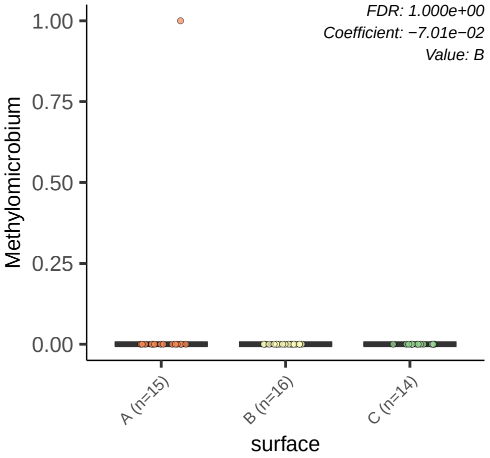
FDR: 1.000e+00
Coefficient: −7.01e−02
Value: B
Methylomicrobium
1.00
0.75
0.50
0.25
0.00
A (n=15)
B (n=16)
C (n=14)
surface

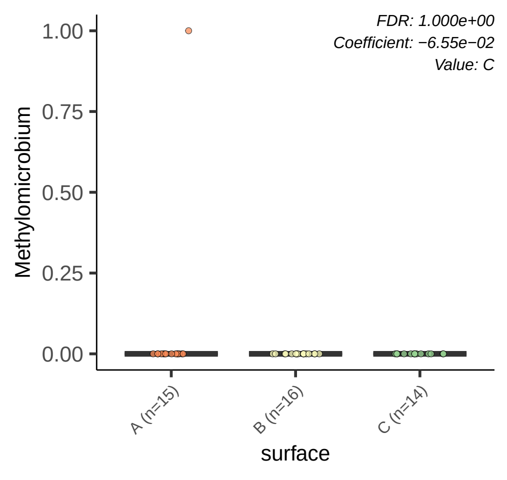

FDR: 1.000e+00
Coefficient: −6.55e−02
Value: C
Methylomicrobium
1.00
0.75
0.50
0.25
0.00
A (n=15)
B (n=16)
C (n=14)
surface

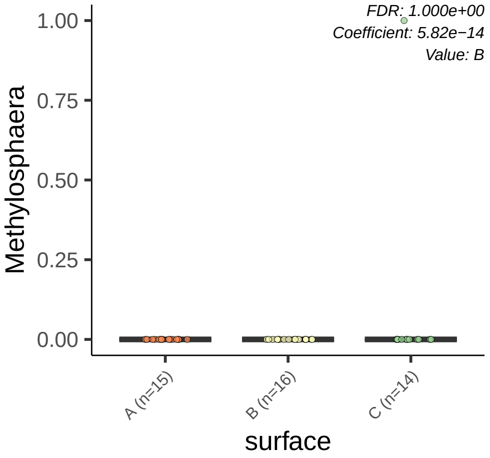
FDR: 1.000e+00
Coefficient: 5.82e−14
Value: B
1.00
0.75
0.50
0.25
0.00
Methylosphaera
A (n=15)
B (n=16)
C (n=14)
surface

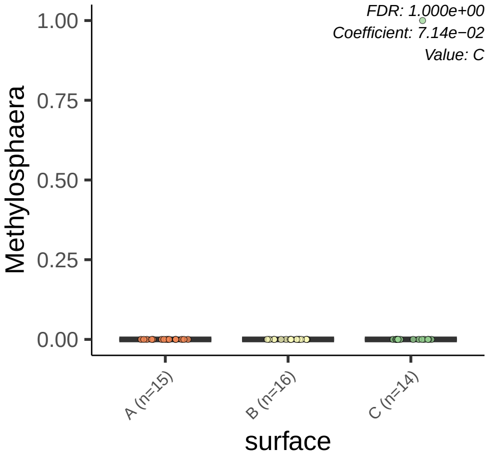
FDR: 1.000e+00
Coefficient: 7.14e−02
Value: C
Methylosphaera
1.00
0.75
0.50
0.25
0.00
A (n=15)
B (n=16)
C (n=14)
surface

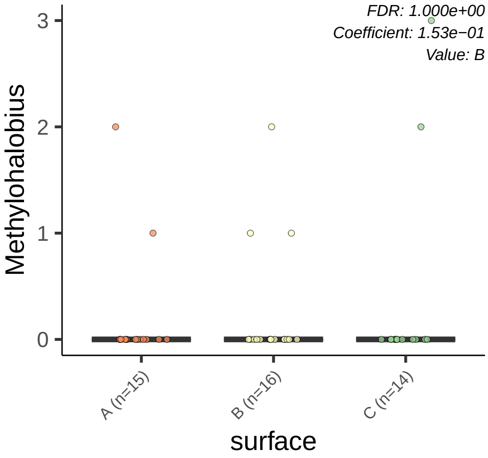

FDR: 1.000e+00
Coefficient: 1.53e−01
Value: B
Methylohalobius
0
1
2
3
A (n=15)
B (n=16)
C (n=14)
surface

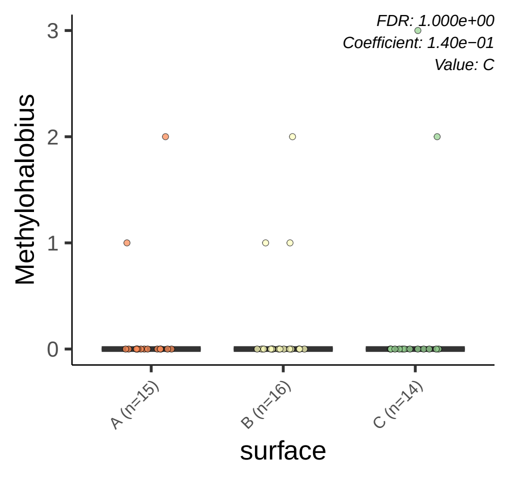

FDR: 1.000e+00
Coefficient: 1.40e−01
Value: C
Methylohalobius
0
1
2
3
A (n=15)
B (n=16)
C (n=14)
surface

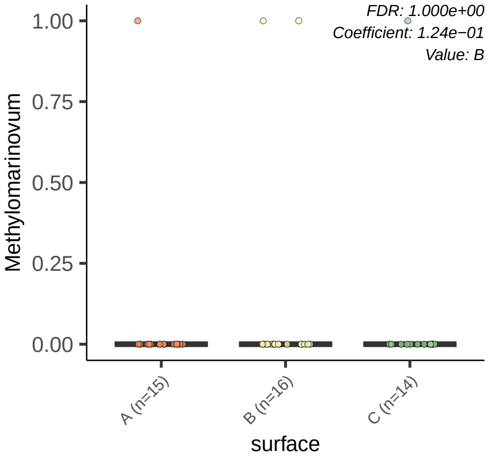

FDR: 1.000e+00
Coefficient: 1.24e−01
Value: B
1.00
0.75
0.50
0.25
0.00
Methylomarinovum
A (n=15)
B (n=16)
C (n=14)
surface

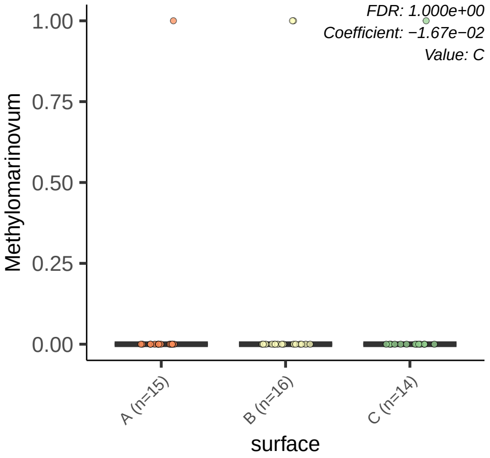
FDR: 1.000e+00
Coefficient: −1.67e−02
Value: C
1.00
0.75
0.50
0.25
0.00
Methylomarinovum
A (n=15)
B (n=16)
C (n=14)
surface

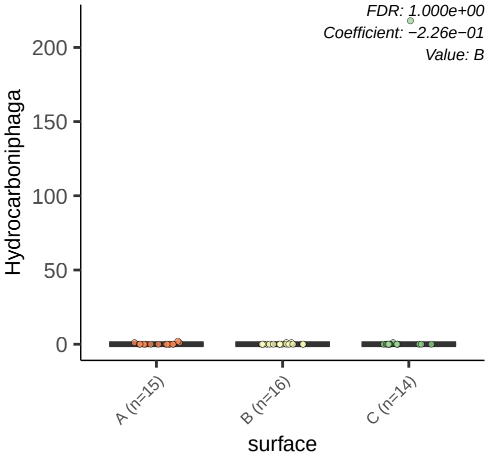
FDR: 1.000e+00
Coefficient: −2.26e−01
Value: B
Hydrocarboniphaga
200
150
100
50
0
A (n=15)
B (n=16)
C (n=14)
surface

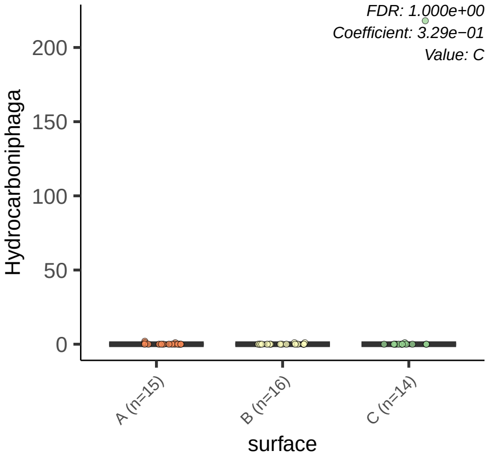

FDR: 1.000e+00
Coefficient: 3.29e−01
Value: C
Hydrocarboniphaga
200
150
100
50
0
A (n=15)
B (n=16)
C (n=14)
surface

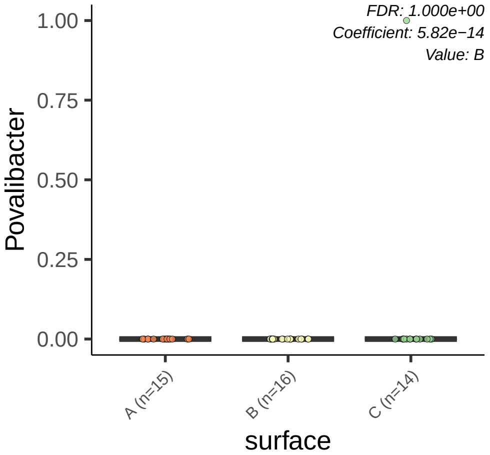

FDR: 1.000e+00
Coefficient: 5.82e−14
Value: B
Povalibacter
1.00
0.75
0.50
0.25
0.00
A (n=15)
B (n=16)
C (n=14)
surface

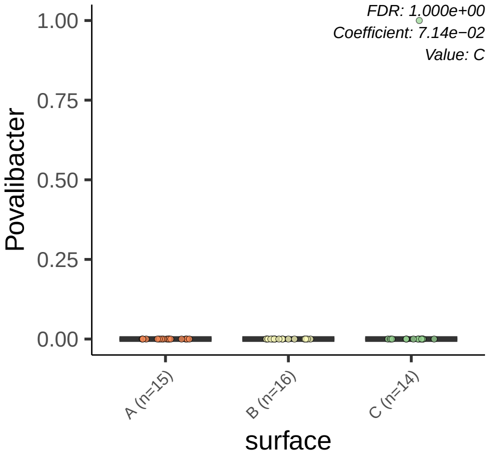
FDR: 1.000e+00
Coefficient: 7.14e−02
Value: C
1.00
0.75
0.50
0.25
0.00
Povalibacter
A (n=15)
B (n=16)
C (n=14)
surface

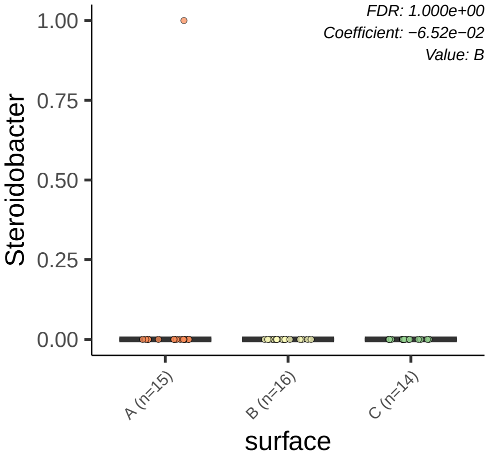

FDR: 1.000e+00
Coefficient: −6.52e−02
Value: B
Steroidobacter
1.00
0.75
0.50
0.25
0.00
A (n=15)
B (n=16)
C (n=14)
surface

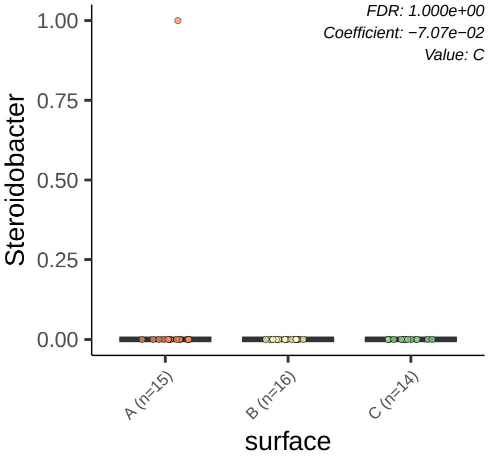

FDR: 1.000e+00
Coefficient: −7.07e−02
Value: C
1.00
0.75
0.50
0.25
0.00
Steroidobacter
A (n=15)
B (n=16)
C (n=14)
surface

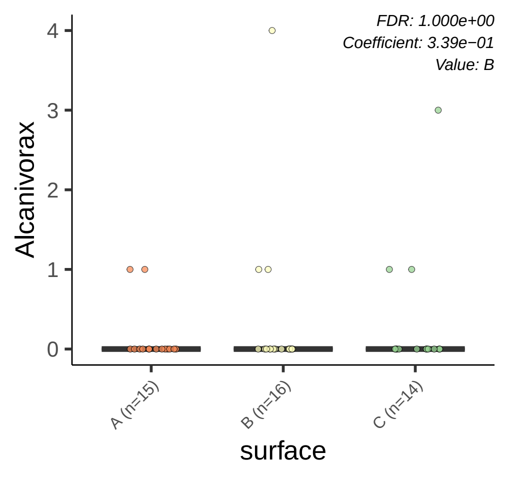
FDR: 1.000e+00
Coefficient: 3.39e−01
Value: B
Alcanivorax
0
1
2
3
4
A (n=15)
B (n=16)
C (n=14)
surface

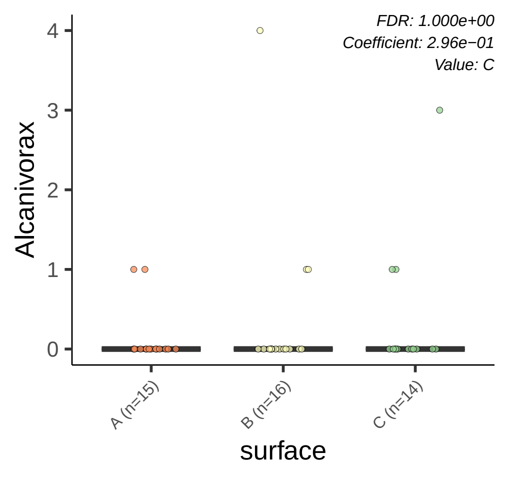

FDR: 1.000e+00
Coefficient: 2.96e−01
Value: C
Alcanivorax
0
1
2
3
4
A (n=15)
B (n=16)
C (n=14)
surface

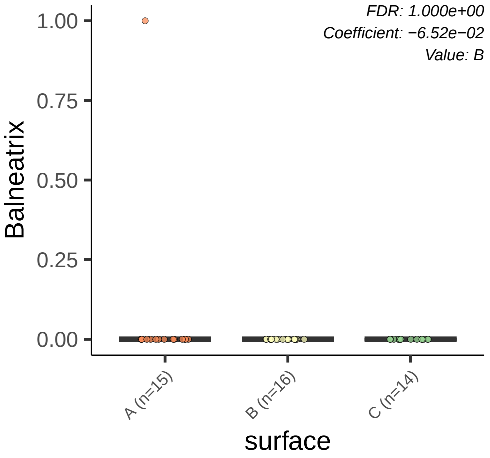
FDR: 1.000e+00
Coefficient: −6.52e−02
Value: B
Balneatrix
1.00
0.75
0.50
0.25
0.00
A (n=15)
B (n=16)
C (n=14)
surface

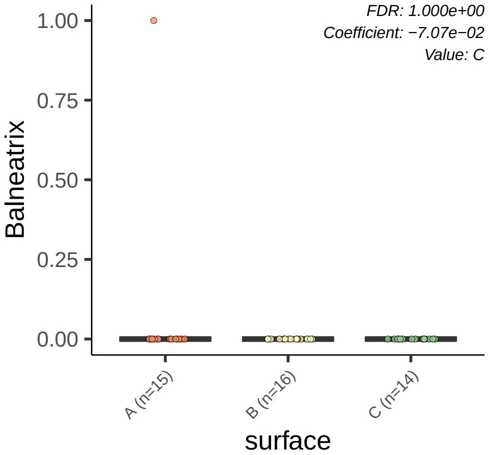
FDR: 1.000e+00
Coefficient: −7.07e−02
Value: C
Balneatrix
1.00
0.75
0.50
0.25
0.00
A (n=15)
B (n=16)
C (n=14)
surface

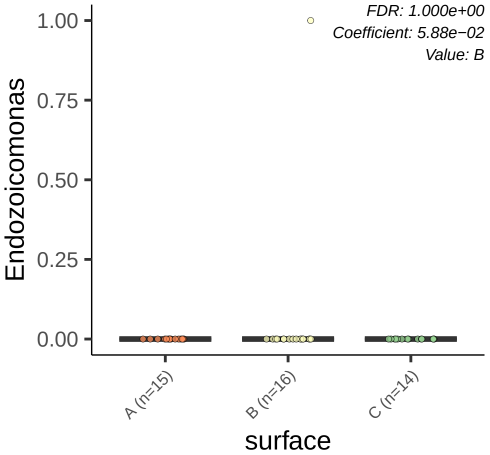
FDR: 1.000e+00
Coefficient: 5.88e−02
Value: B
Endozoicomonas
1.00
0.75
0.50
0.25
0.00
A (n=15)
B (n=16)
C (n=14)
surface

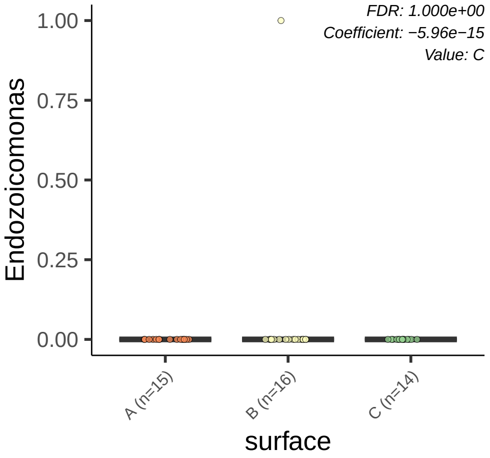
FDR: 1.000e+00
Coefficient: −5.96e−15
Value: C
1.00
0.75
0.50
0.25
0.00
Endozoicomonas
A (n=15)
B (n=16)
C (n=14)
surface

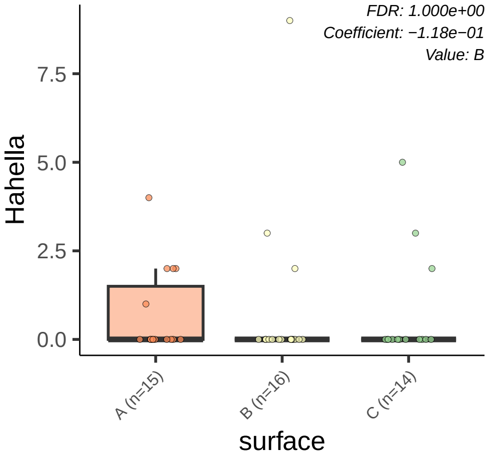

FDR: 1.000e+00
Coefficient: −1.18e−01
Value: B
Hahella
7.5
5.0
2.5
0.0
A (n=15)
B (n=16)
C (n=14)
surface

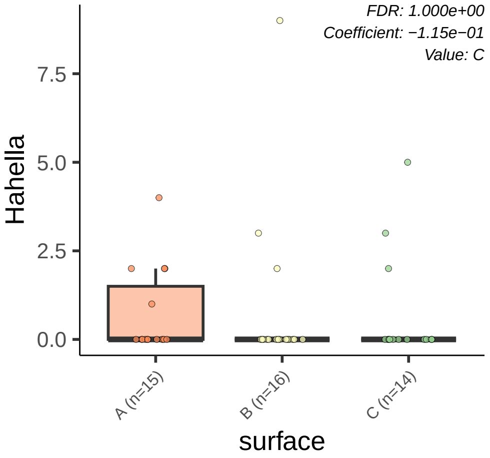
FDR: 1.000e+00
Coefficient: −1.15e−01
Value: C
Hahella
7.5
5.0
2.5
0.0
A (n=15)
B (n=16)
C (n=14)
surface

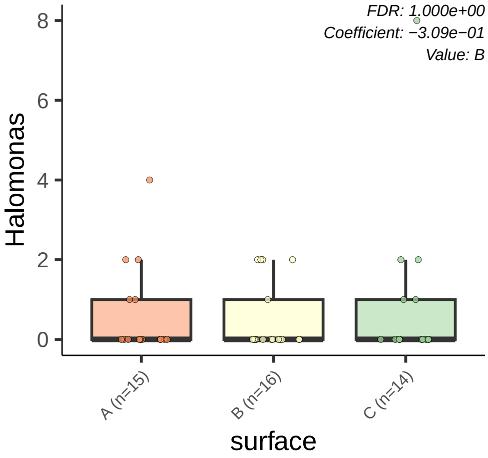
FDR: 1.000e+00
Coefficient: −3.09e−01
Value: B
Halomonas
8
6
4
2
0
A (n=15)
B (n=16)
C (n=14)
surface

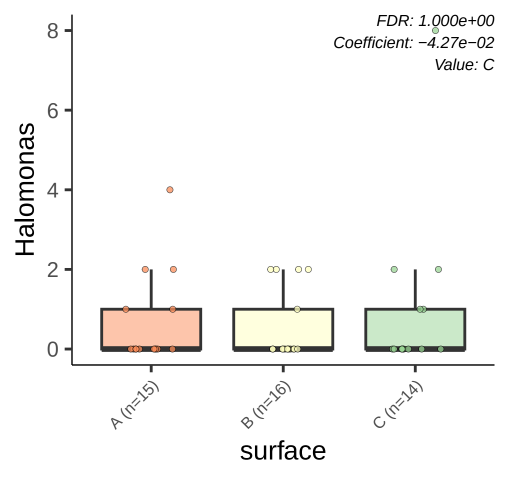
FDR: 1.000e+00
Coefficient: −4.27e−02
Value: C
Halomonas
8
6
4
2
0
A (n=15)
B (n=16)
C (n=14)
surface

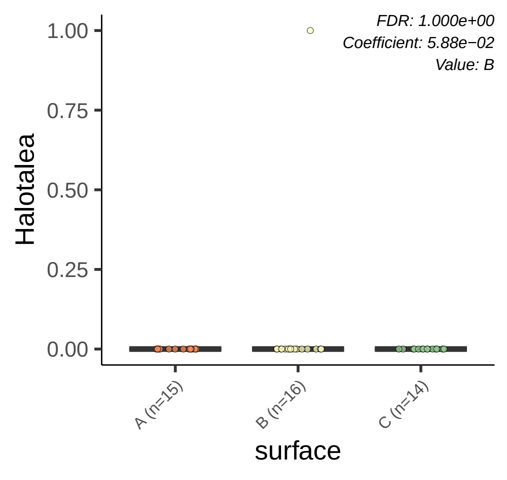

FDR: 1.000e+00
Coefficient: 5.88e−02
Value: B
Halotalea
1.00
0.75
0.50
0.25
0.00
A (n=15)
B (n=16)
C (n=14)
surface

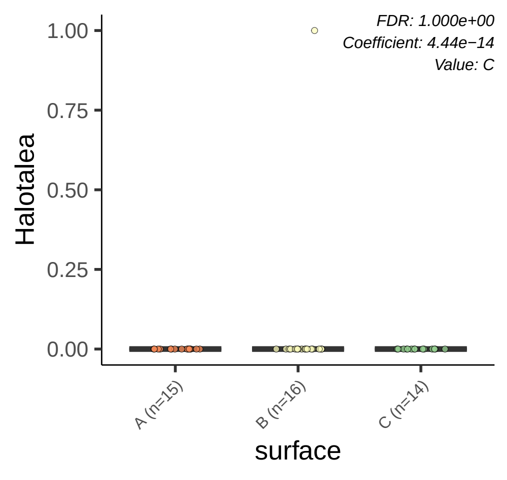

FDR: 1.000e+00
Coefficient: 4.44e−14
Value: C
1.00
0.75
0.50
0.25
0.00
Halotalea
A (n=15)
B (n=16)
C (n=14)
surface

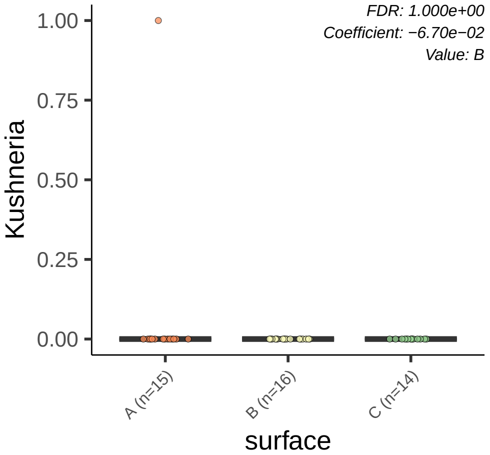
FDR: 1.000e+00
Coefficient: −6.70e−02
Value: B
Kushneria
1.00
0.75
0.50
0.25
0.00
A (n=15)
B (n=16)
C (n=14)
surface

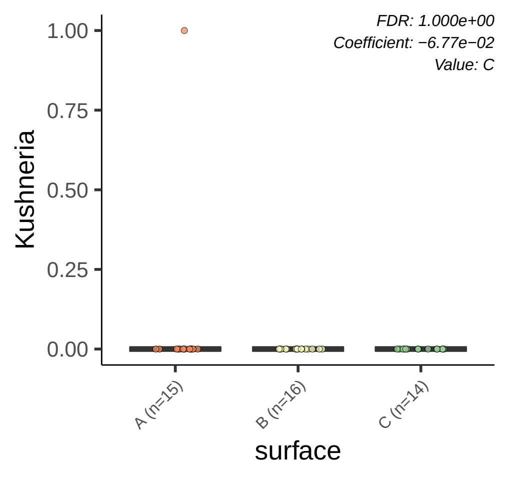
FDR: 1.000e+00
Coefficient: −6.77e−02
Value: C
Kushneria
1.00
0.75
0.50
0.25
0.00
A (n=15)
B (n=16)
C (n=14)
surface

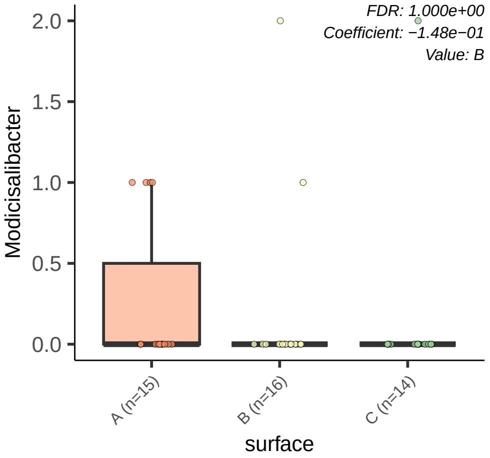

FDR: 1.000e+00
Coefficient: −1.48e−01
Value: B
Modicisalibacter
2.0
1.5
1.0
0.5
0.0
A (n=15)
B (n=16)
C (n=14)
surface

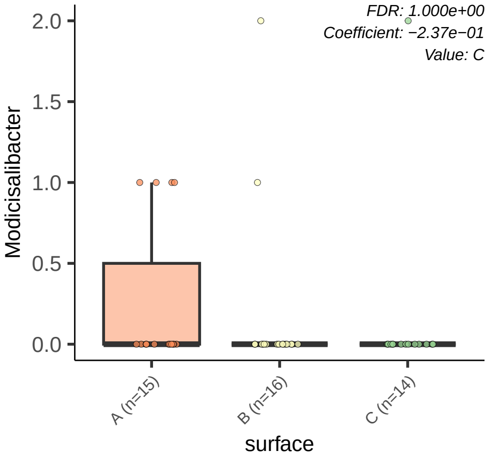
FDR: 1.000e+00
Coefficient: −2.37e−01
Value: C
Modicisalibacter
2.0
1.5
1.0
0.5
0.0
A (n=15)
B (n=16)
C (n=14)
surface

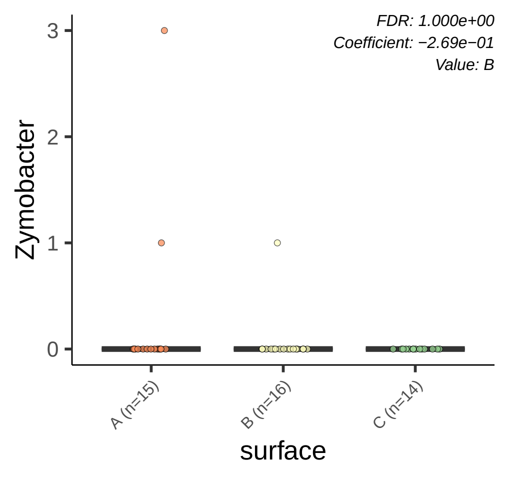
FDR: 1.000e+00
Coefficient: −2.69e−01
Value: B
Zymobacter
3
2
1
0
A (n=15)
B (n=16)
C (n=14)
surface

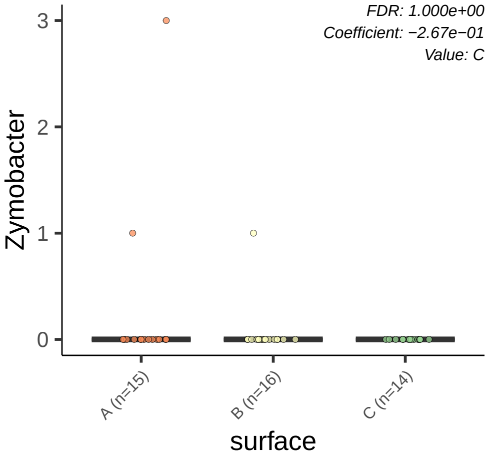
FDR: 1.000e+00
Coefficient: −2.67e−01
Value: C
Zymobacter
0
1
2
3
A (n=15)
B (n=16)
C (n=14)
surface

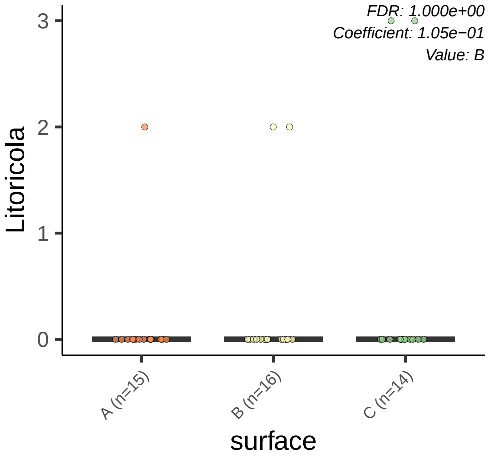

FDR: 1.000e+00
Coefficient: 1.05e−01
Value: B
Litoricola
3
2
1
0
A (n=15)
B (n=16)
C (n=14)
surface

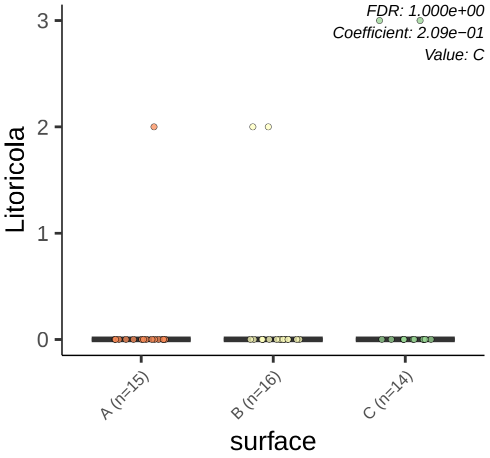
FDR: 1.000e+00
Coefficient: 2.09e−01
Value: C
Litoricola
3
2
1
0
A (n=15)
B (n=16)
C (n=14)
surface

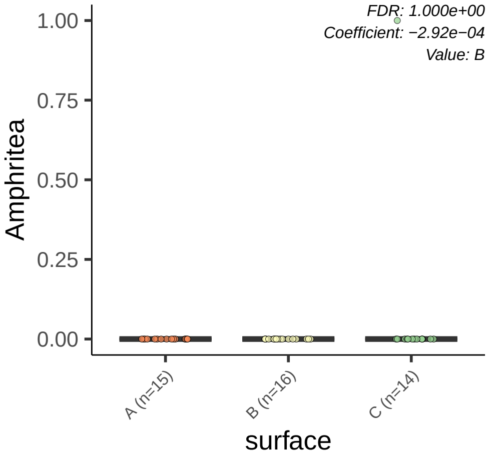
FDR: 1.000e+00
Coefficient: −2.92e−04
Value: B
1.00
0.75
0.50
0.25
0.00
Amphritea
A (n=15)
B (n=16)
C (n=14)
surface

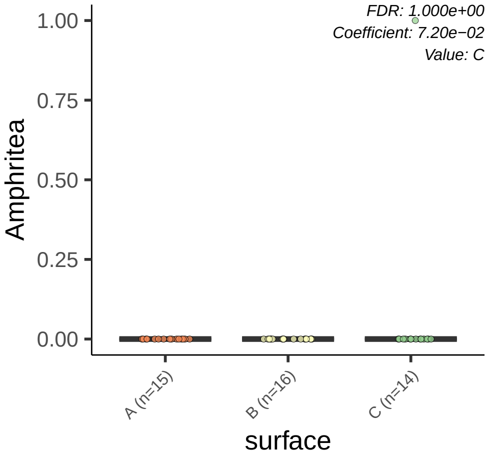
FDR: 1.000e+00
Coefficient: 7.20e−02
Value: C
1.00
0.75
0.50
0.25
0.00
Amphritea
A (n=15)
B (n=16)
C (n=14)
surface

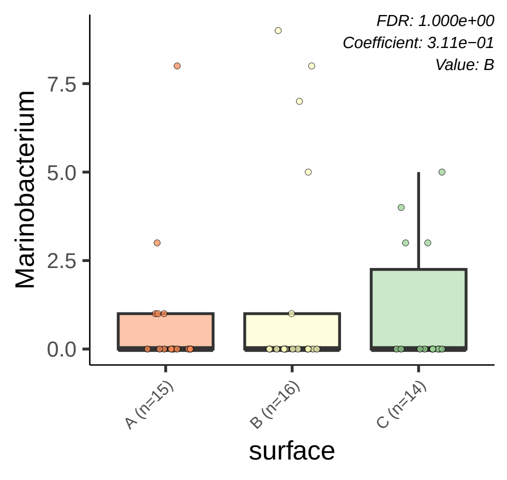
FDR: 1.000e+00
Coefficient: 3.11e−01
Value: B
Marinobacterium
7.5
5.0
2.5
0.0
A (n=15)
B (n=16)
C (n=14)
surface

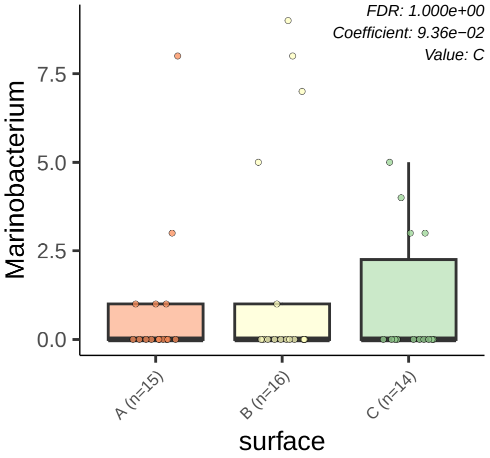
FDR: 1.000e+00
Coefficient: 9.36e−02
Value: C
Marinobacterium
7.5
5.0
2.5
0.0
A (n=15)
B (n=16)
C (n=14)
surface

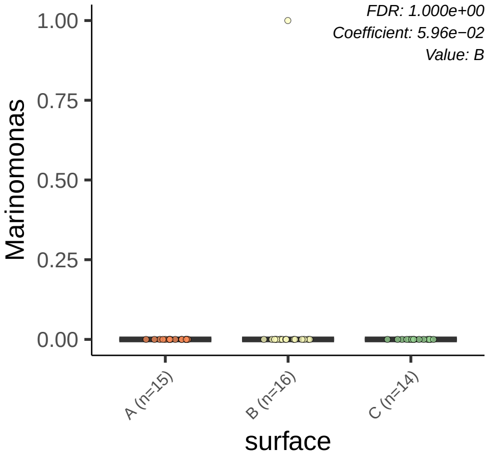
FDR: 1.000e+00
Coefficient: 5.96e−02
Value: B
Marinomonas
1.00
0.75
0.50
0.25
0.00
A (n=15)
B (n=16)
C (n=14)
surface

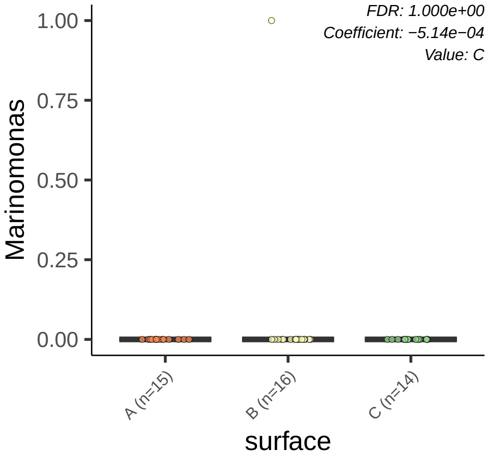
FDR: 1.000e+00
Coefficient: −5.14e−04
Value: C
Marinomonas
1.00
0.75
0.50
0.25
0.00
A (n=15)
B (n=16)
C (n=14)
surface

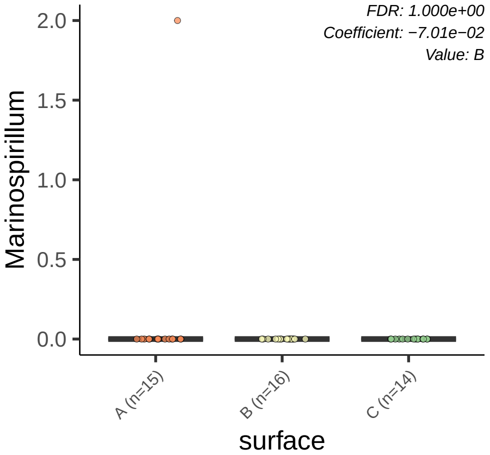
FDR: 1.000e+00
Coefficient: −7.01e−02
Value: B
Marinospirillum
2.0
1.5
1.0
0.5
0.0
A (n=15)
B (n=16)
C (n=14)
surface

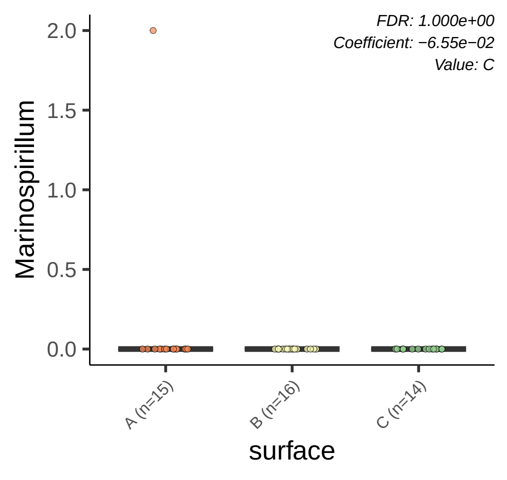
FDR: 1.000e+00
Coefficient: −6.55e−02
Value: C
Marinospirillum
2.0
1.5
1.0
0.5
0.0
A (n=15)
B (n=16)
C (n=14)
surface

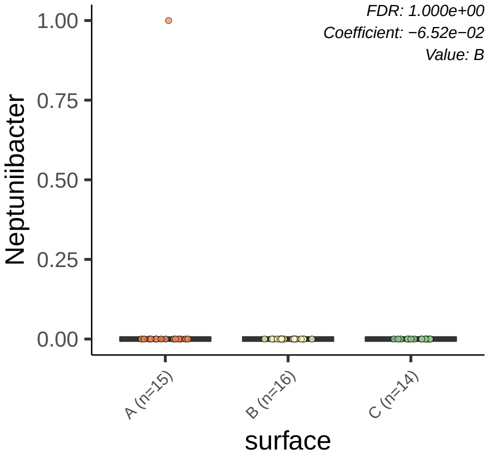
FDR: 1.000e+00
Coefficient: −6.52e−02
Value: B
Neptuniibacter
1.00
0.75
0.50
0.25
0.00
A (n=15)
B (n=16)
C (n=14)
surface

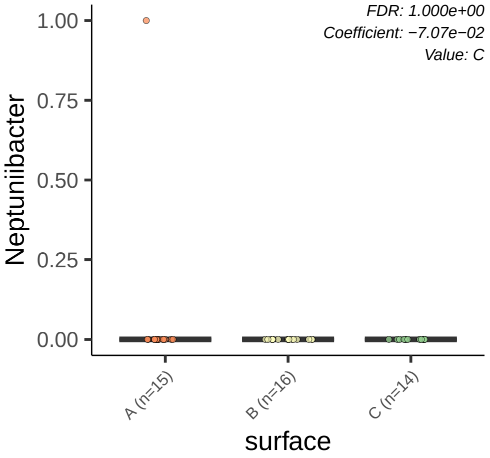
FDR: 1.000e+00
Coefficient: −7.07e−02
Value: C
Neptuniibacter
1.00
0.75
0.50
0.25
0.00
A (n=15)
B (n=16)
C (n=14)
surface

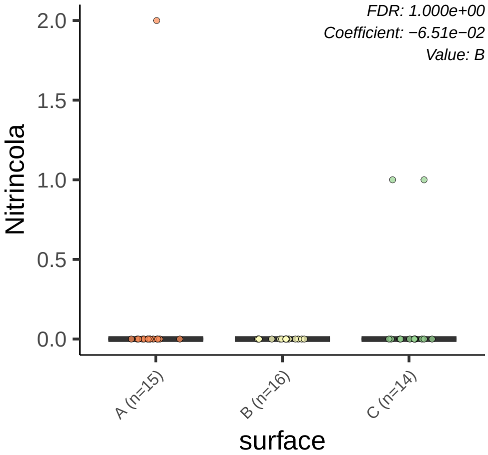
FDR: 1.000e+00
Coefficient: −6.51e−02
Value: B
Nitrincola
2.0
1.5
1.0
0.5
0.0
A (n=15)
B (n=16)
C (n=14)
surface

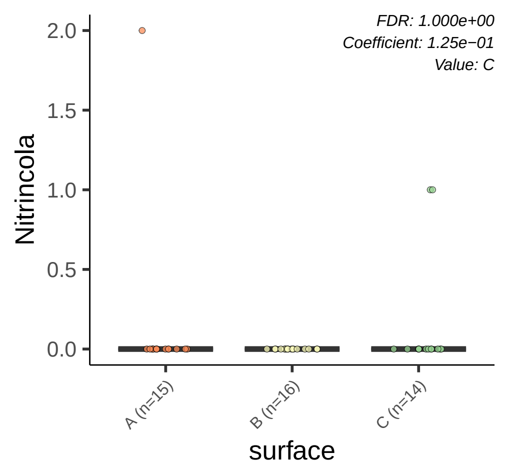

FDR: 1.000e+00
Coefficient: 1.25e−01
Value: C
Nitrincola
2.0
1.5
1.0
0.5
0.0
A (n=15)
B (n=16)
C (n=14)
surface

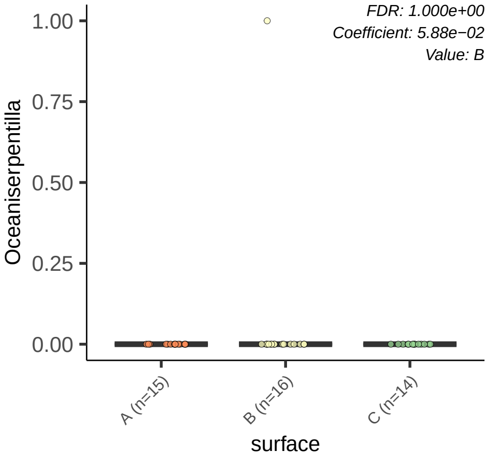
FDR: 1.000e+00
Coefficient: 5.88e−02
Value: B
Oceaniserpentilla
1.00
0.75
0.50
0.25
0.00
A (n=15)
B (n=16)
C (n=14)
surface

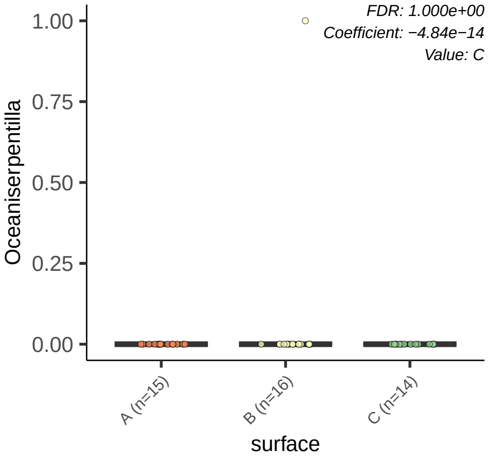

FDR: 1.000e+00
Coefficient: −4.84e−14
Value: C
1.00
0.75
0.50
0.25
0.00
Oceaniserpentilla
A (n=15)
B (n=16)
C (n=14)
surface

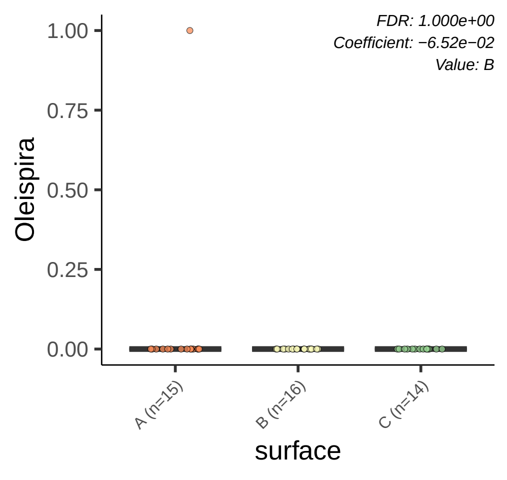
FDR: 1.000e+00
Coefficient: −6.52e−02
Value: B
Oleispira
1.00
0.75
0.50
0.25
0.00
A (n=15)
B (n=16)
C (n=14)
surface

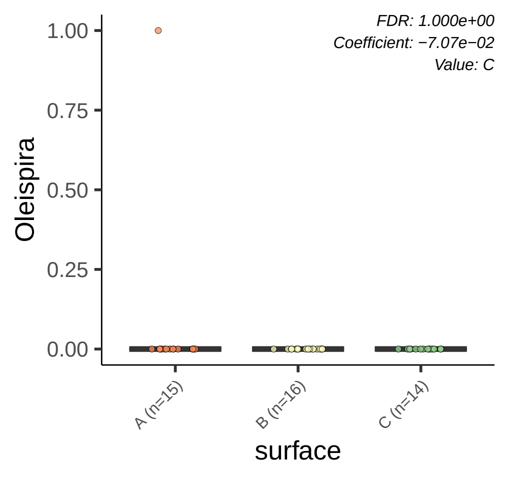

FDR: 1.000e+00
Coefficient: −7.07e−02
Value: C
1.00
0.75
0.50
0.25
0.00
Oleispira
A (n=15)
B (n=16)
C (n=14)
surface

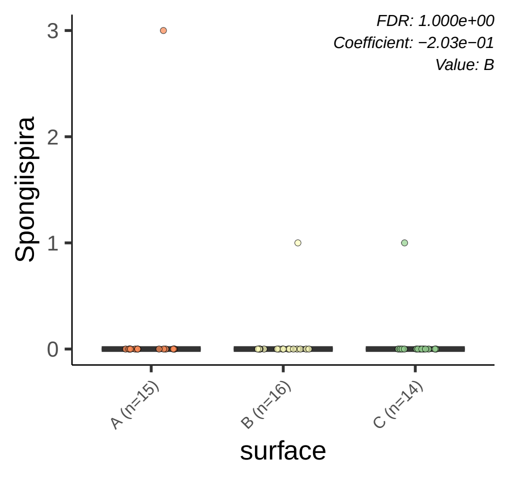
FDR: 1.000e+00
Coefficient: −2.03e−01
Value: B
Spongiispira
3
2
1
0
A (n=15)
B (n=16)
C (n=14)
surface

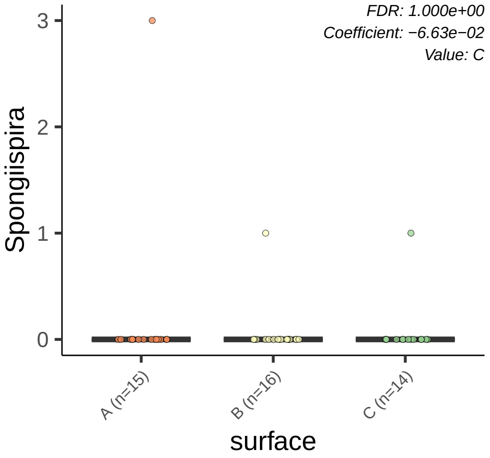
FDR: 1.000e+00
Coefficient: −6.63e−02
Value: C
Spongiispira
3
2
1
0
A (n=15)
B (n=16)
C (n=14)
surface

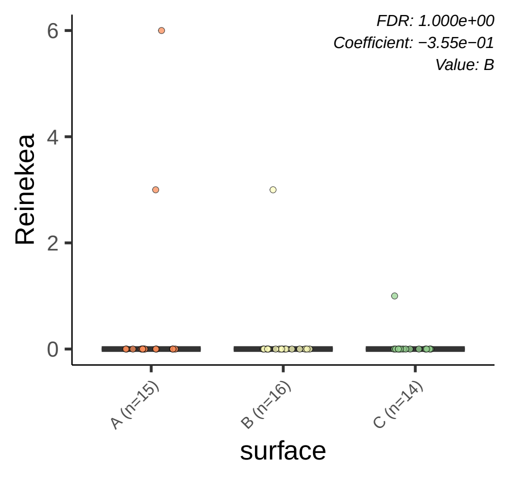

FDR: 1.000e+00
Coefficient: −3.55e−01
Value: B
Reinekea
0
2
4
6
A (n=15)
B (n=16)
C (n=14)
surface

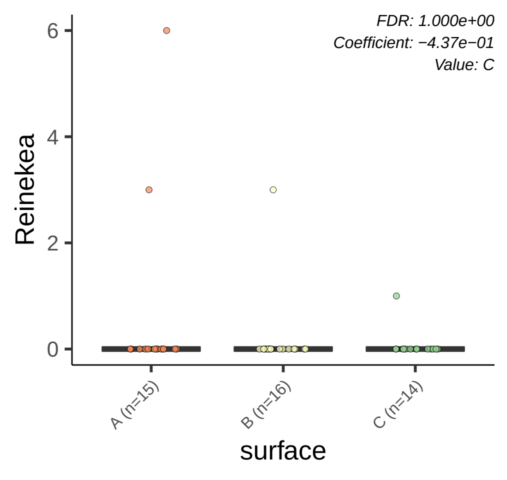
FDR: 1.000e+00
Coefficient: −4.37e−01
Value: C
Reinekea
6
4
2
0
A (n=15)
B (n=16)
C (n=14)
surface

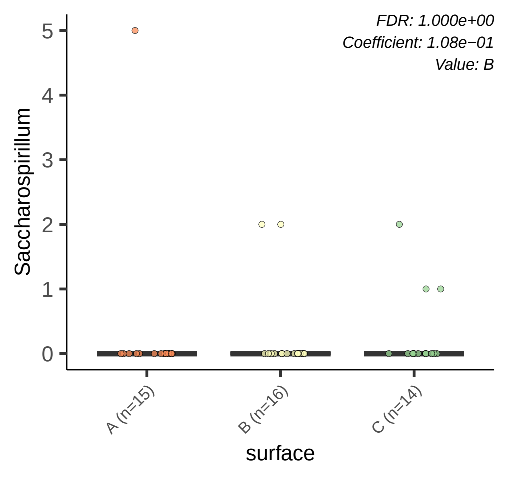
FDR: 1.000e+00
Coefficient: 1.08e−01
Value: B
Saccharospirillum
0
1
2
3
4
5
A (n=15)
B (n=16)
C (n=14)
surface

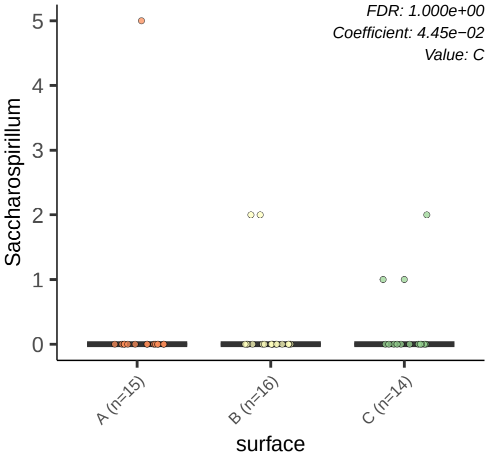

FDR: 1.000e+00
Coefficient: 4.45e−02
Value: C
Saccharospirillum
5
4
3
2
1
0
A (n=15)
B (n=16)
C (n=14)
surface

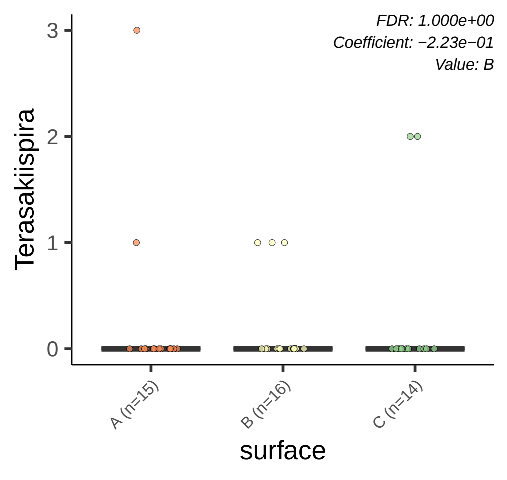

FDR: 1.000e+00
Coefficient: −2.23e−01
Value: B
Terasakiispira
3
2
1
0
A (n=15)
B (n=16)
C (n=14)
surface

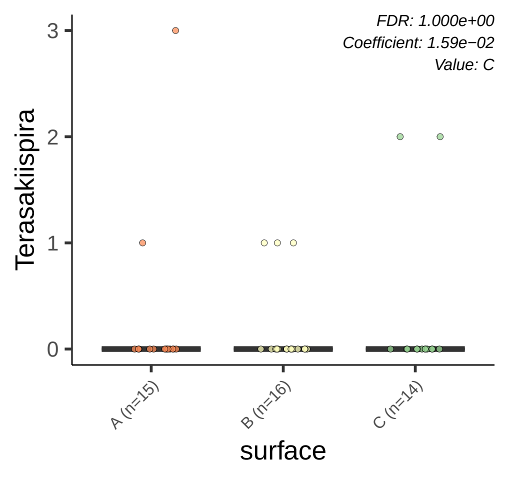
FDR: 1.000e+00
Coefficient: 1.59e−02
Value: C
Terasakiispira
0
1
2
3
A (n=15)
B (n=16)
C (n=14)
surface

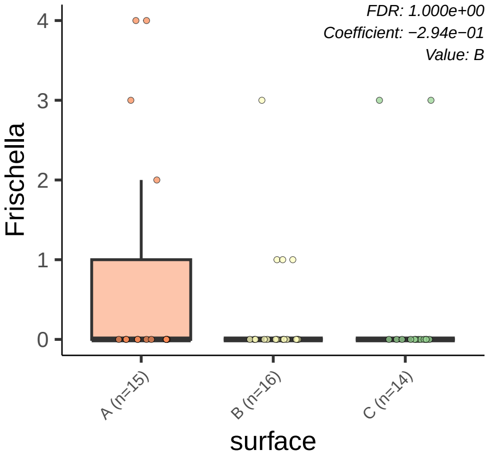

FDR: 1.000e+00
Coefficient: −2.94e−01
Value: B
Frischella
4
3
2
1
0
A (n=15)
B (n=16)
C (n=14)
surface

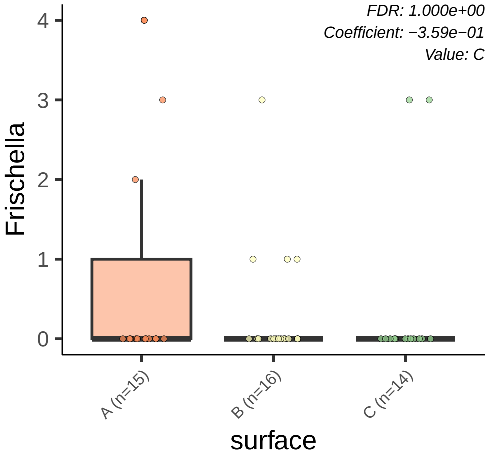
FDR: 1.000e+00
Coefficient: −3.59e−01
Value: C
Frischella
0
1
2
3
4
A (n=15)
B (n=16)
C (n=14)
surface

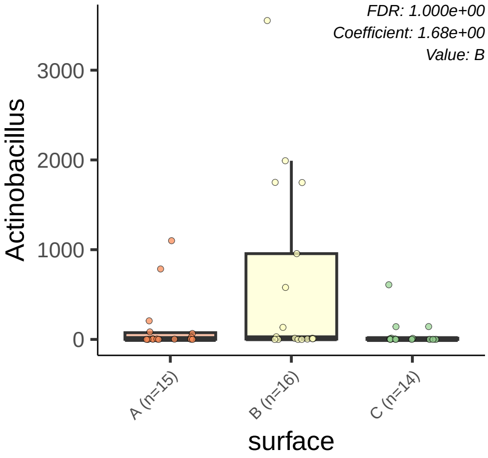

FDR: 1.000e+00
Coefficient: 1.68e+00
Value: B
Actinobacillus
3000
2000
1000
0
A (n=15)
B (n=16)
C (n=14)
surface

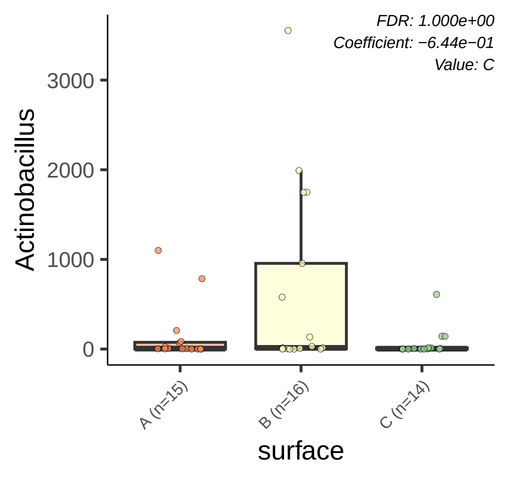
FDR: 1.000e+00
Coefficient: −6.44e−01
Value: C
Actinobacillus
3000
2000
1000
0
A (n=15)
B (n=16)
C (n=14)
surface

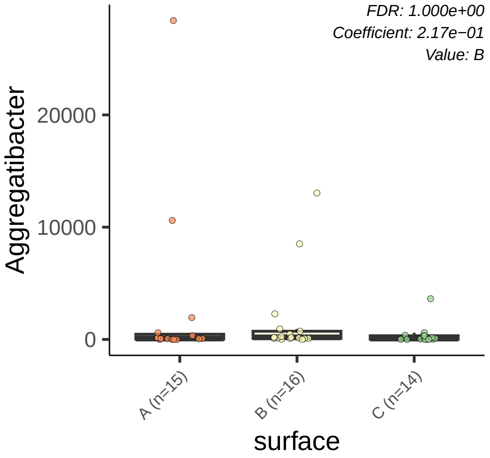
FDR: 1.000e+00
Coefficient: 2.17e−01
Value: B
Aggregatibacter
20000
10000
0
A (n=15)
B (n=16)
C (n=14)
surface

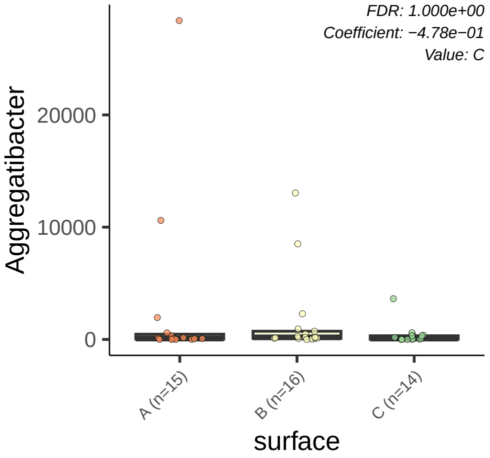

FDR: 1.000e+00
Coefficient: −4.78e−01
Value: C
Aggregatibacter
20000
10000
0
A (n=15)
B (n=16)
C (n=14)
surface

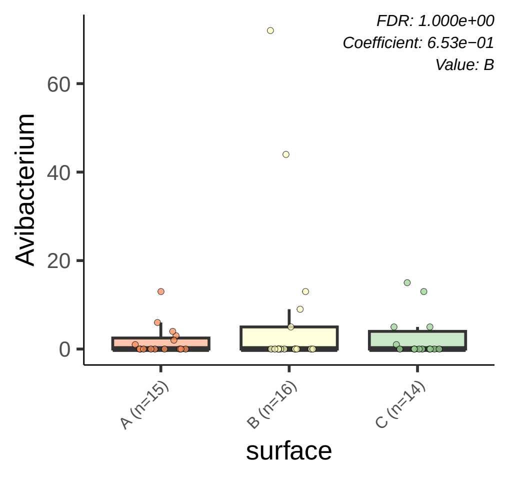

FDR: 1.000e+00
Coefficient: 6.53e−01
Value: B
Avibacterium
0
20
40
60
A (n=15)
B (n=16)
C (n=14)
surface

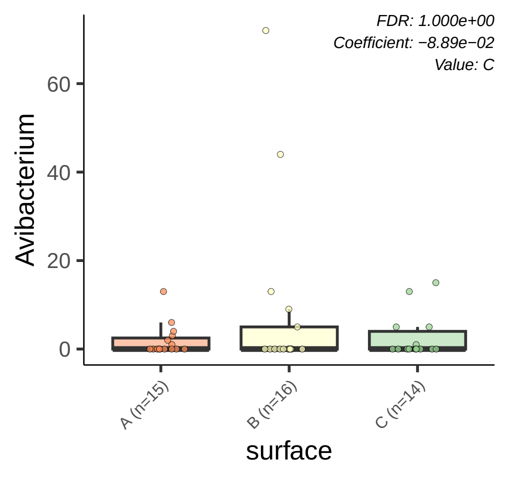
FDR: 1.000e+00
Coefficient: −8.89e−02
Value: C
Avibacterium
60
40
20
0
A (n=15)
B (n=16)
C (n=14)
surface

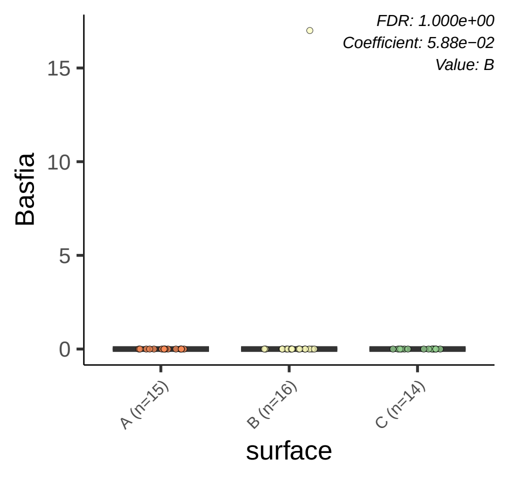

FDR: 1.000e+00
Coefficient: 5.88e−02
Value: B
Basfia
0
5
10
15
A (n=15)
B (n=16)
C (n=14)
surface

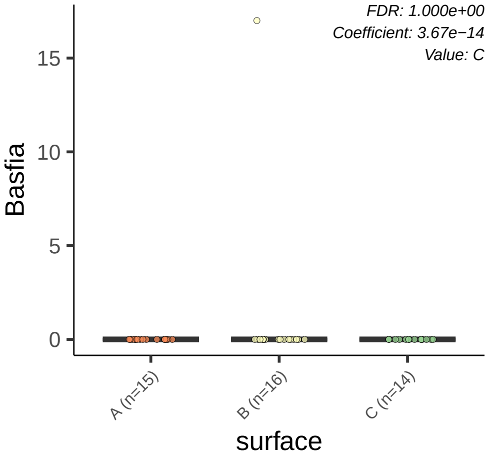
FDR: 1.000e+00
Coefficient: 3.67e−14
Value: C
Basfia
15
10
5
0
A (n=15)
B (n=16)
C (n=14)
surface

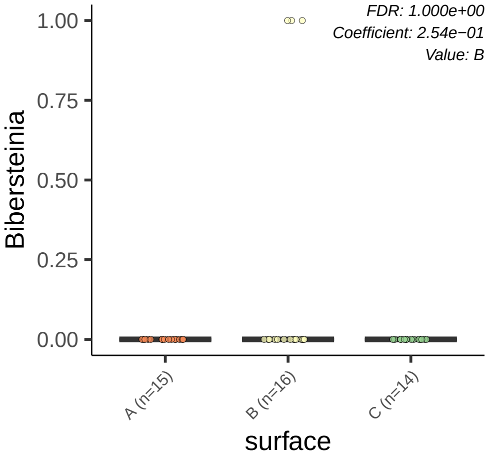
FDR: 1.000e+00
Coefficient: 2.54e−01
Value: B
Bibersteinia
1.00
0.75
0.50
0.25
0.00
A (n=15)
B (n=16)
C (n=14)
surface

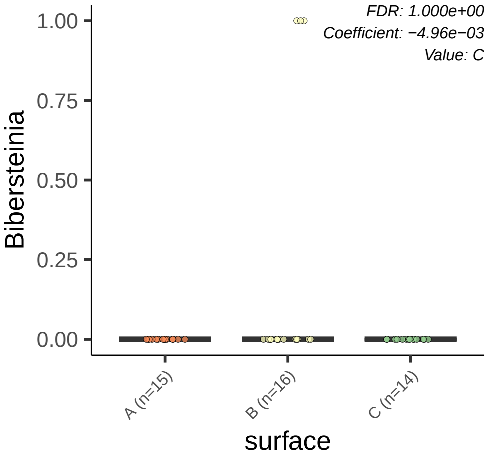
FDR: 1.000e+00
Coefficient: −4.96e−03
Value: C
Bibersteinia
1.00
0.75
0.50
0.25
0.00
A (n=15)
B (n=16)
C (n=14)
surface

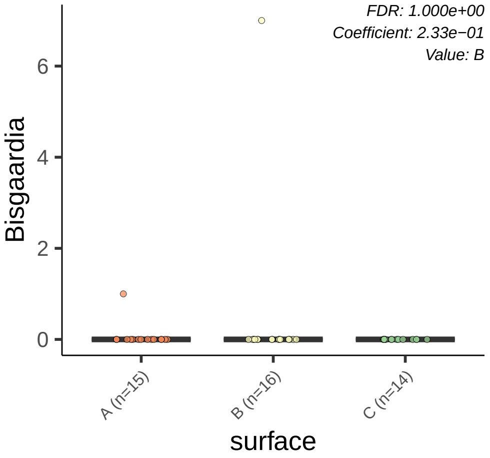
FDR: 1.000e+00
Coefficient: 2.33e−01
Value: B
Bisgaardia
0
2
4
6
A (n=15)
B (n=16)
C (n=14)
surface

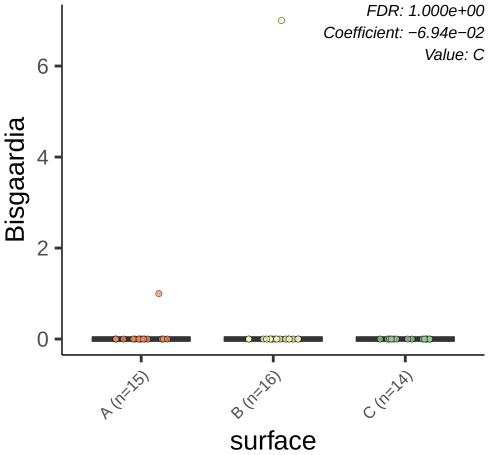
FDR: 1.000e+00
Coefficient: −6.94e−02
Value: C
Bisgaardia
0
2
4
6
A (n=15)
B (n=16)
C (n=14)
surface

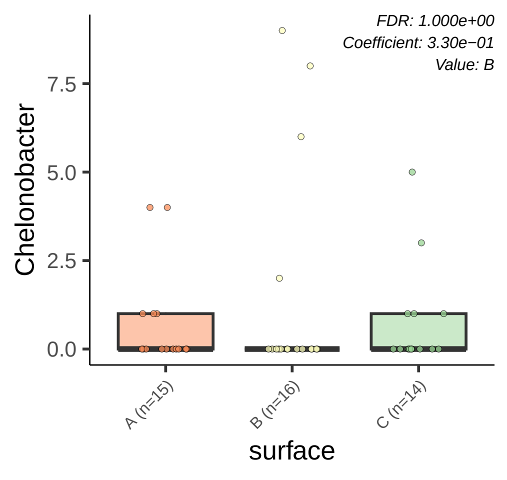
FDR: 1.000e+00
Coefficient: 3.30e−01
Value: B
Chelonobacter
7.5
5.0
2.5
0.0
A (n=15)
B (n=16)
C (n=14)
surface

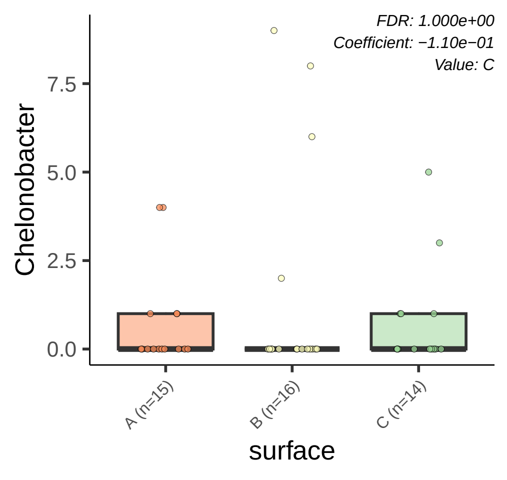
FDR: 1.000e+00
Coefficient: −1.10e−01
Value: C
Chelonobacter
7.5
5.0
2.5
0.0
A (n=15)
B (n=16)
C (n=14)
surface

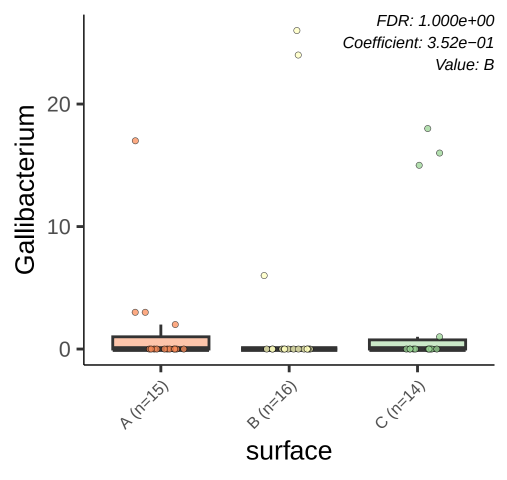
FDR: 1.000e+00
Coefficient: 3.52e−01
Value: B
Gallibacterium
20
10
0
A (n=15)
B (n=16)
C (n=14)
surface

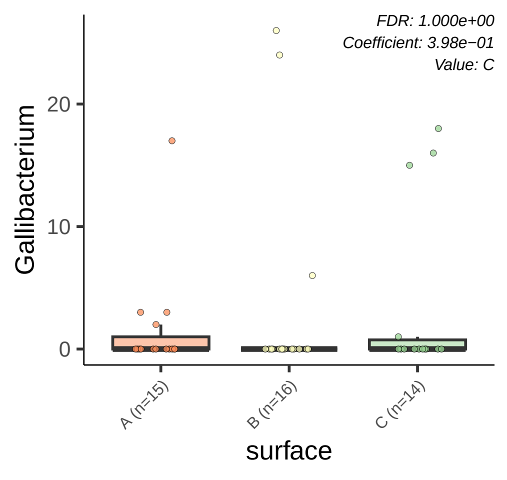

FDR: 1.000e+00
Coefficient: 3.98e−01
Value: C
Gallibacterium
20
10
0
A (n=15)
B (n=16)
C (n=14)
surface

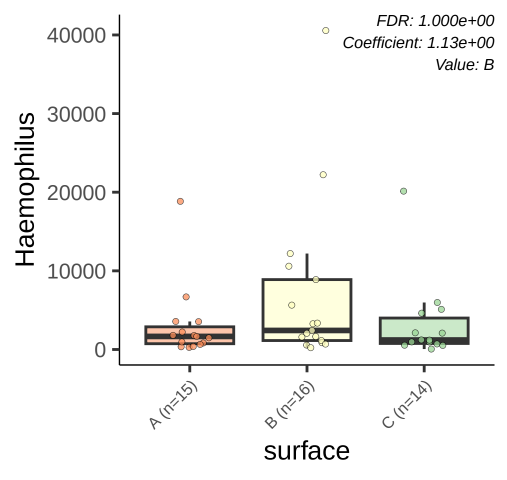

FDR: 1.000e+00
Coefficient: 1.13e+00
Value: B
40000
30000
20000
10000
0
Haemophilus
A (n=15)
B (n=16)
C (n=14)
surface

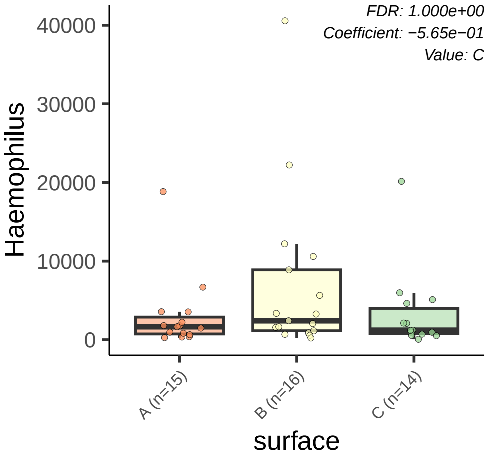

FDR: 1.000e+00
Coefficient: −5.65e−01
Value: C
Haemophilus
40000
30000
20000
10000
0
A (n=15)
B (n=16)
C (n=14)
surface

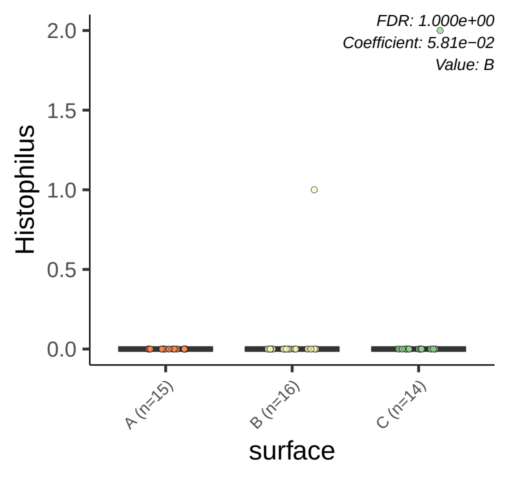
FDR: 1.000e+00
Coefficient: 5.81e−02
Value: B
Histophilus
2.0
1.5
1.0
0.5
0.0
A (n=15)
B (n=16)
C (n=14)
surface

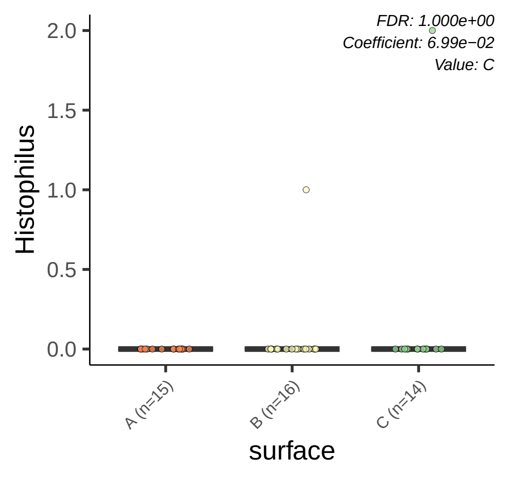
FDR: 1.000e+00
Coefficient: 6.99e−02
Value: C
Histophilus
2.0
1.5
1.0
0.5
0.0
A (n=15)
B (n=16)
C (n=14)
surface

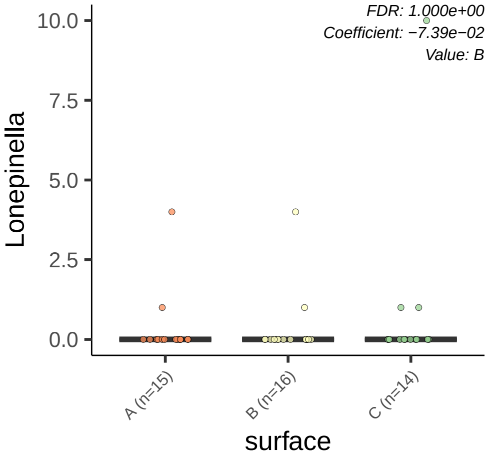

FDR: 1.000e+00
Coefficient: −7.39e−02
Value: B
Lonepinella
10.0
7.5
5.0
2.5
0.0
A (n=15)
B (n=16)
C (n=14)
surface

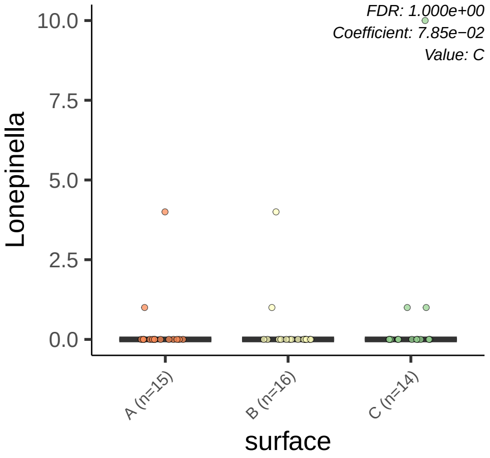

FDR: 1.000e+00
Coefficient: 7.85e−02
Value: C
10.0
7.5
5.0
2.5
0.0
Lonepinella
A (n=15)
B (n=16)
C (n=14)
surface

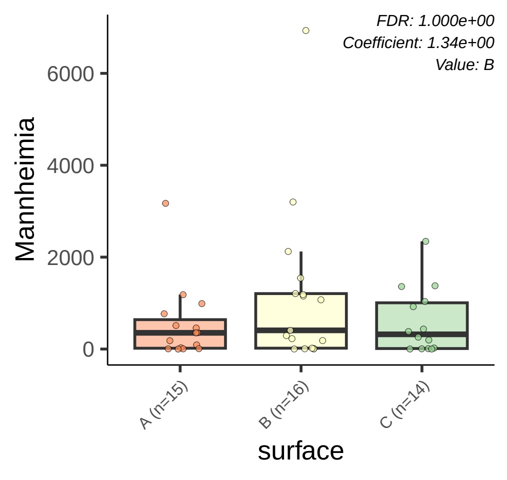
FDR: 1.000e+00
Coefficient: 1.34e+00
Value: B
Mannheimia
6000
4000
2000
0
A (n=15)
B (n=16)
C (n=14)
surface

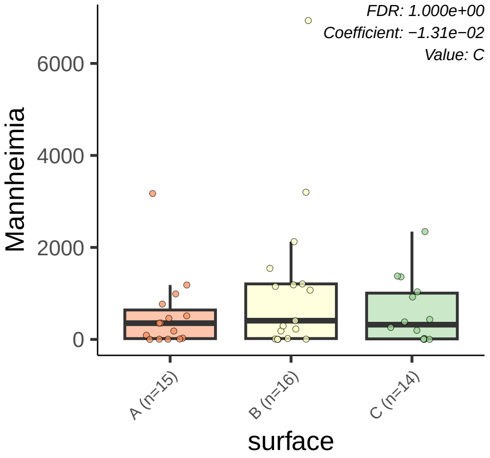
FDR: 1.000e+00
Coefficient: −1.31e−02
Value: C
Mannheimia
6000
4000
2000
0
A (n=15)
B (n=16)
C (n=14)
surface

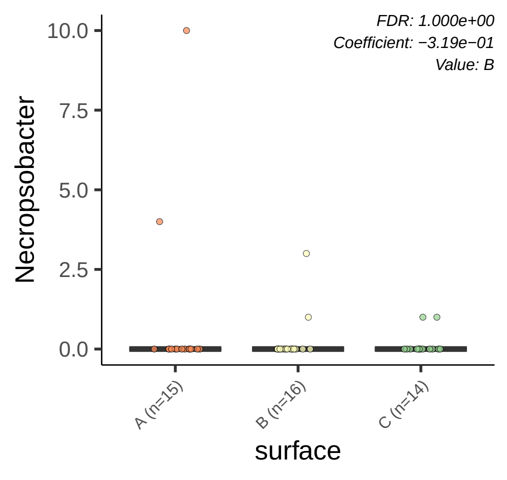

FDR: 1.000e+00
Coefficient: −3.19e−01
Value: B
Necropsobacter
10.0
7.5
5.0
2.5
0.0
A (n=15)
B (n=16)
C (n=14)
surface

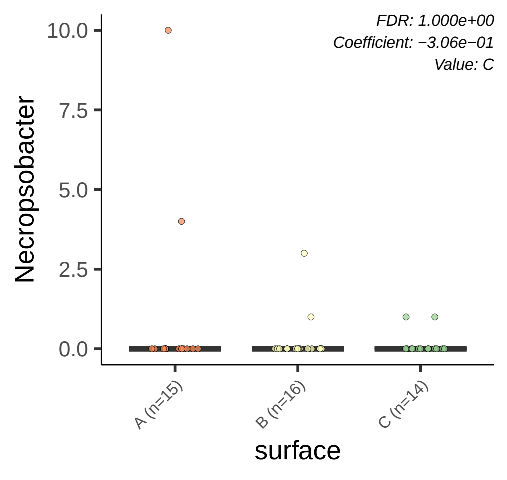

FDR: 1.000e+00
Coefficient: −3.06e−01
Value: C
Necropsobacter
10.0
7.5
5.0
2.5
0.0
A (n=15)
B (n=16)
C (n=14)
surface

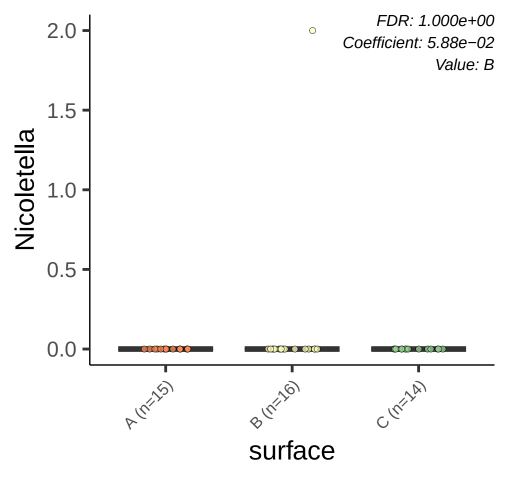
FDR: 1.000e+00
Coefficient: 5.88e−02
Value: B
Nicoletella
2.0
1.5
1.0
0.5
0.0
A (n=15)
B (n=16)
C (n=14)
surface

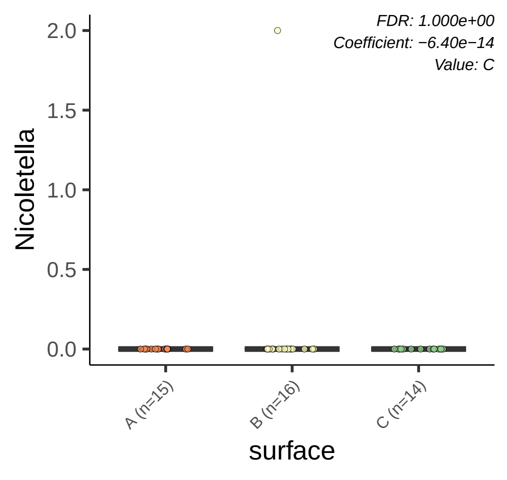
FDR: 1.000e+00
Coefficient: −6.40e−14
Value: C
Nicoletella
2.0
1.5
1.0
0.5
0.0
A (n=15)
B (n=16)
C (n=14)
surface

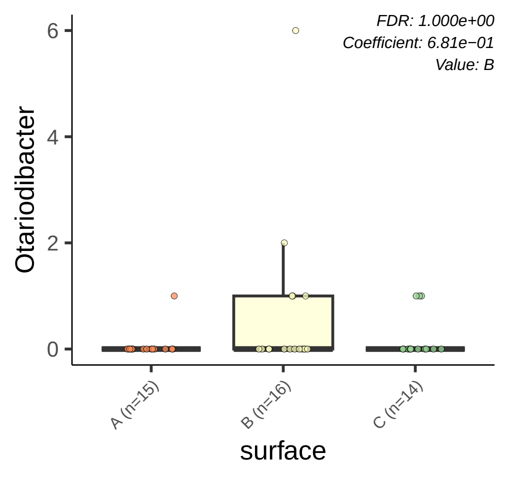

FDR: 1.000e+00
Coefficient: 6.81e−01
Value: B
Otariodibacter
0
2
4
6
A (n=15)
B (n=16)
C (n=14)
surface

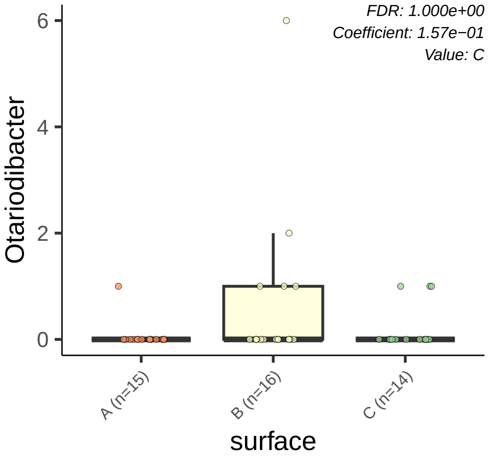

FDR: 1.000e+00
Coefficient: 1.57e−01
Value: C
Otariodibacter
0
2
4
6
A (n=15)
B (n=16)
C (n=14)
surface

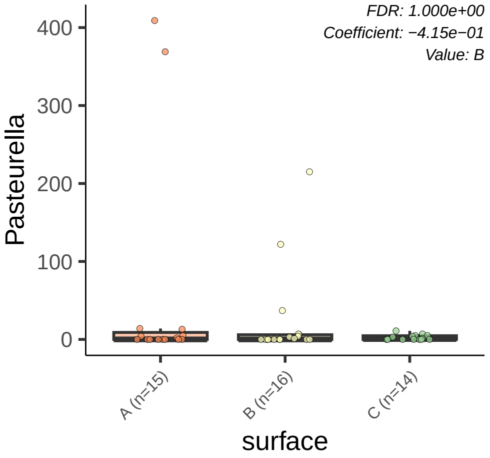
FDR: 1.000e+00
Coefficient: −4.15e−01
Value: B
Pasteurella
400
300
200
100
0
A (n=15)
B (n=16)
C (n=14)
surface

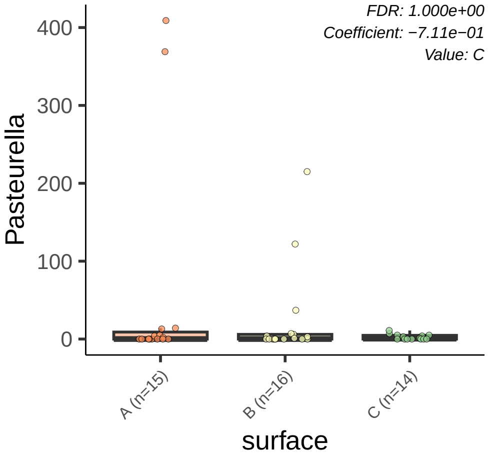

FDR: 1.000e+00
Coefficient: −7.11e−01
Value: C
Pasteurella
400
300
200
100
0
A (n=15)
B (n=16)
C (n=14)
surface

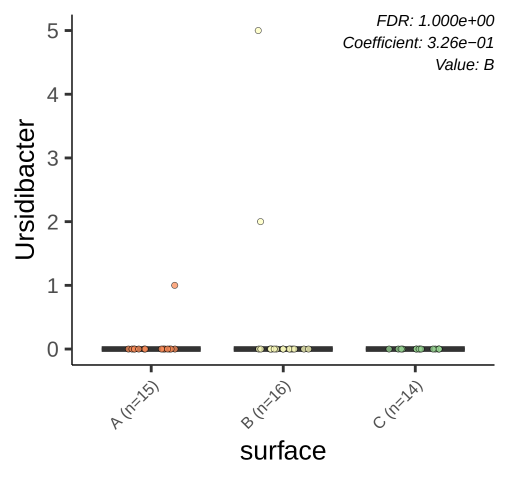
FDR: 1.000e+00
Coefficient: 3.26e−01
Value: B
Ursidibacter
5
4
3
2
1
0
A (n=15)
B (n=16)
C (n=14)
surface

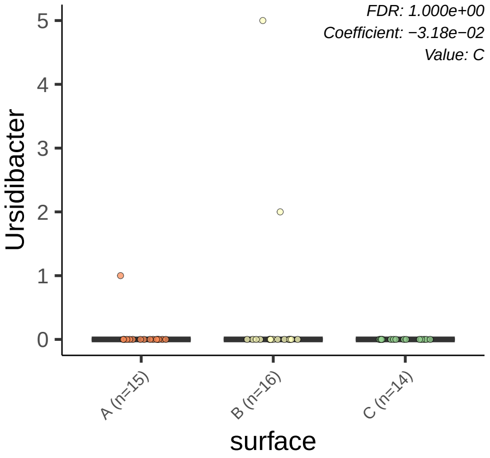
FDR: 1.000e+00
Coefficient: −3.18e−02
Value: C
Ursidibacter
0
1
2
3
4
5
A (n=15)
B (n=16)
C (n=14)
surface

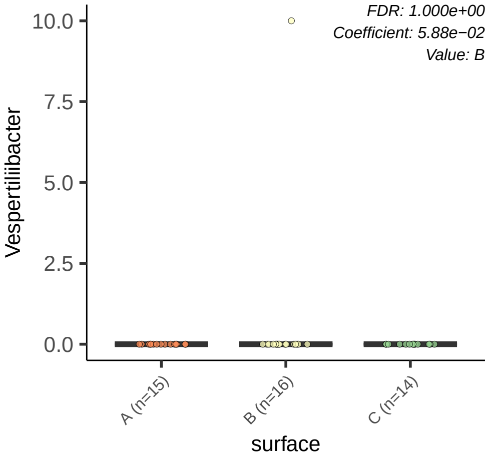
FDR: 1.000e+00
Coefficient: 5.88e−02
Value: B
10.0
7.5
5.0
2.5
0.0
Vespertiliibacter
A (n=15)
B (n=16)
C (n=14)
surface

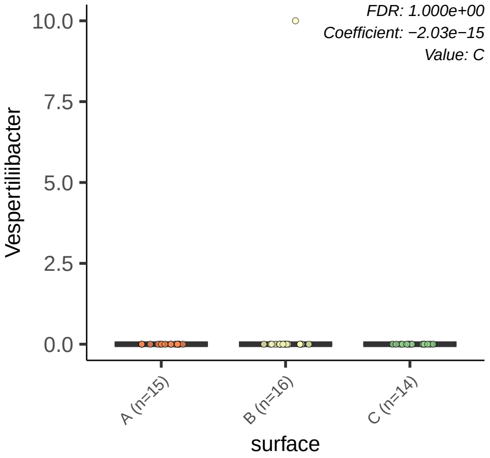

FDR: 1.000e+00
Coefficient: −2.03e−15
Value: C
10.0
7.5
5.0
2.5
0.0
Vespertiliibacter
A (n=15)
B (n=16)
C (n=14)
surface

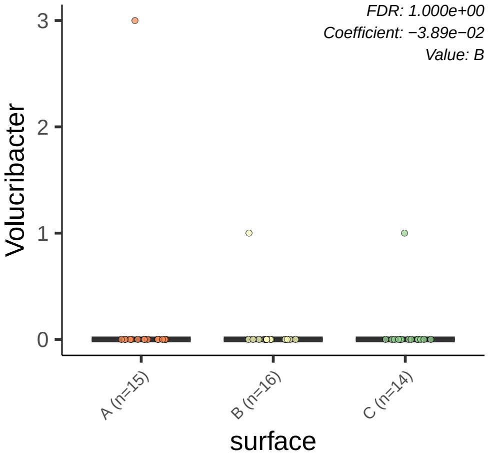
FDR: 1.000e+00
Coefficient: −3.89e−02
Value: B
Volucribacter
3
2
1
0
A (n=15)
B (n=16)
C (n=14)
surface

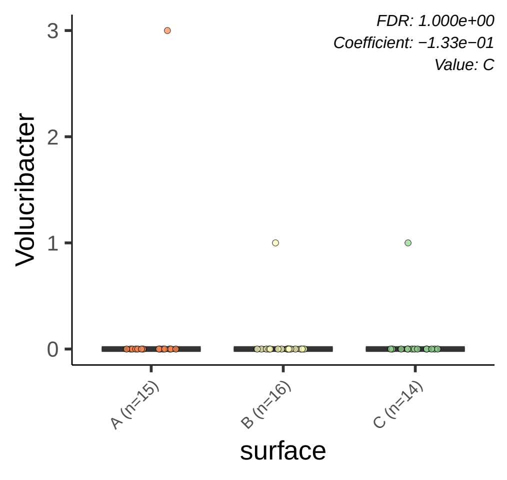
FDR: 1.000e+00
Coefficient: −1.33e−01
Value: C
Volucribacter
3
2
1
0
A (n=15)
B (n=16)
C (n=14)
surface

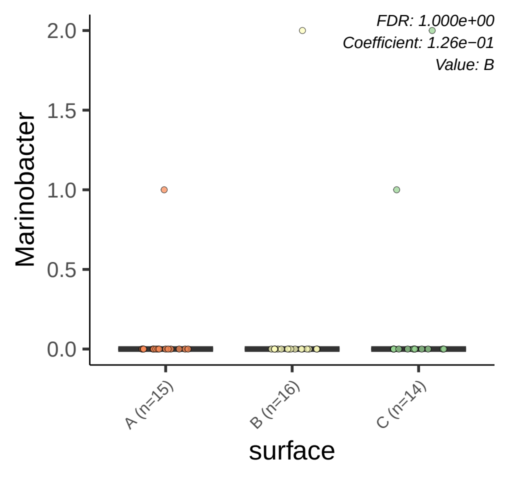
FDR: 1.000e+00
Coefficient: 1.26e−01
Value: B
Marinobacter
2.0
1.5
1.0
0.5
0.0
A (n=15)
B (n=16)
C (n=14)
surface

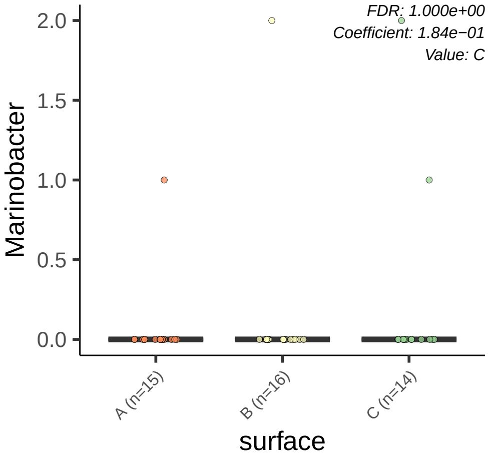
FDR: 1.000e+00
Coefficient: 1.84e−01
Value: C
Marinobacter
2.0
1.5
1.0
0.5
0.0
A (n=15)
B (n=16)
C (n=14)
surface

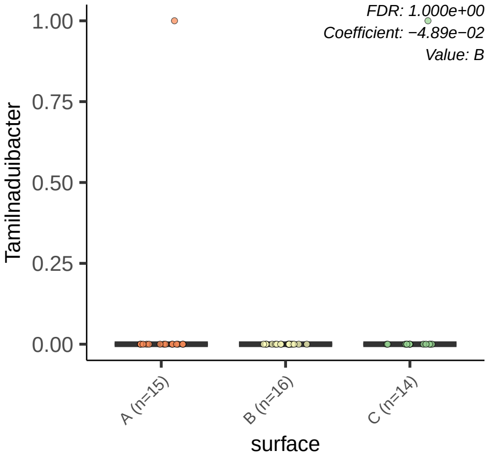
FDR: 1.000e+00
Coefficient: −4.89e−02
Value: B
Tamilnaduibacter
1.00
0.75
0.50
0.25
0.00
A (n=15)
B (n=16)
C (n=14)
surface

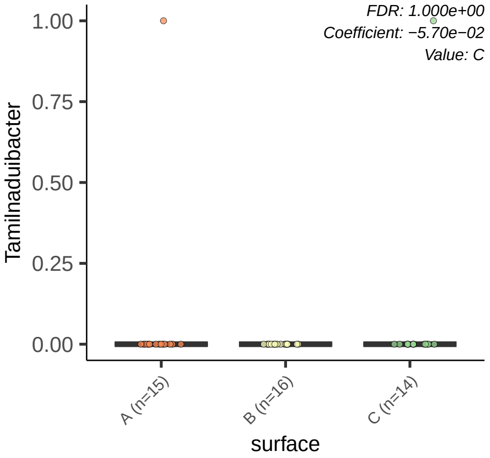
FDR: 1.000e+00
Coefficient: −5.70e−02
Value: C
Tamilnaduibacter
1.00
0.75
0.50
0.25
0.00
A (n=15)
B (n=16)
C (n=14)
surface

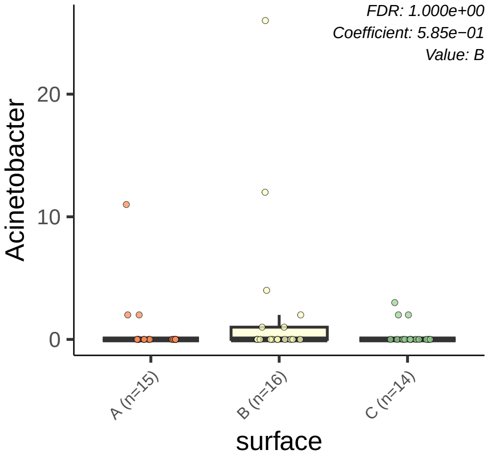
FDR: 1.000e+00
Coefficient: 5.85e−01
Value: B
Acinetobacter
0
10
20
A (n=15)
B (n=16)
C (n=14)
surface

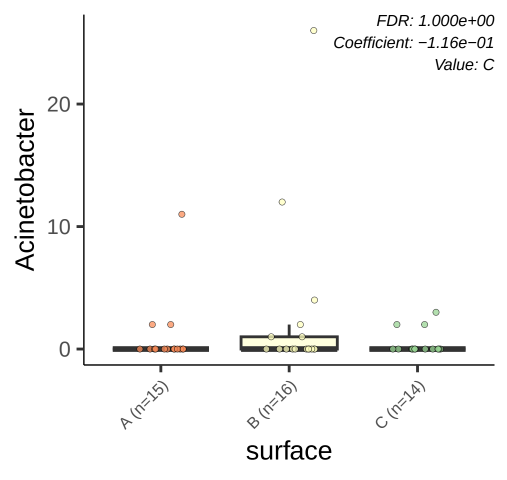

FDR: 1.000e+00
Coefficient: −1.16e−01
Value: C
Acinetobacter
20
10
0
A (n=15)
B (n=16)
C (n=14)
surface

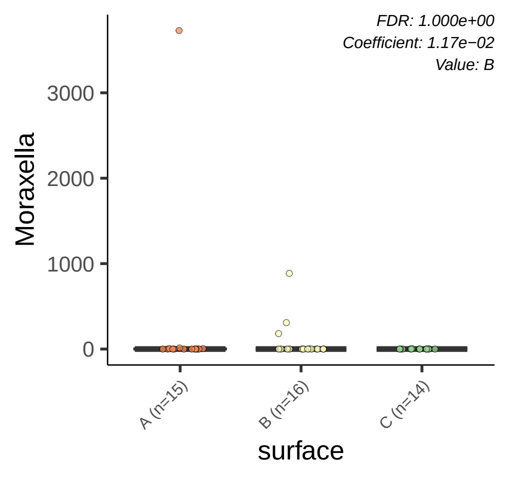
FDR: 1.000e+00
Coefficient: 1.17e−02
Value: B
Moraxella
3000
2000
1000
0
A (n=15)
B (n=16)
C (n=14)
surface

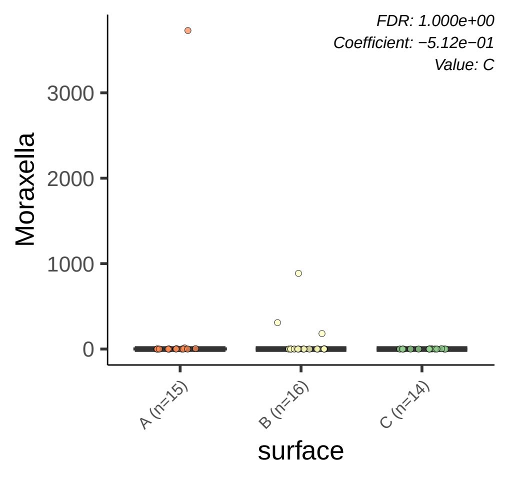
FDR: 1.000e+00
Coefficient: −5.12e−01
Value: C
Moraxella
3000
2000
1000
0
A (n=15)
B (n=16)
C (n=14)
surface

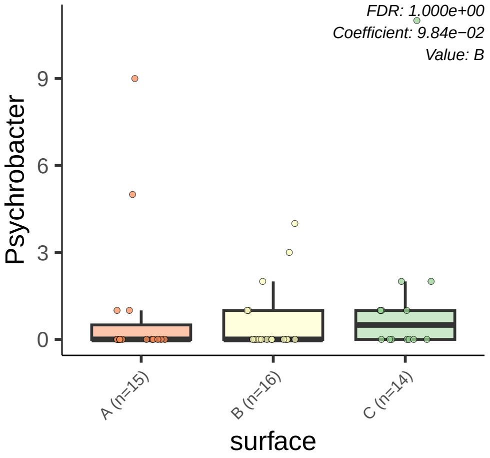

FDR: 1.000e+00
Coefficient: 9.84e−02
Value: B
Psychrobacter
9
6
3
0
A (n=15)
B (n=16)
C (n=14)
surface

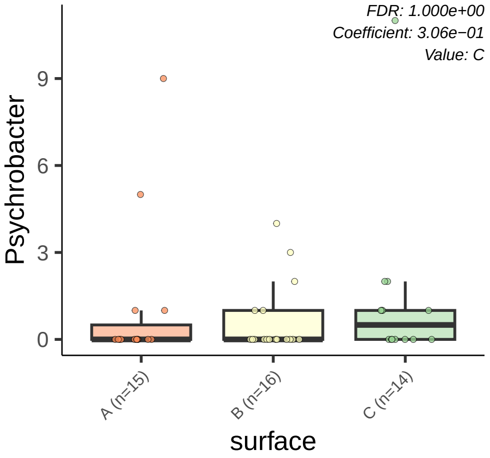

FDR: 1.000e+00
Coefficient: 3.06e−01
Value: C
Psychrobacter
9
6
3
0
A (n=15)
B (n=16)
C (n=14)
surface

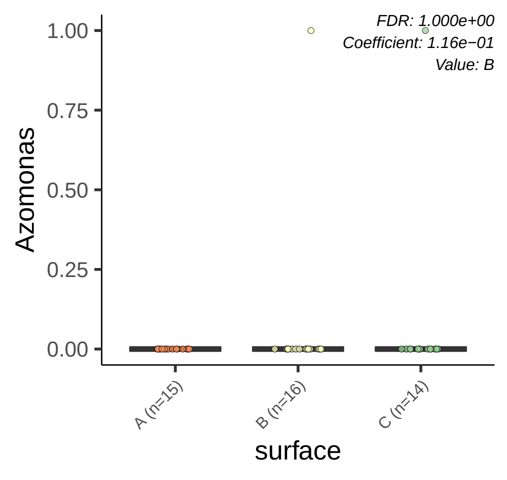

FDR: 1.000e+00
Coefficient: 1.16e−01
Value: B
Azomonas
1.00
0.75
0.50
0.25
0.00
A (n=15)
B (n=16)
C (n=14)
surface

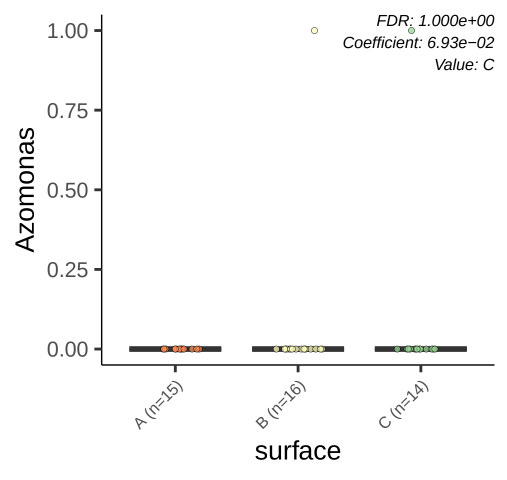
FDR: 1.000e+00
Coefficient: 6.93e−02
Value: C
Azomonas
1.00
0.75
0.50
0.25
0.00
A (n=15)
B (n=16)
C (n=14)
surface

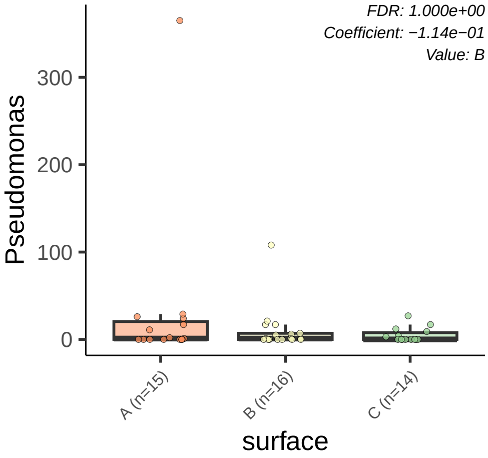
FDR: 1.000e+00
Coefficient: −1.14e−01
Value: B
Pseudomonas
300
200
100
0
A (n=15)
B (n=16)
C (n=14)
surface

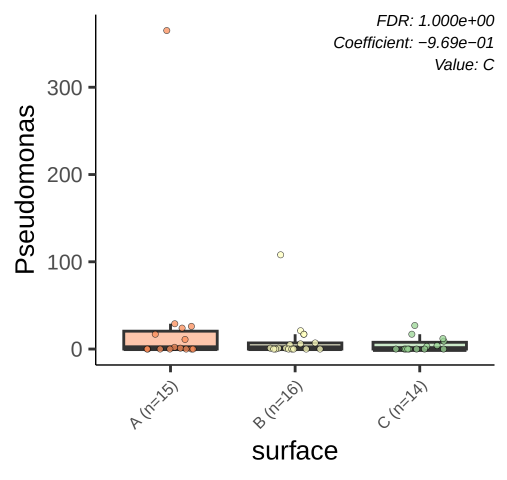
FDR: 1.000e+00
Coefficient: −9.69e−01
Value: C
Pseudomonas
300
200
100
0
A (n=15)
B (n=16)
C (n=14)
surface

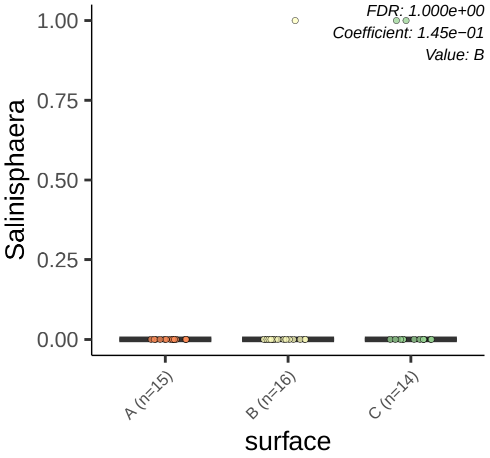
FDR: 1.000e+00
Coefficient: 1.45e−01
Value: B
Salinisphaera
1.00
0.75
0.50
0.25
0.00
A (n=15)
B (n=16)
C (n=14)
surface

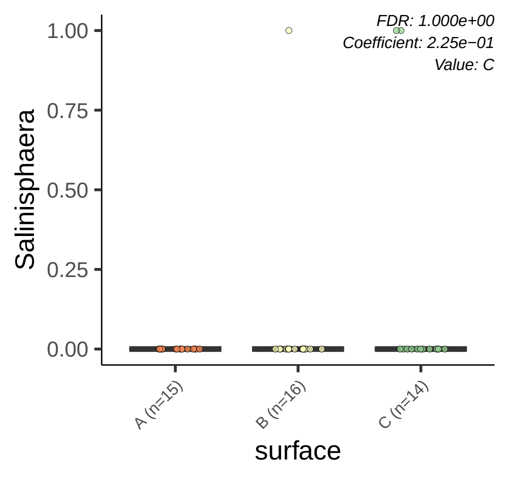
FDR: 1.000e+00
Coefficient: 2.25e−01
Value: C
Salinisphaera
1.00
0.75
0.50
0.25
0.00
A (n=15)
B (n=16)
C (n=14)
surface

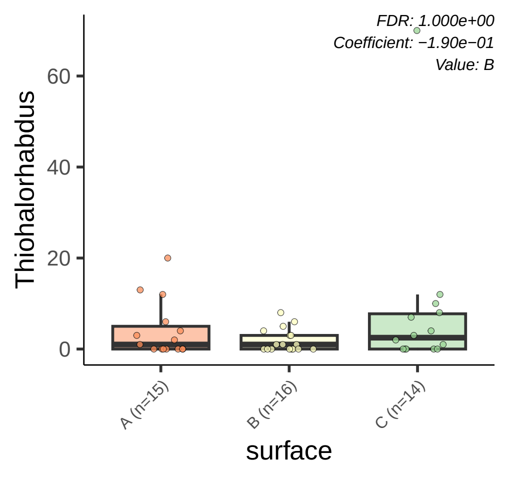

FDR: 1.000e+00
Coefficient: −1.90e−01
Value: B
Thiohalorhabdus
0
20
40
60
A (n=15)
B (n=16)
C (n=14)
surface

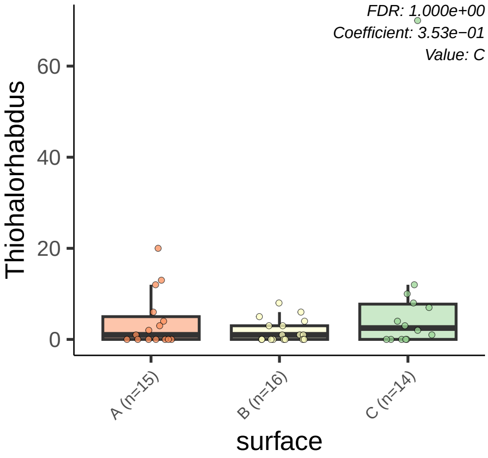
FDR: 1.000e+00
Coefficient: 3.53e−01
Value: C
Thiohalorhabdus
60
40
20
0
A (n=15)
B (n=16)
C (n=14)
surface

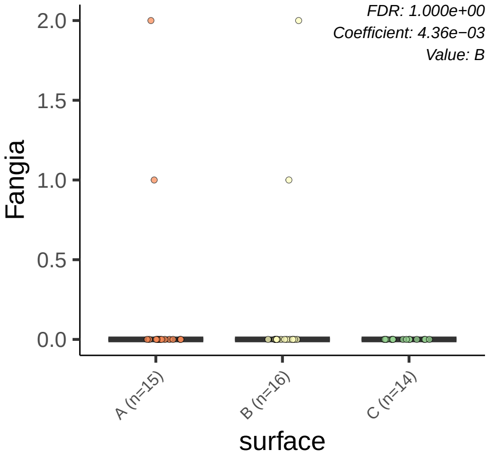

FDR: 1.000e+00
Coefficient: 4.36e−03
Value: B
Fangia
2.0
1.5
1.0
0.5
0.0
A (n=15)
B (n=16)
C (n=14)
surface

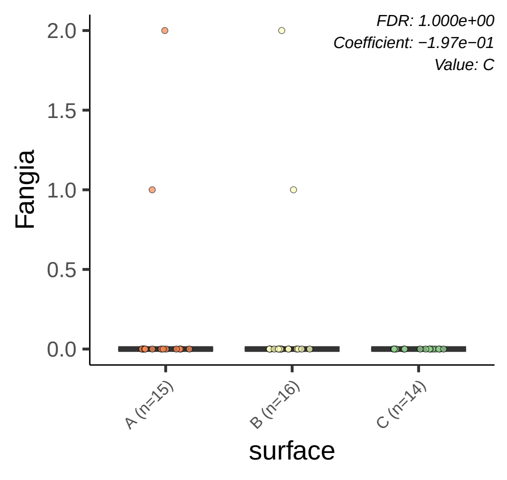
FDR: 1.000e+00
Coefficient: −1.97e−01
Value: C
Fangia
2.0
1.5
1.0
0.5
0.0
A (n=15)
B (n=16)
C (n=14)
surface

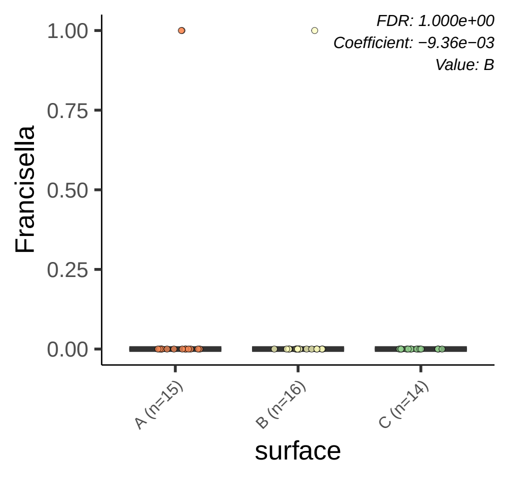
FDR: 1.000e+00
Coefficient: −9.36e−03
Value: B
Francisella
1.00
0.75
0.50
0.25
0.00
A (n=15)
B (n=16)
C (n=14)
surface

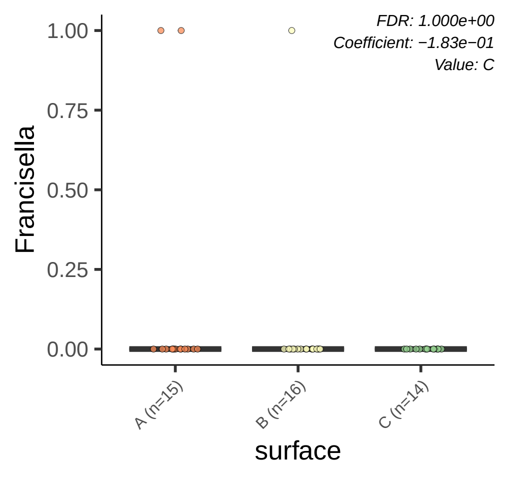
FDR: 1.000e+00
Coefficient: −1.83e−01
Value: C
1.00
0.75
0.50
0.25
0.00
Francisella
A (n=15)
B (n=16)
C (n=14)
surface

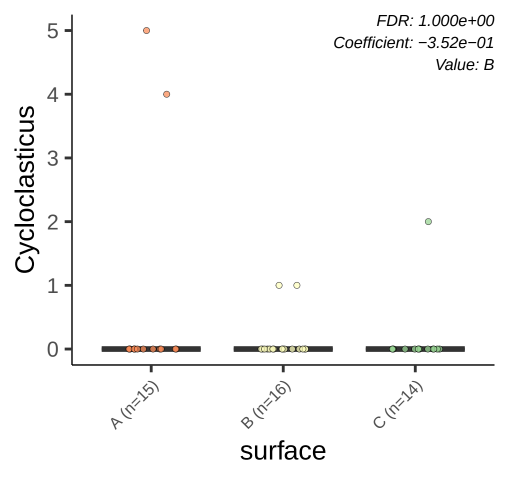

FDR: 1.000e+00
Coefficient: −3.52e−01
Value: B
Cycloclasticus
0
1
2
3
4
5
A (n=15)
B (n=16)
C (n=14)
surface

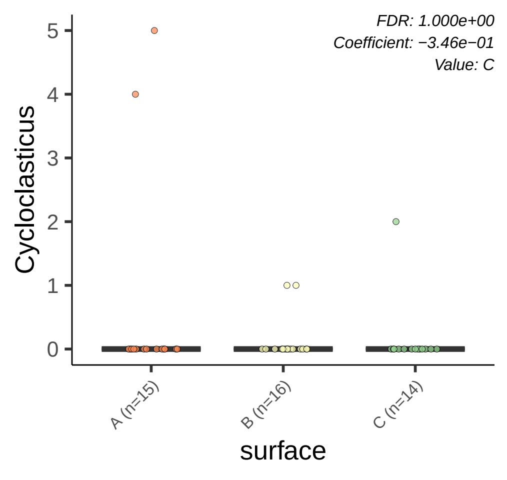
FDR: 1.000e+00
Coefficient: −3.46e−01
Value: C
Cycloclasticus
0
1
2
3
4
5
A (n=15)
B (n=16)
C (n=14)
surface

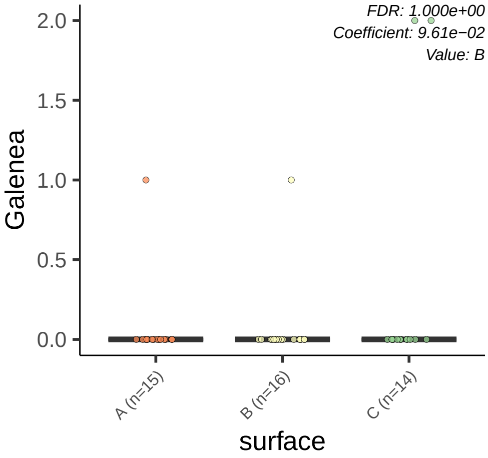

FDR: 1.000e+00
Coefficient: 9.61e−02
Value: B
Galenea
2.0
1.5
1.0
0.5
0.0
A (n=15)
B (n=16)
C (n=14)
surface

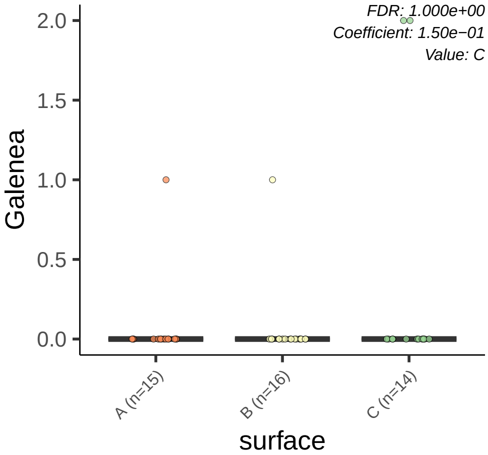

FDR: 1.000e+00
Coefficient: 1.50e−01
Value: C
Galenea
2.0
1.5
1.0
0.5
0.0
A (n=15)
B (n=16)
C (n=14)
surface

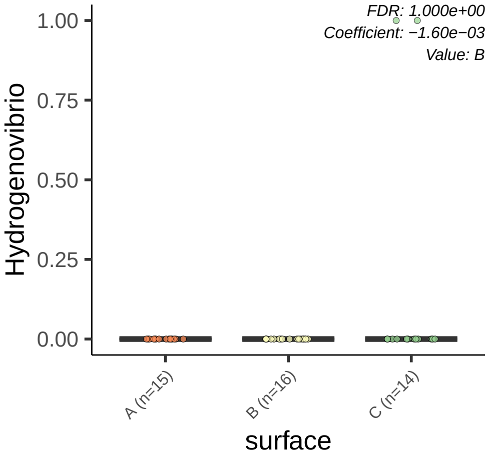
FDR: 1.000e+00
Coefficient: −1.60e−03
Value: B
Hydrogenovibrio
1.00
0.75
0.50
0.25
0.00
A (n=15)
B (n=16)
C (n=14)
surface

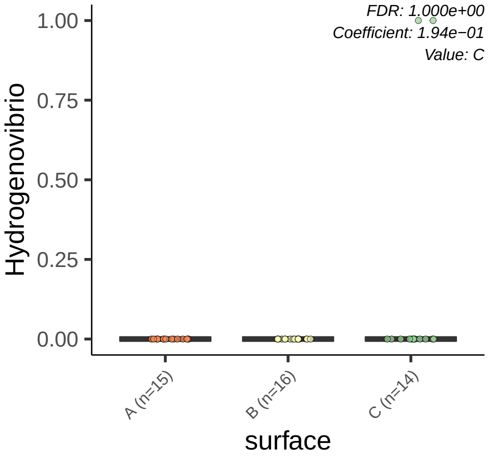
FDR: 1.000e+00
Coefficient: 1.94e−01
Value: C
Hydrogenovibrio
1.00
0.75
0.50
0.25
0.00
A (n=15)
B (n=16)
C (n=14)
surface

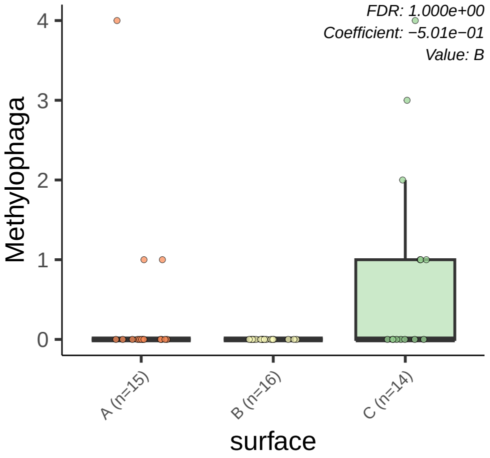
FDR: 1.000e+00
Coefficient: −5.01e−01
Value: B
Methylophaga
4
3
2
1
0
A (n=15)
B (n=16)
C (n=14)
surface

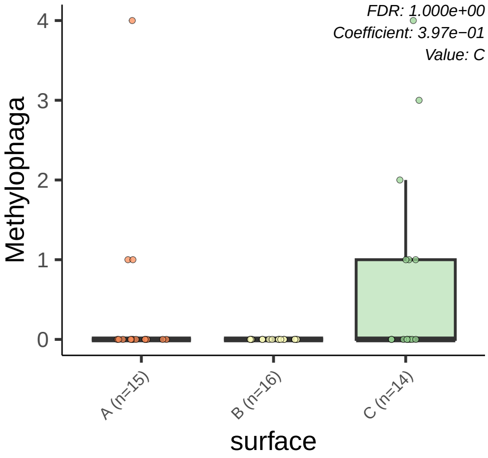
FDR: 1.000e+00
Coefficient: 3.97e−01
Value: C
Methylophaga
0
1
2
3
4
A (n=15)
B (n=16)
C (n=14)
surface

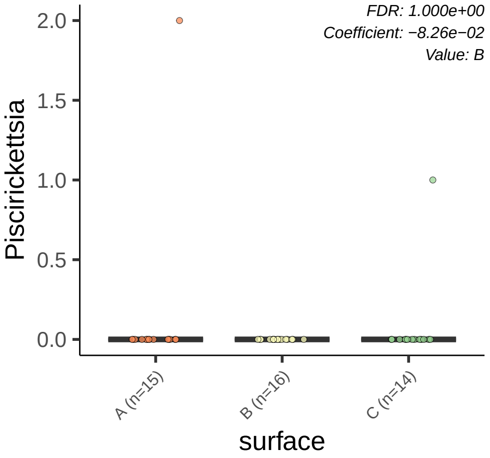
FDR: 1.000e+00
Coefficient: −8.26e−02
Value: B
Piscirickettsia
2.0
1.5
1.0
0.5
0.0
A (n=15)
B (n=16)
C (n=14)
surface

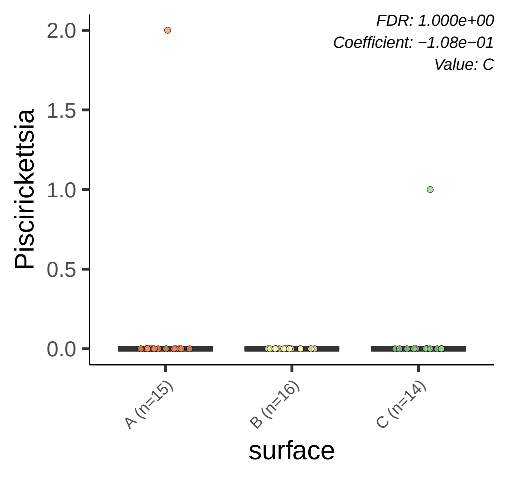

FDR: 1.000e+00
Coefficient: −1.08e−01
Value: C
Piscirickettsia
2.0
1.5
1.0
0.5
0.0
A (n=15)
B (n=16)
C (n=14)
surface

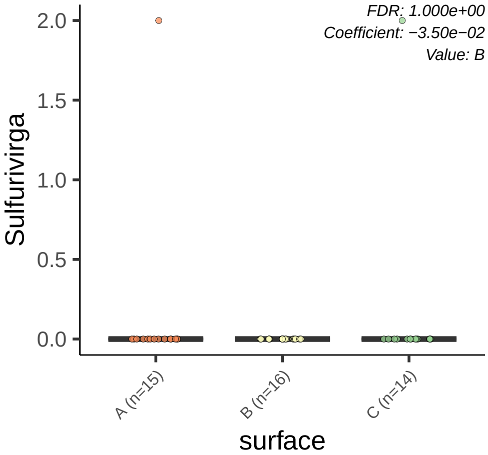

FDR: 1.000e+00
Coefficient: −3.50e−02
Value: B
Sulfurivirga
2.0
1.5
1.0
0.5
0.0
A (n=15)
B (n=16)
C (n=14)
surface

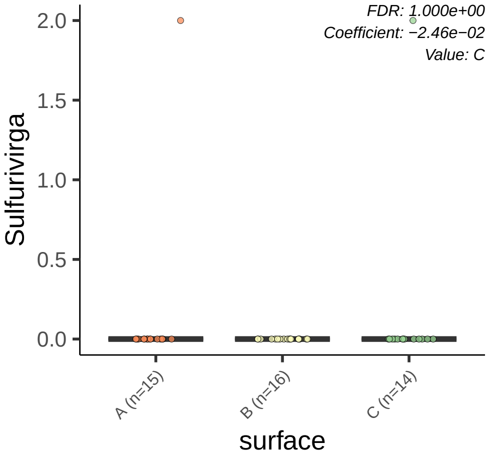

FDR: 1.000e+00
Coefficient: −2.46e−02
Value: C
Sulfurivirga
2.0
1.5
1.0
0.5
0.0
A (n=15)
B (n=16)
C (n=14)
surface

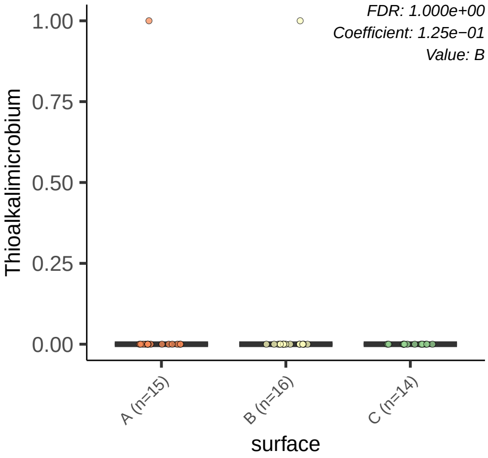

FDR: 1.000e+00
Coefficient: 1.25e−01
Value: B
Thioalkalimicrobium
1.00
0.75
0.50
0.25
0.00
A (n=15)
B (n=16)
C (n=14)
surface

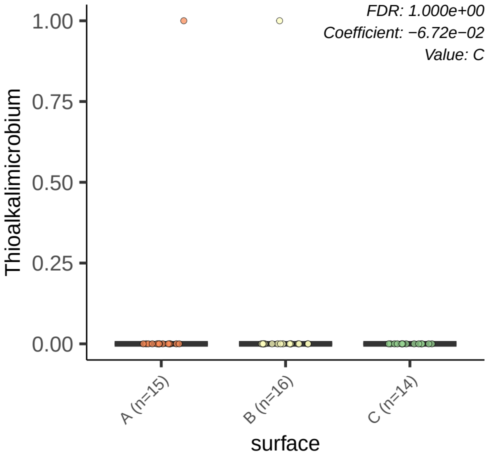
FDR: 1.000e+00
Coefficient: −6.72e−02
Value: C
Thioalkalimicrobium
1.00
0.75
0.50
0.25
0.00
A (n=15)
B (n=16)
C (n=14)
surface

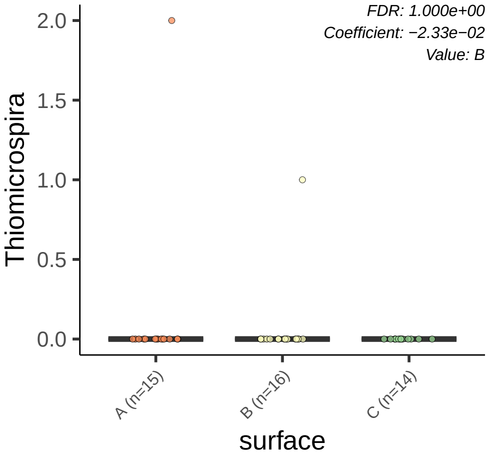

FDR: 1.000e+00
Coefficient: −2.33e−02
Value: B
Thiomicrospira
2.0
1.5
1.0
0.5
0.0
A (n=15)
B (n=16)
C (n=14)
surface

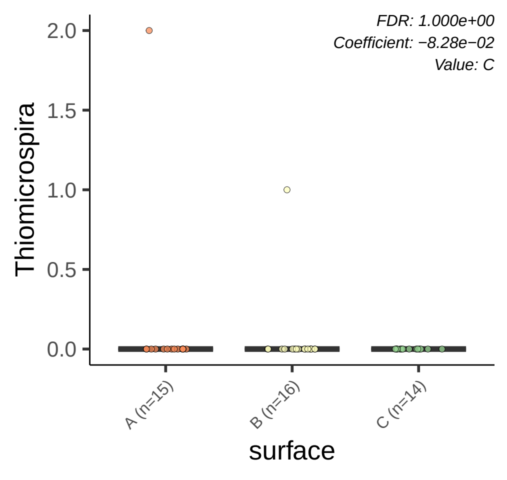
FDR: 1.000e+00
Coefficient: −8.28e−02
Value: C
Thiomicrospira
2.0
1.5
1.0
0.5
0.0
A (n=15)
B (n=16)
C (n=14)
surface

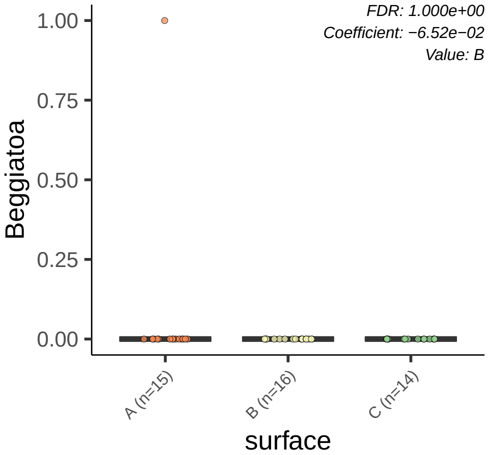
FDR: 1.000e+00
Coefficient: −6.52e−02
Value: B
Beggiatoa
1.00
0.75
0.50
0.25
0.00
A (n=15)
B (n=16)
C (n=14)
surface

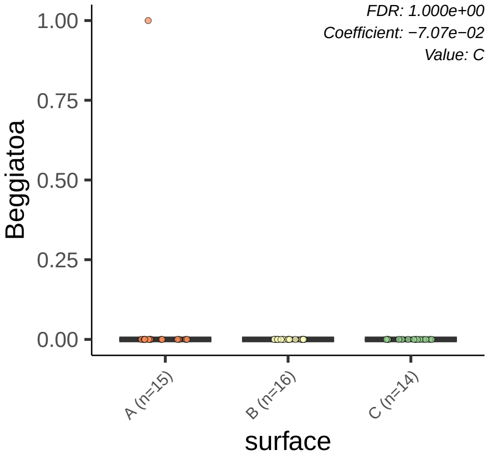
FDR: 1.000e+00
Coefficient: −7.07e−02
Value: C
Beggiatoa
1.00
0.75
0.50
0.25
0.00
A (n=15)
B (n=16)
C (n=14)
surface

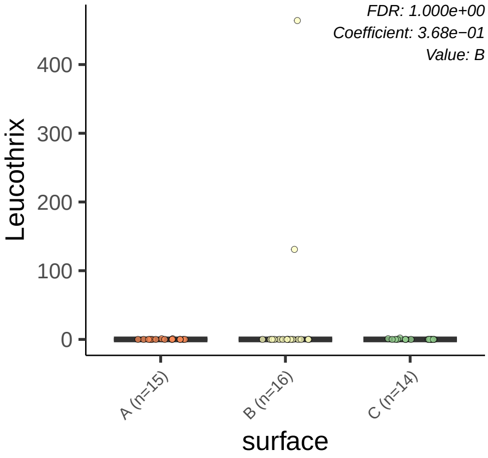
FDR: 1.000e+00
Coefficient: 3.68e−01
Value: B
Leucothrix
400
300
200
100
0
A (n=15)
B (n=16)
C (n=14)
surface

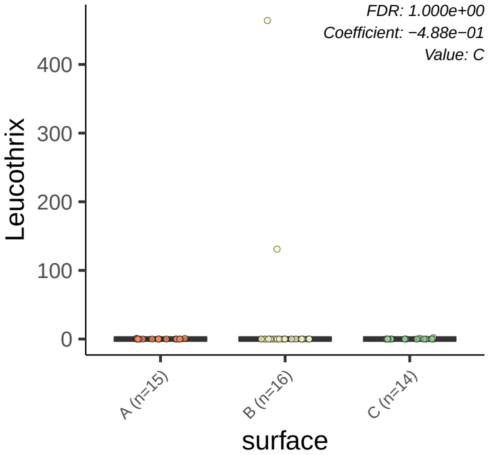

FDR: 1.000e+00
Coefficient: −4.88e−01
Value: C
Leucothrix
400
300
200
100
0
A (n=15)
B (n=16)
C (n=14)
surface

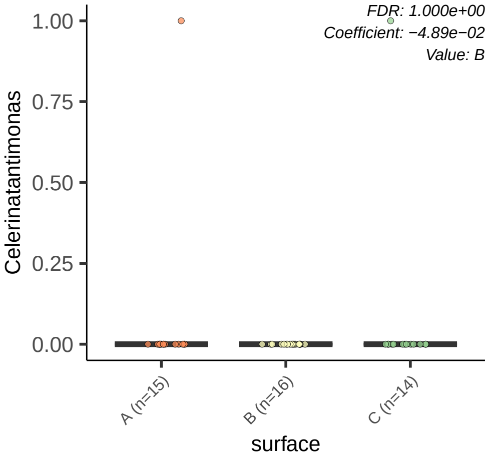

FDR: 1.000e+00
Coefficient: −4.89e−02
Value: B
Celerinatantimonas
1.00
0.75
0.50
0.25
0.00
A (n=15)
B (n=16)
C (n=14)
surface

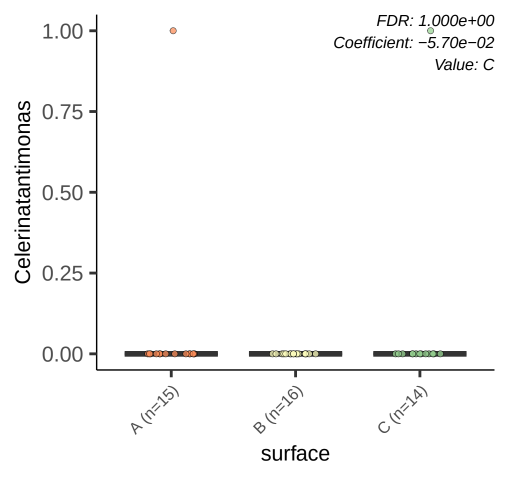

FDR: 1.000e+00
Coefficient: −5.70e−02
Value: C
Celerinatantimonas
1.00
0.75
0.50
0.25
0.00
A (n=15)
B (n=16)
C (n=14)
surface

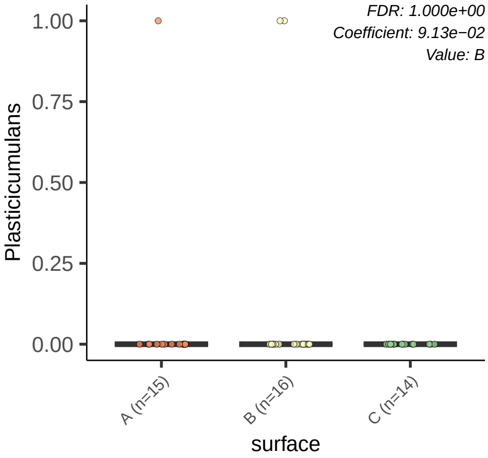

FDR: 1.000e+00
Coefficient: 9.13e−02
Value: B
1.00
0.75
0.50
0.25
0.00
Plasticicumulans
A (n=15)
B (n=16)
C (n=14)
surface

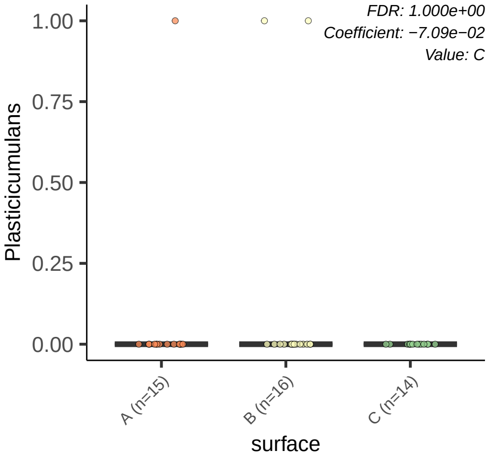
FDR: 1.000e+00
Coefficient: −7.09e−02
Value: C
Plasticicumulans
1.00
0.75
0.50
0.25
0.00
A (n=15)
B (n=16)
C (n=14)
surface

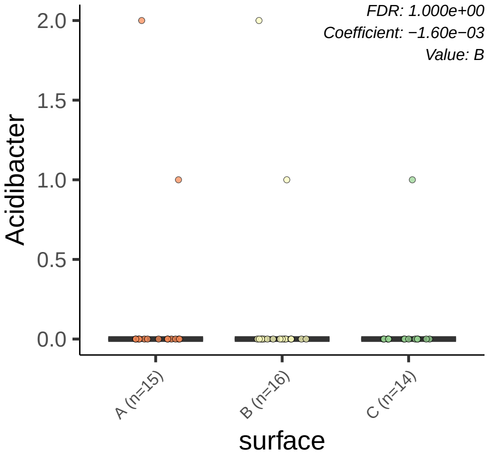
FDR: 1.000e+00
Coefficient: −1.60e−03
Value: B
Acidibacter
2.0
1.5
1.0
0.5
0.0
A (n=15)
B (n=16)
C (n=14)
surface

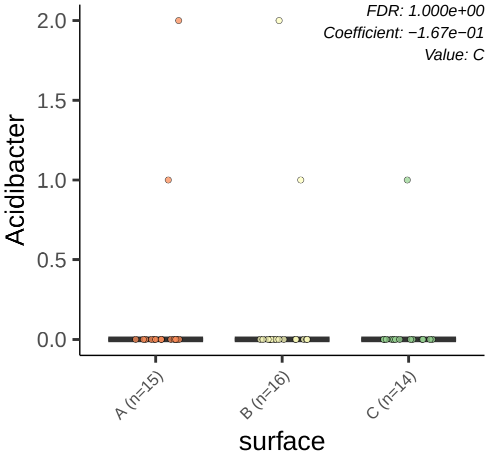

FDR: 1.000e+00
Coefficient: −1.67e−01
Value: C
Acidibacter
2.0
1.5
1.0
0.5
0.0
A (n=15)
B (n=16)
C (n=14)
surface

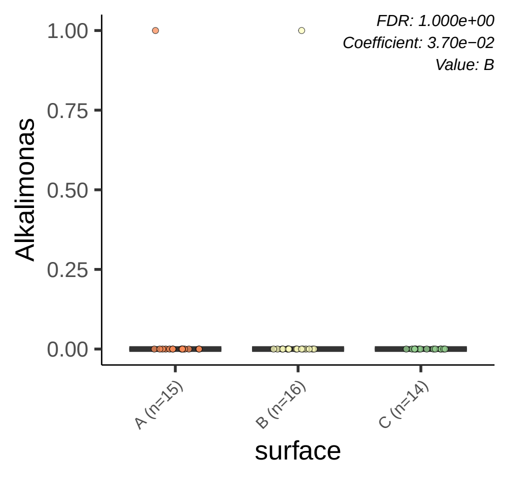
FDR: 1.000e+00
Coefficient: 3.70e−02
Value: B
Alkalimonas
1.00
0.75
0.50
0.25
0.00
A (n=15)
B (n=16)
C (n=14)
surface

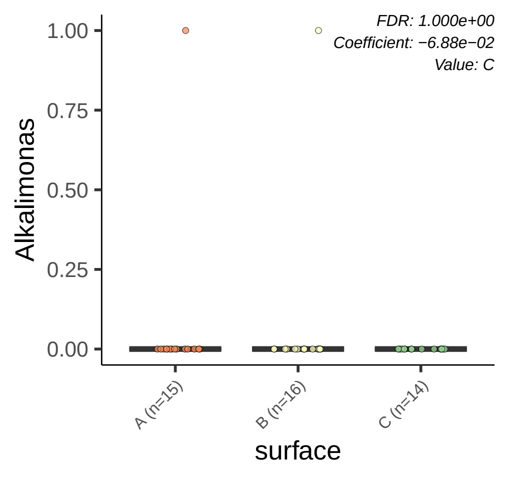

FDR: 1.000e+00
Coefficient: −6.88e−02
Value: C
Alkalimonas
1.00
0.75
0.50
0.25
0.00
A (n=15)
B (n=16)
C (n=14)
surface

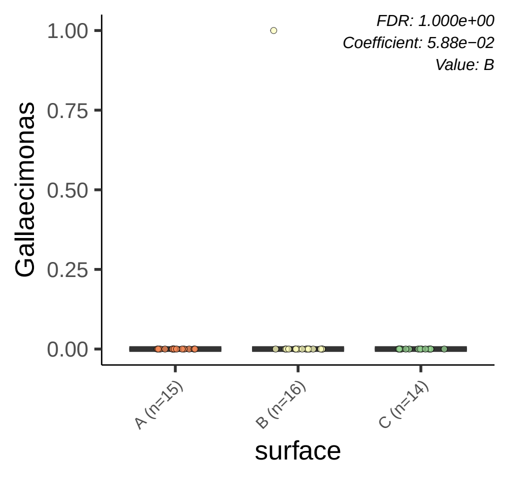
FDR: 1.000e+00
Coefficient: 5.88e−02
Value: B
Gallaecimonas
1.00
0.75
0.50
0.25
0.00
A (n=15)
B (n=16)
C (n=14)
surface

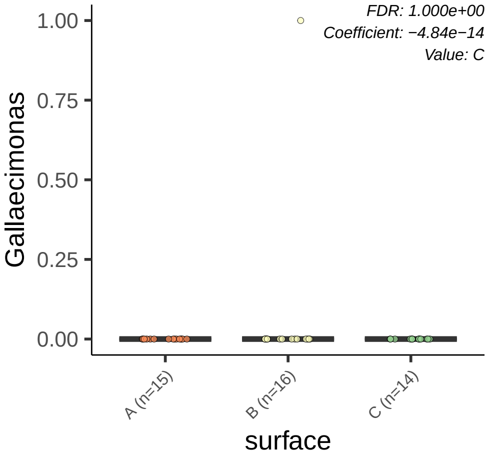

FDR: 1.000e+00
Coefficient: −4.84e−14
Value: C
Gallaecimonas
1.00
0.75
0.50
0.25
0.00
A (n=15)
B (n=16)
C (n=14)
surface

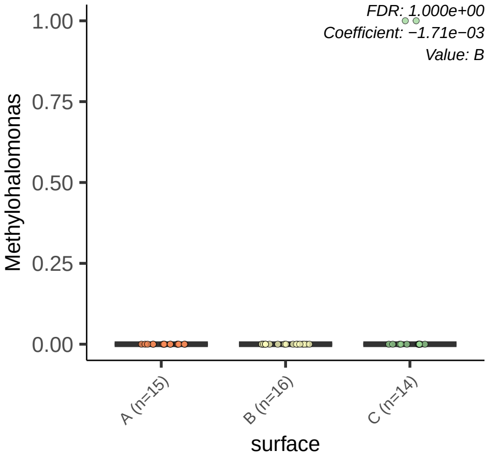

FDR: 1.000e+00
Coefficient: −1.71e−03
Value: B
Methylohalomonas
1.00
0.75
0.50
0.25
0.00
A (n=15)
B (n=16)
C (n=14)
surface

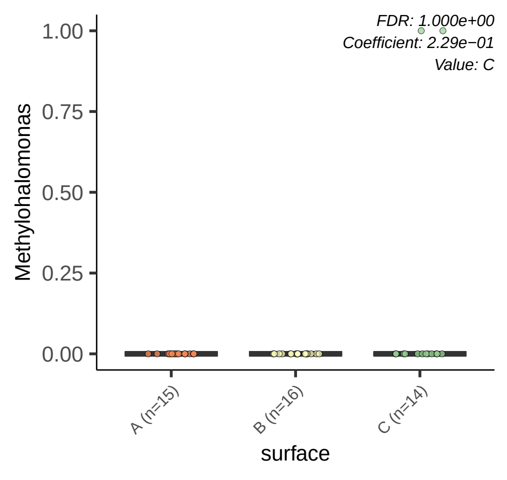
FDR: 1.000e+00
Coefficient: 2.29e−01
Value: C
Methylohalomonas
1.00
0.75
0.50
0.25
0.00
A (n=15)
B (n=16)
C (n=14)
surface

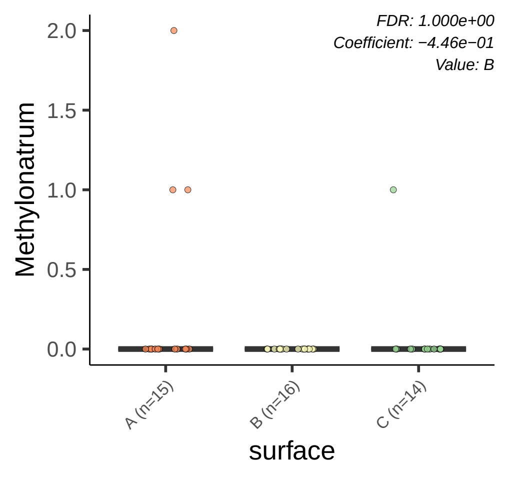

FDR: 1.000e+00
Coefficient: −4.46e−01
Value: B
Methylonatrum
2.0
1.5
1.0
0.5
0.0
A (n=15)
B (n=16)
C (n=14)
surface

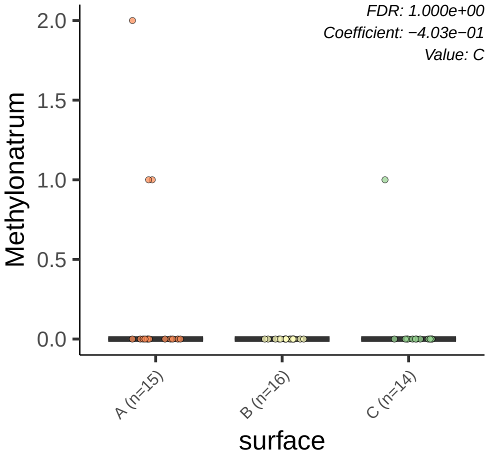
FDR: 1.000e+00
Coefficient: −4.03e−01
Value: C
Methylonatrum
2.0
1.5
1.0
0.5
0.0
A (n=15)
B (n=16)
C (n=14)
surface

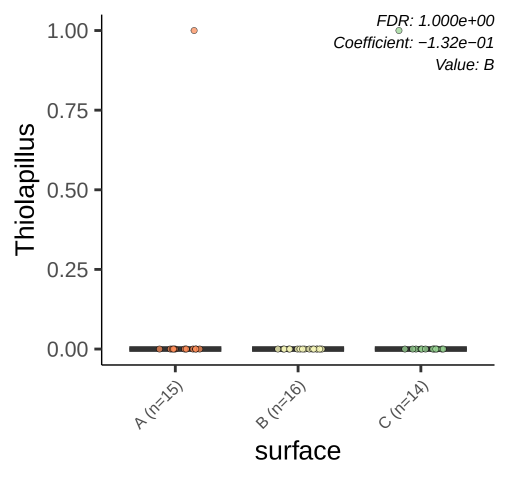
FDR: 1.000e+00
Coefficient: −1.32e−01
Value: B
Thiolapillus
1.00
0.75
0.50
0.25
0.00
A (n=15)
B (n=16)
C (n=14)
surface

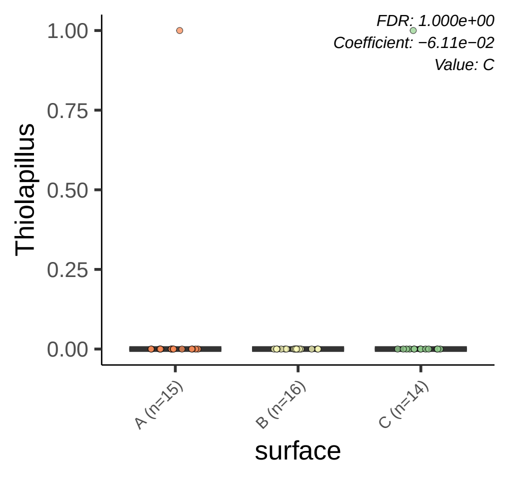

FDR: 1.000e+00
Coefficient: −6.11e−02
Value: C
Thiolapillus
1.00
0.75
0.50
0.25
0.00
A (n=15)
B (n=16)
C (n=14)
surface

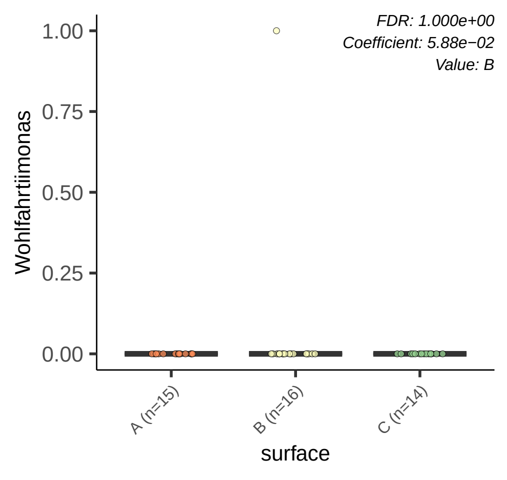
FDR: 1.000e+00
Coefficient: 5.88e−02
Value: B
Wohlfahrtiimonas
1.00
0.75
0.50
0.25
0.00
A (n=15)
B (n=16)
C (n=14)
surface

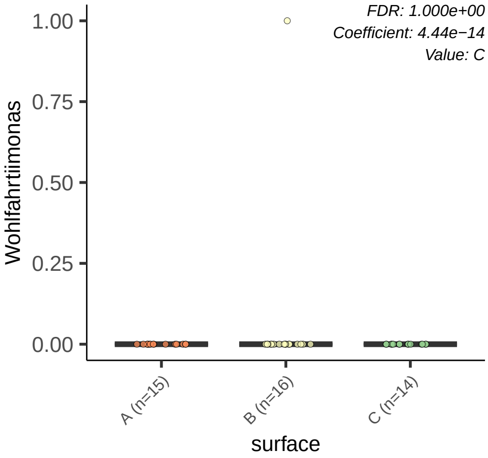

FDR: 1.000e+00
Coefficient: 4.44e−14
Value: C
Wohlfahrtiimonas
1.00
0.75
0.50
0.25
0.00
A (n=15)
B (n=16)
C (n=14)
surface

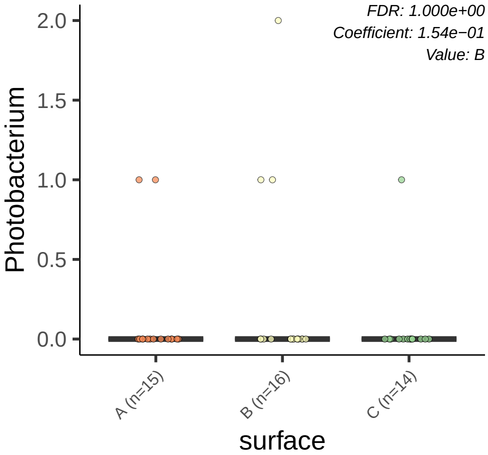
FDR: 1.000e+00
Coefficient: 1.54e−01
Value: B
Photobacterium
2.0
1.5
1.0
0.5
0.0
A (n=15)
B (n=16)
C (n=14)
surface

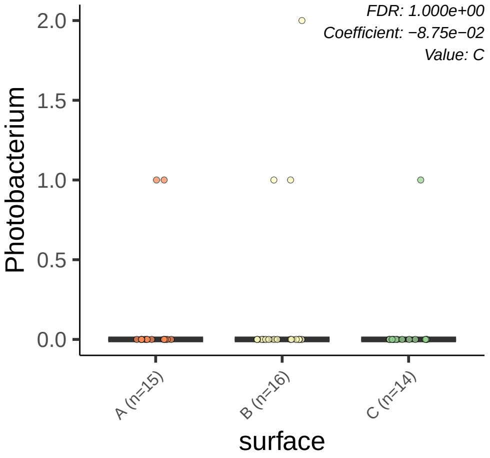
FDR: 1.000e+00
Coefficient: −8.75e−02
Value: C
2.0
1.5
1.0
0.5
0.0
Photobacterium
A (n=15)
B (n=16)
C (n=14)
surface

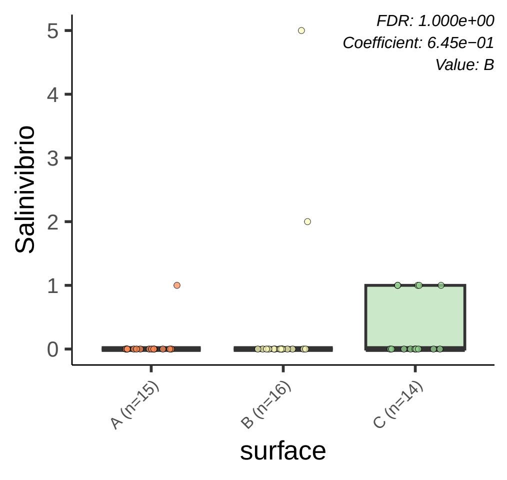
FDR: 1.000e+00
Coefficient: 6.45e−01
Value: B
Salinivibrio
0
1
2
3
4
5
A (n=15)
B (n=16)
C (n=14)
surface

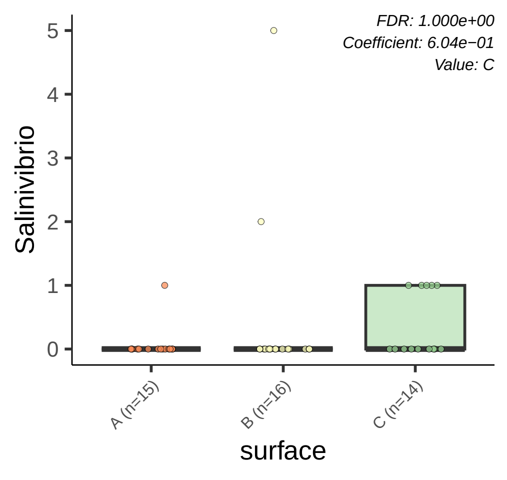

FDR: 1.000e+00
Coefficient: 6.04e−01
Value: C
Salinivibrio
0
1
2
3
4
5
A (n=15)
B (n=16)
C (n=14)
surface

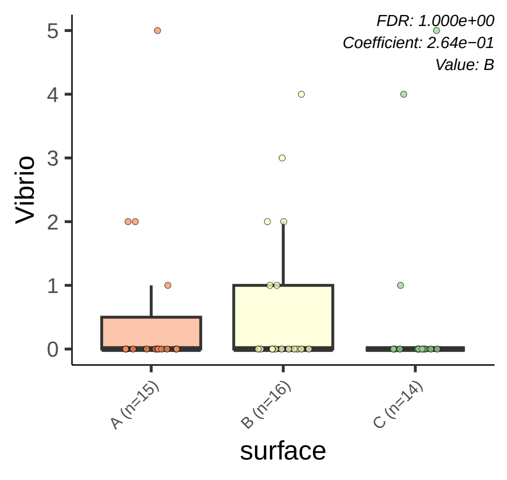
FDR: 1.000e+00
Coefficient: 2.64e−01
Value: B
Vibrio
5
4
3
2
1
0
A (n=15)
B (n=16)
C (n=14)
surface

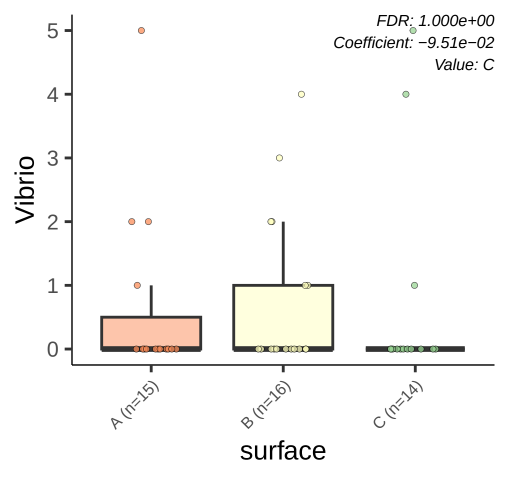
FDR: 1.000e+00
Coefficient: −9.51e−02
Value: C
Vibrio
5
4
3
2
1
0
A (n=15)
B (n=16)
C (n=14)
surface

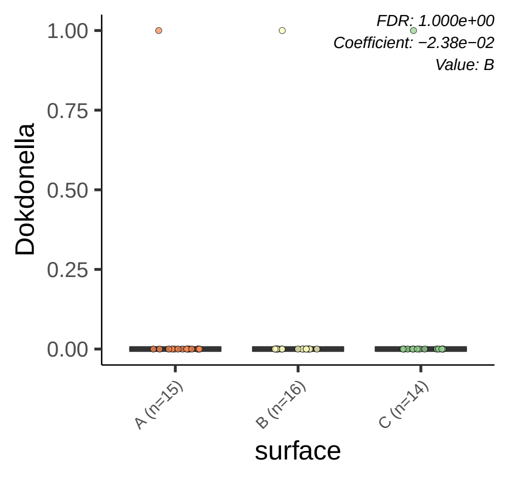
FDR: 1.000e+00
Coefficient: −2.38e−02
Value: B
Dokdonella
1.00
0.75
0.50
0.25
0.00
A (n=15)
B (n=16)
C (n=14)
surface

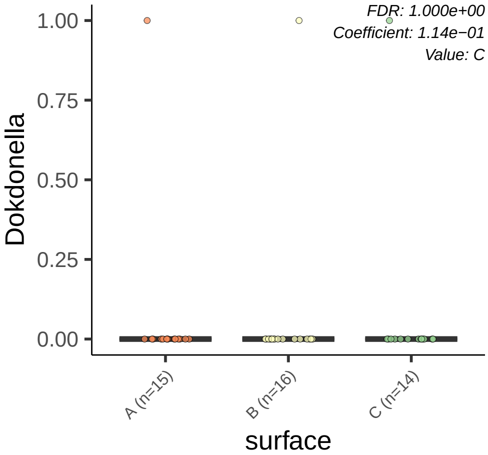

FDR: 1.000e+00
Coefficient: 1.14e−01
Value: C
Dokdonella
1.00
0.75
0.50
0.25
0.00
A (n=15)
B (n=16)
C (n=14)
surface

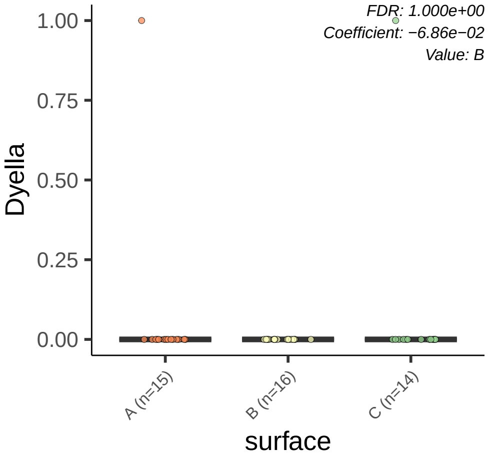

FDR: 1.000e+00
Coefficient: −6.86e−02
Value: B
Dyella
1.00
0.75
0.50
0.25
0.00
A (n=15)
B (n=16)
C (n=14)
surface

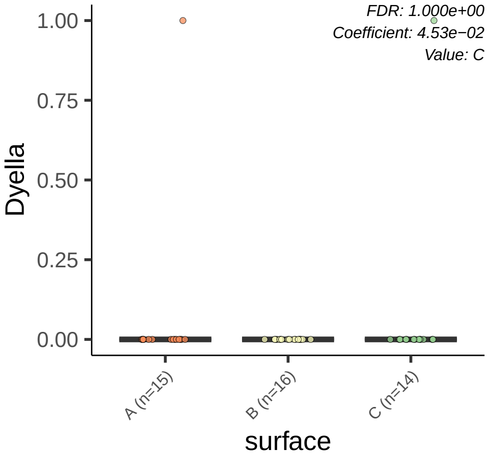
FDR: 1.000e+00
Coefficient: 4.53e−02
Value: C
Dyella
1.00
0.75
0.50
0.25
0.00
A (n=15)
B (n=16)
C (n=14)
surface

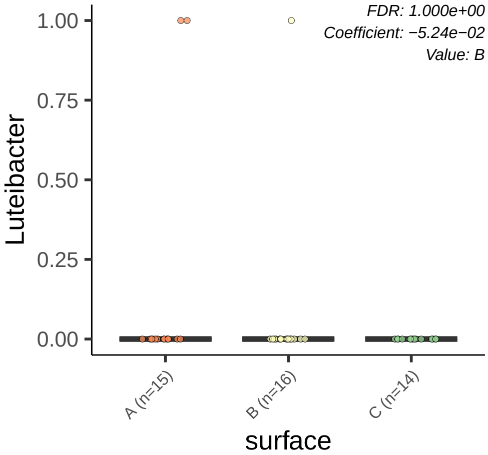
FDR: 1.000e+00
Coefficient: −5.24e−02
Value: B
Luteibacter
1.00
0.75
0.50
0.25
0.00
A (n=15)
B (n=16)
C (n=14)
surface

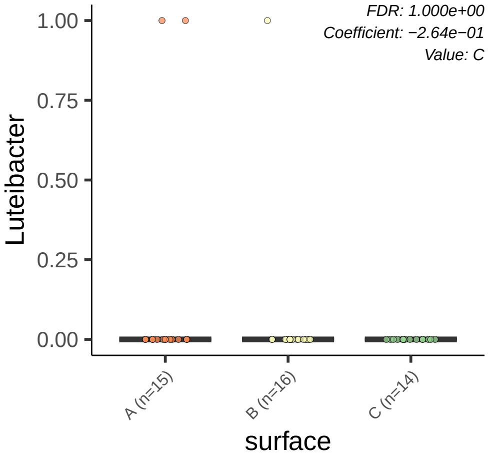
FDR: 1.000e+00
Coefficient: −2.64e−01
Value: C
1.00
0.75
0.50
0.25
0.00
Luteibacter
A (n=15)
B (n=16)
C (n=14)
surface

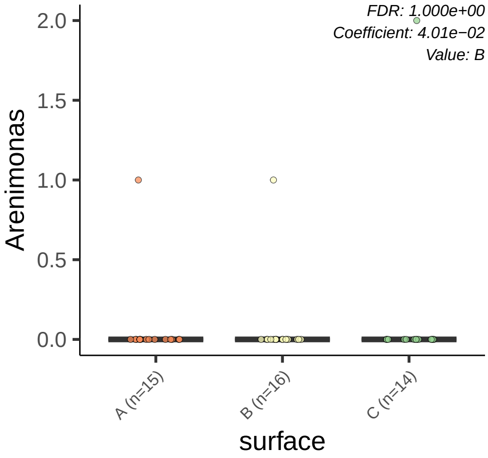

FDR: 1.000e+00
Coefficient: 4.01e−02
Value: B
Arenimonas
2.0
1.5
1.0
0.5
0.0
A (n=15)
B (n=16)
C (n=14)
surface

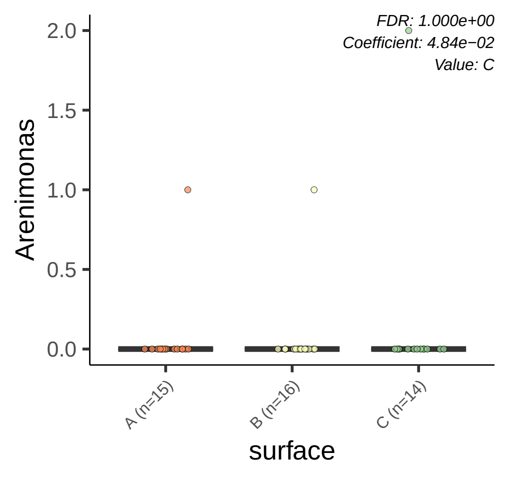
FDR: 1.000e+00
Coefficient: 4.84e−02
Value: C
Arenimonas
2.0
1.5
1.0
0.5
0.0
A (n=15)
B (n=16)
C (n=14)
surface

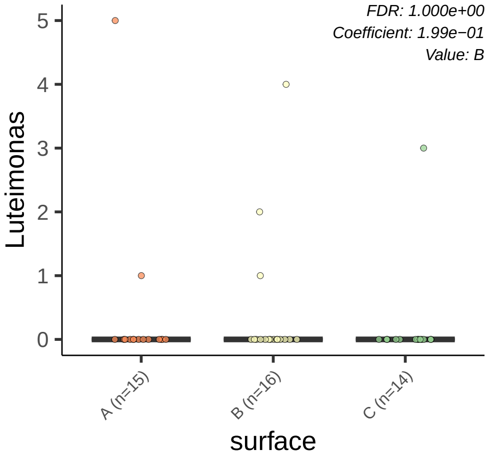
FDR: 1.000e+00
Coefficient: 1.99e−01
Value: B
Luteimonas
5
4
3
2
1
0
A (n=15)
B (n=16)
C (n=14)
surface

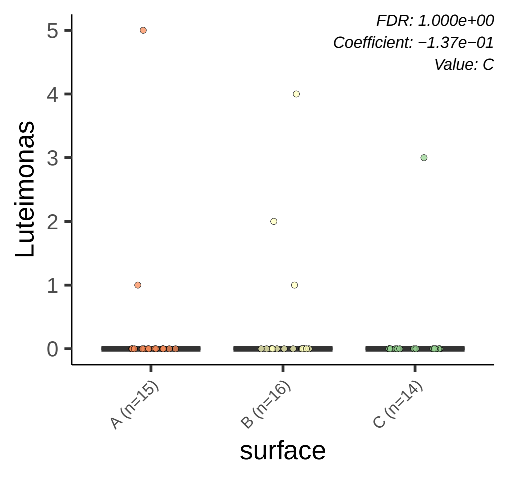

FDR: 1.000e+00
Coefficient: −1.37e−01
Value: C
Luteimonas
0
1
2
3
4
5
A (n=15)
B (n=16)
C (n=14)
surface

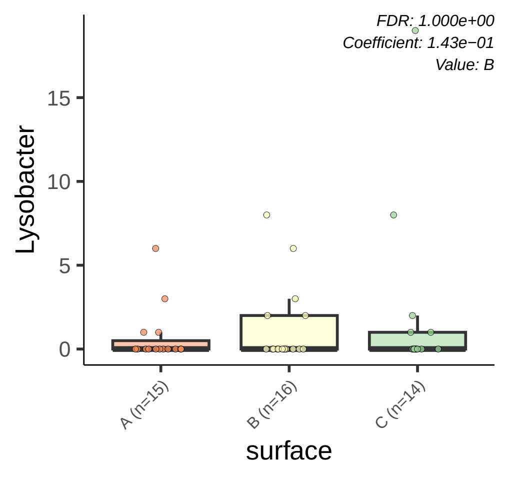

FDR: 1.000e+00
Coefficient: 1.43e−01
Value: B
Lysobacter
15
10
5
0
A (n=15)
B (n=16)
C (n=14)
surface

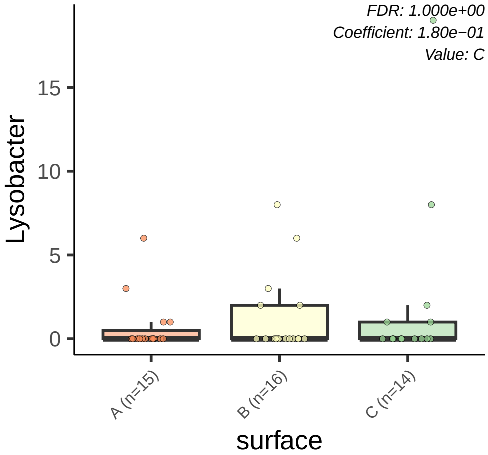

FDR: 1.000e+00
Coefficient: 1.80e−01
Value: C
Lysobacter
15
10
5
0
A (n=15)
B (n=16)
C (n=14)
surface

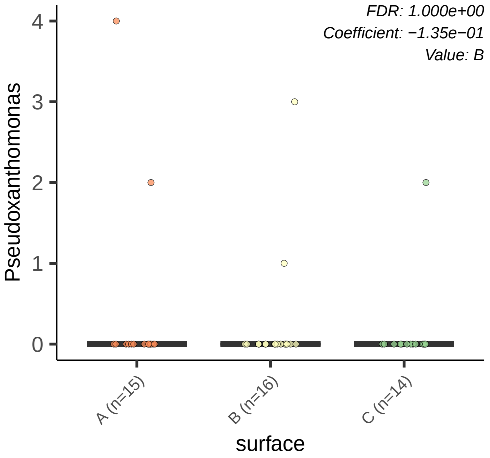

FDR: 1.000e+00
Coefficient: −1.35e−01
Value: B
Pseudoxanthomonas
4
3
2
1
0
A (n=15)
B (n=16)
C (n=14)
surface

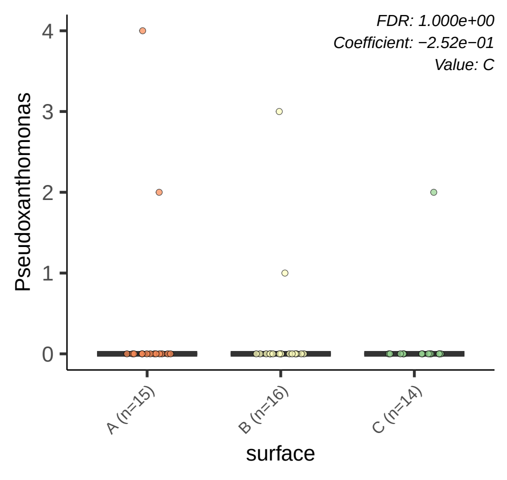

FDR: 1.000e+00
Coefficient: −2.52e−01
Value: C
Pseudoxanthomonas
4
3
2
1
0
A (n=15)
B (n=16)
C (n=14)
surface

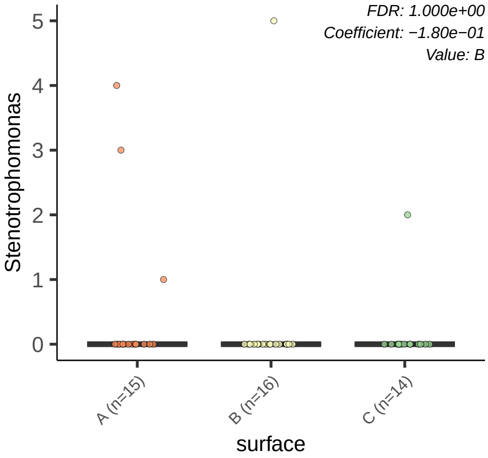

FDR: 1.000e+00
Coefficient: −1.80e−01
Value: B
Stenotrophomonas
0
1
2
3
4
5
A (n=15)
B (n=16)
C (n=14)
surface

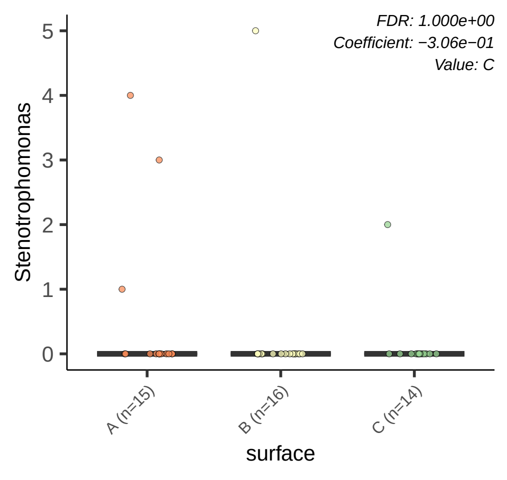

FDR: 1.000e+00
Coefficient: −3.06e−01
Value: C
Stenotrophomonas
0
1
2
3
4
5
A (n=15)
B (n=16)
C (n=14)
surface

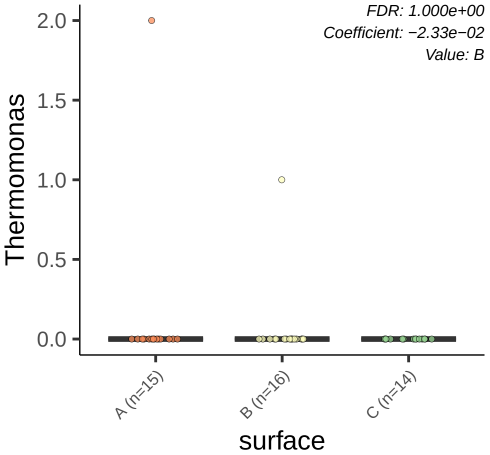

FDR: 1.000e+00
Coefficient: −2.33e−02
Value: B
Thermomonas
2.0
1.5
1.0
0.5
0.0
A (n=15)
B (n=16)
C (n=14)
surface

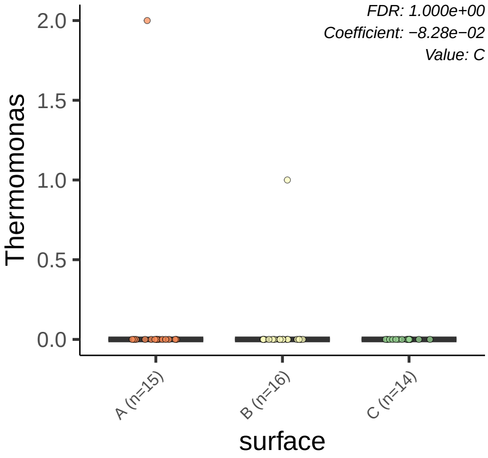

FDR: 1.000e+00
Coefficient: −8.28e−02
Value: C
Thermomonas
2.0
1.5
1.0
0.5
0.0
A (n=15)
B (n=16)
C (n=14)
surface

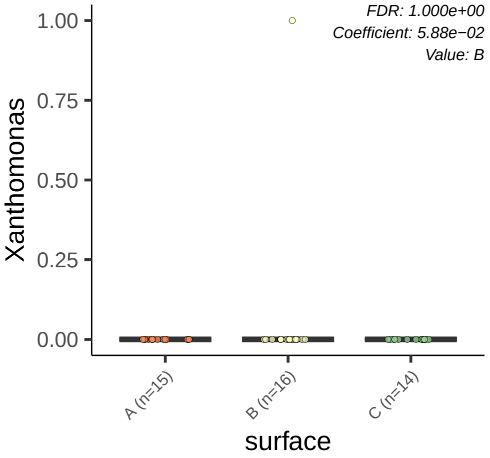

FDR: 1.000e+00
Coefficient: 5.88e−02
Value: B
1.00
0.75
0.50
0.25
0.00
Xanthomonas
A (n=15)
B (n=16)
C (n=14)
surface

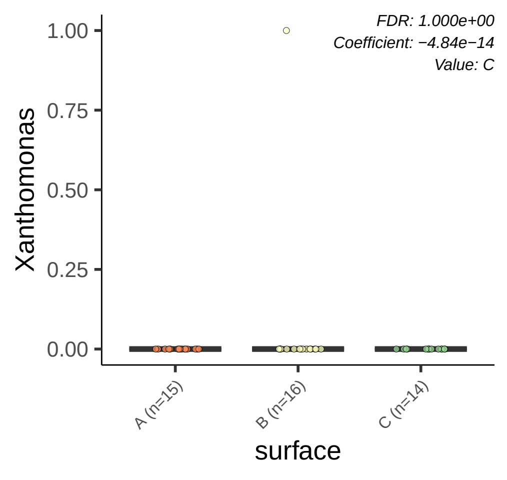
FDR: 1.000e+00
Coefficient: −4.84e−14
Value: C
1.00
0.75
0.50
0.25
0.00
Xanthomonas
A (n=15)
B (n=16)
C (n=14)
surface

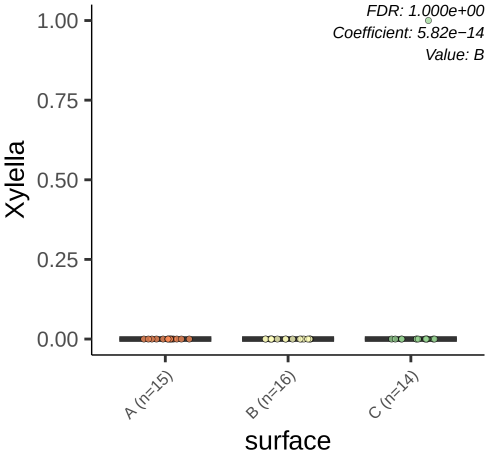
FDR: 1.000e+00
Coefficient: 5.82e−14
Value: B
Xylella
1.00
0.75
0.50
0.25
0.00
A (n=15)
B (n=16)
C (n=14)
surface

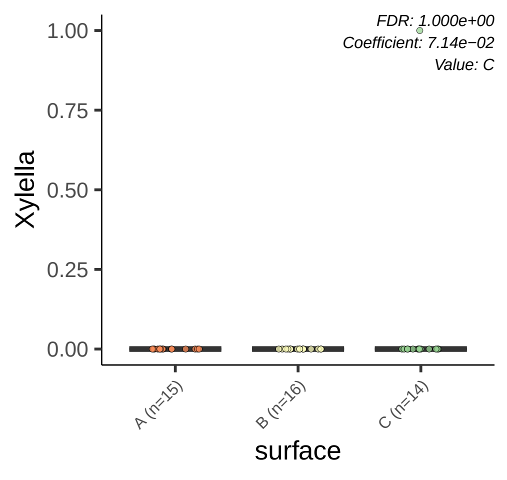
FDR: 1.000e+00
Coefficient: 7.14e−02
Value: C
1.00
0.75
0.50
0.25
0.00
Xylella
A (n=15)
B (n=16)
C (n=14)
surface

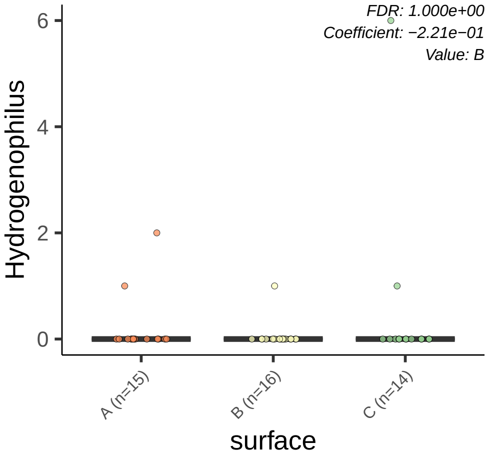
FDR: 1.000e+00
Coefficient: −2.21e−01
Value: B
Hydrogenophilus
0
2
4
6
A (n=15)
B (n=16)
C (n=14)
surface

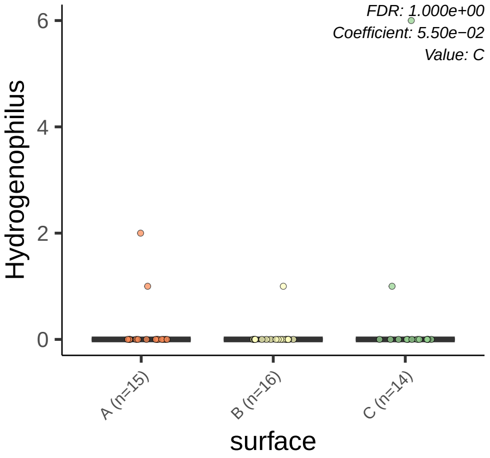
FDR: 1.000e+00
Coefficient: 5.50e−02
Value: C
Hydrogenophilus
6
4
2
0
A (n=15)
B (n=16)
C (n=14)
surface

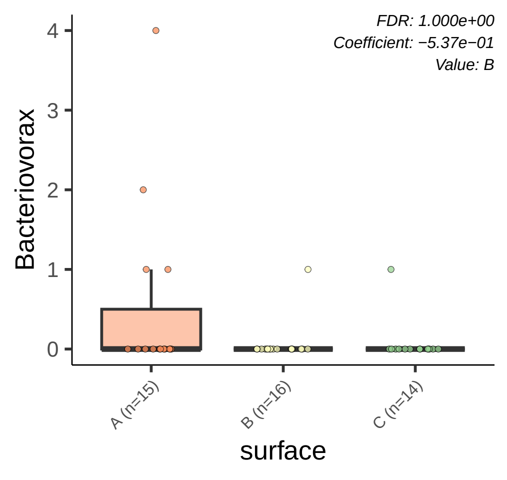

FDR: 1.000e+00
Coefficient: −5.37e−01
Value: B
Bacteriovorax
0
1
2
3
4
A (n=15)
B (n=16)
C (n=14)
surface

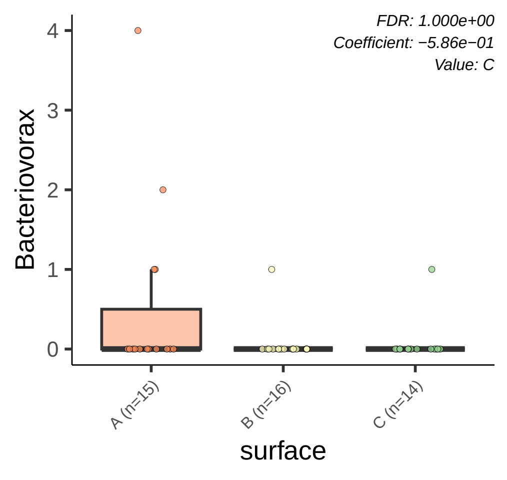
FDR: 1.000e+00
Coefficient: −5.86e−01
Value: C
Bacteriovorax
4
3
2
1
0
A (n=15)
B (n=16)
C (n=14)
surface

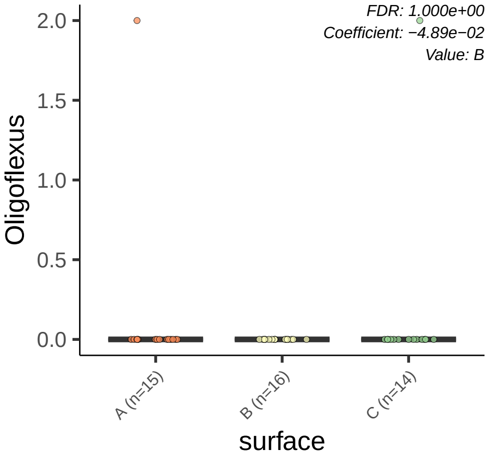

FDR: 1.000e+00
Coefficient: −4.89e−02
Value: B
Oligoflexus
2.0
1.5
1.0
0.5
0.0
A (n=15)
B (n=16)
C (n=14)
surface

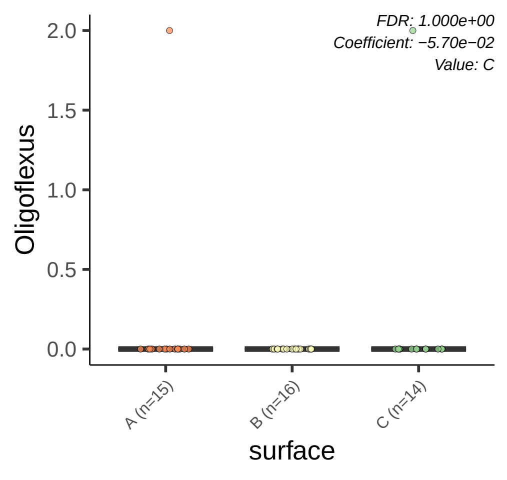

FDR: 1.000e+00
Coefficient: −5.70e−02
Value: C
Oligoflexus
2.0
1.5
1.0
0.5
0.0
A (n=15)
B (n=16)
C (n=14)
surface

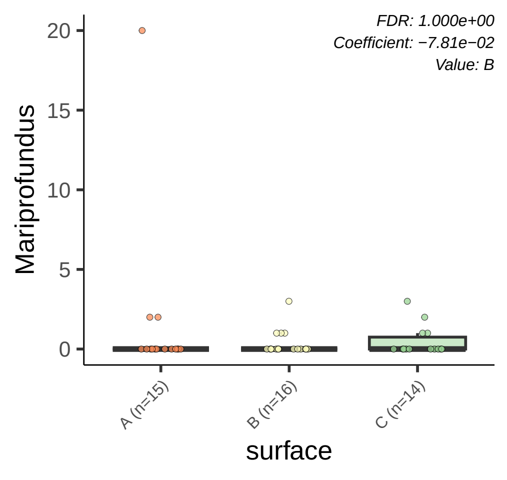

FDR: 1.000e+00
Coefficient: −7.81e−02
Value: B
Mariprofundus
20
15
10
5
0
A (n=15)
B (n=16)
C (n=14)
surface

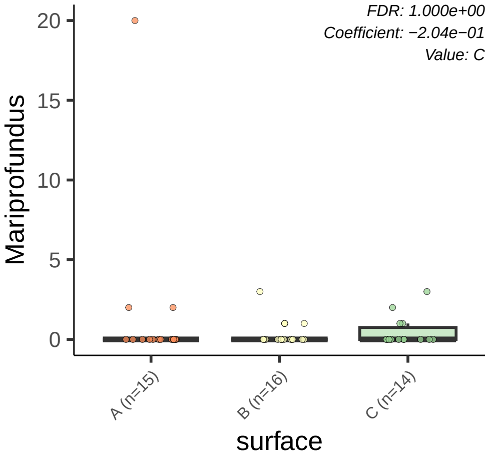
FDR: 1.000e+00
Coefficient: −2.04e−01
Value: C
Mariprofundus
20
15
10
5
0
A (n=15)
B (n=16)
C (n=14)
surface

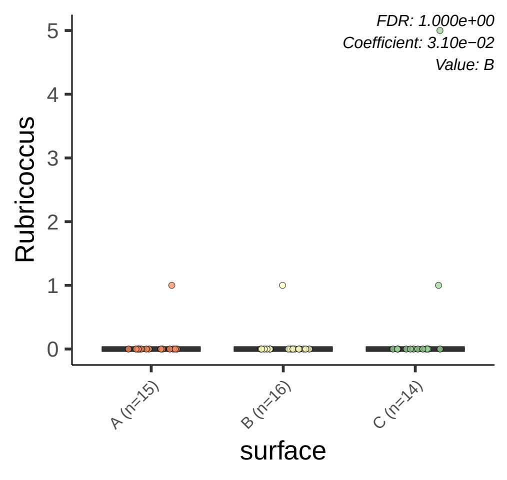
FDR: 1.000e+00
Coefficient: 3.10e−02
Value: B
Rubricoccus
0
1
2
3
4
5
A (n=15)
B (n=16)
C (n=14)
surface

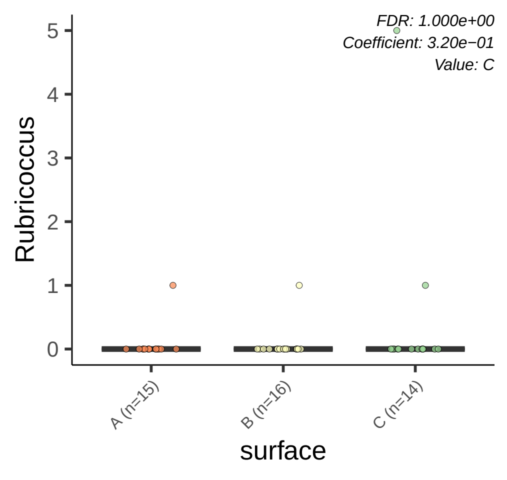
FDR: 1.000e+00
Coefficient: 3.20e−01
Value: C
Rubricoccus
0
1
2
3
4
5
A (n=15)
B (n=16)
C (n=14)
surface

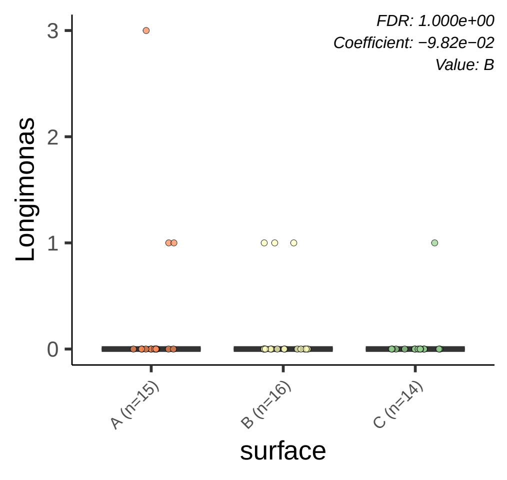
FDR: 1.000e+00
Coefficient: −9.82e−02
Value: B
Longimonas
3
2
1
0
A (n=15)
B (n=16)
C (n=14)
surface

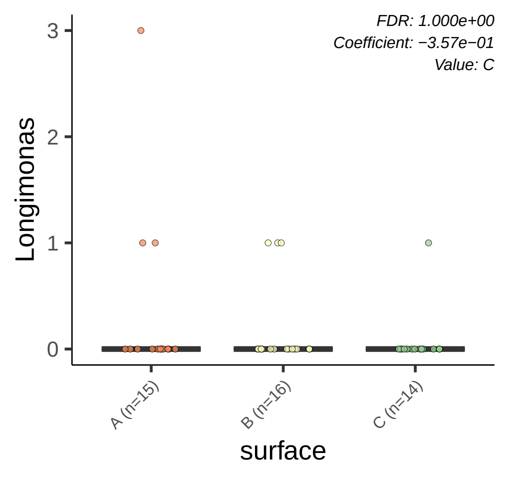

FDR: 1.000e+00
Coefficient: −3.57e−01
Value: C
Longimonas
3
2
1
0
A (n=15)
B (n=16)
C (n=14)
surface

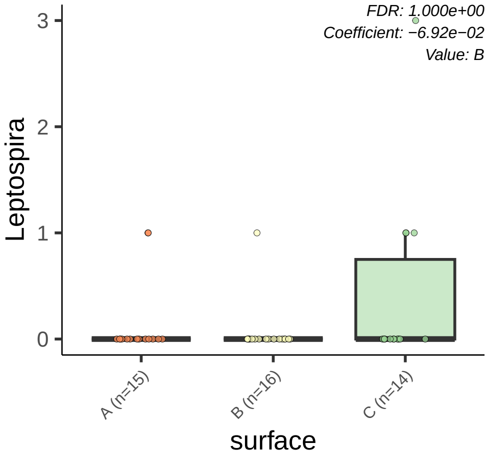

FDR: 1.000e+00
Coefficient: −6.92e−02
Value: B
Leptospira
3
2
1
0
A (n=15)
B (n=16)
C (n=14)
surface

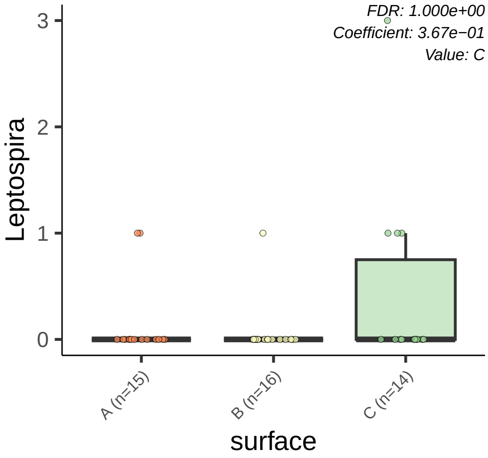

FDR: 1.000e+00
Coefficient: 3.67e−01
Value: C
Leptospira
3
2
1
0
A (n=15)
B (n=16)
C (n=14)
surface

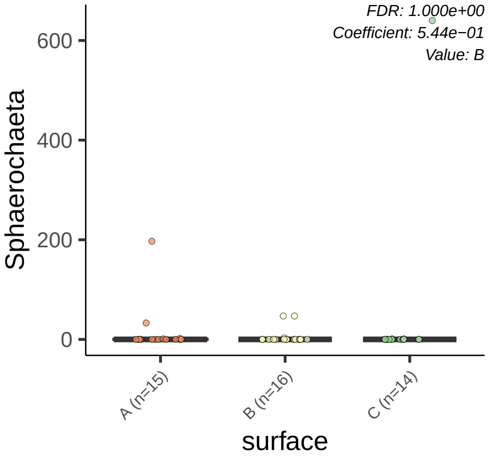

FDR: 1.000e+00
Coefficient: 5.44e−01
Value: B
Sphaerochaeta
600
400
200
0
A (n=15)
B (n=16)
C (n=14)
surface

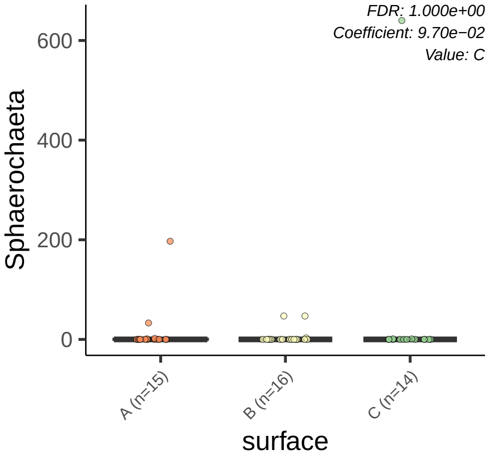
FDR: 1.000e+00
Coefficient: 9.70e−02
Value: C
Sphaerochaeta
600
400
200
0
A (n=15)
B (n=16)
C (n=14)
surface

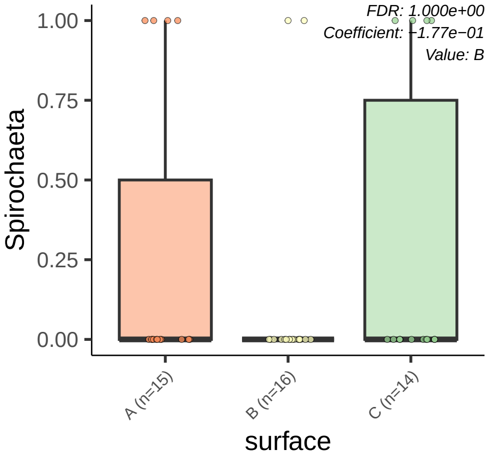
FDR: 1.000e+00
Coefficient: −1.77e−01
Value: B
Spirochaeta
1.00
0.75
0.50
0.25
0.00
A (n=15)
B (n=16)
C (n=14)
surface

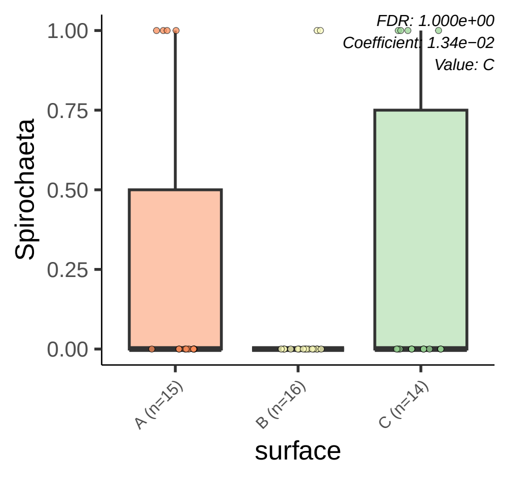
FDR: 1.000e+00
Coefficient: 1.34e−02
Value: C
Spirochaeta
1.00
0.75
0.50
0.25
0.00
A (n=15)
B (n=16)
C (n=14)
surface

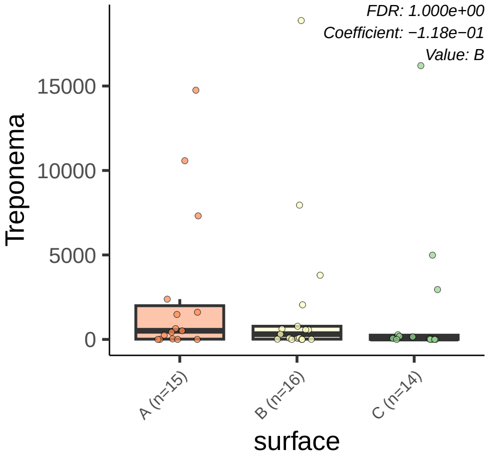

FDR: 1.000e+00
Coefficient: −1.18e−01
Value: B
Treponema
15000
10000
5000
0
A (n=15)
B (n=16)
C (n=14)
surface

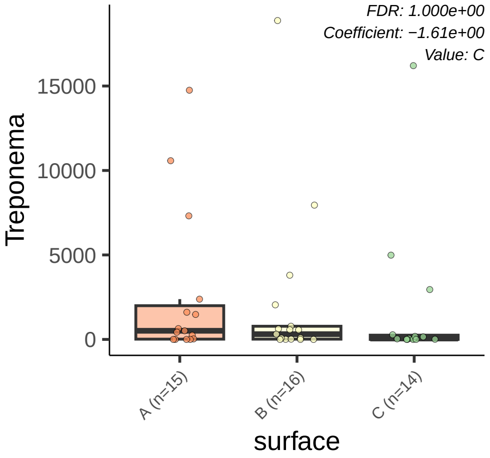

FDR: 1.000e+00
Coefficient: −1.61e+00
Value: C
Treponema
15000
10000
5000
0
A (n=15)
B (n=16)
C (n=14)
surface

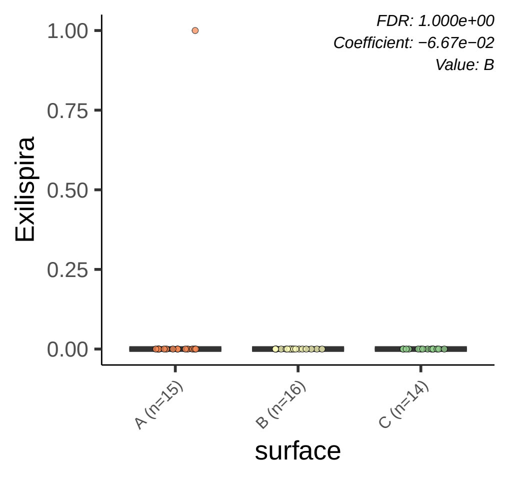
FDR: 1.000e+00
Coefficient: −6.67e−02
Value: B
Exilispira
1.00
0.75
0.50
0.25
0.00
A (n=15)
B (n=16)
C (n=14)
surface

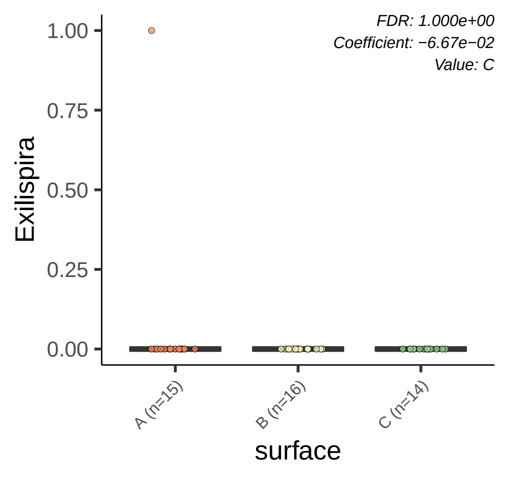
FDR: 1.000e+00
Coefficient: −6.67e−02
Value: C
1.00
0.75
0.50
0.25
0.00
Exilispira
A (n=15)
B (n=16)
C (n=14)
surface

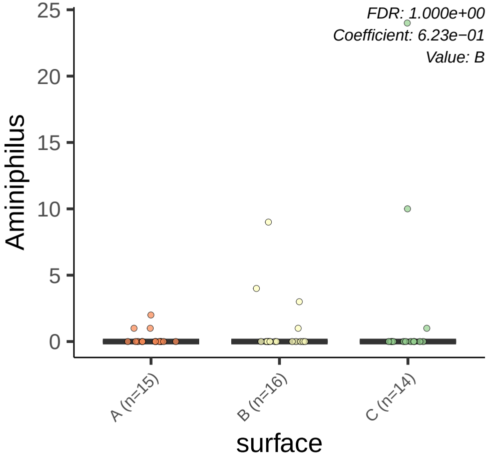

FDR: 1.000e+00
Coefficient: 6.23e−01
Value: B
Aminiphilus
25
20
15
10
5
0
A (n=15)
B (n=16)
C (n=14)
surface

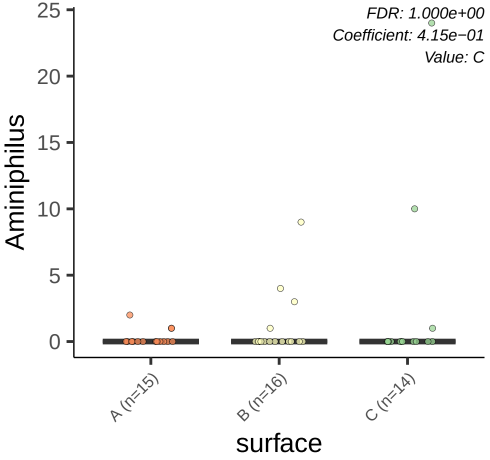
FDR: 1.000e+00
Coefficient: 4.15e−01
Value: C
Aminiphilus
25
20
15
10
5
0
A (n=15)
B (n=16)
C (n=14)
surface

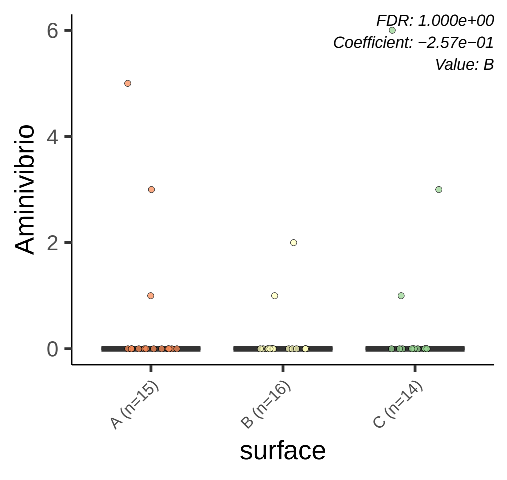

FDR: 1.000e+00
Coefficient: −2.57e−01
Value: B
Aminivibrio
6
4
2
0
A (n=15)
B (n=16)
C (n=14)
surface

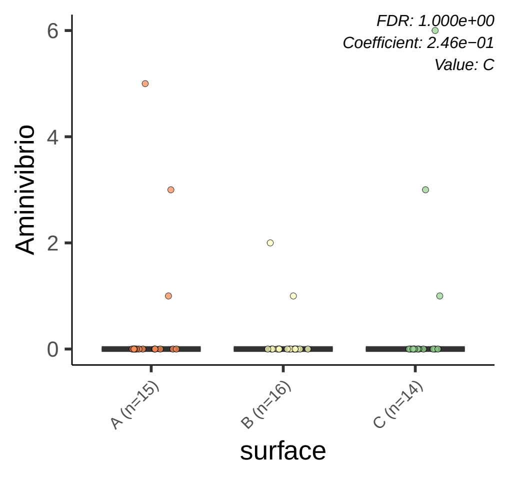
FDR: 1.000e+00
Coefficient: 2.46e−01
Value: C
Aminivibrio
6
4
2
0
A (n=15)
B (n=16)
C (n=14)
surface

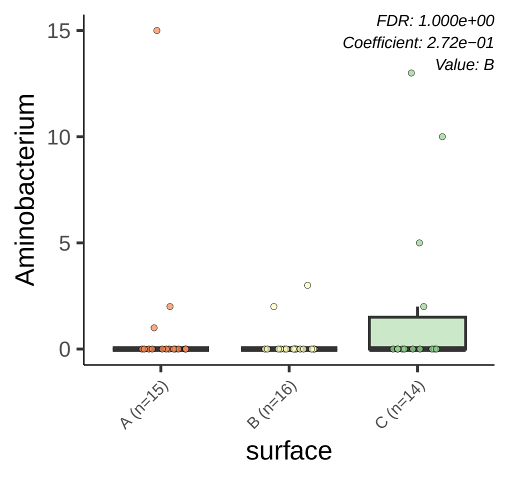
FDR: 1.000e+00
Coefficient: 2.72e−01
Value: B
Aminobacterium
15
10
5
0
A (n=15)
B (n=16)
C (n=14)
surface

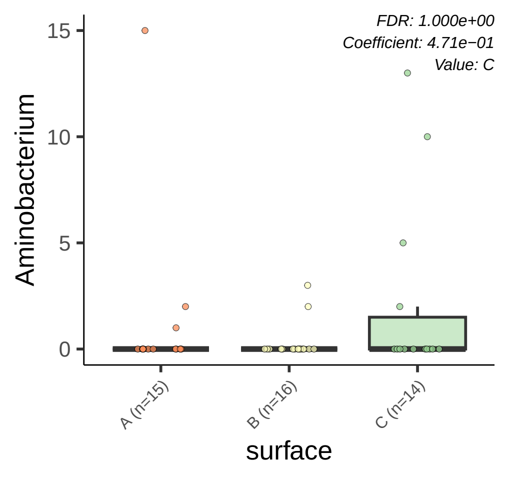
FDR: 1.000e+00
Coefficient: 4.71e−01
Value: C
Aminobacterium
15
10
5
0
A (n=15)
B (n=16)
C (n=14)
surface

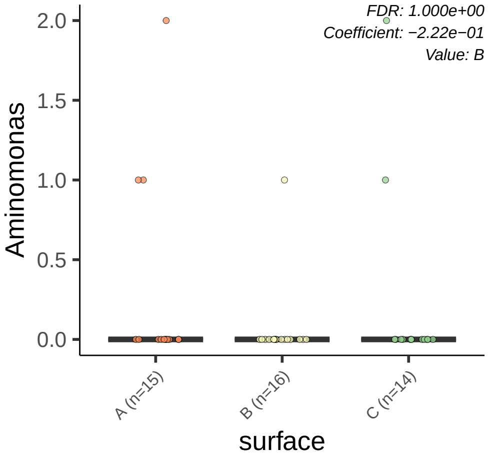

FDR: 1.000e+00
Coefficient: −2.22e−01
Value: B
Aminomonas
2.0
1.5
1.0
0.5
0.0
A (n=15)
B (n=16)
C (n=14)
surface

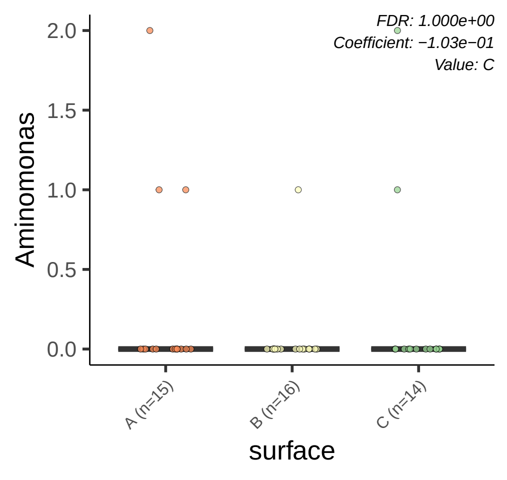
FDR: 1.000e+00
Coefficient: −1.03e−01
Value: C
Aminomonas
2.0
1.5
1.0
0.5
0.0
A (n=15)
B (n=16)
C (n=14)
surface

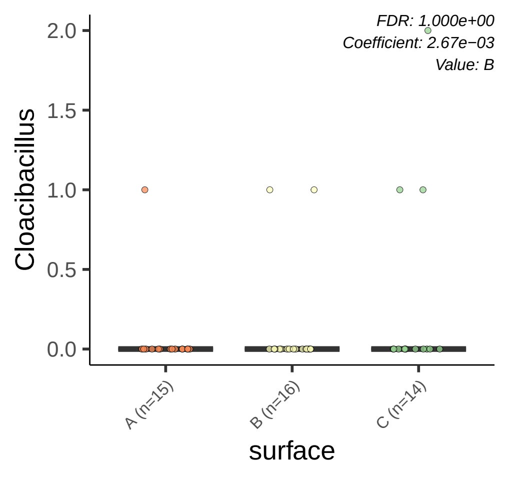
FDR: 1.000e+00
Coefficient: 2.67e−03
Value: B
Cloacibacillus
2.0
1.5
1.0
0.5
0.0
A (n=15)
B (n=16)
C (n=14)
surface

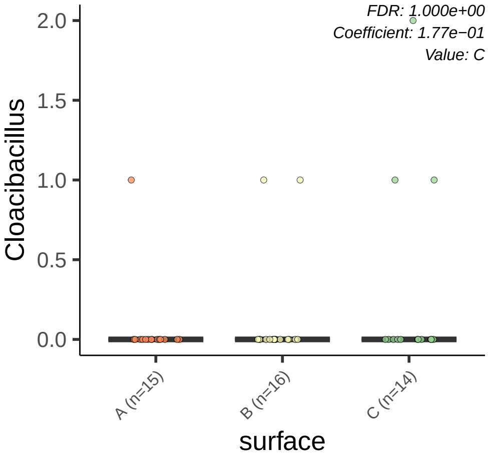

FDR: 1.000e+00
Coefficient: 1.77e−01
Value: C
Cloacibacillus
2.0
1.5
1.0
0.5
0.0
A (n=15)
B (n=16)
C (n=14)
surface

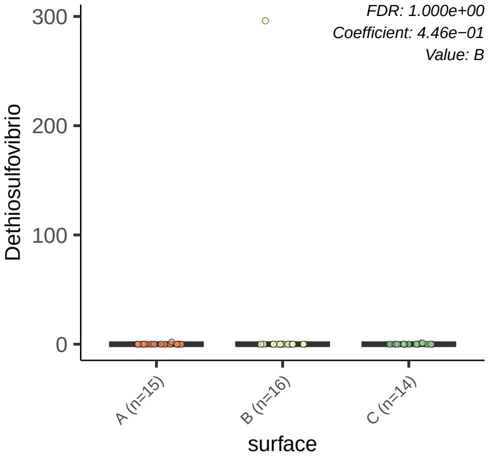
FDR: 1.000e+00
Coefficient: 4.46e−01
Value: B
Dethiosulfovibrio
300
200
100
0
A (n=15)
B (n=16)
C (n=14)
surface

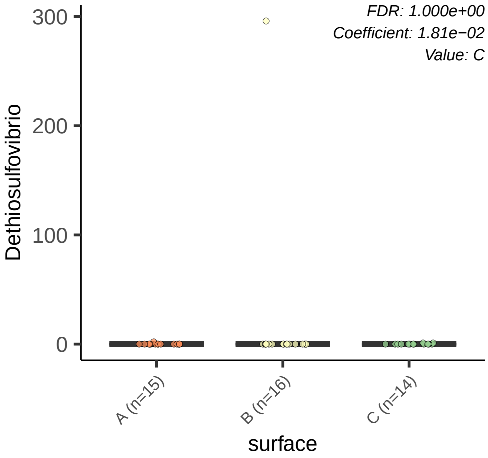
FDR: 1.000e+00
Coefficient: 1.81e−02
Value: C
300
200
100
0
Dethiosulfovibrio
A (n=15)
B (n=16)
C (n=14)
surface

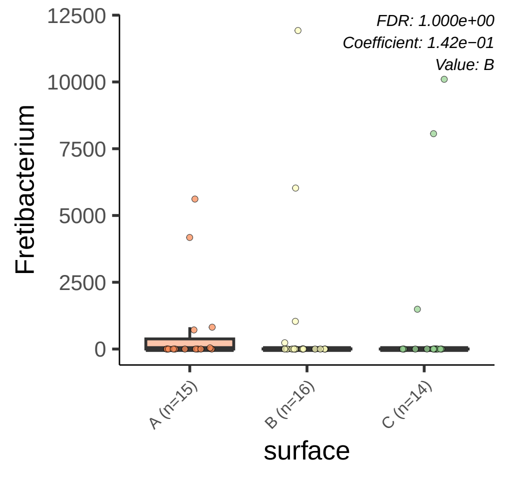

FDR: 1.000e+00
Coefficient: 1.42e−01
Value: B
Fretibacterium
12500
10000
7500
5000
2500
0
A (n=15)
B (n=16)
C (n=14)
surface

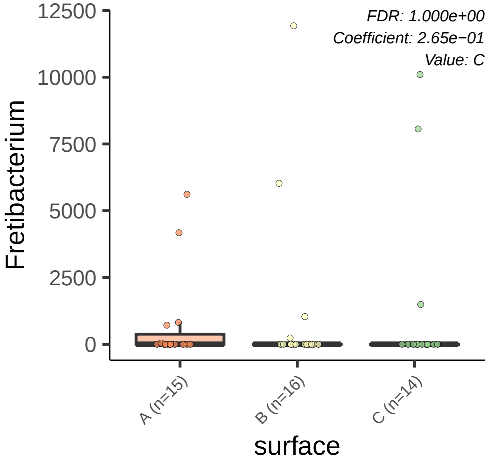
FDR: 1.000e+00
Coefficient: 2.65e−01
Value: C
Fretibacterium
12500
10000
7500
5000
2500
0
A (n=15)
B (n=16)
C (n=14)
surface

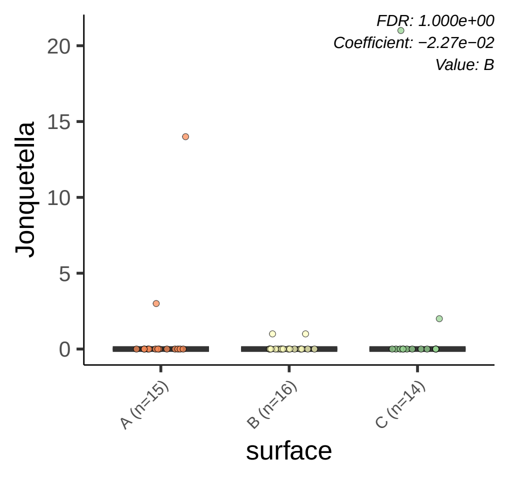
FDR: 1.000e+00
Coefficient: −2.27e−02
Value: B
Jonquetella
20
15
10
5
0
A (n=15)
B (n=16)
C (n=14)
surface

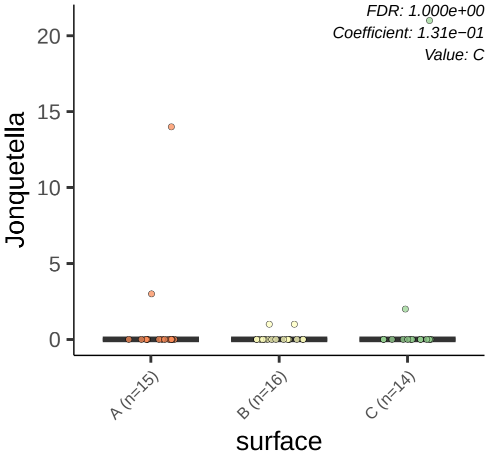
FDR: 1.000e+00
Coefficient: 1.31e−01
Value: C
Jonquetella
20
15
10
5
0
A (n=15)
B (n=16)
C (n=14)
surface

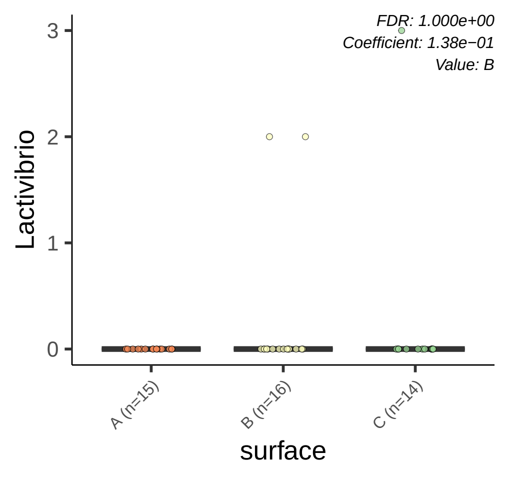
FDR: 1.000e+00
Coefficient: 1.38e−01
Value: B
Lactivibrio
3
2
1
0
A (n=15)
B (n=16)
C (n=14)
surface

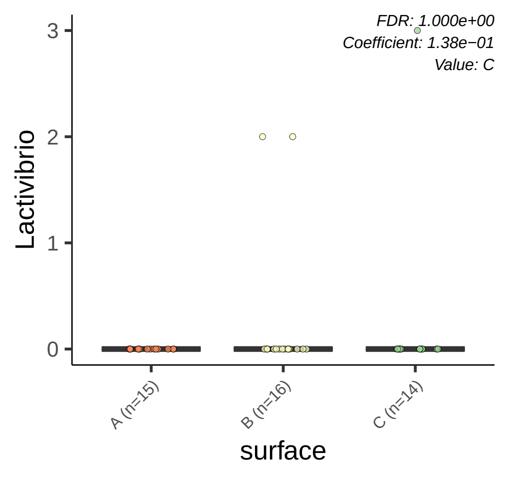
FDR: 1.000e+00
Coefficient: 1.38e−01
Value: C
Lactivibrio
3
2
1
0
A (n=15)
B (n=16)
C (n=14)
surface

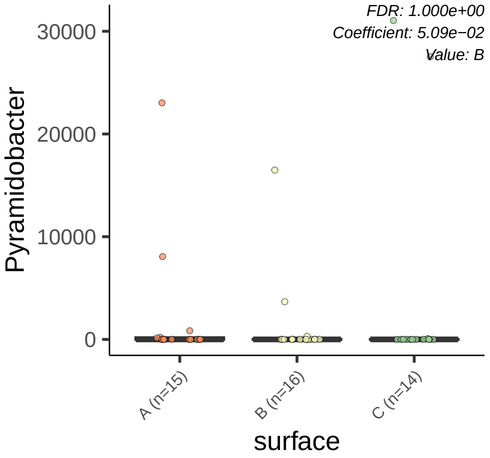
FDR: 1.000e+00
Coefficient: 5.09e−02
Value: B
Pyramidobacter
30000
20000
10000
0
A (n=15)
B (n=16)
C (n=14)
surface

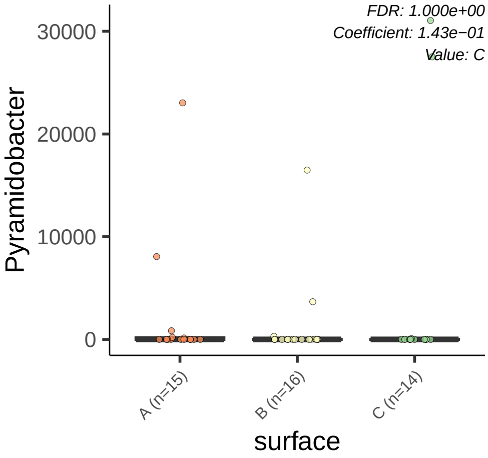
FDR: 1.000e+00
Coefficient: 1.43e−01
Value: C
Pyramidobacter
30000
20000
10000
0
A (n=15)
B (n=16)
C (n=14)
surface

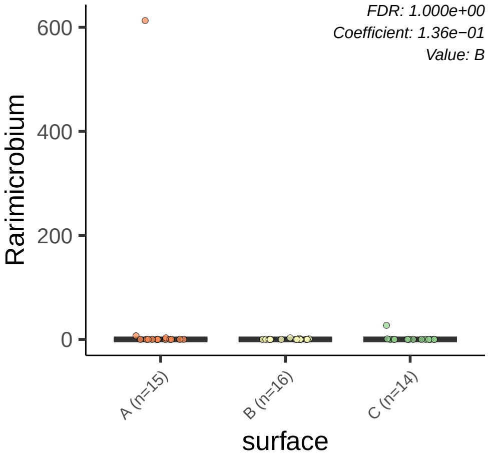

FDR: 1.000e+00
Coefficient: 1.36e−01
Value: B
Rarimicrobium
600
400
200
0
A (n=15)
B (n=16)
C (n=14)
surface

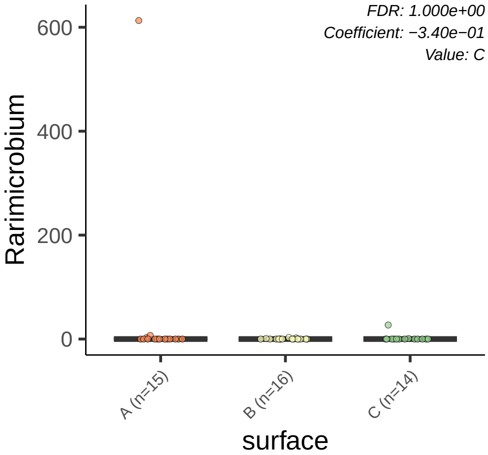

FDR: 1.000e+00
Coefficient: −3.40e−01
Value: C
Rarimicrobium
600
400
200
0
A (n=15)
B (n=16)
C (n=14)
surface

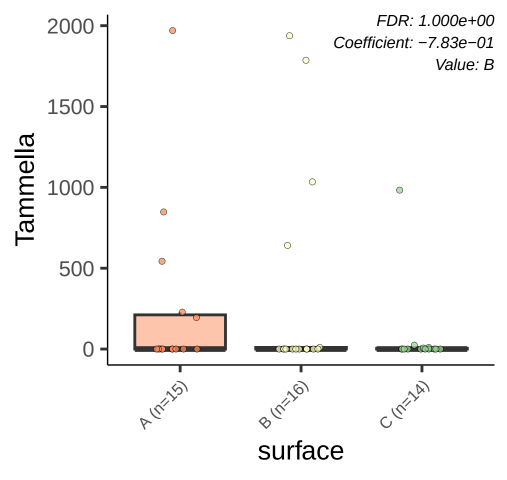

FDR: 1.000e+00
Coefficient: −7.83e−01
Value: B
Tammella
2000
1500
1000
500
0
A (n=15)
B (n=16)
C (n=14)
surface

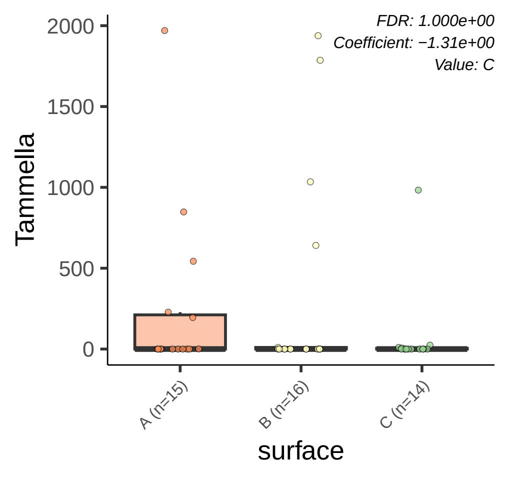
FDR: 1.000e+00
Coefficient: −1.31e+00
Value: C
Tammella
2000
1500
1000
500
0
A (n=15)
B (n=16)
C (n=14)
surface

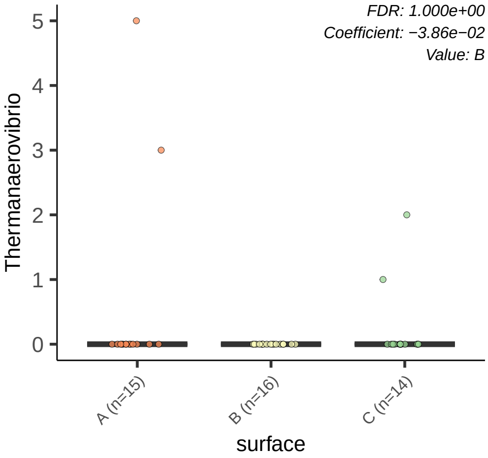
FDR: 1.000e+00
Coefficient: −3.86e−02
Value: B
Thermanaerovibrio
0
1
2
3
4
5
A (n=15)
B (n=16)
C (n=14)
surface

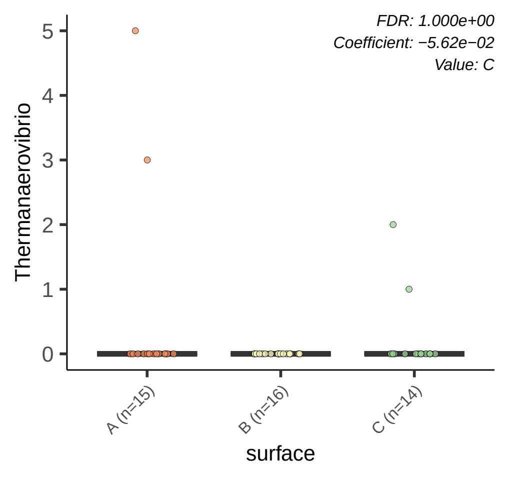
FDR: 1.000e+00
Coefficient: −5.62e−02
Value: C
Thermanaerovibrio
0
1
2
3
4
5
A (n=15)
B (n=16)
C (n=14)
surface

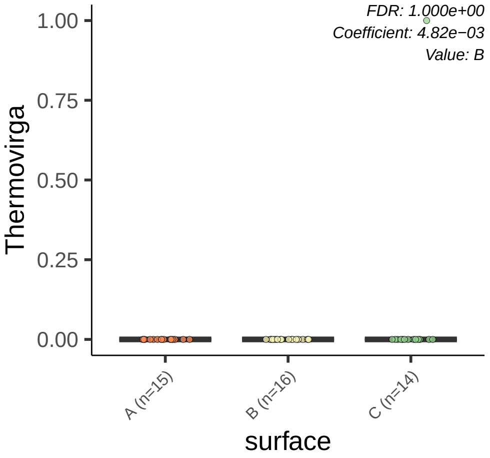
FDR: 1.000e+00
Coefficient: 4.82e−03
Value: B
Thermovirga
1.00
0.75
0.50
0.25
0.00
A (n=15)
B (n=16)
C (n=14)
surface

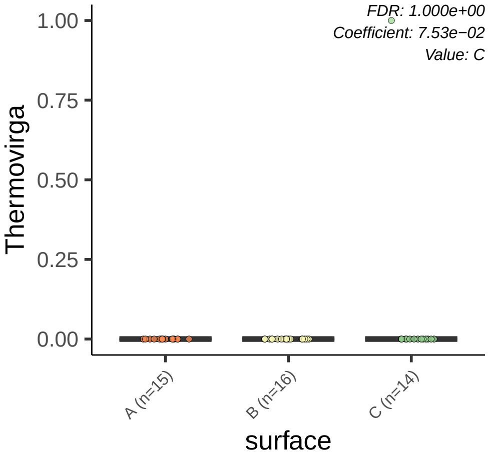

FDR: 1.000e+00
Coefficient: 7.53e−02
Value: C
Thermovirga
1.00
0.75
0.50
0.25
0.00
A (n=15)
B (n=16)
C (n=14)
surface

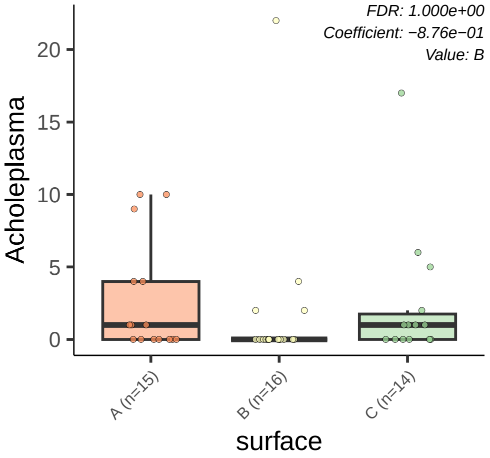
FDR: 1.000e+00
Coefficient: −8.76e−01
Value: B
Acholeplasma
20
15
10
5
0
A (n=15)
B (n=16)
C (n=14)
surface

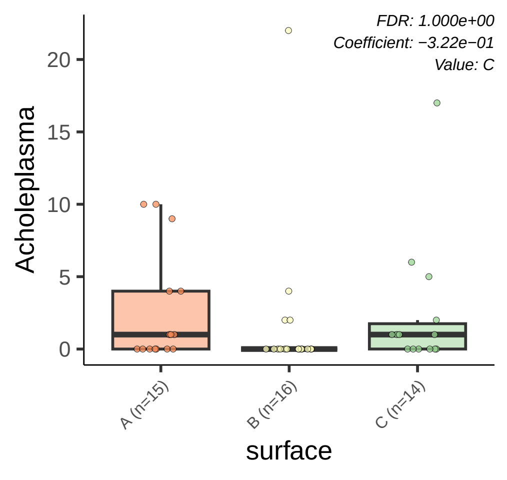

FDR: 1.000e+00
Coefficient: −3.22e−01
Value: C
Acholeplasma
20
15
10
5
0
A (n=15)
B (n=16)
C (n=14)
surface

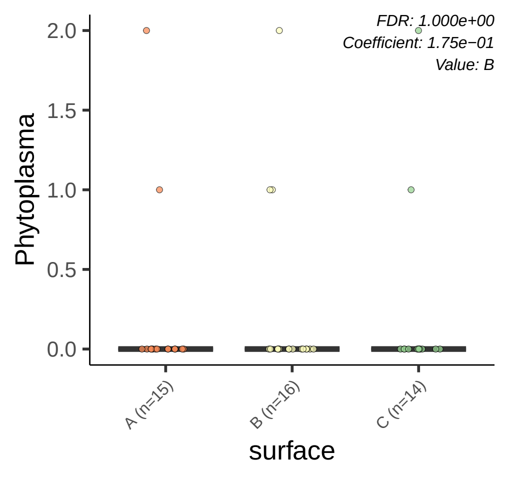
FDR: 1.000e+00
Coefficient: 1.75e−01
Value: B
Phytoplasma
2.0
1.5
1.0
0.5
0.0
A (n=15)
B (n=16)
C (n=14)
surface

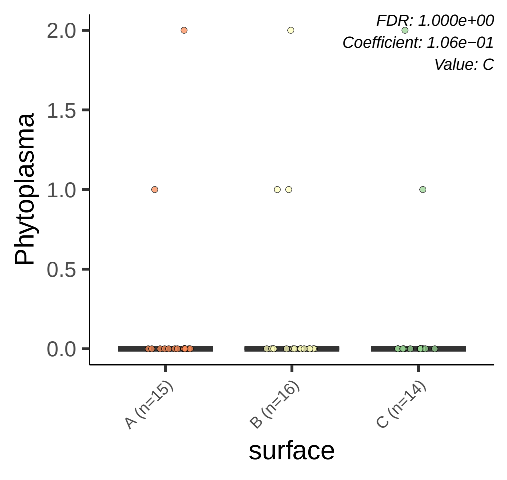
FDR: 1.000e+00
Coefficient: 1.06e−01
Value: C
Phytoplasma
2.0
1.5
1.0
0.5
0.0
A (n=15)
B (n=16)
C (n=14)
surface

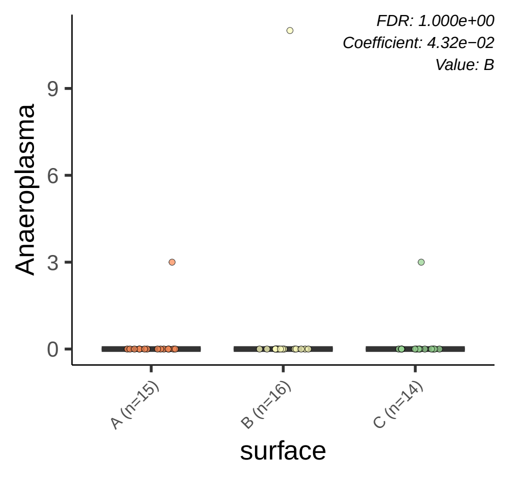

FDR: 1.000e+00
Coefficient: 4.32e−02
Value: B
Anaeroplasma
9
6
3
0
A (n=15)
B (n=16)
C (n=14)
surface

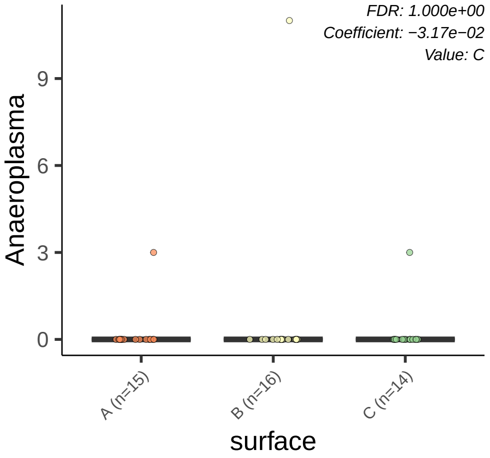
FDR: 1.000e+00
Coefficient: −3.17e−02
Value: C
Anaeroplasma
9
6
3
0
A (n=15)
B (n=16)
C (n=14)
surface

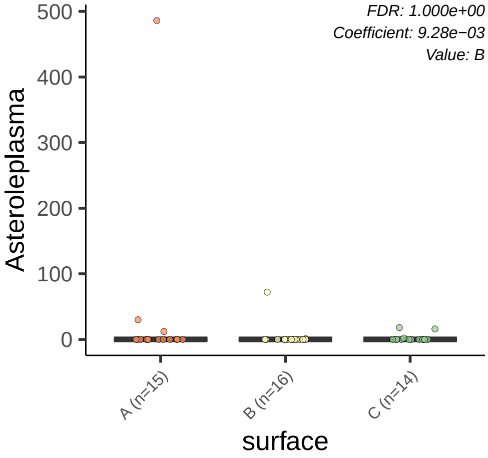
FDR: 1.000e+00
Coefficient: 9.28e−03
Value: B
Asteroleplasma
500
400
300
200
100
0
A (n=15)
B (n=16)
C (n=14)
surface

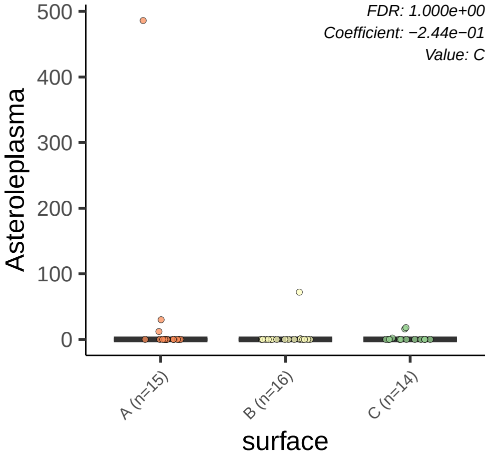
FDR: 1.000e+00
Coefficient: −2.44e−01
Value: C
Asteroleplasma
500
400
300
200
100
0
A (n=15)
B (n=16)
C (n=14)
surface

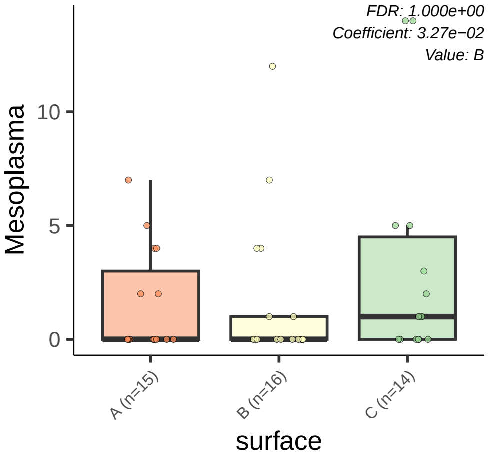
FDR: 1.000e+00
Coefficient: 3.27e−02
Value: B
Mesoplasma
10
5
0
A (n=15)
B (n=16)
C (n=14)
surface

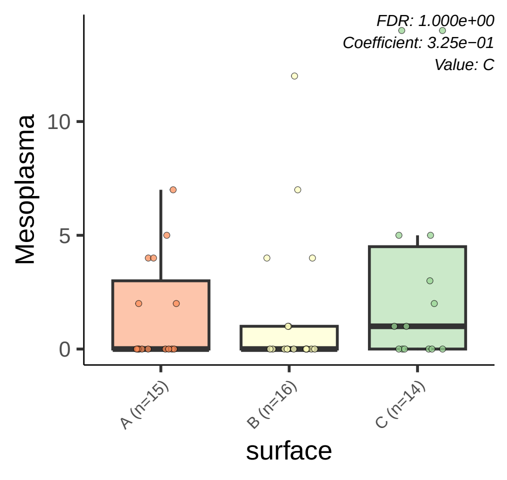

FDR: 1.000e+00
Coefficient: 3.25e−01
Value: C
Mesoplasma
0
5
10
A (n=15)
B (n=16)
C (n=14)
surface

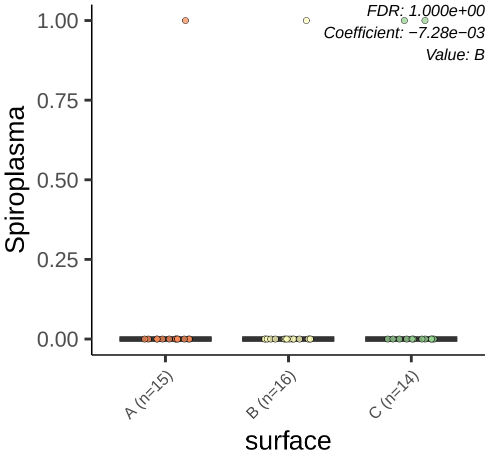
FDR: 1.000e+00
Coefficient: −7.28e−03
Value: B
Spiroplasma
1.00
0.75
0.50
0.25
0.00
A (n=15)
B (n=16)
C (n=14)
surface

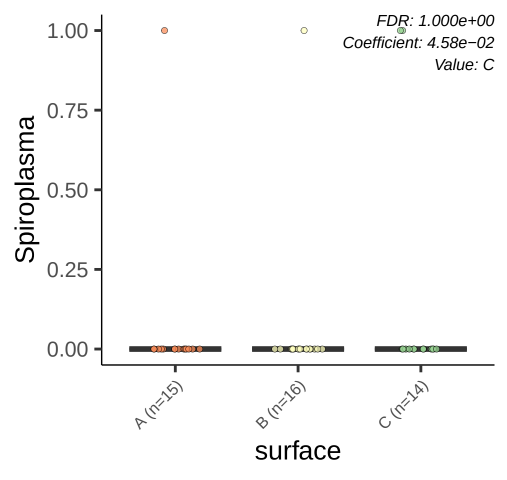

FDR: 1.000e+00
Coefficient: 4.58e−02
Value: C
Spiroplasma
1.00
0.75
0.50
0.25
0.00
A (n=15)
B (n=16)
C (n=14)
surface

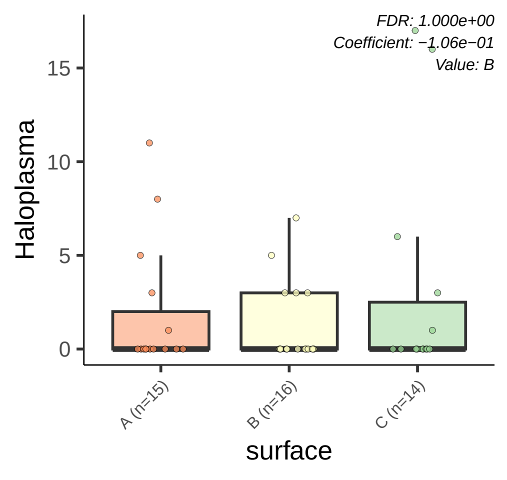
FDR: 1.000e+00
Coefficient: −1.06e−01
Value: B
Haloplasma
0
5
10
15
A (n=15)
B (n=16)
C (n=14)
surface

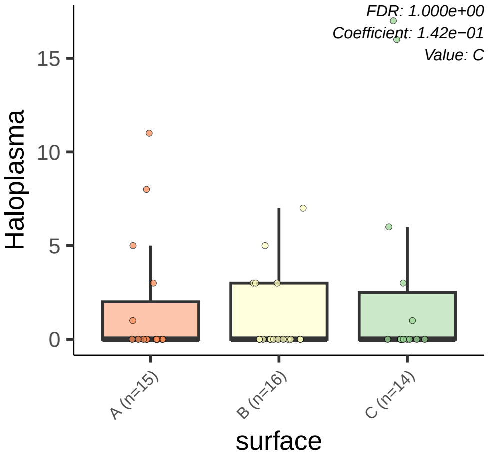

FDR: 1.000e+00
Coefficient: 1.42e−01
Value: C
Haloplasma
0
5
10
15
A (n=15)
B (n=16)
C (n=14)
surface

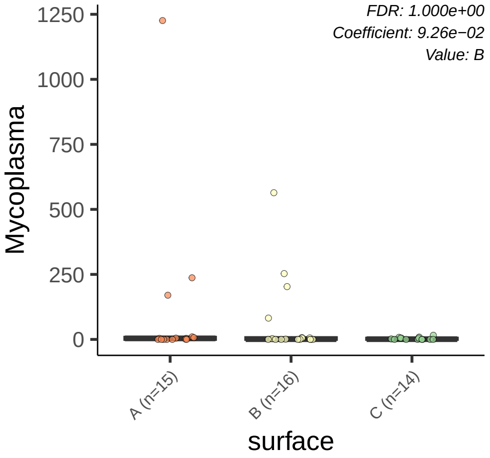

FDR: 1.000e+00
Coefficient: 9.26e−02
Value: B
Mycoplasma
1250
1000
750
500
250
0
A (n=15)
B (n=16)
C (n=14)
surface

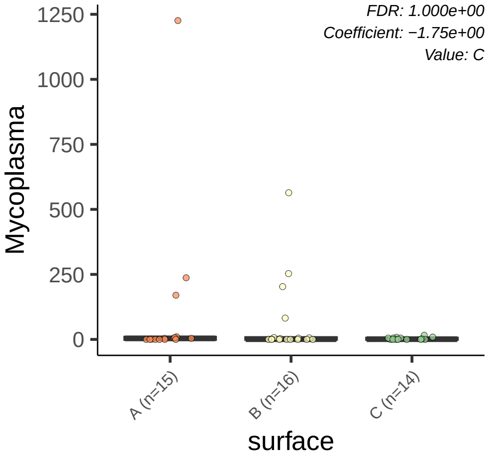

FDR: 1.000e+00
Coefficient: −1.75e+00
Value: C
Mycoplasma
1250
1000
750
500
250
0
A (n=15)
B (n=16)
C (n=14)
surface

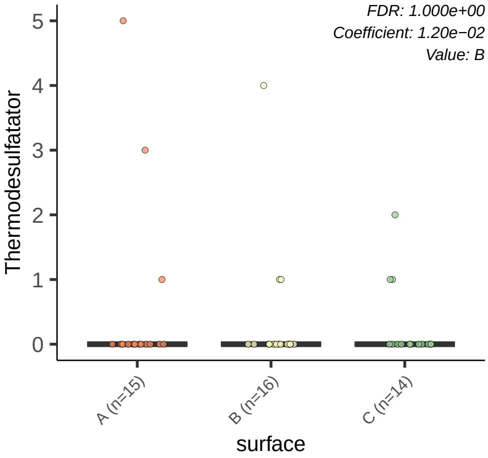

FDR: 1.000e+00
Coefficient: 1.20e−02
Value: B
Thermodesulfatator
0
1
2
3
4
5
A (n=15)
B (n=16)
C (n=14)
surface

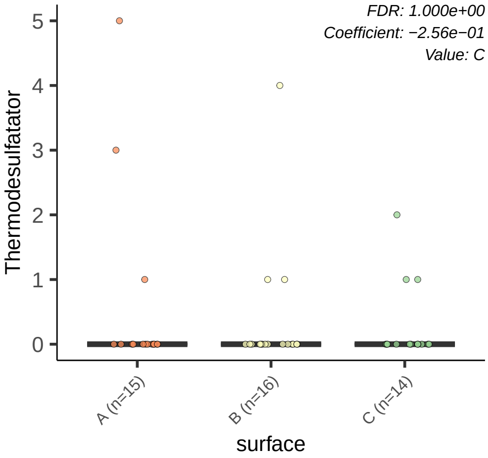
FDR: 1.000e+00
Coefficient: −2.56e−01
Value: C
Thermodesulfatator
0
1
2
3
4
5
A (n=15)
B (n=16)
C (n=14)
surface

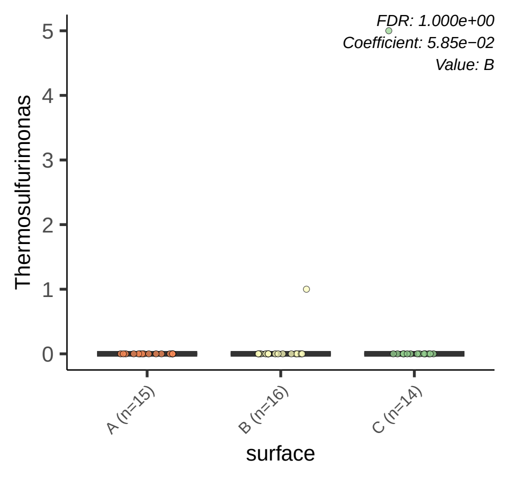
FDR: 1.000e+00
Coefficient: 5.85e−02
Value: B
Thermosulfurimonas
0
1
2
3
4
5
A (n=15)
B (n=16)
C (n=14)
surface

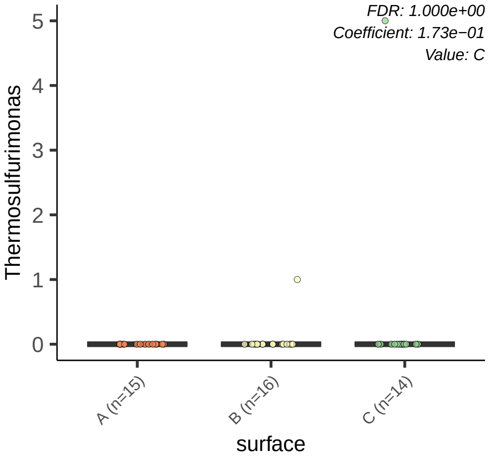
FDR: 1.000e+00
Coefficient: 1.73e−01
Value: C
Thermosulfurimonas
5
4
3
2
1
0
A (n=15)
B (n=16)
C (n=14)
surface

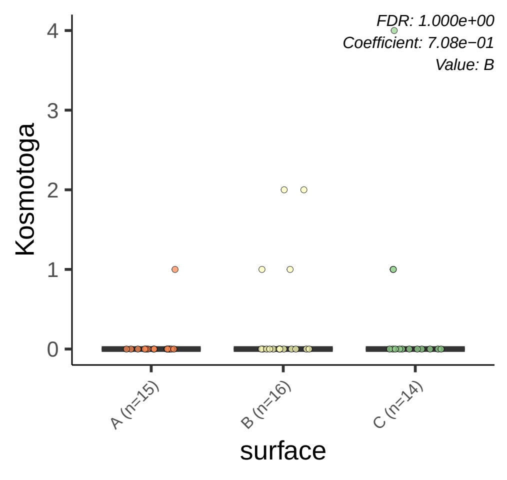
FDR: 1.000e+00
Coefficient: 7.08e−01
Value: B
Kosmotoga
4
3
2
1
0
A (n=15)
B (n=16)
C (n=14)
surface

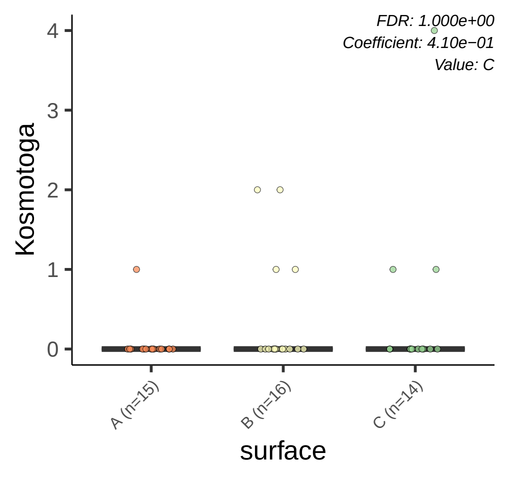
FDR: 1.000e+00
Coefficient: 4.10e−01
Value: C
Kosmotoga
0
1
2
3
4
A (n=15)
B (n=16)
C (n=14)
surface

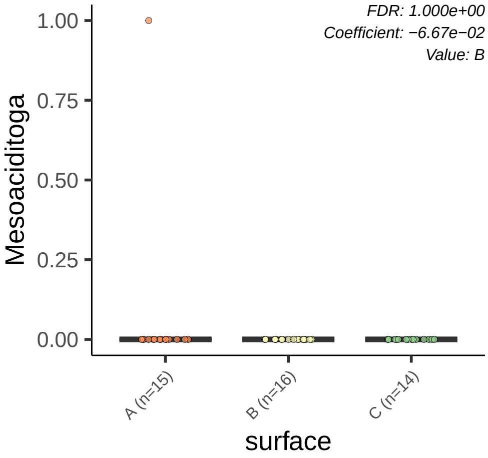

FDR: 1.000e+00
Coefficient: −6.67e−02
Value: B
Mesoaciditoga
1.00
0.75
0.50
0.25
0.00
A (n=15)
B (n=16)
C (n=14)
surface

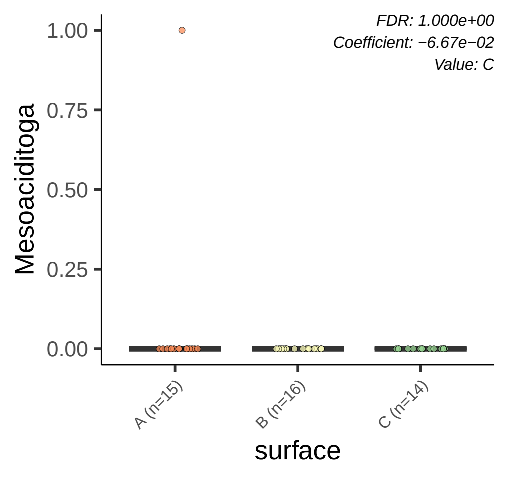
FDR: 1.000e+00
Coefficient: −6.67e−02
Value: C
Mesoaciditoga
1.00
0.75
0.50
0.25
0.00
A (n=15)
B (n=16)
C (n=14)
surface

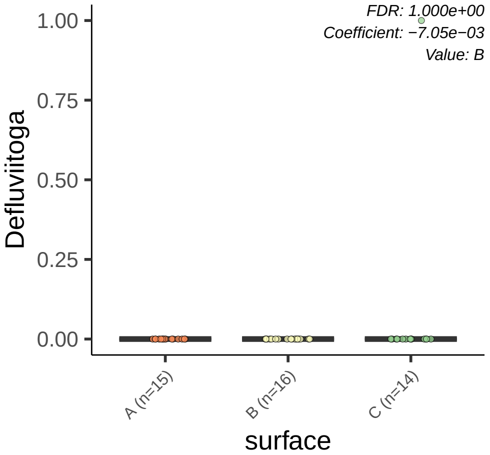
FDR: 1.000e+00
Coefficient: −7.05e−03
Value: B
1.00
0.75
0.50
0.25
0.00
Defluviitoga
A (n=15)
B (n=16)
C (n=14)
surface

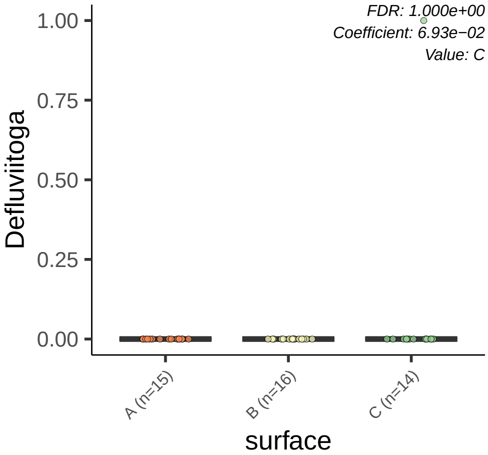

FDR: 1.000e+00
Coefficient: 6.93e−02
Value: C
Defluviitoga
1.00
0.75
0.50
0.25
0.00
A (n=15)
B (n=16)
C (n=14)
surface

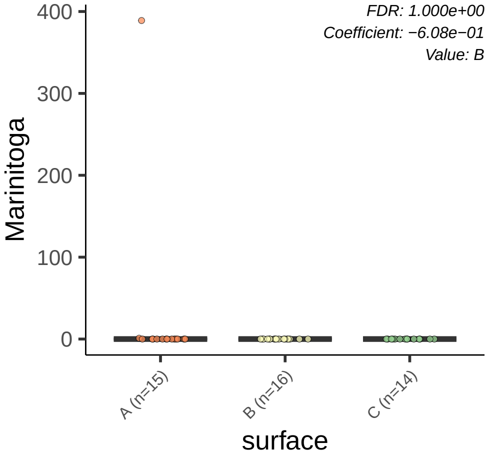
FDR: 1.000e+00
Coefficient: −6.08e−01
Value: B
400
300
200
100
0
Marinitoga
A (n=15)
B (n=16)
C (n=14)
surface

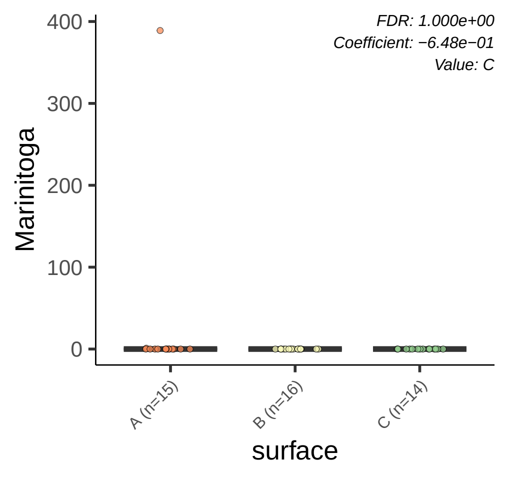
FDR: 1.000e+00
Coefficient: −6.48e−01
Value: C
400
300
200
100
0
Marinitoga
A (n=15)
B (n=16)
C (n=14)
surface

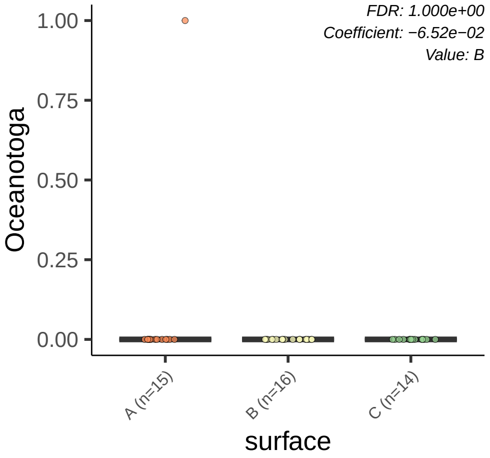

FDR: 1.000e+00
Coefficient: −6.52e−02
Value: B
Oceanotoga
1.00
0.75
0.50
0.25
0.00
A (n=15)
B (n=16)
C (n=14)
surface

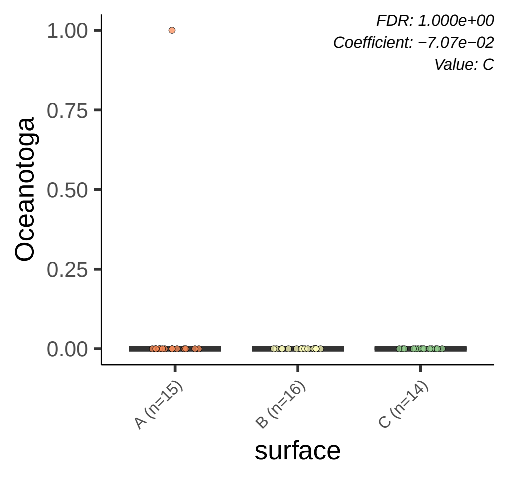

FDR: 1.000e+00
Coefficient: −7.07e−02
Value: C
Oceanotoga
1.00
0.75
0.50
0.25
0.00
A (n=15)
B (n=16)
C (n=14)
surface

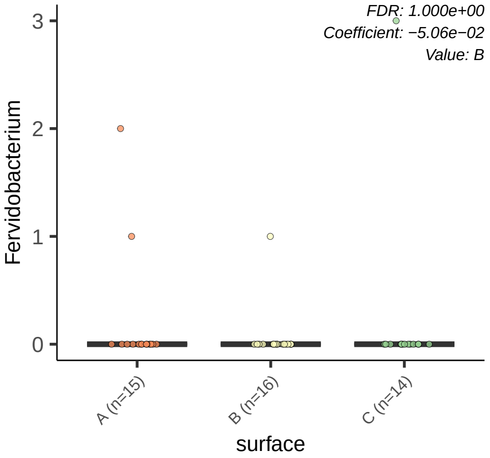
FDR: 1.000e+00
Coefficient: −5.06e−02
Value: B
Fervidobacterium
0
1
2
3
A (n=15)
B (n=16)
C (n=14)
surface

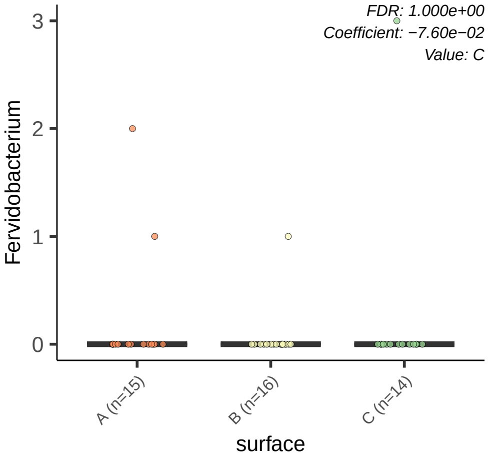
FDR: 1.000e+00
Coefficient: −7.60e−02
Value: C
Fervidobacterium
3
2
1
0
A (n=15)
B (n=16)
C (n=14)
surface

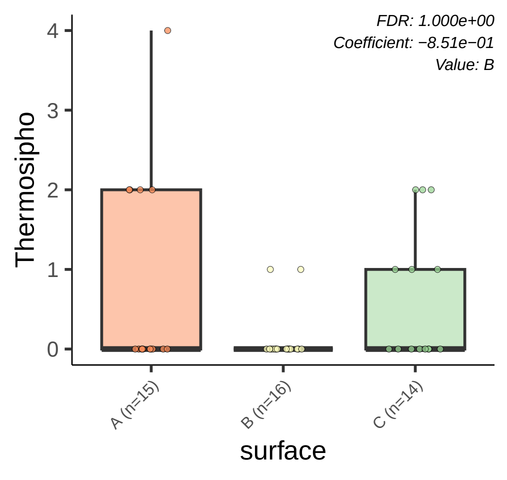
FDR: 1.000e+00
Coefficient: −8.51e−01
Value: B
Thermosipho
4
3
2
1
0
A (n=15)
B (n=16)
C (n=14)
surface

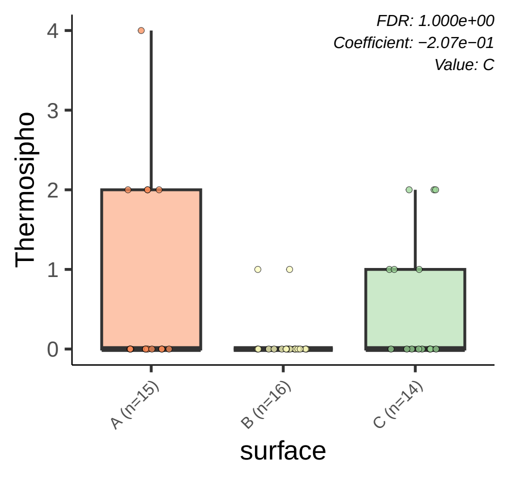
FDR: 1.000e+00
Coefficient: −2.07e−01
Value: C
Thermosipho
0
1
2
3
4
A (n=15)
B (n=16)
C (n=14)
surface

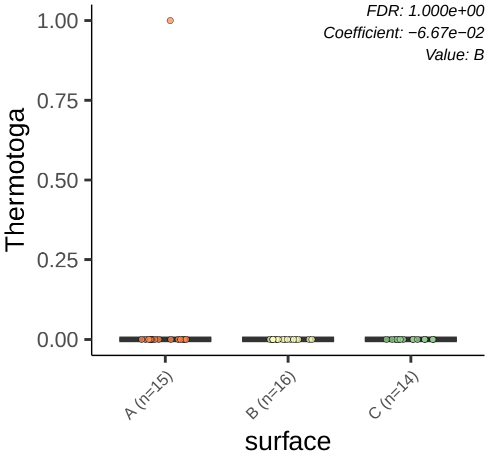
FDR: 1.000e+00
Coefficient: −6.67e−02
Value: B
Thermotoga
1.00
0.75
0.50
0.25
0.00
A (n=15)
B (n=16)
C (n=14)
surface

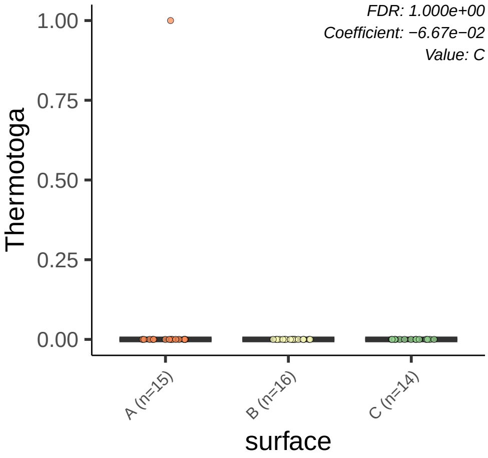

FDR: 1.000e+00
Coefficient: −6.67e−02
Value: C
1.00
0.75
0.50
0.25
0.00
Thermotoga
A (n=15)
B (n=16)
C (n=14)
surface

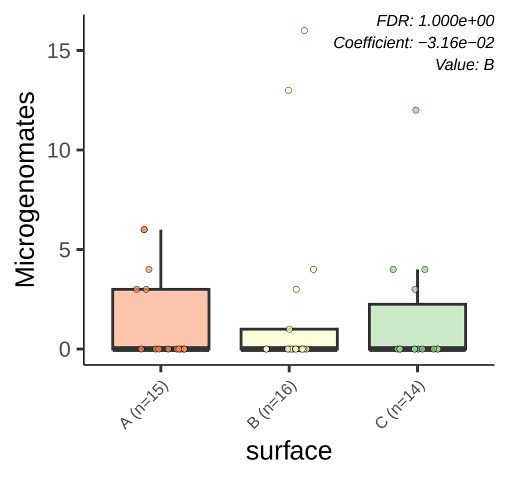
FDR: 1.000e+00
Coefficient: −3.16e−02
Value: B
Microgenomates
0
5
10
15
A (n=15)
B (n=16)
C (n=14)
surface

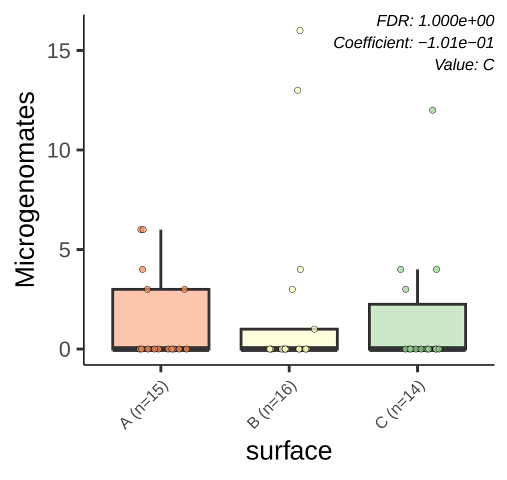

FDR: 1.000e+00
Coefficient: −1.01e−01
Value: C
Microgenomates
0
5
10
15
A (n=15)
B (n=16)
C (n=14)
surface

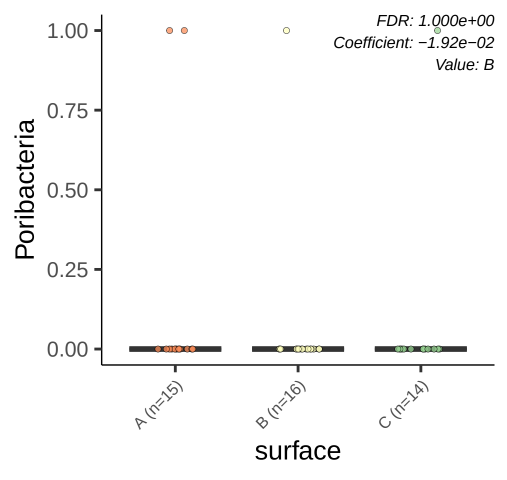

FDR: 1.000e+00
Coefficient: −1.92e−02
Value: B
Poribacteria
1.00
0.75
0.50
0.25
0.00
A (n=15)
B (n=16)
C (n=14)
surface

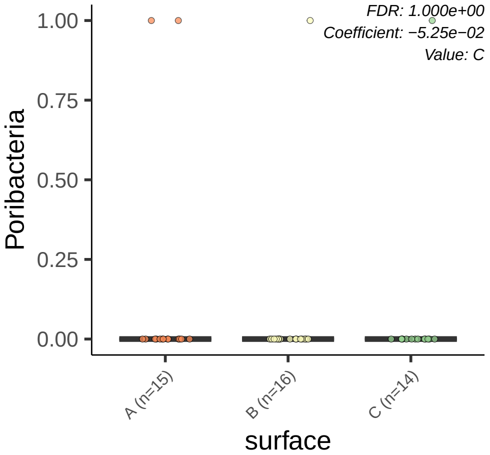

FDR: 1.000e+00
Coefficient: −5.25e−02
Value: C
1.00
0.75
0.50
0.25
0.00
Poribacteria
A (n=15)
B (n=16)
C (n=14)
surface

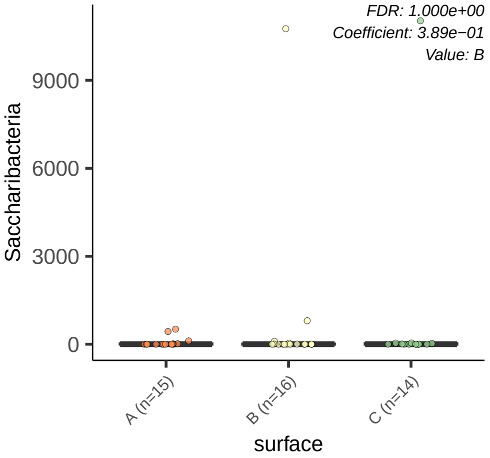
FDR: 1.000e+00
Coefficient: 3.89e−01
Value: B
Saccharibacteria
9000
6000
3000
0
A (n=15)
B (n=16)
C (n=14)
surface

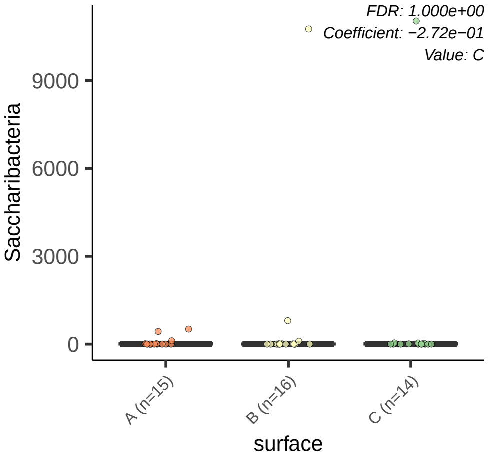
FDR: 1.000e+00
Coefficient: −2.72e−01
Value: C
Saccharibacteria
9000
6000
3000
0
A (n=15)
B (n=16)
C (n=14)
surface

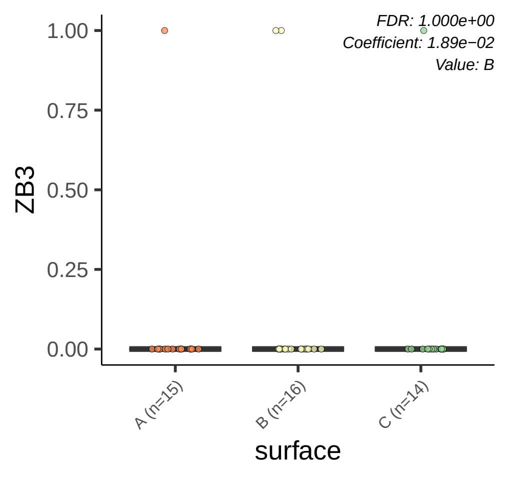

FDR: 1.000e+00
Coefficient: 1.89e−02
Value: B
ZB3
1.00
0.75
0.50
0.25
0.00
A (n=15)
B (n=16)
C (n=14)
surface

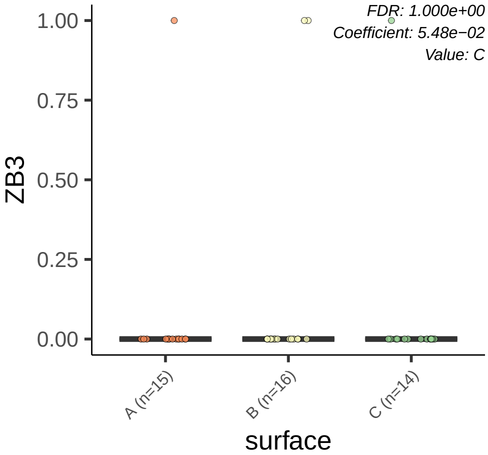
FDR: 1.000e+00
Coefficient: 5.48e−02
Value: C
1.00
0.75
0.50
0.25
0.00
ZB3
A (n=15)
B (n=16)
C (n=14)
surface

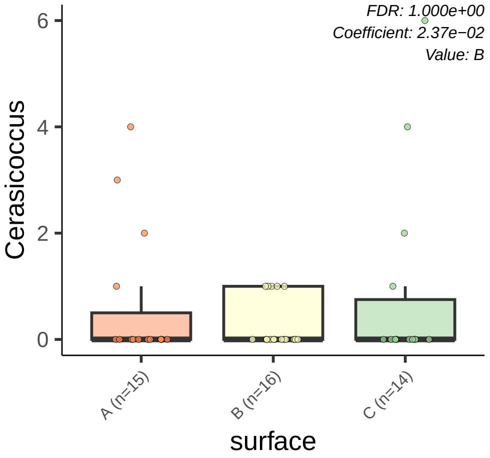

FDR: 1.000e+00
Coefficient: 2.37e−02
Value: B
Cerasicoccus
0
2
4
6
A (n=15)
B (n=16)
C (n=14)
surface

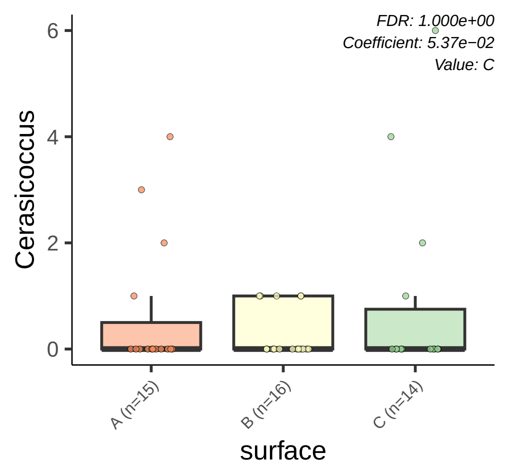
FDR: 1.000e+00
Coefficient: 5.37e−02
Value: C
Cerasicoccus
0
2
4
6
A (n=15)
B (n=16)
C (n=14)
surface

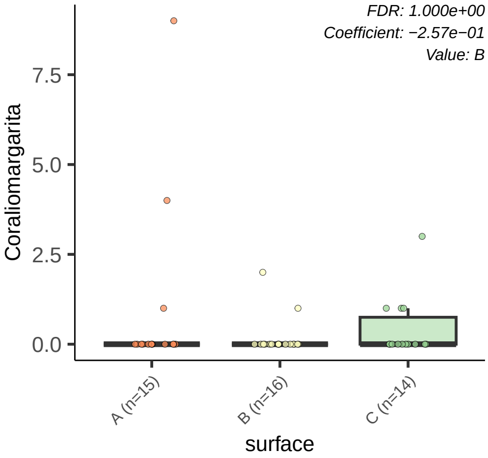

FDR: 1.000e+00
Coefficient: −2.57e−01
Value: B
Coraliomargarita
7.5
5.0
2.5
0.0
A (n=15)
B (n=16)
C (n=14)
surface

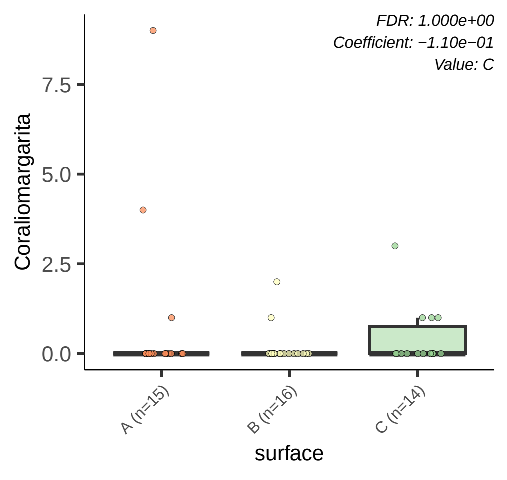

FDR: 1.000e+00
Coefficient: −1.10e−01
Value: C
Coraliomargarita
7.5
5.0
2.5
0.0
A (n=15)
B (n=16)
C (n=14)
surface

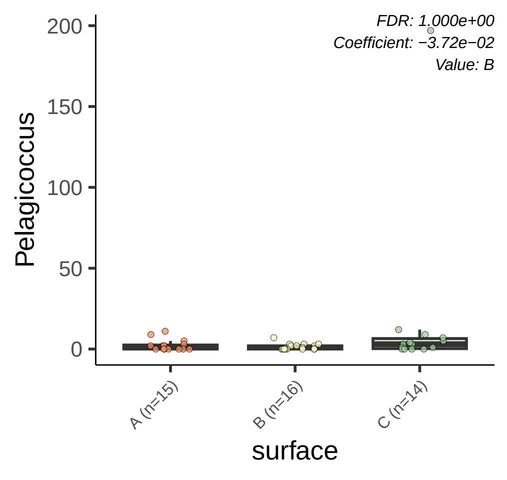

FDR: 1.000e+00
Coefficient: −3.72e−02
Value: B
200
150
100
50
0
Pelagicoccus
A (n=15)
B (n=16)
C (n=14)
surface

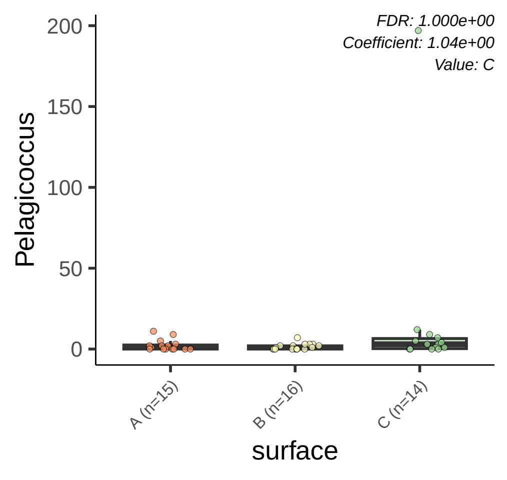

FDR: 1.000e+00
Coefficient: 1.04e+00
Value: C
Pelagicoccus
200
150
100
50
0
A (n=15)
B (n=16)
C (n=14)
surface

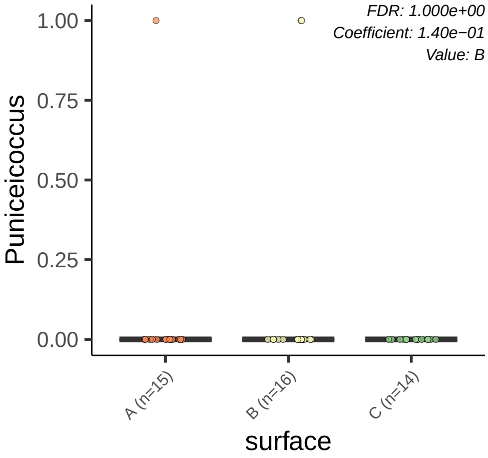
FDR: 1.000e+00
Coefficient: 1.40e−01
Value: B
1.00
0.75
0.50
0.25
0.00
Puniceicoccus
A (n=15)
B (n=16)
C (n=14)
surface

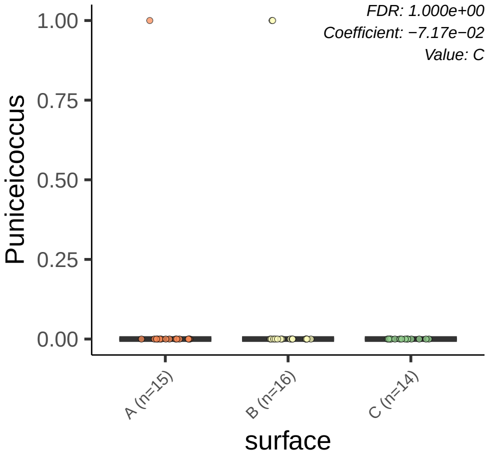
FDR: 1.000e+00
Coefficient: −7.17e−02
Value: C
Puniceicoccus
1.00
0.75
0.50
0.25
0.00
A (n=15)
B (n=16)
C (n=14)
surface

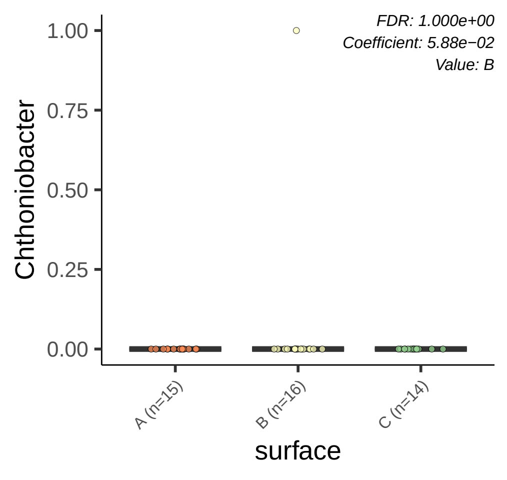
FDR: 1.000e+00
Coefficient: 5.88e−02
Value: B
Chthoniobacter
1.00
0.75
0.50
0.25
0.00
A (n=15)
B (n=16)
C (n=14)
surface

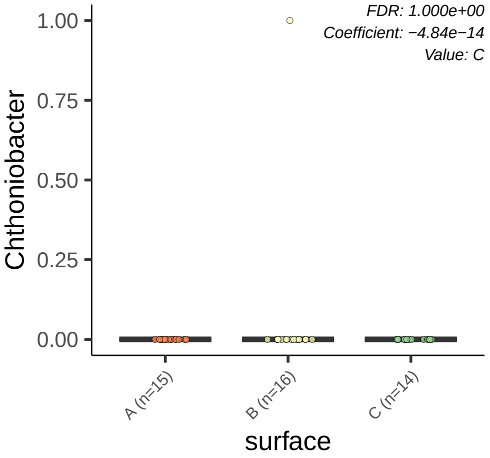

FDR: 1.000e+00
Coefficient: −4.84e−14
Value: C
Chthoniobacter
1.00
0.75
0.50
0.25
0.00
A (n=15)
B (n=16)
C (n=14)
surface

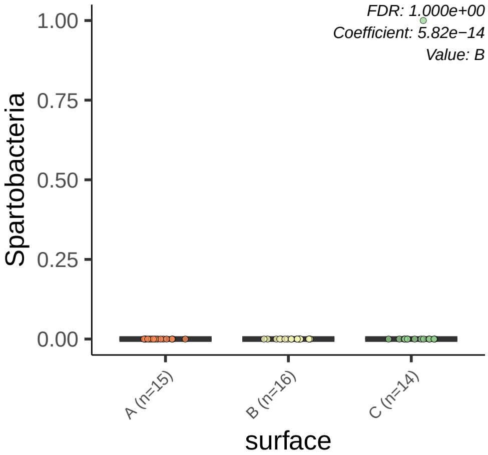
FDR: 1.000e+00
Coefficient: 5.82e−14
Value: B
Spartobacteria
1.00
0.75
0.50
0.25
0.00
A (n=15)
B (n=16)
C (n=14)
surface

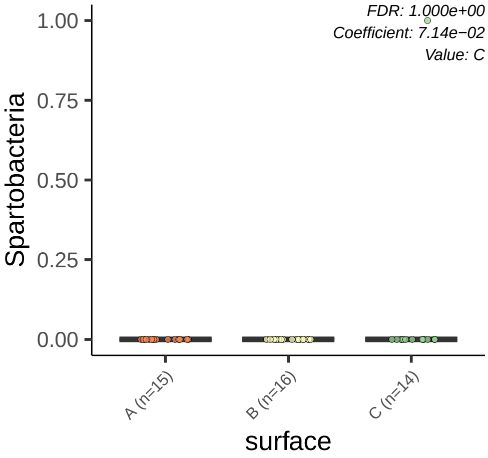

FDR: 1.000e+00
Coefficient: 7.14e−02
Value: C
Spartobacteria
1.00
0.75
0.50
0.25
0.00
A (n=15)
B (n=16)
C (n=14)
surface

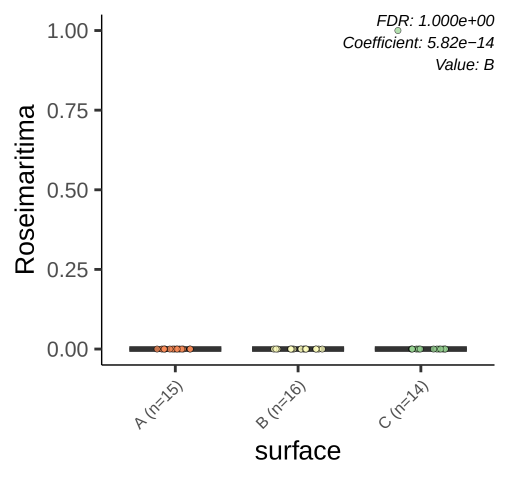
FDR: 1.000e+00
Coefficient: 5.82e−14
Value: B
Roseimaritima
1.00
0.75
0.50
0.25
0.00
A (n=15)
B (n=16)
C (n=14)
surface

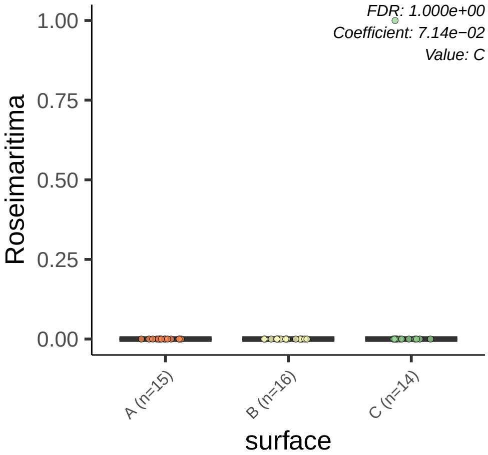
FDR: 1.000e+00
Coefficient: 7.14e−02
Value: C
Roseimaritima
1.00
0.75
0.50
0.25
0.00
A (n=15)
B (n=16)
C (n=14)
surface

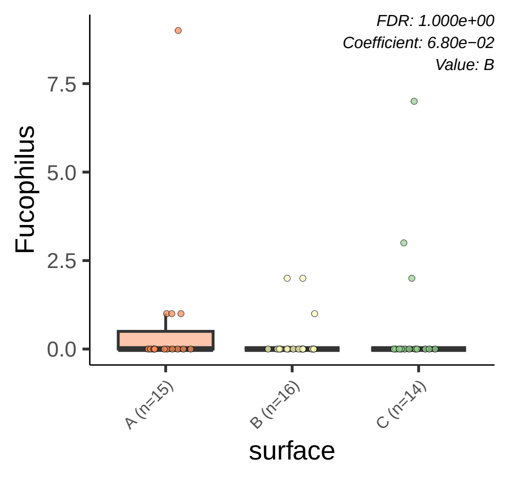

FDR: 1.000e+00
Coefficient: 6.80e−02
Value: B
Fucophilus
7.5
5.0
2.5
0.0
A (n=15)
B (n=16)
C (n=14)
surface

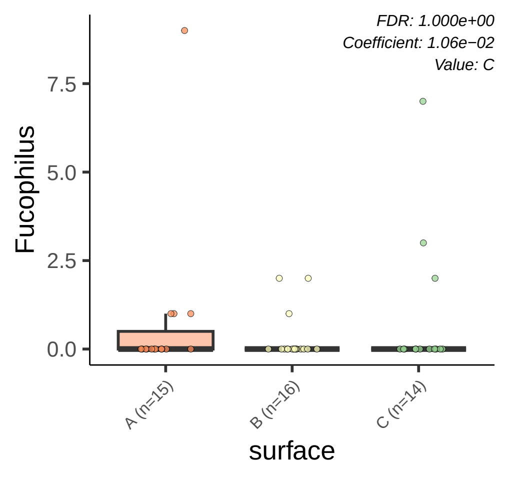
FDR: 1.000e+00
Coefficient: 1.06e−02
Value: C
Fucophilus
7.5
5.0
2.5
0.0
A (n=15)
B (n=16)
C (n=14)
surface

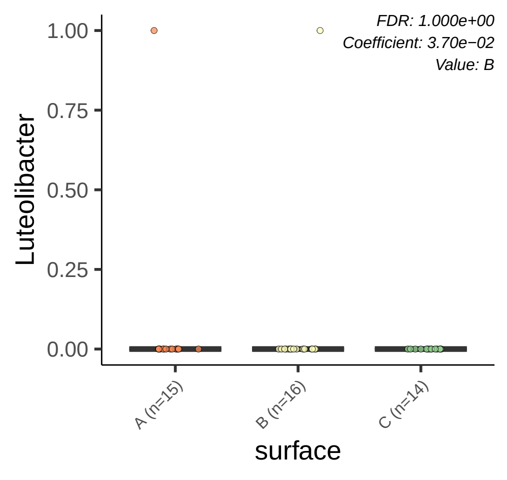

FDR: 1.000e+00
Coefficient: 3.70e−02
Value: B
Luteolibacter
1.00
0.75
0.50
0.25
0.00
A (n=15)
B (n=16)
C (n=14)
surface

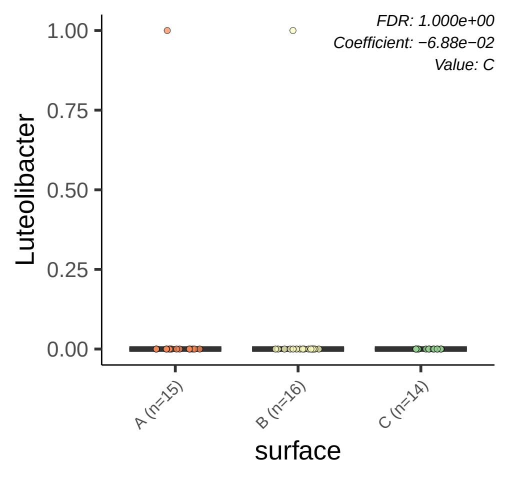

FDR: 1.000e+00
Coefficient: −6.88e−02
Value: C
Luteolibacter
1.00
0.75
0.50
0.25
0.00
A (n=15)
B (n=16)
C (n=14)
surface

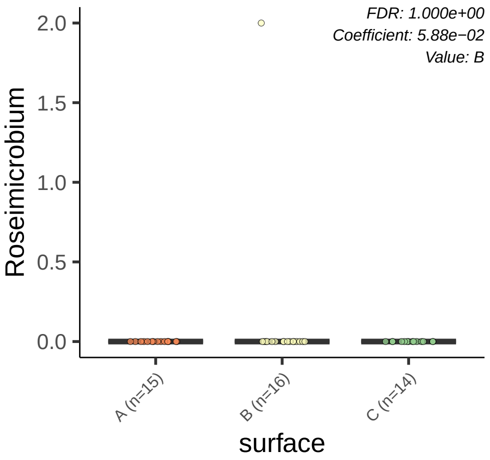
FDR: 1.000e+00
Coefficient: 5.88e−02
Value: B
Roseimicrobium
2.0
1.5
1.0
0.5
0.0
A (n=15)
B (n=16)
C (n=14)
surface

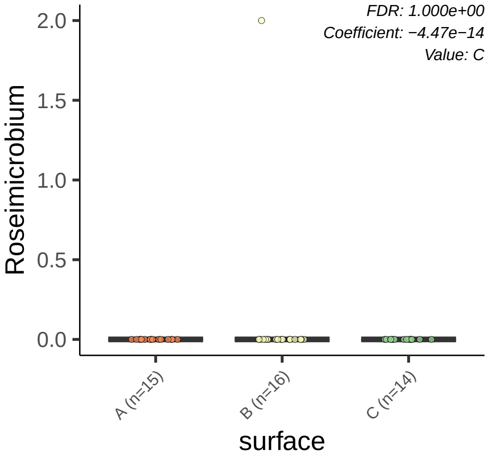

FDR: 1.000e+00
Coefficient: −4.47e−14
Value: C
Roseimicrobium
2.0
1.5
1.0
0.5
0.0
A (n=15)
B (n=16)
C (n=14)
surface

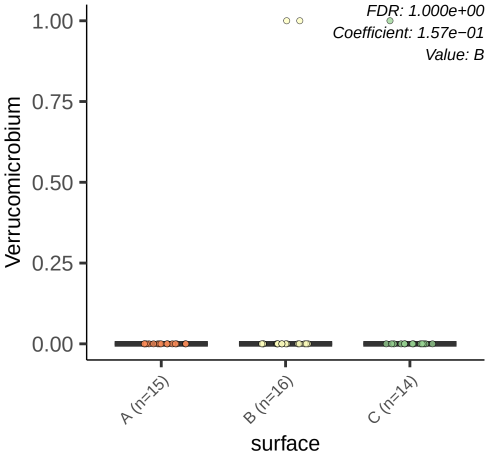
FDR: 1.000e+00
Coefficient: 1.57e−01
Value: B
Verrucomicrobium
1.00
0.75
0.50
0.25
0.00
A (n=15)
B (n=16)
C (n=14)
surface

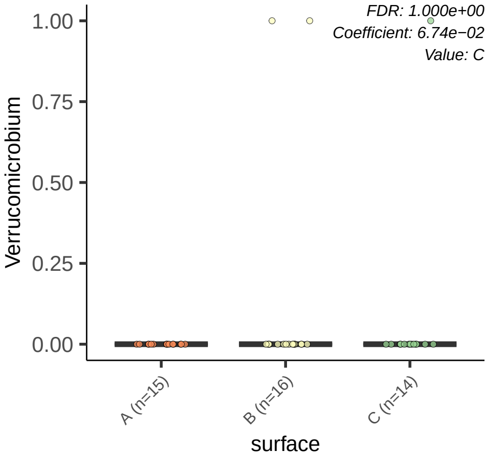

FDR: 1.000e+00
Coefficient: 6.74e−02
Value: C
1.00
0.75
0.50
0.25
0.00
Verrucomicrobium
A (n=15)
B (n=16)
C (n=14)
surface

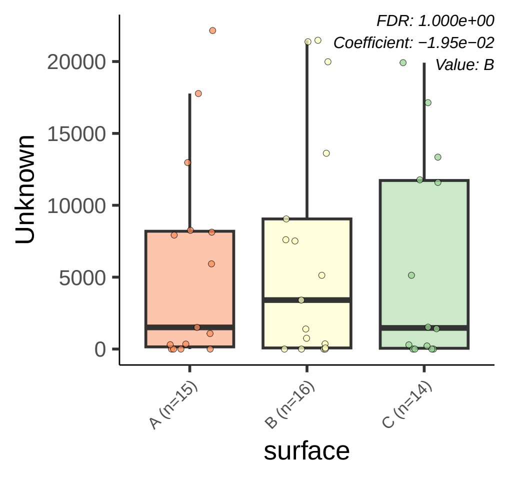
FDR: 1.000e+00
Coefficient: −1.95e−02
Value: B
Unknown
20000
15000
10000
5000
0
A (n=15)
B (n=16)
C (n=14)
surface

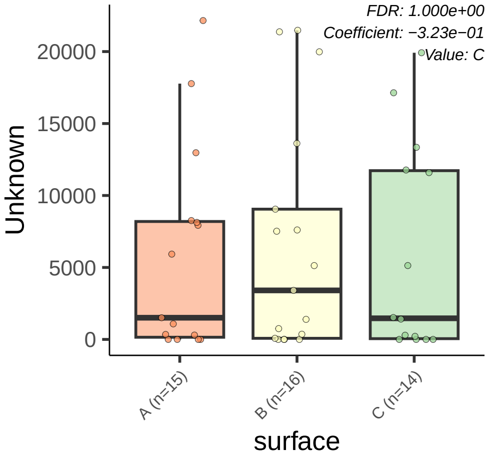

FDR: 1.000e+00
Coefficient: −3.23e−01
Value: C
Unknown
0
5000
10000
15000
20000
A (n=15)
B (n=16)
C (n=14)
surface

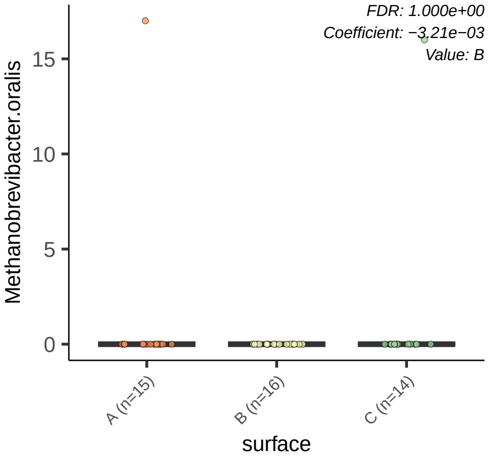
FDR: 1.000e+00
Coefficient: −3.21e−03
Value: B
Methanobrevibacter.oralis
15
10
5
0
A (n=15)
B (n=16)
C (n=14)
surface

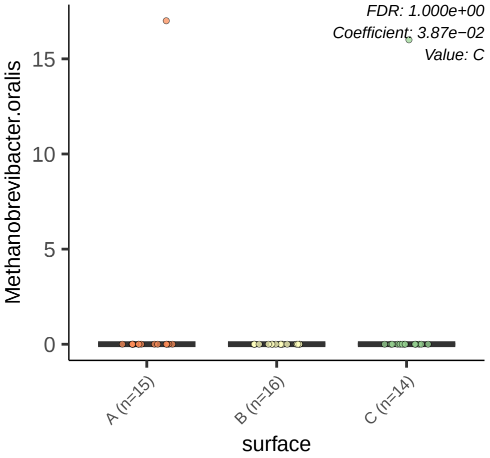
FDR: 1.000e+00
Coefficient: 3.87e−02
Value: C
Methanobrevibacter.oralis
0
5
10
15
A (n=15)
B (n=16)
C (n=14)
surface

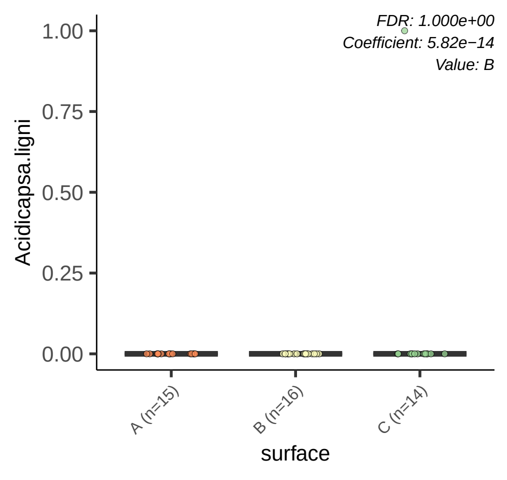
FDR: 1.000e+00
Coefficient: 5.82e−14
Value: B
Acidicapsa.ligni
1.00
0.75
0.50
0.25
0.00
A (n=15)
B (n=16)
C (n=14)
surface

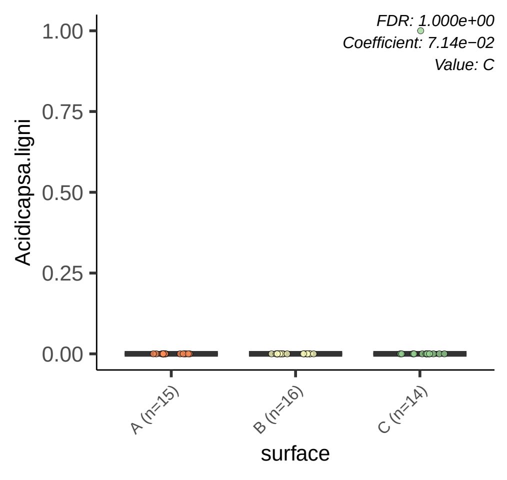
FDR: 1.000e+00
Coefficient: 7.14e−02
Value: C
1.00
0.75
0.50
0.25
0.00
Acidicapsa.ligni
A (n=15)
B (n=16)
C (n=14)
surface

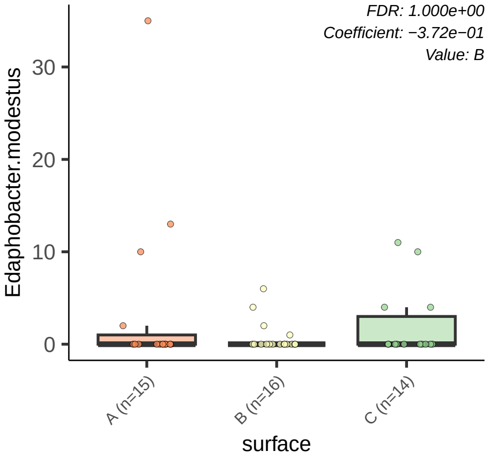
FDR: 1.000e+00
Coefficient: −3.72e−01
Value: B
Edaphobacter.modestus
30
20
10
0
A (n=15)
B (n=16)
C (n=14)
surface

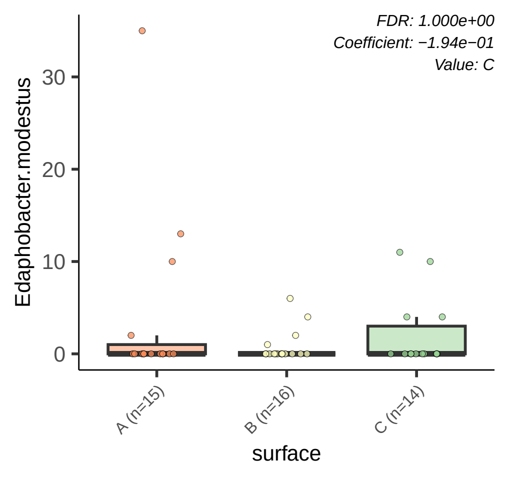
FDR: 1.000e+00
Coefficient: −1.94e−01
Value: C
Edaphobacter.modestus
30
20
10
0
A (n=15)
B (n=16)
C (n=14)
surface

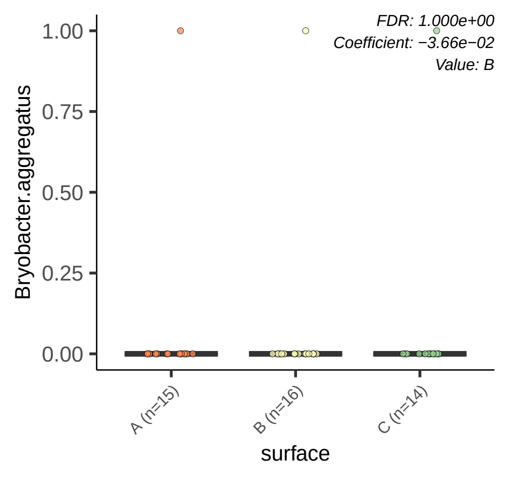
FDR: 1.000e+00
Coefficient: −3.66e−02
Value: B
Bryobacter.aggregatus
1.00
0.75
0.50
0.25
0.00
A (n=15)
B (n=16)
C (n=14)
surface

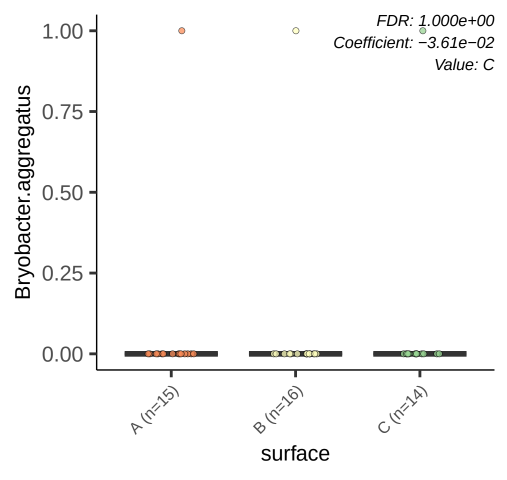
FDR: 1.000e+00
Coefficient: −3.61e−02
Value: C
Bryobacter.aggregatus
1.00
0.75
0.50
0.25
0.00
A (n=15)
B (n=16)
C (n=14)
surface

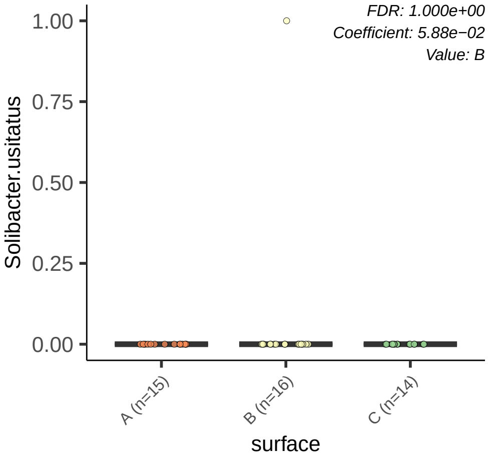

FDR: 1.000e+00
Coefficient: 5.88e−02
Value: B
Solibacter.usitatus
1.00
0.75
0.50
0.25
0.00
A (n=15)
B (n=16)
C (n=14)
surface

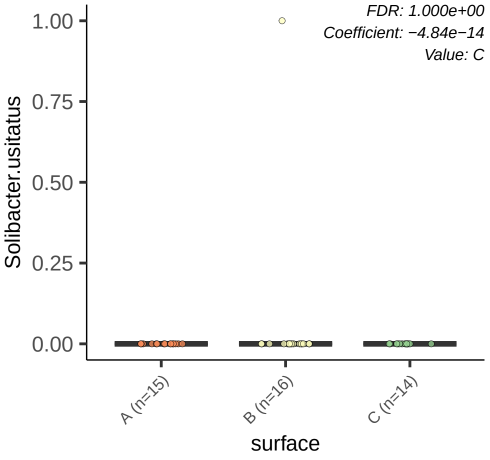
FDR: 1.000e+00
Coefficient: −4.84e−14
Value: C
Solibacter.usitatus
1.00
0.75
0.50
0.25
0.00
A (n=15)
B (n=16)
C (n=14)
surface

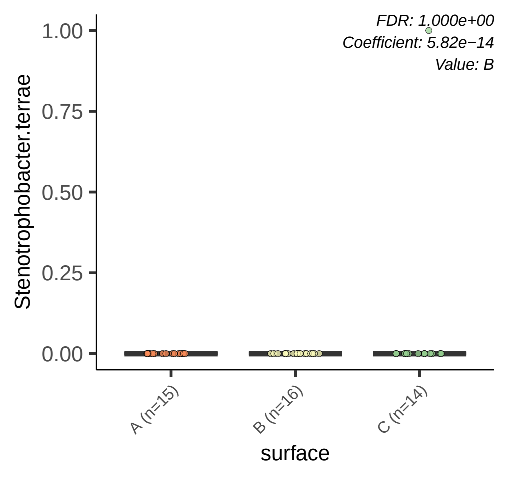
FDR: 1.000e+00
Coefficient: 5.82e−14
Value: B
Stenotrophobacter.terrae
1.00
0.75
0.50
0.25
0.00
A (n=15)
B (n=16)
C (n=14)
surface

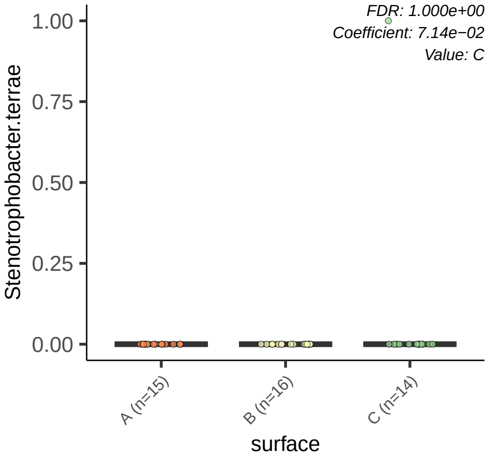
FDR: 1.000e+00
Coefficient: 7.14e−02
Value: C
Stenotrophobacter.terrae
1.00
0.75
0.50
0.25
0.00
A (n=15)
B (n=16)
C (n=14)
surface

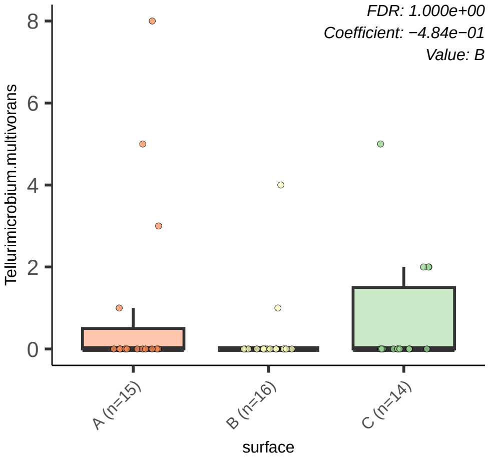

FDR: 1.000e+00
Coefficient: −4.84e−01
Value: B
Tellurimicrobium.multivorans
8
6
4
2
0
A (n=15)
B (n=16)
C (n=14)
surface

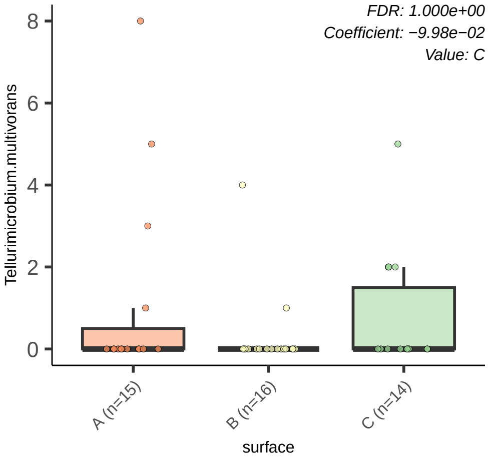

FDR: 1.000e+00
Coefficient: −9.98e−02
Value: C
Tellurimicrobium.multivorans
8
6
4
2
0
A (n=15)
B (n=16)
C (n=14)
surface

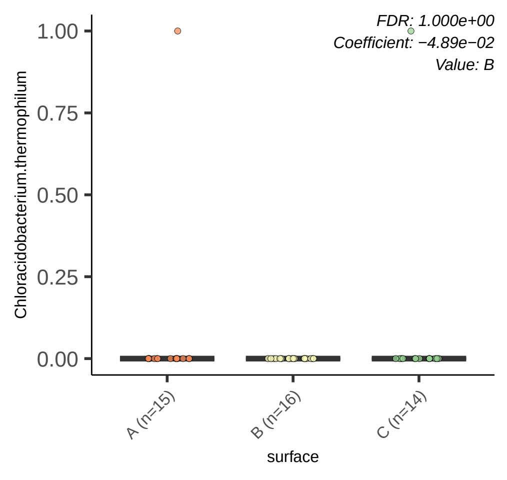
FDR: 1.000e+00
Coefficient: −4.89e−02
Value: B
Chloracidobacterium.thermophilum
1.00
0.75
0.50
0.25
0.00
A (n=15)
B (n=16)
C (n=14)
surface

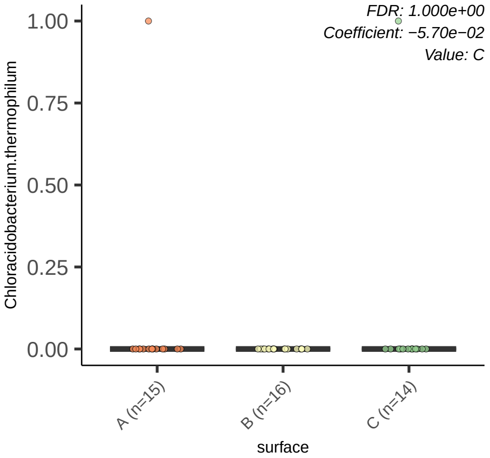

FDR: 1.000e+00
Coefficient: −5.70e−02
Value: C
Chloracidobacterium.thermophilum
1.00
0.75
0.50
0.25
0.00
A (n=15)
B (n=16)
C (n=14)
surface

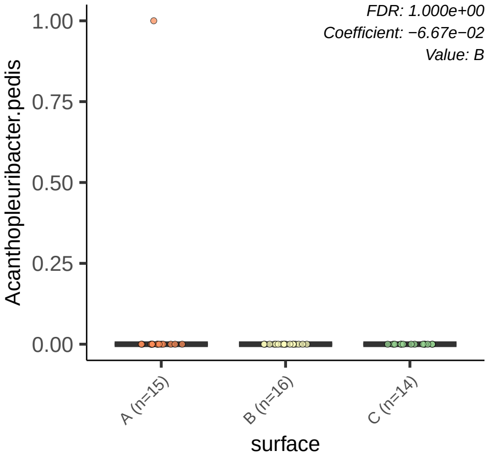

FDR: 1.000e+00
Coefficient: −6.67e−02
Value: B
Acanthopleuribacter.pedis
1.00
0.75
0.50
0.25
0.00
A (n=15)
B (n=16)
C (n=14)
surface

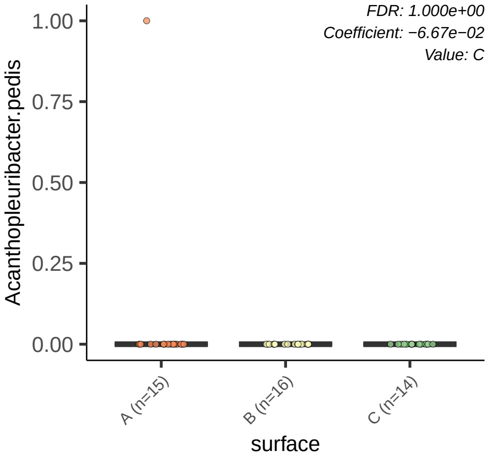
FDR: 1.000e+00
Coefficient: −6.67e−02
Value: C
Acanthopleuribacter.pedis
1.00
0.75
0.50
0.25
0.00
A (n=15)
B (n=16)
C (n=14)
surface

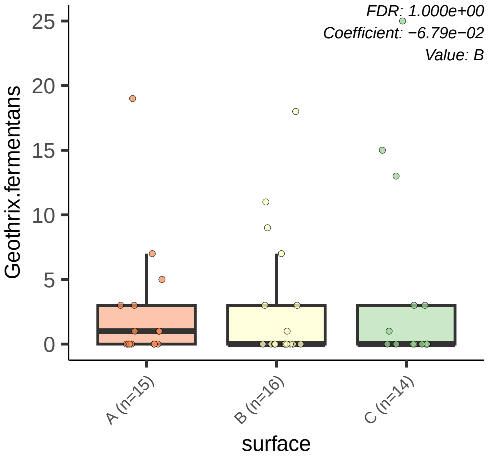

FDR: 1.000e+00
Coefficient: −6.79e−02
Value: B
Geothrix.fermentans
25
20
15
10
5
0
A (n=15)
B (n=16)
C (n=14)
surface

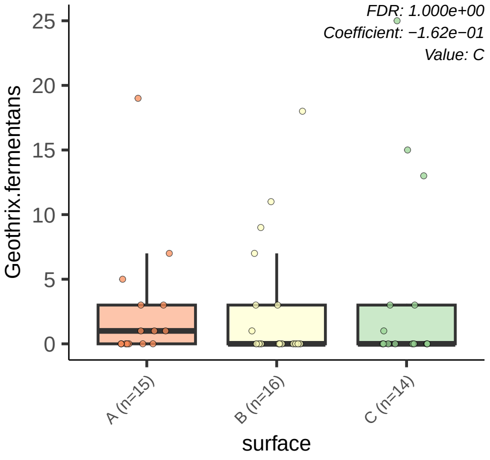

FDR: 1.000e+00
Coefficient: −1.62e−01
Value: C
Geothrix.fermentans
0
5
10
15
20
25
A (n=15)
B (n=16)
C (n=14)
surface

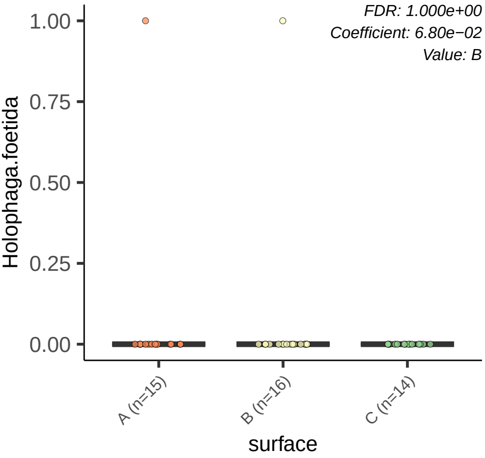

FDR: 1.000e+00
Coefficient: 6.80e−02
Value: B
Holophaga.foetida
1.00
0.75
0.50
0.25
0.00
A (n=15)
B (n=16)
C (n=14)
surface

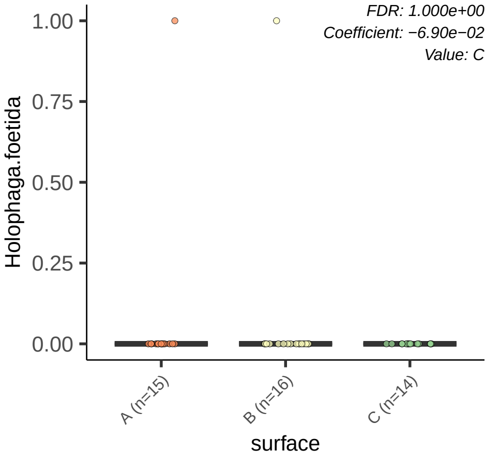
FDR: 1.000e+00
Coefficient: −6.90e−02
Value: C
Holophaga.foetida
1.00
0.75
0.50
0.25
0.00
A (n=15)
B (n=16)
C (n=14)
surface

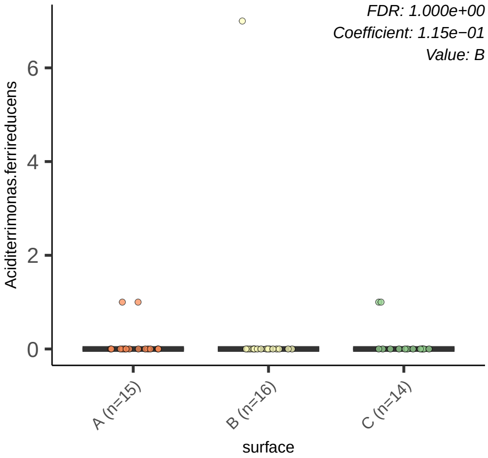

FDR: 1.000e+00
Coefficient: 1.15e−01
Value: B
Aciditerrimonas.ferrireducens
0
2
4
6
A (n=15)
B (n=16)
C (n=14)
surface

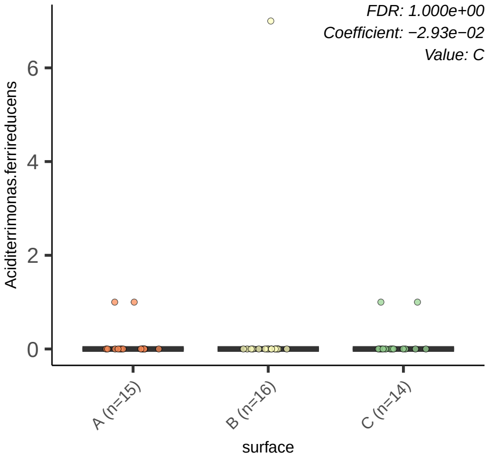

FDR: 1.000e+00
Coefficient: −2.93e−02
Value: C
Aciditerrimonas.ferrireducens
0
2
4
6
A (n=15)
B (n=16)
C (n=14)
surface

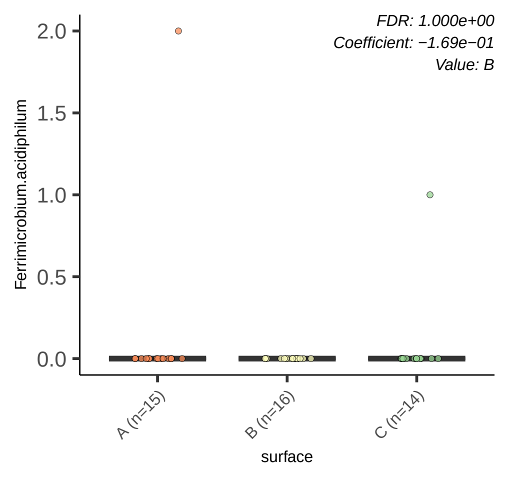

FDR: 1.000e+00
Coefficient: −1.69e−01
Value: B
Ferrimicrobium.acidiphilum
2.0
1.5
1.0
0.5
0.0
A (n=15)
B (n=16)
C (n=14)
surface

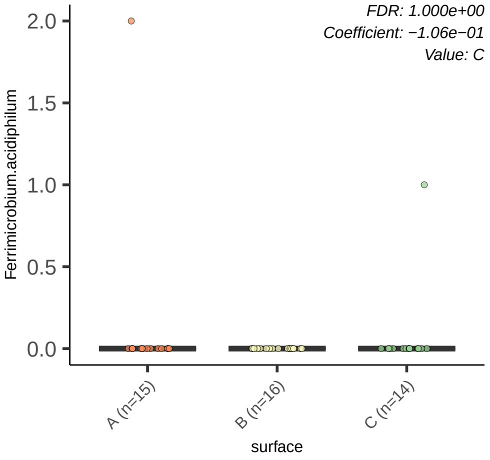
FDR: 1.000e+00
Coefficient: −1.06e−01
Value: C
Ferrimicrobium.acidiphilum
2.0
1.5
1.0
0.5
0.0
A (n=15)
B (n=16)
C (n=14)
surface

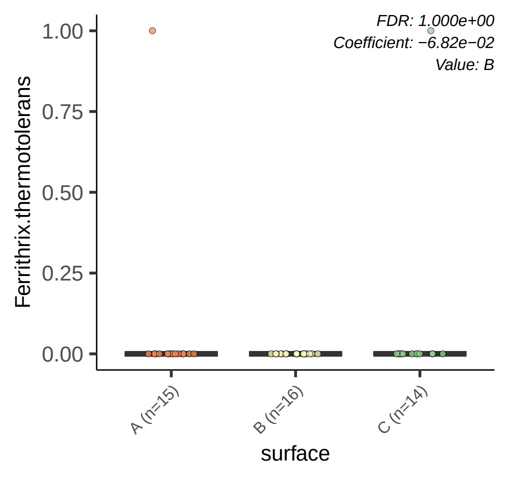

FDR: 1.000e+00
Coefficient: −6.82e−02
Value: B
Ferrithrix.thermotolerans
1.00
0.75
0.50
0.25
0.00
A (n=15)
B (n=16)
C (n=14)
surface

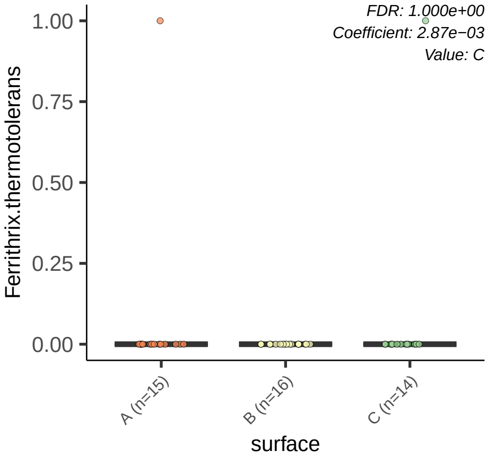
FDR: 1.000e+00
Coefficient: 2.87e−03
Value: C
Ferrithrix.thermotolerans
1.00
0.75
0.50
0.25
0.00
A (n=15)
B (n=16)
C (n=14)
surface

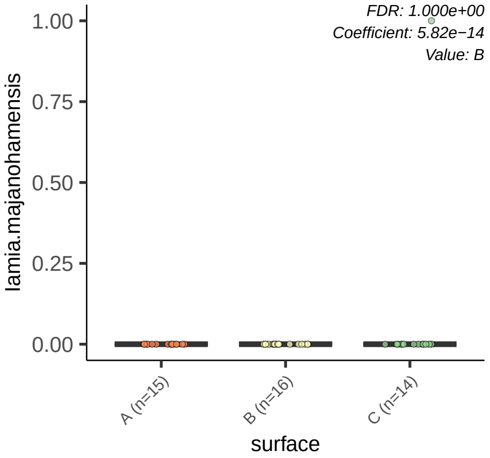

FDR: 1.000e+00
Coefficient: 5.82e−14
Value: B
lamia.majanohamensis
1.00
0.75
0.50
0.25
0.00
A (n=15)
B (n=16)
C (n=14)
surface

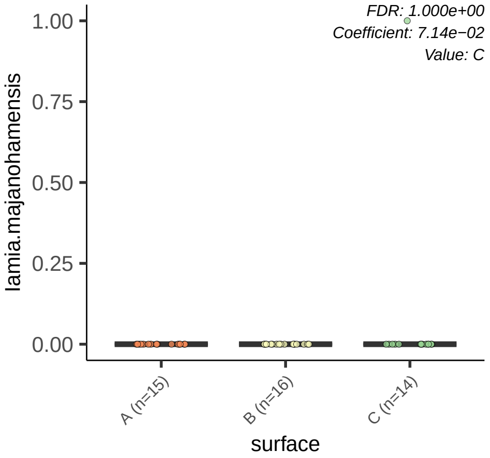

FDR: 1.000e+00
Coefficient: 7.14e−02
Value: C
lamia.majanohamensis
1.00
0.75
0.50
0.25
0.00
A (n=15)
B (n=16)
C (n=14)
surface

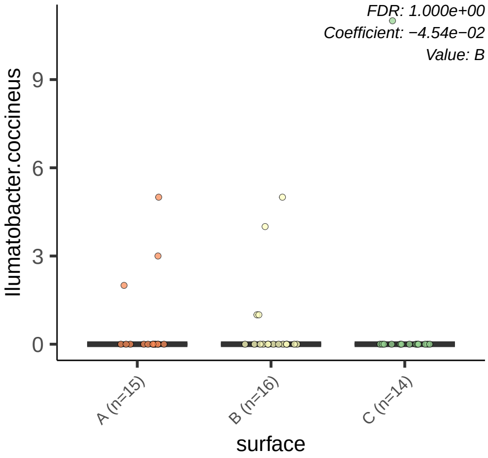

FDR: 1.000e+00
Coefficient: −4.54e−02
Value: B
Ilumatobacter.coccineus
0
3
6
9
A (n=15)
B (n=16)
C (n=14)
surface

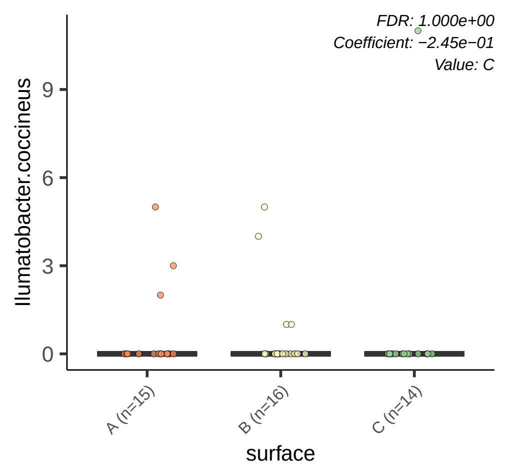

FDR: 1.000e+00
Coefficient: −2.45e−01
Value: C
Ilumatobacter.coccineus
9
6
3
0
A (n=15)
B (n=16)
C (n=14)
surface

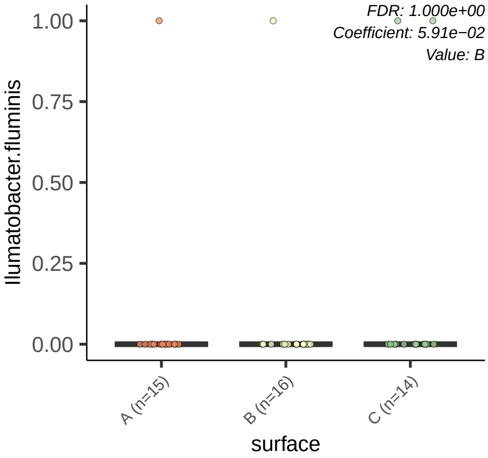
FDR: 1.000e+00
Coefficient: 5.91e−02
Value: B
Ilumatobacter.fluminis
1.00
0.75
0.50
0.25
0.00
A (n=15)
B (n=16)
C (n=14)
surface

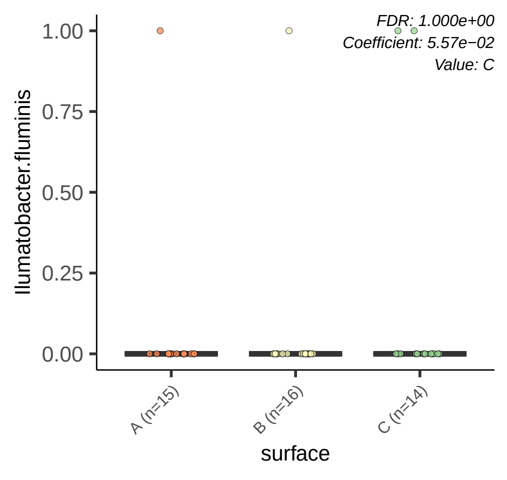
FDR: 1.000e+00
Coefficient: 5.57e−02
Value: C
Ilumatobacter.fluminis
1.00
0.75
0.50
0.25
0.00
A (n=15)
B (n=16)
C (n=14)
surface

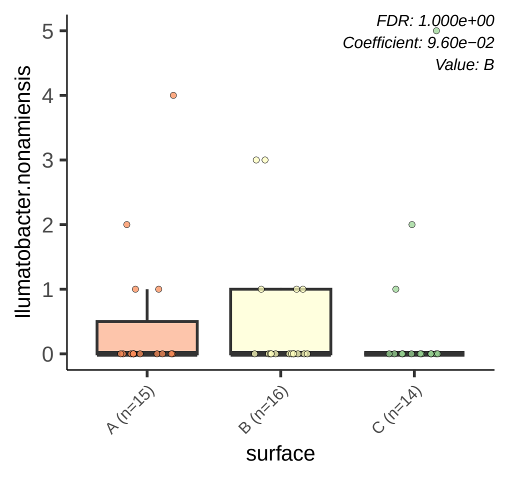

FDR: 1.000e+00
Coefficient: 9.60e−02
Value: B
Ilumatobacter.nonamiensis
0
1
2
3
4
5
A (n=15)
B (n=16)
C (n=14)
surface

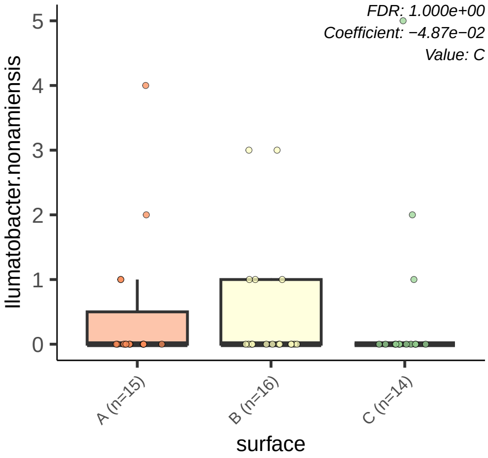
FDR: 1.000e+00
Coefficient: −4.87e−02
Value: C
Ilumatobacter.nonamiensis
5
4
3
2
1
0
A (n=15)
B (n=16)
C (n=14)
surface

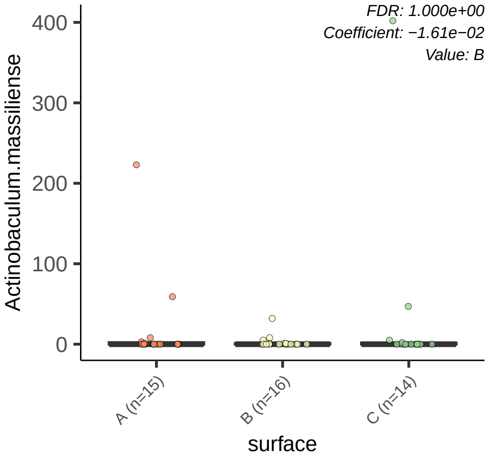
FDR: 1.000e+00
Coefficient: −1.61e−02
Value: B
Actinobaculum.massiliense
400
300
200
100
0
A (n=15)
B (n=16)
C (n=14)
surface

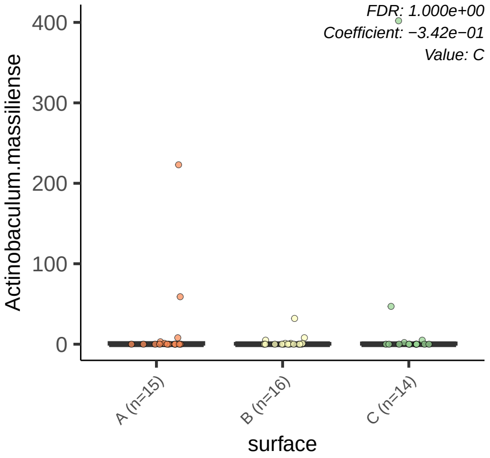
FDR: 1.000e+00
Coefficient: −3.42e−01
Value: C
Actinobaculum.massiliense
400
300
200
100
0
A (n=15)
B (n=16)
C (n=14)
surface

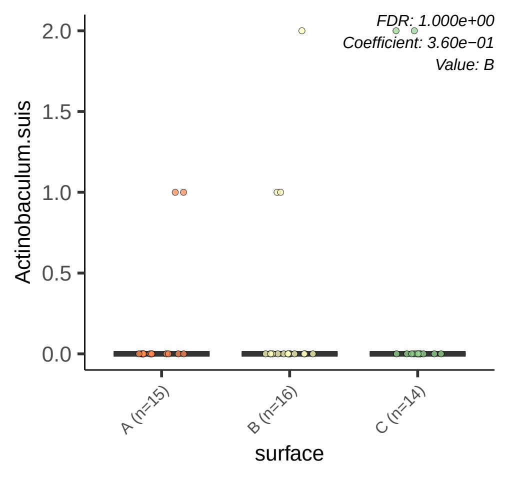

FDR: 1.000e+00
Coefficient: 3.60e−01
Value: B
Actinobaculum.suis
2.0
1.5
1.0
0.5
0.0
A (n=15)
B (n=16)
C (n=14)
surface

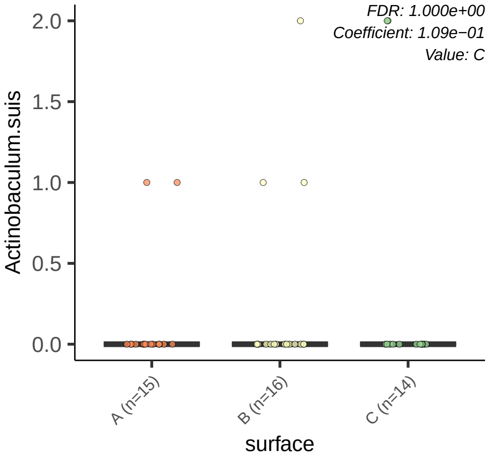
FDR: 1.000e+00
Coefficient: 1.09e−01
Value: C
Actinobaculum.suis
2.0
1.5
1.0
0.5
0.0
A (n=15)
B (n=16)
C (n=14)
surface

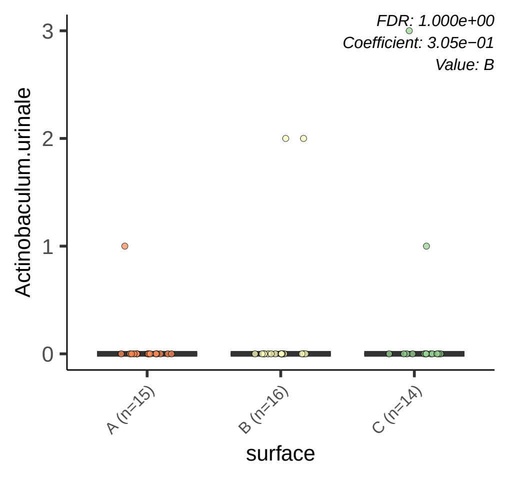

FDR: 1.000e+00
Coefficient: 3.05e−01
Value: B
Actinobaculum.urinale
0
1
2
3
A (n=15)
B (n=16)
C (n=14)
surface

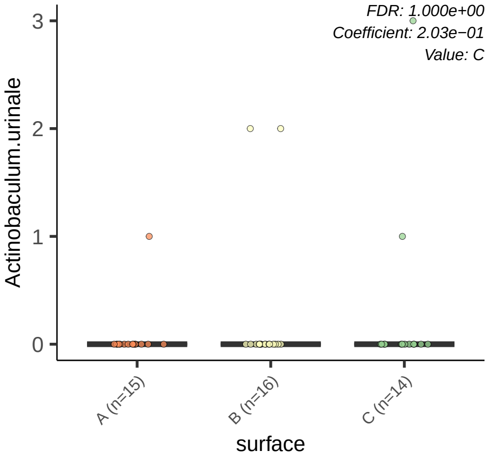

FDR: 1.000e+00
Coefficient: 2.03e−01
Value: C
Actinobaculum.urinale
0
1
2
3
A (n=15)
B (n=16)
C (n=14)
surface

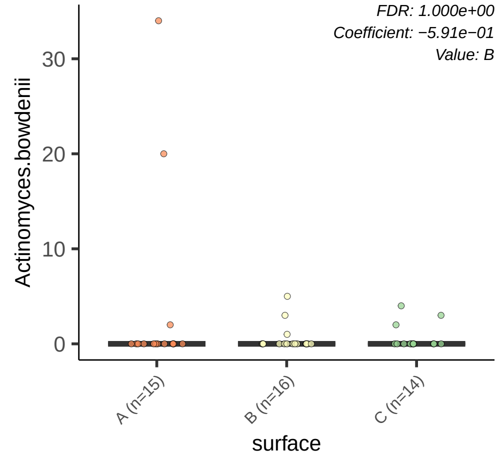
FDR: 1.000e+00
Coefficient: −5.91e−01
Value: B
Actinomyces.bowdenii
30
20
10
0
A (n=15)
B (n=16)
C (n=14)
surface

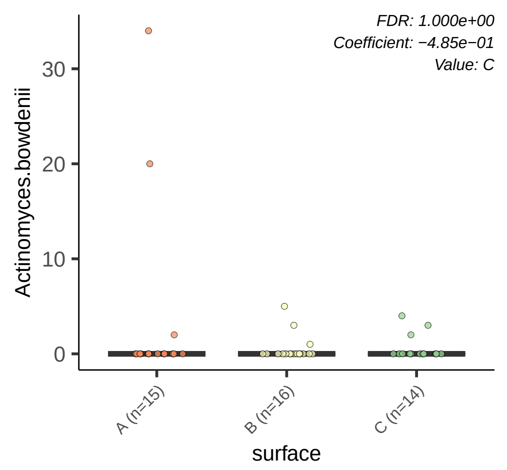

FDR: 1.000e+00
Coefficient: −4.85e−01
Value: C
Actinomyces.bowdenii
30
20
10
0
A (n=15)
B (n=16)
C (n=14)
surface

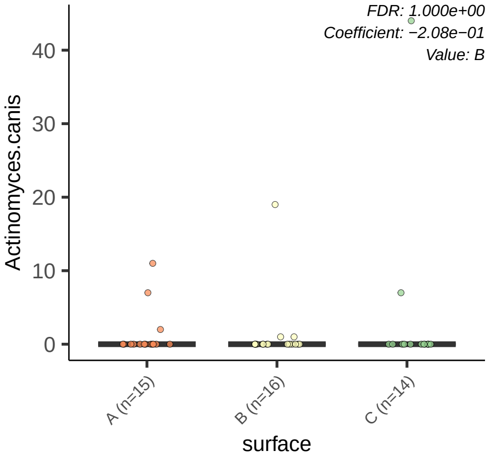

FDR: 1.000e+00
Coefficient: −2.08e−01
Value: B
Actinomyces.canis
0
10
20
30
40
A (n=15)
B (n=16)
C (n=14)
surface

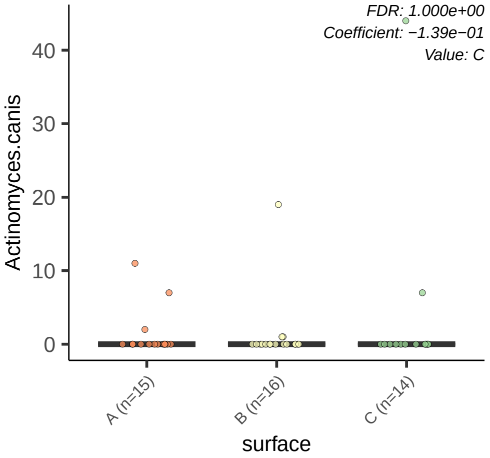

FDR: 1.000e+00
Coefficient: −1.39e−01
Value: C
Actinomyces.canis
0
10
20
30
40
A (n=15)
B (n=16)
C (n=14)
surface

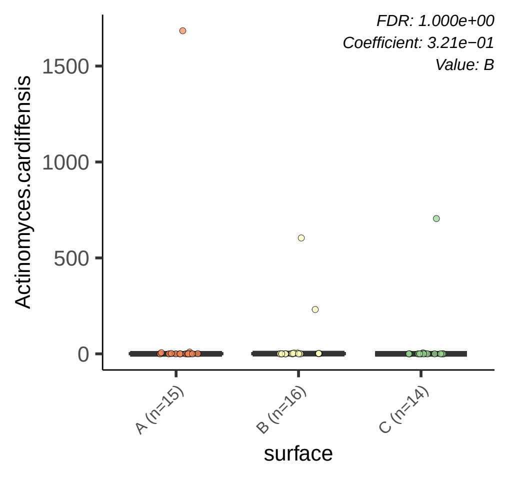
FDR: 1.000e+00
Coefficient: 3.21e−01
Value: B
Actinomyces.cardiffensis
1500
1000
500
0
A (n=15)
B (n=16)
C (n=14)
surface

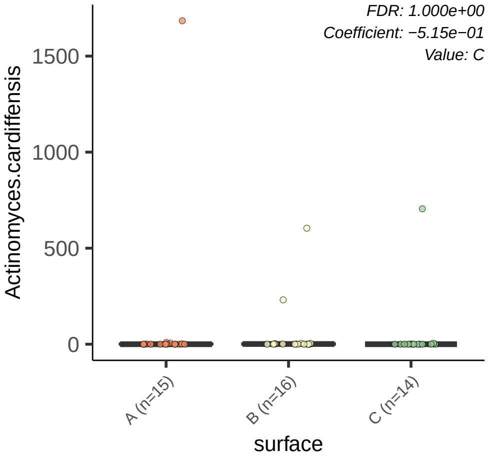
FDR: 1.000e+00
Coefficient: −5.15e−01
Value: C
1500
1000
500
0
Actinomyces.cardiffensis
A (n=15)
B (n=16)
C (n=14)
surface

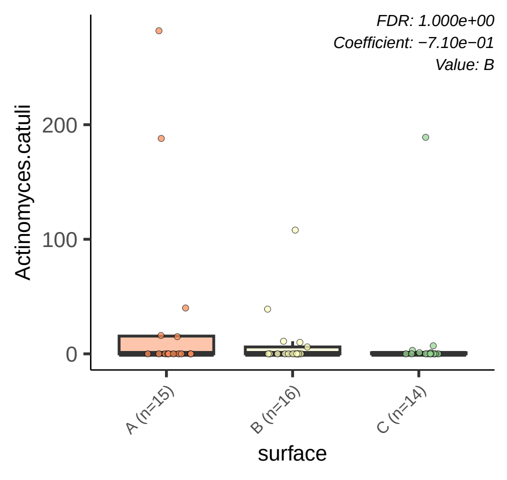

FDR: 1.000e+00
Coefficient: −7.10e−01
Value: B
Actinomyces.catuli
200
100
0
A (n=15)
B (n=16)
C (n=14)
surface

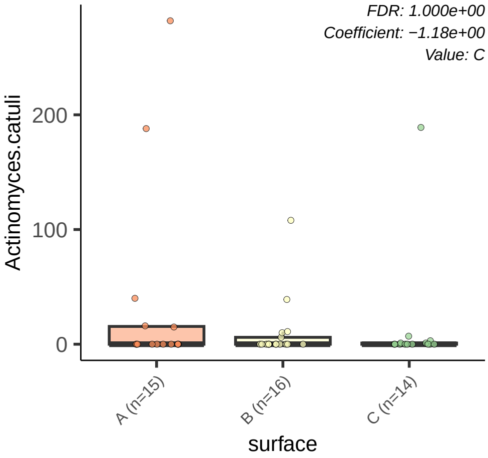
FDR: 1.000e+00
Coefficient: −1.18e+00
Value: C
Actinomyces.catuli
200
100
0
A (n=15)
B (n=16)
C (n=14)
surface

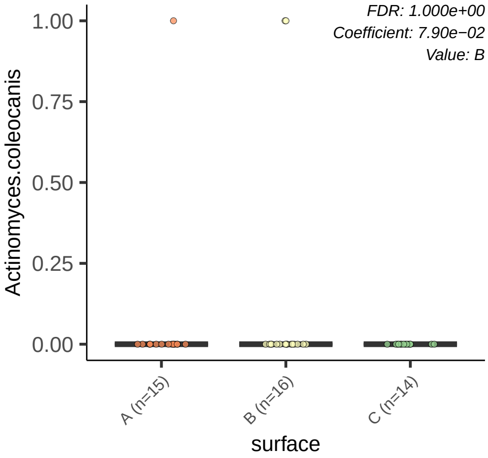

FDR: 1.000e+00
Coefficient: 7.90e−02
Value: B
Actinomyces.coleocanis
1.00
0.75
0.50
0.25
0.00
A (n=15)
B (n=16)
C (n=14)
surface

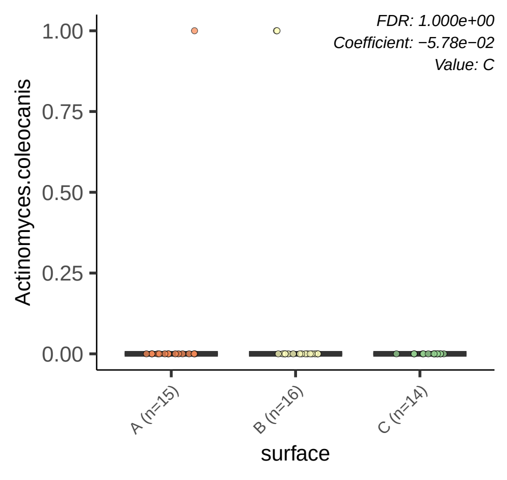

FDR: 1.000e+00
Coefficient: −5.78e−02
Value: C
Actinomyces.coleocanis
1.00
0.75
0.50
0.25
0.00
A (n=15)
B (n=16)
C (n=14)
surface

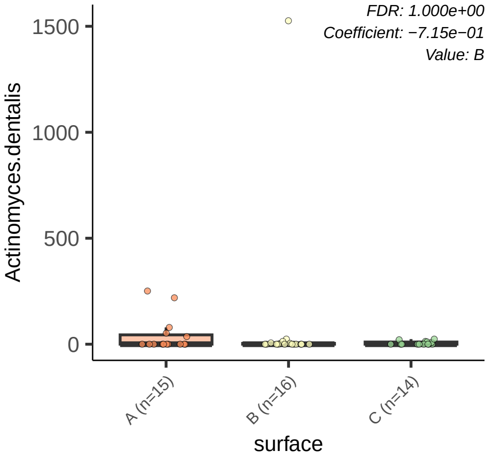

FDR: 1.000e+00
Coefficient: −7.15e−01
Value: B
Actinomyces.dentalis
1500
1000
500
0
A (n=15)
B (n=16)
C (n=14)
surface

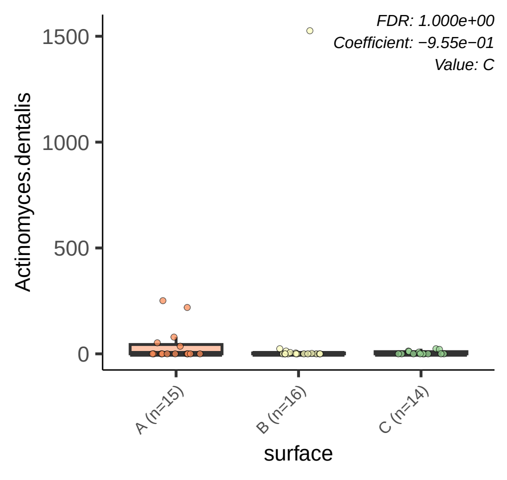
FDR: 1.000e+00
Coefficient: −9.55e−01
Value: C
Actinomyces.dentalis
1500
1000
500
0
A (n=15)
B (n=16)
C (n=14)
surface

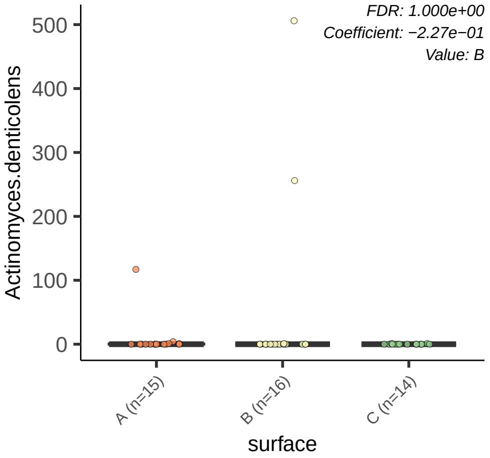
FDR: 1.000e+00
Coefficient: −2.27e−01
Value: B
Actinomyces.denticolens
500
400
300
200
100
0
A (n=15)
B (n=16)
C (n=14)
surface

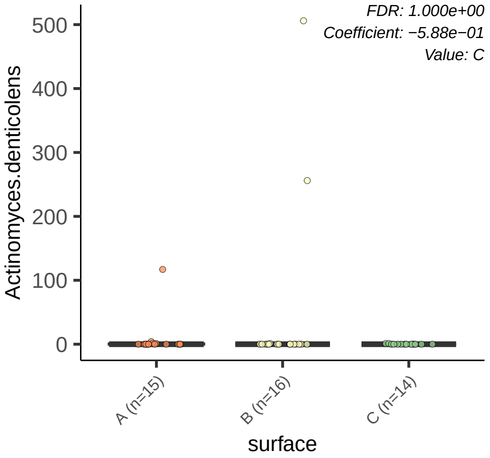
FDR: 1.000e+00
Coefficient: −5.88e−01
Value: C
Actinomyces.denticolens
0
100
200
300
400
500
A (n=15)
B (n=16)
C (n=14)
surface

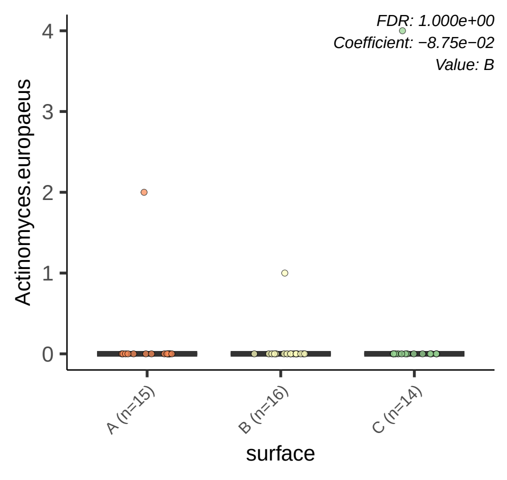
FDR: 1.000e+00
Coefficient: −8.75e−02
Value: B
Actinomyces.europaeus
4
3
2
1
0
A (n=15)
B (n=16)
C (n=14)
surface

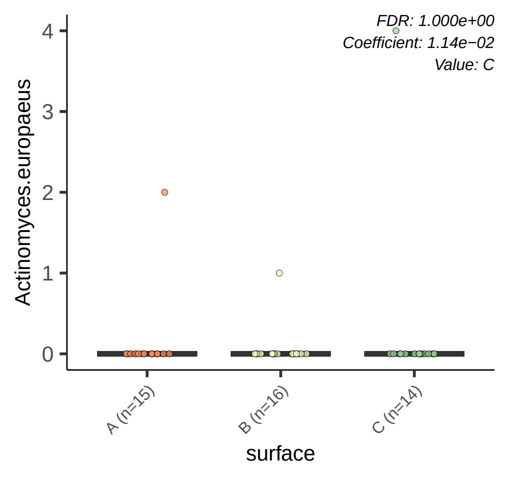
FDR: 1.000e+00
Coefficient: 1.14e−02
Value: C
Actinomyces.europaeus
0
1
2
3
4
A (n=15)
B (n=16)
C (n=14)
surface

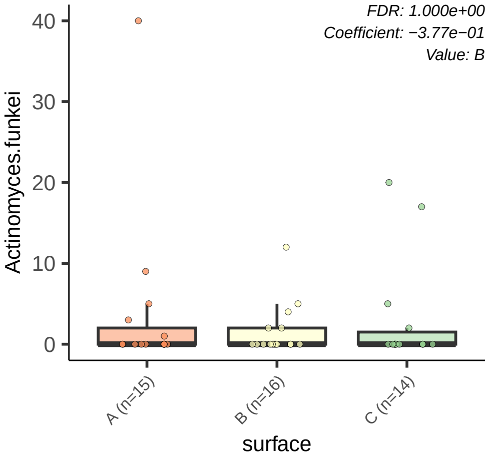

FDR: 1.000e+00
Coefficient: −3.77e−01
Value: B
Actinomyces.funkei
40
30
20
10
0
A (n=15)
B (n=16)
C (n=14)
surface

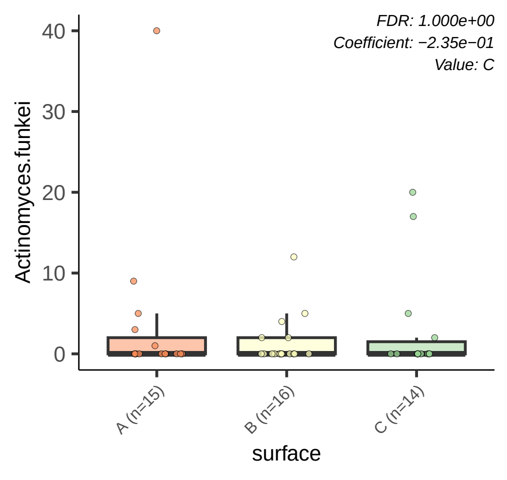
FDR: 1.000e+00
Coefficient: −2.35e−01
Value: C
Actinomyces.funkei
0
10
20
30
40
A (n=15)
B (n=16)
C (n=14)
surface

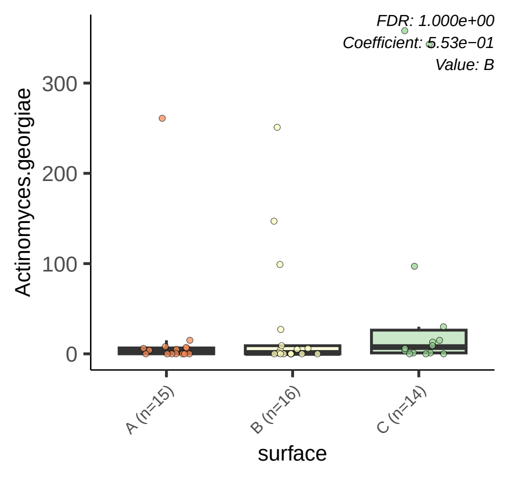
FDR: 1.000e+00
Coefficient: 5.53e−01
Value: B
Actinomyces.georgiae
300
200
100
0
A (n=15)
B (n=16)
C (n=14)
surface

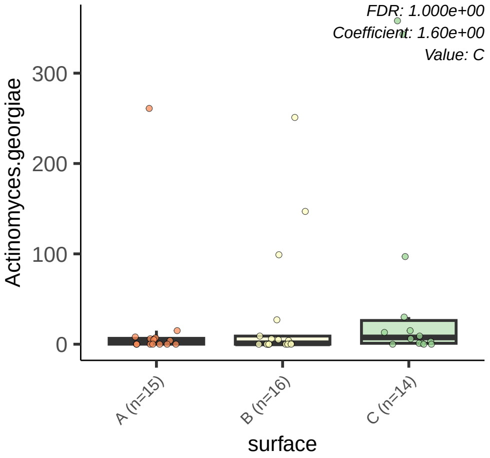
FDR: 1.000e+00
Coefficient: 1.60e+00
Value: C
Actinomyces.georgiae
300
200
100
0
A (n=15)
B (n=16)
C (n=14)
surface

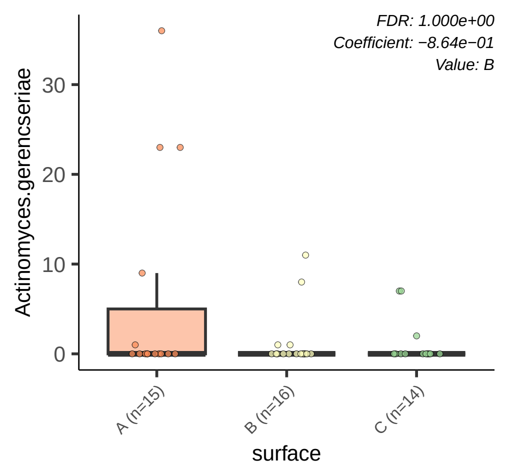

FDR: 1.000e+00
Coefficient: −8.64e−01
Value: B
Actinomyces.gerencseriae
30
20
10
0
A (n=15)
B (n=16)
C (n=14)
surface

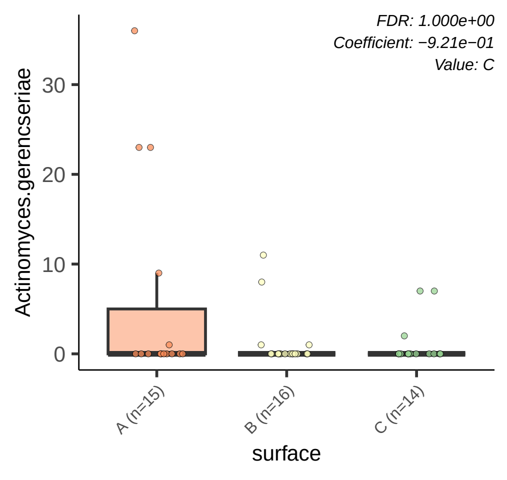

FDR: 1.000e+00
Coefficient: −9.21e−01
Value: C
Actinomyces.gerencseriae
30
20
10
0
A (n=15)
B (n=16)
C (n=14)
surface

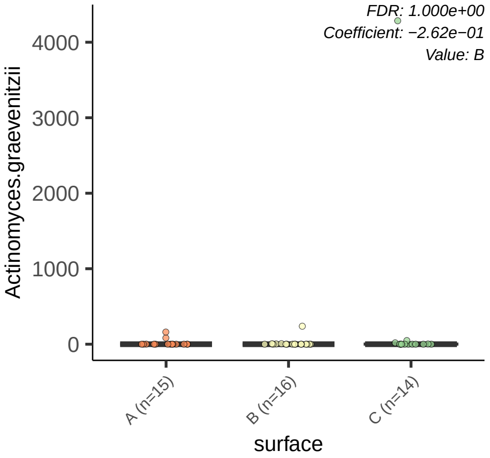
FDR: 1.000e+00
Coefficient: −2.62e−01
Value: B
Actinomyces.graevenitzii
4000
3000
2000
1000
0
A (n=15)
B (n=16)
C (n=14)
surface

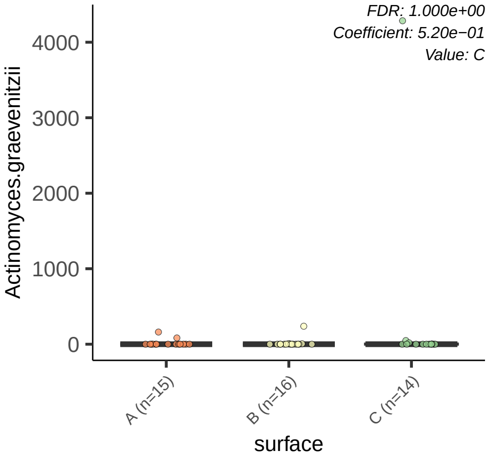
FDR: 1.000e+00
Coefficient: 5.20e−01
Value: C
4000
3000
2000
1000
0
Actinomyces.graevenitzii
A (n=15)
B (n=16)
C (n=14)
surface

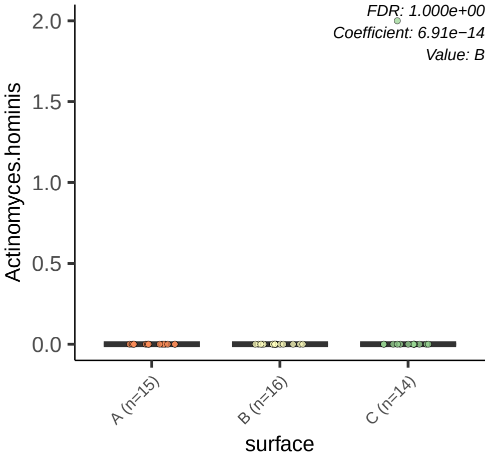

FDR: 1.000e+00
Coefficient: 6.91e−14
Value: B
Actinomyces.hominis
2.0
1.5
1.0
0.5
0.0
A (n=15)
B (n=16)
C (n=14)
surface

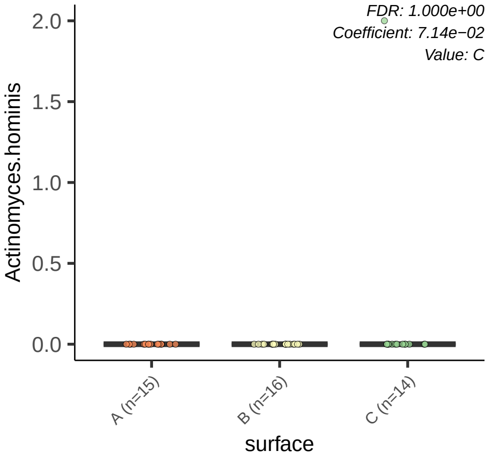
FDR: 1.000e+00
Coefficient: 7.14e−02
Value: C
Actinomyces.hominis
2.0
1.5
1.0
0.5
0.0
A (n=15)
B (n=16)
C (n=14)
surface

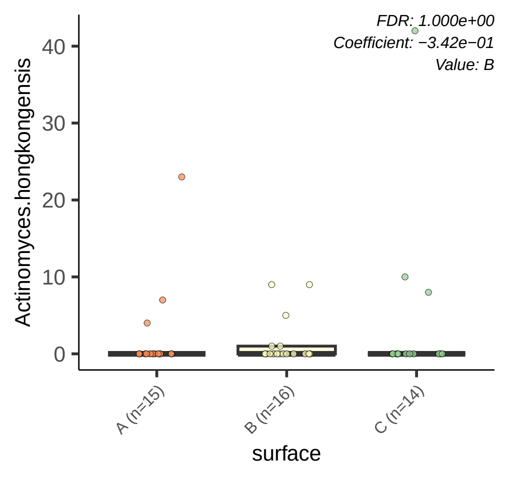
FDR: 1.000e+00
Coefficient: −3.42e−01
Value: B
Actinomyces.hongkongensis
0
10
20
30
40
A (n=15)
B (n=16)
C (n=14)
surface

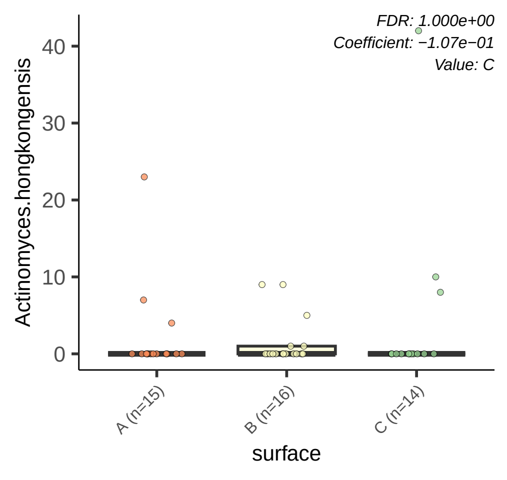

FDR: 1.000e+00
Coefficient: −1.07e−01
Value: C
Actinomyces.hongkongensis
0
10
20
30
40
A (n=15)
B (n=16)
C (n=14)
surface

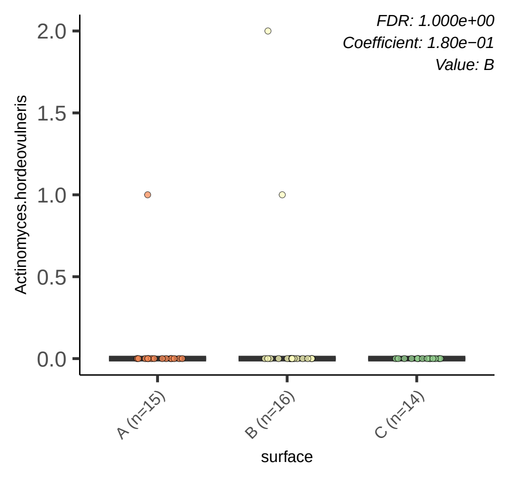

FDR: 1.000e+00
Coefficient: 1.80e−01
Value: B
Actinomyces.hordeovulneris
2.0
1.5
1.0
0.5
0.0
A (n=15)
B (n=16)
C (n=14)
surface

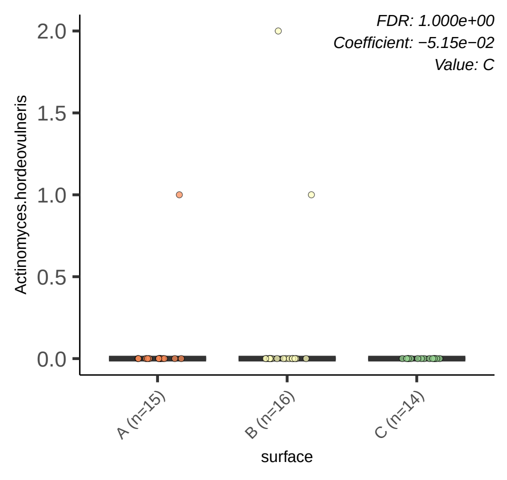
FDR: 1.000e+00
Coefficient: −5.15e−02
Value: C
Actinomyces.hordeovulneris
2.0
1.5
1.0
0.5
0.0
A (n=15)
B (n=16)
C (n=14)
surface

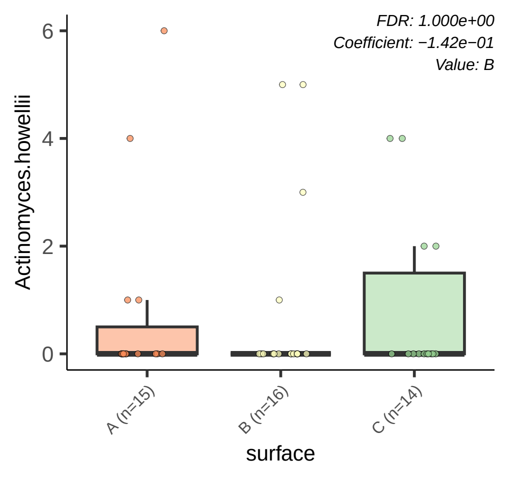
FDR: 1.000e+00
Coefficient: −1.42e−01
Value: B
Actinomyces.howellii
0
2
4
6
A (n=15)
B (n=16)
C (n=14)
surface

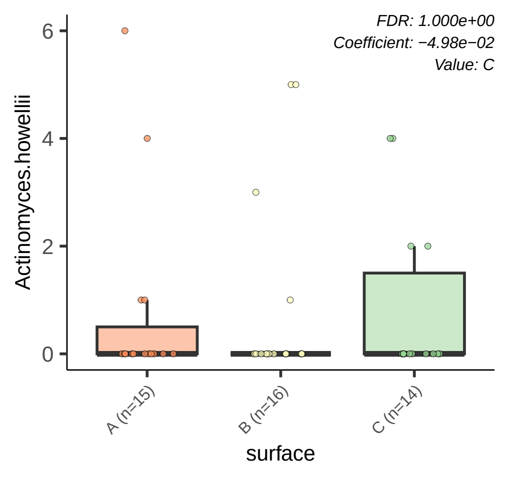
FDR: 1.000e+00
Coefficient: −4.98e−02
Value: C
Actinomyces.howellii
0
2
4
6
A (n=15)
B (n=16)
C (n=14)
surface

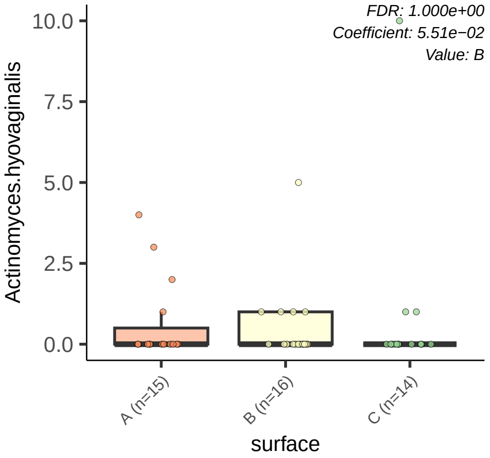

FDR: 1.000e+00
Coefficient: 5.51e−02
Value: B
Actinomyces.hyovaginalis
10.0
7.5
5.0
2.5
0.0
A (n=15)
B (n=16)
C (n=14)
surface

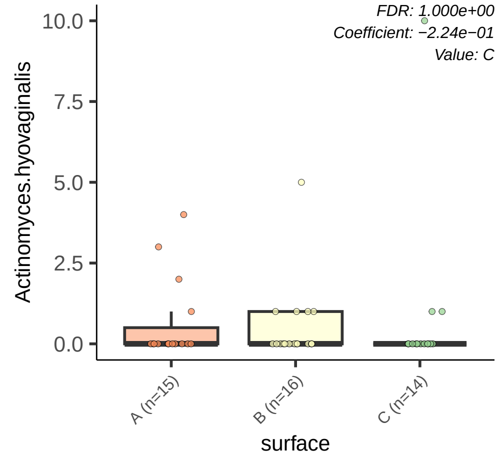

FDR: 1.000e+00
Coefficient: −2.24e−01
Value: C
Actinomyces.hyovaginalis
10.0
7.5
5.0
2.5
0.0
A (n=15)
B (n=16)
C (n=14)
surface

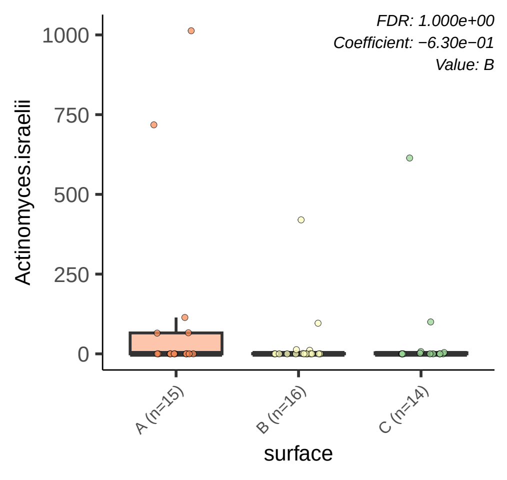
FDR: 1.000e+00
Coefficient: −6.30e−01
Value: B
Actinomyces.israelii
1000
750
500
250
0
A (n=15)
B (n=16)
C (n=14)
surface

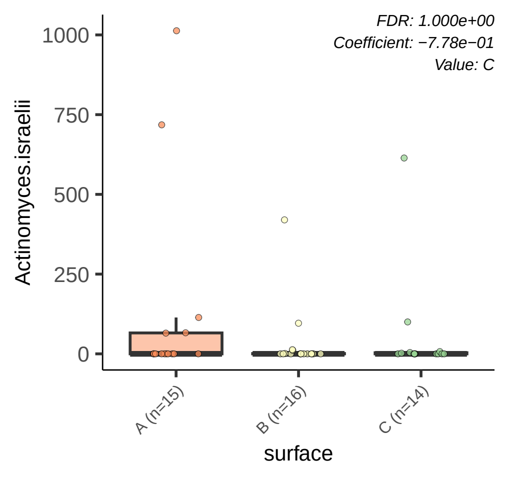

FDR: 1.000e+00
Coefficient: −7.78e−01
Value: C
Actinomyces.israelii
1000
750
500
250
0
A (n=15)
B (n=16)
C (n=14)
surface

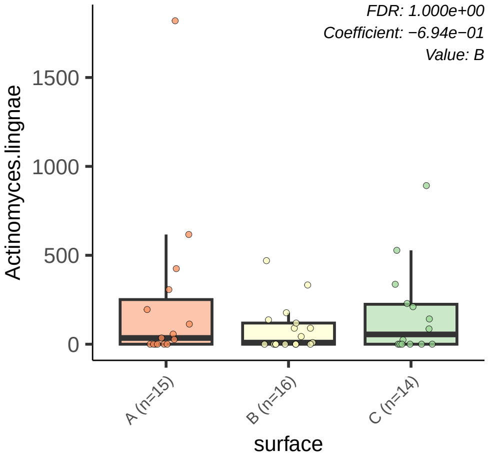

FDR: 1.000e+00
Coefficient: −6.94e−01
Value: B
Actinomyces.lingnae
1500
1000
500
0
A (n=15)
B (n=16)
C (n=14)
surface

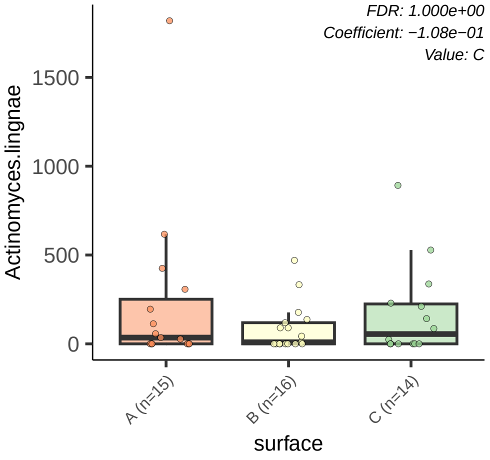
FDR: 1.000e+00
Coefficient: −1.08e−01
Value: C
Actinomyces.lingnae
1500
1000
500
0
A (n=15)
B (n=16)
C (n=14)
surface

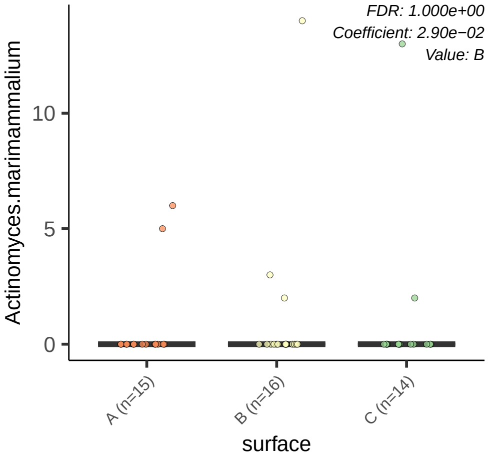

FDR: 1.000e+00
Coefficient: 2.90e−02
Value: B
Actinomyces.marimammalium
0
5
10
A (n=15)
B (n=16)
C (n=14)
surface

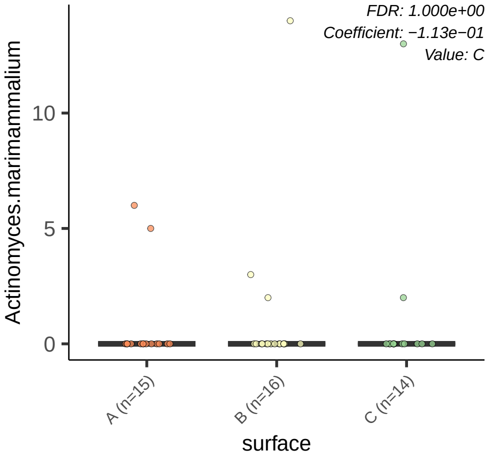

FDR: 1.000e+00
Coefficient: −1.13e−01
Value: C
Actinomyces.marimammalium
0
5
10
A (n=15)
B (n=16)
C (n=14)
surface

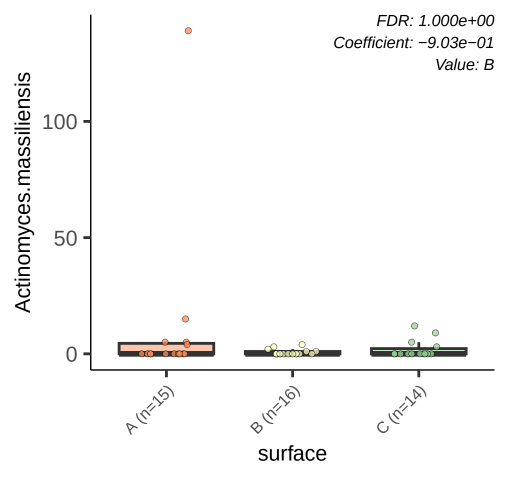
FDR: 1.000e+00
Coefficient: −9.03e−01
Value: B
Actinomyces.massiliensis
100
50
0
A (n=15)
B (n=16)
C (n=14)
surface

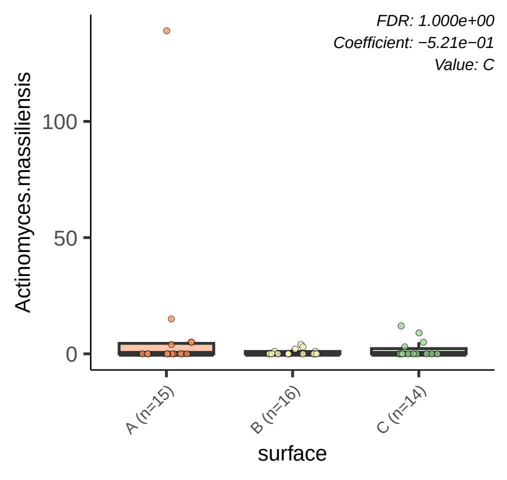

FDR: 1.000e+00
Coefficient: −5.21e−01
Value: C
Actinomyces.massiliensis
100
50
0
A (n=15)
B (n=16)
C (n=14)
surface

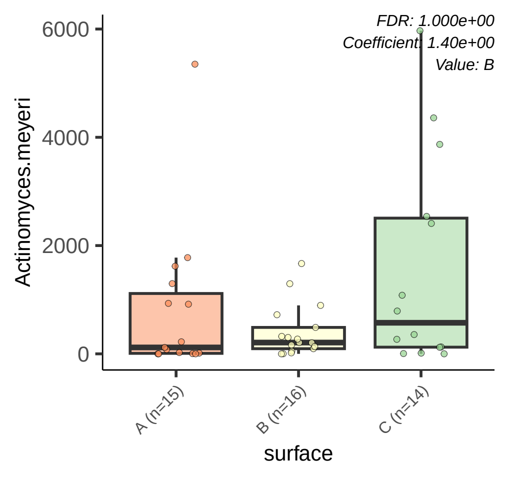

FDR: 1.000e+00
Coefficient: 1.40e+00
Value: B
Actinomyces.meyeri
6000
4000
2000
0
A (n=15)
B (n=16)
C (n=14)
surface

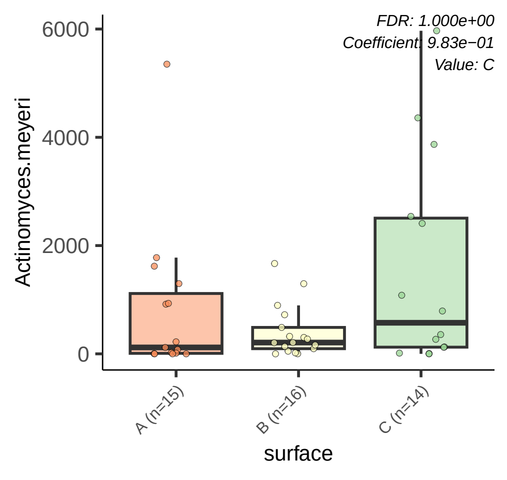

FDR: 1.000e+00
Coefficient: 9.83e−01
Value: C
6000
4000
2000
0
Actinomyces.meyeri
A (n=15)
B (n=16)
C (n=14)
surface

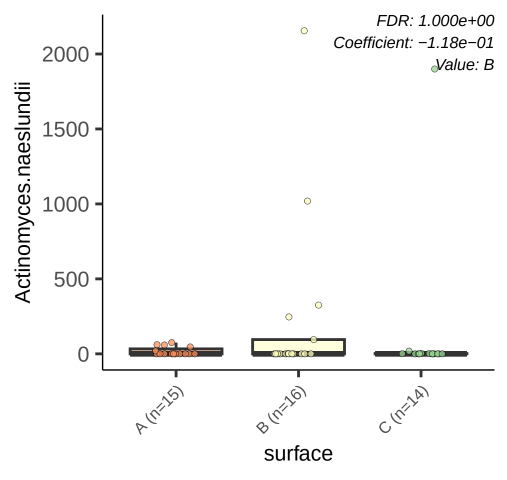
FDR: 1.000e+00
Coefficient: −1.18e−01
Value: B
Actinomyces.naeslundii
2000
1500
1000
500
0
A (n=15)
B (n=16)
C (n=14)
surface

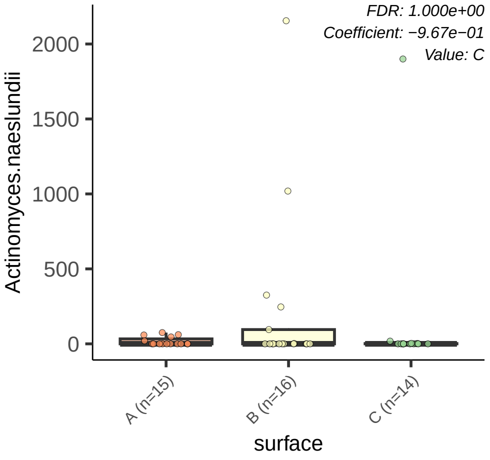

FDR: 1.000e+00
Coefficient: −9.67e−01
Value: C
Actinomyces.naeslundii
2000
1500
1000
500
0
A (n=15)
B (n=16)
C (n=14)
surface

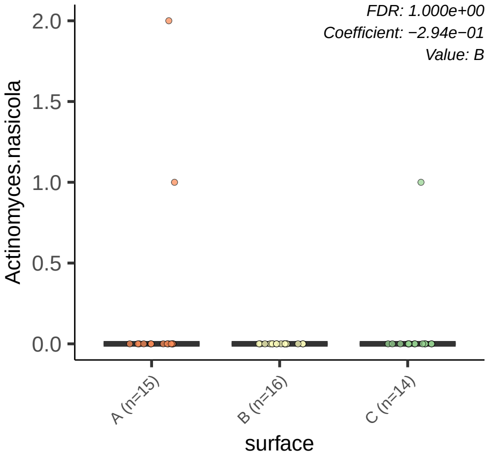

FDR: 1.000e+00
Coefficient: −2.94e−01
Value: B
Actinomyces.nasicola
2.0
1.5
1.0
0.5
0.0
A (n=15)
B (n=16)
C (n=14)
surface

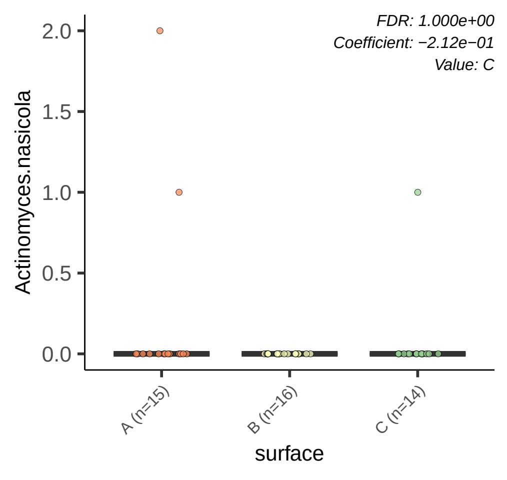

FDR: 1.000e+00
Coefficient: −2.12e−01
Value: C
Actinomyces.nasicola
2.0
1.5
1.0
0.5
0.0
A (n=15)
B (n=16)
C (n=14)
surface

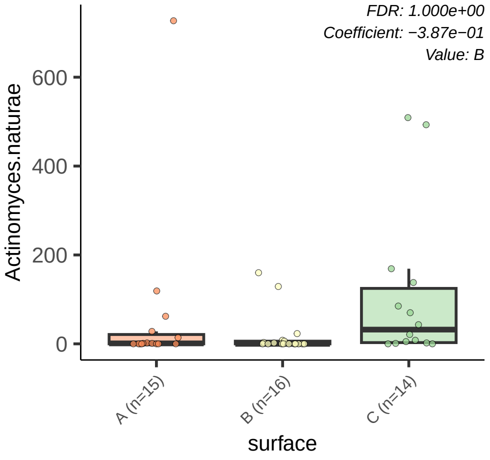

FDR: 1.000e+00
Coefficient: −3.87e−01
Value: B
Actinomyces.naturae
0
200
400
600
A (n=15)
B (n=16)
C (n=14)
surface

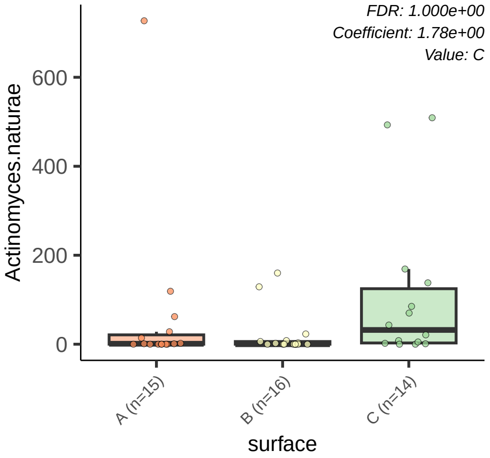

FDR: 1.000e+00
Coefficient: 1.78e+00
Value: C
Actinomyces.naturae
600
400
200
0
A (n=15)
B (n=16)
C (n=14)
surface

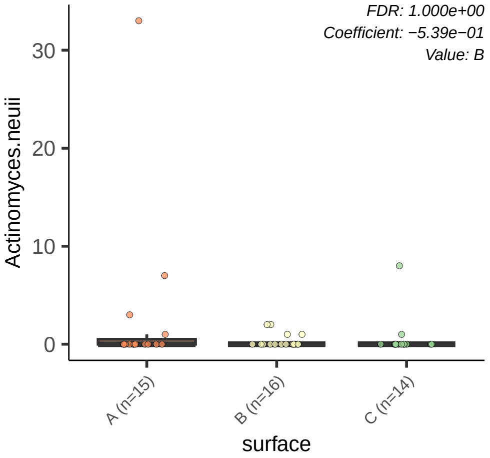

FDR: 1.000e+00
Coefficient: −5.39e−01
Value: B
Actinomyces.neuii
0
10
20
30
A (n=15)
B (n=16)
C (n=14)
surface

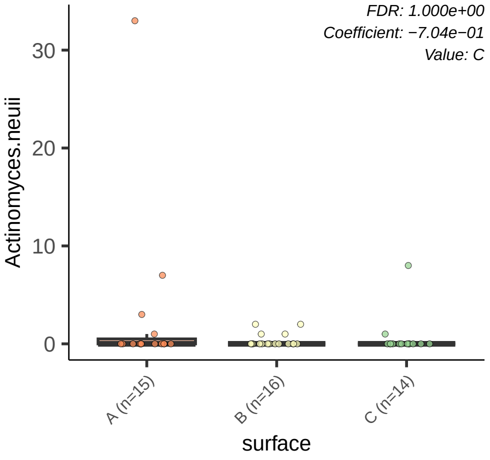
FDR: 1.000e+00
Coefficient: −7.04e−01
Value: C
Actinomyces.neuii
30
20
10
0
A (n=15)
B (n=16)
C (n=14)
surface

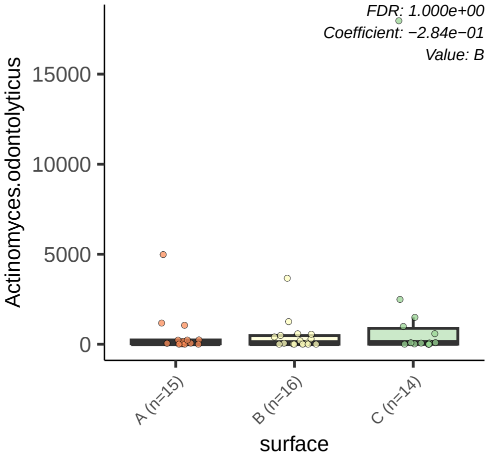

FDR: 1.000e+00
Coefficient: −2.84e−01
Value: B
Actinomyces.odontolyticus
15000
10000
5000
0
A (n=15)
B (n=16)
C (n=14)
surface

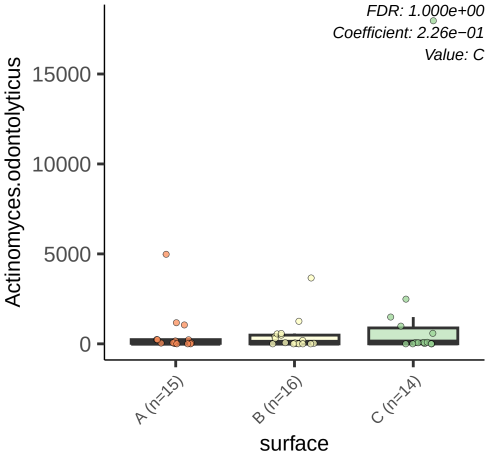
FDR: 1.000e+00
Coefficient: 2.26e−01
Value: C
Actinomyces.odontolyticus
15000
10000
5000
0
A (n=15)
B (n=16)
C (n=14)
surface

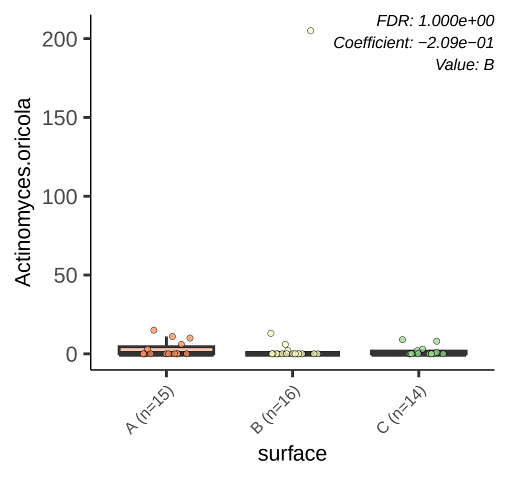

FDR: 1.000e+00
Coefficient: −2.09e−01
Value: B
Actinomyces.oricola
200
150
100
50
0
A (n=15)
B (n=16)
C (n=14)
surface

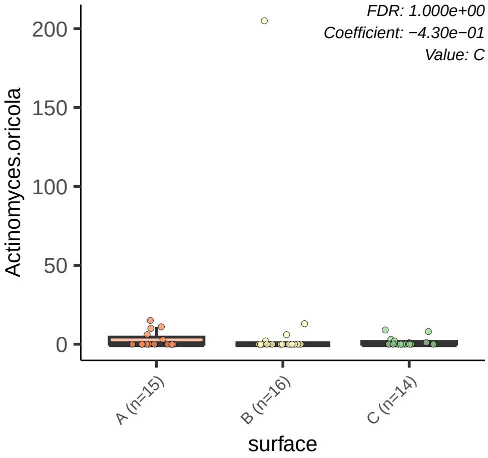
FDR: 1.000e+00
Coefficient: −4.30e−01
Value: C
Actinomyces.oricola
200
150
100
50
0
A (n=15)
B (n=16)
C (n=14)
surface

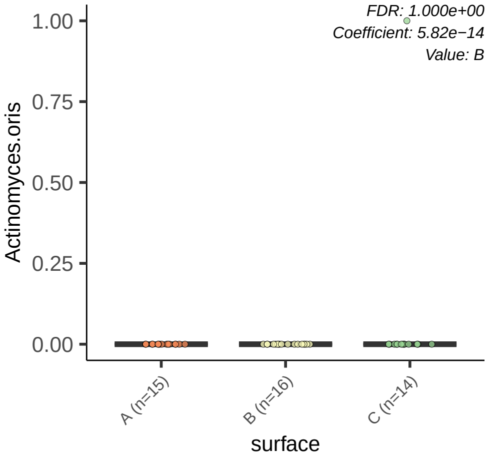
FDR: 1.000e+00
Coefficient: 5.82e−14
Value: B
Actinomyces.oris
1.00
0.75
0.50
0.25
0.00
A (n=15)
B (n=16)
C (n=14)
surface

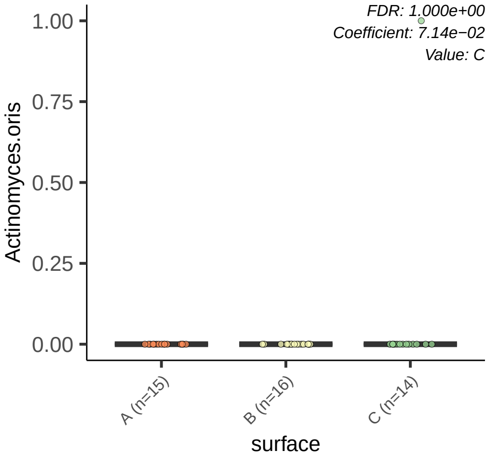

FDR: 1.000e+00
Coefficient: 7.14e−02
Value: C
1.00
0.75
0.50
0.25
0.00
Actinomyces.oris
A (n=15)
B (n=16)
C (n=14)
surface

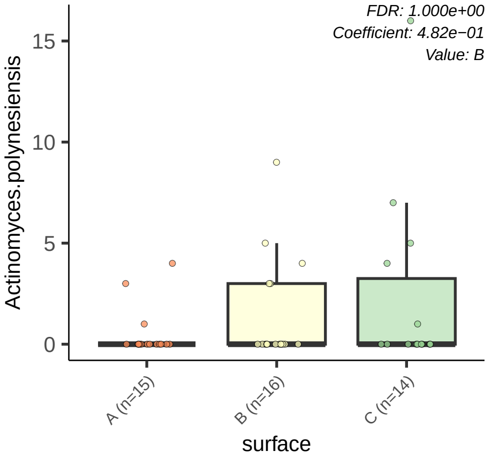
FDR: 1.000e+00
Coefficient: 4.82e−01
Value: B
Actinomyces.polynesiensis
15
10
5
0
A (n=15)
B (n=16)
C (n=14)
surface

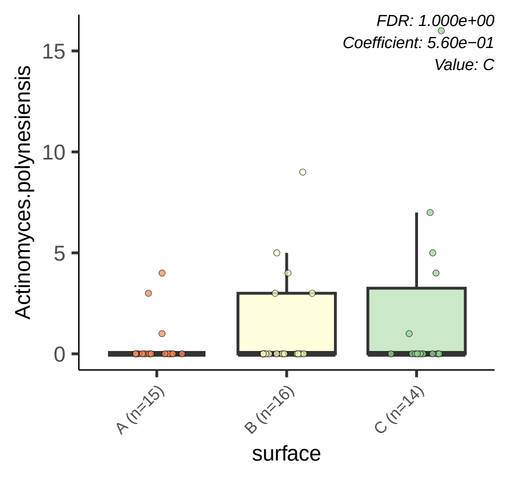
FDR: 1.000e+00
Coefficient: 5.60e−01
Value: C
Actinomyces.polynesiensis
15
10
5
0
A (n=15)
B (n=16)
C (n=14)
surface

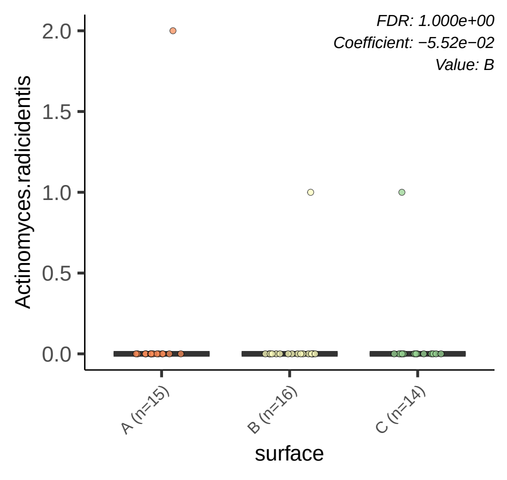
FDR: 1.000e+00
Coefficient: −5.52e−02
Value: B
Actinomyces.radicidentis
2.0
1.5
1.0
0.5
0.0
A (n=15)
B (n=16)
C (n=14)
surface

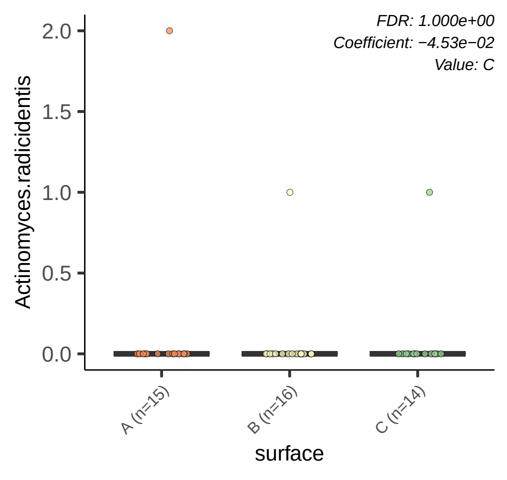
FDR: 1.000e+00
Coefficient: −4.53e−02
Value: C
Actinomyces.radicidentis
2.0
1.5
1.0
0.5
0.0
A (n=15)
B (n=16)
C (n=14)
surface

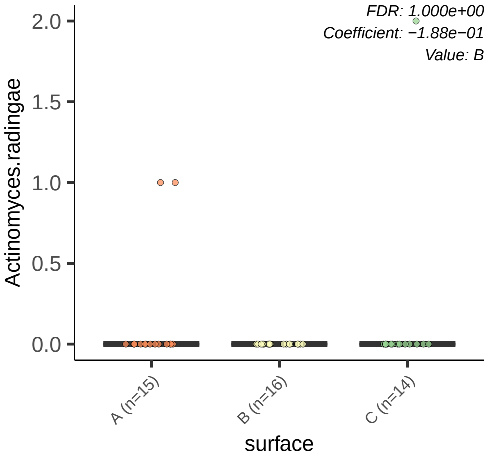
FDR: 1.000e+00
Coefficient: −1.88e−01
Value: B
Actinomyces.radingae
2.0
1.5
1.0
0.5
0.0
A (n=15)
B (n=16)
C (n=14)
surface

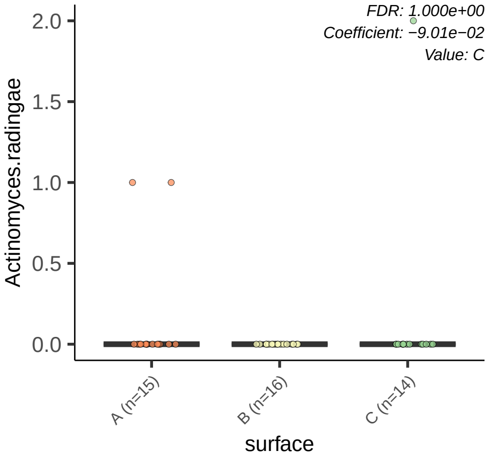

FDR: 1.000e+00
Coefficient: −9.01e−02
Value: C
Actinomyces.radingae
2.0
1.5
1.0
0.5
0.0
A (n=15)
B (n=16)
C (n=14)
surface

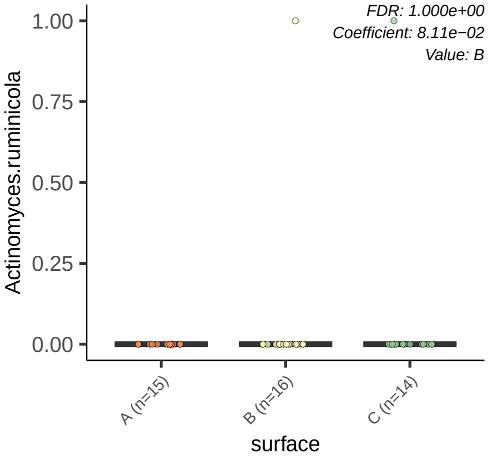

FDR: 1.000e+00
Coefficient: 8.11e−02
Value: B
1.00
0.75
0.50
0.25
0.00
Actinomyces.ruminicola
A (n=15)
B (n=16)
C (n=14)
surface

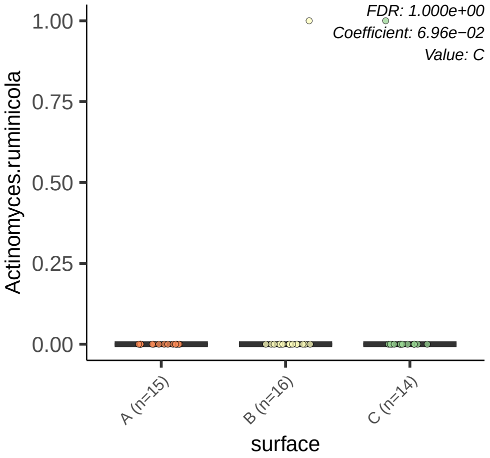
FDR: 1.000e+00
Coefficient: 6.96e−02
Value: C
Actinomyces.ruminicola
1.00
0.75
0.50
0.25
0.00
A (n=15)
B (n=16)
C (n=14)
surface

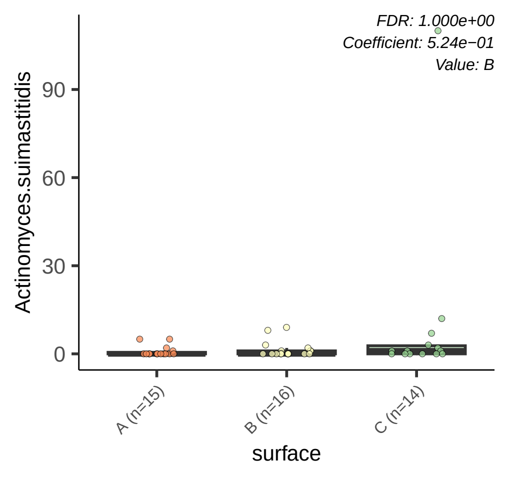
FDR: 1.000e+00
Coefficient: 5.24e−01
Value: B
Actinomyces.suimastitidis
90
60
30
0
A (n=15)
B (n=16)
C (n=14)
surface

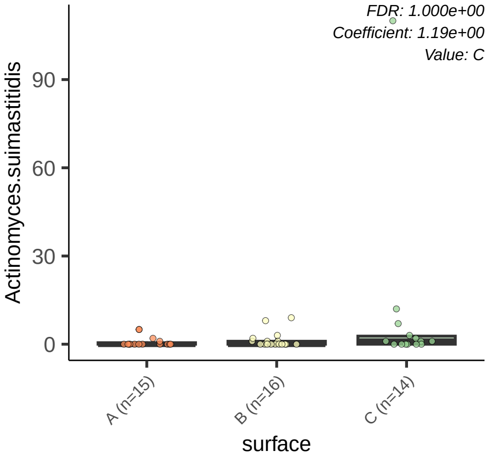
FDR: 1.000e+00
Coefficient: 1.19e+00
Value: C
Actinomyces.suimastitidis
90
60
30
0
A (n=15)
B (n=16)
C (n=14)
surface

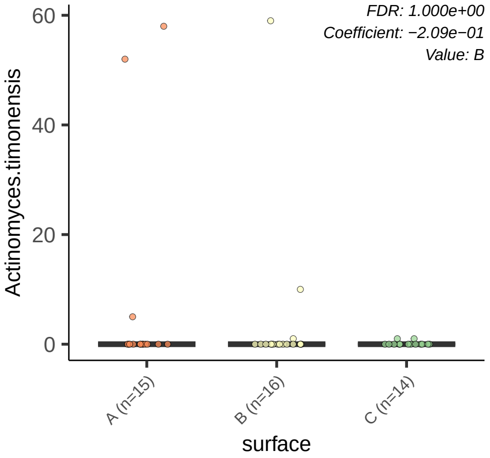
FDR: 1.000e+00
Coefficient: −2.09e−01
Value: B
Actinomyces.timonensis
60
40
20
0
A (n=15)
B (n=16)
C (n=14)
surface

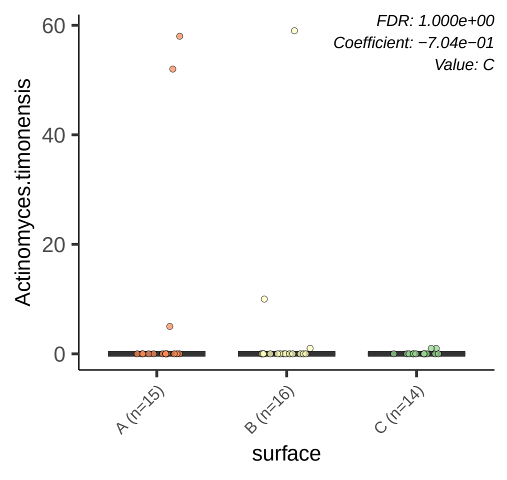
FDR: 1.000e+00
Coefficient: −7.04e−01
Value: C
Actinomyces.timonensis
0
20
40
60
A (n=15)
B (n=16)
C (n=14)
surface

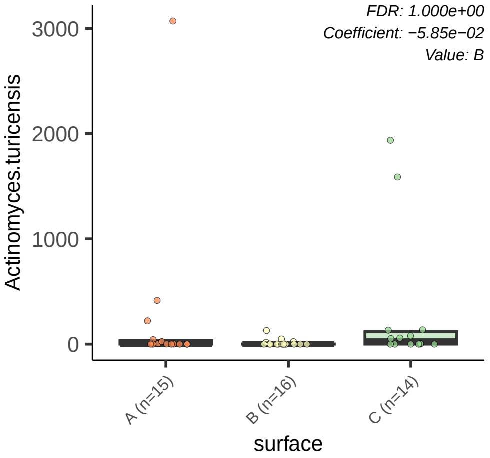
FDR: 1.000e+00
Coefficient: −5.85e−02
Value: B
3000
2000
1000
0
Actinomyces.turicensis
A (n=15)
B (n=16)
C (n=14)
surface

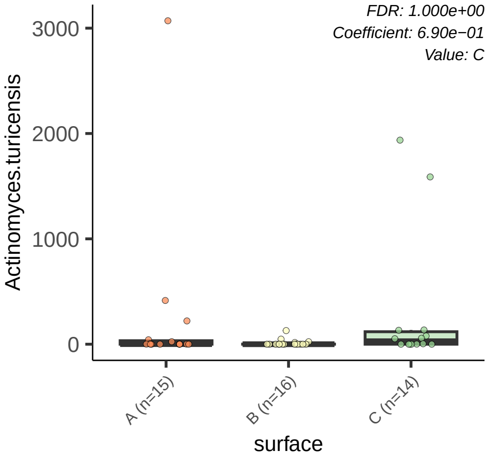
FDR: 1.000e+00
Coefficient: 6.90e−01
Value: C
3000
2000
1000
0
Actinomyces.turicensis
A (n=15)
B (n=16)
C (n=14)
surface

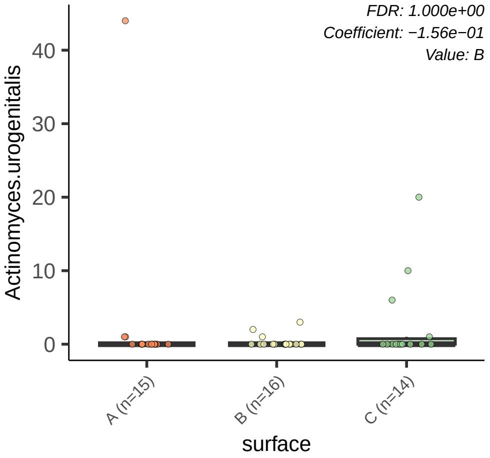
FDR: 1.000e+00
Coefficient: −1.56e−01
Value: B
Actinomyces.urogenitalis
0
10
20
30
40
A (n=15)
B (n=16)
C (n=14)
surface

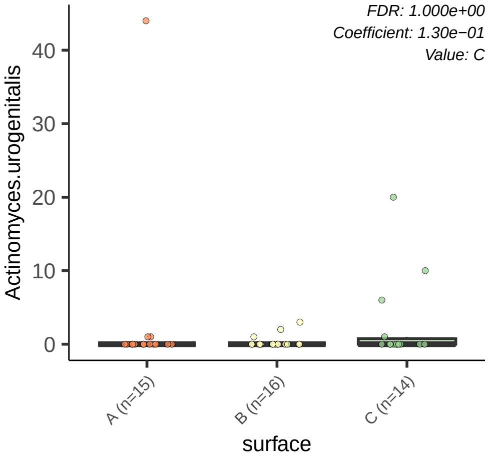

FDR: 1.000e+00
Coefficient: 1.30e−01
Value: C
Actinomyces.urogenitalis
0
10
20
30
40
A (n=15)
B (n=16)
C (n=14)
surface

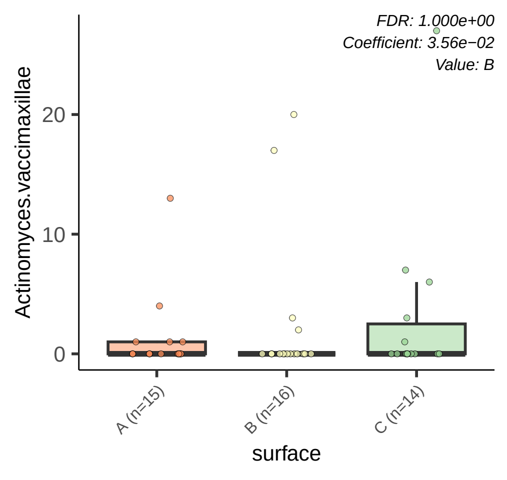
FDR: 1.000e+00
Coefficient: 3.56e−02
Value: B
Actinomyces.vaccimaxillae
20
10
0
A (n=15)
B (n=16)
C (n=14)
surface

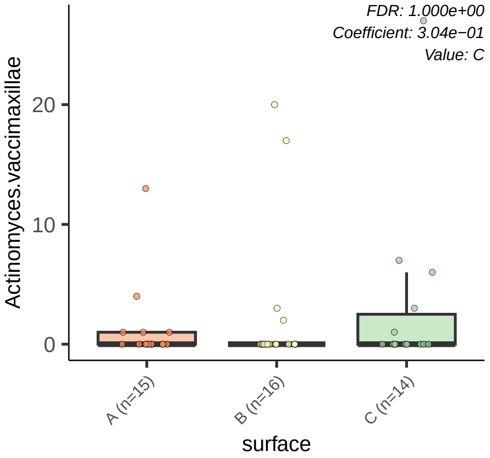

FDR: 1.000e+00
Coefficient: 3.04e−01
Value: C
Actinomyces.vaccimaxillae
20
10
0
A (n=15)
B (n=16)
C (n=14)
surface

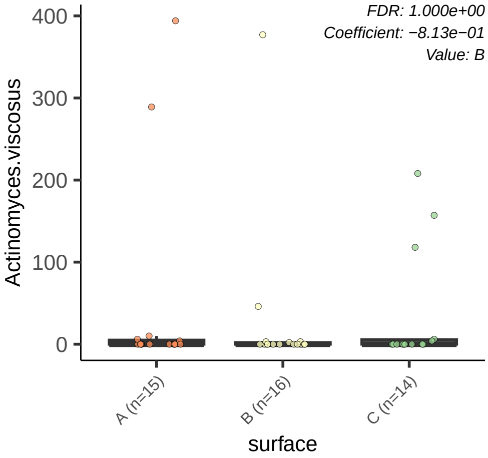

FDR: 1.000e+00
Coefficient: −8.13e−01
Value: B
Actinomyces.viscosus
400
300
200
100
0
A (n=15)
B (n=16)
C (n=14)
surface

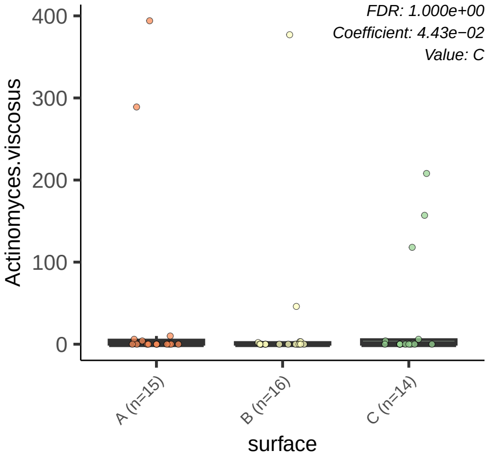

FDR: 1.000e+00
Coefficient: 4.43e−02
Value: C
Actinomyces.viscosus
400
300
200
100
0
A (n=15)
B (n=16)
C (n=14)
surface

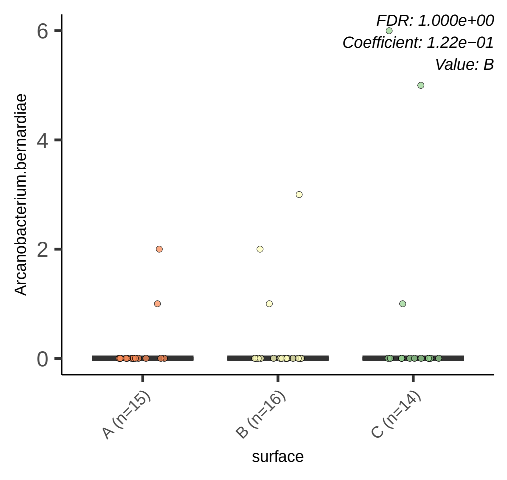
FDR: 1.000e+00
Coefficient: 1.22e−01
Value: B
Arcanobacterium.bernardiae
6
4
2
0
A (n=15)
B (n=16)
C (n=14)
surface

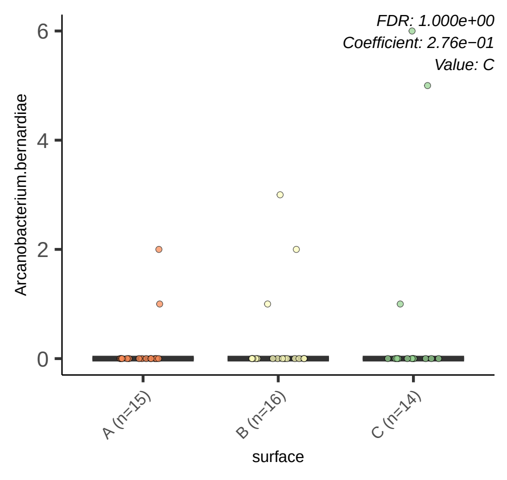
FDR: 1.000e+00
Coefficient: 2.76e−01
Value: C
Arcanobacterium.bernardiae
6
4
2
0
A (n=15)
B (n=16)
C (n=14)
surface

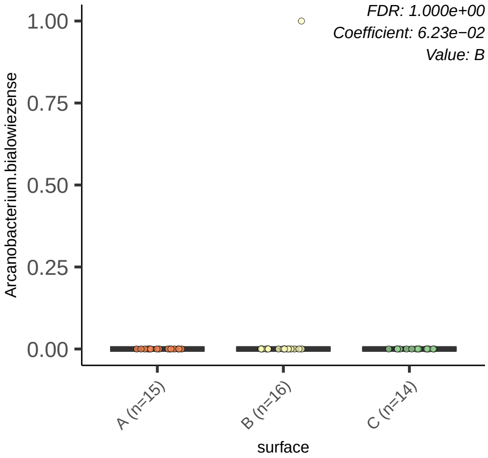
FDR: 1.000e+00
Coefficient: 6.23e−02
Value: B
Arcanobacterium.bialowiezense
1.00
0.75
0.50
0.25
0.00
A (n=15)
B (n=16)
C (n=14)
surface

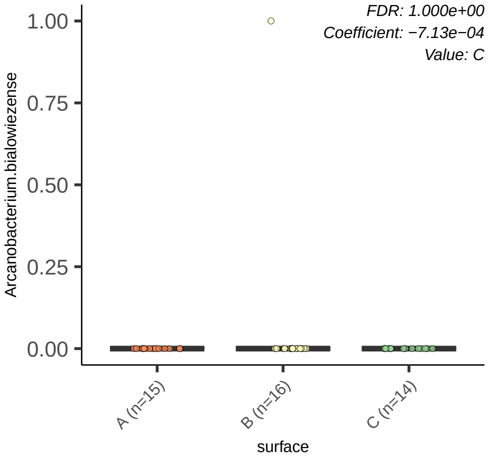
FDR: 1.000e+00
Coefficient: −7.13e−04
Value: C
Arcanobacterium.bialowiezense
1.00
0.75
0.50
0.25
0.00
A (n=15)
B (n=16)
C (n=14)
surface

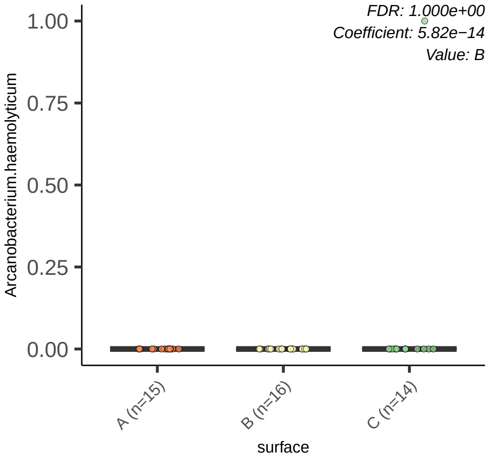
FDR: 1.000e+00
Coefficient: 5.82e−14
Value: B
Arcanobacterium.haemolyticum
1.00
0.75
0.50
0.25
0.00
A (n=15)
B (n=16)
C (n=14)
surface

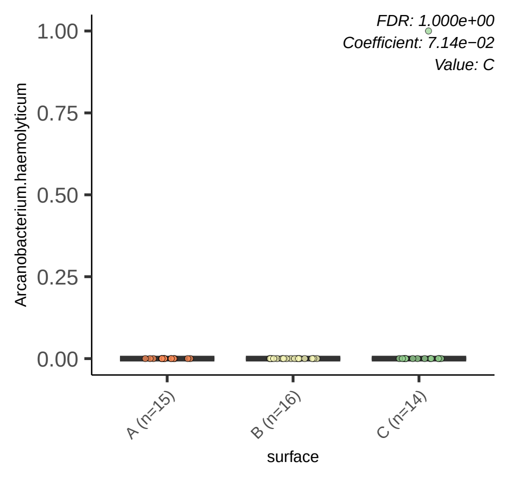
FDR: 1.000e+00
Coefficient: 7.14e−02
Value: C
1.00
0.75
0.50
0.25
0.00
Arcanobacterium.haemolyticum
A (n=15)
B (n=16)
C (n=14)
surface

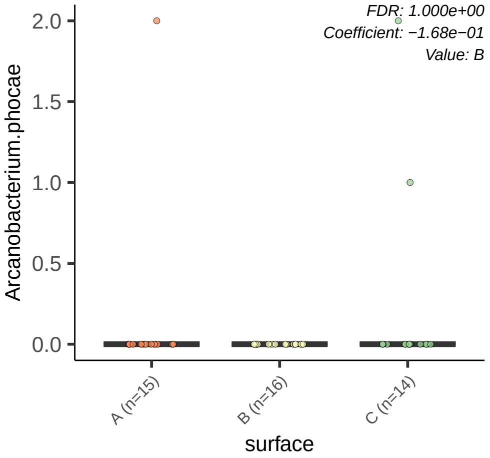

FDR: 1.000e+00
Coefficient: −1.68e−01
Value: B
Arcanobacterium.phocae
2.0
1.5
1.0
0.5
0.0
A (n=15)
B (n=16)
C (n=14)
surface

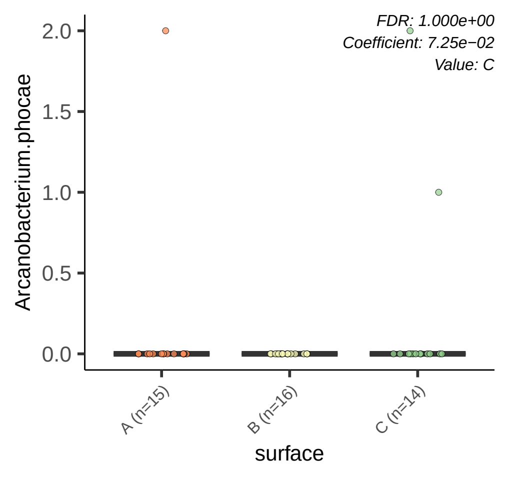

FDR: 1.000e+00
Coefficient: 7.25e−02
Value: C
Arcanobacterium.phocae
2.0
1.5
1.0
0.5
0.0
A (n=15)
B (n=16)
C (n=14)
surface

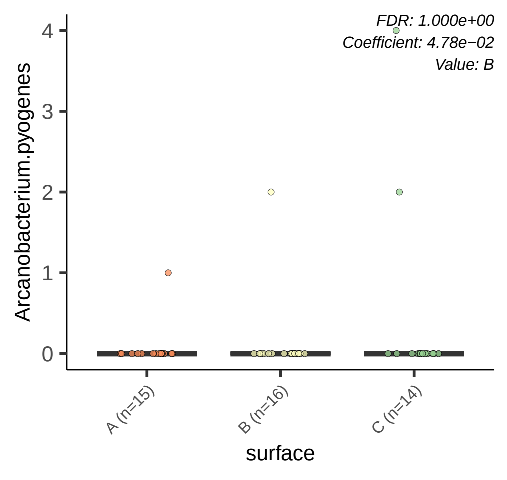

FDR: 1.000e+00
Coefficient: 4.78e−02
Value: B
Arcanobacterium.pyogenes
0
1
2
3
4
A (n=15)
B (n=16)
C (n=14)
surface

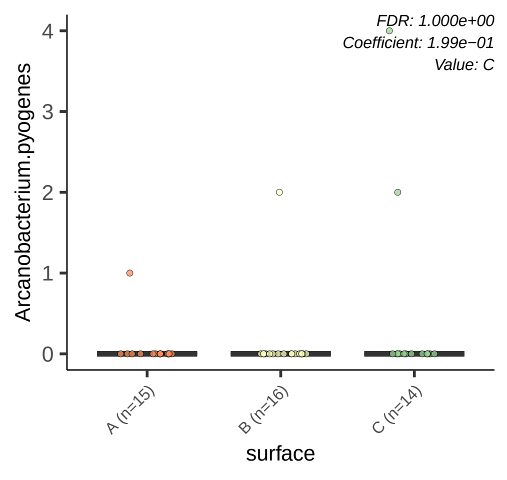
FDR: 1.000e+00
Coefficient: 1.99e−01
Value: C
Arcanobacterium.pyogenes
0
1
2
3
4
A (n=15)
B (n=16)
C (n=14)
surface

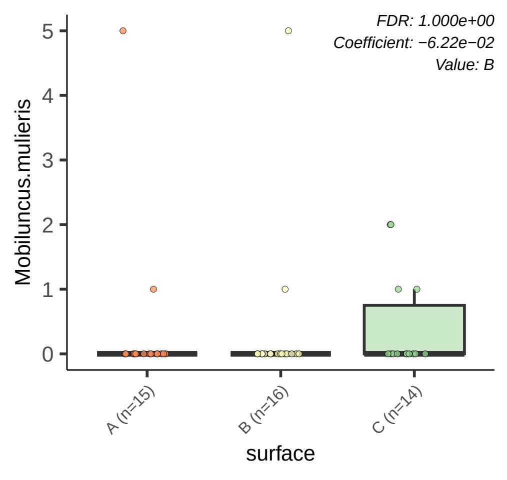
FDR: 1.000e+00
Coefficient: −6.22e−02
Value: B
Mobiluncus.mulieris
0
1
2
3
4
5
A (n=15)
B (n=16)
C (n=14)
surface

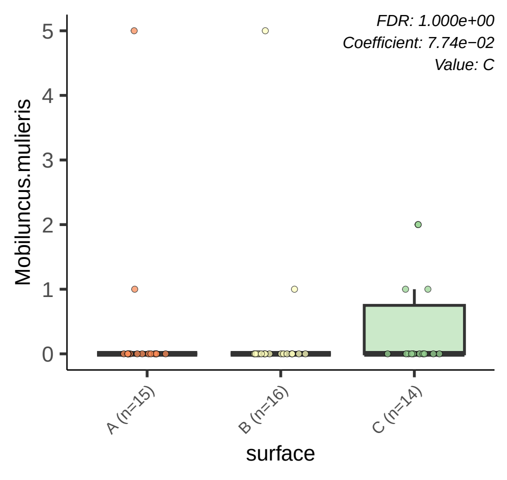
FDR: 1.000e+00
Coefficient: 7.74e−02
Value: C
Mobiluncus.mulieris
0
1
2
3
4
5
A (n=15)
B (n=16)
C (n=14)
surface

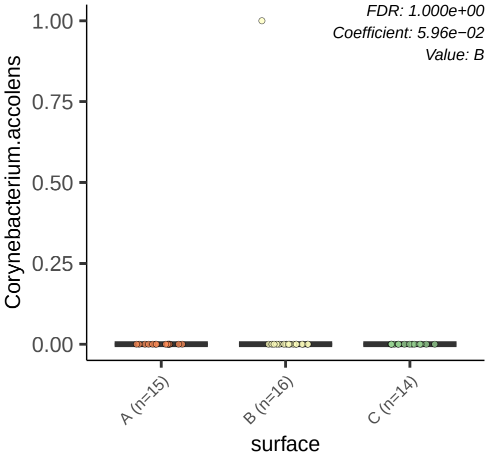
FDR: 1.000e+00
Coefficient: 5.96e−02
Value: B
Corynebacterium.accolens
1.00
0.75
0.50
0.25
0.00
A (n=15)
B (n=16)
C (n=14)
surface

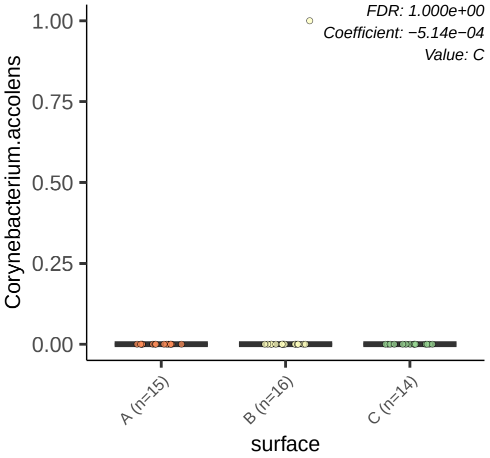
FDR: 1.000e+00
Coefficient: −5.14e−04
Value: C
1.00
0.75
0.50
0.25
0.00
Corynebacterium.accolens
A (n=15)
B (n=16)
C (n=14)
surface

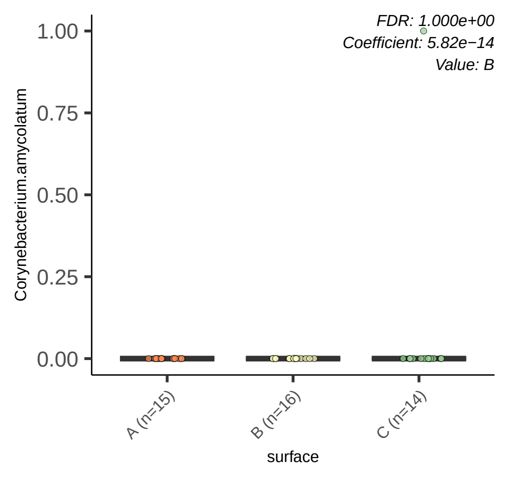
FDR: 1.000e+00
Coefficient: 5.82e−14
Value: B
1.00
0.75
0.50
0.25
0.00
Corynebacterium.amycolatum
A (n=15)
B (n=16)
C (n=14)
surface

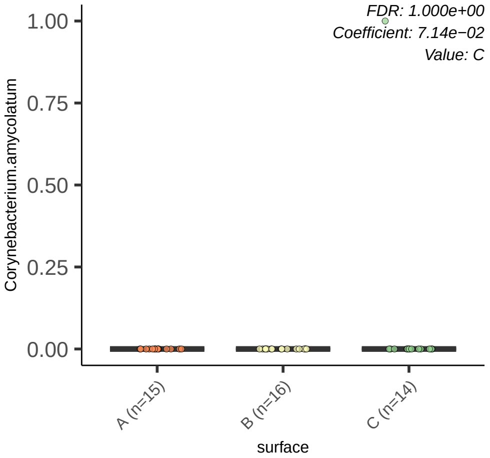
FDR: 1.000e+00
Coefficient: 7.14e−02
Value: C
1.00
0.75
0.50
0.25
0.00
Corynebacterium.amycolatum
A (n=15)
B (n=16)
C (n=14)
surface

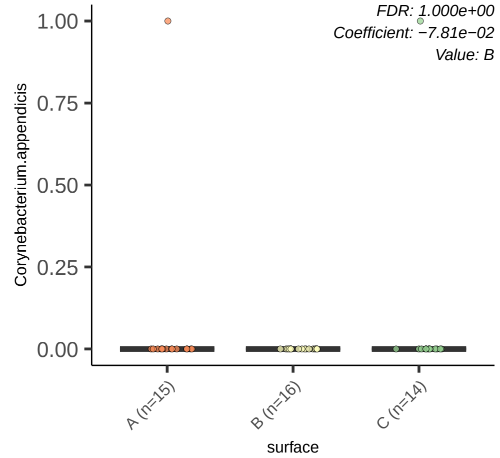
FDR: 1.000e+00
Coefficient: −7.81e−02
Value: B
1.00
0.75
0.50
0.25
0.00
Corynebacterium.appendicis
A (n=15)
B (n=16)
C (n=14)
surface

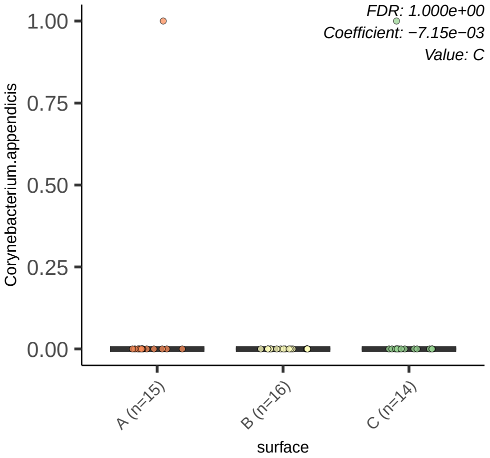

FDR: 1.000e+00
Coefficient: −7.15e−03
Value: C
1.00
0.75
0.50
0.25
0.00
Corynebacterium.appendicis
A (n=15)
B (n=16)
C (n=14)
surface

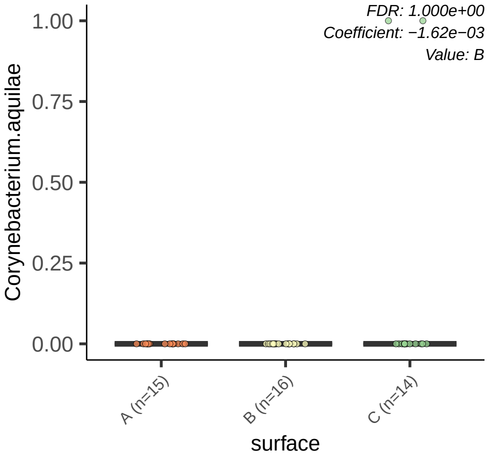

FDR: 1.000e+00
Coefficient: −1.62e−03
Value: B
Corynebacterium.aquilae
1.00
0.75
0.50
0.25
0.00
A (n=15)
B (n=16)
C (n=14)
surface

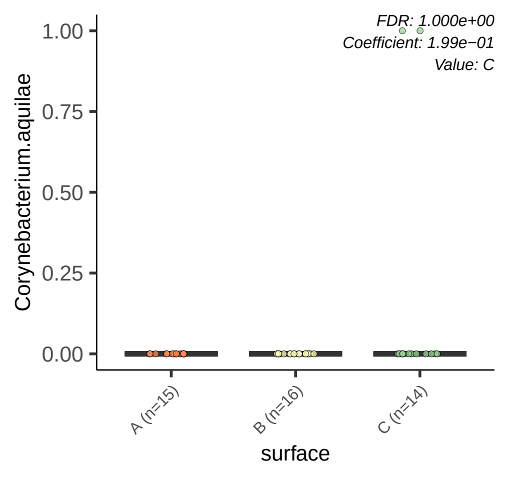
FDR: 1.000e+00
Coefficient: 1.99e−01
Value: C
Corynebacterium.aquilae
1.00
0.75
0.50
0.25
0.00
A (n=15)
B (n=16)
C (n=14)
surface

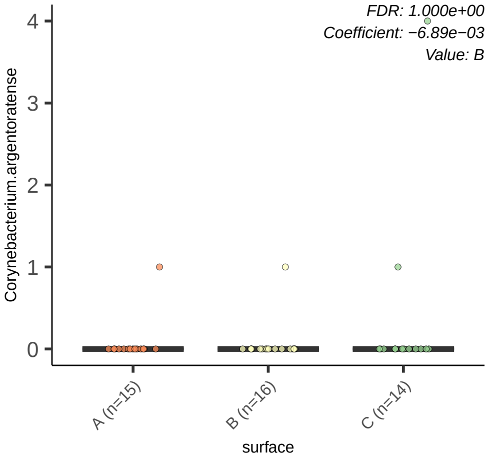
FDR: 1.000e+00
Coefficient: −6.89e−03
Value: B
Corynebacterium.argentoratense
4
3
2
1
0
A (n=15)
B (n=16)
C (n=14)
surface

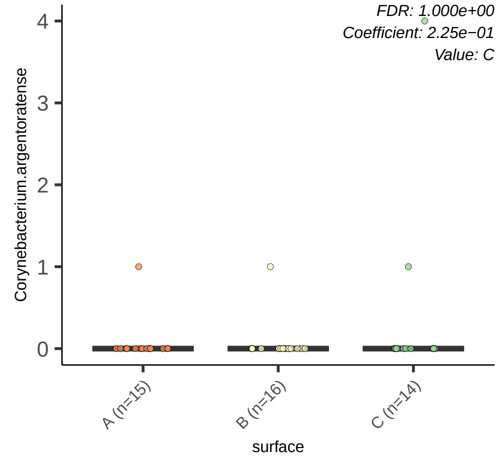

FDR: 1.000e+00
Coefficient: 2.25e−01
Value: C
Corynebacterium.argentoratense
4
3
2
1
0
A (n=15)
B (n=16)
C (n=14)
surface

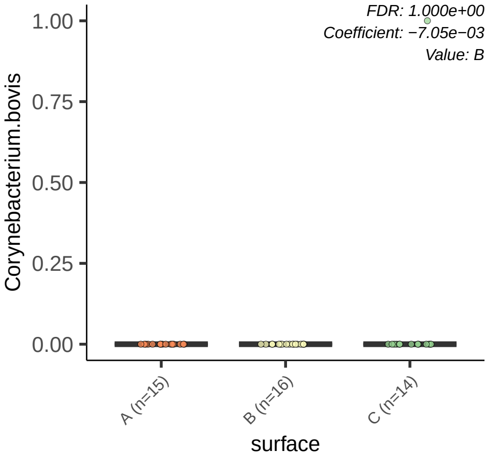
FDR: 1.000e+00
Coefficient: −7.05e−03
Value: B
Corynebacterium.bovis
1.00
0.75
0.50
0.25
0.00
A (n=15)
B (n=16)
C (n=14)
surface

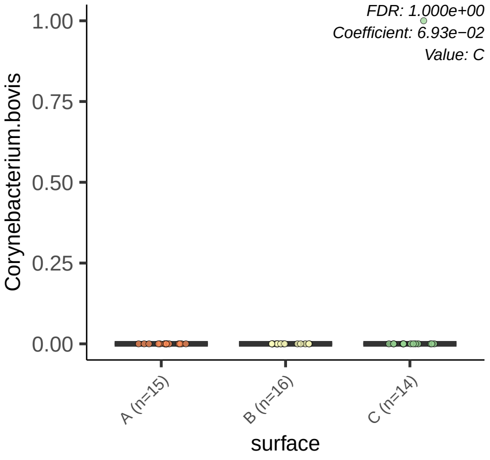
FDR: 1.000e+00
Coefficient: 6.93e−02
Value: C
Corynebacterium.bovis
1.00
0.75
0.50
0.25
0.00
A (n=15)
B (n=16)
C (n=14)
surface

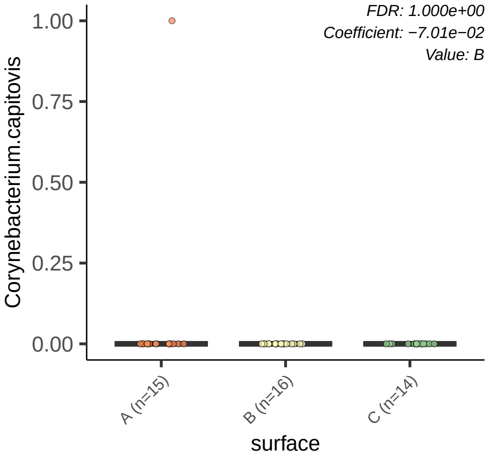

FDR: 1.000e+00
Coefficient: −7.01e−02
Value: B
Corynebacterium.capitovis
1.00
0.75
0.50
0.25
0.00
A (n=15)
B (n=16)
C (n=14)
surface

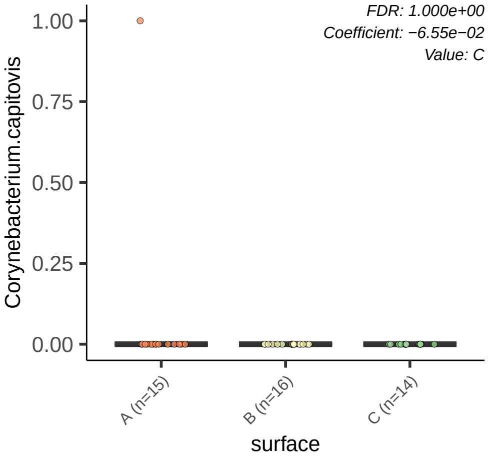

FDR: 1.000e+00
Coefficient: −6.55e−02
Value: C
Corynebacterium.capitovis
1.00
0.75
0.50
0.25
0.00
A (n=15)
B (n=16)
C (n=14)
surface

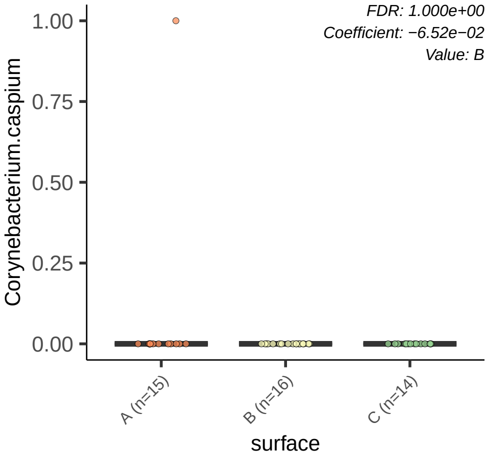
FDR: 1.000e+00
Coefficient: −6.52e−02
Value: B
Corynebacterium.caspium
1.00
0.75
0.50
0.25
0.00
A (n=15)
B (n=16)
C (n=14)
surface

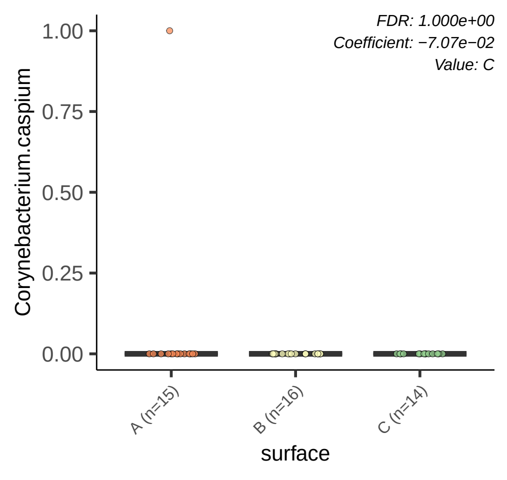
FDR: 1.000e+00
Coefficient: −7.07e−02
Value: C
Corynebacterium.caspium
1.00
0.75
0.50
0.25
0.00
A (n=15)
B (n=16)
C (n=14)
surface

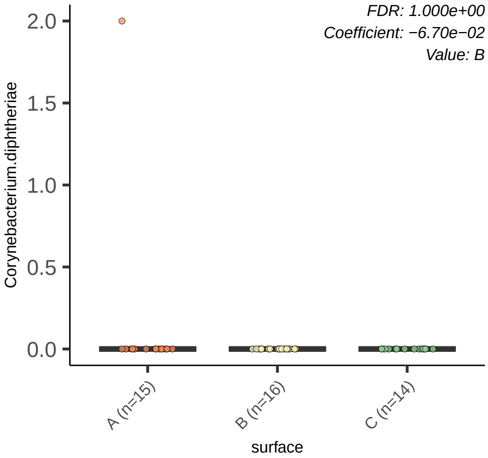

FDR: 1.000e+00
Coefficient: −6.70e−02
Value: B
Corynebacterium.diphtheriae
2.0
1.5
1.0
0.5
0.0
A (n=15)
B (n=16)
C (n=14)
surface

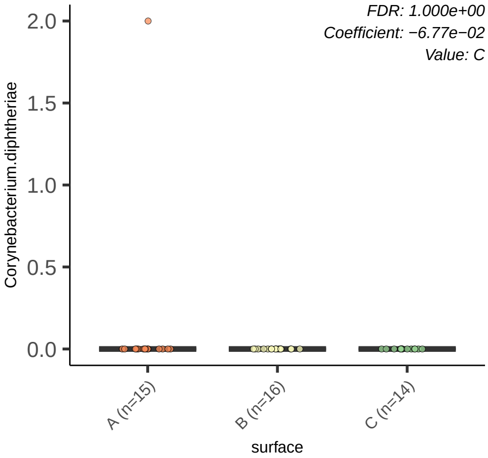
FDR: 1.000e+00
Coefficient: −6.77e−02
Value: C
Corynebacterium.diphtheriae
2.0
1.5
1.0
0.5
0.0
A (n=15)
B (n=16)
C (n=14)
surface

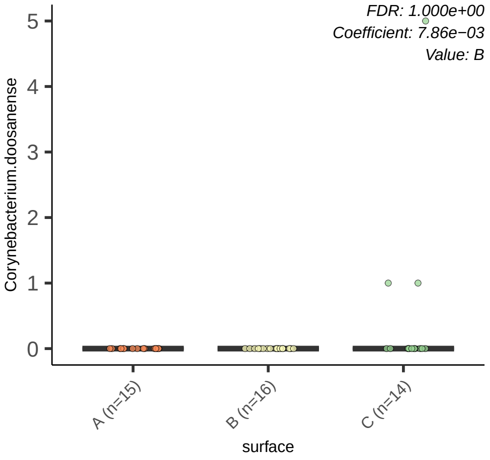
FDR: 1.000e+00
Coefficient: 7.86e−03
Value: B
Corynebacterium.doosanense
5
4
3
2
1
0
A (n=15)
B (n=16)
C (n=14)
surface

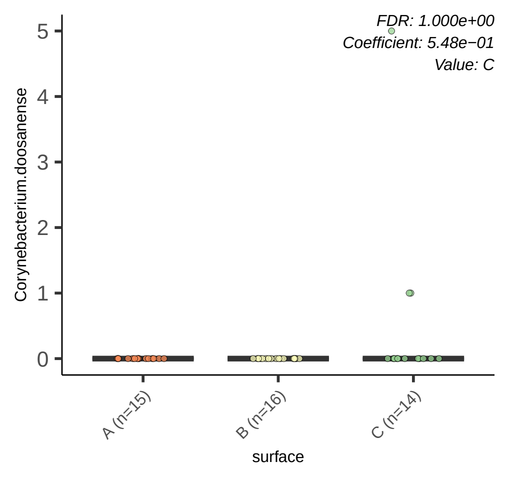

FDR: 1.000e+00
Coefficient: 5.48e−01
Value: C
Corynebacterium.doosanense
5
4
3
2
1
0
A (n=15)
B (n=16)
C (n=14)
surface

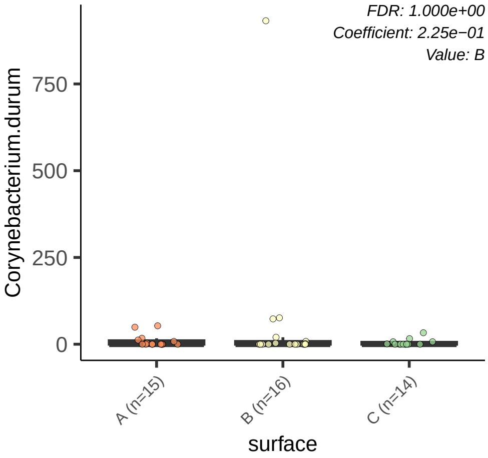
FDR: 1.000e+00
Coefficient: 2.25e−01
Value: B
Corynebacterium.durum
750
500
250
0
A (n=15)
B (n=16)
C (n=14)
surface

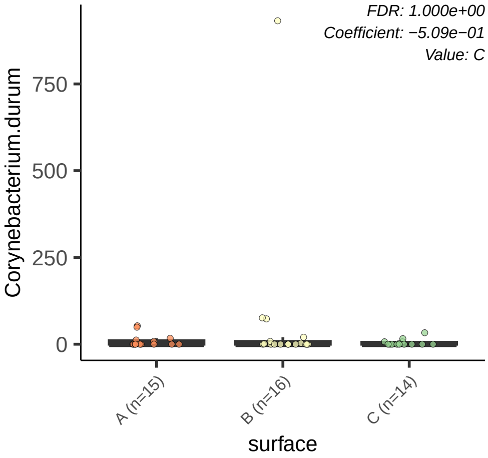
FDR: 1.000e+00
Coefficient: −5.09e−01
Value: C
Corynebacterium.durum
750
500
250
0
A (n=15)
B (n=16)
C (n=14)
surface

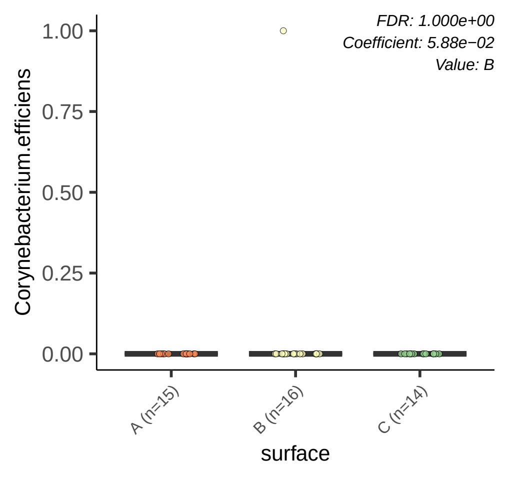
FDR: 1.000e+00
Coefficient: 5.88e−02
Value: B
Corynebacterium.efficiens
1.00
0.75
0.50
0.25
0.00
A (n=15)
B (n=16)
C (n=14)
surface

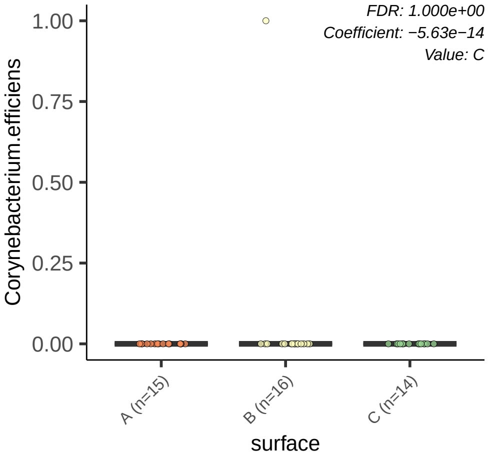

FDR: 1.000e+00
Coefficient: −5.63e−14
Value: C
Corynebacterium.efficiens
1.00
0.75
0.50
0.25
0.00
A (n=15)
B (n=16)
C (n=14)
surface

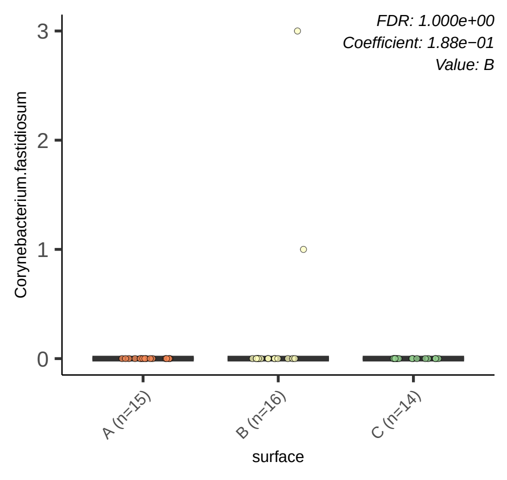
FDR: 1.000e+00
Coefficient: 1.88e−01
Value: B
Corynebacterium.fastidiosum
3
2
1
0
A (n=15)
B (n=16)
C (n=14)
surface

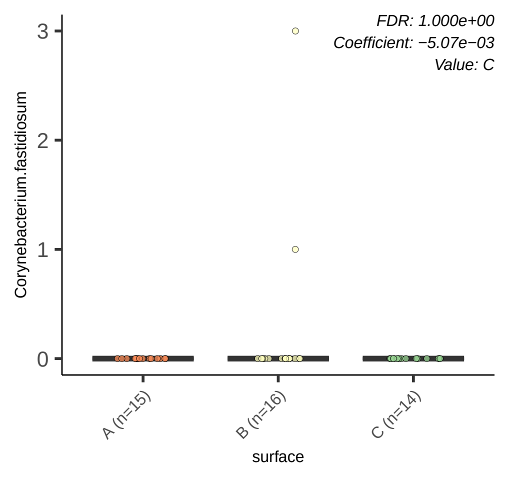
FDR: 1.000e+00
Coefficient: −5.07e−03
Value: C
Corynebacterium.fastidiosum
3
2
1
0
A (n=15)
B (n=16)
C (n=14)
surface

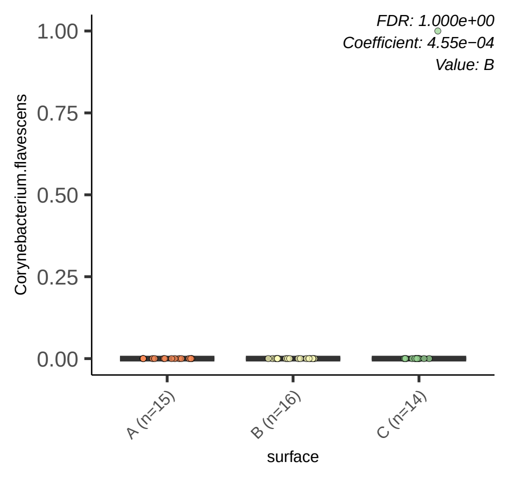
FDR: 1.000e+00
Coefficient: 4.55e−04
Value: B
1.00
0.75
0.50
0.25
0.00
Corynebacterium.flavescens
A (n=15)
B (n=16)
C (n=14)
surface

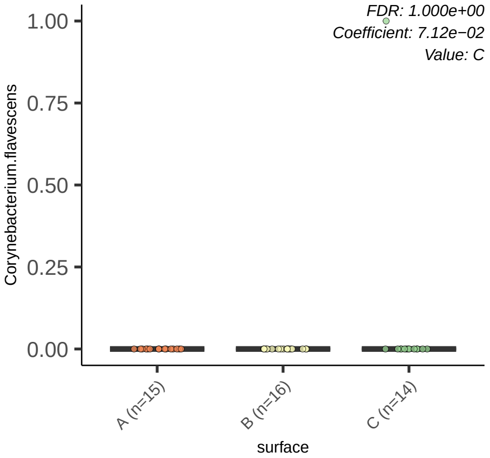
FDR: 1.000e+00
Coefficient: 7.12e−02
Value: C
Corynebacterium.flavescens
1.00
0.75
0.50
0.25
0.00
A (n=15)
B (n=16)
C (n=14)
surface

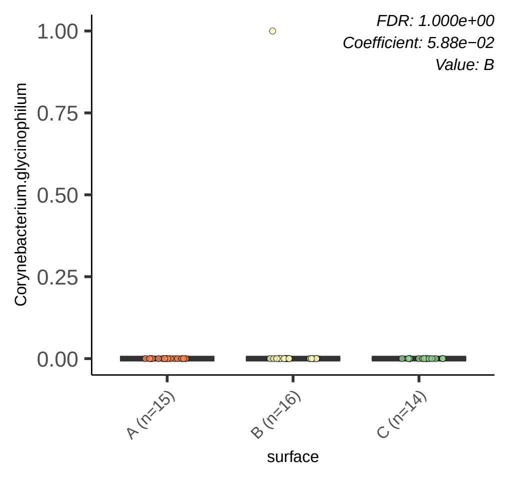

FDR: 1.000e+00
Coefficient: 5.88e−02
Value: B
Corynebacterium.glycinophilum
1.00
0.75
0.50
0.25
0.00
A (n=15)
B (n=16)
C (n=14)
surface

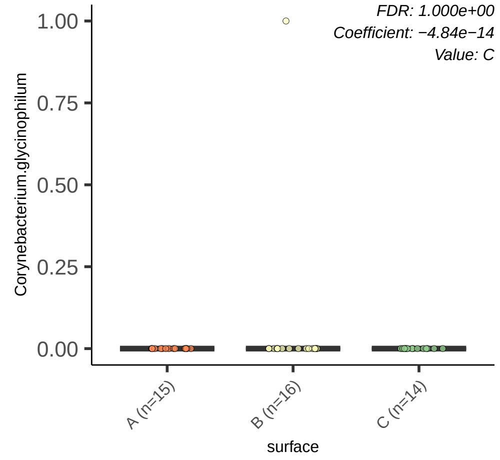
FDR: 1.000e+00
Coefficient: −4.84e−14
Value: C
Corynebacterium.glycinophilum
1.00
0.75
0.50
0.25
0.00
A (n=15)
B (n=16)
C (n=14)
surface

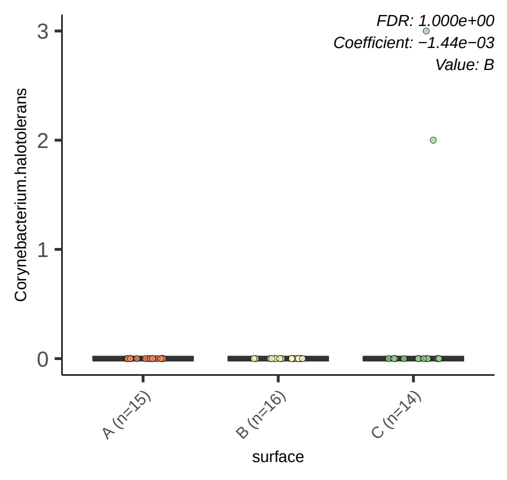

FDR: 1.000e+00
Coefficient: −1.44e−03
Value: B
Corynebacterium.halotolerans
3
2
1
0
A (n=15)
B (n=16)
C (n=14)
surface

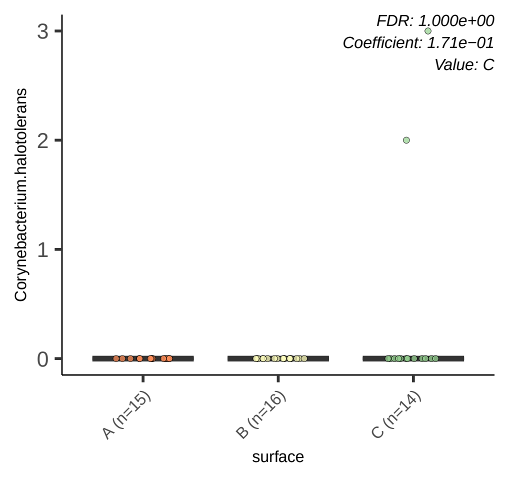

FDR: 1.000e+00
Coefficient: 1.71e−01
Value: C
Corynebacterium.halotolerans
3
2
1
0
A (n=15)
B (n=16)
C (n=14)
surface

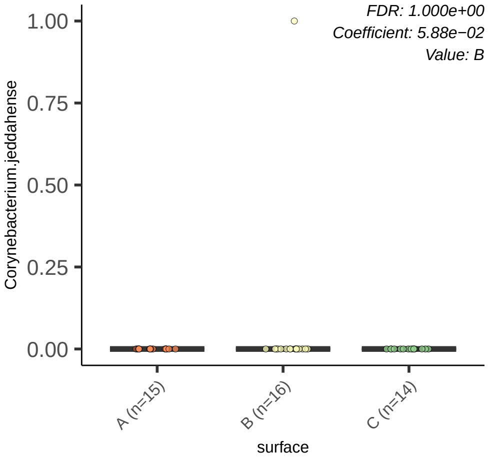
FDR: 1.000e+00
Coefficient: 5.88e−02
Value: B
1.00
0.75
0.50
0.25
0.00
Corynebacterium.jeddahense
A (n=15)
B (n=16)
C (n=14)
surface

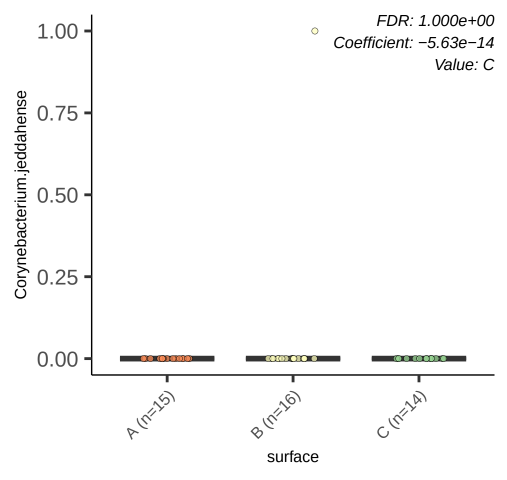

FDR: 1.000e+00
Coefficient: −5.63e−14
Value: C
1.00
0.75
0.50
0.25
0.00
Corynebacterium.jeddahense
A (n=15)
B (n=16)
C (n=14)
surface

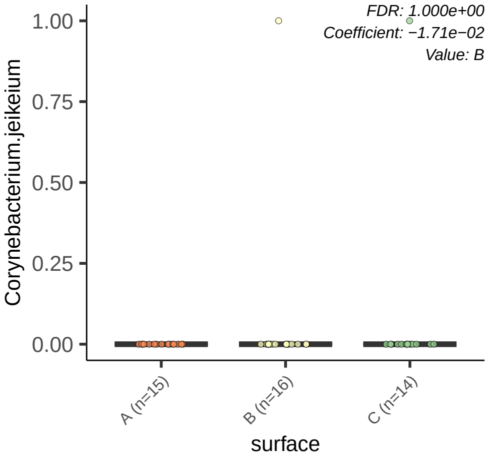

FDR: 1.000e+00
Coefficient: −1.71e−02
Value: B
Corynebacterium.jeikeium
1.00
0.75
0.50
0.25
0.00
A (n=15)
B (n=16)
C (n=14)
surface

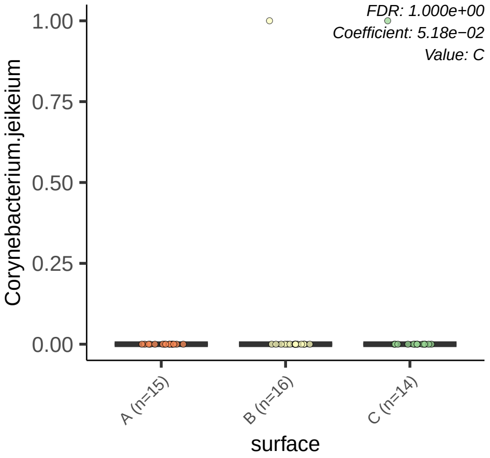

FDR: 1.000e+00
Coefficient: 5.18e−02
Value: C
Corynebacterium.jeikeium
1.00
0.75
0.50
0.25
0.00
A (n=15)
B (n=16)
C (n=14)
surface

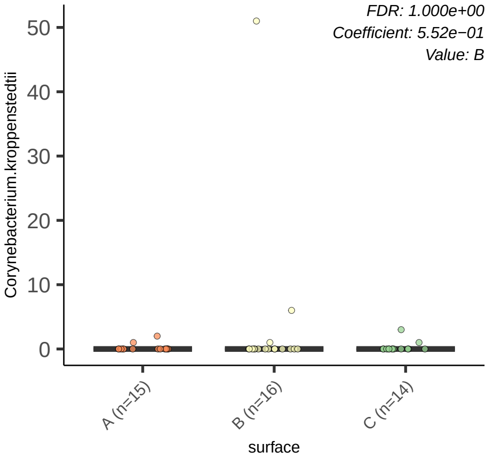

FDR: 1.000e+00
Coefficient: 5.52e−01
Value: B
Corynebacterium.kroppenstedtii
50
40
30
20
10
0
A (n=15)
B (n=16)
C (n=14)
surface

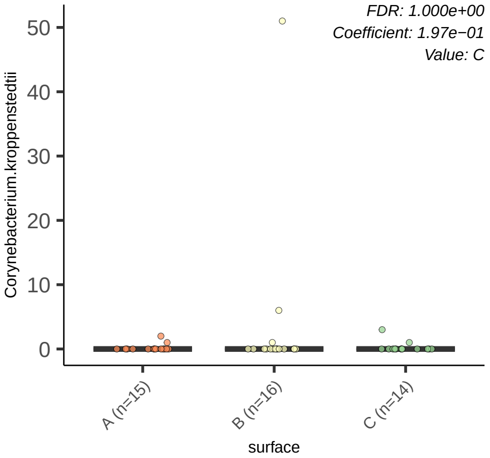

FDR: 1.000e+00
Coefficient: 1.97e−01
Value: C
Corynebacterium.kroppenstedtii
50
40
30
20
10
0
A (n=15)
B (n=16)
C (n=14)
surface

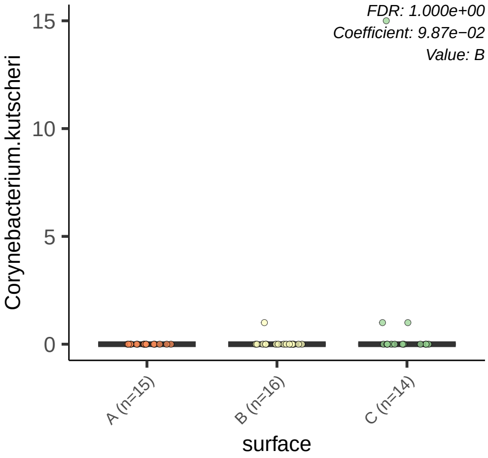

FDR: 1.000e+00
Coefficient: 9.87e−02
Value: B
Corynebacterium.kutscheri
15
10
5
0
A (n=15)
B (n=16)
C (n=14)
surface

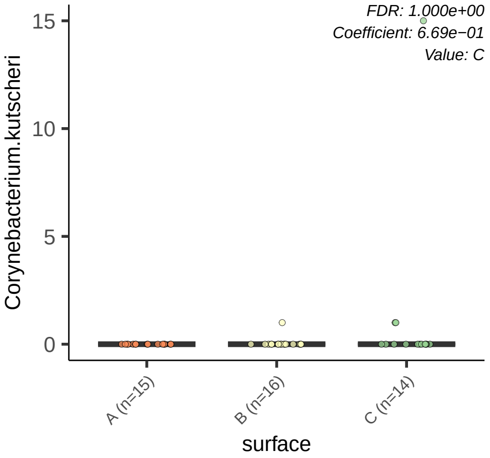

FDR: 1.000e+00
Coefficient: 6.69e−01
Value: C
Corynebacterium.kutscheri
15
10
5
0
A (n=15)
B (n=16)
C (n=14)
surface

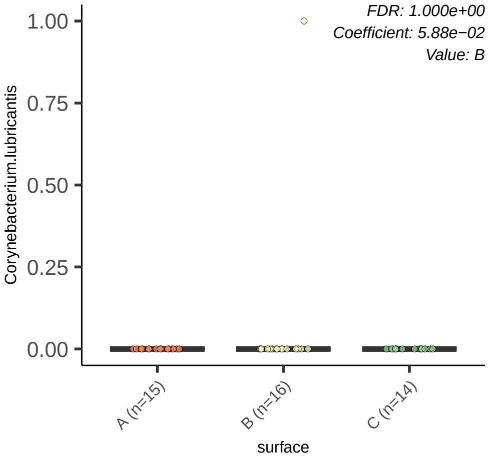

FDR: 1.000e+00
Coefficient: 5.88e−02
Value: B
1.00
0.75
0.50
0.25
0.00
Corynebacterium.lubricantis
A (n=15)
B (n=16)
C (n=14)
surface

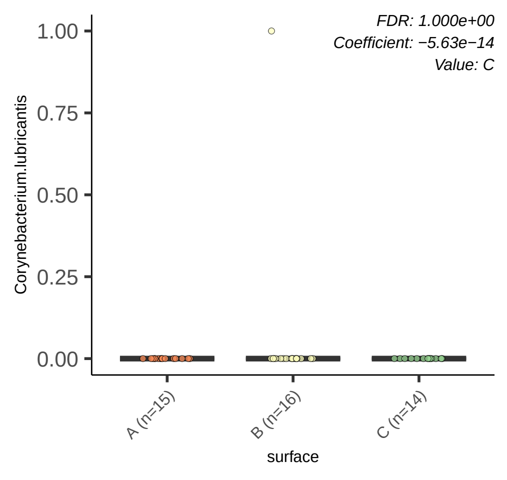
FDR: 1.000e+00
Coefficient: −5.63e−14
Value: C
1.00
0.75
0.50
0.25
0.00
Corynebacterium.lubricantis
A (n=15)
B (n=16)
C (n=14)
surface

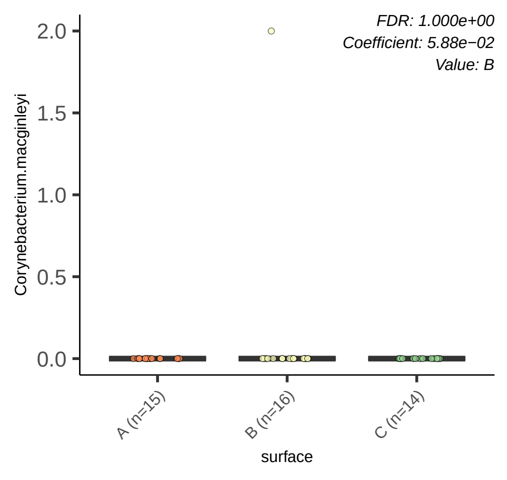

FDR: 1.000e+00
Coefficient: 5.88e−02
Value: B
2.0
1.5
1.0
0.5
0.0
Corynebacterium.macginleyi
A (n=15)
B (n=16)
C (n=14)
surface

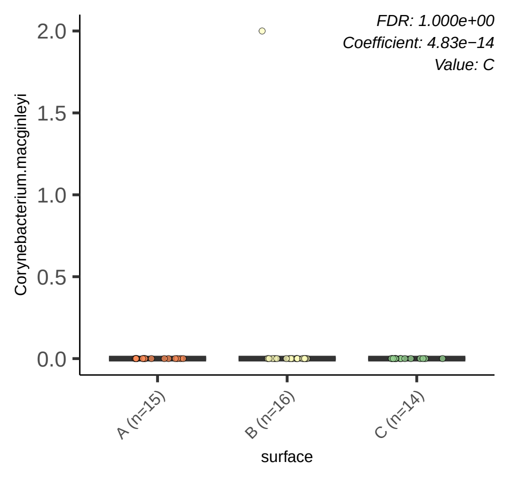
FDR: 1.000e+00
Coefficient: 4.83e−14
Value: C
2.0
1.5
1.0
0.5
0.0
Corynebacterium.macginleyi
A (n=15)
B (n=16)
C (n=14)
surface

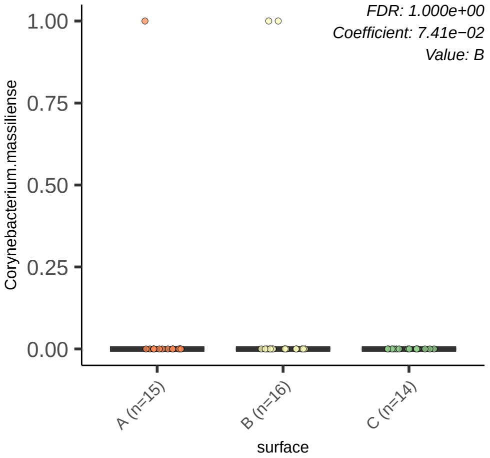
FDR: 1.000e+00
Coefficient: 7.41e−02
Value: B
Corynebacterium.massiliense
1.00
0.75
0.50
0.25
0.00
A (n=15)
B (n=16)
C (n=14)
surface

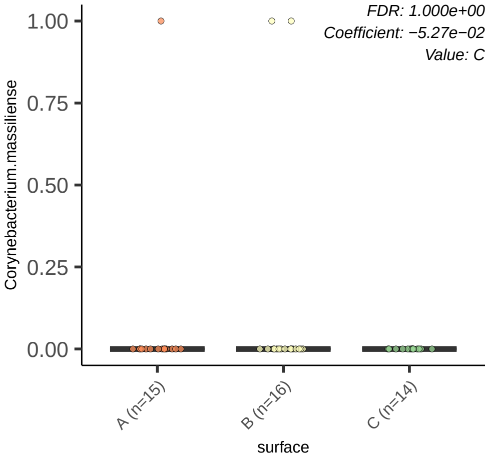

FDR: 1.000e+00
Coefficient: −5.27e−02
Value: C
1.00
0.75
0.50
0.25
0.00
Corynebacterium.massiliense
A (n=15)
B (n=16)
C (n=14)
surface

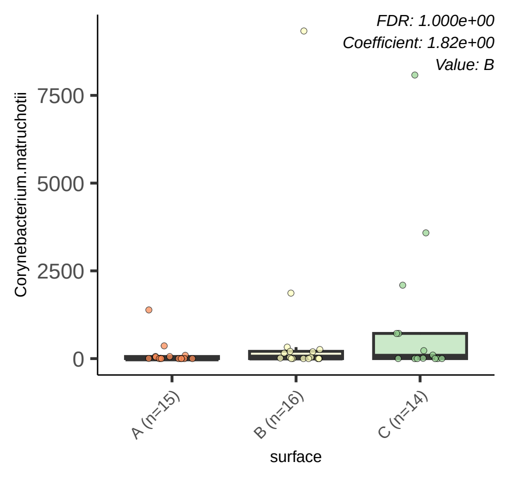
FDR: 1.000e+00
Coefficient: 1.82e+00
Value: B
Corynebacterium.matruchotii
7500
5000
2500
0
A (n=15)
B (n=16)
C (n=14)
surface

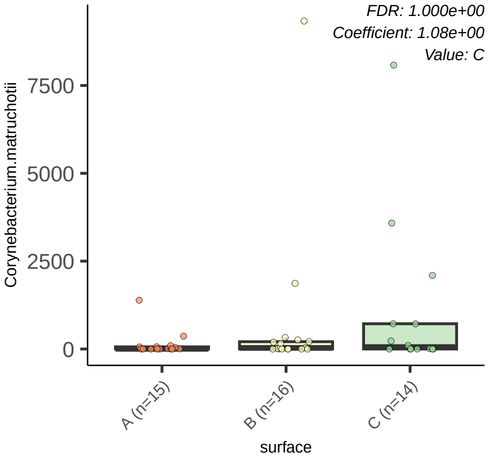

FDR: 1.000e+00
Coefficient: 1.08e+00
Value: C
Corynebacterium.matruchotii
7500
5000
2500
0
A (n=15)
B (n=16)
C (n=14)
surface

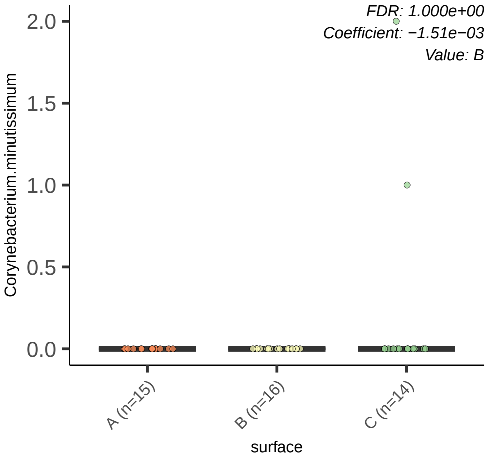
FDR: 1.000e+00
Coefficient: −1.51e−03
Value: B
Corynebacterium.minutissimum
2.0
1.5
1.0
0.5
0.0
A (n=15)
B (n=16)
C (n=14)
surface

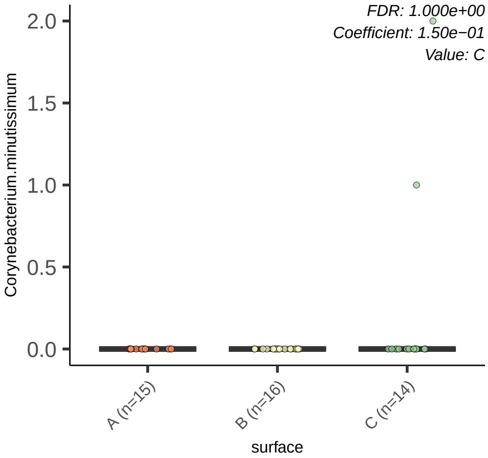

FDR: 1.000e+00
Coefficient: 1.50e−01
Value: C
Corynebacterium.minutissimum
2.0
1.5
1.0
0.5
0.0
A (n=15)
B (n=16)
C (n=14)
surface

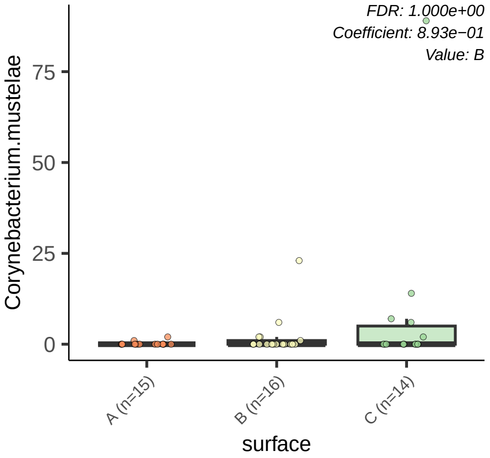
FDR: 1.000e+00
Coefficient: 8.93e−01
Value: B
Corynebacterium.mustelae
75
50
25
0
A (n=15)
B (n=16)
C (n=14)
surface

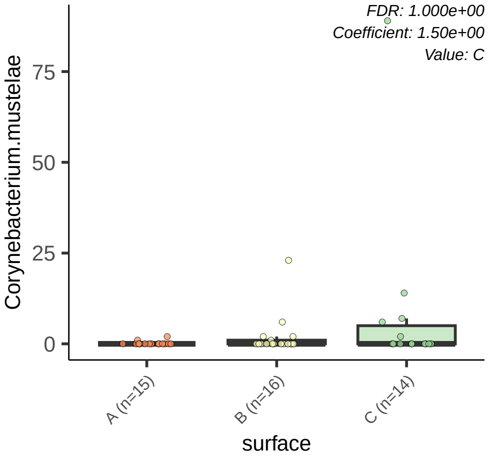

FDR: 1.000e+00
Coefficient: 1.50e+00
Value: C
Corynebacterium.mustelae
75
50
25
0
A (n=15)
B (n=16)
C (n=14)
surface

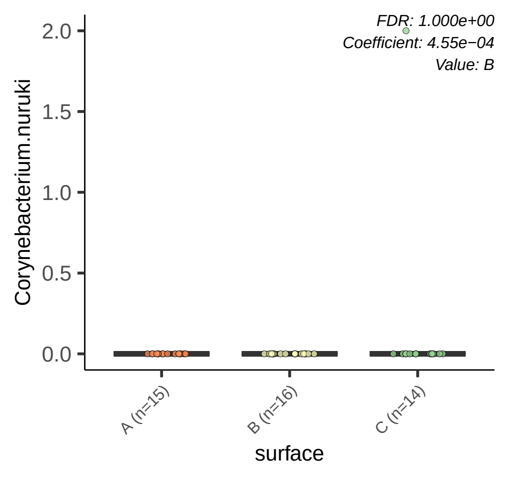
FDR: 1.000e+00
Coefficient: 4.55e−04
Value: B
Corynebacterium.nuruki
2.0
1.5
1.0
0.5
0.0
A (n=15)
B (n=16)
C (n=14)
surface

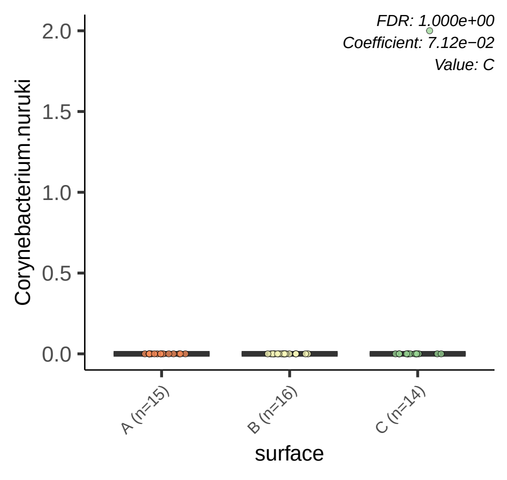

FDR: 1.000e+00
Coefficient: 7.12e−02
Value: C
Corynebacterium.nuruki
2.0
1.5
1.0
0.5
0.0
A (n=15)
B (n=16)
C (n=14)
surface

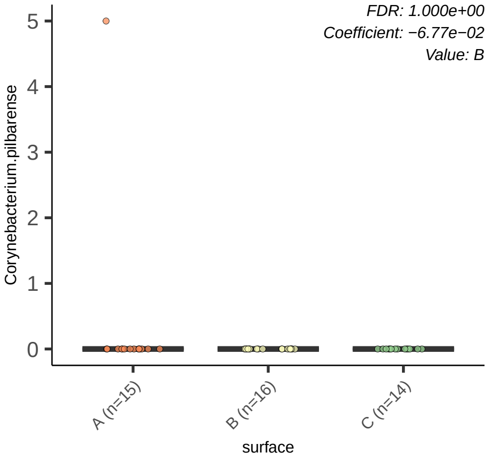
FDR: 1.000e+00
Coefficient: −6.77e−02
Value: B
Corynebacterium.pilbarense
0
1
2
3
4
5
A (n=15)
B (n=16)
C (n=14)
surface

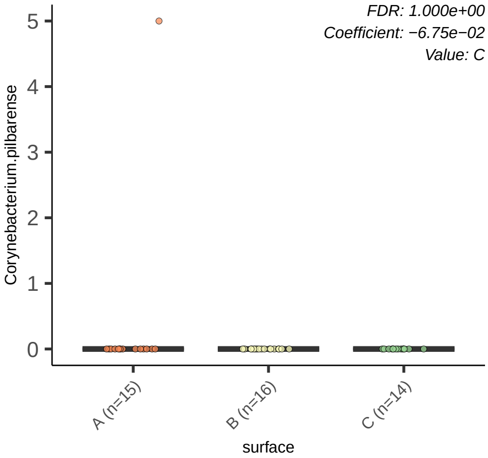
FDR: 1.000e+00
Coefficient: −6.75e−02
Value: C
Corynebacterium.pilbarense
5
4
3
2
1
0
A (n=15)
B (n=16)
C (n=14)
surface

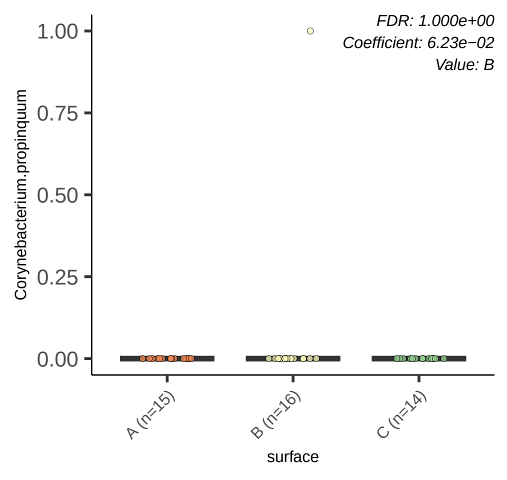
FDR: 1.000e+00
Coefficient: 6.23e−02
Value: B
Corynebacterium.propinquum
1.00
0.75
0.50
0.25
0.00
A (n=15)
B (n=16)
C (n=14)
surface

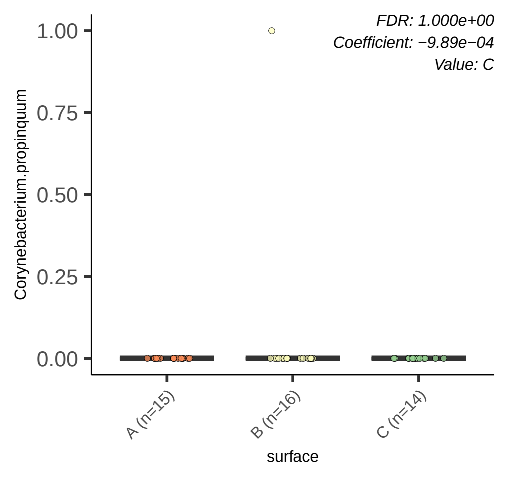
FDR: 1.000e+00
Coefficient: −9.89e−04
Value: C
Corynebacterium.propinquum
1.00
0.75
0.50
0.25
0.00
A (n=15)
B (n=16)
C (n=14)
surface

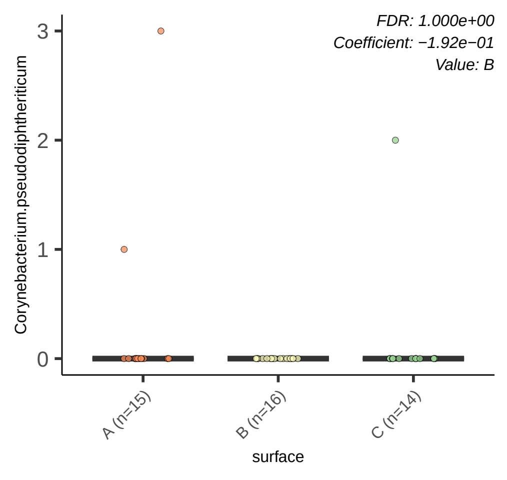

FDR: 1.000e+00
Coefficient: −1.92e−01
Value: B
Corynebacterium.pseudodiphtheriticum
3
2
1
0
A (n=15)
B (n=16)
C (n=14)
surface

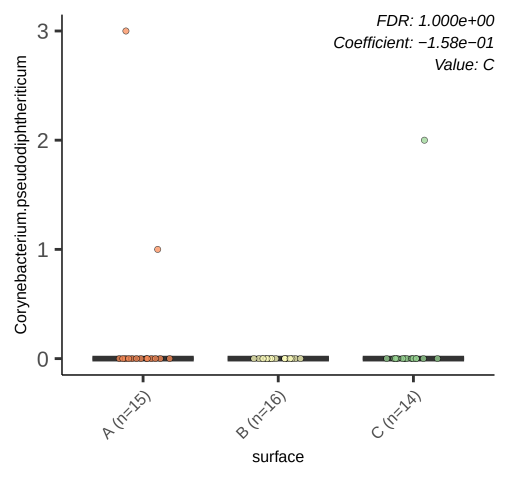

FDR: 1.000e+00
Coefficient: −1.58e−01
Value: C
Corynebacterium.pseudodiphtheriticum
3
2
1
0
A (n=15)
B (n=16)
C (n=14)
surface

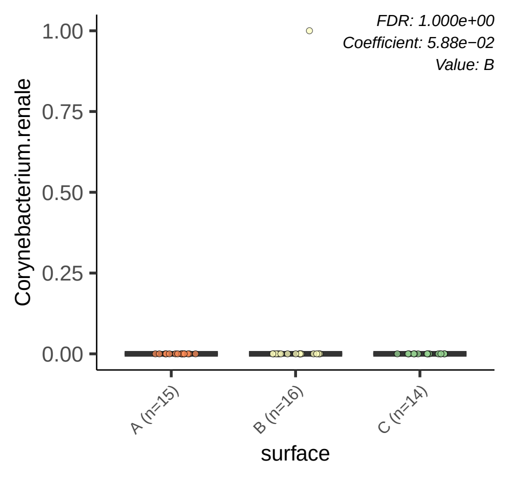
FDR: 1.000e+00
Coefficient: 5.88e−02
Value: B
Corynebacterium.renale
1.00
0.75
0.50
0.25
0.00
A (n=15)
B (n=16)
C (n=14)
surface

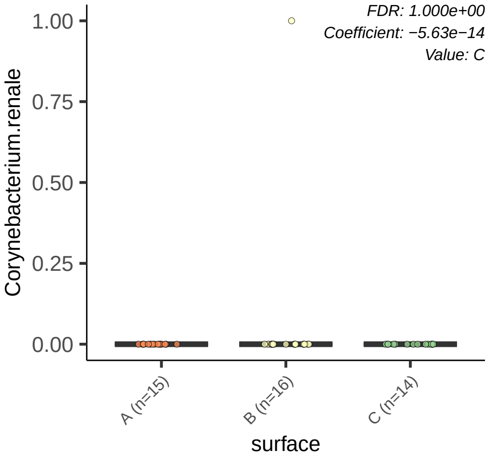
FDR: 1.000e+00
Coefficient: −5.63e−14
Value: C
Corynebacterium.renale
1.00
0.75
0.50
0.25
0.00
A (n=15)
B (n=16)
C (n=14)
surface

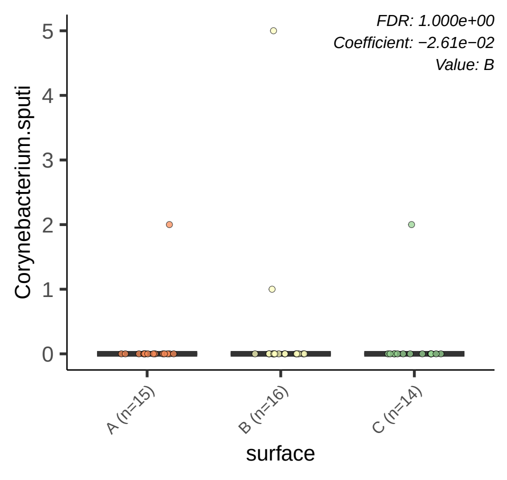
FDR: 1.000e+00
Coefficient: −2.61e−02
Value: B
Corynebacterium.sputi
0
1
2
3
4
5
A (n=15)
B (n=16)
C (n=14)
surface

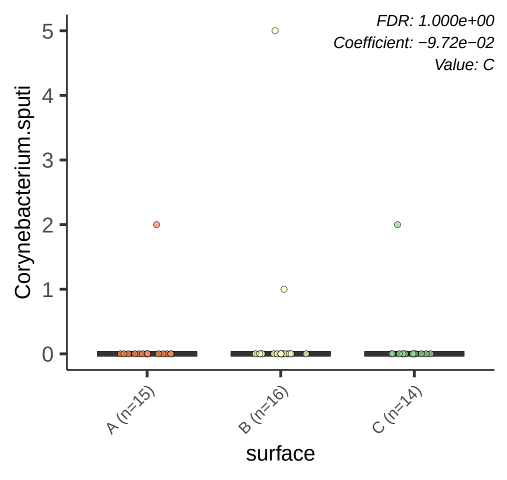
FDR: 1.000e+00
Coefficient: −9.72e−02
Value: C
Corynebacterium.sputi
0
1
2
3
4
5
A (n=15)
B (n=16)
C (n=14)
surface

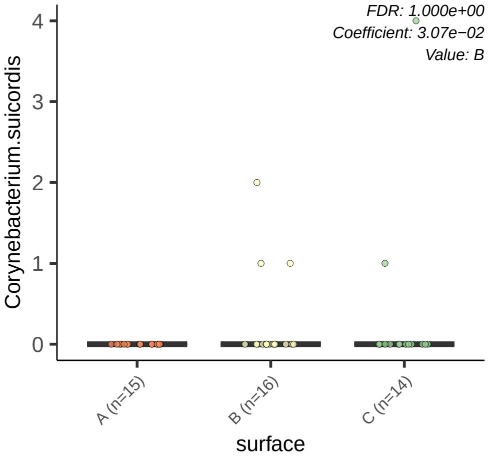

FDR: 1.000e+00
Coefficient: 3.07e−02
Value: B
Corynebacterium.suicordis
0
1
2
3
4
A (n=15)
B (n=16)
C (n=14)
surface

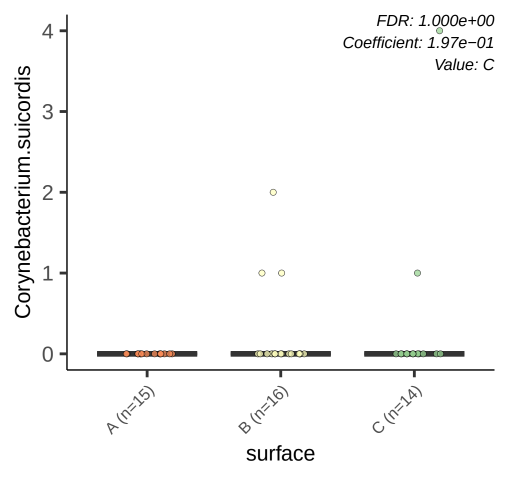

FDR: 1.000e+00
Coefficient: 1.97e−01
Value: C
Corynebacterium.suicordis
0
1
2
3
4
A (n=15)
B (n=16)
C (n=14)
surface

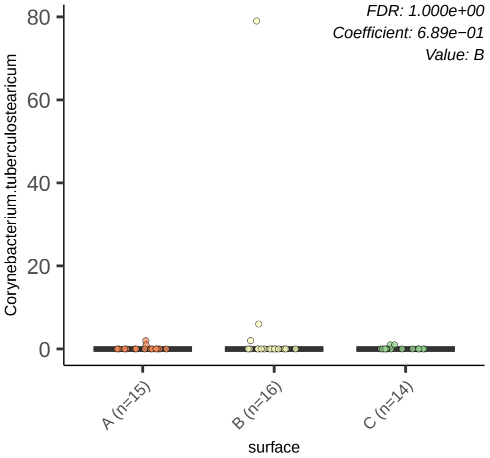
FDR: 1.000e+00
Coefficient: 6.89e−01
Value: B
Corynebacterium.tuberculostearicum
80
60
40
20
0
A (n=15)
B (n=16)
C (n=14)
surface

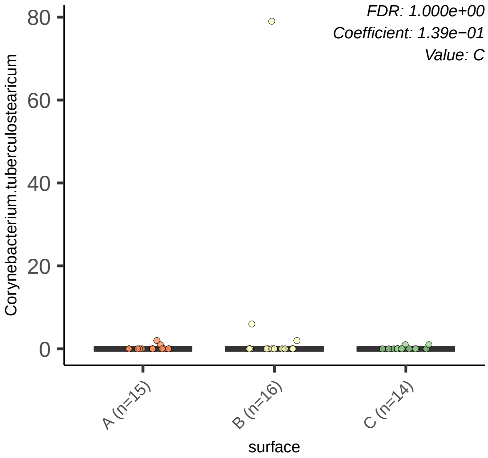
FDR: 1.000e+00
Coefficient: 1.39e−01
Value: C
Corynebacterium.tuberculostearicum
80
60
40
20
0
A (n=15)
B (n=16)
C (n=14)
surface

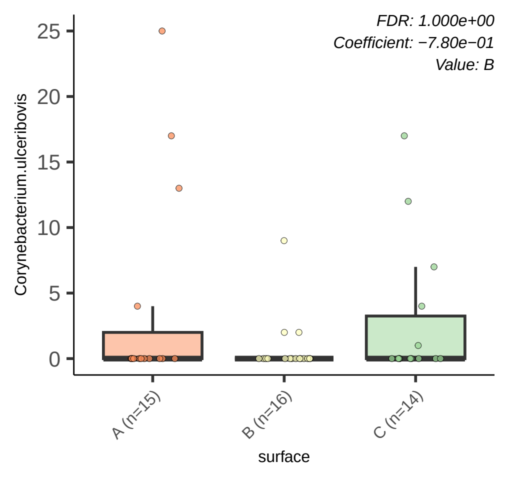

FDR: 1.000e+00
Coefficient: −7.80e−01
Value: B
Corynebacterium.ulceribovis
0
5
10
15
20
25
A (n=15)
B (n=16)
C (n=14)
surface

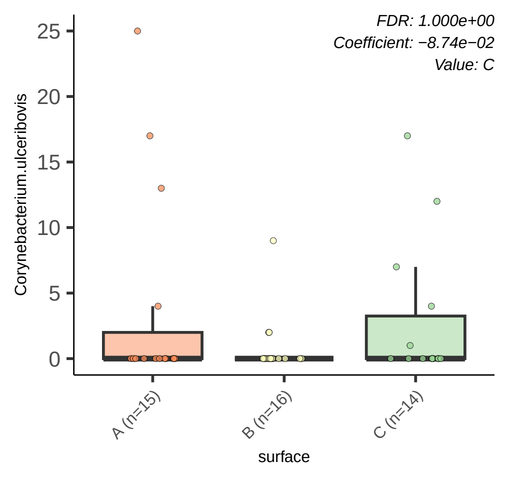
FDR: 1.000e+00
Coefficient: −8.74e−02
Value: C
Corynebacterium.ulceribovis
0
5
10
15
20
25
A (n=15)
B (n=16)
C (n=14)
surface

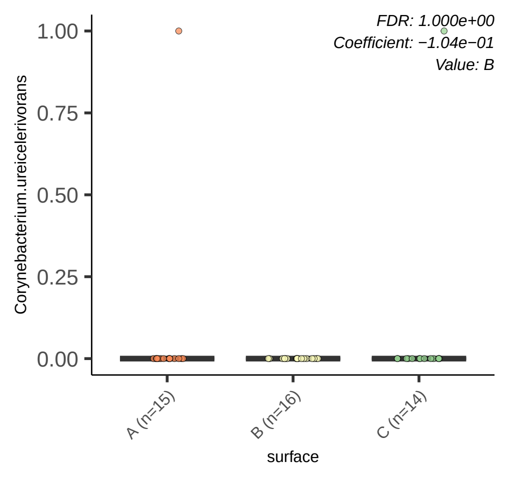
FDR: 1.000e+00
Coefficient: −1.04e−01
Value: B
1.00
0.75
0.50
0.25
0.00
Corynebacterium.ureicelerivorans
A (n=15)
B (n=16)
C (n=14)
surface

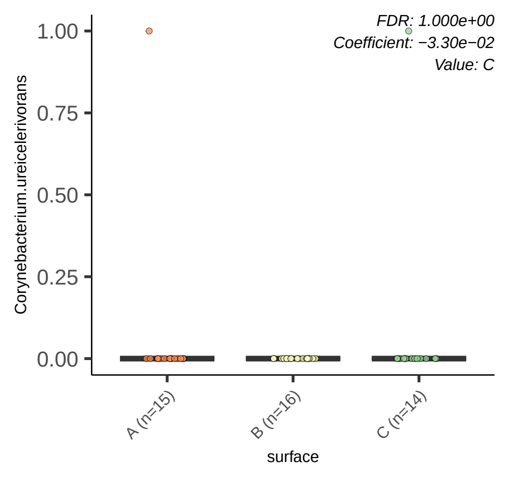

FDR: 1.000e+00
Coefficient: −3.30e−02
Value: C
1.00
0.75
0.50
0.25
0.00
Corynebacterium.ureicelerivorans
A (n=15)
B (n=16)
C (n=14)
surface

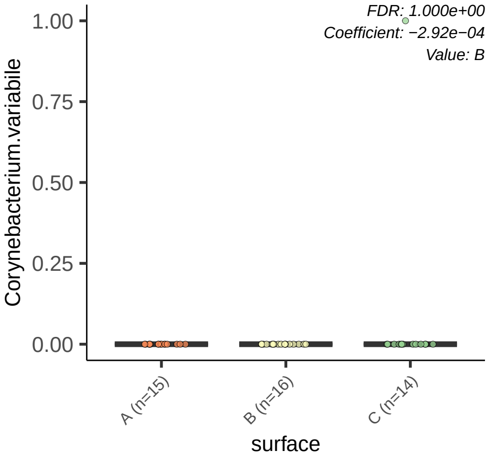
FDR: 1.000e+00
Coefficient: −2.92e−04
Value: B
Corynebacterium.variabile
1.00
0.75
0.50
0.25
0.00
A (n=15)
B (n=16)
C (n=14)
surface

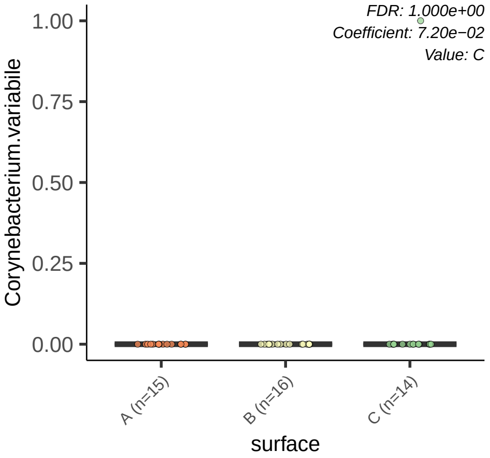
FDR: 1.000e+00
Coefficient: 7.20e−02
Value: C
Corynebacterium.variabile
1.00
0.75
0.50
0.25
0.00
A (n=15)
B (n=16)
C (n=14)
surface

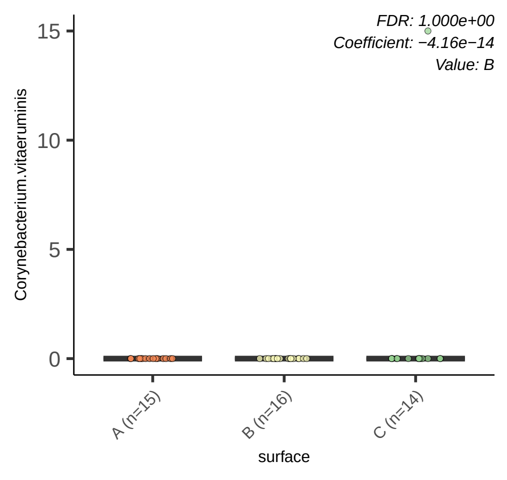
FDR: 1.000e+00
Coefficient: −4.16e−14
Value: B
Corynebacterium.vitaeruminis
15
10
5
0
A (n=15)
B (n=16)
C (n=14)
surface

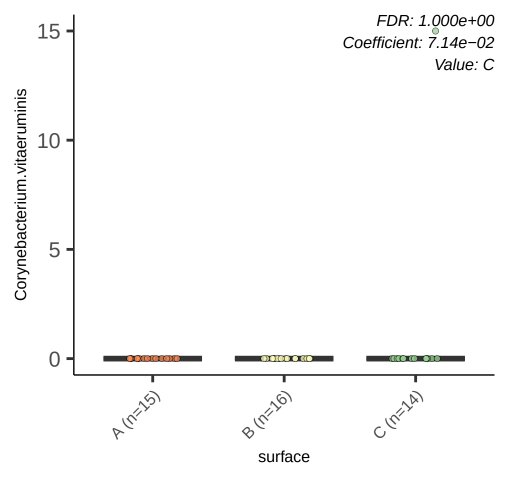
FDR: 1.000e+00
Coefficient: 7.14e−02
Value: C
Corynebacterium.vitaeruminis
15
10
5
0
A (n=15)
B (n=16)
C (n=14)
surface

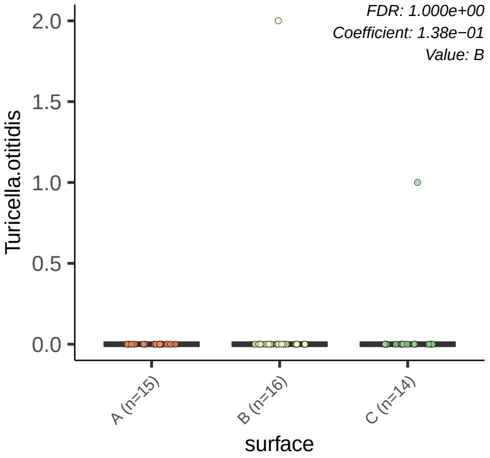

FDR: 1.000e+00
Coefficient: 1.38e−01
Value: B
Turicella.otitidis
2.0
1.5
1.0
0.5
0.0
A (n=15)
B (n=16)
C (n=14)
surface

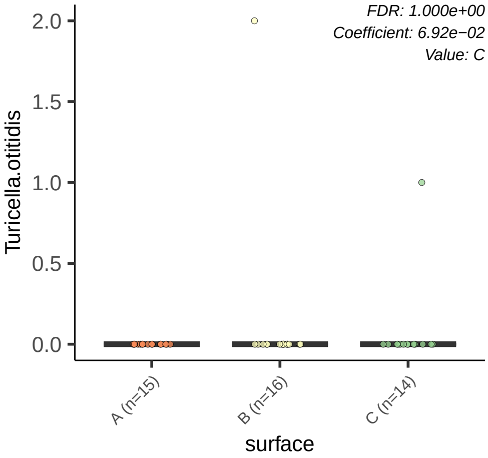

FDR: 1.000e+00
Coefficient: 6.92e−02
Value: C
Turicella.otitidis
2.0
1.5
1.0
0.5
0.0
A (n=15)
B (n=16)
C (n=14)
surface

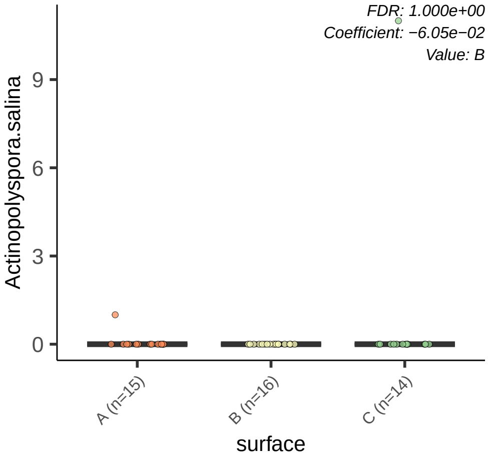
FDR: 1.000e+00
Coefficient: −6.05e−02
Value: B
Actinopolyspora.salina
9
6
3
0
A (n=15)
B (n=16)
C (n=14)
surface

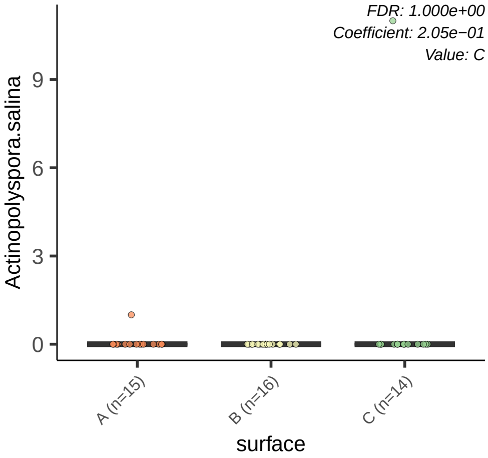

FDR: 1.000e+00
Coefficient: 2.05e−01
Value: C
Actinopolyspora.salina
0
3
6
9
A (n=15)
B (n=16)
C (n=14)
surface

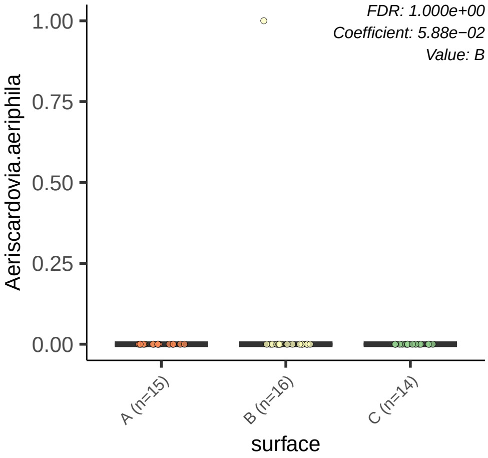
FDR: 1.000e+00
Coefficient: 5.88e−02
Value: B
Aeriscardovia.aeriphila
1.00
0.75
0.50
0.25
0.00
A (n=15)
B (n=16)
C (n=14)
surface

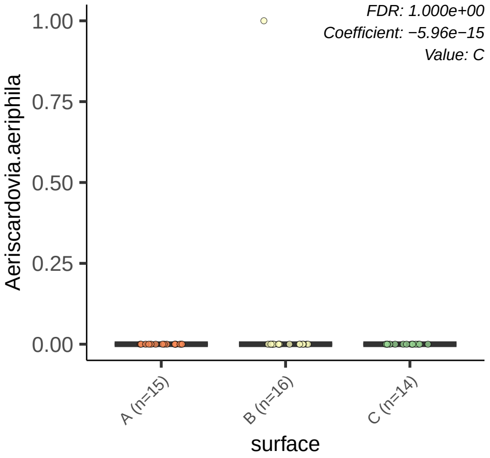

FDR: 1.000e+00
Coefficient: −5.96e−15
Value: C
Aeriscardovia.aeriphila
1.00
0.75
0.50
0.25
0.00
A (n=15)
B (n=16)
C (n=14)
surface

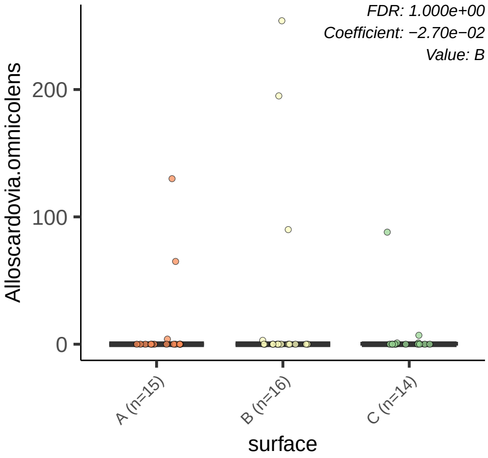

FDR: 1.000e+00
Coefficient: −2.70e−02
Value: B
Alloscardovia.omnicolens
200
100
0
A (n=15)
B (n=16)
C (n=14)
surface

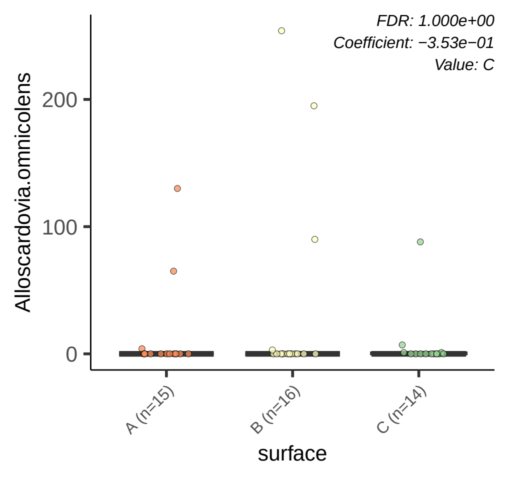

FDR: 1.000e+00
Coefficient: −3.53e−01
Value: C
Alloscardovia.omnicolens
200
100
0
A (n=15)
B (n=16)
C (n=14)
surface

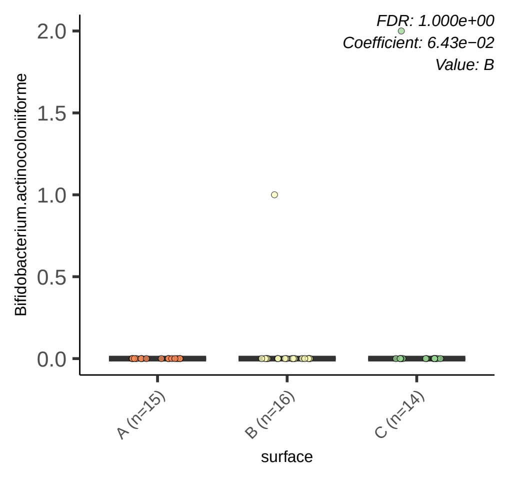

FDR: 1.000e+00
Coefficient: 6.43e−02
Value: B
Bifidobacterium.actinocoloniiforme
2.0
1.5
1.0
0.5
0.0
A (n=15)
B (n=16)
C (n=14)
surface

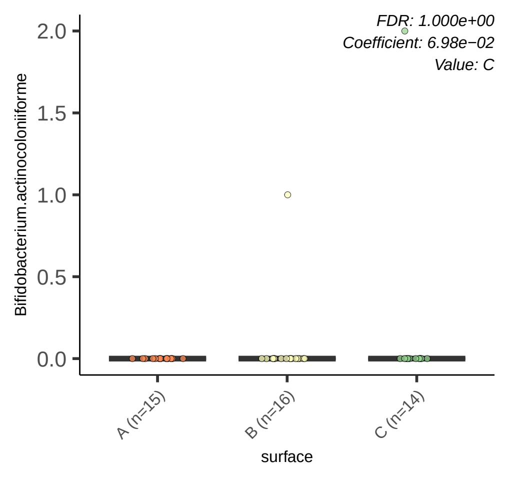
FDR: 1.000e+00
Coefficient: 6.98e−02
Value: C
Bifidobacterium.actinocoloniiforme
2.0
1.5
1.0
0.5
0.0
A (n=15)
B (n=16)
C (n=14)
surface

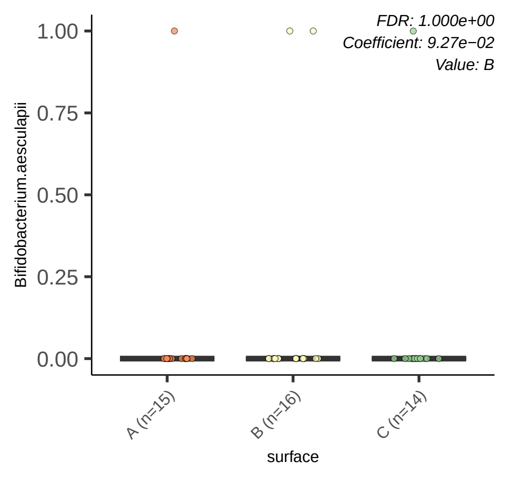

FDR: 1.000e+00
Coefficient: 9.27e−02
Value: B
Bifidobacterium.aesculapii
1.00
0.75
0.50
0.25
0.00
A (n=15)
B (n=16)
C (n=14)
surface

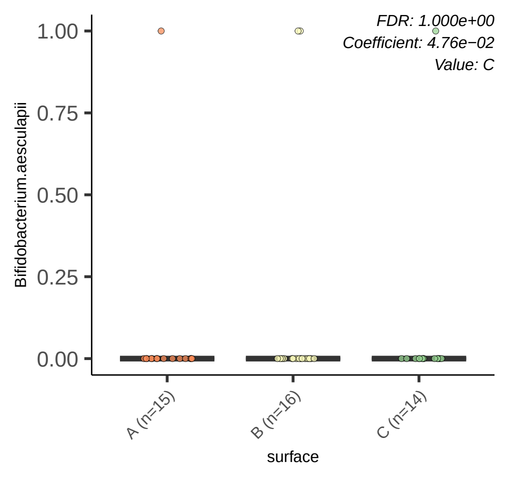
FDR: 1.000e+00
Coefficient: 4.76e−02
Value: C
1.00
0.75
0.50
0.25
0.00
Bifidobacterium.aesculapii
A (n=15)
B (n=16)
C (n=14)
surface

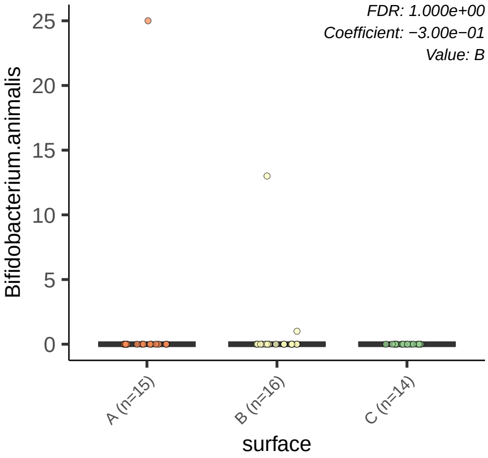

FDR: 1.000e+00
Coefficient: −3.00e−01
Value: B
Bifidobacterium.animalis
25
20
15
10
5
0
A (n=15)
B (n=16)
C (n=14)
surface

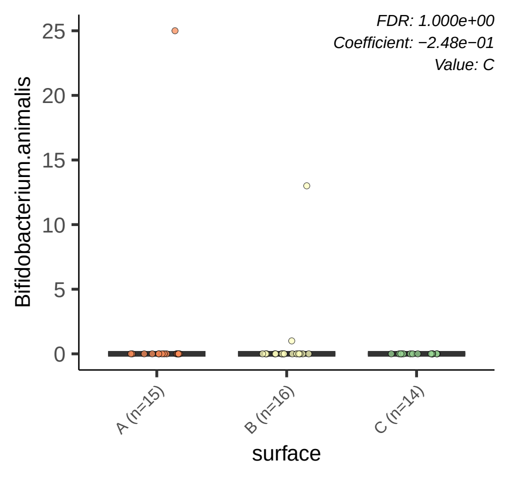
FDR: 1.000e+00
Coefficient: −2.48e−01
Value: C
Bifidobacterium.animalis
0
5
10
15
20
25
A (n=15)
B (n=16)
C (n=14)
surface

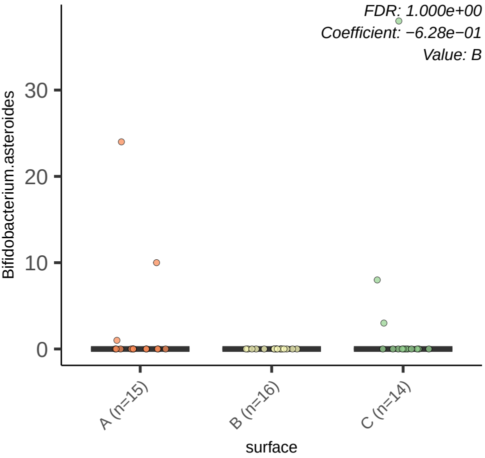

FDR: 1.000e+00
Coefficient: −6.28e−01
Value: B
Bifidobacterium.asteroides
30
20
10
0
A (n=15)
B (n=16)
C (n=14)
surface

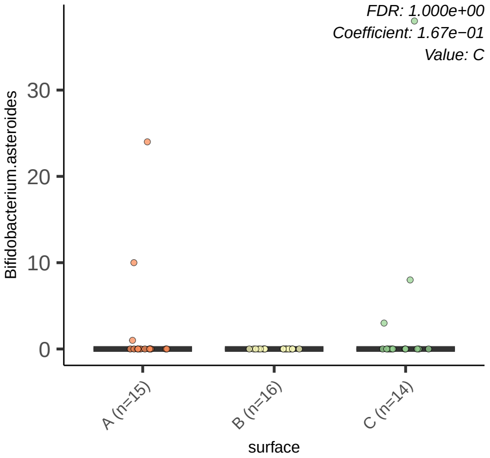

FDR: 1.000e+00
Coefficient: 1.67e−01
Value: C
Bifidobacterium.asteroides
0
10
20
30
A (n=15)
B (n=16)
C (n=14)
surface

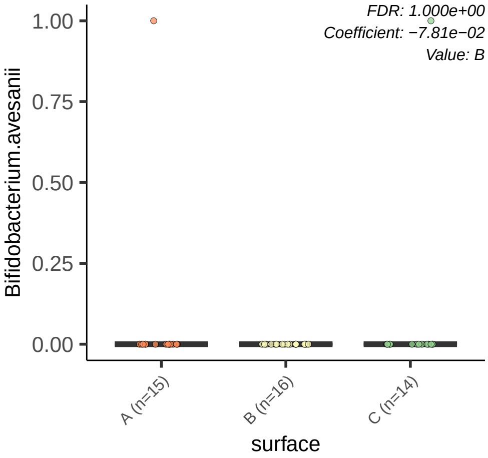

FDR: 1.000e+00
Coefficient: −7.81e−02
Value: B
Bifidobacterium.avesanii
1.00
0.75
0.50
0.25
0.00
A (n=15)
B (n=16)
C (n=14)
surface

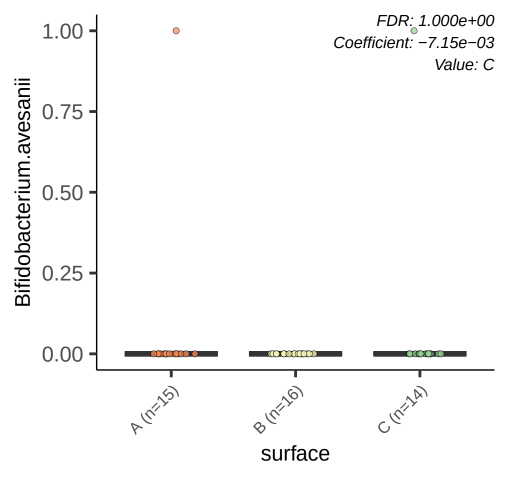

FDR: 1.000e+00
Coefficient: −7.15e−03
Value: C
Bifidobacterium.avesanii
1.00
0.75
0.50
0.25
0.00
A (n=15)
B (n=16)
C (n=14)
surface

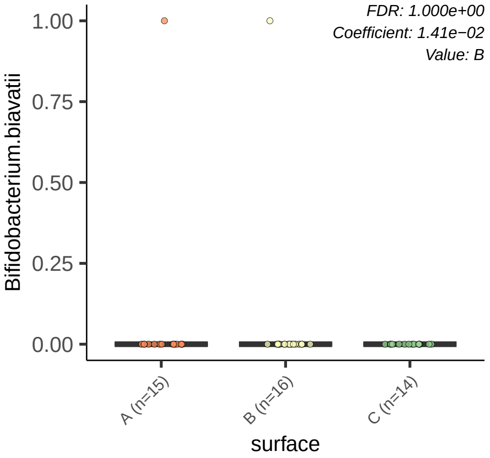
FDR: 1.000e+00
Coefficient: 1.41e−02
Value: B
Bifidobacterium.biavatii
1.00
0.75
0.50
0.25
0.00
A (n=15)
B (n=16)
C (n=14)
surface

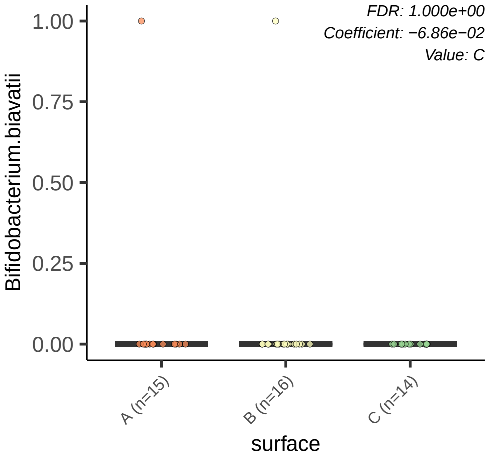
FDR: 1.000e+00
Coefficient: −6.86e−02
Value: C
Bifidobacterium.biavatii
1.00
0.75
0.50
0.25
0.00
A (n=15)
B (n=16)
C (n=14)
surface

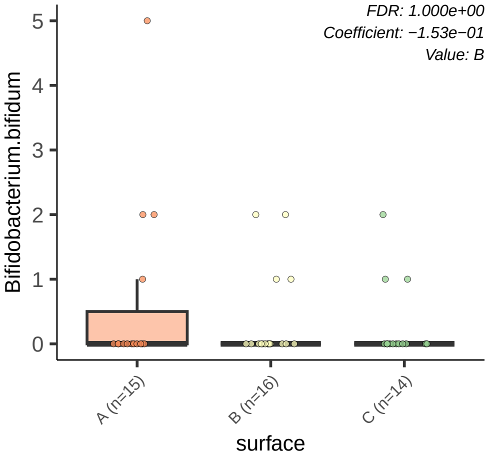

FDR: 1.000e+00
Coefficient: −1.53e−01
Value: B
Bifidobacterium.bifidum
5
4
3
2
1
0
A (n=15)
B (n=16)
C (n=14)
surface

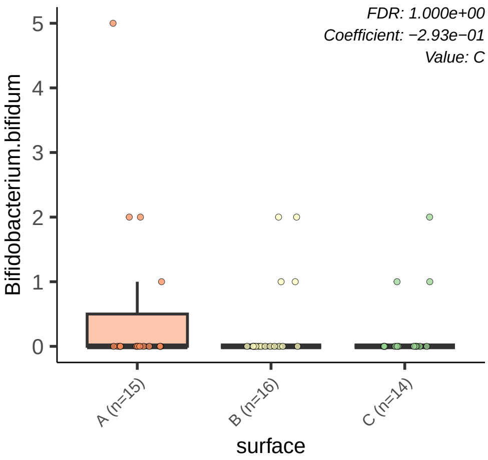
FDR: 1.000e+00
Coefficient: −2.93e−01
Value: C
Bifidobacterium.bifidum
5
4
3
2
1
0
A (n=15)
B (n=16)
C (n=14)
surface

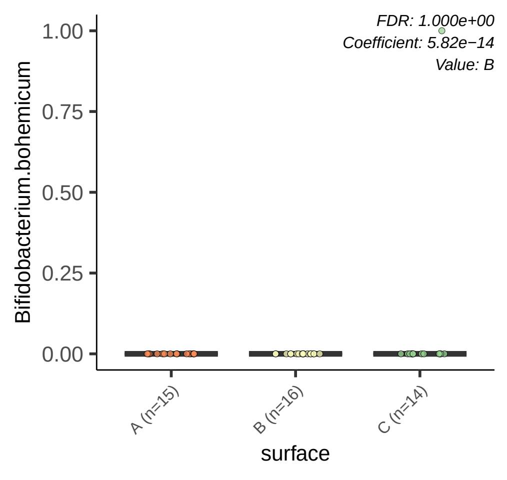
FDR: 1.000e+00
Coefficient: 5.82e−14
Value: B
Bifidobacterium.bohemicum
1.00
0.75
0.50
0.25
0.00
A (n=15)
B (n=16)
C (n=14)
surface

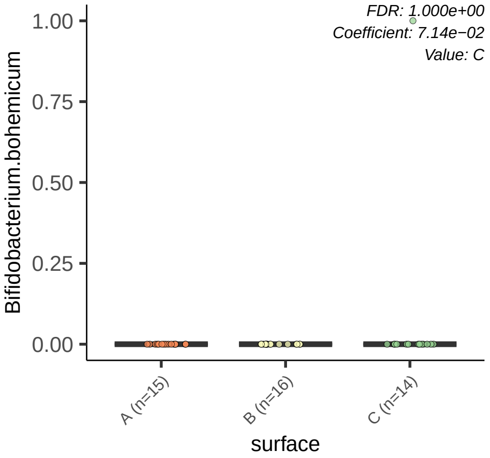
FDR: 1.000e+00
Coefficient: 7.14e−02
Value: C
Bifidobacterium.bohemicum
1.00
0.75
0.50
0.25
0.00
A (n=15)
B (n=16)
C (n=14)
surface

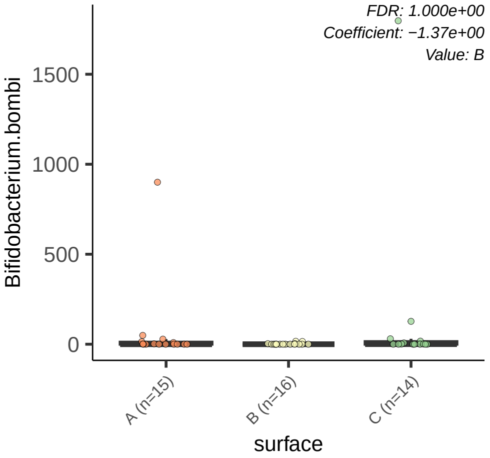

FDR: 1.000e+00
Coefficient: −1.37e+00
Value: B
Bifidobacterium.bombi
1500
1000
500
0
A (n=15)
B (n=16)
C (n=14)
surface

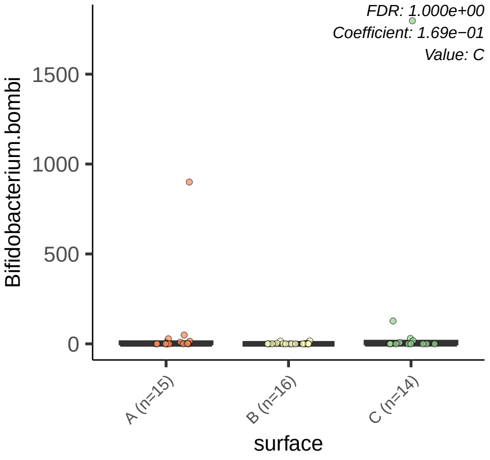
FDR: 1.000e+00
Coefficient: 1.69e−01
Value: C
Bifidobacterium.bombi
1500
1000
500
0
A (n=15)
B (n=16)
C (n=14)
surface

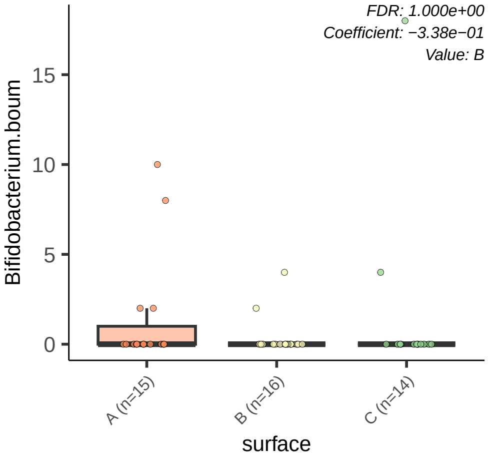
FDR: 1.000e+00
Coefficient: −3.38e−01
Value: B
Bifidobacterium.boum
15
10
5
0
A (n=15)
B (n=16)
C (n=14)
surface

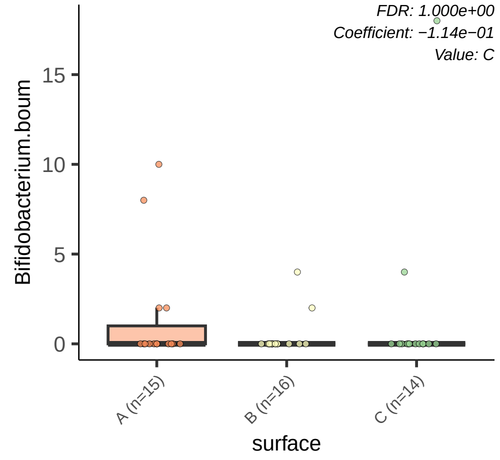
FDR: 1.000e+00
Coefficient: −1.14e−01
Value: C
Bifidobacterium.boum
15
10
5
0
A (n=15)
B (n=16)
C (n=14)
surface

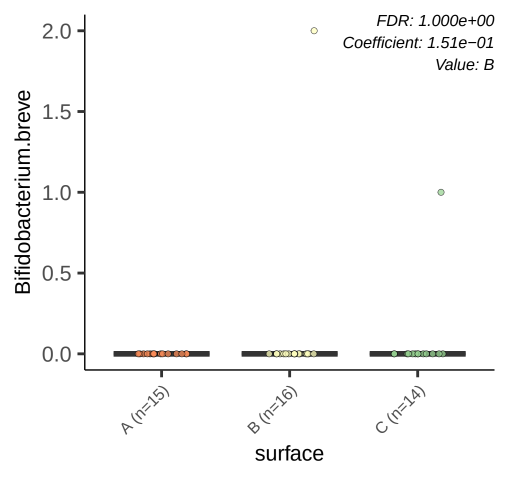
FDR: 1.000e+00
Coefficient: 1.51e−01
Value: B
Bifidobacterium.breve
2.0
1.5
1.0
0.5
0.0
A (n=15)
B (n=16)
C (n=14)
surface

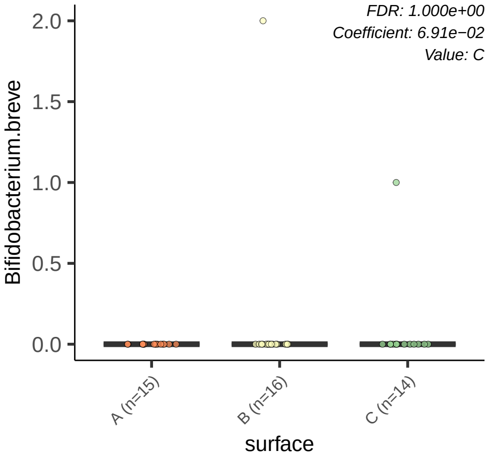
FDR: 1.000e+00
Coefficient: 6.91e−02
Value: C
Bifidobacterium.breve
2.0
1.5
1.0
0.5
0.0
A (n=15)
B (n=16)
C (n=14)
surface

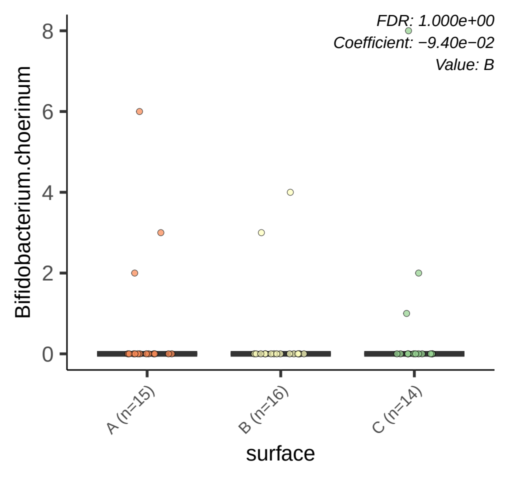
FDR: 1.000e+00
Coefficient: −9.40e−02
Value: B
Bifidobacterium.choerinum
0
2
4
6
8
A (n=15)
B (n=16)
C (n=14)
surface

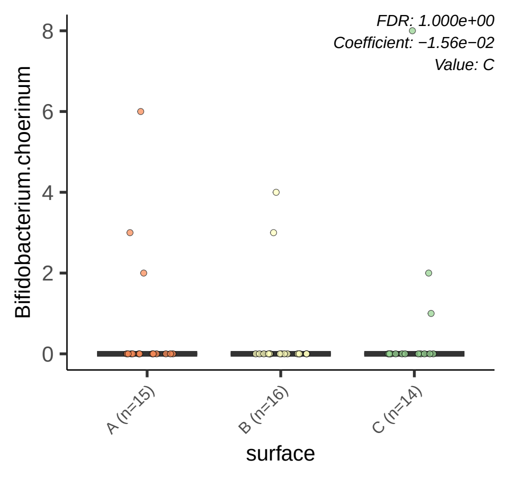

FDR: 1.000e+00
Coefficient: −1.56e−02
Value: C
Bifidobacterium.choerinum
8
6
4
2
0
A (n=15)
B (n=16)
C (n=14)
surface

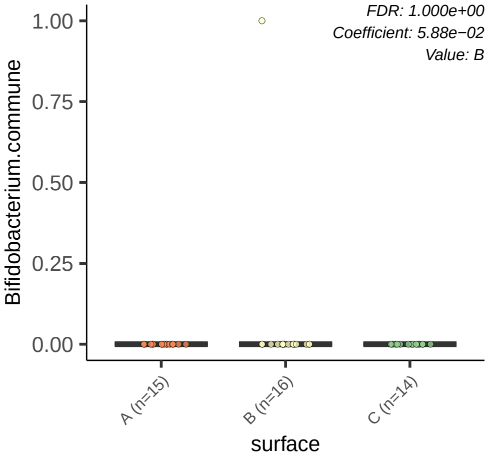
FDR: 1.000e+00
Coefficient: 5.88e−02
Value: B
Bifidobacterium.commune
1.00
0.75
0.50
0.25
0.00
A (n=15)
B (n=16)
C (n=14)
surface

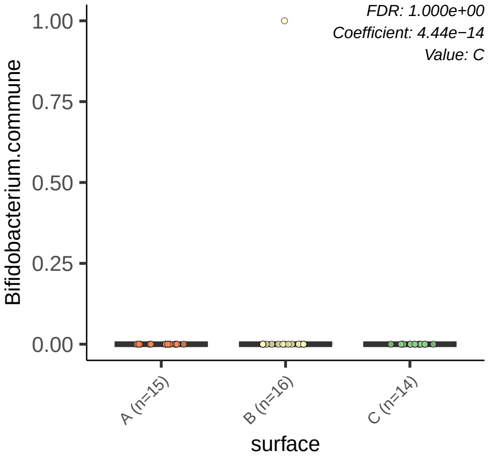
FDR: 1.000e+00
Coefficient: 4.44e−14
Value: C
Bifidobacterium.commune
1.00
0.75
0.50
0.25
0.00
A (n=15)
B (n=16)
C (n=14)
surface

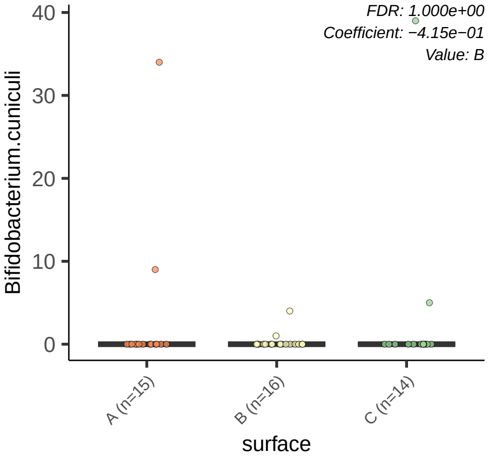
FDR: 1.000e+00
Coefficient: −4.15e−01
Value: B
Bifidobacterium.cuniculi
40
30
20
10
0
A (n=15)
B (n=16)
C (n=14)
surface

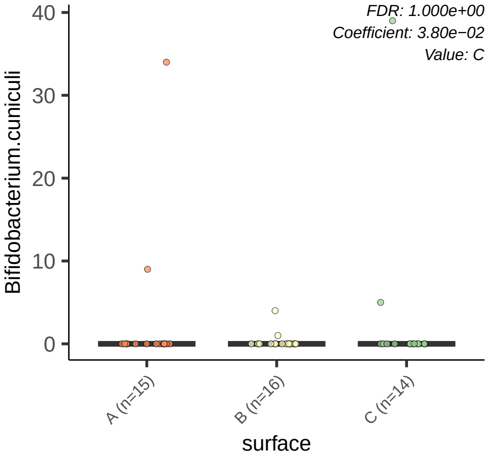

FDR: 1.000e+00
Coefficient: 3.80e−02
Value: C
Bifidobacterium.cuniculi
0
10
20
30
40
A (n=15)
B (n=16)
C (n=14)
surface

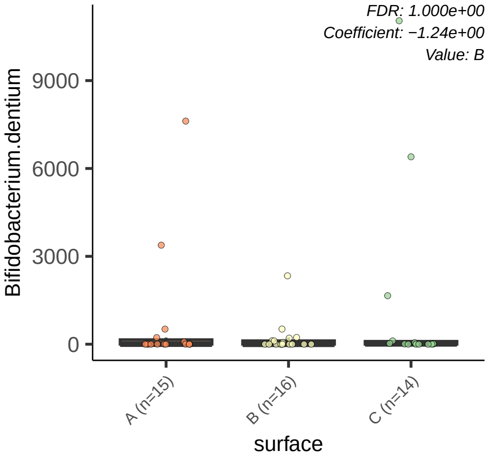

FDR: 1.000e+00
Coefficient: −1.24e+00
Value: B
Bifidobacterium.dentium
9000
6000
3000
0
A (n=15)
B (n=16)
C (n=14)
surface

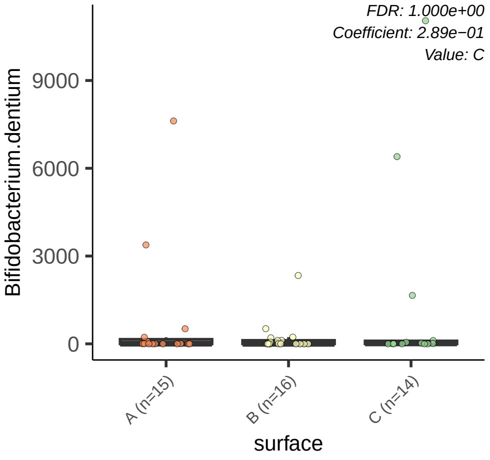

FDR: 1.000e+00
Coefficient: 2.89e−01
Value: C
Bifidobacterium.dentium
9000
6000
3000
0
A (n=15)
B (n=16)
C (n=14)
surface

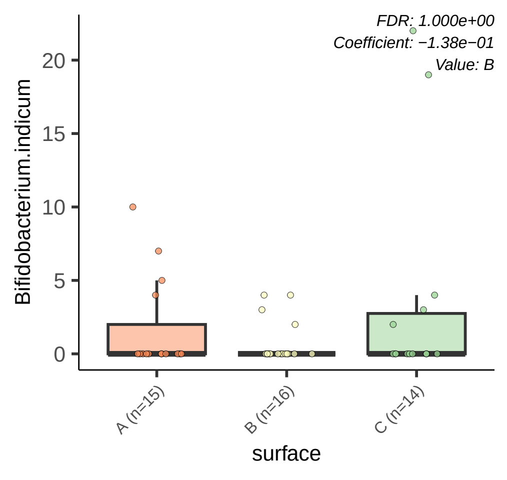

FDR: 1.000e+00
Coefficient: −1.38e−01
Value: B
Bifidobacterium.indicum
0
5
10
15
20
A (n=15)
B (n=16)
C (n=14)
surface

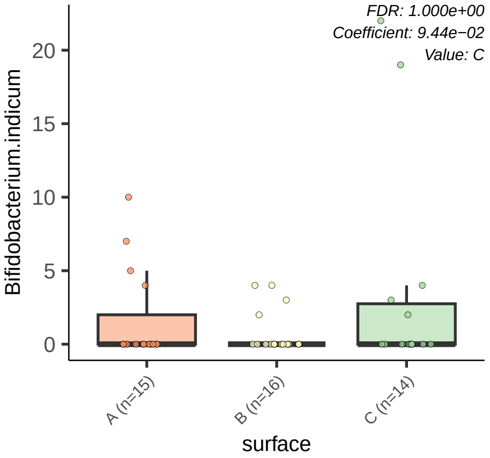
FDR: 1.000e+00
Coefficient: 9.44e−02
Value: C
20
15
10
5
0
Bifidobacterium.indicum
A (n=15)
B (n=16)
C (n=14)
surface

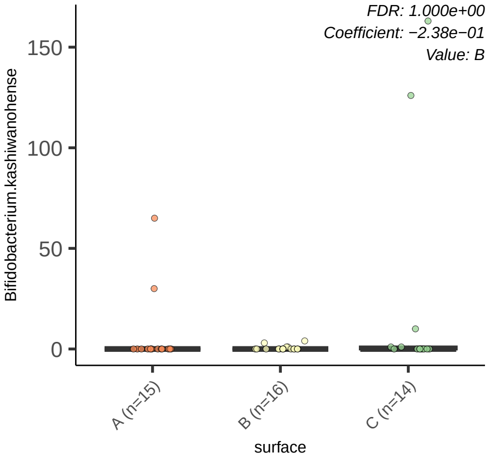
FDR: 1.000e+00
Coefficient: −2.38e−01
Value: B
Bifidobacterium.kashiwanohense
150
100
50
0
A (n=15)
B (n=16)
C (n=14)
surface

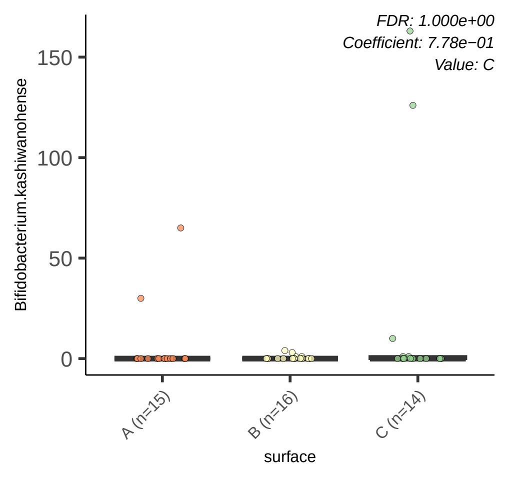

FDR: 1.000e+00
Coefficient: 7.78e−01
Value: C
Bifidobacterium.kashiwanohense
150
100
50
0
A (n=15)
B (n=16)
C (n=14)
surface

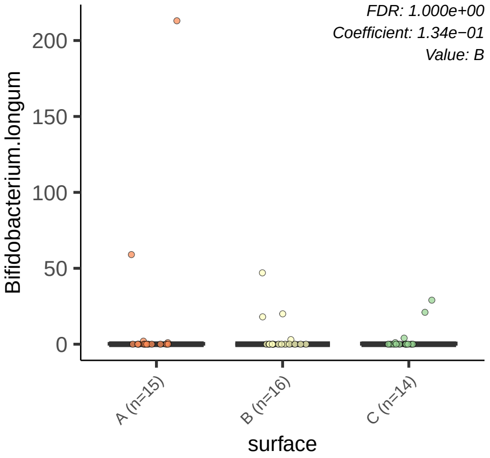
FDR: 1.000e+00
Coefficient: 1.34e−01
Value: B
Bifidobacterium.longum
200
150
100
50
0
A (n=15)
B (n=16)
C (n=14)
surface

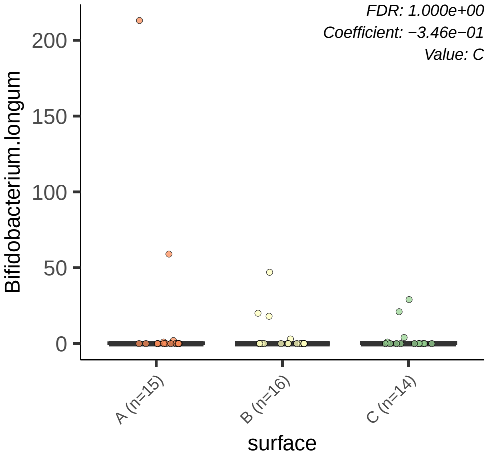
FDR: 1.000e+00
Coefficient: −3.46e−01
Value: C
Bifidobacterium.longum
200
150
100
50
0
A (n=15)
B (n=16)
C (n=14)
surface

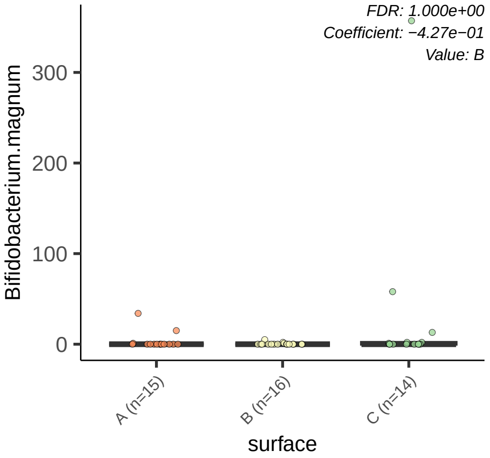

FDR: 1.000e+00
Coefficient: −4.27e−01
Value: B
Bifidobacterium.magnum
300
200
100
0
A (n=15)
B (n=16)
C (n=14)
surface

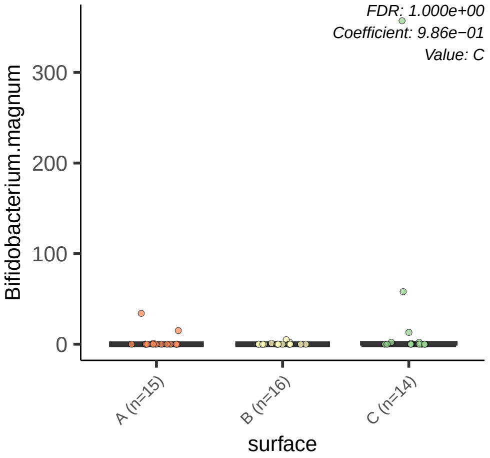

FDR: 1.000e+00
Coefficient: 9.86e−01
Value: C
Bifidobacterium.magnum
300
200
100
0
A (n=15)
B (n=16)
C (n=14)
surface

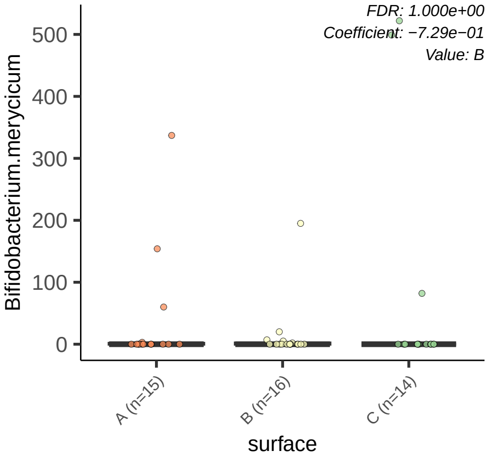
FDR: 1.000e+00
Coefficient: −7.29e−01
Value: B
Bifidobacterium.merycicum
500
400
300
200
100
0
A (n=15)
B (n=16)
C (n=14)
surface

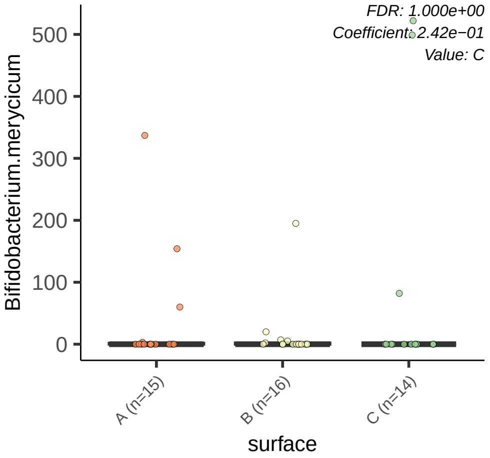
FDR: 1.000e+00
Coefficient: 2.42e−01
Value: C
Bifidobacterium.merycicum
0
100
200
300
400
500
A (n=15)
B (n=16)
C (n=14)
surface

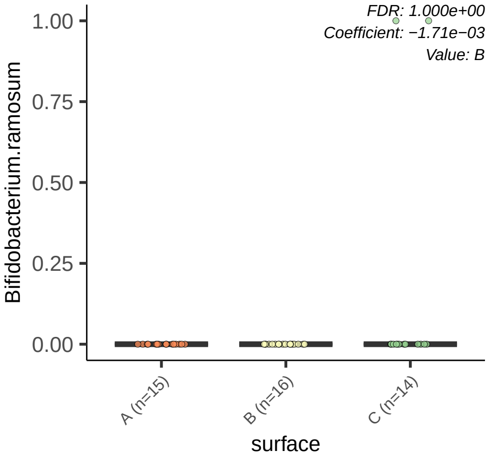

FDR: 1.000e+00
Coefficient: −1.71e−03
Value: B
Bifidobacterium.ramosum
1.00
0.75
0.50
0.25
0.00
A (n=15)
B (n=16)
C (n=14)
surface

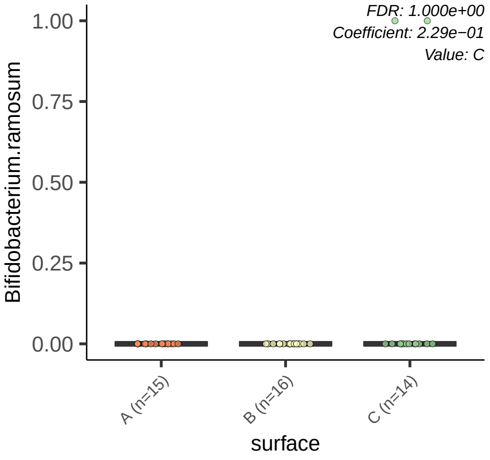

FDR: 1.000e+00
Coefficient: 2.29e−01
Value: C
Bifidobacterium.ramosum
1.00
0.75
0.50
0.25
0.00
A (n=15)
B (n=16)
C (n=14)
surface

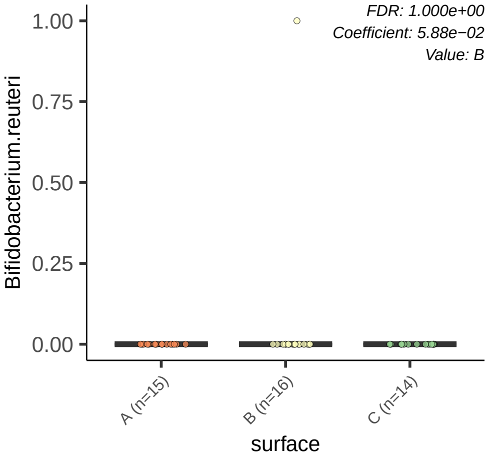
FDR: 1.000e+00
Coefficient: 5.88e−02
Value: B
Bifidobacterium.reuteri
1.00
0.75
0.50
0.25
0.00
A (n=15)
B (n=16)
C (n=14)
surface

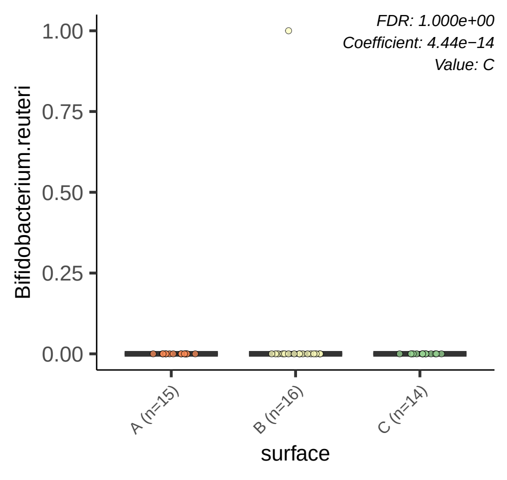
FDR: 1.000e+00
Coefficient: 4.44e−14
Value: C
Bifidobacterium.reuteri
1.00
0.75
0.50
0.25
0.00
A (n=15)
B (n=16)
C (n=14)
surface

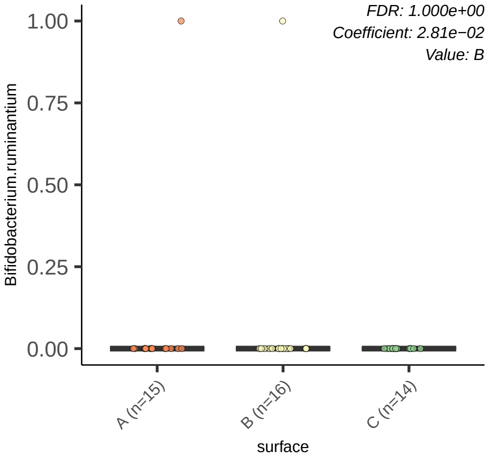
FDR: 1.000e+00
Coefficient: 2.81e−02
Value: B
Bifidobacterium.ruminantium
1.00
0.75
0.50
0.25
0.00
A (n=15)
B (n=16)
C (n=14)
surface

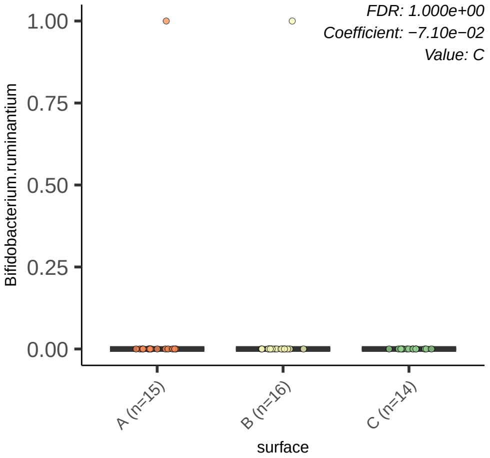
FDR: 1.000e+00
Coefficient: −7.10e−02
Value: C
1.00
0.75
0.50
0.25
0.00
Bifidobacterium.ruminantium
A (n=15)
B (n=16)
C (n=14)
surface

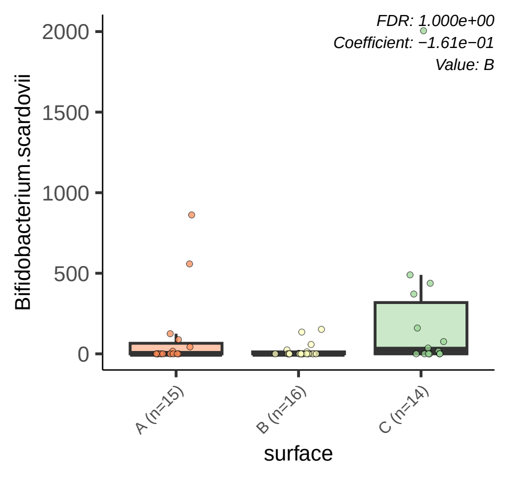

FDR: 1.000e+00
Coefficient: −1.61e−01
Value: B
2000
1500
1000
500
0
Bifidobacterium.scardovii
A (n=15)
B (n=16)
C (n=14)
surface

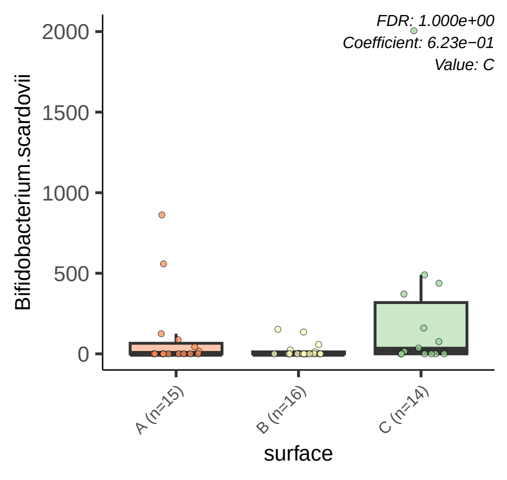
FDR: 1.000e+00
Coefficient: 6.23e−01
Value: C
2000
1500
1000
500
0
Bifidobacterium.scardovii
A (n=15)
B (n=16)
C (n=14)
surface

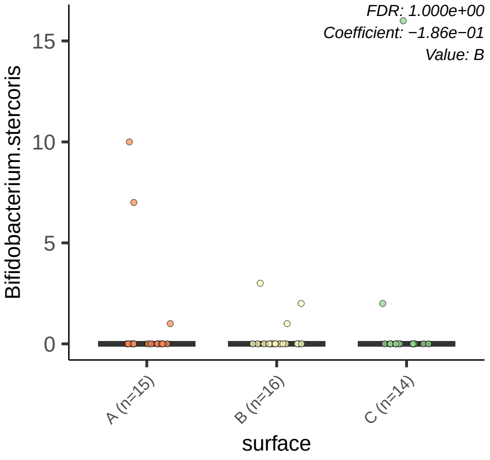
FDR: 1.000e+00
Coefficient: −1.86e−01
Value: B
Bifidobacterium.stercoris
15
10
5
0
A (n=15)
B (n=16)
C (n=14)
surface

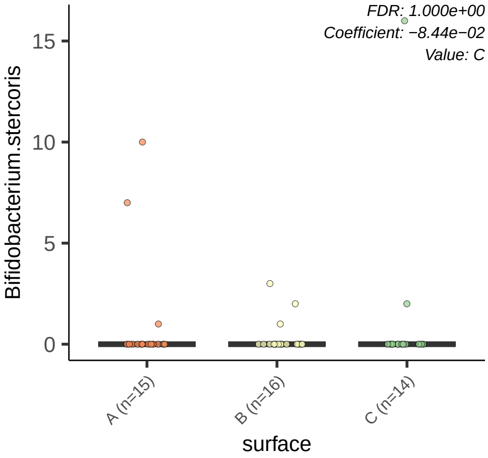

FDR: 1.000e+00
Coefficient: −8.44e−02
Value: C
Bifidobacterium.stercoris
15
10
5
0
A (n=15)
B (n=16)
C (n=14)
surface

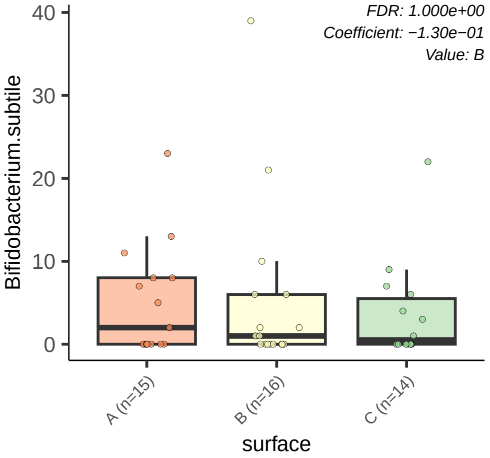

FDR: 1.000e+00
Coefficient: −1.30e−01
Value: B
Bifidobacterium.subtile
0
10
20
30
40
A (n=15)
B (n=16)
C (n=14)
surface

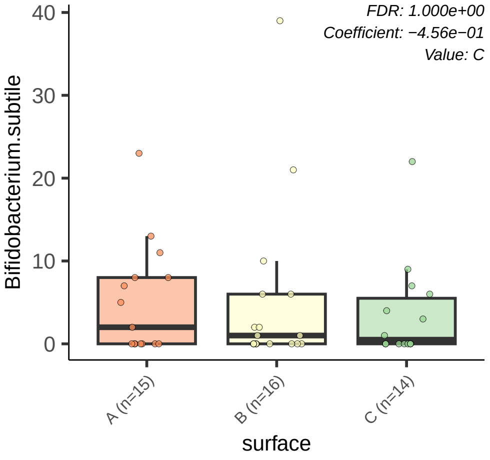

FDR: 1.000e+00
Coefficient: −4.56e−01
Value: C
40
30
20
10
0
Bifidobacterium.subtile
A (n=15)
B (n=16)
C (n=14)
surface

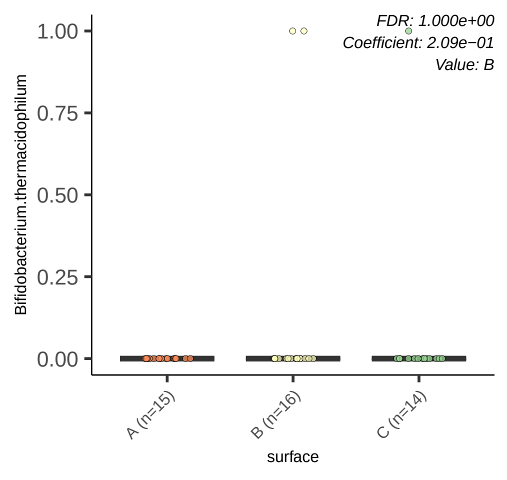
FDR: 1.000e+00
Coefficient: 2.09e−01
Value: B
Bifidobacterium.thermacidophilum
1.00
0.75
0.50
0.25
0.00
A (n=15)
B (n=16)
C (n=14)
surface

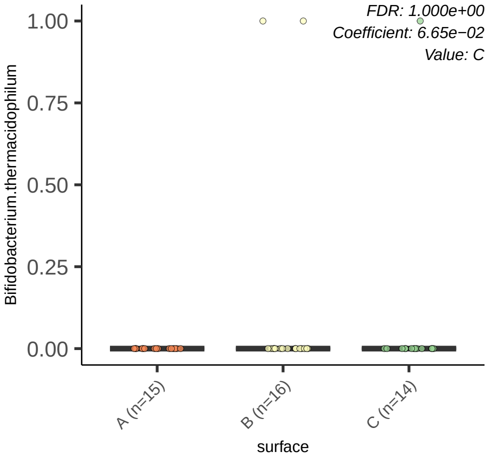

FDR: 1.000e+00
Coefficient: 6.65e−02
Value: C
1.00
0.75
0.50
0.25
0.00
Bifidobacterium.thermacidophilum
A (n=15)
B (n=16)
C (n=14)
surface

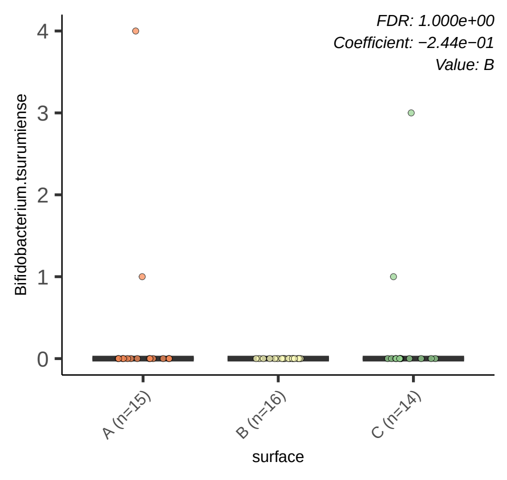
FDR: 1.000e+00
Coefficient: −2.44e−01
Value: B
Bifidobacterium.tsurumiense
0
1
2
3
4
A (n=15)
B (n=16)
C (n=14)
surface

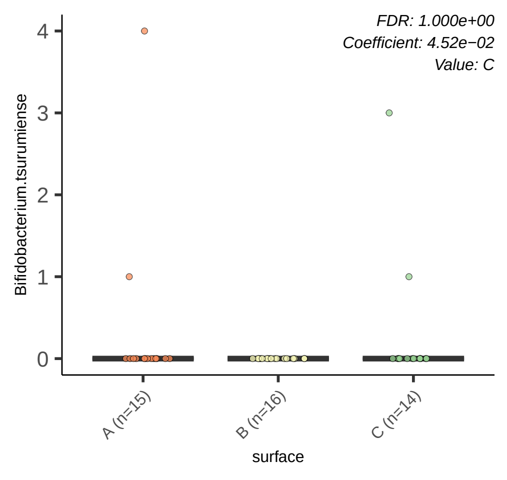
FDR: 1.000e+00
Coefficient: 4.52e−02
Value: C
Bifidobacterium.tsurumiense
0
1
2
3
4
A (n=15)
B (n=16)
C (n=14)
surface

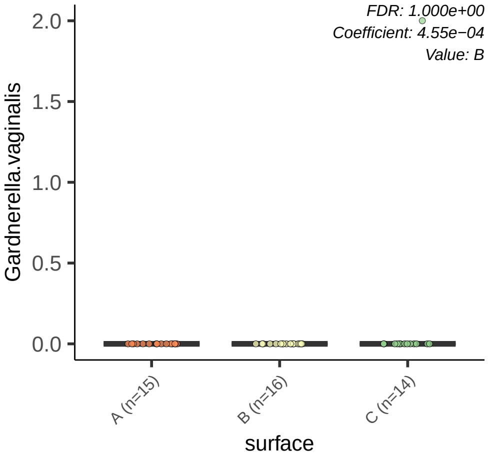
FDR: 1.000e+00
Coefficient: 4.55e−04
Value: B
Gardnerella.vaginalis
2.0
1.5
1.0
0.5
0.0
A (n=15)
B (n=16)
C (n=14)
surface

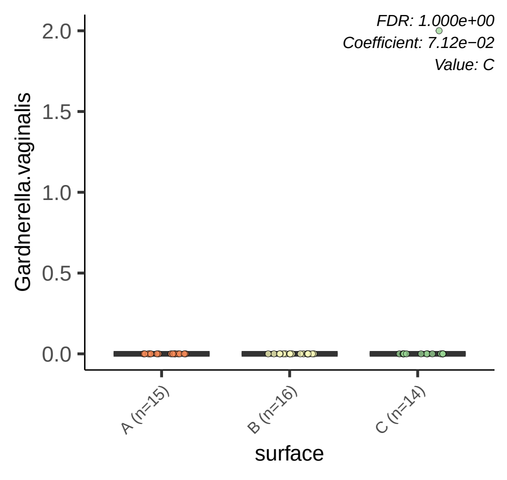
FDR: 1.000e+00
Coefficient: 7.12e−02
Value: C
Gardnerella.vaginalis
2.0
1.5
1.0
0.5
0.0
A (n=15)
B (n=16)
C (n=14)
surface

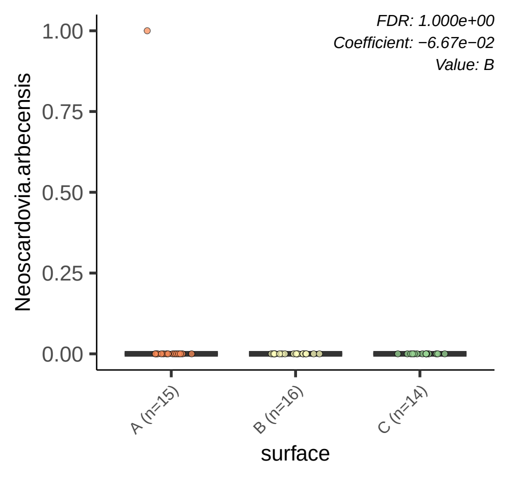
FDR: 1.000e+00
Coefficient: −6.67e−02
Value: B
Neoscardovia.arbecensis
1.00
0.75
0.50
0.25
0.00
A (n=15)
B (n=16)
C (n=14)
surface

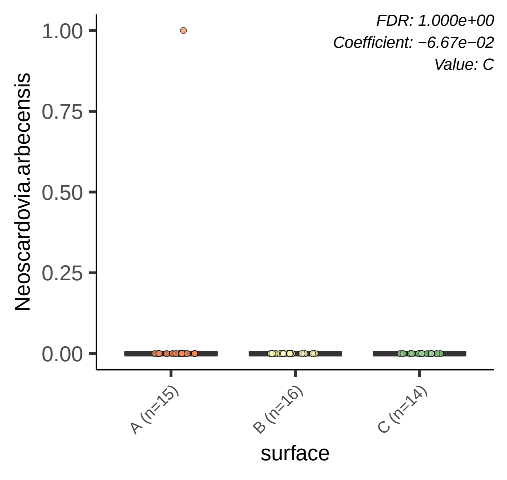

FDR: 1.000e+00
Coefficient: −6.67e−02
Value: C
Neoscardovia.arbecensis
1.00
0.75
0.50
0.25
0.00
A (n=15)
B (n=16)
C (n=14)
surface

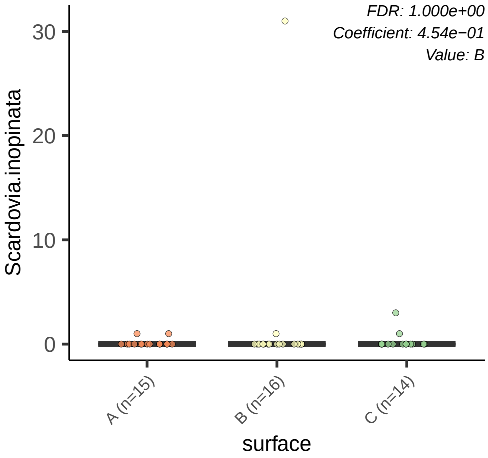

FDR: 1.000e+00
Coefficient: 4.54e−01
Value: B
Scardovia.inopinata
30
20
10
0
A (n=15)
B (n=16)
C (n=14)
surface

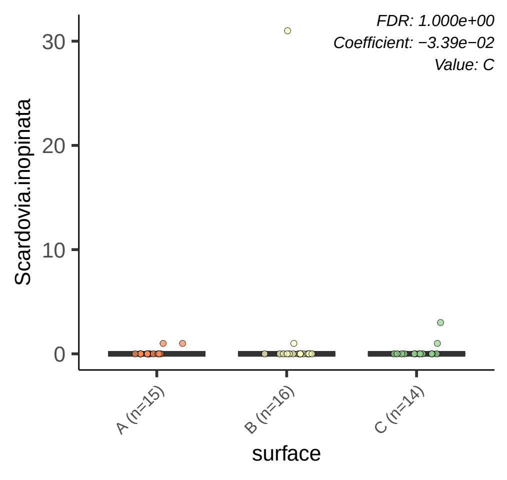

FDR: 1.000e+00
Coefficient: −3.39e−02
Value: C
Scardovia.inopinata
30
20
10
0
A (n=15)
B (n=16)
C (n=14)
surface

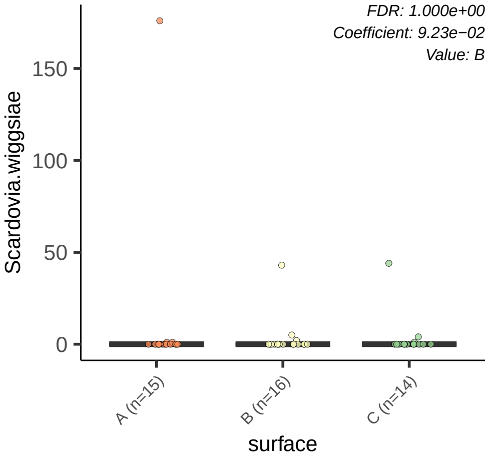
FDR: 1.000e+00
Coefficient: 9.23e−02
Value: B
Scardovia.wiggsiae
150
100
50
0
A (n=15)
B (n=16)
C (n=14)
surface

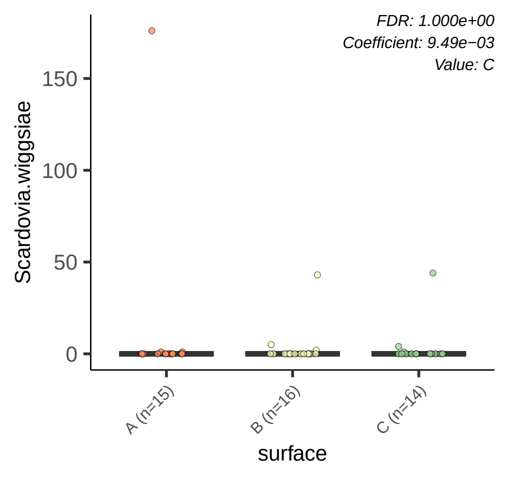

FDR: 1.000e+00
Coefficient: 9.49e−03
Value: C
Scardovia.wiggsiae
150
100
50
0
A (n=15)
B (n=16)
C (n=14)
surface

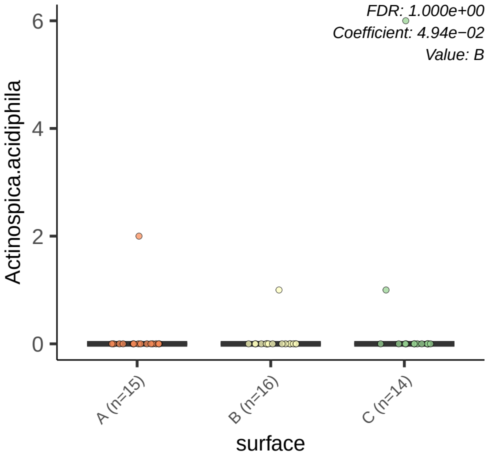
FDR: 1.000e+00
Coefficient: 4.94e−02
Value: B
Actinospica.acidiphila
0
2
4
6
A (n=15)
B (n=16)
C (n=14)
surface

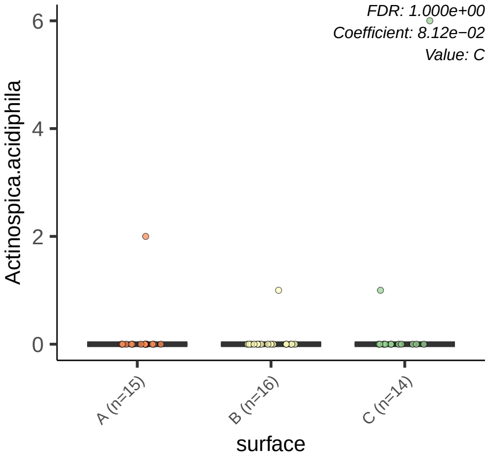
FDR: 1.000e+00
Coefficient: 8.12e−02
Value: C
Actinospica.acidiphila
6
4
2
0
A (n=15)
B (n=16)
C (n=14)
surface

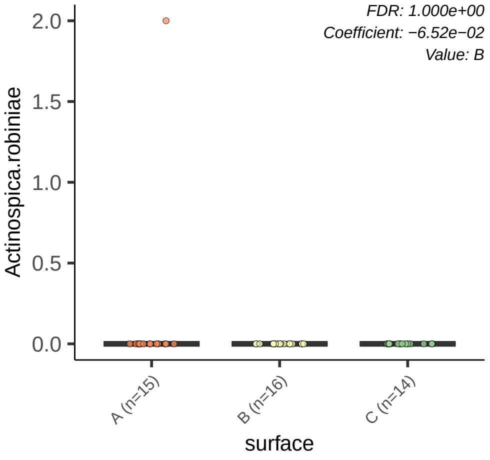
FDR: 1.000e+00
Coefficient: −6.52e−02
Value: B
Actinospica.robiniae
2.0
1.5
1.0
0.5
0.0
A (n=15)
B (n=16)
C (n=14)
surface

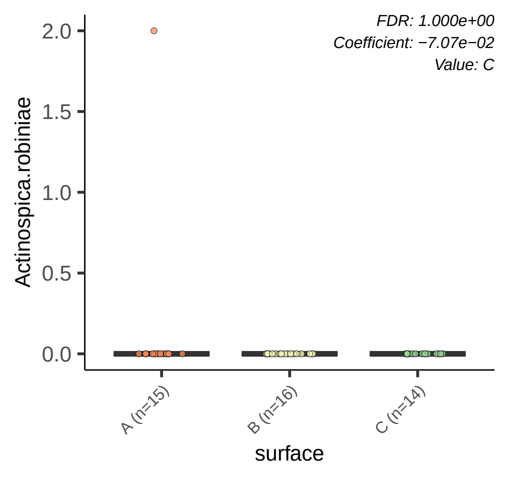
FDR: 1.000e+00
Coefficient: −7.07e−02
Value: C
Actinospica.robiniae
2.0
1.5
1.0
0.5
0.0
A (n=15)
B (n=16)
C (n=14)
surface

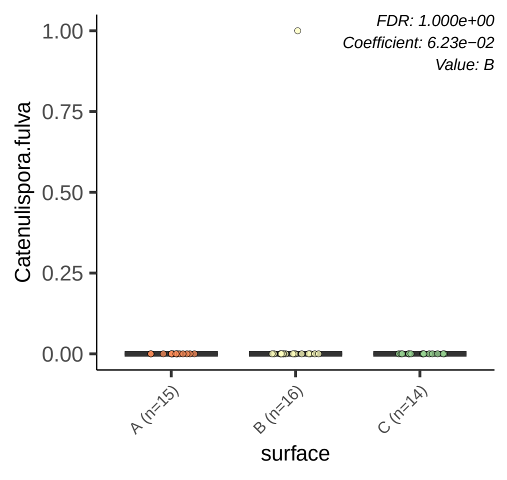

FDR: 1.000e+00
Coefficient: 6.23e−02
Value: B
Catenulispora.fulva
1.00
0.75
0.50
0.25
0.00
A (n=15)
B (n=16)
C (n=14)
surface

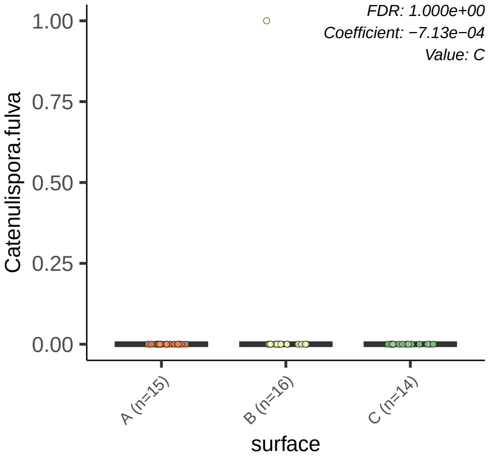

FDR: 1.000e+00
Coefficient: −7.13e−04
Value: C
Catenulispora.fulva
1.00
0.75
0.50
0.25
0.00
A (n=15)
B (n=16)
C (n=14)
surface

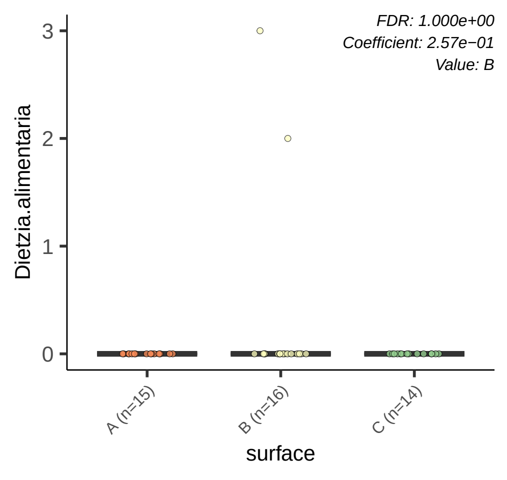
FDR: 1.000e+00
Coefficient: 2.57e−01
Value: B
Dietzia.alimentaria
3
2
1
0
A (n=15)
B (n=16)
C (n=14)
surface

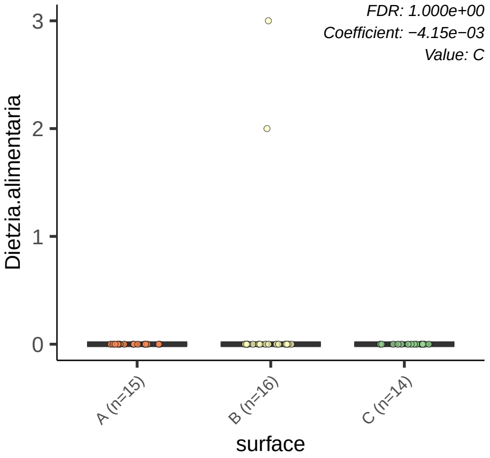
FDR: 1.000e+00
Coefficient: −4.15e−03
Value: C
Dietzia.alimentaria
3
2
1
0
A (n=15)
B (n=16)
C (n=14)
surface

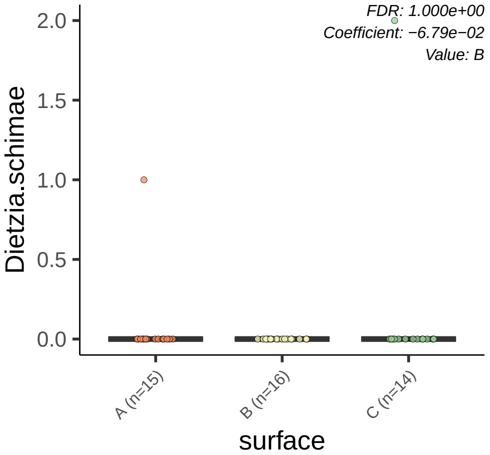
FDR: 1.000e+00
Coefficient: −6.79e−02
Value: B
Dietzia.schimae
2.0
1.5
1.0
0.5
0.0
A (n=15)
B (n=16)
C (n=14)
surface

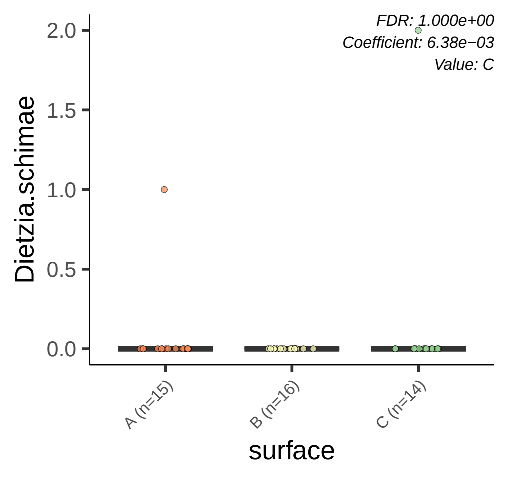

FDR: 1.000e+00
Coefficient: 6.38e−03
Value: C
Dietzia.schimae
2.0
1.5
1.0
0.5
0.0
A (n=15)
B (n=16)
C (n=14)
surface

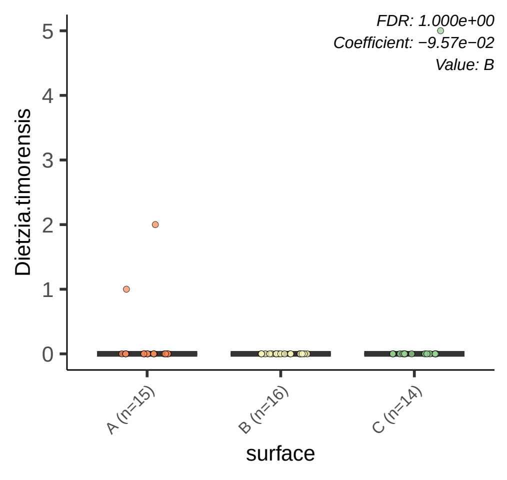
FDR: 1.000e+00
Coefficient: −9.57e−02
Value: B
Dietzia.timorensis
0
1
2
3
4
5
A (n=15)
B (n=16)
C (n=14)
surface

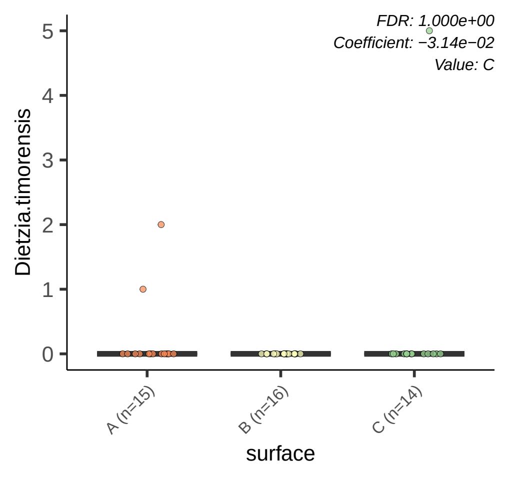
FDR: 1.000e+00
Coefficient: −3.14e−02
Value: C
Dietzia.timorensis
0
1
2
3
4
5
A (n=15)
B (n=16)
C (n=14)
surface

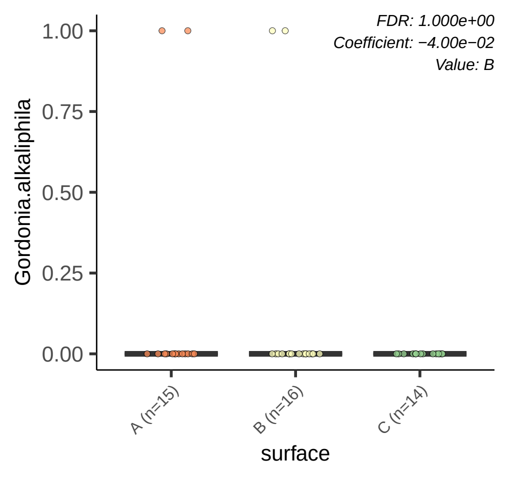
FDR: 1.000e+00
Coefficient: −4.00e−02
Value: B
Gordonia.alkaliphila
1.00
0.75
0.50
0.25
0.00
A (n=15)
B (n=16)
C (n=14)
surface

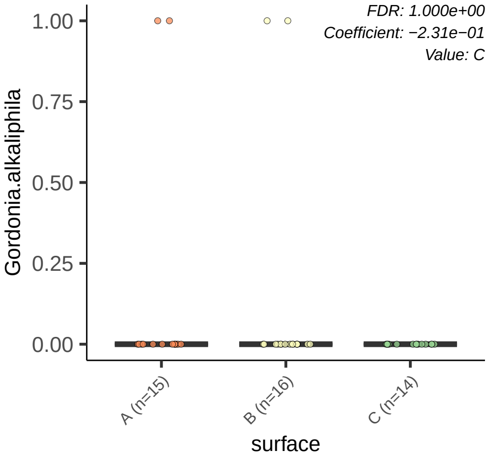

FDR: 1.000e+00
Coefficient: −2.31e−01
Value: C
Gordonia.alkaliphila
1.00
0.75
0.50
0.25
0.00
A (n=15)
B (n=16)
C (n=14)
surface

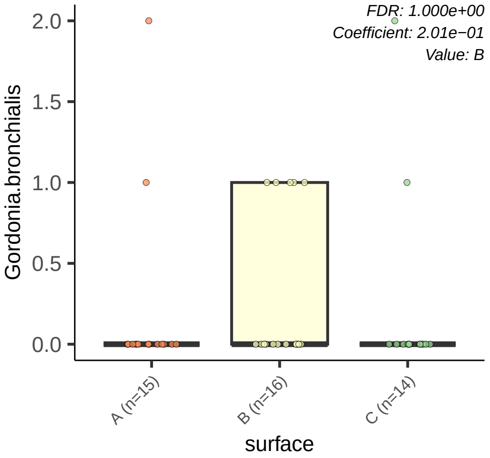

FDR: 1.000e+00
Coefficient: 2.01e−01
Value: B
Gordonia.bronchialis
2.0
1.5
1.0
0.5
0.0
A (n=15)
B (n=16)
C (n=14)
surface

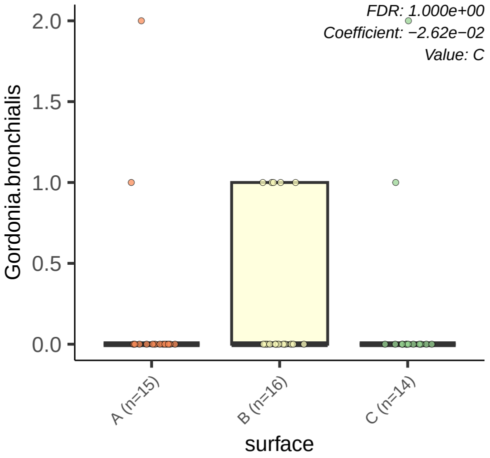

FDR: 1.000e+00
Coefficient: −2.62e−02
Value: C
Gordonia.bronchialis
2.0
1.5
1.0
0.5
0.0
A (n=15)
B (n=16)
C (n=14)
surface

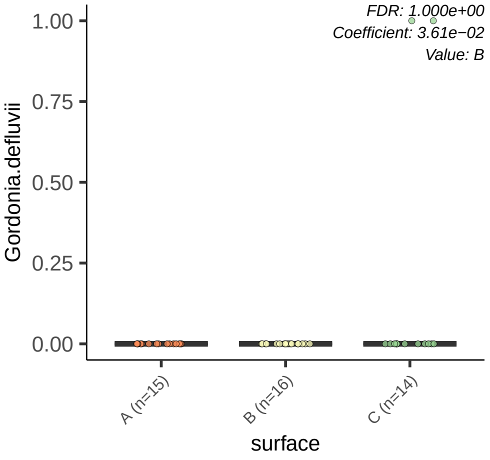

FDR: 1.000e+00
Coefficient: 3.61e−02
Value: B
Gordonia.defluvii
1.00
0.75
0.50
0.25
0.00
A (n=15)
B (n=16)
C (n=14)
surface

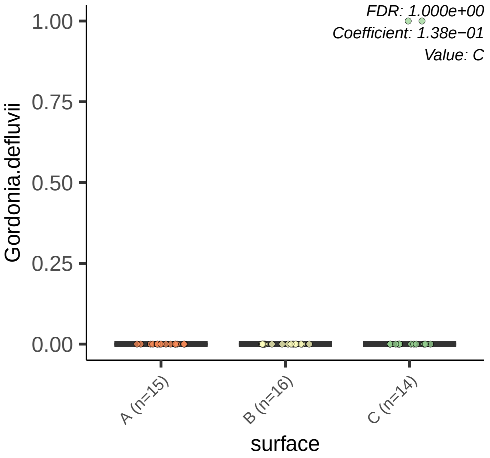
FDR: 1.000e+00
Coefficient: 1.38e−01
Value: C
Gordonia.defluvii
1.00
0.75
0.50
0.25
0.00
A (n=15)
B (n=16)
C (n=14)
surface

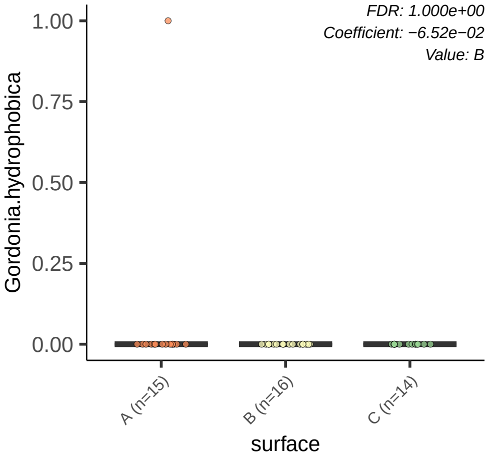

FDR: 1.000e+00
Coefficient: −6.52e−02
Value: B
Gordonia.hydrophobica
1.00
0.75
0.50
0.25
0.00
A (n=15)
B (n=16)
C (n=14)
surface

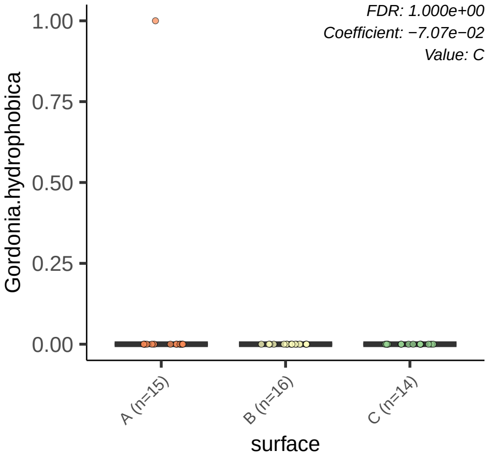
FDR: 1.000e+00
Coefficient: −7.07e−02
Value: C
Gordonia.hydrophobica
1.00
0.75
0.50
0.25
0.00
A (n=15)
B (n=16)
C (n=14)
surface

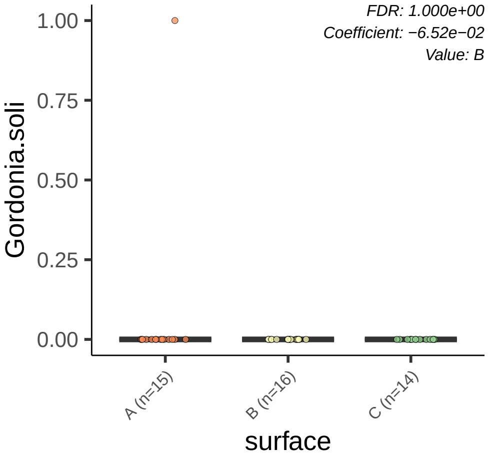

FDR: 1.000e+00
Coefficient: −6.52e−02
Value: B
Gordonia.soli
1.00
0.75
0.50
0.25
0.00
A (n=15)
B (n=16)
C (n=14)
surface

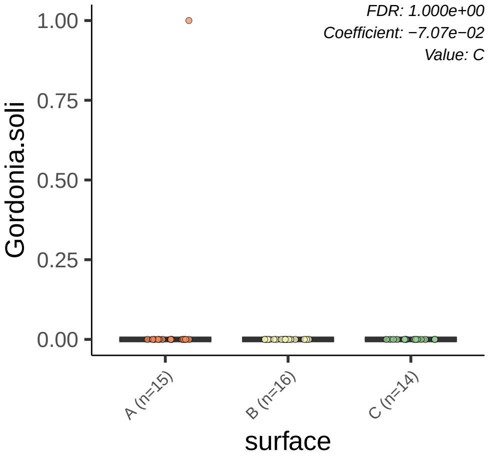
FDR: 1.000e+00
Coefficient: −7.07e−02
Value: C
Gordonia.soli
1.00
0.75
0.50
0.25
0.00
A (n=15)
B (n=16)
C (n=14)
surface

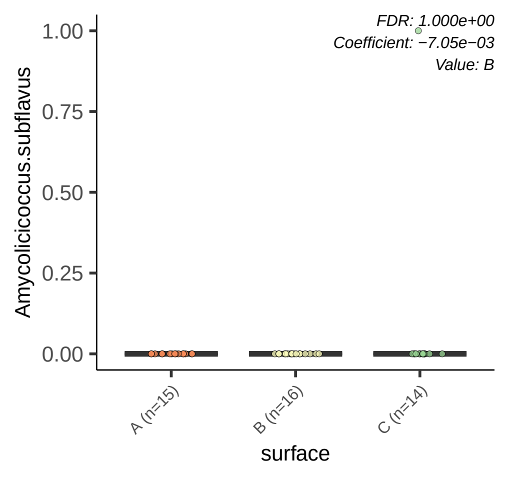

FDR: 1.000e+00
Coefficient: −7.05e−03
Value: B
Amycolicicoccus.subflavus
1.00
0.75
0.50
0.25
0.00
A (n=15)
B (n=16)
C (n=14)
surface

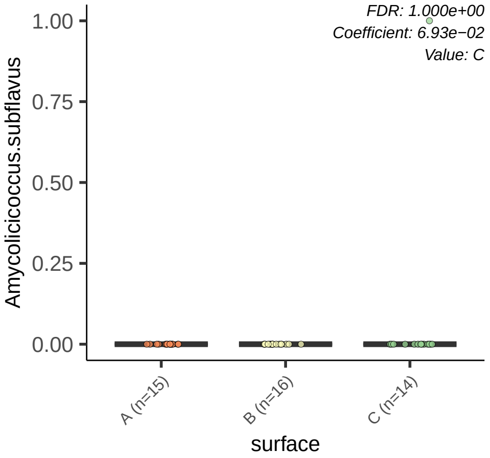

FDR: 1.000e+00
Coefficient: 6.93e−02
Value: C
Amycolicicoccus.subflavus
1.00
0.75
0.50
0.25
0.00
A (n=15)
B (n=16)
C (n=14)
surface

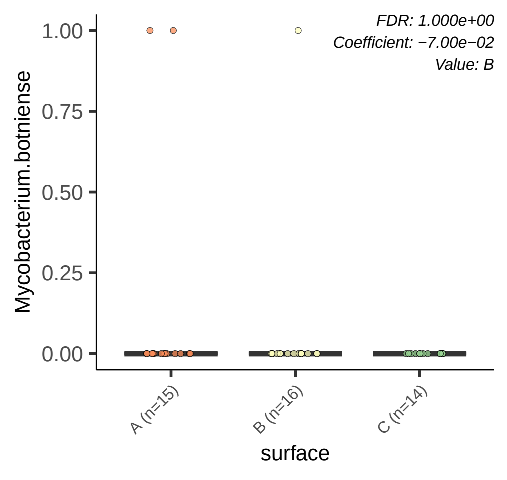

FDR: 1.000e+00
Coefficient: −7.00e−02
Value: B
Mycobacterium.botniense
1.00
0.75
0.50
0.25
0.00
A (n=15)
B (n=16)
C (n=14)
surface

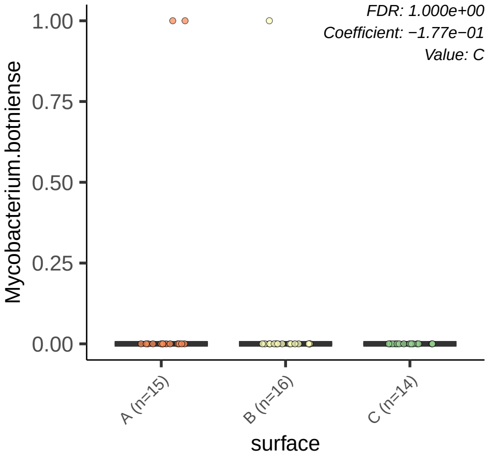
FDR: 1.000e+00
Coefficient: −1.77e−01
Value: C
Mycobacterium.botniense
1.00
0.75
0.50
0.25
0.00
A (n=15)
B (n=16)
C (n=14)
surface

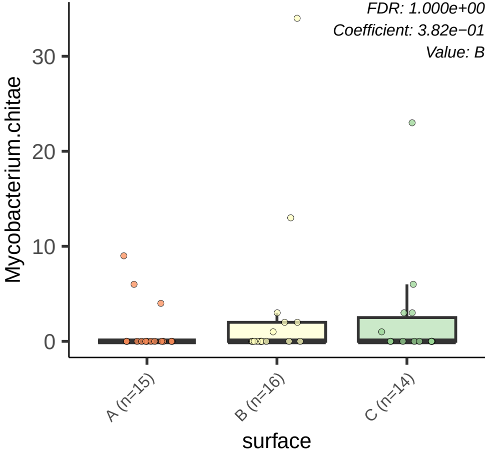
FDR: 1.000e+00
Coefficient: 3.82e−01
Value: B
Mycobacterium.chitae
0
10
20
30
A (n=15)
B (n=16)
C (n=14)
surface

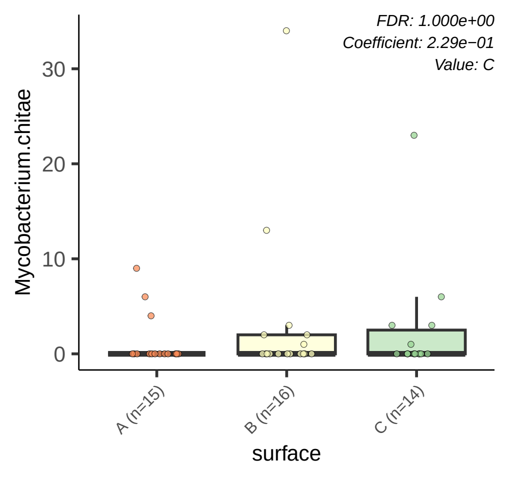

FDR: 1.000e+00
Coefficient: 2.29e−01
Value: C
Mycobacterium.chitae
30
20
10
0
A (n=15)
B (n=16)
C (n=14)
surface

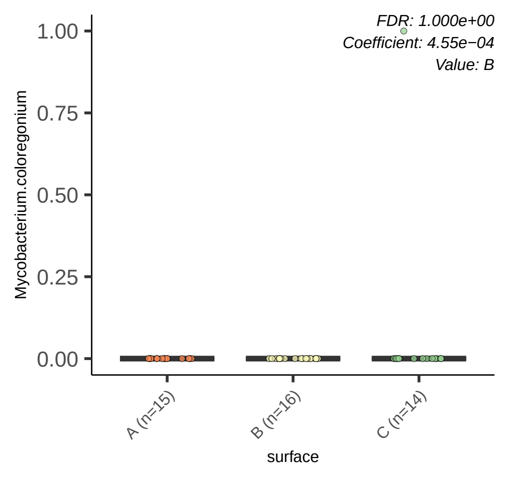
FDR: 1.000e+00
Coefficient: 4.55e−04
Value: B
Mycobacterium.coloregonium
1.00
0.75
0.50
0.25
0.00
A (n=15)
B (n=16)
C (n=14)
surface

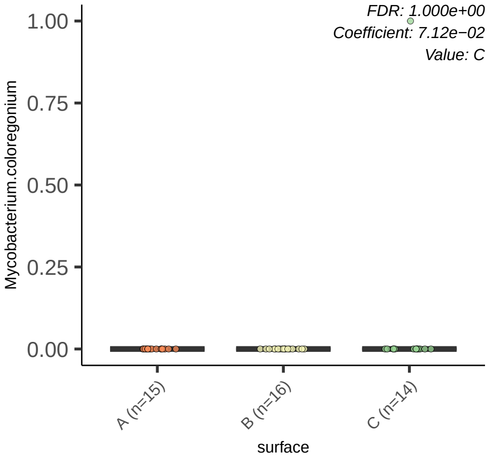
FDR: 1.000e+00
Coefficient: 7.12e−02
Value: C
1.00
0.75
0.50
0.25
0.00
Mycobacterium.coloregonium
A (n=15)
B (n=16)
C (n=14)
surface

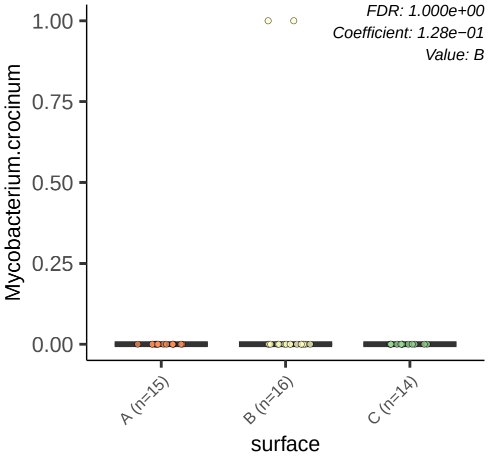

FDR: 1.000e+00
Coefficient: 1.28e−01
Value: B
Mycobacterium.crocinum
1.00
0.75
0.50
0.25
0.00
A (n=15)
B (n=16)
C (n=14)
surface

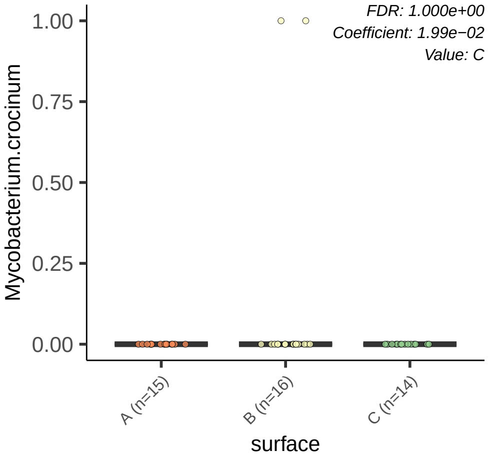

FDR: 1.000e+00
Coefficient: 1.99e−02
Value: C
Mycobacterium.crocinum
1.00
0.75
0.50
0.25
0.00
A (n=15)
B (n=16)
C (n=14)
surface

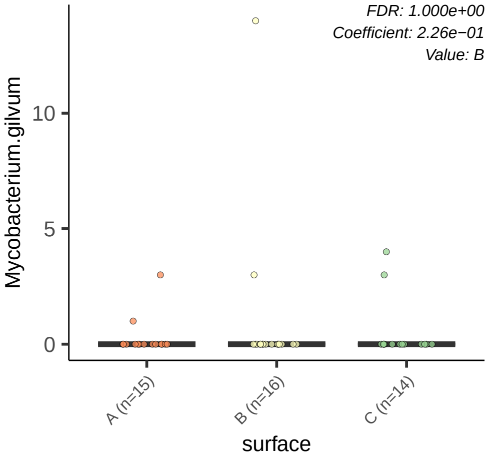
FDR: 1.000e+00
Coefficient: 2.26e−01
Value: B
Mycobacterium.gilvum
0
5
10
A (n=15)
B (n=16)
C (n=14)
surface

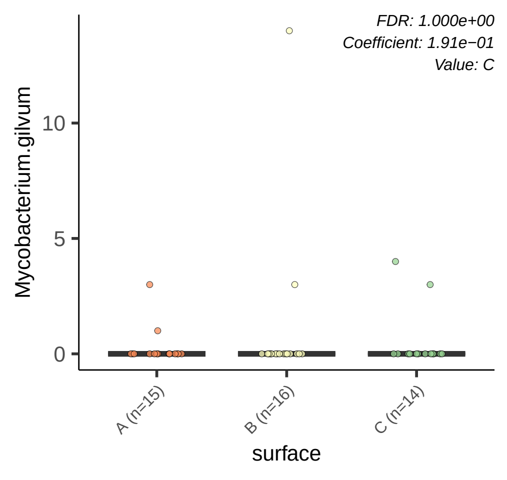
FDR: 1.000e+00
Coefficient: 1.91e−01
Value: C
Mycobacterium.gilvum
0
5
10
A (n=15)
B (n=16)
C (n=14)
surface

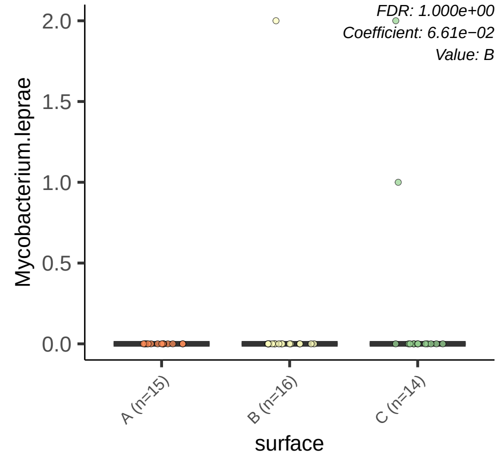

FDR: 1.000e+00
Coefficient: 6.61e−02
Value: B
Mycobacterium.leprae
2.0
1.5
1.0
0.5
0.0
A (n=15)
B (n=16)
C (n=14)
surface

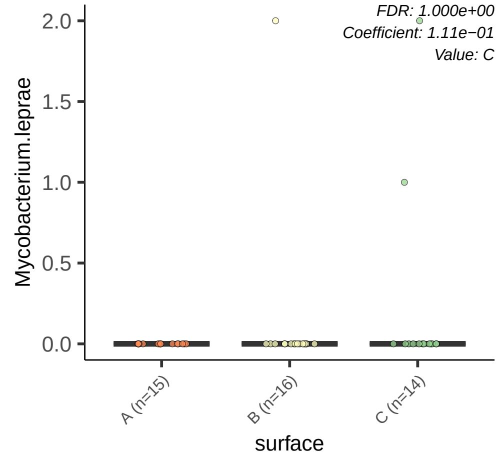
FDR: 1.000e+00
Coefficient: 1.11e−01
Value: C
Mycobacterium.leprae
2.0
1.5
1.0
0.5
0.0
A (n=15)
B (n=16)
C (n=14)
surface

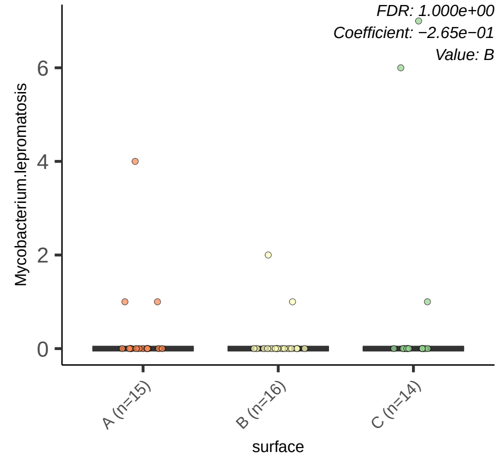

FDR: 1.000e+00
Coefficient: −2.65e−01
Value: B
Mycobacterium.lepromatosis
0
2
4
6
A (n=15)
B (n=16)
C (n=14)
surface

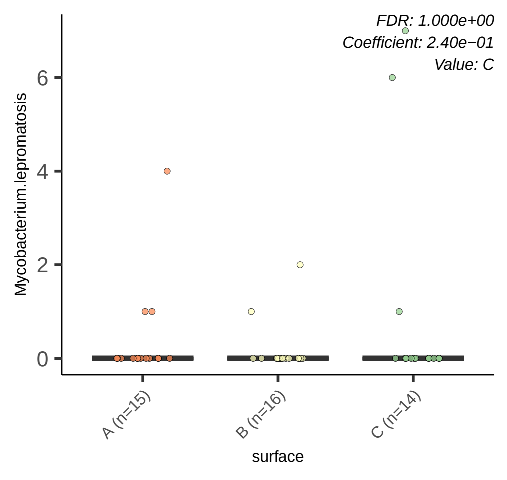

FDR: 1.000e+00
Coefficient: 2.40e−01
Value: C
Mycobacterium.lepromatosis
0
2
4
6
A (n=15)
B (n=16)
C (n=14)
surface

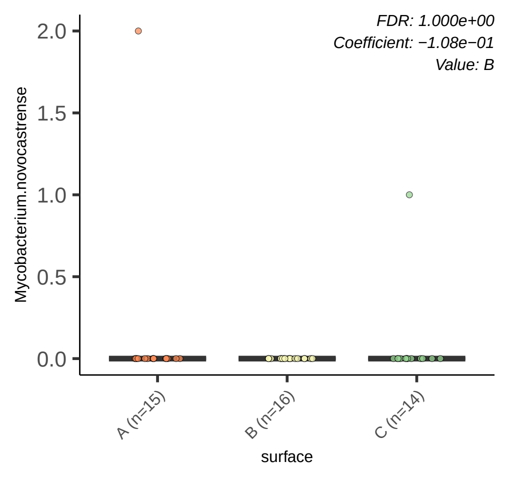
FDR: 1.000e+00
Coefficient: −1.08e−01
Value: B
Mycobacterium.novocastrense
2.0
1.5
1.0
0.5
0.0
A (n=15)
B (n=16)
C (n=14)
surface

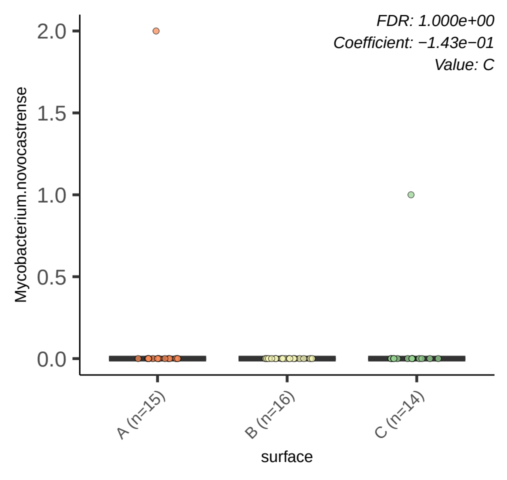
FDR: 1.000e+00
Coefficient: −1.43e−01
Value: C
Mycobacterium.novocastrense
2.0
1.5
1.0
0.5
0.0
A (n=15)
B (n=16)
C (n=14)
surface

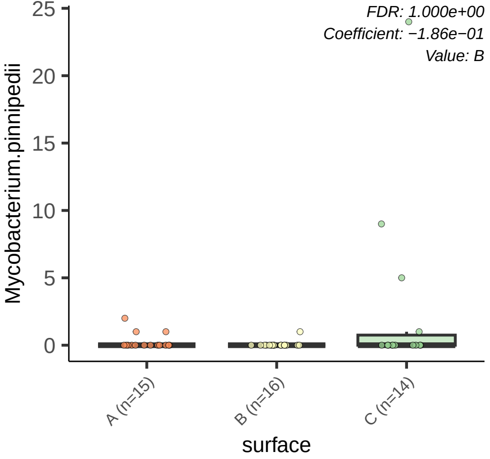
FDR: 1.000e+00
Coefficient: −1.86e−01
Value: B
Mycobacterium.pinnipedii
25
20
15
10
5
0
A (n=15)
B (n=16)
C (n=14)
surface

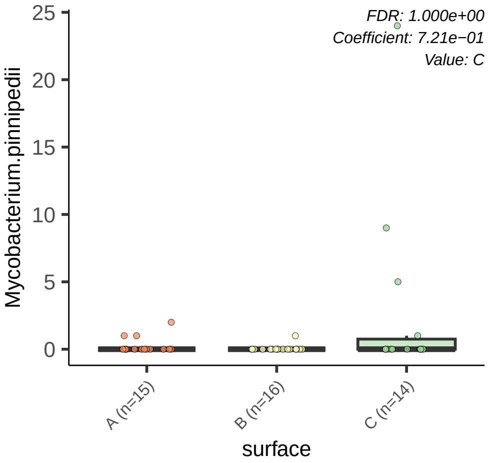
FDR: 1.000e+00
Coefficient: 7.21e−01
Value: C
Mycobacterium.pinnipedii
25
20
15
10
5
0
A (n=15)
B (n=16)
C (n=14)
surface

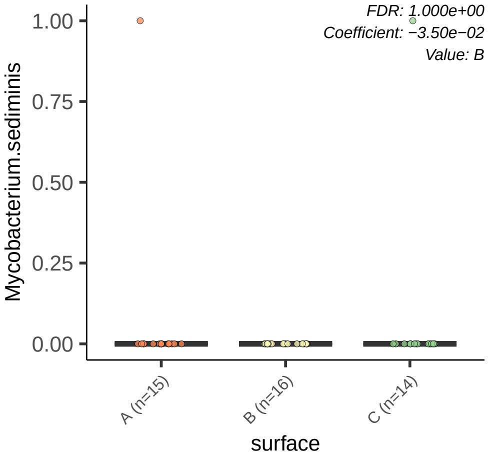
FDR: 1.000e+00
Coefficient: −3.50e−02
Value: B
Mycobacterium.sedimini s
1.00
0.75
0.50
0.25
0.00
A (n=15)
B (n=16)
C (n=14)
surface

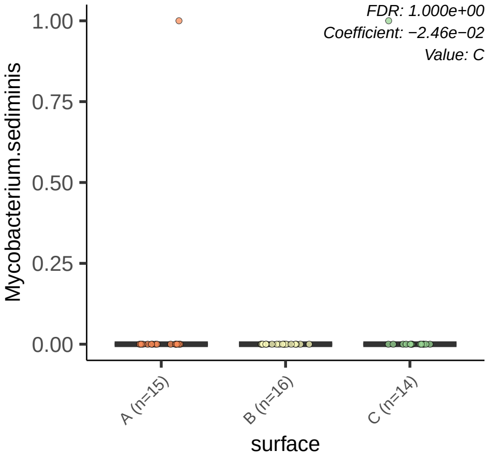

FDR: 1.000e+00
Coefficient: −2.46e−02
Value: C
Mycobacterium.sedimins
1.00
0.75
0.50
0.25
0.00
A (n=15)
B (n=16)
C (n=14)
surface

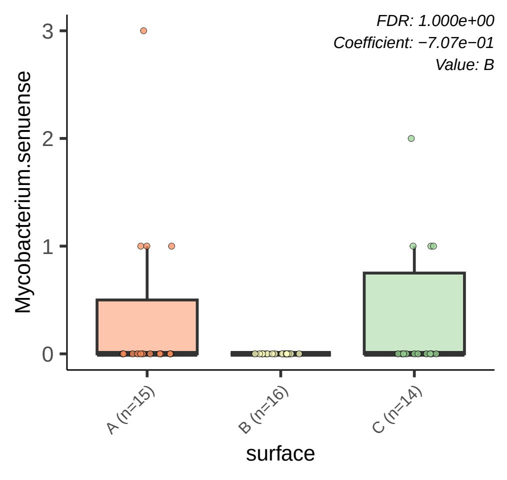
FDR: 1.000e+00
Coefficient: −7.07e−01
Value: B
Mycobacterium.senuense
3
2
1
0
A (n=15)
B (n=16)
C (n=14)
surface

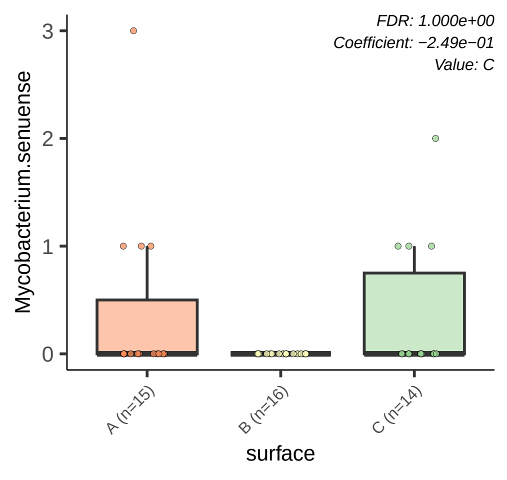
FDR: 1.000e+00
Coefficient: −2.49e−01
Value: C
Mycobacterium.senuense
3
2
1
0
A (n=15)
B (n=16)
C (n=14)
surface

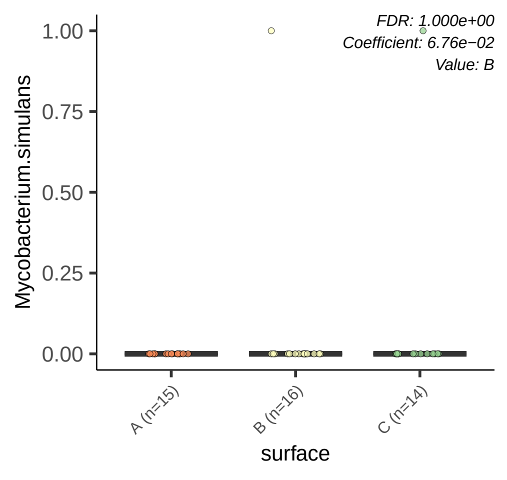
FDR: 1.000e+00
Coefficient: 6.76e−02
Value: B
Mycobacterium.simulans
1.00
0.75
0.50
0.25
0.00
A (n=15)
B (n=16)
C (n=14)
surface

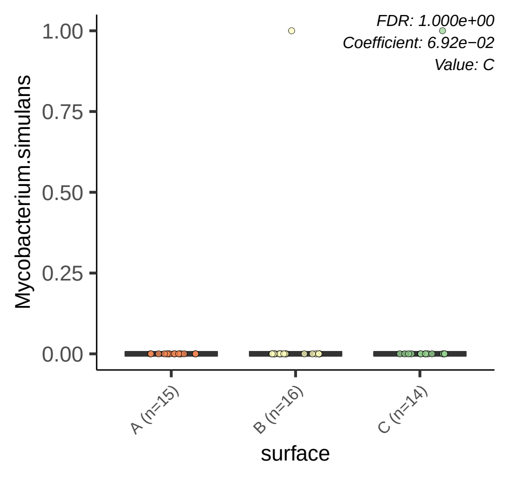
FDR: 1.000e+00
Coefficient: 6.92e−02
Value: C
Mycobacterium.simulans
1.00
0.75
0.50
0.25
0.00
A (n=15)
B (n=16)
C (n=14)
surface

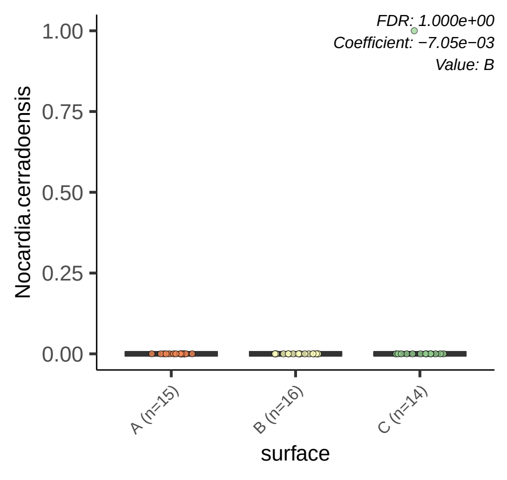
FDR: 1.000e+00
Coefficient: −7.05e−03
Value: B
Nocardia.cerradoensis
1.00
0.75
0.50
0.25
0.00
A (n=15)
B (n=16)
C (n=14)
surface

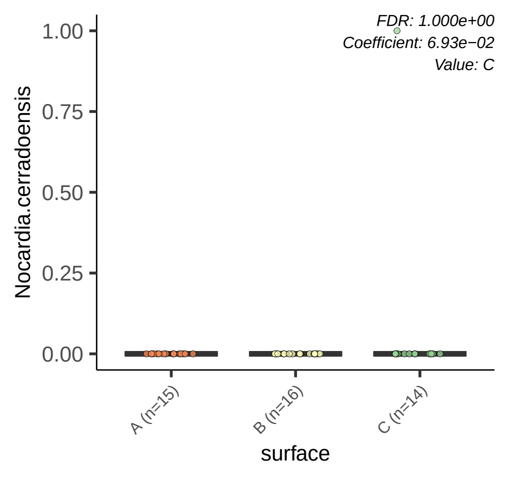
FDR: 1.000e+00
Coefficient: 6.93e−02
Value: C
Nocardia.cerradoensis
1.00
0.75
0.50
0.25
0.00
A (n=15)
B (n=16)
C (n=14)
surface

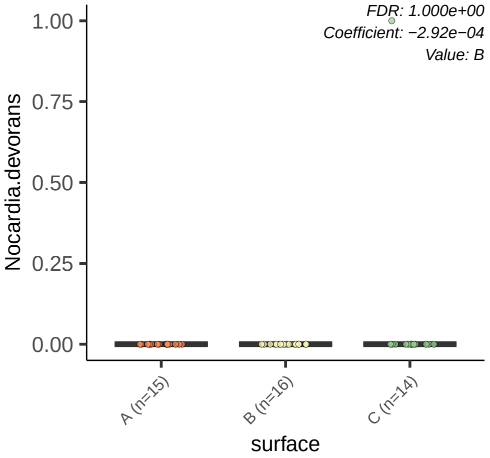

FDR: 1.000e+00
Coefficient: −2.92e−04
Value: B
Nocardia.devorans
1.00
0.75
0.50
0.25
0.00
A (n=15)
B (n=16)
C (n=14)
surface

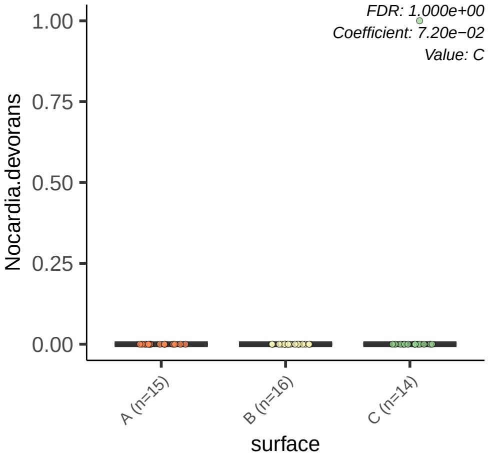
FDR: 1.000e+00
Coefficient: 7.20e−02
Value: C
Nocardia.devorans
1.00
0.75
0.50
0.25
0.00
A (n=15)
B (n=16)
C (n=14)
surface

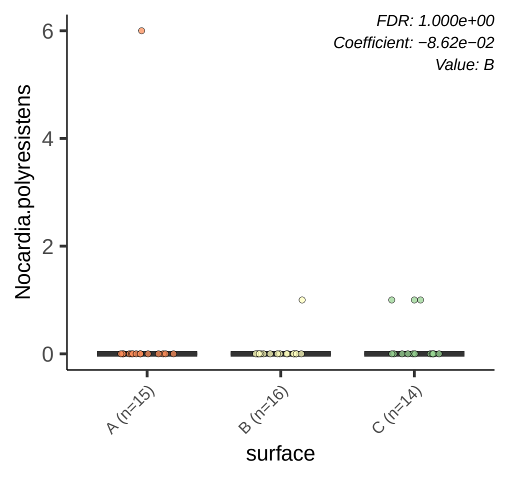
FDR: 1.000e+00
Coefficient: −8.62e−02
Value: B
Nocardia.polyresistens
0
2
4
6
A (n=15)
B (n=16)
C (n=14)
surface

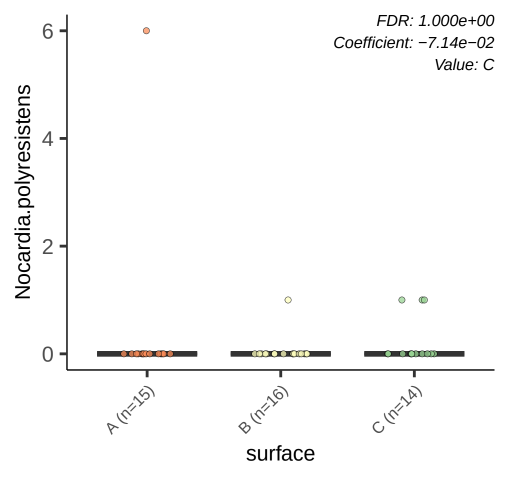

FDR: 1.000e+00
Coefficient: −7.14e−02
Value: C
Nocardia.polyresistens
6
4
2
0
A (n=15)
B (n=16)
C (n=14)
surface

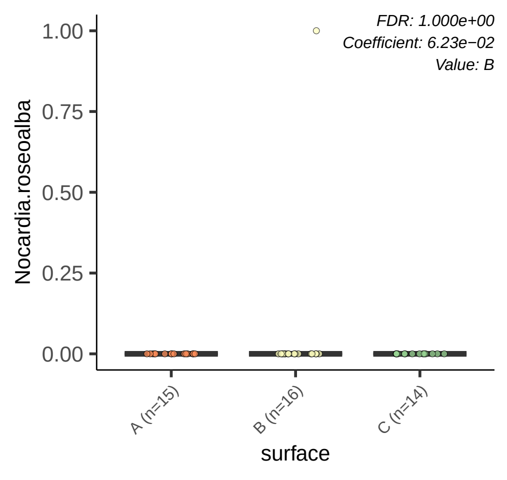
FDR: 1.000e+00
Coefficient: 6.23e−02
Value: B
Nocardia.roseoalba
1.00
0.75
0.50
0.25
0.00
A (n=15)
B (n=16)
C (n=14)
surface

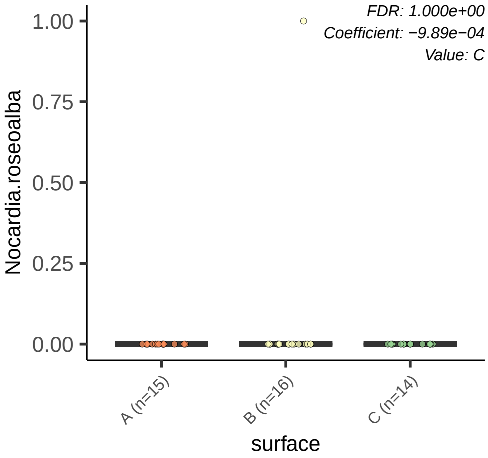

FDR: 1.000e+00
Coefficient: −9.89e−04
Value: C
Nocardia.roseoalba
1.00
0.75
0.50
0.25
0.00
A (n=15)
B (n=16)
C (n=14)
surface

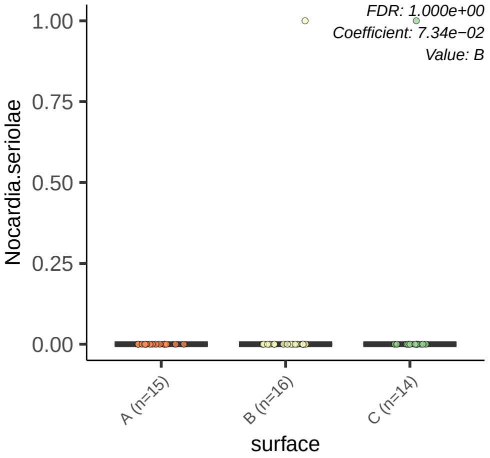
FDR: 1.000e+00
Coefficient: 7.34e−02
Value: B
Nocardia.seriolae
1.00
0.75
0.50
0.25
0.00
A (n=15)
B (n=16)
C (n=14)
surface

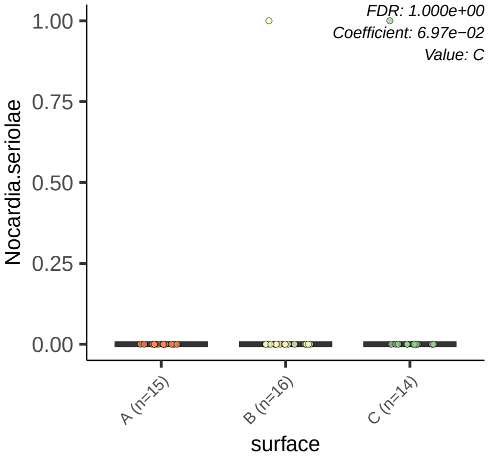
FDR: 1.000e+00
Coefficient: 6.97e−02
Value: C
Nocardia.seriolae
1.00
0.75
0.50
0.25
0.00
A (n=15)
B (n=16)
C (n=14)
surface

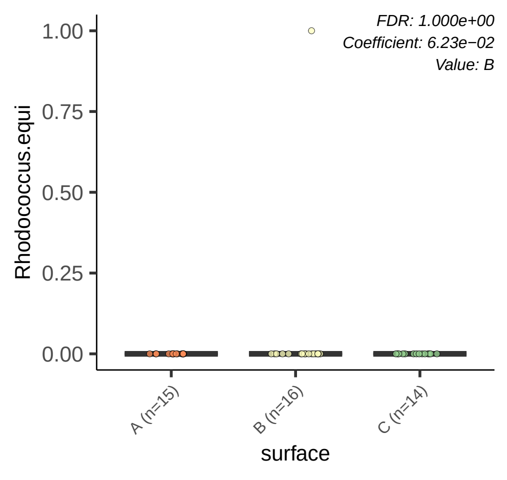

FDR: 1.000e+00
Coefficient: 6.23e−02
Value: B
Rhodococcus.equi
1.00
0.75
0.50
0.25
0.00
A (n=15)
B (n=16)
C (n=14)
surface

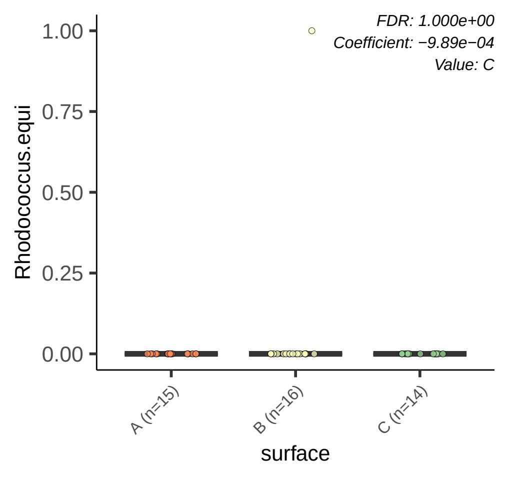
FDR: 1.000e+00
Coefficient: −9.89e−04
Value: C
Rhodococcus.equi
1.00
0.75
0.50
0.25
0.00
A (n=15)
B (n=16)
C (n=14)
surface

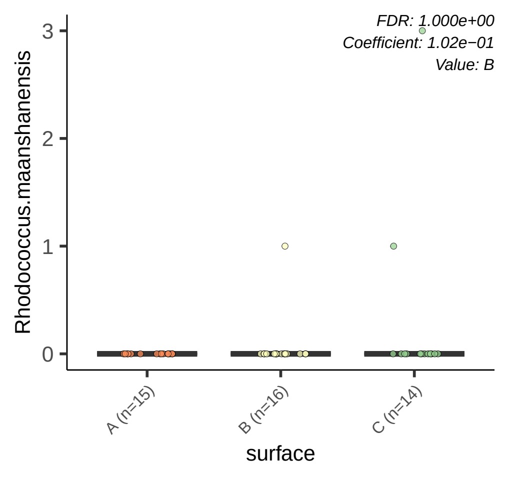

FDR: 1.000e+00
Coefficient: 1.02e−01
Value: B
Rhodococcus.maanshanensis
3
2
1
0
A (n=15)
B (n=16)
C (n=14)
surface

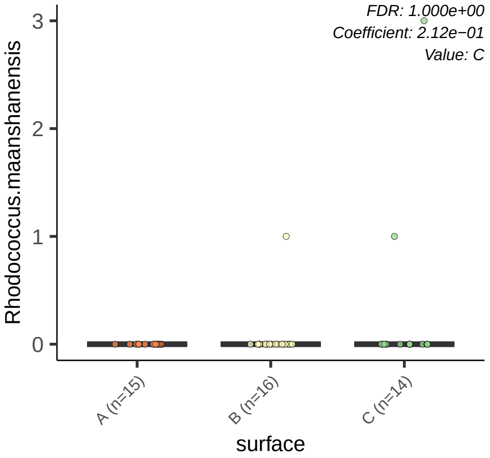

FDR: 1.000e+00
Coefficient: 2.12e−01
Value: C
Rhodococcus.maanshanensis
0
1
2
3
A (n=15)
B (n=16)
C (n=14)
surface

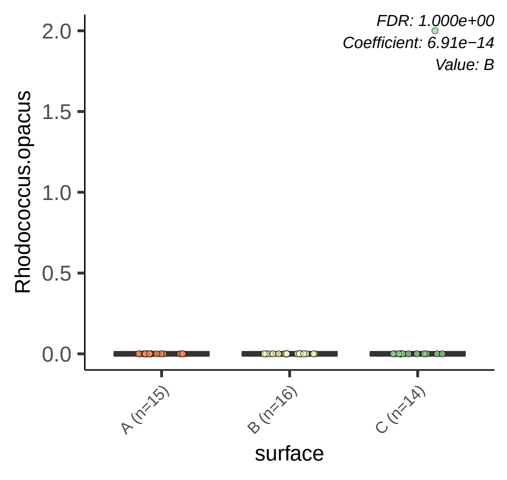
FDR: 1.000e+00
Coefficient: 6.91e−14
Value: B
2.0
1.5
1.0
0.5
0.0
Rhodococcus.opacus
A (n=15)
B (n=16)
C (n=14)
surface

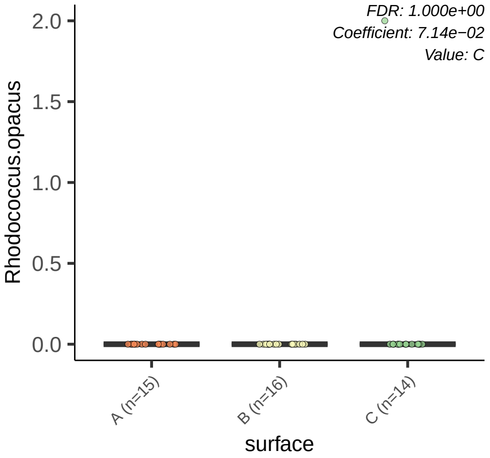

FDR: 1.000e+00
Coefficient: 7.14e−02
Value: C
Rhodococcus.opacus
2.0
1.5
1.0
0.5
0.0
A (n=15)
B (n=16)
C (n=14)
surface

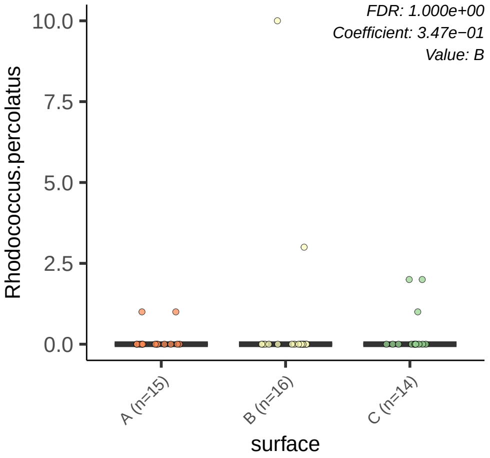

FDR: 1.000e+00
Coefficient: 3.47e−01
Value: B
10.0
7.5
5.0
2.5
0.0
Rhodococcus.percolatus
A (n=15)
B (n=16)
C (n=14)
surface

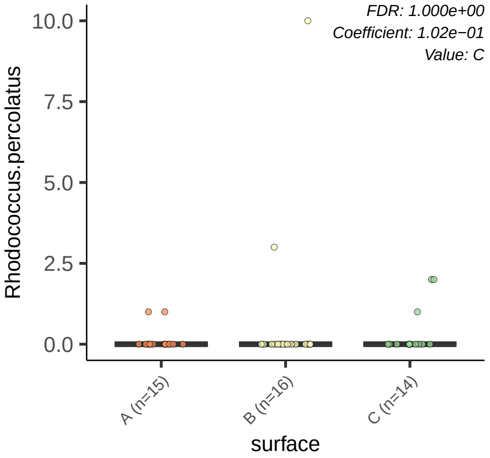
FDR: 1.000e+00
Coefficient: 1.02e−01
Value: C
Rhodococcus.percolatus
10.0
7.5
5.0
2.5
0.0
A (n=15)
B (n=16)
C (n=14)
surface

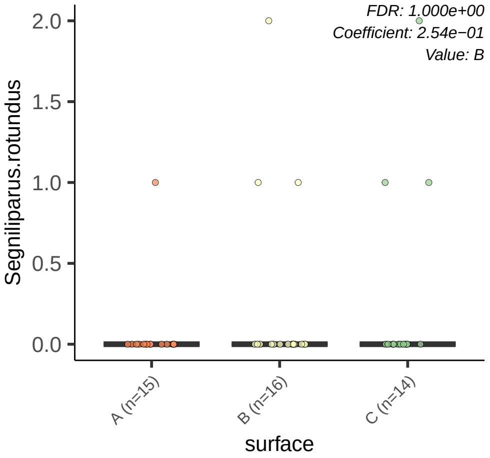
FDR: 1.000e+00
Coefficient: 2.54e−01
Value: B
Segniliparus.rotundus
2.0
1.5
1.0
0.5
0.0
A (n=15)
B (n=16)
C (n=14)
surface

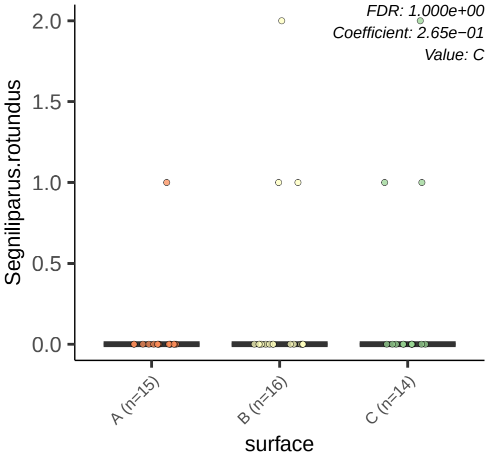

FDR: 1.000e+00
Coefficient: 2.65e−01
Value: C
Segniliparus.rotundus
2.0
1.5
1.0
0.5
0.0
A (n=15)
B (n=16)
C (n=14)
surface

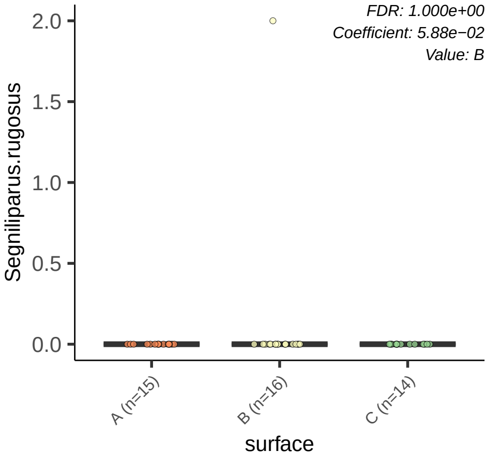
FDR: 1.000e+00
Coefficient: 5.88e−02
Value: B
Segniliparus.rugosus
2.0
1.5
1.0
0.5
0.0
A (n=15)
B (n=16)
C (n=14)
surface

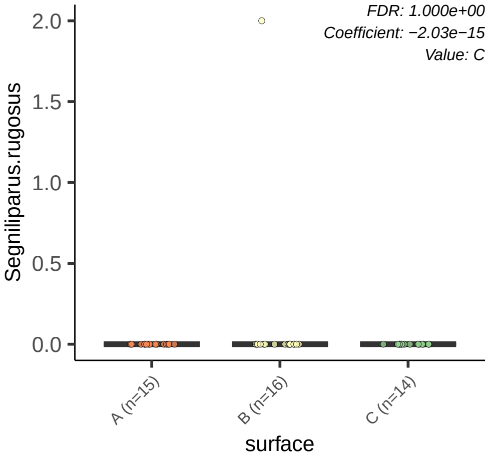

FDR: 1.000e+00
Coefficient: −2.03e−15
Value: C
2.0
1.5
1.0
0.5
0.0
Segniliparus.rugosus
A (n=15)
B (n=16)
C (n=14)
surface

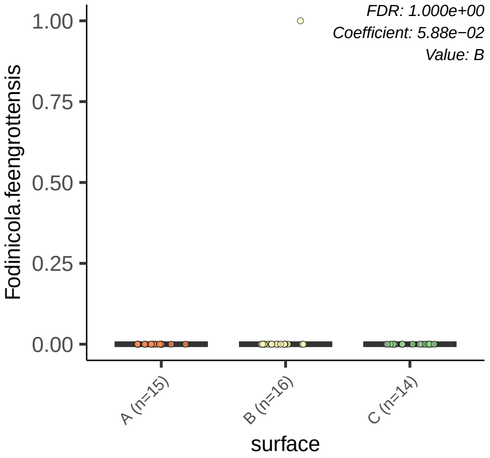
FDR: 1.000e+00
Coefficient: 5.88e−02
Value: B
Fodinicola.feengrottensis
1.00
0.75
0.50
0.25
0.00
A (n=15)
B (n=16)
C (n=14)
surface

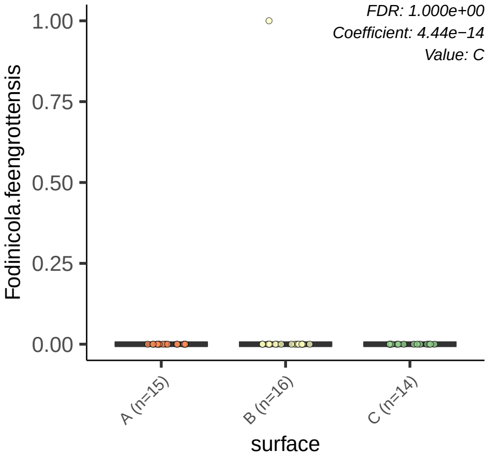
FDR: 1.000e+00
Coefficient: 4.44e−14
Value: C
Fodinicola.feengrottensis
1.00
0.75
0.50
0.25
0.00
A (n=15)
B (n=16)
C (n=14)
surface

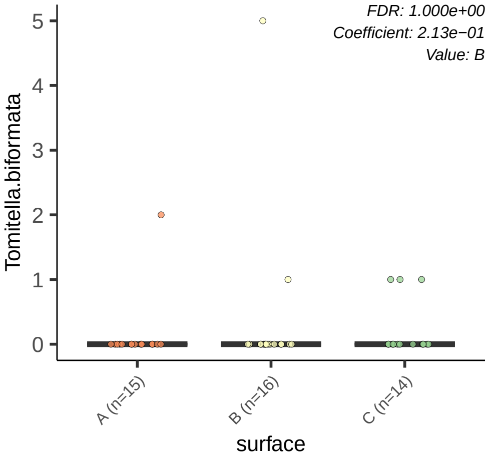
FDR: 1.000e+00
Coefficient: 2.13e−01
Value: B
Tomitella.biformata
0
1
2
3
4
5
A (n=15)
B (n=16)
C (n=14)
surface

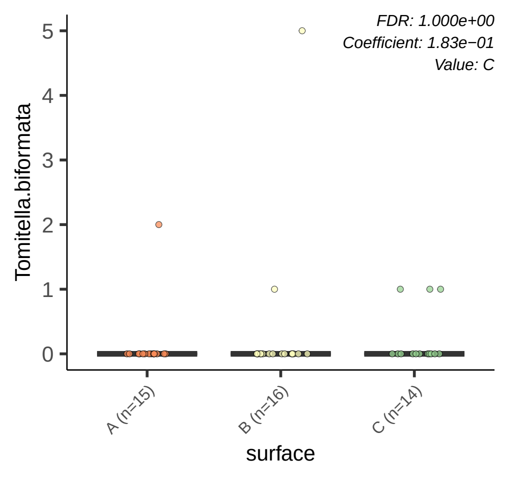

FDR: 1.000e+00
Coefficient: 1.83e−01
Value: C
Tomitella.biformata
0
1
2
3
4
5
A (n=15)
B (n=16)
C (n=14)
surface

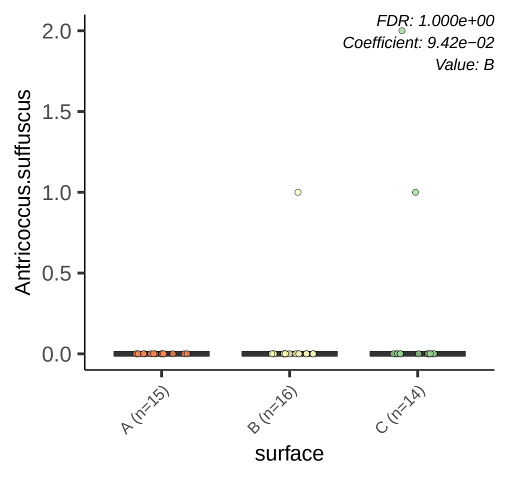
FDR: 1.000e+00
Coefficient: 9.42e−02
Value: B
Antricoccus.suffuscus
2.0
1.5
1.0
0.5
0.0
A (n=15)
B (n=16)
C (n=14)
surface

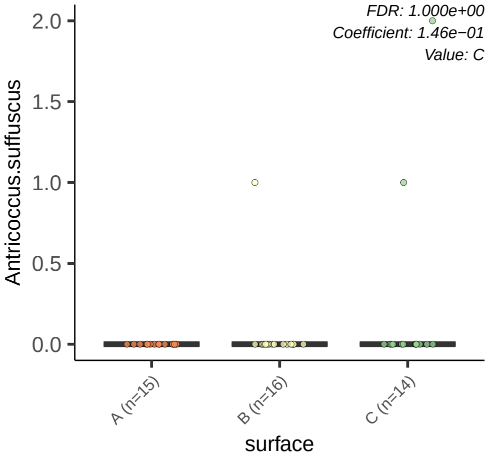

FDR: 1.000e+00
Coefficient: 1.46e−01
Value: C
Antricoccus.suffuscus
2.0
1.5
1.0
0.5
0.0
A (n=15)
B (n=16)
C (n=14)
surface

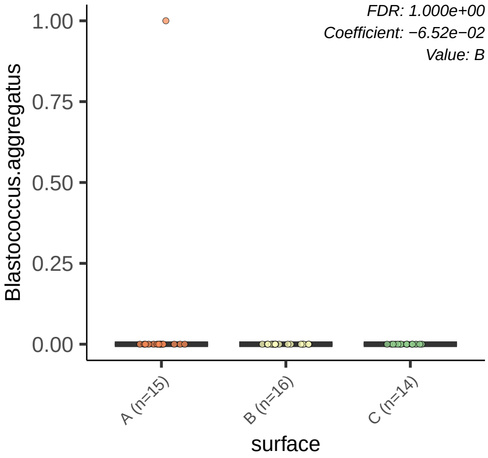

FDR: 1.000e+00
Coefficient: −6.52e−02
Value: B
Blastococcus.aggregatus
1.00
0.75
0.50
0.25
0.00
A (n=15)
B (n=16)
C (n=14)
surface

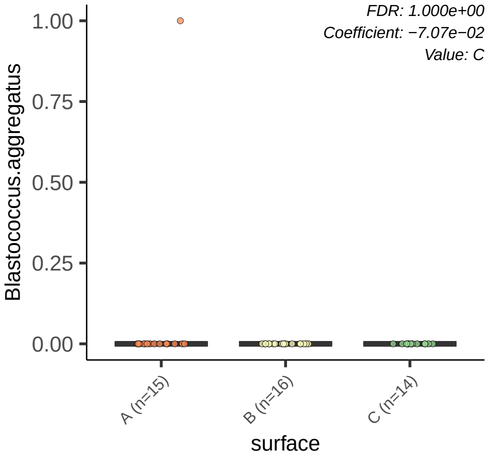

FDR: 1.000e+00
Coefficient: −7.07e−02
Value: C
Blastococcus.aggregatus
1.00
0.75
0.50
0.25
0.00
A (n=15)
B (n=16)
C (n=14)
surface

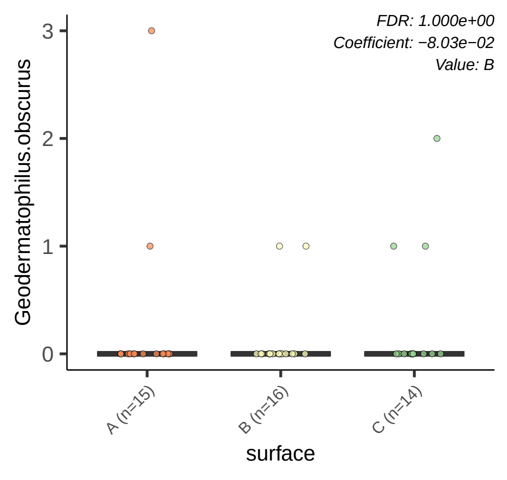
FDR: 1.000e+00
Coefficient: −8.03e−02
Value: B
Geodermatophilus.obscurus
0
1
2
3
A (n=15)
B (n=16)
C (n=14)
surface

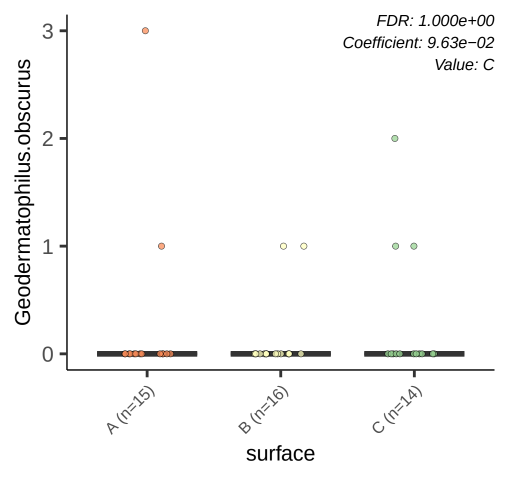
FDR: 1.000e+00
Coefficient: 9.63e−02
Value: C
Geodermatophilus.obscurus
3
2
1
0
A (n=15)
B (n=16)
C (n=14)
surface

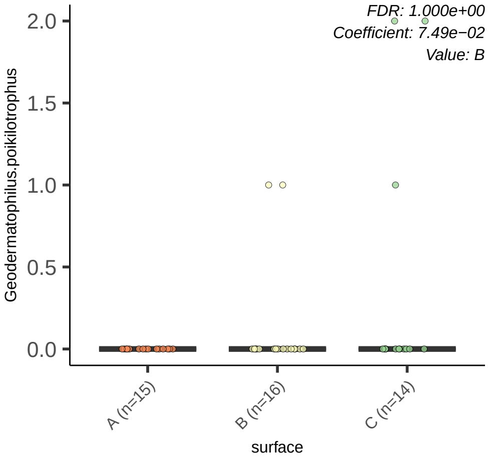

FDR: 1.000e+00
Coefficient: 7.49e−02
Value: B
Geodermatophilus.poikilotrophus
2.0
1.5
1.0
0.5
0.0
A (n=15)
B (n=16)
C (n=14)
surface

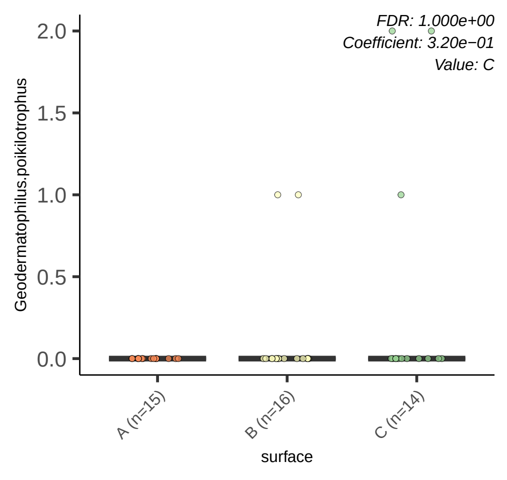
FDR: 1.000e+00
Coefficient: 3.20e−01
Value: C
Geodermatophilus.poikilotrophus
2.0
1.5
1.0
0.5
0.0
A (n=15)
B (n=16)
C (n=14)
surface

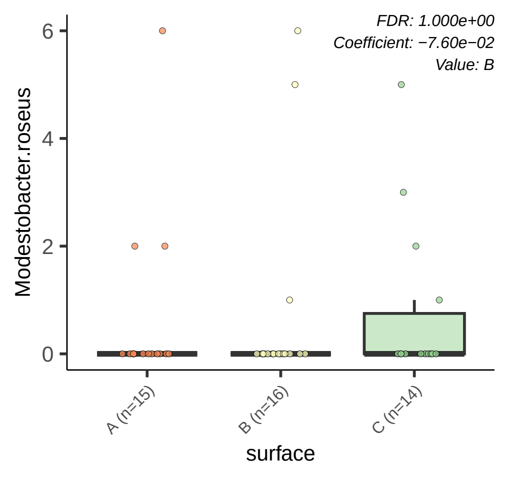
FDR: 1.000e+00
Coefficient: −7.60e−02
Value: B
Modestobacter.roseus
0
2
4
6
A (n=15)
B (n=16)
C (n=14)
surface

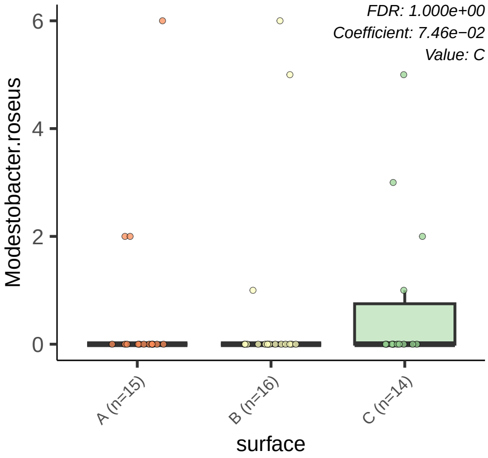
FDR: 1.000e+00
Coefficient: 7.46e−02
Value: C
Modestobacter.roseus
0
2
4
6
A (n=15)
B (n=16)
C (n=14)
surface

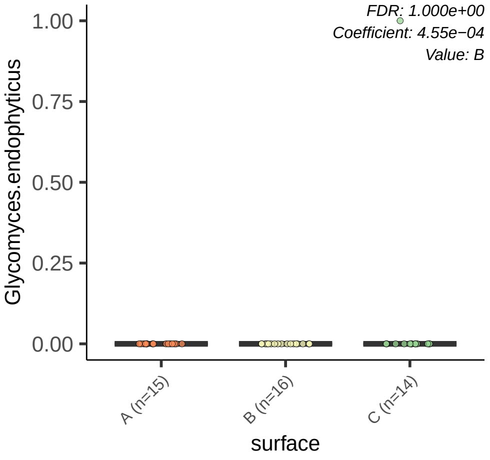

FDR: 1.000e+00
Coefficient: 4.55e−04
Value: B
Glycomyces.endophyticus
1.00
0.75
0.50
0.25
0.00
A (n=15)
B (n=16)
C (n=14)
surface

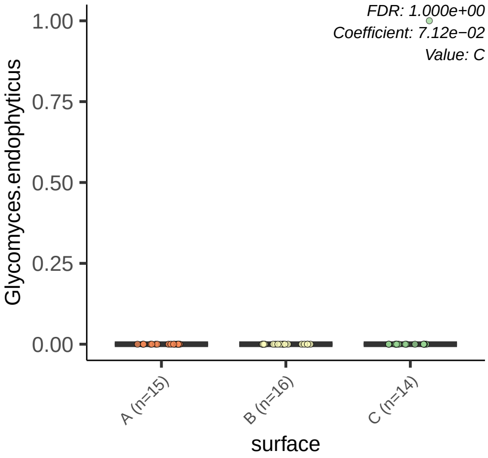

FDR: 1.000e+00
Coefficient: 7.12e−02
Value: C
Glycomyces.endophyticus
1.00
0.75
0.50
0.25
0.00
A (n=15)
B (n=16)
C (n=14)
surface

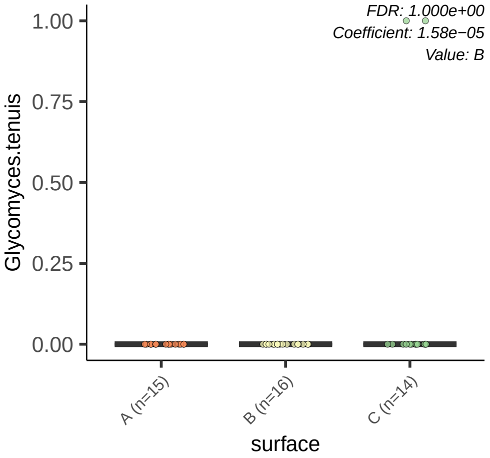
FDR: 1.000e+00
Coefficient: 1.58e−05
Value: B
Glycomyces.tenuis
1.00
0.75
0.50
0.25
0.00
A (n=15)
B (n=16)
C (n=14)
surface

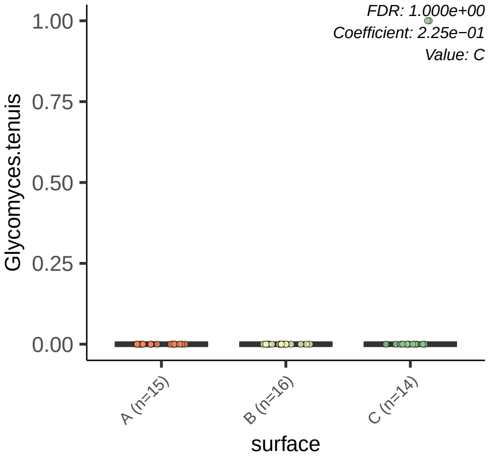
FDR: 1.000e+00
Coefficient: 2.25e−01
Value: C
Glycomyces.tenuis
1.00
0.75
0.50
0.25
0.00
A (n=15)
B (n=16)
C (n=14)
surface

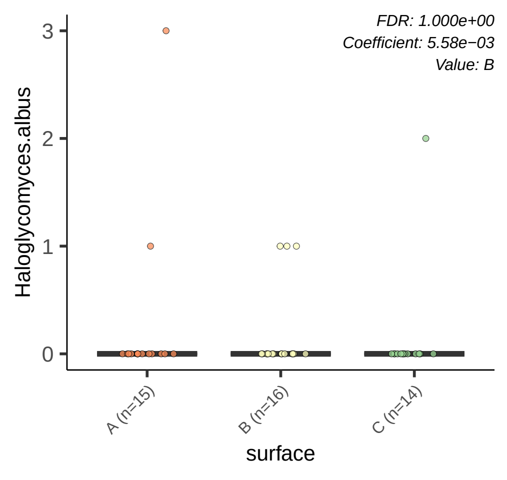
FDR: 1.000e+00
Coefficient: 5.58e−03
Value: B
Haloglycomyces.albus
3
2
1
0
A (n=15)
B (n=16)
C (n=14)
surface

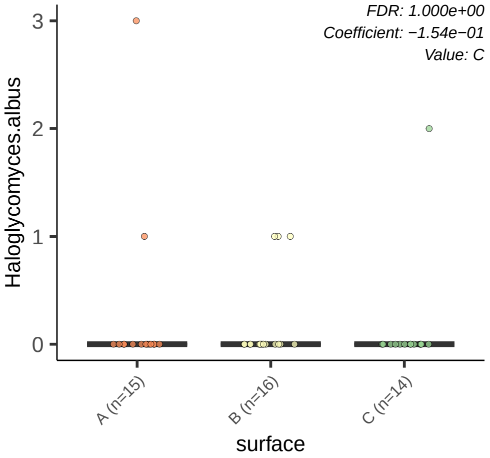

FDR: 1.000e+00
Coefficient: −1.54e−01
Value: C
Haloglycomyces.albus
3
2
1
0
A (n=15)
B (n=16)
C (n=14)
surface

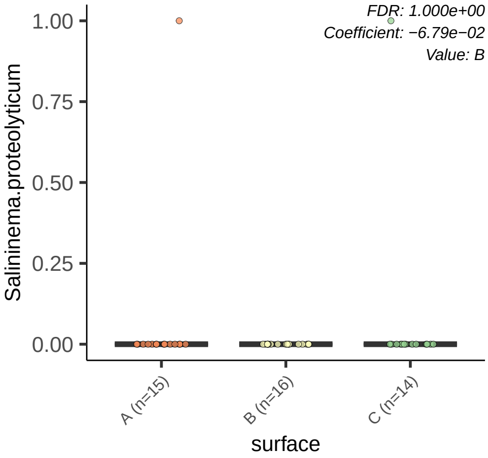

FDR: 1.000e+00
Coefficient: −6.79e−02
Value: B
Salininema.proteolyticum
1.00
0.75
0.50
0.25
0.00
A (n=15)
B (n=16)
C (n=14)
surface

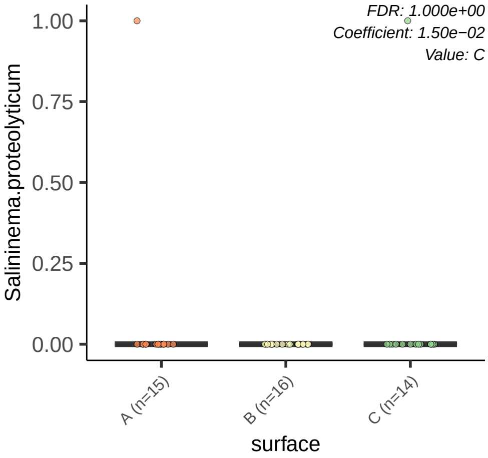
FDR: 1.000e+00
Coefficient: 1.50e−02
Value: C
Salininema.proteolyticum
1.00
0.75
0.50
0.25
0.00
A (n=15)
B (n=16)
C (n=14)
surface

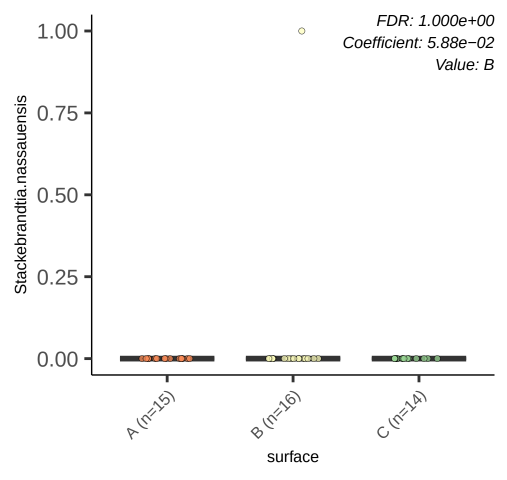
FDR: 1.000e+00
Coefficient: 5.88e−02
Value: B
1.00
0.75
0.50
0.25
0.00
Stackebrandtia.nassauensis
A (n=15)
B (n=16)
C (n=14)
surface

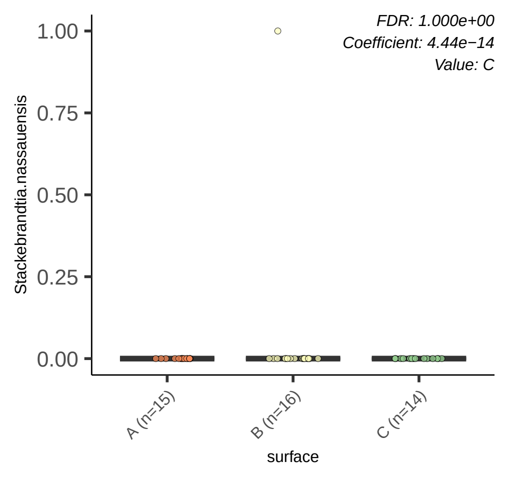

FDR: 1.000e+00
Coefficient: 4.44e−14
Value: C
1.00
0.75
0.50
0.25
0.00
Stackebrandtia.nassauensis
A (n=15)
B (n=16)
C (n=14)
surface

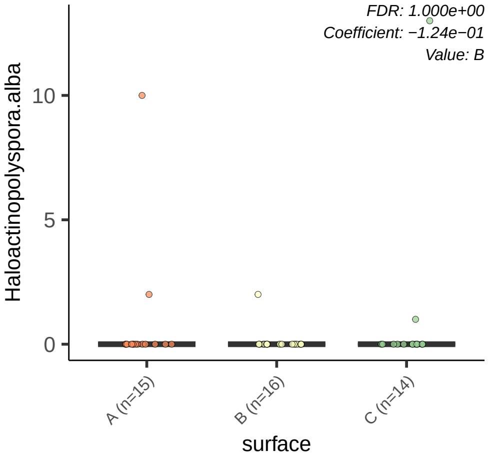
FDR: 1.000e+00
Coefficient: −1.24e−01
Value: B
Haloactinopolyspora.alba
10
5
0
A (n=15)
B (n=16)
C (n=14)
surface

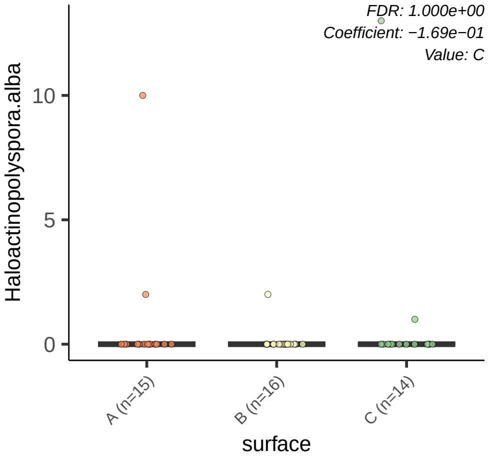

FDR: 1.000e+00
Coefficient: −1.69e−01
Value: C
Haloactinopolyspora.alba
10
5
0
A (n=15)
B (n=16)
C (n=14)
surface

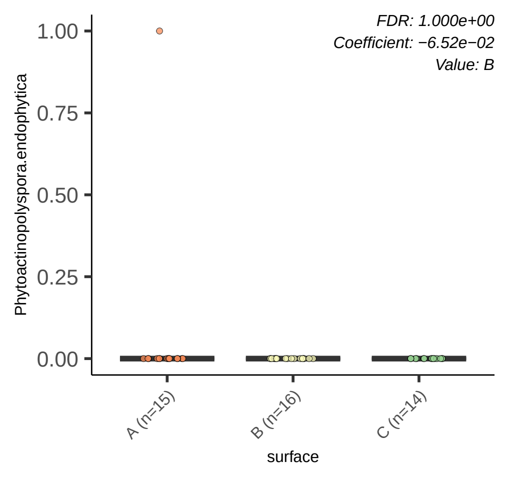
FDR: 1.000e+00
Coefficient: −6.52e−02
Value: B
Phytoactinopolyspora.endophytica
1.00
0.75
0.50
0.25
0.00
A (n=15)
B (n=16)
C (n=14)
surface

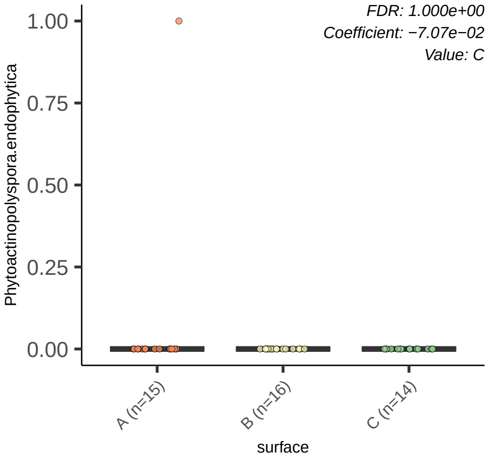
FDR: 1.000e+00
Coefficient: −7.07e−02
Value: C
1.00
0.75
0.50
0.25
0.00
Phytoactinopolyspora.endophytica
A (n=15)
B (n=16)
C (n=14)
surface

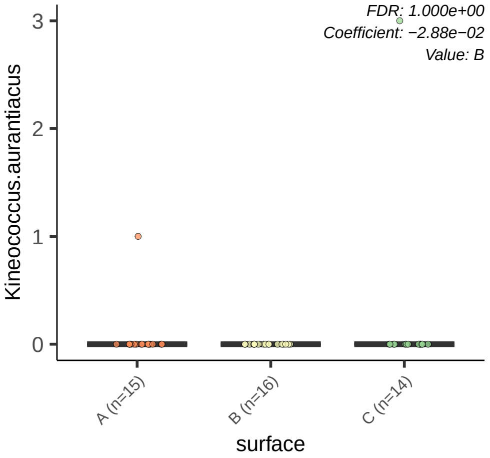

FDR: 1.000e+00
Coefficient: −2.88e−02
Value: B
Kineococcus.aurantiacus
3
2
1
0
A (n=15)
B (n=16)
C (n=14)
surface

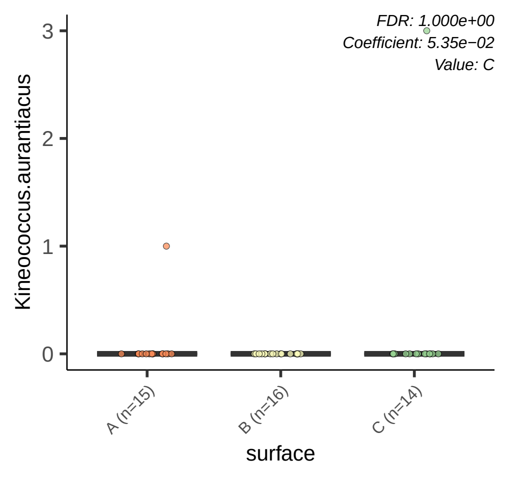

FDR: 1.000e+00
Coefficient: 5.35e−02
Value: C
Kineococcus.aurantiacus
3
2
1
0
A (n=15)
B (n=16)
C (n=14)
surface

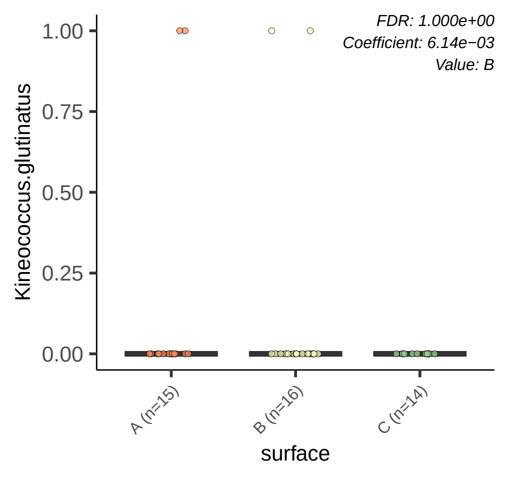
FDR: 1.000e+00
Coefficient: 6.14e−03
Value: B
Kineococcus.glutinatus
1.00
0.75
0.50
0.25
0.00
A (n=15)
B (n=16)
C (n=14)
surface

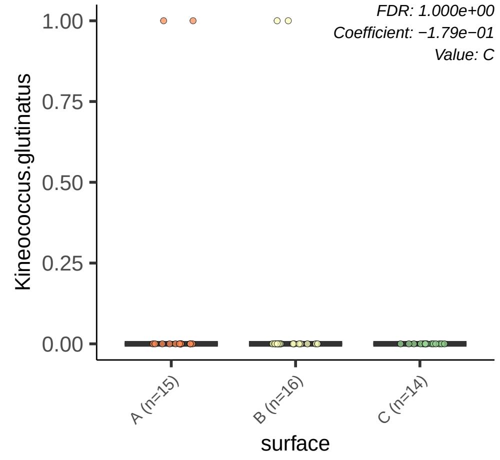
FDR: 1.000e+00
Coefficient: −1.79e−01
Value: C
1.00
0.75
0.50
0.25
0.00
Kineococcus.glutinatus
A (n=15)
B (n=16)
C (n=14)
surface

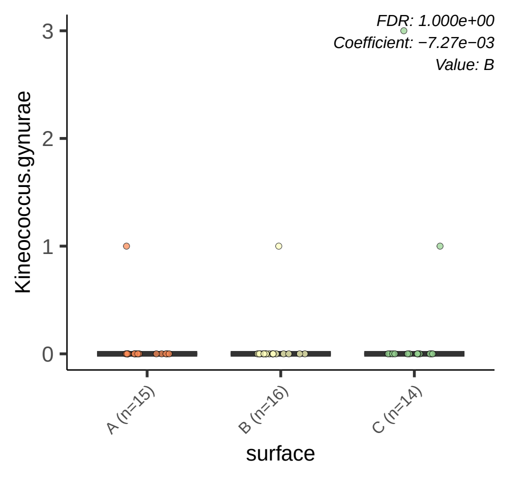

FDR: 1.000e+00
Coefficient: −7.27e−03
Value: B
Kineococcus.gynurae
0
1
2
3
A (n=15)
B (n=16)
C (n=14)
surface

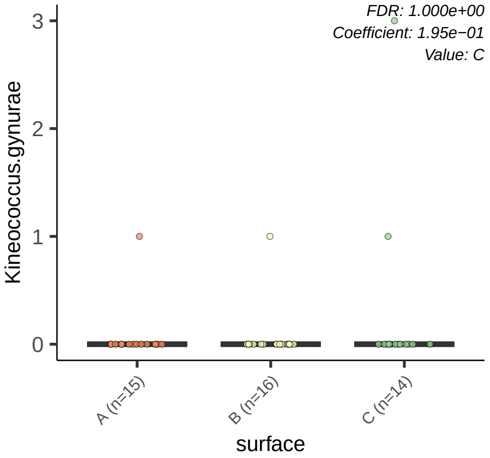

FDR: 1.000e+00
Coefficient: 1.95e−01
Value: C
Kineococcus.gynurae
0
1
2
3
A (n=15)
B (n=16)
C (n=14)
surface

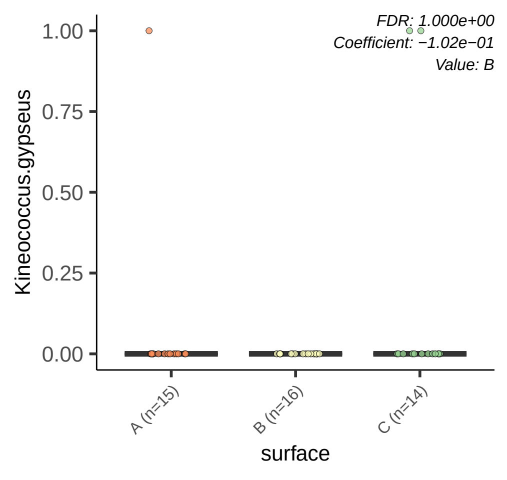
FDR: 1.000e+00
Coefficient: −1.02e−01
Value: B
Kineococcus.gypseus
1.00
0.75
0.50
0.25
0.00
A (n=15)
B (n=16)
C (n=14)
surface

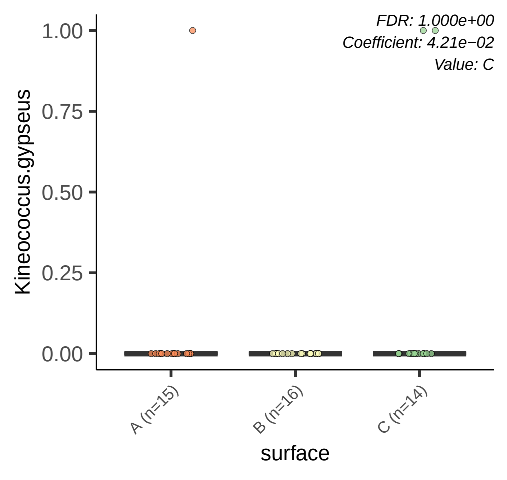

FDR: 1.000e+00
Coefficient: 4.21e−02
Value: C
Kineococcus.gypseus
1.00
0.75
0.50
0.25
0.00
A (n=15)
B (n=16)
C (n=14)
surface

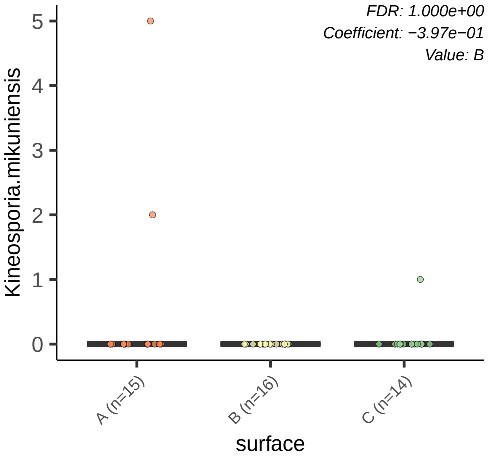
FDR: 1.000e+00
Coefficient: −3.97e−01
Value: B
Kineosporia.mikuniensis
0
1
2
3
4
5
A (n=15)
B (n=16)
C (n=14)
surface

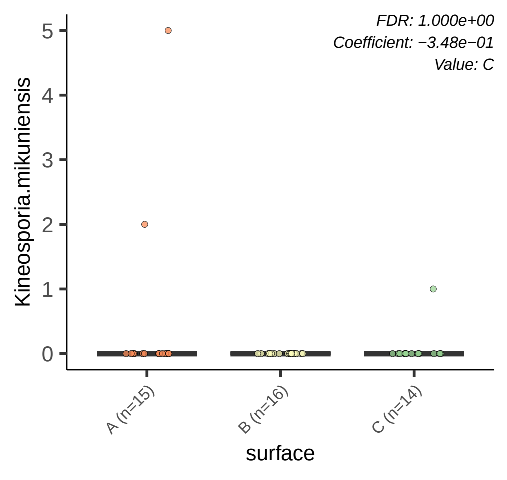
FDR: 1.000e+00
Coefficient: −3.48e−01
Value: C
Kineosporia.mikuniensis
0
1
2
3
4
5
A (n=15)
B (n=16)
C (n=14)
surface

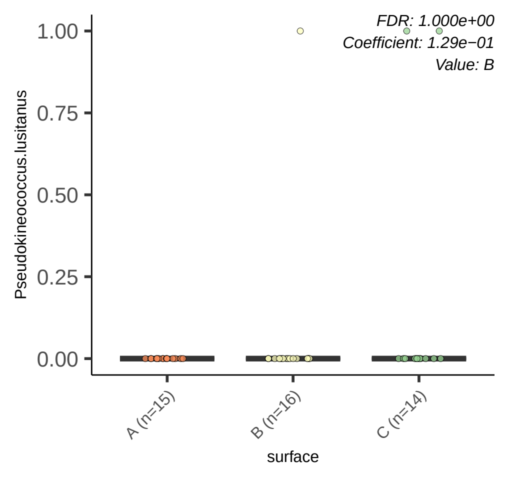
FDR: 1.000e+00
Coefficient: 1.29e−01
Value: B
Pseudokineococcus.lusitanus
1.00
0.75
0.50
0.25
0.00
A (n=15)
B (n=16)
C (n=14)
surface

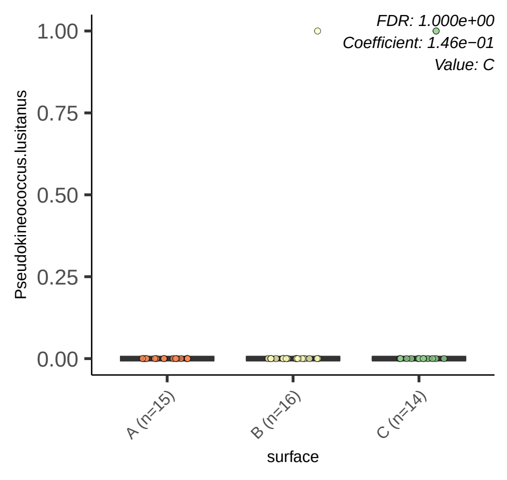
FDR: 1.000e+00
Coefficient: 1.46e−01
Value: C
Pseudokineococcus.lusitanus
1.00
0.75
0.50
0.25
0.00
A (n=15)
B (n=16)
C (n=14)
surface

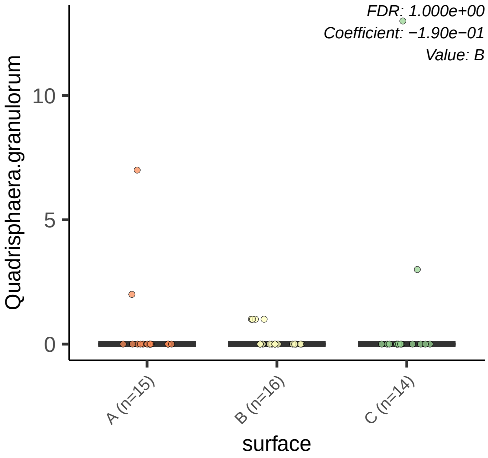

FDR: 1.000e+00
Coefficient: −1.90e−01
Value: B
Quadrisphaera.granulorum
10
5
0
A (n=15)
B (n=16)
C (n=14)
surface

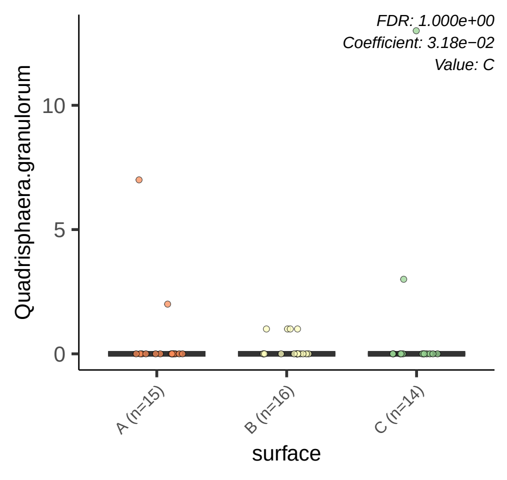
FDR: 1.000e+00
Coefficient: 3.18e−02
Value: C
Quadrisphaera.granulorum
10
5
0
A (n=15)
B (n=16)
C (n=14)
surface

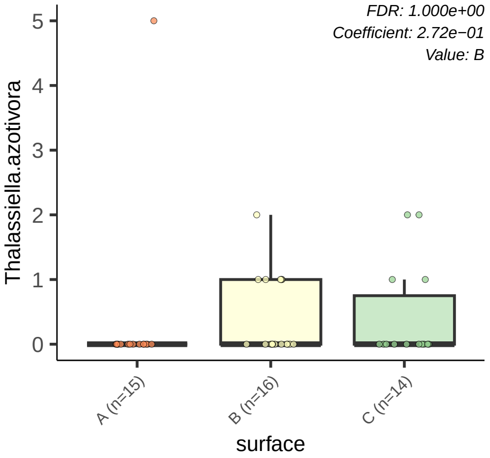

FDR: 1.000e+00
Coefficient: 2.72e−01
Value: B
Thalassiella.azotivora
0
1
2
3
4
5
A (n=15)
B (n=16)
C (n=14)
surface

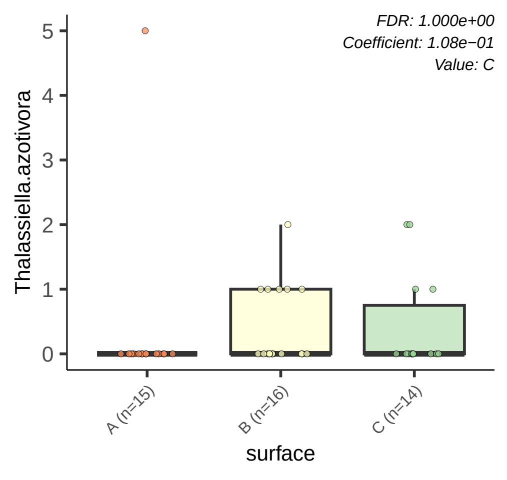
FDR: 1.000e+00
Coefficient: 1.08e−01
Value: C
Thalassiella.azotivora
0
1
2
3
4
5
A (n=15)
B (n=16)
C (n=14)
surface

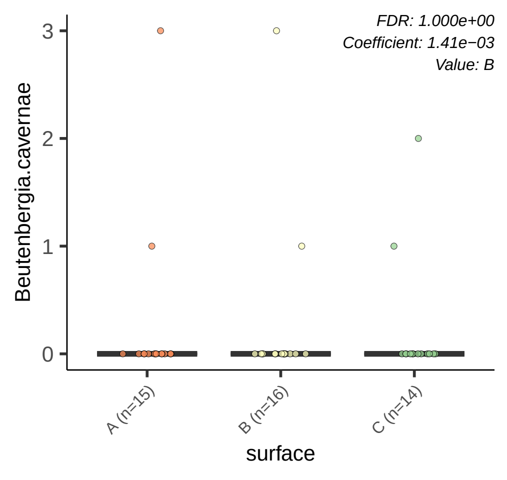
FDR: 1.000e+00
Coefficient: 1.41e−03
Value: B
Beutenbergia.cavernae
0
1
2
3
A (n=15)
B (n=16)
C (n=14)
surface

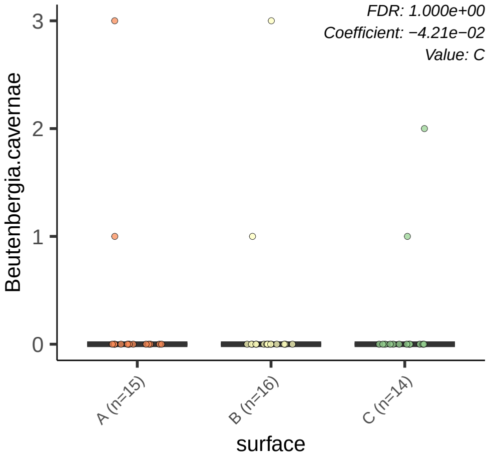
FDR: 1.000e+00
Coefficient: −4.21e−02
Value: C
Beutenbergia.cavernae
3
2
1
0
A (n=15)
B (n=16)
C (n=14)
surface

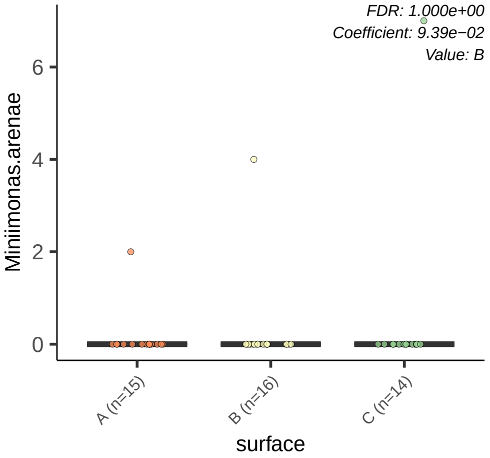

FDR: 1.000e+00
Coefficient: 9.39e−02
Value: B
Miniimonas.arenae
0
2
4
6
A (n=15)
B (n=16)
C (n=14)
surface

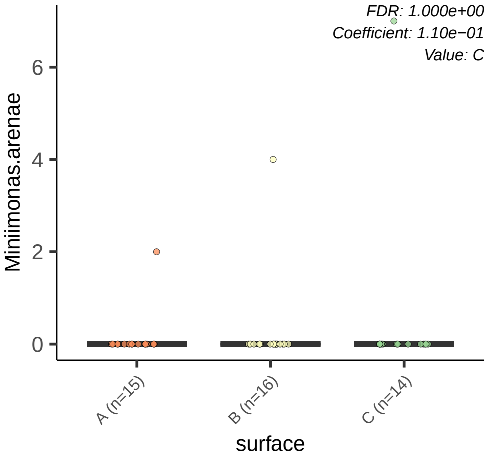

FDR: 1.000e+00
Coefficient: 1.10e−01
Value: C
Miniimonas.arenae
0
2
4
6
A (n=15)
B (n=16)
C (n=14)
surface

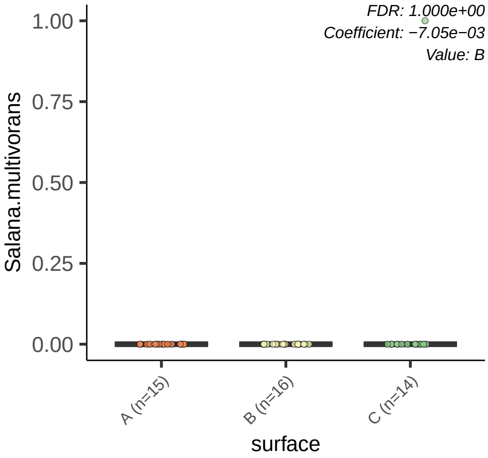

FDR: 1.000e+00
Coefficient: −7.05e−03
Value: B
Salana.multivorans
1.00
0.75
0.50
0.25
0.00
A (n=15)
B (n=16)
C (n=14)
surface

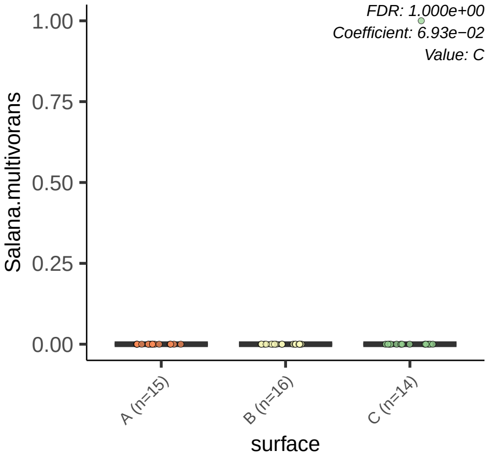
FDR: 1.000e+00
Coefficient: 6.93e−02
Value: C
Salana.multivorans
1.00
0.75
0.50
0.25
0.00
A (n=15)
B (n=16)
C (n=14)
surface

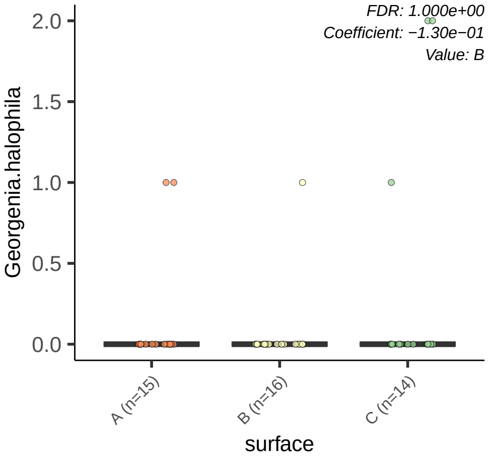

FDR: 1.000e+00
Coefficient: −1.30e−01
Value: B
Georgenia.halophila
2.0
1.5
1.0
0.5
0.0
A (n=15)
B (n=16)
C (n=14)
surface

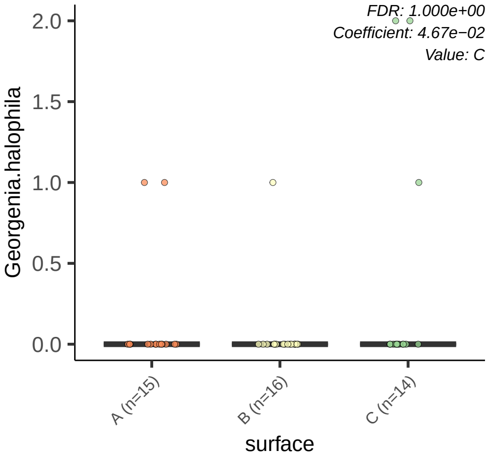
FDR: 1.000e+00
Coefficient: 4.67e−02
Value: C
Georgenia.halophila
2.0
1.5
1.0
0.5
0.0
A (n=15)
B (n=16)
C (n=14)
surface

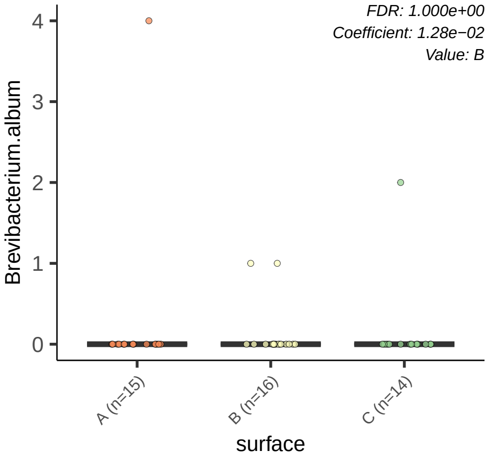

FDR: 1.000e+00
Coefficient: 1.28e−02
Value: B
Brevibacterium.album
0
1
2
3
4
A (n=15)
B (n=16)
C (n=14)
surface

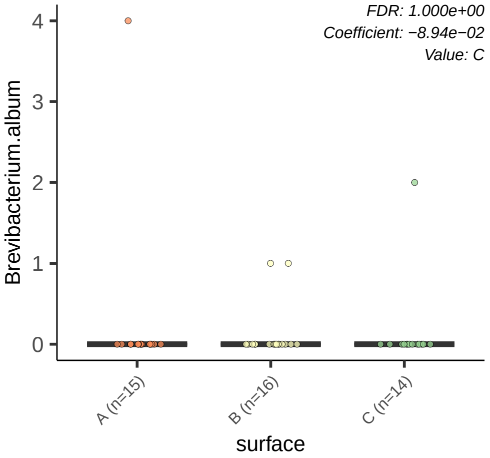

FDR: 1.000e+00
Coefficient: −8.94e−02
Value: C
Brevibacterium.album
0
1
2
3
4
A (n=15)
B (n=16)
C (n=14)
surface

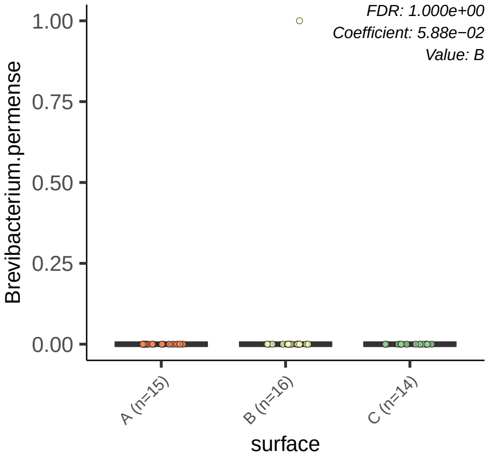
FDR: 1.000e+00
Coefficient: 5.88e−02
Value: B
Brevibacterium.permense
1.00
0.75
0.50
0.25
0.00
A (n=15)
B (n=16)
C (n=14)
surface

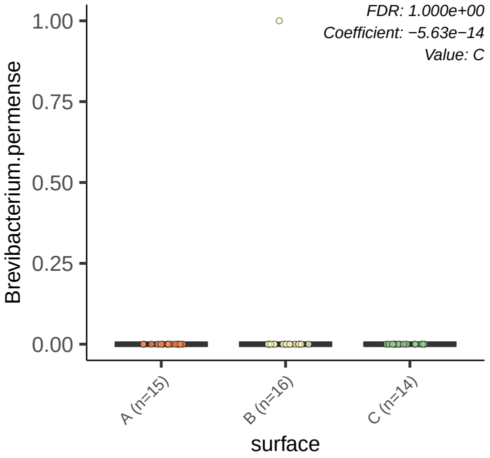
FDR: 1.000e+00
Coefficient: −5.63e−14
Value: C
Brevibacterium.permense
1.00
0.75
0.50
0.25
0.00
A (n=15)
B (n=16)
C (n=14)
surface

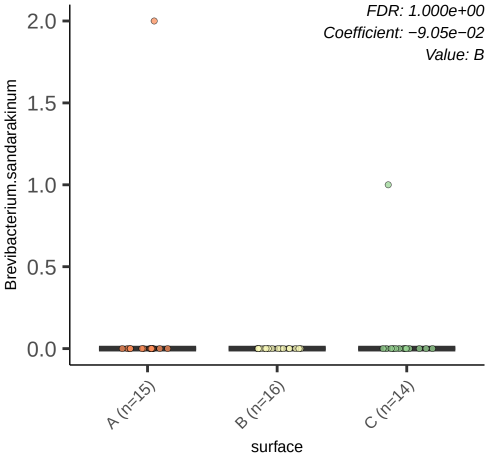
FDR: 1.000e+00
Coefficient: −9.05e−02
Value: B
Brevibacterium.sandarakinum
2.0
1.5
1.0
0.5
0.0
A (n=15)
B (n=16)
C (n=14)
surface

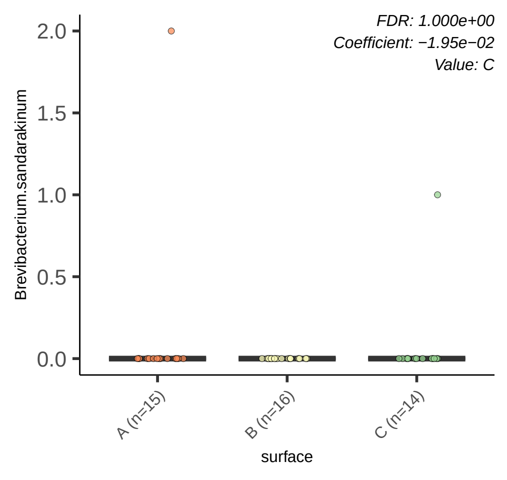

FDR: 1.000e+00
Coefficient: −1.95e−02
Value: C
Brevibacterium.sandarakinum
2.0
1.5
1.0
0.5
0.0
A (n=15)
B (n=16)
C (n=14)
surface

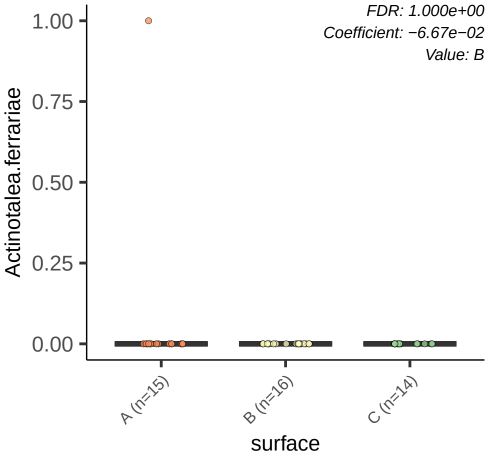
FDR: 1.000e+00
Coefficient: −6.67e−02
Value: B
Actinotalea.ferrariae
1.00
0.75
0.50
0.25
0.00
A (n=15)
B (n=16)
C (n=14)
surface

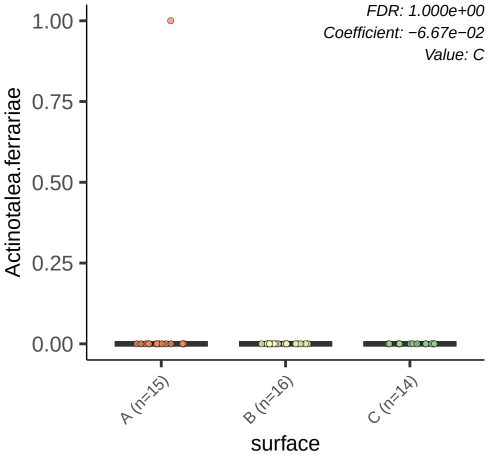
FDR: 1.000e+00
Coefficient: −6.67e−02
Value: C
Actinotalea.ferrariae
1.00
0.75
0.50
0.25
0.00
A (n=15)
B (n=16)
C (n=14)
surface

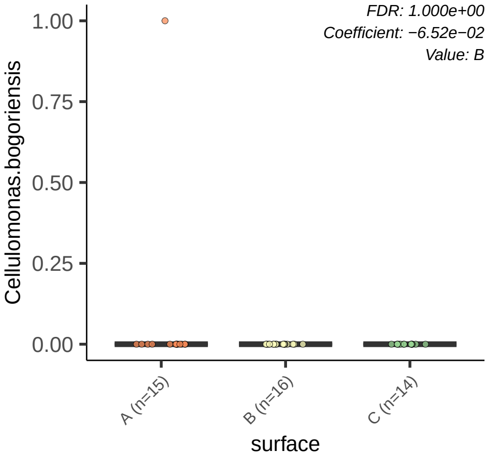
FDR: 1.000e+00
Coefficient: −6.52e−02
Value: B
Cellulomonas.bogoriensis
1.00
0.75
0.50
0.25
0.00
A (n=15)
B (n=16)
C (n=14)
surface

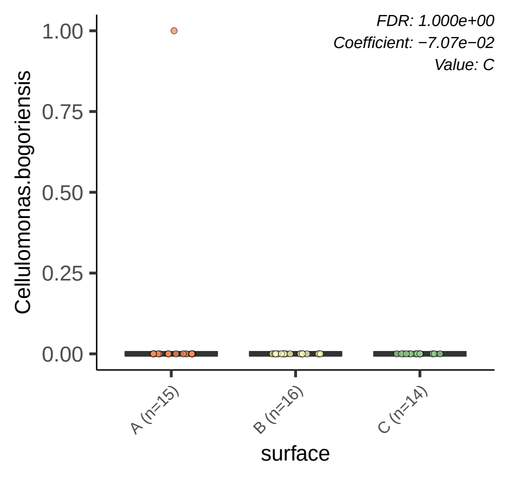

FDR: 1.000e+00
Coefficient: −7.07e−02
Value: C
Cellulomonas.bogoriensis
1.00
0.75
0.50
0.25
0.00
A (n=15)
B (n=16)
C (n=14)
surface

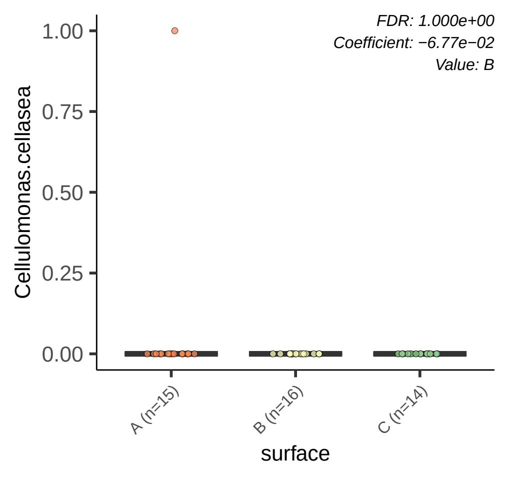
FDR: 1.000e+00
Coefficient: −6.77e−02
Value: B
Cellulomonas.cellasea
1.00
0.75
0.50
0.25
0.00
A (n=15)
B (n=16)
C (n=14)
surface

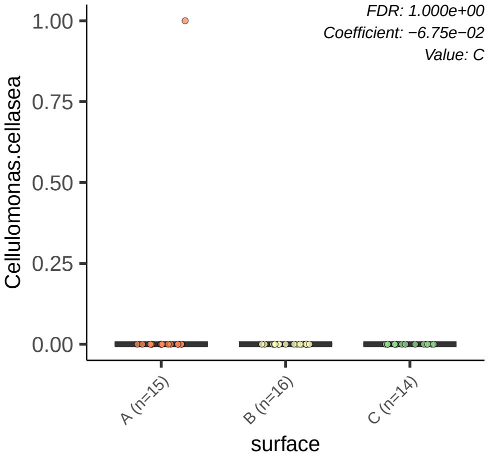

FDR: 1.000e+00
Coefficient: −6.75e−02
Value: C
Cellulomonas.cellasea
1.00
0.75
0.50
0.25
0.00
A (n=15)
B (n=16)
C (n=14)
surface

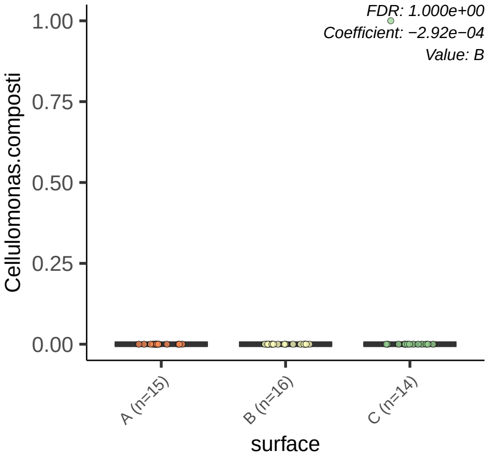

FDR: 1.000e+00
Coefficient: −2.92e−04
Value: B
Cellulomonas.composti
1.00
0.75
0.50
0.25
0.00
A (n=15)
B (n=16)
C (n=14)
surface

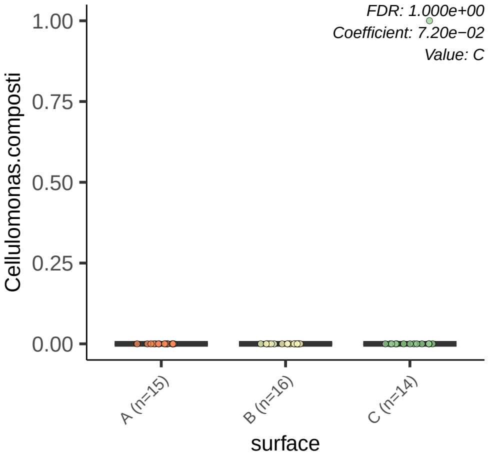
FDR: 1.000e+00
Coefficient: 7.20e−02
Value: C
Cellulomonas.composti
1.00
0.75
0.50
0.25
0.00
A (n=15)
B (n=16)
C (n=14)
surface

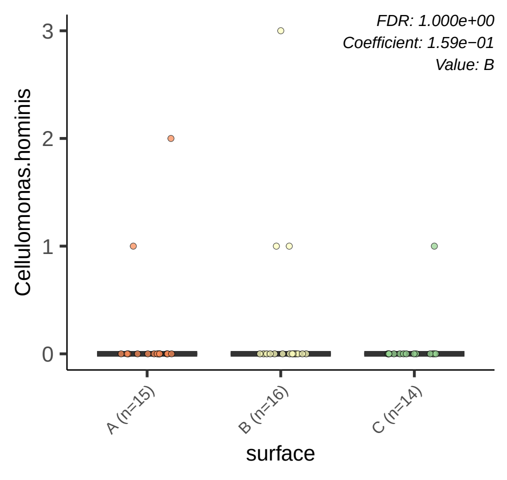

FDR: 1.000e+00
Coefficient: 1.59e−01
Value: B
Cellulomonas.hominis
0
1
2
3
A (n=15)
B (n=16)
C (n=14)
surface

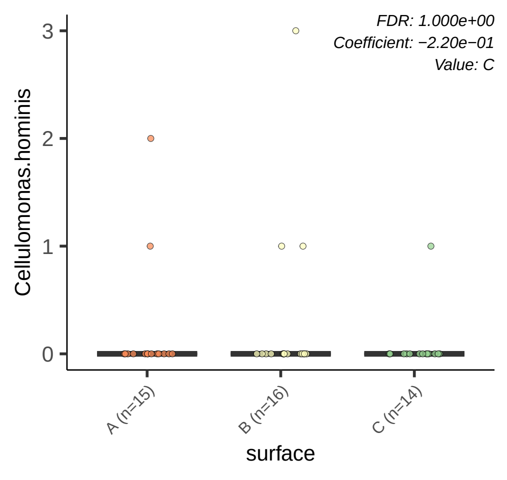
FDR: 1.000e+00
Coefficient: −2.20e−01
Value: C
Cellulomonas.hominis
0
1
2
3
A (n=15)
B (n=16)
C (n=14)
surface

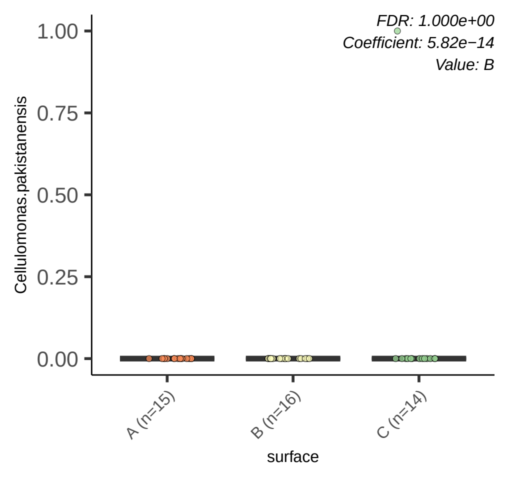
FDR: 1.000e+00
Coefficient: 5.82e−14
Value: B
1.00
0.75
0.50
0.25
0.00
Cellulomonas.pakistanensis
A (n=15)
B (n=16)
C (n=14)
surface

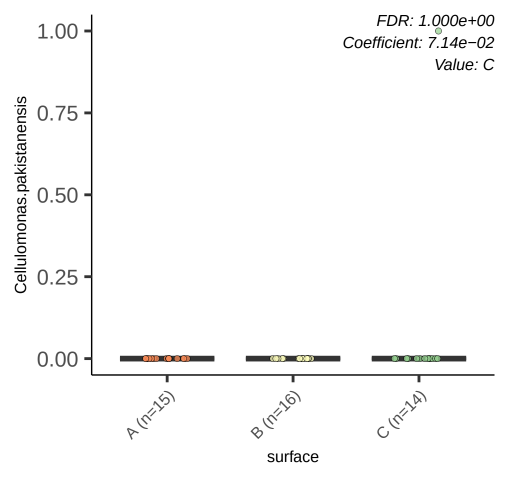
FDR: 1.000e+00
Coefficient: 7.14e−02
Value: C
1.00
0.75
0.50
0.25
0.00
Cellulomonas.pakistanensis
A (n=15)
B (n=16)
C (n=14)
surface

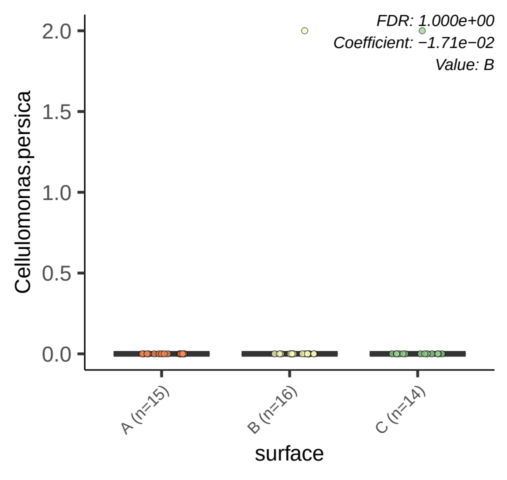
FDR: 1.000e+00
Coefficient: −1.71e−02
Value: B
Cellulomonas.persica
2.0
1.5
1.0
0.5
0.0
A (n=15)
B (n=16)
C (n=14)
surface

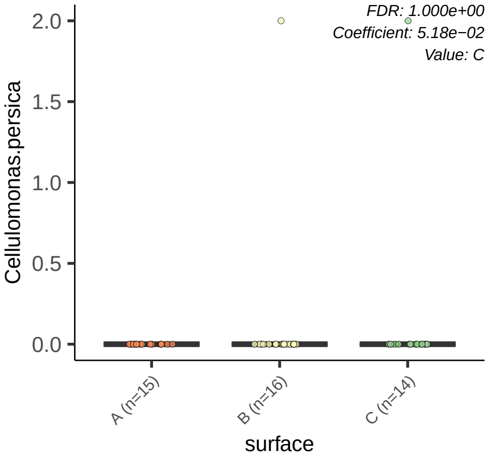
FDR: 1.000e+00
Coefficient: 5.18e−02
Value: C
Cellulomonas.persica
2.0
1.5
1.0
0.5
0.0
A (n=15)
B (n=16)
C (n=14)
surface

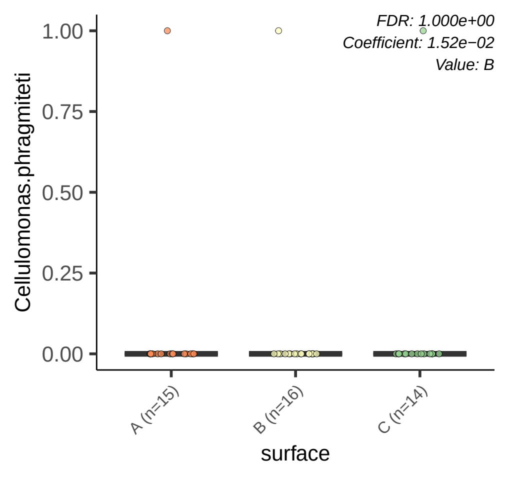

FDR: 1.000e+00
Coefficient: 1.52e−02
Value: B
Cellulomonas.phragmiteti
1.00
0.75
0.50
0.25
0.00
A (n=15)
B (n=16)
C (n=14)
surface

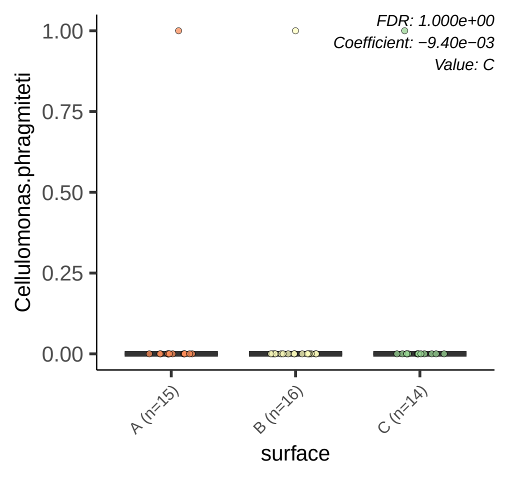
FDR: 1.000e+00
Coefficient: −9.40e−03
Value: C
Cellulomonas.phragmiteti
1.00
0.75
0.50
0.25
0.00
A (n=15)
B (n=16)
C (n=14)
surface

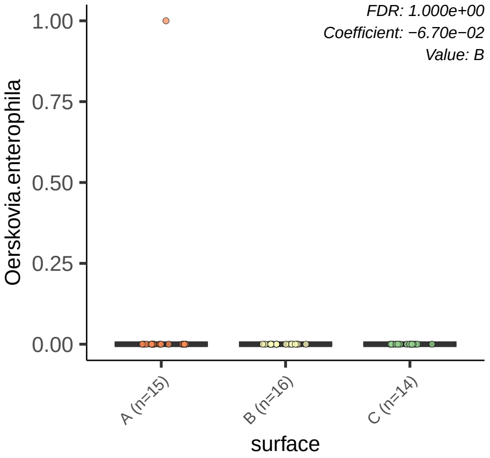
FDR: 1.000e+00
Coefficient: −6.70e−02
Value: B
Oerskovia.enterophila
1.00
0.75
0.50
0.25
0.00
A (n=15)
B (n=16)
C (n=14)
surface

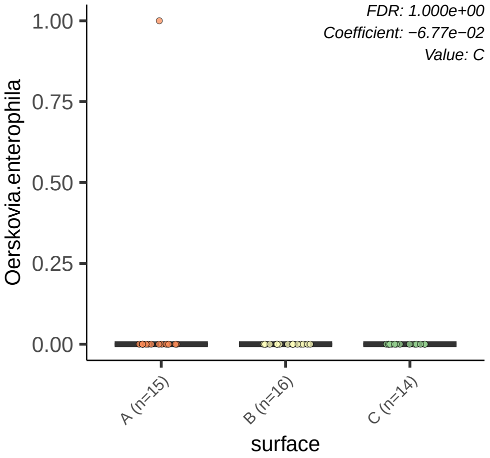

FDR: 1.000e+00
Coefficient: −6.77e−02
Value: C
Oerskovia.enterophila
1.00
0.75
0.50
0.25
0.00
A (n=15)
B (n=16)
C (n=14)
surface

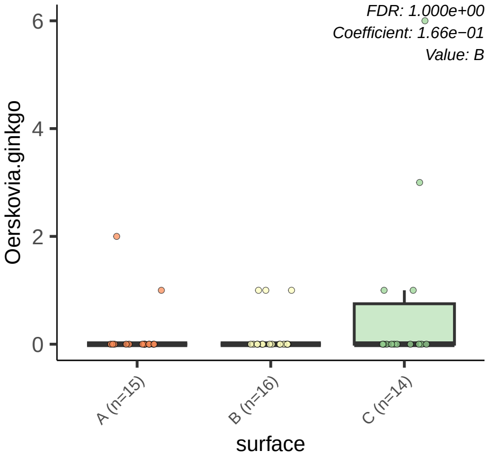

FDR: 1.000e+00
Coefficient: 1.66e−01
Value: B
Oerskovia.ginkgo
0
2
4
6
A (n=15)
B (n=16)
C (n=14)
surface

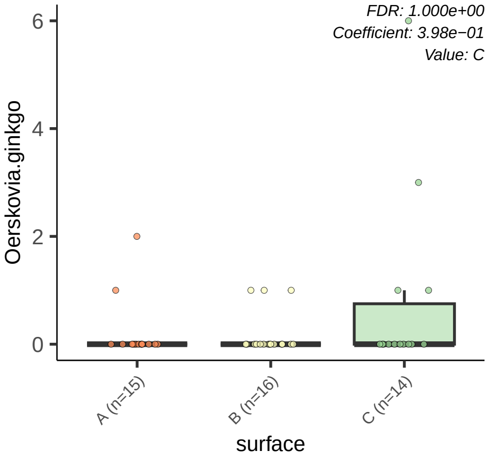

FDR: 1.000e+00
Coefficient: 3.98e−01
Value: C
Oerskovia.ginkgo
0
2
4
6
A (n=15)
B (n=16)
C (n=14)
surface

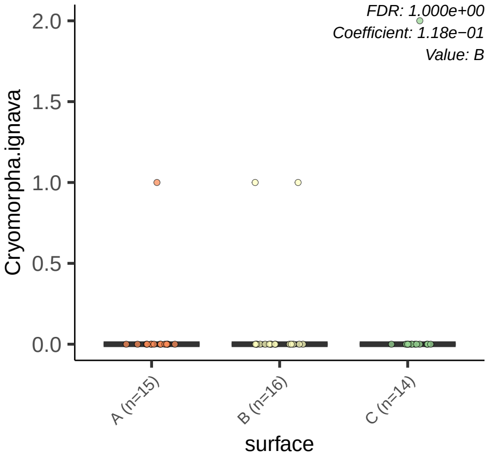

FDR: 1.000e+00
Coefficient: 1.18e−01
Value: B
Cryomorpha.ignava
2.0
1.5
1.0
0.5
0.0
A (n=15)
B (n=16)
C (n=14)
surface

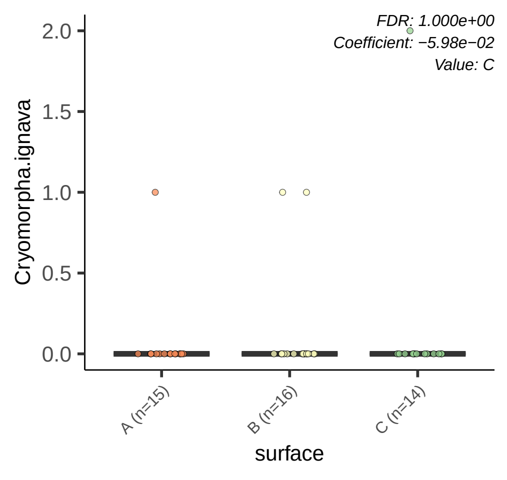

FDR: 1.000e+00
Coefficient: −5.98e−02
Value: C
Cryomorpha.ignava
2.0
1.5
1.0
0.5
0.0
A (n=15)
B (n=16)
C (n=14)
surface

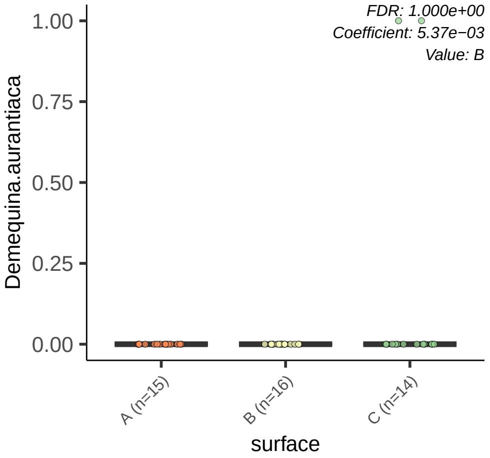
FDR: 1.000e+00
Coefficient: 5.37e−03
Value: B
1.00
0.75
0.50
0.25
0.00
Demequina.aurantiaca
A (n=15)
B (n=16)
C (n=14)
surface

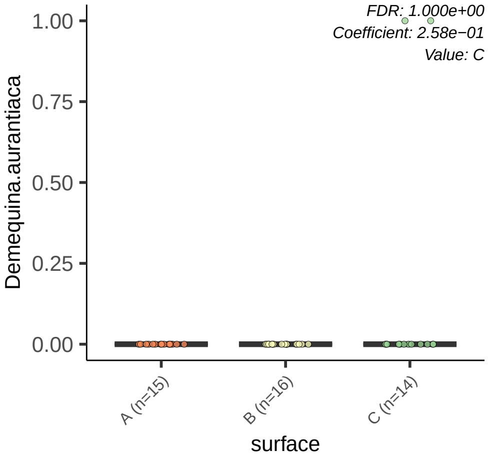
FDR: 1.000e+00
Coefficient: 2.58e−01
Value: C
Demequina.aurantiaca
1.00
0.75
0.50
0.25
0.00
A (n=15)
B (n=16)
C (n=14)
surface

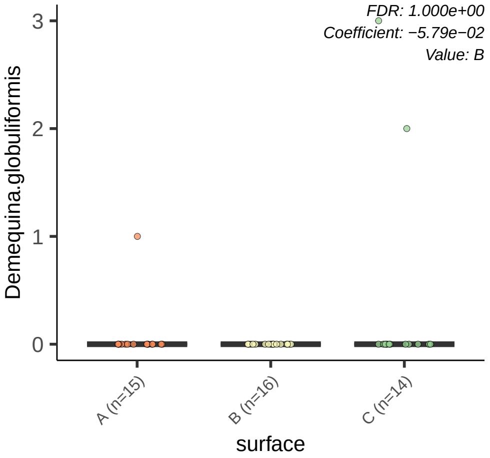
FDR: 1.000e+00
Coefficient: −5.79e−02
Value: B
Demequina.globuliformis
3
2
1
0
A (n=15)
B (n=16)
C (n=14)
surface

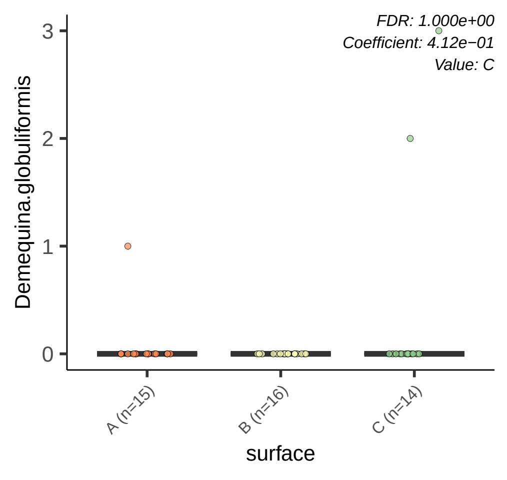

FDR: 1.000e+00
Coefficient: 4.12e−01
Value: C
Demequina.globuliformis
3
2
1
0
A (n=15)
B (n=16)
C (n=14)
surface

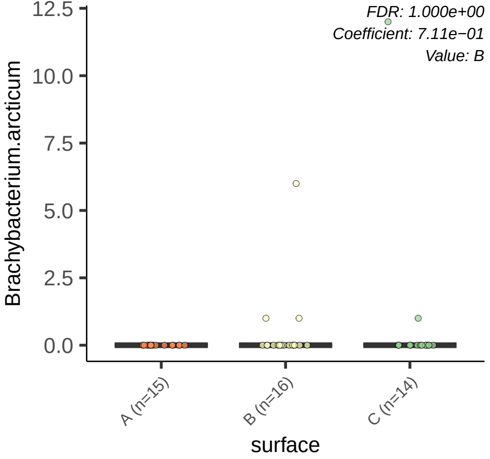

FDR: 1.000e+00
Coefficient: 7.11e−01
Value: B
Brachybacterium.arcticum
12.5
10.0
7.5
5.0
2.5
0.0
A (n=15)
B (n=16)
C (n=14)
surface

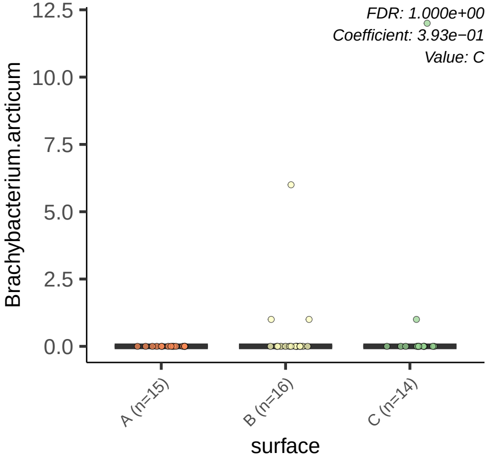
FDR: 1.000e+00
Coefficient: 3.93e−01
Value: C
Brachybacterium.arcticum
12.5
10.0
7.5
5.0
2.5
0.0
A (n=15)
B (n=16)
C (n=14)
surface

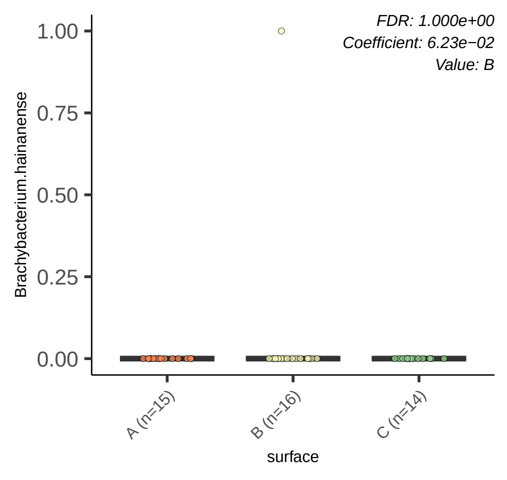
FDR: 1.000e+00
Coefficient: 6.23e−02
Value: B
Brachybacterium.hainanense
1.00
0.75
0.50
0.25
0.00
A (n=15)
B (n=16)
C (n=14)
surface

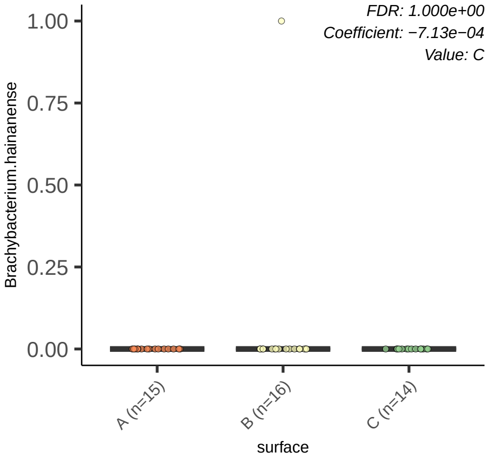

FDR: 1.000e+00
Coefficient: −7.13e−04
Value: C
Brachybacterium.hainanense
1.00
0.75
0.50
0.25
0.00
A (n=15)
B (n=16)
C (n=14)
surface

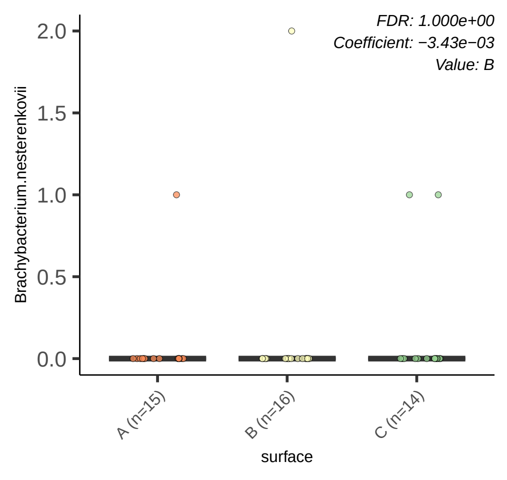

FDR: 1.000e+00
Coefficient: −3.43e−03
Value: B
2.0
1.5
1.0
0.5
0.0
Brachybacterium.nesterenkovii
A (n=15)
B (n=16)
C (n=14)
surface

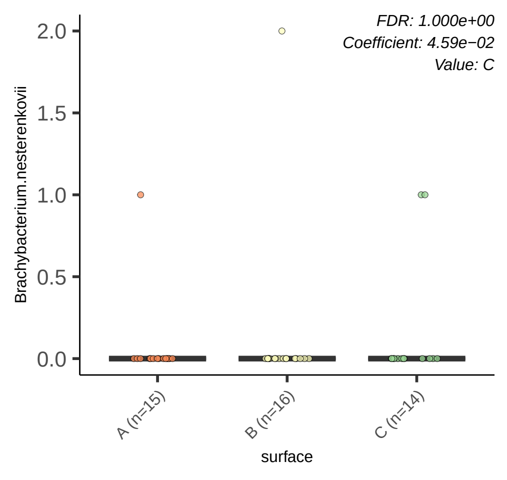
FDR: 1.000e+00
Coefficient: 4.59e−02
Value: C
Brachybacterium.nesterenkovii
2.0
1.5
1.0
0.5
0.0
A (n=15)
B (n=16)
C (n=14)
surface

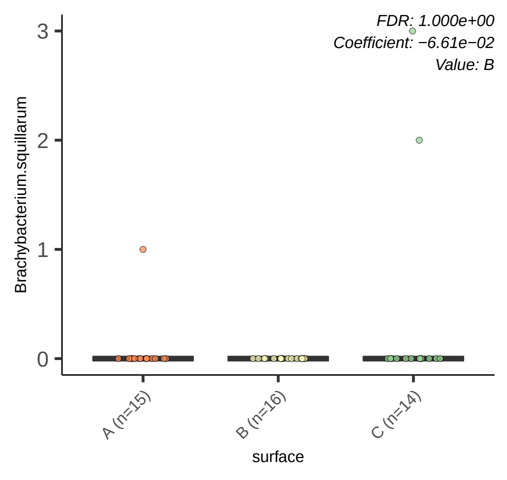

FDR: 1.000e+00
Coefficient: −6.61e−02
Value: B
Brachybacterium.squillarum
3
2
1
0
A (n=15)
B (n=16)
C (n=14)
surface

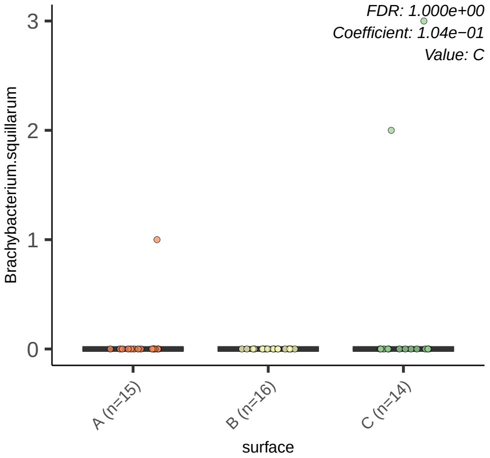
FDR: 1.000e+00
Coefficient: 1.04e−01
Value: C
Brachybacterium.squillarum
3
2
1
0
A (n=15)
B (n=16)
C (n=14)
surface

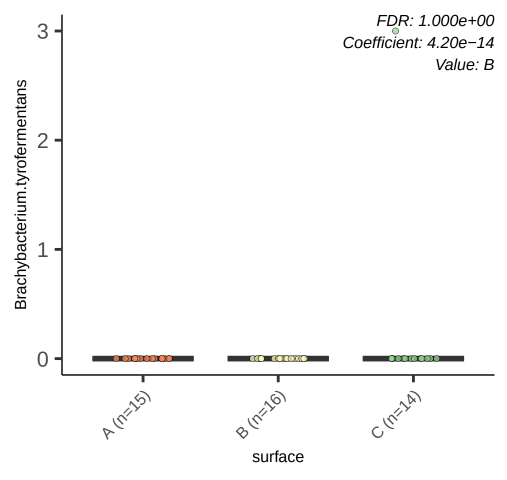
FDR: 1.000e+00
Coefficient: 4.20e−14
Value: B
Brachybacterium.tyrofermentans
3
2
1
0
A (n=15)
B (n=16)
C (n=14)
surface

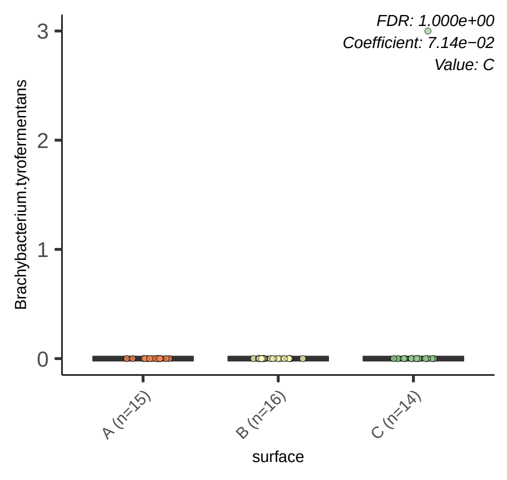
FDR: 1.000e+00
Coefficient: 7.14e−02
Value: C
Brachybacterium.tyrofermentans
3
2
1
0
A (n=15)
B (n=16)
C (n=14)
surface

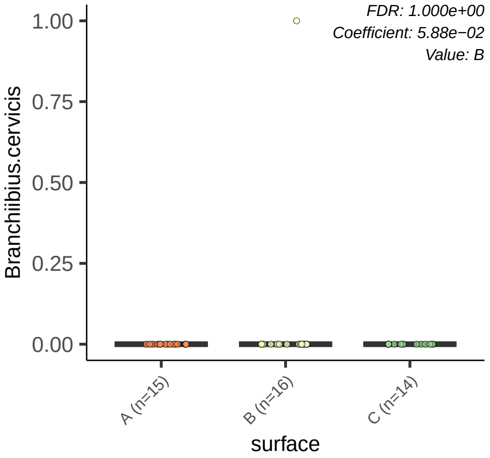

FDR: 1.000e+00
Coefficient: 5.88e−02
Value: B
1.00
0.75
0.50
0.25
0.00
Branchiibius.cervicis
A (n=15)
B (n=16)
C (n=14)
surface

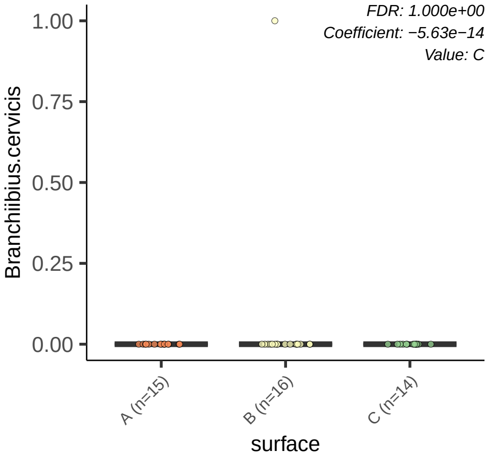
FDR: 1.000e+00
Coefficient: −5.63e−14
Value: C
1.00
0.75
0.50
0.25
0.00
Branchiibius.cervicis
A (n=15)
B (n=16)
C (n=14)
surface

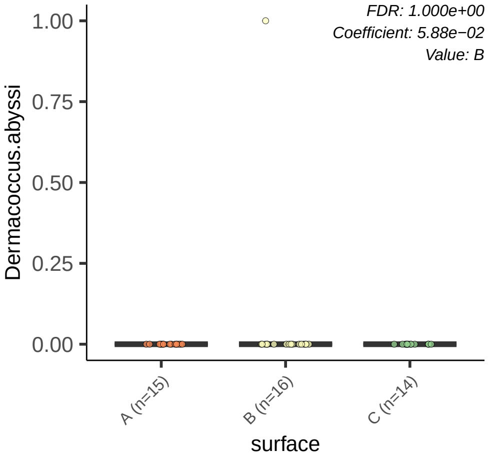
FDR: 1.000e+00
Coefficient: 5.88e−02
Value: B
Dermacoccus.abyssi
1.00
0.75
0.50
0.25
0.00
A (n=15)
B (n=16)
C (n=14)
surface

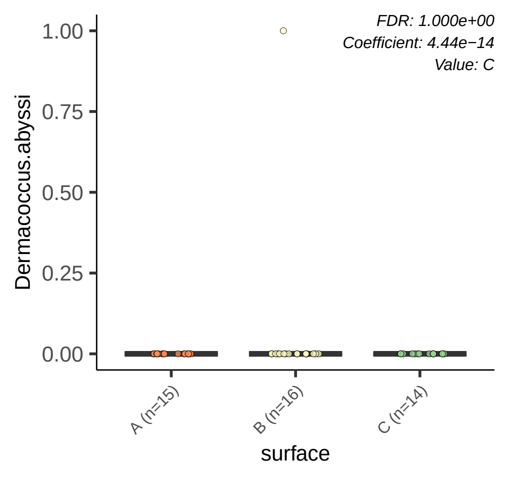

FDR: 1.000e+00
Coefficient: 4.44e−14
Value: C
Dermacoccus.abyssi
1.00
0.75
0.50
0.25
0.00
A (n=15)
B (n=16)
C (n=14)
surface

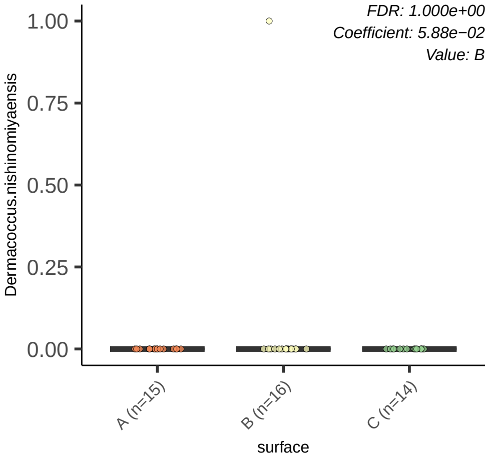
FDR: 1.000e+00
Coefficient: 5.88e−02
Value: B
1.00
0.75
0.50
0.25
0.00
Dermacoccus.nishinomiyaensis
A (n=15)
B (n=16)
C (n=14)
surface

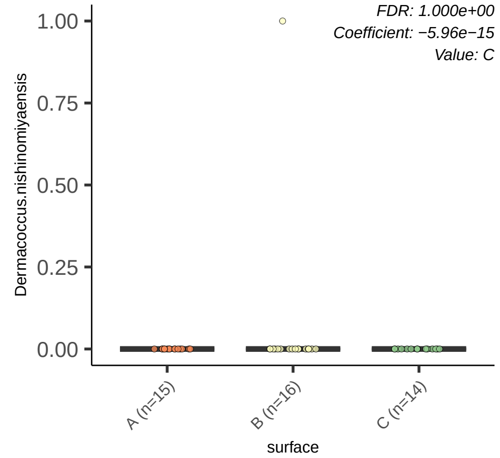

FDR: 1.000e+00
Coefficient: −5.96e−15
Value: C
Dermacoccus.nishinomiyaensis
1.00
0.75
0.50
0.25
0.00
A (n=15)
B (n=16)
C (n=14)
surface

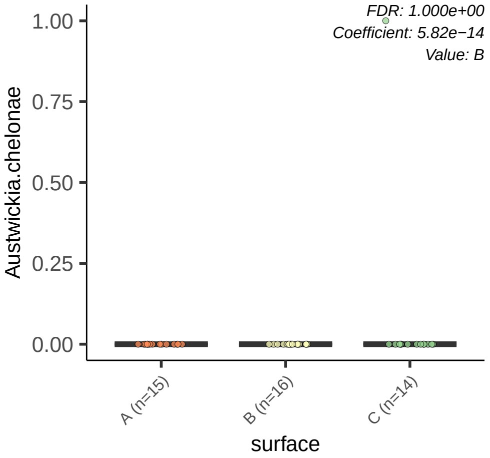

FDR: 1.000e+00
Coefficient: 5.82e−14
Value: B
Austwickia.chelonae
1.00
0.75
0.50
0.25
0.00
A (n=15)
B (n=16)
C (n=14)
surface

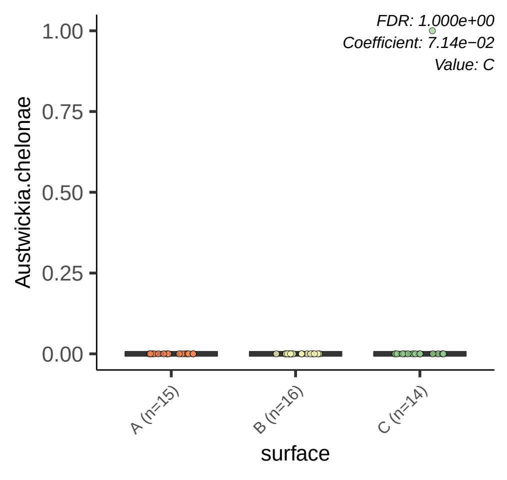
FDR: 1.000e+00
Coefficient: 7.14e−02
Value: C
1.00
0.75
0.50
0.25
0.00
Austwickia.chelonae
A (n=15)
B (n=16)
C (n=14)
surface

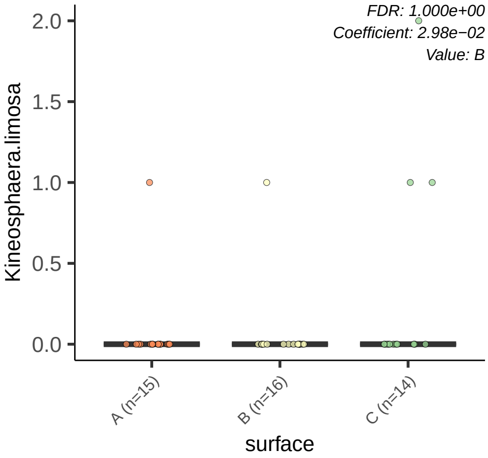

FDR: 1.000e+00
Coefficient: 2.98e−02
Value: B
Kineosphaera.limosa
2.0
1.5
1.0
0.5
0.0
A (n=15)
B (n=16)
C (n=14)
surface

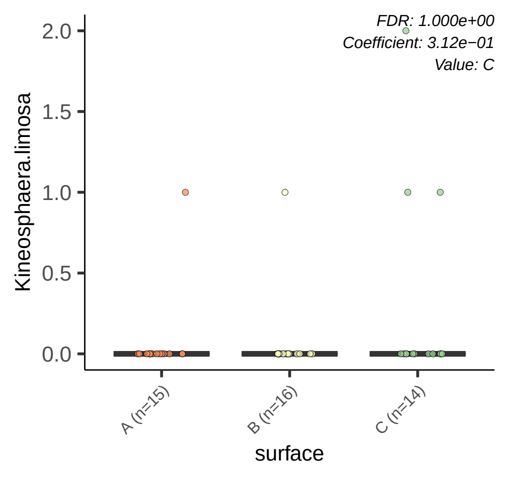
FDR: 1.000e+00
Coefficient: 3.12e−01
Value: C
Kineosphaera.limosa
2.0
1.5
1.0
0.5
0.0
A (n=15)
B (n=16)
C (n=14)
surface

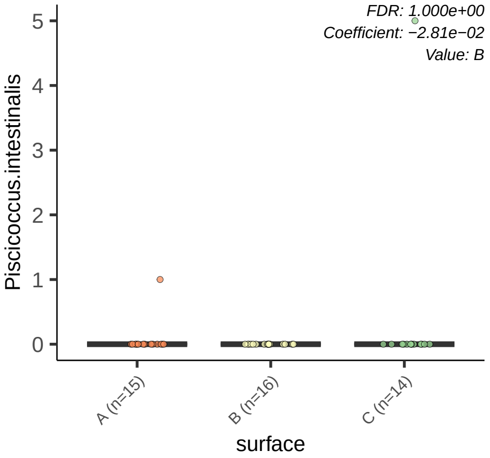
FDR: 1.000e+00
Coefficient: −2.81e−02
Value: B
Piscicoccus.intestinalis
0
1
2
3
4
5
A (n=15)
B (n=16)
C (n=14)
surface

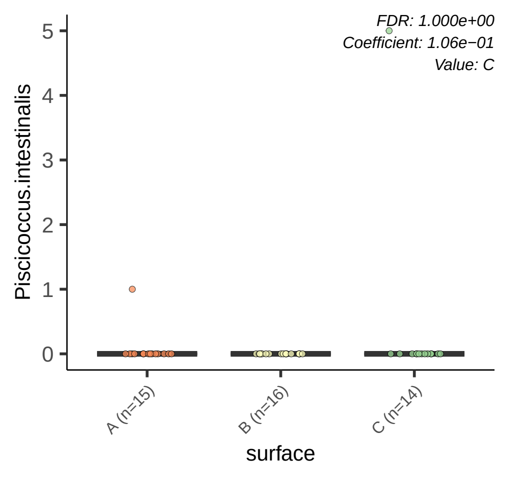

FDR: 1.000e+00
Coefficient: 1.06e−01
Value: C
Piscicoccus.intestinalis
0
1
2
3
4
5
A (n=15)
B (n=16)
C (n=14)
surface

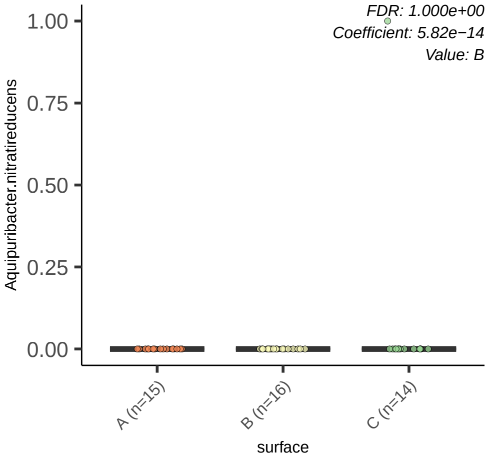
FDR: 1.000e+00
Coefficient: 5.82e−14
Value: B
1.00
0.75
0.50
0.25
0.00
Aquipuribacter.nitratireducens
A (n=15)
B (n=16)
C (n=14)
surface

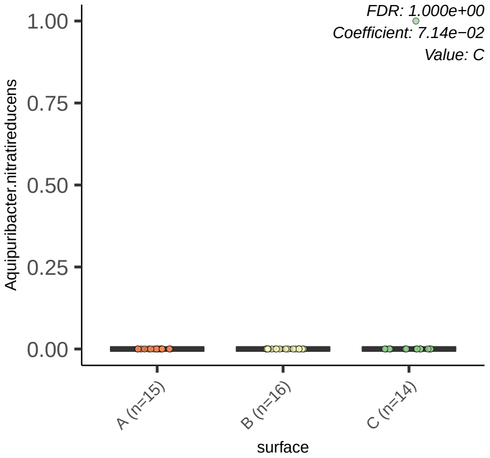

FDR: 1.000e+00
Coefficient: 7.14e−02
Value: C
Aquipuribacter.nitratireducens
1.00
0.75
0.50
0.25
0.00
A (n=15)
B (n=16)
C (n=14)
surface

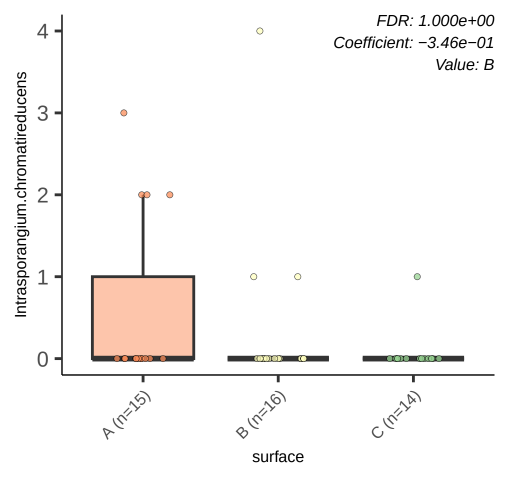
FDR: 1.000e+00
Coefficient: −3.46e−01
Value: B
Intrasporangium.chromatireducens
0
1
2
3
4
A (n=15)
B (n=16)
C (n=14)
surface

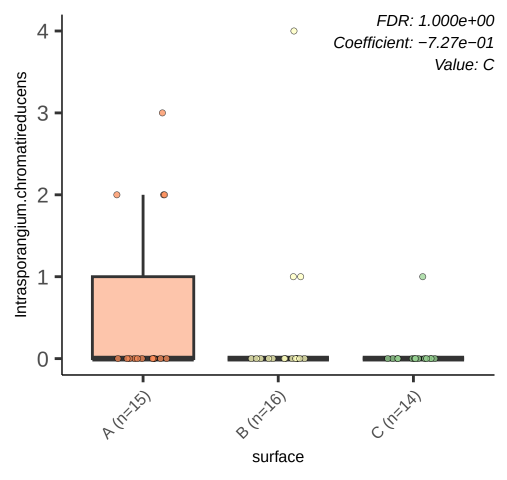

FDR: 1.000e+00
Coefficient: −7.27e−01
Value: C
Intrasporangium.chromatireducens
0
1
2
3
4
A (n=15)
B (n=16)
C (n=14)
surface

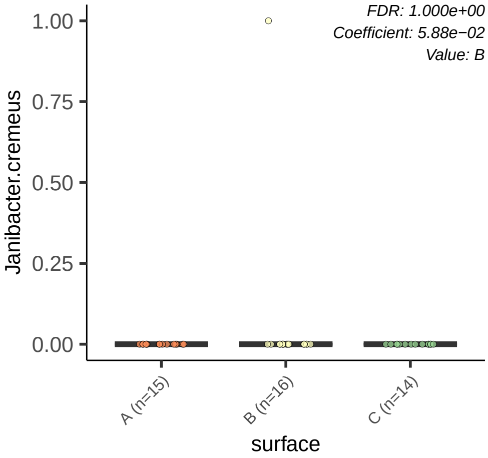

FDR: 1.000e+00
Coefficient: 5.88e−02
Value: B
Janibacter.cremeus
1.00
0.75
0.50
0.25
0.00
A (n=15)
B (n=16)
C (n=14)
surface

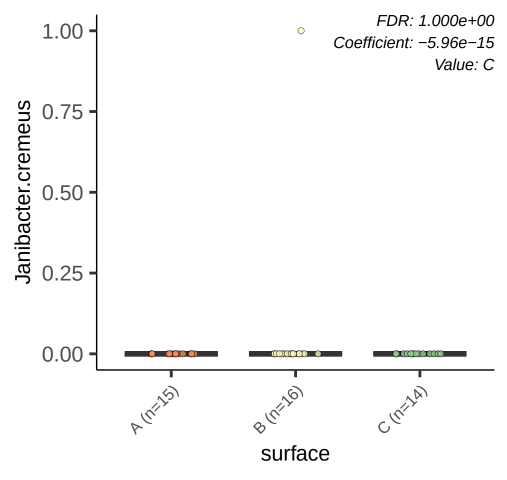
FDR: 1.000e+00
Coefficient: −5.96e−15
Value: C
Janibacter.cremeus
1.00
0.75
0.50
0.25
0.00
A (n=15)
B (n=16)
C (n=14)
surface

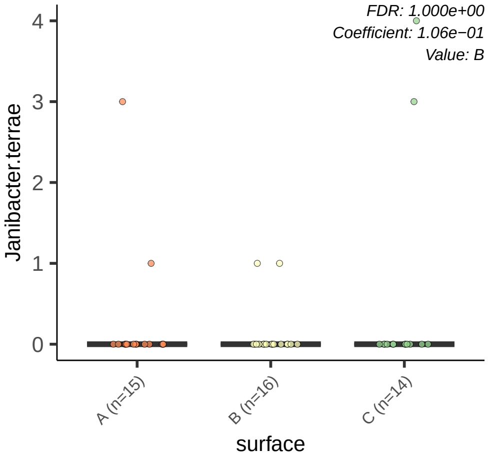

FDR: 1.000e+00
Coefficient: 1.06e−01
Value: B
Janibacter.terrae
0
1
2
3
4
A (n=15)
B (n=16)
C (n=14)
surface

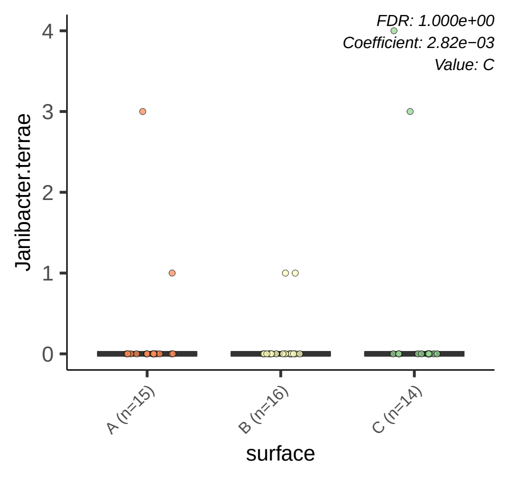
FDR: 1.000e+00
Coefficient: 2.82e−03
Value: C
Janibacter.terrae
4
3
2
1
0
A (n=15)
B (n=16)
C (n=14)
surface

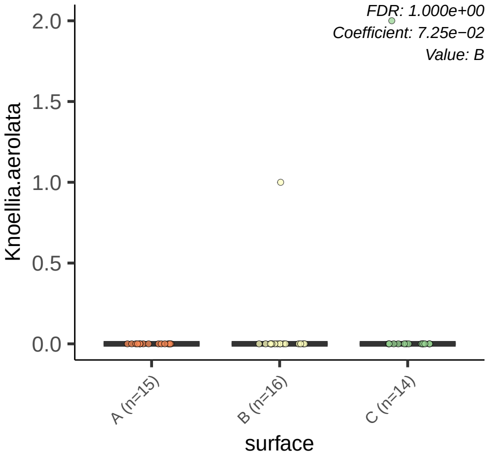

FDR: 1.000e+00
Coefficient: 7.25e−02
Value: B
Knoellia.aerolata
2.0
1.5
1.0
0.5
0.0
A (n=15)
B (n=16)
C (n=14)
surface

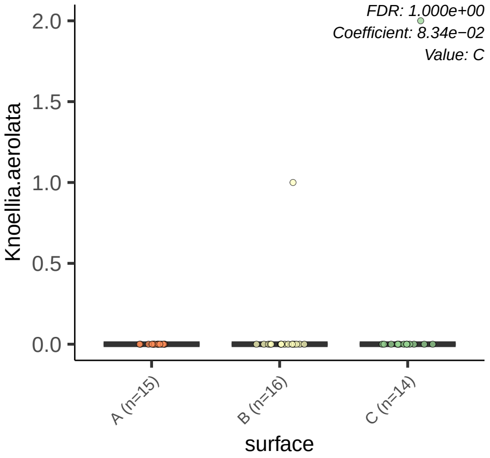

FDR: 1.000e+00
Coefficient: 8.34e−02
Value: C
Knoellia.aerolata
2.0
1.5
1.0
0.5
0.0
A (n=15)
B (n=16)
C (n=14)
surface

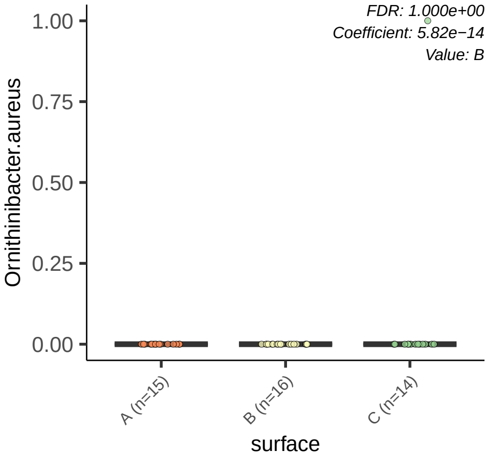

FDR: 1.000e+00
Coefficient: 5.82e−14
Value: B
Ornithinibacter.aureus
1.00
0.75
0.50
0.25
0.00
A (n=15)
B (n=16)
C (n=14)
surface

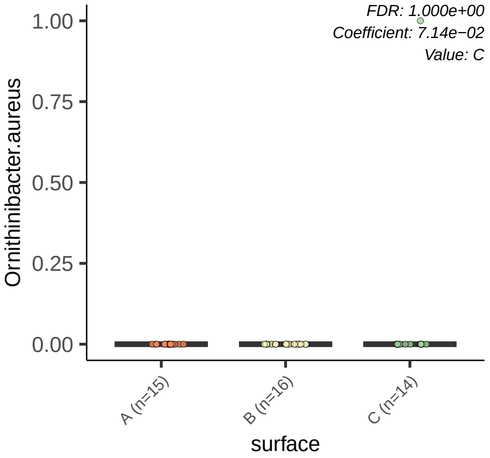
FDR: 1.000e+00
Coefficient: 7.14e−02
Value: C
Ornithinibacter.aureus
1.00
0.75
0.50
0.25
0.00
A (n=15)
B (n=16)
C (n=14)
surface

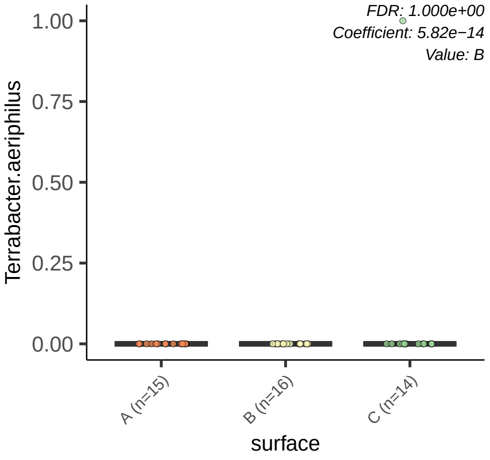
FDR: 1.000e+00
Coefficient: 5.82e−14
Value: B
Terrabacter.aeriphilus
1.00
0.75
0.50
0.25
0.00
A (n=15)
B (n=16)
C (n=14)
surface

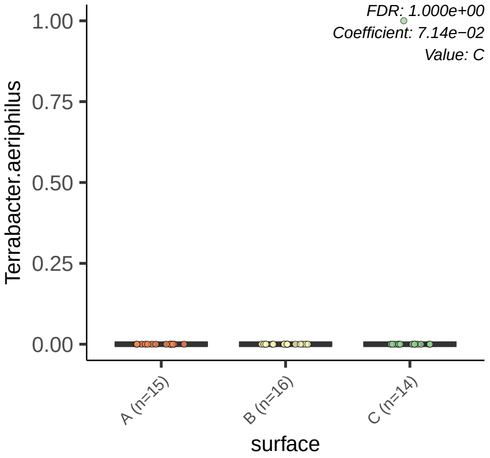

FDR: 1.000e+00
Coefficient: 7.14e−02
Value: C
1.00
0.75
0.50
0.25
0.00
Terrabacter.aeriphilus
A (n=15)
B (n=16)
C (n=14)
surface

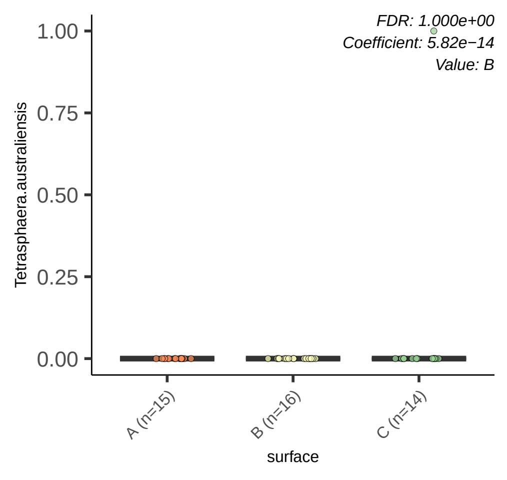
FDR: 1.000e+00
Coefficient: 5.82e−14
Value: B
Tetrasphaera.australiensis
1.00
0.75
0.50
0.25
0.00
A (n=15)
B (n=16)
C (n=14)
surface

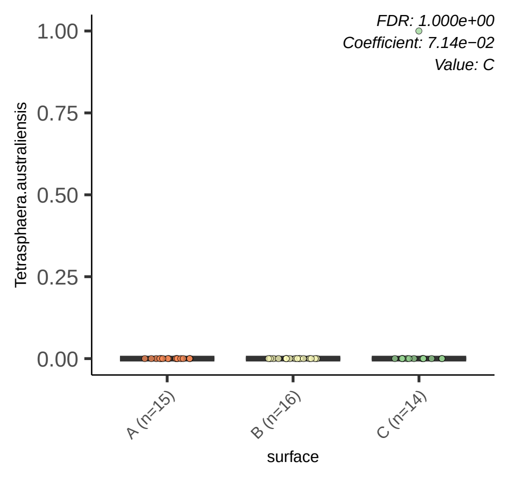
FDR: 1.000e+00
Coefficient: 7.14e−02
Value: C
1.00
0.75
0.50
0.25
0.00
Tetrasphaera.australiensis
A (n=15)
B (n=16)
C (n=14)
surface

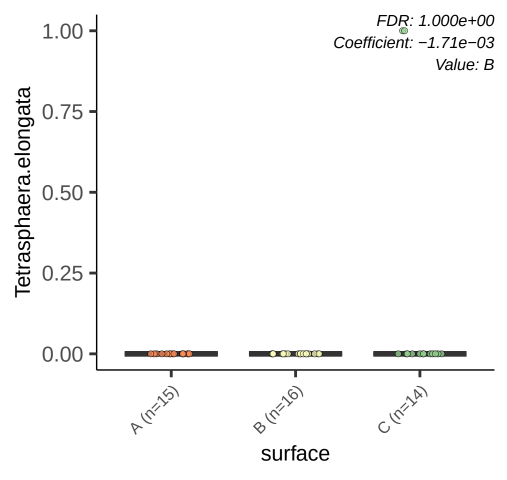

FDR: 1.000e+00
Coefficient: −1.71e−03
Value: B
Tetrasphaera.elongata
1.00
0.75
0.50
0.25
0.00
A (n=15)
B (n=16)
C (n=14)
surface

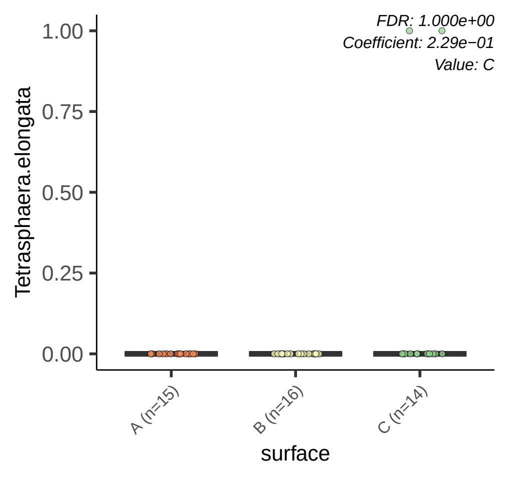

FDR: 1.000e+00
Coefficient: 2.29e−01
Value: C
1.00
0.75
0.50
0.25
0.00
Tetrasphaera.elongata
A (n=15)
B (n=16)
C (n=14)
surface

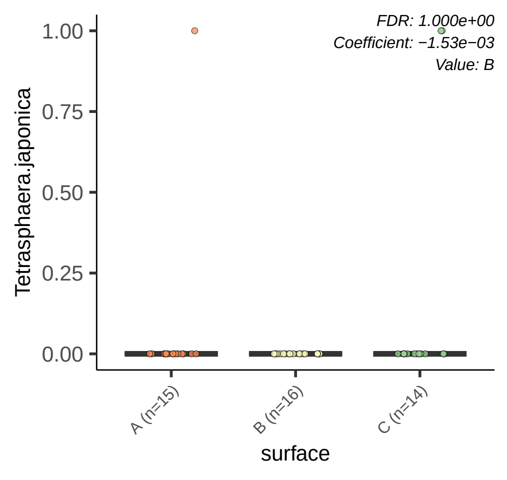
FDR: 1.000e+00
Coefficient: −1.53e−03
Value: B
Tetrasphaera.japonica
1.00
0.75
0.50
0.25
0.00
A (n=15)
B (n=16)
C (n=14)
surface

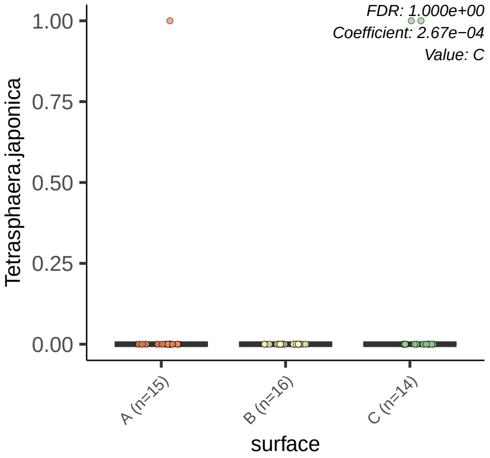
FDR: 1.000e+00
Coefficient: 2.67e−04
Value: C
Tetrasphaera.japonica
1.00
0.75
0.50
0.25
0.00
A (n=15)
B (n=16)
C (n=14)
surface

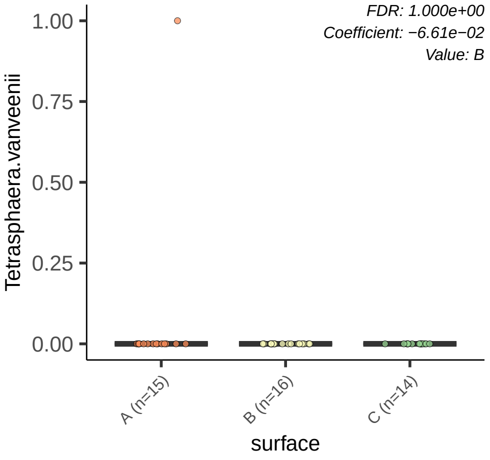

FDR: 1.000e+00
Coefficient: −6.61e−02
Value: B
Tetrasphaera.vanveenii
1.00
0.75
0.50
0.25
0.00
A (n=15)
B (n=16)
C (n=14)
surface

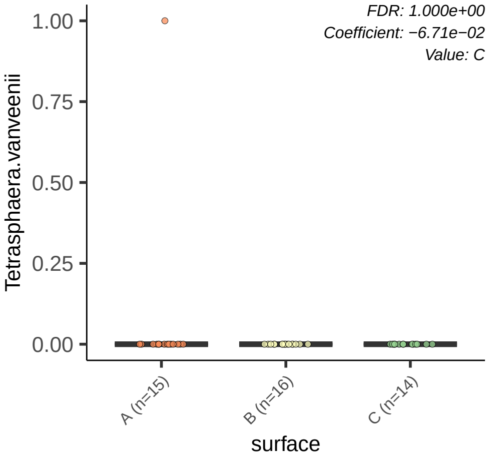
FDR: 1.000e+00
Coefficient: −6.71e−02
Value: C
Tetrasphaera.vanveenii
1.00
0.75
0.50
0.25
0.00
A (n=15)
B (n=16)
C (n=14)
surface

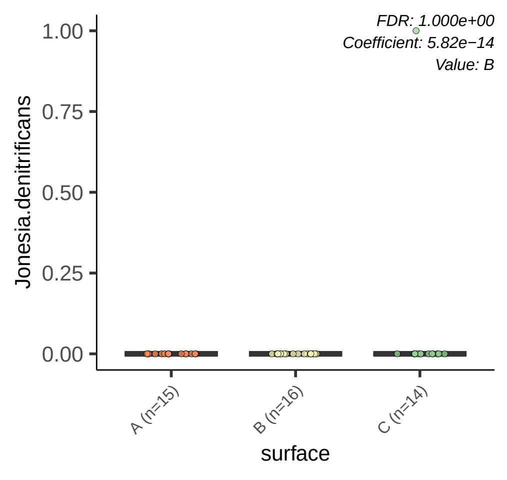
FDR: 1.000e+00
Coefficient: 5.82e−14
Value: B
Jonesia.denitrificans
1.00
0.75
0.50
0.25
0.00
A (n=15)
B (n=16)
C (n=14)
surface

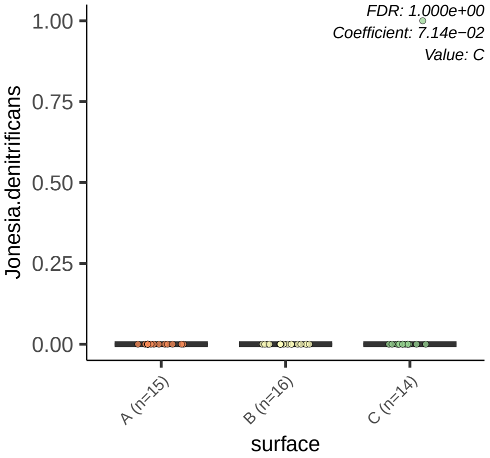
FDR: 1.000e+00
Coefficient: 7.14e−02
Value: C
Jonesia.denitrificans
1.00
0.75
0.50
0.25
0.00
A (n=15)
B (n=16)
C (n=14)
surface

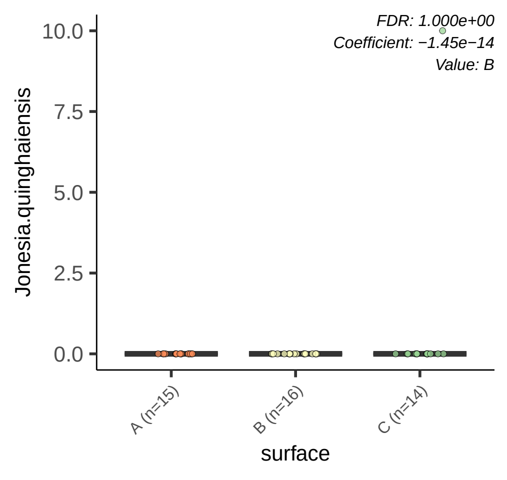

FDR: 1.000e+00
Coefficient: −1.45e−14
Value: B
Jonesia.quinghaiensis
10.0
7.5
5.0
2.5
0.0
A (n=15)
B (n=16)
C (n=14)
surface

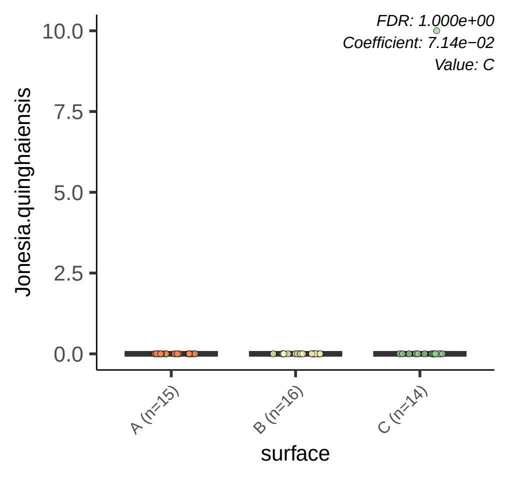

FDR: 1.000e+00
Coefficient: 7.14e−02
Value: C
10.0
7.5
5.0
2.5
0.0
Jonesia.quinghaiensis
A (n=15)
B (n=16)
C (n=14)
surface

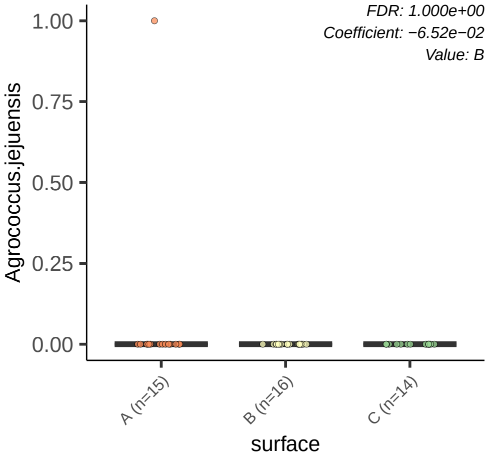
FDR: 1.000e+00
Coefficient: −6.52e−02
Value: B
1.00
0.75
0.50
0.25
0.00
Agrococcus.jejuensis
A (n=15)
B (n=16)
C (n=14)
surface

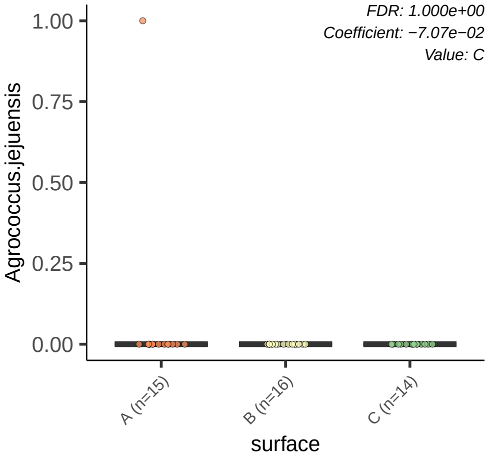
FDR: 1.000e+00
Coefficient: −7.07e−02
Value: C
Agrococcus.jejuensis
1.00
0.75
0.50
0.25
0.00
A (n=15)
B (n=16)
C (n=14)
surface

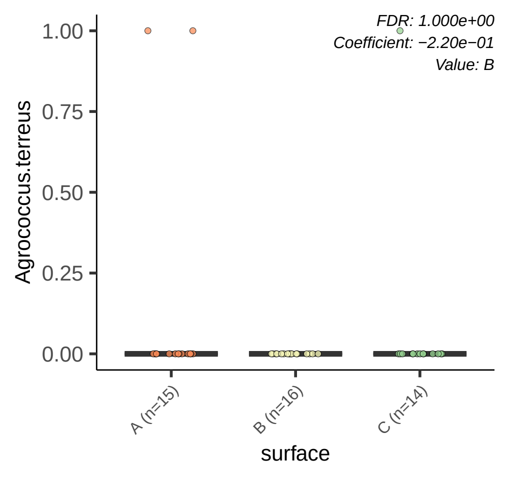
FDR: 1.000e+00
Coefficient: −2.20e−01
Value: B
1.00
0.75
0.50
0.25
0.00
Agrococcus.terreus
A (n=15)
B (n=16)
C (n=14)
surface

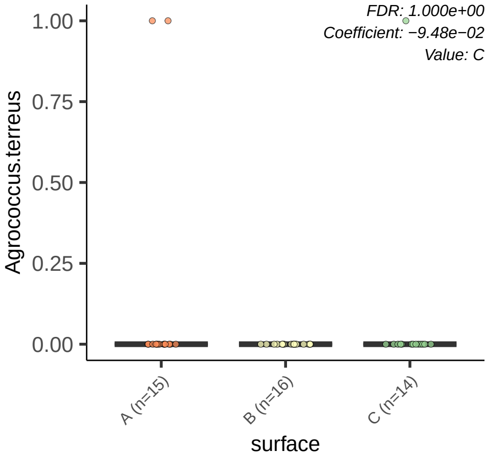
FDR: 1.000e+00
Coefficient: −9.48e−02
Value: C
1.00
0.75
0.50
0.25
0.00
Agrococcus.terreus
A (n=15)
B (n=16)
C (n=14)
surface

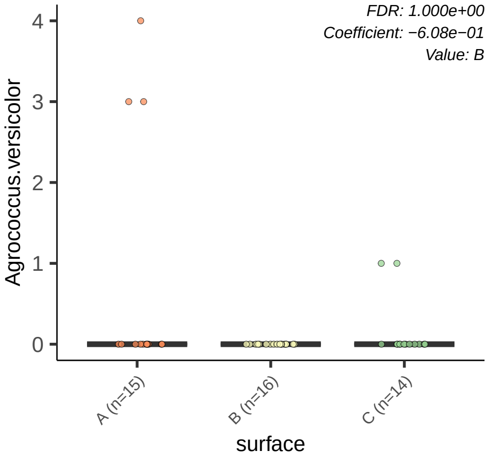

FDR: 1.000e+00
Coefficient: −6.08e−01
Value: B
Agrococcus.versicolor
4
3
2
1
0
A (n=15)
B (n=16)
C (n=14)
surface

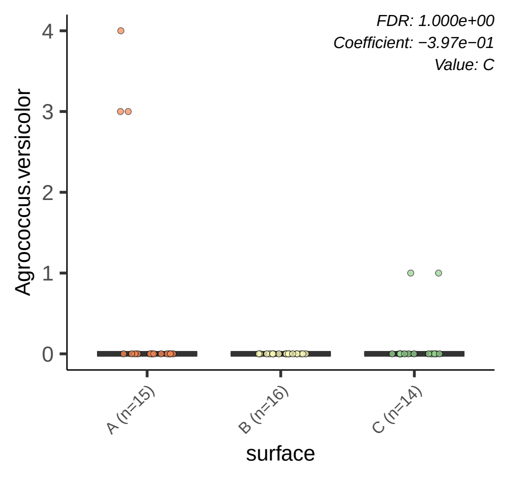
FDR: 1.000e+00
Coefficient: −3.97e−01
Value: C
Agrococcus.versicolor
4
3
2
1
0
A (n=15)
B (n=16)
C (n=14)
surface

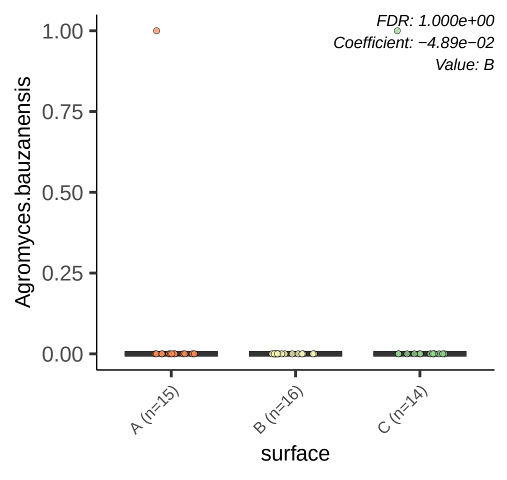

FDR: 1.000e+00
Coefficient: −4.89e−02
Value: B
Agromyces.bauzanensis
1.00
0.75
0.50
0.25
0.00
A (n=15)
B (n=16)
C (n=14)
surface

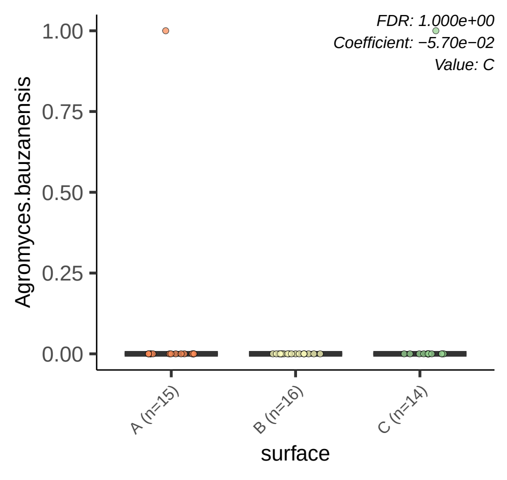
FDR: 1.000e+00
Coefficient: −5.70e−02
Value: C
Agromyces.bauzanensis
1.00
0.75
0.50
0.25
0.00
A (n=15)
B (n=16)
C (n=14)
surface

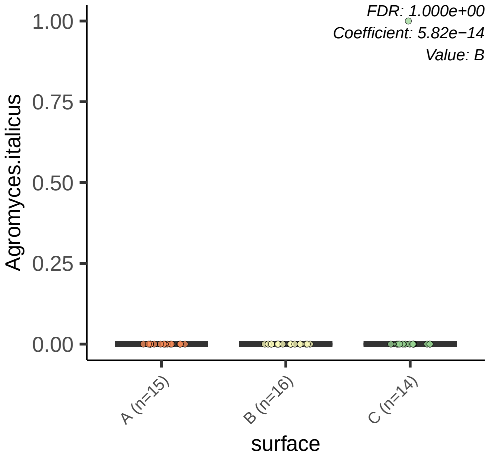

FDR: 1.000e+00
Coefficient: 5.82e−14
Value: B
1.00
0.75
0.50
0.25
0.00
Agromyces.italicus
A (n=15)
B (n=16)
C (n=14)
surface

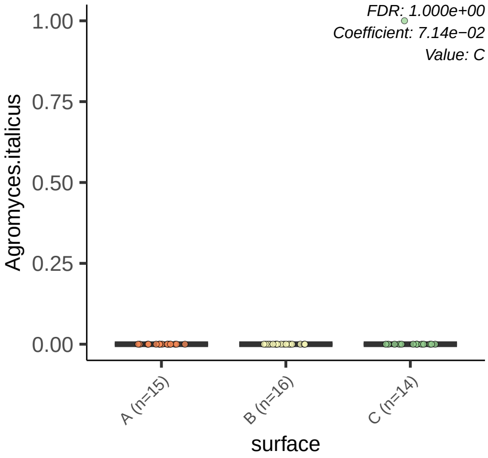
FDR: 1.000e+00
Coefficient: 7.14e−02
Value: C
1.00
0.75
0.50
0.25
0.00
Agromyces.italicus
A (n=15)
B (n=16)
C (n=14)
surface

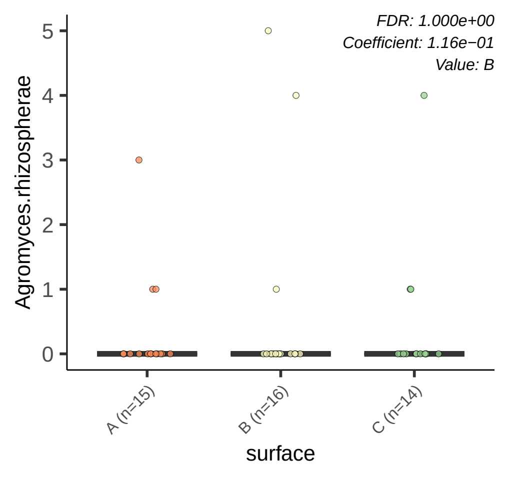

FDR: 1.000e+00
Coefficient: 1.16e−01
Value: B
Agromyces.rhizospherae
0
1
2
3
4
5
A (n=15)
B (n=16)
C (n=14)
surface

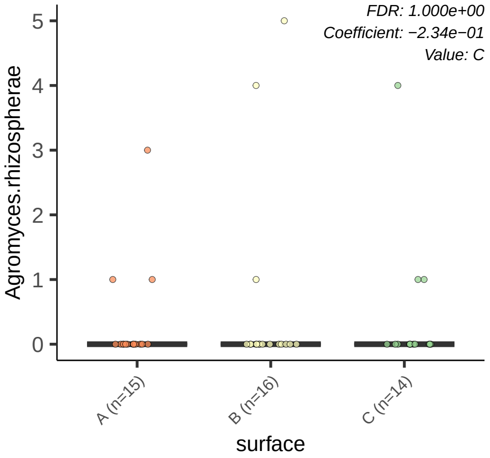

FDR: 1.000e+00
Coefficient: −2.34e−01
Value: C
Agromyces.rhizospherae
0
1
2
3
4
5
A (n=15)
B (n=16)
C (n=14)
surface

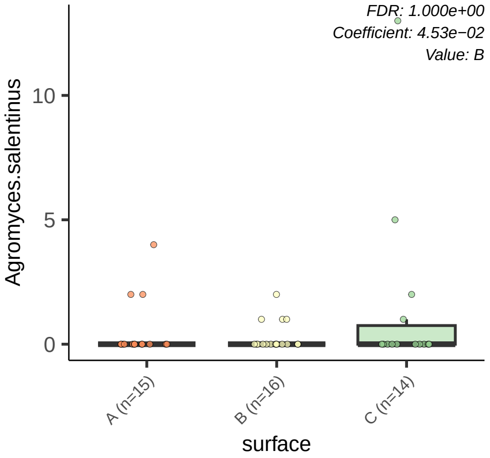

FDR: 1.000e+00
Coefficient: 4.53e−02
Value: B
Agromyces.salentinus
0
5
10
A (n=15)
B (n=16)
C (n=14)
surface

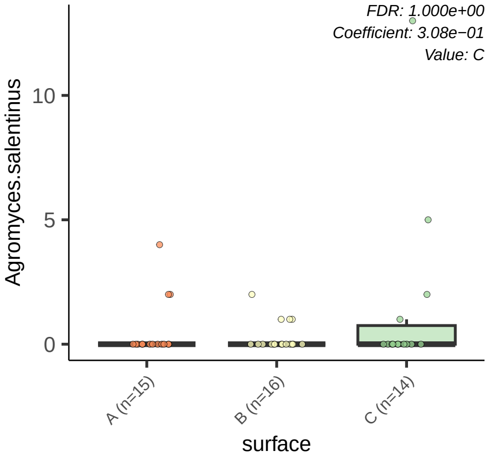
FDR: 1.000e+00
Coefficient: 3.08e−01
Value: C
Agromyces.salentinus
10
5
0
A (n=15)
B (n=16)
C (n=14)
surface

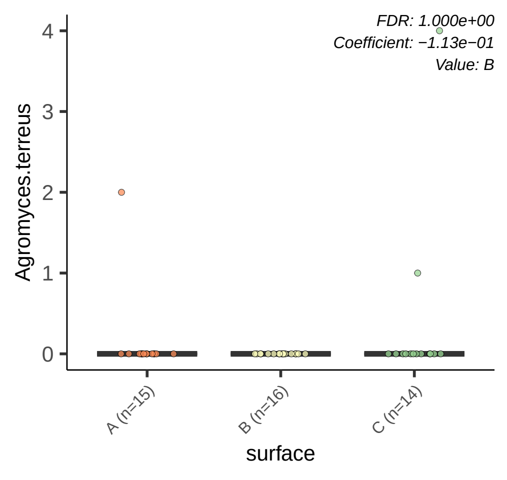
FDR: 1.000e+00
Coefficient: −1.13e−01
Value: B
Agromyces.terreus
0
1
2
3
4
A (n=15)
B (n=16)
C (n=14)
surface

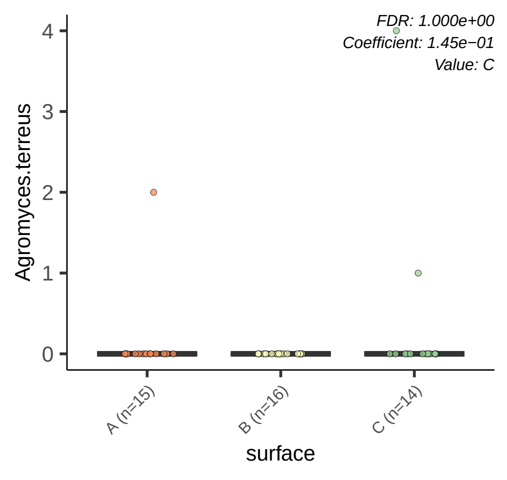

FDR: 1.000e+00
Coefficient: 1.45e−01
Value: C
Agromyces.terreus
0
1
2
3
4
A (n=15)
B (n=16)
C (n=14)
surface

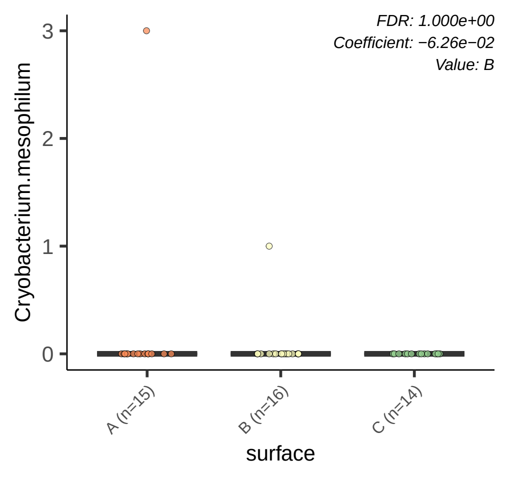
FDR: 1.000e+00
Coefficient: −6.26e−02
Value: B
Cryobacterium.mesophilum
3
2
1
0
A (n=15)
B (n=16)
C (n=14)
surface

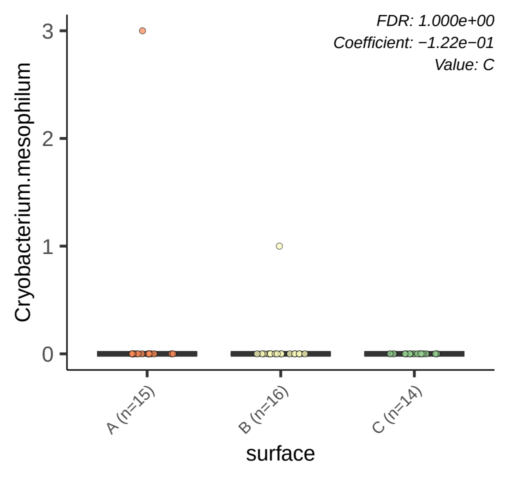

FDR: 1.000e+00
Coefficient: −1.22e−01
Value: C
Cryobacterium.mesophilum
0
1
2
3
A (n=15)
B (n=16)
C (n=14)
surface

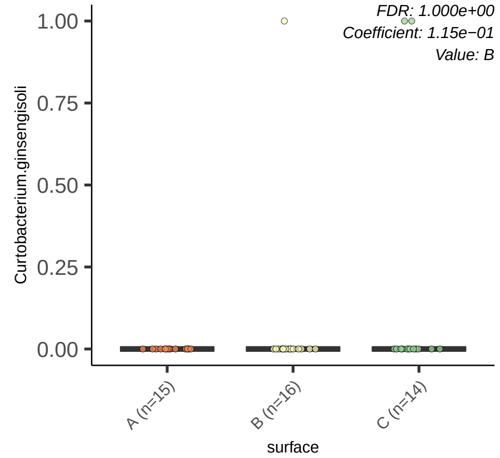
FDR: 1.000e+00
Coefficient: 1.15e−01
Value: B
1.00
0.75
0.50
0.25
0.00
Curtobacterium.ginsengisoli
A (n=15)
B (n=16)
C (n=14)
surface

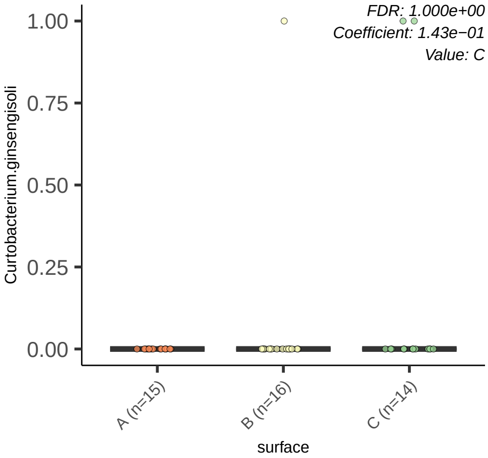

FDR: 1.000e+00
Coefficient: 1.43e−01
Value: C
1.00
0.75
0.50
0.25
0.00
Curtobacterium.ginsengisoli
A (n=15)
B (n=16)
C (n=14)
surface

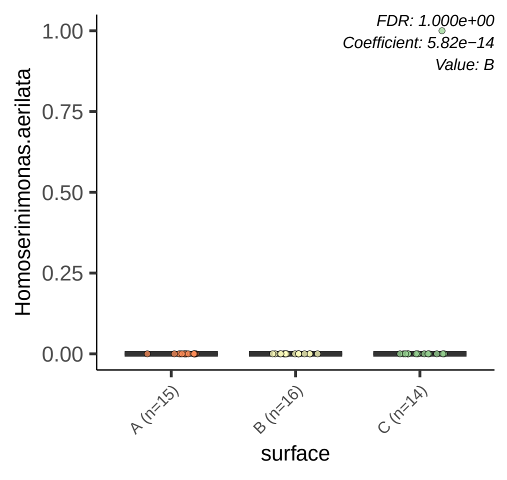
FDR: 1.000e+00
Coefficient: 5.82e−14
Value: B
Homoserinimonas.aerilata
1.00
0.75
0.50
0.25
0.00
A (n=15)
B (n=16)
C (n=14)
surface

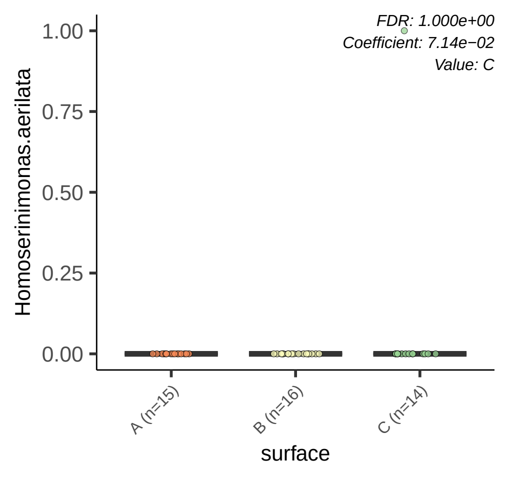
FDR: 1.000e+00
Coefficient: 7.14e−02
Value: C
Homoserinimonas.aerilata
1.00
0.75
0.50
0.25
0.00
A (n=15)
B (n=16)
C (n=14)
surface

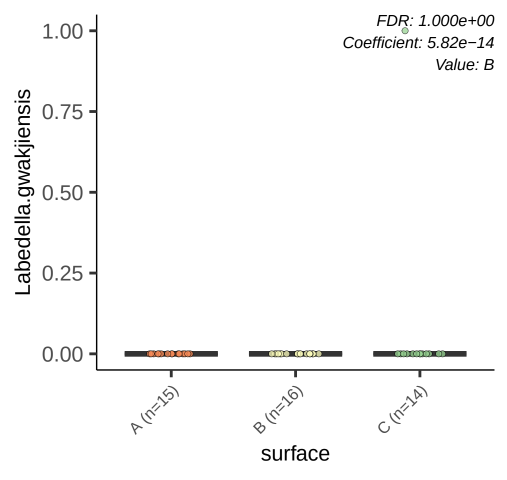
FDR: 1.000e+00
Coefficient: 5.82e−14
Value: B
Labedella.gwakjiensis
1.00
0.75
0.50
0.25
0.00
A (n=15)
B (n=16)
C (n=14)
surface

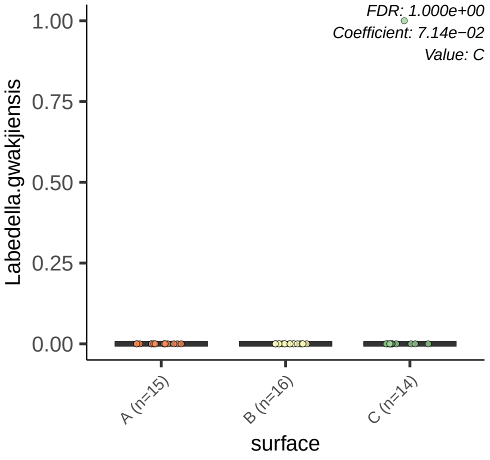

FDR: 1.000e+00
Coefficient: 7.14e−02
Value: C
1.00
0.75
0.50
0.25
0.00
Labedella.gwakjiensis
A (n=15)
B (n=16)
C (n=14)
surface

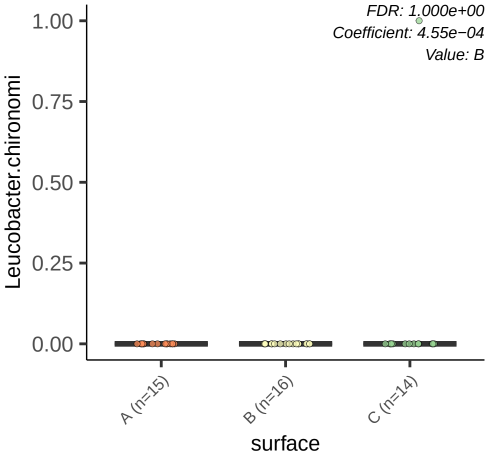
FDR: 1.000e+00
Coefficient: 4.55e−04
Value: B
Leucobacter.chironomi
1.00
0.75
0.50
0.25
0.00
A (n=15)
B (n=16)
C (n=14)
surface

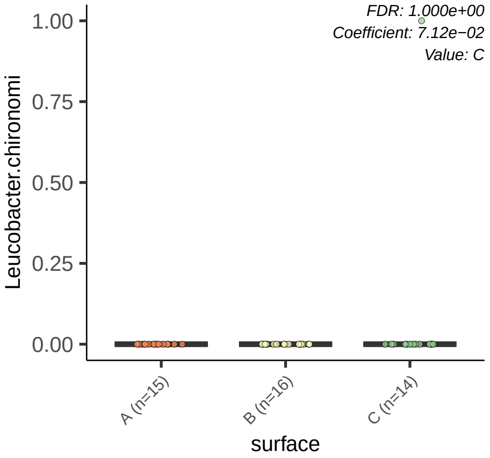

FDR: 1.000e+00
Coefficient: 7.12e−02
Value: C
Leucobacter.chironomi
1.00
0.75
0.50
0.25
0.00
A (n=15)
B (n=16)
C (n=14)
surface

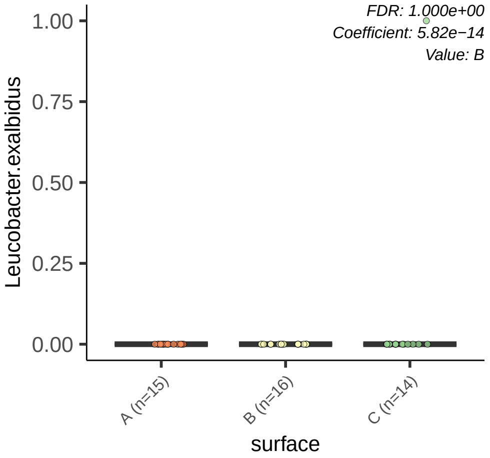

FDR: 1.000e+00
Coefficient: 5.82e−14
Value: B
Leucobacter.exalbidus
1.00
0.75
0.50
0.25
0.00
A (n=15)
B (n=16)
C (n=14)
surface

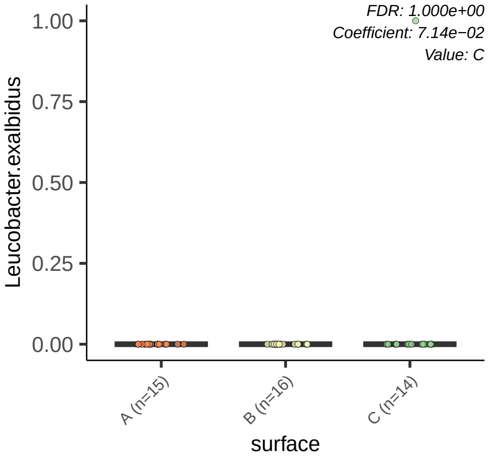

FDR: 1.000e+00
Coefficient: 7.14e−02
Value: C
Leucobacter.exalbidus
1.00
0.75
0.50
0.25
0.00
A (n=15)
B (n=16)
C (n=14)
surface

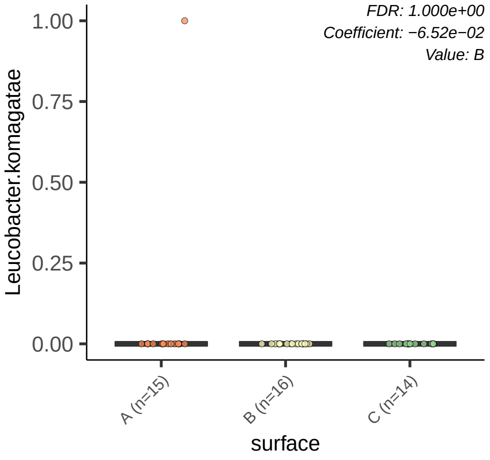
FDR: 1.000e+00
Coefficient: −6.52e−02
Value: B
Leucobacter.komagatae
1.00
0.75
0.50
0.25
0.00
A (n=15)
B (n=16)
C (n=14)
surface

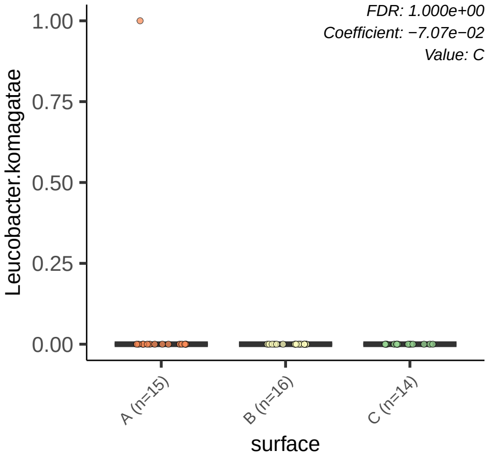
FDR: 1.000e+00
Coefficient: −7.07e−02
Value: C
Leucobacter.komagatae
1.00
0.75
0.50
0.25
0.00
A (n=15)
B (n=16)
C (n=14)
surface

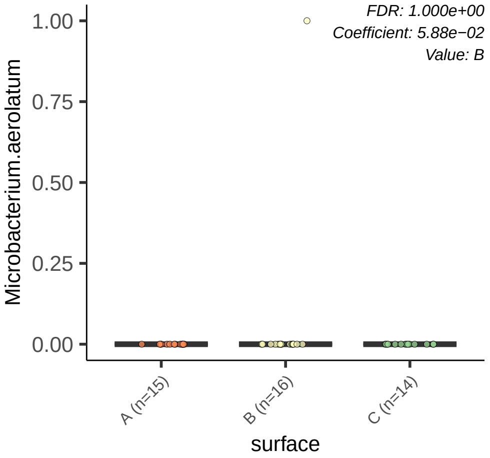
FDR: 1.000e+00
Coefficient: 5.88e−02
Value: B
Microbacterium.aerolatum
1.00
0.75
0.50
0.25
0.00
A (n=15)
B (n=16)
C (n=14)
surface

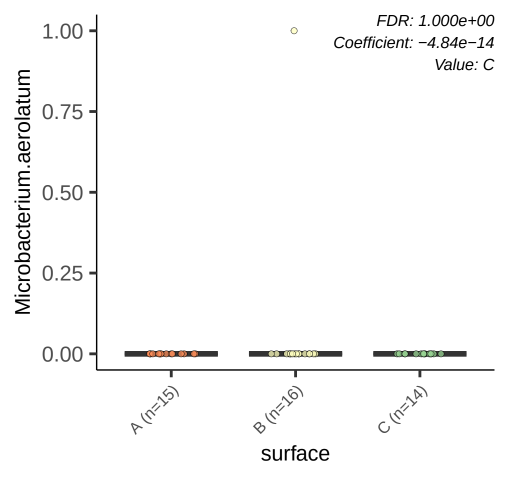

FDR: 1.000e+00
Coefficient: −4.84e−14
Value: C
Microbacterium.aerolatum
1.00
0.75
0.50
0.25
0.00
A (n=15)
B (n=16)
C (n=14)
surface

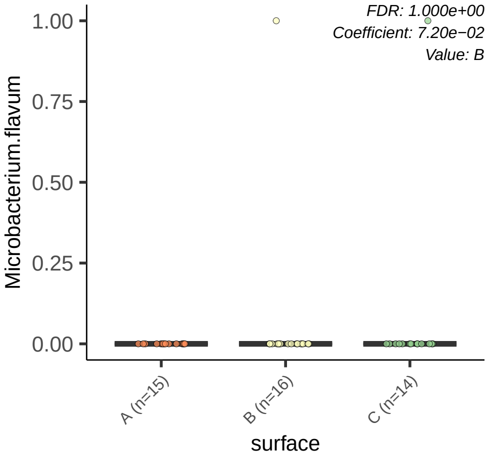
FDR: 1.000e+00
Coefficient: 7.20e−02
Value: B
Microbacterium.flavum
1.00
0.75
0.50
0.25
0.00
A (n=15)
B (n=16)
C (n=14)
surface

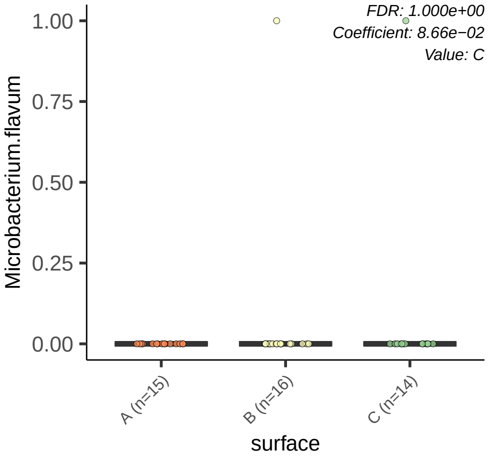

FDR: 1.000e+00
Coefficient: 8.66e−02
Value: C
Microbacterium.flavum
1.00
0.75
0.50
0.25
0.00
A (n=15)
B (n=16)
C (n=14)
surface

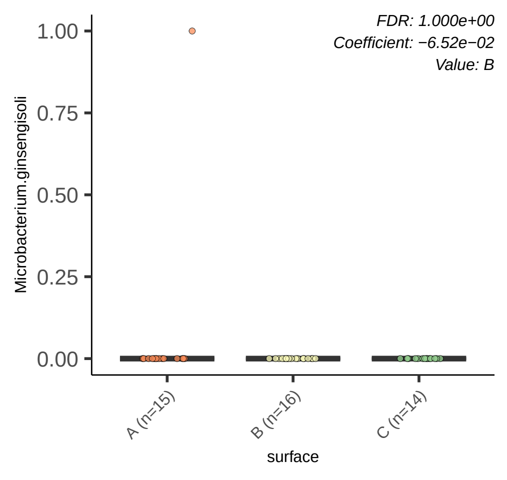
FDR: 1.000e+00
Coefficient: −6.52e−02
Value: B
1.00
0.75
0.50
0.25
0.00
Microbacterium.ginsengisoli
A (n=15)
B (n=16)
C (n=14)
surface

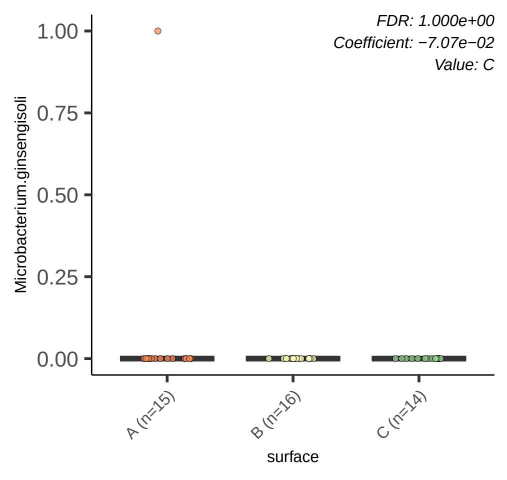
FDR: 1.000e+00
Coefficient: −7.07e−02
Value: C
1.00
0.75
0.50
0.25
0.00
Microbacterium.ginsengisoli
A (n=15)
B (n=16)
C (n=14)
surface

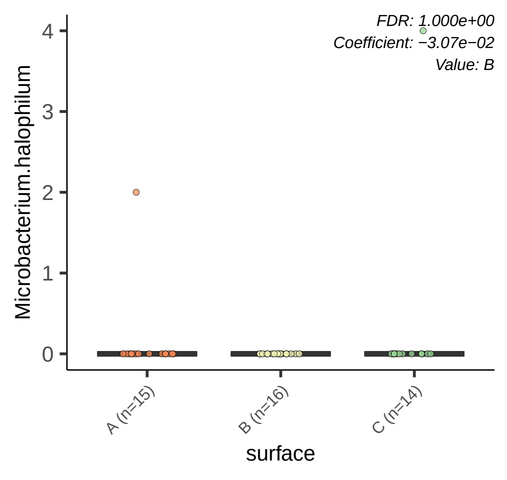

FDR: 1.000e+00
Coefficient: −3.07e−02
Value: B
Microbacterium.halophilum
0
1
2
3
4
A (n=15)
B (n=16)
C (n=14)
surface

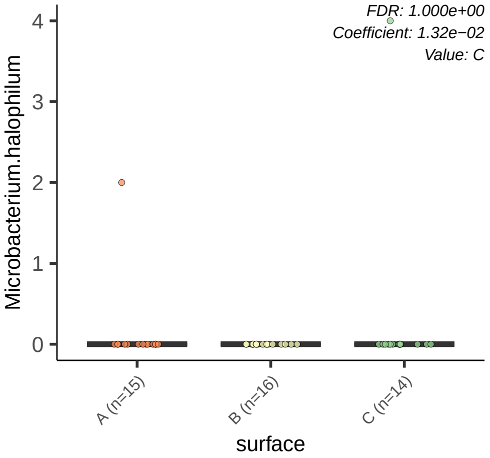

FDR: 1.000e+00
Coefficient: 1.32e−02
Value: C
Microbacterium.halophilum
0
1
2
3
4
A (n=15)
B (n=16)
C (n=14)
surface

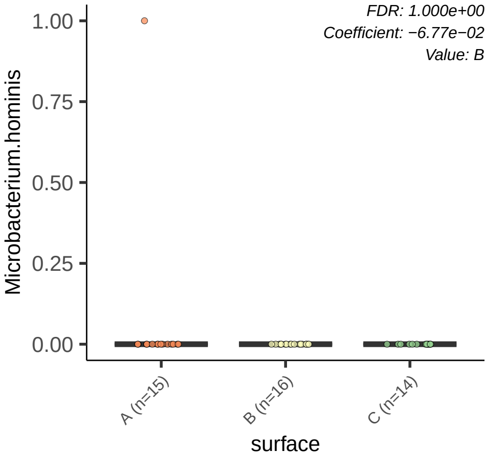

FDR: 1.000e+00
Coefficient: −6.77e−02
Value: B
Microbacterium.hominis
1.00
0.75
0.50
0.25
0.00
A (n=15)
B (n=16)
C (n=14)
surface

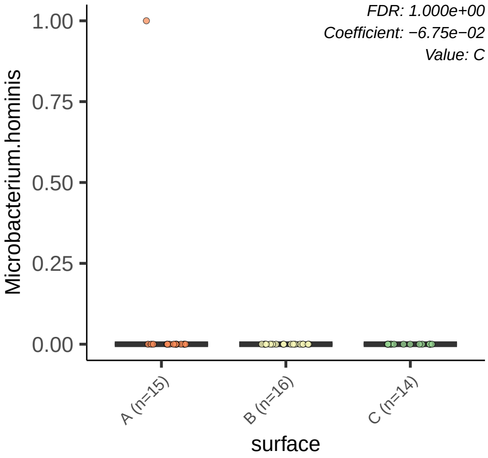

FDR: 1.000e+00
Coefficient: −6.75e−02
Value: C
Microbacterium.hominis
1.00
0.75
0.50
0.25
0.00
A (n=15)
B (n=16)
C (n=14)
surface

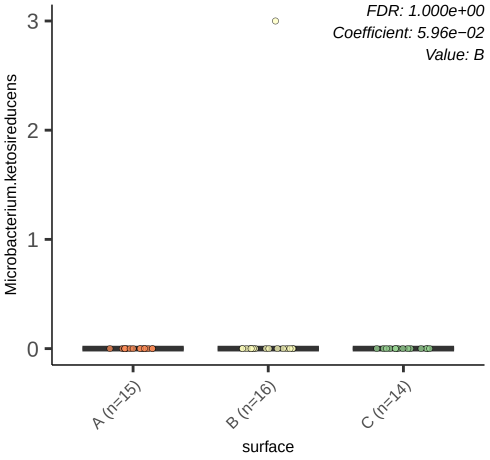

FDR: 1.000e+00
Coefficient: 5.96e−02
Value: B
Microbacterium.ketosireducens
3
2
1
0
A (n=15)
B (n=16)
C (n=14)
surface

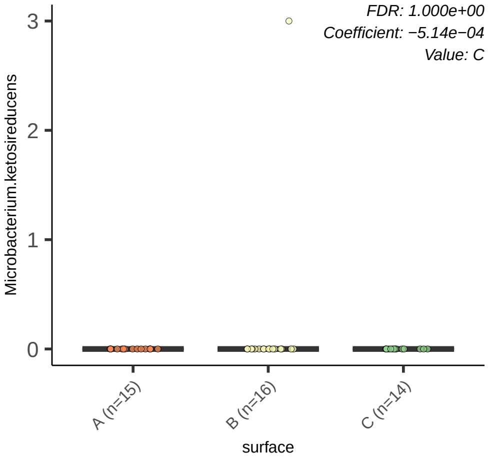
FDR: 1.000e+00
Coefficient: −5.14e−04
Value: C
Microbacterium.ketosireducens
3
2
1
0
A (n=15)
B (n=16)
C (n=14)
surface

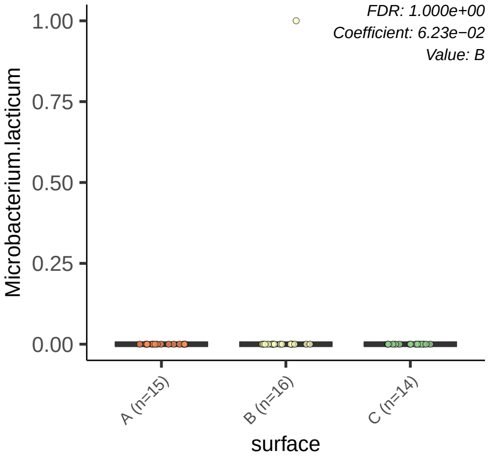
FDR: 1.000e+00
Coefficient: 6.23e−02
Value: B
Microbacterium.lacticum
1.00
0.75
0.50
0.25
0.00
A (n=15)
B (n=16)
C (n=14)
surface

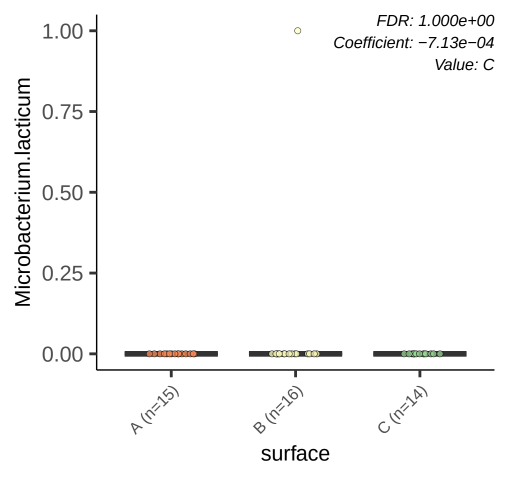

FDR: 1.000e+00
Coefficient: −7.13e−04
Value: C
Microbacterium.lacticum
1.00
0.75
0.50
0.25
0.00
A (n=15)
B (n=16)
C (n=14)
surface

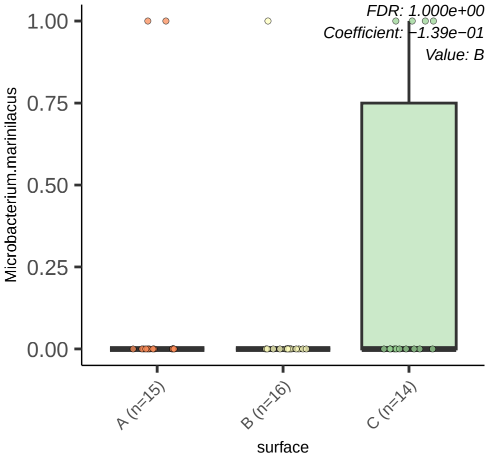

FDR: 1.000e+00
Coefficient: −1.39e−01
Value: B
Microbacterium.marinilacus
1.00
0.75
0.50
0.25
0.00
A (n=15)
B (n=16)
C (n=14)
surface

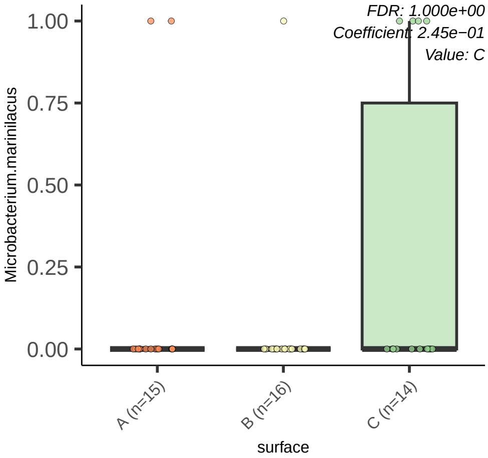

FDR: 1.000e+00
Coefficient: 2.45e−01
Value: C
Microbacterium.marinilacus
1.00
0.75
0.50
0.25
0.00
A (n=15)
B (n=16)
C (n=14)
surface

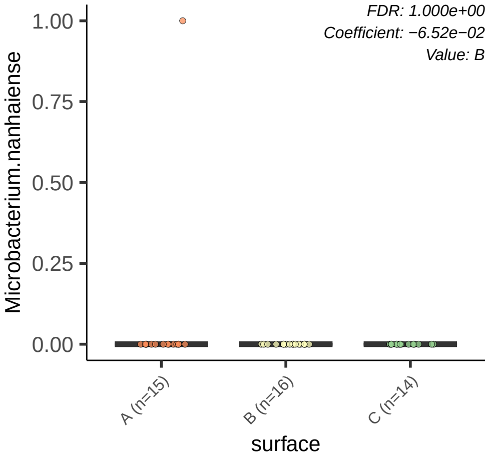

FDR: 1.000e+00
Coefficient: −6.52e−02
Value: B
Microbacterium.nanhaiense
1.00
0.75
0.50
0.25
0.00
A (n=15)
B (n=16)
C (n=14)
surface

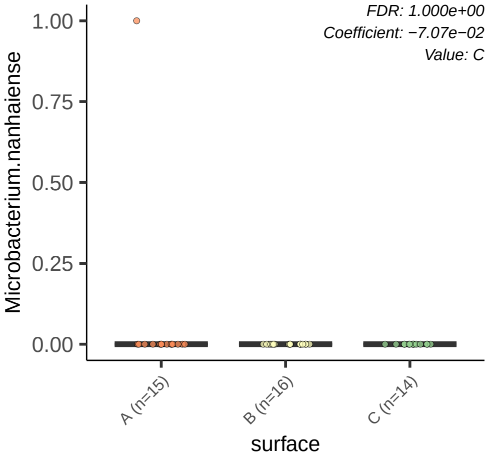
FDR: 1.000e+00
Coefficient: −7.07e−02
Value: C
Microbacterium.nanhaiense
1.00
0.75
0.50
0.25
0.00
A (n=15)
B (n=16)
C (n=14)
surface

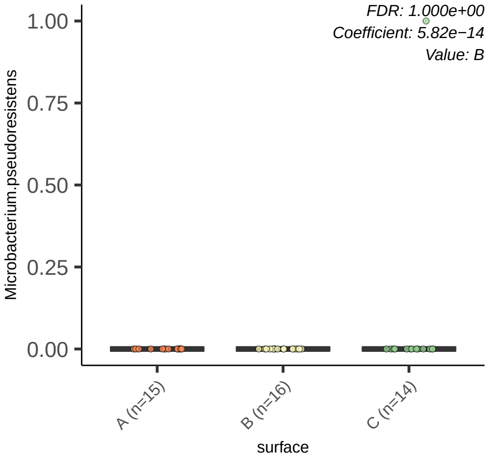
FDR: 1.000e+00
Coefficient: 5.82e−14
Value: B
Microbacterium.pseudoresistens
1.00
0.75
0.50
0.25
0.00
A (n=15)
B (n=16)
C (n=14)
surface

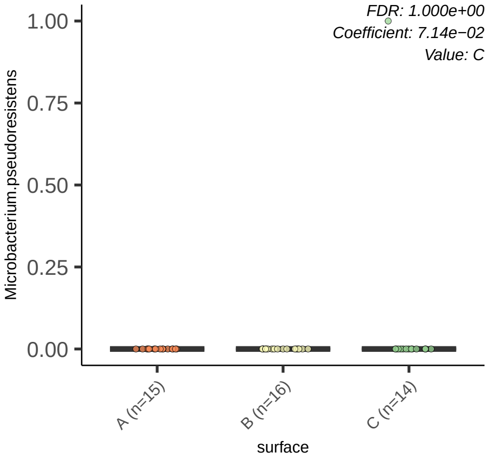
FDR: 1.000e+00
Coefficient: 7.14e−02
Value: C
Microbacterium.pseudoresistens
1.00
0.75
0.50
0.25
0.00
A (n=15)
B (n=16)
C (n=14)
surface

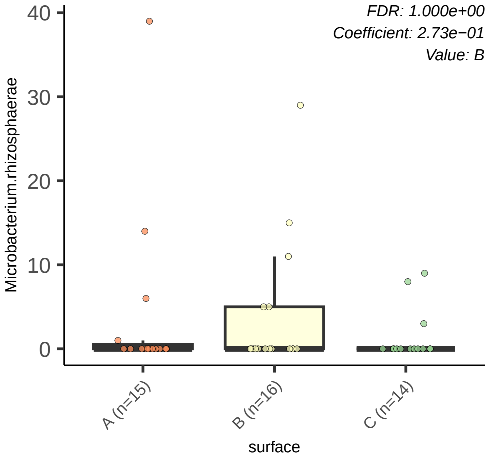

FDR: 1.000e+00
Coefficient: 2.73e−01
Value: B
Microbacterium.rhizosphaerae
0
10
20
30
40
A (n=15)
B (n=16)
C (n=14)
surface

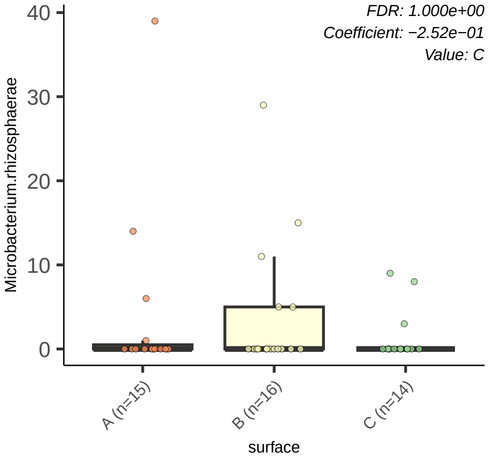

FDR: 1.000e+00
Coefficient: −2.52e−01
Value: C
Microbacterium.rhizosphaerae
40
30
20
10
0
A (n=15)
B (n=16)
C (n=14)
surface

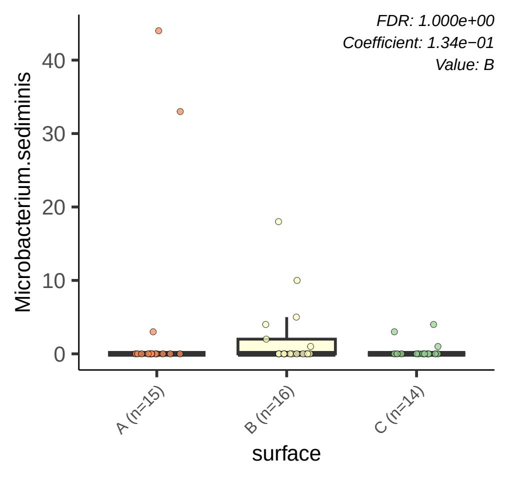

FDR: 1.000e+00
Coefficient: 1.34e−01
Value: B
Microbacterium.sediminis
0
10
20
30
40
A (n=15)
B (n=16)
C (n=14)
surface

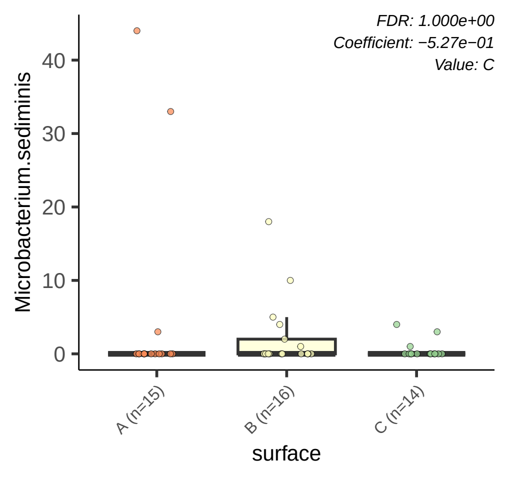

FDR: 1.000e+00
Coefficient: −5.27e−01
Value: C
Microbacterium.sedimninis
0
10
20
30
40
A (n=15)
B (n=16)
C (n=14)
surface

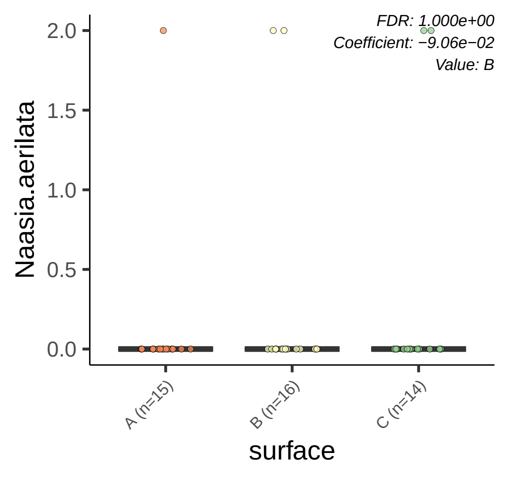
FDR: 1.000e+00
Coefficient: −9.06e−02
Value: B
Naasia.aerilata
2.0
1.5
1.0
0.5
0.0
A (n=15)
B (n=16)
C (n=14)
surface

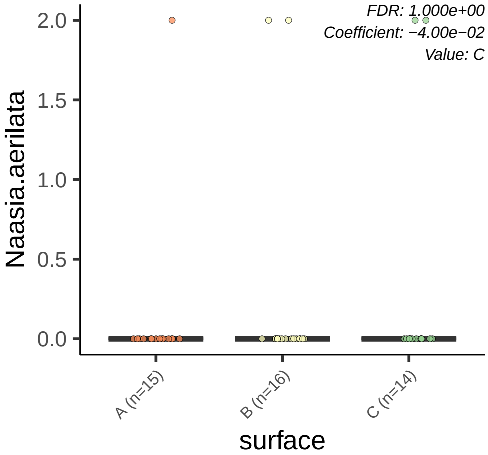

FDR: 1.000e+00
Coefficient: −4.00e−02
Value: C
Naasia.aerilata
2.0
1.5
1.0
0.5
0.0
A (n=15)
B (n=16)
C (n=14)
surface

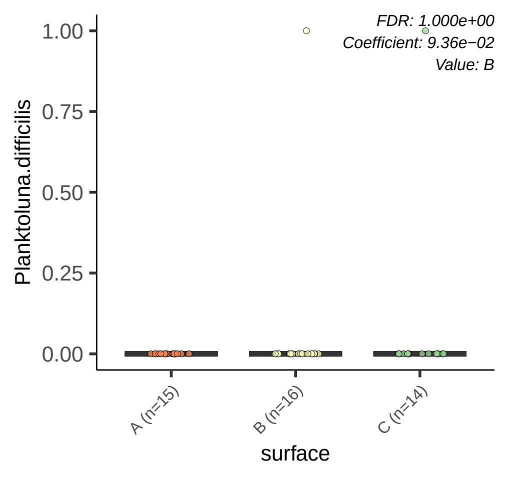

FDR: 1.000e+00
Coefficient: 9.36e−02
Value: B
Planktoluna.difficilis
1.00
0.75
0.50
0.25
0.00
A (n=15)
B (n=16)
C (n=14)
surface

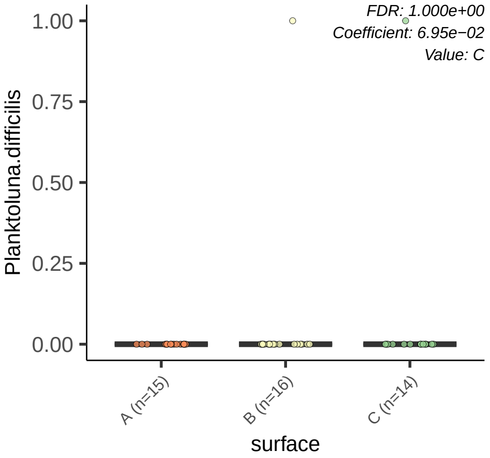

FDR: 1.000e+00
Coefficient: 6.95e−02
Value: C
Planktoluna.difficilis
1.00
0.75
0.50
0.25
0.00
A (n=15)
B (n=16)
C (n=14)
surface

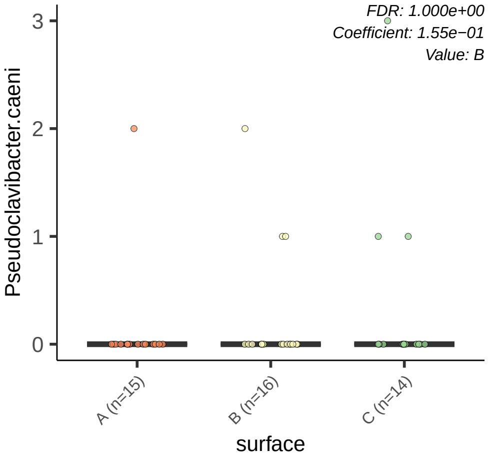

FDR: 1.000e+00
Coefficient: 1.55e−01
Value: B
Pseudoclavibacter.caeni
3
2
1
0
A (n=15)
B (n=16)
C (n=14)
surface

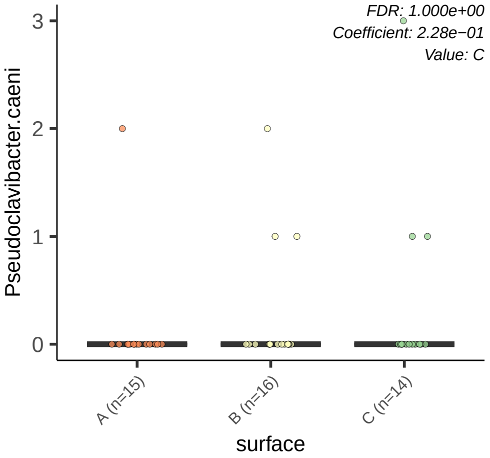
FDR: 1.000e+00
Coefficient: 2.28e−01
Value: C
Pseudoclavibacter.caeni
0
1
2
3
A (n=15)
B (n=16)
C (n=14)
surface

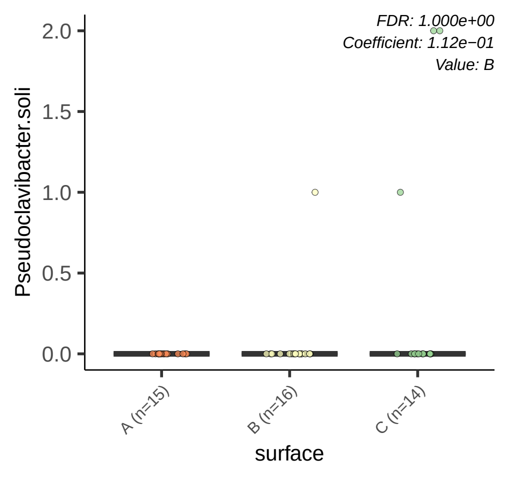

FDR: 1.000e+00
Coefficient: 1.12e−01
Value: B
Pseudoclavibacter.soli
2.0
1.5
1.0
0.5
0.0
A (n=15)
B (n=16)
C (n=14)
surface

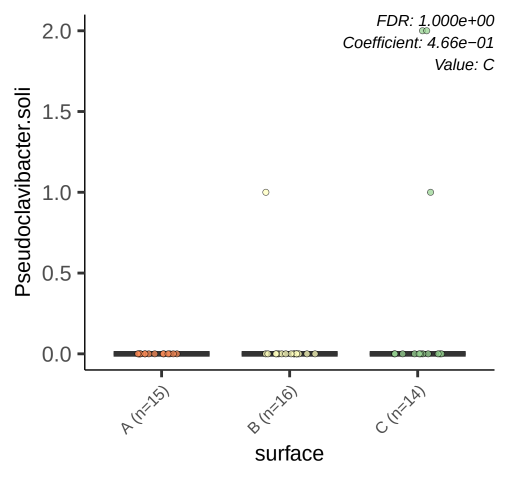
FDR: 1.000e+00
Coefficient: 4.66e−01
Value: C
Pseudoclavibacter.soli
2.0
1.5
1.0
0.5
0.0
A (n=15)
B (n=16)
C (n=14)
surface

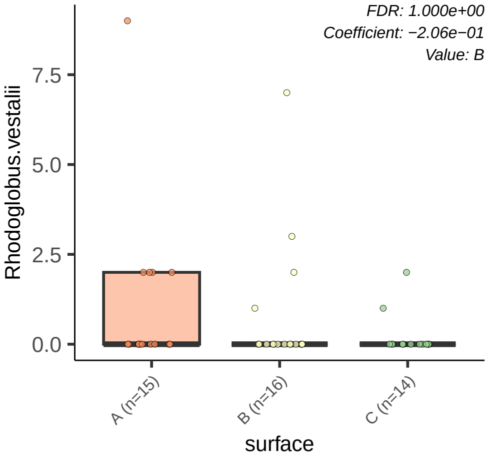

FDR: 1.000e+00
Coefficient: −2.06e−01
Value: B
Rhodoglobus.vestalii
7.5
5.0
2.5
0.0
A (n=15)
B (n=16)
C (n=14)
surface

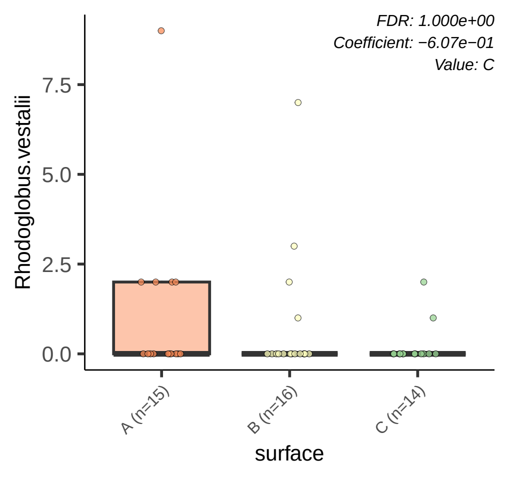
FDR: 1.000e+00
Coefficient: −6.07e−01
Value: C
Rhodoglobus.vestalii
7.5
5.0
2.5
0.0
A (n=15)
B (n=16)
C (n=14)
surface

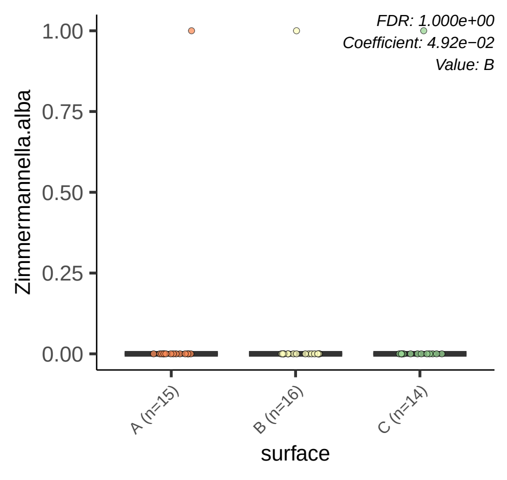
FDR: 1.000e+00
Coefficient: 4.92e−02
Value: B
Zimmermannella.alba
1.00
0.75
0.50
0.25
0.00
A (n=15)
B (n=16)
C (n=14)
surface

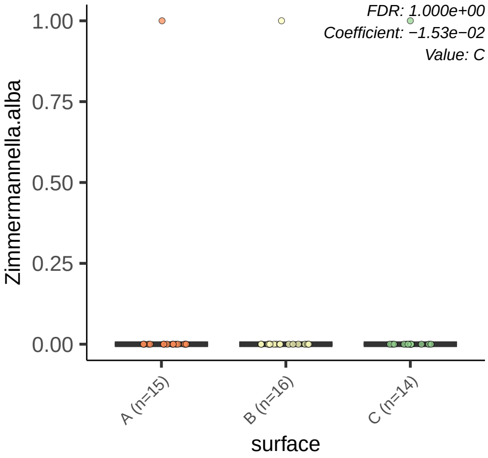

FDR: 1.000e+00
Coefficient: −1.53e−02
Value: C
Zimmermannella.alba
1.00
0.75
0.50
0.25
0.00
A (n=15)
B (n=16)
C (n=14)
surface

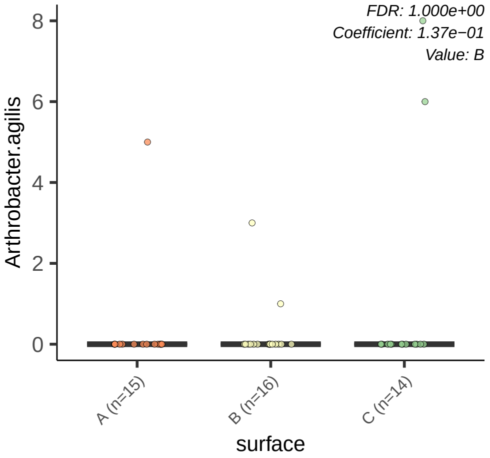

FDR: 1.000e+00
Coefficient: 1.37e−01
Value: B
Arthrobacter.agilis
0
2
4
6
8
A (n=15)
B (n=16)
C (n=14)
surface

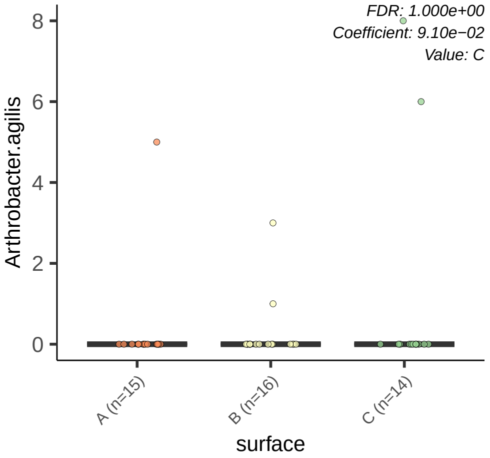

FDR: 1.000e+00
Coefficient: 9.10e−02
Value: C
Arthrobacter.agilis
8
6
4
2
0
A (n=15)
B (n=16)
C (n=14)
surface

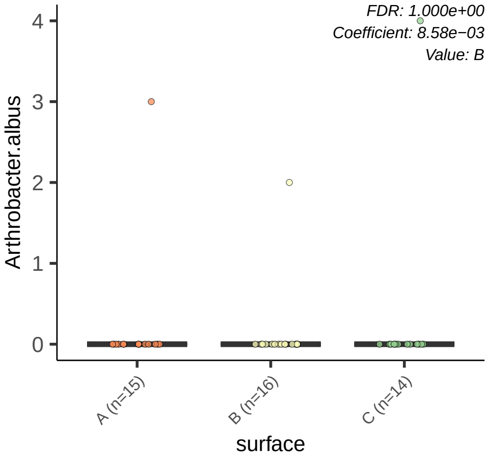

FDR: 1.000e+00
Coefficient: 8.58e−03
Value: B
Arthrobacter.albus
0
1
2
3
4
A (n=15)
B (n=16)
C (n=14)
surface

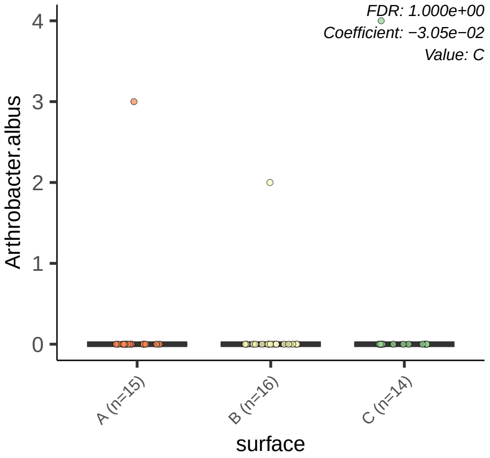

FDR: 1.000e+00
Coefficient: −3.05e−02
Value: C
Arthrobacter.albus
0
1
2
3
4
A (n=15)
B (n=16)
C (n=14)
surface

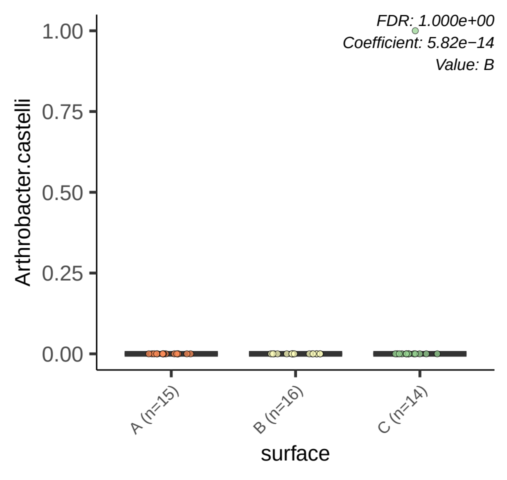

FDR: 1.000e+00
Coefficient: 5.82e−14
Value: B
Arthrobacter.castelli
1.00
0.75
0.50
0.25
0.00
A (n=15)
B (n=16)
C (n=14)
surface

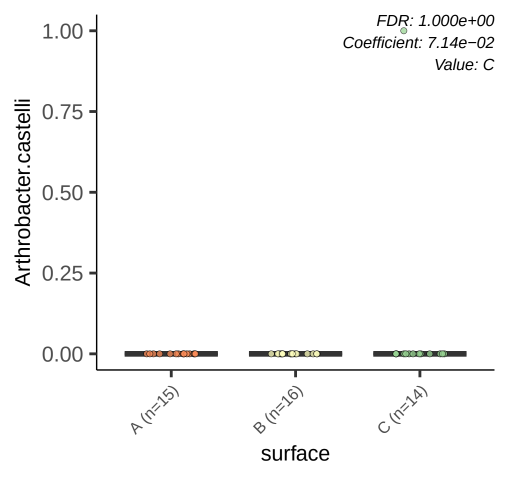
FDR: 1.000e+00
Coefficient: 7.14e−02
Value: C
Arthrobacter.castelli
1.00
0.75
0.50
0.25
0.00
A (n=15)
B (n=16)
C (n=14)
surface

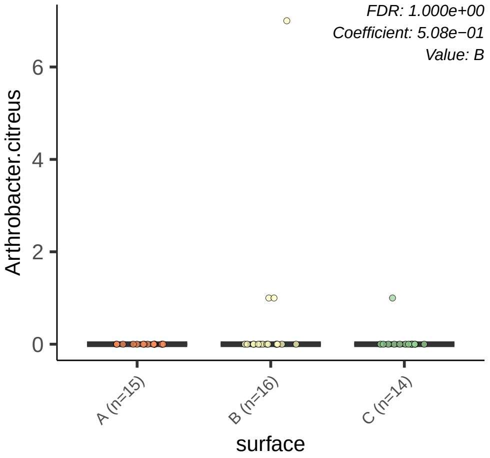
FDR: 1.000e+00
Coefficient: 5.08e−01
Value: B
Arthrobacter.citreus
0
2
4
6
A (n=15)
B (n=16)
C (n=14)
surface

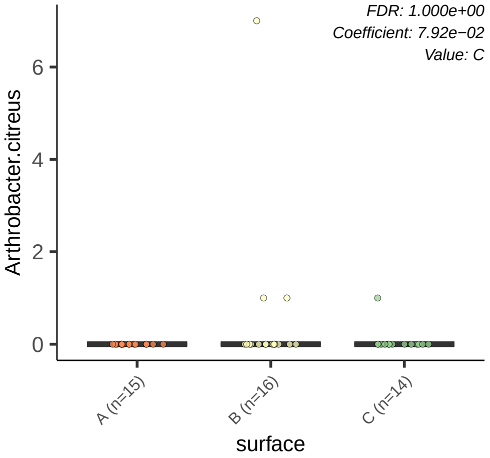
FDR: 1.000e+00
Coefficient: 7.92e−02
Value: C
Arthrobacter.citreus
0
2
4
6
A (n=15)
B (n=16)
C (n=14)
surface

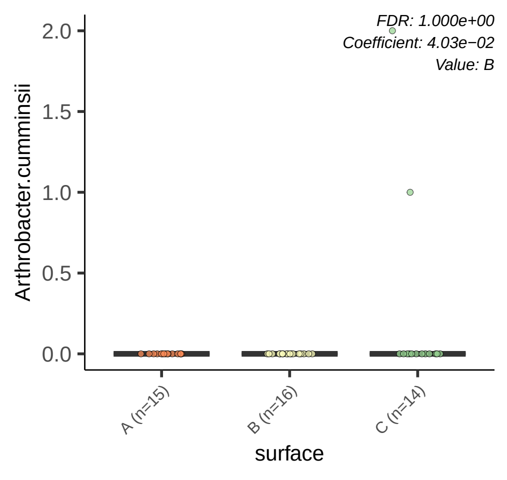
FDR: 1.000e+00
Coefficient: 4.03e−02
Value: B
Arthrobacter.cumminsii
2.0
1.5
1.0
0.5
0.0
A (n=15)
B (n=16)
C (n=14)
surface

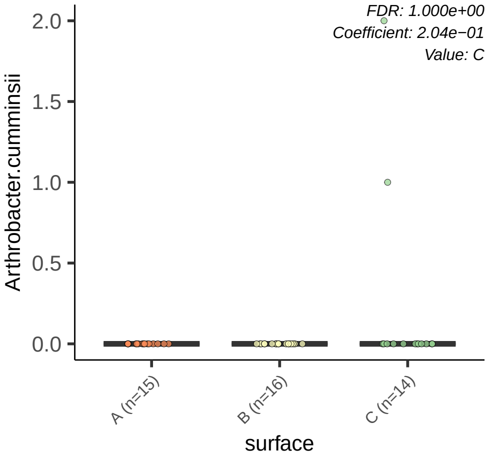
FDR: 1.000e+00
Coefficient: 2.04e−01
Value: C
Arthrobacter.cumminsii
2.0
1.5
1.0
0.5
0.0
A (n=15)
B (n=16)
C (n=14)
surface

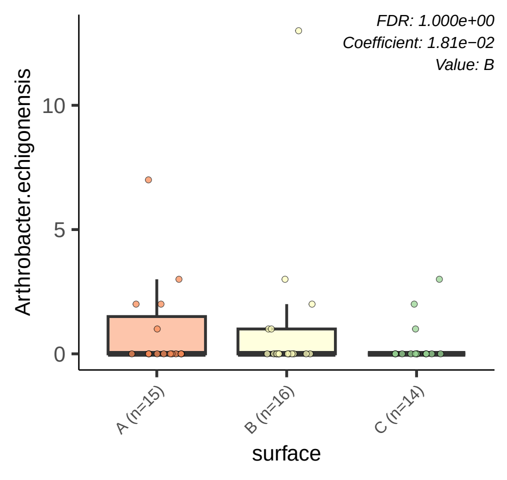

FDR: 1.000e+00
Coefficient: 1.81e−02
Value: B
Arthrobacter.echigonensis
0
5
10
A (n=15)
B (n=16)
C (n=14)
surface

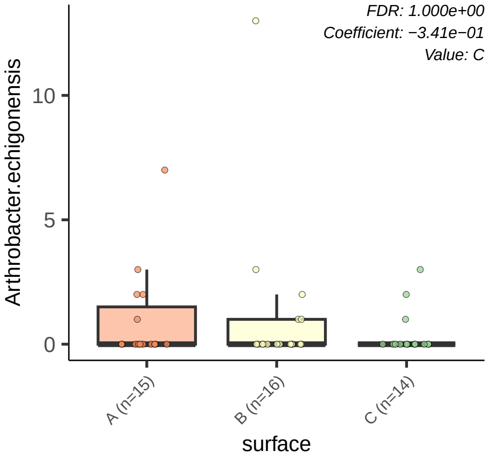
FDR: 1.000e+00
Coefficient: −3.41e−01
Value: C
Arthrobacter.echigonensis
10
5
0
A (n=15)
B (n=16)
C (n=14)
surface

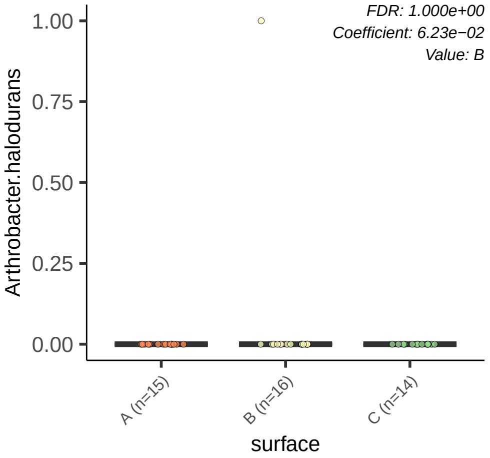
FDR: 1.000e+00
Coefficient: 6.23e−02
Value: B
Arthrobacter.halodurans
1.00
0.75
0.50
0.25
0.00
A (n=15)
B (n=16)
C (n=14)
surface

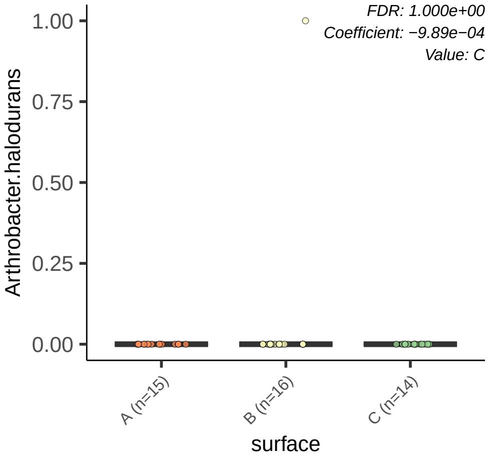

FDR: 1.000e+00
Coefficient: −9.89e−04
Value: C
Arthrobacter.halodurans
1.00
0.75
0.50
0.25
0.00
A (n=15)
B (n=16)
C (n=14)
surface

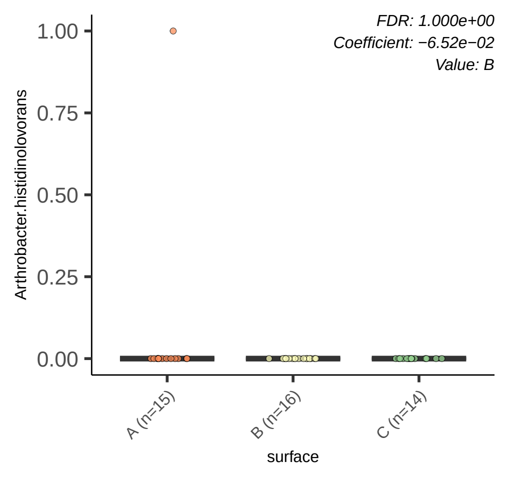
FDR: 1.000e+00
Coefficient: −6.52e−02
Value: B
1.00
0.75
0.50
0.25
0.00
Arthrobacter.histidinolovorans
A (n=15)
B (n=16)
C (n=14)
surface

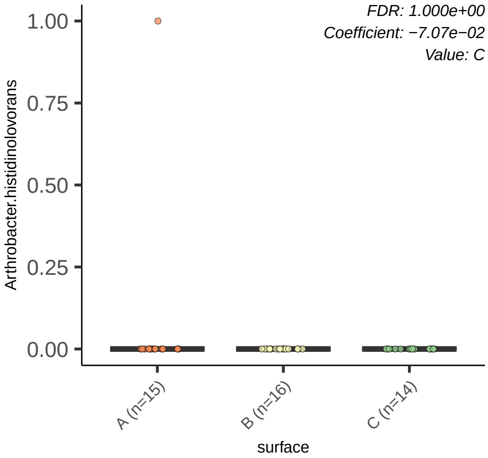
FDR: 1.000e+00
Coefficient: −7.07e−02
Value: C
Arthrobacter.histidinolovorans
1.00
0.75
0.50
0.25
0.00
A (n=15)
B (n=16)
C (n=14)
surface

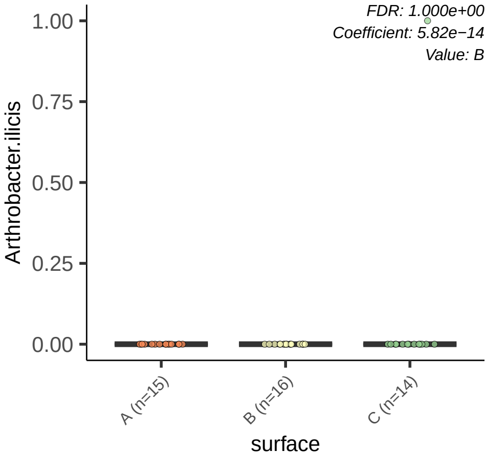
FDR: 1.000e+00
Coefficient: 5.82e−14
Value: B
1.00
0.75
0.50
0.25
0.00
Arthrobacter.ilicis
A (n=15)
B (n=16)
C (n=14)
surface

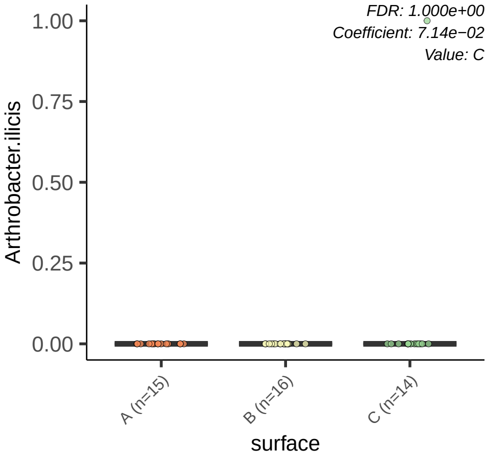

FDR: 1.000e+00
Coefficient: 7.14e−02
Value: C
Arthrobacter.ilicis
1.00
0.75
0.50
0.25
0.00
A (n=15)
B (n=16)
C (n=14)
surface

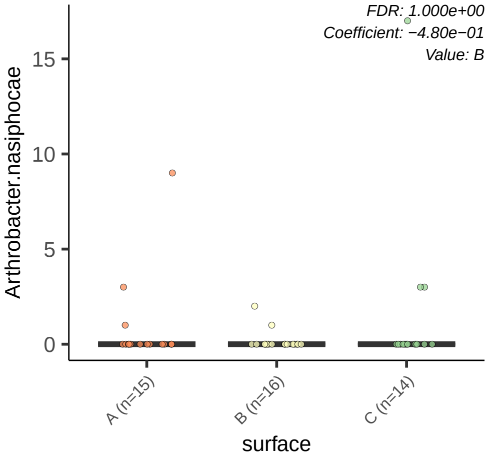
FDR: 1.000e+00
Coefficient: −4.80e−01
Value: B
Arthrobacter.nasiphocae
15
10
5
0
A (n=15)
B (n=16)
C (n=14)
surface

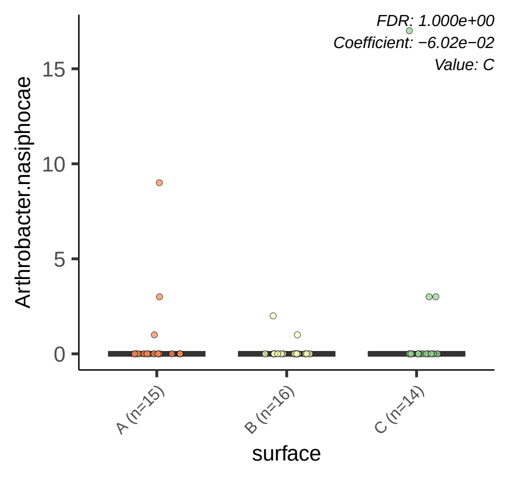
FDR: 1.000e+00
Coefficient: −6.02e−02
Value: C
Arthrobacter.nasiphocae
15
10
5
0
A (n=15)
B (n=16)
C (n=14)
surface

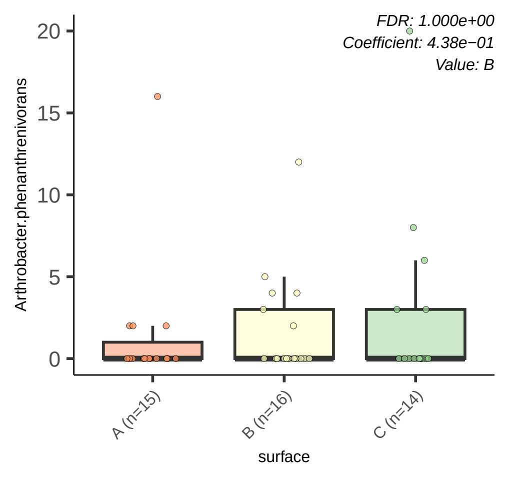
FDR: 1.000e+00
Coefficient: 4.38e−01
Value: B
Arthrobacter.phenanthrenivorans
20
15
10
5
0
A (n=15)
B (n=16)
C (n=14)
surface

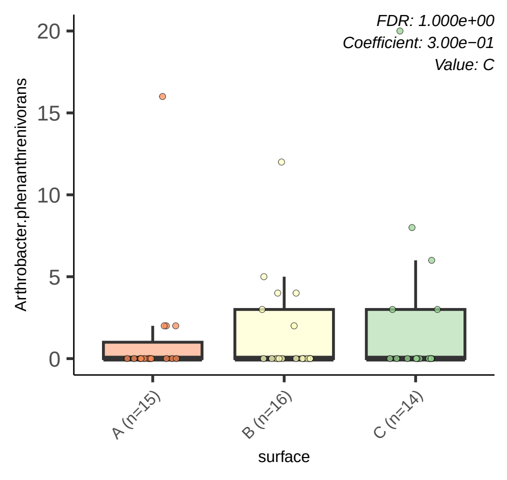
FDR: 1.000e+00
Coefficient: 3.00e−01
Value: C
Arthrobacter.phenanthrenivorans
0
5
10
15
20
A (n=15)
B (n=16)
C (n=14)
surface

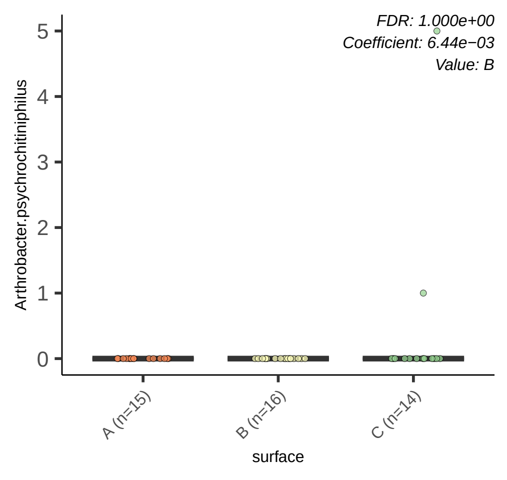
FDR: 1.000e+00
Coefficient: 6.44e−03
Value: B
Arthrobacter.psychrochitiniphilus
5
4
3
2
1
0
A (n=15)
B (n=16)
C (n=14)
surface

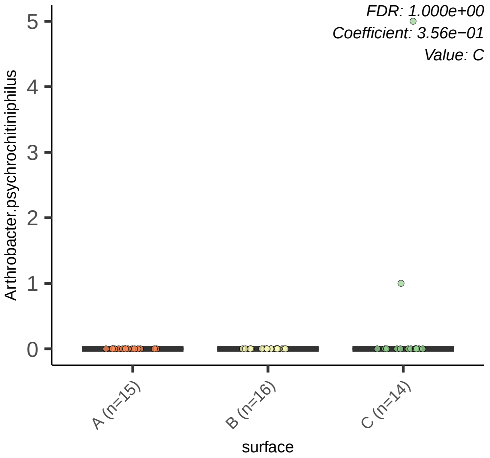

FDR: 1.000e+00
Coefficient: 3.56e−01
Value: C
Arthrobacter.psychrochitiniphilus
5
4
3
2
1
0
A (n=15)
B (n=16)
C (n=14)
surface

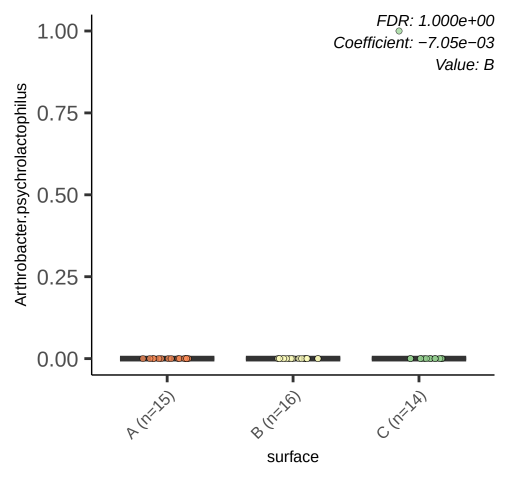
FDR: 1.000e+00
Coefficient: −7.05e−03
Value: B
1.00
0.75
0.50
0.25
0.00
Arthrobacter.psychrolactophilus
A (n=15)
B (n=16)
C (n=14)
surface

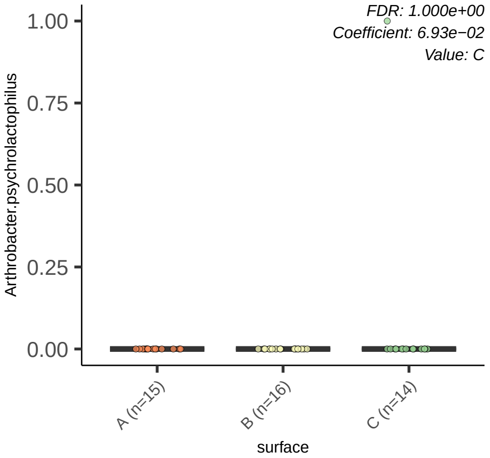

FDR: 1.000e+00
Coefficient: 6.93e−02
Value: C
1.00
0.75
0.50
0.25
0.00
Arthrobacter.psychrolactophilus
A (n=15)
B (n=16)
C (n=14)
surface

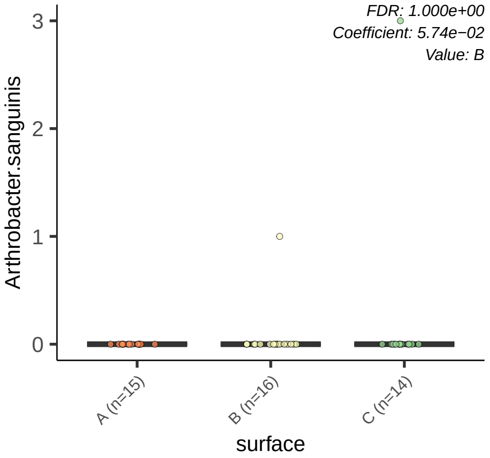
FDR: 1.000e+00
Coefficient: 5.74e−02
Value: B
Arthrobacter.sanguinis
3
2
1
0
A (n=15)
B (n=16)
C (n=14)
surface

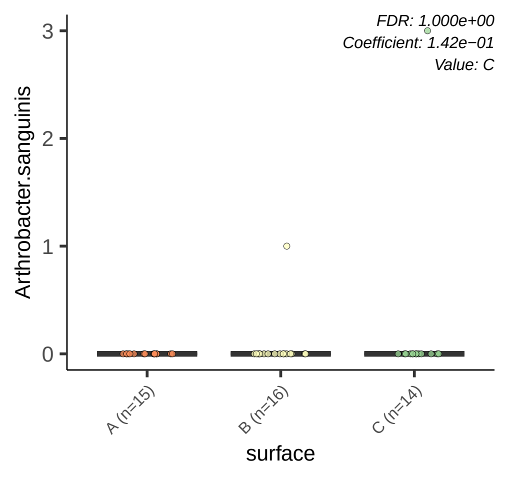
FDR: 1.000e+00
Coefficient: 1.42e−01
Value: C
Arthrobacter.sanguinis
3
2
1
0
A (n=15)
B (n=16)
C (n=14)
surface

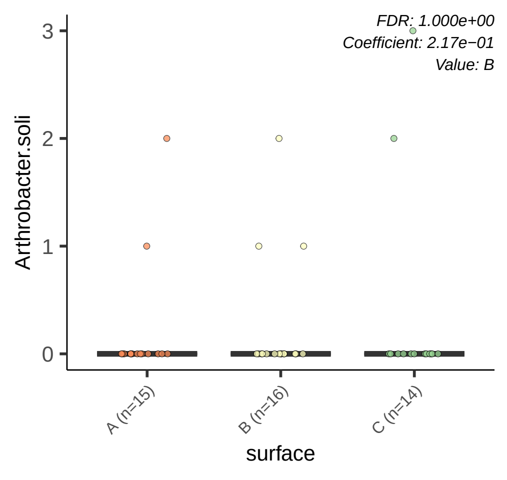
FDR: 1.000e+00
Coefficient: 2.17e−01
Value: B
Arthrobacter.soli
3
2
1
0
A (n=15)
B (n=16)
C (n=14)
surface

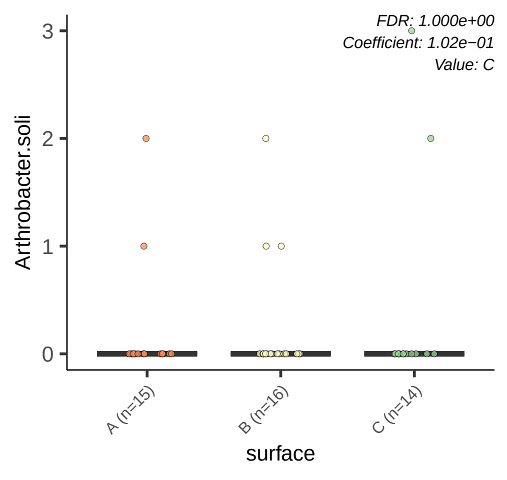

FDR: 1.000e+00
Coefficient: 1.02e−01
Value: C
Arthrobacter.soli
0
1
2
3
A (n=15)
B (n=16)
C (n=14)
surface

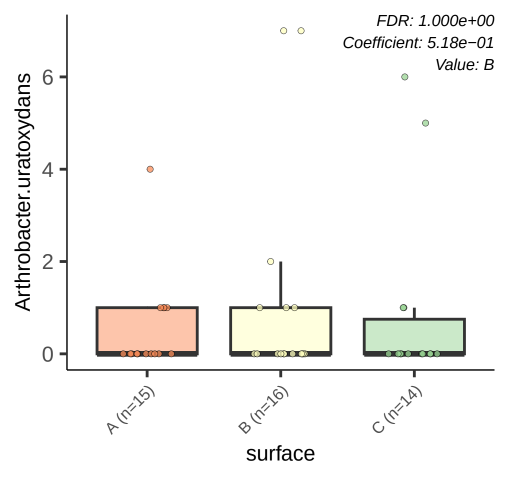
FDR: 1.000e+00
Coefficient: 5.18e−01
Value: B
Arthrobacter.uratoxydans
0
2
4
6
A (n=15)
B (n=16)
C (n=14)
surface

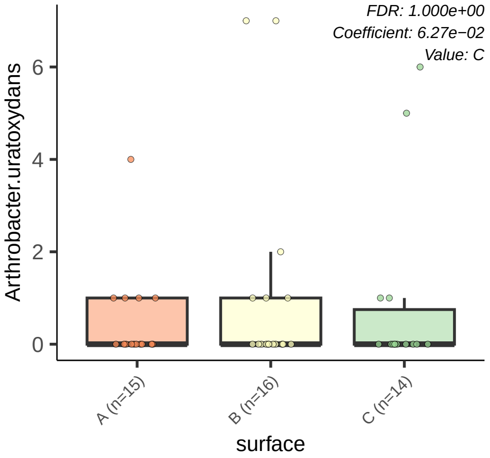
FDR: 1.000e+00
Coefficient: 6.27e−02
Value: C
Arthrobacter.uratoxydans
0
2
4
6
A (n=15)
B (n=16)
C (n=14)
surface

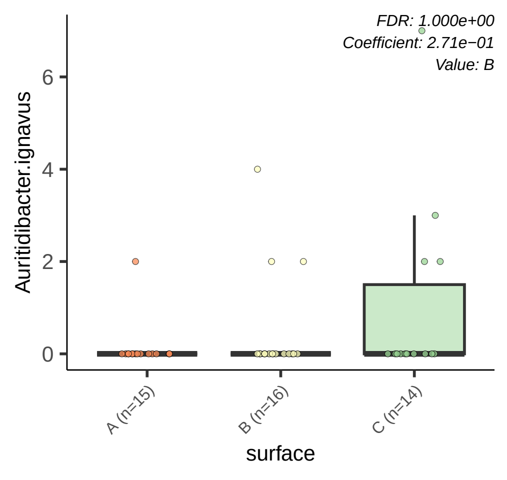

FDR: 1.000e+00
Coefficient: 2.71e−01
Value: B
Auritidibacter.ignavus
0
2
4
6
A (n=15)
B (n=16)
C (n=14)
surface

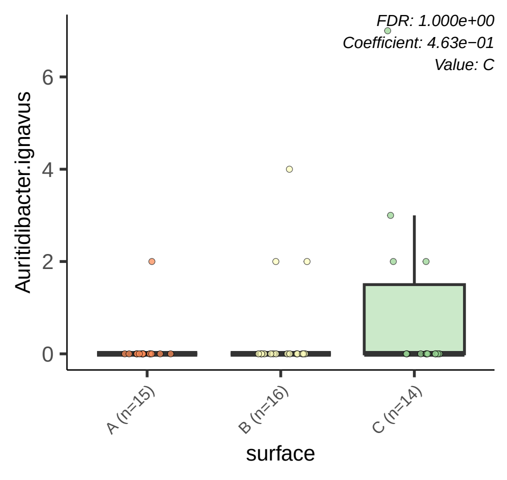
FDR: 1.000e+00
Coefficient: 4.63e−01
Value: C
Auritidibacter.ignavus
0
2
4
6
A (n=15)
B (n=16)
C (n=14)
surface

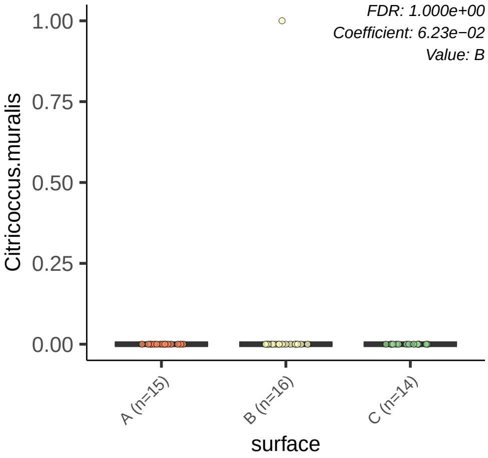

FDR: 1.000e+00
Coefficient: 6.23e−02
Value: B
Citricoccus.muralis
1.00
0.75
0.50
0.25
0.00
A (n=15)
B (n=16)
C (n=14)
surface

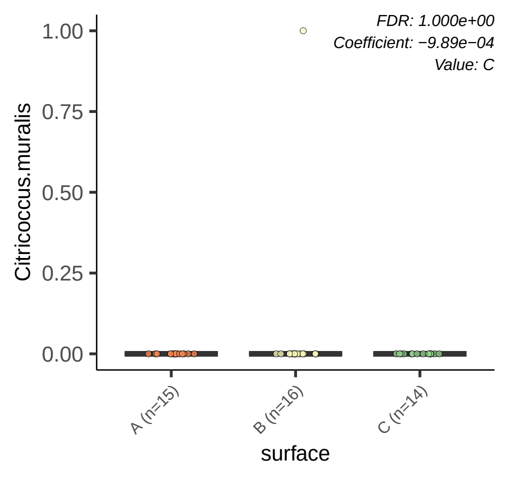

FDR: 1.000e+00
Coefficient: −9.89e−04
Value: C
Citricoccus.muralis
1.00
0.75
0.50
0.25
0.00
A (n=15)
B (n=16)
C (n=14)
surface

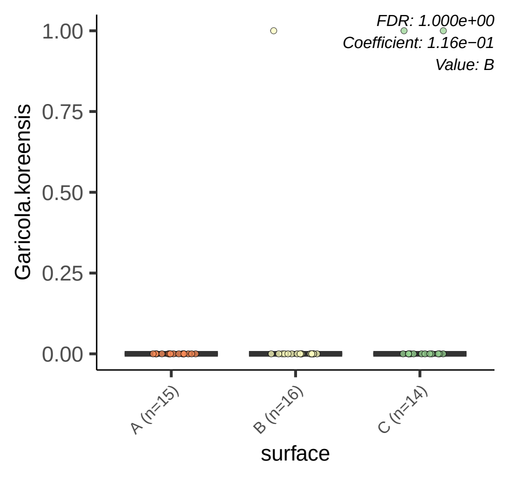
FDR: 1.000e+00
Coefficient: 1.16e−01
Value: B
Garicola.koreensis
1.00
0.75
0.50
0.25
0.00
A (n=15)
B (n=16)
C (n=14)
surface

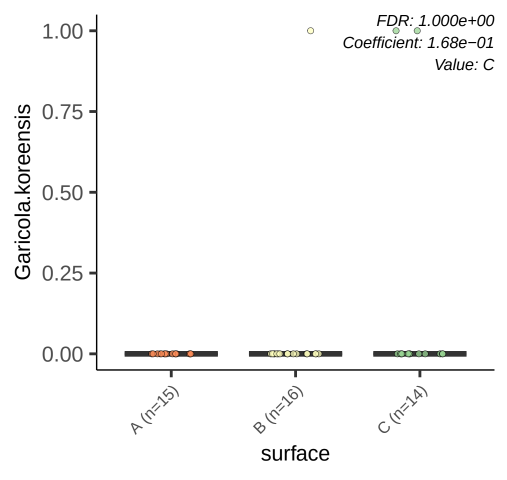
FDR: 1.000e+00
Coefficient: 1.68e−01
Value: C
Garicola.koreensis
1.00
0.75
0.50
0.25
0.00
A (n=15)
B (n=16)
C (n=14)
surface

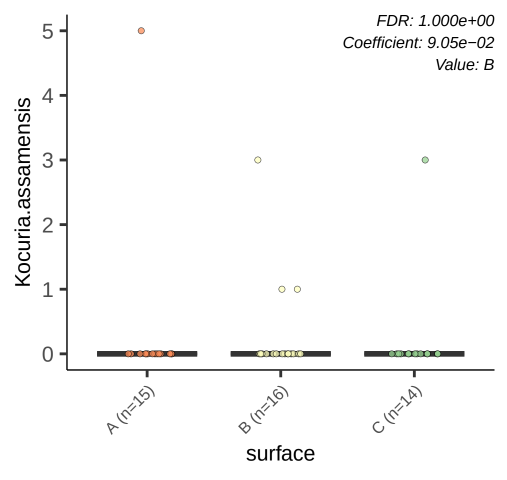
FDR: 1.000e+00
Coefficient: 9.05e−02
Value: B
Kocuria.assamensis
0
1
2
3
4
5
A (n=15)
B (n=16)
C (n=14)
surface

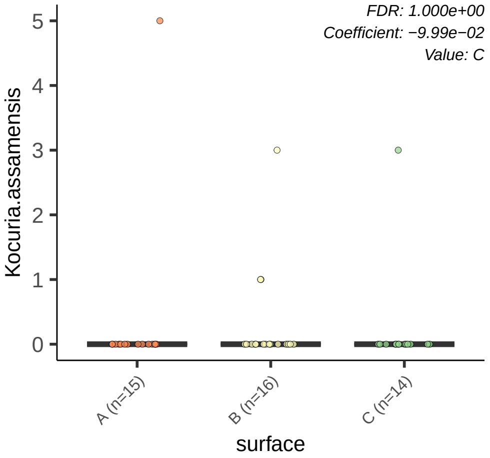

FDR: 1.000e+00
Coefficient: −9.99e−02
Value: C
Kocuria.assamensis
0
1
2
3
4
5
A (n=15)
B (n=16)
C (n=14)
surface

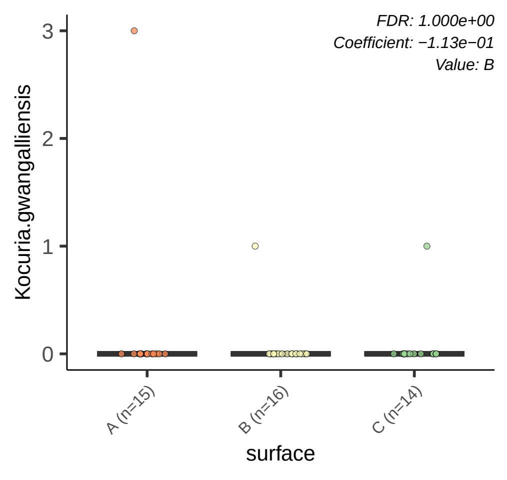

FDR: 1.000e+00
Coefficient: −1.13e−01
Value: B
Kocuria.gwangalliensis
3
2
1
0
A (n=15)
B (n=16)
C (n=14)
surface

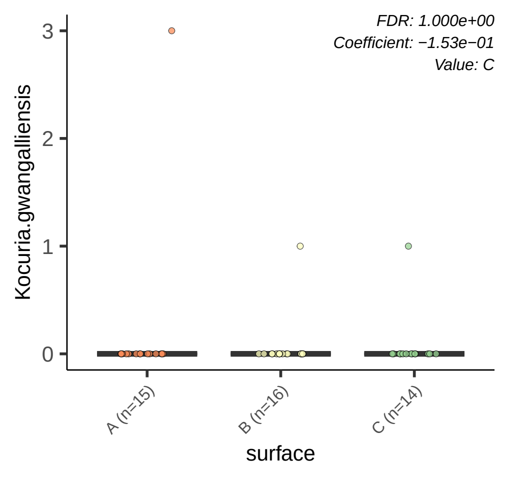

FDR: 1.000e+00
Coefficient: −1.53e−01
Value: C
Kocuria.gwangalliensis
0
1
2
3
A (n=15)
B (n=16)
C (n=14)
surface

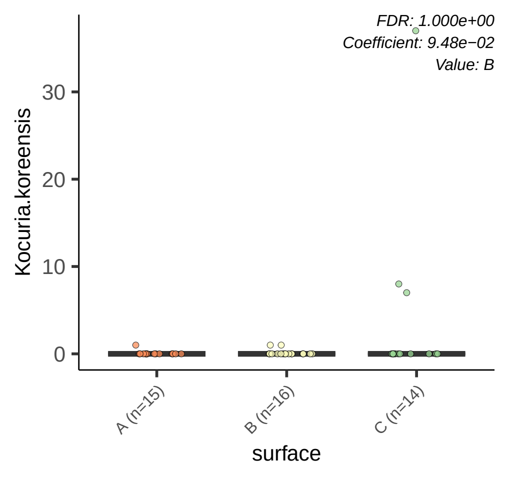

FDR: 1.000e+00
Coefficient: 9.48e−02
Value: B
30
20
10
0
Kocuria.koreensis
A (n=15)
B (n=16)
C (n=14)
surface

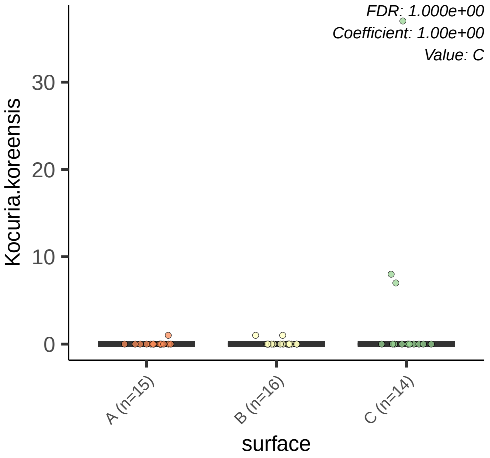
FDR: 1.000e+00
Coefficient: 1.00e+00
Value: C
Kocuria.koreensis
0
10
20
30
A (n=15)
B (n=16)
C (n=14)
surface

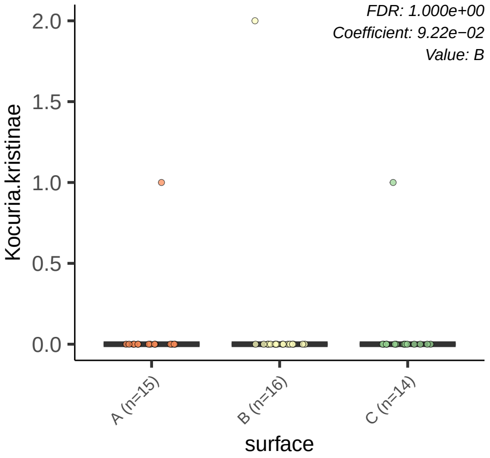
FDR: 1.000e+00
Coefficient: 9.22e−02
Value: B
Kocuria.kristinae
2.0
1.5
1.0
0.5
0.0
A (n=15)
B (n=16)
C (n=14)
surface

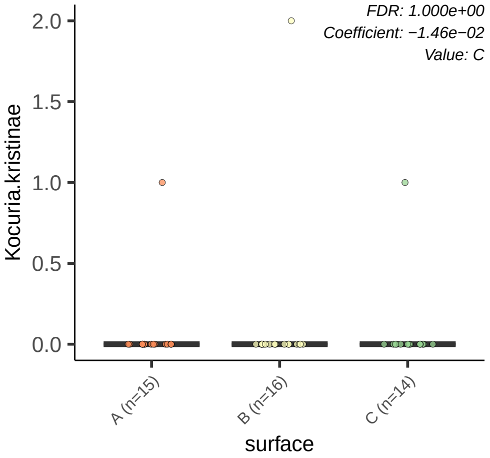
FDR: 1.000e+00
Coefficient: −1.46e−02
Value: C
Kocuria.kristinae
2.0
1.5
1.0
0.5
0.0
A (n=15)
B (n=16)
C (n=14)
surface

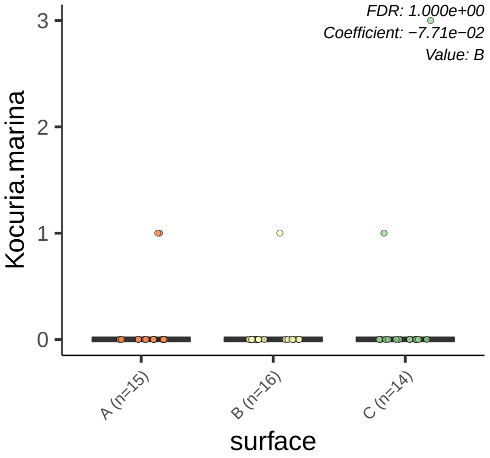

FDR: 1.000e+00
Coefficient: −7.71e−02
Value: B
Kocuria.marina
3
2
1
0
A (n=15)
B (n=16)
C (n=14)
surface

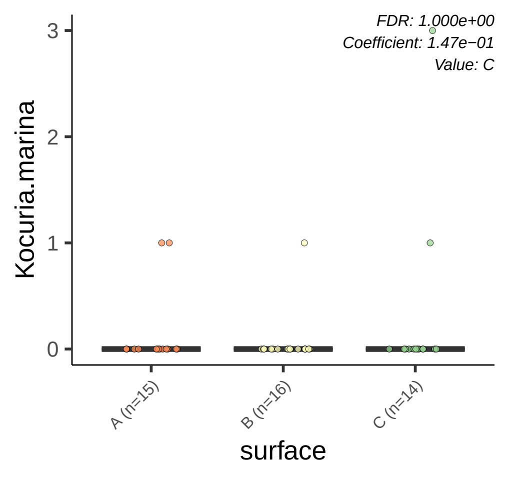

FDR: 1.000e+00
Coefficient: 1.47e−01
Value: C
Kocuria.marina
3
2
1
0
A (n=15)
B (n=16)
C (n=14)
surface

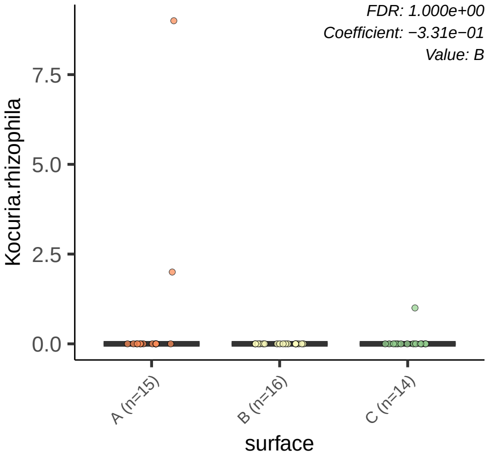

FDR: 1.000e+00
Coefficient: −3.31e−01
Value: B
Kocuria.rhizophila
7.5
5.0
2.5
0.0
A (n=15)
B (n=16)
C (n=14)
surface

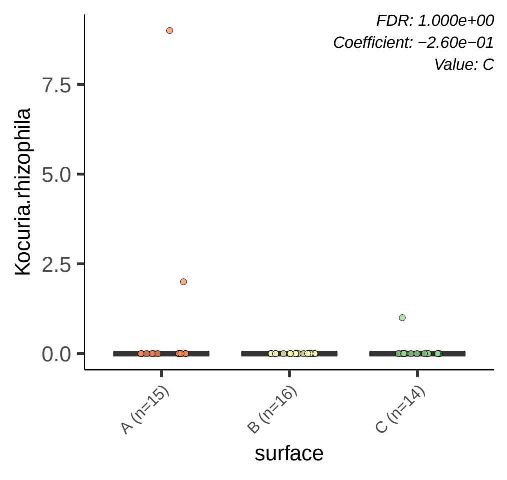

FDR: 1.000e+00
Coefficient: −2.60e−01
Value: C
Kocuria.rhizophila
7.5
5.0
2.5
0.0
A (n=15)
B (n=16)
C (n=14)
surface

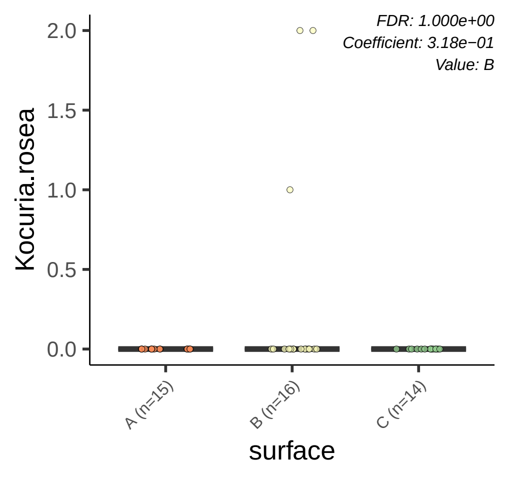
FDR: 1.000e+00
Coefficient: 3.18e−01
Value: B
Kocuria.rosea
2.0
1.5
1.0
0.5
0.0
A (n=15)
B (n=16)
C (n=14)
surface

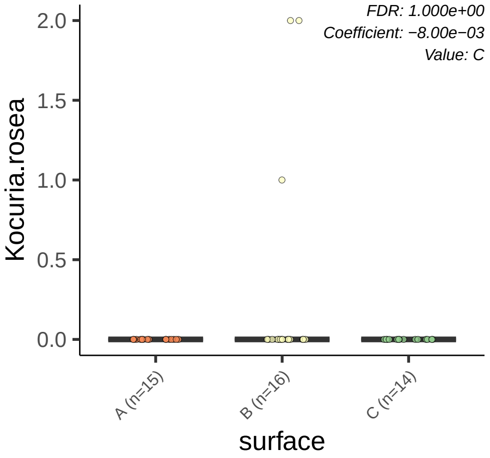

FDR: 1.000e+00
Coefficient: −8.00e−03
Value: C
Kocuria.rosea
2.0
1.5
1.0
0.5
0.0
A (n=15)
B (n=16)
C (n=14)
surface

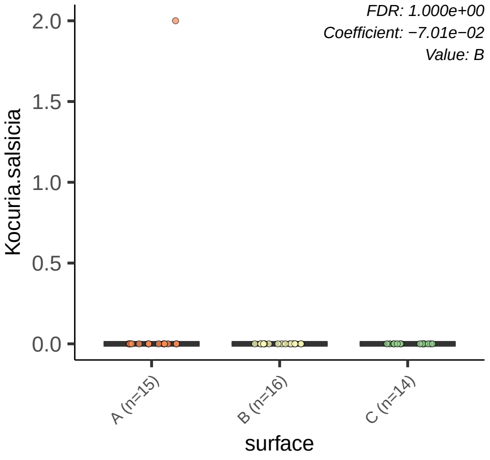

FDR: 1.000e+00
Coefficient: −7.01e−02
Value: B
Kocuria.salsicia
2.0
1.5
1.0
0.5
0.0
A (n=15)
B (n=16)
C (n=14)
surface

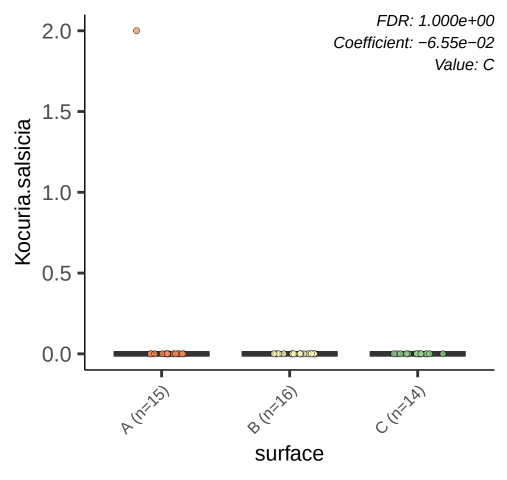
FDR: 1.000e+00
Coefficient: −6.55e−02
Value: C
Kocuria.salsicia
2.0
1.5
1.0
0.5
0.0
A (n=15)
B (n=16)
C (n=14)
surface

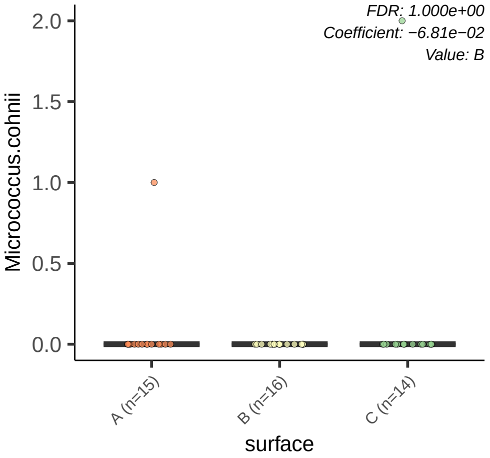
FDR: 1.000e+00
Coefficient: −6.81e−02
Value: B
Micrococcus.cohnii
2.0
1.5
1.0
0.5
0.0
A (n=15)
B (n=16)
C (n=14)
surface

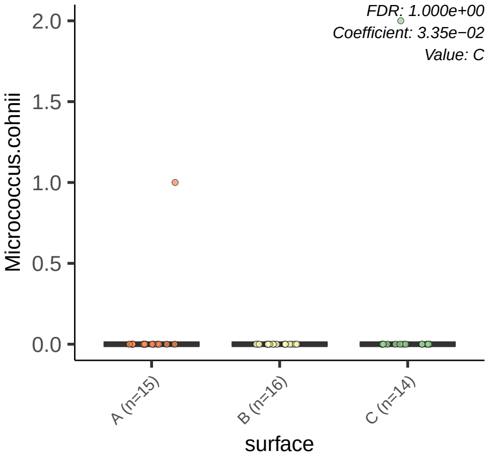

FDR: 1.000e+00
Coefficient: 3.35e−02
Value: C
Micrococcus.cohnii
2.0
1.5
1.0
0.5
0.0
A (n=15)
B (n=16)
C (n=14)
surface

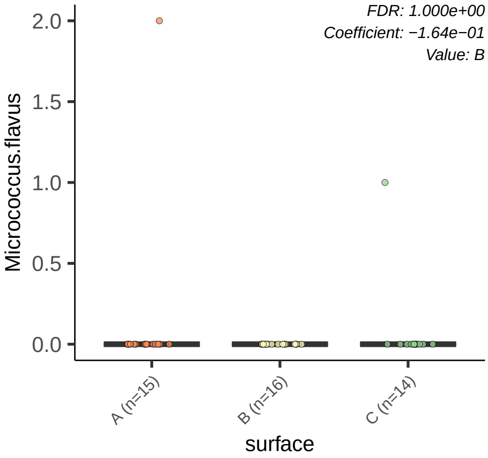
FDR: 1.000e+00
Coefficient: −1.64e−01
Value: B
Micrococcus.flavus
2.0
1.5
1.0
0.5
0.0
A (n=15)
B (n=16)
C (n=14)
surface

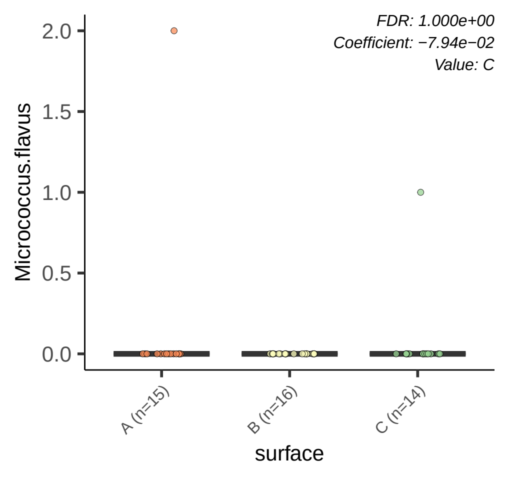

FDR: 1.000e+00
Coefficient: −7.94e−02
Value: C
Micrococcus.flavus
2.0
1.5
1.0
0.5
0.0
A (n=15)
B (n=16)
C (n=14)
surface

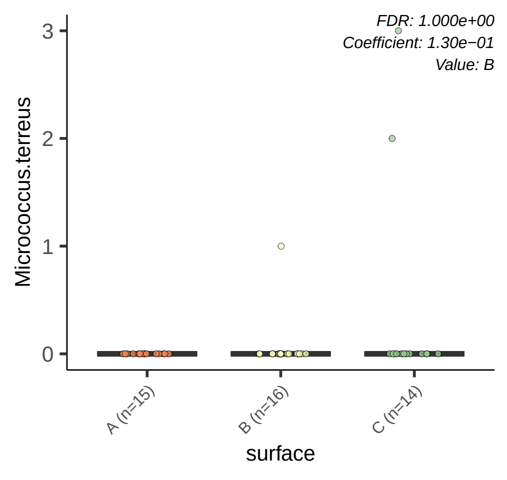
FDR: 1.000e+00
Coefficient: 1.30e−01
Value: B
Micrococcus.terreus
3
2
1
0
A (n=15)
B (n=16)
C (n=14)
surface

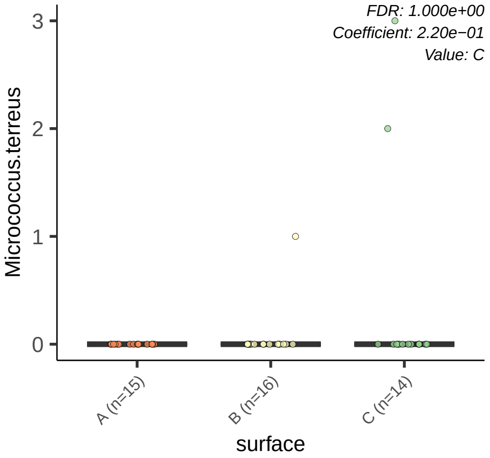
FDR: 1.000e+00
Coefficient: 2.20e−01
Value: C
Micrococcus.terreus
3
2
1
0
A (n=15)
B (n=16)
C (n=14)
surface

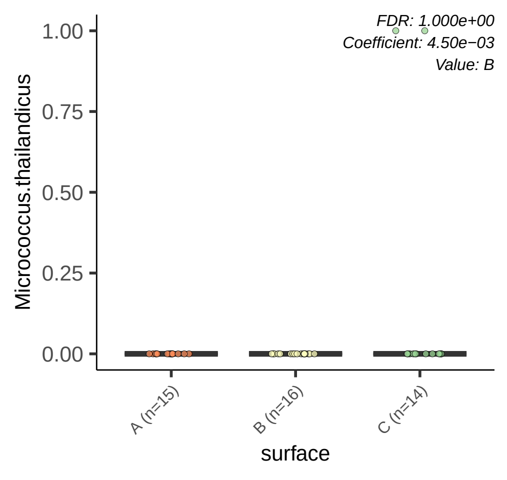

FDR: 1.000e+00
Coefficient: 4.50e−03
Value: B
Micrococcus.thailandicus
1.00
0.75
0.50
0.25
0.00
A (n=15)
B (n=16)
C (n=14)
surface

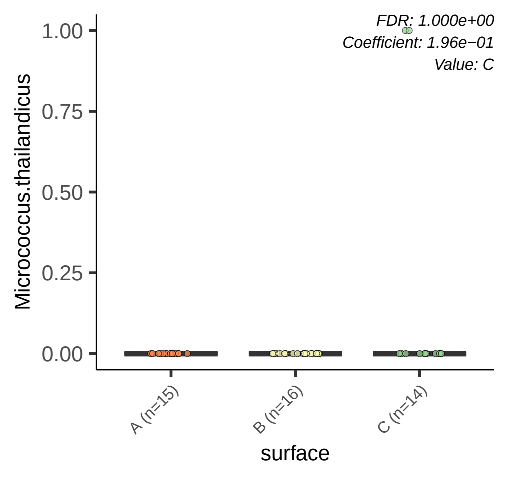
FDR: 1.000e+00
Coefficient: 1.96e−01
Value: C
Micrococcus.thailandicus
1.00
0.75
0.50
0.25
0.00
A (n=15)
B (n=16)
C (n=14)
surface

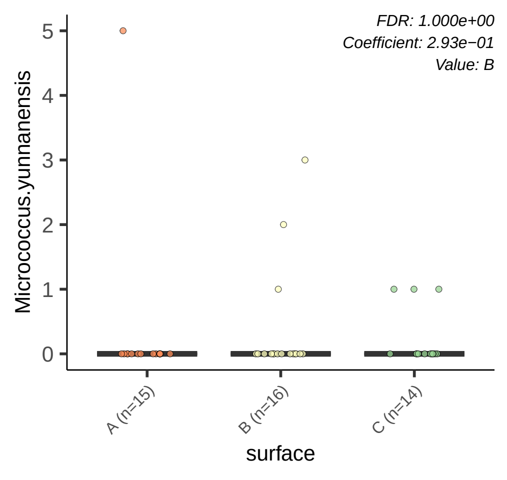

FDR: 1.000e+00
Coefficient: 2.93e−01
Value: B
Micrococcus.yunnanensis
0
1
2
3
4
5
A (n=15)
B (n=16)
C (n=14)
surface

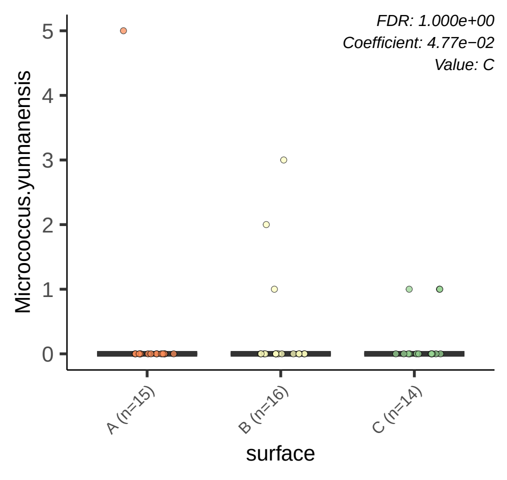

FDR: 1.000e+00
Coefficient: 4.77e−02
Value: C
Micrococcus.yunnanensis
0
1
2
3
4
5
A (n=15)
B (n=16)
C (n=14)
surface

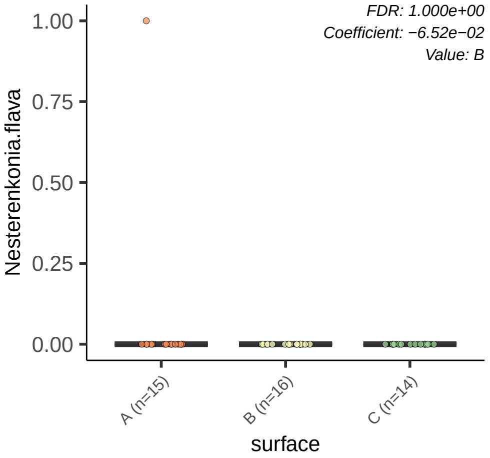
FDR: 1.000e+00
Coefficient: −6.52e−02
Value: B
Nesterenkonia.flava
1.00
0.75
0.50
0.25
0.00
A (n=15)
B (n=16)
C (n=14)
surface

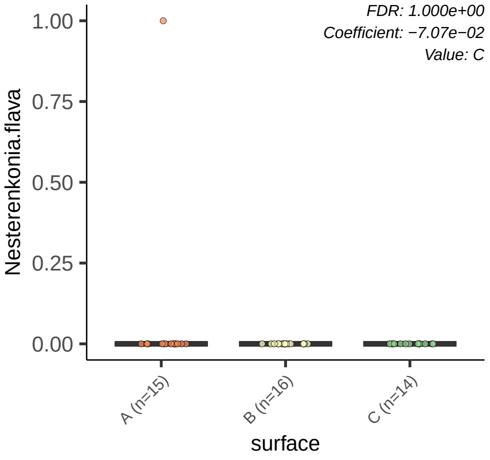
FDR: 1.000e+00
Coefficient: −7.07e−02
Value: C
Nesterenkonia.flava
1.00
0.75
0.50
0.25
0.00
A (n=15)
B (n=16)
C (n=14)
surface

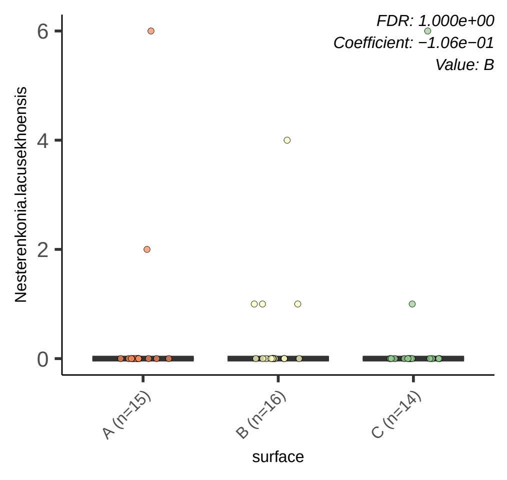

FDR: 1.000e+00
Coefficient: −1.06e−01
Value: B
Nesterenkonia.lacusekhoensis
0
2
4
6
A (n=15)
B (n=16)
C (n=14)
surface

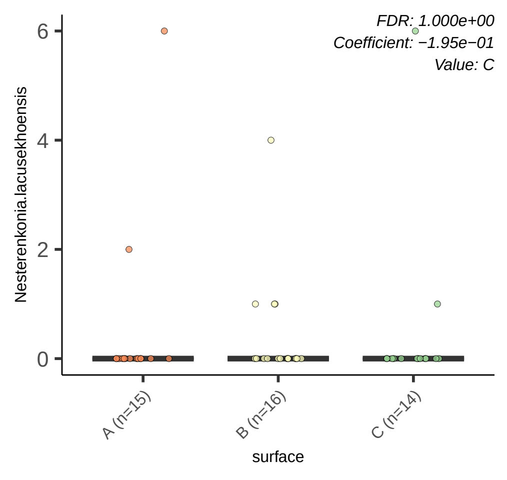
FDR: 1.000e+00
Coefficient: −1.95e−01
Value: C
Nesterenkonia.lacusekhoensis
0
2
4
6
A (n=15)
B (n=16)
C (n=14)
surface

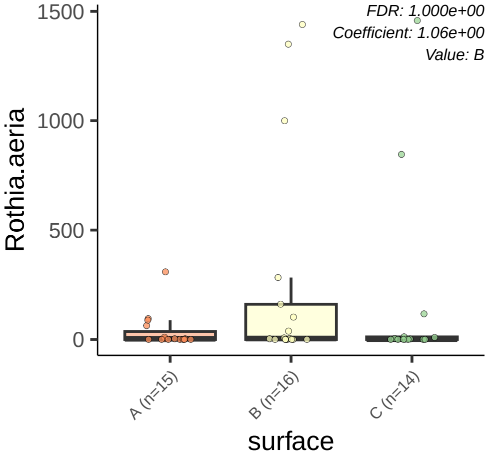
FDR: 1.000e+00
Coefficient: 1.06e+00
Value: B
Rothia.aeria
1500
1000
500
0
A (n=15)
B (n=16)
C (n=14)
surface

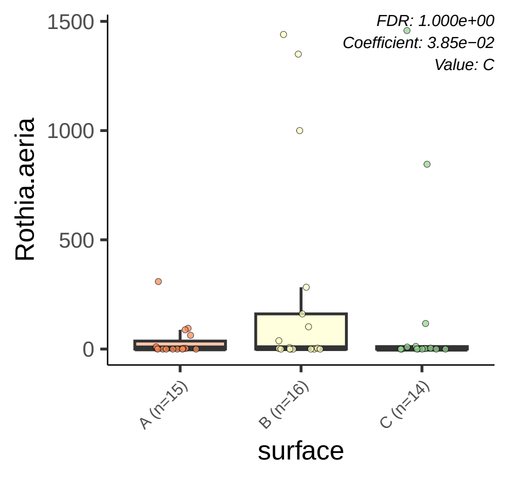
FDR: 1.000e+00
Coefficient: 3.85e−02
Value: C
Rothia.aeria
1500
1000
500
0
A (n=15)
B (n=16)
C (n=14)
surface

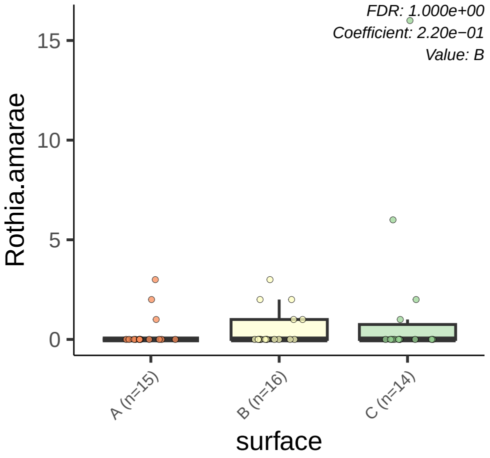
FDR: 1.000e+00
Coefficient: 2.20e−01
Value: B
Rothia.amarae
0
5
10
15
A (n=15)
B (n=16)
C (n=14)
surface

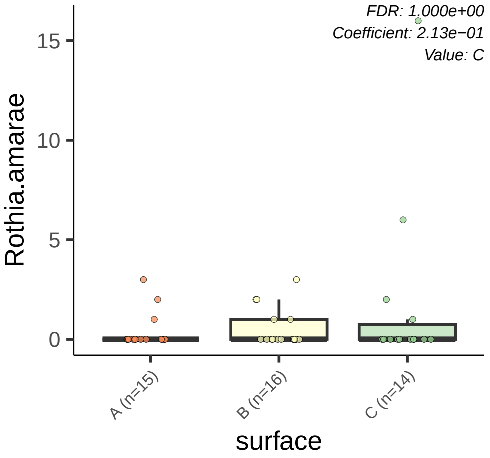

FDR: 1.000e+00
Coefficient: 2.13e−01
Value: C
Rothia.amarae
15
10
5
0
A (n=15)
B (n=16)
C (n=14)
surface

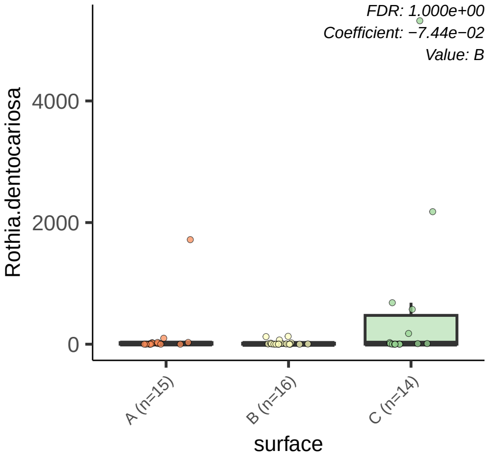

FDR: 1.000e+00
Coefficient: −7.44e−02
Value: B
Rothia.dentocariosa
4000
2000
0
A (n=15)
B (n=16)
C (n=14)
surface

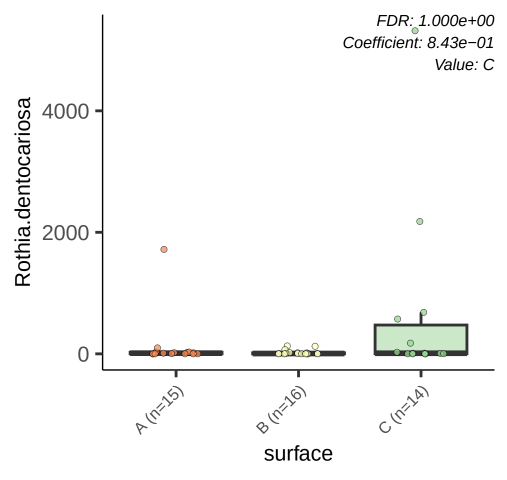
FDR: 1.000e+00
Coefficient: 8.43e−01
Value: C
Rothia.dentocariosa
4000
2000
0
A (n=15)
B (n=16)
C (n=14)
surface

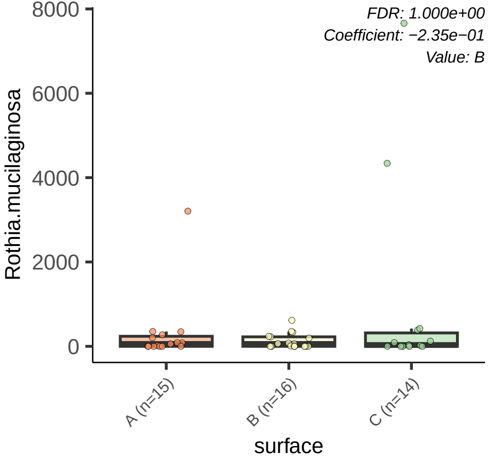

FDR: 1.000e+00
Coefficient: −2.35e−01
Value: B
Rothia.mucilaginosa
8000
6000
4000
2000
0
A (n=15)
B (n=16)
C (n=14)
surface

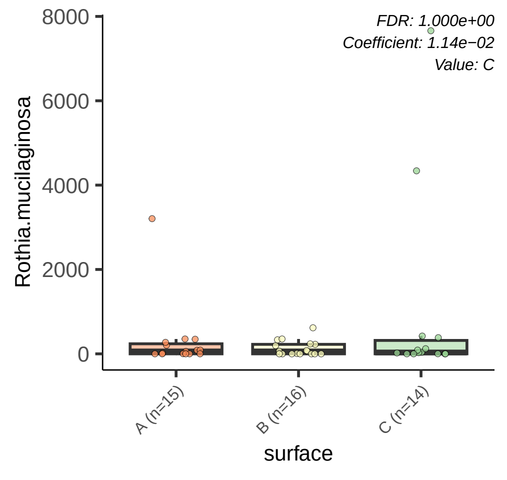

FDR: 1.000e+00
Coefficient: 1.14e−02
Value: C
8000
6000
4000
2000
0
Rothia.mucilaginosa
A (n=15)
B (n=16)
C (n=14)
surface

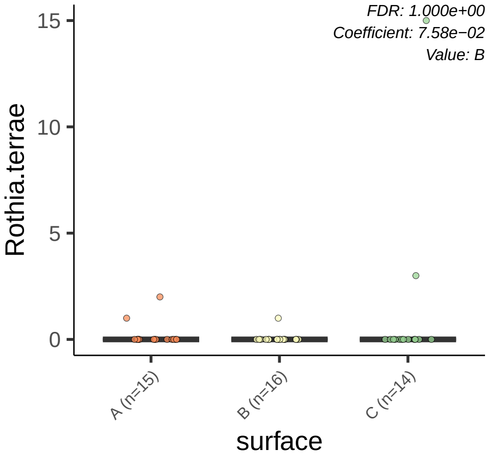

FDR: 1.000e+00
Coefficient: 7.58e−02
Value: B
Rothia.terrae
15
10
5
0
A (n=15)
B (n=16)
C (n=14)
surface

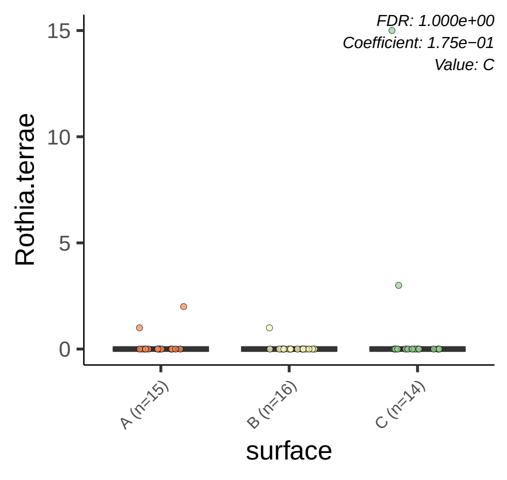

FDR: 1.000e+00
Coefficient: 1.75e−01
Value: C
Rothia.terrae
15
10
5
0
A (n=15)
B (n=16)
C (n=14)
surface

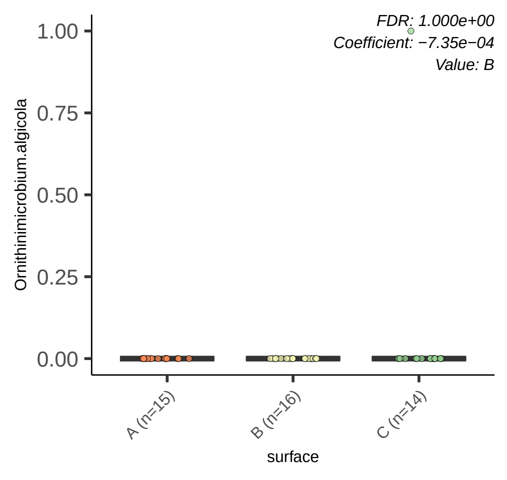

FDR: 1.000e+00
Coefficient: −7.35e−04
Value: B
1.00
0.75
0.50
0.25
0.00
Ornithinimicrobium.algicola
A (n=15)
B (n=16)
C (n=14)
surface

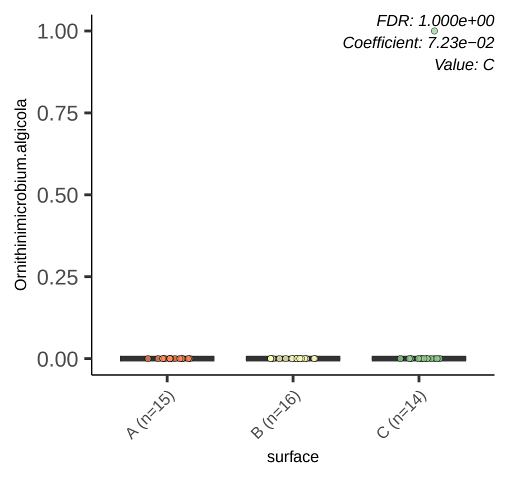

FDR: 1.000e+00
Coefficient: 7.23e−02
Value: C
1.00
0.75
0.50
0.25
0.00
Ornithinimicrobium.algicola
A (n=15)
B (n=16)
C (n=14)
surface

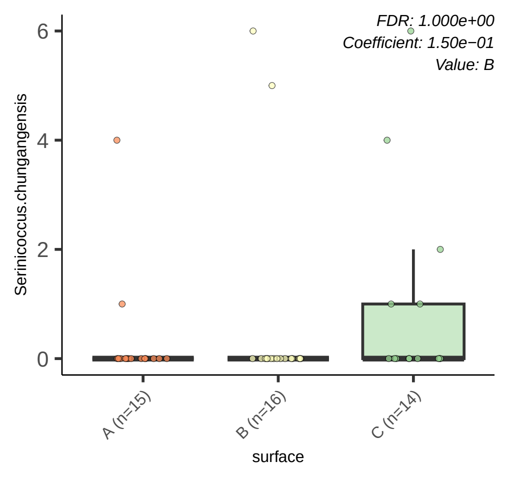
FDR: 1.000e+00
Coefficient: 1.50e−01
Value: B
Serinicoccus.chungangensis
0
2
4
6
A (n=15)
B (n=16)
C (n=14)
surface

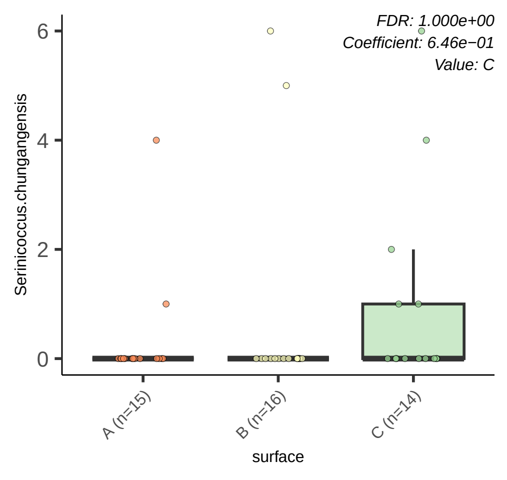

FDR: 1.000e+00
Coefficient: 6.46e−01
Value: C
Serinicoccus.chungangensis
0
2
4
6
A (n=15)
B (n=16)
C (n=14)
surface

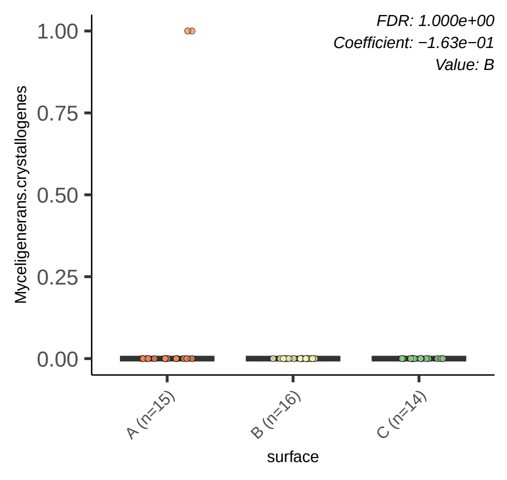

FDR: 1.000e+00
Coefficient: −1.63e−01
Value: B
Myceligenerans.crystallogenes
1.00
0.75
0.50
0.25
0.00
A (n=15)
B (n=16)
C (n=14)
surface

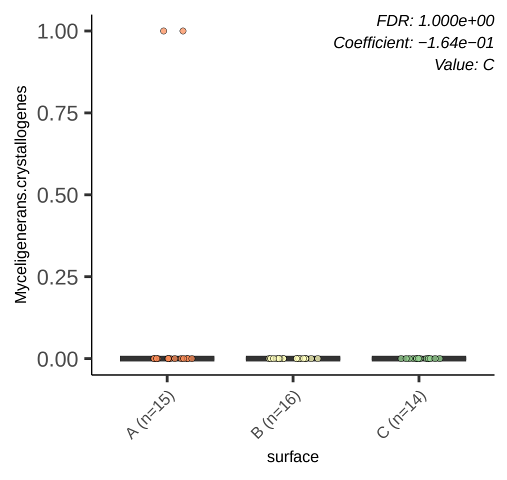

FDR: 1.000e+00
Coefficient: −1.64e−01
Value: C
Myceligenerans.crystallogenes
1.00
0.75
0.50
0.25
0.00
A (n=15)
B (n=16)
C (n=14)
surface

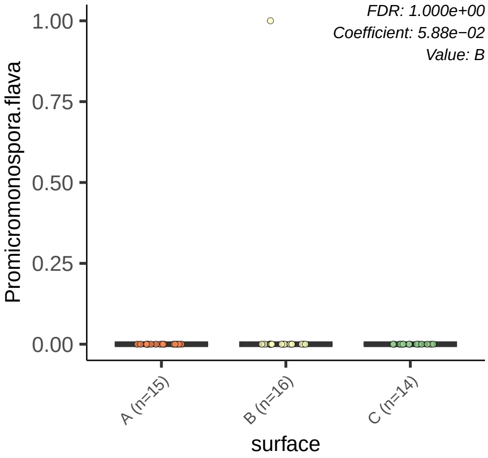
FDR: 1.000e+00
Coefficient: 5.88e−02
Value: B
Promicromonospora.flava
1.00
0.75
0.50
0.25
0.00
A (n=15)
B (n=16)
C (n=14)
surface

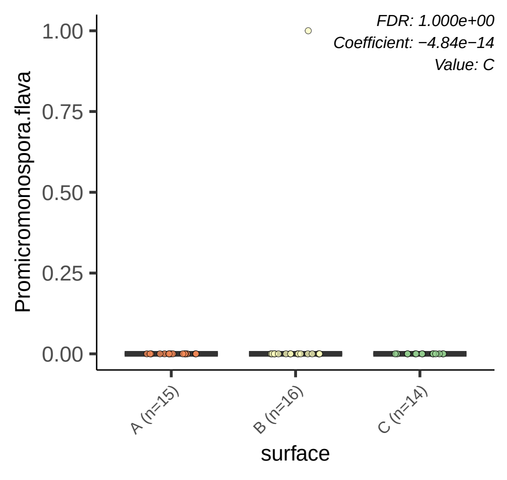

FDR: 1.000e+00
Coefficient: −4.84e−14
Value: C
Promicromonospora.flava
1.00
0.75
0.50
0.25
0.00
A (n=15)
B (n=16)
C (n=14)
surface

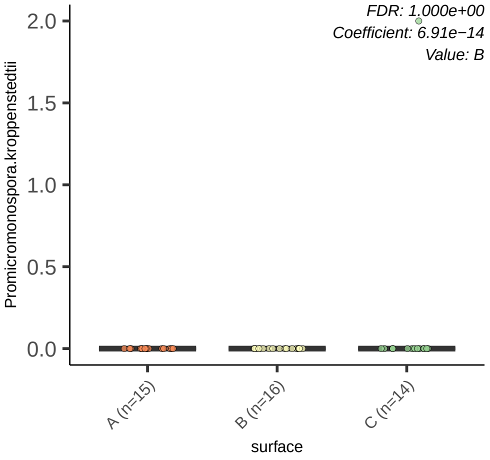
FDR: 1.000e+00
Coefficient: 6.91e−14
Value: B
Promicromonospora.kroppenstedtii
2.0
1.5
1.0
0.5
0.0
A (n=15)
B (n=16)
C (n=14)
surface

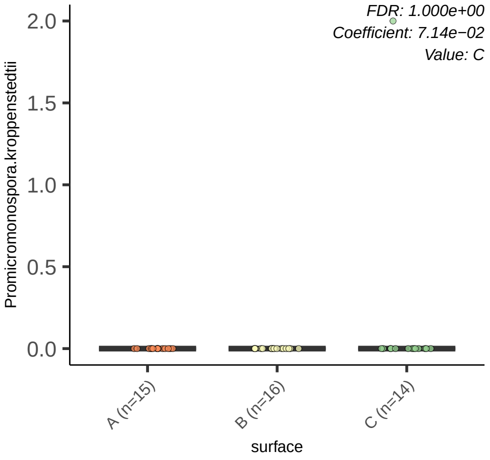
FDR: 1.000e+00
Coefficient: 7.14e−02
Value: C
Promicromonospora.kroppenstedtii
2.0
1.5
1.0
0.5
0.0
A (n=15)
B (n=16)
C (n=14)
surface

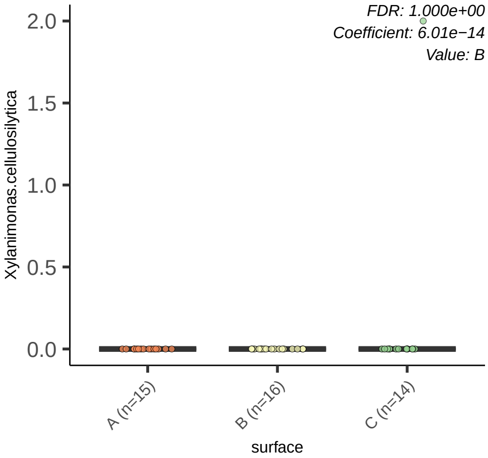
FDR: 1.000e+00
Coefficient: 6.01e−14
Value: B
Xylanimonas.cellulosilytica
2.0
1.5
1.0
0.5
0.0
A (n=15)
B (n=16)
C (n=14)
surface

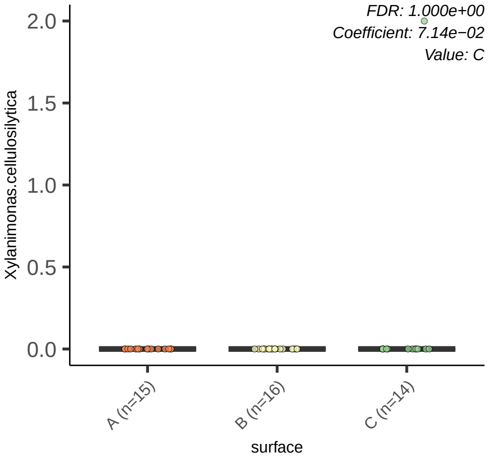

FDR: 1.000e+00
Coefficient: 7.14e−02
Value: C
Xylanimonas.cellulosilytica
2.0
1.5
1.0
0.5
0.0
A (n=15)
B (n=16)
C (n=14)
surface

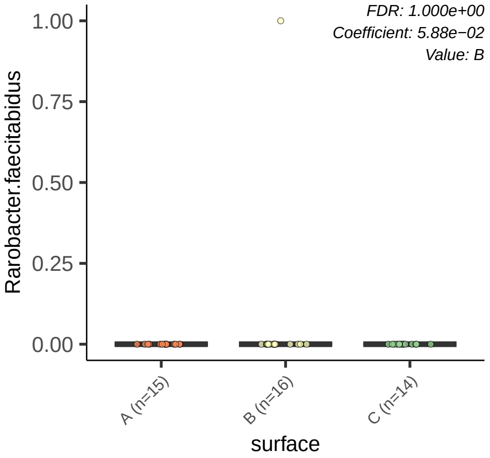
FDR: 1.000e+00
Coefficient: 5.88e−02
Value: B
Rarobacter.faecitabidus
1.00
0.75
0.50
0.25
0.00
A (n=15)
B (n=16)
C (n=14)
surface

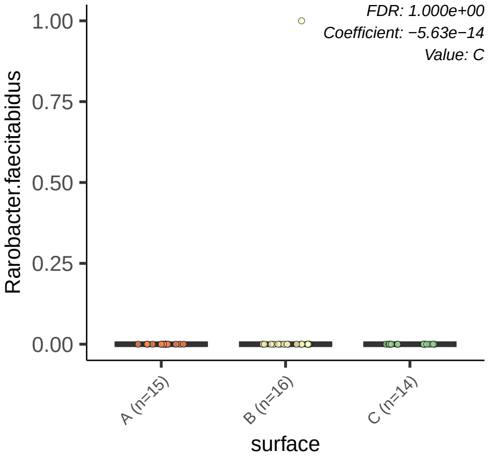
FDR: 1.000e+00
Coefficient: −5.63e−14
Value: C
Rarobacter.faecitabidus
1.00
0.75
0.50
0.25
0.00
A (n=15)
B (n=16)
C (n=14)
surface

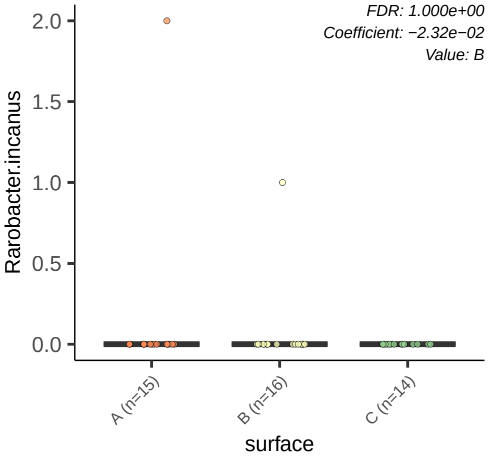
FDR: 1.000e+00
Coefficient: −2.32e−02
Value: B
Rarobacter.incanus
2.0
1.5
1.0
0.5
0.0
A (n=15)
B (n=16)
C (n=14)
surface

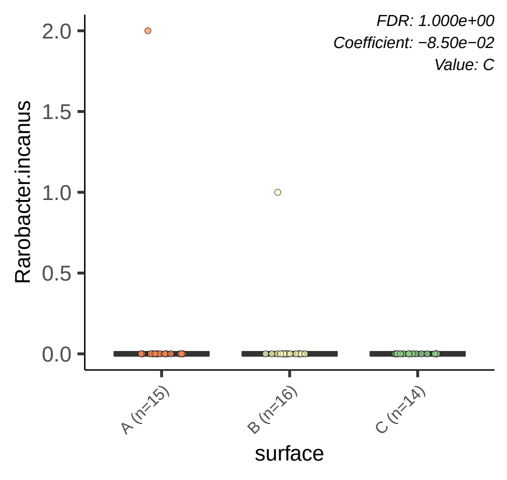
FDR: 1.000e+00
Coefficient: −8.50e−02
Value: C
Rarobacter.incanus
2.0
1.5
1.0
0.5
0.0
A (n=15)
B (n=16)
C (n=14)
surface

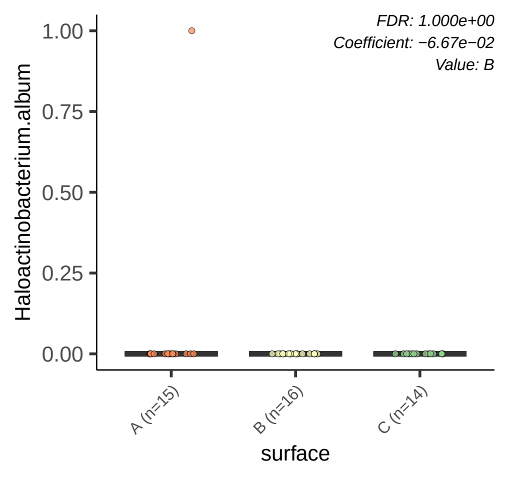
FDR: 1.000e+00
Coefficient: −6.67e−02
Value: B
Haloactinobacterium.album
1.00
0.75
0.50
0.25
0.00
A (n=15)
B (n=16)
C (n=14)
surface

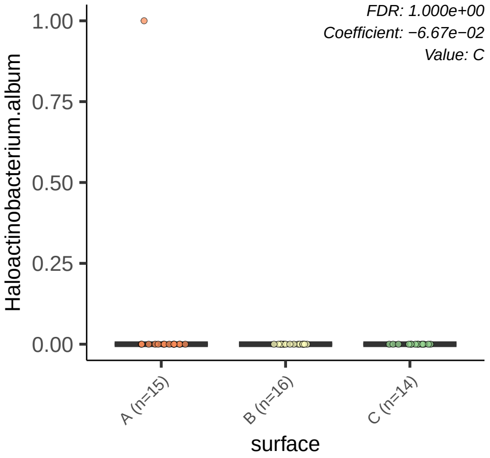

FDR: 1.000e+00
Coefficient: −6.67e−02
Value: C
Haloactinobacterium.album
1.00
0.75
0.50
0.25
0.00
A (n=15)
B (n=16)
C (n=14)
surface

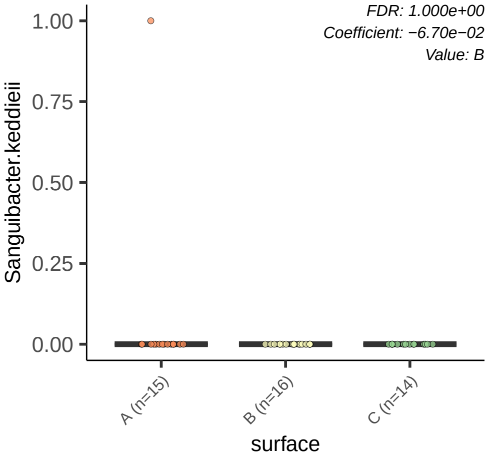

FDR: 1.000e+00
Coefficient: −6.70e−02
Value: B
Sanguibacter.keddieii
1.00
0.75
0.50
0.25
0.00
A (n=15)
B (n=16)
C (n=14)
surface

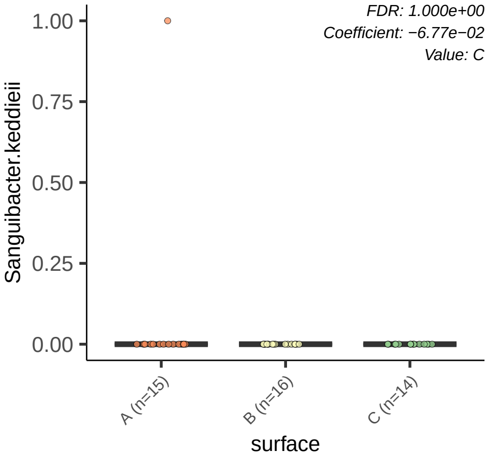

FDR: 1.000e+00
Coefficient: −6.77e−02
Value: C
Sanguibacter.keddieii
1.00
0.75
0.50
0.25
0.00
A (n=15)
B (n=16)
C (n=14)
surface

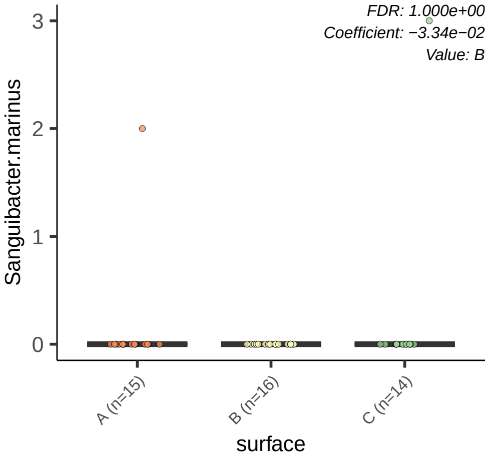

FDR: 1.000e+00
Coefficient: −3.34e−02
Value: B
Sanguibacter.marinus
3
2
1
0
A (n=15)
B (n=16)
C (n=14)
surface

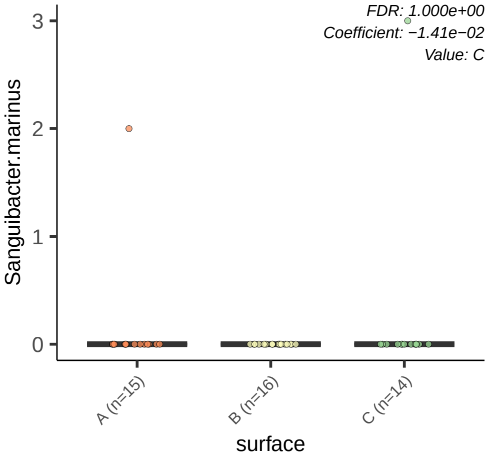
FDR: 1.000e+00
Coefficient: −1.41e−02
Value: C
Sanguibacter.marinus
3
2
1
0
A (n=15)
B (n=16)
C (n=14)
surface

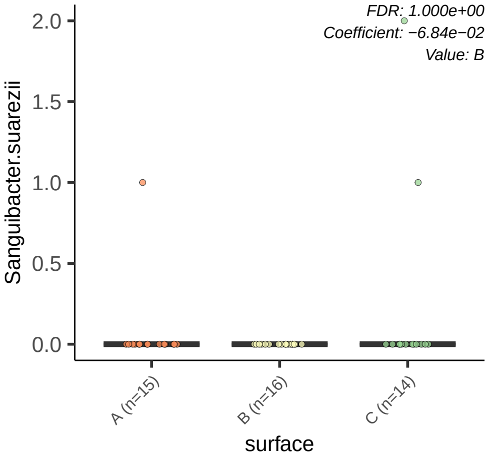
FDR: 1.000e+00
Coefficient: −6.84e−02
Value: B
Sanguibacter.suarezii
2.0
1.5
1.0
0.5
0.0
A (n=15)
B (n=16)
C (n=14)
surface

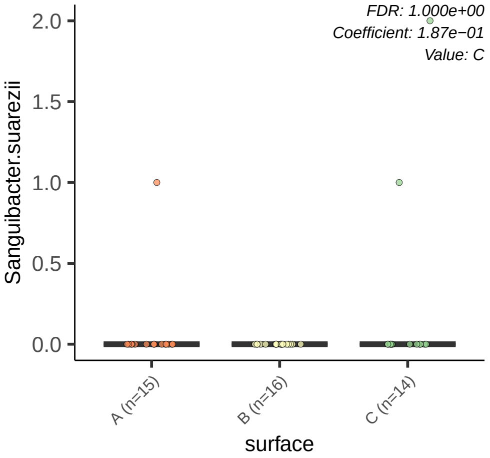
FDR: 1.000e+00
Coefficient: 1.87e−01
Value: C
Sanguibacter.suarezii
2.0
1.5
1.0
0.5
0.0
A (n=15)
B (n=16)
C (n=14)
surface

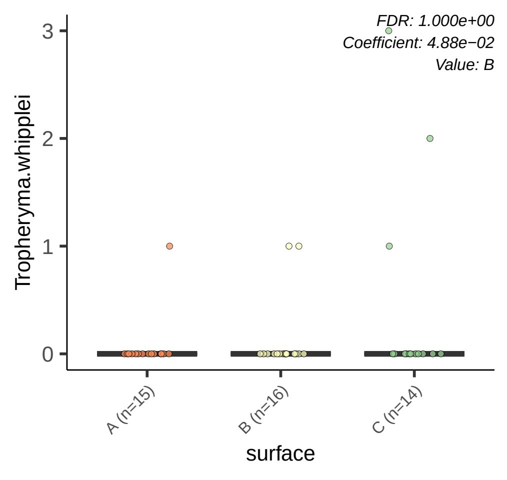

FDR: 1.000e+00
Coefficient: 4.88e−02
Value: B
Tropheryma.whipplei
0
1
2
3
A (n=15)
B (n=16)
C (n=14)
surface

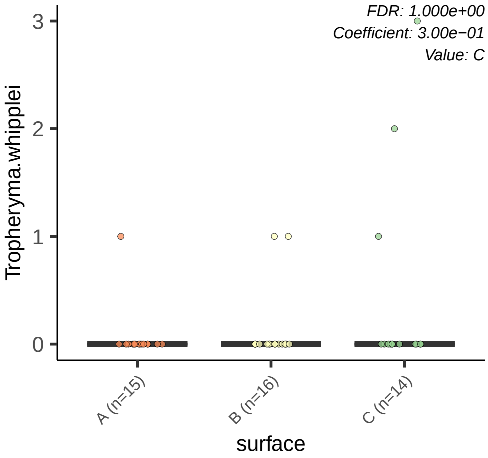
FDR: 1.000e+00
Coefficient: 3.00e−01
Value: C
Tropheryma.whipplei
0
1
2
3
A (n=15)
B (n=16)
C (n=14)
surface

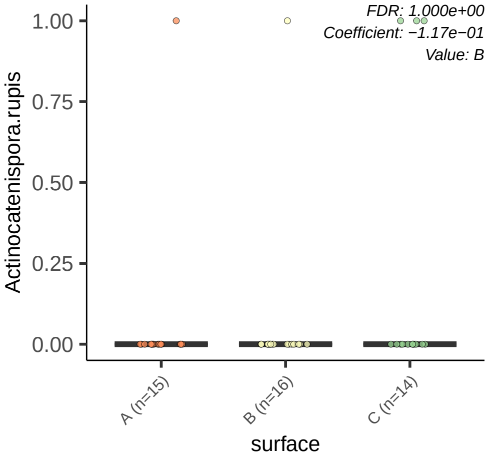
FDR: 1.000e+00
Coefficient: −1.17e−01
Value: B
Actinocatenispora.rupis
1.00
0.75
0.50
0.25
0.00
A (n=15)
B (n=16)
C (n=14)
surface

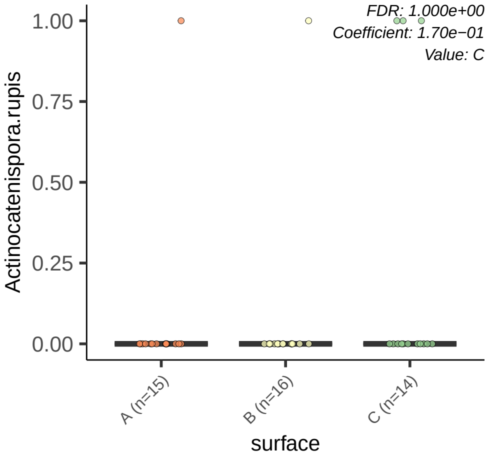
FDR: 1.000e+00
Coefficient: 1.70e−01
Value: C
Actinocatenispora.rupis
1.00
0.75
0.50
0.25
0.00
A (n=15)
B (n=16)
C (n=14)
surface

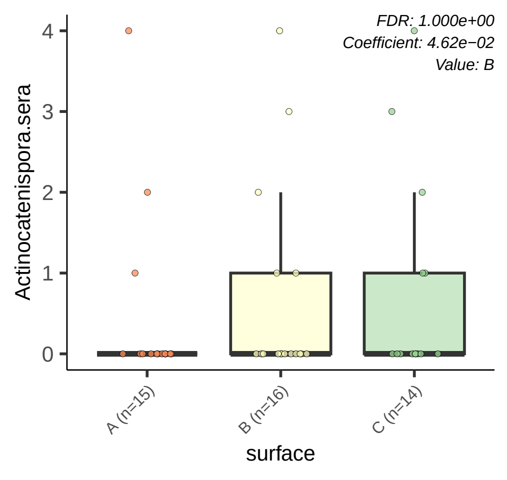
FDR: 1.000e+00
Coefficient: 4.62e−02
Value: B
Actinocatenispora.sera
0
1
2
3
4
A (n=15)
B (n=16)
C (n=14)
surface

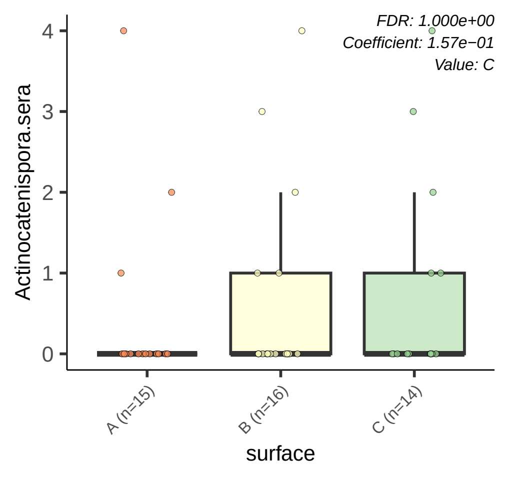

FDR: 1.000e+00
Coefficient: 1.57e−01
Value: C
Actinocatenispora.sera
0
1
2
3
4
A (n=15)
B (n=16)
C (n=14)
surface

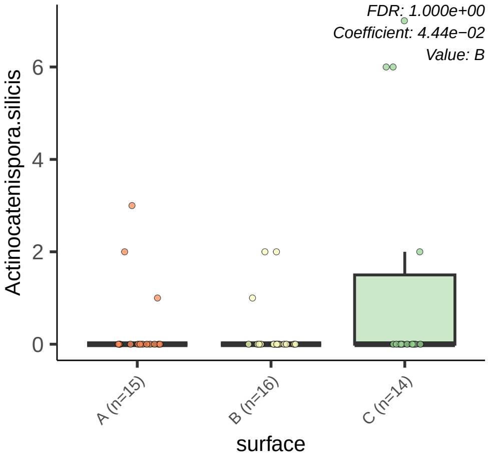

FDR: 1.000e+00
Coefficient: 4.44e−02
Value: B
Actinocatenispora.silicis
0
2
4
6
A (n=15)
B (n=16)
C (n=14)
surface

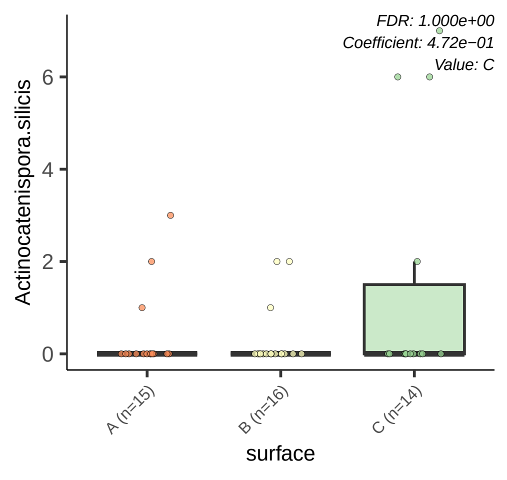

FDR: 1.000e+00
Coefficient: 4.72e−01
Value: C
Actinocatenispora.silicis
0
2
4
6
A (n=15)
B (n=16)
C (n=14)
surface

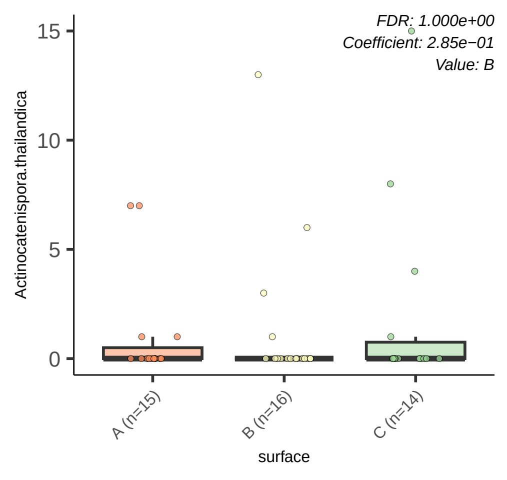
FDR: 1.000e+00
Coefficient: 2.85e−01
Value: B
Actinocatenispora.thailandica
15
10
5
0
A (n=15)
B (n=16)
C (n=14)
surface

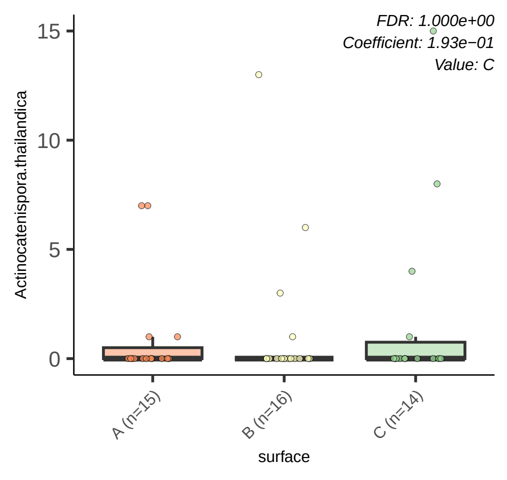
FDR: 1.000e+00
Coefficient: 1.93e−01
Value: C
Actinocatenispora.thailandica
0
5
10
15
A (n=15)
B (n=16)
C (n=14)
surface

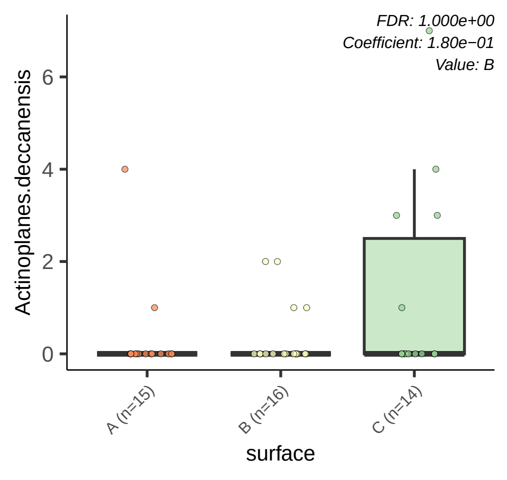
FDR: 1.000e+00
Coefficient: 1.80e−01
Value: B
Actinoplanes.deccanensis
0
2
4
6
A (n=15)
B (n=16)
C (n=14)
surface

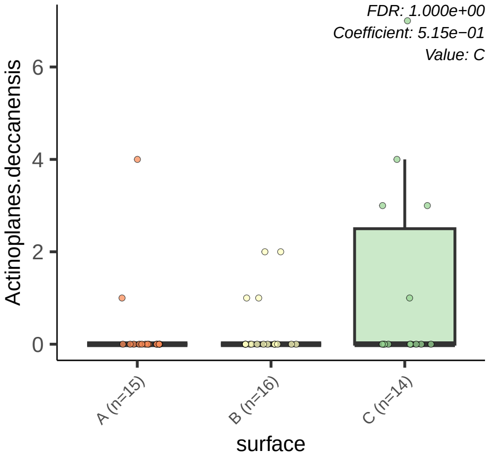

FDR: 1.000e+00
Coefficient: 5.15e−01
Value: C
Actinoplanes.deccanensis
0
2
4
6
A (n=15)
B (n=16)
C (n=14)
surface

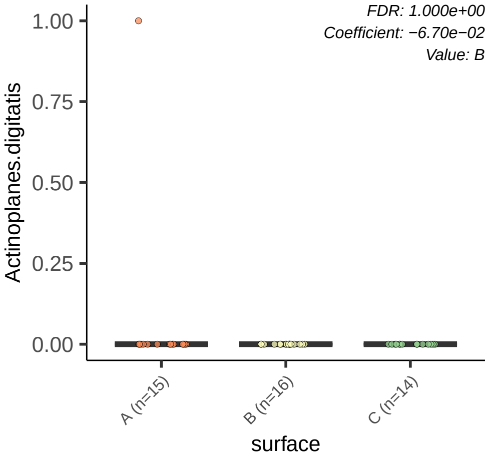
FDR: 1.000e+00
Coefficient: −6.70e−02
Value: B
Actinoplanes.digitatis
1.00
0.75
0.50
0.25
0.00
A (n=15)
B (n=16)
C (n=14)
surface

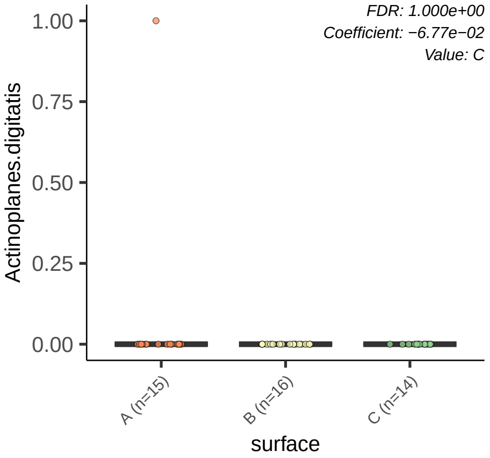
FDR: 1.000e+00
Coefficient: −6.77e−02
Value: C
Actinoplanes.digitatis
1.00
0.75
0.50
0.25
0.00
A (n=15)
B (n=16)
C (n=14)
surface

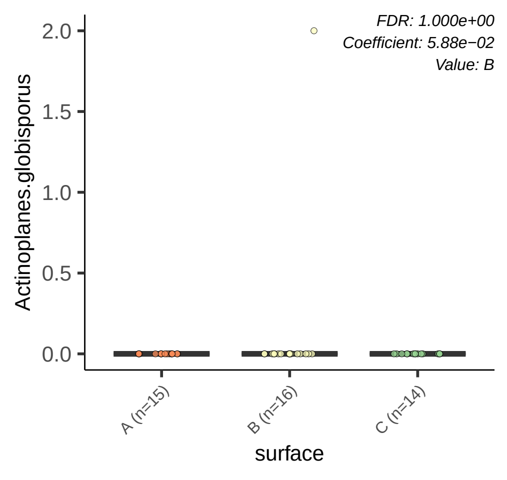

FDR: 1.000e+00
Coefficient: 5.88e−02
Value: B
Actinoplanes.globisporus
2.0
1.5
1.0
0.5
0.0
A (n=15)
B (n=16)
C (n=14)
surface

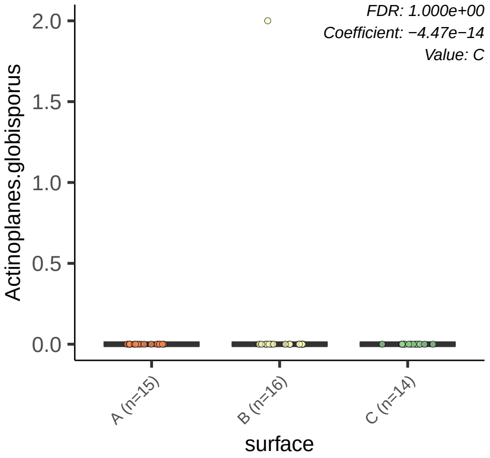

FDR: 1.000e+00
Coefficient: −4.47e−14
Value: C
Actinoplanes.globisporus
2.0
1.5
1.0
0.5
0.0
A (n=15)
B (n=16)
C (n=14)
surface

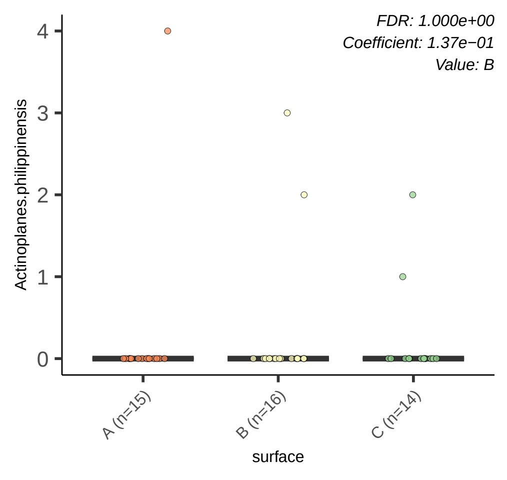
FDR: 1.000e+00
Coefficient: 1.37e−01
Value: B
Actinoplanes.philippinensis
4
3
2
1
0
A (n=15)
B (n=16)
C (n=14)
surface

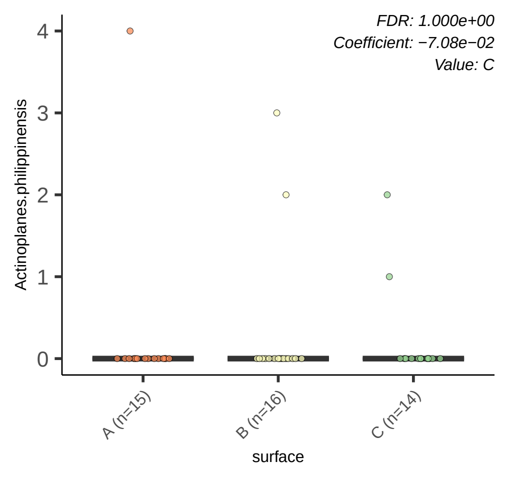
FDR: 1.000e+00
Coefficient: −7.08e−02
Value: C
Actinoplanes.philippinensis
4
3
2
1
0
A (n=15)
B (n=16)
C (n=14)
surface

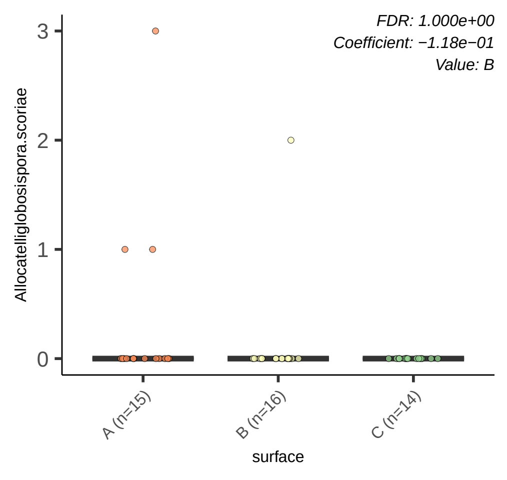
FDR: 1.000e+00
Coefficient: −1.18e−01
Value: B
Allocatelliglobosispora.scoriae
3
2
1
0
A (n=15)
B (n=16)
C (n=14)
surface

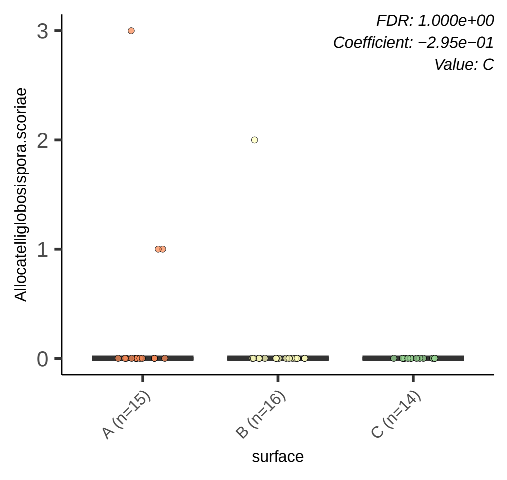
FDR: 1.000e+00
Coefficient: −2.95e−01
Value: C
Allocatelliglobosispora.scoriae
3
2
1
0
A (n=15)
B (n=16)
C (n=14)
surface

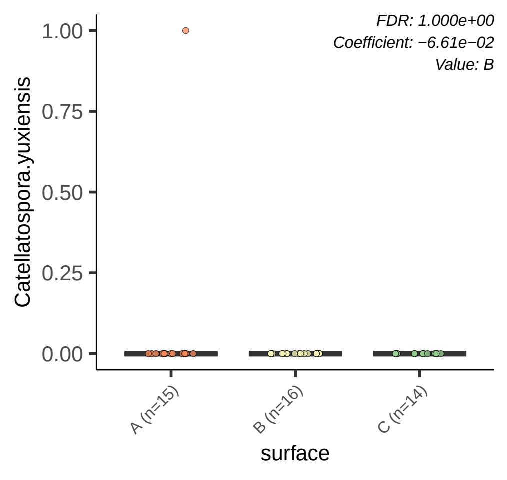
FDR: 1.000e+00
Coefficient: −6.61e−02
Value: B
Catellatospora.yuxiensis
1.00
0.75
0.50
0.25
0.00
A (n=15)
B (n=16)
C (n=14)
surface

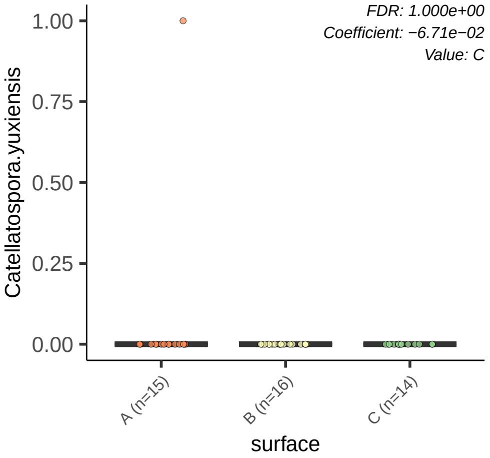
FDR: 1.000e+00
Coefficient: −6.71e−02
Value: C
1.00
0.75
0.50
0.25
0.00
Catellatospora.yuxiensis
A (n=15)
B (n=16)
C (n=14)
surface

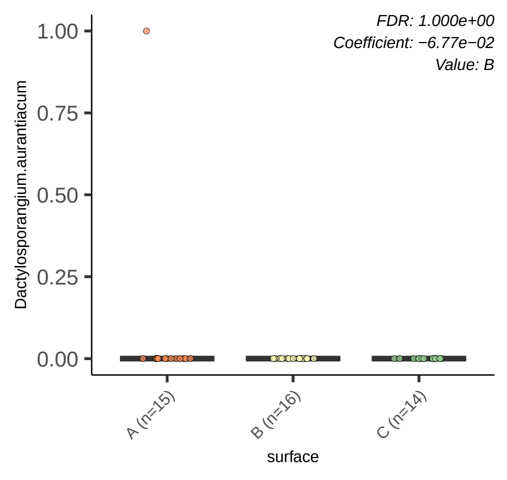

FDR: 1.000e+00
Coefficient: −6.77e−02
Value: B
1.00
0.75
0.50
0.25
0.00
Dactylosporangium.aurantiacum
A (n=15)
B (n=16)
C (n=14)
surface

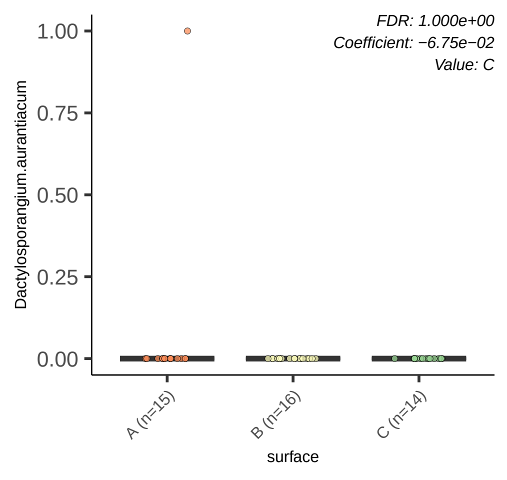
FDR: 1.000e+00
Coefficient: −6.75e−02
Value: C
1.00
0.75
0.50
0.25
0.00
Dactylosporangium.aurantiacum
A (n=15)
B (n=16)
C (n=14)
surface

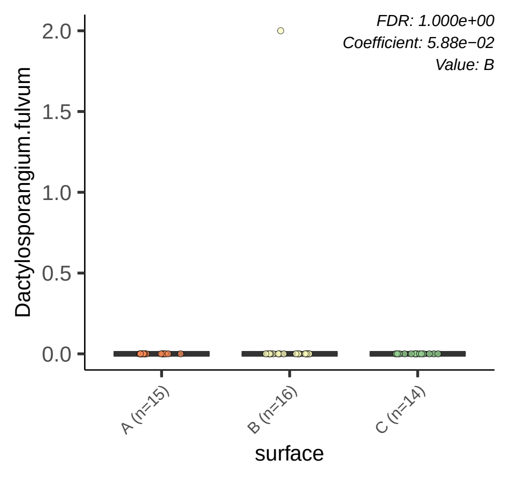
FDR: 1.000e+00
Coefficient: 5.88e−02
Value: B
Dactylosporangium.fulvum
2.0
1.5
1.0
0.5
0.0
A (n=15)
B (n=16)
C (n=14)
surface

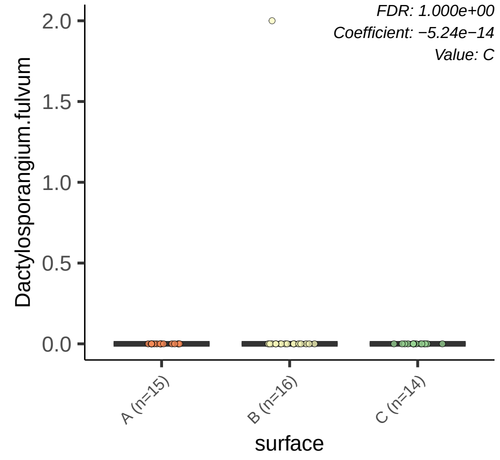

FDR: 1.000e+00
Coefficient: −5.24e−14
Value: C
Dactylosporangium.fulvum
2.0
1.5
1.0
0.5
0.0
A (n=15)
B (n=16)
C (n=14)
surface

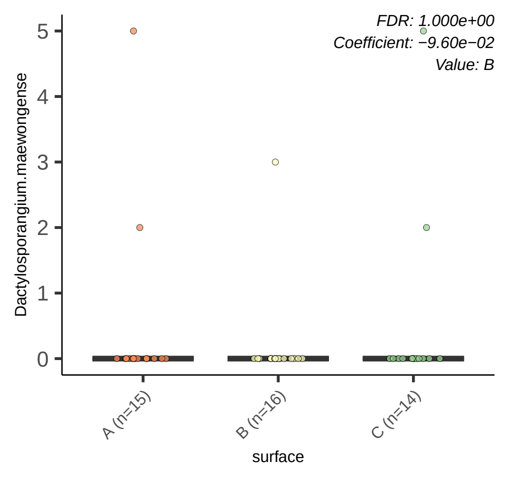
FDR: 1.000e+00
Coefficient: −9.60e−02
Value: B
Dactylosporangium.maewongense
0
1
2
3
4
5
A (n=15)
B (n=16)
C (n=14)
surface

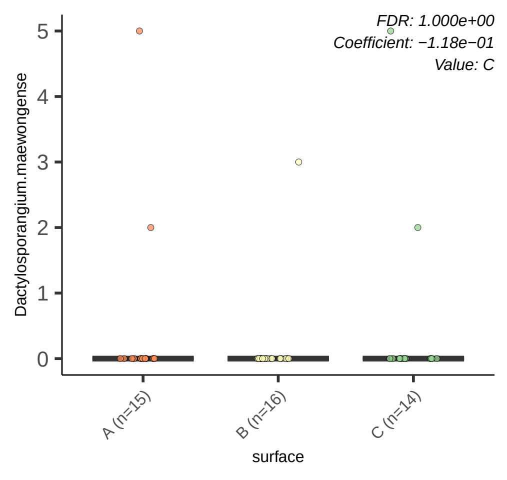
FDR: 1.000e+00
Coefficient: −1.18e−01
Value: C
Dactylosporangium.maewongense
0
1
2
3
4
5
A (n=15)
B (n=16)
C (n=14)
surface

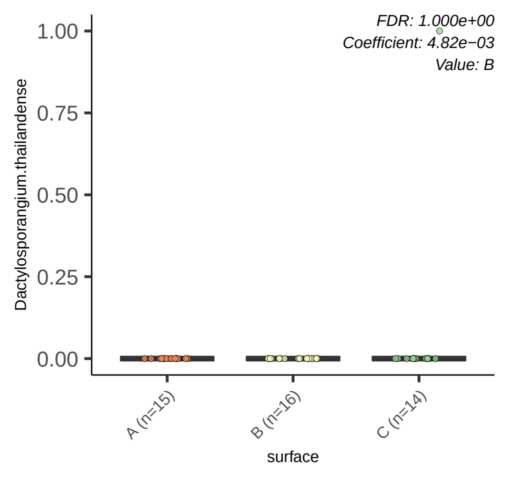
FDR: 1.000e+00
Coefficient: 4.82e−03
Value: B
1.00
0.75
0.50
0.25
0.00
Dactylosporangium.thailandense
A (n=15)
B (n=16)
C (n=14)
surface

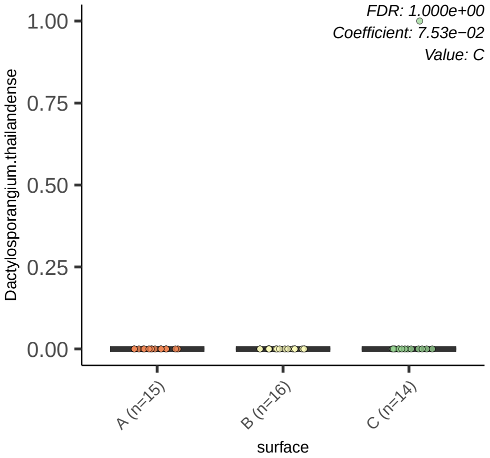

FDR: 1.000e+00
Coefficient: 7.53e−02
Value: C
1.00
0.75
0.50
0.25
0.00
Dactylosporangium.thailandense
A (n=15)
B (n=16)
C (n=14)
surface

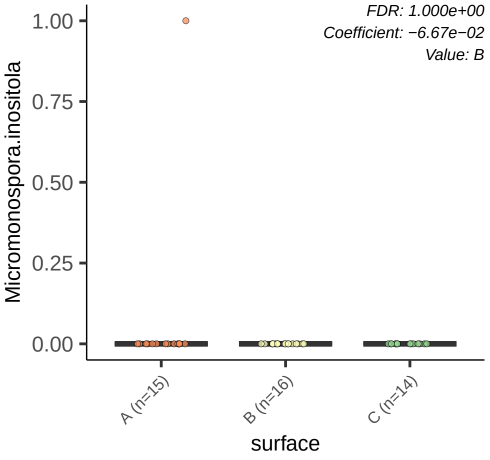
FDR: 1.000e+00
Coefficient: −6.67e−02
Value: B
Micromonospora.inositola
1.00
0.75
0.50
0.25
0.00
A (n=15)
B (n=16)
C (n=14)
surface

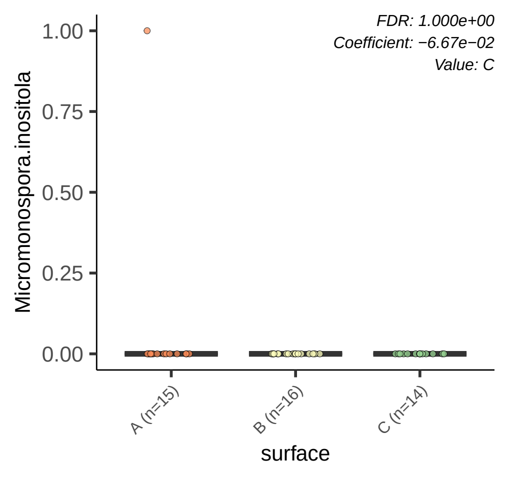

FDR: 1.000e+00
Coefficient: −6.67e−02
Value: C
Micromonospora.inositola
1.00
0.75
0.50
0.25
0.00
A (n=15)
B (n=16)
C (n=14)
surface

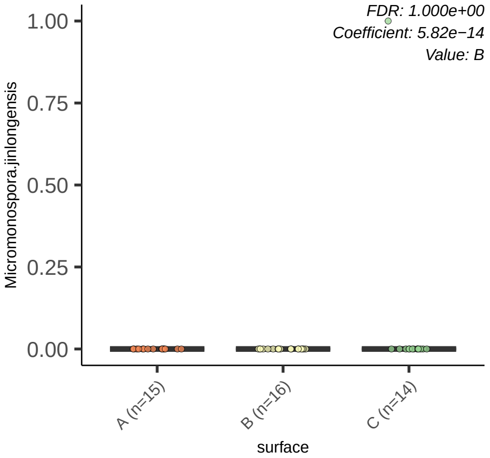

FDR: 1.000e+00
Coefficient: 5.82e−14
Value: B
Micromonospora.jinlongensis
1.00
0.75
0.50
0.25
0.00
A (n=15)
B (n=16)
C (n=14)
surface

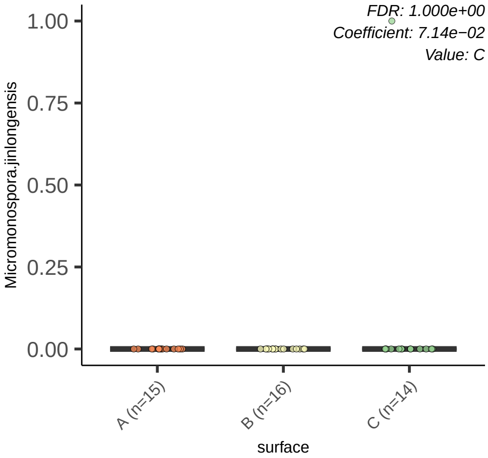
FDR: 1.000e+00
Coefficient: 7.14e−02
Value: C
Micromonospora.jinlongensis
1.00
0.75
0.50
0.25
0.00
A (n=15)
B (n=16)
C (n=14)
surface

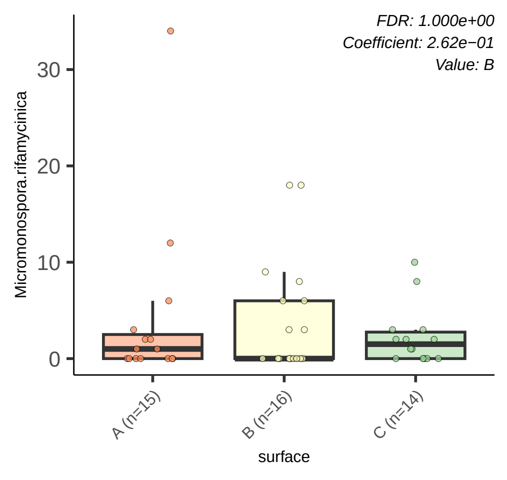
FDR: 1.000e+00
Coefficient: 2.62e−01
Value: B
Micromonospora.rifamycinica
30
20
10
0
A (n=15)
B (n=16)
C (n=14)
surface

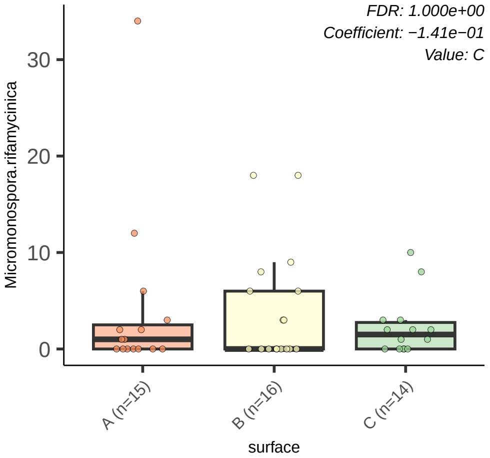

FDR: 1.000e+00
Coefficient: −1.41e−01
Value: C
Micromonospora.rifamycinica
0
10
20
30
A (n=15)
B (n=16)
C (n=14)
surface

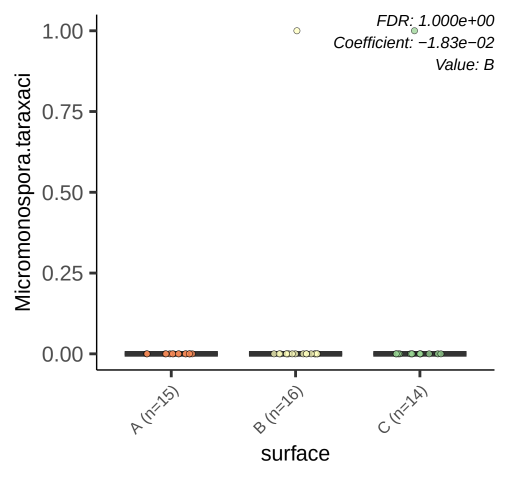
FDR: 1.000e+00
Coefficient: −1.83e−02
Value: B
Micromonospora.taraxaci
1.00
0.75
0.50
0.25
0.00
A (n=15)
B (n=16)
C (n=14)
surface

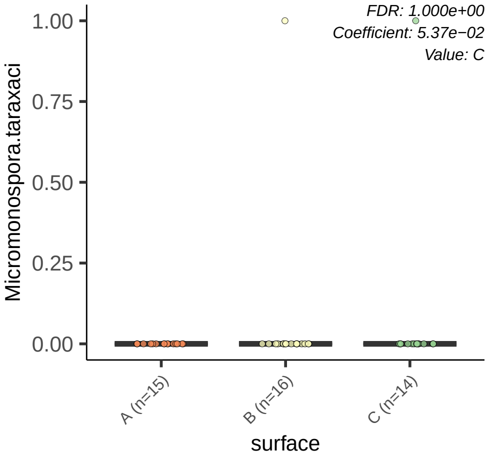
FDR: 1.000e+00
Coefficient: 5.37e−02
Value: C
Micromonospora.taraxaci
1.00
0.75
0.50
0.25
0.00
A (n=15)
B (n=16)
C (n=14)
surface

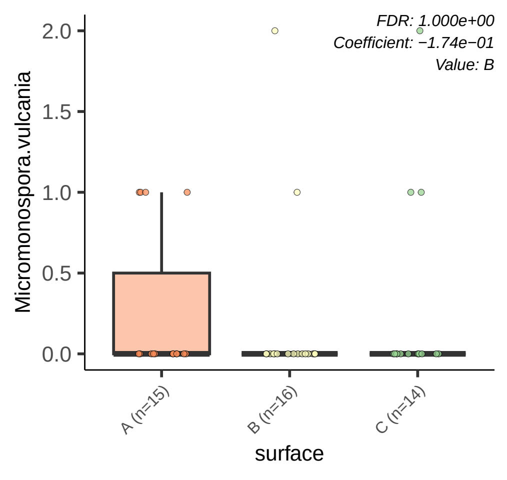

FDR: 1.000e+00
Coefficient: −1.74e−01
Value: B
Micromonospora.vulcania
2.0
1.5
1.0
0.5
0.0
A (n=15)
B (n=16)
C (n=14)
surface

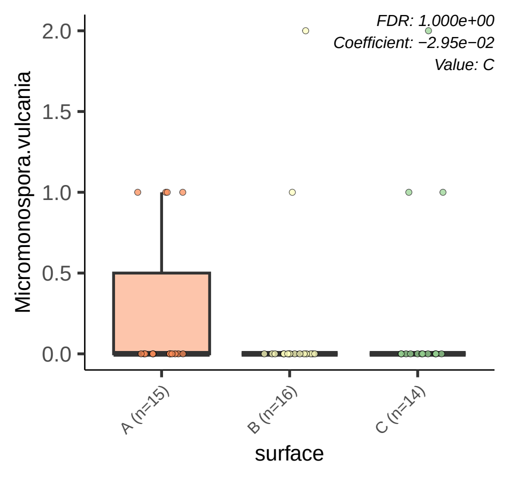
FDR: 1.000e+00
Coefficient: −2.95e−02
Value: C
Micromonospora.vulcania
2.0
1.5
1.0
0.5
0.0
A (n=15)
B (n=16)
C (n=14)
surface

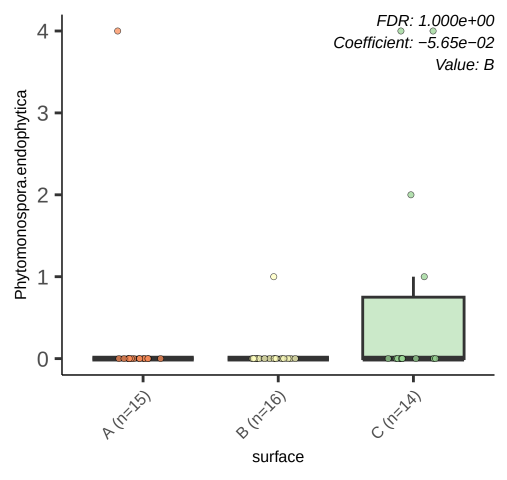
FDR: 1.000e+00
Coefficient: −5.65e−02
Value: B
Phytomonospora.endophytica
0
1
2
3
4
A (n=15)
B (n=16)
C (n=14)
surface

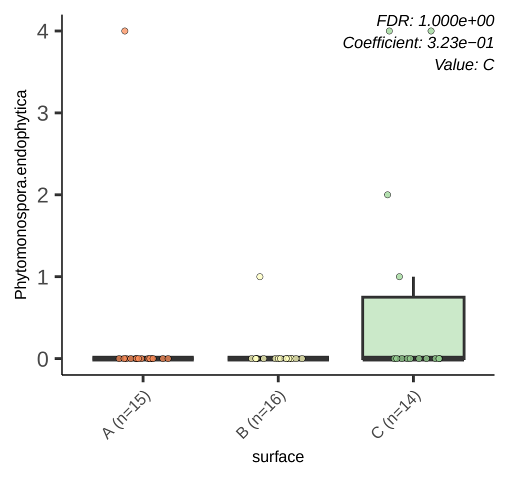
FDR: 1.000e+00
Coefficient: 3.23e−01
Value: C
Phytomonospora.endophytica
0
1
2
3
4
A (n=15)
B (n=16)
C (n=14)
surface

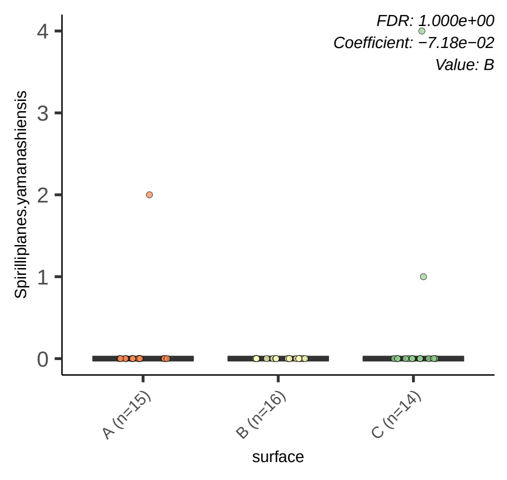
FDR: 1.000e+00
Coefficient: −7.18e−02
Value: B
Spirilliplanes.yamanashiensis
0
1
2
3
4
A (n=15)
B (n=16)
C (n=14)
surface

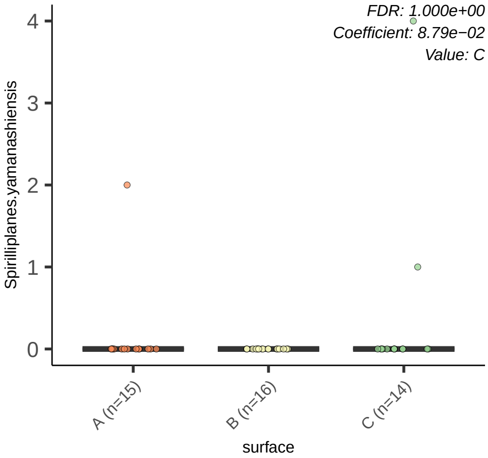

FDR: 1.000e+00
Coefficient: 8.79e−02
Value: C
Spirilliplanes.yamanashiensis
0
1
2
3
4
A (n=15)
B (n=16)
C (n=14)
surface

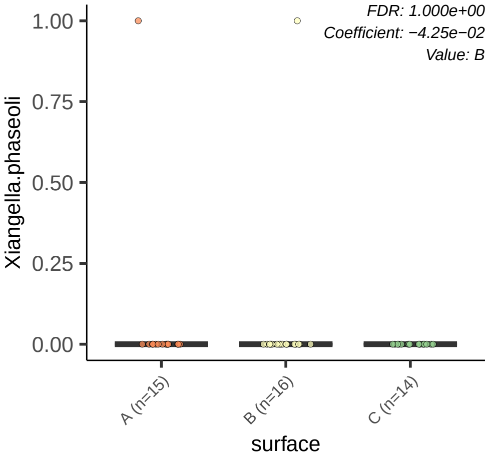
FDR: 1.000e+00
Coefficient: −4.25e−02
Value: B
Xiangella.phaseoli
1.00
0.75
0.50
0.25
0.00
A (n=15)
B (n=16)
C (n=14)
surface

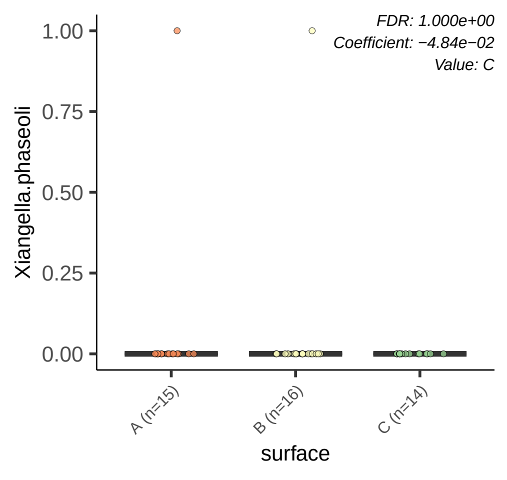
FDR: 1.000e+00
Coefficient: −4.84e−02
Value: C
Xiangella.phaseoli
1.00
0.75
0.50
0.25
0.00
A (n=15)
B (n=16)
C (n=14)
surface

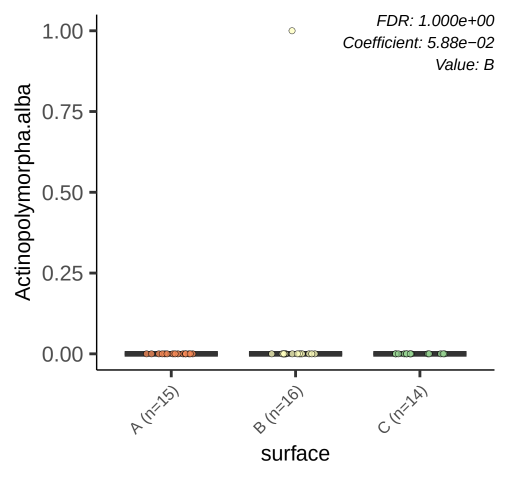
FDR: 1.000e+00
Coefficient: 5.88e−02
Value: B
Actinopolymorpha.alba
1.00
0.75
0.50
0.25
0.00
A (n=15)
B (n=16)
C (n=14)
surface

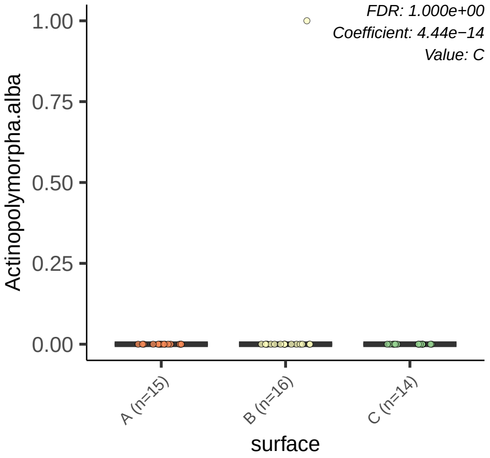
FDR: 1.000e+00
Coefficient: 4.44e−14
Value: C
Actinopolymorpha.alba
1.00
0.75
0.50
0.25
0.00
A (n=15)
B (n=16)
C (n=14)
surface

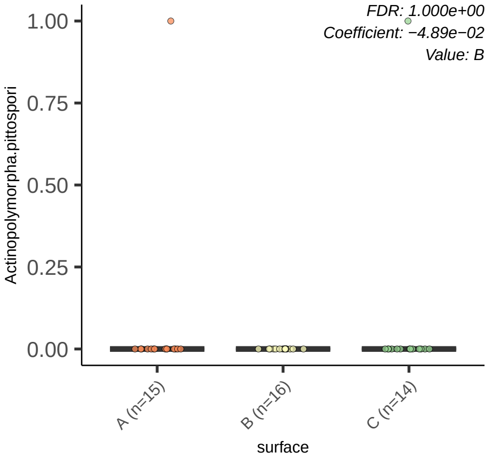
FDR: 1.000e+00
Coefficient: −4.89e−02
Value: B
1.00
0.75
0.50
0.25
0.00
Actinopolymorpha.pittospori
A (n=15)
B (n=16)
C (n=14)
surface

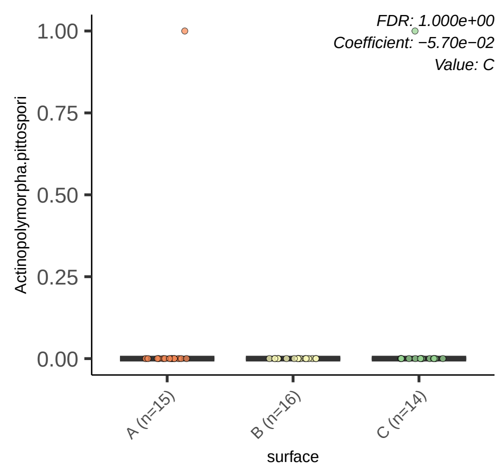
FDR: 1.000e+00
Coefficient: −5.70e−02
Value: C
Actinopolymorpha.pittospori
1.00
0.75
0.50
0.25
0.00
A (n=15)
B (n=16)
C (n=14)
surface

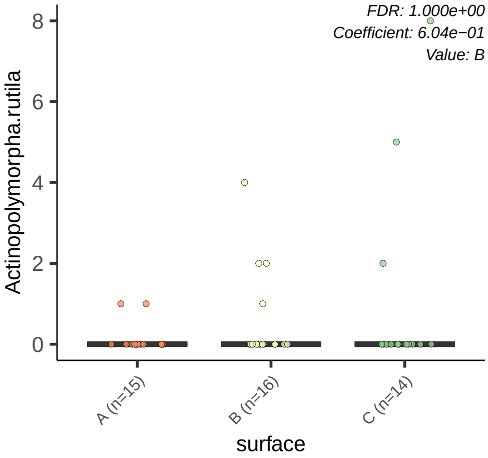
FDR: 1.000e+00
Coefficient: 6.04e−01
Value: B
Actinopolymorpha.rutila
0
2
4
6
8
A (n=15)
B (n=16)
C (n=14)
surface

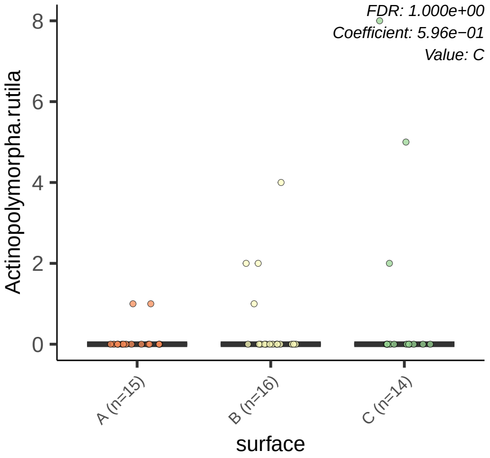
FDR: 1.000e+00
Coefficient: 5.96e−01
Value: C
Actinopolymorpha.rutila
0
2
4
6
8
A (n=15)
B (n=16)
C (n=14)
surface

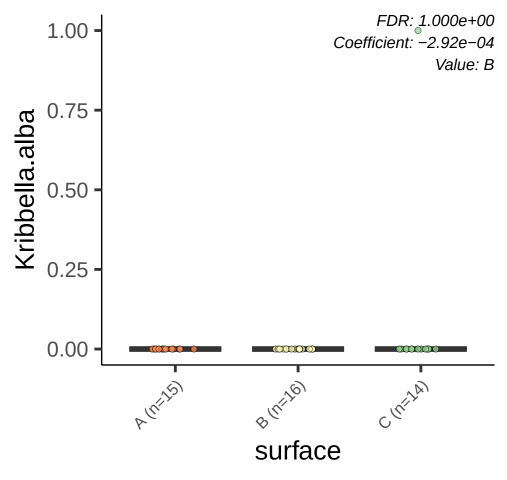
FDR: 1.000e+00
Coefficient: −2.92e−04
Value: B
Kribbella.alba
1.00
0.75
0.50
0.25
0.00
A (n=15)
B (n=16)
C (n=14)
surface

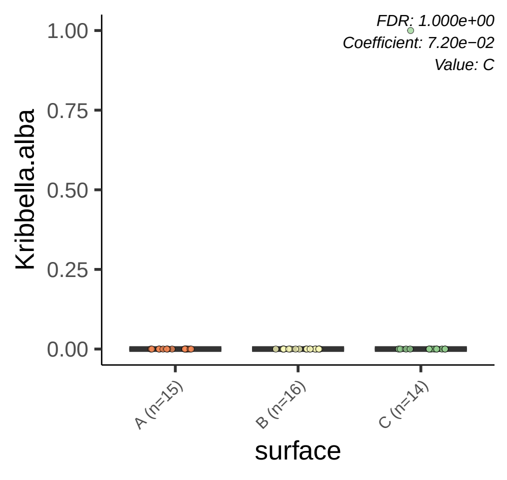
FDR: 1.000e+00
Coefficient: 7.20e−02
Value: C
Kribbella.alba
1.00
0.75
0.50
0.25
0.00
A (n=15)
B (n=16)
C (n=14)
surface

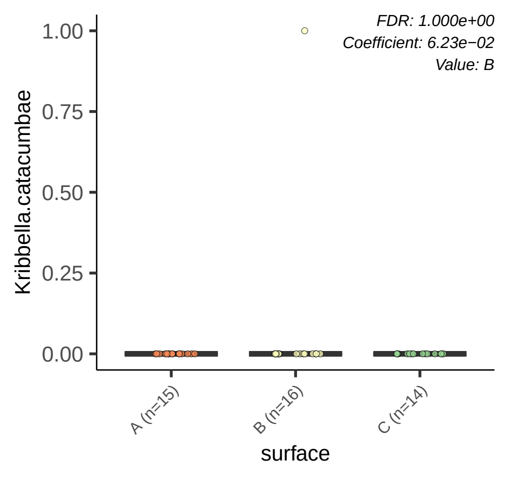

FDR: 1.000e+00
Coefficient: 6.23e−02
Value: B
Kribbella.catacumbae
1.00
0.75
0.50
0.25
0.00
A (n=15)
B (n=16)
C (n=14)
surface

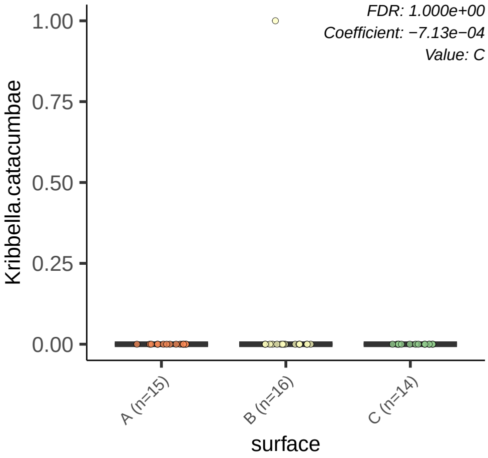

FDR: 1.000e+00
Coefficient: −7.13e−04
Value: C
Kribbella.catacumbae
1.00
0.75
0.50
0.25
0.00
A (n=15)
B (n=16)
C (n=14)
surface

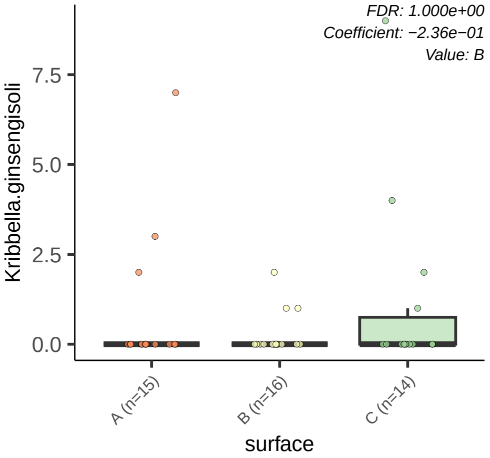

FDR: 1.000e+00
Coefficient: −2.36e−01
Value: B
Kribbella.ginsengisoli
7.5
5.0
2.5
0.0
A (n=15)
B (n=16)
C (n=14)
surface

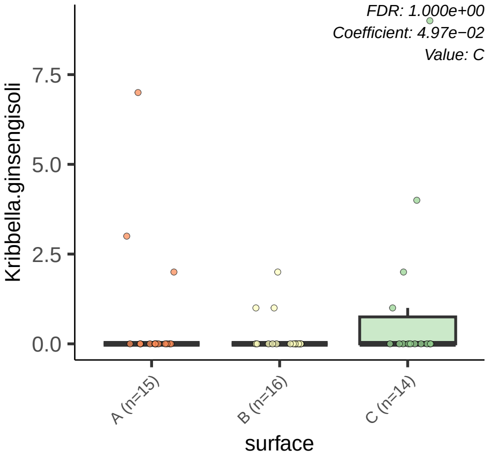

FDR: 1.000e+00
Coefficient: 4.97e−02
Value: C
7.5
5.0
2.5
0.0
Kribbella.ginsengisoli
A (n=15)
B (n=16)
C (n=14)
surface

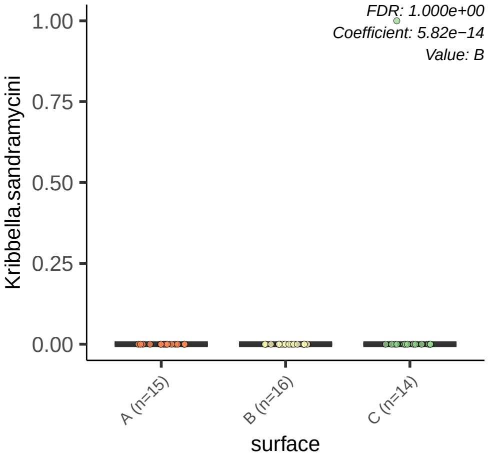
FDR: 1.000e+00
Coefficient: 5.82e−14
Value: B
Kribbella.sandramycini
1.00
0.75
0.50
0.25
0.00
A (n=15)
B (n=16)
C (n=14)
surface

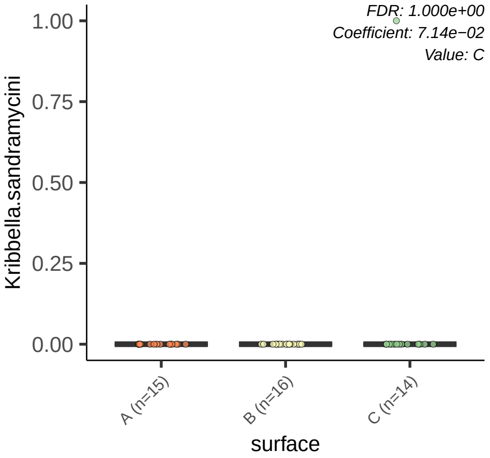

FDR: 1.000e+00
Coefficient: 7.14e−02
Value: C
1.00
0.75
0.50
0.25
0.00
Kribbella.sandramycini
A (n=15)
B (n=16)
C (n=14)
surface

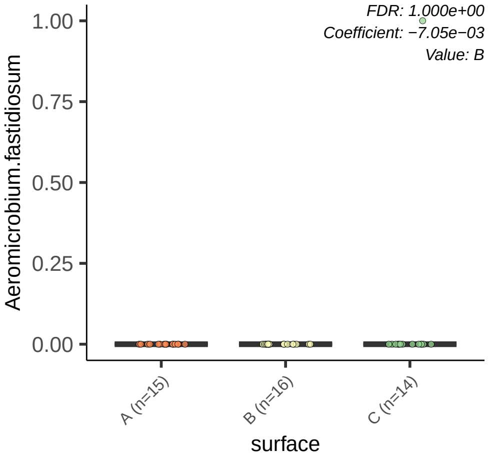

FDR: 1.000e+00
Coefficient: −7.05e−03
Value: B
Aeromicrobium.fastidiosum
1.00
0.75
0.50
0.25
0.00
A (n=15)
B (n=16)
C (n=14)
surface

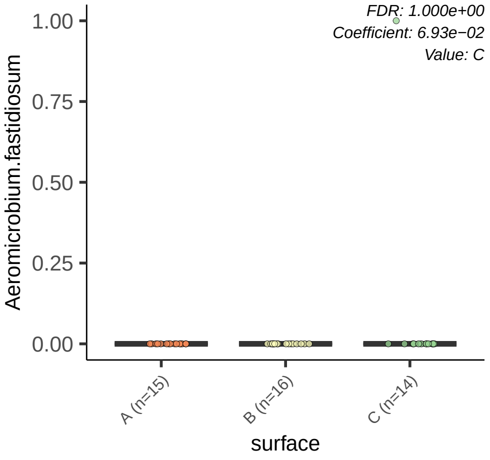

FDR: 1.000e+00
Coefficient: 6.93e−02
Value: C
1.00
0.75
0.50
0.25
0.00
Aeromicrobium.fastidiosum
A (n=15)
B (n=16)
C (n=14)
surface

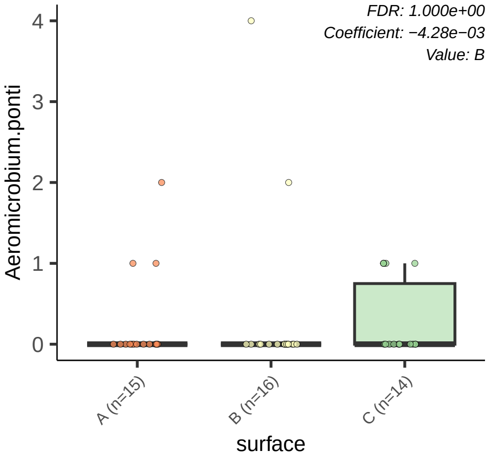

FDR: 1.000e+00
Coefficient: −4.28e−03
Value: B
Aeromicrobium.ponti
4
3
2
1
0
A (n=15)
B (n=16)
C (n=14)
surface

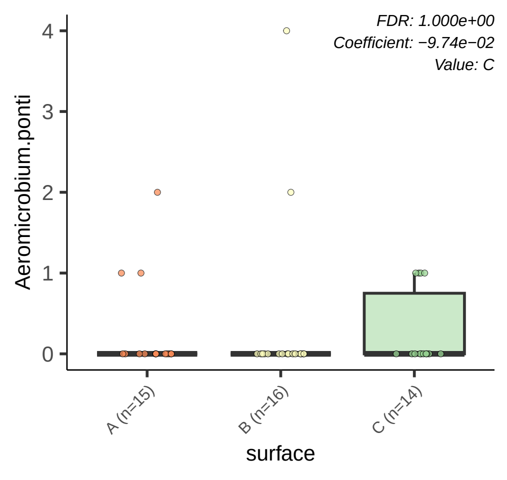
FDR: 1.000e+00
Coefficient: −9.74e−02
Value: C
Aeromicrobium.ponti
4
3
2
1
0
A (n=15)
B (n=16)
C (n=14)
surface

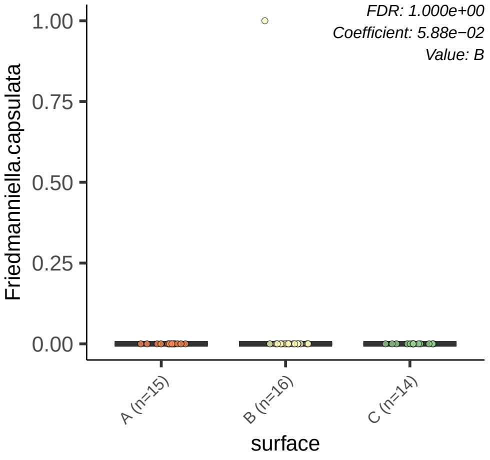
FDR: 1.000e+00
Coefficient: 5.88e−02
Value: B
Friedmanniella.capsulata
1.00
0.75
0.50
0.25
0.00
A (n=15)
B (n=16)
C (n=14)
surface

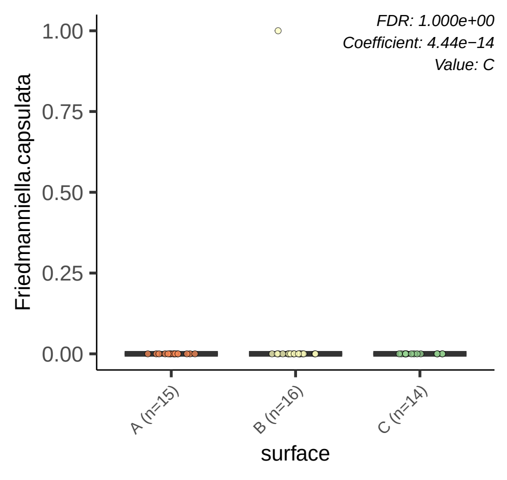

FDR: 1.000e+00
Coefficient: 4.44e−14
Value: C
Friedmanniella.capsulata
1.00
0.75
0.50
0.25
0.00
A (n=15)
B (n=16)
C (n=14)
surface

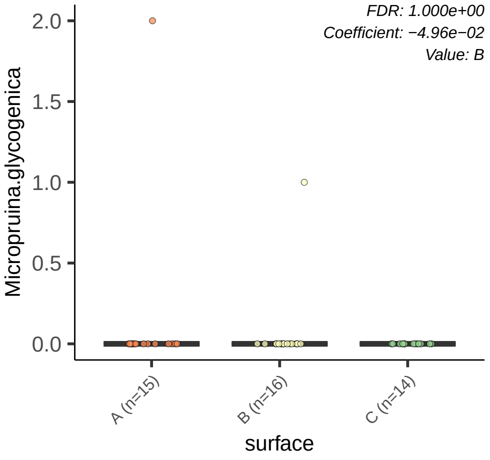

FDR: 1.000e+00
Coefficient: −4.96e−02
Value: B
Micropruina.glycogenica
2.0
1.5
1.0
0.5
0.0
A (n=15)
B (n=16)
C (n=14)
surface

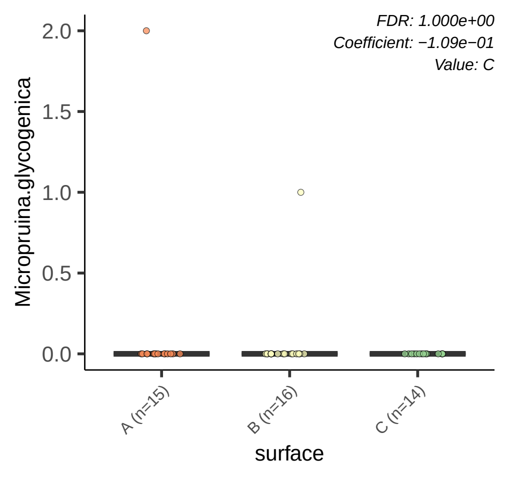
FDR: 1.000e+00
Coefficient: −1.09e−01
Value: C
Micropruina.glycogenica
2.0
1.5
1.0
0.5
0.0
A (n=15)
B (n=16)
C (n=14)
surface

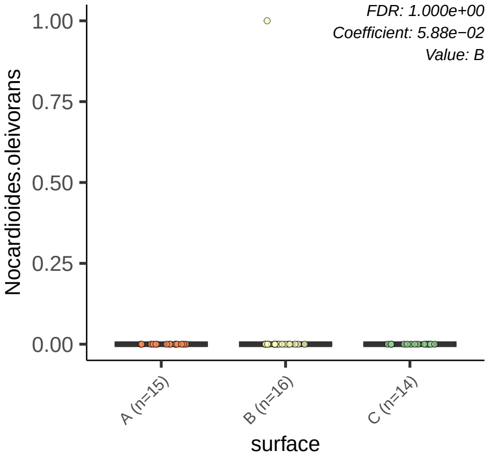

FDR: 1.000e+00
Coefficient: 5.88e−02
Value: B
Nocardioides.oleivorans
1.00
0.75
0.50
0.25
0.00
A (n=15)
B (n=16)
C (n=14)
surface

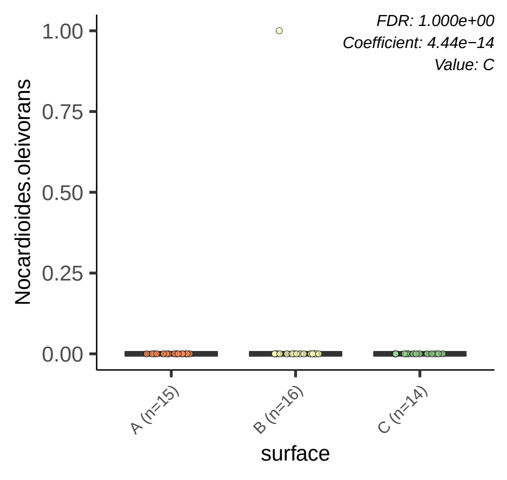
FDR: 1.000e+00
Coefficient: 4.44e−14
Value: C
Nocardioides.oleivorans
1.00
0.75
0.50
0.25
0.00
A (n=15)
B (n=16)
C (n=14)
surface

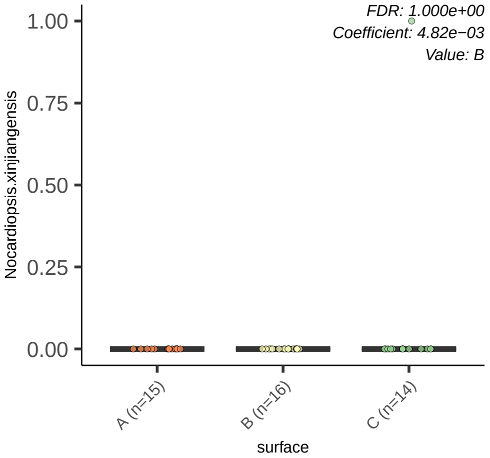

FDR: 1.000e+00
Coefficient: 4.82e−03
Value: B
1.00
0.75
0.50
0.25
0.00
Nocardiopsis.xinjiangensis
A (n=15)
B (n=16)
C (n=14)
surface

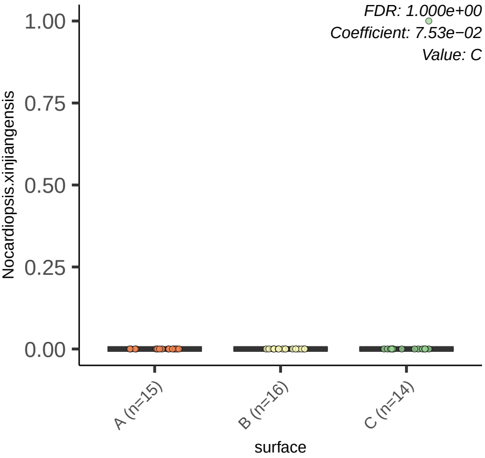

FDR: 1.000e+00
Coefficient: 7.53e−02
Value: C
1.00
0.75
0.50
0.25
0.00
Nocardiopsis.xinjiangensis
A (n=15)
B (n=16)
C (n=14)
surface

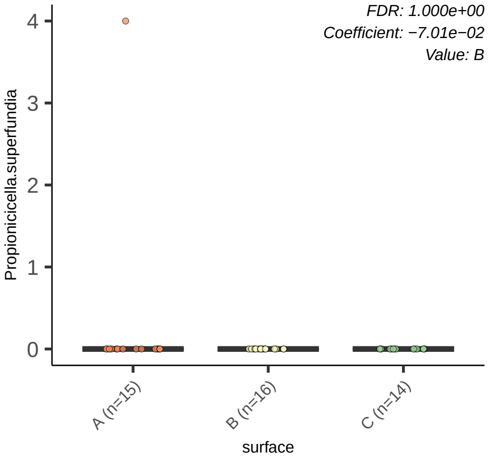
FDR: 1.000e+00
Coefficient: −7.01e−02
Value: B
Propionicicella.superfundia
4
3
2
1
0
A (n=15)
B (n=16)
C (n=14)
surface

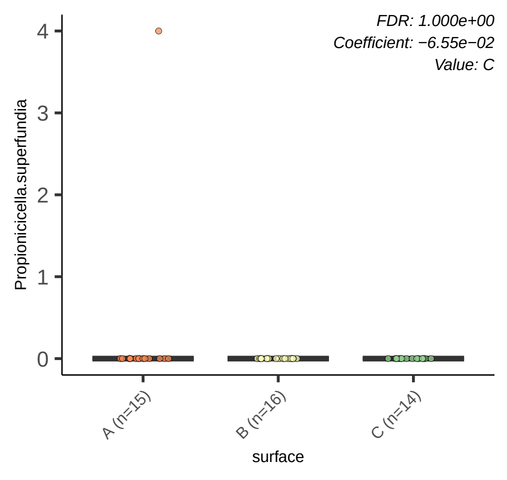

FDR: 1.000e+00
Coefficient: −6.55e−02
Value: C
Propionicicella.superfundia
4
3
2
1
0
A (n=15)
B (n=16)
C (n=14)
surface

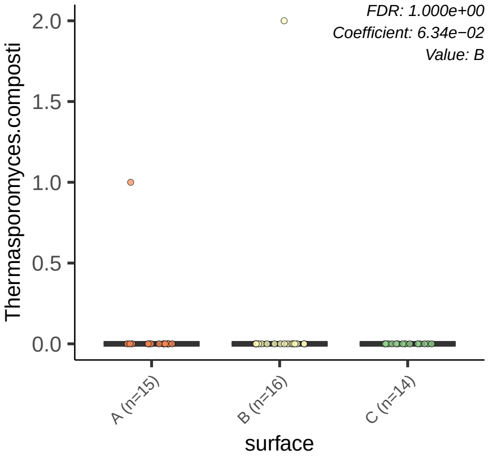
FDR: 1.000e+00
Coefficient: 6.34e−02
Value: B
Thermasporomyces.composti
2.0
1.5
1.0
0.5
0.0
A (n=15)
B (n=16)
C (n=14)
surface

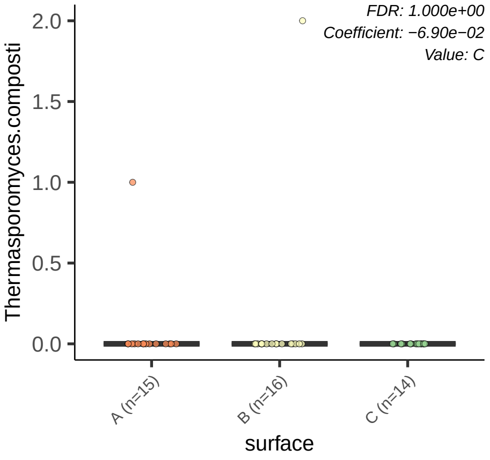

FDR: 1.000e+00
Coefficient: −6.90e−02
Value: C
Thermasporomyces.composti
2.0
1.5
1.0
0.5
0.0
A (n=15)
B (n=16)
C (n=14)
surface

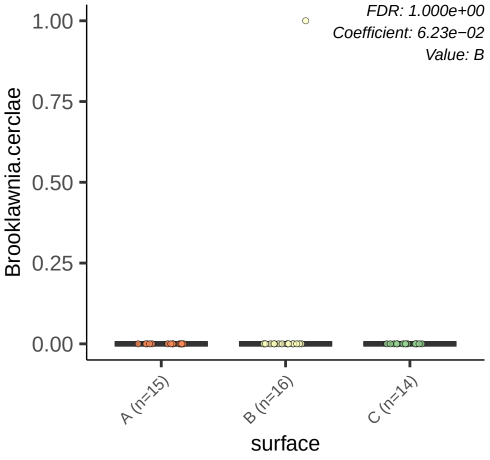

FDR: 1.000e+00
Coefficient: 6.23e−02
Value: B
1.00
0.75
0.50
0.25
0.00
Brooklawnia.cerclae
A (n=15)
B (n=16)
C (n=14)
surface

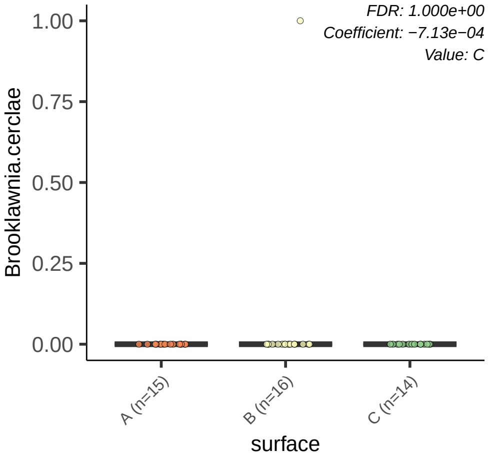
FDR: 1.000e+00
Coefficient: −7.13e−04
Value: C
Brooklawnia.cerclae
1.00
0.75
0.50
0.25
0.00
A (n=15)
B (n=16)
C (n=14)
surface

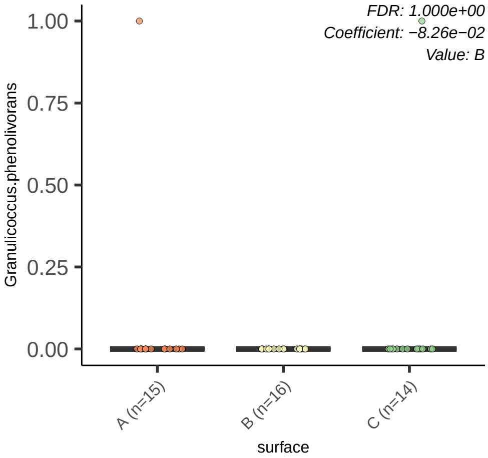

FDR: 1.000e+00
Coefficient: −8.26e−02
Value: B
Granulicoccus.phenolivorans
1.00
0.75
0.50
0.25
0.00
A (n=15)
B (n=16)
C (n=14)
surface

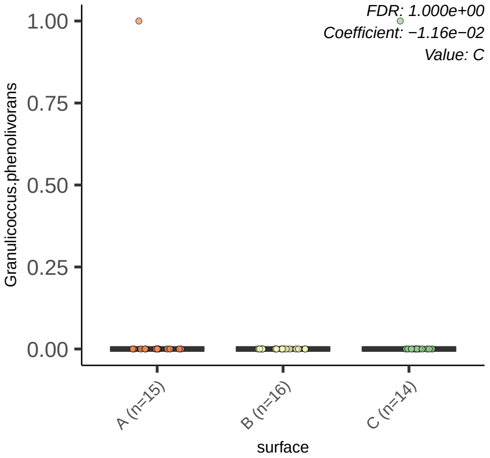
FDR: 1.000e+00
Coefficient: −1.16e−02
Value: C
Granulicoccus.phenolivorans
1.00
0.75
0.50
0.25
0.00
A (n=15)
B (n=16)
C (n=14)
surface

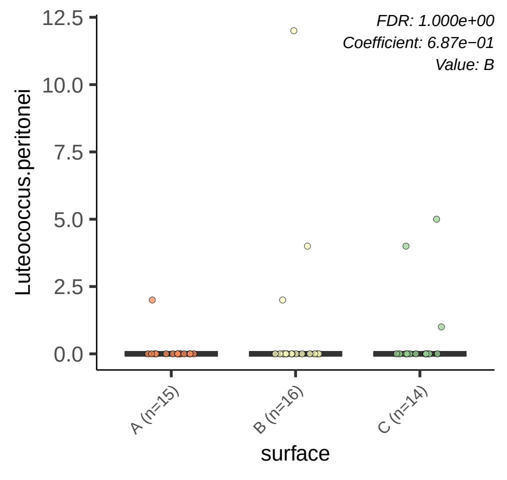
FDR: 1.000e+00
Coefficient: 6.87e−01
Value: B
Luteococcus.peritonei
12.5
10.0
7.5
5.0
2.5
0.0
A (n=15)
B (n=16)
C (n=14)
surface

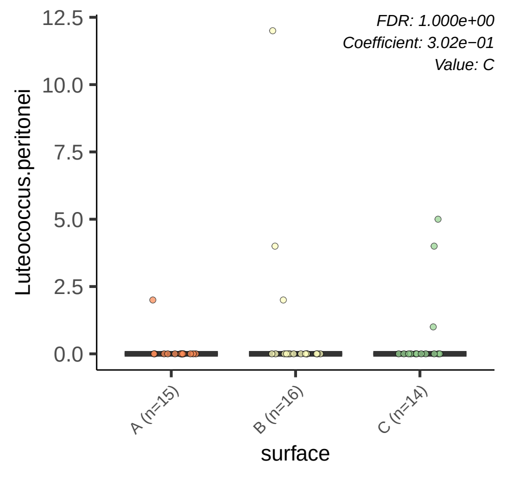

FDR: 1.000e+00
Coefficient: 3.02e−01
Value: C
Luteococcus.peritonei
12.5
10.0
7.5
5.0
2.5
0.0
A (n=15)
B (n=16)
C (n=14)
surface

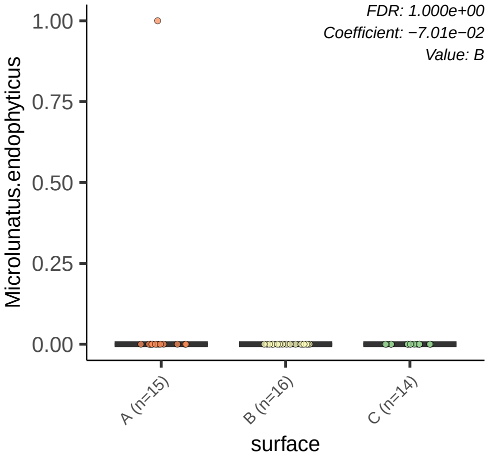
FDR: 1.000e+00
Coefficient: −7.01e−02
Value: B
Microlunatus.endophyticus
1.00
0.75
0.50
0.25
0.00
A (n=15)
B (n=16)
C (n=14)
surface

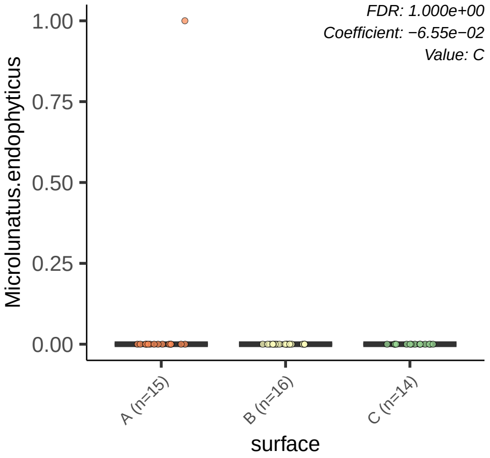
FDR: 1.000e+00
Coefficient: −6.55e−02
Value: C
Microlunatus.endophyticus
1.00
0.75
0.50
0.25
0.00
A (n=15)
B (n=16)
C (n=14)
surface

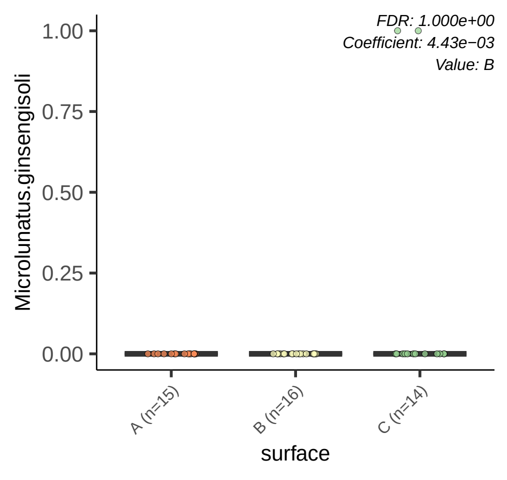

FDR: 1.000e+00
Coefficient: 4.43e−03
Value: B
Microlunatus.ginsengisoli
1.00
0.75
0.50
0.25
0.00
A (n=15)
B (n=16)
C (n=14)
surface

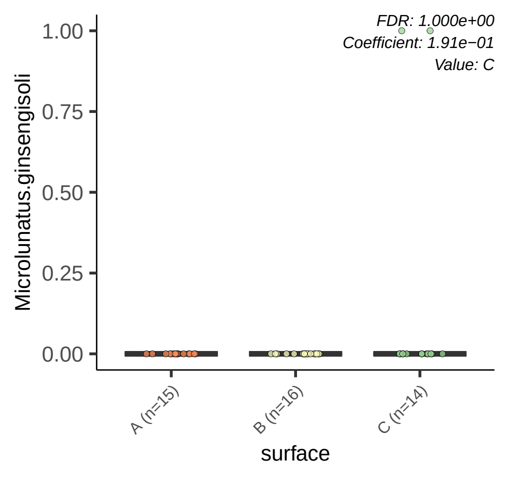

FDR: 1.000e+00
Coefficient: 1.91e−01
Value: C
Microlunatus.ginsengisoli
1.00
0.75
0.50
0.25
0.00
A (n=15)
B (n=16)
C (n=14)
surface

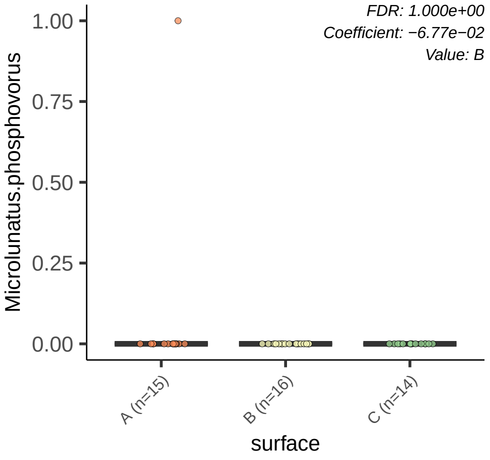

FDR: 1.000e+00
Coefficient: −6.77e−02
Value: B
Microlunatus.phosphovorus
1.00
0.75
0.50
0.25
0.00
A (n=15)
B (n=16)
C (n=14)
surface

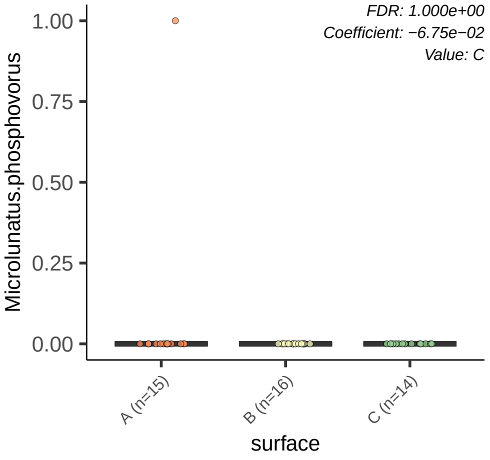
FDR: 1.000e+00
Coefficient: −6.75e−02
Value: C
Microlunatus.phosphovorus
1.00
0.75
0.50
0.25
0.00
A (n=15)
B (n=16)
C (n=14)
surface

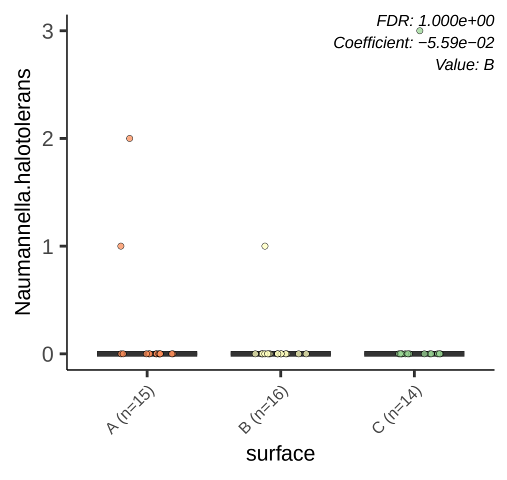
FDR: 1.000e+00
Coefficient: −5.59e−02
Value: B
Naumannella.halotolerans
3
2
1
0
A (n=15)
B (n=16)
C (n=14)
surface

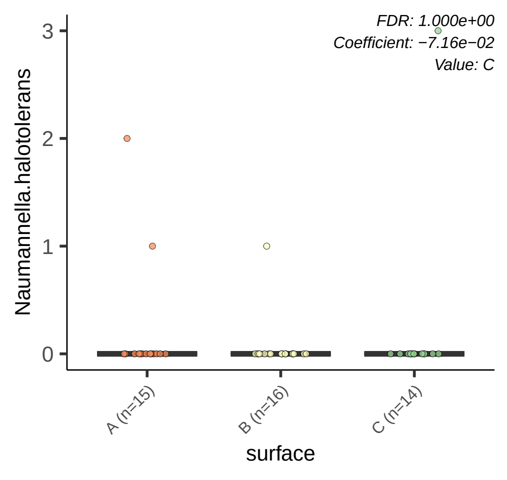
FDR: 1.000e+00
Coefficient: −7.16e−02
Value: C
Naumannella.halotolerans
3
2
1
0
A (n=15)
B (n=16)
C (n=14)
surface

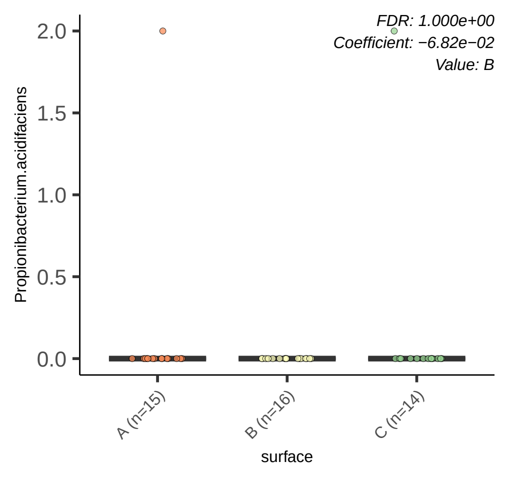

FDR: 1.000e+00
Coefficient: −6.82e−02
Value: B
Propionibacterium.acidifaciens
2.0
1.5
1.0
0.5
0.0
A (n=15)
B (n=16)
C (n=14)
surface

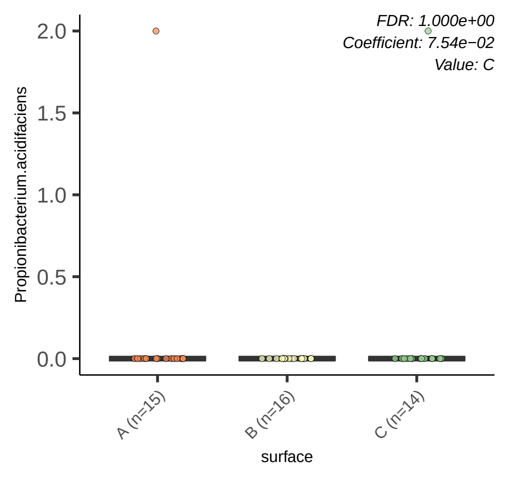
FDR: 1.000e+00
Coefficient: 7.54e−02
Value: C
Propionibacterium.acidifaciens
2.0
1.5
1.0
0.5
0.0
A (n=15)
B (n=16)
C (n=14)
surface

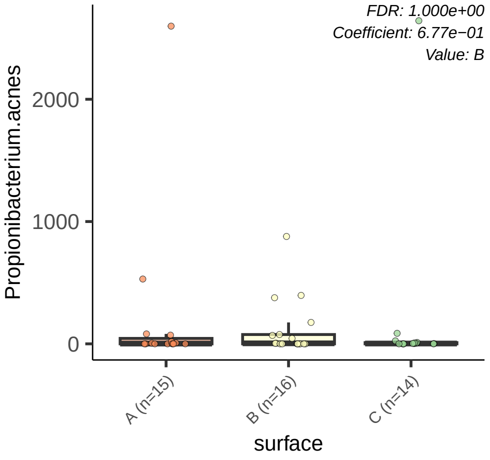

FDR: 1.000e+00
Coefficient: 6.77e−01
Value: B
Propionibacterium.acnes
2000
1000
0
A (n=15)
B (n=16)
C (n=14)
surface

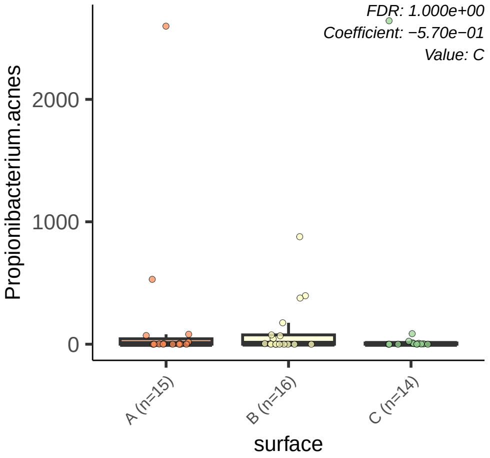

FDR: 1.000e+00
Coefficient: −5.70e−01
Value: C
Propionibacterium.acnes
2000
1000
0
A (n=15)
B (n=16)
C (n=14)
surface

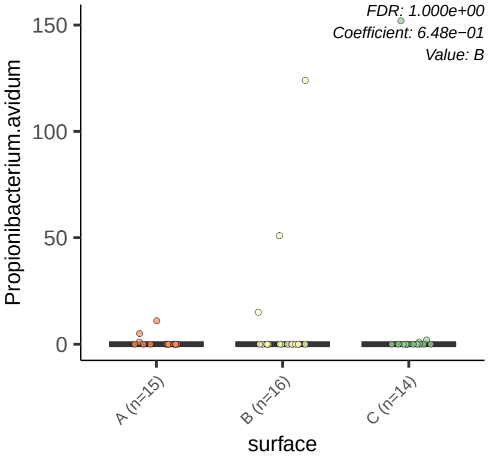
FDR: 1.000e+00
Coefficient: 6.48e−01
Value: B
Propionibacterium.avidum
150
100
50
0
A (n=15)
B (n=16)
C (n=14)
surface

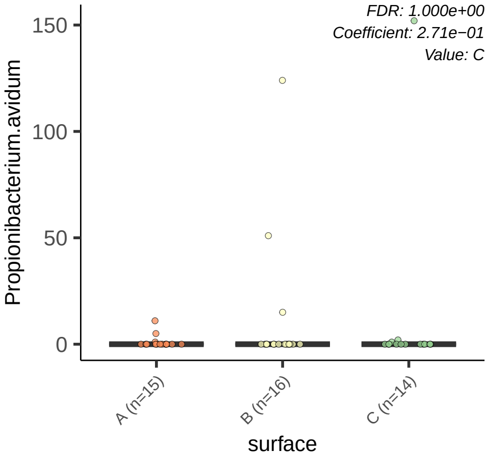
FDR: 1.000e+00
Coefficient: 2.71e−01
Value: C
Propionibacterium.avidum
150
100
50
0
A (n=15)
B (n=16)
C (n=14)
surface

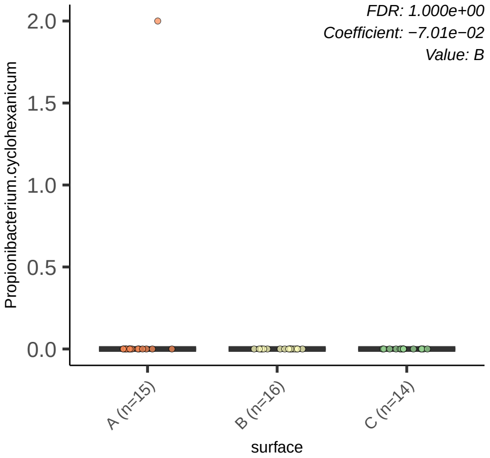

FDR: 1.000e+00
Coefficient: −7.01e−02
Value: B
Propionibacterium.cyclohexanicum
2.0
1.5
1.0
0.5
0.0
A (n=15)
B (n=16)
C (n=14)
surface

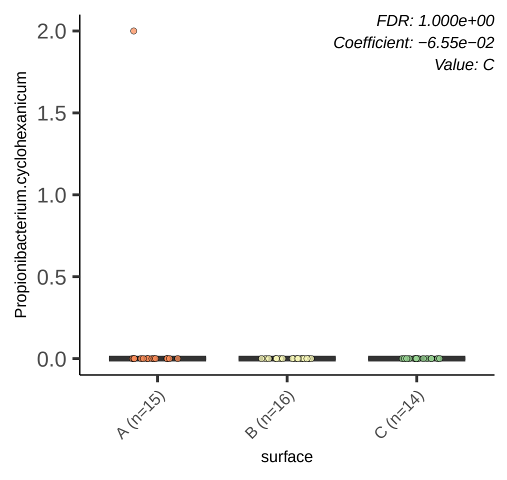
FDR: 1.000e+00
Coefficient: −6.55e−02
Value: C
Propionibacterium.cyclohexanicum
2.0
1.5
1.0
0.5
0.0
A (n=15)
B (n=16)
C (n=14)
surface

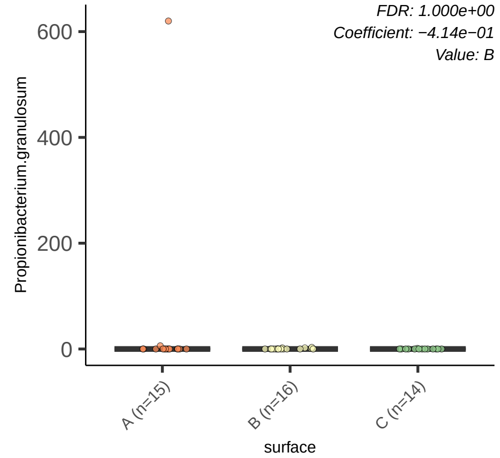
FDR: 1.000e+00
Coefficient: −4.14e−01
Value: B
Propionibacterium.granulosum
600
400
200
0
A (n=15)
B (n=16)
C (n=14)
surface

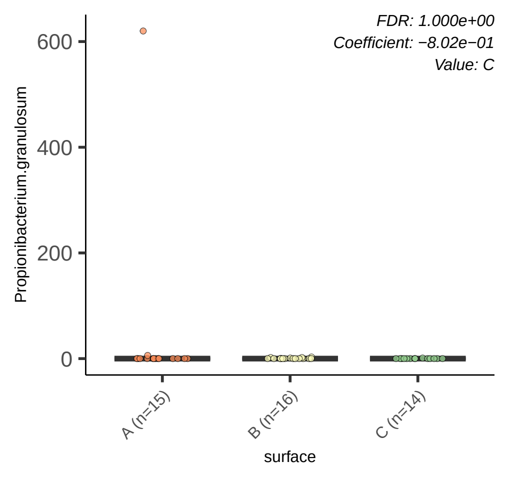
FDR: 1.000e+00
Coefficient: −8.02e−01
Value: C
Propionibacterium.granulosum
600
400
200
0
A (n=15)
B (n=16)
C (n=14)
surface

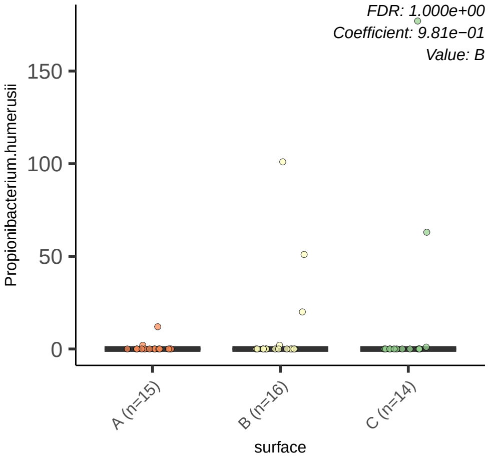

FDR: 1.000e+00
Coefficient: 9.81e−01
Value: B
Propionibacterium.humerusii
0
50
100
150
A (n=15)
B (n=16)
C (n=14)
surface

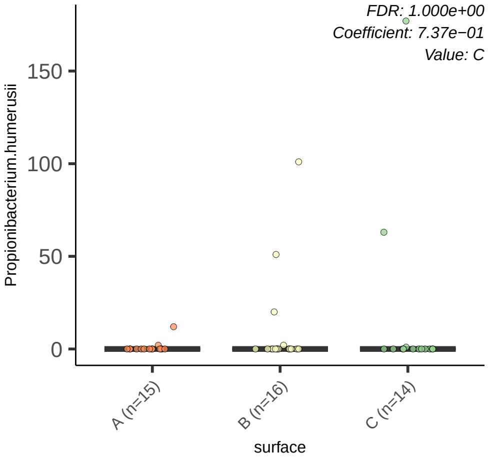

FDR: 1.000e+00
Coefficient: 7.37e−01
Value: C
150
100
50
0
Propionibacterium.humerusii
A (n=15)
B (n=16)
C (n=14)
surface

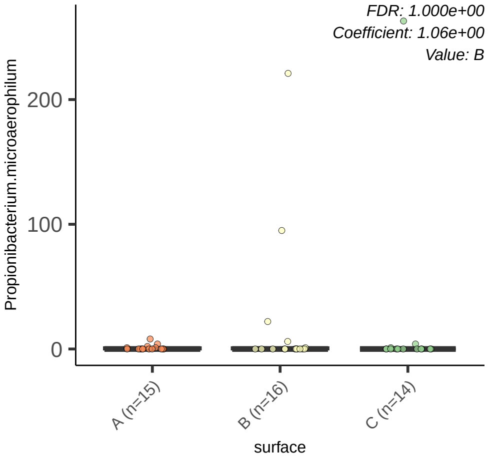
FDR: 1.000e+00
Coefficient: 1.06e+00
Value: B
Propionibacterium.microaerophilum
200
100
0
A (n=15)
B (n=16)
C (n=14)
surface

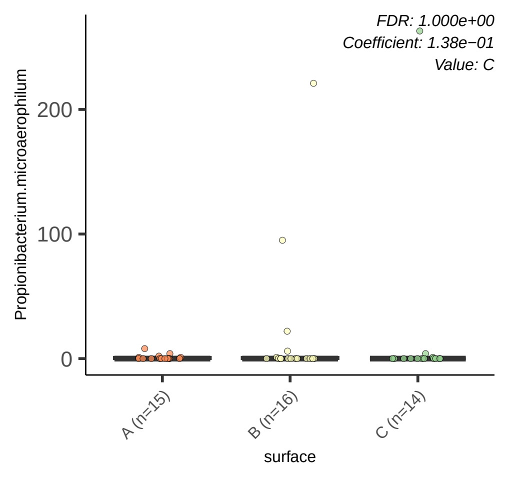
FDR: 1.000e+00
Coefficient: 1.38e−01
Value: C
Propionibacterium.microaerophilum
200
100
0
A (n=15)
B (n=16)
C (n=14)
surface

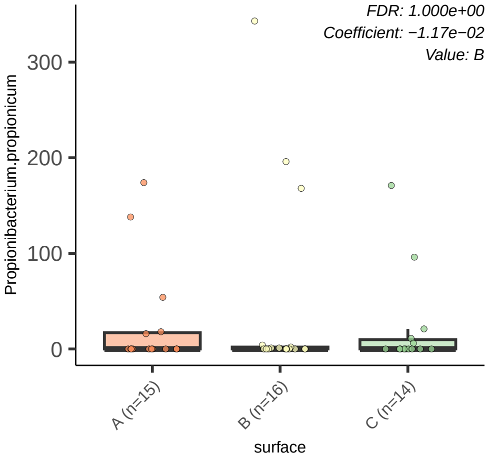
FDR: 1.000e+00
Coefficient: −1.17e−02
Value: B
Propionibacterium.propionicum
300
200
100
0
A (n=15)
B (n=16)
C (n=14)
surface

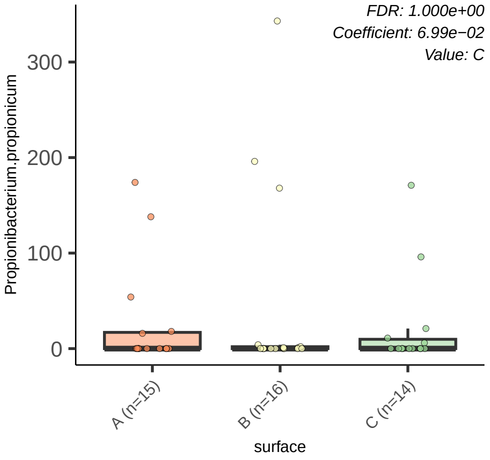
FDR: 1.000e+00
Coefficient: 6.99e−02
Value: C
Propionibacterium.propionicum
0
100
200
300
A (n=15)
B (n=16)
C (n=14)
surface

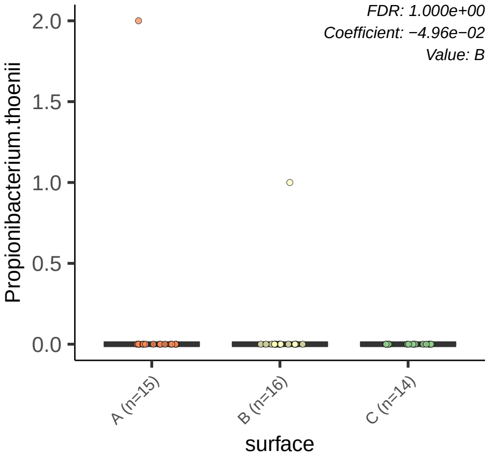
FDR: 1.000e+00
Coefficient: −4.96e−02
Value: B
Propionibacterium.thoenii
2.0
1.5
1.0
0.5
0.0
A (n=15)
B (n=16)
C (n=14)
surface

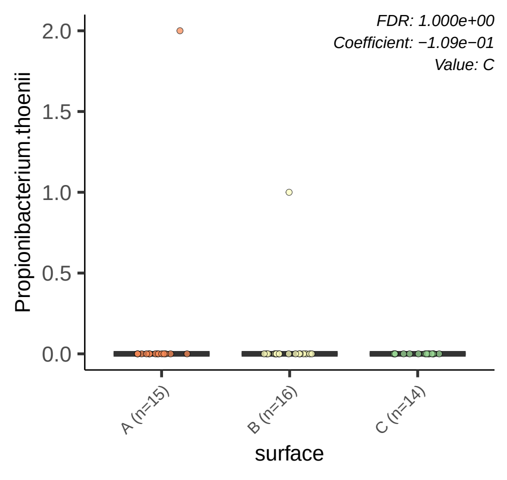
FDR: 1.000e+00
Coefficient: −1.09e−01
Value: C
Propionibacterium.thoenii
2.0
1.5
1.0
0.5
0.0
A (n=15)
B (n=16)
C (n=14)
surface

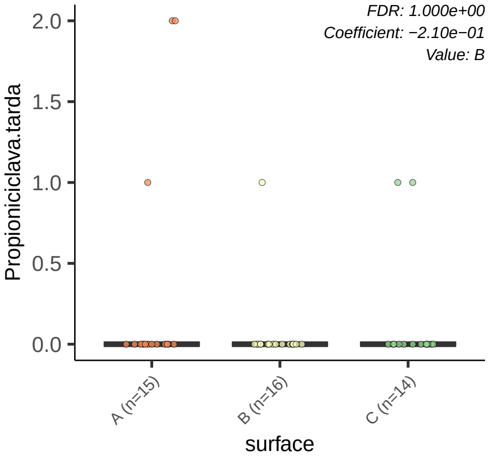

FDR: 1.000e+00
Coefficient: −2.10e−01
Value: B
Propioniciclava.tarda
2.0
1.5
1.0
0.5
0.0
A (n=15)
B (n=16)
C (n=14)
surface

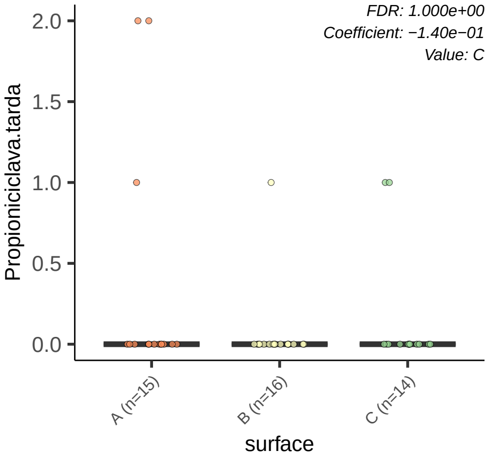
FDR: 1.000e+00
Coefficient: −1.40e−01
Value: C
Propioniciclava.tarda
2.0
1.5
1.0
0.5
0.0
A (n=15)
B (n=16)
C (n=14)
surface

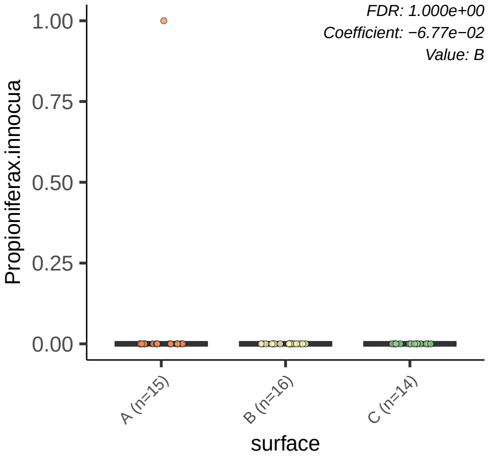

FDR: 1.000e+00
Coefficient: −6.77e−02
Value: B
Propioniferax.innocua
1.00
0.75
0.50
0.25
0.00
A (n=15)
B (n=16)
C (n=14)
surface

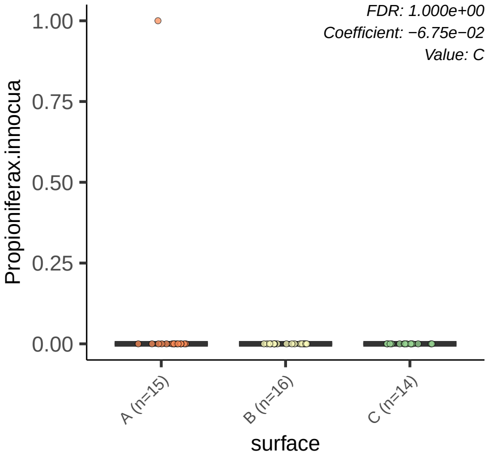

FDR: 1.000e+00
Coefficient: −6.75e−02
Value: C
Propioniferax.innocua
1.00
0.75
0.50
0.25
0.00
A (n=15)
B (n=16)
C (n=14)
surface

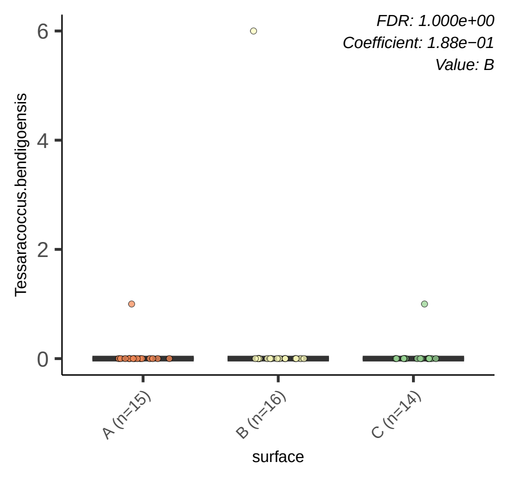
FDR: 1.000e+00
Coefficient: 1.88e−01
Value: B
Tessaracoccus.bendigoensis
0
2
4
6
A (n=15)
B (n=16)
C (n=14)
surface

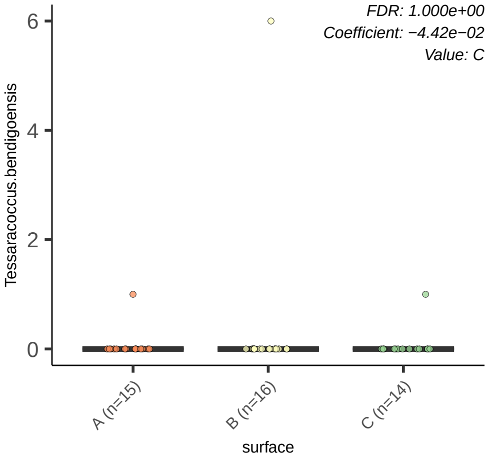
FDR: 1.000e+00
Coefficient: −4.42e−02
Value: C
Tessaracoccus.bendigoensis
0
2
4
6
A (n=15)
B (n=16)
C (n=14)
surface

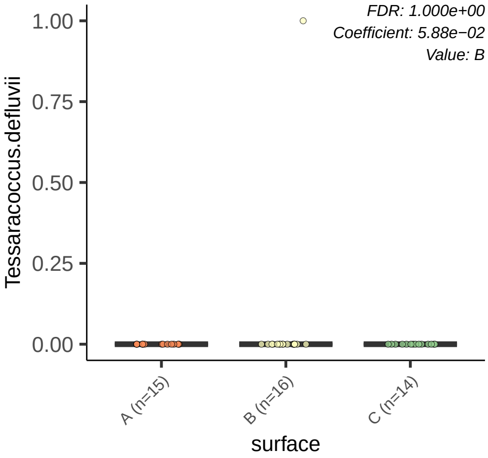

FDR: 1.000e+00
Coefficient: 5.88e−02
Value: B
Tessaracoccus.defluvii
1.00
0.75
0.50
0.25
0.00
A (n=15)
B (n=16)
C (n=14)
surface

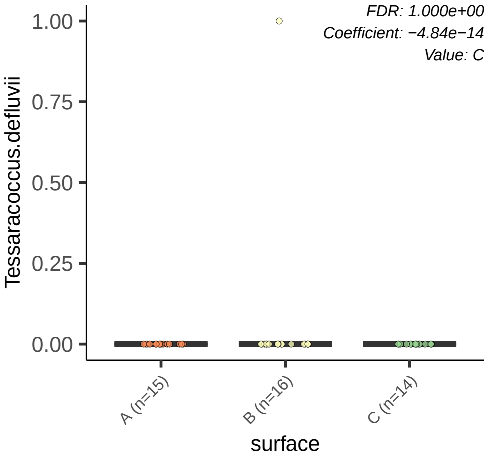
FDR: 1.000e+00
Coefficient: −4.84e−14
Value: C
Tessaracoccus.defluvii
1.00
0.75
0.50
0.25
0.00
A (n=15)
B (n=16)
C (n=14)
surface

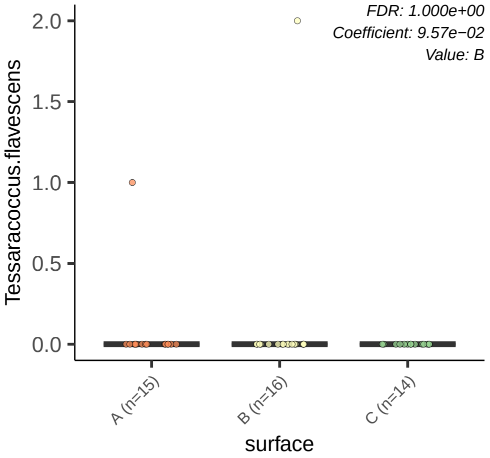
FDR: 1.000e+00
Coefficient: 9.57e−02
Value: B
Tessaracoccus.flavescens
2.0
1.5
1.0
0.5
0.0
A (n=15)
B (n=16)
C (n=14)
surface

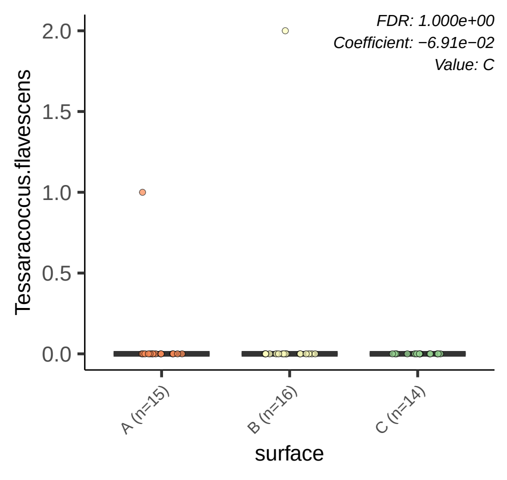
FDR: 1.000e+00
Coefficient: −6.91e−02
Value: C
Tessaracoccus.flavescens
2.0
1.5
1.0
0.5
0.0
A (n=15)
B (n=16)
C (n=14)
surface

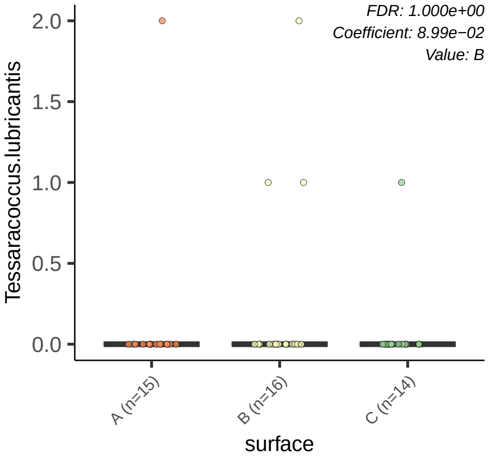

FDR: 1.000e+00
Coefficient: 8.99e−02
Value: B
Tessaracoccus.lubricantis
2.0
1.5
1.0
0.5
0.0
A (n=15)
B (n=16)
C (n=14)
surface

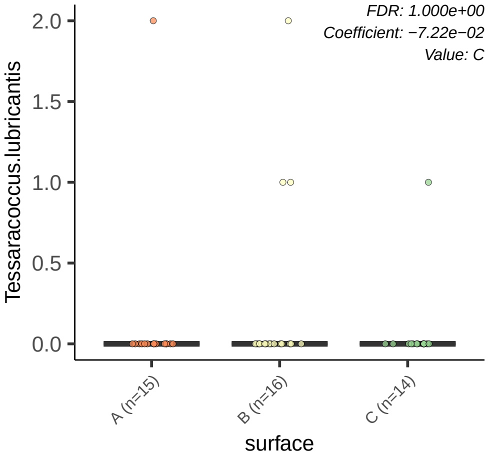
FDR: 1.000e+00
Coefficient: −7.22e−02
Value: C
Tessaracoccus.lubricantis
2.0
1.5
1.0
0.5
0.0
A (n=15)
B (n=16)
C (n=14)
surface

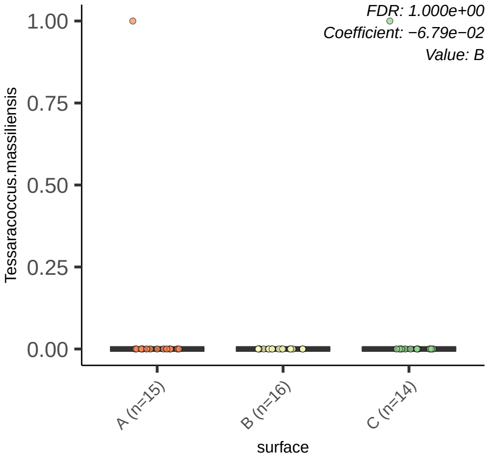
FDR: 1.000e+00
Coefficient: −6.79e−02
Value: B
1.00
0.75
0.50
0.25
0.00
Tessaracoccus.massiliensis
A (n=15)
B (n=16)
C (n=14)
surface

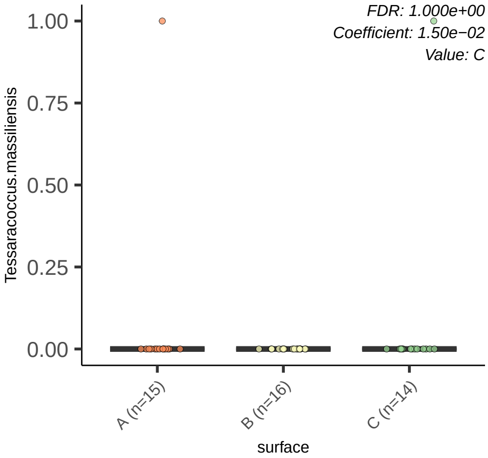
FDR: 1.000e+00
Coefficient: 1.50e−02
Value: C
1.00
0.75
0.50
0.25
0.00
Tessaracoccus.massiliensis
A (n=15)
B (n=16)
C (n=14)
surface

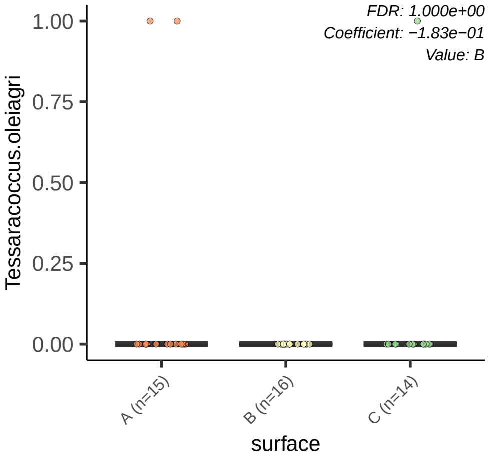

FDR: 1.000e+00
Coefficient: −1.83e−01
Value: B
Tessaracoccus.oleiagri
1.00
0.75
0.50
0.25
0.00
A (n=15)
B (n=16)
C (n=14)
surface

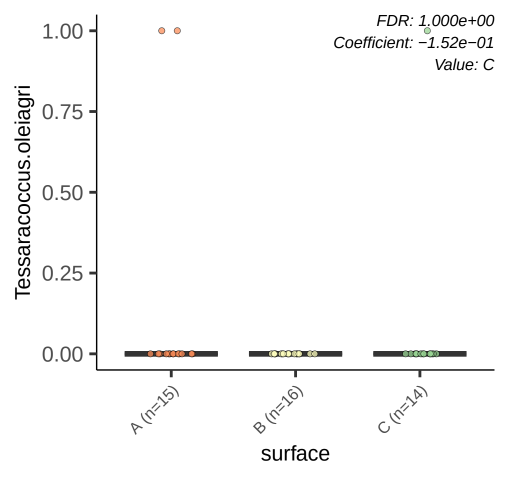

FDR: 1.000e+00
Coefficient: −1.52e−01
Value: C
Tessaracoccus.oleiagri
1.00
0.75
0.50
0.25
0.00
A (n=15)
B (n=16)
C (n=14)
surface

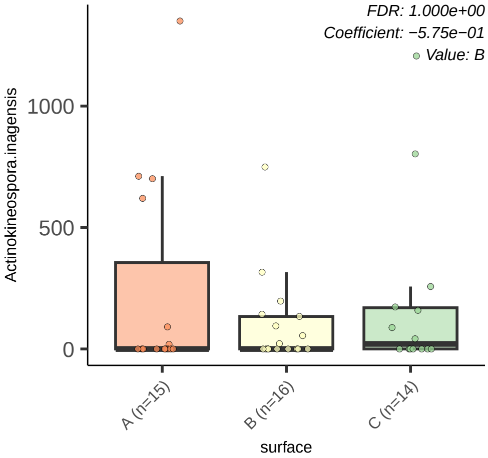
FDR: 1.000e+00
Coefficient: −5.75e−01
Value: B
Actinokineospora.inagensis
1000
500
0
A (n=15)
B (n=16)
C (n=14)
surface

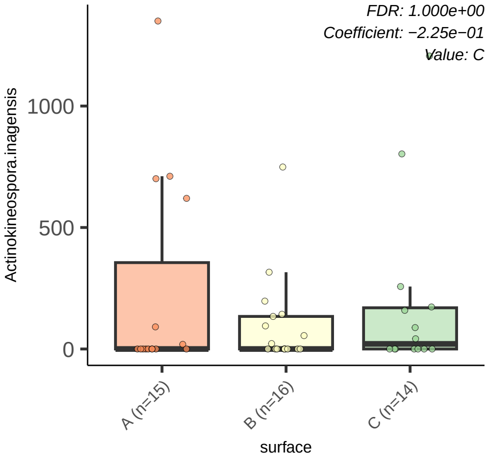
FDR: 1.000e+00
Coefficient: −2.25e−01
Value: C
Actinokineospora.inagensis
1000
500
0
A (n=15)
B (n=16)
C (n=14)
surface

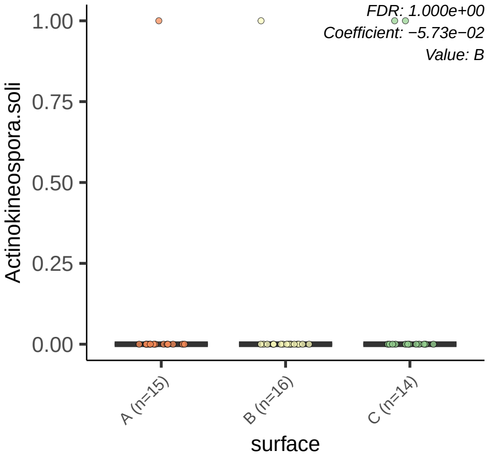

FDR: 1.000e+00
Coefficient: −5.73e−02
Value: B
Actinokineospora.soli
1.00
0.75
0.50
0.25
0.00
A (n=15)
B (n=16)
C (n=14)
surface

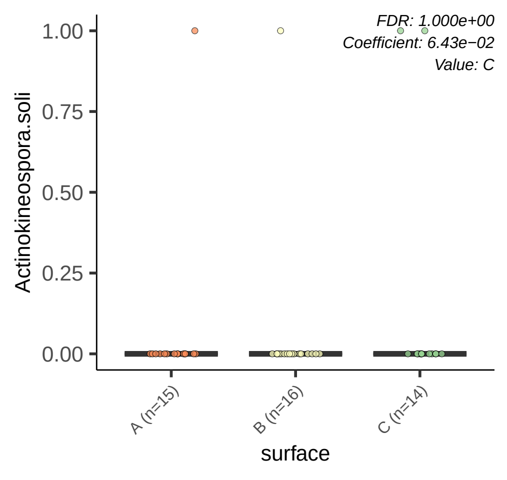

FDR: 1.000e+00
Coefficient: 6.43e−02
Value: C
Actinokineospora.soli
1.00
0.75
0.50
0.25
0.00
A (n=15)
B (n=16)
C (n=14)
surface

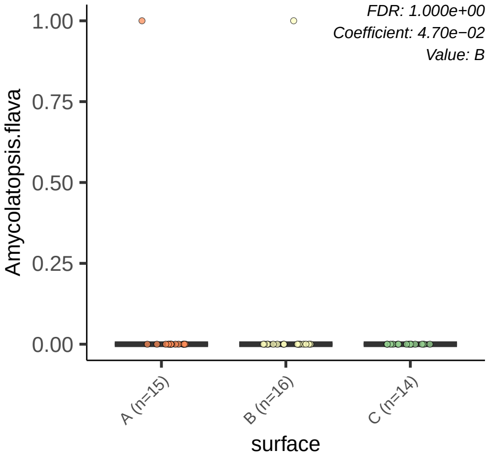

FDR: 1.000e+00
Coefficient: 4.70e−02
Value: B
Amycolatopsis.flava
1.00
0.75
0.50
0.25
0.00
A (n=15)
B (n=16)
C (n=14)
surface

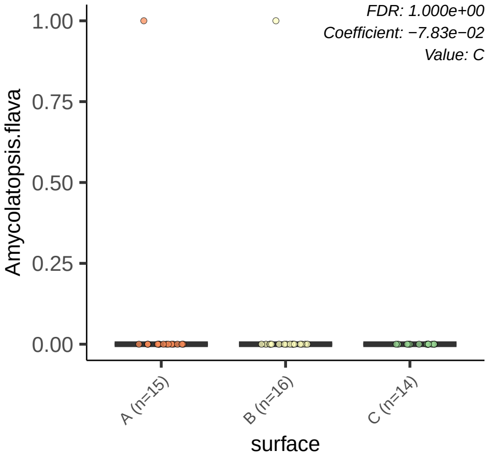
FDR: 1.000e+00
Coefficient: −7.83e−02
Value: C
Amycolatopsis.flava
1.00
0.75
0.50
0.25
0.00
A (n=15)
B (n=16)
C (n=14)
surface

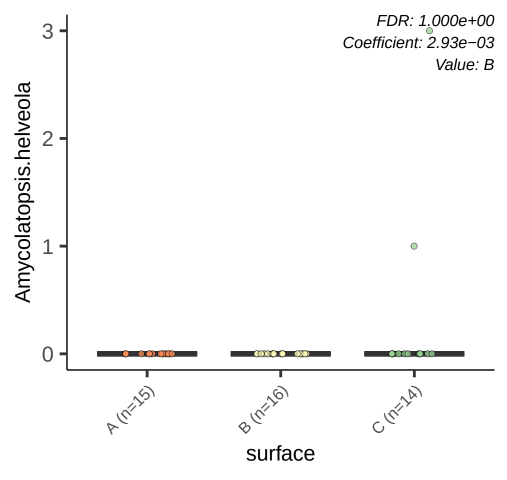

FDR: 1.000e+00
Coefficient: 2.93e−03
Value: B
Amycolatopsis.helveola
3
2
1
0
A (n=15)
B (n=16)
C (n=14)
surface

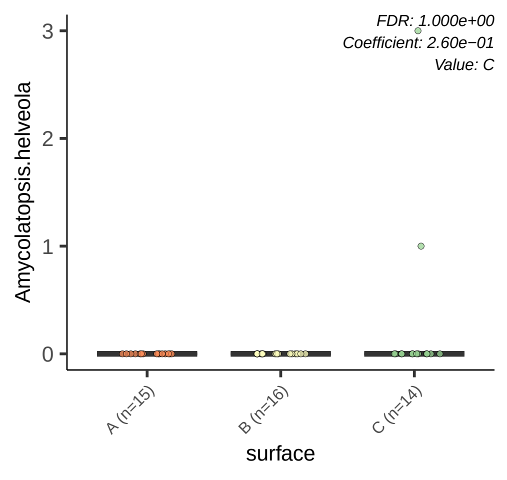

FDR: 1.000e+00
Coefficient: 2.60e−01
Value: C
Amycolatopsis.helveola
0
1
2
3
A (n=15)
B (n=16)
C (n=14)
surface

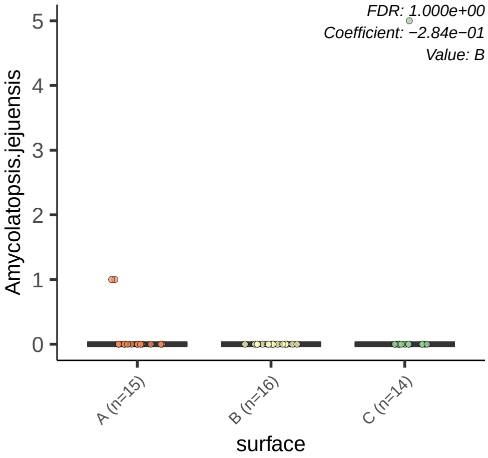
FDR: 1.000e+00
Coefficient: −2.84e−01
Value: B
Amycolatopsis.jejuensis
0
1
2
3
4
5
A (n=15)
B (n=16)
C (n=14)
surface

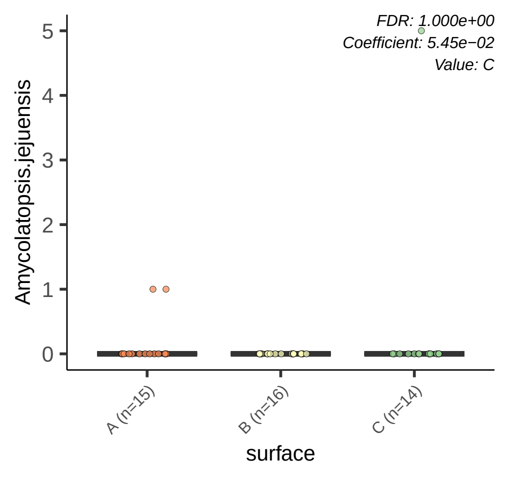
FDR: 1.000e+00
Coefficient: 5.45e−02
Value: C
Amycolatopsis.jejuensis
5
4
3
2
1
0
A (n=15)
B (n=16)
C (n=14)
surface

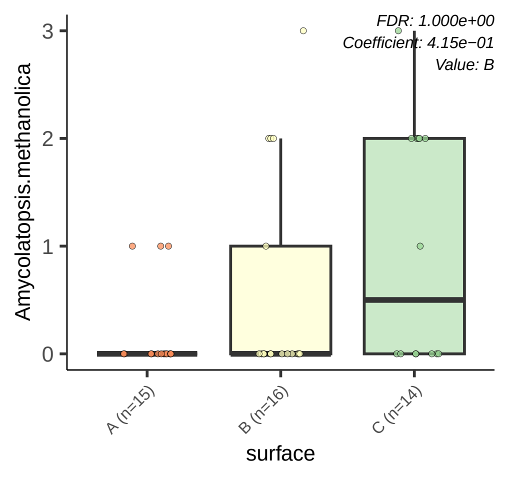
FDR: 1.000e+00
Coefficient: 4.15e−01
Value: B
Amycolatopsis.methanolica
0
1
2
3
A (n=15)
B (n=16)
C (n=14)
surface

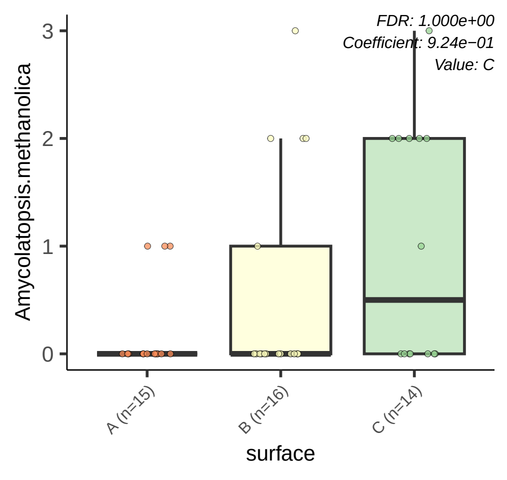

FDR: 1.000e+00
Coefficient: 9.24e−01
Value: C
Amycolatopsis.methanolica
0
1
2
3
A (n=15)
B (n=16)
C (n=14)
surface

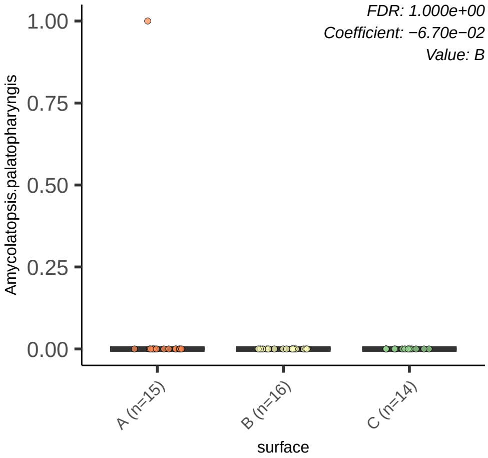
FDR: 1.000e+00
Coefficient: −6.70e−02
Value: B
Amycolatopsis.palatopharyngis
1.00
0.75
0.50
0.25
0.00
A (n=15)
B (n=16)
C (n=14)
surface

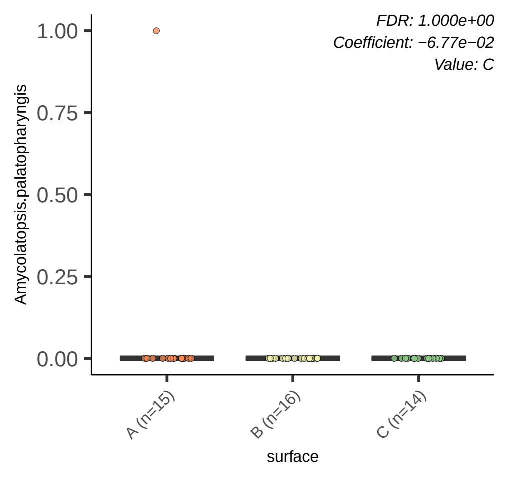
FDR: 1.000e+00
Coefficient: −6.77e−02
Value: C
Amycolatopsis.palatopharyngis
1.00
0.75
0.50
0.25
0.00
A (n=15)
B (n=16)
C (n=14)
surface

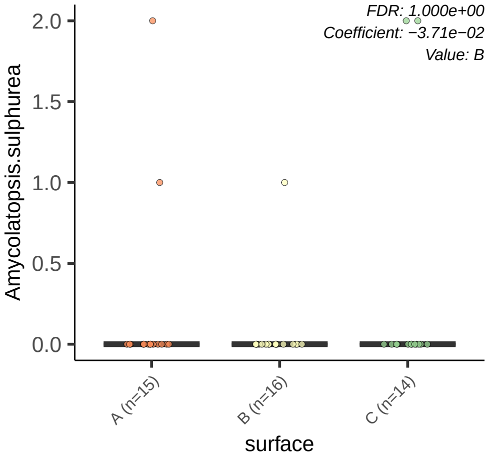
FDR: 1.000e+00
Coefficient: −3.71e−02
Value: B
Amycolatopsis.sulphurea
2.0
1.5
1.0
0.5
0.0
A (n=15)
B (n=16)
C (n=14)
surface

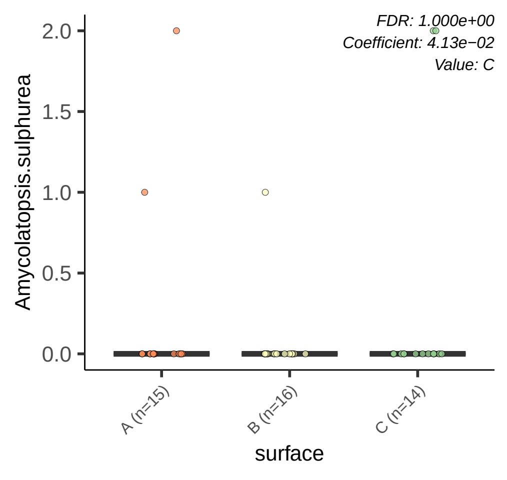
FDR: 1.000e+00
Coefficient: 4.13e−02
Value: C
Amycolatopsis.sulphurea
2.0
1.5
1.0
0.5
0.0
A (n=15)
B (n=16)
C (n=14)
surface

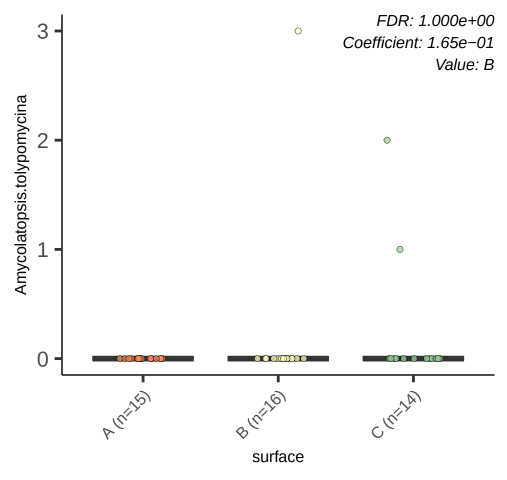

FDR: 1.000e+00
Coefficient: 1.65e−01
Value: B
Amycolatopsis.tolypomycina
3
2
1
0
A (n=15)
B (n=16)
C (n=14)
surface

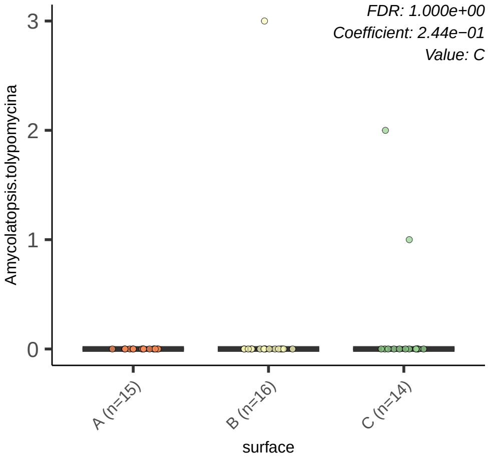

FDR: 1.000e+00
Coefficient: 2.44e−01
Value: C
Amycolatopsis.tolypomycina
3
2
1
0
A (n=15)
B (n=16)
C (n=14)
surface

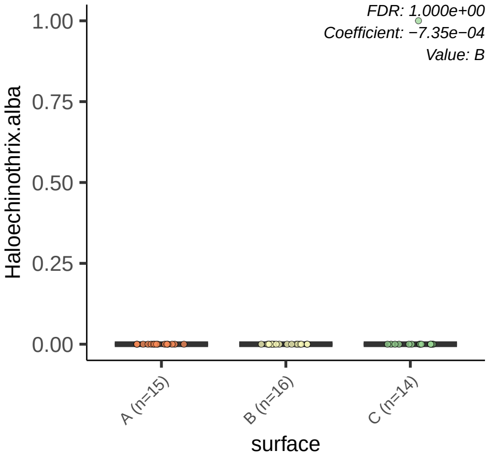
FDR: 1.000e+00
Coefficient: −7.35e−04
Value: B
Haloechinothrix.alba
1.00
0.75
0.50
0.25
0.00
A (n=15)
B (n=16)
C (n=14)
surface

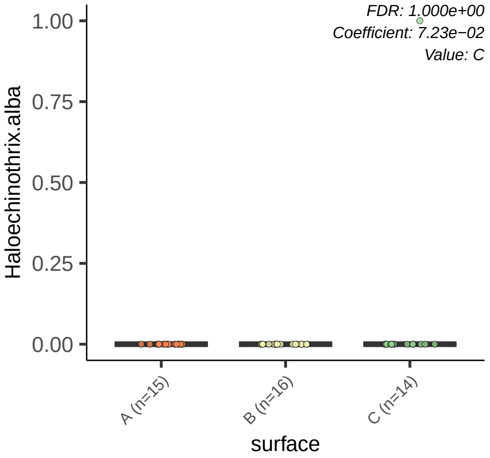
FDR: 1.000e+00
Coefficient: 7.23e−02
Value: C
Haloechinothrix.alba
1.00
0.75
0.50
0.25
0.00
A (n=15)
B (n=16)
C (n=14)
surface

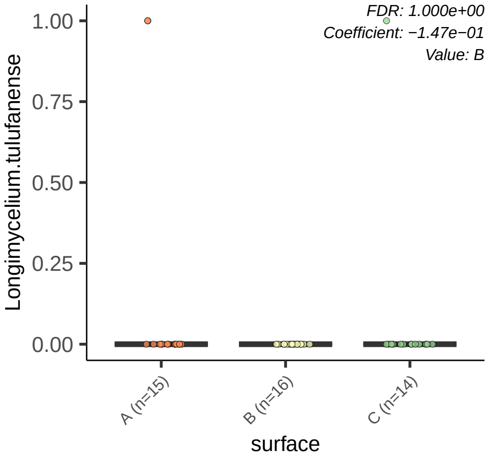
FDR: 1.000e+00
Coefficient: −1.47e−01
Value: B
Longimycelium.tulufanense
1.00
0.75
0.50
0.25
0.00
A (n=15)
B (n=16)
C (n=14)
surface

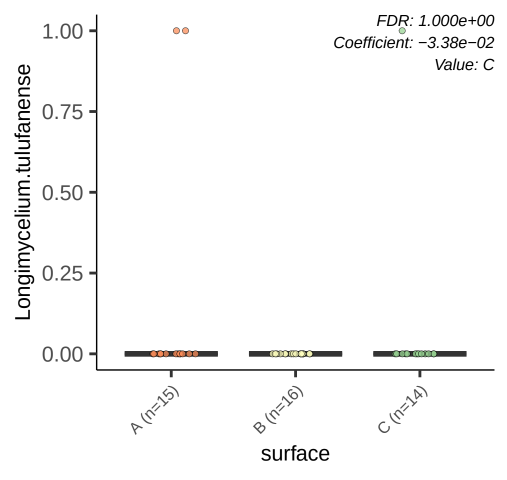

FDR: 1.000e+00
Coefficient: −3.38e−02
Value: C
1.00
0.75
0.50
0.25
0.00
Longimycelium.tulufanense
A (n=15)
B (n=16)
C (n=14)
surface

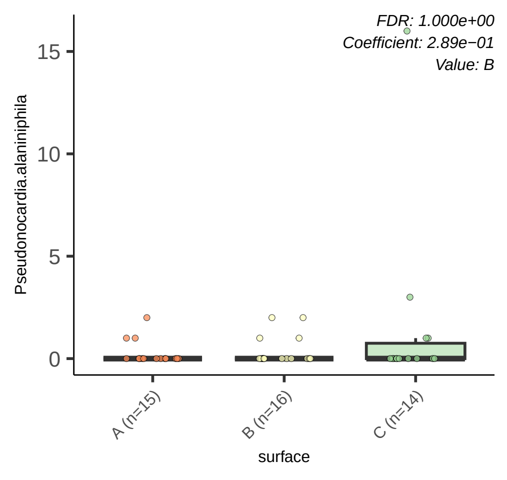

FDR: 1.000e+00
Coefficient: 2.89e−01
Value: B
Pseudonocardia.alaniniphila
15
10
5
0
A (n=15)
B (n=16)
C (n=14)
surface

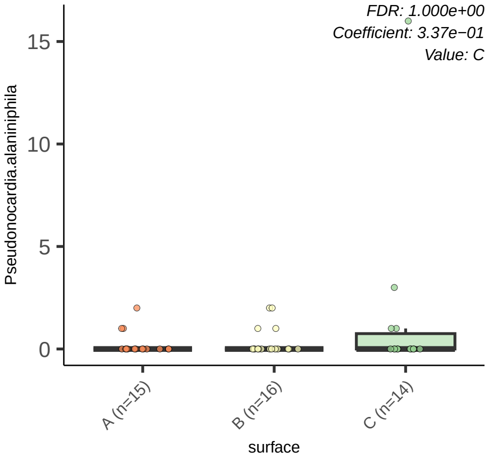

FDR: 1.000e+00
Coefficient: 3.37e−01
Value: C
Pseudonocardia.alaniniphila
15
10
5
0
A (n=15)
B (n=16)
C (n=14)
surface

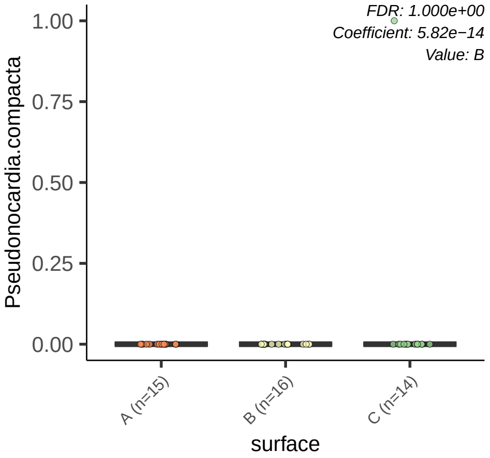

FDR: 1.000e+00
Coefficient: 5.82e−14
Value: B
Pseudonocardia.compacta
1.00
0.75
0.50
0.25
0.00
A (n=15)
B (n=16)
C (n=14)
surface

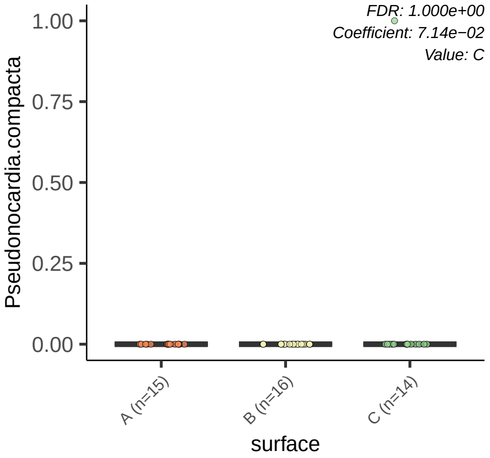
FDR: 1.000e+00
Coefficient: 7.14e−02
Value: C
Pseudonocardia.compacta
1.00
0.75
0.50
0.25
0.00
A (n=15)
B (n=16)
C (n=14)
surface

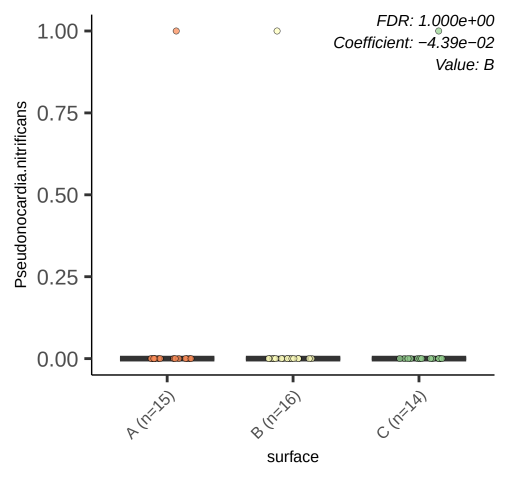
FDR: 1.000e+00
Coefficient: −4.39e−02
Value: B
1.00
0.75
0.50
0.25
0.00
Pseudonocardia.nitrificans
A (n=15)
B (n=16)
C (n=14)
surface

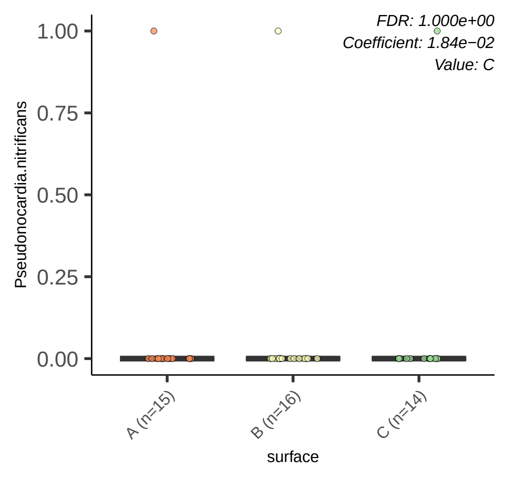
FDR: 1.000e+00
Coefficient: 1.84e−02
Value: C
1.00
0.75
0.50
0.25
0.00
Pseudonocardia.nitrificans
A (n=15)
B (n=16)
C (n=14)
surface

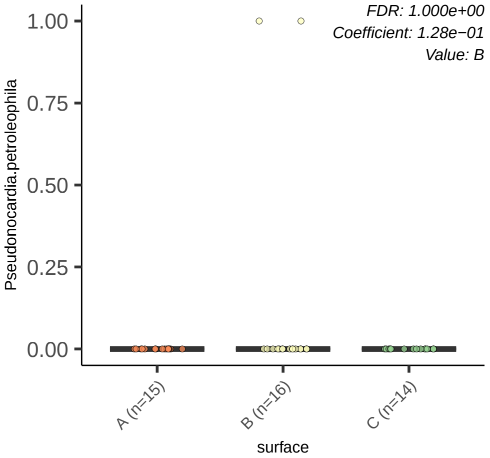
FDR: 1.000e+00
Coefficient: 1.28e−01
Value: B
Pseudonocardia.petroleophila
1.00
0.75
0.50
0.25
0.00
A (n=15)
B (n=16)
C (n=14)
surface

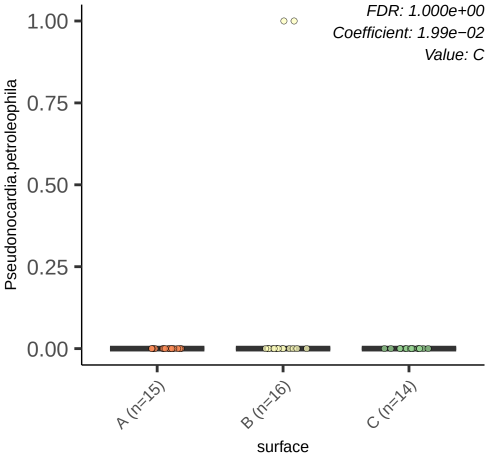
FDR: 1.000e+00
Coefficient: 1.99e−02
Value: C
1.00
0.75
0.50
0.25
0.00
Pseudonocardia.petroleophila
A (n=15)
B (n=16)
C (n=14)
surface

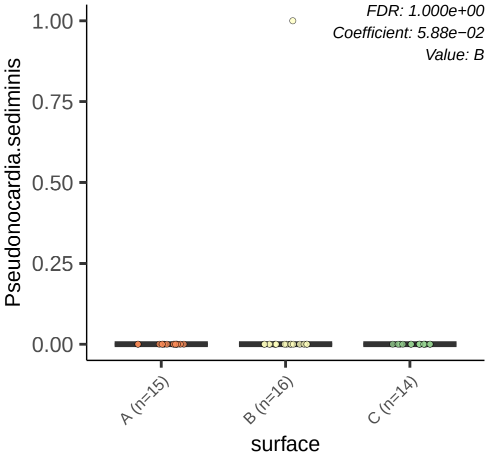
FDR: 1.000e+00
Coefficient: 5.88e−02
Value: B
Pseudonocardia.sediminis
1.00
0.75
0.50
0.25
0.00
A (n=15)
B (n=16)
C (n=14)
surface

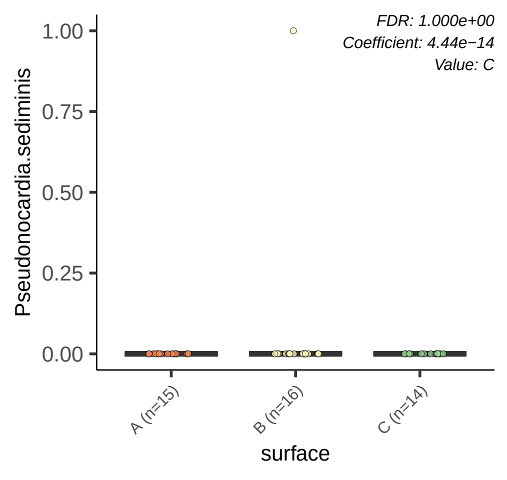

FDR: 1.000e+00
Coefficient: 4.44e−14
Value: C
Pseudonocardia.sedíminis
1.00
0.75
0.50
0.25
0.00
A (n=15)
B (n=16)
C (n=14)
surface

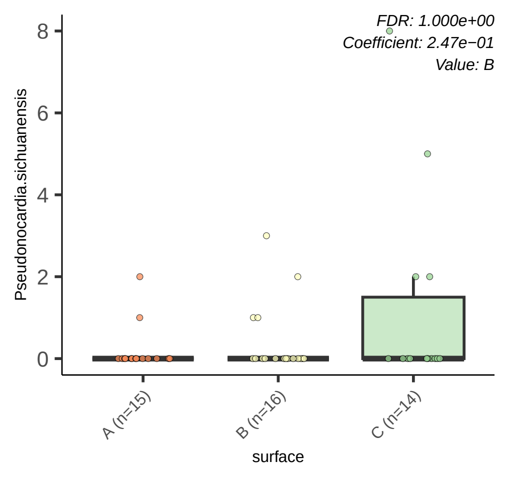
FDR: 1.000e+00
Coefficient: 2.47e−01
Value: B
Pseudonocardia.sichuanensis
0
2
4
6
8
A (n=15)
B (n=16)
C (n=14)
surface

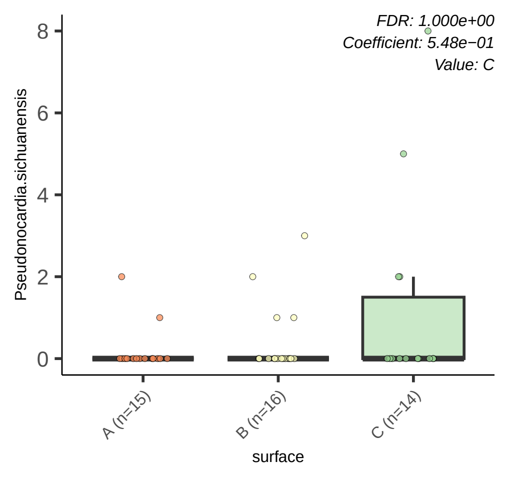
FDR: 1.000e+00
Coefficient: 5.48e−01
Value: C
Pseudonocardia.sichuanensis
0
2
4
6
8
A (n=15)
B (n=16)
C (n=14)
surface

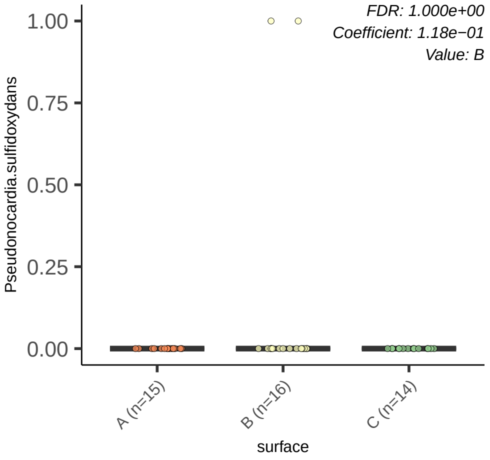

FDR: 1.000e+00
Coefficient: 1.18e−01
Value: B
Pseudonocardia.sulfidoxydans
1.00
0.75
0.50
0.25
0.00
A (n=15)
B (n=16)
C (n=14)
surface

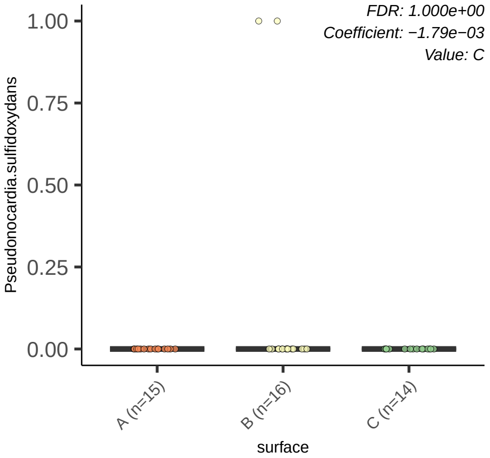
FDR: 1.000e+00
Coefficient: −1.79e−03
Value: C
Pseudonocardia.sulfidoxydans
1.00
0.75
0.50
0.25
0.00
A (n=15)
B (n=16)
C (n=14)
surface

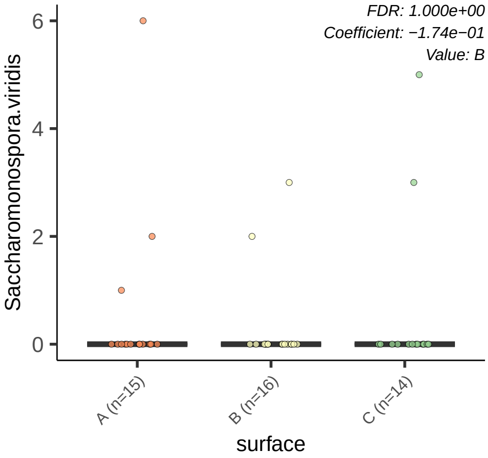
FDR: 1.000e+00
Coefficient: −1.74e−01
Value: B
Saccharomonospora.viridis
0
2
4
6
A (n=15)
B (n=16)
C (n=14)
surface

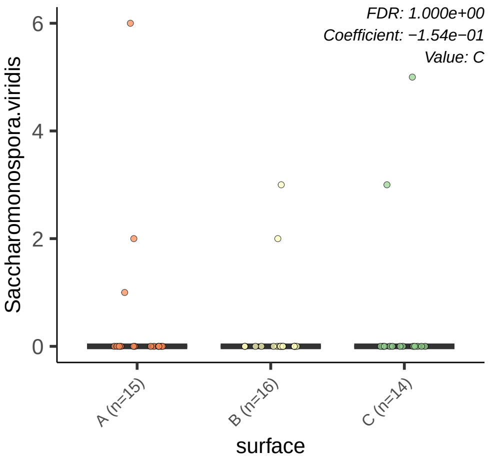

FDR: 1.000e+00
Coefficient: −1.54e−01
Value: C
Saccharomonospora.viridis
0
2
4
6
A (n=15)
B (n=16)
C (n=14)
surface

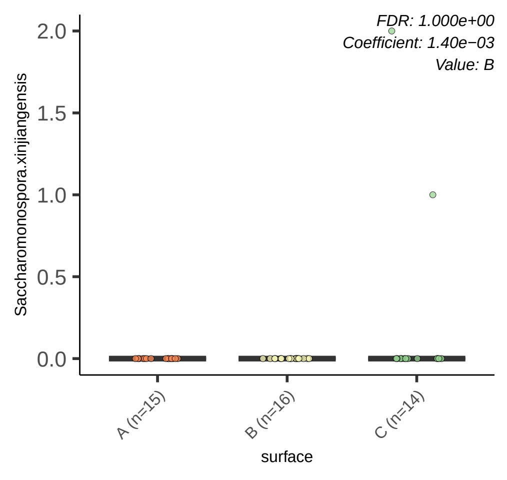
FDR: 1.000e+00
Coefficient: 1.40e−03
Value: B
Saccharomonospora.xinjiangensis
2.0
1.5
1.0
0.5
0.0
A (n=15)
B (n=16)
C (n=14)
surface

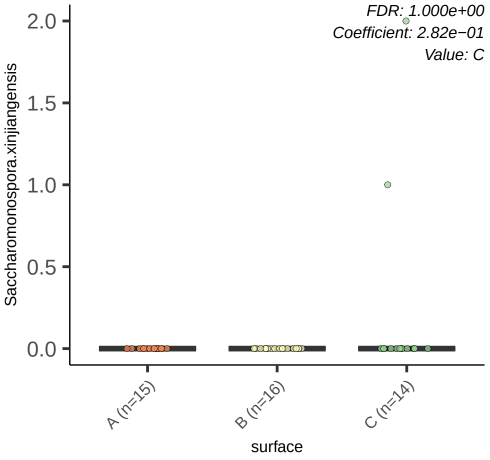

FDR: 1.000e+00
Coefficient: 2.82e−01
Value: C
Saccharomonospora.xinjiangensis
2.0
1.5
1.0
0.5
0.0
A (n=15)
B (n=16)
C (n=14)
surface

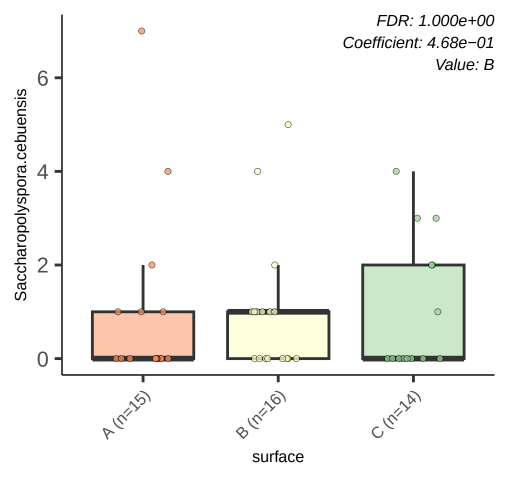

FDR: 1.000e+00
Coefficient: 4.68e−01
Value: B
Saccharopolyspora.cebuensis
0
2
4
6
A (n=15)
B (n=16)
C (n=14)
surface

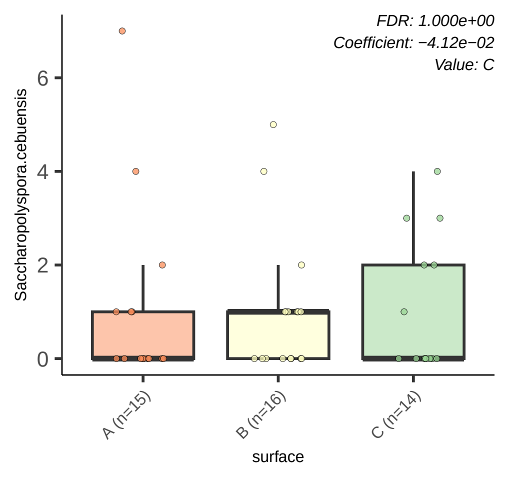
FDR: 1.000e+00
Coefficient: −4.12e−02
Value: C
Saccharopolyspora.cebuensis
0
2
4
6
A (n=15)
B (n=16)
C (n=14)
surface

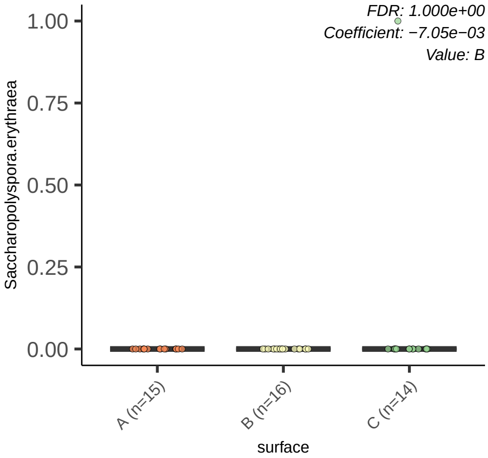
FDR: 1.000e+00
Coefficient: −7.05e−03
Value: B
1.00
0.75
0.50
0.25
0.00
Saccharopolyspora.erythraea
A (n=15)
B (n=16)
C (n=14)
surface

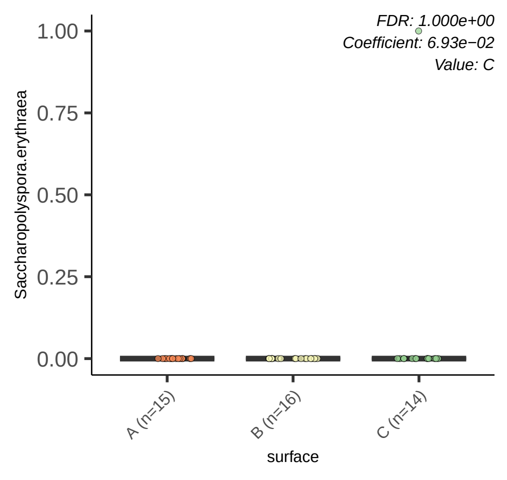

FDR: 1.000e+00
Coefficient: 6.93e−02
Value: C
1.00
0.75
0.50
0.25
0.00
Saccharopolyspora.erythraea
A (n=15)
B (n=16)
C (n=14)
surface

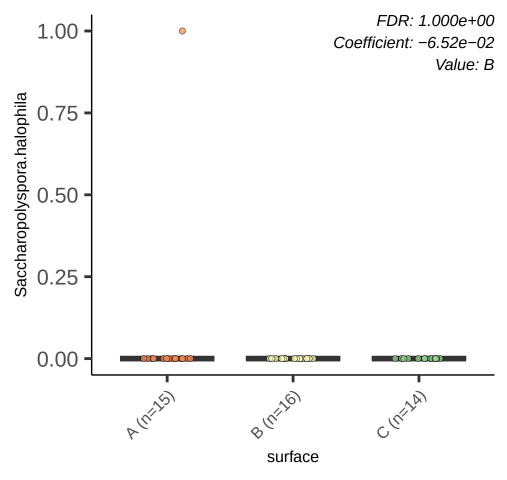

FDR: 1.000e+00
Coefficient: −6.52e−02
Value: B
Saccharopolyspora.halophila
1.00
0.75
0.50
0.25
0.00
A (n=15)
B (n=16)
C (n=14)
surface

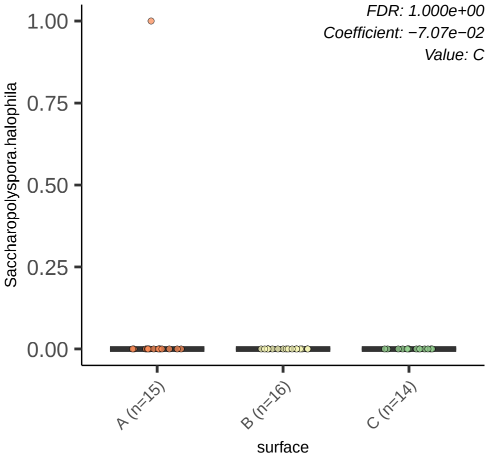

FDR: 1.000e+00
Coefficient: −7.07e−02
Value: C
Saccharopolyspora.halophila
1.00
0.75
0.50
0.25
0.00
A (n=15)
B (n=16)
C (n=14)
surface

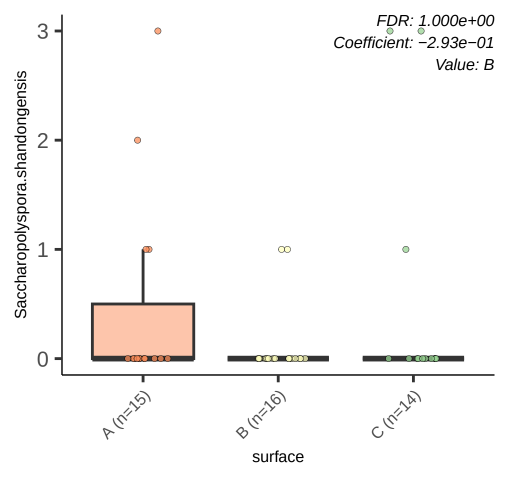

FDR: 1.000e+00
Coefficient: −2.93e−01
Value: B
Saccharopolyspora.shandongensis
3
2
1
0
A (n=15)
B (n=16)
C (n=14)
surface

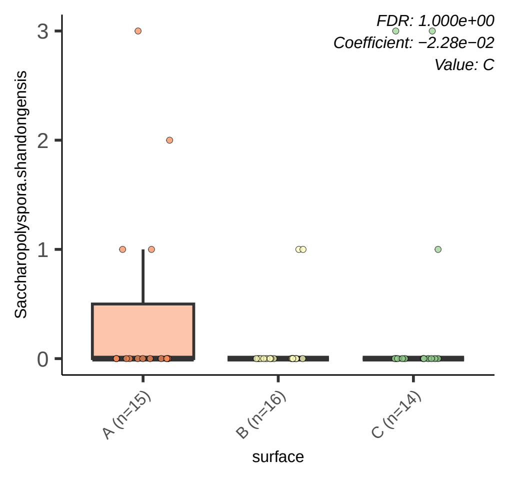

FDR: 1.000e+00
Coefficient: −2.28e−02
Value: C
3
2
1
0
Saccharopolyspora.shandongensis
A (n=15)
B (n=16)
C (n=14)
surface

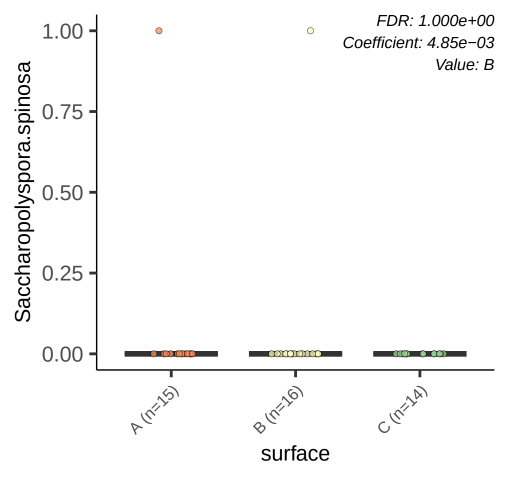

FDR: 1.000e+00
Coefficient: 4.85e−03
Value: B
Saccharopolyspora.spinosa
1.00
0.75
0.50
0.25
0.00
A (n=15)
B (n=16)
C (n=14)
surface

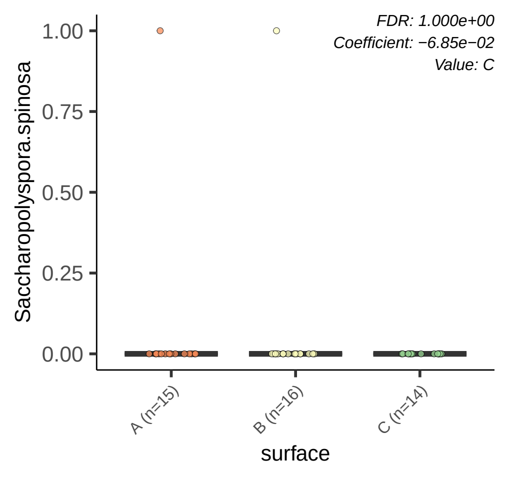

FDR: 1.000e+00
Coefficient: −6.85e−02
Value: C
Saccharopolyspora.spinosa
1.00
0.75
0.50
0.25
0.00
A (n=15)
B (n=16)
C (n=14)
surface

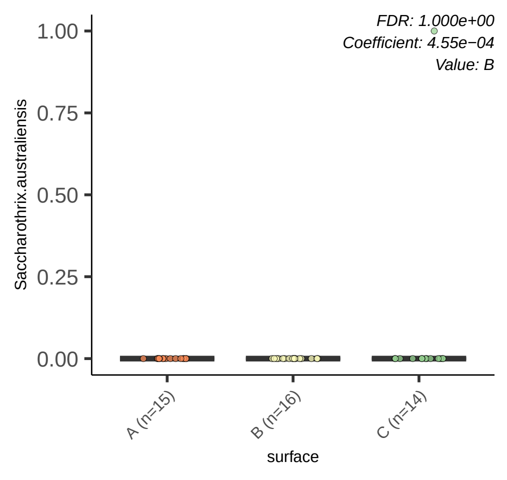
FDR: 1.000e+00
Coefficient: 4.55e−04
Value: B
1.00
0.75
0.50
0.25
0.00
Saccharothrix.australiensis
A (n=15)
B (n=16)
C (n=14)
surface

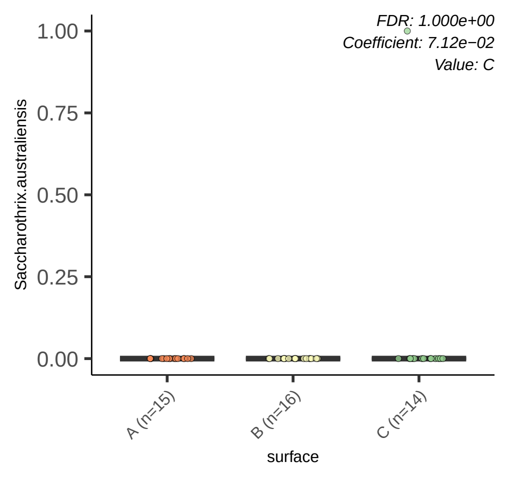
FDR: 1.000e+00
Coefficient: 7.12e−02
Value: C
Saccharothrix.australiensis
1.00
0.75
0.50
0.25
0.00
A (n=15)
B (n=16)
C (n=14)
surface

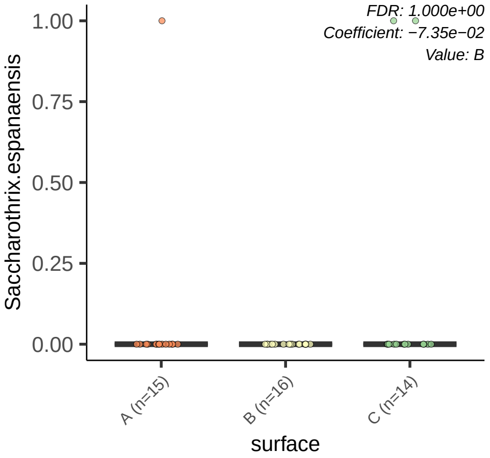
FDR: 1.000e+00
Coefficient: −7.35e−02
Value: B
Saccharothrix.espanaensis
1.00
0.75
0.50
0.25
0.00
A (n=15)
B (n=16)
C (n=14)
surface

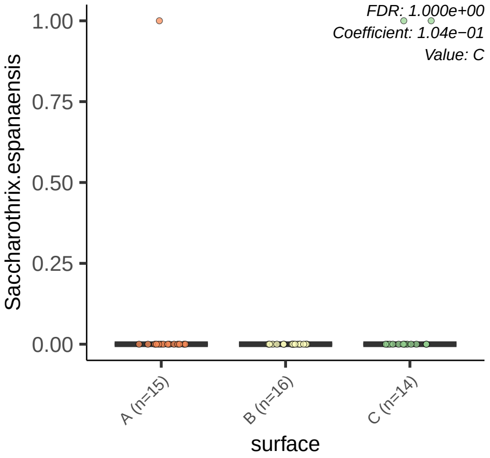
FDR: 1.000e+00
Coefficient: 1.04e−01
Value: C
Saccharothrix.espanaensis
1.00
0.75
0.50
0.25
0.00
A (n=15)
B (n=16)
C (n=14)
surface

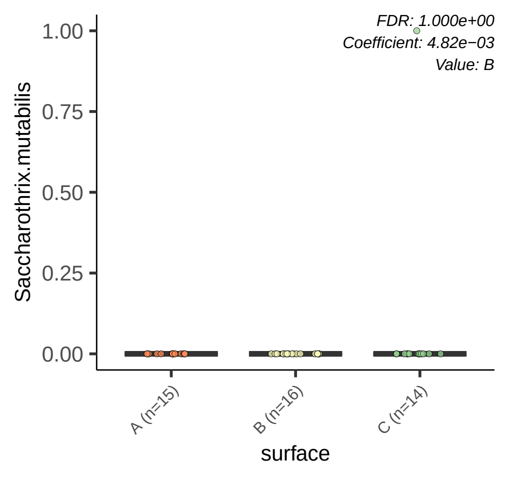
FDR: 1.000e+00
Coefficient: 4.82e−03
Value: B
Saccharothrix.mutabilis
1.00
0.75
0.50
0.25
0.00
A (n=15)
B (n=16)
C (n=14)
surface

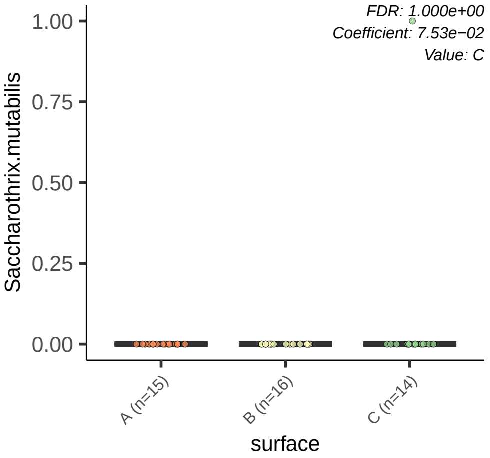
FDR: 1.000e+00
Coefficient: 7.53e−02
Value: C
Saccharothrix.mutabilis
1.00
0.75
0.50
0.25
0.00
A (n=15)
B (n=16)
C (n=14)
surface

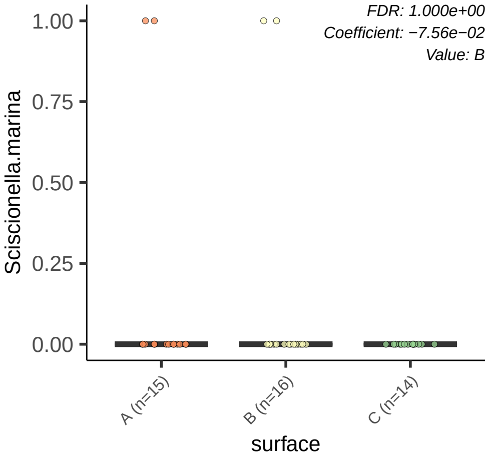

FDR: 1.000e+00
Coefficient: −7.56e−02
Value: B
Sciscionella.marina
1.00
0.75
0.50
0.25
0.00
A (n=15)
B (n=16)
C (n=14)
surface

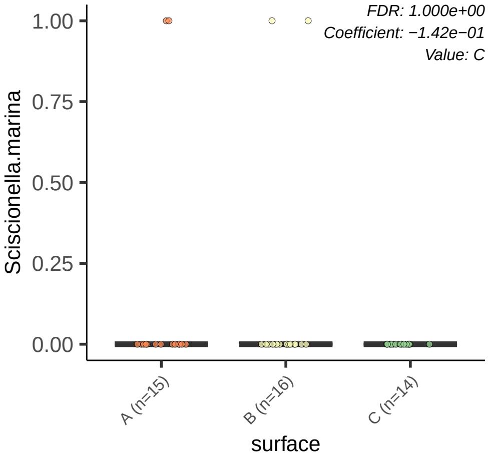

FDR: 1.000e+00
Coefficient: −1.42e−01
Value: C
Sciscionella.marina
1.00
0.75
0.50
0.25
0.00
A (n=15)
B (n=16)
C (n=14)
surface

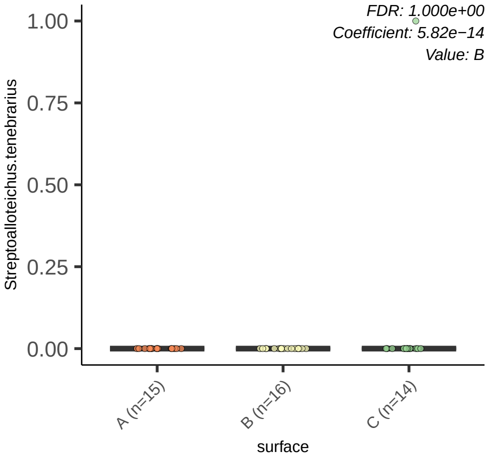
FDR: 1.000e+00
Coefficient: 5.82e−14
Value: B
1.00
0.75
0.50
0.25
0.00
Streptoalloteichus.tenebrarius
A (n=15)
B (n=16)
C (n=14)
surface

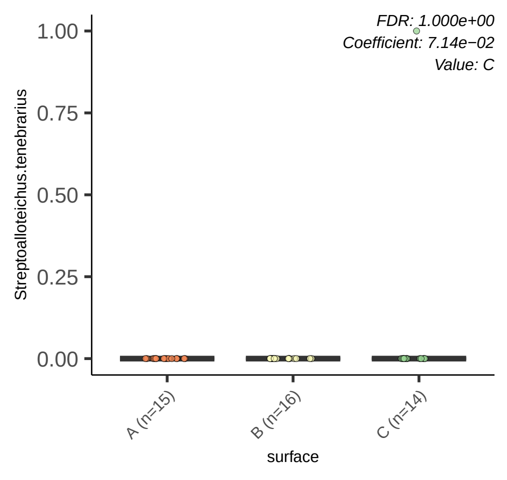

FDR: 1.000e+00
Coefficient: 7.14e−02
Value: C
1.00
0.75
0.50
0.25
0.00
Streptoalloteichus.tenebrarius
A (n=15)
B (n=16)
C (n=14)
surface

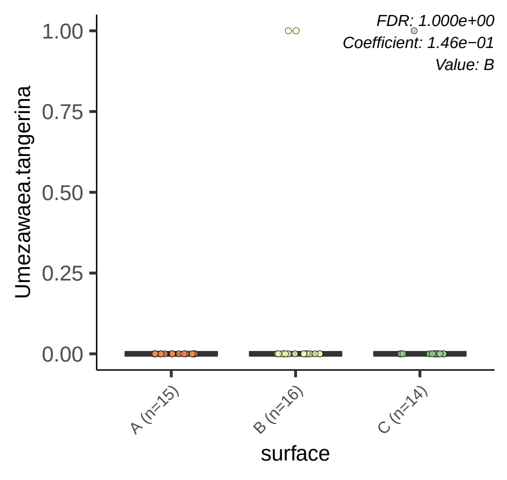

FDR: 1.000e+00
Coefficient: 1.46e−01
Value: B
Umezawaea.tangerina
1.00
0.75
0.50
0.25
0.00
A (n=15)
B (n=16)
C (n=14)
surface

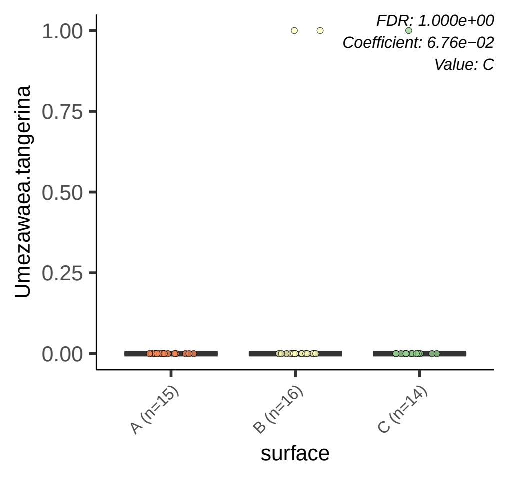
FDR: 1.000e+00
Coefficient: 6.76e−02
Value: C
Umezawaea.tangerina
1.00
0.75
0.50
0.25
0.00
A (n=15)
B (n=16)
C (n=14)
surface

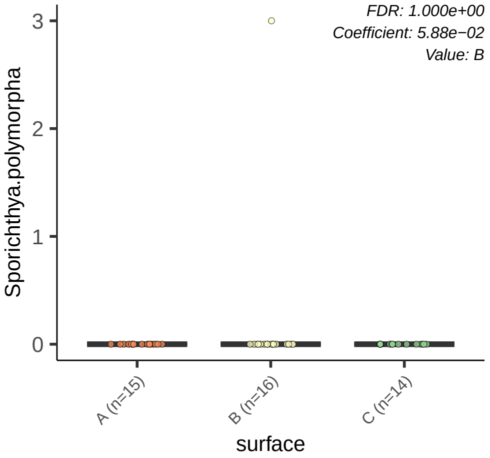
FDR: 1.000e+00
Coefficient: 5.88e−02
Value: B
Sporichthya.polymorpha
3
2
1
0
A (n=15)
B (n=16)
C (n=14)
surface

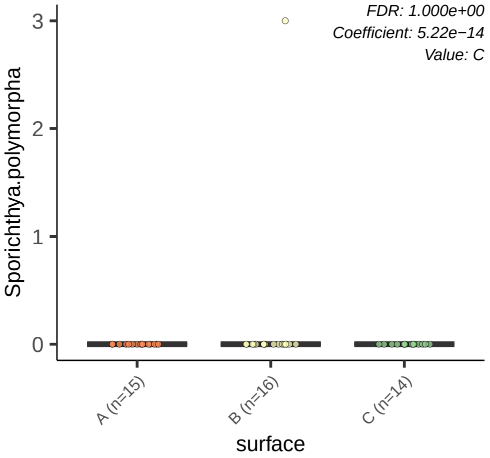
FDR: 1.000e+00
Coefficient: 5.22e−14
Value: C
Sporichthya.polymorpha
3
2
1
0
A (n=15)
B (n=16)
C (n=14)
surface

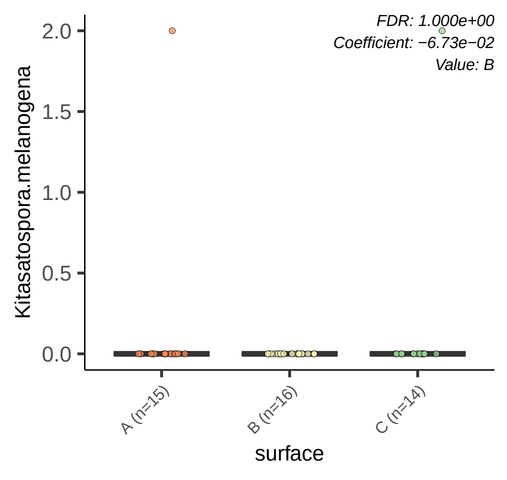

FDR: 1.000e+00
Coefficient: −6.73e−02
Value: B
Kitasatospora.melanogena
2.0
1.5
1.0
0.5
0.0
A (n=15)
B (n=16)
C (n=14)
surface

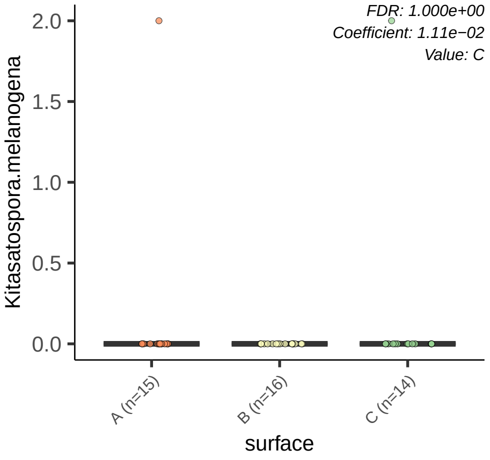
FDR: 1.000e+00
Coefficient: 1.11e−02
Value: C
Kitasatospora.melanogena
2.0
1.5
1.0
0.5
0.0
A (n=15)
B (n=16)
C (n=14)
surface

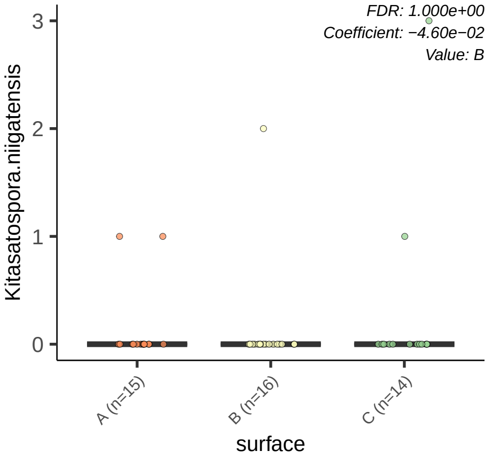
FDR: 1.000e+00
Coefficient: −4.60e−02
Value: B
Kitasatospora.niigatensis
0
1
2
3
A (n=15)
B (n=16)
C (n=14)
surface

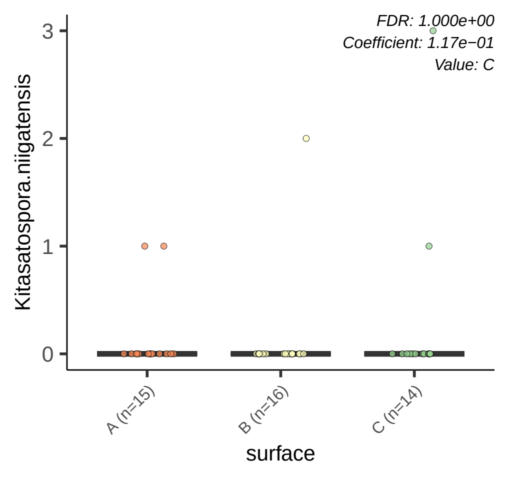

FDR: 1.000e+00
Coefficient: 1.17e−01
Value: C
Kitasatospora.niigatensis
3
2
1
0
A (n=15)
B (n=16)
C (n=14)
surface

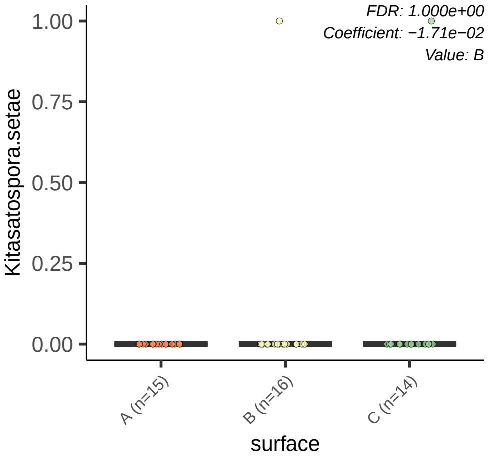

FDR: 1.000e+00
Coefficient: −1.71e−02
Value: B
Kitasatospora.setae
1.00
0.75
0.50
0.25
0.00
A (n=15)
B (n=16)
C (n=14)
surface

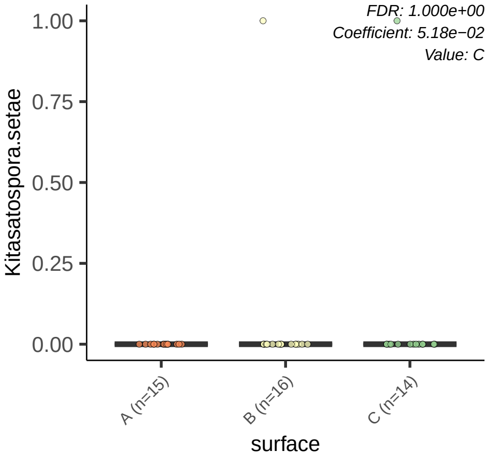
FDR: 1.000e+00
Coefficient: 5.18e−02
Value: C
Kitasatospora.setae
1.00
0.75
0.50
0.25
0.00
A (n=15)
B (n=16)
C (n=14)
surface

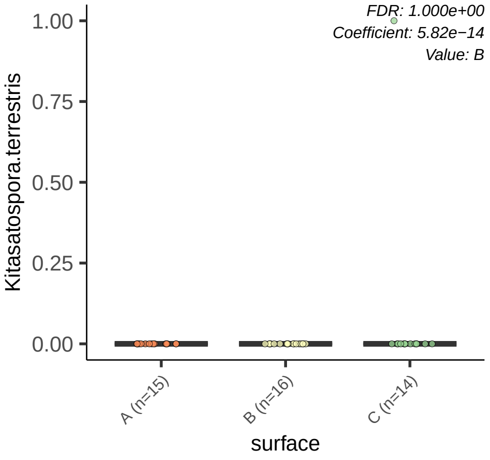
FDR: 1.000e+00
Coefficient: 5.82e−14
Value: B
Kitasatospora.terrestris
1.00
0.75
0.50
0.25
0.00
A (n=15)
B (n=16)
C (n=14)
surface

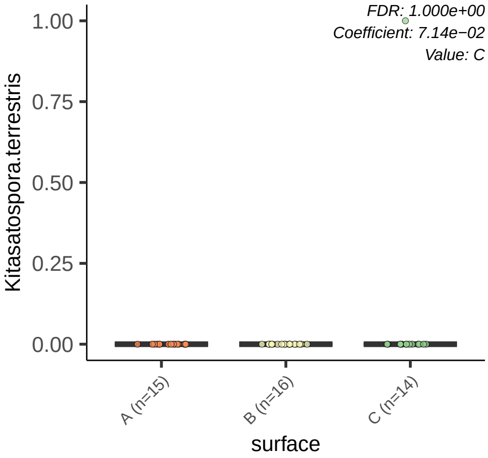

FDR: 1.000e+00
Coefficient: 7.14e−02
Value: C
Kitasatospora.terrestris
1.00
0.75
0.50
0.25
0.00
A (n=15)
B (n=16)
C (n=14)
surface

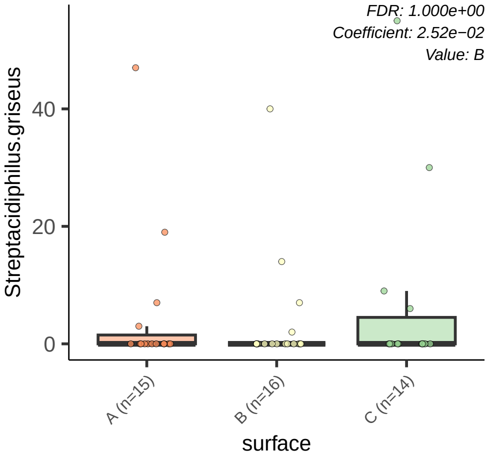

FDR: 1.000e+00
Coefficient: 2.52e−02
Value: B
Streptacidiphilus.griseus
0
20
40
A (n=15)
B (n=16)
C (n=14)
surface

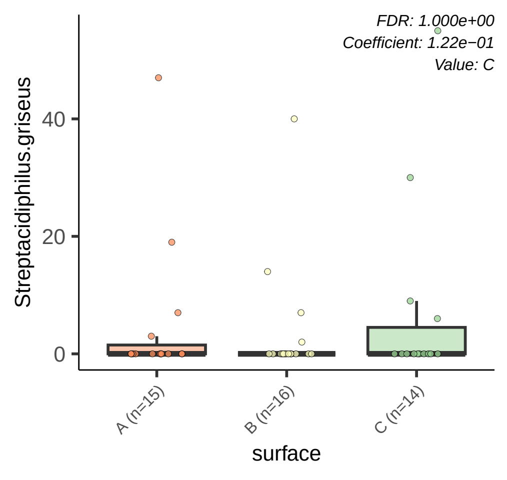

FDR: 1.000e+00
Coefficient: 1.22e−01
Value: C
Streptacidiphilus.griseus
0
20
40
A (n=15)
B (n=16)
C (n=14)
surface

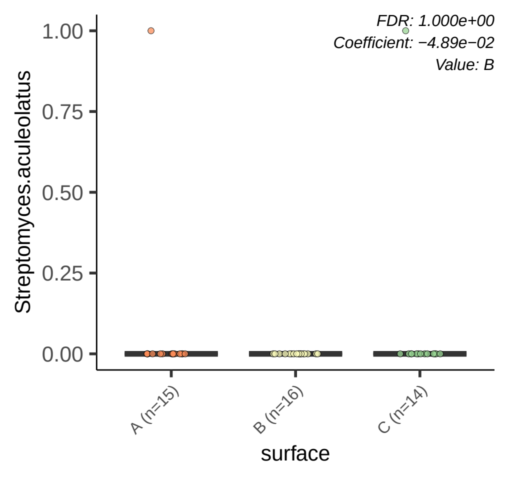
FDR: 1.000e+00
Coefficient: −4.89e−02
Value: B
Streptomyces.aculeolatus
1.00
0.75
0.50
0.25
0.00
A (n=15)
B (n=16)
C (n=14)
surface

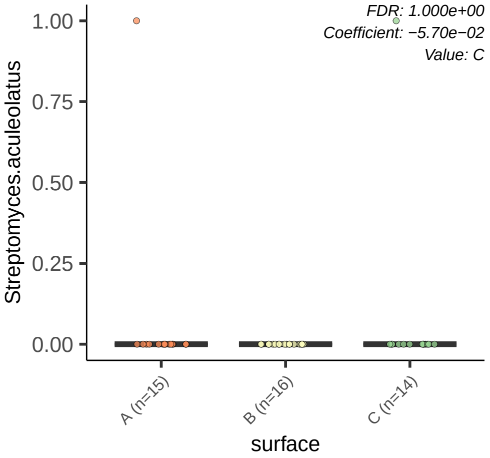

FDR: 1.000e+00
Coefficient: −5.70e−02
Value: C
Streptomyces.aculeolatus
1.00
0.75
0.50
0.25
0.00
A (n=15)
B (n=16)
C (n=14)
surface

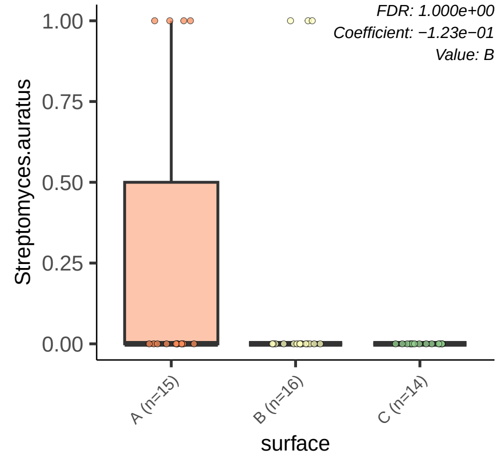
FDR: 1.000e+00
Coefficient: −1.23e−01
Value: B
Streptomyces.auratus
1.00
0.75
0.50
0.25
0.00
A (n=15)
B (n=16)
C (n=14)
surface

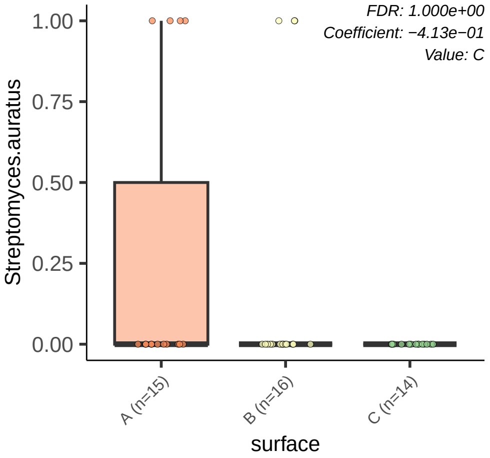
FDR: 1.000e+00
Coefficient: −4.13e−01
Value: C
Streptomyces.auratus
1.00
0.75
0.50
0.25
0.00
A (n=15)
B (n=16)
C (n=14)
surface

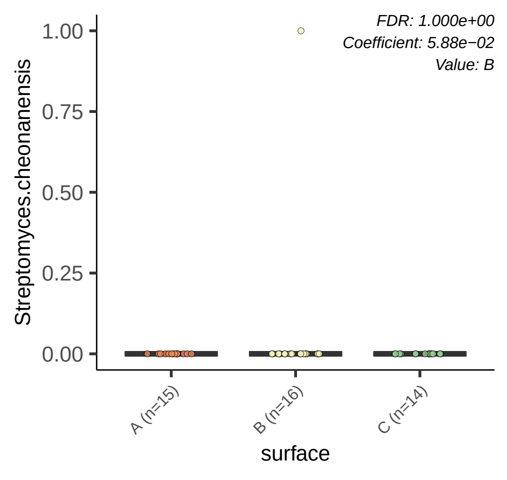

FDR: 1.000e+00
Coefficient: 5.88e−02
Value: B
Streptomyces.cheonanensis
1.00
0.75
0.50
0.25
0.00
A (n=15)
B (n=16)
C (n=14)
surface

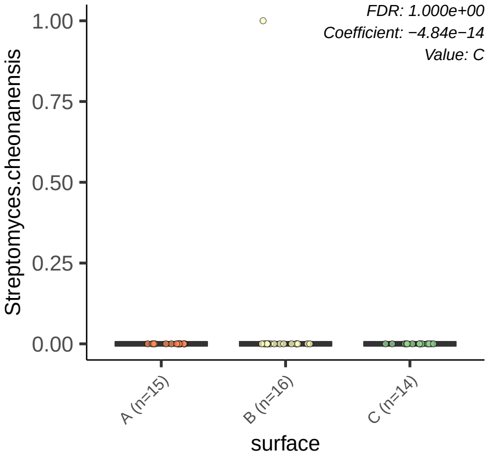
FDR: 1.000e+00
Coefficient: −4.84e−14
Value: C
Streptomyces.cheonanensis
1.00
0.75
0.50
0.25
0.00
A (n=15)
B (n=16)
C (n=14)
surface

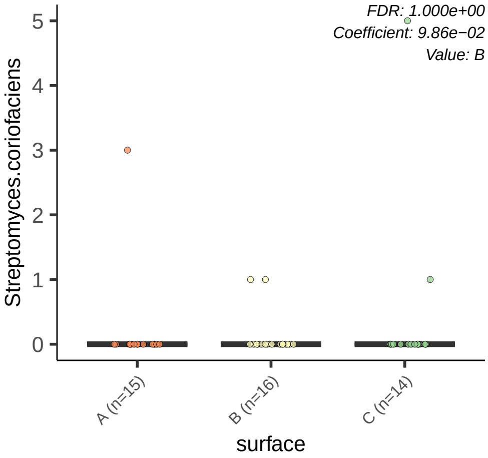
FDR: 1.000e+00
Coefficient: 9.86e−02
Value: B
Streptomyces.coriofaciens
0
1
2
3
4
5
A (n=15)
B (n=16)
C (n=14)
surface

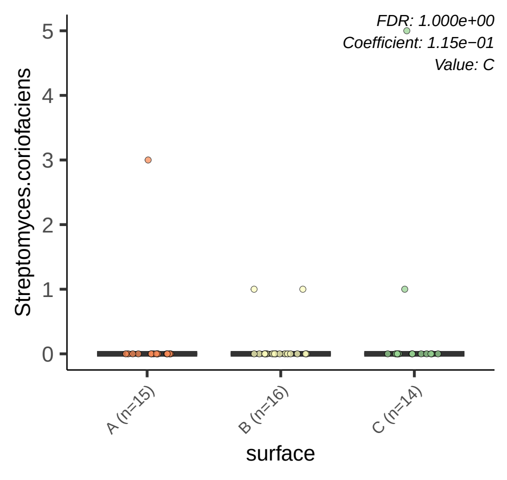
FDR: 1.000e+00
Coefficient: 1.15e−01
Value: C
Streptomyces.coriofaciens
0
1
2
3
4
5
A (n=15)
B (n=16)
C (n=14)
surface

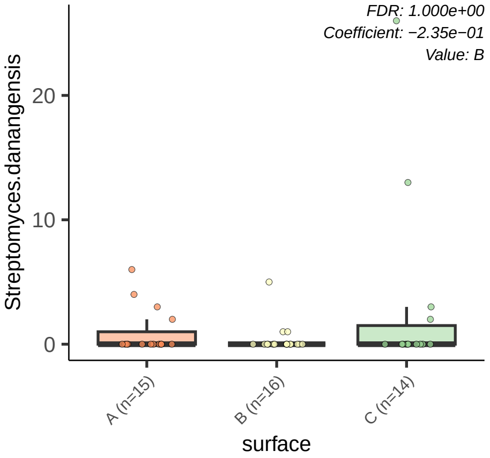

FDR: 1.000e+00
Coefficient: −2.35e−01
Value: B
Streptomyces.danangensis
0
10
20
A (n=15)
B (n=16)
C (n=14)
surface

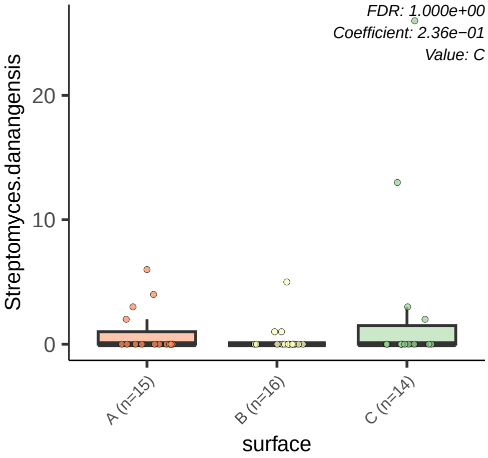
FDR: 1.000e+00
Coefficient: 2.36e−01
Value: C
Streptomyces.danangensis
0
10
20
A (n=15)
B (n=16)
C (n=14)
surface

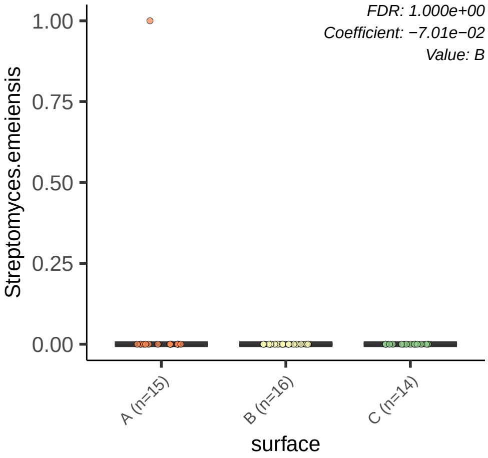
FDR: 1.000e+00
Coefficient: −7.01e−02
Value: B
Streptomyces.emeiensis
1.00
0.75
0.50
0.25
0.00
A (n=15)
B (n=16)
C (n=14)
surface

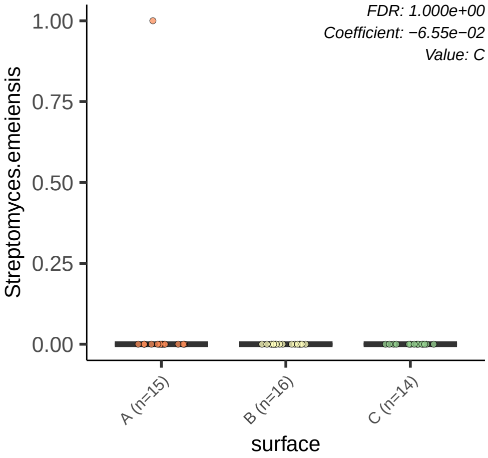
FDR: 1.000e+00
Coefficient: −6.55e−02
Value: C
Streptomyces.emeiensis
1.00
0.75
0.50
0.25
0.00
A (n=15)
B (n=16)
C (n=14)
surface

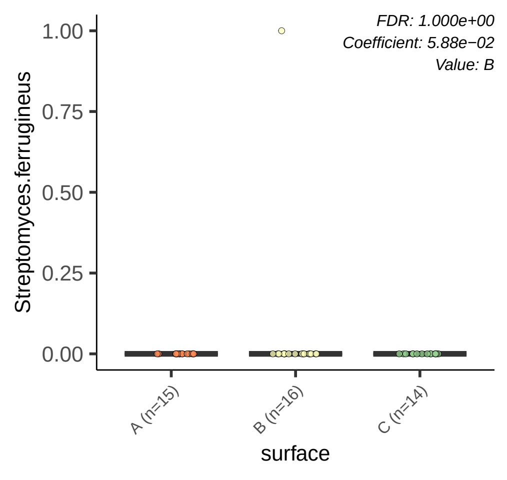
FDR: 1.000e+00
Coefficient: 5.88e−02
Value: B
Streptomyces.ferrugineus
1.00
0.75
0.50
0.25
0.00
A (n=15)
B (n=16)
C (n=14)
surface

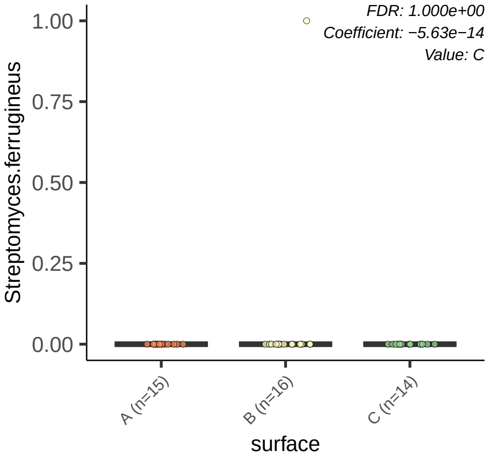

FDR: 1.000e+00
Coefficient: −5.63e−14
Value: C
Streptomyces.ferrugineus
1.00
0.75
0.50
0.25
0.00
A (n=15)
B (n=16)
C (n=14)
surface

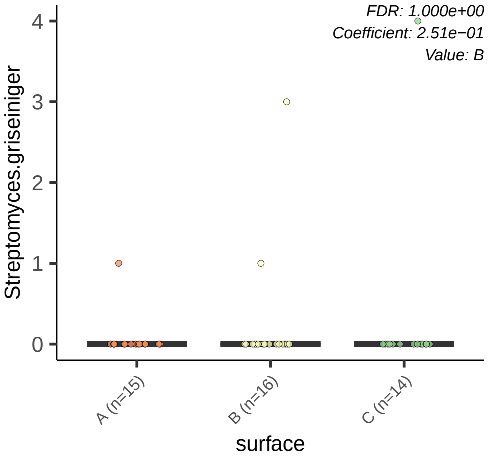
FDR: 1.000e+00
Coefficient: 2.51e−01
Value: B
Streptomyces.griseiniger
0
1
2
3
4
A (n=15)
B (n=16)
C (n=14)
surface

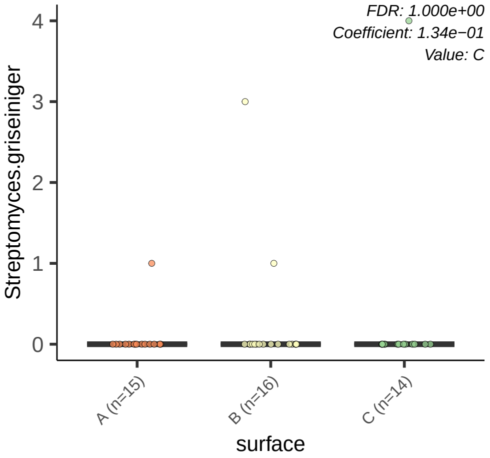

FDR: 1.000e+00
Coefficient: 1.34e−01
Value: C
Streptomyces.griseiniger
0
1
2
3
4
A (n=15)
B (n=16)
C (n=14)
surface

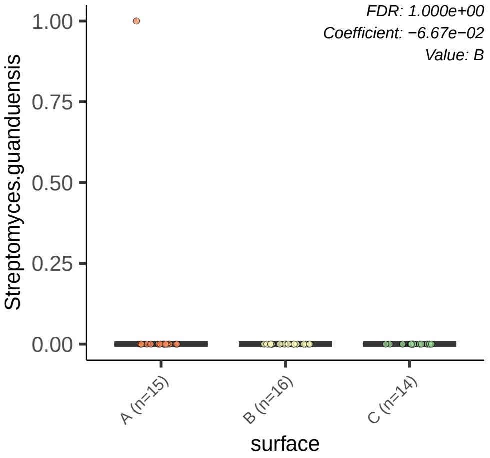

FDR: 1.000e+00
Coefficient: −6.67e−02
Value: B
Streptomyces.guanduensis
1.00
0.75
0.50
0.25
0.00
A (n=15)
B (n=16)
C (n=14)
surface

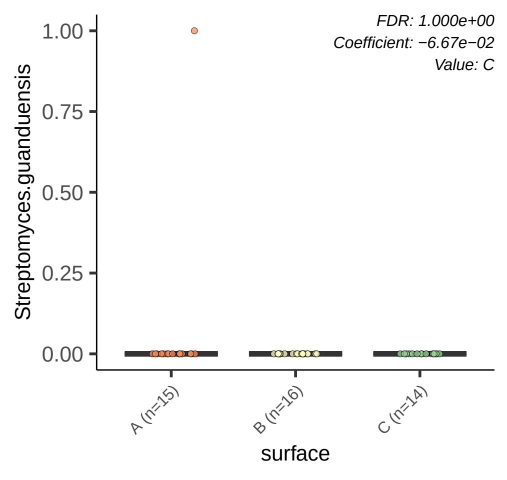
FDR: 1.000e+00
Coefficient: −6.67e−02
Value: C
Streptomyces.guanduensis
1.00
0.75
0.50
0.25
0.00
A (n=15)
B (n=16)
C (n=14)
surface

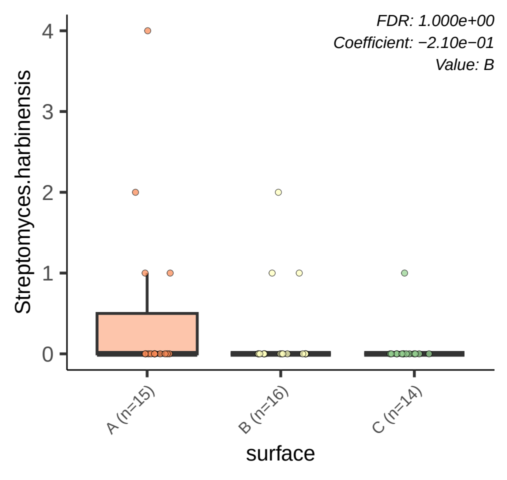
FDR: 1.000e+00
Coefficient: −2.10e−01
Value: B
Streptomyces.harbinensis
0
1
2
3
4
A (n=15)
B (n=16)
C (n=14)
surface

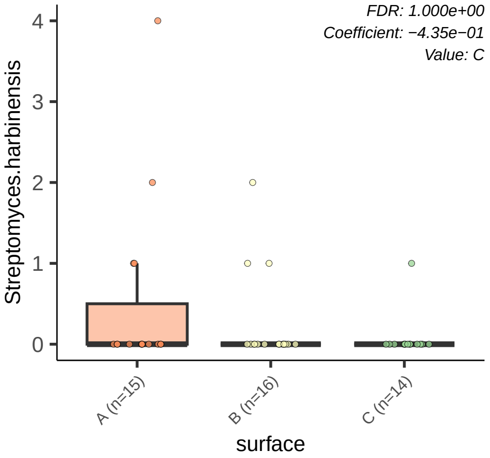
FDR: 1.000e+00
Coefficient: −4.35e−01
Value: C
Streptomyces.harbinensis
0
1
2
3
4
A (n=15)
B (n=16)
C (n=14)
surface

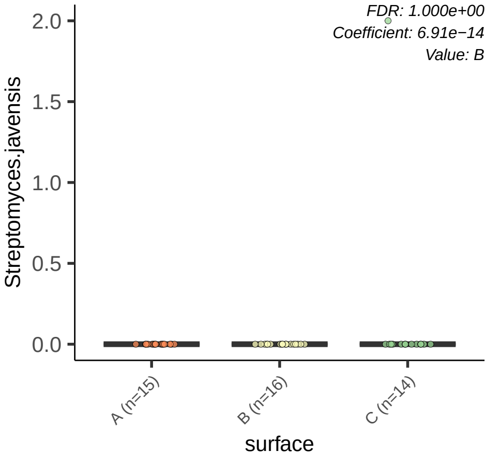
FDR: 1.000e+00
Coefficient: 6.91e−14
Value: B
Streptomyces.javensis
2.0
1.5
1.0
0.5
0.0
A (n=15)
B (n=16)
C (n=14)
surface

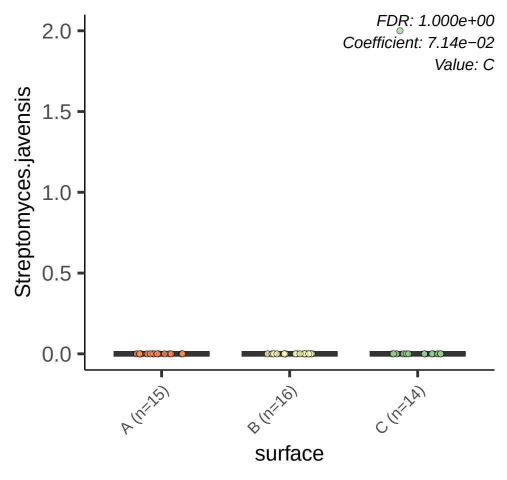

FDR: 1.000e+00
Coefficient: 7.14e−02
Value: C
2.0
1.5
1.0
0.5
0.0
Streptomyces.javensis
A (n=15)
B (n=16)
C (n=14)
surface

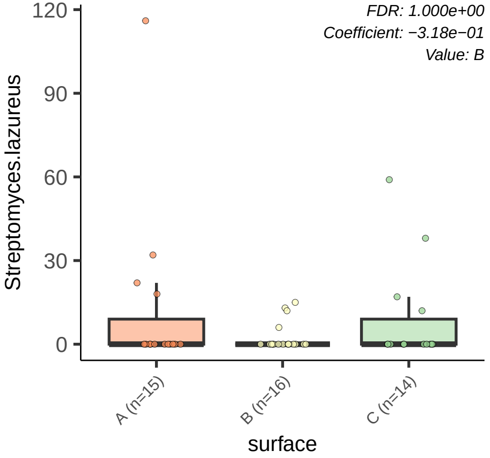

FDR: 1.000e+00
Coefficient: −3.18e−01
Value: B
Streptomyces.lazureus
120
90
60
30
0
A (n=15)
B (n=16)
C (n=14)
surface

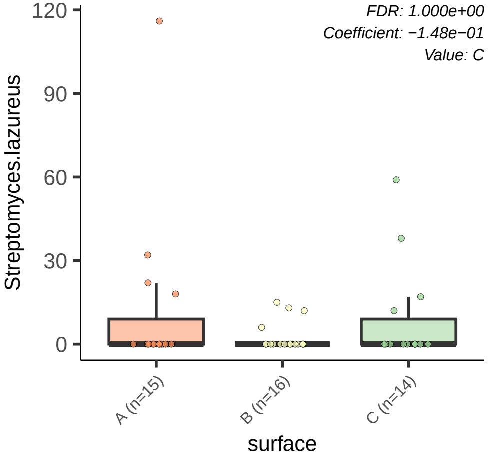
FDR: 1.000e+00
Coefficient: −1.48e−01
Value: C
Streptomyces.lazureus
120
90
60
30
0
A (n=15)
B (n=16)
C (n=14)
surface

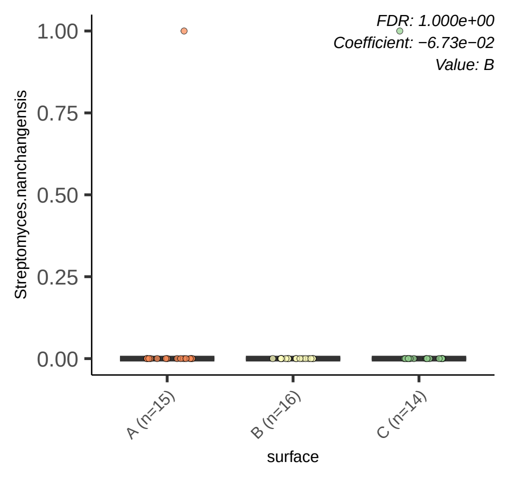
FDR: 1.000e+00
Coefficient: −6.73e−02
Value: B
1.00
0.75
0.50
0.25
0.00
Streptomyces.nanchangensis
A (n=15)
B (n=16)
C (n=14)
surface

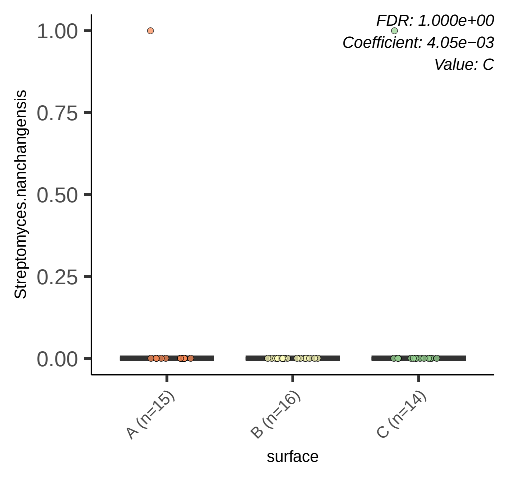

FDR: 1.000e+00
Coefficient: 4.05e−03
Value: C
Streptomyces.nanchangensis
1.00
0.75
0.50
0.25
0.00
A (n=15)
B (n=16)
C (n=14)
surface

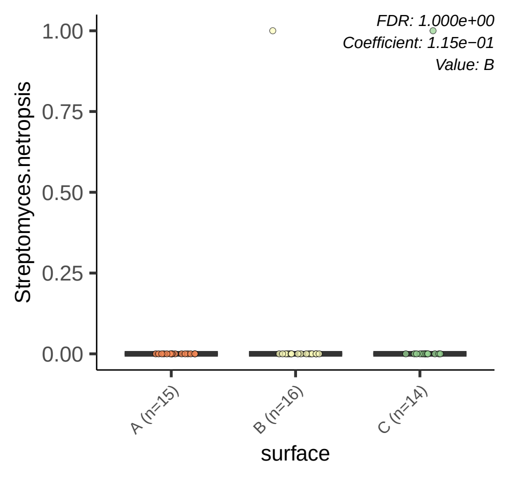
FDR: 1.000e+00
Coefficient: 1.15e−01
Value: B
Streptomyces.netropsis
1.00
0.75
0.50
0.25
0.00
A (n=15)
B (n=16)
C (n=14)
surface

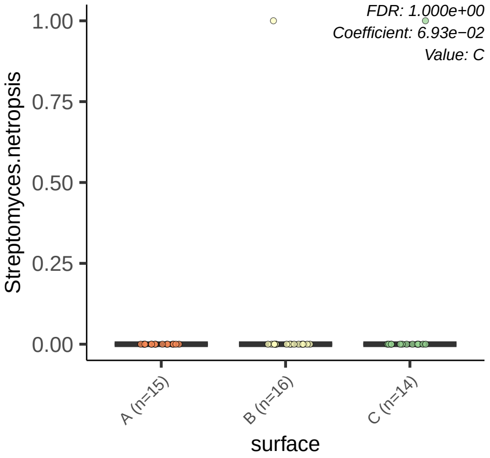
FDR: 1.000e+00
Coefficient: 6.93e−02
Value: C
Streptomyces.netropsis
1.00
0.75
0.50
0.25
0.00
A (n=15)
B (n=16)
C (n=14)
surface

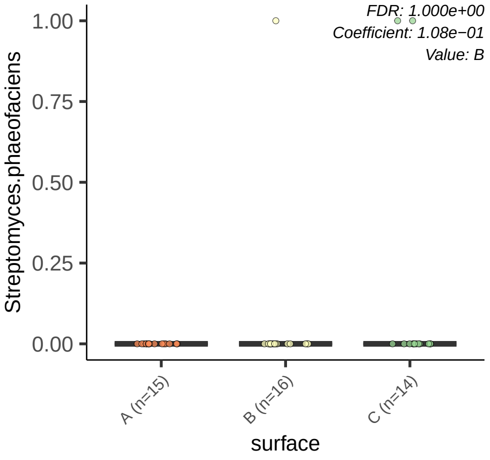

FDR: 1.000e+00
Coefficient: 1.08e−01
Value: B
1.00
0.75
0.50
0.25
0.00
Streptomyces.phaeofaciens
A (n=15)
B (n=16)
C (n=14)
surface

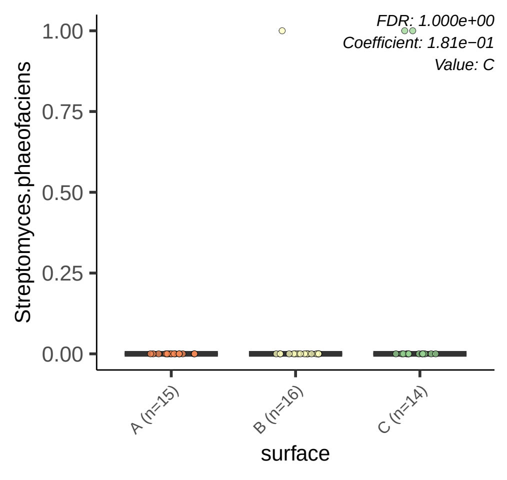

FDR: 1.000e+00
Coefficient: 1.81e−01
Value: C
Streptomyces.phaeofaciens
1.00
0.75
0.50
0.25
0.00
A (n=15)
B (n=16)
C (n=14)
surface

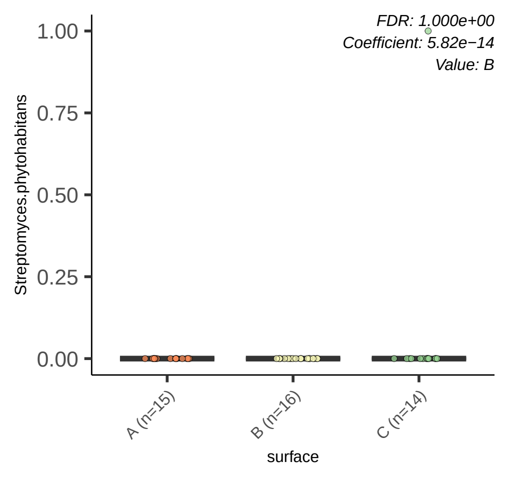

FDR: 1.000e+00
Coefficient: 5.82e−14
Value: B
Streptomyces.phytohabitans
1.00
0.75
0.50
0.25
0.00
A (n=15)
B (n=16)
C (n=14)
surface

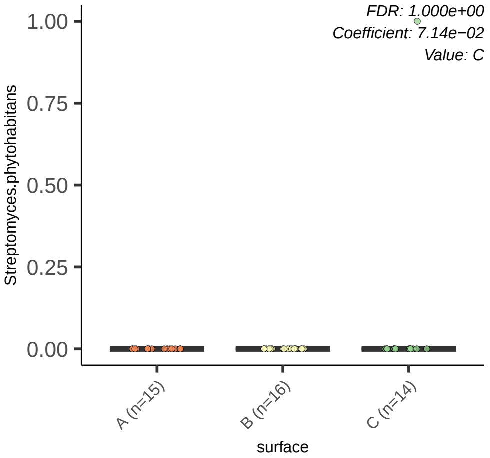

FDR: 1.000e+00
Coefficient: 7.14e−02
Value: C
Streptomyces.phytohabitans
1.00
0.75
0.50
0.25
0.00
A (n=15)
B (n=16)
C (n=14)
surface

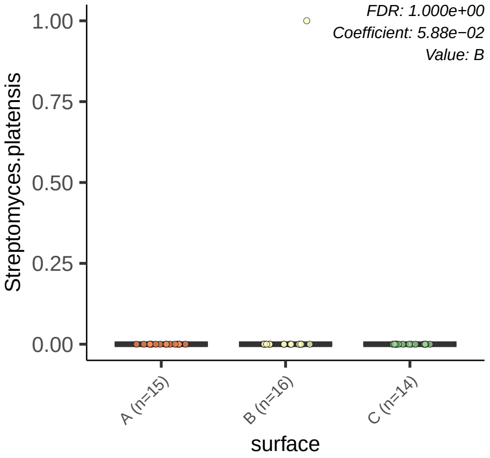
FDR: 1.000e+00
Coefficient: 5.88e−02
Value: B
Streptomyces.platensis
1.00
0.75
0.50
0.25
0.00
A (n=15)
B (n=16)
C (n=14)
surface

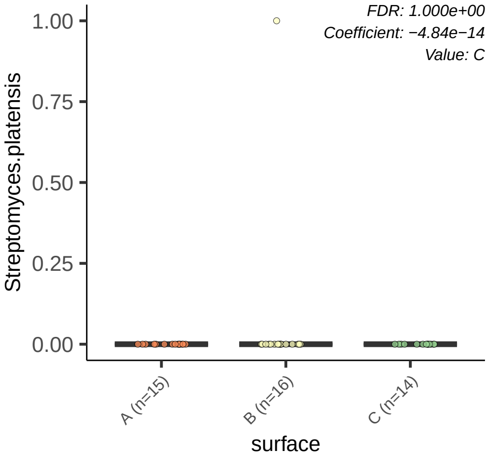
FDR: 1.000e+00
Coefficient: −4.84e−14
Value: C
Streptomyces.platensis
1.00
0.75
0.50
0.25
0.00
A (n=15)
B (n=16)
C (n=14)
surface

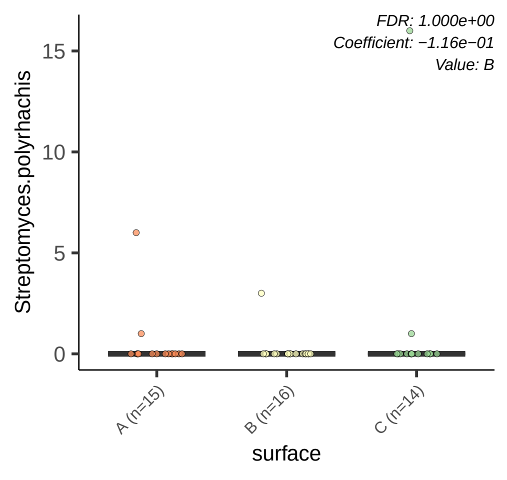
FDR: 1.000e+00
Coefficient: −1.16e−01
Value: B
Streptomyces.polyrhachis
15
10
5
0
A (n=15)
B (n=16)
C (n=14)
surface

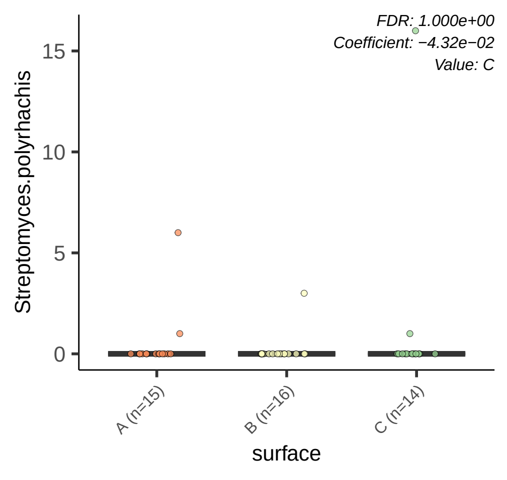
FDR: 1.000e+00
Coefficient: −4.32e−02
Value: C
Streptomyces.polyrhachis
15
10
5
0
A (n=15)
B (n=16)
C (n=14)
surface

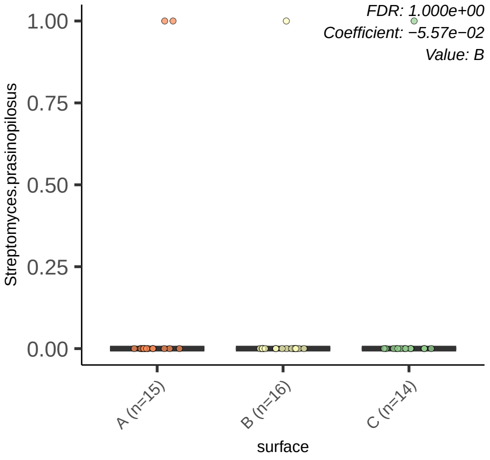
FDR: 1.000e+00
Coefficient: −5.57e−02
Value: B
Streptomyces.prasinopilosus
1.00
0.75
0.50
0.25
0.00
A (n=15)
B (n=16)
C (n=14)
surface

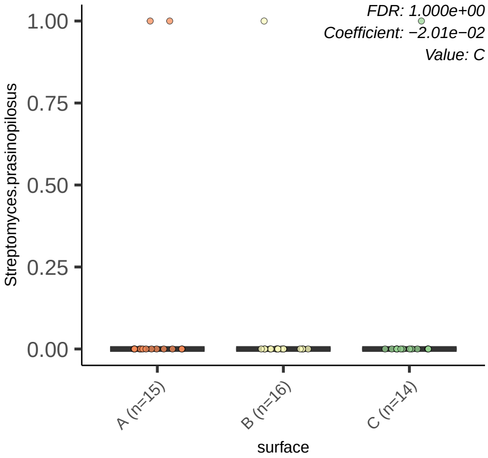

FDR: 1.000e+00
Coefficient: −2.01e−02
Value: C
Streptomyces.prasinopilosus
1.00
0.75
0.50
0.25
0.00
A (n=15)
B (n=16)
C (n=14)
surface

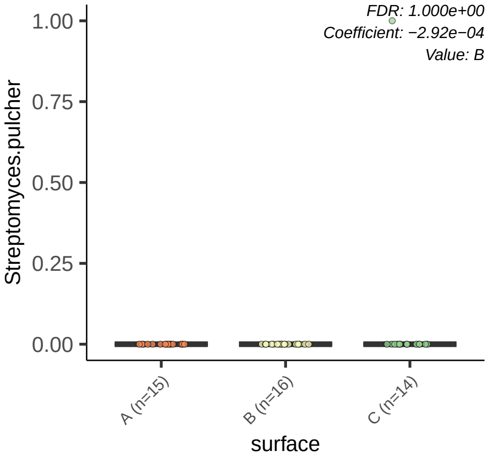

FDR: 1.000e+00
Coefficient: −2.92e−04
Value: B
Streptomyces.pulcher
1.00
0.75
0.50
0.25
0.00
A (n=15)
B (n=16)
C (n=14)
surface

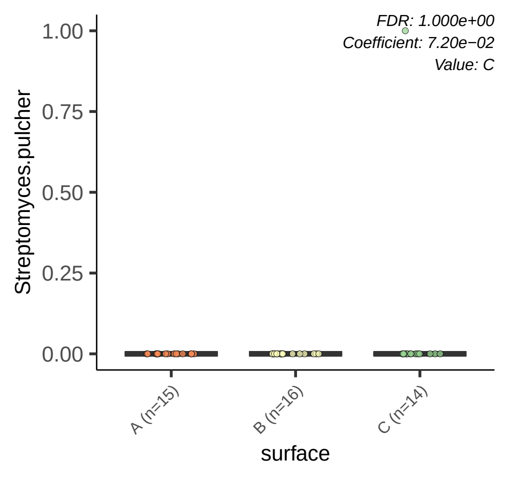
FDR: 1.000e+00
Coefficient: 7.20e−02
Value: C
Streptomyces.pulcher
1.00
0.75
0.50
0.25
0.00
A (n=15)
B (n=16)
C (n=14)
surface

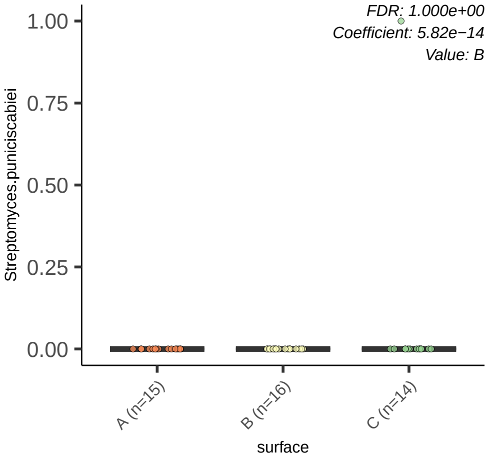
FDR: 1.000e+00
Coefficient: 5.82e−14
Value: B
1.00
0.75
0.50
0.25
0.00
Streptomyces.puniciscabiei
A (n=15)
B (n=16)
C (n=14)
surface

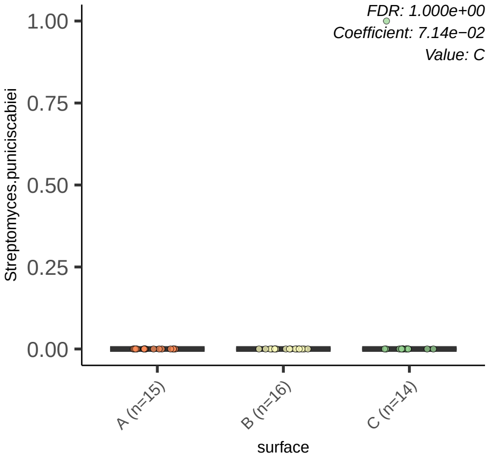

FDR: 1.000e+00
Coefficient: 7.14e−02
Value: C
1.00
0.75
0.50
0.25
0.00
Streptomyces.puniciscabiei
A (n=15)
B (n=16)
C (n=14)
surface

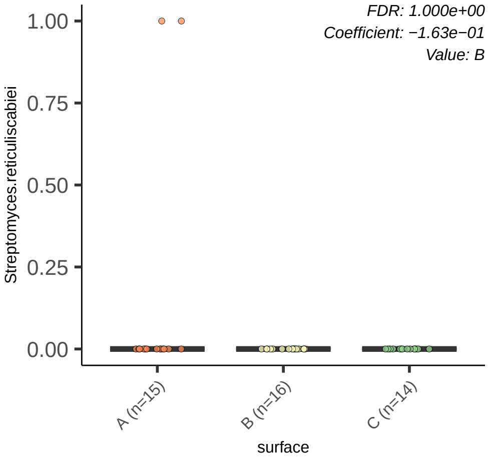

FDR: 1.000e+00
Coefficient: −1.63e−01
Value: B
1.00
0.75
0.50
0.25
0.00
Streptomyces.reticuliscabiei
A (n=15)
B (n=16)
C (n=14)
surface

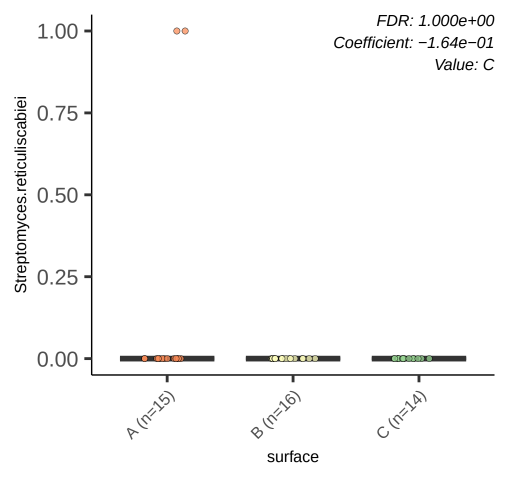

FDR: 1.000e+00
Coefficient: −1.64e−01
Value: C
1.00
0.75
0.50
0.25
0.00
Streptomyces.reticuliscabiei
A (n=15)
B (n=16)
C (n=14)
surface

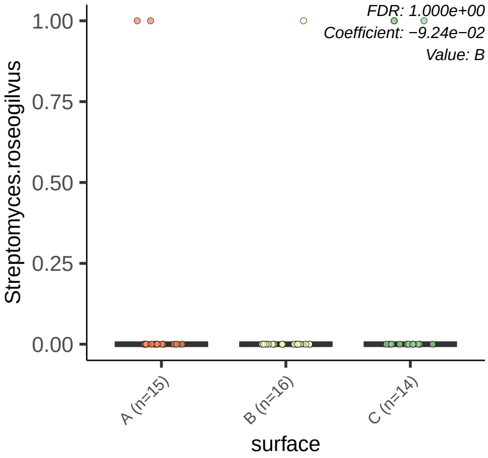

FDR: 1.000e+00
Coefficient: −9.24e−02
Value: B
Streptomyces.roseogilvus
1.00
0.75
0.50
0.25
0.00
A (n=15)
B (n=16)
C (n=14)
surface

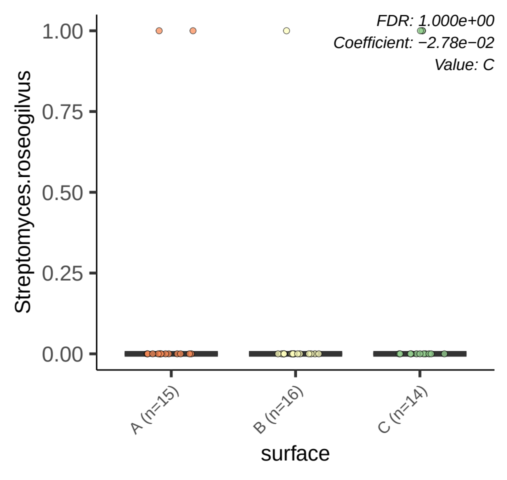
FDR: 1.000e+00
Coefficient: −2.78e−02
Value: C
Streptomyces.roseogilvus
1.00
0.75
0.50
0.25
0.00
A (n=15)
B (n=16)
C (n=14)
surface

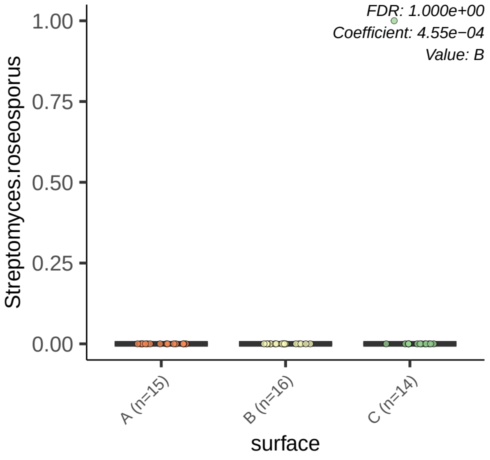
FDR: 1.000e+00
Coefficient: 4.55e−04
Value: B
Streptomyces.roseosporus
1.00
0.75
0.50
0.25
0.00
A (n=15)
B (n=16)
C (n=14)
surface

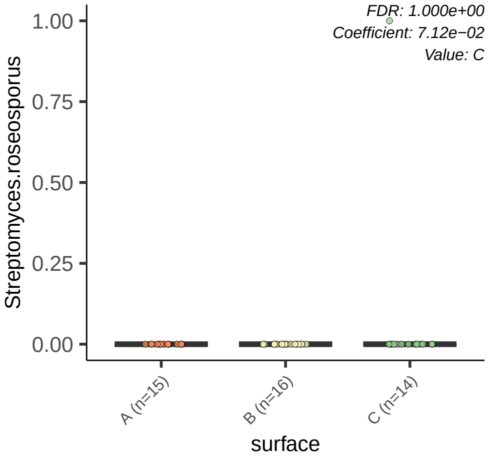

FDR: 1.000e+00
Coefficient: 7.12e−02
Value: C
Streptomyces.roseosporus
1.00
0.75
0.50
0.25
0.00
A (n=15)
B (n=16)
C (n=14)
surface

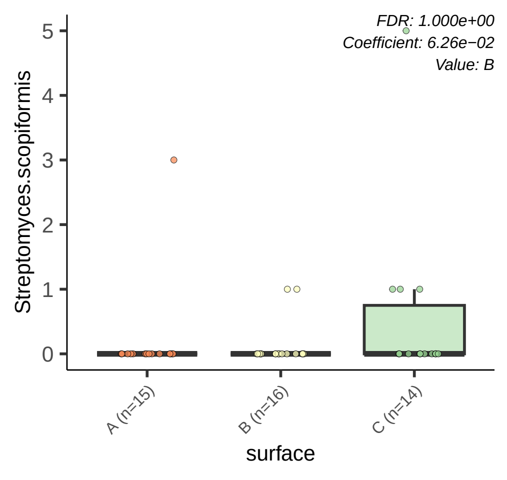

FDR: 1.000e+00
Coefficient: 6.26e−02
Value: B
Streptomyces.scopiformis
0
1
2
3
4
5
A (n=15)
B (n=16)
C (n=14)
surface

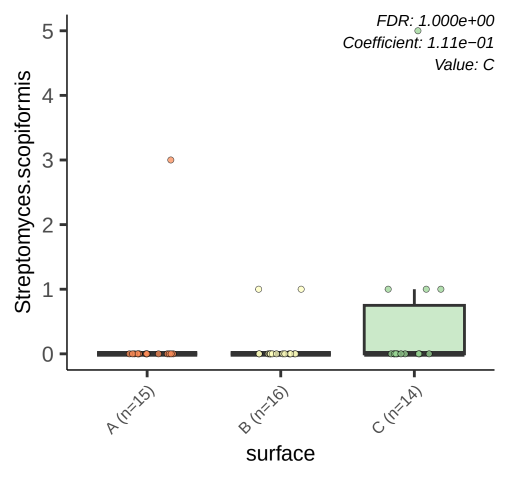

FDR: 1.000e+00
Coefficient: 1.11e−01
Value: C
Streptomyces.scopiformis
0
1
2
3
4
5
A (n=15)
B (n=16)
C (n=14)
surface

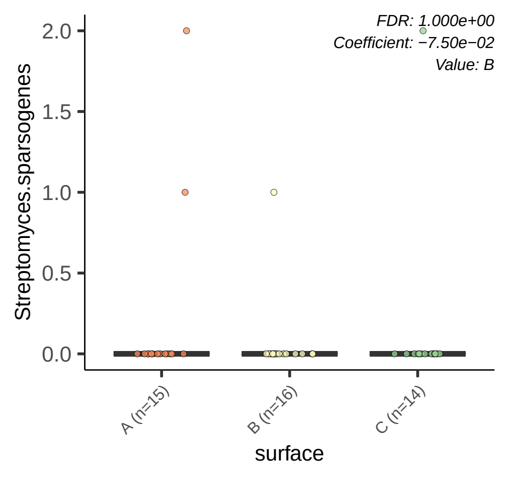

FDR: 1.000e+00
Coefficient: −7.50e−02
Value: B
Streptomyces.sparsogenes
2.0
1.5
1.0
0.5
0.0
A (n=15)
B (n=16)
C (n=14)
surface

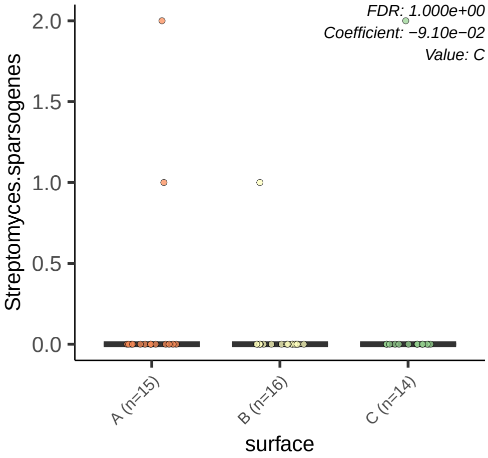

FDR: 1.000e+00
Coefficient: −9.10e−02
Value: C
Streptomyces.sparsogenes
2.0
1.5
1.0
0.5
0.0
A (n=15)
B (n=16)
C (n=14)
surface

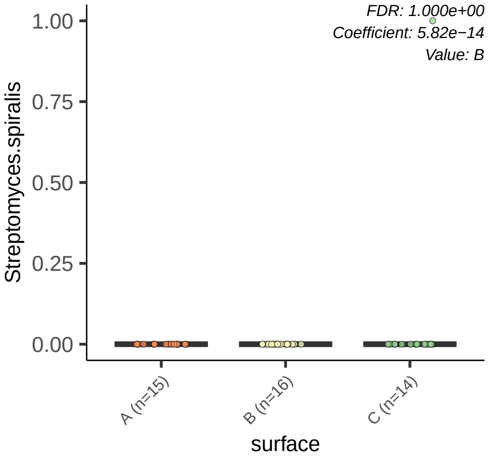
FDR: 1.000e+00
Coefficient: 5.82e−14
Value: B
Streptomyces.spiralis
1.00
0.75
0.50
0.25
0.00
A (n=15)
B (n=16)
C (n=14)
surface

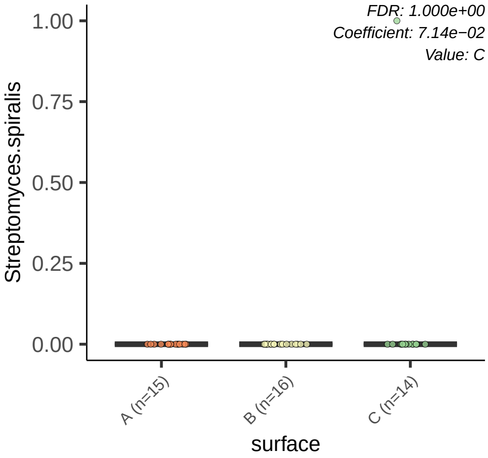

FDR: 1.000e+00
Coefficient: 7.14e−02
Value: C
1.00
0.75
0.50
0.25
0.00
Streptomyces.spiralis
A (n=15)
B (n=16)
C (n=14)
surface

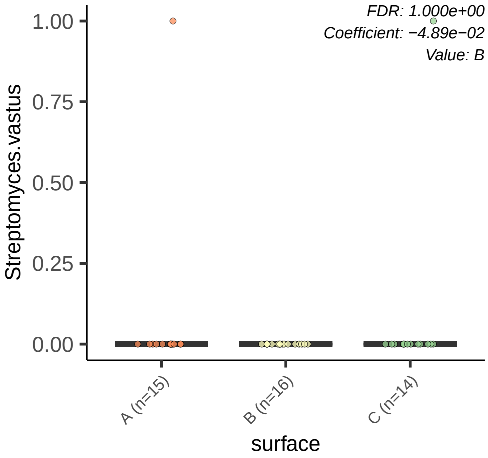

FDR: 1.000e+00
Coefficient: −4.89e−02
Value: B
Streptomyces.vastus
1.00
0.75
0.50
0.25
0.00
A (n=15)
B (n=16)
C (n=14)
surface

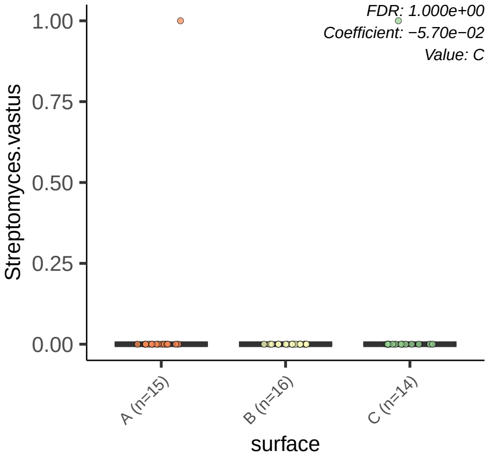
FDR: 1.000e+00
Coefficient: −5.70e−02
Value: C
Streptomyces.vastus
1.00
0.75
0.50
0.25
0.00
A (n=15)
B (n=16)
C (n=14)
surface

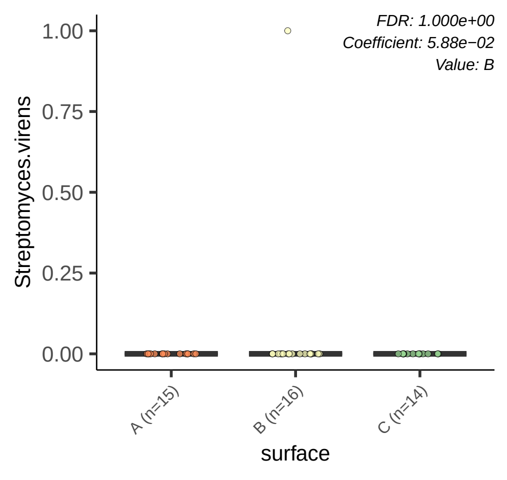
FDR: 1.000e+00
Coefficient: 5.88e−02
Value: B
Streptomyces.virens
1.00
0.75
0.50
0.25
0.00
A (n=15)
B (n=16)
C (n=14)
surface

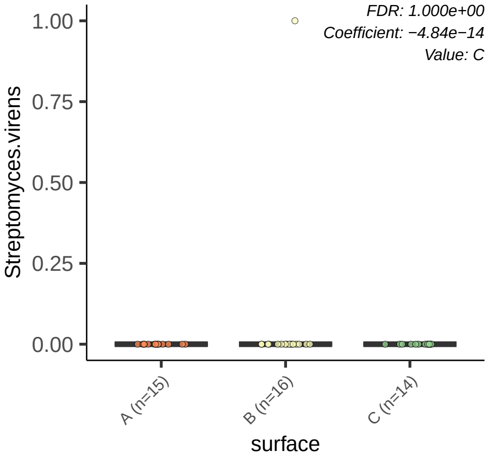
FDR: 1.000e+00
Coefficient: −4.84e−14
Value: C
Streptomyces.virens
1.00
0.75
0.50
0.25
0.00
A (n=15)
B (n=16)
C (n=14)
surface

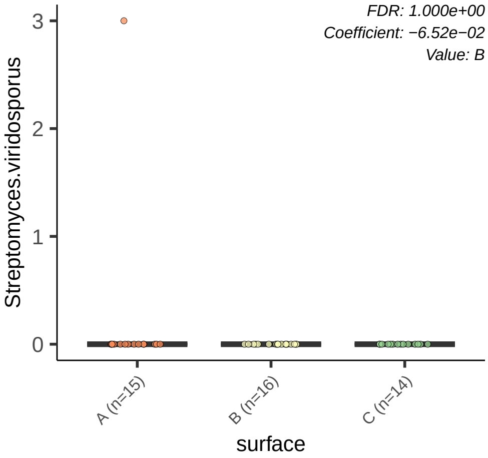
FDR: 1.000e+00
Coefficient: −6.52e−02
Value: B
Streptomyces.viridosporus
3
2
1
0
A (n=15)
B (n=16)
C (n=14)
surface

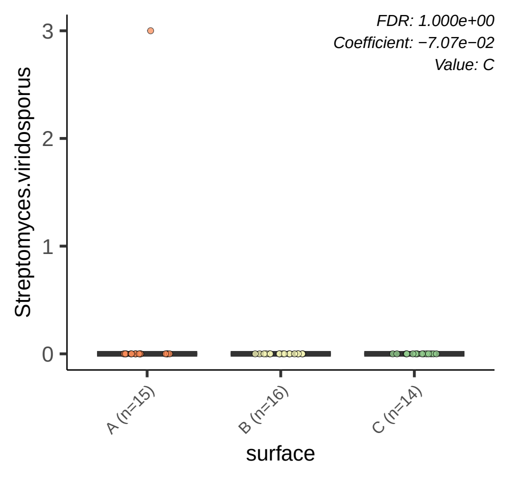
FDR: 1.000e+00
Coefficient: −7.07e−02
Value: C
Streptomyces.viridosporus
3
2
1
0
A (n=15)
B (n=16)
C (n=14)
surface

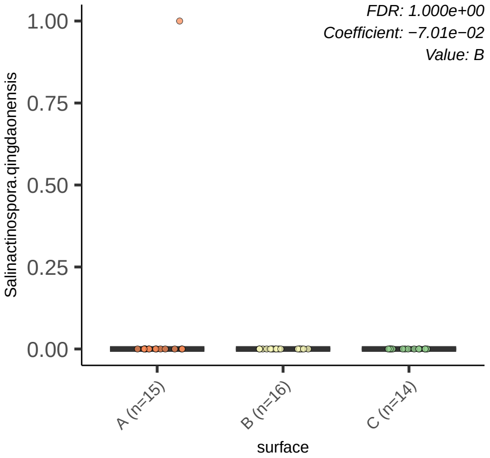

FDR: 1.000e+00
Coefficient: −7.01e−02
Value: B
Salinactinospora.qingdaonensis
1.00
0.75
0.50
0.25
0.00
A (n=15)
B (n=16)
C (n=14)
surface

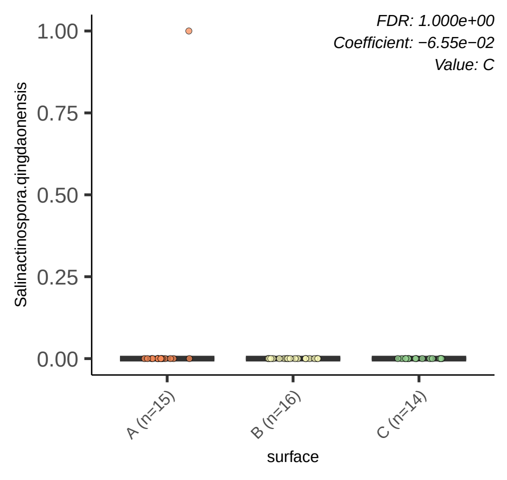

FDR: 1.000e+00
Coefficient: −6.55e−02
Value: C
1.00
0.75
0.50
0.25
0.00
Salinactinospora.qingdaonensis
A (n=15)
B (n=16)
C (n=14)
surface

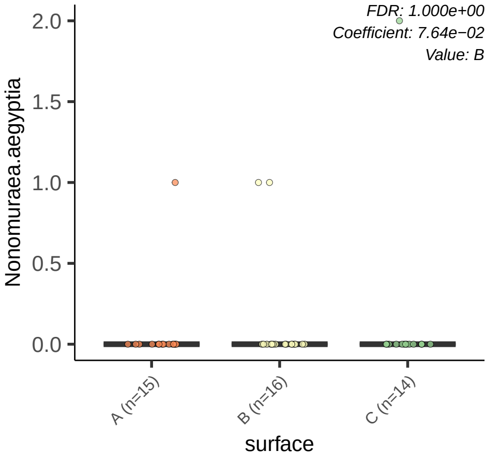

FDR: 1.000e+00
Coefficient: 7.64e−02
Value: B
Nonomuraea.aegyptia
2.0
1.5
1.0
0.5
0.0
A (n=15)
B (n=16)
C (n=14)
surface

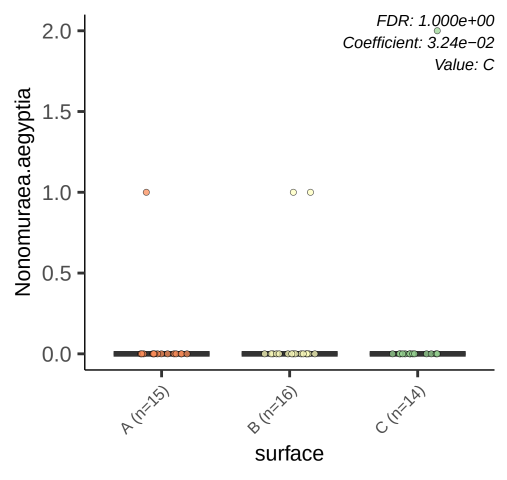
FDR: 1.000e+00
Coefficient: 3.24e−02
Value: C
Nonomuraea.aegyptia
2.0
1.5
1.0
0.5
0.0
A (n=15)
B (n=16)
C (n=14)
surface

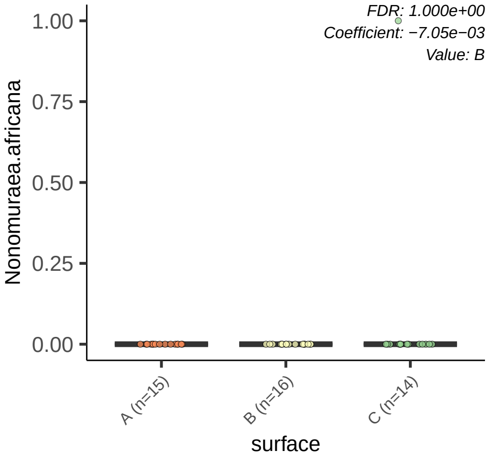

FDR: 1.000e+00
Coefficient: −7.05e−03
Value: B
1.00
0.75
0.50
0.25
0.00
Nonomuraea.africana
A (n=15)
B (n=16)
C (n=14)
surface

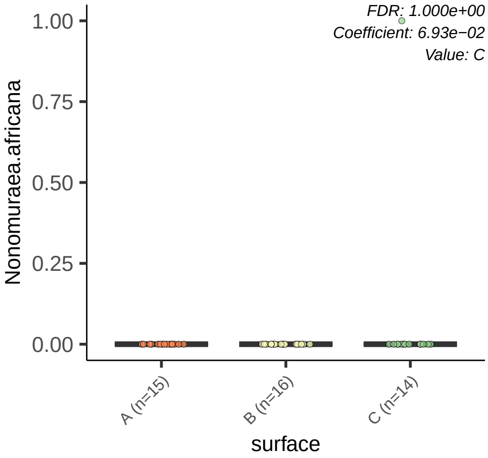

FDR: 1.000e+00
Coefficient: 6.93e−02
Value: C
Nonomuraea.africana
1.00
0.75
0.50
0.25
0.00
A (n=15)
B (n=16)
C (n=14)
surface

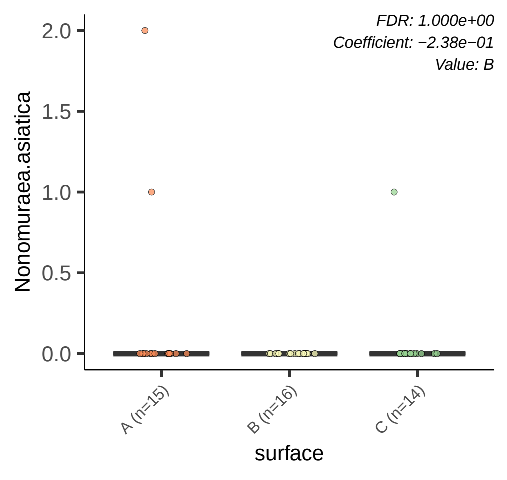
FDR: 1.000e+00
Coefficient: −2.38e−01
Value: B
Nonomuraea.asiatica
2.0
1.5
1.0
0.5
0.0
A (n=15)
B (n=16)
C (n=14)
surface

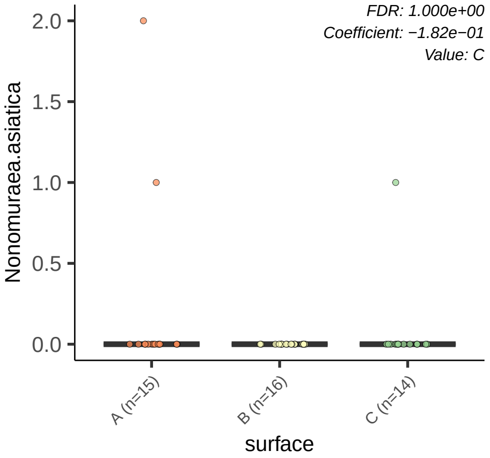
FDR: 1.000e+00
Coefficient: −1.82e−01
Value: C
Nonomuraea.asiatica
2.0
1.5
1.0
0.5
0.0
A (n=15)
B (n=16)
C (n=14)
surface

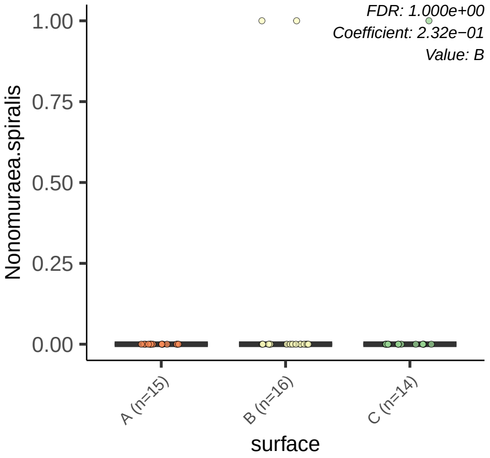
FDR: 1.000e+00
Coefficient: 2.32e−01
Value: B
Nonomuraea.spiralis
1.00
0.75
0.50
0.25
0.00
A (n=15)
B (n=16)
C (n=14)
surface

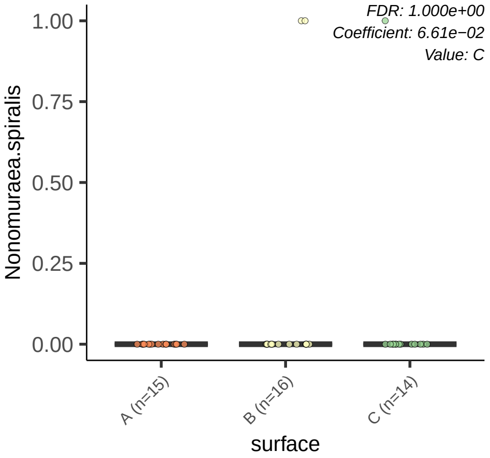

FDR: 1.000e+00
Coefficient: 6.61e−02
Value: C
Nonomuraea.spiralis
1.00
0.75
0.50
0.25
0.00
A (n=15)
B (n=16)
C (n=14)
surface

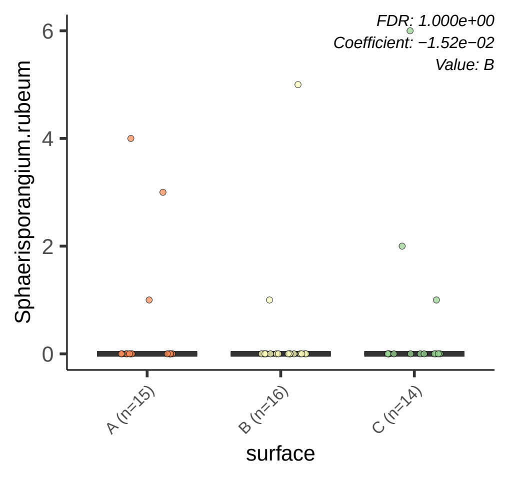
FDR: 1.000e+00
Coefficient: −1.52e−02
Value: B
Sphaerisporangium.rubeum
0
2
4
6
A (n=15)
B (n=16)
C (n=14)
surface

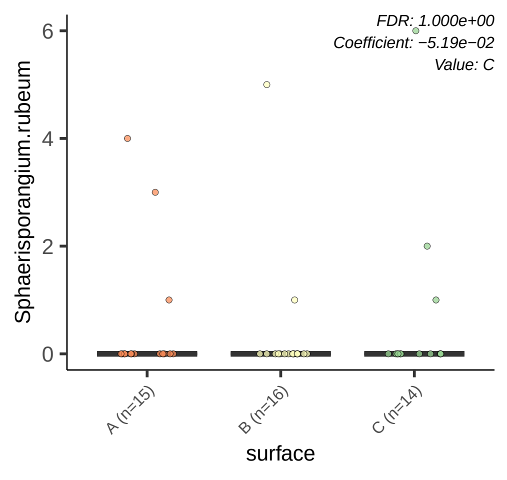
FDR: 1.000e+00
Coefficient: −5.19e−02
Value: C
Sphaerisporangium.rubeum
0
2
4
6
A (n=15)
B (n=16)
C (n=14)
surface

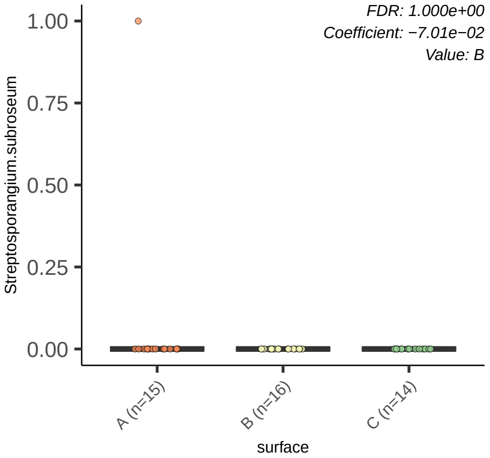
FDR: 1.000e+00
Coefficient: −7.01e−02
Value: B
1.00
0.75
0.50
0.25
0.00
Streptosporangium.subroseum
A (n=15)
B (n=16)
C (n=14)
surface

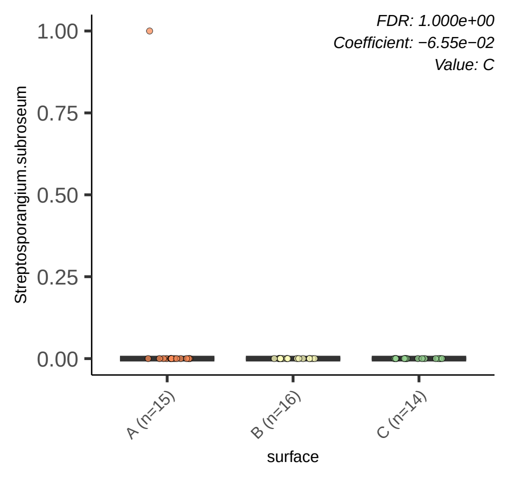

FDR: 1.000e+00
Coefficient: −6.55e−02
Value: C
Streptosporangium.subroseum
1.00
0.75
0.50
0.25
0.00
A (n=15)
B (n=16)
C (n=14)
surface

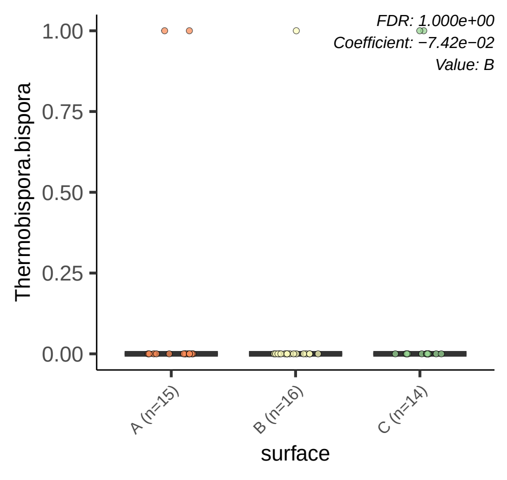
FDR: 1.000e+00
Coefficient: −7.42e−02
Value: B
Thermobispora.bispora
1.00
0.75
0.50
0.25
0.00
A (n=15)
B (n=16)
C (n=14)
surface

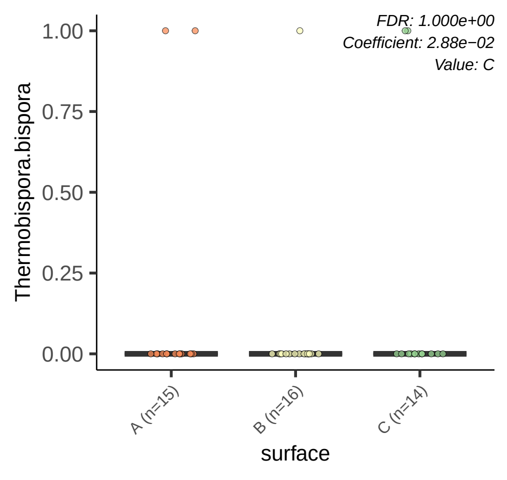
FDR: 1.000e+00
Coefficient: 2.88e−02
Value: C
Thermobispora.bispora
1.00
0.75
0.50
0.25
0.00
A (n=15)
B (n=16)
C (n=14)
surface

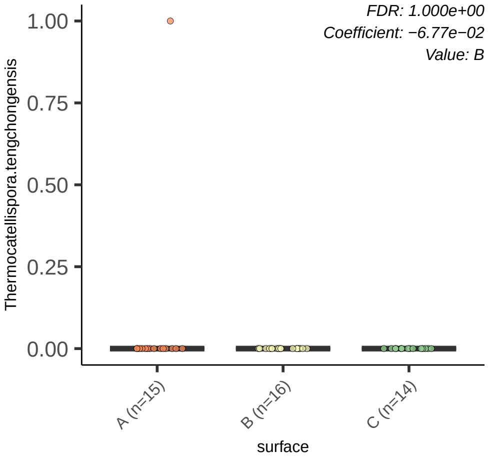
FDR: 1.000e+00
Coefficient: −6.77e−02
Value: B
Thermocatellispora.tengchongensis
1.00
0.75
0.50
0.25
0.00
A (n=15)
B (n=16)
C (n=14)
surface

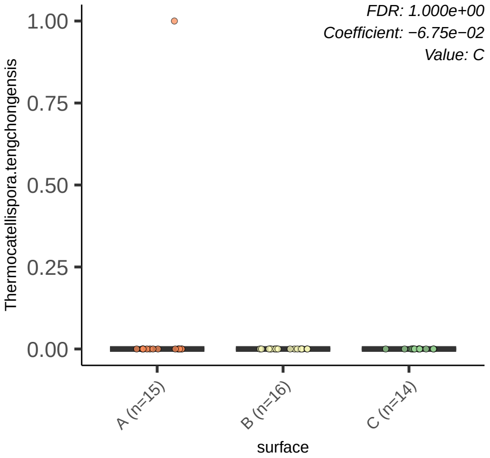

FDR: 1.000e+00
Coefficient: −6.75e−02
Value: C
Thermocatellispora.tengchongensis
1.00
0.75
0.50
0.25
0.00
A (n=15)
B (n=16)
C (n=14)
surface

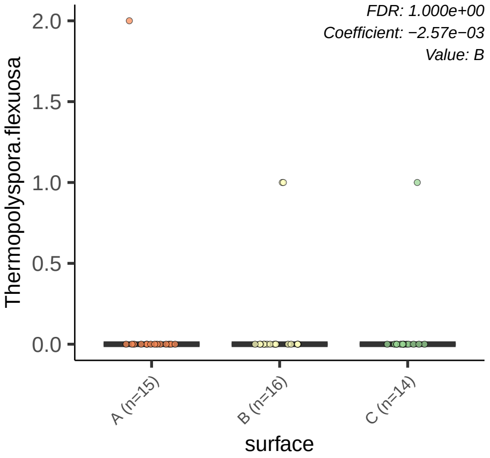

FDR: 1.000e+00
Coefficient: −2.57e−03
Value: B
Thermopolyspora.flexuosa
2.0
1.5
1.0
0.5
0.0
A (n=15)
B (n=16)
C (n=14)
surface

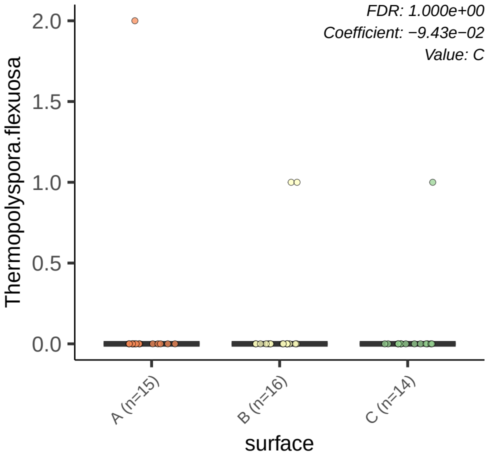

FDR: 1.000e+00
Coefficient: −9.43e−02
Value: C
Thermopolyspora.flexuosa
2.0
1.5
1.0
0.5
0.0
A (n=15)
B (n=16)
C (n=14)
surface

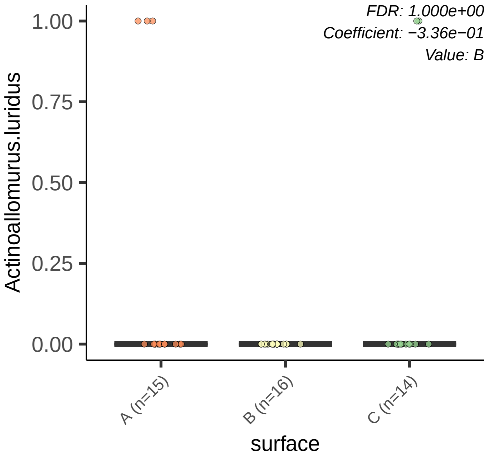
FDR: 1.000e+00
Coefficient: −3.36e−01
Value: B
1.00
0.75
0.50
0.25
0.00
Actinoallomurus.luridus
A (n=15)
B (n=16)
C (n=14)
surface

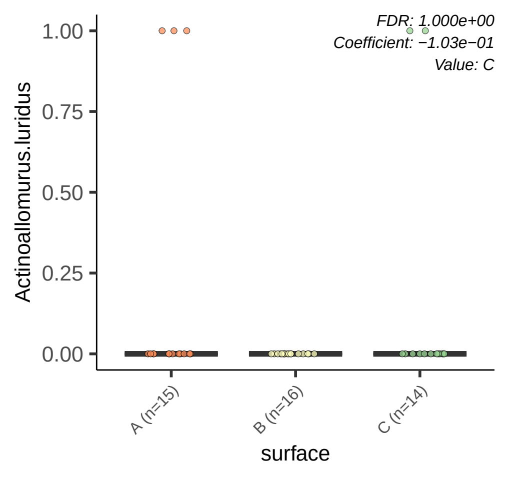

FDR: 1.000e+00
Coefficient: −1.03e−01
Value: C
Actinoallomurus.luridus
1.00
0.75
0.50
0.25
0.00
A (n=15)
B (n=16)
C (n=14)
surface

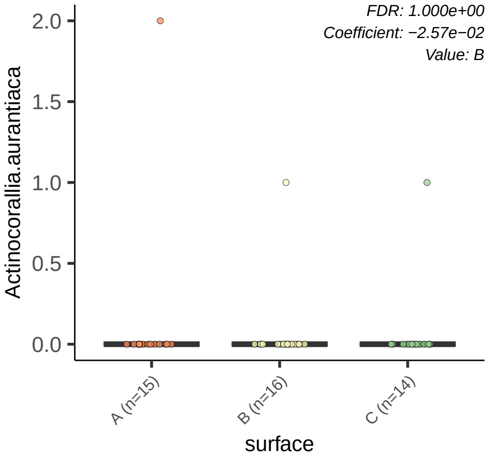

FDR: 1.000e+00
Coefficient: −2.57e−02
Value: B
Actinocorallia.aurantiaca
2.0
1.5
1.0
0.5
0.0
A (n=15)
B (n=16)
C (n=14)
surface

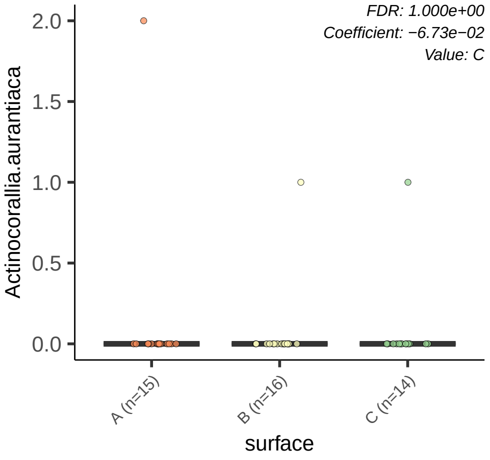
FDR: 1.000e+00
Coefficient: −6.73e−02
Value: C
Actinocorallia.aurantiaca
2.0
1.5
1.0
0.5
0.0
A (n=15)
B (n=16)
C (n=14)
surface

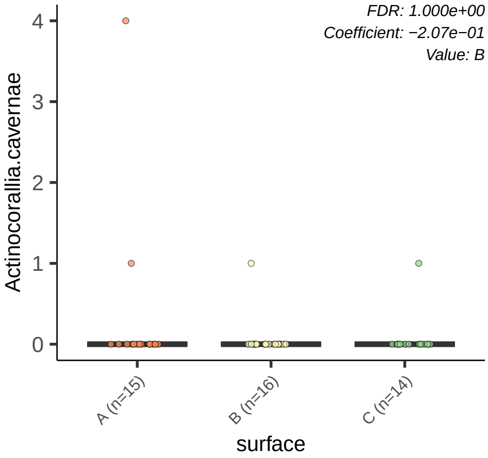
FDR: 1.000e+00
Coefficient: −2.07e−01
Value: B
Actinocorallia.cavernae
0
1
2
3
4
A (n=15)
B (n=16)
C (n=14)
surface

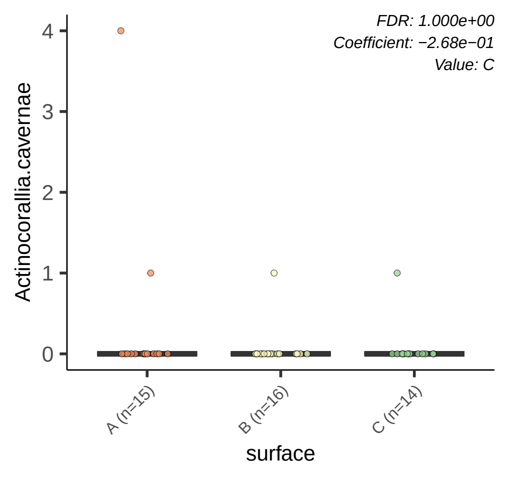

FDR: 1.000e+00
Coefficient: −2.68e−01
Value: C
Actinocorallia.cavernae
4
3
2
1
0
A (n=15)
B (n=16)
C (n=14)
surface

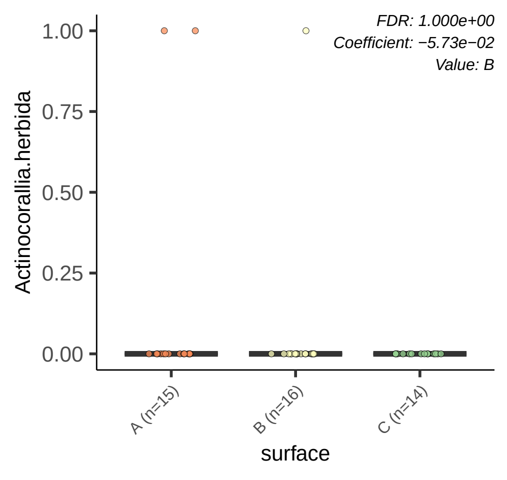
FDR: 1.000e+00
Coefficient: −5.73e−02
Value: B
Actinocorallia.herbida
1.00
0.75
0.50
0.25
0.00
A (n=15)
B (n=16)
C (n=14)
surface

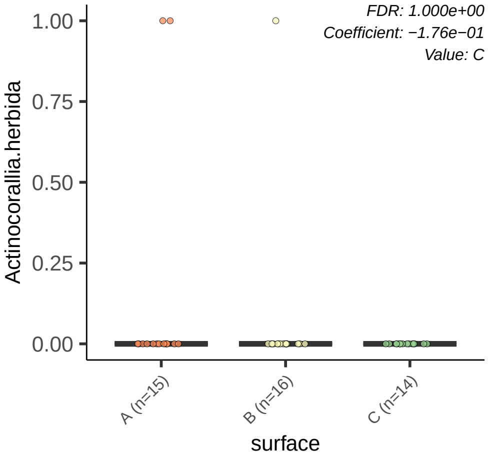
FDR: 1.000e+00
Coefficient: −1.76e−01
Value: C
Actinocorallia.herbida
1.00
0.75
0.50
0.25
0.00
A (n=15)
B (n=16)
C (n=14)
surface

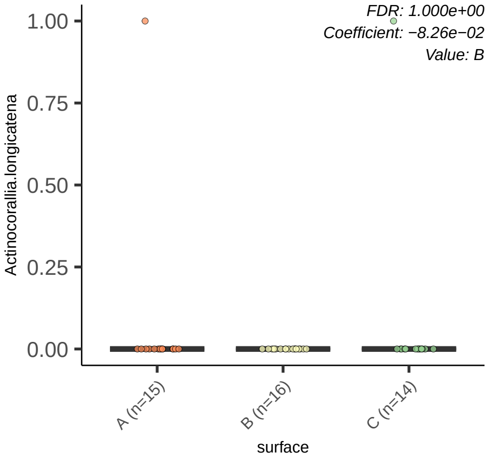

FDR: 1.000e+00
Coefficient: −8.26e−02
Value: B
Actinocorallia.longicatena
1.00
0.75
0.50
0.25
0.00
A (n=15)
B (n=16)
C (n=14)
surface

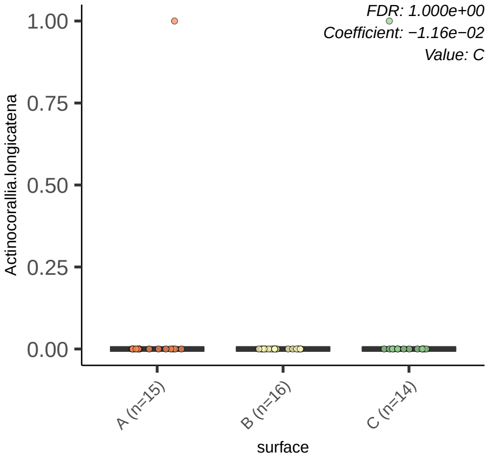

FDR: 1.000e+00
Coefficient: −1.16e−02
Value: C
1.00
0.75
0.50
0.25
0.00
Actinocorallia.longicatena
A (n=15)
B (n=16)
C (n=14)
surface

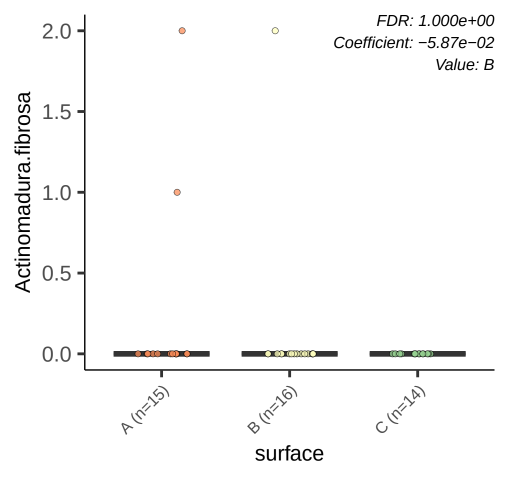
FDR: 1.000e+00
Coefficient: −5.87e−02
Value: B
Actinomadura.fibrosa
2.0
1.5
1.0
0.5
0.0
A (n=15)
B (n=16)
C (n=14)
surface

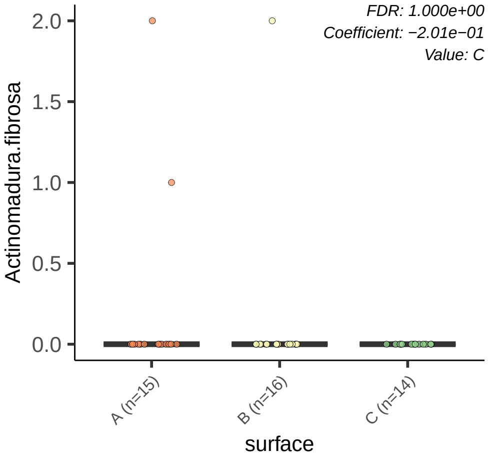
FDR: 1.000e+00
Coefficient: −2.01e−01
Value: C
Actinomadura.fibrosa
2.0
1.5
1.0
0.5
0.0
A (n=15)
B (n=16)
C (n=14)
surface

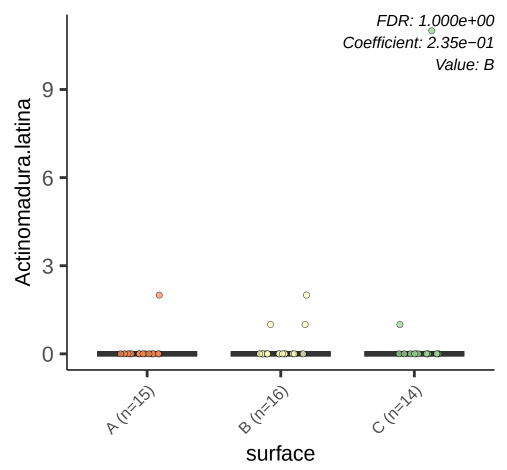
FDR: 1.000e+00
Coefficient: 2.35e−01
Value: B
Actinomadura.latina
9
6
3
0
A (n=15)
B (n=16)
C (n=14)
surface

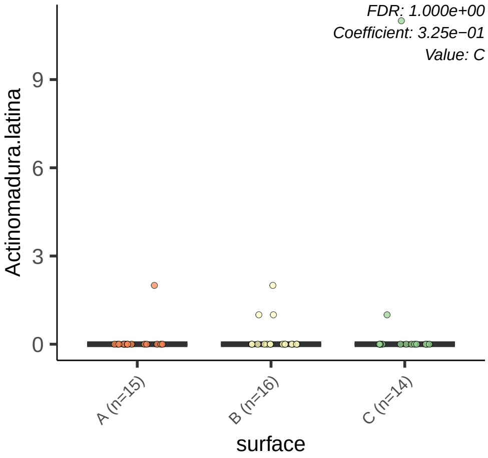

FDR: 1.000e+00
Coefficient: 3.25e−01
Value: C
Actinomadura.latina
0
3
6
9
A (n=15)
B (n=16)
C (n=14)
surface

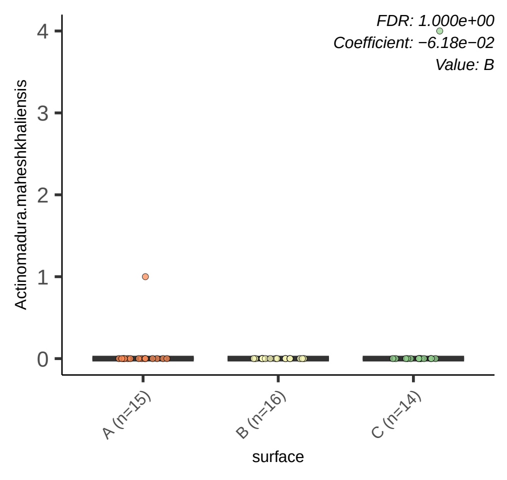
FDR: 1.000e+00
Coefficient: −6.18e−02
Value: B
4
3
2
1
0
Actinomadura.maheshkhaliensis
A (n=15)
B (n=16)
C (n=14)
surface

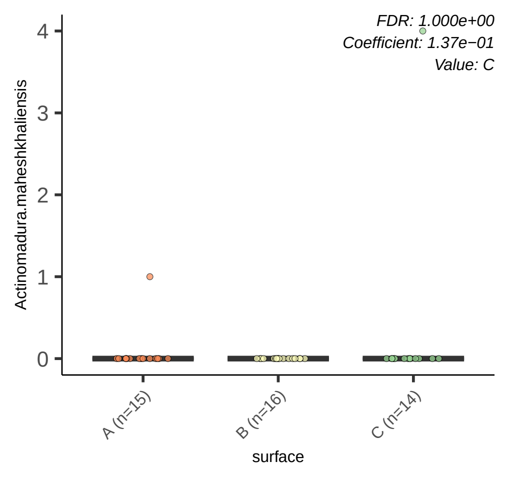

FDR: 1.000e+00
Coefficient: 1.37e−01
Value: C
Actinomadura.maheshkhaliensis
4
3
2
1
0
A (n=15)
B (n=16)
C (n=14)
surface

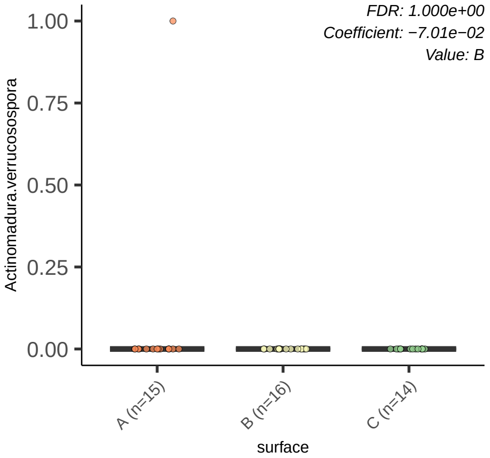
FDR: 1.000e+00
Coefficient: −7.01e−02
Value: B
Actinomadura.verrucosospora
1.00
0.75
0.50
0.25
0.00
A (n=15)
B (n=16)
C (n=14)
surface

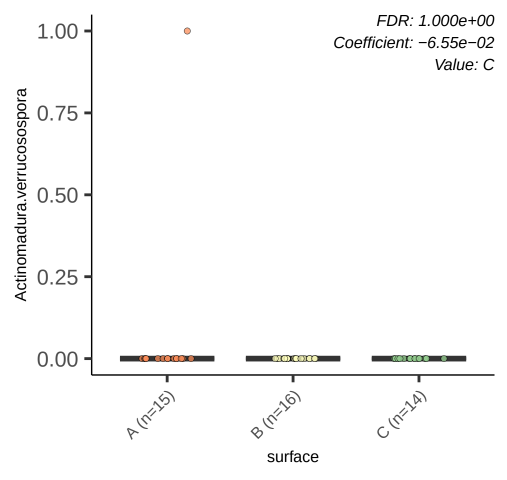
FDR: 1.000e+00
Coefficient: −6.55e−02
Value: C
1.00
0.75
0.50
0.25
0.00
Actinomadura.verrucosospora
A (n=15)
B (n=16)
C (n=14)
surface

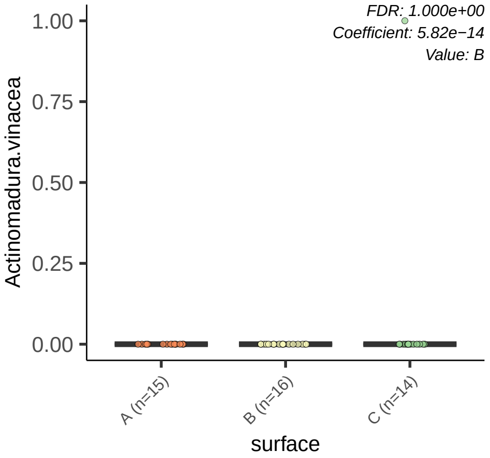
FDR: 1.000e+00
Coefficient: 5.82e−14
Value: B
1.00
0.75
0.50
0.25
0.00
Actinomadura.vinacea
A (n=15)
B (n=16)
C (n=14)
surface

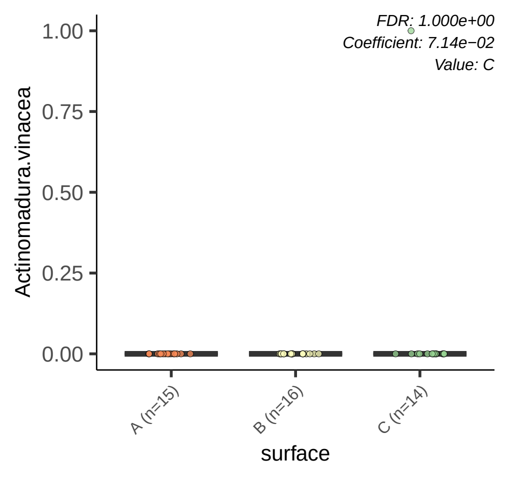

FDR: 1.000e+00
Coefficient: 7.14e−02
Value: C
Actinomadura.vinacea
1.00
0.75
0.50
0.25
0.00
A (n=15)
B (n=16)
C (n=14)
surface

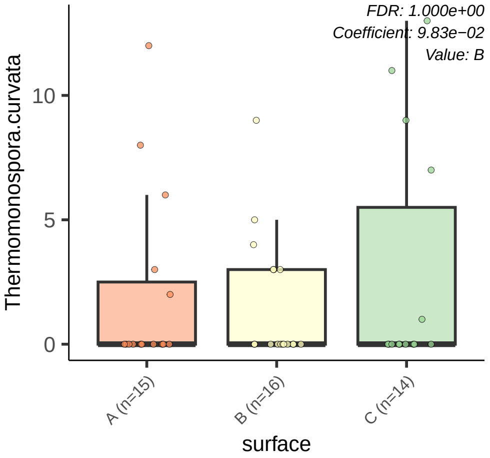

FDR: 1.000e+00
Coefficient: 9.83e−02
Value: B
Thermomonospora.curvata
0
5
10
A (n=15)
B (n=16)
C (n=14)
surface

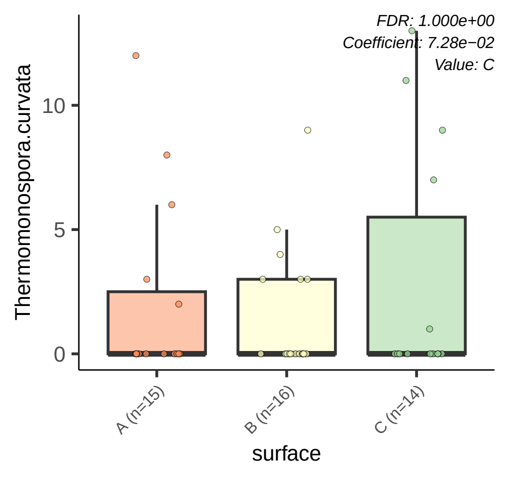

FDR: 1.000e+00
Coefficient: 7.28e−02
Value: C
Thermomonospora.curvata
0
5
10
A (n=15)
B (n=16)
C (n=14)
surface

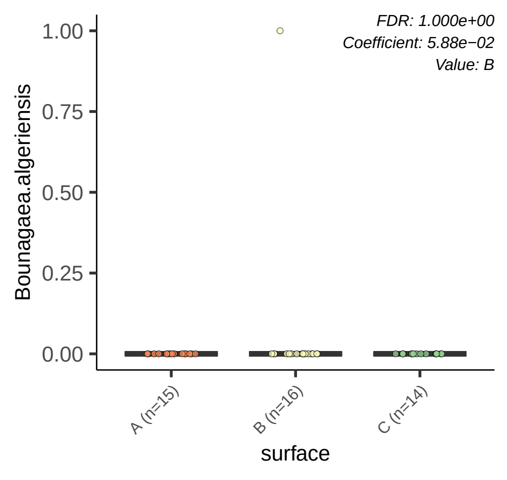

FDR: 1.000e+00
Coefficient: 5.88e−02
Value: B
Bounagaea.algeriensis
1.00
0.75
0.50
0.25
0.00
A (n=15)
B (n=16)
C (n=14)
surface

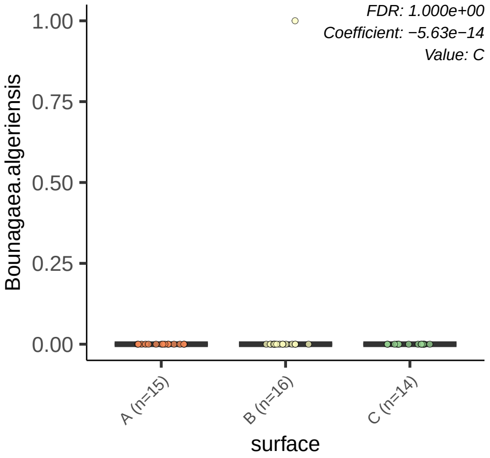

FDR: 1.000e+00
Coefficient: −5.63e−14
Value: C
Bounagaea.algeriensis
1.00
0.75
0.50
0.25
0.00
A (n=15)
B (n=16)
C (n=14)
surface

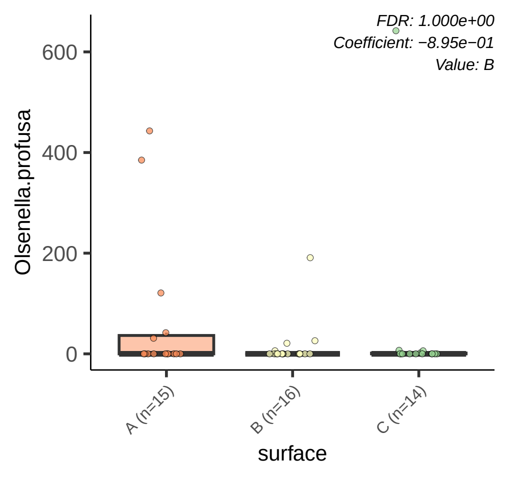
FDR: 1.000e+00
Coefficient: −8.95e−01
Value: B
Olsenella.profusa
600
400
200
0
A (n=15)
B (n=16)
C (n=14)
surface

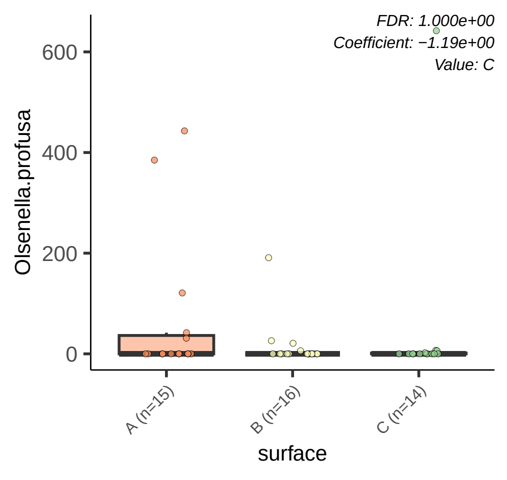
FDR: 1.000e+00
Coefficient: −1.19e+00
Value: C
Olsenella.profusa
600
400
200
0
A (n=15)
B (n=16)
C (n=14)
surface

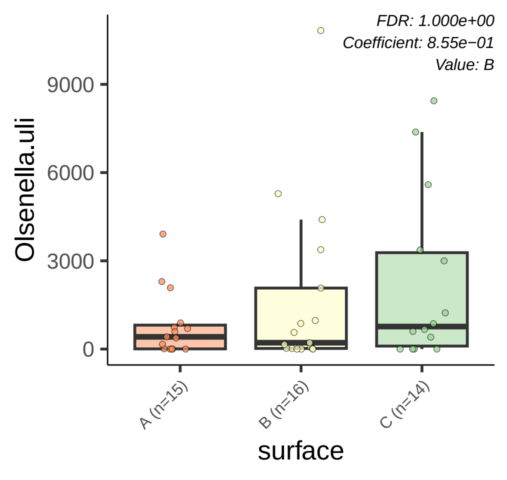

FDR: 1.000e+00
Coefficient: 8.55e−01
Value: B
Olsenella.uli
9000
6000
3000
0
A (n=15)
B (n=16)
C (n=14)
surface

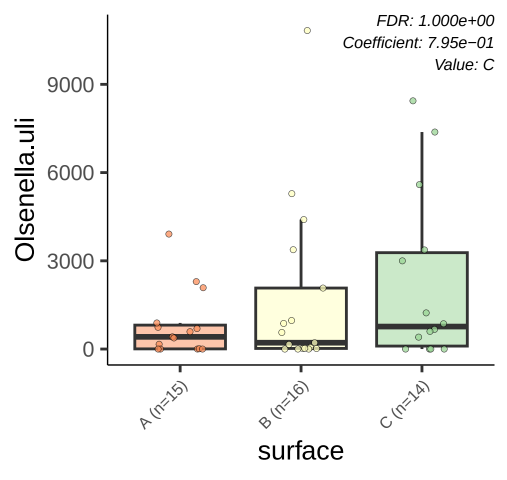
FDR: 1.000e+00
Coefficient: 7.95e−01
Value: C
Olsenella.uli
9000
6000
3000
0
A (n=15)
B (n=16)
C (n=14)
surface

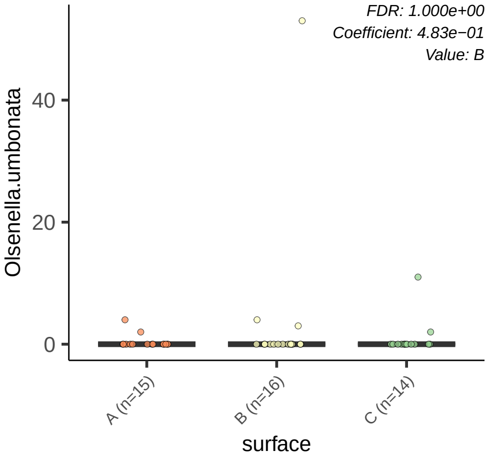
FDR: 1.000e+00
Coefficient: 4.83e−01
Value: B
Olsenella.umbonata
0
20
40
A (n=15)
B (n=16)
C (n=14)
surface

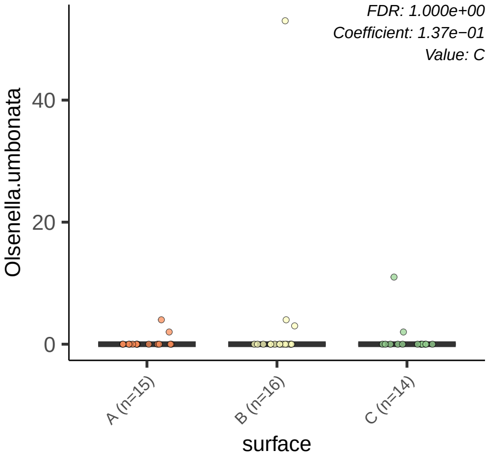
FDR: 1.000e+00
Coefficient: 1.37e−01
Value: C
Olsenella.umbonata
0
20
40
A (n=15)
B (n=16)
C (n=14)
surface

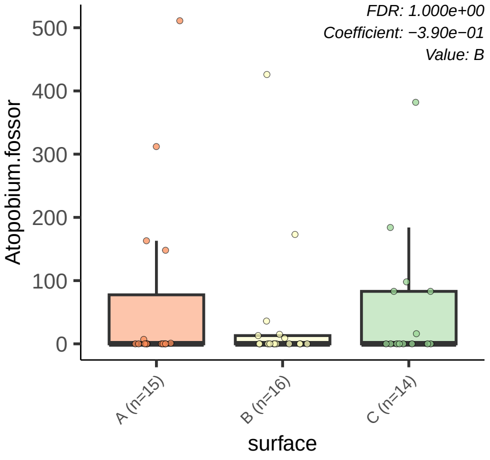

FDR: 1.000e+00
Coefficient: −3.90e−01
Value: B
Atopobium.fossor
0
100
200
300
400
500
A (n=15)
B (n=16)
C (n=14)
surface

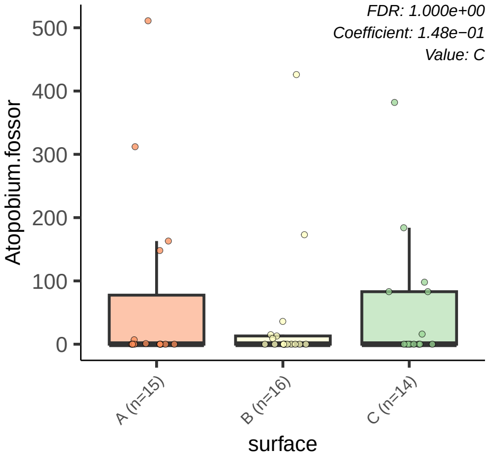

FDR: 1.000e+00
Coefficient: 1.48e−01
Value: C
Atopobium.fossor
500
400
300
200
100
0
A (n=15)
B (n=16)
C (n=14)
surface

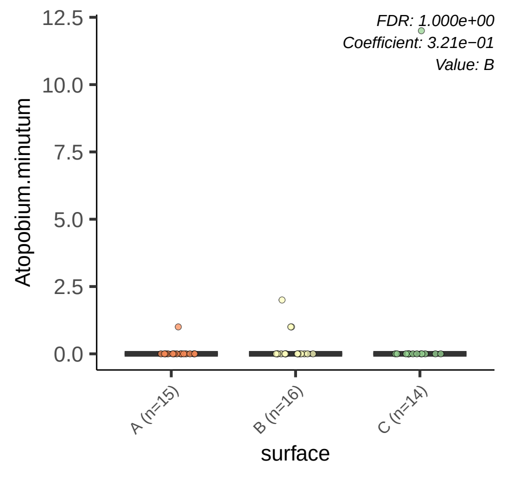

FDR: 1.000e+00
Coefficient: 3.21e−01
Value: B
12.5
10.0
7.5
5.0
2.5
0.0
Atopobium.minutum
A (n=15)
B (n=16)
C (n=14)
surface

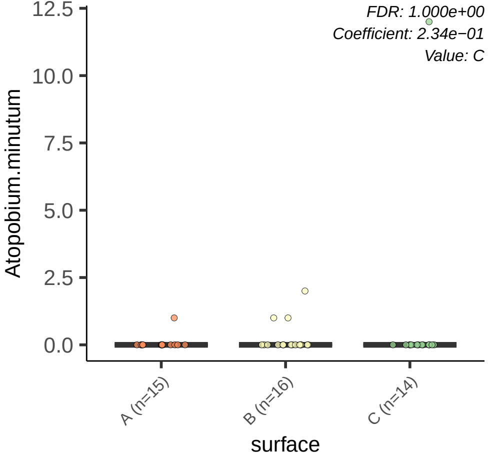

FDR: 1.000e+00
Coefficient: 2.34e−01
Value: C
12.5
10.0
7.5
5.0
2.5
0.0
Atopobium.minutum
A (n=15)
B (n=16)
C (n=14)
surface

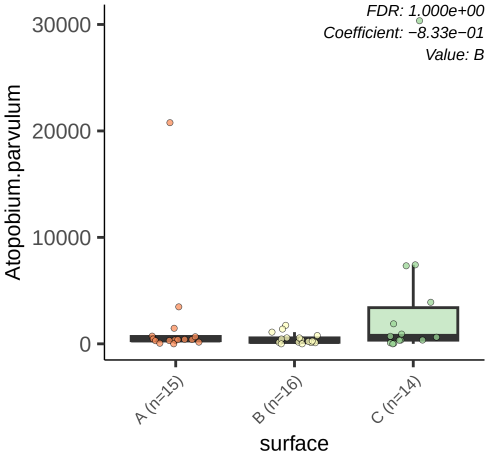

FDR: 1.000e+00
Coefficient: −8.33e−01
Value: B
Atopobium.parvulum
30000
20000
10000
0
A (n=15)
B (n=16)
C (n=14)
surface

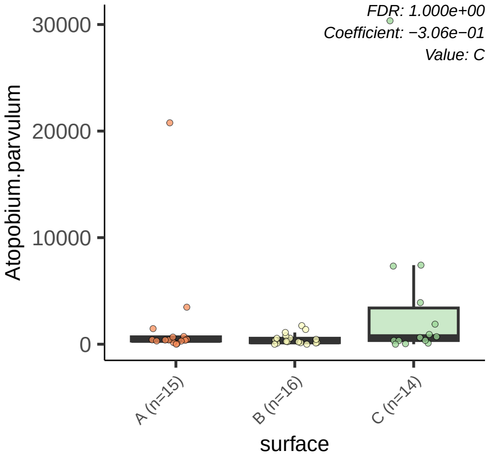

FDR: 1.000e+00
Coefficient: −3.06e−01
Value: C
Atopobium.parvulum
30000
20000
10000
0
A (n=15)
B (n=16)
C (n=14)
surface

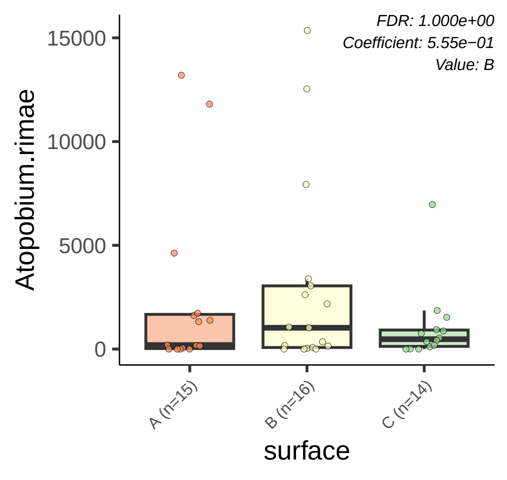

FDR: 1.000e+00
Coefficient: 5.55e−01
Value: B
Atopobium.rimae
15000
10000
5000
0
A (n=15)
B (n=16)
C (n=14)
surface

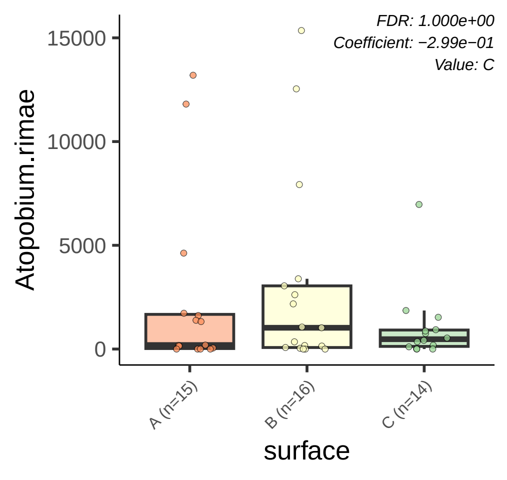

FDR: 1.000e+00
Coefficient: −2.99e−01
Value: C
Atopobium.rimae
15000
10000
5000
0
A (n=15)
B (n=16)
C (n=14)
surface

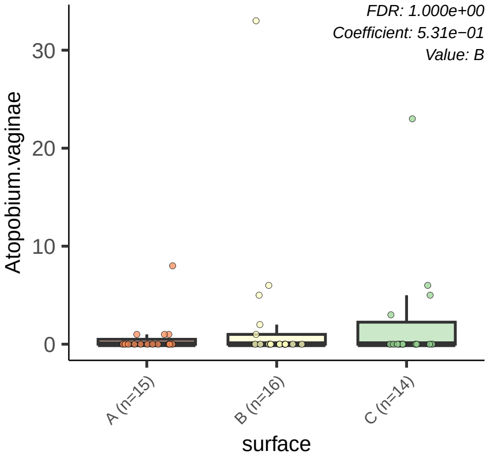

FDR: 1.000e+00
Coefficient: 5.31e−01
Value: B
Atopobium.vaginae
0
10
20
30
A (n=15)
B (n=16)
C (n=14)
surface

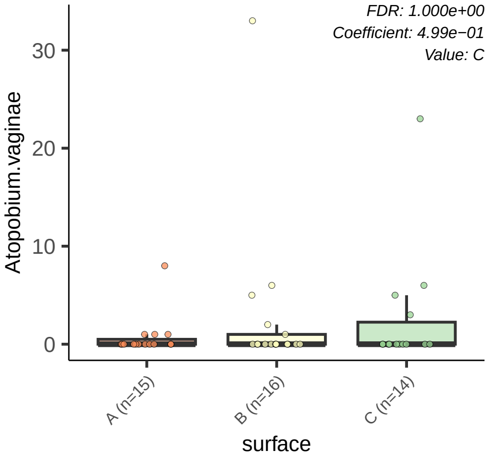
FDR: 1.000e+00
Coefficient: 4.99e−01
Value: C
Atopobium.vaginae
0
10
20
30
A (n=15)
B (n=16)
C (n=14)
surface

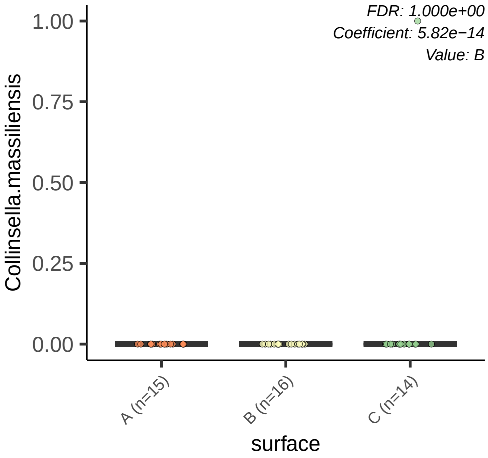
FDR: 1.000e+00
Coefficient: 5.82e−14
Value: B
Collinsella.massiliensis
1.00
0.75
0.50
0.25
0.00
A (n=15)
B (n=16)
C (n=14)
surface

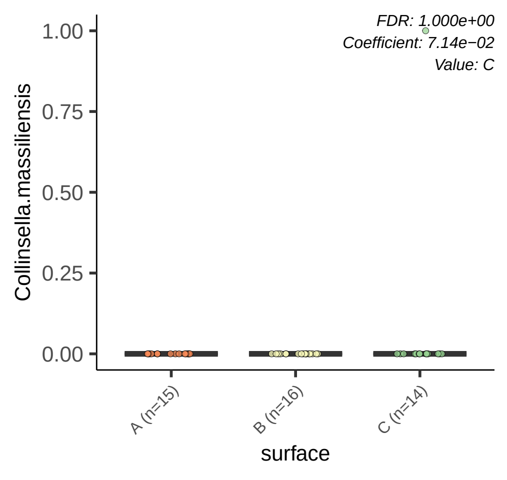
FDR: 1.000e+00
Coefficient: 7.14e−02
Value: C
Collinsella.massiliensis
1.00
0.75
0.50
0.25
0.00
A (n=15)
B (n=16)
C (n=14)
surface

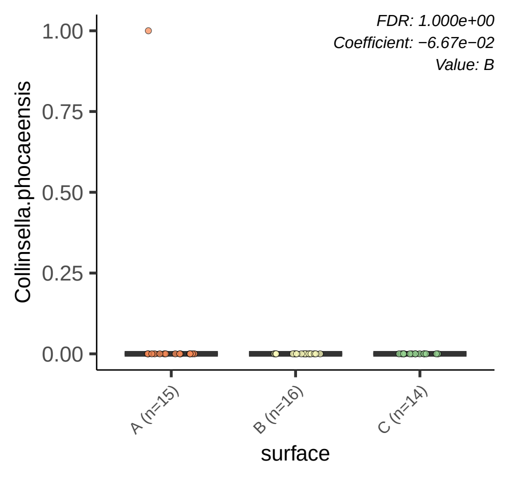

FDR: 1.000e+00
Coefficient: −6.67e−02
Value: B
1.00
0.75
0.50
0.25
0.00
Collinsella.phocaeensis
A (n=15)
B (n=16)
C (n=14)
surface

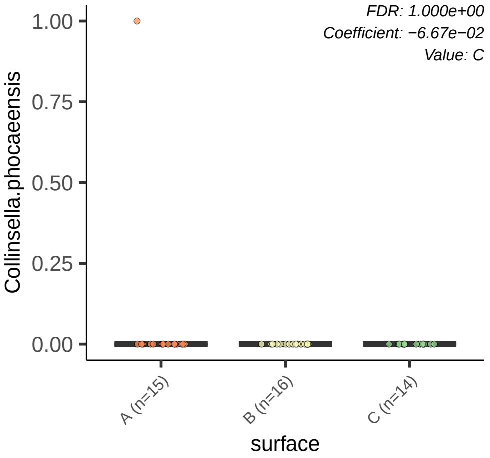

FDR: 1.000e+00
Coefficient: −6.67e−02
Value: C
Collinsella.phocaeensis
1.00
0.75
0.50
0.25
0.00
A (n=15)
B (n=16)
C (n=14)
surface

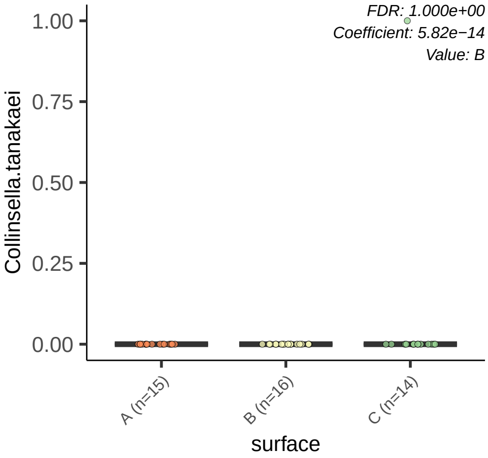
FDR: 1.000e+00
Coefficient: 5.82e−14
Value: B
Collinsella.tanakaei
1.00
0.75
0.50
0.25
0.00
A (n=15)
B (n=16)
C (n=14)
surface

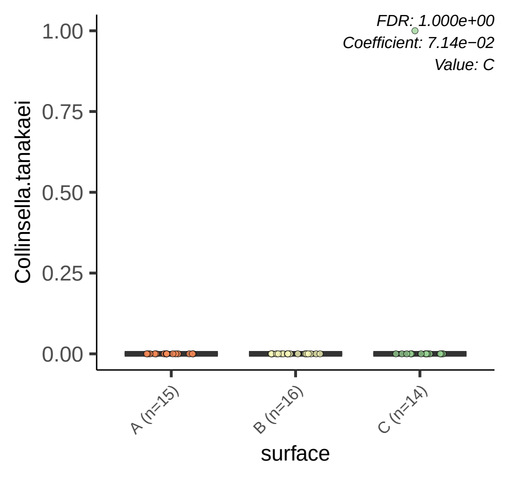
FDR: 1.000e+00
Coefficient: 7.14e−02
Value: C
1.00
0.75
0.50
0.25
0.00
Collinsella.tanakaei
A (n=15)
B (n=16)
C (n=14)
surface

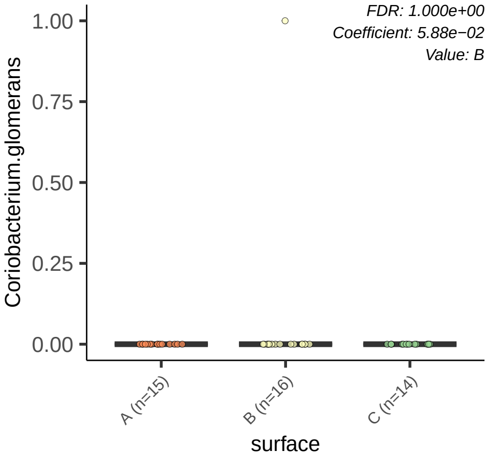
FDR: 1.000e+00
Coefficient: 5.88e−02
Value: B
Coriobacterium.glomerans
1.00
0.75
0.50
0.25
0.00
A (n=15)
B (n=16)
C (n=14)
surface

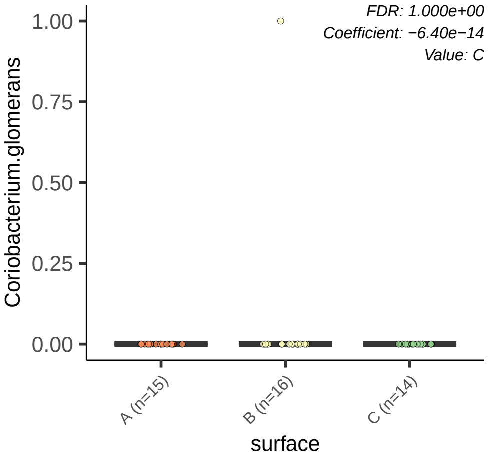

FDR: 1.000e+00
Coefficient: −6.40e−14
Value: C
Coriobacterium.glomerans
1.00
0.75
0.50
0.25
0.00
A (n=15)
B (n=16)
C (n=14)
surface

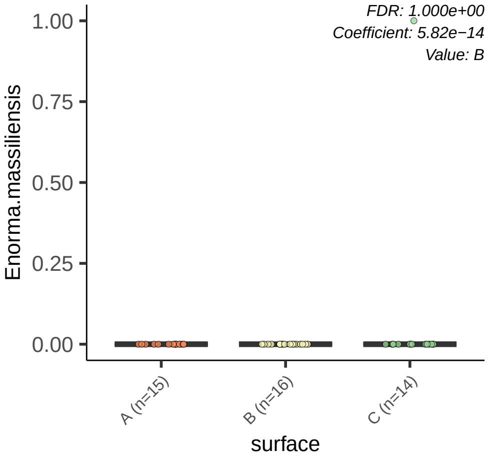
FDR: 1.000e+00
Coefficient: 5.82e−14
Value: B
Enorma.massiliensis
1.00
0.75
0.50
0.25
0.00
A (n=15)
B (n=16)
C (n=14)
surface

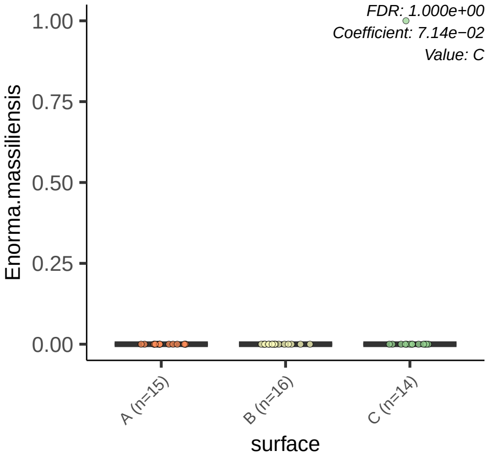

FDR: 1.000e+00
Coefficient: 7.14e−02
Value: C
Enorma.massiliensis
1.00
0.75
0.50
0.25
0.00
A (n=15)
B (n=16)
C (n=14)
surface

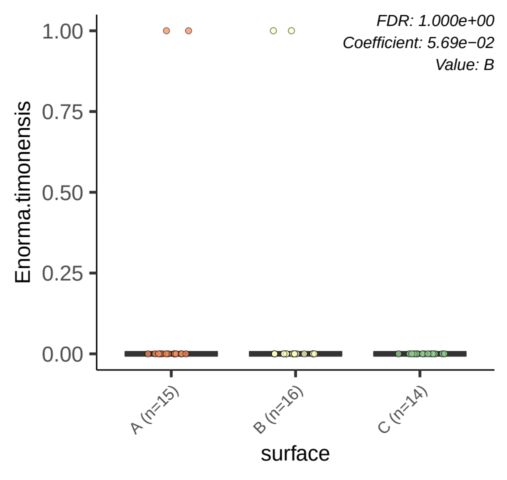

FDR: 1.000e+00
Coefficient: 5.69e−02
Value: B
Enorma.timonensis
1.00
0.75
0.50
0.25
0.00
A (n=15)
B (n=16)
C (n=14)
surface

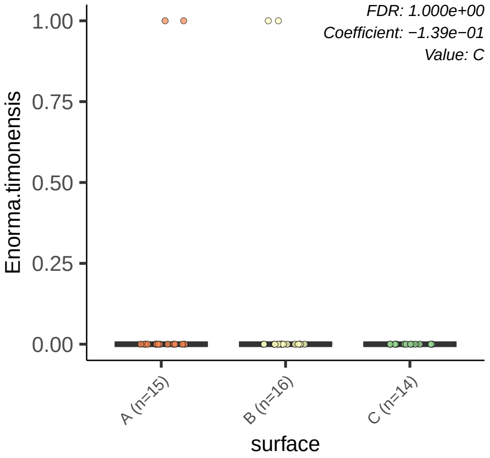
FDR: 1.000e+00
Coefficient: −1.39e−01
Value: C
Enorma.timonensis
1.00
0.75
0.50
0.25
0.00
A (n=15)
B (n=16)
C (n=14)
surface

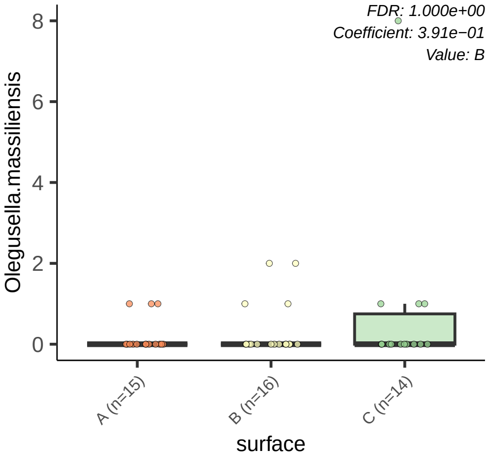

FDR: 1.000e+00
Coefficient: 3.91e−01
Value: B
Olegusella.massiliensis
8
6
4
2
0
A (n=15)
B (n=16)
C (n=14)
surface

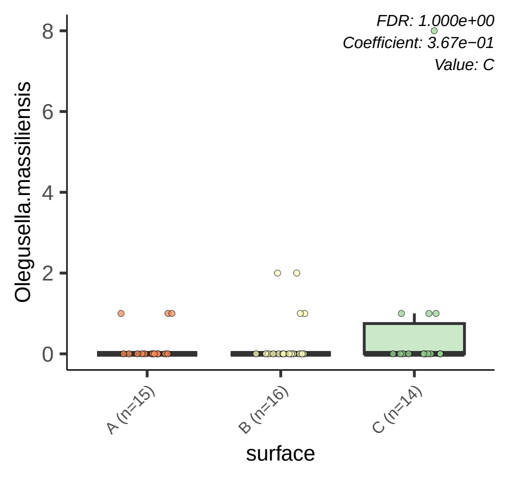
FDR: 1.000e+00
Coefficient: 3.67e−01
Value: C
8
6
4
2
0
Olegusella.massiliensis
A (n=15)
B (n=16)
C (n=14)
surface

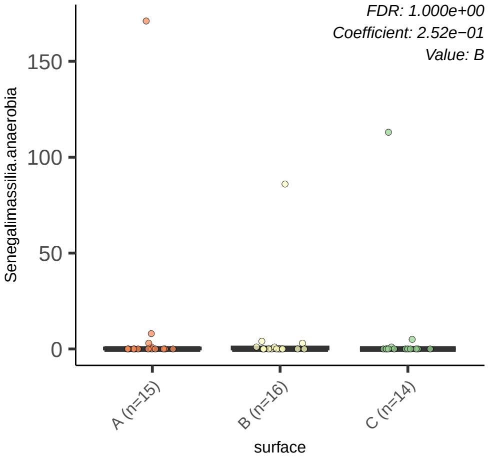
FDR: 1.000e+00
Coefficient: 2.52e−01
Value: B
Senegalimassilia.anaerobia
150
100
50
0
A (n=15)
B (n=16)
C (n=14)
surface

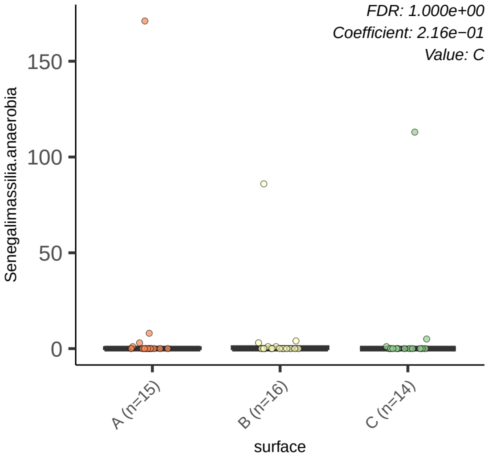

FDR: 1.000e+00
Coefficient: 2.16e−01
Value: C
Senegalimassilia.anaerobia
150
100
50
0
A (n=15)
B (n=16)
C (n=14)
surface

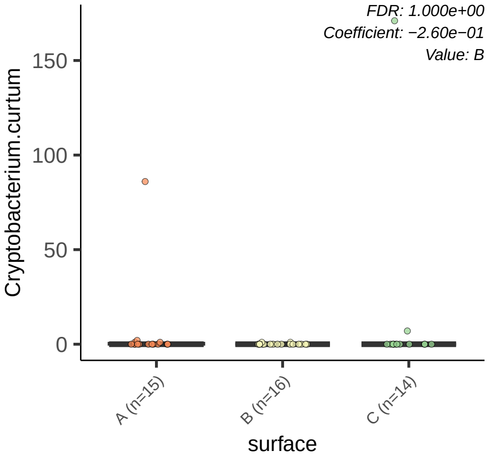

FDR: 1.000e+00
Coefficient: −2.60e−01
Value: B
Cryptobacterium.curtum
150
100
50
0
A (n=15)
B (n=16)
C (n=14)
surface

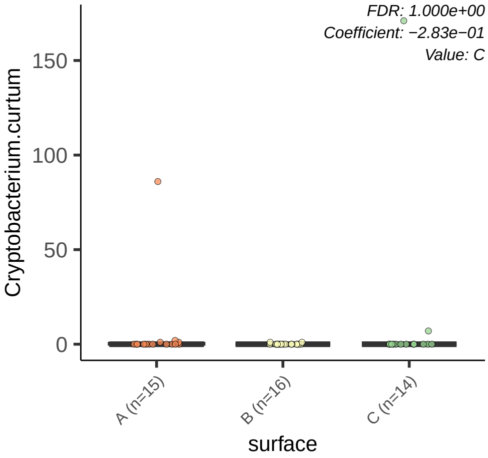
FDR: 1.000e+00
Coefficient: −2.83e−01
Value: C
Cryptobacterium.curtum
150
100
50
0
A (n=15)
B (n=16)
C (n=14)
surface

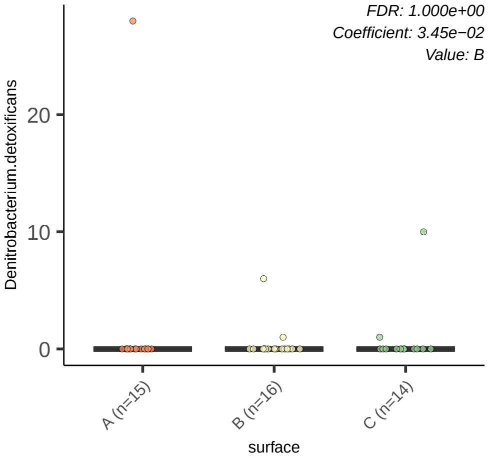

FDR: 1.000e+00
Coefficient: 3.45e−02
Value: B
Denitrobacterium.detoxificans
20
10
0
A (n=15)
B (n=16)
C (n=14)
surface

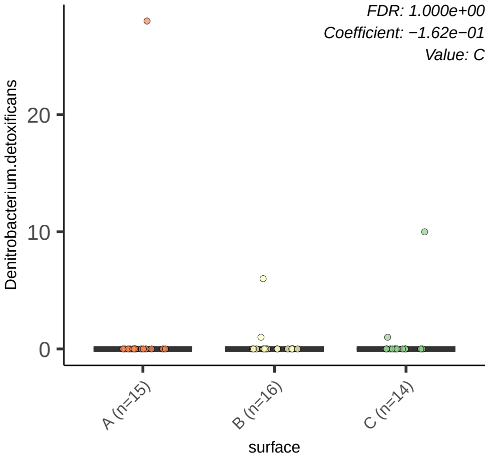

FDR: 1.000e+00
Coefficient: −1.62e−01
Value: C
Denitrobacterium.detoxificans
20
10
0
A (n=15)
B (n=16)
C (n=14)
surface

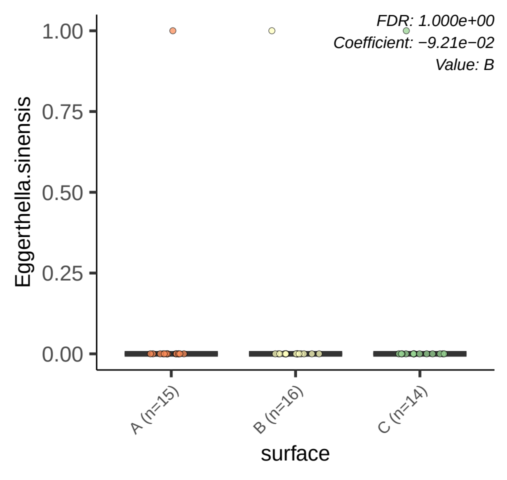
FDR: 1.000e+00
Coefficient: −9.21e−02
Value: B
Eggerthella.sinensis
1.00
0.75
0.50
0.25
0.00
A (n=15)
B (n=16)
C (n=14)
surface

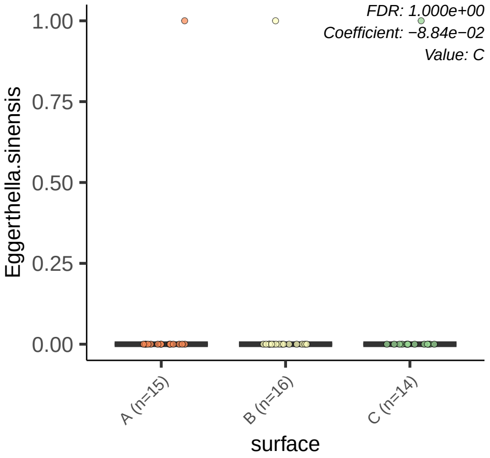
FDR: 1.000e+00
Coefficient: −8.84e−02
Value: C
Eggerthella.sinensis
1.00
0.75
0.50
0.25
0.00
A (n=15)
B (n=16)
C (n=14)
surface

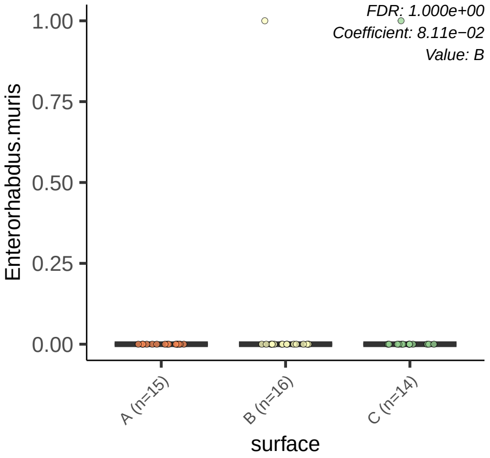

FDR: 1.000e+00
Coefficient: 8.11e−02
Value: B
Enterorhabdus.muris
1.00
0.75
0.50
0.25
0.00
A (n=15)
B (n=16)
C (n=14)
surface

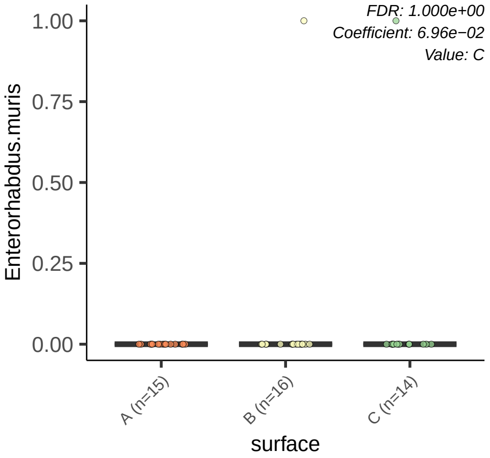
FDR: 1.000e+00
Coefficient: 6.96e−02
Value: C
Enterorhabdus.muris
1.00
0.75
0.50
0.25
0.00
A (n=15)
B (n=16)
C (n=14)
surface

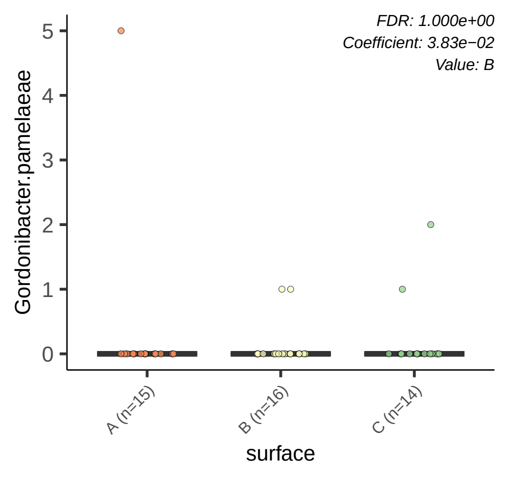
FDR: 1.000e+00
Coefficient: 3.83e−02
Value: B
Gordonibacter.pamelaeae
0
1
2
3
4
5
A (n=15)
B (n=16)
C (n=14)
surface

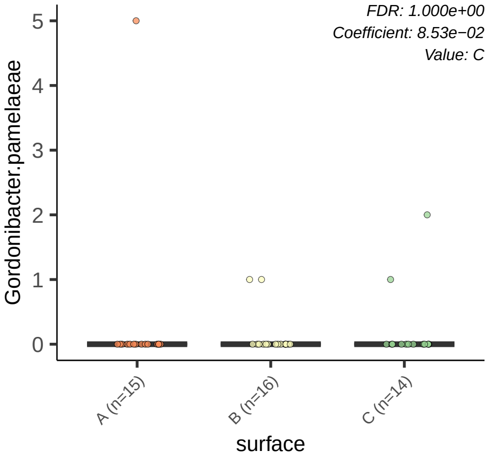
FDR: 1.000e+00
Coefficient: 8.53e−02
Value: C
Gordonibacter.pamelaeae
0
1
2
3
4
5
A (n=15)
B (n=16)
C (n=14)
surface

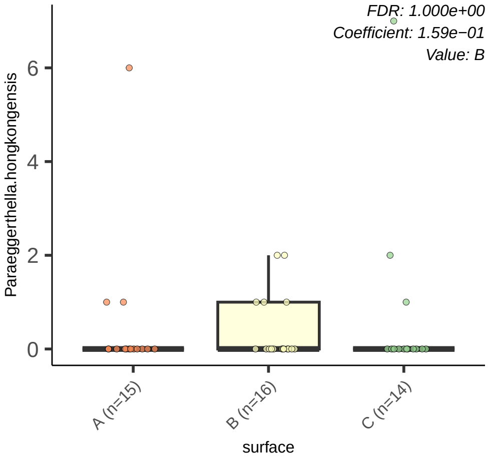

FDR: 1.000e+00
Coefficient: 1.59e−01
Value: B
Paraeggerthella.hongkongensis
0
2
4
6
A (n=15)
B (n=16)
C (n=14)
surface

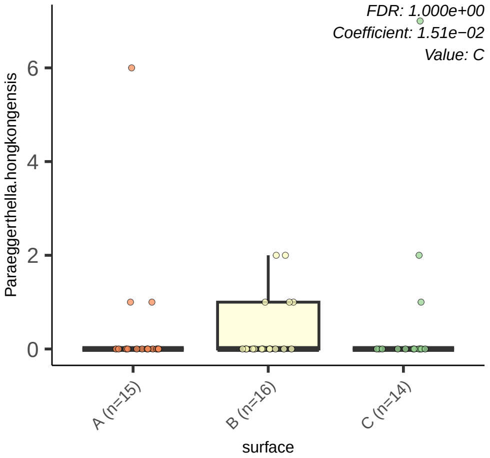
FDR: 1.000e+00
Coefficient: 1.51e−02
Value: C
Paraeggerthella.hongkongensis
0
2
4
6
A (n=15)
B (n=16)
C (n=14)
surface

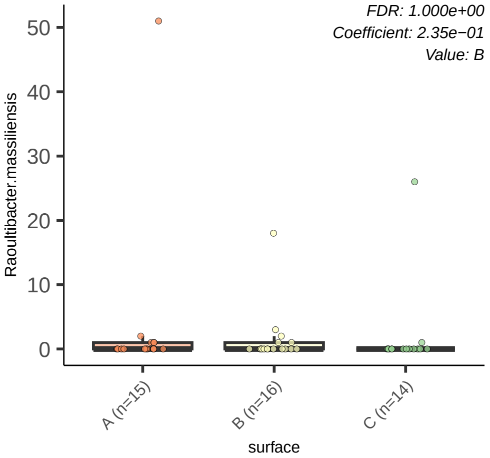

FDR: 1.000e+00
Coefficient: 2.35e−01
Value: B
Raoultibacter.massiliensis
0
10
20
30
40
50
A (n=15)
B (n=16)
C (n=14)
surface

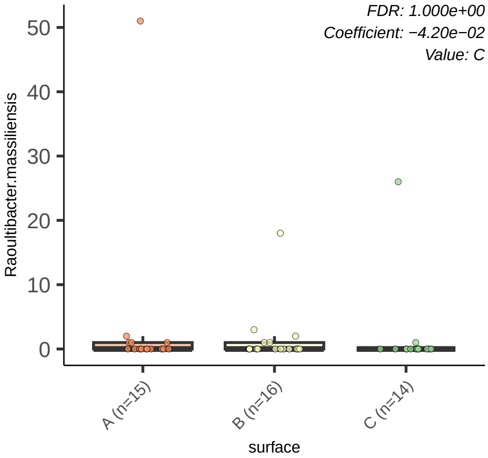

FDR: 1.000e+00
Coefficient: −4.20e−02
Value: C
Raoultibacter.massiliensis
50
40
30
20
10
0
A (n=15)
B (n=16)
C (n=14)
surface

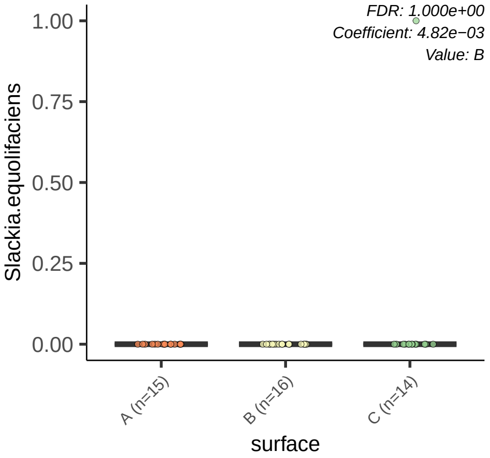
FDR: 1.000e+00
Coefficient: 4.82e−03
Value: B
Slackia.equolifaciens
1.00
0.75
0.50
0.25
0.00
A (n=15)
B (n=16)
C (n=14)
surface

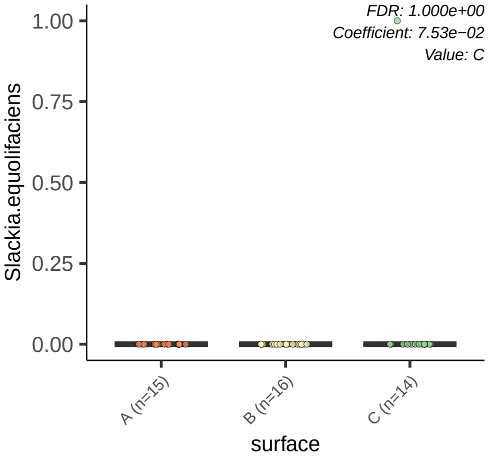
FDR: 1.000e+00
Coefficient: 7.53e−02
Value: C
Slackia.equolifaciens
1.00
0.75
0.50
0.25
0.00
A (n=15)
B (n=16)
C (n=14)
surface

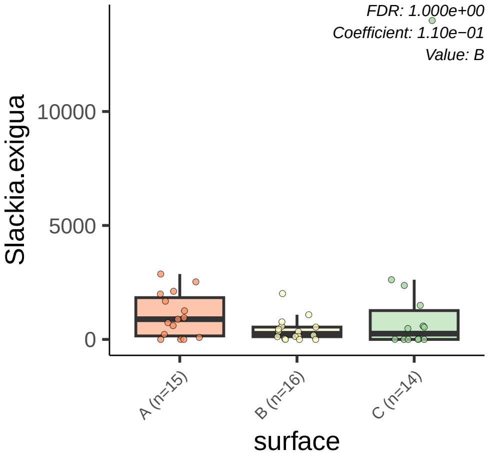
FDR: 1.000e+00
Coefficient: 1.10e−01
Value: B
Slackia.exigua
10000
5000
0
A (n=15)
B (n=16)
C (n=14)
surface

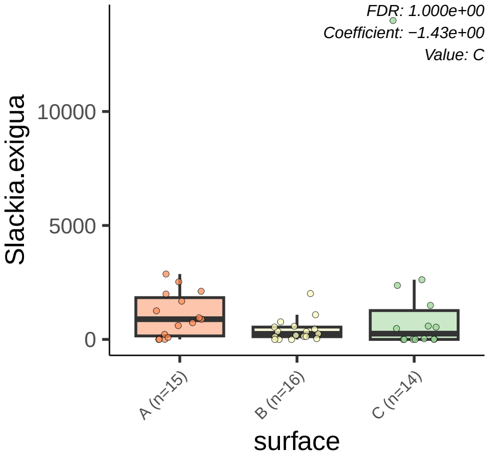

FDR: 1.000e+00
Coefficient: −1.43e+00
Value: C
Slackia.exigua
10000
5000
0
A (n=15)
B (n=16)
C (n=14)
surface

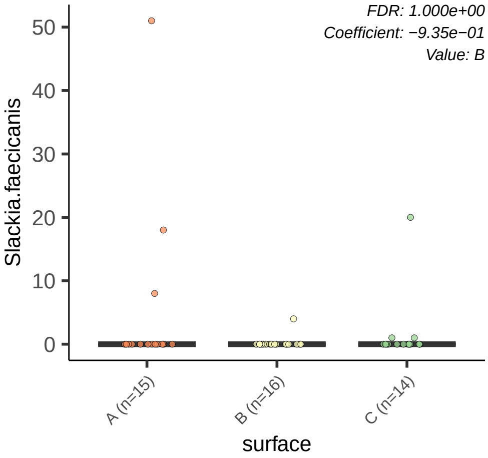
FDR: 1.000e+00
Coefficient: −9.35e−01
Value: B
Slackia.faecicanis
50
40
30
20
10
0
A (n=15)
B (n=16)
C (n=14)
surface

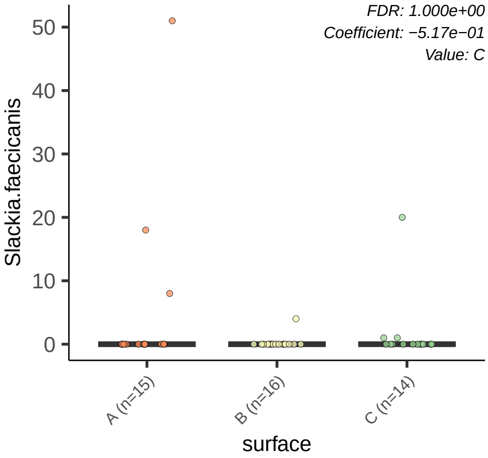

FDR: 1.000e+00
Coefficient: −5.17e−01
Value: C
Slackia.faecicanis
50
40
30
20
10
0
A (n=15)
B (n=16)
C (n=14)
surface

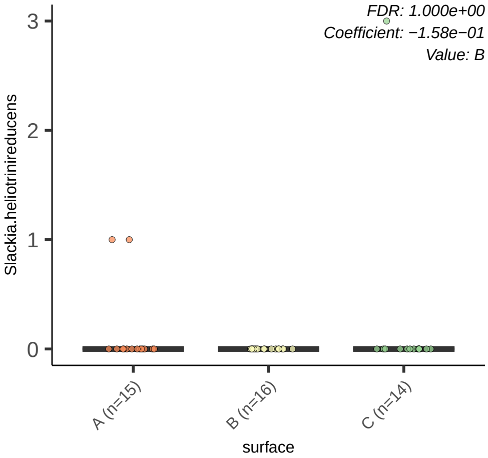
FDR: 1.000e+00
Coefficient: −1.58e−01
Value: B
Slackia.heliotrinireducens
3
2
1
0
A (n=15)
B (n=16)
C (n=14)
surface

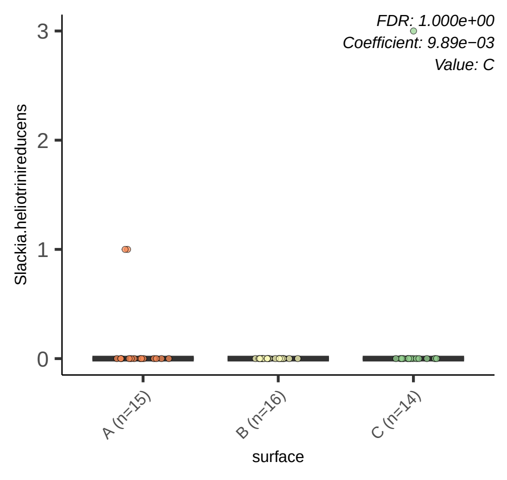

FDR: 1.000e+00
Coefficient: 9.89e−03
Value: C
Slackia.heliotrinireducens
0
1
2
3
A (n=15)
B (n=16)
C (n=14)
surface

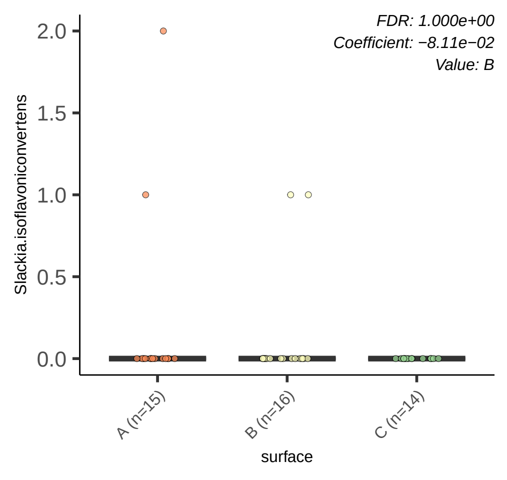

FDR: 1.000e+00
Coefficient: −8.11e−02
Value: B
Slackia.isoflavoniconvertens
2.0
1.5
1.0
0.5
0.0
A (n=15)
B (n=16)
C (n=14)
surface

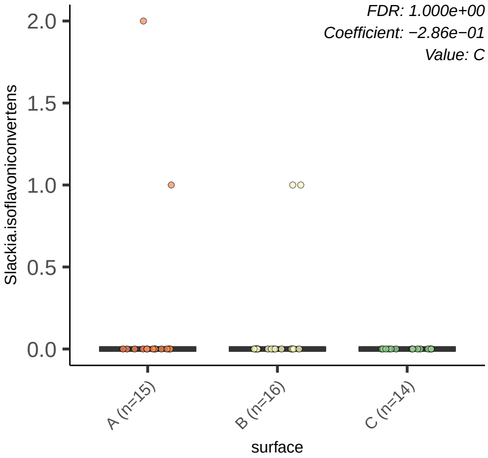

FDR: 1.000e+00
Coefficient: −2.86e−01
Value: C
Slackia.isoflavoniconvertens
2.0
1.5
1.0
0.5
0.0
A (n=15)
B (n=16)
C (n=14)
surface

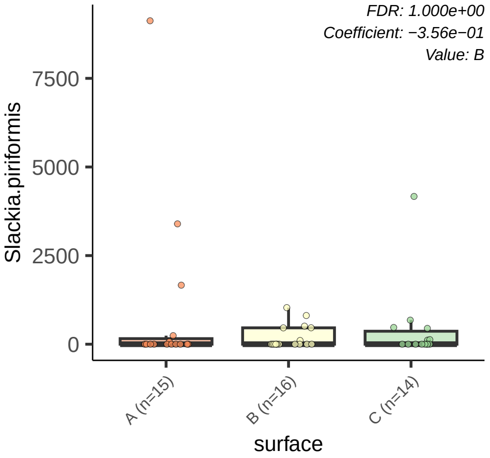
FDR: 1.000e+00
Coefficient: −3.56e−01
Value: B
Slackia.piriformis
7500
5000
2500
0
A (n=15)
B (n=16)
C (n=14)
surface

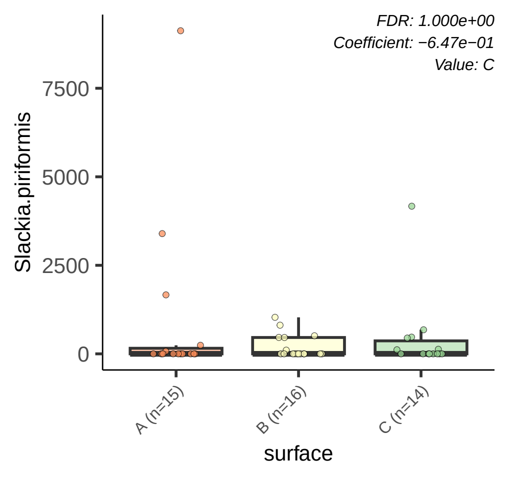
FDR: 1.000e+00
Coefficient: −6.47e−01
Value: C
Slackia.piriformis
7500
5000
2500
0
A (n=15)
B (n=16)
C (n=14)
surface

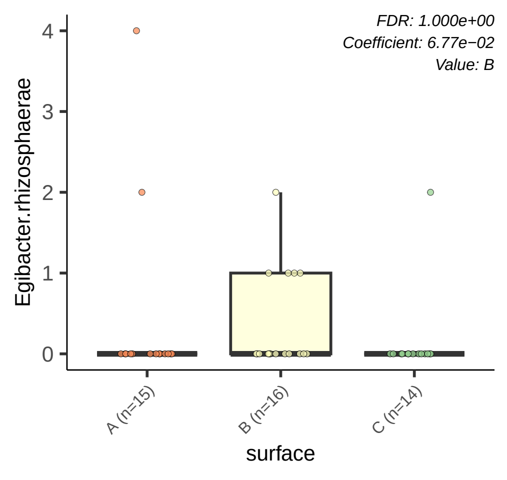
FDR: 1.000e+00
Coefficient: 6.77e−02
Value: B
Egibacter.rhizosphaerae
0
1
2
3
4
A (n=15)
B (n=16)
C (n=14)
surface

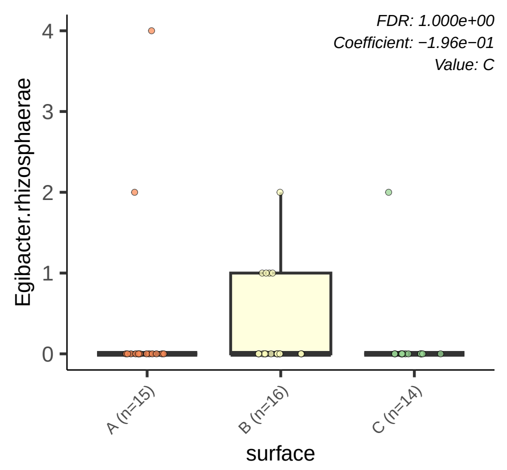

FDR: 1.000e+00
Coefficient: −1.96e−01
Value: C
Egibacter.rhizosphaerae
4
3
2
1
0
A (n=15)
B (n=16)
C (n=14)
surface

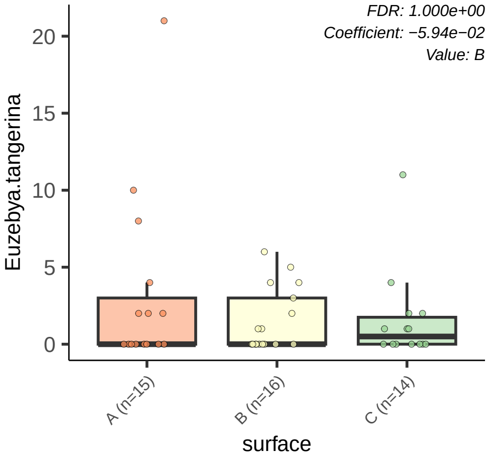

FDR: 1.000e+00
Coefficient: −5.94e−02
Value: B
Euzebya.tangerina
20
15
10
5
0
A (n=15)
B (n=16)
C (n=14)
surface

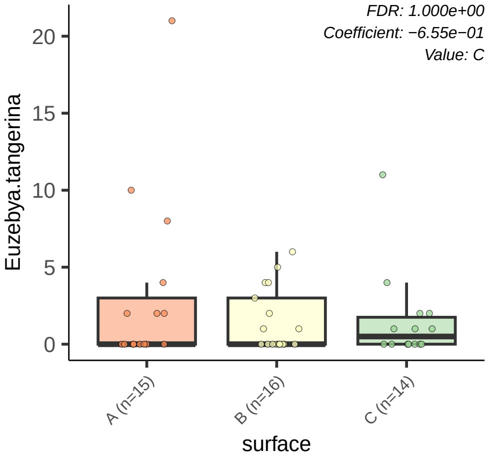
FDR: 1.000e+00
Coefficient: −6.55e−01
Value: C
Euzebya.tangerina
20
15
10
5
0
A (n=15)
B (n=16)
C (n=14)
surface

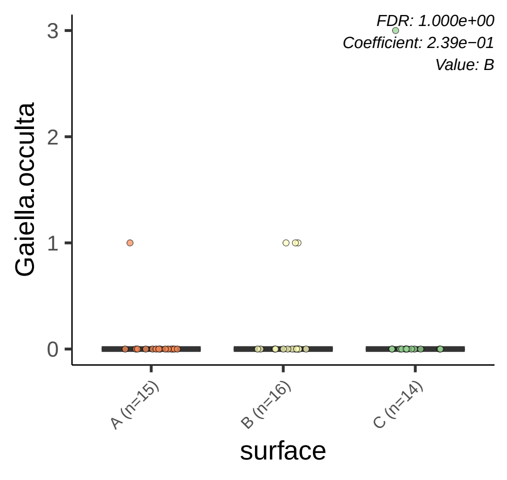

FDR: 1.000e+00
Coefficient: 2.39e−01
Value: B
Gaiella.occulta
3
2
1
0
A (n=15)
B (n=16)
C (n=14)
surface

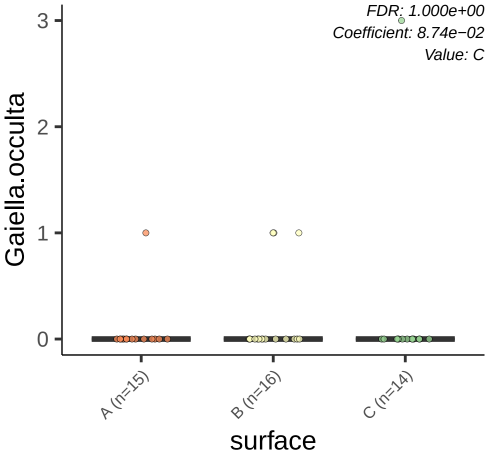
FDR: 1.000e+00
Coefficient: 8.74e−02
Value: C
Gaiella.occulta
3
2
1
0
A (n=15)
B (n=16)
C (n=14)
surface

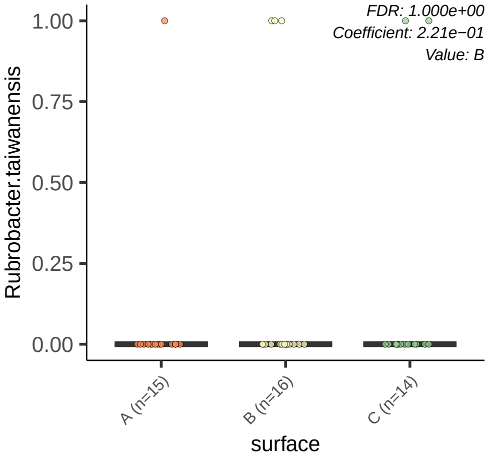
FDR: 1.000e+00
Coefficient: 2.21e−01
Value: B
Rubrobacter.taiwanensis
1.00
0.75
0.50
0.25
0.00
A (n=15)
B (n=16)
C (n=14)
surface

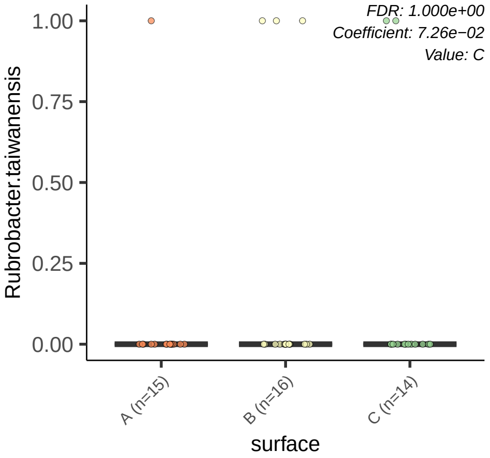

FDR: 1.000e+00
Coefficient: 7.26e−02
Value: C
Rubrobacter.taiwanensis
1.00
0.75
0.50
0.25
0.00
A (n=15)
B (n=16)
C (n=14)
surface

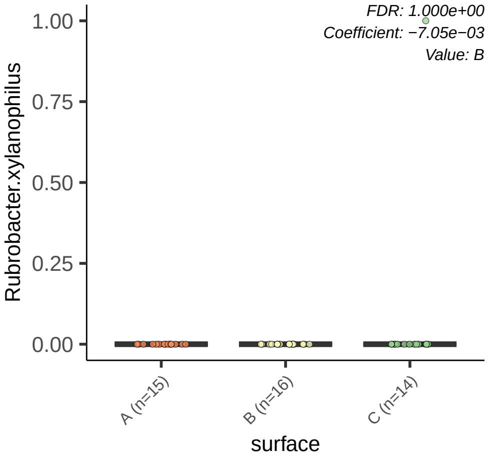
FDR: 1.000e+00
Coefficient: −7.05e−03
Value: B
Rubrobacter.xylanophilus
1.00
0.75
0.50
0.25
0.00
A (n=15)
B (n=16)
C (n=14)
surface

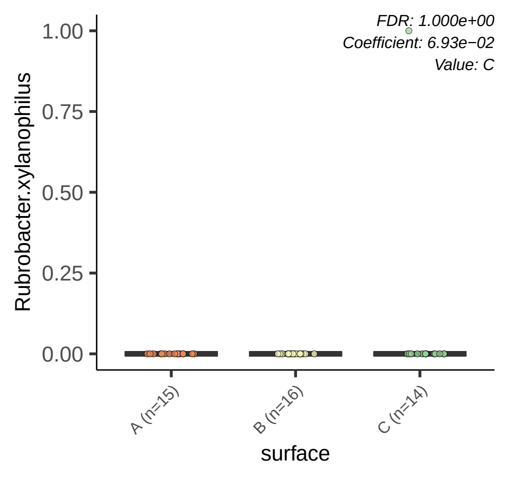

FDR: 1.000e+00
Coefficient: 6.93e−02
Value: C
Rubrobacter.xylanophilus
1.00
0.75
0.50
0.25
0.00
A (n=15)
B (n=16)
C (n=14)
surface

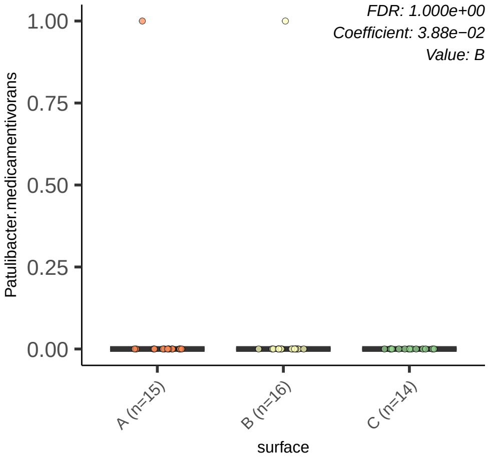
FDR: 1.000e+00
Coefficient: 3.88e−02
Value: B
Patulibacter.medicamentivorans
1.00
0.75
0.50
0.25
0.00
A (n=15)
B (n=16)
C (n=14)
surface

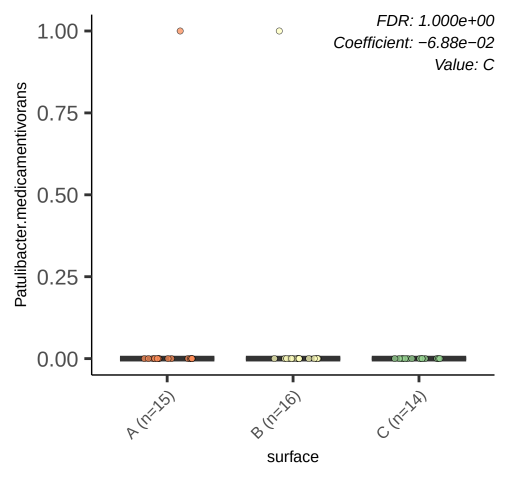

FDR: 1.000e+00
Coefficient: −6.88e−02
Value: C
Patulibacter.medicamentivorans
1.00
0.75
0.50
0.25
0.00
A (n=15)
B (n=16)
C (n=14)
surface

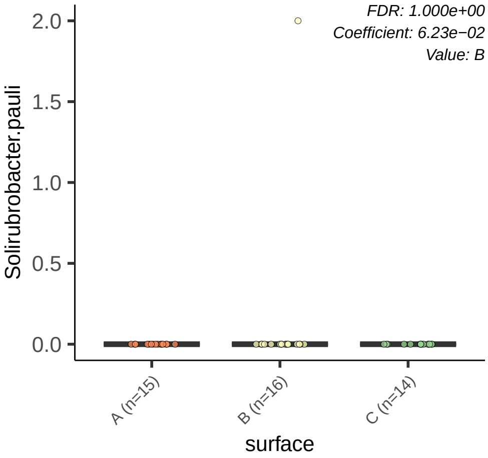
FDR: 1.000e+00
Coefficient: 6.23e−02
Value: B
Solirubrobacter.pauli
2.0
1.5
1.0
0.5
0.0
A (n=15)
B (n=16)
C (n=14)
surface

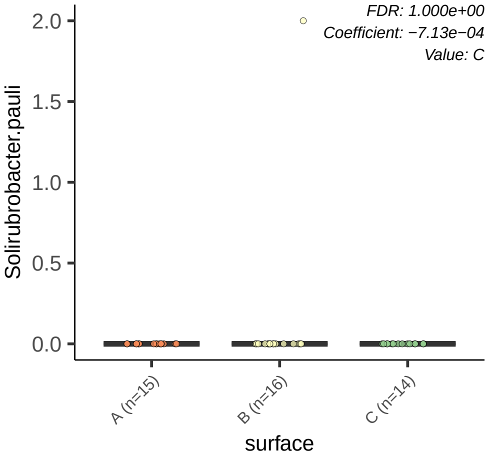
FDR: 1.000e+00
Coefficient: −7.13e−04
Value: C
Solirubrobacter.pauli
2.0
1.5
1.0
0.5
0.0
A (n=15)
B (n=16)
C (n=14)
surface

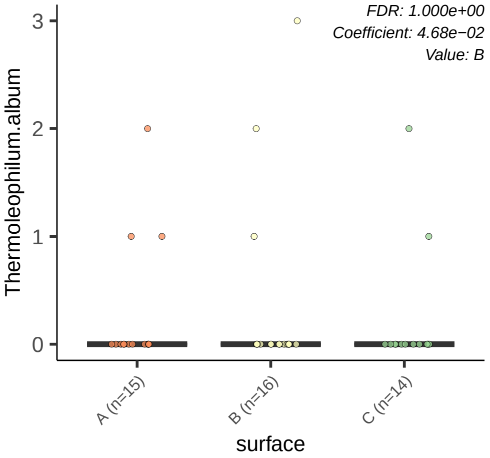
FDR: 1.000e+00
Coefficient: 4.68e−02
Value: B
Thermoleophilum.album
3
2
1
0
A (n=15)
B (n=16)
C (n=14)
surface

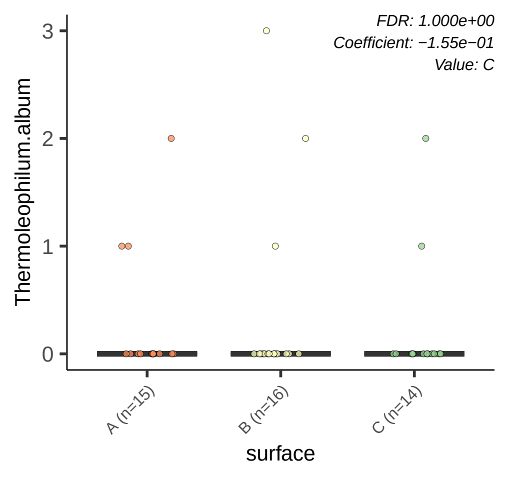
FDR: 1.000e+00
Coefficient: −1.55e−01
Value: C
Thermoleophilum.album
3
2
1
0
A (n=15)
B (n=16)
C (n=14)
surface

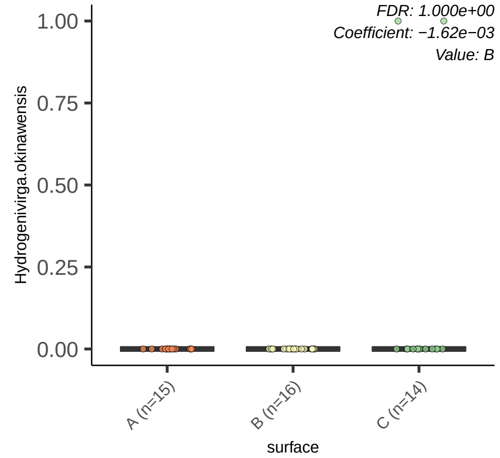
FDR: 1.000e+00
Coefficient: −1.62e−03
Value: B
Hydrogenivirga.okinawensis
1.00
0.75
0.50
0.25
0.00
A (n=15)
B (n=16)
C (n=14)
surface

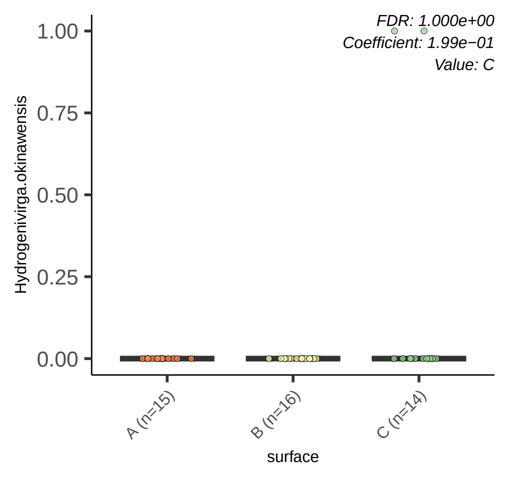
FDR: 1.000e+00
Coefficient: 1.99e−01
Value: C
Hydrogenivirga.okinawensis
1.00
0.75
0.50
0.25
0.00
A (n=15)
B (n=16)
C (n=14)
surface

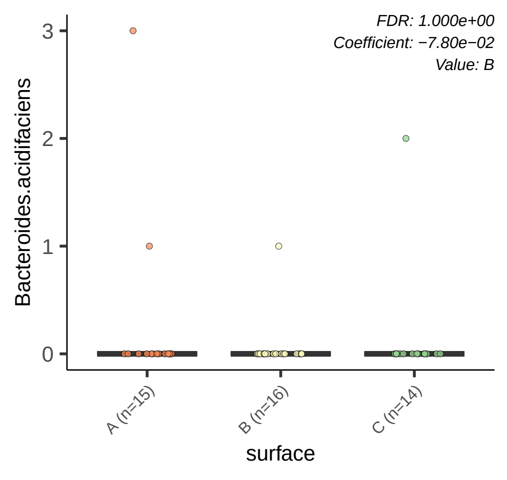
FDR: 1.000e+00
Coefficient: −7.80e−02
Value: B
Bacteroides.acidifaciens
3
2
1
0
A (n=15)
B (n=16)
C (n=14)
surface

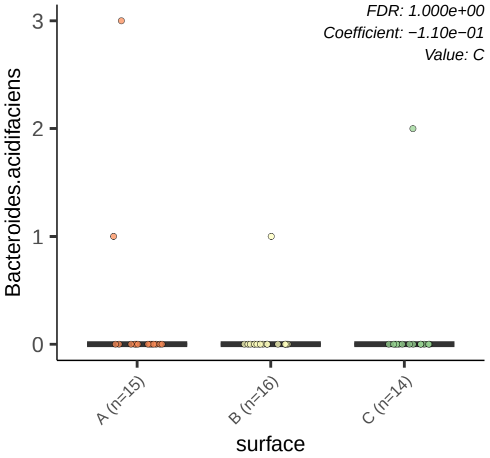

FDR: 1.000e+00
Coefficient: −1.10e−01
Value: C
Bacteroides.acidifaciens
3
2
1
0
A (n=15)
B (n=16)
C (n=14)
surface

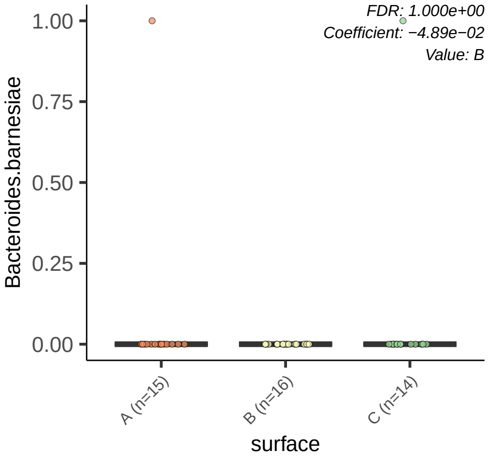
FDR: 1.000e+00
Coefficient: −4.89e−02
Value: B
1.00
0.75
0.50
0.25
0.00
Bacteroides.barnesiae
A (n=15)
B (n=16)
C (n=14)
surface

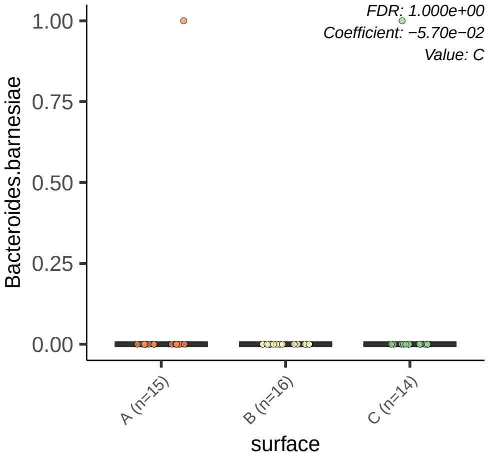
FDR: 1.000e+00
Coefficient: −5.70e−02
Value: C
Bacteroides.barnesiae
1.00
0.75
0.50
0.25
0.00
A (n=15)
B (n=16)
C (n=14)
surface

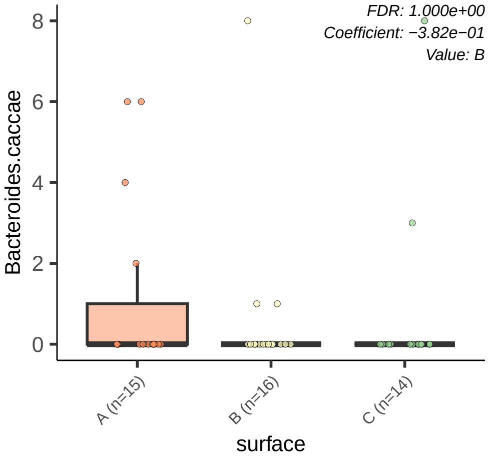

FDR: 1.000e+00
Coefficient: −3.82e−01
Value: B
Bacteroides.caccae
0
2
4
6
8
A (n=15)
B (n=16)
C (n=14)
surface

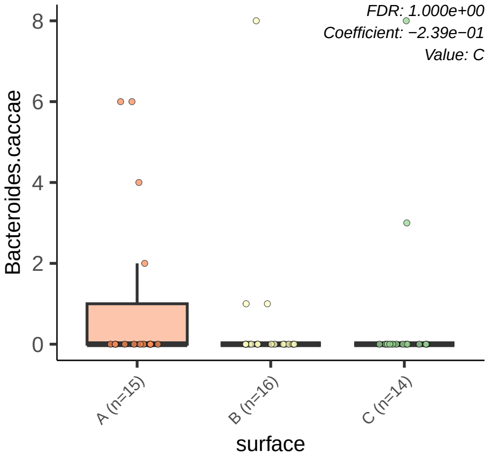

FDR: 1.000e+00
Coefficient: −2.39e−01
Value: C
Bacteroides.caccae
0
2
4
6
8
A (n=15)
B (n=16)
C (n=14)
surface

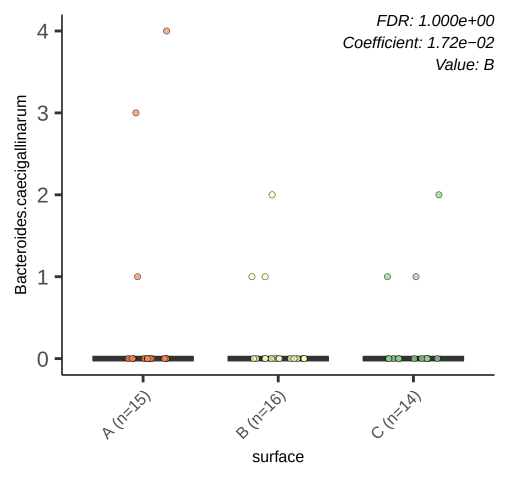

FDR: 1.000e+00
Coefficient: 1.72e−02
Value: B
Bacteroides.caecigallinarum
4
3
2
1
0
A (n=15)
B (n=16)
C (n=14)
surface

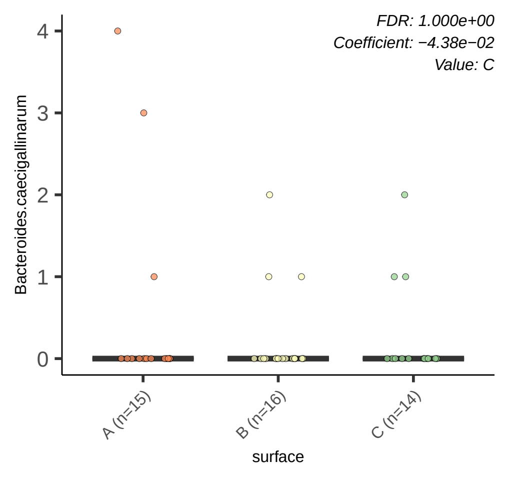
FDR: 1.000e+00
Coefficient: −4.38e−02
Value: C
Bacteroides.caecigallinarum
0
1
2
3
4
A (n=15)
B (n=16)
C (n=14)
surface

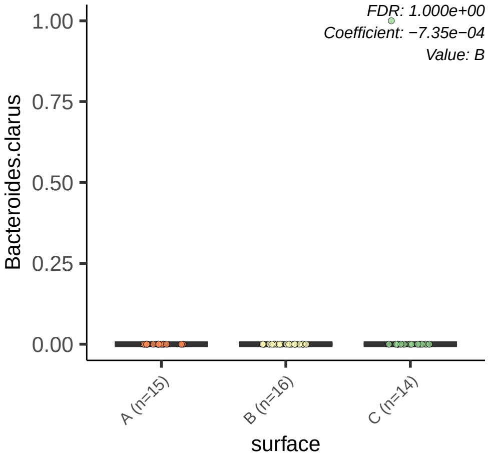
FDR: 1.000e+00
Coefficient: −7.35e−04
Value: B
Bacteroides.clarus
1.00
0.75
0.50
0.25
0.00
A (n=15)
B (n=16)
C (n=14)
surface

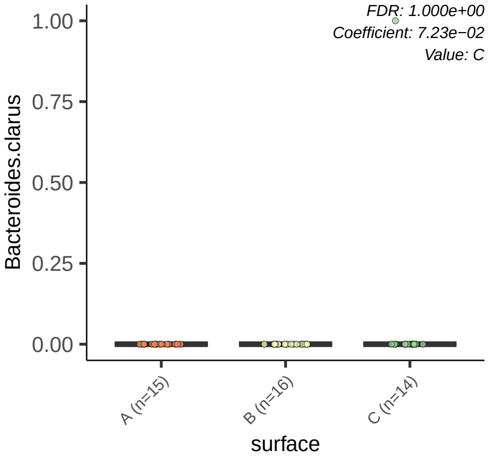
FDR: 1.000e+00
Coefficient: 7.23e−02
Value: C
Bacteroides.clarus
1.00
0.75
0.50
0.25
0.00
A (n=15)
B (n=16)
C (n=14)
surface

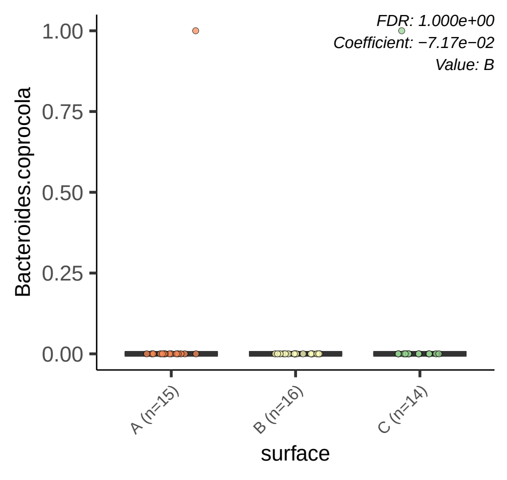
FDR: 1.000e+00
Coefficient: −7.17e−02
Value: B
Bacteroides.coprocola
1.00
0.75
0.50
0.25
0.00
A (n=15)
B (n=16)
C (n=14)
surface

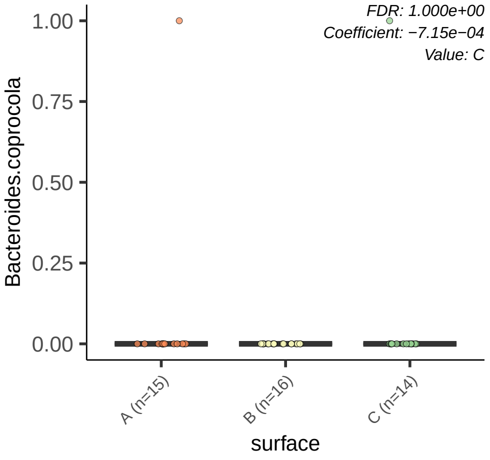
FDR: 1.000e+00
Coefficient: −7.15e−04
Value: C
Bacteroides.coprocola
1.00
0.75
0.50
0.25
0.00
A (n=15)
B (n=16)
C (n=14)
surface

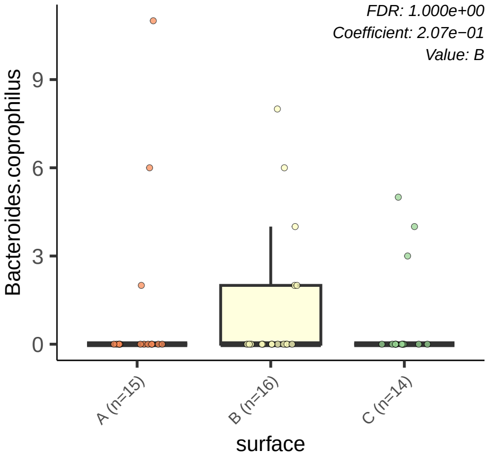

FDR: 1.000e+00
Coefficient: 2.07e−01
Value: B
Bacteroides.coprophilus
0
3
6
9
A (n=15)
B (n=16)
C (n=14)
surface

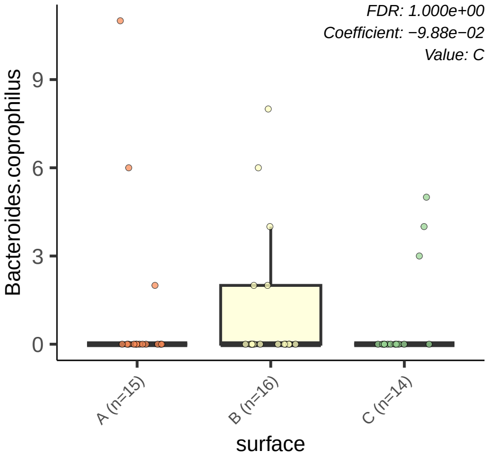
FDR: 1.000e+00
Coefficient: −9.88e−02
Value: C
Bacteroides.coprophilus
0
3
6
9
A (n=15)
B (n=16)
C (n=14)
surface

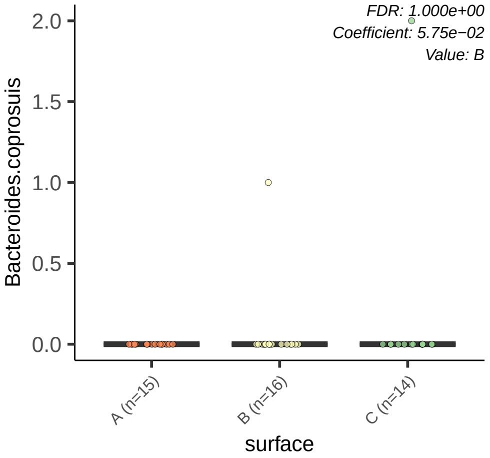

FDR: 1.000e+00
Coefficient: 5.75e−02
Value: B
Bacteroides.coprosuis
2.0
1.5
1.0
0.5
0.0
A (n=15)
B (n=16)
C (n=14)
surface

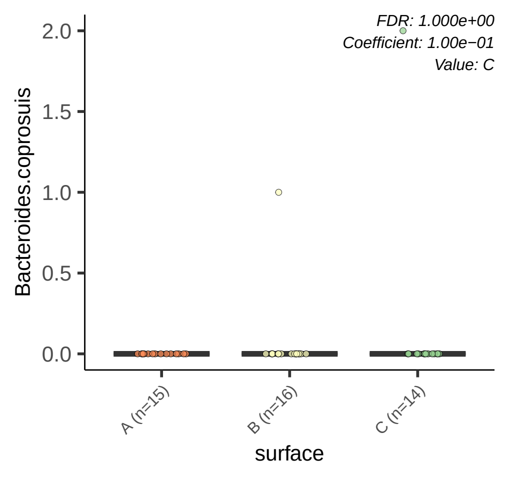
FDR: 1.000e+00
Coefficient: 1.00e−01
Value: C
Bacteroides.coprosuis
2.0
1.5
1.0
0.5
0.0
A (n=15)
B (n=16)
C (n=14)
surface

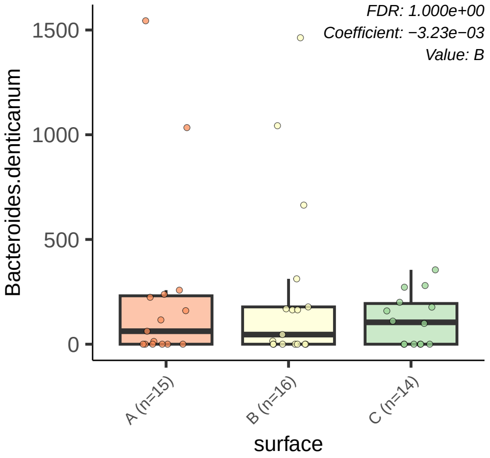
FDR: 1.000e+00
Coefficient: −3.23e−03
Value: B
Bacteroides.denticanum
1500
1000
500
0
A (n=15)
B (n=16)
C (n=14)
surface

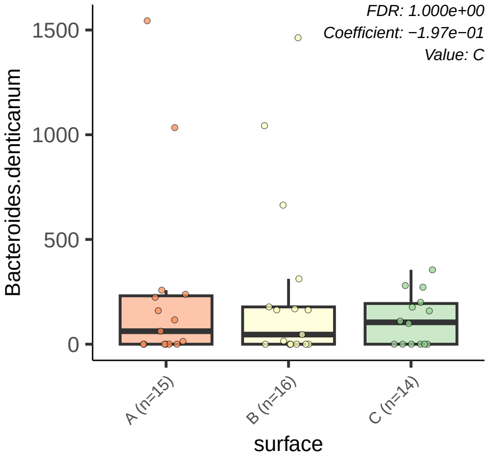
FDR: 1.000e+00
Coefficient: −1.97e−01
Value: C
Bacteroides.denticanum
1500
1000
500
0
A (n=15)
B (n=16)
C (n=14)
surface

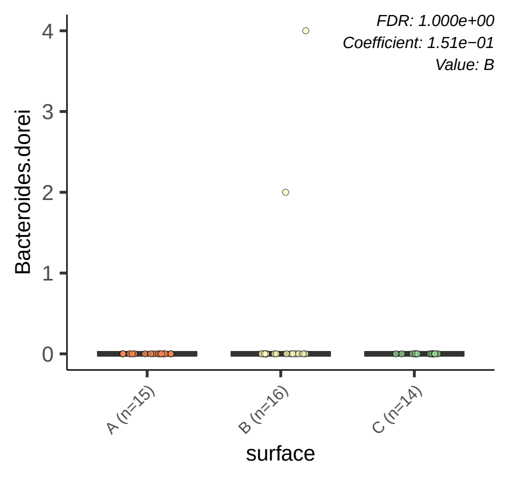
FDR: 1.000e+00
Coefficient: 1.51e−01
Value: B
Bacteroides.dorei
4
3
2
1
0
A (n=15)
B (n=16)
C (n=14)
surface

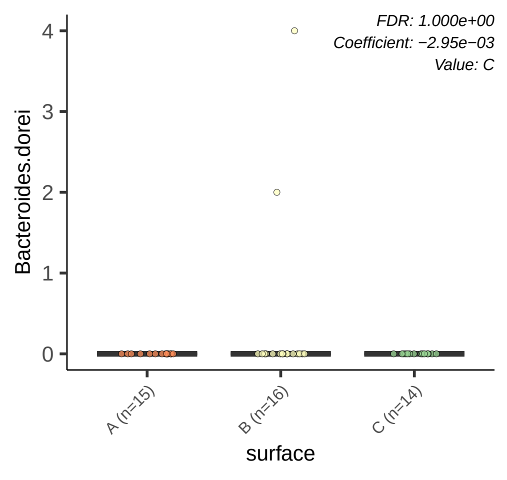
FDR: 1.000e+00
Coefficient: −2.95e−03
Value: C
Bacteroides.dorei
0
1
2
3
4
A (n=15)
B (n=16)
C (n=14)
surface

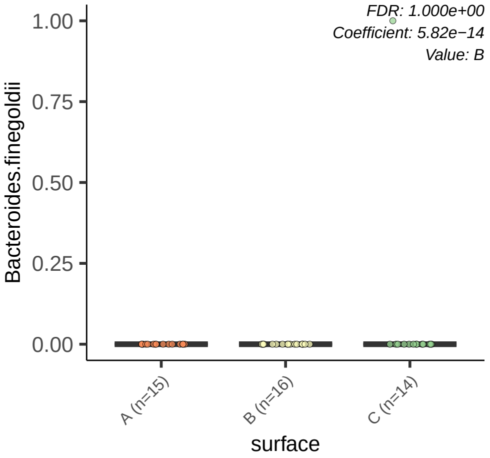

FDR: 1.000e+00
Coefficient: 5.82e−14
Value: B
Bacteroides.finegoldii
1.00
0.75
0.50
0.25
0.00
A (n=15)
B (n=16)
C (n=14)
surface

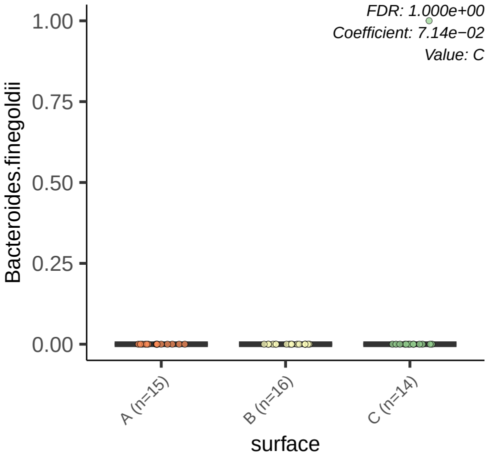
FDR: 1.000e+00
Coefficient: 7.14e−02
Value: C
Bacteroides.finegoldii
1.00
0.75
0.50
0.25
0.00
A (n=15)
B (n=16)
C (n=14)
surface

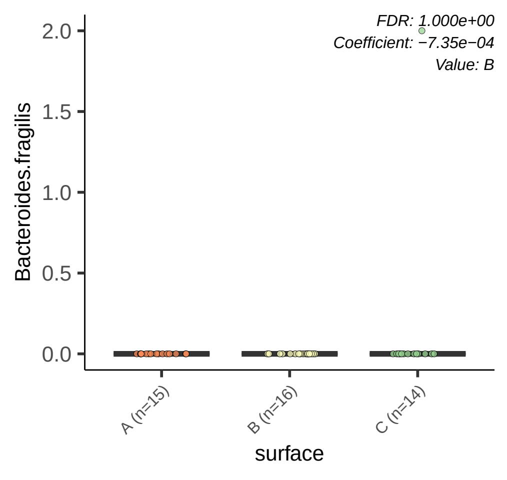
FDR: 1.000e+00
Coefficient: −7.35e−04
Value: B
Bacteroides.fragilis
2.0
1.5
1.0
0.5
0.0
A (n=15)
B (n=16)
C (n=14)
surface

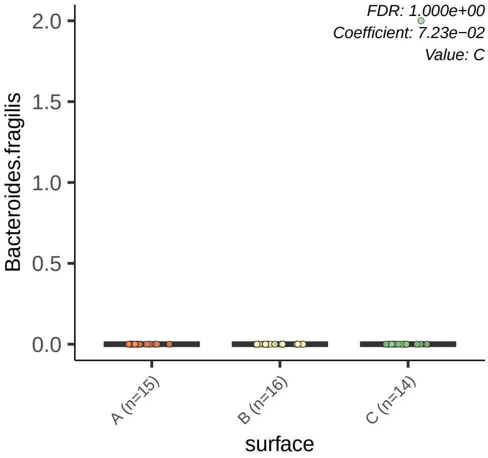

FDR: 1.000e+00
Coefficient: 7.23e−02
Value: C
Bacteroides.fragilis
2.0
1.5
1.0
0.5
0.0
A (n=15)
B (n=16)
C (n=14)
surface

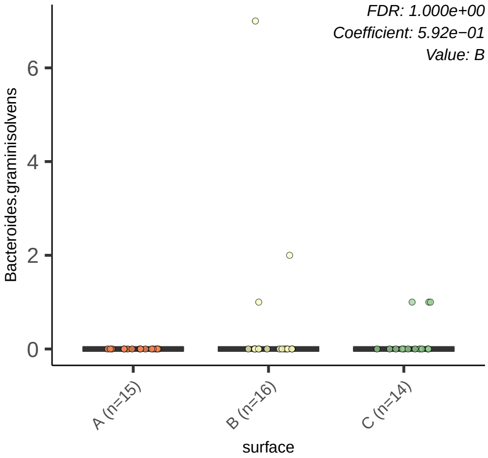

FDR: 1.000e+00
Coefficient: 5.92e−01
Value: B
Bacteroides.graminisolvens
0
2
4
6
A (n=15)
B (n=16)
C (n=14)
surface

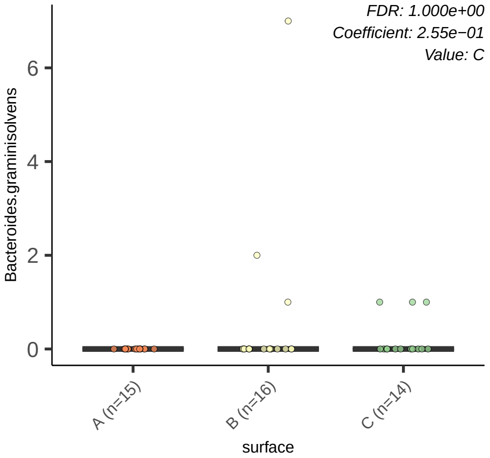
FDR: 1.000e+00
Coefficient: 2.55e−01
Value: C
Bacteroides.graminisolvens
0
2
4
6
A (n=15)
B (n=16)
C (n=14)
surface

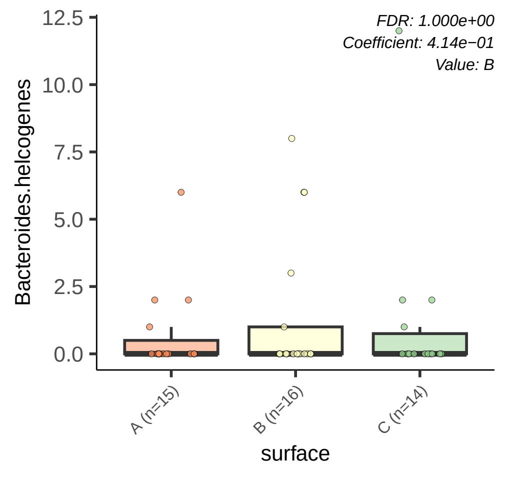
FDR: 1.000e+00
Coefficient: 4.14e−01
Value: B
Bacteroides.helcogenes
12.5
10.0
7.5
5.0
2.5
0.0
A (n=15)
B (n=16)
C (n=14)
surface

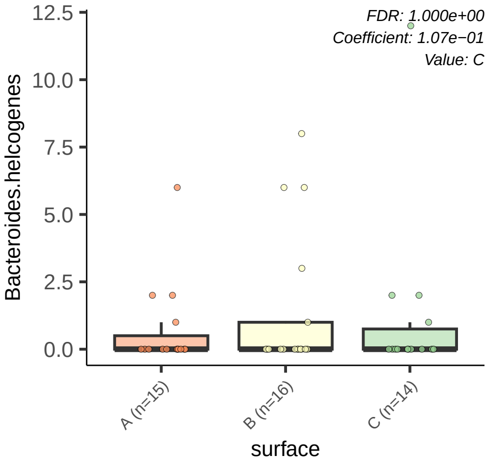
FDR: 1.000e+00
Coefficient: 1.07e−01
Value: C
12.5
10.0
7.5
5.0
2.5
0.0
Bacteroides.helcogenes
A (n=15)
B (n=16)
C (n=14)
surface

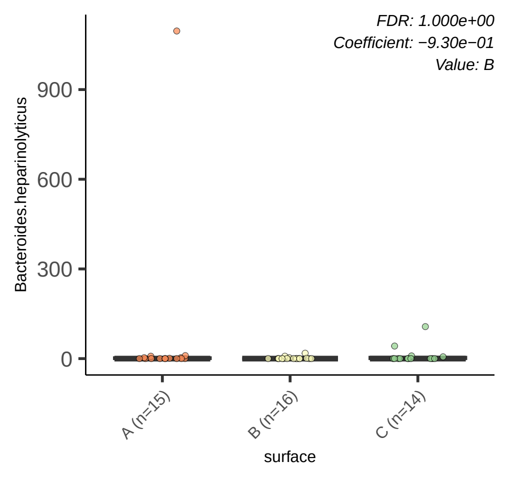
FDR: 1.000e+00
Coefficient: −9.30e−01
Value: B
Bacteroides.heparinolyticus
900
600
300
0
A (n=15)
B (n=16)
C (n=14)
surface

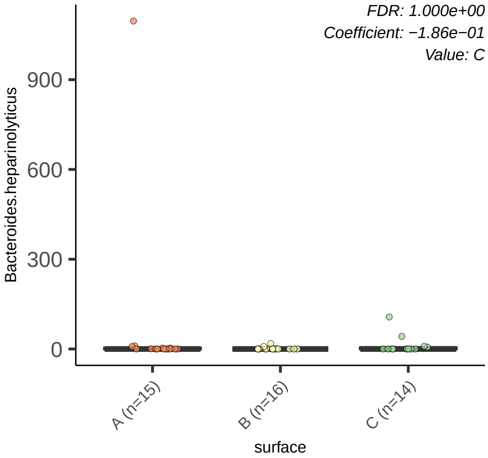

FDR: 1.000e+00
Coefficient: −1.86e−01
Value: C
900
600
300
0
Bacteroides.heparinolyticus
A (n=15)
B (n=16)
C (n=14)
surface

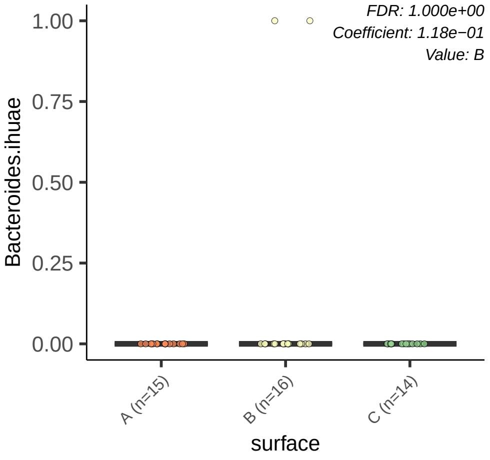
FDR: 1.000e+00
Coefficient: 1.18e−01
Value: B
Bacteroides.ihuae
1.00
0.75
0.50
0.25
0.00
A (n=15)
B (n=16)
C (n=14)
surface

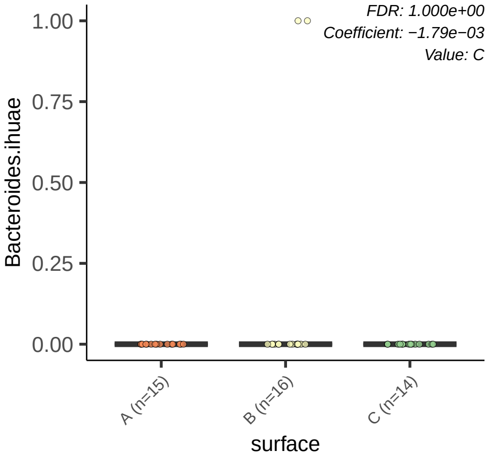
FDR: 1.000e+00
Coefficient: −1.79e−03
Value: C
Bacteroides.ihuae
1.00
0.75
0.50
0.25
0.00
A (n=15)
B (n=16)
C (n=14)
surface

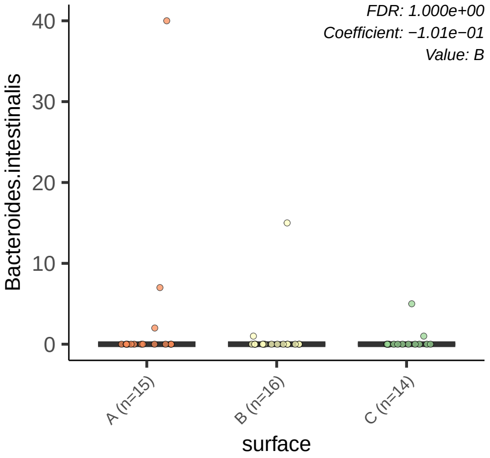
FDR: 1.000e+00
Coefficient: −1.01e−01
Value: B
Bacteroides.intestinalis
40
30
20
10
0
A (n=15)
B (n=16)
C (n=14)
surface

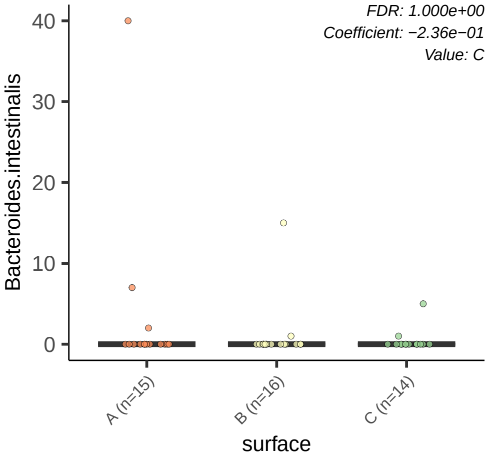
FDR: 1.000e+00
Coefficient: −2.36e−01
Value: C
Bacteroides.intestinalis
40
30
20
10
0
A (n=15)
B (n=16)
C (n=14)
surface

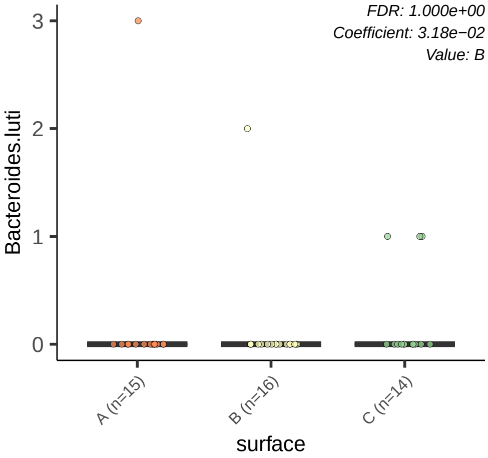

FDR: 1.000e+00
Coefficient: 3.18e−02
Value: B
Bacteroides.luti
3
2
1
0
A (n=15)
B (n=16)
C (n=14)
surface

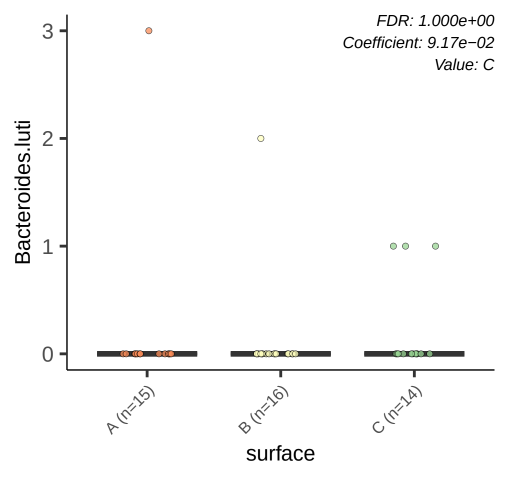

FDR: 1.000e+00
Coefficient: 9.17e−02
Value: C
Bacteroides.luti
3
2
1
0
A (n=15)
B (n=16)
C (n=14)
surface

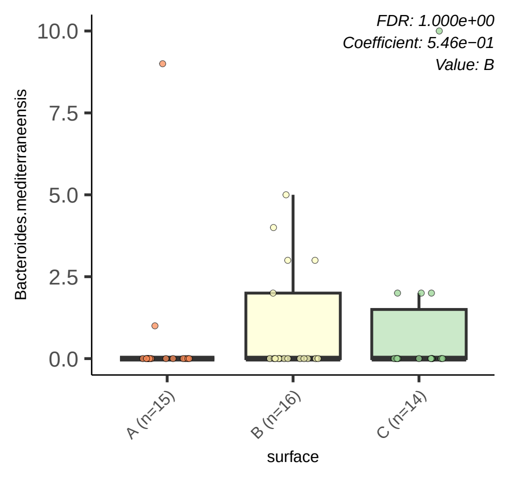
FDR: 1.000e+00
Coefficient: 5.46e−01
Value: B
Bacteroides.mediterraneensis
10.0
7.5
5.0
2.5
0.0
A (n=15)
B (n=16)
C (n=14)
surface

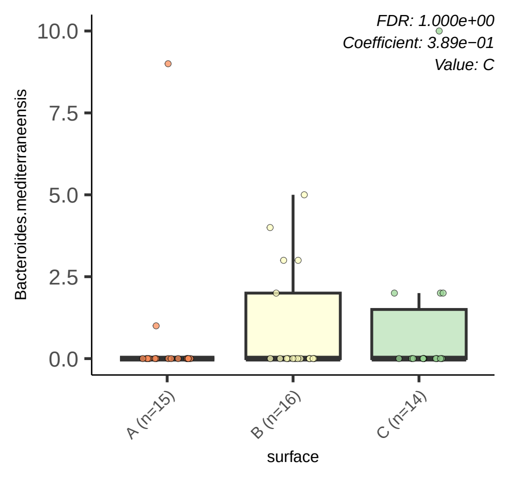
FDR: 1.000e+00
Coefficient: 3.89e−01
Value: C
Bacteroides.mediterraneensis
10.0
7.5
5.0
2.5
0.0
A (n=15)
B (n=16)
C (n=14)
surface

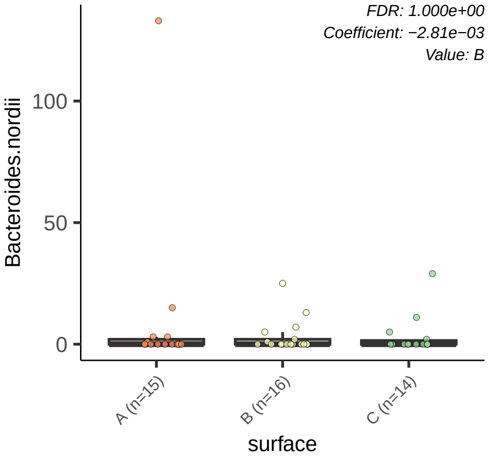
FDR: 1.000e+00
Coefficient: −2.81e−03
Value: B
Bacteroides.nordii
100
50
0
A (n=15)
B (n=16)
C (n=14)
surface

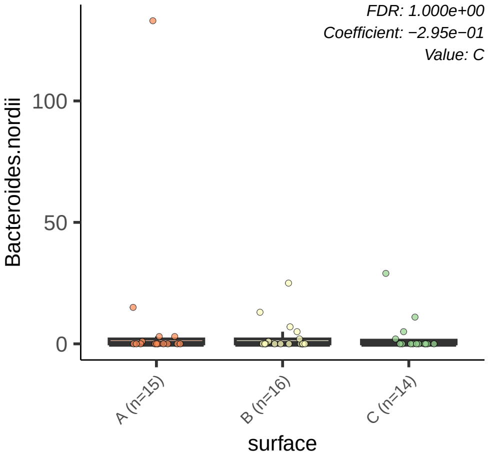

FDR: 1.000e+00
Coefficient: −2.95e−01
Value: C
Bacteroides.nordii
100
50
0
A (n=15)
B (n=16)
C (n=14)
surface

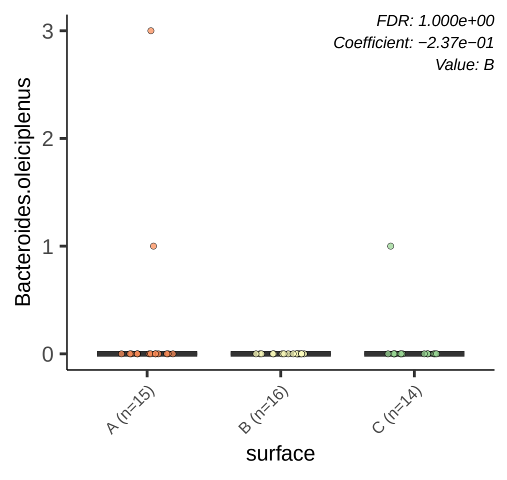
FDR: 1.000e+00
Coefficient: −2.37e−01
Value: B
Bacteroides.oleiciplenus
3
2
1
0
A (n=15)
B (n=16)
C (n=14)
surface

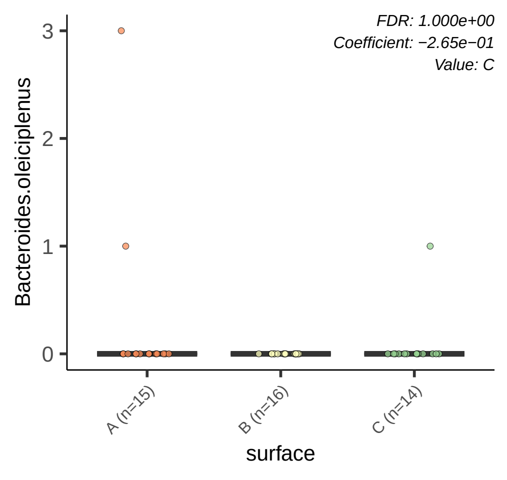

FDR: 1.000e+00
Coefficient: −2.65e−01
Value: C
Bacteroides.oleiciplenus
3
2
1
0
A (n=15)
B (n=16)
C (n=14)
surface

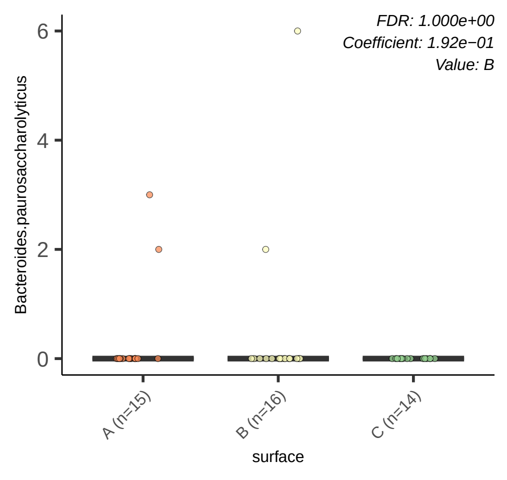
FDR: 1.000e+00
Coefficient: 1.92e−01
Value: B
Bacteroides.paurosaccharolyticus
6
4
2
0
A (n=15)
B (n=16)
C (n=14)
surface

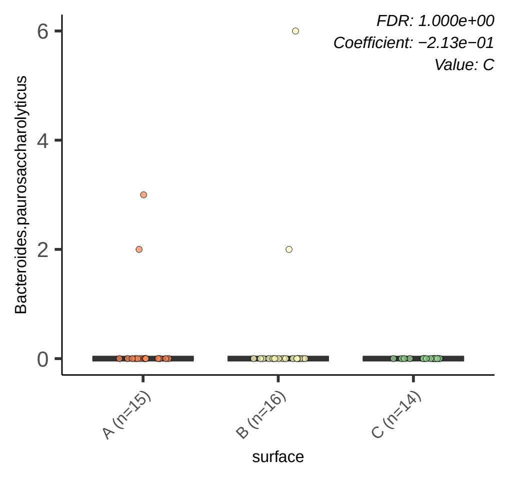
FDR: 1.000e+00
Coefficient: −2.13e−01
Value: C
Bacteroides.paurosaccharolyticus
6
4
2
0
A (n=15)
B (n=16)
C (n=14)
surface

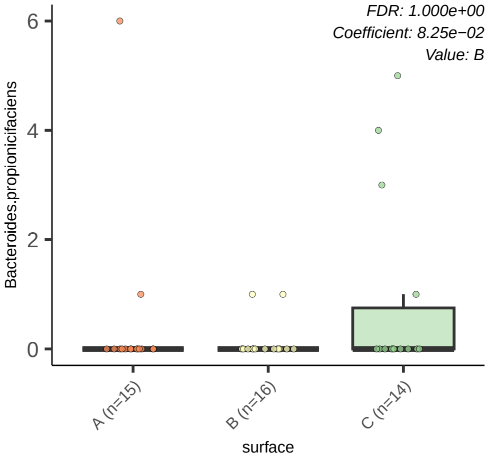
FDR: 1.000e+00
Coefficient: 8.25e−02
Value: B
Bacteroides.propionicifaciens
6
4
2
0
A (n=15)
B (n=16)
C (n=14)
surface

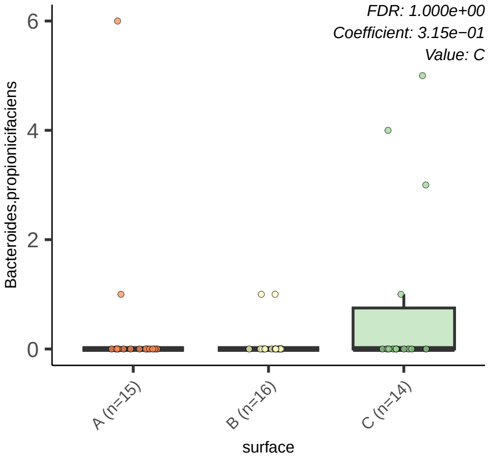

FDR: 1.000e+00
Coefficient: 3.15e−01
Value: C
6
4
2
0
Bacteroides.propionicifaciens
A (n=15)
B (n=16)
C (n=14)
surface

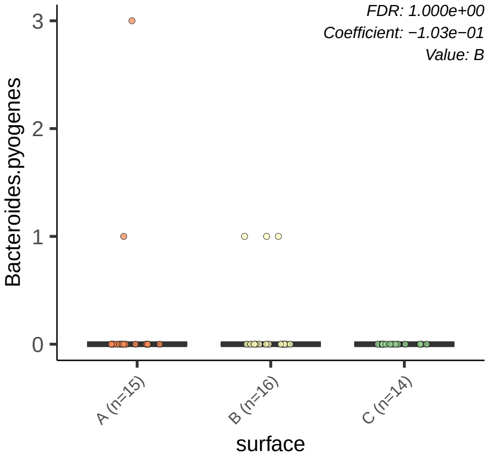

FDR: 1.000e+00
Coefficient: −1.03e−01
Value: B
Bacteroides.pyogenes
0
1
2
3
A (n=15)
B (n=16)
C (n=14)
surface

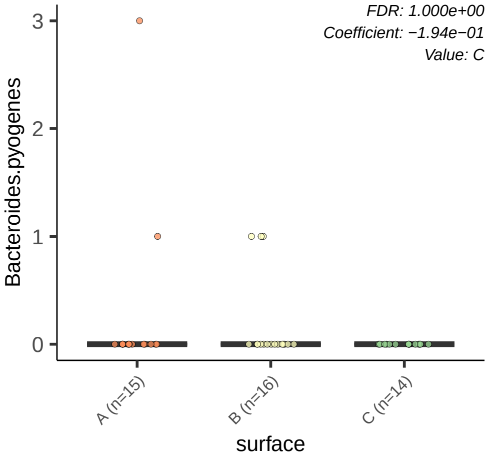
FDR: 1.000e+00
Coefficient: −1.94e−01
Value: C
Bacteroides.pyogenes
3
2
1
0
A (n=15)
B (n=16)
C (n=14)
surface

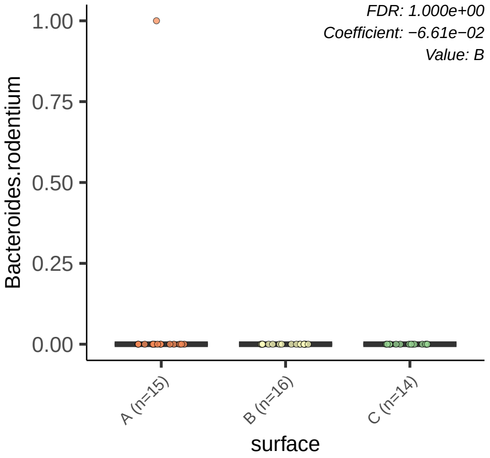

FDR: 1.000e+00
Coefficient: −6.61e−02
Value: B
Bacteroides.rodentium
1.00
0.75
0.50
0.25
0.00
A (n=15)
B (n=16)
C (n=14)
surface

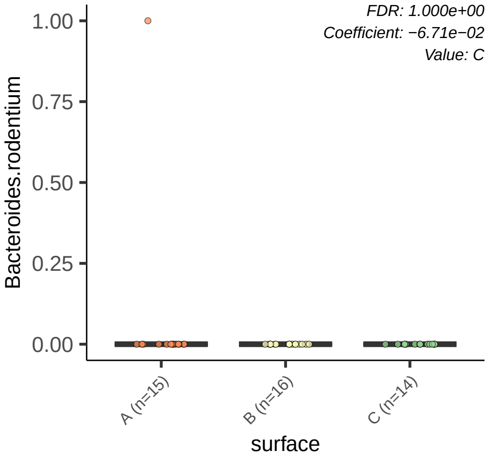

FDR: 1.000e+00
Coefficient: −6.71e−02
Value: C
Bacteroides.rodentium
1.00
0.75
0.50
0.25
0.00
A (n=15)
B (n=16)
C (n=14)
surface

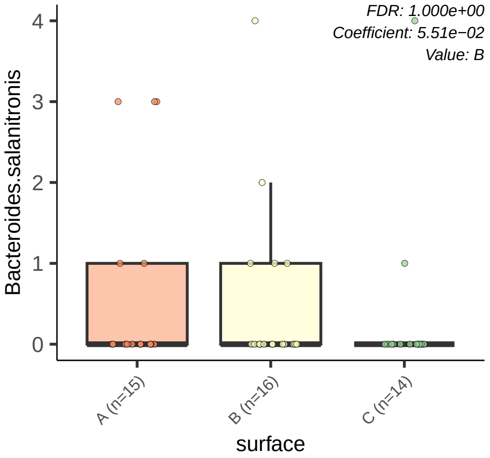

FDR: 1.000e+00
Coefficient: 5.51e−02
Value: B
Bacteroides.salanitronis
0
1
2
3
4
A (n=15)
B (n=16)
C (n=14)
surface

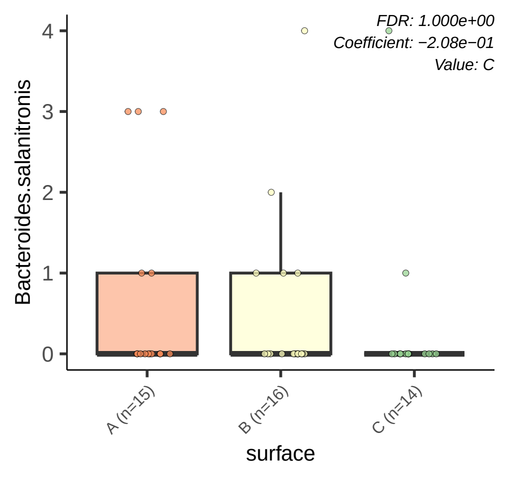
FDR: 1.000e+00
Coefficient: −2.08e−01
Value: C
Bacteroides.salanitronis
0
1
2
3
4
A (n=15)
B (n=16)
C (n=14)
surface

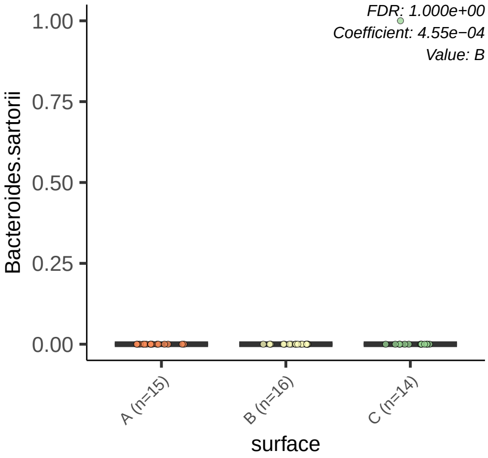
FDR: 1.000e+00
Coefficient: 4.55e−04
Value: B
Bacteroides.sartorii
1.00
0.75
0.50
0.25
0.00
A (n=15)
B (n=16)
C (n=14)
surface

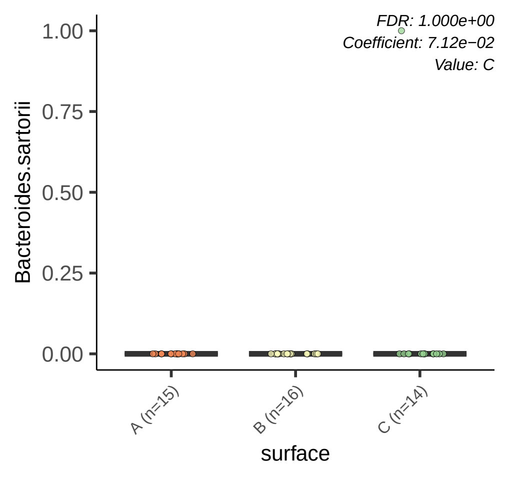
FDR: 1.000e+00
Coefficient: 7.12e−02
Value: C
Bacteroides.sartorii
1.00
0.75
0.50
0.25
0.00
A (n=15)
B (n=16)
C (n=14)
surface

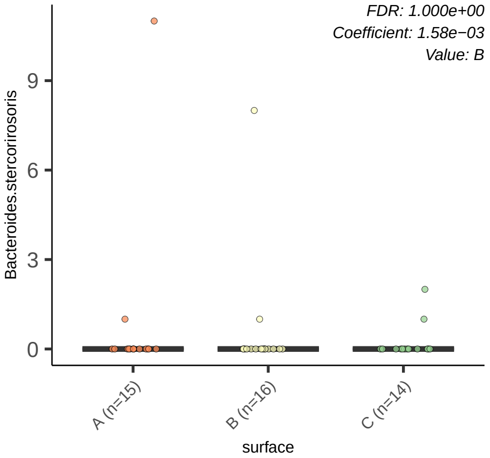

FDR: 1.000e+00
Coefficient: 1.58e−03
Value: B
Bacteroides.stercorirosoris
0
3
6
9
A (n=15)
B (n=16)
C (n=14)
surface

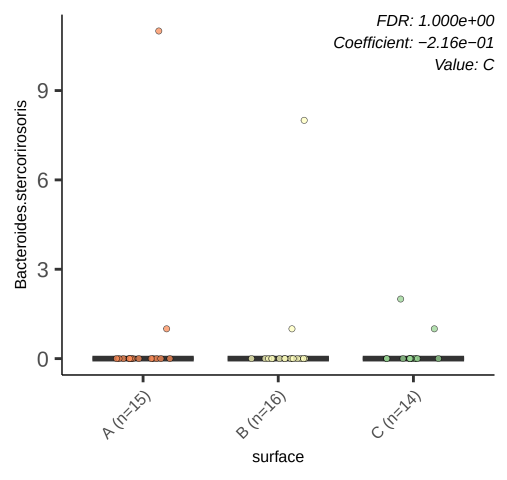

FDR: 1.000e+00
Coefficient: −2.16e−01
Value: C
Bacteroides.stercorirosoris
0
3
6
9
A (n=15)
B (n=16)
C (n=14)
surface

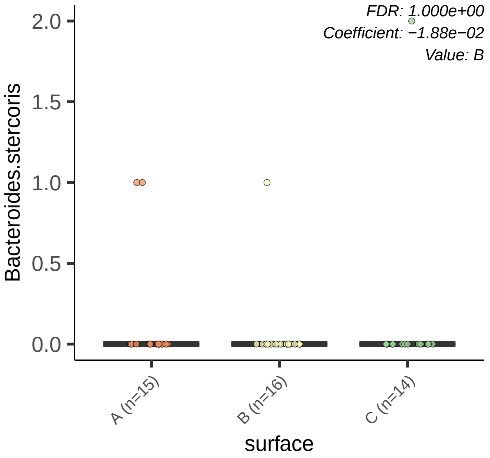

FDR: 1.000e+00
Coefficient: −1.88e−02
Value: B
Bacteroides.stercoris
2.0
1.5
1.0
0.5
0.0
A (n=15)
B (n=16)
C (n=14)
surface

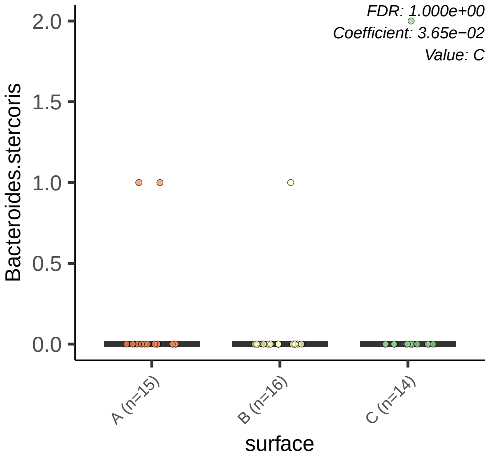
FDR: 1.000e+00
Coefficient: 3.65e−02
Value: C
Bacteroides.stercoris
2.0
1.5
1.0
0.5
0.0
A (n=15)
B (n=16)
C (n=14)
surface

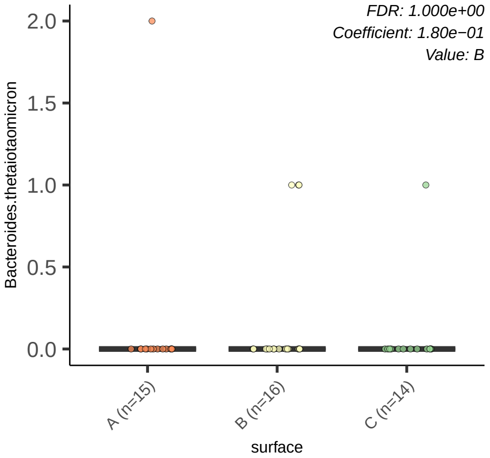
FDR: 1.000e+00
Coefficient: 1.80e−01
Value: B
Bacteroides.thetaiotaomicron
2.0
1.5
1.0
0.5
0.0
A (n=15)
B (n=16)
C (n=14)
surface

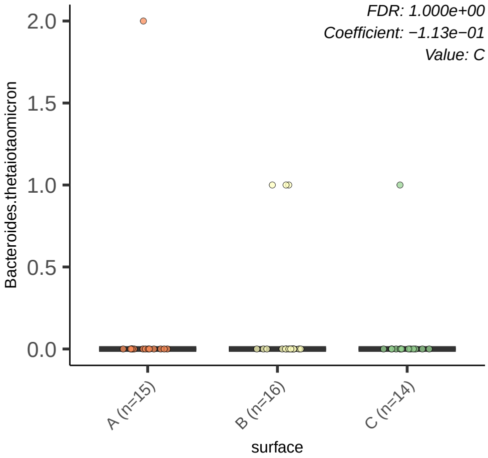
FDR: 1.000e+00
Coefficient: −1.13e−01
Value: C
2.0
1.5
1.0
0.5
0.0
Bacteroides.thetaiotaomicron
A (n=15)
B (n=16)
C (n=14)
surface

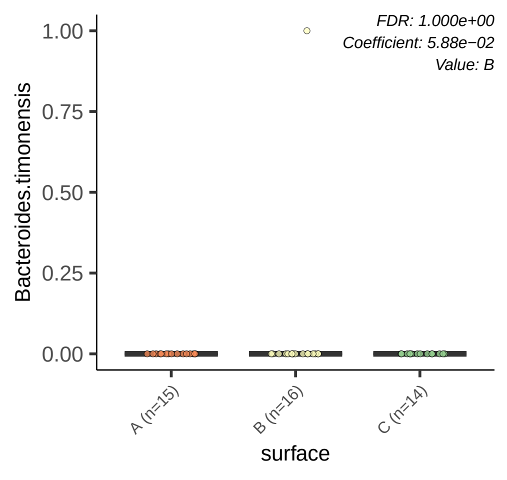
FDR: 1.000e+00
Coefficient: 5.88e−02
Value: B
Bacteroides.timonensis
1.00
0.75
0.50
0.25
0.00
A (n=15)
B (n=16)
C (n=14)
surface

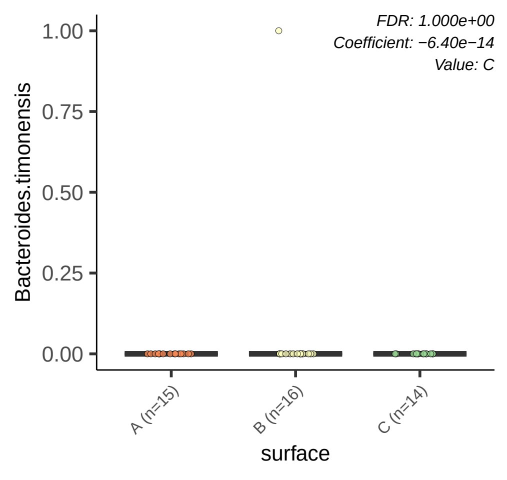

FDR: 1.000e+00
Coefficient: −6.40e−14
Value: C
Bacteroides.timonensis
1.00
0.75
0.50
0.25
0.00
A (n=15)
B (n=16)
C (n=14)
surface

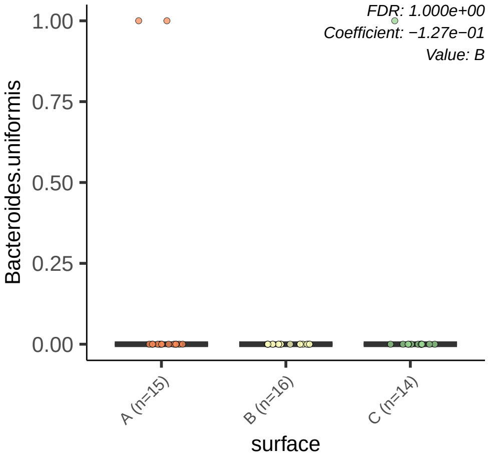
FDR: 1.000e+00
Coefficient: −1.27e−01
Value: B
Bacteroides.uniformis
1.00
0.75
0.50
0.25
0.00
A (n=15)
B (n=16)
C (n=14)
surface

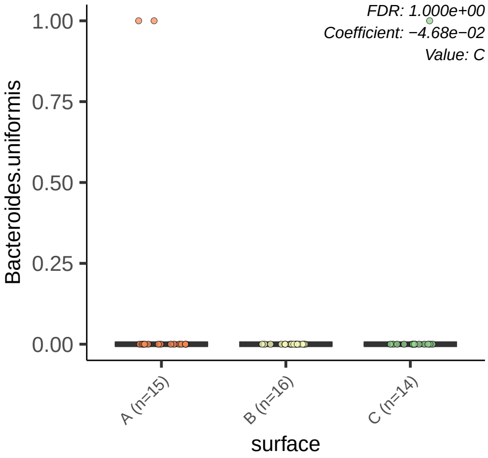
FDR: 1.000e+00
Coefficient: −4.68e−02
Value: C
1.00
0.75
0.50
0.25
0.00
Bacteroides.uniformis
A (n=15)
B (n=16)
C (n=14)
surface

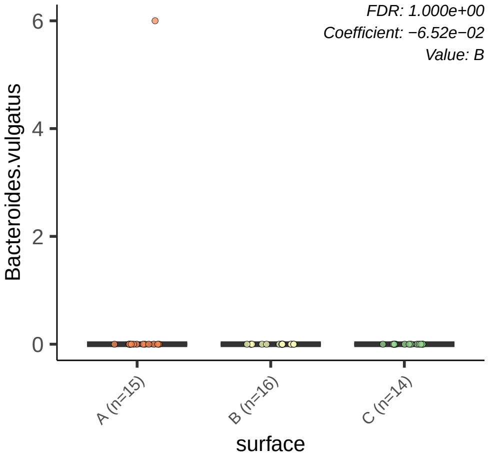
FDR: 1.000e+00
Coefficient: −6.52e−02
Value: B
Bacteroides.vulgatus
6
4
2
0
A (n=15)
B (n=16)
C (n=14)
surface

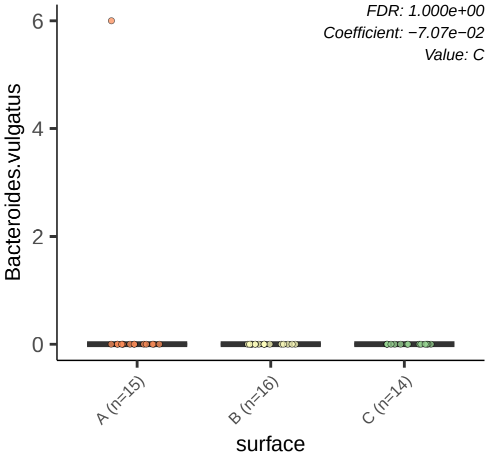
FDR: 1.000e+00
Coefficient: −7.07e−02
Value: C
Bacteroides.vulgatus
0
2
4
6
A (n=15)
B (n=16)
C (n=14)
surface

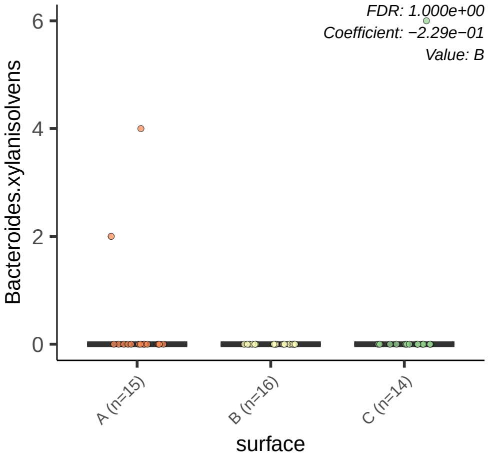
FDR: 1.000e+00
Coefficient: −2.29e−01
Value: B
Bacteroides.xylanisolvens
6
4
2
0
A (n=15)
B (n=16)
C (n=14)
surface

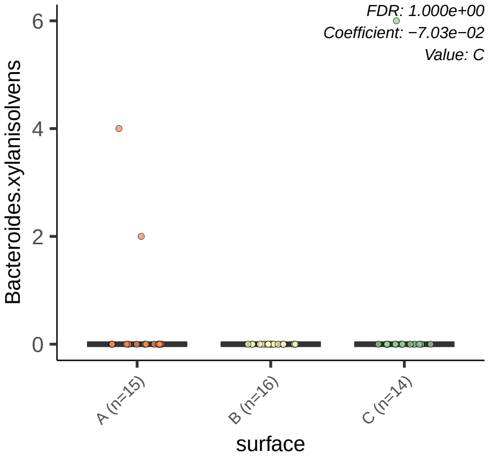
FDR: 1.000e+00
Coefficient: −7.03e−02
Value: C
Bacteroides.xylanisolvens
6
4
2
0
A (n=15)
B (n=16)
C (n=14)
surface

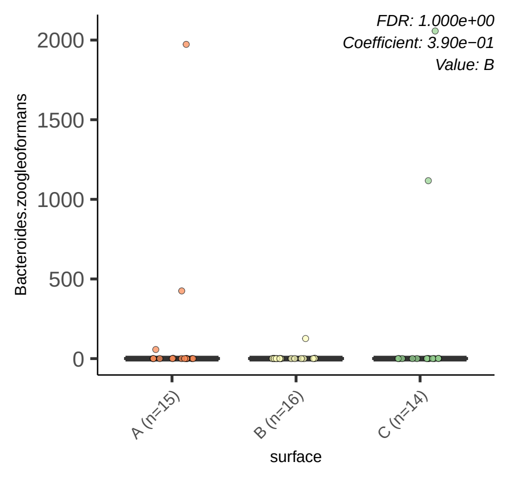
FDR: 1.000e+00
Coefficient: 3.90e−01
Value: B
Bacteroides.zoogleoformans
2000
1500
1000
500
0
A (n=15)
B (n=16)
C (n=14)
surface

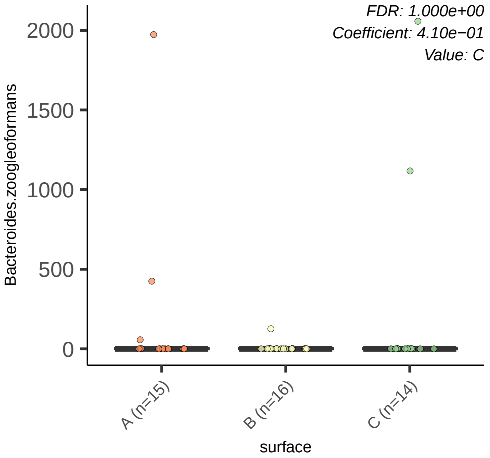

FDR: 1.000e+00
Coefficient: 4.10e−01
Value: C
2000
1500
1000
500
0
Bacteroides.zoogleoformans
A (n=15)
B (n=16)
C (n=14)
surface

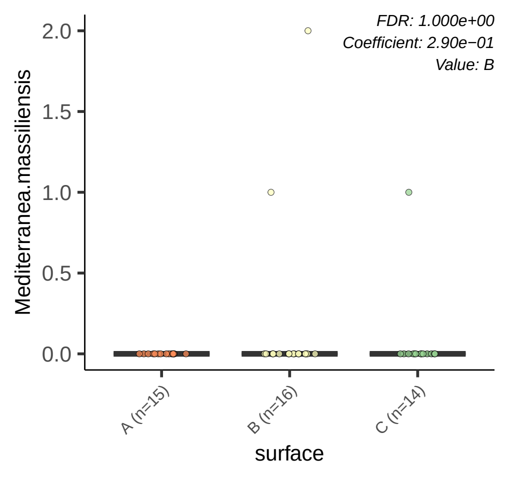

FDR: 1.000e+00
Coefficient: 2.90e−01
Value: B
Mediterranea.massiliensis
2.0
1.5
1.0
0.5
0.0
A (n=15)
B (n=16)
C (n=14)
surface

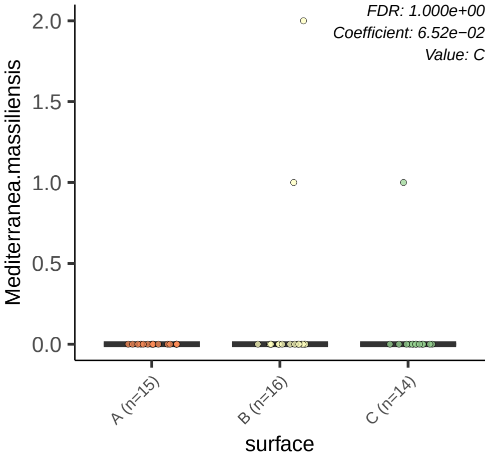
FDR: 1.000e+00
Coefficient: 6.52e−02
Value: C
Mediterranea.massiliensis
2.0
1.5
1.0
0.5
0.0
A (n=15)
B (n=16)
C (n=14)
surface

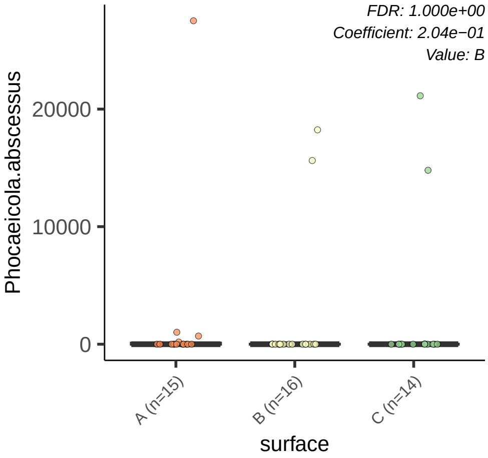

FDR: 1.000e+00
Coefficient: 2.04e−01
Value: B
Phocaeicola.abscessus
20000
10000
0
A (n=15)
B (n=16)
C (n=14)
surface

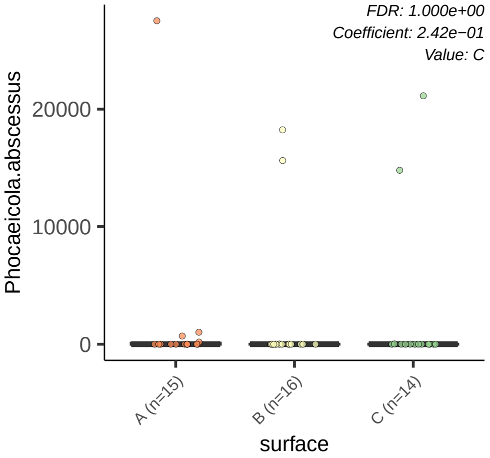
FDR: 1.000e+00
Coefficient: 2.42e−01
Value: C
Phocaeicola.abscessus
20000
10000
0
A (n=15)
B (n=16)
C (n=14)
surface

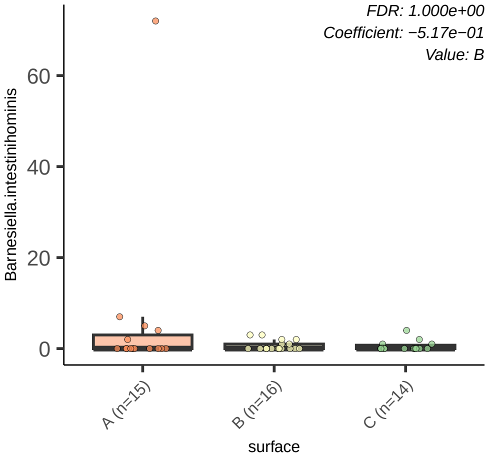
FDR: 1.000e+00
Coefficient: −5.17e−01
Value: B
Barnesiella.intestinihominis
0
20
40
60
A (n=15)
B (n=16)
C (n=14)
surface

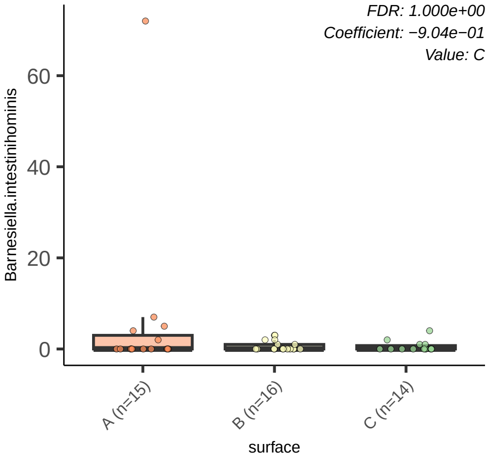
FDR: 1.000e+00
Coefficient: −9.04e−01
Value: C
Barnesiella.intestinihominis
0
20
40
60
A (n=15)
B (n=16)
C (n=14)
surface

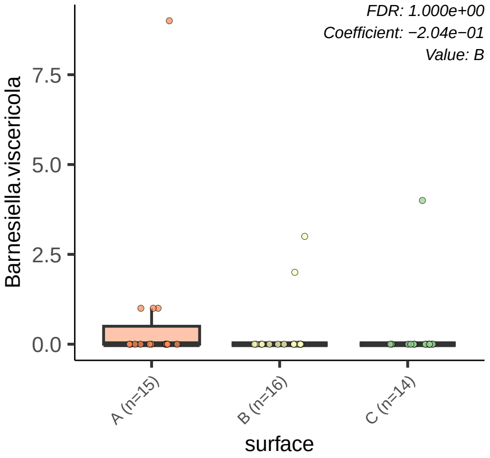
FDR: 1.000e+00
Coefficient: −2.04e−01
Value: B
Barnesiella.viscericola
7.5
5.0
2.5
0.0
A (n=15)
B (n=16)
C (n=14)
surface

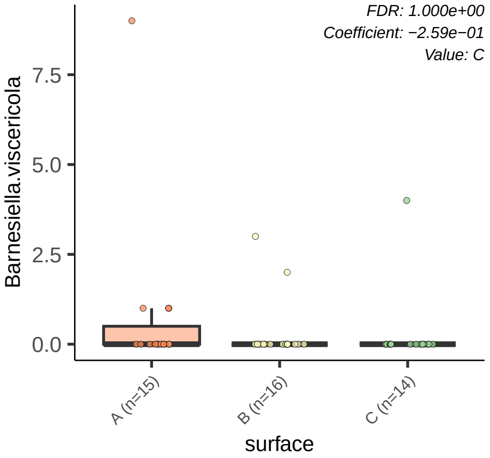
FDR: 1.000e+00
Coefficient: −2.59e−01
Value: C
Barnesiella.viscericola
7.5
5.0
2.5
0.0
A (n=15)
B (n=16)
C (n=14)
surface

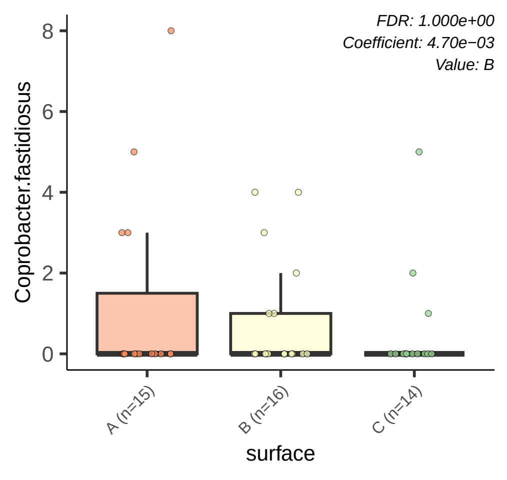
FDR: 1.000e+00
Coefficient: 4.70e−03
Value: B
Coprobacter.fastidiosus
0
2
4
6
8
A (n=15)
B (n=16)
C (n=14)
surface

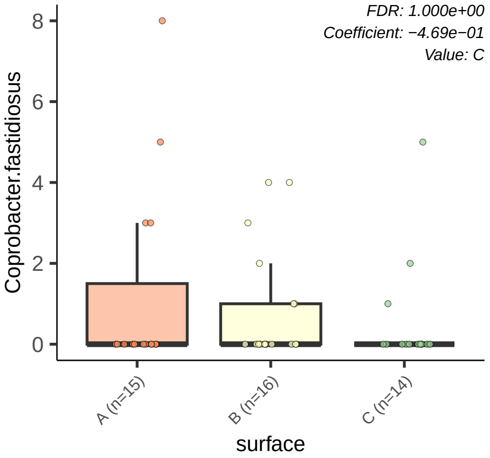

FDR: 1.000e+00
Coefficient: −4.69e−01
Value: C
Coprobacter.fastidiosus
8
6
4
2
0
A (n=15)
B (n=16)
C (n=14)
surface

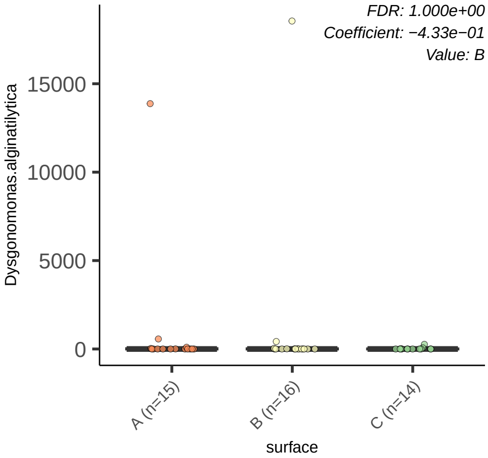

FDR: 1.000e+00
Coefficient: −4.33e−01
Value: B
Dysgonomonas.alginatilytica
15000
10000
5000
0
A (n=15)
B (n=16)
C (n=14)
surface

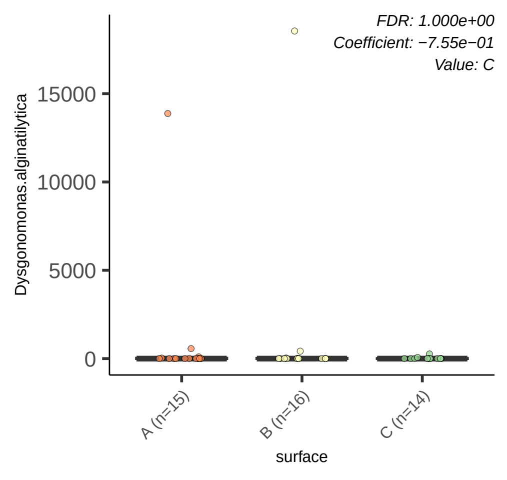

FDR: 1.000e+00
Coefficient: −7.55e−01
Value: C
Dysgonomonas.alginatilytica
15000
10000
5000
0
A (n=15)
B (n=16)
C (n=14)
surface

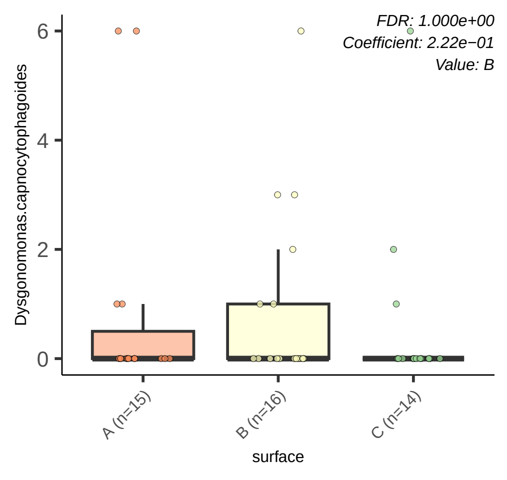
FDR: 1.000e+00
Coefficient: 2.22e−01
Value: B
Dysgonomonas.capnocytophagoides
0
2
4
6
A (n=15)
B (n=16)
C (n=14)
surface

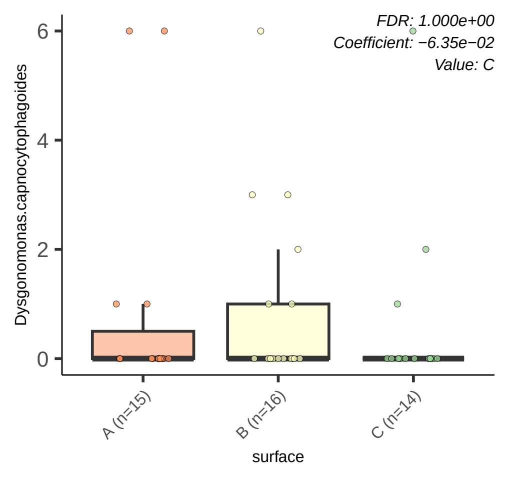

FDR: 1.000e+00
Coefficient: −6.35e−02
Value: C
Dysgonomonas.capnocytophagoides
6
4
2
0
A (n=15)
B (n=16)
C (n=14)
surface

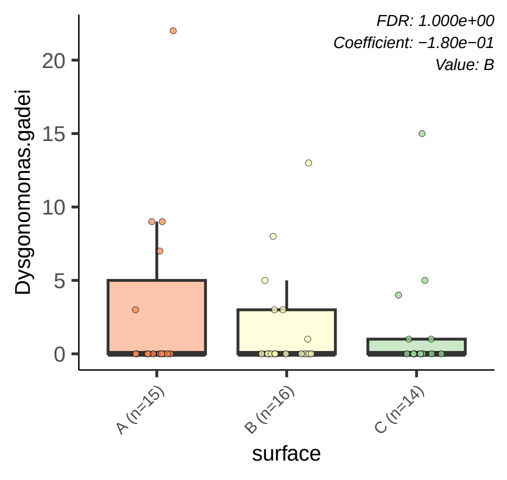

FDR: 1.000e+00
Coefficient: −1.80e−01
Value: B
Dysgonomonas.gadei
0
5
10
15
20
A (n=15)
B (n=16)
C (n=14)
surface

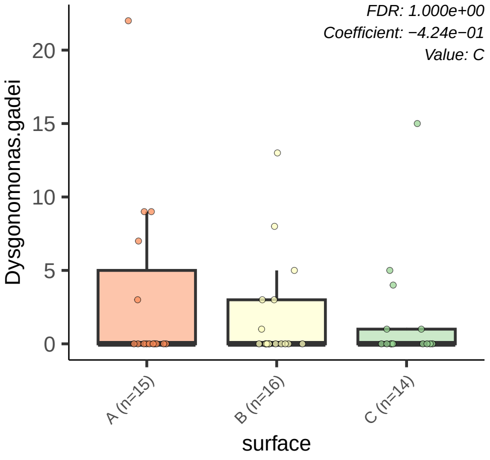
FDR: 1.000e+00
Coefficient: −4.24e−01
Value: C
Dysgonomonas.gadei
0
5
10
15
20
A (n=15)
B (n=16)
C (n=14)
surface

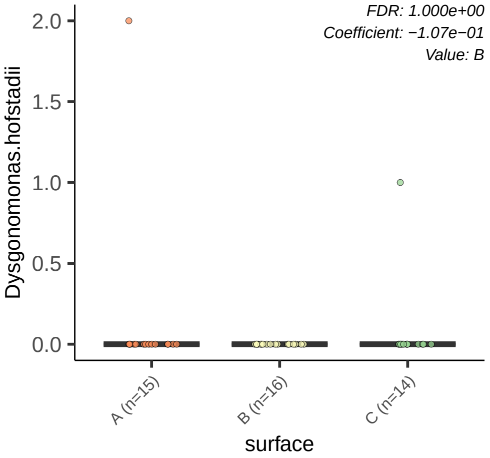

FDR: 1.000e+00
Coefficient: −1.07e−01
Value: B
Dysgonomonas.hofstadii
2.0
1.5
1.0
0.5
0.0
A (n=15)
B (n=16)
C (n=14)
surface

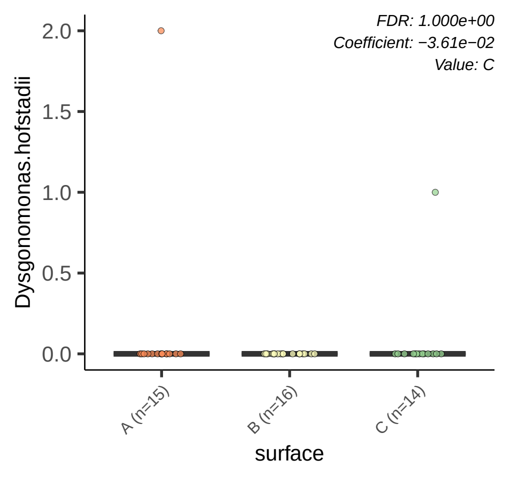
FDR: 1.000e+00
Coefficient: −3.61e−02
Value: C
Dysgonomonas.hofstadii
2.0
1.5
1.0
0.5
0.0
A (n=15)
B (n=16)
C (n=14)
surface

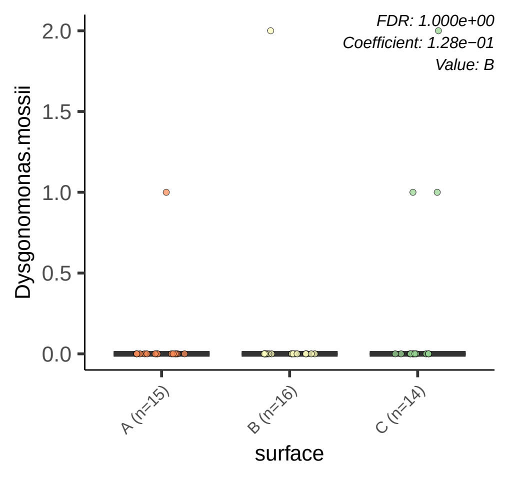
FDR: 1.000e+00
Coefficient: 1.28e−01
Value: B
Dysgonomonas.mossii
2.0
1.5
1.0
0.5
0.0
A (n=15)
B (n=16)
C (n=14)
surface

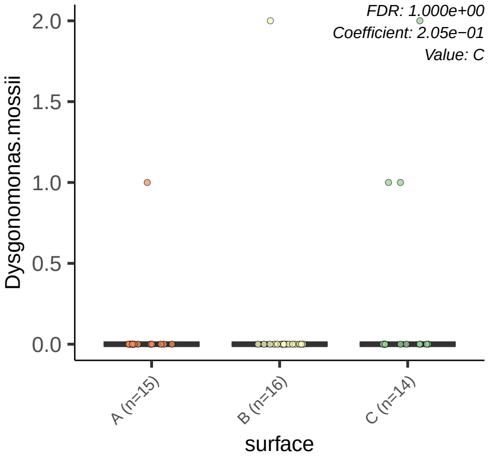

FDR: 1.000e+00
Coefficient: 2.05e−01
Value: C
Dysgonomonas.mossii
2.0
1.5
1.0
0.5
0.0
A (n=15)
B (n=16)
C (n=14)
surface

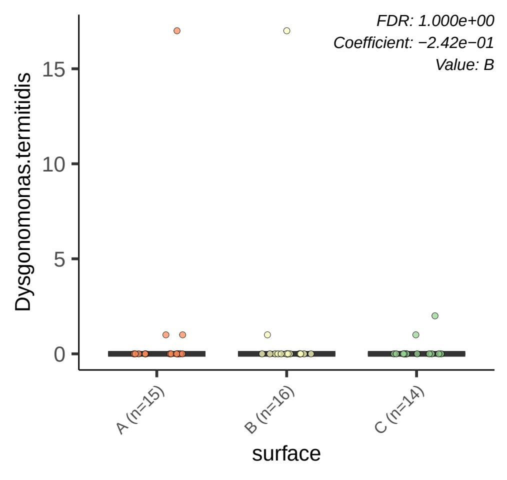
FDR: 1.000e+00
Coefficient: −2.42e−01
Value: B
Dysgonomonas.termitidis
0
5
10
15
A (n=15)
B (n=16)
C (n=14)
surface

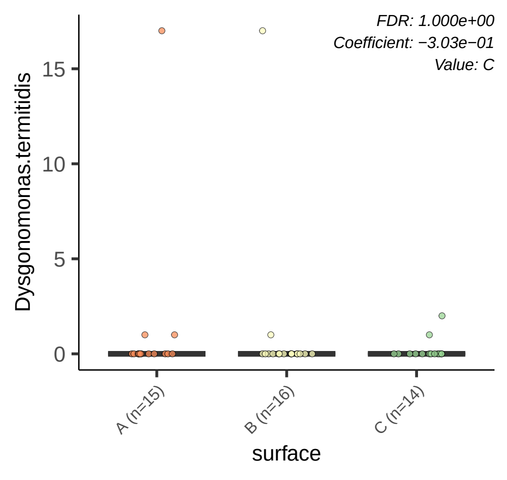

FDR: 1.000e+00
Coefficient: −3.03e−01
Value: C
Dysgonomonas.termitidis
0
5
10
15
A (n=15)
B (n=16)
C (n=14)
surface

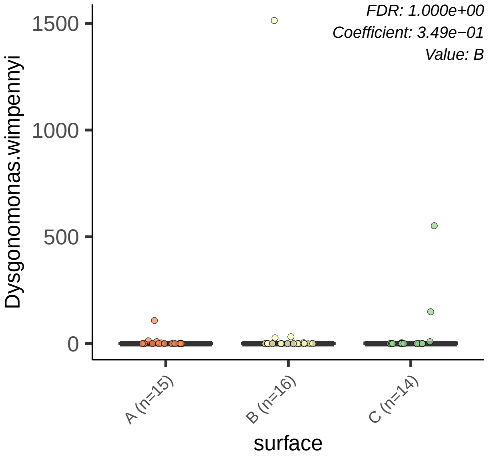
FDR: 1.000e+00
Coefficient: 3.49e−01
Value: B
Dysgonomonas.wimpennyi
1500
1000
500
0
A (n=15)
B (n=16)
C (n=14)
surface

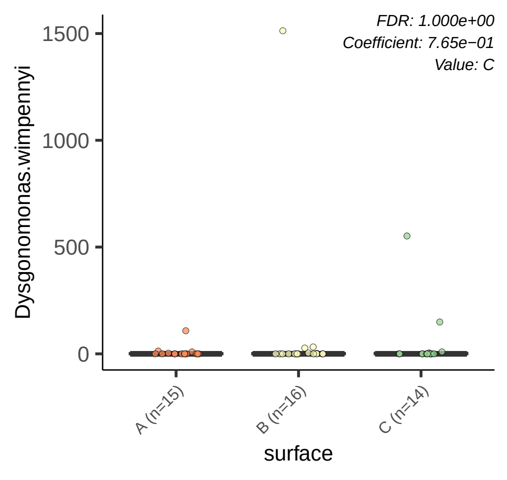

FDR: 1.000e+00
Coefficient: 7.65e−01
Value: C
Dysgonomonas.wimpennyi
1500
1000
500
0
A (n=15)
B (n=16)
C (n=14)
surface

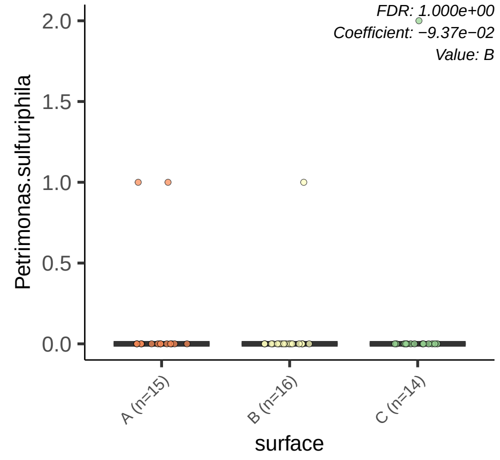
FDR: 1.000e+00
Coefficient: −9.37e−02
Value: B
Petrimonas.sulfuriphila
2.0
1.5
1.0
0.5
0.0
A (n=15)
B (n=16)
C (n=14)
surface

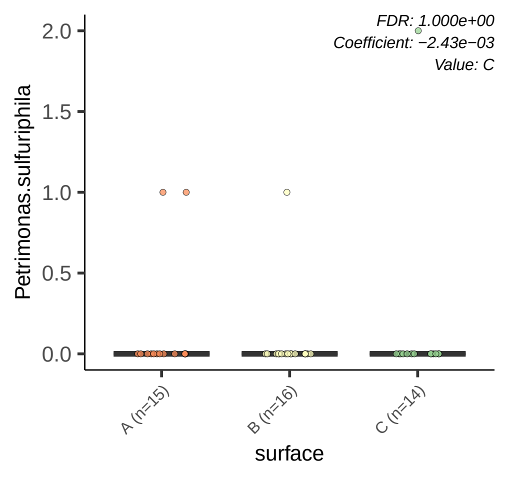
FDR: 1.000e+00
Coefficient: −2.43e−03
Value: C
Petrimonas.sulfuriphila
2.0
1.5
1.0
0.5
0.0
A (n=15)
B (n=16)
C (n=14)
surface

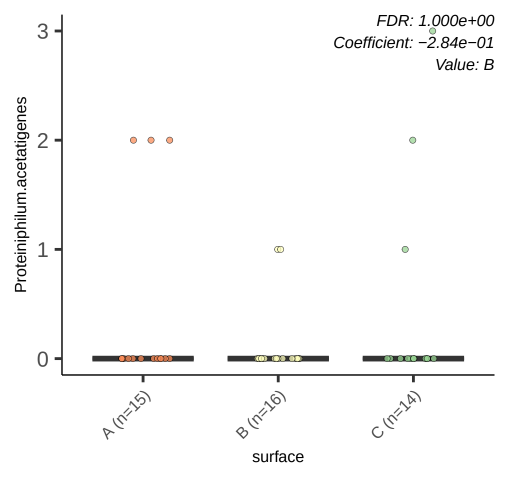
FDR: 1.000e+00
Coefficient: −2.84e−01
Value: B
Proteiniphilum.acetatigenes
0
1
2
3
A (n=15)
B (n=16)
C (n=14)
surface

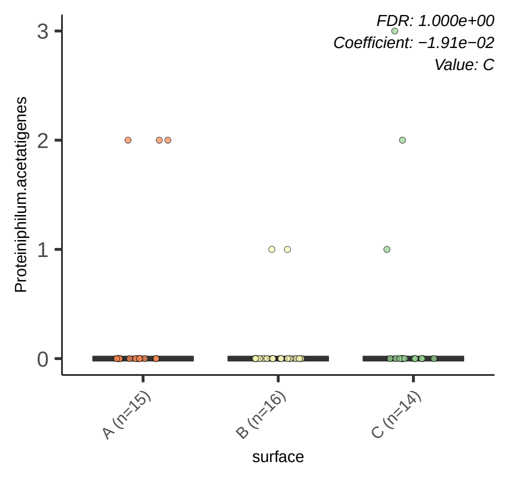

FDR: 1.000e+00
Coefficient: −1.91e−02
Value: C
Proteiniphilum.acetatigenes
3
2
1
0
A (n=15)
B (n=16)
C (n=14)
surface

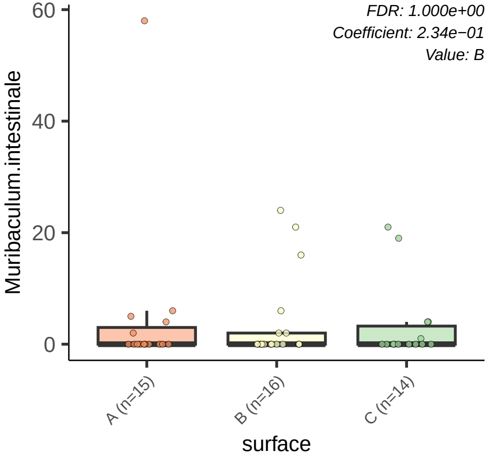

FDR: 1.000e+00
Coefficient: 2.34e−01
Value: B
Muribaculum.intestinale
60
40
20
0
A (n=15)
B (n=16)
C (n=14)
surface

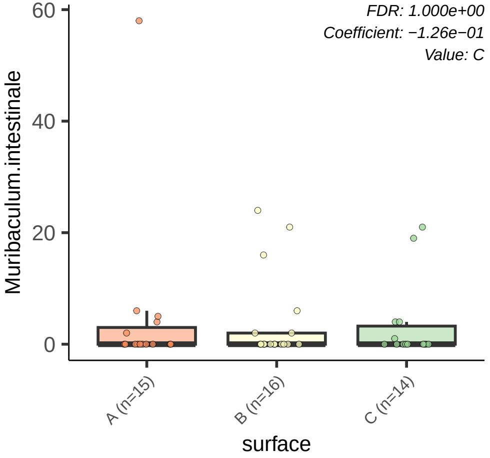

FDR: 1.000e+00
Coefficient: −1.26e−01
Value: C
60
40
20
0
Muribaculum.intestinale
A (n=15)
B (n=16)
C (n=14)
surface

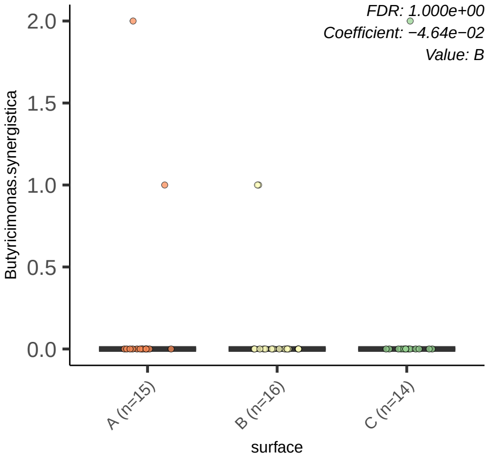
FDR: 1.000e+00
Coefficient: −4.64e−02
Value: B
Butyricimonas.synergistica
2.0
1.5
1.0
0.5
0.0
A (n=15)
B (n=16)
C (n=14)
surface

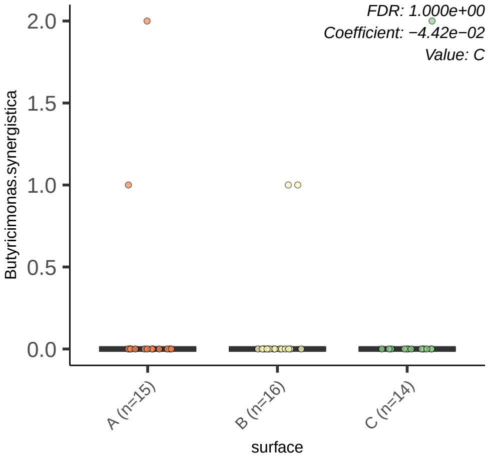
FDR: 1.000e+00
Coefficient: −4.42e−02
Value: C
Butyricimonas.synergistica
2.0
1.5
1.0
0.5
0.0
A (n=15)
B (n=16)
C (n=14)
surface

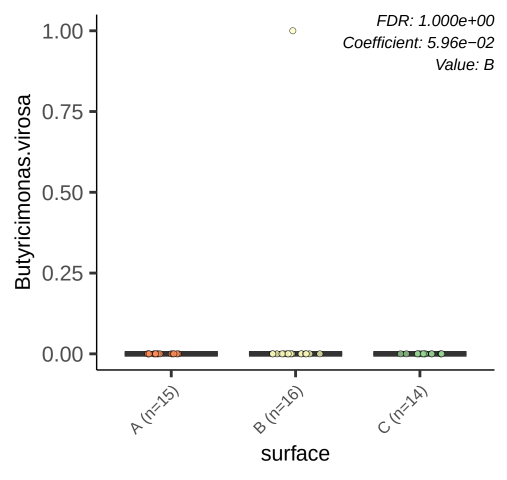
FDR: 1.000e+00
Coefficient: 5.96e−02
Value: B
Butyricimonas.virosa
1.00
0.75
0.50
0.25
0.00
A (n=15)
B (n=16)
C (n=14)
surface

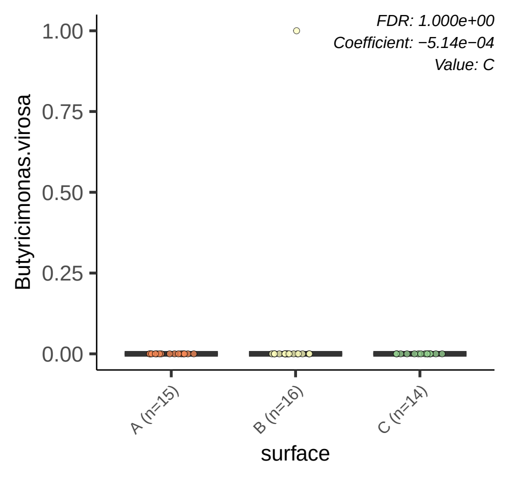
FDR: 1.000e+00
Coefficient: −5.14e−04
Value: C
Butyricimonas.virosa
1.00
0.75
0.50
0.25
0.00
A (n=15)
B (n=16)
C (n=14)
surface

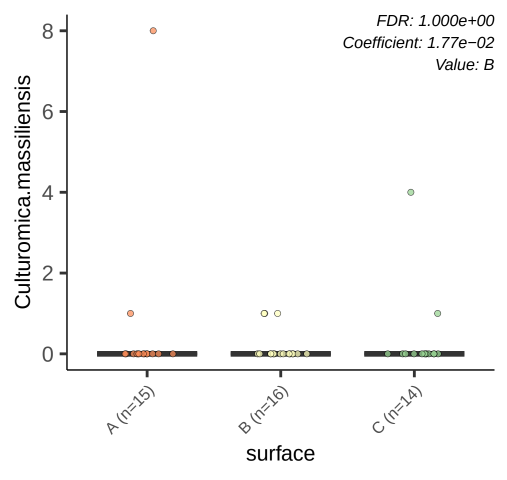
FDR: 1.000e+00
Coefficient: 1.77e−02
Value: B
Culturomica.massiliensis
0
2
4
6
8
A (n=15)
B (n=16)
C (n=14)
surface

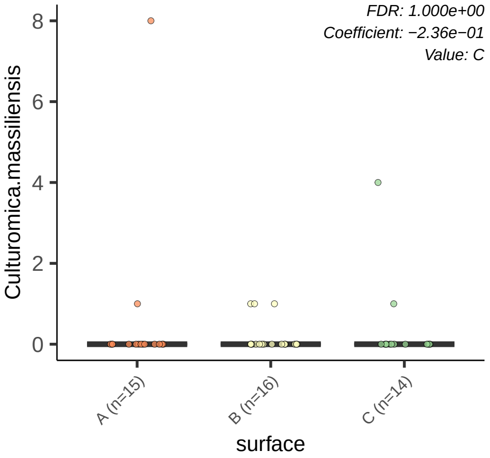

FDR: 1.000e+00
Coefficient: −2.36e−01
Value: C
Culturomica.massiliensis
0
2
4
6
8
A (n=15)
B (n=16)
C (n=14)
surface

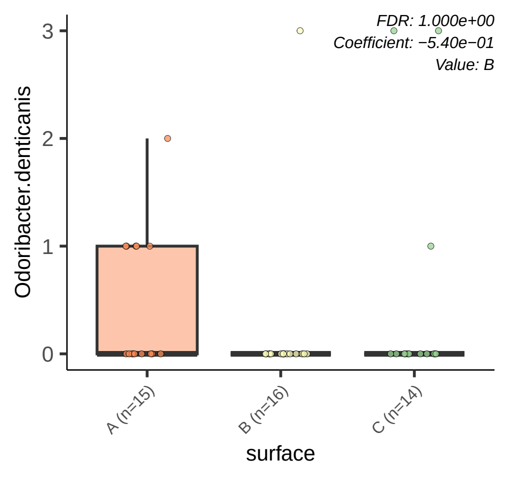
FDR: 1.000e+00
Coefficient: −5.40e−01
Value: B
Odoribacter.denticanis
3
2
1
0
A (n=15)
B (n=16)
C (n=14)
surface

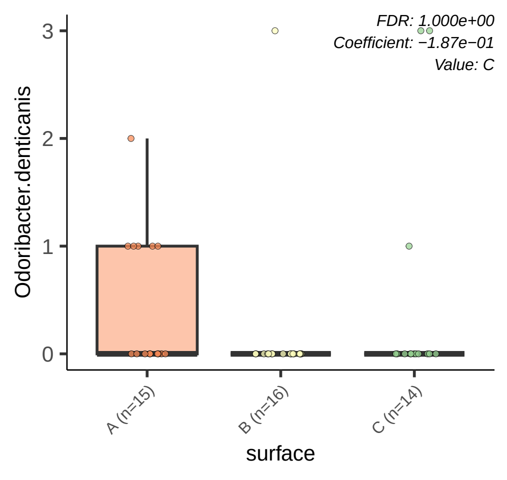
FDR: 1.000e+00
Coefficient: −1.87e−01
Value: C
Odoribacter.denticanis
0
1
2
3
A (n=15)
B (n=16)
C (n=14)
surface

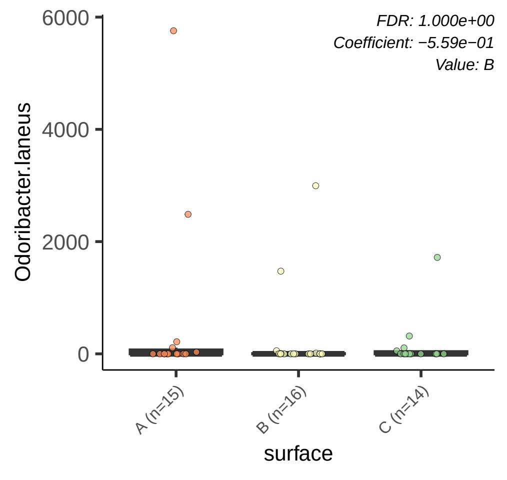

FDR: 1.000e+00
Coefficient: −5.59e−01
Value: B
6000
4000
2000
0
Odoribacter.laneus
A (n=15)
B (n=16)
C (n=14)
surface

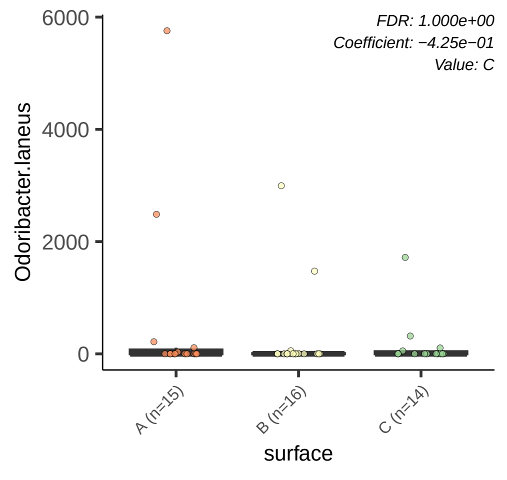
FDR: 1.000e+00
Coefficient: −4.25e−01
Value: C
Odoribacter.laneus
6000
4000
2000
0
A (n=15)
B (n=16)
C (n=14)
surface

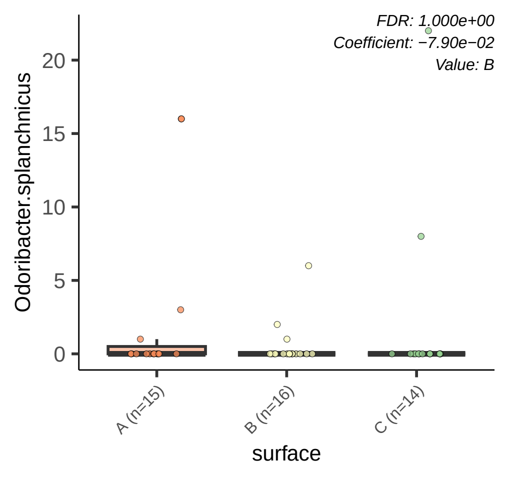

FDR: 1.000e+00
Coefficient: −7.90e−02
Value: B
Odoribacter.splanchnicus
0
5
10
15
20
A (n=15)
B (n=16)
C (n=14)
surface

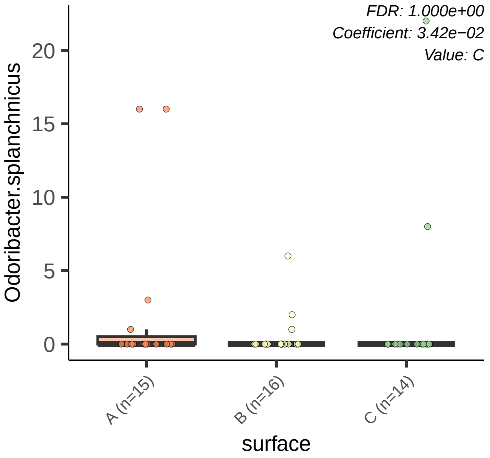

FDR: 1.000e+00
Coefficient: 3.42e−02
Value: C
Odoribacter.splanchnicus
20
15
10
5
0
A (n=15)
B (n=16)
C (n=14)
surface

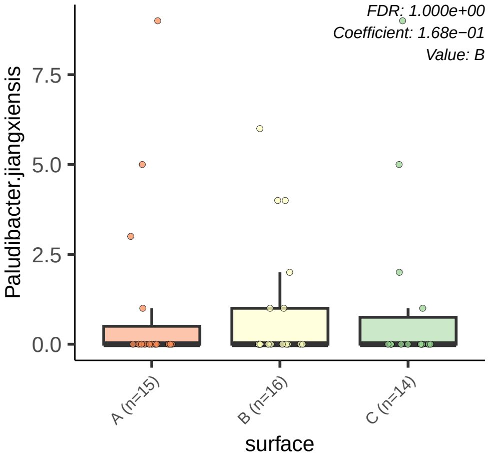
FDR: 1.000e+00
Coefficient: 1.68e−01
Value: B
Paludibacter.jiangxiensis
7.5
5.0
2.5
0.0
A (n=15)
B (n=16)
C (n=14)
surface

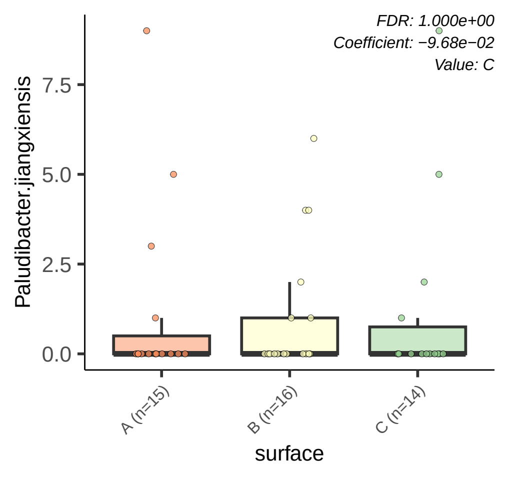
FDR: 1.000e+00
Coefficient: −9.68e−02
Value: C
Paludibacter.jiangxiensis
7.5
5.0
2.5
0.0
A (n=15)
B (n=16)
C (n=14)
surface

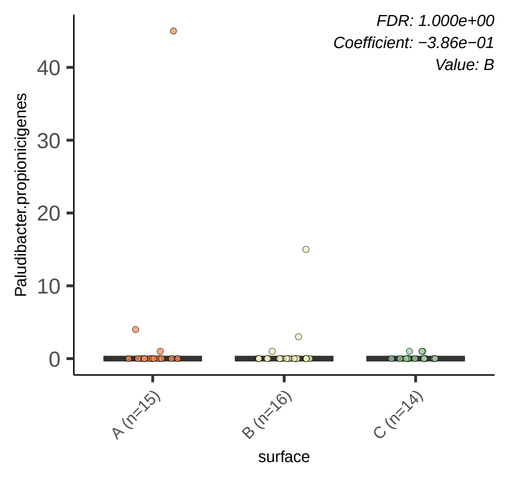
FDR: 1.000e+00
Coefficient: −3.86e−01
Value: B
Paludibacter.propionicigenes
40
30
20
10
0
A (n=15)
B (n=16)
C (n=14)
surface

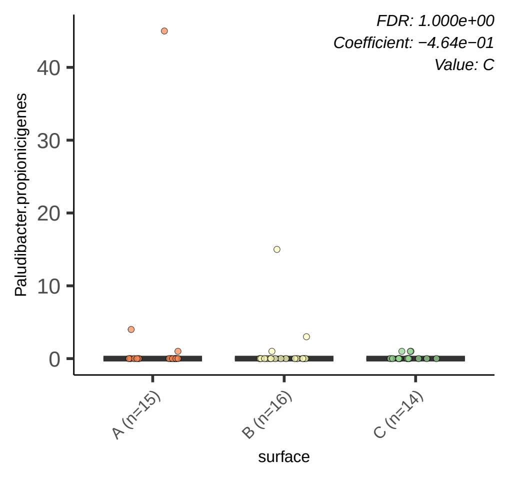

FDR: 1.000e+00
Coefficient: −4.64e−01
Value: C
Paludibacter.propionicigenes
40
30
20
10
0
A (n=15)
B (n=16)
C (n=14)
surface

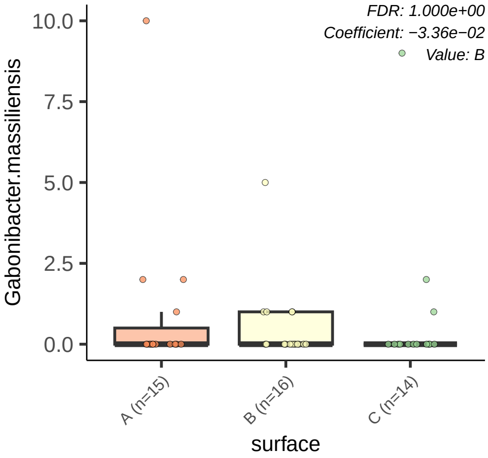
FDR: 1.000e+00
Coefficient: −3.36e−02
Value: B
Gabonibacter.massiliensis
10.0
7.5
5.0
2.5
0.0
A (n=15)
B (n=16)
C (n=14)
surface

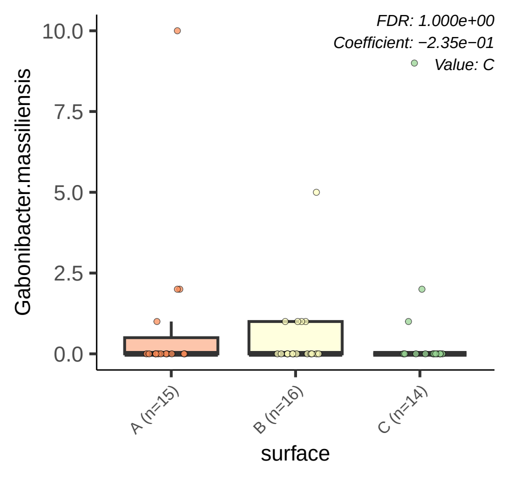

FDR: 1.000e+00
Coefficient: −2.35e−01
Value: C
Gabonibacter.massiliensis
10.0
7.5
5.0
2.5
0.0
A (n=15)
B (n=16)
C (n=14)
surface

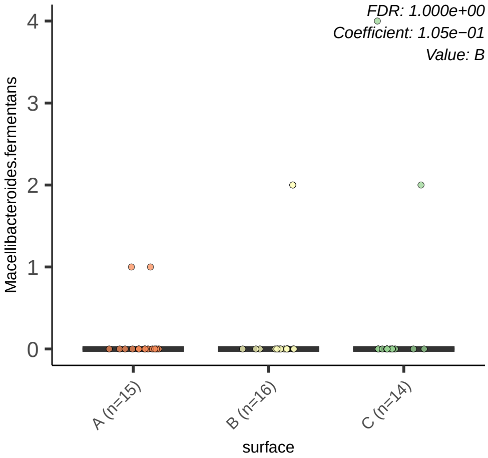

FDR: 1.000e+00
Coefficient: 1.05e−01
Value: B
Macellibacteroides.fermentans
0
1
2
3
4
A (n=15)
B (n=16)
C (n=14)
surface

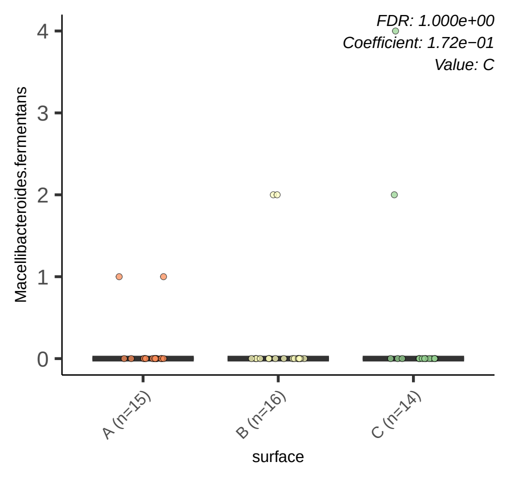

FDR: 1.000e+00
Coefficient: 1.72e−01
Value: C
Macellibacteroides.fermentans
4
3
2
1
0
A (n=15)
B (n=16)
C (n=14)
surface

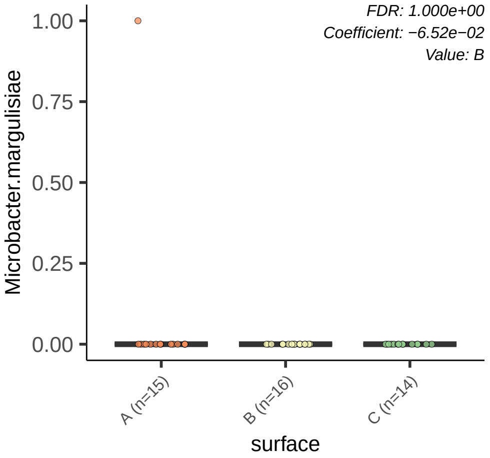
FDR: 1.000e+00
Coefficient: −6.52e−02
Value: B
Microbacter.margulisiae
1.00
0.75
0.50
0.25
0.00
A (n=15)
B (n=16)
C (n=14)
surface

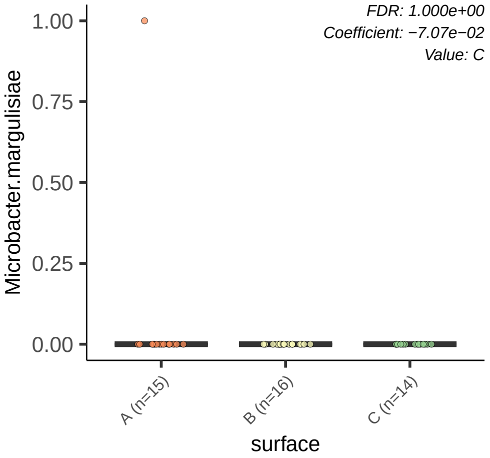
FDR: 1.000e+00
Coefficient: −7.07e−02
Value: C
Microbacter.margulisiae
1.00
0.75
0.50
0.25
0.00
A (n=15)
B (n=16)
C (n=14)
surface

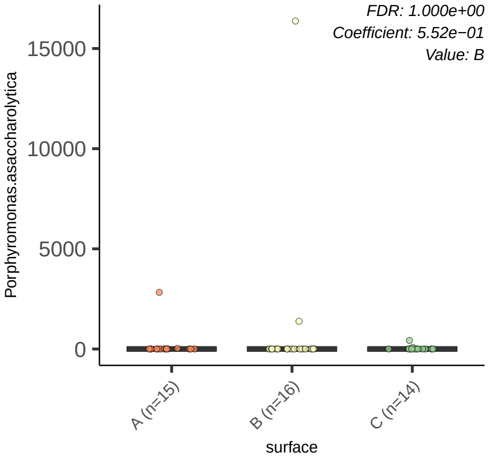

FDR: 1.000e+00
Coefficient: 5.52e−01
Value: B
Porphyromonas.asaccharolytica
15000
10000
5000
0
A (n=15)
B (n=16)
C (n=14)
surface

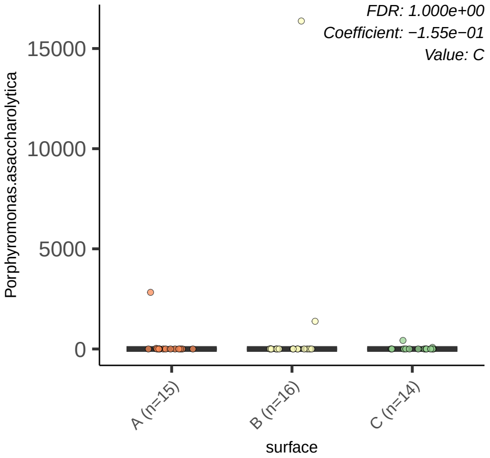

FDR: 1.000e+00
Coefficient: −1.55e−01
Value: C
15000
10000
5000
0
Porphyromonas.asaccharolytica
A (n=15)
B (n=16)
C (n=14)
surface

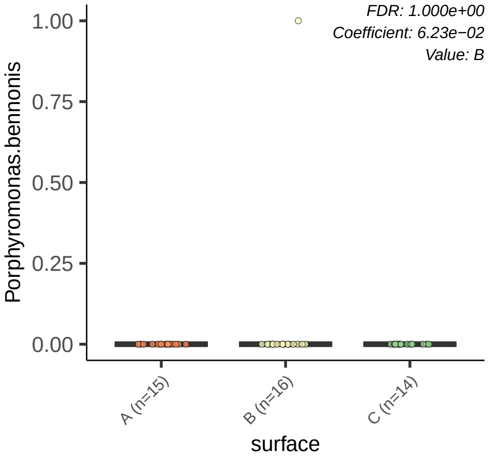
FDR: 1.000e+00
Coefficient: 6.23e−02
Value: B
Porphyromonas.bennonis
1.00
0.75
0.50
0.25
0.00
A (n=15)
B (n=16)
C (n=14)
surface

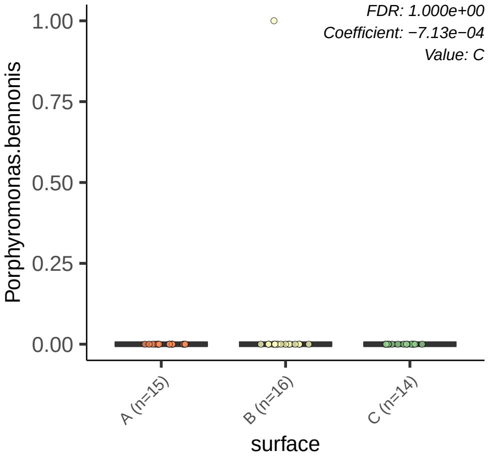

FDR: 1.000e+00
Coefficient: −7.13e−04
Value: C
Porphyromonas.bennonis
1.00
0.75
0.50
0.25
0.00
A (n=15)
B (n=16)
C (n=14)
surface

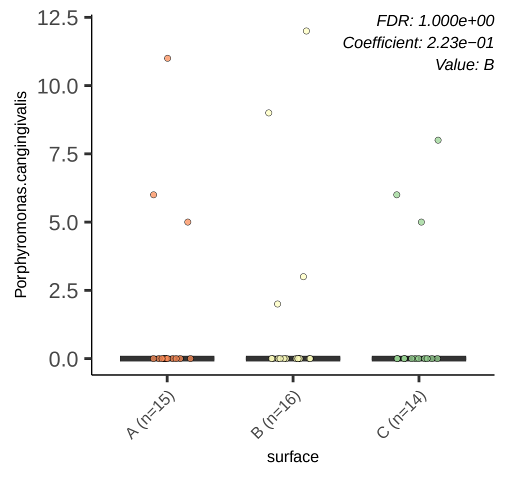
FDR: 1.000e+00
Coefficient: 2.23e−01
Value: B
Porphyromonas.cangingivalis
12.5
10.0
7.5
5.0
2.5
0.0
A (n=15)
B (n=16)
C (n=14)
surface

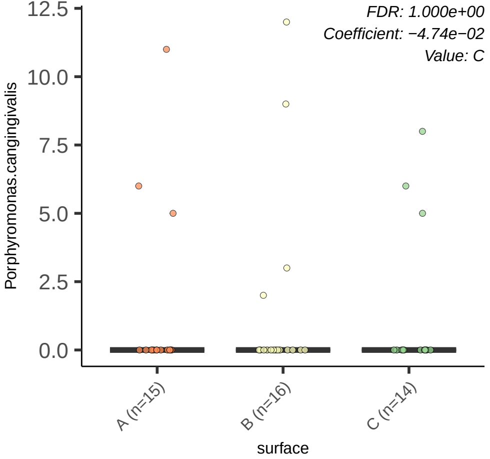
FDR: 1.000e+00
Coefficient: −4.74e−02
Value: C
Porphyromonas.cangingivalis
12.5
10.0
7.5
5.0
2.5
0.0
A (n=15)
B (n=16)
C (n=14)
surface

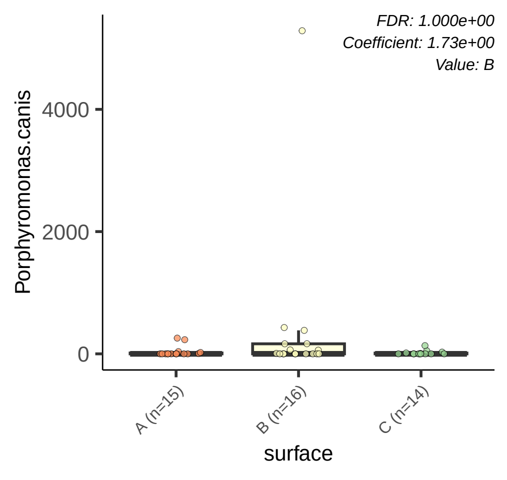

FDR: 1.000e+00
Coefficient: 1.73e+00
Value: B
Porphyromonas.canis
4000
2000
0
A (n=15)
B (n=16)
C (n=14)
surface

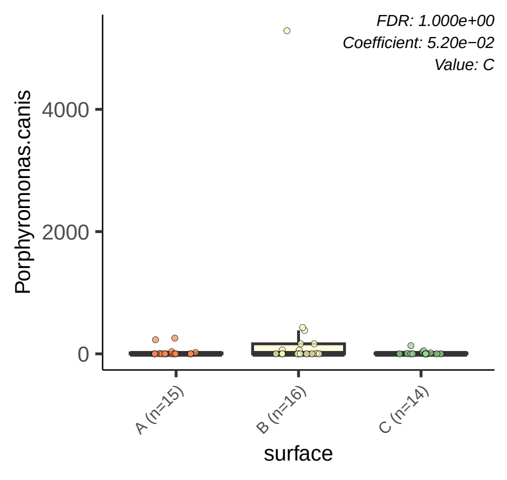
FDR: 1.000e+00
Coefficient: 5.20e−02
Value: C
Porphyromonas.canis
4000
2000
0
A (n=15)
B (n=16)
C (n=14)
surface

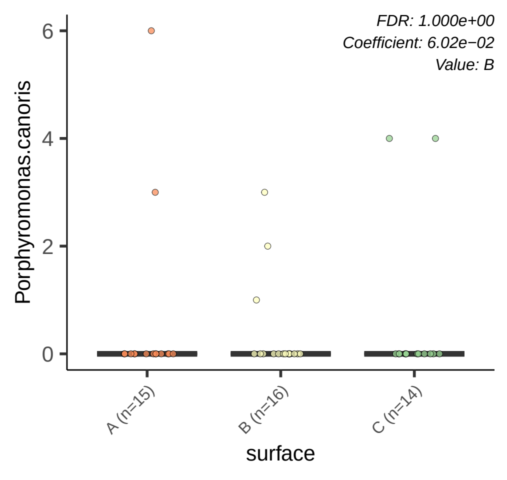

FDR: 1.000e+00
Coefficient: 6.02e−02
Value: B
Porphyromonas.canoris
0
2
4
6
A (n=15)
B (n=16)
C (n=14)
surface

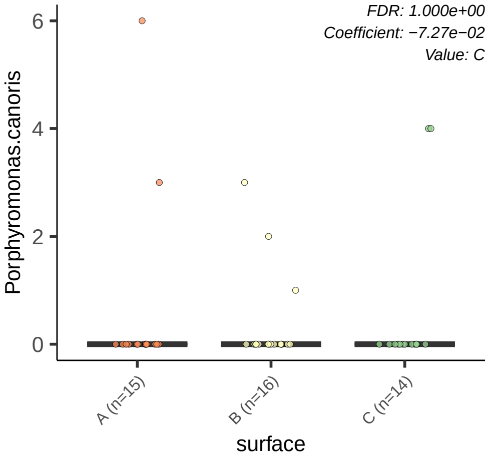

FDR: 1.000e+00
Coefficient: −7.27e−02
Value: C
Porphyromonas.canoris
6
4
2
0
A (n=15)
B (n=16)
C (n=14)
surface

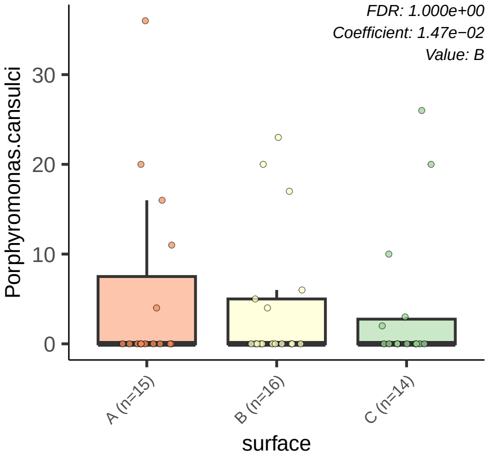
FDR: 1.000e+00
Coefficient: 1.47e−02
Value: B
Porphyromonas.cansulci
0
10
20
30
A (n=15)
B (n=16)
C (n=14)
surface

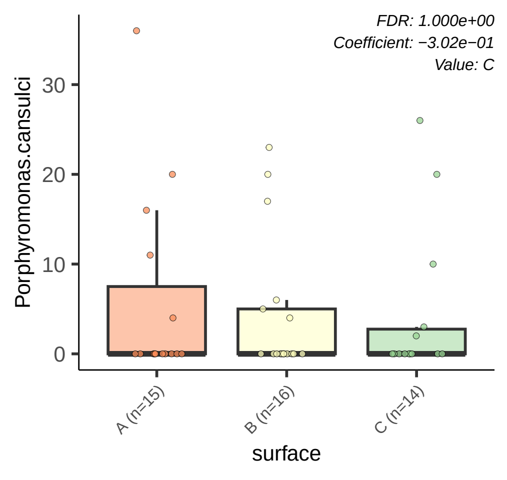
FDR: 1.000e+00
Coefficient: −3.02e−01
Value: C
Porphyromonas.cansulci
30
20
10
0
A (n=15)
B (n=16)
C (n=14)
surface

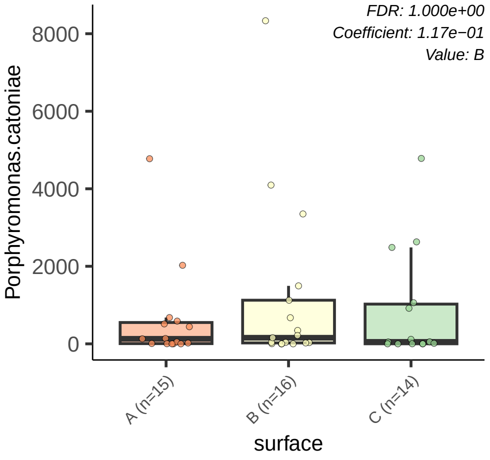
FDR: 1.000e+00
Coefficient: 1.17e−01
Value: B
Porphyromonas.catoniae
8000
6000
4000
2000
0
A (n=15)
B (n=16)
C (n=14)
surface

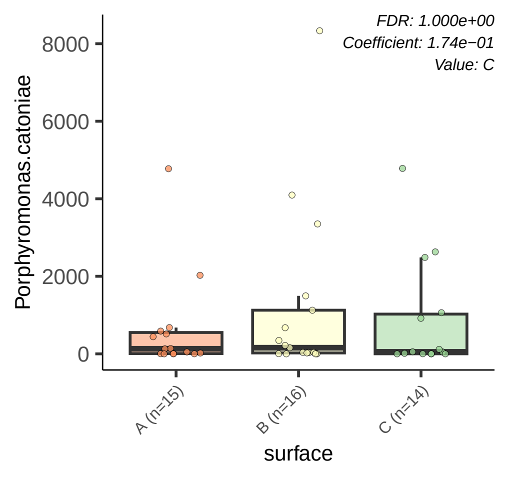

FDR: 1.000e+00
Coefficient: 1.74e−01
Value: C
8000
6000
4000
2000
0
Porphyromonas.catoniae
A (n=15)
B (n=16)
C (n=14)
surface

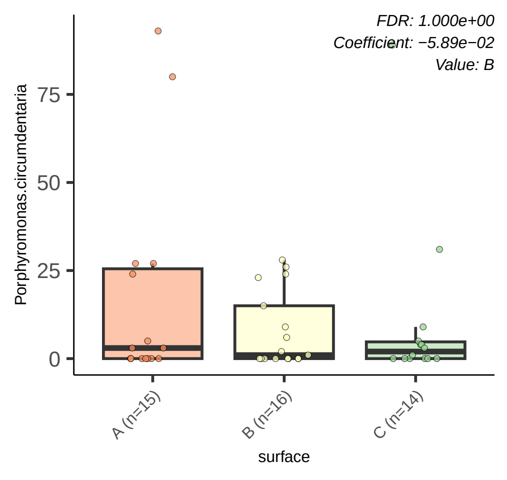
FDR: 1.000e+00
Coefficient: −5.89e−02
Value: B
Porphyromonas.circumdentaria
0
25
50
75
A (n=15)
B (n=16)
C (n=14)
surface

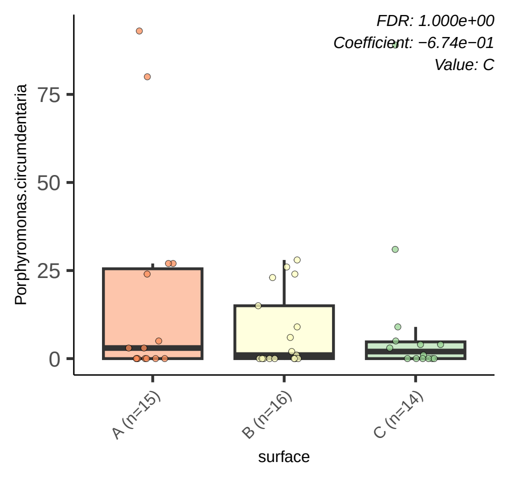

FDR: 1.000e+00
Coefficient: −6.74e−01
Value: C
Porphyromonas.circumdentaria
0
25
50
75
A (n=15)
B (n=16)
C (n=14)
surface

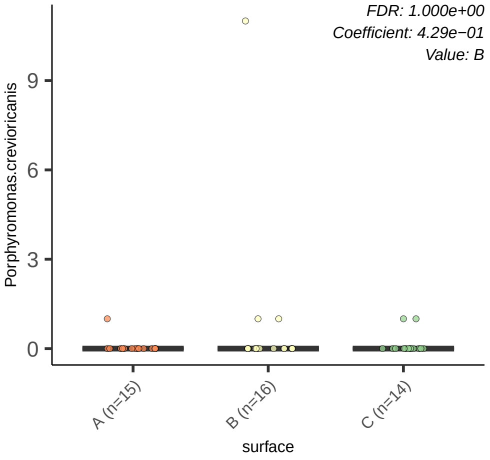

FDR: 1.000e+00
Coefficient: 4.29e−01
Value: B
Porphyromonas.crevioricanis
0
3
6
9
A (n=15)
B (n=16)
C (n=14)
surface

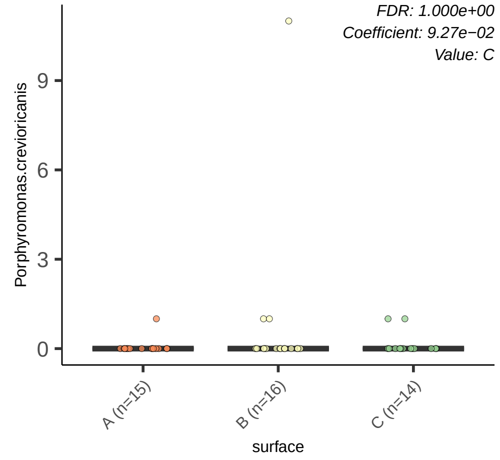

FDR: 1.000e+00
Coefficient: 9.27e−02
Value: C
Porphyromonas.crevioricanis
9
6
3
0
A (n=15)
B (n=16)
C (n=14)
surface

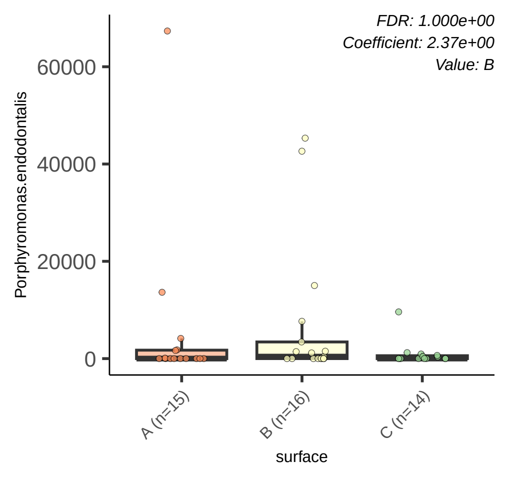
FDR: 1.000e+00
Coefficient: 2.37e+00
Value: B
60000
40000
20000
0
Porphyromonas.endodontalis
A (n=15)
B (n=16)
C (n=14)
surface

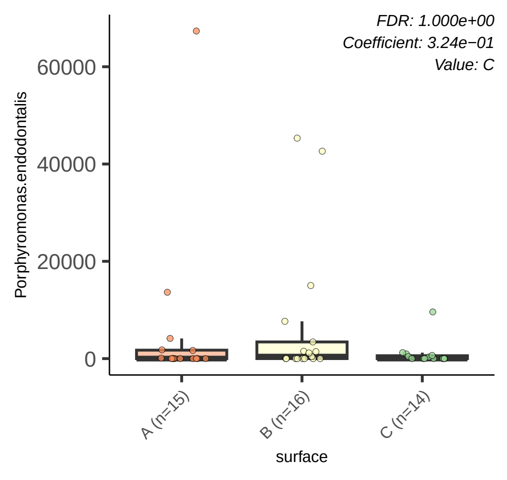

FDR: 1.000e+00
Coefficient: 3.24e−01
Value: C
Porphyromonas.endodontalis
60000
40000
20000
0
A (n=15)
B (n=16)
C (n=14)
surface

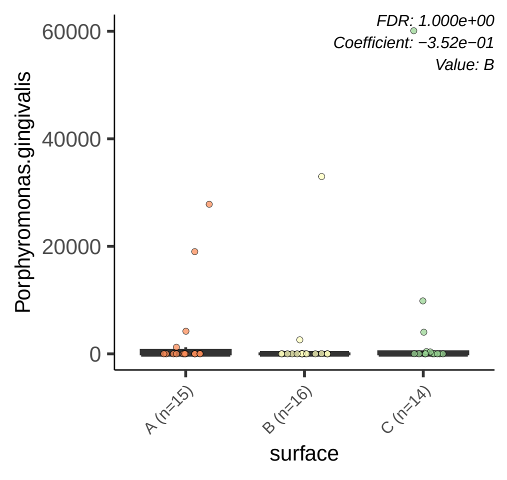
FDR: 1.000e+00
Coefficient: −3.52e−01
Value: B
Porphyromonas.gingivalis
60000
40000
20000
0
A (n=15)
B (n=16)
C (n=14)
surface

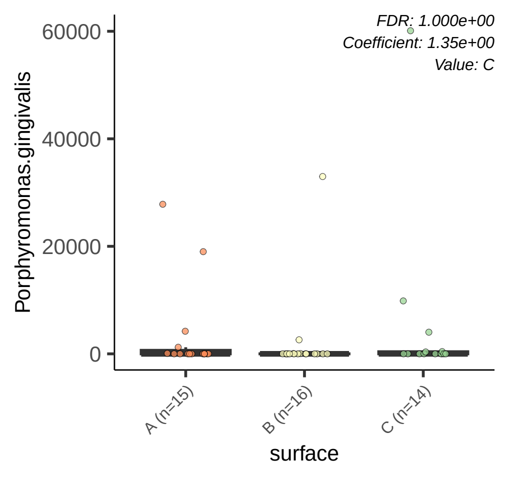
FDR: 1.000e+00
Coefficient: 1.35e+00
Value: C
Porphyromonas.gingivalis
60000
40000
20000
0
A (n=15)
B (n=16)
C (n=14)
surface

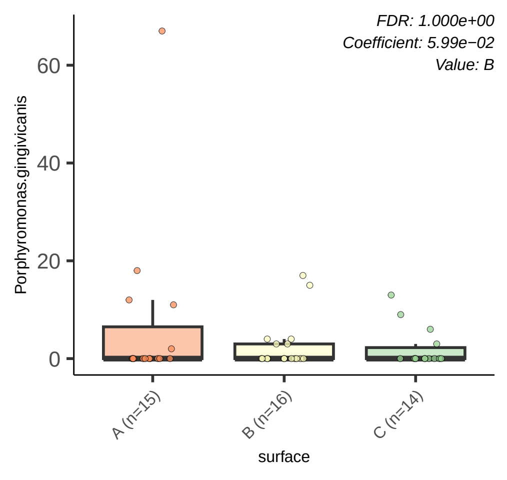
FDR: 1.000e+00
Coefficient: 5.99e−02
Value: B
Porphyromonas.gingivicanis
0
20
40
60
A (n=15)
B (n=16)
C (n=14)
surface

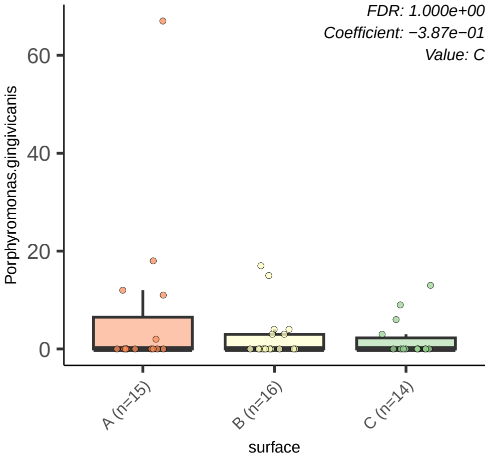

FDR: 1.000e+00
Coefficient: −3.87e−01
Value: C
Porphyromonas.gingivicanis
60
40
20
0
A (n=15)
B (n=16)
C (n=14)
surface

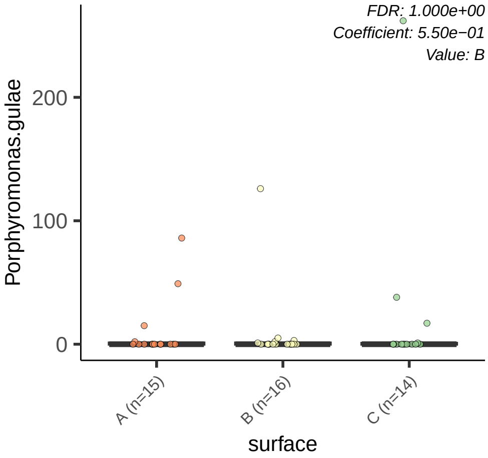
FDR: 1.000e+00
Coefficient: 5.50e−01
Value: B
Porphyromonas.gulae
200
100
0
A (n=15)
B (n=16)
C (n=14)
surface

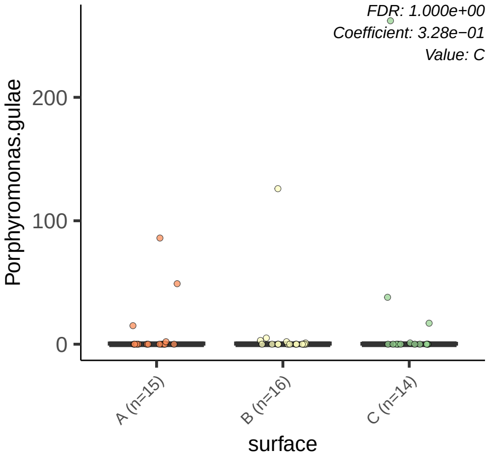

FDR: 1.000e+00
Coefficient: 3.28e−01
Value: C
Porphyromonas.gulae
200
100
0
A (n=15)
B (n=16)
C (n=14)
surface

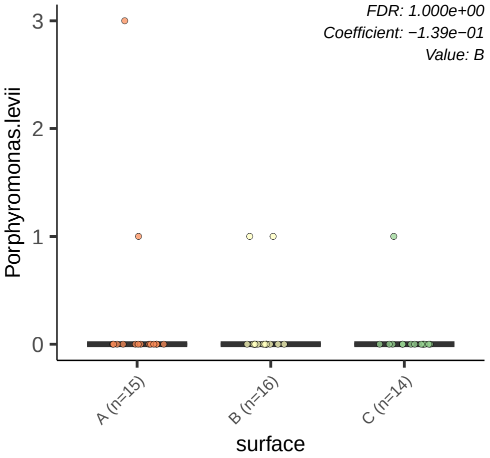
FDR: 1.000e+00
Coefficient: −1.39e−01
Value: B
Porphyromonas.levii
3
2
1
0
A (n=15)
B (n=16)
C (n=14)
surface

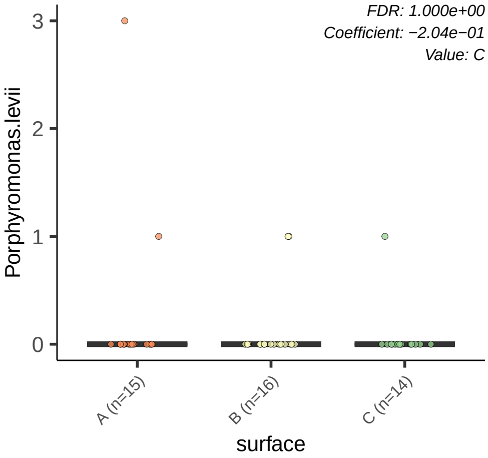

FDR: 1.000e+00
Coefficient: −2.04e−01
Value: C
Porphyromonas.levii
3
2
1
0
A (n=15)
B (n=16)
C (n=14)
surface

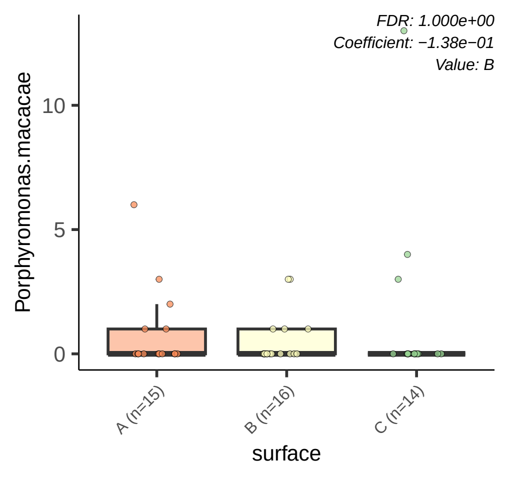
FDR: 1.000e+00
Coefficient: −1.38e−01
Value: B
Porphyromonas.macacae
10
5
0
A (n=15)
B (n=16)
C (n=14)
surface

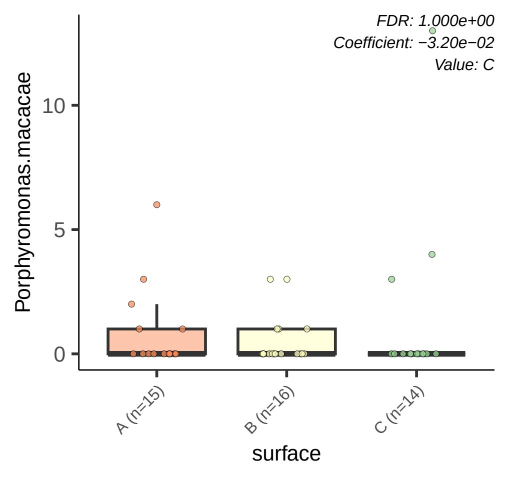
FDR: 1.000e+00
Coefficient: −3.20e−02
Value: C
Porphyromonas.macacae
10
5
0
A (n=15)
B (n=16)
C (n=14)
surface

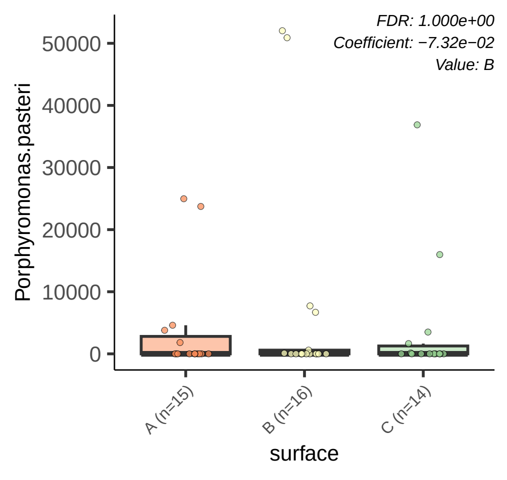
FDR: 1.000e+00
Coefficient: −7.32e−02
Value: B
Porphyromonas.pasteri
50000
40000
30000
20000
10000
0
A (n=15)
B (n=16)
C (n=14)
surface

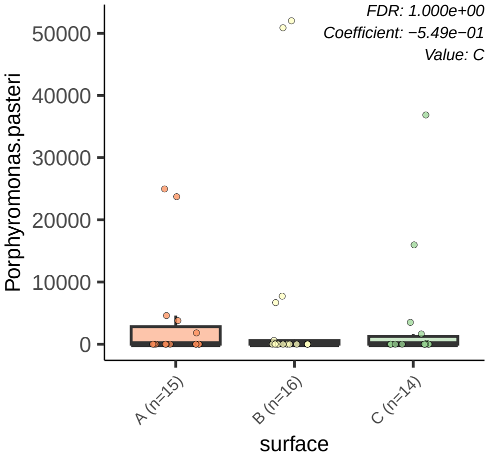

FDR: 1.000e+00
Coefficient: −5.49e−01
Value: C
Porphyromonas.pasteri
50000
40000
30000
20000
10000
0
A (n=15)
B (n=16)
C (n=14)
surface

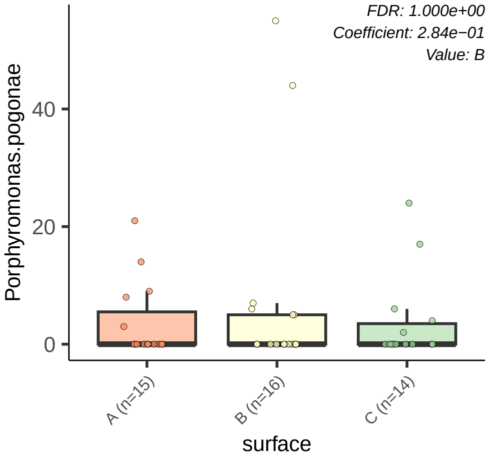

FDR: 1.000e+00
Coefficient: 2.84e−01
Value: B
Porphyromonas.pogonae
0
20
40
A (n=15)
B (n=16)
C (n=14)
surface

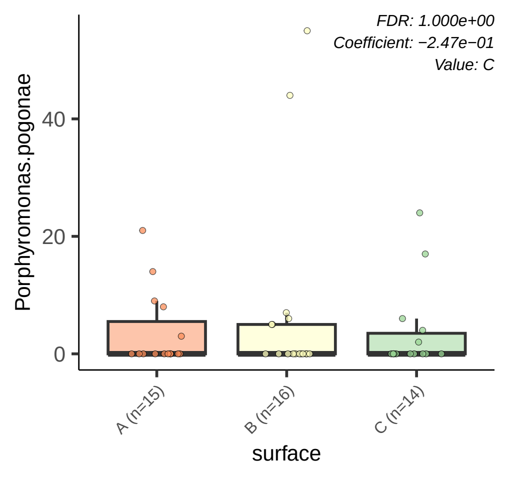
FDR: 1.000e+00
Coefficient: −2.47e−01
Value: C
Porphyromonas.pogonae
0
20
40
A (n=15)
B (n=16)
C (n=14)
surface

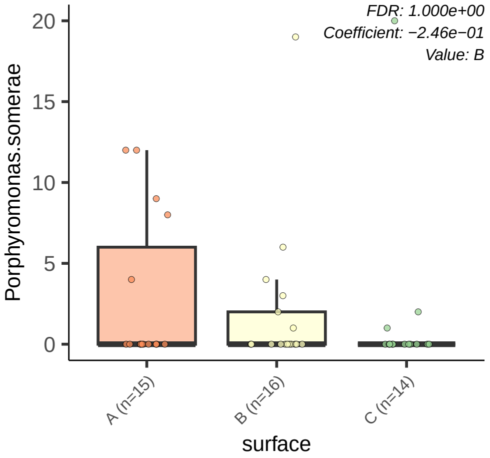

FDR: 1.000e+00
Coefficient: −2.46e−01
Value: B
Porphyromonas.somerae
20
15
10
5
0
A (n=15)
B (n=16)
C (n=14)
surface

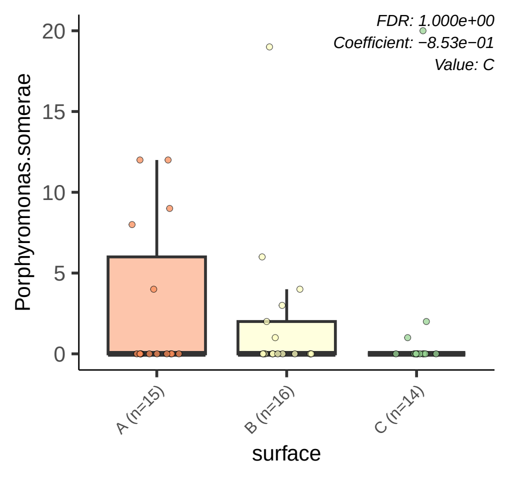
FDR: 1.000e+00
Coefficient: −8.53e−01
Value: C
Porphyromonas.somerae
20
15
10
5
0
A (n=15)
B (n=16)
C (n=14)
surface

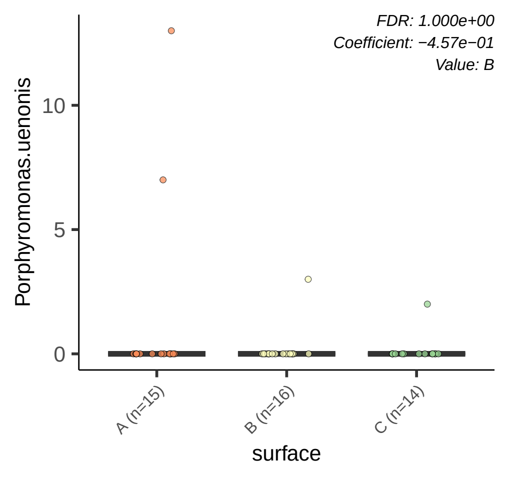
FDR: 1.000e+00
Coefficient: −4.57e−01
Value: B
Porphyromonas.uenonis
10
5
0
A (n=15)
B (n=16)
C (n=14)
surface

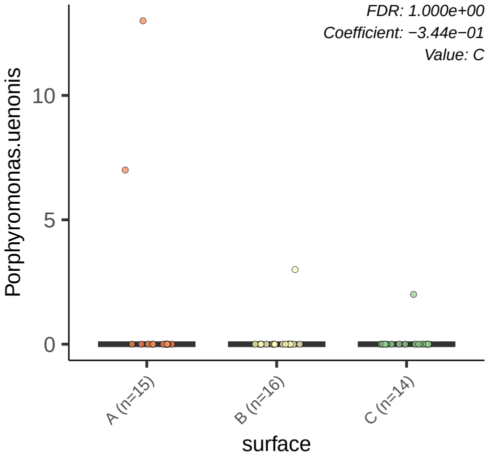

FDR: 1.000e+00
Coefficient: −3.44e−01
Value: C
Porphyromonas.uenonis
10
5
0
A (n=15)
B (n=16)
C (n=14)
surface

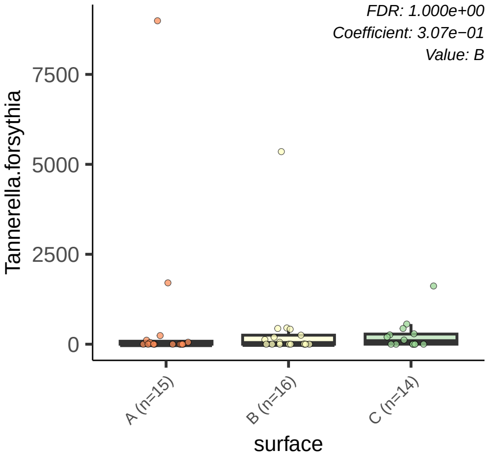
FDR: 1.000e+00
Coefficient: 3.07e−01
Value: B
Tannerella.forsythia
7500
5000
2500
0
A (n=15)
B (n=16)
C (n=14)
surface

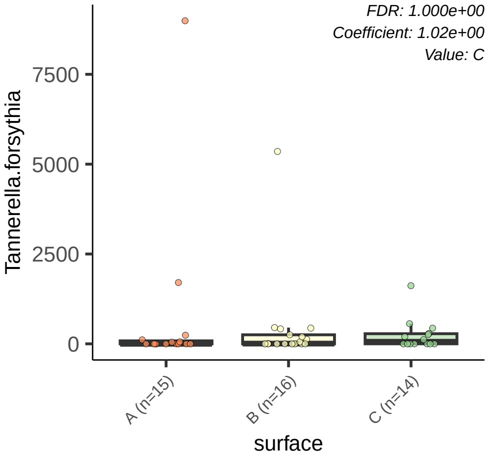

FDR: 1.000e+00
Coefficient: 1.02e+00
Value: C
Tannerella.forsythia
7500
5000
2500
0
A (n=15)
B (n=16)
C (n=14)
surface

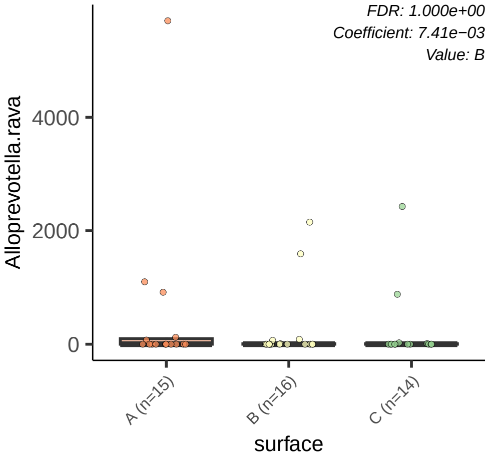
FDR: 1.000e+00
Coefficient: 7.41e−03
Value: B
Alloprevotella.rava
4000
2000
0
A (n=15)
B (n=16)
C (n=14)
surface

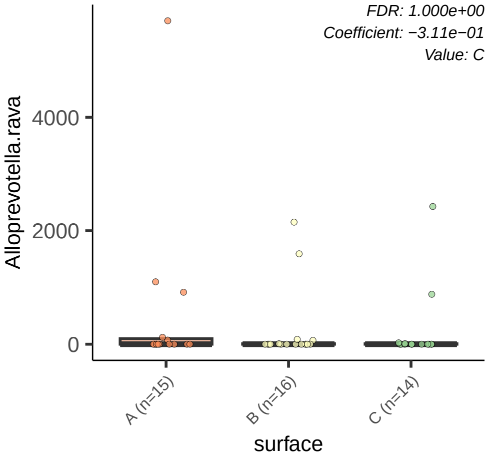

FDR: 1.000e+00
Coefficient: −3.11e−01
Value: C
Alloprevotella.rava
4000
2000
0
A (n=15)
B (n=16)
C (n=14)
surface

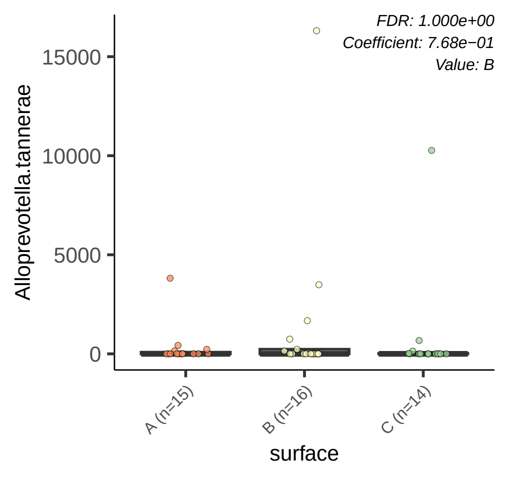
FDR: 1.000e+00
Coefficient: 7.68e−01
Value: B
Alloprevotella.tannerae
15000
10000
5000
0
A (n=15)
B (n=16)
C (n=14)
surface

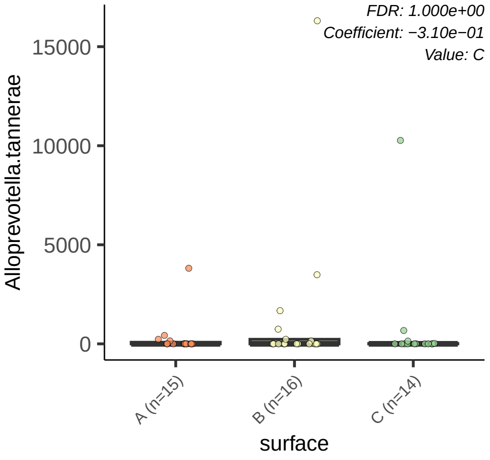
FDR: 1.000e+00
Coefficient: −3.10e−01
Value: C
Alloprevotella.tannerae
15000
10000
5000
0
A (n=15)
B (n=16)
C (n=14)
surface

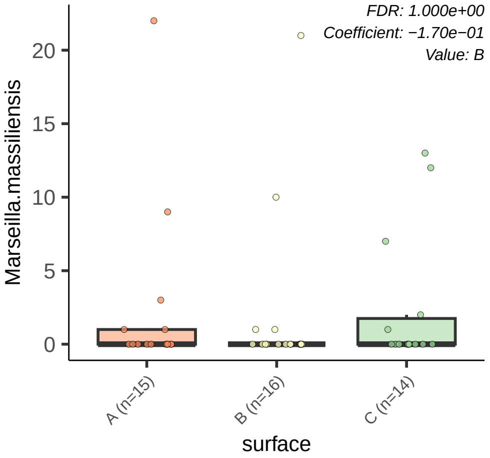
FDR: 1.000e+00
Coefficient: −1.70e−01
Value: B
Marseilla.massiliensis
20
15
10
5
0
A (n=15)
B (n=16)
C (n=14)
surface

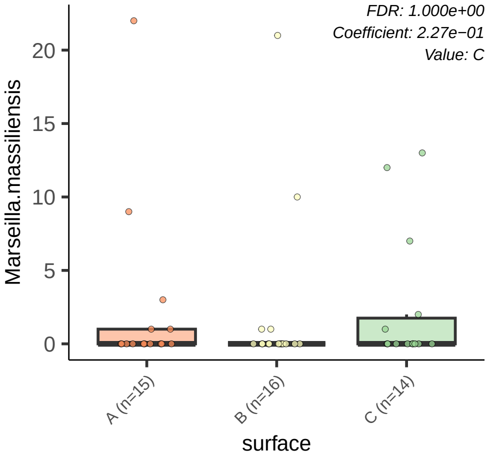

FDR: 1.000e+00
Coefficient: 2.27e−01
Value: C
Marseilla.massiliensis
20
15
10
5
0
A (n=15)
B (n=16)
C (n=14)
surface

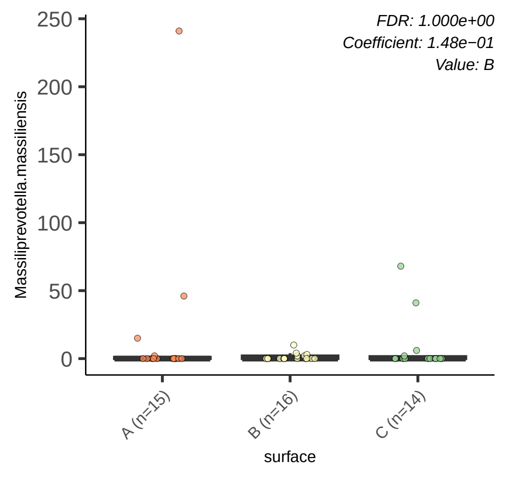

FDR: 1.000e+00
Coefficient: 1.48e−01
Value: B
250
200
150
100
50
0
Massiliprevotella.massiliensis
A (n=15)
B (n=16)
C (n=14)
surface

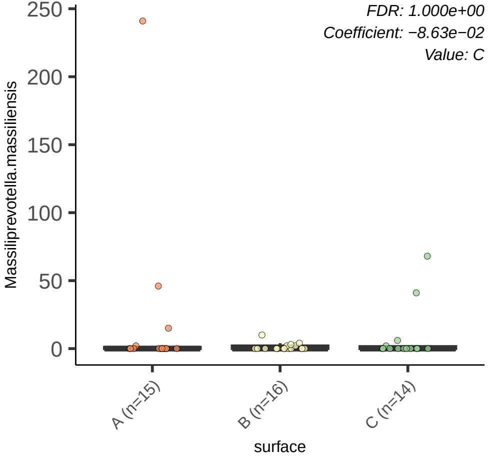
FDR: 1.000e+00
Coefficient: −8.63e−02
Value: C
Massiliprevotella.massiliensis
250
200
150
100
50
0
A (n=15)
B (n=16)
C (n=14)
surface

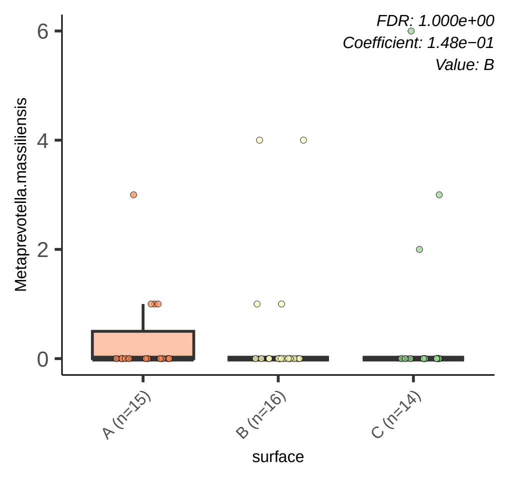
FDR: 1.000e+00
Coefficient: 1.48e−01
Value: B
Metaprevotella.massiliensis
0
2
4
6
A (n=15)
B (n=16)
C (n=14)
surface

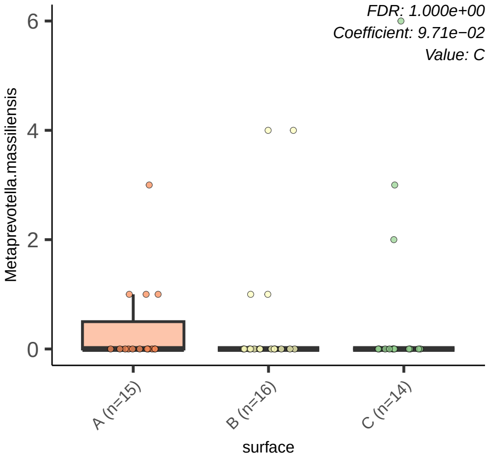

FDR: 1.000e+00
Coefficient: 9.71e−02
Value: C
Metaprevotella.massiliensis
0
2
4
6
A (n=15)
B (n=16)
C (n=14)
surface

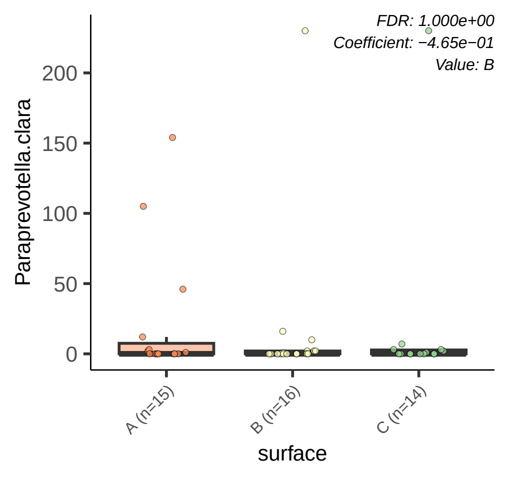
FDR: 1.000e+00
Coefficient: −4.65e−01
Value: B
Paraprevotella.clara
200
150
100
50
0
A (n=15)
B (n=16)
C (n=14)
surface

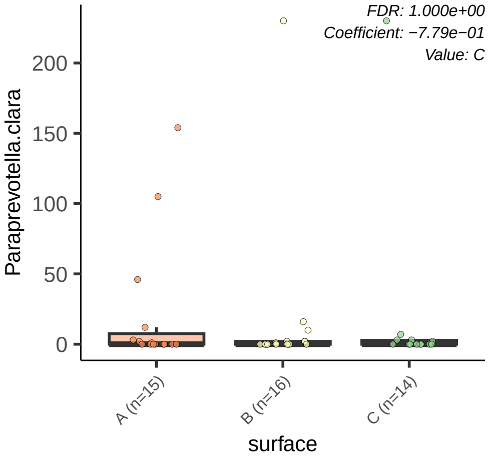

FDR: 1.000e+00
Coefficient: −7.79e−01
Value: C
Paraprevotella.clara
200
150
100
50
0
A (n=15)
B (n=16)
C (n=14)
surface

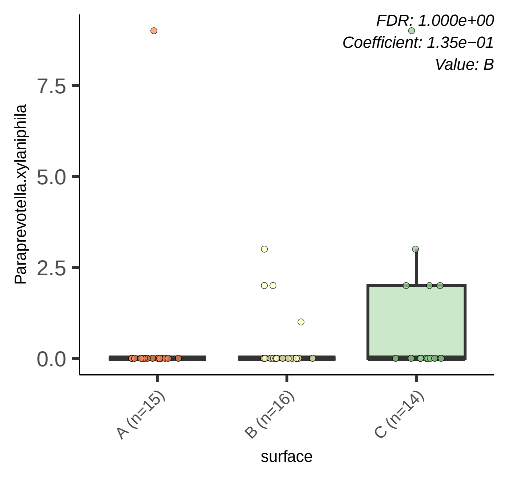
FDR: 1.000e+00
Coefficient: 1.35e−01
Value: B
Paraprevotella.xylaniphila
7.5
5.0
2.5
0.0
A (n=15)
B (n=16)
C (n=14)
surface

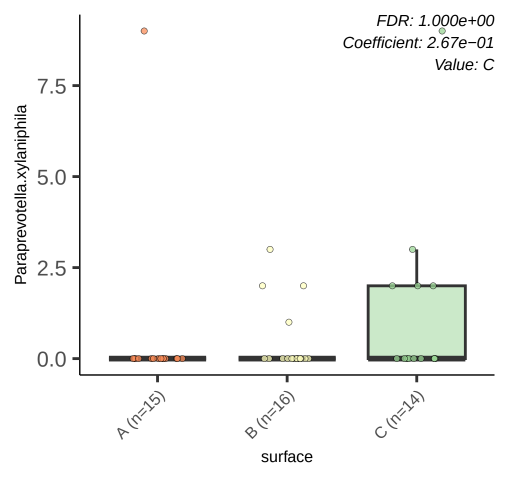
FDR: 1.000e+00
Coefficient: 2.67e−01
Value: C
Paraprevotella.xylaniphila
7.5
5.0
2.5
0.0
A (n=15)
B (n=16)
C (n=14)
surface

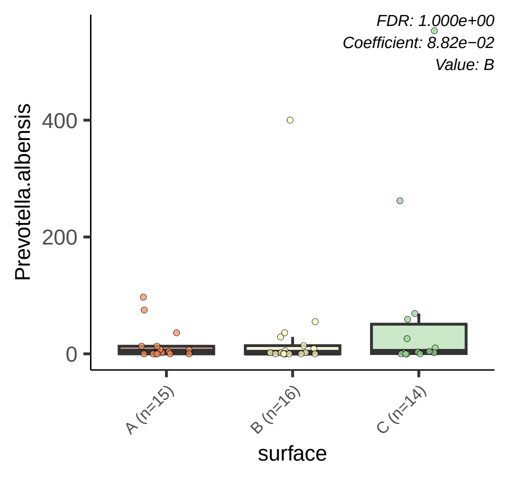

FDR: 1.000e+00
Coefficient: 8.82e−02
Value: B
Prevotella.albensis
400
200
0
A (n=15)
B (n=16)
C (n=14)
surface

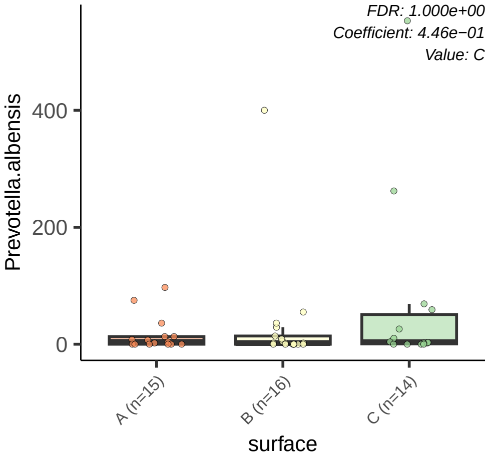

FDR: 1.000e+00
Coefficient: 4.46e−01
Value: C
Prevotella.albensis
400
200
0
A (n=15)
B (n=16)
C (n=14)
surface

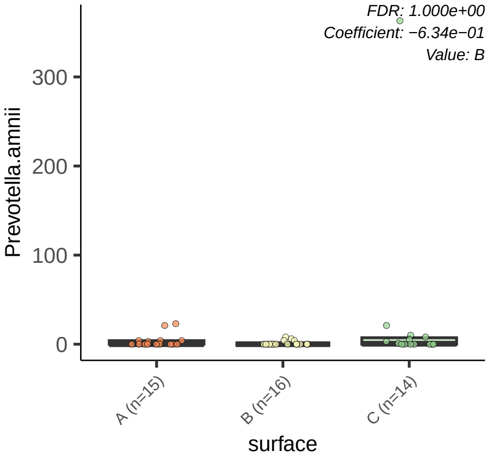

FDR: 1.000e+00
Coefficient: −6.34e−01
Value: B
Prevotella.amnii
300
200
100
0
A (n=15)
B (n=16)
C (n=14)
surface

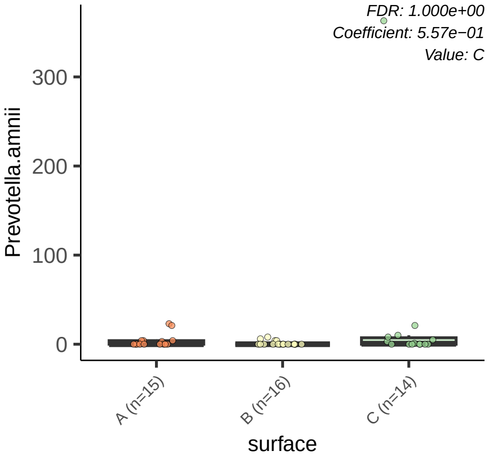

FDR: 1.000e+00
Coefficient: 5.57e−01
Value: C
Prevotella.amnii
300
200
100
0
A (n=15)
B (n=16)
C (n=14)
surface

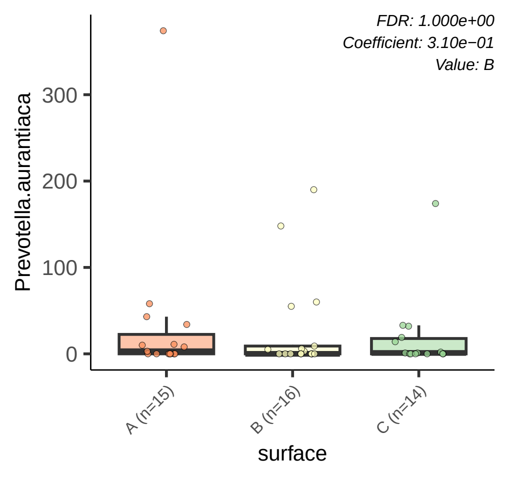
FDR: 1.000e+00
Coefficient: 3.10e−01
Value: B
Prevotella.aurantiaca
300
200
100
0
A (n=15)
B (n=16)
C (n=14)
surface

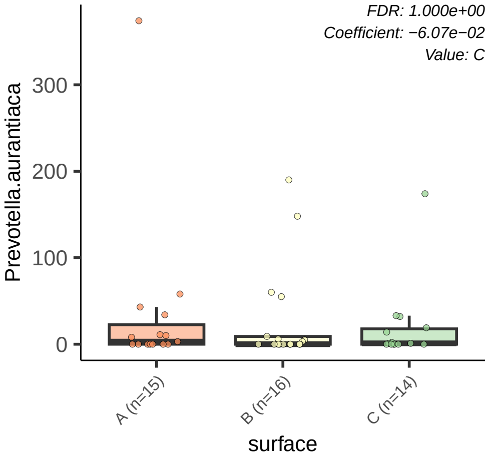
FDR: 1.000e+00
Coefficient: −6.07e−02
Value: C
Prevotella.aurantiaca
300
200
100
0
A (n=15)
B (n=16)
C (n=14)
surface

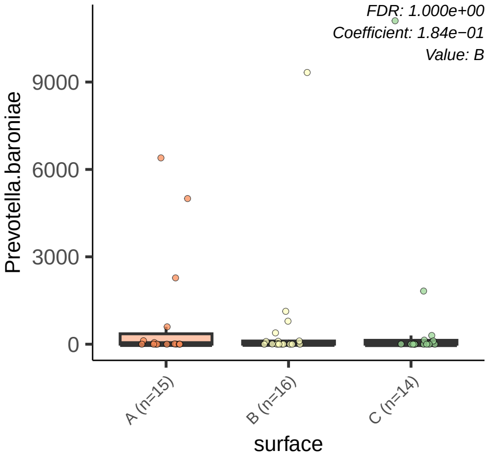
FDR: 1.000e+00
Coefficient: 1.84e−01
Value: B
Prevotella.baroniae
9000
6000
3000
0
A (n=15)
B (n=16)
C (n=14)
surface

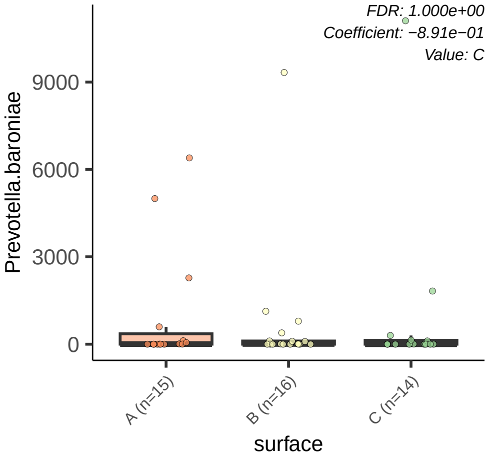
FDR: 1.000e+00
Coefficient: −8.91e−01
Value: C
9000
6000
3000
0
Prevotella.baroniae
A (n=15)
B (n=16)
C (n=14)
surface

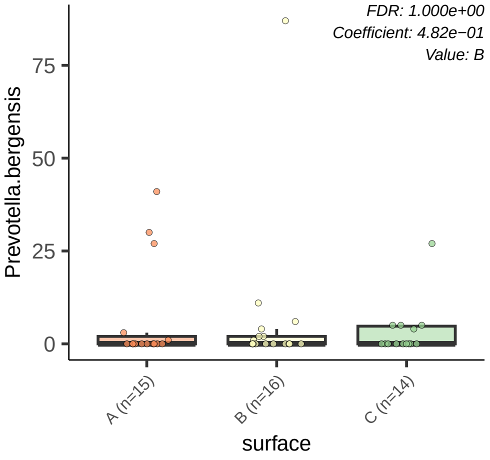
FDR: 1.000e+00
Coefficient: 4.82e−01
Value: B
Prevotella.bergensis
75
50
25
0
A (n=15)
B (n=16)
C (n=14)
surface

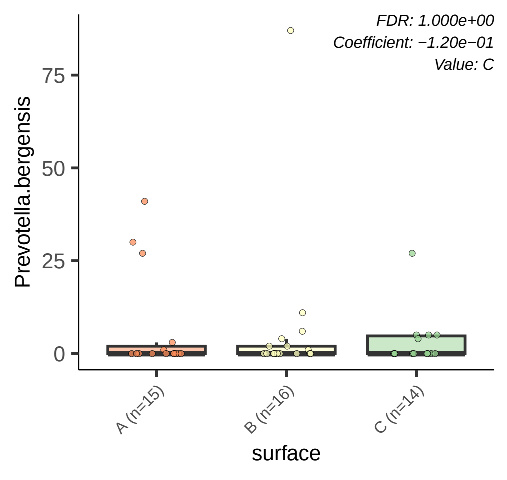
FDR: 1.000e+00
Coefficient: −1.20e−01
Value: C
Prevotella.bergensis
75
50
25
0
A (n=15)
B (n=16)
C (n=14)
surface

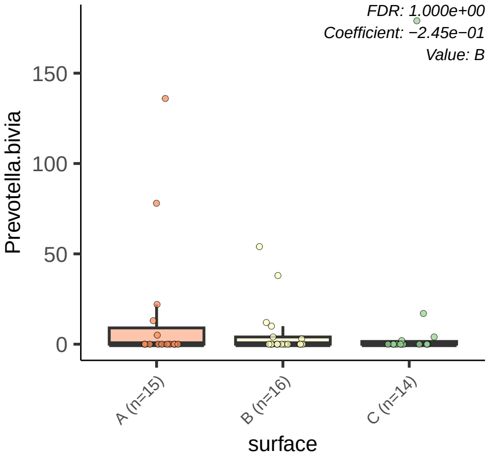
FDR: 1.000e+00
Coefficient: −2.45e−01
Value: B
Prevotella.bivia
150
100
50
0
A (n=15)
B (n=16)
C (n=14)
surface

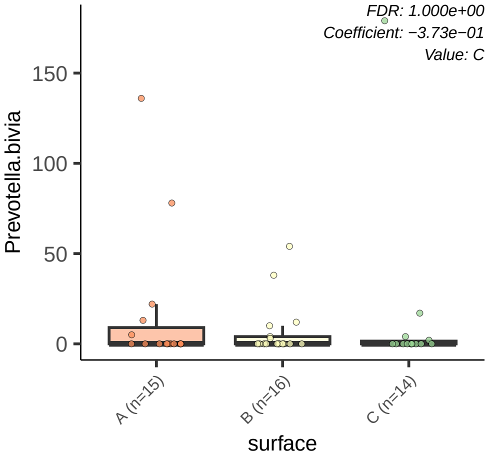

FDR: 1.000e+00
Coefficient: −3.73e−01
Value: C
Prevotella.bivia
150
100
50
0
A (n=15)
B (n=16)
C (n=14)
surface

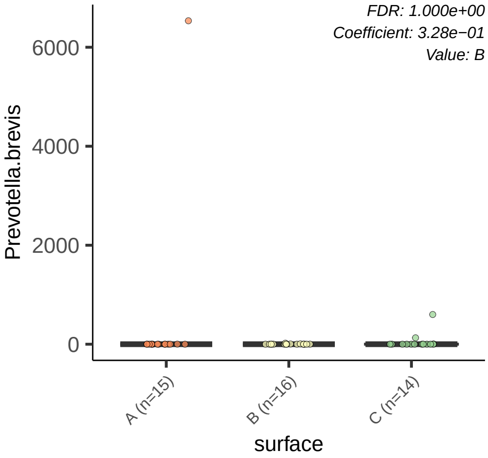
FDR: 1.000e+00
Coefficient: 3.28e−01
Value: B
Prevotella.brevis
6000
4000
2000
0
A (n=15)
B (n=16)
C (n=14)
surface

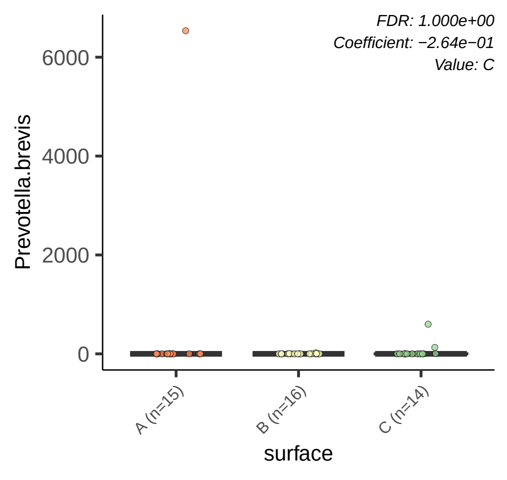

FDR: 1.000e+00
Coefficient: −2.64e−01
Value: C
Prevotella.brevis
6000
4000
2000
0
A (n=15)
B (n=16)
C (n=14)
surface

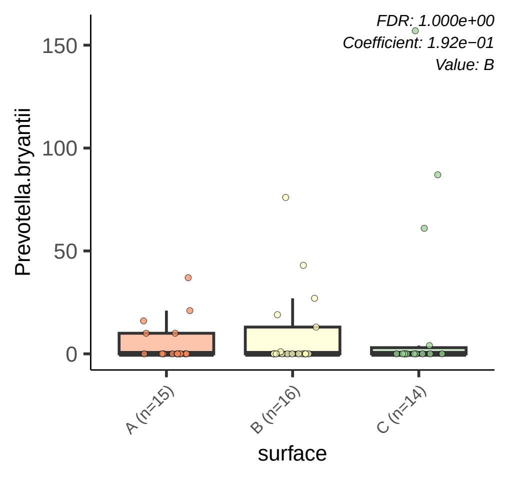
FDR: 1.000e+00
Coefficient: 1.92e−01
Value: B
Prevotella.bryantii
150
100
50
0
A (n=15)
B (n=16)
C (n=14)
surface

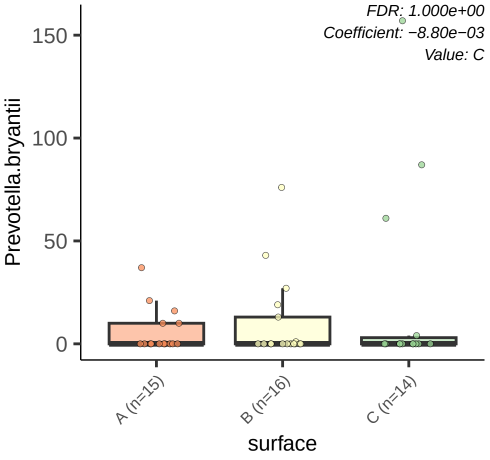

FDR: 1.000e+00
Coefficient: −8.80e−03
Value: C
150
100
50
0
Prevotella.bryantii
A (n=15)
B (n=16)
C (n=14)
surface

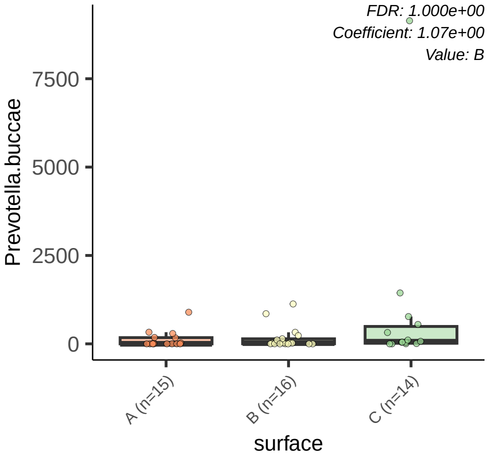
FDR: 1.000e+00
Coefficient: 1.07e+00
Value: B
Prevotella.buccae
7500
5000
2500
0
A (n=15)
B (n=16)
C (n=14)
surface

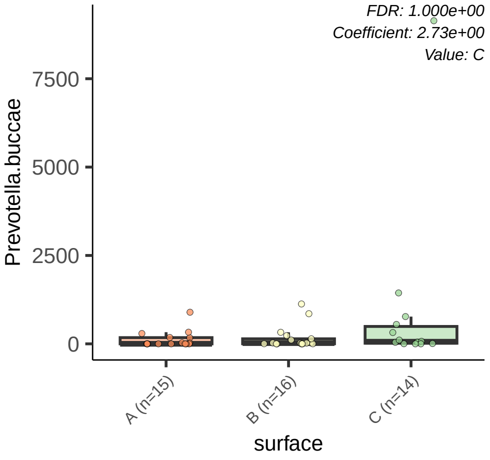

FDR: 1.000e+00
Coefficient: 2.73e+00
Value: C
Prevotella.buccae
7500
5000
2500
0
A (n=15)
B (n=16)
C (n=14)
surface

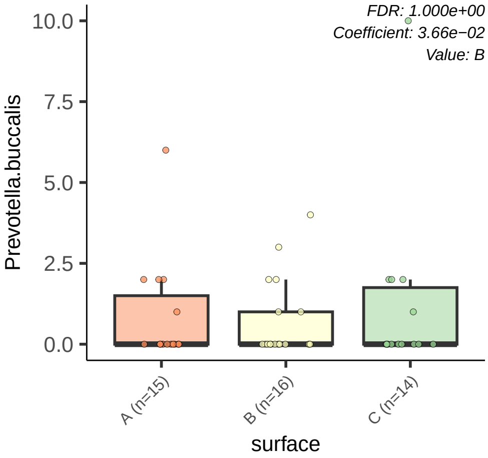

FDR: 1.000e+00
Coefficient: 3.66e−02
Value: B
Prevotella.buccalis
10.0
7.5
5.0
2.5
0.0
A (n=15)
B (n=16)
C (n=14)
surface

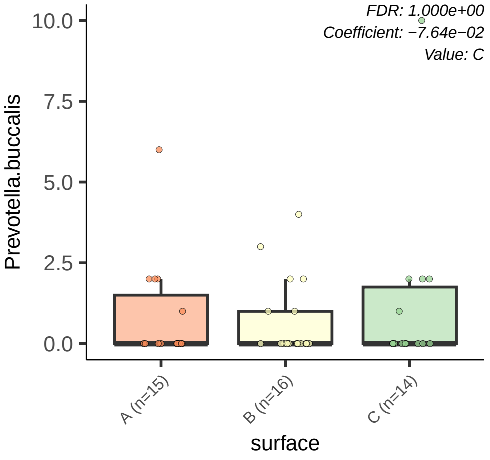

FDR: 1.000e+00
Coefficient: −7.64e−02
Value: C
Prevotella.buccalis
10.0
7.5
5.0
2.5
0.0
A (n=15)
B (n=16)
C (n=14)
surface

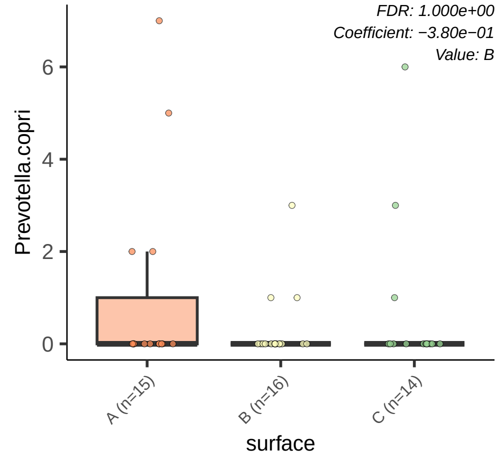

FDR: 1.000e+00
Coefficient: −3.80e−01
Value: B
Prevotella.copri
0
2
4
6
A (n=15)
B (n=16)
C (n=14)
surface

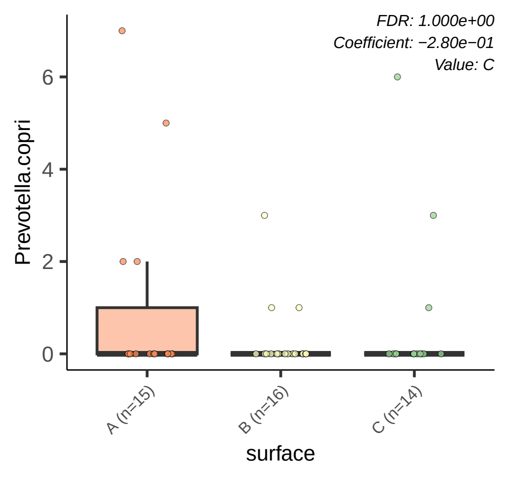

FDR: 1.000e+00
Coefficient: −2.80e−01
Value: C
Prevotella.copri
0
2
4
6
A (n=15)
B (n=16)
C (n=14)
surface

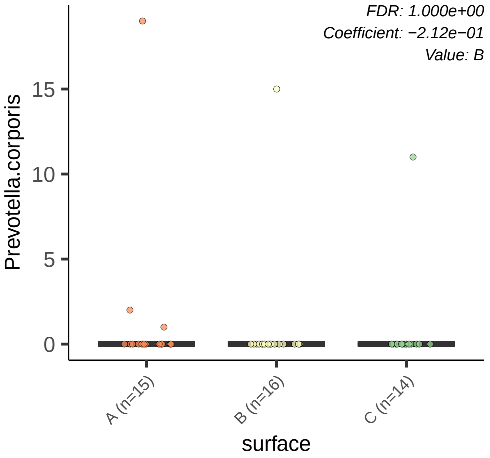

FDR: 1.000e+00
Coefficient: −2.12e−01
Value: B
Prevotella.corporis
0
5
10
15
A (n=15)
B (n=16)
C (n=14)
surface

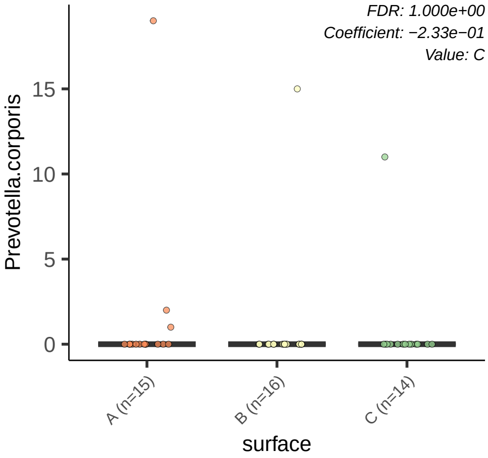
FDR: 1.000e+00
Coefficient: −2.33e−01
Value: C
Prevotella.corporis
0
5
10
15
A (n=15)
B (n=16)
C (n=14)
surface

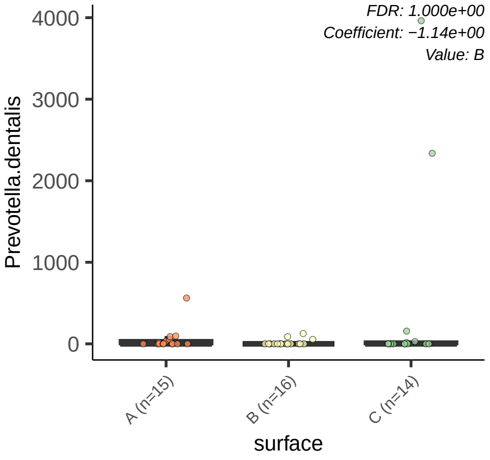
FDR: 1.000e+00
Coefficient: −1.14e+00
Value: B
4000
3000
2000
1000
0
Prevotella.dentalis
A (n=15)
B (n=16)
C (n=14)
surface

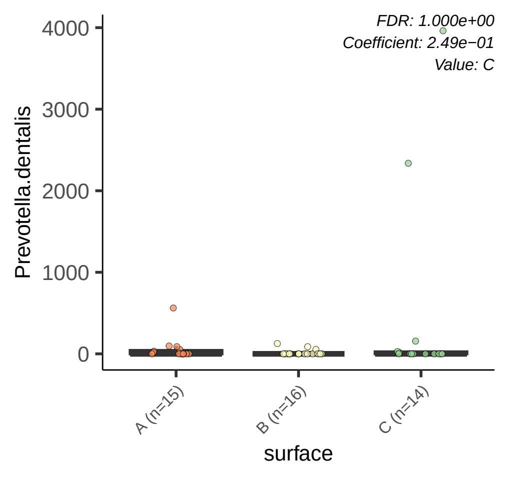

FDR: 1.000e+00
Coefficient: 2.49e−01
Value: C
4000
3000
2000
1000
0
Prevotella.dentalis
A (n=15)
B (n=16)
C (n=14)
surface

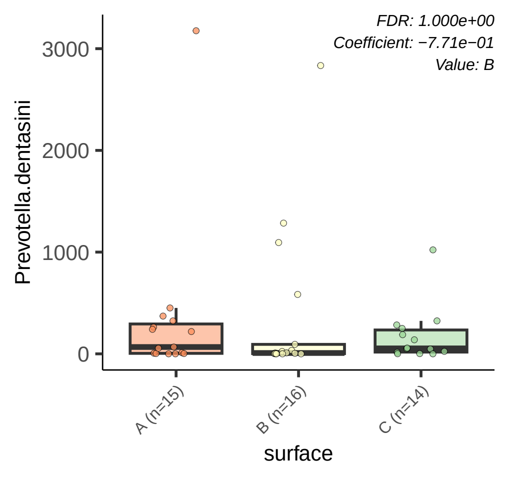
FDR: 1.000e+00
Coefficient: −7.71e−01
Value: B
Prevotella.dentasini
3000
2000
1000
0
A (n=15)
B (n=16)
C (n=14)
surface

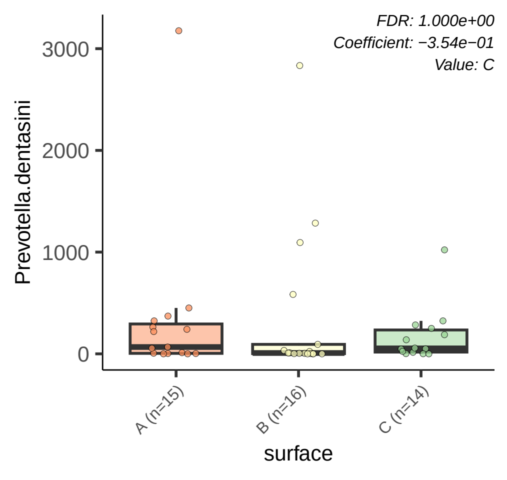
FDR: 1.000e+00
Coefficient: −3.54e−01
Value: C
Prevotella.dentasini
3000
2000
1000
0
A (n=15)
B (n=16)
C (n=14)
surface

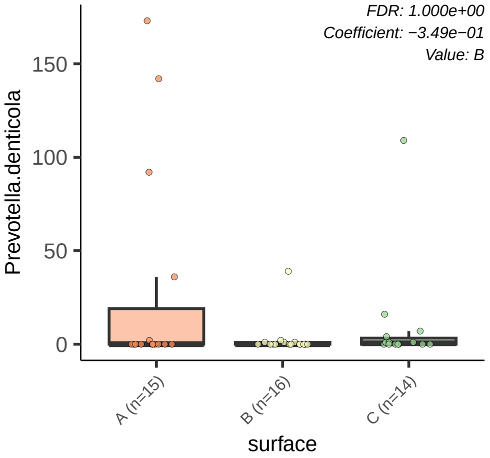
FDR: 1.000e+00
Coefficient: −3.49e−01
Value: B
Prevotella.denticola
150
100
50
0
A (n=15)
B (n=16)
C (n=14)
surface

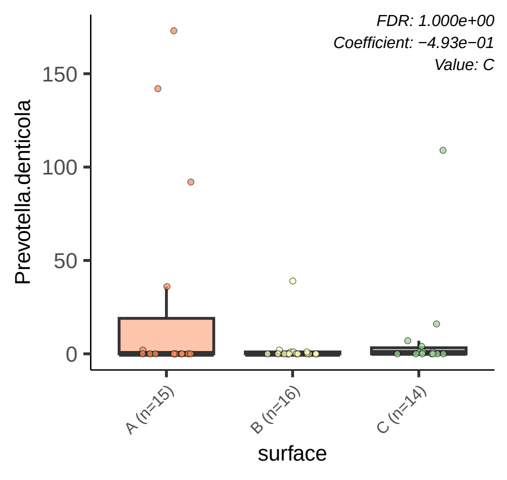
FDR: 1.000e+00
Coefficient: −4.93e−01
Value: C
Prevotella.denticola
150
100
50
0
A (n=15)
B (n=16)
C (n=14)
surface

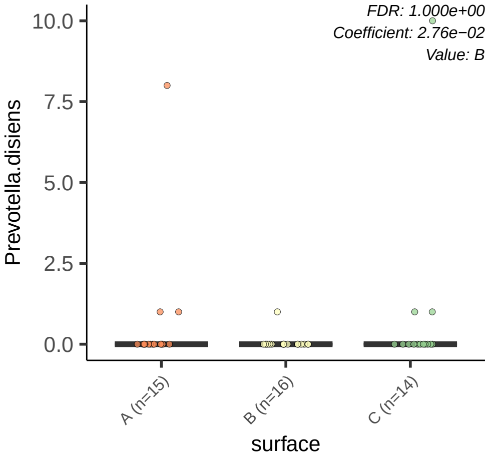
FDR: 1.000e+00
Coefficient: 2.76e−02
Value: B
Prevotella.disiens
10.0
7.5
5.0
2.5
0.0
A (n=15)
B (n=16)
C (n=14)
surface

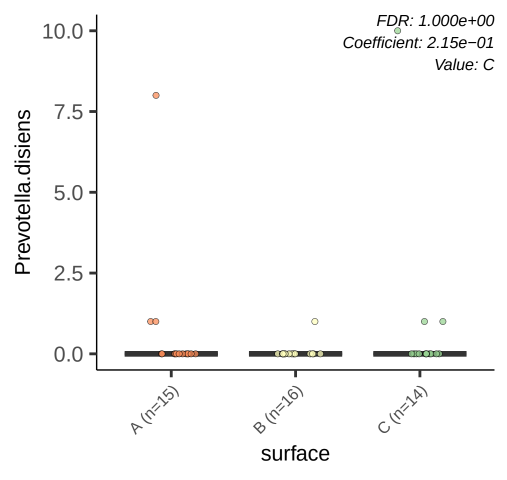
FDR: 1.000e+00
Coefficient: 2.15e−01
Value: C
10.0
7.5
5.0
2.5
0.0
Prevotella.disiens
A (n=15)
B (n=16)
C (n=14)
surface

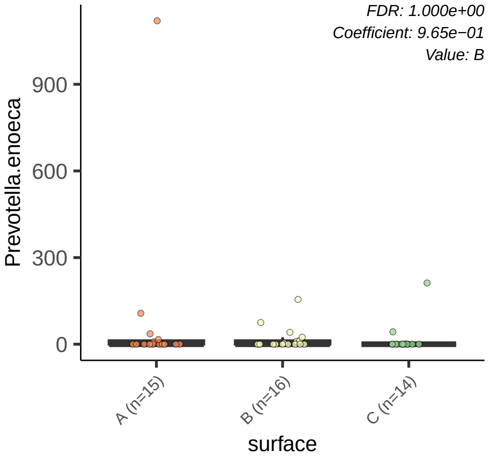
FDR: 1.000e+00
Coefficient: 9.65e−01
Value: B
Prevotella.enoeca
900
600
300
0
A (n=15)
B (n=16)
C (n=14)
surface

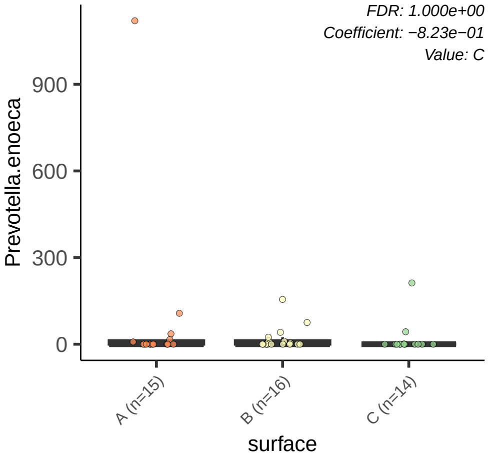
FDR: 1.000e+00
Coefficient: −8.23e−01
Value: C
Prevotella.enoeca
900
600
300
0
A (n=15)
B (n=16)
C (n=14)
surface

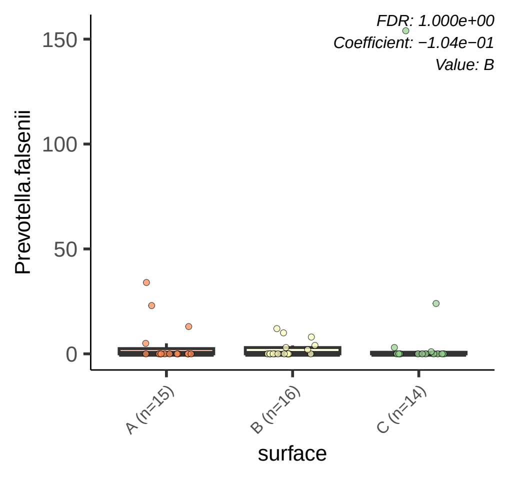
FDR: 1.000e+00
Coefficient: −1.04e−01
Value: B
Prevotella.falsenii
150
100
50
0
A (n=15)
B (n=16)
C (n=14)
surface

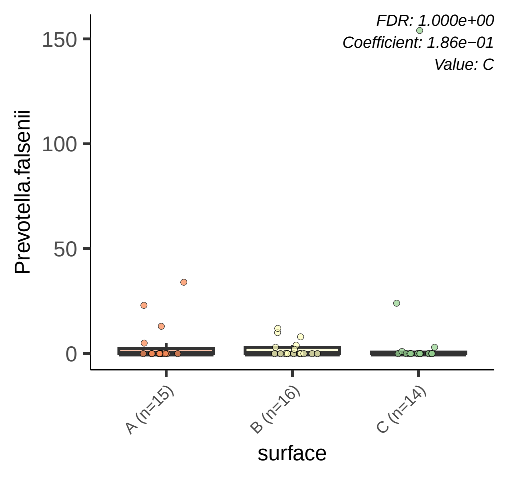
FDR: 1.000e+00
Coefficient: 1.86e−01
Value: C
150
100
50
0
Prevotella.falsenii
A (n=15)
B (n=16)
C (n=14)
surface

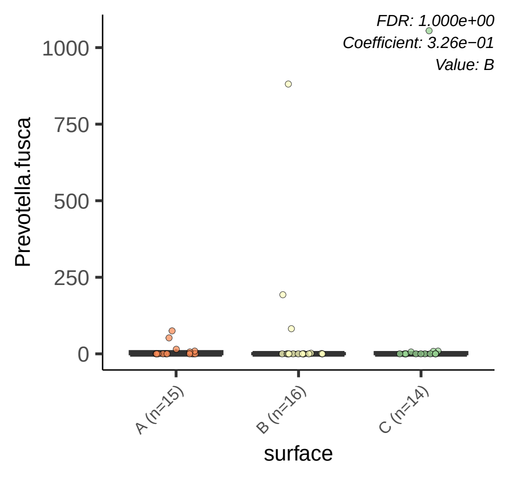

FDR: 1.000e+00
Coefficient: 3.26e−01
Value: B
Prevotella.fusca
1000
750
500
250
0
A (n=15)
B (n=16)
C (n=14)
surface

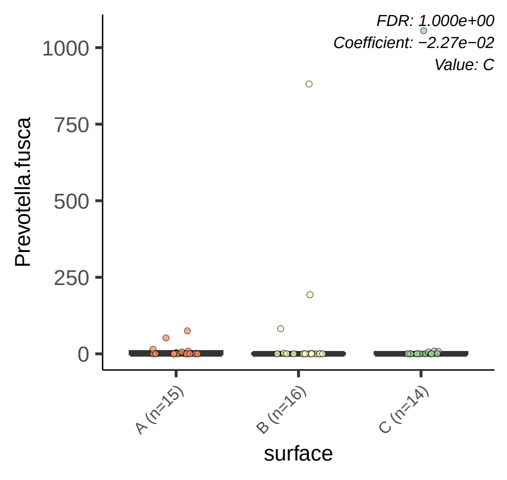

FDR: 1.000e+00
Coefficient: −2.27e−02
Value: C
1000
750
500
250
0
Prevotella.fusca
A (n=15)
B (n=16)
C (n=14)
surface

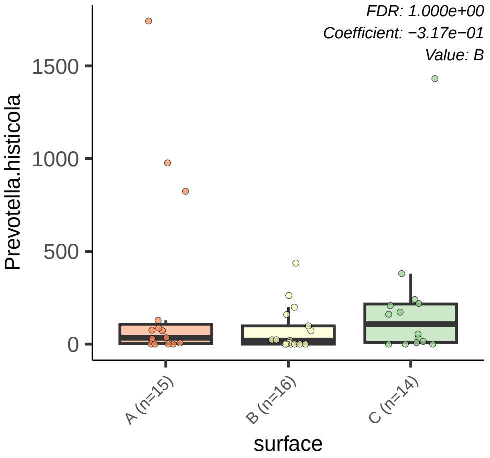

FDR: 1.000e+00
Coefficient: −3.17e−01
Value: B
Prevotella.histicola
0
500
1000
1500
A (n=15)
B (n=16)
C (n=14)
surface

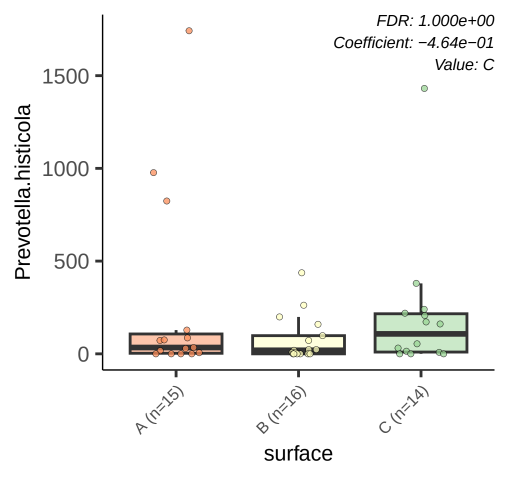
FDR: 1.000e+00
Coefficient: −4.64e−01
Value: C
Prevotella.histicola
1500
1000
500
0
A (n=15)
B (n=16)
C (n=14)
surface

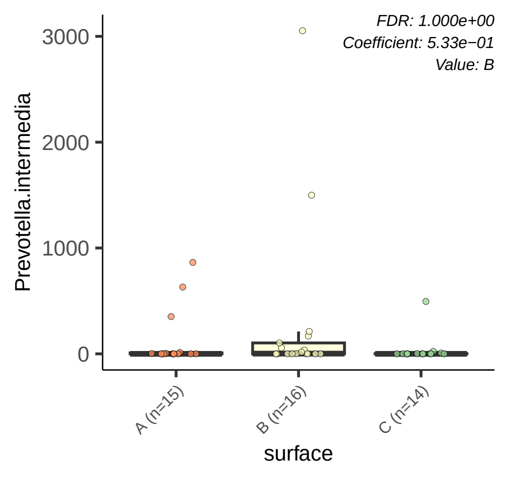

FDR: 1.000e+00
Coefficient: 5.33e−01
Value: B
Prevotella.intermedia
3000
2000
1000
0
A (n=15)
B (n=16)
C (n=14)
surface

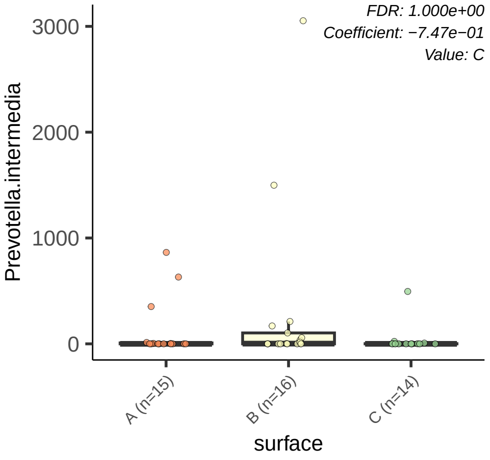
FDR: 1.000e+00
Coefficient: −7.47e−01
Value: C
Prevotella.intermedia
3000
2000
1000
0
A (n=15)
B (n=16)
C (n=14)
surface

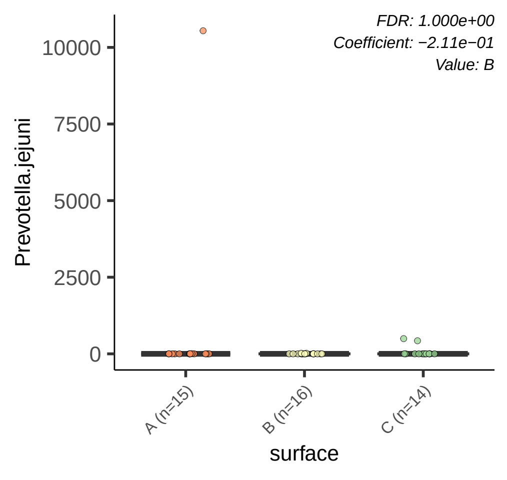

FDR: 1.000e+00
Coefficient: −2.11e−01
Value: B
Prevotella.jejuni
10000
7500
5000
2500
0
A (n=15)
B (n=16)
C (n=14)
surface

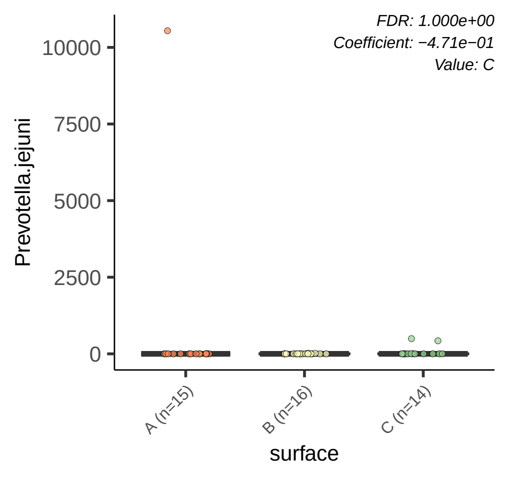

FDR: 1.000e+00
Coefficient: −4.71e−01
Value: C
10000
7500
5000
2500
0
Prevotella.jejuni
A (n=15)
B (n=16)
C (n=14)
surface

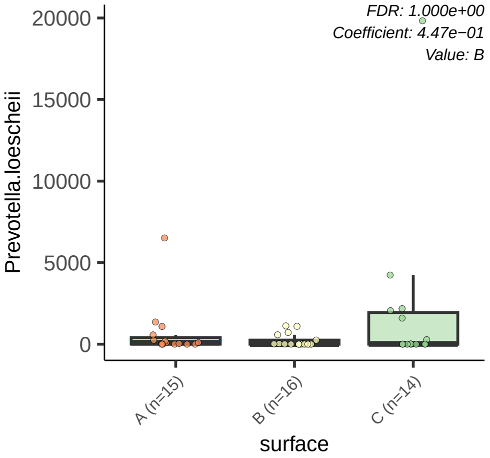

FDR: 1.000e+00
Coefficient: 4.47e−01
Value: B
Prevotella.loescheii
20000
15000
10000
5000
0
A (n=15)
B (n=16)
C (n=14)
surface

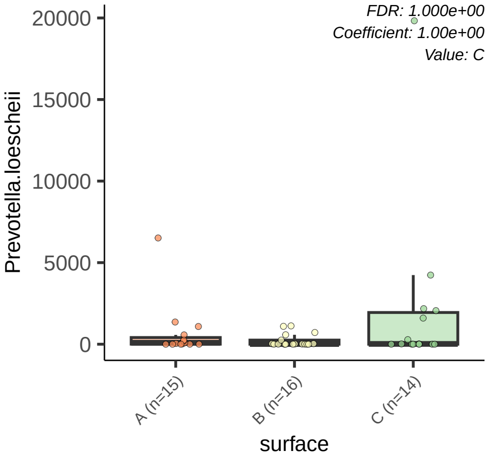
FDR: 1.000e+00
Coefficient: 1.00e+00
Value: C
Prevotella.loescheii
20000
15000
10000
5000
0
A (n=15)
B (n=16)
C (n=14)
surface

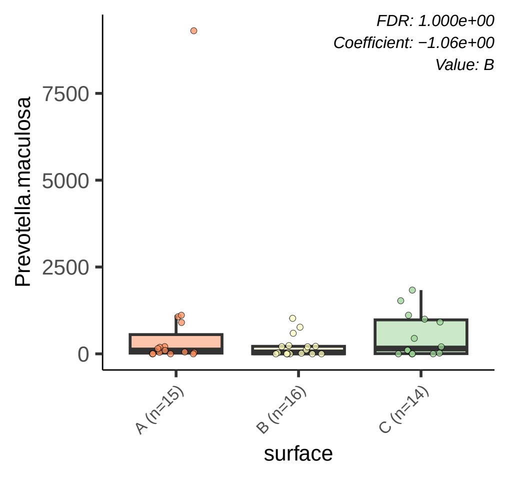

FDR: 1.000e+00
Coefficient: −1.06e+00
Value: B
Prevotella.maculosa
7500
5000
2500
0
A (n=15)
B (n=16)
C (n=14)
surface

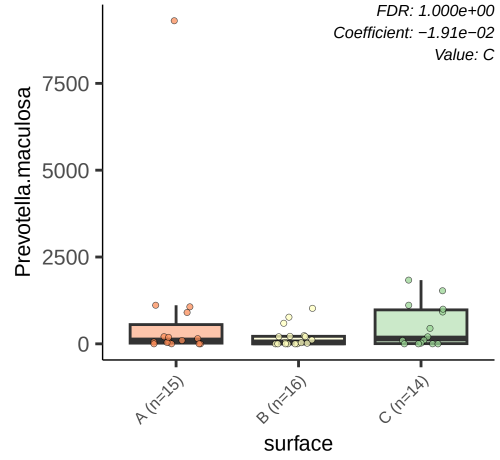
FDR: 1.000e+00
Coefficient: −1.91e−02
Value: C
Prevotella.maculosa
7500
5000
2500
0
A (n=15)
B (n=16)
C (n=14)
surface

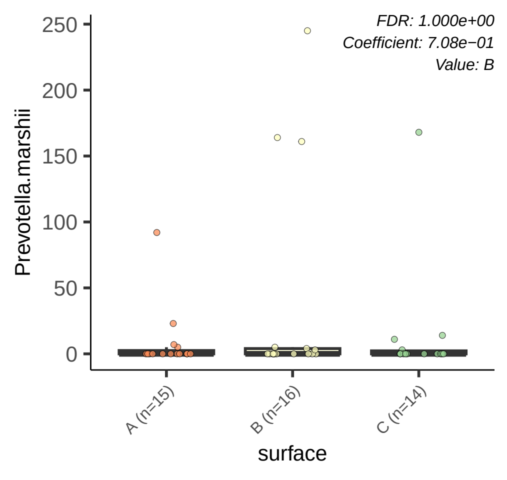
FDR: 1.000e+00
Coefficient: 7.08e−01
Value: B
250
200
150
100
50
0
Prevotella.marshii
A (n=15)
B (n=16)
C (n=14)
surface

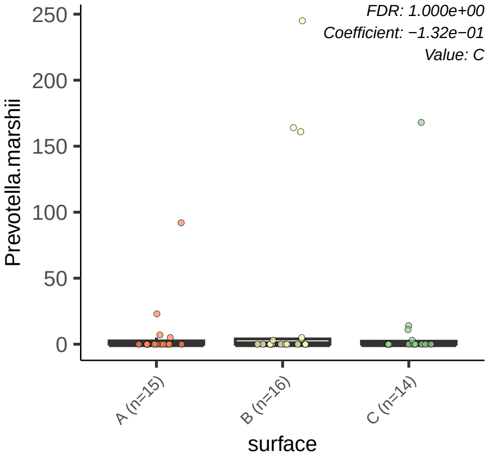

FDR: 1.000e+00
Coefficient: −1.32e−01
Value: C
Prevotella.marshii
250
200
150
100
50
0
A (n=15)
B (n=16)
C (n=14)
surface

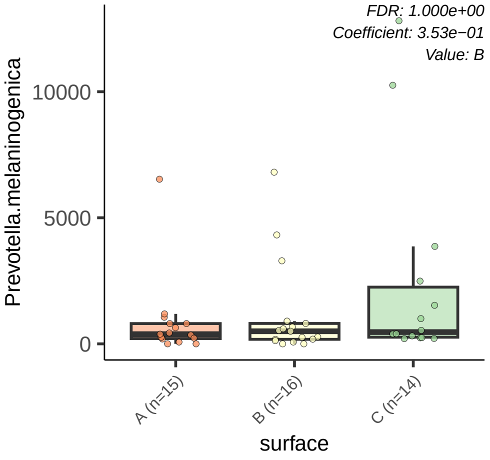
FDR: 1.000e+00
Coefficient: 3.53e−01
Value: B
Prevotella.melaninogenica
10000
5000
0
A (n=15)
B (n=16)
C (n=14)
surface

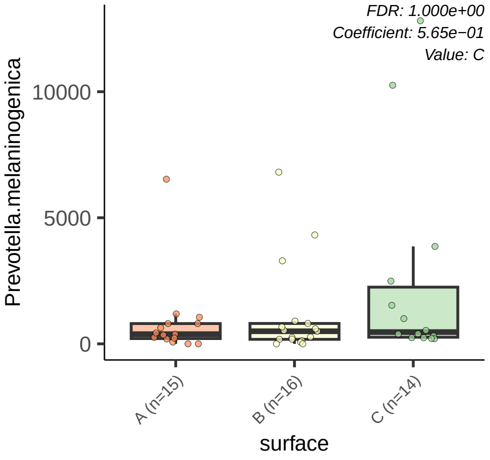

FDR: 1.000e+00
Coefficient: 5.65e−01
Value: C
Prevotella.melaninogenica
10000
5000
0
A (n=15)
B (n=16)
C (n=14)
surface

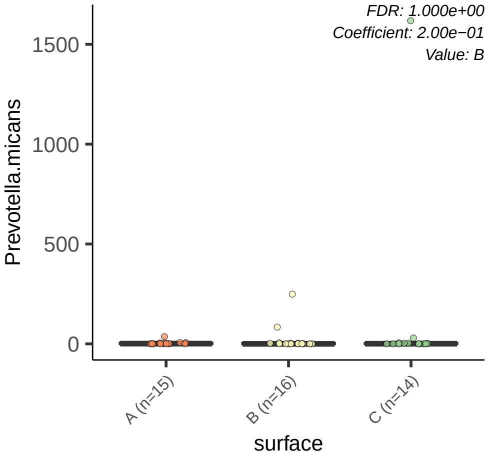
FDR: 1.000e+00
Coefficient: 2.00e−01
Value: B
Prevotella.micans
1500
1000
500
0
A (n=15)
B (n=16)
C (n=14)
surface

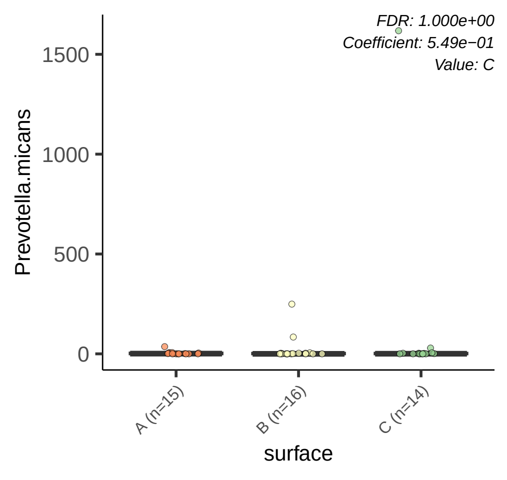
FDR: 1.000e+00
Coefficient: 5.49e−01
Value: C
Prevotella.micans
1500
1000
500
0
A (n=15)
B (n=16)
C (n=14)
surface

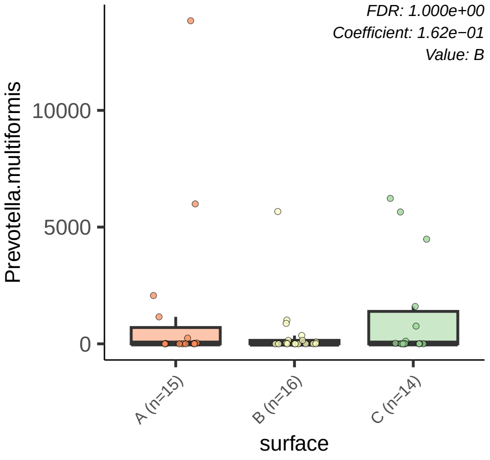
FDR: 1.000e+00
Coefficient: 1.62e−01
Value: B
Prevotella.multiformis
10000
5000
0
A (n=15)
B (n=16)
C (n=14)
surface

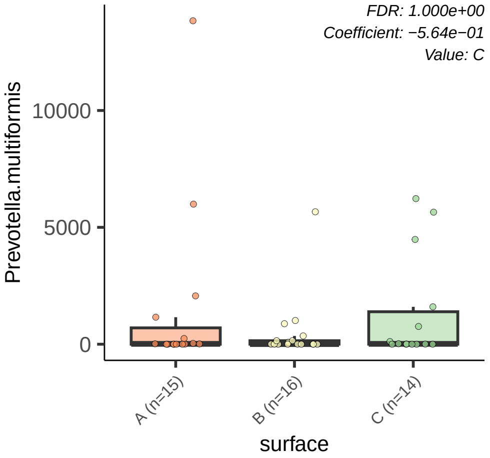

FDR: 1.000e+00
Coefficient: −5.64e−01
Value: C
Prevotella.multiformis
10000
5000
0
A (n=15)
B (n=16)
C (n=14)
surface

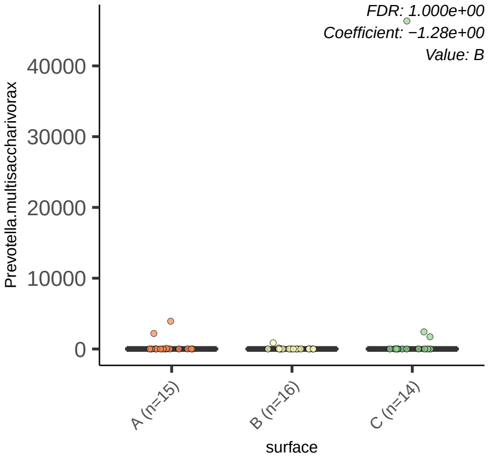
FDR: 1.000e+00
Coefficient: −1.28e+00
Value: B
Prevotella.multisaccharivorax
40000
30000
20000
10000
0
A (n=15)
B (n=16)
C (n=14)
surface

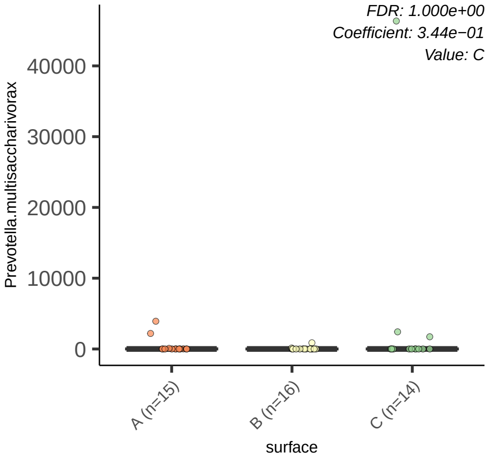
FDR: 1.000e+00
Coefficient: 3.44e−01
Value: C
Prevotella.multisaccharivorax
40000
30000
20000
10000
0
A (n=15)
B (n=16)
C (n=14)
surface

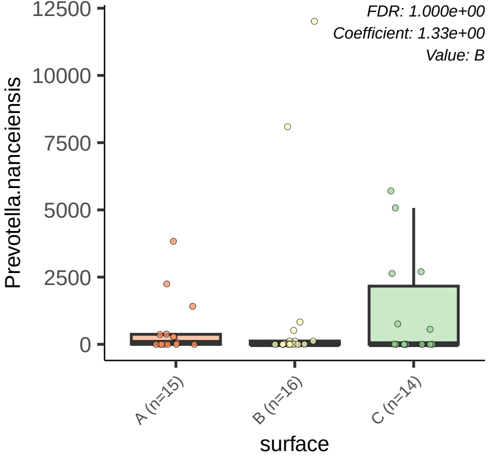
FDR: 1.000e+00
Coefficient: 1.33e+00
Value: B
12500
10000
7500
5000
2500
0
Prevotella.nanceiensis
A (n=15)
B (n=16)
C (n=14)
surface

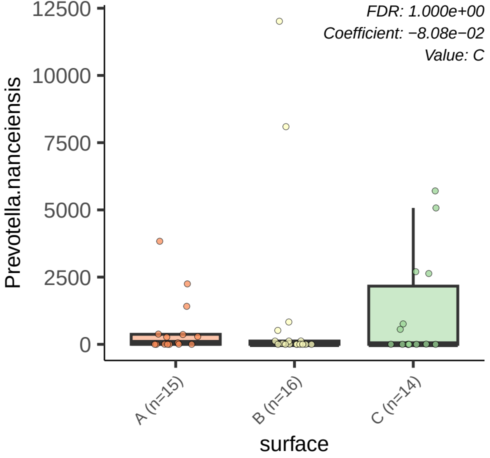

FDR: 1.000e+00
Coefficient: −8.08e−02
Value: C
Prevotella.nanceiensis
12500
10000
7500
5000
2500
0
A (n=15)
B (n=16)
C (n=14)
surface

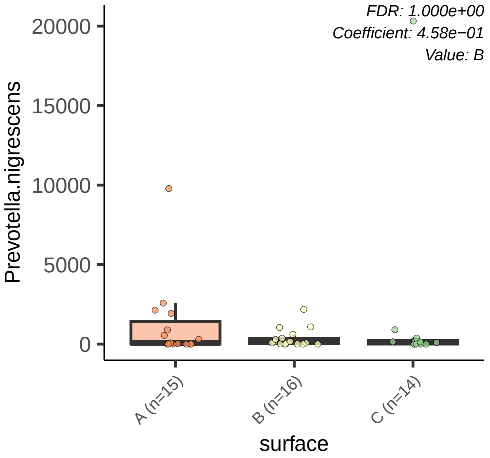
FDR: 1.000e+00
Coefficient: 4.58e−01
Value: B
Prevotella.nigrescens
20000
15000
10000
5000
0
A (n=15)
B (n=16)
C (n=14)
surface

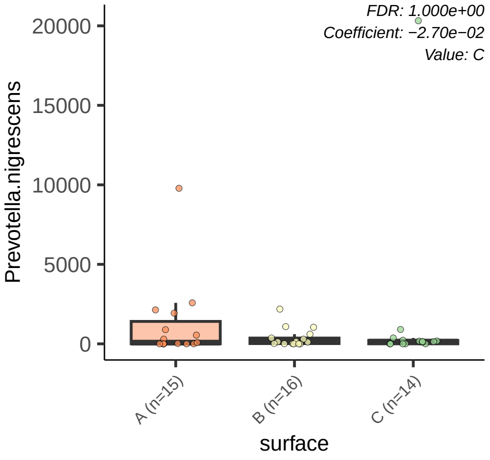
FDR: 1.000e+00
Coefficient: −2.70e−02
Value: C
20000
15000
10000
5000
0
Prevotella.nigrescens
A (n=15)
B (n=16)
C (n=14)
surface

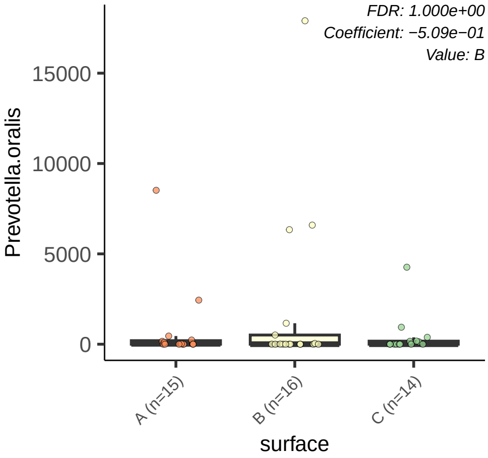

FDR: 1.000e+00
Coefficient: −5.09e−01
Value: B
Prevotella.oralis
15000
10000
5000
0
A (n=15)
B (n=16)
C (n=14)
surface

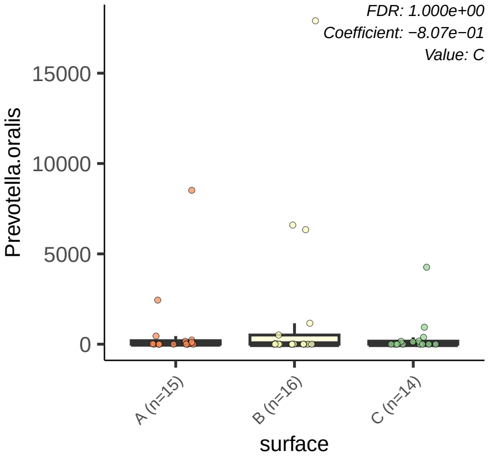
FDR: 1.000e+00
Coefficient: −8.07e−01
Value: C
Prevotella.oralis
15000
10000
5000
0
A (n=15)
B (n=16)
C (n=14)
surface

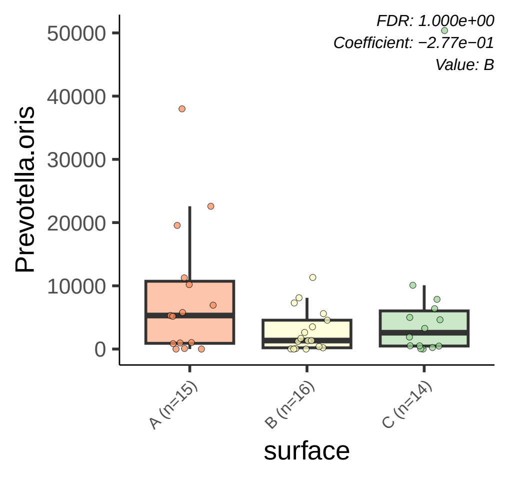

FDR: 1.000e+00
Coefficient: −2.77e−01
Value: B
Prevotella.oris
50000
40000
30000
20000
10000
0
A (n=15)
B (n=16)
C (n=14)
surface

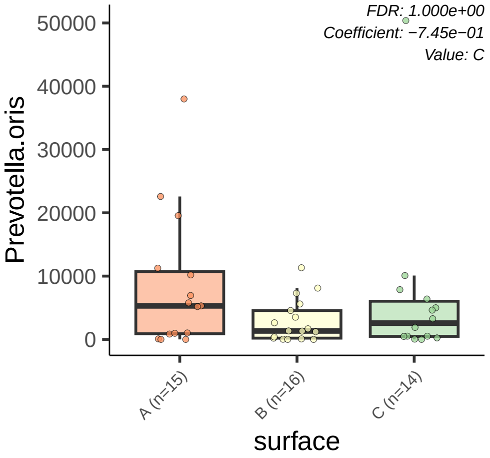
FDR: 1.000e+00
Coefficient: −7.45e−01
Value: C
Prevotella.oris
50000
40000
30000
20000
10000
0
A (n=15)
B (n=16)
C (n=14)
surface

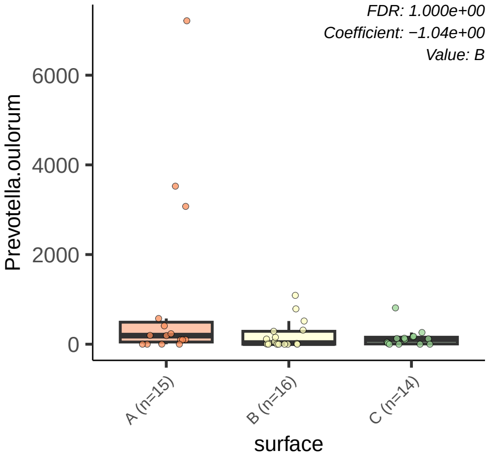
FDR: 1.000e+00
Coefficient: −1.04e+00
Value: B
Prevotella.oulorum
6000
4000
2000
0
A (n=15)
B (n=16)
C (n=14)
surface

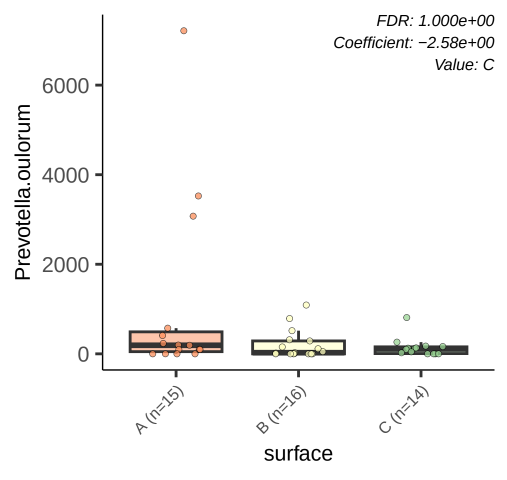
FDR: 1.000e+00
Coefficient: −2.58e+00
Value: C
Prevotella.oulorum
6000
4000
2000
0
A (n=15)
B (n=16)
C (n=14)
surface

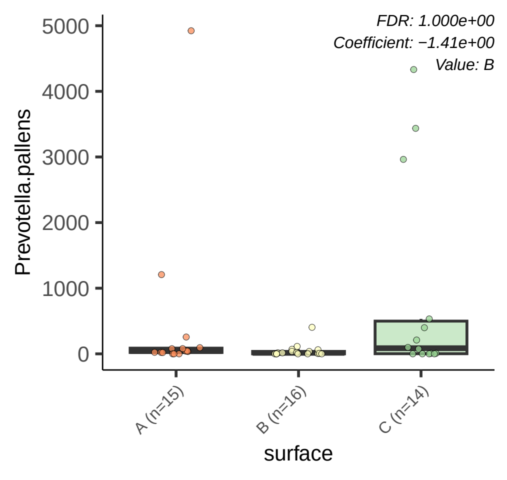
FDR: 1.000e+00
Coefficient: −1.41e+00
Value: B
Prevotella.pallens
5000
4000
3000
2000
1000
0
A (n=15)
B (n=16)
C (n=14)
surface

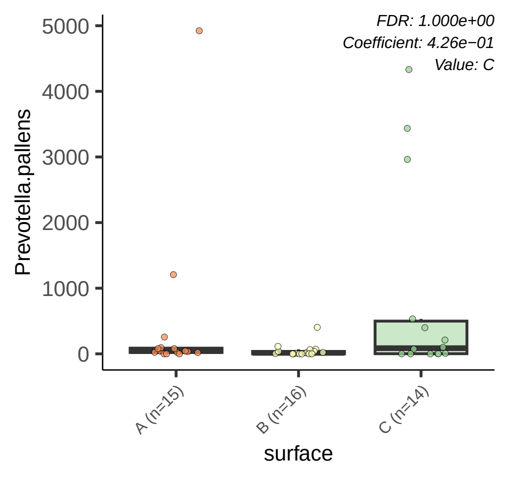
FDR: 1.000e+00
Coefficient: 4.26e−01
Value: C
5000
4000
3000
2000
1000
0
Prevotella.pallens
A (n=15)
B (n=16)
C (n=14)
surface

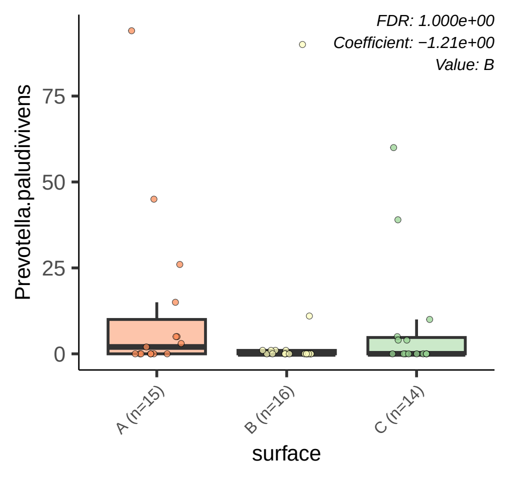

FDR: 1.000e+00
Coefficient: −1.21e+00
Value: B
Prevotella.paludivivens
75
50
25
0
A (n=15)
B (n=16)
C (n=14)
surface

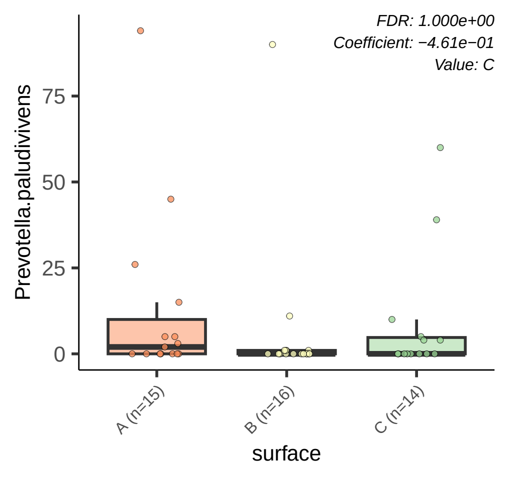

FDR: 1.000e+00
Coefficient: −4.61e−01
Value: C
Prevotella.paludivivens
75
50
25
0
A (n=15)
B (n=16)
C (n=14)
surface

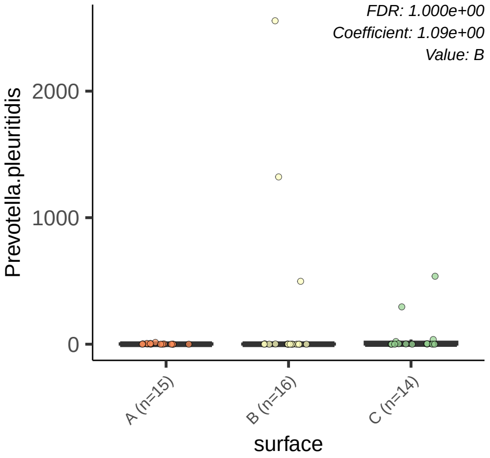
FDR: 1.000e+00
Coefficient: 1.09e+00
Value: B
Prevotella.pleuritidis
2000
1000
0
A (n=15)
B (n=16)
C (n=14)
surface

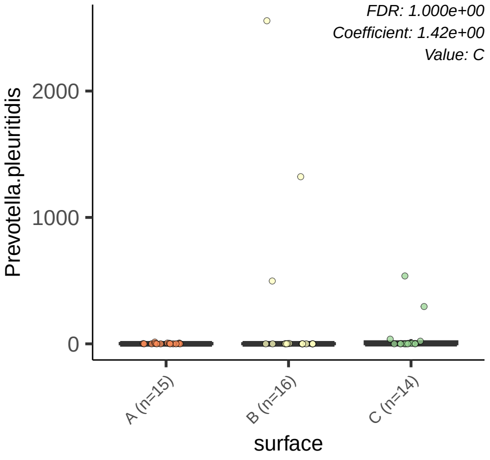

FDR: 1.000e+00
Coefficient: 1.42e+00
Value: C
2000
1000
0
Prevotella.pleuritidis
A (n=15)
B (n=16)
C (n=14)
surface

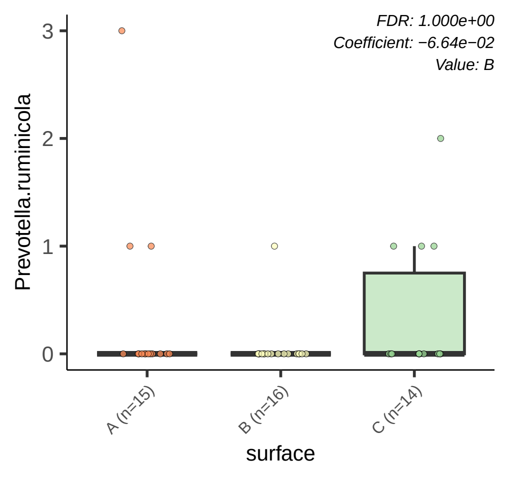
FDR: 1.000e+00
Coefficient: −6.64e−02
Value: B
Prevotella.ruminicola
3
2
1
0
A (n=15)
B (n=16)
C (n=14)
surface

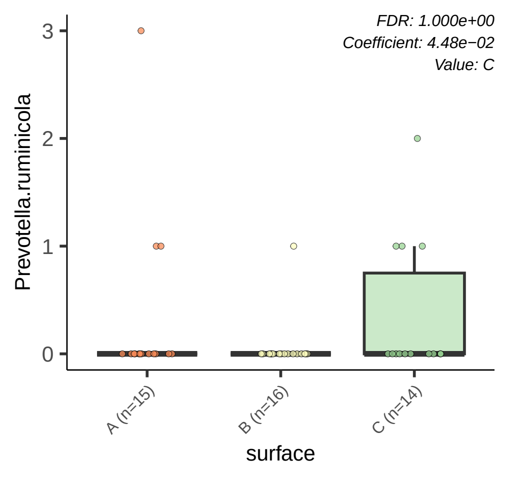

FDR: 1.000e+00
Coefficient: 4.48e−02
Value: C
Prevotella.ruminicola
3
2
1
0
A (n=15)
B (n=16)
C (n=14)
surface

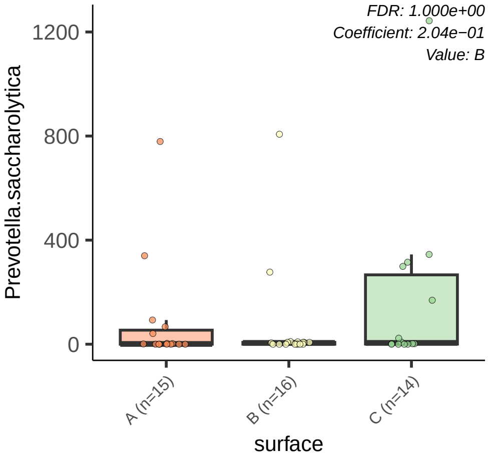

FDR: 1.000e+00
Coefficient: 2.04e−01
Value: B
Prevotella.saccharolytica
1200
800
400
0
A (n=15)
B (n=16)
C (n=14)
surface

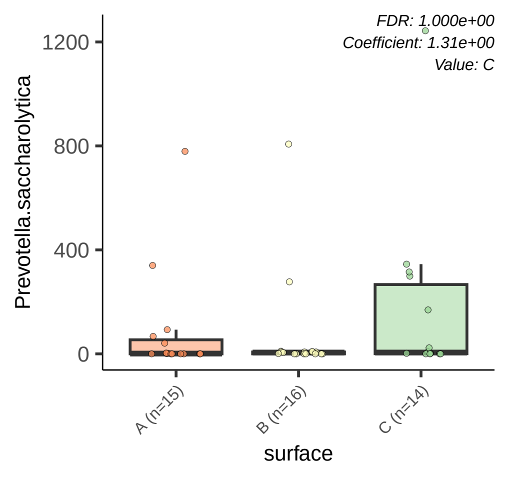

FDR: 1.000e+00
Coefficient: 1.31e+00
Value: C
Prevotella.saccharolytica
1200
800
400
0
A (n=15)
B (n=16)
C (n=14)
surface

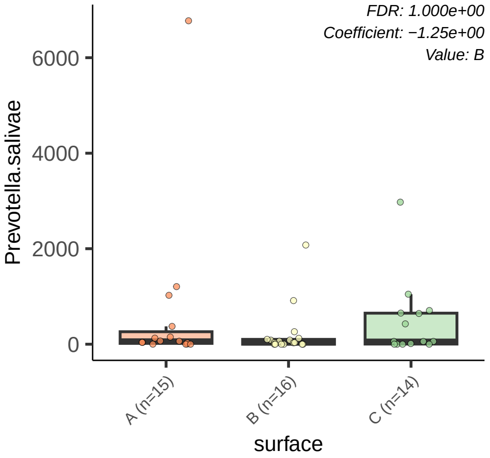
FDR: 1.000e+00
Coefficient: −1.25e+00
Value: B
Prevotella.salivae
6000
4000
2000
0
A (n=15)
B (n=16)
C (n=14)
surface

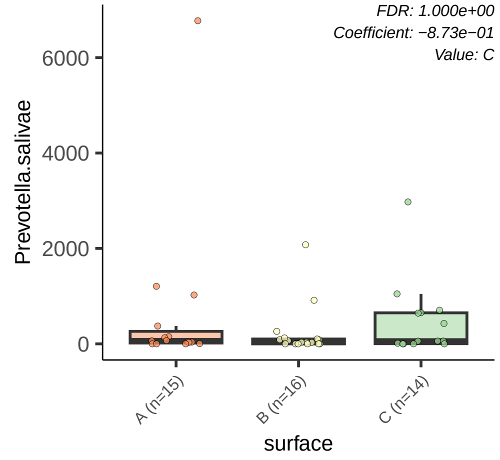
FDR: 1.000e+00
Coefficient: −8.73e−01
Value: C
Prevotella.salivae
6000
4000
2000
0
A (n=15)
B (n=16)
C (n=14)
surface

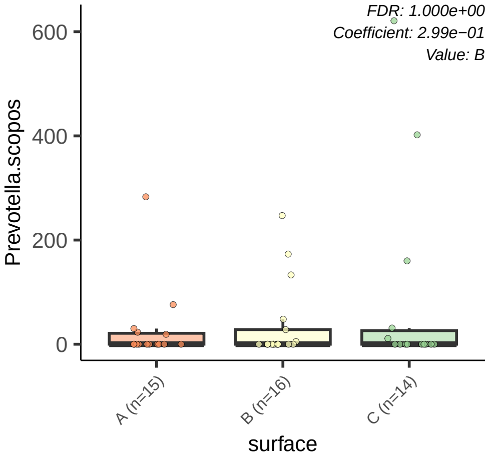

FDR: 1.000e+00
Coefficient: 2.99e−01
Value: B
Prevotella.scopos
600
400
200
0
A (n=15)
B (n=16)
C (n=14)
surface

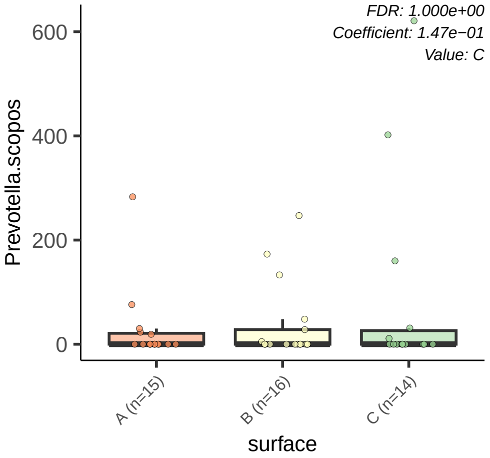
FDR: 1.000e+00
Coefficient: 1.47e−01
Value: C
Prevotella.scopos
600
400
200
0
A (n=15)
B (n=16)
C (n=14)
surface

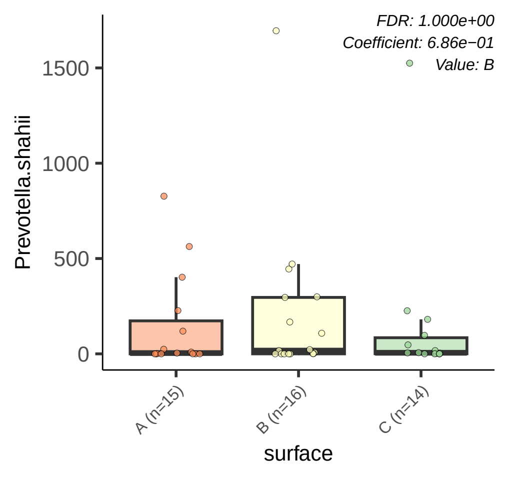
FDR: 1.000e+00
Coefficient: 6.86e−01
Value: B
Prevotella.shahii
1500
1000
500
0
A (n=15)
B (n=16)
C (n=14)
surface

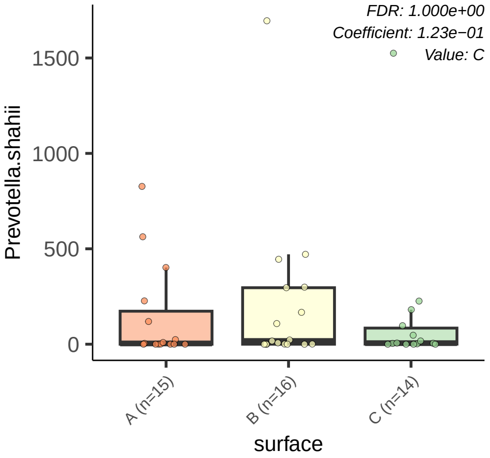

FDR: 1.000e+00
Coefficient: 1.23e−01
Value: C
Prevotella.shahii
1500
1000
500
0
A (n=15)
B (n=16)
C (n=14)
surface

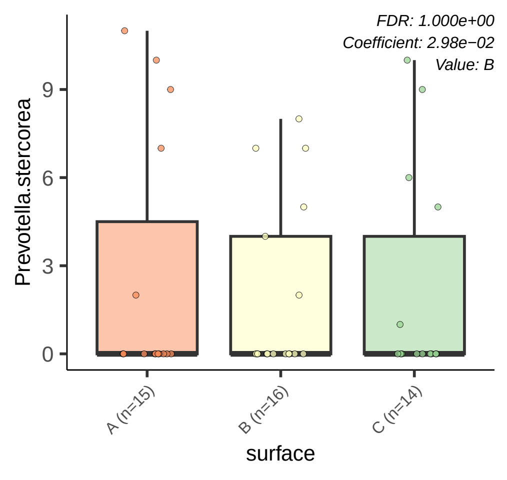

FDR: 1.000e+00
Coefficient: 2.98e−02
Value: B
Prevotella.stercorea
0
3
6
9
A (n=15)
B (n=16)
C (n=14)
surface

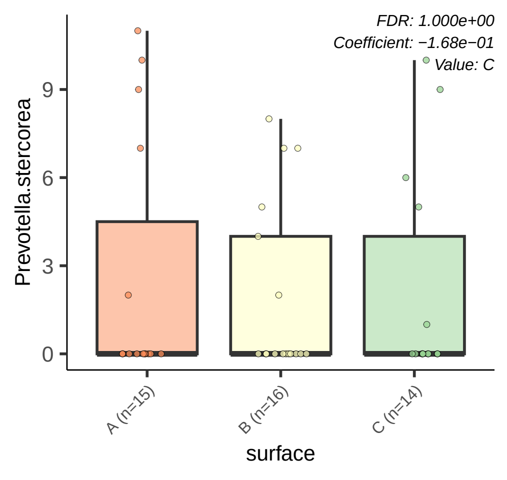

FDR: 1.000e+00
Coefficient: −1.68e−01
Value: C
Prevotella.stercorea
0
3
6
9
A (n=15)
B (n=16)
C (n=14)
surface

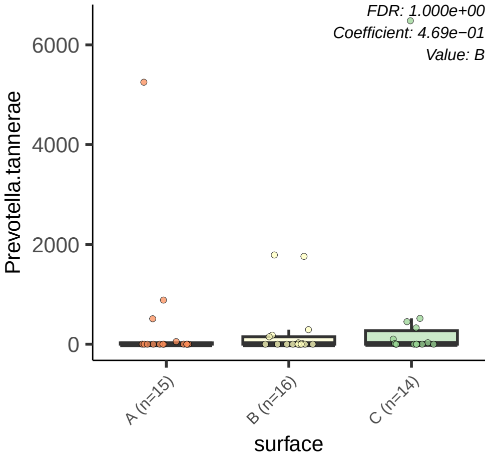
FDR: 1.000e+00
Coefficient: 4.69e−01
Value: B
Prevotella.tannerae
6000
4000
2000
0
A (n=15)
B (n=16)
C (n=14)
surface

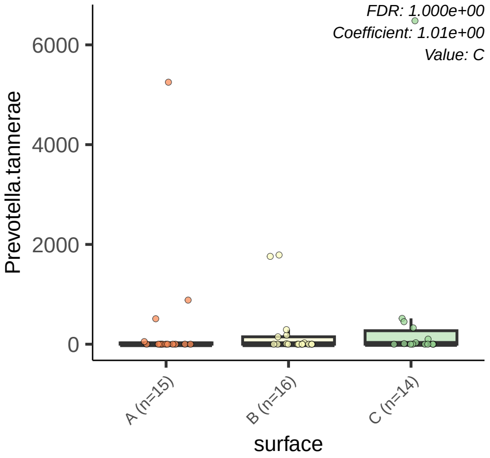

FDR: 1.000e+00
Coefficient: 1.01e+00
Value: C
Prevotella.tannerae
6000
4000
2000
0
A (n=15)
B (n=16)
C (n=14)
surface

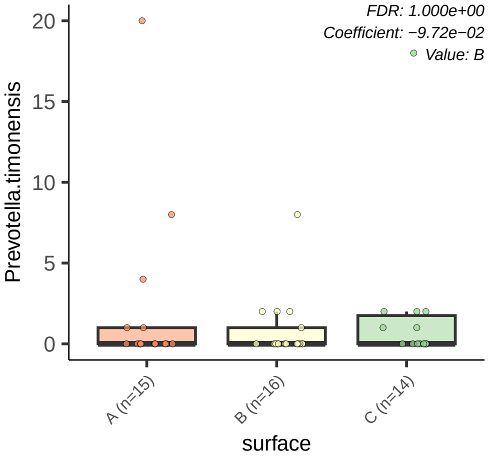

FDR: 1.000e+00
Coefficient: −9.72e−02
Value: B
Prevotella.timonensis
20
15
10
5
0
A (n=15)
B (n=16)
C (n=14)
surface

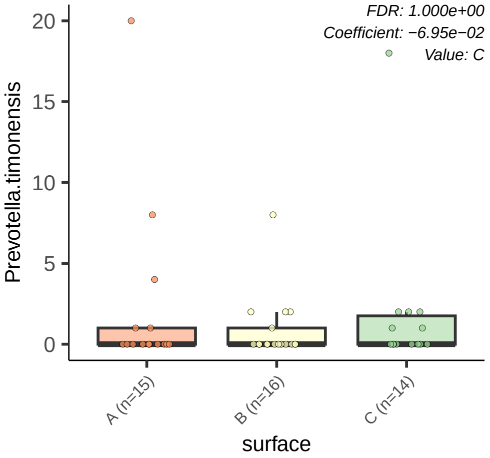

FDR: 1.000e+00
Coefficient: −6.95e−02
Value: C
Prevotella.timonensis
20
15
10
5
0
A (n=15)
B (n=16)
C (n=14)
surface

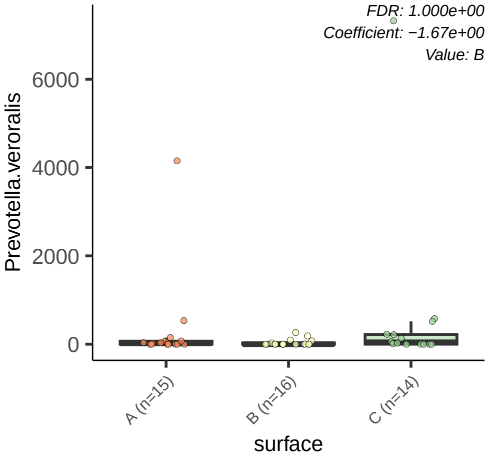

FDR: 1.000e+00
Coefficient: −1.67e+00
Value: B
6000
4000
2000
0
Prevotella.veroralis
A (n=15)
B (n=16)
C (n=14)
surface

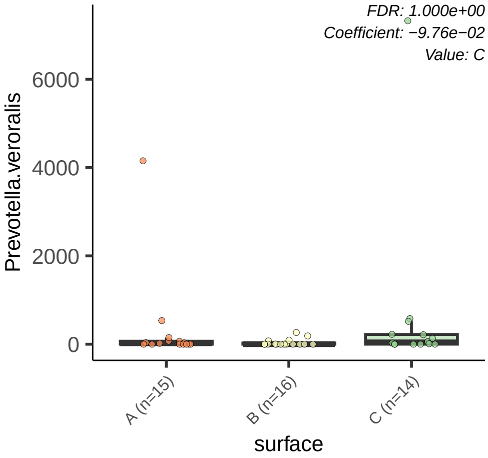
FDR: 1.000e+00
Coefficient: −9.76e−02
Value: C
Prevotella.veroralis
6000
4000
2000
0
A (n=15)
B (n=16)
C (n=14)
surface

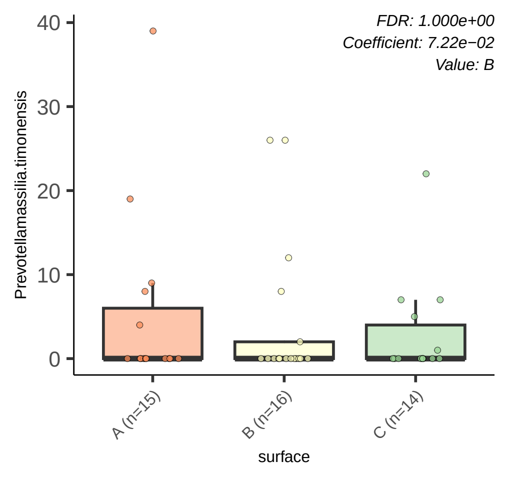
FDR: 1.000e+00
Coefficient: 7.22e−02
Value: B
Prevotellamassilia.timonensis
0
10
20
30
40
A (n=15)
B (n=16)
C (n=14)
surface

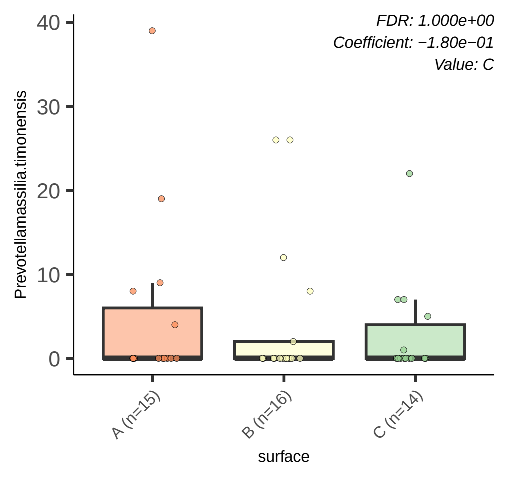

FDR: 1.000e+00
Coefficient: −1.80e−01
Value: C
Prevotellamassilia.timonensis
0
10
20
30
40
A (n=15)
B (n=16)
C (n=14)
surface

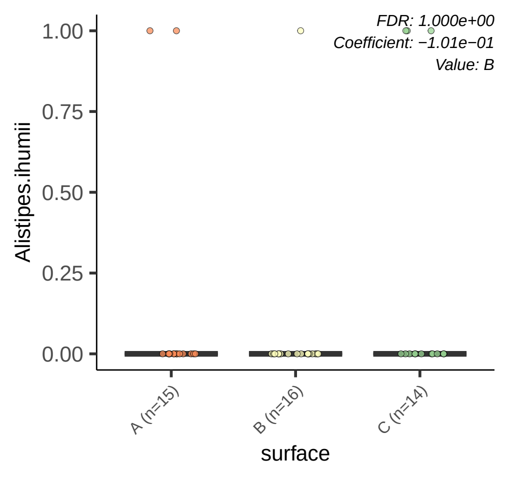
FDR: 1.000e+00
Coefficient: −1.01e−01
Value: B
Alistipes.ihumii
1.00
0.75
0.50
0.25
0.00
A (n=15)
B (n=16)
C (n=14)
surface

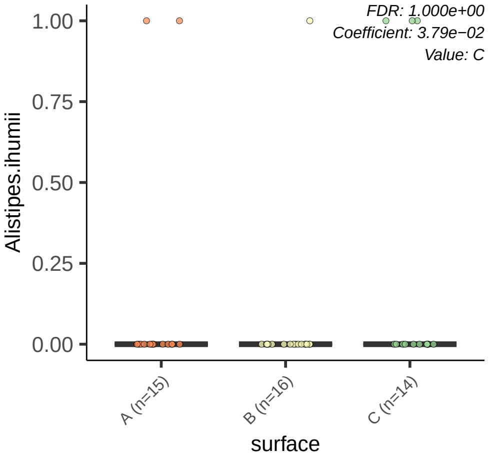
FDR: 1.000e+00
Coefficient: 3.79e−02
Value: C
1.00
0.75
0.50
0.25
0.00
Alistipes.ihumii
A (n=15)
B (n=16)
C (n=14)
surface

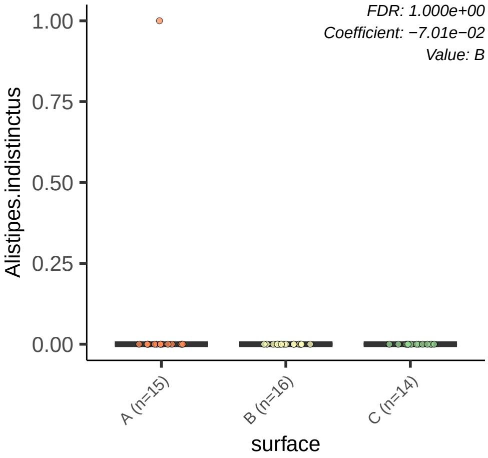

FDR: 1.000e+00
Coefficient: −7.01e−02
Value: B
1.00
0.75
0.50
0.25
0.00
Alistipes.indistinctus
A (n=15)
B (n=16)
C (n=14)
surface

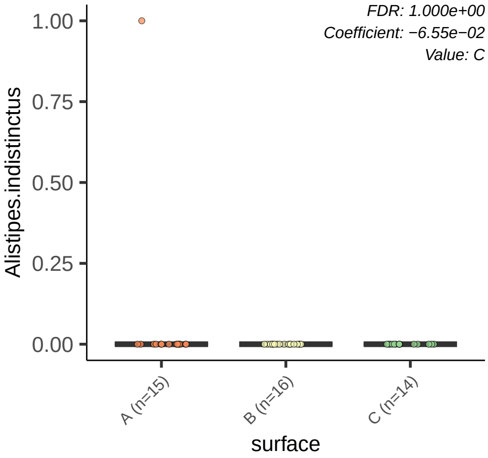

FDR: 1.000e+00
Coefficient: −6.55e−02
Value: C
Alistipes.indistinctus
1.00
0.75
0.50
0.25
0.00
A (n=15)
B (n=16)
C (n=14)
surface

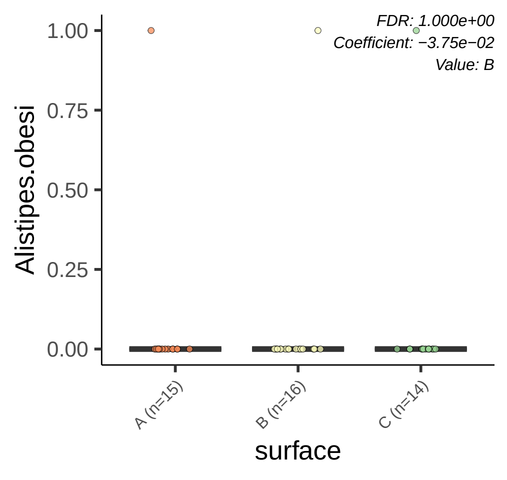
FDR: 1.000e+00
Coefficient: −3.75e−02
Value: B
Alistipes.obesi
1.00
0.75
0.50
0.25
0.00
A (n=15)
B (n=16)
C (n=14)
surface

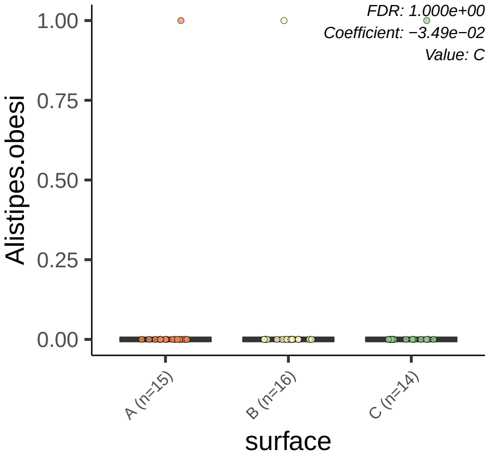

FDR: 1.000e+00
Coefficient: −3.49e−02
Value: C
Alistipes.obesi
1.00
0.75
0.50
0.25
0.00
A (n=15)
B (n=16)
C (n=14)
surface

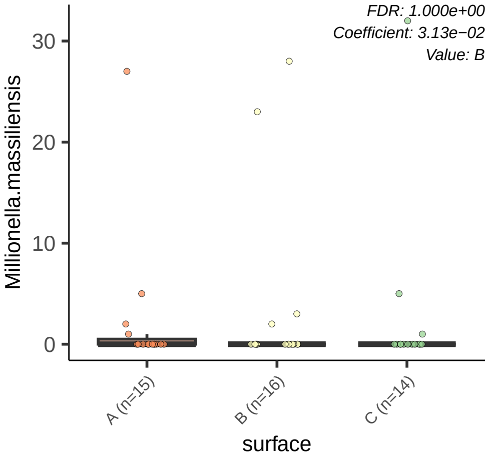
FDR: 1.000e+00
Coefficient: 3.13e−02
Value: B
Millionella.massiliensis
30
20
10
0
A (n=15)
B (n=16)
C (n=14)
surface

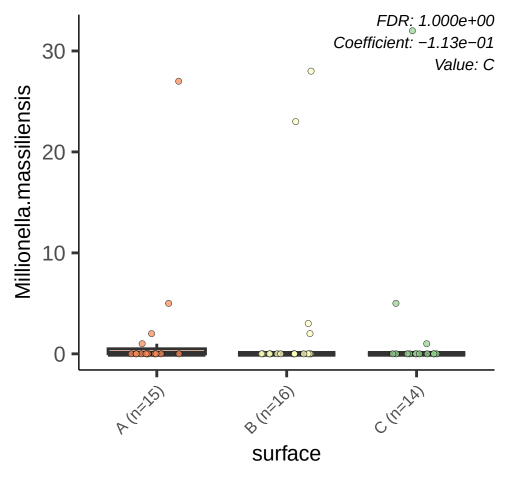

FDR: 1.000e+00
Coefficient: −1.13e−01
Value: C
Millionella.massiliensis
30
20
10
0
A (n=15)
B (n=16)
C (n=14)
surface

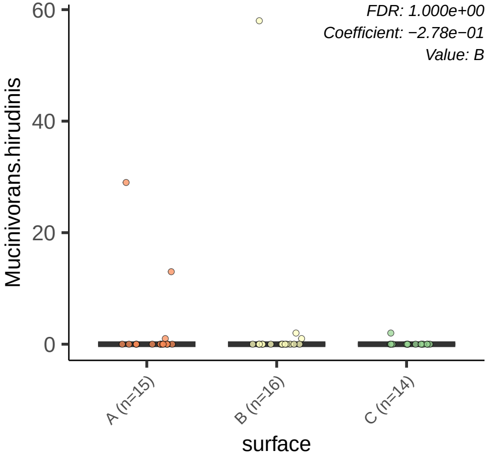
FDR: 1.000e+00
Coefficient: −2.78e−01
Value: B
Mucinivorans.hirudinis
60
40
20
0
A (n=15)
B (n=16)
C (n=14)
surface

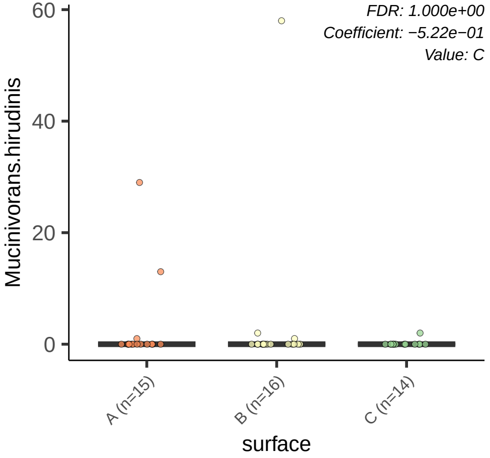
FDR: 1.000e+00
Coefficient: −5.22e−01
Value: C
Mucinivorans.hirudinis
60
40
20
0
A (n=15)
B (n=16)
C (n=14)
surface

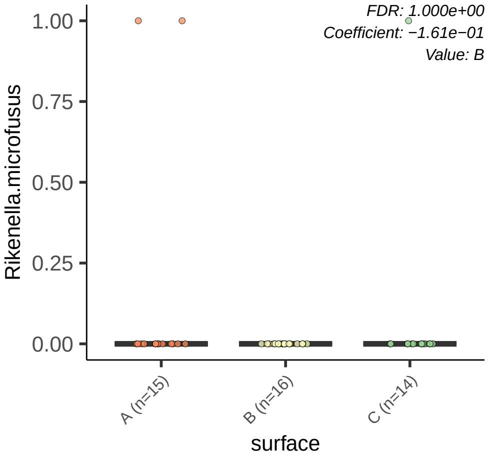

FDR: 1.000e+00
Coefficient: −1.61e−01
Value: B
Rikenella.microfusus
1.00
0.75
0.50
0.25
0.00
A (n=15)
B (n=16)
C (n=14)
surface

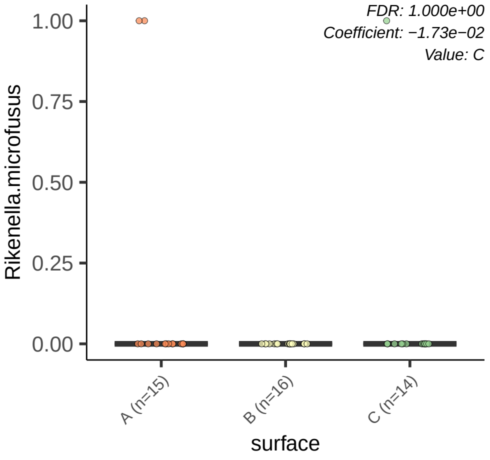

FDR: 1.000e+00
Coefficient: −1.73e−02
Value: C
Rikenella.microfusus
1.00
0.75
0.50
0.25
0.00
A (n=15)
B (n=16)
C (n=14)
surface

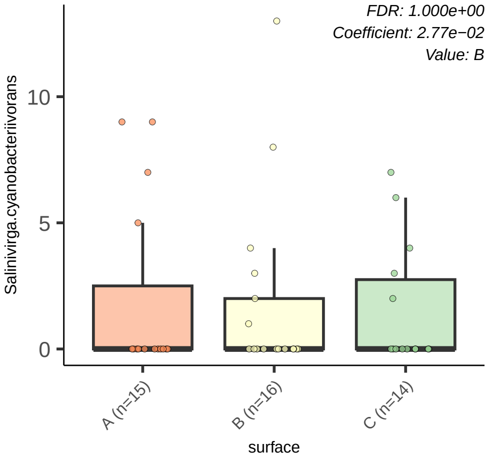

FDR: 1.000e+00
Coefficient: 2.77e−02
Value: B
Salinivirga.cyanobacteriivorans
10
5
0
A (n=15)
B (n=16)
C (n=14)
surface

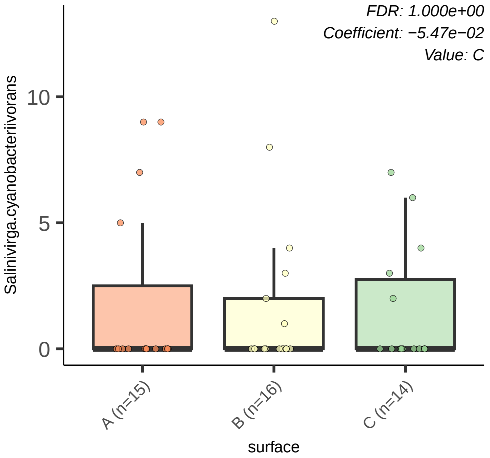
FDR: 1.000e+00
Coefficient: −5.47e−02
Value: C
Saliniviraga.cyanobacteriivorans
10
5
0
A (n=15)
B (n=16)
C (n=14)
surface

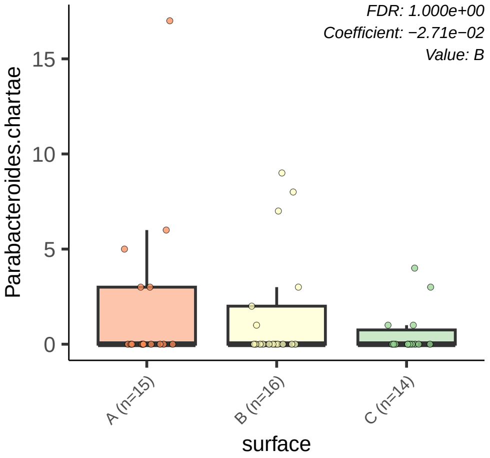
FDR: 1.000e+00
Coefficient: −2.71e−02
Value: B
Parabacteroides.chartae
15
10
5
0
A (n=15)
B (n=16)
C (n=14)
surface

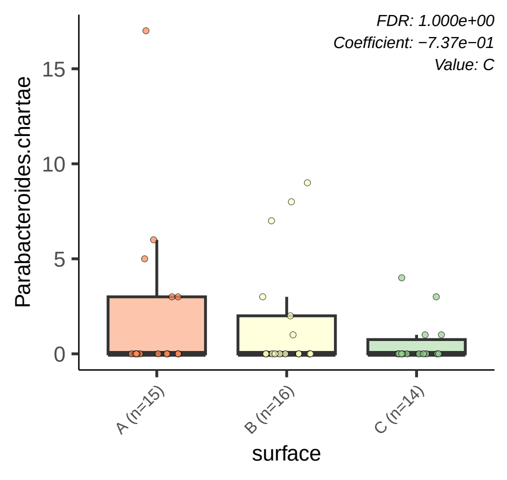
FDR: 1.000e+00
Coefficient: −7.37e−01
Value: C
Parabacteroides.chartae
15
10
5
0
A (n=15)
B (n=16)
C (n=14)
surface

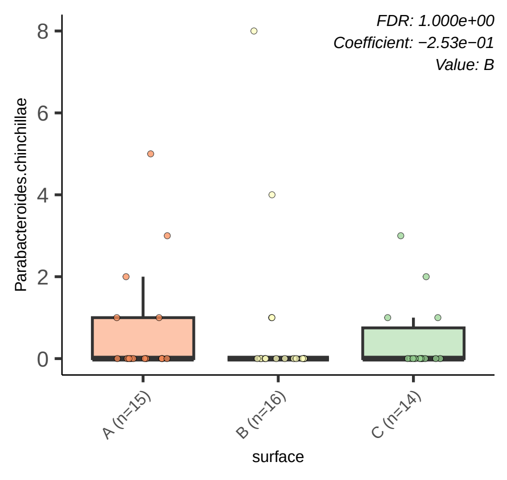

FDR: 1.000e+00
Coefficient: −2.53e−01
Value: B
Parabacteroides.chinchillae
8
6
4
2
0
A (n=15)
B (n=16)
C (n=14)
surface

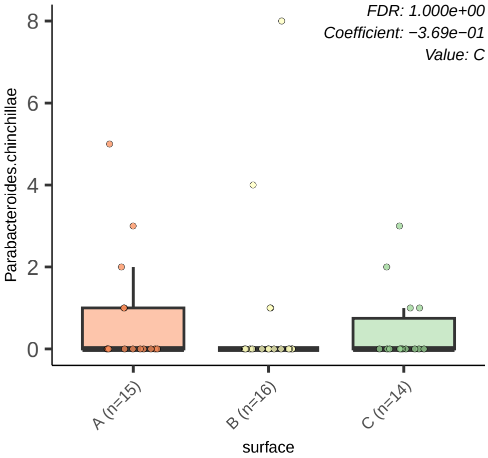
FDR: 1.000e+00
Coefficient: −3.69e−01
Value: C
Parabacteroides.chinchillae
8
6
4
2
0
A (n=15)
B (n=16)
C (n=14)
surface

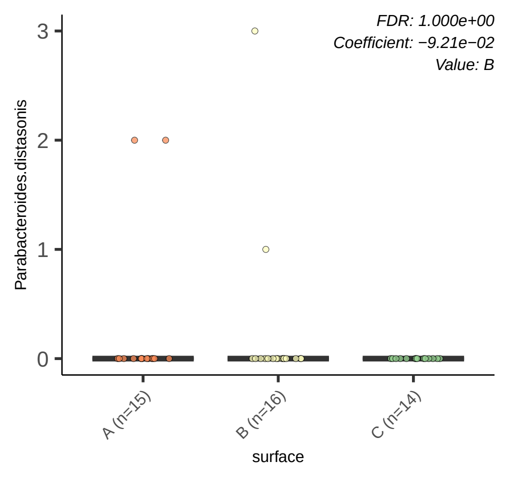
FDR: 1.000e+00
Coefficient: −9.21e−02
Value: B
Parabacteroides.distasonis
3
2
1
0
A (n=15)
B (n=16)
C (n=14)
surface

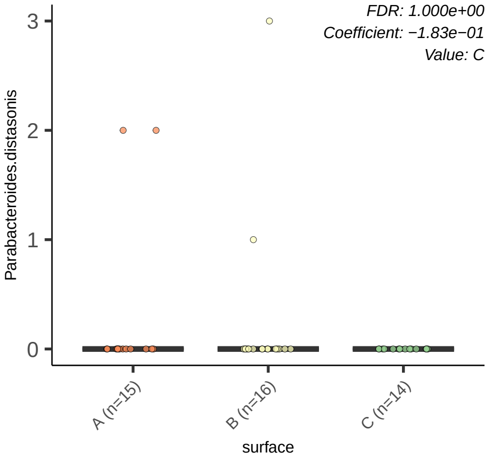
FDR: 1.000e+00
Coefficient: −1.83e−01
Value: C
Parabacteroides.distasonis
3
2
1
0
A (n=15)
B (n=16)
C (n=14)
surface

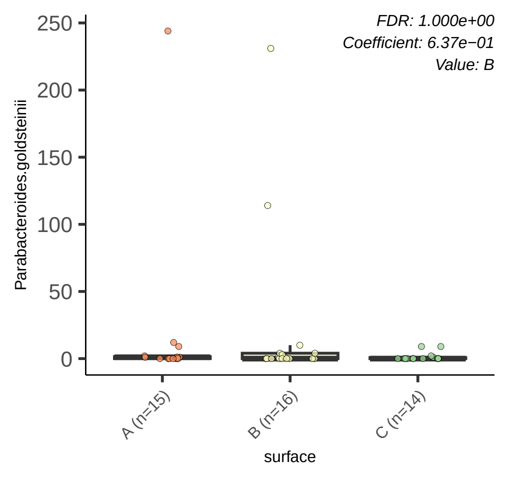
FDR: 1.000e+00
Coefficient: 6.37e−01
Value: B
Parabacteroides.goldsteinii
250
200
150
100
50
0
A (n=15)
B (n=16)
C (n=14)
surface

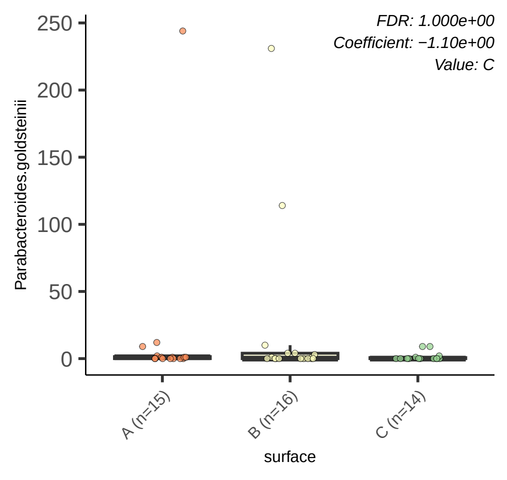

FDR: 1.000e+00
Coefficient: −1.10e+00
Value: C
250
200
150
100
50
0
Parabacteroides.goldsteinii
A (n=15)
B (n=16)
C (n=14)
surface

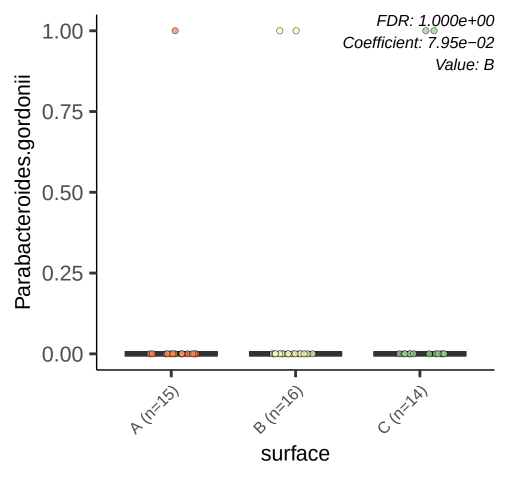
FDR: 1.000e+00
Coefficient: 7.95e−02
Value: B
Parabacteroides.gordonii
1.00
0.75
0.50
0.25
0.00
A (n=15)
B (n=16)
C (n=14)
surface

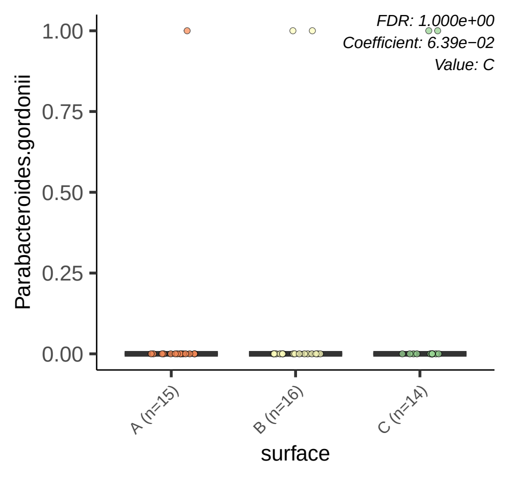
FDR: 1.000e+00
Coefficient: 6.39e−02
Value: C
Parabacteroides.gordonii
1.00
0.75
0.50
0.25
0.00
A (n=15)
B (n=16)
C (n=14)
surface

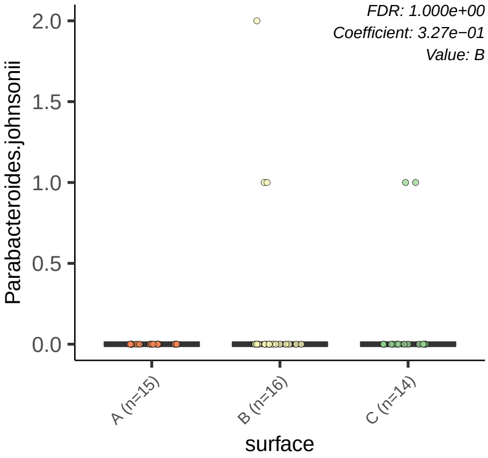

FDR: 1.000e+00
Coefficient: 3.27e−01
Value: B
Parabacteroides.johnsonii
2.0
1.5
1.0
0.5
0.0
A (n=15)
B (n=16)
C (n=14)
surface

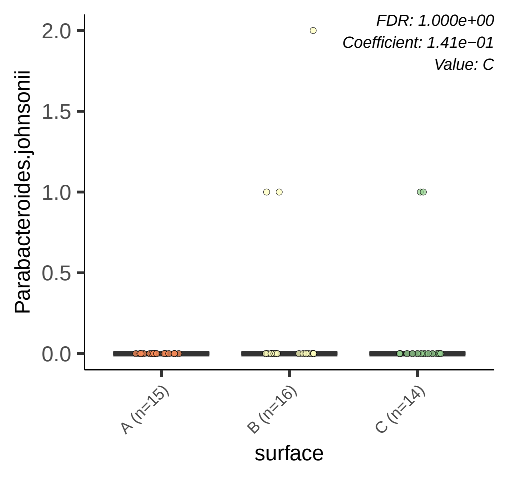
FDR: 1.000e+00
Coefficient: 1.41e−01
Value: C
Parabacteroides.johnsonii
2.0
1.5
1.0
0.5
0.0
A (n=15)
B (n=16)
C (n=14)
surface

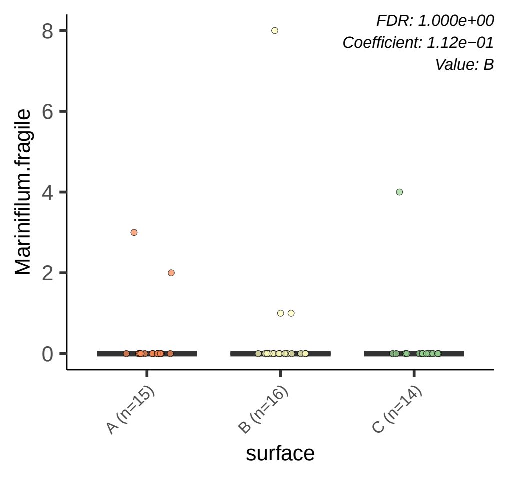
FDR: 1.000e+00
Coefficient: 1.12e−01
Value: B
Marinifilum.fragile
0
2
4
6
8
A (n=15)
B (n=16)
C (n=14)
surface

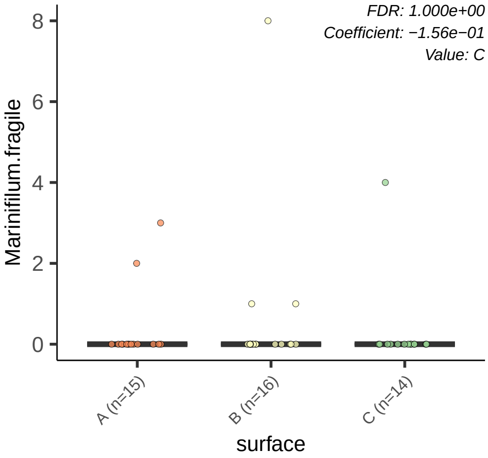
FDR: 1.000e+00
Coefficient: −1.56e−01
Value: C
Marinifilum.fragile
0
2
4
6
8
A (n=15)
B (n=16)
C (n=14)
surface

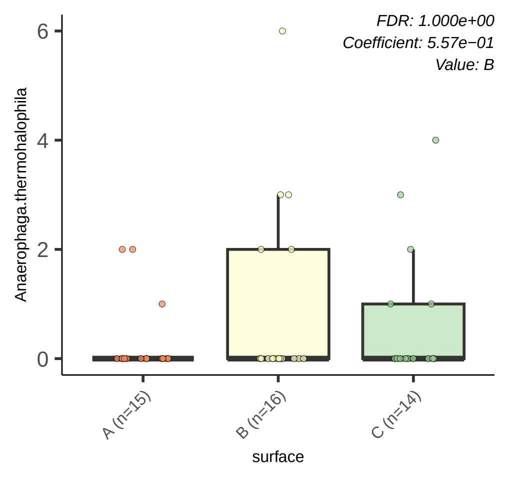
FDR: 1.000e+00
Coefficient: 5.57e−01
Value: B
Anaerophaga.thermohalophila
0
2
4
6
A (n=15)
B (n=16)
C (n=14)
surface

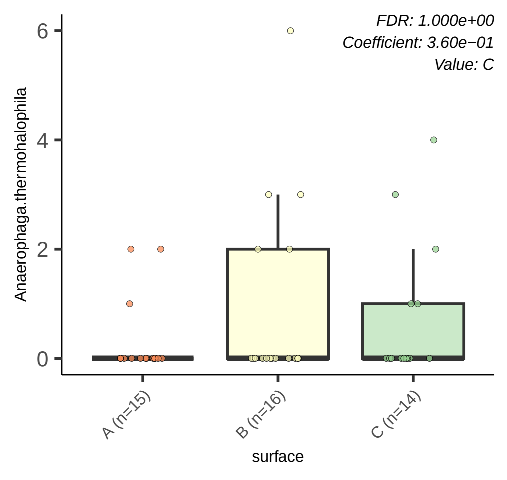
FDR: 1.000e+00
Coefficient: 3.60e−01
Value: C
Anaerophaga.thermohalophila
0
2
4
6
A (n=15)
B (n=16)
C (n=14)
surface

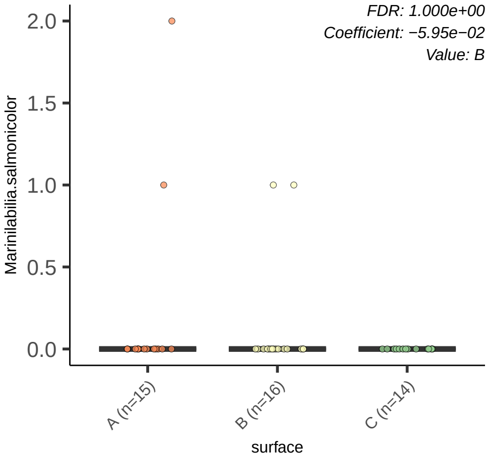
FDR: 1.000e+00
Coefficient: −5.95e−02
Value: B
2.0
1.5
1.0
0.5
0.0
Marinilabilia.salmonicolor
A (n=15)
B (n=16)
C (n=14)
surface

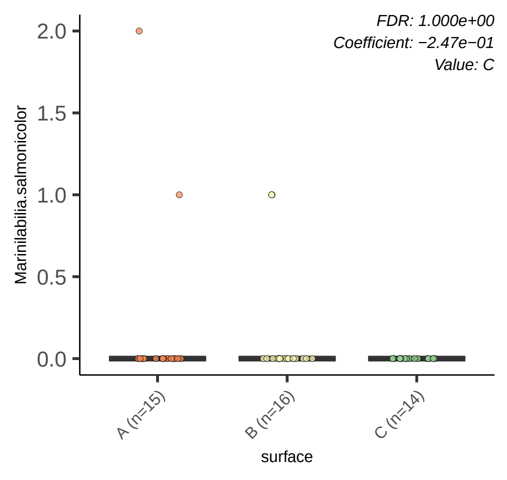
FDR: 1.000e+00
Coefficient: −2.47e−01
Value: C
Marinilabilia.salmonicolor
2.0
1.5
1.0
0.5
0.0
A (n=15)
B (n=16)
C (n=14)
surface

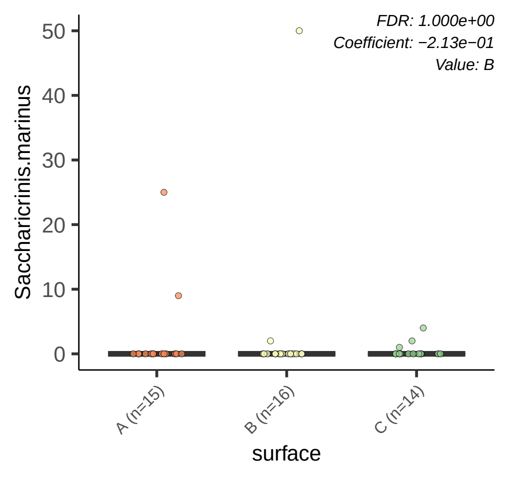
FDR: 1.000e+00
Coefficient: −2.13e−01
Value: B
Saccharicrinis.marinus
0
10
20
30
40
50
A (n=15)
B (n=16)
C (n=14)
surface

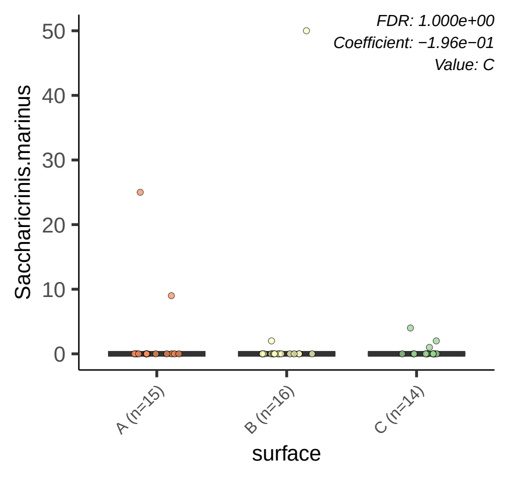
FDR: 1.000e+00
Coefficient: −1.96e−01
Value: C
Saccharicrinis.marinus
50
40
30
20
10
0
A (n=15)
B (n=16)
C (n=14)
surface

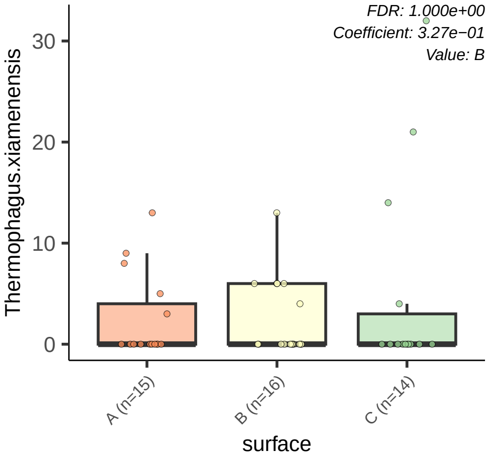

FDR: 1.000e+00
Coefficient: 3.27e−01
Value: B
Thermophagus.xiamenensis
30
20
10
0
A (n=15)
B (n=16)
C (n=14)
surface

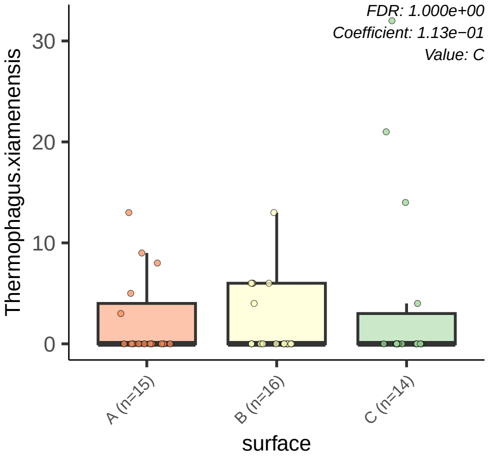
FDR: 1.000e+00
Coefficient: 1.13e−01
Value: C
Thermophagus.xiamenensis
30
20
10
0
A (n=15)
B (n=16)
C (n=14)
surface

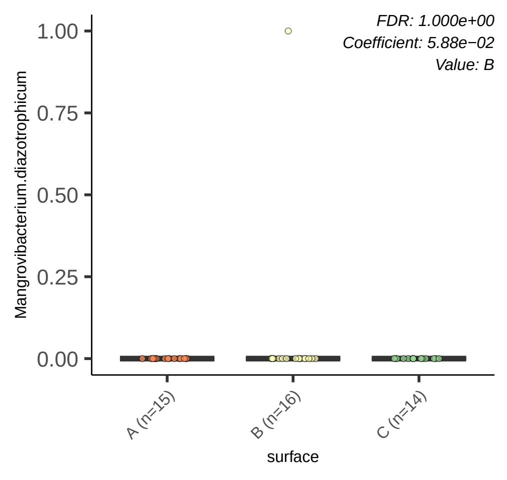

FDR: 1.000e+00
Coefficient: 5.88e−02
Value: B
Mangrovibacterium.diazotrophicum
1.00
0.75
0.50
0.25
0.00
A (n=15)
B (n=16)
C (n=14)
surface

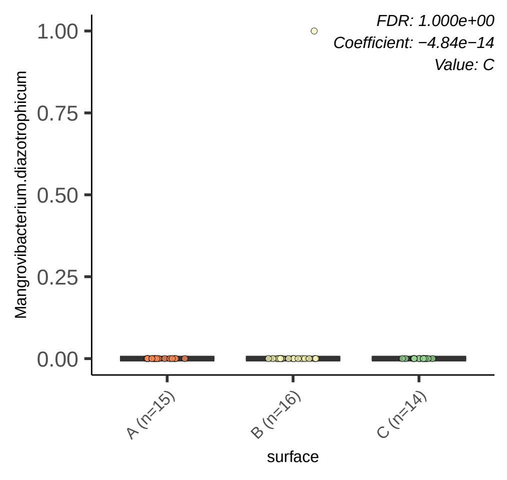
FDR: 1.000e+00
Coefficient: −4.84e−14
Value: C
Mangrovibacterium.diazotrophicum
1.00
0.75
0.50
0.25
0.00
A (n=15)
B (n=16)
C (n=14)
surface

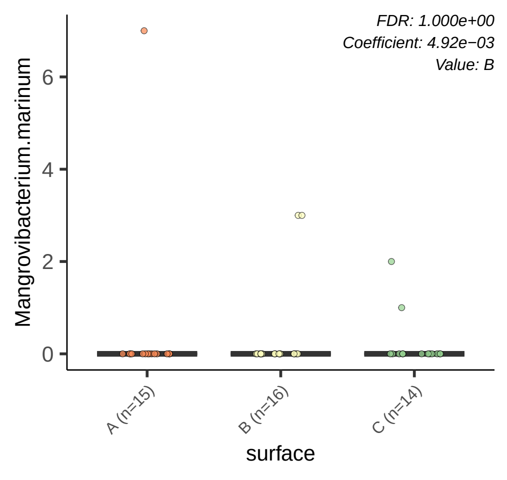
FDR: 1.000e+00
Coefficient: 4.92e−03
Value: B
Mangrovibacterium.marinum
0
2
4
6
A (n=15)
B (n=16)
C (n=14)
surface

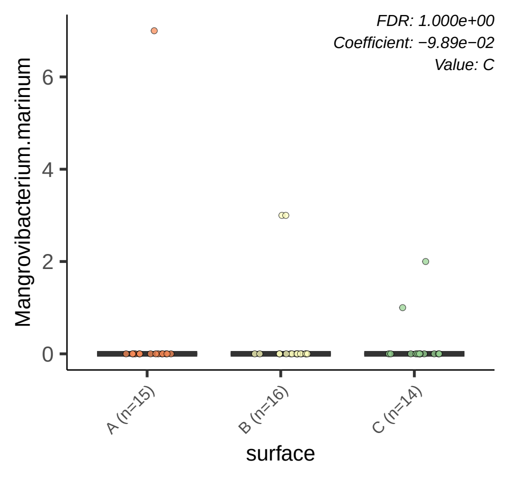
FDR: 1.000e+00
Coefficient: −9.89e−02
Value: C
Mangrovibacterium.marinum
0
2
4
6
A (n=15)
B (n=16)
C (n=14)
surface

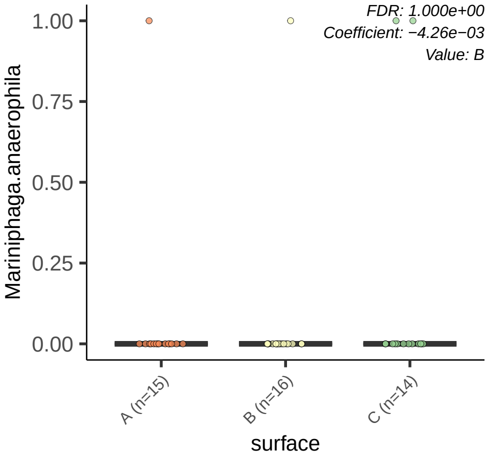
FDR: 1.000e+00
Coefficient: −4.26e−03
Value: B
Mariniphaga.anaerophila
1.00
0.75
0.50
0.25
0.00
A (n=15)
B (n=16)
C (n=14)
surface

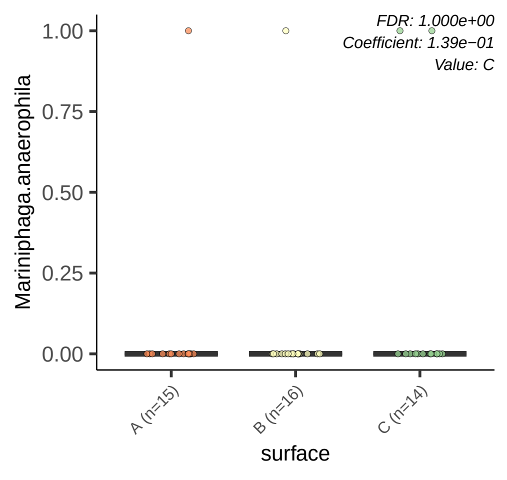

FDR: 1.000e+00
Coefficient: 1.39e−01
Value: C
1.00
0.75
0.50
0.25
0.00
Mariniphaga.anaerophila
A (n=15)
B (n=16)
C (n=14)
surface

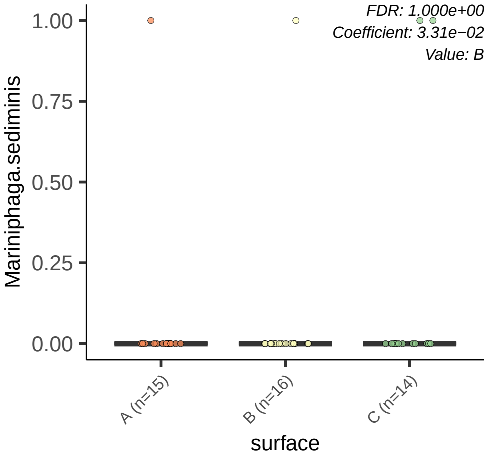

FDR: 1.000e+00
Coefficient: 3.31e−02
Value: B
Mariniphaga.sedimimis
1.00
0.75
0.50
0.25
0.00
A (n=15)
B (n=16)
C (n=14)
surface

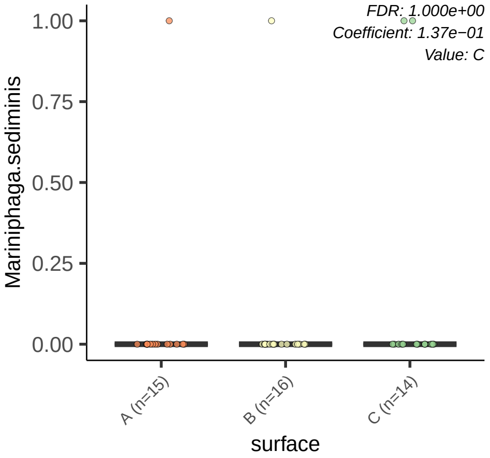
FDR: 1.000e+00
Coefficient: 1.37e−01
Value: C
Mariniphaga.sediminis
1.00
0.75
0.50
0.25
0.00
A (n=15)
B (n=16)
C (n=14)
surface

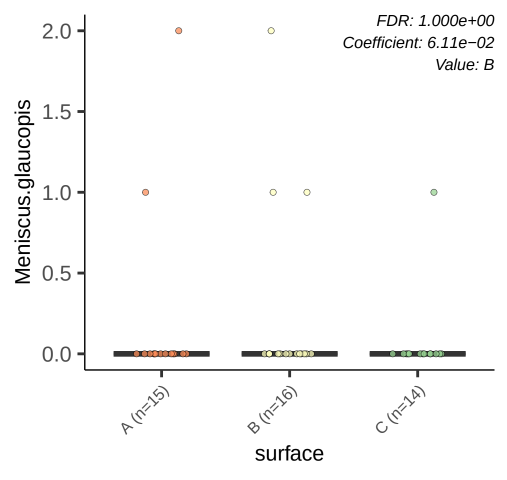
FDR: 1.000e+00
Coefficient: 6.11e−02
Value: B
Meniscus.glaucopis
2.0
1.5
1.0
0.5
0.0
A (n=15)
B (n=16)
C (n=14)
surface

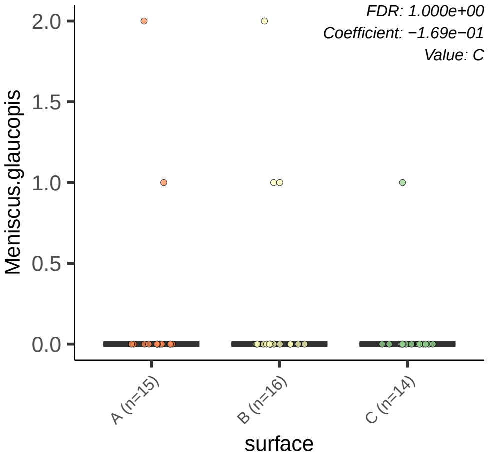

FDR: 1.000e+00
Coefficient: −1.69e−01
Value: C
Meniscus.glaucopis
2.0
1.5
1.0
0.5
0.0
A (n=15)
B (n=16)
C (n=14)
surface

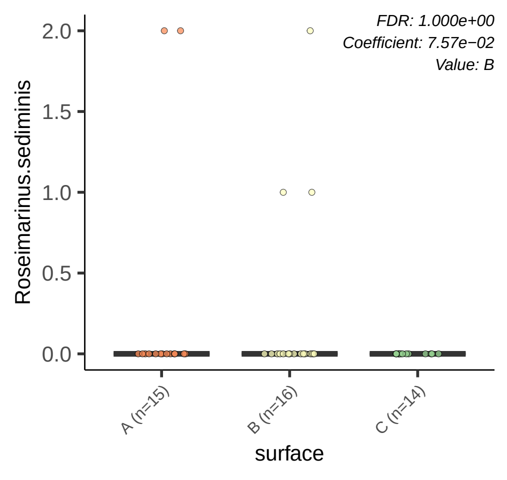
FDR: 1.000e+00
Coefficient: 7.57e−02
Value: B
Roseimarinus.sedimins
2.0
1.5
1.0
0.5
0.0
A (n=15)
B (n=16)
C (n=14)
surface

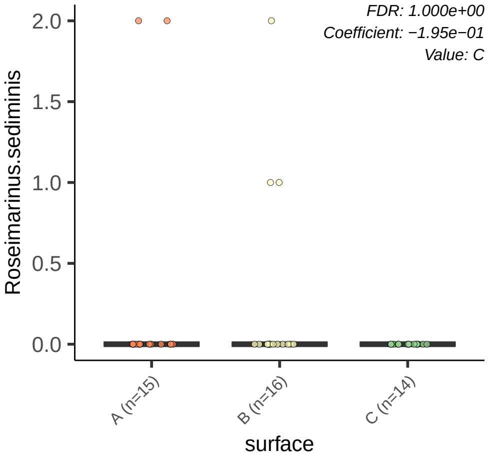

FDR: 1.000e+00
Coefficient: −1.95e−01
Value: C
Roseimarinus.sedimininis
2.0
1.5
1.0
0.5
0.0
A (n=15)
B (n=16)
C (n=14)
surface

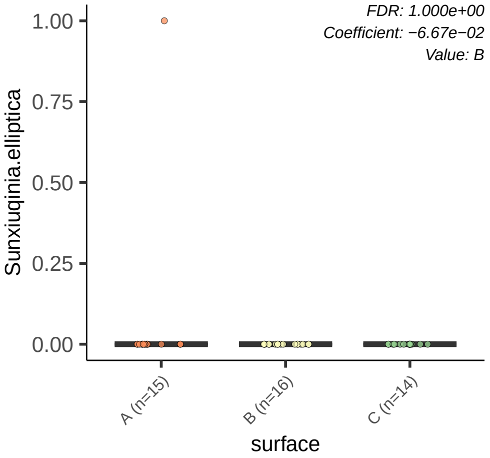

FDR: 1.000e+00
Coefficient: −6.67e−02
Value: B
Sunxiuqinia.elliptica
1.00
0.75
0.50
0.25
0.00
A (n=15)
B (n=16)
C (n=14)
surface

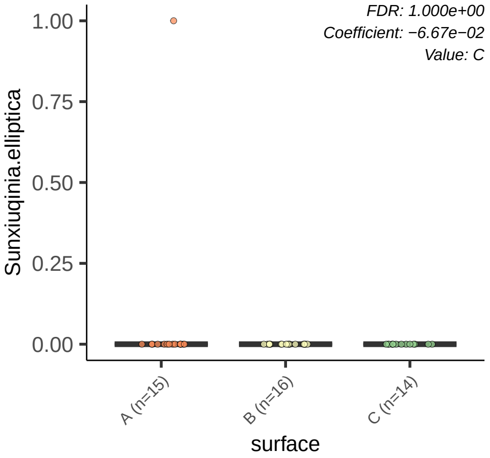
FDR: 1.000e+00
Coefficient: −6.67e−02
Value: C
Sunxiuqinia.elliptica
1.00
0.75
0.50
0.25
0.00
A (n=15)
B (n=16)
C (n=14)
surface

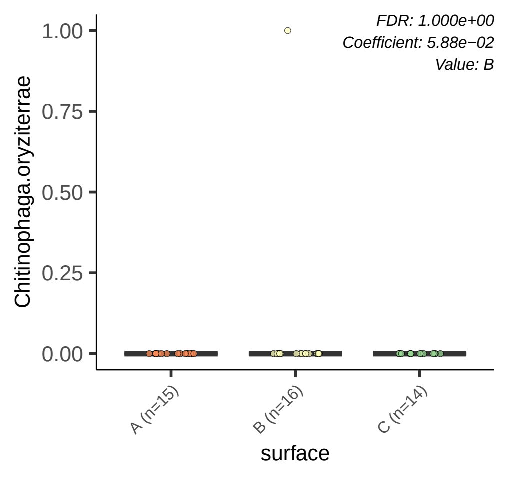
FDR: 1.000e+00
Coefficient: 5.88e−02
Value: B
Chitinophaga.oryziterrae
1.00
0.75
0.50
0.25
0.00
A (n=15)
B (n=16)
C (n=14)
surface

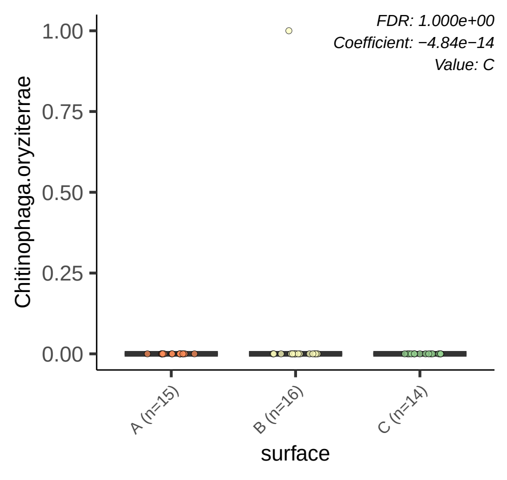
FDR: 1.000e+00
Coefficient: −4.84e−14
Value: C
Chitinophaga.oryziterrae
1.00
0.75
0.50
0.25
0.00
A (n=15)
B (n=16)
C (n=14)
surface

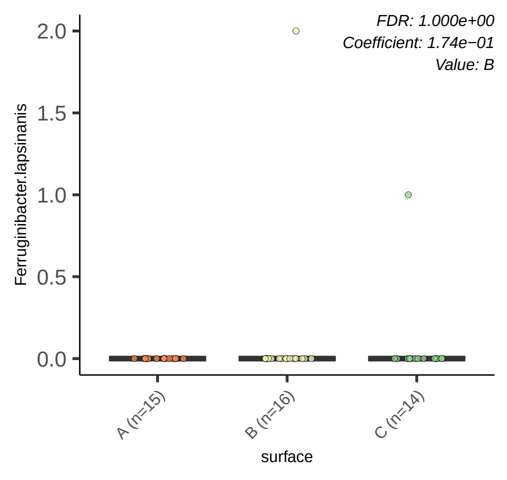
FDR: 1.000e+00
Coefficient: 1.74e−01
Value: B
Ferruginibacter.lapsinanis
2.0
1.5
1.0
0.5
0.0
A (n=15)
B (n=16)
C (n=14)
surface

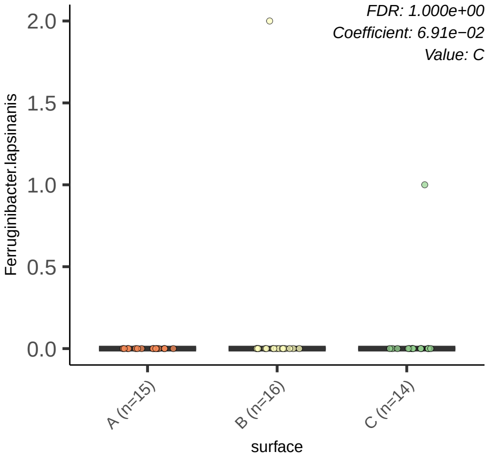
FDR: 1.000e+00
Coefficient: 6.91e−02
Value: C
Ferruginibacter.lapsinanis
2.0
1.5
1.0
0.5
0.0
A (n=15)
B (n=16)
C (n=14)
surface

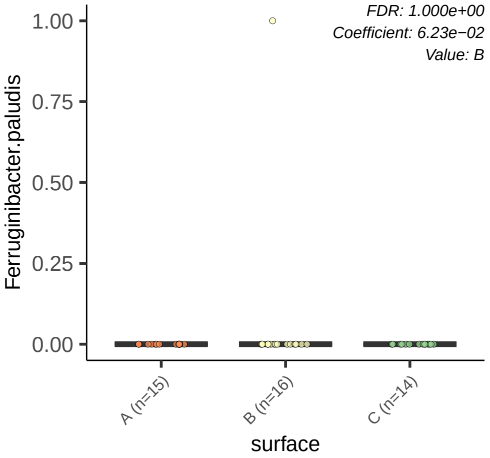

FDR: 1.000e+00
Coefficient: 6.23e−02
Value: B
Ferruginibacter.paludis
1.00
0.75
0.50
0.25
0.00
A (n=15)
B (n=16)
C (n=14)
surface

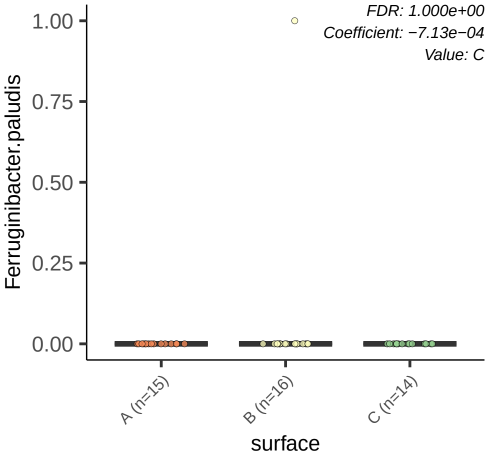
FDR: 1.000e+00
Coefficient: −7.13e−04
Value: C
Ferruginibacter.paludis
1.00
0.75
0.50
0.25
0.00
A (n=15)
B (n=16)
C (n=14)
surface

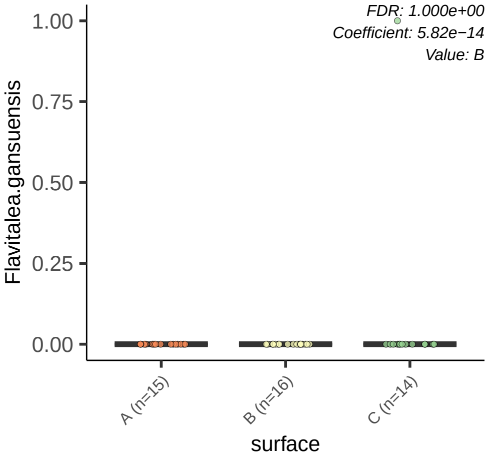
FDR: 1.000e+00
Coefficient: 5.82e−14
Value: B
1.00
0.75
0.50
0.25
0.00
Flavitalea.gansuensis
A (n=15)
B (n=16)
C (n=14)
surface

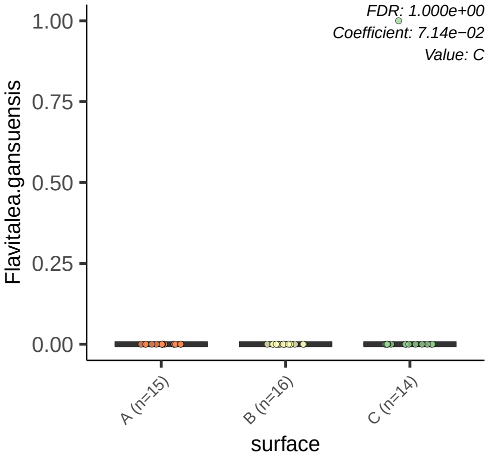

FDR: 1.000e+00
Coefficient: 7.14e−02
Value: C
1.00
0.75
0.50
0.25
0.00
Flavitalea.gansuensis
A (n=15)
B (n=16)
C (n=14)
surface

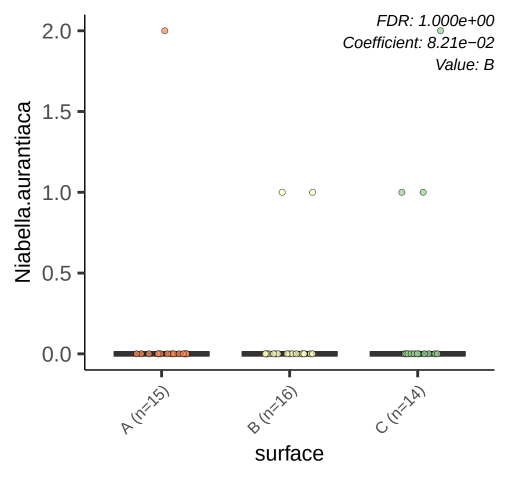
FDR: 1.000e+00
Coefficient: 8.21e−02
Value: B
Niabella.aurantiaca
2.0
1.5
1.0
0.5
0.0
A (n=15)
B (n=16)
C (n=14)
surface

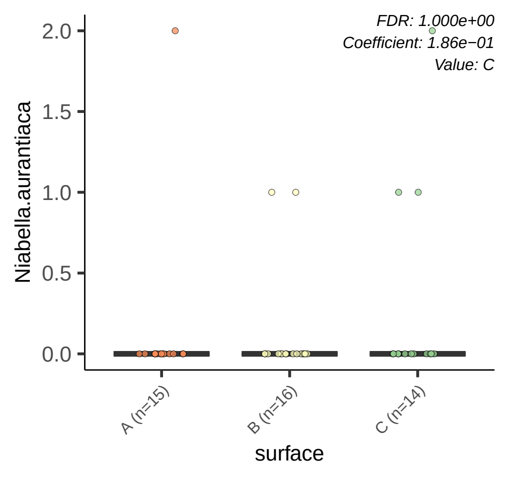
FDR: 1.000e+00
Coefficient: 1.86e−01
Value: C
Niabella.aurantiaca
2.0
1.5
1.0
0.5
0.0
A (n=15)
B (n=16)
C (n=14)
surface

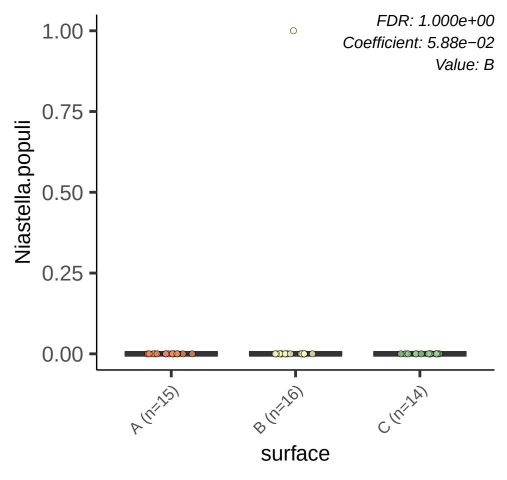

FDR: 1.000e+00
Coefficient: 5.88e−02
Value: B
Niastella.populi
1.00
0.75
0.50
0.25
0.00
A (n=15)
B (n=16)
C (n=14)
surface

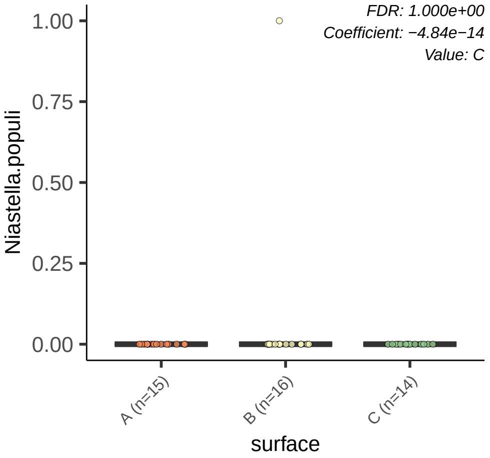
FDR: 1.000e+00
Coefficient: −4.84e−14
Value: C
Niastella.populi
1.00
0.75
0.50
0.25
0.00
A (n=15)
B (n=16)
C (n=14)
surface

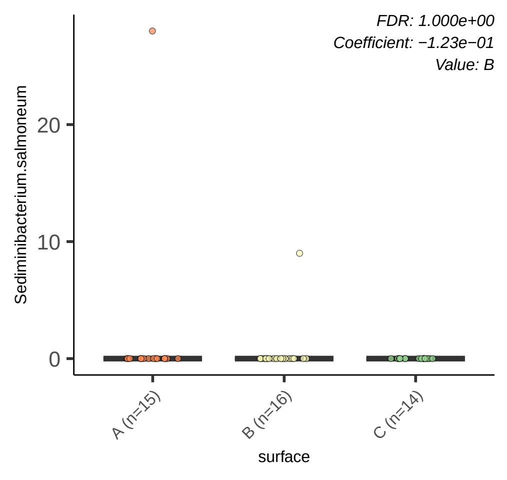

FDR: 1.000e+00
Coefficient: −1.23e−01
Value: B
Sediminibacterium.salmoneum
20
10
0
A (n=15)
B (n=16)
C (n=14)
surface

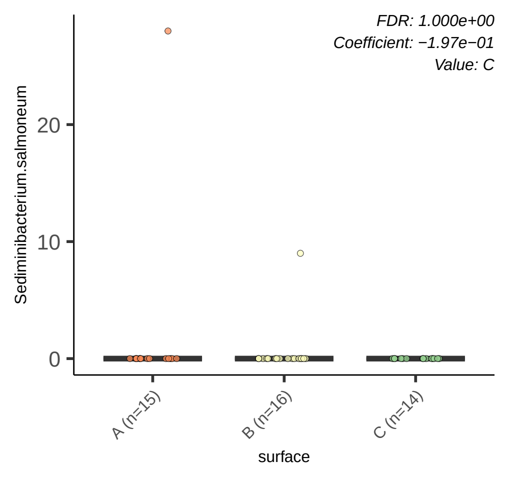
FDR: 1.000e+00
Coefficient: −1.97e−01
Value: C
Sediminibacterium.salmoneum
20
10
0
A (n=15)
B (n=16)
C (n=14)
surface

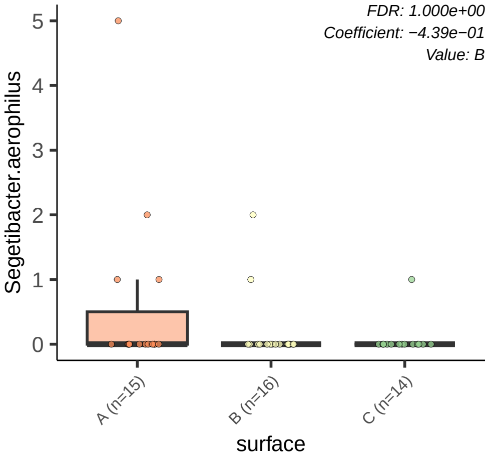
FDR: 1.000e+00
Coefficient: −4.39e−01
Value: B
Segetibacter.aerophilus
0
1
2
3
4
5
A (n=15)
B (n=16)
C (n=14)
surface

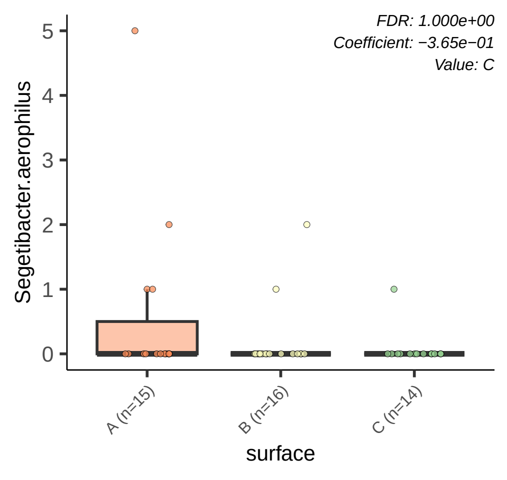
FDR: 1.000e+00
Coefficient: −3.65e−01
Value: C
Segetibacter.aerophilus
0
1
2
3
4
5
A (n=15)
B (n=16)
C (n=14)
surface

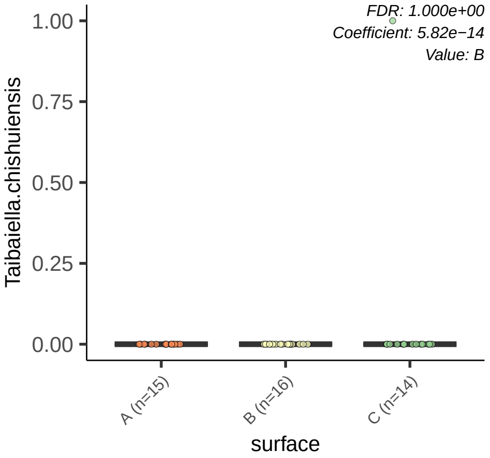

FDR: 1.000e+00
Coefficient: 5.82e−14
Value: B
Taibaiella.chishuiensis
1.00
0.75
0.50
0.25
0.00
A (n=15)
B (n=16)
C (n=14)
surface

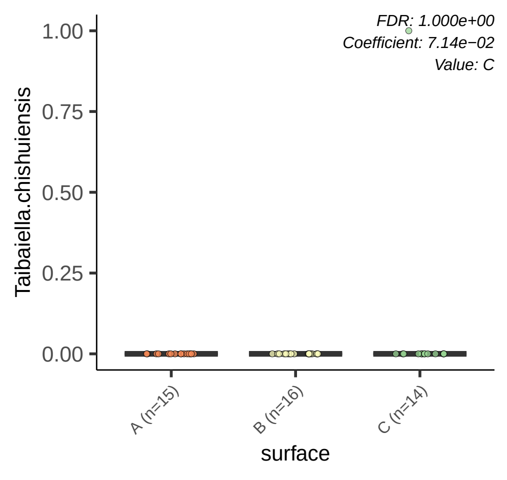

FDR: 1.000e+00
Coefficient: 7.14e−02
Value: C
Taibaiella.chishuiensis
1.00
0.75
0.50
0.25
0.00
A (n=15)
B (n=16)
C (n=14)
surface

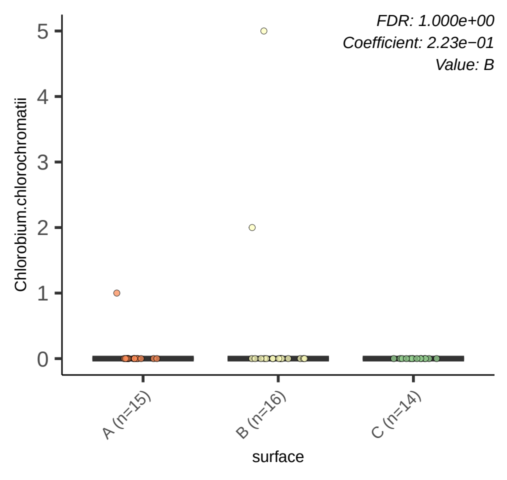

FDR: 1.000e+00
Coefficient: 2.23e−01
Value: B
Chlorobium.chlorochromatii
0
1
2
3
4
5
A (n=15)
B (n=16)
C (n=14)
surface

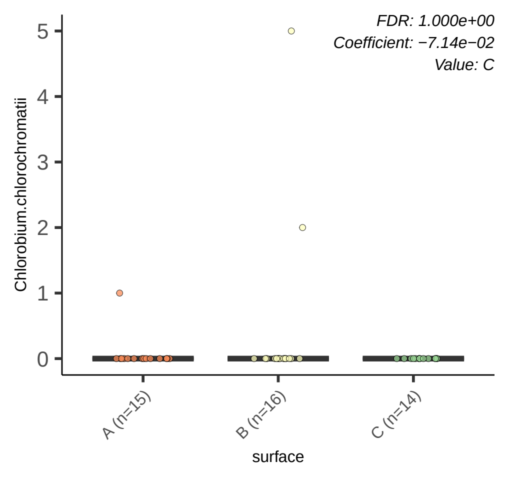
FDR: 1.000e+00
Coefficient: −7.14e−02
Value: C
Chlorobium.chlorochromatii
0
1
2
3
4
5
A (n=15)
B (n=16)
C (n=14)
surface

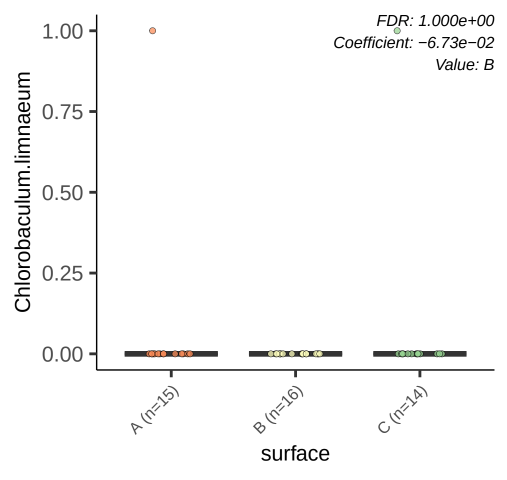
FDR: 1.000e+00
Coefficient: −6.73e−02
Value: B
Chlorobaculum.limnaeum
1.00
0.75
0.50
0.25
0.00
A (n=15)
B (n=16)
C (n=14)
surface

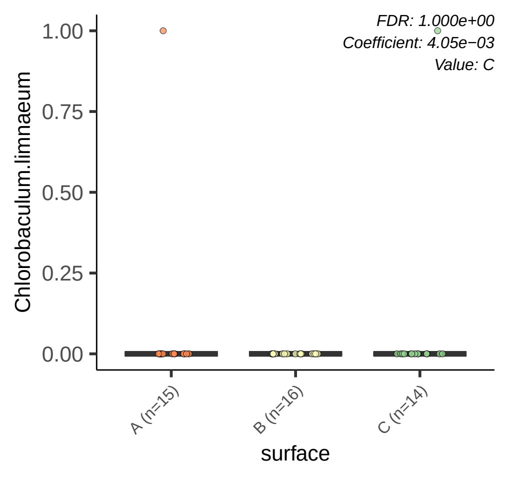

FDR: 1.000e+00
Coefficient: 4.05e−03
Value: C
Chlorobaculum.limnaeum
1.00
0.75
0.50
0.25
0.00
A (n=15)
B (n=16)
C (n=14)
surface

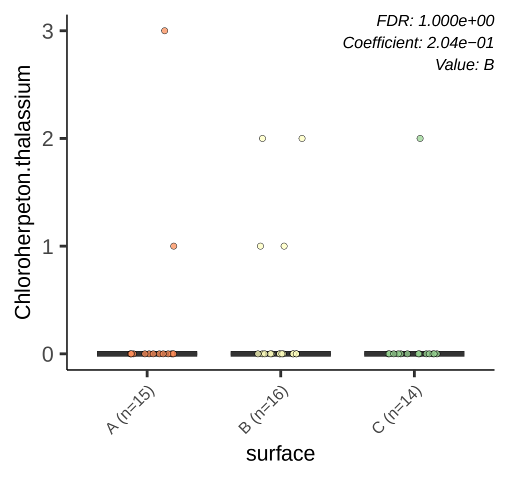
FDR: 1.000e+00
Coefficient: 2.04e−01
Value: B
Chloroherpeton.thalassium
3
2
1
0
A (n=15)
B (n=16)
C (n=14)
surface

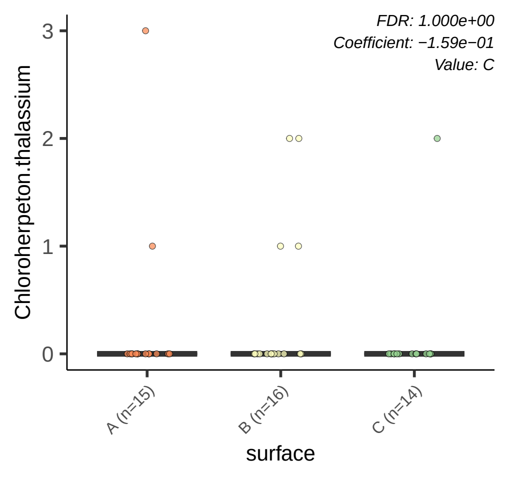

FDR: 1.000e+00
Coefficient: −1.59e−01
Value: C
Chloroherpeton.thalassium
3
2
1
0
A (n=15)
B (n=16)
C (n=14)
surface

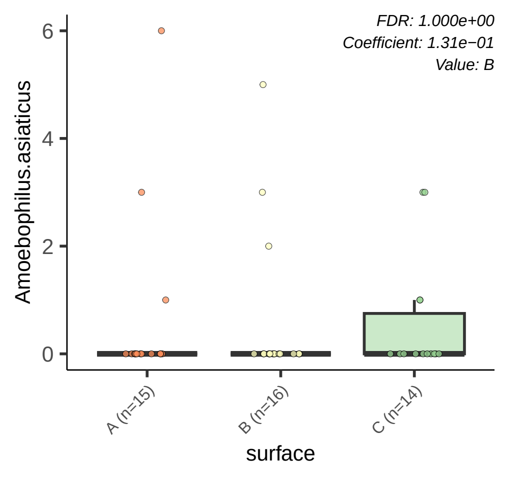

FDR: 1.000e+00
Coefficient: 1.31e−01
Value: B
Amoebophilus.asiaticus
6
4
2
0
A (n=15)
B (n=16)
C (n=14)
surface

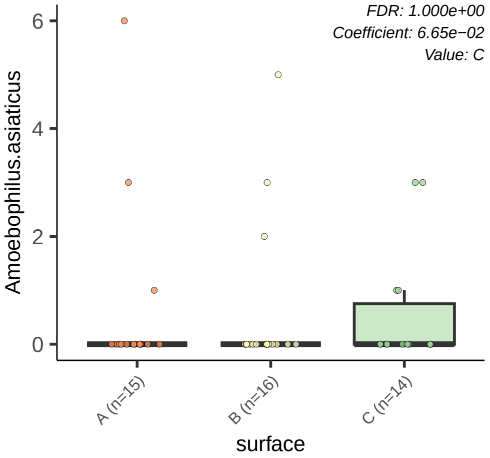

FDR: 1.000e+00
Coefficient: 6.65e−02
Value: C
Amoebophilus.asiaticus
0
2
4
6
A (n=15)
B (n=16)
C (n=14)
surface

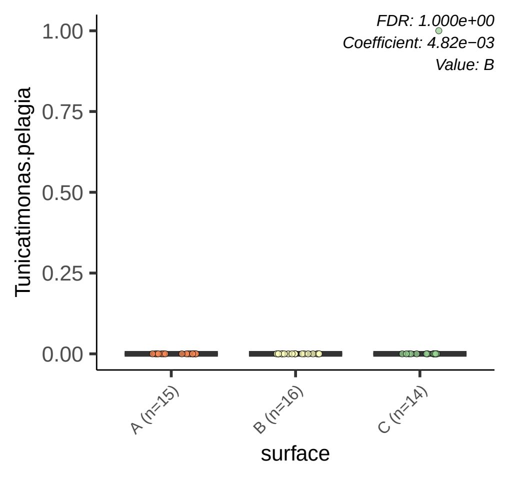

FDR: 1.000e+00
Coefficient: 4.82e−03
Value: B
Tunicatimonas.pelagia
1.00
0.75
0.50
0.25
0.00
A (n=15)
B (n=16)
C (n=14)
surface

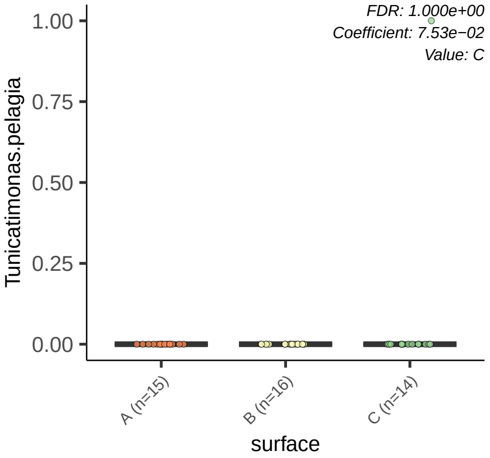
FDR: 1.000e+00
Coefficient: 7.53e−02
Value: C
Tunicatimonas.pelagia
1.00
0.75
0.50
0.25
0.00
A (n=15)
B (n=16)
C (n=14)
surface

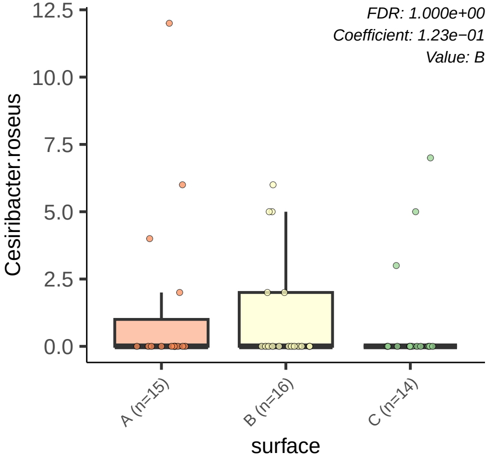
FDR: 1.000e+00
Coefficient: 1.23e−01
Value: B
Cesiribacter.roseus
12.5
10.0
7.5
5.0
2.5
0.0
A (n=15)
B (n=16)
C (n=14)
surface

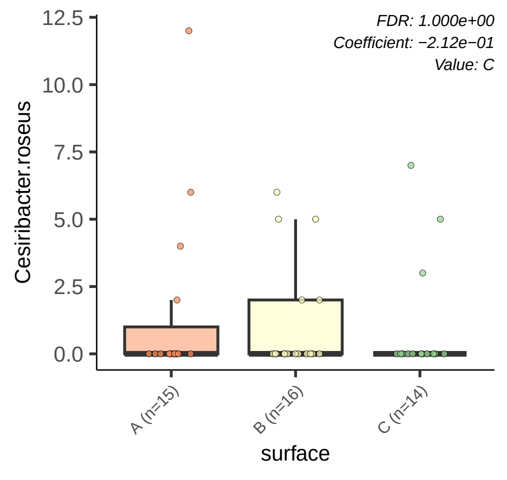

FDR: 1.000e+00
Coefficient: −2.12e−01
Value: C
Cesiribacter.roseus
12.5
10.0
7.5
5.0
2.5
0.0
A (n=15)
B (n=16)
C (n=14)
surface

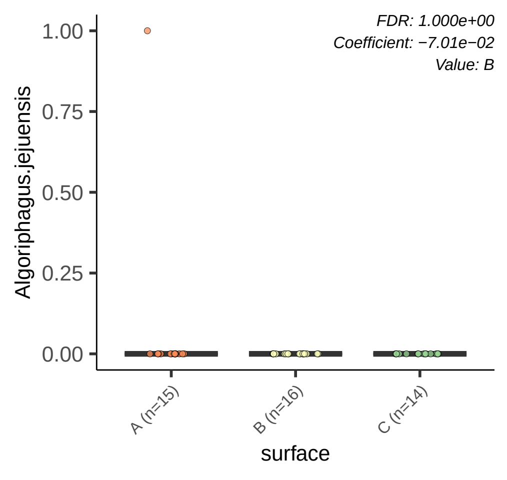
FDR: 1.000e+00
Coefficient: −7.01e−02
Value: B
Algoriphagus.jejuensis
1.00
0.75
0.50
0.25
0.00
A (n=15)
B (n=16)
C (n=14)
surface

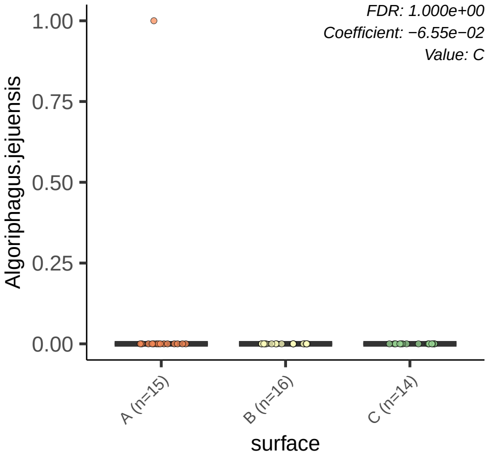

FDR: 1.000e+00
Coefficient: −6.55e−02
Value: C
Algoriphagus.jejuensis
1.00
0.75
0.50
0.25
0.00
A (n=15)
B (n=16)
C (n=14)
surface

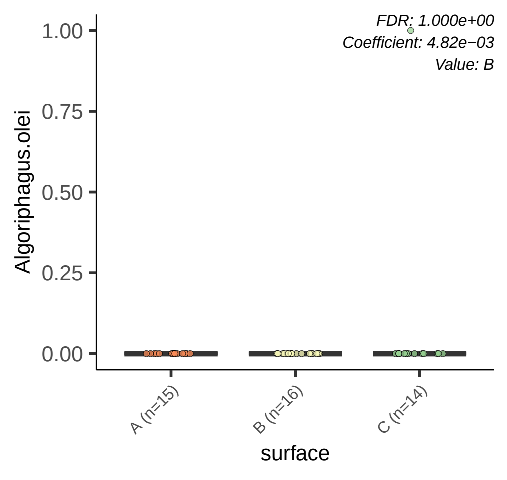
FDR: 1.000e+00
Coefficient: 4.82e−03
Value: B
Algoriphagus.olei
1.00
0.75
0.50
0.25
0.00
A (n=15)
B (n=16)
C (n=14)
surface

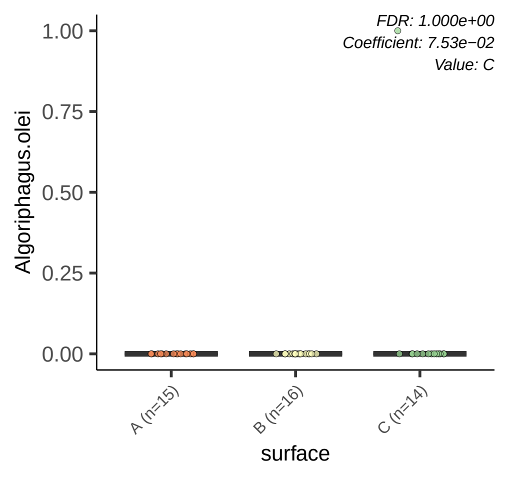
FDR: 1.000e+00
Coefficient: 7.53e−02
Value: C
Algoriphagus.olei
1.00
0.75
0.50
0.25
0.00
A (n=15)
B (n=16)
C (n=14)
surface

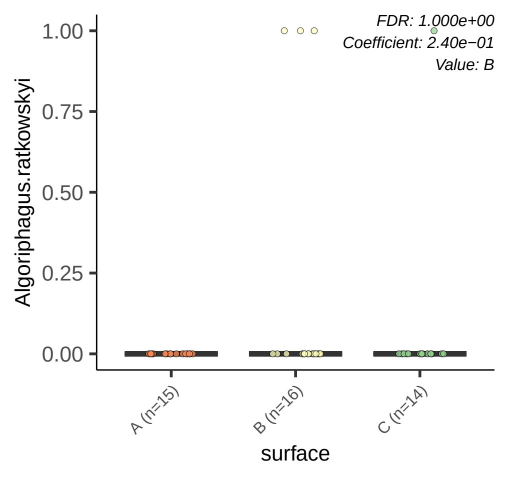

FDR: 1.000e+00
Coefficient: 2.40e−01
Value: B
1.00
0.75
0.50
0.25
0.00
Algoriphagus.ratkowskyi
A (n=15)
B (n=16)
C (n=14)
surface

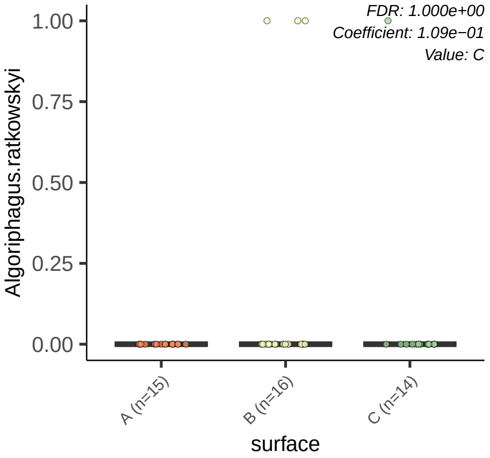

FDR: 1.000e+00
Coefficient: 1.09e−01
Value: C
Algoriphagus.ratkowskyi
1.00
0.75
0.50
0.25
0.00
A (n=15)
B (n=16)
C (n=14)
surface

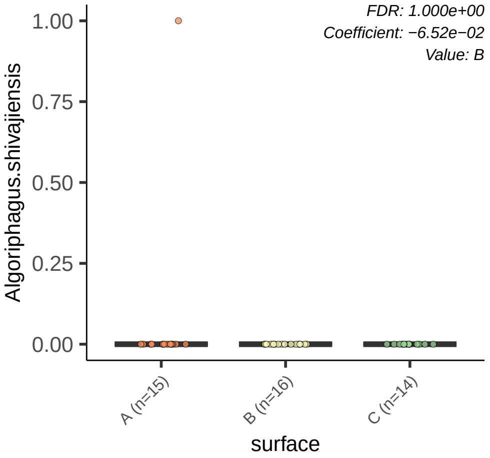
FDR: 1.000e+00
Coefficient: −6.52e−02
Value: B
Algoriphagus.shivajiensis
1.00
0.75
0.50
0.25
0.00
A (n=15)
B (n=16)
C (n=14)
surface

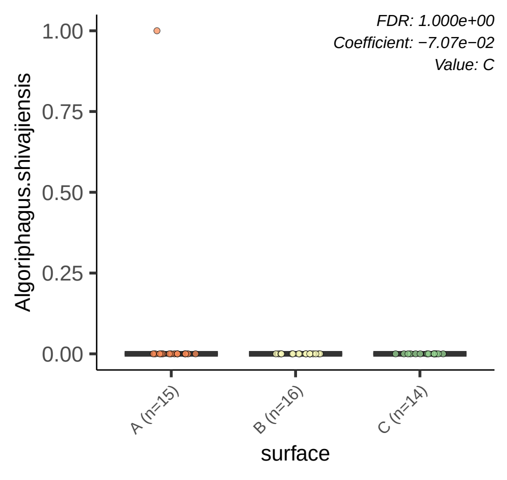
FDR: 1.000e+00
Coefficient: −7.07e−02
Value: C
Algoriphagus.shivajiensis
1.00
0.75
0.50
0.25
0.00
A (n=15)
B (n=16)
C (n=14)
surface

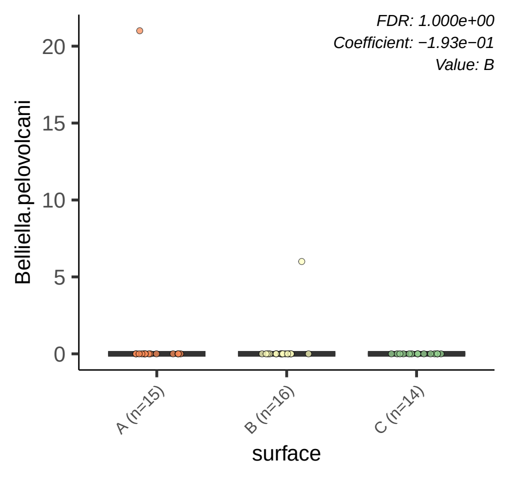
FDR: 1.000e+00
Coefficient: −1.93e−01
Value: B
Belliella.pelovolcani
20
15
10
5
0
A (n=15)
B (n=16)
C (n=14)
surface

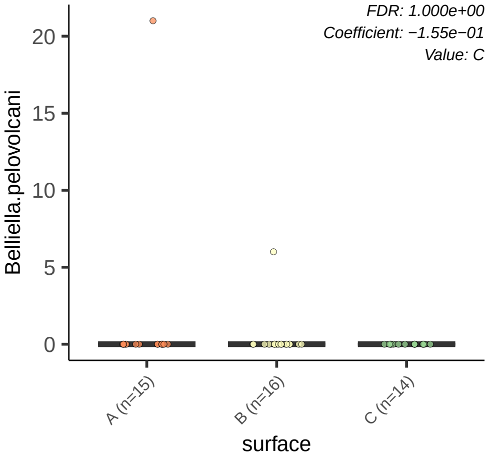

FDR: 1.000e+00
Coefficient: −1.55e−01
Value: C
Belliella.pelovolcani
20
15
10
5
0
A (n=15)
B (n=16)
C (n=14)
surface

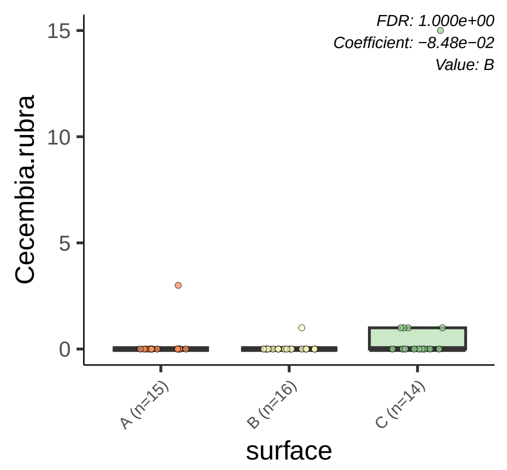

FDR: 1.000e+00
Coefficient: −8.48e−02
Value: B
Cecembia.rubra
15
10
5
0
A (n=15)
B (n=16)
C (n=14)
surface

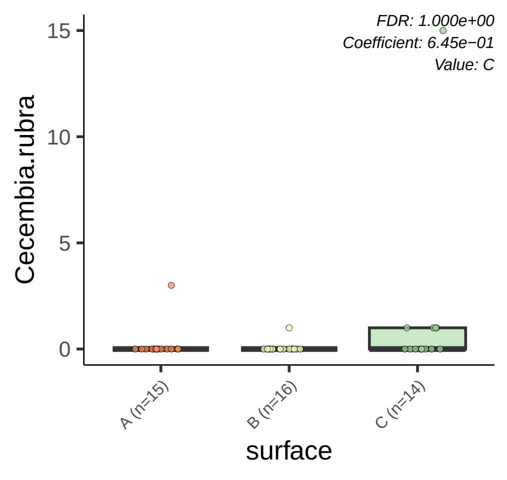

FDR: 1.000e+00
Coefficient: 6.45e−01
Value: C
Cecembia.rubra
15
10
5
0
A (n=15)
B (n=16)
C (n=14)
surface

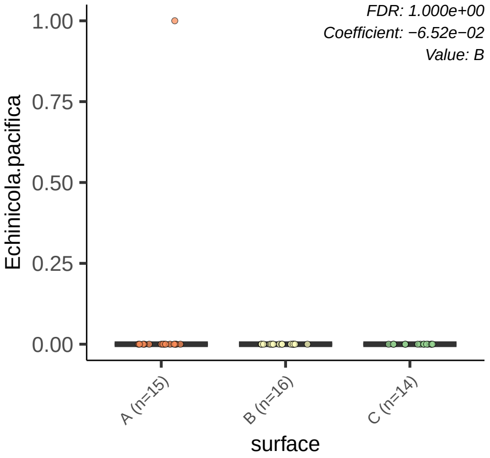

FDR: 1.000e+00
Coefficient: −6.52e−02
Value: B
Echinicola.pacifica
1.00
0.75
0.50
0.25
0.00
A (n=15)
B (n=16)
C (n=14)
surface

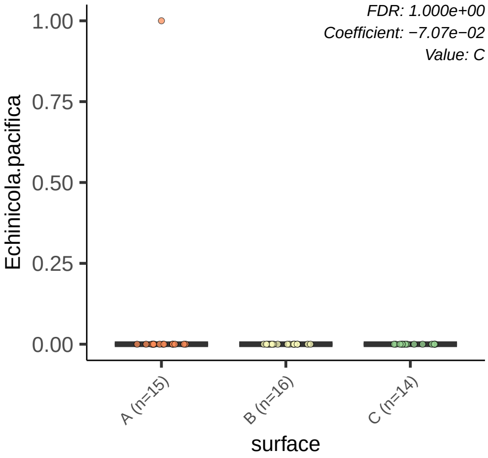
FDR: 1.000e+00
Coefficient: −7.07e−02
Value: C
Echinicola.pacifica
1.00
0.75
0.50
0.25
0.00
A (n=15)
B (n=16)
C (n=14)
surface

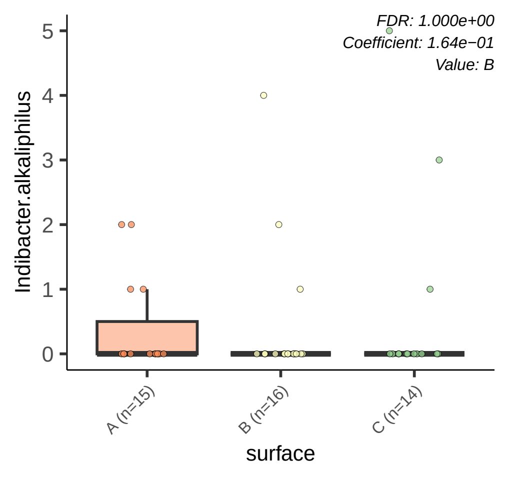

FDR: 1.000e+00
Coefficient: 1.64e−01
Value: B
Indibacter.alkaliphilus
0
1
2
3
4
5
A (n=15)
B (n=16)
C (n=14)
surface

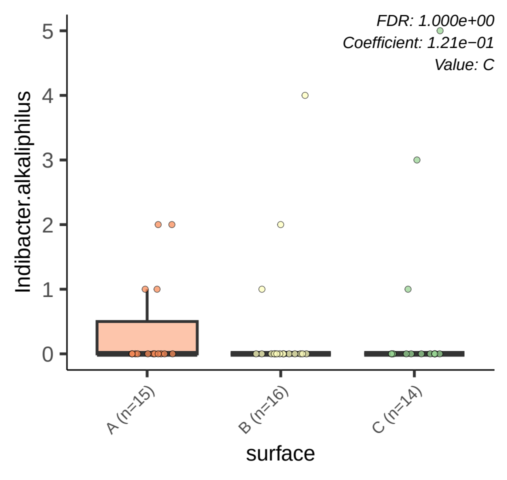
FDR: 1.000e+00
Coefficient: 1.21e−01
Value: C
Indibacter.alkaliphilus
0
1
2
3
4
5
A (n=15)
B (n=16)
C (n=14)
surface

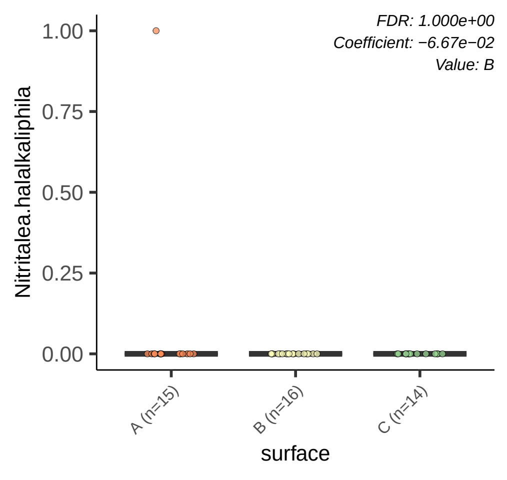

FDR: 1.000e+00
Coefficient: −6.67e−02
Value: B
Nitritalea.halalkaliphila
1.00
0.75
0.50
0.25
0.00
A (n=15)
B (n=16)
C (n=14)
surface

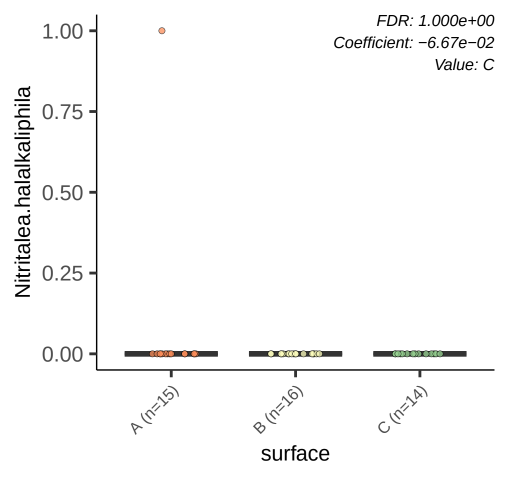
FDR: 1.000e+00
Coefficient: −6.67e−02
Value: C
Nitritalea.halalkaliphila
1.00
0.75
0.50
0.25
0.00
A (n=15)
B (n=16)
C (n=14)
surface

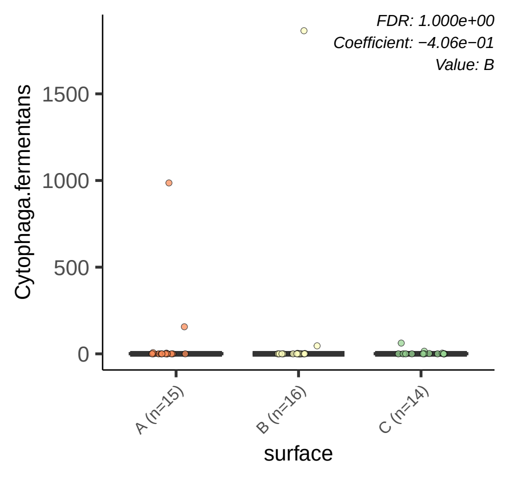
FDR: 1.000e+00
Coefficient: −4.06e−01
Value: B
Cytophaga.fermentans
1500
1000
500
0
A (n=15)
B (n=16)
C (n=14)
surface

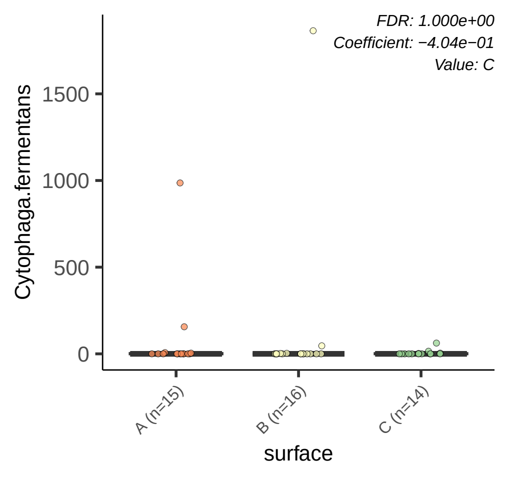
FDR: 1.000e+00
Coefficient: −4.04e−01
Value: C
Cytophaga.fermentans
1500
1000
500
0
A (n=15)
B (n=16)
C (n=14)
surface

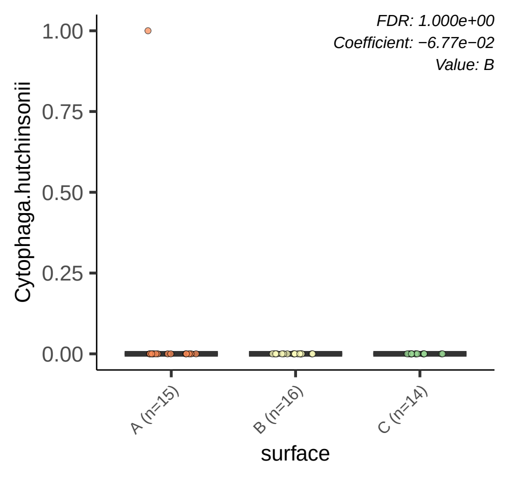
FDR: 1.000e+00
Coefficient: −6.77e−02
Value: B
1.00
0.75
0.50
0.25
0.00
Cytophaga.hutchinsonii
A (n=15)
B (n=16)
C (n=14)
surface

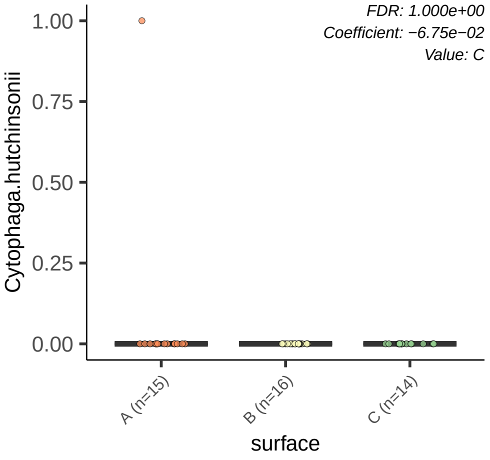

FDR: 1.000e+00
Coefficient: −6.75e−02
Value: C
Cytophaga.hutchinsonii
1.00
0.75
0.50
0.25
0.00
A (n=15)
B (n=16)
C (n=14)
surface

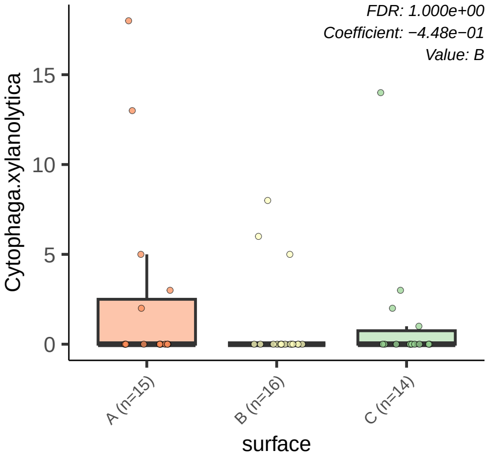

FDR: 1.000e+00
Coefficient: −4.48e−01
Value: B
Cytophaga.xylanolytica
15
10
5
0
A (n=15)
B (n=16)
C (n=14)
surface

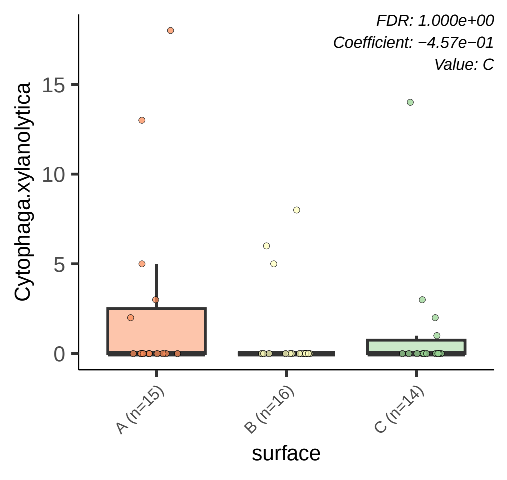

FDR: 1.000e+00
Coefficient: −4.57e−01
Value: C
Cytophaga.xylanolytica
0
5
10
15
A (n=15)
B (n=16)
C (n=14)
surface

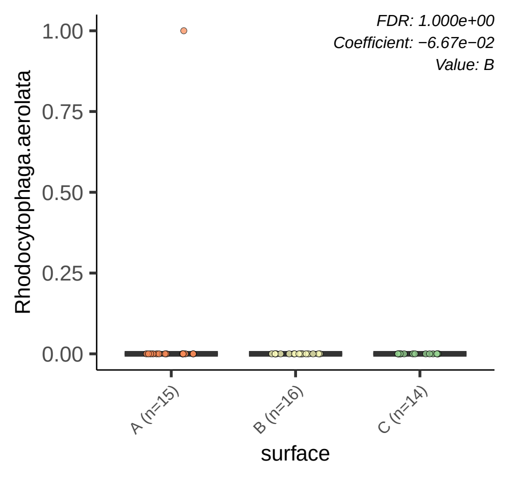
FDR: 1.000e+00
Coefficient: −6.67e−02
Value: B
Rhodocytophaga.aerolata
1.00
0.75
0.50
0.25
0.00
A (n=15)
B (n=16)
C (n=14)
surface

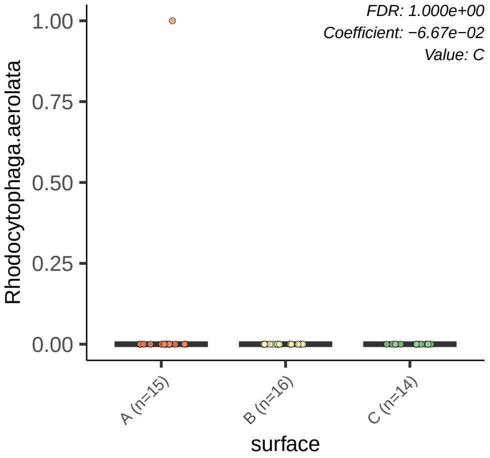

FDR: 1.000e+00
Coefficient: −6.67e−02
Value: C
Rhodocytophaga.aerolata
1.00
0.75
0.50
0.25
0.00
A (n=15)
B (n=16)
C (n=14)
surface

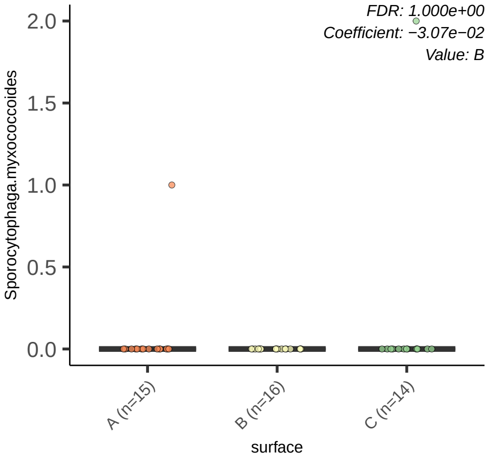
FDR: 1.000e+00
Coefficient: −3.07e−02
Value: B
Sporocytophaga.myxococcoides
2.0
1.5
1.0
0.5
0.0
A (n=15)
B (n=16)
C (n=14)
surface

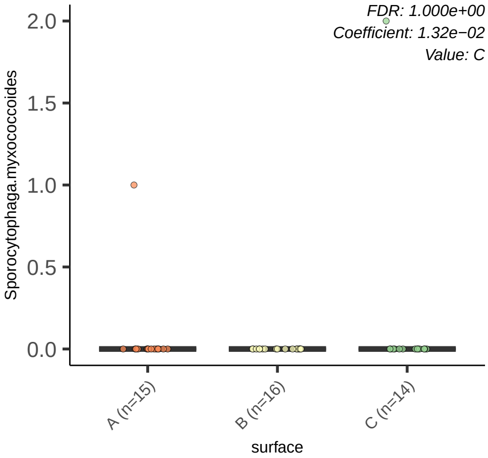

FDR: 1.000e+00
Coefficient: 1.32e−02
Value: C
2.0
1.5
1.0
0.5
0.0
Sporocytophaga.myxococcoides
A (n=15)
B (n=16)
C (n=14)
surface

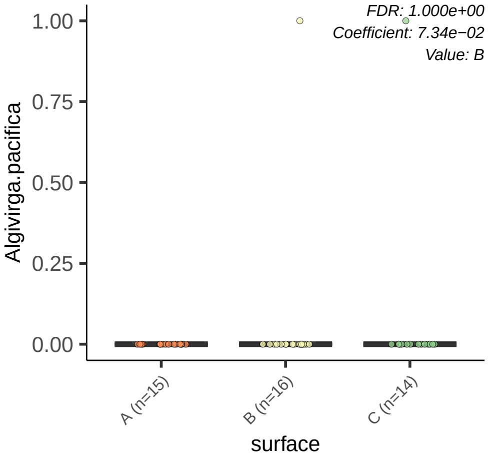
FDR: 1.000e+00
Coefficient: 7.34e−02
Value: B
Algivirga.pacifica
1.00
0.75
0.50
0.25
0.00
A (n=15)
B (n=16)
C (n=14)
surface

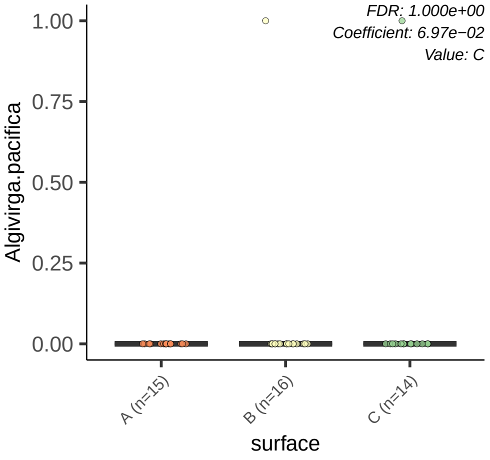
FDR: 1.000e+00
Coefficient: 6.97e−02
Value: C
Algivirga.pacifica
1.00
0.75
0.50
0.25
0.00
A (n=15)
B (n=16)
C (n=14)
surface

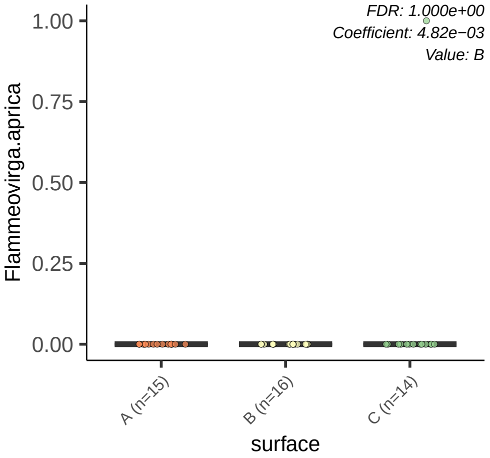
FDR: 1.000e+00
Coefficient: 4.82e−03
Value: B
Flammeovirga.aprica
1.00
0.75
0.50
0.25
0.00
A (n=15)
B (n=16)
C (n=14)
surface

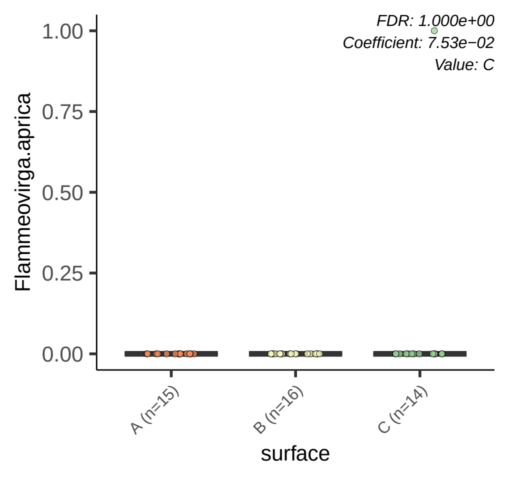
FDR: 1.000e+00
Coefficient: 7.53e−02
Value: C
Flammeovirga.aprica
1.00
0.75
0.50
0.25
0.00
A (n=15)
B (n=16)
C (n=14)
surface

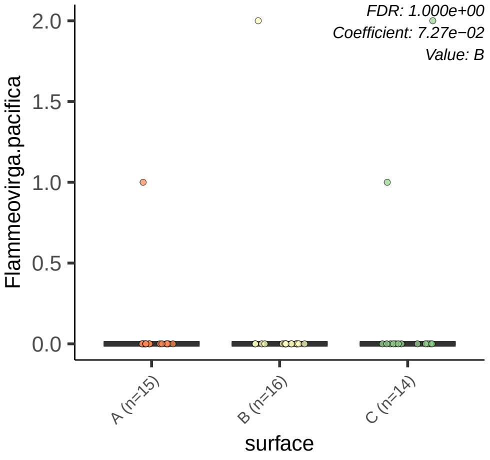
FDR: 1.000e+00
Coefficient: 7.27e−02
Value: B
2.0
1.5
1.0
0.5
0.0
Flammeovirga.pacifica
A (n=15)
B (n=16)
C (n=14)
surface

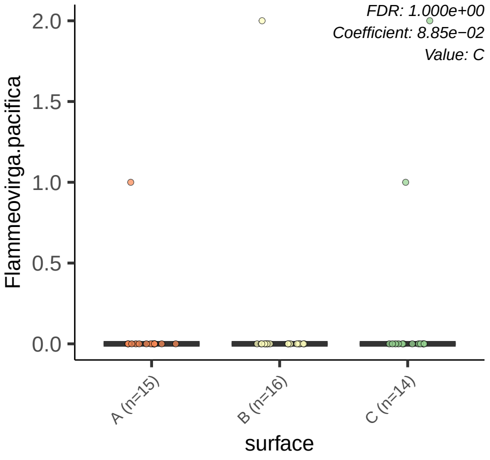

FDR: 1.000e+00
Coefficient: 8.85e−02
Value: C
Flammeovirga.pacifica
2.0
1.5
1.0
0.5
0.0
A (n=15)
B (n=16)
C (n=14)
surface

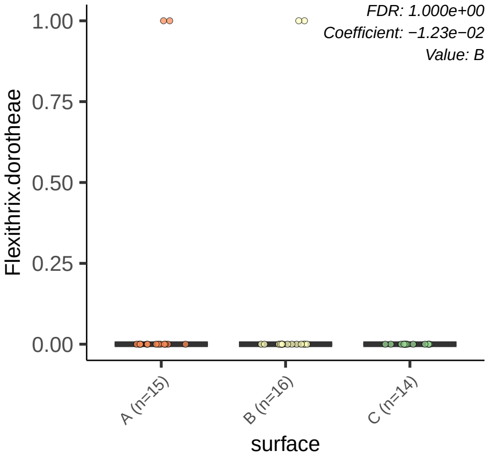
FDR: 1.000e+00
Coefficient: −1.23e−02
Value: B
Flexithrix.dorotheae
1.00
0.75
0.50
0.25
0.00
A (n=15)
B (n=16)
C (n=14)
surface

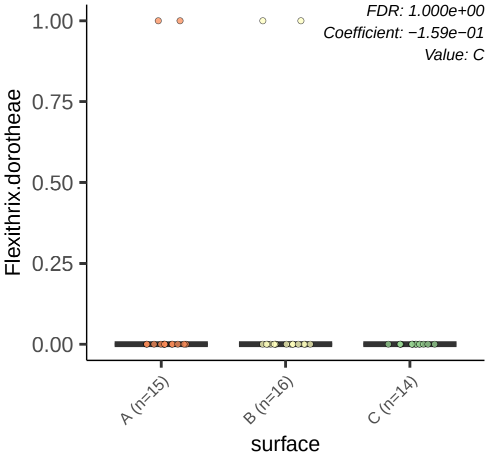
FDR: 1.000e+00
Coefficient: −1.59e−01
Value: C
Flexithrix.dorotheae
1.00
0.75
0.50
0.25
0.00
A (n=15)
B (n=16)
C (n=14)
surface

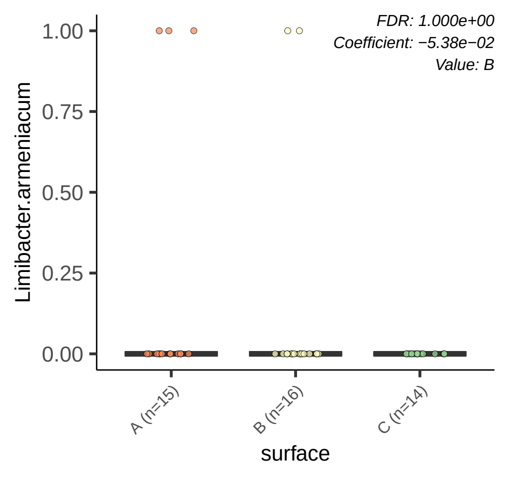
FDR: 1.000e+00
Coefficient: −5.38e−02
Value: B
Limibacter.armeniacum
1.00
0.75
0.50
0.25
0.00
A (n=15)
B (n=16)
C (n=14)
surface

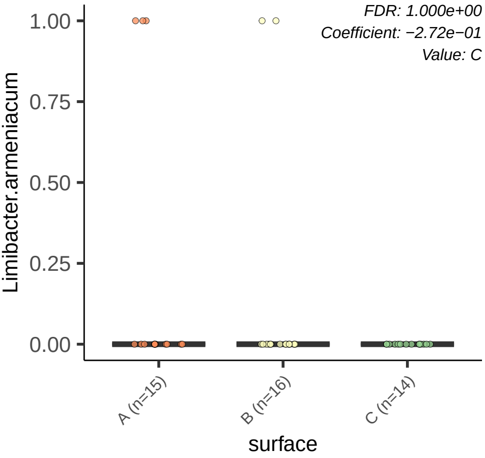
FDR: 1.000e+00
Coefficient: −2.72e−01
Value: C
Limibacter.armeniacum
1.00
0.75
0.50
0.25
0.00
A (n=15)
B (n=16)
C (n=14)
surface

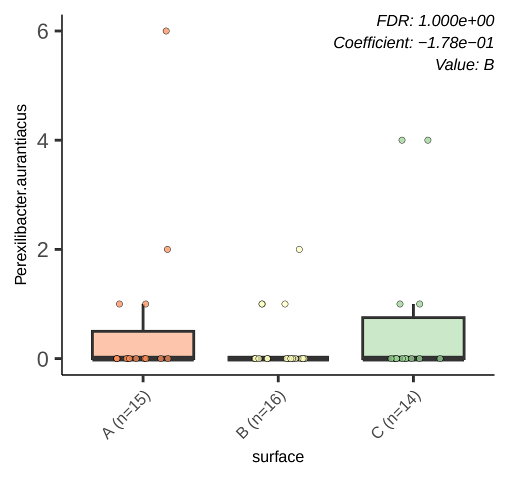

FDR: 1.000e+00
Coefficient: −1.78e−01
Value: B
Perexilibacter.aurantiacus
0
2
4
6
A (n=15)
B (n=16)
C (n=14)
surface

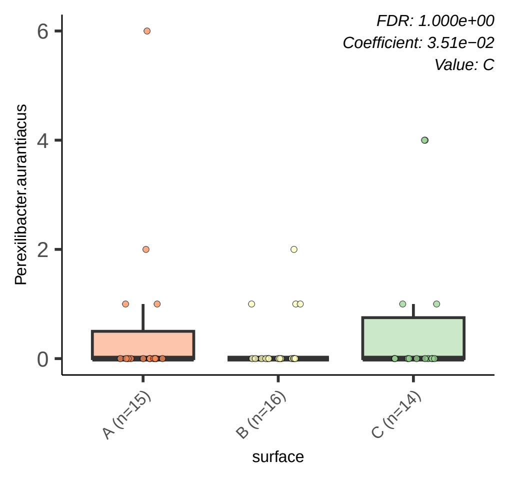
FDR: 1.000e+00
Coefficient: 3.51e−02
Value: C
Perexilibacter.aurantiacus
6
4
2
0
A (n=15)
B (n=16)
C (n=14)
surface

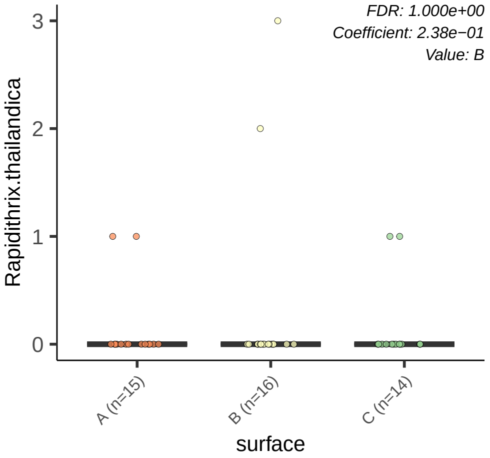

FDR: 1.000e+00
Coefficient: 2.38e−01
Value: B
Rapidithrix.thailandica
3
2
1
0
A (n=15)
B (n=16)
C (n=14)
surface

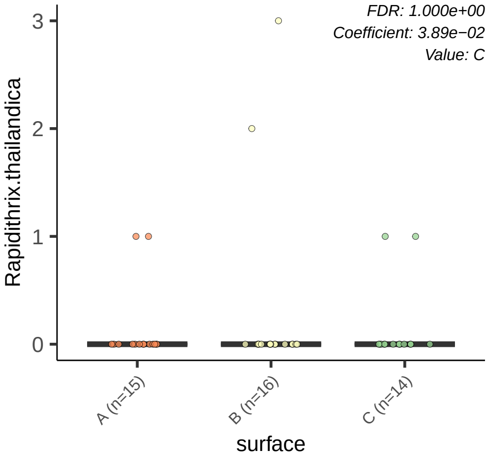

FDR: 1.000e+00
Coefficient: 3.89e−02
Value: C
Rapidithrix.thailandica
3
2
1
0
A (n=15)
B (n=16)
C (n=14)
surface

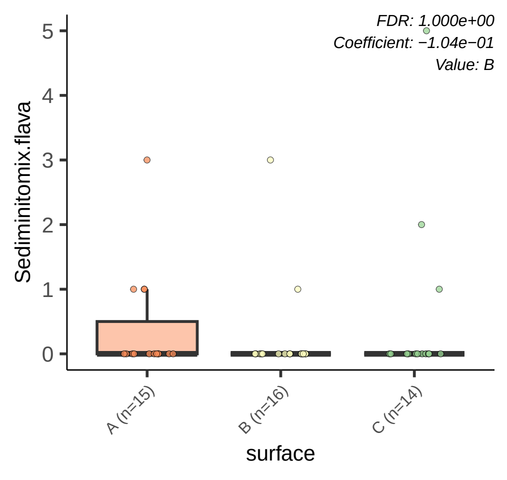

FDR: 1.000e+00
Coefficient: −1.04e−01
Value: B
Sediminitomix.flava
5
4
3
2
1
0
A (n=15)
B (n=16)
C (n=14)
surface

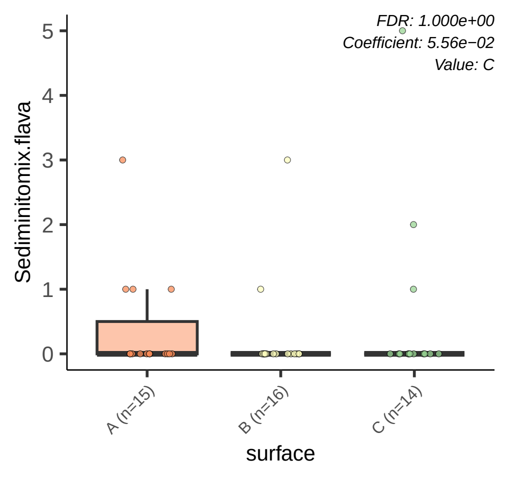

FDR: 1.000e+00
Coefficient: 5.56e−02
Value: C
Sediminitomix.flava
0
1
2
3
4
5
A (n=15)
B (n=16)
C (n=14)
surface

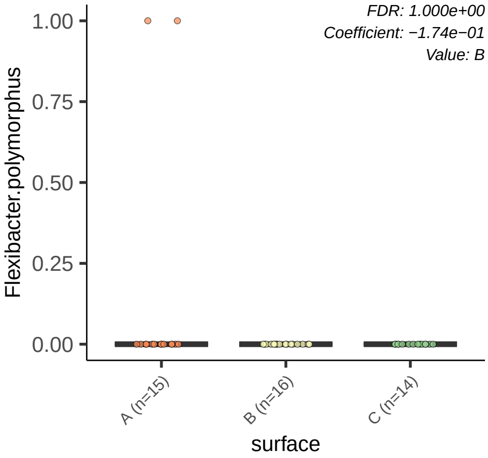
FDR: 1.000e+00
Coefficient: −1.74e−01
Value: B
Flexibacter.polymorphus
1.00
0.75
0.50
0.25
0.00
A (n=15)
B (n=16)
C (n=14)
surface

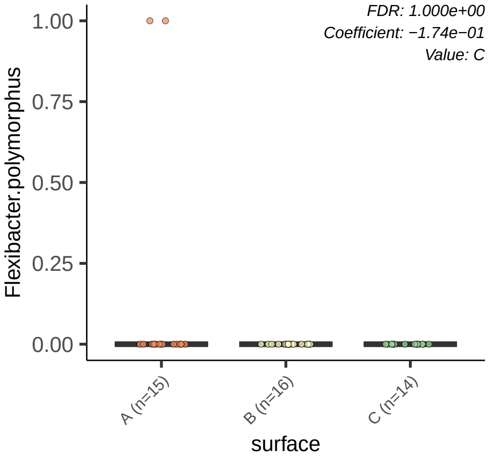
FDR: 1.000e+00
Coefficient: −1.74e−01
Value: C
Flexibacter.polymorphus
1.00
0.75
0.50
0.25
0.00
A (n=15)
B (n=16)
C (n=14)
surface

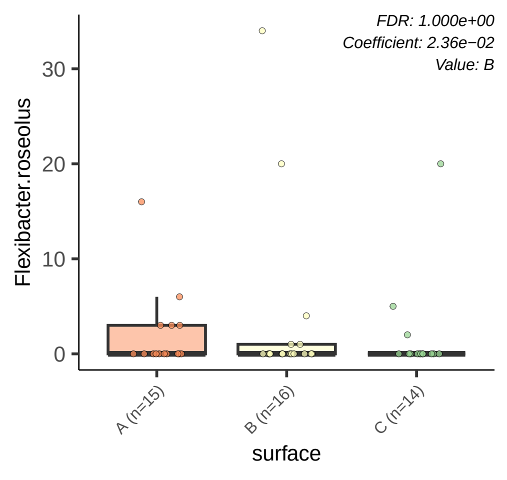

FDR: 1.000e+00
Coefficient: 2.36e−02
Value: B
Flexibacter.roseolus
0
10
20
30
A (n=15)
B (n=16)
C (n=14)
surface

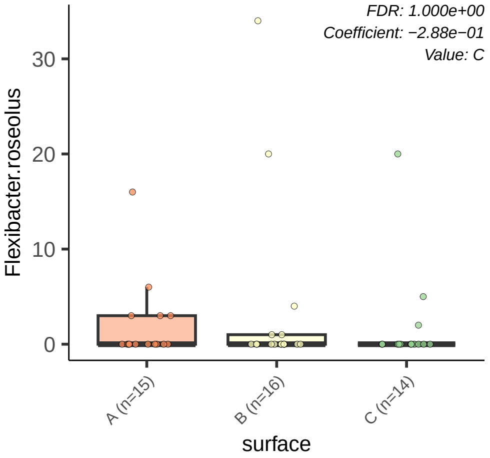

FDR: 1.000e+00
Coefficient: −2.88e−01
Value: C
Flexibacter.roseolus
0
10
20
30
A (n=15)
B (n=16)
C (n=14)
surface

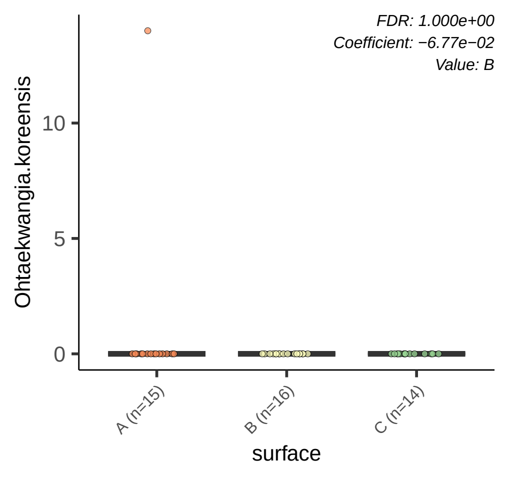

FDR: 1.000e+00
Coefficient: −6.77e−02
Value: B
Ohtaekwangia.koreensis
0
5
10
A (n=15)
B (n=16)
C (n=14)
surface

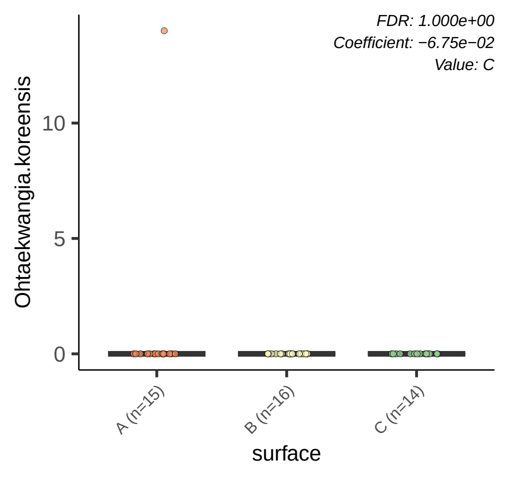
FDR: 1.000e+00
Coefficient: −6.75e−02
Value: C
Ohtaekwangia.koreensis
10
5
0
A (n=15)
B (n=16)
C (n=14)
surface

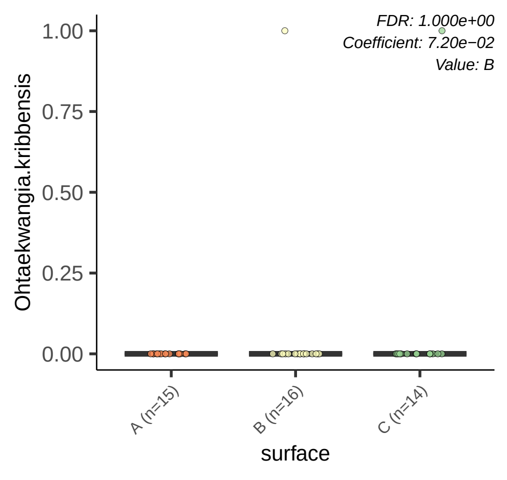
FDR: 1.000e+00
Coefficient: 7.20e−02
Value: B
Ohtaekwangia.kribbensis
1.00
0.75
0.50
0.25
0.00
A (n=15)
B (n=16)
C (n=14)
surface

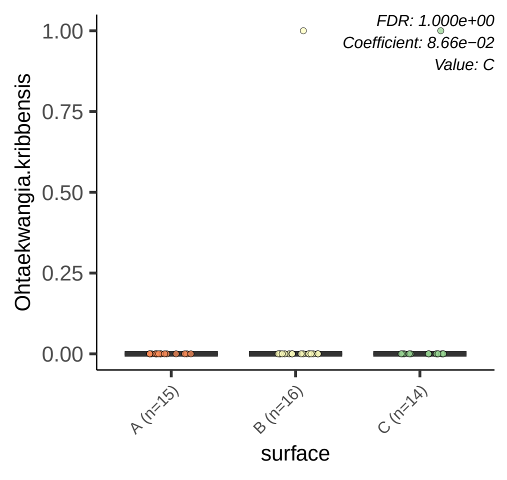

FDR: 1.000e+00
Coefficient: 8.66e−02
Value: C
Ohtaekwangia.kribbensis
1.00
0.75
0.50
0.25
0.00
A (n=15)
B (n=16)
C (n=14)
surface

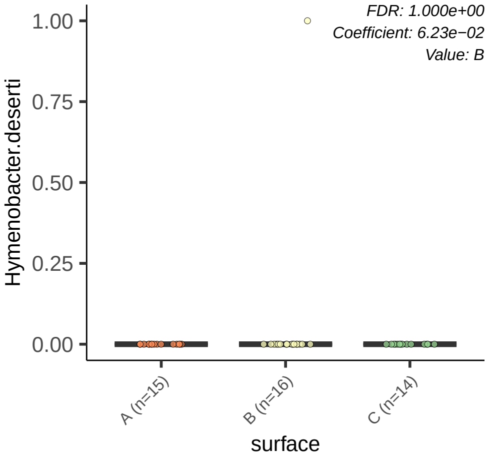

FDR: 1.000e+00
Coefficient: 6.23e−02
Value: B
1.00
0.75
0.50
0.25
0.00
Hymenobacter.deserti
A (n=15)
B (n=16)
C (n=14)
surface

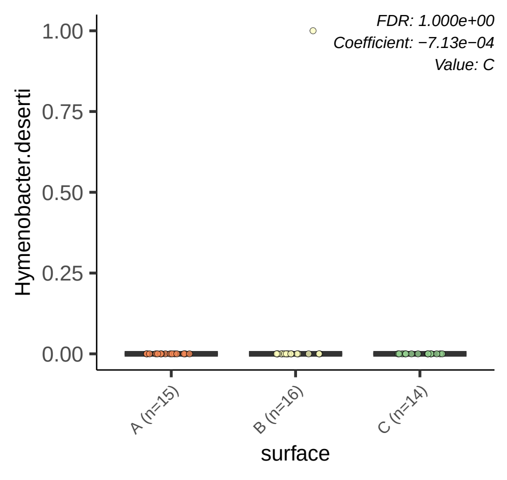

FDR: 1.000e+00
Coefficient: −7.13e−04
Value: C
1.00
0.75
0.50
0.25
0.00
Hymenobacter.deserti
A (n=15)
B (n=16)
C (n=14)
surface

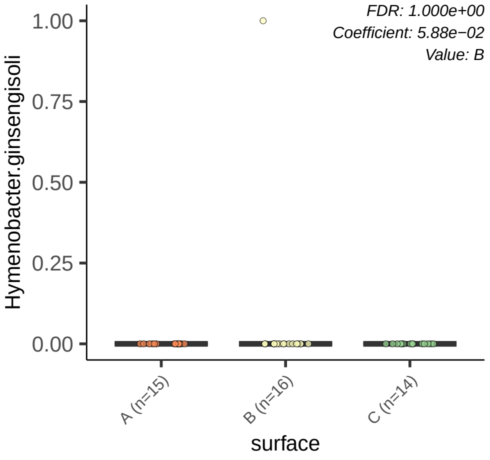
FDR: 1.000e+00
Coefficient: 5.88e−02
Value: B
Hymenobacter.ginsengisoli
1.00
0.75
0.50
0.25
0.00
A (n=15)
B (n=16)
C (n=14)
surface

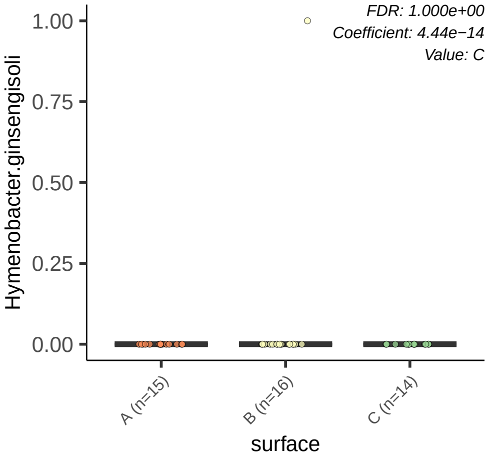
FDR: 1.000e+00
Coefficient: 4.44e−14
Value: C
Hymenobacter.ginsengisoli
1.00
0.75
0.50
0.25
0.00
A (n=15)
B (n=16)
C (n=14)
surface

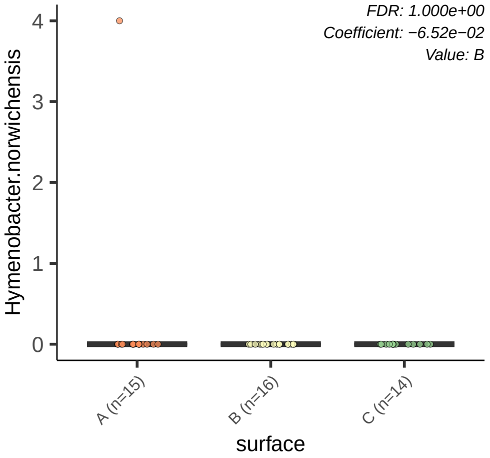
FDR: 1.000e+00
Coefficient: −6.52e−02
Value: B
Hymenobacter.norwichensis
4
3
2
1
0
A (n=15)
B (n=16)
C (n=14)
surface

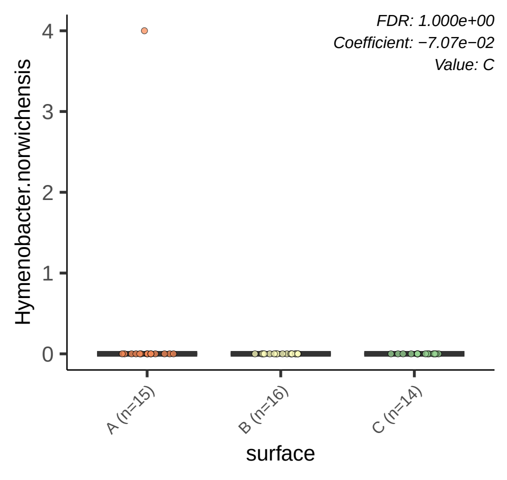
FDR: 1.000e+00
Coefficient: −7.07e−02
Value: C
Hymenobacter.norwichensis
4
3
2
1
0
A (n=15)
B (n=16)
C (n=14)
surface

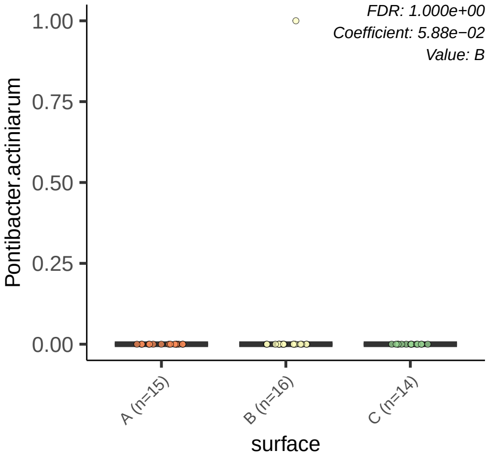
FDR: 1.000e+00
Coefficient: 5.88e−02
Value: B
Pontibacter.actiniarum
1.00
0.75
0.50
0.25
0.00
A (n=15)
B (n=16)
C (n=14)
surface

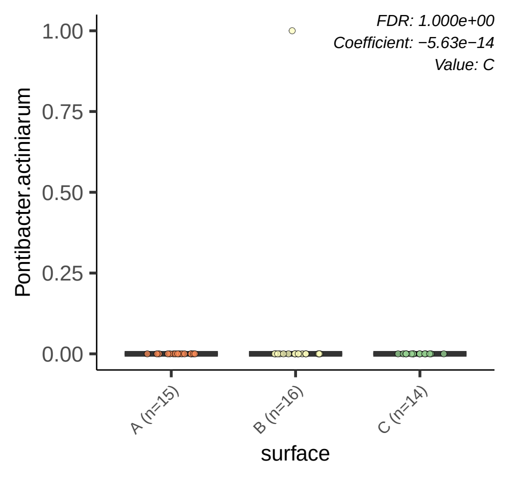

FDR: 1.000e+00
Coefficient: −5.63e−14
Value: C
Pontibacter.actiniarum
1.00
0.75
0.50
0.25
0.00
A (n=15)
B (n=16)
C (n=14)
surface

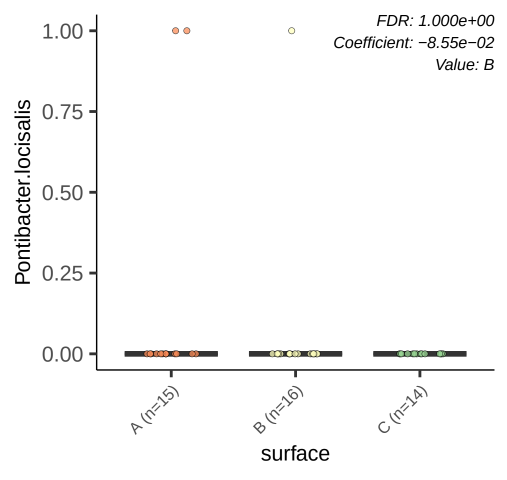

FDR: 1.000e+00
Coefficient: −8.55e−02
Value: B
Pontibacter.locisalis
1.00
0.75
0.50
0.25
0.00
A (n=15)
B (n=16)
C (n=14)
surface

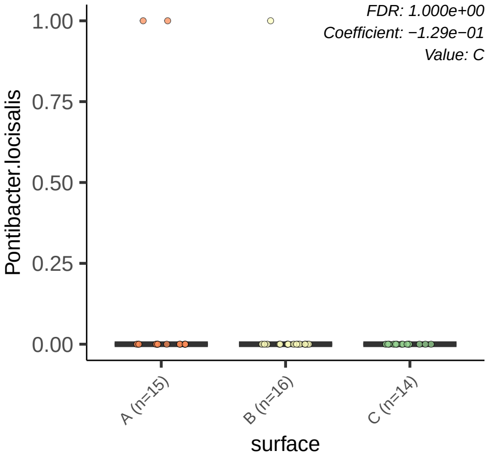
FDR: 1.000e+00
Coefficient: −1.29e−01
Value: C
Pontibacter.locisalis
1.00
0.75
0.50
0.25
0.00
A (n=15)
B (n=16)
C (n=14)
surface

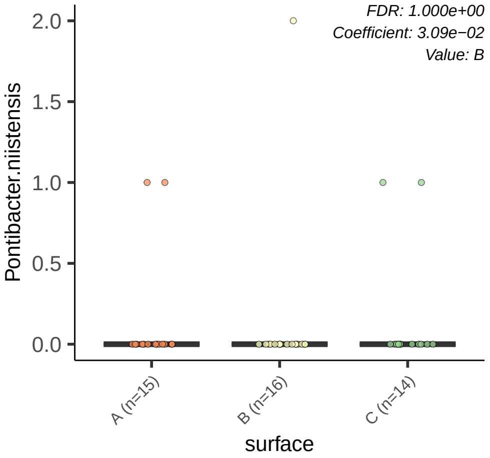

FDR: 1.000e+00
Coefficient: 3.09e−02
Value: B
Pontibacter.niistensis
2.0
1.5
1.0
0.5
0.0
A (n=15)
B (n=16)
C (n=14)
surface

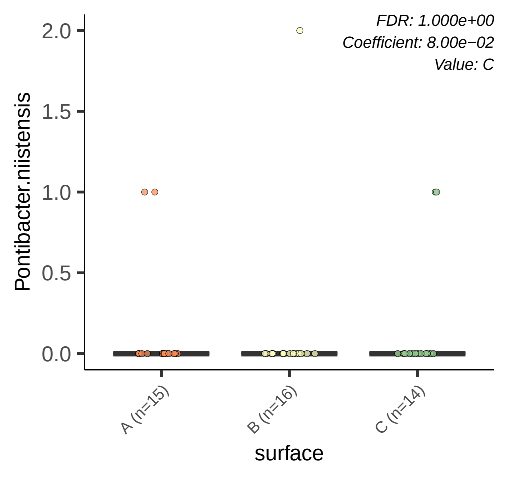
FDR: 1.000e+00
Coefficient: 8.00e−02
Value: C
Pontibacter.niistensis
2.0
1.5
1.0
0.5
0.0
A (n=15)
B (n=16)
C (n=14)
surface

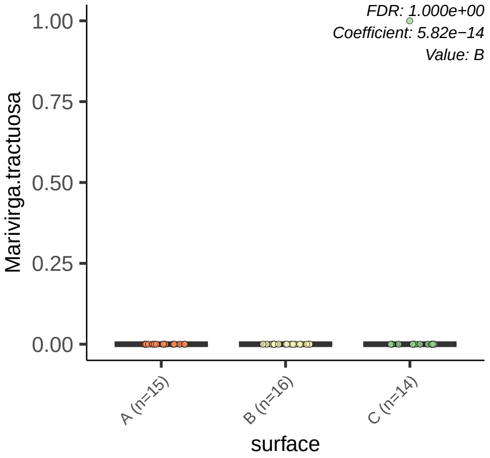

FDR: 1.000e+00
Coefficient: 5.82e−14
Value: B
1.00
0.75
0.50
0.25
0.00
Marivirga.tractuosa
A (n=15)
B (n=16)
C (n=14)
surface

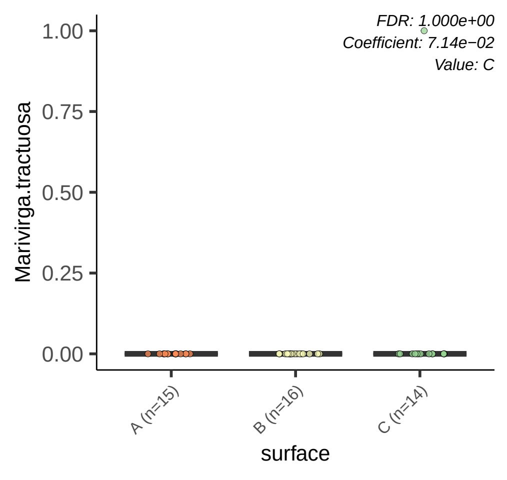
FDR: 1.000e+00
Coefficient: 7.14e−02
Value: C
Marivirga.tractuosa
1.00
0.75
0.50
0.25
0.00
A (n=15)
B (n=16)
C (n=14)
surface

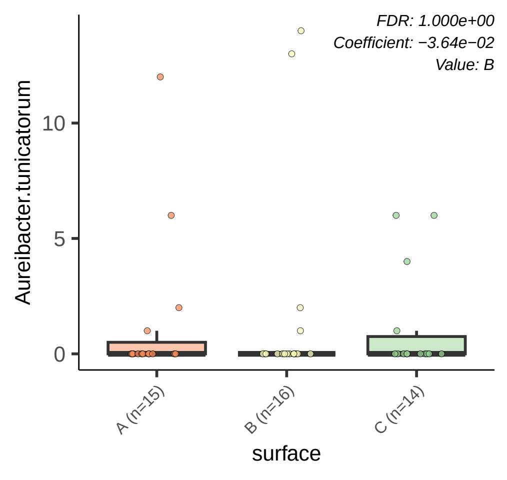

FDR: 1.000e+00
Coefficient: −3.64e−02
Value: B
Aureibacter.tunicatorum
0
5
10
A (n=15)
B (n=16)
C (n=14)
surface

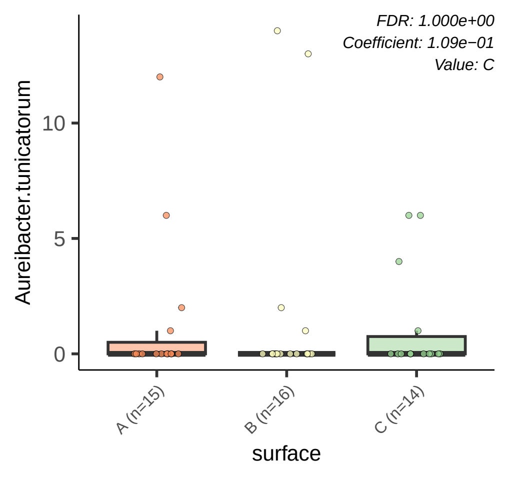

FDR: 1.000e+00
Coefficient: 1.09e−01
Value: C
Aureibacter.tunicatorum
0
5
10
A (n=15)
B (n=16)
C (n=14)
surface

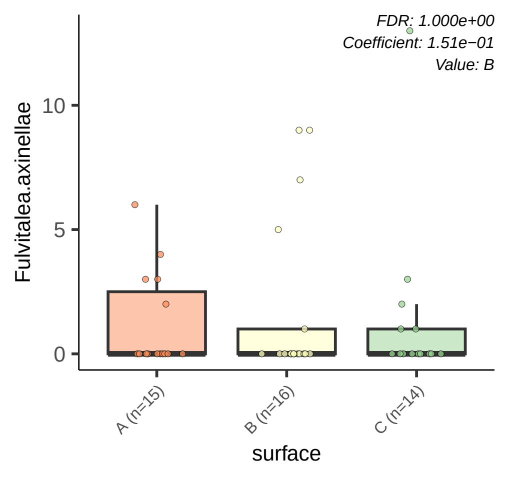
FDR: 1.000e+00
Coefficient: 1.51e−01
Value: B
Fulvitalea.axinellae
10
5
0
A (n=15)
B (n=16)
C (n=14)
surface

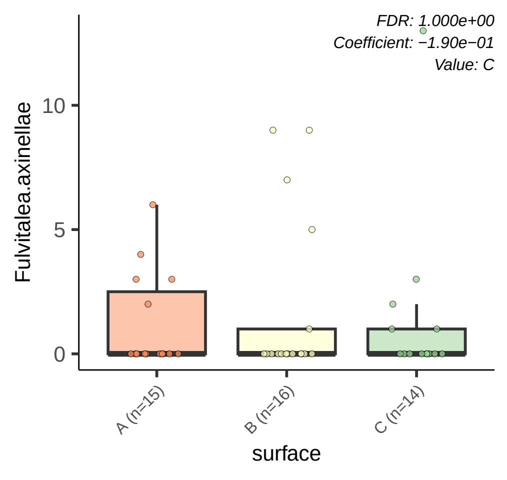

FDR: 1.000e+00
Coefficient: −1.90e−01
Value: C
Fulvitalea.axinellae
10
5
0
A (n=15)
B (n=16)
C (n=14)
surface

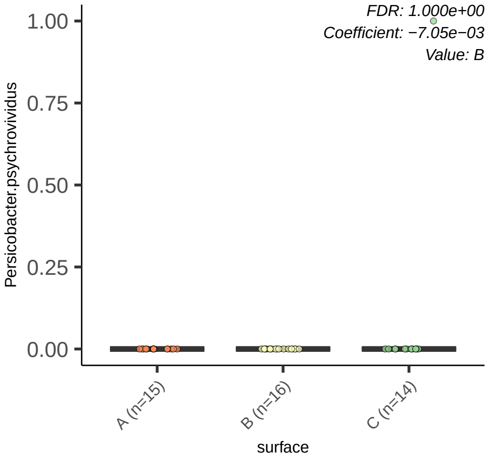
FDR: 1.000e+00
Coefficient: −7.05e−03
Value: B
1.00
0.75
0.50
0.25
0.00
Persicobacter.psychrovividus
A (n=15)
B (n=16)
C (n=14)
surface

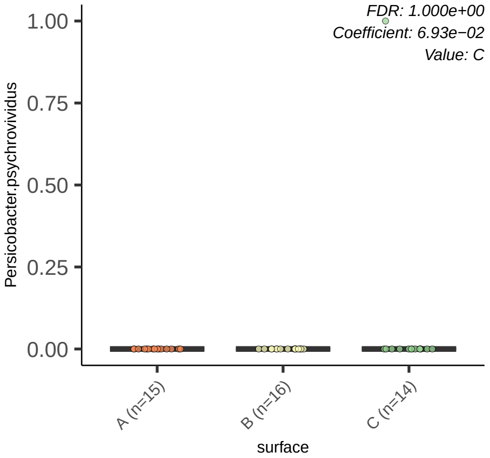
FDR: 1.000e+00
Coefficient: 6.93e−02
Value: C
Persicobacter.psychrovividus
1.00
0.75
0.50
0.25
0.00
A (n=15)
B (n=16)
C (n=14)
surface

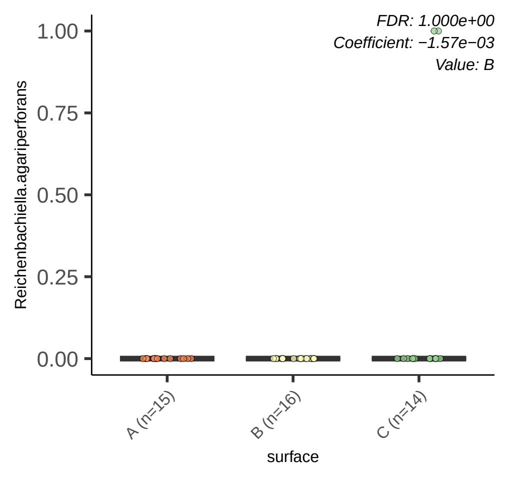

FDR: 1.000e+00
Coefficient: −1.57e−03
Value: B
1.00
0.75
0.50
0.25
0.00
Reichenbachiella.agariperforans
A (n=15)
B (n=16)
C (n=14)
surface

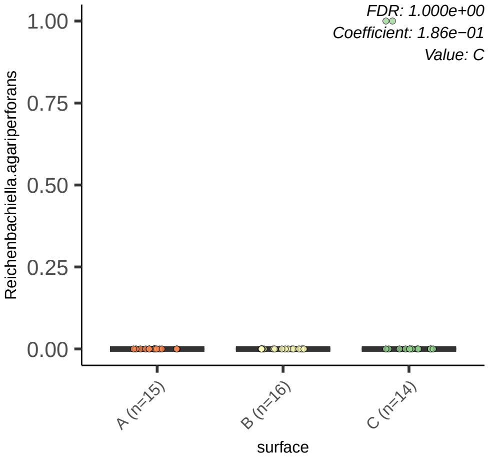
FDR: 1.000e+00
Coefficient: 1.86e−01
Value: C
Reichenbachiella.agariperforans
1.00
0.75
0.50
0.25
0.00
A (n=15)
B (n=16)
C (n=14)
surface

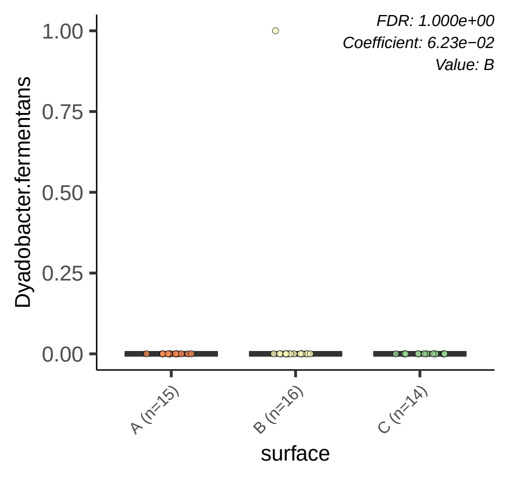
FDR: 1.000e+00
Coefficient: 6.23e−02
Value: B
Dyadobacter.fermentans
1.00
0.75
0.50
0.25
0.00
A (n=15)
B (n=16)
C (n=14)
surface

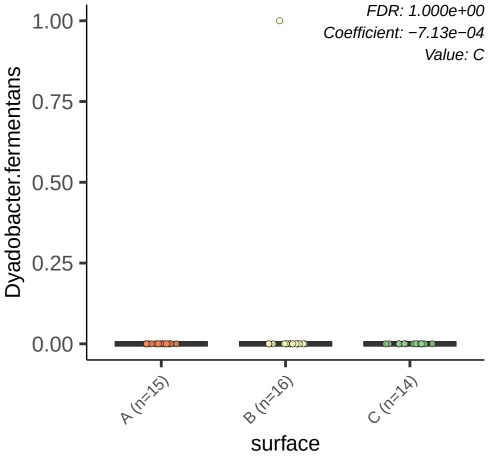

FDR: 1.000e+00
Coefficient: −7.13e−04
Value: C
Dyadobacter.fermentans
1.00
0.75
0.50
0.25
0.00
A (n=15)
B (n=16)
C (n=14)
surface

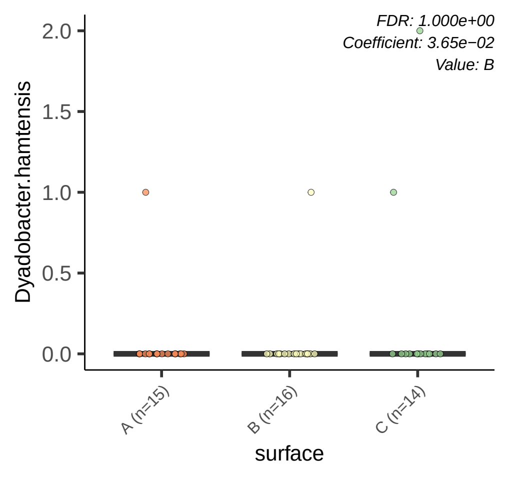
FDR: 1.000e+00
Coefficient: 3.65e−02
Value: B
Dyadobacter.hamtensis
2.0
1.5
1.0
0.5
0.0
A (n=15)
B (n=16)
C (n=14)
surface

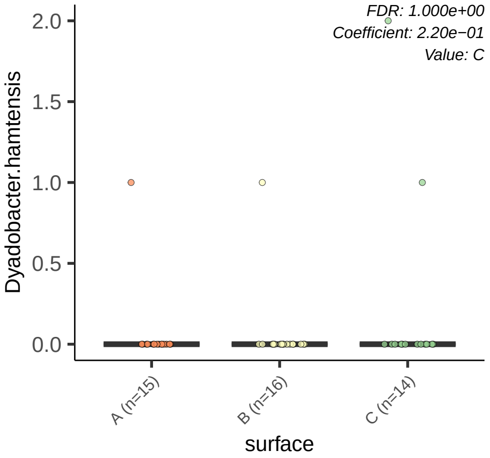
FDR: 1.000e+00
Coefficient: 2.20e−01
Value: C
Dyadobacter.hamtensis
2.0
1.5
1.0
0.5
0.0
A (n=15)
B (n=16)
C (n=14)
surface

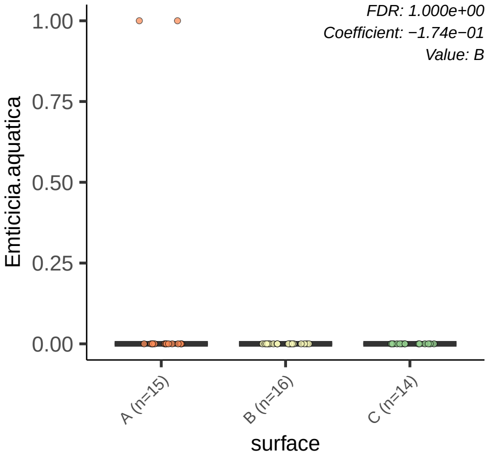

FDR: 1.000e+00
Coefficient: −1.74e−01
Value: B
Emticicia.aquatica
1.00
0.75
0.50
0.25
0.00
A (n=15)
B (n=16)
C (n=14)
surface

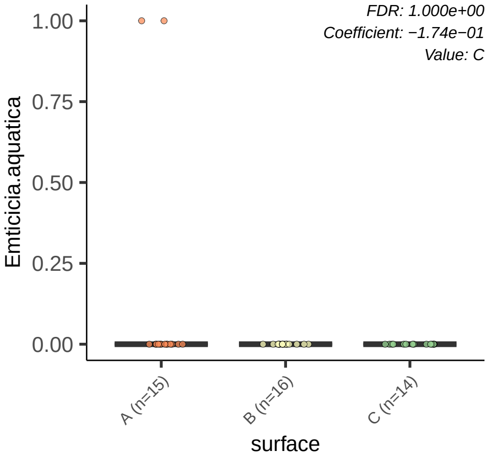
FDR: 1.000e+00
Coefficient: −1.74e−01
Value: C
Emticicia.aquatica
1.00
0.75
0.50
0.25
0.00
A (n=15)
B (n=16)
C (n=14)
surface

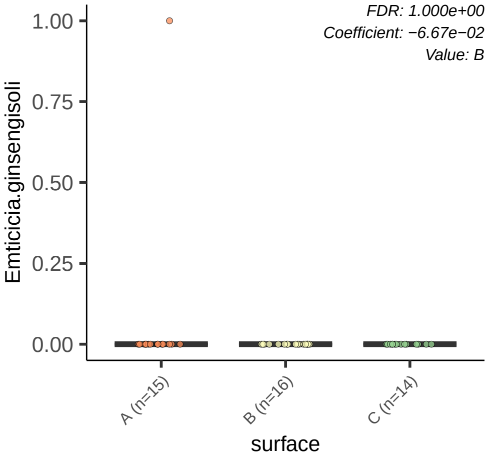

FDR: 1.000e+00
Coefficient: −6.67e−02
Value: B
Emticicia.ginsengisoli
1.00
0.75
0.50
0.25
0.00
A (n=15)
B (n=16)
C (n=14)
surface

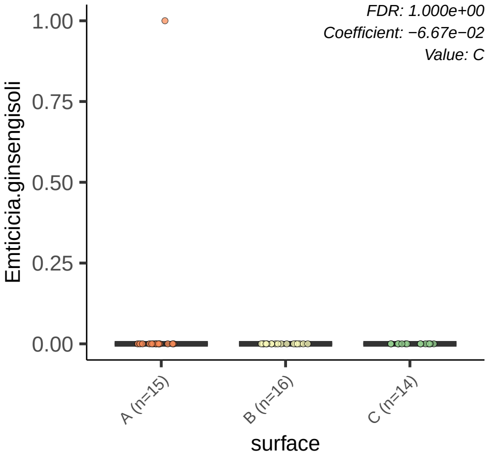
FDR: 1.000e+00
Coefficient: −6.67e−02
Value: C
Emticicia.ginsengisoli
1.00
0.75
0.50
0.25
0.00
A (n=15)
B (n=16)
C (n=14)
surface

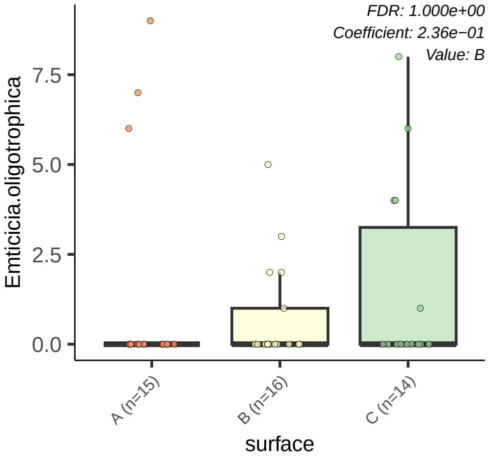

FDR: 1.000e+00
Coefficient: 2.36e−01
Value: B
Emticicia.oligotrophica
7.5
5.0
2.5
0.0
A (n=15)
B (n=16)
C (n=14)
surface

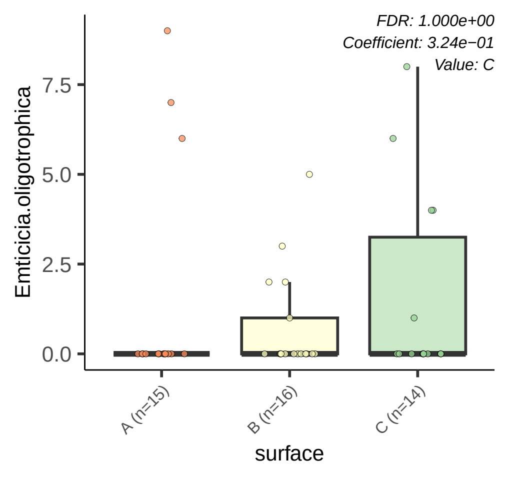
FDR: 1.000e+00
Coefficient: 3.24e−01
Value: C
Emticicia.oligotrophica
7.5
5.0
2.5
0.0
A (n=15)
B (n=16)
C (n=14)
surface

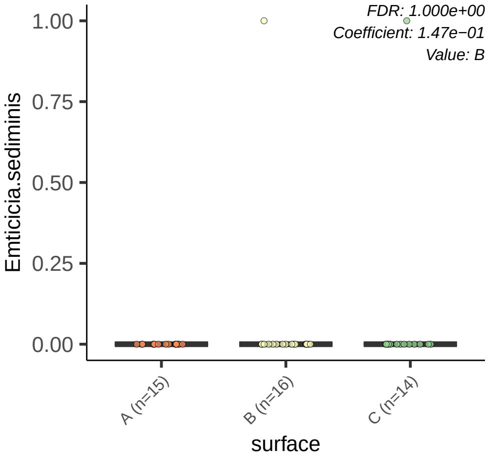
FDR: 1.000e+00
Coefficient: 1.47e−01
Value: B
1.00
0.75
0.50
0.25
0.00
Emticicia.sedaminis
A (n=15)
B (n=16)
C (n=14)
surface

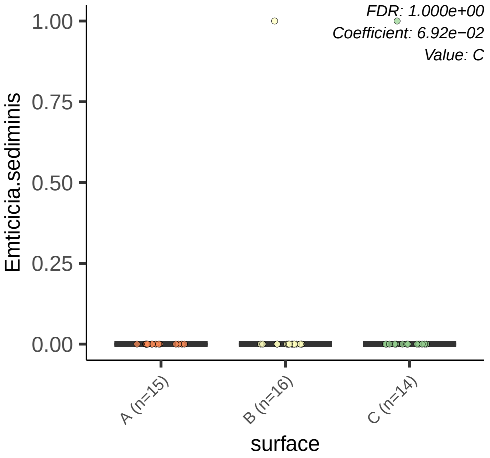
FDR: 1.000e+00
Coefficient: 6.92e−02
Value: C
Emticicia.sediminis
1.00
0.75
0.50
0.25
0.00
A (n=15)
B (n=16)
C (n=14)
surface

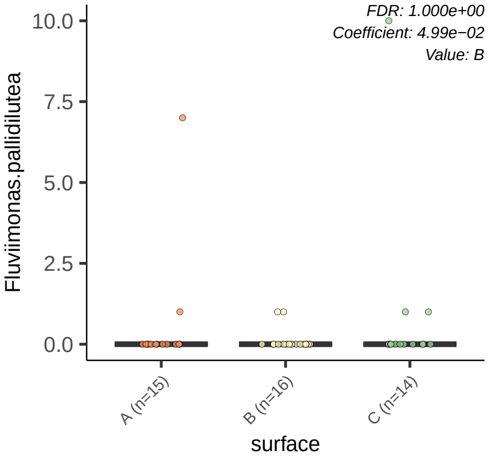
FDR: 1.000e+00
Coefficient: 4.99e−02
Value: B
Fluviimonas.pallidilutea
10.0
7.5
5.0
2.5
0.0
A (n=15)
B (n=16)
C (n=14)
surface

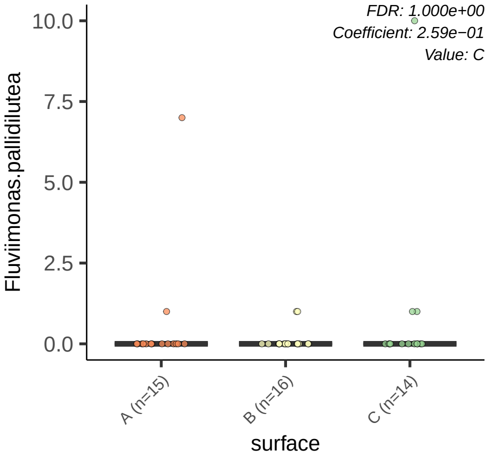

FDR: 1.000e+00
Coefficient: 2.59e−01
Value: C
10.0
7.5
5.0
2.5
0.0
Fluviimonas.pallidilutea
A (n=15)
B (n=16)
C (n=14)
surface

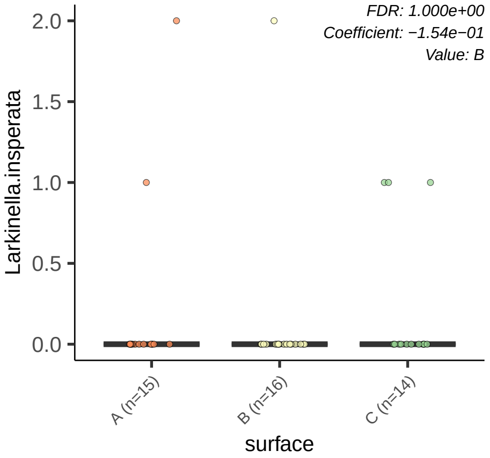

FDR: 1.000e+00
Coefficient: −1.54e−01
Value: B
Larkinella.insperata
2.0
1.5
1.0
0.5
0.0
A (n=15)
B (n=16)
C (n=14)
surface

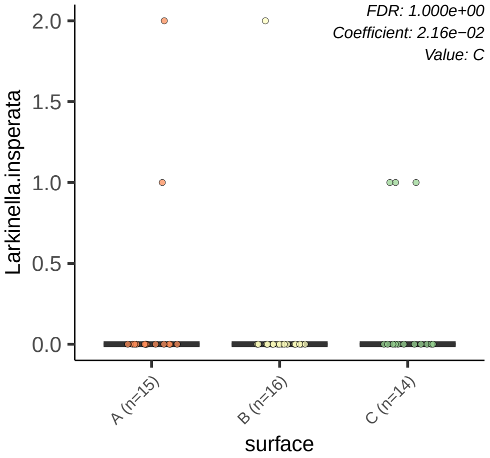
FDR: 1.000e+00
Coefficient: 2.16e−02
Value: C
Larkinella.insperata
2.0
1.5
1.0
0.5
0.0
A (n=15)
B (n=16)
C (n=14)
surface

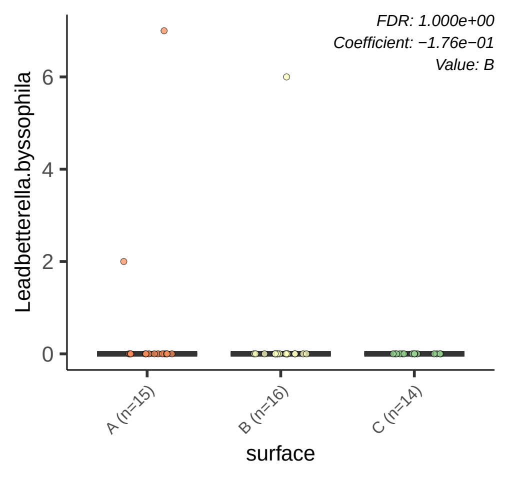
FDR: 1.000e+00
Coefficient: −1.76e−01
Value: B
Leadbetterella.byssophila
0
2
4
6
A (n=15)
B (n=16)
C (n=14)
surface

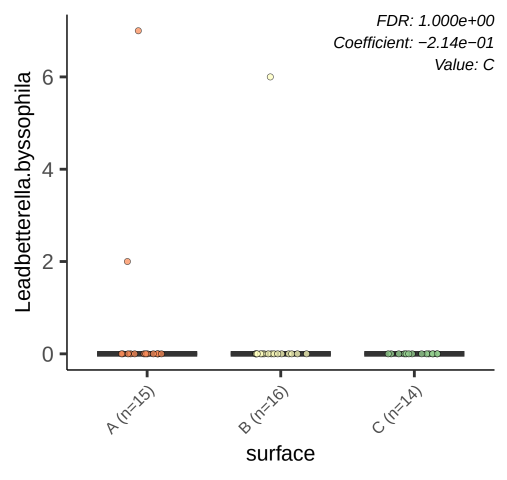

FDR: 1.000e+00
Coefficient: −2.14e−01
Value: C
Leadbetterella.byssophila
0
2
4
6
A (n=15)
B (n=16)
C (n=14)
surface

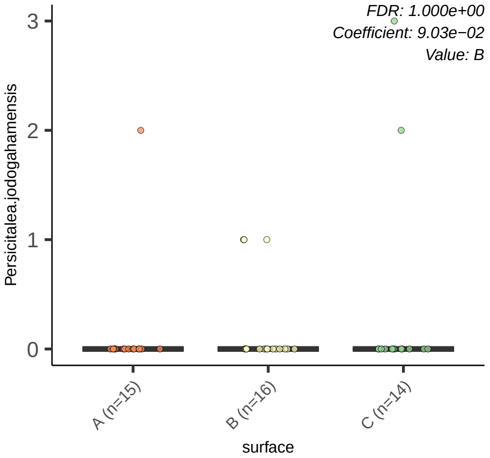
FDR: 1.000e+00
Coefficient: 9.03e−02
Value: B
Persicitalea.jodogahamensis
3
2
1
0
A (n=15)
B (n=16)
C (n=14)
surface

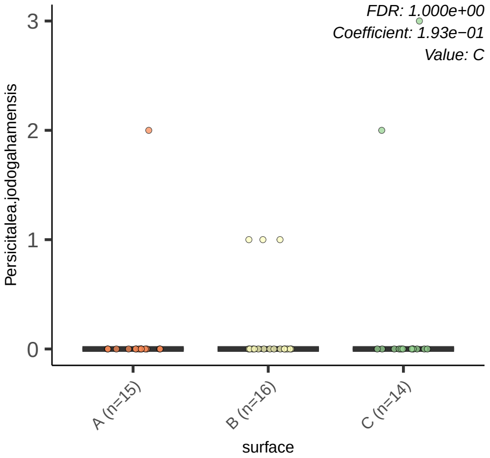
FDR: 1.000e+00
Coefficient: 1.93e−01
Value: C
Persicitalea.jodogahamensis
3
2
1
0
A (n=15)
B (n=16)
C (n=14)
surface

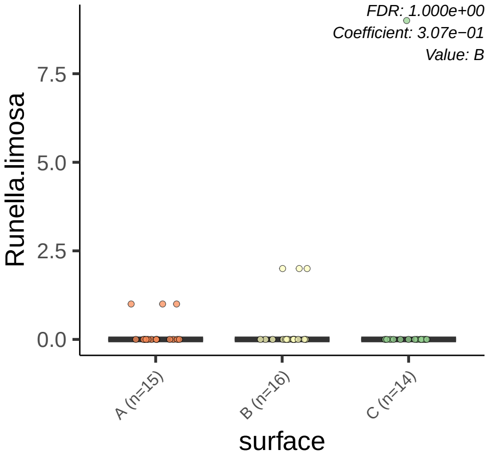
FDR: 1.000e+00
Coefficient: 3.07e−01
Value: B
Runella.limosa
7.5
5.0
2.5
0.0
A (n=15)
B (n=16)
C (n=14)
surface

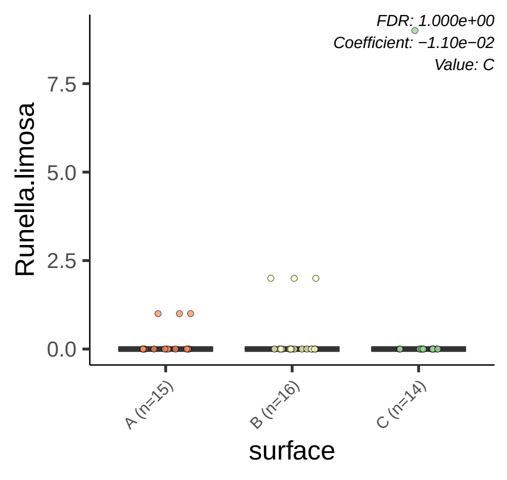
FDR: 1.000e+00
Coefficient: −1.10e−02
Value: C
Runella.limosa
7.5
5.0
2.5
0.0
A (n=15)
B (n=16)
C (n=14)
surface

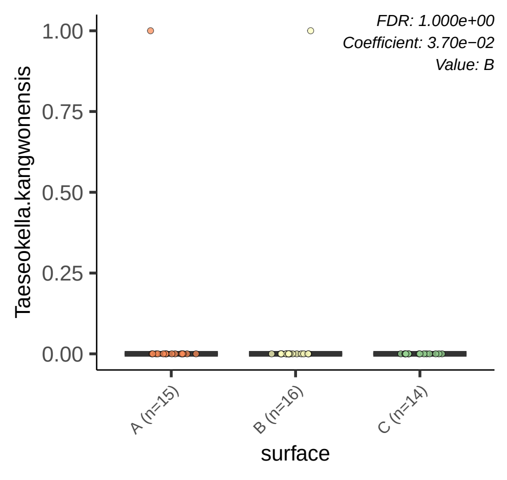

FDR: 1.000e+00
Coefficient: 3.70e−02
Value: B
Taeseokella.kangwonensis
1.00
0.75
0.50
0.25
0.00
A (n=15)
B (n=16)
C (n=14)
surface

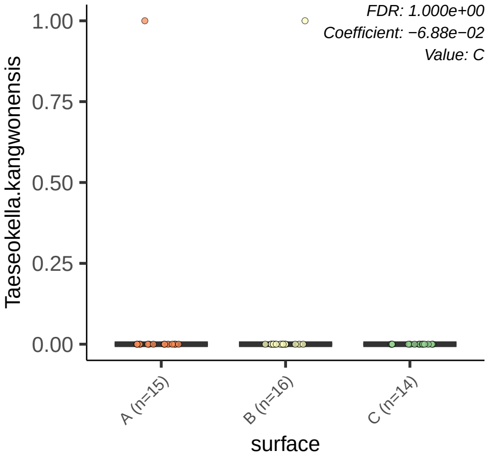

FDR: 1.000e+00
Coefficient: −6.88e−02
Value: C
Taeseokella.kangwonensis
1.00
0.75
0.50
0.25
0.00
A (n=15)
B (n=16)
C (n=14)
surface

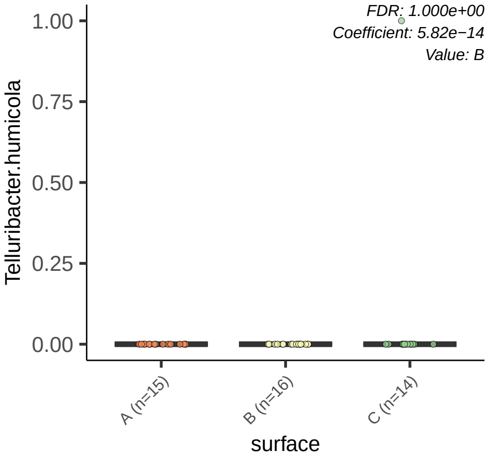

FDR: 1.000e+00
Coefficient: 5.82e−14
Value: B
Telluribacter.humicola
1.00
0.75
0.50
0.25
0.00
A (n=15)
B (n=16)
C (n=14)
surface

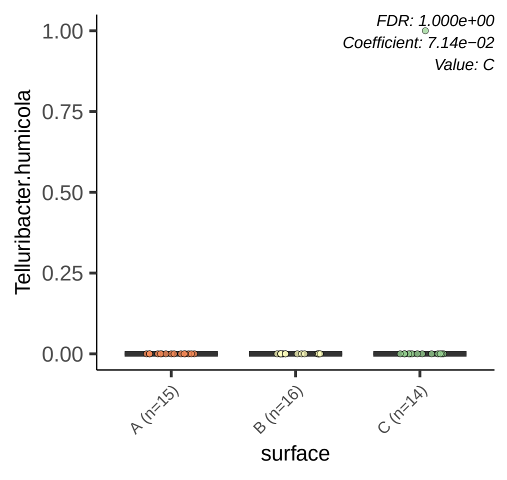
FDR: 1.000e+00
Coefficient: 7.14e−02
Value: C
Telluribacter.humicola
1.00
0.75
0.50
0.25
0.00
A (n=15)
B (n=16)
C (n=14)
surface

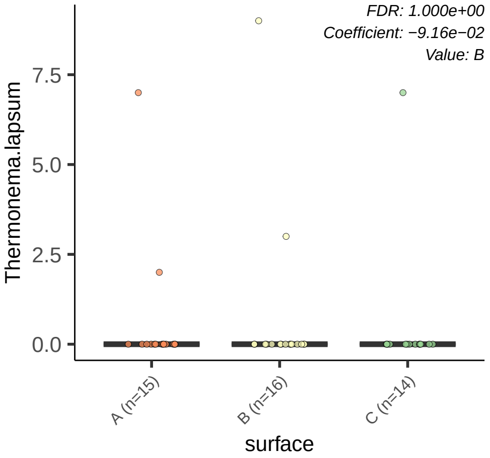
FDR: 1.000e+00
Coefficient: −9.16e−02
Value: B
Thermonema.lapsum
7.5
5.0
2.5
0.0
A (n=15)
B (n=16)
C (n=14)
surface

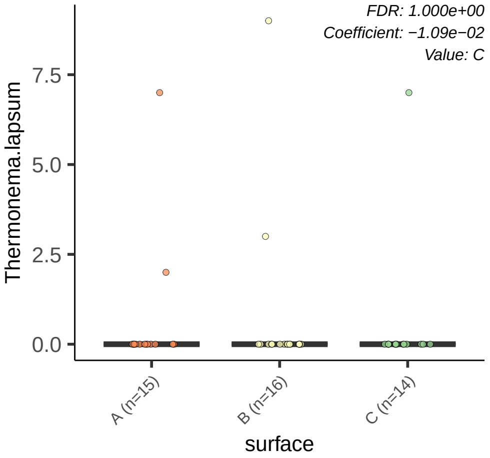
FDR: 1.000e+00
Coefficient: −1.09e−02
Value: C
Thermonema.lapsum
7.5
5.0
2.5
0.0
A (n=15)
B (n=16)
C (n=14)
surface

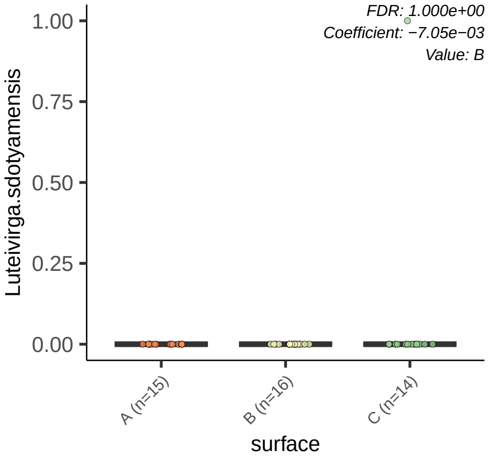

FDR: 1.000e+00
Coefficient: −7.05e−03
Value: B
Luteivirga.sdotyamensis
1.00
0.75
0.50
0.25
0.00
A (n=15)
B (n=16)
C (n=14)
surface

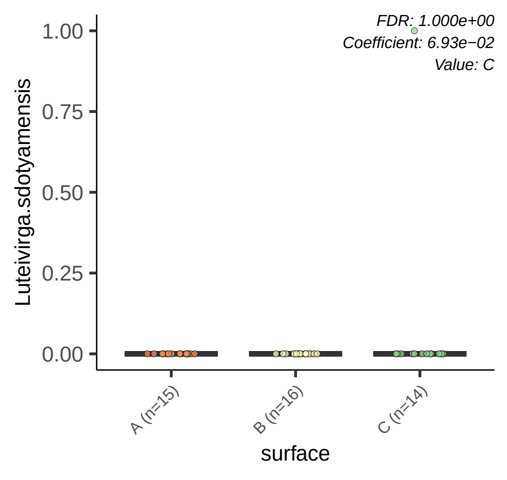
FDR: 1.000e+00
Coefficient: 6.93e−02
Value: C
Luteivirga.sdotyamensis
1.00
0.75
0.50
0.25
0.00
A (n=15)
B (n=16)
C (n=14)
surface

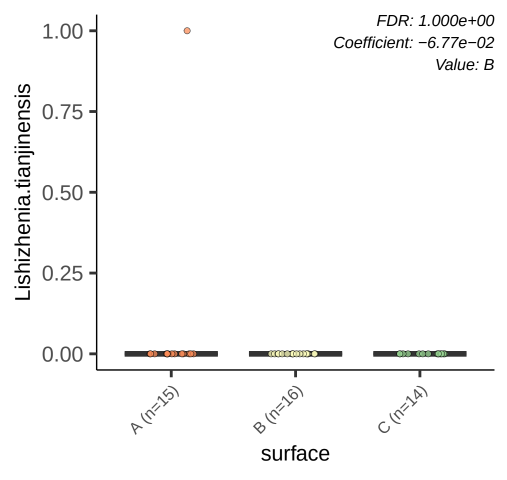

FDR: 1.000e+00
Coefficient: −6.77e−02
Value: B
Lishizhenia.tianjinensis
1.00
0.75
0.50
0.25
0.00
A (n=15)
B (n=16)
C (n=14)
surface

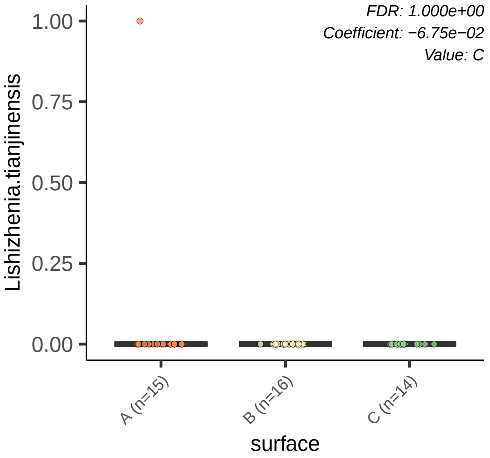
FDR: 1.000e+00
Coefficient: −6.75e−02
Value: C
Lishizhenia.tianjinensis
1.00
0.75
0.50
0.25
0.00
A (n=15)
B (n=16)
C (n=14)
surface

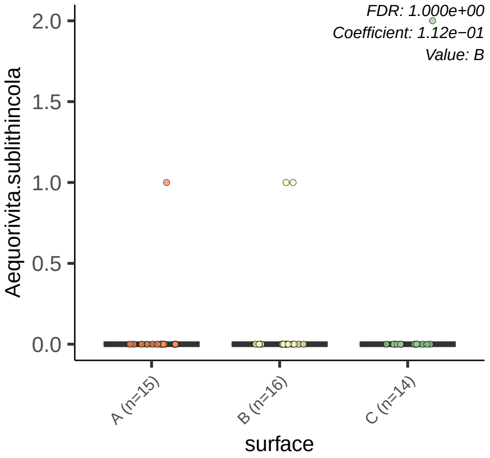
FDR: 1.000e+00
Coefficient: 1.12e−01
Value: B
Aequorivita.sublithincola
2.0
1.5
1.0
0.5
0.0
A (n=15)
B (n=16)
C (n=14)
surface

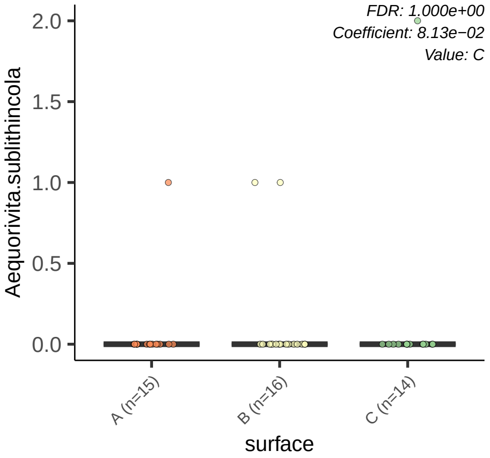
FDR: 1.000e+00
Coefficient: 8.13e−02
Value: C
Aequorivita.sublithincola
2.0
1.5
1.0
0.5
0.0
A (n=15)
B (n=16)
C (n=14)
surface

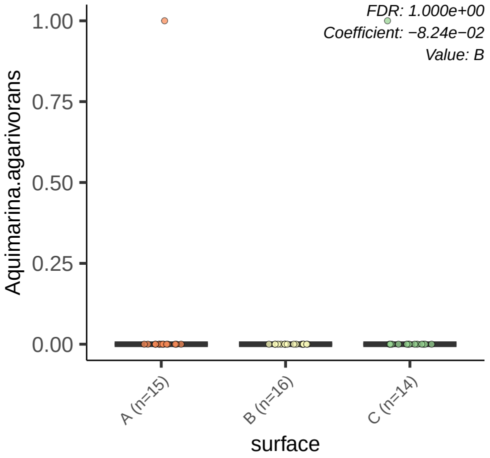
FDR: 1.000e+00
Coefficient: −8.24e−02
Value: B
Aquimarina.agarivorans
1.00
0.75
0.50
0.25
0.00
A (n=15)
B (n=16)
C (n=14)
surface

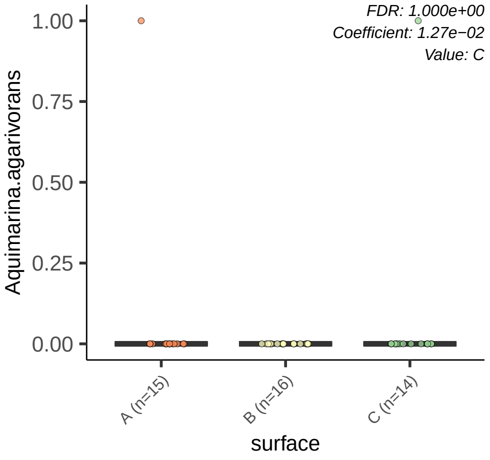
FDR: 1.000e+00
Coefficient: 1.27e−02
Value: C
Aquimarina.agarivorans
1.00
0.75
0.50
0.25
0.00
A (n=15)
B (n=16)
C (n=14)
surface

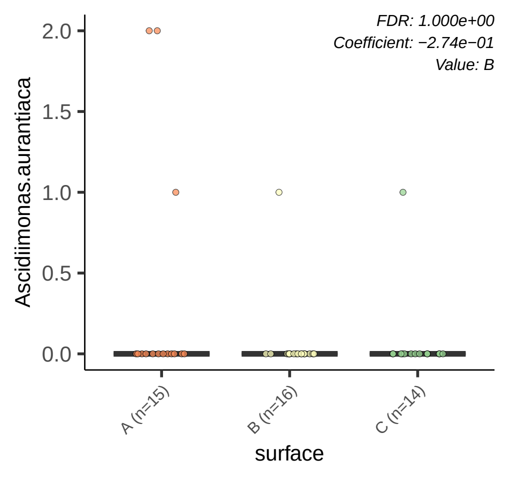
FDR: 1.000e+00
Coefficient: −2.74e−01
Value: B
Ascidiimonas.aurantiaca
2.0
1.5
1.0
0.5
0.0
A (n=15)
B (n=16)
C (n=14)
surface

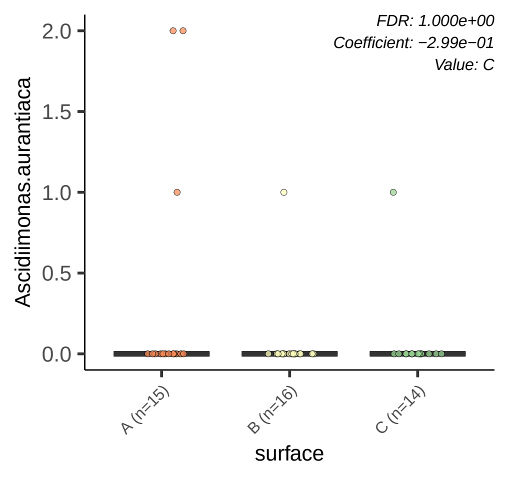

FDR: 1.000e+00
Coefficient: −2.99e−01
Value: C
Ascidiimonas.aurantiaca
2.0
1.5
1.0
0.5
0.0
A (n=15)
B (n=16)
C (n=14)
surface

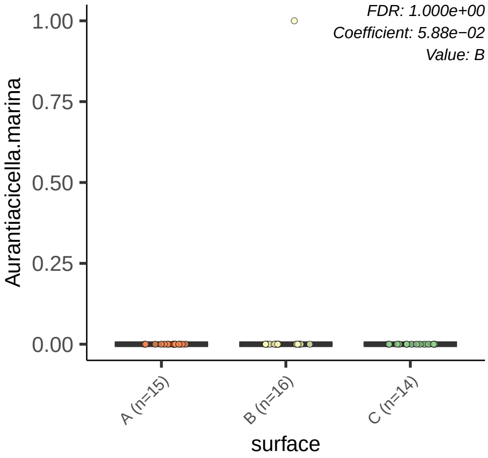

FDR: 1.000e+00
Coefficient: 5.88e−02
Value: B
Aurantiacicella.marina
1.00
0.75
0.50
0.25
0.00
A (n=15)
B (n=16)
C (n=14)
surface

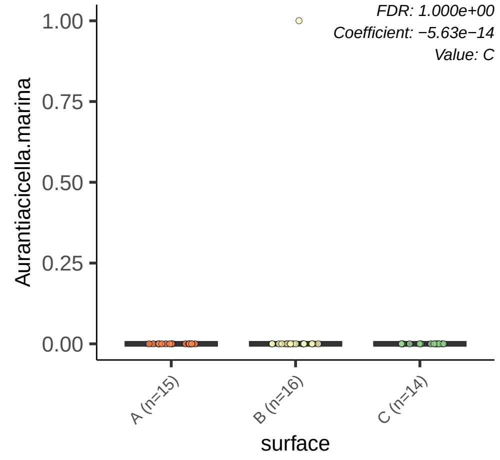
FDR: 1.000e+00
Coefficient: −5.63e−14
Value: C
Aurantiacicella.marina
1.00
0.75
0.50
0.25
0.00
A (n=15)
B (n=16)
C (n=14)
surface

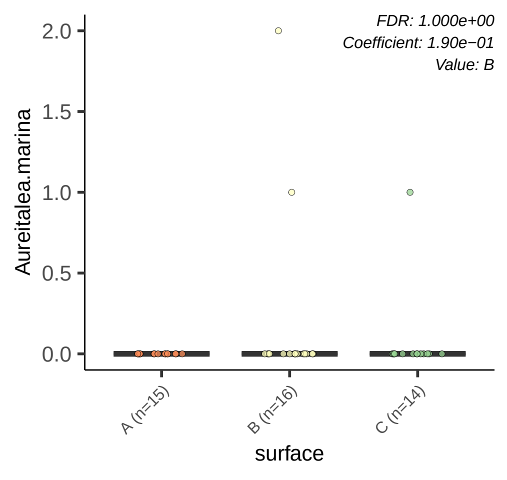

FDR: 1.000e+00
Coefficient: 1.90e−01
Value: B
Aureitalea.marina
2.0
1.5
1.0
0.5
0.0
A (n=15)
B (n=16)
C (n=14)
surface

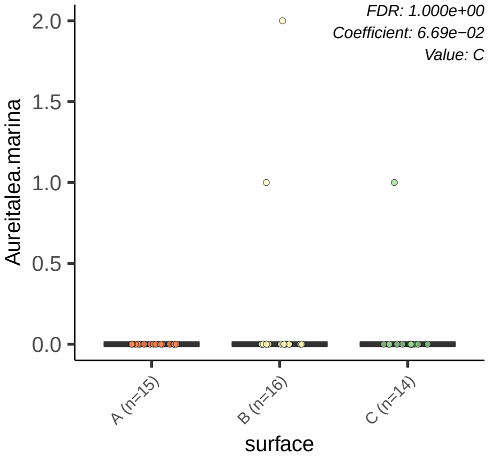
FDR: 1.000e+00
Coefficient: 6.69e−02
Value: C
Aureitalea.marina
2.0
1.5
1.0
0.5
0.0
A (n=15)
B (n=16)
C (n=14)
surface

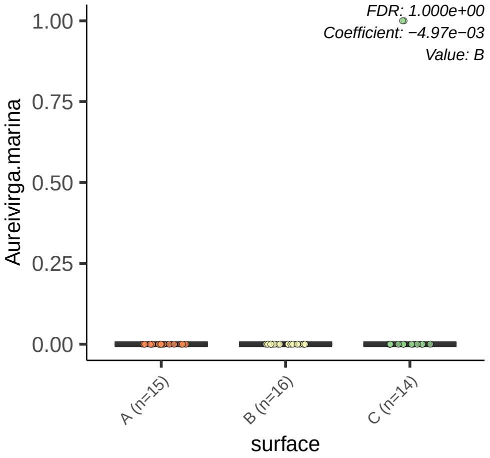

FDR: 1.000e+00
Coefficient: −4.97e−03
Value: B
1.00
0.75
0.50
0.25
0.00
Aureivirga.marina
A (n=15)
B (n=16)
C (n=14)
surface

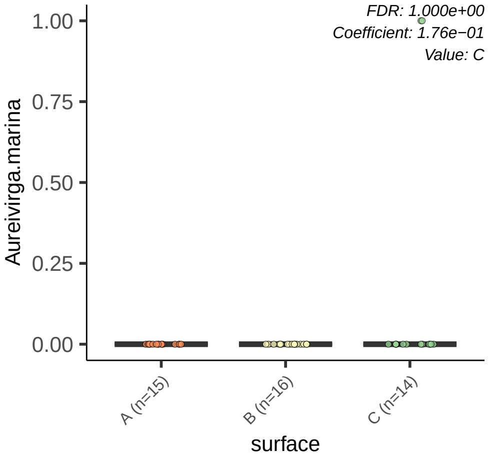
FDR: 1.000e+00
Coefficient: 1.76e−01
Value: C
Aureivirga.marina
1.00
0.75
0.50
0.25
0.00
A (n=15)
B (n=16)
C (n=14)
surface

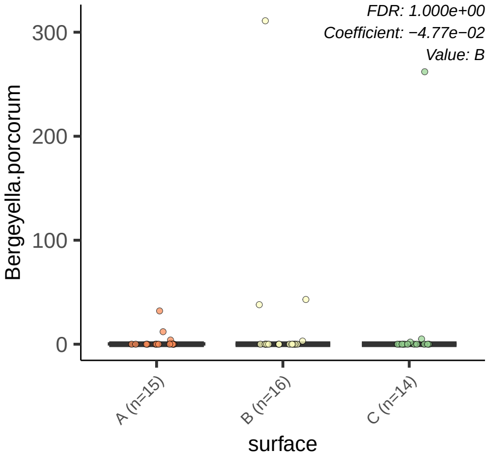
FDR: 1.000e+00
Coefficient: −4.77e−02
Value: B
Bergeyella.porcorum
300
200
100
0
A (n=15)
B (n=16)
C (n=14)
surface

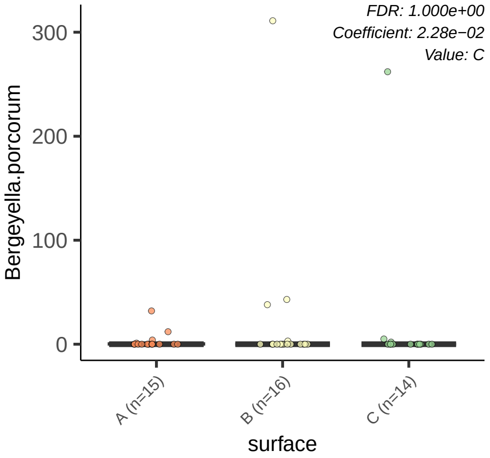

FDR: 1.000e+00
Coefficient: 2.28e−02
Value: C
Bergeyella.porcorum
300
200
100
0
A (n=15)
B (n=16)
C (n=14)
surface

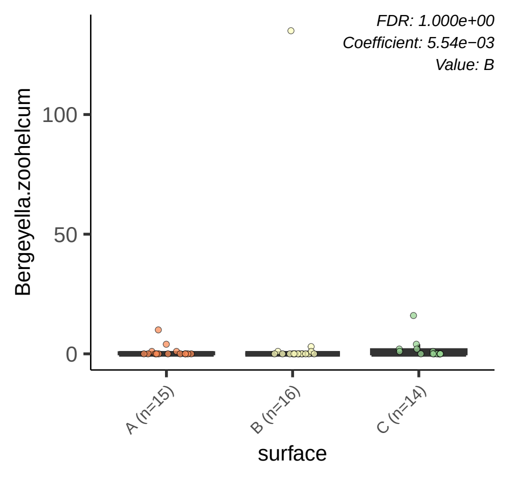

FDR: 1.000e+00
Coefficient: 5.54e−03
Value: B
Bergeyella.zoohelcum
100
50
0
A (n=15)
B (n=16)
C (n=14)
surface

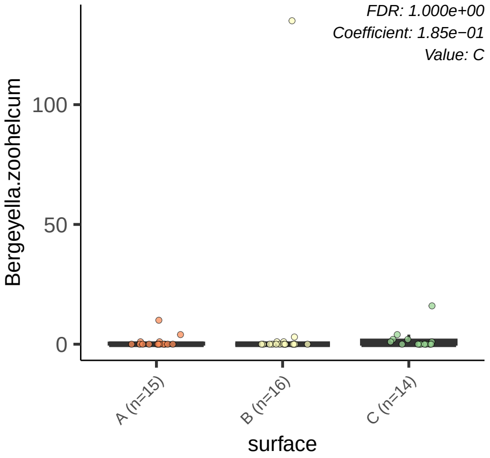

FDR: 1.000e+00
Coefficient: 1.85e−01
Value: C
Bergeyella.zoohelcum
100
50
0
A (n=15)
B (n=16)
C (n=14)
surface

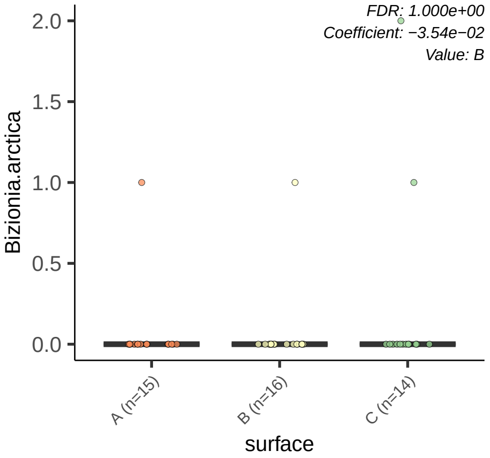

FDR: 1.000e+00
Coefficient: −3.54e−02
Value: B
Bizionia.arctica
2.0
1.5
1.0
0.5
0.0
A (n=15)
B (n=16)
C (n=14)
surface

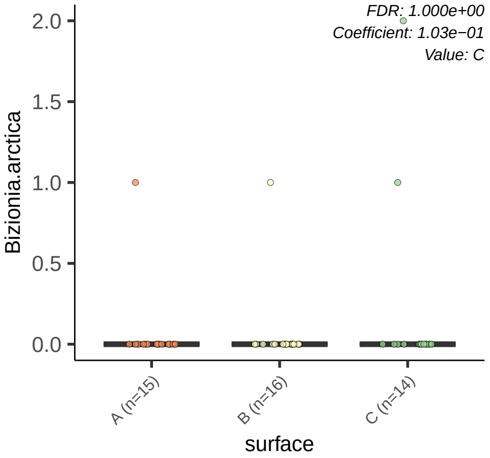
FDR: 1.000e+00
Coefficient: 1.03e−01
Value: C
Bizionia.arctica
2.0
1.5
1.0
0.5
0.0
A (n=15)
B (n=16)
C (n=14)
surface

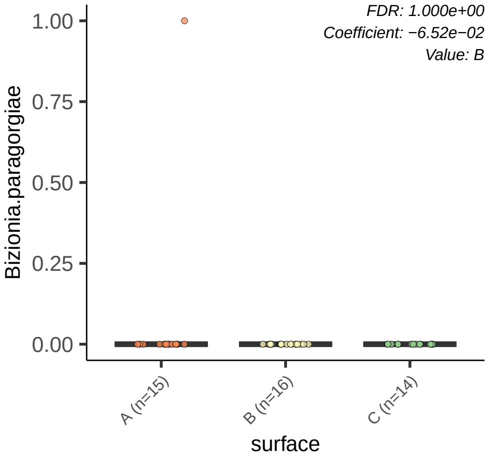
FDR: 1.000e+00
Coefficient: −6.52e−02
Value: B
Bizionia.paragorgiae
1.00
0.75
0.50
0.25
0.00
A (n=15)
B (n=16)
C (n=14)
surface

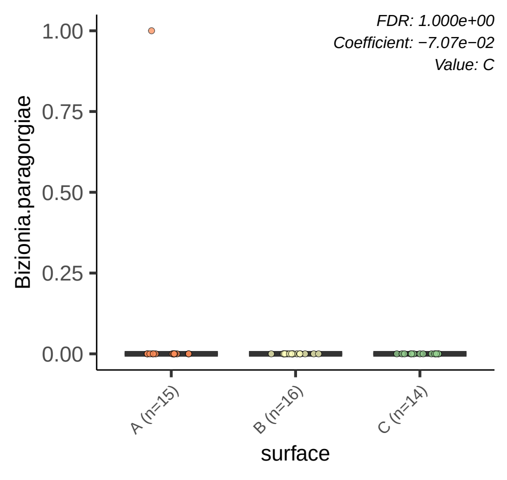

FDR: 1.000e+00
Coefficient: −7.07e−02
Value: C
Bizionia.paragorgiae
1.00
0.75
0.50
0.25
0.00
A (n=15)
B (n=16)
C (n=14)
surface

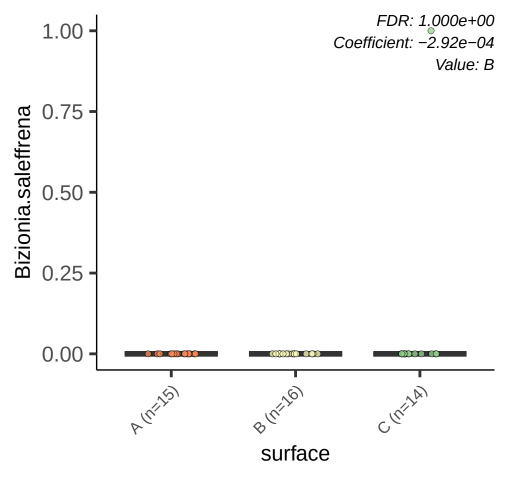

FDR: 1.000e+00
Coefficient: −2.92e−04
Value: B
Bizionia.saleffrena
1.00
0.75
0.50
0.25
0.00
A (n=15)
B (n=16)
C (n=14)
surface

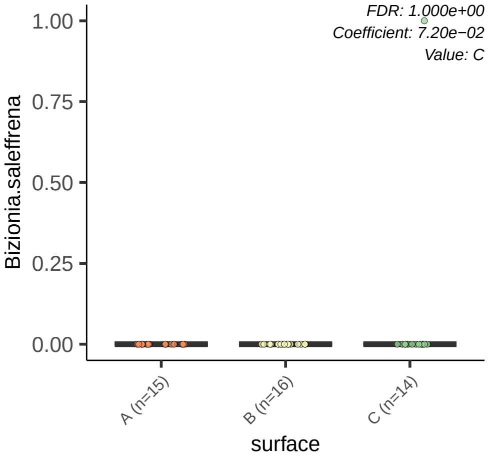

FDR: 1.000e+00
Coefficient: 7.20e−02
Value: C
Bizionia.saleffrena
1.00
0.75
0.50
0.25
0.00
A (n=15)
B (n=16)
C (n=14)
surface

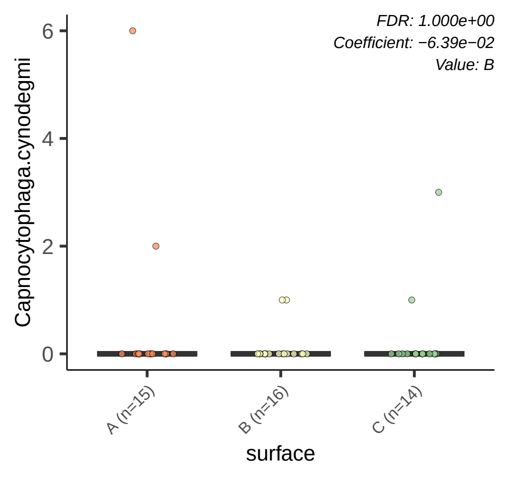
FDR: 1.000e+00
Coefficient: −6.39e−02
Value: B
Capnocytophaga.cynodegmi
6
4
2
0
A (n=15)
B (n=16)
C (n=14)
surface

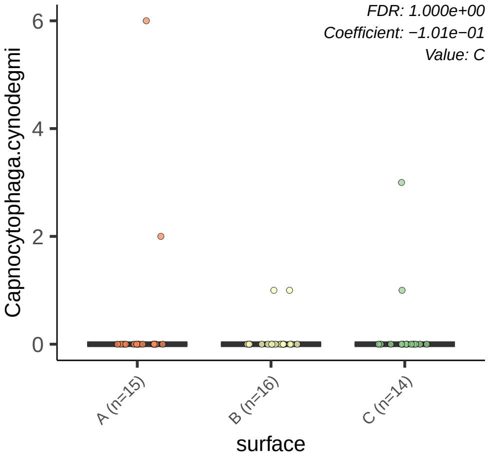

FDR: 1.000e+00
Coefficient: −1.01e−01
Value: C
Capnocytophaga.cynodegmi
6
4
2
0
A (n=15)
B (n=16)
C (n=14)
surface

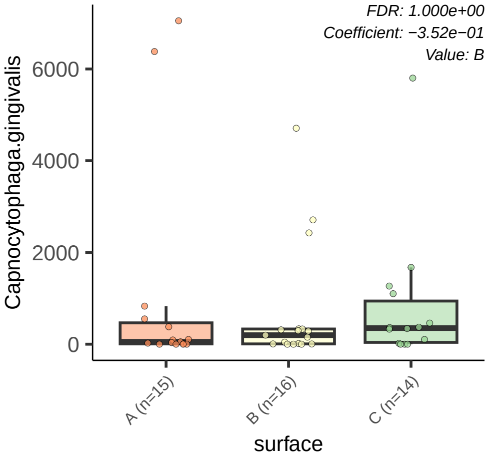

FDR: 1.000e+00
Coefficient: −3.52e−01
Value: B
Capnocytophaga.gingivalis
6000
4000
2000
0
A (n=15)
B (n=16)
C (n=14)
surface

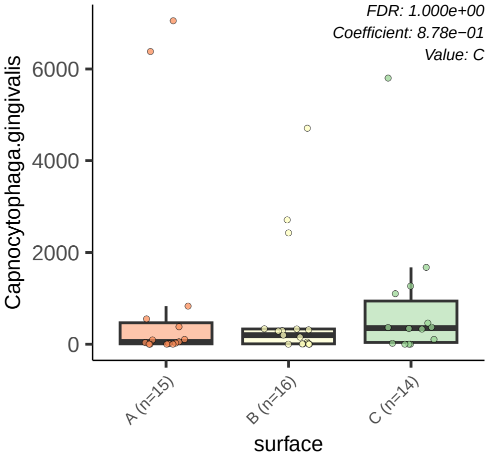
FDR: 1.000e+00
Coefficient: 8.78e−01
Value: C
Capnocytophaga.gingivalis
6000
4000
2000
0
A (n=15)
B (n=16)
C (n=14)
surface

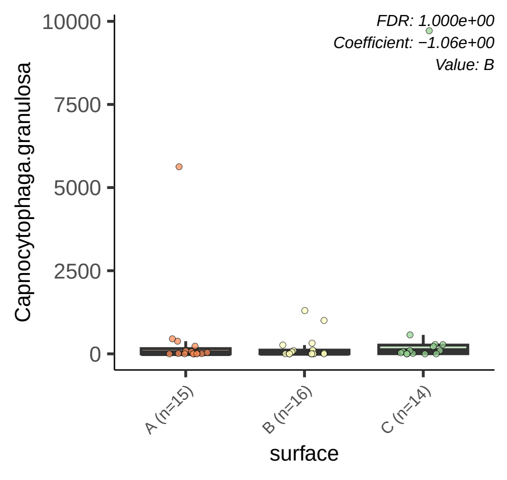
FDR: 1.000e+00
Coefficient: −1.06e+00
Value: B
Capnocytophaga.granulosa
10000
7500
5000
2500
0
A (n=15)
B (n=16)
C (n=14)
surface

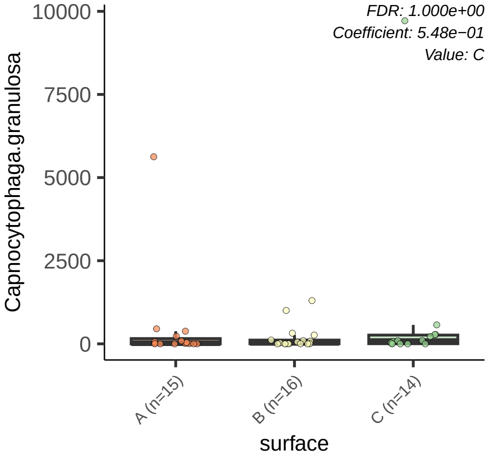
FDR: 1.000e+00
Coefficient: 5.48e−01
Value: C
10000
7500
5000
2500
0
Capnocytophaga.granulosa
A (n=15)
B (n=16)
C (n=14)
surface

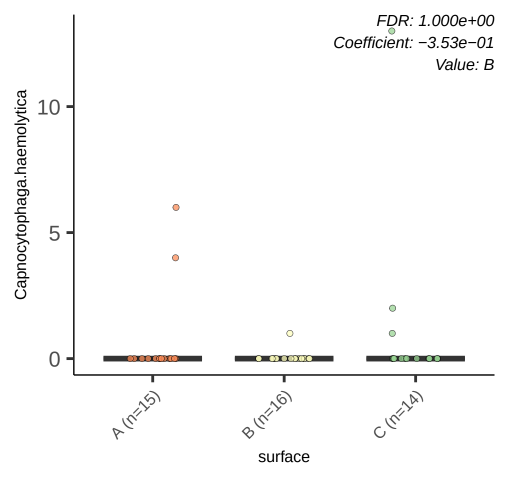

FDR: 1.000e+00
Coefficient: −3.53e−01
Value: B
Capnocytophaga.haemolytica
10
5
0
A (n=15)
B (n=16)
C (n=14)
surface

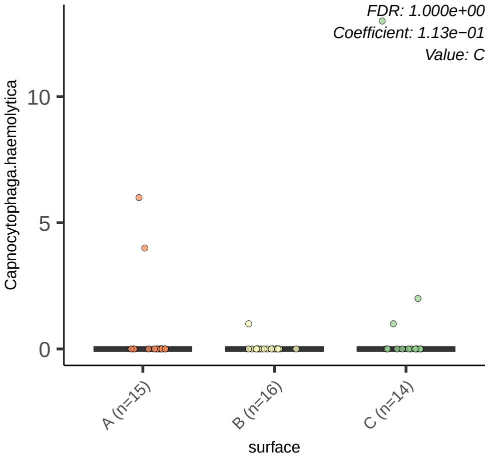
FDR: 1.000e+00
Coefficient: 1.13e−01
Value: C
Capnocytophaga.haemolytica
0
5
10
A (n=15)
B (n=16)
C (n=14)
surface

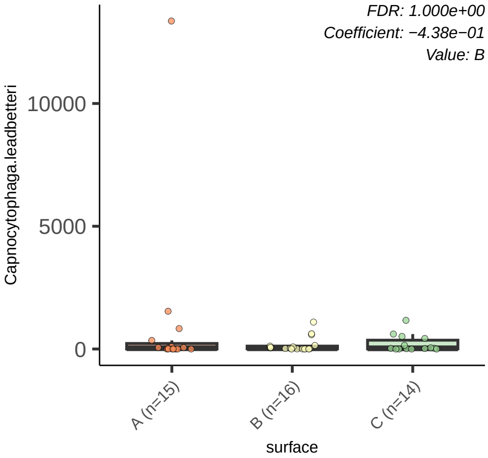

FDR: 1.000e+00
Coefficient: −4.38e−01
Value: B
Capnocytophaga.leadbetteri
10000
5000
0
A (n=15)
B (n=16)
C (n=14)
surface

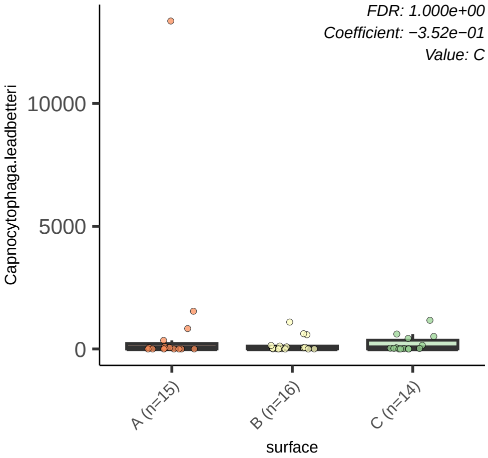

FDR: 1.000e+00
Coefficient: −3.52e−01
Value: C
Capnocytophaga.leadbetteri
10000
5000
0
A (n=15)
B (n=16)
C (n=14)
surface

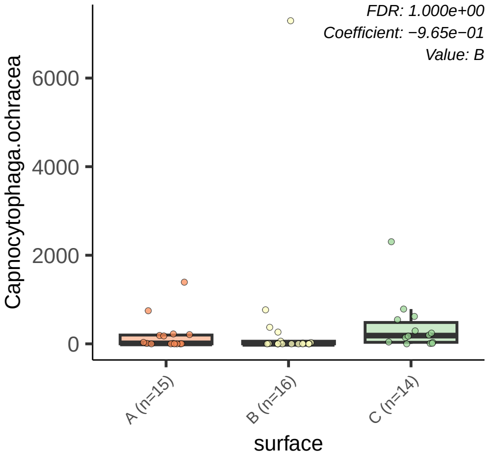
FDR: 1.000e+00
Coefficient: −9.65e−01
Value: B
Capnocytophaga.ochracea
6000
4000
2000
0
A (n=15)
B (n=16)
C (n=14)
surface

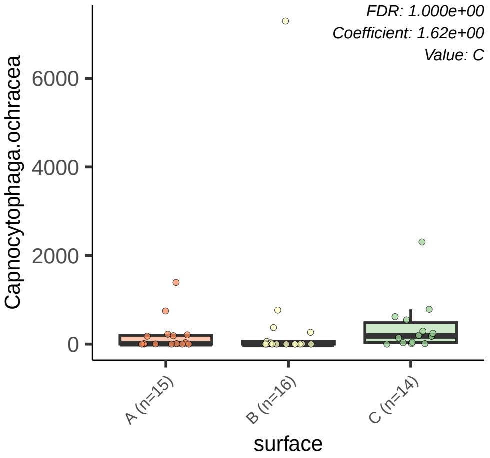
FDR: 1.000e+00
Coefficient: 1.62e+00
Value: C
6000
4000
2000
0
Capnocytophaga.ochracea
A (n=15)
B (n=16)
C (n=14)
surface

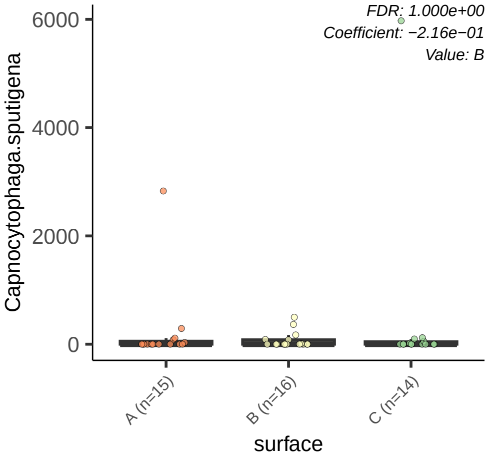
FDR: 1.000e+00
Coefficient: −2.16e−01
Value: B
Capnocytophaga.sputigena
6000
4000
2000
0
A (n=15)
B (n=16)
C (n=14)
surface

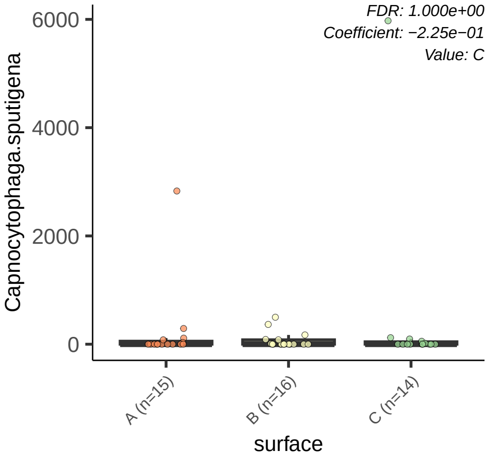

FDR: 1.000e+00
Coefficient: −2.25e−01
Value: C
Capnocytophaga.sputigena
6000
4000
2000
0
A (n=15)
B (n=16)
C (n=14)
surface

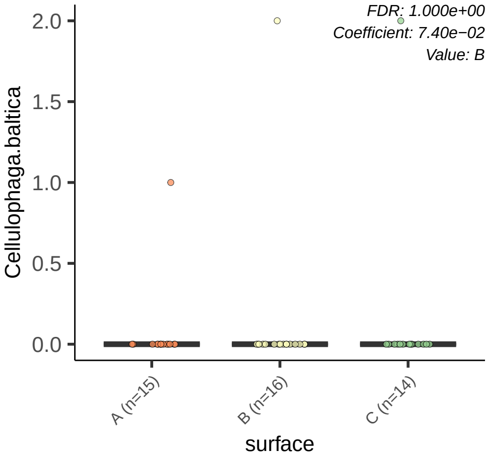
FDR: 1.000e+00
Coefficient: 7.40e−02
Value: B
Cellulophaga.baltica
2.0
1.5
1.0
0.5
0.0
A (n=15)
B (n=16)
C (n=14)
surface

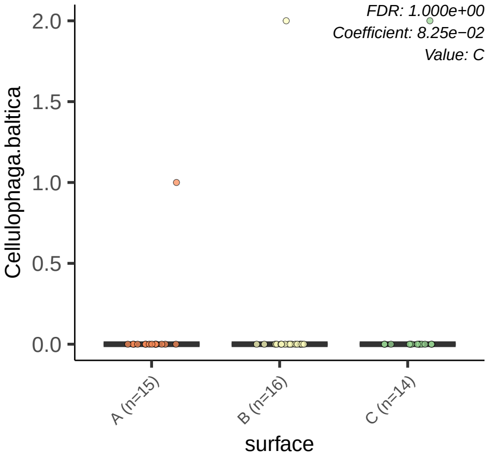

FDR: 1.000e+00
Coefficient: 8.25e−02
Value: C
Cellulophaga.baltica
2.0
1.5
1.0
0.5
0.0
A (n=15)
B (n=16)
C (n=14)
surface

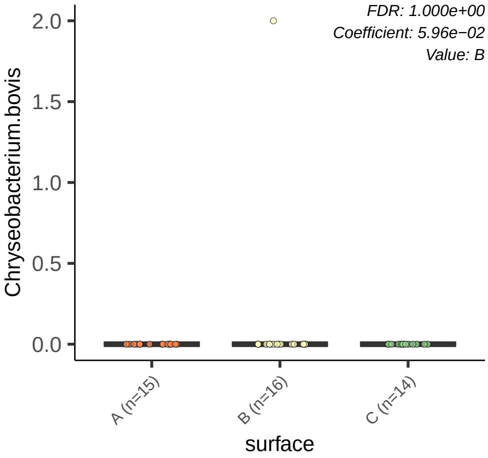
FDR: 1.000e+00
Coefficient: 5.96e−02
Value: B
Chryseobacterium.bovis
2.0
1.5
1.0
0.5
0.0
A (n=15)
B (n=16)
C (n=14)
surface

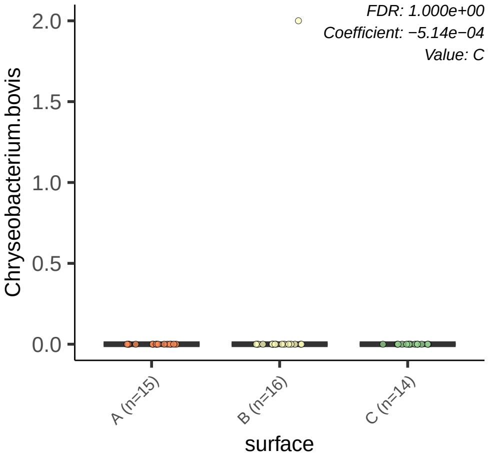
FDR: 1.000e+00
Coefficient: −5.14e−04
Value: C
Chryseobacterium.bovis
2.0
1.5
1.0
0.5
0.0
A (n=15)
B (n=16)
C (n=14)
surface

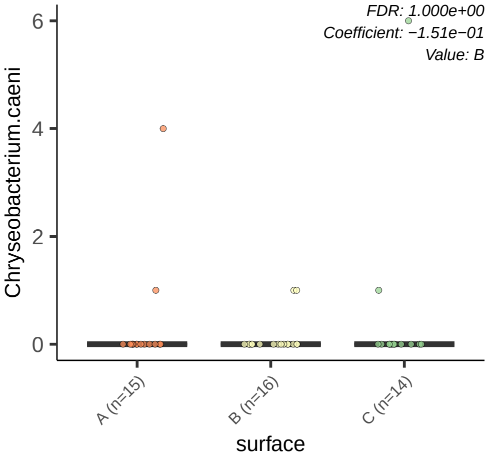

FDR: 1.000e+00
Coefficient: −1.51e−01
Value: B
Chryseobacterium.caeni
0
2
4
6
A (n=15)
B (n=16)
C (n=14)
surface

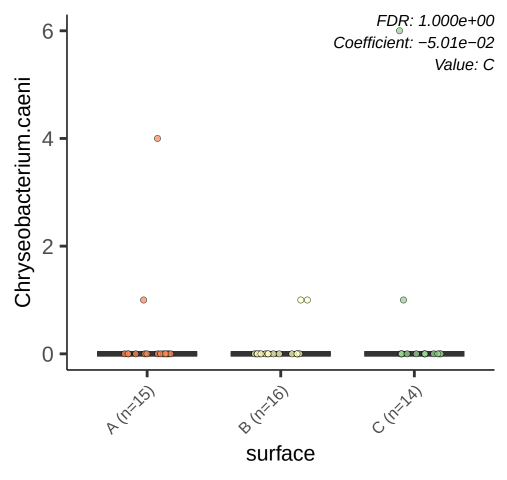
FDR: 1.000e+00
Coefficient: −5.01e−02
Value: C
Chryseobacterium.caeni
0
2
4
6
A (n=15)
B (n=16)
C (n=14)
surface

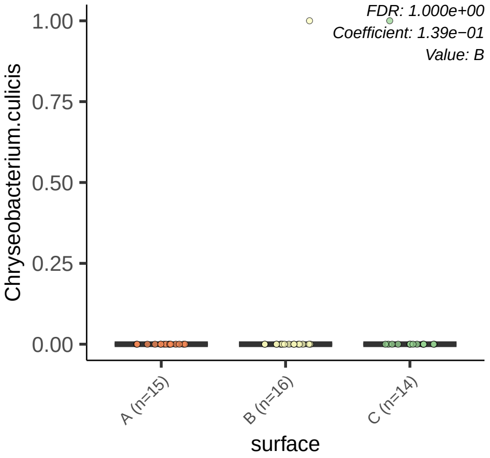

FDR: 1.000e+00
Coefficient: 1.39e−01
Value: B
Chryseobacterium.culicis
1.00
0.75
0.50
0.25
0.00
A (n=15)
B (n=16)
C (n=14)
surface

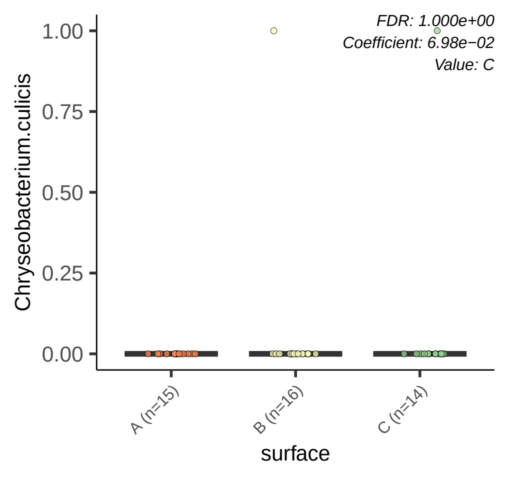
FDR: 1.000e+00
Coefficient: 6.98e−02
Value: C
Chryseobacterium.culicis
1.00
0.75
0.50
0.25
0.00
A (n=15)
B (n=16)
C (n=14)
surface

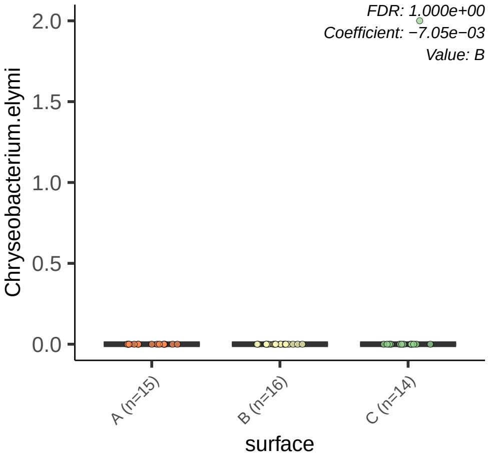
FDR: 1.000e+00
Coefficient: −7.05e−03
Value: B
Chryseobacterium.elymi
2.0
1.5
1.0
0.5
0.0
A (n=15)
B (n=16)
C (n=14)
surface

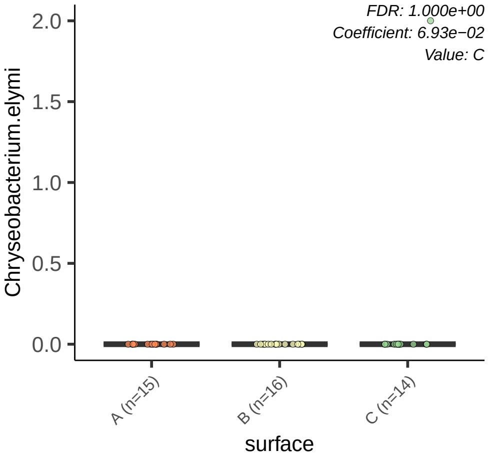

FDR: 1.000e+00
Coefficient: 6.93e−02
Value: C
Chryseobacterium.elymi
2.0
1.5
1.0
0.5
0.0
A (n=15)
B (n=16)
C (n=14)
surface

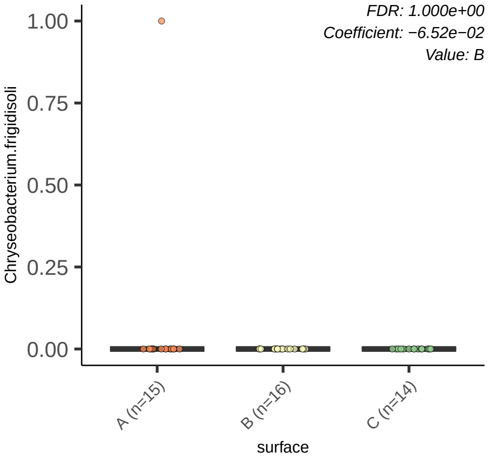
FDR: 1.000e+00
Coefficient: −6.52e−02
Value: B
1.00
0.75
0.50
0.25
0.00
Chryseobacterium.frigidisoli
A (n=15)
B (n=16)
C (n=14)
surface

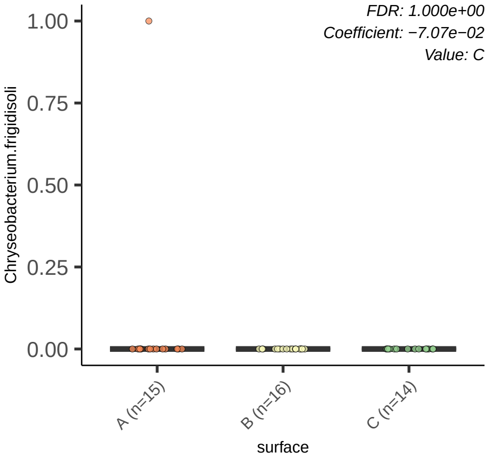

FDR: 1.000e+00
Coefficient: −7.07e−02
Value: C
Chryseobacterium.frigidisoli
1.00
0.75
0.50
0.25
0.00
A (n=15)
B (n=16)
C (n=14)
surface

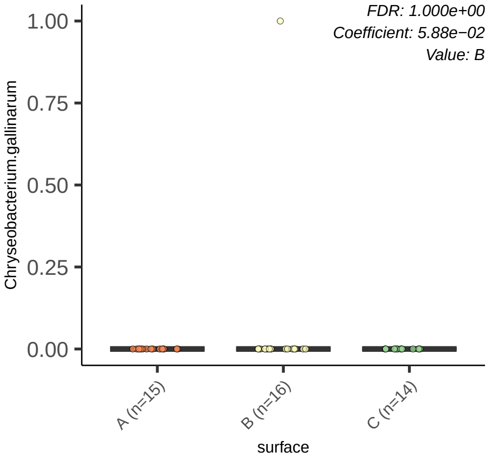
FDR: 1.000e+00
Coefficient: 5.88e−02
Value: B
Chryseobacterium.gallinarum
1.00
0.75
0.50
0.25
0.00
A (n=15)
B (n=16)
C (n=14)
surface

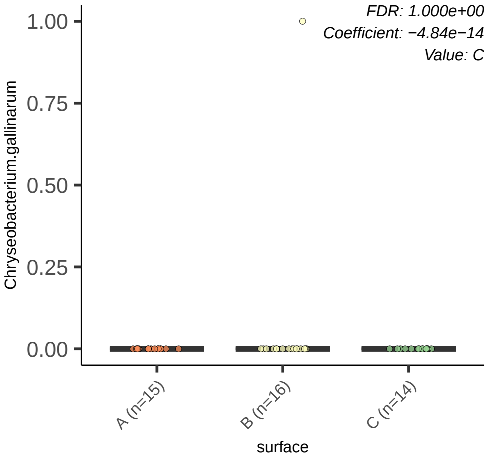
FDR: 1.000e+00
Coefficient: −4.84e−14
Value: C
Chryseobacterium.gallinarum
1.00
0.75
0.50
0.25
0.00
A (n=15)
B (n=16)
C (n=14)
surface

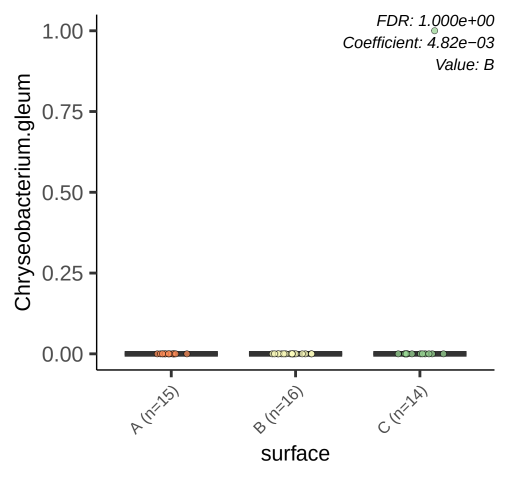

FDR: 1.000e+00
Coefficient: 4.82e−03
Value: B
Chryseobacterium.gleum
1.00
0.75
0.50
0.25
0.00
A (n=15)
B (n=16)
C (n=14)
surface

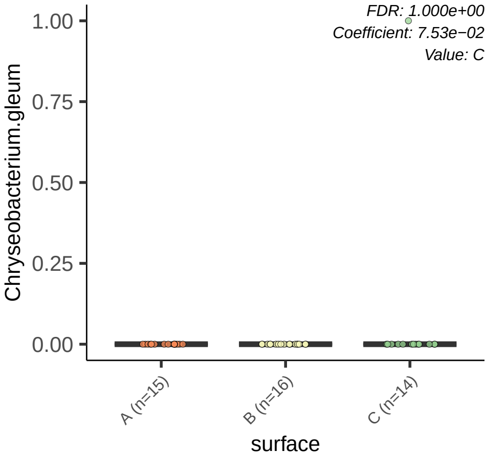
FDR: 1.000e+00
Coefficient: 7.53e−02
Value: C
Chryseobacterium.gleum
1.00
0.75
0.50
0.25
0.00
A (n=15)
B (n=16)
C (n=14)
surface

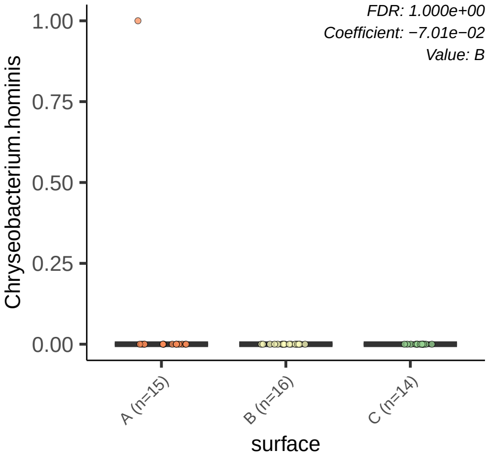

FDR: 1.000e+00
Coefficient: −7.01e−02
Value: B
1.00
0.75
0.50
0.25
0.00
Chryseobacterium.hominis
A (n=15)
B (n=16)
C (n=14)
surface

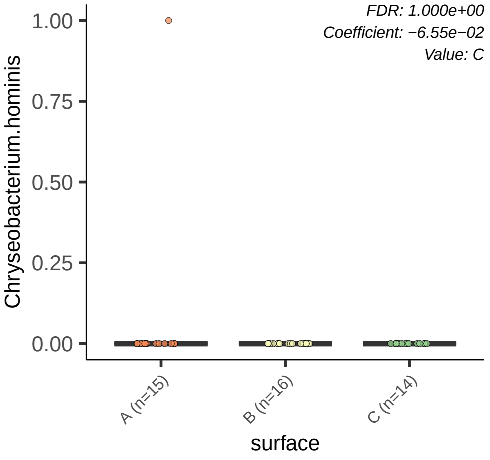

FDR: 1.000e+00
Coefficient: −6.55e−02
Value: C
Chryseobacterium.hominis
1.00
0.75
0.50
0.25
0.00
A (n=15)
B (n=16)
C (n=14)
surface

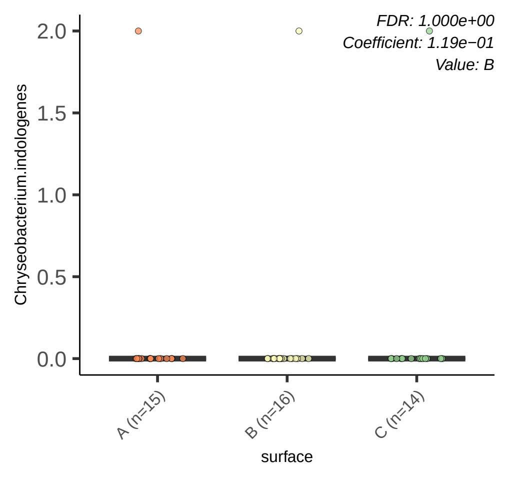

FDR: 1.000e+00
Coefficient: 1.19e−01
Value: B
Chryseobacterium.indologenes
2.0
1.5
1.0
0.5
0.0
A (n=15)
B (n=16)
C (n=14)
surface

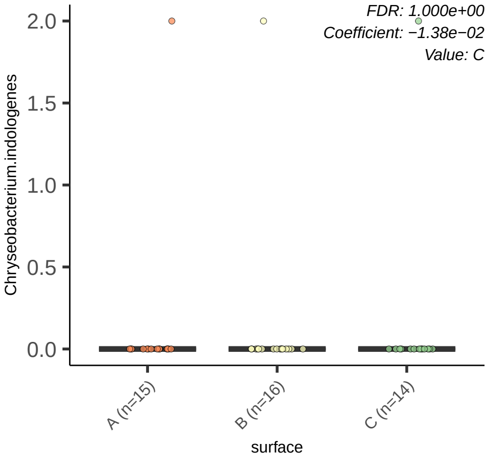
FDR: 1.000e+00
Coefficient: −1.38e−02
Value: C
Chryseobacterium.indologenes
2.0
1.5
1.0
0.5
0.0
A (n=15)
B (n=16)
C (n=14)
surface

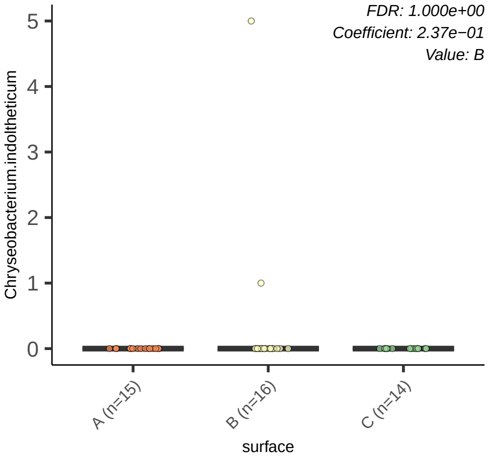
FDR: 1.000e+00
Coefficient: 2.37e−01
Value: B
Chryseobacterium.indoltheticum
5
4
3
2
1
0
A (n=15)
B (n=16)
C (n=14)
surface

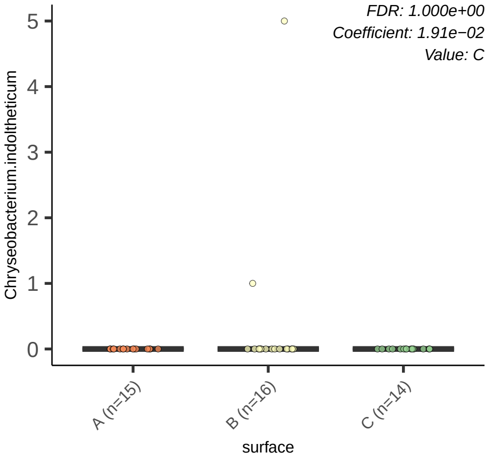
FDR: 1.000e+00
Coefficient: 1.91e−02
Value: C
Chryseobacterium.indoltheticum
0
1
2
3
4
5
A (n=15)
B (n=16)
C (n=14)
surface

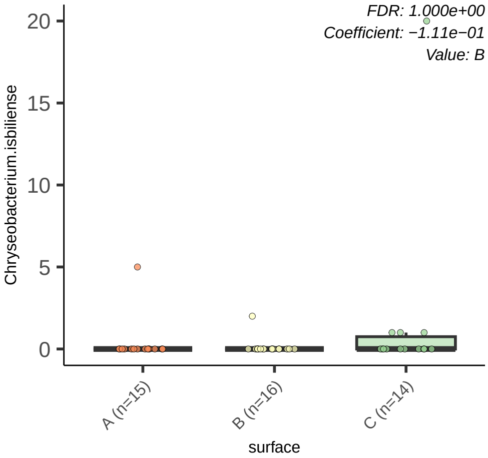

FDR: 1.000e+00
Coefficient: −1.11e−01
Value: B
Chryseobacterium.isbiliense
20
15
10
5
0
A (n=15)
B (n=16)
C (n=14)
surface

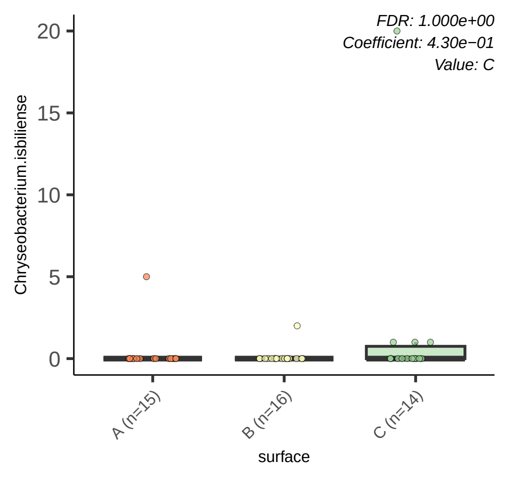
FDR: 1.000e+00
Coefficient: 4.30e−01
Value: C
Chryseobacterium.isbiliense
20
15
10
5
0
A (n=15)
B (n=16)
C (n=14)
surface

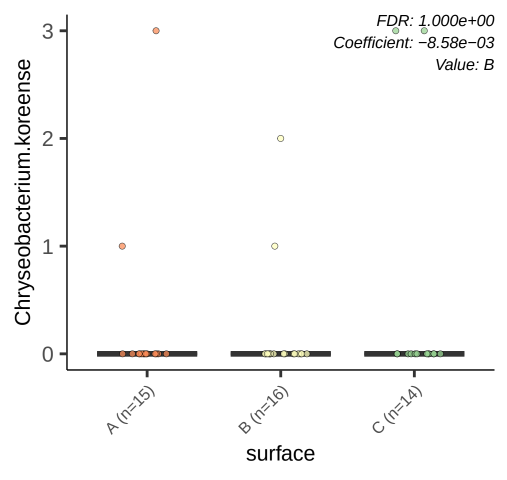

FDR: 1.000e+00
Coefficient: −8.58e−03
Value: B
Chryseobacterium.koreense
3
2
1
0
A (n=15)
B (n=16)
C (n=14)
surface

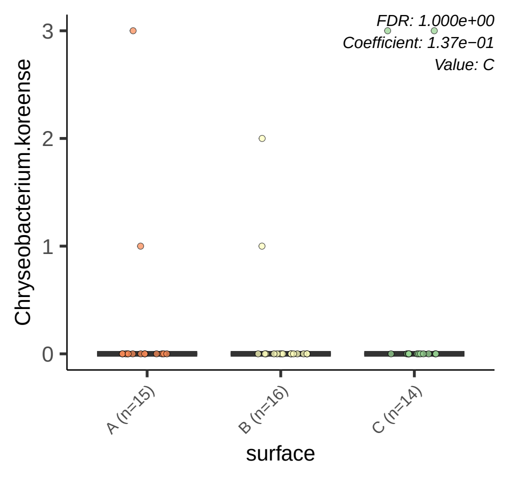
FDR: 1.000e+00
Coefficient: 1.37e−01
Value: C
Chryseobacterium.koreense
0
1
2
3
A (n=15)
B (n=16)
C (n=14)
surface

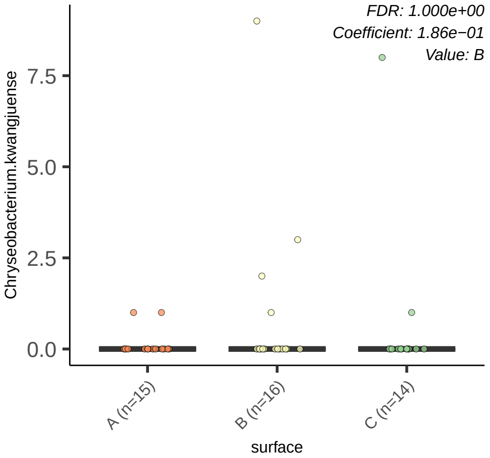

FDR: 1.000e+00
Coefficient: 1.86e−01
Value: B
Chryseobacterium.kwangjuense
7.5
5.0
2.5
0.0
A (n=15)
B (n=16)
C (n=14)
surface

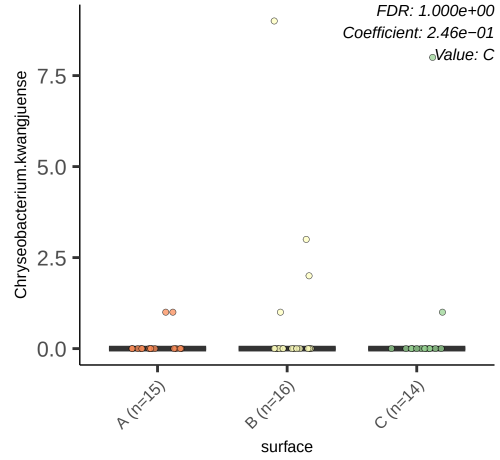

FDR: 1.000e+00
Coefficient: 2.46e−01
Value: C
Chryseobacterium.kwangjuense
7.5
5.0
2.5
0.0
A (n=15)
B (n=16)
C (n=14)
surface

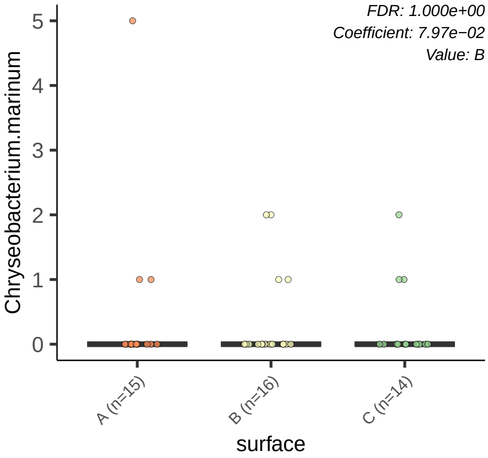

FDR: 1.000e+00
Coefficient: 7.97e−02
Value: B
Chryseobacterium.marinum
0
1
2
3
4
5
A (n=15)
B (n=16)
C (n=14)
surface

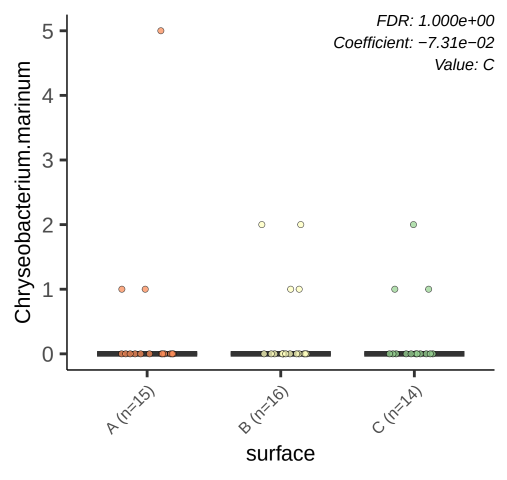
FDR: 1.000e+00
Coefficient: −7.31e−02
Value: C
Chryseobacterium.marinum
0
1
2
3
4
5
A (n=15)
B (n=16)
C (n=14)
surface

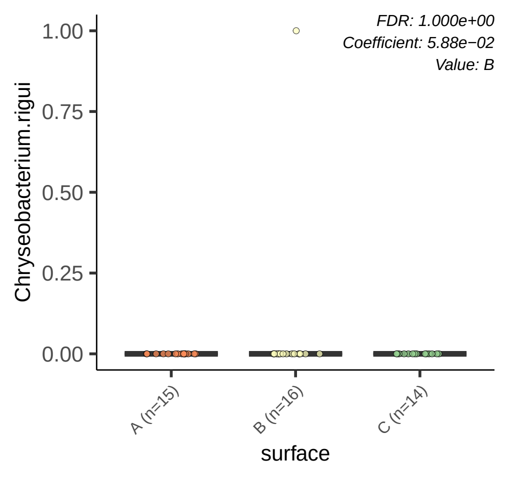
FDR: 1.000e+00
Coefficient: 5.88e−02
Value: B
Chryseobacterium.rigui
1.00
0.75
0.50
0.25
0.00
A (n=15)
B (n=16)
C (n=14)
surface

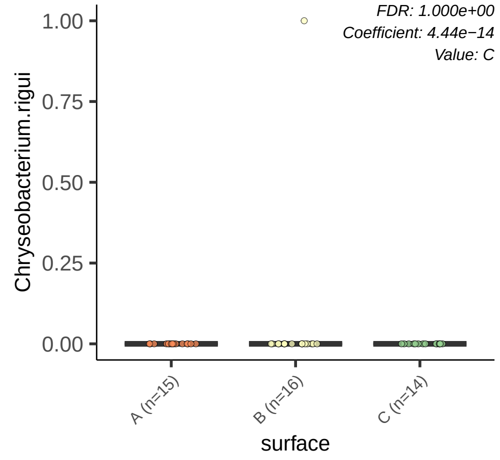

FDR: 1.000e+00
Coefficient: 4.44e−14
Value: C
Chryseobacterium.rigui
1.00
0.75
0.50
0.25
0.00
A (n=15)
B (n=16)
C (n=14)
surface

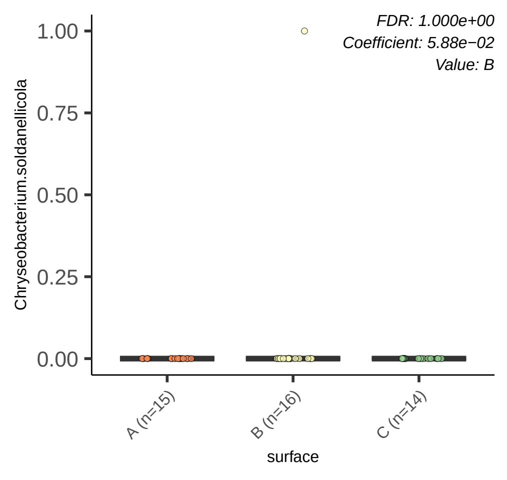
FDR: 1.000e+00
Coefficient: 5.88e−02
Value: B
1.00
0.75
0.50
0.25
0.00
Chryseobacterium.soldanellicola
A (n=15)
B (n=16)
C (n=14)
surface

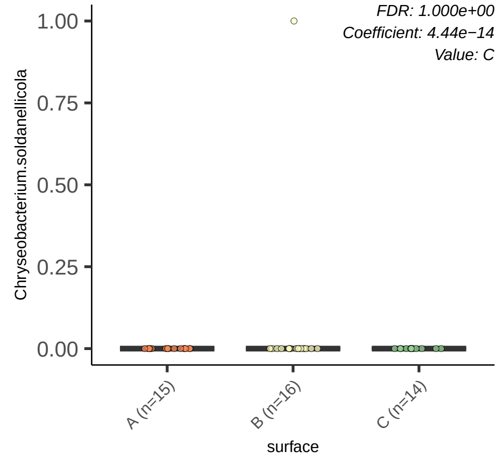
FDR: 1.000e+00
Coefficient: 4.44e−14
Value: C
1.00
0.75
0.50
0.25
0.00
Chryseobacterium.soldanellicola
A (n=15)
B (n=16)
C (n=14)
surface

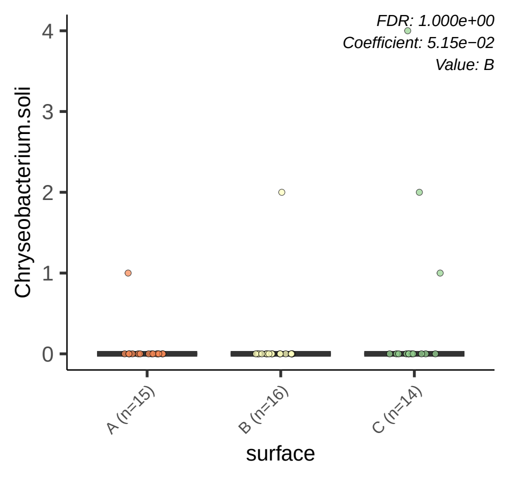
FDR: 1.000e+00
Coefficient: 5.15e−02
Value: B
Chryseobacterium.soli
0
1
2
3
4
A (n=15)
B (n=16)
C (n=14)
surface

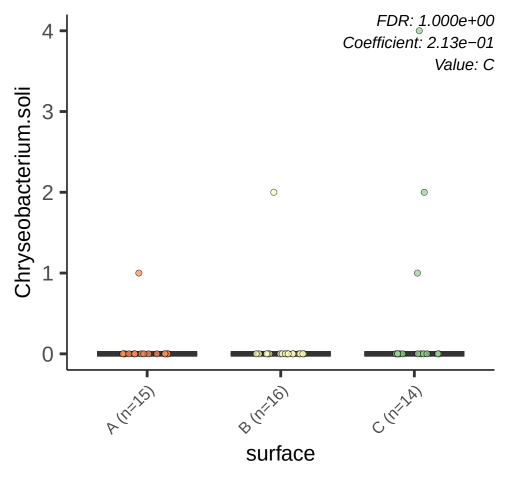
FDR: 1.000e+00
Coefficient: 2.13e−01
Value: C
Chryseobacterium.soli
4
3
2
1
0
A (n=15)
B (n=16)
C (n=14)
surface

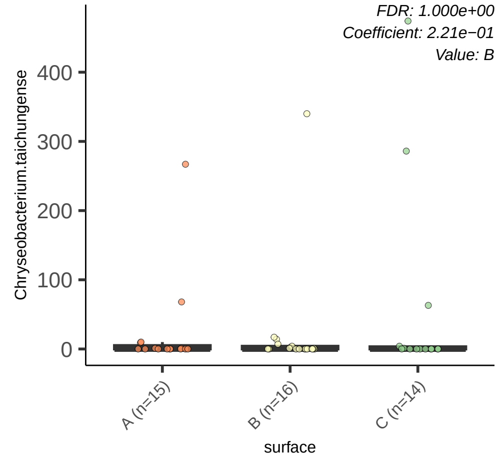

FDR: 1.000e+00
Coefficient: 2.21e−01
Value: B
Chryseobacterium.taichungense
400
300
200
100
0
A (n=15)
B (n=16)
C (n=14)
surface

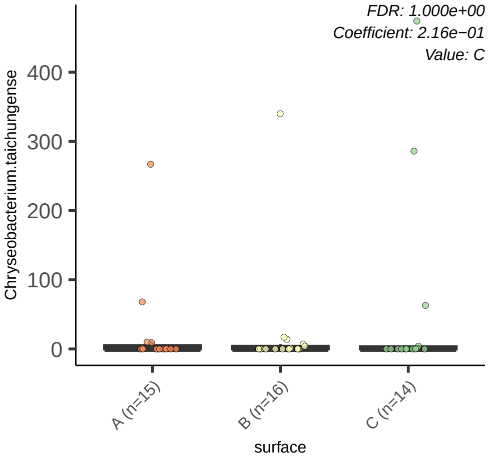

FDR: 1.000e+00
Coefficient: 2.16e−01
Value: C
400
300
200
100
0
Chryseobacterium.taichungense
A (n=15)
B (n=16)
C (n=14)
surface

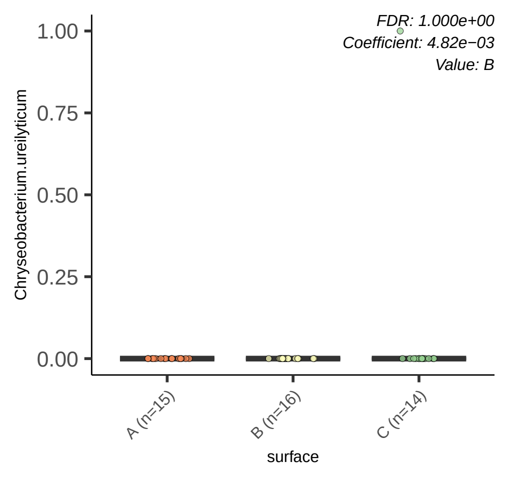
FDR: 1.000e+00
Coefficient: 4.82e−03
Value: B
1.00
0.75
0.50
0.25
0.00
Chryseobacterium.ureilyticum
A (n=15)
B (n=16)
C (n=14)
surface

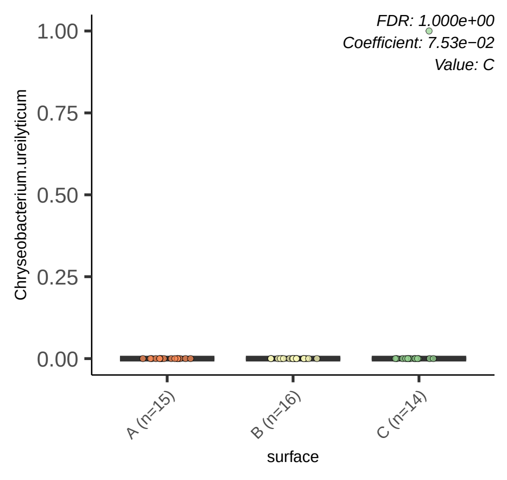
FDR: 1.000e+00
Coefficient: 7.53e−02
Value: C
Chryseobacterium.ureilyticum
1.00
0.75
0.50
0.25
0.00
A (n=15)
B (n=16)
C (n=14)
surface

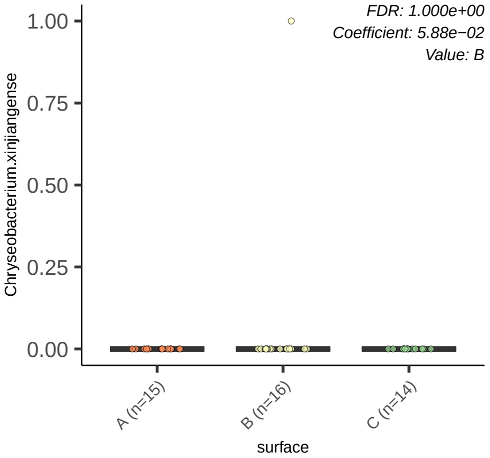
FDR: 1.000e+00
Coefficient: 5.88e−02
Value: B
Chryseobacterium.xinjiangense
1.00
0.75
0.50
0.25
0.00
A (n=15)
B (n=16)
C (n=14)
surface

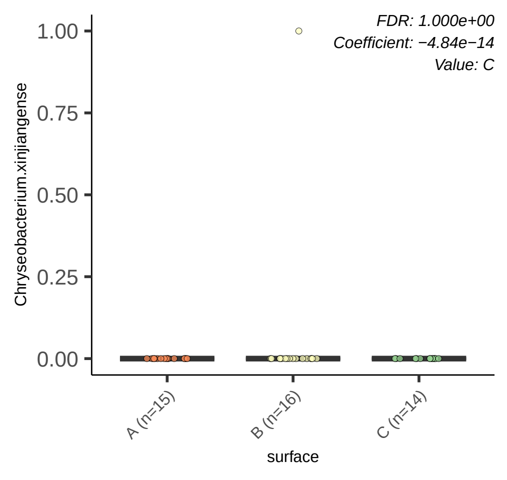
FDR: 1.000e+00
Coefficient: −4.84e−14
Value: C
1.00
0.75
0.50
0.25
0.00
Chryseobacterium.xinjiangense
A (n=15)
B (n=16)
C (n=14)
surface

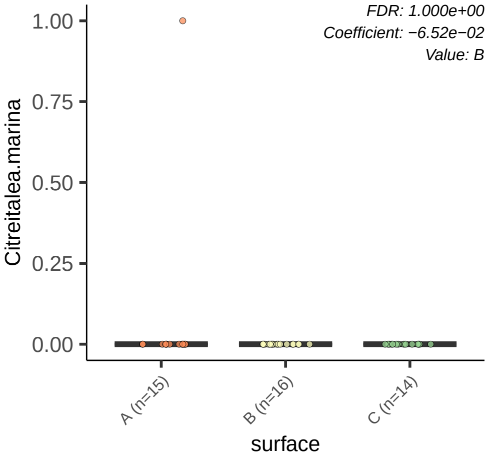
FDR: 1.000e+00
Coefficient: −6.52e−02
Value: B
Citreitalea.marina
1.00
0.75
0.50
0.25
0.00
A (n=15)
B (n=16)
C (n=14)
surface

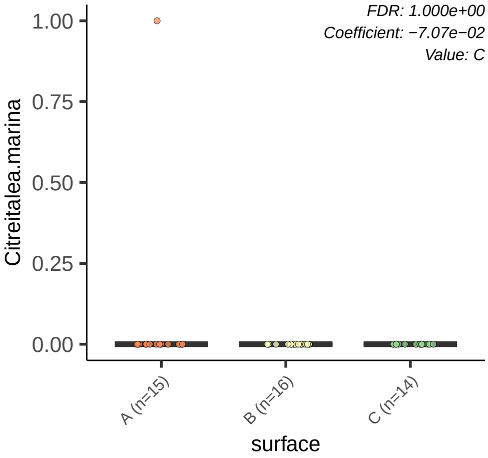

FDR: 1.000e+00
Coefficient: −7.07e−02
Value: C
Citreitalea.marina
1.00
0.75
0.50
0.25
0.00
A (n=15)
B (n=16)
C (n=14)
surface

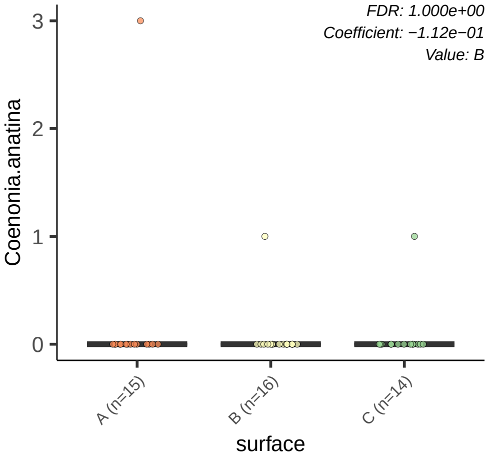
FDR: 1.000e+00
Coefficient: −1.12e−01
Value: B
Coenonia.anatina
3
2
1
0
A (n=15)
B (n=16)
C (n=14)
surface

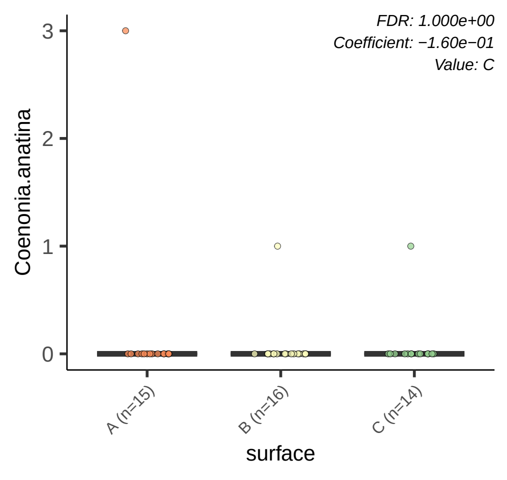
FDR: 1.000e+00
Coefficient: −1.60e−01
Value: C
Coenonia.anatina
3
2
1
0
A (n=15)
B (n=16)
C (n=14)
surface

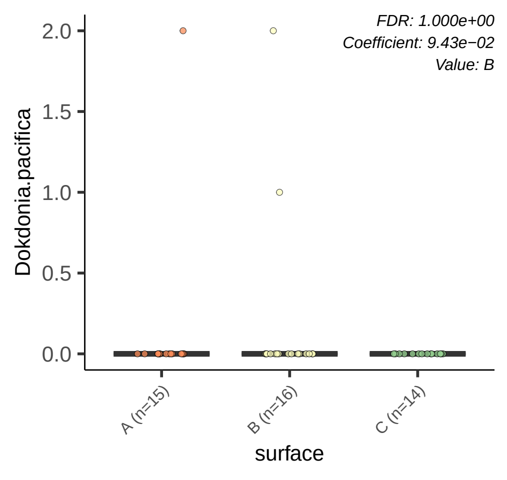
FDR: 1.000e+00
Coefficient: 9.43e−02
Value: B
Dokdonia.pacifica
2.0
1.5
1.0
0.5
0.0
A (n=15)
B (n=16)
C (n=14)
surface

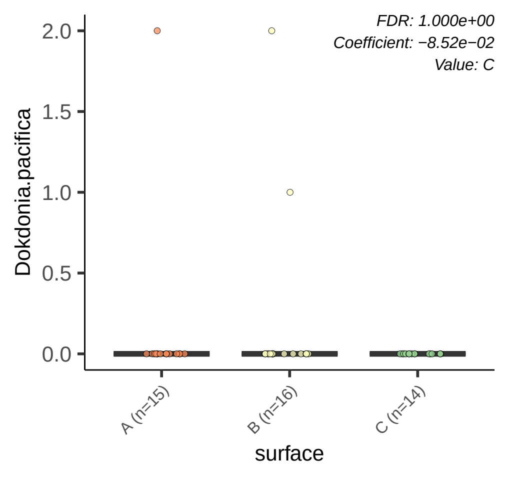
FDR: 1.000e+00
Coefficient: −8.52e−02
Value: C
Dokdonia.pacifica
2.0
1.5
1.0
0.5
0.0
A (n=15)
B (n=16)
C (n=14)
surface

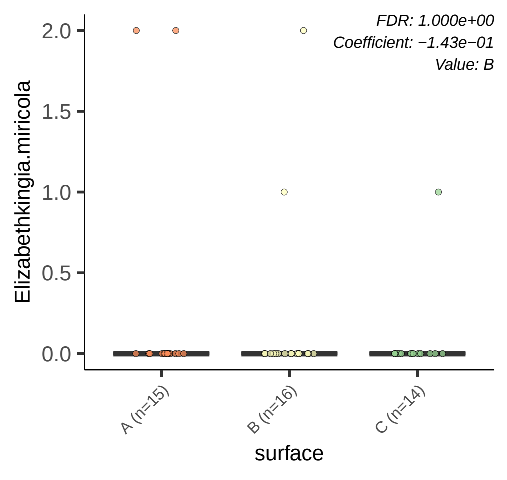

FDR: 1.000e+00
Coefficient: −1.43e−01
Value: B
Elizabethkingia.miricola
2.0
1.5
1.0
0.5
0.0
A (n=15)
B (n=16)
C (n=14)
surface

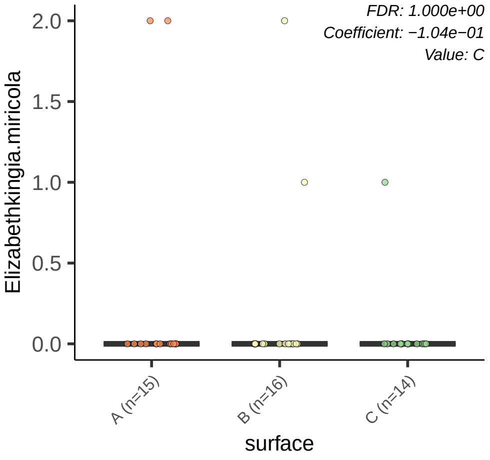
FDR: 1.000e+00
Coefficient: −1.04e−01
Value: C
Elizabethkingia.miricola
2.0
1.5
1.0
0.5
0.0
A (n=15)
B (n=16)
C (n=14)
surface

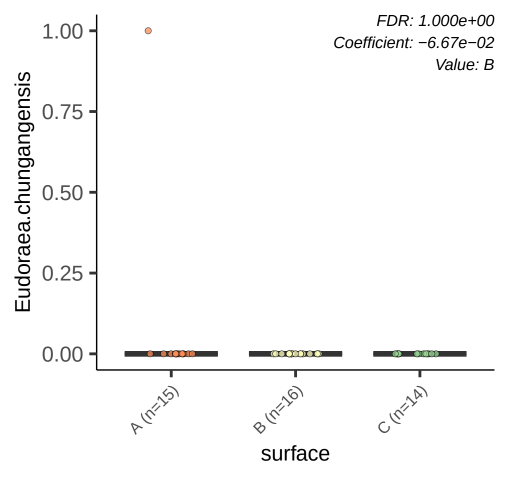
FDR: 1.000e+00
Coefficient: −6.67e−02
Value: B
Eudoraea.chungangensis
1.00
0.75
0.50
0.25
0.00
A (n=15)
B (n=16)
C (n=14)
surface

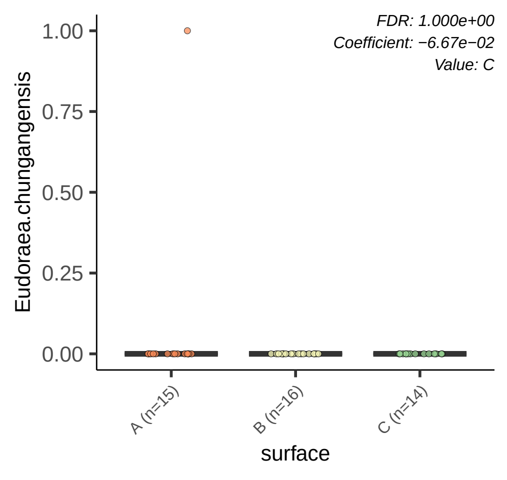
FDR: 1.000e+00
Coefficient: −6.67e−02
Value: C
Eudoraea.chungangensis
1.00
0.75
0.50
0.25
0.00
A (n=15)
B (n=16)
C (n=14)
surface

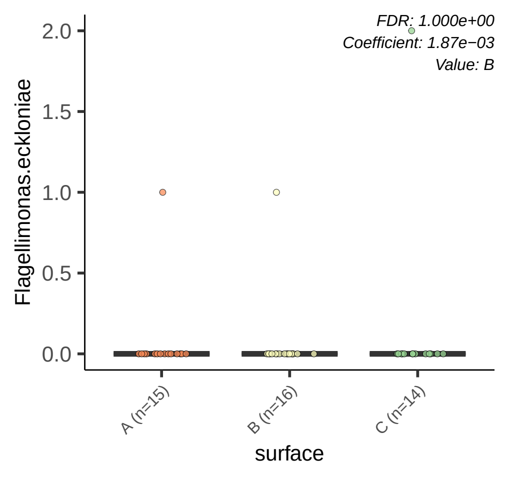
FDR: 1.000e+00
Coefficient: 1.87e−03
Value: B
Flagellimonas.eckloniae
2.0
1.5
1.0
0.5
0.0
A (n=15)
B (n=16)
C (n=14)
surface

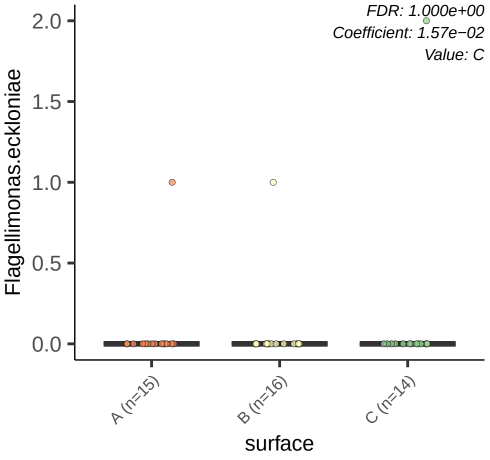

FDR: 1.000e+00
Coefficient: 1.57e−02
Value: C
Flagellimonas.ecklonia
2.0
1.5
1.0
0.5
0.0
A (n=15)
B (n=16)
C (n=14)
surface

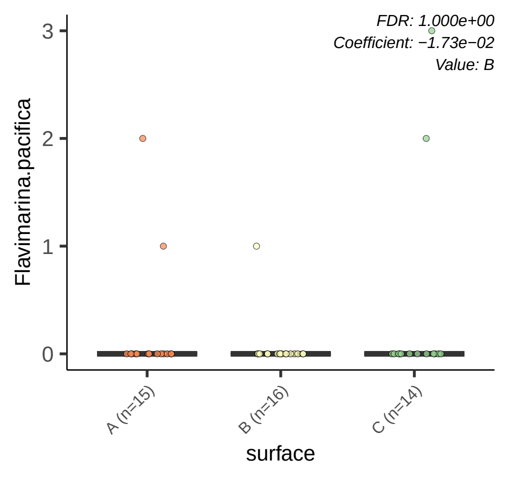

FDR: 1.000e+00
Coefficient: −1.73e−02
Value: B
Flavimarina.pacifica
3
2
1
0
A (n=15)
B (n=16)
C (n=14)
surface

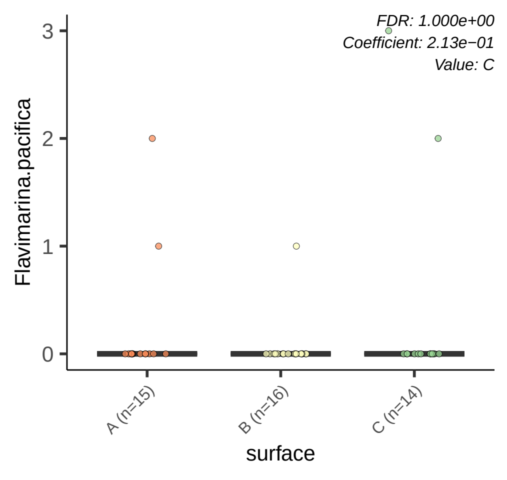

FDR: 1.000e+00
Coefficient: 2.13e−01
Value: C
Flavimarina.pacifica
0
1
2
3
A (n=15)
B (n=16)
C (n=14)
surface

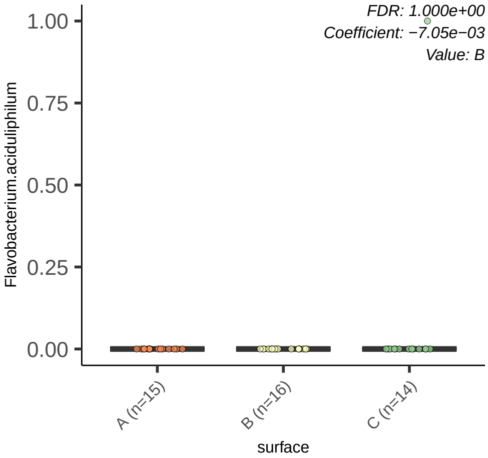
FDR: 1.000e+00
Coefficient: −7.05e−03
Value: B
1.00
0.75
0.50
0.25
0.00
Flavobacterium.aciduliphilum
A (n=15)
B (n=16)
C (n=14)
surface

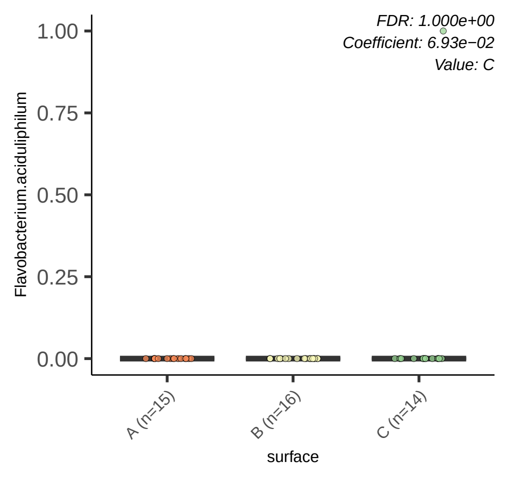

FDR: 1.000e+00
Coefficient: 6.93e−02
Value: C
1.00
0.75
0.50
0.25
0.00
Flavobacterium.aciduliphilum
A (n=15)
B (n=16)
C (n=14)
surface

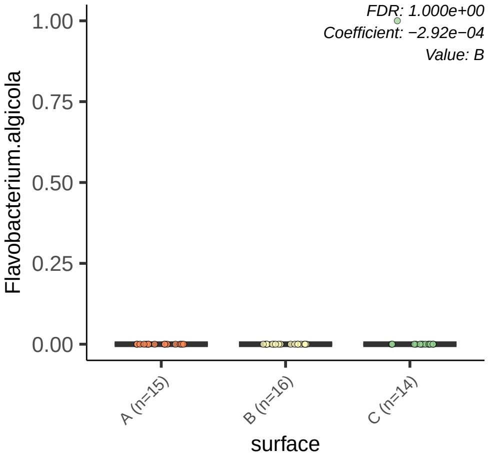
FDR: 1.000e+00
Coefficient: −2.92e−04
Value: B
Flavobacterium.algicola
1.00
0.75
0.50
0.25
0.00
A (n=15)
B (n=16)
C (n=14)
surface

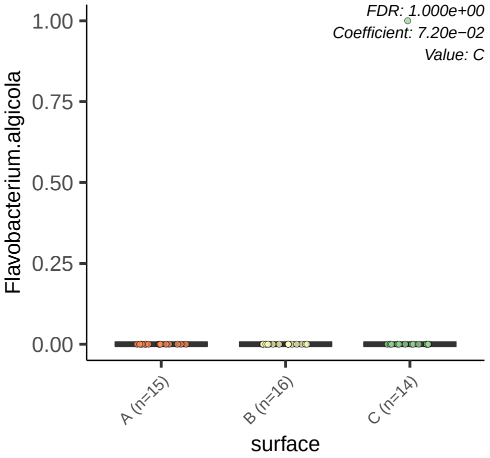

FDR: 1.000e+00
Coefficient: 7.20e−02
Value: C
1.00
0.75
0.50
0.25
0.00
Flavobacterium.algicola
A (n=15)
B (n=16)
C (n=14)
surface

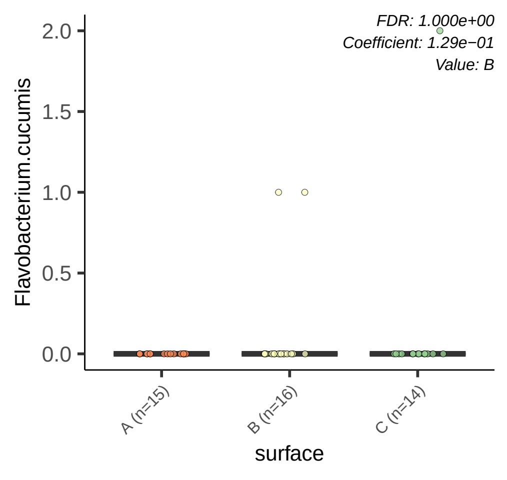
FDR: 1.000e+00
Coefficient: 1.29e−01
Value: B
Flavobacterium.cucumis
2.0
1.5
1.0
0.5
0.0
A (n=15)
B (n=16)
C (n=14)
surface

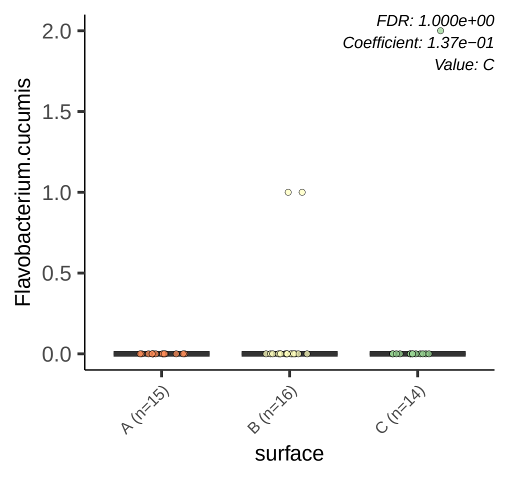
FDR: 1.000e+00
Coefficient: 1.37e−01
Value: C
Flavobacterium.cucumis
2.0
1.5
1.0
0.5
0.0
A (n=15)
B (n=16)
C (n=14)
surface

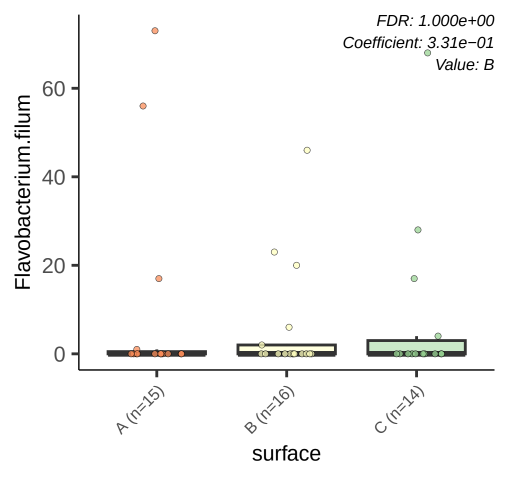
FDR: 1.000e+00
Coefficient: 3.31e−01
Value: B
Flavobacterium.filum
60
40
20
0
A (n=15)
B (n=16)
C (n=14)
surface

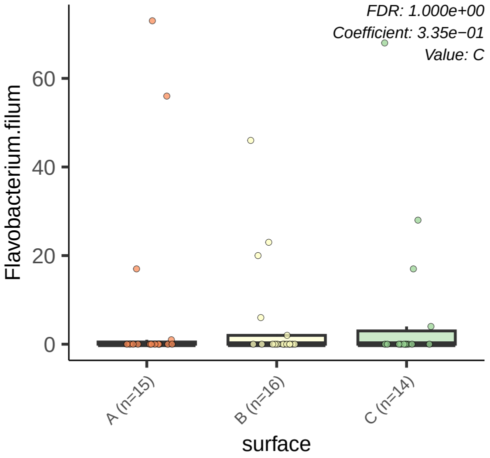

FDR: 1.000e+00
Coefficient: 3.35e−01
Value: C
Flavobacterium.filum
0
20
40
60
A (n=15)
B (n=16)
C (n=14)
surface

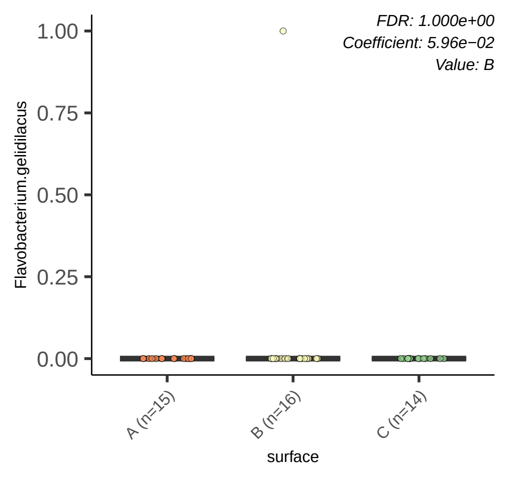
FDR: 1.000e+00
Coefficient: 5.96e−02
Value: B
Flavobacterium.gelidilacus
1.00
0.75
0.50
0.25
0.00
A (n=15)
B (n=16)
C (n=14)
surface

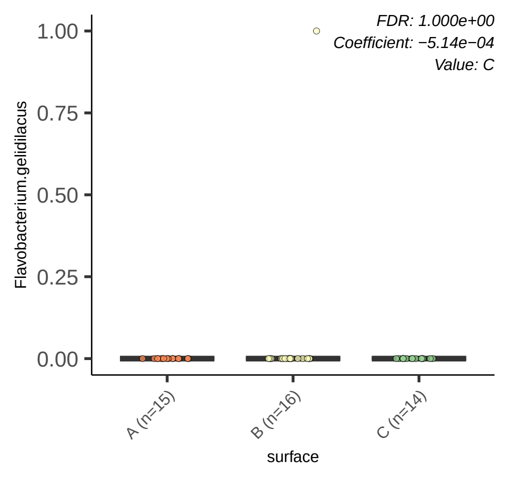
FDR: 1.000e+00
Coefficient: −5.14e−04
Value: C
1.00
0.75
0.50
0.25
0.00
Flavobacterium.gelidilacus
A (n=15)
B (n=16)
C (n=14)
surface

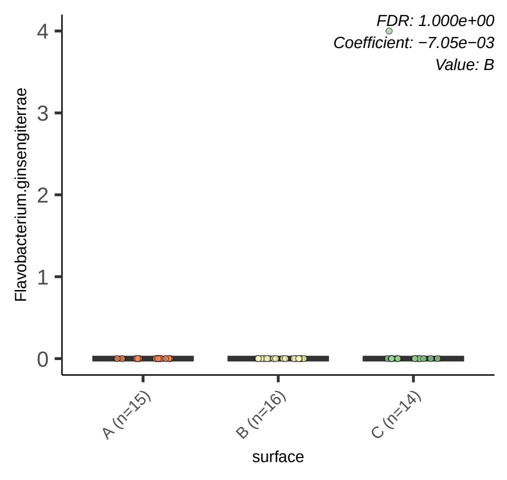
FDR: 1.000e+00
Coefficient: −7.05e−03
Value: B
Flavobacterium.ginsengiterrae
4
3
2
1
0
A (n=15)
B (n=16)
C (n=14)
surface

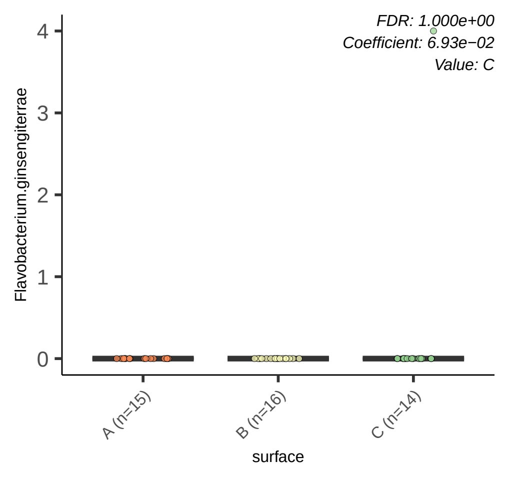
FDR: 1.000e+00
Coefficient: 6.93e−02
Value: C
Flavobacterium.ginsengiterrae
4
3
2
1
0
A (n=15)
B (n=16)
C (n=14)
surface

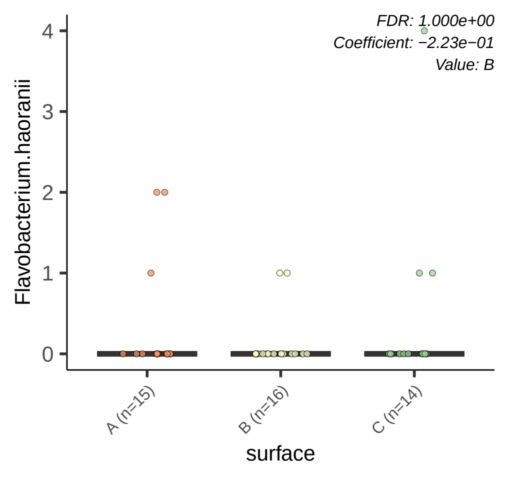
FDR: 1.000e+00
Coefficient: −2.23e−01
Value: B
Flavobacterium.haoranii
0
1
2
3
4
A (n=15)
B (n=16)
C (n=14)
surface

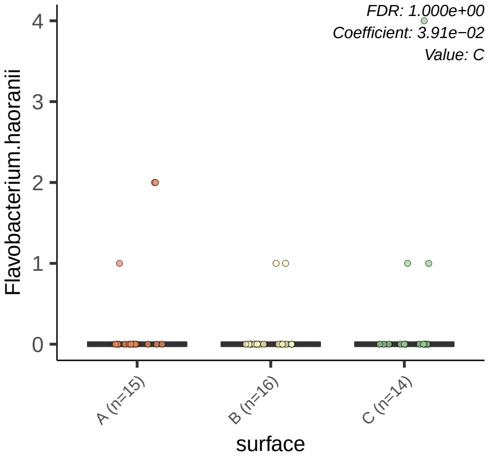
FDR: 1.000e+00
Coefficient: 3.91e−02
Value: C
Flavobacterium.haoranii
0
1
2
3
4
A (n=15)
B (n=16)
C (n=14)
surface

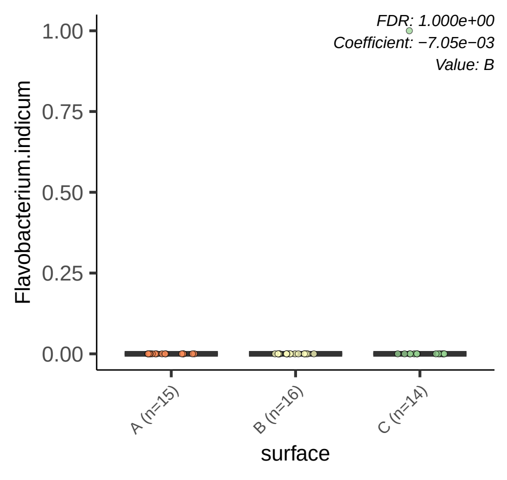
FDR: 1.000e+00
Coefficient: −7.05e−03
Value: B
Flavobacterium.indicum
1.00
0.75
0.50
0.25
0.00
A (n=15)
B (n=16)
C (n=14)
surface

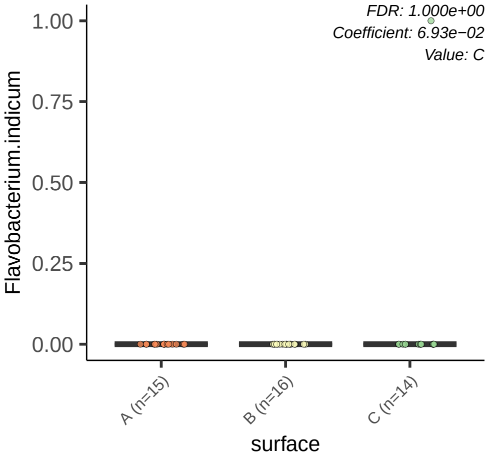
FDR: 1.000e+00
Coefficient: 6.93e−02
Value: C
Flavobacterium.indicum
1.00
0.75
0.50
0.25
0.00
A (n=15)
B (n=16)
C (n=14)
surface

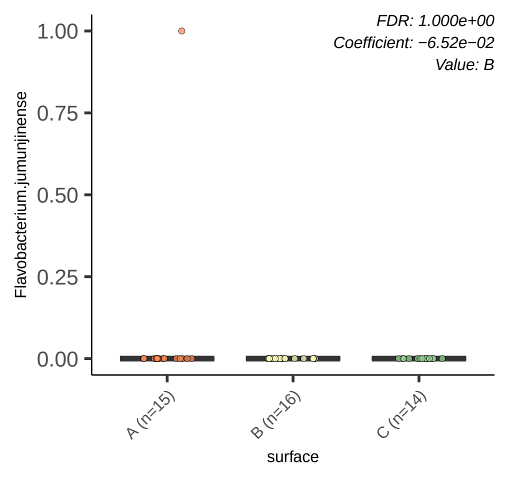
FDR: 1.000e+00
Coefficient: −6.52e−02
Value: B
Flavobacterium.jumunjinense
1.00
0.75
0.50
0.25
0.00
A (n=15)
B (n=16)
C (n=14)
surface

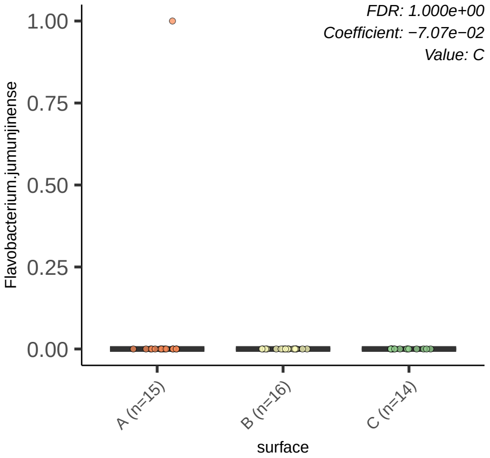
FDR: 1.000e+00
Coefficient: −7.07e−02
Value: C
1.00
0.75
0.50
0.25
0.00
Flavobacterium.jumunjinense
A (n=15)
B (n=16)
C (n=14)
surface

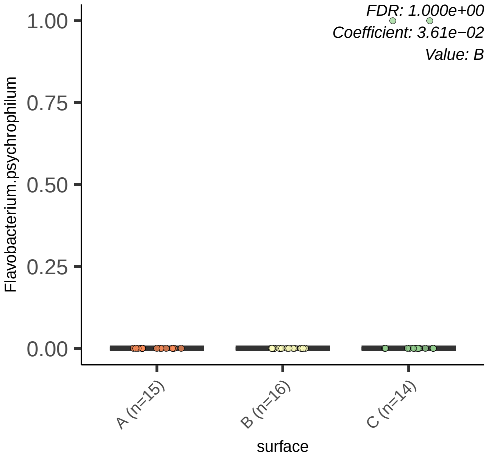
FDR: 1.000e+00
Coefficient: 3.61e−02
Value: B
1.00
0.75
0.50
0.25
0.00
Flavobacterium.psychrophilum
A (n=15)
B (n=16)
C (n=14)
surface

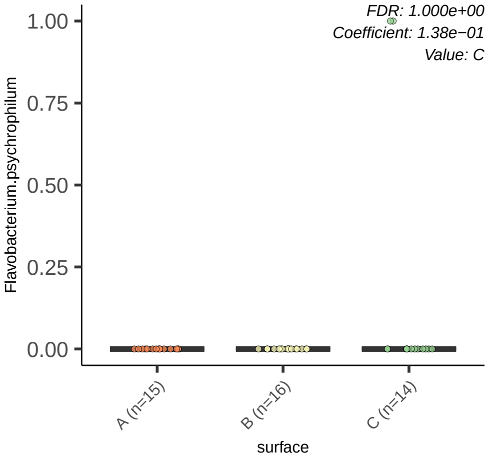
FDR: 1.000e+00
Coefficient: 1.38e−01
Value: C
1.00
0.75
0.50
0.25
0.00
Flavobacterium.psychrophilum
A (n=15)
B (n=16)
C (n=14)
surface

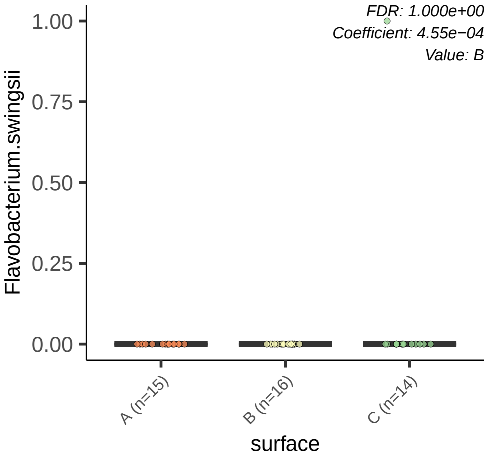
FDR: 1.000e+00
Coefficient: 4.55e−04
Value: B
Flavobacterium.swingsii
1.00
0.75
0.50
0.25
0.00
A (n=15)
B (n=16)
C (n=14)
surface

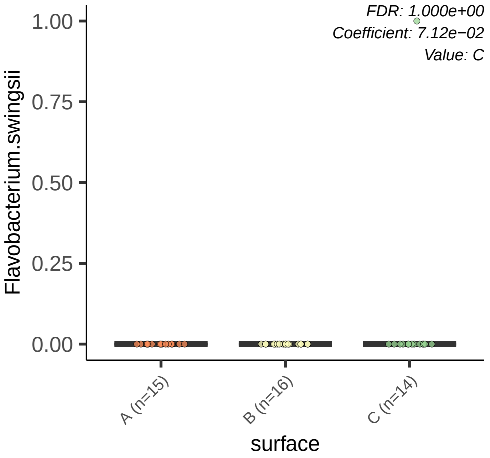
FDR: 1.000e+00
Coefficient: 7.12e−02
Value: C
Flavobacterium.swingsii
1.00
0.75
0.50
0.25
0.00
A (n=15)
B (n=16)
C (n=14)
surface

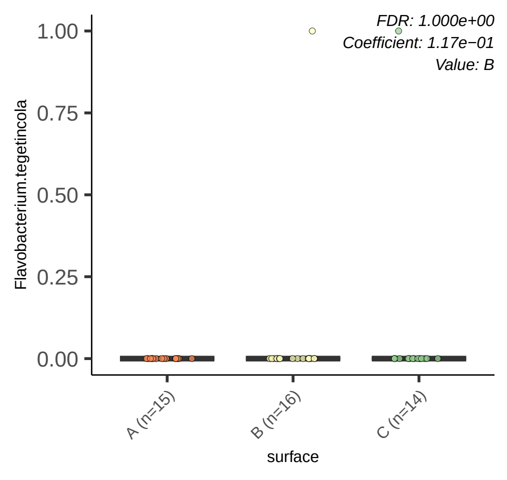
FDR: 1.000e+00
Coefficient: 1.17e−01
Value: B
1.00
0.75
0.50
0.25
0.00
Flavobacterium.tegetincola
A (n=15)
B (n=16)
C (n=14)
surface

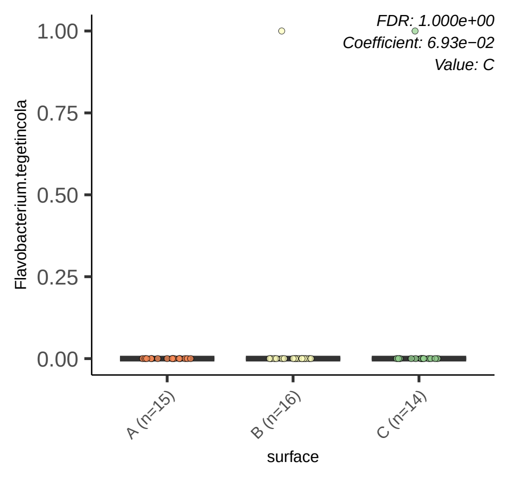
FDR: 1.000e+00
Coefficient: 6.93e−02
Value: C
1.00
0.75
0.50
0.25
0.00
Flavobacterium.tegetincola
A (n=15)
B (n=16)
C (n=14)
surface

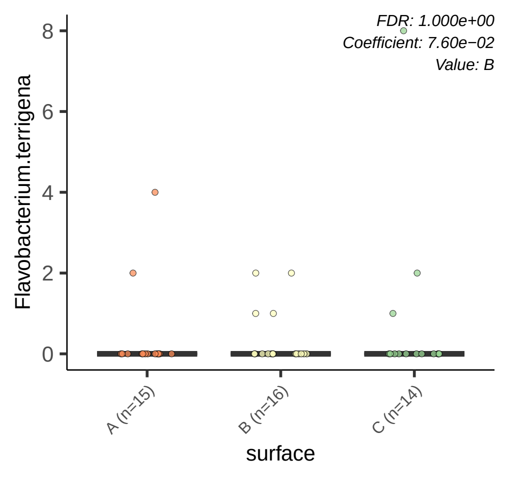
FDR: 1.000e+00
Coefficient: 7.60e−02
Value: B
Flavobacterium.terrigena
8
6
4
2
0
A (n=15)
B (n=16)
C (n=14)
surface

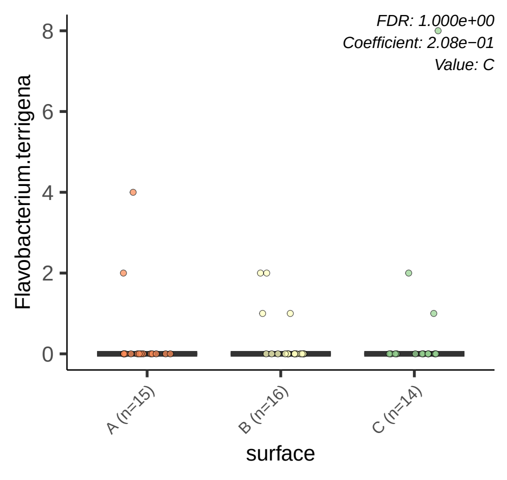
FDR: 1.000e+00
Coefficient: 2.08e−01
Value: C
Flavobacterium.terrigena
0
2
4
6
8
A (n=15)
B (n=16)
C (n=14)
surface

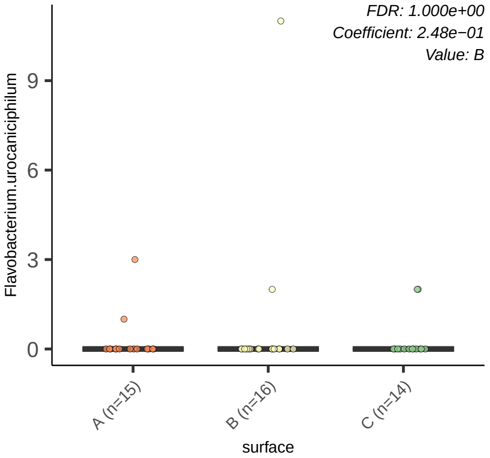
FDR: 1.000e+00
Coefficient: 2.48e−01
Value: B
Flavobacterium.urocaniciphilum
9
6
3
0
A (n=15)
B (n=16)
C (n=14)
surface

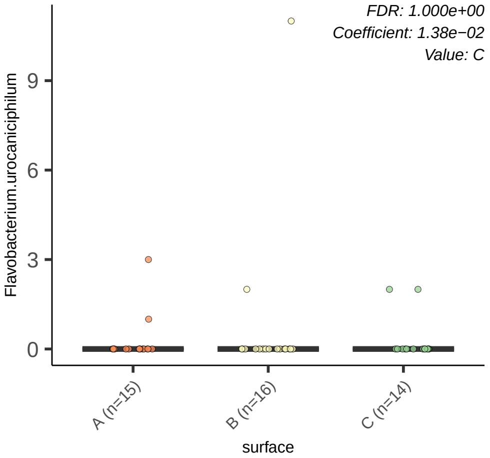
FDR: 1.000e+00
Coefficient: 1.38e−02
Value: C
Flavobacterium.urocaniciphilum
9
6
3
0
A (n=15)
B (n=16)
C (n=14)
surface

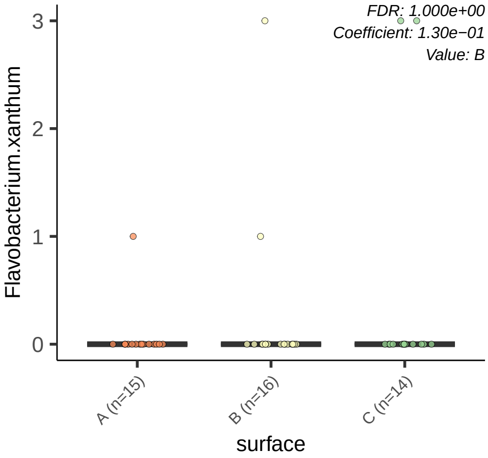
FDR: 1.000e+00
Coefficient: 1.30e−01
Value: B
Flavobacterium.xanthum
0
1
2
3
A (n=15)
B (n=16)
C (n=14)
surface

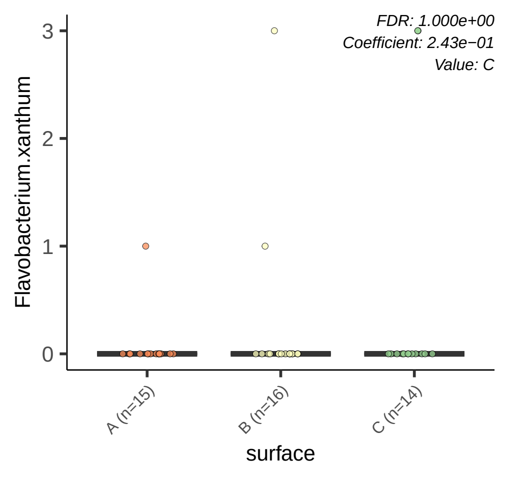
FDR: 1.000e+00
Coefficient: 2.43e−01
Value: C
Flavobacterium.xanthum
3
2
1
0
A (n=15)
B (n=16)
C (n=14)
surface

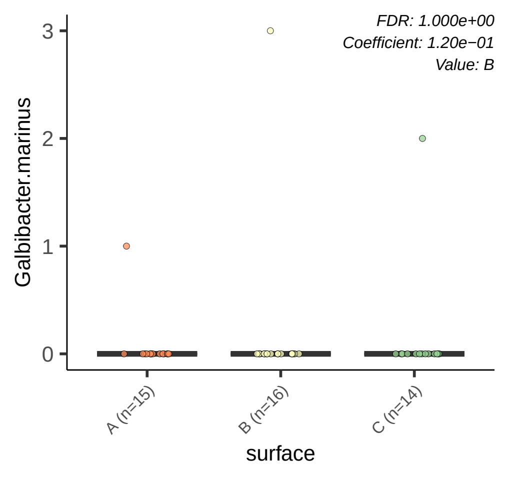

FDR: 1.000e+00
Coefficient: 1.20e−01
Value: B
Galbibacter.marinus
3
2
1
0
A (n=15)
B (n=16)
C (n=14)
surface

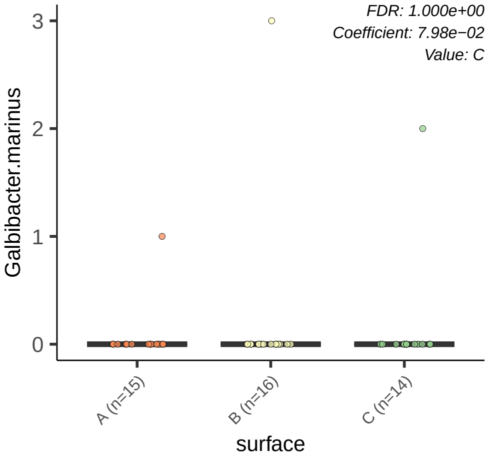

FDR: 1.000e+00
Coefficient: 7.98e−02
Value: C
Galbibacter.marinus
0
1
2
3
A (n=15)
B (n=16)
C (n=14)
surface

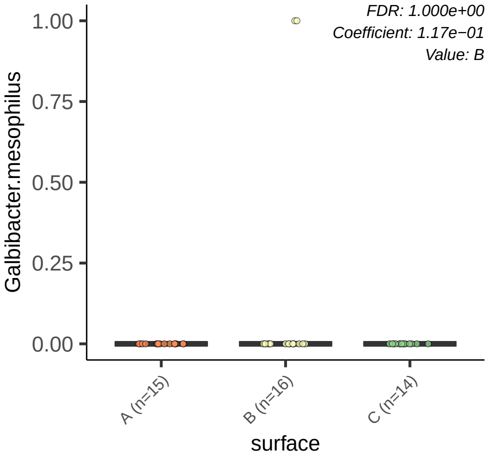
FDR: 1.000e+00
Coefficient: 1.17e−01
Value: B
Galbibacter.mesophilus
1.00
0.75
0.50
0.25
0.00
A (n=15)
B (n=16)
C (n=14)
surface

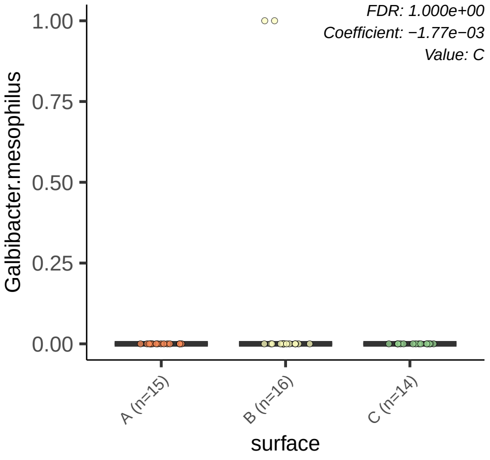

FDR: 1.000e+00
Coefficient: −1.77e−03
Value: C
Galbibacter.mesophilus
1.00
0.75
0.50
0.25
0.00
A (n=15)
B (n=16)
C (n=14)
surface

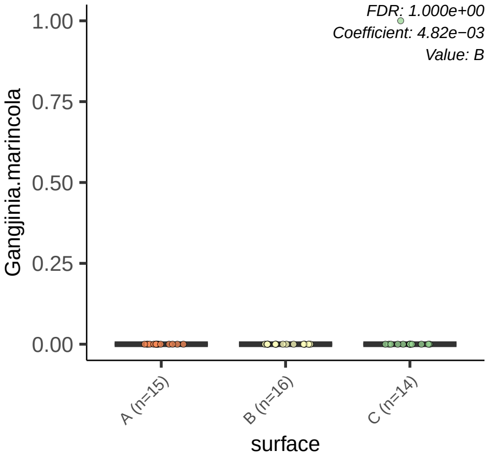
FDR: 1.000e+00
Coefficient: 4.82e−03
Value: B
1.00
0.75
0.50
0.25
0.00
Gangjinia.marincola
A (n=15)
B (n=16)
C (n=14)
surface

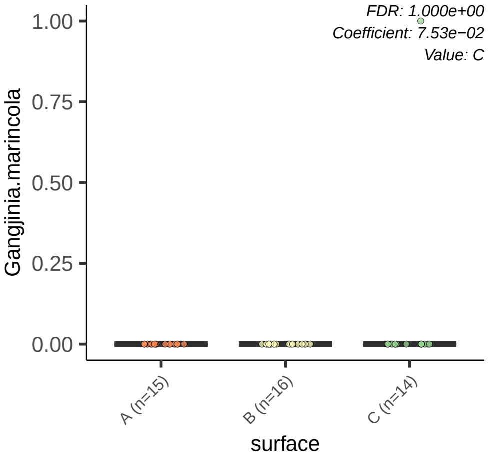
FDR: 1.000e+00
Coefficient: 7.53e−02
Value: C
Gangjinia.marincola
1.00
0.75
0.50
0.25
0.00
A (n=15)
B (n=16)
C (n=14)
surface

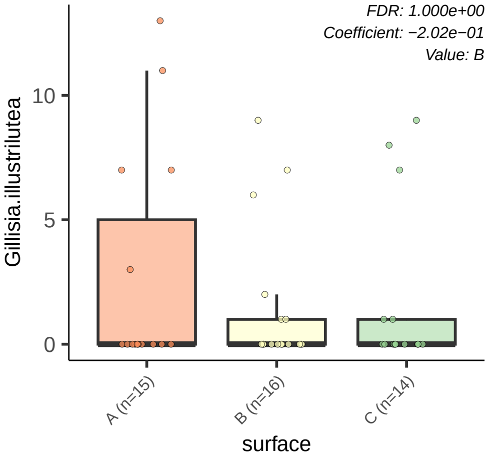

FDR: 1.000e+00
Coefficient: −2.02e−01
Value: B
Gillisia.illustrilutea
10
5
0
A (n=15)
B (n=16)
C (n=14)
surface

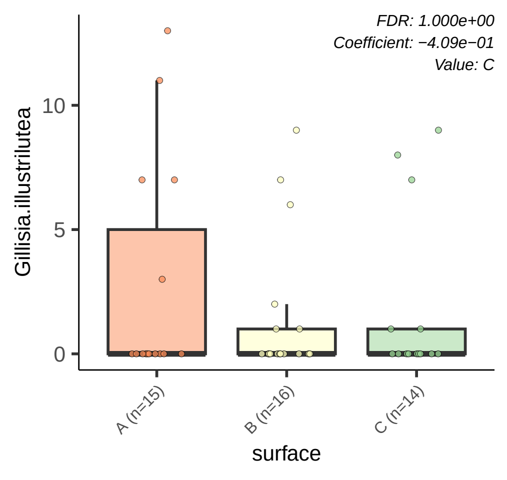
FDR: 1.000e+00
Coefficient: −4.09e−01
Value: C
Gillisia.illustrilutea
10
5
0
A (n=15)
B (n=16)
C (n=14)
surface

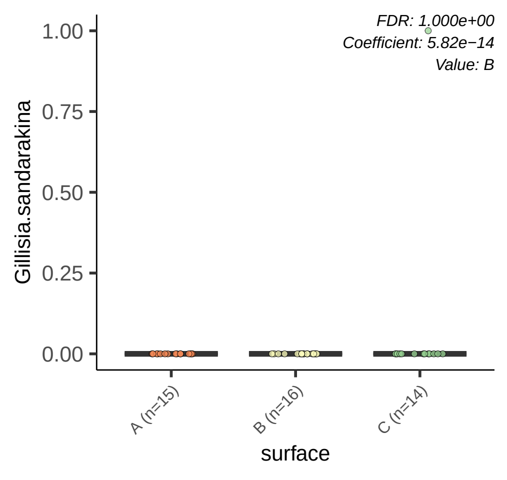
FDR: 1.000e+00
Coefficient: 5.82e−14
Value: B
Gillisia.sandarakina
1.00
0.75
0.50
0.25
0.00
A (n=15)
B (n=16)
C (n=14)
surface

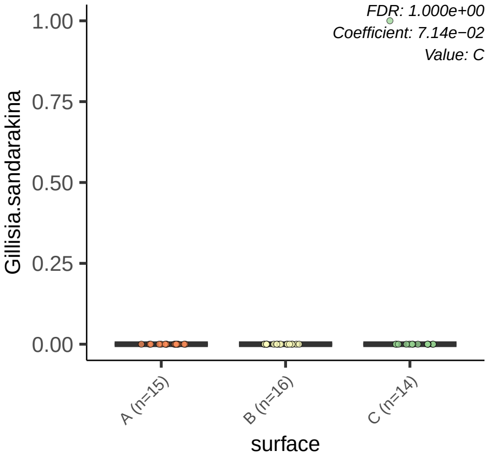
FDR: 1.000e+00
Coefficient: 7.14e−02
Value: C
Gillisia.sandarakina
1.00
0.75
0.50
0.25
0.00
A (n=15)
B (n=16)
C (n=14)
surface

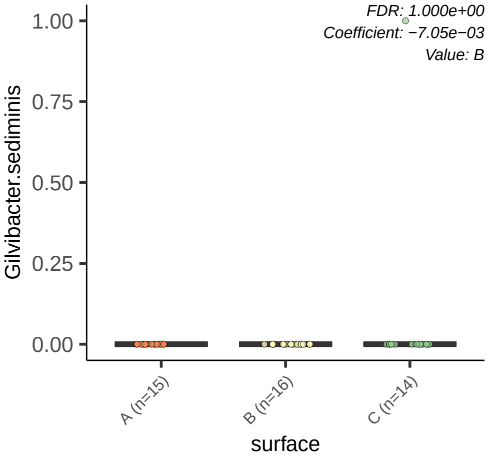
FDR: 1.000e+00
Coefficient: −7.05e−03
Value: B
Gilvibacter.sedimimis
1.00
0.75
0.50
0.25
0.00
A (n=15)
B (n=16)
C (n=14)
surface

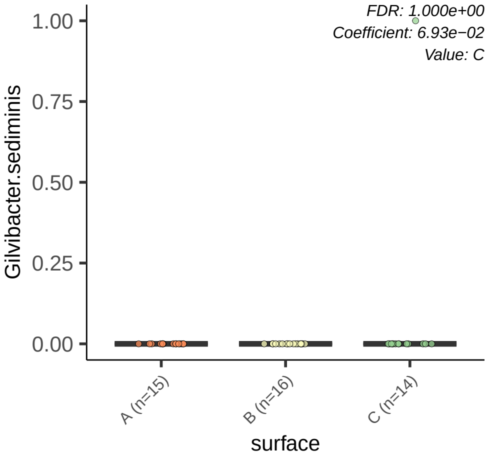

FDR: 1.000e+00
Coefficient: 6.93e−02
Value: C
Gilvibacter.sedimini s
1.00
0.75
0.50
0.25
0.00
A (n=15)
B (n=16)
C (n=14)
surface

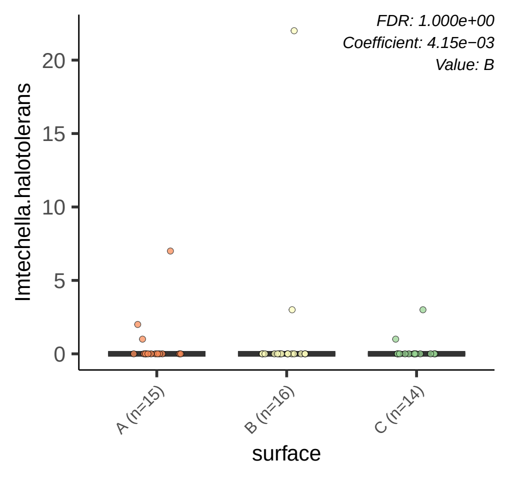
FDR: 1.000e+00
Coefficient: 4.15e−03
Value: B
Imtechella.halotolerans
0
5
10
15
20
A (n=15)
B (n=16)
C (n=14)
surface

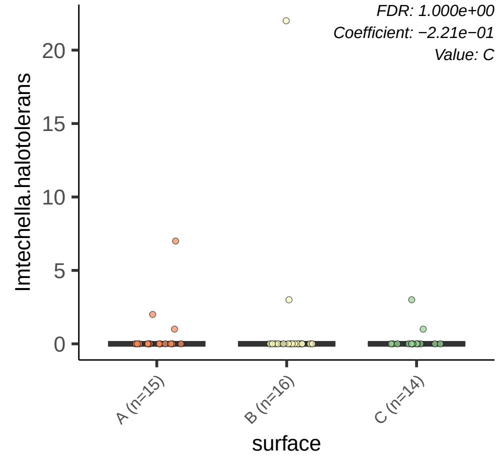

FDR: 1.000e+00
Coefficient: −2.21e−01
Value: C
Imtechella.halotolerans
0
5
10
15
20
A (n=15)
B (n=16)
C (n=14)
surface

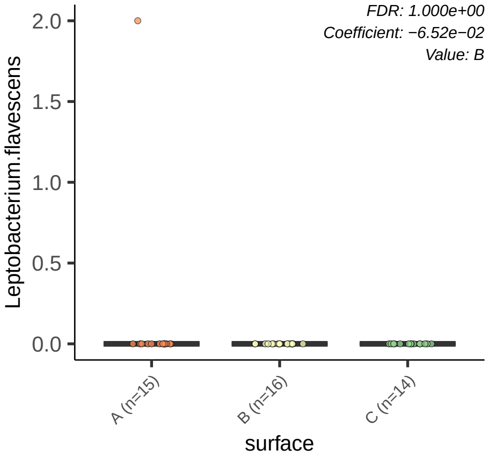

FDR: 1.000e+00
Coefficient: −6.52e−02
Value: B
Leptobacterium.flavescens
2.0
1.5
1.0
0.5
0.0
A (n=15)
B (n=16)
C (n=14)
surface

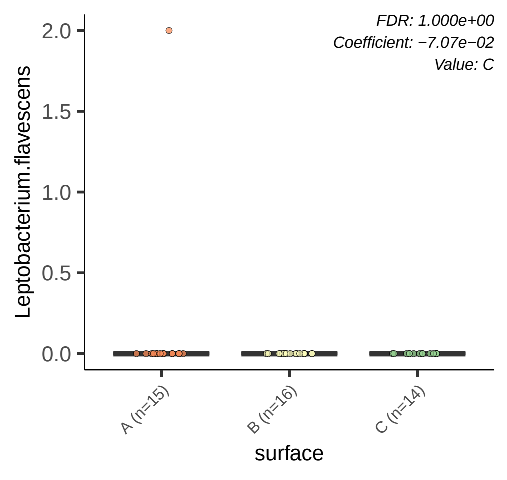
FDR: 1.000e+00
Coefficient: −7.07e−02
Value: C
Leptobacterium.flavescens
2.0
1.5
1.0
0.5
0.0
A (n=15)
B (n=16)
C (n=14)
surface

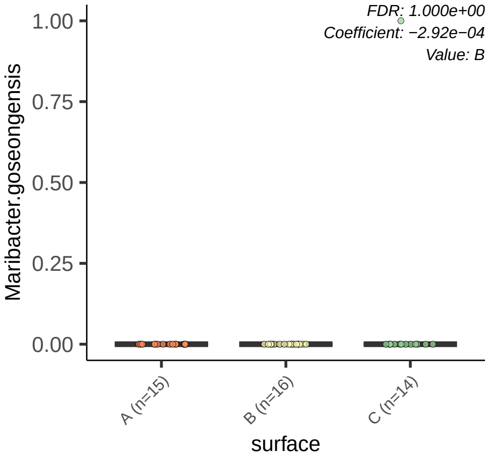
FDR: 1.000e+00
Coefficient: −2.92e−04
Value: B
Maribacter.goseongensis
1.00
0.75
0.50
0.25
0.00
A (n=15)
B (n=16)
C (n=14)
surface

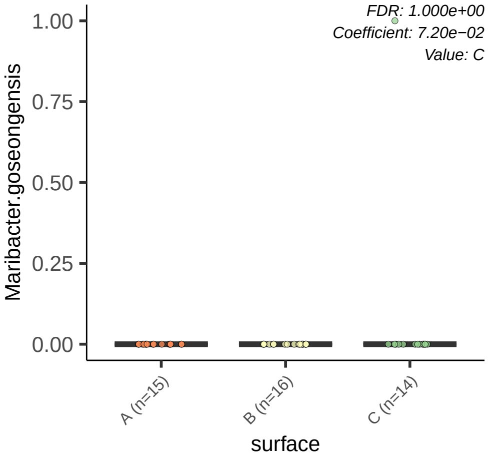
FDR: 1.000e+00
Coefficient: 7.20e−02
Value: C
Maribacter.goseongensis
1.00
0.75
0.50
0.25
0.00
A (n=15)
B (n=16)
C (n=14)
surface

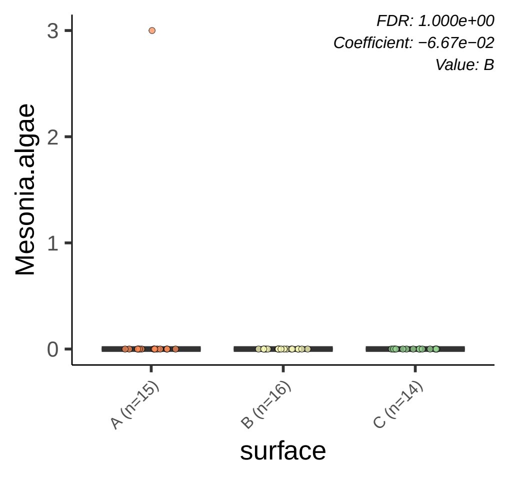
FDR: 1.000e+00
Coefficient: −6.67e−02
Value: B
Mesonia.algae
3
2
1
0
A (n=15)
B (n=16)
C (n=14)
surface

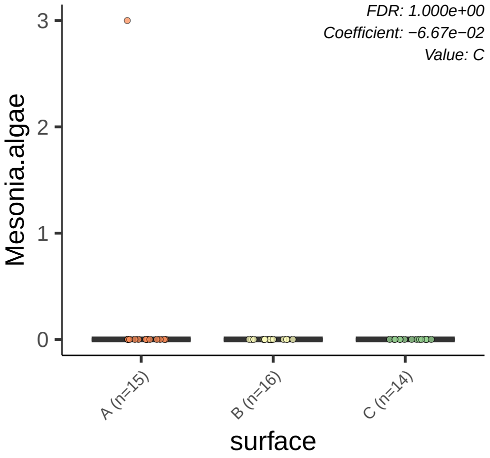

FDR: 1.000e+00
Coefficient: −6.67e−02
Value: C
Mesonia.algae
3
2
1
0
A (n=15)
B (n=16)
C (n=14)
surface

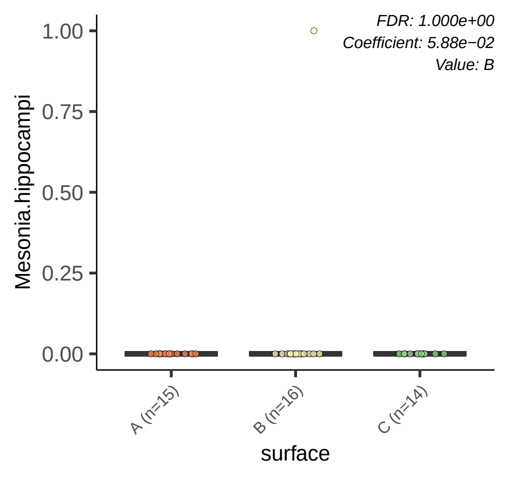
FDR: 1.000e+00
Coefficient: 5.88e−02
Value: B
Mesonia.hippocampi
1.00
0.75
0.50
0.25
0.00
A (n=15)
B (n=16)
C (n=14)
surface

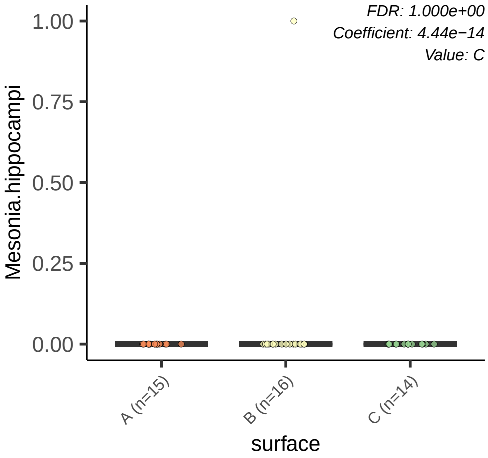
FDR: 1.000e+00
Coefficient: 4.44e−14
Value: C
Mesonia.hippocampi
1.00
0.75
0.50
0.25
0.00
A (n=15)
B (n=16)
C (n=14)
surface

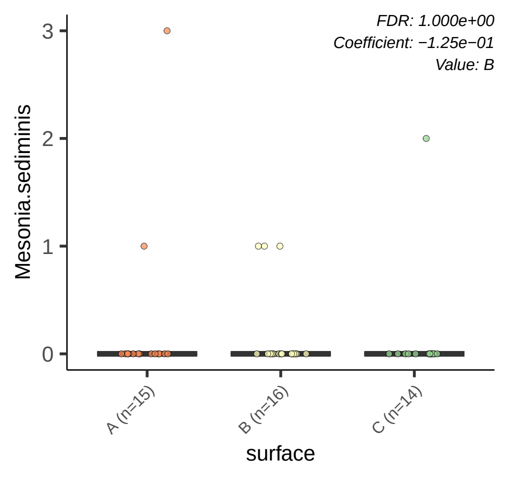

FDR: 1.000e+00
Coefficient: −1.25e−01
Value: B
Mesonia.sedimінis
0
1
2
3
A (n=15)
B (n=16)
C (n=14)
surface

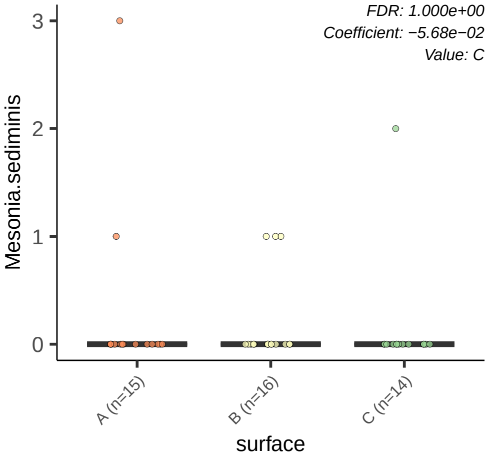

FDR: 1.000e+00
Coefficient: −5.68e−02
Value: C
Mesonia.sedimini
3
2
1
0
A (n=15)
B (n=16)
C (n=14)
surface

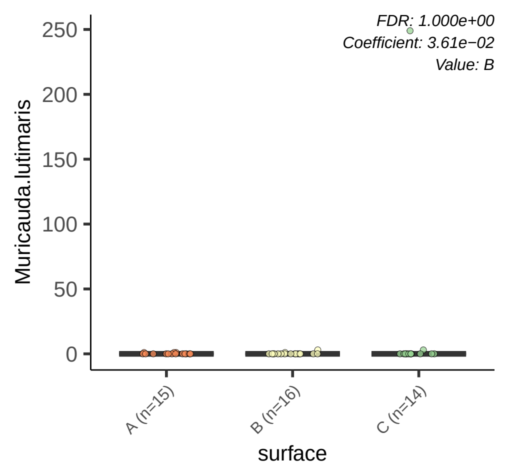

FDR: 1.000e+00
Coefficient: 3.61e−02
Value: B
Muricauda.lutimaris
250
200
150
100
50
0
A (n=15)
B (n=16)
C (n=14)
surface

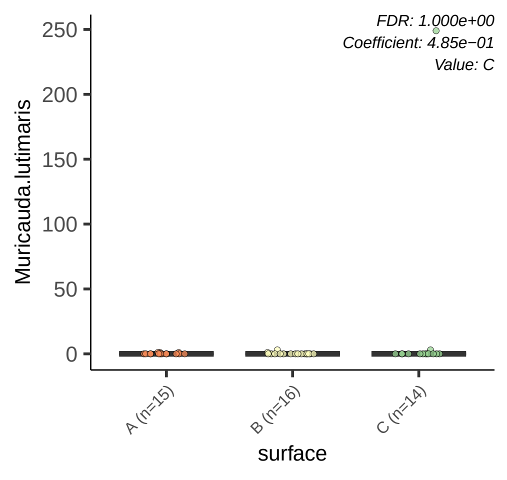

FDR: 1.000e+00
Coefficient: 4.85e−01
Value: C
Muricauda.lutimaris
250
200
150
100
50
0
A (n=15)
B (n=16)
C (n=14)
surface

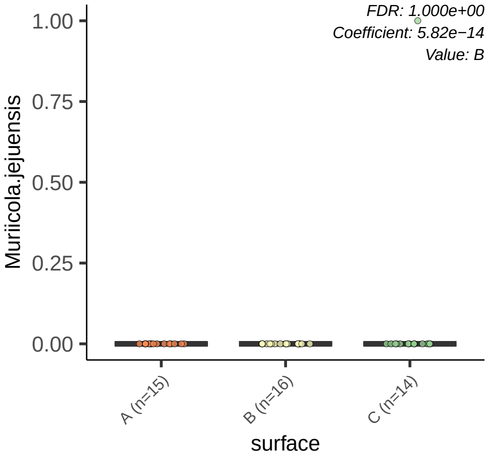

FDR: 1.000e+00
Coefficient: 5.82e−14
Value: B
Muriicola.jejuensis
1.00
0.75
0.50
0.25
0.00
A (n=15)
B (n=16)
C (n=14)
surface

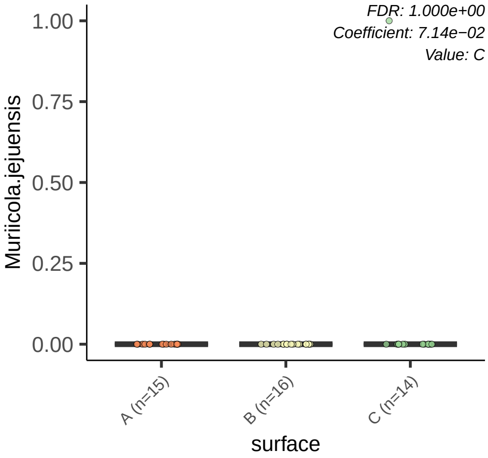

FDR: 1.000e+00
Coefficient: 7.14e−02
Value: C
Muriicola.jejuensis
1.00
0.75
0.50
0.25
0.00
A (n=15)
B (n=16)
C (n=14)
surface

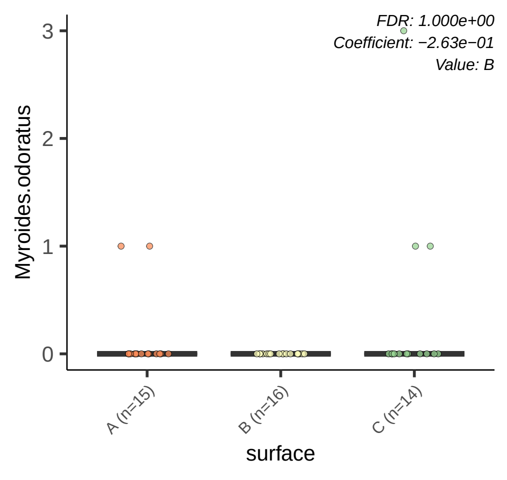

FDR: 1.000e+00
Coefficient: −2.63e−01
Value: B
Myroides.odoratus
3
2
1
0
A (n=15)
B (n=16)
C (n=14)
surface

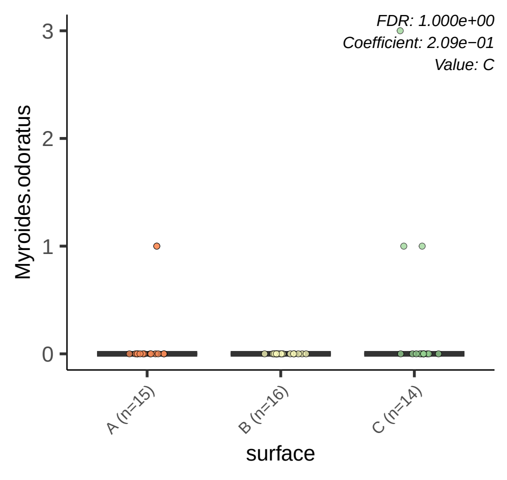
FDR: 1.000e+00
Coefficient: 2.09e−01
Value: C
Myroides.odoratus
0
1
2
3
A (n=15)
B (n=16)
C (n=14)
surface

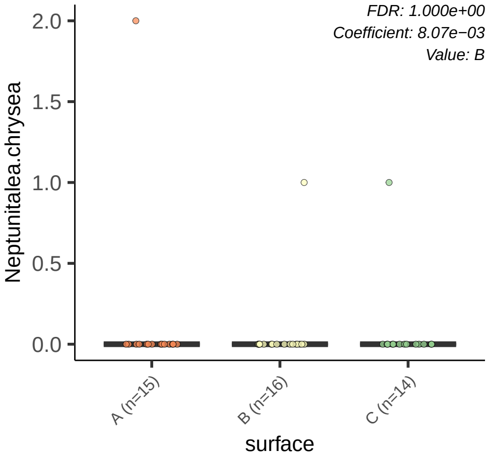
FDR: 1.000e+00
Coefficient: 8.07e−03
Value: B
Neptunitalea.chrysea
2.0
1.5
1.0
0.5
0.0
A (n=15)
B (n=16)
C (n=14)
surface

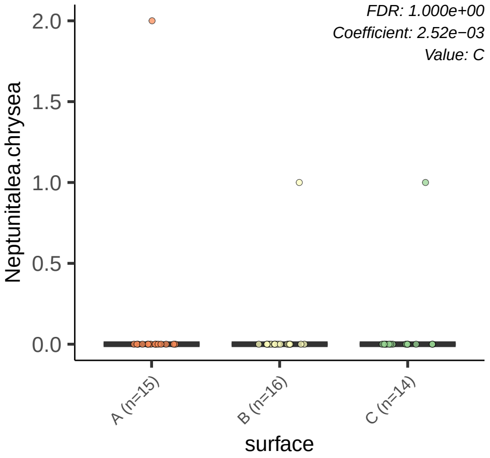

FDR: 1.000e+00
Coefficient: 2.52e−03
Value: C
Neptunitalea.chrysea
2.0
1.5
1.0
0.5
0.0
A (n=15)
B (n=16)
C (n=14)
surface

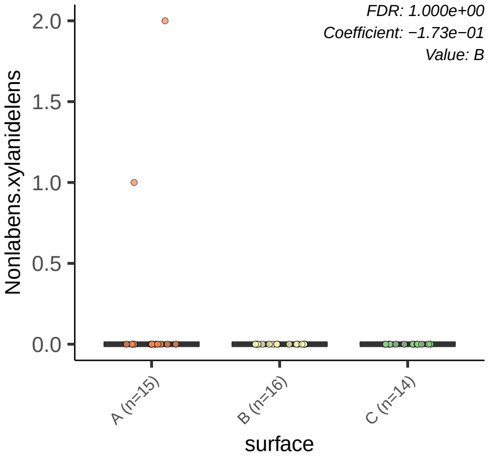

FDR: 1.000e+00
Coefficient: −1.73e−01
Value: B
Nonlabens.xylanidelens
2.0
1.5
1.0
0.5
0.0
A (n=15)
B (n=16)
C (n=14)
surface

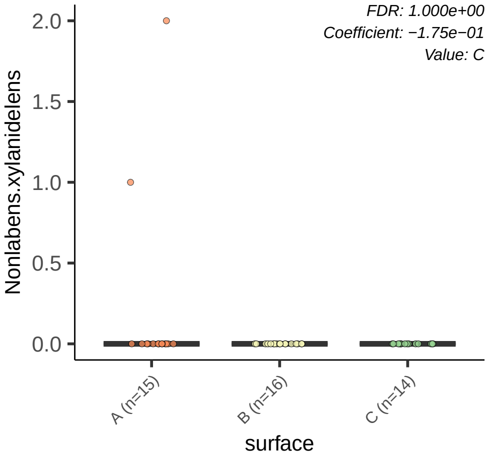

FDR: 1.000e+00
Coefficient: −1.75e−01
Value: C
Nonlabens.xylanidelens
2.0
1.5
1.0
0.5
0.0
A (n=15)
B (n=16)
C (n=14)
surface

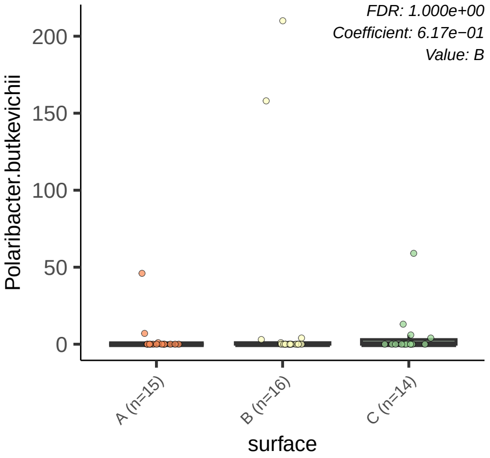

FDR: 1.000e+00
Coefficient: 6.17e−01
Value: B
Polaribacter.butkevichii
200
150
100
50
0
A (n=15)
B (n=16)
C (n=14)
surface

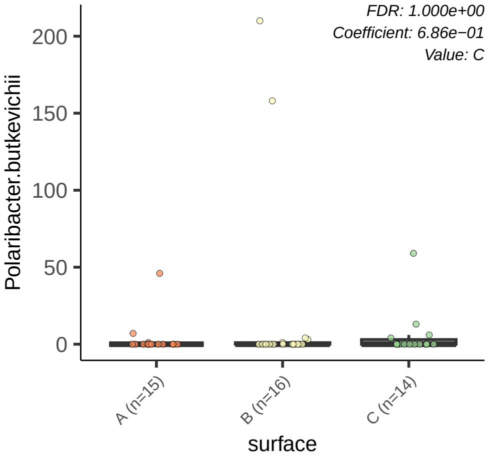

FDR: 1.000e+00
Coefficient: 6.86e−01
Value: C
200
150
100
50
0
Polaribacter.butkevichii
A (n=15)
B (n=16)
C (n=14)
surface

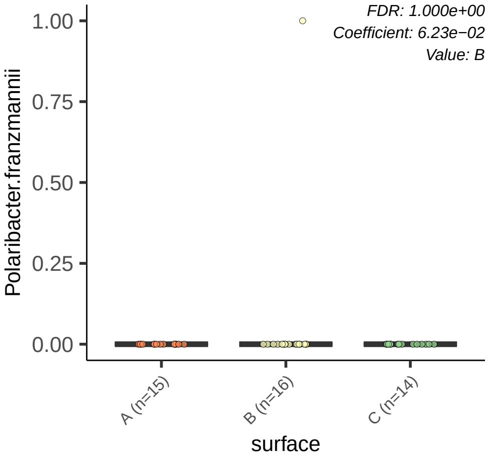

FDR: 1.000e+00
Coefficient: 6.23e−02
Value: B
Polaribacter.franzmannii
1.00
0.75
0.50
0.25
0.00
A (n=15)
B (n=16)
C (n=14)
surface

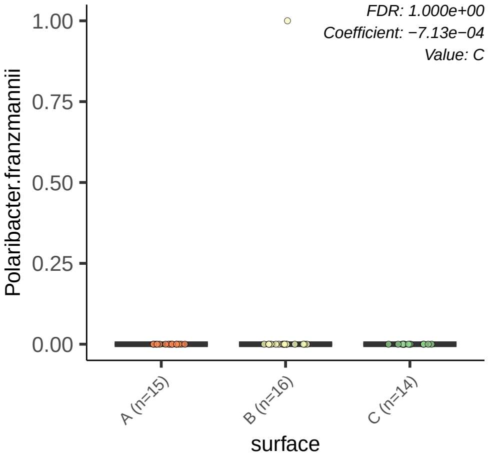
FDR: 1.000e+00
Coefficient: −7.13e−04
Value: C
Polaribacter.franzmannii
1.00
0.75
0.50
0.25
0.00
A (n=15)
B (n=16)
C (n=14)
surface

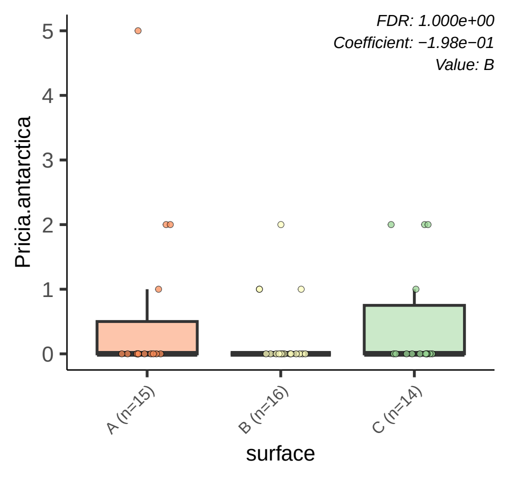

FDR: 1.000e+00
Coefficient: −1.98e−01
Value: B
Pricia.antarctica
5
4
3
2
1
0
A (n=15)
B (n=16)
C (n=14)
surface

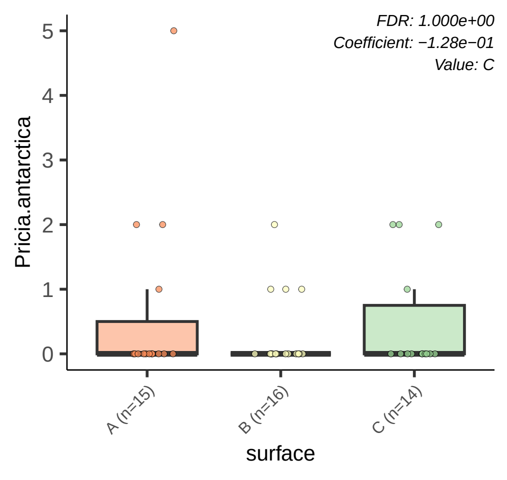

FDR: 1.000e+00
Coefficient: −1.28e−01
Value: C
Pricia.antarctica
0
1
2
3
4
5
A (n=15)
B (n=16)
C (n=14)
surface

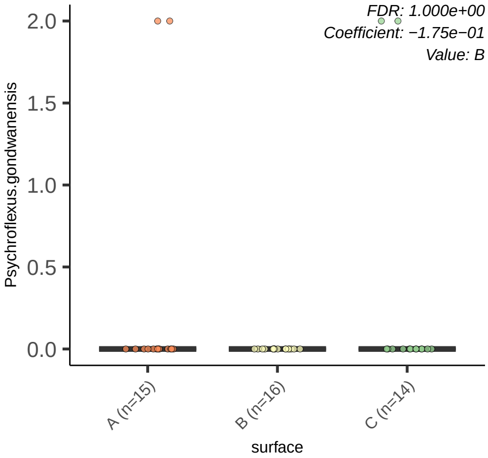

FDR: 1.000e+00
Coefficient: −1.75e−01
Value: B
2.0
1.5
1.0
0.5
0.0
Psychroflexus.gondwanensis
A (n=15)
B (n=16)
C (n=14)
surface

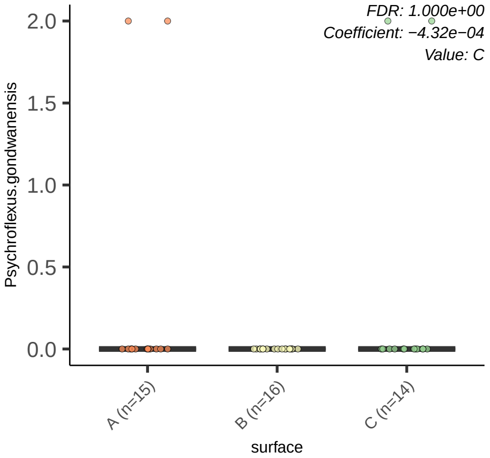
FDR: 1.000e+00
Coefficient: −4.32e−04
Value: C
Psychroflexus.gondwanensis
2.0
1.5
1.0
0.5
0.0
A (n=15)
B (n=16)
C (n=14)
surface

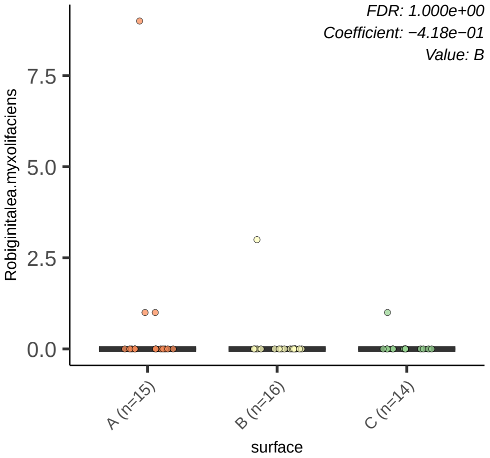

FDR: 1.000e+00
Coefficient: −4.18e−01
Value: B
Robiginitalea.myxolifaciens
7.5
5.0
2.5
0.0
A (n=15)
B (n=16)
C (n=14)
surface

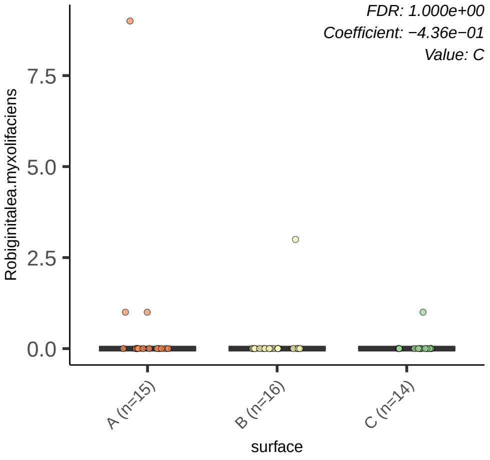
FDR: 1.000e+00
Coefficient: −4.36e−01
Value: C
Robiginitalea.myxolifaciens
7.5
5.0
2.5
0.0
A (n=15)
B (n=16)
C (n=14)
surface

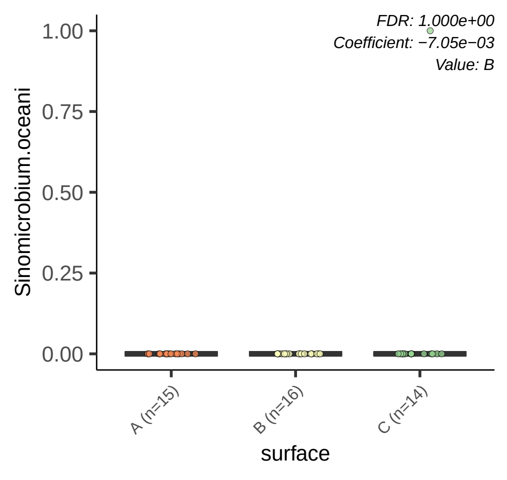

FDR: 1.000e+00
Coefficient: −7.05e−03
Value: B
1.00
0.75
0.50
0.25
0.00
Sinomicrobium.oceani
A (n=15)
B (n=16)
C (n=14)
surface

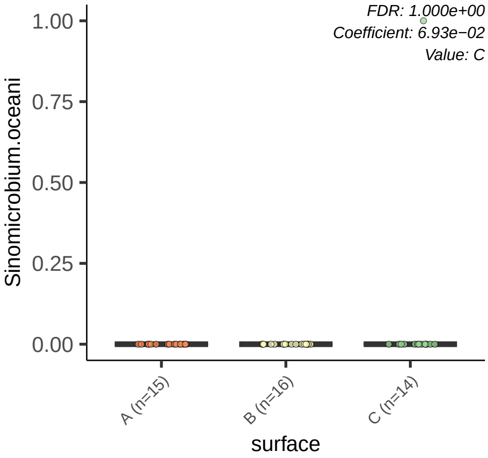

FDR: 1.000e+00
Coefficient: 6.93e−02
Value: C
Sinomicrobium.oceani
1.00
0.75
0.50
0.25
0.00
A (n=15)
B (n=16)
C (n=14)
surface

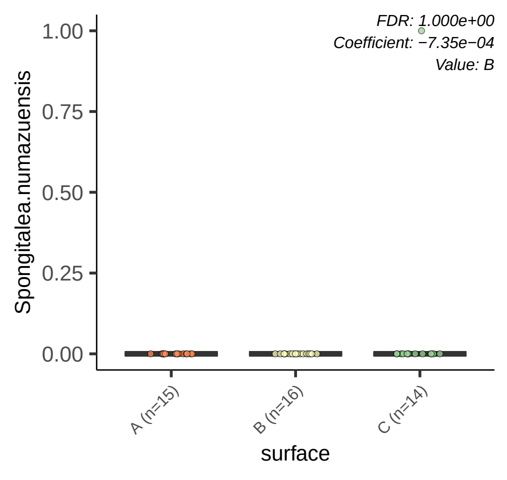
FDR: 1.000e+00
Coefficient: −7.35e−04
Value: B
Spongitalea.numazuensis
1.00
0.75
0.50
0.25
0.00
A (n=15)
B (n=16)
C (n=14)
surface

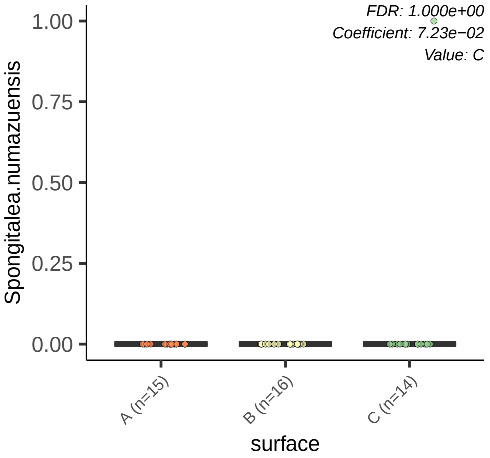

FDR: 1.000e+00
Coefficient: 7.23e−02
Value: C
Spongitalea.numazuensis
1.00
0.75
0.50
0.25
0.00
A (n=15)
B (n=16)
C (n=14)
surface

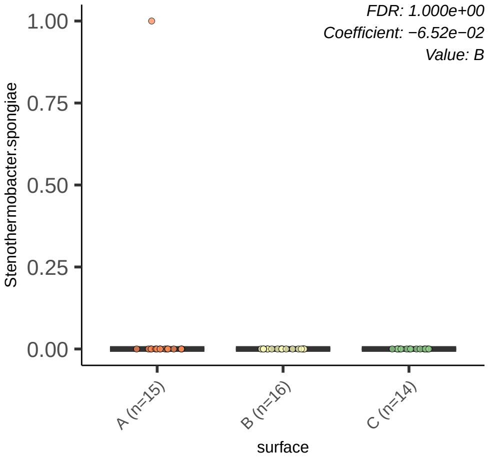
FDR: 1.000e+00
Coefficient: −6.52e−02
Value: B
Stenothermobacter.spongiae
1.00
0.75
0.50
0.25
0.00
A (n=15)
B (n=16)
C (n=14)
surface

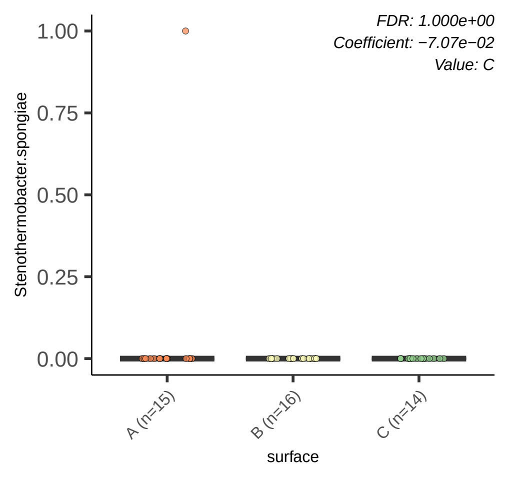

FDR: 1.000e+00
Coefficient: −7.07e−02
Value: C
1.00
0.75
0.50
0.25
0.00
Stenothermobacter.spongiae
A (n=15)
B (n=16)
C (n=14)
surface

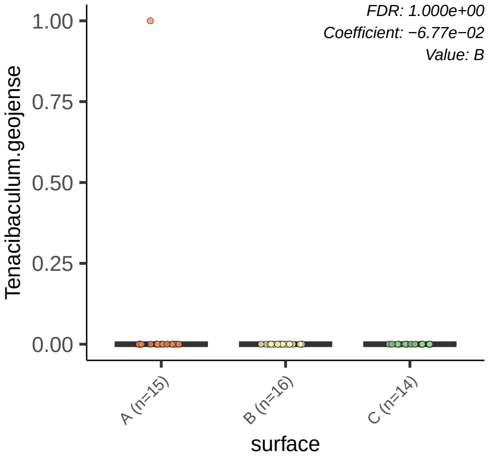
FDR: 1.000e+00
Coefficient: −6.77e−02
Value: B
Tenacibaculum.geojense
1.00
0.75
0.50
0.25
0.00
A (n=15)
B (n=16)
C (n=14)
surface

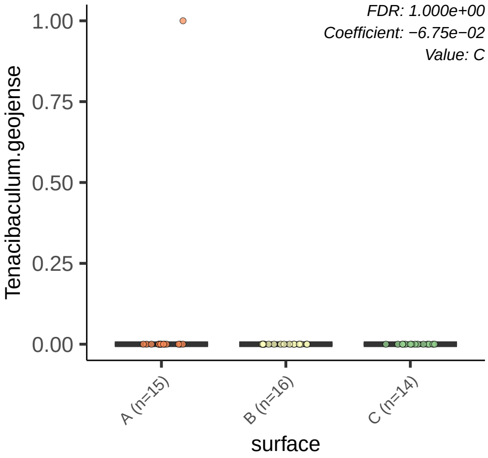

FDR: 1.000e+00
Coefficient: −6.75e−02
Value: C
Tenacibaculum.geojense
1.00
0.75
0.50
0.25
0.00
A (n=15)
B (n=16)
C (n=14)
surface

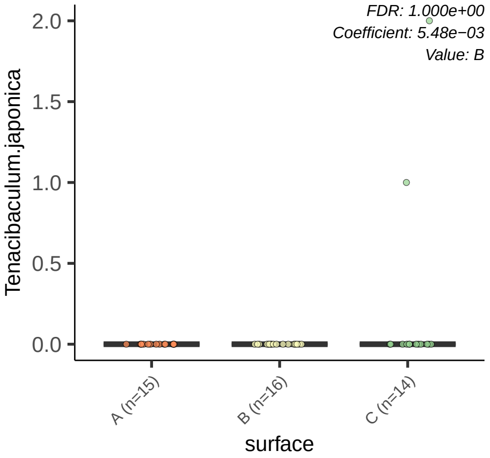
FDR: 1.000e+00
Coefficient: 5.48e−03
Value: B
2.0
1.5
1.0
0.5
0.0
Tenacibaculum.japonica
A (n=15)
B (n=16)
C (n=14)
surface

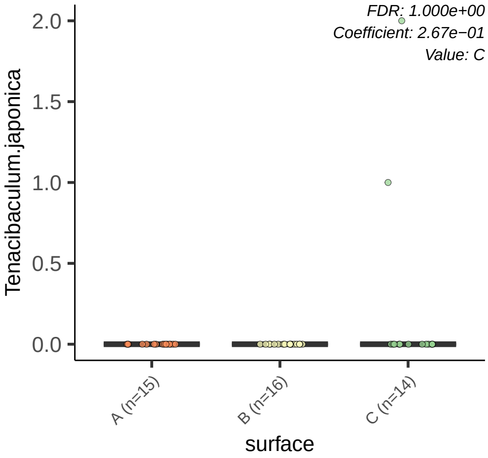

FDR: 1.000e+00
Coefficient: 2.67e−01
Value: C
Tenacibaculum.japonica
2.0
1.5
1.0
0.5
0.0
A (n=15)
B (n=16)
C (n=14)
surface

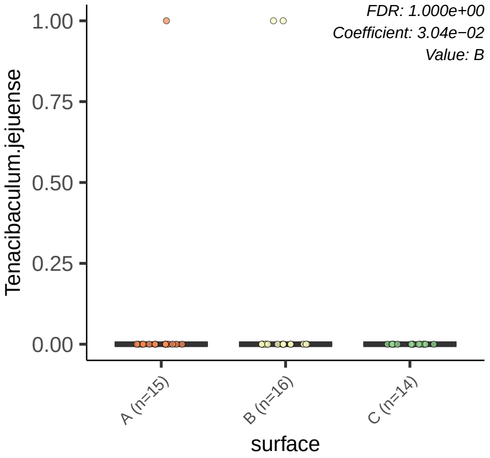
FDR: 1.000e+00
Coefficient: 3.04e−02
Value: B
Tenacibaculum.jejuense
1.00
0.75
0.50
0.25
0.00
A (n=15)
B (n=16)
C (n=14)
surface

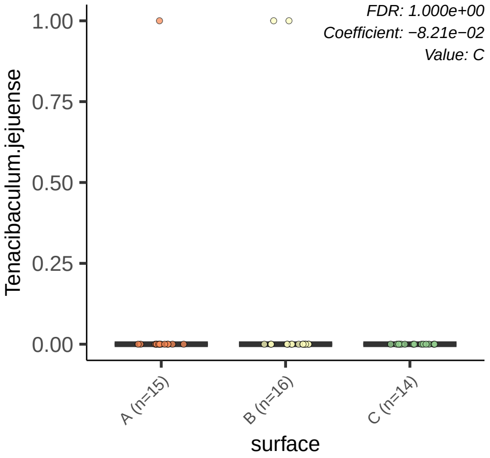

FDR: 1.000e+00
Coefficient: −8.21e−02
Value: C
Tenacibaculum.jejuense
1.00
0.75
0.50
0.25
0.00
A (n=15)
B (n=16)
C (n=14)
surface

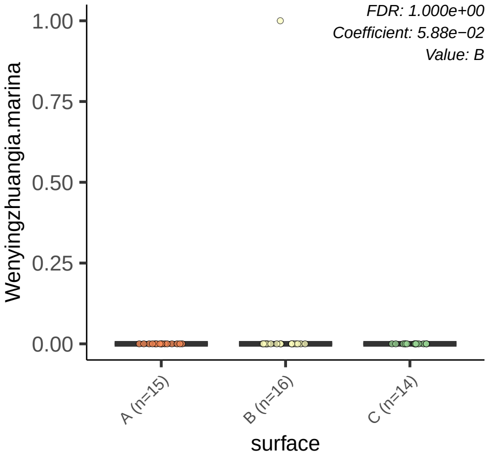

FDR: 1.000e+00
Coefficient: 5.88e−02
Value: B
Wenyingzhuangia.marina
1.00
0.75
0.50
0.25
0.00
A (n=15)
B (n=16)
C (n=14)
surface

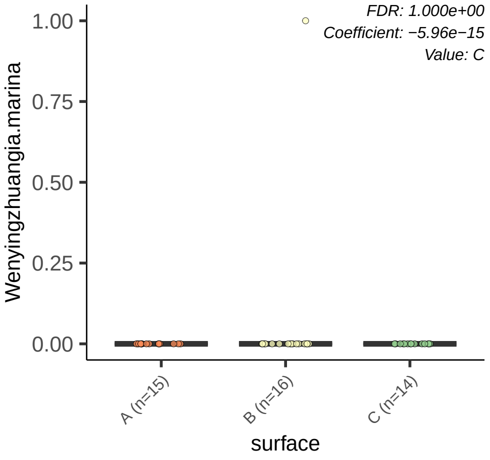
FDR: 1.000e+00
Coefficient: −5.96e−15
Value: C
Wenyingzhuangia.marina
1.00
0.75
0.50
0.25
0.00
A (n=15)
B (n=16)
C (n=14)
surface

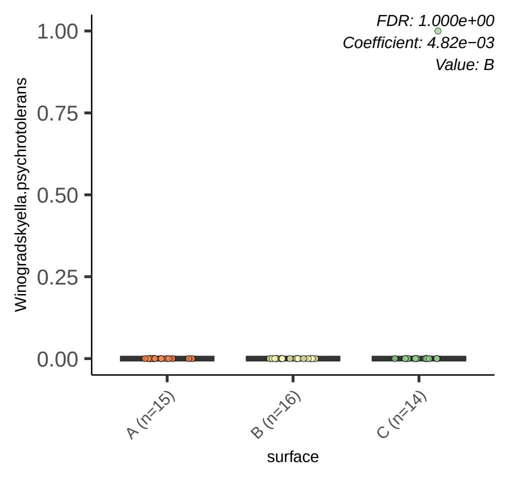
FDR: 1.000e+00
Coefficient: 4.82e−03
Value: B
Winogradskyella.psychrotolerans
1.00
0.75
0.50
0.25
0.00
A (n=15)
B (n=16)
C (n=14)
surface

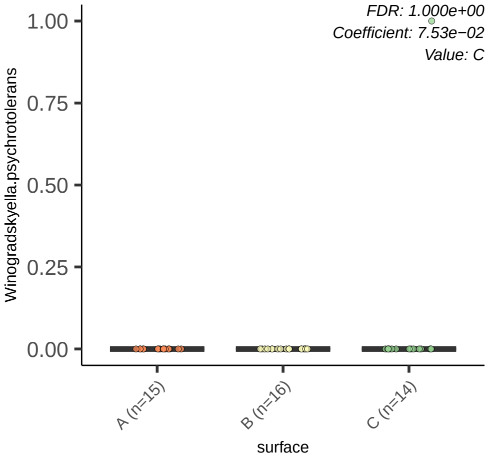

FDR: 1.000e+00
Coefficient: 7.53e−02
Value: C
Winogradskyella.psychrotolerans
1.00
0.75
0.50
0.25
0.00
A (n=15)
B (n=16)
C (n=14)
surface

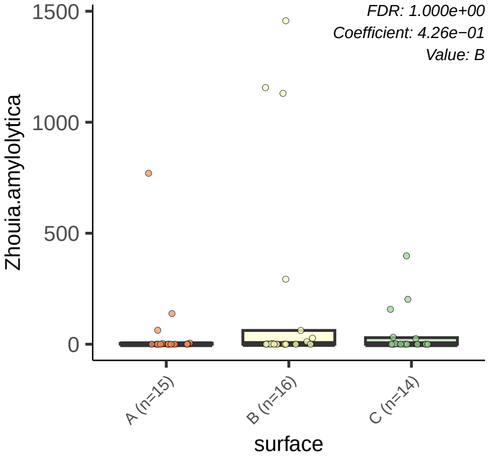
FDR: 1.000e+00
Coefficient: 4.26e−01
Value: B
Zhouia.amylolytica
1500
1000
500
0
A (n=15)
B (n=16)
C (n=14)
surface

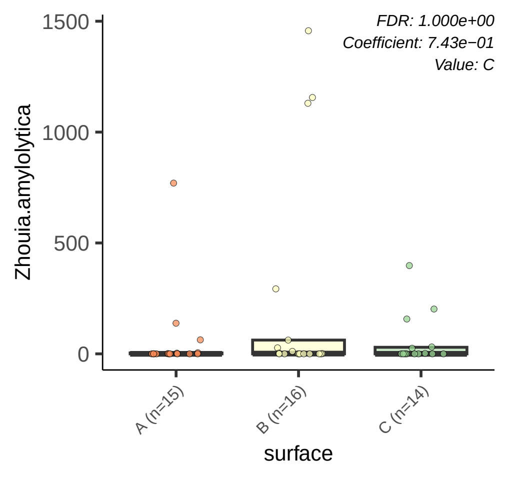
FDR: 1.000e+00
Coefficient: 7.43e−01
Value: C
Zhouia.amylolytica
1500
1000
500
0
A (n=15)
B (n=16)
C (n=14)
surface

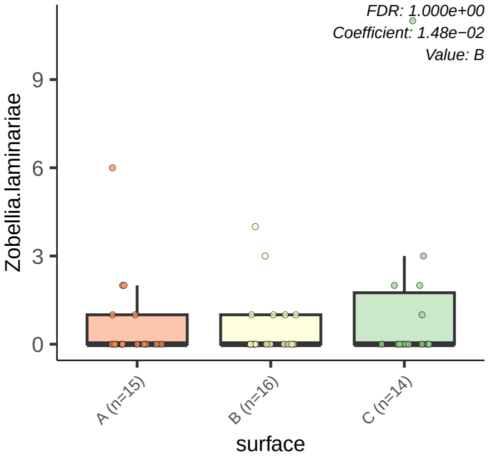
FDR: 1.000e+00
Coefficient: 1.48e−02
Value: B
Zobellia.laminariae
0
3
6
9
A (n=15)
B (n=16)
C (n=14)
surface

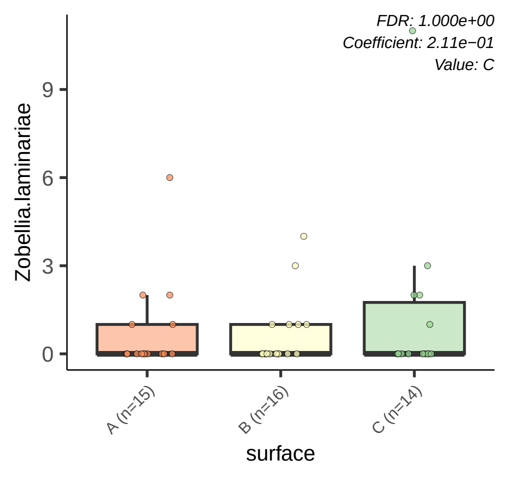

FDR: 1.000e+00
Coefficient: 2.11e−01
Value: C
Zobellia.laminariae
0
3
6
9
A (n=15)
B (n=16)
C (n=14)
surface

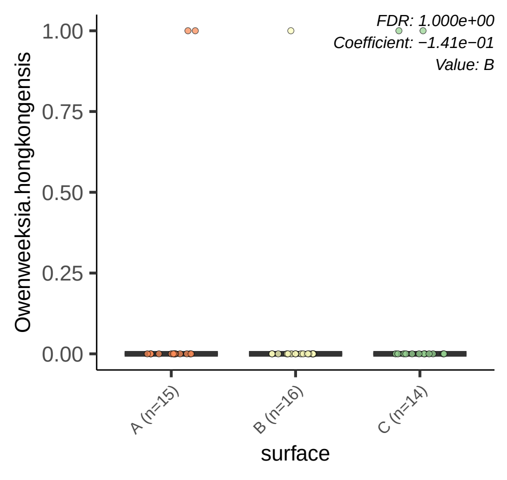
FDR: 1.000e+00
Coefficient: −1.41e−01
Value: B
Owenweeksia.hongkongensis
1.00
0.75
0.50
0.25
0.00
A (n=15)
B (n=16)
C (n=14)
surface

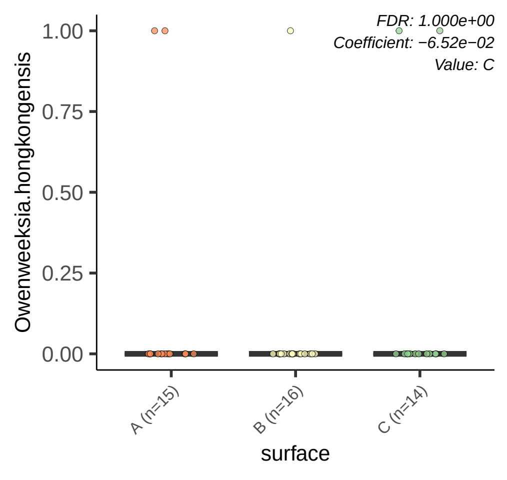
FDR: 1.000e+00
Coefficient: −6.52e−02
Value: C
Owenweeksia.hongkongensis
1.00
0.75
0.50
0.25
0.00
A (n=15)
B (n=16)
C (n=14)
surface

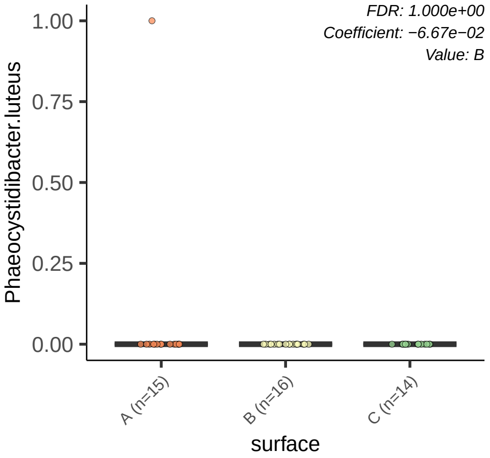

FDR: 1.000e+00
Coefficient: −6.67e−02
Value: B
Phaeocystidibacter.luteus
1.00
0.75
0.50
0.25
0.00
A (n=15)
B (n=16)
C (n=14)
surface

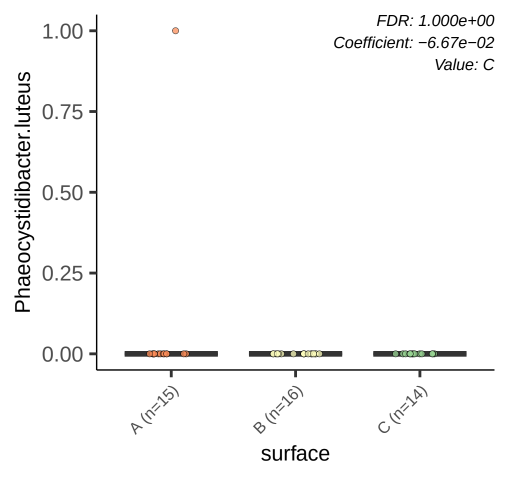
FDR: 1.000e+00
Coefficient: −6.67e−02
Value: C
Phaeocystidibacter.luteus
1.00
0.75
0.50
0.25
0.00
A (n=15)
B (n=16)
C (n=14)
surface

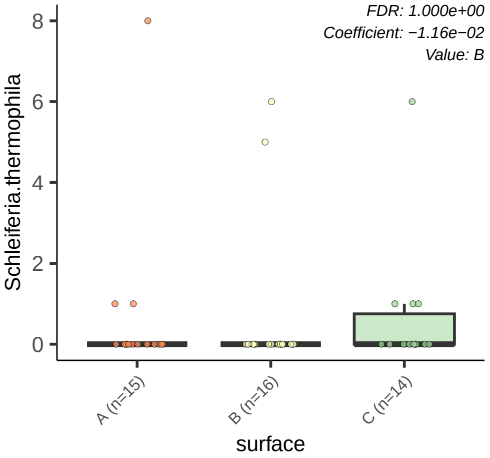

FDR: 1.000e+00
Coefficient: −1.16e−02
Value: B
Schleiferia.thermophila
8
6
4
2
0
A (n=15)
B (n=16)
C (n=14)
surface

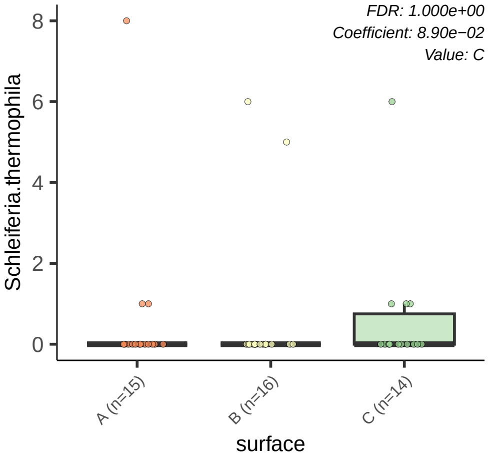

FDR: 1.000e+00
Coefficient: 8.90e−02
Value: C
Schleiferia.thermophila
8
6
4
2
0
A (n=15)
B (n=16)
C (n=14)
surface

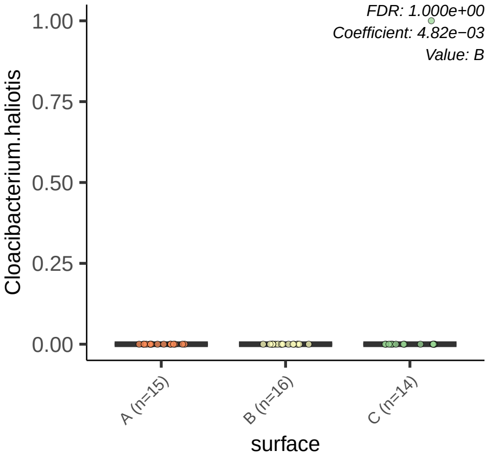
FDR: 1.000e+00
Coefficient: 4.82e−03
Value: B
1.00
0.75
0.50
0.25
0.00
Cloacibacterium.haliotis
A (n=15)
B (n=16)
C (n=14)
surface

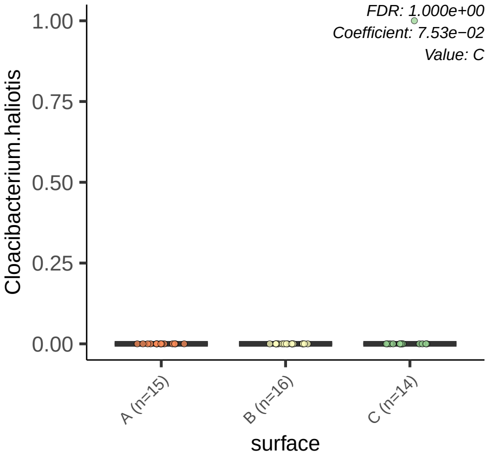
FDR: 1.000e+00
Coefficient: 7.53e−02
Value: C
Cloacibacterium.haliotis
1.00
0.75
0.50
0.25
0.00
A (n=15)
B (n=16)
C (n=14)
surface

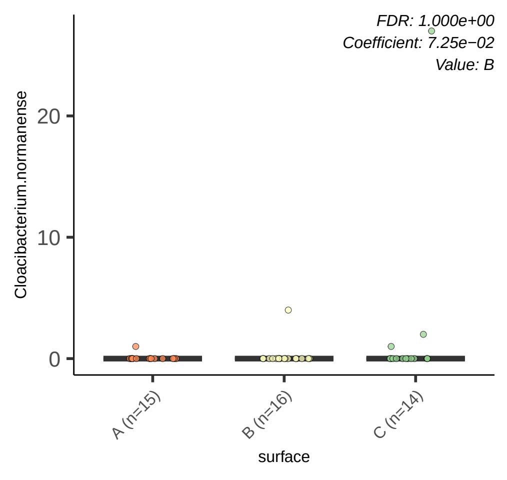
FDR: 1.000e+00
Coefficient: 7.25e−02
Value: B
Cloacibacterium.normanense
0
10
20
A (n=15)
B (n=16)
C (n=14)
surface

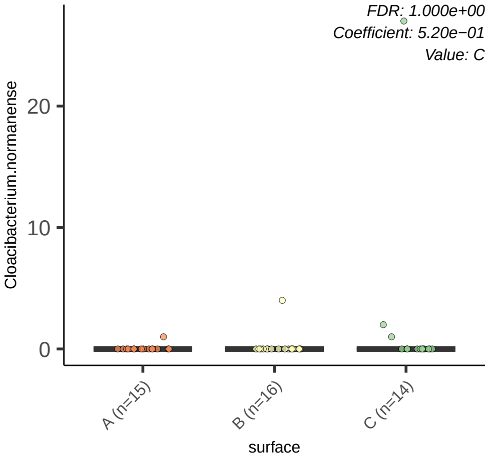

FDR: 1.000e+00
Coefficient: 5.20e−01
Value: C
Cloacibacterium.normanense
0
10
20
A (n=15)
B (n=16)
C (n=14)
surface

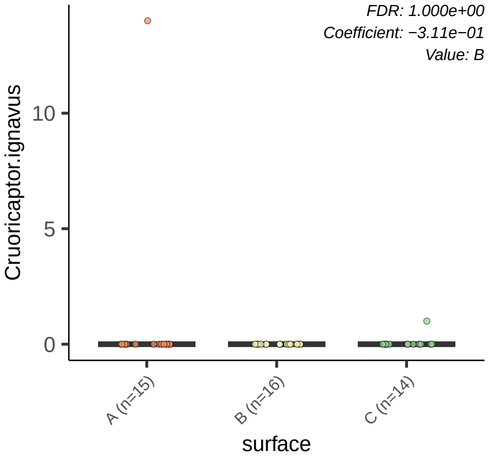

FDR: 1.000e+00
Coefficient: −3.11e−01
Value: B
Cruoricaptor.ignavus
10
5
0
A (n=15)
B (n=16)
C (n=14)
surface

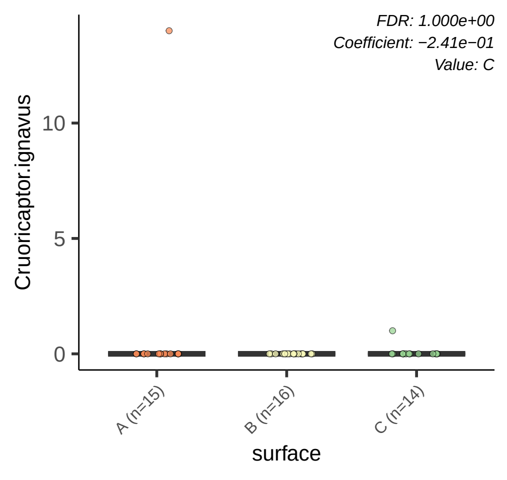
FDR: 1.000e+00
Coefficient: −2.41e−01
Value: C
Cruoricaptor.ignavus
10
5
0
A (n=15)
B (n=16)
C (n=14)
surface

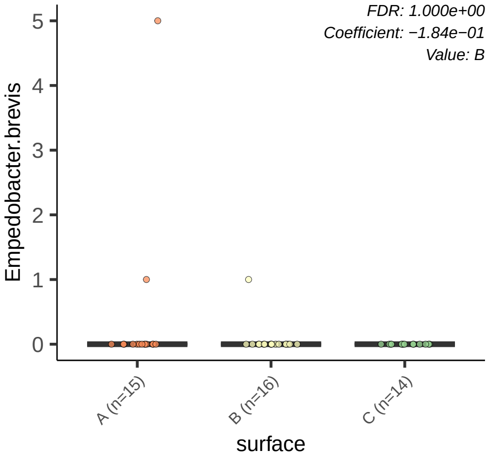

FDR: 1.000e+00
Coefficient: −1.84e−01
Value: B
Empedobacter.brevis
5
4
3
2
1
0
A (n=15)
B (n=16)
C (n=14)
surface

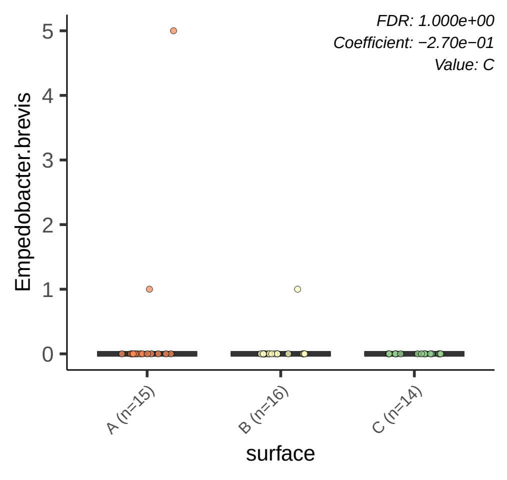

FDR: 1.000e+00
Coefficient: −2.70e−01
Value: C
Empedobacter.brevis
0
1
2
3
4
5
A (n=15)
B (n=16)
C (n=14)
surface

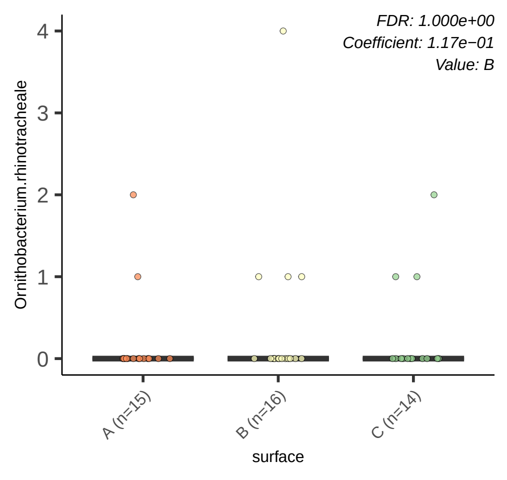
FDR: 1.000e+00
Coefficient: 1.17e−01
Value: B
Ornithobacterium.rhinotracheale
4
3
2
1
0
A (n=15)
B (n=16)
C (n=14)
surface

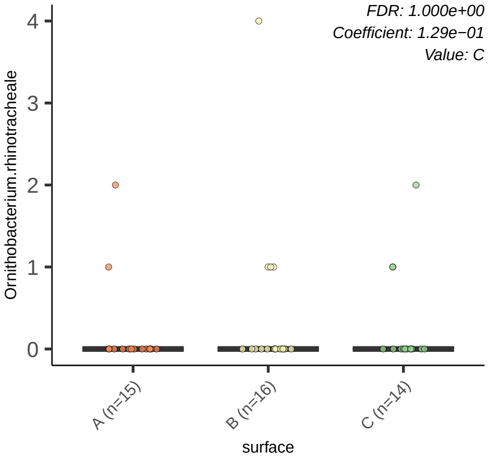

FDR: 1.000e+00
Coefficient: 1.29e−01
Value: C
Ornithobacterium.rhinotracheale
0
1
2
3
4
A (n=15)
B (n=16)
C (n=14)
surface

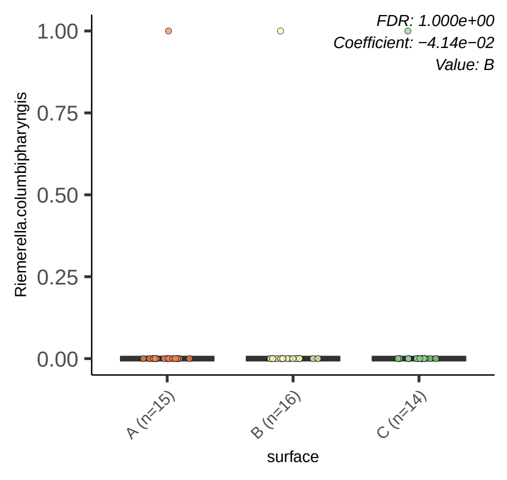
FDR: 1.000e+00
Coefficient: −4.14e−02
Value: B
Riemerella.columbipharyngis
1.00
0.75
0.50
0.25
0.00
A (n=15)
B (n=16)
C (n=14)
surface

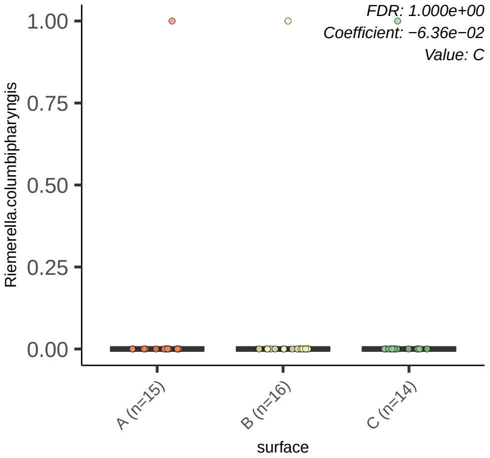
FDR: 1.000e+00
Coefficient: −6.36e−02
Value: C
Riemerella.columbipharyngis
1.00
0.75
0.50
0.25
0.00
A (n=15)
B (n=16)
C (n=14)
surface

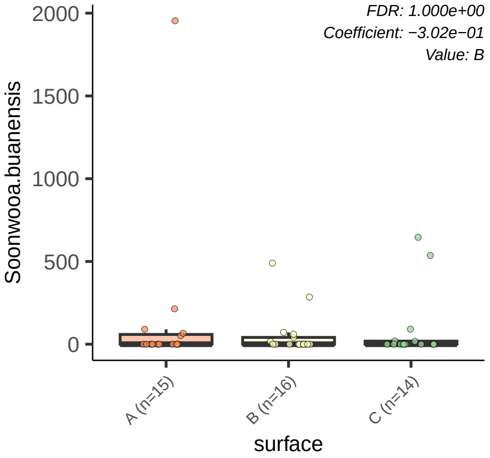
FDR: 1.000e+00
Coefficient: −3.02e−01
Value: B
Soonwooa.buanensis
2000
1500
1000
500
0
A (n=15)
B (n=16)
C (n=14)
surface

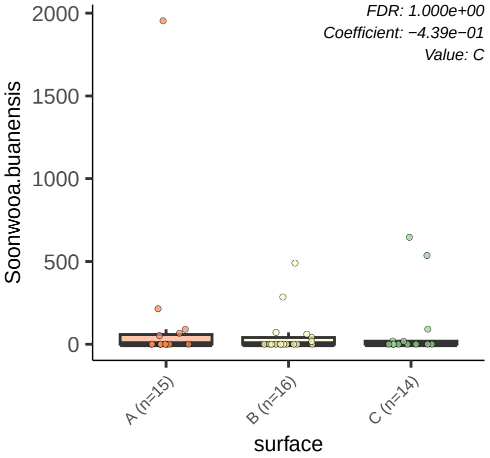

FDR: 1.000e+00
Coefficient: −4.39e−01
Value: C
Soonwooa.buanensis
2000
1500
1000
500
0
A (n=15)
B (n=16)
C (n=14)
surface

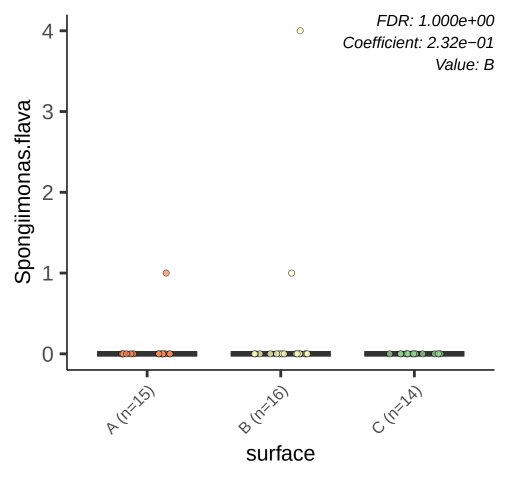

FDR: 1.000e+00
Coefficient: 2.32e−01
Value: B
Spongiimonas.flava
0
1
2
3
4
A (n=15)
B (n=16)
C (n=14)
surface

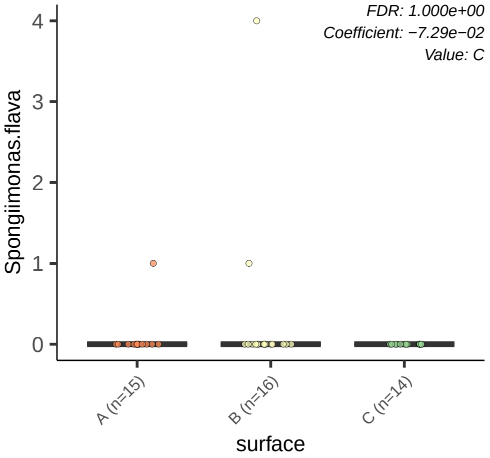

FDR: 1.000e+00
Coefficient: −7.29e−02
Value: C
Spongiimonas.flava
4
3
2
1
0
A (n=15)
B (n=16)
C (n=14)
surface

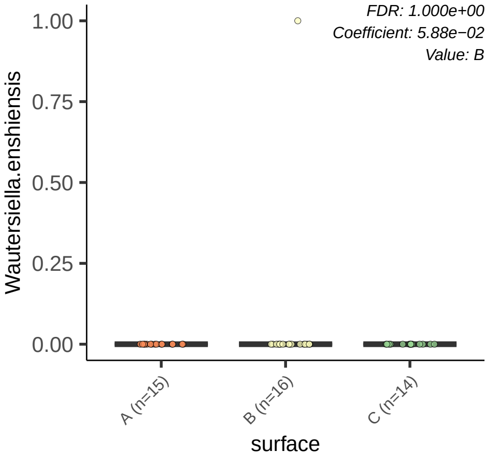

FDR: 1.000e+00
Coefficient: 5.88e−02
Value: B
Wautersiella.enshiensis
1.00
0.75
0.50
0.25
0.00
A (n=15)
B (n=16)
C (n=14)
surface

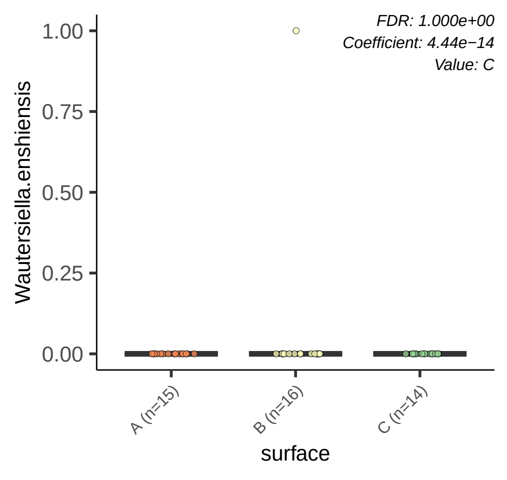

FDR: 1.000e+00
Coefficient: 4.44e−14
Value: C
Wautersiella.enshiensis
1.00
0.75
0.50
0.25
0.00
A (n=15)
B (n=16)
C (n=14)
surface

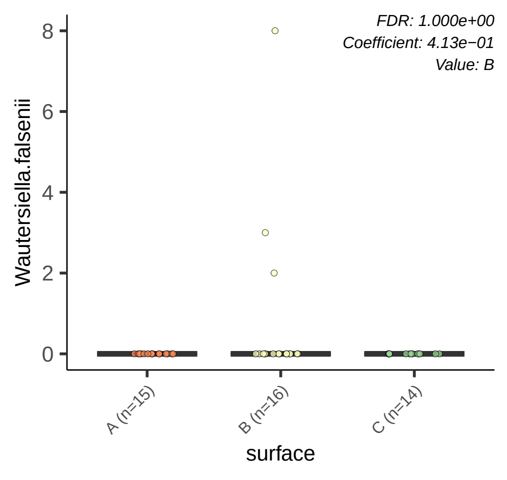

FDR: 1.000e+00
Coefficient: 4.13e−01
Value: B
Wautersiella.falsenii
0
2
4
6
8
A (n=15)
B (n=16)
C (n=14)
surface

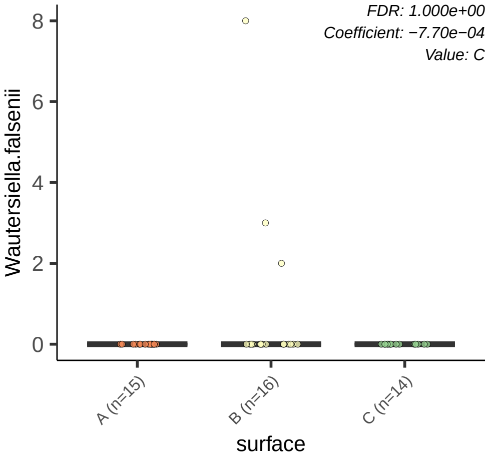
FDR: 1.000e+00
Coefficient: −7.70e−04
Value: C
Wautersiella.falsenii
8
6
4
2
0
A (n=15)
B (n=16)
C (n=14)
surface

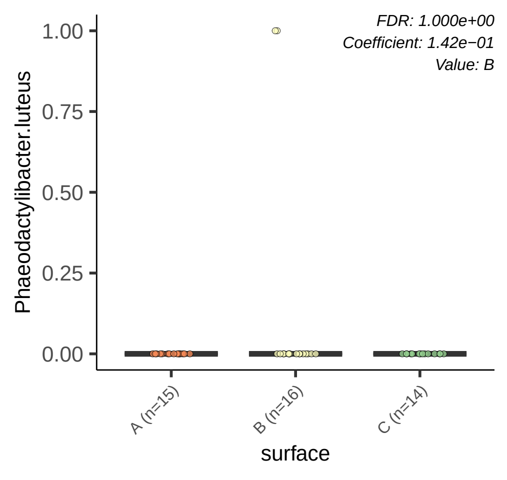

FDR: 1.000e+00
Coefficient: 1.42e−01
Value: B
Phaeodactylibacter.luteus
1.00
0.75
0.50
0.25
0.00
A (n=15)
B (n=16)
C (n=14)
surface

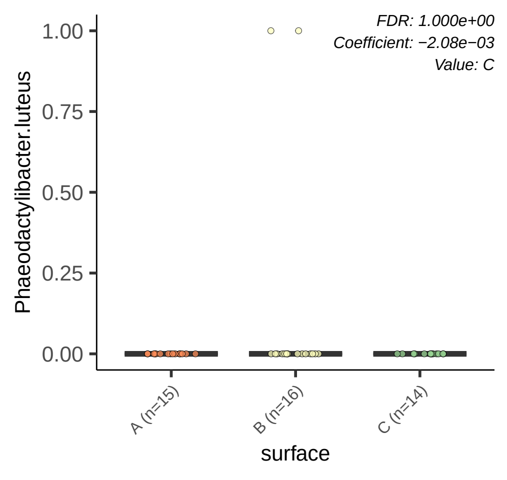

FDR: 1.000e+00
Coefficient: −2.08e−03
Value: C
Phaeodactylibacter.luteus
1.00
0.75
0.50
0.25
0.00
A (n=15)
B (n=16)
C (n=14)
surface

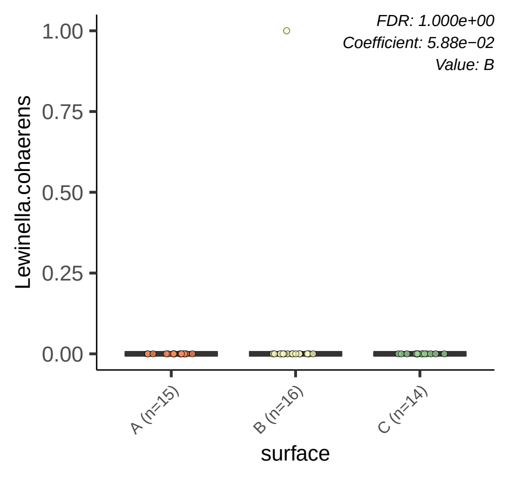
FDR: 1.000e+00
Coefficient: 5.88e−02
Value: B
Lewinella.cohaerens
1.00
0.75
0.50
0.25
0.00
A (n=15)
B (n=16)
C (n=14)
surface

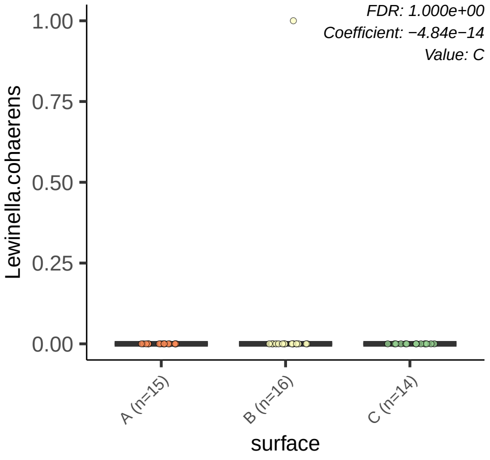
FDR: 1.000e+00
Coefficient: −4.84e−14
Value: C
Lewinella.cohaerens
1.00
0.75
0.50
0.25
0.00
A (n=15)
B (n=16)
C (n=14)
surface

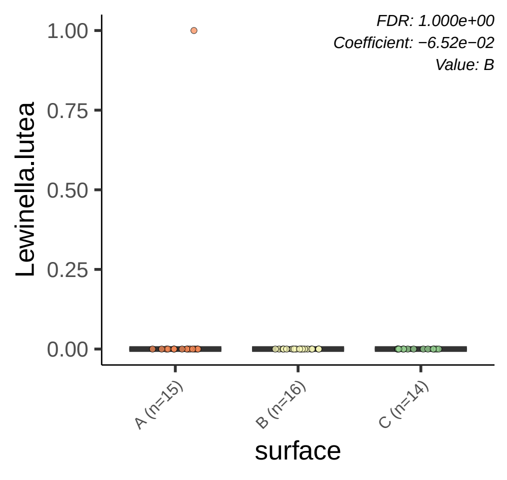

FDR: 1.000e+00
Coefficient: −6.52e−02
Value: B
Lewinella.lutea
1.00
0.75
0.50
0.25
0.00
A (n=15)
B (n=16)
C (n=14)
surface

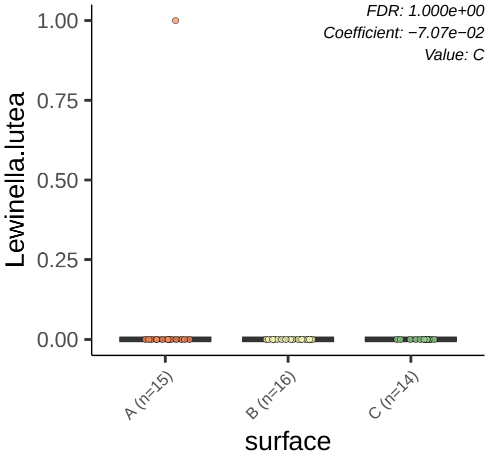
FDR: 1.000e+00
Coefficient: −7.07e−02
Value: C
Lewinella.lutea
1.00
0.75
0.50
0.25
0.00
A (n=15)
B (n=16)
C (n=14)
surface

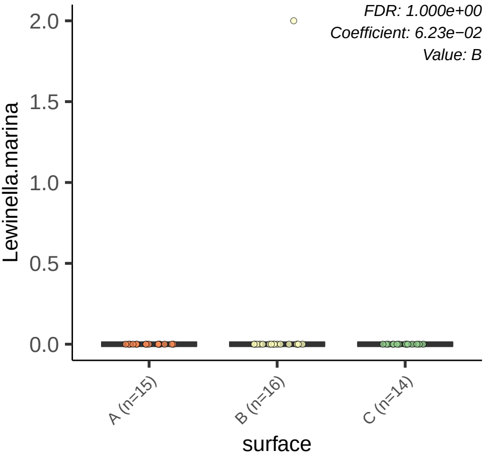

FDR: 1.000e+00
Coefficient: 6.23e−02
Value: B
Lewinella.marina
2.0
1.5
1.0
0.5
0.0
A (n=15)
B (n=16)
C (n=14)
surface

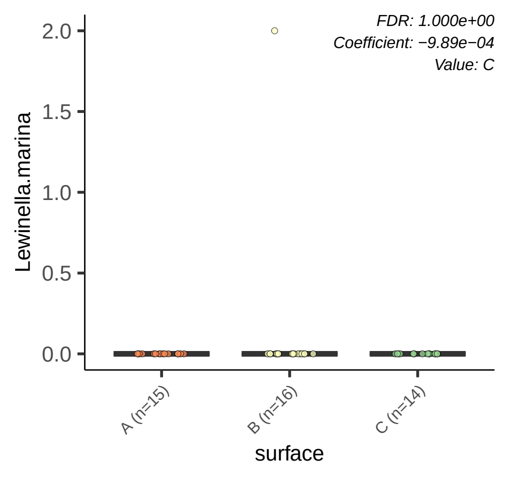
FDR: 1.000e+00
Coefficient: −9.89e−04
Value: C
Lewinella.marina
2.0
1.5
1.0
0.5
0.0
A (n=15)
B (n=16)
C (n=14)
surface

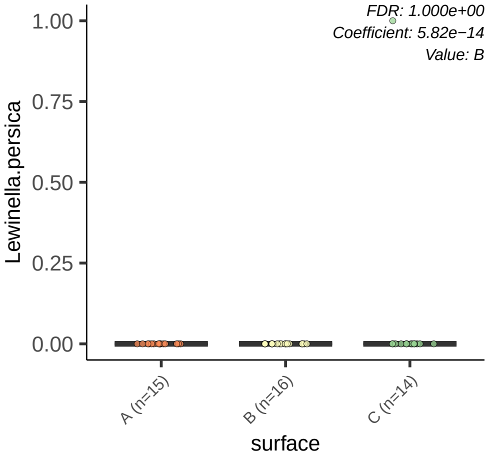

FDR: 1.000e+00
Coefficient: 5.82e−14
Value: B
Lewinella.persica
1.00
0.75
0.50
0.25
0.00
A (n=15)
B (n=16)
C (n=14)
surface

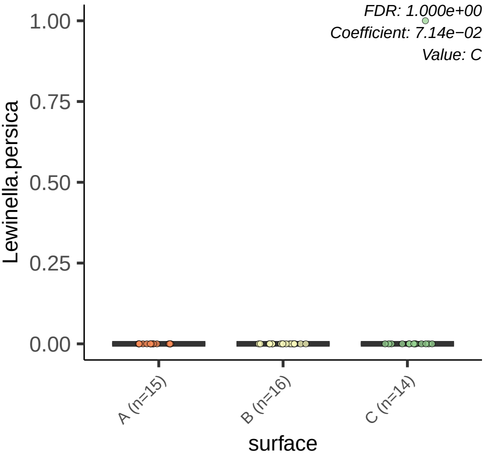
FDR: 1.000e+00
Coefficient: 7.14e−02
Value: C
Lewinella.persica
1.00
0.75
0.50
0.25
0.00
A (n=15)
B (n=16)
C (n=14)
surface

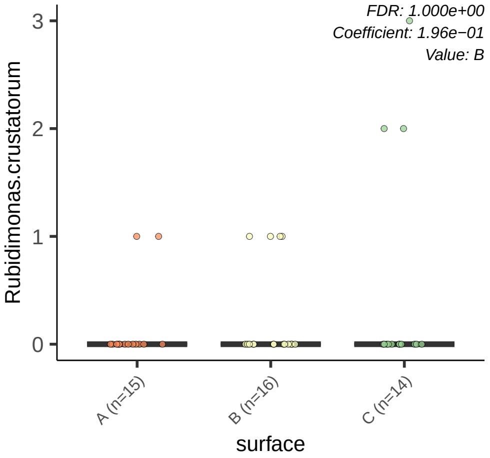

FDR: 1.000e+00
Coefficient: 1.96e−01
Value: B
Rubidimonas.crustatorum
3
2
1
0
A (n=15)
B (n=16)
C (n=14)
surface

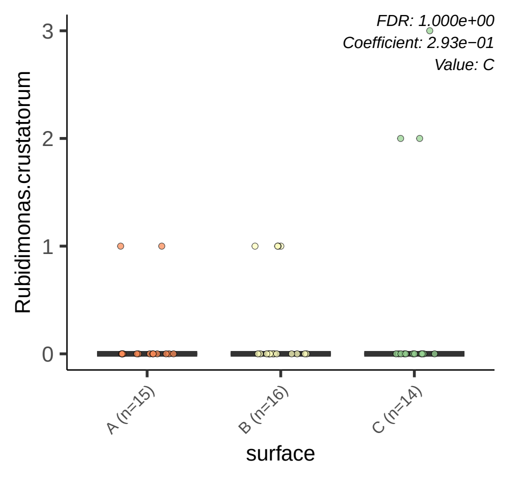
FDR: 1.000e+00
Coefficient: 2.93e−01
Value: C
Rubidimonas.crustatorum
0
1
2
3
A (n=15)
B (n=16)
C (n=14)
surface

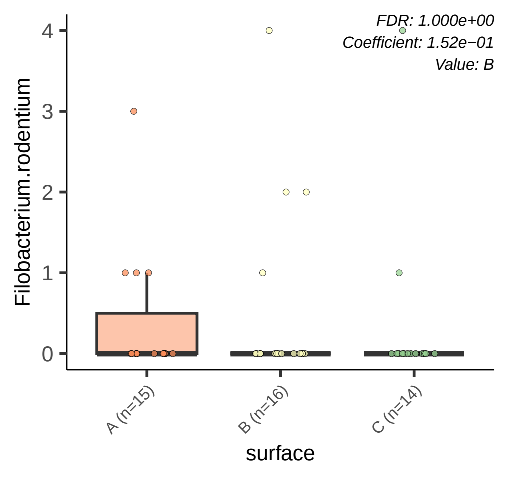
FDR: 1.000e+00
Coefficient: 1.52e−01
Value: B
Filobacterium.rodentium
0
1
2
3
4
A (n=15)
B (n=16)
C (n=14)
surface

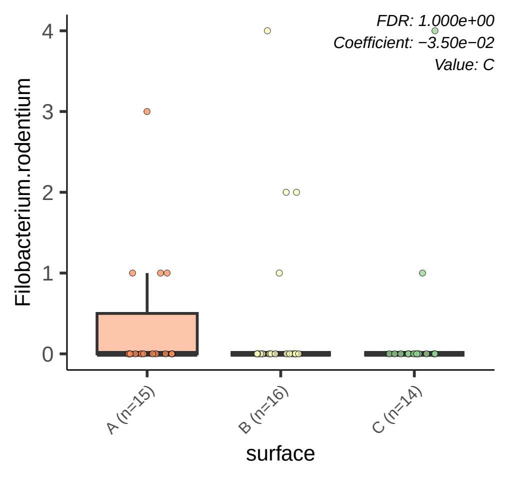
FDR: 1.000e+00
Coefficient: −3.50e−02
Value: C
Filobacterium.rodentium
0
1
2
3
4
A (n=15)
B (n=16)
C (n=14)
surface

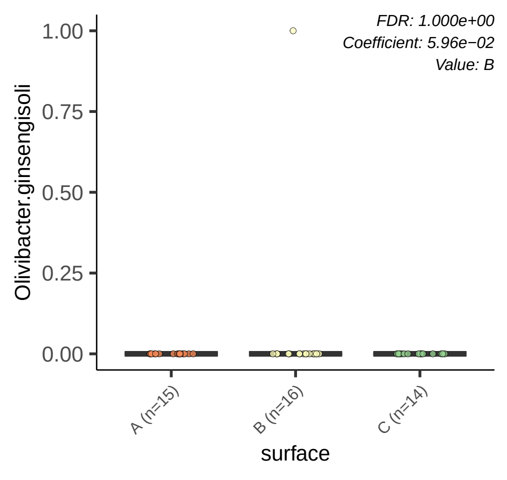
FDR: 1.000e+00
Coefficient: 5.96e−02
Value: B
Olivibacter.ginsengisoli
1.00
0.75
0.50
0.25
0.00
A (n=15)
B (n=16)
C (n=14)
surface

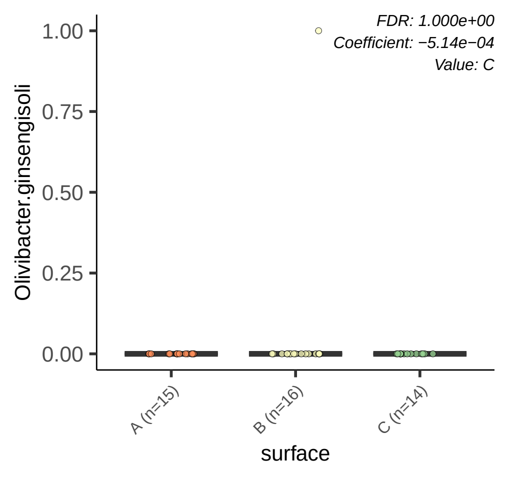

FDR: 1.000e+00
Coefficient: −5.14e−04
Value: C
Olivibacter.ginsengisoli
1.00
0.75
0.50
0.25
0.00
A (n=15)
B (n=16)
C (n=14)
surface

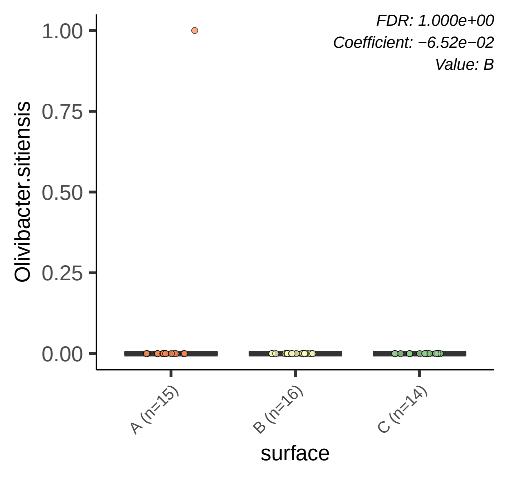

FDR: 1.000e+00
Coefficient: −6.52e−02
Value: B
Olivibacter.sitiensis
1.00
0.75
0.50
0.25
0.00
A (n=15)
B (n=16)
C (n=14)
surface

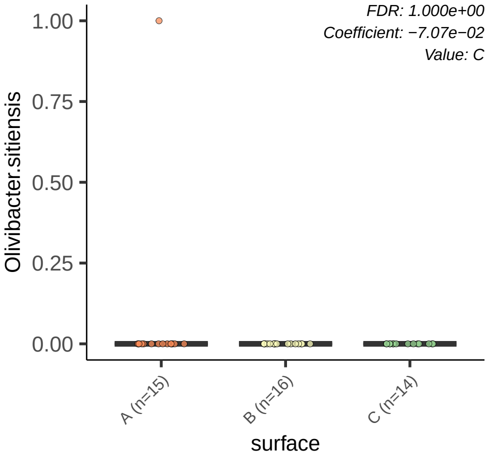

FDR: 1.000e+00
Coefficient: −7.07e−02
Value: C
Olivibacter.sitiensis
1.00
0.75
0.50
0.25
0.00
A (n=15)
B (n=16)
C (n=14)
surface

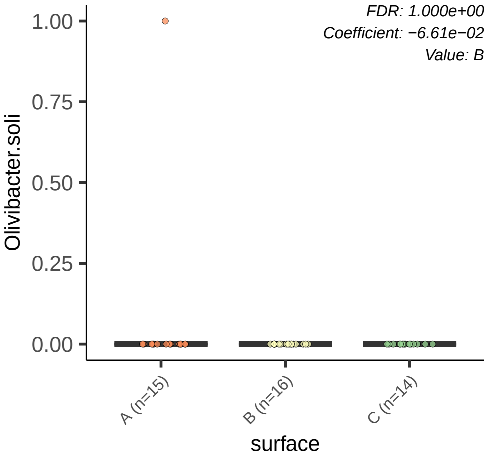

FDR: 1.000e+00
Coefficient: −6.61e−02
Value: B
1.00
0.75
0.50
0.25
0.00
Olivibacter.soli
A (n=15)
B (n=16)
C (n=14)
surface

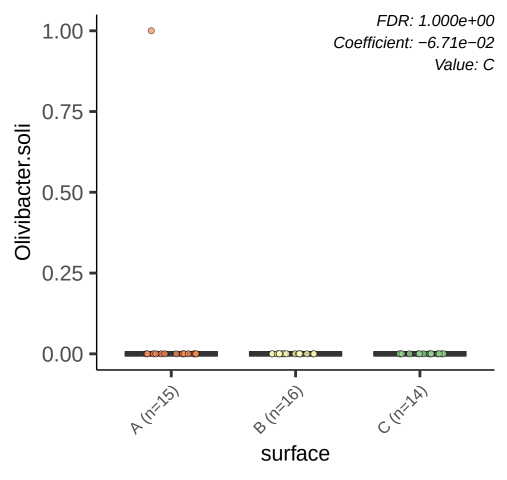

FDR: 1.000e+00
Coefficient: −6.71e−02
Value: C
Olivibacter.soli
1.00
0.75
0.50
0.25
0.00
A (n=15)
B (n=16)
C (n=14)
surface

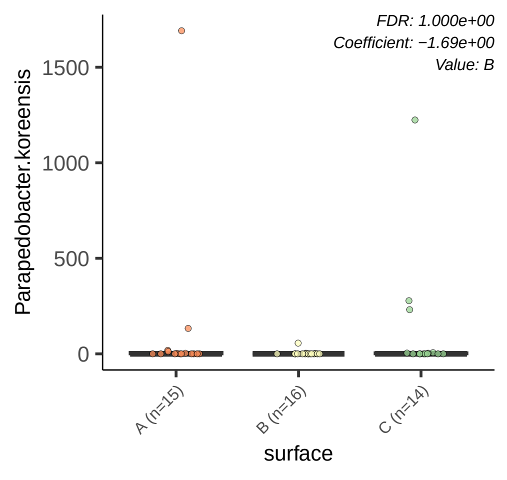

FDR: 1.000e+00
Coefficient: −1.69e+00
Value: B
Parapedobacter.koreensis
1500
1000
500
0
A (n=15)
B (n=16)
C (n=14)
surface

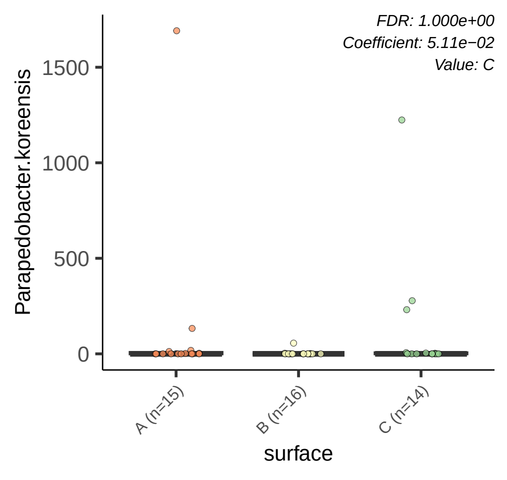
FDR: 1.000e+00
Coefficient: 5.11e−02
Value: C
Parapedobacter.koreensis
1500
1000
500
0
A (n=15)
B (n=16)
C (n=14)
surface

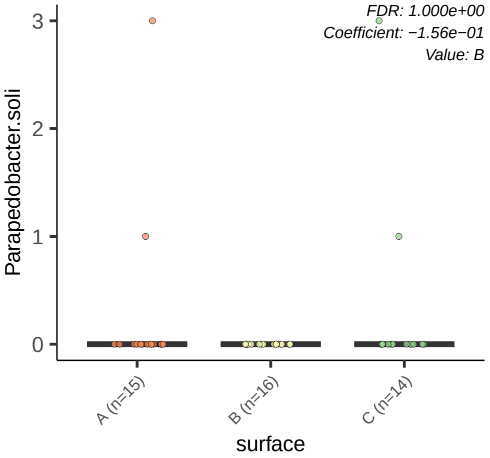

FDR: 1.000e+00
Coefficient: −1.56e−01
Value: B
Parapedobacter.soli
3
2
1
0
A (n=15)
B (n=16)
C (n=14)
surface

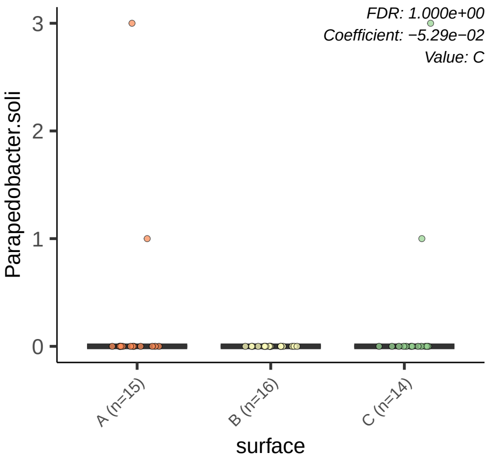
FDR: 1.000e+00
Coefficient: −5.29e−02
Value: C
Parapedobacter.soli
3
2
1
0
A (n=15)
B (n=16)
C (n=14)
surface

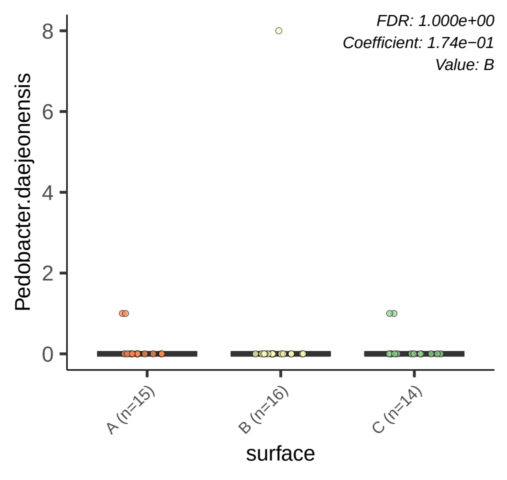

FDR: 1.000e+00
Coefficient: 1.74e−01
Value: B
Pedobacter.daejeonensis
0
2
4
6
8
A (n=15)
B (n=16)
C (n=14)
surface

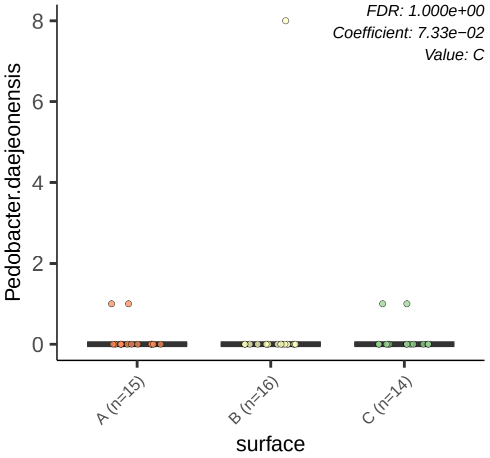
FDR: 1.000e+00
Coefficient: 7.33e−02
Value: C
Pedobacter.daejeonensis
0
2
4
6
8
A (n=15)
B (n=16)
C (n=14)
surface

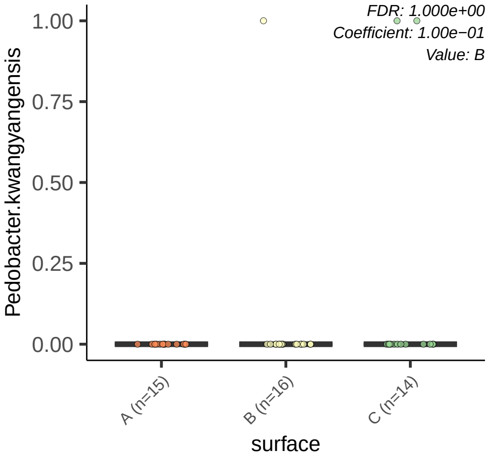

FDR: 1.000e+00
Coefficient: 1.00e−01
Value: B
Pedobacter.kwangyangensis
1.00
0.75
0.50
0.25
0.00
A (n=15)
B (n=16)
C (n=14)
surface

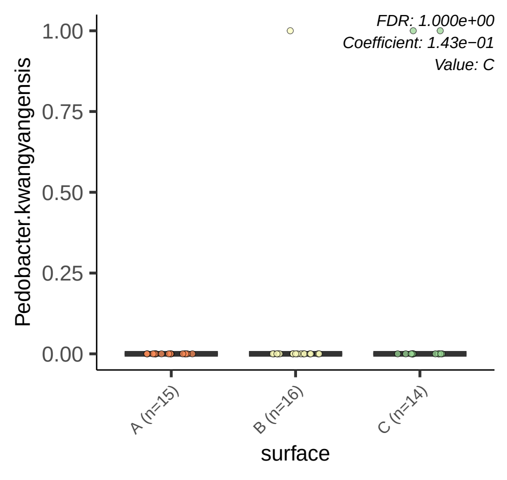

FDR: 1.000e+00
Coefficient: 1.43e−01
Value: C
Pedobacter.kwangyangensis
1.00
0.75
0.50
0.25
0.00
A (n=15)
B (n=16)
C (n=14)
surface

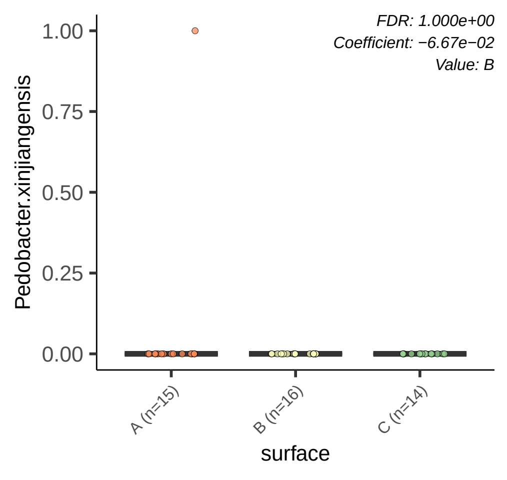

FDR: 1.000e+00
Coefficient: −6.67e−02
Value: B
Pedobacter.xinjiangensis
1.00
0.75
0.50
0.25
0.00
A (n=15)
B (n=16)
C (n=14)
surface

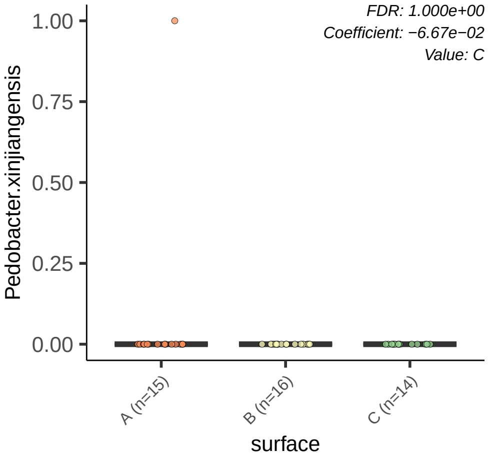
FDR: 1.000e+00
Coefficient: −6.67e−02
Value: C
Pedobacter.xinjiangensis
1.00
0.75
0.50
0.25
0.00
A (n=15)
B (n=16)
C (n=14)
surface

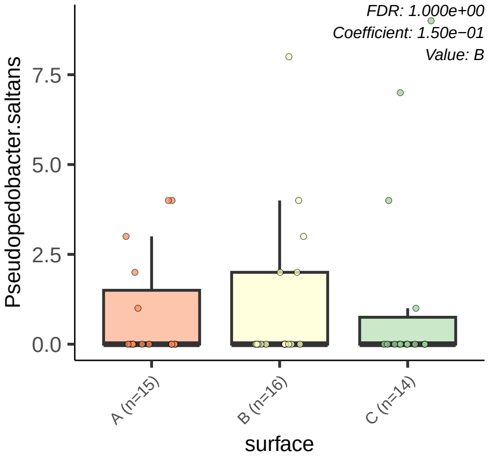

FDR: 1.000e+00
Coefficient: 1.50e−01
Value: B
Pseudopedobacter.saltans
7.5
5.0
2.5
0.0
A (n=15)
B (n=16)
C (n=14)
surface

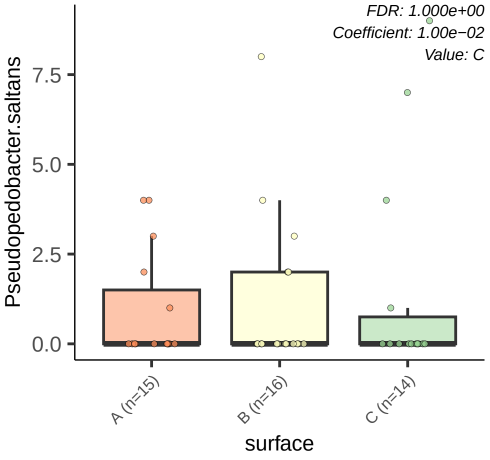
FDR: 1.000e+00
Coefficient: 1.00e−02
Value: C
Pseudopedobacter.saltans
7.5
5.0
2.5
0.0
A (n=15)
B (n=16)
C (n=14)
surface

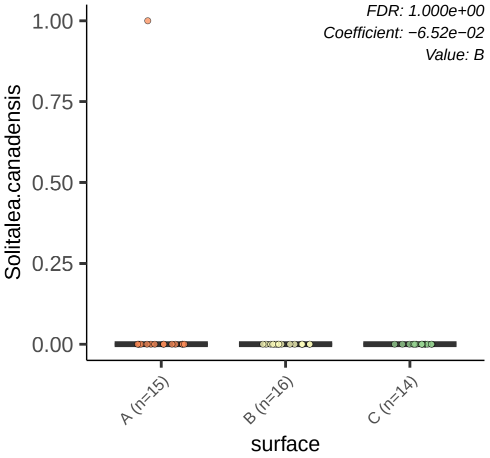
FDR: 1.000e+00
Coefficient: −6.52e−02
Value: B
Solitalea.canadensis
1.00
0.75
0.50
0.25
0.00
A (n=15)
B (n=16)
C (n=14)
surface

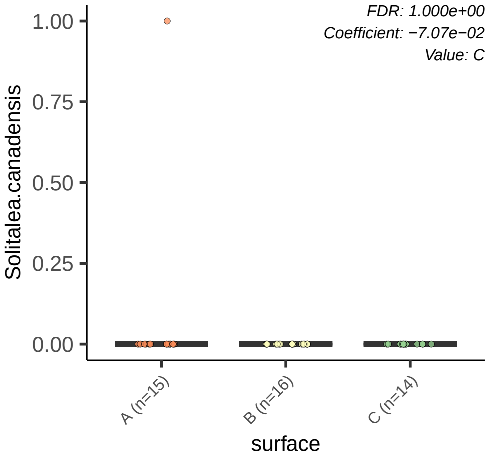
FDR: 1.000e+00
Coefficient: −7.07e−02
Value: C
Solitalea.canadensis
1.00
0.75
0.50
0.25
0.00
A (n=15)
B (n=16)
C (n=14)
surface

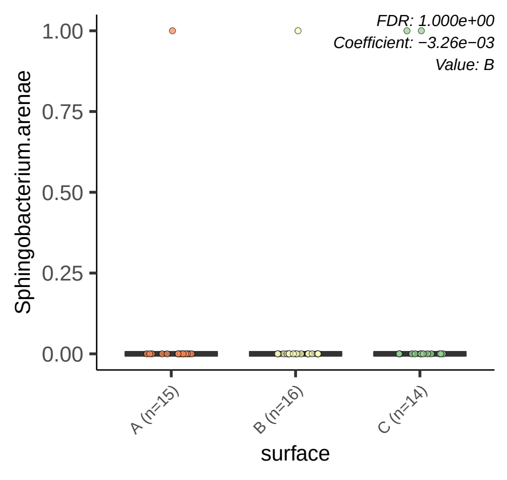
FDR: 1.000e+00
Coefficient: −3.26e−03
Value: B
Sphingobacterium.arenae
1.00
0.75
0.50
0.25
0.00
A (n=15)
B (n=16)
C (n=14)
surface

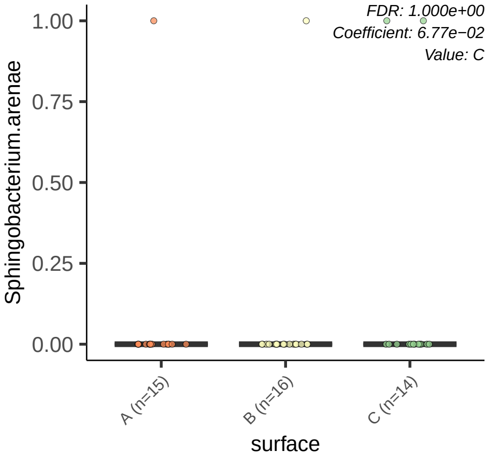

FDR: 1.000e+00
Coefficient: 6.77e−02
Value: C
Sphingobacterium.arenae
1.00
0.75
0.50
0.25
0.00
A (n=15)
B (n=16)
C (n=14)
surface

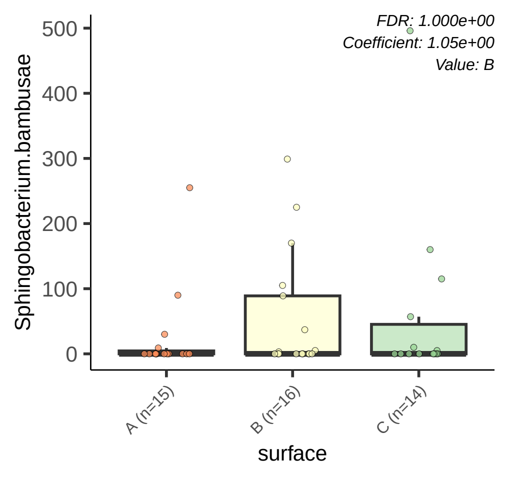

FDR: 1.000e+00
Coefficient: 1.05e+00
Value: B
Sphingobacterium.bambusae
0
100
200
300
400
500
A (n=15)
B (n=16)
C (n=14)
surface

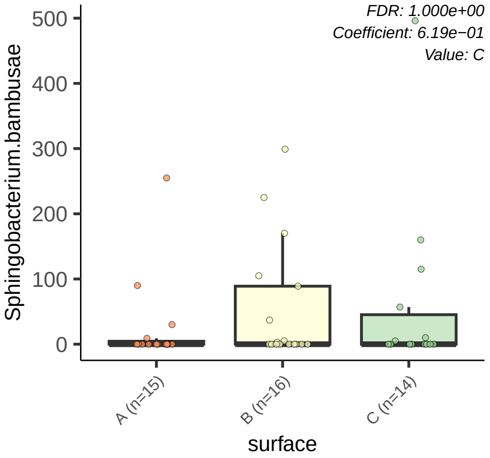
FDR: 1.000e+00
Coefficient: 6.19e−01
Value: C
500
400
300
200
100
0
Sphingobacterium.bambusae
A (n=15)
B (n=16)
C (n=14)
surface

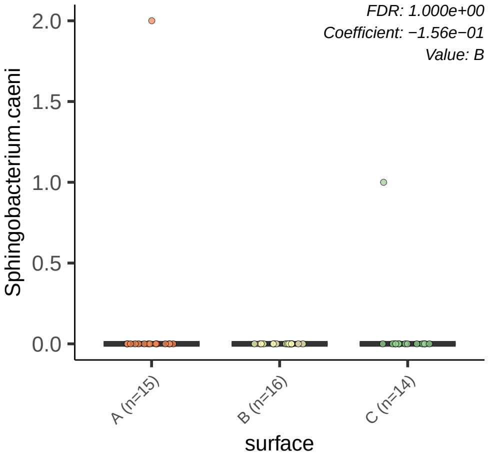
FDR: 1.000e+00
Coefficient: −1.56e−01
Value: B
Sphingobacterium.caeni
2.0
1.5
1.0
0.5
0.0
A (n=15)
B (n=16)
C (n=14)
surface

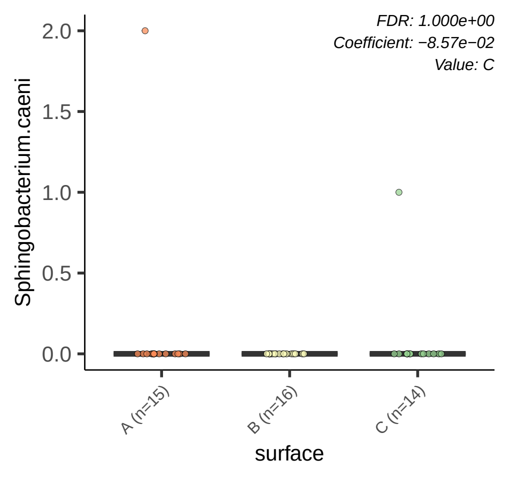

FDR: 1.000e+00
Coefficient: −8.57e−02
Value: C
Sphingobacterium.caeni
2.0
1.5
1.0
0.5
0.0
A (n=15)
B (n=16)
C (n=14)
surface

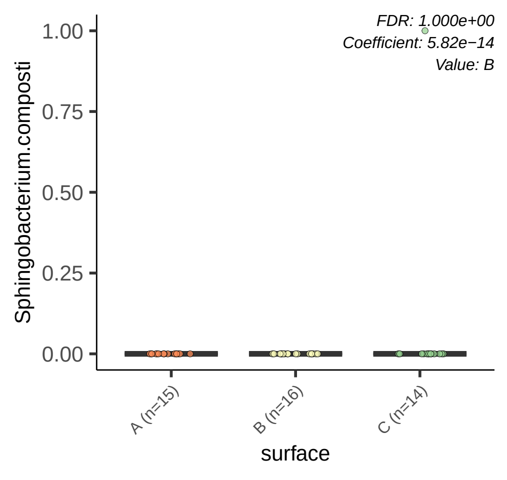

FDR: 1.000e+00
Coefficient: 5.82e−14
Value: B
Sphingobacterium.composti
1.00
0.75
0.50
0.25
0.00
A (n=15)
B (n=16)
C (n=14)
surface

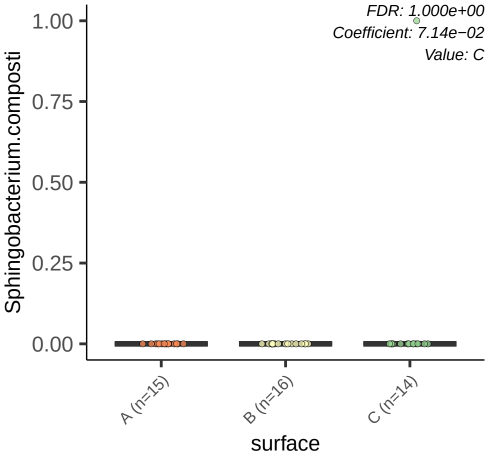

FDR: 1.000e+00
Coefficient: 7.14e−02
Value: C
Sphingobacterium.composti
1.00
0.75
0.50
0.25
0.00
A (n=15)
B (n=16)
C (n=14)
surface

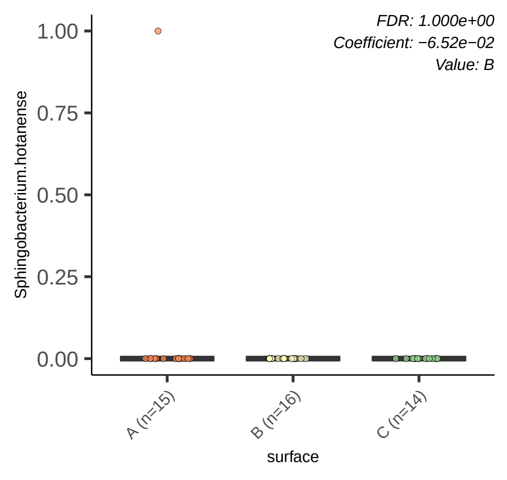
FDR: 1.000e+00
Coefficient: −6.52e−02
Value: B
1.00
0.75
0.50
0.25
0.00
Sphingobacterium.hotanense
A (n=15)
B (n=16)
C (n=14)
surface

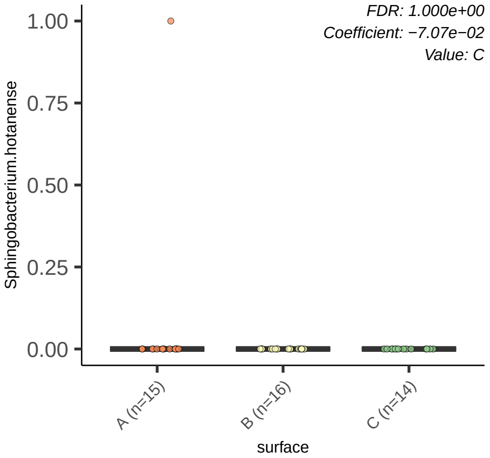
FDR: 1.000e+00
Coefficient: −7.07e−02
Value: C
Sphingobacterium.hotanense
1.00
0.75
0.50
0.25
0.00
A (n=15)
B (n=16)
C (n=14)
surface

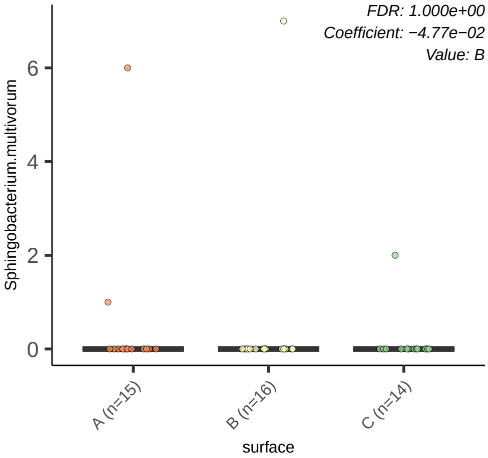

FDR: 1.000e+00
Coefficient: −4.77e−02
Value: B
Sphingobacterium.multivorum
0
2
4
6
A (n=15)
B (n=16)
C (n=14)
surface

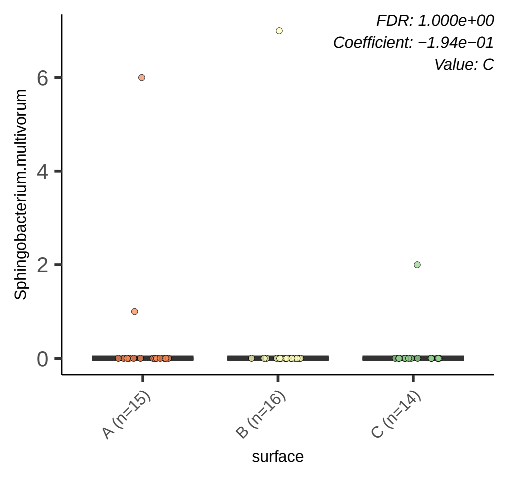

FDR: 1.000e+00
Coefficient: −1.94e−01
Value: C
Sphingobacterium.multivorum
0
2
4
6
A (n=15)
B (n=16)
C (n=14)
surface

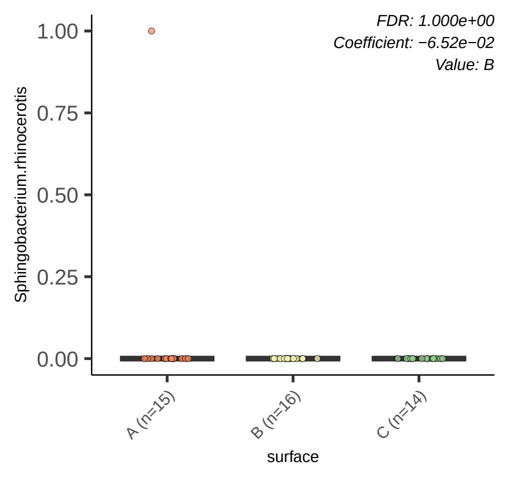

FDR: 1.000e+00
Coefficient: −6.52e−02
Value: B
Sphingobacterium.rhinocerotis
1.00
0.75
0.50
0.25
0.00
A (n=15)
B (n=16)
C (n=14)
surface

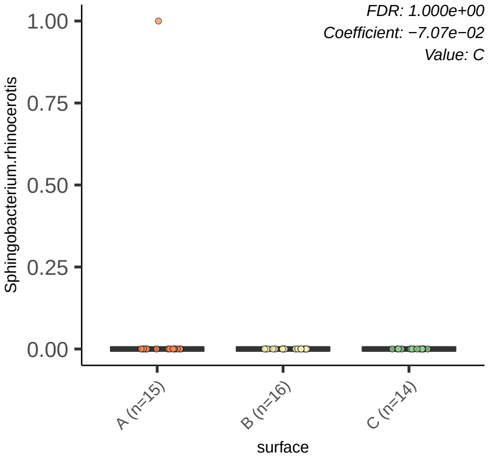
FDR: 1.000e+00
Coefficient: −7.07e−02
Value: C
Sphingobacterium.rhinocerotis
1.00
0.75
0.50
0.25
0.00
A (n=15)
B (n=16)
C (n=14)
surface

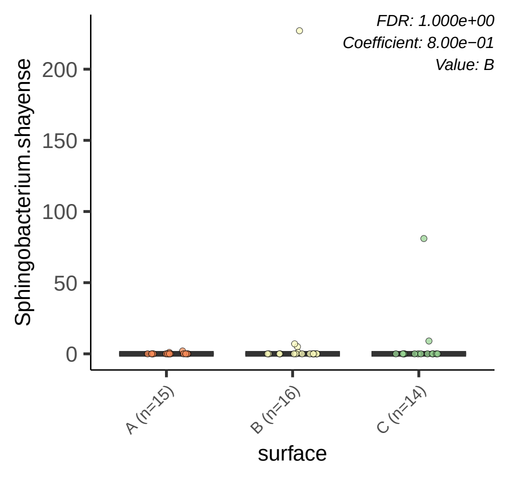
FDR: 1.000e+00
Coefficient: 8.00e−01
Value: B
Sphingobacterium.shayense
200
150
100
50
0
A (n=15)
B (n=16)
C (n=14)
surface

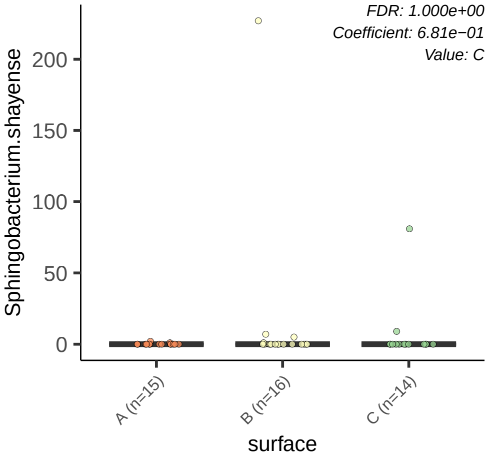

FDR: 1.000e+00
Coefficient: 6.81e−01
Value: C
Sphingobacterium.shayense
200
150
100
50
0
A (n=15)
B (n=16)
C (n=14)
surface

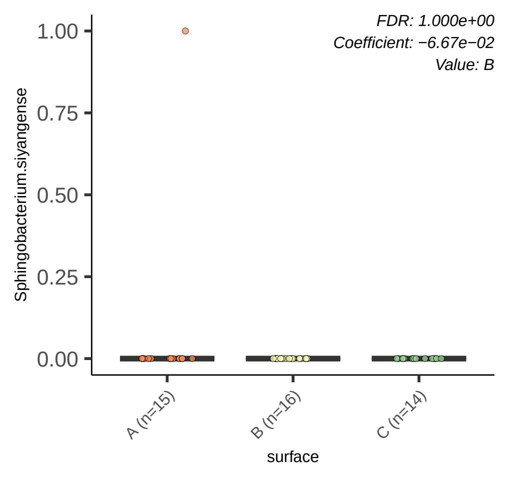
FDR: 1.000e+00
Coefficient: −6.67e−02
Value: B
Sphingobacterium.siyangense
1.00
0.75
0.50
0.25
0.00
A (n=15)
B (n=16)
C (n=14)
surface

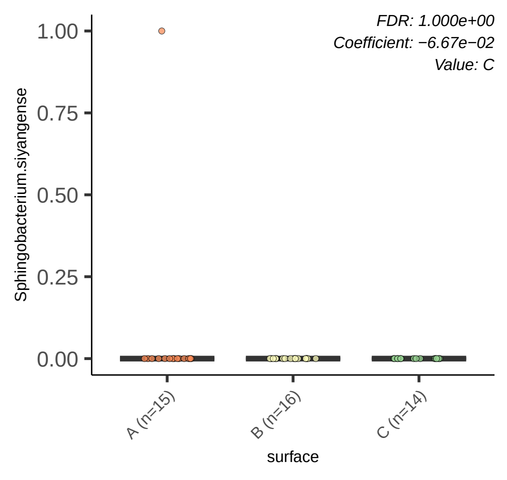
FDR: 1.000e+00
Coefficient: −6.67e−02
Value: C
1.00
0.75
0.50
0.25
0.00
Sphingobacterium.siyangense
A (n=15)
B (n=16)
C (n=14)
surface

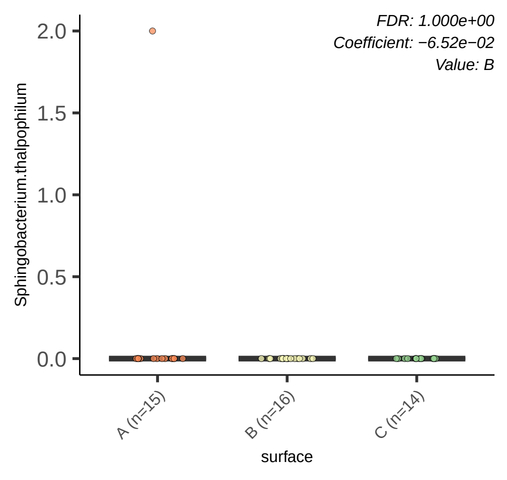
FDR: 1.000e+00
Coefficient: −6.52e−02
Value: B
Sphingobacterium.thalpophilum
2.0
1.5
1.0
0.5
0.0
A (n=15)
B (n=16)
C (n=14)
surface

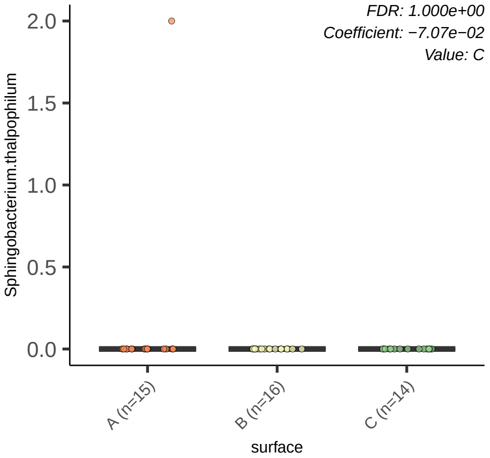

FDR: 1.000e+00
Coefficient: −7.07e−02
Value: C
Sphingobacterium.thalpophilum
2.0
1.5
1.0
0.5
0.0
A (n=15)
B (n=16)
C (n=14)
surface

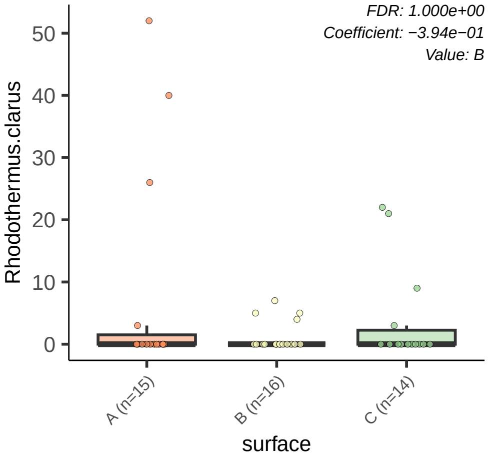

FDR: 1.000e+00
Coefficient: −3.94e−01
Value: B
Rhodothermus.clarus
0
10
20
30
40
50
A (n=15)
B (n=16)
C (n=14)
surface

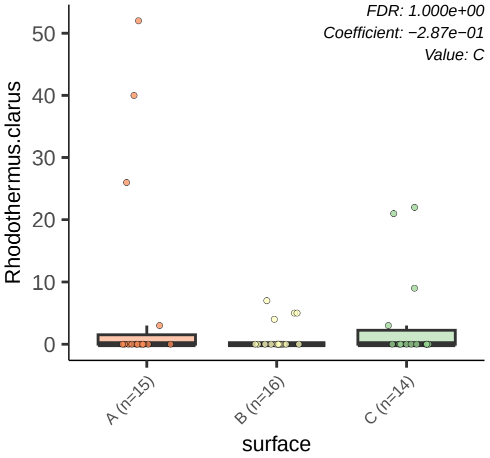
FDR: 1.000e+00
Coefficient: −2.87e−01
Value: C
Rhodothermus.clarus
0
10
20
30
40
50
A (n=15)
B (n=16)
C (n=14)
surface

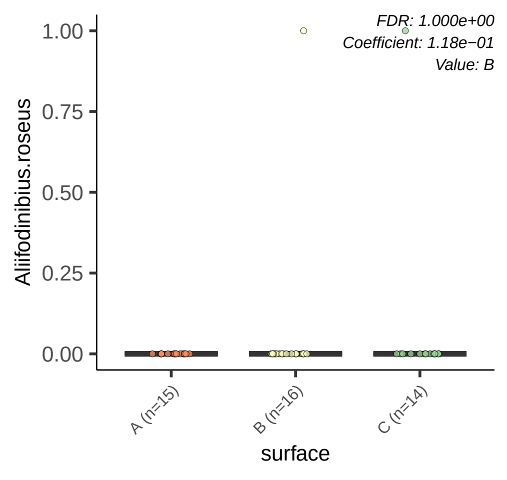

FDR: 1.000e+00
Coefficient: 1.18e−01
Value: B
Aliifodinibius.roseus
1.00
0.75
0.50
0.25
0.00
A (n=15)
B (n=16)
C (n=14)
surface

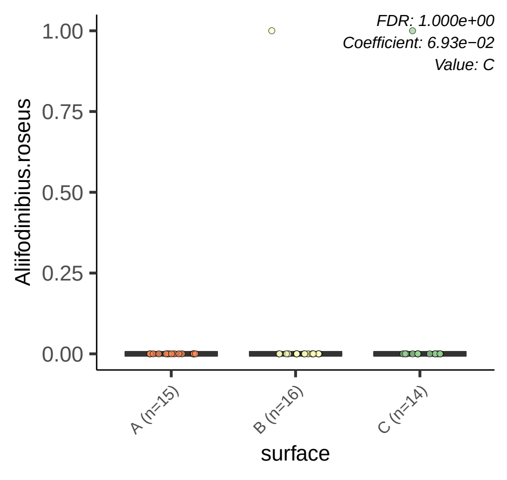

FDR: 1.000e+00
Coefficient: 6.93e−02
Value: C
1.00
0.75
0.50
0.25
0.00
Aliifodinibius.roseus
A (n=15)
B (n=16)
C (n=14)
surface

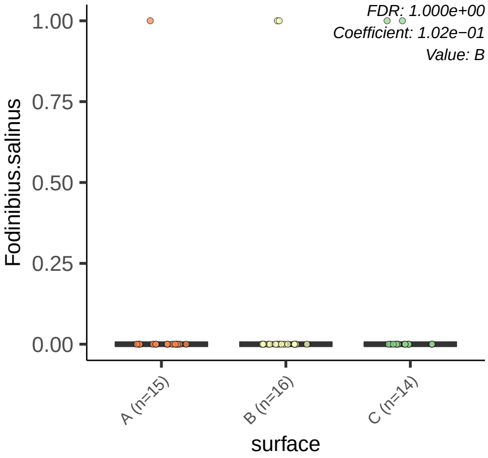

FDR: 1.000e+00
Coefficient: 1.02e−01
Value: B
Fodinibius.salinus
1.00
0.75
0.50
0.25
0.00
A (n=15)
B (n=16)
C (n=14)
surface

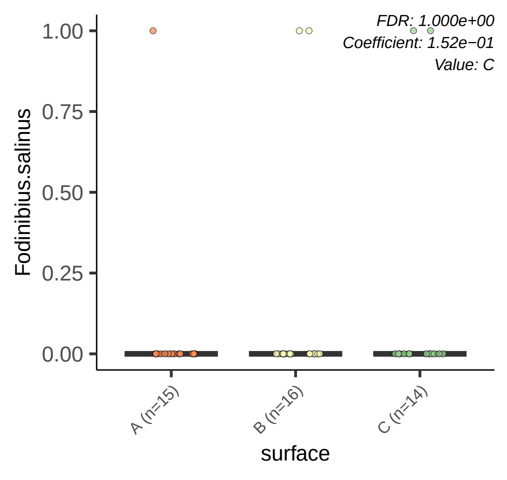
FDR: 1.000e+00
Coefficient: 1.52e−01
Value: C
Fodinibius.salinus
1.00
0.75
0.50
0.25
0.00
A (n=15)
B (n=16)
C (n=14)
surface

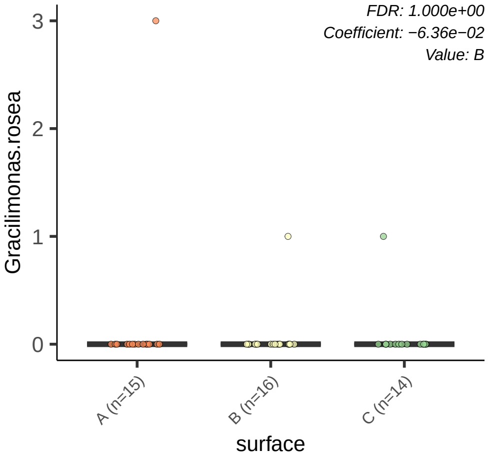
FDR: 1.000e+00
Coefficient: −6.36e−02
Value: B
Gracilimonas.rosea
3
2
1
0
A (n=15)
B (n=16)
C (n=14)
surface

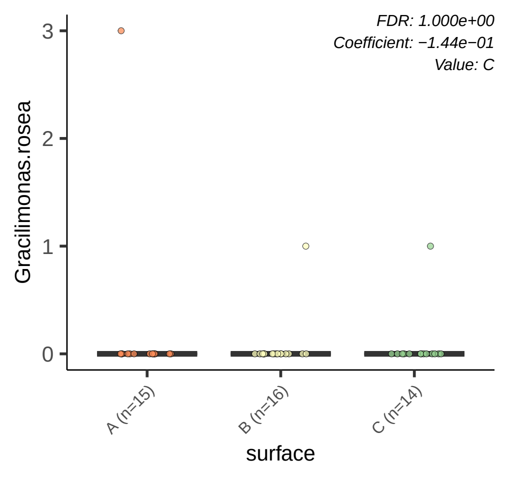

FDR: 1.000e+00
Coefficient: −1.44e−01
Value: C
Gracilimonas.rosea
3
2
1
0
A (n=15)
B (n=16)
C (n=14)
surface

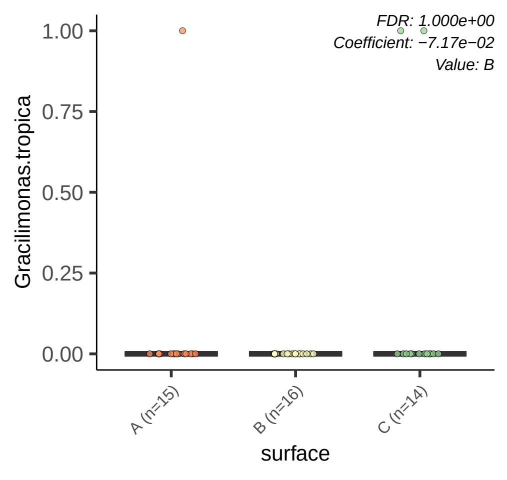
FDR: 1.000e+00
Coefficient: −7.17e−02
Value: B
Gracilimonas.tropica
1.00
0.75
0.50
0.25
0.00
A (n=15)
B (n=16)
C (n=14)
surface

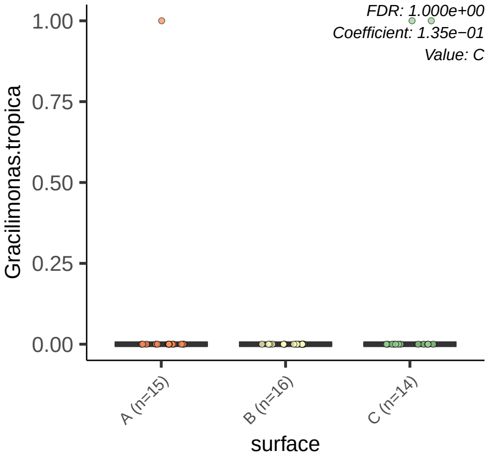
FDR: 1.000e+00
Coefficient: 1.35e−01
Value: C
Gracilimonas.tropica
1.00
0.75
0.50
0.25
0.00
A (n=15)
B (n=16)
C (n=14)
surface

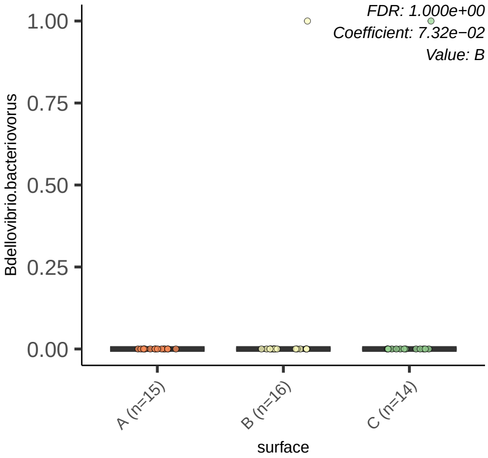

FDR: 1.000e+00
Coefficient: 7.32e−02
Value: B
1.00
0.75
0.50
0.25
0.00
Bdellovibrio.bacteriovorus
A (n=15)
B (n=16)
C (n=14)
surface

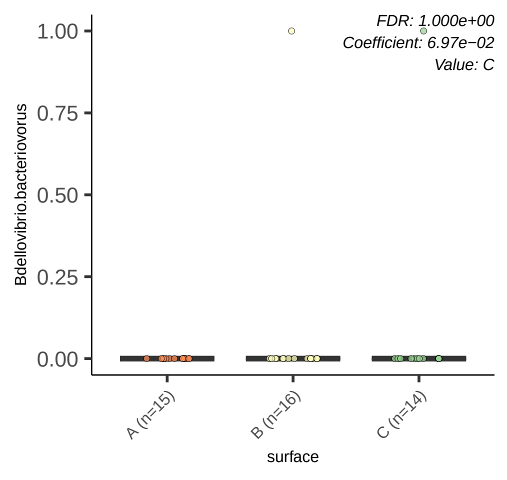
FDR: 1.000e+00
Coefficient: 6.97e−02
Value: C
1.00
0.75
0.50
0.25
0.00
Bdellovibrio.bacteriovorus
A (n=15)
B (n=16)
C (n=14)
surface

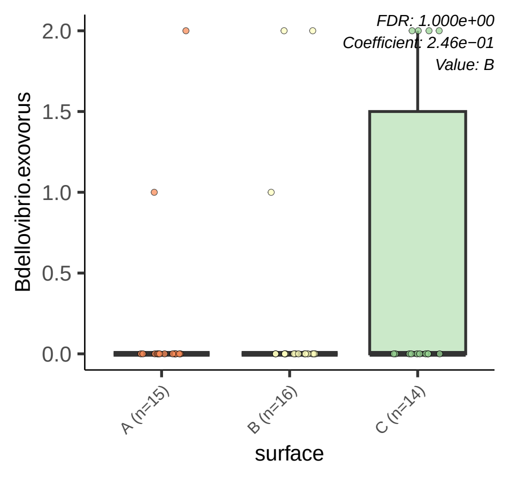
FDR: 1.000e+00
Coefficient: 2.46e−01
Value: B
Bdellovibrio.exovorus
2.0
1.5
1.0
0.5
0.0
A (n=15)
B (n=16)
C (n=14)
surface

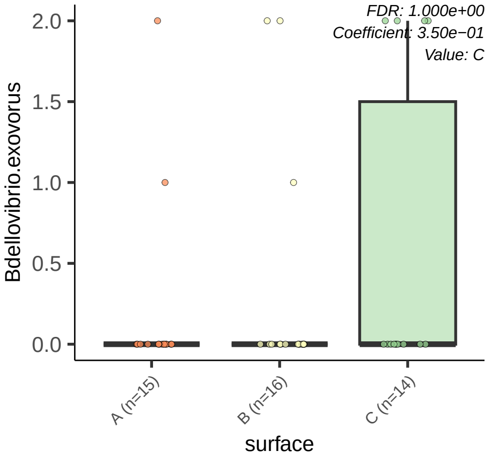
FDR: 1.000e+00
Coefficient: 3.50e−01
Value: C
2.0
1.5
1.0
0.5
0.0
Bdellovibrio.exovorus
A (n=15)
B (n=16)
C (n=14)
surface

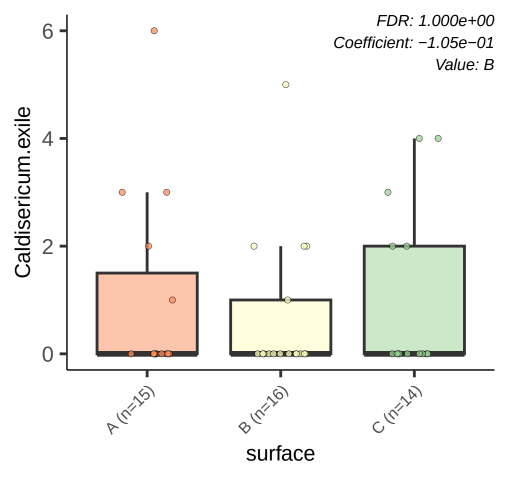
FDR: 1.000e+00
Coefficient: −1.05e−01
Value: B
Caldisericum.exile
6
4
2
0
A (n=15)
B (n=16)
C (n=14)
surface

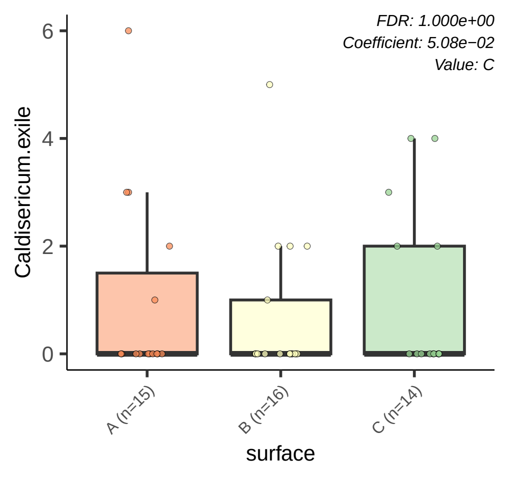
FDR: 1.000e+00
Coefficient: 5.08e−02
Value: C
Caldisericum.exile
0
2
4
6
A (n=15)
B (n=16)
C (n=14)
surface

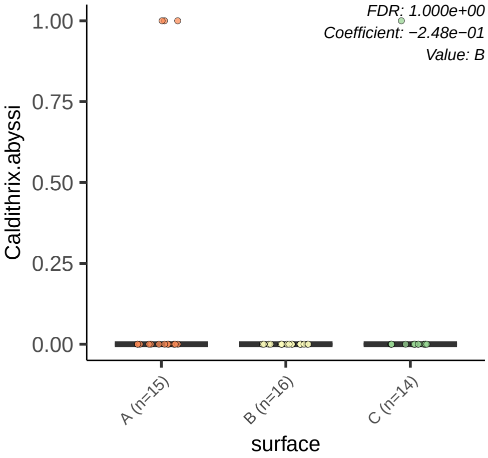
FDR: 1.000e+00
Coefficient: −2.48e−01
Value: B
Caldithrix.abyssi
1.00
0.75
0.50
0.25
0.00
A (n=15)
B (n=16)
C (n=14)
surface

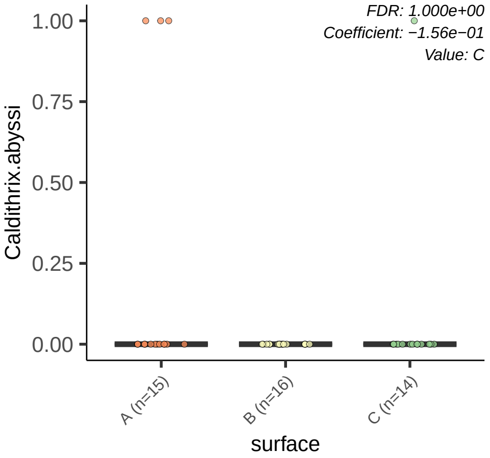

FDR: 1.000e+00
Coefficient: −1.56e−01
Value: C
Caldithrix.abyssi
1.00
0.75
0.50
0.25
0.00
A (n=15)
B (n=16)
C (n=14)
surface

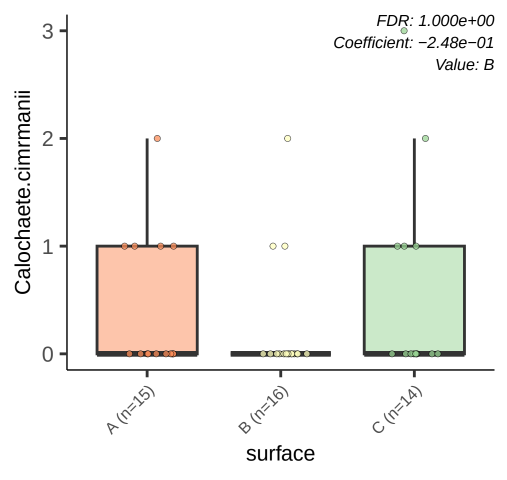
FDR: 1.000e+00
Coefficient: −2.48e−01
Value: B
Calochaete.cimrmanii
0
1
2
3
A (n=15)
B (n=16)
C (n=14)
surface

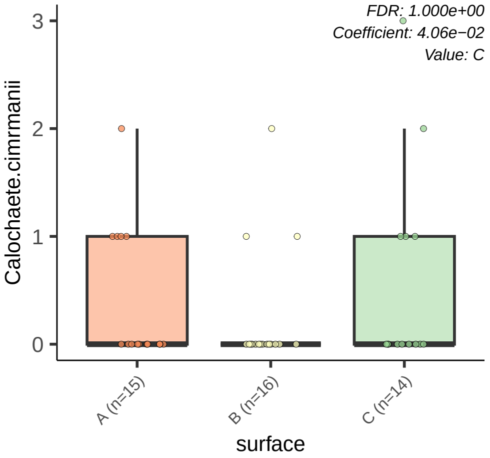

FDR: 1.000e+00
Coefficient: 4.06e−02
Value: C
Calochaete.cimrmanii
3
2
1
0
A (n=15)
B (n=16)
C (n=14)
surface

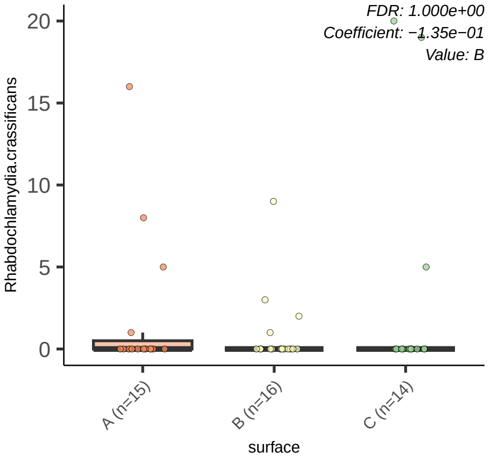
FDR: 1.000e+00
Coefficient: −1.35e−01
Value: B
Rhabdochlamydia.crassificans
20
15
10
5
0
A (n=15)
B (n=16)
C (n=14)
surface

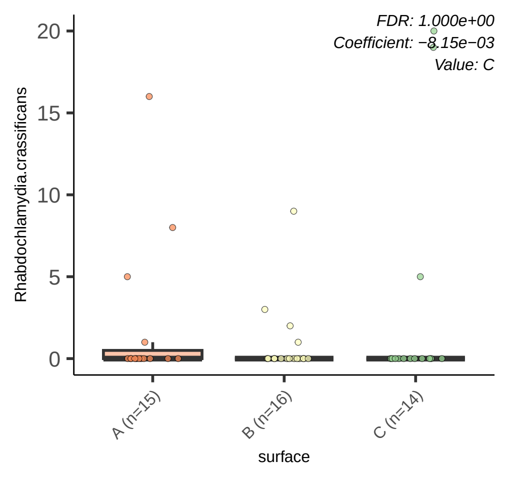
FDR: 1.000e+00
Coefficient: −8.15e−03
Value: C
Rhabdochlamydia.crassificans
20
15
10
5
0
A (n=15)
B (n=16)
C (n=14)
surface

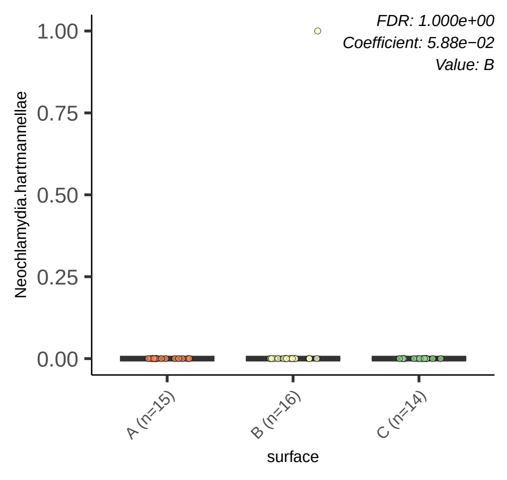

FDR: 1.000e+00
Coefficient: 5.88e−02
Value: B
1.00
0.75
0.50
0.25
0.00
Neochlamydia.hartmannellae
A (n=15)
B (n=16)
C (n=14)
surface

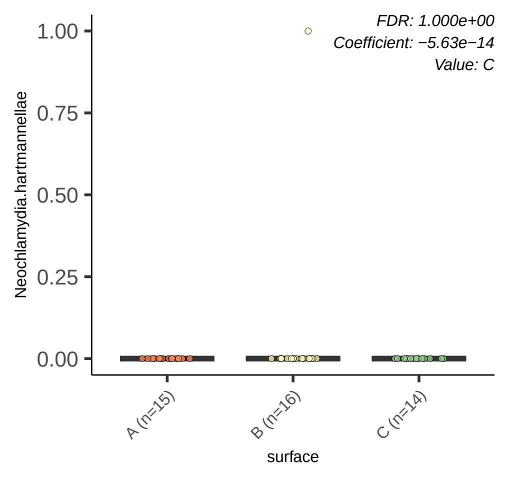
FDR: 1.000e+00
Coefficient: −5.63e−14
Value: C
Neochlamydia.hartmannellae
1.00
0.75
0.50
0.25
0.00
A (n=15)
B (n=16)
C (n=14)
surface

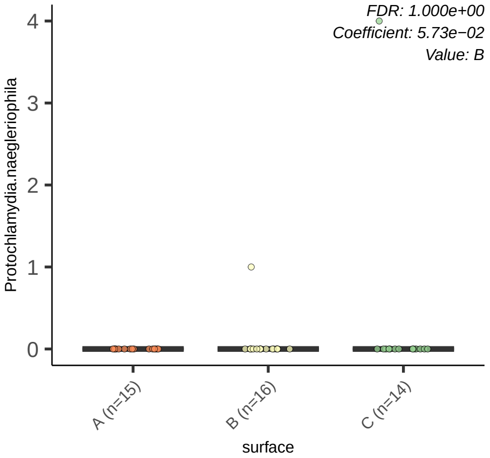

FDR: 1.000e+00
Coefficient: 5.73e−02
Value: B
Protochlamydia.naegleriophila
4
3
2
1
0
A (n=15)
B (n=16)
C (n=14)
surface

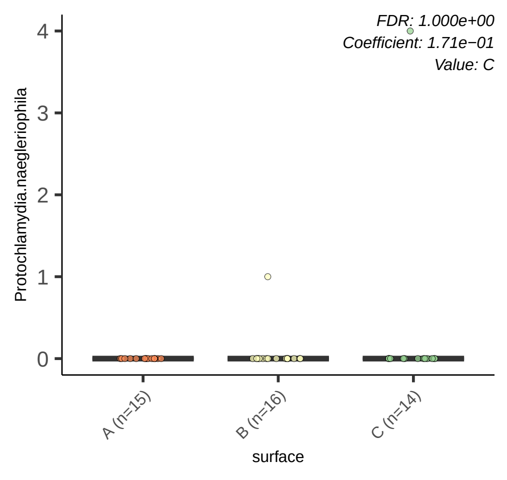

FDR: 1.000e+00
Coefficient: 1.71e−01
Value: C
Protochlamydia.naegleriophila
0
1
2
3
4
A (n=15)
B (n=16)
C (n=14)
surface

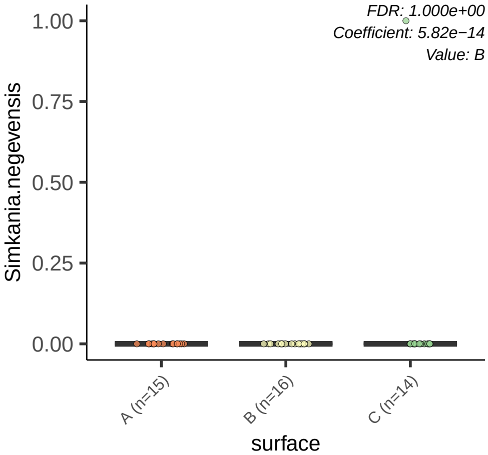
FDR: 1.000e+00
Coefficient: 5.82e−14
Value: B
Simkania.negevensis
1.00
0.75
0.50
0.25
0.00
A (n=15)
B (n=16)
C (n=14)
surface

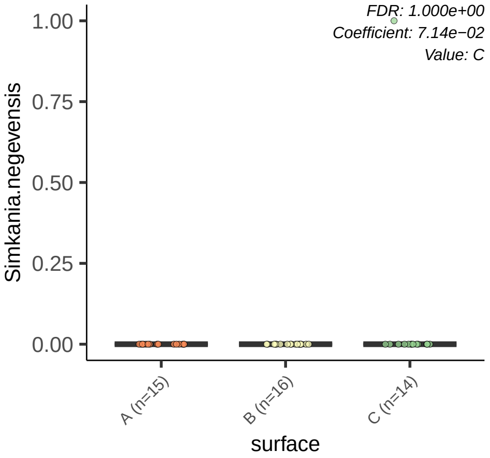
FDR: 1.000e+00
Coefficient: 7.14e−02
Value: C
1.00
0.75
0.50
0.25
0.00
Simkania.negevensis
A (n=15)
B (n=16)
C (n=14)
surface

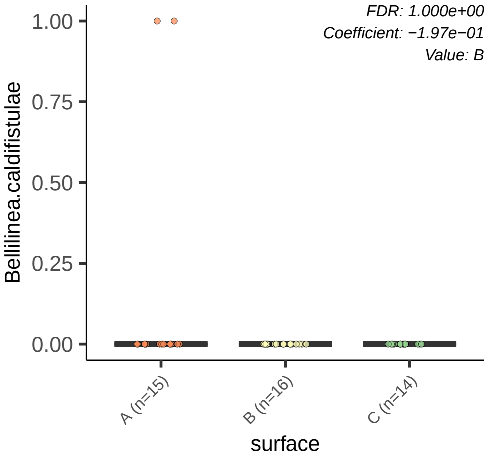

FDR: 1.000e+00
Coefficient: −1.97e−01
Value: B
1.00
0.75
0.50
0.25
0.00
Bellilinea.caldifistulae
A (n=15)
B (n=16)
C (n=14)
surface

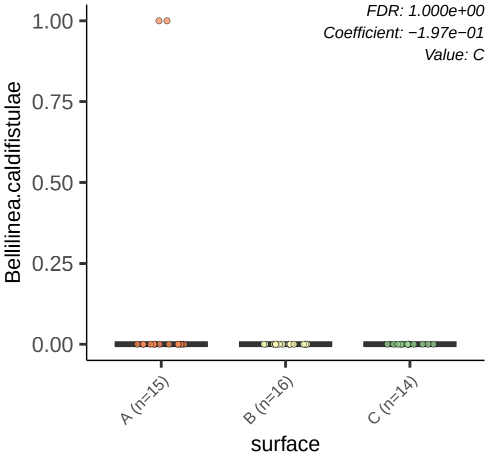

FDR: 1.000e+00
Coefficient: −1.97e−01
Value: C
1.00
0.75
0.50
0.25
0.00
Bellilinea.caldifistulae
A (n=15)
B (n=16)
C (n=14)
surface

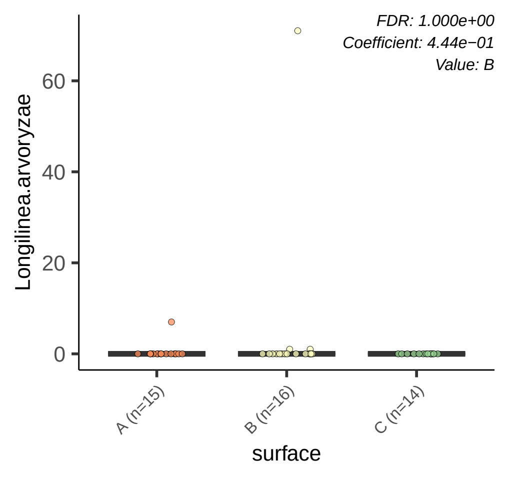
FDR: 1.000e+00
Coefficient: 4.44e−01
Value: B
Longilinea.arvoryzae
60
40
20
0
A (n=15)
B (n=16)
C (n=14)
surface

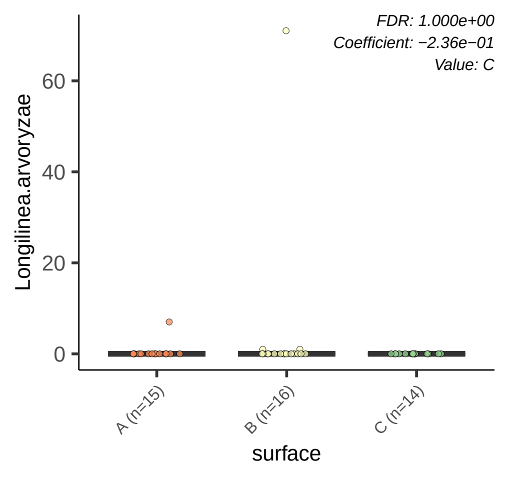
FDR: 1.000e+00
Coefficient: −2.36e−01
Value: C
Longilinea.arvoryzae
0
20
40
60
A (n=15)
B (n=16)
C (n=14)
surface

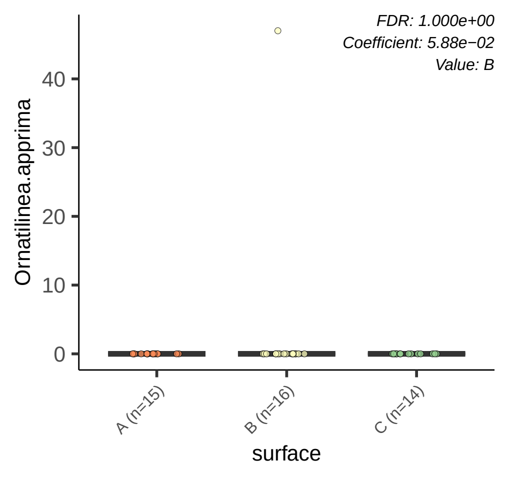
FDR: 1.000e+00
Coefficient: 5.88e−02
Value: B
Ornatilinea.apprima
0
10
20
30
40
A (n=15)
B (n=16)
C (n=14)
surface

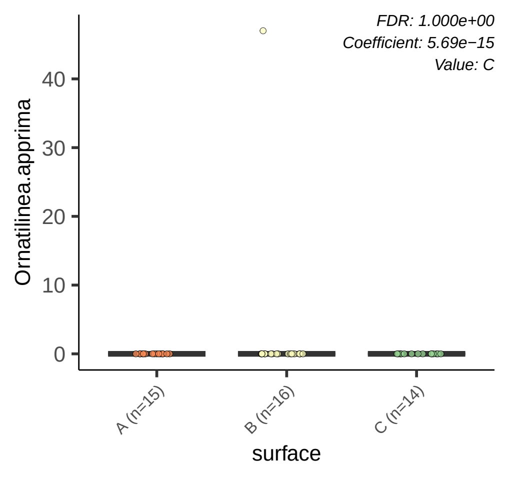
FDR: 1.000e+00
Coefficient: 5.69e−15
Value: C
Ornatilinea.apprima
0
10
20
30
40
A (n=15)
B (n=16)
C (n=14)
surface

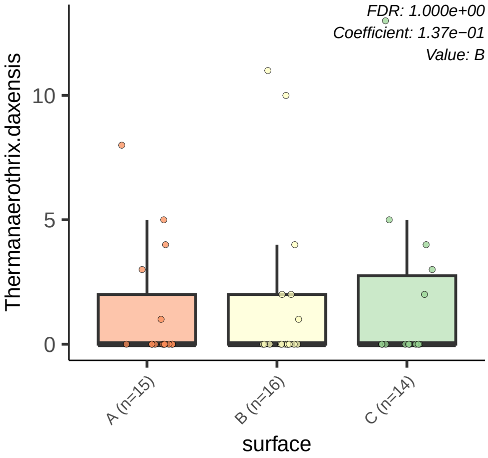

FDR: 1.000e+00
Coefficient: 1.37e−01
Value: B
Thermanaerothrix.daxensis
10
5
0
A (n=15)
B (n=16)
C (n=14)
surface

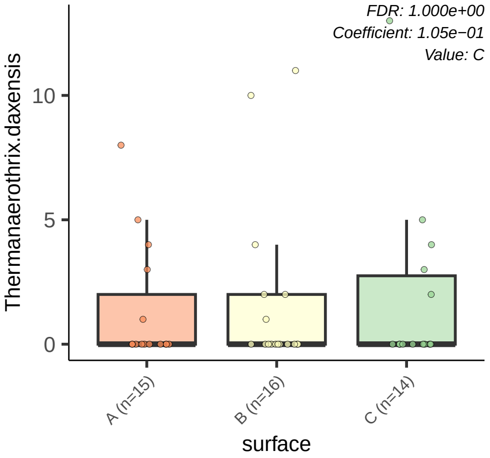

FDR: 1.000e+00
Coefficient: 1.05e−01
Value: C
Thermanaerothrix.daxensis
10
5
0
A (n=15)
B (n=16)
C (n=14)
surface

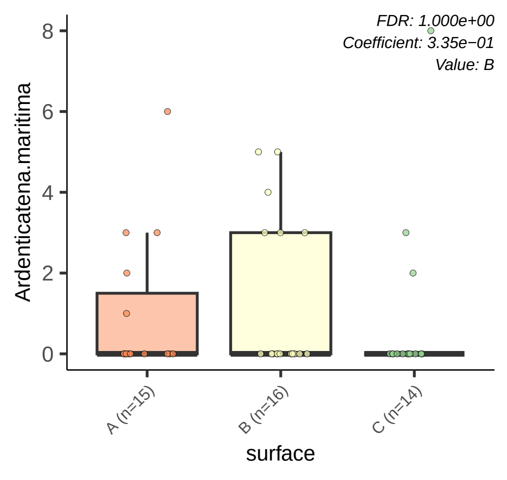

FDR: 1.000e+00
Coefficient: 3.35e−01
Value: B
Ardenticatena.maritima
0
2
4
6
8
A (n=15)
B (n=16)
C (n=14)
surface

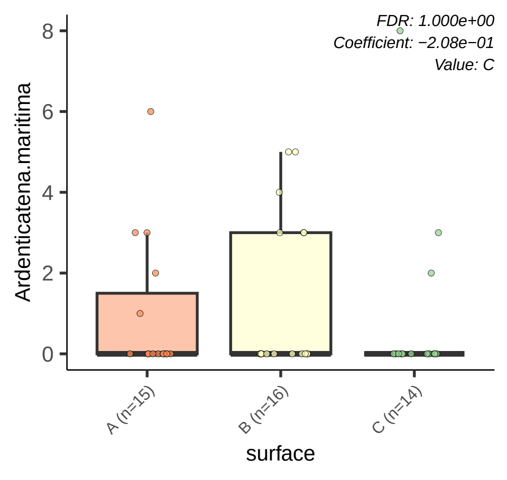
FDR: 1.000e+00
Coefficient: −2.08e−01
Value: C
Ardenticatena.maritima
0
2
4
6
8
A (n=15)
B (n=16)
C (n=14)
surface

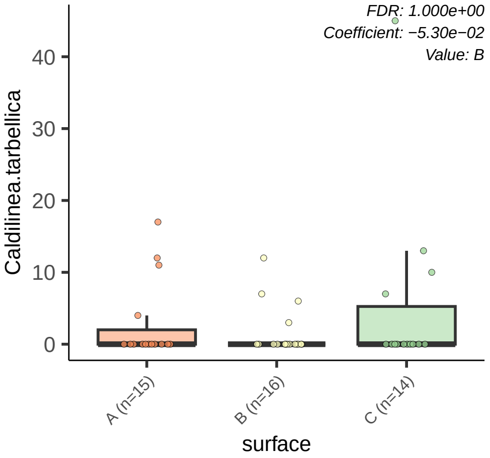

FDR: 1.000e+00
Coefficient: −5.30e−02
Value: B
Caldilinea.tarbellica
0
10
20
30
40
A (n=15)
B (n=16)
C (n=14)
surface

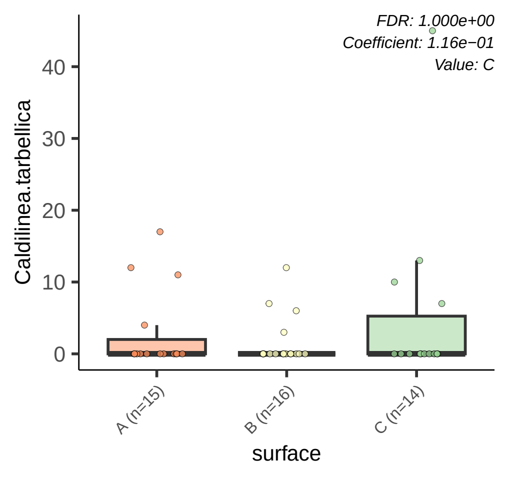
FDR: 1.000e+00
Coefficient: 1.16e−01
Value: C
Caldilinea.tarbellica
0
10
20
30
40
A (n=15)
B (n=16)
C (n=14)
surface

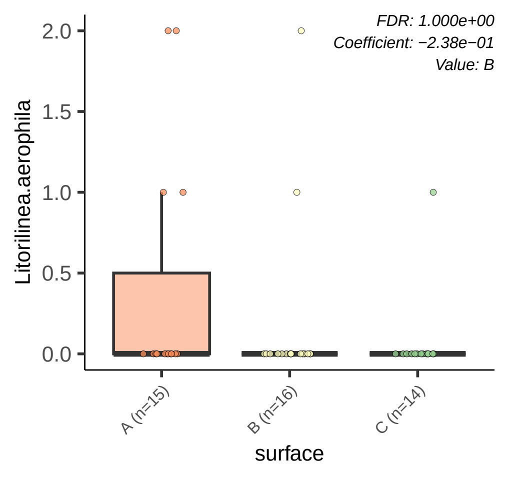
FDR: 1.000e+00
Coefficient: −2.38e−01
Value: B
Litorilinea.aerophila
2.0
1.5
1.0
0.5
0.0
A (n=15)
B (n=16)
C (n=14)
surface

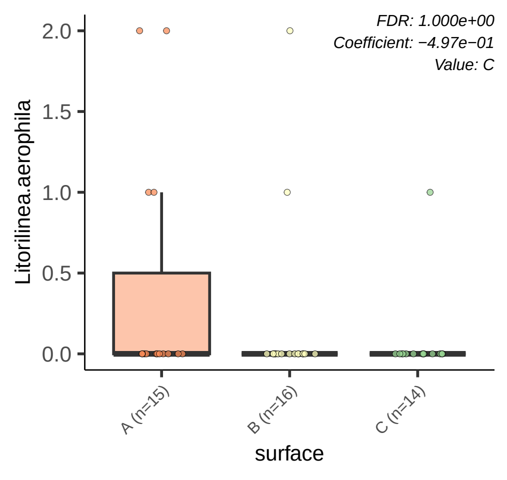
FDR: 1.000e+00
Coefficient: −4.97e−01
Value: C
Litorilinea.aerophila
2.0
1.5
1.0
0.5
0.0
A (n=15)
B (n=16)
C (n=14)
surface

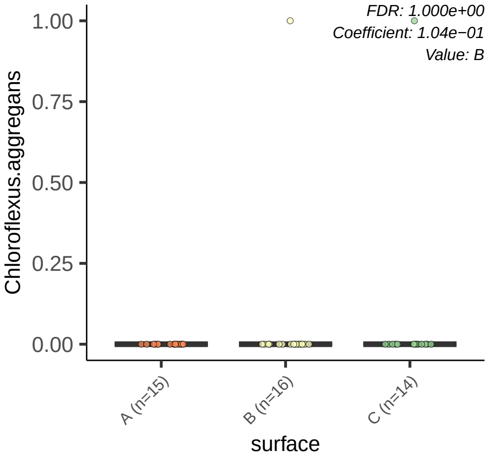
FDR: 1.000e+00
Coefficient: 1.04e−01
Value: B
Chloroflexus.aggregans
1.00
0.75
0.50
0.25
0.00
A (n=15)
B (n=16)
C (n=14)
surface

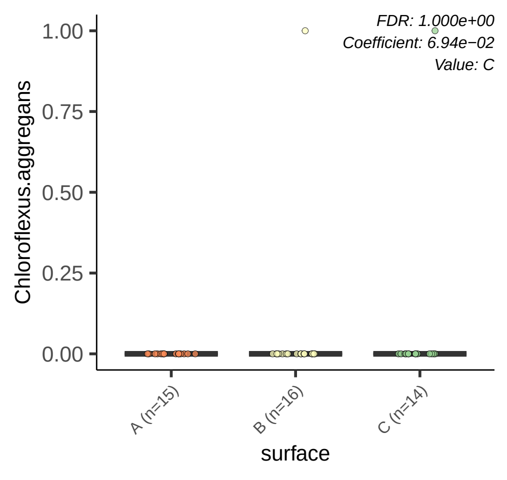
FDR: 1.000e+00
Coefficient: 6.94e−02
Value: C
Chloroflexus.aggregans
1.00
0.75
0.50
0.25
0.00
A (n=15)
B (n=16)
C (n=14)
surface

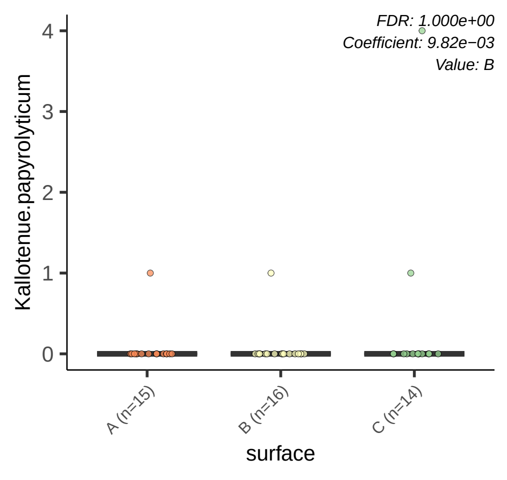
FDR: 1.000e+00
Coefficient: 9.82e−03
Value: B
Kallotenue.papyrolyticum
0
1
2
3
4
A (n=15)
B (n=16)
C (n=14)
surface

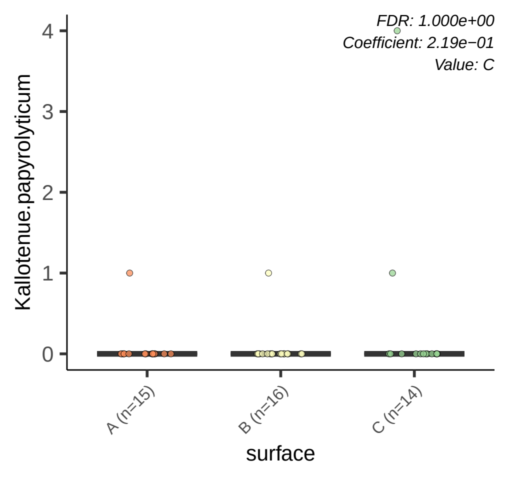

FDR: 1.000e+00
Coefficient: 2.19e−01
Value: C
Kallotenue.papyrolyticum
0
1
2
3
4
A (n=15)
B (n=16)
C (n=14)
surface

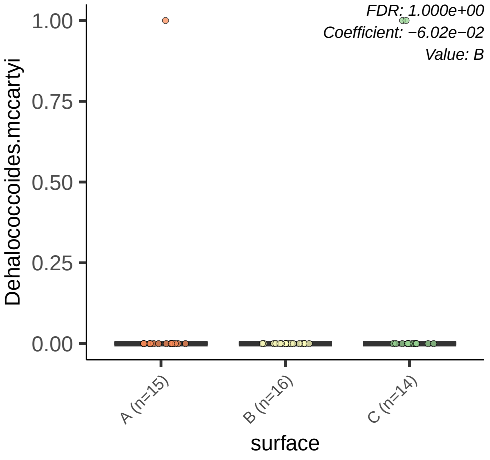
FDR: 1.000e+00
Coefficient: −6.02e−02
Value: B
Dehalococcoides.mccartyi
1.00
0.75
0.50
0.25
0.00
A (n=15)
B (n=16)
C (n=14)
surface

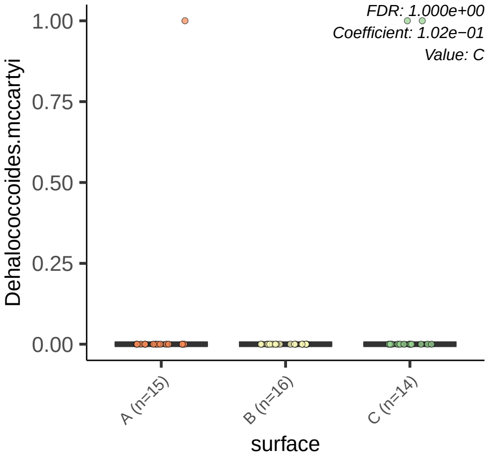

FDR: 1.000e+00
Coefficient: 1.02e−01
Value: C
Dehalococcoides.mccartyi
1.00
0.75
0.50
0.25
0.00
A (n=15)
B (n=16)
C (n=14)
surface

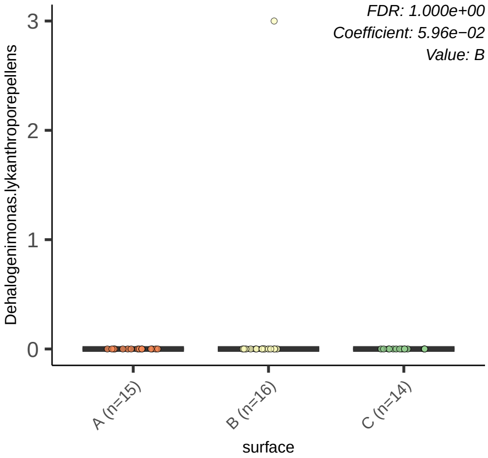
FDR: 1.000e+00
Coefficient: 5.96e−02
Value: B
Dehalogenimonas.lykanthroporepellens
3
2
1
0
A (n=15)
B (n=16)
C (n=14)
surface

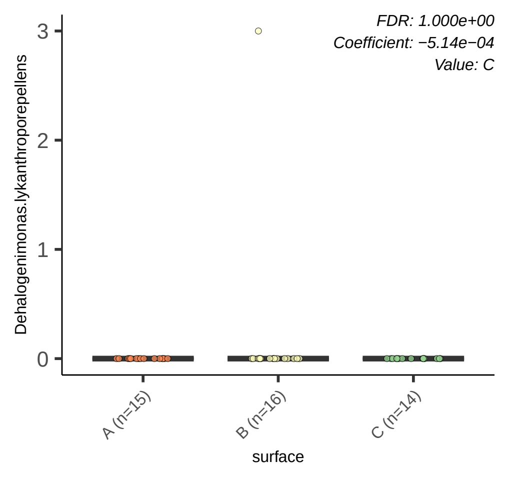
FDR: 1.000e+00
Coefficient: −5.14e−04
Value: C
Dehalogenimonas.lykanthroporepellens
3
2
1
0
A (n=15)
B (n=16)
C (n=14)
surface

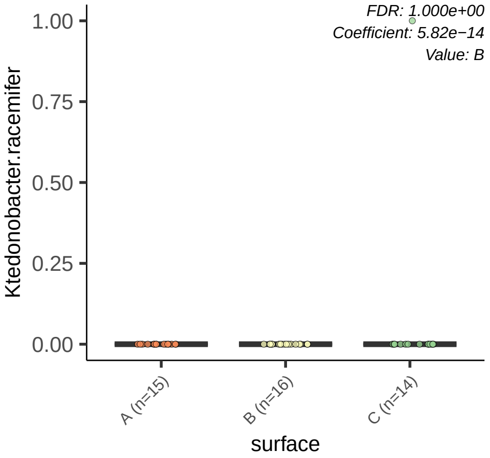
FDR: 1.000e+00
Coefficient: 5.82e−14
Value: B
Ktedonobacter.racemifer
1.00
0.75
0.50
0.25
0.00
A (n=15)
B (n=16)
C (n=14)
surface

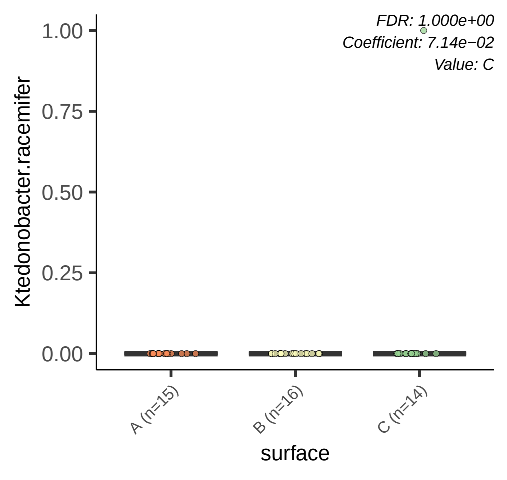

FDR: 1.000e+00
Coefficient: 7.14e−02
Value: C
Ktedonobacter.racemifer
1.00
0.75
0.50
0.25
0.00
A (n=15)
B (n=16)
C (n=14)
surface

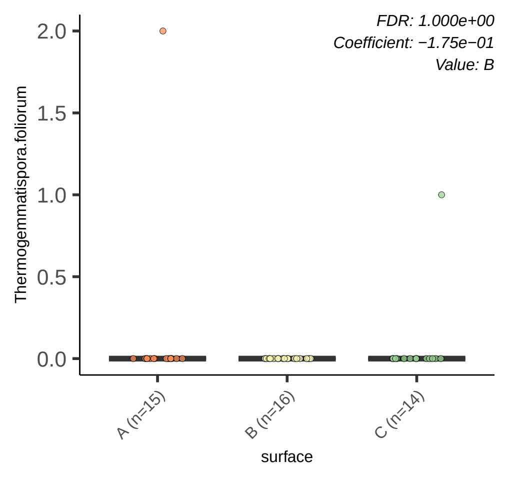

FDR: 1.000e+00
Coefficient: −1.75e−01
Value: B
Thermogemmatispora.foliorum
2.0
1.5
1.0
0.5
0.0
A (n=15)
B (n=16)
C (n=14)
surface

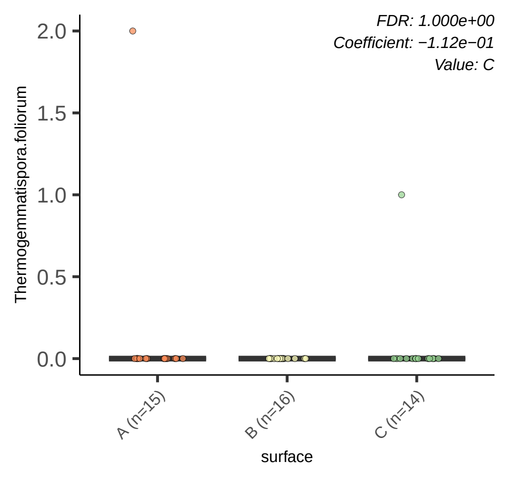
FDR: 1.000e+00
Coefficient: −1.12e−01
Value: C
Thermogemmatispora.foliorum
2.0
1.5
1.0
0.5
0.0
A (n=15)
B (n=16)
C (n=14)
surface

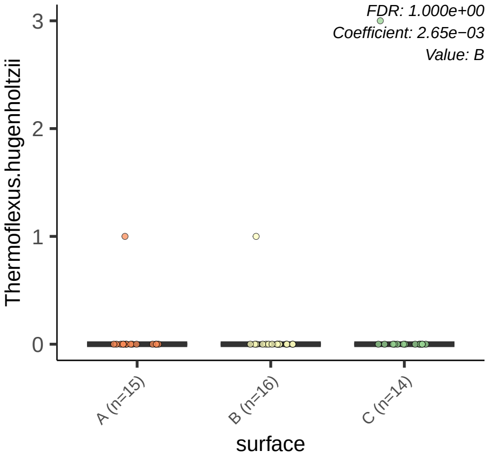

FDR: 1.000e+00
Coefficient: 2.65e−03
Value: B
Thermoflexus.hugenholtzii
0
1
2
3
A (n=15)
B (n=16)
C (n=14)
surface

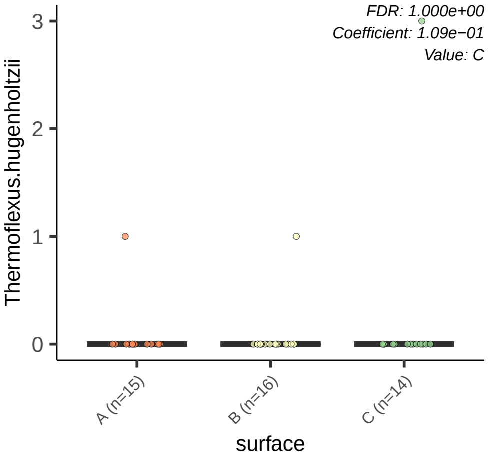
FDR: 1.000e+00
Coefficient: 1.09e−01
Value: C
Thermoflexus.hugenholtzii
3
2
1
0
A (n=15)
B (n=16)
C (n=14)
surface

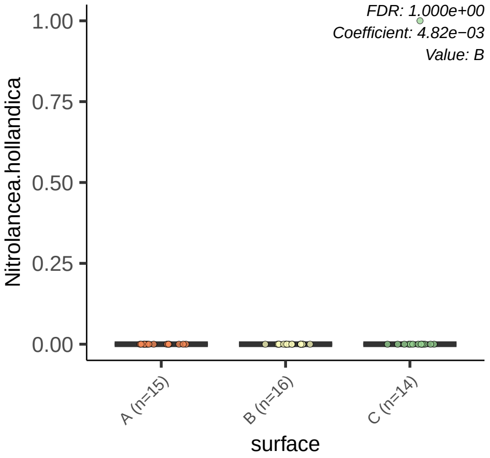
FDR: 1.000e+00
Coefficient: 4.82e−03
Value: B
Nitrolancea.hollandica
1.00
0.75
0.50
0.25
0.00
A (n=15)
B (n=16)
C (n=14)
surface

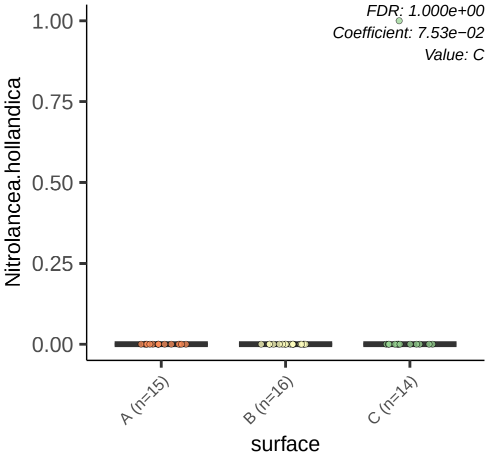

FDR: 1.000e+00
Coefficient: 7.53e−02
Value: C
Nitrolancea.hollandica
1.00
0.75
0.50
0.25
0.00
A (n=15)
B (n=16)
C (n=14)
surface

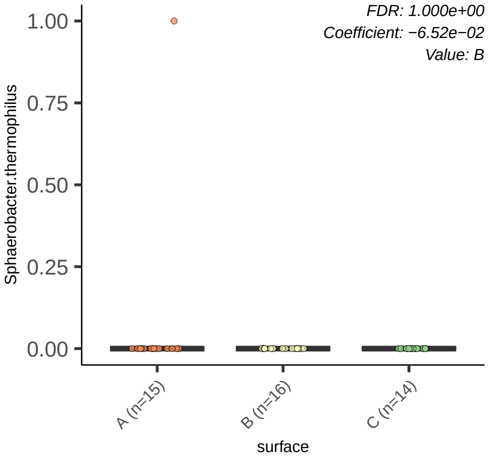

FDR: 1.000e+00
Coefficient: −6.52e−02
Value: B
1.00
0.75
0.50
0.25
0.00
Sphaerobacter.thermophilus
A (n=15)
B (n=16)
C (n=14)
surface

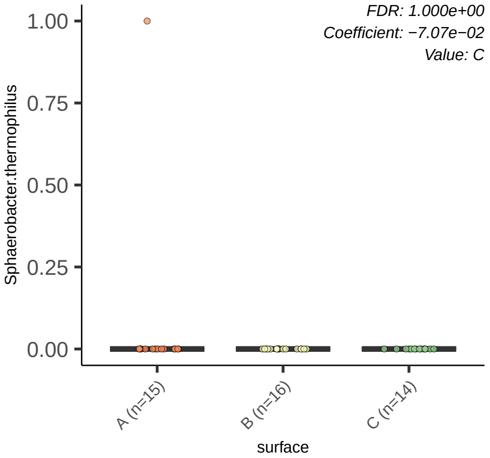
FDR: 1.000e+00
Coefficient: −7.07e−02
Value: C
1.00
0.75
0.50
0.25
0.00
Sphaerobacter.thermophilus
A (n=15)
B (n=16)
C (n=14)
surface

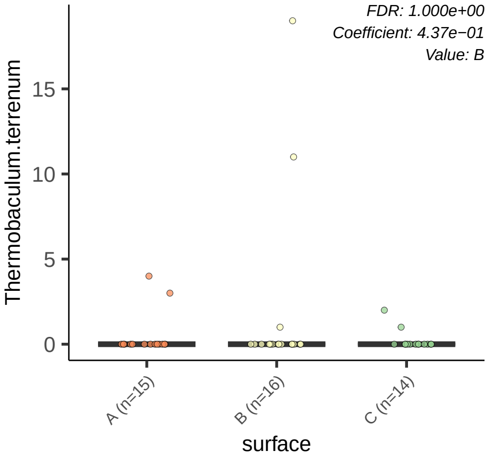
FDR: 1.000e+00
Coefficient: 4.37e−01
Value: B
Thermobaculum.terrenum
0
5
10
15
A (n=15)
B (n=16)
C (n=14)
surface

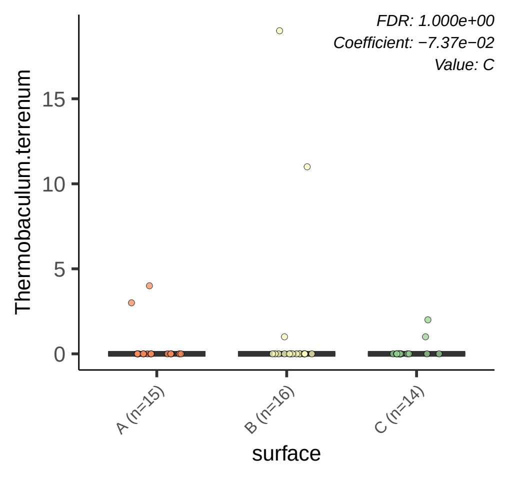

FDR: 1.000e+00
Coefficient: −7.37e−02
Value: C
Thermobaculum.terrenum
15
10
5
0
A (n=15)
B (n=16)
C (n=14)
surface

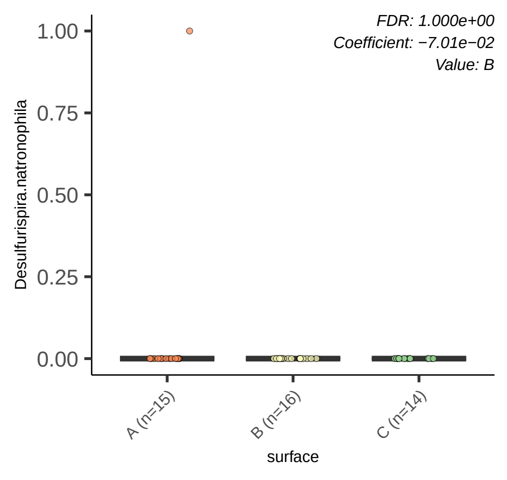

FDR: 1.000e+00
Coefficient: −7.01e−02
Value: B
1.00
0.75
0.50
0.25
0.00
Desulfurispira.natronophila
A (n=15)
B (n=16)
C (n=14)
surface

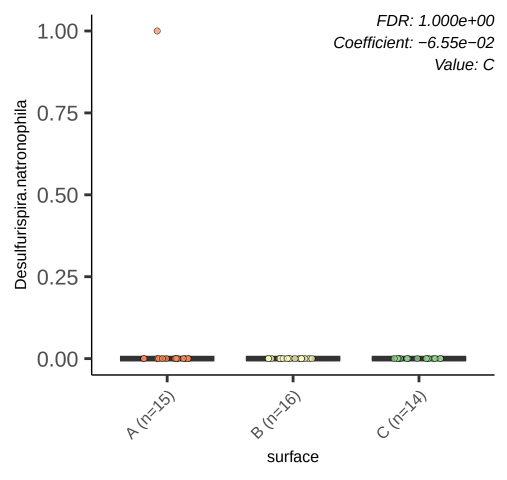

FDR: 1.000e+00
Coefficient: −6.55e−02
Value: C
1.00
0.75
0.50
0.25
0.00
Desulfurispira.natronophila
A (n=15)
B (n=16)
C (n=14)
surface

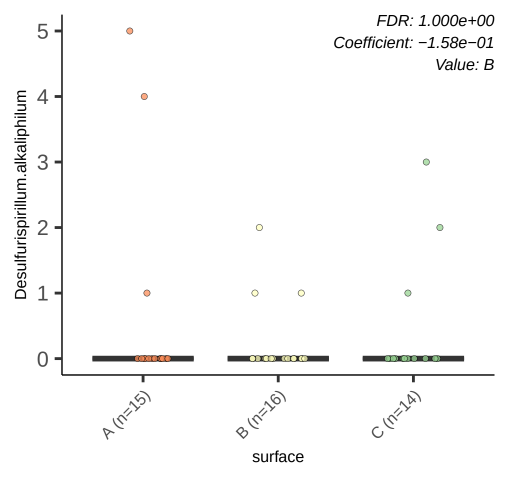

FDR: 1.000e+00
Coefficient: −1.58e−01
Value: B
Desulfurispirillum.alkaliphilum
0
1
2
3
4
5
A (n=15)
B (n=16)
C (n=14)
surface

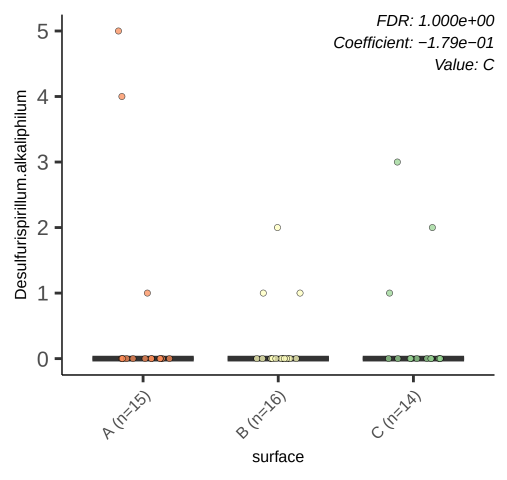

FDR: 1.000e+00
Coefficient: −1.79e−01
Value: C
Desulfurispirillum.alkaliphilum
0
1
2
3
4
5
A (n=15)
B (n=16)
C (n=14)
surface

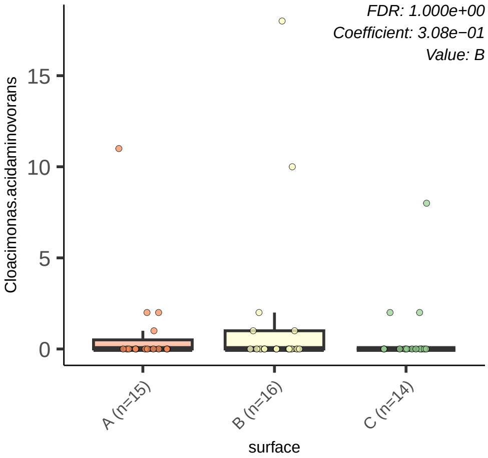

FDR: 1.000e+00
Coefficient: 3.08e−01
Value: B
Cloacimonas.acidaminovorans
0
5
10
15
A (n=15)
B (n=16)
C (n=14)
surface

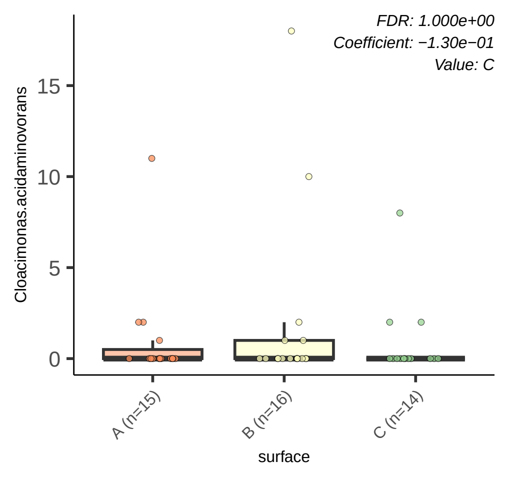
FDR: 1.000e+00
Coefficient: −1.30e−01
Value: C
Cloacimonas.acidaminovorans
0
5
10
15
A (n=15)
B (n=16)
C (n=14)
surface

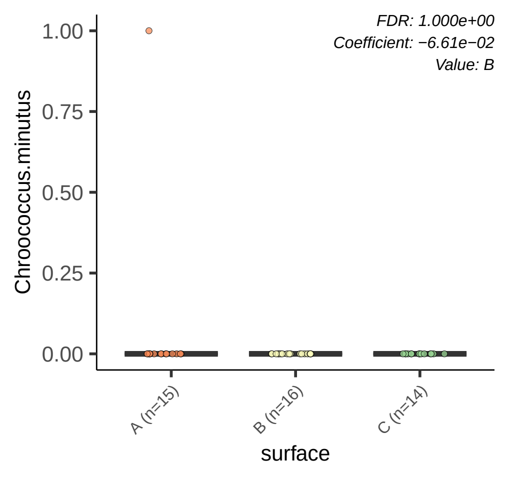

FDR: 1.000e+00
Coefficient: −6.61e−02
Value: B
Chroococcus.minutus
1.00
0.75
0.50
0.25
0.00
A (n=15)
B (n=16)
C (n=14)
surface

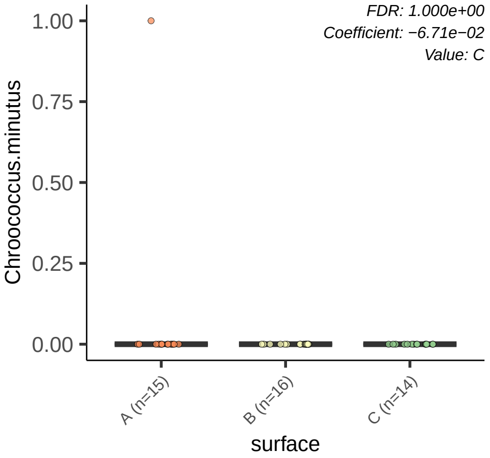
FDR: 1.000e+00
Coefficient: −6.71e−02
Value: C
Chroococcus.minutus
1.00
0.75
0.50
0.25
0.00
A (n=15)
B (n=16)
C (n=14)
surface

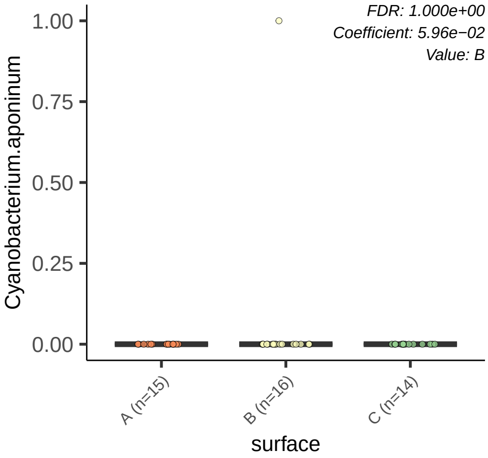
FDR: 1.000e+00
Coefficient: 5.96e−02
Value: B
Cyanobacterium.aponinum
1.00
0.75
0.50
0.25
0.00
A (n=15)
B (n=16)
C (n=14)
surface

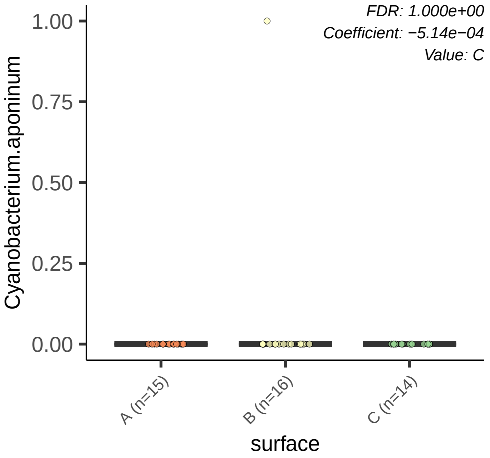
FDR: 1.000e+00
Coefficient: −5.14e−04
Value: C
Cyanobacterium.aponinum
1.00
0.75
0.50
0.25
0.00
A (n=15)
B (n=16)
C (n=14)
surface

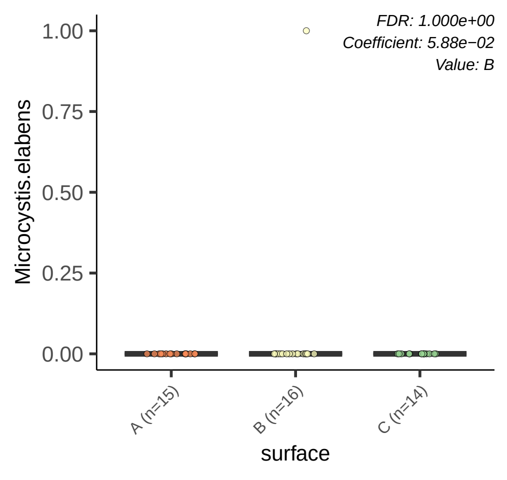

FDR: 1.000e+00
Coefficient: 5.88e−02
Value: B
Microcystis.elabens
1.00
0.75
0.50
0.25
0.00
A (n=15)
B (n=16)
C (n=14)
surface

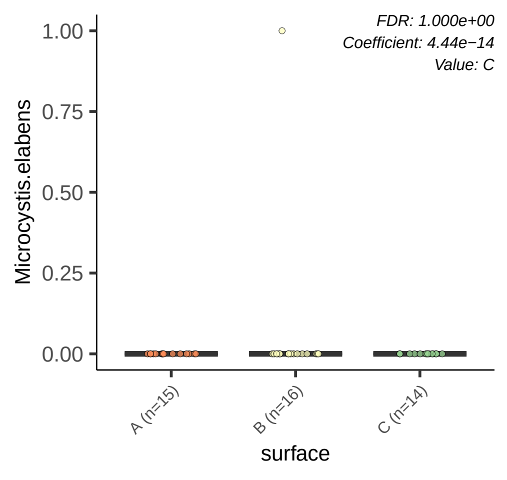

FDR: 1.000e+00
Coefficient: 4.44e−14
Value: C
Microcystis.elabens
1.00
0.75
0.50
0.25
0.00
A (n=15)
B (n=16)
C (n=14)
surface

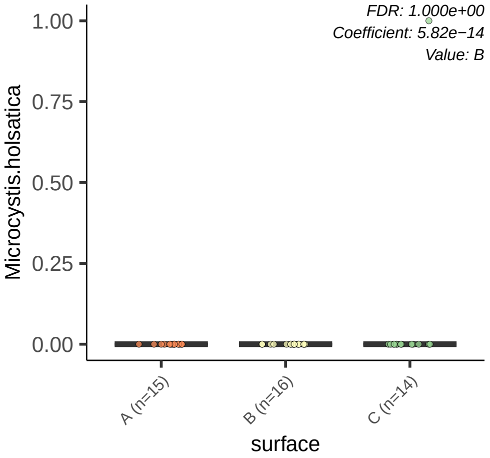
FDR: 1.000e+00
Coefficient: 5.82e−14
Value: B
Microcystis.holsatica
1.00
0.75
0.50
0.25
0.00
A (n=15)
B (n=16)
C (n=14)
surface

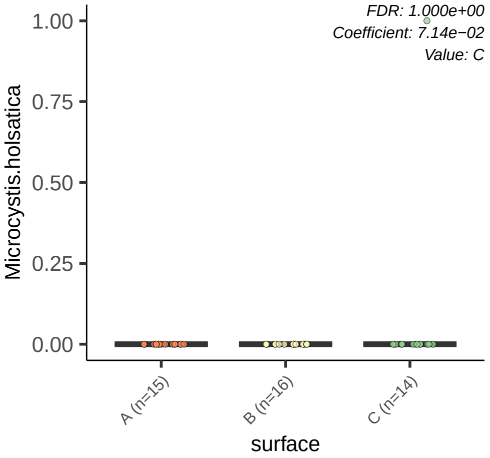

FDR: 1.000e+00
Coefficient: 7.14e−02
Value: C
Microcystis.holsatica
1.00
0.75
0.50
0.25
0.00
A (n=15)
B (n=16)
C (n=14)
surface

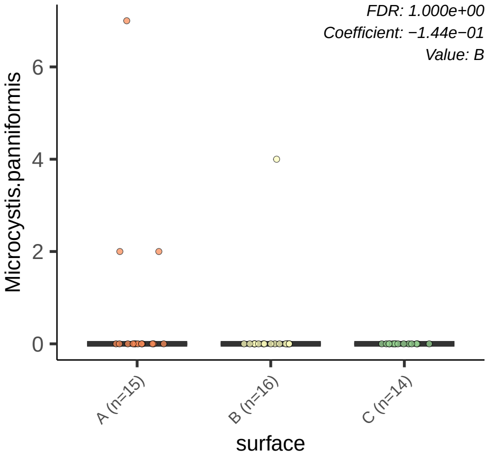

FDR: 1.000e+00
Coefficient: −1.44e−01
Value: B
Microcystis.panniformis
0
2
4
6
A (n=15)
B (n=16)
C (n=14)
surface

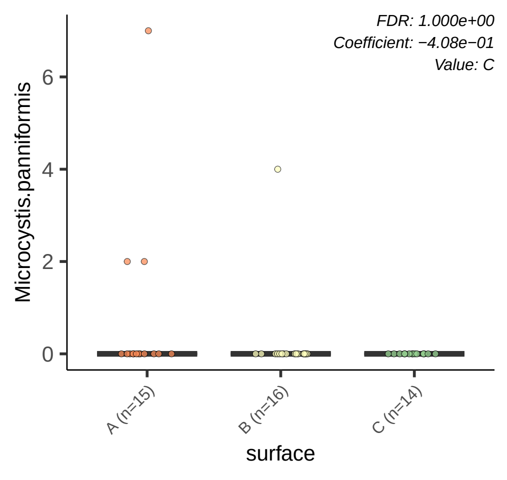
FDR: 1.000e+00
Coefficient: −4.08e−01
Value: C
Microcystis.panniformis
0
2
4
6
A (n=15)
B (n=16)
C (n=14)
surface

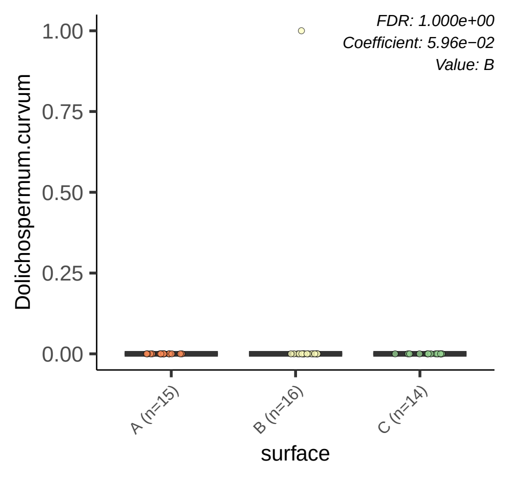

FDR: 1.000e+00
Coefficient: 5.96e−02
Value: B
Dolichospermum.curvum
1.00
0.75
0.50
0.25
0.00
A (n=15)
B (n=16)
C (n=14)
surface

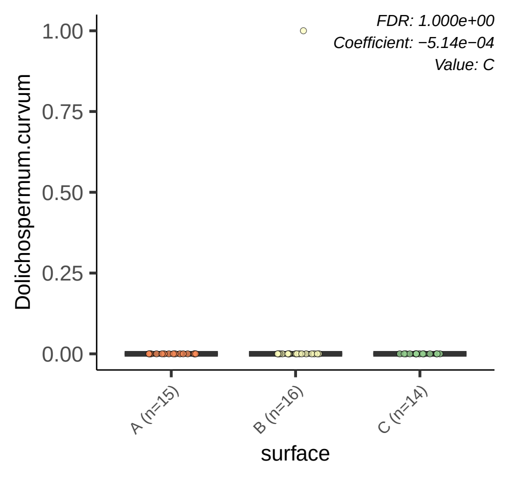

FDR: 1.000e+00
Coefficient: −5.14e−04
Value: C
Dolichospermum.curvum
1.00
0.75
0.50
0.25
0.00
A (n=15)
B (n=16)
C (n=14)
surface

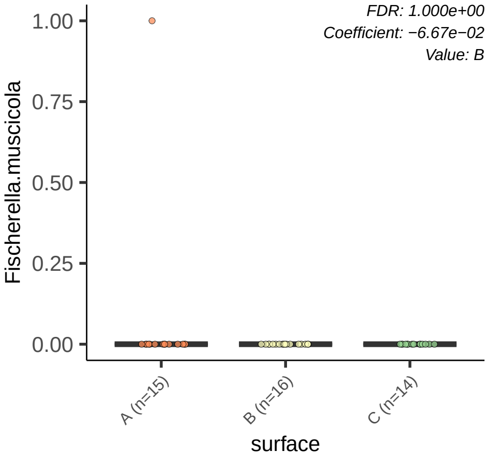
FDR: 1.000e+00
Coefficient: −6.67e−02
Value: B
Fischerella.muscicola
1.00
0.75
0.50
0.25
0.00
A (n=15)
B (n=16)
C (n=14)
surface

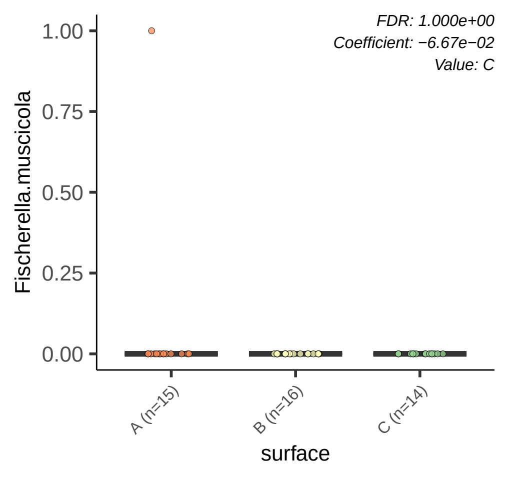
FDR: 1.000e+00
Coefficient: −6.67e−02
Value: C
Fischerella.muscicola
1.00
0.75
0.50
0.25
0.00
A (n=15)
B (n=16)
C (n=14)
surface

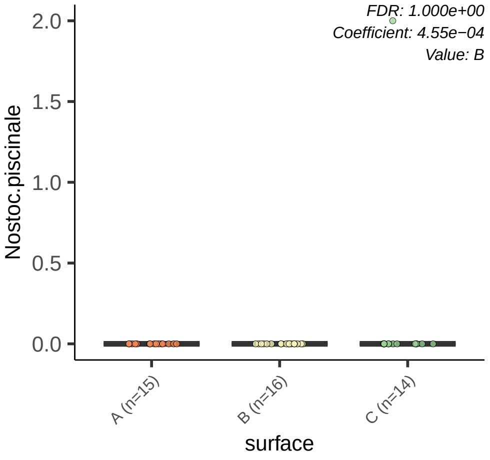

FDR: 1.000e+00
Coefficient: 4.55e−04
Value: B
Nostoc.piscinale
2.0
1.5
1.0
0.5
0.0
A (n=15)
B (n=16)
C (n=14)
surface

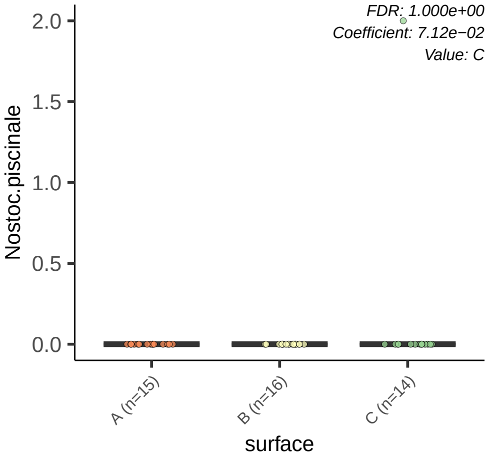
FDR: 1.000e+00
Coefficient: 7.12e−02
Value: C
Nostoc.piscinale
2.0
1.5
1.0
0.5
0.0
A (n=15)
B (n=16)
C (n=14)
surface

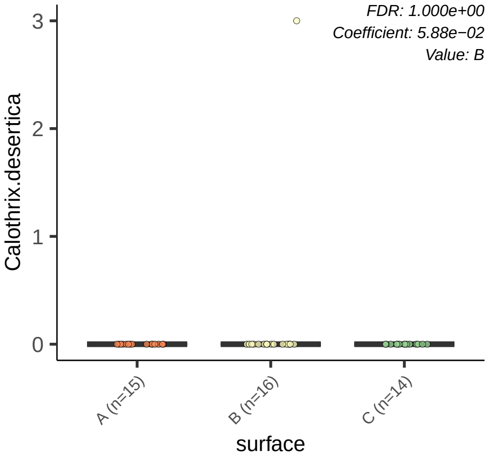

FDR: 1.000e+00
Coefficient: 5.88e−02
Value: B
Calothrix.desertica
0
1
2
3
A (n=15)
B (n=16)
C (n=14)
surface

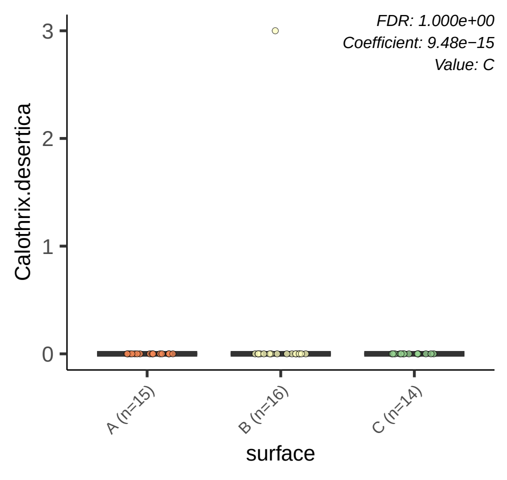
FDR: 1.000e+00
Coefficient: 9.48e−15
Value: C
Calothrix.desertica
0
1
2
3
A (n=15)
B (n=16)
C (n=14)
surface

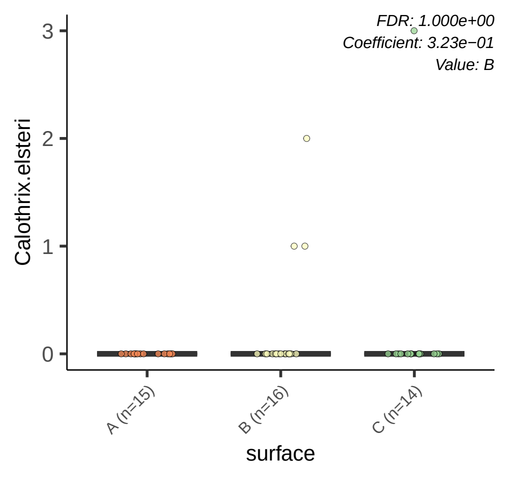
FDR: 1.000e+00
Coefficient: 3.23e−01
Value: B
Calothrix.elsteri
3
2
1
0
A (n=15)
B (n=16)
C (n=14)
surface

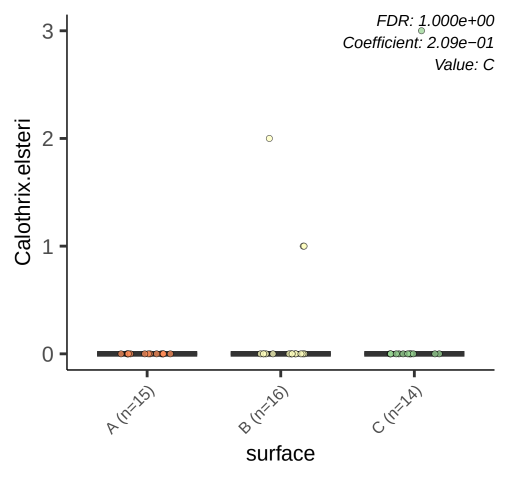
FDR: 1.000e+00
Coefficient: 2.09e−01
Value: C
Calothrix.elsteri
3
2
1
0
A (n=15)
B (n=16)
C (n=14)
surface

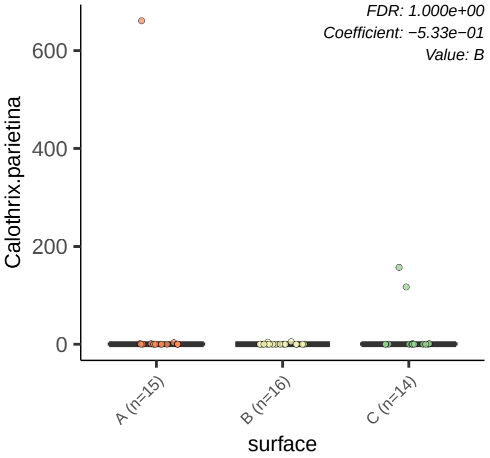
FDR: 1.000e+00
Coefficient: −5.33e−01
Value: B
Calothrix.parietina
600
400
200
0
A (n=15)
B (n=16)
C (n=14)
surface

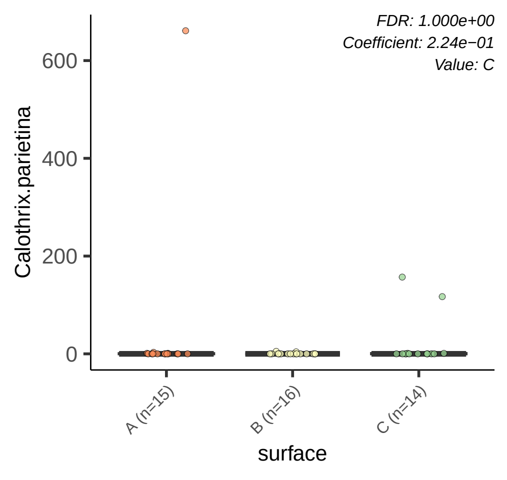

FDR: 1.000e+00
Coefficient: 2.24e−01
Value: C
Calothrix.parietina
600
400
200
0
A (n=15)
B (n=16)
C (n=14)
surface

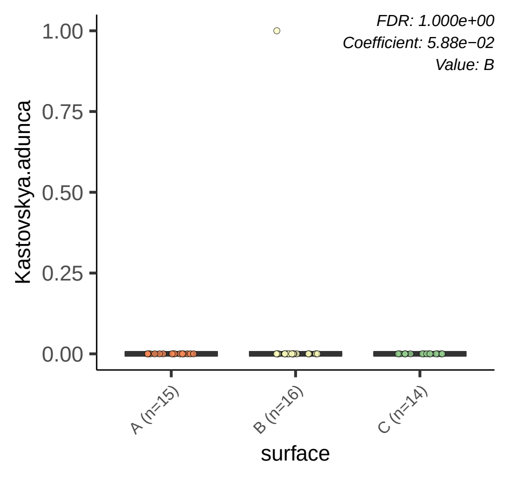

FDR: 1.000e+00
Coefficient: 5.88e−02
Value: B
Kastovskya.adunca
1.00
0.75
0.50
0.25
0.00
A (n=15)
B (n=16)
C (n=14)
surface

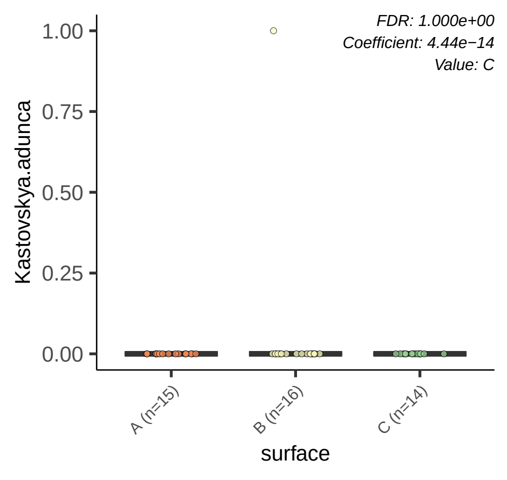

FDR: 1.000e+00
Coefficient: 4.44e−14
Value: C
Kastovskya.adunca
1.00
0.75
0.50
0.25
0.00
A (n=15)
B (n=16)
C (n=14)
surface

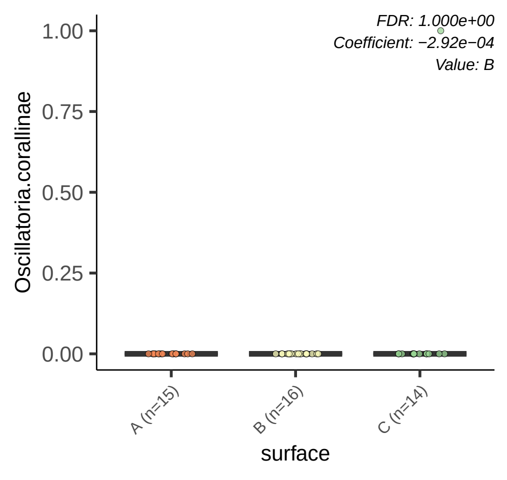
FDR: 1.000e+00
Coefficient: −2.92e−04
Value: B
Oscillatoria.corallinae
1.00
0.75
0.50
0.25
0.00
A (n=15)
B (n=16)
C (n=14)
surface

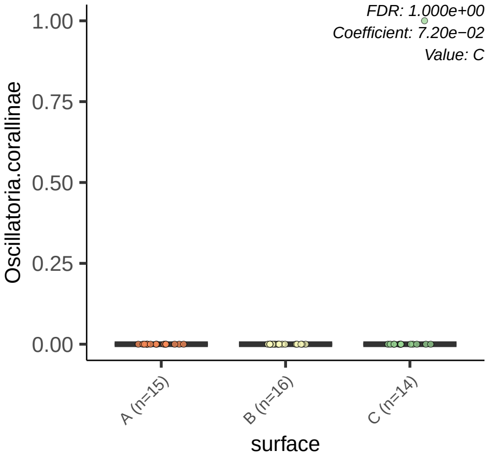

FDR: 1.000e+00
Coefficient: 7.20e−02
Value: C
Oscillatoria.corallinae
1.00
0.75
0.50
0.25
0.00
A (n=15)
B (n=16)
C (n=14)
surface

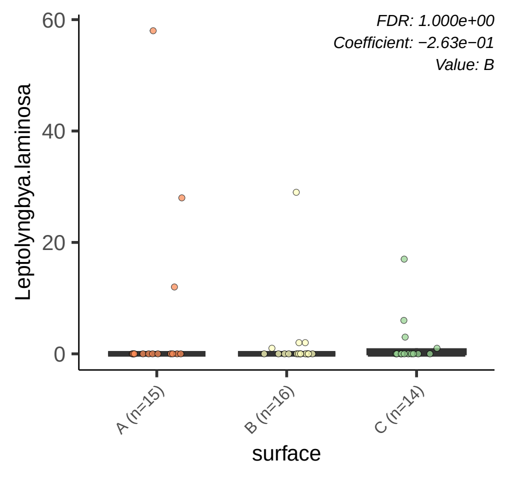
FDR: 1.000e+00
Coefficient: −2.63e−01
Value: B
Leptolyngbya.laminosa
60
40
20
0
A (n=15)
B (n=16)
C (n=14)
surface

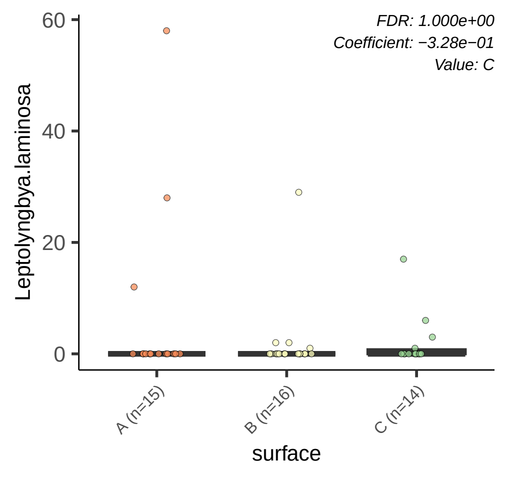
FDR: 1.000e+00
Coefficient: −3.28e−01
Value: C
Leptolyngbya.laminosa
60
40
20
0
A (n=15)
B (n=16)
C (n=14)
surface

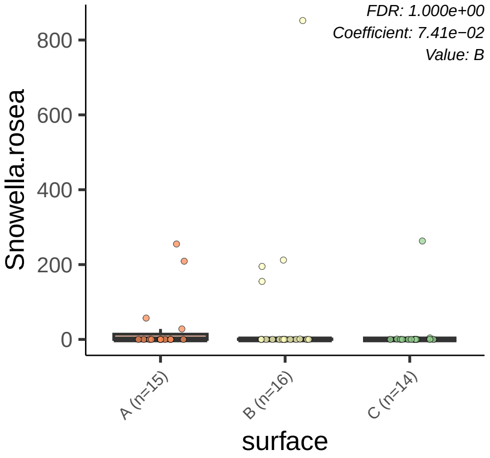

FDR: 1.000e+00
Coefficient: 7.41e−02
Value: B
Snowella.rosea
800
600
400
200
0
A (n=15)
B (n=16)
C (n=14)
surface

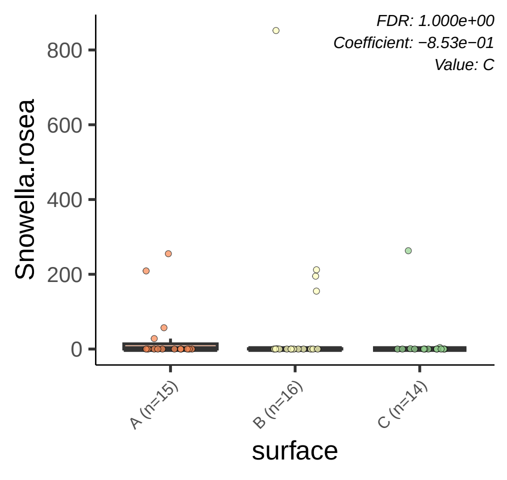
FDR: 1.000e+00
Coefficient: −8.53e−01
Value: C
Snowella.rosea
800
600
400
200
0
A (n=15)
B (n=16)
C (n=14)
surface

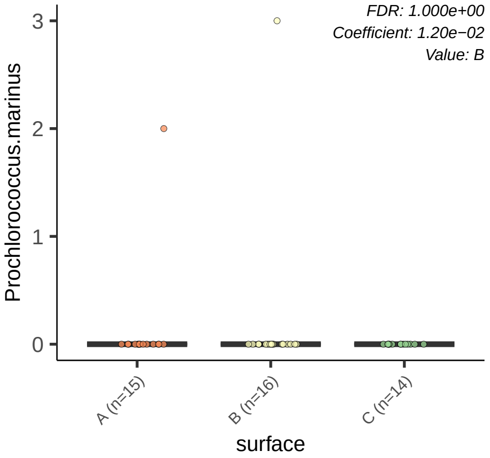

FDR: 1.000e+00
Coefficient: 1.20e−02
Value: B
Prochlorococcus.marinus
3
2
1
0
A (n=15)
B (n=16)
C (n=14)
surface

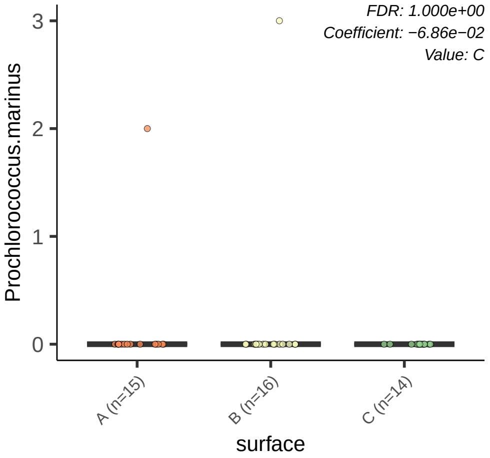
FDR: 1.000e+00
Coefficient: −6.86e−02
Value: C
Prochlorococcus.marinus
3
2
1
0
A (n=15)
B (n=16)
C (n=14)
surface

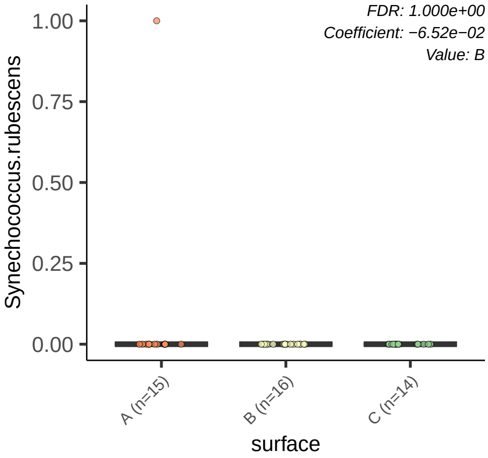

FDR: 1.000e+00
Coefficient: −6.52e−02
Value: B
Synechococcus.rubescens
1.00
0.75
0.50
0.25
0.00
A (n=15)
B (n=16)
C (n=14)
surface

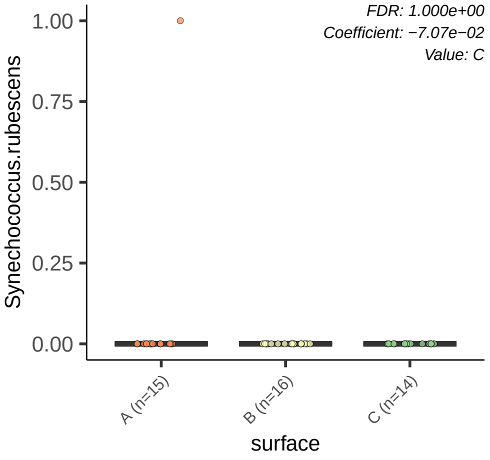
FDR: 1.000e+00
Coefficient: −7.07e−02
Value: C
Synechococcus.rubescens
1.00
0.75
0.50
0.25
0.00
A (n=15)
B (n=16)
C (n=14)
surface

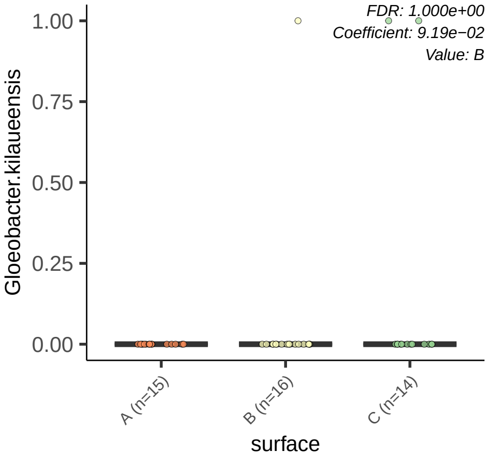
FDR: 1.000e+00
Coefficient: 9.19e−02
Value: B
Gloeobacter.kilaueensis
1.00
0.75
0.50
0.25
0.00
A (n=15)
B (n=16)
C (n=14)
surface

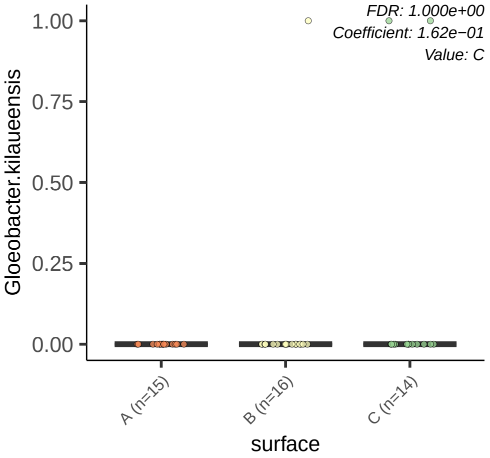

FDR: 1.000e+00
Coefficient: 1.62e−01
Value: C
Gloeobacter.kilaueensis
1.00
0.75
0.50
0.25
0.00
A (n=15)
B (n=16)
C (n=14)
surface

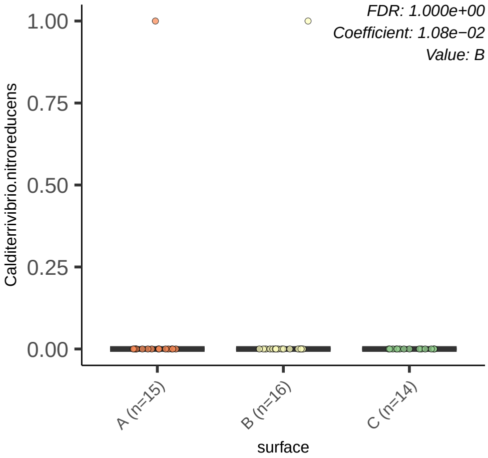

FDR: 1.000e+00
Coefficient: 1.08e−02
Value: B
Calditerrivibrio.nitroreducens
1.00
0.75
0.50
0.25
0.00
A (n=15)
B (n=16)
C (n=14)
surface

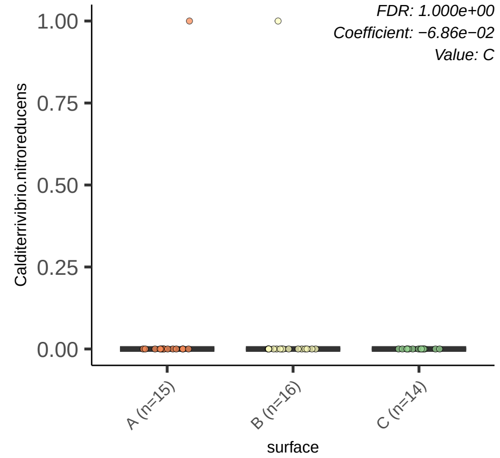

FDR: 1.000e+00
Coefficient: −6.86e−02
Value: C
Calditerrivibrio.nitroreducens
1.00
0.75
0.50
0.25
0.00
A (n=15)
B (n=16)
C (n=14)
surface

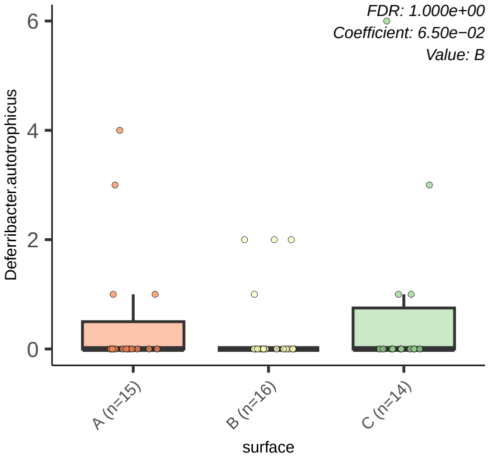

FDR: 1.000e+00
Coefficient: 6.50e−02
Value: B
Deferribacter.autotrophicus
0
2
4
6
A (n=15)
B (n=16)
C (n=14)
surface

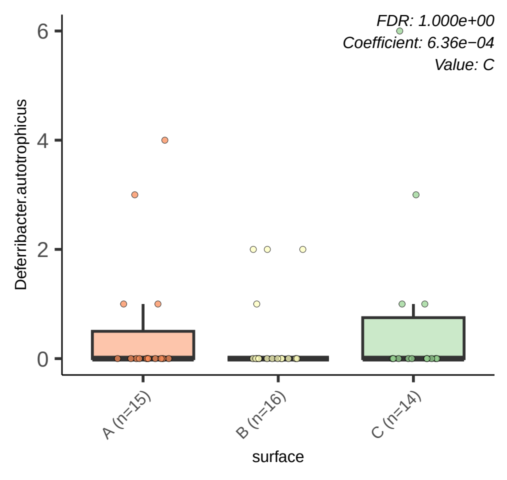

FDR: 1.000e+00
Coefficient: 6.36e−04
Value: C
6
4
2
0
Deferribacter.autotrophicus
A (n=15)
B (n=16)
C (n=14)
surface

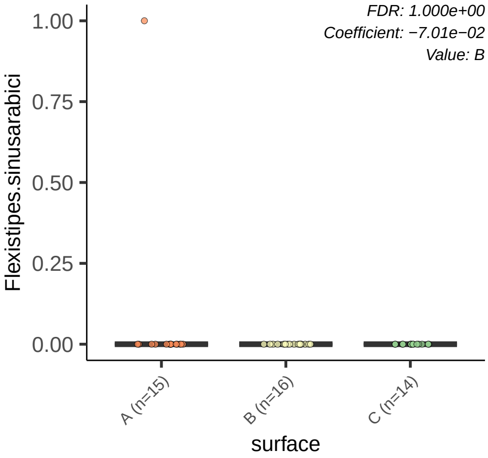
FDR: 1.000e+00
Coefficient: −7.01e−02
Value: B
Flexistipes.sinusarabici
1.00
0.75
0.50
0.25
0.00
A (n=15)
B (n=16)
C (n=14)
surface

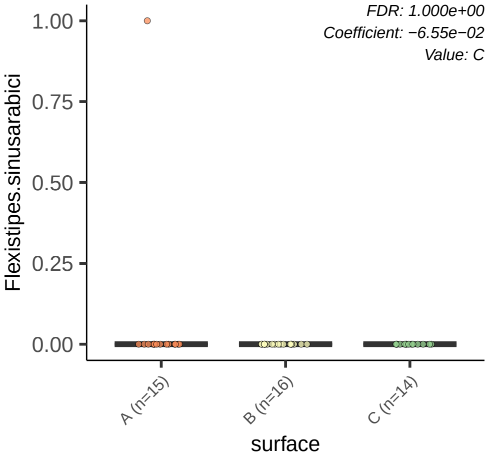

FDR: 1.000e+00
Coefficient: −6.55e−02
Value: C
Flexistipes.sinusarabici
1.00
0.75
0.50
0.25
0.00
A (n=15)
B (n=16)
C (n=14)
surface

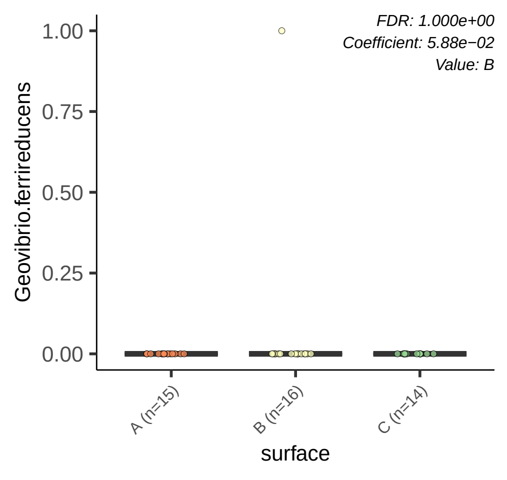
FDR: 1.000e+00
Coefficient: 5.88e−02
Value: B
Geovibrio.ferrireducens
1.00
0.75
0.50
0.25
0.00
A (n=15)
B (n=16)
C (n=14)
surface

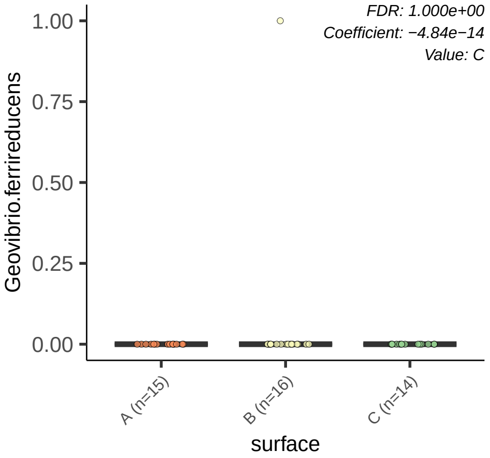
FDR: 1.000e+00
Coefficient: −4.84e−14
Value: C
Geovibrio.ferrireducens
1.00
0.75
0.50
0.25
0.00
A (n=15)
B (n=16)
C (n=14)
surface

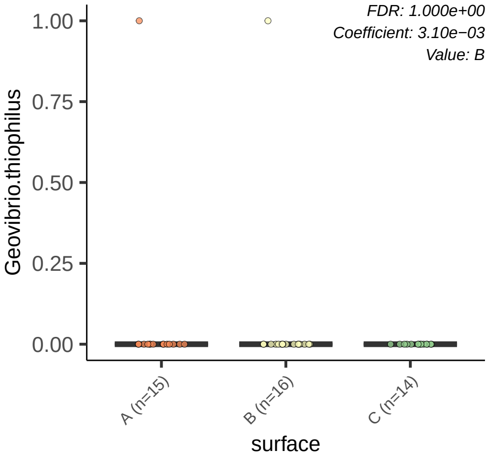

FDR: 1.000e+00
Coefficient: 3.10e−03
Value: B
Geovibrio.thiophilus
1.00
0.75
0.50
0.25
0.00
A (n=15)
B (n=16)
C (n=14)
surface

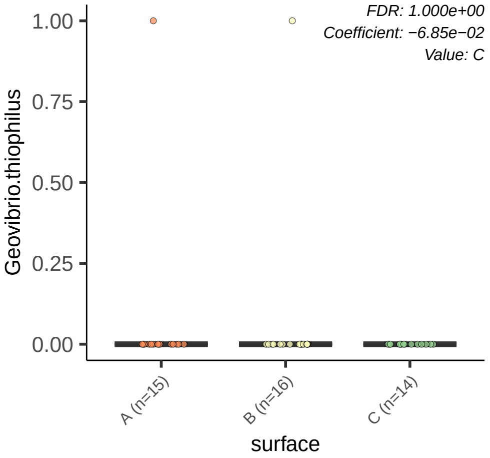
FDR: 1.000e+00
Coefficient: −6.85e−02
Value: C
Geovibrio.thiophilus
1.00
0.75
0.50
0.25
0.00
A (n=15)
B (n=16)
C (n=14)
surface

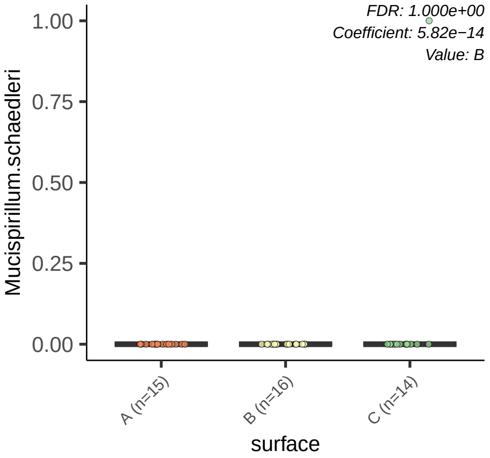

FDR: 1.000e+00
Coefficient: 5.82e−14
Value: B
Mucispirillum.schaedleri
1.00
0.75
0.50
0.25
0.00
A (n=15)
B (n=16)
C (n=14)
surface

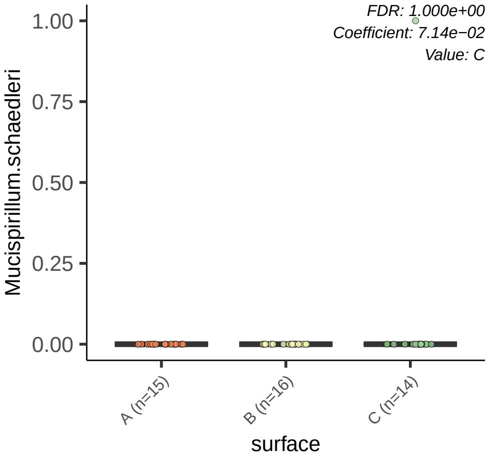
FDR: 1.000e+00
Coefficient: 7.14e−02
Value: C
Mucispirillum.schaedleri
1.00
0.75
0.50
0.25
0.00
A (n=15)
B (n=16)
C (n=14)
surface

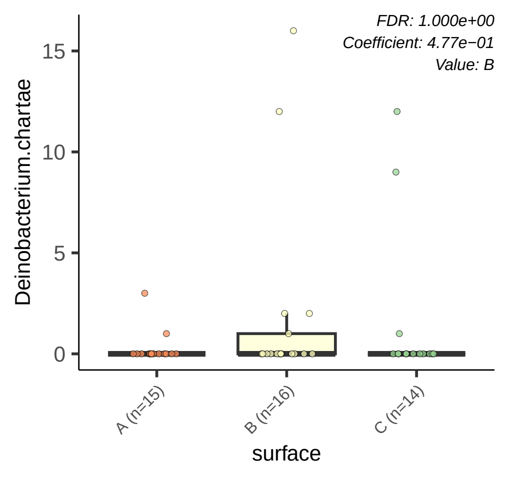
FDR: 1.000e+00
Coefficient: 4.77e−01
Value: B
Deinobacterium.chartae
15
10
5
0
A (n=15)
B (n=16)
C (n=14)
surface

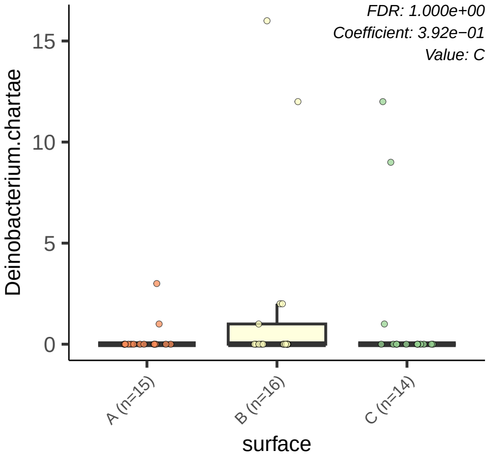

FDR: 1.000e+00
Coefficient: 3.92e−01
Value: C
Deinobacterium.chartae
0
5
10
15
A (n=15)
B (n=16)
C (n=14)
surface

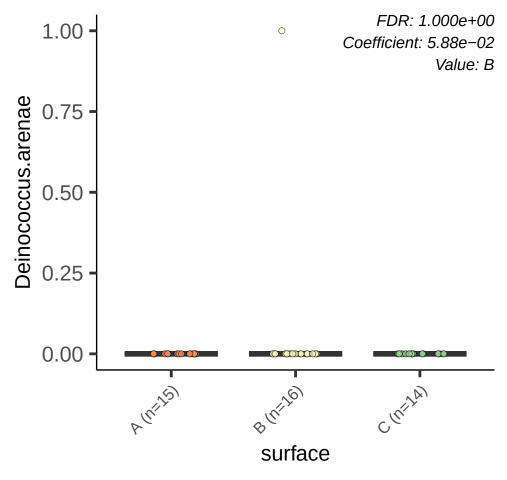
FDR: 1.000e+00
Coefficient: 5.88e−02
Value: B
Deinococcus.arenae
1.00
0.75
0.50
0.25
0.00
A (n=15)
B (n=16)
C (n=14)
surface

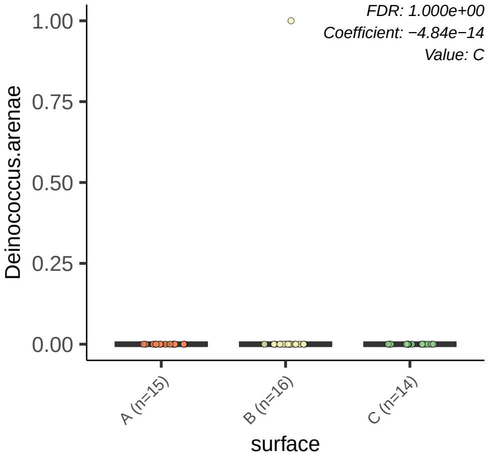

FDR: 1.000e+00
Coefficient: −4.84e−14
Value: C
Deinococcus.arenae
1.00
0.75
0.50
0.25
0.00
A (n=15)
B (n=16)
C (n=14)
surface

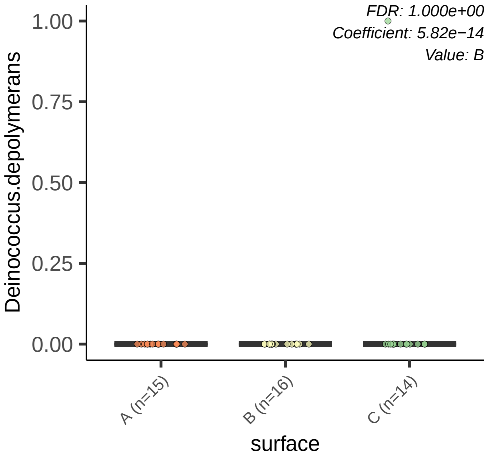

FDR: 1.000e+00
Coefficient: 5.82e−14
Value: B
Deinococcus.depolymerans
1.00
0.75
0.50
0.25
0.00
A (n=15)
B (n=16)
C (n=14)
surface

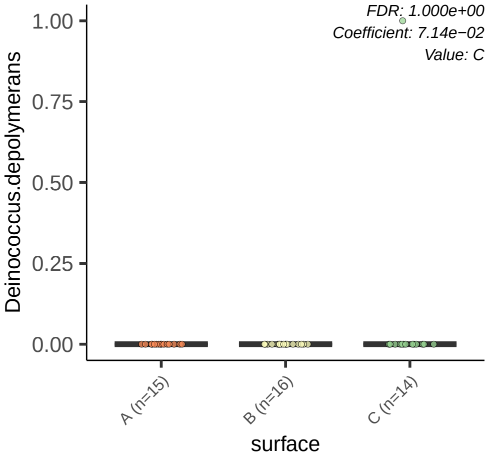
FDR: 1.000e+00
Coefficient: 7.14e−02
Value: C
Deinococcus.depolymerans
1.00
0.75
0.50
0.25
0.00
A (n=15)
B (n=16)
C (n=14)
surface

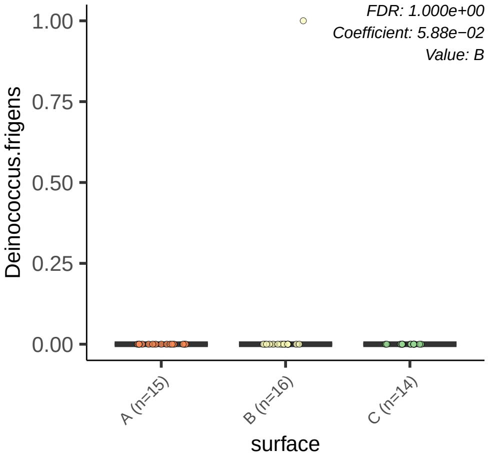

FDR: 1.000e+00
Coefficient: 5.88e−02
Value: B
Deinococcus.frigens
1.00
0.75
0.50
0.25
0.00
A (n=15)
B (n=16)
C (n=14)
surface

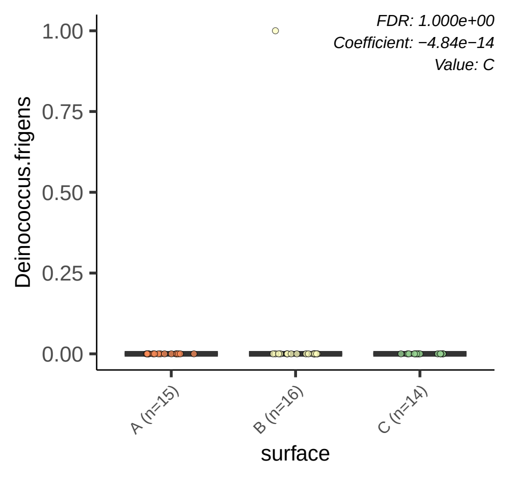
FDR: 1.000e+00
Coefficient: −4.84e−14
Value: C
Deinococcus.frigens
1.00
0.75
0.50
0.25
0.00
A (n=15)
B (n=16)
C (n=14)
surface

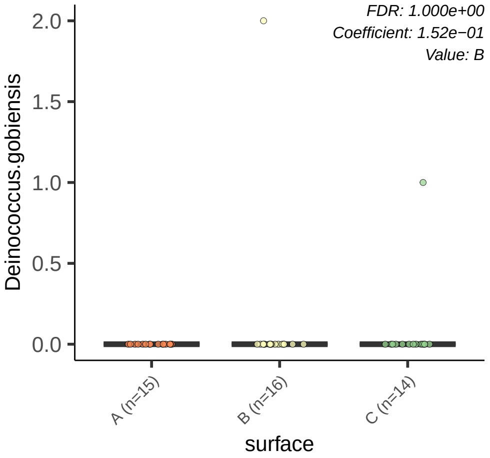

FDR: 1.000e+00
Coefficient: 1.52e−01
Value: B
Deinococcus.gobiensis
2.0
1.5
1.0
0.5
0.0
A (n=15)
B (n=16)
C (n=14)
surface

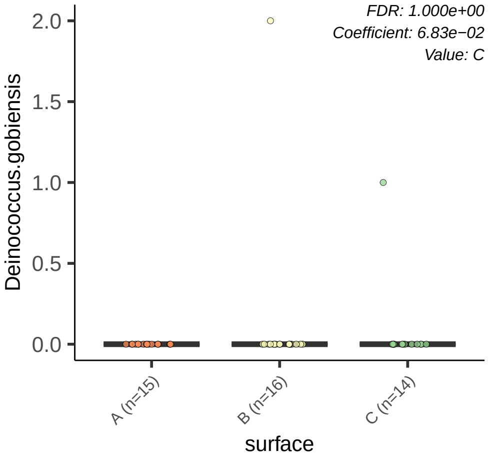

FDR: 1.000e+00
Coefficient: 6.83e−02
Value: C
Deinococcus.gobiensis
2.0
1.5
1.0
0.5
0.0
A (n=15)
B (n=16)
C (n=14)
surface

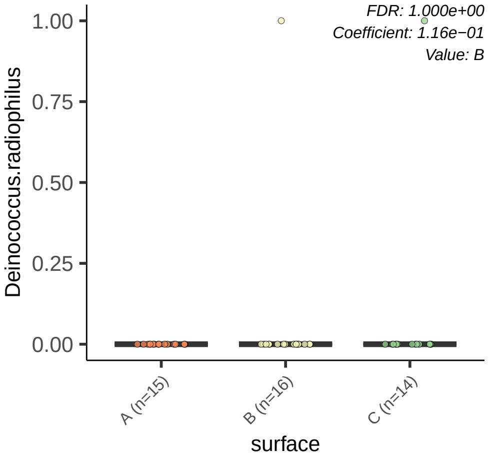
FDR: 1.000e+00
Coefficient: 1.16e−01
Value: B
Deinococcus.radiophilus
1.00
0.75
0.50
0.25
0.00
A (n=15)
B (n=16)
C (n=14)
surface

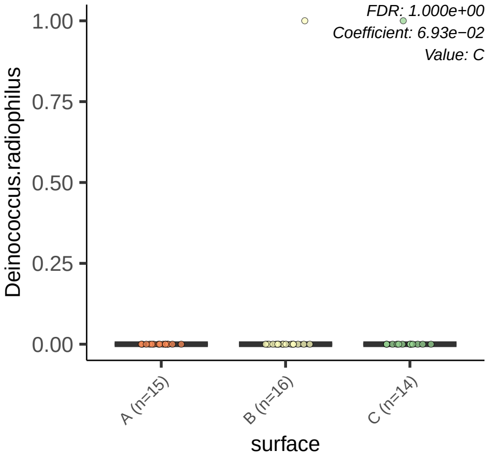
FDR: 1.000e+00
Coefficient: 6.93e−02
Value: C
Deinococcus.radiophilus
1.00
0.75
0.50
0.25
0.00
A (n=15)
B (n=16)
C (n=14)
surface

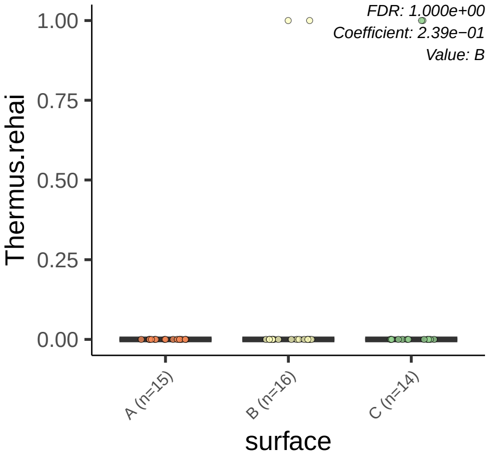
FDR: 1.000e+00
Coefficient: 2.39e−01
Value: B
1.00
0.75
0.50
0.25
0.00
Thermus.rehai
A (n=15)
B (n=16)
C (n=14)
surface

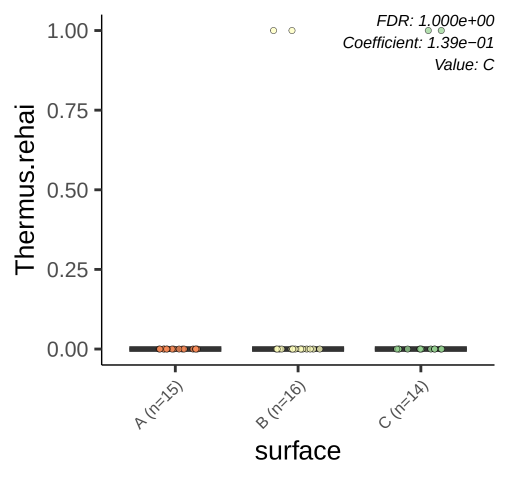
FDR: 1.000e+00
Coefficient: 1.39e−01
Value: C
Thermus.rehai
1.00
0.75
0.50
0.25
0.00
A (n=15)
B (n=16)
C (n=14)
surface

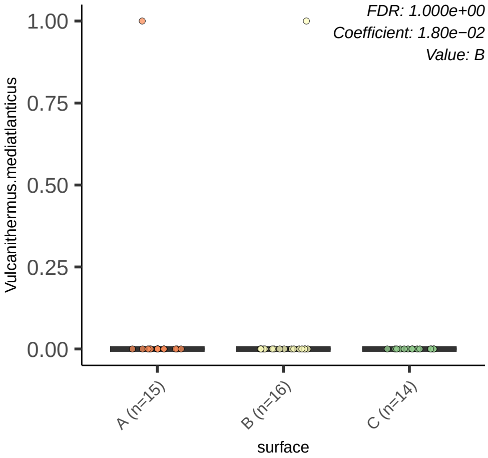

FDR: 1.000e+00
Coefficient: 1.80e−02
Value: B
1.00
0.75
0.50
0.25
0.00
Vulcanithermus.mediatlanticus
A (n=15)
B (n=16)
C (n=14)
surface

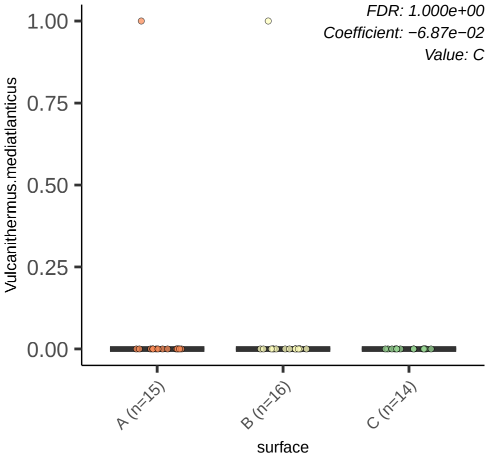

FDR: 1.000e+00
Coefficient: −6.87e−02
Value: C
1.00
0.75
0.50
0.25
0.00
Vulcanithermus.mediatlanticus
A (n=15)
B (n=16)
C (n=14)
surface

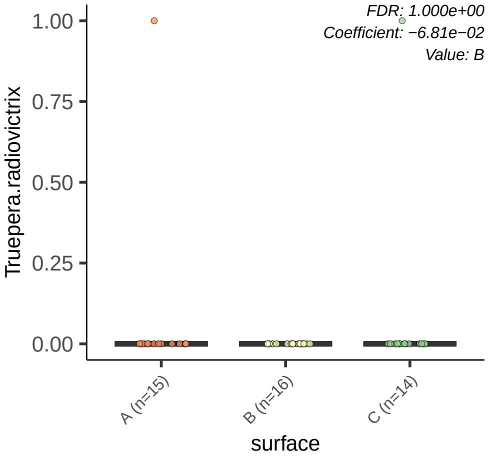

FDR: 1.000e+00
Coefficient: −6.81e−02
Value: B
Truepera.radiovictrix
1.00
0.75
0.50
0.25
0.00
A (n=15)
B (n=16)
C (n=14)
surface

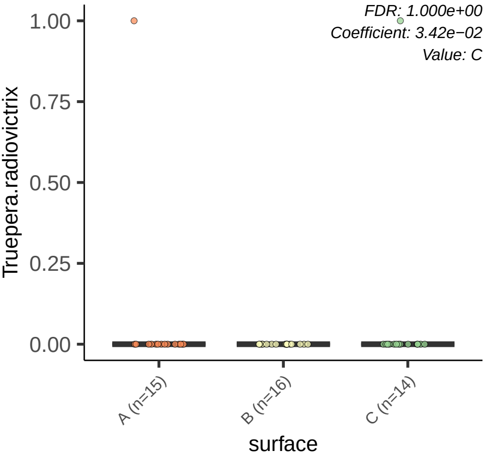

FDR: 1.000e+00
Coefficient: 3.42e−02
Value: C
1.00
0.75
0.50
0.25
0.00
Truepera.radiovictrix
A (n=15)
B (n=16)
C (n=14)
surface

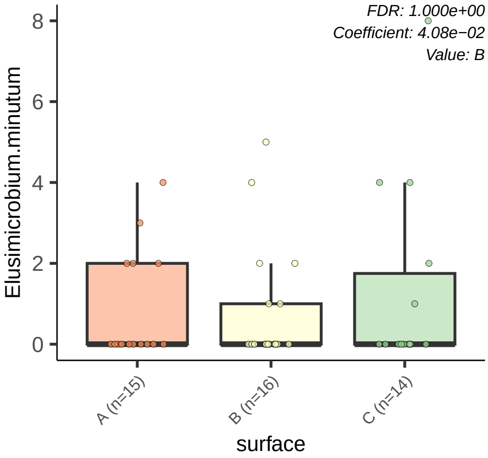

FDR: 1.000e+00
Coefficient: 4.08e−02
Value: B
Elusimicrobium.minutum
0
2
4
6
8
A (n=15)
B (n=16)
C (n=14)
surface

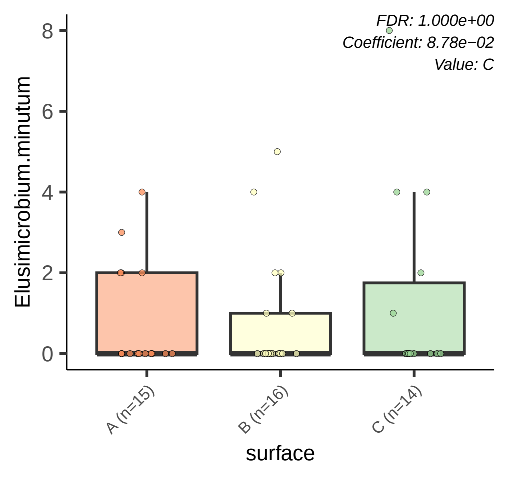

FDR: 1.000e+00
Coefficient: 8.78e−02
Value: C
Elusimicrobium.minutum
0
2
4
6
8
A (n=15)
B (n=16)
C (n=14)
surface

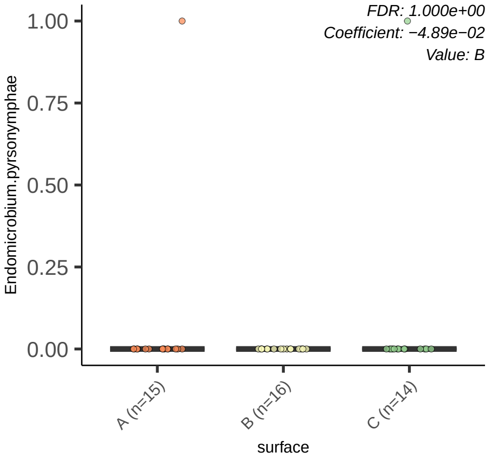

FDR: 1.000e+00
Coefficient: −4.89e−02
Value: B
1.00
0.75
0.50
0.25
0.00
Endomicrobium.pyrsonymphae
A (n=15)
B (n=16)
C (n=14)
surface

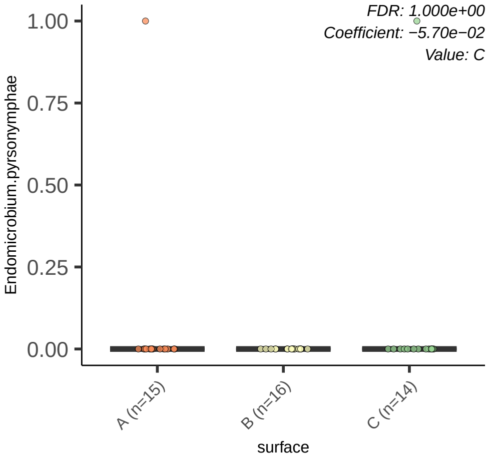

FDR: 1.000e+00
Coefficient: −5.70e−02
Value: C
1.00
0.75
0.50
0.25
0.00
Endomicrobium.pyrsonymphae
A (n=15)
B (n=16)
C (n=14)
surface

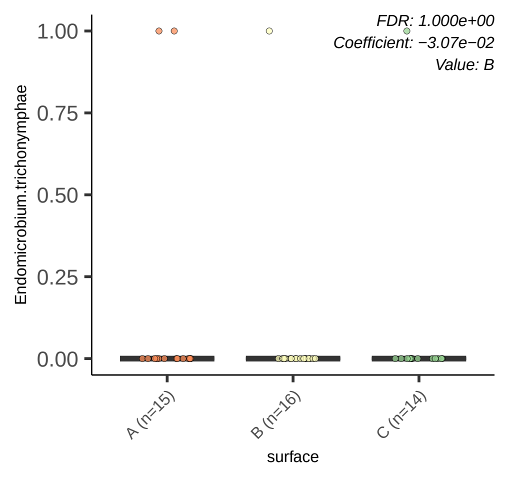

FDR: 1.000e+00
Coefficient: −3.07e−02
Value: B
Endomicrobium.trichonymphae
1.00
0.75
0.50
0.25
0.00
A (n=15)
B (n=16)
C (n=14)
surface

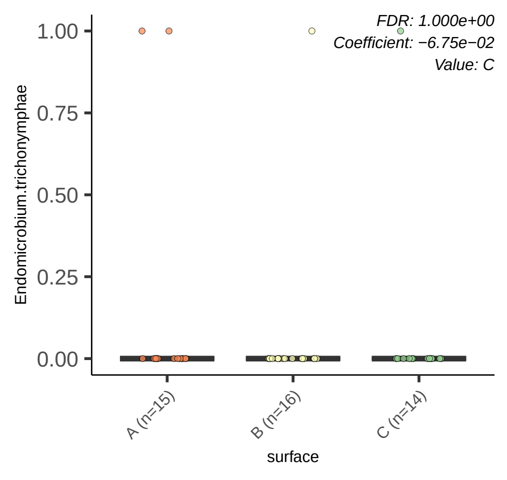
FDR: 1.000e+00
Coefficient: −6.75e−02
Value: C
Endomicrobium.trichonymphae
1.00
0.75
0.50
0.25
0.00
A (n=15)
B (n=16)
C (n=14)
surface

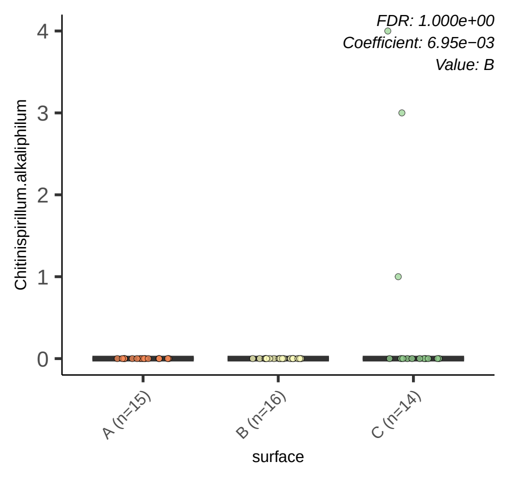
FDR: 1.000e+00
Coefficient: 6.95e−03
Value: B
Chitinispirillum.alkaliphilum
0
1
2
3
4
A (n=15)
B (n=16)
C (n=14)
surface

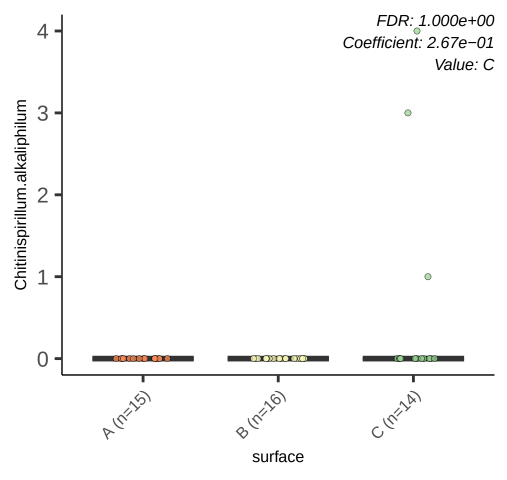

FDR: 1.000e+00
Coefficient: 2.67e−01
Value: C
Chitinispirillum.alkaliphilum
0
1
2
3
4
A (n=15)
B (n=16)
C (n=14)
surface

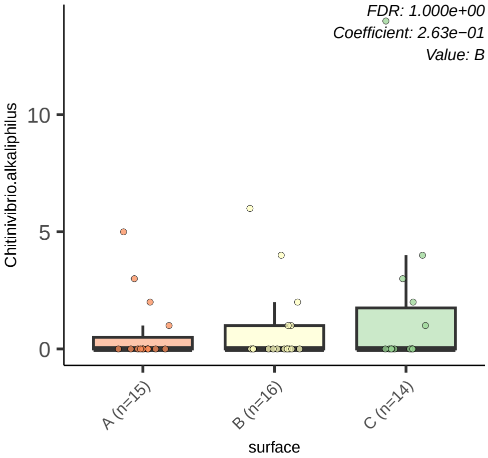

FDR: 1.000e+00
Coefficient: 2.63e−01
Value: B
Chitinivibrio.alkaliphilus
10
5
0
A (n=15)
B (n=16)
C (n=14)
surface

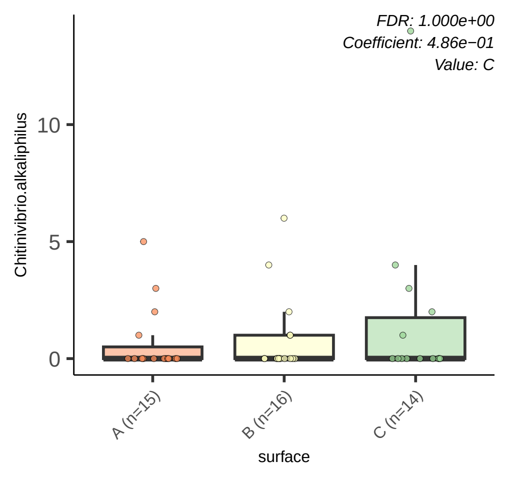

FDR: 1.000e+00
Coefficient: 4.86e−01
Value: C
Chitinivibrio.alkaliphilus
10
5
0
A (n=15)
B (n=16)
C (n=14)
surface

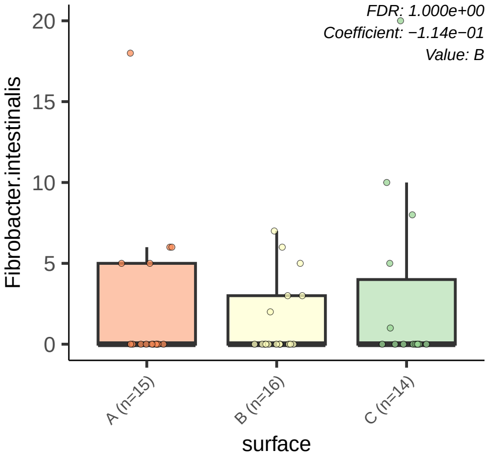
FDR: 1.000e+00
Coefficient: −1.14e−01
Value: B
Fibrobacter.intestinalis
20
15
10
5
0
A (n=15)
B (n=16)
C (n=14)
surface

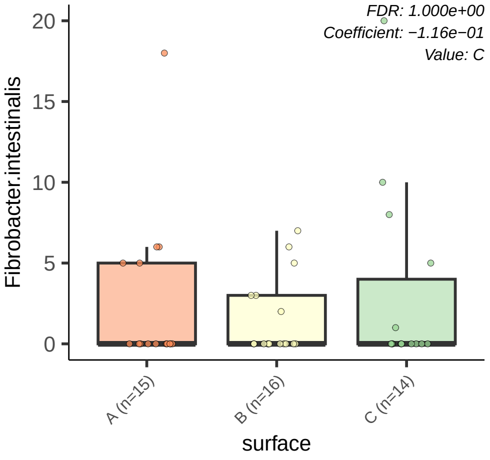

FDR: 1.000e+00
Coefficient: −1.16e−01
Value: C
Fibrobacter.intestinalis
20
15
10
5
0
A (n=15)
B (n=16)
C (n=14)
surface

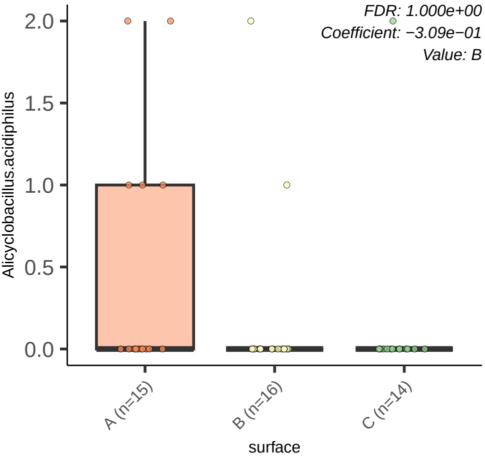

FDR: 1.000e+00
Coefficient: −3.09e−01
Value: B
Alicyclobacillus.acidiphilus
2.0
1.5
1.0
0.5
0.0
A (n=15)
B (n=16)
C (n=14)
surface

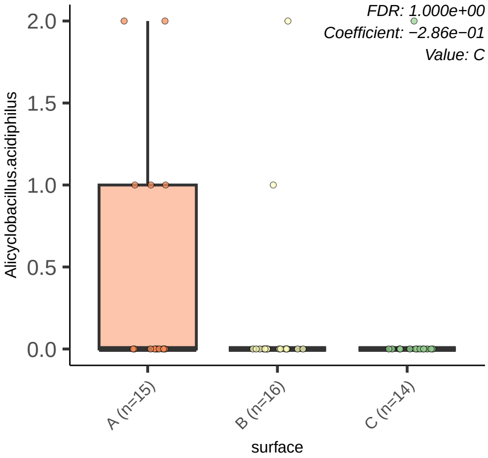
FDR: 1.000e+00
Coefficient: −2.86e−01
Value: C
Alicyclobacillus.acidiphilus
2.0
1.5
1.0
0.5
0.0
A (n=15)
B (n=16)
C (n=14)
surface

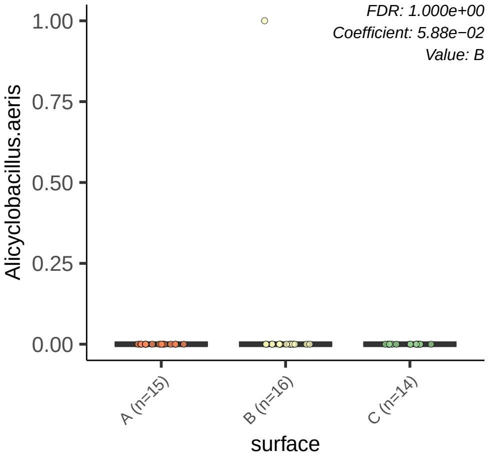
FDR: 1.000e+00
Coefficient: 5.88e−02
Value: B
Alicyclobacillus.aeris
1.00
0.75
0.50
0.25
0.00
A (n=15)
B (n=16)
C (n=14)
surface

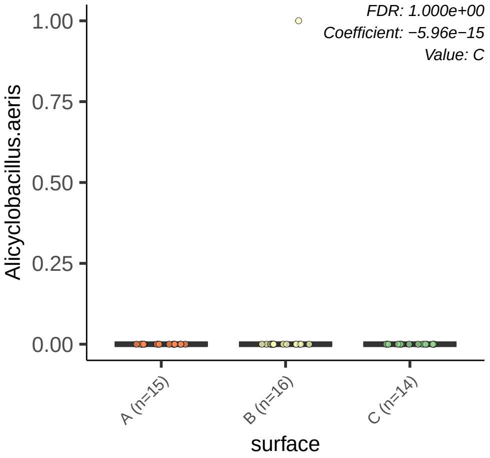
FDR: 1.000e+00
Coefficient: −5.96e−15
Value: C
Alicyclobacillus.aeris
1.00
0.75
0.50
0.25
0.00
A (n=15)
B (n=16)
C (n=14)
surface

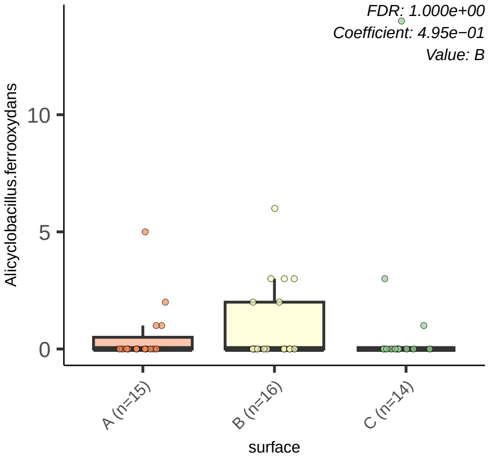

FDR: 1.000e+00
Coefficient: 4.95e−01
Value: B
Alicyclobacillus.ferrooxydans
10
5
0
A (n=15)
B (n=16)
C (n=14)
surface

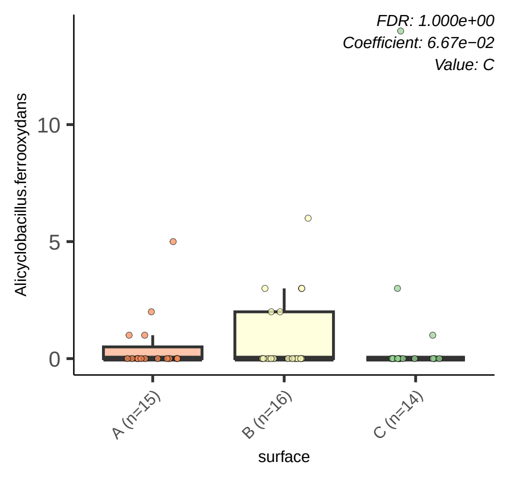
FDR: 1.000e+00
Coefficient: 6.67e−02
Value: C
Alicyclobacillus.ferrooxydans
10
5
0
A (n=15)
B (n=16)
C (n=14)
surface

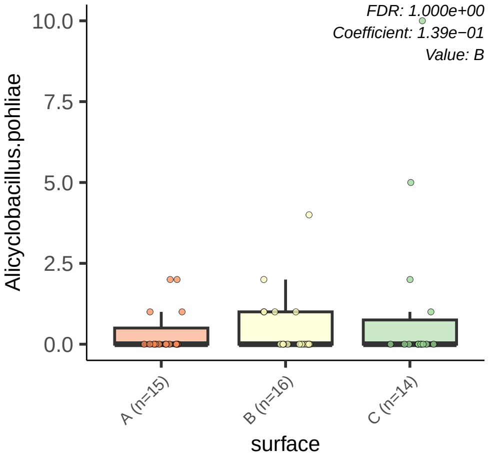

FDR: 1.000e+00
Coefficient: 1.39e−01
Value: B
Alicyclobacillus.pohliae
10.0
7.5
5.0
2.5
0.0
A (n=15)
B (n=16)
C (n=14)
surface

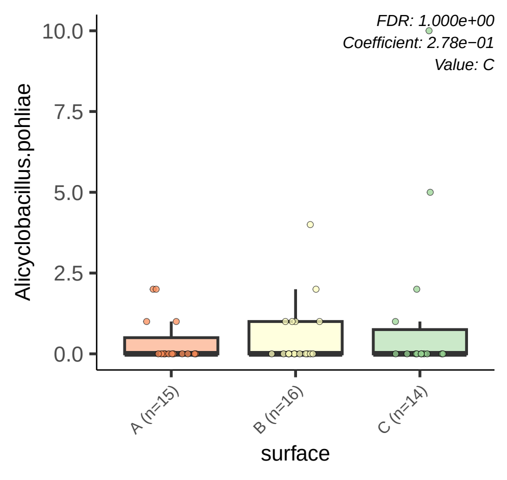
FDR: 1.000e+00
Coefficient: 2.78e−01
Value: C
Alicyclobacillus.pohliae
10.0
7.5
5.0
2.5
0.0
A (n=15)
B (n=16)
C (n=14)
surface

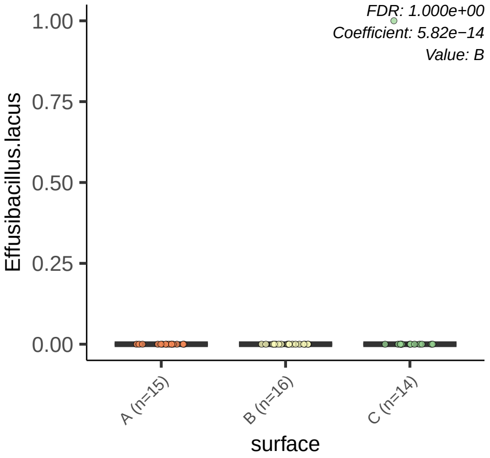
FDR: 1.000e+00
Coefficient: 5.82e−14
Value: B
Effusibacillus.lacus
1.00
0.75
0.50
0.25
0.00
A (n=15)
B (n=16)
C (n=14)
surface

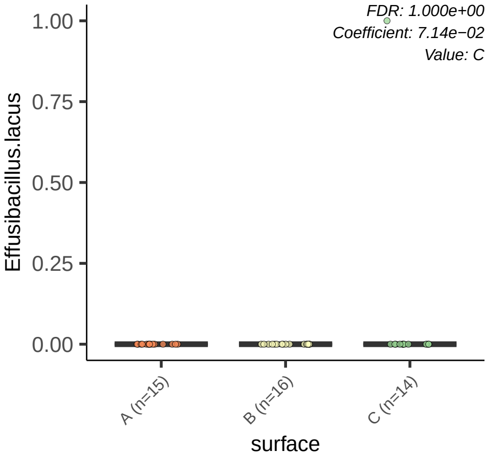
FDR: 1.000e+00
Coefficient: 7.14e−02
Value: C
1.00
0.75
0.50
0.25
0.00
Effusibacillus.lacus
A (n=15)
B (n=16)
C (n=14)
surface

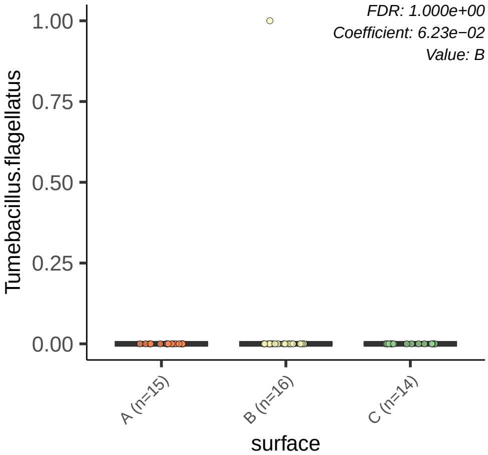

FDR: 1.000e+00
Coefficient: 6.23e−02
Value: B
Tumebacillus.flagellatus
1.00
0.75
0.50
0.25
0.00
A (n=15)
B (n=16)
C (n=14)
surface

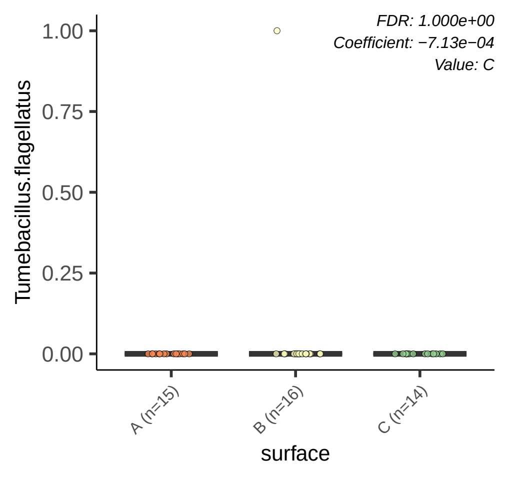
FDR: 1.000e+00
Coefficient: −7.13e−04
Value: C
Tumebacillus.flagellatus
1.00
0.75
0.50
0.25
0.00
A (n=15)
B (n=16)
C (n=14)
surface

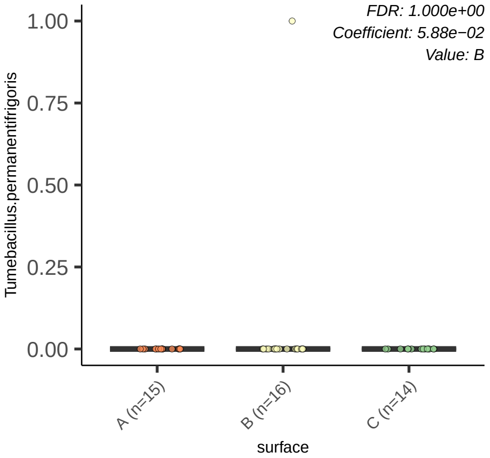

FDR: 1.000e+00
Coefficient: 5.88e−02
Value: B
Tumebacillus.permanentifrigoris
1.00
0.75
0.50
0.25
0.00
A (n=15)
B (n=16)
C (n=14)
surface

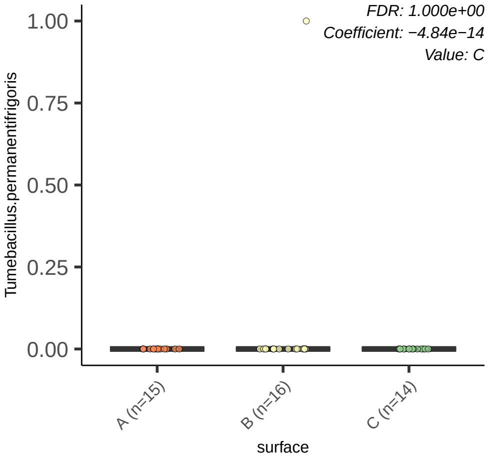

FDR: 1.000e+00
Coefficient: −4.84e−14
Value: C
Tumebacillus.permanentifrigoris
1.00
0.75
0.50
0.25
0.00
A (n=15)
B (n=16)
C (n=14)
surface

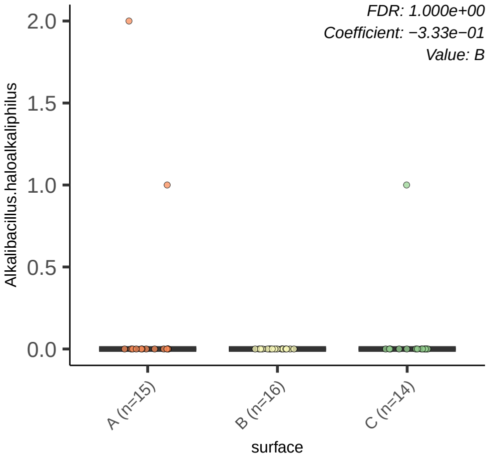
FDR: 1.000e+00
Coefficient: −3.33e−01
Value: B
2.0
1.5
1.0
0.5
0.0
Alkalibacillus.haloalkaliphilus
A (n=15)
B (n=16)
C (n=14)
surface

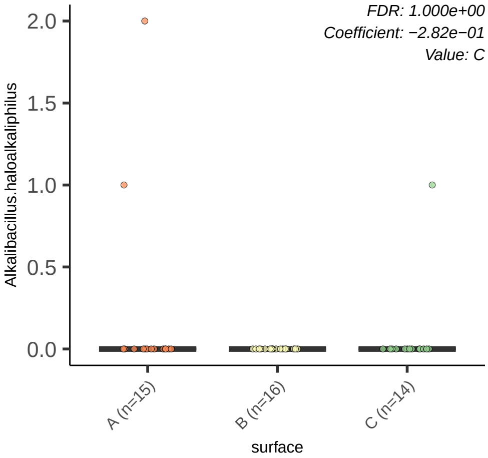
FDR: 1.000e+00
Coefficient: −2.82e−01
Value: C
Alkalibacillus.haloalkaliphilus
2.0
1.5
1.0
0.5
0.0
A (n=15)
B (n=16)
C (n=14)
surface

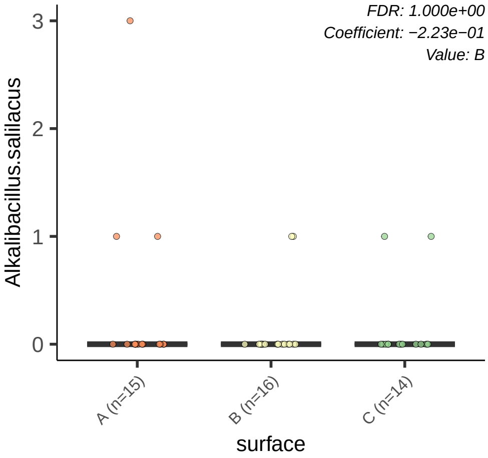
FDR: 1.000e+00
Coefficient: −2.23e−01
Value: B
Alkalibacillus.salilacus
3
2
1
0
A (n=15)
B (n=16)
C (n=14)
surface

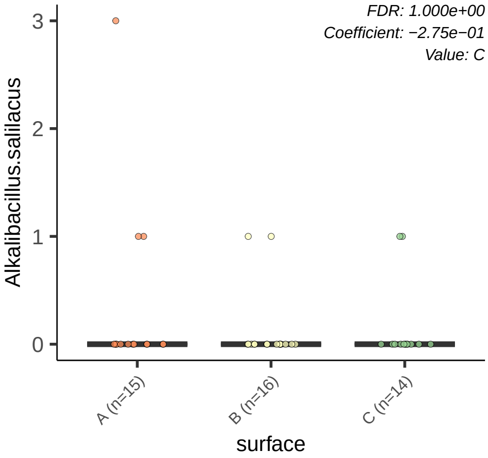
FDR: 1.000e+00
Coefficient: −2.75e−01
Value: C
Alkalibacillus.salilacus
3
2
1
0
A (n=15)
B (n=16)
C (n=14)
surface

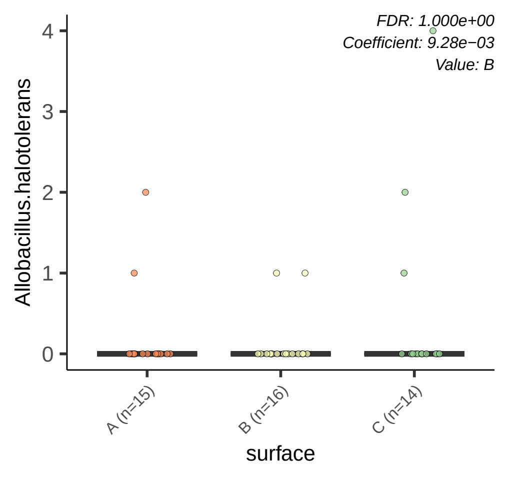

FDR: 1.000e+00
Coefficient: 9.28e−03
Value: B
Allobacillus.halotolerans
4
3
2
1
0
A (n=15)
B (n=16)
C (n=14)
surface

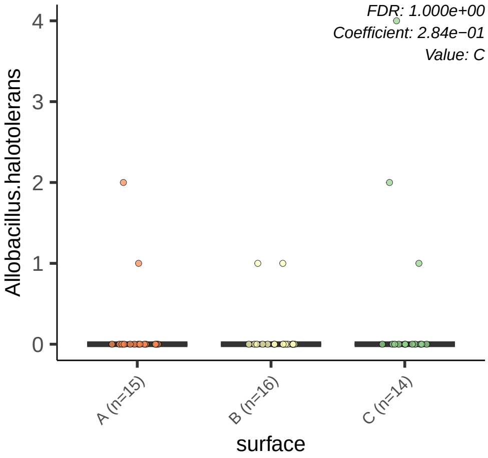
FDR: 1.000e+00
Coefficient: 2.84e−01
Value: C
Allobacillus.halotolerans
0
1
2
3
4
A (n=15)
B (n=16)
C (n=14)
surface

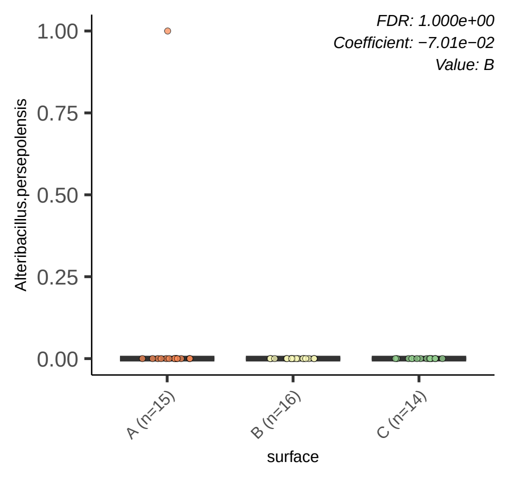
FDR: 1.000e+00
Coefficient: −7.01e−02
Value: B
Alteribacillus.persepolensis
1.00
0.75
0.50
0.25
0.00
A (n=15)
B (n=16)
C (n=14)
surface

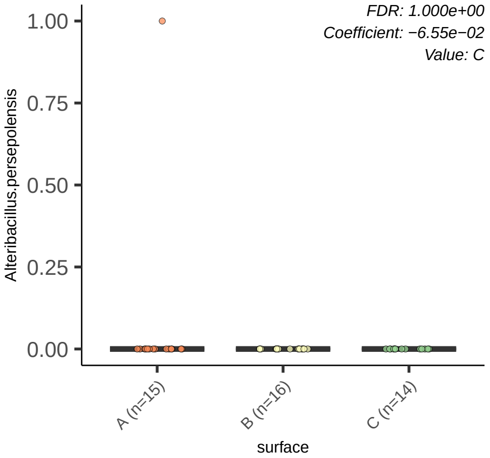
FDR: 1.000e+00
Coefficient: −6.55e−02
Value: C
1.00
0.75
0.50
0.25
0.00
Alteribacillus.persepolensis
A (n=15)
B (n=16)
C (n=14)
surface

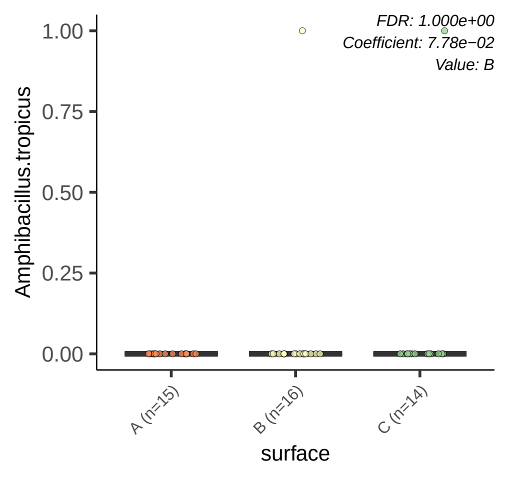

FDR: 1.000e+00
Coefficient: 7.78e−02
Value: B
Amphibacillus.tropicus
1.00
0.75
0.50
0.25
0.00
A (n=15)
B (n=16)
C (n=14)
surface

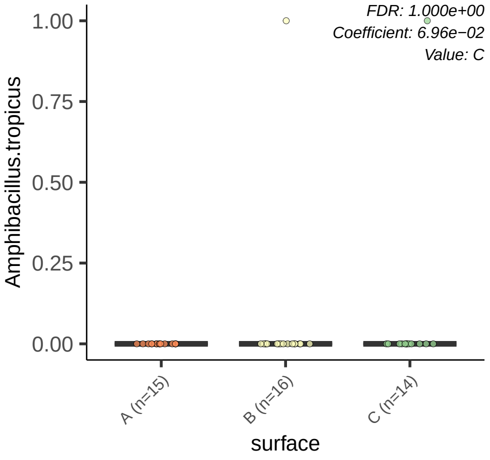
FDR: 1.000e+00
Coefficient: 6.96e−02
Value: C
Amphibacillus.tropicus
1.00
0.75
0.50
0.25
0.00
A (n=15)
B (n=16)
C (n=14)
surface

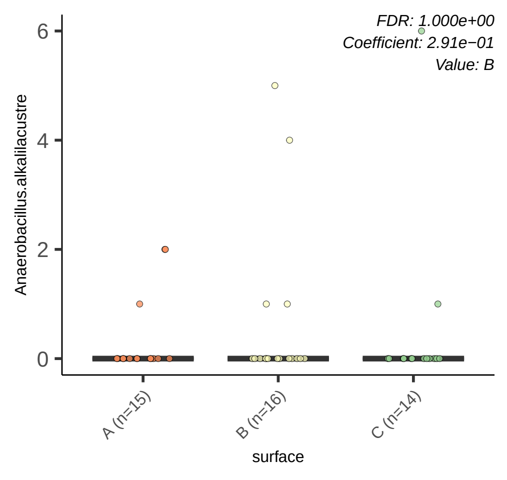

FDR: 1.000e+00
Coefficient: 2.91e−01
Value: B
Anaerobacillus.alkaliacustre
0
2
4
6
A (n=15)
B (n=16)
C (n=14)
surface

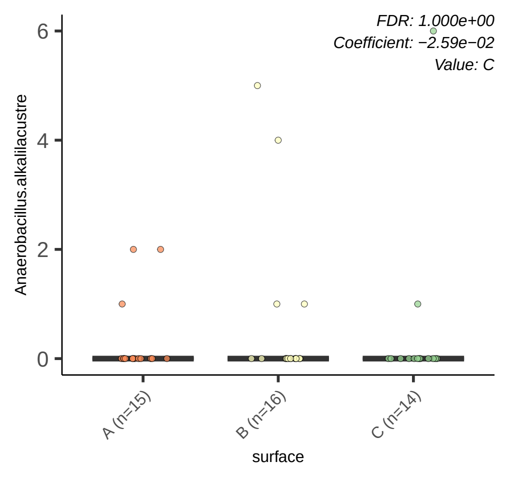

FDR: 1.000e+00
Coefficient: −2.59e−02
Value: C
Anaerobacillus.alkaliacustre
0
2
4
6
A (n=15)
B (n=16)
C (n=14)
surface

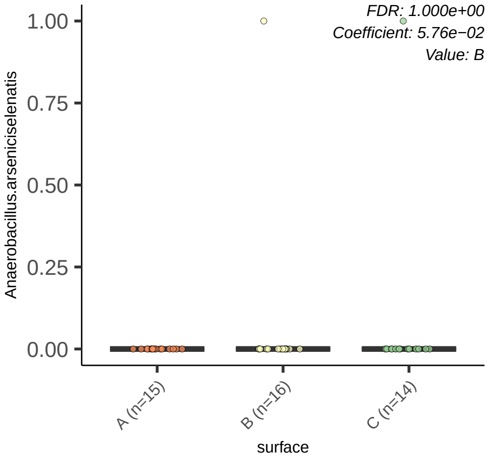

FDR: 1.000e+00
Coefficient: 5.76e−02
Value: B
1.00
0.75
0.50
0.25
0.00
Anaerobacillus.arseniciselenatis
A (n=15)
B (n=16)
C (n=14)
surface

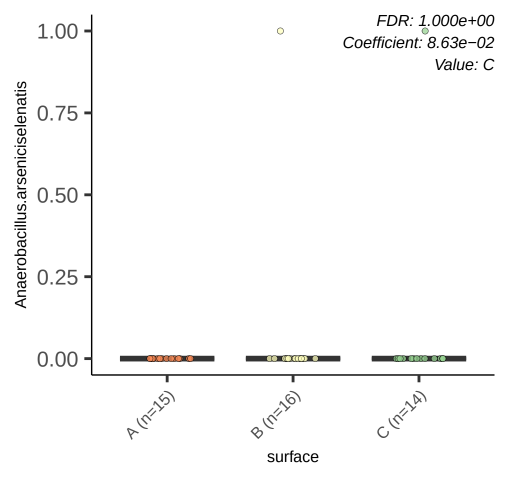
FDR: 1.000e+00
Coefficient: 8.63e−02
Value: C
1.00
0.75
0.50
0.25
0.00
Anaerobacillus.arseniciselenatis
A (n=15)
B (n=16)
C (n=14)
surface

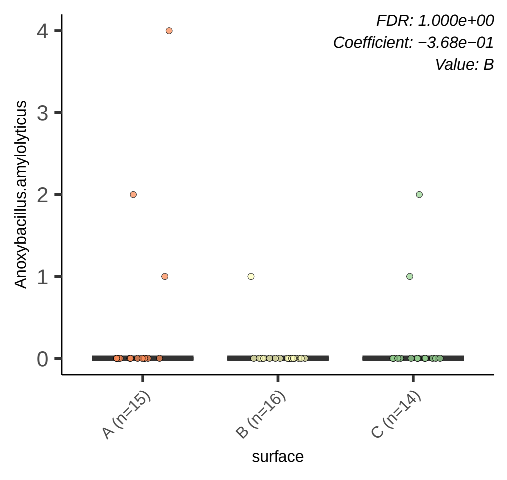
FDR: 1.000e+00
Coefficient: −3.68e−01
Value: B
Anoxybacillus.amylolyticus
4
3
2
1
0
A (n=15)
B (n=16)
C (n=14)
surface

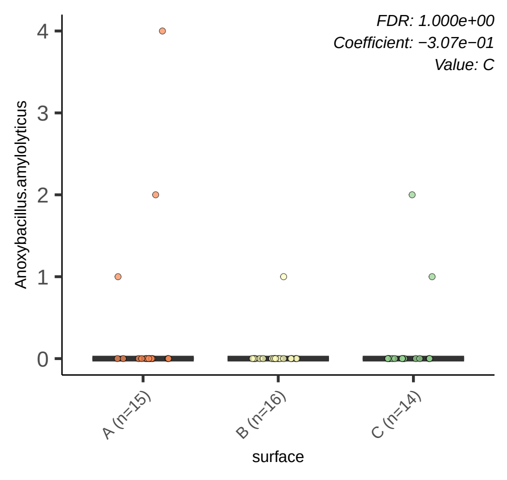
FDR: 1.000e+00
Coefficient: −3.07e−01
Value: C
Anoxybacillus.amylolyticus
0
1
2
3
4
A (n=15)
B (n=16)
C (n=14)
surface

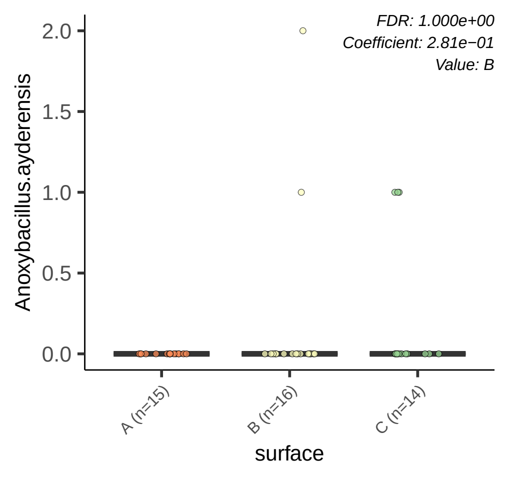

FDR: 1.000e+00
Coefficient: 2.81e−01
Value: B
Anoxybacillus.ayderensis
2.0
1.5
1.0
0.5
0.0
A (n=15)
B (n=16)
C (n=14)
surface

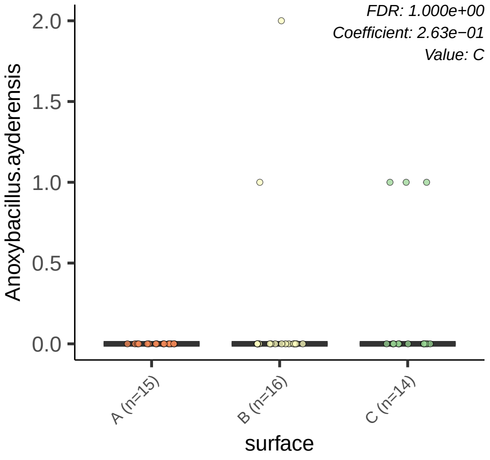
FDR: 1.000e+00
Coefficient: 2.63e−01
Value: C
Anoxybacillus.ayderensis
2.0
1.5
1.0
0.5
0.0
A (n=15)
B (n=16)
C (n=14)
surface

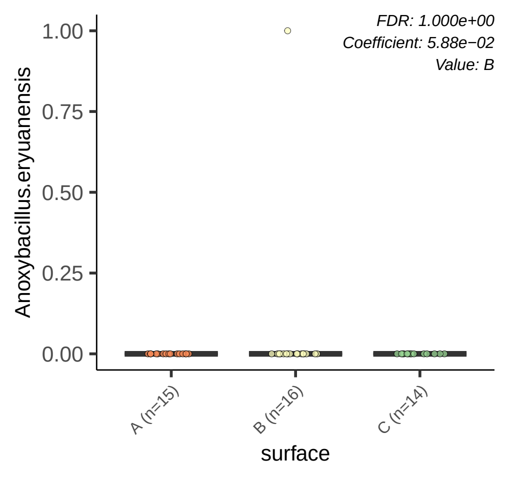

FDR: 1.000e+00
Coefficient: 5.88e−02
Value: B
Anoxybacillus.eryuanensis
1.00
0.75
0.50
0.25
0.00
A (n=15)
B (n=16)
C (n=14)
surface

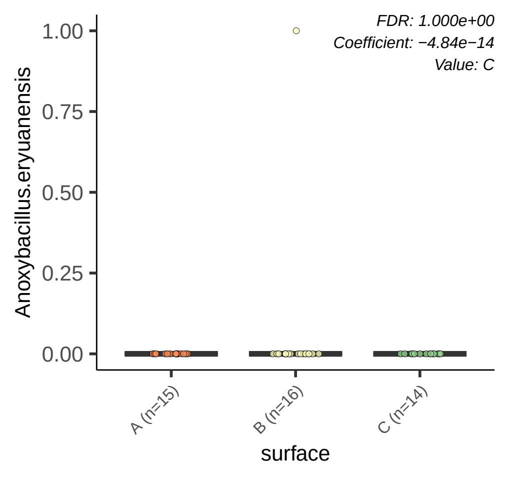
FDR: 1.000e+00
Coefficient: −4.84e−14
Value: C
Anoxybacillus.eryuanensis
1.00
0.75
0.50
0.25
0.00
A (n=15)
B (n=16)
C (n=14)
surface

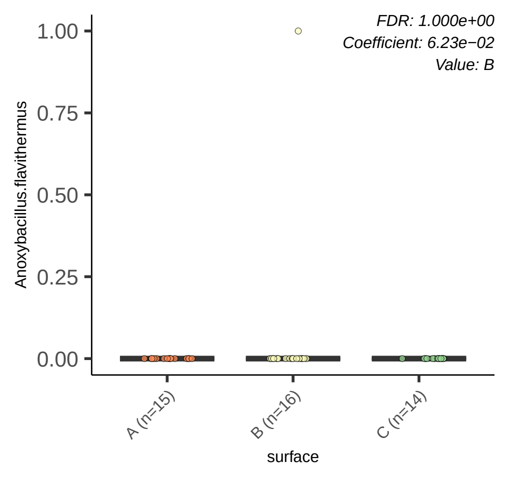
FDR: 1.000e+00
Coefficient: 6.23e−02
Value: B
1.00
0.75
0.50
0.25
0.00
Anoxybacillus.flavithermus
A (n=15)
B (n=16)
C (n=14)
surface

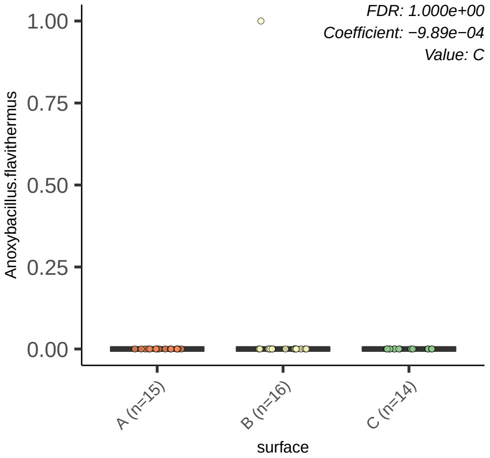
FDR: 1.000e+00
Coefficient: −9.89e−04
Value: C
Anoxybacillus.flavithermus
1.00
0.75
0.50
0.25
0.00
A (n=15)
B (n=16)
C (n=14)
surface

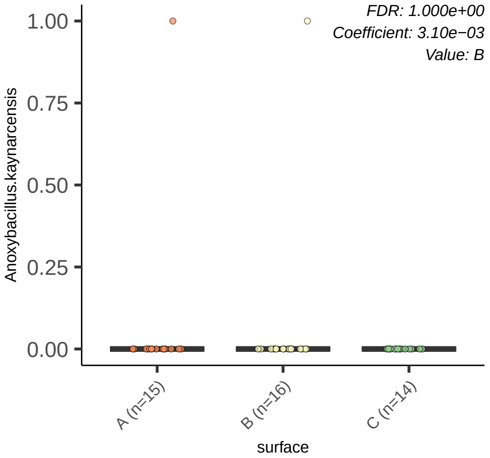

FDR: 1.000e+00
Coefficient: 3.10e−03
Value: B
Anoxybacillus.kaynarcensis
1.00
0.75
0.50
0.25
0.00
A (n=15)
B (n=16)
C (n=14)
surface

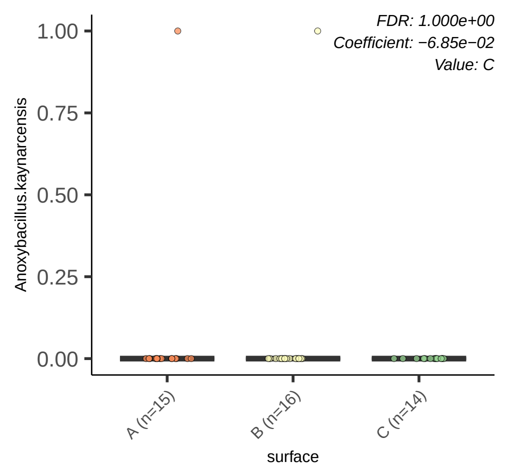

FDR: 1.000e+00
Coefficient: −6.85e−02
Value: C
Anoxybacillus.kaynarcensis
1.00
0.75
0.50
0.25
0.00
A (n=15)
B (n=16)
C (n=14)
surface

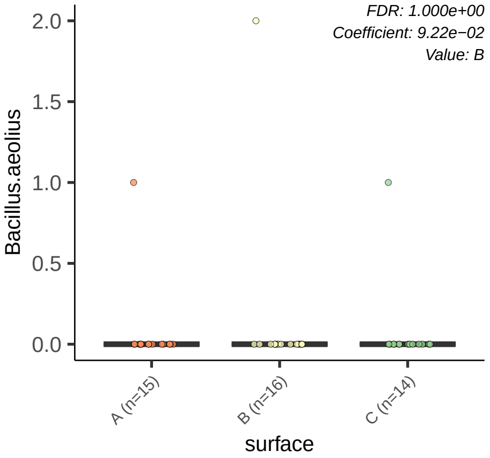
FDR: 1.000e+00
Coefficient: 9.22e−02
Value: B
2.0
1.5
1.0
0.5
0.0
Bacillus.aeolius
A (n=15)
B (n=16)
C (n=14)
surface

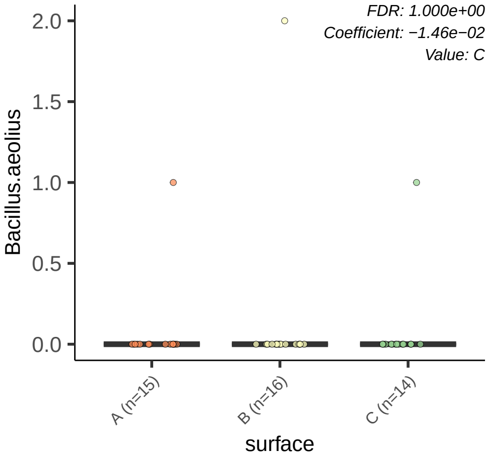
FDR: 1.000e+00
Coefficient: −1.46e−02
Value: C
Bacillus.aeolius
2.0
1.5
1.0
0.5
0.0
A (n=15)
B (n=16)
C (n=14)
surface

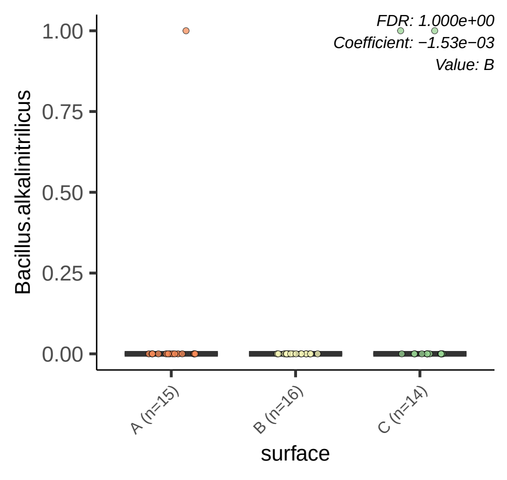

FDR: 1.000e+00
Coefficient: −1.53e−03
Value: B
Bacillus.alkalinitrilicus
1.00
0.75
0.50
0.25
0.00
A (n=15)
B (n=16)
C (n=14)
surface

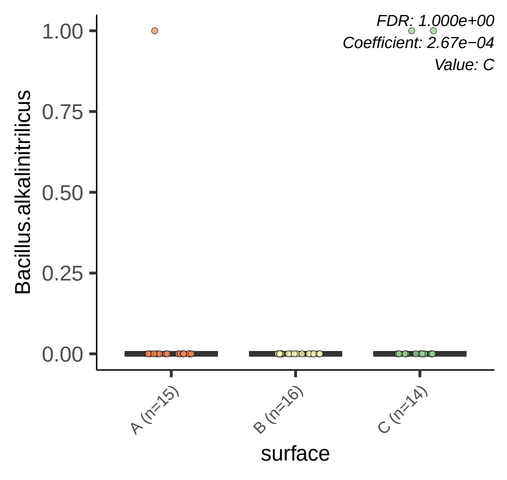
FDR: 1.000e+00
Coefficient: 2.67e−04
Value: C
Bacillus.alkalinitrilicus
1.00
0.75
0.50
0.25
0.00
A (n=15)
B (n=16)
C (n=14)
surface

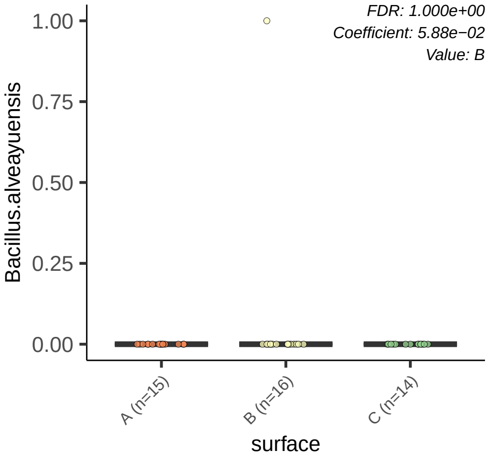
FDR: 1.000e+00
Coefficient: 5.88e−02
Value: B
Bacillus.alveayuensis
1.00
0.75
0.50
0.25
0.00
A (n=15)
B (n=16)
C (n=14)
surface

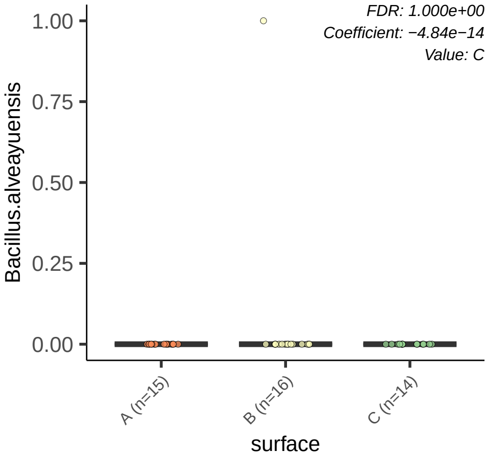
FDR: 1.000e+00
Coefficient: −4.84e−14
Value: C
Bacillus.alveayuensis
1.00
0.75
0.50
0.25
0.00
A (n=15)
B (n=16)
C (n=14)
surface

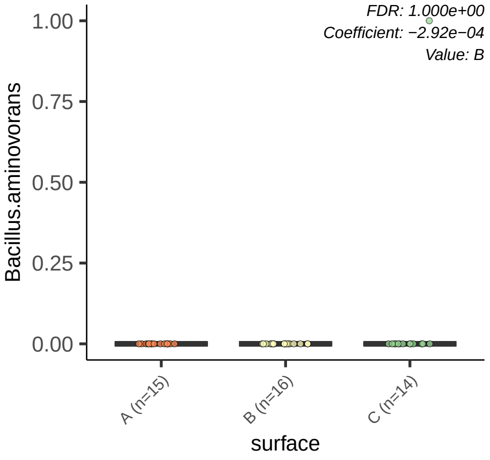
FDR: 1.000e+00
Coefficient: −2.92e−04
Value: B
Bacillus.aminovorans
1.00
0.75
0.50
0.25
0.00
A (n=15)
B (n=16)
C (n=14)
surface

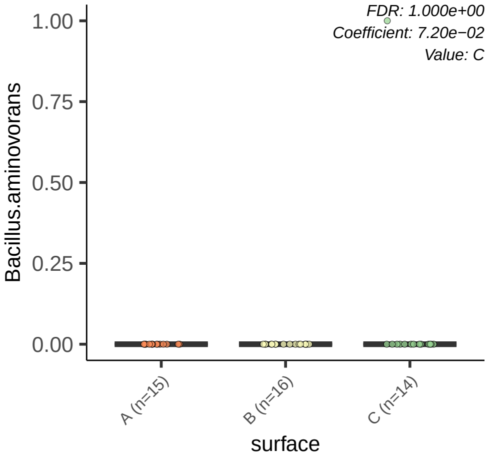

FDR: 1.000e+00
Coefficient: 7.20e−02
Value: C
Bacillus.aminovorans
1.00
0.75
0.50
0.25
0.00
A (n=15)
B (n=16)
C (n=14)
surface

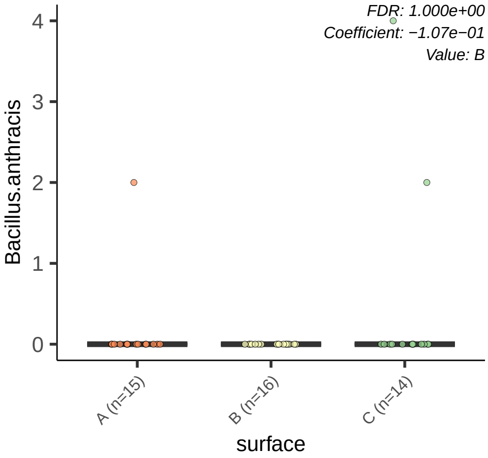
FDR: 1.000e+00
Coefficient: −1.07e−01
Value: B
Bacillus.anthracis
0
1
2
3
4
A (n=15)
B (n=16)
C (n=14)
surface

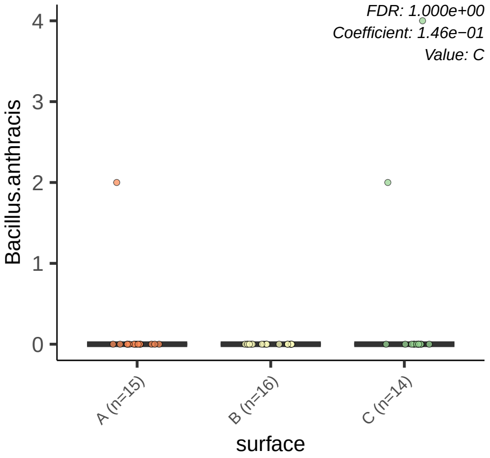

FDR: 1.000e+00
Coefficient: 1.46e−01
Value: C
Bacillus.anthracis
0
1
2
3
4
A (n=15)
B (n=16)
C (n=14)
surface

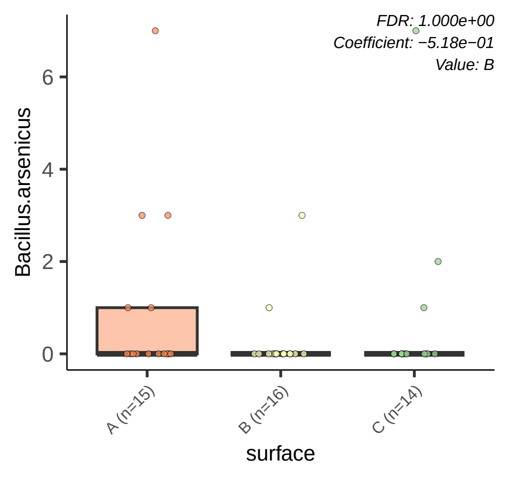
FDR: 1.000e+00
Coefficient: −5.18e−01
Value: B
Bacillus.arsenicus
0
2
4
6
A (n=15)
B (n=16)
C (n=14)
surface

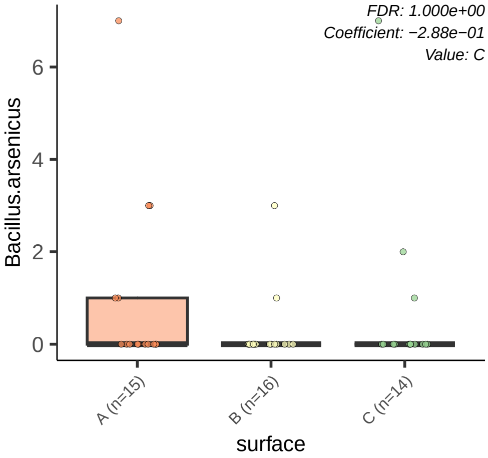

FDR: 1.000e+00
Coefficient: −2.88e−01
Value: C
Bacillus.arsenicus
0
2
4
6
A (n=15)
B (n=16)
C (n=14)
surface

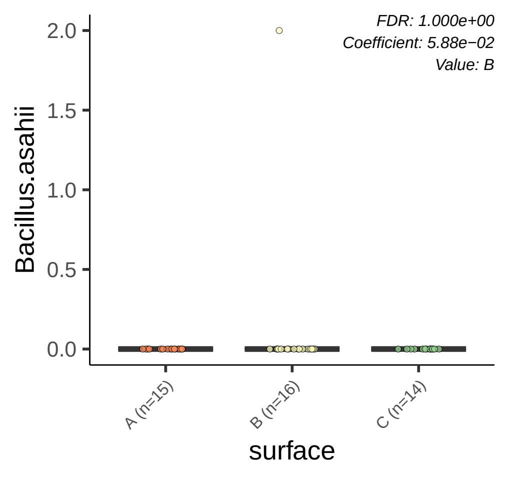

FDR: 1.000e+00
Coefficient: 5.88e−02
Value: B
2.0
1.5
1.0
0.5
0.0
Bacillus.asahii
A (n=15)
B (n=16)
C (n=14)
surface

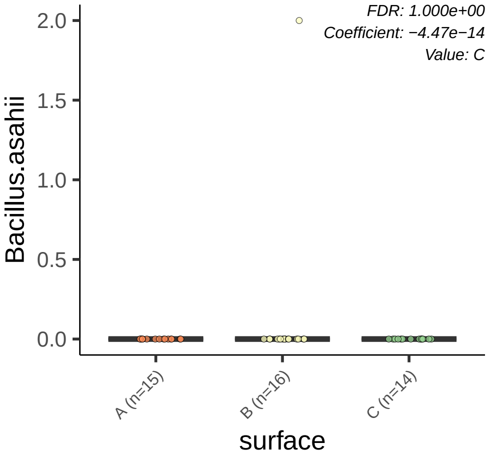

FDR: 1.000e+00
Coefficient: −4.47e−14
Value: C
Bacillus.asahii
2.0
1.5
1.0
0.5
0.0
A (n=15)
B (n=16)
C (n=14)
surface

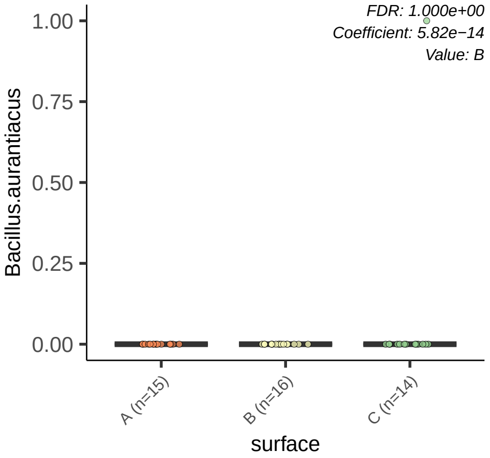
FDR: 1.000e+00
Coefficient: 5.82e−14
Value: B
1.00
0.75
0.50
0.25
0.00
Bacillus.aurantiacus
A (n=15)
B (n=16)
C (n=14)
surface

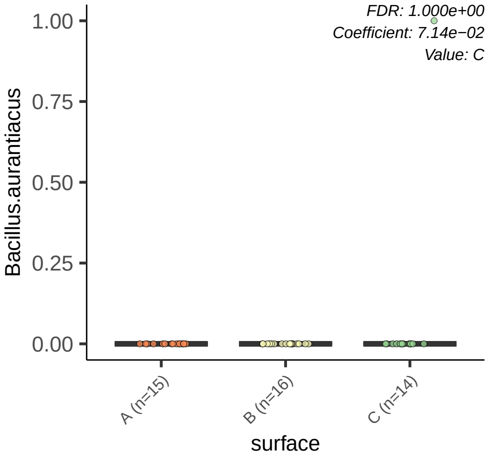
FDR: 1.000e+00
Coefficient: 7.14e−02
Value: C
Bacillus.aurantiacus
1.00
0.75
0.50
0.25
0.00
A (n=15)
B (n=16)
C (n=14)
surface

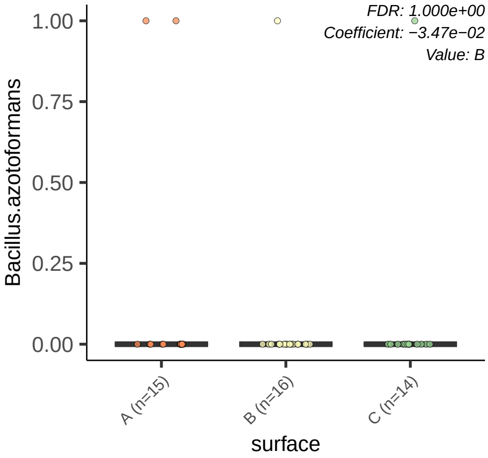
FDR: 1.000e+00
Coefficient: −3.47e−02
Value: B
1.00
0.75
0.50
0.25
0.00
Bacillus.azotoformans
A (n=15)
B (n=16)
C (n=14)
surface

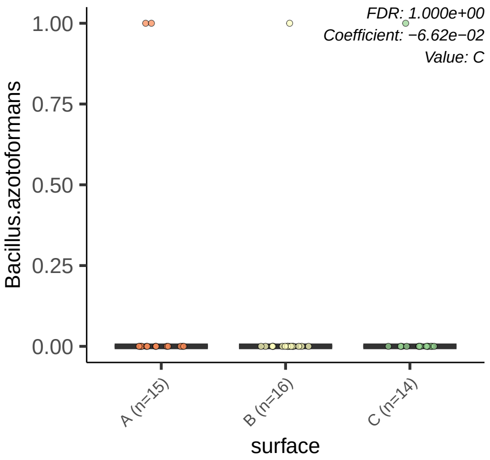

FDR: 1.000e+00
Coefficient: −6.62e−02
Value: C
Bacillus.azotoformans
1.00
0.75
0.50
0.25
0.00
A (n=15)
B (n=16)
C (n=14)
surface

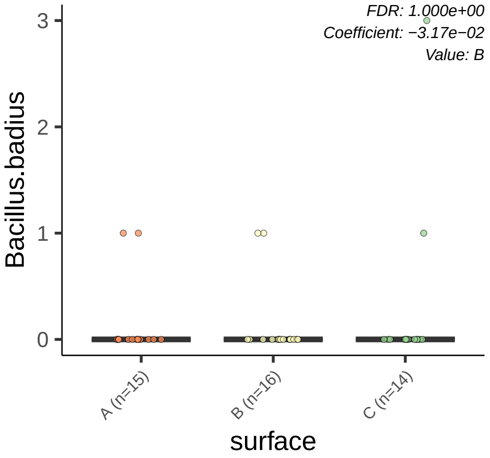
FDR: 1.000e+00
Coefficient: −3.17e−02
Value: B
Bacillus.badius
3
2
1
0
A (n=15)
B (n=16)
C (n=14)
surface

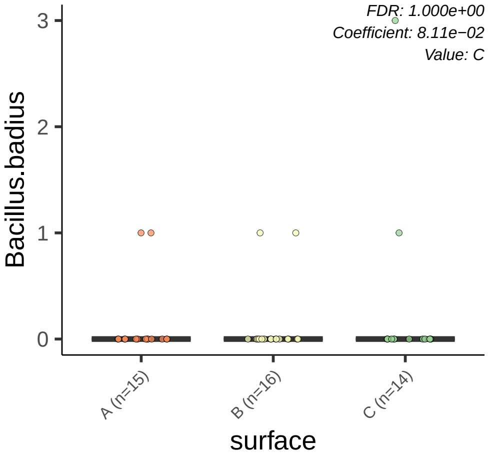
FDR: 1.000e+00
Coefficient: 8.11e−02
Value: C
Bacillus.badius
0
1
2
3
A (n=15)
B (n=16)
C (n=14)
surface

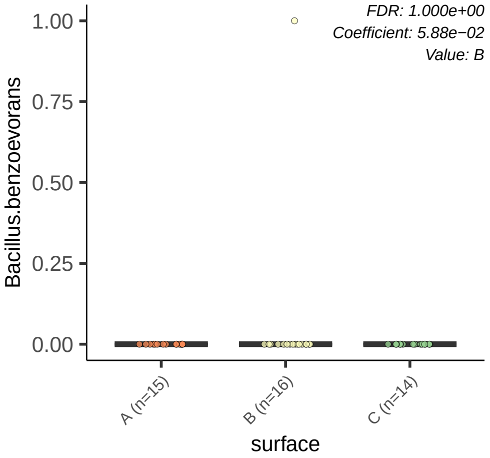

FDR: 1.000e+00
Coefficient: 5.88e−02
Value: B
Bacillus.benzoevorans
1.00
0.75
0.50
0.25
0.00
A (n=15)
B (n=16)
C (n=14)
surface

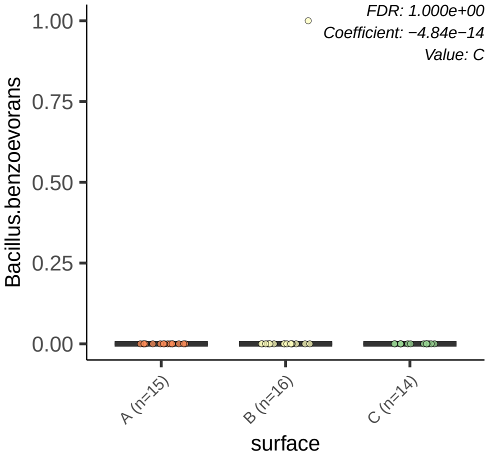
FDR: 1.000e+00
Coefficient: −4.84e−14
Value: C
Bacillus.benzoevorans
1.00
0.75
0.50
0.25
0.00
A (n=15)
B (n=16)
C (n=14)
surface

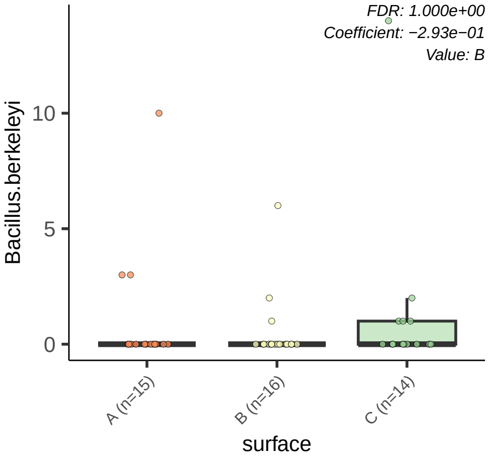
FDR: 1.000e+00
Coefficient: −2.93e−01
Value: B
Bacillus.berkeleyi
10
5
0
A (n=15)
B (n=16)
C (n=14)
surface

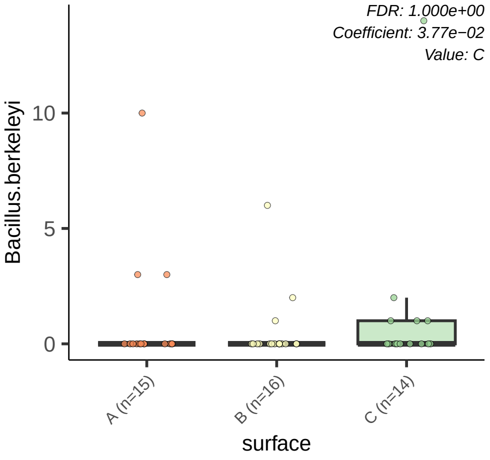

FDR: 1.000e+00
Coefficient: 3.77e−02
Value: C
Bacillus.berkeleyi
10
5
0
A (n=15)
B (n=16)
C (n=14)
surface

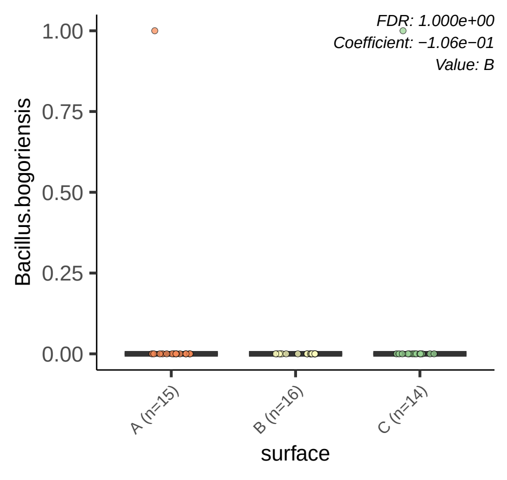

FDR: 1.000e+00
Coefficient: −1.06e−01
Value: B
Bacillus.bogoriensis
1.00
0.75
0.50
0.25
0.00
A (n=15)
B (n=16)
C (n=14)
surface

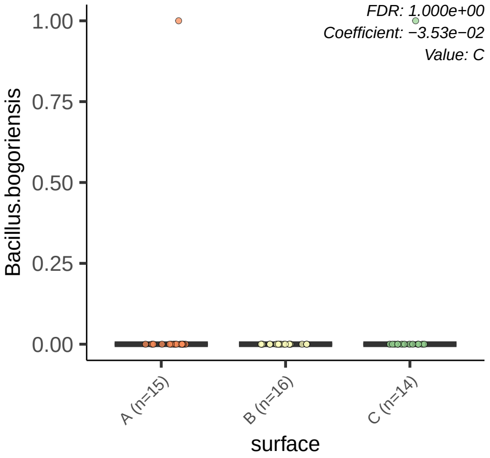

FDR: 1.000e+00
Coefficient: −3.53e−02
Value: C
Bacillus.bogoriensis
1.00
0.75
0.50
0.25
0.00
A (n=15)
B (n=16)
C (n=14)
surface

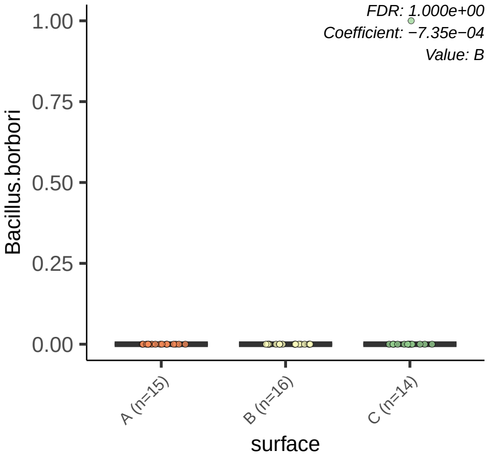
FDR: 1.000e+00
Coefficient: −7.35e−04
Value: B
Bacillus.borbori
1.00
0.75
0.50
0.25
0.00
A (n=15)
B (n=16)
C (n=14)
surface

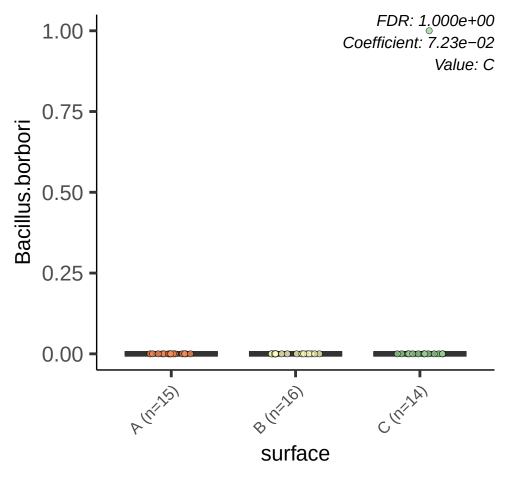
FDR: 1.000e+00
Coefficient: 7.23e−02
Value: C
Bacillus.borbori
1.00
0.75
0.50
0.25
0.00
A (n=15)
B (n=16)
C (n=14)
surface

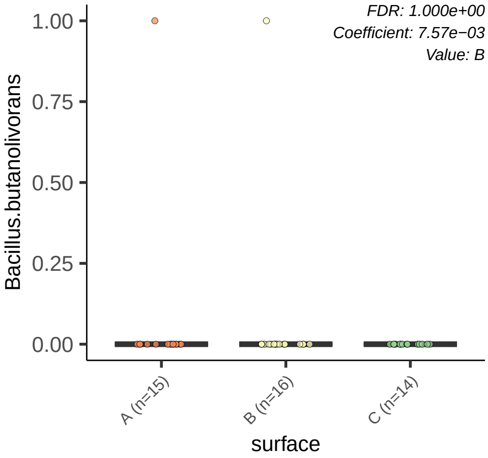
FDR: 1.000e+00
Coefficient: 7.57e−03
Value: B
Bacillus.butanolivorans
1.00
0.75
0.50
0.25
0.00
A (n=15)
B (n=16)
C (n=14)
surface

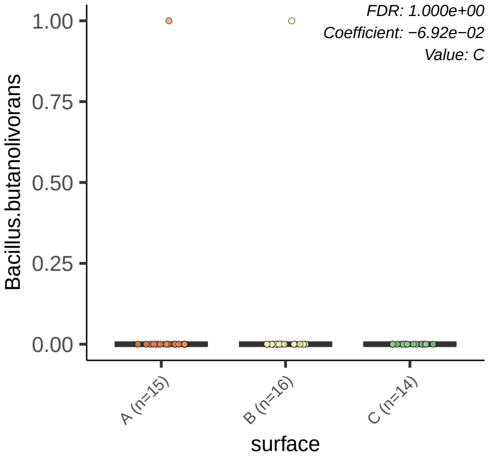

FDR: 1.000e+00
Coefficient: −6.92e−02
Value: C
Bacillus.butanolivorans
1.00
0.75
0.50
0.25
0.00
A (n=15)
B (n=16)
C (n=14)
surface

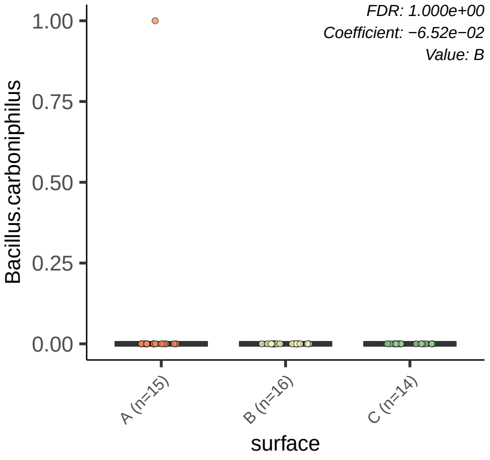

FDR: 1.000e+00
Coefficient: −6.52e−02
Value: B
Bacillus.carboniphilus
1.00
0.75
0.50
0.25
0.00
A (n=15)
B (n=16)
C (n=14)
surface

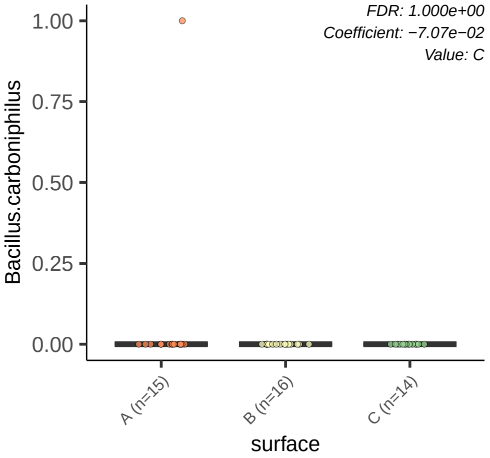
FDR: 1.000e+00
Coefficient: −7.07e−02
Value: C
Bacillus.carboniphilus
1.00
0.75
0.50
0.25
0.00
A (n=15)
B (n=16)
C (n=14)
surface

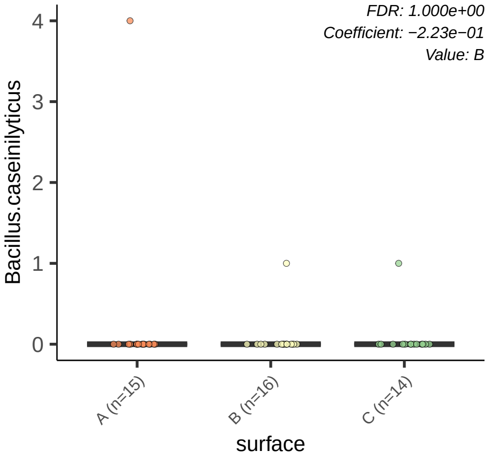

FDR: 1.000e+00
Coefficient: −2.23e−01
Value: B
Bacillus.caseinilyticus
0
1
2
3
4
A (n=15)
B (n=16)
C (n=14)
surface

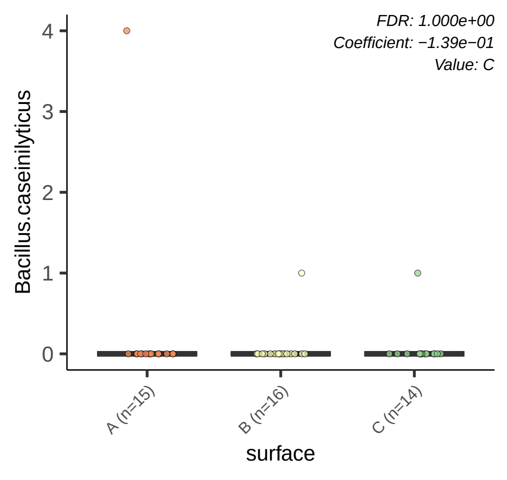
FDR: 1.000e+00
Coefficient: −1.39e−01
Value: C
Bacillus.caseinilyticus
0
1
2
3
4
A (n=15)
B (n=16)
C (n=14)
surface

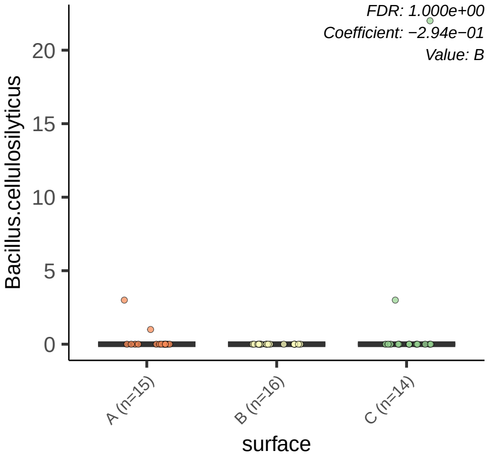
FDR: 1.000e+00
Coefficient: −2.94e−01
Value: B
Bacillus.cellulosilyticus
20
15
10
5
0
A (n=15)
B (n=16)
C (n=14)
surface

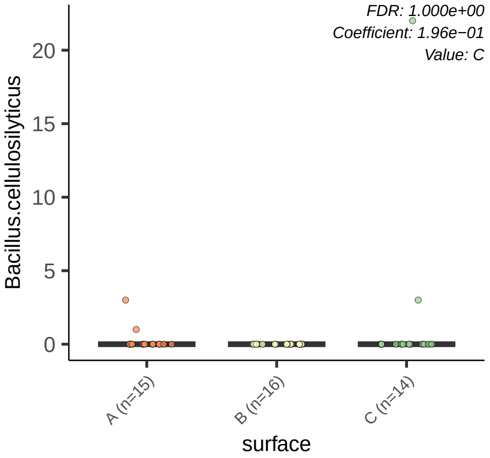
FDR: 1.000e+00
Coefficient: 1.96e−01
Value: C
Bacillus.cellulosilyticus
20
15
10
5
0
A (n=15)
B (n=16)
C (n=14)
surface

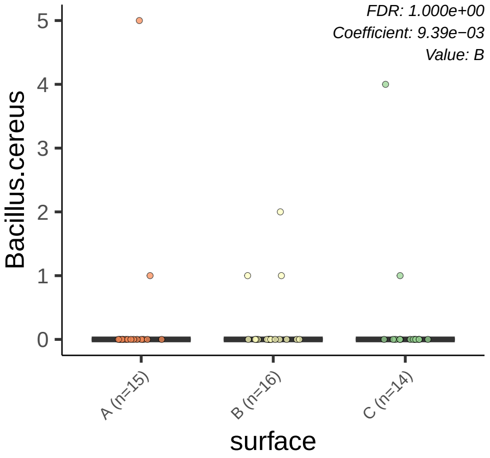

FDR: 1.000e+00
Coefficient: 9.39e−03
Value: B
Bacillus.cereus
0
1
2
3
4
5
A (n=15)
B (n=16)
C (n=14)
surface

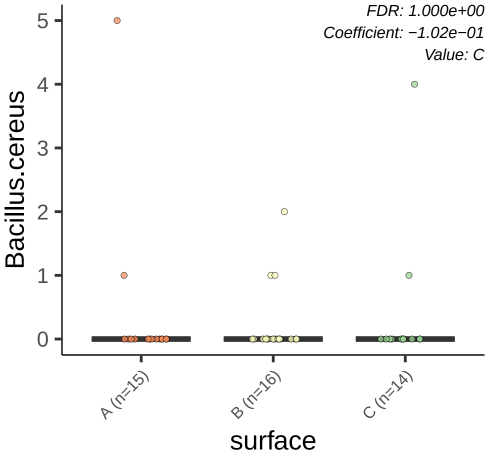

FDR: 1.000e+00
Coefficient: −1.02e−01
Value: C
Bacillus.cereus
0
1
2
3
4
5
A (n=15)
B (n=16)
C (n=14)
surface

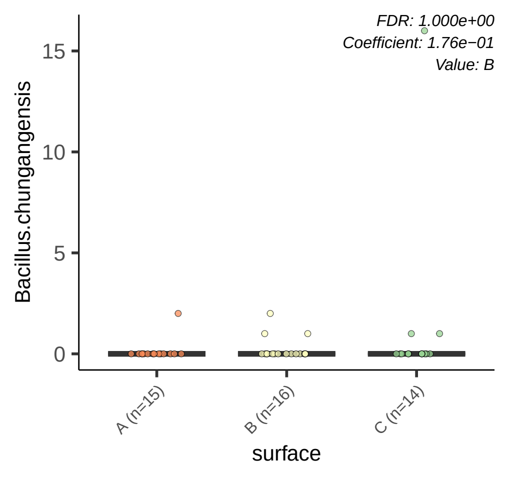
FDR: 1.000e+00
Coefficient: 1.76e−01
Value: B
Bacillus.chungangensis
0
5
10
15
A (n=15)
B (n=16)
C (n=14)
surface

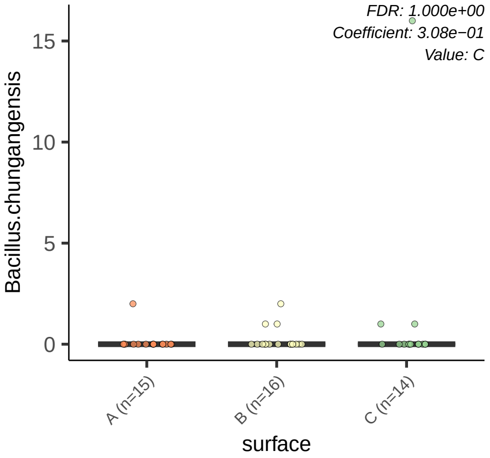

FDR: 1.000e+00
Coefficient: 3.08e−01
Value: C
Bacillus.chungangensis
15
10
5
0
A (n=15)
B (n=16)
C (n=14)
surface

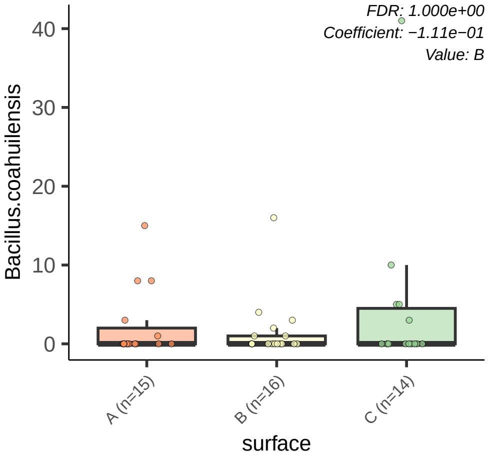

FDR: 1.000e+00
Coefficient: −1.11e−01
Value: B
Bacillus.coahuilensis
40
30
20
10
0
A (n=15)
B (n=16)
C (n=14)
surface

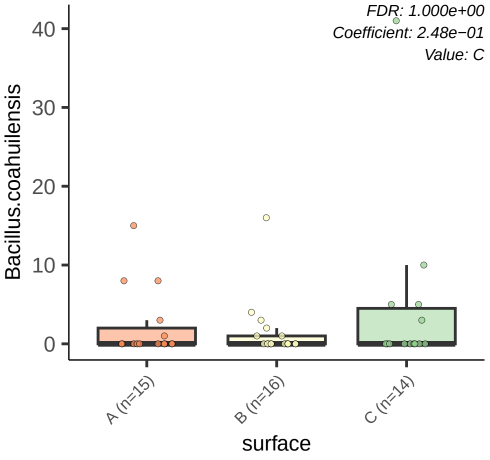

FDR: 1.000e+00
Coefficient: 2.48e−01
Value: C
40
30
20
10
0
Bacillus.coahuilensis
A (n=15)
B (n=16)
C (n=14)
surface

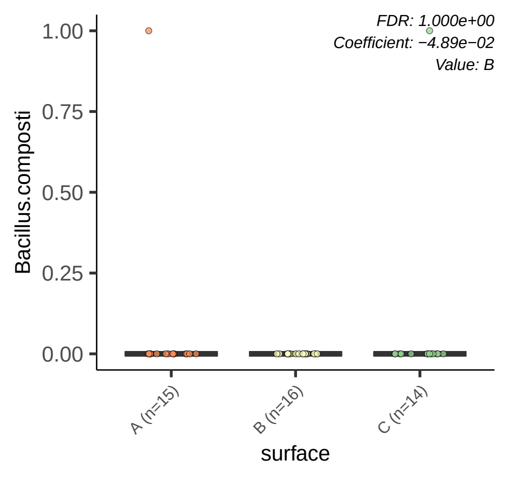

FDR: 1.000e+00
Coefficient: −4.89e−02
Value: B
Bacillus.composti
1.00
0.75
0.50
0.25
0.00
A (n=15)
B (n=16)
C (n=14)
surface

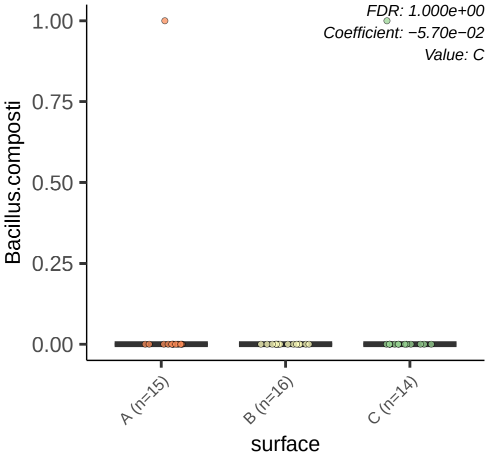

FDR: 1.000e+00
Coefficient: −5.70e−02
Value: C
Bacillus.composti
1.00
0.75
0.50
0.25
0.00
A (n=15)
B (n=16)
C (n=14)
surface

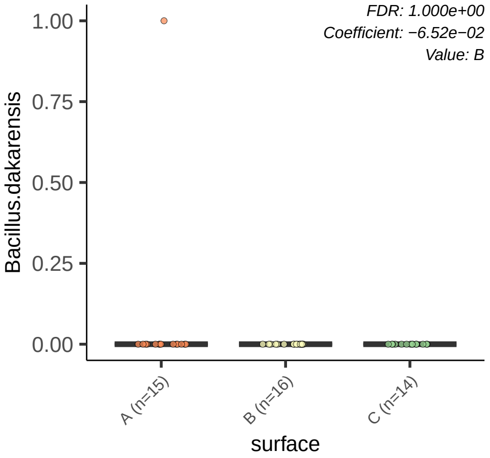
FDR: 1.000e+00
Coefficient: −6.52e−02
Value: B
Bacillus.dakarensis
1.00
0.75
0.50
0.25
0.00
A (n=15)
B (n=16)
C (n=14)
surface

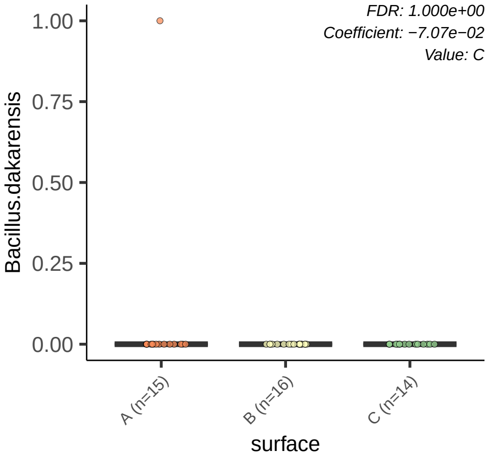
FDR: 1.000e+00
Coefficient: −7.07e−02
Value: C
Bacillus.dakarensis
1.00
0.75
0.50
0.25
0.00
A (n=15)
B (n=16)
C (n=14)
surface

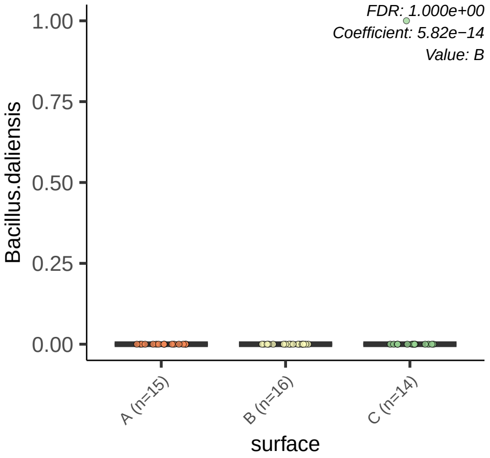

FDR: 1.000e+00
Coefficient: 5.82e−14
Value: B
1.00
0.75
0.50
0.25
0.00
Bacillus.daliensis
A (n=15)
B (n=16)
C (n=14)
surface

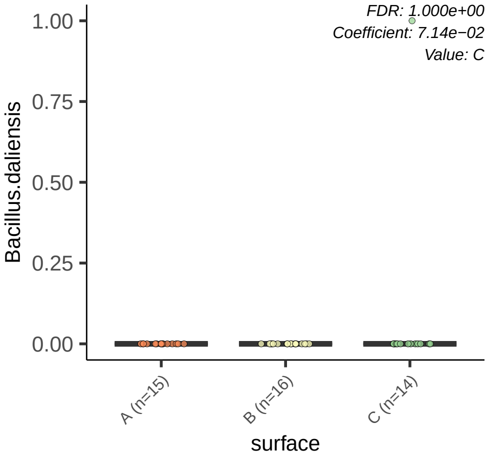
FDR: 1.000e+00
Coefficient: 7.14e−02
Value: C
Bacillus.daliensis
1.00
0.75
0.50
0.25
0.00
A (n=15)
B (n=16)
C (n=14)
surface

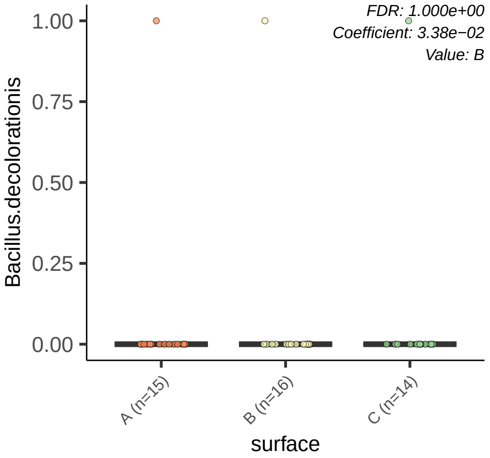

FDR: 1.000e+00
Coefficient: 3.38e−02
Value: B
1.00
0.75
0.50
0.25
0.00
Bacillus.decolorationis
A (n=15)
B (n=16)
C (n=14)
surface

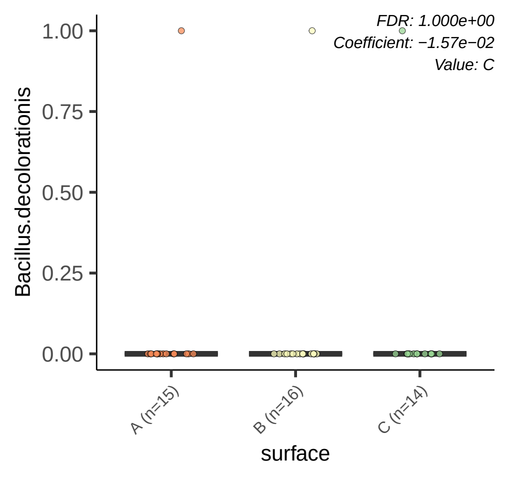
FDR: 1.000e+00
Coefficient: −1.57e−02
Value: C
Bacillus.decolorationis
1.00
0.75
0.50
0.25
0.00
A (n=15)
B (n=16)
C (n=14)
surface

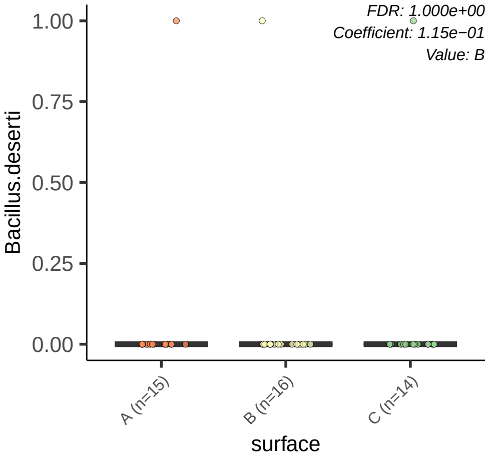
FDR: 1.000e+00
Coefficient: 1.15e−01
Value: B
Bacillus.deserti
1.00
0.75
0.50
0.25
0.00
A (n=15)
B (n=16)
C (n=14)
surface

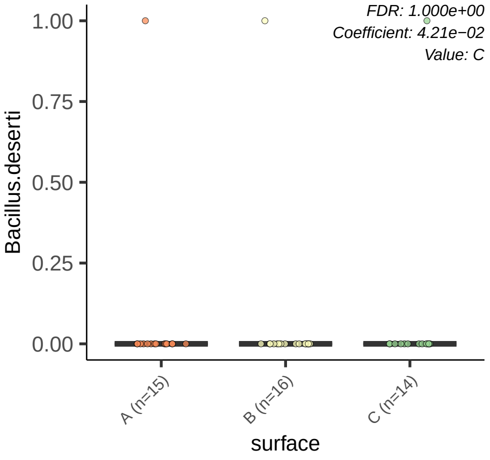

FDR: 1.000e+00
Coefficient: 4.21e−02
Value: C
Bacillus.deserti
1.00
0.75
0.50
0.25
0.00
A (n=15)
B (n=16)
C (n=14)
surface

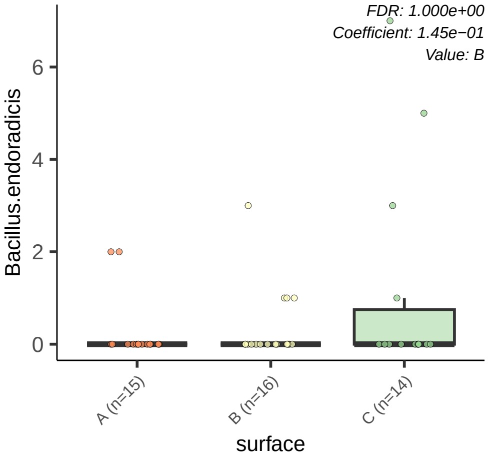

FDR: 1.000e+00
Coefficient: 1.45e−01
Value: B
Bacillus.endoradicis
0
2
4
6
A (n=15)
B (n=16)
C (n=14)
surface

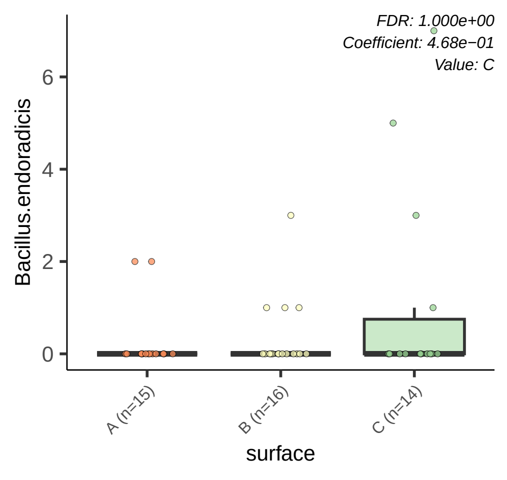
FDR: 1.000e+00
Coefficient: 4.68e−01
Value: C
Bacillus.endoradicis
0
2
4
6
A (n=15)
B (n=16)
C (n=14)
surface

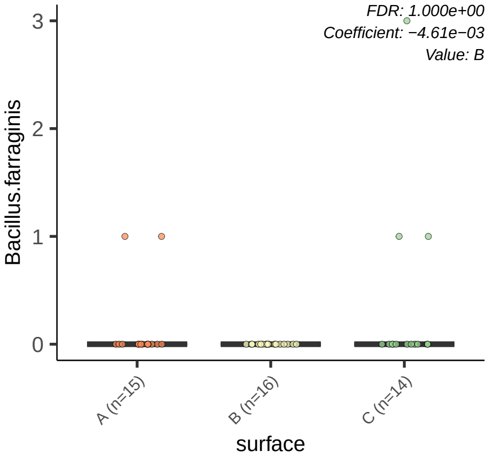

FDR: 1.000e+00
Coefficient: −4.61e−03
Value: B
Bacillus.farraginis
0
1
2
3
A (n=15)
B (n=16)
C (n=14)
surface

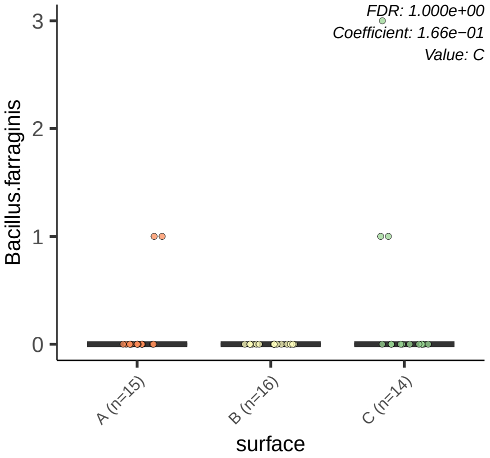
FDR: 1.000e+00
Coefficient: 1.66e−01
Value: C
Bacillus.farraginis
3
2
1
0
A (n=15)
B (n=16)
C (n=14)
surface

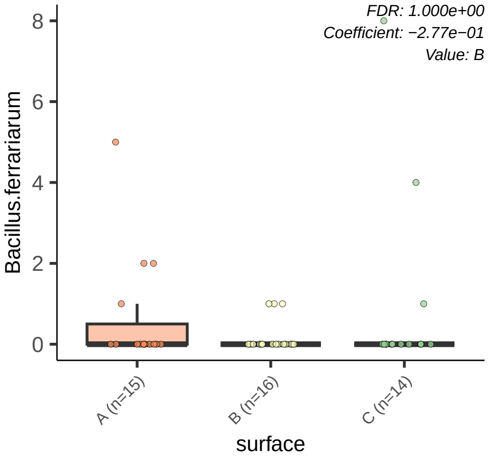
FDR: 1.000e+00
Coefficient: −2.77e−01
Value: B
Bacillus.ferrariarum
0
2
4
6
8
A (n=15)
B (n=16)
C (n=14)
surface

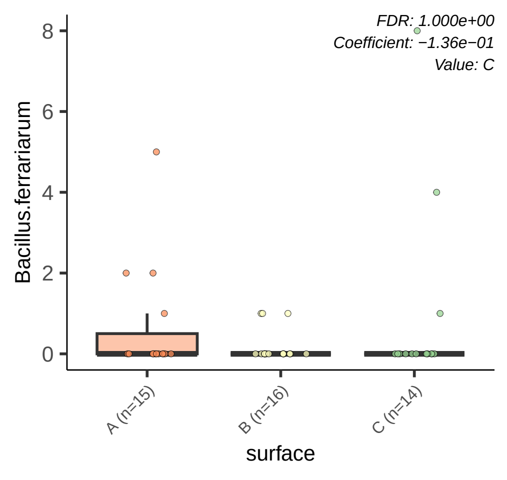
FDR: 1.000e+00
Coefficient: −1.36e−01
Value: C
Bacillus.ferrariarum
0
2
4
6
8
A (n=15)
B (n=16)
C (n=14)
surface

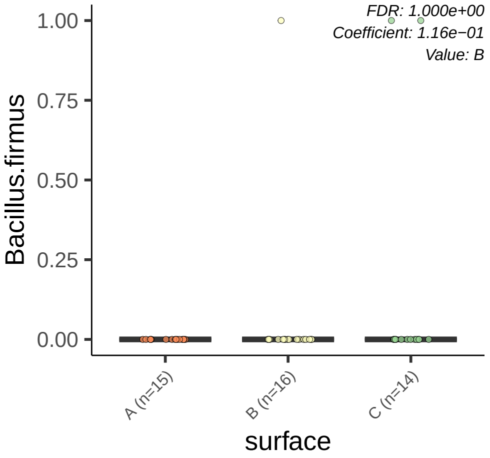
FDR: 1.000e+00
Coefficient: 1.16e−01
Value: B
1.00
0.75
0.50
0.25
0.00
Bacillus.firmus
A (n=15)
B (n=16)
C (n=14)
surface

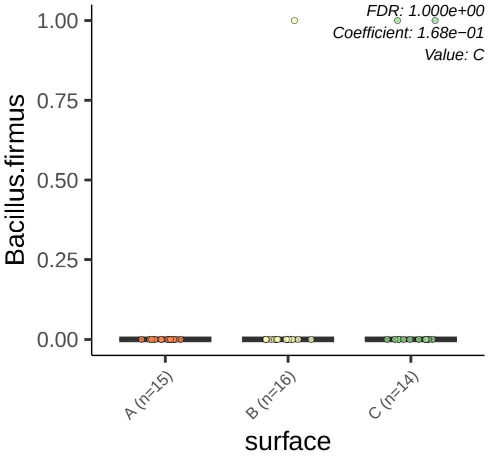
FDR: 1.000e+00
Coefficient: 1.68e−01
Value: C
Bacillus.firmus
1.00
0.75
0.50
0.25
0.00
A (n=15)
B (n=16)
C (n=14)
surface

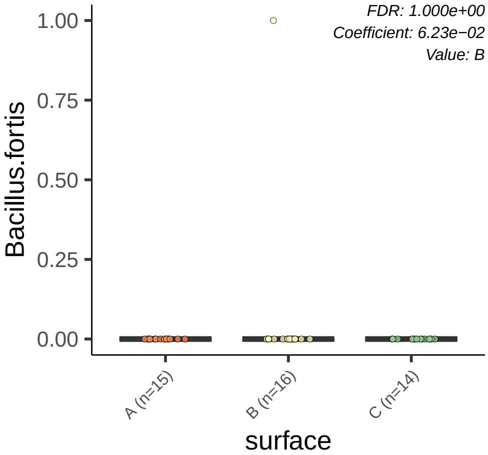

FDR: 1.000e+00
Coefficient: 6.23e−02
Value: B
1.00
0.75
0.50
0.25
0.00
Bacillus.fortis
A (n=15)
B (n=16)
C (n=14)
surface

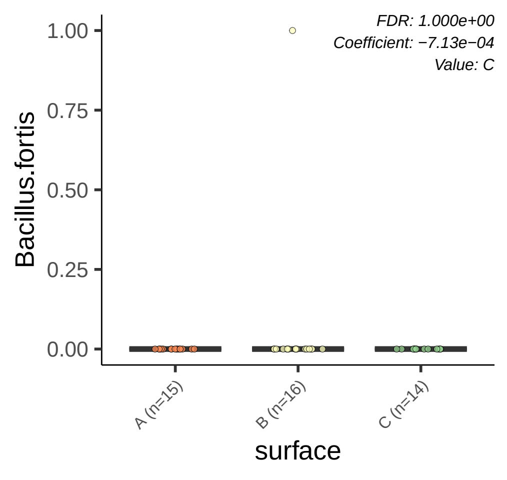
FDR: 1.000e+00
Coefficient: −7.13e−04
Value: C
Bacillus.fortis
1.00
0.75
0.50
0.25
0.00
A (n=15)
B (n=16)
C (n=14)
surface

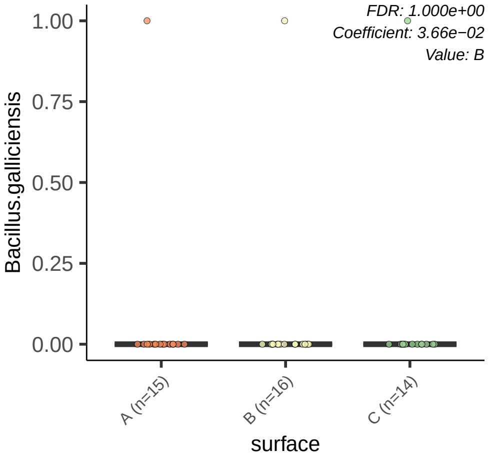

FDR: 1.000e+00
Coefficient: 3.66e−02
Value: B
Bacillus.galliciensis
1.00
0.75
0.50
0.25
0.00
A (n=15)
B (n=16)
C (n=14)
surface

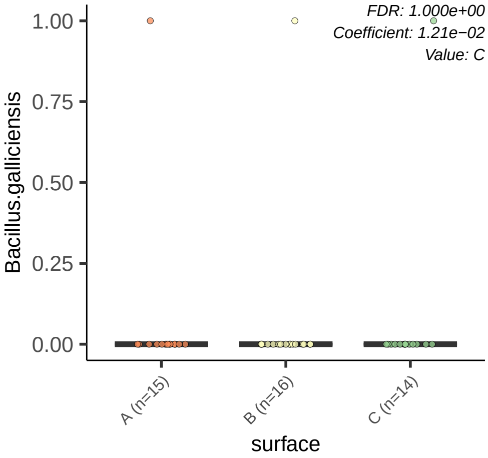
FDR: 1.000e+00
Coefficient: 1.21e−02
Value: C
Bacillus.galliciensis
1.00
0.75
0.50
0.25
0.00
A (n=15)
B (n=16)
C (n=14)
surface

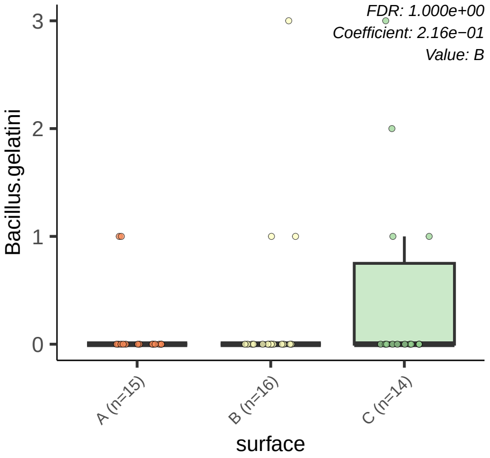
FDR: 1.000e+00
Coefficient: 2.16e−01
Value: B
Bacillus.gelatini
0
1
2
3
A (n=15)
B (n=16)
C (n=14)
surface

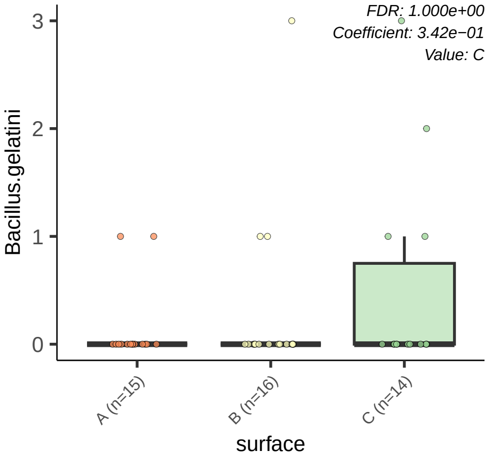

FDR: 1.000e+00
Coefficient: 3.42e−01
Value: C
Bacillus.gelatini
0
1
2
3
A (n=15)
B (n=16)
C (n=14)
surface

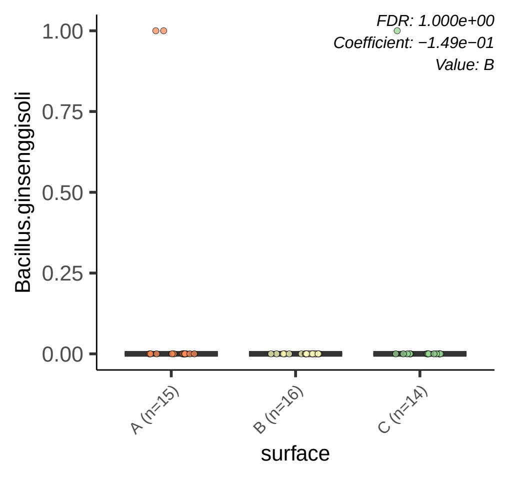
FDR: 1.000e+00
Coefficient: −1.49e−01
Value: B
Bacillus.ginsenggisoli
1.00
0.75
0.50
0.25
0.00
A (n=15)
B (n=16)
C (n=14)
surface

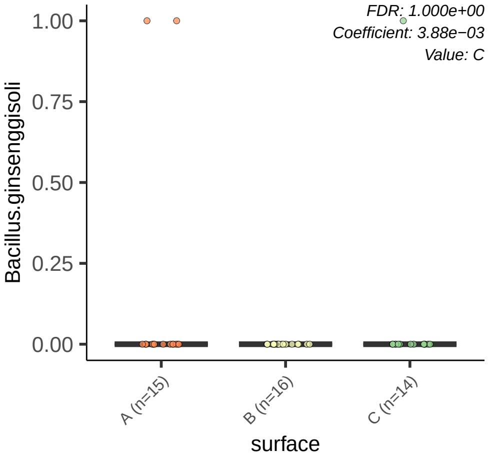

FDR: 1.000e+00
Coefficient: 3.88e−03
Value: C
Bacillus.ginsenggisoli
1.00
0.75
0.50
0.25
0.00
A (n=15)
B (n=16)
C (n=14)
surface

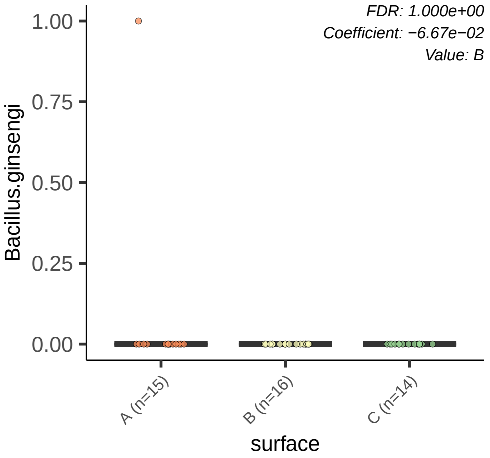

FDR: 1.000e+00
Coefficient: −6.67e−02
Value: B
Bacillus.ginsengi
1.00
0.75
0.50
0.25
0.00
A (n=15)
B (n=16)
C (n=14)
surface

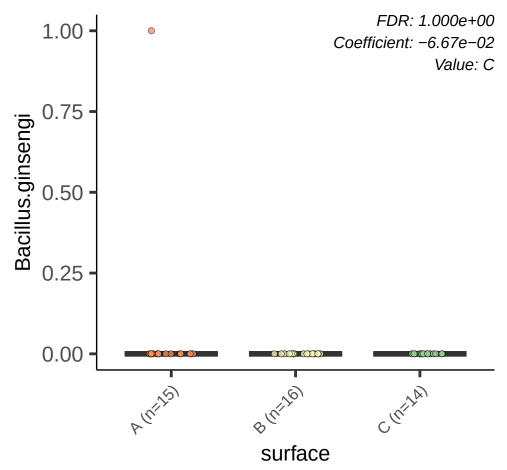
FDR: 1.000e+00
Coefficient: −6.67e−02
Value: C
Bacillus.ginsengi
1.00
0.75
0.50
0.25
0.00
A (n=15)
B (n=16)
C (n=14)
surface

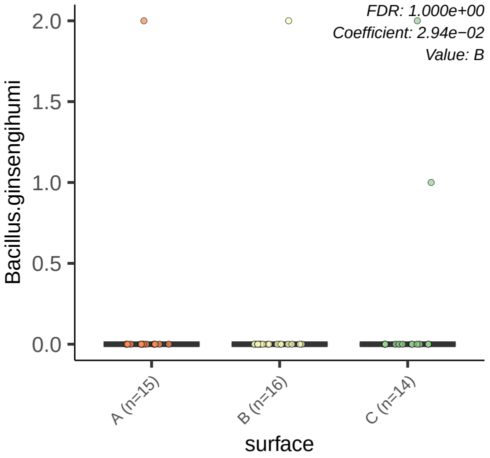
FDR: 1.000e+00
Coefficient: 2.94e−02
Value: B
Bacillus.ginsengihumi
2.0
1.5
1.0
0.5
0.0
A (n=15)
B (n=16)
C (n=14)
surface

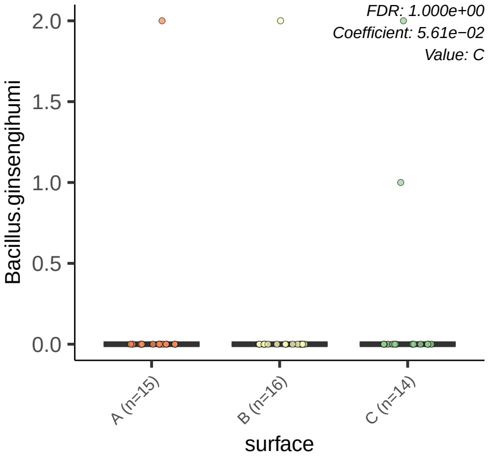

FDR: 1.000e+00
Coefficient: 5.61e−02
Value: C
Bacillus.ginsengihumi
2.0
1.5
1.0
0.5
0.0
A (n=15)
B (n=16)
C (n=14)
surface

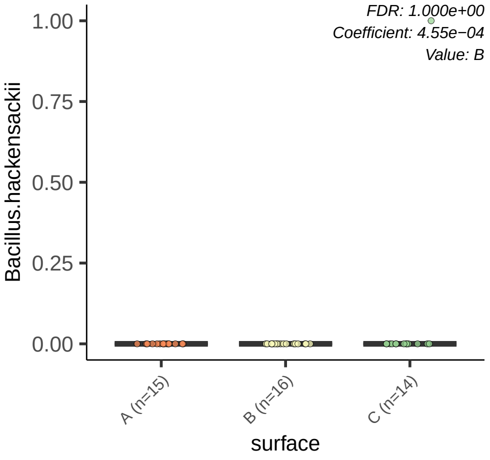

FDR: 1.000e+00
Coefficient: 4.55e−04
Value: B
1.00
0.75
0.50
0.25
0.00
Bacillus.hackensackii
A (n=15)
B (n=16)
C (n=14)
surface

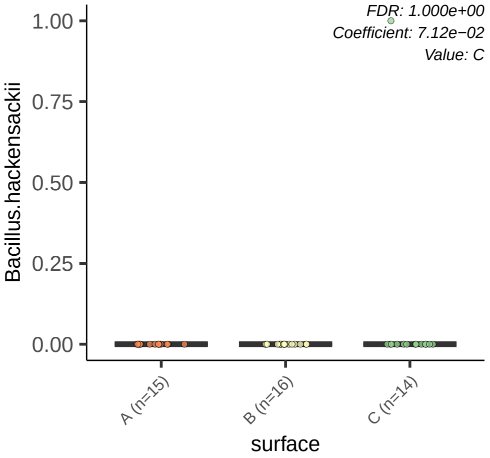
FDR: 1.000e+00
Coefficient: 7.12e−02
Value: C
1.00
0.75
0.50
0.25
0.00
Bacillus.hackensackii
A (n=15)
B (n=16)
C (n=14)
surface

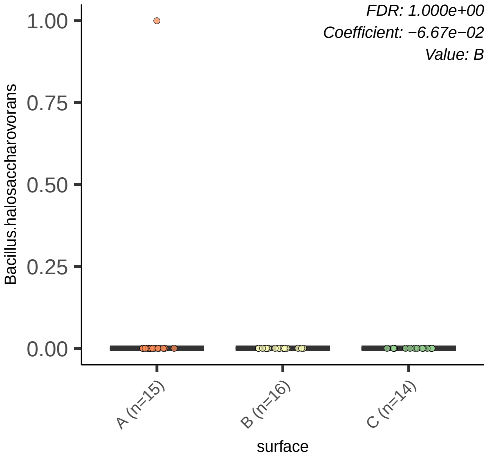
FDR: 1.000e+00
Coefficient: −6.67e−02
Value: B
Bacillus.halosaccharovorans
1.00
0.75
0.50
0.25
0.00
A (n=15)
B (n=16)
C (n=14)
surface

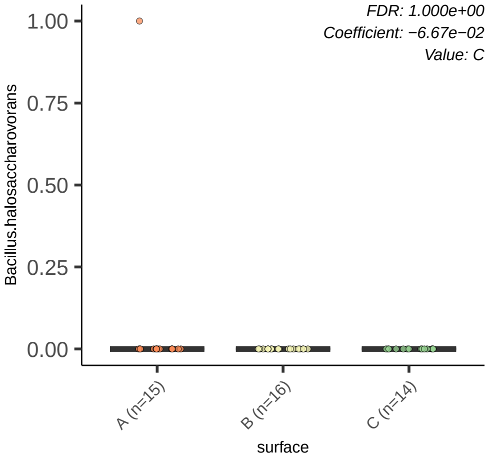

FDR: 1.000e+00
Coefficient: −6.67e−02
Value: C
1.00
0.75
0.50
0.25
0.00
Bacillus.halosaccharovorans
A (n=15)
B (n=16)
C (n=14)
surface

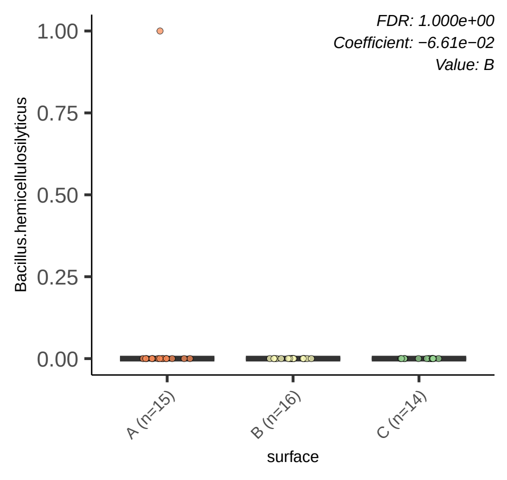
FDR: 1.000e+00
Coefficient: −6.61e−02
Value: B
Bacillus.hemicellulosilyticus
1.00
0.75
0.50
0.25
0.00
A (n=15)
B (n=16)
C (n=14)
surface

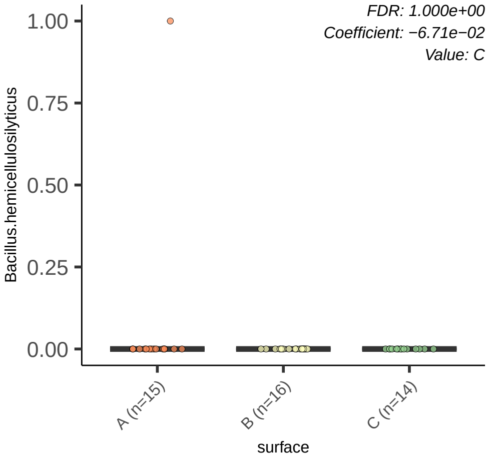
FDR: 1.000e+00
Coefficient: −6.71e−02
Value: C
Bacillus.hemicellulosilyticus
1.00
0.75
0.50
0.25
0.00
A (n=15)
B (n=16)
C (n=14)
surface

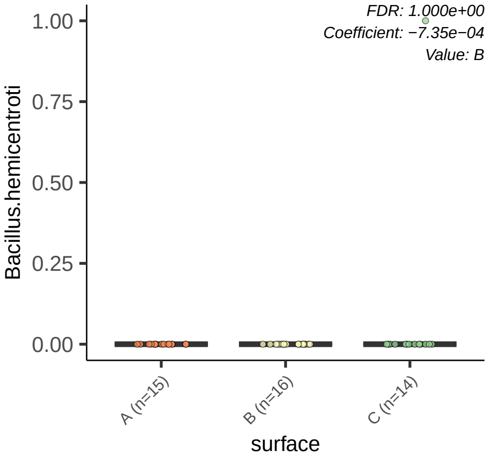

FDR: 1.000e+00
Coefficient: −7.35e−04
Value: B
Bacillus.hemicentroti
1.00
0.75
0.50
0.25
0.00
A (n=15)
B (n=16)
C (n=14)
surface

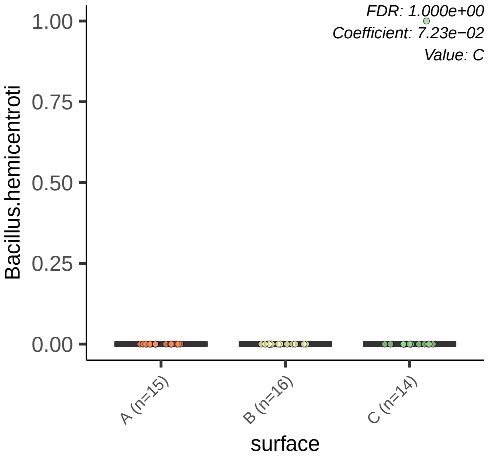
FDR: 1.000e+00
Coefficient: 7.23e−02
Value: C
Bacillus.hemicentroti
1.00
0.75
0.50
0.25
0.00
A (n=15)
B (n=16)
C (n=14)
surface

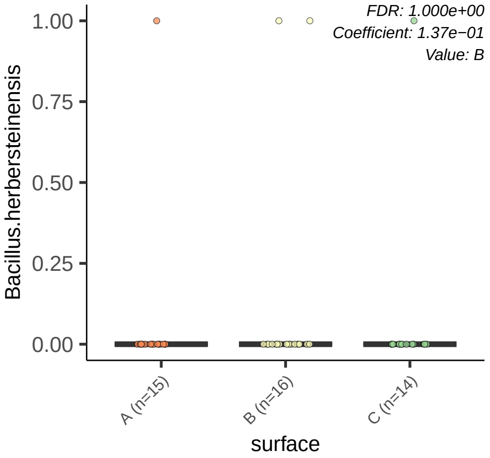

FDR: 1.000e+00
Coefficient: 1.37e−01
Value: B
Bacillus.herbersteinensis
1.00
0.75
0.50
0.25
0.00
A (n=15)
B (n=16)
C (n=14)
surface

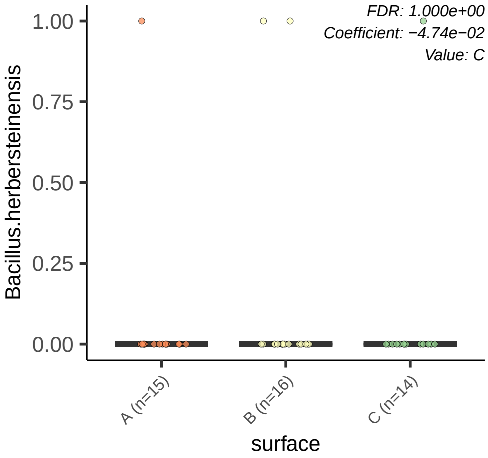

FDR: 1.000e+00
Coefficient: −4.74e−02
Value: C
Bacillus.herbersteinensis
1.00
0.75
0.50
0.25
0.00
A (n=15)
B (n=16)
C (n=14)
surface

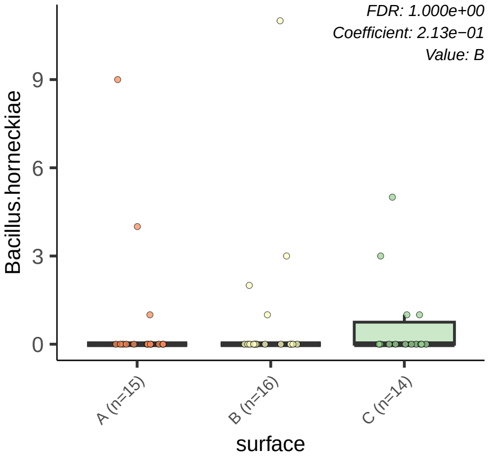
FDR: 1.000e+00
Coefficient: 2.13e−01
Value: B
Bacillus.horneckiae
0
3
6
9
A (n=15)
B (n=16)
C (n=14)
surface

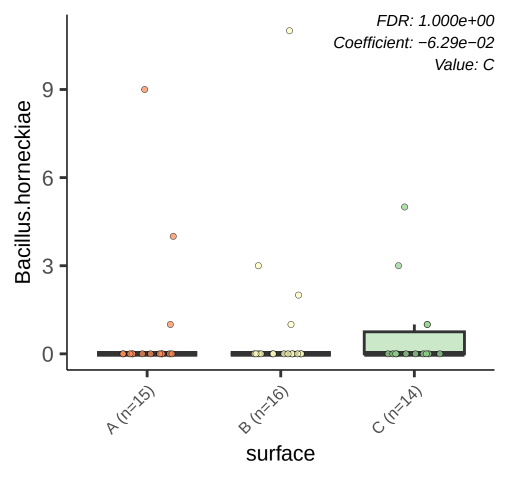

FDR: 1.000e+00
Coefficient: −6.29e−02
Value: C
Bacillus.horneckiae
0
3
6
9
A (n=15)
B (n=16)
C (n=14)
surface

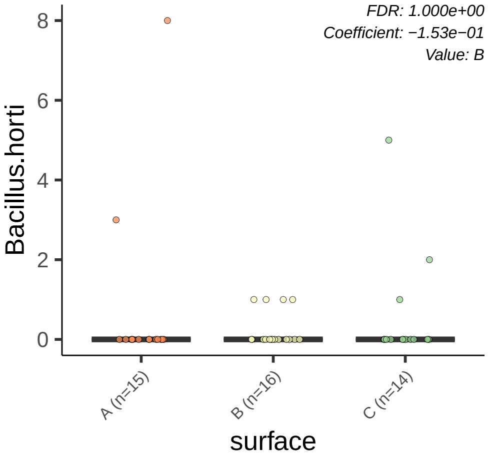

FDR: 1.000e+00
Coefficient: −1.53e−01
Value: B
Bacillus.horti
8
6
4
2
0
A (n=15)
B (n=16)
C (n=14)
surface

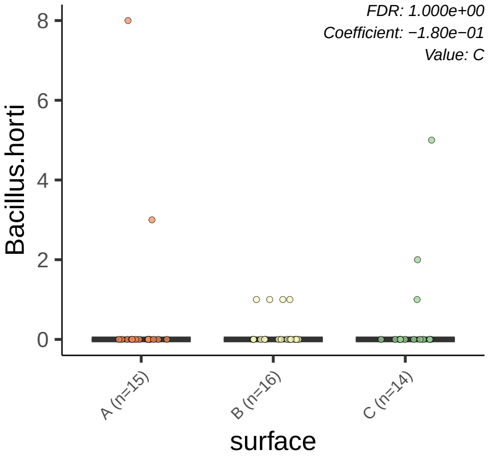
FDR: 1.000e+00
Coefficient: −1.80e−01
Value: C
Bacillus.horti
8
6
4
2
0
A (n=15)
B (n=16)
C (n=14)
surface

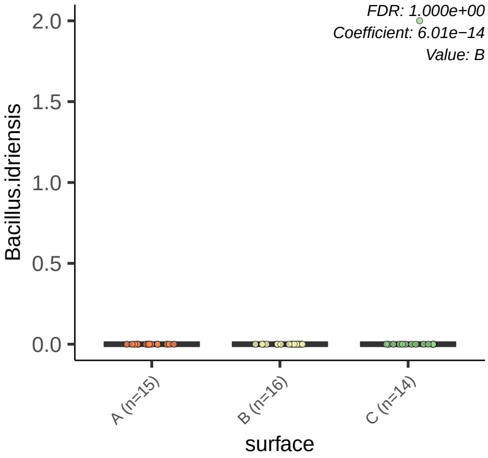
FDR: 1.000e+00
Coefficient: 6.01e−14
Value: B
Bacillus.idriensis
2.0
1.5
1.0
0.5
0.0
A (n=15)
B (n=16)
C (n=14)
surface

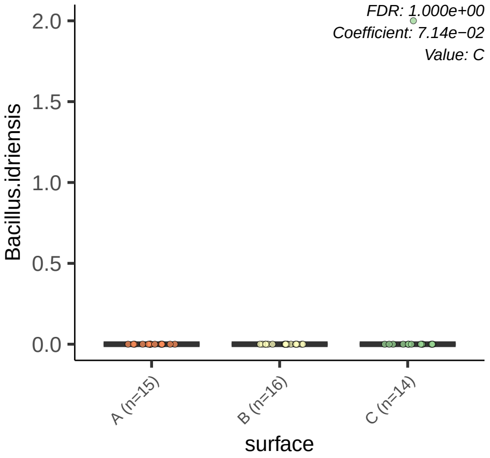
FDR: 1.000e+00
Coefficient: 7.14e−02
Value: C
Bacillus.idriensis
2.0
1.5
1.0
0.5
0.0
A (n=15)
B (n=16)
C (n=14)
surface

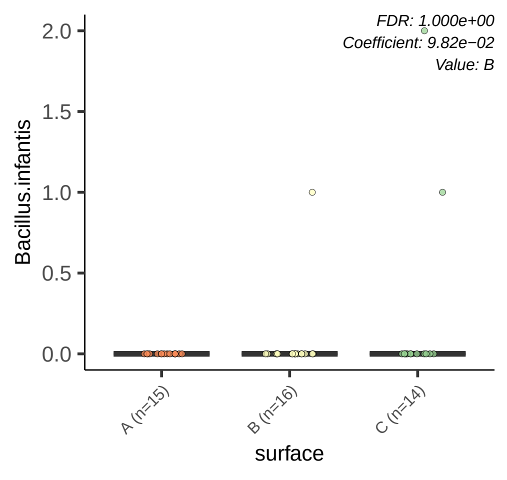
FDR: 1.000e+00
Coefficient: 9.82e−02
Value: B
Bacillus.infantis
2.0
1.5
1.0
0.5
0.0
A (n=15)
B (n=16)
C (n=14)
surface

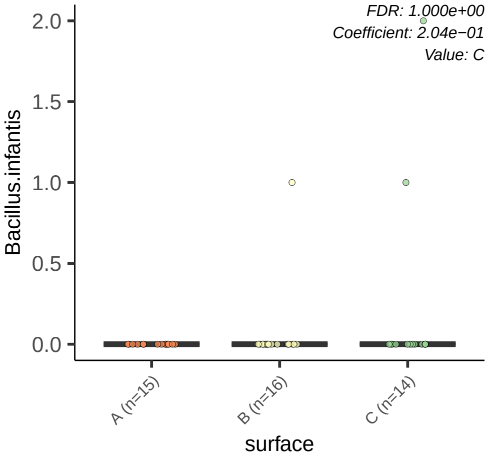

FDR: 1.000e+00
Coefficient: 2.04e−01
Value: C
2.0
1.5
1.0
0.5
0.0
Bacillus.infantis
A (n=15)
B (n=16)
C (n=14)
surface

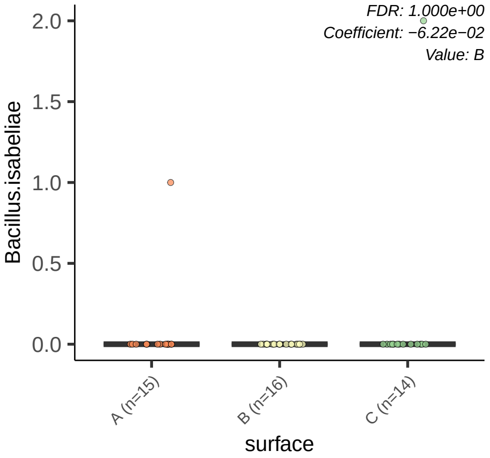
FDR: 1.000e+00
Coefficient: −6.22e−02
Value: B
Bacillus.isabeliae
2.0
1.5
1.0
0.5
0.0
A (n=15)
B (n=16)
C (n=14)
surface

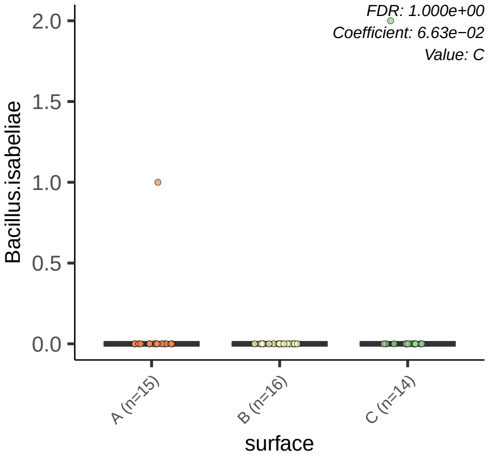
FDR: 1.000e+00
Coefficient: 6.63e−02
Value: C
Bacillus.isabeliae
2.0
1.5
1.0
0.5
0.0
A (n=15)
B (n=16)
C (n=14)
surface

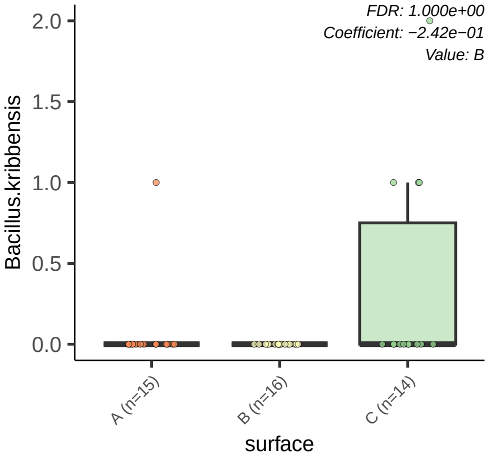
FDR: 1.000e+00
Coefficient: −2.42e−01
Value: B
Bacillus.kribbensis
2.0
1.5
1.0
0.5
0.0
A (n=15)
B (n=16)
C (n=14)
surface

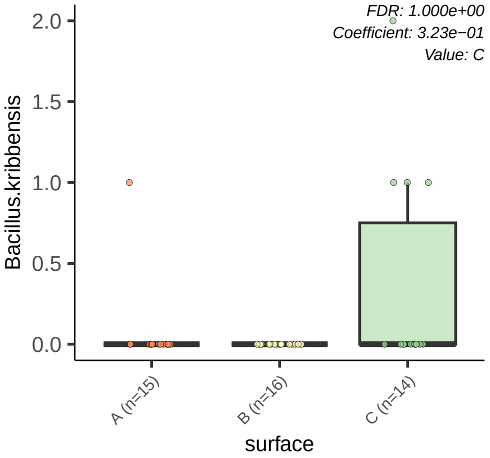
FDR: 1.000e+00
Coefficient: 3.23e−01
Value: C
2.0
1.5
1.0
0.5
0.0
Bacillus.kribbensis
A (n=15)
B (n=16)
C (n=14)
surface

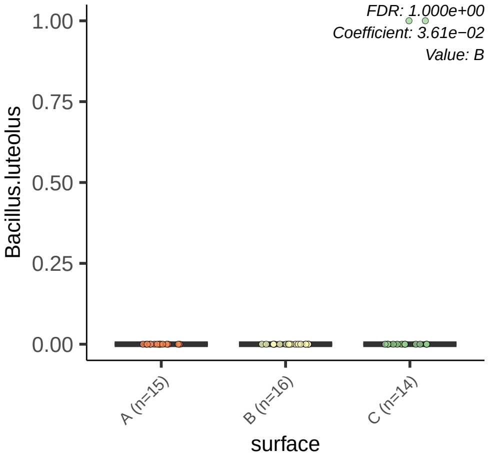

FDR: 1.000e+00
Coefficient: 3.61e−02
Value: B
1.00
0.75
0.50
0.25
0.00
Bacillus.luteolus
A (n=15)
B (n=16)
C (n=14)
surface

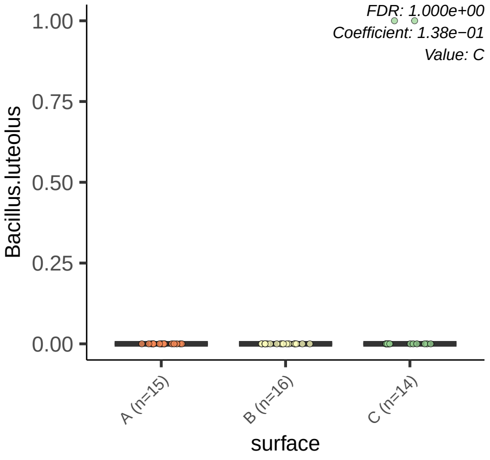
FDR: 1.000e+00
Coefficient: 1.38e−01
Value: C
1.00
0.75
0.50
0.25
0.00
Bacillus.luteolus
A (n=15)
B (n=16)
C (n=14)
surface

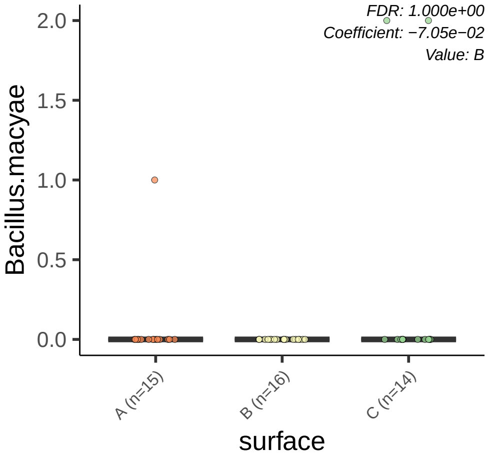

FDR: 1.000e+00
Coefficient: −7.05e−02
Value: B
Bacillus.macyae
2.0
1.5
1.0
0.5
0.0
A (n=15)
B (n=16)
C (n=14)
surface

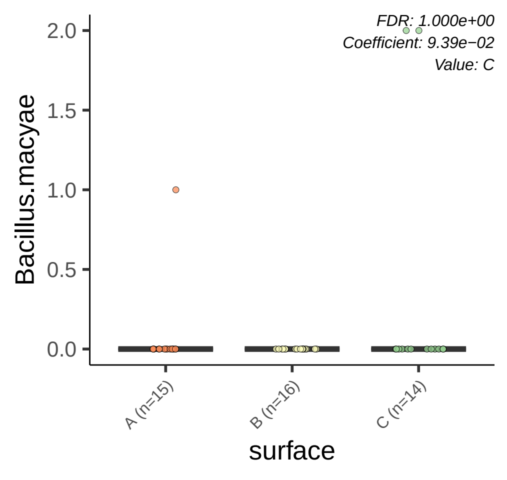
FDR: 1.000e+00
Coefficient: 9.39e−02
Value: C
Bacillus.macyae
2.0
1.5
1.0
0.5
0.0
A (n=15)
B (n=16)
C (n=14)
surface

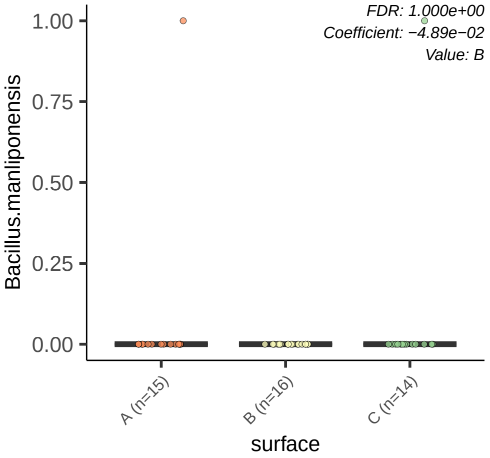
FDR: 1.000e+00
Coefficient: −4.89e−02
Value: B
Bacillus.manliponensis
1.00
0.75
0.50
0.25
0.00
A (n=15)
B (n=16)
C (n=14)
surface

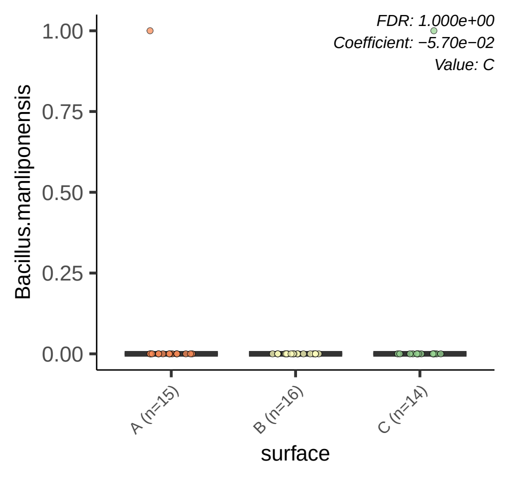
FDR: 1.000e+00
Coefficient: −5.70e−02
Value: C
Bacillus.manliponensis
1.00
0.75
0.50
0.25
0.00
A (n=15)
B (n=16)
C (n=14)
surface

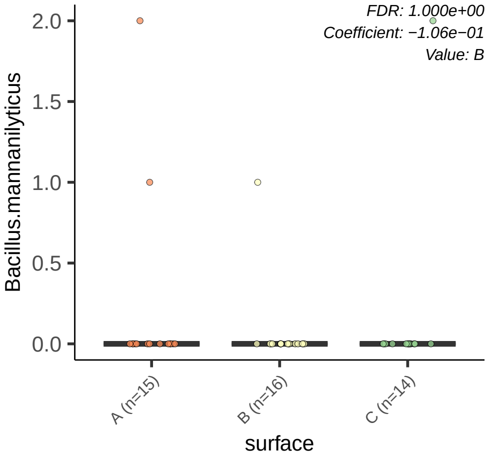
FDR: 1.000e+00
Coefficient: −1.06e−01
Value: B
Bacillus.mannanilyticus
2.0
1.5
1.0
0.5
0.0
A (n=15)
B (n=16)
C (n=14)
surface

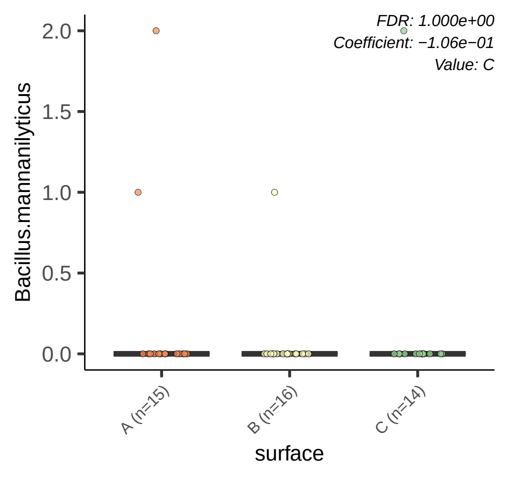

FDR: 1.000e+00
Coefficient: −1.06e−01
Value: C
Bacillus.mannanilyticus
2.0
1.5
1.0
0.5
0.0
A (n=15)
B (n=16)
C (n=14)
surface

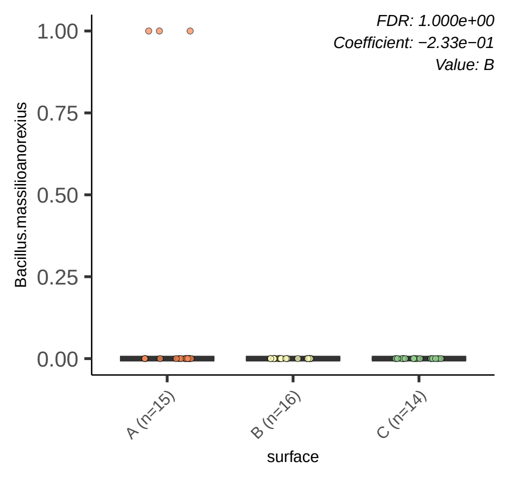
FDR: 1.000e+00
Coefficient: −2.33e−01
Value: B
Bacillus.massilioanorexius
1.00
0.75
0.50
0.25
0.00
A (n=15)
B (n=16)
C (n=14)
surface

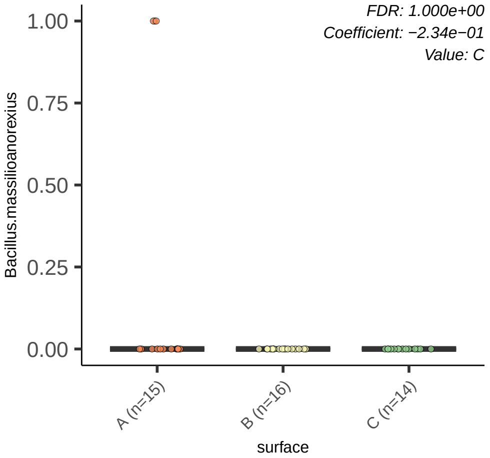
FDR: 1.000e+00
Coefficient: −2.34e−01
Value: C
Bacillus.massilioanorexius
1.00
0.75
0.50
0.25
0.00
A (n=15)
B (n=16)
C (n=14)
surface

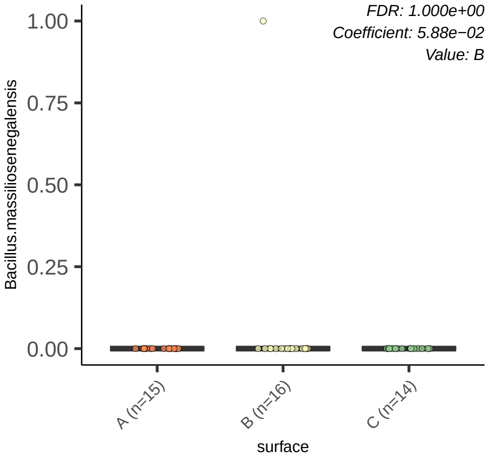
FDR: 1.000e+00
Coefficient: 5.88e−02
Value: B
1.00
0.75
0.50
0.25
0.00
Bacillus.massiliosenegalensis
A (n=15)
B (n=16)
C (n=14)
surface

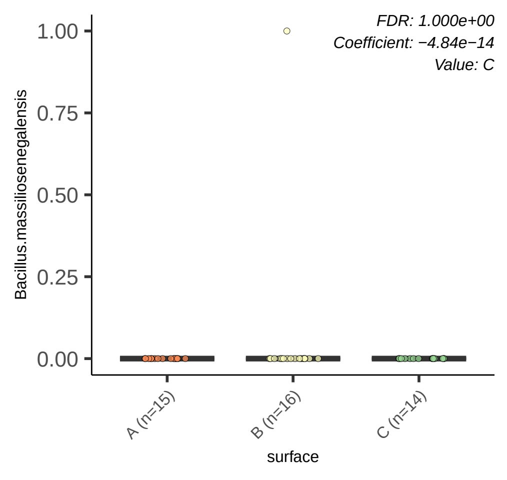
FDR: 1.000e+00
Coefficient: −4.84e−14
Value: C
1.00
0.75
0.50
0.25
0.00
Bacillus.massiliosenegalensis
A (n=15)
B (n=16)
C (n=14)
surface

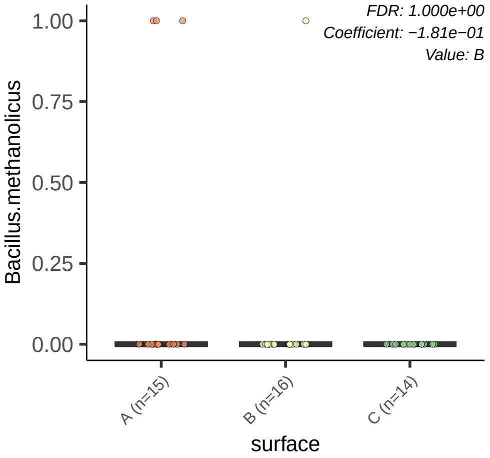
FDR: 1.000e+00
Coefficient: −1.81e−01
Value: B
Bacillus.methanolicus
1.00
0.75
0.50
0.25
0.00
A (n=15)
B (n=16)
C (n=14)
surface

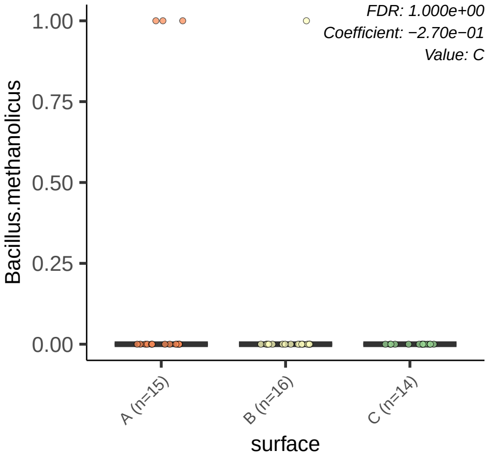
FDR: 1.000e+00
Coefficient: −2.70e−01
Value: C
Bacillus.methanolicus
1.00
0.75
0.50
0.25
0.00
A (n=15)
B (n=16)
C (n=14)
surface

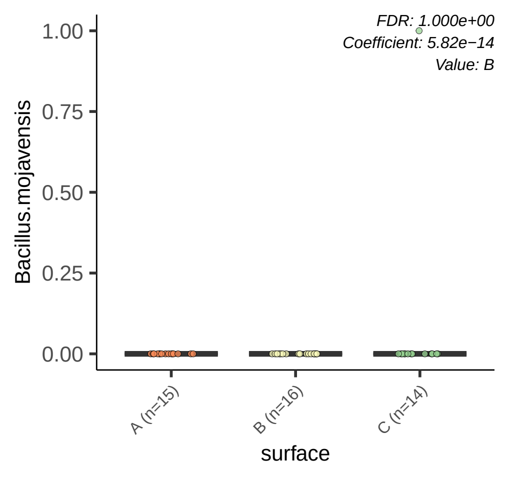
FDR: 1.000e+00
Coefficient: 5.82e−14
Value: B
1.00
0.75
0.50
0.25
0.00
Bacillus.mojavensis
A (n=15)
B (n=16)
C (n=14)
surface

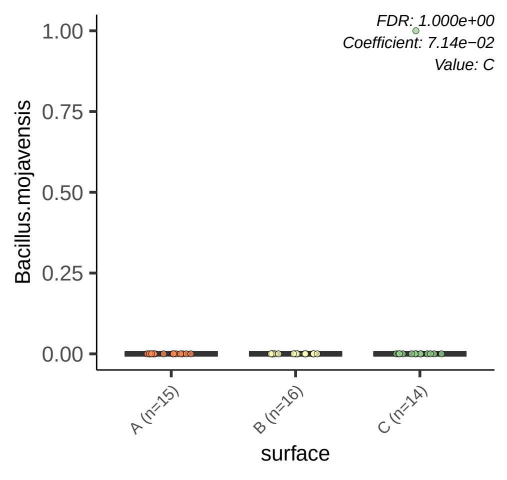
FDR: 1.000e+00
Coefficient: 7.14e−02
Value: C
Bacillus.mojavensis
1.00
0.75
0.50
0.25
0.00
A (n=15)
B (n=16)
C (n=14)
surface

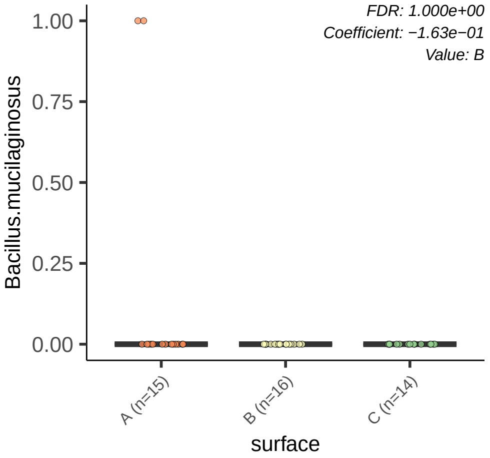
FDR: 1.000e+00
Coefficient: −1.63e−01
Value: B
Bacillus.mucilaginosus
1.00
0.75
0.50
0.25
0.00
A (n=15)
B (n=16)
C (n=14)
surface

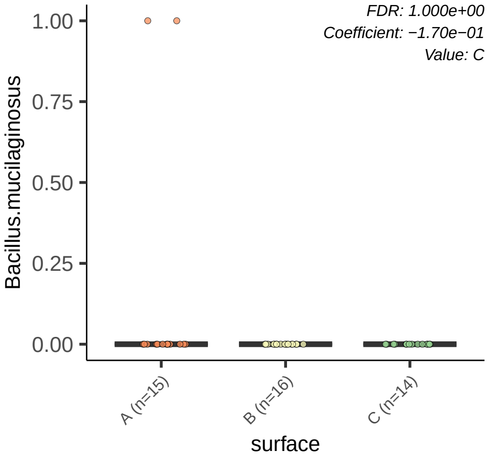

FDR: 1.000e+00
Coefficient: −1.70e−01
Value: C
Bacillus.mucilaginosus
1.00
0.75
0.50
0.25
0.00
A (n=15)
B (n=16)
C (n=14)
surface

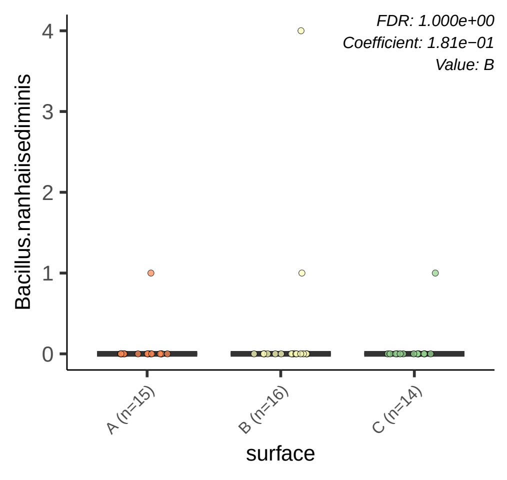

FDR: 1.000e+00
Coefficient: 1.81e−01
Value: B
Bacillus.nanhaisediminis
4
3
2
1
0
A (n=15)
B (n=16)
C (n=14)
surface

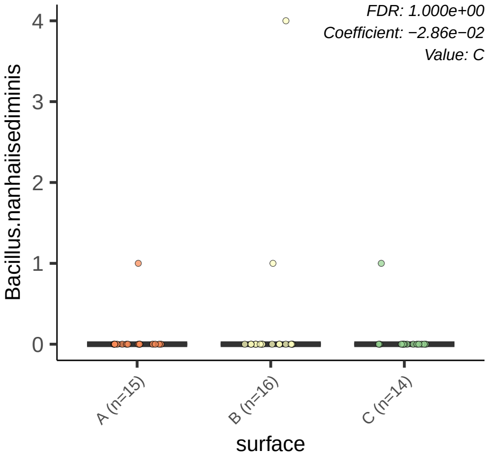

FDR: 1.000e+00
Coefficient: −2.86e−02
Value: C
Bacillus.nanhaisediminis
0
1
2
3
4
A (n=15)
B (n=16)
C (n=14)
surface

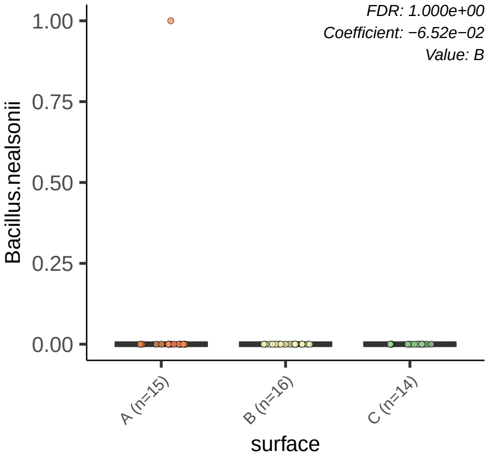
FDR: 1.000e+00
Coefficient: −6.52e−02
Value: B
Bacillus.nealsonii
1.00
0.75
0.50
0.25
0.00
A (n=15)
B (n=16)
C (n=14)
surface

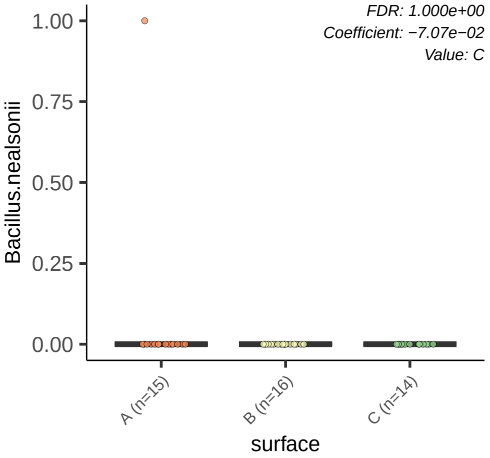
FDR: 1.000e+00
Coefficient: −7.07e−02
Value: C
1.00
0.75
0.50
0.25
0.00
Bacillus.nealsonii
A (n=15)
B (n=16)
C (n=14)
surface

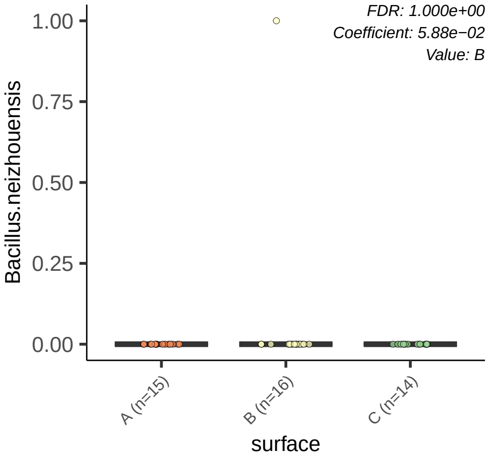

FDR: 1.000e+00
Coefficient: 5.88e−02
Value: B
Bacillus.neizhouensis
1.00
0.75
0.50
0.25
0.00
A (n=15)
B (n=16)
C (n=14)
surface

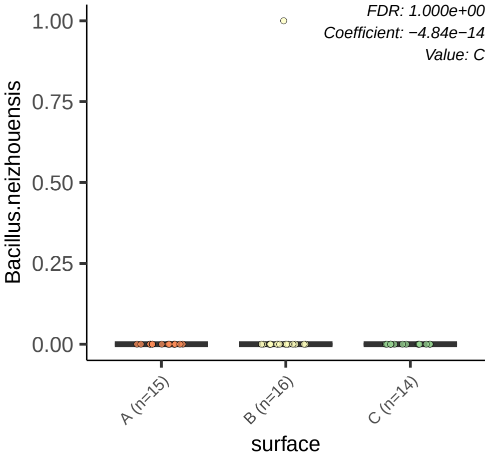

FDR: 1.000e+00
Coefficient: −4.84e−14
Value: C
Bacillus.neizhouensis
1.00
0.75
0.50
0.25
0.00
A (n=15)
B (n=16)
C (n=14)
surface

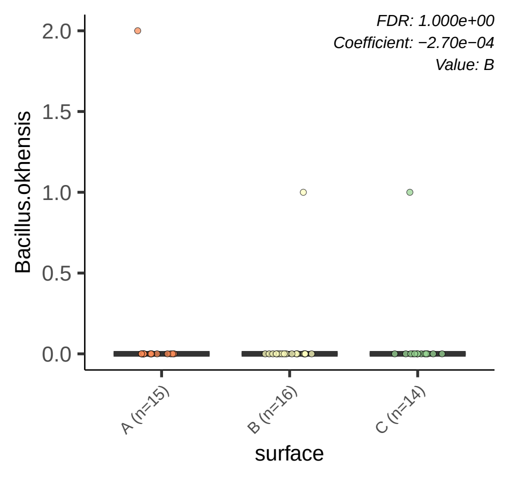
FDR: 1.000e+00
Coefficient: −2.70e−04
Value: B
Bacillus.okhensis
2.0
1.5
1.0
0.5
0.0
A (n=15)
B (n=16)
C (n=14)
surface

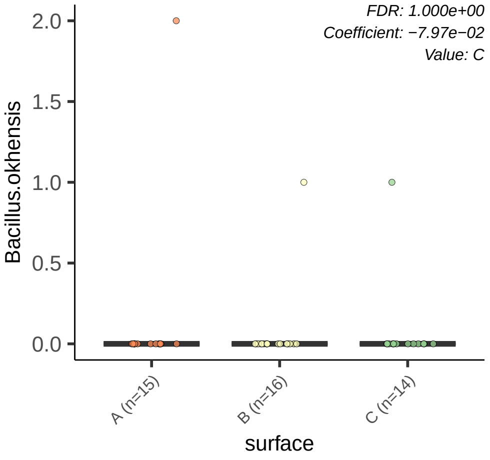
FDR: 1.000e+00
Coefficient: −7.97e−02
Value: C
Bacillus.okhensis
2.0
1.5
1.0
0.5
0.0
A (n=15)
B (n=16)
C (n=14)
surface

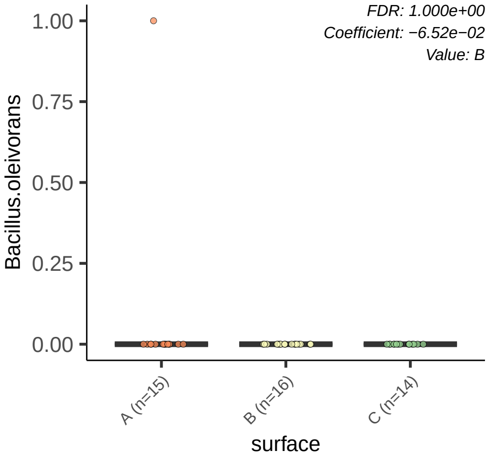
FDR: 1.000e+00
Coefficient: −6.52e−02
Value: B
1.00
0.75
0.50
0.25
0.00
Bacillus.oleivorans
A (n=15)
B (n=16)
C (n=14)
surface

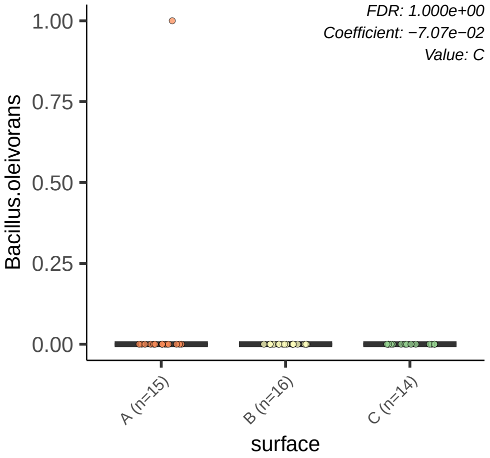
FDR: 1.000e+00
Coefficient: −7.07e−02
Value: C
Bacillus.oleivorans
1.00
0.75
0.50
0.25
0.00
A (n=15)
B (n=16)
C (n=14)
surface

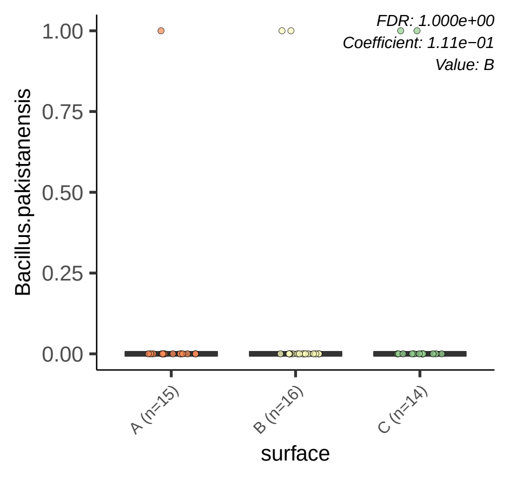
FDR: 1.000e+00
Coefficient: 1.11e−01
Value: B
Bacillus.pakistanensis
1.00
0.75
0.50
0.25
0.00
A (n=15)
B (n=16)
C (n=14)
surface

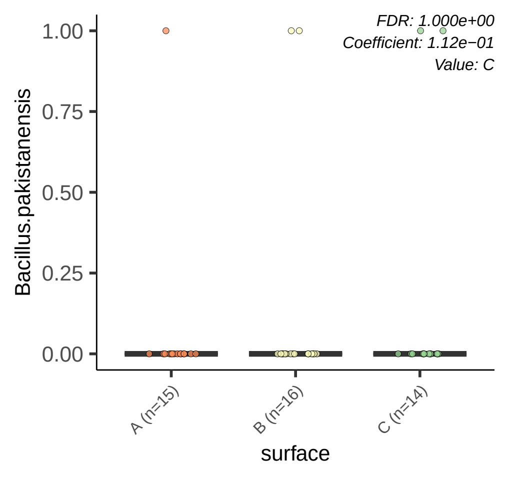
FDR: 1.000e+00
Coefficient: 1.12e−01
Value: C
Bacillus.pakistanensis
1.00
0.75
0.50
0.25
0.00
A (n=15)
B (n=16)
C (n=14)
surface

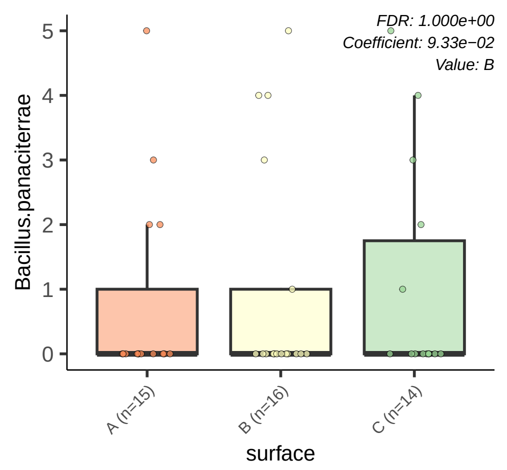

FDR: 1.000e+00
Coefficient: 9.33e−02
Value: B
Bacillus.panaciterrae
0
1
2
3
4
5
A (n=15)
B (n=16)
C (n=14)
surface

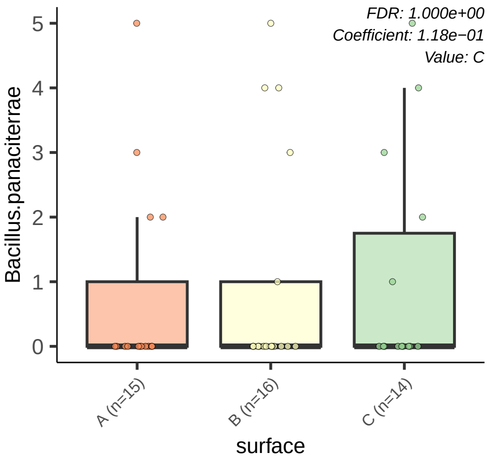
FDR: 1.000e+00
Coefficient: 1.18e−01
Value: C
Bacillus.panaciterrae
0
1
2
3
4
5
A (n=15)
B (n=16)
C (n=14)
surface

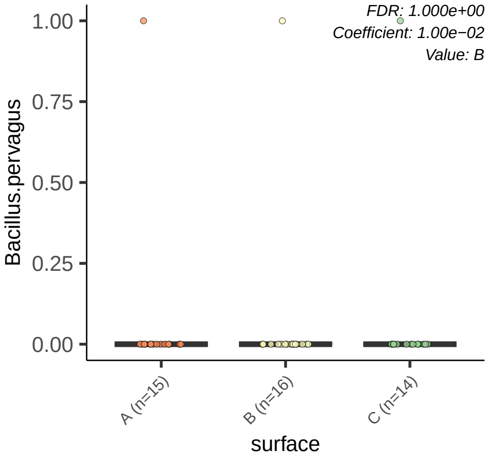
FDR: 1.000e+00
Coefficient: 1.00e−02
Value: B
Bacillus.pervagus
1.00
0.75
0.50
0.25
0.00
A (n=15)
B (n=16)
C (n=14)
surface

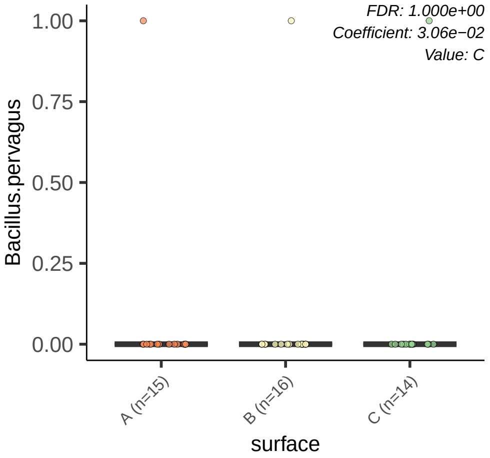

FDR: 1.000e+00
Coefficient: 3.06e−02
Value: C
Bacillus.pervagus
1.00
0.75
0.50
0.25
0.00
A (n=15)
B (n=16)
C (n=14)
surface

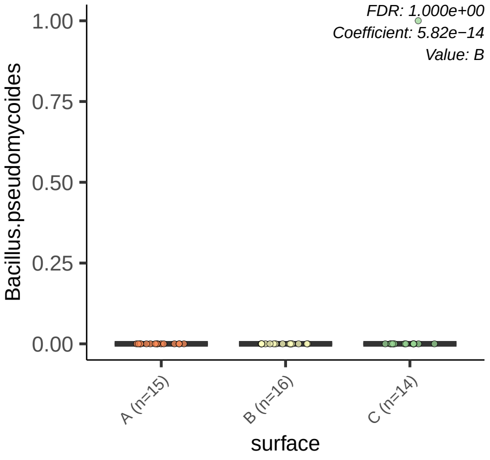
FDR: 1.000e+00
Coefficient: 5.82e−14
Value: B
Bacillus.pseudomycoides
1.00
0.75
0.50
0.25
0.00
A (n=15)
B (n=16)
C (n=14)
surface

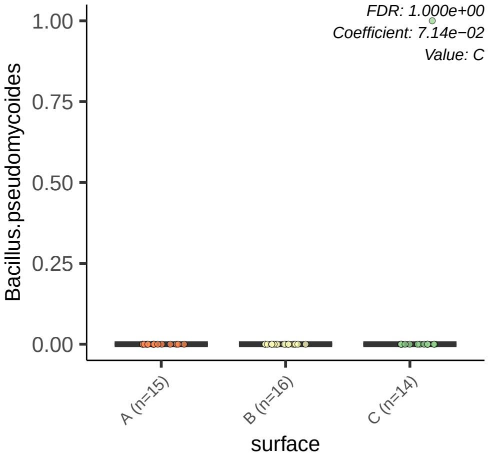

FDR: 1.000e+00
Coefficient: 7.14e−02
Value: C
Bacillus.pseudomycoides
1.00
0.75
0.50
0.25
0.00
A (n=15)
B (n=16)
C (n=14)
surface

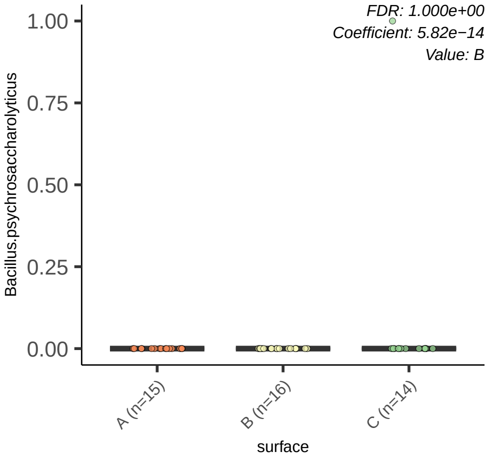

FDR: 1.000e+00
Coefficient: 5.82e−14
Value: B
1.00
0.75
0.50
0.25
0.00
Bacillus.psychrosaccharolyticus
A (n=15)
B (n=16)
C (n=14)
surface

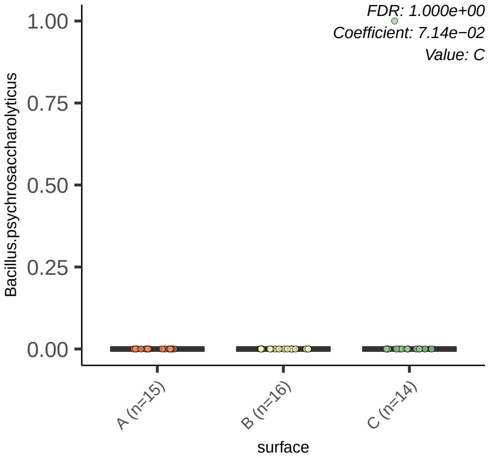
FDR: 1.000e+00
Coefficient: 7.14e−02
Value: C
Bacillus.psychrosaccharolyticus
1.00
0.75
0.50
0.25
0.00
A (n=15)
B (n=16)
C (n=14)
surface

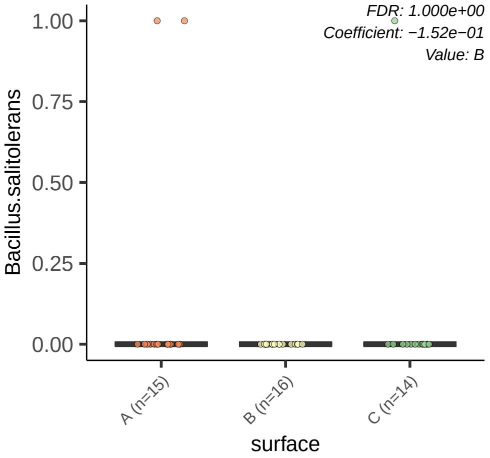
FDR: 1.000e+00
Coefficient: −1.52e−01
Value: B
Bacillus.salitolerans
1.00
0.75
0.50
0.25
0.00
A (n=15)
B (n=16)
C (n=14)
surface

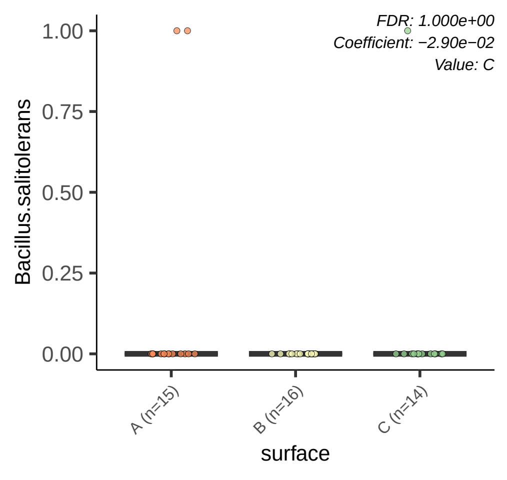
FDR: 1.000e+00
Coefficient: −2.90e−02
Value: C
Bacillus.salitolerans
1.00
0.75
0.50
0.25
0.00
A (n=15)
B (n=16)
C (n=14)
surface

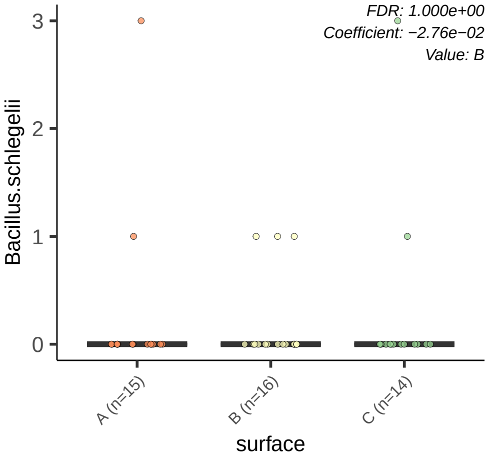

FDR: 1.000e+00
Coefficient: −2.76e−02
Value: B
Bacillus.schlegelii
3
2
1
0
A (n=15)
B (n=16)
C (n=14)
surface

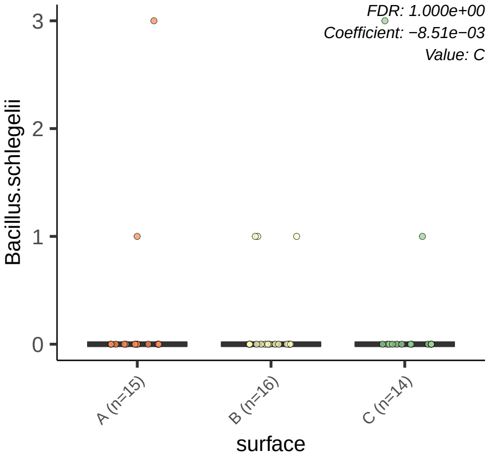

FDR: 1.000e+00
Coefficient: −8.51e−03
Value: C
Bacillus.schlegelii
3
2
1
0
A (n=15)
B (n=16)
C (n=14)
surface

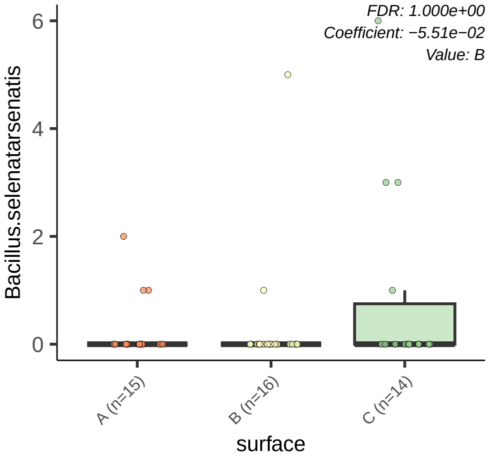
FDR: 1.000e+00
Coefficient: −5.51e−02
Value: B
Bacillus.selenatarsenatis
0
2
4
6
A (n=15)
B (n=16)
C (n=14)
surface

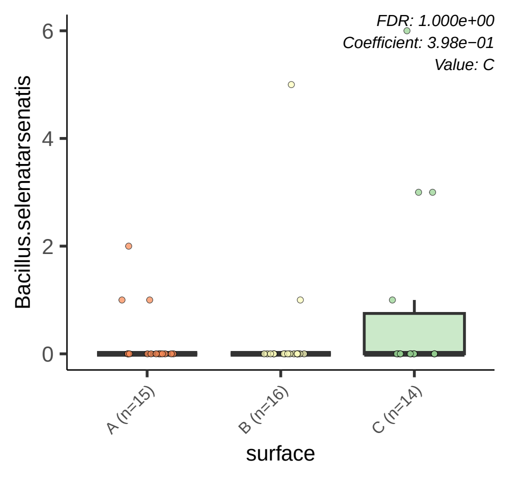

FDR: 1.000e+00
Coefficient: 3.98e−01
Value: C
Bacillus.selenatarsenatis
6
4
2
0
A (n=15)
B (n=16)
C (n=14)
surface

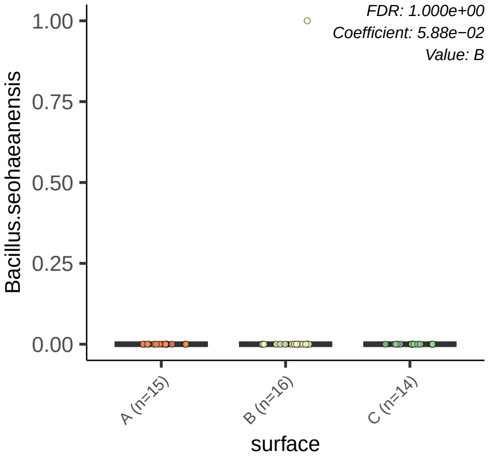
FDR: 1.000e+00
Coefficient: 5.88e−02
Value: B
Bacillus.seohaeanensis
1.00
0.75
0.50
0.25
0.00
A (n=15)
B (n=16)
C (n=14)
surface

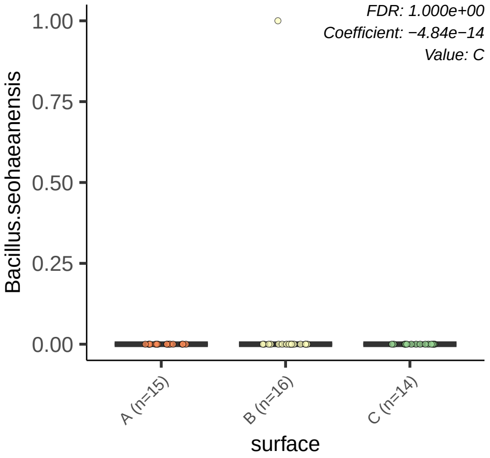

FDR: 1.000e+00
Coefficient: −4.84e−14
Value: C
Bacillus.seohaeanensis
1.00
0.75
0.50
0.25
0.00
A (n=15)
B (n=16)
C (n=14)
surface

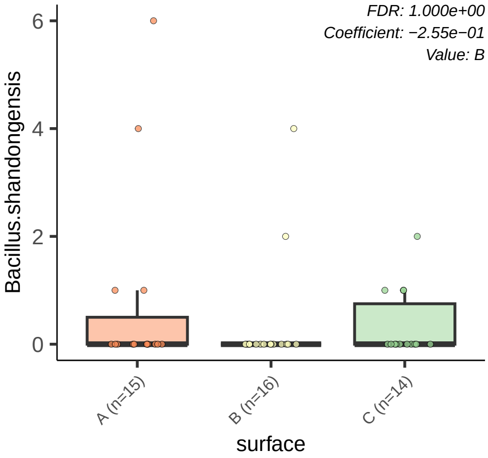
FDR: 1.000e+00
Coefficient: −2.55e−01
Value: B
Bacillus.shandongensis
0
2
4
6
A (n=15)
B (n=16)
C (n=14)
surface

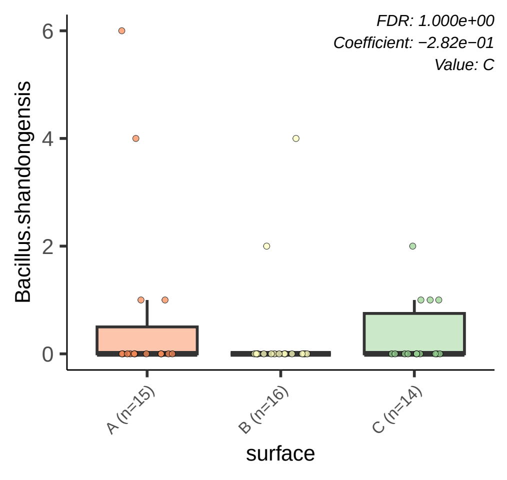
FDR: 1.000e+00
Coefficient: −2.82e−01
Value: C
Bacillus.shandongensis
0
2
4
6
A (n=15)
B (n=16)
C (n=14)
surface

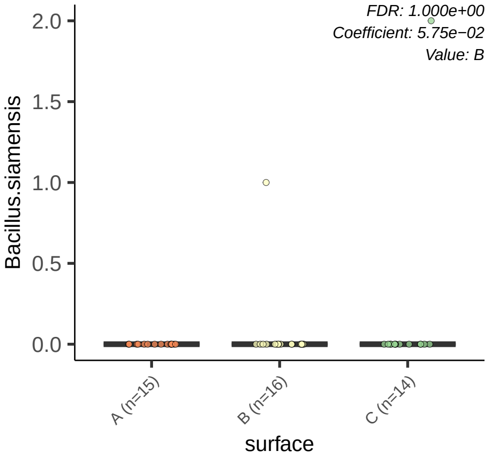

FDR: 1.000e+00
Coefficient: 5.75e−02
Value: B
2.0
1.5
1.0
0.5
0.0
Bacillus.siamensis
A (n=15)
B (n=16)
C (n=14)
surface

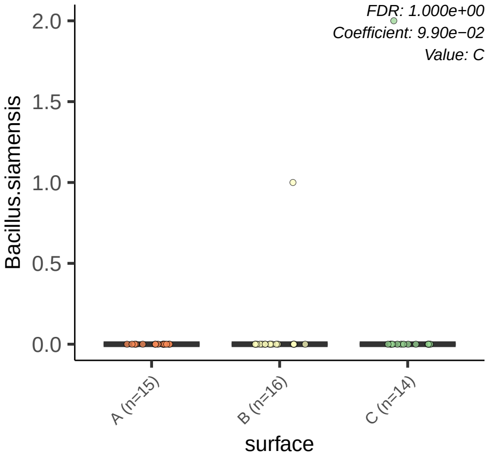

FDR: 1.000e+00
Coefficient: 9.90e−02
Value: C
2.0
1.5
1.0
0.5
0.0
Bacillus.siamensis
A (n=15)
B (n=16)
C (n=14)
surface

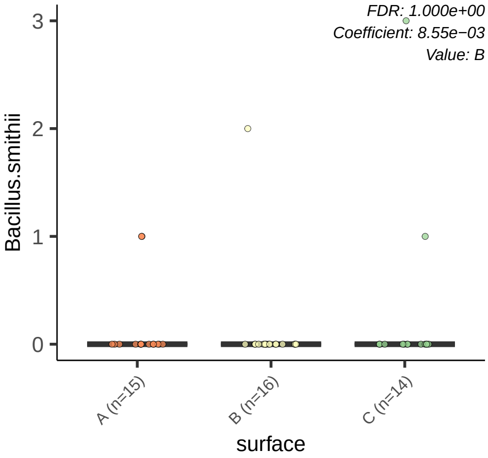
FDR: 1.000e+00
Coefficient: 8.55e−03
Value: B
Bacillus.smithii
3
2
1
0
A (n=15)
B (n=16)
C (n=14)
surface

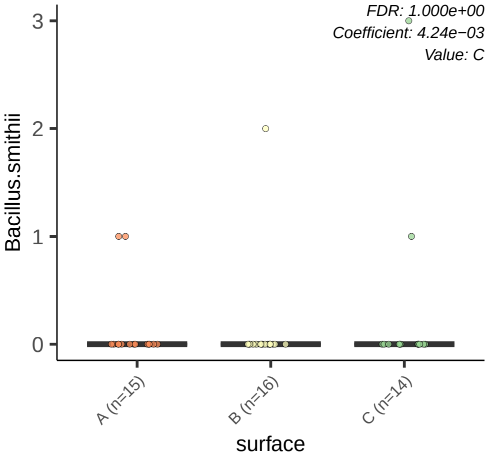

FDR: 1.000e+00
Coefficient: 4.24e−03
Value: C
Bacillus.smithii
0
1
2
3
A (n=15)
B (n=16)
C (n=14)
surface

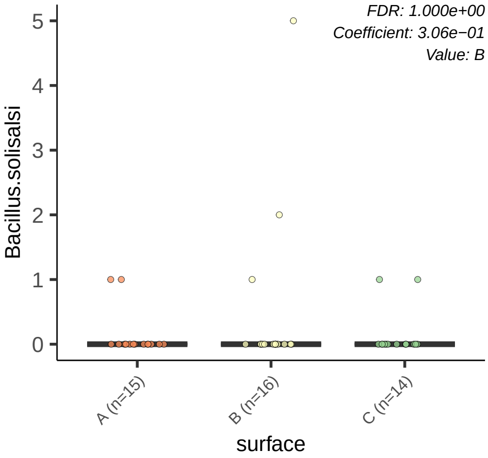

FDR: 1.000e+00
Coefficient: 3.06e−01
Value: B
Bacillus.solisalsi
0
1
2
3
4
5
A (n=15)
B (n=16)
C (n=14)
surface

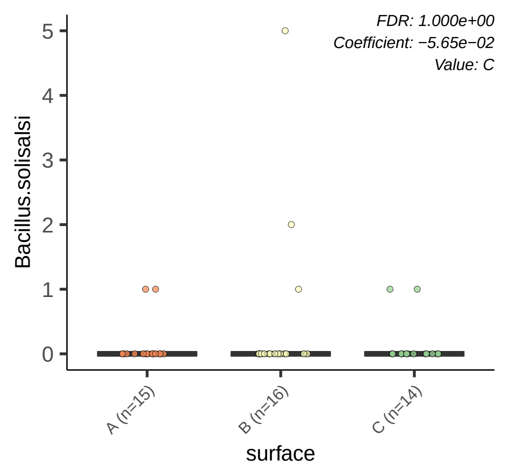
FDR: 1.000e+00
Coefficient: −5.65e−02
Value: C
Bacillus.solisalsi
0
1
2
3
4
5
A (n=15)
B (n=16)
C (n=14)
surface

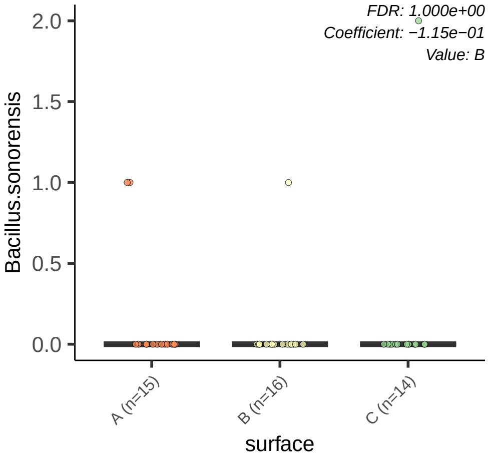

FDR: 1.000e+00
Coefficient: −1.15e−01
Value: B
Bacillus.sonorensis
2.0
1.5
1.0
0.5
0.0
A (n=15)
B (n=16)
C (n=14)
surface

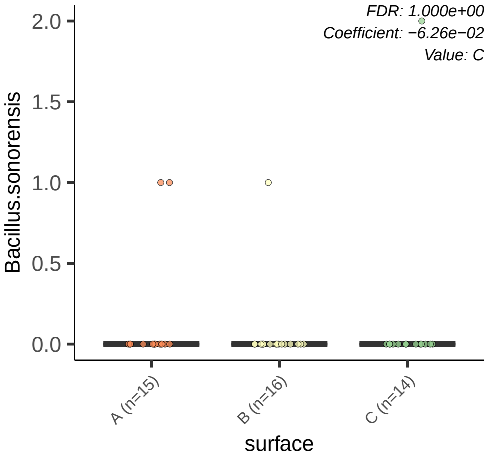

FDR: 1.000e+00
Coefficient: −6.26e−02
Value: C
Bacillus.sonorensis
2.0
1.5
1.0
0.5
0.0
A (n=15)
B (n=16)
C (n=14)
surface

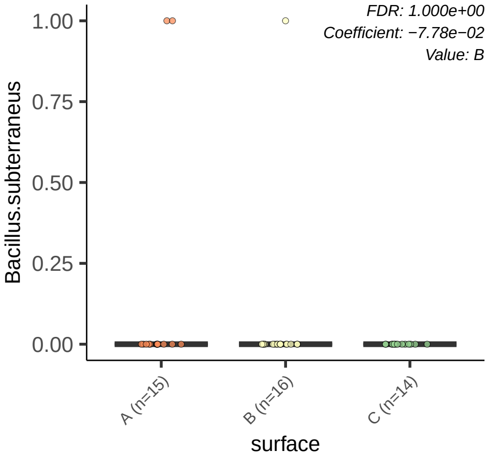

FDR: 1.000e+00
Coefficient: −7.78e−02
Value: B
1.00
0.75
0.50
0.25
0.00
Bacillus.subterraneus
A (n=15)
B (n=16)
C (n=14)
surface

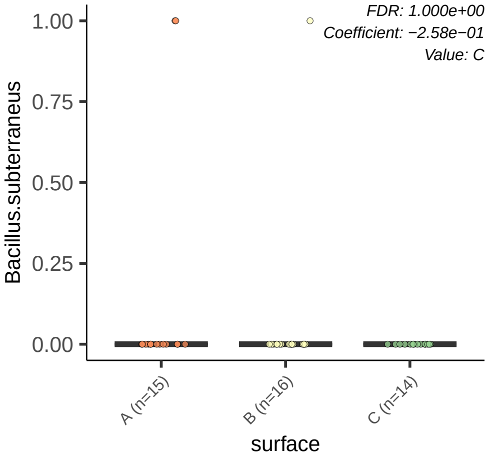

FDR: 1.000e+00
Coefficient: −2.58e−01
Value: C
Bacillus.subterraneus
1.00
0.75
0.50
0.25
0.00
A (n=15)
B (n=16)
C (n=14)
surface

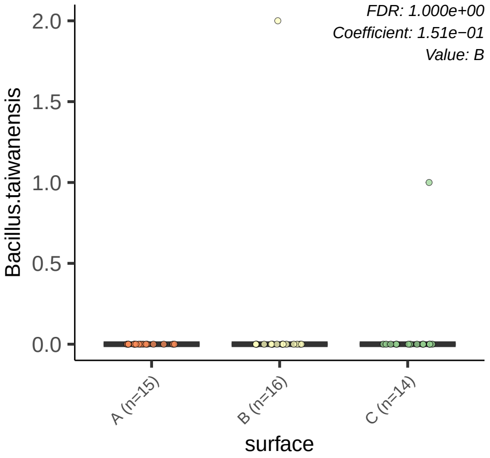

FDR: 1.000e+00
Coefficient: 1.51e−01
Value: B
Bacillus.taiwanensis
2.0
1.5
1.0
0.5
0.0
A (n=15)
B (n=16)
C (n=14)
surface

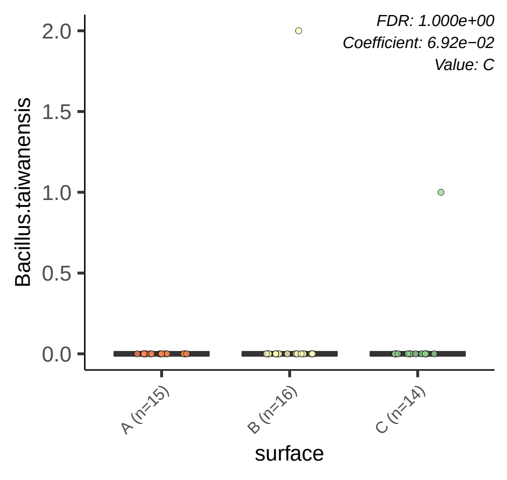
FDR: 1.000e+00
Coefficient: 6.92e−02
Value: C
Bacillus.taiwanensis
2.0
1.5
1.0
0.5
0.0
A (n=15)
B (n=16)
C (n=14)
surface

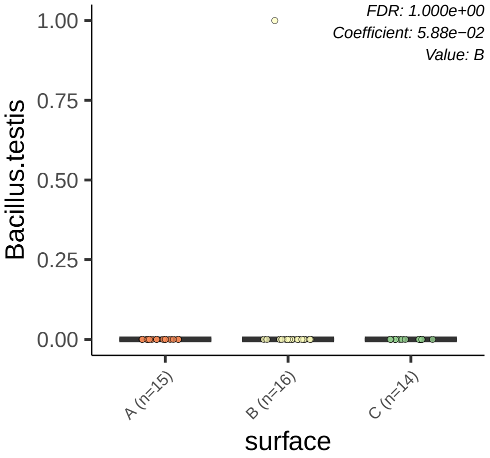
FDR: 1.000e+00
Coefficient: 5.88e−02
Value: B
Bacillus.testis
1.00
0.75
0.50
0.25
0.00
A (n=15)
B (n=16)
C (n=14)
surface

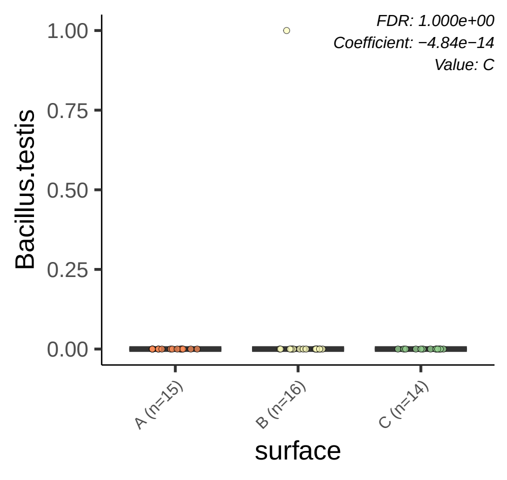
FDR: 1.000e+00
Coefficient: −4.84e−14
Value: C
Bacillus.testis
1.00
0.75
0.50
0.25
0.00
A (n=15)
B (n=16)
C (n=14)
surface

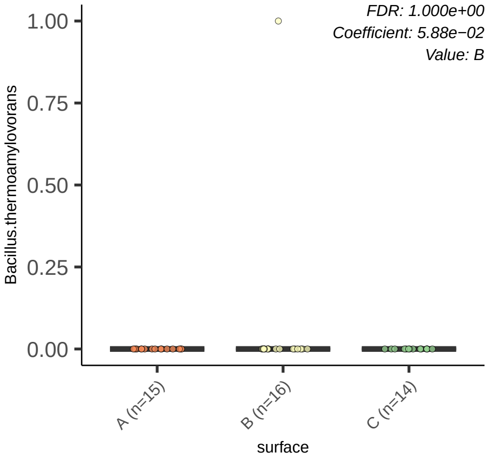

FDR: 1.000e+00
Coefficient: 5.88e−02
Value: B
Bacillus.thermoamylovorans
1.00
0.75
0.50
0.25
0.00
A (n=15)
B (n=16)
C (n=14)
surface

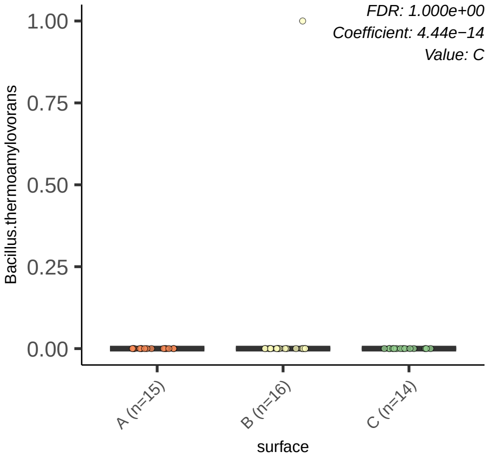
FDR: 1.000e+00
Coefficient: 4.44e−14
Value: C
Bacillus.thermoamylovorans
1.00
0.75
0.50
0.25
0.00
A (n=15)
B (n=16)
C (n=14)
surface

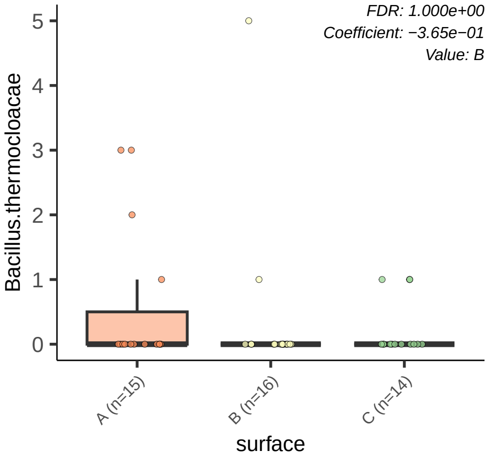
FDR: 1.000e+00
Coefficient: −3.65e−01
Value: B
Bacillus.thermocloacae
0
1
2
3
4
5
A (n=15)
B (n=16)
C (n=14)
surface

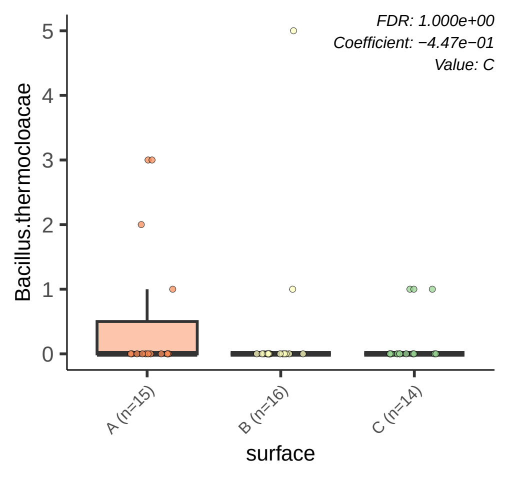

FDR: 1.000e+00
Coefficient: −4.47e−01
Value: C
Bacillus.thermocloacae
0
1
2
3
4
5
A (n=15)
B (n=16)
C (n=14)
surface

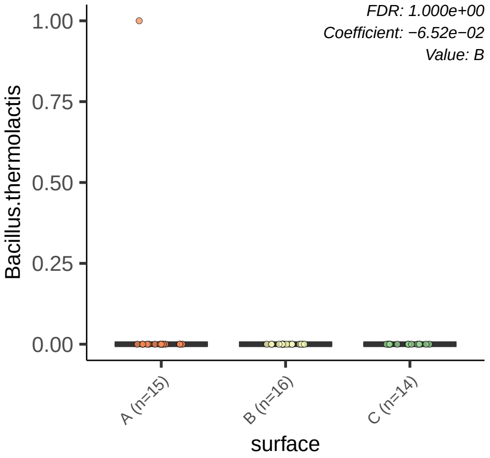
FDR: 1.000e+00
Coefficient: −6.52e−02
Value: B
Bacillus.thermolactis
1.00
0.75
0.50
0.25
0.00
A (n=15)
B (n=16)
C (n=14)
surface

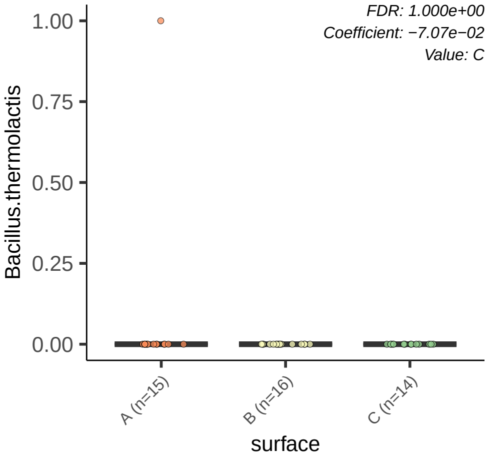
FDR: 1.000e+00
Coefficient: −7.07e−02
Value: C
Bacillus.thermolactis
1.00
0.75
0.50
0.25
0.00
A (n=15)
B (n=16)
C (n=14)
surface

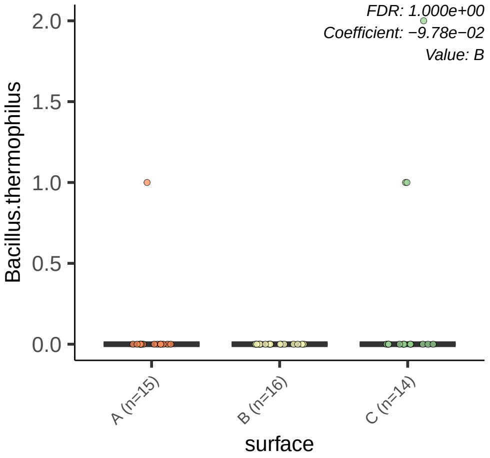
FDR: 1.000e+00
Coefficient: −9.78e−02
Value: B
Bacillus.thermophilus
2.0
1.5
1.0
0.5
0.0
A (n=15)
B (n=16)
C (n=14)
surface

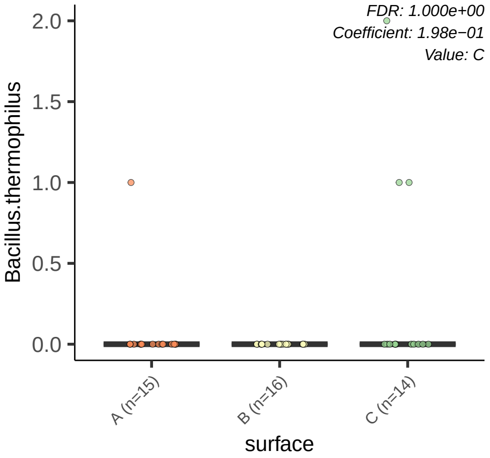
FDR: 1.000e+00
Coefficient: 1.98e−01
Value: C
Bacillus.thermophilus
2.0
1.5
1.0
0.5
0.0
A (n=15)
B (n=16)
C (n=14)
surface

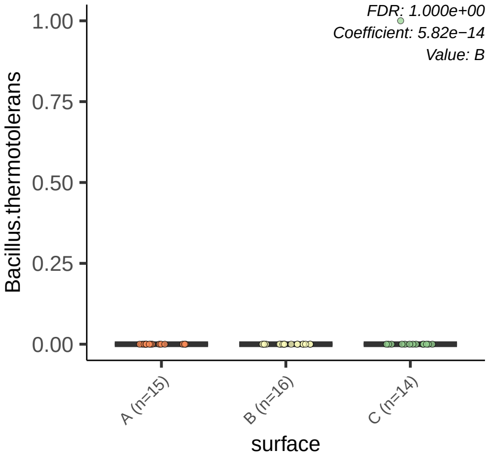
FDR: 1.000e+00
Coefficient: 5.82e−14
Value: B
1.00
0.75
0.50
0.25
0.00
Bacillus.thermotolerans
A (n=15)
B (n=16)
C (n=14)
surface

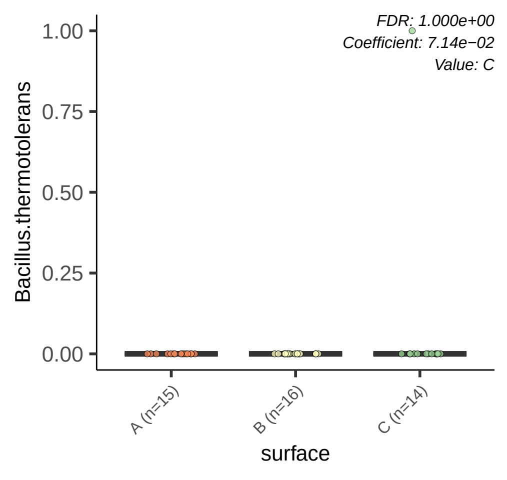

FDR: 1.000e+00
Coefficient: 7.14e−02
Value: C
1.00
0.75
0.50
0.25
0.00
Bacillus.thermotolerans
A (n=15)
B (n=16)
C (n=14)
surface

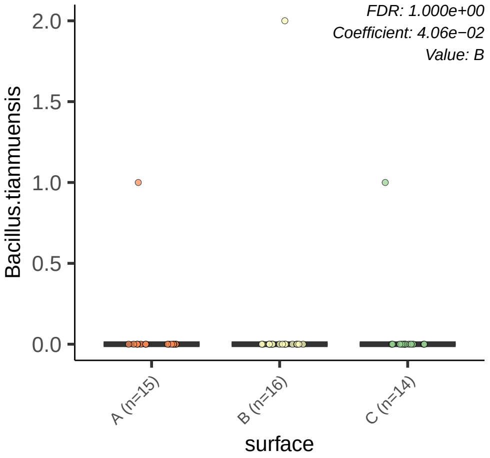

FDR: 1.000e+00
Coefficient: 4.06e−02
Value: B
Bacillus.tianmuensis
2.0
1.5
1.0
0.5
0.0
A (n=15)
B (n=16)
C (n=14)
surface

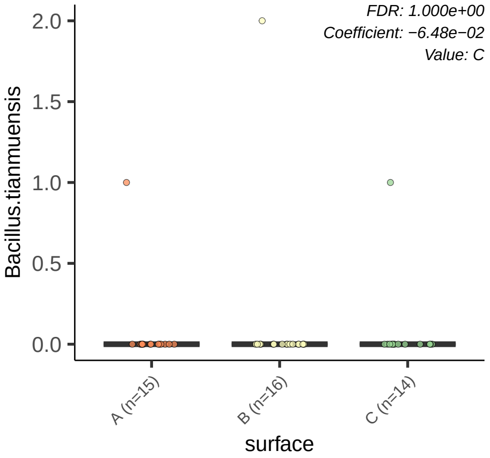
FDR: 1.000e+00
Coefficient: −6.48e−02
Value: C
Bacillus.tianmuensis
2.0
1.5
1.0
0.5
0.0
A (n=15)
B (n=16)
C (n=14)
surface

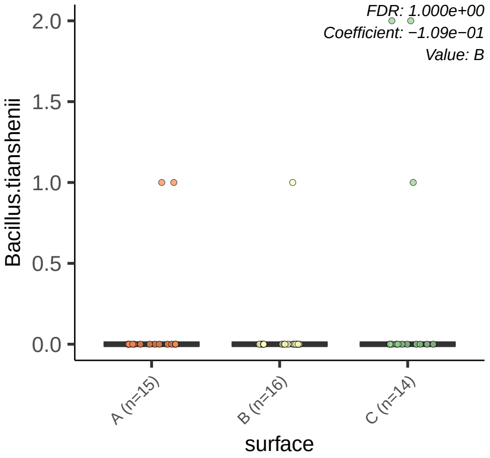

FDR: 1.000e+00
Coefficient: −1.09e−01
Value: B
Bacillus.tianshenii
2.0
1.5
1.0
0.5
0.0
A (n=15)
B (n=16)
C (n=14)
surface

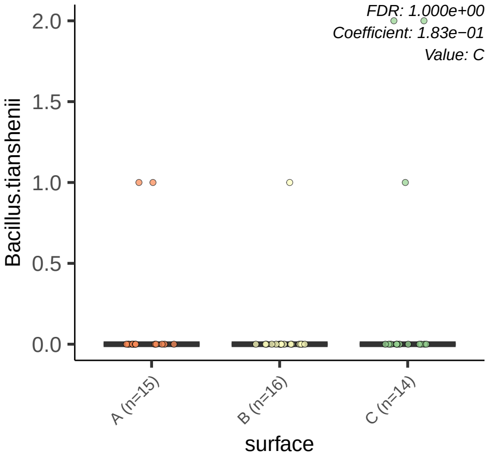

FDR: 1.000e+00
Coefficient: 1.83e−01
Value: C
2.0
1.5
1.0
0.5
0.0
Bacillus.tianshenii
A (n=15)
B (n=16)
C (n=14)
surface

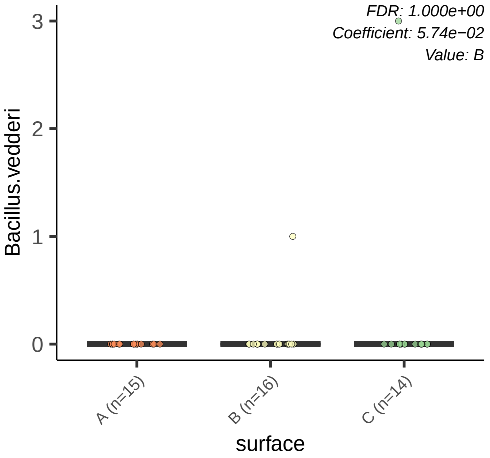

FDR: 1.000e+00
Coefficient: 5.74e−02
Value: B
Bacillus.vedderi
0
1
2
3
A (n=15)
B (n=16)
C (n=14)
surface

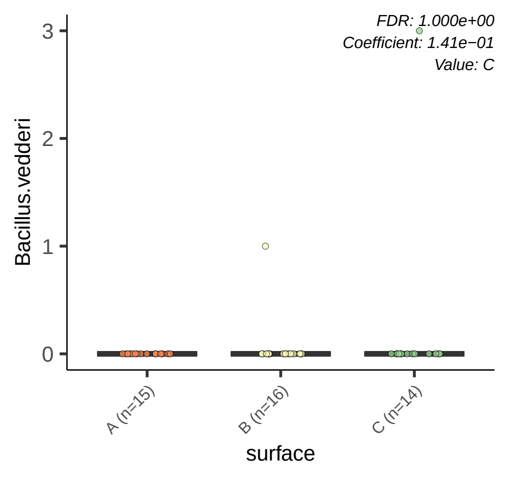
FDR: 1.000e+00
Coefficient: 1.41e−01
Value: C
Bacillus.vedderi
0
1
2
3
A (n=15)
B (n=16)
C (n=14)
surface

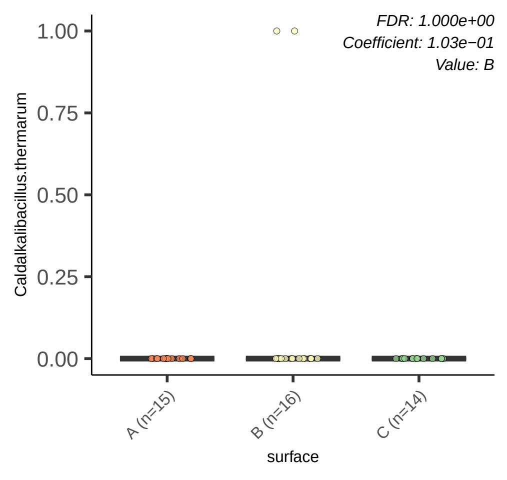
FDR: 1.000e+00
Coefficient: 1.03e−01
Value: B
1.00
0.75
0.50
0.25
0.00
Caldalkalibacillus.thermarum
A (n=15)
B (n=16)
C (n=14)
surface

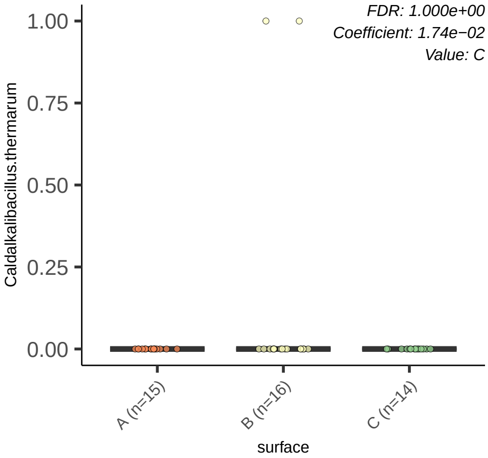

FDR: 1.000e+00
Coefficient: 1.74e−02
Value: C
Caldalkalibacillus.thermarum
1.00
0.75
0.50
0.25
0.00
A (n=15)
B (n=16)
C (n=14)
surface

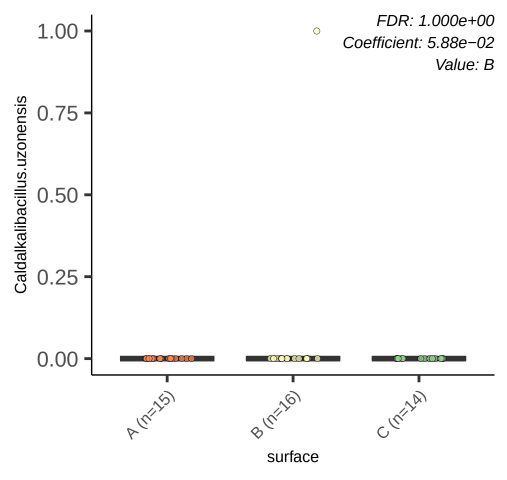
FDR: 1.000e+00
Coefficient: 5.88e−02
Value: B
Caldalkalibacillus.uzonensis
1.00
0.75
0.50
0.25
0.00
A (n=15)
B (n=16)
C (n=14)
surface

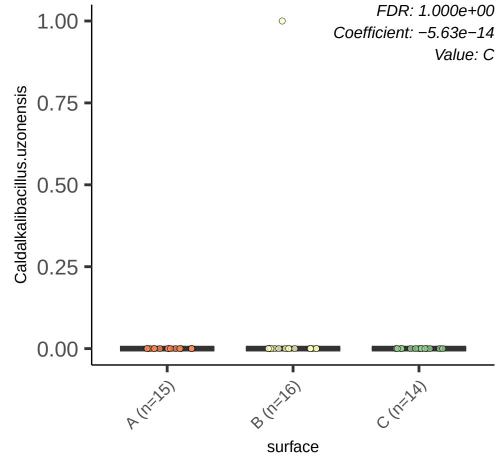
FDR: 1.000e+00
Coefficient: −5.63e−14
Value: C
Caldalkalibacillus.uzonensis
1.00
0.75
0.50
0.25
0.00
A (n=15)
B (n=16)
C (n=14)
surface

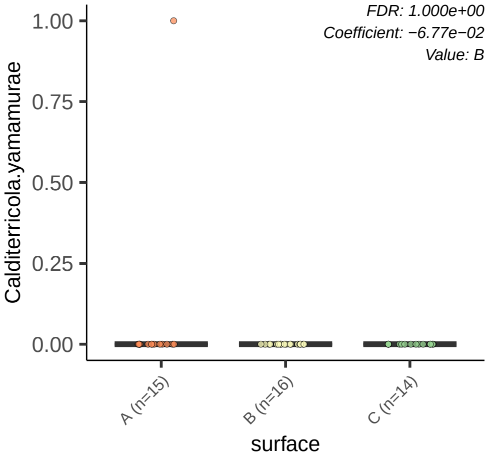

FDR: 1.000e+00
Coefficient: −6.77e−02
Value: B
Calditerricola.yamamurae
1.00
0.75
0.50
0.25
0.00
A (n=15)
B (n=16)
C (n=14)
surface

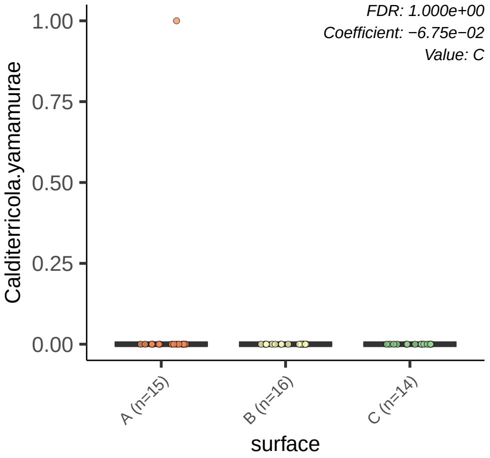
FDR: 1.000e+00
Coefficient: −6.75e−02
Value: C
Calditerricola.yamamurae
1.00
0.75
0.50
0.25
0.00
A (n=15)
B (n=16)
C (n=14)
surface

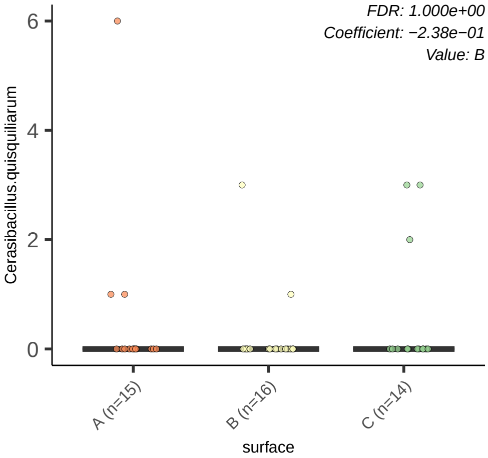

FDR: 1.000e+00
Coefficient: −2.38e−01
Value: B
Cerasibacillus.quisquiliarum
6
4
2
0
A (n=15)
B (n=16)
C (n=14)
surface

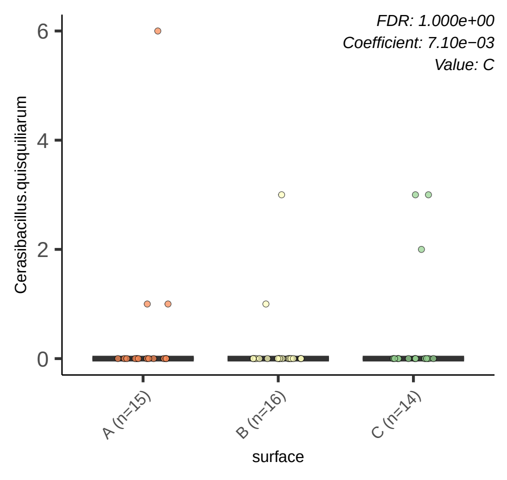
FDR: 1.000e+00
Coefficient: 7.10e−03
Value: C
Cerasibacillus.quisquiliarum
0
2
4
6
A (n=15)
B (n=16)
C (n=14)
surface

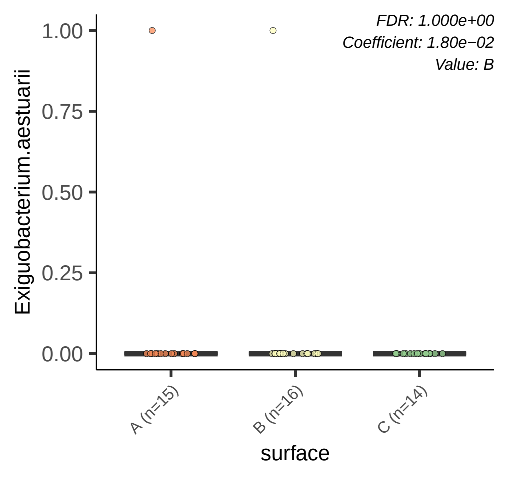
FDR: 1.000e+00
Coefficient: 1.80e−02
Value: B
Exiguobacterium.aestuarii
1.00
0.75
0.50
0.25
0.00
A (n=15)
B (n=16)
C (n=14)
surface

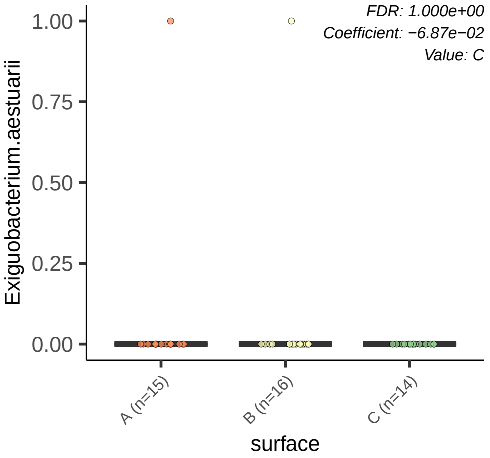
FDR: 1.000e+00
Coefficient: −6.87e−02
Value: C
Exiguobacterium.aestuarii
1.00
0.75
0.50
0.25
0.00
A (n=15)
B (n=16)
C (n=14)
surface

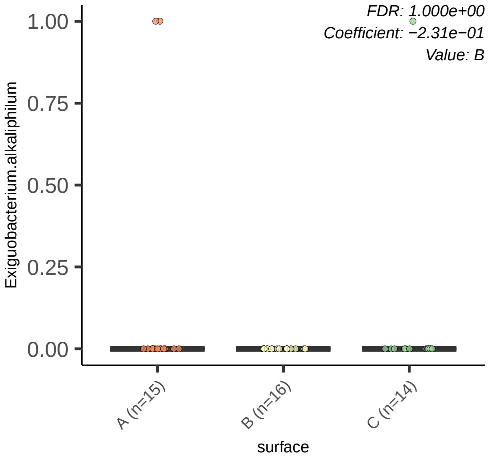

FDR: 1.000e+00
Coefficient: −2.31e−01
Value: B
1.00
0.75
0.50
0.25
0.00
Exiguobacterium.alkaliphilum
A (n=15)
B (n=16)
C (n=14)
surface

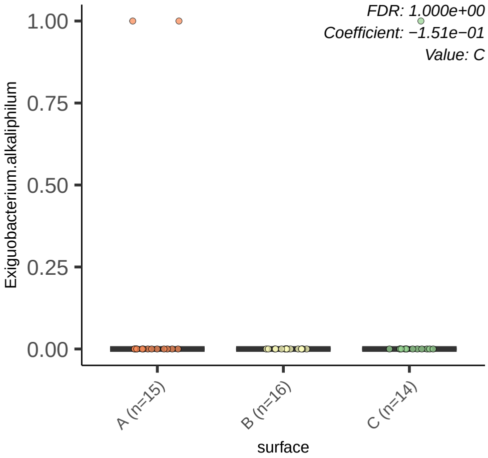

FDR: 1.000e+00
Coefficient: −1.51e−01
Value: C
1.00
0.75
0.50
0.25
0.00
Exiguobacterium.alkaliphilum
A (n=15)
B (n=16)
C (n=14)
surface

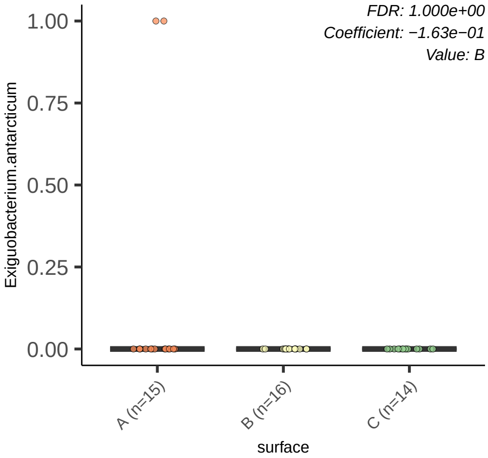
FDR: 1.000e+00
Coefficient: −1.63e−01
Value: B
Exiguobacterium.antarcticum
1.00
0.75
0.50
0.25
0.00
A (n=15)
B (n=16)
C (n=14)
surface

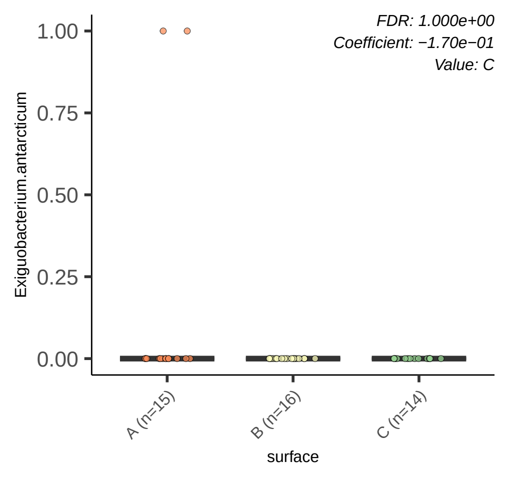

FDR: 1.000e+00
Coefficient: −1.70e−01
Value: C
1.00
0.75
0.50
0.25
0.00
Exiguobacterium.antarcticum
A (n=15)
B (n=16)
C (n=14)
surface

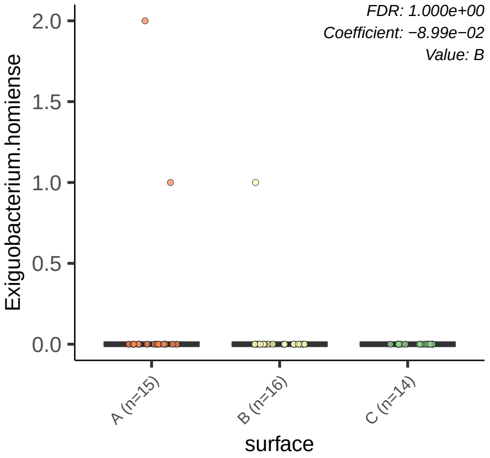
FDR: 1.000e+00
Coefficient: −8.99e−02
Value: B
Exiguobacterium.homiense
2.0
1.5
1.0
0.5
0.0
A (n=15)
B (n=16)
C (n=14)
surface

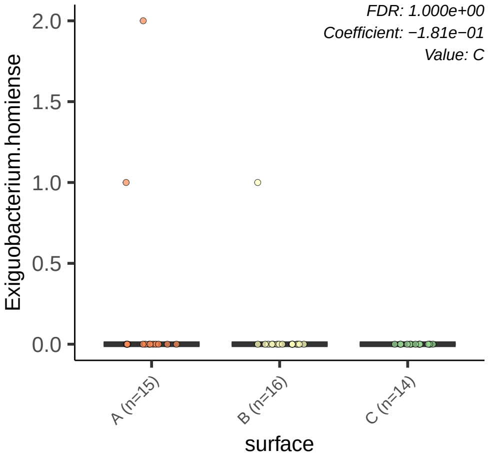
FDR: 1.000e+00
Coefficient: −1.81e−01
Value: C
Exiguobacterium.homiense
2.0
1.5
1.0
0.5
0.0
A (n=15)
B (n=16)
C (n=14)
surface

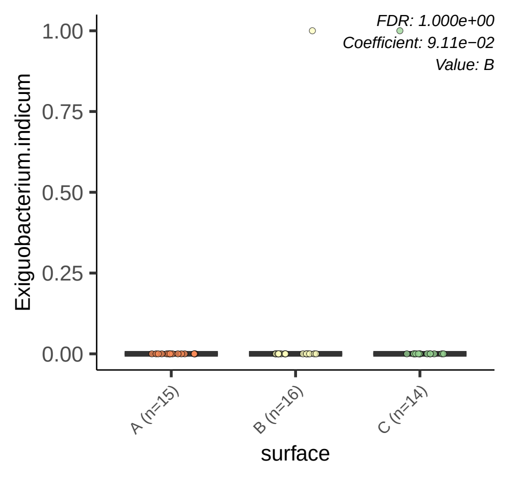
FDR: 1.000e+00
Coefficient: 9.11e−02
Value: B
Exiguobacterium.indicum
1.00
0.75
0.50
0.25
0.00
A (n=15)
B (n=16)
C (n=14)
surface

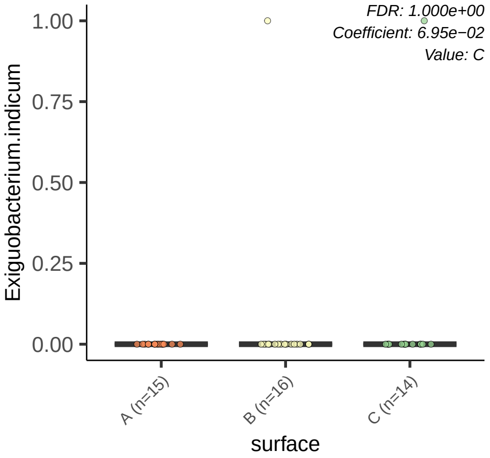
FDR: 1.000e+00
Coefficient: 6.95e−02
Value: C
Exiguobacterium.indicum
1.00
0.75
0.50
0.25
0.00
A (n=15)
B (n=16)
C (n=14)
surface

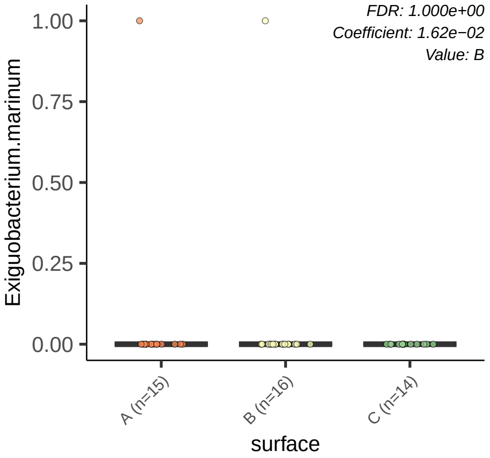

FDR: 1.000e+00
Coefficient: 1.62e−02
Value: B
Exiguobacterium.marinum
1.00
0.75
0.50
0.25
0.00
A (n=15)
B (n=16)
C (n=14)
surface

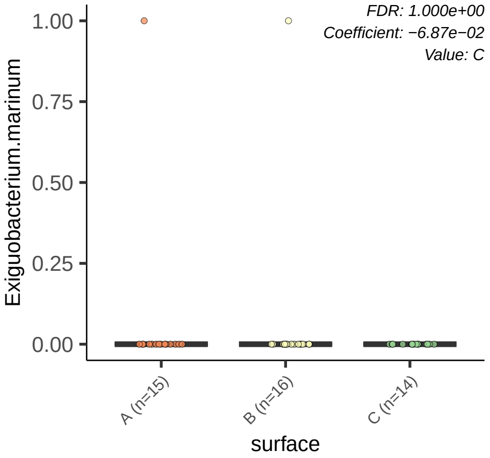
FDR: 1.000e+00
Coefficient: −6.87e−02
Value: C
Exiguobacterium.marinum
1.00
0.75
0.50
0.25
0.00
A (n=15)
B (n=16)
C (n=14)
surface

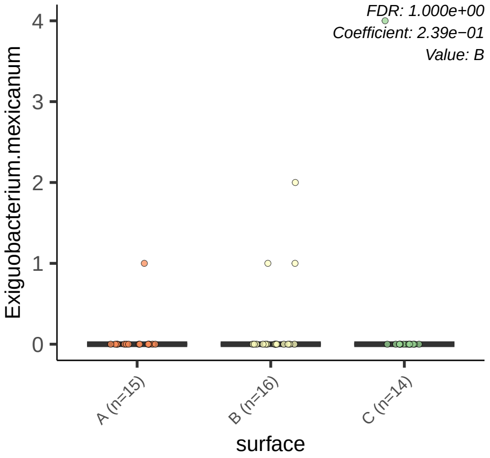

FDR: 1.000e+00
Coefficient: 2.39e−01
Value: B
Exiguobacterium.mexicanum
0
1
2
3
4
A (n=15)
B (n=16)
C (n=14)
surface

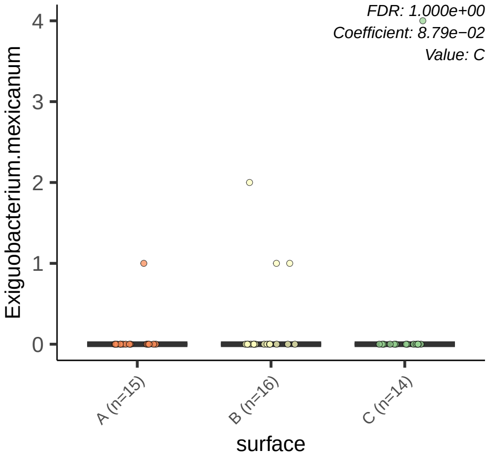

FDR: 1.000e+00
Coefficient: 8.79e−02
Value: C
Exiguobacterium.mexicanum
4
3
2
1
0
A (n=15)
B (n=16)
C (n=14)
surface

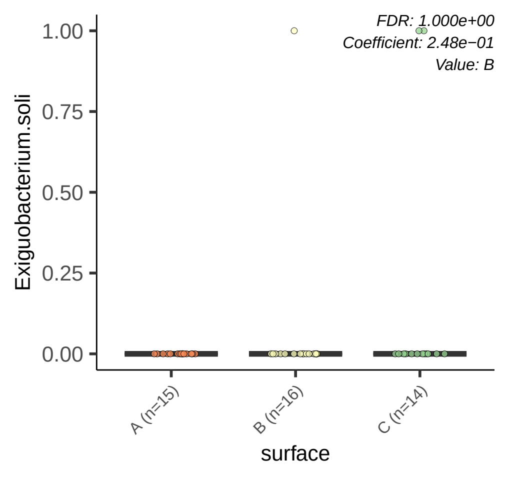
FDR: 1.000e+00
Coefficient: 2.48e−01
Value: B
Exiguobacterium.soli
1.00
0.75
0.50
0.25
0.00
A (n=15)
B (n=16)
C (n=14)
surface

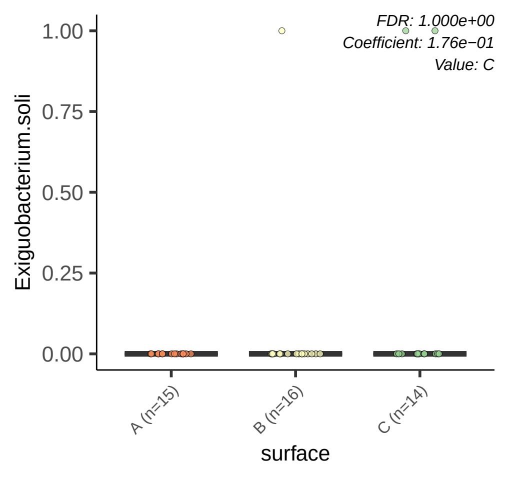

FDR: 1.000e+00
Coefficient: 1.76e−01
Value: C
1.00
0.75
0.50
0.25
0.00
Exiguobacterium.soli
A (n=15)
B (n=16)
C (n=14)
surface

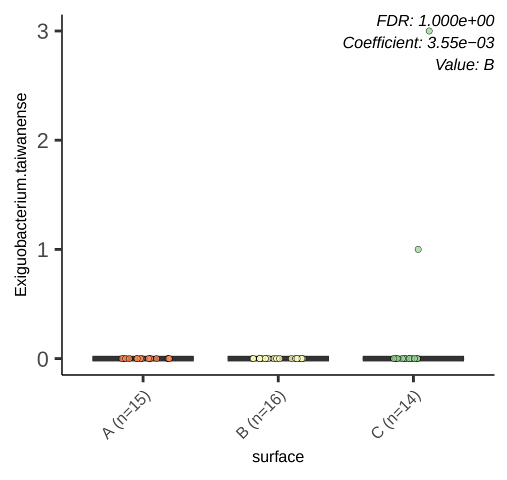

FDR: 1.000e+00
Coefficient: 3.55e−03
Value: B
Exiguobacterium.taiwanense
3
2
1
0
A (n=15)
B (n=16)
C (n=14)
surface

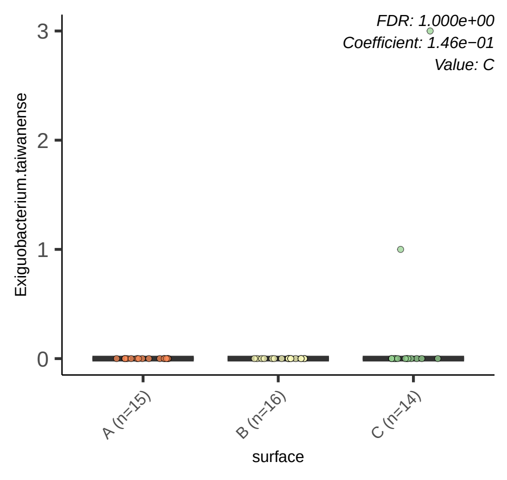

FDR: 1.000e+00
Coefficient: 1.46e−01
Value: C
Exiguobacterium.taiwanense
0
1
2
3
A (n=15)
B (n=16)
C (n=14)
surface

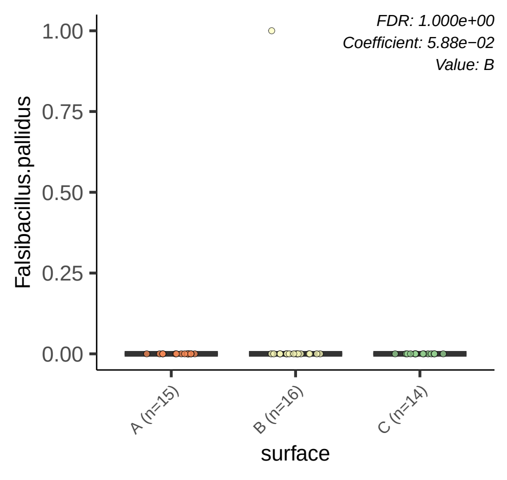
FDR: 1.000e+00
Coefficient: 5.88e−02
Value: B
Falsibacillus.pallidus
1.00
0.75
0.50
0.25
0.00
A (n=15)
B (n=16)
C (n=14)
surface

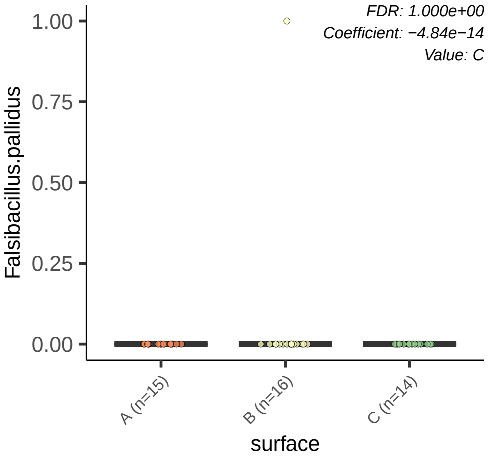

FDR: 1.000e+00
Coefficient: −4.84e−14
Value: C
Falsibacillus.pallidus
1.00
0.75
0.50
0.25
0.00
A (n=15)
B (n=16)
C (n=14)
surface

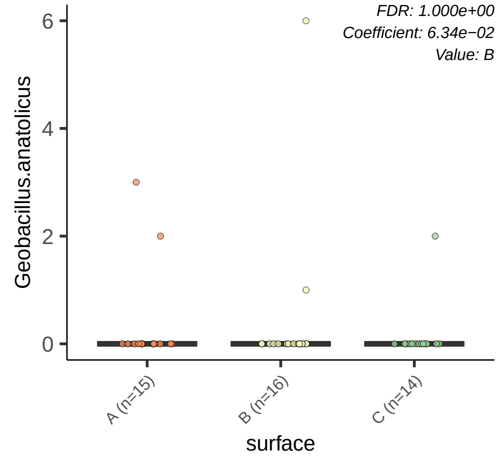

FDR: 1.000e+00
Coefficient: 6.34e−02
Value: B
Geobacillus.anatolicus
0
2
4
6
A (n=15)
B (n=16)
C (n=14)
surface

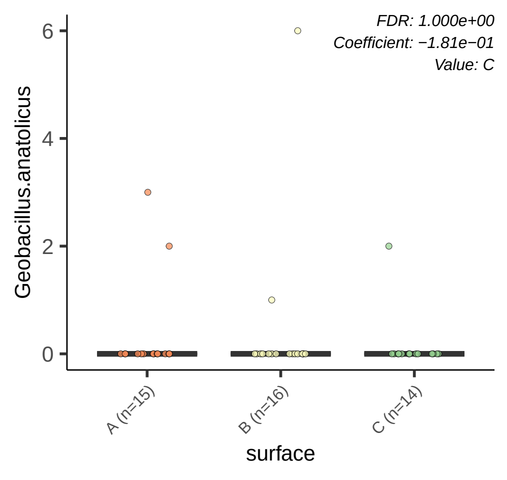
FDR: 1.000e+00
Coefficient: −1.81e−01
Value: C
Geobacillus.anatolicus
0
2
4
6
A (n=15)
B (n=16)
C (n=14)
surface

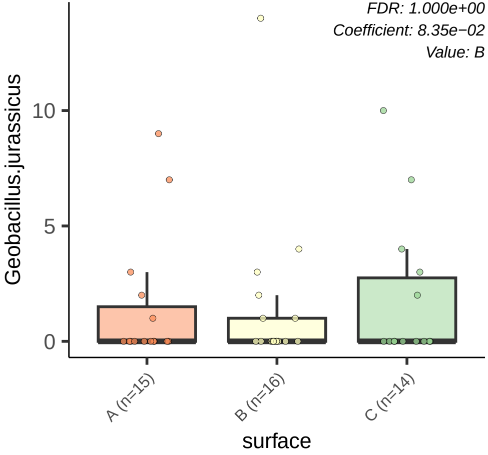
FDR: 1.000e+00
Coefficient: 8.35e−02
Value: B
Geobacillus.jurassicus
0
5
10
A (n=15)
B (n=16)
C (n=14)
surface

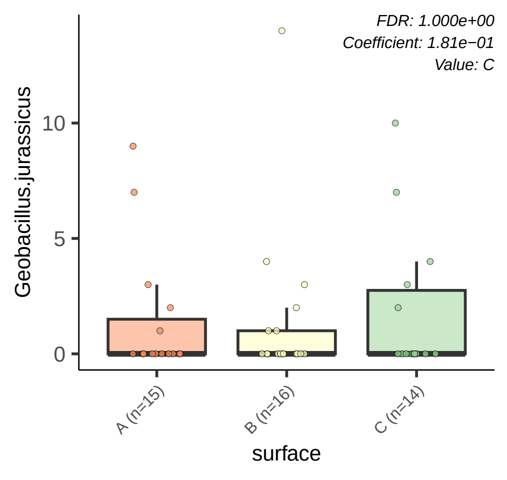
FDR: 1.000e+00
Coefficient: 1.81e−01
Value: C
Geobacillus.jurassicus
10
5
0
A (n=15)
B (n=16)
C (n=14)
surface

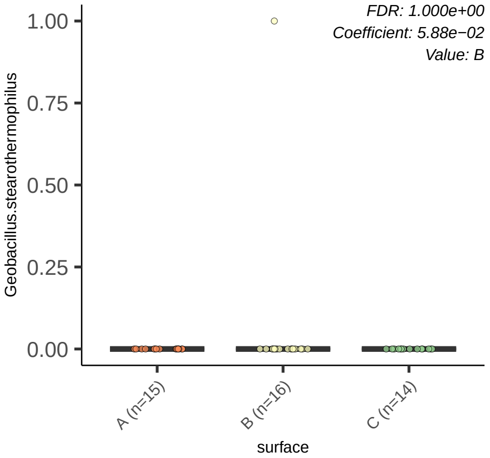

FDR: 1.000e+00
Coefficient: 5.88e−02
Value: B
1.00
0.75
0.50
0.25
0.00
Geobacillus.stearothermophilus
A (n=15)
B (n=16)
C (n=14)
surface

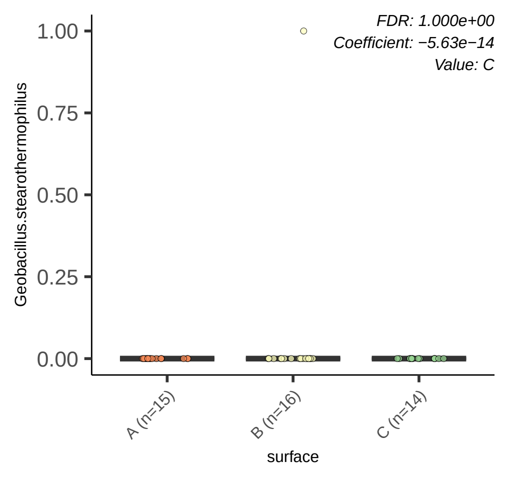
FDR: 1.000e+00
Coefficient: −5.63e−14
Value: C
Geobacillus.stearothermophilus
1.00
0.75
0.50
0.25
0.00
A (n=15)
B (n=16)
C (n=14)
surface

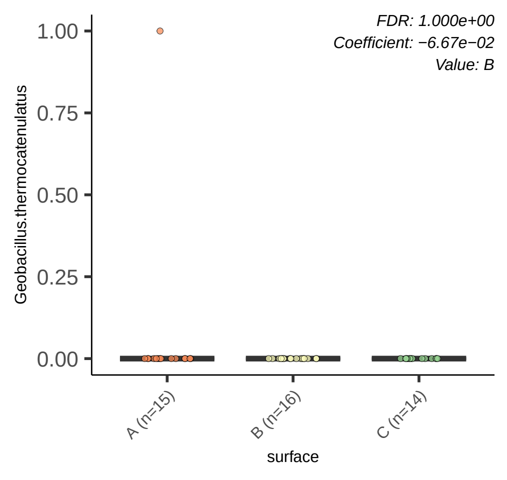
FDR: 1.000e+00
Coefficient: −6.67e−02
Value: B
Geobacillus.thermocatenulatus
1.00
0.75
0.50
0.25
0.00
A (n=15)
B (n=16)
C (n=14)
surface

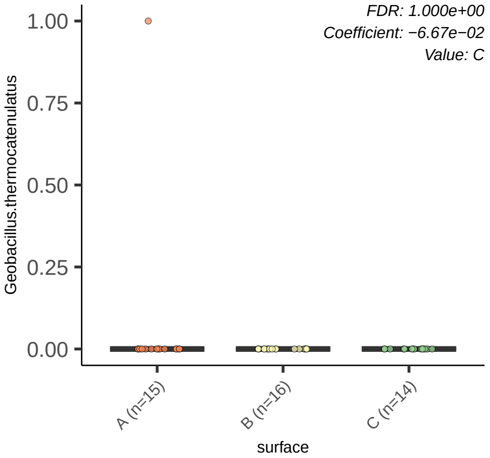
FDR: 1.000e+00
Coefficient: −6.67e−02
Value: C
Geobacillus.thermocatenulatus
1.00
0.75
0.50
0.25
0.00
A (n=15)
B (n=16)
C (n=14)
surface

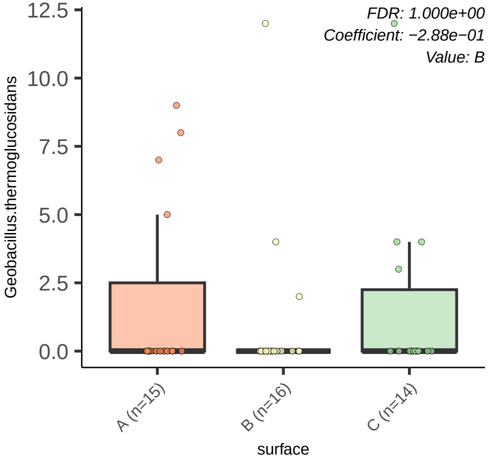
FDR: 1.000e+00
Coefficient: −2.88e−01
Value: B
Geobacillus.thermoglucosidans
12.5
10.0
7.5
5.0
2.5
0.0
A (n=15)
B (n=16)
C (n=14)
surface

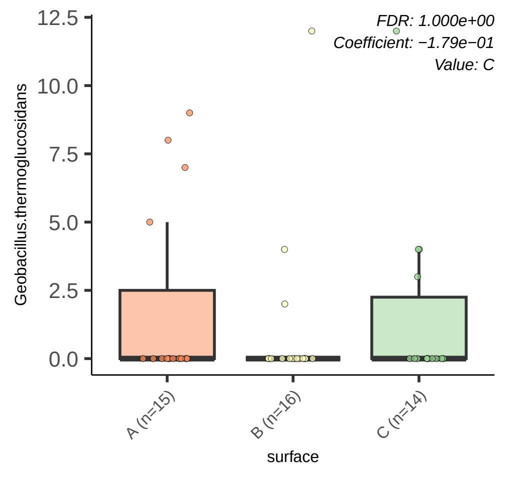

FDR: 1.000e+00
Coefficient: −1.79e−01
Value: C
Geobacillus.thermoglucosidans
12.5
10.0
7.5
5.0
2.5
0.0
A (n=15)
B (n=16)
C (n=14)
surface

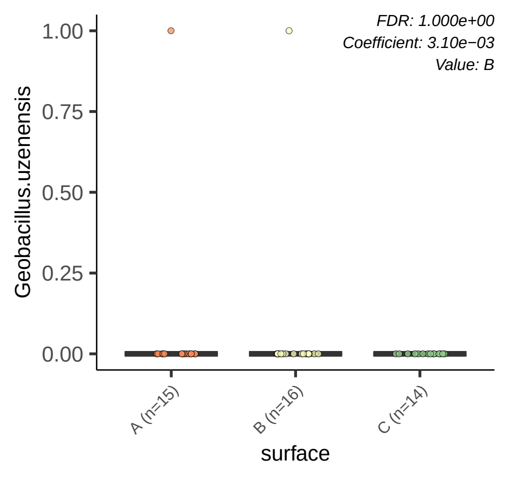
FDR: 1.000e+00
Coefficient: 3.10e−03
Value: B
Geobacillus.uzenensis
1.00
0.75
0.50
0.25
0.00
A (n=15)
B (n=16)
C (n=14)
surface

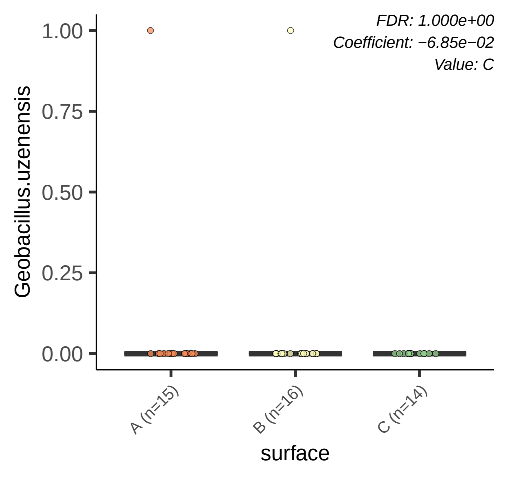
FDR: 1.000e+00
Coefficient: −6.85e−02
Value: C
Geobacillus.uzenensis
1.00
0.75
0.50
0.25
0.00
A (n=15)
B (n=16)
C (n=14)
surface

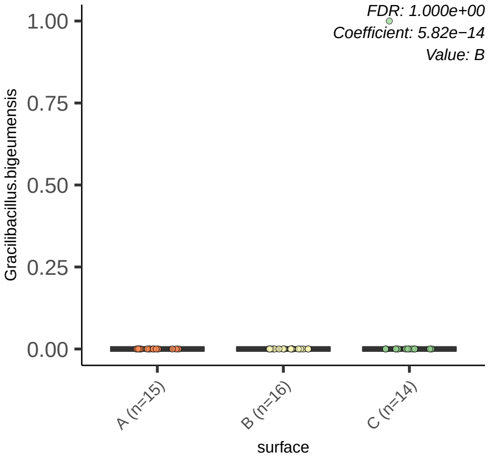

FDR: 1.000e+00
Coefficient: 5.82e−14
Value: B
1.00
0.75
0.50
0.25
0.00
Gracilibacillus.bigeumensis
A (n=15)
B (n=16)
C (n=14)
surface

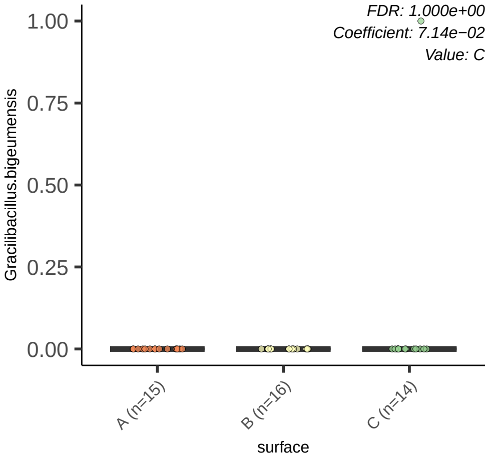
FDR: 1.000e+00
Coefficient: 7.14e−02
Value: C
1.00
0.75
0.50
0.25
0.00
Gracilibacillus.bigeumensis
A (n=15)
B (n=16)
C (n=14)
surface

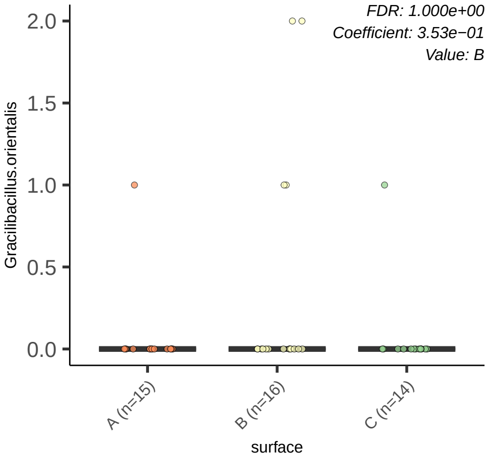
FDR: 1.000e+00
Coefficient: 3.53e−01
Value: B
Gracilibacillus.orientalis
2.0
1.5
1.0
0.5
0.0
A (n=15)
B (n=16)
C (n=14)
surface

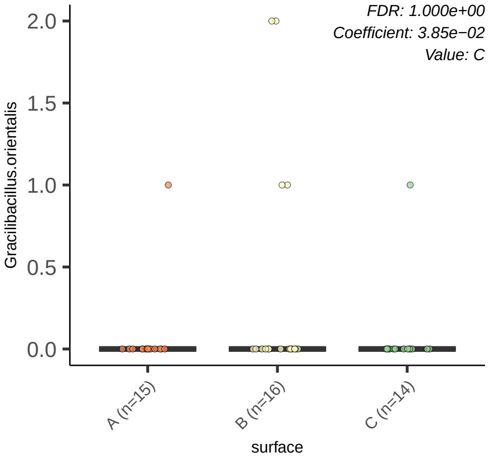

FDR: 1.000e+00
Coefficient: 3.85e−02
Value: C
Gracilibacillus.orientalis
2.0
1.5
1.0
0.5
0.0
A (n=15)
B (n=16)
C (n=14)
surface

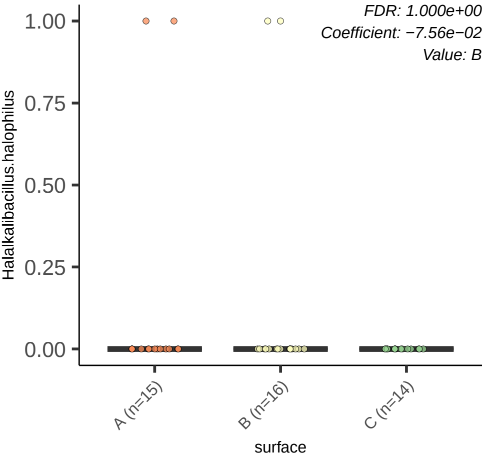

FDR: 1.000e+00
Coefficient: −7.56e−02
Value: B
1.00
0.75
0.50
0.25
0.00
Halalkalibacillus.halophilus
A (n=15)
B (n=16)
C (n=14)
surface

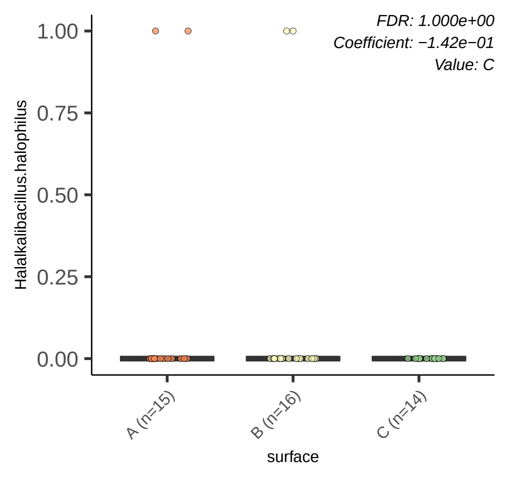

FDR: 1.000e+00
Coefficient: −1.42e−01
Value: C
1.00
0.75
0.50
0.25
0.00
Halalkalibacillus.halophilus
A (n=15)
B (n=16)
C (n=14)
surface

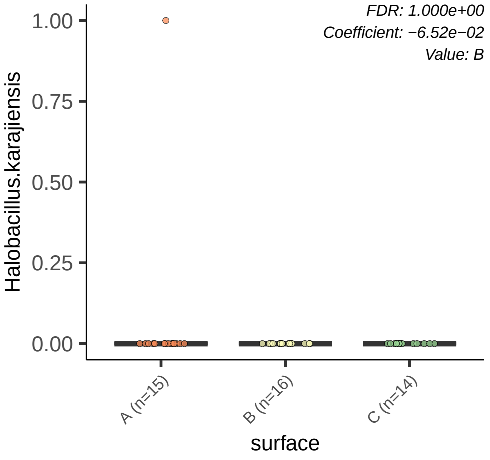
FDR: 1.000e+00
Coefficient: −6.52e−02
Value: B
1.00
0.75
0.50
0.25
0.00
Halobacillus.karajiensis
A (n=15)
B (n=16)
C (n=14)
surface

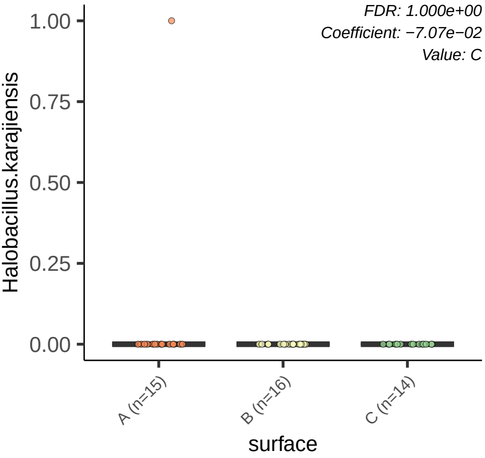
FDR: 1.000e+00
Coefficient: −7.07e−02
Value: C
Halobacillus.karajiensis
1.00
0.75
0.50
0.25
0.00
A (n=15)
B (n=16)
C (n=14)
surface

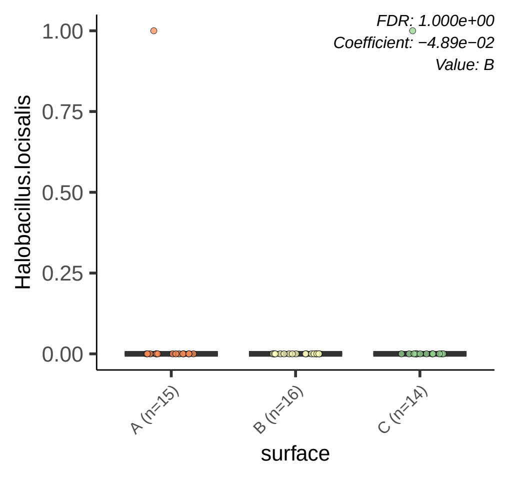
FDR: 1.000e+00
Coefficient: −4.89e−02
Value: B
Halobacillus.locisalis
1.00
0.75
0.50
0.25
0.00
A (n=15)
B (n=16)
C (n=14)
surface

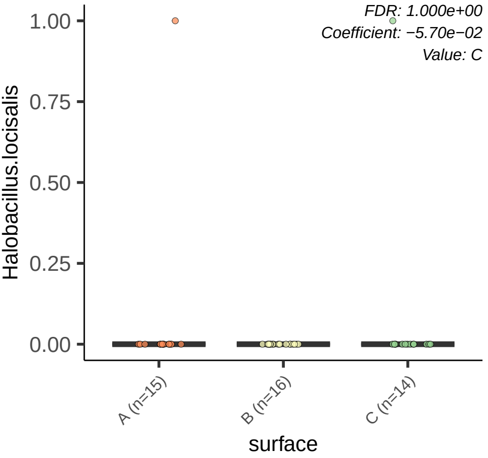
FDR: 1.000e+00
Coefficient: −5.70e−02
Value: C
Halobacillus.locisalis
1.00
0.75
0.50
0.25
0.00
A (n=15)
B (n=16)
C (n=14)
surface

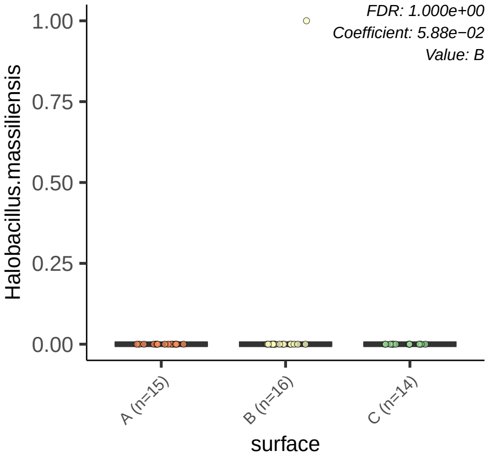

FDR: 1.000e+00
Coefficient: 5.88e−02
Value: B
Halobacillus.massiliensis
1.00
0.75
0.50
0.25
0.00
A (n=15)
B (n=16)
C (n=14)
surface

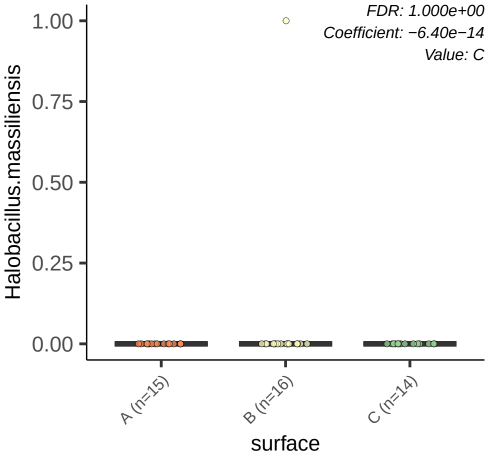

FDR: 1.000e+00
Coefficient: −6.40e−14
Value: C
Halobacillus.massiliensis
1.00
0.75
0.50
0.25
0.00
A (n=15)
B (n=16)
C (n=14)
surface

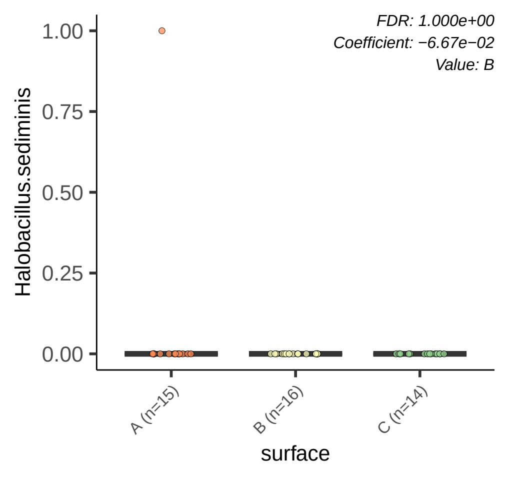

FDR: 1.000e+00
Coefficient: −6.67e−02
Value: B
Halobacillus.sedimінis
1.00
0.75
0.50
0.25
0.00
A (n=15)
B (n=16)
C (n=14)
surface

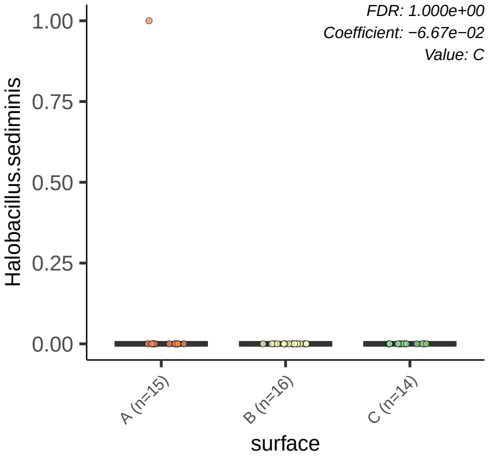
FDR: 1.000e+00
Coefficient: −6.67e−02
Value: C
Halobacillus.sedimins
1.00
0.75
0.50
0.25
0.00
A (n=15)
B (n=16)
C (n=14)
surface

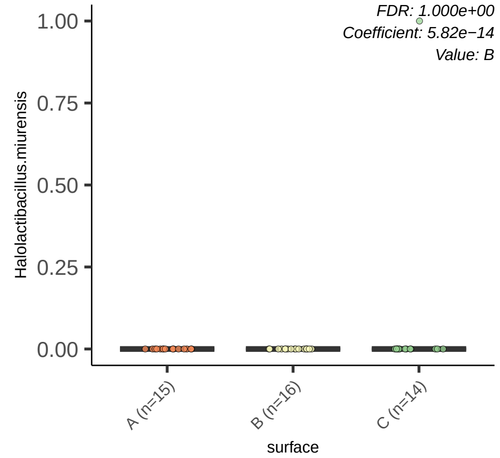

FDR: 1.000e+00
Coefficient: 5.82e−14
Value: B
1.00
0.75
0.50
0.25
0.00
Halolactibacillus.miurensis
A (n=15)
B (n=16)
C (n=14)
surface

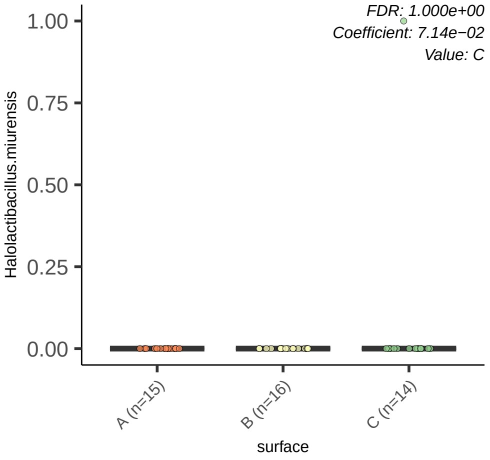
FDR: 1.000e+00
Coefficient: 7.14e−02
Value: C
Halolactibacillus.miurensis
1.00
0.75
0.50
0.25
0.00
A (n=15)
B (n=16)
C (n=14)
surface

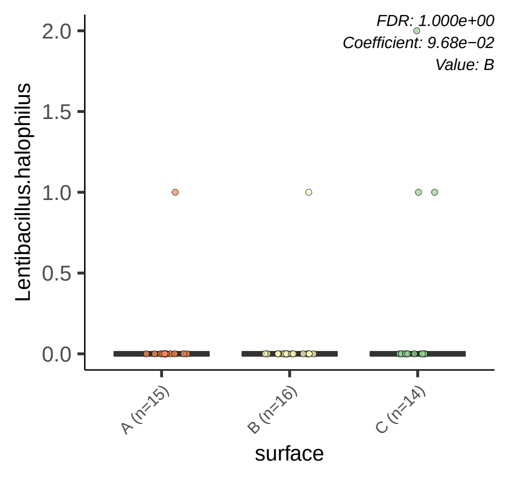

FDR: 1.000e+00
Coefficient: 9.68e−02
Value: B
Lentibacillus.halophilus
2.0
1.5
1.0
0.5
0.0
A (n=15)
B (n=16)
C (n=14)
surface

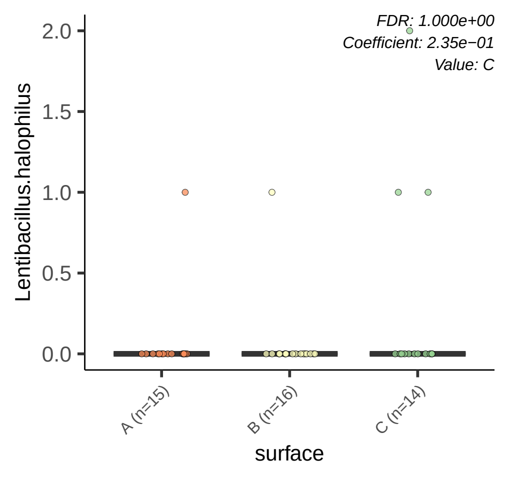
FDR: 1.000e+00
Coefficient: 2.35e−01
Value: C
Lentibacillus.halophilus
2.0
1.5
1.0
0.5
0.0
A (n=15)
B (n=16)
C (n=14)
surface

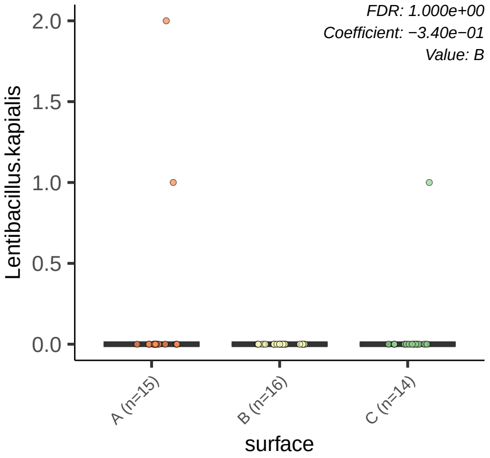

FDR: 1.000e+00
Coefficient: −3.40e−01
Value: B
Lentibacillus.kapialis
2.0
1.5
1.0
0.5
0.0
A (n=15)
B (n=16)
C (n=14)
surface

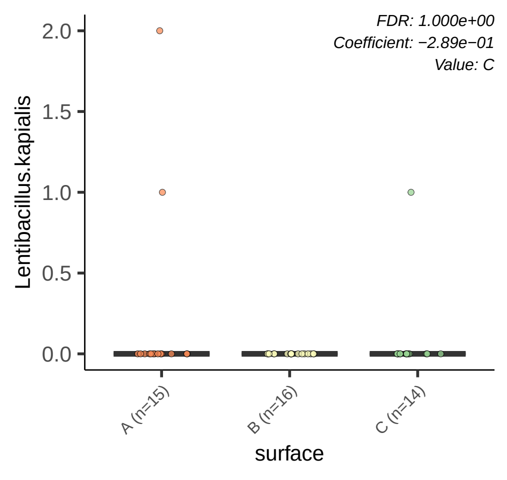

FDR: 1.000e+00
Coefficient: −2.89e−01
Value: C
Lentibacillus.kapialis
2.0
1.5
1.0
0.5
0.0
A (n=15)
B (n=16)
C (n=14)
surface

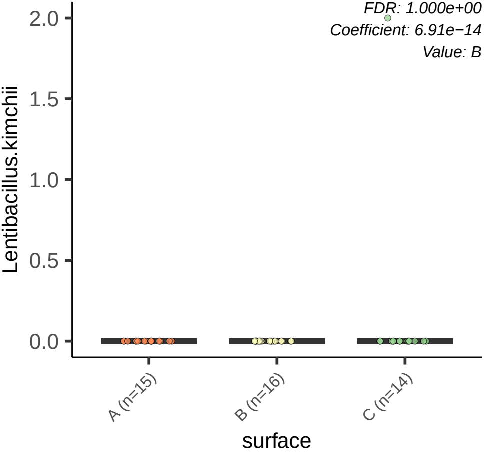

FDR: 1.000e+00
Coefficient: 6.91e−14
Value: B
Lentibacillus.kimchii
2.0
1.5
1.0
0.5
0.0
A (n=15)
B (n=16)
C (n=14)
surface

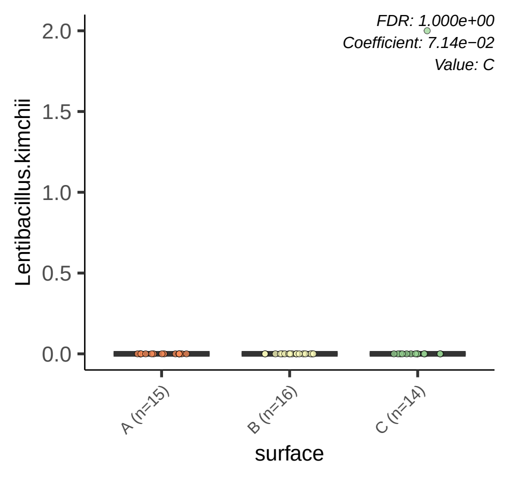

FDR: 1.000e+00
Coefficient: 7.14e−02
Value: C
Lentibacillus.kimchii
2.0
1.5
1.0
0.5
0.0
A (n=15)
B (n=16)
C (n=14)
surface

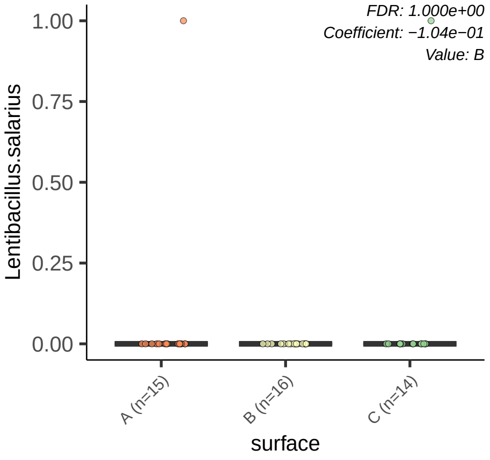
FDR: 1.000e+00
Coefficient: −1.04e−01
Value: B
Lentibacillus.salarius
1.00
0.75
0.50
0.25
0.00
A (n=15)
B (n=16)
C (n=14)
surface

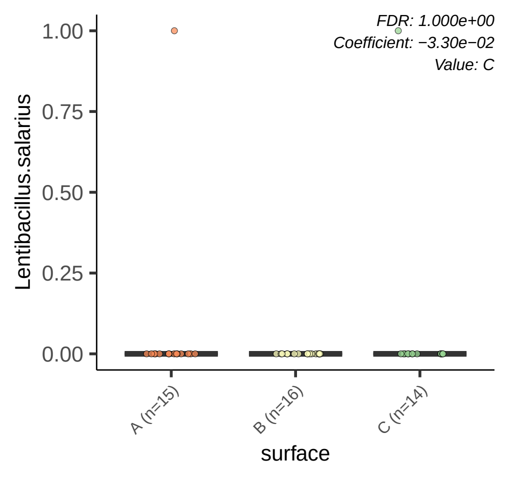
FDR: 1.000e+00
Coefficient: −3.30e−02
Value: C
Lentibacillus.salarius
1.00
0.75
0.50
0.25
0.00
A (n=15)
B (n=16)
C (n=14)
surface

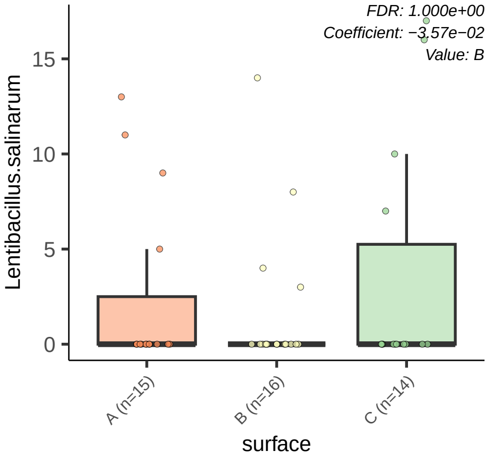

FDR: 1.000e+00
Coefficient: −3.57e−02
Value: B
Lentibacillus.salinarum
15
10
5
0
A (n=15)
B (n=16)
C (n=14)
surface

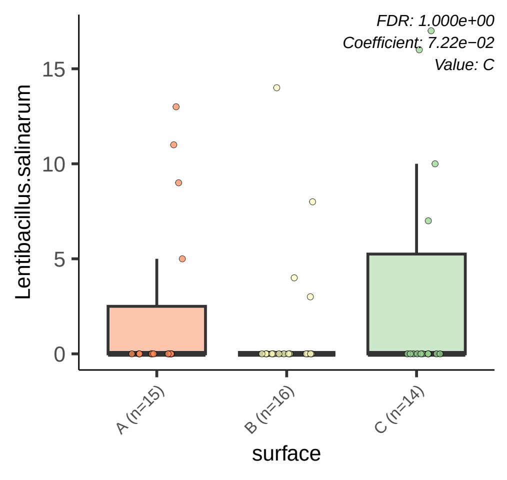

FDR: 1.000e+00
Coefficient: 7.22e−02
Value: C
Lentibacillus.salinarum
0
5
10
15
A (n=15)
B (n=16)
C (n=14)
surface

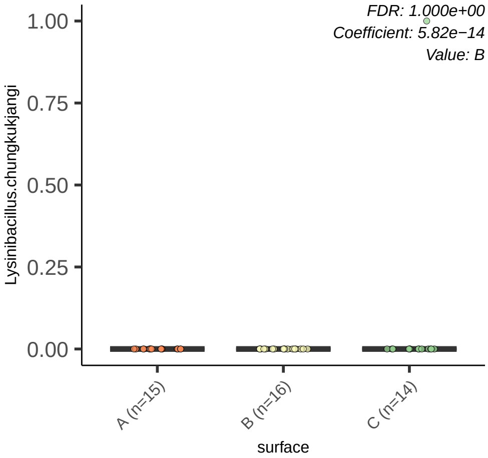
FDR: 1.000e+00
Coefficient: 5.82e−14
Value: B
Lysinibacillus.chungkukjangi
1.00
0.75
0.50
0.25
0.00
A (n=15)
B (n=16)
C (n=14)
surface

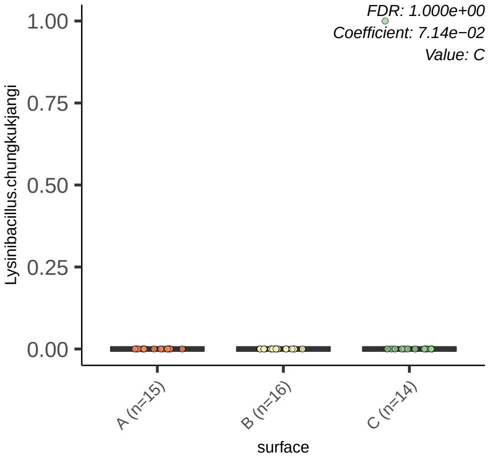

FDR: 1.000e+00
Coefficient: 7.14e−02
Value: C
1.00
0.75
0.50
0.25
0.00
Lysinibacillus.chungkukjangi
A (n=15)
B (n=16)
C (n=14)
surface

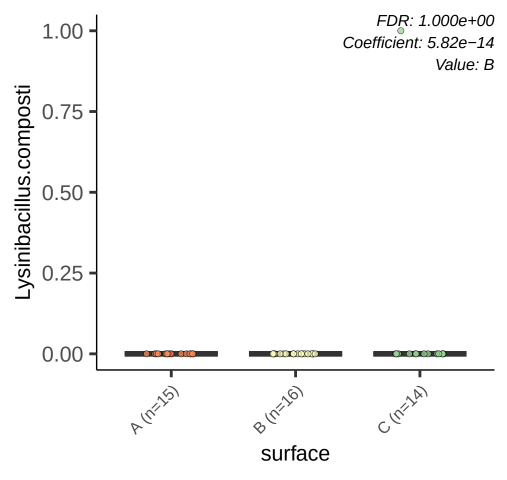

FDR: 1.000e+00
Coefficient: 5.82e−14
Value: B
Lysinibacillus.composti
1.00
0.75
0.50
0.25
0.00
A (n=15)
B (n=16)
C (n=14)
surface

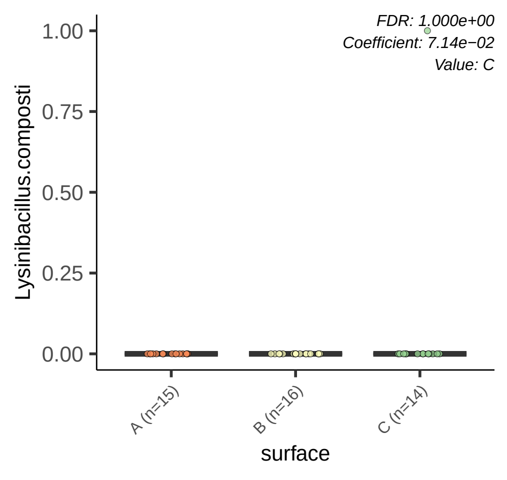

FDR: 1.000e+00
Coefficient: 7.14e−02
Value: C
Lysinibacillus.composti
1.00
0.75
0.50
0.25
0.00
A (n=15)
B (n=16)
C (n=14)
surface

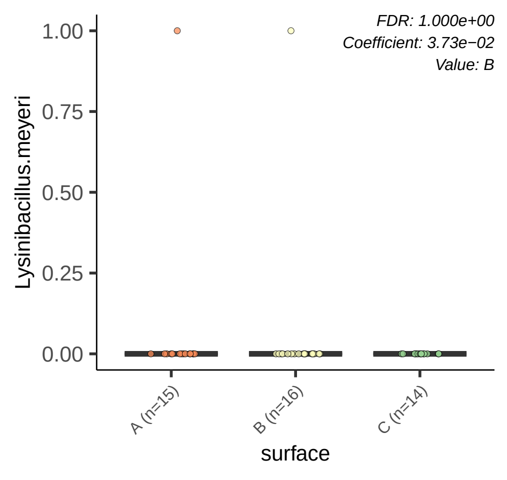

FDR: 1.000e+00
Coefficient: 3.73e−02
Value: B
Lysinibacillus.meyeri
1.00
0.75
0.50
0.25
0.00
A (n=15)
B (n=16)
C (n=14)
surface

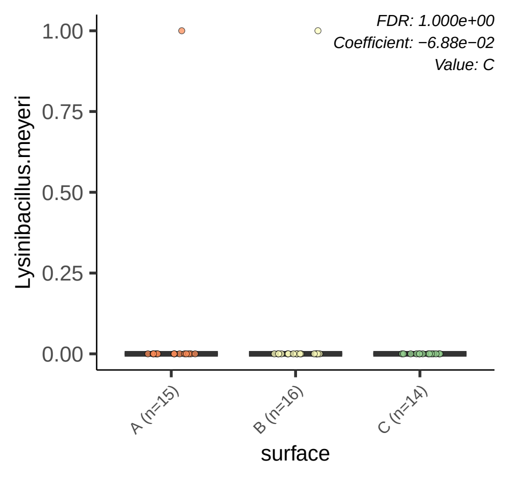

FDR: 1.000e+00
Coefficient: −6.88e−02
Value: C
Lysinibacillus.meyeri
1.00
0.75
0.50
0.25
0.00
A (n=15)
B (n=16)
C (n=14)
surface

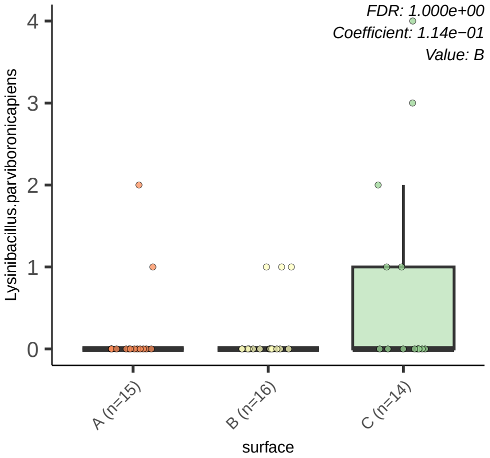

FDR: 1.000e+00
Coefficient: 1.14e−01
Value: B
Lysinibacillus.parviboronicapiens
4
3
2
1
0
A (n=15)
B (n=16)
C (n=14)
surface

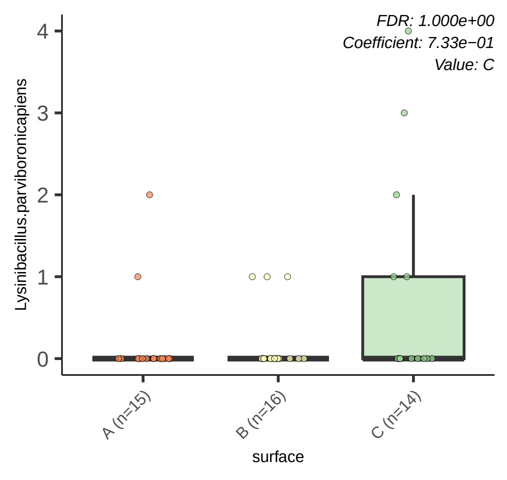
FDR: 1.000e+00
Coefficient: 7.33e−01
Value: C
Lysinibacillus.parviboronicapiens
4
3
2
1
0
A (n=15)
B (n=16)
C (n=14)
surface

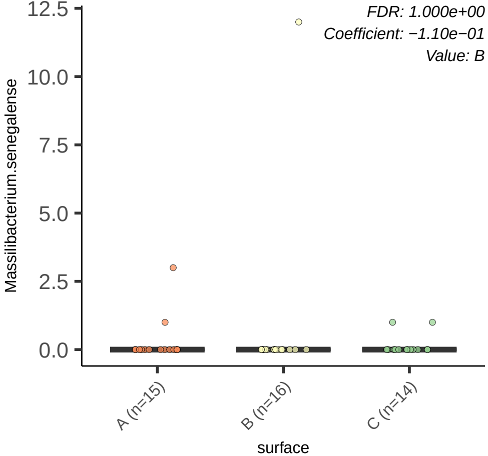

FDR: 1.000e+00
Coefficient: −1.10e−01
Value: B
12.5
10.0
7.5
5.0
2.5
0.0
Massilibacterium.senegalense
A (n=15)
B (n=16)
C (n=14)
surface

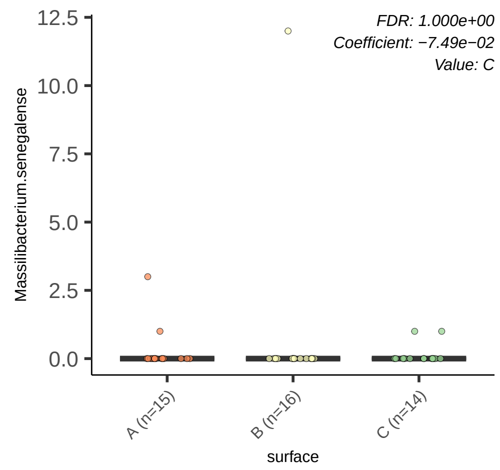
FDR: 1.000e+00
Coefficient: −7.49e−02
Value: C
12.5
10.0
7.5
5.0
2.5
0.0
Massilibacterium.senegalense
A (n=15)
B (n=16)
C (n=14)
surface

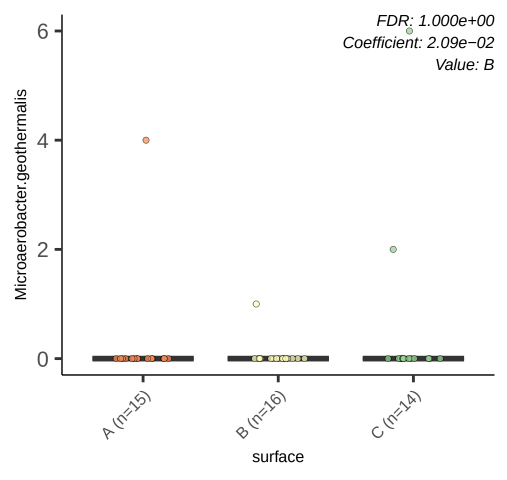
FDR: 1.000e+00
Coefficient: 2.09e−02
Value: B
Microaerobacter.geothermalis
6
4
2
0
A (n=15)
B (n=16)
C (n=14)
surface

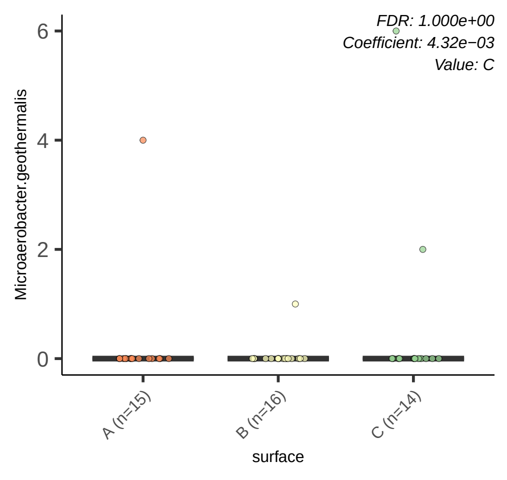
FDR: 1.000e+00
Coefficient: 4.32e−03
Value: C
Microaerobacter.geothermalis
0
2
4
6
A (n=15)
B (n=16)
C (n=14)
surface

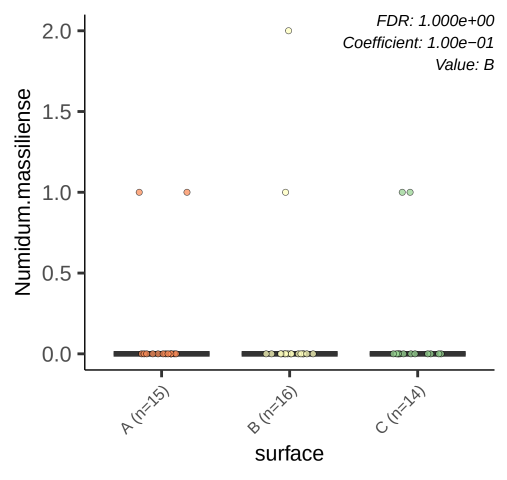
FDR: 1.000e+00
Coefficient: 1.00e−01
Value: B
Numidum.massiliense
2.0
1.5
1.0
0.5
0.0
A (n=15)
B (n=16)
C (n=14)
surface

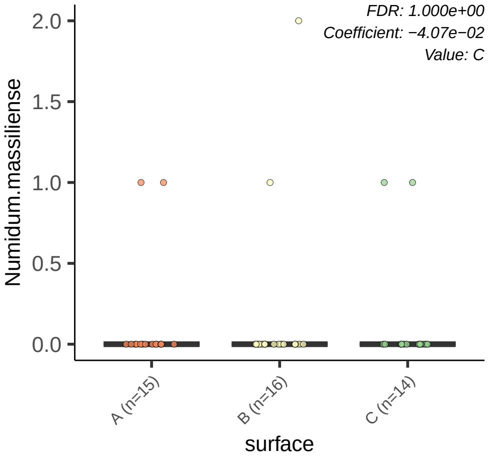

FDR: 1.000e+00
Coefficient: −4.07e−02
Value: C
Numidum.massiliense
2.0
1.5
1.0
0.5
0.0
A (n=15)
B (n=16)
C (n=14)
surface

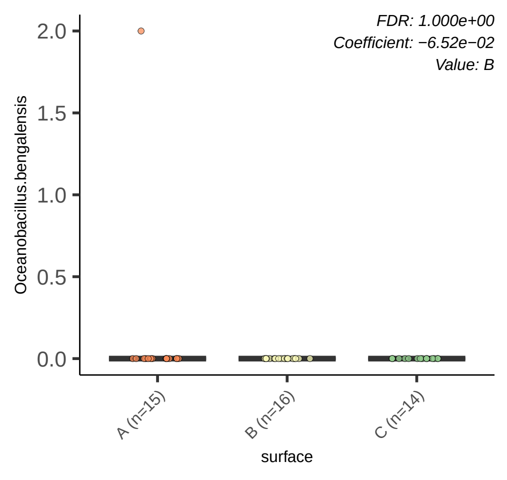

FDR: 1.000e+00
Coefficient: −6.52e−02
Value: B
Oceanobacillus.bengalensis
2.0
1.5
1.0
0.5
0.0
A (n=15)
B (n=16)
C (n=14)
surface

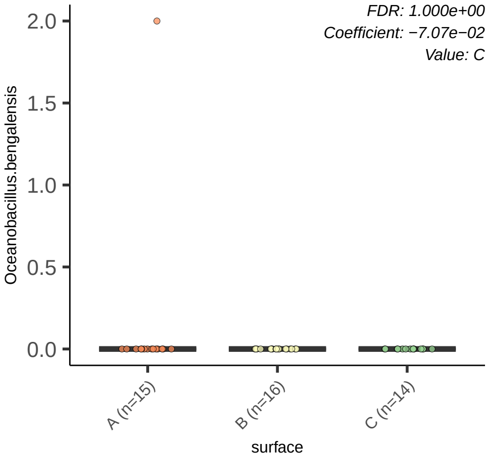
FDR: 1.000e+00
Coefficient: −7.07e−02
Value: C
Oceanobacillus.bengalensis
2.0
1.5
1.0
0.5
0.0
A (n=15)
B (n=16)
C (n=14)
surface

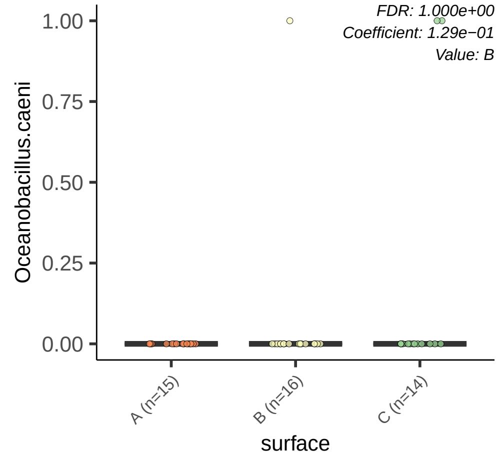
FDR: 1.000e+00
Coefficient: 1.29e−01
Value: B
Oceanobacillus.caeni
1.00
0.75
0.50
0.25
0.00
A (n=15)
B (n=16)
C (n=14)
surface

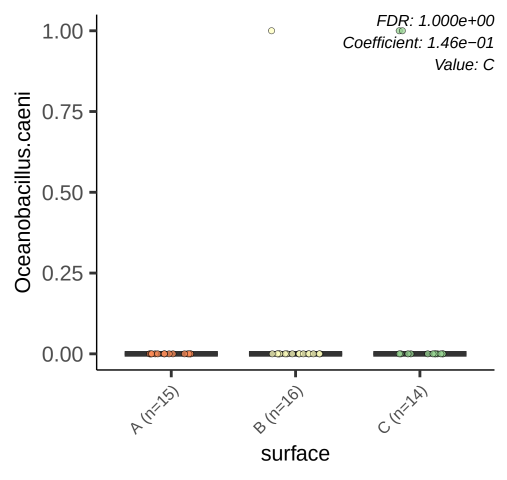
FDR: 1.000e+00
Coefficient: 1.46e−01
Value: C
Oceanobacillus.caeni
1.00
0.75
0.50
0.25
0.00
A (n=15)
B (n=16)
C (n=14)
surface

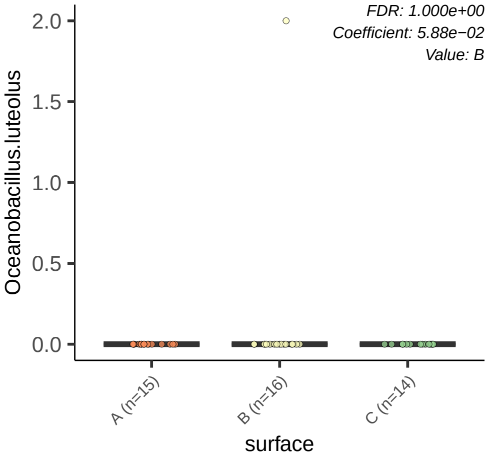

FDR: 1.000e+00
Coefficient: 5.88e−02
Value: B
Oceanobacillus.luteolus
2.0
1.5
1.0
0.5
0.0
A (n=15)
B (n=16)
C (n=14)
surface

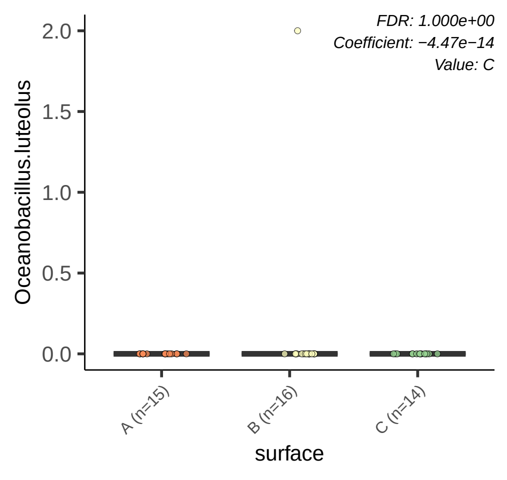

FDR: 1.000e+00
Coefficient: −4.47e−14
Value: C
Oceanobacillus.luteolus
2.0
1.5
1.0
0.5
0.0
A (n=15)
B (n=16)
C (n=14)
surface

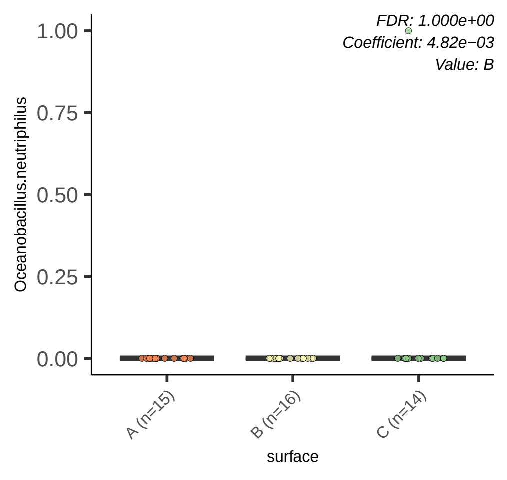
FDR: 1.000e+00
Coefficient: 4.82e−03
Value: B
Oceanobacillus.neutriphilus
1.00
0.75
0.50
0.25
0.00
A (n=15)
B (n=16)
C (n=14)
surface

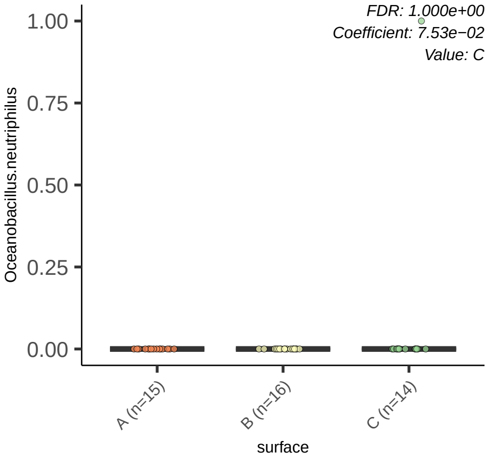
FDR: 1.000e+00
Coefficient: 7.53e−02
Value: C
1.00
0.75
0.50
0.25
0.00
Oceanobacillus.neutriphilus
A (n=15)
B (n=16)
C (n=14)
surface

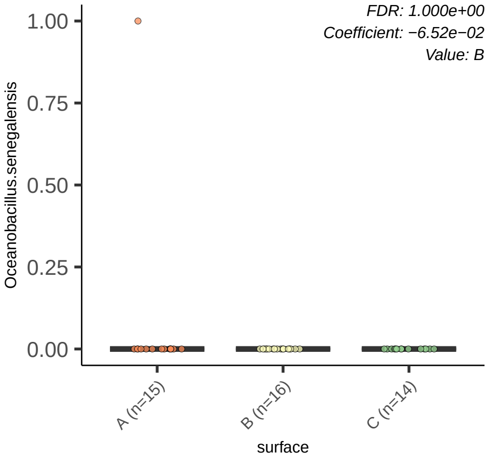

FDR: 1.000e+00
Coefficient: −6.52e−02
Value: B
1.00
0.75
0.50
0.25
0.00
Oceanobacillus.senegalensis
A (n=15)
B (n=16)
C (n=14)
surface

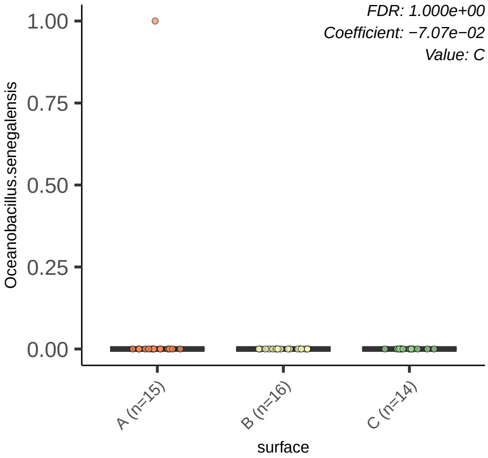
FDR: 1.000e+00
Coefficient: −7.07e−02
Value: C
1.00
0.75
0.50
0.25
0.00
Oceanobacillus.senegalensis
A (n=15)
B (n=16)
C (n=14)
surface

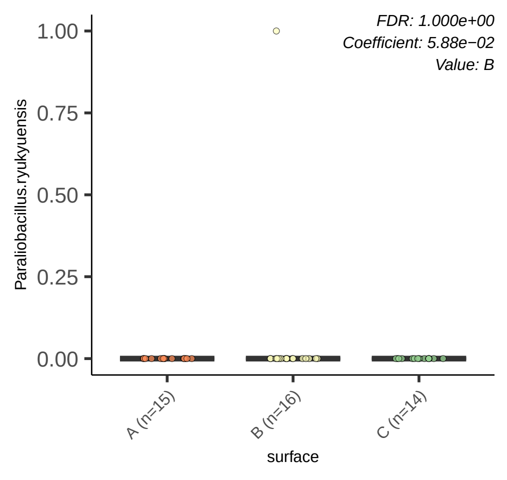
FDR: 1.000e+00
Coefficient: 5.88e−02
Value: B
1.00
0.75
0.50
0.25
0.00
Paraliobacillus.ryukyuensis
A (n=15)
B (n=16)
C (n=14)
surface

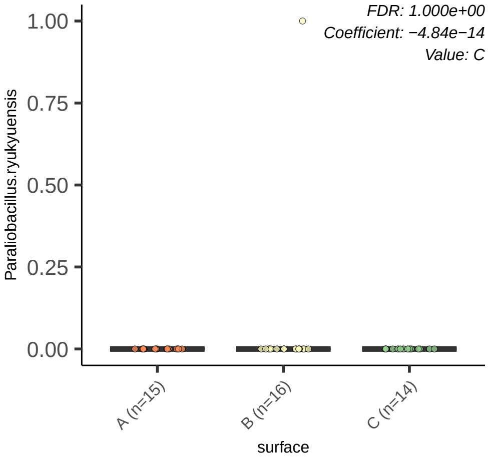

FDR: 1.000e+00
Coefficient: −4.84e−14
Value: C
Paraliobacillus.ryukyuensis
1.00
0.75
0.50
0.25
0.00
A (n=15)
B (n=16)
C (n=14)
surface

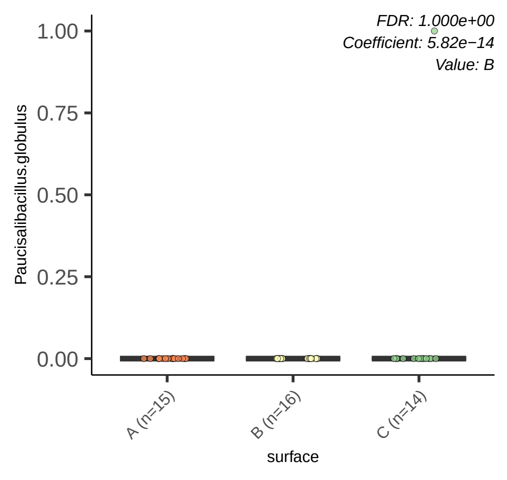
FDR: 1.000e+00
Coefficient: 5.82e−14
Value: B
1.00
0.75
0.50
0.25
0.00
Paucisalibacillus.globulus
A (n=15)
B (n=16)
C (n=14)
surface

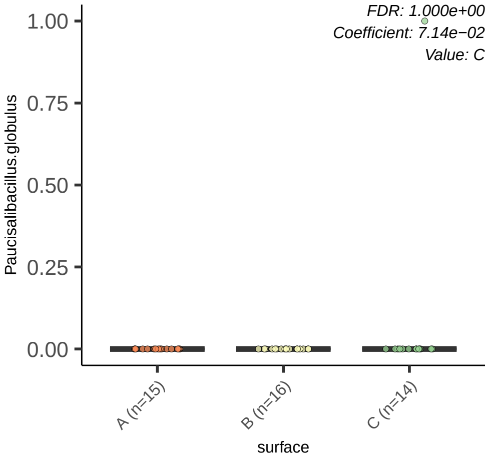
FDR: 1.000e+00
Coefficient: 7.14e−02
Value: C
1.00
0.75
0.50
0.25
0.00
Paucisalibacillus.globulus
A (n=15)
B (n=16)
C (n=14)
surface

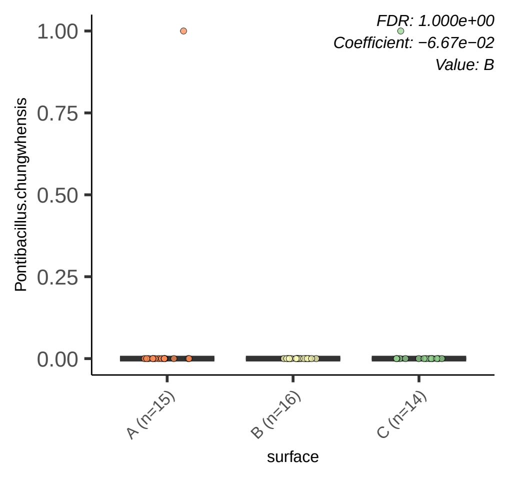

FDR: 1.000e+00
Coefficient: −6.67e−02
Value: B
1.00
0.75
0.50
0.25
0.00
Pontibacillus.chungwhensis
A (n=15)
B (n=16)
C (n=14)
surface

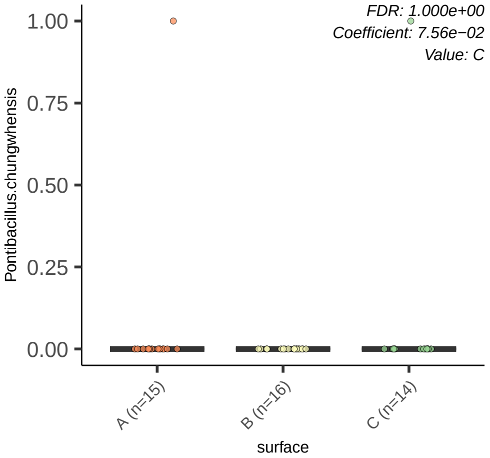
FDR: 1.000e+00
Coefficient: 7.56e−02
Value: C
Pontibacillus.chungwhensis
1.00
0.75
0.50
0.25
0.00
A (n=15)
B (n=16)
C (n=14)
surface

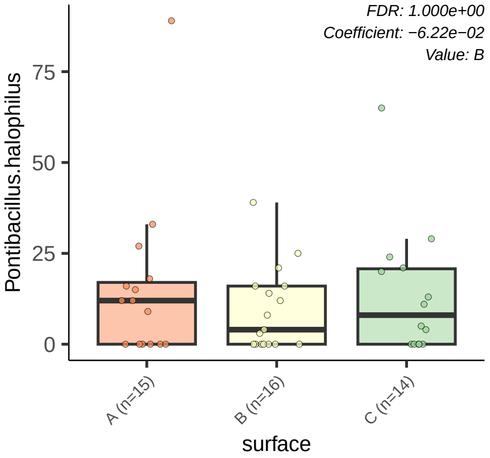

FDR: 1.000e+00
Coefficient: −6.22e−02
Value: B
Pontibacillus.halophilus
75
50
25
0
A (n=15)
B (n=16)
C (n=14)
surface

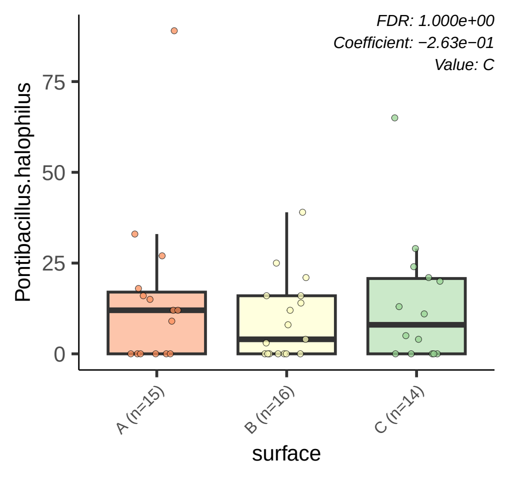
FDR: 1.000e+00
Coefficient: −2.63e−01
Value: C
Pontibacillus.halophilus
0
25
50
75
A (n=15)
B (n=16)
C (n=14)
surface

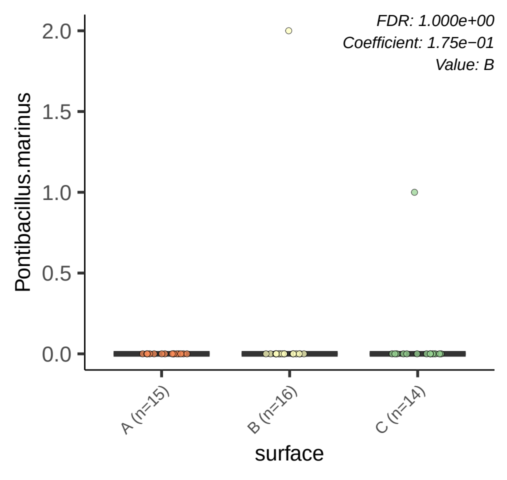
FDR: 1.000e+00
Coefficient: 1.75e−01
Value: B
2.0
1.5
1.0
0.5
0.0
Pontibacillus.marinus
A (n=15)
B (n=16)
C (n=14)
surface

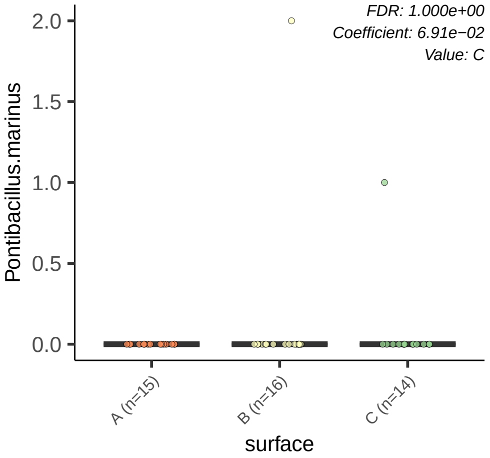
FDR: 1.000e+00
Coefficient: 6.91e−02
Value: C
Pontibacillus.marinus
2.0
1.5
1.0
0.5
0.0
A (n=15)
B (n=16)
C (n=14)
surface

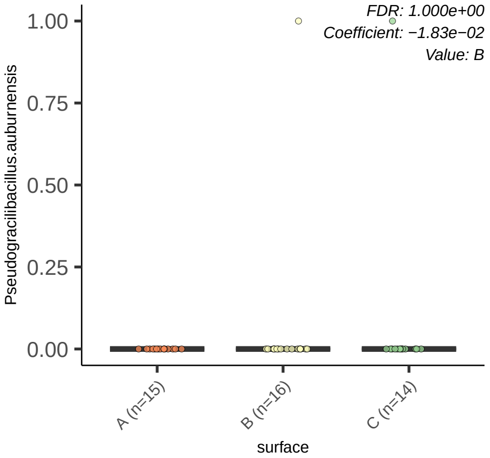
FDR: 1.000e+00
Coefficient: −1.83e−02
Value: B
Pseudogracilibacillus.auburnensis
1.00
0.75
0.50
0.25
0.00
A (n=15)
B (n=16)
C (n=14)
surface

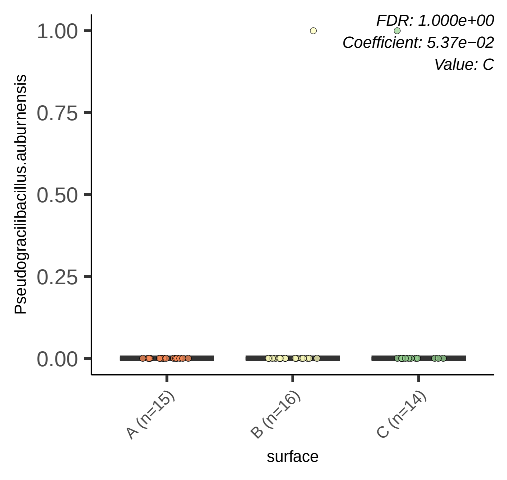
FDR: 1.000e+00
Coefficient: 5.37e−02
Value: C
1.00
0.75
0.50
0.25
0.00
Pseudogracilibacillus.auburnensis
A (n=15)
B (n=16)
C (n=14)
surface

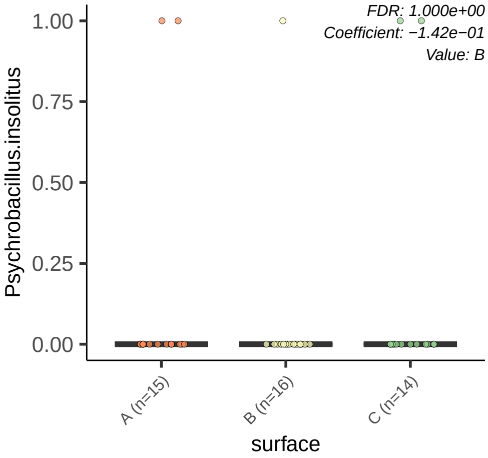
FDR: 1.000e+00
Coefficient: −1.42e−01
Value: B
Psychrobacillus.insolitus
1.00
0.75
0.50
0.25
0.00
A (n=15)
B (n=16)
C (n=14)
surface

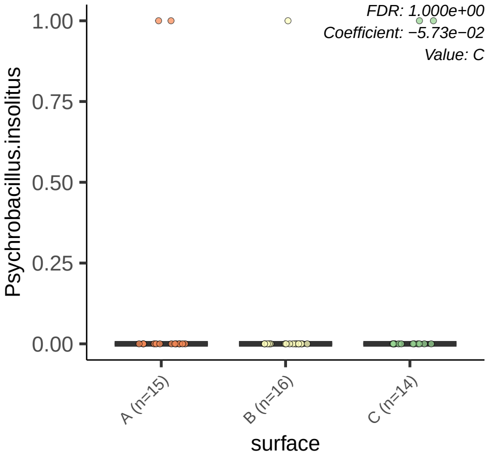
FDR: 1.000e+00
Coefficient: −5.73e−02
Value: C
Psychrobacillus.insolitus
1.00
0.75
0.50
0.25
0.00
A (n=15)
B (n=16)
C (n=14)
surface

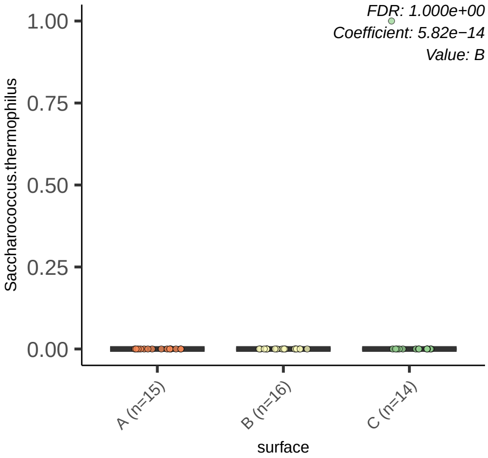

FDR: 1.000e+00
Coefficient: 5.82e−14
Value: B
1.00
0.75
0.50
0.25
0.00
Saccharococcus.thermophilus
A (n=15)
B (n=16)
C (n=14)
surface

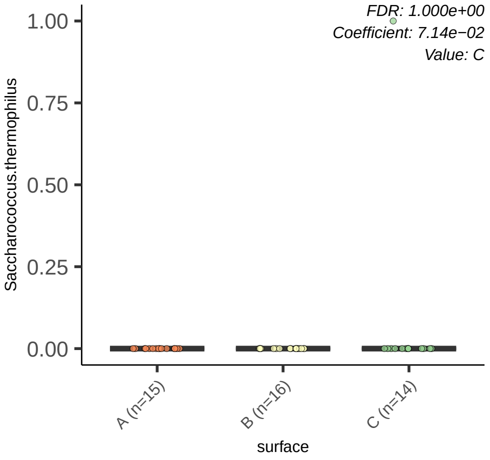
FDR: 1.000e+00
Coefficient: 7.14e−02
Value: C
Saccharococcus.thermophilus
1.00
0.75
0.50
0.25
0.00
A (n=15)
B (n=16)
C (n=14)
surface

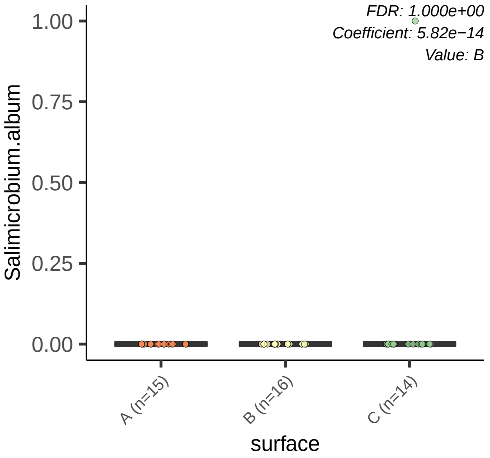
FDR: 1.000e+00
Coefficient: 5.82e−14
Value: B
Salimicrobium.album
1.00
0.75
0.50
0.25
0.00
A (n=15)
B (n=16)
C (n=14)
surface

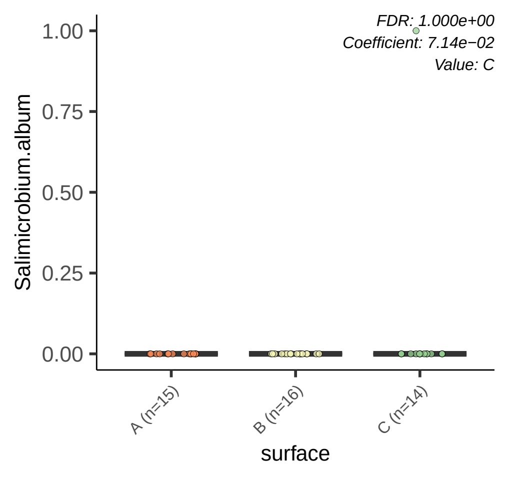
FDR: 1.000e+00
Coefficient: 7.14e−02
Value: C
1.00
0.75
0.50
0.25
0.00
Salimicrobium.album
A (n=15)
B (n=16)
C (n=14)
surface

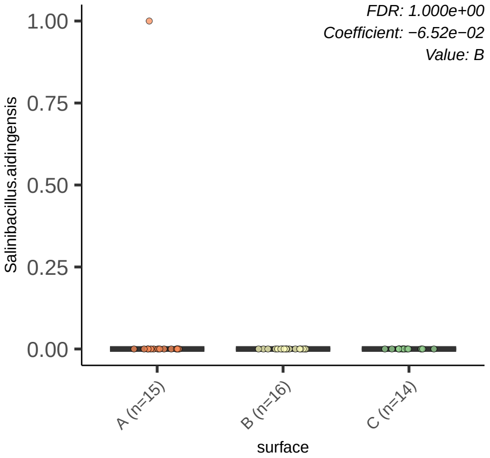
FDR: 1.000e+00
Coefficient: −6.52e−02
Value: B
Salinibacillus.aidingensis
1.00
0.75
0.50
0.25
0.00
A (n=15)
B (n=16)
C (n=14)
surface

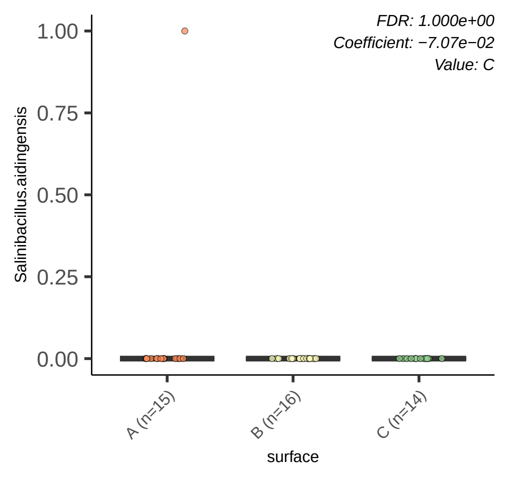
FDR: 1.000e+00
Coefficient: −7.07e−02
Value: C
Salinibacillus.aidingensis
1.00
0.75
0.50
0.25
0.00
A (n=15)
B (n=16)
C (n=14)
surface

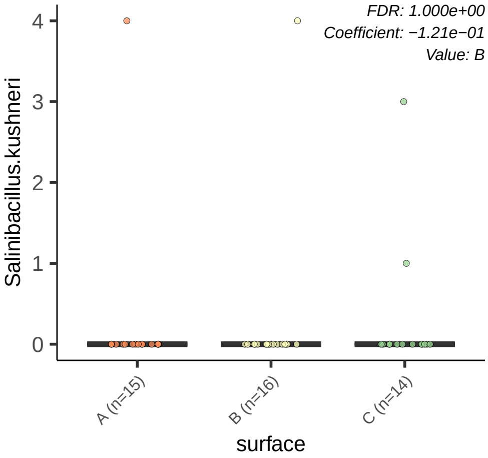

FDR: 1.000e+00
Coefficient: −1.21e−01
Value: B
Salinibacillus.kushneri
0
1
2
3
4
A (n=15)
B (n=16)
C (n=14)
surface

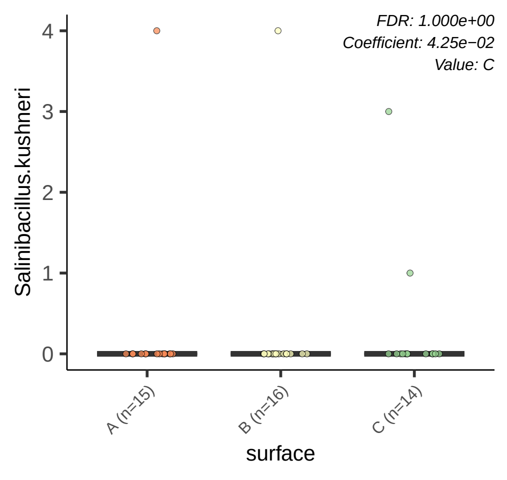
FDR: 1.000e+00
Coefficient: 4.25e−02
Value: C
Salinibacillus.kushneri
4
3
2
1
0
A (n=15)
B (n=16)
C (n=14)
surface

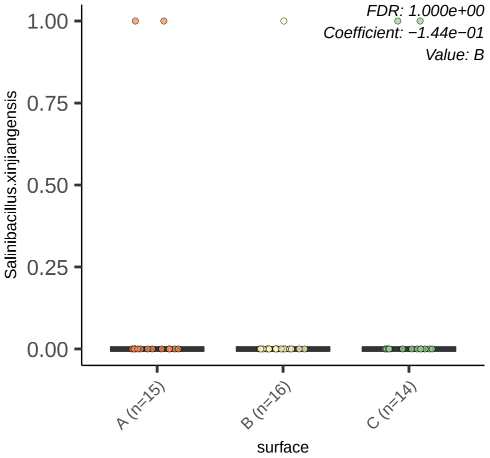

FDR: 1.000e+00
Coefficient: −1.44e−01
Value: B
Salinibacillus.xinjiangensis
1.00
0.75
0.50
0.25
0.00
A (n=15)
B (n=16)
C (n=14)
surface

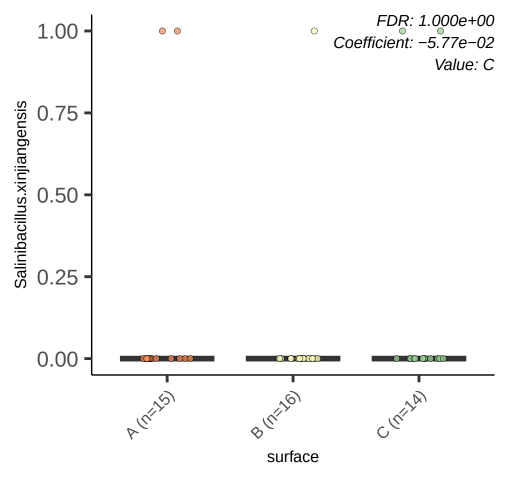
FDR: 1.000e+00
Coefficient: −5.77e−02
Value: C
1.00
0.75
0.50
0.25
0.00
Salinibacillus.xinjiangensis
A (n=15)
B (n=16)
C (n=14)
surface

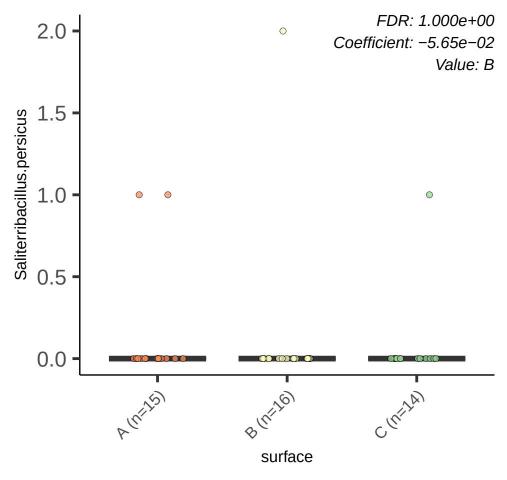
FDR: 1.000e+00
Coefficient: −5.65e−02
Value: B
Saliterribacillus.persicus
2.0
1.5
1.0
0.5
0.0
A (n=15)
B (n=16)
C (n=14)
surface

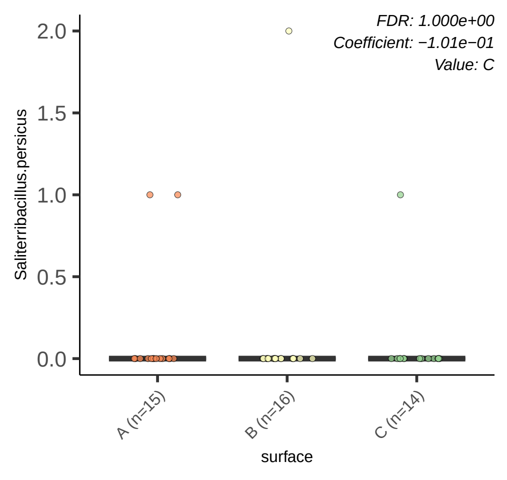

FDR: 1.000e+00
Coefficient: −1.01e−01
Value: C
Saliterribacillus.persicus
2.0
1.5
1.0
0.5
0.0
A (n=15)
B (n=16)
C (n=14)
surface

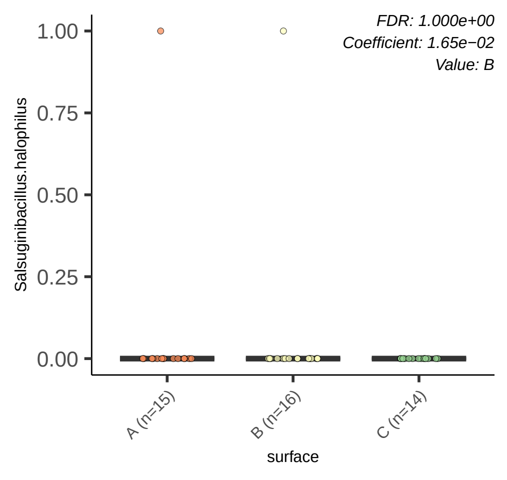
FDR: 1.000e+00
Coefficient: 1.65e−02
Value: B
Salsuginibacillus.halophilus
1.00
0.75
0.50
0.25
0.00
A (n=15)
B (n=16)
C (n=14)
surface

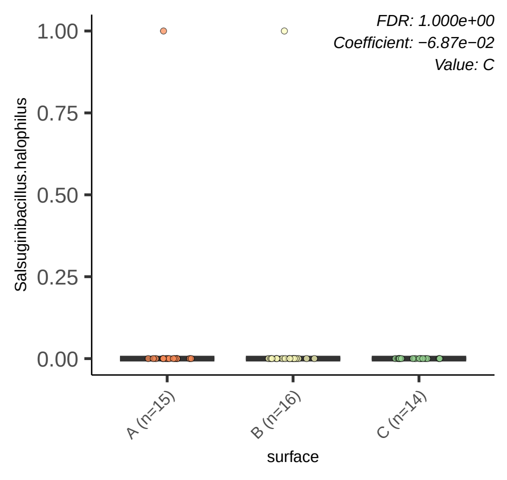

FDR: 1.000e+00
Coefficient: −6.87e−02
Value: C
Salsuginibacillus.halophilus
1.00
0.75
0.50
0.25
0.00
A (n=15)
B (n=16)
C (n=14)
surface

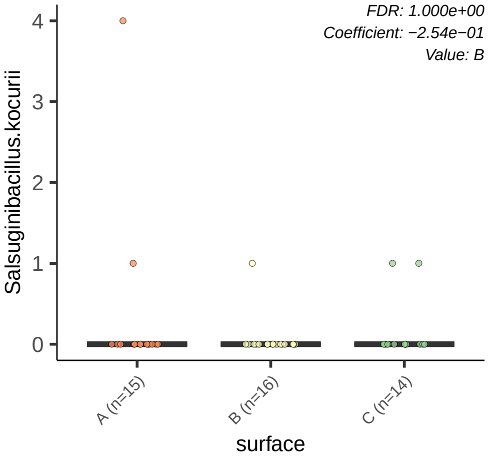

FDR: 1.000e+00
Coefficient: −2.54e−01
Value: B
Salsuginibacillus.kocurii
0
1
2
3
4
A (n=15)
B (n=16)
C (n=14)
surface

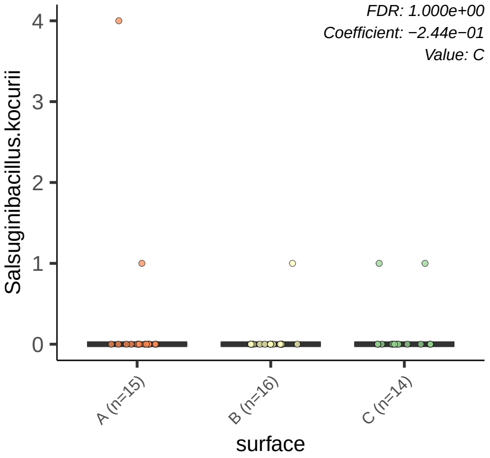

FDR: 1.000e+00
Coefficient: −2.44e−01
Value: C
Salsuginibacillus.kocurii
4
3
2
1
0
A (n=15)
B (n=16)
C (n=14)
surface

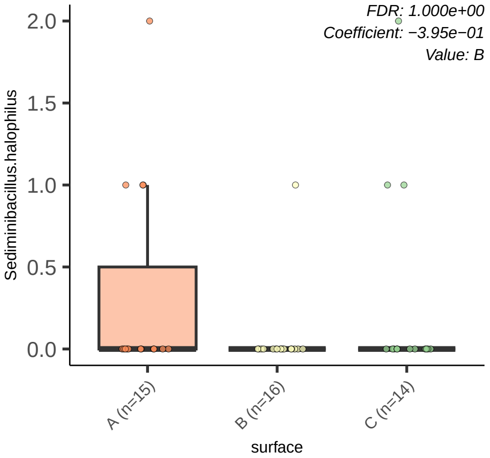

FDR: 1.000e+00
Coefficient: −3.95e−01
Value: B
Sediminibacillus.halophilus
2.0
1.5
1.0
0.5
0.0
A (n=15)
B (n=16)
C (n=14)
surface

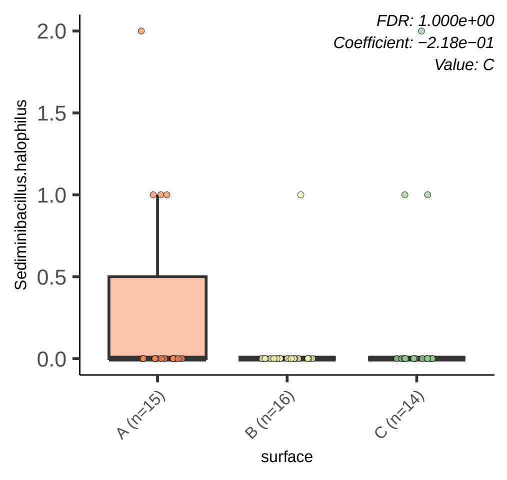

FDR: 1.000e+00
Coefficient: −2.18e−01
Value: C
Sediminibacillus.halophilus
2.0
1.5
1.0
0.5
0.0
A (n=15)
B (n=16)
C (n=14)
surface

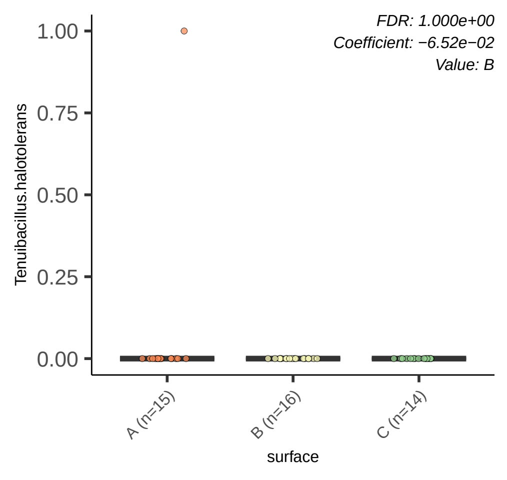

FDR: 1.000e+00
Coefficient: −6.52e−02
Value: B
1.00
0.75
0.50
0.25
0.00
Tenuibacillus.halotolerans
A (n=15)
B (n=16)
C (n=14)
surface

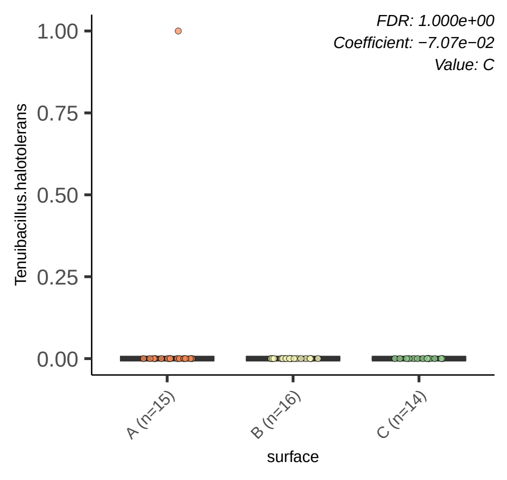
FDR: 1.000e+00
Coefficient: −7.07e−02
Value: C
1.00
0.75
0.50
0.25
0.00
Tenuibacillus.halotolerans
A (n=15)
B (n=16)
C (n=14)
surface

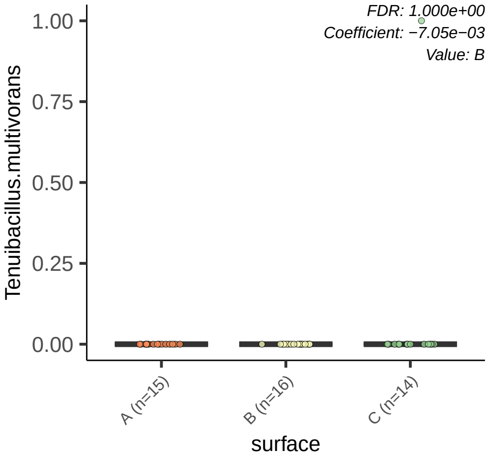

FDR: 1.000e+00
Coefficient: −7.05e−03
Value: B
Tenuibacillus.multivorans
1.00
0.75
0.50
0.25
0.00
A (n=15)
B (n=16)
C (n=14)
surface

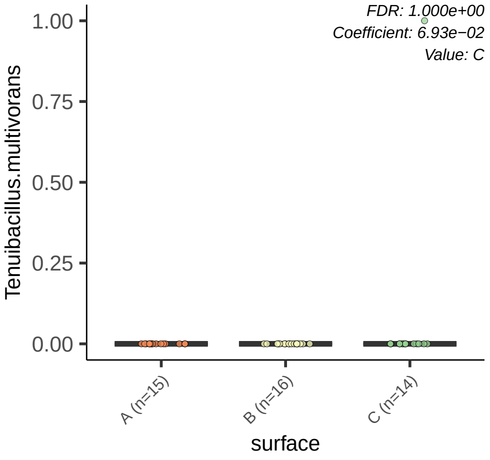
FDR: 1.000e+00
Coefficient: 6.93e−02
Value: C
Tenuibacillus.multivorans
1.00
0.75
0.50
0.25
0.00
A (n=15)
B (n=16)
C (n=14)
surface

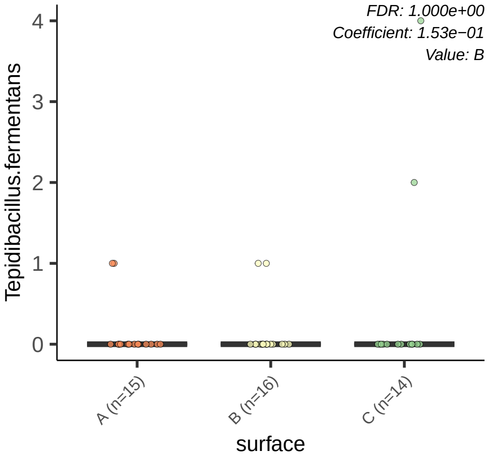

FDR: 1.000e+00
Coefficient: 1.53e−01
Value: B
Tepidibacillus.fermentans
0
1
2
3
4
A (n=15)
B (n=16)
C (n=14)
surface

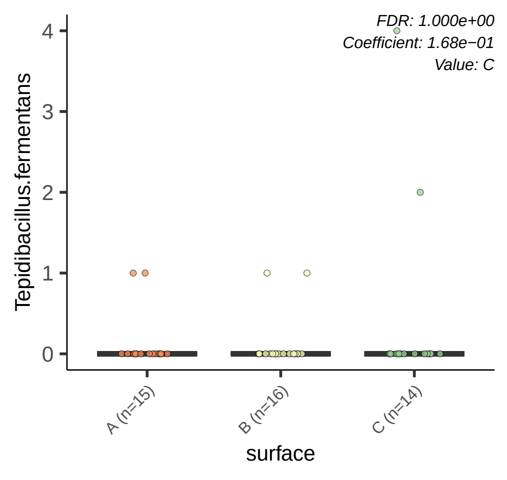

FDR: 1.000e+00
Coefficient: 1.68e−01
Value: C
Tepidibacillus.fermentans
0
1
2
3
4
A (n=15)
B (n=16)
C (n=14)
surface

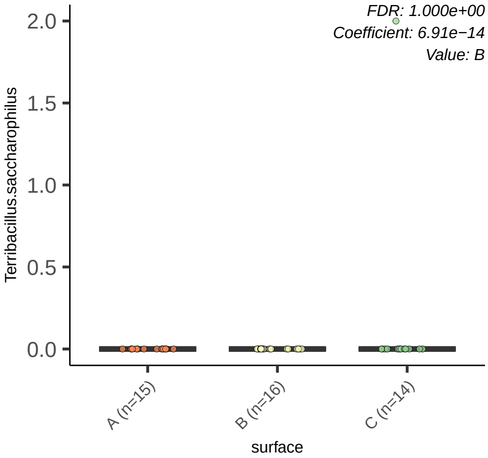

FDR: 1.000e+00
Coefficient: 6.91e−14
Value: B
Terribacillus.saccharophilus
2.0
1.5
1.0
0.5
0.0
A (n=15)
B (n=16)
C (n=14)
surface

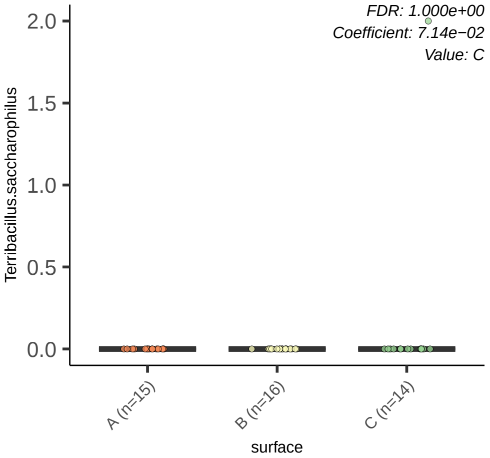
FDR: 1.000e+00
Coefficient: 7.14e−02
Value: C
2.0
1.5
1.0
0.5
0.0
Terribacillus.saccharophilus
A (n=15)
B (n=16)
C (n=14)
surface

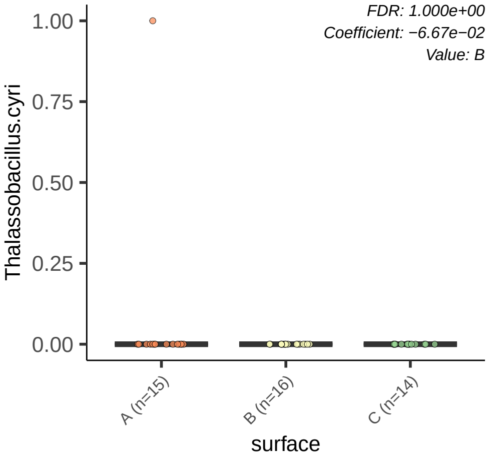

FDR: 1.000e+00
Coefficient: −6.67e−02
Value: B
Thalassobacillus.cyri
1.00
0.75
0.50
0.25
0.00
A (n=15)
B (n=16)
C (n=14)
surface

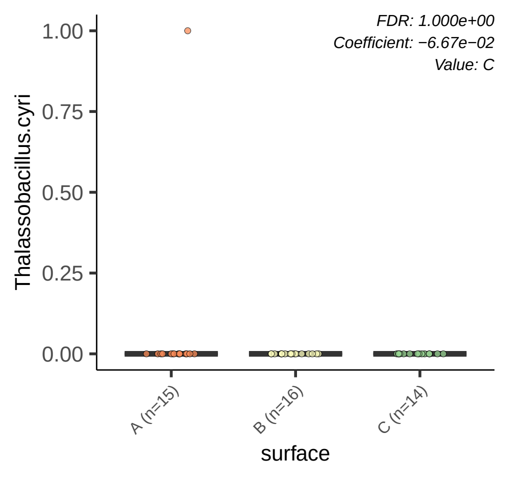
FDR: 1.000e+00
Coefficient: −6.67e−02
Value: C
Thalassobacillus.cyri
1.00
0.75
0.50
0.25
0.00
A (n=15)
B (n=16)
C (n=14)
surface

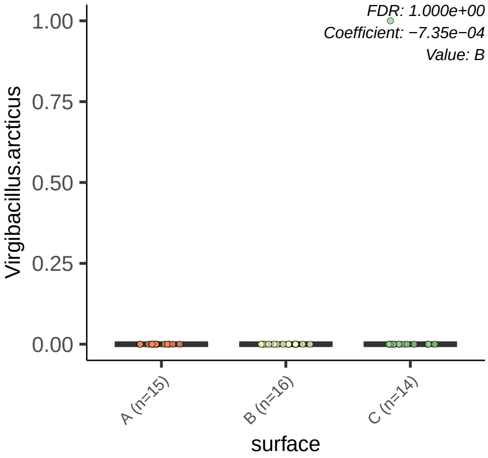

FDR: 1.000e+00
Coefficient: −7.35e−04
Value: B
1.00
0.75
0.50
0.25
0.00
Virgibacillus.arcticus
A (n=15)
B (n=16)
C (n=14)
surface

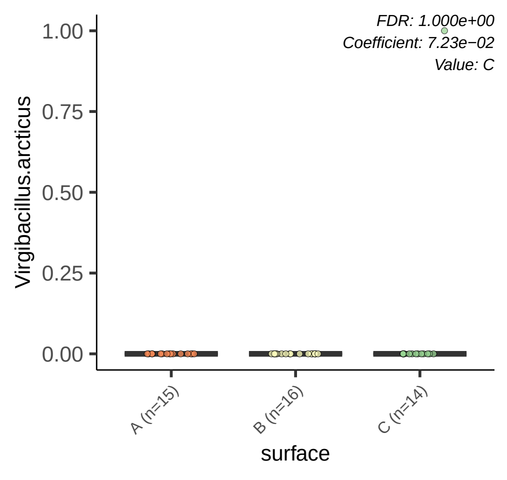

FDR: 1.000e+00
Coefficient: 7.23e−02
Value: C
Virgibacillus.arcticus
1.00
0.75
0.50
0.25
0.00
A (n=15)
B (n=16)
C (n=14)
surface

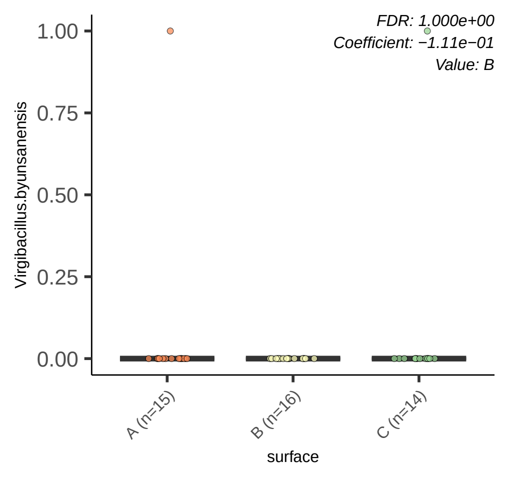

FDR: 1.000e+00
Coefficient: −1.11e−01
Value: B
Virgibacillus.byunsanensis
1.00
0.75
0.50
0.25
0.00
A (n=15)
B (n=16)
C (n=14)
surface

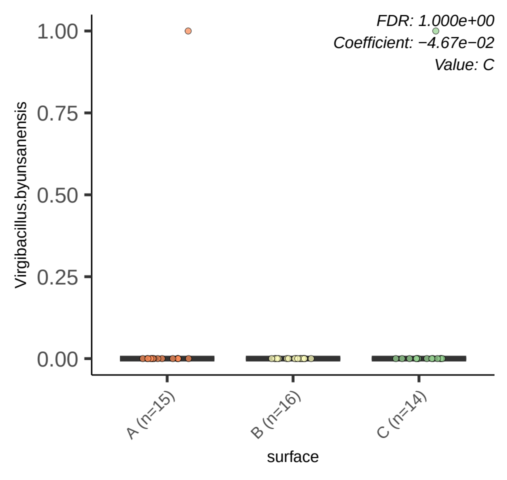

FDR: 1.000e+00
Coefficient: −4.67e−02
Value: C
Virgibacillus.byunsanensis
1.00
0.75
0.50
0.25
0.00
A (n=15)
B (n=16)
C (n=14)
surface

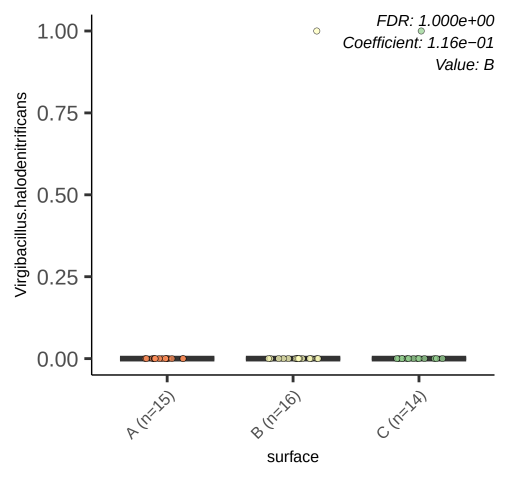
FDR: 1.000e+00
Coefficient: 1.16e−01
Value: B
Virgibacillus.halodenitrificans
1.00
0.75
0.50
0.25
0.00
A (n=15)
B (n=16)
C (n=14)
surface

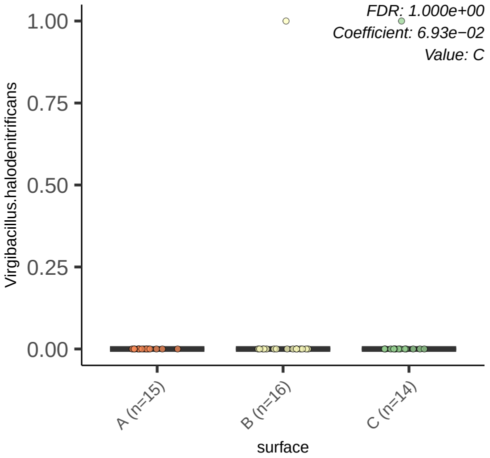
FDR: 1.000e+00
Coefficient: 6.93e−02
Value: C
1.00
0.75
0.50
0.25
0.00
Virgibacillus.halodenitrificans
A (n=15)
B (n=16)
C (n=14)
surface

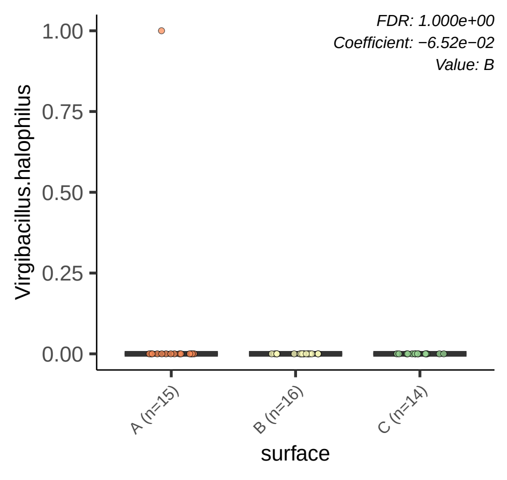

FDR: 1.000e+00
Coefficient: −6.52e−02
Value: B
Virgibacillus.halophilus
1.00
0.75
0.50
0.25
0.00
A (n=15)
B (n=16)
C (n=14)
surface

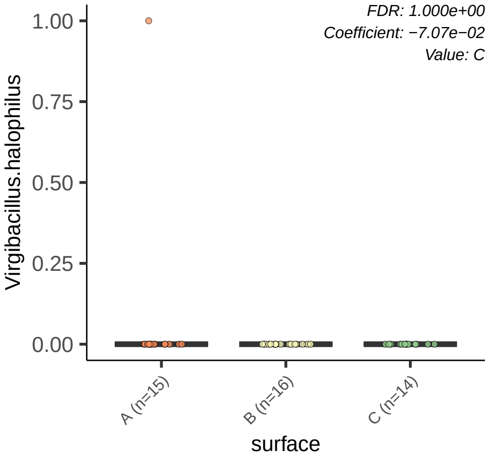
FDR: 1.000e+00
Coefficient: −7.07e−02
Value: C
Virgibacillus.halophilus
1.00
0.75
0.50
0.25
0.00
A (n=15)
B (n=16)
C (n=14)
surface

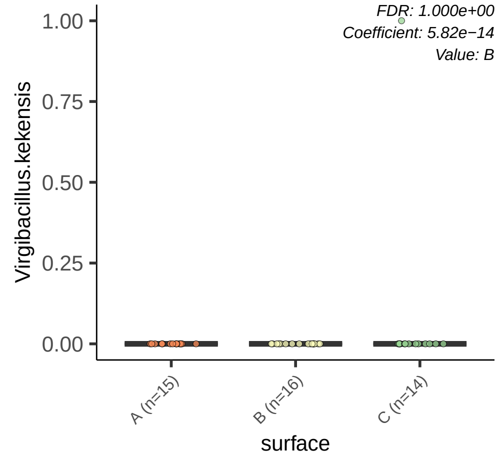

FDR: 1.000e+00
Coefficient: 5.82e−14
Value: B
Virgibacillus.kekensis
1.00
0.75
0.50
0.25
0.00
A (n=15)
B (n=16)
C (n=14)
surface

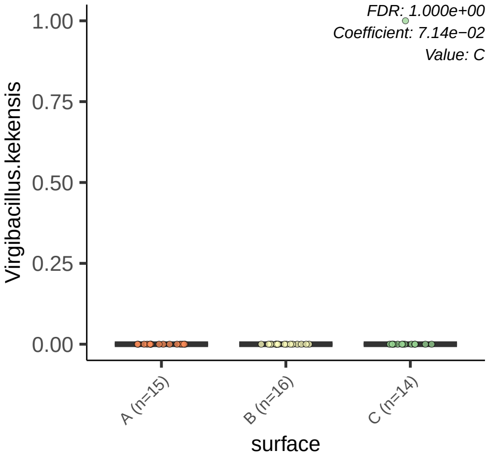

FDR: 1.000e+00
Coefficient: 7.14e−02
Value: C
1.00
0.75
0.50
0.25
0.00
Virgibacillus.kekensis
A (n=15)
B (n=16)
C (n=14)
surface

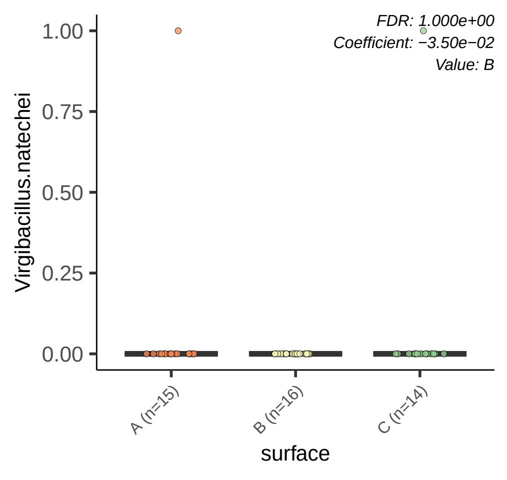
FDR: 1.000e+00
Coefficient: −3.50e−02
Value: B
Virgibacillus.natechei
1.00
0.75
0.50
0.25
0.00
A (n=15)
B (n=16)
C (n=14)
surface

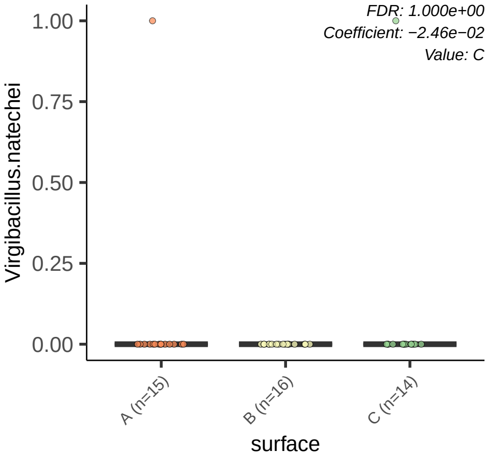
FDR: 1.000e+00
Coefficient: −2.46e−02
Value: C
Virgibacillus.natechei
1.00
0.75
0.50
0.25
0.00
A (n=15)
B (n=16)
C (n=14)
surface

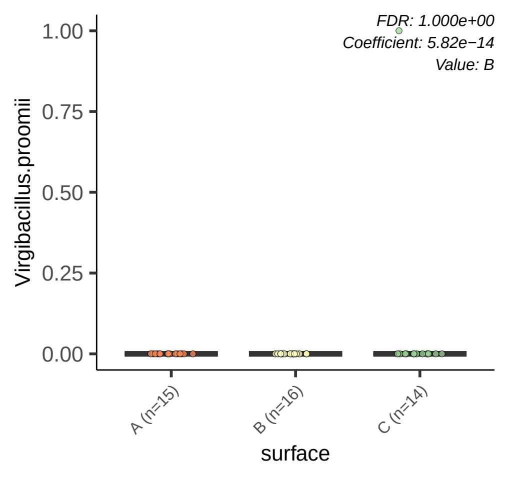
FDR: 1.000e+00
Coefficient: 5.82e−14
Value: B
Virgibacillus.proomii
1.00
0.75
0.50
0.25
0.00
A (n=15)
B (n=16)
C (n=14)
surface

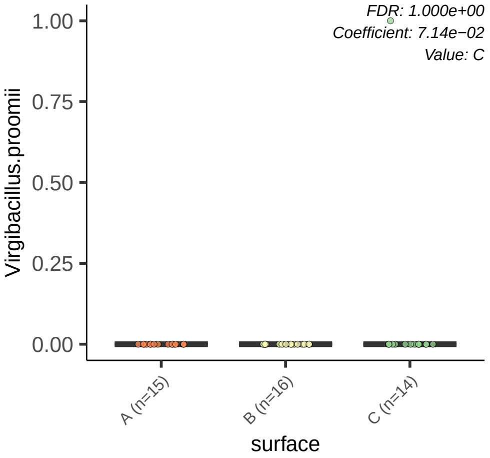

FDR: 1.000e+00
Coefficient: 7.14e−02
Value: C
1.00
0.75
0.50
0.25
0.00
Virgibacillus.proomii
A (n=15)
B (n=16)
C (n=14)
surface

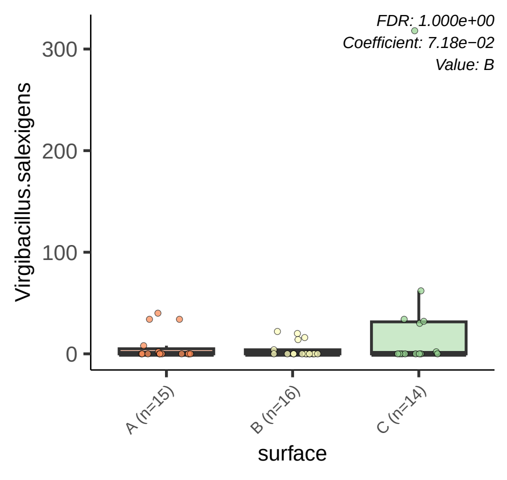

FDR: 1.000e+00
Coefficient: 7.18e−02
Value: B
Virgibacillus.salexigens
300
200
100
0
A (n=15)
B (n=16)
C (n=14)
surface

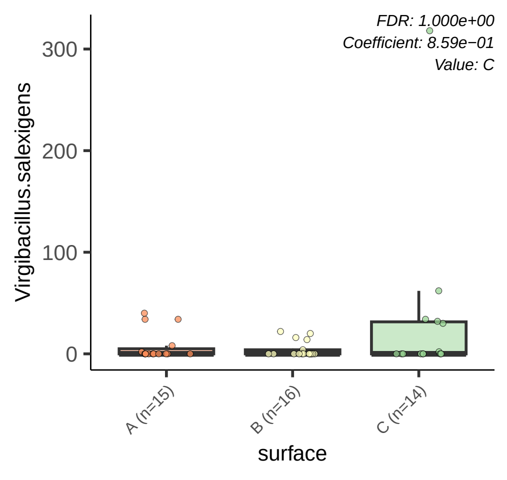

FDR: 1.000e+00
Coefficient: 8.59e−01
Value: C
300
200
100
0
Virgibacillus.salexigens
A (n=15)
B (n=16)
C (n=14)
surface

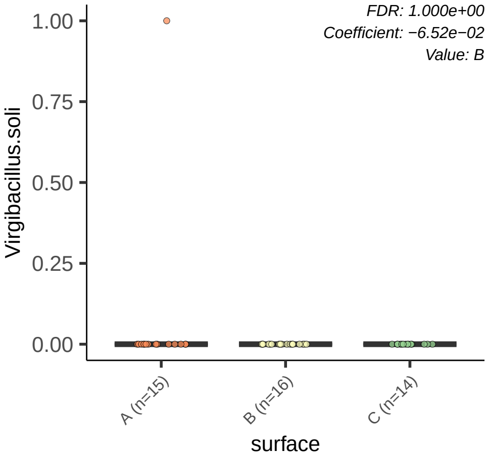
FDR: 1.000e+00
Coefficient: −6.52e−02
Value: B
Virgibacillus.soli
1.00
0.75
0.50
0.25
0.00
A (n=15)
B (n=16)
C (n=14)
surface

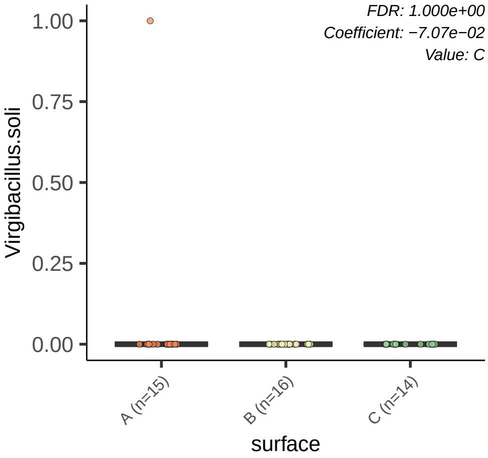

FDR: 1.000e+00
Coefficient: −7.07e−02
Value: C
Virgibacillus.soli
1.00
0.75
0.50
0.25
0.00
A (n=15)
B (n=16)
C (n=14)
surface

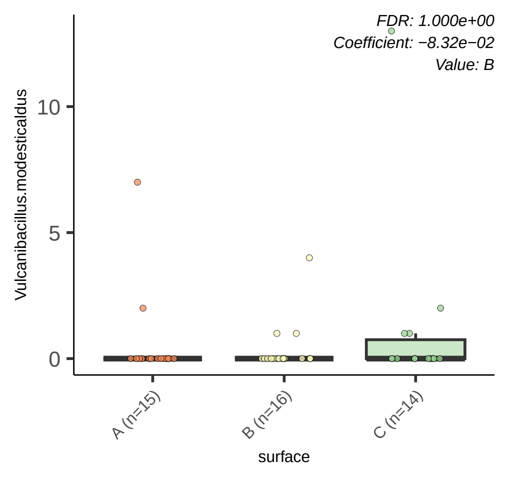
FDR: 1.000e+00
Coefficient: −8.32e−02
Value: B
Vulcanibacillus.modesticaldus
10
5
0
A (n=15)
B (n=16)
C (n=14)
surface

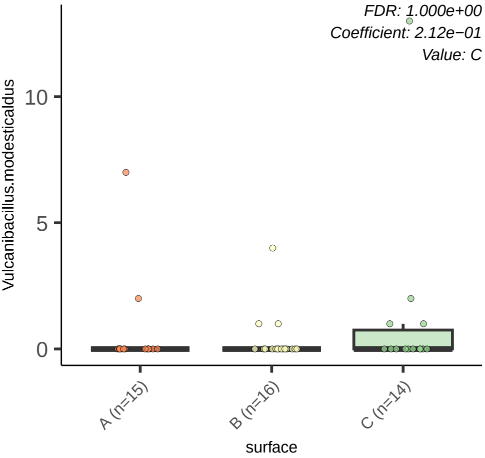
FDR: 1.000e+00
Coefficient: 2.12e−01
Value: C
Vulcanibacillus.modesticaldus
10
5
0
A (n=15)
B (n=16)
C (n=14)
surface

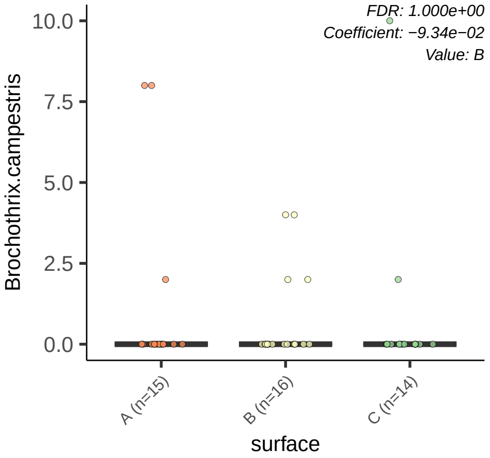
FDR: 1.000e+00
Coefficient: −9.34e−02
Value: B
Brochothrix.campestris
10.0
7.5
5.0
2.5
0.0
A (n=15)
B (n=16)
C (n=14)
surface

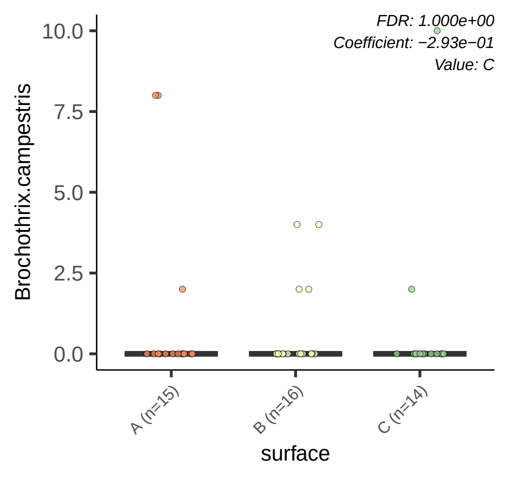
FDR: 1.000e+00
Coefficient: −2.93e−01
Value: C
Brochothrix.campestris
10.0
7.5
5.0
2.5
0.0
A (n=15)
B (n=16)
C (n=14)
surface

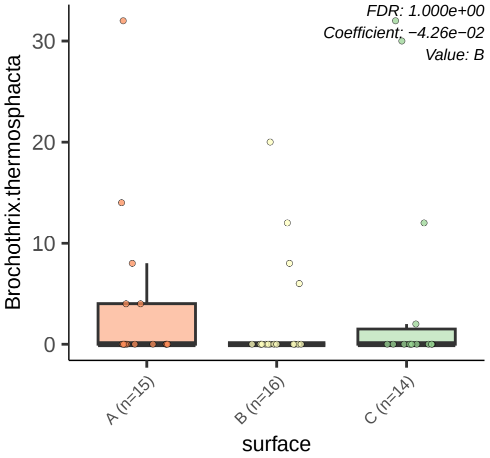
FDR: 1.000e+00
Coefficient: −4.26e−02
Value: B
Brochothrix.thermosphacta
0
10
20
30
A (n=15)
B (n=16)
C (n=14)
surface

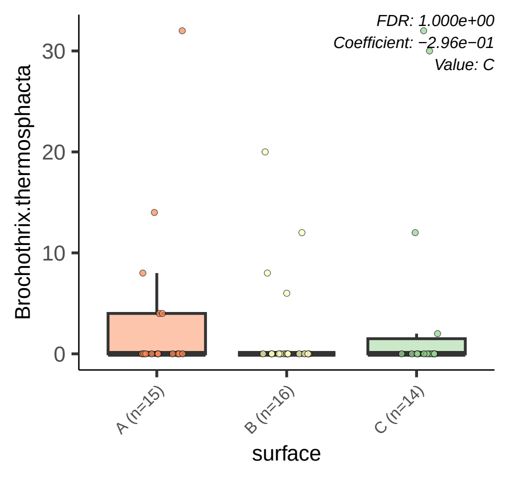

FDR: 1.000e+00
Coefficient: −2.96e−01
Value: C
Brochothrix.thermosphacta
30
20
10
0
A (n=15)
B (n=16)
C (n=14)
surface

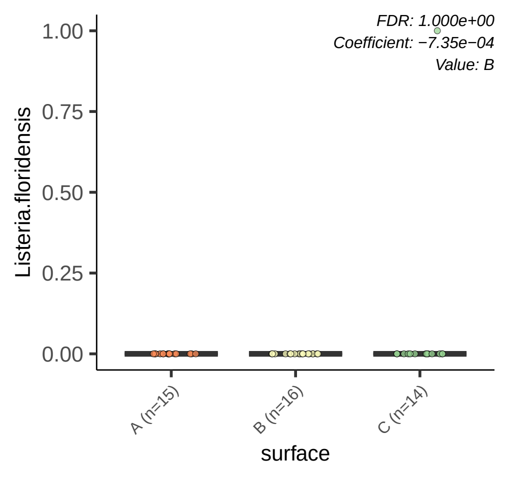
FDR: 1.000e+00
Coefficient: −7.35e−04
Value: B
Listeria.floridensis
1.00
0.75
0.50
0.25
0.00
A (n=15)
B (n=16)
C (n=14)
surface

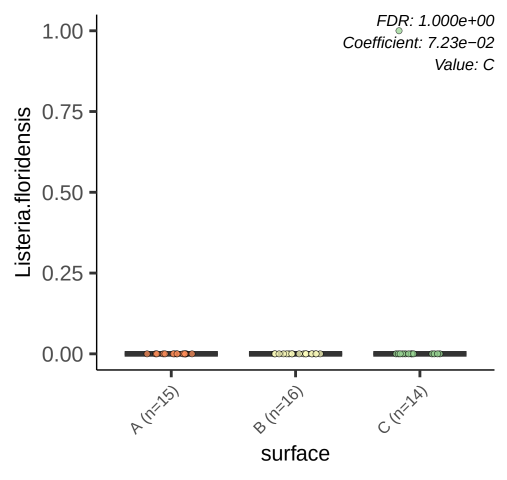

FDR: 1.000e+00
Coefficient: 7.23e−02
Value: C
1.00
0.75
0.50
0.25
0.00
Listeria.floridensis
A (n=15)
B (n=16)
C (n=14)
surface

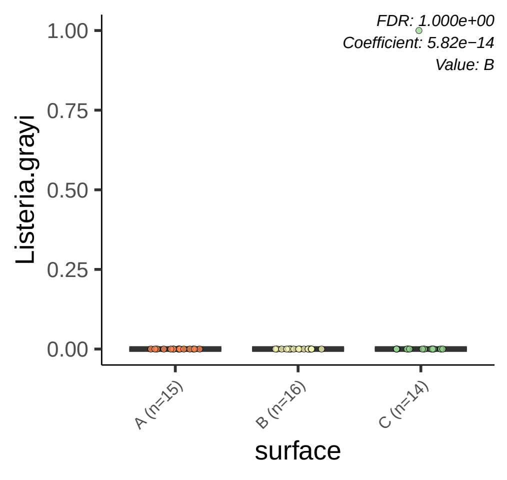
FDR: 1.000e+00
Coefficient: 5.82e−14
Value: B
Listeria.grayi
1.00
0.75
0.50
0.25
0.00
A (n=15)
B (n=16)
C (n=14)
surface

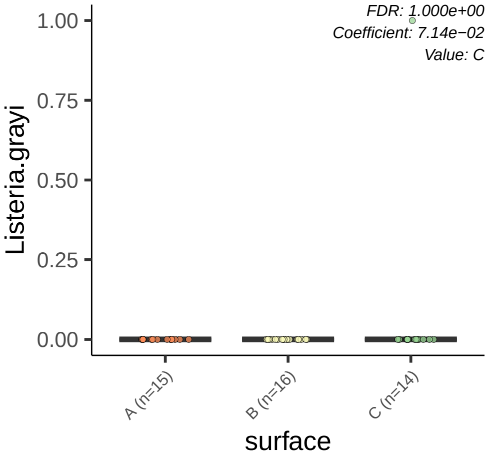
FDR: 1.000e+00
Coefficient: 7.14e−02
Value: C
Listeria.grayi
1.00
0.75
0.50
0.25
0.00
A (n=15)
B (n=16)
C (n=14)
surface

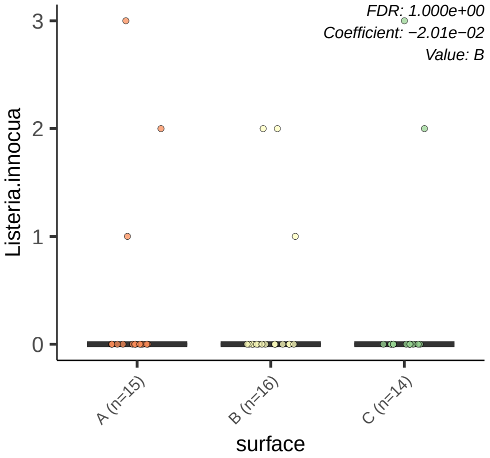

FDR: 1.000e+00
Coefficient: −2.01e−02
Value: B
Listeria.innocua
3
2
1
0
A (n=15)
B (n=16)
C (n=14)
surface

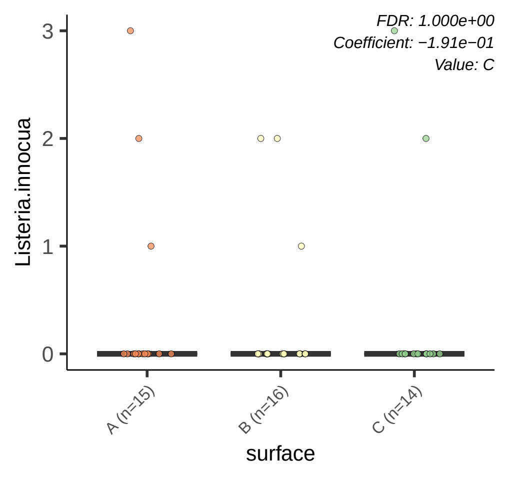

FDR: 1.000e+00
Coefficient: −1.91e−01
Value: C
Listeria.innocua
3
2
1
0
A (n=15)
B (n=16)
C (n=14)
surface

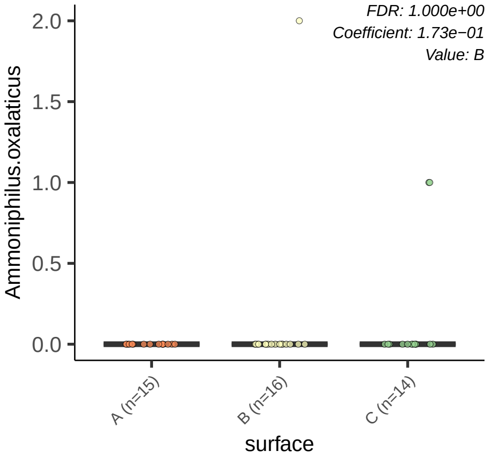

FDR: 1.000e+00
Coefficient: 1.73e−01
Value: B
Ammoniphilus.oxalaticus
2.0
1.5
1.0
0.5
0.0
A (n=15)
B (n=16)
C (n=14)
surface

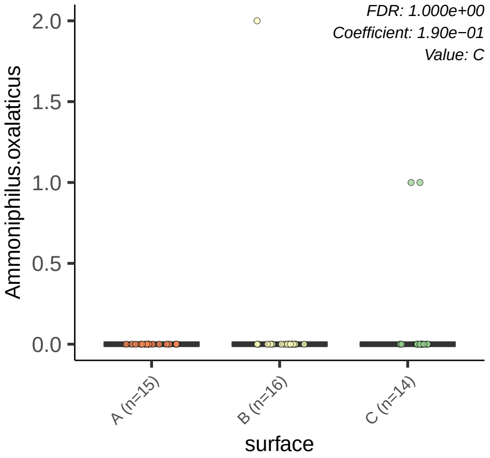
FDR: 1.000e+00
Coefficient: 1.90e−01
Value: C
Ammoniphilus.oxalaticus
2.0
1.5
1.0
0.5
0.0
A (n=15)
B (n=16)
C (n=14)
surface

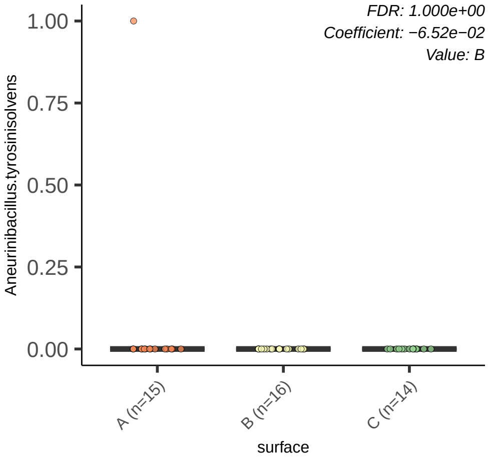
FDR: 1.000e+00
Coefficient: −6.52e−02
Value: B
Aneurinibacillus.tyrosinisolvens
1.00
0.75
0.50
0.25
0.00
A (n=15)
B (n=16)
C (n=14)
surface

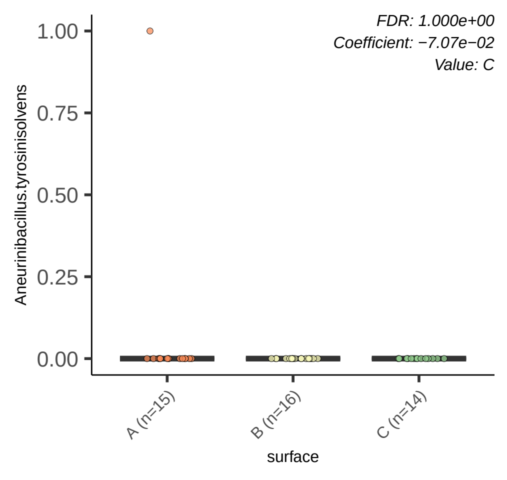
FDR: 1.000e+00
Coefficient: −7.07e−02
Value: C
Aneurinibacillus.tyrosinisolvens
1.00
0.75
0.50
0.25
0.00
A (n=15)
B (n=16)
C (n=14)
surface

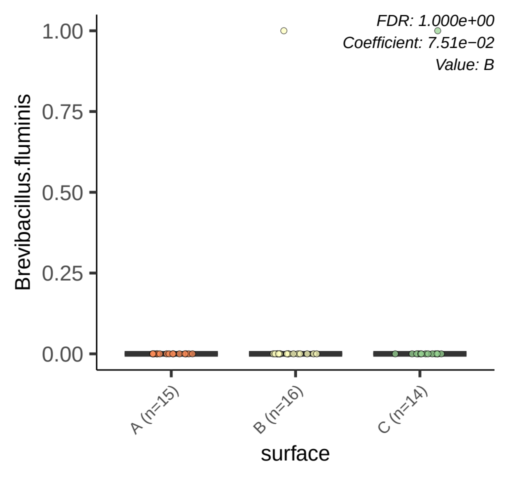
FDR: 1.000e+00
Coefficient: 7.51e−02
Value: B
Brevibacillus.fluminis
1.00
0.75
0.50
0.25
0.00
A (n=15)
B (n=16)
C (n=14)
surface

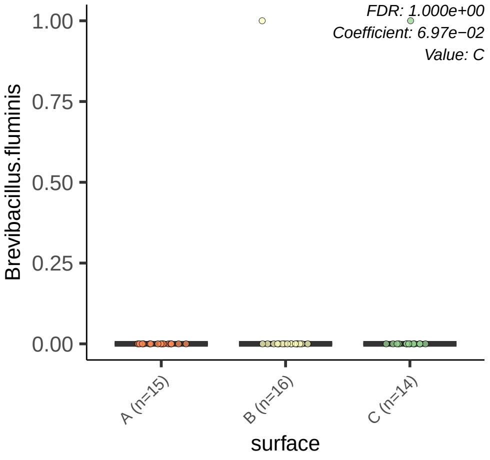
FDR: 1.000e+00
Coefficient: 6.97e−02
Value: C
1.00
0.75
0.50
0.25
0.00
Brevibacillus.fluminis
A (n=15)
B (n=16)
C (n=14)
surface

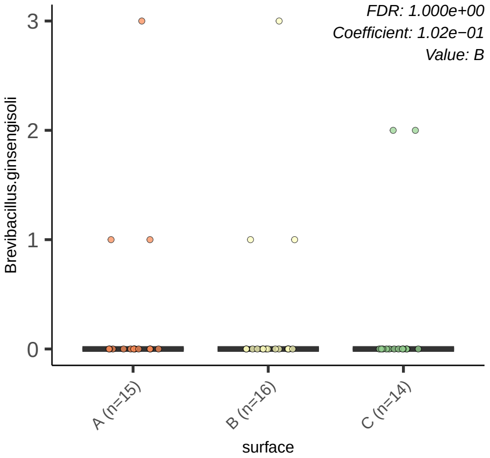

FDR: 1.000e+00
Coefficient: 1.02e−01
Value: B
Brevibacillus.ginsengisoli
0
1
2
3
A (n=15)
B (n=16)
C (n=14)
surface

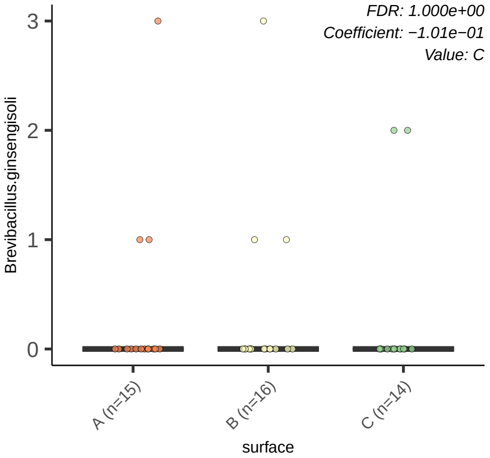
FDR: 1.000e+00
Coefficient: −1.01e−01
Value: C
Brevibacillus.ginsengisoli
3
2
1
0
A (n=15)
B (n=16)
C (n=14)
surface

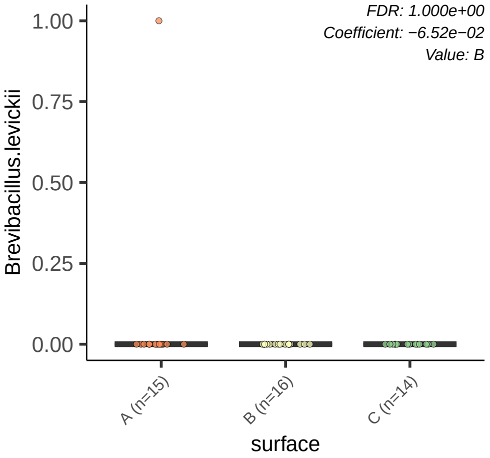

FDR: 1.000e+00
Coefficient: −6.52e−02
Value: B
Brevibacillus.levickii
1.00
0.75
0.50
0.25
0.00
A (n=15)
B (n=16)
C (n=14)
surface

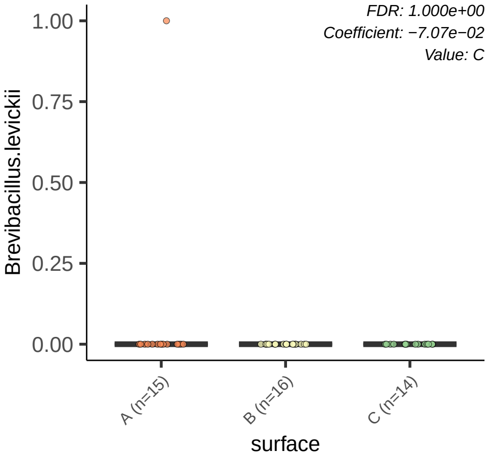

FDR: 1.000e+00
Coefficient: −7.07e−02
Value: C
Brevibacillus.levickii
1.00
0.75
0.50
0.25
0.00
A (n=15)
B (n=16)
C (n=14)
surface

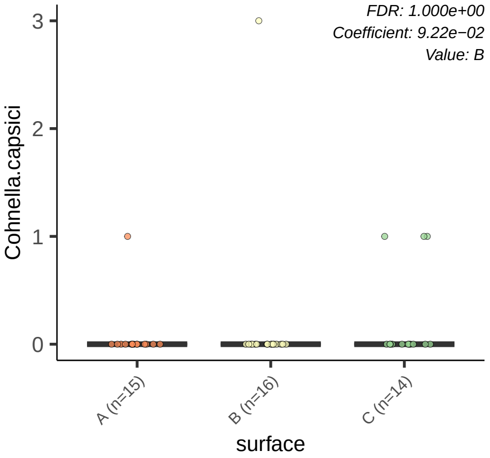
FDR: 1.000e+00
Coefficient: 9.22e−02
Value: B
Cohnella.capsici
3
2
1
0
A (n=15)
B (n=16)
C (n=14)
surface

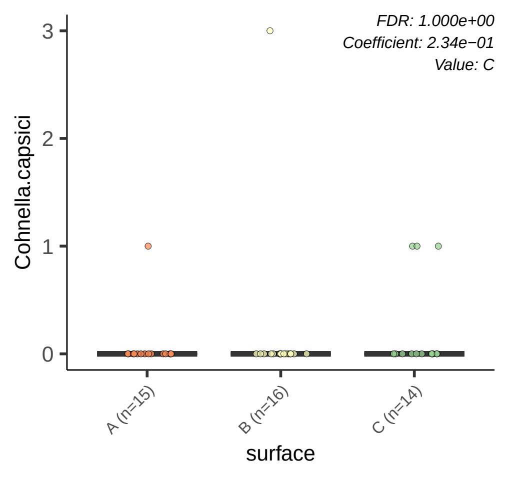

FDR: 1.000e+00
Coefficient: 2.34e−01
Value: C
Cohnella.capsici
3
2
1
0
A (n=15)
B (n=16)
C (n=14)
surface

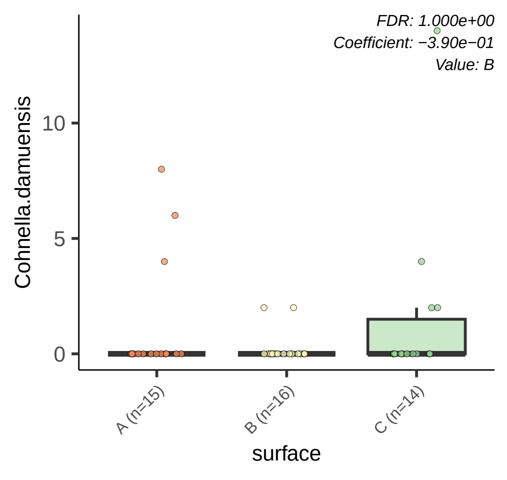
FDR: 1.000e+00
Coefficient: −3.90e−01
Value: B
Cohnella.damuensis
10
5
0
A (n=15)
B (n=16)
C (n=14)
surface

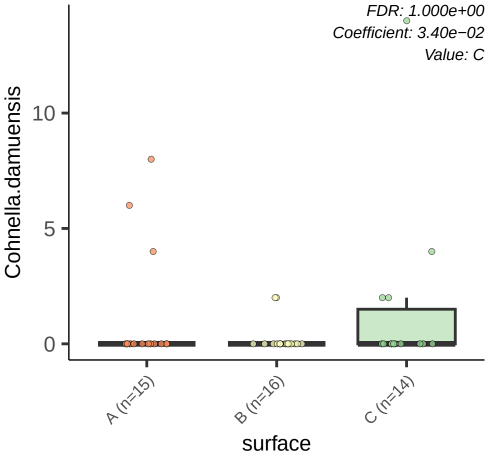

FDR: 1.000e+00
Coefficient: 3.40e−02
Value: C
Cohnella.damuensis
0
5
10
A (n=15)
B (n=16)
C (n=14)
surface

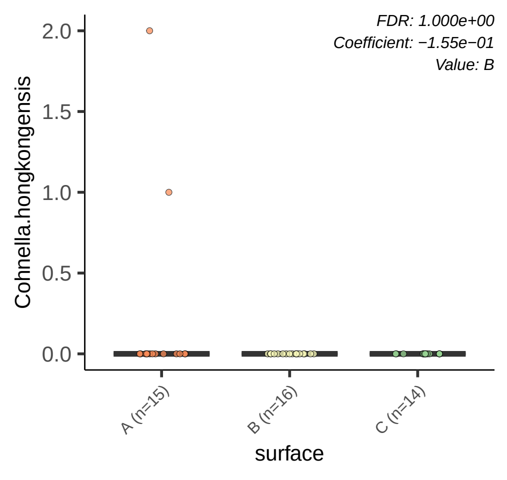

FDR: 1.000e+00
Coefficient: −1.55e−01
Value: B
Cohnella.hongkongensis
2.0
1.5
1.0
0.5
0.0
A (n=15)
B (n=16)
C (n=14)
surface

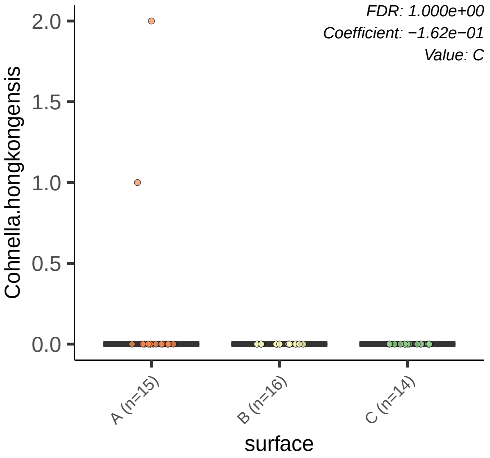

FDR: 1.000e+00
Coefficient: −1.62e−01
Value: C
Cohnella.hongkongensis
2.0
1.5
1.0
0.5
0.0
A (n=15)
B (n=16)
C (n=14)
surface

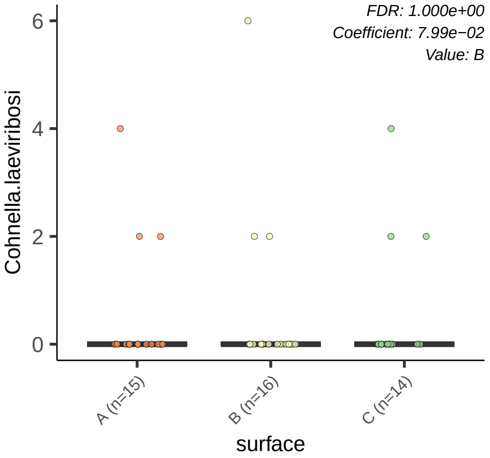
FDR: 1.000e+00
Coefficient: 7.99e−02
Value: B
Cohnella.laeviribosi
0
2
4
6
A (n=15)
B (n=16)
C (n=14)
surface

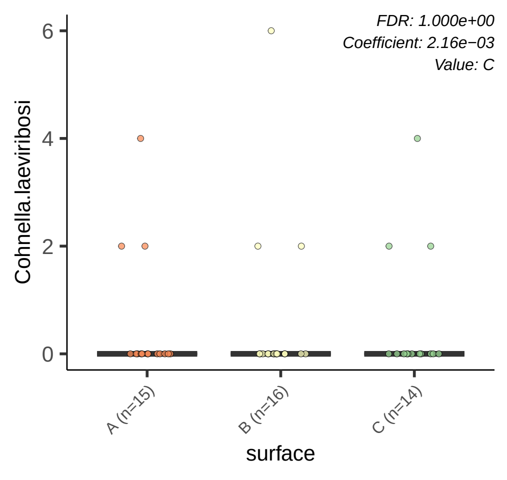

FDR: 1.000e+00
Coefficient: 2.16e−03
Value: C
Cohnella.laeviribosi
6
4
2
0
A (n=15)
B (n=16)
C (n=14)
surface

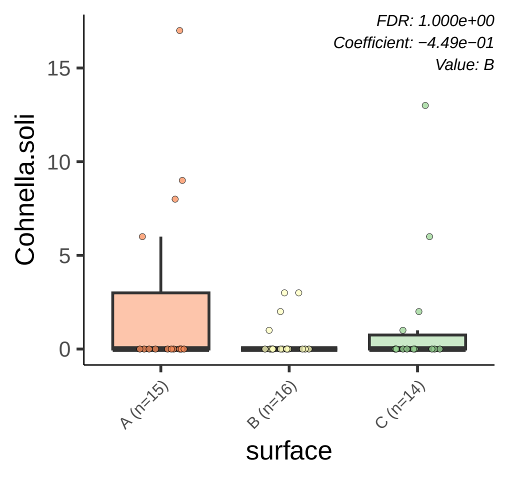

FDR: 1.000e+00
Coefficient: −4.49e−01
Value: B
Cohnella.soli
0
5
10
15
A (n=15)
B (n=16)
C (n=14)
surface

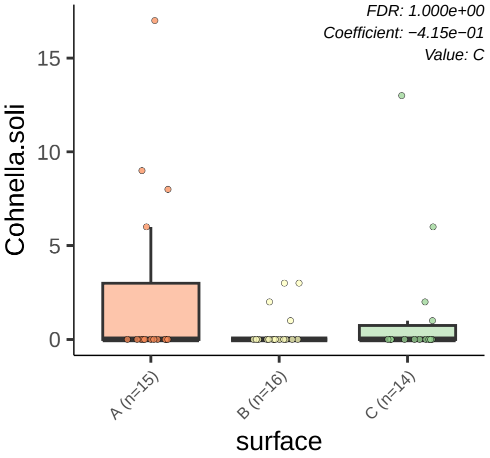

FDR: 1.000e+00
Coefficient: −4.15e−01
Value: C
Cohnella.soli
0
5
10
15
A (n=15)
B (n=16)
C (n=14)
surface

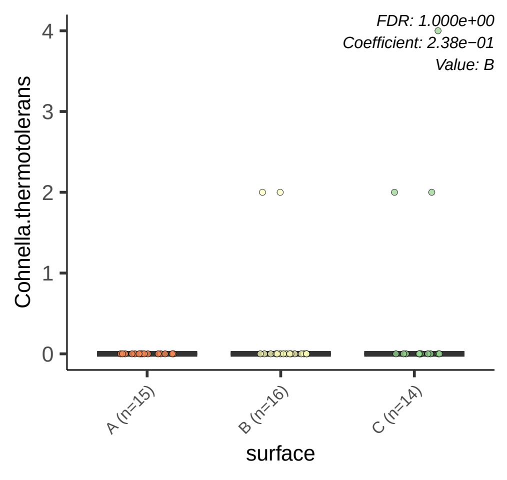
FDR: 1.000e+00
Coefficient: 2.38e−01
Value: B
Cohnella.thermotolerans
0
1
2
3
4
A (n=15)
B (n=16)
C (n=14)
surface

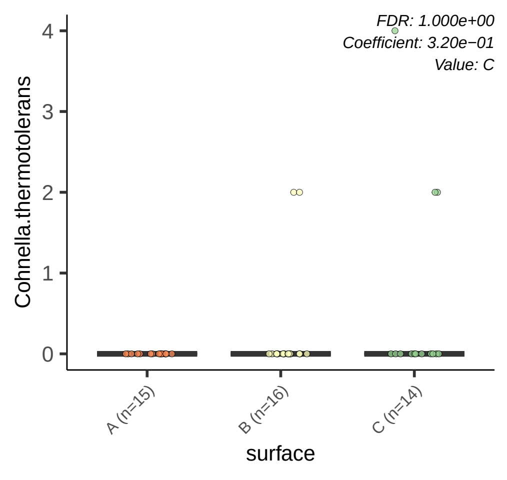
FDR: 1.000e+00
Coefficient: 3.20e−01
Value: C
Cohnella.thermotolerans
0
1
2
3
4
A (n=15)
B (n=16)
C (n=14)
surface

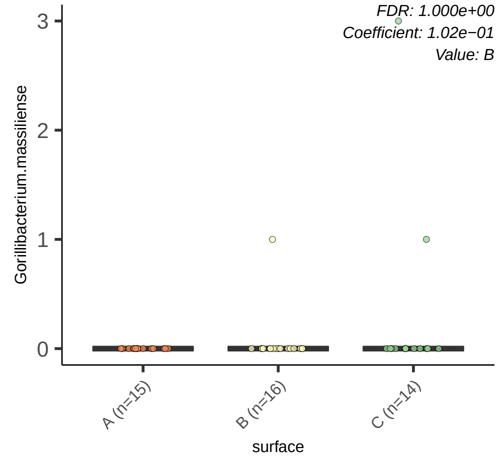

FDR: 1.000e+00
Coefficient: 1.02e−01
Value: B
Gorillibacterium.massiliense
3
2
1
0
A (n=15)
B (n=16)
C (n=14)
surface

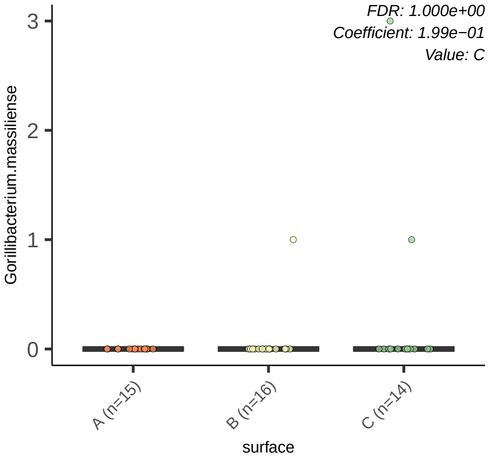
FDR: 1.000e+00
Coefficient: 1.99e−01
Value: C
Gorillibacterium.massiliense
3
2
1
0
A (n=15)
B (n=16)
C (n=14)
surface

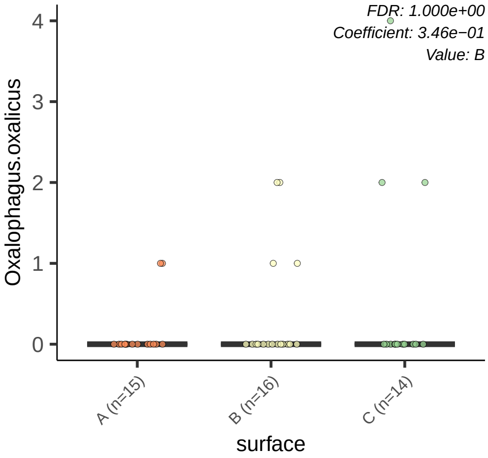
FDR: 1.000e+00
Coefficient: 3.46e−01
Value: B
Oxalophagus.oxalicus
0
1
2
3
4
A (n=15)
B (n=16)
C (n=14)
surface

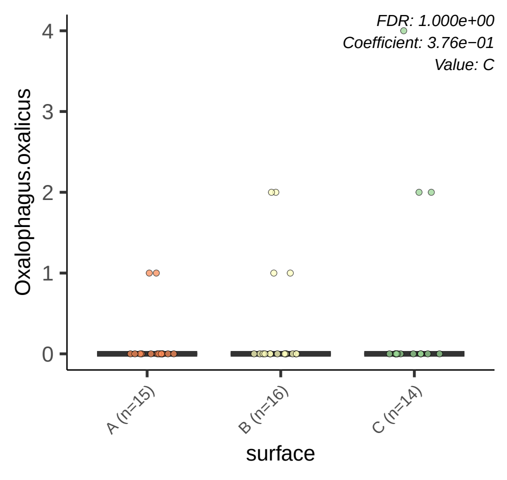
FDR: 1.000e+00
Coefficient: 3.76e−01
Value: C
Oxalophagus.oxalicus
0
1
2
3
4
A (n=15)
B (n=16)
C (n=14)
surface

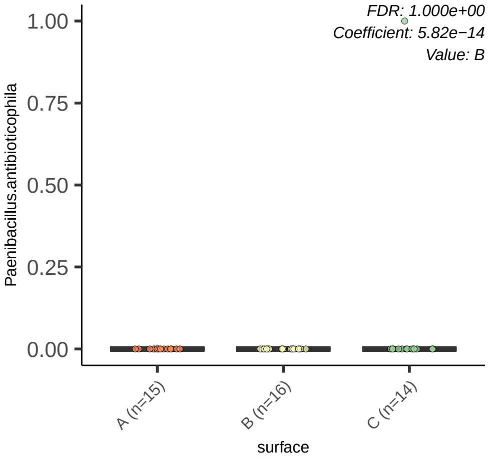
FDR: 1.000e+00
Coefficient: 5.82e−14
Value: B
1.00
0.75
0.50
0.25
0.00
Paenibacillus.antibioticophila
A (n=15)
B (n=16)
C (n=14)
surface

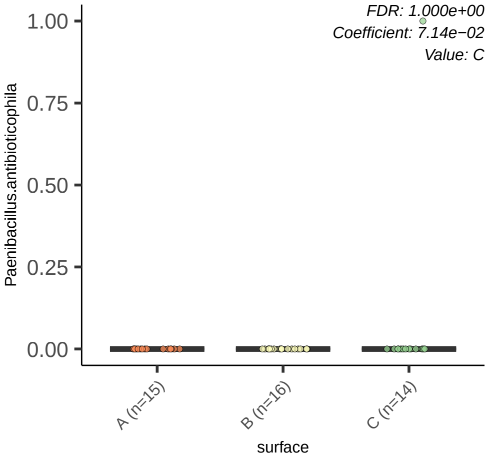
FDR: 1.000e+00
Coefficient: 7.14e−02
Value: C
Paenibacillus.antibioticophila
1.00
0.75
0.50
0.25
0.00
A (n=15)
B (n=16)
C (n=14)
surface

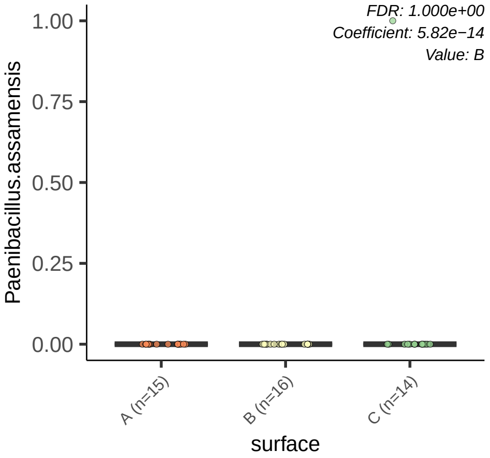
FDR: 1.000e+00
Coefficient: 5.82e−14
Value: B
Paenibacillus.assamensis
1.00
0.75
0.50
0.25
0.00
A (n=15)
B (n=16)
C (n=14)
surface

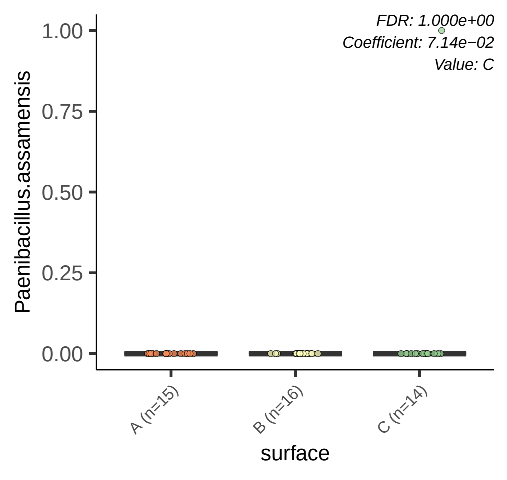

FDR: 1.000e+00
Coefficient: 7.14e−02
Value: C
Paenibacillus.assamensis
1.00
0.75
0.50
0.25
0.00
A (n=15)
B (n=16)
C (n=14)
surface

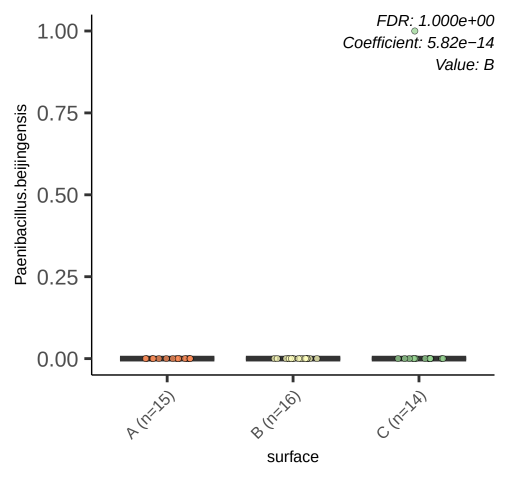

FDR: 1.000e+00
Coefficient: 5.82e−14
Value: B
Paenibacillus.beijingensis
1.00
0.75
0.50
0.25
0.00
A (n=15)
B (n=16)
C (n=14)
surface

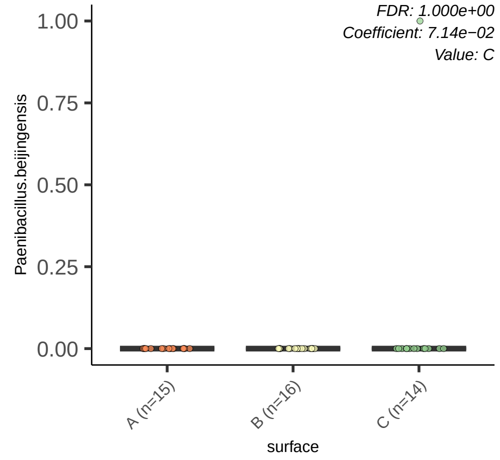

FDR: 1.000e+00
Coefficient: 7.14e−02
Value: C
Paenibacillus.beijingensis
1.00
0.75
0.50
0.25
0.00
A (n=15)
B (n=16)
C (n=14)
surface

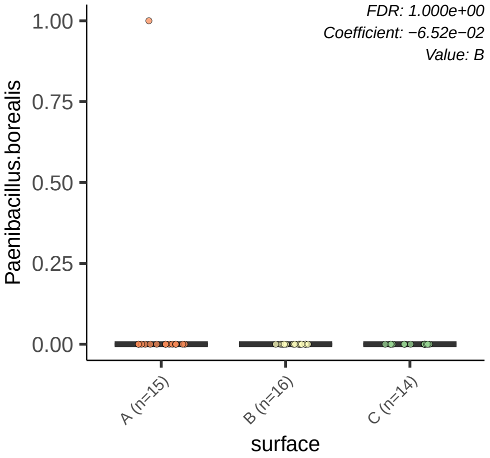

FDR: 1.000e+00
Coefficient: −6.52e−02
Value: B
Paenibacillus.borealis
1.00
0.75
0.50
0.25
0.00
A (n=15)
B (n=16)
C (n=14)
surface

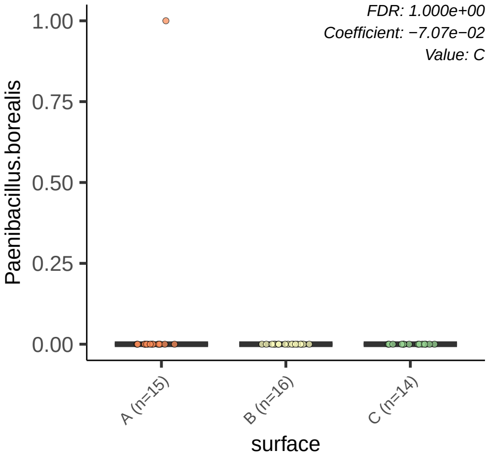
FDR: 1.000e+00
Coefficient: −7.07e−02
Value: C
Paenibacillus.borealis
1.00
0.75
0.50
0.25
0.00
A (n=15)
B (n=16)
C (n=14)
surface

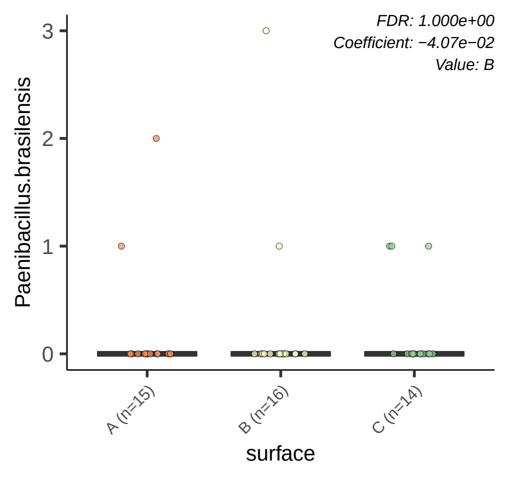

FDR: 1.000e+00
Coefficient: −4.07e−02
Value: B
Paenibacillus.brasilensis
3
2
1
0
A (n=15)
B (n=16)
C (n=14)
surface

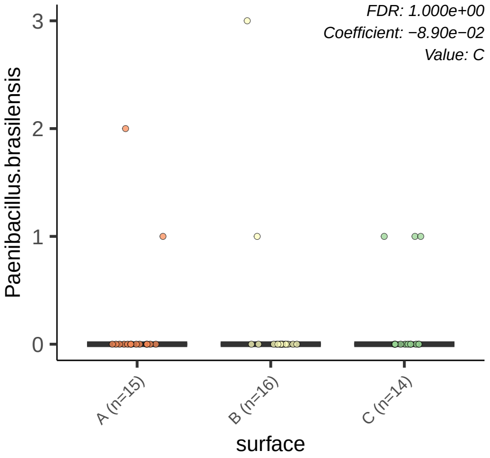
FDR: 1.000e+00
Coefficient: −8.90e−02
Value: C
Paenibacillus.brasilensis
0
1
2
3
A (n=15)
B (n=16)
C (n=14)
surface

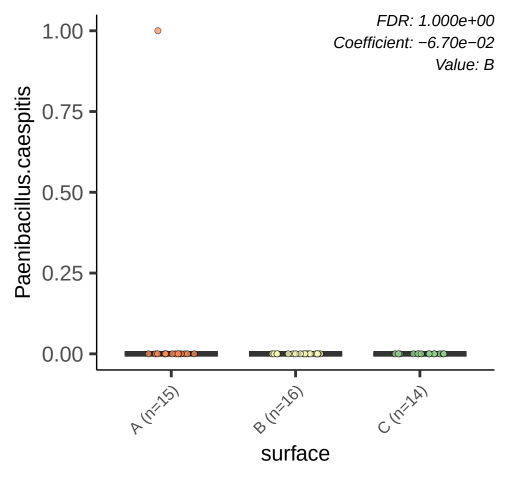
FDR: 1.000e+00
Coefficient: −6.70e−02
Value: B
Paenibacillus.caespitis
1.00
0.75
0.50
0.25
0.00
A (n=15)
B (n=16)
C (n=14)
surface

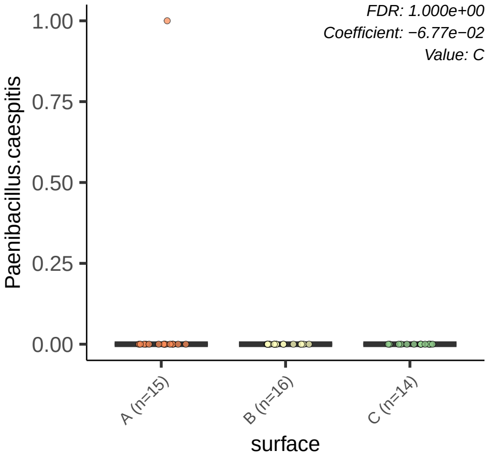
FDR: 1.000e+00
Coefficient: −6.77e−02
Value: C
Paenibacillus.caespitis
1.00
0.75
0.50
0.25
0.00
A (n=15)
B (n=16)
C (n=14)
surface

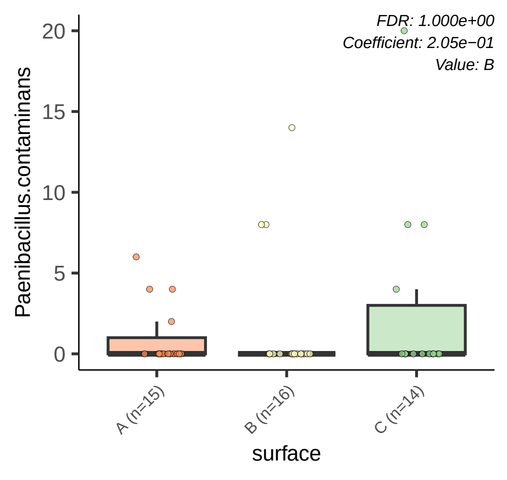

FDR: 1.000e+00
Coefficient: 2.05e−01
Value: B
Paenibacillus.contaminans
20
15
10
5
0
A (n=15)
B (n=16)
C (n=14)
surface

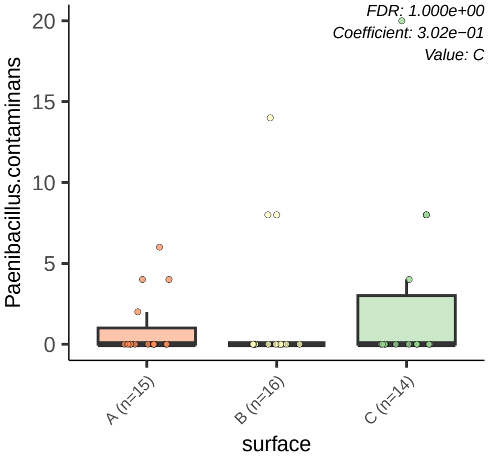
FDR: 1.000e+00
Coefficient: 3.02e−01
Value: C
Paenibacillus.contaminans
0
5
10
15
20
A (n=15)
B (n=16)
C (n=14)
surface

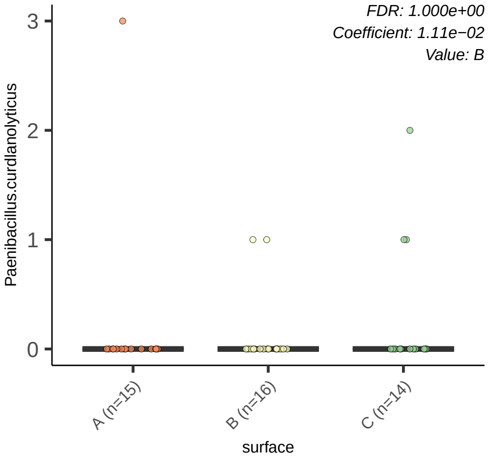

FDR: 1.000e+00
Coefficient: 1.11e−02
Value: B
Paenibacillus.curdlanolyticus
3
2
1
0
A (n=15)
B (n=16)
C (n=14)
surface

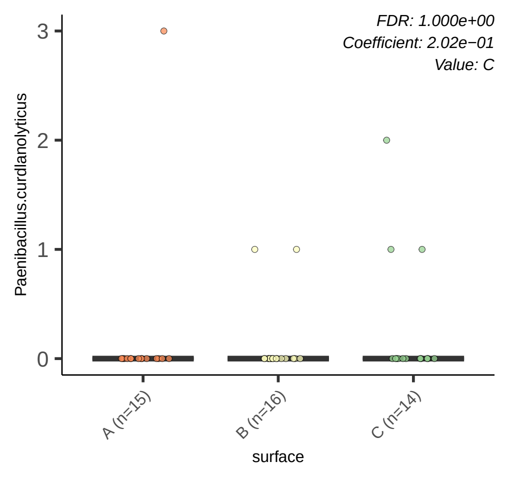
FDR: 1.000e+00
Coefficient: 2.02e−01
Value: C
Paenibacillus.curdlanolyticus
3
2
1
0
A (n=15)
B (n=16)
C (n=14)
surface

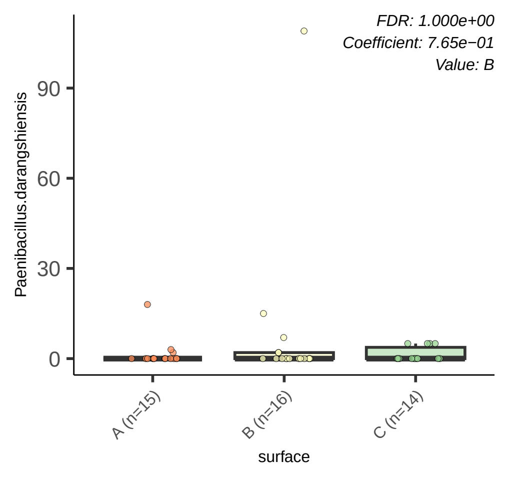

FDR: 1.000e+00
Coefficient: 7.65e−01
Value: B
Paenibacillus.darangshiensis
90
60
30
0
A (n=15)
B (n=16)
C (n=14)
surface

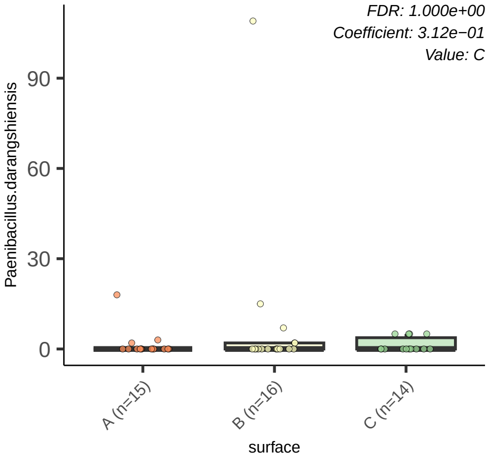

FDR: 1.000e+00
Coefficient: 3.12e−01
Value: C
Paenibacillus.darangshiensis
90
60
30
0
A (n=15)
B (n=16)
C (n=14)
surface

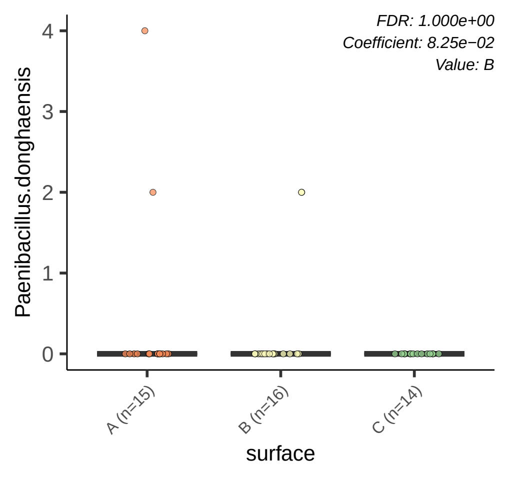
FDR: 1.000e+00
Coefficient: 8.25e−02
Value: B
Paenibacillus.donghaensis
0
1
2
3
4
A (n=15)
B (n=16)
C (n=14)
surface

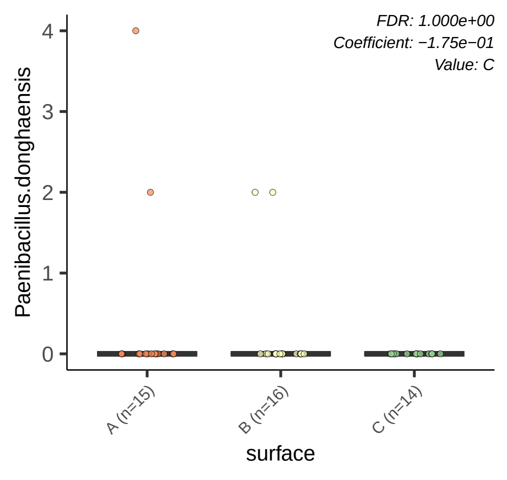

FDR: 1.000e+00
Coefficient: −1.75e−01
Value: C
Paenibacillus.donghaensis
4
3
2
1
0
A (n=15)
B (n=16)
C (n=14)
surface

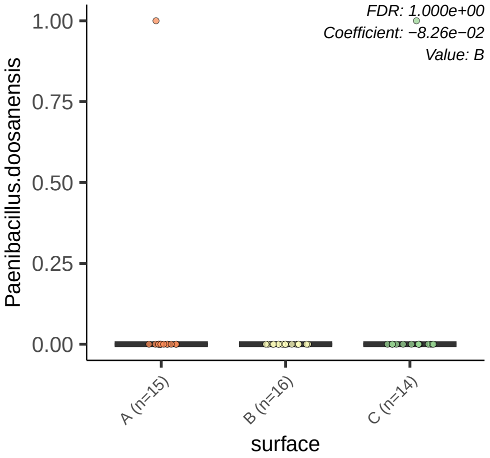
FDR: 1.000e+00
Coefficient: −8.26e−02
Value: B
Paenibacillus.doosanensis
1.00
0.75
0.50
0.25
0.00
A (n=15)
B (n=16)
C (n=14)
surface

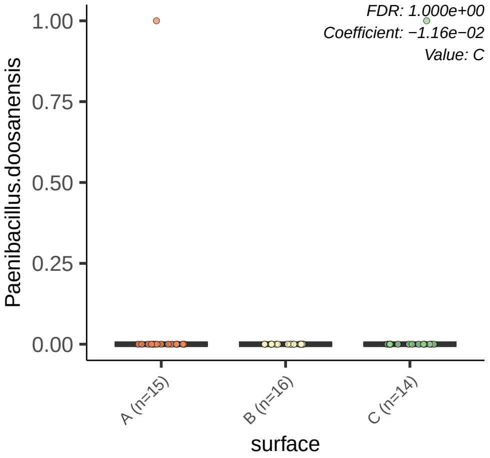

FDR: 1.000e+00
Coefficient: −1.16e−02
Value: C
Paenibacillus.doosanensis
1.00
0.75
0.50
0.25
0.00
A (n=15)
B (n=16)
C (n=14)
surface

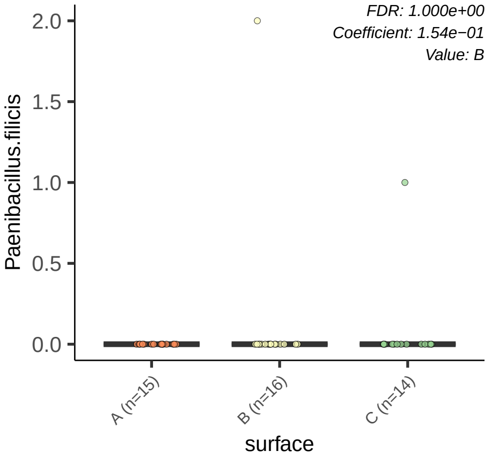

FDR: 1.000e+00
Coefficient: 1.54e−01
Value: B
Paenibacillus.filicis
2.0
1.5
1.0
0.5
0.0
A (n=15)
B (n=16)
C (n=14)
surface

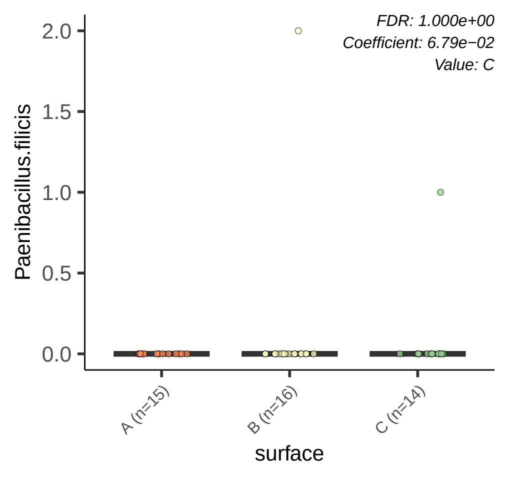

FDR: 1.000e+00
Coefficient: 6.79e−02
Value: C
Paenibacillus.filicis
2.0
1.5
1.0
0.5
0.0
A (n=15)
B (n=16)
C (n=14)
surface

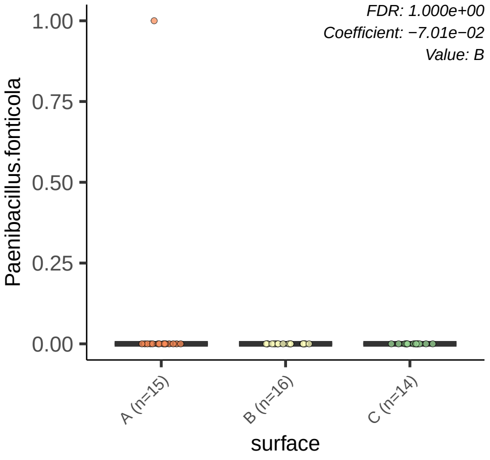
FDR: 1.000e+00
Coefficient: −7.01e−02
Value: B
Paenibacillus.fonticola
1.00
0.75
0.50
0.25
0.00
A (n=15)
B (n=16)
C (n=14)
surface

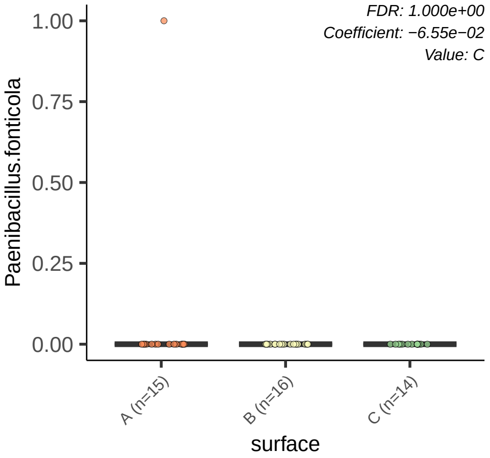
FDR: 1.000e+00
Coefficient: −6.55e−02
Value: C
Paenibacillus.fonticola
1.00
0.75
0.50
0.25
0.00
A (n=15)
B (n=16)
C (n=14)
surface

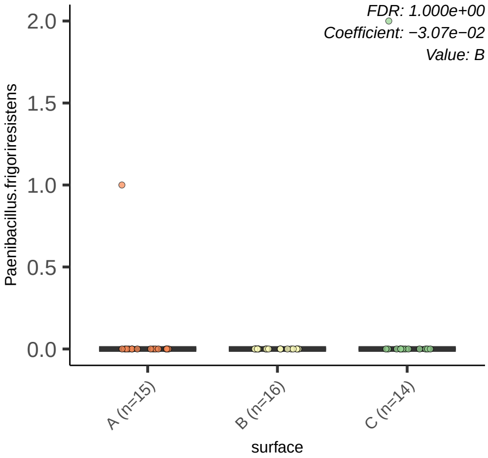

FDR: 1.000e+00
Coefficient: −3.07e−02
Value: B
Paenibacillus.frigoriresistens
2.0
1.5
1.0
0.5
0.0
A (n=15)
B (n=16)
C (n=14)
surface

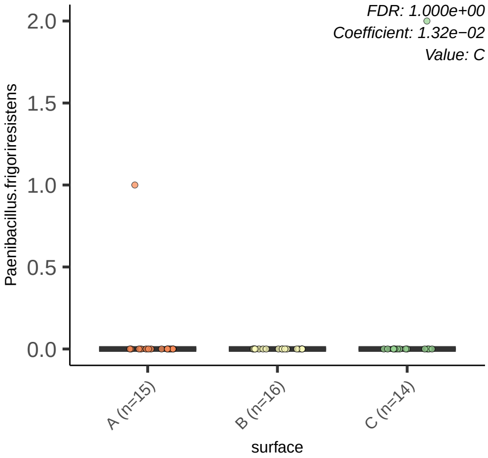
FDR: 1.000e+00
Coefficient: 1.32e−02
Value: C
Paenibacillus.frigoriresistens
2.0
1.5
1.0
0.5
0.0
A (n=15)
B (n=16)
C (n=14)
surface

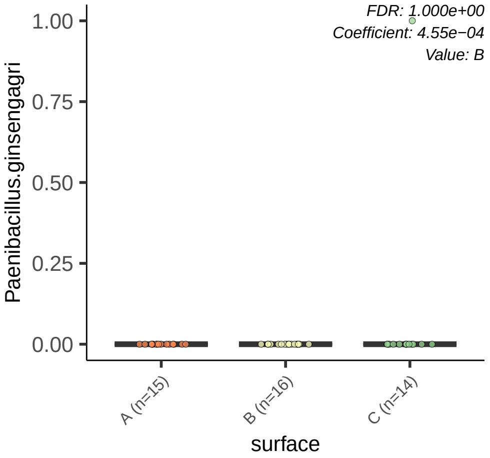
FDR: 1.000e+00
Coefficient: 4.55e−04
Value: B
Paenibacillus.ginsengagri
1.00
0.75
0.50
0.25
0.00
A (n=15)
B (n=16)
C (n=14)
surface

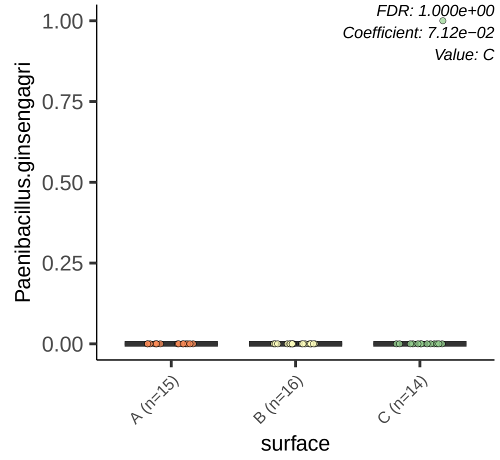
FDR: 1.000e+00
Coefficient: 7.12e−02
Value: C
1.00
0.75
0.50
0.25
0.00
Paenibacillus.ginsengagri
A (n=15)
B (n=16)
C (n=14)
surface

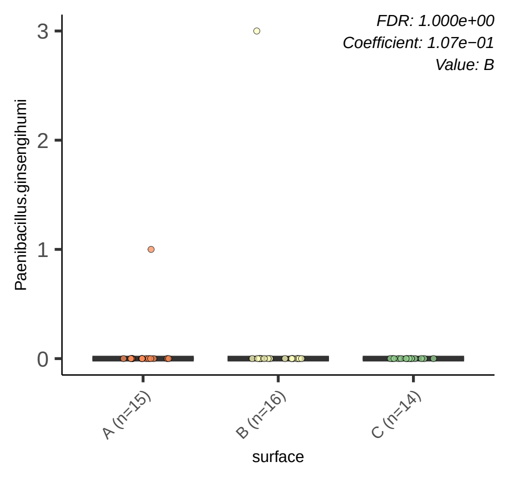

FDR: 1.000e+00
Coefficient: 1.07e−01
Value: B
Paenibacillus.ginsengihumi
3
2
1
0
A (n=15)
B (n=16)
C (n=14)
surface

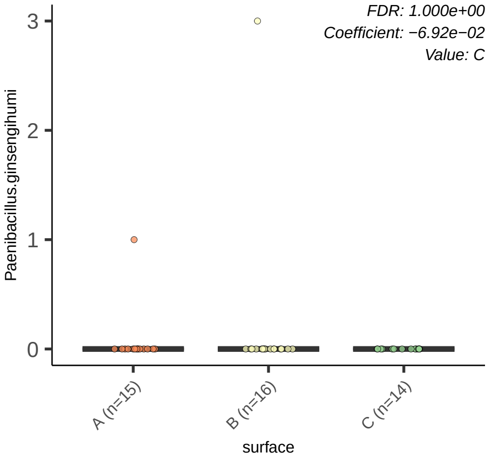
FDR: 1.000e+00
Coefficient: −6.92e−02
Value: C
Paenibacillus.ginsengihumi
3
2
1
0
A (n=15)
B (n=16)
C (n=14)
surface

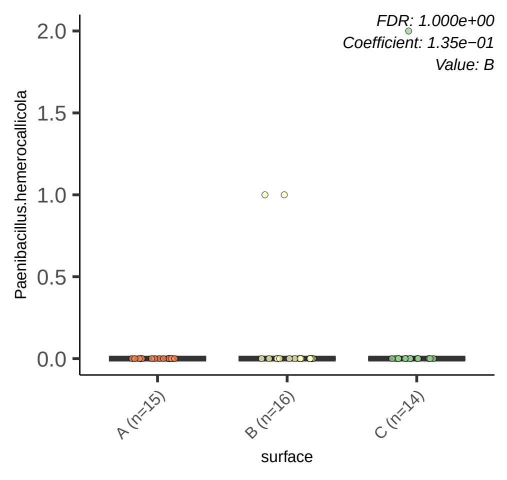
FDR: 1.000e+00
Coefficient: 1.35e−01
Value: B
Paenibacillus.hemerocallicola
2.0
1.5
1.0
0.5
0.0
A (n=15)
B (n=16)
C (n=14)
surface

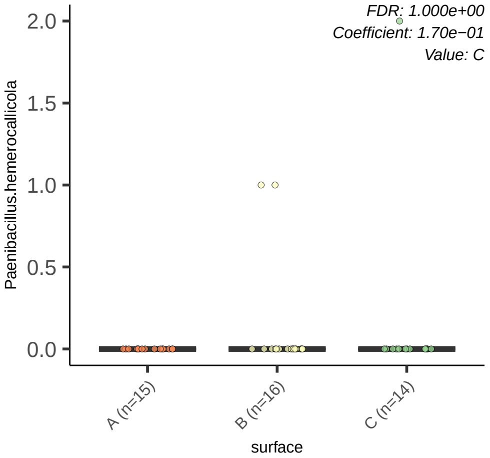

FDR: 1.000e+00
Coefficient: 1.70e−01
Value: C
Paenibacillus.hemerocallicola
2.0
1.5
1.0
0.5
0.0
A (n=15)
B (n=16)
C (n=14)
surface

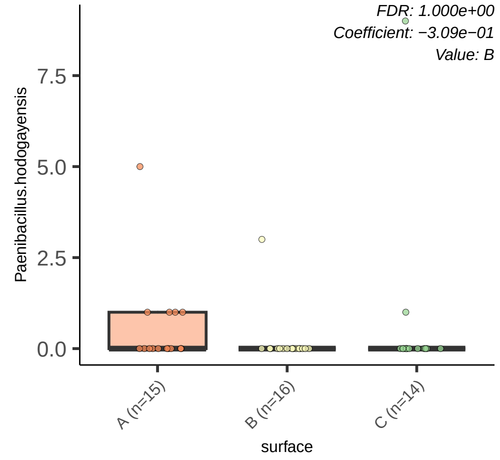

FDR: 1.000e+00
Coefficient: −3.09e−01
Value: B
Paenibacillus.hodogayensis
7.5
5.0
2.5
0.0
A (n=15)
B (n=16)
C (n=14)
surface

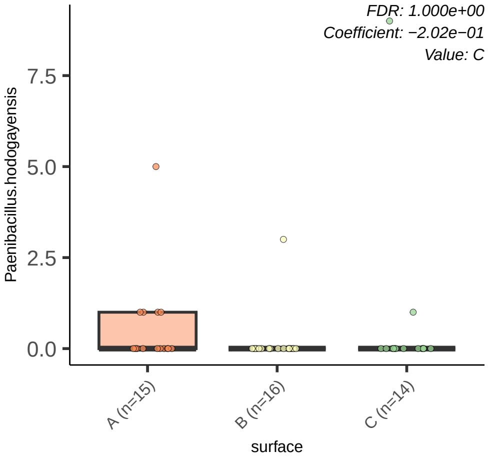

FDR: 1.000e+00
Coefficient: −2.02e−01
Value: C
Paenibacillus.hodogayensis
7.5
5.0
2.5
0.0
A (n=15)
B (n=16)
C (n=14)
surface

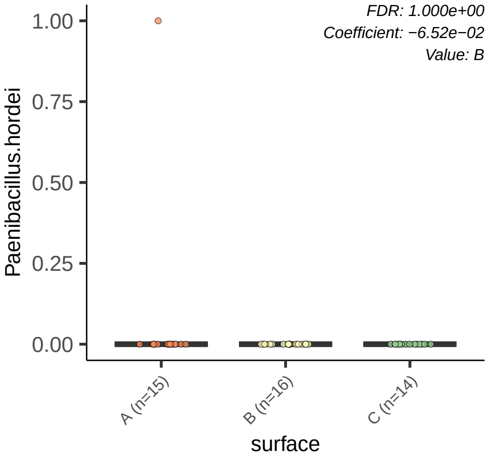

FDR: 1.000e+00
Coefficient: −6.52e−02
Value: B
Paenibacillus.hordei
1.00
0.75
0.50
0.25
0.00
A (n=15)
B (n=16)
C (n=14)
surface

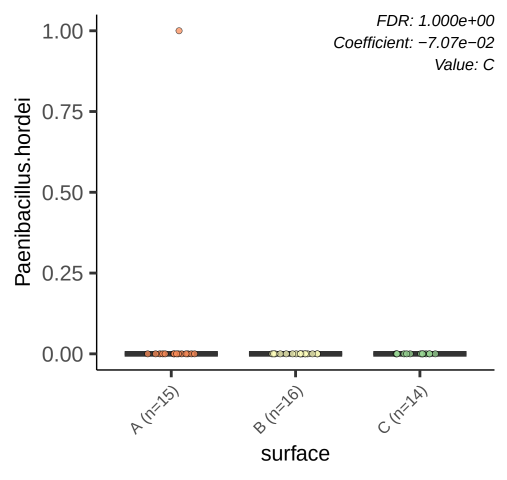

FDR: 1.000e+00
Coefficient: −7.07e−02
Value: C
Paenibacillus.hordei
1.00
0.75
0.50
0.25
0.00
A (n=15)
B (n=16)
C (n=14)
surface

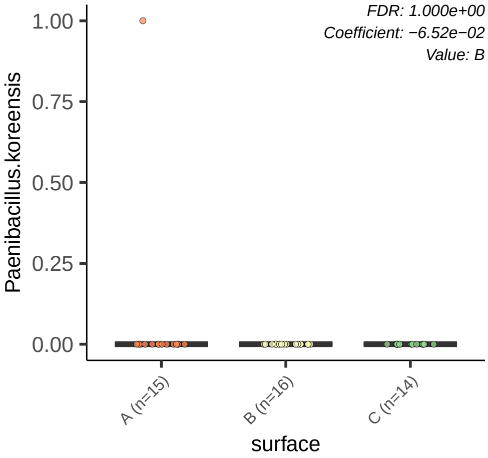
FDR: 1.000e+00
Coefficient: −6.52e−02
Value: B
Paenibacillus.koreensis
1.00
0.75
0.50
0.25
0.00
A (n=15)
B (n=16)
C (n=14)
surface

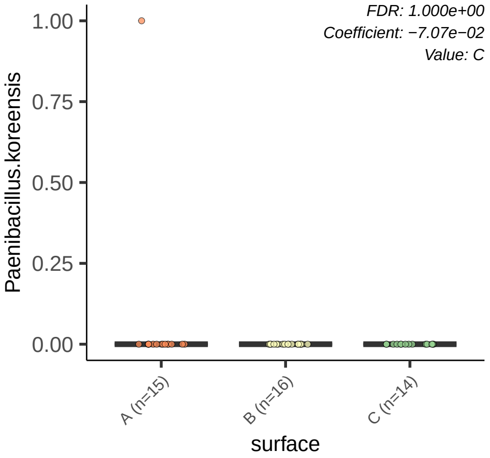

FDR: 1.000e+00
Coefficient: −7.07e−02
Value: C
Paenibacillus.koreensis
1.00
0.75
0.50
0.25
0.00
A (n=15)
B (n=16)
C (n=14)
surface

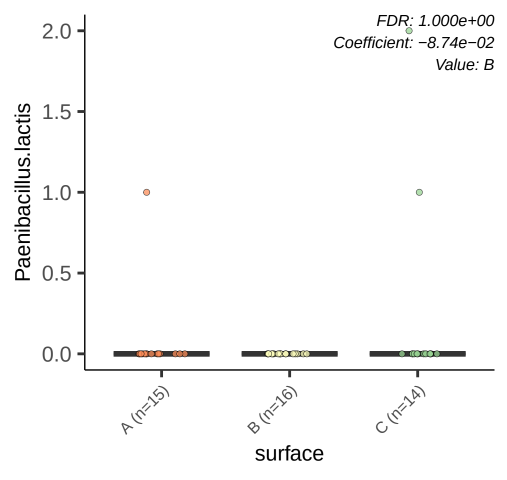

FDR: 1.000e+00
Coefficient: −8.74e−02
Value: B
Paenibacillus.lactis
2.0
1.5
1.0
0.5
0.0
A (n=15)
B (n=16)
C (n=14)
surface

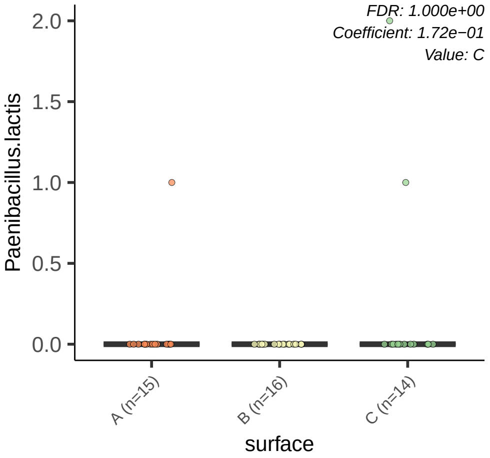

FDR: 1.000e+00
Coefficient: 1.72e−01
Value: C
Paenibacillus.lactis
2.0
1.5
1.0
0.5
0.0
A (n=15)
B (n=16)
C (n=14)
surface

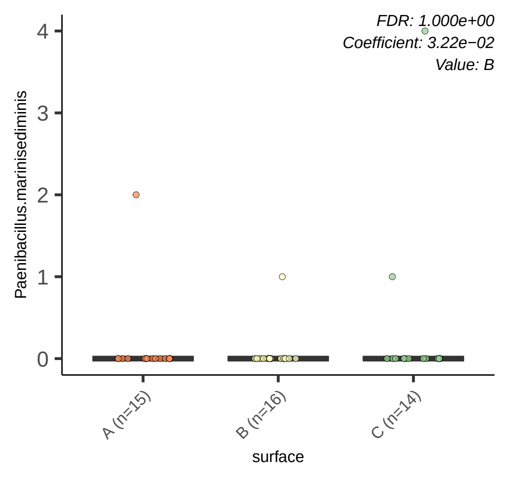

FDR: 1.000e+00
Coefficient: 3.22e−02
Value: B
Paenibacillus.marinisediminis
0
1
2
3
4
A (n=15)
B (n=16)
C (n=14)
surface

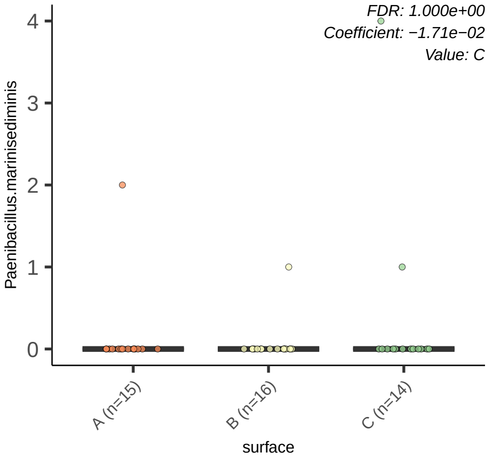
FDR: 1.000e+00
Coefficient: −1.71e−02
Value: C
Paenibacillus.marinisediminis
4
3
2
1
0
A (n=15)
B (n=16)
C (n=14)
surface

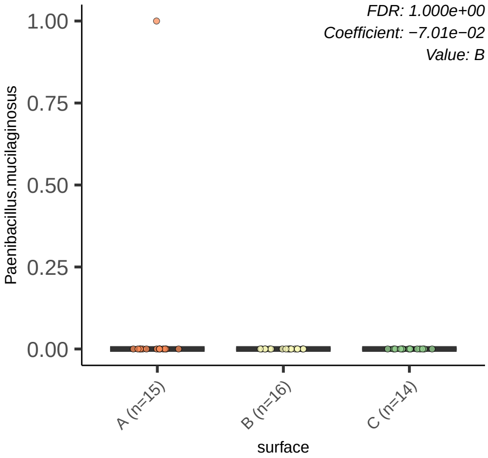
FDR: 1.000e+00
Coefficient: −7.01e−02
Value: B
Paenibacillus.mucilaginosus
1.00
0.75
0.50
0.25
0.00
A (n=15)
B (n=16)
C (n=14)
surface

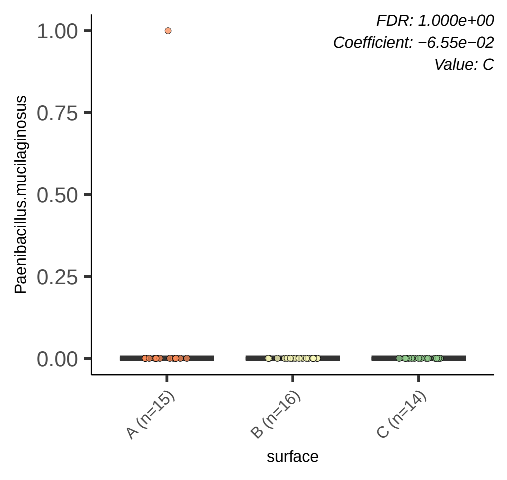
FDR: 1.000e+00
Coefficient: −6.55e−02
Value: C
Paenibacillus.mucilaginosus
1.00
0.75
0.50
0.25
0.00
A (n=15)
B (n=16)
C (n=14)
surface

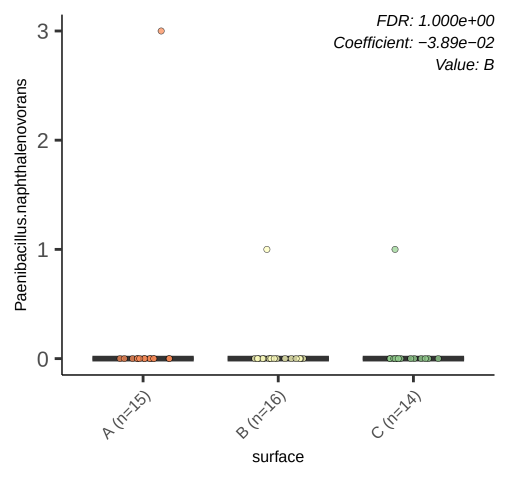

FDR: 1.000e+00
Coefficient: −3.89e−02
Value: B
Paenibacillus.naphthalenovorans
3
2
1
0
A (n=15)
B (n=16)
C (n=14)
surface

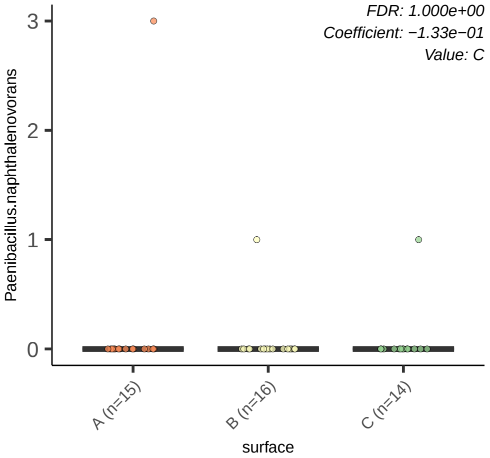
FDR: 1.000e+00
Coefficient: −1.33e−01
Value: C
Paenibacillus.naphthalenovorans
3
2
1
0
A (n=15)
B (n=16)
C (n=14)
surface

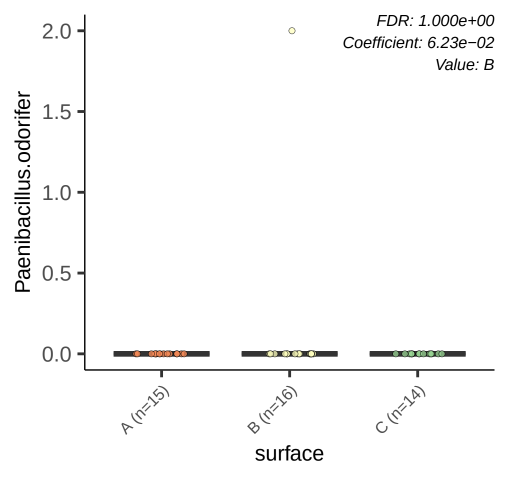
FDR: 1.000e+00
Coefficient: 6.23e−02
Value: B
Paenibacillus.odorifer
2.0
1.5
1.0
0.5
0.0
A (n=15)
B (n=16)
C (n=14)
surface

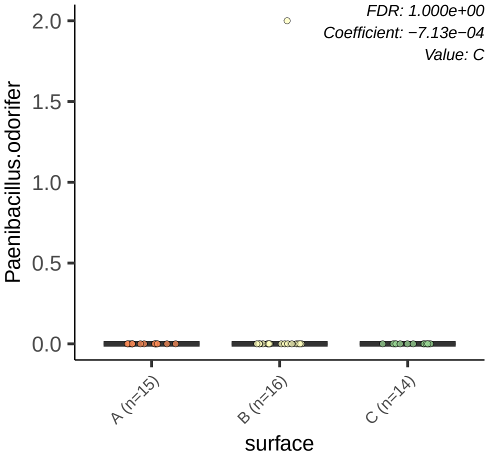

FDR: 1.000e+00
Coefficient: −7.13e−04
Value: C
Paenibacillus.odorifer
2.0
1.5
1.0
0.5
0.0
A (n=15)
B (n=16)
C (n=14)
surface

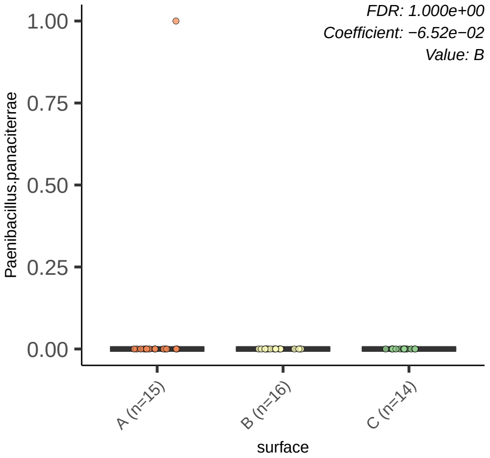
FDR: 1.000e+00
Coefficient: −6.52e−02
Value: B
Paenibacillus.panaciterrae
1.00
0.75
0.50
0.25
0.00
A (n=15)
B (n=16)
C (n=14)
surface

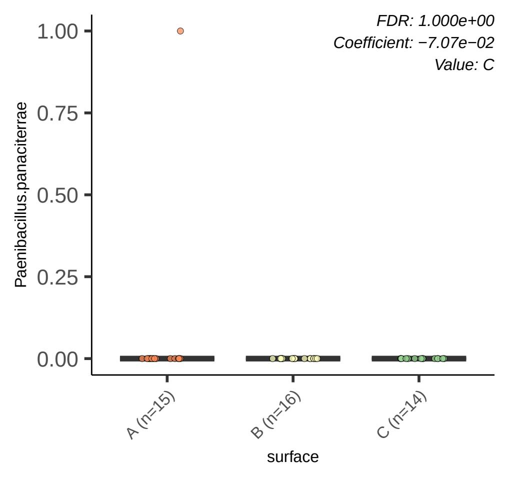
FDR: 1.000e+00
Coefficient: −7.07e−02
Value: C
Paenibacillus.panaciterrae
1.00
0.75
0.50
0.25
0.00
A (n=15)
B (n=16)
C (n=14)
surface

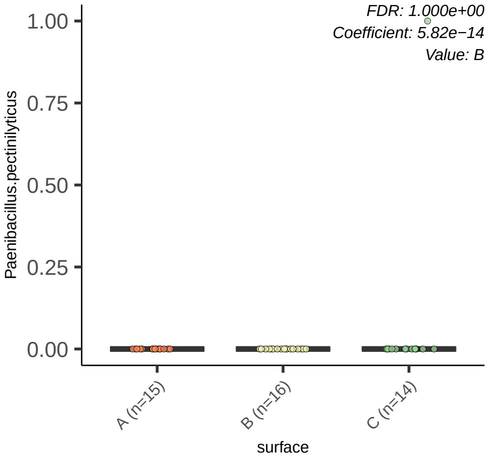

FDR: 1.000e+00
Coefficient: 5.82e−14
Value: B
1.00
0.75
0.50
0.25
0.00
Paenibacillus.pectinilyticus
A (n=15)
B (n=16)
C (n=14)
surface

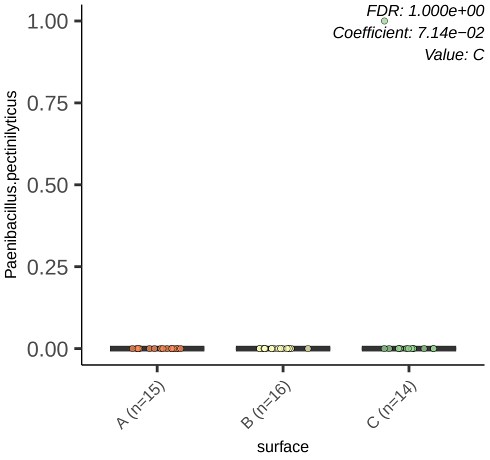
FDR: 1.000e+00
Coefficient: 7.14e−02
Value: C
1.00
0.75
0.50
0.25
0.00
Paenibacillus.pectinilyticus
A (n=15)
B (n=16)
C (n=14)
surface

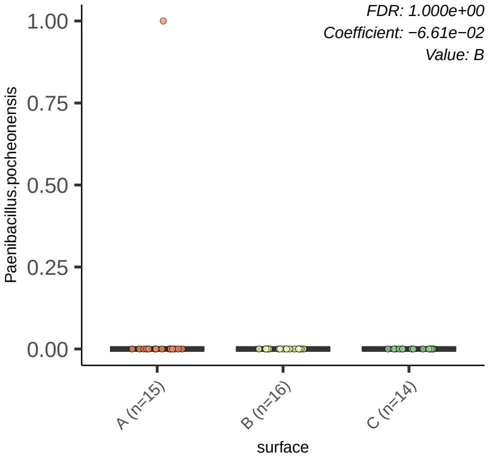
FDR: 1.000e+00
Coefficient: −6.61e−02
Value: B
1.00
0.75
0.50
0.25
0.00
Paenibacillus.pocheonensis
A (n=15)
B (n=16)
C (n=14)
surface

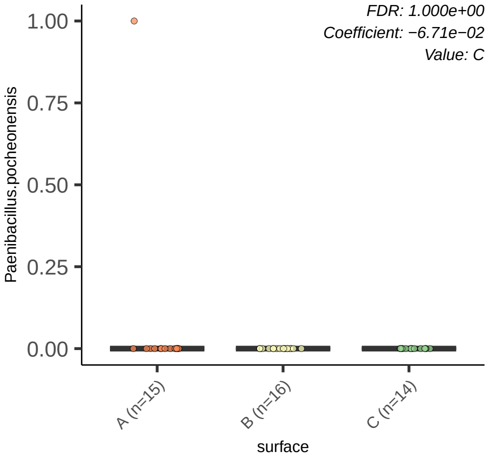

FDR: 1.000e+00
Coefficient: −6.71e−02
Value: C
Paenibacillus.pocheonensis
1.00
0.75
0.50
0.25
0.00
A (n=15)
B (n=16)
C (n=14)
surface

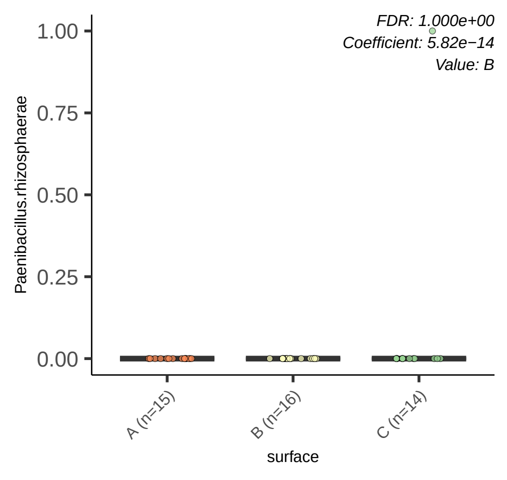

FDR: 1.000e+00
Coefficient: 5.82e−14
Value: B
Paenibacillus.rhizosphaerae
1.00
0.75
0.50
0.25
0.00
A (n=15)
B (n=16)
C (n=14)
surface

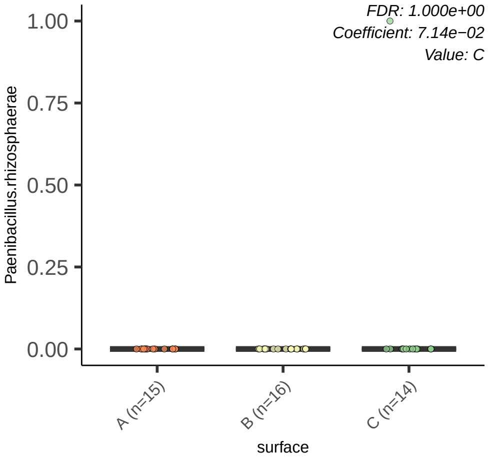

FDR: 1.000e+00
Coefficient: 7.14e−02
Value: C
Paenibacillus.rhizosphaerae
1.00
0.75
0.50
0.25
0.00
A (n=15)
B (n=16)
C (n=14)
surface

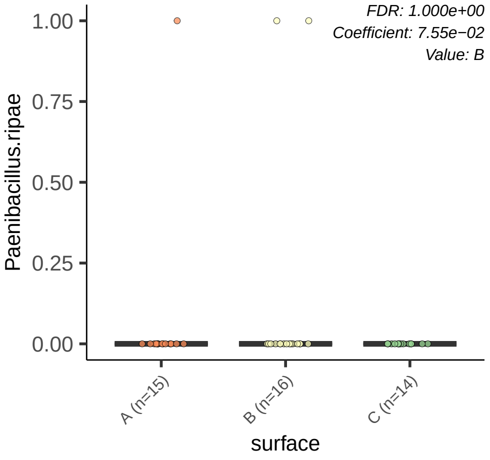
FDR: 1.000e+00
Coefficient: 7.55e−02
Value: B
Paenibacillus.ripae
1.00
0.75
0.50
0.25
0.00
A (n=15)
B (n=16)
C (n=14)
surface

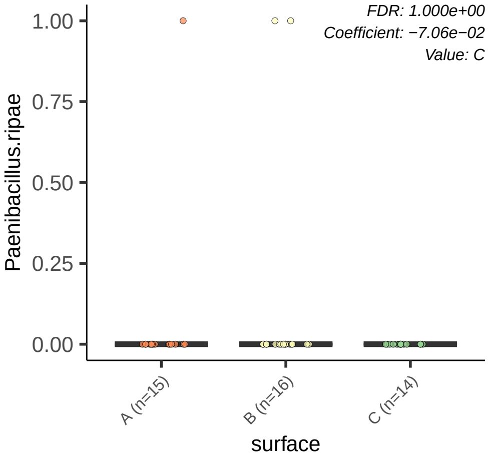

FDR: 1.000e+00
Coefficient: −7.06e−02
Value: C
Paenibacillus.ripae
1.00
0.75
0.50
0.25
0.00
A (n=15)
B (n=16)
C (n=14)
surface

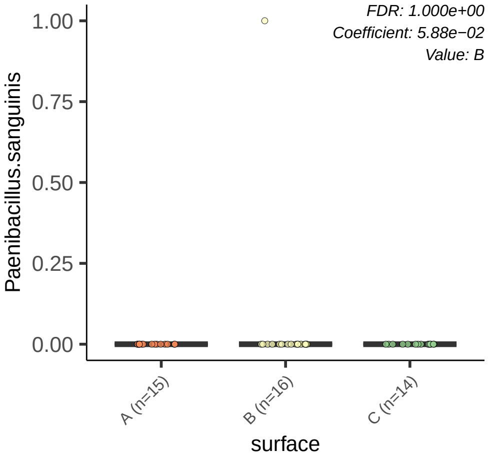

FDR: 1.000e+00
Coefficient: 5.88e−02
Value: B
Paenibacillus.sanguinis
1.00
0.75
0.50
0.25
0.00
A (n=15)
B (n=16)
C (n=14)
surface

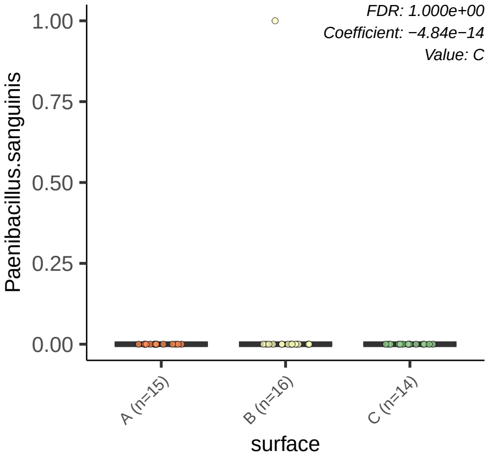

FDR: 1.000e+00
Coefficient: −4.84e−14
Value: C
Paenibacillus.sanguinis
1.00
0.75
0.50
0.25
0.00
A (n=15)
B (n=16)
C (n=14)
surface

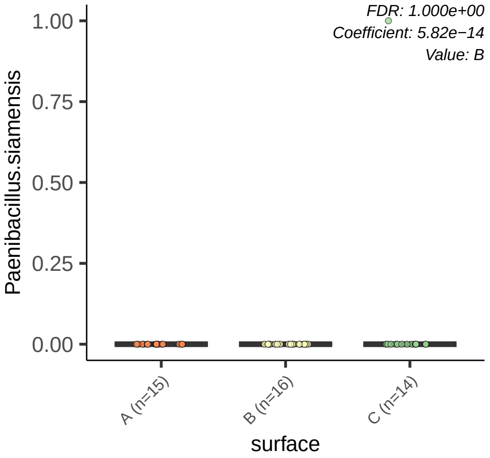
FDR: 1.000e+00
Coefficient: 5.82e−14
Value: B
Paenibacillus.siamensis
1.00
0.75
0.50
0.25
0.00
A (n=15)
B (n=16)
C (n=14)
surface

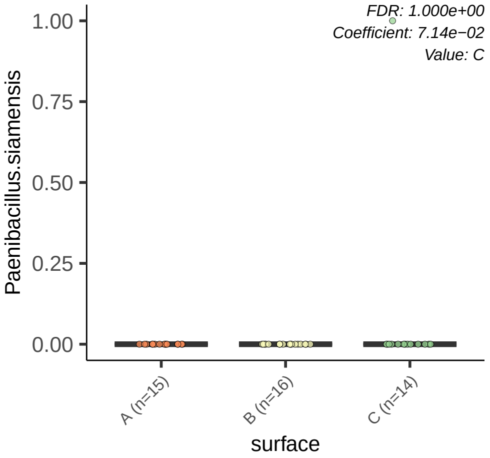

FDR: 1.000e+00
Coefficient: 7.14e−02
Value: C
Paenibacillus.siamensis
1.00
0.75
0.50
0.25
0.00
A (n=15)
B (n=16)
C (n=14)
surface

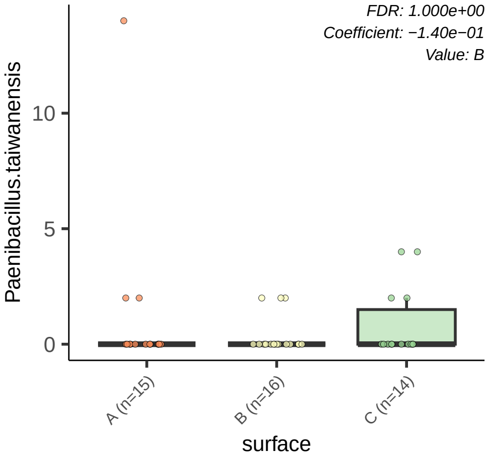
FDR: 1.000e+00
Coefficient: −1.40e−01
Value: B
Paenibacillus.taiwanensis
10
5
0
A (n=15)
B (n=16)
C (n=14)
surface

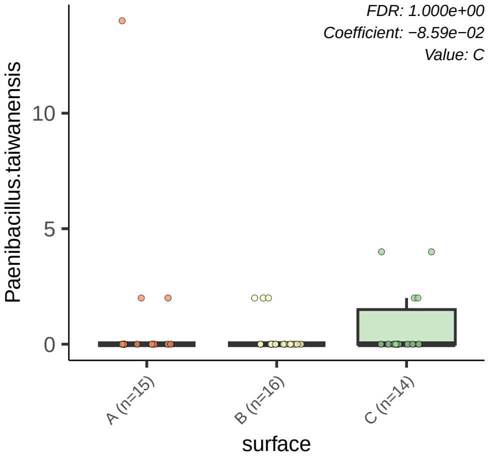
FDR: 1.000e+00
Coefficient: −8.59e−02
Value: C
Paenibacillus.taiwanensis
0
5
10
A (n=15)
B (n=16)
C (n=14)
surface

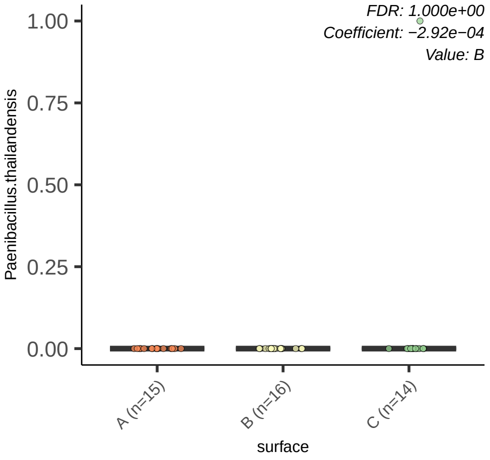

FDR: 1.000e+00
Coefficient: −2.92e−04
Value: B
Paenibacillus.thailandensis
1.00
0.75
0.50
0.25
0.00
A (n=15)
B (n=16)
C (n=14)
surface

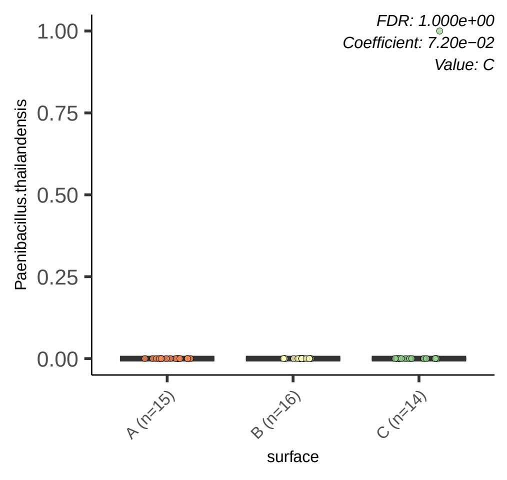

FDR: 1.000e+00
Coefficient: 7.20e−02
Value: C
1.00
0.75
0.50
0.25
0.00
Paenibacillus.thailandensis
A (n=15)
B (n=16)
C (n=14)
surface

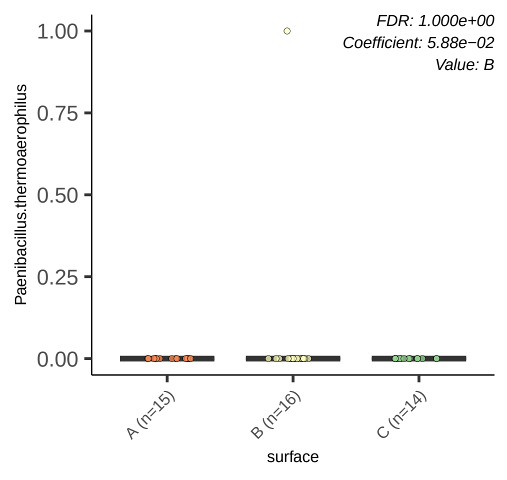

FDR: 1.000e+00
Coefficient: 5.88e−02
Value: B
Paenibacillus.thermoaerophilus
1.00
0.75
0.50
0.25
0.00
A (n=15)
B (n=16)
C (n=14)
surface

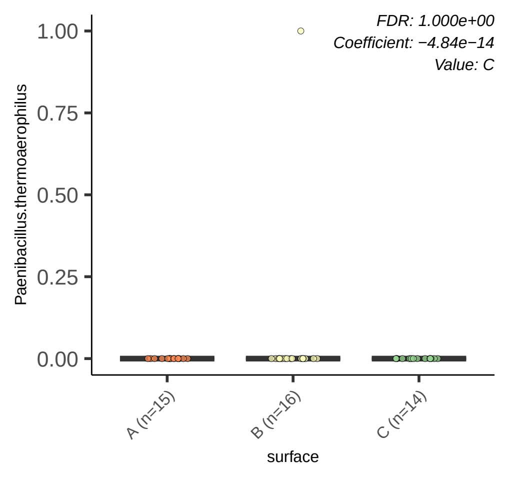
FDR: 1.000e+00
Coefficient: −4.84e−14
Value: C
Paenibacillus.thermoaerophilus
1.00
0.75
0.50
0.25
0.00
A (n=15)
B (n=16)
C (n=14)
surface

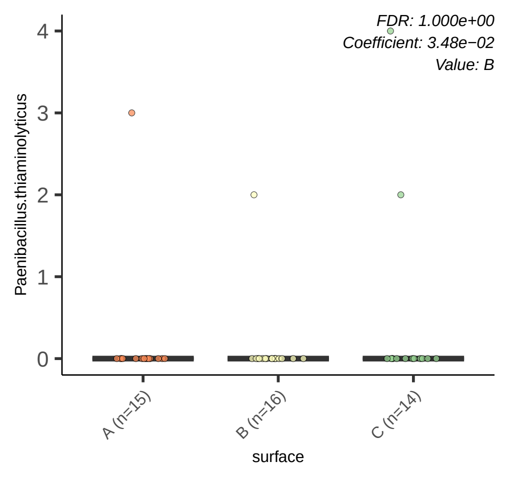
FDR: 1.000e+00
Coefficient: 3.48e−02
Value: B
Paenibacillus.thiaminolyticus
0
1
2
3
4
A (n=15)
B (n=16)
C (n=14)
surface

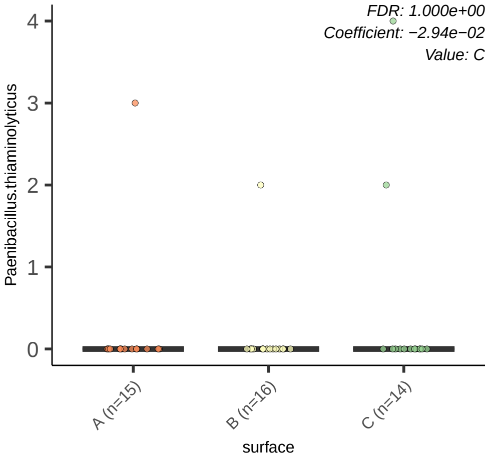

FDR: 1.000e+00
Coefficient: −2.94e−02
Value: C
Paenibacillus.thiaminolyticus
0
1
2
3
4
A (n=15)
B (n=16)
C (n=14)
surface

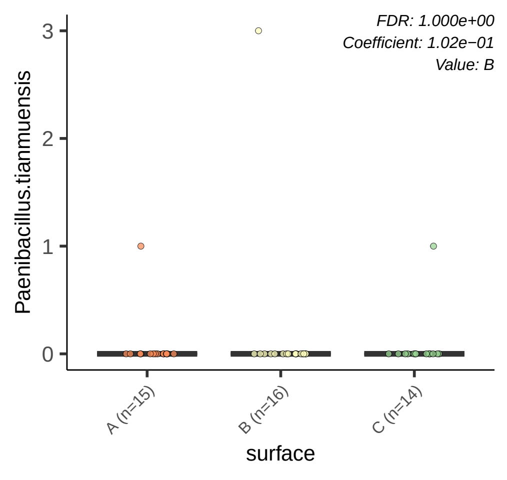

FDR: 1.000e+00
Coefficient: 1.02e−01
Value: B
Paenibacillus.tianmuensis
3
2
1
0
A (n=15)
B (n=16)
C (n=14)
surface

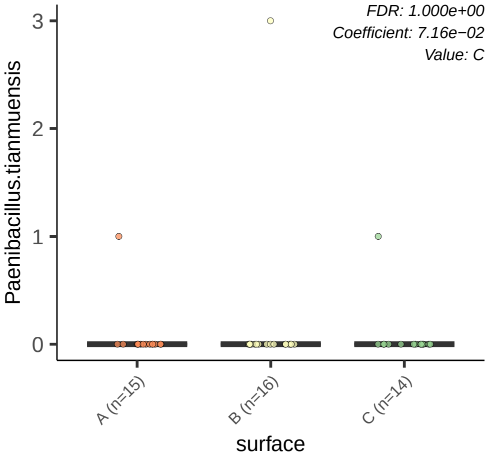

FDR: 1.000e+00
Coefficient: 7.16e−02
Value: C
Paenibacillus.tianmuensis
3
2
1
0
A (n=15)
B (n=16)
C (n=14)
surface

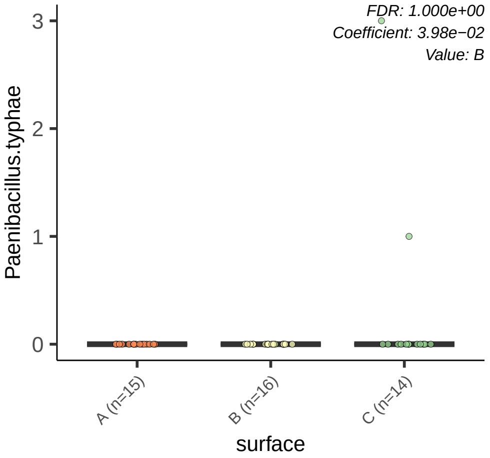
FDR: 1.000e+00
Coefficient: 3.98e−02
Value: B
Paenibacillus.typhae
3
2
1
0
A (n=15)
B (n=16)
C (n=14)
surface

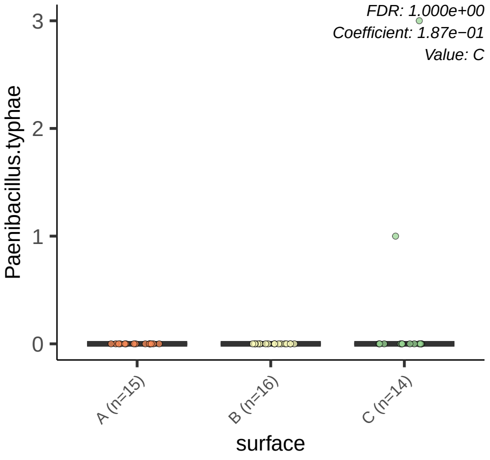
FDR: 1.000e+00
Coefficient: 1.87e−01
Value: C
Paenibacillus.typhae
3
2
1
0
A (n=15)
B (n=16)
C (n=14)
surface

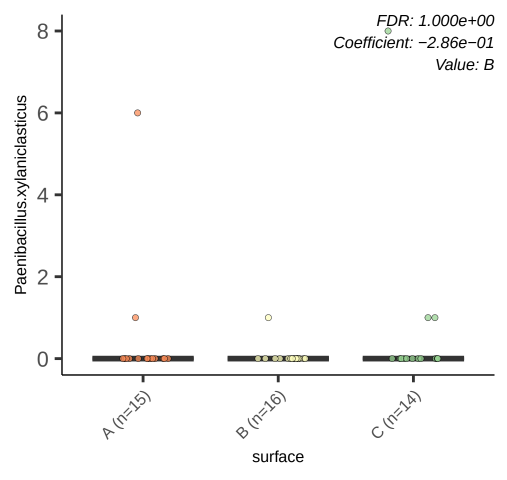

FDR: 1.000e+00
Coefficient: −2.86e−01
Value: B
Paenibacillus.xylaniclasticus
8
6
4
2
0
A (n=15)
B (n=16)
C (n=14)
surface

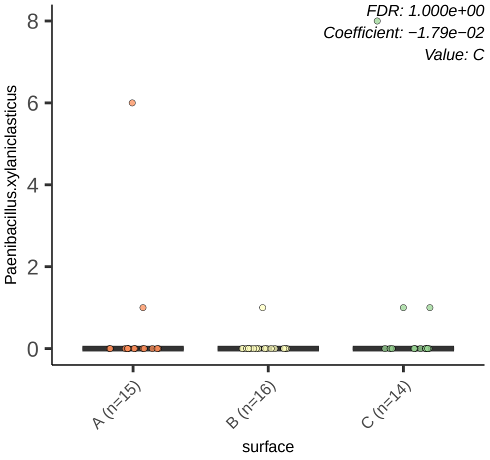

FDR: 1.000e+00
Coefficient: −1.79e−02
Value: C
Paenibacillus.xylaniclasticus
0
2
4
6
8
A (n=15)
B (n=16)
C (n=14)
surface

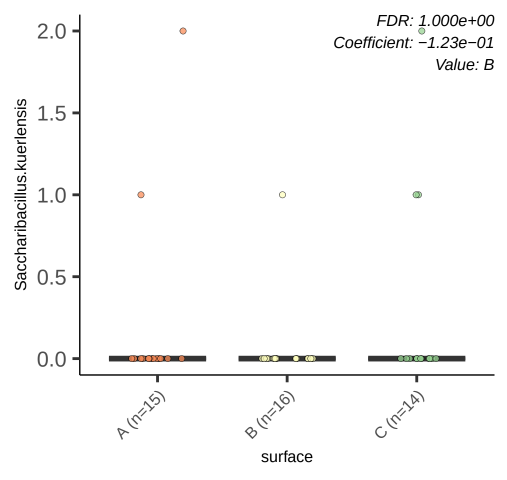
FDR: 1.000e+00
Coefficient: −1.23e−01
Value: B
Saccharibacillus.kuerlensis
2.0
1.5
1.0
0.5
0.0
A (n=15)
B (n=16)
C (n=14)
surface

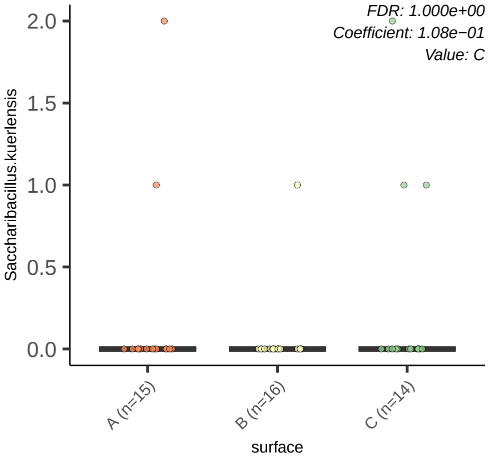

FDR: 1.000e+00
Coefficient: 1.08e−01
Value: C
Saccharibacillus.kuerlensis
2.0
1.5
1.0
0.5
0.0
A (n=15)
B (n=16)
C (n=14)
surface

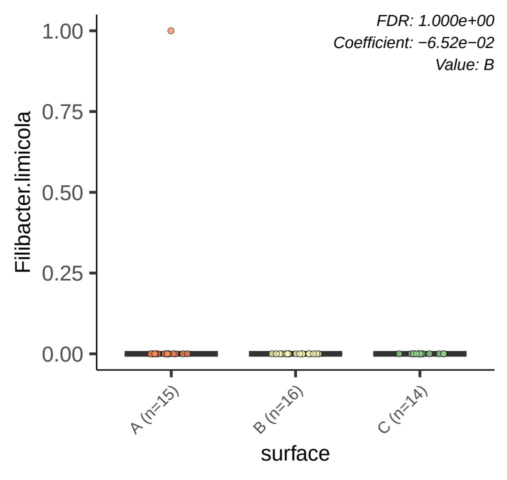
FDR: 1.000e+00
Coefficient: −6.52e−02
Value: B
Filibacter.limicola
1.00
0.75
0.50
0.25
0.00
A (n=15)
B (n=16)
C (n=14)
surface

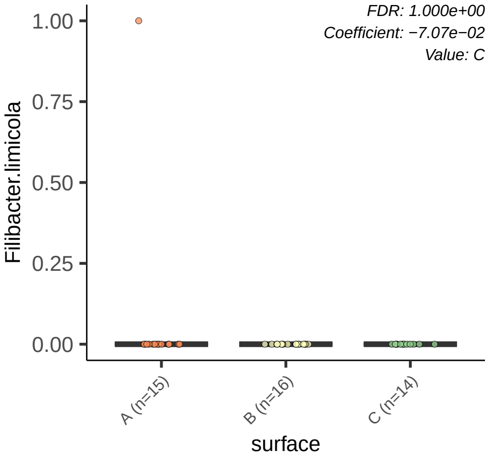
FDR: 1.000e+00
Coefficient: −7.07e−02
Value: C
Filibacter.limicola
1.00
0.75
0.50
0.25
0.00
A (n=15)
B (n=16)
C (n=14)
surface

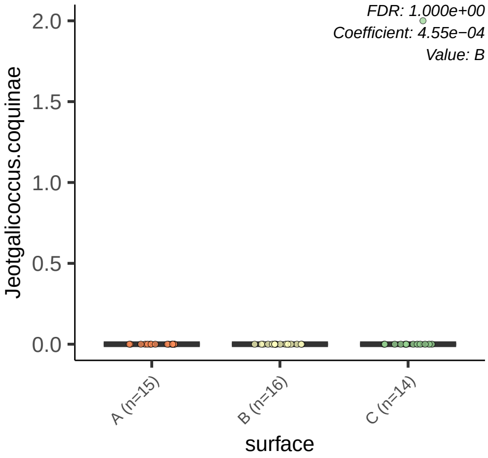

FDR: 1.000e+00
Coefficient: 4.55e−04
Value: B
Jeotgalicoccus.coquinae
2.0
1.5
1.0
0.5
0.0
A (n=15)
B (n=16)
C (n=14)
surface

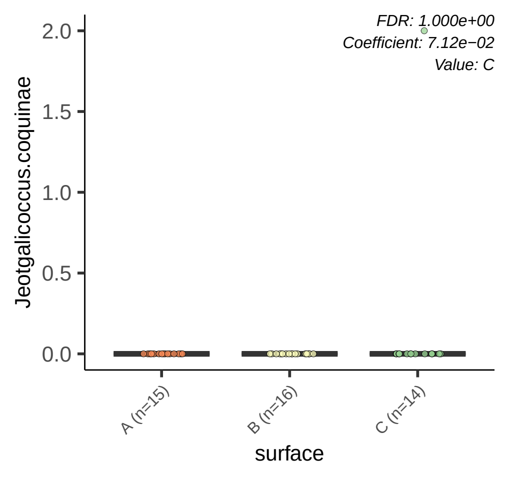
FDR: 1.000e+00
Coefficient: 7.12e−02
Value: C
Jeotgalicoccus.coquinae
2.0
1.5
1.0
0.5
0.0
A (n=15)
B (n=16)
C (n=14)
surface

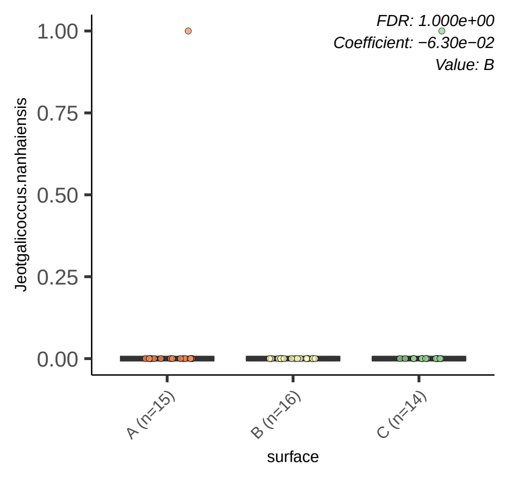

FDR: 1.000e+00
Coefficient: −6.30e−02
Value: B
Jeotgalicoccus.nanhaiensis
1.00
0.75
0.50
0.25
0.00
A (n=15)
B (n=16)
C (n=14)
surface

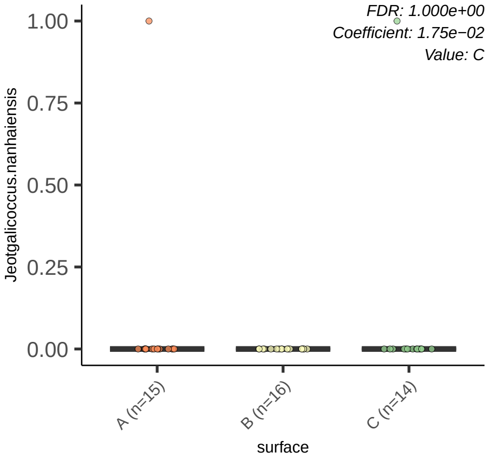

FDR: 1.000e+00
Coefficient: 1.75e−02
Value: C
Jeotgalicoccus.nanhaiensis
1.00
0.75
0.50
0.25
0.00
A (n=15)
B (n=16)
C (n=14)
surface

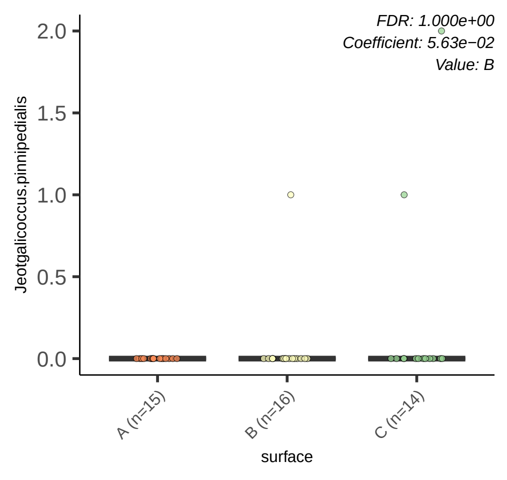

FDR: 1.000e+00
Coefficient: 5.63e−02
Value: B
Jeotgalicoccus.pinnipedialis
2.0
1.5
1.0
0.5
0.0
A (n=15)
B (n=16)
C (n=14)
surface

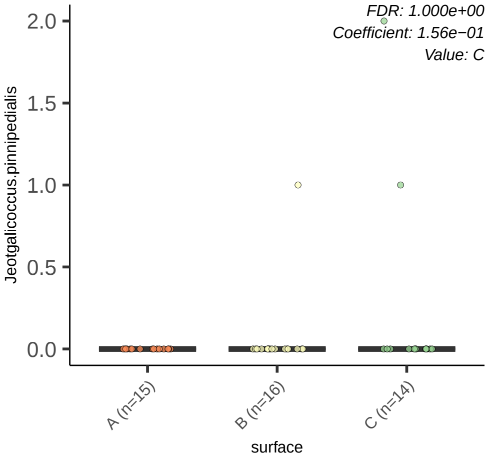
FDR: 1.000e+00
Coefficient: 1.56e−01
Value: C
Jeotgalicoccus.pinnipedialis
2.0
1.5
1.0
0.5
0.0
A (n=15)
B (n=16)
C (n=14)
surface

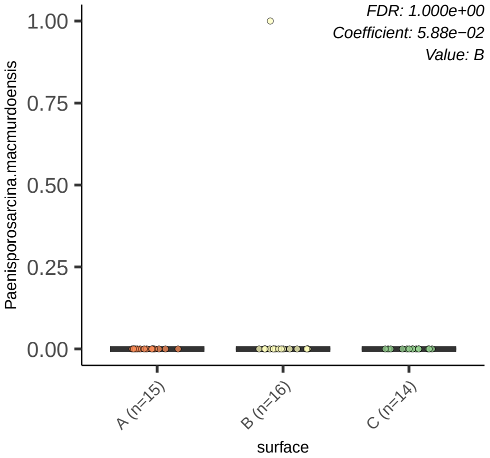

FDR: 1.000e+00
Coefficient: 5.88e−02
Value: B
Paenisporosarcina.macmurdoensis
1.00
0.75
0.50
0.25
0.00
A (n=15)
B (n=16)
C (n=14)
surface

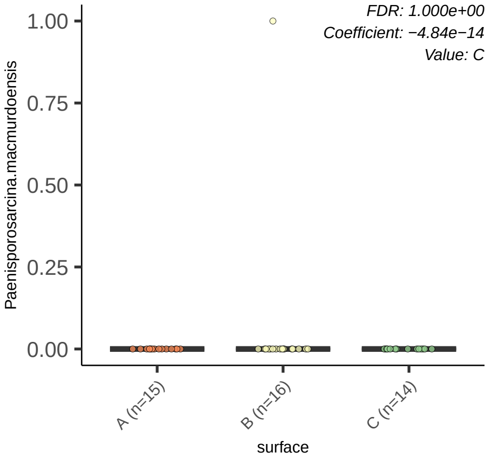
FDR: 1.000e+00
Coefficient: −4.84e−14
Value: C
Paenisporosarcina.macmurdoensis
1.00
0.75
0.50
0.25
0.00
A (n=15)
B (n=16)
C (n=14)
surface

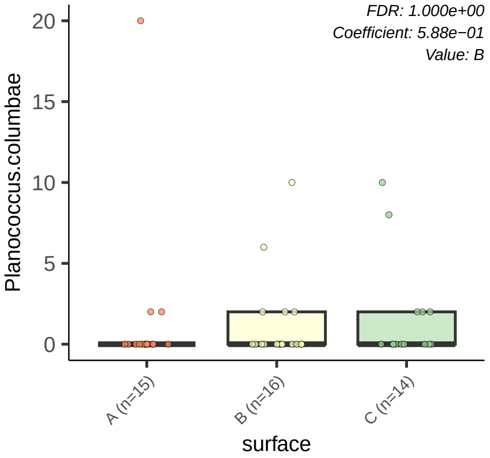

FDR: 1.000e+00
Coefficient: 5.88e−01
Value: B
Planococcus.columbae
20
15
10
5
0
A (n=15)
B (n=16)
C (n=14)
surface

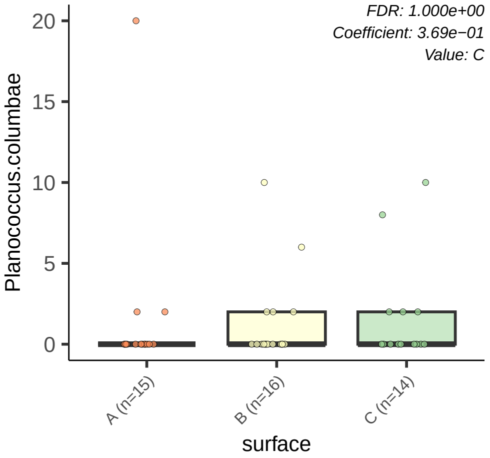
FDR: 1.000e+00
Coefficient: 3.69e−01
Value: C
Planococcus.columbae
0
5
10
15
20
A (n=15)
B (n=16)
C (n=14)
surface

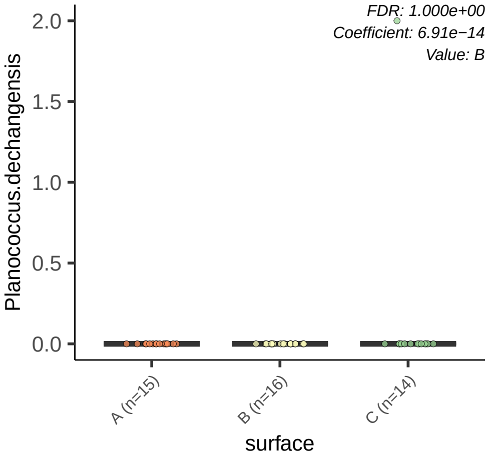
FDR: 1.000e+00
Coefficient: 6.91e−14
Value: B
Planococcus.dechangensis
2.0
1.5
1.0
0.5
0.0
A (n=15)
B (n=16)
C (n=14)
surface

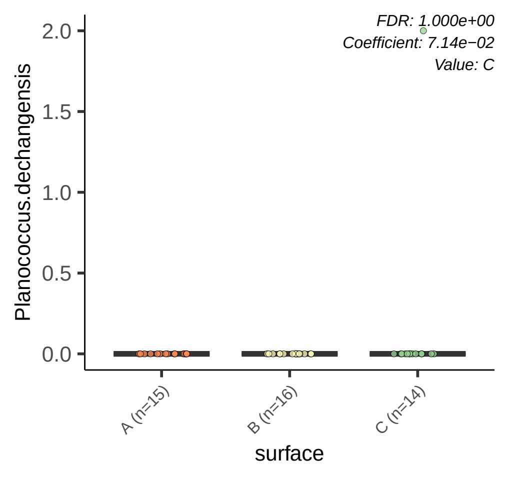
FDR: 1.000e+00
Coefficient: 7.14e−02
Value: C
Planococcus.dechangensis
2.0
1.5
1.0
0.5
0.0
A (n=15)
B (n=16)
C (n=14)
surface

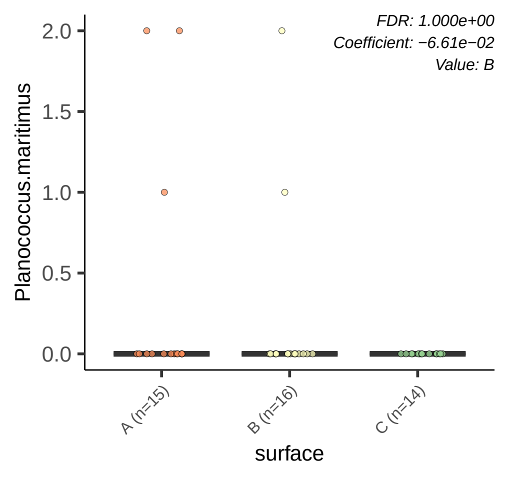
FDR: 1.000e+00
Coefficient: −6.61e−02
Value: B
Planococcus.maritimus
2.0
1.5
1.0
0.5
0.0
A (n=15)
B (n=16)
C (n=14)
surface

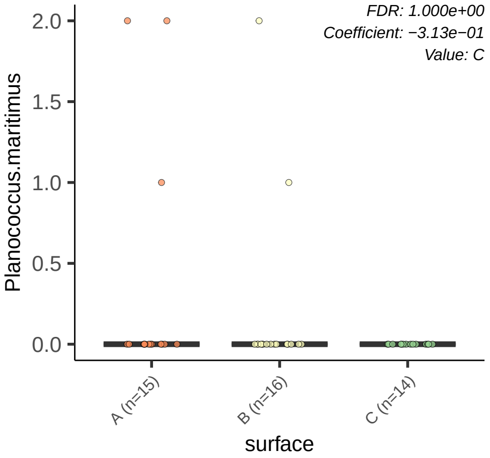

FDR: 1.000e+00
Coefficient: −3.13e−01
Value: C
Planococcus.maritimus
2.0
1.5
1.0
0.5
0.0
A (n=15)
B (n=16)
C (n=14)
surface

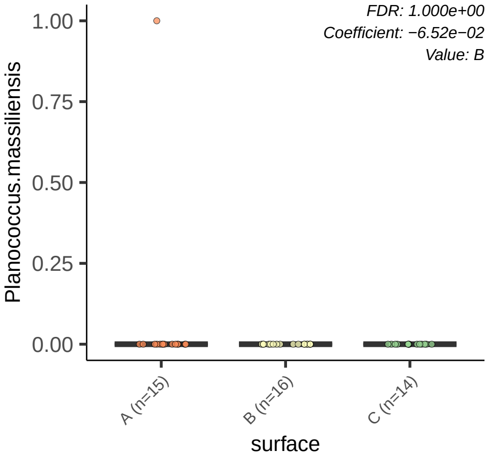
FDR: 1.000e+00
Coefficient: −6.52e−02
Value: B
Planococcus.massiliensis
1.00
0.75
0.50
0.25
0.00
A (n=15)
B (n=16)
C (n=14)
surface

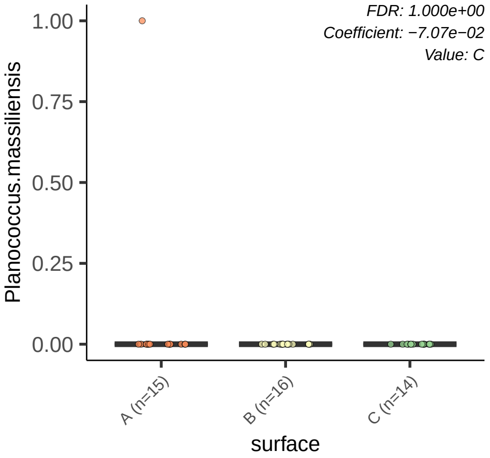
FDR: 1.000e+00
Coefficient: −7.07e−02
Value: C
Planococcus.massiliensis
1.00
0.75
0.50
0.25
0.00
A (n=15)
B (n=16)
C (n=14)
surface

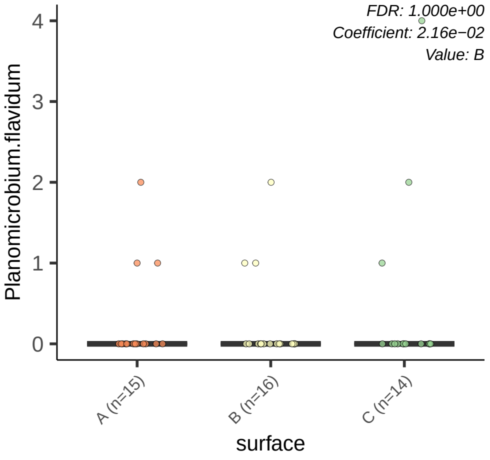
FDR: 1.000e+00
Coefficient: 2.16e−02
Value: B
Planomicrobium.flavidum
0
1
2
3
4
A (n=15)
B (n=16)
C (n=14)
surface

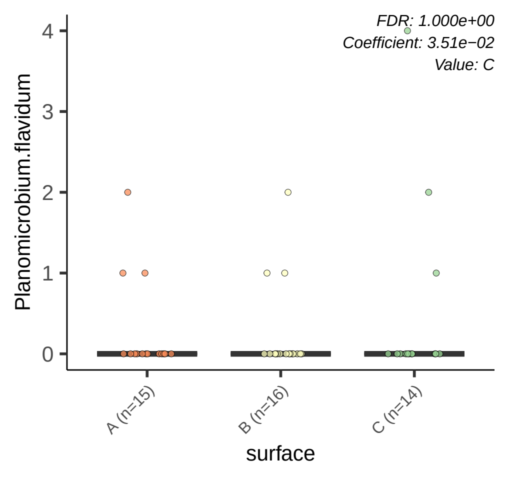
FDR: 1.000e+00
Coefficient: 3.51e−02
Value: C
Planomicrobium.flavidum
4
3
2
1
0
A (n=15)
B (n=16)
C (n=14)
surface

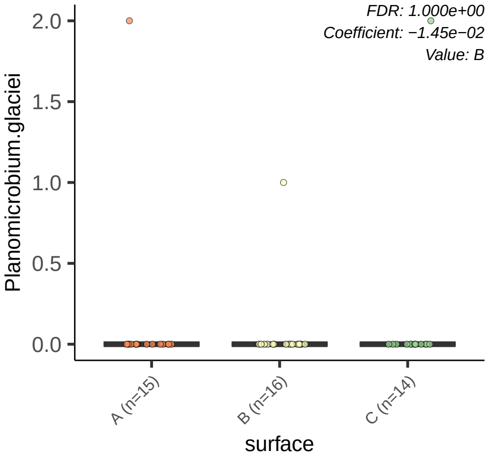

FDR: 1.000e+00
Coefficient: −1.45e−02
Value: B
Planomicrobium.glaciei
2.0
1.5
1.0
0.5
0.0
A (n=15)
B (n=16)
C (n=14)
surface

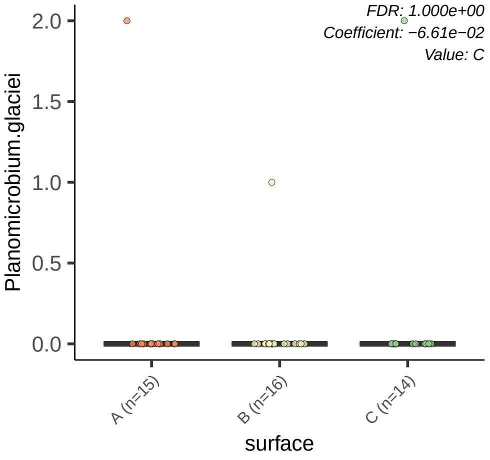

FDR: 1.000e+00
Coefficient: −6.61e−02
Value: C
Planomicrobium.glaciei
2.0
1.5
1.0
0.5
0.0
A (n=15)
B (n=16)
C (n=14)
surface

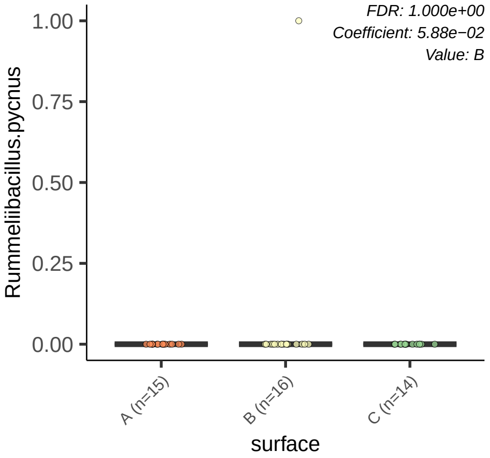

FDR: 1.000e+00
Coefficient: 5.88e−02
Value: B
Rummeliibacillus.pycnus
1.00
0.75
0.50
0.25
0.00
A (n=15)
B (n=16)
C (n=14)
surface

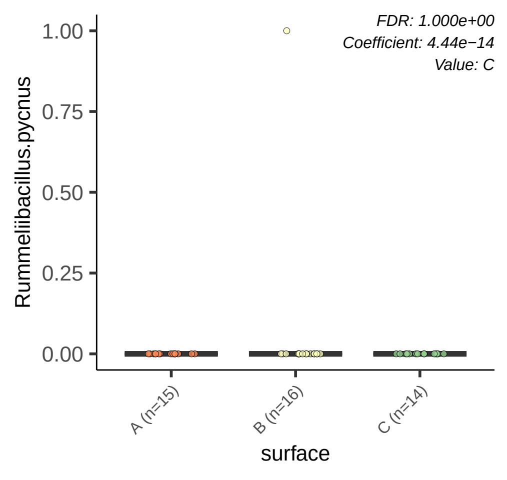

FDR: 1.000e+00
Coefficient: 4.44e−14
Value: C
Rummeliibacillus.pycnus
1.00
0.75
0.50
0.25
0.00
A (n=15)
B (n=16)
C (n=14)
surface

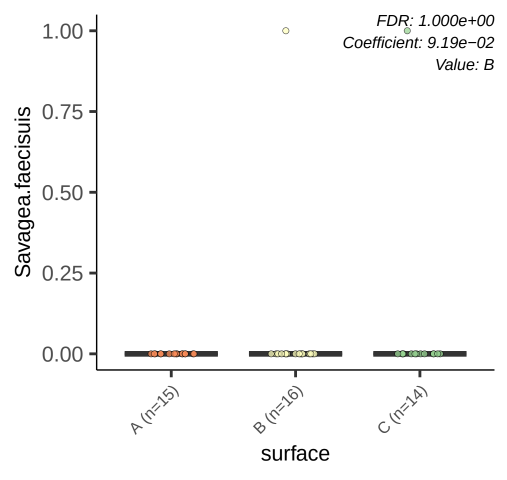
FDR: 1.000e+00
Coefficient: 9.19e−02
Value: B
Savagea.faecisuis
1.00
0.75
0.50
0.25
0.00
A (n=15)
B (n=16)
C (n=14)
surface

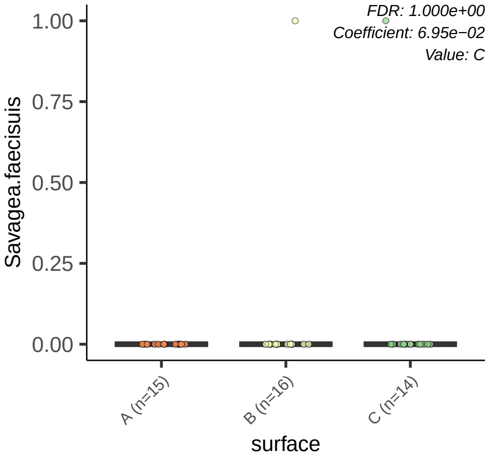

FDR: 1.000e+00
Coefficient: 6.95e−02
Value: C
Savagea.faecisuis
1.00
0.75
0.50
0.25
0.00
A (n=15)
B (n=16)
C (n=14)
surface

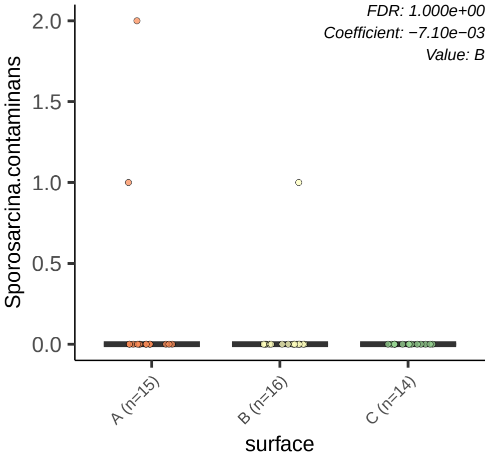
FDR: 1.000e+00
Coefficient: −7.10e−03
Value: B
Sporosarcina.contaminans
2.0
1.5
1.0
0.5
0.0
A (n=15)
B (n=16)
C (n=14)
surface

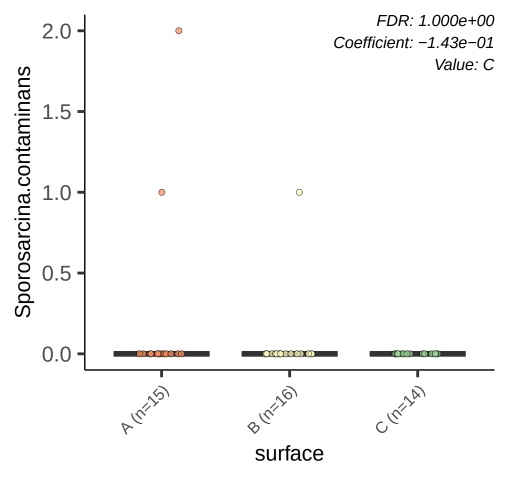
FDR: 1.000e+00
Coefficient: −1.43e−01
Value: C
Sporosarcina.contaminans
2.0
1.5
1.0
0.5
0.0
A (n=15)
B (n=16)
C (n=14)
surface

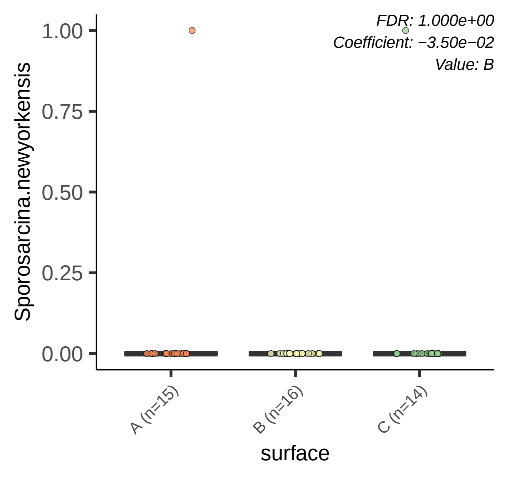
FDR: 1.000e+00
Coefficient: −3.50e−02
Value: B
Sporosarcina.newyorkensis
1.00
0.75
0.50
0.25
0.00
A (n=15)
B (n=16)
C (n=14)
surface

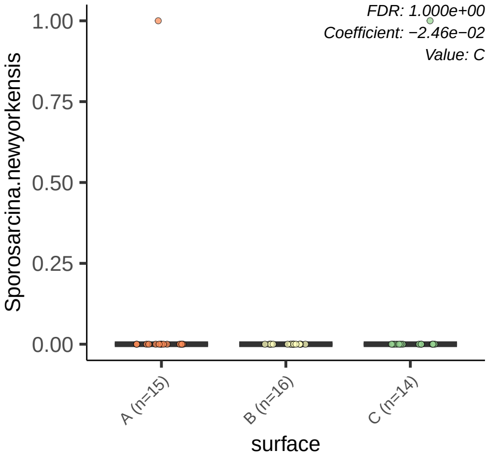

FDR: 1.000e+00
Coefficient: −2.46e−02
Value: C
Sporosarcina.newyorkensis
1.00
0.75
0.50
0.25
0.00
A (n=15)
B (n=16)
C (n=14)
surface

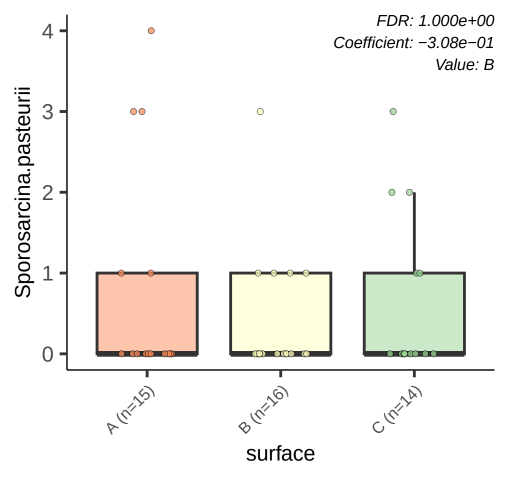

FDR: 1.000e+00
Coefficient: −3.08e−01
Value: B
Sporosarcina.pasteurii
4
3
2
1
0
A (n=15)
B (n=16)
C (n=14)
surface

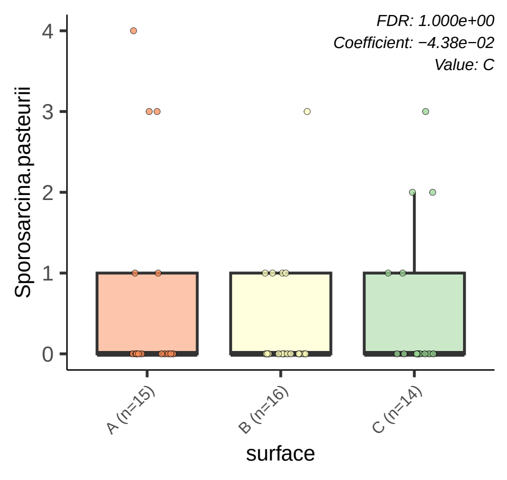

FDR: 1.000e+00
Coefficient: −4.38e−02
Value: C
Sporosarcina.pasteurii
0
1
2
3
4
A (n=15)
B (n=16)
C (n=14)
surface

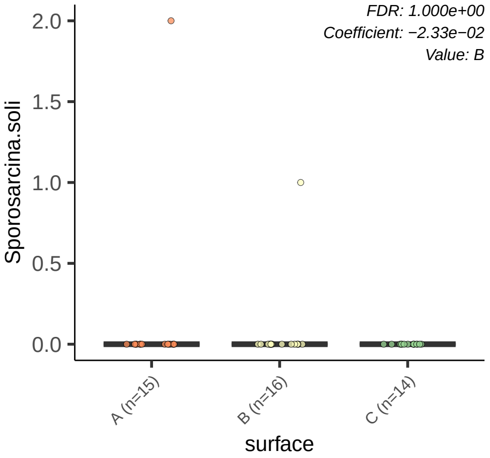
FDR: 1.000e+00
Coefficient: −2.33e−02
Value: B
Sporosarcina.soli
2.0
1.5
1.0
0.5
0.0
A (n=15)
B (n=16)
C (n=14)
surface

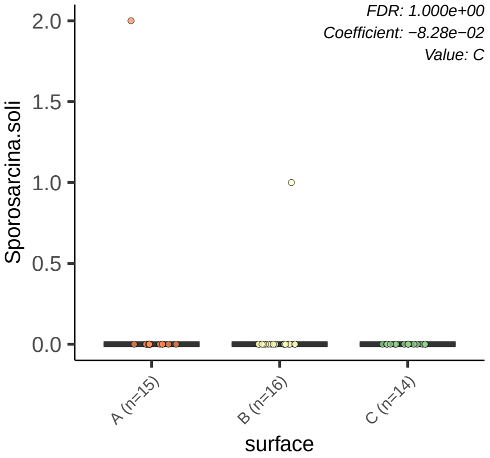

FDR: 1.000e+00
Coefficient: −8.28e−02
Value: C
Sporosarcina.soli
2.0
1.5
1.0
0.5
0.0
A (n=15)
B (n=16)
C (n=14)
surface

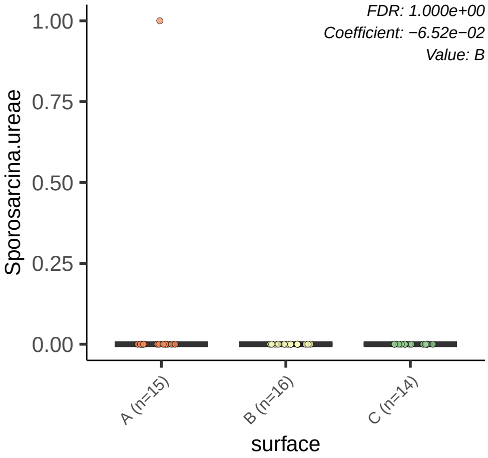
FDR: 1.000e+00
Coefficient: −6.52e−02
Value: B
Sporosarcina.ureae
1.00
0.75
0.50
0.25
0.00
A (n=15)
B (n=16)
C (n=14)
surface

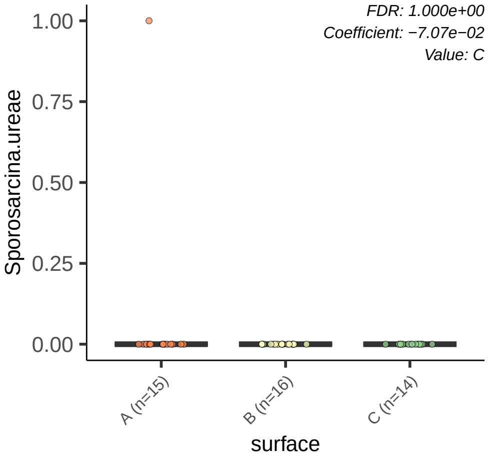
FDR: 1.000e+00
Coefficient: −7.07e−02
Value: C
1.00
0.75
0.50
0.25
0.00
Sporosarcina.ureae
A (n=15)
B (n=16)
C (n=14)
surface

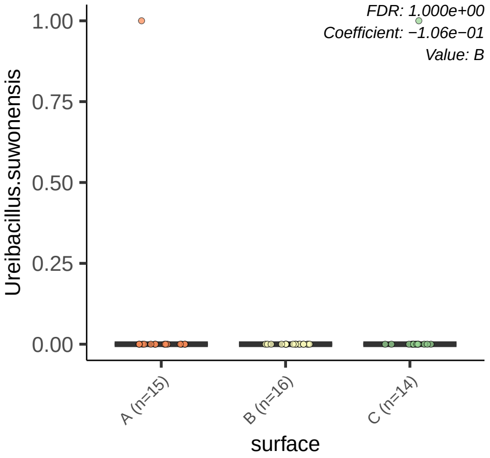
FDR: 1.000e+00
Coefficient: −1.06e−01
Value: B
Ureibacillus.suwonensis
1.00
0.75
0.50
0.25
0.00
A (n=15)
B (n=16)
C (n=14)
surface

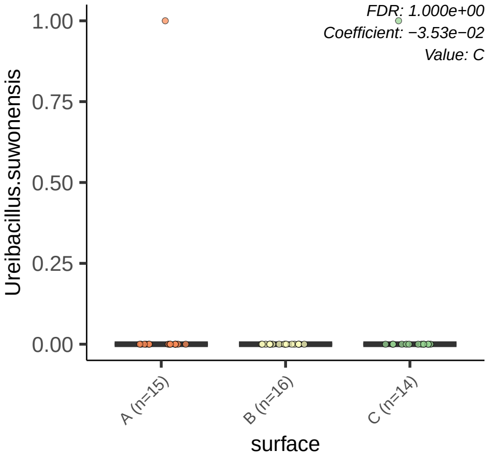

FDR: 1.000e+00
Coefficient: −3.53e−02
Value: C
Ureibacillus.suwonensis
1.00
0.75
0.50
0.25
0.00
A (n=15)
B (n=16)
C (n=14)
surface

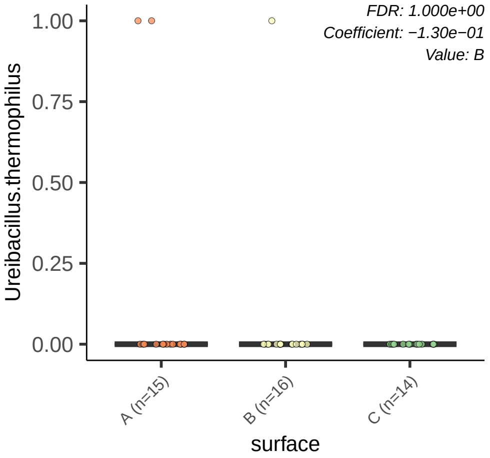

FDR: 1.000e+00
Coefficient: −1.30e−01
Value: B
Ureibacillus.thermophilus
1.00
0.75
0.50
0.25
0.00
A (n=15)
B (n=16)
C (n=14)
surface

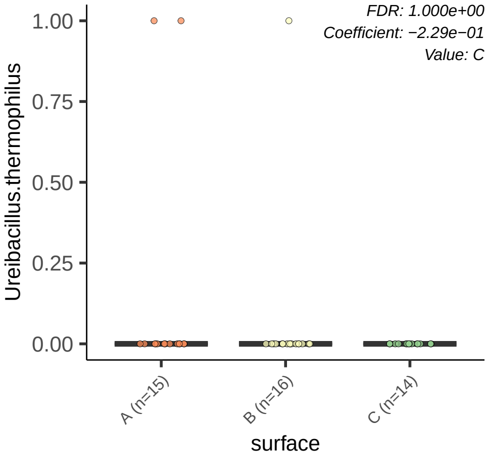
FDR: 1.000e+00
Coefficient: −2.29e−01
Value: C
Ureibacillus.thermophilus
1.00
0.75
0.50
0.25
0.00
A (n=15)
B (n=16)
C (n=14)
surface

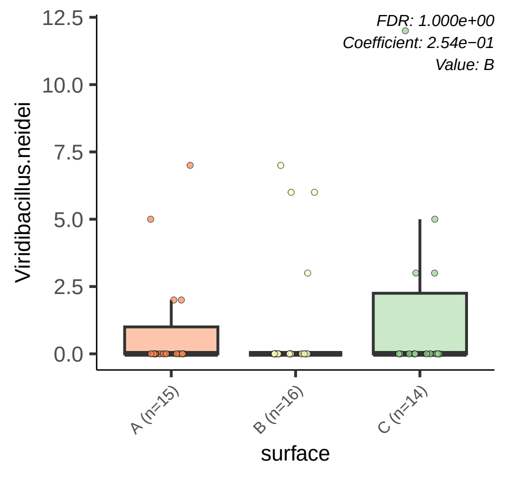

FDR: 1.000e+00
Coefficient: 2.54e−01
Value: B
Viridibacillus.neidei
12.5
10.0
7.5
5.0
2.5
0.0
A (n=15)
B (n=16)
C (n=14)
surface

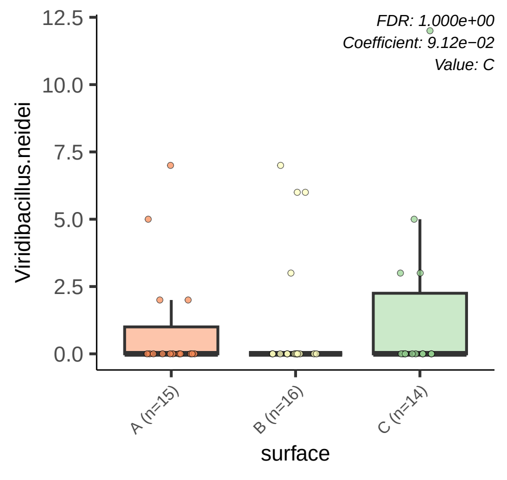

FDR: 1.000e+00
Coefficient: 9.12e−02
Value: C
Viridibacillus.neidei
12.5
10.0
7.5
5.0
2.5
0.0
A (n=15)
B (n=16)
C (n=14)
surface

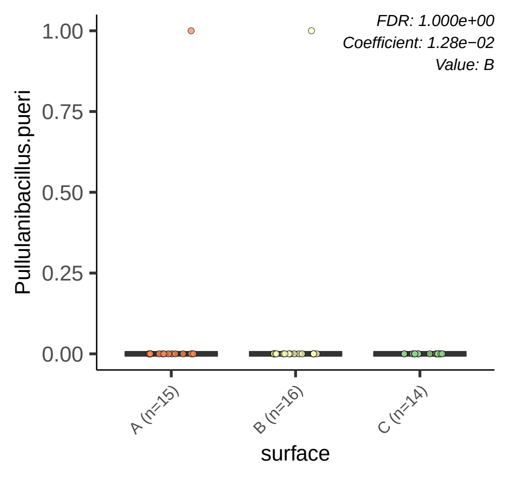

FDR: 1.000e+00
Coefficient: 1.28e−02
Value: B
Pullulanibacillus.pueri
1.00
0.75
0.50
0.25
0.00
A (n=15)
B (n=16)
C (n=14)
surface

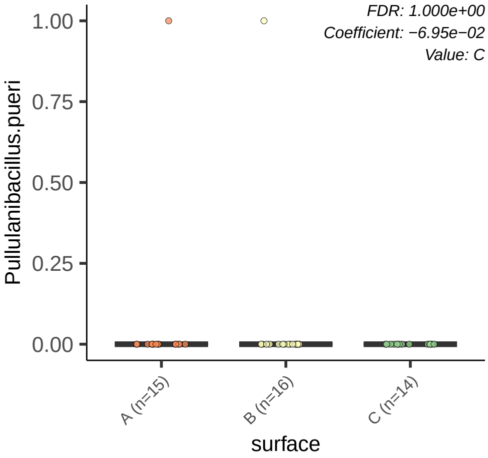

FDR: 1.000e+00
Coefficient: −6.95e−02
Value: C
Pullulanibacillus.pueri
1.00
0.75
0.50
0.25
0.00
A (n=15)
B (n=16)
C (n=14)
surface

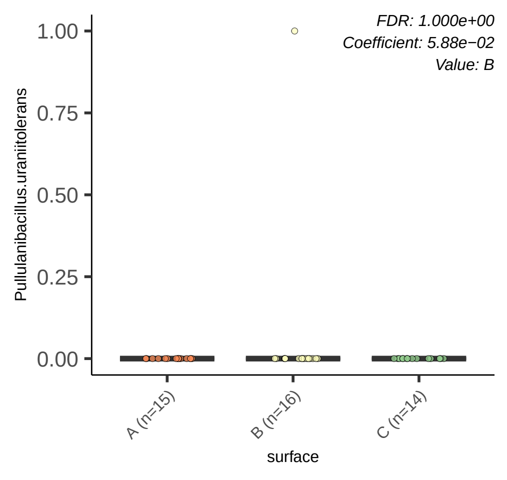

FDR: 1.000e+00
Coefficient: 5.88e−02
Value: B
Pullulanibacillus.uraniitolerans
1.00
0.75
0.50
0.25
0.00
A (n=15)
B (n=16)
C (n=14)
surface

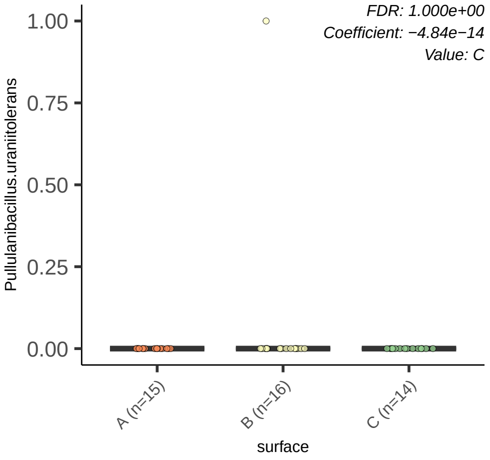
FDR: 1.000e+00
Coefficient: −4.84e−14
Value: C
Pullulanibacillus.uranitolerans
1.00
0.75
0.50
0.25
0.00
A (n=15)
B (n=16)
C (n=14)
surface

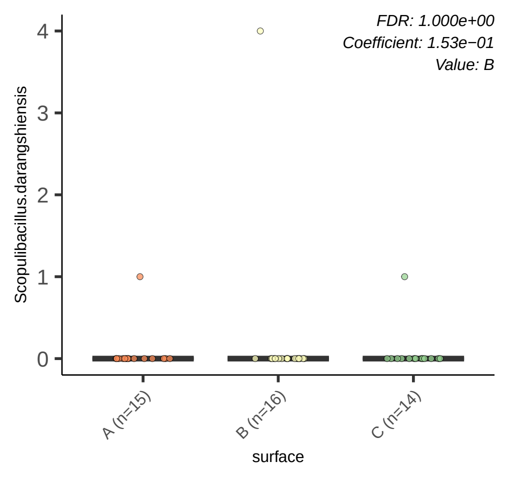
FDR: 1.000e+00
Coefficient: 1.53e−01
Value: B
Scopulibacillus.darangshiensis
0
1
2
3
4
A (n=15)
B (n=16)
C (n=14)
surface

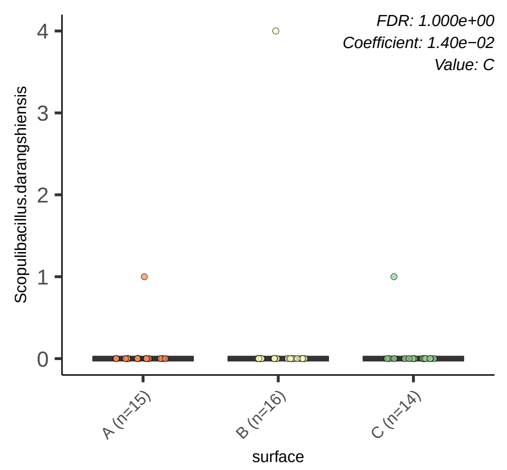

FDR: 1.000e+00
Coefficient: 1.40e−02
Value: C
Scopulibacillus.darangshiensis
4
3
2
1
0
A (n=15)
B (n=16)
C (n=14)
surface

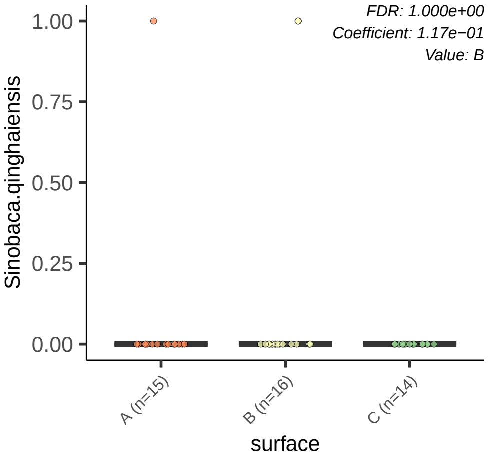

FDR: 1.000e+00
Coefficient: 1.17e−01
Value: B
Sinobaca.qinghaiensis
1.00
0.75
0.50
0.25
0.00
A (n=15)
B (n=16)
C (n=14)
surface

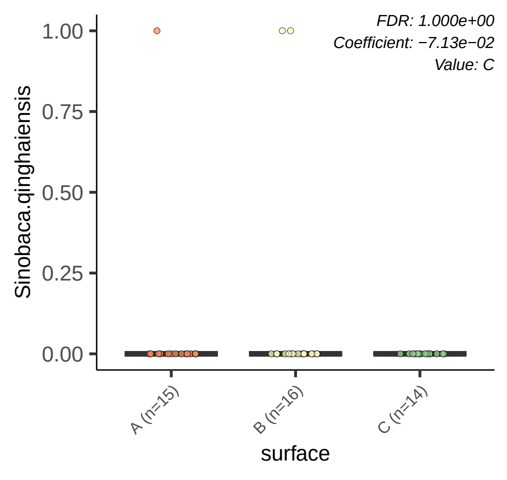

FDR: 1.000e+00
Coefficient: −7.13e−02
Value: C
Sinobaca.qinghaiensis
1.00
0.75
0.50
0.25
0.00
A (n=15)
B (n=16)
C (n=14)
surface

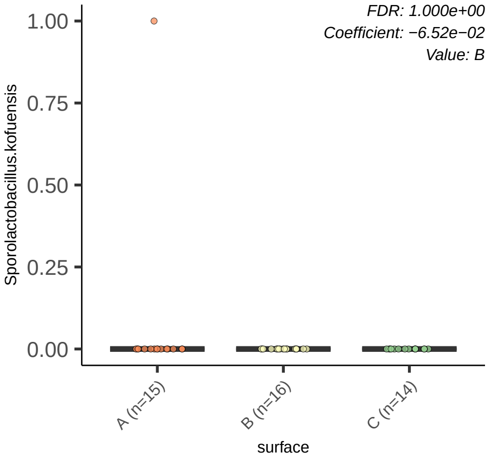

FDR: 1.000e+00
Coefficient: −6.52e−02
Value: B
Sporolactobacillus.kofuensis
1.00
0.75
0.50
0.25
0.00
A (n=15)
B (n=16)
C (n=14)
surface

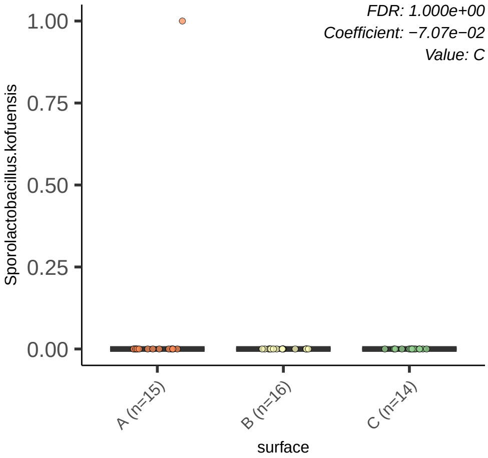

FDR: 1.000e+00
Coefficient: −7.07e−02
Value: C
1.00
0.75
0.50
0.25
0.00
Sporolactobacillus.kofuensis
A (n=15)
B (n=16)
C (n=14)
surface

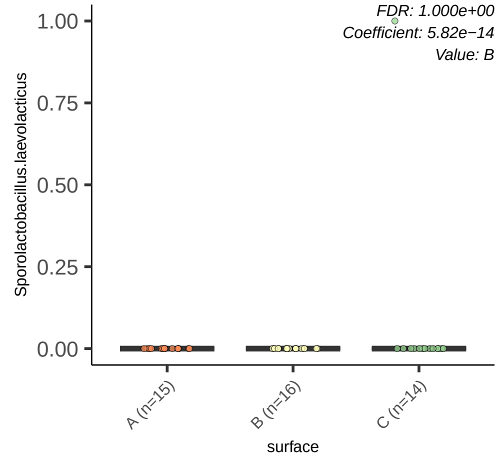
FDR: 1.000e+00
Coefficient: 5.82e−14
Value: B
Sporolactobacillus.laevolacticus
1.00
0.75
0.50
0.25
0.00
A (n=15)
B (n=16)
C (n=14)
surface

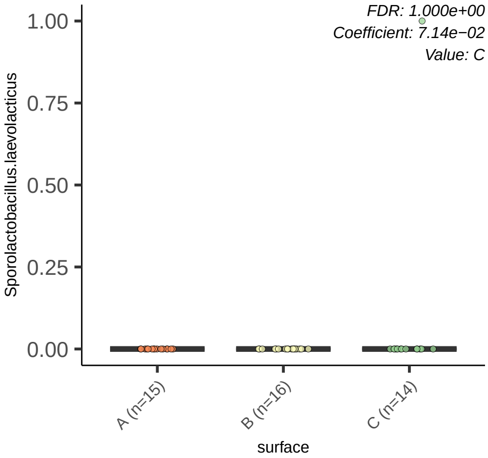
FDR: 1.000e+00
Coefficient: 7.14e−02
Value: C
Sporolactobacillus.laevolacticus
1.00
0.75
0.50
0.25
0.00
A (n=15)
B (n=16)
C (n=14)
surface

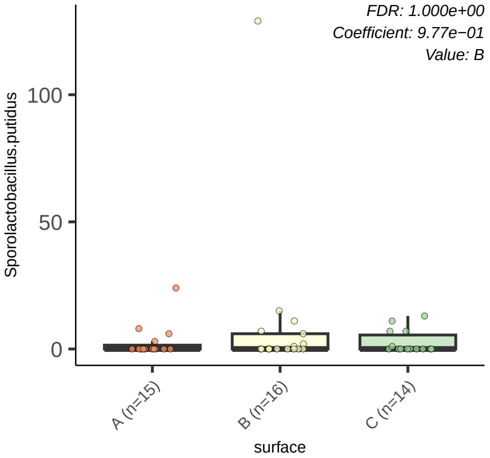
FDR: 1.000e+00
Coefficient: 9.77e−01
Value: B
Sporolactobacillus.putidus
100
50
0
A (n=15)
B (n=16)
C (n=14)
surface

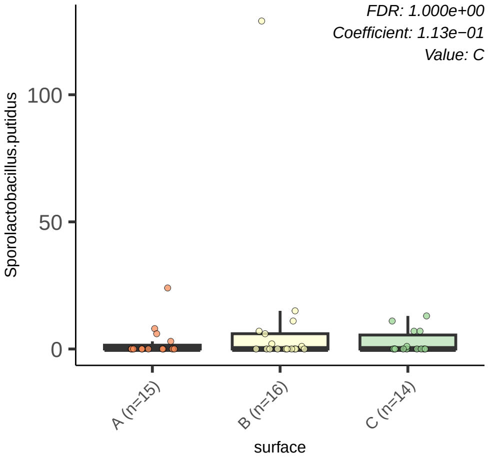
FDR: 1.000e+00
Coefficient: 1.13e−01
Value: C
Sporolactobacillus.putidus
100
50
0
A (n=15)
B (n=16)
C (n=14)
surface

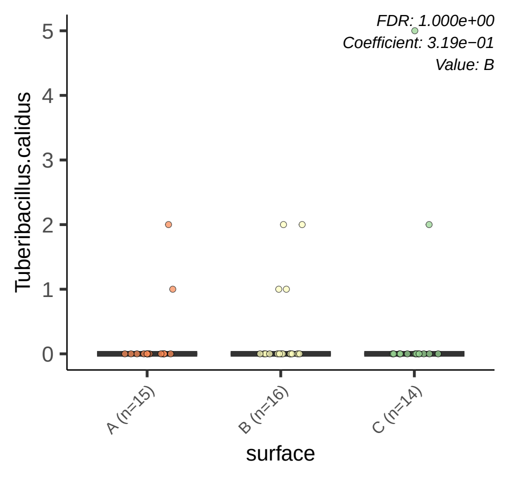

FDR: 1.000e+00
Coefficient: 3.19e−01
Value: B
Tuberibacillus.calidus
0
1
2
3
4
5
A (n=15)
B (n=16)
C (n=14)
surface

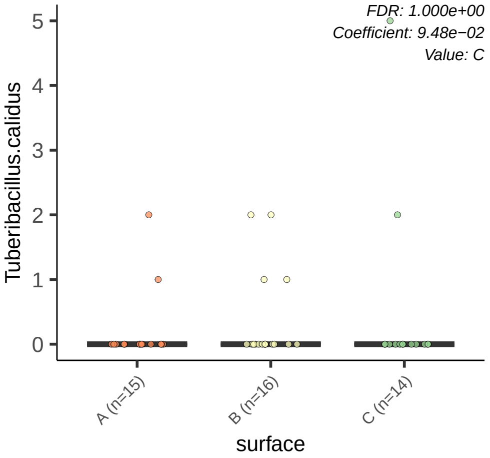

FDR: 1.000e+00
Coefficient: 9.48e−02
Value: C
Tuberibacillus.calidus
0
1
2
3
4
5
A (n=15)
B (n=16)
C (n=14)
surface

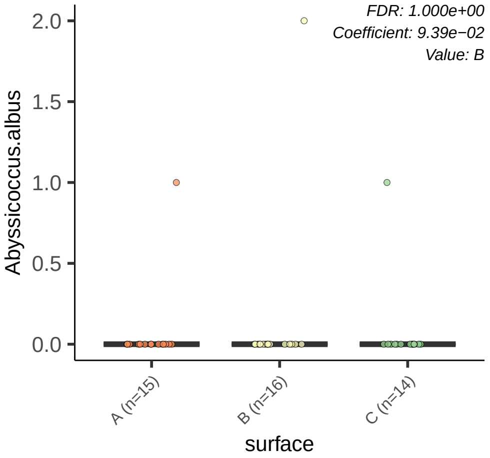
FDR: 1.000e+00
Coefficient: 9.39e−02
Value: B
Abyssicoccus.albus
2.0
1.5
1.0
0.5
0.0
A (n=15)
B (n=16)
C (n=14)
surface

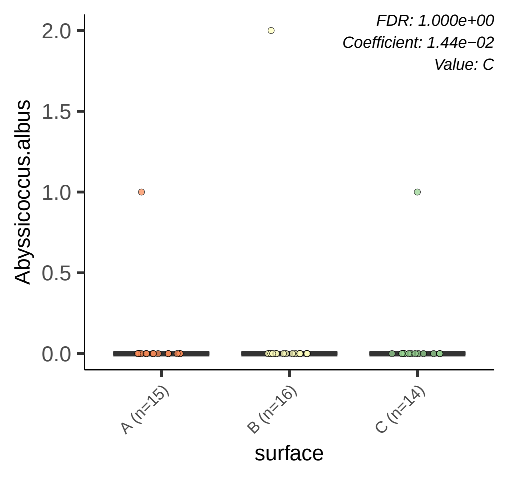

FDR: 1.000e+00
Coefficient: 1.44e−02
Value: C
Abyssicoccus.albus
2.0
1.5
1.0
0.5
0.0
A (n=15)
B (n=16)
C (n=14)
surface

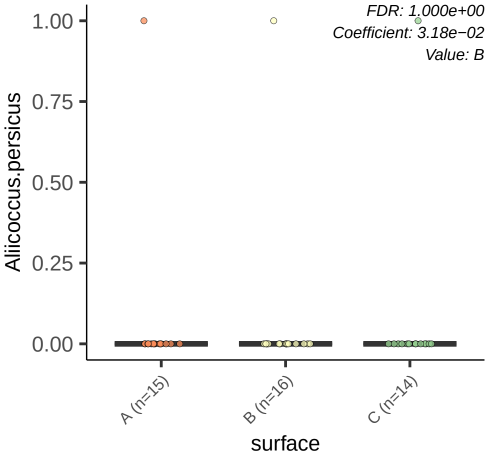

FDR: 1.000e+00
Coefficient: 3.18e−02
Value: B
1.00
0.75
0.50
0.25
0.00
Aliicoccus.persicus
A (n=15)
B (n=16)
C (n=14)
surface

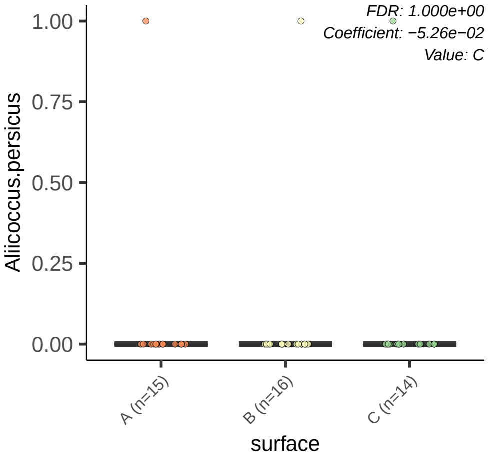

FDR: 1.000e+00
Coefficient: −5.26e−02
Value: C
Aliicoccus.persicus
1.00
0.75
0.50
0.25
0.00
A (n=15)
B (n=16)
C (n=14)
surface

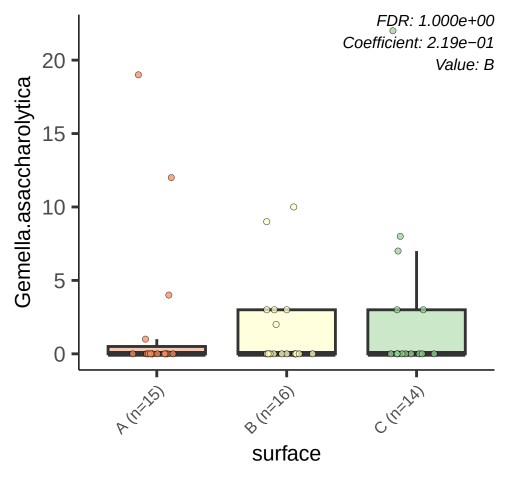

FDR: 1.000e+00
Coefficient: 2.19e−01
Value: B
Gemella.asaccharolytica
20
15
10
5
0
A (n=15)
B (n=16)
C (n=14)
surface

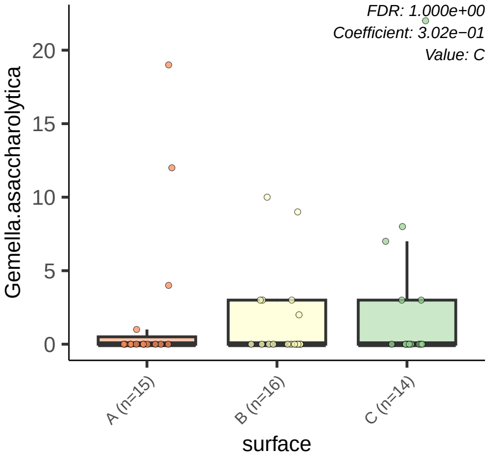
FDR: 1.000e+00
Coefficient: 3.02e−01
Value: C
Gemella.asaccharolytica
0
5
10
15
20
A (n=15)
B (n=16)
C (n=14)
surface

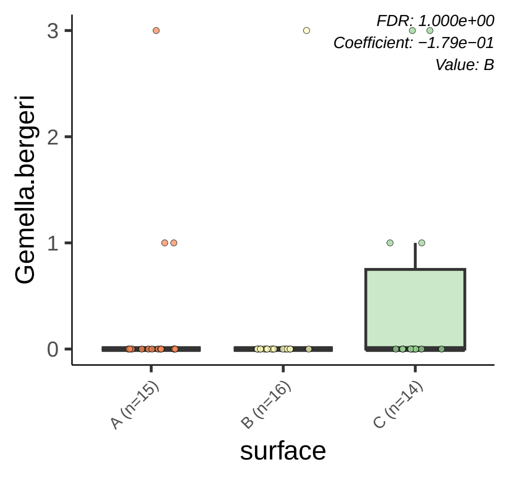

FDR: 1.000e+00
Coefficient: −1.79e−01
Value: B
Gemella.bergeri
3
2
1
0
A (n=15)
B (n=16)
C (n=14)
surface

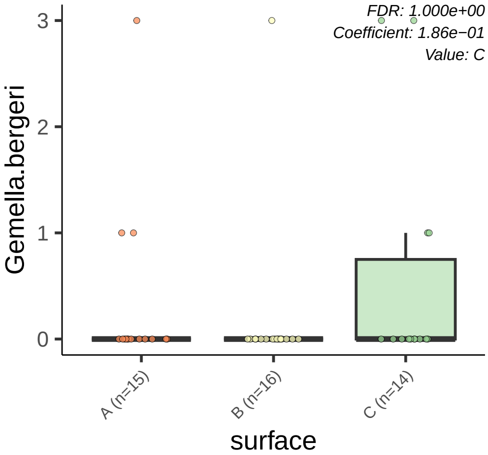

FDR: 1.000e+00
Coefficient: 1.86e−01
Value: C
Gemella.bergeri
0
1
2
3
A (n=15)
B (n=16)
C (n=14)
surface

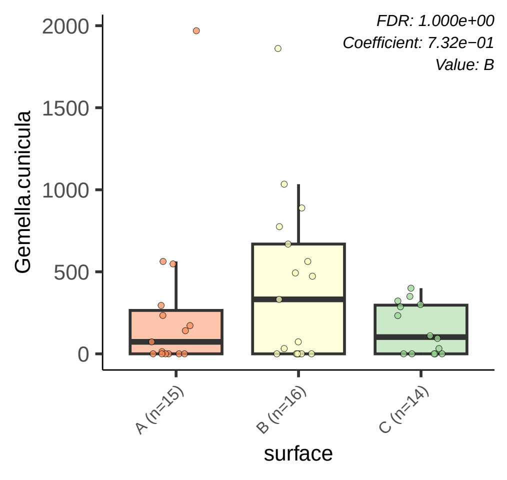

FDR: 1.000e+00
Coefficient: 7.32e−01
Value: B
Gemella.cunicula
2000
1500
1000
500
0
A (n=15)
B (n=16)
C (n=14)
surface

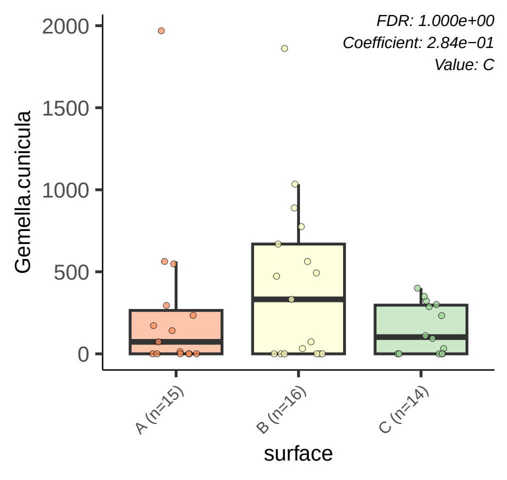

FDR: 1.000e+00
Coefficient: 2.84e−01
Value: C
2000
1500
1000
500
0
Gemella.cunicula
A (n=15)
B (n=16)
C (n=14)
surface

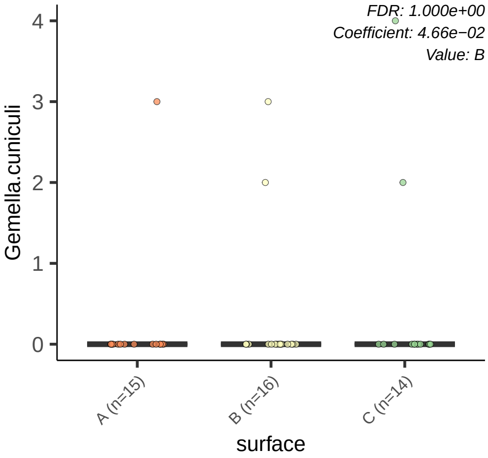
FDR: 1.000e+00
Coefficient: 4.66e−02
Value: B
Gemella.cuniculi
0
1
2
3
4
A (n=15)
B (n=16)
C (n=14)
surface

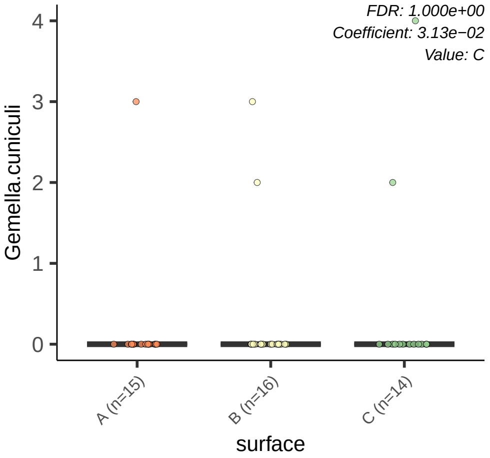

FDR: 1.000e+00
Coefficient: 3.13e−02
Value: C
Gemella.cuniculi
0
1
2
3
4
A (n=15)
B (n=16)
C (n=14)
surface

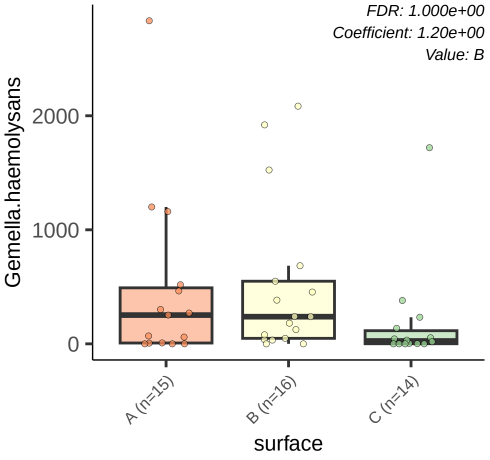

FDR: 1.000e+00
Coefficient: 1.20e+00
Value: B
Gemella.haemolysans
2000
1000
0
A (n=15)
B (n=16)
C (n=14)
surface

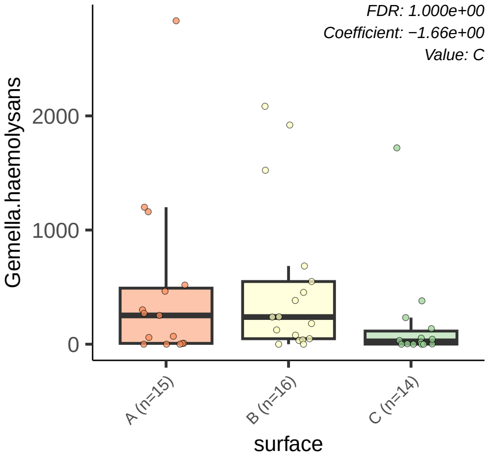
FDR: 1.000e+00
Coefficient: −1.66e+00
Value: C
Gemella.haemolysans
0
1000
2000
A (n=15)
B (n=16)
C (n=14)
surface

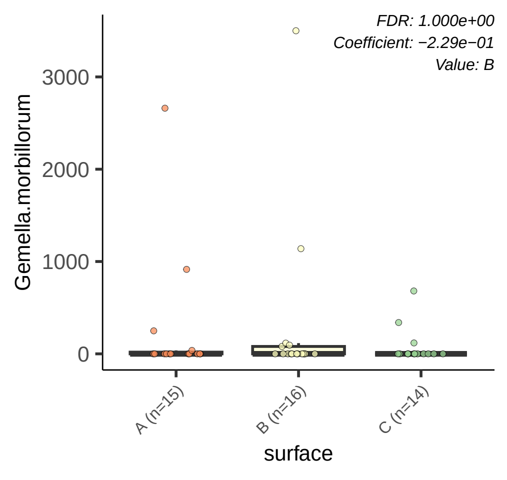
FDR: 1.000e+00
Coefficient: −2.29e−01
Value: B
Gemella.morbillorum
3000
2000
1000
0
A (n=15)
B (n=16)
C (n=14)
surface

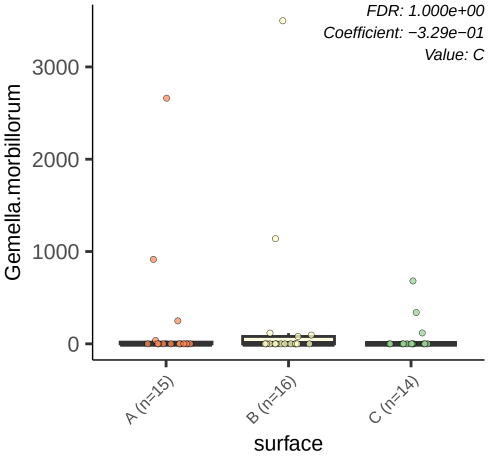

FDR: 1.000e+00
Coefficient: −3.29e−01
Value: C
Gemella.morbillorum
0
1000
2000
3000
A (n=15)
B (n=16)
C (n=14)
surface

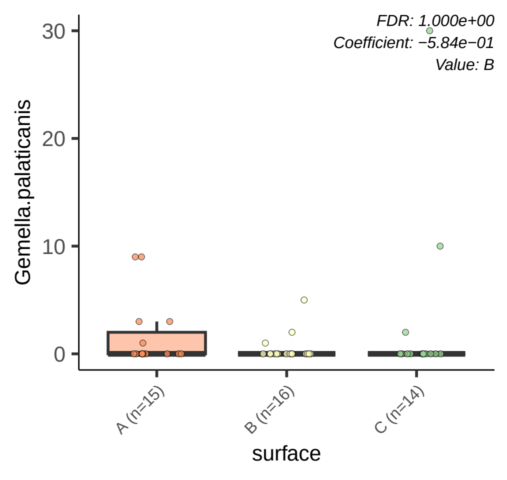
FDR: 1.000e+00
Coefficient: −5.84e−01
Value: B
Gemella.palaticanis
30
20
10
0
A (n=15)
B (n=16)
C (n=14)
surface

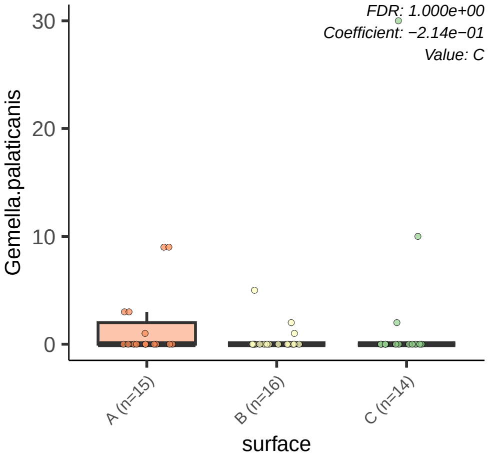

FDR: 1.000e+00
Coefficient: −2.14e−01
Value: C
Gemella.palaticanis
30
20
10
0
A (n=15)
B (n=16)
C (n=14)
surface

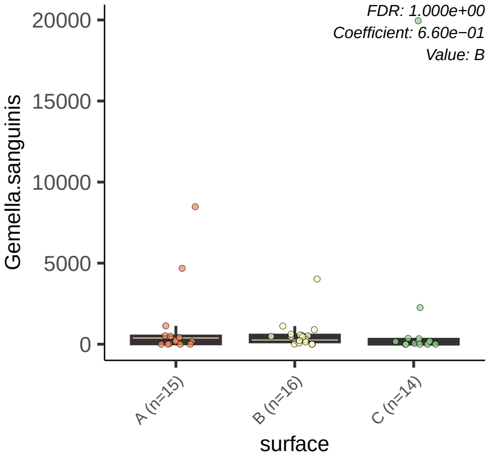
FDR: 1.000e+00
Coefficient: 6.60e−01
Value: B
Gemella.sanguinis
20000
15000
10000
5000
0
A (n=15)
B (n=16)
C (n=14)
surface

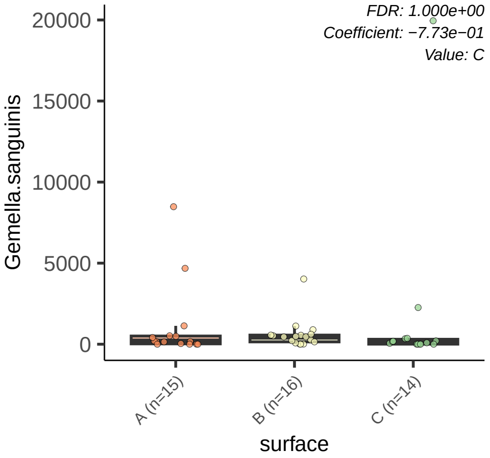
FDR: 1.000e+00
Coefficient: −7.73e−01
Value: C
Gemella.sanguinis
20000
15000
10000
5000
0
A (n=15)
B (n=16)
C (n=14)
surface

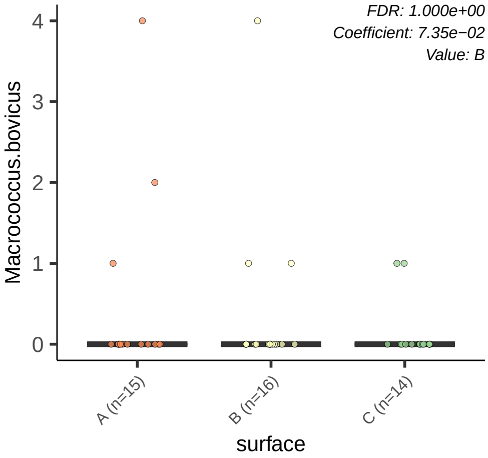

FDR: 1.000e+00
Coefficient: 7.35e−02
Value: B
Macrococcus.bovicus
4
3
2
1
0
A (n=15)
B (n=16)
C (n=14)
surface

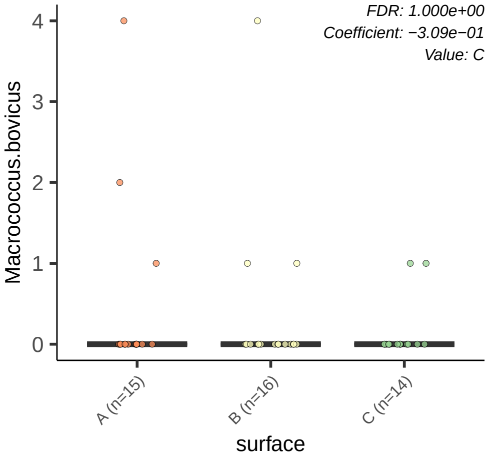

FDR: 1.000e+00
Coefficient: −3.09e−01
Value: C
Macrococcus.bovicus
0
1
2
3
4
A (n=15)
B (n=16)
C (n=14)
surface

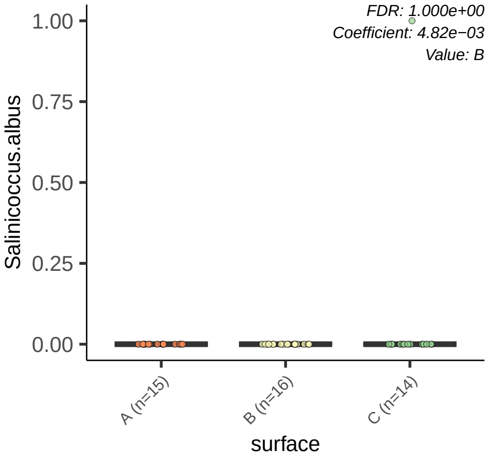
FDR: 1.000e+00
Coefficient: 4.82e−03
Value: B
Salinicoccus.albus
1.00
0.75
0.50
0.25
0.00
A (n=15)
B (n=16)
C (n=14)
surface

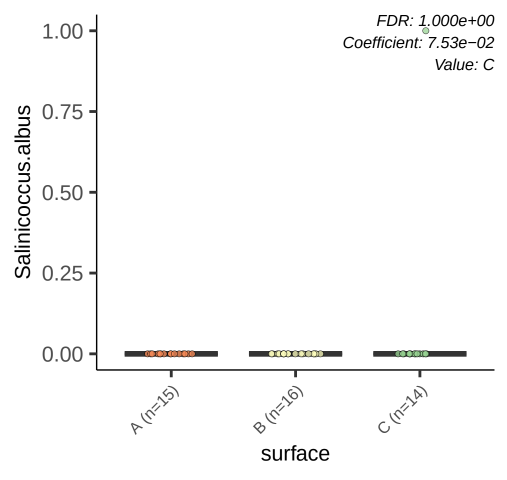

FDR: 1.000e+00
Coefficient: 7.53e−02
Value: C
Salinicoccus.albus
1.00
0.75
0.50
0.25
0.00
A (n=15)
B (n=16)
C (n=14)
surface

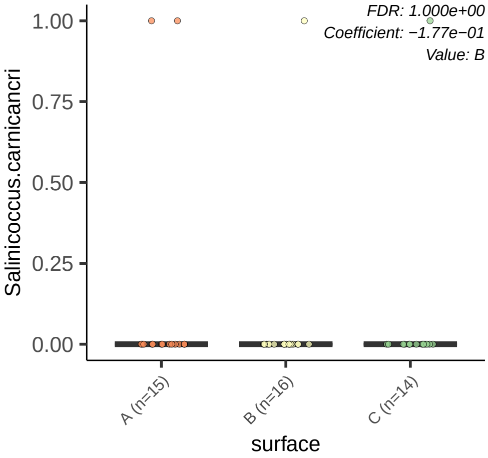
FDR: 1.000e+00
Coefficient: −1.77e−01
Value: B
Salinicoccus.carnicancri
1.00
0.75
0.50
0.25
0.00
A (n=15)
B (n=16)
C (n=14)
surface

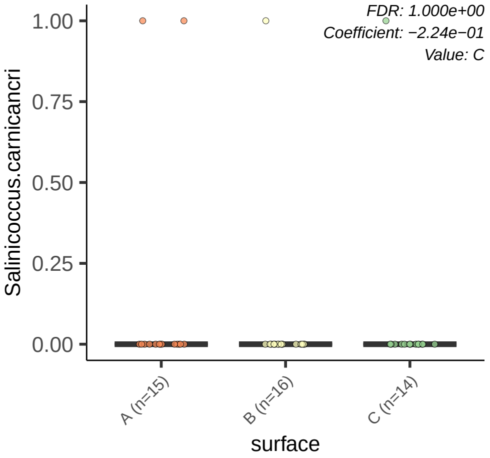
FDR: 1.000e+00
Coefficient: −2.24e−01
Value: C
1.00
0.75
0.50
0.25
0.00
Salinicoccus.carnicancri
A (n=15)
B (n=16)
C (n=14)
surface

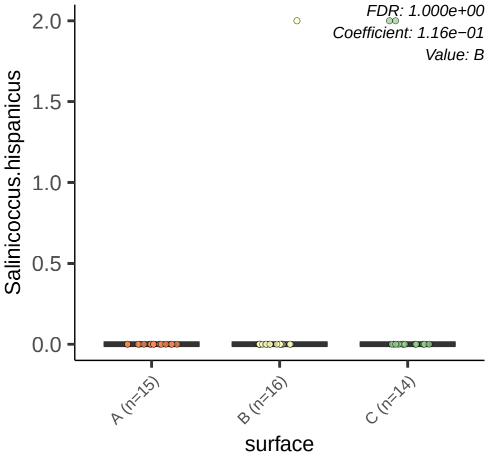
FDR: 1.000e+00
Coefficient: 1.16e−01
Value: B
Salinicoccus.hispanicus
2.0
1.5
1.0
0.5
0.0
A (n=15)
B (n=16)
C (n=14)
surface

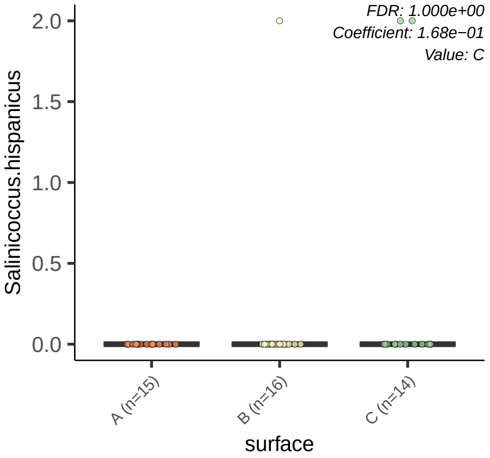

FDR: 1.000e+00
Coefficient: 1.68e−01
Value: C
Salinicoccus.hispanicus
2.0
1.5
1.0
0.5
0.0
A (n=15)
B (n=16)
C (n=14)
surface

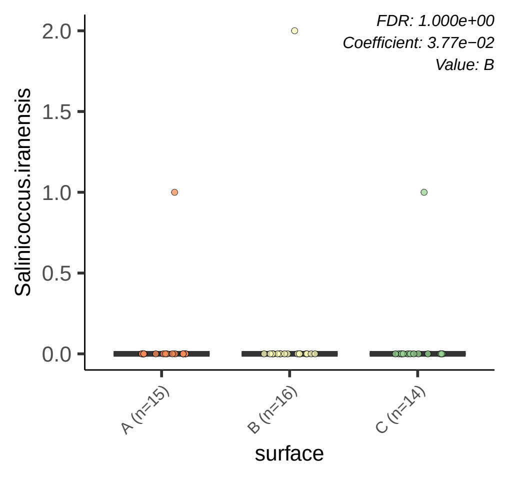

FDR: 1.000e+00
Coefficient: 3.77e−02
Value: B
Salinicoccus.iranensis
2.0
1.5
1.0
0.5
0.0
A (n=15)
B (n=16)
C (n=14)
surface

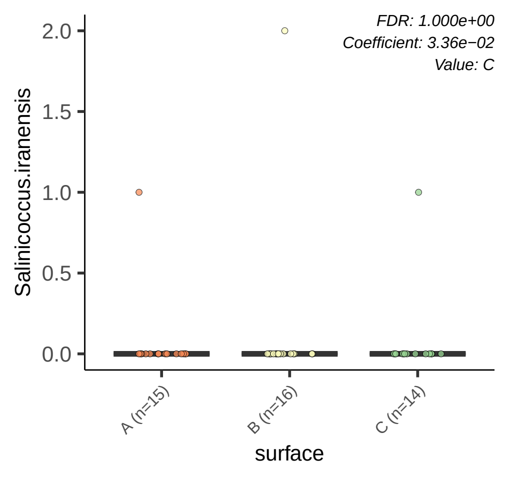

FDR: 1.000e+00
Coefficient: 3.36e−02
Value: C
Salinicoccus.iranensis
2.0
1.5
1.0
0.5
0.0
A (n=15)
B (n=16)
C (n=14)
surface

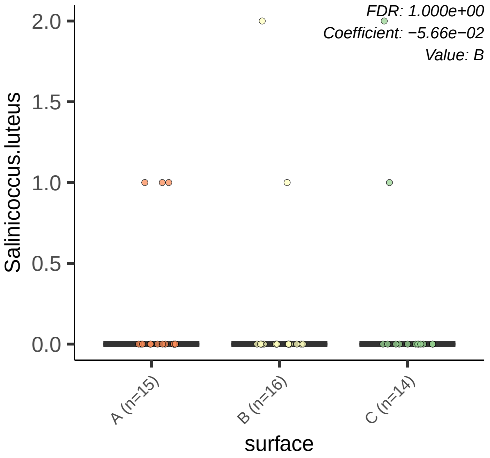
FDR: 1.000e+00
Coefficient: −5.66e−02
Value: B
Salinicoccus.luteus
2.0
1.5
1.0
0.5
0.0
A (n=15)
B (n=16)
C (n=14)
surface

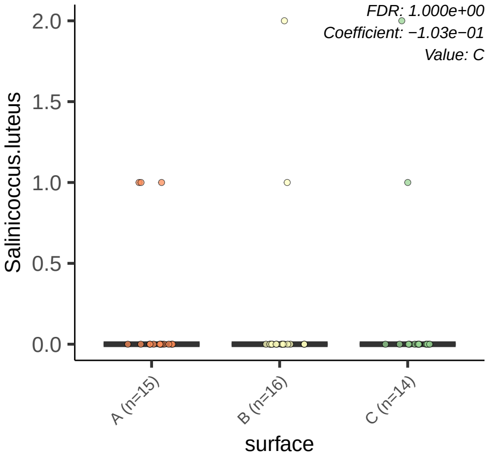

FDR: 1.000e+00
Coefficient: −1.03e−01
Value: C
Salinicoccus.luteus
2.0
1.5
1.0
0.5
0.0
A (n=15)
B (n=16)
C (n=14)
surface

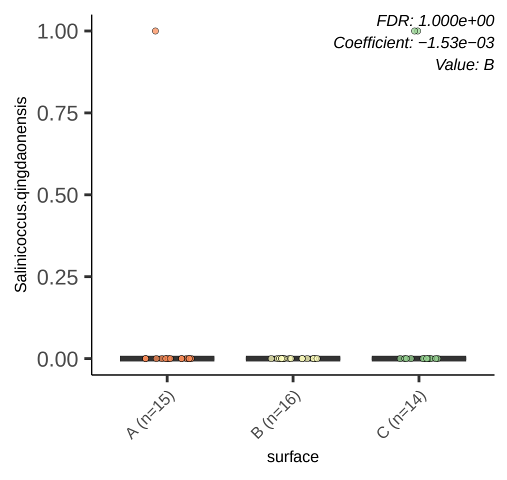
FDR: 1.000e+00
Coefficient: −1.53e−03
Value: B
Salinicoccus.qingdaonensis
1.00
0.75
0.50
0.25
0.00
A (n=15)
B (n=16)
C (n=14)
surface

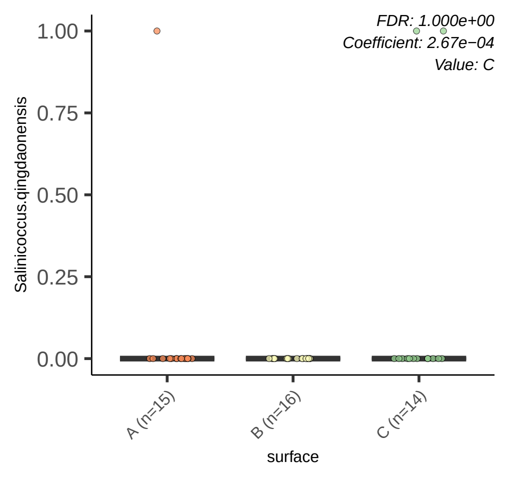
FDR: 1.000e+00
Coefficient: 2.67e−04
Value: C
Salinicoccus.qingdaonensis
1.00
0.75
0.50
0.25
0.00
A (n=15)
B (n=16)
C (n=14)
surface

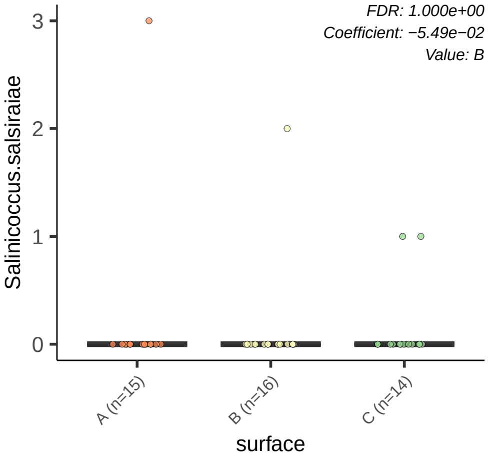
FDR: 1.000e+00
Coefficient: −5.49e−02
Value: B
Salinicoccus.salsiraiae
3
2
1
0
A (n=15)
B (n=16)
C (n=14)
surface

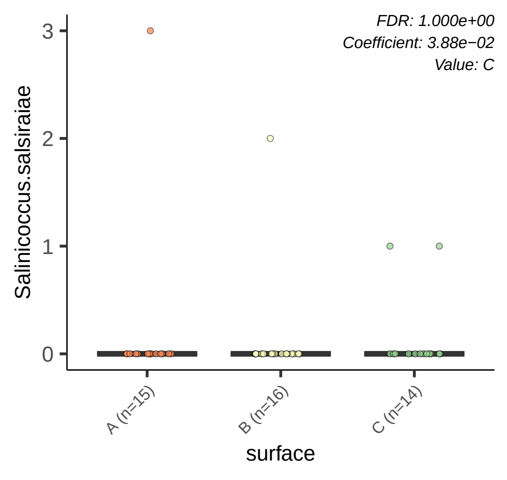

FDR: 1.000e+00
Coefficient: 3.88e−02
Value: C
Salinicoccus.salsiraiae
3
2
1
0
A (n=15)
B (n=16)
C (n=14)
surface

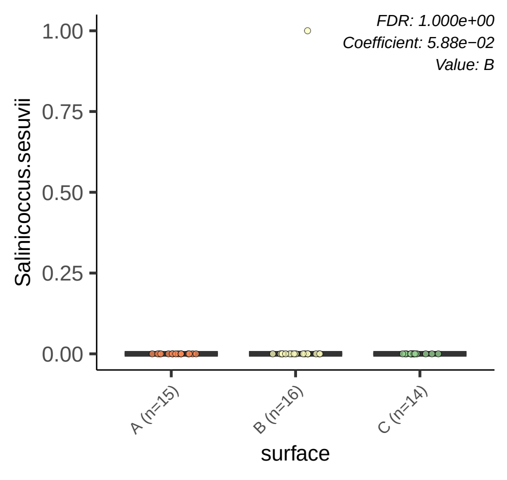

FDR: 1.000e+00
Coefficient: 5.88e−02
Value: B
Salinicoccus.sesuvii
1.00
0.75
0.50
0.25
0.00
A (n=15)
B (n=16)
C (n=14)
surface

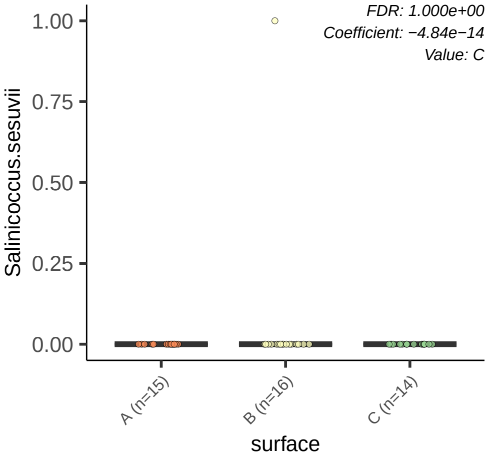

FDR: 1.000e+00
Coefficient: −4.84e−14
Value: C
Salinicoccus.sesuvii
1.00
0.75
0.50
0.25
0.00
A (n=15)
B (n=16)
C (n=14)
surface

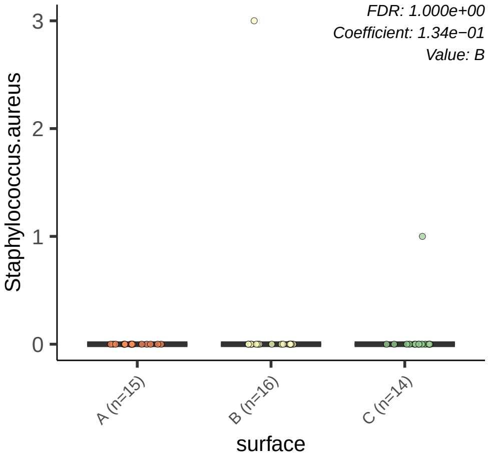

FDR: 1.000e+00
Coefficient: 1.34e−01
Value: B
Staphylococcus.aureus
3
2
1
0
A (n=15)
B (n=16)
C (n=14)
surface

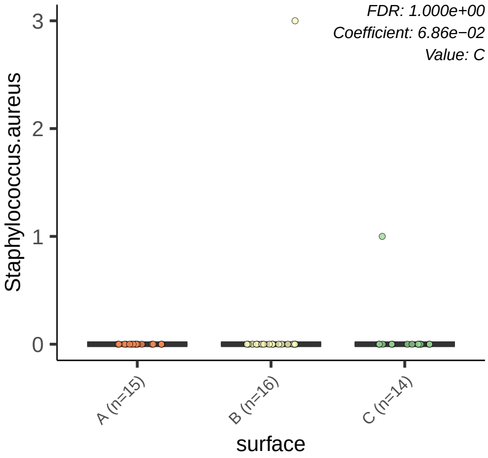

FDR: 1.000e+00
Coefficient: 6.86e−02
Value: C
Staphylococcus.aureus
3
2
1
0
A (n=15)
B (n=16)
C (n=14)
surface

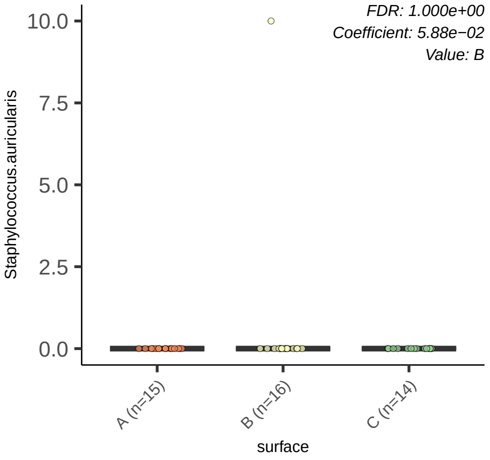

FDR: 1.000e+00
Coefficient: 5.88e−02
Value: B
10.0
7.5
5.0
2.5
0.0
Staphylococcus.auricularis
A (n=15)
B (n=16)
C (n=14)
surface

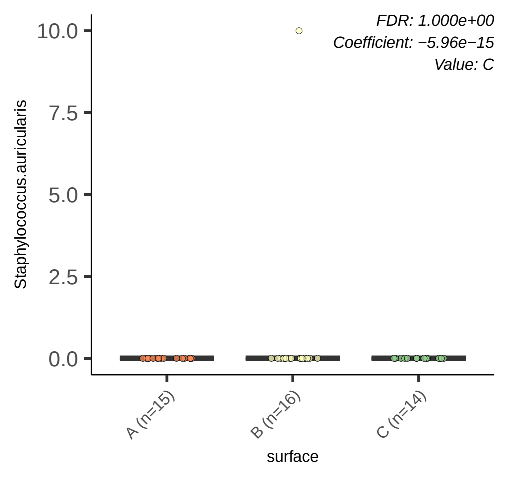
FDR: 1.000e+00
Coefficient: −5.96e−15
Value: C
Staphylococcus.auricularis
10.0
7.5
5.0
2.5
0.0
A (n=15)
B (n=16)
C (n=14)
surface

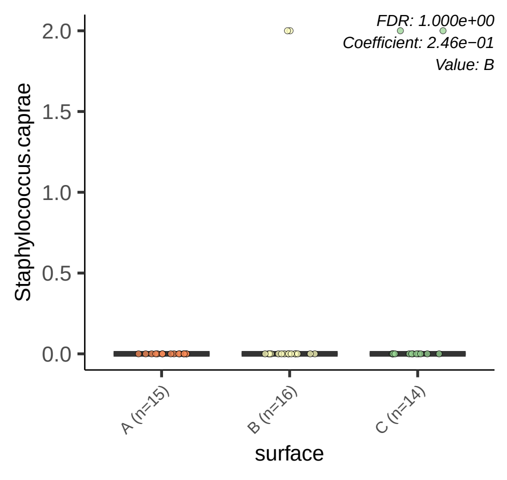
FDR: 1.000e+00
Coefficient: 2.46e−01
Value: B
Staphylococcus.caprae
2.0
1.5
1.0
0.5
0.0
A (n=15)
B (n=16)
C (n=14)
surface

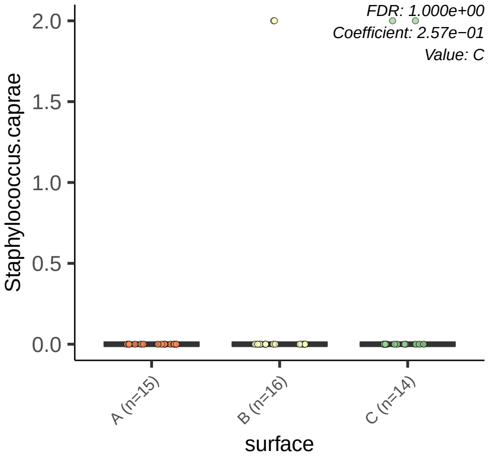

FDR: 1.000e+00
Coefficient: 2.57e−01
Value: C
Staphylococcus.caprae
2.0
1.5
1.0
0.5
0.0
A (n=15)
B (n=16)
C (n=14)
surface

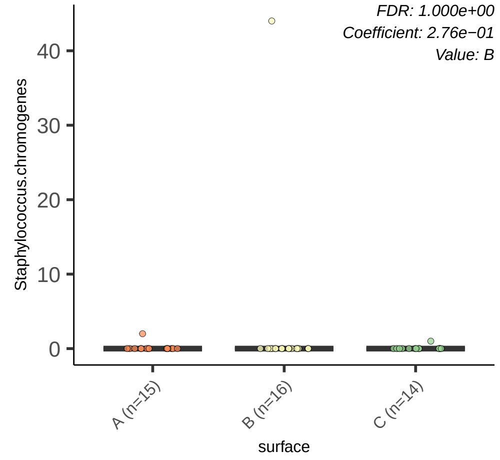

FDR: 1.000e+00
Coefficient: 2.76e−01
Value: B
Staphylococcus.chromogenes
40
30
20
10
0
A (n=15)
B (n=16)
C (n=14)
surface

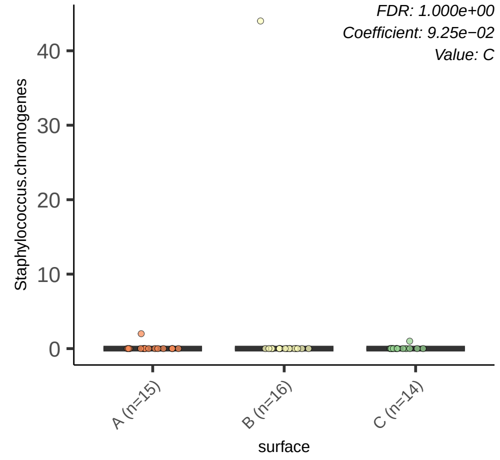

FDR: 1.000e+00
Coefficient: 9.25e−02
Value: C
Staphylococcus.chromogenes
40
30
20
10
0
A (n=15)
B (n=16)
C (n=14)
surface

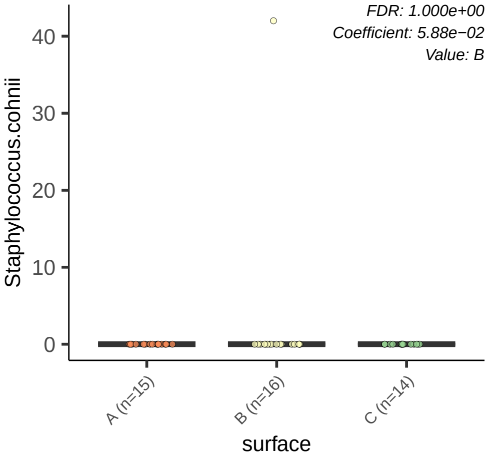
FDR: 1.000e+00
Coefficient: 5.88e−02
Value: B
Staphylococcus.cohnii
40
30
20
10
0
A (n=15)
B (n=16)
C (n=14)
surface

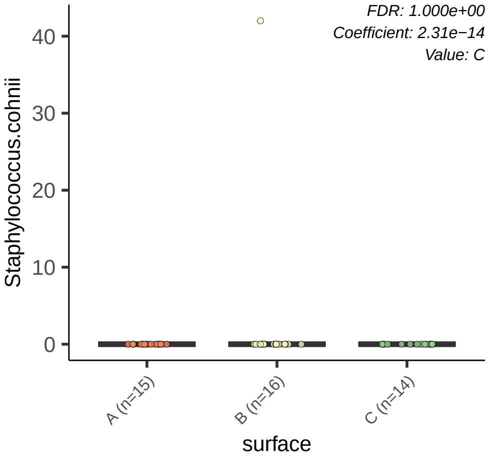

FDR: 1.000e+00
Coefficient: 2.31e−14
Value: C
Staphylococcus.cohnii
40
30
20
10
0
A (n=15)
B (n=16)
C (n=14)
surface

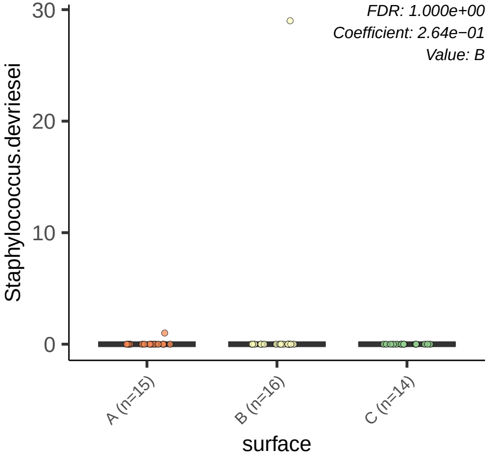

FDR: 1.000e+00
Coefficient: 2.64e−01
Value: B
Staphylococcus.devriesei
30
20
10
0
A (n=15)
B (n=16)
C (n=14)
surface

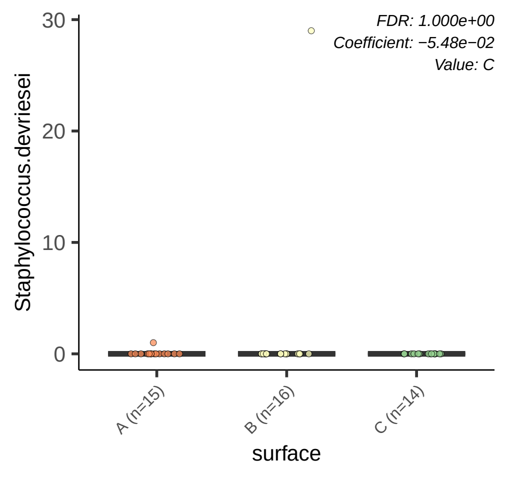

FDR: 1.000e+00
Coefficient: −5.48e−02
Value: C
Staphylococcus.devriesei
30
20
10
0
A (n=15)
B (n=16)
C (n=14)
surface

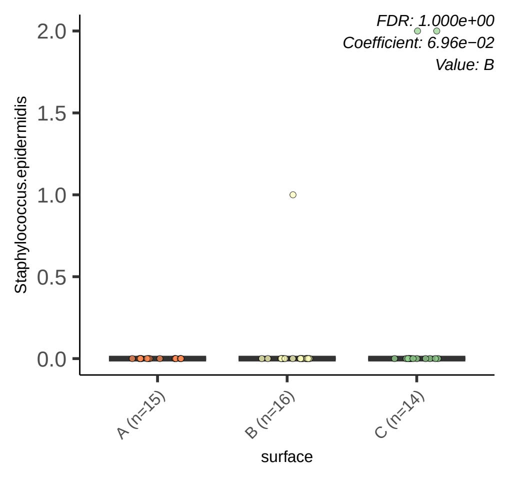

FDR: 1.000e+00
Coefficient: 6.96e−02
Value: B
2.0
1.5
1.0
0.5
0.0
Staphylococcus.epidermidis
A (n=15)
B (n=16)
C (n=14)
surface

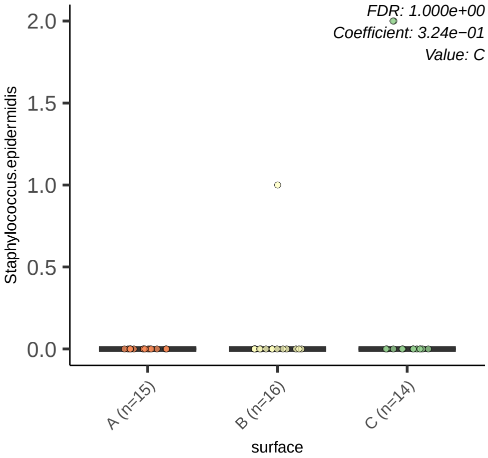

FDR: 1.000e+00
Coefficient: 3.24e−01
Value: C
Staphylococcus.epidermidis
2.0
1.5
1.0
0.5
0.0
A (n=15)
B (n=16)
C (n=14)
surface

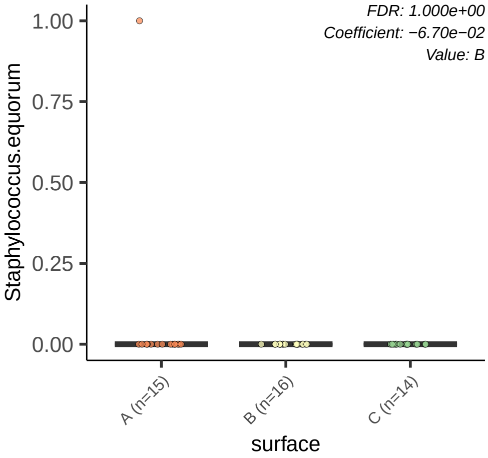
FDR: 1.000e+00
Coefficient: −6.70e−02
Value: B
Staphylococcus.equorum
1.00
0.75
0.50
0.25
0.00
A (n=15)
B (n=16)
C (n=14)
surface

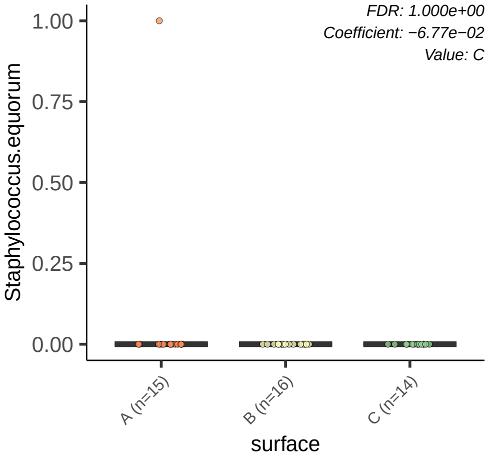
FDR: 1.000e+00
Coefficient: −6.77e−02
Value: C
Staphylococcus.equorum
1.00
0.75
0.50
0.25
0.00
A (n=15)
B (n=16)
C (n=14)
surface

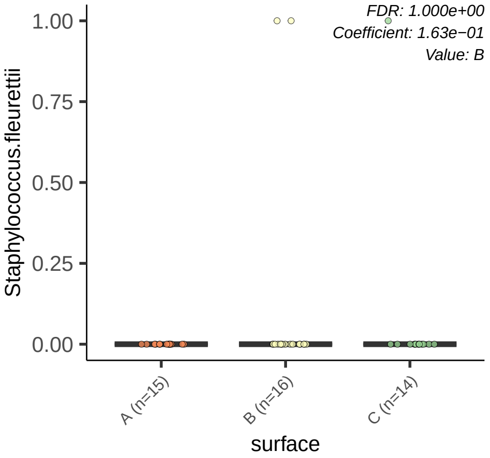
FDR: 1.000e+00
Coefficient: 1.63e−01
Value: B
Staphylococcus.fleuretii
1.00
0.75
0.50
0.25
0.00
A (n=15)
B (n=16)
C (n=14)
surface

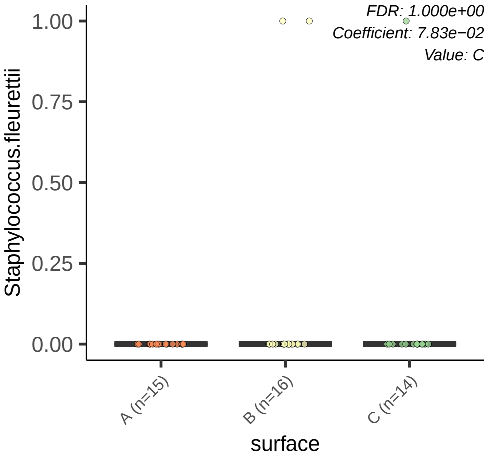
FDR: 1.000e+00
Coefficient: 7.83e−02
Value: C
Staphylococcus.fleuretii
1.00
0.75
0.50
0.25
0.00
A (n=15)
B (n=16)
C (n=14)
surface

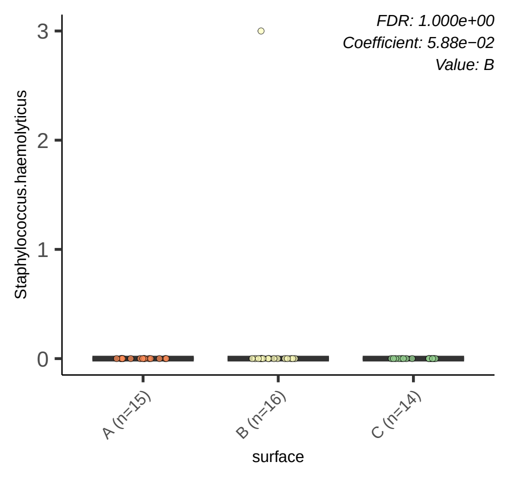

FDR: 1.000e+00
Coefficient: 5.88e−02
Value: B
Staphylococcus.haemolyticus
3
2
1
0
A (n=15)
B (n=16)
C (n=14)
surface

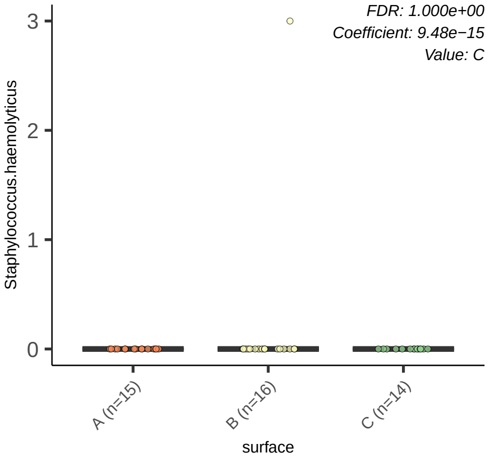

FDR: 1.000e+00
Coefficient: 9.48e−15
Value: C
Staphylococcus.haemolyticus
3
2
1
0
A (n=15)
B (n=16)
C (n=14)
surface

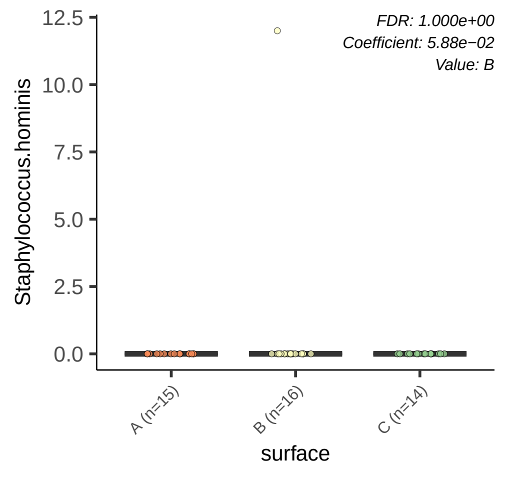

FDR: 1.000e+00
Coefficient: 5.88e−02
Value: B
12.5
10.0
7.5
5.0
2.5
0.0
Staphylococcus.hominis
A (n=15)
B (n=16)
C (n=14)
surface

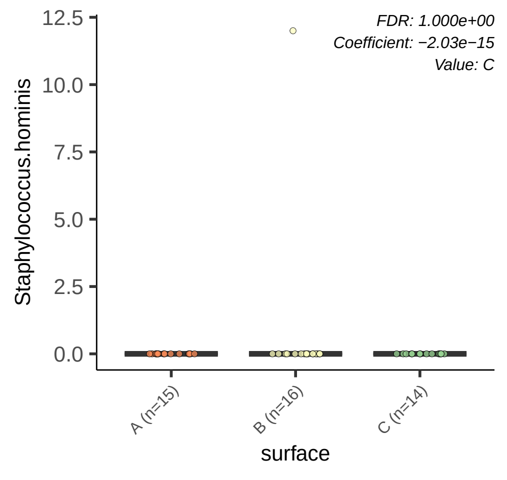

FDR: 1.000e+00
Coefficient: −2.03e−15
Value: C
Staphylococcus.hominis
12.5
10.0
7.5
5.0
2.5
0.0
A (n=15)
B (n=16)
C (n=14)
surface

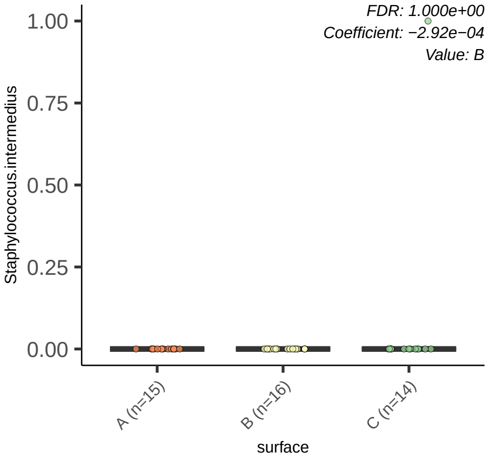
FDR: 1.000e+00
Coefficient: −2.92e−04
Value: B
Staphylococcus.intermedius
1.00
0.75
0.50
0.25
0.00
A (n=15)
B (n=16)
C (n=14)
surface

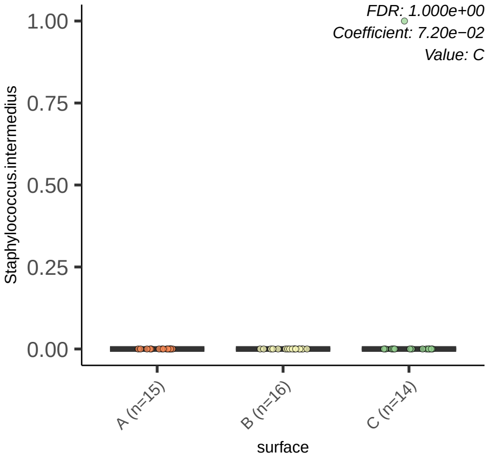

FDR: 1.000e+00
Coefficient: 7.20e−02
Value: C
1.00
0.75
0.50
0.25
0.00
Staphylococcus.intermedius
A (n=15)
B (n=16)
C (n=14)
surface

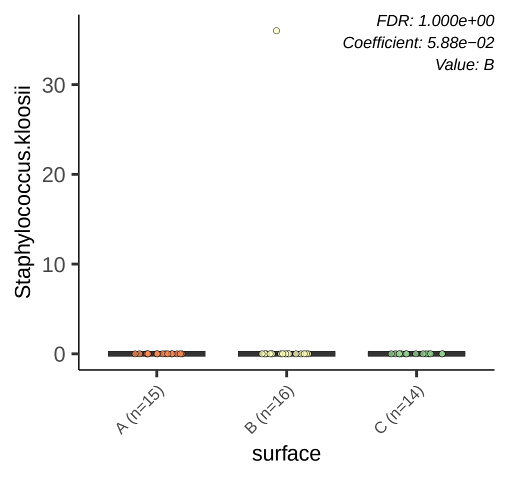
FDR: 1.000e+00
Coefficient: 5.88e−02
Value: B
Staphylococcus.kloosii
30
20
10
0
A (n=15)
B (n=16)
C (n=14)
surface

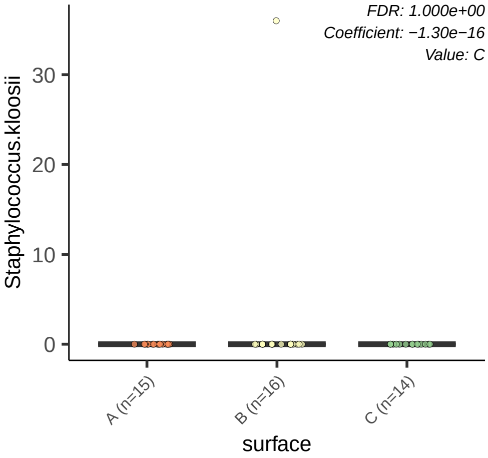

FDR: 1.000e+00
Coefficient: −1.30e−16
Value: C
Staphylococcus.kloosii
30
20
10
0
A (n=15)
B (n=16)
C (n=14)
surface

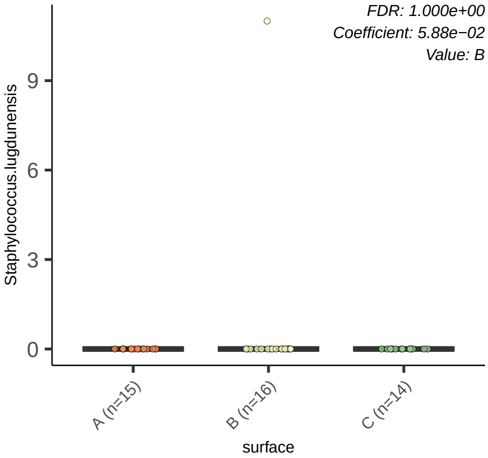

FDR: 1.000e+00
Coefficient: 5.88e−02
Value: B
Staphylococcus.lugdunensis
9
6
3
0
A (n=15)
B (n=16)
C (n=14)
surface

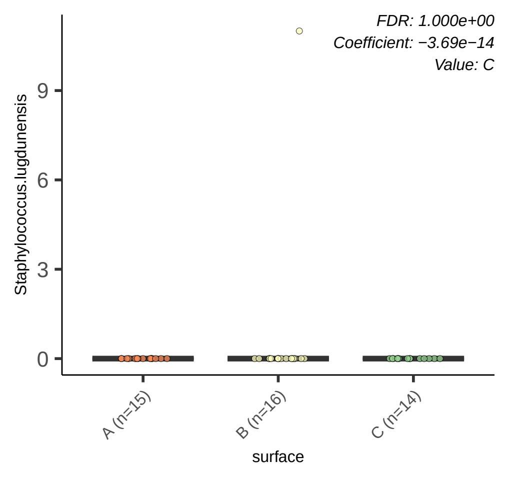

FDR: 1.000e+00
Coefficient: −3.69e−14
Value: C
Staphylococcus.lugdunensis
9
6
3
0
A (n=15)
B (n=16)
C (n=14)
surface

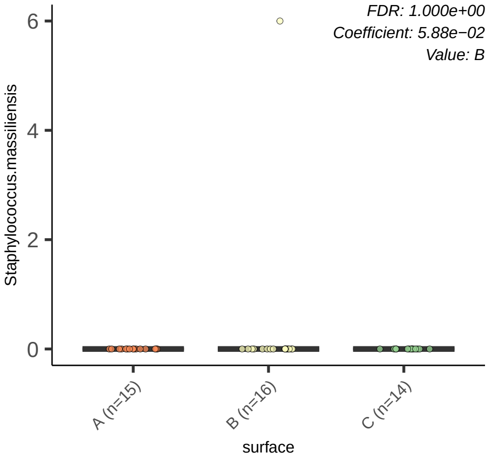
FDR: 1.000e+00
Coefficient: 5.88e−02
Value: B
Staphylococcus.massiliensis
0
2
4
6
A (n=15)
B (n=16)
C (n=14)
surface

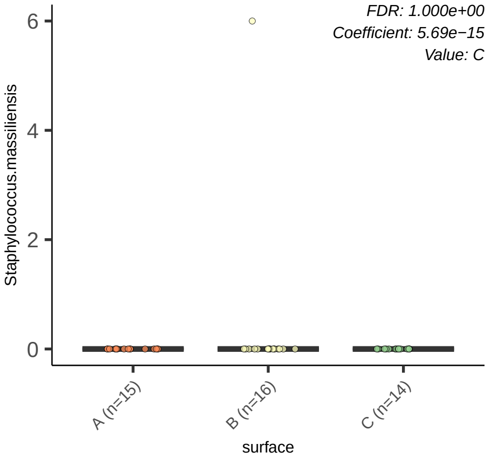
FDR: 1.000e+00
Coefficient: 5.69e−15
Value: C
6
4
2
0
Staphylococcus.massiliensis
A (n=15)
B (n=16)
C (n=14)
surface

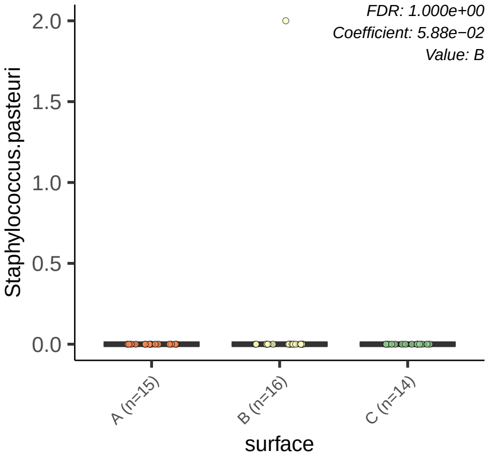
FDR: 1.000e+00
Coefficient: 5.88e−02
Value: B
Staphylococcus.pasteuri
2.0
1.5
1.0
0.5
0.0
A (n=15)
B (n=16)
C (n=14)
surface

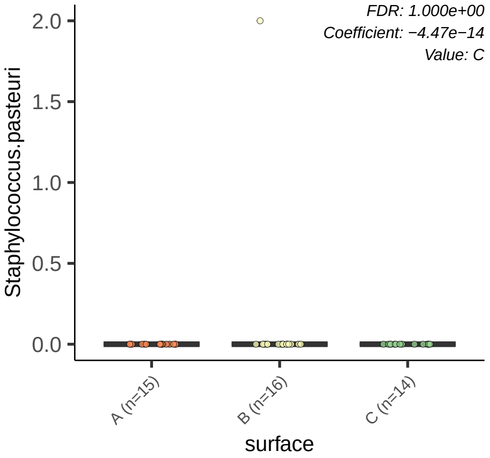
FDR: 1.000e+00
Coefficient: −4.47e−14
Value: C
Staphylococcus.pasteuri
2.0
1.5
1.0
0.5
0.0
A (n=15)
B (n=16)
C (n=14)
surface

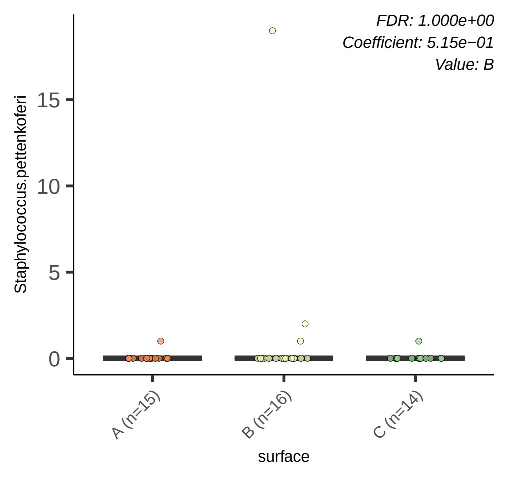
FDR: 1.000e+00
Coefficient: 5.15e−01
Value: B
Staphylococcus.pettenkoferi
0
5
10
15
A (n=15)
B (n=16)
C (n=14)
surface

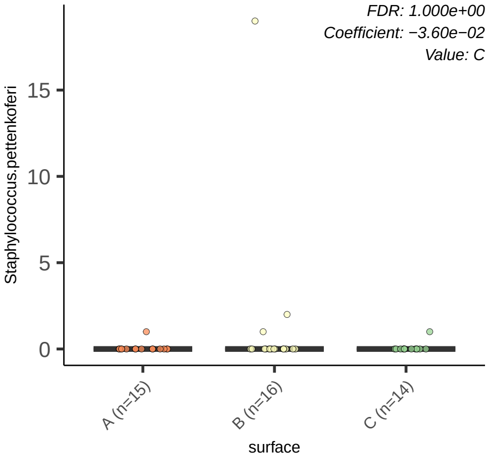

FDR: 1.000e+00
Coefficient: −3.60e−02
Value: C
Staphylococcus.pettenkoferi
15
10
5
0
A (n=15)
B (n=16)
C (n=14)
surface

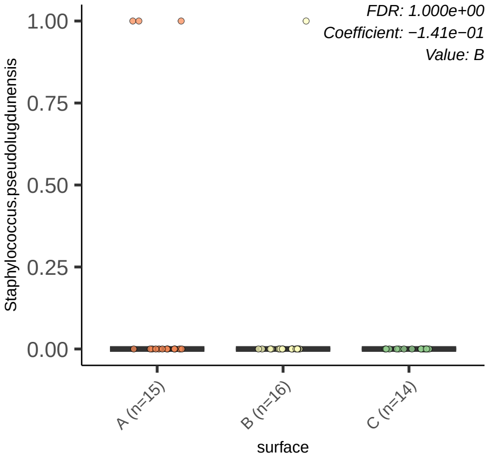
FDR: 1.000e+00
Coefficient: −1.41e−01
Value: B
Staphylococcus.pseudolugdunensis
1.00
0.75
0.50
0.25
0.00
A (n=15)
B (n=16)
C (n=14)
surface

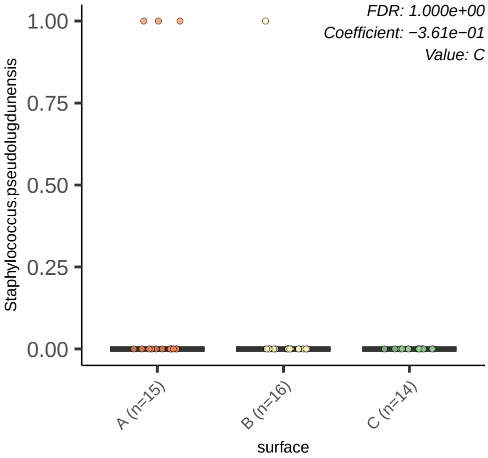
FDR: 1.000e+00
Coefficient: −3.61e−01
Value: C
Staphylococcus.pseudolugdunensis
1.00
0.75
0.50
0.25
0.00
A (n=15)
B (n=16)
C (n=14)
surface

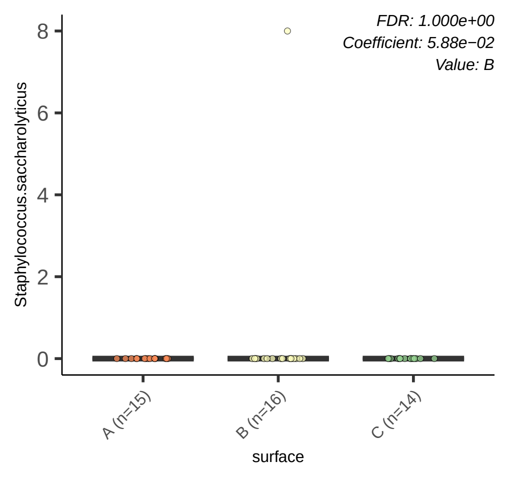
FDR: 1.000e+00
Coefficient: 5.88e−02
Value: B
Staphylococcus.saccharolyticus
0
2
4
6
8
A (n=15)
B (n=16)
C (n=14)
surface

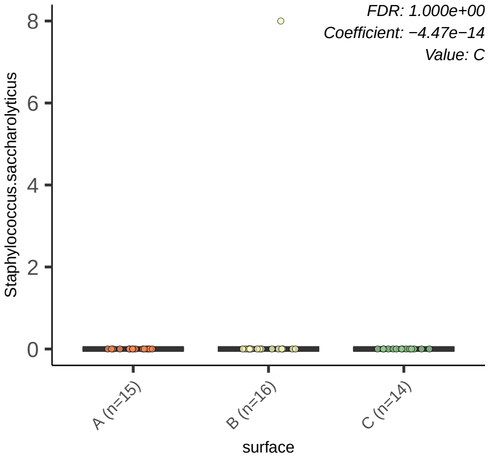
FDR: 1.000e+00
Coefficient: −4.47e−14
Value: C
Staphylococcus.saccharolyticus
8
6
4
2
0
A (n=15)
B (n=16)
C (n=14)
surface

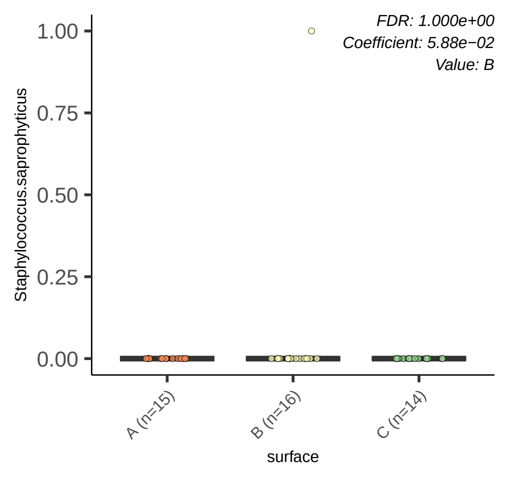
FDR: 1.000e+00
Coefficient: 5.88e−02
Value: B
Staphylococcus.saprophyticus
1.00
0.75
0.50
0.25
0.00
A (n=15)
B (n=16)
C (n=14)
surface

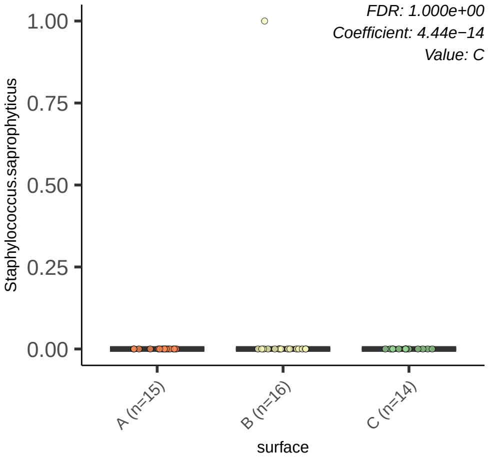
FDR: 1.000e+00
Coefficient: 4.44e−14
Value: C
Staphylococcus.saprophyticus
1.00
0.75
0.50
0.25
0.00
A (n=15)
B (n=16)
C (n=14)
surface

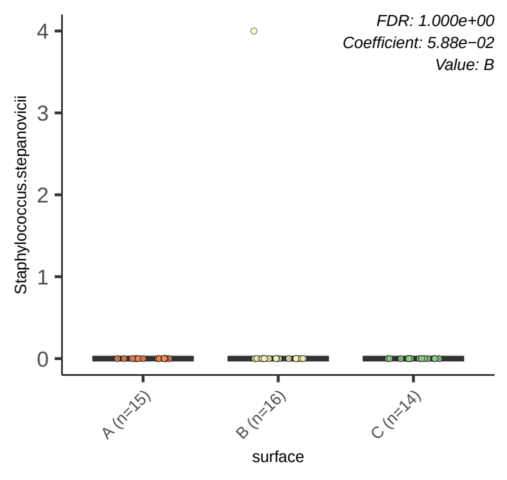

FDR: 1.000e+00
Coefficient: 5.88e−02
Value: B
Staphylococcus.stepanovicii
0
1
2
3
4
A (n=15)
B (n=16)
C (n=14)
surface

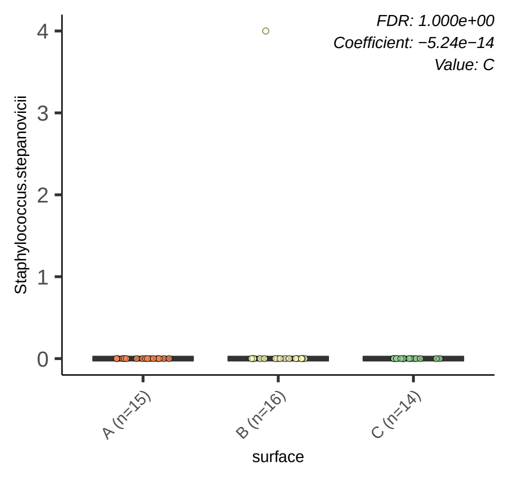
FDR: 1.000e+00
Coefficient: −5.24e−14
Value: C
Staphylococcus.stepanovicii
0
1
2
3
4
A (n=15)
B (n=16)
C (n=14)
surface

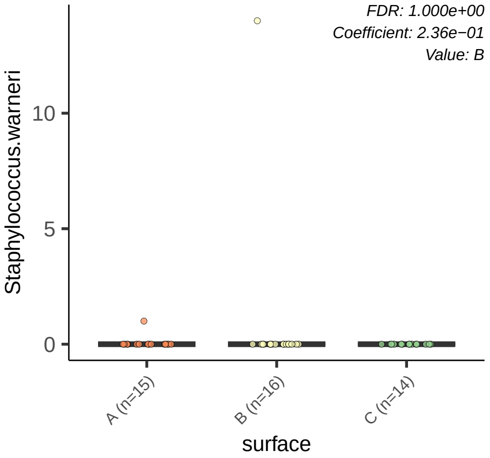
FDR: 1.000e+00
Coefficient: 2.36e−01
Value: B
Staphylococcus.warneri
0
5
10
A (n=15)
B (n=16)
C (n=14)
surface

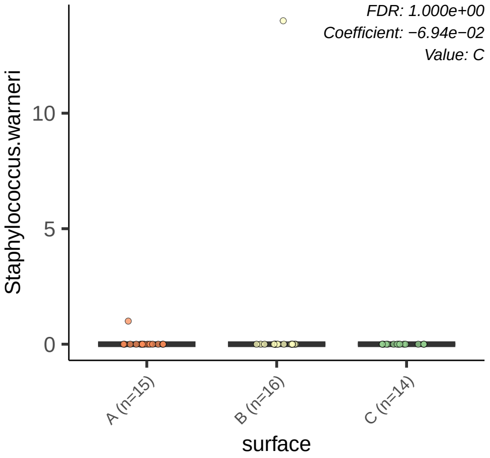

FDR: 1.000e+00
Coefficient: −6.94e−02
Value: C
Staphylococcus.warneri
10
5
0
A (n=15)
B (n=16)
C (n=14)
surface

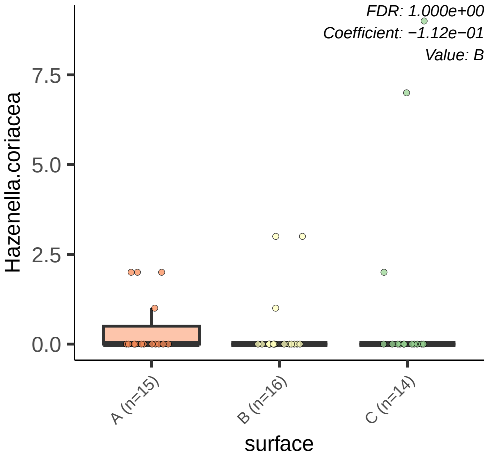

FDR: 1.000e+00
Coefficient: −1.12e−01
Value: B
Hazenella.coriacea
7.5
5.0
2.5
0.0
A (n=15)
B (n=16)
C (n=14)
surface

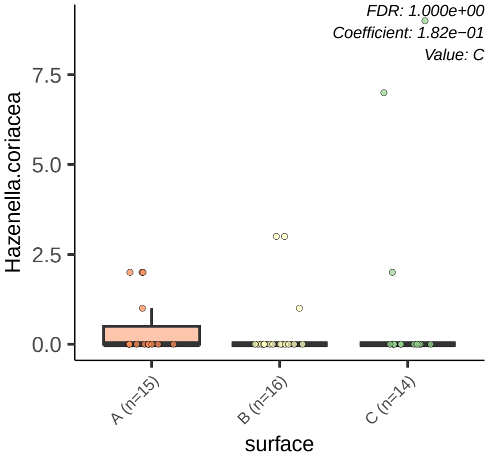

FDR: 1.000e+00
Coefficient: 1.82e−01
Value: C
Hazenella.coriacea
7.5
5.0
2.5
0.0
A (n=15)
B (n=16)
C (n=14)
surface

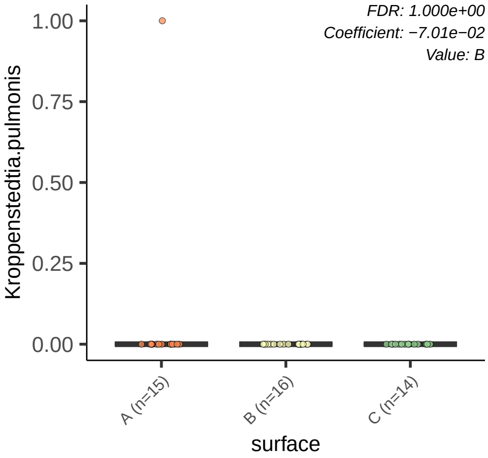
FDR: 1.000e+00
Coefficient: −7.01e−02
Value: B
Kroppenstedtia.pulmonis
1.00
0.75
0.50
0.25
0.00
A (n=15)
B (n=16)
C (n=14)
surface

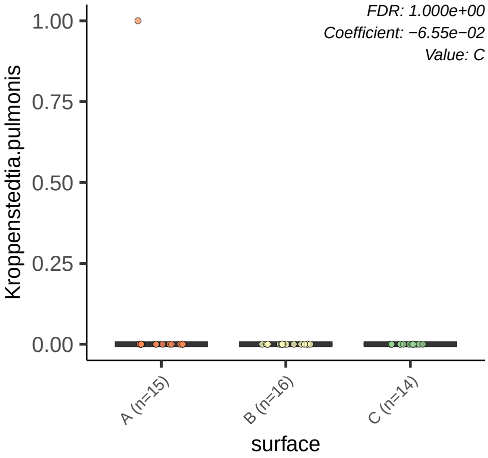

FDR: 1.000e+00
Coefficient: −6.55e−02
Value: C
1.00
0.75
0.50
0.25
0.00
Kroppenstedtia.pulmonis
A (n=15)
B (n=16)
C (n=14)
surface

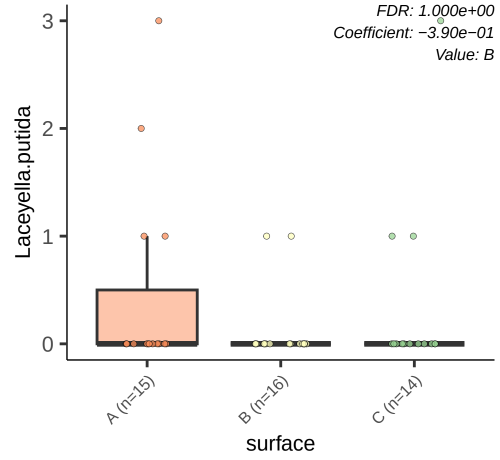
FDR: 1.000e+00
Coefficient: −3.90e−01
Value: B
Laceyella.putida
3
2
1
0
A (n=15)
B (n=16)
C (n=14)
surface

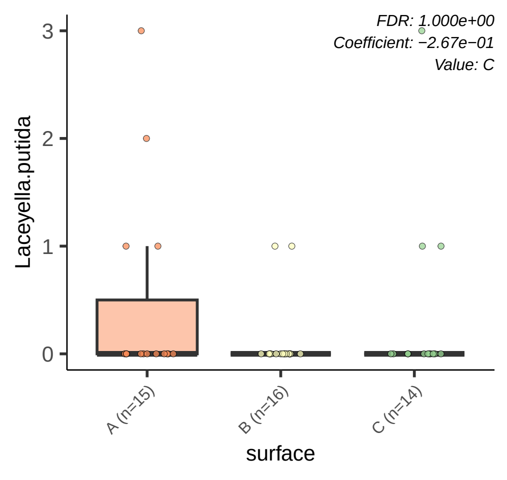

FDR: 1.000e+00
Coefficient: −2.67e−01
Value: C
Laceyella.putida
0
1
2
3
A (n=15)
B (n=16)
C (n=14)
surface

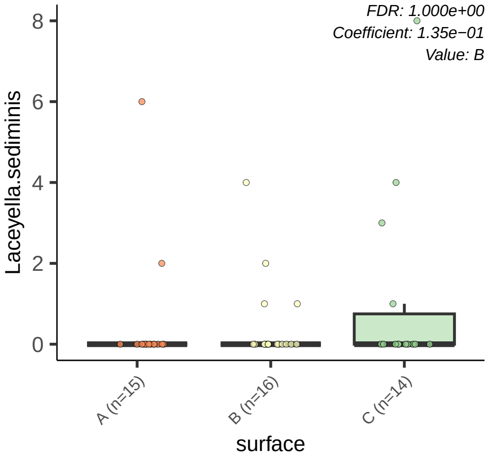
FDR: 1.000e+00
Coefficient: 1.35e−01
Value: B
Laceyella.sedimimis
8
6
4
2
0
A (n=15)
B (n=16)
C (n=14)
surface

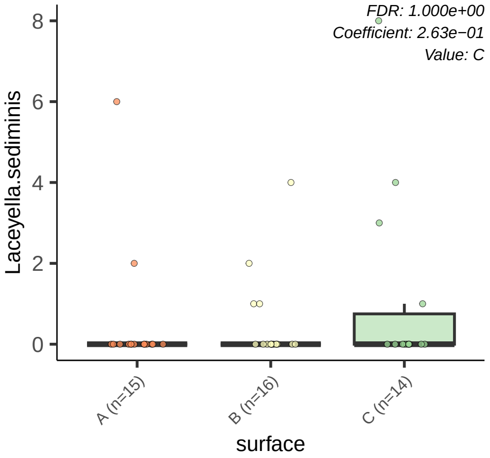
FDR: 1.000e+00
Coefficient: 2.63e−01
Value: C
Laceyella.sedíminis
8
6
4
2
0
A (n=15)
B (n=16)
C (n=14)
surface

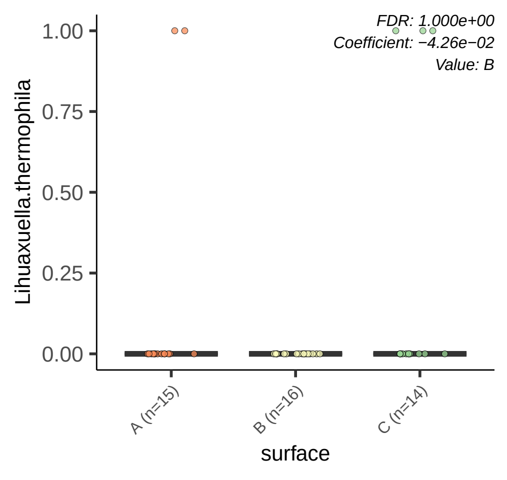
FDR: 1.000e+00
Coefficient: −4.26e−02
Value: B
Lihuaxuella.thermophila
1.00
0.75
0.50
0.25
0.00
A (n=15)
B (n=16)
C (n=14)
surface

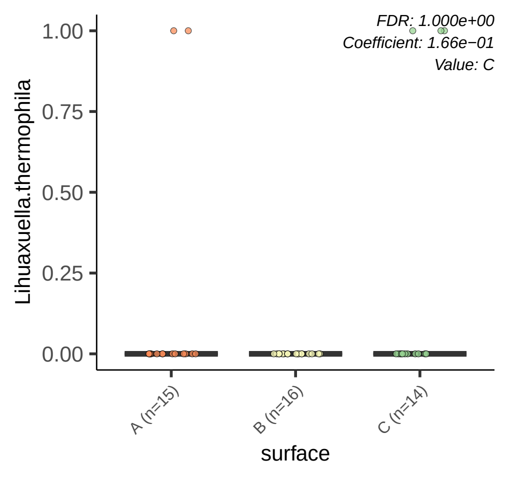
FDR: 1.000e+00
Coefficient: 1.66e−01
Value: C
Lihuaxuella.thermophila
1.00
0.75
0.50
0.25
0.00
A (n=15)
B (n=16)
C (n=14)
surface

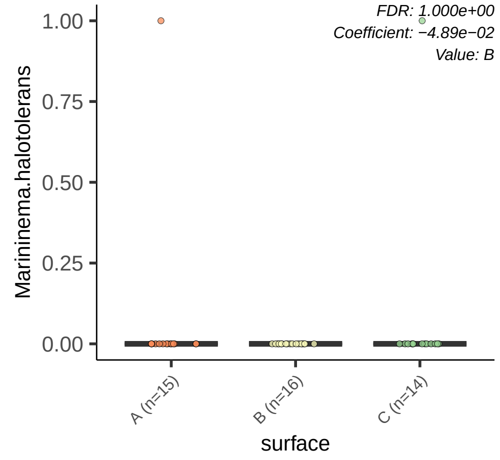

FDR: 1.000e+00
Coefficient: −4.89e−02
Value: B
Marininema.halotolerans
1.00
0.75
0.50
0.25
0.00
A (n=15)
B (n=16)
C (n=14)
surface

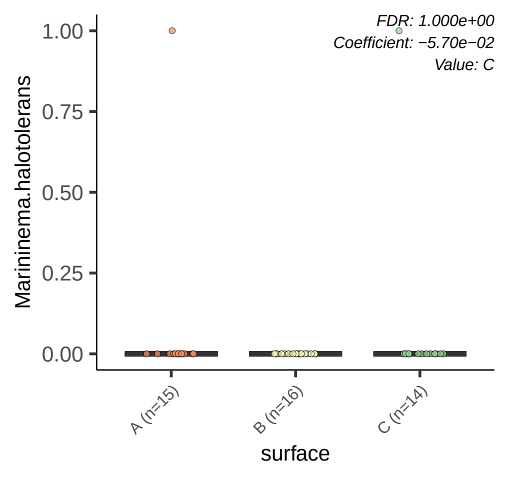

FDR: 1.000e+00
Coefficient: −5.70e−02
Value: C
Marininema.halotolerans
1.00
0.75
0.50
0.25
0.00
A (n=15)
B (n=16)
C (n=14)
surface

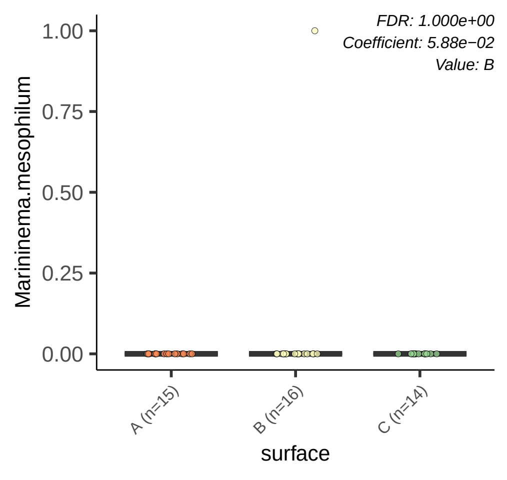

FDR: 1.000e+00
Coefficient: 5.88e−02
Value: B
Marininema.mesophilum
1.00
0.75
0.50
0.25
0.00
A (n=15)
B (n=16)
C (n=14)
surface

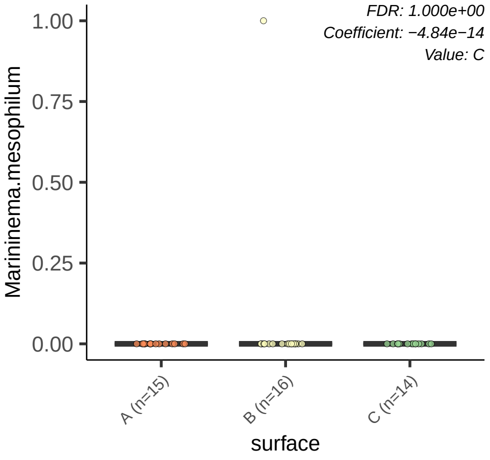
FDR: 1.000e+00
Coefficient: −4.84e−14
Value: C
Marininema.mesophilum
1.00
0.75
0.50
0.25
0.00
A (n=15)
B (n=16)
C (n=14)
surface

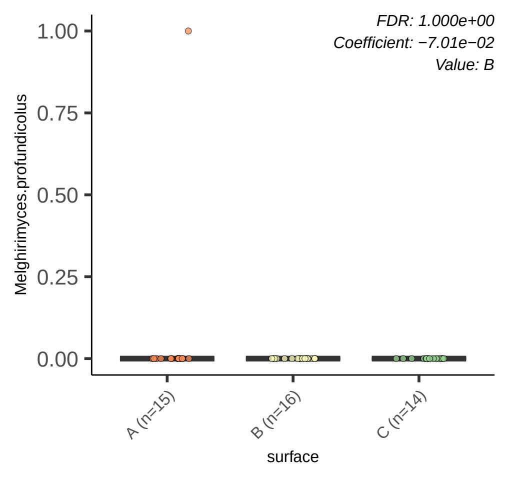
FDR: 1.000e+00
Coefficient: −7.01e−02
Value: B
Melghirimyces.profundicolus
1.00
0.75
0.50
0.25
0.00
A (n=15)
B (n=16)
C (n=14)
surface

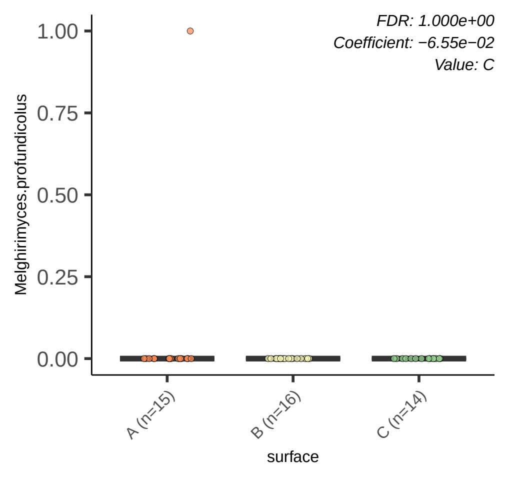

FDR: 1.000e+00
Coefficient: −6.55e−02
Value: C
Melghirimyces.profundicolus
1.00
0.75
0.50
0.25
0.00
A (n=15)
B (n=16)
C (n=14)
surface

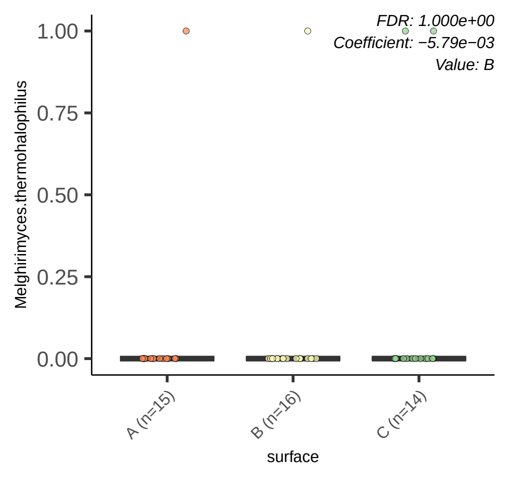

FDR: 1.000e+00
Coefficient: −5.79e−03
Value: B
1.00
0.75
0.50
0.25
0.00
Melghirimyces.thermohalophilus
A (n=15)
B (n=16)
C (n=14)
surface

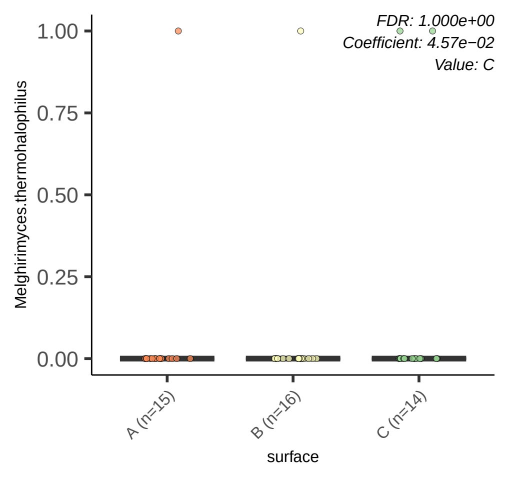
FDR: 1.000e+00
Coefficient: 4.57e−02
Value: C
Melghirimyces.thermohalophilus
1.00
0.75
0.50
0.25
0.00
A (n=15)
B (n=16)
C (n=14)
surface

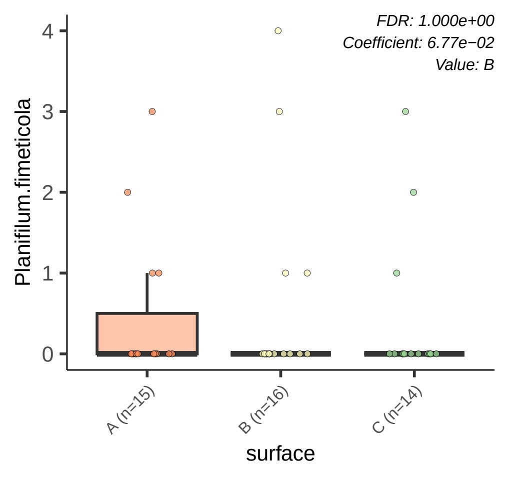
FDR: 1.000e+00
Coefficient: 6.77e−02
Value: B
Planifilum.fimeticola
0
1
2
3
4
A (n=15)
B (n=16)
C (n=14)
surface

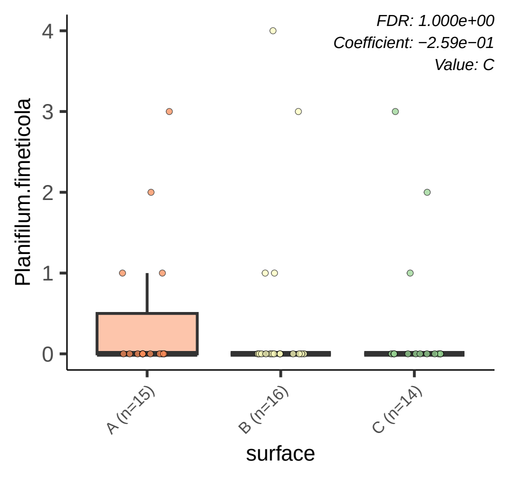
FDR: 1.000e+00
Coefficient: −2.59e−01
Value: C
Planifilum.fimeticola
0
1
2
3
4
A (n=15)
B (n=16)
C (n=14)
surface

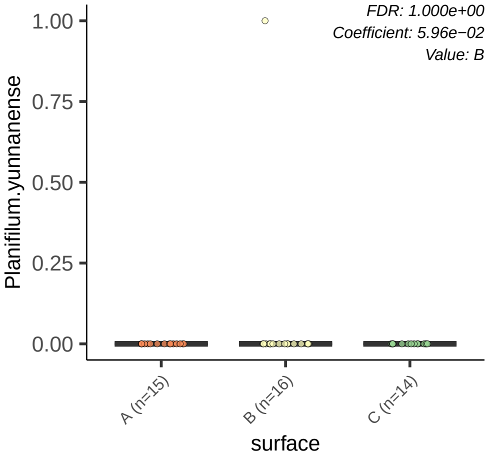

FDR: 1.000e+00
Coefficient: 5.96e−02
Value: B
Planifilum.yunnanense
1.00
0.75
0.50
0.25
0.00
A (n=15)
B (n=16)
C (n=14)
surface

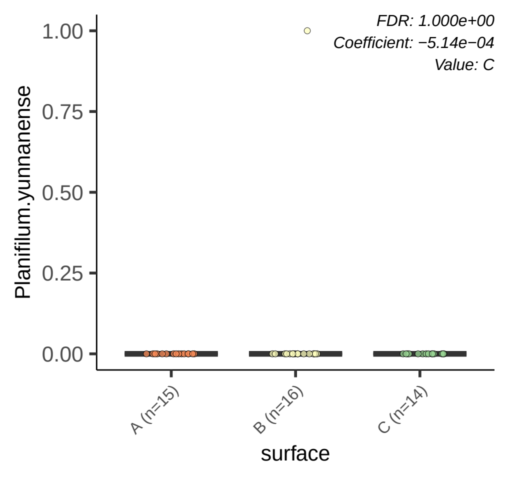

FDR: 1.000e+00
Coefficient: −5.14e−04
Value: C
Planifilum.yunnanense
1.00
0.75
0.50
0.25
0.00
A (n=15)
B (n=16)
C (n=14)
surface

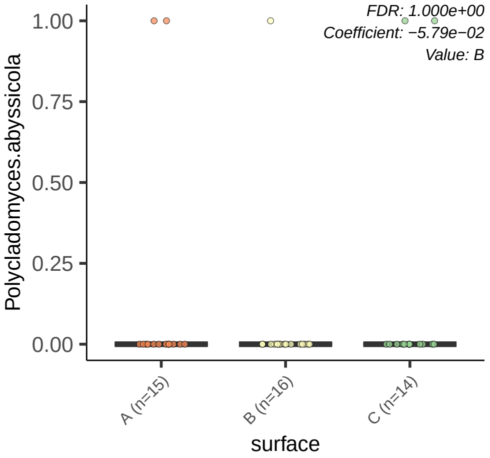
FDR: 1.000e+00
Coefficient: −5.79e−02
Value: B
Polycladomyces.abyssicola
1.00
0.75
0.50
0.25
0.00
A (n=15)
B (n=16)
C (n=14)
surface

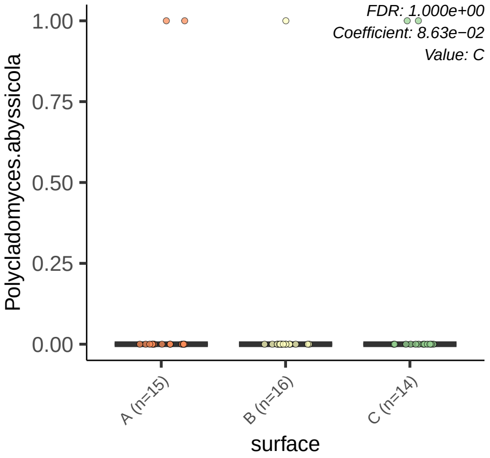
FDR: 1.000e+00
Coefficient: 8.63e−02
Value: C
Polycladomyces.abyssicola
1.00
0.75
0.50
0.25
0.00
A (n=15)
B (n=16)
C (n=14)
surface

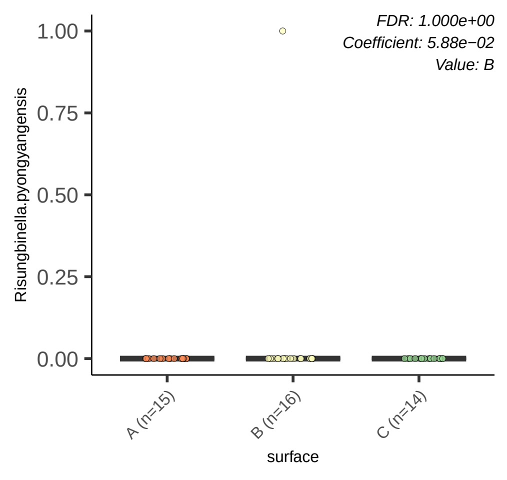
FDR: 1.000e+00
Coefficient: 5.88e−02
Value: B
1.00
0.75
0.50
0.25
0.00
Risungbinella.pyongyangensis
A (n=15)
B (n=16)
C (n=14)
surface

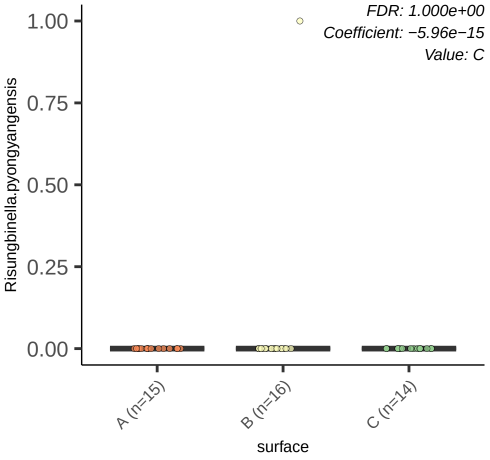

FDR: 1.000e+00
Coefficient: −5.96e−15
Value: C
Risungbinella.pyongyangensis
1.00
0.75
0.50
0.25
0.00
A (n=15)
B (n=16)
C (n=14)
surface

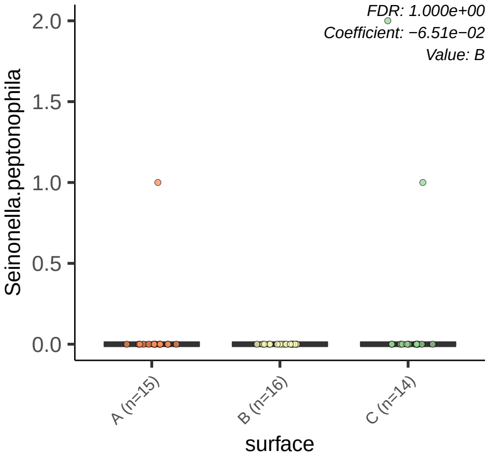

FDR: 1.000e+00
Coefficient: −6.51e−02
Value: B
Seinonella.peptonophila
2.0
1.5
1.0
0.5
0.0
A (n=15)
B (n=16)
C (n=14)
surface

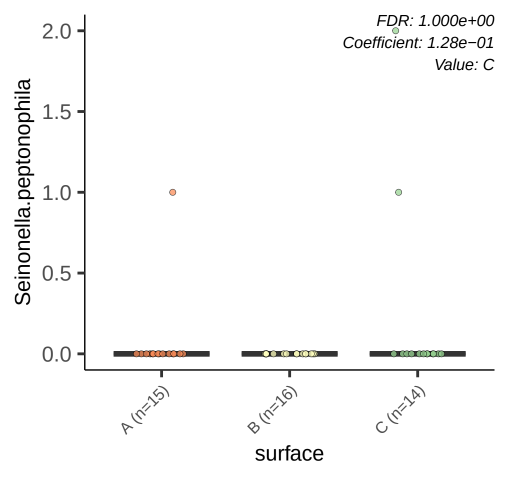
FDR: 1.000e+00
Coefficient: 1.28e−01
Value: C
Seinonella.peptonophila
2.0
1.5
1.0
0.5
0.0
A (n=15)
B (n=16)
C (n=14)
surface

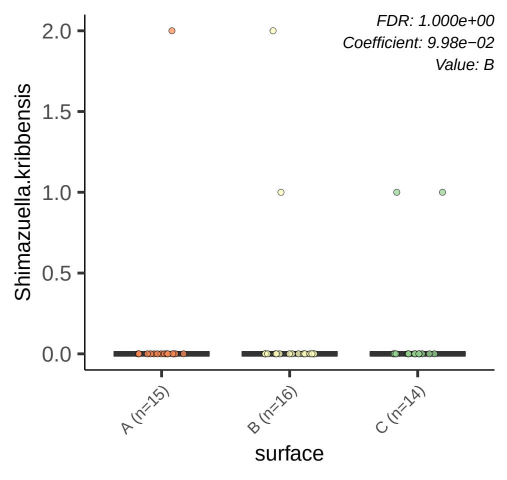
FDR: 1.000e+00
Coefficient: 9.98e−02
Value: B
Shimazuella.kribbensis
2.0
1.5
1.0
0.5
0.0
A (n=15)
B (n=16)
C (n=14)
surface

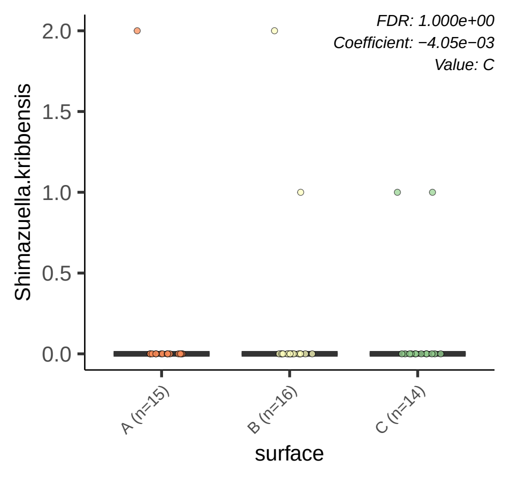

FDR: 1.000e+00
Coefficient: −4.05e−03
Value: C
Shimazuella.kribbensis
2.0
1.5
1.0
0.5
0.0
A (n=15)
B (n=16)
C (n=14)
surface

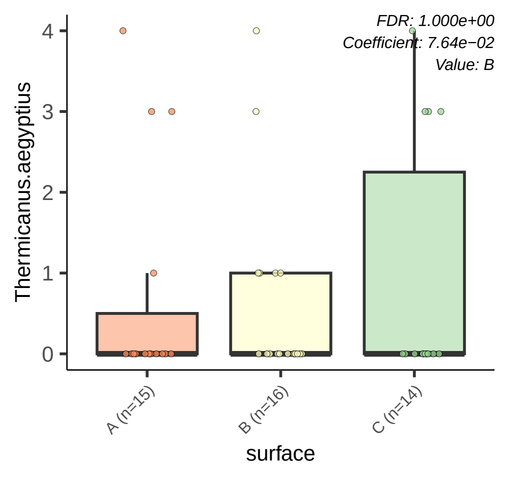

FDR: 1.000e+00
Coefficient: 7.64e−02
Value: B
Thermicanus.aegyptius
0
1
2
3
4
A (n=15)
B (n=16)
C (n=14)
surface

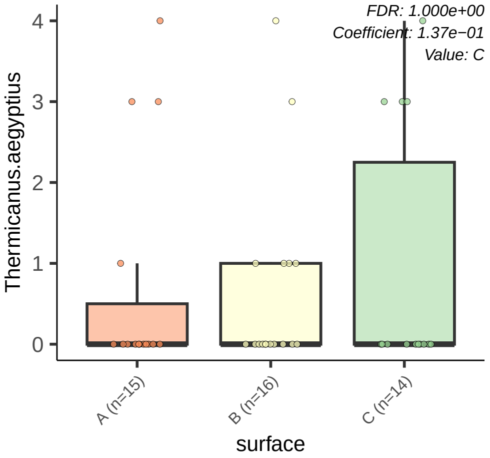
FDR: 1.000e+00
Coefficient: 1.37e−01
Value: C
Thermicanus.aegyptius
0
1
2
3
4
A (n=15)
B (n=16)
C (n=14)
surface

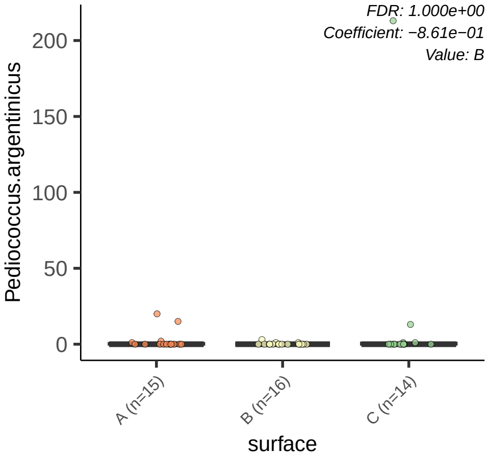
FDR: 1.000e+00
Coefficient: −8.61e−01
Value: B
Pediococcus.argentinicus
200
150
100
50
0
A (n=15)
B (n=16)
C (n=14)
surface

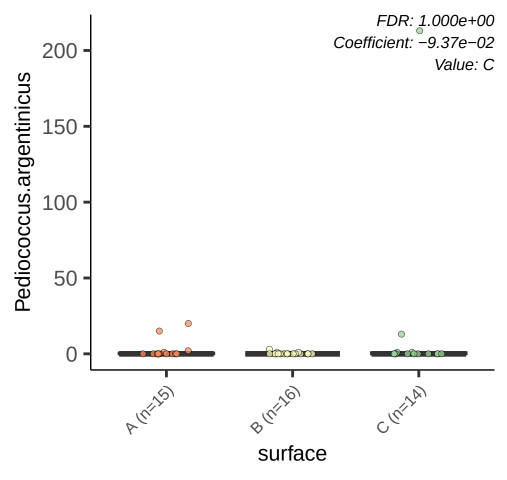
FDR: 1.000e+00
Coefficient: −9.37e−02
Value: C
Pediococcus.argentinicus
200
150
100
50
0
A (n=15)
B (n=16)
C (n=14)
surface

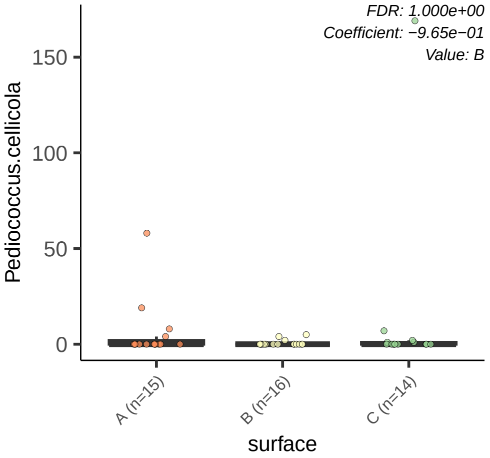

FDR: 1.000e+00
Coefficient: −9.65e−01
Value: B
Pediococcus.cellicola
150
100
50
0
A (n=15)
B (n=16)
C (n=14)
surface

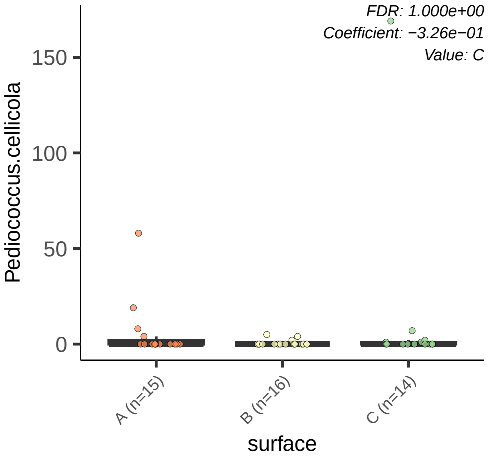
FDR: 1.000e+00
Coefficient: −3.26e−01
Value: C
Pediococcus.cellicola
150
100
50
0
A (n=15)
B (n=16)
C (n=14)
surface

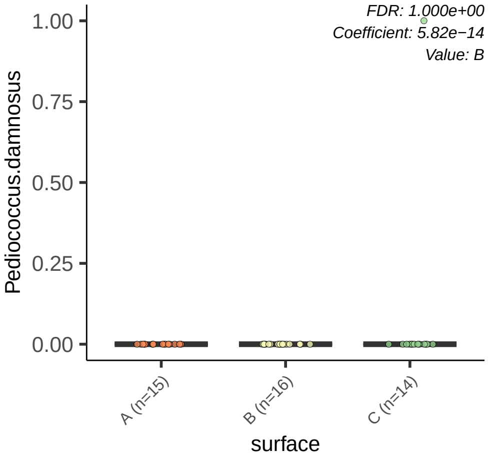
FDR: 1.000e+00
Coefficient: 5.82e−14
Value: B
Pediococcus.damnosus
1.00
0.75
0.50
0.25
0.00
A (n=15)
B (n=16)
C (n=14)
surface

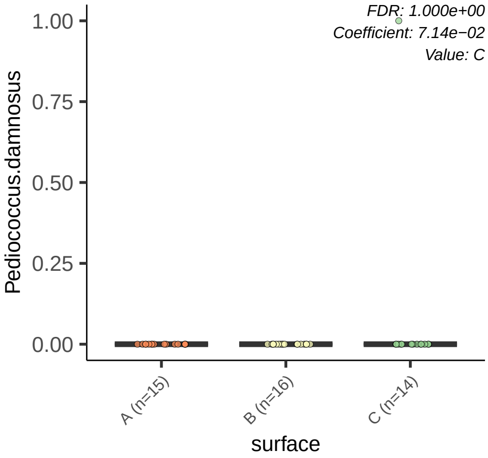
FDR: 1.000e+00
Coefficient: 7.14e−02
Value: C
Pediococcus.damnosus
1.00
0.75
0.50
0.25
0.00
A (n=15)
B (n=16)
C (n=14)
surface

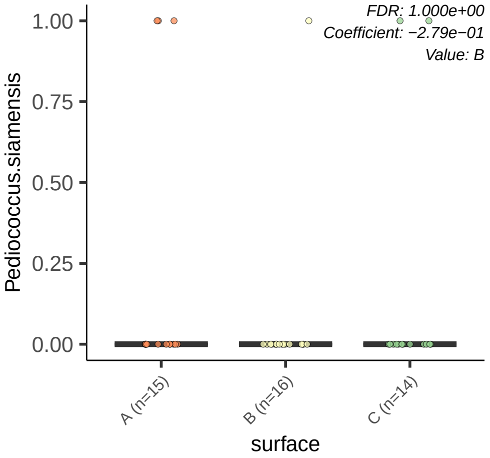
FDR: 1.000e+00
Coefficient: −2.79e−01
Value: B
Pediococcus.siamensis
1.00
0.75
0.50
0.25
0.00
A (n=15)
B (n=16)
C (n=14)
surface

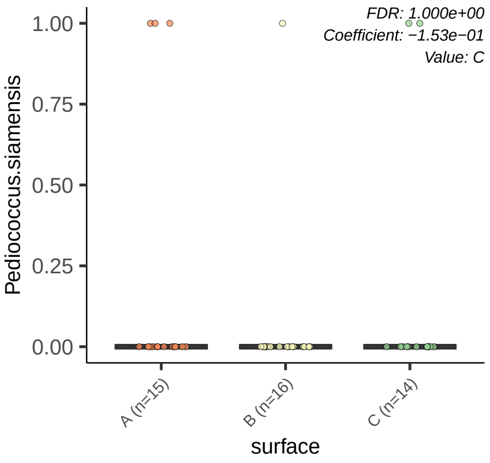

FDR: 1.000e+00
Coefficient: −1.53e−01
Value: C
Pediococcus.siamensis
1.00
0.75
0.50
0.25
0.00
A (n=15)
B (n=16)
C (n=14)
surface

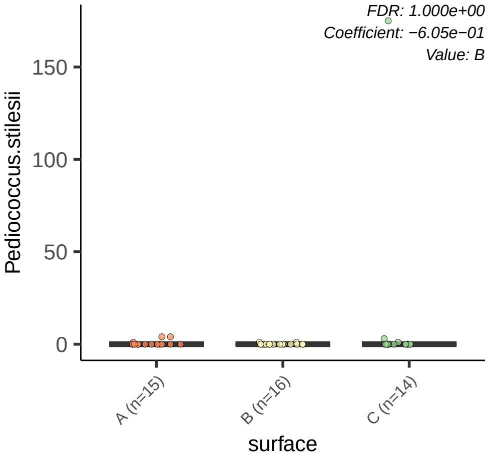
FDR: 1.000e+00
Coefficient: −6.05e−01
Value: B
Pediococcus.stilesii
150
100
50
0
A (n=15)
B (n=16)
C (n=14)
surface

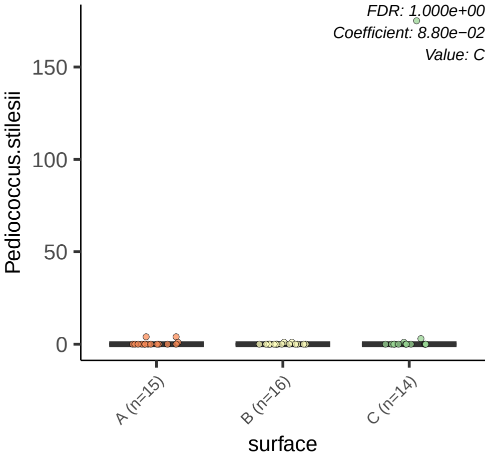
FDR: 1.000e+00
Coefficient: 8.80e−02
Value: C
Pediococcus.stilesii
150
100
50
0
A (n=15)
B (n=16)
C (n=14)
surface

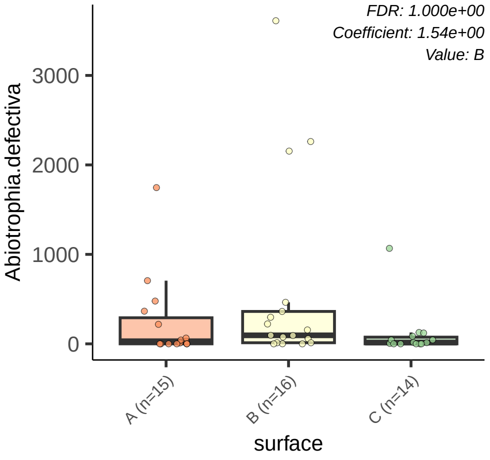

FDR: 1.000e+00
Coefficient: 1.54e+00
Value: B
Abiotrophia.defectiva
3000
2000
1000
0
A (n=15)
B (n=16)
C (n=14)
surface

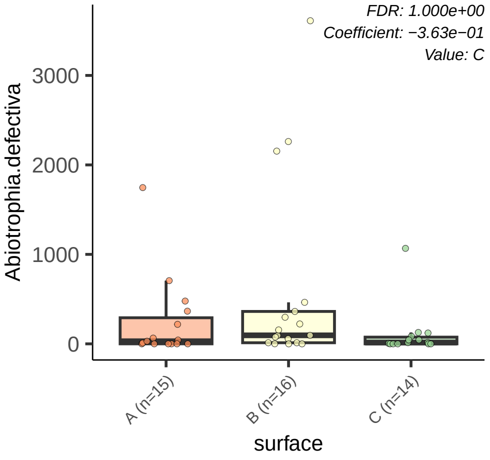

FDR: 1.000e+00
Coefficient: −3.63e−01
Value: C
Abiotrophia.defectiva
3000
2000
1000
0
A (n=15)
B (n=16)
C (n=14)
surface

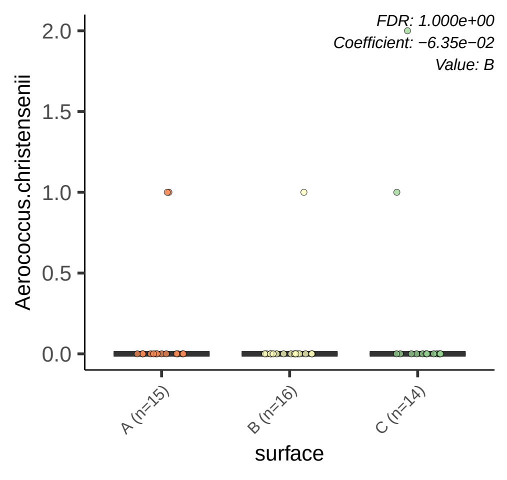
FDR: 1.000e+00
Coefficient: −6.35e−02
Value: B
Aerococcus.christensenii
2.0
1.5
1.0
0.5
0.0
A (n=15)
B (n=16)
C (n=14)
surface

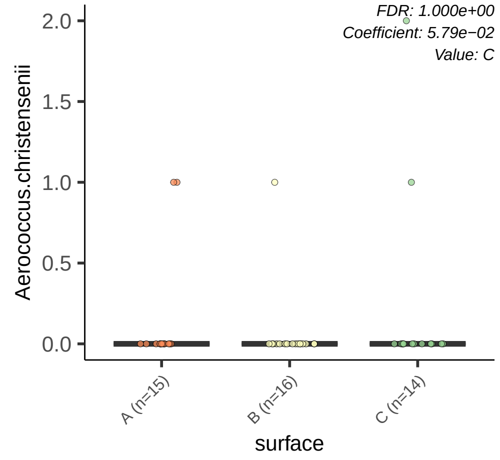
FDR: 1.000e+00
Coefficient: 5.79e−02
Value: C
2.0
1.5
1.0
0.5
0.0
Aerococcus.christensenii
A (n=15)
B (n=16)
C (n=14)
surface

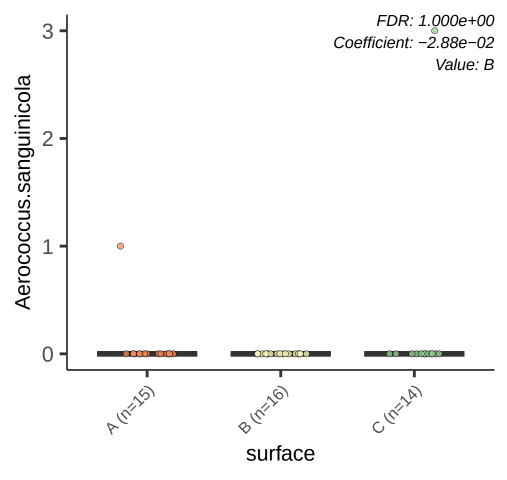
FDR: 1.000e+00
Coefficient: −2.88e−02
Value: B
Aerococcus.sanguinicola
3
2
1
0
A (n=15)
B (n=16)
C (n=14)
surface

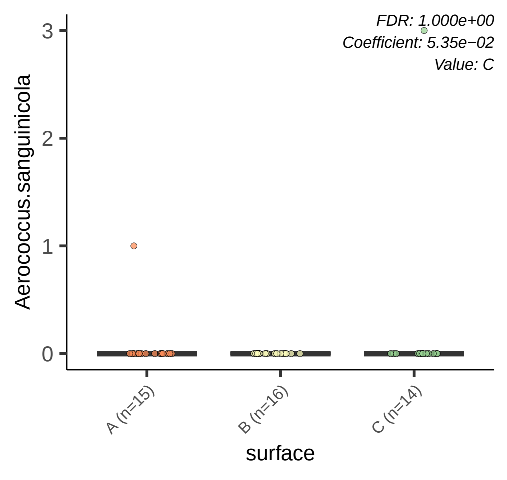

FDR: 1.000e+00
Coefficient: 5.35e−02
Value: C
Aerococcus.sanguinicola
3
2
1
0
A (n=15)
B (n=16)
C (n=14)
surface

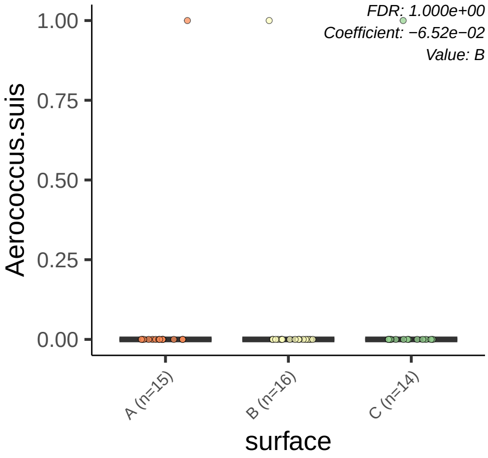

FDR: 1.000e+00
Coefficient: −6.52e−02
Value: B
Aerococcus.suis
1.00
0.75
0.50
0.25
0.00
A (n=15)
B (n=16)
C (n=14)
surface

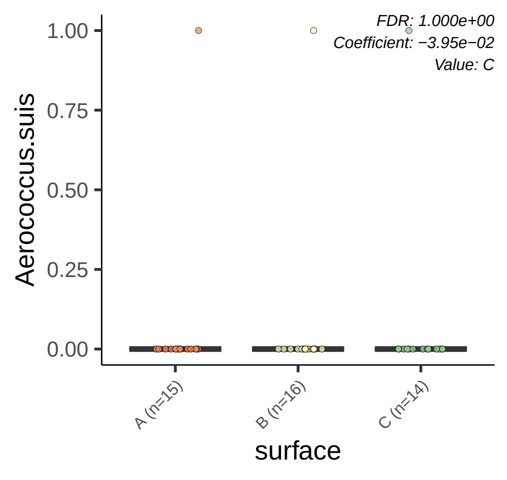
FDR: 1.000e+00
Coefficient: −3.95e−02
Value: C
Aerococcus.suis
1.00
0.75
0.50
0.25
0.00
A (n=15)
B (n=16)
C (n=14)
surface

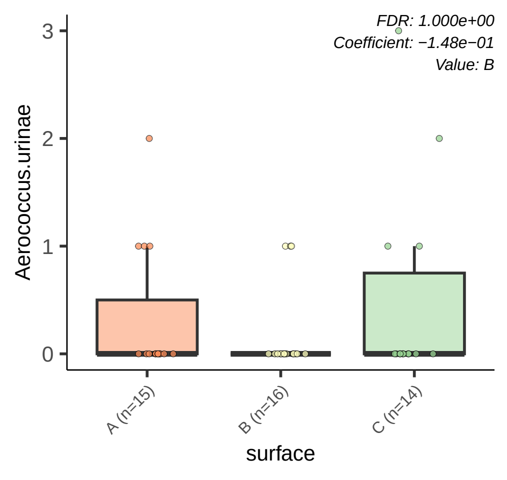
FDR: 1.000e+00
Coefficient: −1.48e−01
Value: B
Aerococcus.urinae
0
1
2
3
A (n=15)
B (n=16)
C (n=14)
surface

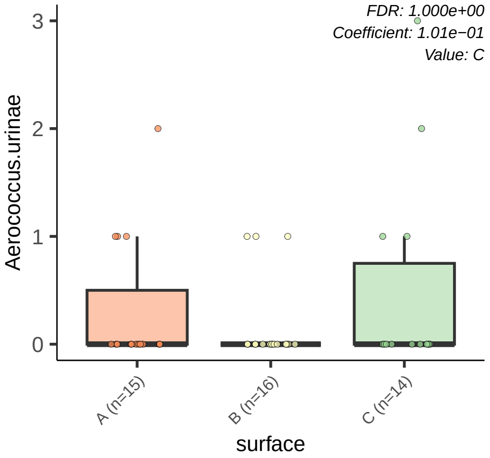
FDR: 1.000e+00
Coefficient: 1.01e−01
Value: C
Aerococcus.urinae
3
2
1
0
A (n=15)
B (n=16)
C (n=14)
surface

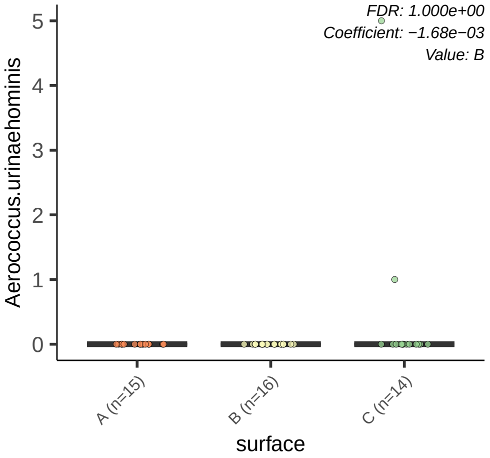
FDR: 1.000e+00
Coefficient: −1.68e−03
Value: B
Aerococcus.urinaehominis
0
1
2
3
4
5
A (n=15)
B (n=16)
C (n=14)
surface

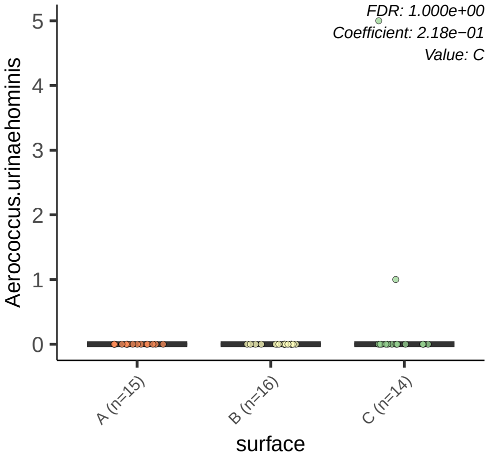
FDR: 1.000e+00
Coefficient: 2.18e−01
Value: C
Aerococcus.urinaehominis
0
1
2
3
4
5
A (n=15)
B (n=16)
C (n=14)
surface

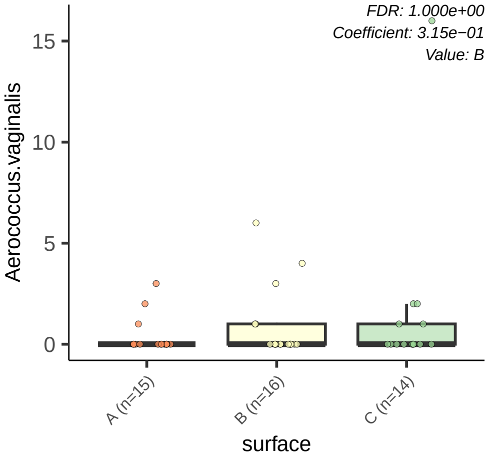
FDR: 1.000e+00
Coefficient: 3.15e−01
Value: B
Aerococcus.vaginalis
0
5
10
15
A (n=15)
B (n=16)
C (n=14)
surface

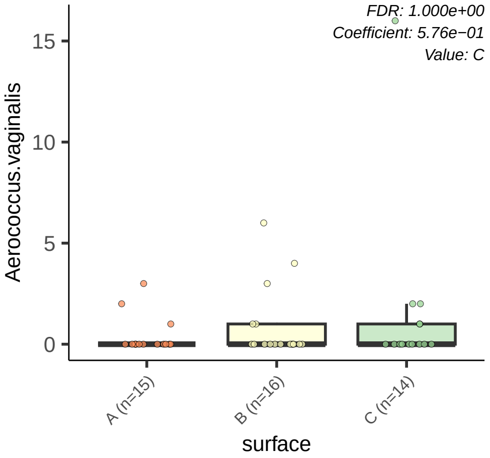

FDR: 1.000e+00
Coefficient: 5.76e−01
Value: C
Aerococcus.vaginalis
0
5
10
15
A (n=15)
B (n=16)
C (n=14)
surface

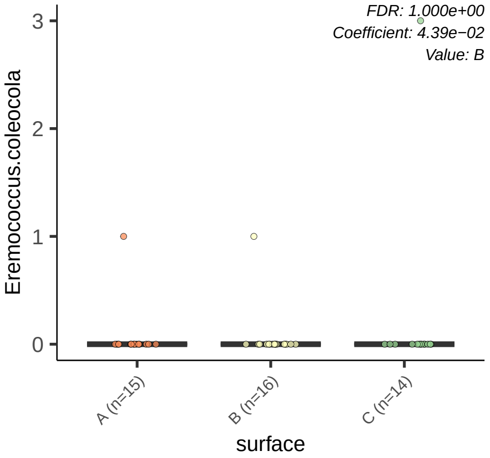

FDR: 1.000e+00
Coefficient: 4.39e−02
Value: B
Eremococcus.coleocola
0
1
2
3
A (n=15)
B (n=16)
C (n=14)
surface

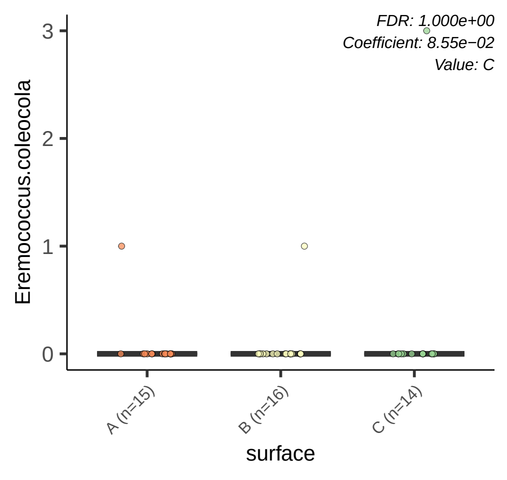

FDR: 1.000e+00
Coefficient: 8.55e−02
Value: C
Eremococcus.coleocola
3
2
1
0
A (n=15)
B (n=16)
C (n=14)
surface

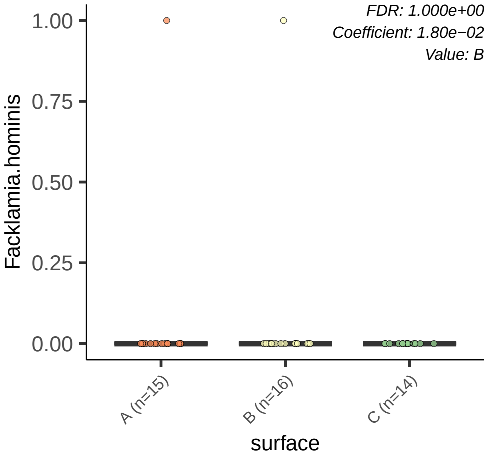
FDR: 1.000e+00
Coefficient: 1.80e−02
Value: B
Facklamia.hominis
1.00
0.75
0.50
0.25
0.00
A (n=15)
B (n=16)
C (n=14)
surface

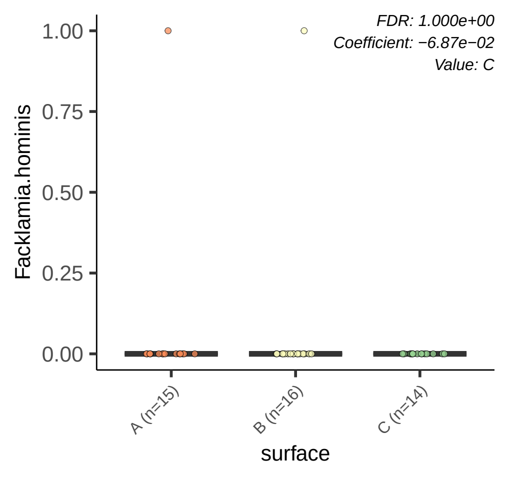
FDR: 1.000e+00
Coefficient: −6.87e−02
Value: C
Facklamia.hominis
1.00
0.75
0.50
0.25
0.00
A (n=15)
B (n=16)
C (n=14)
surface

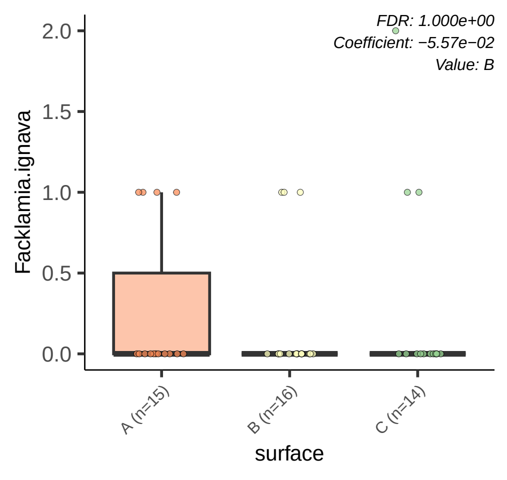

FDR: 1.000e+00
Coefficient: −5.57e−02
Value: B
Facklamia.ignava
2.0
1.5
1.0
0.5
0.0
A (n=15)
B (n=16)
C (n=14)
surface

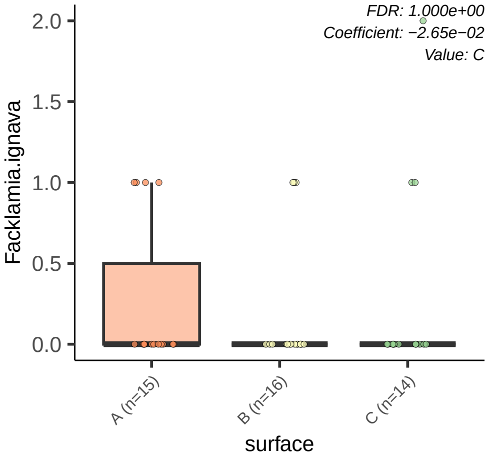

FDR: 1.000e+00
Coefficient: −2.65e−02
Value: C
2.0
1.5
1.0
0.5
0.0
Facklamia.ignava
A (n=15)
B (n=16)
C (n=14)
surface

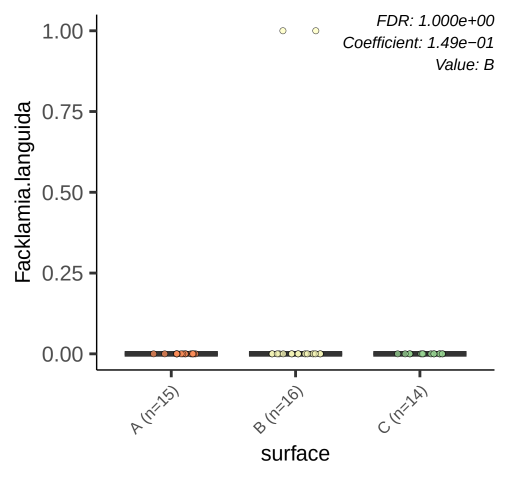

FDR: 1.000e+00
Coefficient: 1.49e−01
Value: B
Facklamia.languida
1.00
0.75
0.50
0.25
0.00
A (n=15)
B (n=16)
C (n=14)
surface

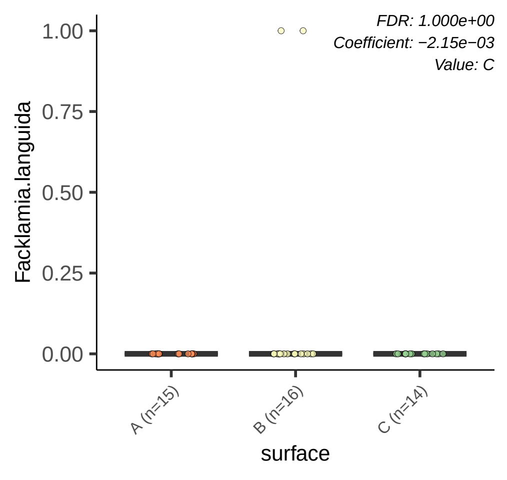
FDR: 1.000e+00
Coefficient: −2.15e−03
Value: C
Facklamia.languida
1.00
0.75
0.50
0.25
0.00
A (n=15)
B (n=16)
C (n=14)
surface

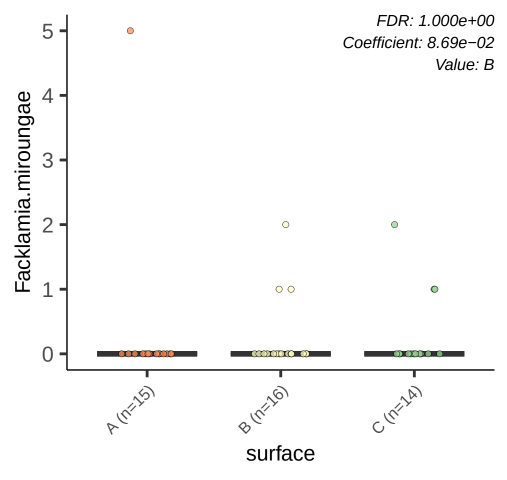
FDR: 1.000e+00
Coefficient: 8.69e−02
Value: B
Facklamia.miroungae
0
1
2
3
4
5
A (n=15)
B (n=16)
C (n=14)
surface

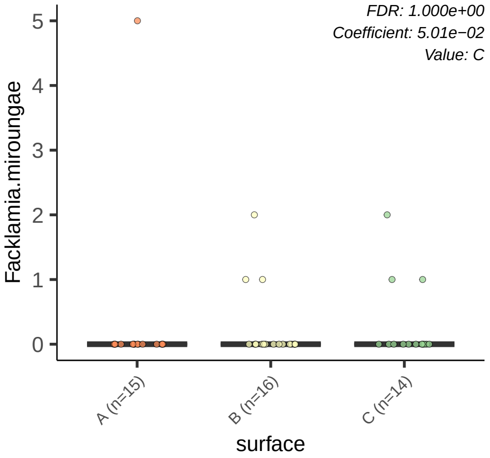

FDR: 1.000e+00
Coefficient: 5.01e−02
Value: C
Facklamia.miroungae
0
1
2
3
4
5
A (n=15)
B (n=16)
C (n=14)
surface

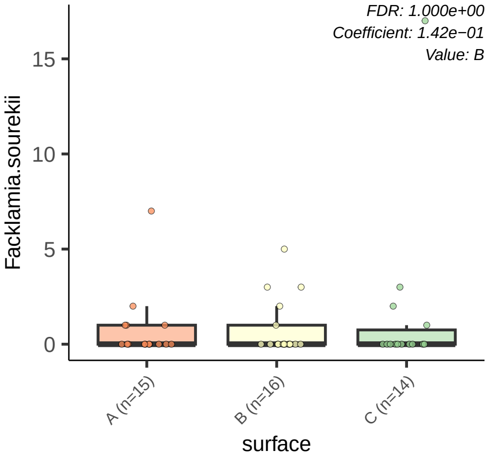

FDR: 1.000e+00
Coefficient: 1.42e−01
Value: B
Facklamia.sourekii
0
5
10
15
A (n=15)
B (n=16)
C (n=14)
surface

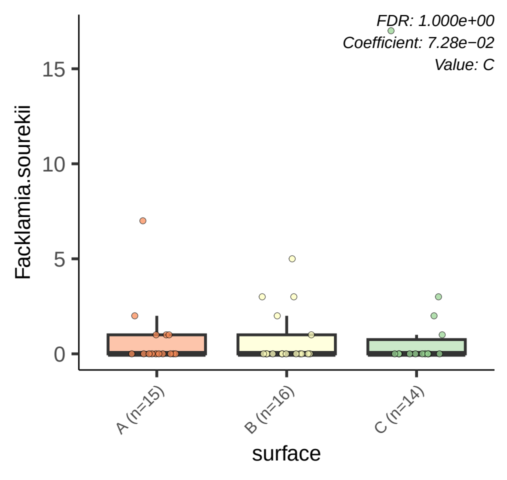
FDR: 1.000e+00
Coefficient: 7.28e−02
Value: C
Facklamia.sourekii
0
5
10
15
A (n=15)
B (n=16)
C (n=14)
surface

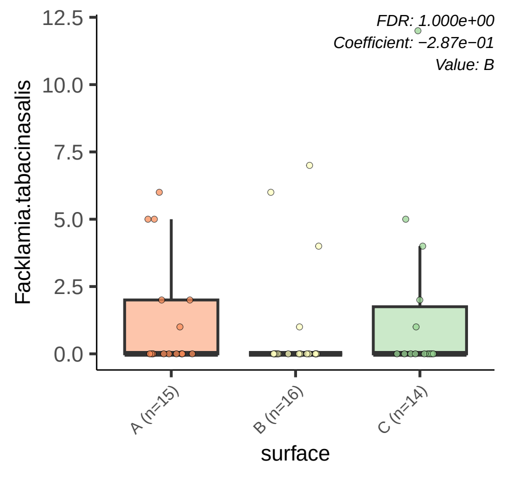

FDR: 1.000e+00
Coefficient: −2.87e−01
Value: B
Facklamia.tabacinasalis
12.5
10.0
7.5
5.0
2.5
0.0
A (n=15)
B (n=16)
C (n=14)
surface

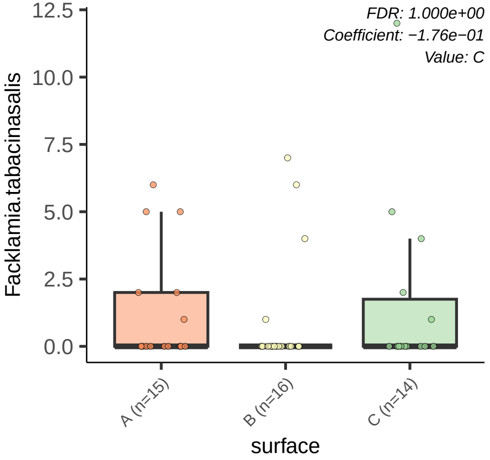

FDR: 1.000e+00
Coefficient: −1.76e−01
Value: C
Facklamia.tabacinasalis
12.5
10.0
7.5
5.0
2.5
0.0
A (n=15)
B (n=16)
C (n=14)
surface

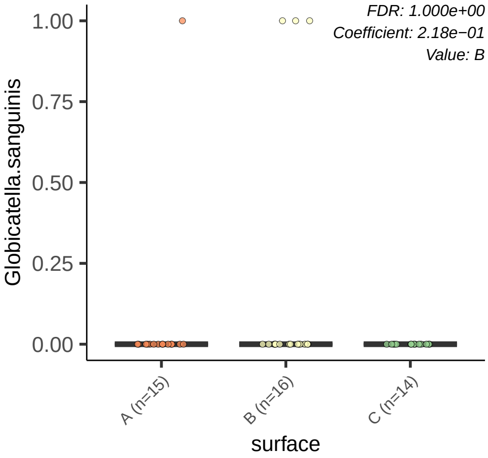

FDR: 1.000e+00
Coefficient: 2.18e−01
Value: B
Globicatella.sanguinis
1.00
0.75
0.50
0.25
0.00
A (n=15)
B (n=16)
C (n=14)
surface

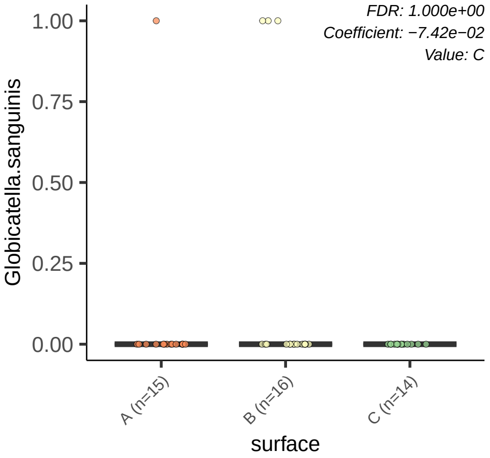
FDR: 1.000e+00
Coefficient: −7.42e−02
Value: C
Globicatella.sanguinis
1.00
0.75
0.50
0.25
0.00
A (n=15)
B (n=16)
C (n=14)
surface

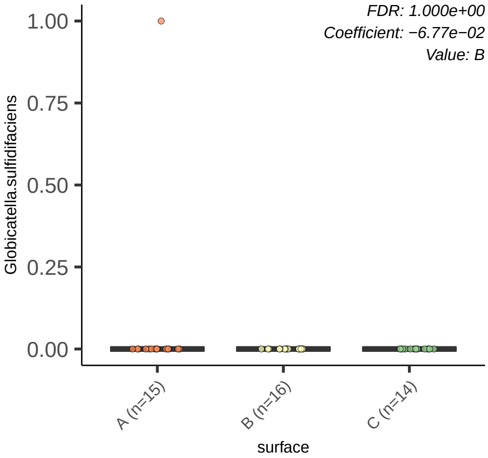
FDR: 1.000e+00
Coefficient: −6.77e−02
Value: B
Globicatella.sulfidifaciens
1.00
0.75
0.50
0.25
0.00
A (n=15)
B (n=16)
C (n=14)
surface

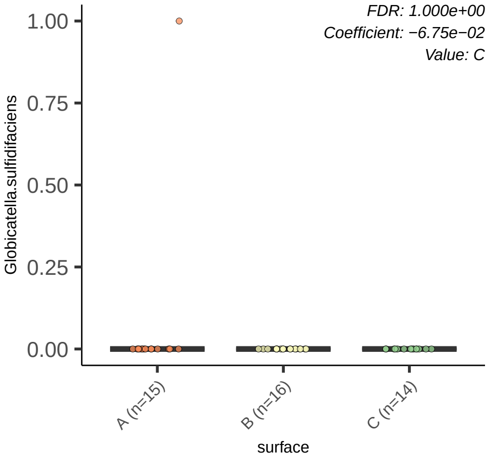

FDR: 1.000e+00
Coefficient: −6.75e−02
Value: C
Globicatella.sulfidifaciens
1.00
0.75
0.50
0.25
0.00
A (n=15)
B (n=16)
C (n=14)
surface

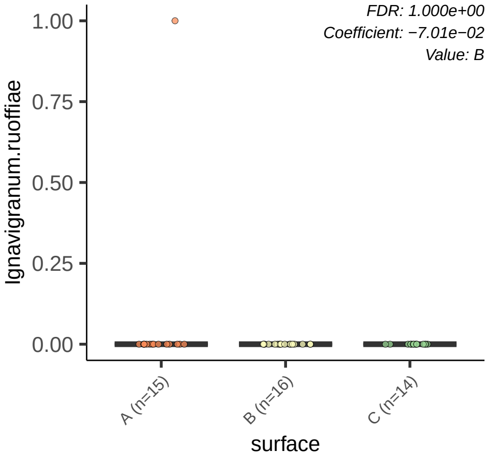
FDR: 1.000e+00
Coefficient: −7.01e−02
Value: B
Ignavigranum.ruoffiae
1.00
0.75
0.50
0.25
0.00
A (n=15)
B (n=16)
C (n=14)
surface

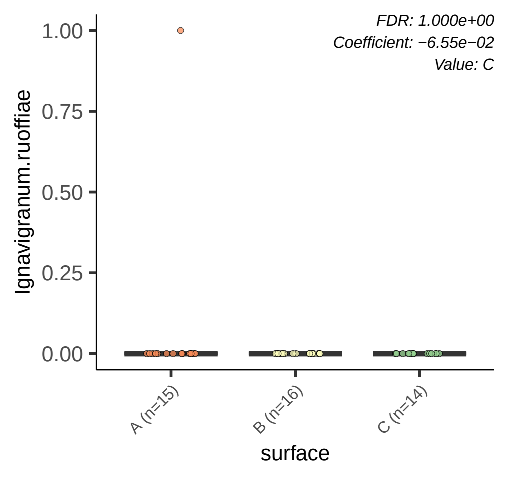
FDR: 1.000e+00
Coefficient: −6.55e−02
Value: C
Ignavigranum.ruoffiae
1.00
0.75
0.50
0.25
0.00
A (n=15)
B (n=16)
C (n=14)
surface

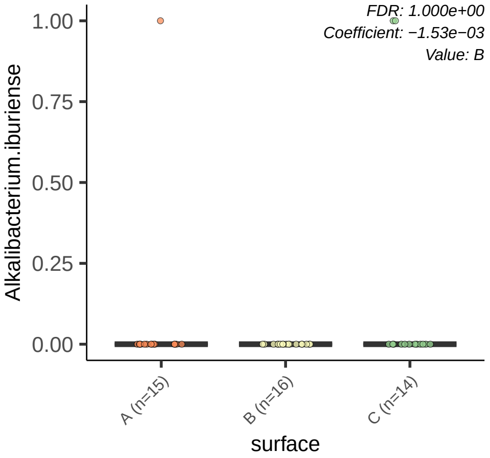

FDR: 1.000e+00
Coefficient: −1.53e−03
Value: B
Alkalibacterium.iburiense
1.00
0.75
0.50
0.25
0.00
A (n=15)
B (n=16)
C (n=14)
surface

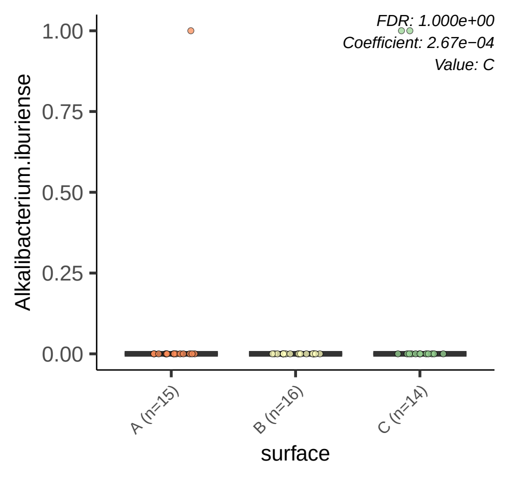
FDR: 1.000e+00
Coefficient: 2.67e−04
Value: C
Alkalibacterium.iburiense
1.00
0.75
0.50
0.25
0.00
A (n=15)
B (n=16)
C (n=14)
surface

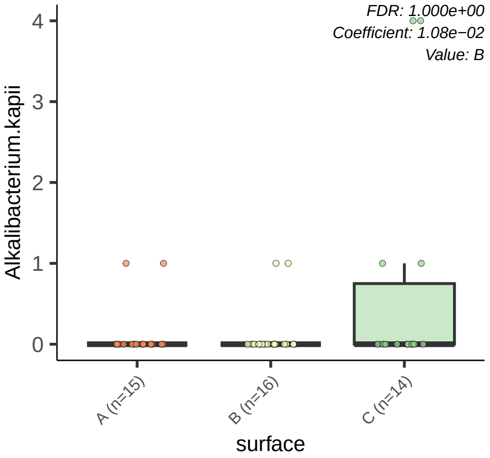

FDR: 1.000e+00
Coefficient: 1.08e−02
Value: B
Alkalibacterium.kapii
4
3
2
1
0
A (n=15)
B (n=16)
C (n=14)
surface

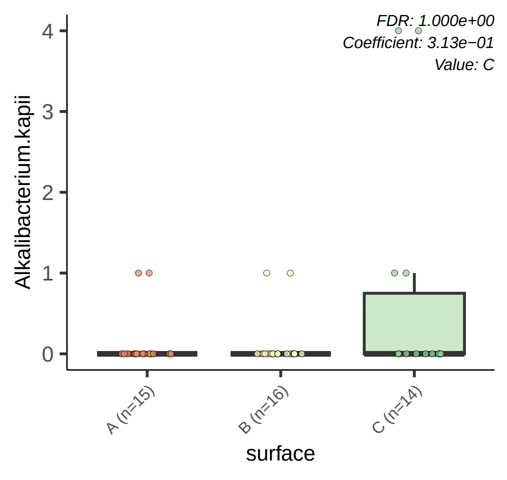
FDR: 1.000e+00
Coefficient: 3.13e−01
Value: C
Alkalibacterium.kapii
0
1
2
3
4
A (n=15)
B (n=16)
C (n=14)
surface

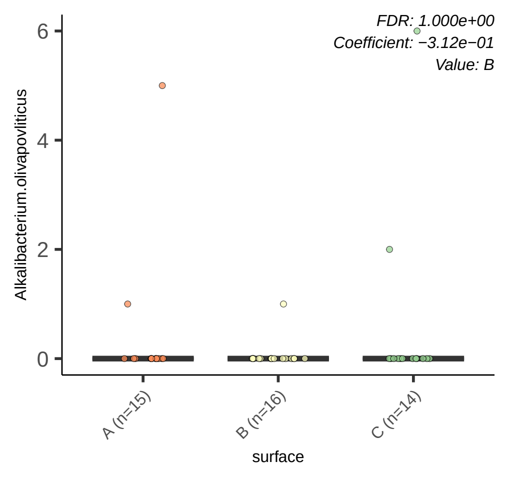
FDR: 1.000e+00
Coefficient: −3.12e−01
Value: B
Alkalibacterium.olivapovliticus
0
2
4
6
A (n=15)
B (n=16)
C (n=14)
surface

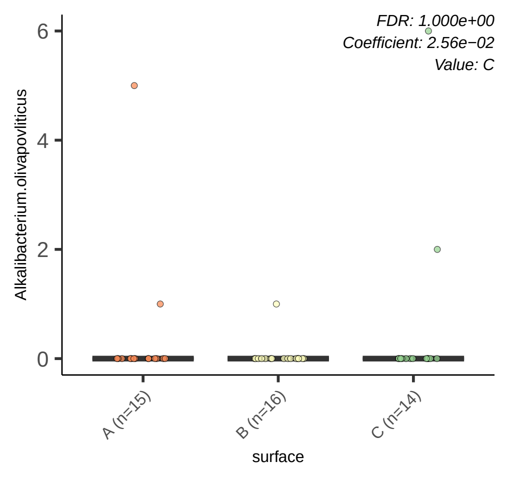
FDR: 1.000e+00
Coefficient: 2.56e−02
Value: C
Alkalibacterium.olivapovliticus
0
2
4
6
A (n=15)
B (n=16)
C (n=14)
surface

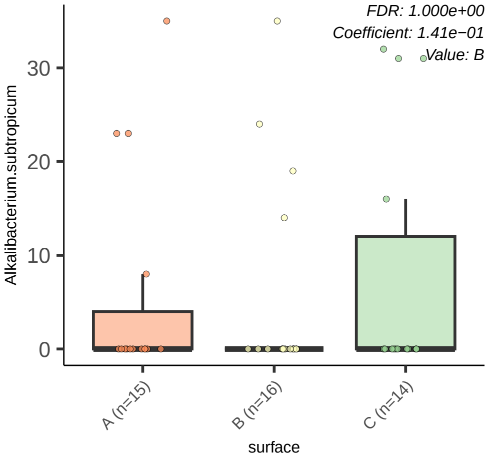

FDR: 1.000e+00
Coefficient: 1.41e−01
Value: B
Alkalibacterium.subtropicum
0
10
20
30
A (n=15)
B (n=16)
C (n=14)
surface

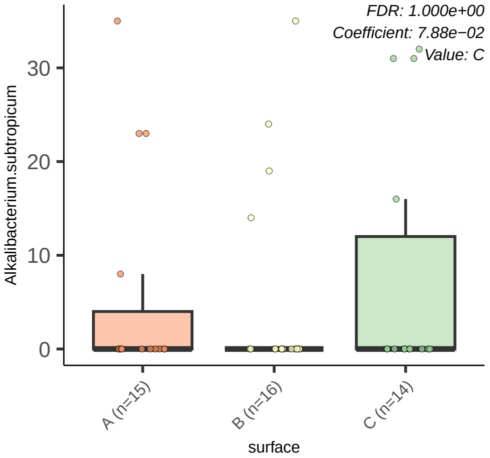
FDR: 1.000e+00
Coefficient: 7.88e−02
Value: C
Alkalibacterium.subtropicum
0
10
20
30
A (n=15)
B (n=16)
C (n=14)
surface

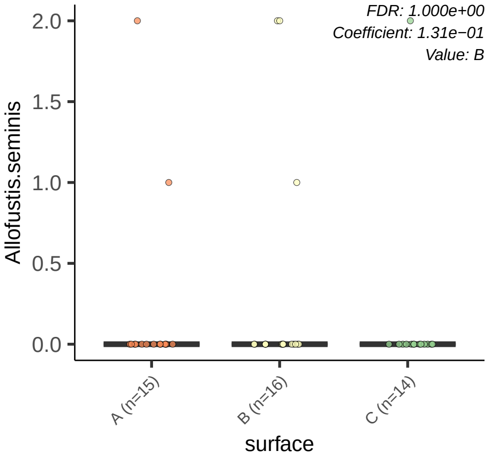

FDR: 1.000e+00
Coefficient: 1.31e−01
Value: B
Allofustis.seminis
2.0
1.5
1.0
0.5
0.0
A (n=15)
B (n=16)
C (n=14)
surface

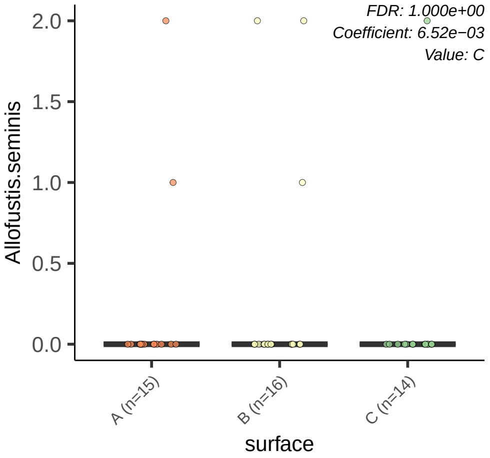
FDR: 1.000e+00
Coefficient: 6.52e−03
Value: C
Allofustis.seminis
2.0
1.5
1.0
0.5
0.0
A (n=15)
B (n=16)
C (n=14)
surface

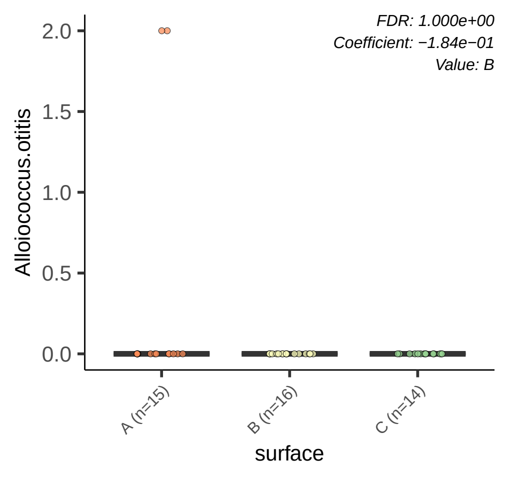

FDR: 1.000e+00
Coefficient: −1.84e−01
Value: B
Alloiococcus.otitis
2.0
1.5
1.0
0.5
0.0
A (n=15)
B (n=16)
C (n=14)
surface

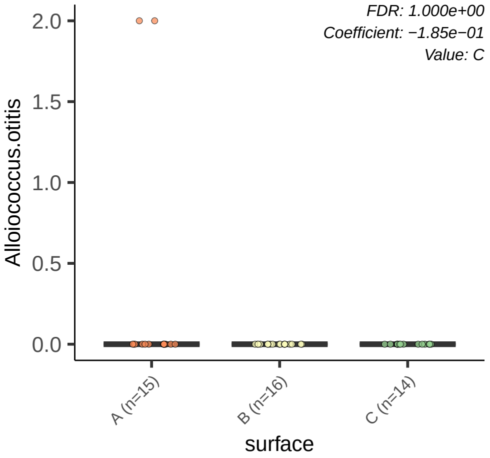

FDR: 1.000e+00
Coefficient: −1.85e−01
Value: C
Alloiococcus.otitis
2.0
1.5
1.0
0.5
0.0
A (n=15)
B (n=16)
C (n=14)
surface

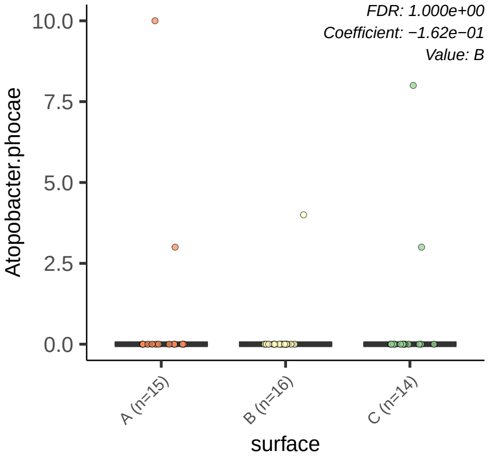

FDR: 1.000e+00
Coefficient: −1.62e−01
Value: B
Atopobacter.phocae
10.0
7.5
5.0
2.5
0.0
A (n=15)
B (n=16)
C (n=14)
surface

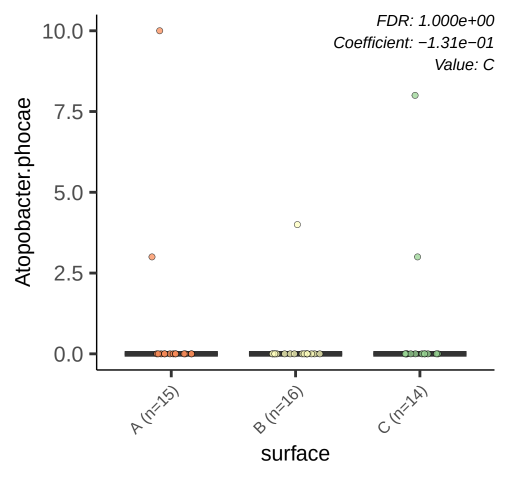
FDR: 1.000e+00
Coefficient: −1.31e−01
Value: C
Atopobacter.phocae
10.0
7.5
5.0
2.5
0.0
A (n=15)
B (n=16)
C (n=14)
surface

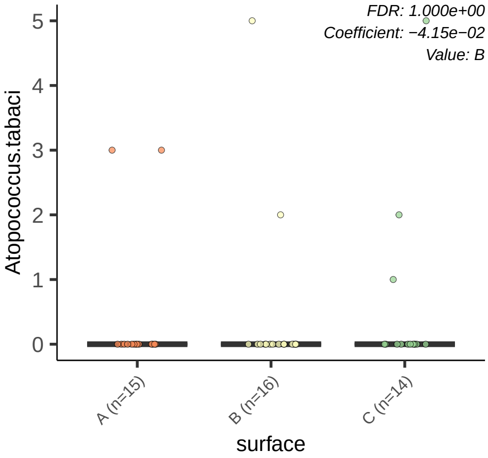
FDR: 1.000e+00
Coefficient: −4.15e−02
Value: B
Atopococcus.tabaci
5
4
3
2
1
0
A (n=15)
B (n=16)
C (n=14)
surface

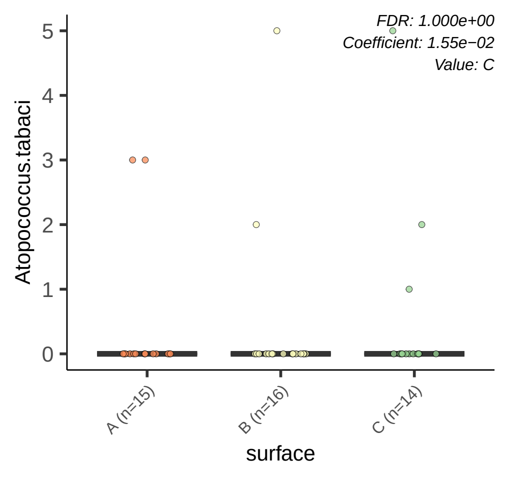
FDR: 1.000e+00
Coefficient: 1.55e−02
Value: C
Atopococcus.tabaci
0
1
2
3
4
5
A (n=15)
B (n=16)
C (n=14)
surface

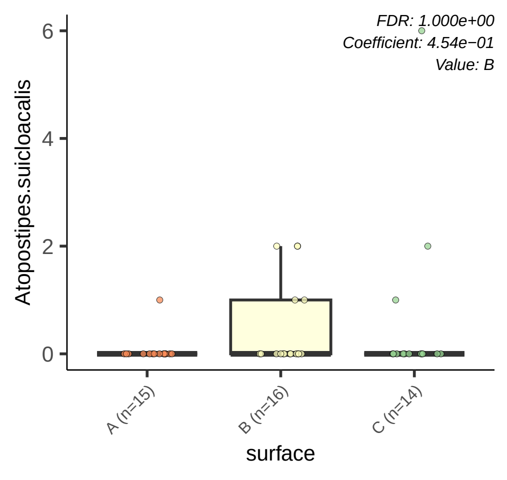
FDR: 1.000e+00
Coefficient: 4.54e−01
Value: B
Atopostipes.suicloacalis
0
2
4
6
A (n=15)
B (n=16)
C (n=14)
surface

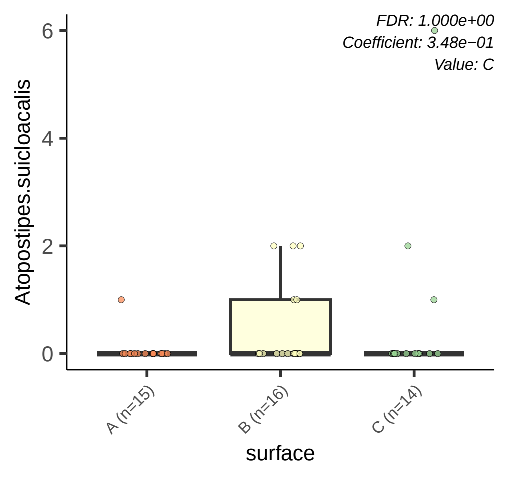
FDR: 1.000e+00
Coefficient: 3.48e−01
Value: C
Atopostipes.suicloacalis
0
2
4
6
A (n=15)
B (n=16)
C (n=14)
surface

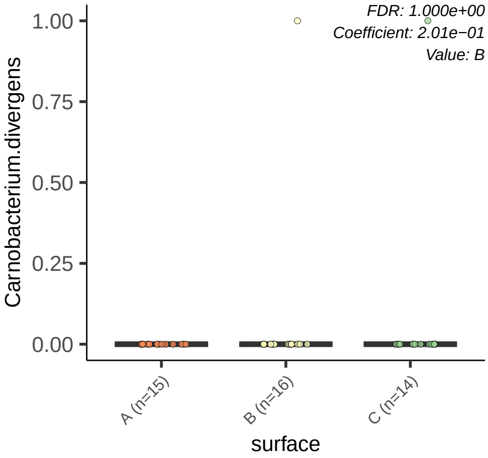
FDR: 1.000e+00
Coefficient: 2.01e−01
Value: B
Carnobacterium.divergens
1.00
0.75
0.50
0.25
0.00
A (n=15)
B (n=16)
C (n=14)
surface

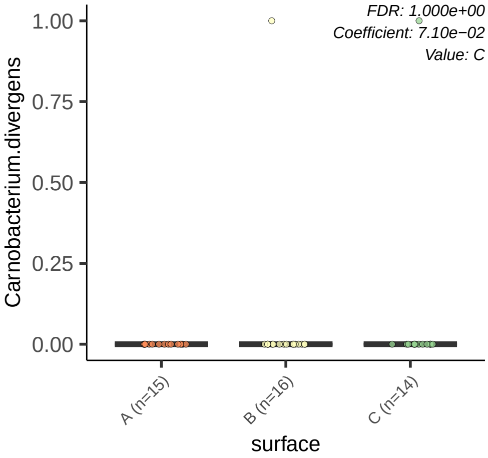
FDR: 1.000e+00
Coefficient: 7.10e−02
Value: C
Carnobacterium.divergens
1.00
0.75
0.50
0.25
0.00
A (n=15)
B (n=16)
C (n=14)
surface

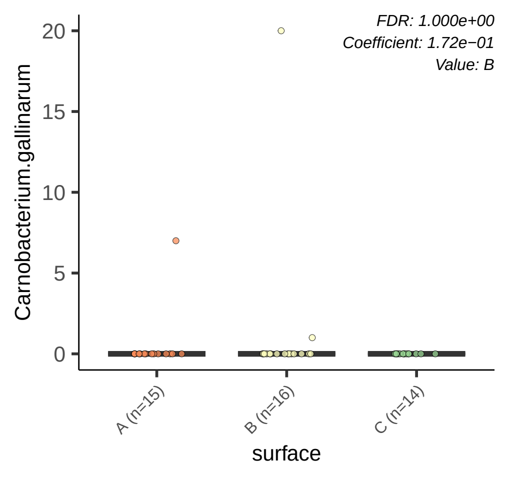

FDR: 1.000e+00
Coefficient: 1.72e−01
Value: B
Carnobacterium.gallinarum
20
15
10
5
0
A (n=15)
B (n=16)
C (n=14)
surface

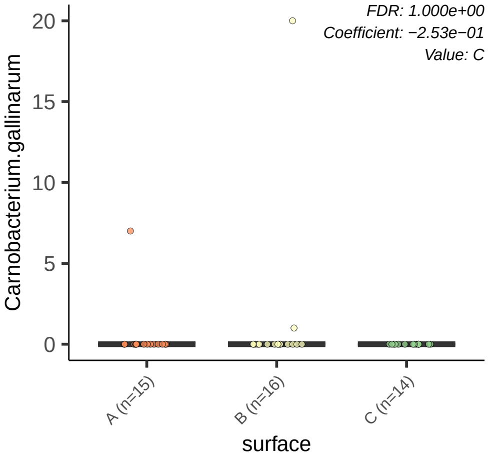
FDR: 1.000e+00
Coefficient: −2.53e−01
Value: C
Carnobacterium.gallinarum
20
15
10
5
0
A (n=15)
B (n=16)
C (n=14)
surface

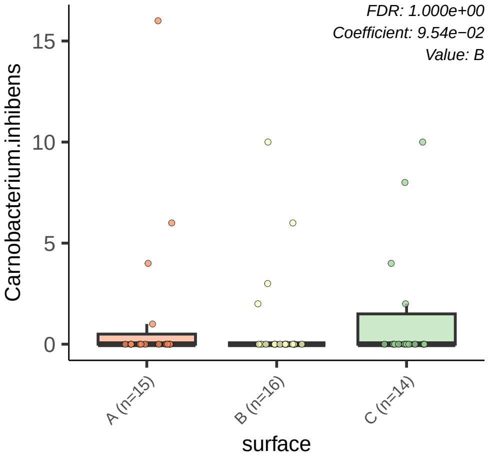

FDR: 1.000e+00
Coefficient: 9.54e−02
Value: B
Carnobacterium.inhibens
15
10
5
0
A (n=15)
B (n=16)
C (n=14)
surface

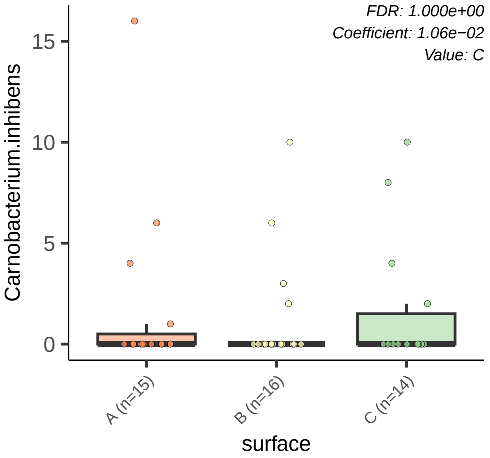

FDR: 1.000e+00
Coefficient: 1.06e−02
Value: C
Carnobacterium.inhibens
15
10
5
0
A (n=15)
B (n=16)
C (n=14)
surface

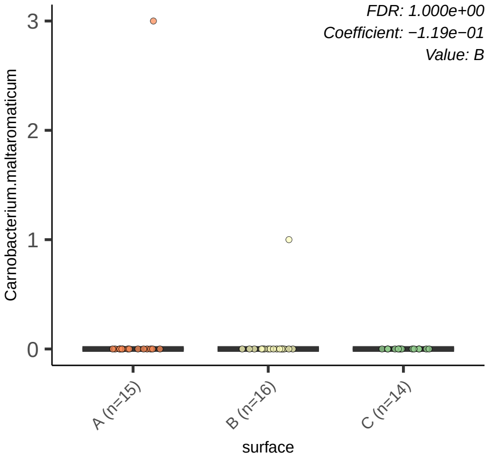
FDR: 1.000e+00
Coefficient: −1.19e−01
Value: B
Carnobacterium.maltaromaticum
3
2
1
0
A (n=15)
B (n=16)
C (n=14)
surface

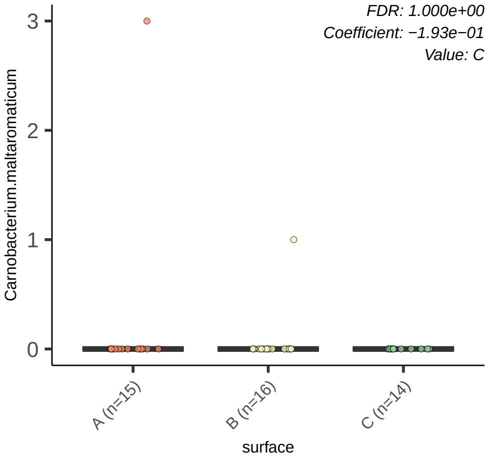
FDR: 1.000e+00
Coefficient: −1.93e−01
Value: C
Carnobacterium.maltaromaticum
3
2
1
0
A (n=15)
B (n=16)
C (n=14)
surface

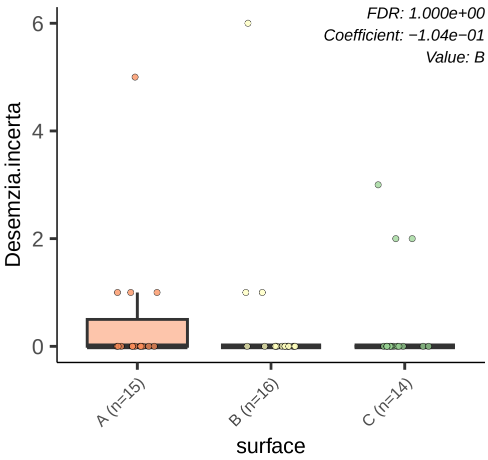
FDR: 1.000e+00
Coefficient: −1.04e−01
Value: B
Desemzia.incerta
0
2
4
6
A (n=15)
B (n=16)
C (n=14)
surface

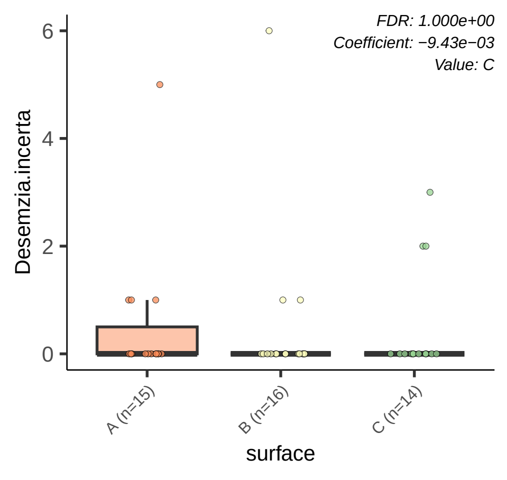
FDR: 1.000e+00
Coefficient: −9.43e−03
Value: C
Desemzia.incerta
0
2
4
6
A (n=15)
B (n=16)
C (n=14)
surface

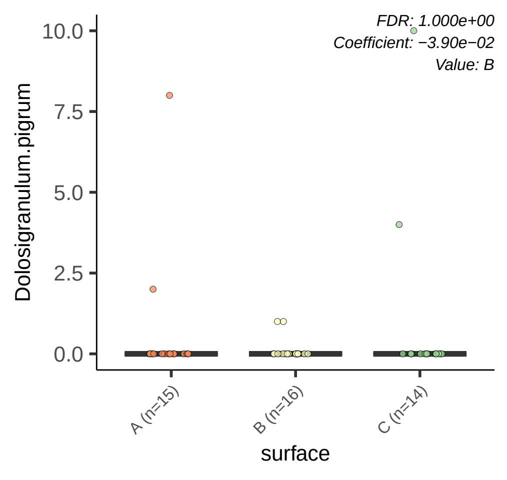
FDR: 1.000e+00
Coefficient: −3.90e−02
Value: B
Dolosigranulum.pigrum
10.0
7.5
5.0
2.5
0.0
A (n=15)
B (n=16)
C (n=14)
surface

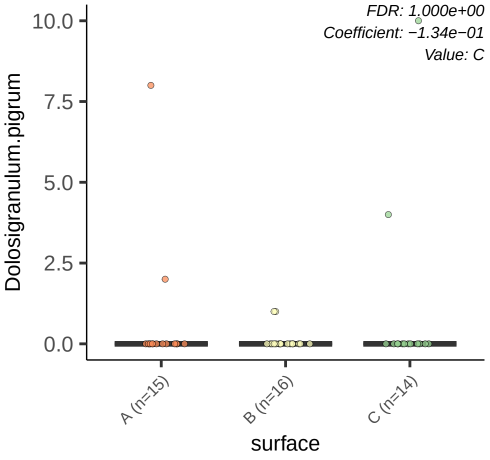

FDR: 1.000e+00
Coefficient: −1.34e−01
Value: C
Dolosigranulum.pigrum
10.0
7.5
5.0
2.5
0.0
A (n=15)
B (n=16)
C (n=14)
surface

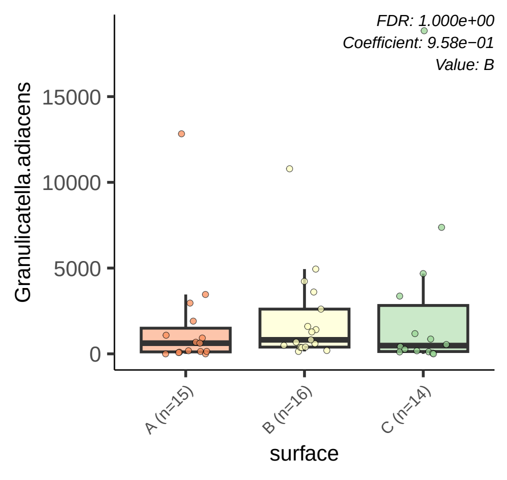

FDR: 1.000e+00
Coefficient: 9.58e−01
Value: B
Granulicatella.adiacens
15000
10000
5000
0
A (n=15)
B (n=16)
C (n=14)
surface

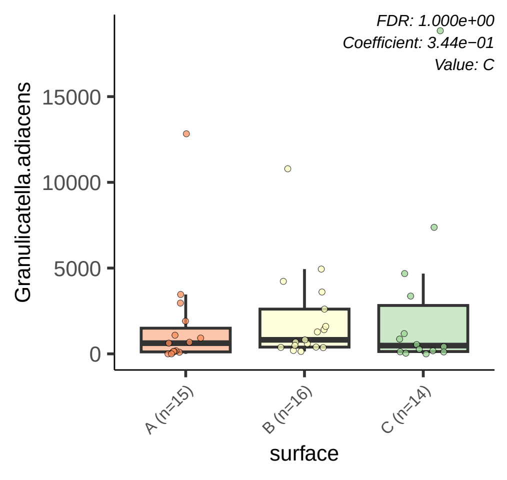
FDR: 1.000e+00
Coefficient: 3.44e−01
Value: C
Granulicatella.adiacens
15000
10000
5000
0
A (n=15)
B (n=16)
C (n=14)
surface

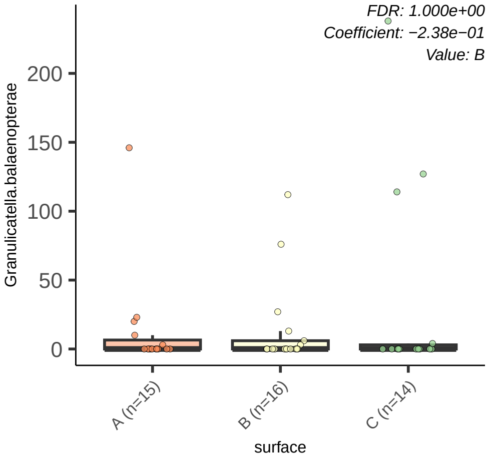

FDR: 1.000e+00
Coefficient: −2.38e−01
Value: B
Granulicatella.balaenopterae
200
150
100
50
0
A (n=15)
B (n=16)
C (n=14)
surface

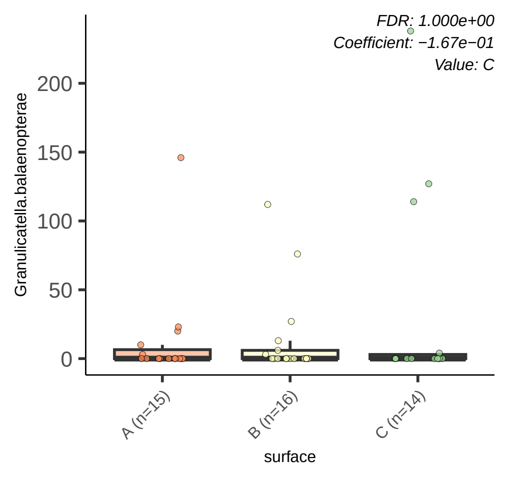
FDR: 1.000e+00
Coefficient: −1.67e−01
Value: C
Granulicatella.balaenopterae
200
150
100
50
0
A (n=15)
B (n=16)
C (n=14)
surface

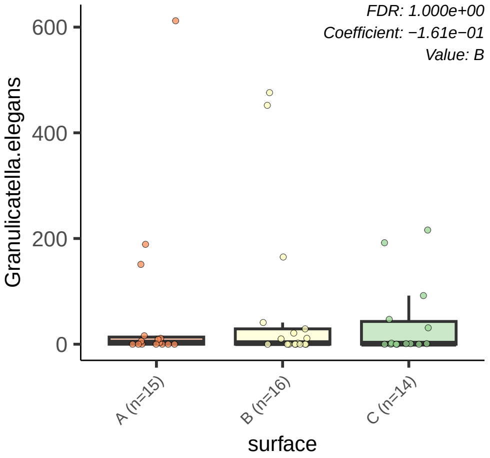
FDR: 1.000e+00
Coefficient: −1.61e−01
Value: B
Granulicatella.elegans
600
400
200
0
A (n=15)
B (n=16)
C (n=14)
surface

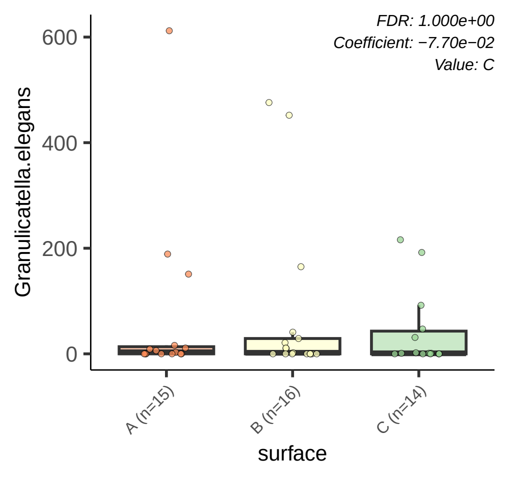
FDR: 1.000e+00
Coefficient: −7.70e−02
Value: C
Granulicatella.elegans
600
400
200
0
A (n=15)
B (n=16)
C (n=14)
surface

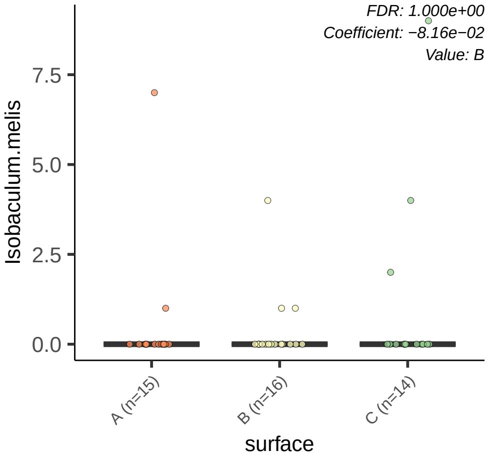

FDR: 1.000e+00
Coefficient: −8.16e−02
Value: B
Isobaculum.melis
7.5
5.0
2.5
0.0
A (n=15)
B (n=16)
C (n=14)
surface

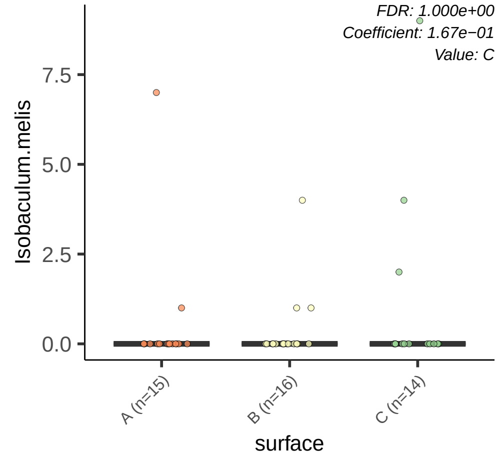
FDR: 1.000e+00
Coefficient: 1.67e−01
Value: C
Isobaculum.melis
7.5
5.0
2.5
0.0
A (n=15)
B (n=16)
C (n=14)
surface

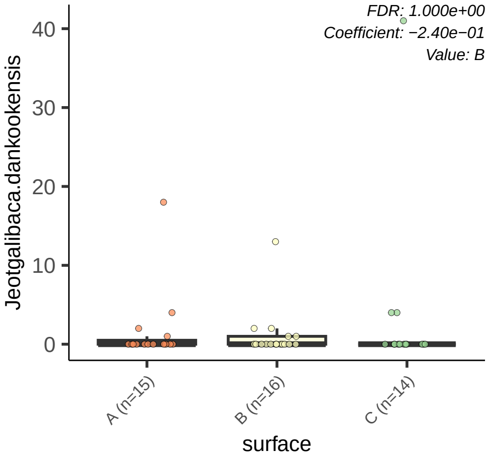
FDR: 1.000e+00
Coefficient: −2.40e−01
Value: B
Jeotgalibaca.dankookensis
40
30
20
10
0
A (n=15)
B (n=16)
C (n=14)
surface

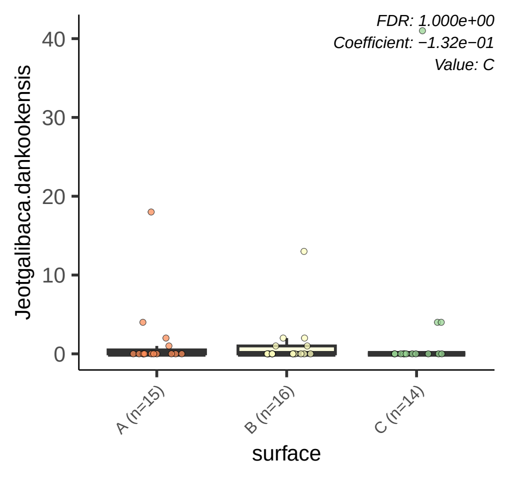
FDR: 1.000e+00
Coefficient: −1.32e−01
Value: C
Jeotgalibaca.dankookensis
0
10
20
30
40
A (n=15)
B (n=16)
C (n=14)
surface

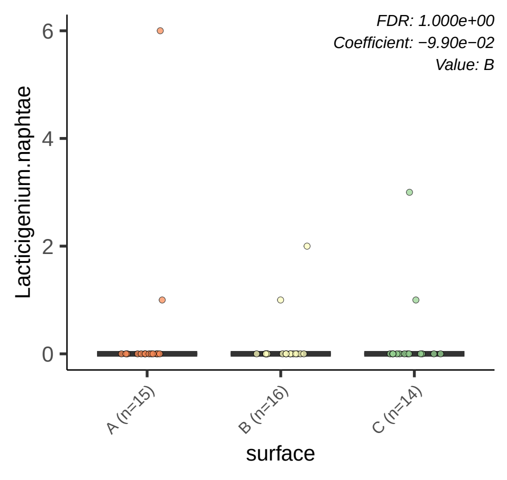

FDR: 1.000e+00
Coefficient: −9.90e−02
Value: B
Lacticigenium.naphtae
0
2
4
6
A (n=15)
B (n=16)
C (n=14)
surface

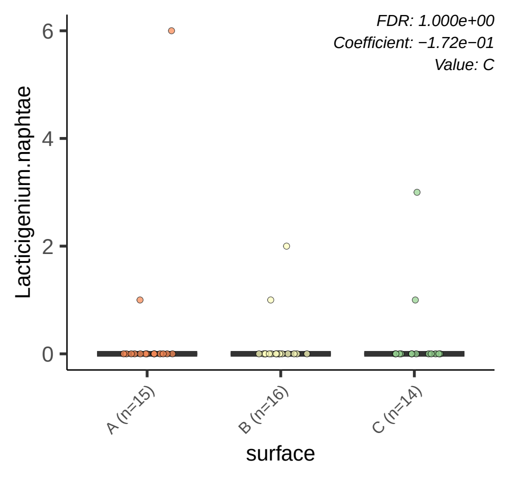

FDR: 1.000e+00
Coefficient: −1.72e−01
Value: C
Lacticigenium.naphtae
0
2
4
6
A (n=15)
B (n=16)
C (n=14)
surface

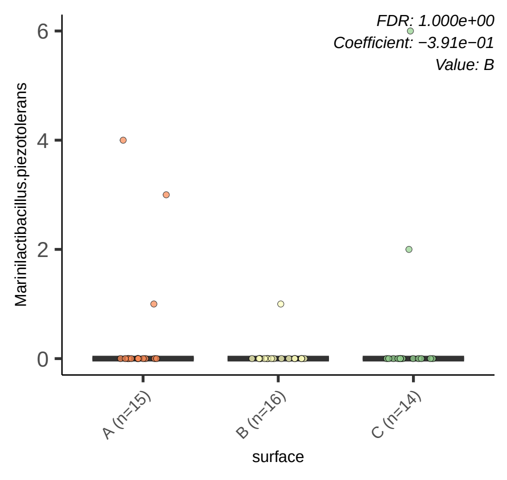
FDR: 1.000e+00
Coefficient: −3.91e−01
Value: B
Marinilactibacillus.piezotolerans
0
2
4
6
A (n=15)
B (n=16)
C (n=14)
surface

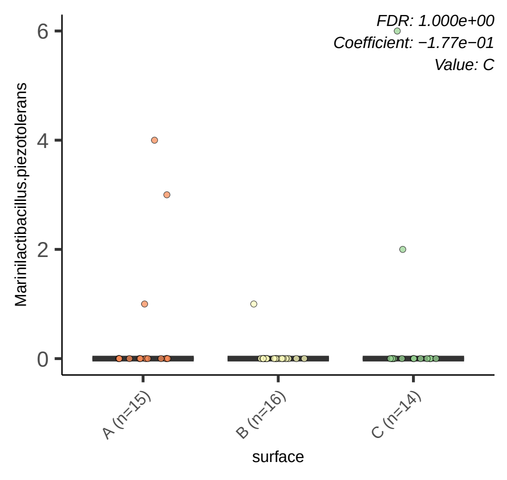

FDR: 1.000e+00
Coefficient: −1.77e−01
Value: C
Marinilactibacillus.piezotolerans
0
2
4
6
A (n=15)
B (n=16)
C (n=14)
surface

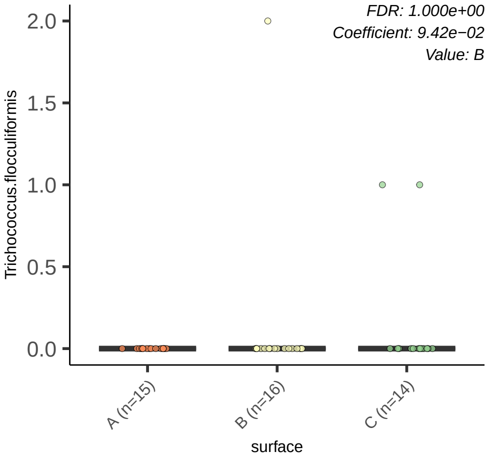

FDR: 1.000e+00
Coefficient: 9.42e−02
Value: B
Trichococcus.flocculiformis
2.0
1.5
1.0
0.5
0.0
A (n=15)
B (n=16)
C (n=14)
surface

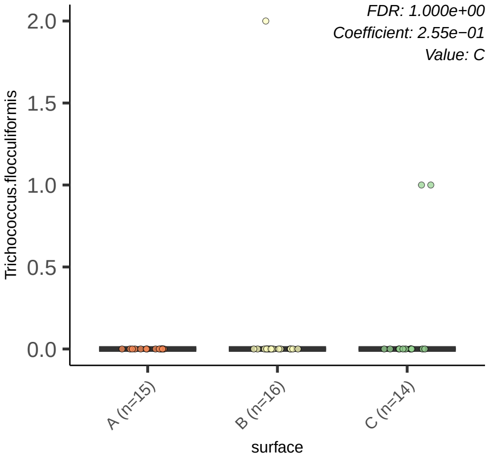
FDR: 1.000e+00
Coefficient: 2.55e−01
Value: C
Trichococcus.flocculiformis
2.0
1.5
1.0
0.5
0.0
A (n=15)
B (n=16)
C (n=14)
surface

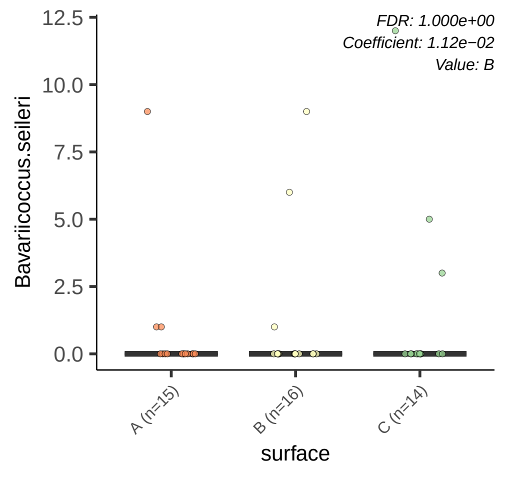

FDR: 1.000e+00
Coefficient: 1.12e−02
Value: B
Bavariicoccus.seileri
12.5
10.0
7.5
5.0
2.5
0.0
A (n=15)
B (n=16)
C (n=14)
surface

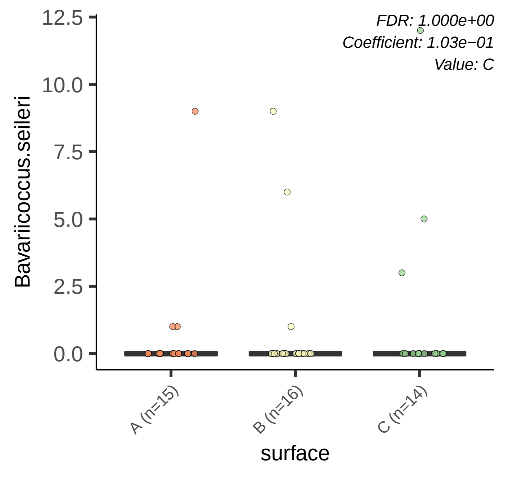

FDR: 1.000e+00
Coefficient: 1.03e−01
Value: C
Bavariicoccus.seileri
12.5
10.0
7.5
5.0
2.5
0.0
A (n=15)
B (n=16)
C (n=14)
surface

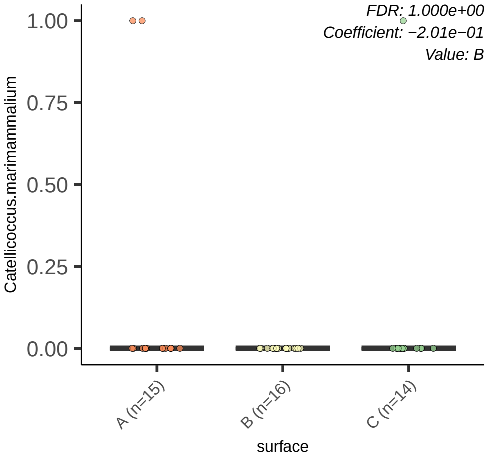
FDR: 1.000e+00
Coefficient: −2.01e−01
Value: B
Catellicoccus.marimammalium
1.00
0.75
0.50
0.25
0.00
A (n=15)
B (n=16)
C (n=14)
surface

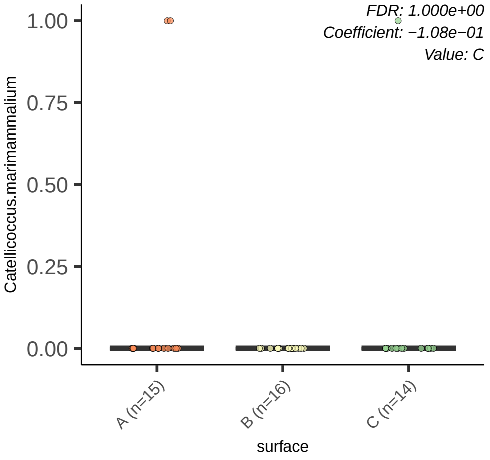
FDR: 1.000e+00
Coefficient: −1.08e−01
Value: C
Catellicoccus.marimammalium
1.00
0.75
0.50
0.25
0.00
A (n=15)
B (n=16)
C (n=14)
surface

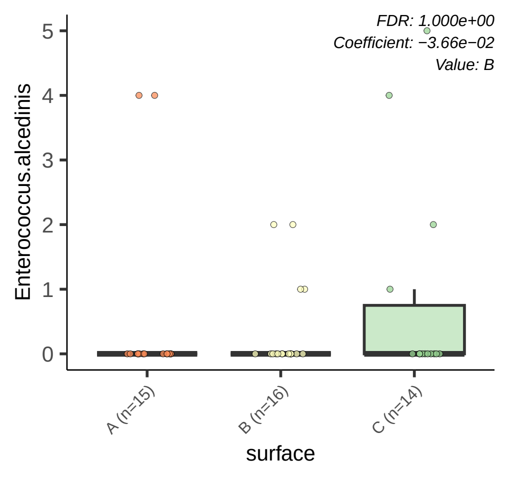
FDR: 1.000e+00
Coefficient: −3.66e−02
Value: B
Enterococcus.alcedinis
0
1
2
3
4
5
A (n=15)
B (n=16)
C (n=14)
surface

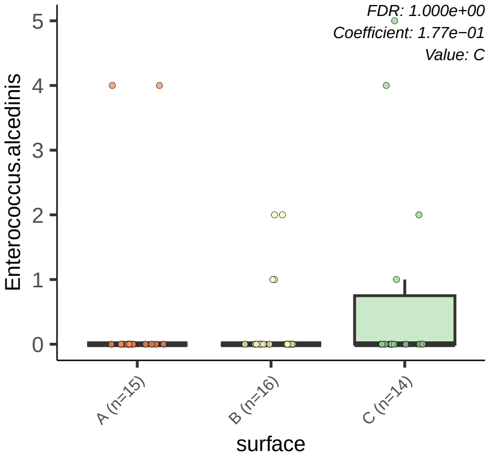

FDR: 1.000e+00
Coefficient: 1.77e−01
Value: C
Enterococcus.alcedinis
0
1
2
3
4
5
A (n=15)
B (n=16)
C (n=14)
surface

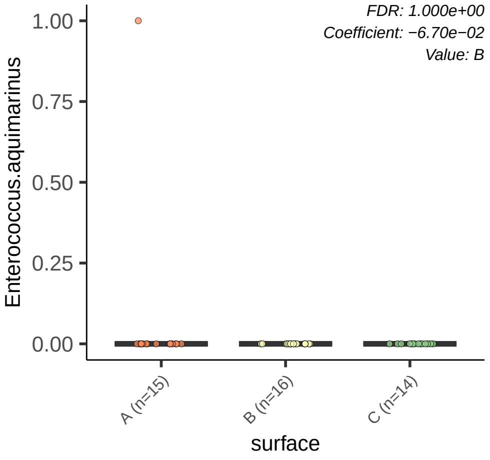

FDR: 1.000e+00
Coefficient: −6.70e−02
Value: B
Enterococcus.aquimarinus
1.00
0.75
0.50
0.25
0.00
A (n=15)
B (n=16)
C (n=14)
surface

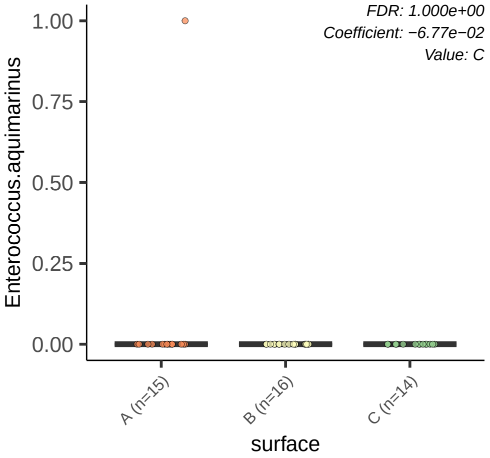

FDR: 1.000e+00
Coefficient: −6.77e−02
Value: C
Enterococcus.aquimarinus
1.00
0.75
0.50
0.25
0.00
A (n=15)
B (n=16)
C (n=14)
surface

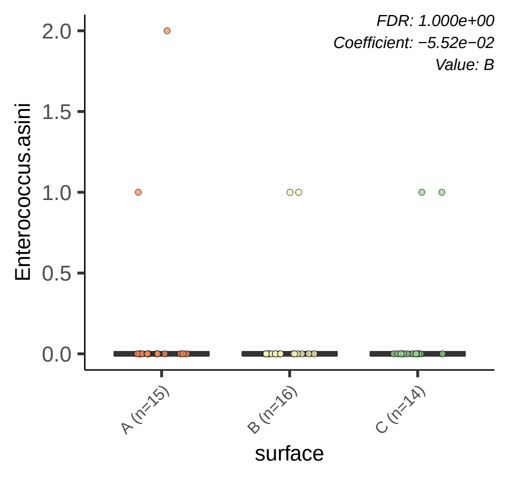

FDR: 1.000e+00
Coefficient: −5.52e−02
Value: B
Enterococcus.asini
2.0
1.5
1.0
0.5
0.0
A (n=15)
B (n=16)
C (n=14)
surface

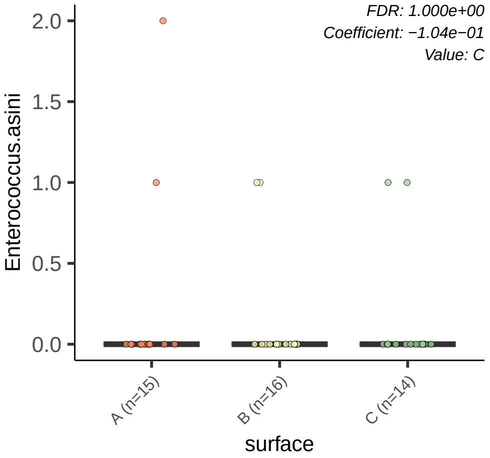
FDR: 1.000e+00
Coefficient: −1.04e−01
Value: C
Enterococcus.asini
2.0
1.5
1.0
0.5
0.0
A (n=15)
B (n=16)
C (n=14)
surface

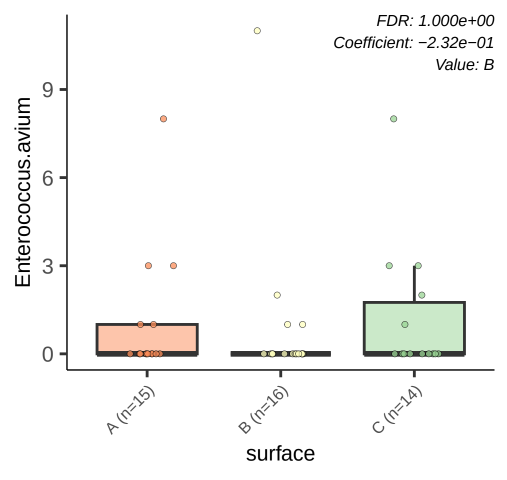
FDR: 1.000e+00
Coefficient: −2.32e−01
Value: B
Enterococcus.avium
0
3
6
9
A (n=15)
B (n=16)
C (n=14)
surface

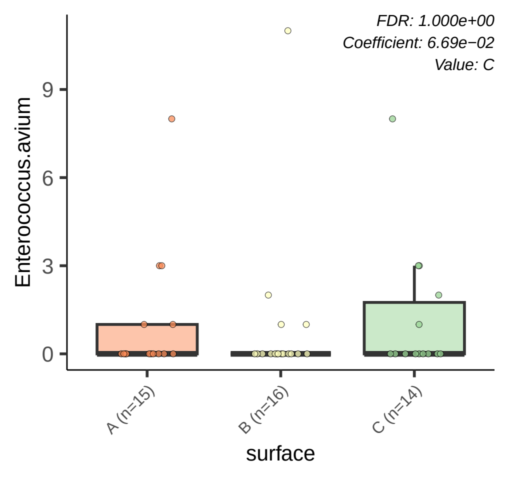
FDR: 1.000e+00
Coefficient: 6.69e−02
Value: C
Enterococcus.avium
0
3
6
9
A (n=15)
B (n=16)
C (n=14)
surface

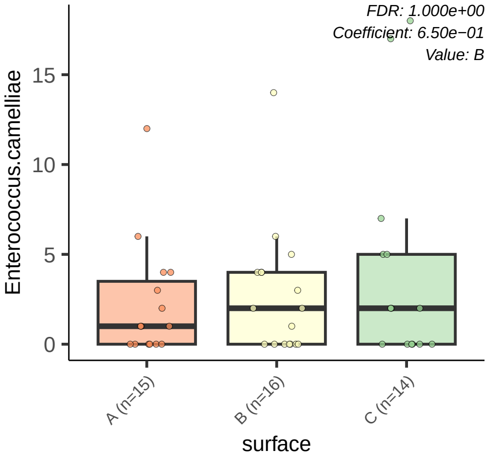
FDR: 1.000e+00
Coefficient: 6.50e−01
Value: B
Enterococcus.camelliae
0
5
10
15
A (n=15)
B (n=16)
C (n=14)
surface

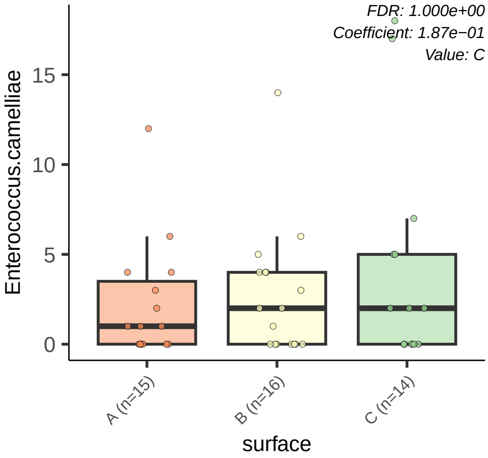
FDR: 1.000e+00
Coefficient: 1.87e−01
Value: C
Enterococcus.camelliae
0
5
10
15
A (n=15)
B (n=16)
C (n=14)
surface

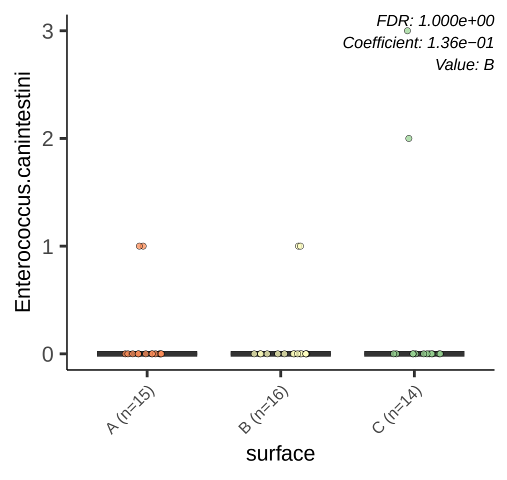
FDR: 1.000e+00
Coefficient: 1.36e−01
Value: B
Enterococcus.canintestini
3
2
1
0
A (n=15)
B (n=16)
C (n=14)
surface

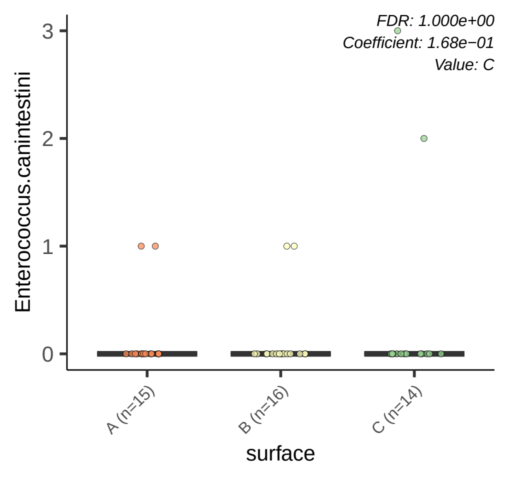
FDR: 1.000e+00
Coefficient: 1.68e−01
Value: C
Enterococcus.canintestini
3
2
1
0
A (n=15)
B (n=16)
C (n=14)
surface

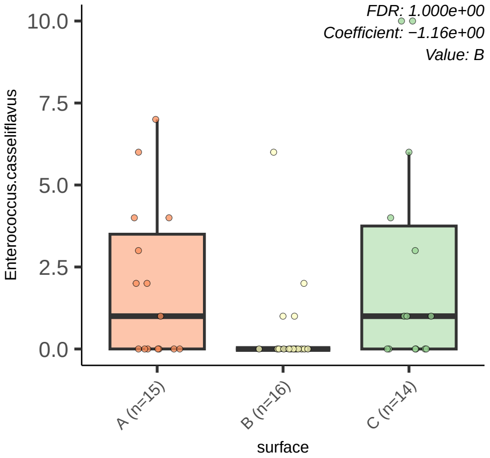
FDR: 1.000e+00
Coefficient: −1.16e+00
Value: B
10.0
7.5
5.0
2.5
0.0
Enterococcus.casseliflavus
A (n=15)
B (n=16)
C (n=14)
surface

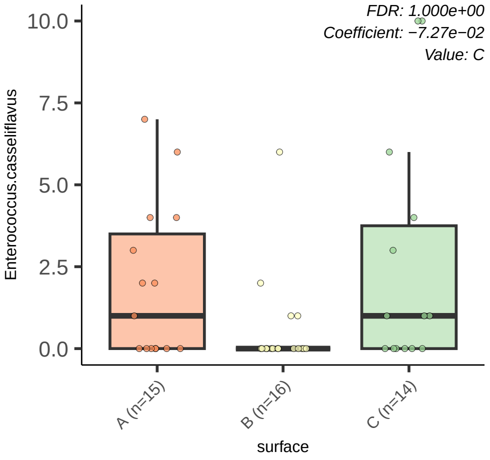
FDR: 1.000e+00
Coefficient: −7.27e−02
Value: C
Enterococcus.casseliflavus
10.0
7.5
5.0
2.5
0.0
A (n=15)
B (n=16)
C (n=14)
surface

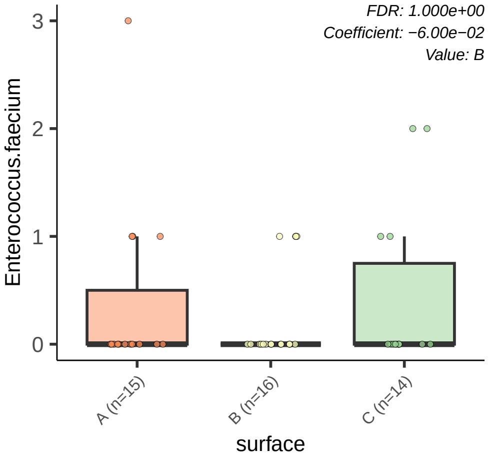
FDR: 1.000e+00
Coefficient: −6.00e−02
Value: B
Enterococcus.faecium
3
2
1
0
A (n=15)
B (n=16)
C (n=14)
surface

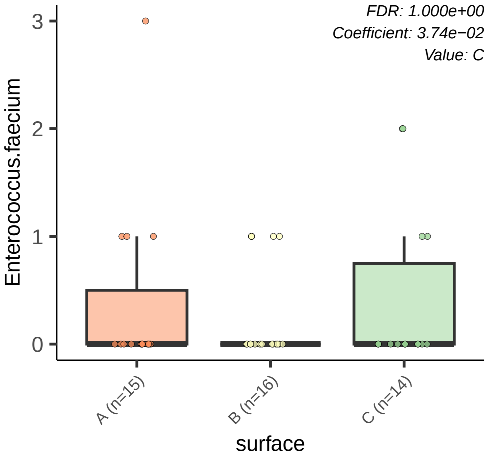
FDR: 1.000e+00
Coefficient: 3.74e−02
Value: C
Enterococcus.faecium
0
1
2
3
A (n=15)
B (n=16)
C (n=14)
surface

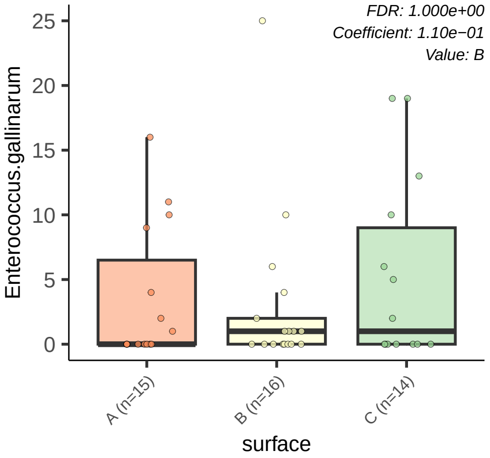

FDR: 1.000e+00
Coefficient: 1.10e−01
Value: B
Enterococcus.gallinarum
0
5
10
15
20
25
A (n=15)
B (n=16)
C (n=14)
surface

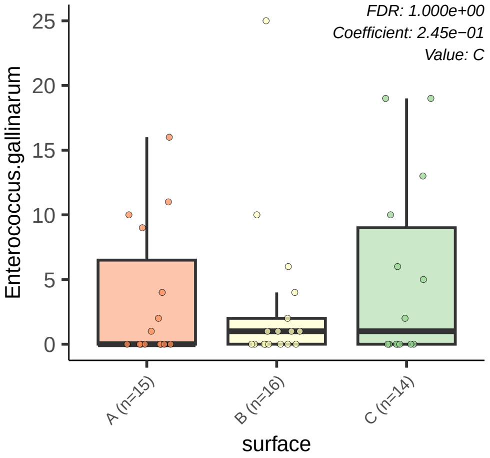

FDR: 1.000e+00
Coefficient: 2.45e−01
Value: C
Enterococcus.gallinarum
25
20
15
10
5
0
A (n=15)
B (n=16)
C (n=14)
surface

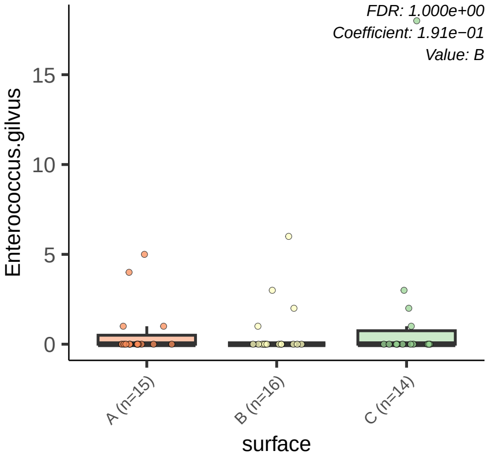
FDR: 1.000e+00
Coefficient: 1.91e−01
Value: B
Enterococcus.gilvus
15
10
5
0
A (n=15)
B (n=16)
C (n=14)
surface

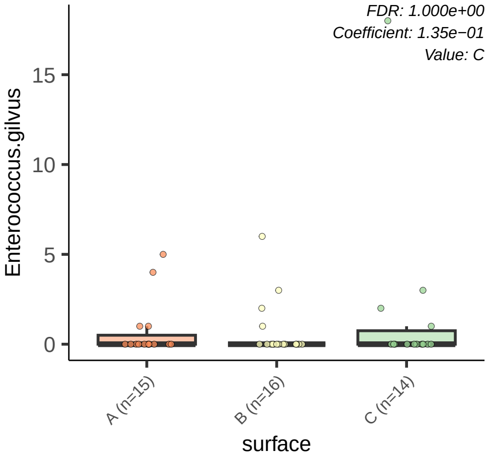
FDR: 1.000e+00
Coefficient: 1.35e−01
Value: C
Enterococcus.gilvus
0
5
10
15
A (n=15)
B (n=16)
C (n=14)
surface

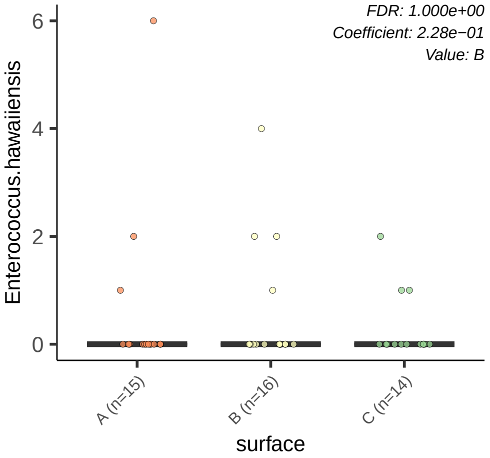

FDR: 1.000e+00
Coefficient: 2.28e−01
Value: B
Enterococcus.hawaiiensis
0
2
4
6
A (n=15)
B (n=16)
C (n=14)
surface

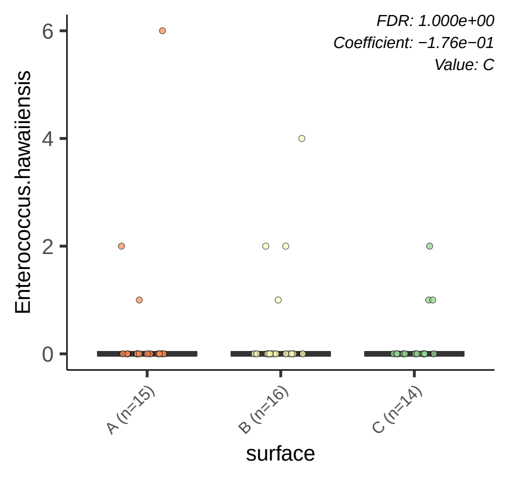

FDR: 1.000e+00
Coefficient: −1.76e−01
Value: C
Enterococcus.hawaiiensis
0
2
4
6
A (n=15)
B (n=16)
C (n=14)
surface

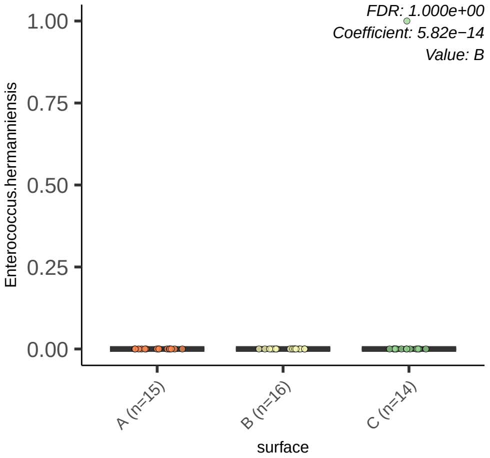

FDR: 1.000e+00
Coefficient: 5.82e−14
Value: B
1.00
0.75
0.50
0.25
0.00
Enterococcus.hermanniensis
A (n=15)
B (n=16)
C (n=14)
surface

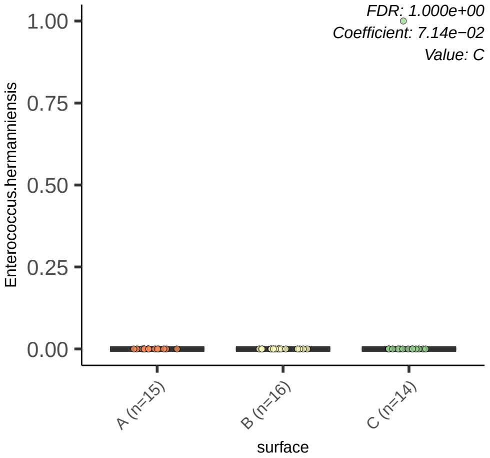
FDR: 1.000e+00
Coefficient: 7.14e−02
Value: C
Enterococcus.hermanniensis
1.00
0.75
0.50
0.25
0.00
A (n=15)
B (n=16)
C (n=14)
surface

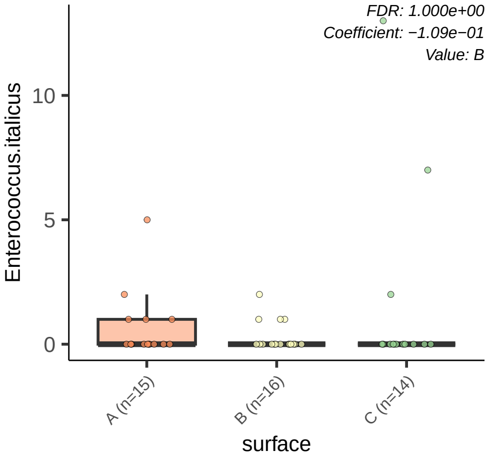
FDR: 1.000e+00
Coefficient: −1.09e−01
Value: B
Enterococcus.italicus
10
5
0
A (n=15)
B (n=16)
C (n=14)
surface

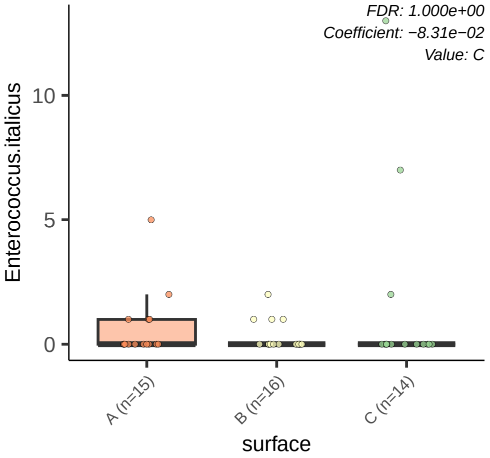
FDR: 1.000e+00
Coefficient: −8.31e−02
Value: C
Enterococcus.italicus
10
5
0
A (n=15)
B (n=16)
C (n=14)
surface

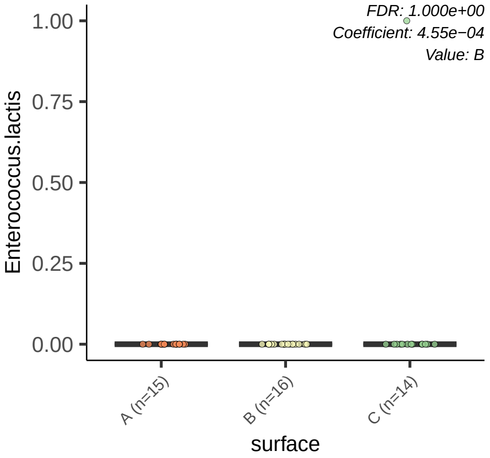
FDR: 1.000e+00
Coefficient: 4.55e−04
Value: B
Enterococcus.lactis
1.00
0.75
0.50
0.25
0.00
A (n=15)
B (n=16)
C (n=14)
surface

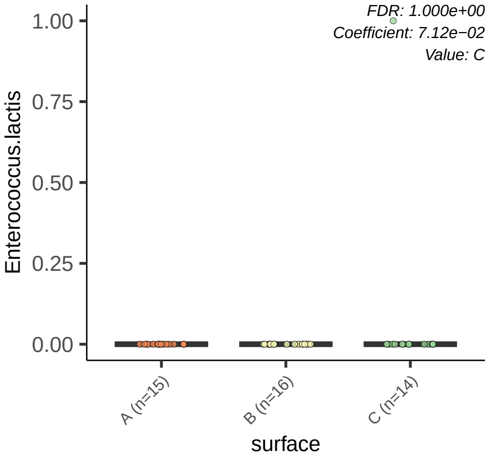
FDR: 1.000e+00
Coefficient: 7.12e−02
Value: C
Enterococcus.lactis
1.00
0.75
0.50
0.25
0.00
A (n=15)
B (n=16)
C (n=14)
surface

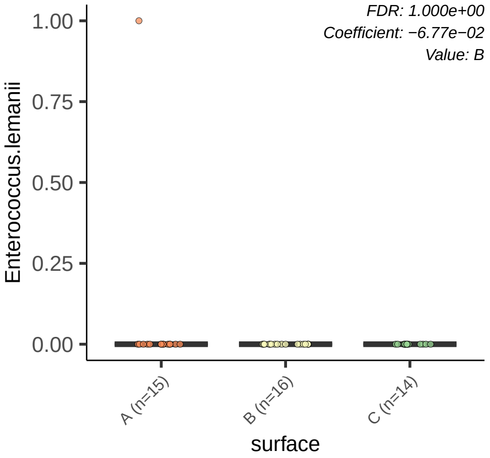

FDR: 1.000e+00
Coefficient: −6.77e−02
Value: B
Enterococcus.lemanii
1.00
0.75
0.50
0.25
0.00
A (n=15)
B (n=16)
C (n=14)
surface

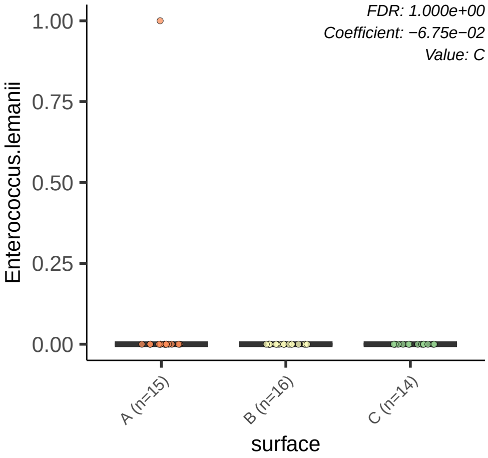

FDR: 1.000e+00
Coefficient: −6.75e−02
Value: C
Enterococcus.lemanii
1.00
0.75
0.50
0.25
0.00
A (n=15)
B (n=16)
C (n=14)
surface

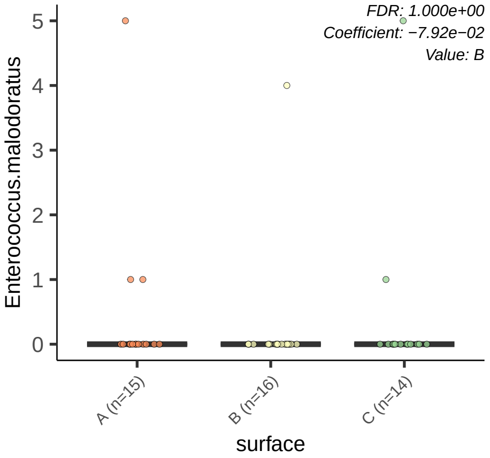

FDR: 1.000e+00
Coefficient: −7.92e−02
Value: B
Enterococcus.malodoratus
0
1
2
3
4
5
A (n=15)
B (n=16)
C (n=14)
surface

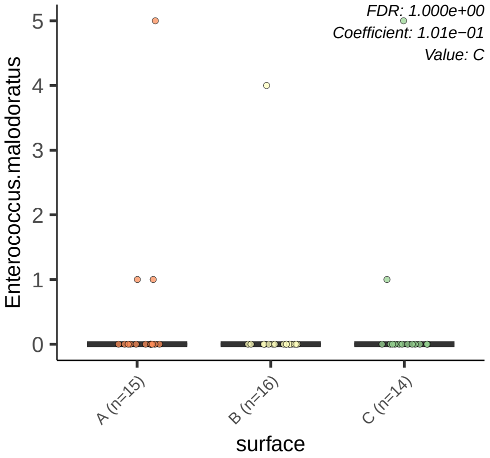
FDR: 1.000e+00
Coefficient: 1.01e−01
Value: C
Enterococcus.malodoratus
0
1
2
3
4
5
A (n=15)
B (n=16)
C (n=14)
surface

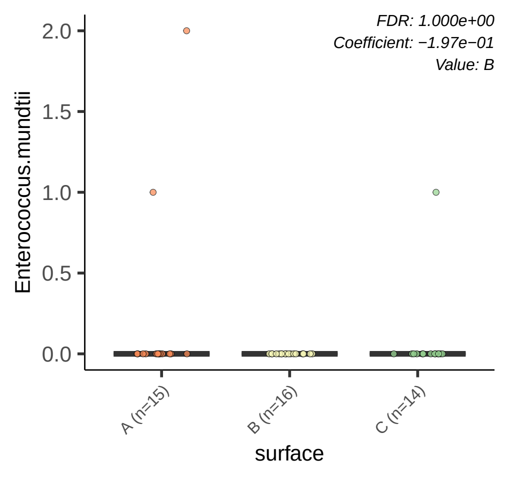
FDR: 1.000e+00
Coefficient: −1.97e−01
Value: B
Enterococcus.mundtii
2.0
1.5
1.0
0.5
0.0
A (n=15)
B (n=16)
C (n=14)
surface

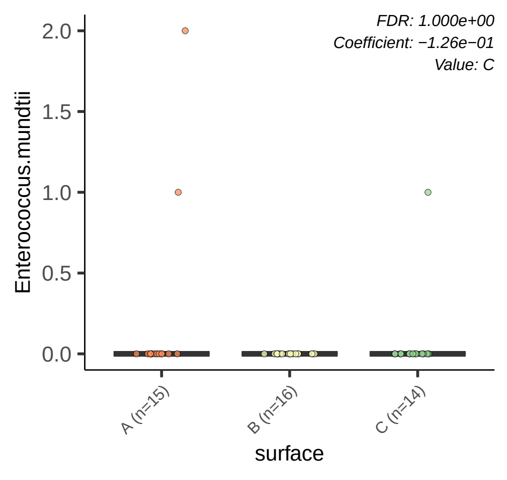
FDR: 1.000e+00
Coefficient: −1.26e−01
Value: C
Enterococcus.mundtii
2.0
1.5
1.0
0.5
0.0
A (n=15)
B (n=16)
C (n=14)
surface

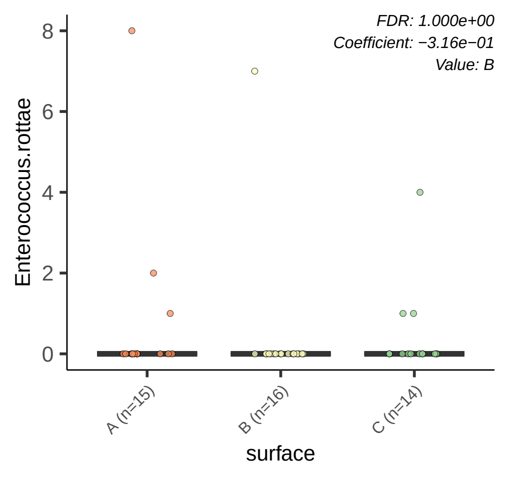

FDR: 1.000e+00
Coefficient: −3.16e−01
Value: B
Enterococcus.rottae
8
6
4
2
0
A (n=15)
B (n=16)
C (n=14)
surface

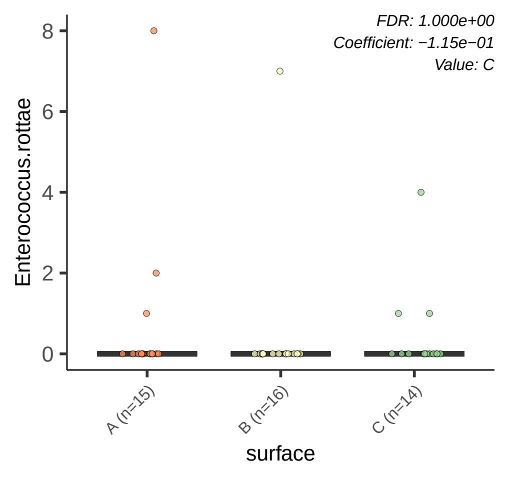

FDR: 1.000e+00
Coefficient: −1.15e−01
Value: C
Enterococcus.rottae
0
2
4
6
8
A (n=15)
B (n=16)
C (n=14)
surface

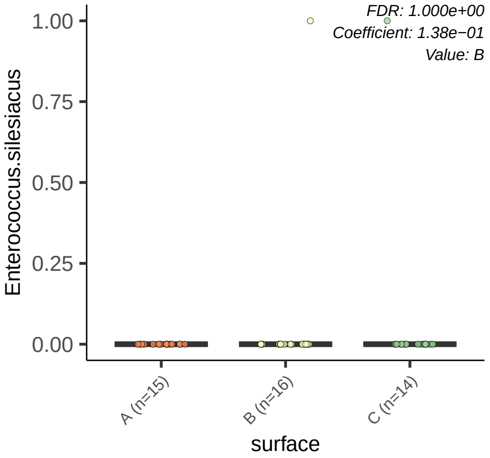
FDR: 1.000e+00
Coefficient: 1.38e−01
Value: B
1.00
0.75
0.50
0.25
0.00
Enterococcus.silesiacus
A (n=15)
B (n=16)
C (n=14)
surface

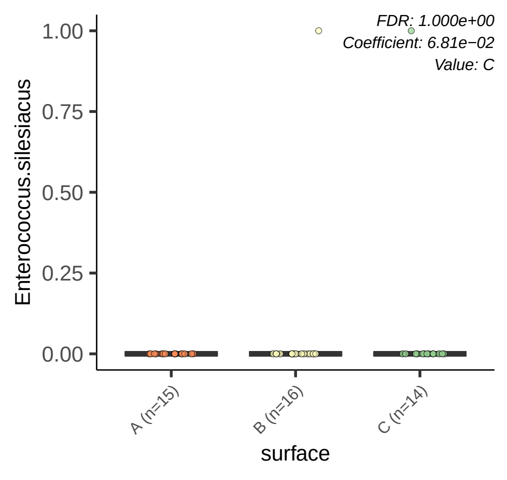
FDR: 1.000e+00
Coefficient: 6.81e−02
Value: C
Enterococcus.silesiacus
1.00
0.75
0.50
0.25
0.00
A (n=15)
B (n=16)
C (n=14)
surface

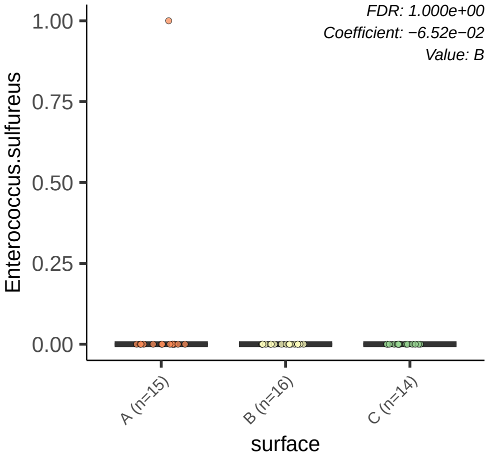
FDR: 1.000e+00
Coefficient: −6.52e−02
Value: B
Enterococcus.sulfureus
1.00
0.75
0.50
0.25
0.00
A (n=15)
B (n=16)
C (n=14)
surface

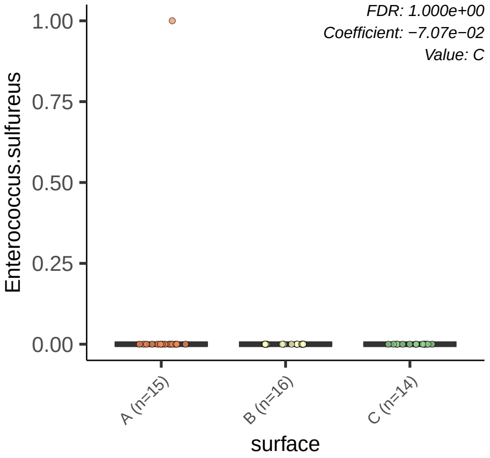
FDR: 1.000e+00
Coefficient: −7.07e−02
Value: C
1.00
0.75
0.50
0.25
0.00
Enterococcus.sulfureus
A (n=15)
B (n=16)
C (n=14)
surface

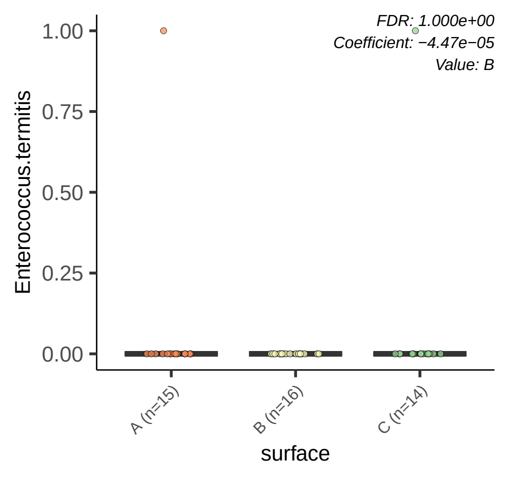
FDR: 1.000e+00
Coefficient: −4.47e−05
Value: B
Enterococcus.termitis
1.00
0.75
0.50
0.25
0.00
A (n=15)
B (n=16)
C (n=14)
surface

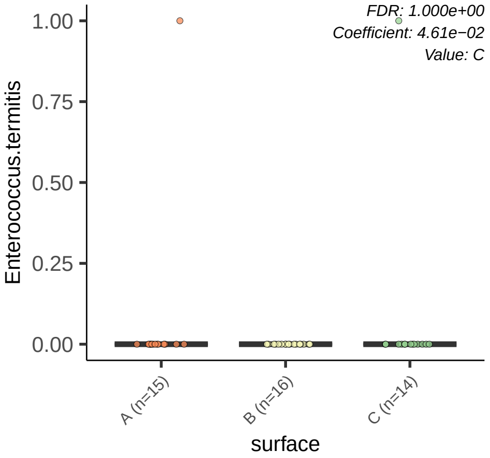
FDR: 1.000e+00
Coefficient: 4.61e−02
Value: C
Enterococcus.termitis
1.00
0.75
0.50
0.25
0.00
A (n=15)
B (n=16)
C (n=14)
surface

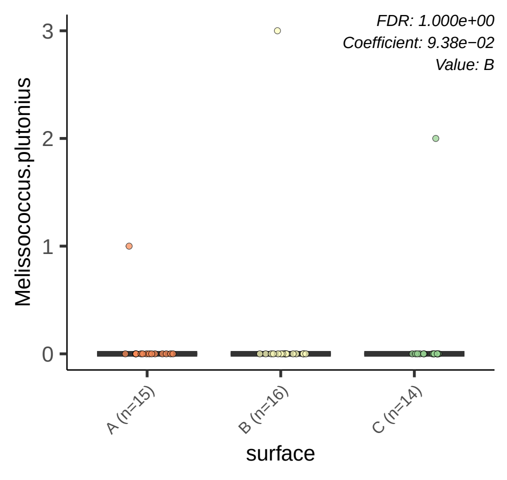
FDR: 1.000e+00
Coefficient: 9.38e−02
Value: B
Melissococcus.plutonius
3
2
1
0
A (n=15)
B (n=16)
C (n=14)
surface

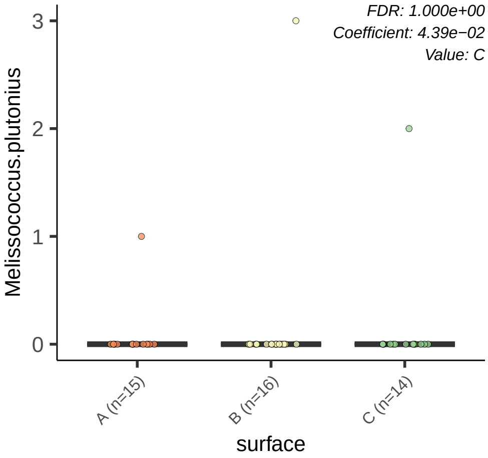

FDR: 1.000e+00
Coefficient: 4.39e−02
Value: C
Melissococcus.plutonius
3
2
1
0
A (n=15)
B (n=16)
C (n=14)
surface

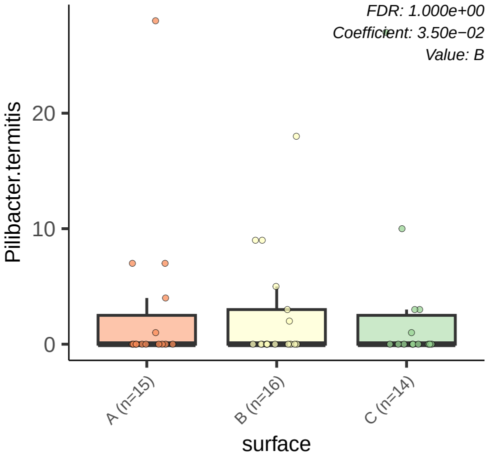
FDR: 1.000e+00
Coefficient: 3.50e−02
Value: B
Pilibacter.termitis
20
10
0
A (n=15)
B (n=16)
C (n=14)
surface

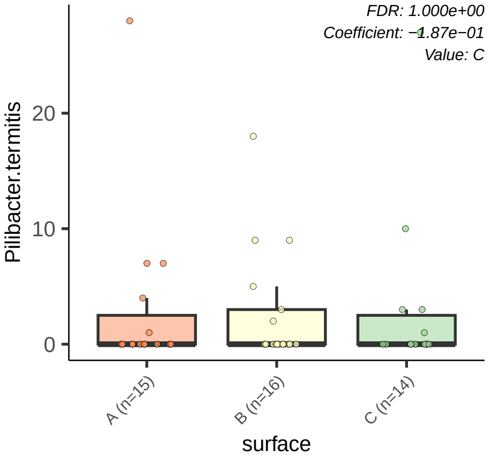
FDR: 1.000e+00
Coefficient: −1.87e−01
Value: C
Pilibacter.termitis
20
10
0
A (n=15)
B (n=16)
C (n=14)
surface

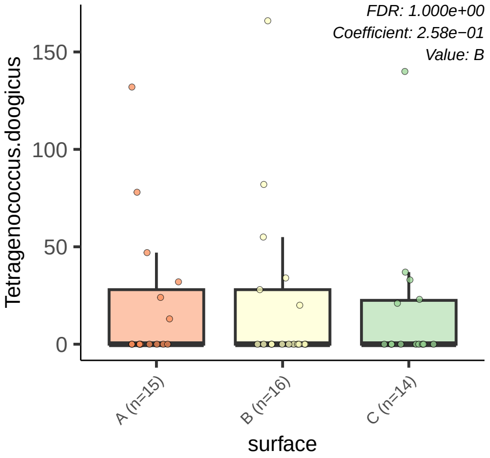
FDR: 1.000e+00
Coefficient: 2.58e−01
Value: B
Tetragenococcus.doogicus
150
100
50
0
A (n=15)
B (n=16)
C (n=14)
surface

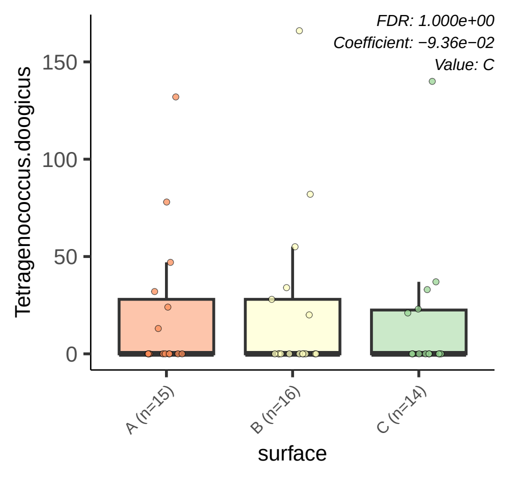

FDR: 1.000e+00
Coefficient: −9.36e−02
Value: C
Tetragenococcus.doogicus
150
100
50
0
A (n=15)
B (n=16)
C (n=14)
surface

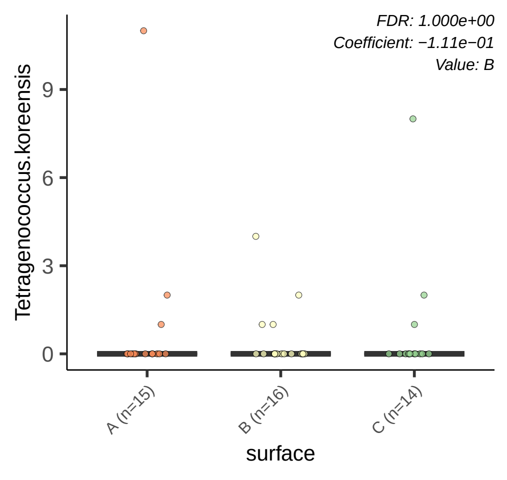

FDR: 1.000e+00
Coefficient: −1.11e−01
Value: B
Tetragenococcus.koreensis
0
3
6
9
A (n=15)
B (n=16)
C (n=14)
surface

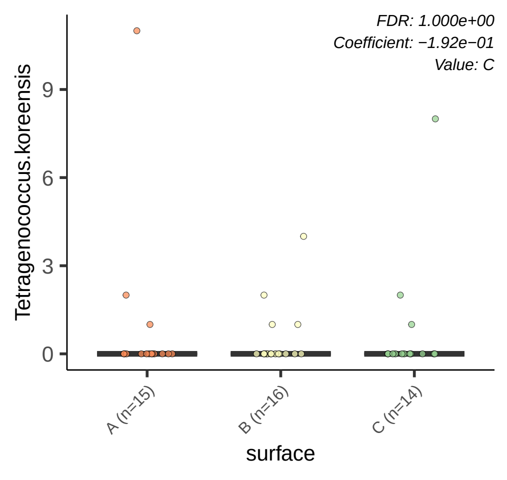
FDR: 1.000e+00
Coefficient: −1.92e−01
Value: C
Tetragenococcus.koreensis
0
3
6
9
A (n=15)
B (n=16)
C (n=14)
surface

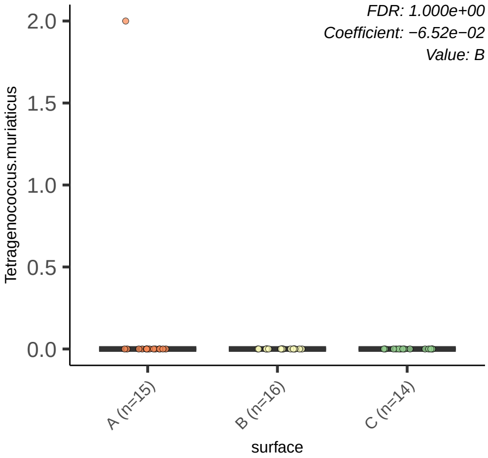

FDR: 1.000e+00
Coefficient: −6.52e−02
Value: B
Tetragenococcus.muriaticus
2.0
1.5
1.0
0.5
0.0
A (n=15)
B (n=16)
C (n=14)
surface

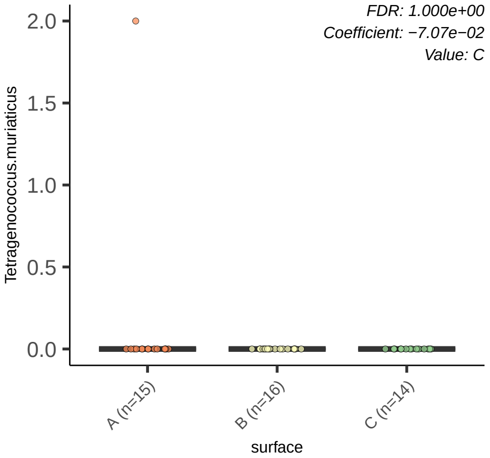
FDR: 1.000e+00
Coefficient: −7.07e−02
Value: C
Tetragenococcus.muriaticus
2.0
1.5
1.0
0.5
0.0
A (n=15)
B (n=16)
C (n=14)
surface

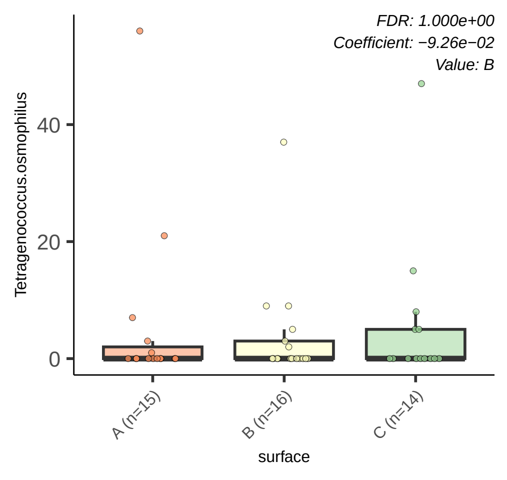

FDR: 1.000e+00
Coefficient: −9.26e−02
Value: B
Tetragenococcus.osmophilus
0
20
40
A (n=15)
B (n=16)
C (n=14)
surface

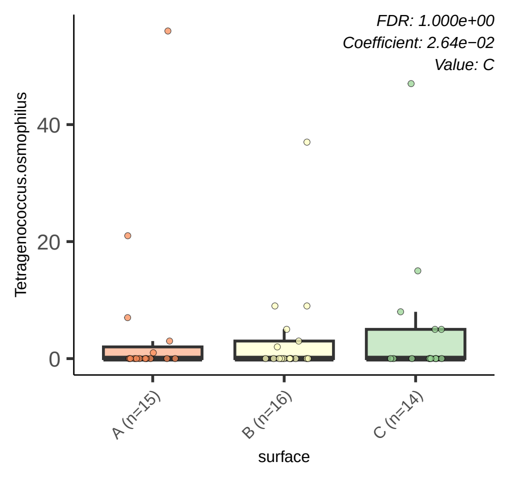
FDR: 1.000e+00
Coefficient: 2.64e−02
Value: C
Tetragenococcus.osmophilus
40
20
0
A (n=15)
B (n=16)
C (n=14)
surface

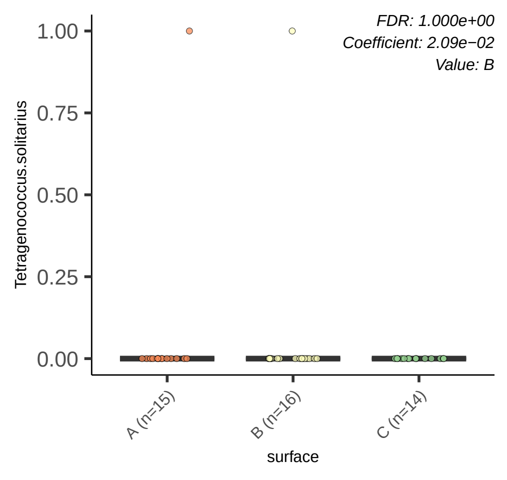
FDR: 1.000e+00
Coefficient: 2.09e−02
Value: B
1.00
0.75
0.50
0.25
0.00
Tetragenococcus.solitarius
A (n=15)
B (n=16)
C (n=14)
surface

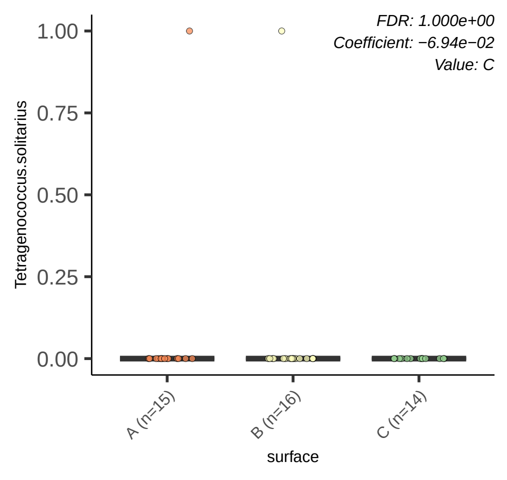
FDR: 1.000e+00
Coefficient: −6.94e−02
Value: C
Tetragenococcus.solitarius
1.00
0.75
0.50
0.25
0.00
A (n=15)
B (n=16)
C (n=14)
surface

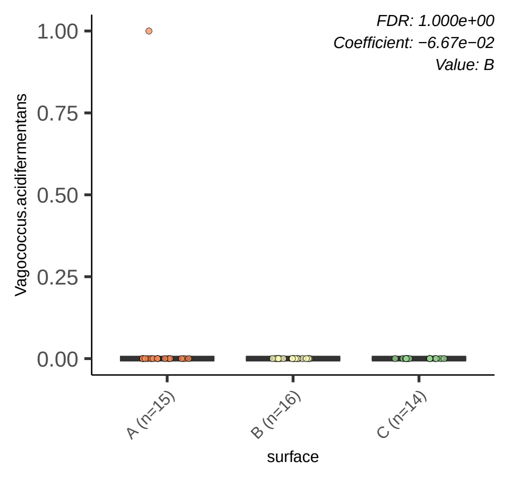

FDR: 1.000e+00
Coefficient: −6.67e−02
Value: B
Vagococcus.acidifermentans
1.00
0.75
0.50
0.25
0.00
A (n=15)
B (n=16)
C (n=14)
surface

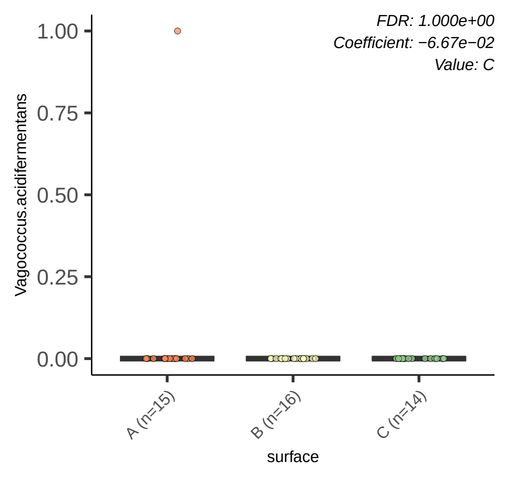
FDR: 1.000e+00
Coefficient: −6.67e−02
Value: C
1.00
0.75
0.50
0.25
0.00
Vagococcus.acidifermentans
A (n=15)
B (n=16)
C (n=14)
surface

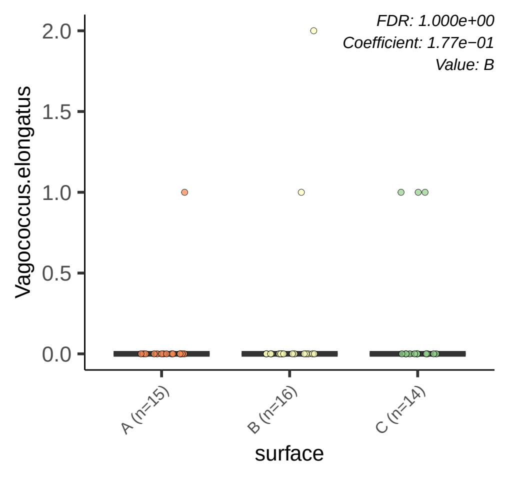
FDR: 1.000e+00
Coefficient: 1.77e−01
Value: B
Vagococcus.elongatus
2.0
1.5
1.0
0.5
0.0
A (n=15)
B (n=16)
C (n=14)
surface

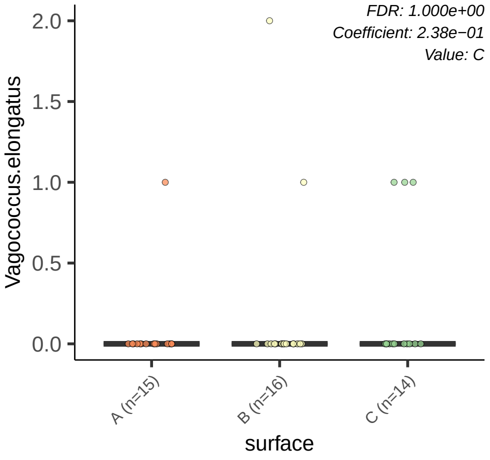

FDR: 1.000e+00
Coefficient: 2.38e−01
Value: C
Vagococcus.elongatus
2.0
1.5
1.0
0.5
0.0
A (n=15)
B (n=16)
C (n=14)
surface

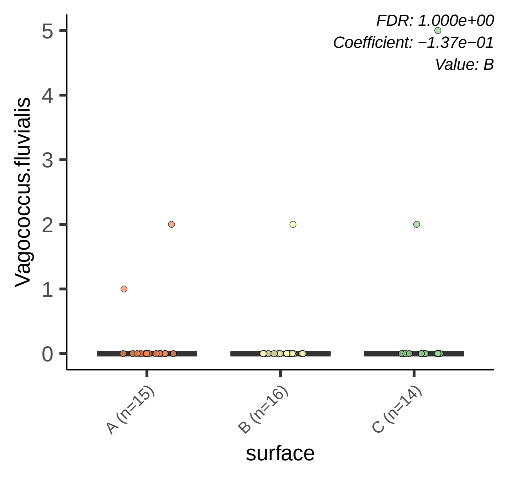
FDR: 1.000e+00
Coefficient: −1.37e−01
Value: B
Vagococcus.fluvialis
5
4
3
2
1
0
A (n=15)
B (n=16)
C (n=14)
surface

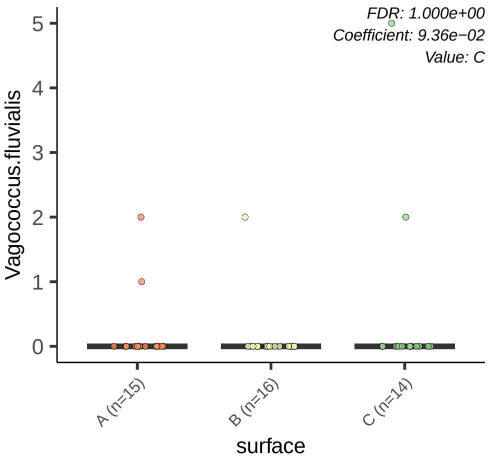

FDR: 1.000e+00
Coefficient: 9.36e−02
Value: C
Vagococcus.fluvialis
0
1
2
3
4
5
A (n=15)
B (n=16)
C (n=14)
surface

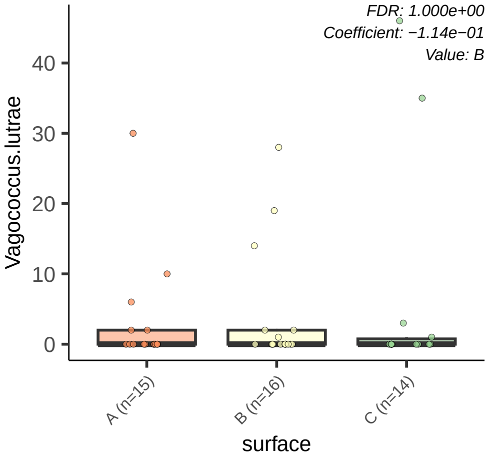

FDR: 1.000e+00
Coefficient: −1.14e−01
Value: B
Vagococcus.lutrae
0
10
20
30
40
A (n=15)
B (n=16)
C (n=14)
surface

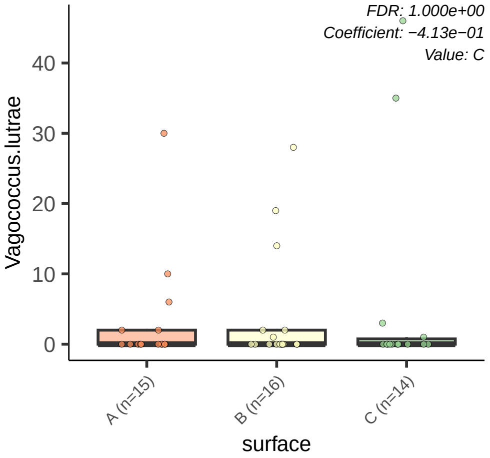
FDR: 1.000e+00
Coefficient: −4.13e−01
Value: C
Vagococcus.lutrae
0
10
20
30
40
A (n=15)
B (n=16)
C (n=14)
surface

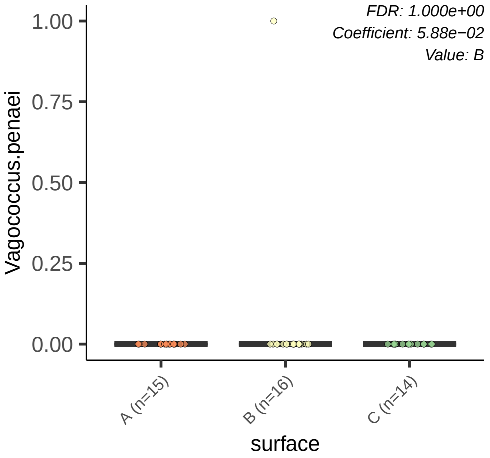
FDR: 1.000e+00
Coefficient: 5.88e−02
Value: B
Vagococcus.penaei
1.00
0.75
0.50
0.25
0.00
A (n=15)
B (n=16)
C (n=14)
surface

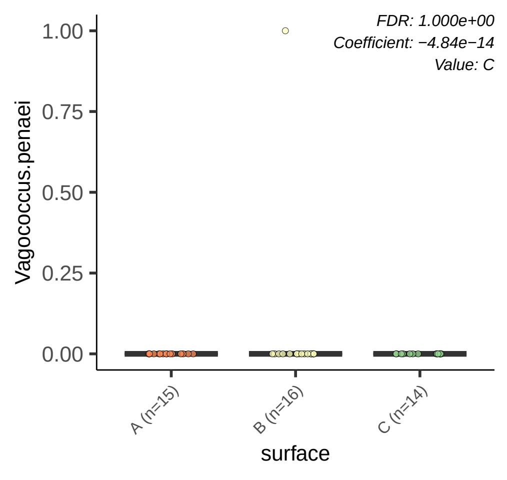
FDR: 1.000e+00
Coefficient: −4.84e−14
Value: C
Vagococcus.penaei
1.00
0.75
0.50
0.25
0.00
A (n=15)
B (n=16)
C (n=14)
surface

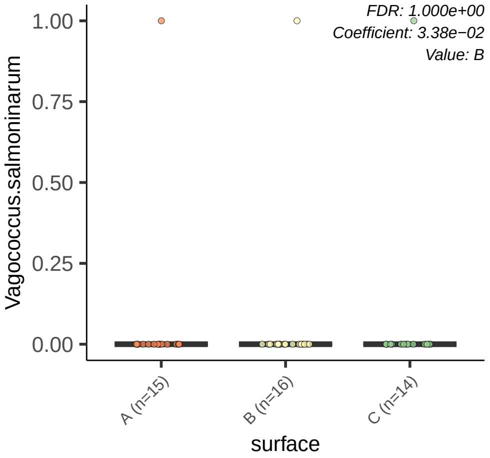
FDR: 1.000e+00
Coefficient: 3.38e−02
Value: B
Vagococcus.salmoninarum
1.00
0.75
0.50
0.25
0.00
A (n=15)
B (n=16)
C (n=14)
surface

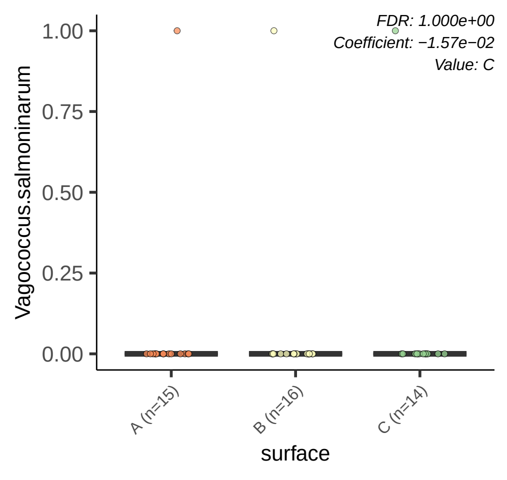

FDR: 1.000e+00
Coefficient: −1.57e−02
Value: C
Vagococcus.salmoninarum
1.00
0.75
0.50
0.25
0.00
A (n=15)
B (n=16)
C (n=14)
surface

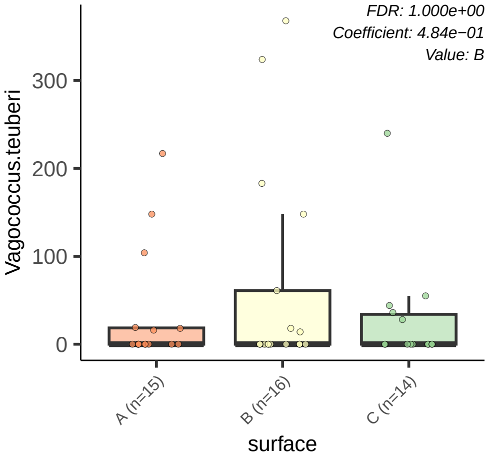
FDR: 1.000e+00
Coefficient: 4.84e−01
Value: B
Vagococcus.teuberi
300
200
100
0
A (n=15)
B (n=16)
C (n=14)
surface

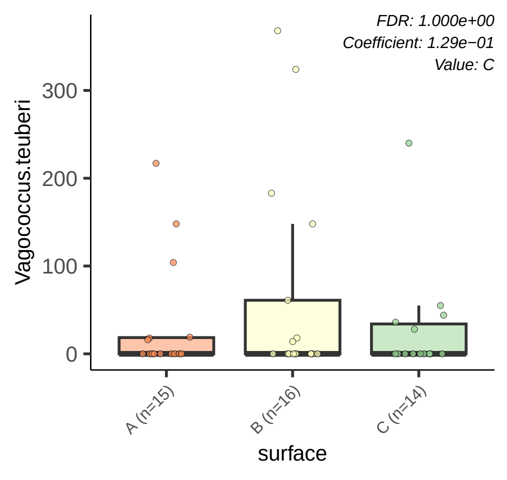
FDR: 1.000e+00
Coefficient: 1.29e−01
Value: C
Vagococcus.teuberi
0
100
200
300
A (n=15)
B (n=16)
C (n=14)
surface

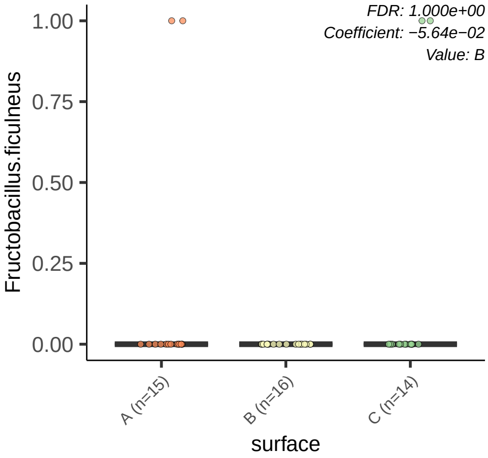

FDR: 1.000e+00
Coefficient: −5.64e−02
Value: B
Fructobacillus.ficulneus
1.00
0.75
0.50
0.25
0.00
A (n=15)
B (n=16)
C (n=14)
surface

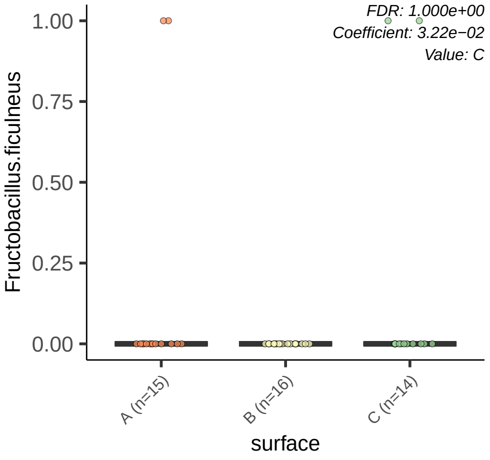

FDR: 1.000e+00
Coefficient: 3.22e−02
Value: C
1.00
0.75
0.50
0.25
0.00
Fructobacillus.ficulneus
A (n=15)
B (n=16)
C (n=14)
surface

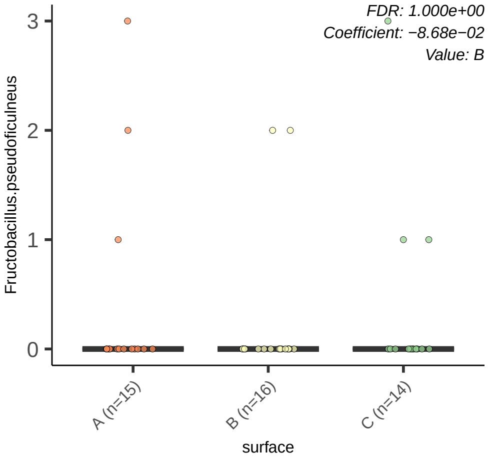

FDR: 1.000e+00
Coefficient: −8.68e−02
Value: B
Fructobacillus.pseudoficulneus
3
2
1
0
A (n=15)
B (n=16)
C (n=14)
surface

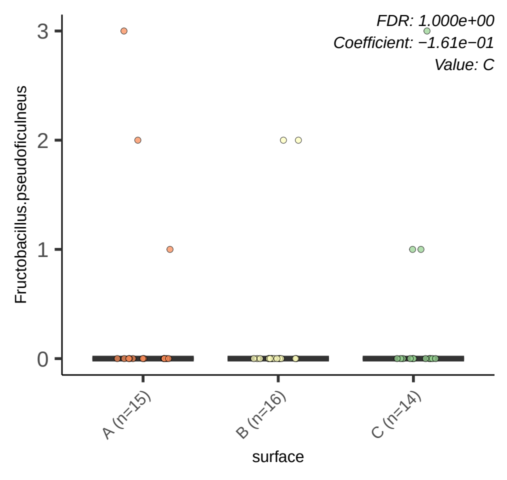

FDR: 1.000e+00
Coefficient: −1.61e−01
Value: C
Fructobacillus.pseudoficulneus
3
2
1
0
A (n=15)
B (n=16)
C (n=14)
surface

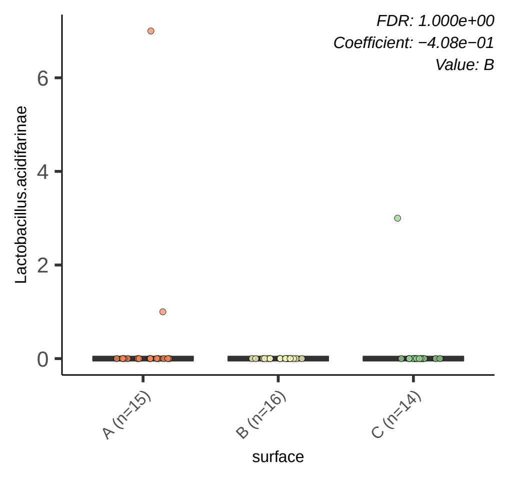

FDR: 1.000e+00
Coefficient: −4.08e−01
Value: B
Lactobacillus.acidifarinae
0
2
4
6
A (n=15)
B (n=16)
C (n=14)
surface

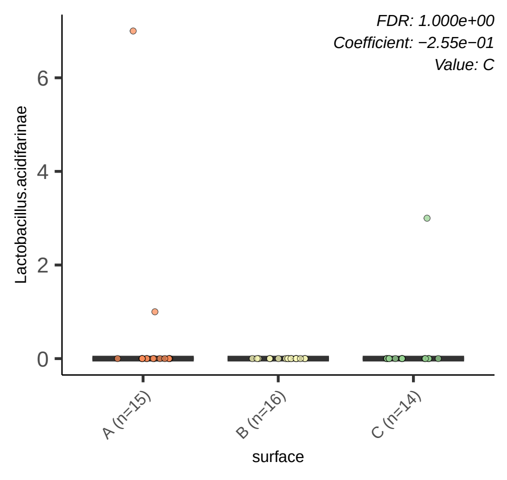
FDR: 1.000e+00
Coefficient: −2.55e−01
Value: C
Lactobacillus.acidifarinae
0
2
4
6
A (n=15)
B (n=16)
C (n=14)
surface

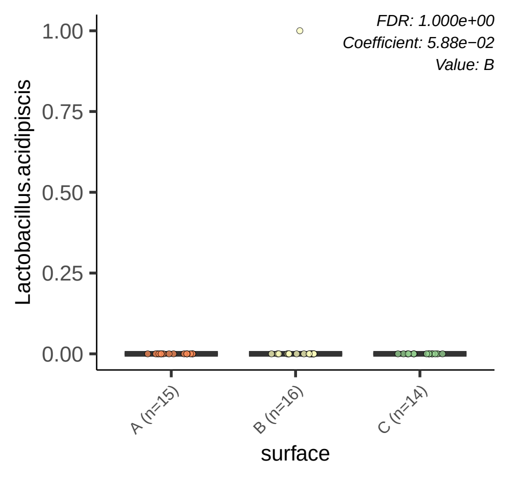
FDR: 1.000e+00
Coefficient: 5.88e−02
Value: B
Lactobacillus.acidipiscis
1.00
0.75
0.50
0.25
0.00
A (n=15)
B (n=16)
C (n=14)
surface

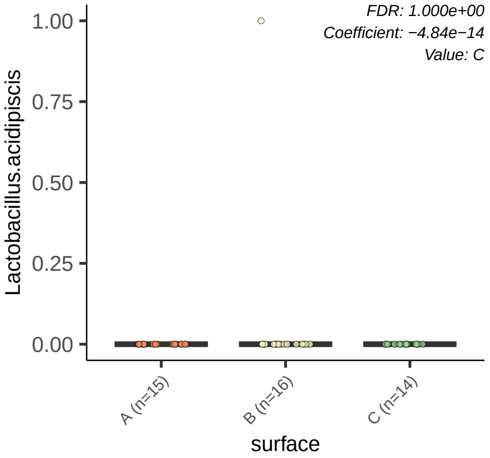

FDR: 1.000e+00
Coefficient: −4.84e−14
Value: C
1.00
0.75
0.50
0.25
0.00
Lactobacillus.acidipiscis
A (n=15)
B (n=16)
C (n=14)
surface

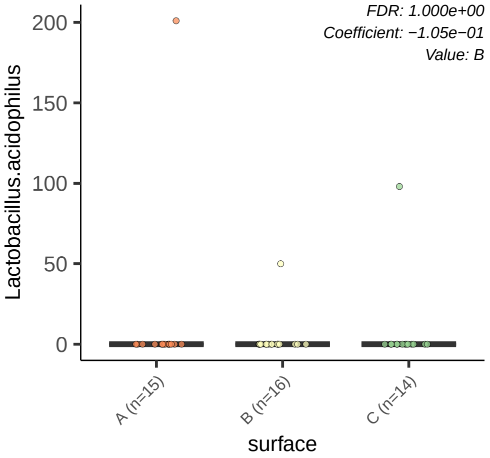

FDR: 1.000e+00
Coefficient: −1.05e−01
Value: B
Lactobacillus.acidophilus
200
150
100
50
0
A (n=15)
B (n=16)
C (n=14)
surface

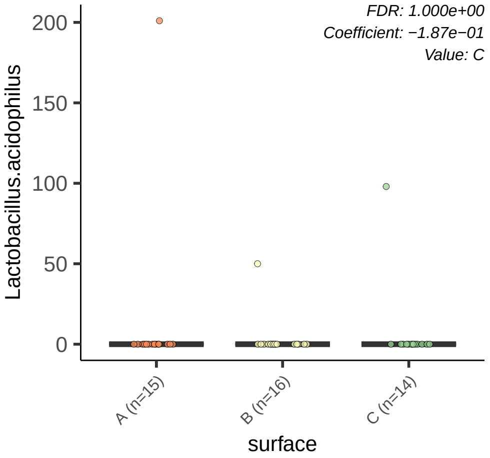
FDR: 1.000e+00
Coefficient: −1.87e−01
Value: C
Lactobacillus.acidophilus
200
150
100
50
0
A (n=15)
B (n=16)
C (n=14)
surface

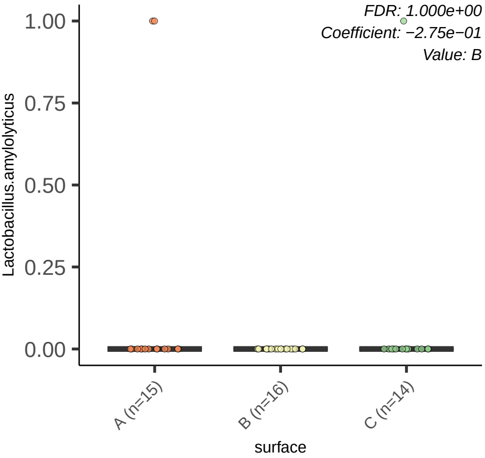
FDR: 1.000e+00
Coefficient: −2.75e−01
Value: B
Lactobacillus.amylolyticus
1.00
0.75
0.50
0.25
0.00
A (n=15)
B (n=16)
C (n=14)
surface

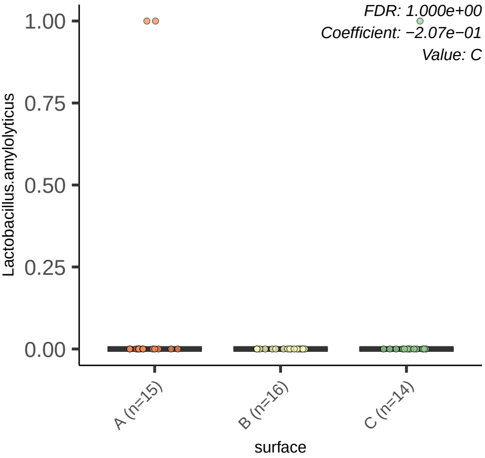
FDR: 1.000e+00
Coefficient: −2.07e−01
Value: C
Lactobacillus.amylolyticus
1.00
0.75
0.50
0.25
0.00
A (n=15)
B (n=16)
C (n=14)
surface

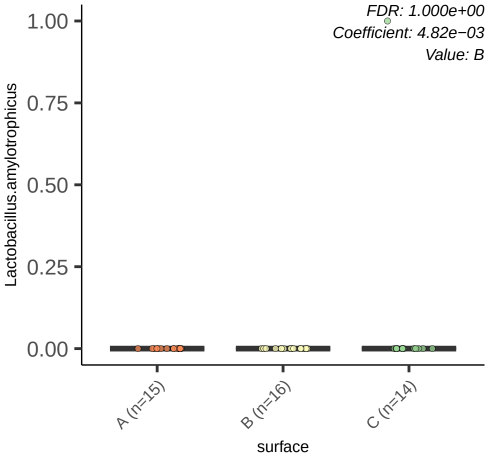

FDR: 1.000e+00
Coefficient: 4.82e−03
Value: B
1.00
0.75
0.50
0.25
0.00
Lactobacillus.amylotrophicus
A (n=15)
B (n=16)
C (n=14)
surface

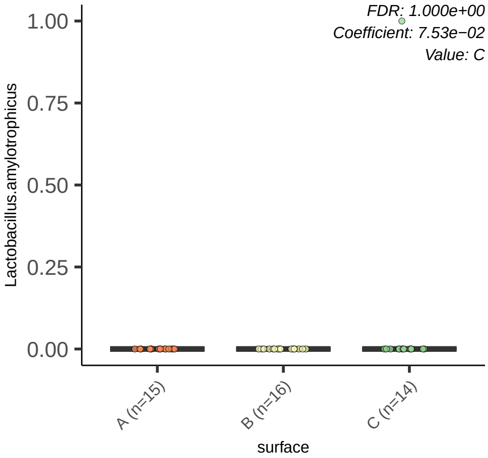
FDR: 1.000e+00
Coefficient: 7.53e−02
Value: C
1.00
0.75
0.50
0.25
0.00
Lactobacillus.amylotrophicus
A (n=15)
B (n=16)
C (n=14)
surface

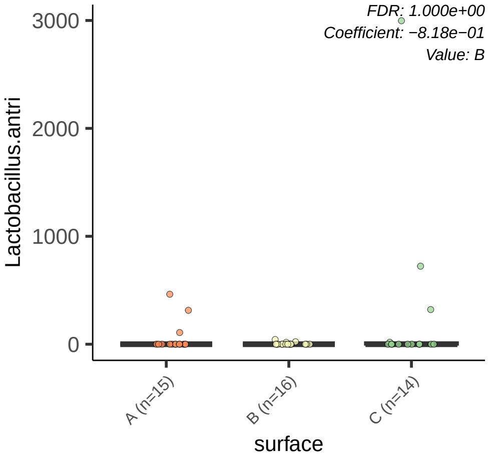
FDR: 1.000e+00
Coefficient: −8.18e−01
Value: B
3000
2000
1000
0
Lactobacillus.antri
A (n=15)
B (n=16)
C (n=14)
surface

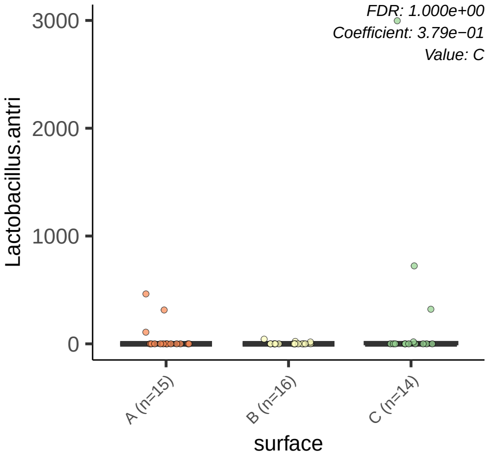
FDR: 1.000e+00
Coefficient: 3.79e−01
Value: C
Lactobacillus.antri
3000
2000
1000
0
A (n=15)
B (n=16)
C (n=14)
surface

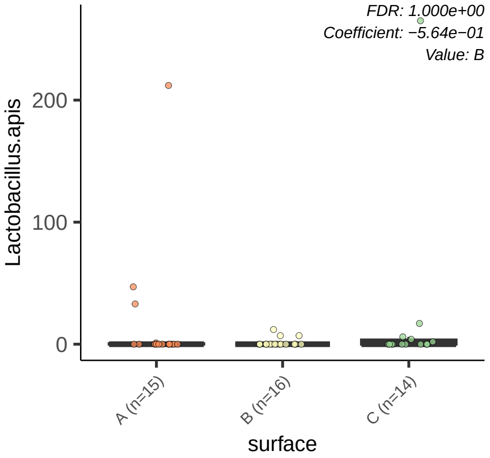
FDR: 1.000e+00
Coefficient: −5.64e−01
Value: B
Lactobacillus.apis
200
100
0
A (n=15)
B (n=16)
C (n=14)
surface

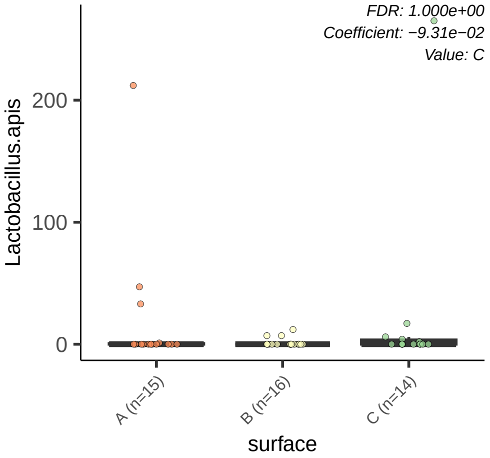

FDR: 1.000e+00
Coefficient: −9.31e−02
Value: C
Lactobacillus.apis
200
100
0
A (n=15)
B (n=16)
C (n=14)
surface

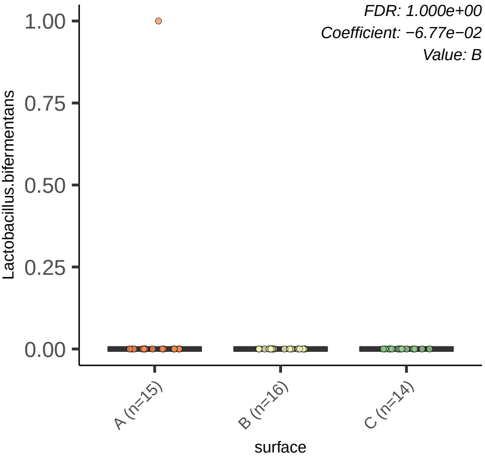
FDR: 1.000e+00
Coefficient: −6.77e−02
Value: B
Lactobacillus.bifermentans
1.00
0.75
0.50
0.25
0.00
A (n=15)
B (n=16)
C (n=14)
surface

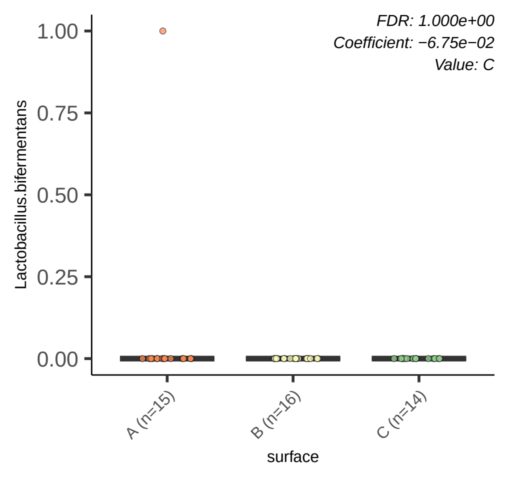
FDR: 1.000e+00
Coefficient: −6.75e−02
Value: C
Lactobacillus.bifermentans
1.00
0.75
0.50
0.25
0.00
A (n=15)
B (n=16)
C (n=14)
surface

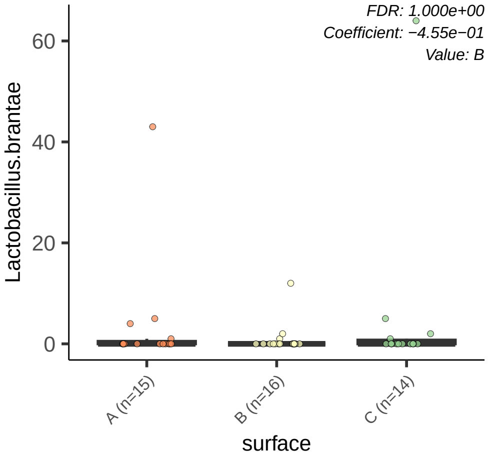
FDR: 1.000e+00
Coefficient: −4.55e−01
Value: B
Lactobacillus.brantae
60
40
20
0
A (n=15)
B (n=16)
C (n=14)
surface

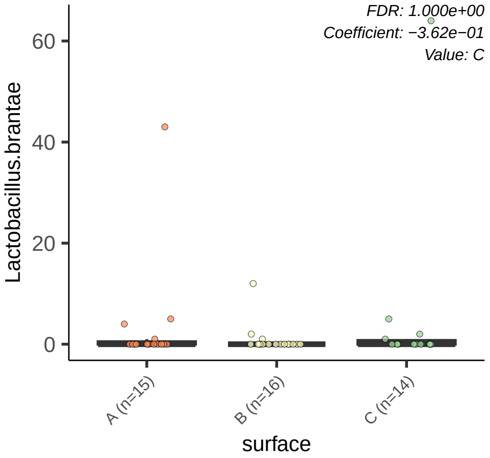

FDR: 1.000e+00
Coefficient: −3.62e−01
Value: C
Lactobacillus.brantae
60
40
20
0
A (n=15)
B (n=16)
C (n=14)
surface

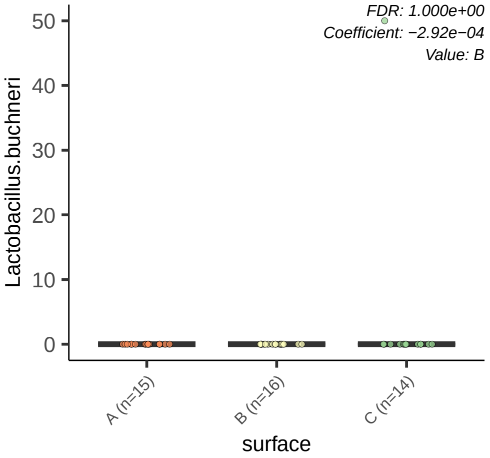

FDR: 1.000e+00
Coefficient: −2.92e−04
Value: B
Lactobacillus.buchneri
50
40
30
20
10
0
A (n=15)
B (n=16)
C (n=14)
surface

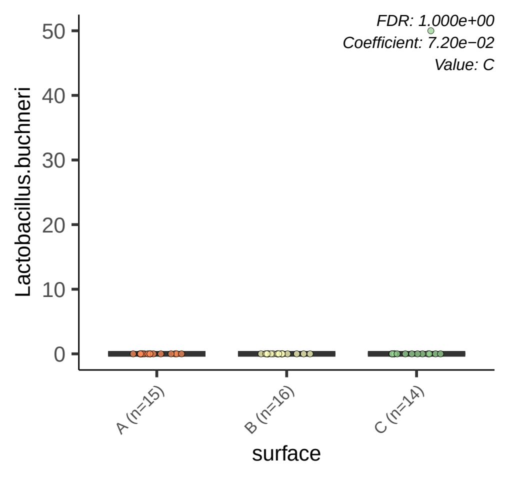
FDR: 1.000e+00
Coefficient: 7.20e−02
Value: C
Lactobacillus.buchneri
50
40
30
20
10
0
A (n=15)
B (n=16)
C (n=14)
surface

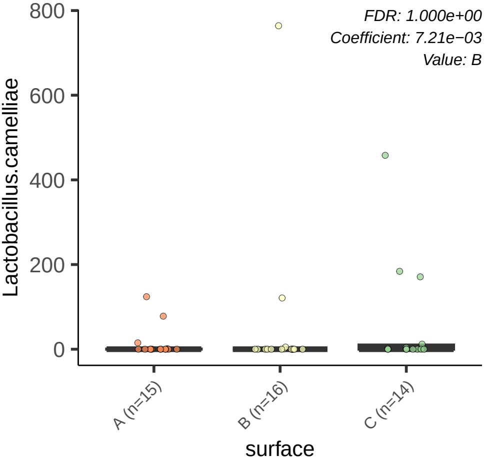
FDR: 1.000e+00
Coefficient: 7.21e−03
Value: B
800
600
400
200
0
Lactobacillus.camelliae
A (n=15)
B (n=16)
C (n=14)
surface

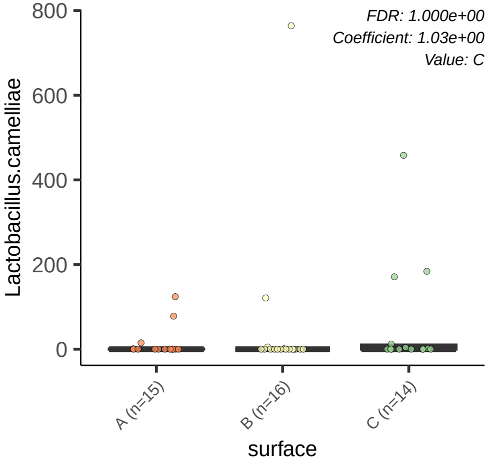
FDR: 1.000e+00
Coefficient: 1.03e+00
Value: C
Lactobacillus.camelliae
800
600
400
200
0
A (n=15)
B (n=16)
C (n=14)
surface

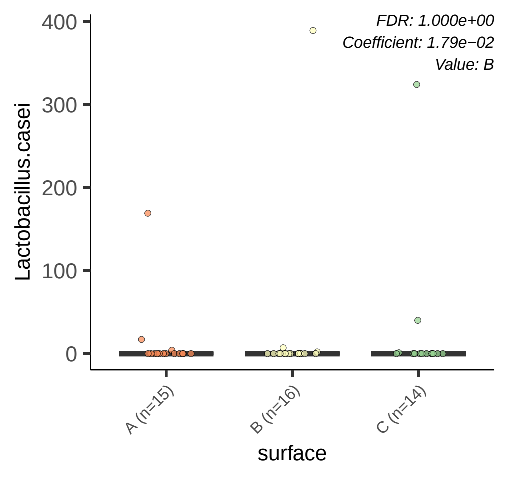

FDR: 1.000e+00
Coefficient: 1.79e−02
Value: B
Lactobacillus.casei
400
300
200
100
0
A (n=15)
B (n=16)
C (n=14)
surface

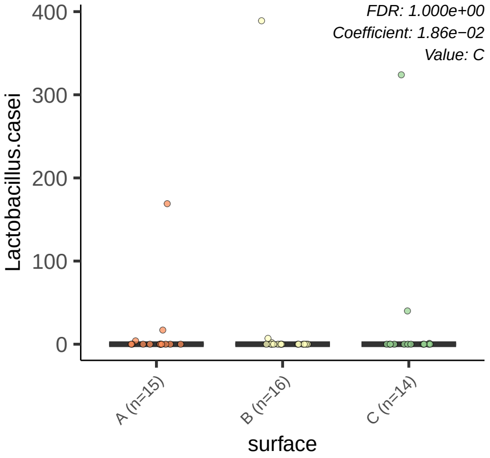
FDR: 1.000e+00
Coefficient: 1.86e−02
Value: C
Lactobacillus.casei
400
300
200
100
0
A (n=15)
B (n=16)
C (n=14)
surface

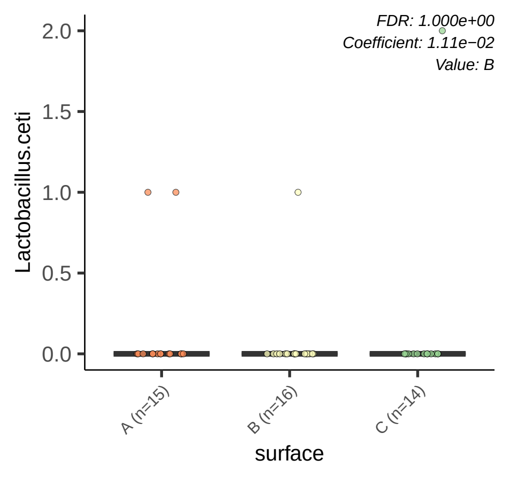
FDR: 1.000e+00
Coefficient: 1.11e−02
Value: B
Lactobacillus.ceti
2.0
1.5
1.0
0.5
0.0
A (n=15)
B (n=16)
C (n=14)
surface

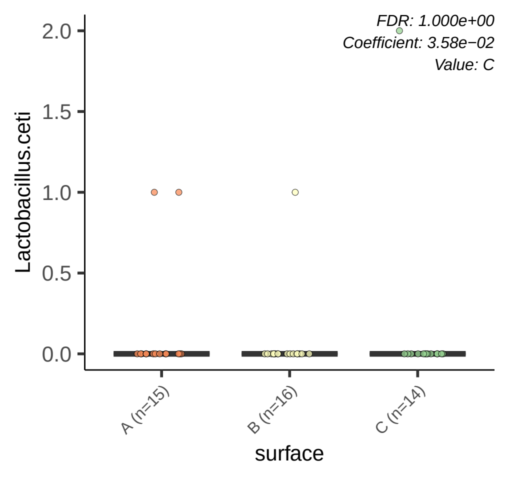
FDR: 1.000e+00
Coefficient: 3.58e−02
Value: C
2.0
1.5
1.0
0.5
0.0
Lactobacillus.ceti
A (n=15)
B (n=16)
C (n=14)
surface

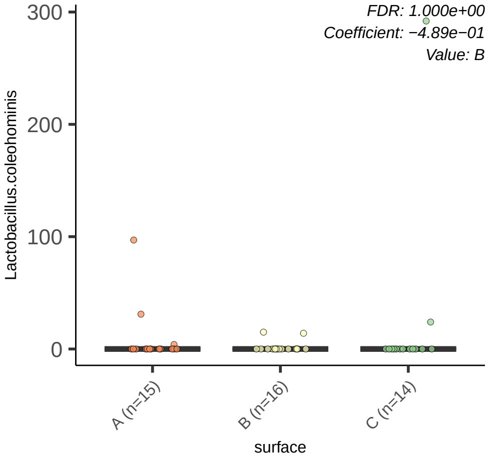
FDR: 1.000e+00
Coefficient: −4.89e−01
Value: B
300
200
100
0
Lactobacillus.coleohominis
A (n=15)
B (n=16)
C (n=14)
surface

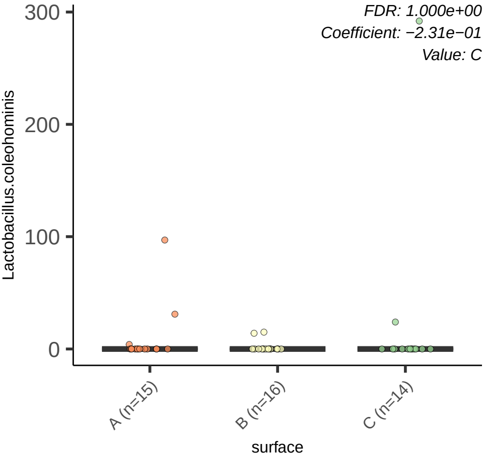

FDR: 1.000e+00
Coefficient: −2.31e−01
Value: C
300
200
100
0
Lactobacillus.coleohominis
A (n=15)
B (n=16)
C (n=14)
surface

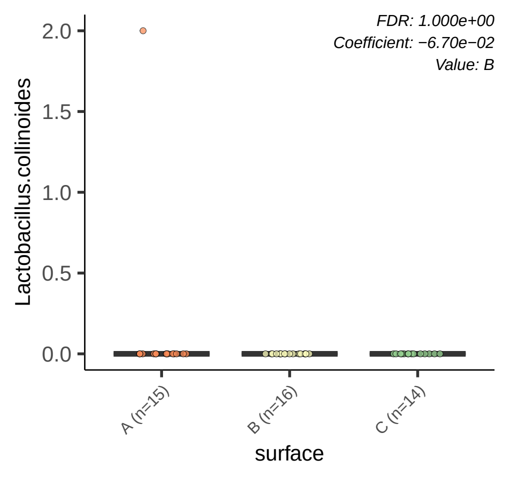

FDR: 1.000e+00
Coefficient: −6.70e−02
Value: B
Lactobacillus.collinoides
2.0
1.5
1.0
0.5
0.0
A (n=15)
B (n=16)
C (n=14)
surface

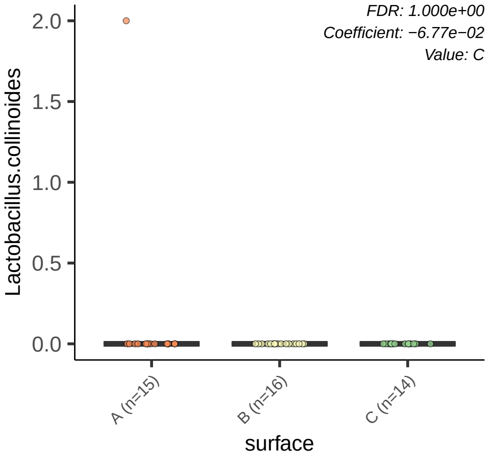

FDR: 1.000e+00
Coefficient: −6.77e−02
Value: C
Lactobacillus.collinoides
2.0
1.5
1.0
0.5
0.0
A (n=15)
B (n=16)
C (n=14)
surface

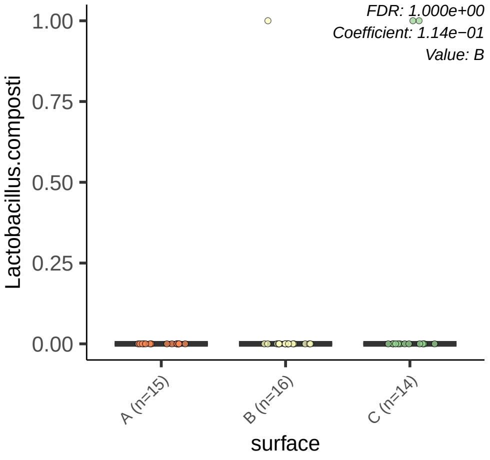
FDR: 1.000e+00
Coefficient: 1.14e−01
Value: B
Lactobacillus.composti
1.00
0.75
0.50
0.25
0.00
A (n=15)
B (n=16)
C (n=14)
surface

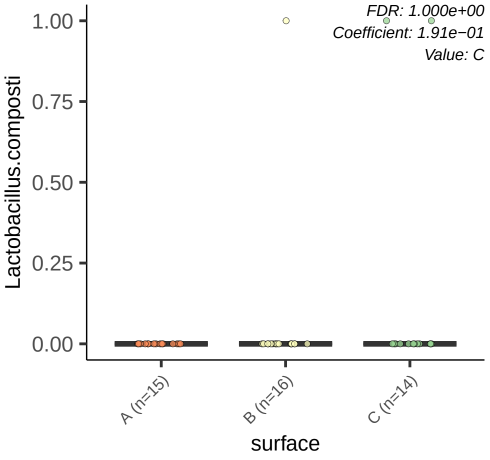

FDR: 1.000e+00
Coefficient: 1.91e−01
Value: C
Lactobacillus.composti
1.00
0.75
0.50
0.25
0.00
A (n=15)
B (n=16)
C (n=14)
surface

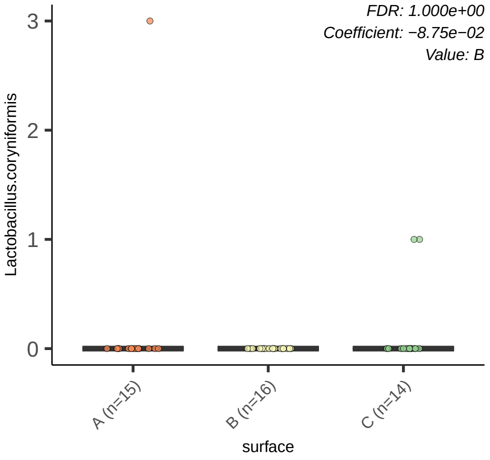

FDR: 1.000e+00
Coefficient: −8.75e−02
Value: B
Lactobacillus.coryniformis
3
2
1
0
A (n=15)
B (n=16)
C (n=14)
surface

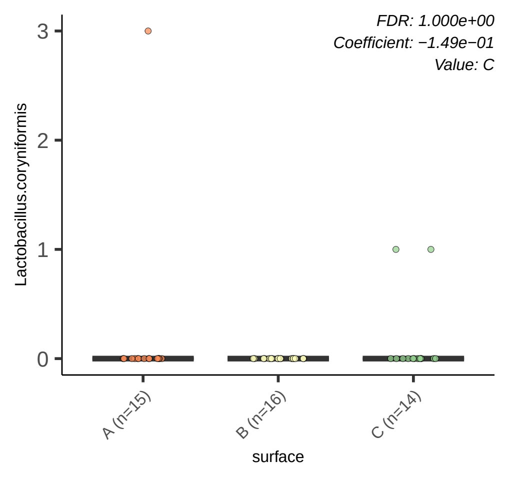
FDR: 1.000e+00
Coefficient: −1.49e−01
Value: C
Lactobacillus.coryniformis
3
2
1
0
A (n=15)
B (n=16)
C (n=14)
surface

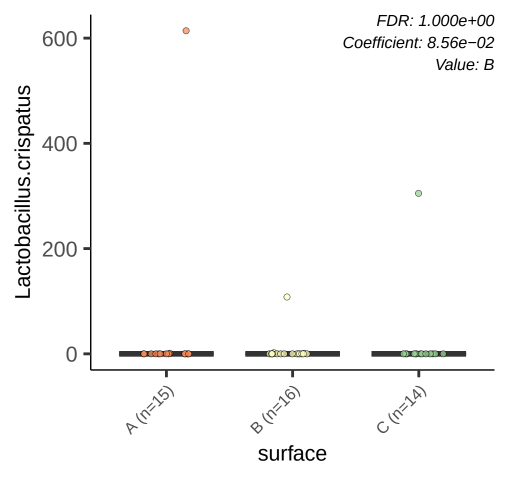
FDR: 1.000e+00
Coefficient: 8.56e−02
Value: B
Lactobacillus.crispatus
600
400
200
0
A (n=15)
B (n=16)
C (n=14)
surface

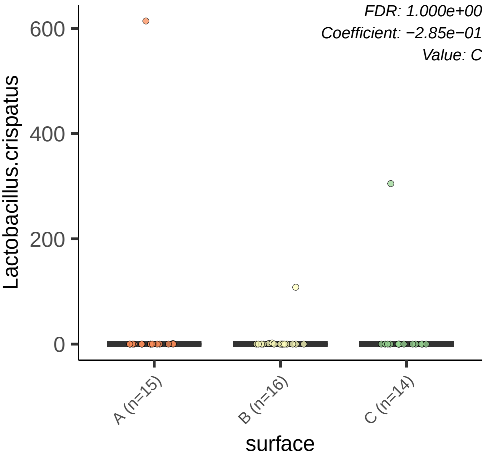

FDR: 1.000e+00
Coefficient: −2.85e−01
Value: C
Lactobacillus.crispatus
600
400
200
0
A (n=15)
B (n=16)
C (n=14)
surface

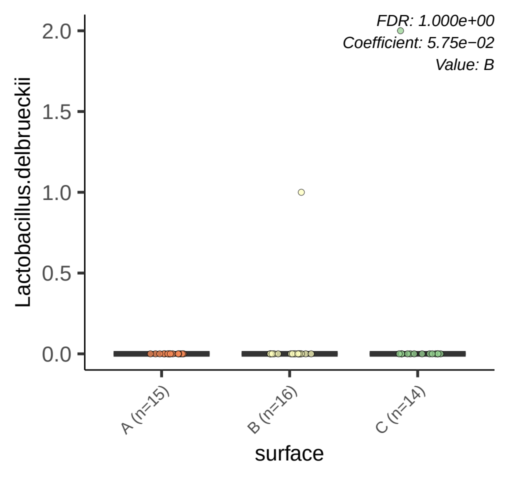
FDR: 1.000e+00
Coefficient: 5.75e−02
Value: B
Lactobacillus.delbrueckii
2.0
1.5
1.0
0.5
0.0
A (n=15)
B (n=16)
C (n=14)
surface

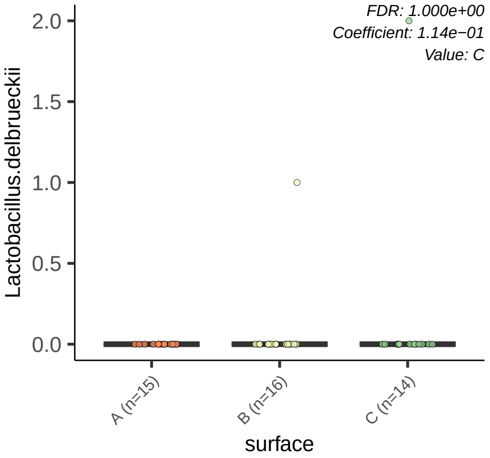
FDR: 1.000e+00
Coefficient: 1.14e−01
Value: C
Lactobacillus.delbrueckii
2.0
1.5
1.0
0.5
0.0
A (n=15)
B (n=16)
C (n=14)
surface

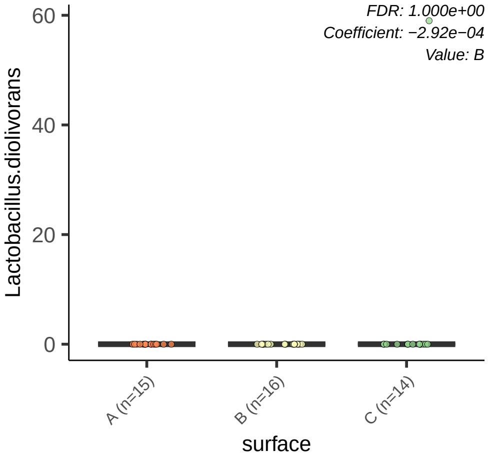

FDR: 1.000e+00
Coefficient: −2.92e−04
Value: B
Lactobacillus.diolivorans
60
40
20
0
A (n=15)
B (n=16)
C (n=14)
surface

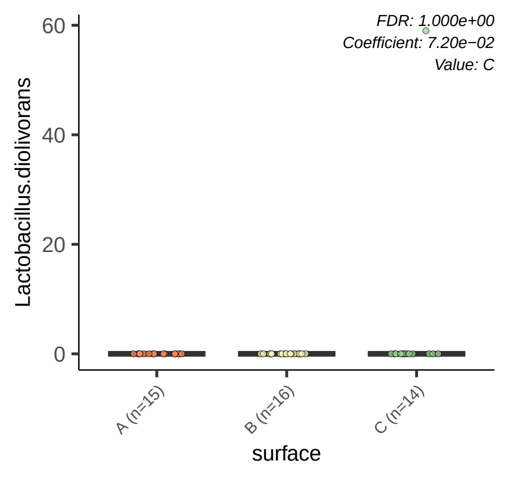

FDR: 1.000e+00
Coefficient: 7.20e−02
Value: C
Lactobacillus.diolivorans
60
40
20
0
A (n=15)
B (n=16)
C (n=14)
surface

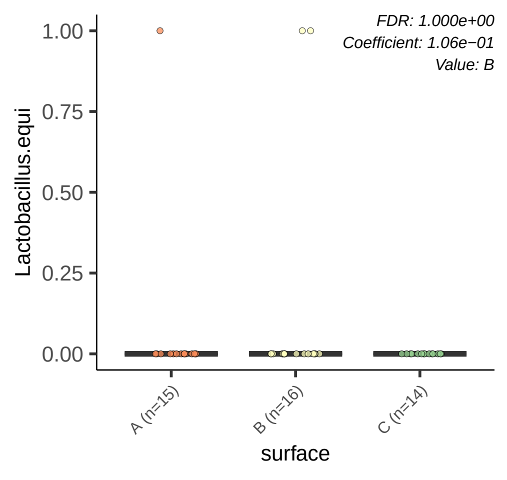
FDR: 1.000e+00
Coefficient: 1.06e−01
Value: B
Lactobacillus.equi
1.00
0.75
0.50
0.25
0.00
A (n=15)
B (n=16)
C (n=14)
surface

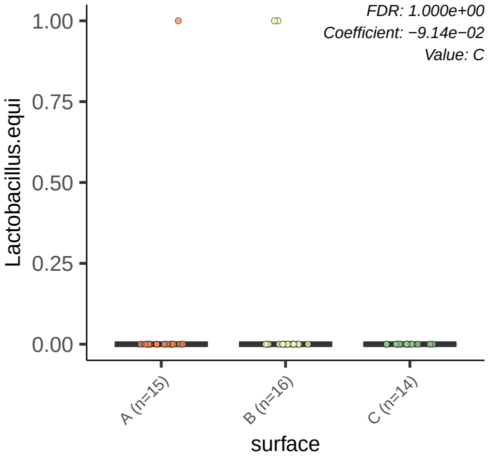
FDR: 1.000e+00
Coefficient: −9.14e−02
Value: C
Lactobacillus.equi
1.00
0.75
0.50
0.25
0.00
A (n=15)
B (n=16)
C (n=14)
surface

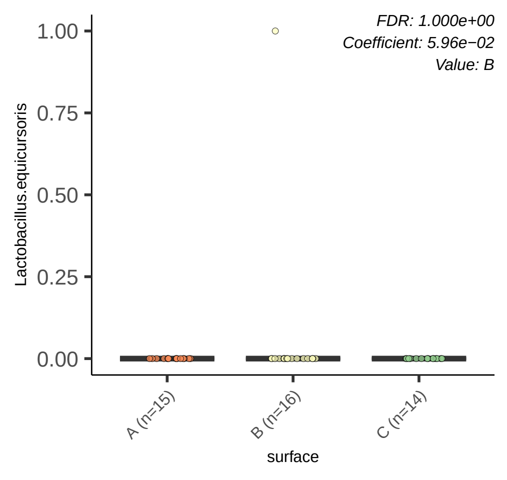
FDR: 1.000e+00
Coefficient: 5.96e−02
Value: B
1.00
0.75
0.50
0.25
0.00
Lactobacillus.equicursoris
A (n=15)
B (n=16)
C (n=14)
surface

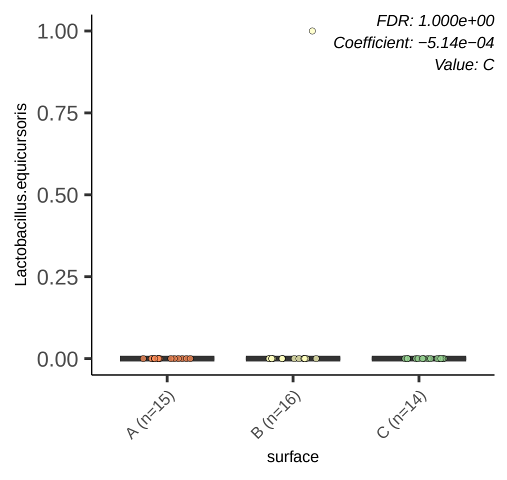
FDR: 1.000e+00
Coefficient: −5.14e−04
Value: C
Lactobacillus.equicursoris
1.00
0.75
0.50
0.25
0.00
A (n=15)
B (n=16)
C (n=14)
surface

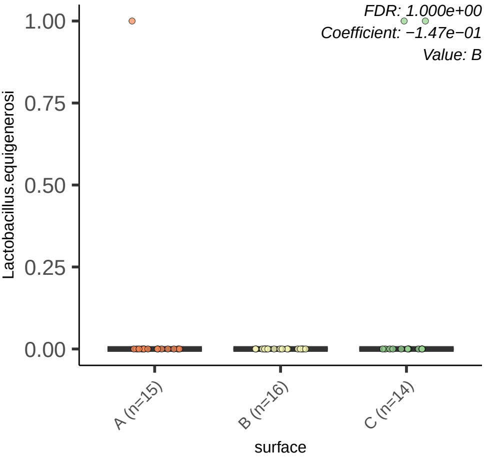

FDR: 1.000e+00
Coefficient: −1.47e−01
Value: B
Lactobacillus.equigenerosi
1.00
0.75
0.50
0.25
0.00
A (n=15)
B (n=16)
C (n=14)
surface

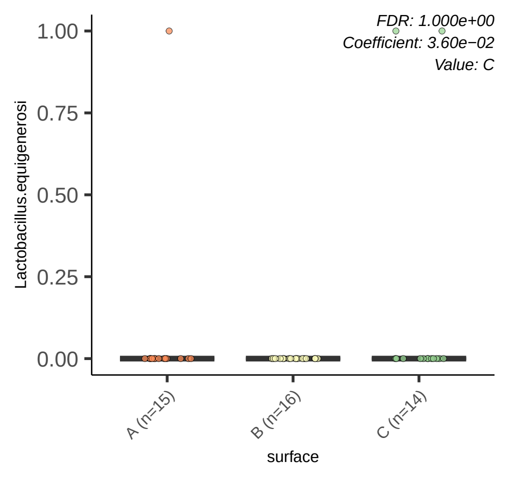
FDR: 1.000e+00
Coefficient: 3.60e−02
Value: C
Lactobacillus.equigenerosi
1.00
0.75
0.50
0.25
0.00
A (n=15)
B (n=16)
C (n=14)
surface

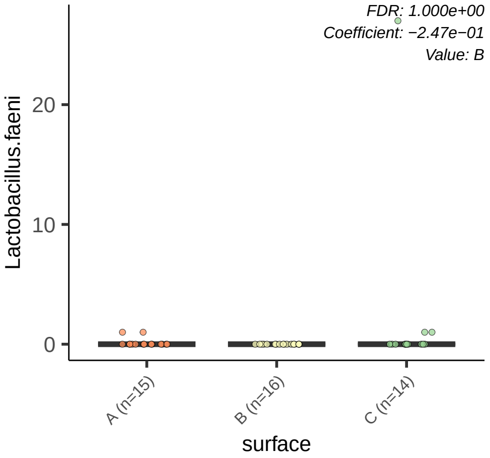
FDR: 1.000e+00
Coefficient: −2.47e−01
Value: B
Lactobacillus.faeni
20
10
0
A (n=15)
B (n=16)
C (n=14)
surface

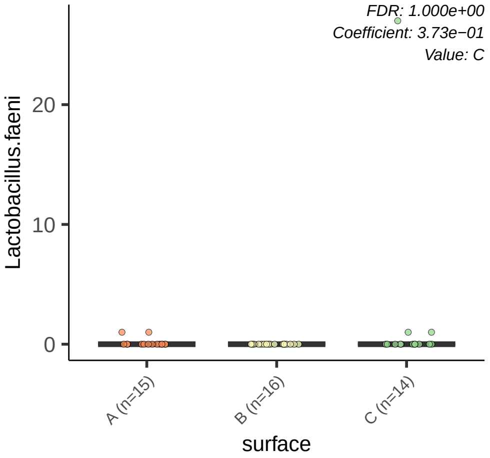
FDR: 1.000e+00
Coefficient: 3.73e−01
Value: C
Lactobacillus.faeni
0
10
20
A (n=15)
B (n=16)
C (n=14)
surface

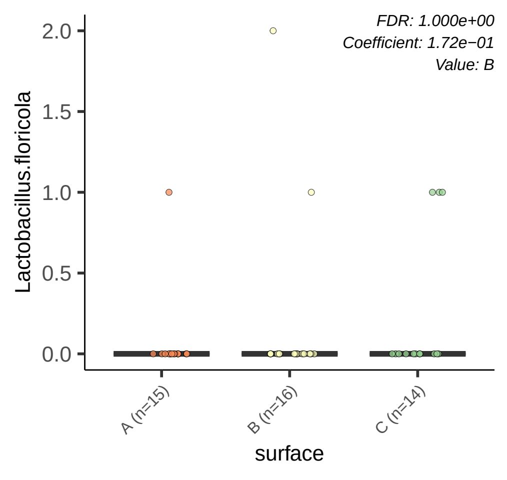
FDR: 1.000e+00
Coefficient: 1.72e−01
Value: B
Lactobacillus.floricola
2.0
1.5
1.0
0.5
0.0
A (n=15)
B (n=16)
C (n=14)
surface

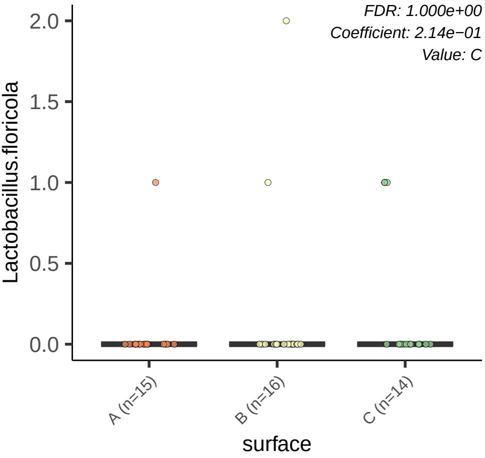

FDR: 1.000e+00
Coefficient: 2.14e−01
Value: C
Lactobacillus.floricola
2.0
1.5
1.0
0.5
0.0
A (n=15)
B (n=16)
C (n=14)
surface

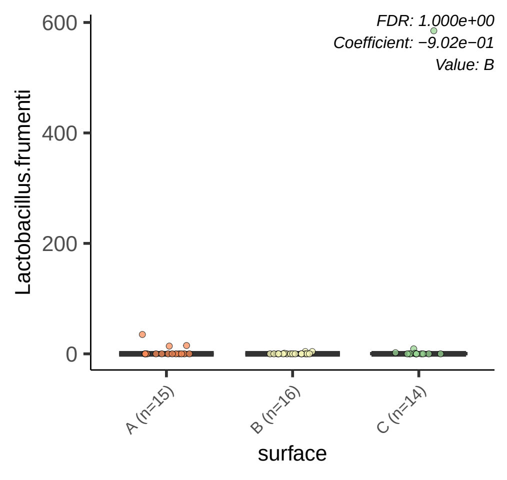

FDR: 1.000e+00
Coefficient: −9.02e−01
Value: B
Lactobacillus.frumenti
600
400
200
0
A (n=15)
B (n=16)
C (n=14)
surface

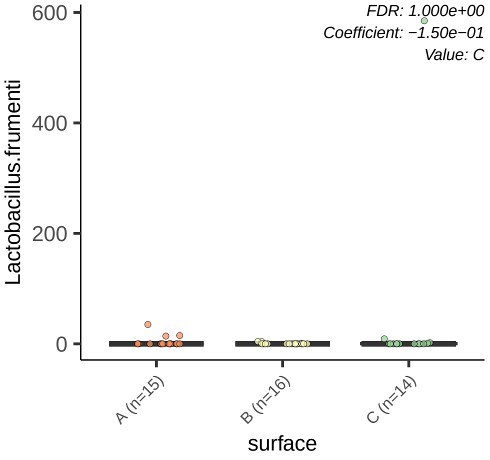

FDR: 1.000e+00
Coefficient: −1.50e−01
Value: C
600
400
200
0
Lactobacillus.frumenti
A (n=15)
B (n=16)
C (n=14)
surface

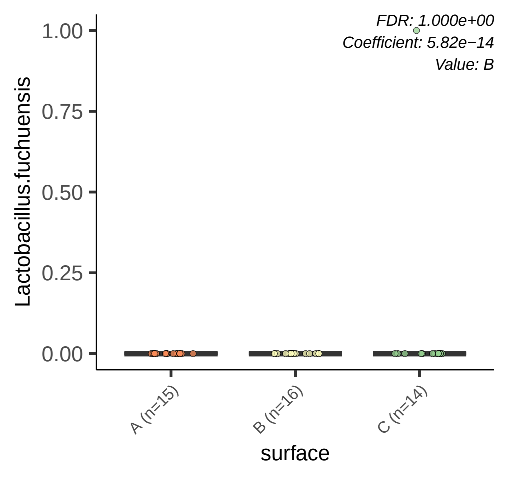
FDR: 1.000e+00
Coefficient: 5.82e−14
Value: B
Lactobacillus.fuchuensis
1.00
0.75
0.50
0.25
0.00
A (n=15)
B (n=16)
C (n=14)
surface

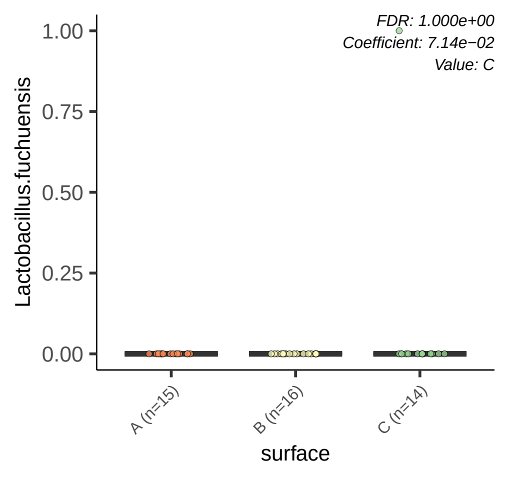
FDR: 1.000e+00
Coefficient: 7.14e−02
Value: C
Lactobacillus.fuchuensis
1.00
0.75
0.50
0.25
0.00
A (n=15)
B (n=16)
C (n=14)
surface

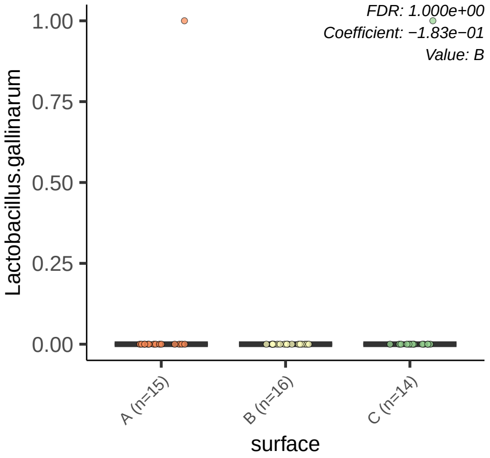

FDR: 1.000e+00
Coefficient: −1.83e−01
Value: B
Lactobacillus.gallinarum
1.00
0.75
0.50
0.25
0.00
A (n=15)
B (n=16)
C (n=14)
surface

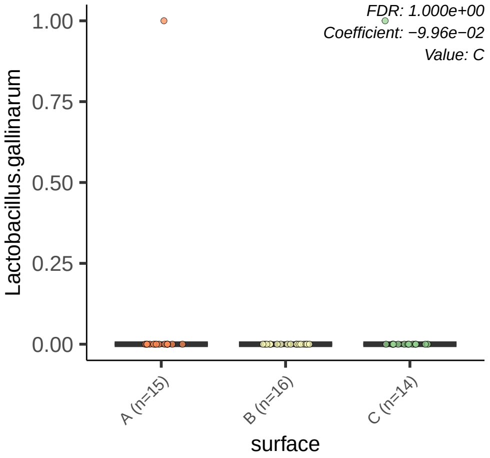

FDR: 1.000e+00
Coefficient: −9.96e−02
Value: C
Lactobacillus.gallinarum
1.00
0.75
0.50
0.25
0.00
A (n=15)
B (n=16)
C (n=14)
surface

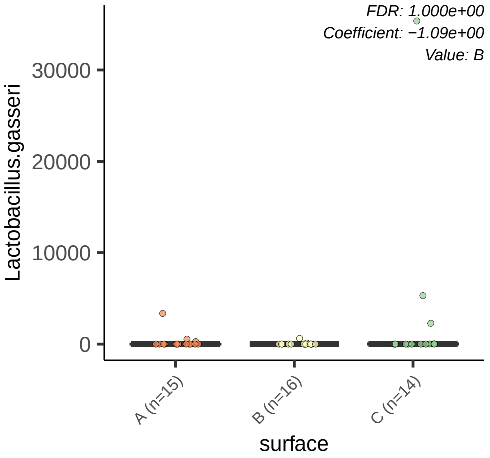
FDR: 1.000e+00
Coefficient: −1.09e+00
Value: B
Lactobacillus.gasseri
30000
20000
10000
0
A (n=15)
B (n=16)
C (n=14)
surface

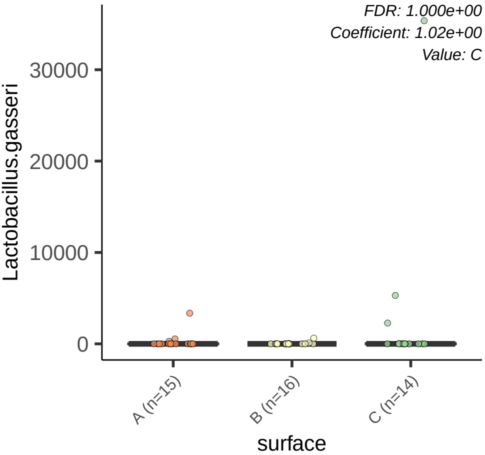
FDR: 1.000e+00
Coefficient: 1.02e+00
Value: C
Lactobacillus.gasseri
30000
20000
10000
0
A (n=15)
B (n=16)
C (n=14)
surface

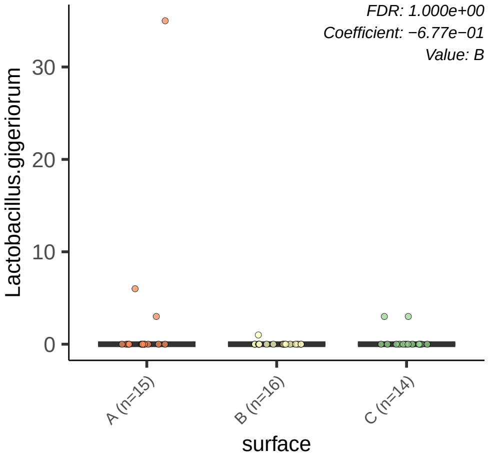
FDR: 1.000e+00
Coefficient: −6.77e−01
Value: B
Lactobacillus.gigeriorum
30
20
10
0
A (n=15)
B (n=16)
C (n=14)
surface

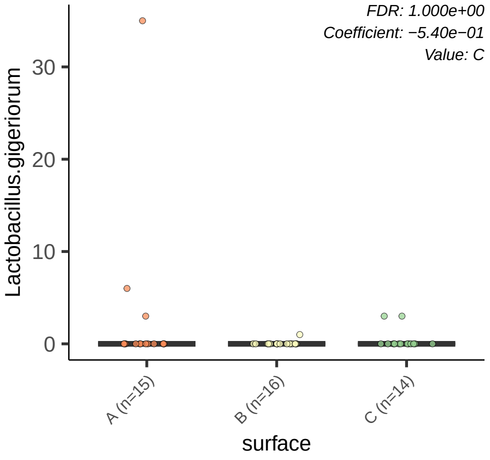

FDR: 1.000e+00
Coefficient: −5.40e−01
Value: C
Lactobacillus.gigeriorum
30
20
10
0
A (n=15)
B (n=16)
C (n=14)
surface

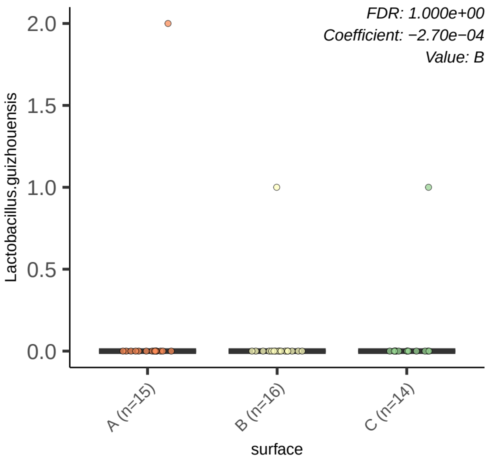
FDR: 1.000e+00
Coefficient: −2.70e−04
Value: B
Lactobacillus.guizhouensis
2.0
1.5
1.0
0.5
0.0
A (n=15)
B (n=16)
C (n=14)
surface

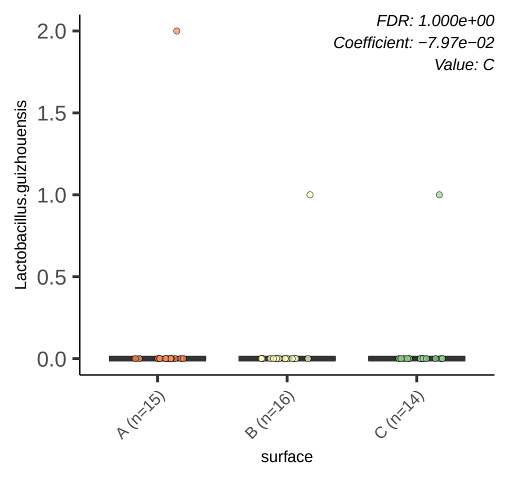

FDR: 1.000e+00
Coefficient: −7.97e−02
Value: C
Lactobacillus.guizhouensis
2.0
1.5
1.0
0.5
0.0
A (n=15)
B (n=16)
C (n=14)
surface

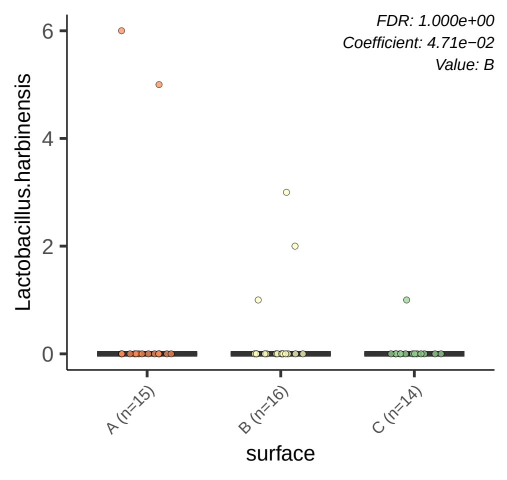

FDR: 1.000e+00
Coefficient: 4.71e−02
Value: B
Lactobacillus.harbinensis
0
2
4
6
A (n=15)
B (n=16)
C (n=14)
surface

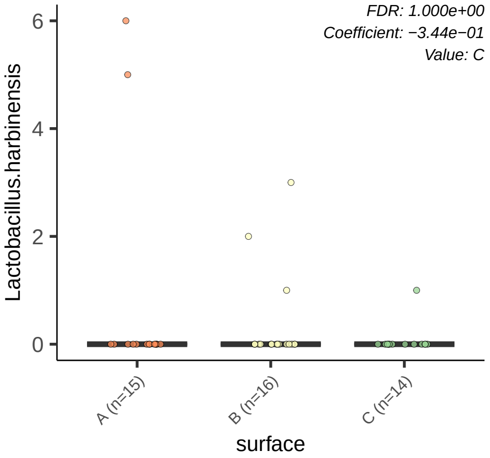
FDR: 1.000e+00
Coefficient: −3.44e−01
Value: C
Lactobacillus.harbinensis
0
2
4
6
A (n=15)
B (n=16)
C (n=14)
surface

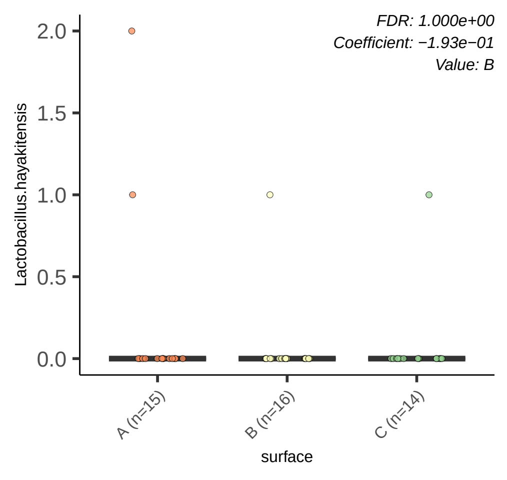
FDR: 1.000e+00
Coefficient: −1.93e−01
Value: B
Lactobacillus.hayakitensis
2.0
1.5
1.0
0.5
0.0
A (n=15)
B (n=16)
C (n=14)
surface

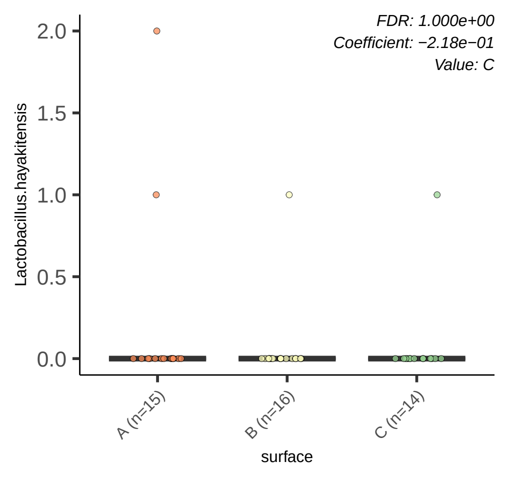
FDR: 1.000e+00
Coefficient: −2.18e−01
Value: C
Lactobacillus.hayakitensis
2.0
1.5
1.0
0.5
0.0
A (n=15)
B (n=16)
C (n=14)
surface

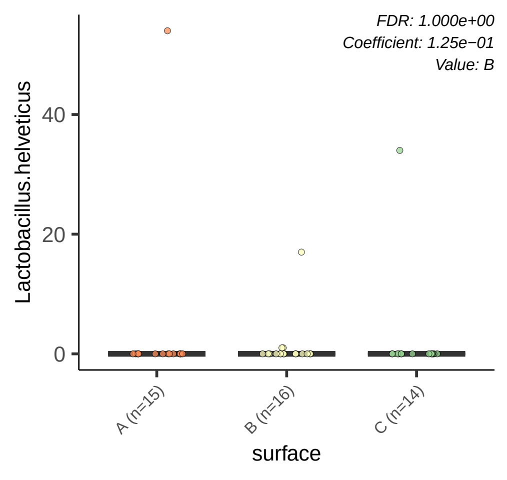

FDR: 1.000e+00
Coefficient: 1.25e−01
Value: B
Lactobacillus.helveticus
40
20
0
A (n=15)
B (n=16)
C (n=14)
surface

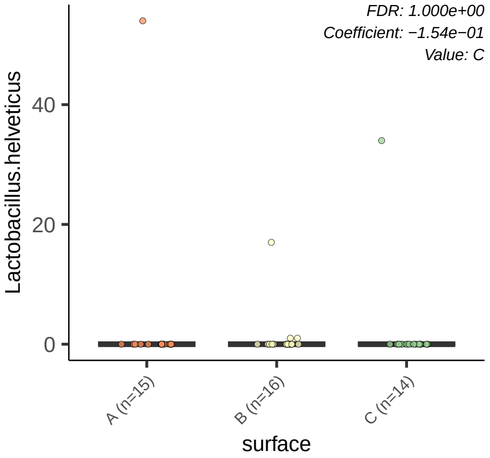
FDR: 1.000e+00
Coefficient: −1.54e−01
Value: C
Lactobacillus.helveticus
40
20
0
A (n=15)
B (n=16)
C (n=14)
surface

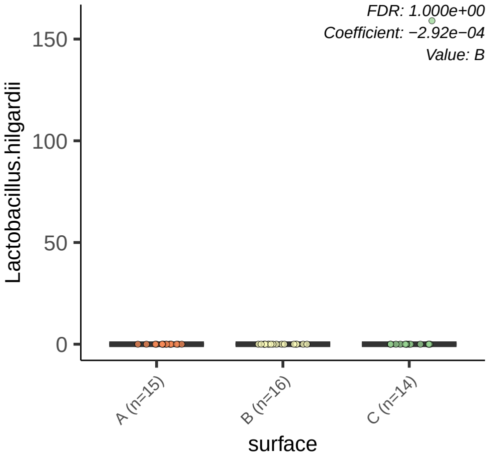

FDR: 1.000e+00
Coefficient: −2.92e−04
Value: B
Lactobacillus.hilgardii
150
100
50
0
A (n=15)
B (n=16)
C (n=14)
surface

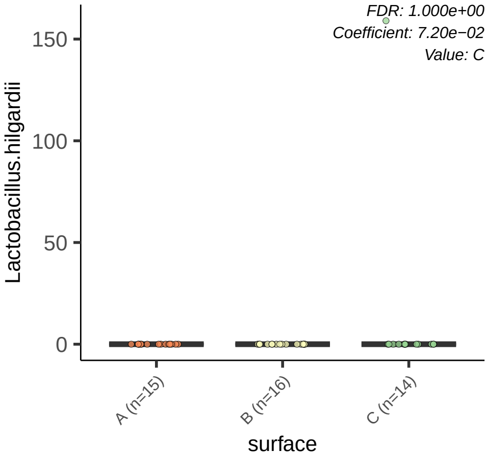
FDR: 1.000e+00
Coefficient: 7.20e−02
Value: C
150
100
50
0
Lactobacillus.hilgardii
A (n=15)
B (n=16)
C (n=14)
surface

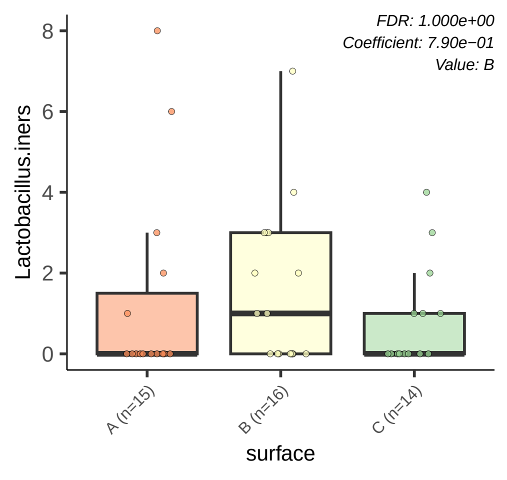
FDR: 1.000e+00
Coefficient: 7.90e−01
Value: B
Lactobacillus.iners
0
2
4
6
8
A (n=15)
B (n=16)
C (n=14)
surface

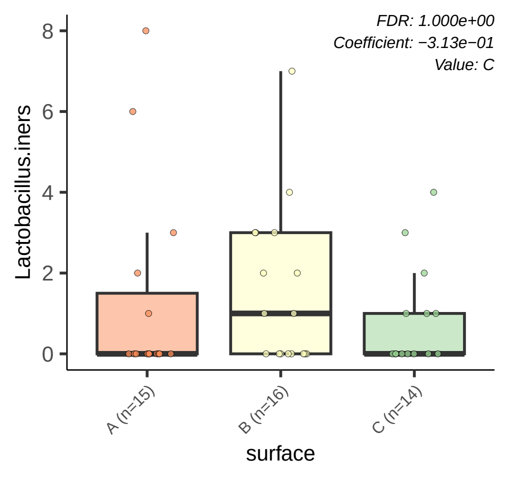

FDR: 1.000e+00
Coefficient: −3.13e−01
Value: C
Lactobacillus.iners
8
6
4
2
0
A (n=15)
B (n=16)
C (n=14)
surface

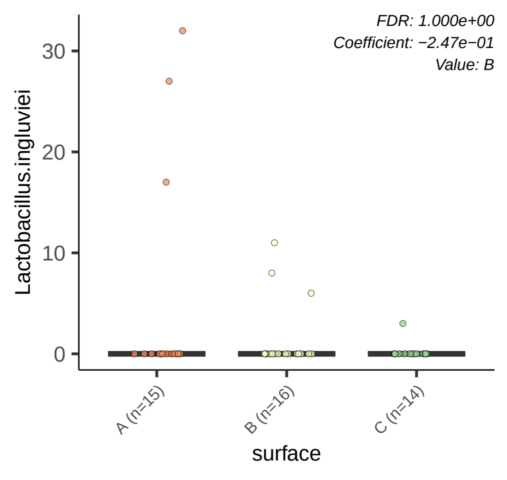

FDR: 1.000e+00
Coefficient: −2.47e−01
Value: B
Lactobacillus.ingluviei
30
20
10
0
A (n=15)
B (n=16)
C (n=14)
surface

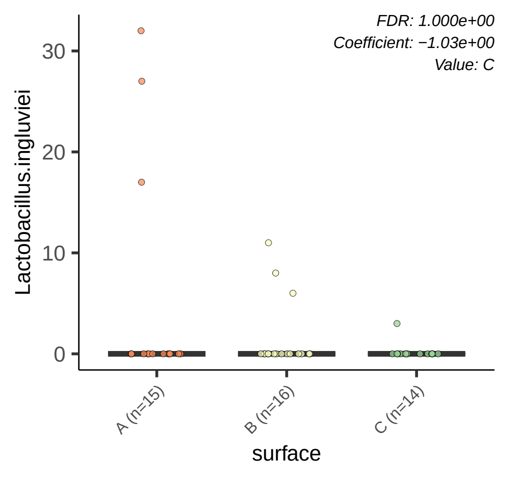
FDR: 1.000e+00
Coefficient: −1.03e+00
Value: C
Lactobacillus.ingluviei
0
10
20
30
A (n=15)
B (n=16)
C (n=14)
surface

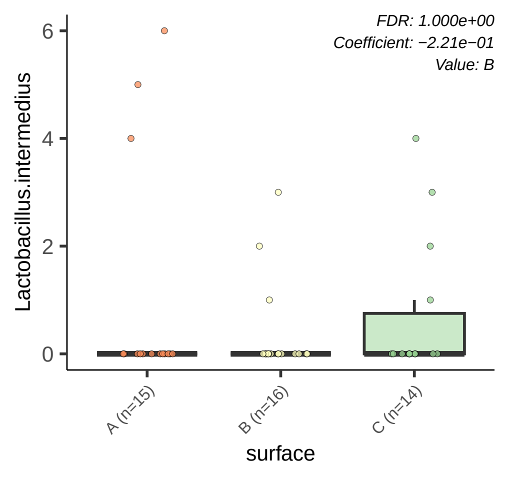
FDR: 1.000e+00
Coefficient: −2.21e−01
Value: B
Lactobacillus.intermedius
0
2
4
6
A (n=15)
B (n=16)
C (n=14)
surface

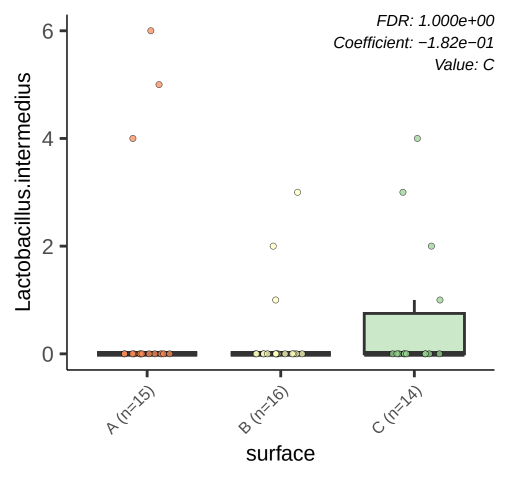
FDR: 1.000e+00
Coefficient: −1.82e−01
Value: C
Lactobacillus.intermedius
6
4
2
0
A (n=15)
B (n=16)
C (n=14)
surface

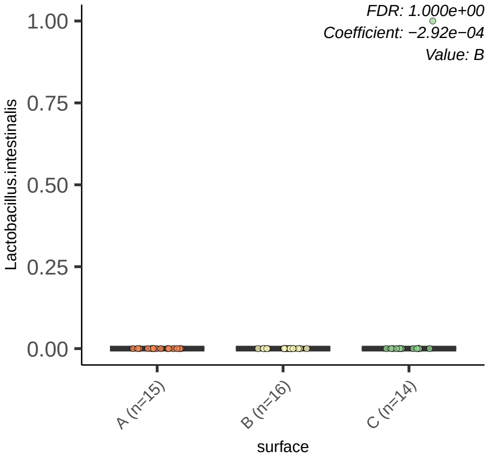
FDR: 1.000e+00
Coefficient: −2.92e−04
Value: B
1.00
0.75
0.50
0.25
0.00
Lactobacillus.intestinalis
A (n=15)
B (n=16)
C (n=14)
surface

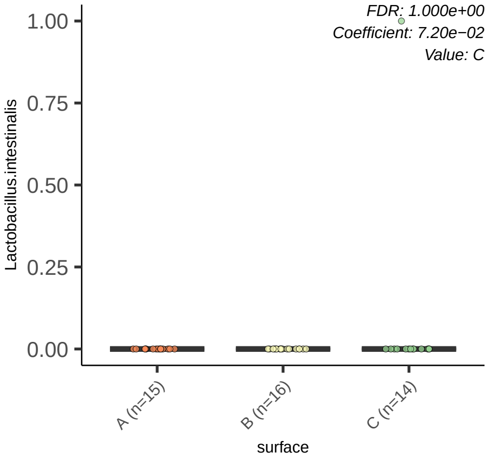
FDR: 1.000e+00
Coefficient: 7.20e−02
Value: C
1.00
0.75
0.50
0.25
0.00
Lactobacillus.intestinalis
A (n=15)
B (n=16)
C (n=14)
surface

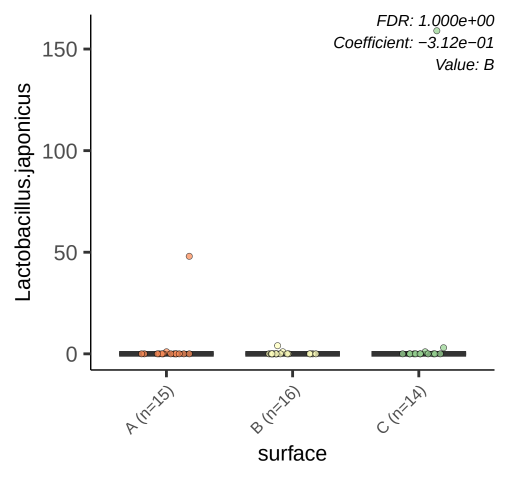
FDR: 1.000e+00
Coefficient: −3.12e−01
Value: B
Lactobacillus.japonicus
150
100
50
0
A (n=15)
B (n=16)
C (n=14)
surface

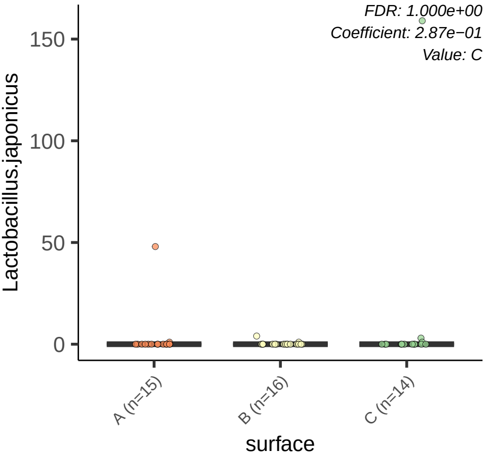

FDR: 1.000e+00
Coefficient: 2.87e−01
Value: C
Lactobacillus.japonicus
150
100
50
0
A (n=15)
B (n=16)
C (n=14)
surface

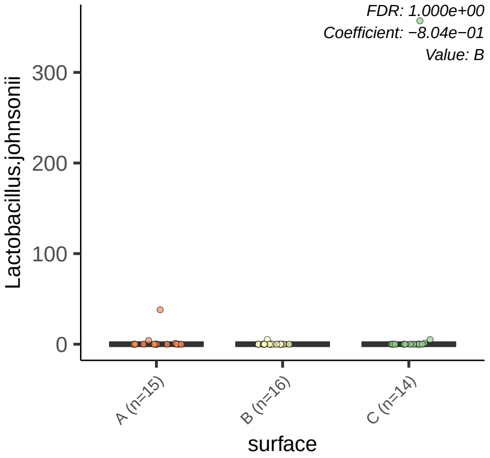

FDR: 1.000e+00
Coefficient: −8.04e−01
Value: B
Lactobacillus.johnsonii
300
200
100
0
A (n=15)
B (n=16)
C (n=14)
surface

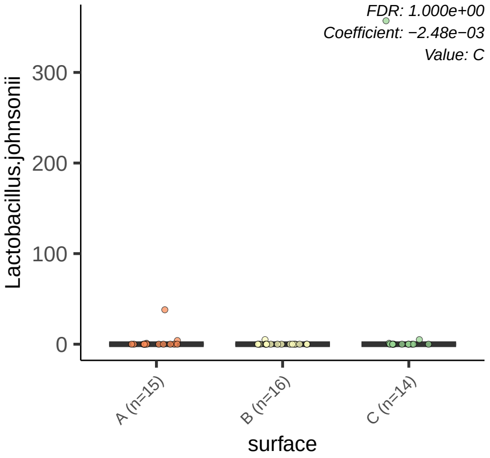

FDR: 1.000e+00
Coefficient: −2.48e−03
Value: C
Lactobacillus.johnsonii
300
200
100
0
A (n=15)
B (n=16)
C (n=14)
surface

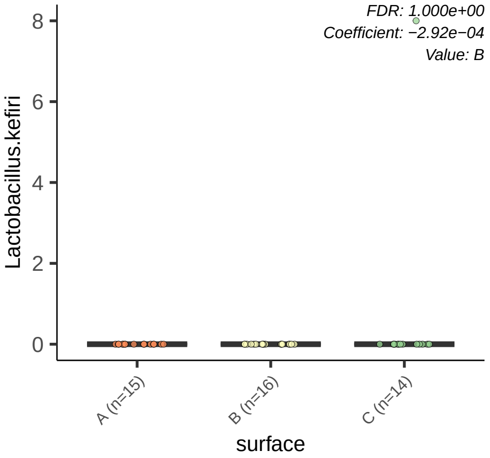

FDR: 1.000e+00
Coefficient: −2.92e−04
Value: B
Lactobacillus.kefiri
8
6
4
2
0
A (n=15)
B (n=16)
C (n=14)
surface

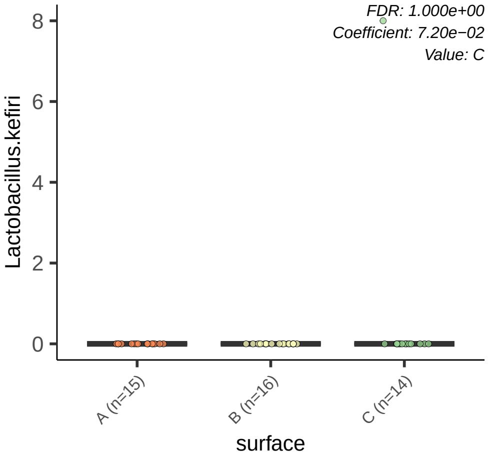

FDR: 1.000e+00
Coefficient: 7.20e−02
Value: C
Lactobacillus.kefiri
8
6
4
2
0
A (n=15)
B (n=16)
C (n=14)
surface

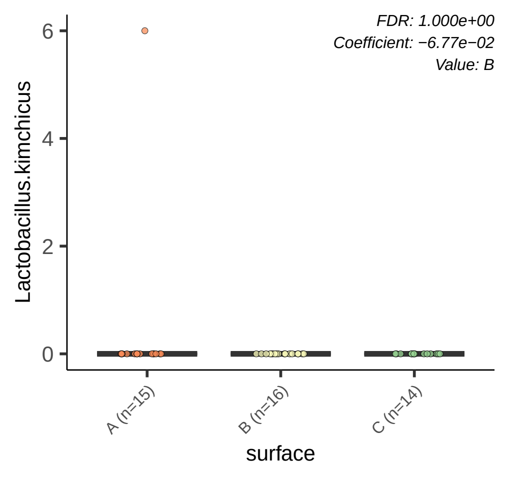
FDR: 1.000e+00
Coefficient: −6.77e−02
Value: B
Lactobacillus.kimchicus
6
4
2
0
A (n=15)
B (n=16)
C (n=14)
surface

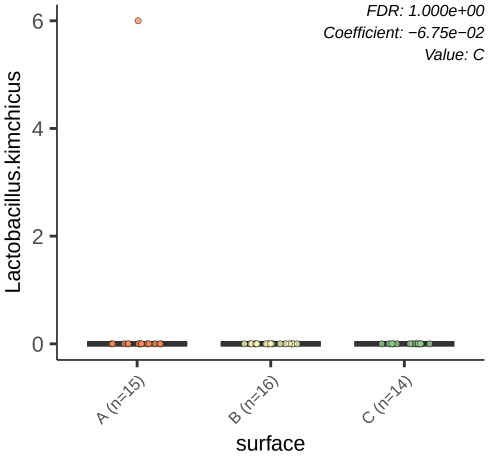

FDR: 1.000e+00
Coefficient: −6.75e−02
Value: C
Lactobacillus.kimchicus
6
4
2
0
A (n=15)
B (n=16)
C (n=14)
surface

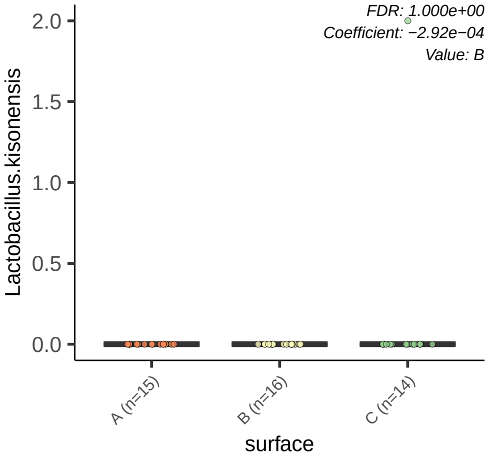
FDR: 1.000e+00
Coefficient: −2.92e−04
Value: B
Lactobacillus.kisonensis
2.0
1.5
1.0
0.5
0.0
A (n=15)
B (n=16)
C (n=14)
surface

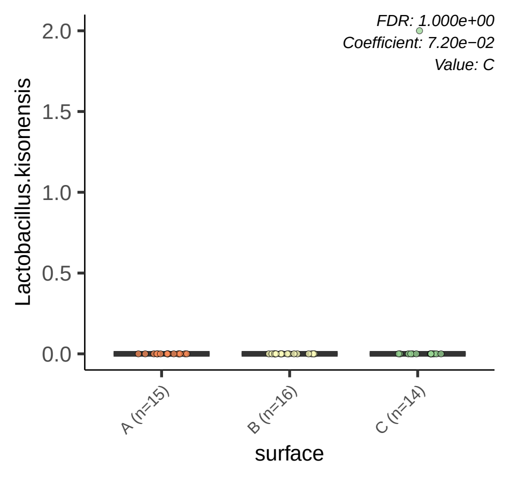

FDR: 1.000e+00
Coefficient: 7.20e−02
Value: C
Lactobacillus.kisonensis
2.0
1.5
1.0
0.5
0.0
A (n=15)
B (n=16)
C (n=14)
surface

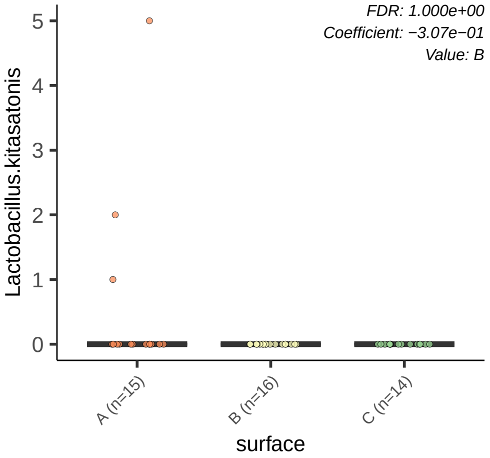
FDR: 1.000e+00
Coefficient: −3.07e−01
Value: B
Lactobacillus.kitasatonis
0
1
2
3
4
5
A (n=15)
B (n=16)
C (n=14)
surface

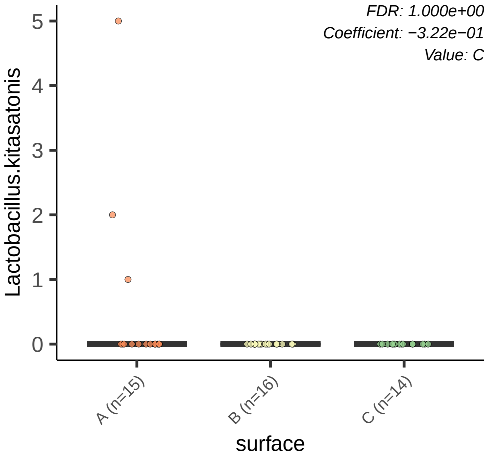

FDR: 1.000e+00
Coefficient: −3.22e−01
Value: C
Lactobacillus.kitasatonis
5
4
3
2
1
0
A (n=15)
B (n=16)
C (n=14)
surface

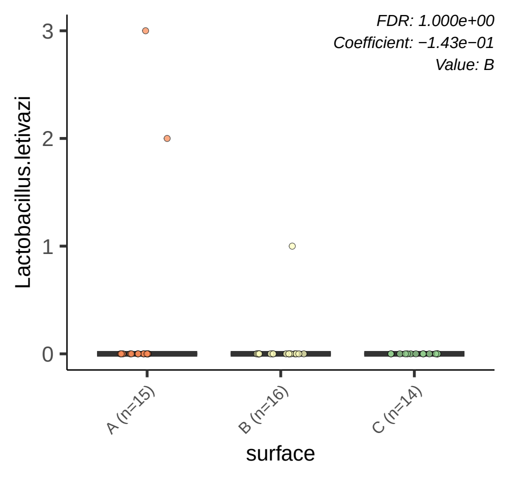
FDR: 1.000e+00
Coefficient: −1.43e−01
Value: B
Lactobacillus.letivazi
0
1
2
3
A (n=15)
B (n=16)
C (n=14)
surface

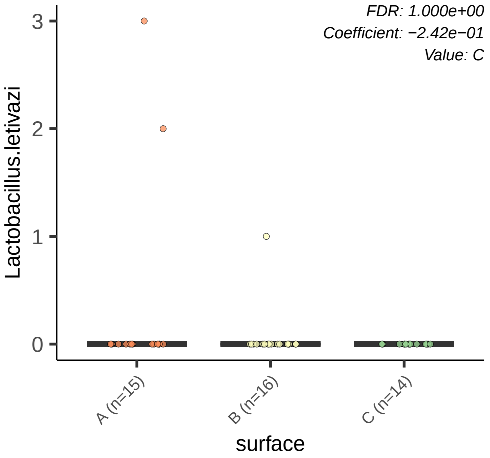
FDR: 1.000e+00
Coefficient: −2.42e−01
Value: C
Lactobacillus.letivazi
3
2
1
0
A (n=15)
B (n=16)
C (n=14)
surface

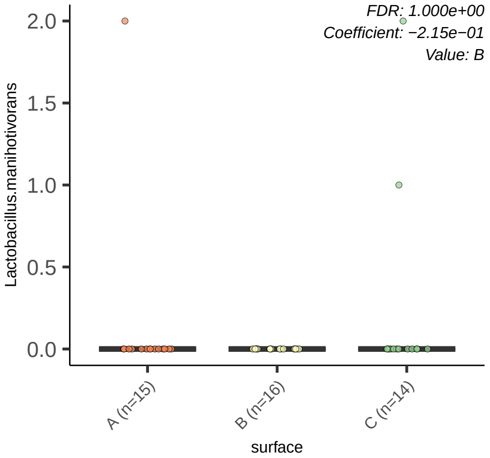
FDR: 1.000e+00
Coefficient: −2.15e−01
Value: B
2.0
1.5
1.0
0.5
0.0
Lactobacillus.manihotivorans
A (n=15)
B (n=16)
C (n=14)
surface

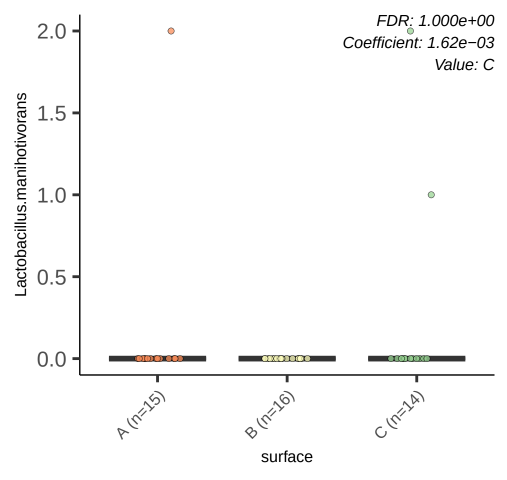

FDR: 1.000e+00
Coefficient: 1.62e−03
Value: C
Lactobacillus.manihotivorans
2.0
1.5
1.0
0.5
0.0
A (n=15)
B (n=16)
C (n=14)
surface

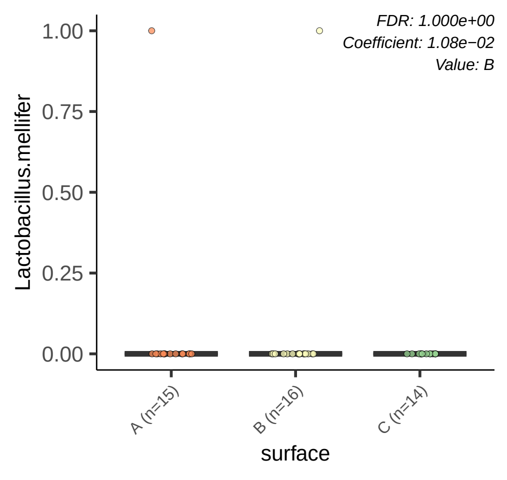
FDR: 1.000e+00
Coefficient: 1.08e−02
Value: B
Lactobacillus.mellifer
1.00
0.75
0.50
0.25
0.00
A (n=15)
B (n=16)
C (n=14)
surface

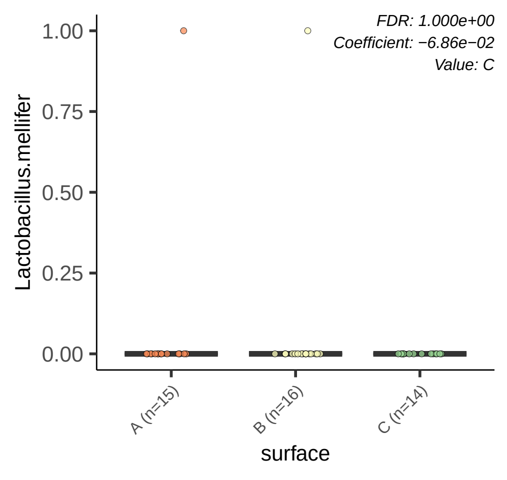

FDR: 1.000e+00
Coefficient: −6.86e−02
Value: C
1.00
0.75
0.50
0.25
0.00
Lactobacillus.mellifer
A (n=15)
B (n=16)
C (n=14)
surface

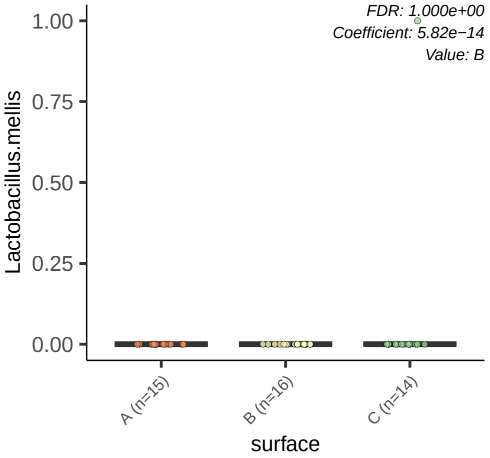

FDR: 1.000e+00
Coefficient: 5.82e−14
Value: B
1.00
0.75
0.50
0.25
0.00
Lactobacillus.mellis
A (n=15)
B (n=16)
C (n=14)
surface

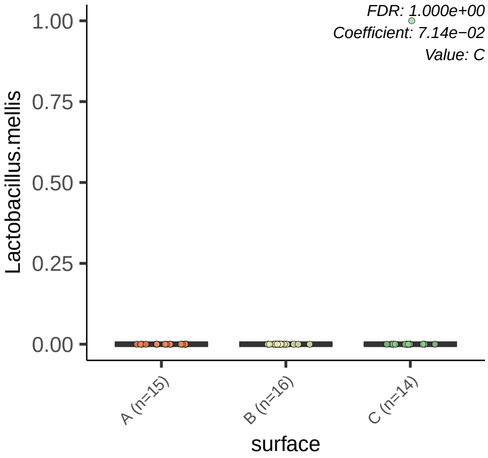

FDR: 1.000e+00
Coefficient: 7.14e−02
Value: C
Lactobacillus.mellis
1.00
0.75
0.50
0.25
0.00
A (n=15)
B (n=16)
C (n=14)
surface

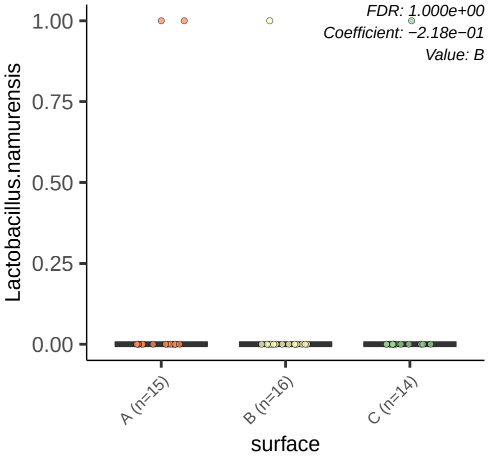
FDR: 1.000e+00
Coefficient: −2.18e−01
Value: B
Lactobacillus.namurensis
1.00
0.75
0.50
0.25
0.00
A (n=15)
B (n=16)
C (n=14)
surface

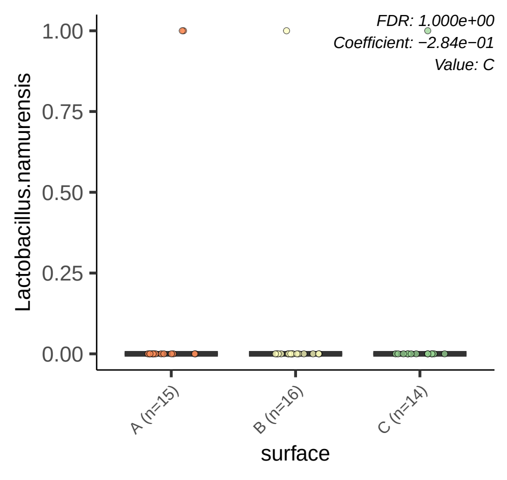
FDR: 1.000e+00
Coefficient: −2.84e−01
Value: C
Lactobacillus.namurensis
1.00
0.75
0.50
0.25
0.00
A (n=15)
B (n=16)
C (n=14)
surface

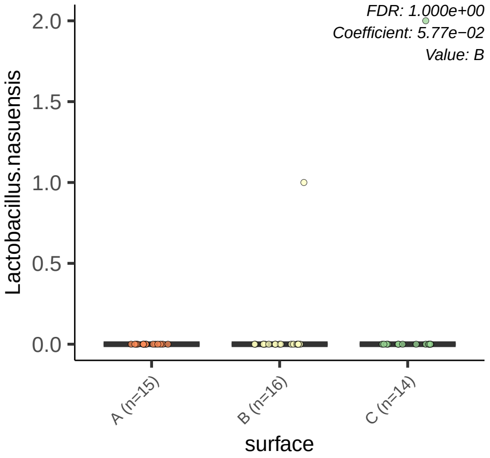
FDR: 1.000e+00
Coefficient: 5.77e−02
Value: B
Lactobacillus.nasuensis
2.0
1.5
1.0
0.5
0.0
A (n=15)
B (n=16)
C (n=14)
surface

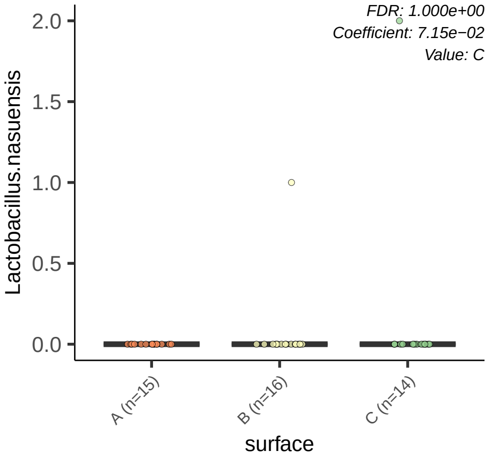
FDR: 1.000e+00
Coefficient: 7.15e−02
Value: C
Lactobacillus.nasuensis
2.0
1.5
1.0
0.5
0.0
A (n=15)
B (n=16)
C (n=14)
surface

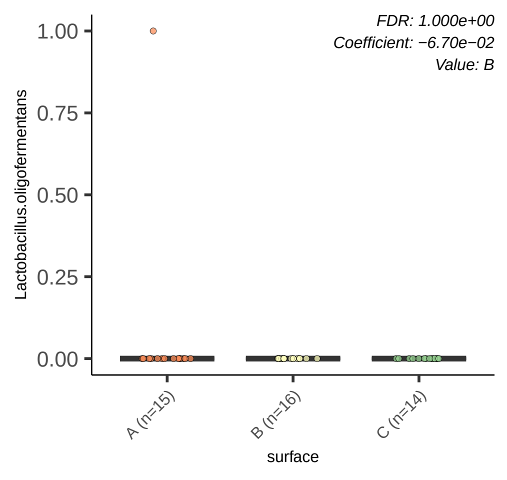

FDR: 1.000e+00
Coefficient: −6.70e−02
Value: B
Lactobacillus.oligofermentans
1.00
0.75
0.50
0.25
0.00
A (n=15)
B (n=16)
C (n=14)
surface

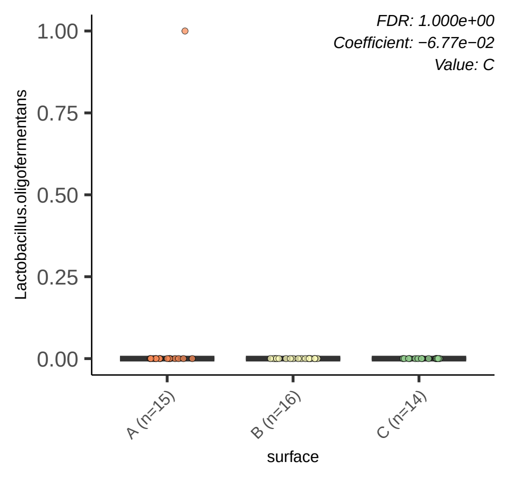
FDR: 1.000e+00
Coefficient: −6.77e−02
Value: C
Lactobacillus.oligofermentans
1.00
0.75
0.50
0.25
0.00
A (n=15)
B (n=16)
C (n=14)
surface

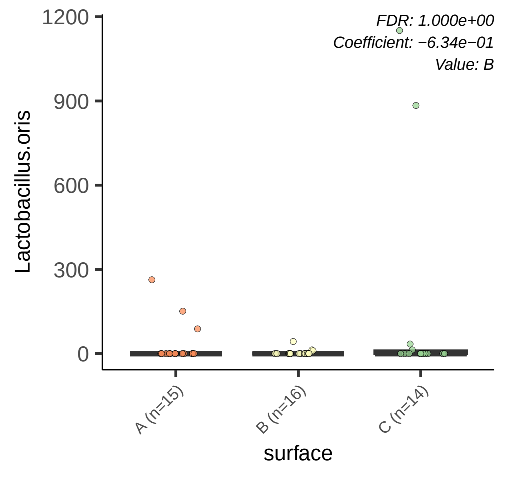
FDR: 1.000e+00
Coefficient: −6.34e−01
Value: B
Lactobacillus.oris
1200
900
600
300
0
A (n=15)
B (n=16)
C (n=14)
surface

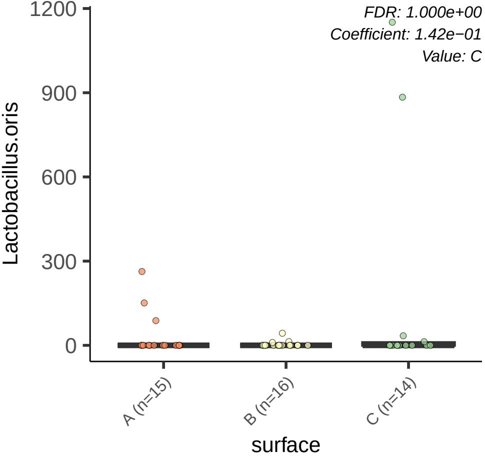

FDR: 1.000e+00
Coefficient: 1.42e−01
Value: C
Lactobacillus.oris
1200
900
600
300
0
A (n=15)
B (n=16)
C (n=14)
surface

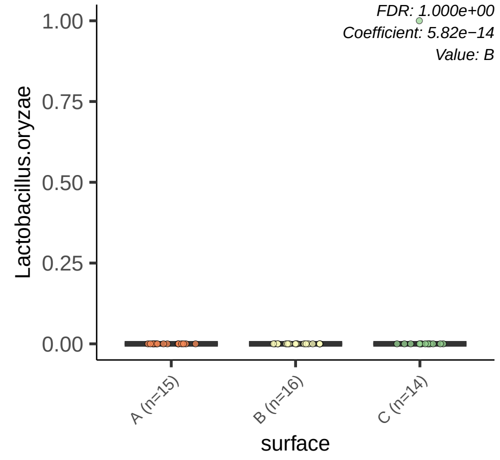
FDR: 1.000e+00
Coefficient: 5.82e−14
Value: B
Lactobacillus.oryzae
1.00
0.75
0.50
0.25
0.00
A (n=15)
B (n=16)
C (n=14)
surface

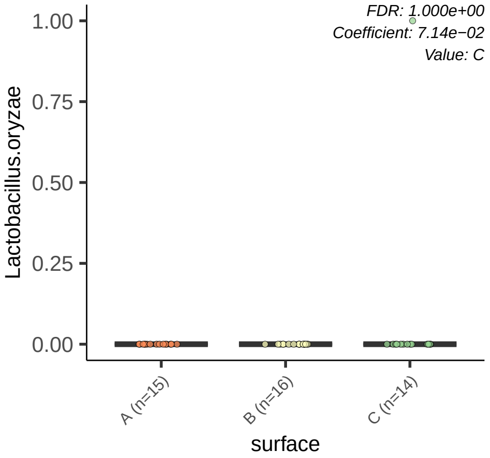

FDR: 1.000e+00
Coefficient: 7.14e−02
Value: C
Lactobacillus.oryzae
1.00
0.75
0.50
0.25
0.00
A (n=15)
B (n=16)
C (n=14)
surface

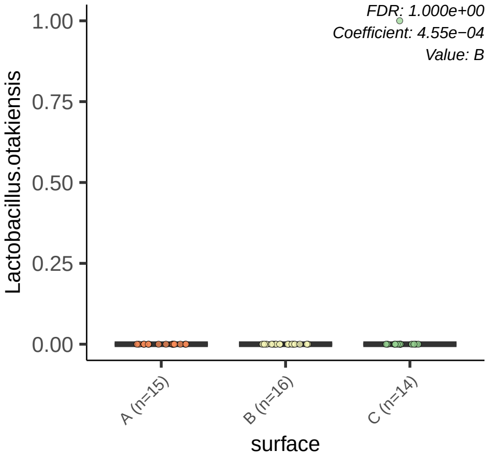
FDR: 1.000e+00
Coefficient: 4.55e−04
Value: B
Lactobacillus.otakiensis
1.00
0.75
0.50
0.25
0.00
A (n=15)
B (n=16)
C (n=14)
surface

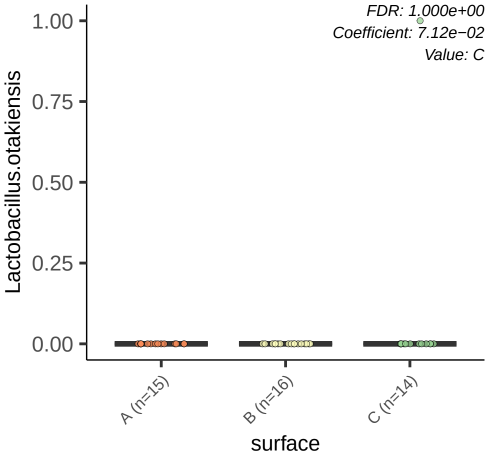

FDR: 1.000e+00
Coefficient: 7.12e−02
Value: C
Lactobacillus.otakiensis
1.00
0.75
0.50
0.25
0.00
A (n=15)
B (n=16)
C (n=14)
surface

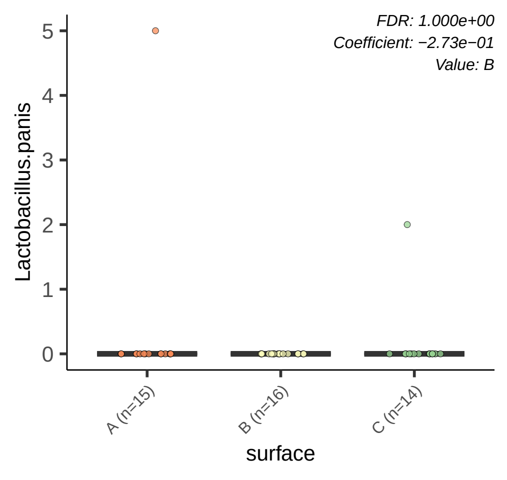
FDR: 1.000e+00
Coefficient: −2.73e−01
Value: B
Lactobacillus.panis
0
1
2
3
4
5
A (n=15)
B (n=16)
C (n=14)
surface

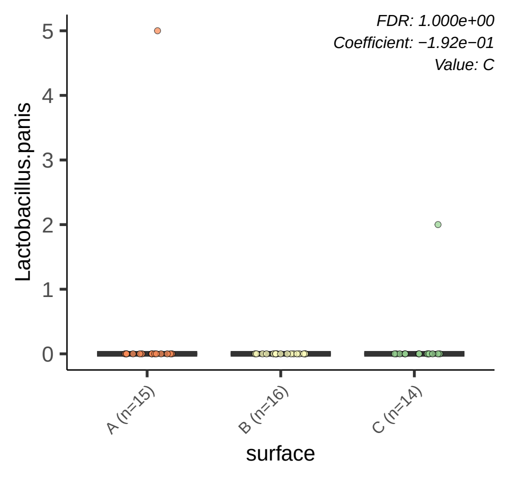

FDR: 1.000e+00
Coefficient: −1.92e−01
Value: C
Lactobacillus.panis
0
1
2
3
4
5
A (n=15)
B (n=16)
C (n=14)
surface

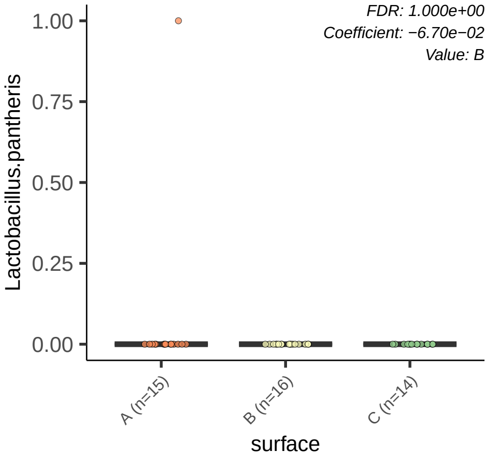

FDR: 1.000e+00
Coefficient: −6.70e−02
Value: B
Lactobacillus.pantheris
1.00
0.75
0.50
0.25
0.00
A (n=15)
B (n=16)
C (n=14)
surface

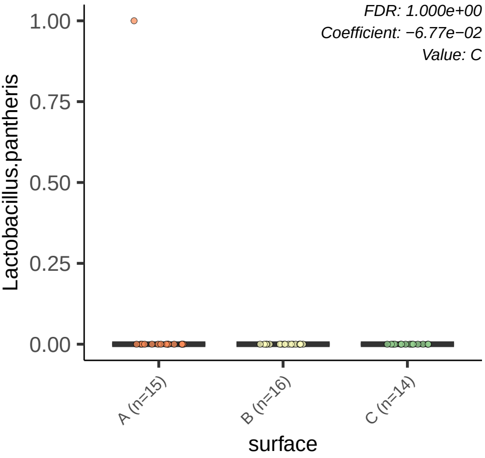
FDR: 1.000e+00
Coefficient: −6.77e−02
Value: C
Lactobacillus.pantheris
1.00
0.75
0.50
0.25
0.00
A (n=15)
B (n=16)
C (n=14)
surface

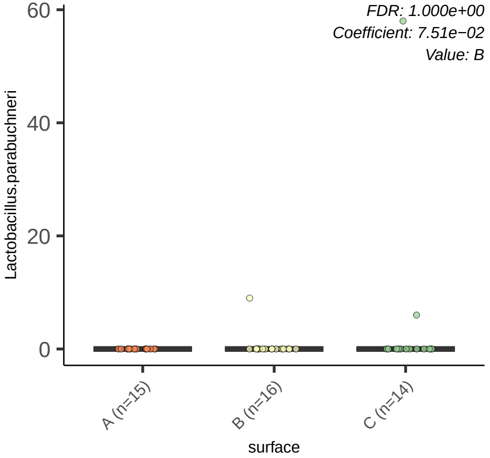

FDR: 1.000e+00
Coefficient: 7.51e−02
Value: B
60
40
20
0
Lactobacillus.parabuchneri
A (n=15)
B (n=16)
C (n=14)
surface

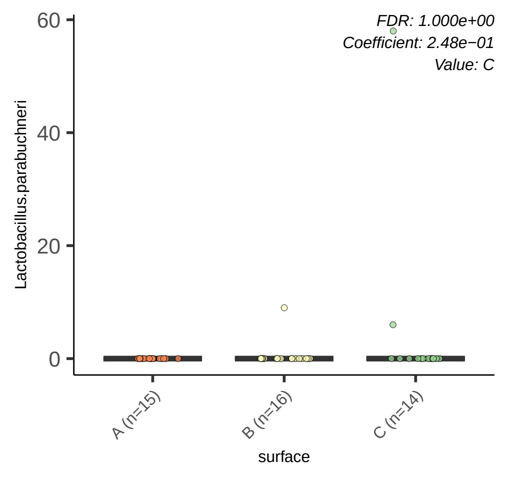
FDR: 1.000e+00
Coefficient: 2.48e−01
Value: C
Lactobacillus.parabuchneri
60
40
20
0
A (n=15)
B (n=16)
C (n=14)
surface

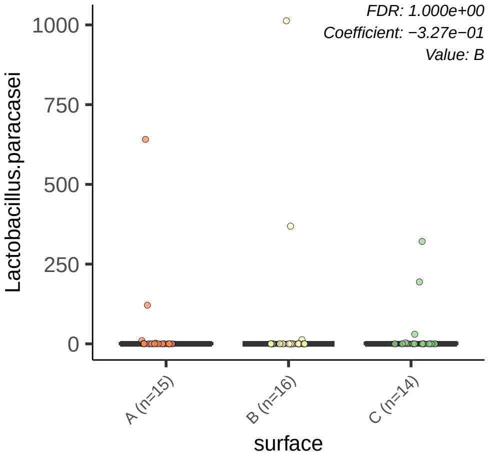
FDR: 1.000e+00
Coefficient: −3.27e−01
Value: B
1000
750
500
250
0
Lactobacillus.paracasei
A (n=15)
B (n=16)
C (n=14)
surface

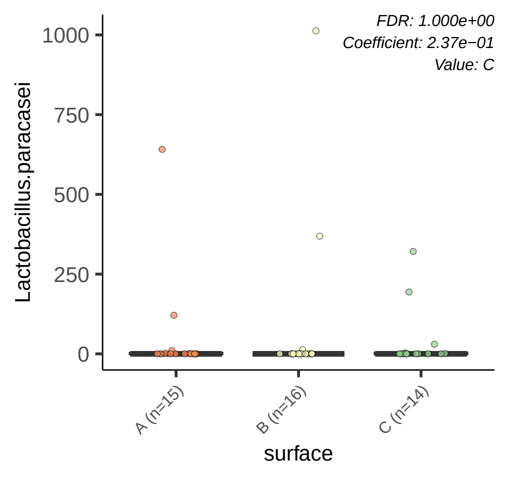

FDR: 1.000e+00
Coefficient: 2.37e−01
Value: C
Lactobacillus.paracasei
1000
750
500
250
0
A (n=15)
B (n=16)
C (n=14)
surface

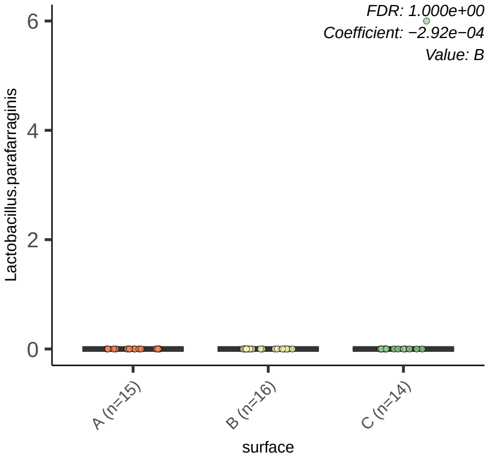

FDR: 1.000e+00
Coefficient: −2.92e−04
Value: B
Lactobacillus.parafarraginis
6
4
2
0
A (n=15)
B (n=16)
C (n=14)
surface

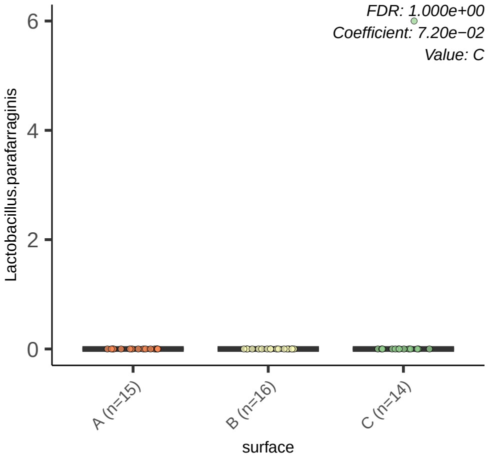
FDR: 1.000e+00
Coefficient: 7.20e−02
Value: C
Lactobacillus.parafarraginis
6
4
2
0
A (n=15)
B (n=16)
C (n=14)
surface

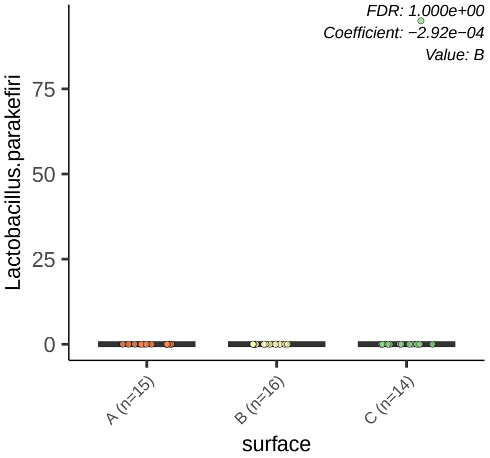

FDR: 1.000e+00
Coefficient: −2.92e−04
Value: B
Lactobacillus.parakefiri
75
50
25
0
A (n=15)
B (n=16)
C (n=14)
surface

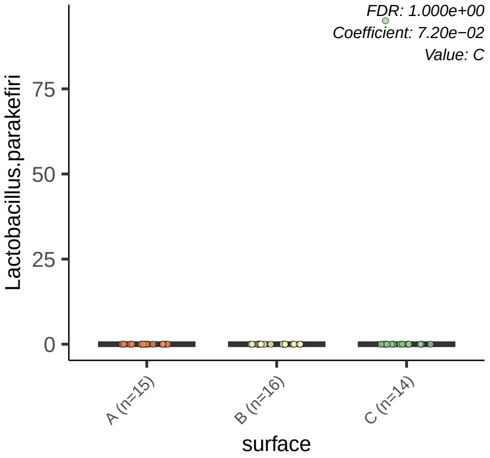
FDR: 1.000e+00
Coefficient: 7.20e−02
Value: C
Lactobacillus.parakefiri
75
50
25
0
A (n=15)
B (n=16)
C (n=14)
surface

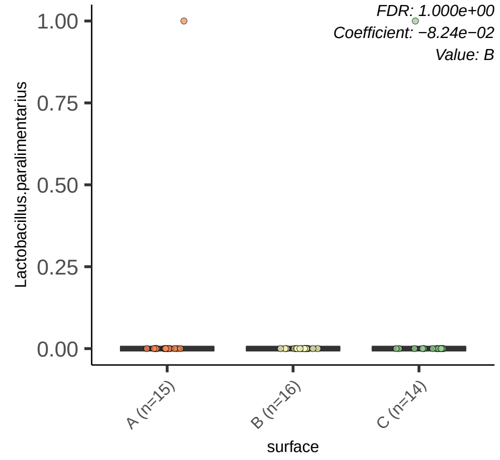

FDR: 1.000e+00
Coefficient: −8.24e−02
Value: B
Lactobacillus.paralimentarius
1.00
0.75
0.50
0.25
0.00
A (n=15)
B (n=16)
C (n=14)
surface

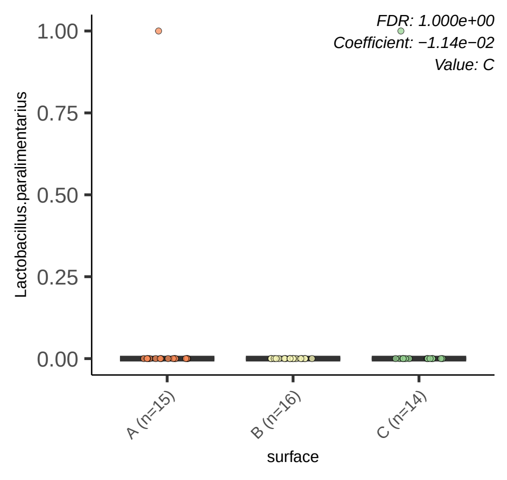
FDR: 1.000e+00
Coefficient: −1.14e−02
Value: C
Lactobacillus.paralimentarius
1.00
0.75
0.50
0.25
0.00
A (n=15)
B (n=16)
C (n=14)
surface

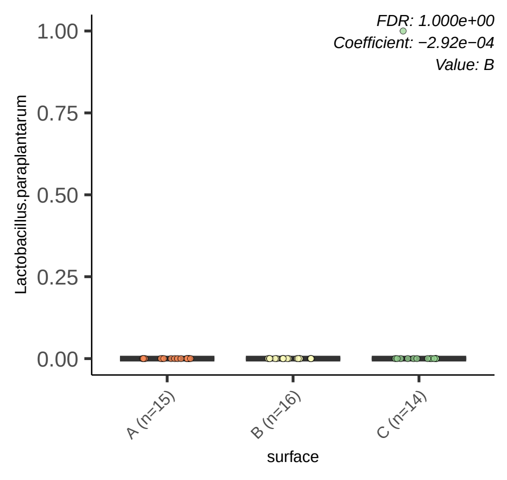

FDR: 1.000e+00
Coefficient: −2.92e−04
Value: B
1.00
0.75
0.50
0.25
0.00
Lactobacillus.paraplantarum
A (n=15)
B (n=16)
C (n=14)
surface

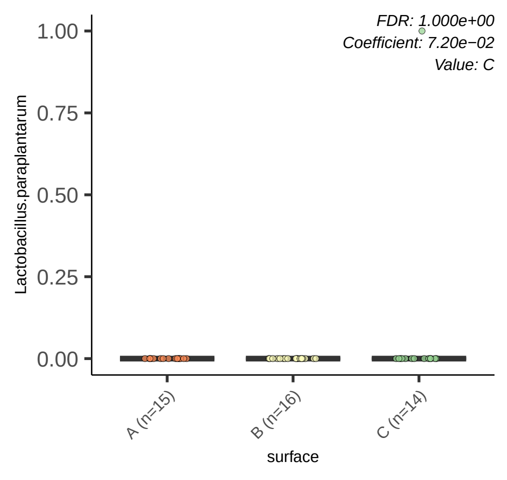

FDR: 1.000e+00
Coefficient: 7.20e−02
Value: C
1.00
0.75
0.50
0.25
0.00
Lactobacillus.paraplantarum
A (n=15)
B (n=16)
C (n=14)
surface

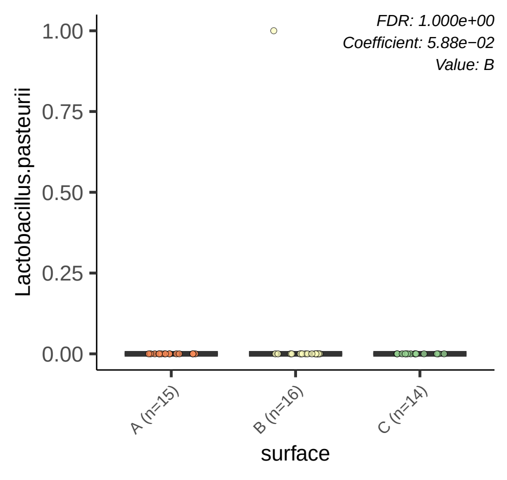

FDR: 1.000e+00
Coefficient: 5.88e−02
Value: B
Lactobacillus.pasteurii
1.00
0.75
0.50
0.25
0.00
A (n=15)
B (n=16)
C (n=14)
surface

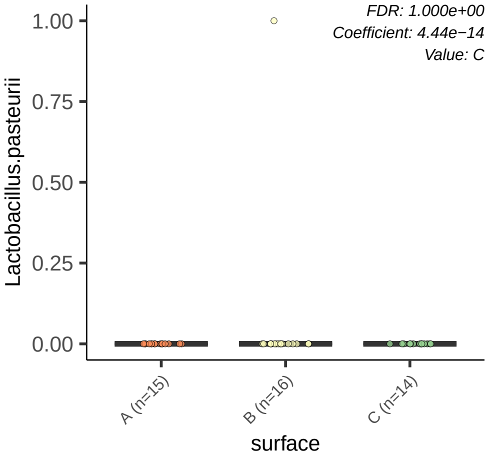
FDR: 1.000e+00
Coefficient: 4.44e−14
Value: C
Lactobacillus.pasteurii
1.00
0.75
0.50
0.25
0.00
A (n=15)
B (n=16)
C (n=14)
surface

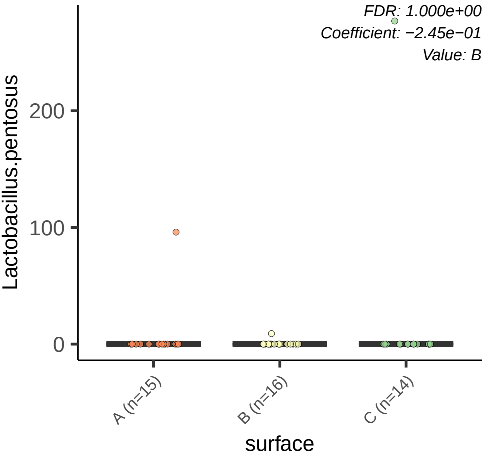
FDR: 1.000e+00
Coefficient: −2.45e−01
Value: B
Lactobacillus.pentosus
200
100
0
A (n=15)
B (n=16)
C (n=14)
surface

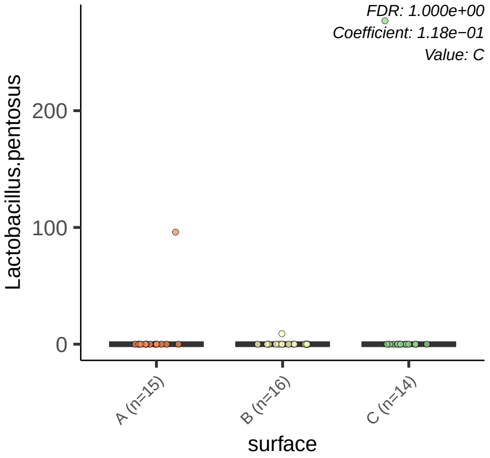
FDR: 1.000e+00
Coefficient: 1.18e−01
Value: C
Lactobacillus.pentosus
200
100
0
A (n=15)
B (n=16)
C (n=14)
surface

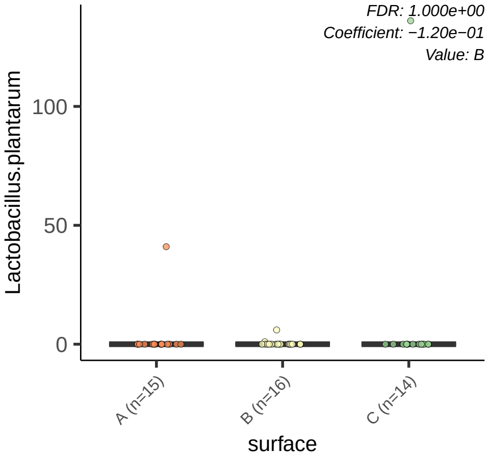
FDR: 1.000e+00
Coefficient: −1.20e−01
Value: B
Lactobacillus.plantarum
100
50
0
A (n=15)
B (n=16)
C (n=14)
surface

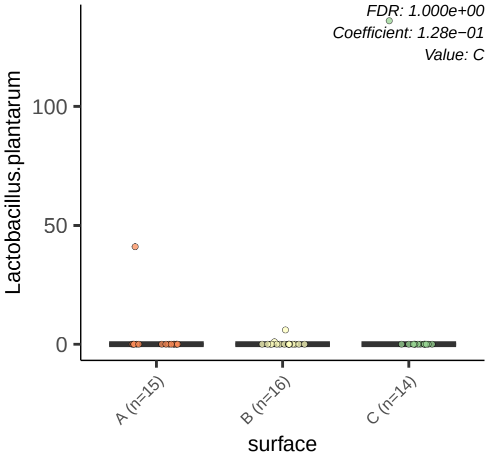

FDR: 1.000e+00
Coefficient: 1.28e−01
Value: C
Lactobacillus.plantarum
100
50
0
A (n=15)
B (n=16)
C (n=14)
surface

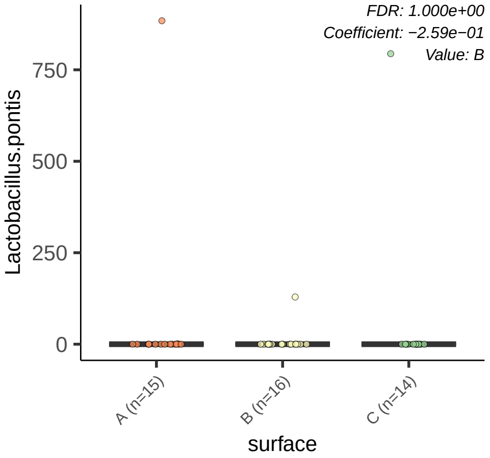

FDR: 1.000e+00
Coefficient: −2.59e−01
Value: B
Lactobacillus.pontis
750
500
250
0
A (n=15)
B (n=16)
C (n=14)
surface

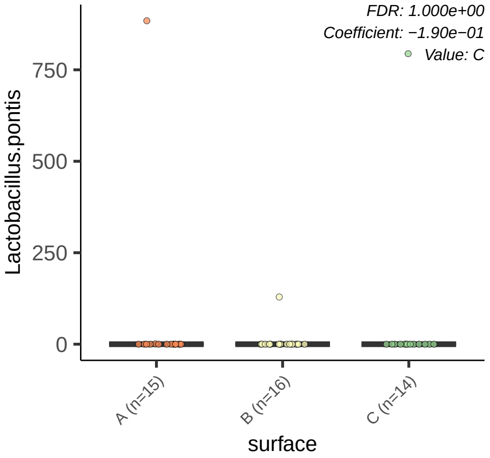

FDR: 1.000e+00
Coefficient: −1.90e−01
Value: C
Lactobacillus.pontis
750
500
250
0
A (n=15)
B (n=16)
C (n=14)
surface

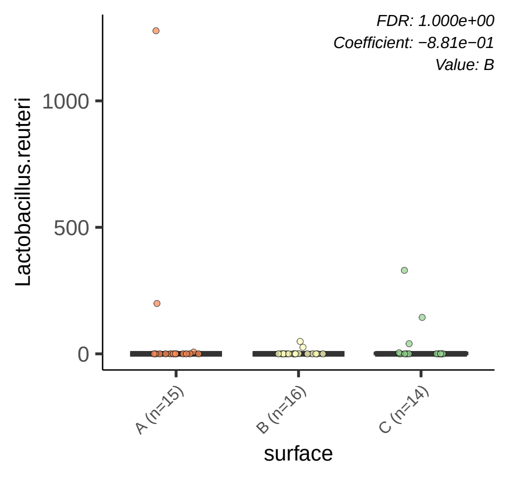
FDR: 1.000e+00
Coefficient: −8.81e−01
Value: B
Lactobacillus.reuteri
1000
500
0
A (n=15)
B (n=16)
C (n=14)
surface

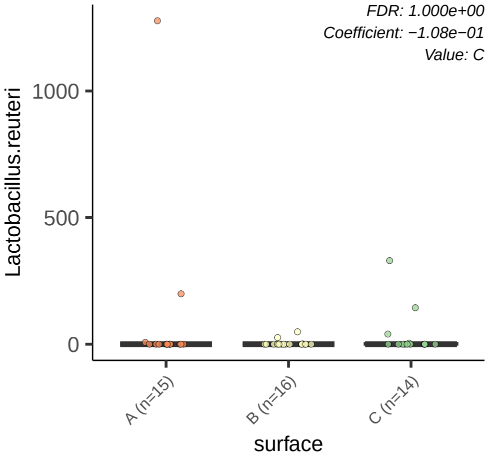
FDR: 1.000e+00
Coefficient: −1.08e−01
Value: C
Lactobacillus.reuteri
1000
500
0
A (n=15)
B (n=16)
C (n=14)
surface

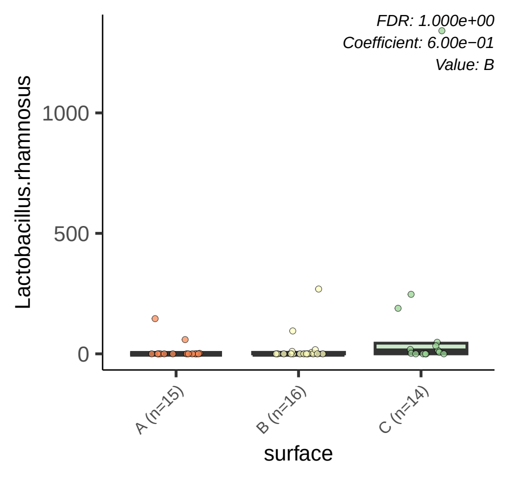

FDR: 1.000e+00
Coefficient: 6.00e−01
Value: B
Lactobacillus.rhamnosus
1000
500
0
A (n=15)
B (n=16)
C (n=14)
surface

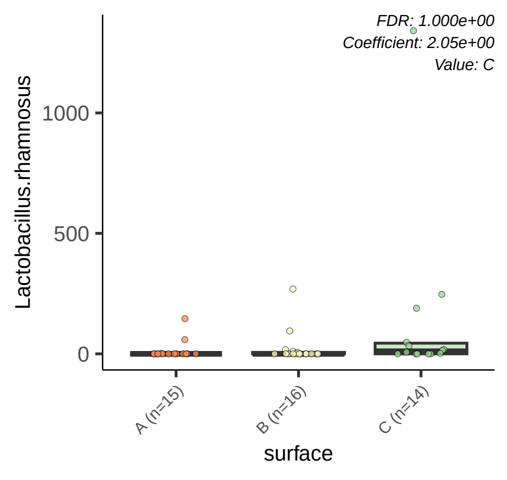

FDR: 1.000e+00
Coefficient: 2.05e+00
Value: C
Lactobacillus.rhamnosus
1000
500
0
A (n=15)
B (n=16)
C (n=14)
surface

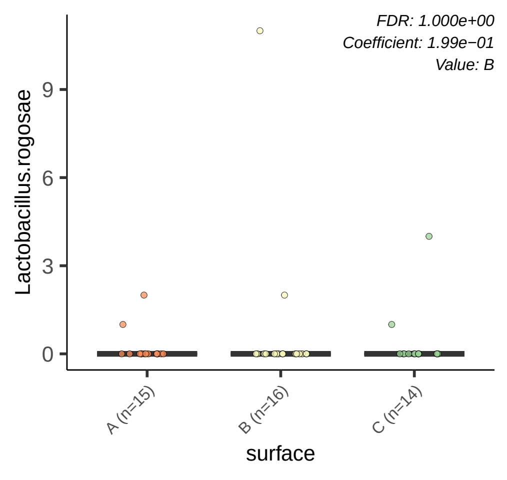

FDR: 1.000e+00
Coefficient: 1.99e−01
Value: B
Lactobacillus.rogosae
0
3
6
9
A (n=15)
B (n=16)
C (n=14)
surface

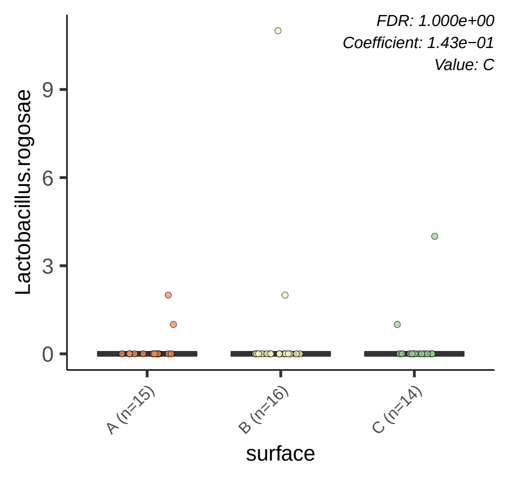

FDR: 1.000e+00
Coefficient: 1.43e−01
Value: C
Lactobacillus.rogosae
0
3
6
9
A (n=15)
B (n=16)
C (n=14)
surface

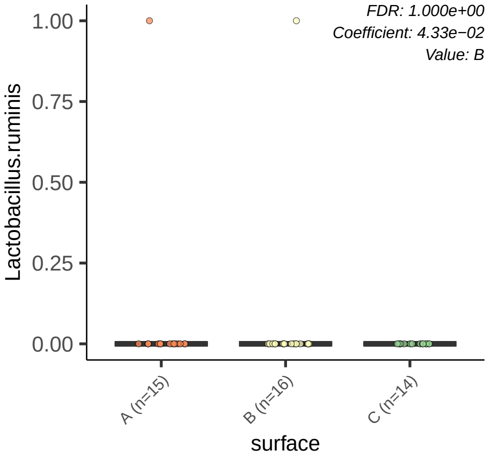
FDR: 1.000e+00
Coefficient: 4.33e−02
Value: B
Lactobacillus.ruminis
1.00
0.75
0.50
0.25
0.00
A (n=15)
B (n=16)
C (n=14)
surface

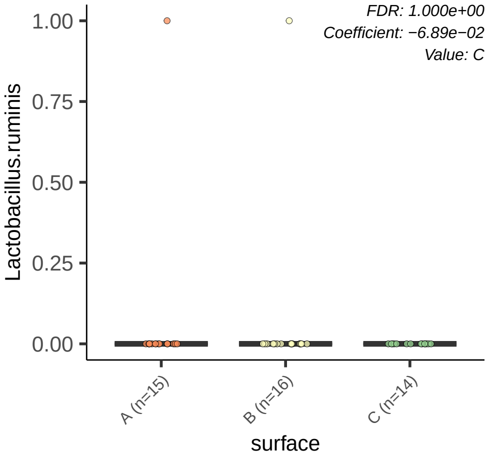
FDR: 1.000e+00
Coefficient: −6.89e−02
Value: C
Lactobacillus.ruminis
1.00
0.75
0.50
0.25
0.00
A (n=15)
B (n=16)
C (n=14)
surface

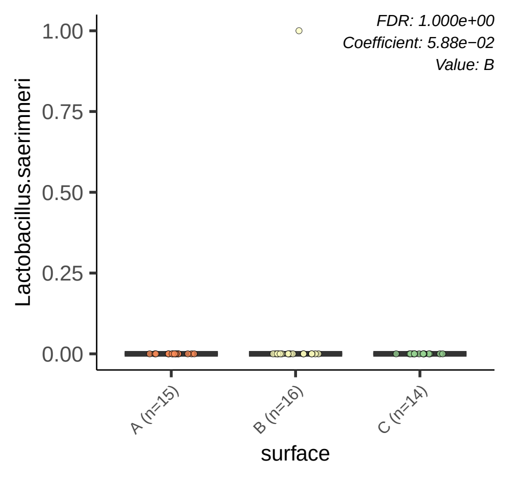
FDR: 1.000e+00
Coefficient: 5.88e−02
Value: B
1.00
0.75
0.50
0.25
0.00
Lactobacillus.saerimneri
A (n=15)
B (n=16)
C (n=14)
surface

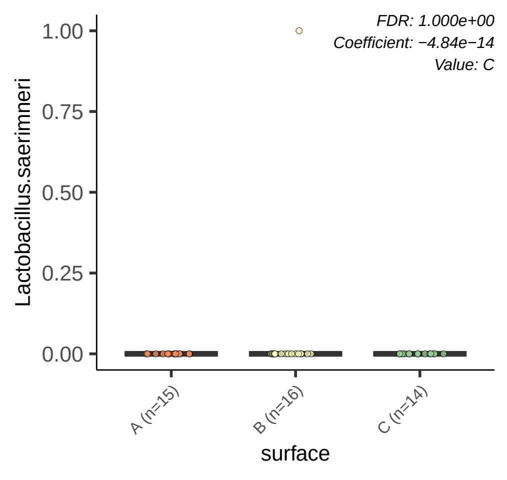
FDR: 1.000e+00
Coefficient: −4.84e−14
Value: C
Lactobacillus.saerimneri
1.00
0.75
0.50
0.25
0.00
A (n=15)
B (n=16)
C (n=14)
surface

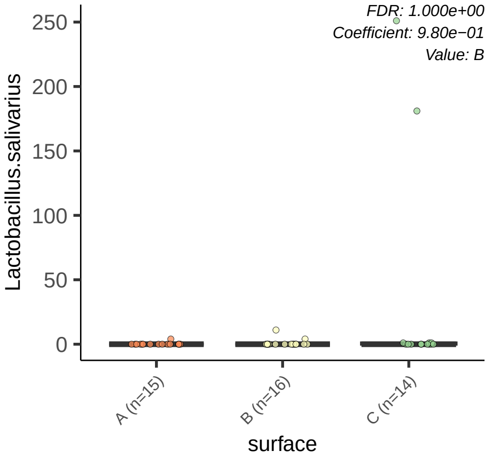

FDR: 1.000e+00
Coefficient: 9.80e−01
Value: B
Lactobacillus.salivarius
250
200
150
100
50
0
A (n=15)
B (n=16)
C (n=14)
surface

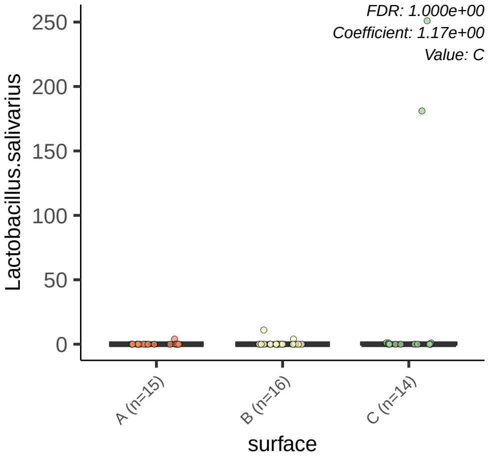

FDR: 1.000e+00
Coefficient: 1.17e+00
Value: C
Lactobacillus.salivarius
250
200
150
100
50
0
A (n=15)
B (n=16)
C (n=14)
surface

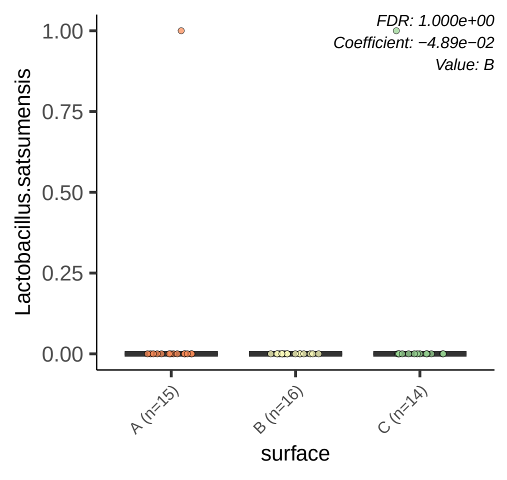
FDR: 1.000e+00
Coefficient: −4.89e−02
Value: B
Lactobacillus.satsumensis
1.00
0.75
0.50
0.25
0.00
A (n=15)
B (n=16)
C (n=14)
surface

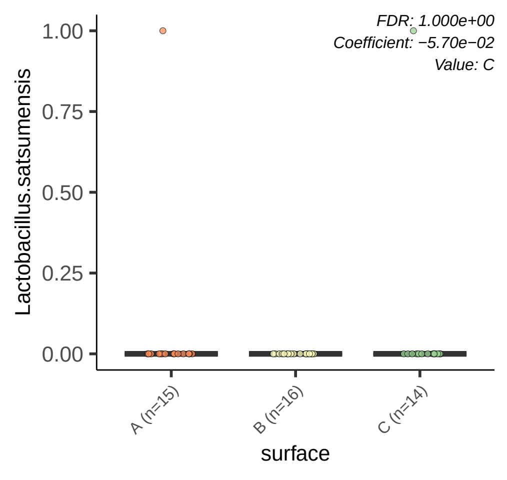

FDR: 1.000e+00
Coefficient: −5.70e−02
Value: C
1.00
0.75
0.50
0.25
0.00
Lactobacillus.satsumensis
A (n=15)
B (n=16)
C (n=14)
surface

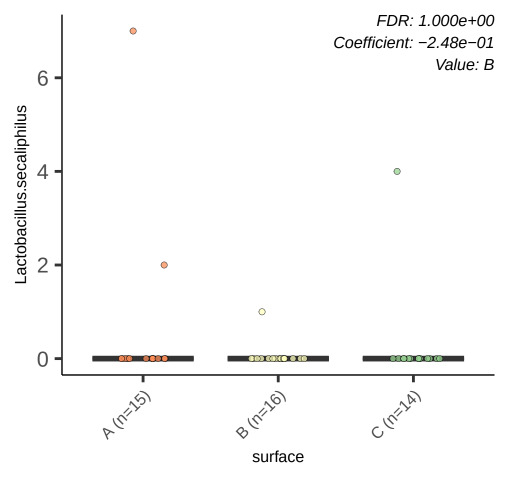

FDR: 1.000e+00
Coefficient: −2.48e−01
Value: B
Lactobacillus.secaliphilus
0
2
4
6
A (n=15)
B (n=16)
C (n=14)
surface

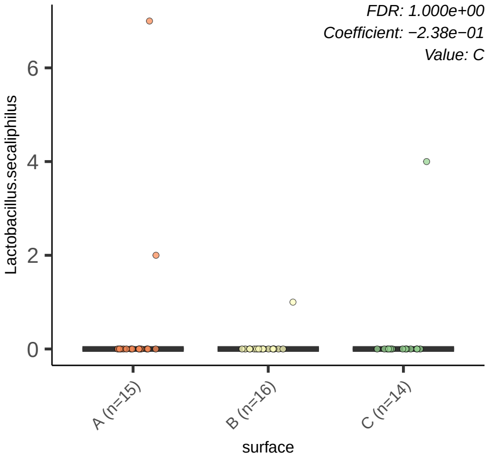
FDR: 1.000e+00
Coefficient: −2.38e−01
Value: C
Lactobacillus.secaliphilus
0
2
4
6
A (n=15)
B (n=16)
C (n=14)
surface

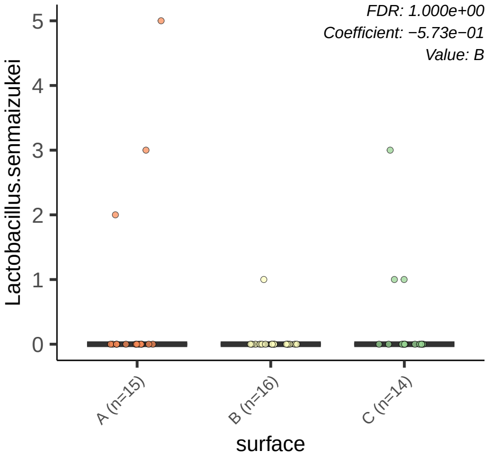

FDR: 1.000e+00
Coefficient: −5.73e−01
Value: B
Lactobacillus.senmaizukei
5
4
3
2
1
0
A (n=15)
B (n=16)
C (n=14)
surface

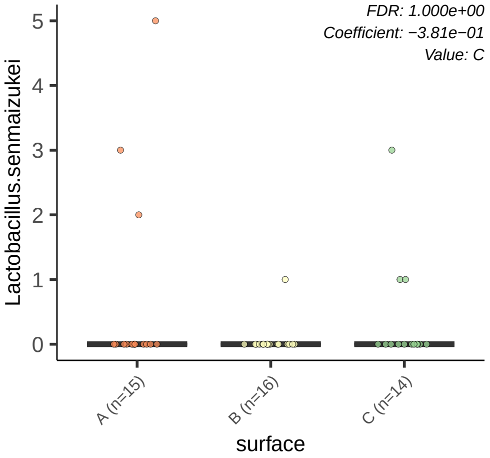

FDR: 1.000e+00
Coefficient: −3.81e−01
Value: C
Lactobacillus.senmaizukei
0
1
2
3
4
5
A (n=15)
B (n=16)
C (n=14)
surface

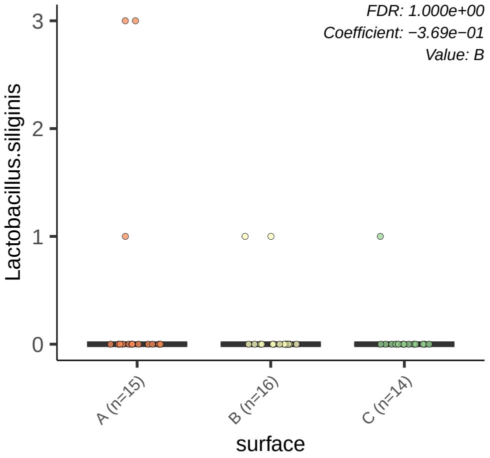
FDR: 1.000e+00
Coefficient: −3.69e−01
Value: B
Lactobacillus.siliginis
0
1
2
3
A (n=15)
B (n=16)
C (n=14)
surface

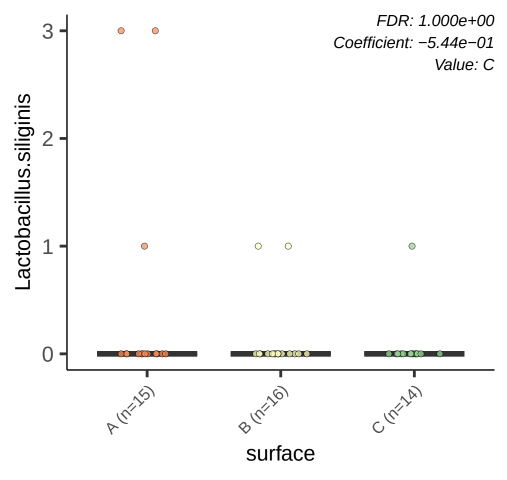
FDR: 1.000e+00
Coefficient: −5.44e−01
Value: C
Lactobacillus.siliginis
3
2
1
0
A (n=15)
B (n=16)
C (n=14)
surface

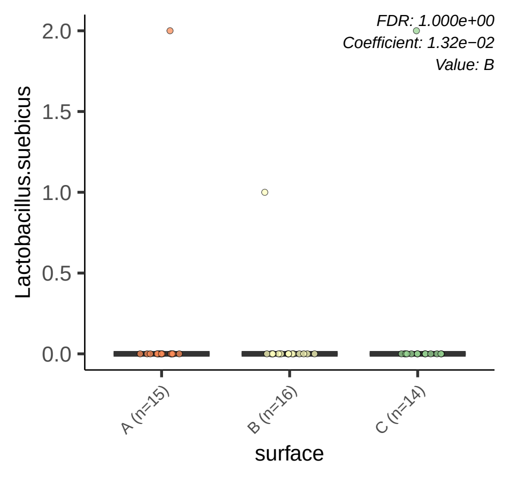
FDR: 1.000e+00
Coefficient: 1.32e−02
Value: B
Lactobacillus.suebicus
2.0
1.5
1.0
0.5
0.0
A (n=15)
B (n=16)
C (n=14)
surface

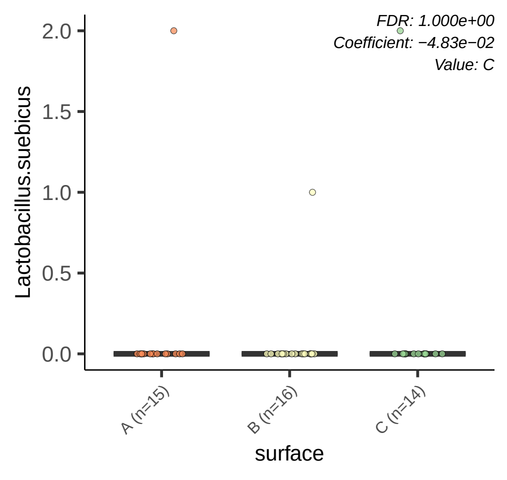
FDR: 1.000e+00
Coefficient: −4.83e−02
Value: C
Lactobacillus.suebicus
2.0
1.5
1.0
0.5
0.0
A (n=15)
B (n=16)
C (n=14)
surface

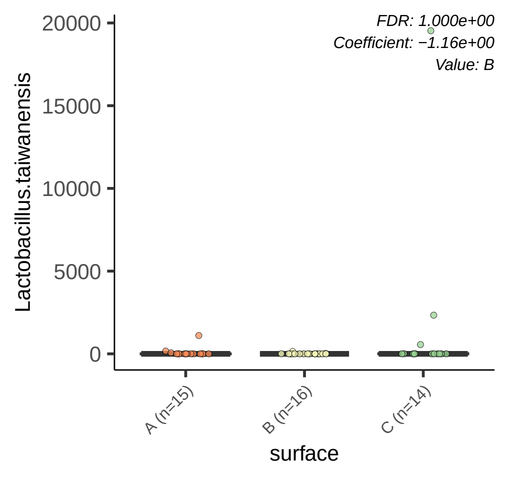

FDR: 1.000e+00
Coefficient: −1.16e+00
Value: B
Lactobacillus.taiwanensis
20000
15000
10000
5000
0
A (n=15)
B (n=16)
C (n=14)
surface

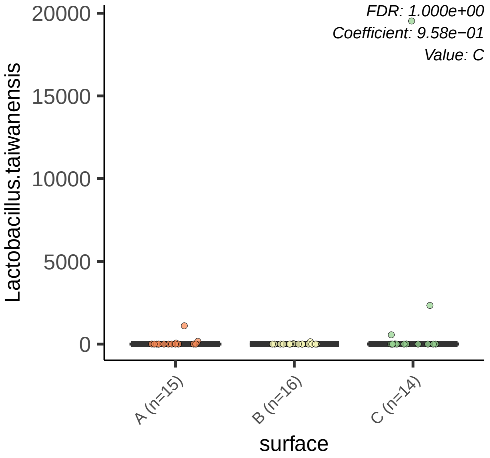

FDR: 1.000e+00
Coefficient: 9.58e−01
Value: C
20000
15000
10000
5000
0
Lactobacillus.taiwanensis
A (n=15)
B (n=16)
C (n=14)
surface

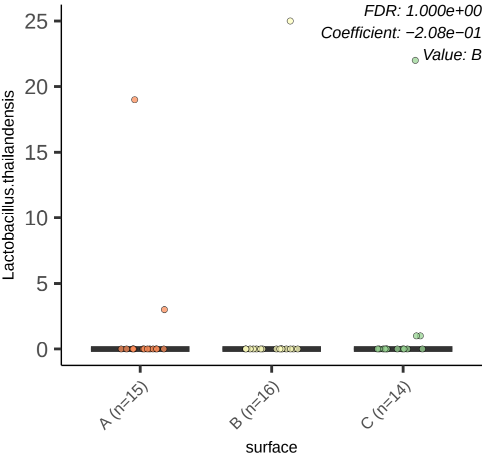
FDR: 1.000e+00
Coefficient: −2.08e−01
Value: B
Lactobacillus.thailandensis
25
20
15
10
5
0
A (n=15)
B (n=16)
C (n=14)
surface

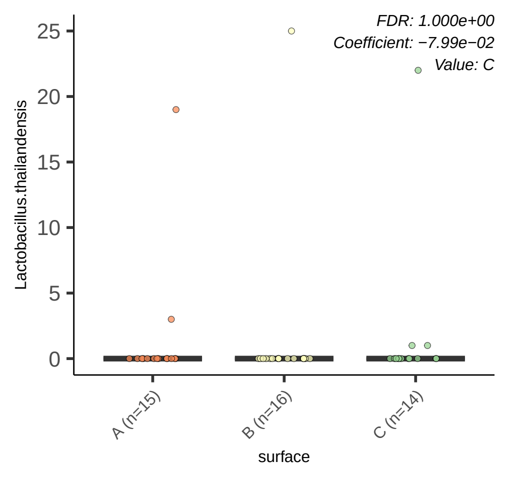
FDR: 1.000e+00
Coefficient: −7.99e−02
Value: C
Lactobacillus.thailandensis
25
20
15
10
5
0
A (n=15)
B (n=16)
C (n=14)
surface

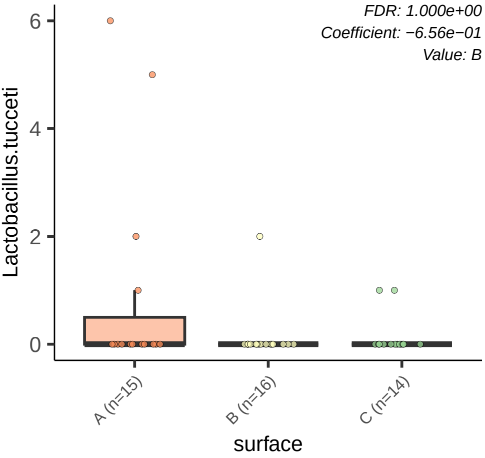

FDR: 1.000e+00
Coefficient: −6.56e−01
Value: B
Lactobacillus.tucceti
0
2
4
6
A (n=15)
B (n=16)
C (n=14)
surface

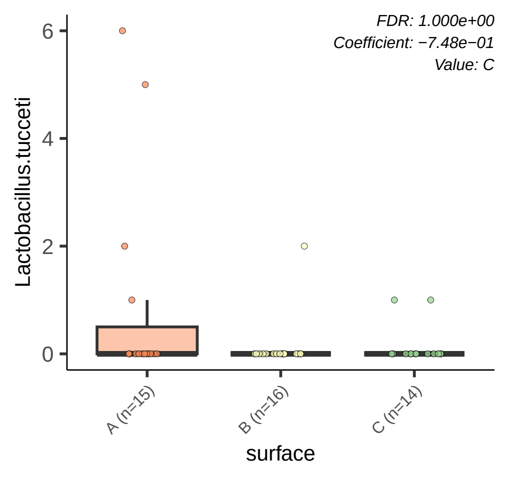
FDR: 1.000e+00
Coefficient: −7.48e−01
Value: C
Lactobacillus.tucceti
0
2
4
6
A (n=15)
B (n=16)
C (n=14)
surface

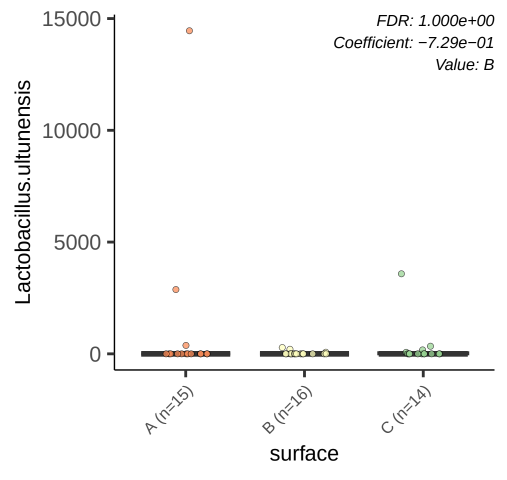

FDR: 1.000e+00
Coefficient: −7.29e−01
Value: B
15000
10000
5000
0
Lactobacillus.ultunensis
A (n=15)
B (n=16)
C (n=14)
surface

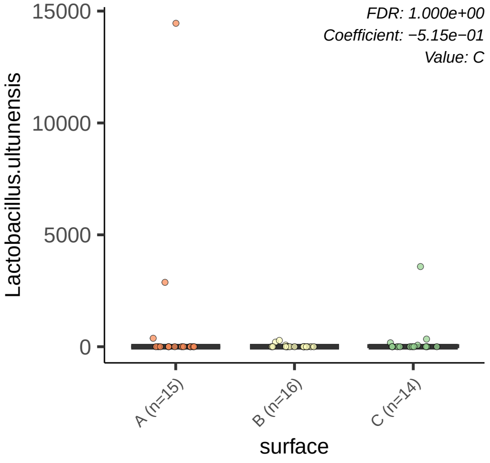
FDR: 1.000e+00
Coefficient: −5.15e−01
Value: C
15000
10000
5000
0
Lactobacillus.ultunensis
A (n=15)
B (n=16)
C (n=14)
surface

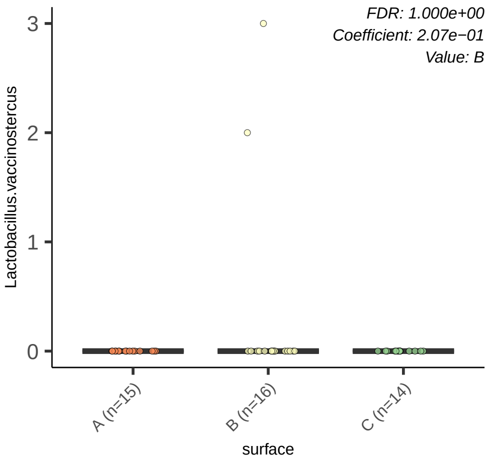

FDR: 1.000e+00
Coefficient: 2.07e−01
Value: B
Lactobacillus.vaccinostercus
3
2
1
0
A (n=15)
B (n=16)
C (n=14)
surface

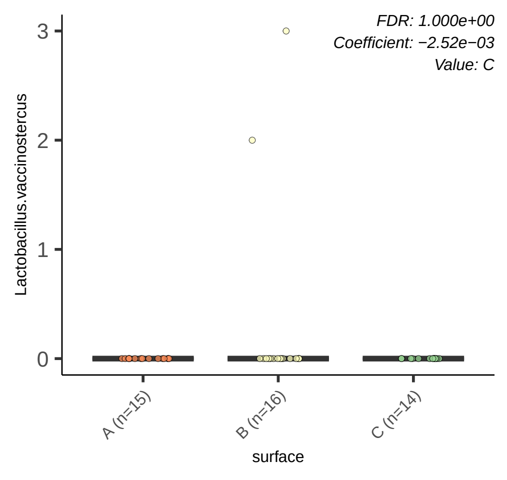
FDR: 1.000e+00
Coefficient: −2.52e−03
Value: C
Lactobacillus.vaccinostercus
3
2
1
0
A (n=15)
B (n=16)
C (n=14)
surface

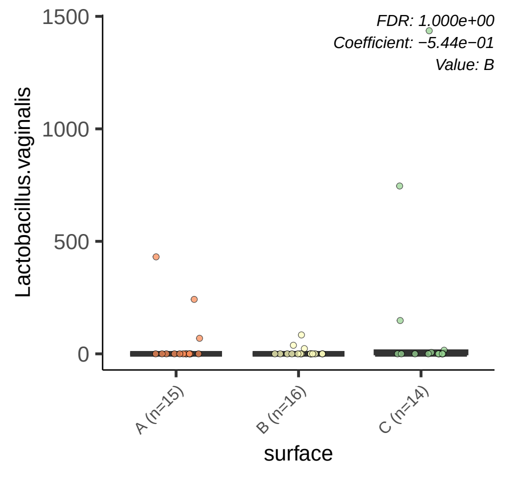

FDR: 1.000e+00
Coefficient: −5.44e−01
Value: B
1500
1000
500
0
Lactobacillus.vaginalis
A (n=15)
B (n=16)
C (n=14)
surface

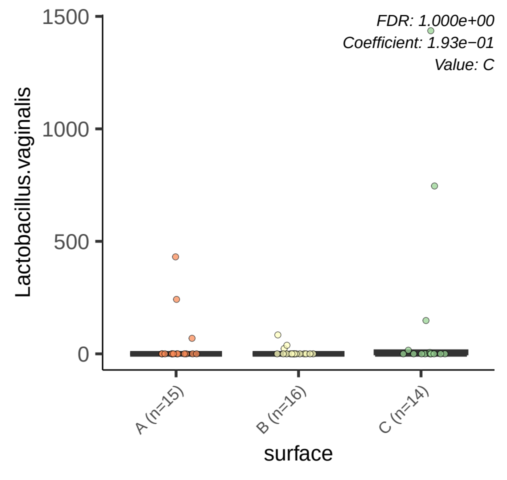

FDR: 1.000e+00
Coefficient: 1.93e−01
Value: C
Lactobacillus.vaginalis
1500
1000
500
0
A (n=15)
B (n=16)
C (n=14)
surface

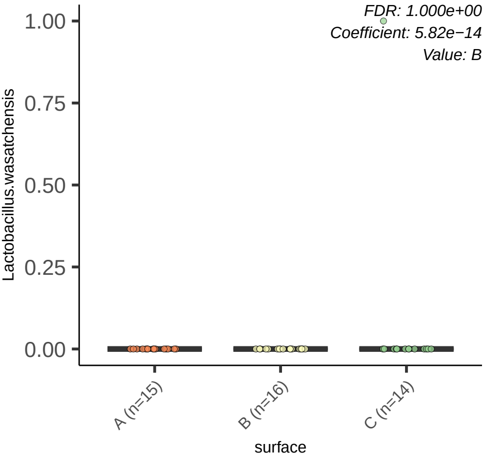
FDR: 1.000e+00
Coefficient: 5.82e−14
Value: B
Lactobacillus.wasatchensis
1.00
0.75
0.50
0.25
0.00
A (n=15)
B (n=16)
C (n=14)
surface

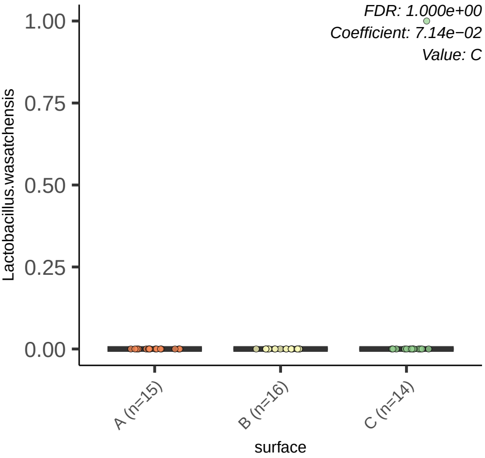
FDR: 1.000e+00
Coefficient: 7.14e−02
Value: C
Lactobacillus.wasatchensis
1.00
0.75
0.50
0.25
0.00
A (n=15)
B (n=16)
C (n=14)
surface

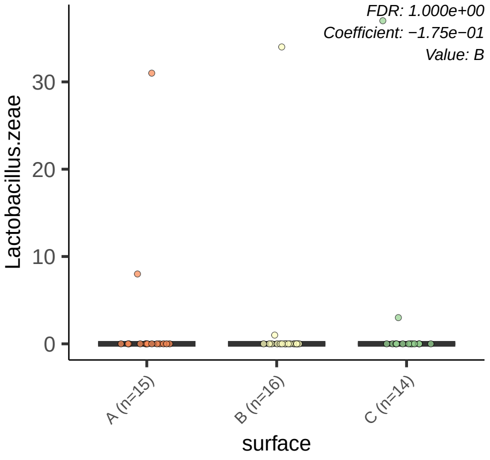
FDR: 1.000e+00
Coefficient: −1.75e−01
Value: B
Lactobacillus.zeae
30
20
10
0
A (n=15)
B (n=16)
C (n=14)
surface

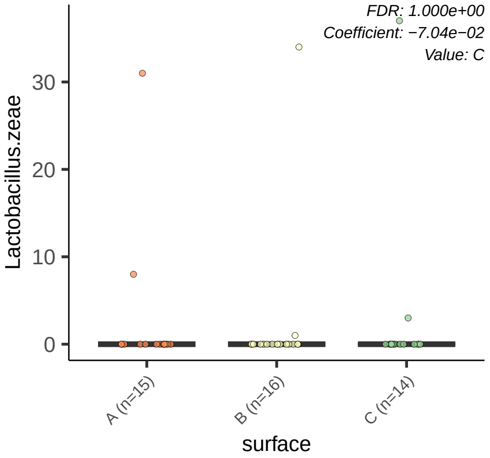
FDR: 1.000e+00
Coefficient: −7.04e−02
Value: C
Lactobacillus.zeae
0
10
20
30
A (n=15)
B (n=16)
C (n=14)
surface

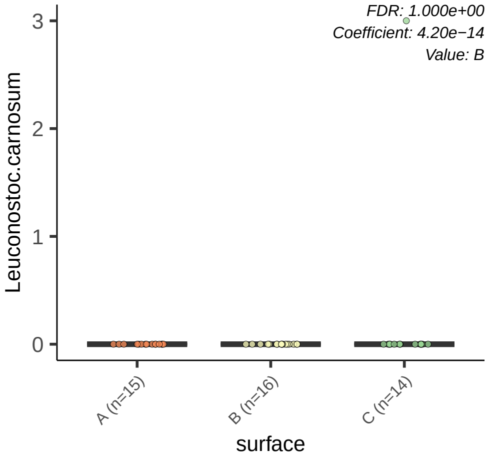
FDR: 1.000e+00
Coefficient: 4.20e−14
Value: B
Leuconostoc.carnosum
3
2
1
0
A (n=15)
B (n=16)
C (n=14)
surface

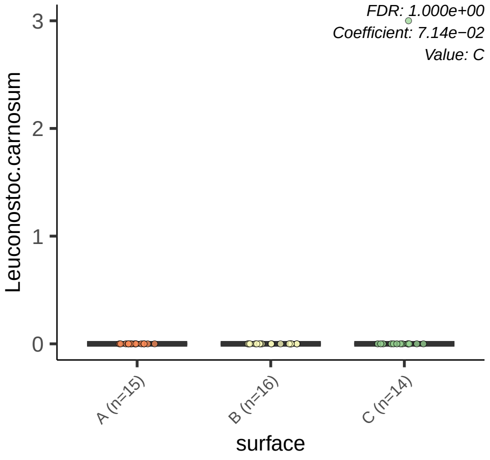
FDR: 1.000e+00
Coefficient: 7.14e−02
Value: C
Leuconostoc.carnosum
0
1
2
3
A (n=15)
B (n=16)
C (n=14)
surface

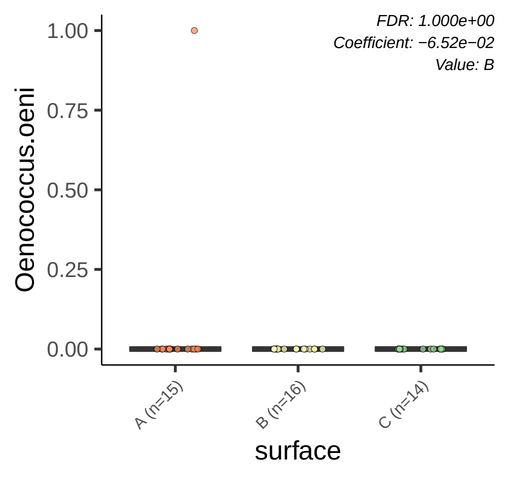
FDR: 1.000e+00
Coefficient: −6.52e−02
Value: B
Oenococcus.oeni
1.00
0.75
0.50
0.25
0.00
A (n=15)
B (n=16)
C (n=14)
surface

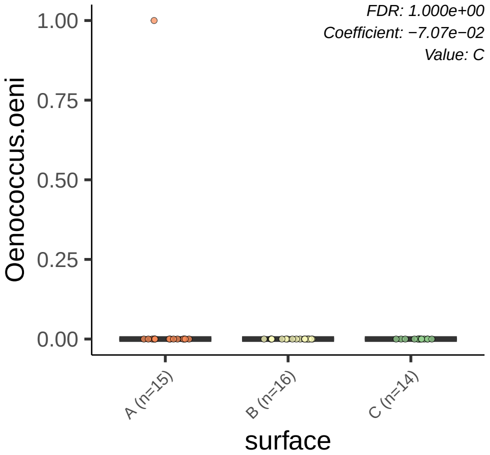

FDR: 1.000e+00
Coefficient: −7.07e−02
Value: C
Oenococcus.oeni
1.00
0.75
0.50
0.25
0.00
A (n=15)
B (n=16)
C (n=14)
surface

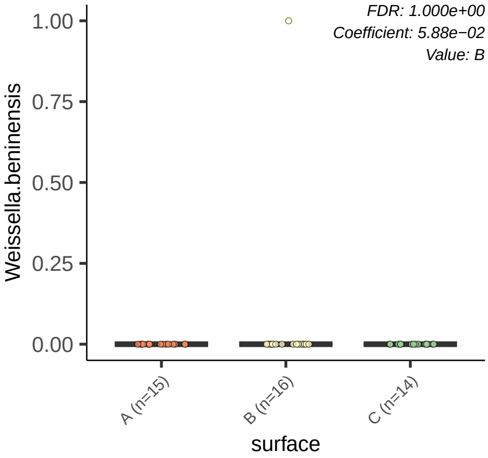
FDR: 1.000e+00
Coefficient: 5.88e−02
Value: B
Weissella.beninensis
1.00
0.75
0.50
0.25
0.00
A (n=15)
B (n=16)
C (n=14)
surface

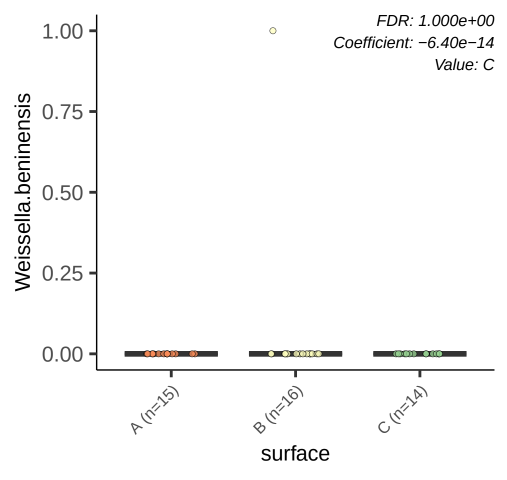
FDR: 1.000e+00
Coefficient: −6.40e−14
Value: C
Weissella.beninensis
1.00
0.75
0.50
0.25
0.00
A (n=15)
B (n=16)
C (n=14)
surface

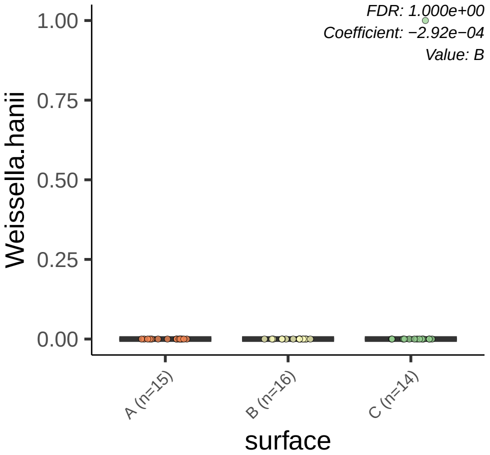

FDR: 1.000e+00
Coefficient: −2.92e−04
Value: B
Weissella.hanii
1.00
0.75
0.50
0.25
0.00
A (n=15)
B (n=16)
C (n=14)
surface

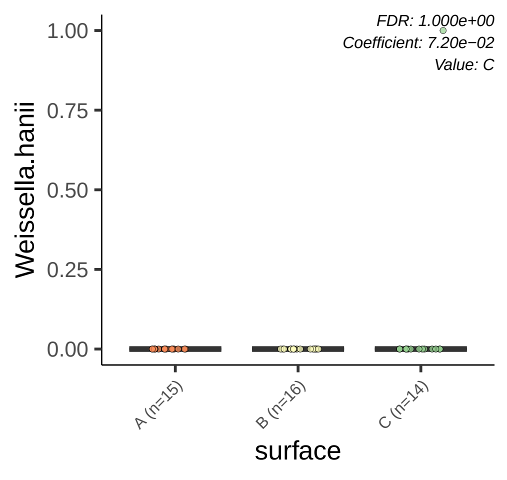
FDR: 1.000e+00
Coefficient: 7.20e−02
Value: C
Weissella.hanii
1.00
0.75
0.50
0.25
0.00
A (n=15)
B (n=16)
C (n=14)
surface

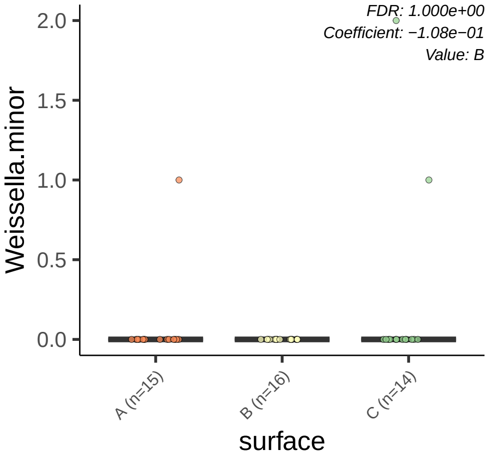
FDR: 1.000e+00
Coefficient: −1.08e−01
Value: B
Weissella.minor
2.0
1.5
1.0
0.5
0.0
A (n=15)
B (n=16)
C (n=14)
surface

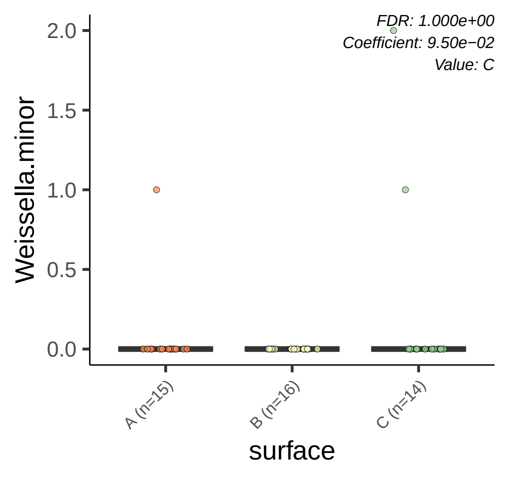
FDR: 1.000e+00
Coefficient: 9.50e−02
Value: C
Weissella.minor
2.0
1.5
1.0
0.5
0.0
A (n=15)
B (n=16)
C (n=14)
surface

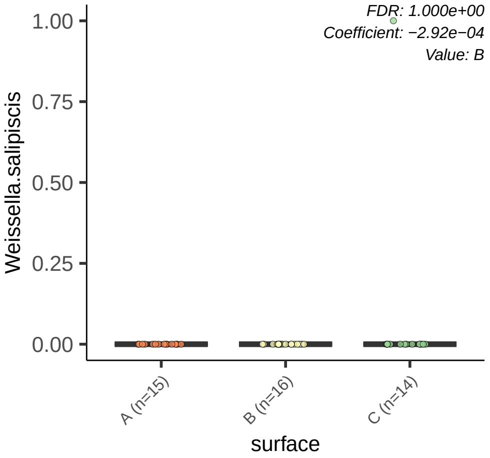
FDR: 1.000e+00
Coefficient: −2.92e−04
Value: B
Weissella.salipiscis
1.00
0.75
0.50
0.25
0.00
A (n=15)
B (n=16)
C (n=14)
surface

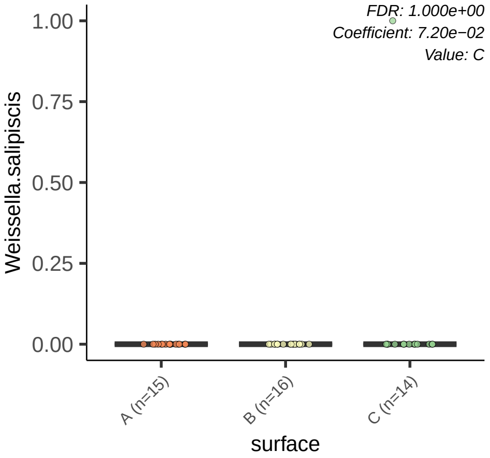

FDR: 1.000e+00
Coefficient: 7.20e−02
Value: C
Weissella.salipiscis
1.00
0.75
0.50
0.25
0.00
A (n=15)
B (n=16)
C (n=14)
surface

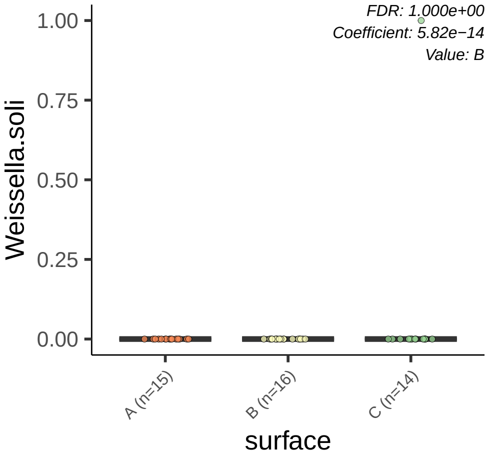

FDR: 1.000e+00
Coefficient: 5.82e−14
Value: B
Weissella.soli
1.00
0.75
0.50
0.25
0.00
A (n=15)
B (n=16)
C (n=14)
surface

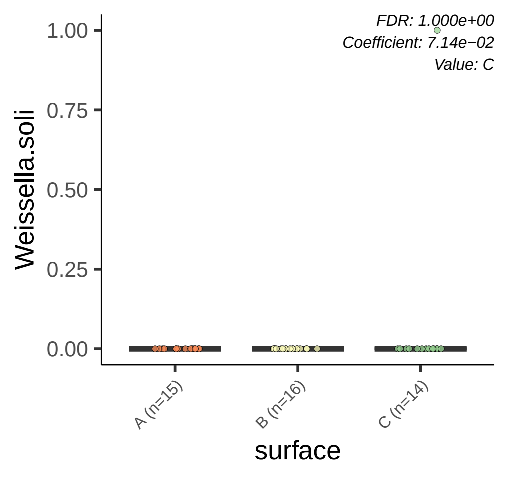
FDR: 1.000e+00
Coefficient: 7.14e−02
Value: C
Weissella.soli
1.00
0.75
0.50
0.25
0.00
A (n=15)
B (n=16)
C (n=14)
surface

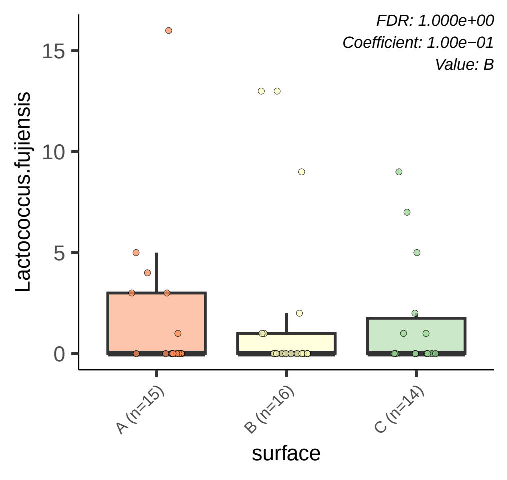

FDR: 1.000e+00
Coefficient: 1.00e−01
Value: B
Lactococcus.fujiensis
15
10
5
0
A (n=15)
B (n=16)
C (n=14)
surface

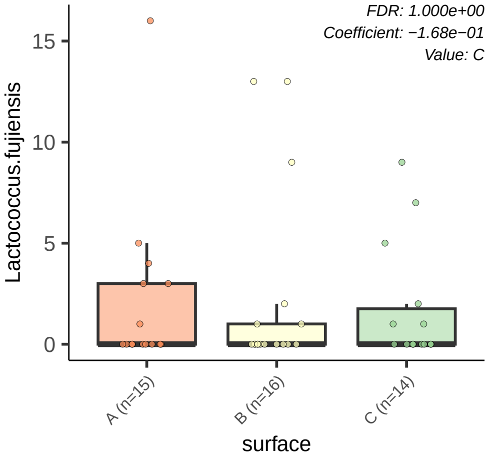
FDR: 1.000e+00
Coefficient: −1.68e−01
Value: C
Lactococcus.fujiensis
15
10
5
0
A (n=15)
B (n=16)
C (n=14)
surface

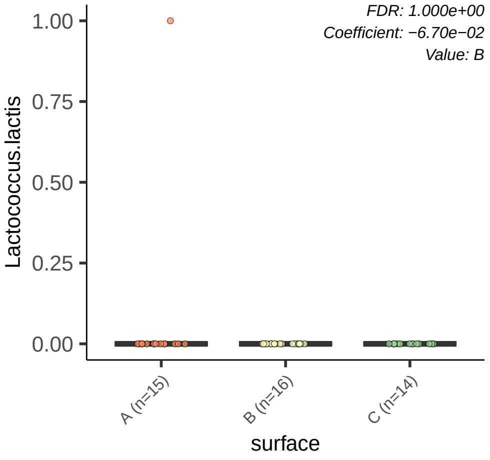

FDR: 1.000e+00
Coefficient: −6.70e−02
Value: B
Lactococcus.lactis
1.00
0.75
0.50
0.25
0.00
A (n=15)
B (n=16)
C (n=14)
surface

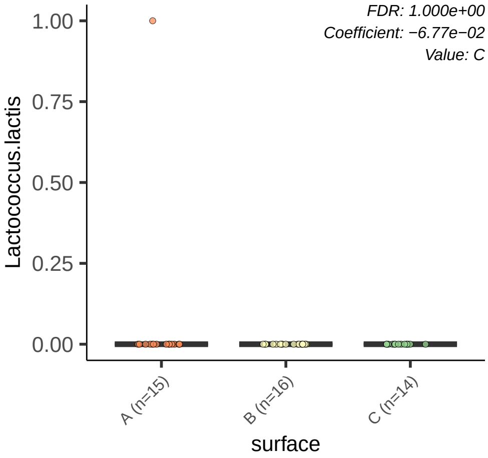

FDR: 1.000e+00
Coefficient: −6.77e−02
Value: C
Lactococcus.lactis
1.00
0.75
0.50
0.25
0.00
A (n=15)
B (n=16)
C (n=14)
surface

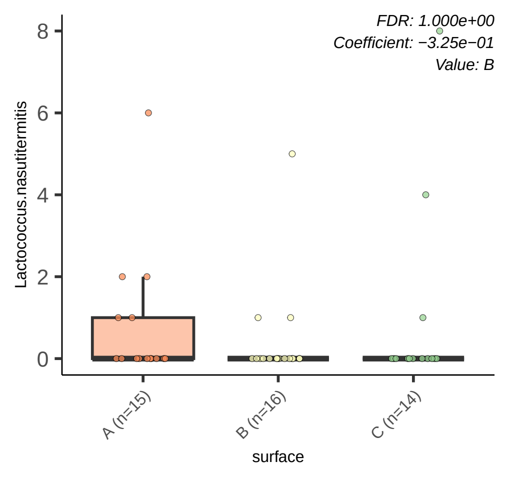

FDR: 1.000e+00
Coefficient: −3.25e−01
Value: B
Lactococcus.nasutitermitis
8
6
4
2
0
A (n=15)
B (n=16)
C (n=14)
surface

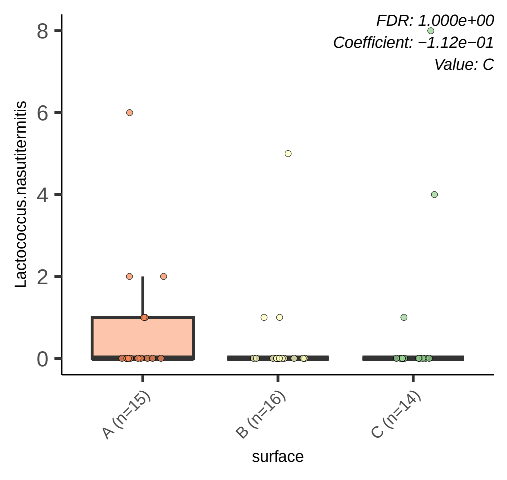
FDR: 1.000e+00
Coefficient: −1.12e−01
Value: C
Lactococcus.nasutitermis
0
2
4
6
8
A (n=15)
B (n=16)
C (n=14)
surface

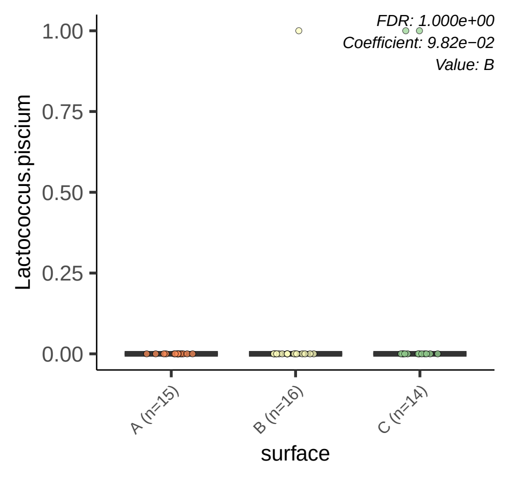
FDR: 1.000e+00
Coefficient: 9.82e−02
Value: B
Lactococcus.piscium
1.00
0.75
0.50
0.25
0.00
A (n=15)
B (n=16)
C (n=14)
surface

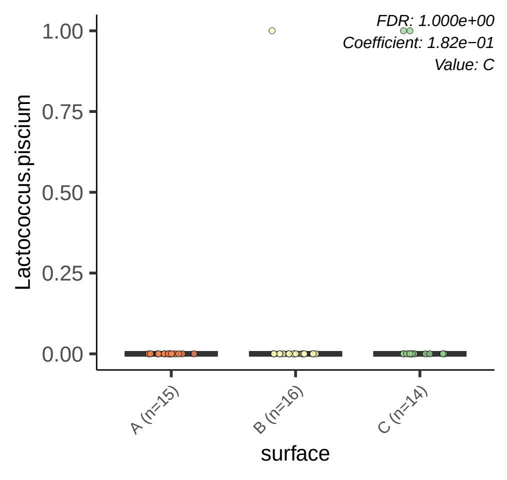

FDR: 1.000e+00
Coefficient: 1.82e−01
Value: C
Lactococcus.piscium
1.00
0.75
0.50
0.25
0.00
A (n=15)
B (n=16)
C (n=14)
surface

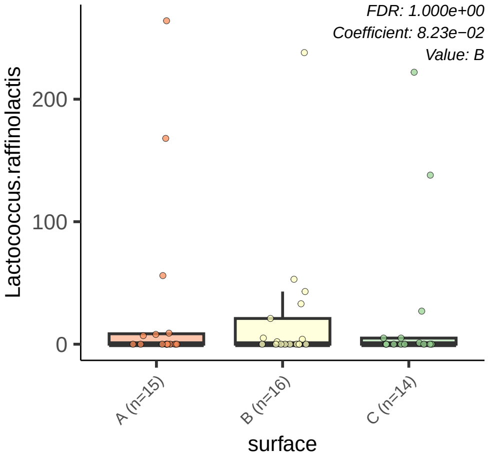
FDR: 1.000e+00
Coefficient: 8.23e−02
Value: B
Lactococcus.raffinolactis
0
100
200
A (n=15)
B (n=16)
C (n=14)
surface

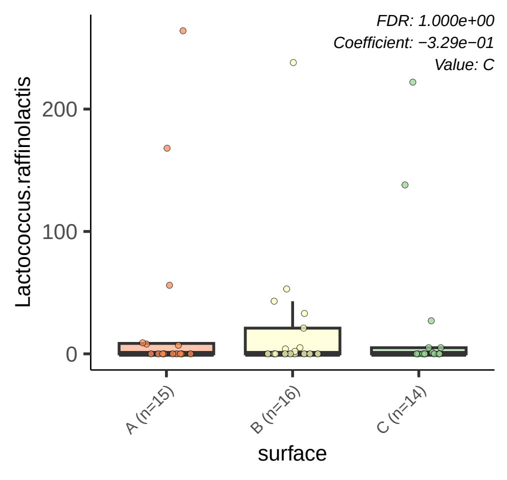

FDR: 1.000e+00
Coefficient: −3.29e−01
Value: C
Lactococcus.raffinolactis
200
100
0
A (n=15)
B (n=16)
C (n=14)
surface

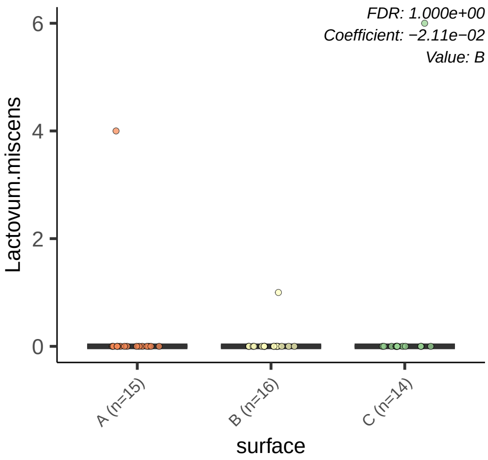

FDR: 1.000e+00
Coefficient: −2.11e−02
Value: B
Lactovum.miscens
0
2
4
6
A (n=15)
B (n=16)
C (n=14)
surface

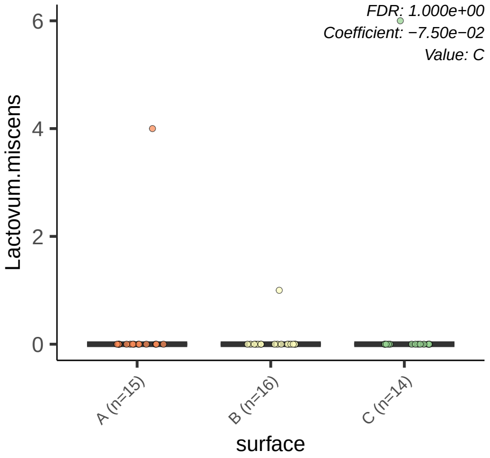

FDR: 1.000e+00
Coefficient: −7.50e−02
Value: C
Lactovum.miscens
6
4
2
0
A (n=15)
B (n=16)
C (n=14)
surface

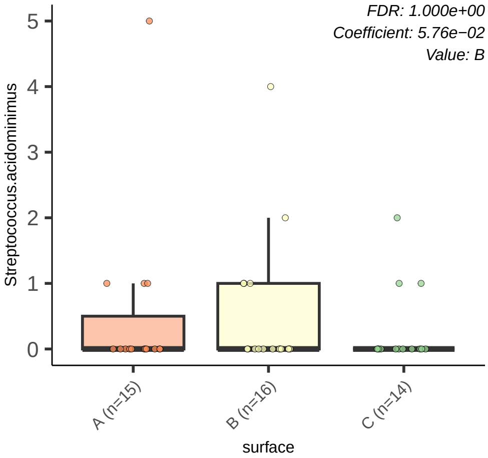

FDR: 1.000e+00
Coefficient: 5.76e−02
Value: B
Streptococcus.acidominimus
0
1
2
3
4
5
A (n=15)
B (n=16)
C (n=14)
surface

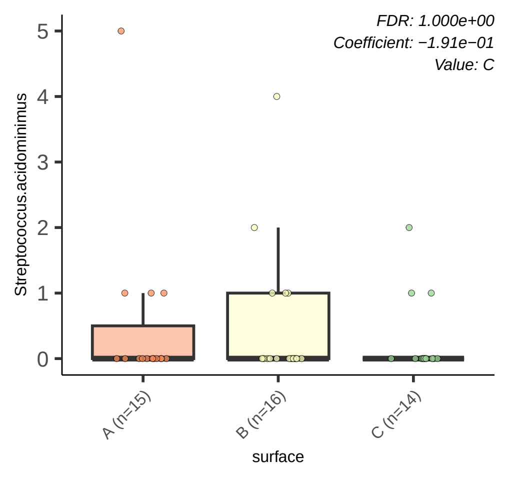
FDR: 1.000e+00
Coefficient: −1.91e−01
Value: C
Streptococcus.acidominimus
0
1
2
3
4
5
A (n=15)
B (n=16)
C (n=14)
surface

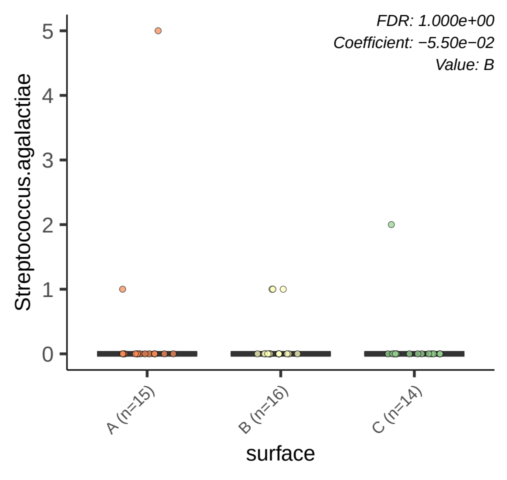
FDR: 1.000e+00
Coefficient: −5.50e−02
Value: B
Streptococcus.agalactiae
0
1
2
3
4
5
A (n=15)
B (n=16)
C (n=14)
surface

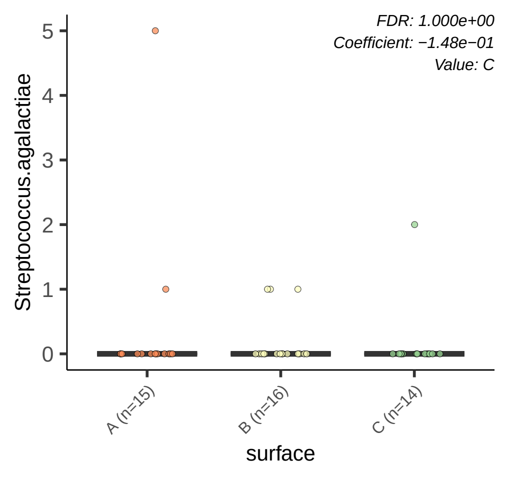
FDR: 1.000e+00
Coefficient: −1.48e−01
Value: C
Streptococcus.agalactiae
5
4
3
2
1
0
A (n=15)
B (n=16)
C (n=14)
surface

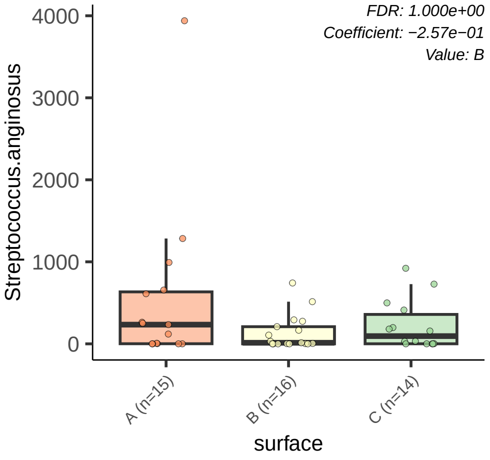

FDR: 1.000e+00
Coefficient: −2.57e−01
Value: B
Streptococcus.anginosus
4000
3000
2000
1000
0
A (n=15)
B (n=16)
C (n=14)
surface

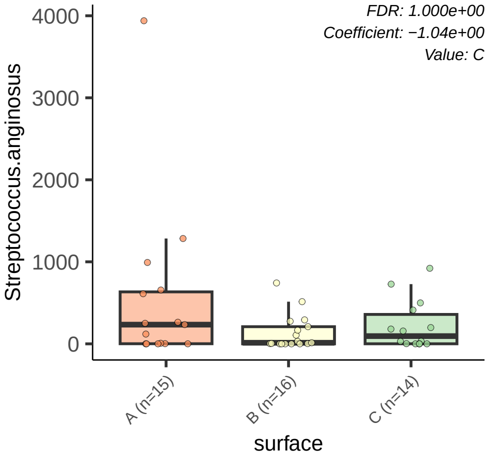

FDR: 1.000e+00
Coefficient: −1.04e+00
Value: C
Streptococcus.anginosus
4000
3000
2000
1000
0
A (n=15)
B (n=16)
C (n=14)
surface

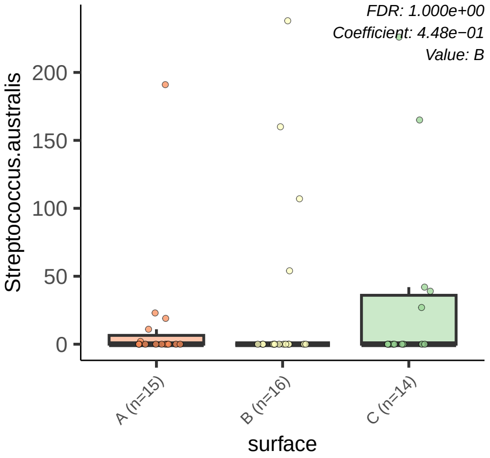

FDR: 1.000e+00
Coefficient: 4.48e−01
Value: B
Streptococcus.australis
0
50
100
150
200
A (n=15)
B (n=16)
C (n=14)
surface

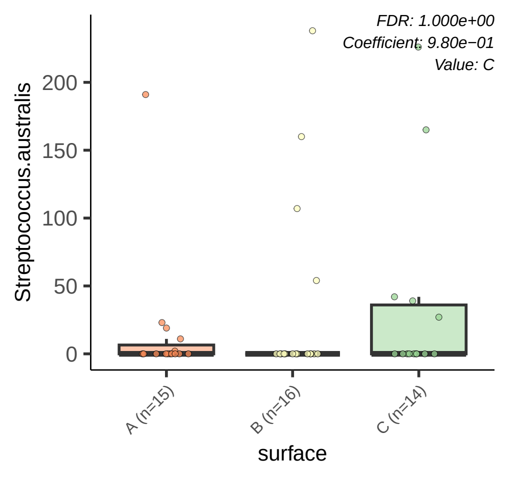
FDR: 1.000e+00
Coefficient: 9.80e−01
Value: C
Streptococcus.australis
200
150
100
50
0
A (n=15)
B (n=16)
C (n=14)
surface

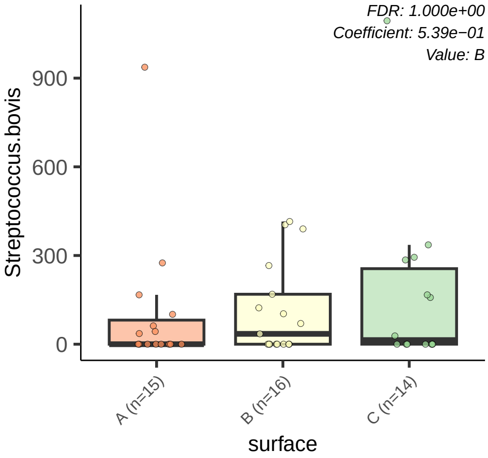

FDR: 1.000e+00
Coefficient: 5.39e−01
Value: B
Streptococcus.bovis
900
600
300
0
A (n=15)
B (n=16)
C (n=14)
surface

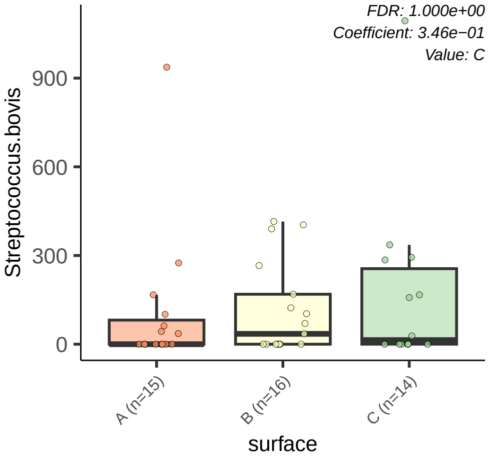

FDR: 1.000e+00
Coefficient: 3.46e−01
Value: C
Streptococcus.bovis
900
600
300
0
A (n=15)
B (n=16)
C (n=14)
surface

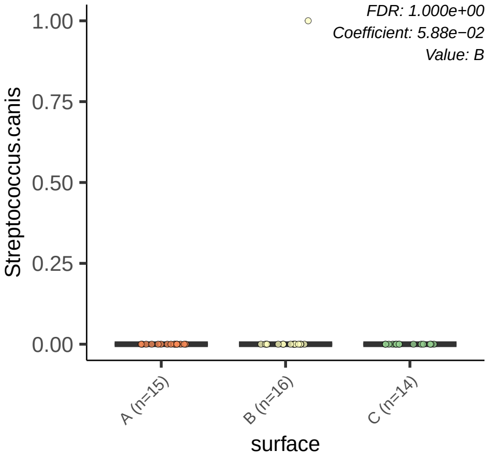
FDR: 1.000e+00
Coefficient: 5.88e−02
Value: B
Streptococcus.canis
1.00
0.75
0.50
0.25
0.00
A (n=15)
B (n=16)
C (n=14)
surface

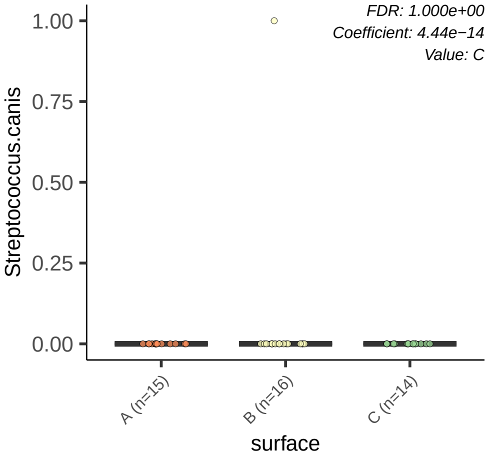

FDR: 1.000e+00
Coefficient: 4.44e−14
Value: C
Streptococcus.canis
1.00
0.75
0.50
0.25
0.00
A (n=15)
B (n=16)
C (n=14)
surface

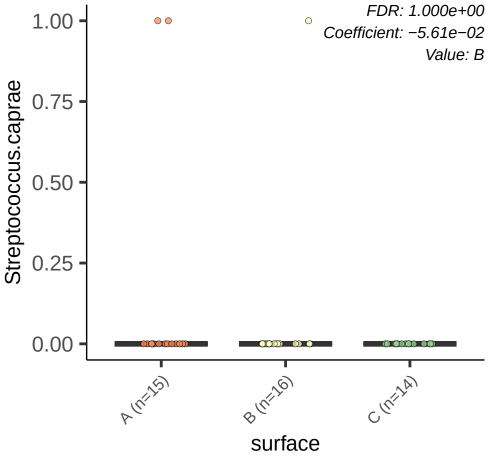
FDR: 1.000e+00
Coefficient: −5.61e−02
Value: B
Streptococcus.caprae
1.00
0.75
0.50
0.25
0.00
A (n=15)
B (n=16)
C (n=14)
surface

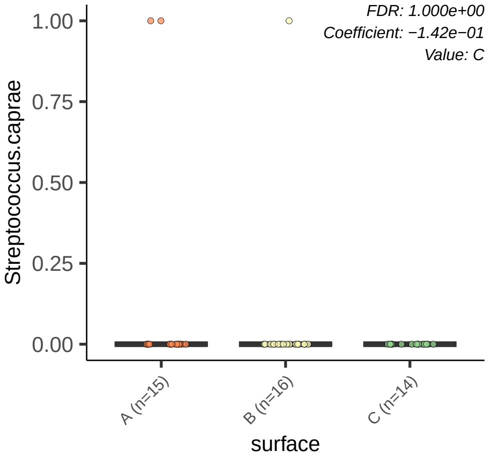
FDR: 1.000e+00
Coefficient: −1.42e−01
Value: C
Streptococcus.caprae
1.00
0.75
0.50
0.25
0.00
A (n=15)
B (n=16)
C (n=14)
surface

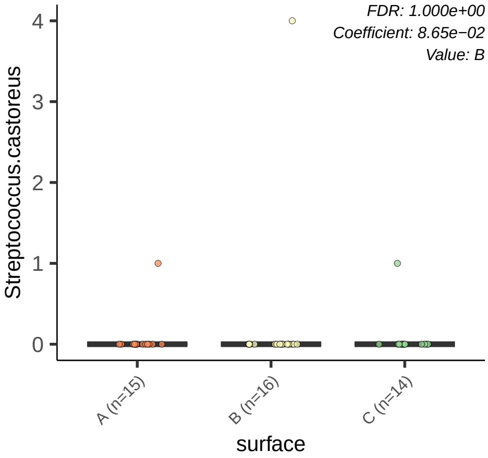
FDR: 1.000e+00
Coefficient: 8.65e−02
Value: B
Streptococcus.castoreus
4
3
2
1
0
A (n=15)
B (n=16)
C (n=14)
surface

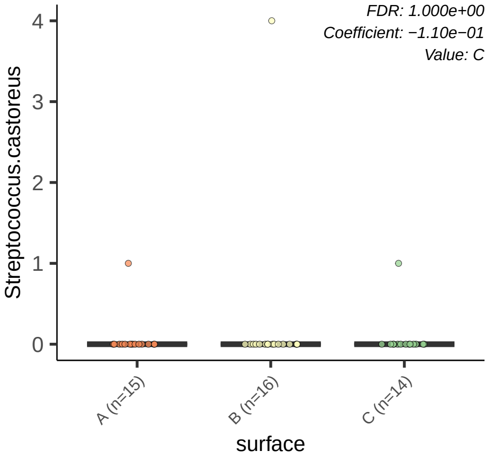

FDR: 1.000e+00
Coefficient: −1.10e−01
Value: C
Streptococcus.castoreus
0
1
2
3
4
A (n=15)
B (n=16)
C (n=14)
surface

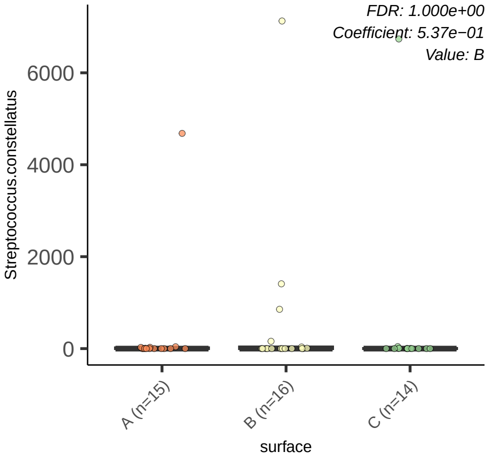

FDR: 1.000e+00
Coefficient: 5.37e−01
Value: B
Streptococcus.constellatus
6000
4000
2000
0
A (n=15)
B (n=16)
C (n=14)
surface

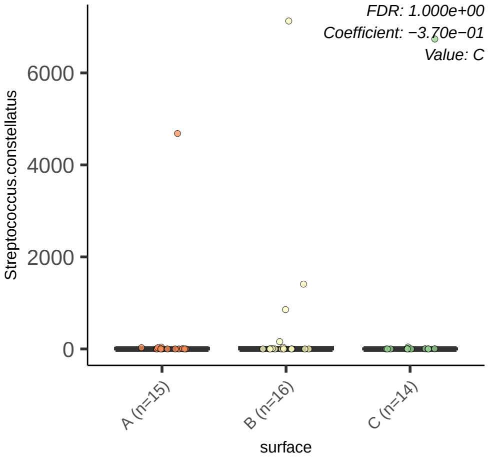
FDR: 1.000e+00
Coefficient: −3.70e−01
Value: C
Streptococcus.constellatus
6000
4000
2000
0
A (n=15)
B (n=16)
C (n=14)
surface

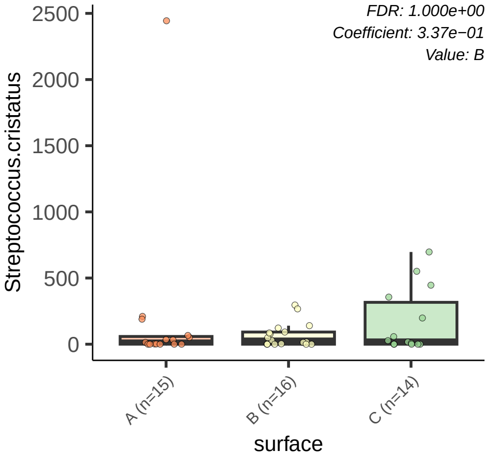
FDR: 1.000e+00
Coefficient: 3.37e−01
Value: B
Streptococcus.cristatus
2500
2000
1500
1000
500
0
A (n=15)
B (n=16)
C (n=14)
surface

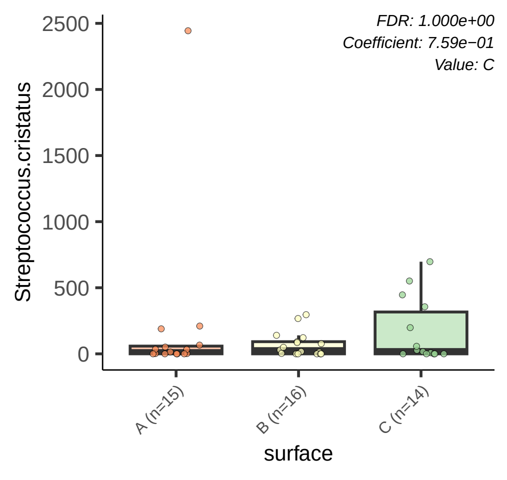

FDR: 1.000e+00
Coefficient: 7.59e−01
Value: C
Streptococcus.cristatus
2500
2000
1500
1000
500
0
A (n=15)
B (n=16)
C (n=14)
surface

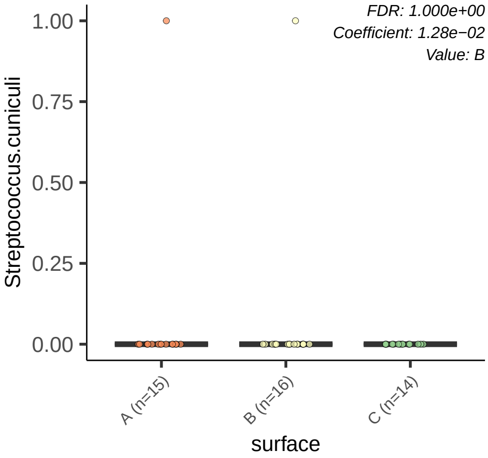

FDR: 1.000e+00
Coefficient: 1.28e−02
Value: B
Streptococcus.cuniculi
1.00
0.75
0.50
0.25
0.00
A (n=15)
B (n=16)
C (n=14)
surface

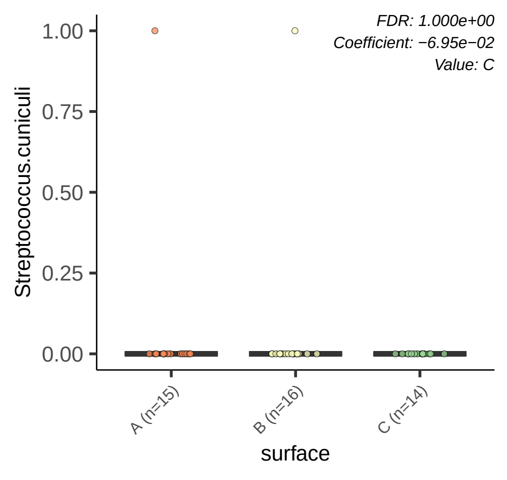
FDR: 1.000e+00
Coefficient: −6.95e−02
Value: C
Streptococcus.cuniculi
1.00
0.75
0.50
0.25
0.00
A (n=15)
B (n=16)
C (n=14)
surface

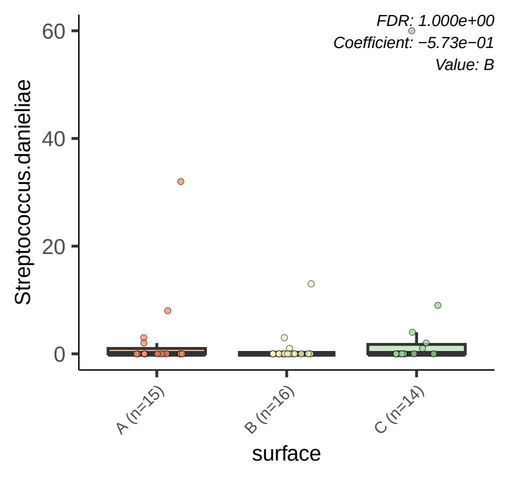
FDR: 1.000e+00
Coefficient: −5.73e−01
Value: B
Streptococcus.danieliae
0
20
40
60
A (n=15)
B (n=16)
C (n=14)
surface

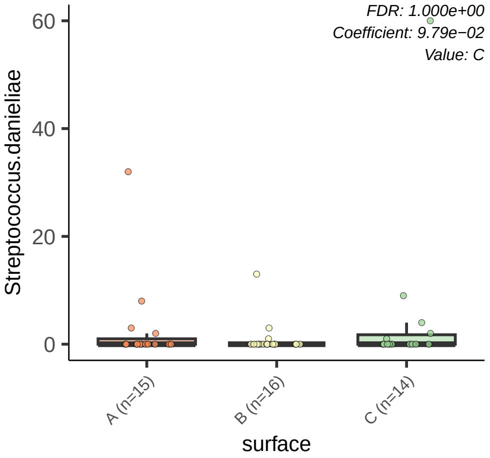
FDR: 1.000e+00
Coefficient: 9.79e−02
Value: C
Streptococcus.danieliae
60
40
20
0
A (n=15)
B (n=16)
C (n=14)
surface

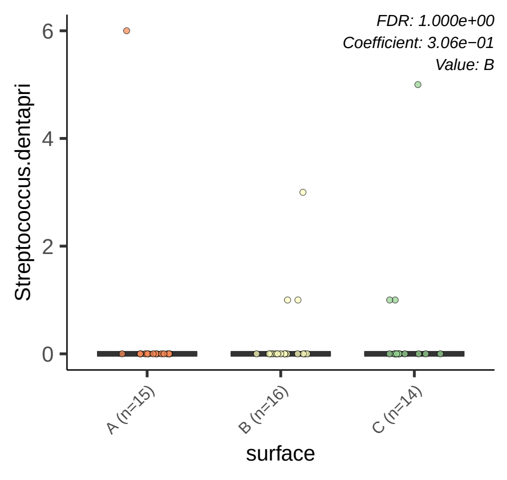
FDR: 1.000e+00
Coefficient: 3.06e−01
Value: B
Streptococcus.dentapri
6
4
2
0
A (n=15)
B (n=16)
C (n=14)
surface

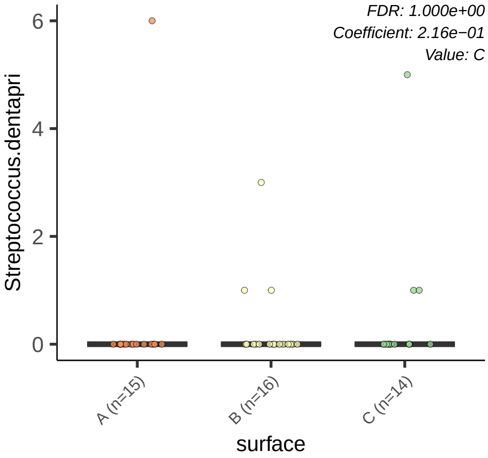

FDR: 1.000e+00
Coefficient: 2.16e−01
Value: C
Streptococcus.dentapri
6
4
2
0
A (n=15)
B (n=16)
C (n=14)
surface

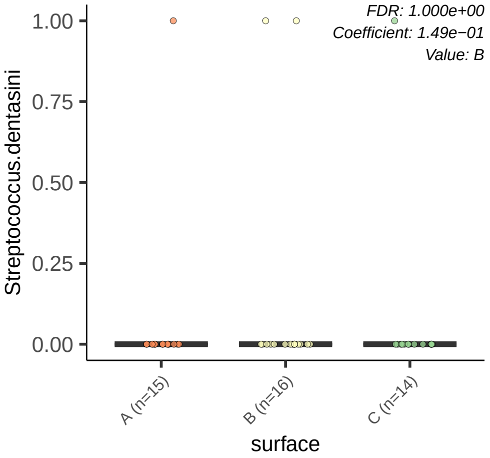

FDR: 1.000e+00
Coefficient: 1.49e−01
Value: B
Streptococcus.dentasini
1.00
0.75
0.50
0.25
0.00
A (n=15)
B (n=16)
C (n=14)
surface

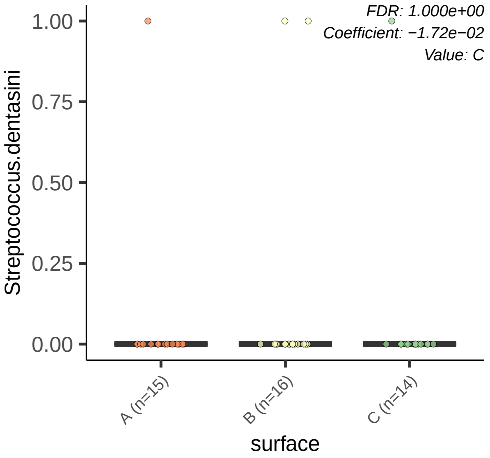
FDR: 1.000e+00
Coefficient: −1.72e−02
Value: C
Streptococcus.dentasini
1.00
0.75
0.50
0.25
0.00
A (n=15)
B (n=16)
C (n=14)
surface

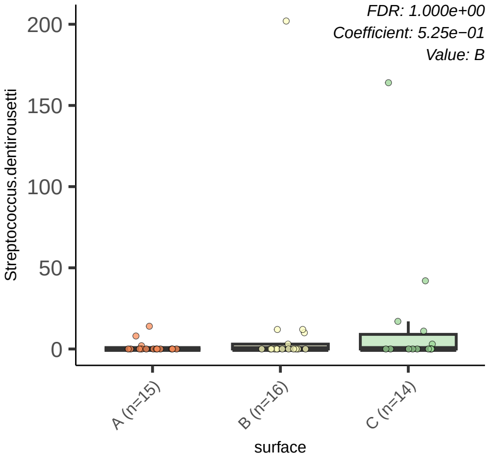

FDR: 1.000e+00
Coefficient: 5.25e−01
Value: B
Streptococcus.dentirousetti
200
150
100
50
0
A (n=15)
B (n=16)
C (n=14)
surface

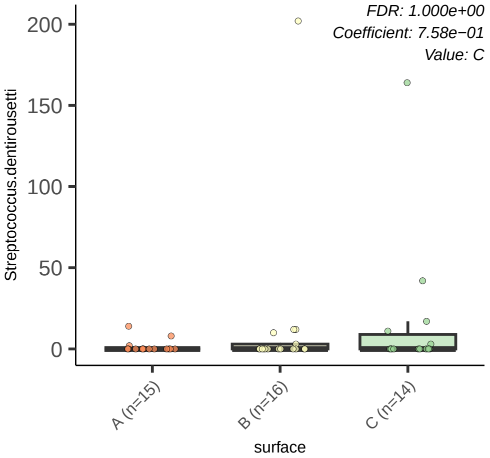

FDR: 1.000e+00
Coefficient: 7.58e−01
Value: C
Streptococcus.dentirousetti
200
150
100
50
0
A (n=15)
B (n=16)
C (n=14)
surface

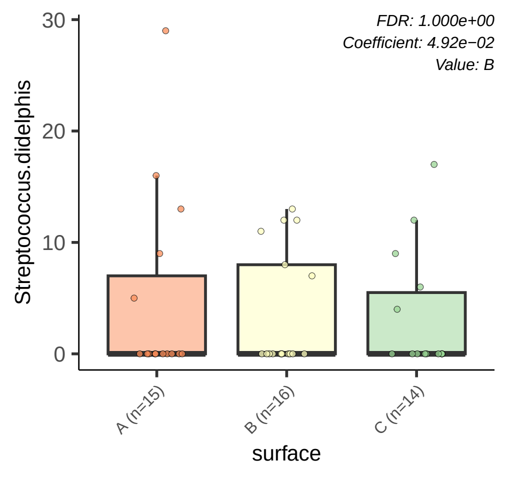
FDR: 1.000e+00
Coefficient: 4.92e−02
Value: B
Streptococcus.didelphis
30
20
10
0
A (n=15)
B (n=16)
C (n=14)
surface

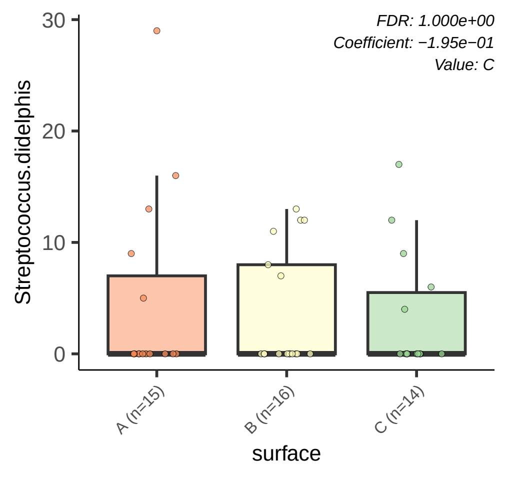

FDR: 1.000e+00
Coefficient: −1.95e−01
Value: C
30
20
10
0
Streptococcus.didelphis
A (n=15)
B (n=16)
C (n=14)
surface

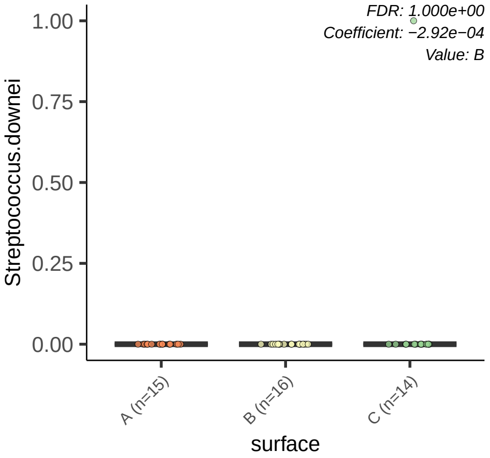
FDR: 1.000e+00
Coefficient: −2.92e−04
Value: B
Streptococcus.downei
1.00
0.75
0.50
0.25
0.00
A (n=15)
B (n=16)
C (n=14)
surface

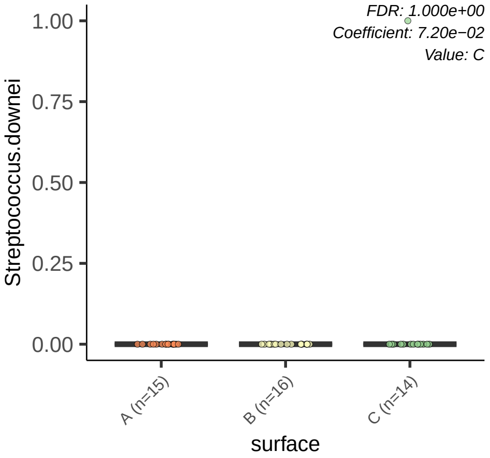
FDR: 1.000e+00
Coefficient: 7.20e−02
Value: C
1.00
0.75
0.50
0.25
0.00
Streptococcus.downei
A (n=15)
B (n=16)
C (n=14)
surface

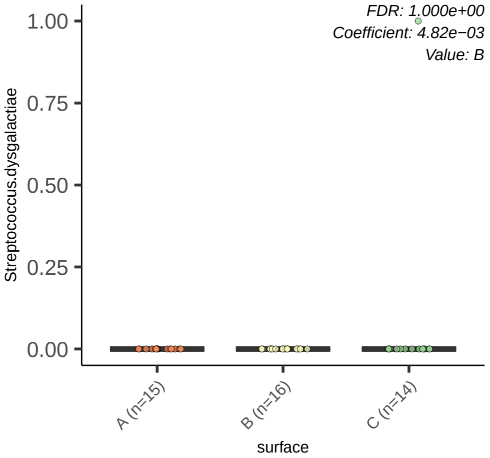
FDR: 1.000e+00
Coefficient: 4.82e−03
Value: B
1.00
0.75
0.50
0.25
0.00
Streptococcus.dysgalactiae
A (n=15)
B (n=16)
C (n=14)
surface

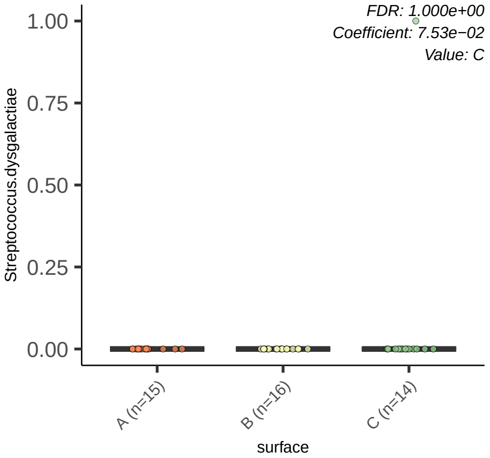
FDR: 1.000e+00
Coefficient: 7.53e−02
Value: C
1.00
0.75
0.50
0.25
0.00
Streptococcus.dysgalactiae
A (n=15)
B (n=16)
C (n=14)
surface

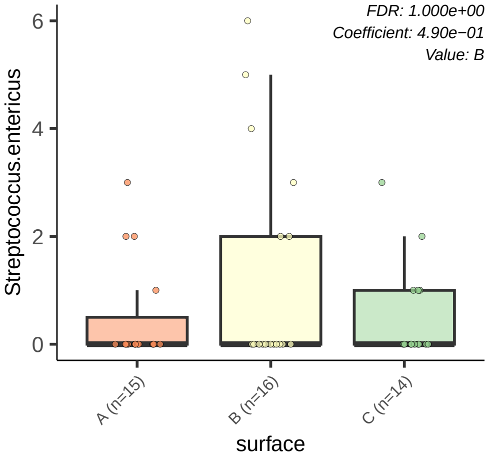
FDR: 1.000e+00
Coefficient: 4.90e−01
Value: B
Streptococcus.enterícus
0
2
4
6
A (n=15)
B (n=16)
C (n=14)
surface

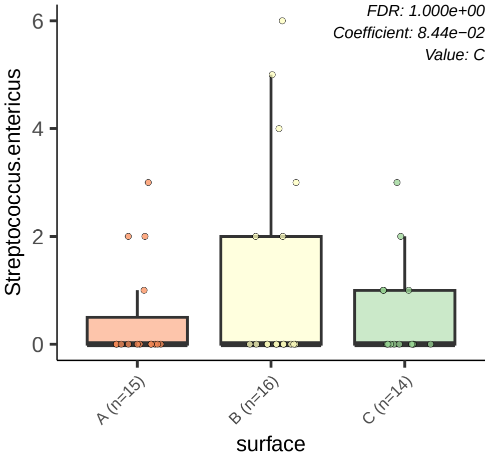
FDR: 1.000e+00
Coefficient: 8.44e−02
Value: C
Streptococcus.entericus
6
4
2
0
A (n=15)
B (n=16)
C (n=14)
surface

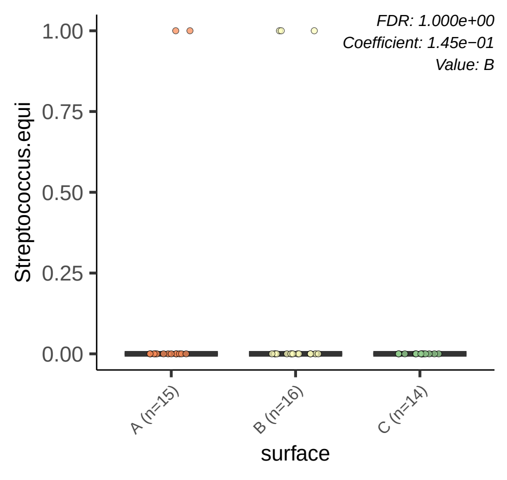

FDR: 1.000e+00
Coefficient: 1.45e−01
Value: B
1.00
0.75
0.50
0.25
0.00
Streptococcus.equi
A (n=15)
B (n=16)
C (n=14)
surface

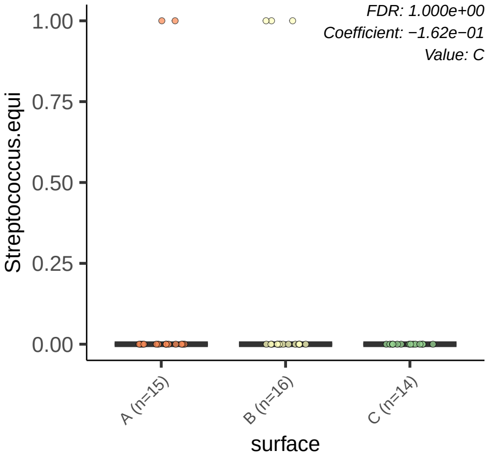

FDR: 1.000e+00
Coefficient: −1.62e−01
Value: C
Streptococcus.equi
1.00
0.75
0.50
0.25
0.00
A (n=15)
B (n=16)
C (n=14)
surface

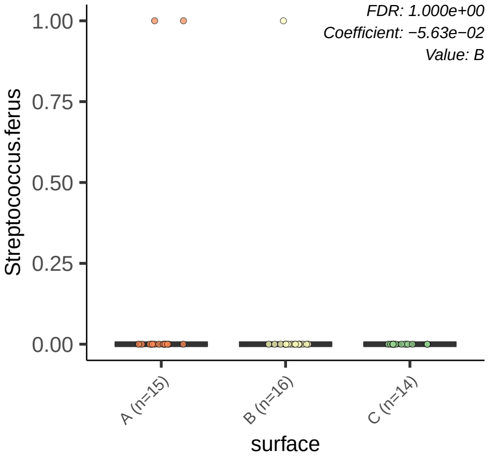

FDR: 1.000e+00
Coefficient: −5.63e−02
Value: B
Streptococcus.ferus
1.00
0.75
0.50
0.25
0.00
A (n=15)
B (n=16)
C (n=14)
surface

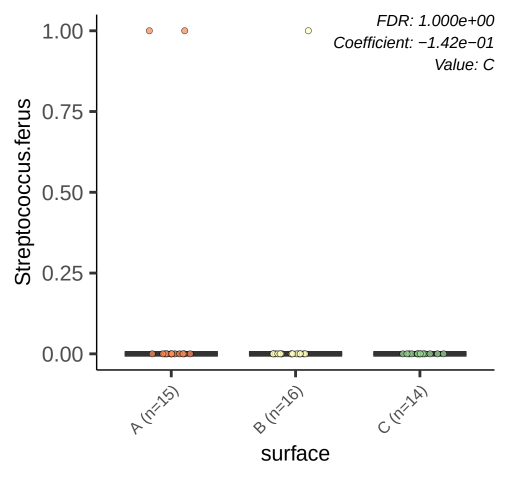
FDR: 1.000e+00
Coefficient: −1.42e−01
Value: C
Streptococcus.ferus
1.00
0.75
0.50
0.25
0.00
A (n=15)
B (n=16)
C (n=14)
surface

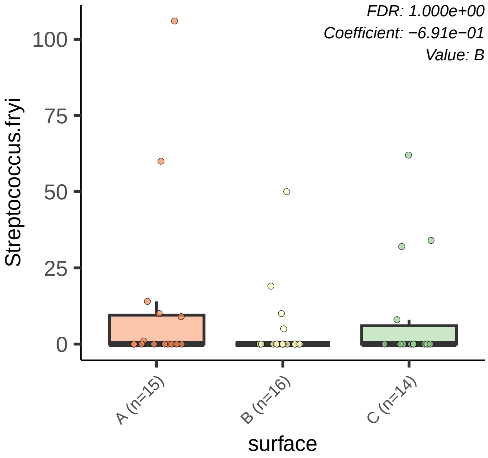
FDR: 1.000e+00
Coefficient: −6.91e−01
Value: B
Streptococcus.fryi
100
75
50
25
0
A (n=15)
B (n=16)
C (n=14)
surface

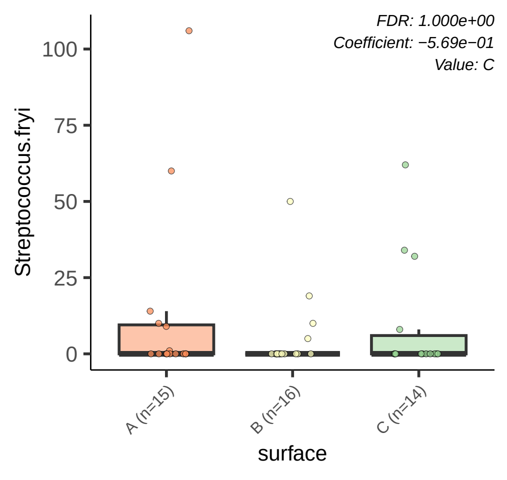

FDR: 1.000e+00
Coefficient: −5.69e−01
Value: C
Streptococcus.fryi
100
75
50
25
0
A (n=15)
B (n=16)
C (n=14)
surface

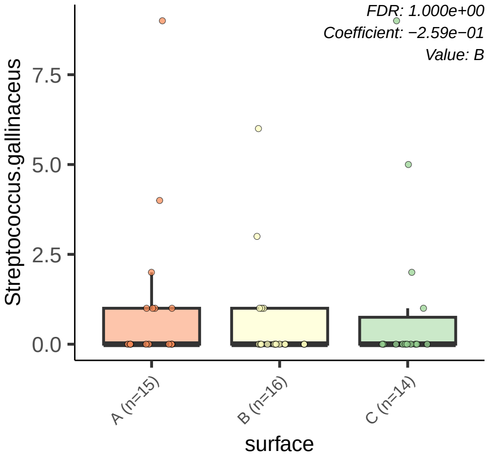
FDR: 1.000e+00
Coefficient: −2.59e−01
Value: B
Streptococcus.gallinaceus
7.5
5.0
2.5
0.0
A (n=15)
B (n=16)
C (n=14)
surface

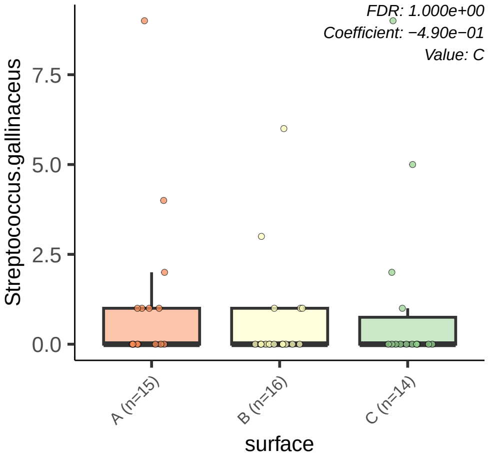

FDR: 1.000e+00
Coefficient: −4.90e−01
Value: C
Streptococcus.gallinaceus
7.5
5.0
2.5
0.0
A (n=15)
B (n=16)
C (n=14)
surface

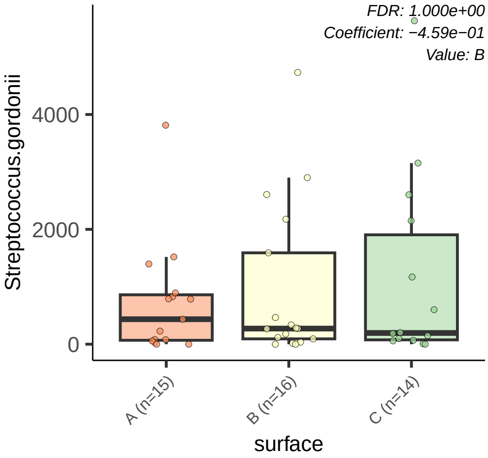

FDR: 1.000e+00
Coefficient: −4.59e−01
Value: B
Streptococcus.gordonii
4000
2000
0
A (n=15)
B (n=16)
C (n=14)
surface

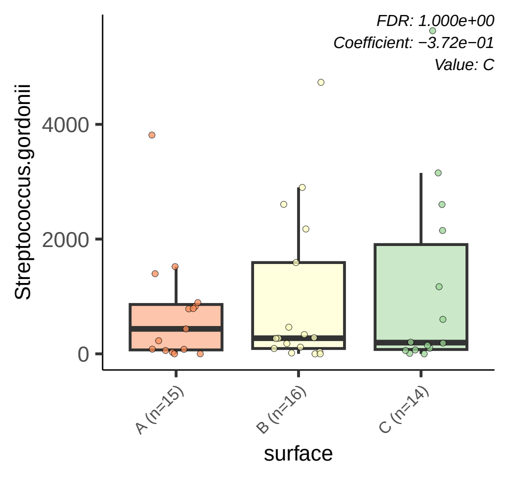

FDR: 1.000e+00
Coefficient: −3.72e−01
Value: C
Streptococcus.gordonii
4000
2000
0
A (n=15)
B (n=16)
C (n=14)
surface

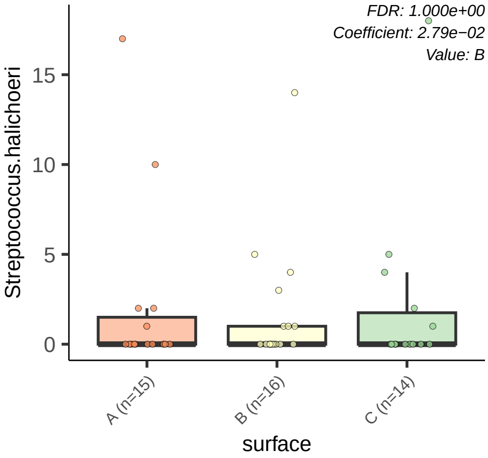

FDR: 1.000e+00
Coefficient: 2.79e−02
Value: B
Streptococcus.halichoeri
15
10
5
0
A (n=15)
B (n=16)
C (n=14)
surface

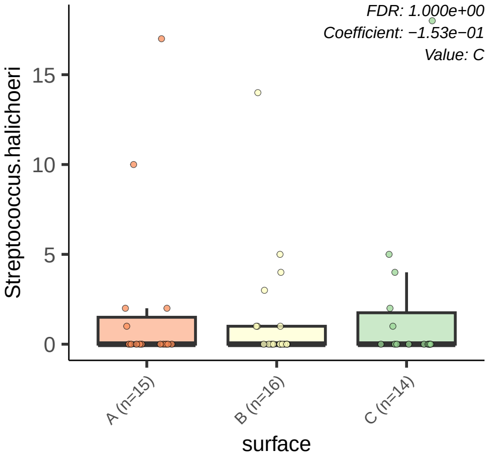
FDR: 1.000e+00
Coefficient: −1.53e−01
Value: C
Streptococcus.halichoeri
0
5
10
15
A (n=15)
B (n=16)
C (n=14)
surface

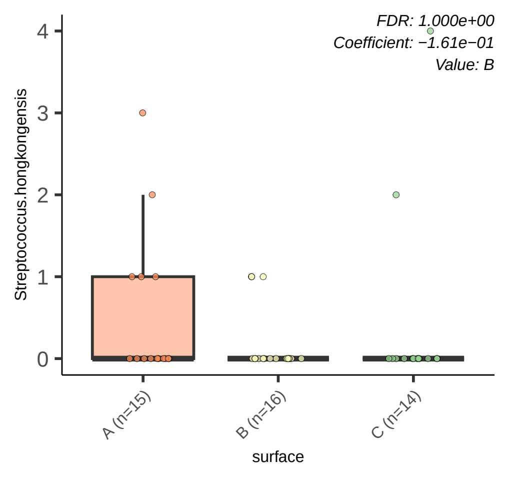
FDR: 1.000e+00
Coefficient: −1.61e−01
Value: B
Streptococcus.hongkongensis
0
1
2
3
4
A (n=15)
B (n=16)
C (n=14)
surface

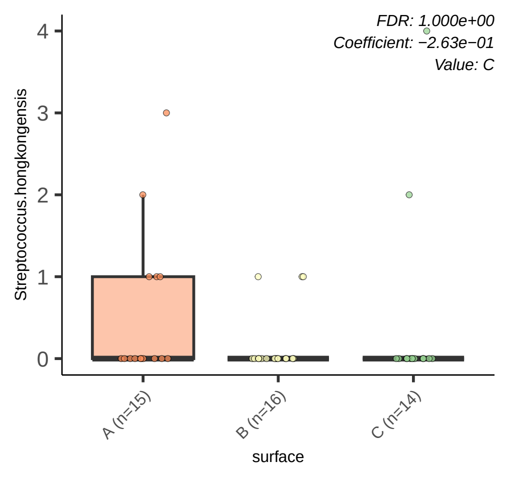

FDR: 1.000e+00
Coefficient: −2.63e−01
Value: C
Streptococcus.hongkongensis
0
1
2
3
4
A (n=15)
B (n=16)
C (n=14)
surface

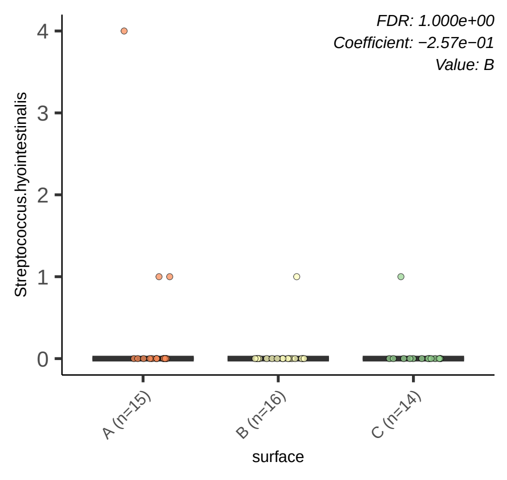

FDR: 1.000e+00
Coefficient: −2.57e−01
Value: B
Streptococcus.hyointestinalis
0
1
2
3
4
A (n=15)
B (n=16)
C (n=14)
surface

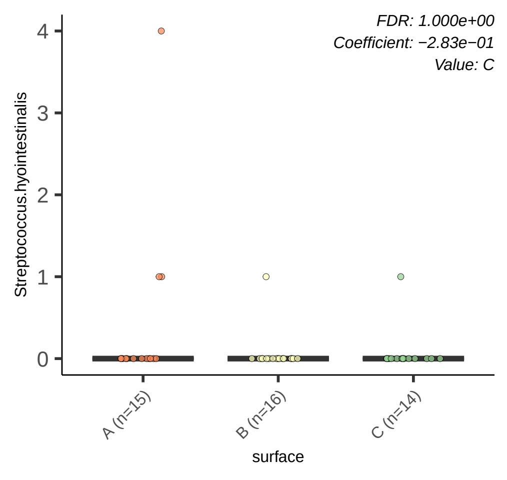
FDR: 1.000e+00
Coefficient: −2.83e−01
Value: C
Streptococcus.hyointestinalis
0
1
2
3
4
A (n=15)
B (n=16)
C (n=14)
surface

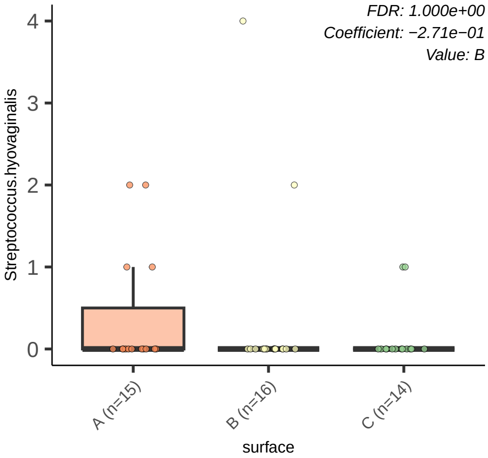
FDR: 1.000e+00
Coefficient: −2.71e−01
Value: B
Streptococcus.hyovaginalis
0
1
2
3
4
A (n=15)
B (n=16)
C (n=14)
surface

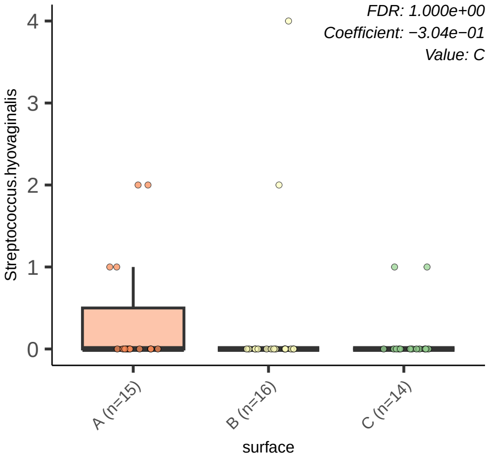
FDR: 1.000e+00
Coefficient: −3.04e−01
Value: C
Streptococcus.hyovaginalis
0
1
2
3
4
A (n=15)
B (n=16)
C (n=14)
surface

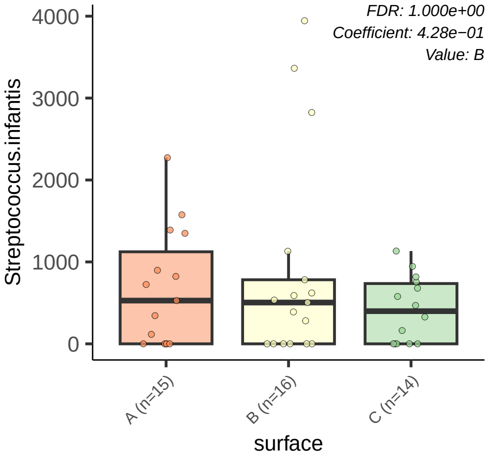
FDR: 1.000e+00
Coefficient: 4.28e−01
Value: B
Streptococcus.infantis
4000
3000
2000
1000
0
A (n=15)
B (n=16)
C (n=14)
surface

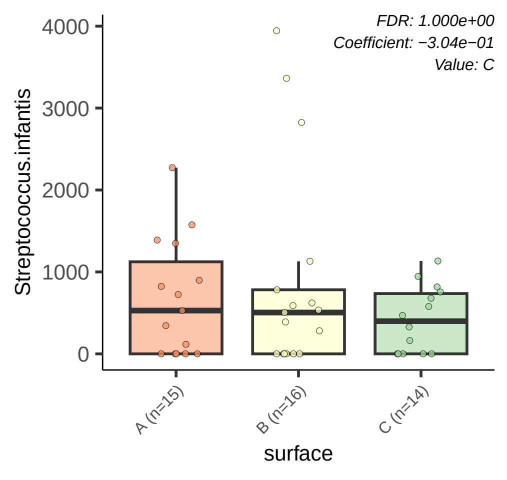

FDR: 1.000e+00
Coefficient: −3.04e−01
Value: C
Streptococcus.infantis
4000
3000
2000
1000
0
A (n=15)
B (n=16)
C (n=14)
surface

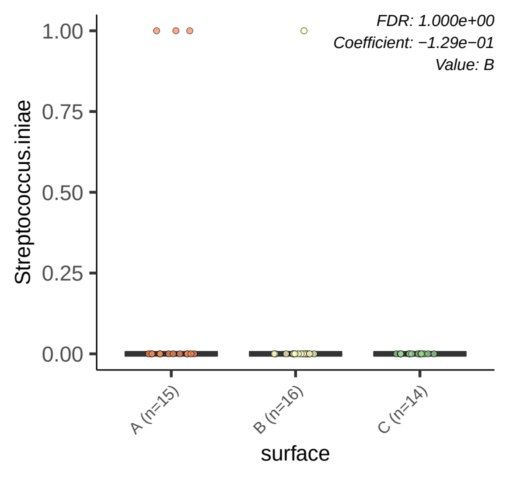

FDR: 1.000e+00
Coefficient: −1.29e−01
Value: B
Streptococcus.iniae
1.00
0.75
0.50
0.25
0.00
A (n=15)
B (n=16)
C (n=14)
surface

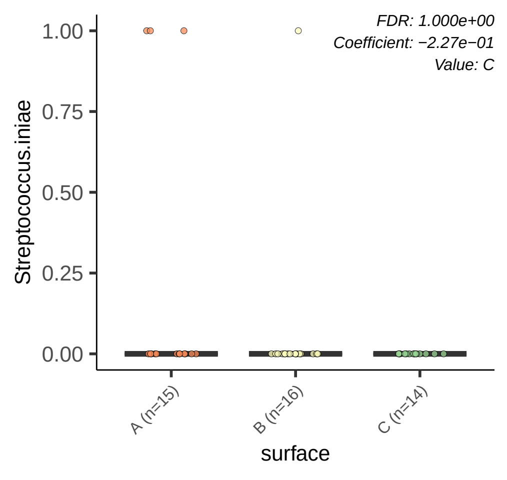

FDR: 1.000e+00
Coefficient: −2.27e−01
Value: C
Streptococcus.iniae
1.00
0.75
0.50
0.25
0.00
A (n=15)
B (n=16)
C (n=14)
surface

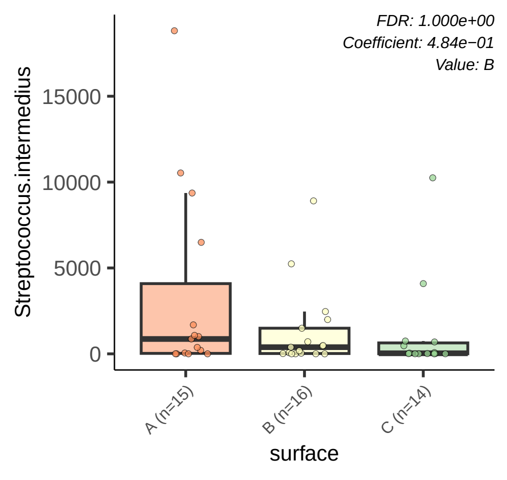

FDR: 1.000e+00
Coefficient: 4.84e−01
Value: B
Streptococcus.intermedius
15000
10000
5000
0
A (n=15)
B (n=16)
C (n=14)
surface

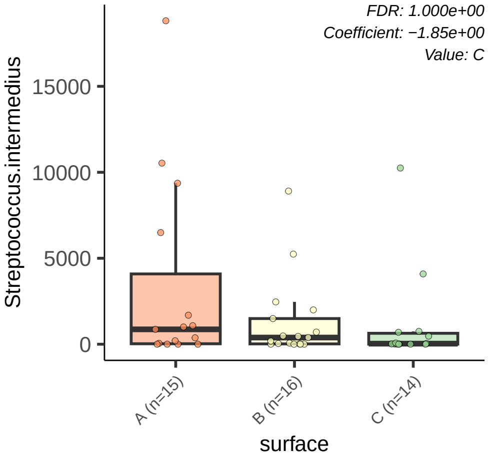
FDR: 1.000e+00
Coefficient: −1.85e+00
Value: C
Streptococcus.intermedius
15000
10000
5000
0
A (n=15)
B (n=16)
C (n=14)
surface

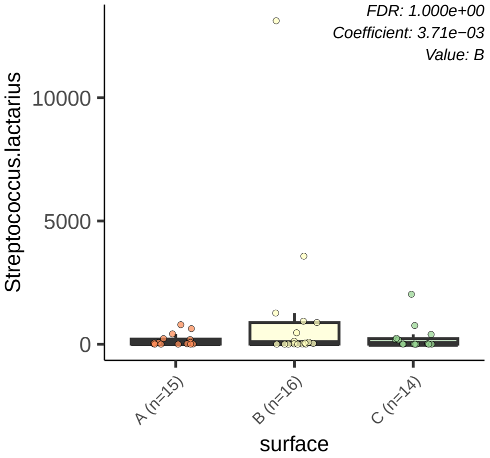
FDR: 1.000e+00
Coefficient: 3.71e−03
Value: B
Streptococcus.lactarius
10000
5000
0
A (n=15)
B (n=16)
C (n=14)
surface

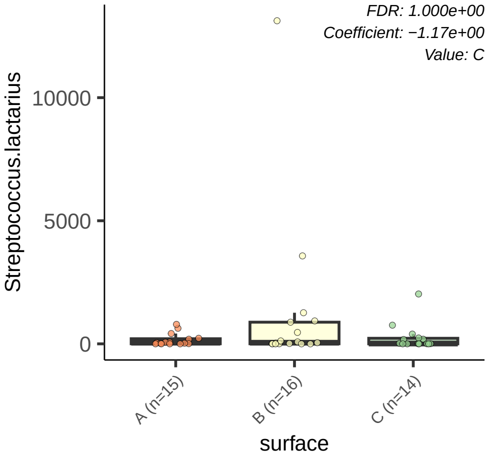
FDR: 1.000e+00
Coefficient: −1.17e+00
Value: C
Streptococcus.lactarius
10000
5000
0
A (n=15)
B (n=16)
C (n=14)
surface

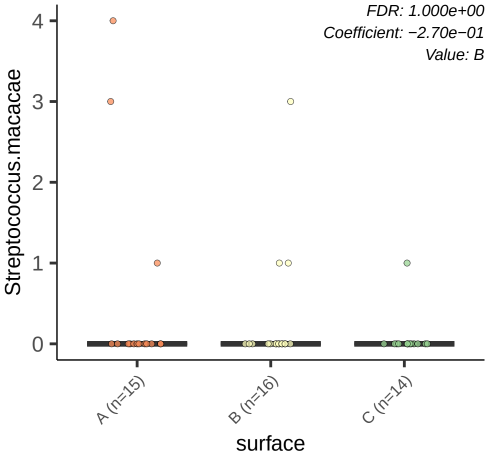
FDR: 1.000e+00
Coefficient: −2.70e−01
Value: B
Streptococcus.macacae
4
3
2
1
0
A (n=15)
B (n=16)
C (n=14)
surface

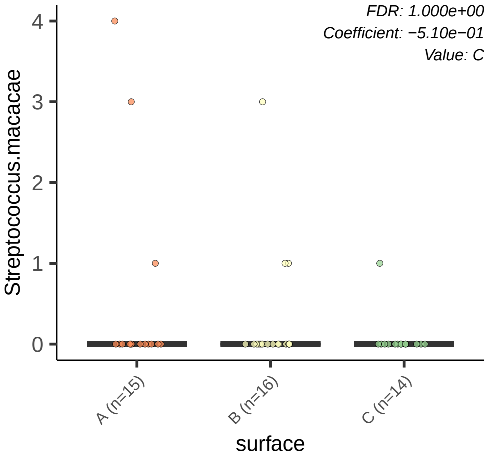

FDR: 1.000e+00
Coefficient: −5.10e−01
Value: C
Streptococcus.macacae
4
3
2
1
0
A (n=15)
B (n=16)
C (n=14)
surface

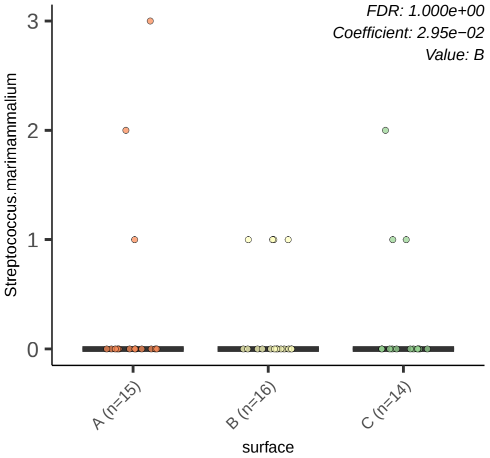
FDR: 1.000e+00
Coefficient: 2.95e−02
Value: B
Streptococcus.marimammalium
3
2
1
0
A (n=15)
B (n=16)
C (n=14)
surface

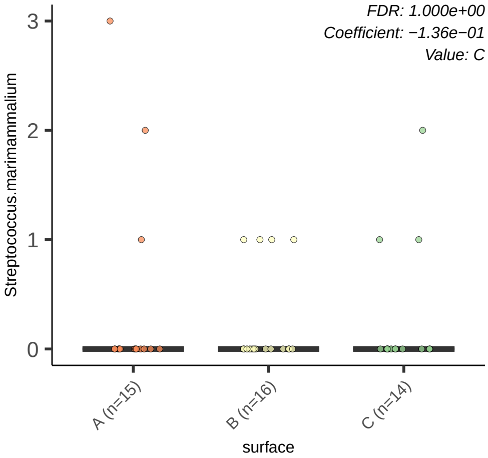
FDR: 1.000e+00
Coefficient: −1.36e−01
Value: C
Streptococcus.marimammalium
3
2
1
0
A (n=15)
B (n=16)
C (n=14)
surface

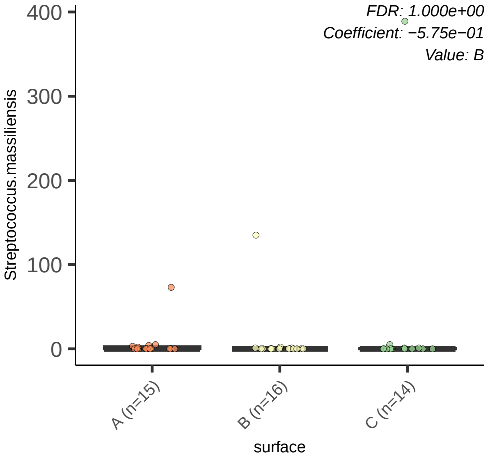
FDR: 1.000e+00
Coefficient: −5.75e−01
Value: B
Streptococcus.massiliensis
400
300
200
100
0
A (n=15)
B (n=16)
C (n=14)
surface

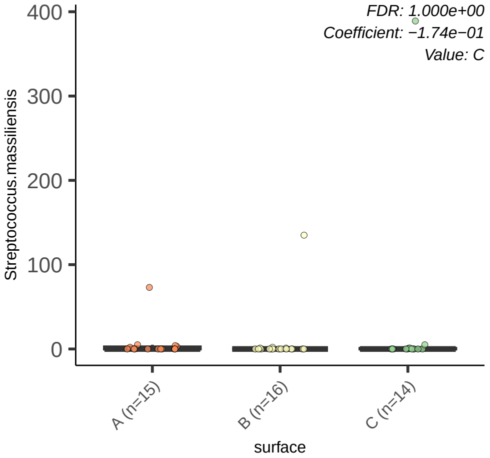
FDR: 1.000e+00
Coefficient: −1.74e−01
Value: C
400
300
200
100
0
Streptococcus.massiliensis
A (n=15)
B (n=16)
C (n=14)
surface

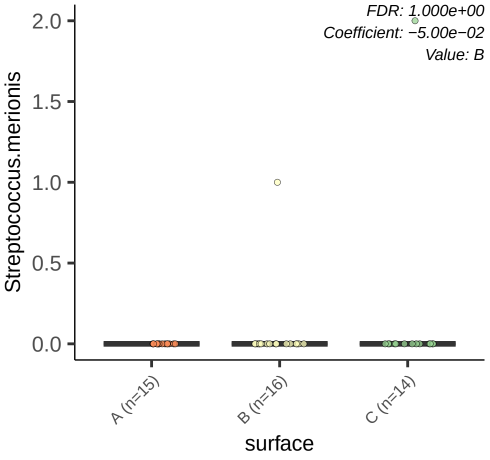
FDR: 1.000e+00
Coefficient: −5.00e−02
Value: B
Streptococcus.merionis
2.0
1.5
1.0
0.5
0.0
A (n=15)
B (n=16)
C (n=14)
surface

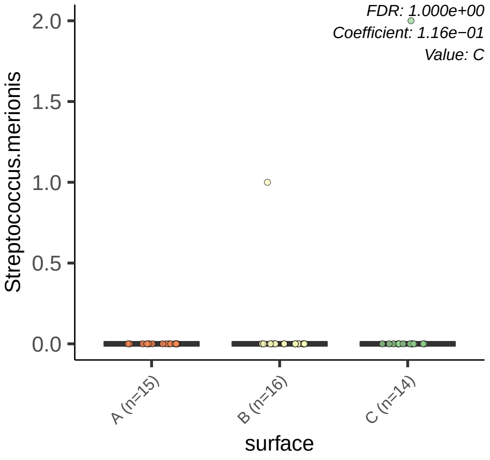
FDR: 1.000e+00
Coefficient: 1.16e−01
Value: C
Streptococcus.merionis
2.0
1.5
1.0
0.5
0.0
A (n=15)
B (n=16)
C (n=14)
surface

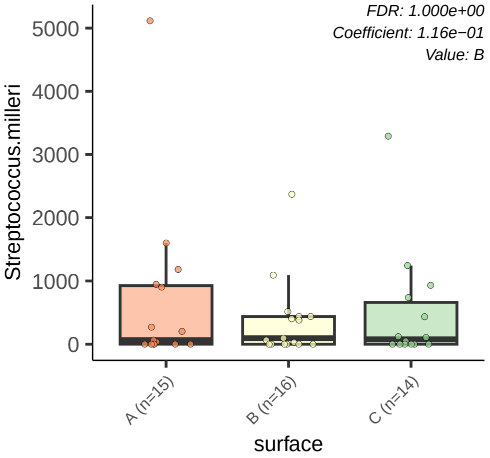
FDR: 1.000e+00
Coefficient: 1.16e−01
Value: B
Streptococcus.milleri
5000
4000
3000
2000
1000
0
A (n=15)
B (n=16)
C (n=14)
surface

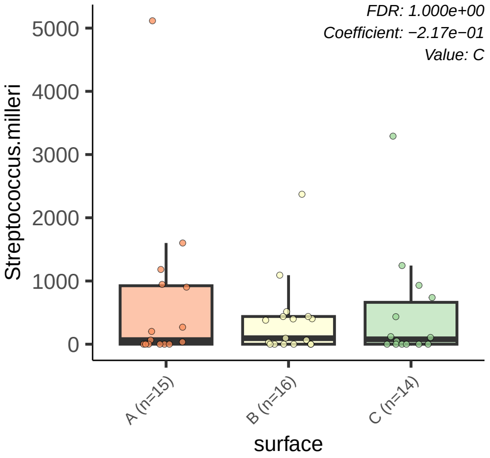

FDR: 1.000e+00
Coefficient: −2.17e−01
Value: C
Streptococcus.milleri
5000
4000
3000
2000
1000
0
A (n=15)
B (n=16)
C (n=14)
surface

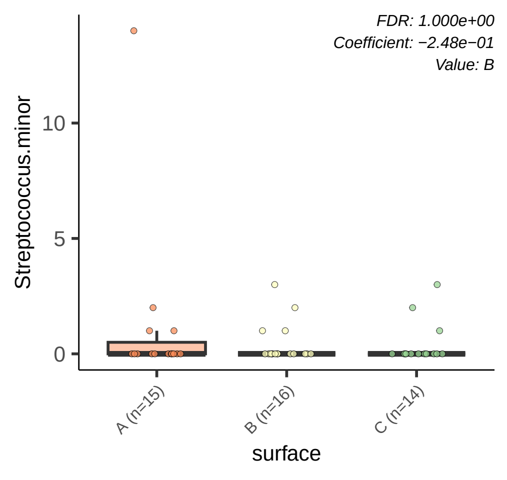

FDR: 1.000e+00
Coefficient: −2.48e−01
Value: B
Streptococcus.minor
0
5
10
A (n=15)
B (n=16)
C (n=14)
surface

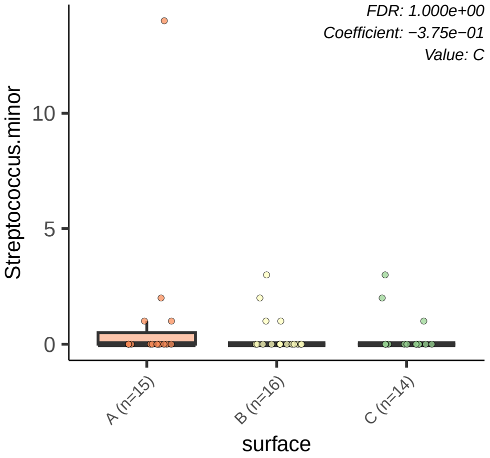
FDR: 1.000e+00
Coefficient: −3.75e−01
Value: C
Streptococcus.minor
10
5
0
A (n=15)
B (n=16)
C (n=14)
surface

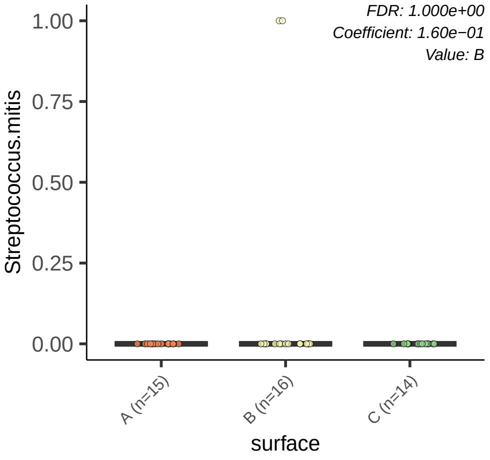
FDR: 1.000e+00
Coefficient: 1.60e−01
Value: B
Streptococcus.mitis
1.00
0.75
0.50
0.25
0.00
A (n=15)
B (n=16)
C (n=14)
surface

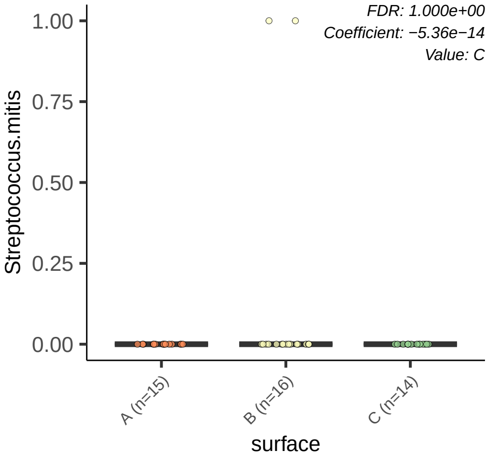
FDR: 1.000e+00
Coefficient: −5.36e−14
Value: C
Streptococcus.mitis
1.00
0.75
0.50
0.25
0.00
A (n=15)
B (n=16)
C (n=14)
surface

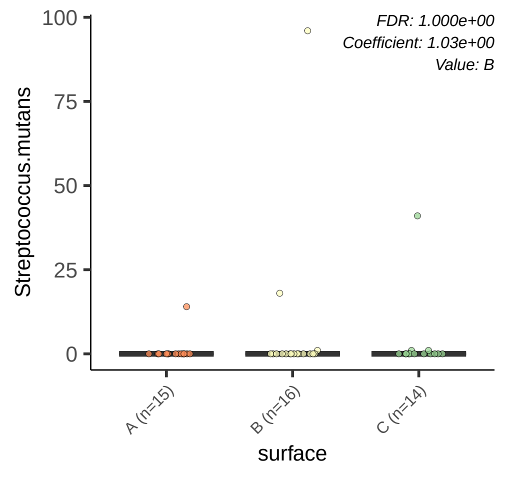
FDR: 1.000e+00
Coefficient: 1.03e+00
Value: B
Streptococcus.mutans
100
75
50
25
0
A (n=15)
B (n=16)
C (n=14)
surface

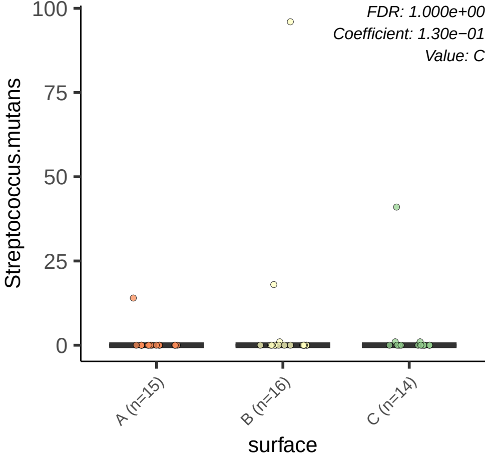
FDR: 1.000e+00
Coefficient: 1.30e−01
Value: C
Streptococcus.mutans
100
75
50
25
0
A (n=15)
B (n=16)
C (n=14)
surface

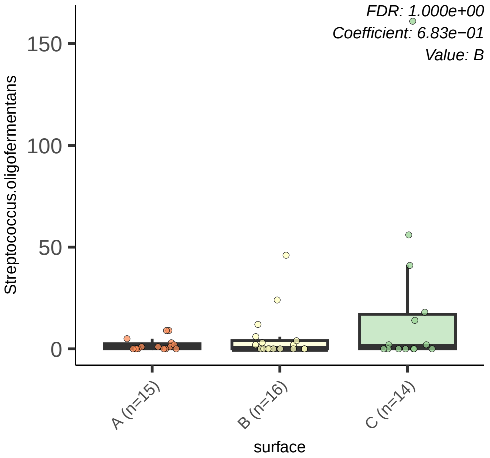
FDR: 1.000e+00
Coefficient: 6.83e−01
Value: B
Streptococcus.oligofermentans
150
100
50
0
A (n=15)
B (n=16)
C (n=14)
surface

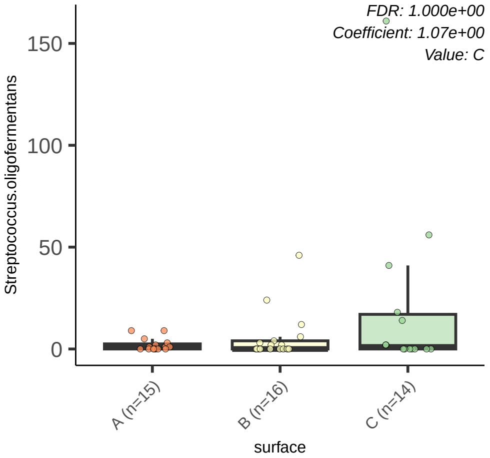
FDR: 1.000e+00
Coefficient: 1.07e+00
Value: C
Streptococcus.oligofermentans
150
100
50
0
A (n=15)
B (n=16)
C (n=14)
surface

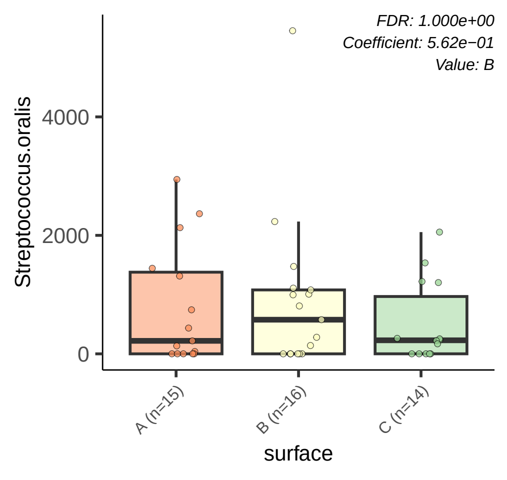
FDR: 1.000e+00
Coefficient: 5.62e−01
Value: B
Streptococcus.oralis
4000
2000
0
A (n=15)
B (n=16)
C (n=14)
surface

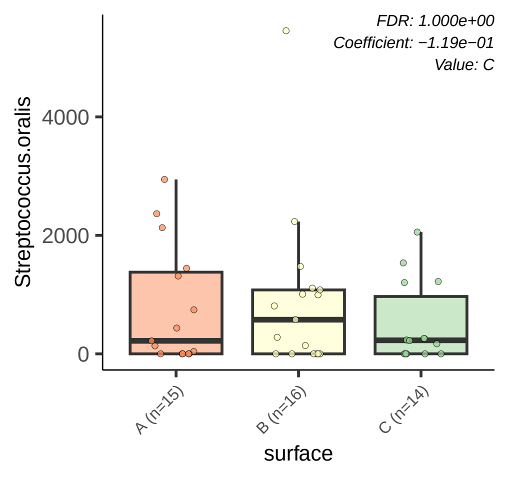
FDR: 1.000e+00
Coefficient: −1.19e−01
Value: C
Streptococcus.oralis
4000
2000
0
A (n=15)
B (n=16)
C (n=14)
surface

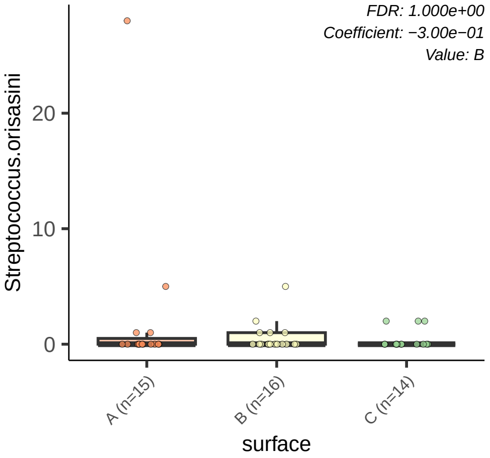
FDR: 1.000e+00
Coefficient: −3.00e−01
Value: B
Streptococcus.orisasini
0
10
20
A (n=15)
B (n=16)
C (n=14)
surface

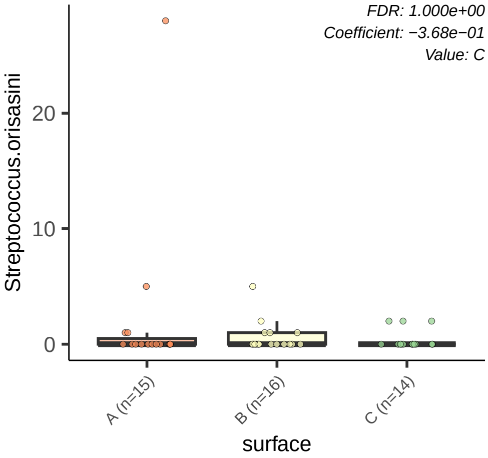

FDR: 1.000e+00
Coefficient: −3.68e−01
Value: C
Streptococcus.orisasini
20
10
0
A (n=15)
B (n=16)
C (n=14)
surface

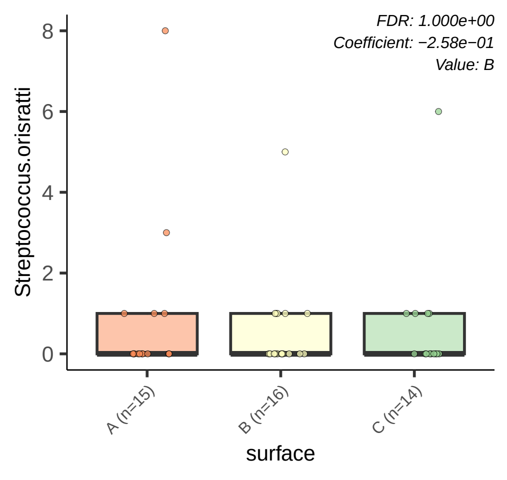

FDR: 1.000e+00
Coefficient: −2.58e−01
Value: B
Streptococcus.orisratti
0
2
4
6
8
A (n=15)
B (n=16)
C (n=14)
surface

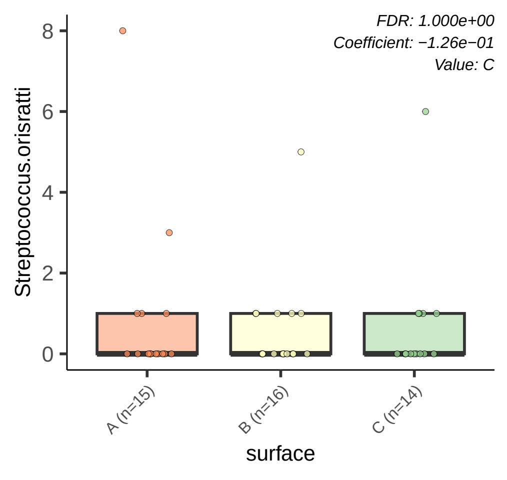

FDR: 1.000e+00
Coefficient: −1.26e−01
Value: C
Streptococcus.orisratti
0
2
4
6
8
A (n=15)
B (n=16)
C (n=14)
surface

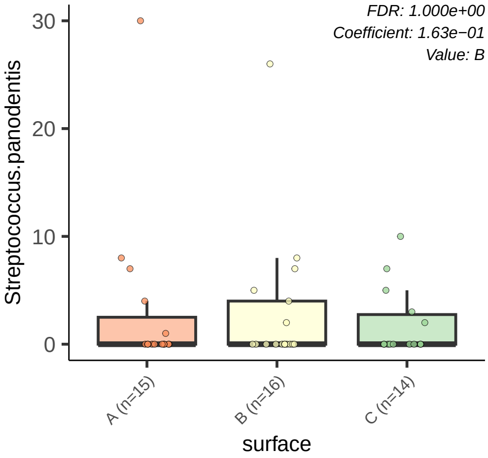

FDR: 1.000e+00
Coefficient: 1.63e−01
Value: B
Streptococcus.panodentis
30
20
10
0
A (n=15)
B (n=16)
C (n=14)
surface

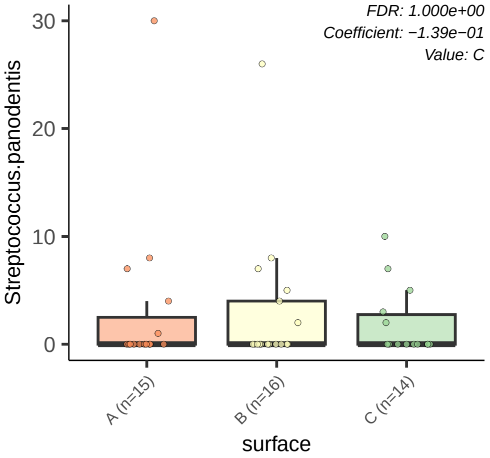

FDR: 1.000e+00
Coefficient: −1.39e−01
Value: C
Streptococcus.panodentis
30
20
10
0
A (n=15)
B (n=16)
C (n=14)
surface

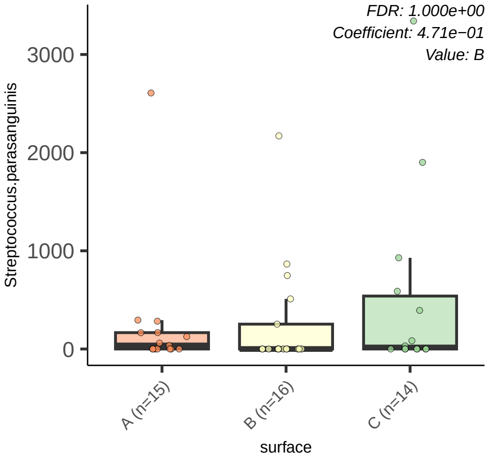

FDR: 1.000e+00
Coefficient: 4.71e−01
Value: B
Streptococcus.parasanguinis
3000
2000
1000
0
A (n=15)
B (n=16)
C (n=14)
surface

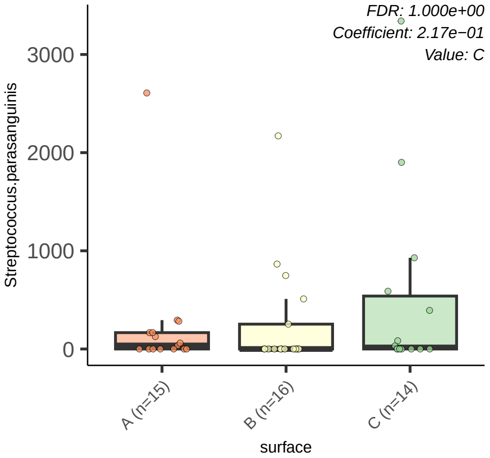
FDR: 1.000e+00
Coefficient: 2.17e−01
Value: C
3000
2000
1000
0
Streptococcus.parasanguinis
A (n=15)
B (n=16)
C (n=14)
surface

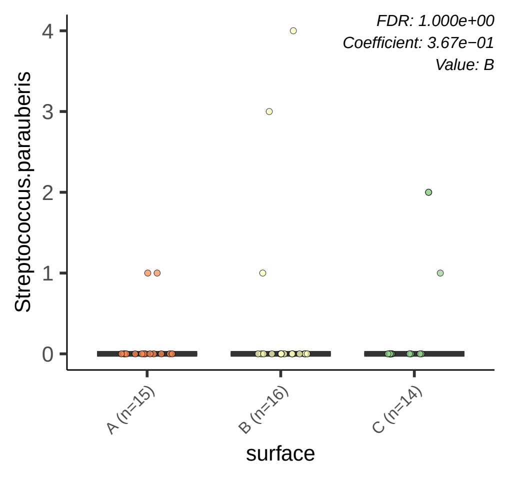

FDR: 1.000e+00
Coefficient: 3.67e−01
Value: B
Streptococcus.parauberis
0
1
2
3
4
A (n=15)
B (n=16)
C (n=14)
surface

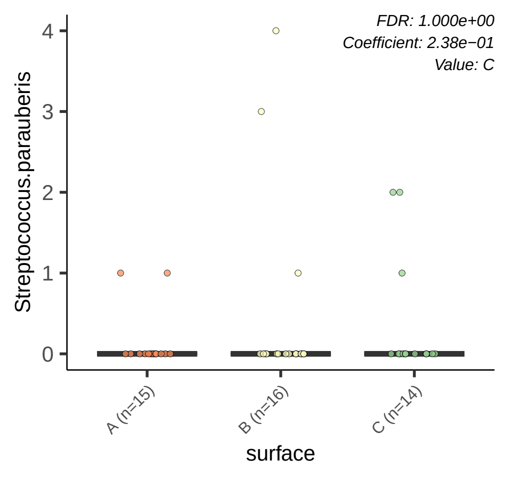
FDR: 1.000e+00
Coefficient: 2.38e−01
Value: C
Streptococcus.parauberis
4
3
2
1
0
A (n=15)
B (n=16)
C (n=14)
surface

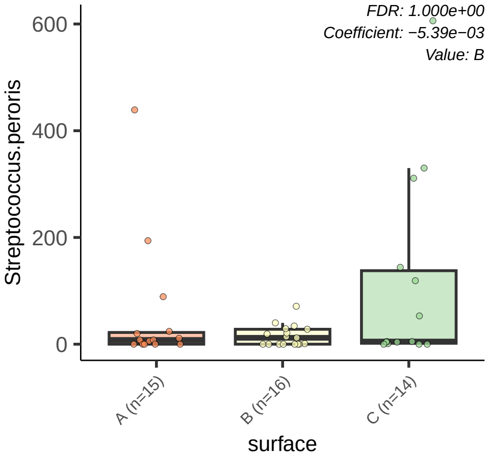

FDR: 1.000e+00
Coefficient: −5.39e−03
Value: B
Streptococcus.peroris
600
400
200
0
A (n=15)
B (n=16)
C (n=14)
surface

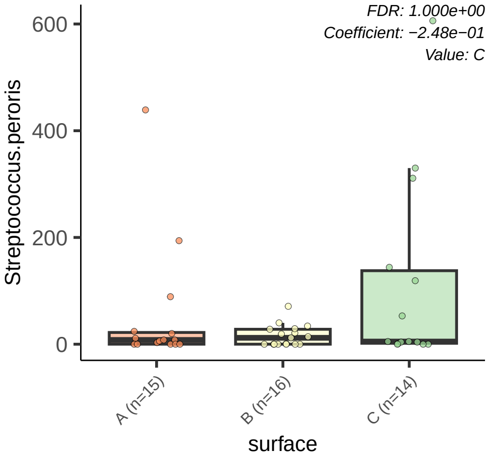
FDR: 1.000e+00
Coefficient: −2.48e−01
Value: C
Streptococcus.peroris
600
400
200
0
A (n=15)
B (n=16)
C (n=14)
surface

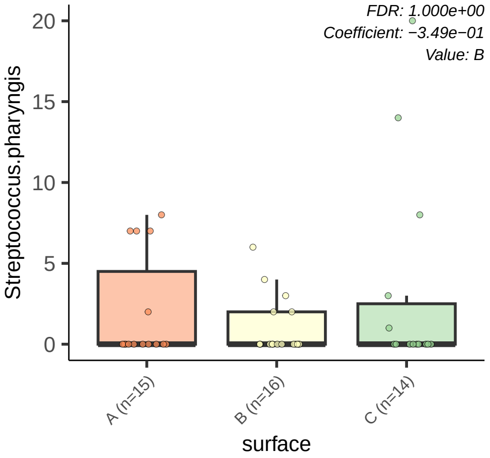
FDR: 1.000e+00
Coefficient: −3.49e−01
Value: B
Streptococcus.pharyngis
0
5
10
15
20
A (n=15)
B (n=16)
C (n=14)
surface

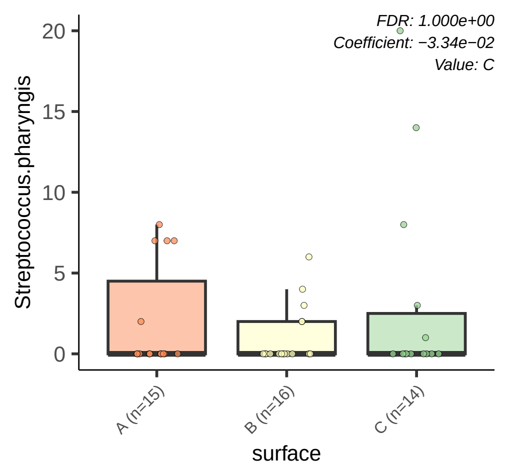
FDR: 1.000e+00
Coefficient: −3.34e−02
Value: C
Streptococcus.pharyngis
20
15
10
5
0
A (n=15)
B (n=16)
C (n=14)
surface

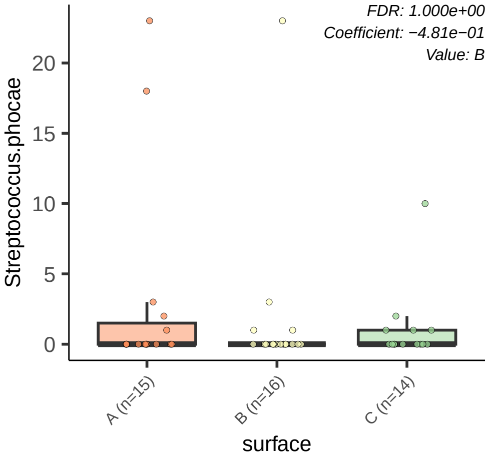

FDR: 1.000e+00
Coefficient: −4.81e−01
Value: B
Streptococcus.phocae
20
15
10
5
0
A (n=15)
B (n=16)
C (n=14)
surface

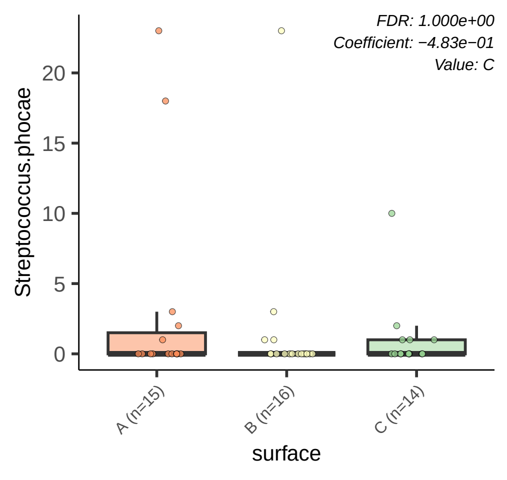

FDR: 1.000e+00
Coefficient: −4.83e−01
Value: C
Streptococcus.phocae
0
5
10
15
20
A (n=15)
B (n=16)
C (n=14)
surface

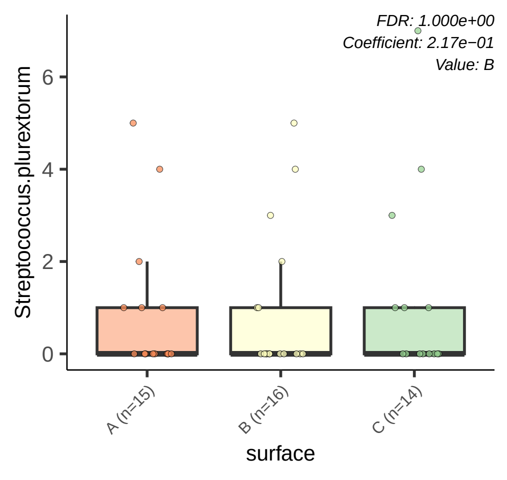
FDR: 1.000e+00
Coefficient: 2.17e−01
Value: B
Streptococcus.plurextorum
0
2
4
6
A (n=15)
B (n=16)
C (n=14)
surface

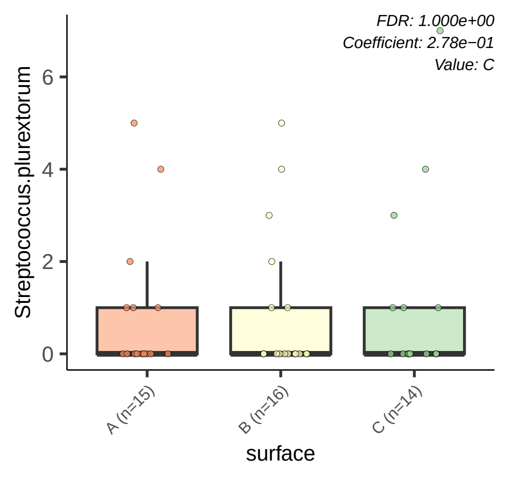
FDR: 1.000e+00
Coefficient: 2.78e−01
Value: C
Streptococcus.plurextorum
0
2
4
6
A (n=15)
B (n=16)
C (n=14)
surface

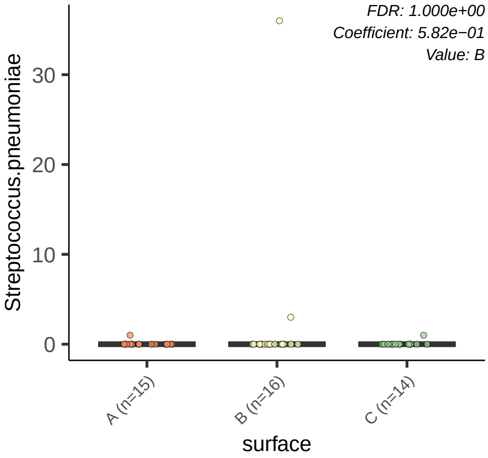
FDR: 1.000e+00
Coefficient: 5.82e−01
Value: B
Streptococcus.pneumoniae
0
10
20
30
A (n=15)
B (n=16)
C (n=14)
surface

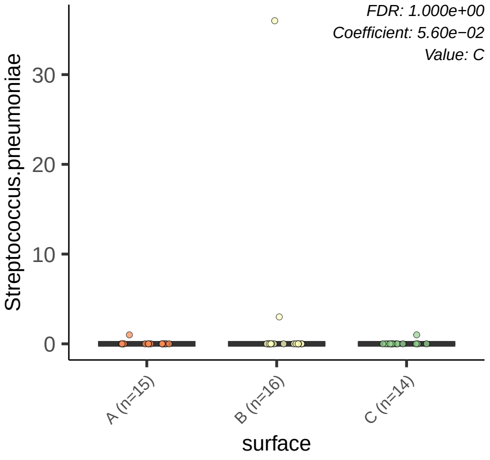
FDR: 1.000e+00
Coefficient: 5.60e−02
Value: C
Streptococcus.pneumoniae
0
10
20
30
A (n=15)
B (n=16)
C (n=14)
surface

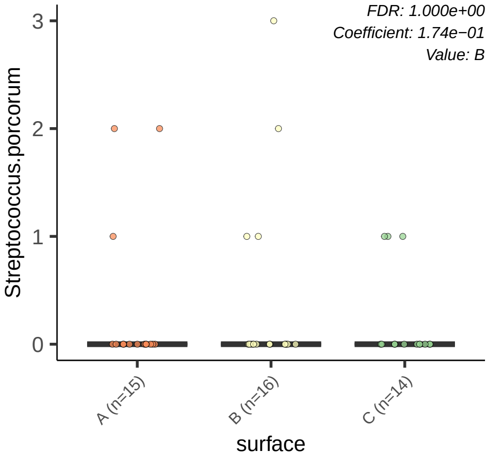

FDR: 1.000e+00
Coefficient: 1.74e−01
Value: B
Streptococcus.porcorum
3
2
1
0
A (n=15)
B (n=16)
C (n=14)
surface

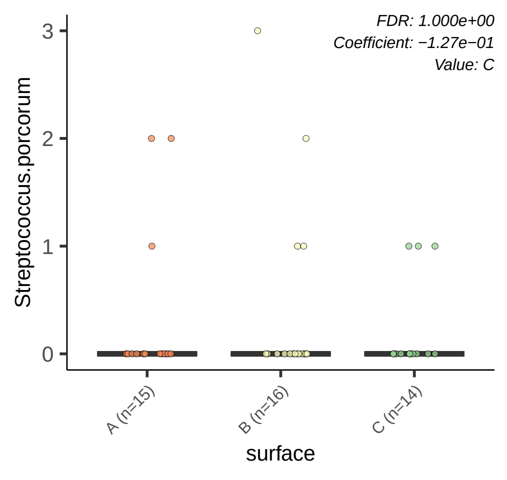
FDR: 1.000e+00
Coefficient: −1.27e−01
Value: C
Streptococcus.porcorum
3
2
1
0
A (n=15)
B (n=16)
C (n=14)
surface

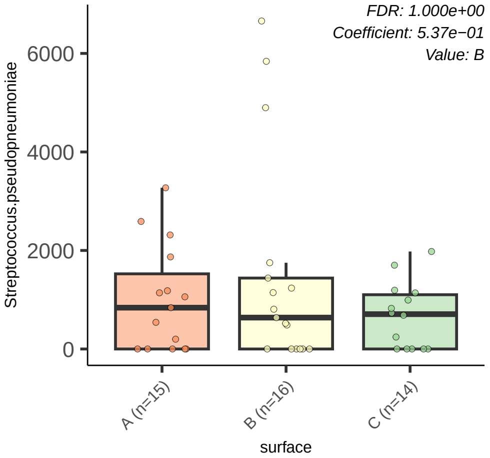

FDR: 1.000e+00
Coefficient: 5.37e−01
Value: B
Streptococcus.pseudopneumoniae
6000
4000
2000
0
A (n=15)
B (n=16)
C (n=14)
surface

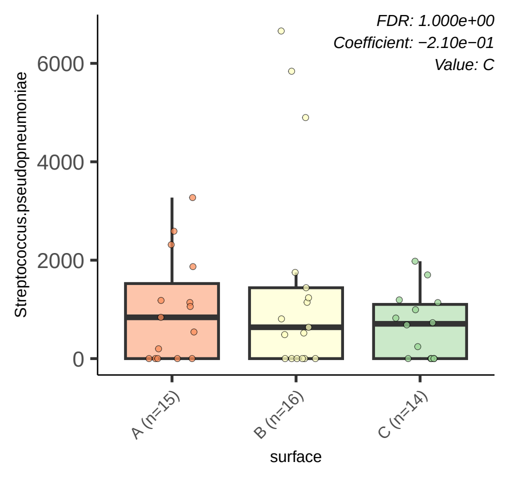
FDR: 1.000e+00
Coefficient: −2.10e−01
Value: C
Streptococcus.pseudopneumoniae
0
2000
4000
6000
A (n=15)
B (n=16)
C (n=14)
surface

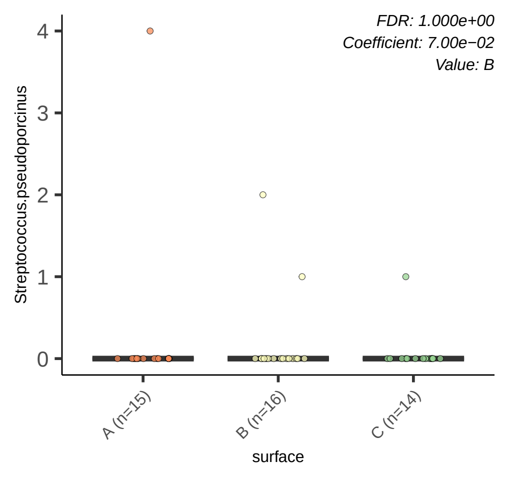
FDR: 1.000e+00
Coefficient: 7.00e−02
Value: B
Streptococcus.pseudoporcinus
4
3
2
1
0
A (n=15)
B (n=16)
C (n=14)
surface

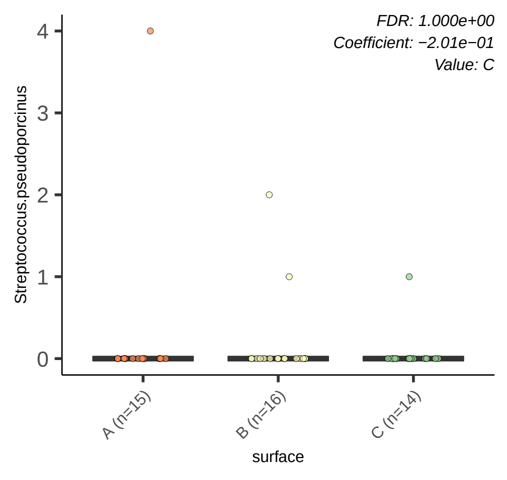
FDR: 1.000e+00
Coefficient: −2.01e−01
Value: C
Streptococcus.pseudoporcinus
4
3
2
1
0
A (n=15)
B (n=16)
C (n=14)
surface

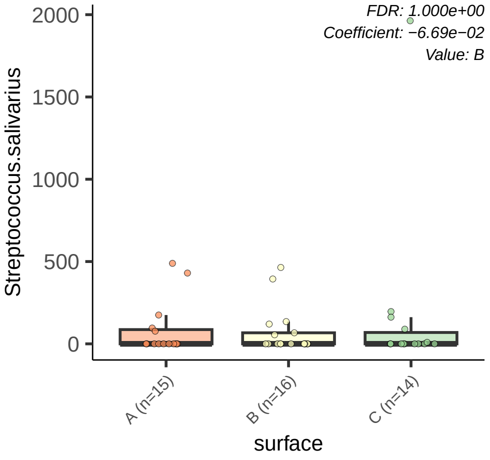
FDR: 1.000e+00
Coefficient: −6.69e−02
Value: B
Streptococcus.salivarius
2000
1500
1000
500
0
A (n=15)
B (n=16)
C (n=14)
surface

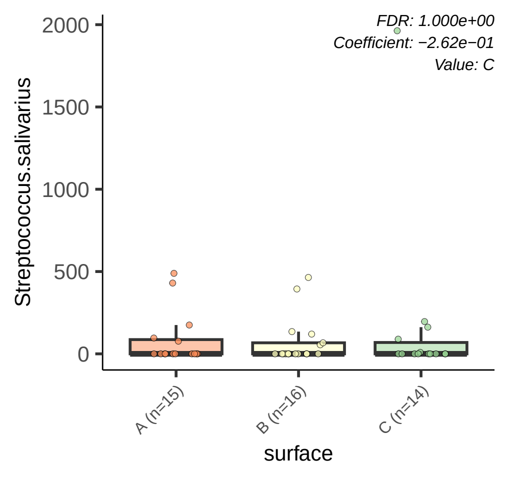
FDR: 1.000e+00
Coefficient: −2.62e−01
Value: C
Streptococcus.salivarius
2000
1500
1000
500
0
A (n=15)
B (n=16)
C (n=14)
surface

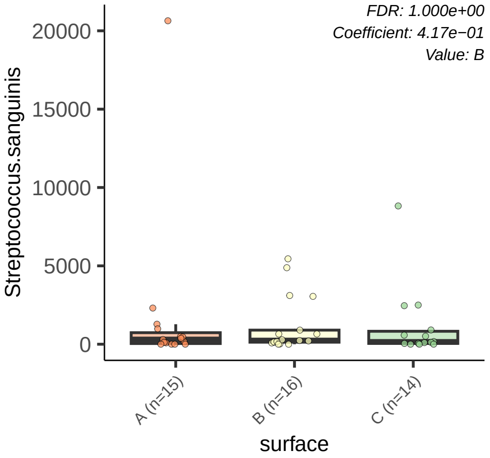

FDR: 1.000e+00
Coefficient: 4.17e−01
Value: B
Streptococcus.sanguinis
20000
15000
10000
5000
0
A (n=15)
B (n=16)
C (n=14)
surface

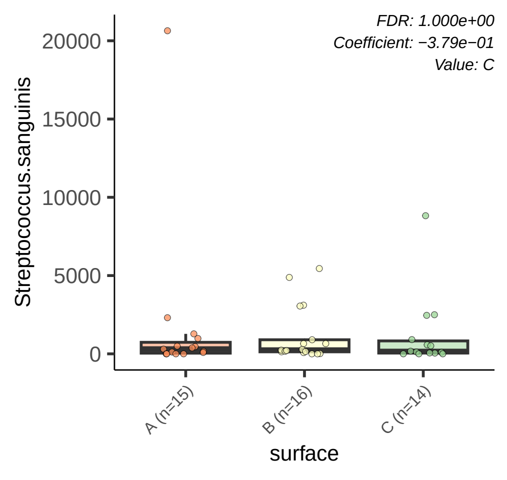

FDR: 1.000e+00
Coefficient: −3.79e−01
Value: C
Streptococcus.sanguinis
20000
15000
10000
5000
0
A (n=15)
B (n=16)
C (n=14)
surface

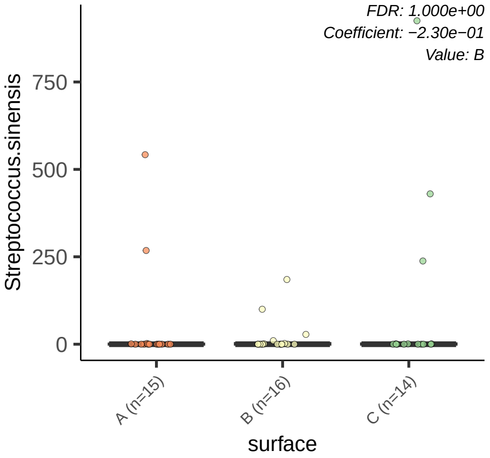

FDR: 1.000e+00
Coefficient: −2.30e−01
Value: B
Streptococcus.sinensis
750
500
250
0
A (n=15)
B (n=16)
C (n=14)
surface

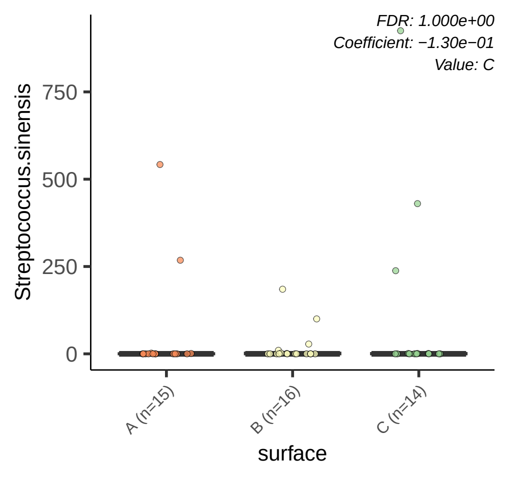

FDR: 1.000e+00
Coefficient: −1.30e−01
Value: C
Streptococcus.sinensis
750
500
250
0
A (n=15)
B (n=16)
C (n=14)
surface

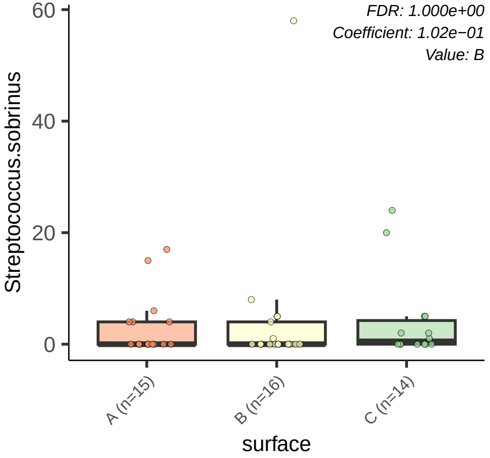

FDR: 1.000e+00
Coefficient: 1.02e−01
Value: B
Streptococcus.sobrinus
0
20
40
60
A (n=15)
B (n=16)
C (n=14)
surface

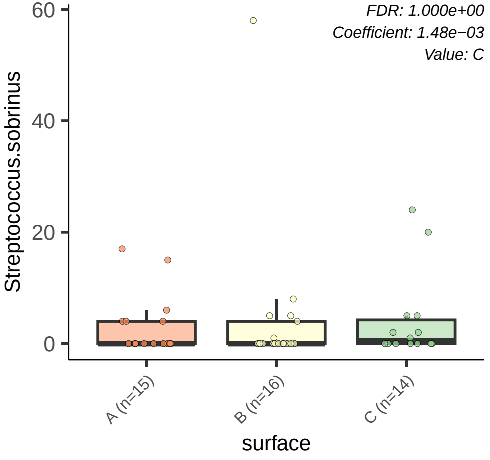

FDR: 1.000e+00
Coefficient: 1.48e−03
Value: C
Streptococcus.sobrinus
60
40
20
0
A (n=15)
B (n=16)
C (n=14)
surface

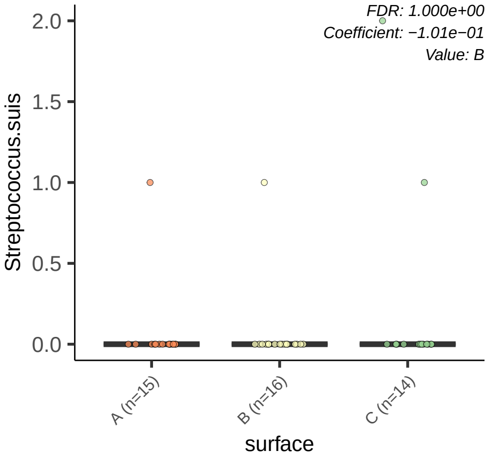
FDR: 1.000e+00
Coefficient: −1.01e−01
Value: B
Streptococcus.suis
2.0
1.5
1.0
0.5
0.0
A (n=15)
B (n=16)
C (n=14)
surface

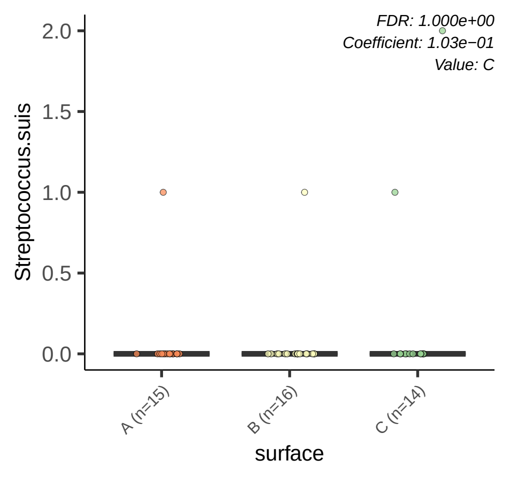

FDR: 1.000e+00
Coefficient: 1.03e−01
Value: C
Streptococcus.suis
2.0
1.5
1.0
0.5
0.0
A (n=15)
B (n=16)
C (n=14)
surface

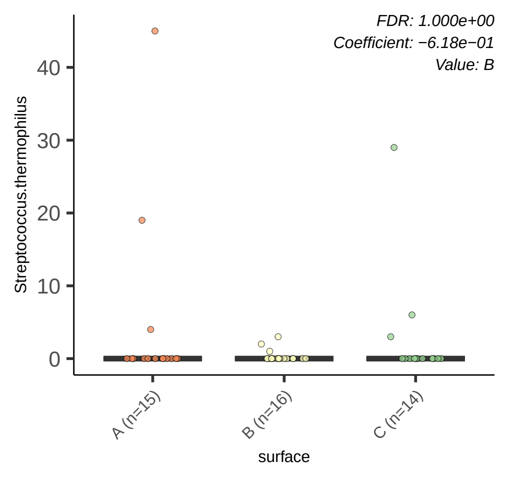

FDR: 1.000e+00
Coefficient: −6.18e−01
Value: B
Streptococcus.thermophilus
40
30
20
10
0
A (n=15)
B (n=16)
C (n=14)
surface

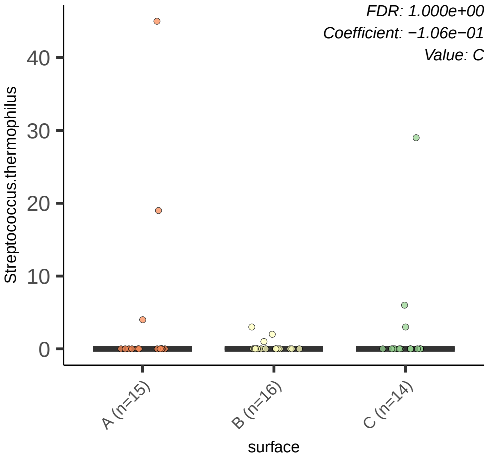

FDR: 1.000e+00
Coefficient: −1.06e−01
Value: C
Streptococcus.thermophilus
40
30
20
10
0
A (n=15)
B (n=16)
C (n=14)
surface

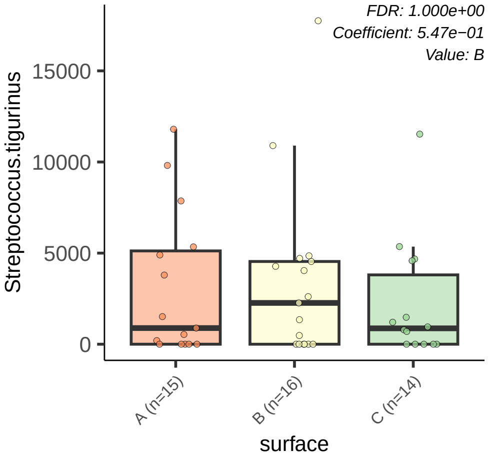
FDR: 1.000e+00
Coefficient: 5.47e−01
Value: B
Streptococcus.tigurinus
15000
10000
5000
0
A (n=15)
B (n=16)
C (n=14)
surface

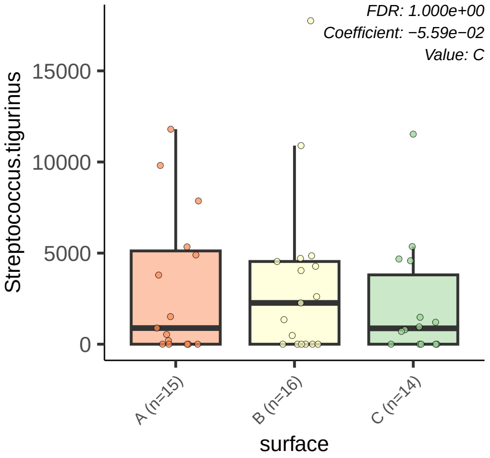
FDR: 1.000e+00
Coefficient: −5.59e−02
Value: C
Streptococcus.tigurinus
15000
10000
5000
0
A (n=15)
B (n=16)
C (n=14)
surface

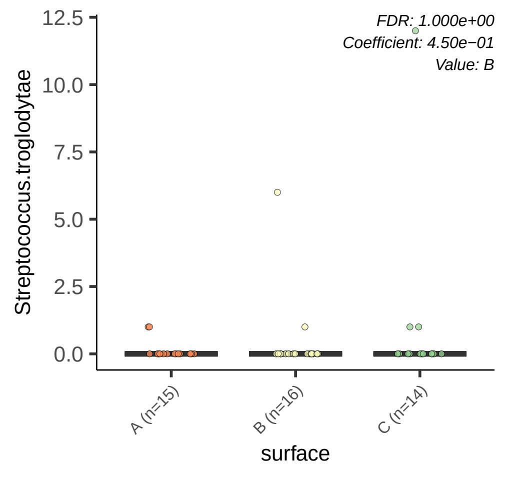

FDR: 1.000e+00
Coefficient: 4.50e−01
Value: B
Streptococcus.troglodytae
12.5
10.0
7.5
5.0
2.5
0.0
A (n=15)
B (n=16)
C (n=14)
surface

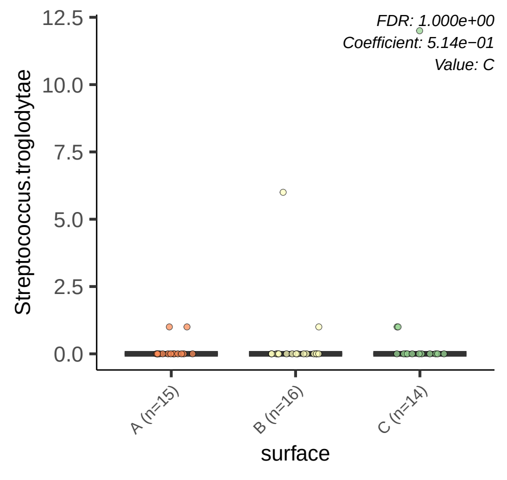

FDR: 1.000e+00
Coefficient: 5.14e−01
Value: C
Streptococcus.troglodytae
12.5
10.0
7.5
5.0
2.5
0.0
A (n=15)
B (n=16)
C (n=14)
surface

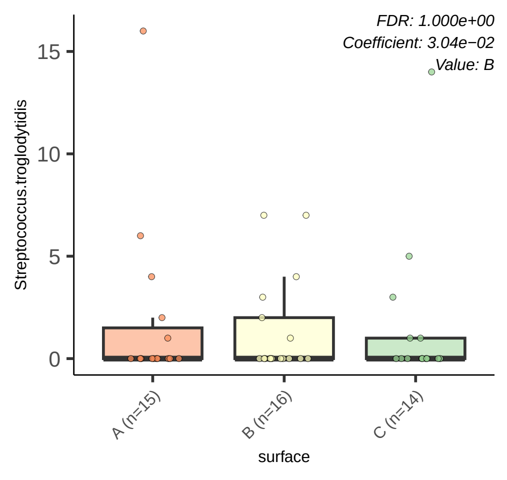

FDR: 1.000e+00
Coefficient: 3.04e−02
Value: B
Streptococcus.troglodytidis
0
5
10
15
A (n=15)
B (n=16)
C (n=14)
surface

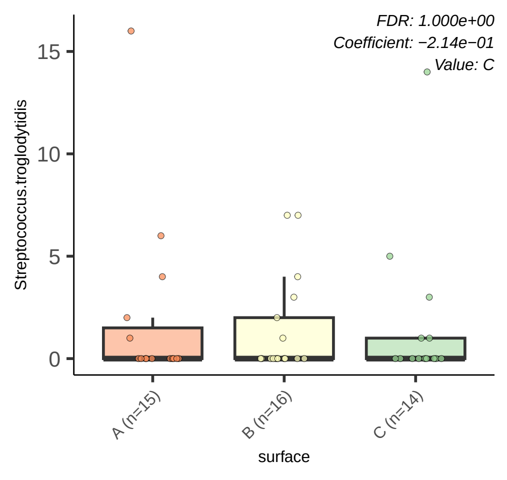
FDR: 1.000e+00
Coefficient: −2.14e−01
Value: C
Streptococcus.troglodytidis
15
10
5
0
A (n=15)
B (n=16)
C (n=14)
surface

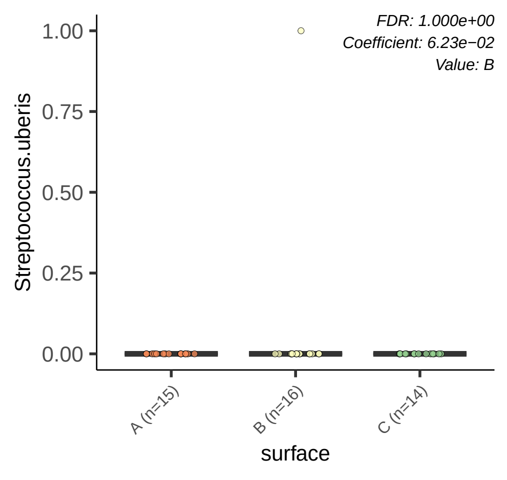
FDR: 1.000e+00
Coefficient: 6.23e−02
Value: B
Streptococcus.uberis
1.00
0.75
0.50
0.25
0.00
A (n=15)
B (n=16)
C (n=14)
surface

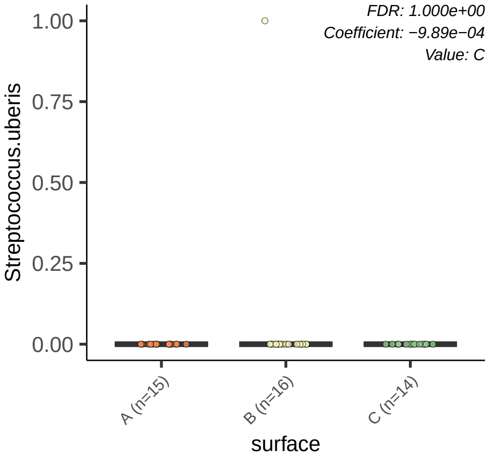
FDR: 1.000e+00
Coefficient: −9.89e−04
Value: C
Streptococcus.uberis
1.00
0.75
0.50
0.25
0.00
A (n=15)
B (n=16)
C (n=14)
surface

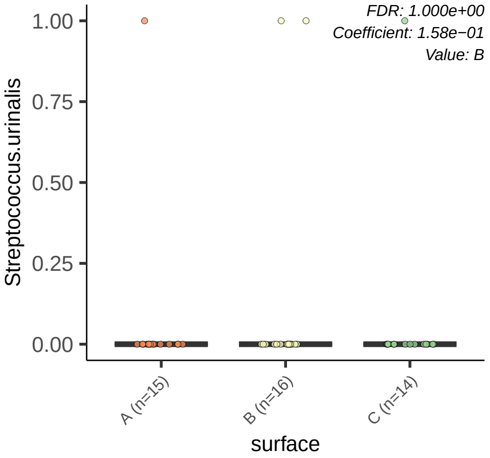

FDR: 1.000e+00
Coefficient: 1.58e−01
Value: B
Streptococcus.urinalis
1.00
0.75
0.50
0.25
0.00
A (n=15)
B (n=16)
C (n=14)
surface

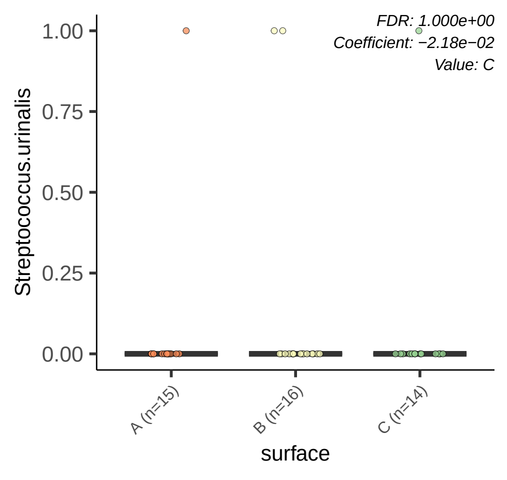
FDR: 1.000e+00
Coefficient: −2.18e−02
Value: C
Streptococcus.urinalis
1.00
0.75
0.50
0.25
0.00
A (n=15)
B (n=16)
C (n=14)
surface

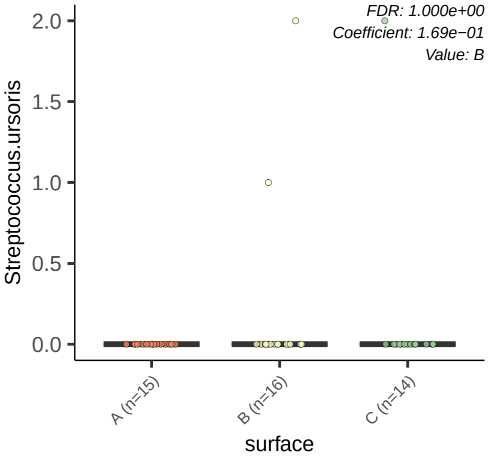
FDR: 1.000e+00
Coefficient: 1.69e−01
Value: B
Streptococcus.ursoris
2.0
1.5
1.0
0.5
0.0
A (n=15)
B (n=16)
C (n=14)
surface

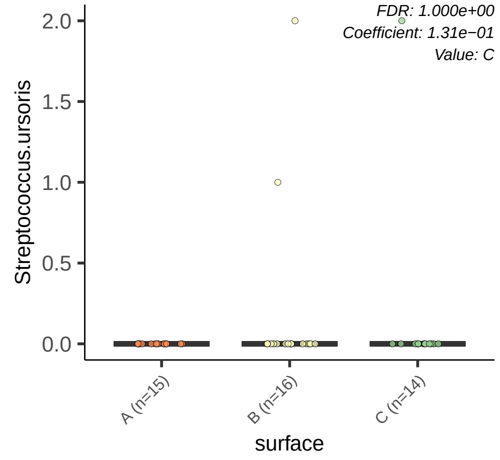

FDR: 1.000e+00
Coefficient: 1.31e−01
Value: C
Streptococcus.ursoris
2.0
1.5
1.0
0.5
0.0
A (n=15)
B (n=16)
C (n=14)
surface

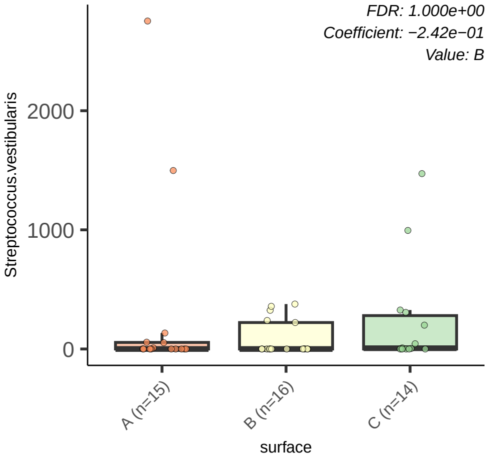

FDR: 1.000e+00
Coefficient: −2.42e−01
Value: B
Streptococcus.vestibularis
2000
1000
0
A (n=15)
B (n=16)
C (n=14)
surface

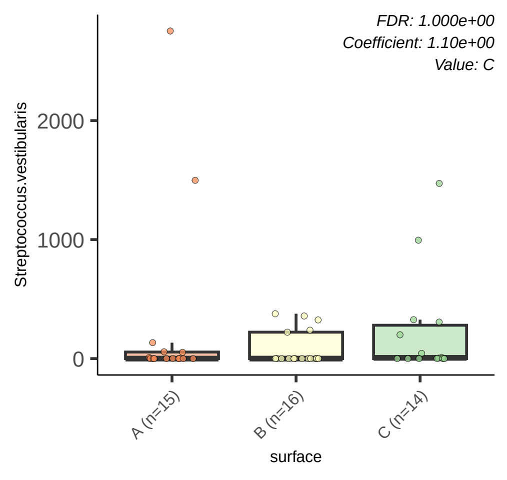
FDR: 1.000e+00
Coefficient: 1.10e+00
Value: C
Streptococcus.vestibularis
2000
1000
0
A (n=15)
B (n=16)
C (n=14)
surface

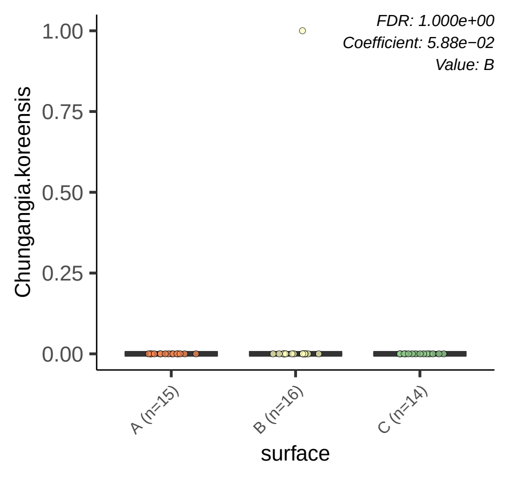
FDR: 1.000e+00
Coefficient: 5.88e−02
Value: B
1.00
0.75
0.50
0.25
0.00
Chungangia.koreensis
A (n=15)
B (n=16)
C (n=14)
surface

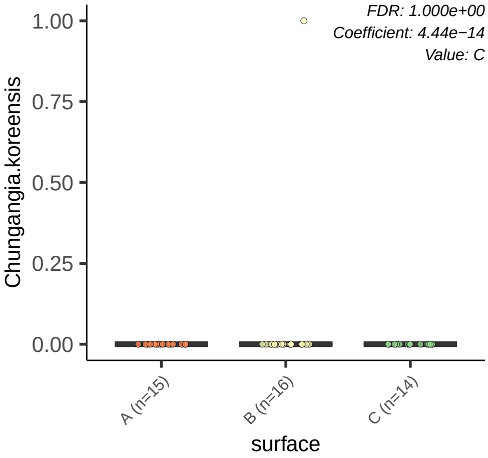

FDR: 1.000e+00
Coefficient: 4.44e−14
Value: C
Chungangia.koreensis
1.00
0.75
0.50
0.25
0.00
A (n=15)
B (n=16)
C (n=14)
surface

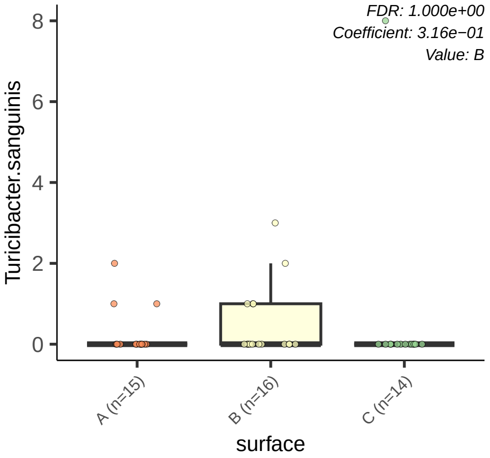
FDR: 1.000e+00
Coefficient: 3.16e−01
Value: B
Turicibacter.sanguinis
8
6
4
2
0
A (n=15)
B (n=16)
C (n=14)
surface

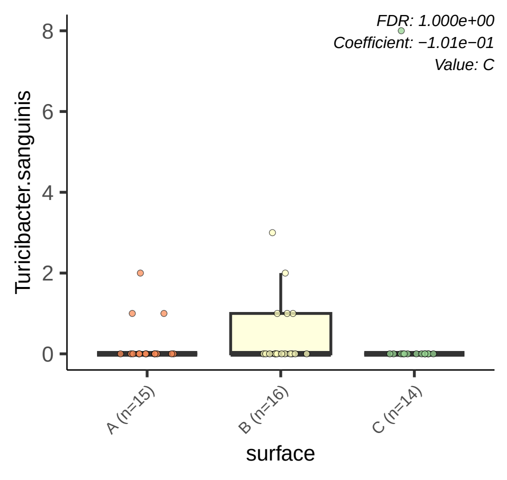
FDR: 1.000e+00
Coefficient: −1.01e−01
Value: C
Turicibacter.sanguinis
8
6
4
2
0
A (n=15)
B (n=16)
C (n=14)
surface

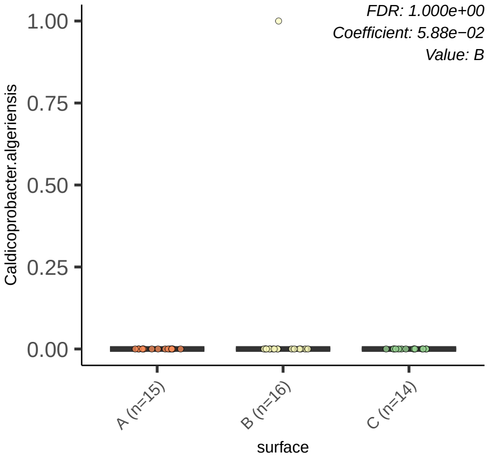
FDR: 1.000e+00
Coefficient: 5.88e−02
Value: B
Caldicoprobacter.algeriensis
1.00
0.75
0.50
0.25
0.00
A (n=15)
B (n=16)
C (n=14)
surface

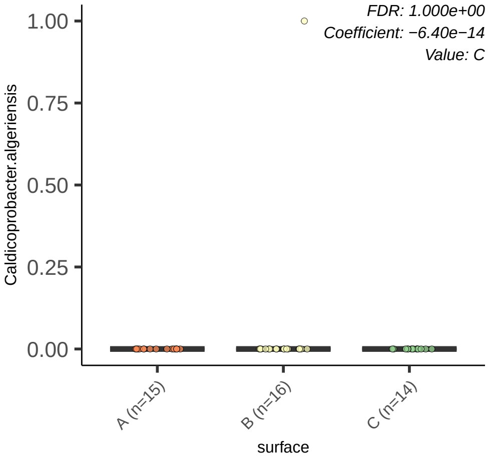

FDR: 1.000e+00
Coefficient: −6.40e−14
Value: C
1.00
0.75
0.50
0.25
0.00
Caldicoprobacter.algeriensis
A (n=15)
B (n=16)
C (n=14)
surface

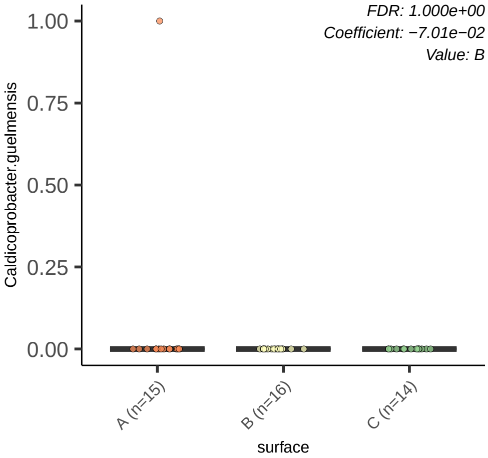
FDR: 1.000e+00
Coefficient: −7.01e−02
Value: B
Caldicoprobacter.guelmensis
1.00
0.75
0.50
0.25
0.00
A (n=15)
B (n=16)
C (n=14)
surface

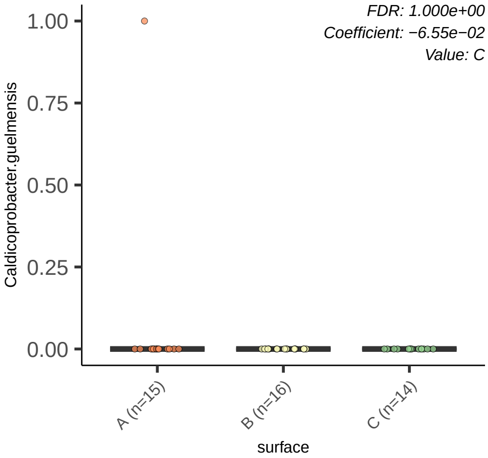
FDR: 1.000e+00
Coefficient: −6.55e−02
Value: C
Caldicoprobacter.guelmensis
1.00
0.75
0.50
0.25
0.00
A (n=15)
B (n=16)
C (n=14)
surface

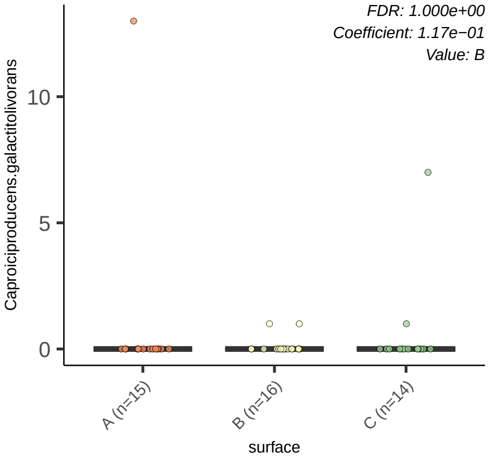

FDR: 1.000e+00
Coefficient: 1.17e−01
Value: B
Caproiciproducens.galactitolivorans
10
5
0
A (n=15)
B (n=16)
C (n=14)
surface

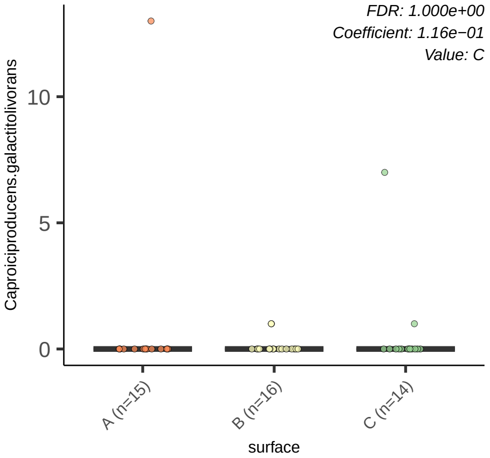
FDR: 1.000e+00
Coefficient: 1.16e−01
Value: C
Caproiciproducens.galactitolivorans
10
5
0
A (n=15)
B (n=16)
C (n=14)
surface

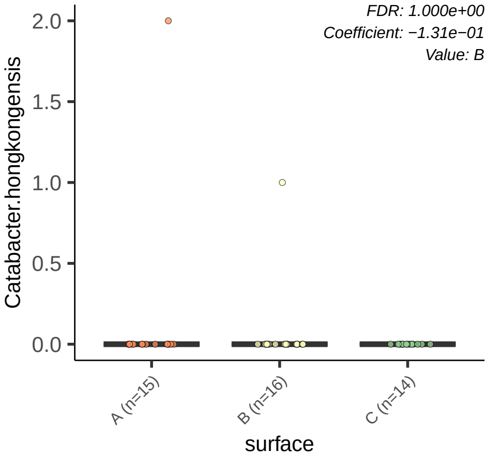
FDR: 1.000e+00
Coefficient: −1.31e−01
Value: B
Catabacter.hongkongensis
2.0
1.5
1.0
0.5
0.0
A (n=15)
B (n=16)
C (n=14)
surface

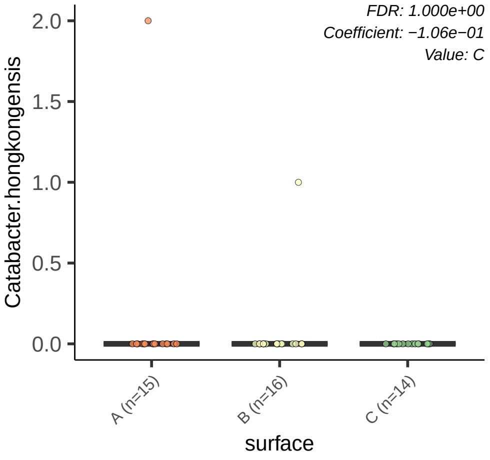

FDR: 1.000e+00
Coefficient: −1.06e−01
Value: C
Catabacter.hongkongensis
2.0
1.5
1.0
0.5
0.0
A (n=15)
B (n=16)
C (n=14)
surface

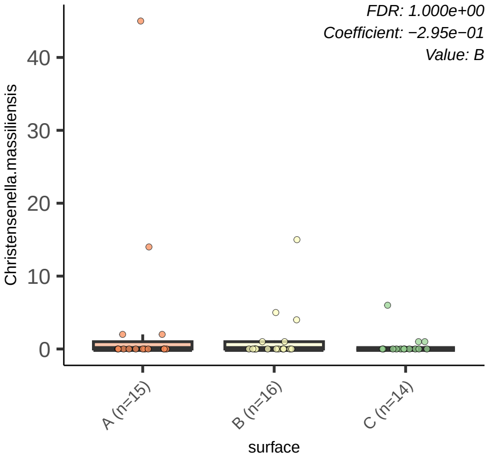

FDR: 1.000e+00
Coefficient: −2.95e−01
Value: B
Christensenella.massiliensis
40
30
20
10
0
A (n=15)
B (n=16)
C (n=14)
surface

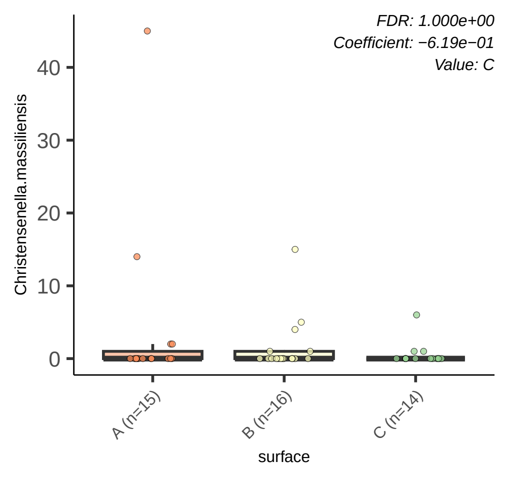

FDR: 1.000e+00
Coefficient: −6.19e−01
Value: C
Christensenella.massiliensis
40
30
20
10
0
A (n=15)
B (n=16)
C (n=14)
surface

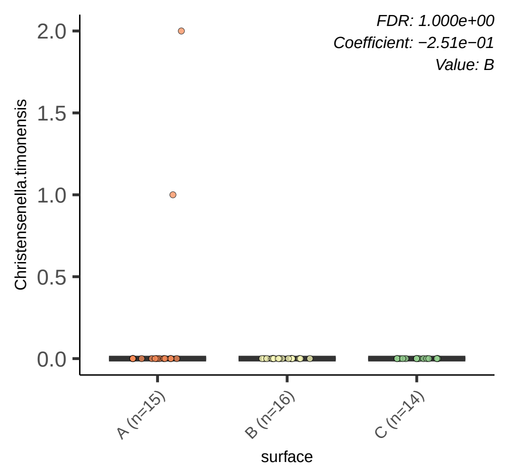
FDR: 1.000e+00
Coefficient: −2.51e−01
Value: B
Christensenella.timonensis
2.0
1.5
1.0
0.5
0.0
A (n=15)
B (n=16)
C (n=14)
surface

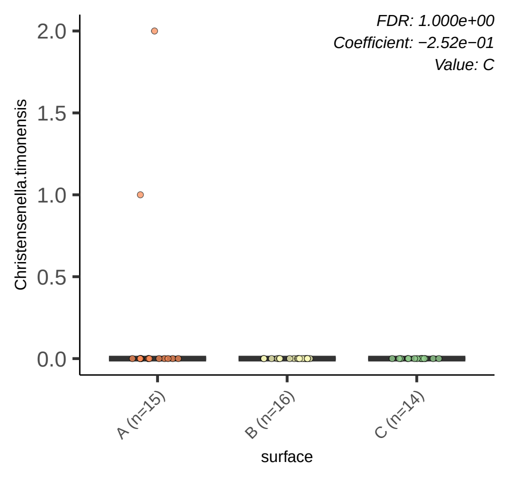
FDR: 1.000e+00
Coefficient: −2.52e−01
Value: C
Christensenella.timonensis
2.0
1.5
1.0
0.5
0.0
A (n=15)
B (n=16)
C (n=14)
surface

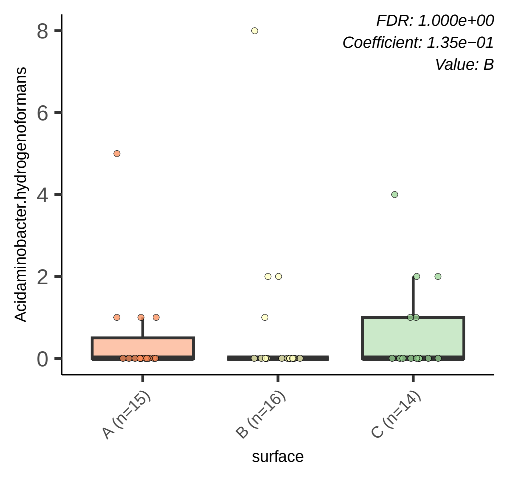
FDR: 1.000e+00
Coefficient: 1.35e−01
Value: B
Acidaminobacter.hydrogenoformans
0
2
4
6
8
A (n=15)
B (n=16)
C (n=14)
surface

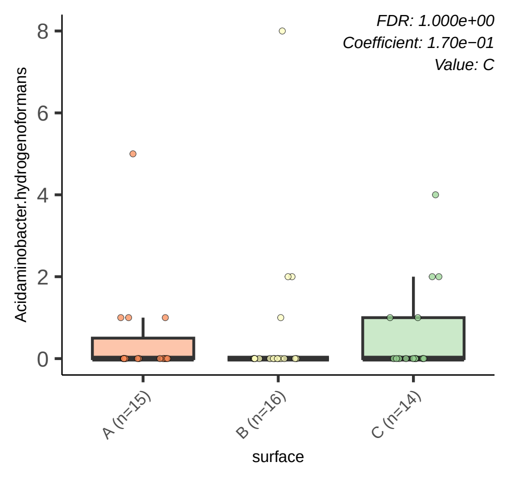

FDR: 1.000e+00
Coefficient: 1.70e−01
Value: C
Acidaminobacter.hydrogenoformans
8
6
4
2
0
A (n=15)
B (n=16)
C (n=14)
surface

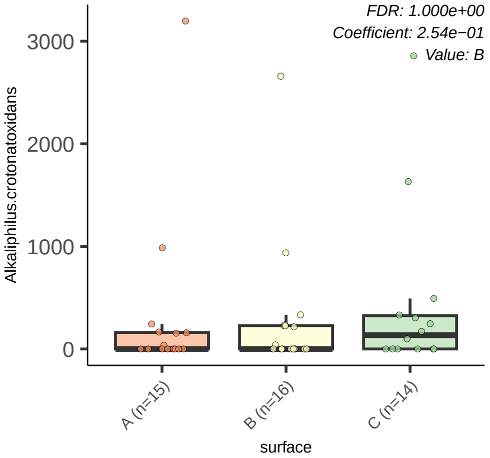
FDR: 1.000e+00
Coefficient: 2.54e−01
Value: B
Alkaliphilus.crotonatoxidans
3000
2000
1000
0
A (n=15)
B (n=16)
C (n=14)
surface

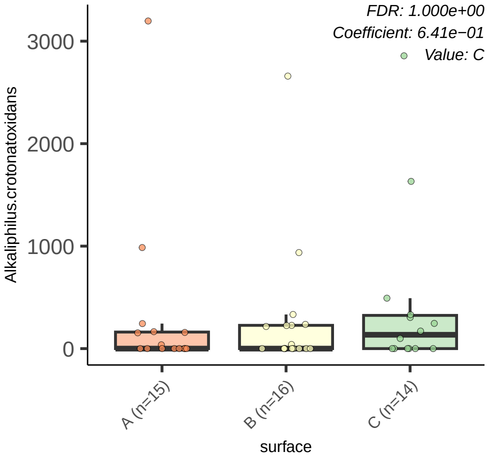

FDR: 1.000e+00
Coefficient: 6.41e−01
Value: C
3000
2000
1000
0
Alkaliphilus.crotonatoxidans
A (n=15)
B (n=16)
C (n=14)
surface

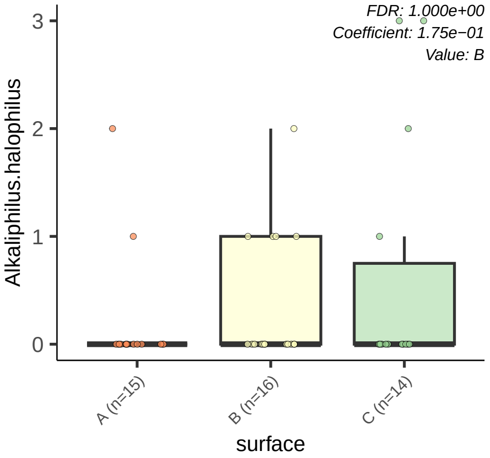

FDR: 1.000e+00
Coefficient: 1.75e−01
Value: B
Alkaliphilus.halophilus
0
1
2
3
A (n=15)
B (n=16)
C (n=14)
surface

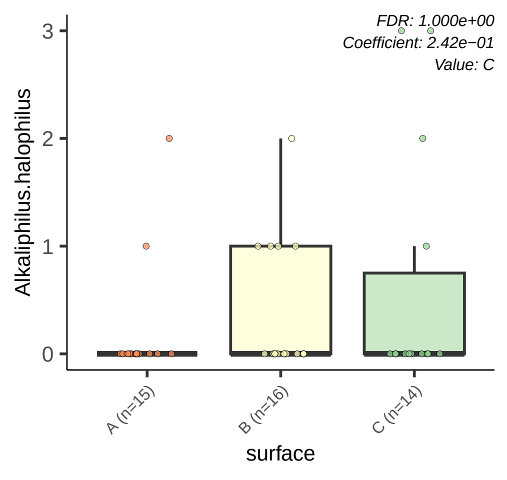

FDR: 1.000e+00
Coefficient: 2.42e−01
Value: C
Alkaliphilus.halophilus
3
2
1
0
A (n=15)
B (n=16)
C (n=14)
surface

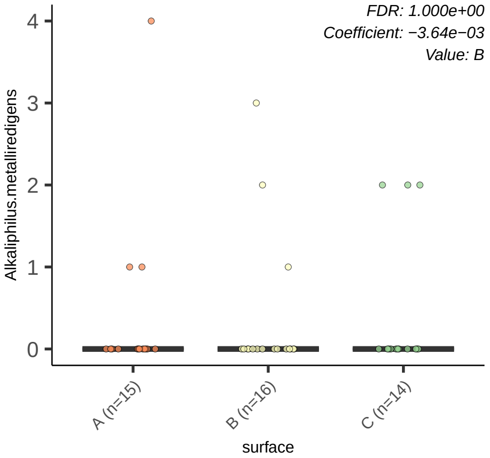
FDR: 1.000e+00
Coefficient: −3.64e−03
Value: B
Alkaliphilus.metalliredigens
0
1
2
3
4
A (n=15)
B (n=16)
C (n=14)
surface

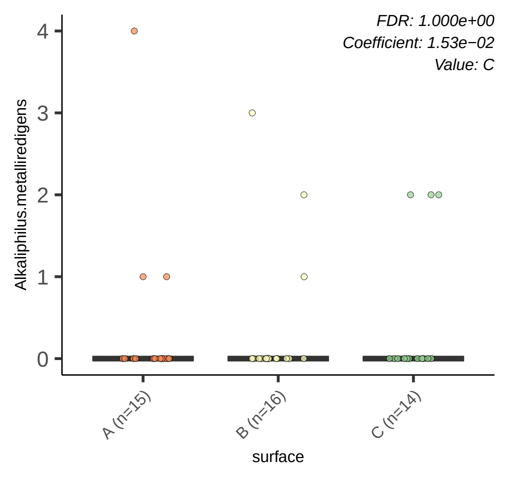
FDR: 1.000e+00
Coefficient: 1.53e−02
Value: C
Alkaliphilus.metalliredigens
4
3
2
1
0
A (n=15)
B (n=16)
C (n=14)
surface

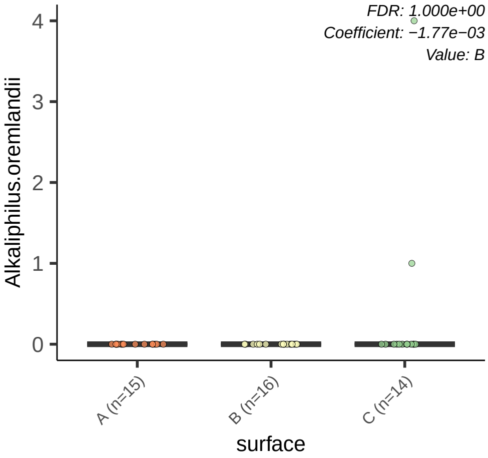

FDR: 1.000e+00
Coefficient: −1.77e−03
Value: B
Alkaliphilus.oremlandii
0
1
2
3
4
A (n=15)
B (n=16)
C (n=14)
surface

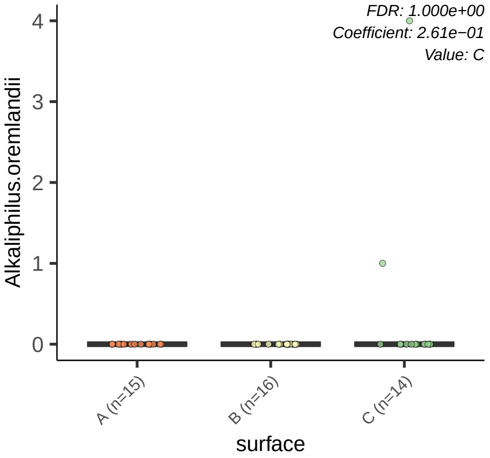

FDR: 1.000e+00
Coefficient: 2.61e−01
Value: C
Alkaliphilus.oremlandii
0
1
2
3
4
A (n=15)
B (n=16)
C (n=14)
surface

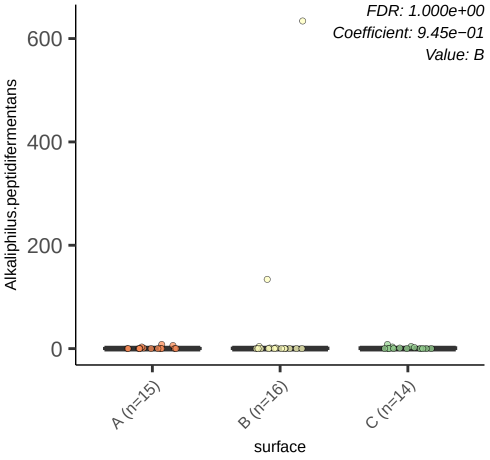

FDR: 1.000e+00
Coefficient: 9.45e−01
Value: B
Alkaliphilus.peptidifermentans
600
400
200
0
A (n=15)
B (n=16)
C (n=14)
surface

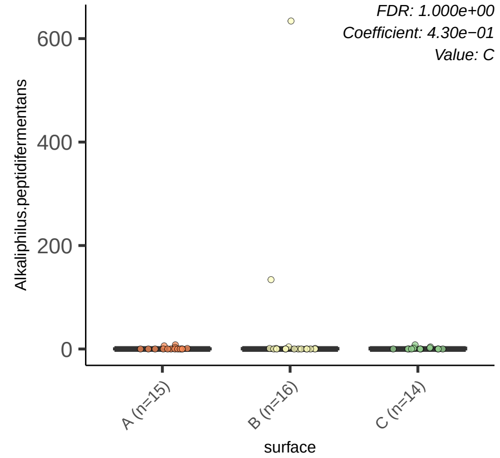
FDR: 1.000e+00
Coefficient: 4.30e−01
Value: C
Alkaliphilus.peptidifermentans
600
400
200
0
A (n=15)
B (n=16)
C (n=14)
surface

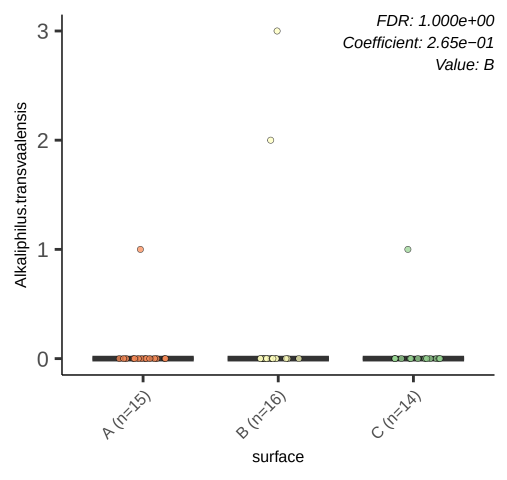

FDR: 1.000e+00
Coefficient: 2.65e−01
Value: B
Alkaliphilus.transvaalensis
3
2
1
0
A (n=15)
B (n=16)
C (n=14)
surface

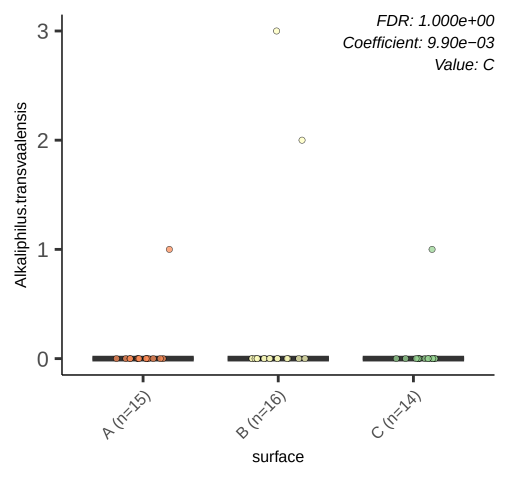

FDR: 1.000e+00
Coefficient: 9.90e−03
Value: C
Alkaliphilus.transvaalensis
3
2
1
0
A (n=15)
B (n=16)
C (n=14)
surface

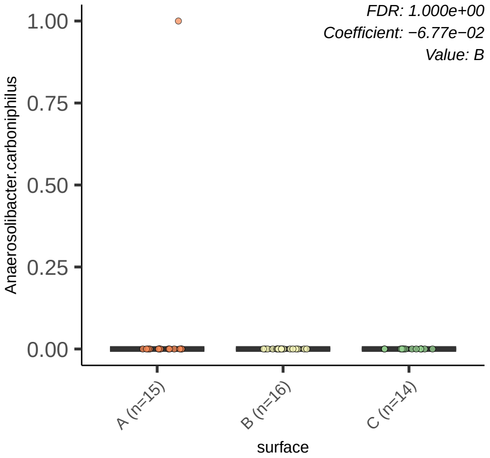

FDR: 1.000e+00
Coefficient: −6.77e−02
Value: B
1.00
0.75
0.50
0.25
0.00
Anaerosolibacter.carboniphilus
A (n=15)
B (n=16)
C (n=14)
surface

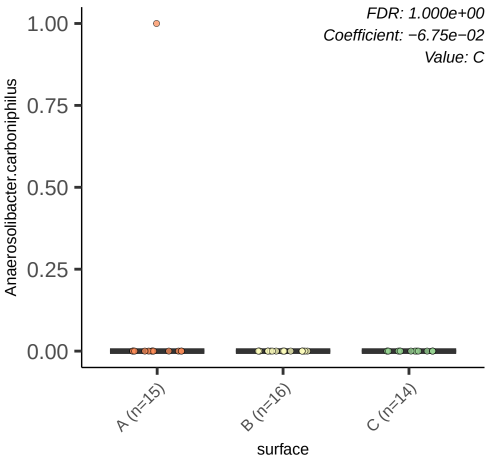
FDR: 1.000e+00
Coefficient: −6.75e−02
Value: C
Anaerosolibacter.carboniphilus
1.00
0.75
0.50
0.25
0.00
A (n=15)
B (n=16)
C (n=14)
surface

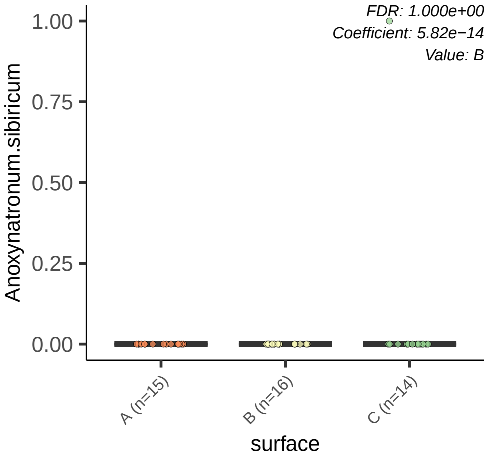

FDR: 1.000e+00
Coefficient: 5.82e−14
Value: B
1.00
0.75
0.50
0.25
0.00
Anoxynatronum.sibiricum
A (n=15)
B (n=16)
C (n=14)
surface

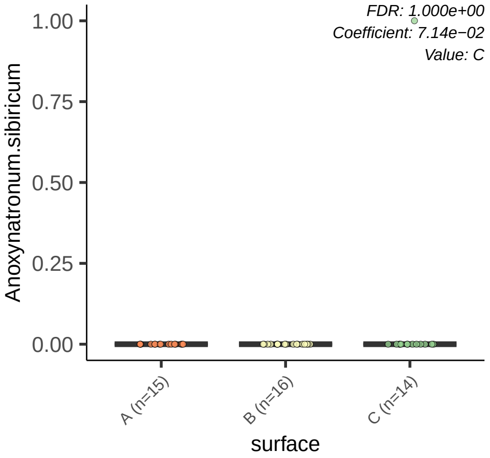
FDR: 1.000e+00
Coefficient: 7.14e−02
Value: C
1.00
0.75
0.50
0.25
0.00
Anoxynatronum.sibiricum
A (n=15)
B (n=16)
C (n=14)
surface

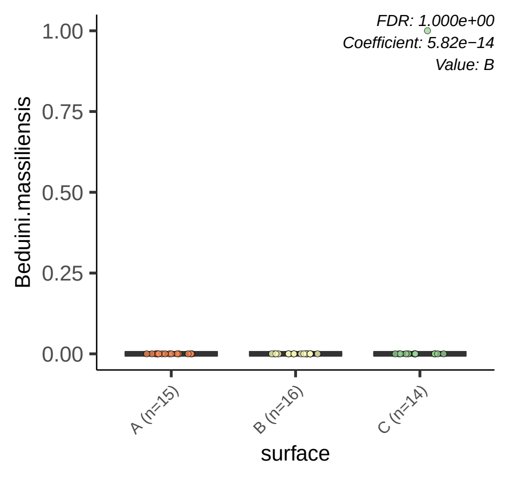
FDR: 1.000e+00
Coefficient: 5.82e−14
Value: B
Beduini.massiliensis
1.00
0.75
0.50
0.25
0.00
A (n=15)
B (n=16)
C (n=14)
surface

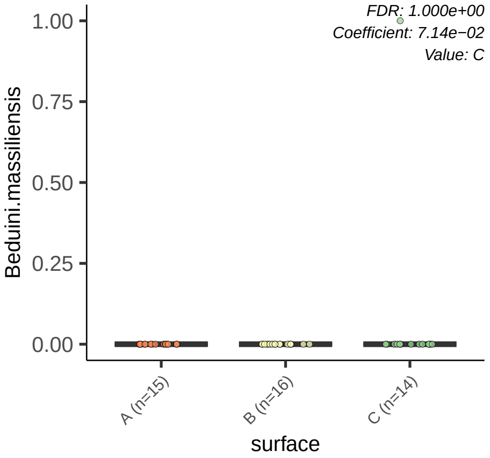
FDR: 1.000e+00
Coefficient: 7.14e−02
Value: C
1.00
0.75
0.50
0.25
0.00
Beduini.massiliensis
A (n=15)
B (n=16)
C (n=14)
surface

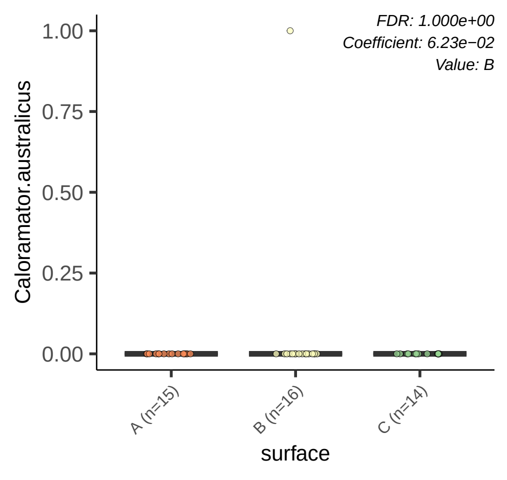

FDR: 1.000e+00
Coefficient: 6.23e−02
Value: B
Caloramator.australicus
1.00
0.75
0.50
0.25
0.00
A (n=15)
B (n=16)
C (n=14)
surface

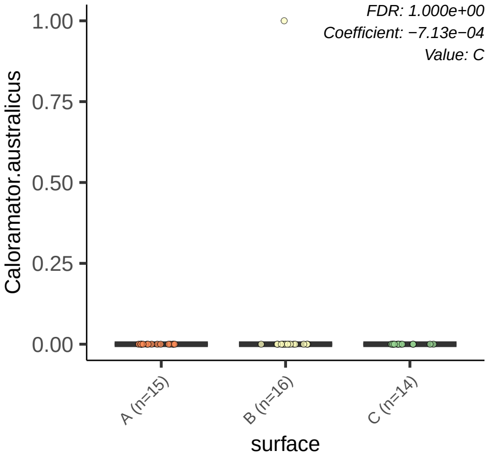
FDR: 1.000e+00
Coefficient: −7.13e−04
Value: C
Caloramator.australicus
1.00
0.75
0.50
0.25
0.00
A (n=15)
B (n=16)
C (n=14)
surface

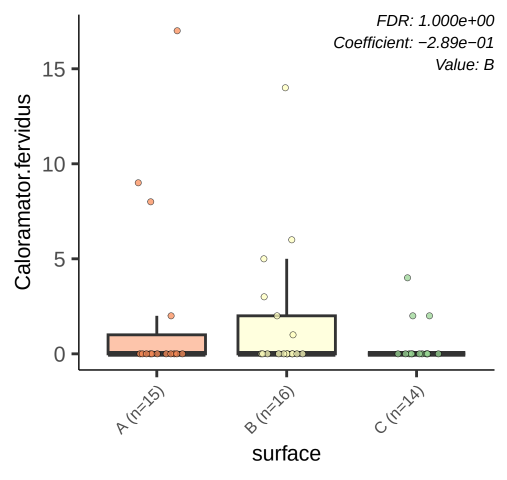
FDR: 1.000e+00
Coefficient: −2.89e−01
Value: B
Caloramator.fervidus
0
5
10
15
A (n=15)
B (n=16)
C (n=14)
surface

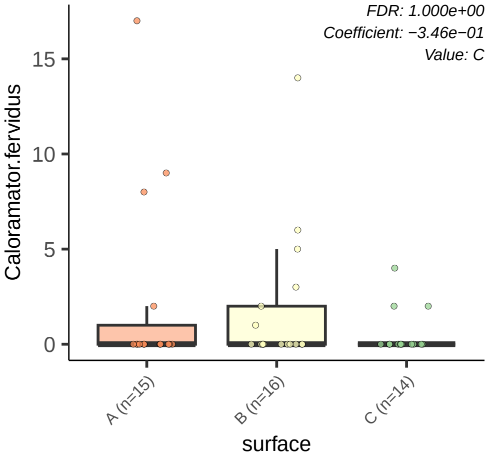
FDR: 1.000e+00
Coefficient: −3.46e−01
Value: C
Caloramator.fervidus
15
10
5
0
A (n=15)
B (n=16)
C (n=14)
surface

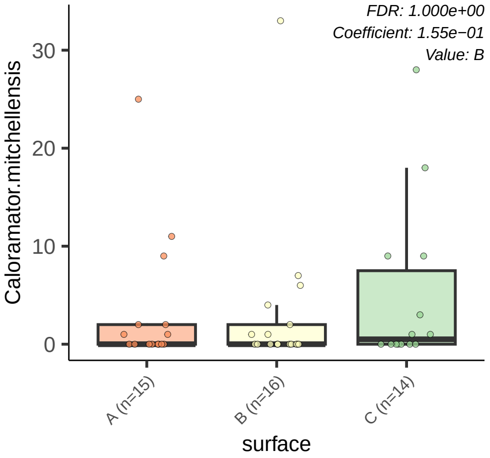

FDR: 1.000e+00
Coefficient: 1.55e−01
Value: B
Caloramator.mitchellensis
0
10
20
30
A (n=15)
B (n=16)
C (n=14)
surface

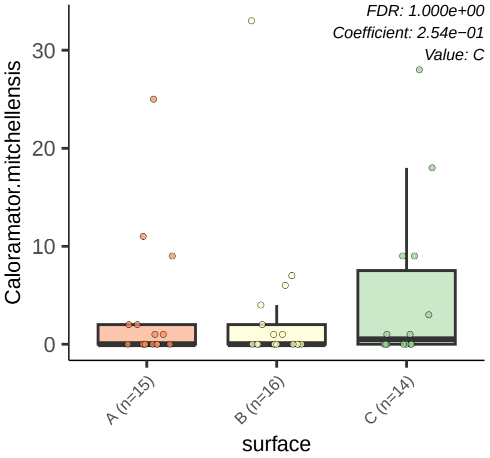

FDR: 1.000e+00
Coefficient: 2.54e−01
Value: C
Caloramator.mitchellensis
0
10
20
30
A (n=15)
B (n=16)
C (n=14)
surface

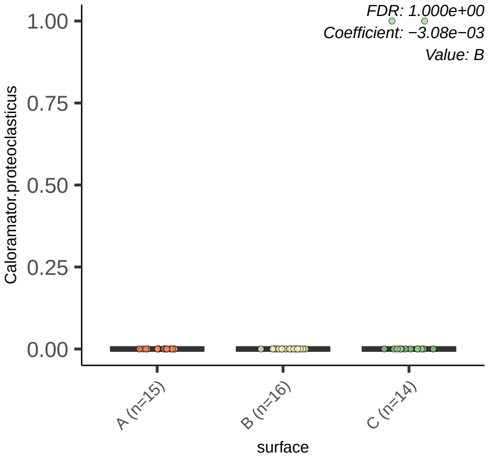
FDR: 1.000e+00
Coefficient: −3.08e−03
Value: B
1.00
0.75
0.50
0.25
0.00
Caloramator.proteoclasticus
A (n=15)
B (n=16)
C (n=14)
surface

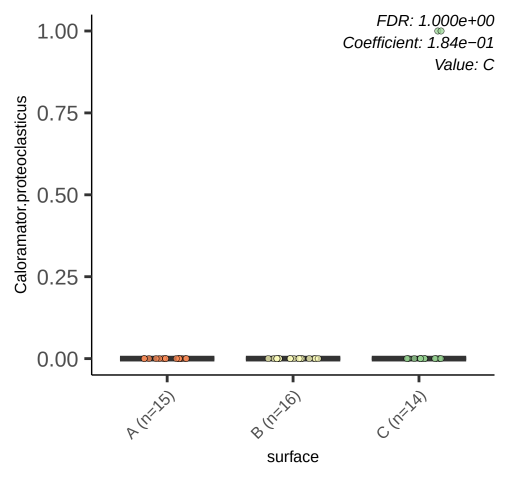

FDR: 1.000e+00
Coefficient: 1.84e−01
Value: C
1.00
0.75
0.50
0.25
0.00
Caloramator.proteoclasticus
A (n=15)
B (n=16)
C (n=14)
surface

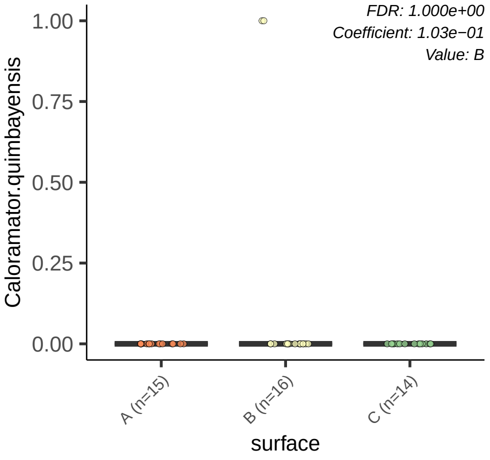

FDR: 1.000e+00
Coefficient: 1.03e−01
Value: B
Caloramator.quimbayensis
1.00
0.75
0.50
0.25
0.00
A (n=15)
B (n=16)
C (n=14)
surface

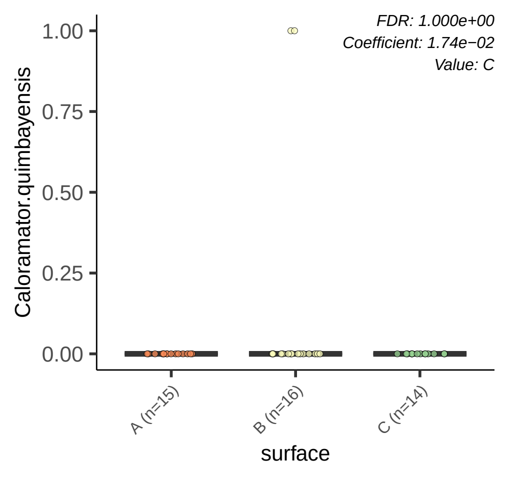
FDR: 1.000e+00
Coefficient: 1.74e−02
Value: C
Caloramator.quimbayensis
1.00
0.75
0.50
0.25
0.00
A (n=15)
B (n=16)
C (n=14)
surface

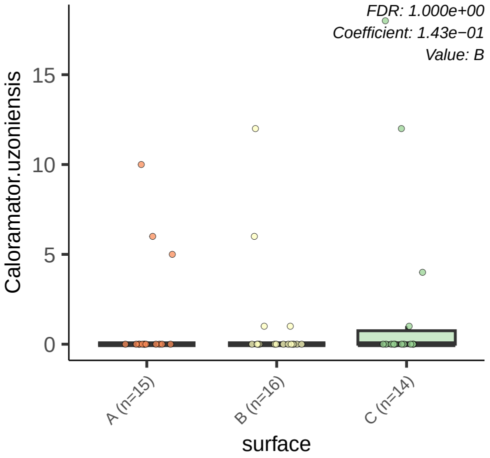
FDR: 1.000e+00
Coefficient: 1.43e−01
Value: B
Caloramator.uzoniensis
15
10
5
0
A (n=15)
B (n=16)
C (n=14)
surface

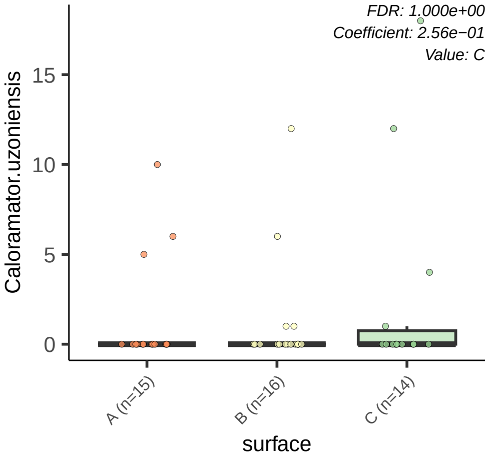

FDR: 1.000e+00
Coefficient: 2.56e−01
Value: C
Caloramator.uzoniensis
0
5
10
15
A (n=15)
B (n=16)
C (n=14)
surface

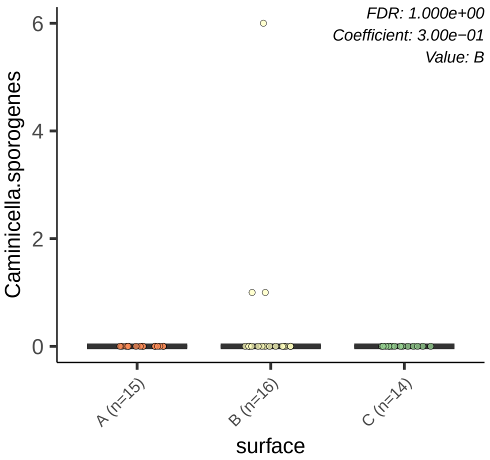
FDR: 1.000e+00
Coefficient: 3.00e−01
Value: B
Caminicella.sporogenes
0
2
4
6
A (n=15)
B (n=16)
C (n=14)
surface

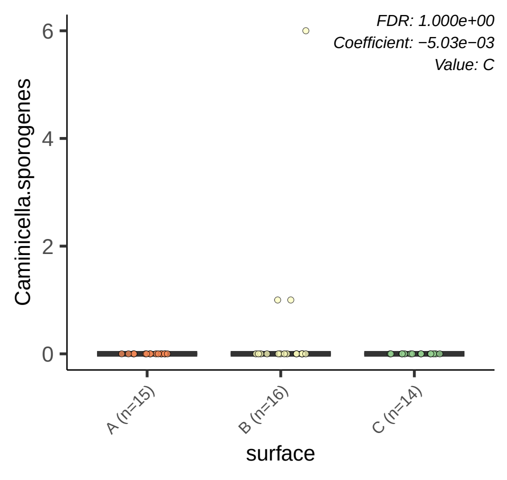

FDR: 1.000e+00
Coefficient: −5.03e−03
Value: C
Caminicella.sporogenes
6
4
2
0
A (n=15)
B (n=16)
C (n=14)
surface

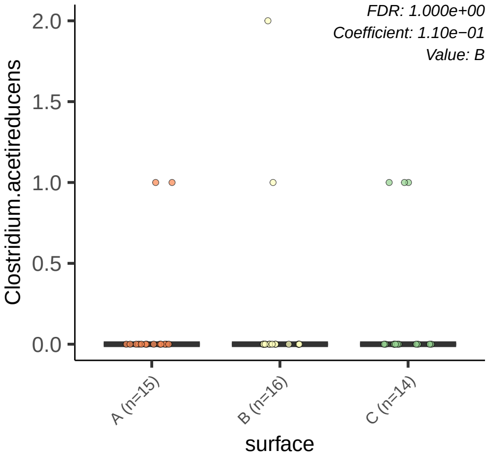

FDR: 1.000e+00
Coefficient: 1.10e−01
Value: B
Clostridium.acetireducens
2.0
1.5
1.0
0.5
0.0
A (n=15)
B (n=16)
C (n=14)
surface

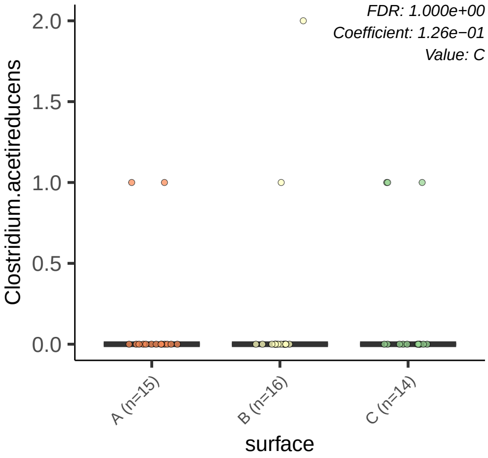

FDR: 1.000e+00
Coefficient: 1.26e−01
Value: C
Clostridium.acetireducens
2.0
1.5
1.0
0.5
0.0
A (n=15)
B (n=16)
C (n=14)
surface

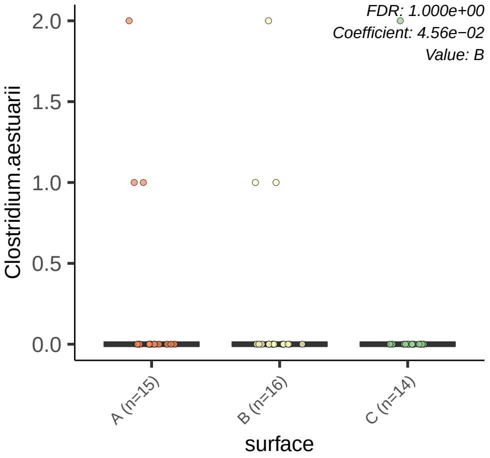

FDR: 1.000e+00
Coefficient: 4.56e−02
Value: B
Clostridium.aestuarii
2.0
1.5
1.0
0.5
0.0
A (n=15)
B (n=16)
C (n=14)
surface

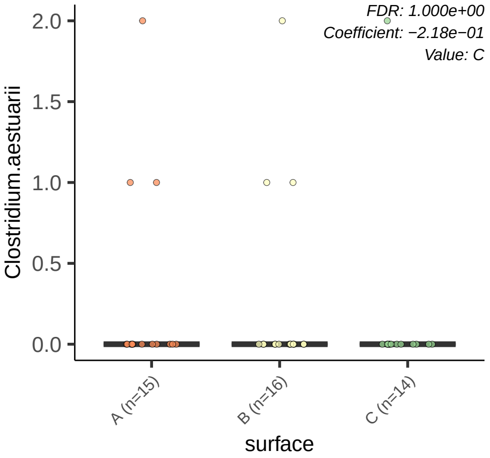

FDR: 1.000e+00
Coefficient: −2.18e−01
Value: C
Clostridium.aestuarii
2.0
1.5
1.0
0.5
0.0
A (n=15)
B (n=16)
C (n=14)
surface

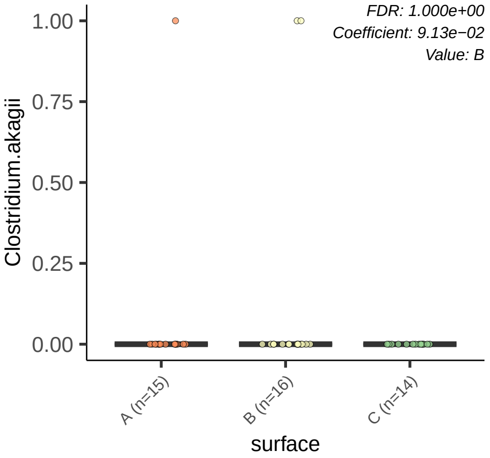
FDR: 1.000e+00
Coefficient: 9.13e−02
Value: B
1.00
0.75
0.50
0.25
0.00
Clostridium.akagii
A (n=15)
B (n=16)
C (n=14)
surface

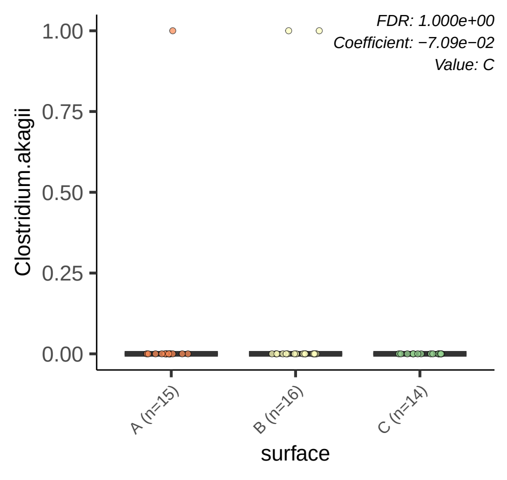
FDR: 1.000e+00
Coefficient: −7.09e−02
Value: C
Clostridium.akagii
1.00
0.75
0.50
0.25
0.00
A (n=15)
B (n=16)
C (n=14)
surface

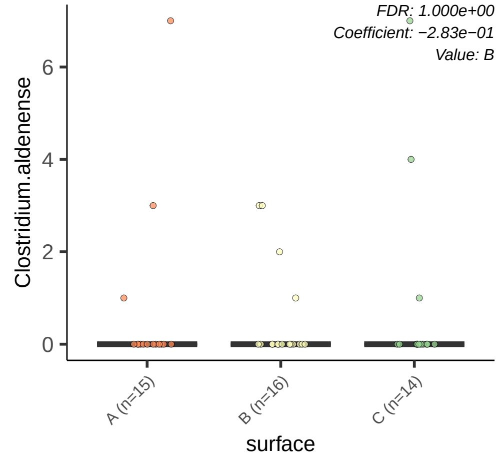
FDR: 1.000e+00
Coefficient: −2.83e−01
Value: B
Clostridium.aldenense
0
2
4
6
A (n=15)
B (n=16)
C (n=14)
surface

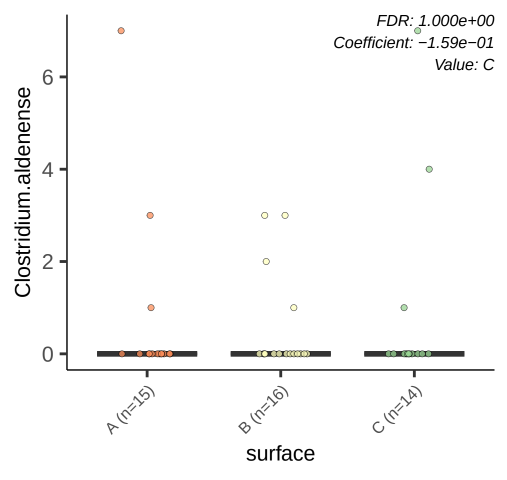
FDR: 1.000e+00
Coefficient: −1.59e−01
Value: C
Clostridium.aldenense
0
2
4
6
A (n=15)
B (n=16)
C (n=14)
surface

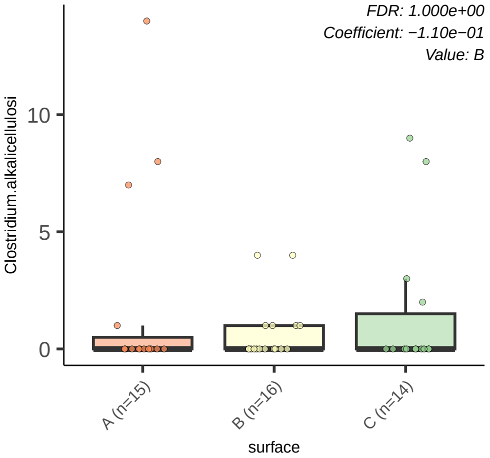
FDR: 1.000e+00
Coefficient: −1.10e−01
Value: B
Clostridium.alkalicellulosi
10
5
0
A (n=15)
B (n=16)
C (n=14)
surface

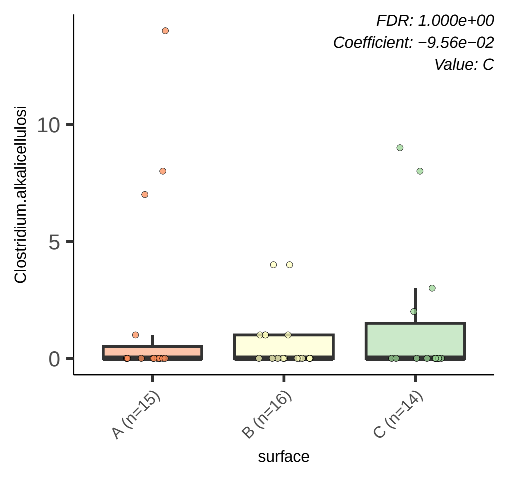
FDR: 1.000e+00
Coefficient: −9.56e−02
Value: C
Clostridium.alkalicellulosi
0
5
10
A (n=15)
B (n=16)
C (n=14)
surface

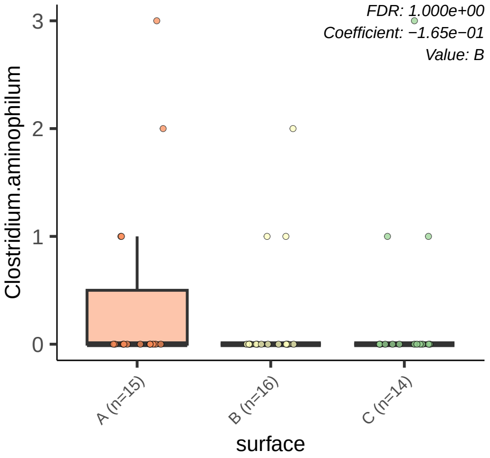
FDR: 1.000e+00
Coefficient: −1.65e−01
Value: B
Clostridium.aminophilum
3
2
1
0
A (n=15)
B (n=16)
C (n=14)
surface

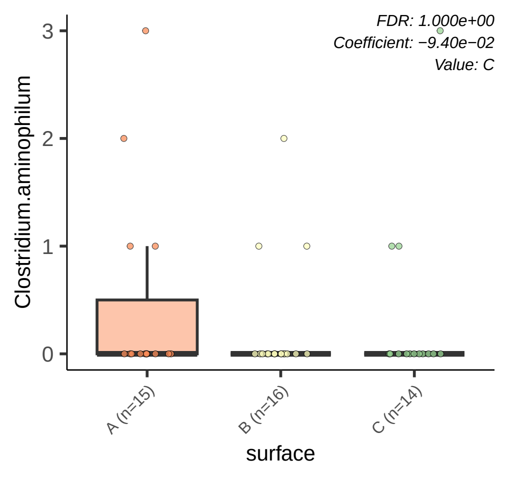
FDR: 1.000e+00
Coefficient: −9.40e−02
Value: C
Clostridium.aminophilum
0
1
2
3
A (n=15)
B (n=16)
C (n=14)
surface

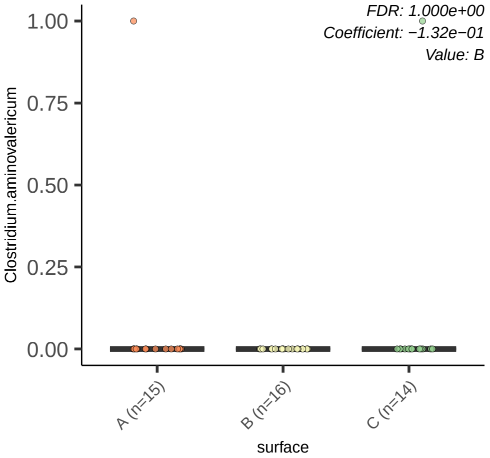
FDR: 1.000e+00
Coefficient: −1.32e−01
Value: B
Clostridium.aminovalericum
1.00
0.75
0.50
0.25
0.00
A (n=15)
B (n=16)
C (n=14)
surface

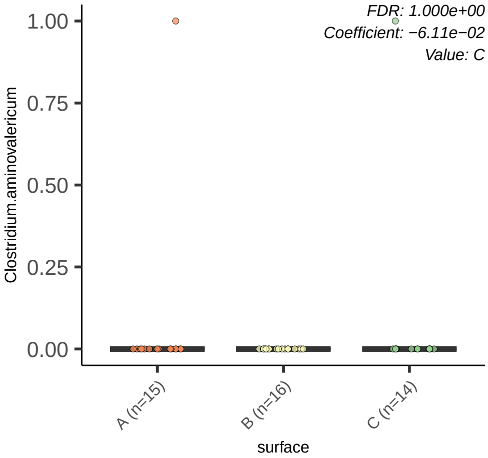
FDR: 1.000e+00
Coefficient: −6.11e−02
Value: C
Clostridium.aminovalericum
1.00
0.75
0.50
0.25
0.00
A (n=15)
B (n=16)
C (n=14)
surface

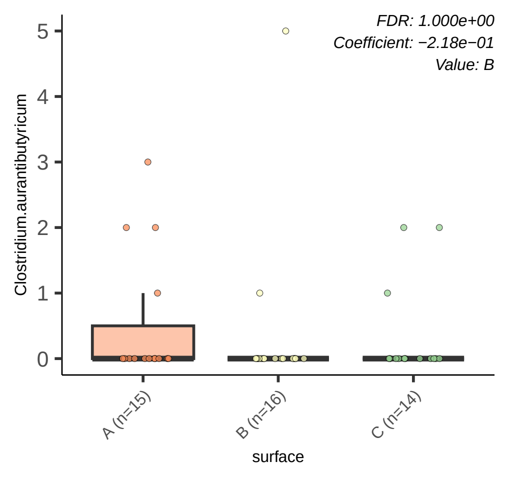

FDR: 1.000e+00
Coefficient: −2.18e−01
Value: B
Clostridium.aurantibutyricum
0
1
2
3
4
5
A (n=15)
B (n=16)
C (n=14)
surface

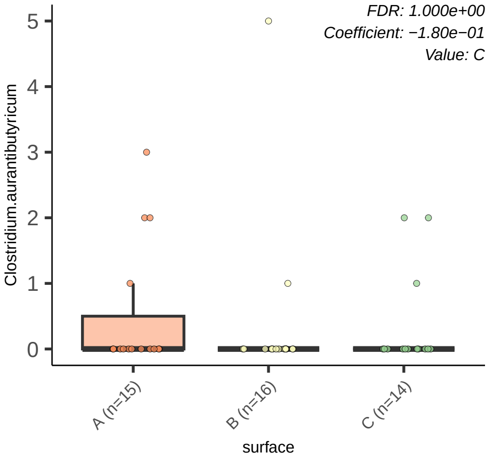
FDR: 1.000e+00
Coefficient: −1.80e−01
Value: C
Clostridium.aurantibutyricum
0
1
2
3
4
5
A (n=15)
B (n=16)
C (n=14)
surface

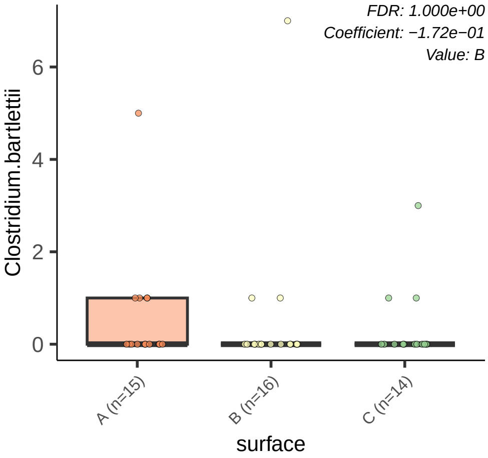

FDR: 1.000e+00
Coefficient: −1.72e−01
Value: B
Clostridium.bartlettii
0
2
4
6
A (n=15)
B (n=16)
C (n=14)
surface

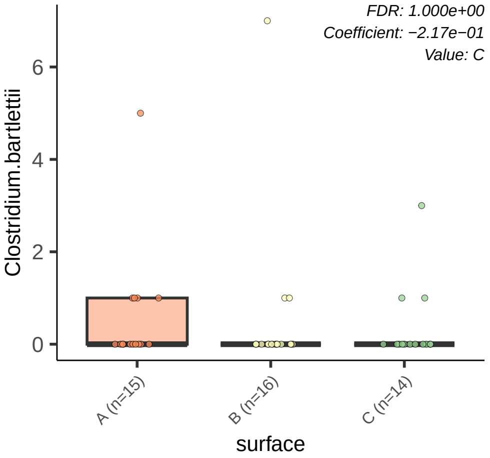

FDR: 1.000e+00
Coefficient: −2.17e−01
Value: C
Clostridium.bartlettii
0
2
4
6
A (n=15)
B (n=16)
C (n=14)
surface

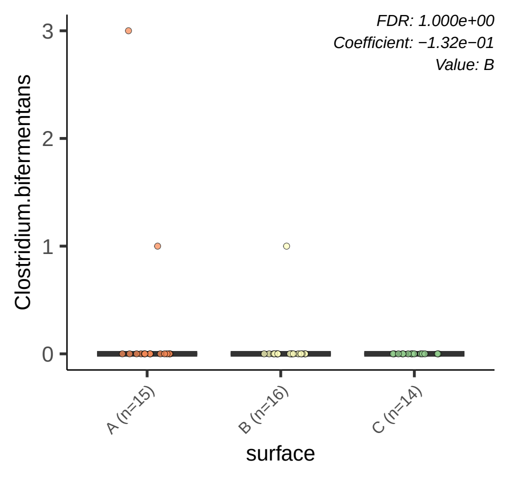
FDR: 1.000e+00
Coefficient: −1.32e−01
Value: B
Clostridium.bifermentans
3
2
1
0
A (n=15)
B (n=16)
C (n=14)
surface

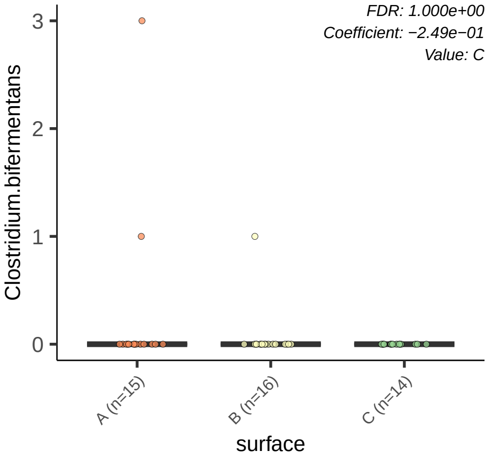

FDR: 1.000e+00
Coefficient: −2.49e−01
Value: C
Clostridium.bifermentans
3
2
1
0
A (n=15)
B (n=16)
C (n=14)
surface

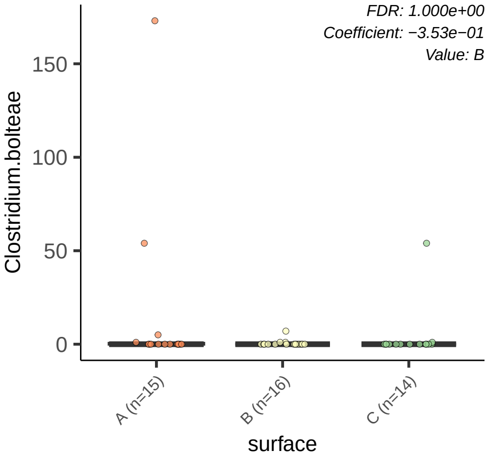
FDR: 1.000e+00
Coefficient: −3.53e−01
Value: B
150
100
50
0
Clostridium.bolteae
A (n=15)
B (n=16)
C (n=14)
surface

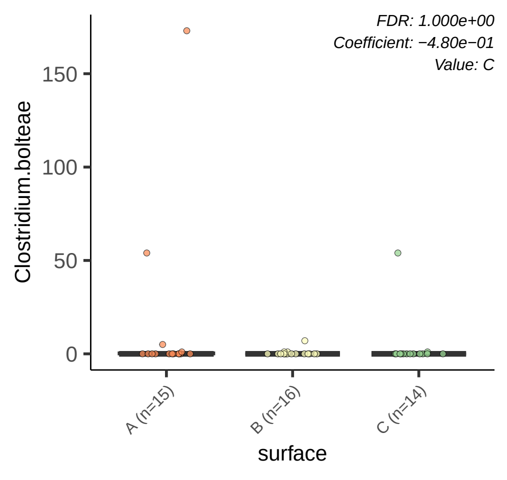
FDR: 1.000e+00
Coefficient: −4.80e−01
Value: C
Clostridium.bolteae
150
100
50
0
A (n=15)
B (n=16)
C (n=14)
surface

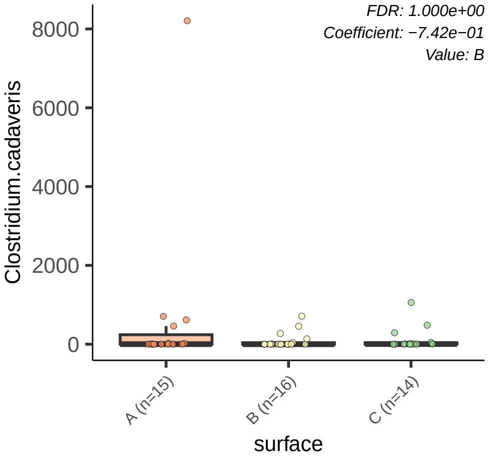
FDR: 1.000e+00
Coefficient: −7.42e−01
Value: B
8000
6000
4000
2000
0
Clostridium.cadaveris
A (n=15)
B (n=16)
C (n=14)
surface

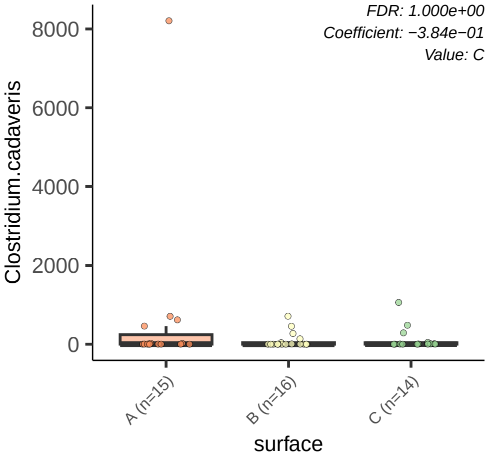

FDR: 1.000e+00
Coefficient: −3.84e−01
Value: C
8000
6000
4000
2000
0
Clostridium.cadaveris
A (n=15)
B (n=16)
C (n=14)
surface

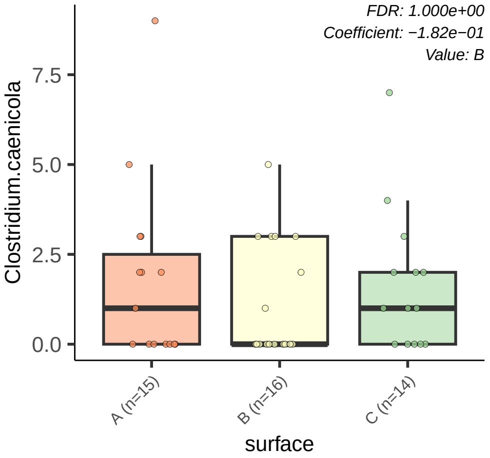
FDR: 1.000e+00
Coefficient: −1.82e−01
Value: B
Clostridium.caenicola
7.5
5.0
2.5
0.0
A (n=15)
B (n=16)
C (n=14)
surface

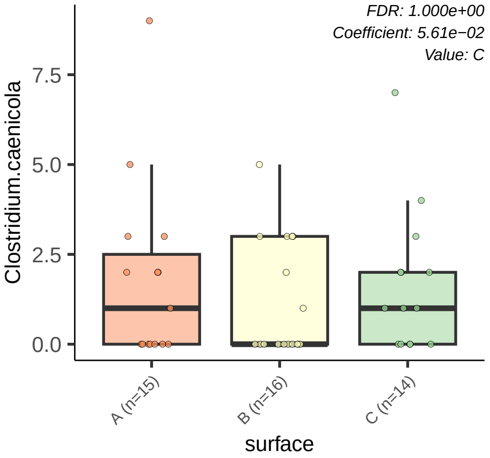

FDR: 1.000e+00
Coefficient: 5.61e−02
Value: C
Clostridium.caenicola
7.5
5.0
2.5
0.0
A (n=15)
B (n=16)
C (n=14)
surface

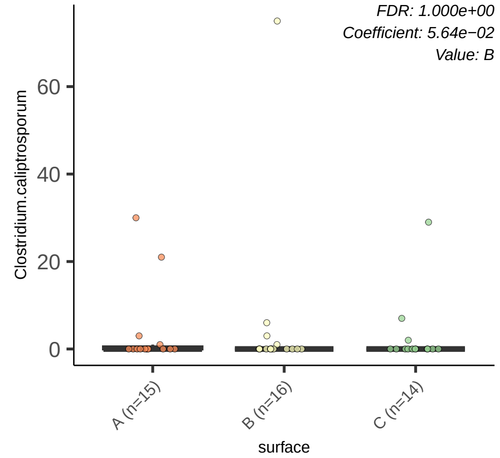
FDR: 1.000e+00
Coefficient: 5.64e−02
Value: B
Clostridium.caliptrosporum
60
40
20
0
A (n=15)
B (n=16)
C (n=14)
surface

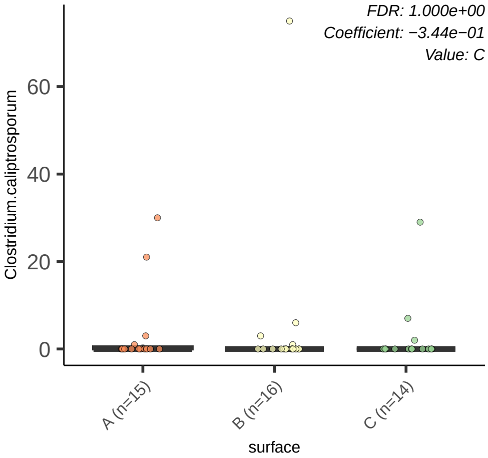
FDR: 1.000e+00
Coefficient: −3.44e−01
Value: C
Clostridium.caliptrosporum
0
20
40
60
A (n=15)
B (n=16)
C (n=14)
surface

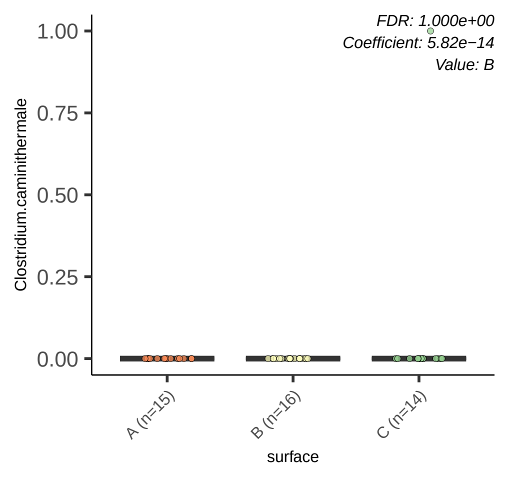
FDR: 1.000e+00
Coefficient: 5.82e−14
Value: B
Clostridium.caminithermale
1.00
0.75
0.50
0.25
0.00
A (n=15)
B (n=16)
C (n=14)
surface

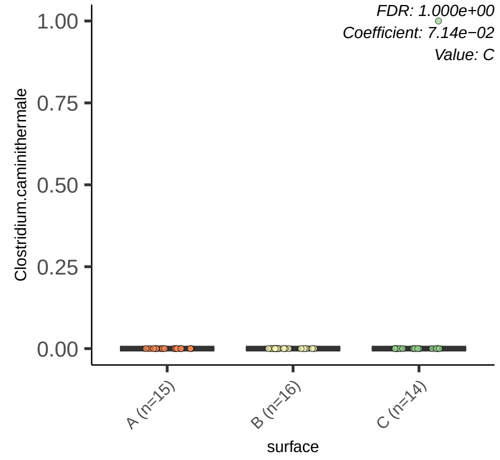
FDR: 1.000e+00
Coefficient: 7.14e−02
Value: C
1.00
0.75
0.50
0.25
0.00
Clostridium.caminithermale
A (n=15)
B (n=16)
C (n=14)
surface

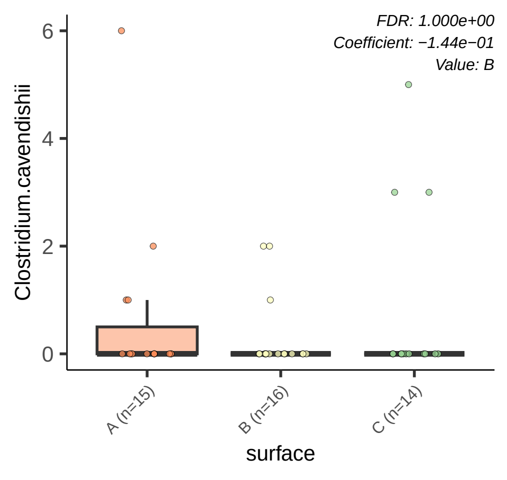
FDR: 1.000e+00
Coefficient: −1.44e−01
Value: B
Clostridium.cavendishii
0
2
4
6
A (n=15)
B (n=16)
C (n=14)
surface

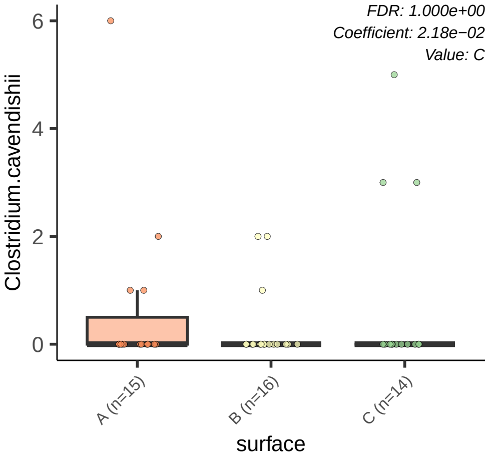

FDR: 1.000e+00
Coefficient: 2.18e−02
Value: C
Clostridium.cavendishii
0
2
4
6
A (n=15)
B (n=16)
C (n=14)
surface

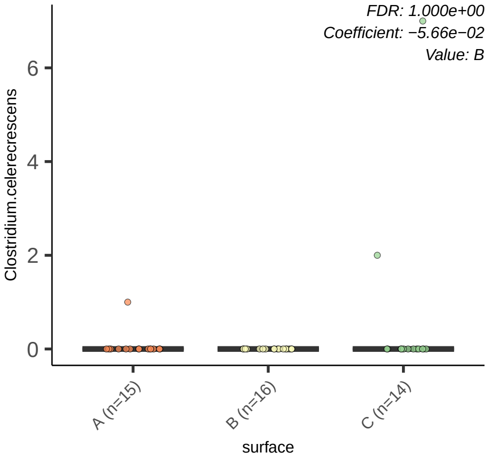

FDR: 1.000e+00
Coefficient: −5.66e−02
Value: B
Clostridium.celerecrescens
0
2
4
6
A (n=15)
B (n=16)
C (n=14)
surface

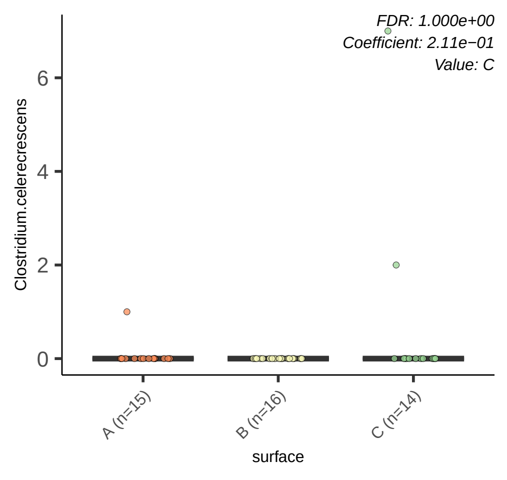
FDR: 1.000e+00
Coefficient: 2.11e−01
Value: C
Clostridium.celerecrescens
0
2
4
6
A (n=15)
B (n=16)
C (n=14)
surface

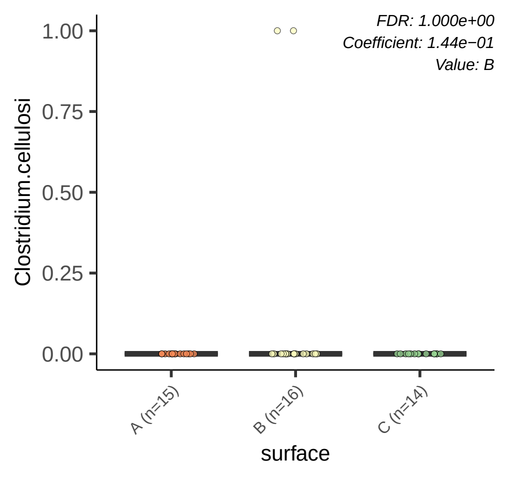
FDR: 1.000e+00
Coefficient: 1.44e−01
Value: B
Clostridium.cellulosi
1.00
0.75
0.50
0.25
0.00
A (n=15)
B (n=16)
C (n=14)
surface

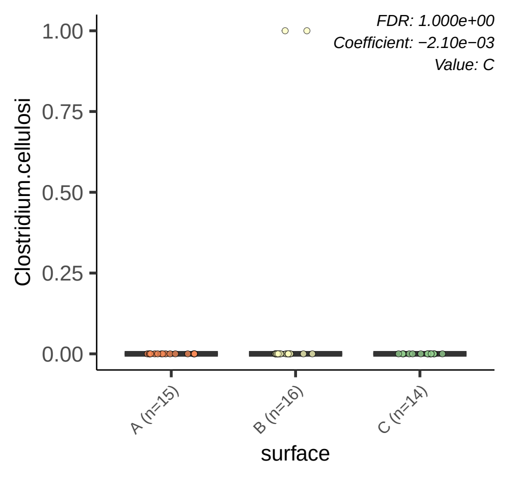

FDR: 1.000e+00
Coefficient: −2.10e−03
Value: C
1.00
0.75
0.50
0.25
0.00
Clostridium.cellulosi
A (n=15)
B (n=16)
C (n=14)
surface

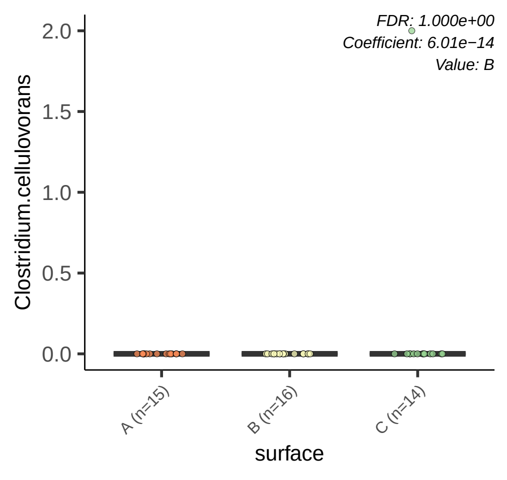
FDR: 1.000e+00
Coefficient: 6.01e−14
Value: B
Clostridium.cellulovorans
2.0
1.5
1.0
0.5
0.0
A (n=15)
B (n=16)
C (n=14)
surface

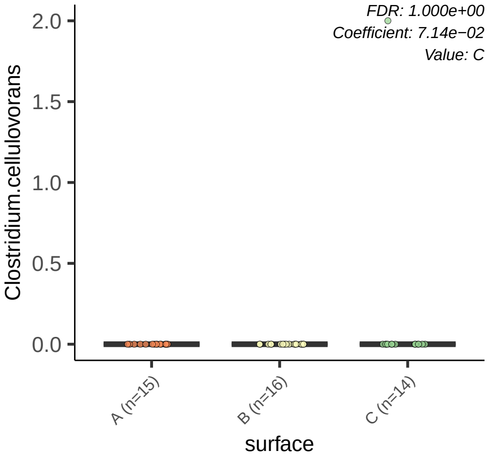

FDR: 1.000e+00
Coefficient: 7.14e−02
Value: C
2.0
1.5
1.0
0.5
0.0
Clostridium.cellulovorans
A (n=15)
B (n=16)
C (n=14)
surface

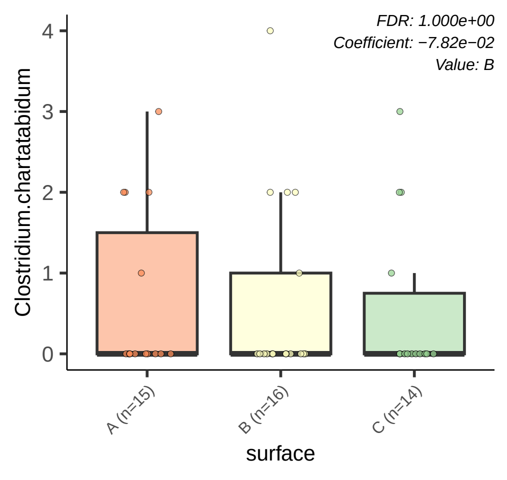
FDR: 1.000e+00
Coefficient: −7.82e−02
Value: B
Clostridium.chartatabidum
0
1
2
3
4
A (n=15)
B (n=16)
C (n=14)
surface

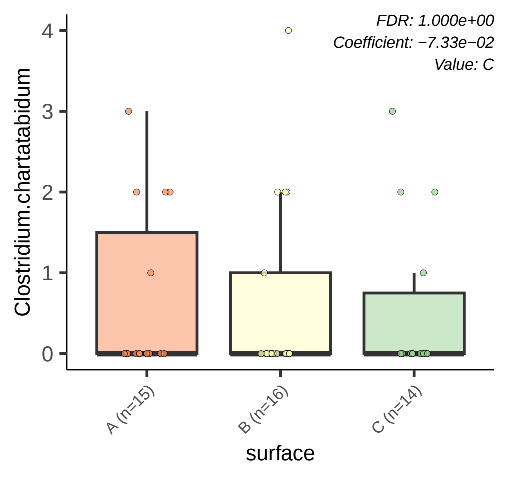
FDR: 1.000e+00
Coefficient: −7.33e−02
Value: C
Clostridium.chartatabidum
0
1
2
3
4
A (n=15)
B (n=16)
C (n=14)
surface

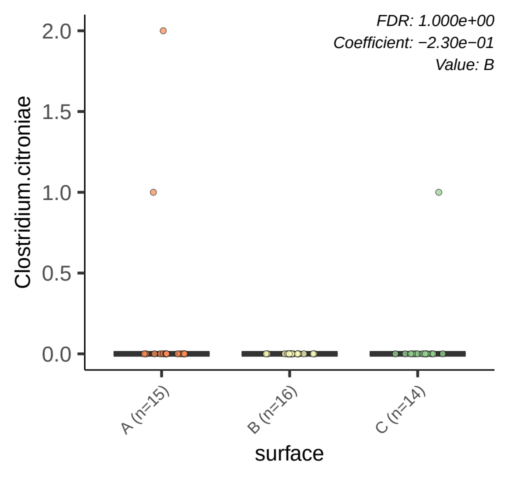
FDR: 1.000e+00
Coefficient: −2.30e−01
Value: B
Clostridium.citroniae
2.0
1.5
1.0
0.5
0.0
A (n=15)
B (n=16)
C (n=14)
surface

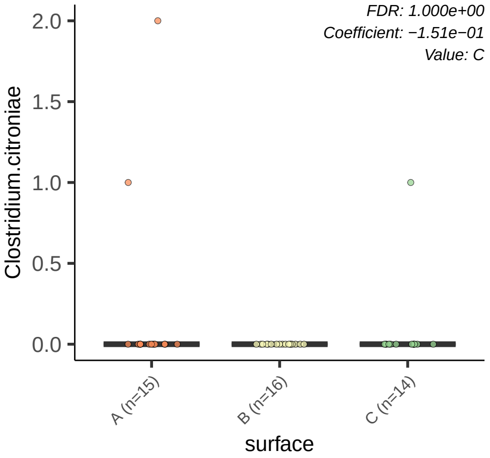
FDR: 1.000e+00
Coefficient: −1.51e−01
Value: C
Clostridium.citroniae
2.0
1.5
1.0
0.5
0.0
A (n=15)
B (n=16)
C (n=14)
surface

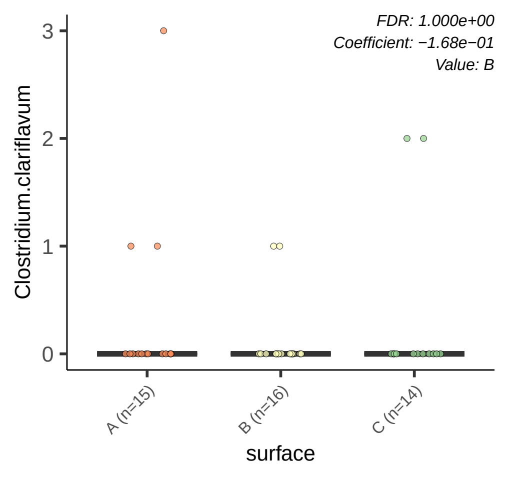

FDR: 1.000e+00
Coefficient: −1.68e−01
Value: B
Clostridium.clariflavum
3
2
1
0
A (n=15)
B (n=16)
C (n=14)
surface

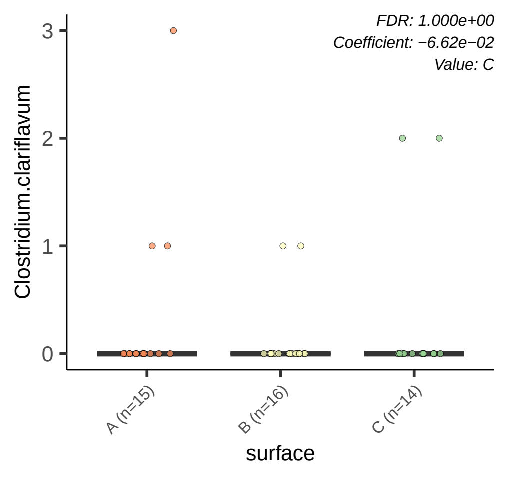

FDR: 1.000e+00
Coefficient: −6.62e−02
Value: C
Clostridium.clariflavum
0
1
2
3
A (n=15)
B (n=16)
C (n=14)
surface

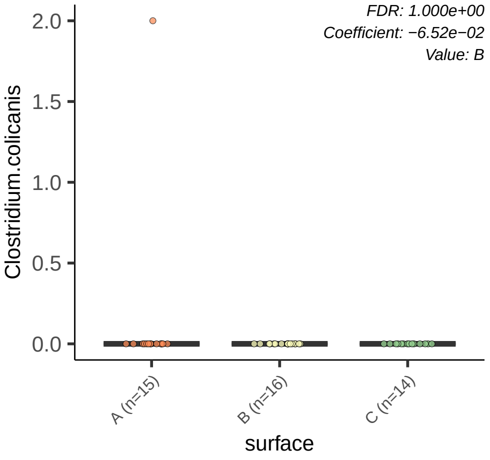
FDR: 1.000e+00
Coefficient: −6.52e−02
Value: B
Clostridium.colicanis
2.0
1.5
1.0
0.5
0.0
A (n=15)
B (n=16)
C (n=14)
surface

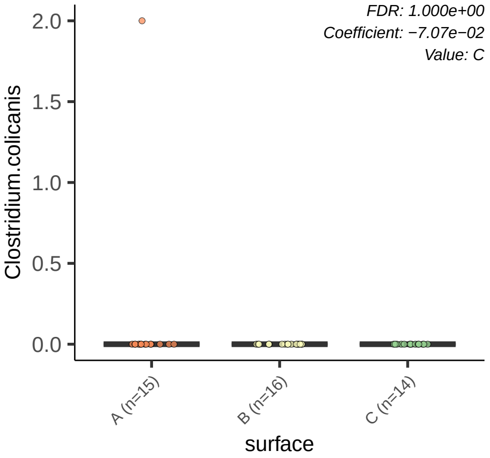
FDR: 1.000e+00
Coefficient: −7.07e−02
Value: C
Clostridium.colicanis
2.0
1.5
1.0
0.5
0.0
A (n=15)
B (n=16)
C (n=14)
surface

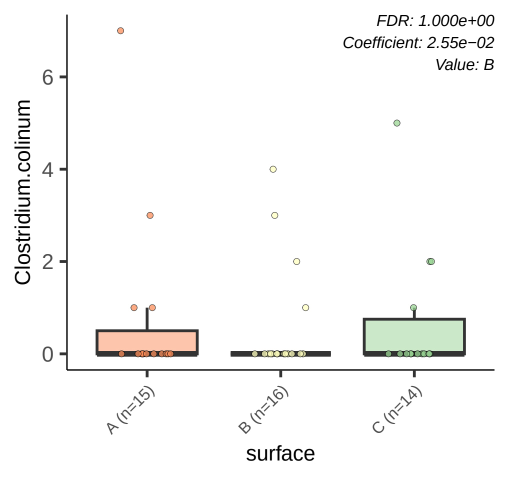
FDR: 1.000e+00
Coefficient: 2.55e−02
Value: B
Clostridium.colinum
0
2
4
6
A (n=15)
B (n=16)
C (n=14)
surface

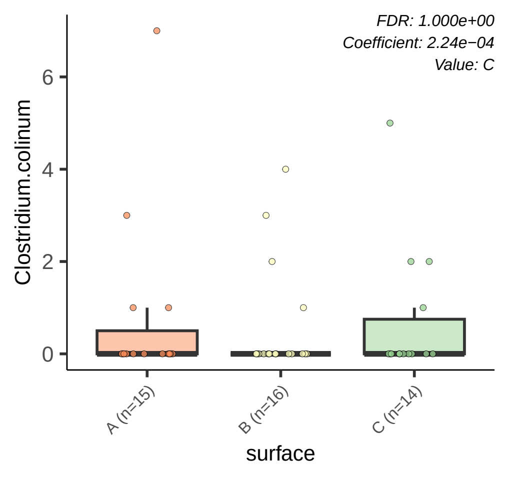
FDR: 1.000e+00
Coefficient: 2.24e−04
Value: C
Clostridium.colinum
0
2
4
6
A (n=15)
B (n=16)
C (n=14)
surface

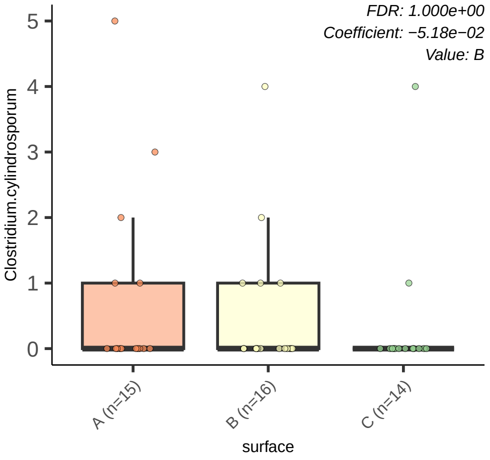
FDR: 1.000e+00
Coefficient: −5.18e−02
Value: B
Clostridium.cylindrosporum
0
1
2
3
4
5
A (n=15)
B (n=16)
C (n=14)
surface

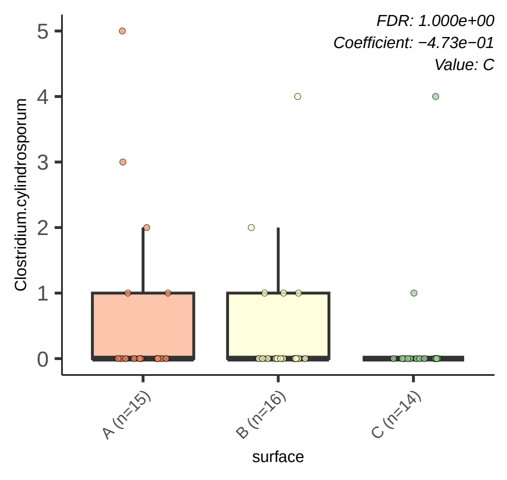

FDR: 1.000e+00
Coefficient: −4.73e−01
Value: C
Clostridium.cylindrosporum
0
1
2
3
4
5
A (n=15)
B (n=16)
C (n=14)
surface

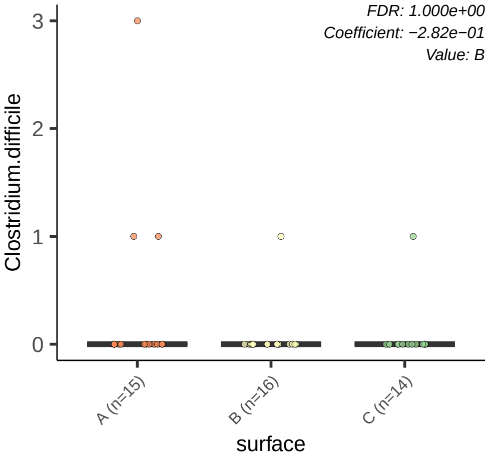
FDR: 1.000e+00
Coefficient: −2.82e−01
Value: B
Clostridium.difficile
3
2
1
0
A (n=15)
B (n=16)
C (n=14)
surface

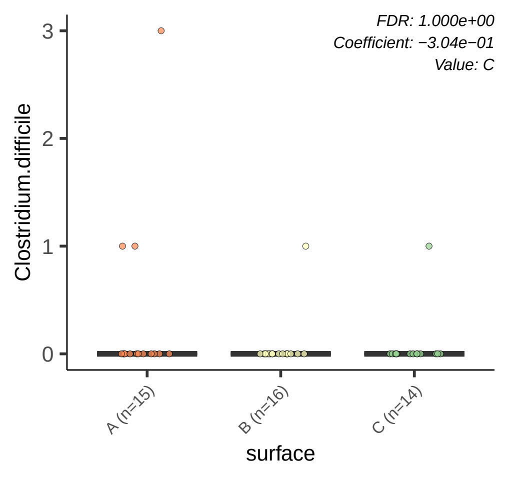
FDR: 1.000e+00
Coefficient: −3.04e−01
Value: C
Clostridium.difficile
0
1
2
3
A (n=15)
B (n=16)
C (n=14)
surface

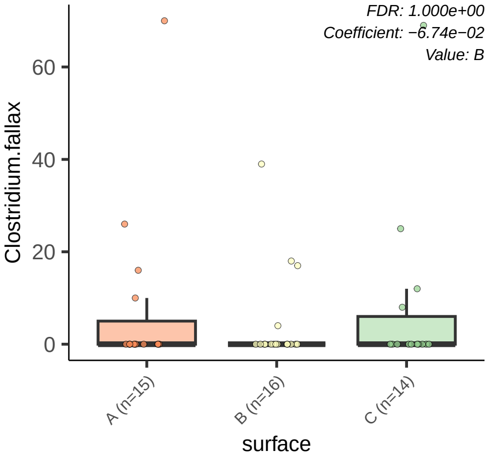

FDR: 1.000e+00
Coefficient: −6.74e−02
Value: B
Clostridium.fallax
0
20
40
60
A (n=15)
B (n=16)
C (n=14)
surface

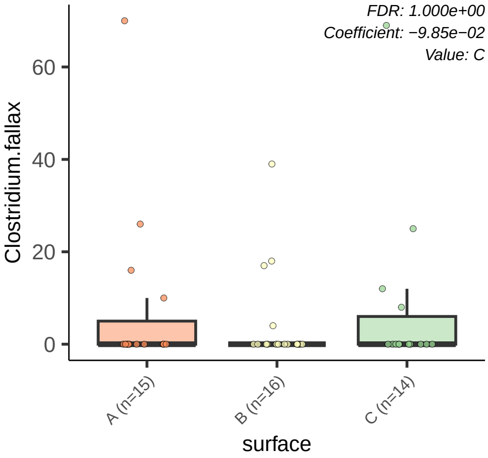
FDR: 1.000e+00
Coefficient: −9.85e−02
Value: C
Clostridium.fallax
60
40
20
0
A (n=15)
B (n=16)
C (n=14)
surface

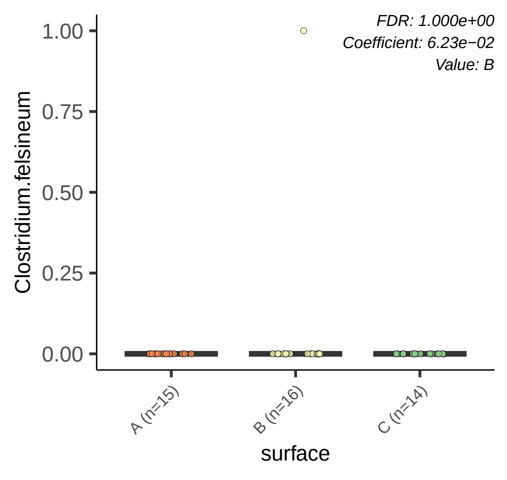
FDR: 1.000e+00
Coefficient: 6.23e−02
Value: B
1.00
0.75
0.50
0.25
0.00
Clostridium.felsineum
A (n=15)
B (n=16)
C (n=14)
surface

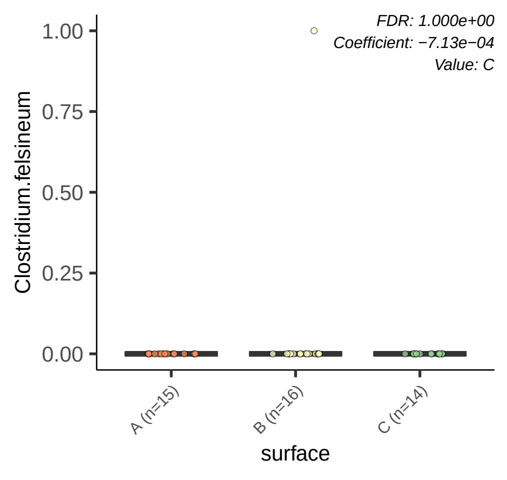
FDR: 1.000e+00
Coefficient: −7.13e−04
Value: C
Clostridium.felsineum
1.00
0.75
0.50
0.25
0.00
A (n=15)
B (n=16)
C (n=14)
surface

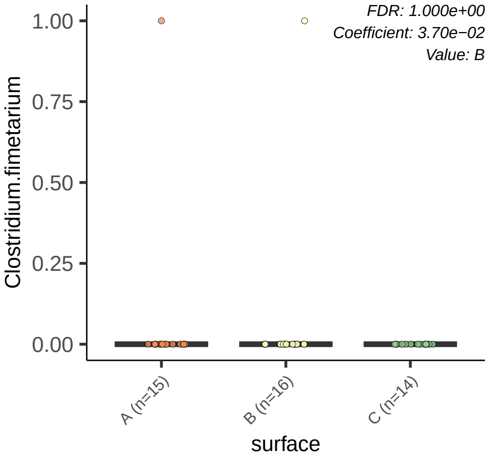
FDR: 1.000e+00
Coefficient: 3.70e−02
Value: B
Clostridium.fimetarium
1.00
0.75
0.50
0.25
0.00
A (n=15)
B (n=16)
C (n=14)
surface

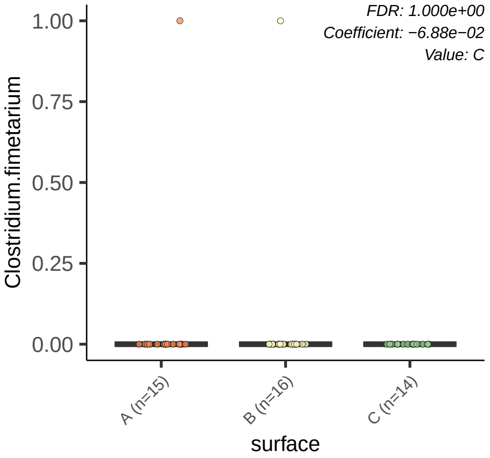
FDR: 1.000e+00
Coefficient: −6.88e−02
Value: C
Clostridium.fimetarium
1.00
0.75
0.50
0.25
0.00
A (n=15)
B (n=16)
C (n=14)
surface

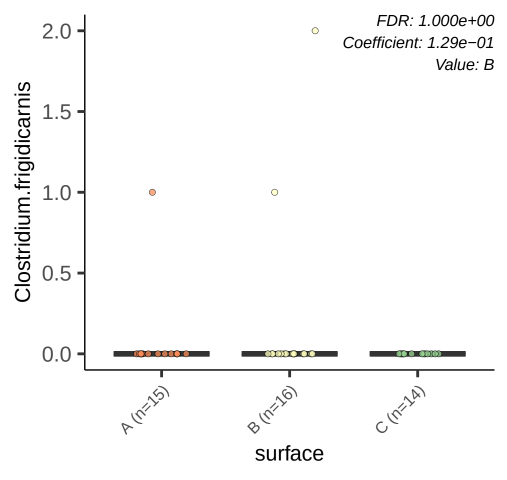
FDR: 1.000e+00
Coefficient: 1.29e−01
Value: B
Clostridium.frigidicarnis
2.0
1.5
1.0
0.5
0.0
A (n=15)
B (n=16)
C (n=14)
surface

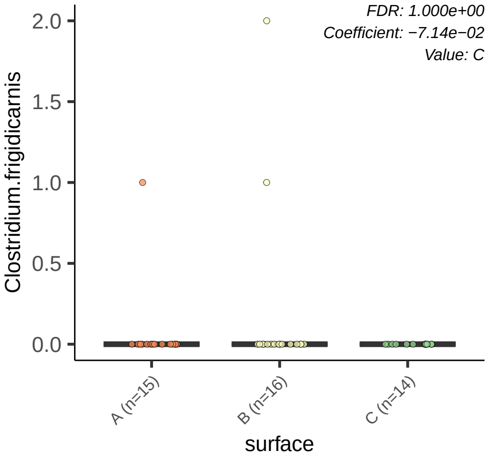
FDR: 1.000e+00
Coefficient: −7.14e−02
Value: C
Clostridium.frigidicarnis
2.0
1.5
1.0
0.5
0.0
A (n=15)
B (n=16)
C (n=14)
surface

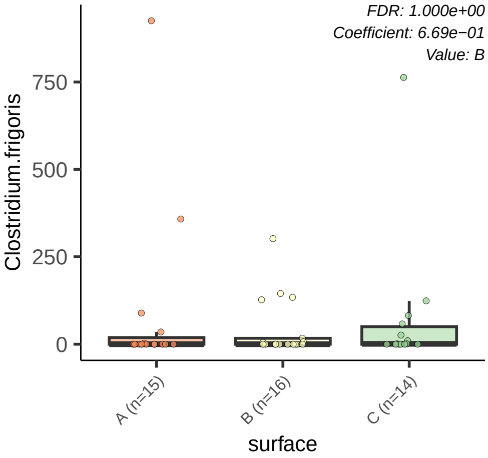

FDR: 1.000e+00
Coefficient: 6.69e−01
Value: B
Clostridium.frigoris
750
500
250
0
A (n=15)
B (n=16)
C (n=14)
surface

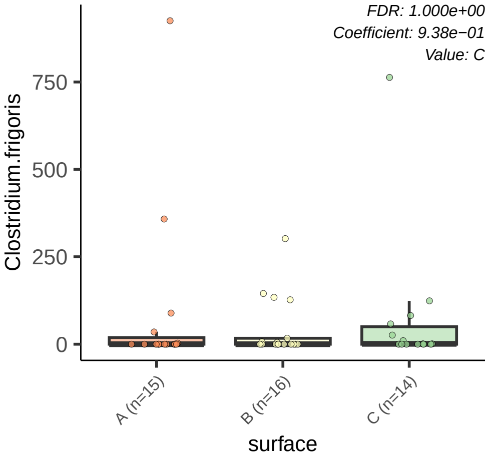

FDR: 1.000e+00
Coefficient: 9.38e−01
Value: C
Clostridium.frigoris
750
500
250
0
A (n=15)
B (n=16)
C (n=14)
surface

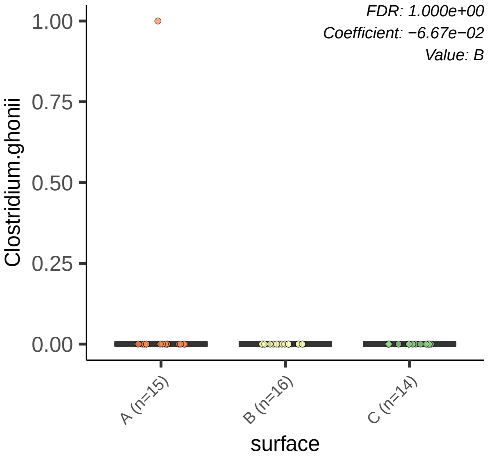
FDR: 1.000e+00
Coefficient: −6.67e−02
Value: B
Clostridium.ghonii
1.00
0.75
0.50
0.25
0.00
A (n=15)
B (n=16)
C (n=14)
surface

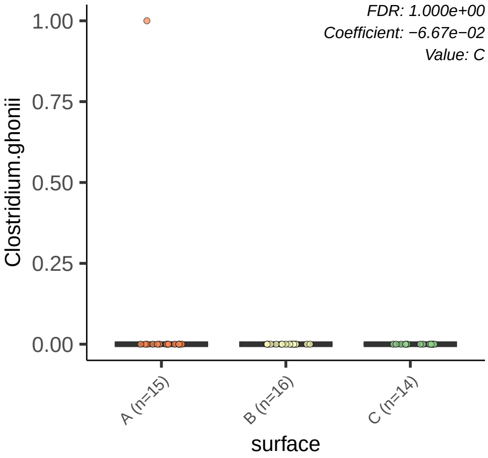
FDR: 1.000e+00
Coefficient: −6.67e−02
Value: C
Clostridium.ghonii
1.00
0.75
0.50
0.25
0.00
A (n=15)
B (n=16)
C (n=14)
surface

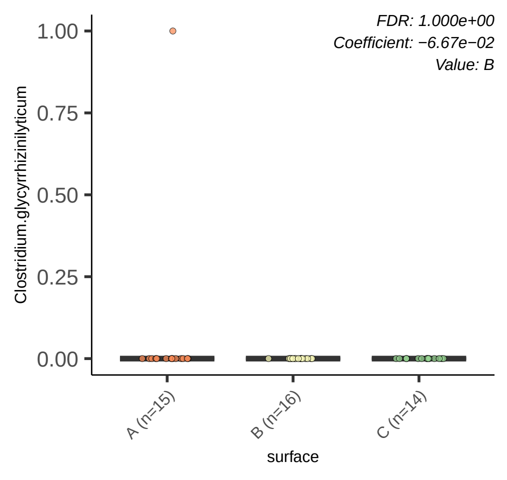
FDR: 1.000e+00
Coefficient: −6.67e−02
Value: B
1.00
0.75
0.50
0.25
0.00
Clostridium.glycyrrhizinilyticum
A (n=15)
B (n=16)
C (n=14)
surface

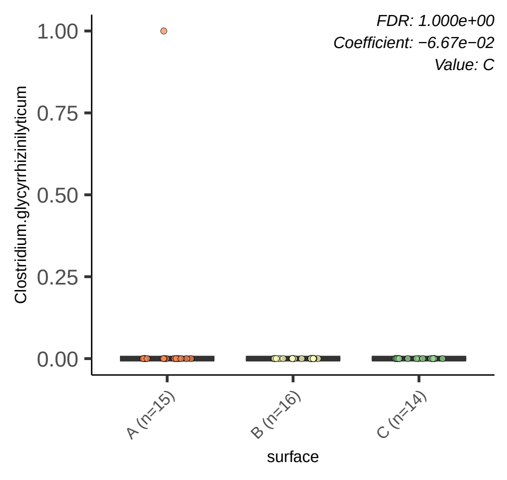
FDR: 1.000e+00
Coefficient: −6.67e−02
Value: C
1.00
0.75
0.50
0.25
0.00
Clostridium.glycyrrhizinilyticum
A (n=15)
B (n=16)
C (n=14)
surface

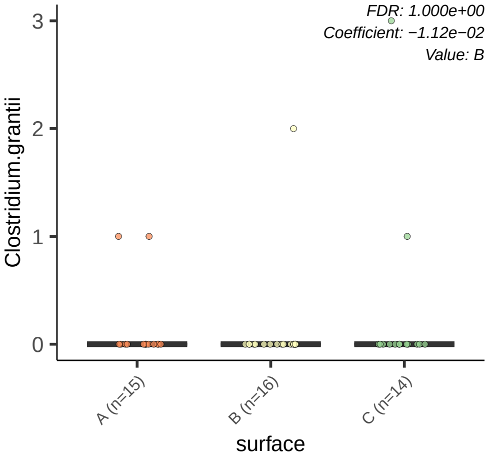
FDR: 1.000e+00
Coefficient: −1.12e−02
Value: B
Clostridium.grantii
3
2
1
0
A (n=15)
B (n=16)
C (n=14)
surface

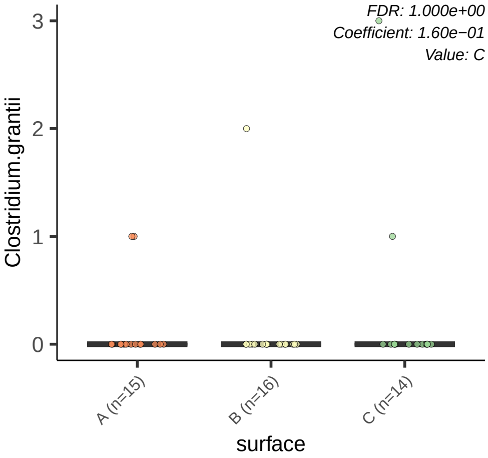
FDR: 1.000e+00
Coefficient: 1.60e−01
Value: C
Clostridium.grantii
3
2
1
0
A (n=15)
B (n=16)
C (n=14)
surface

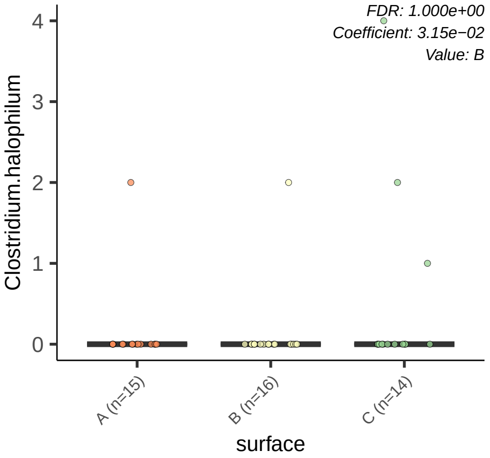
FDR: 1.000e+00
Coefficient: 3.15e−02
Value: B
Clostridium.halophilum
4
3
2
1
0
A (n=15)
B (n=16)
C (n=14)
surface

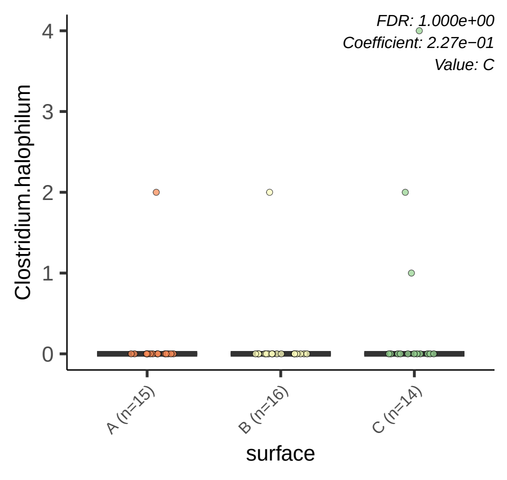

FDR: 1.000e+00
Coefficient: 2.27e−01
Value: C
Clostridium.halophilum
0
1
2
3
4
A (n=15)
B (n=16)
C (n=14)
surface

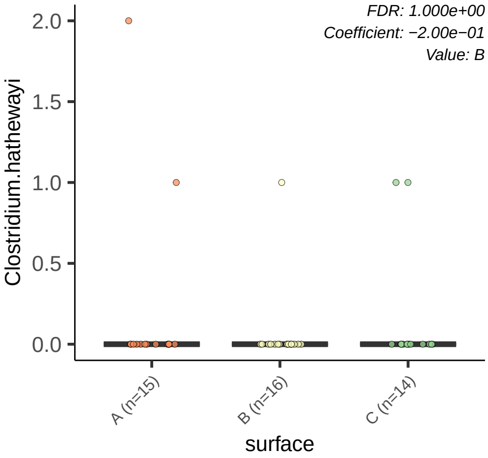
FDR: 1.000e+00
Coefficient: −2.00e−01
Value: B
Clostridium.hathewayi
2.0
1.5
1.0
0.5
0.0
A (n=15)
B (n=16)
C (n=14)
surface

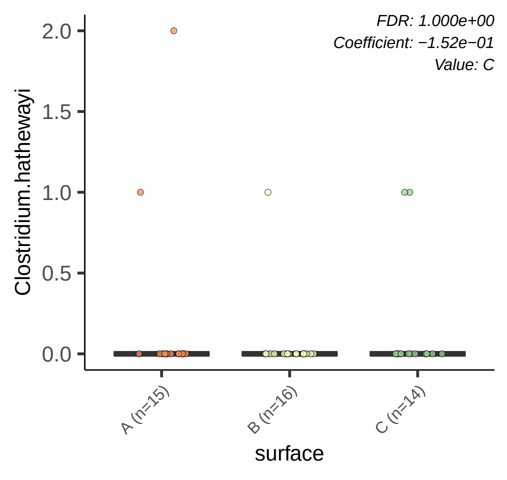
FDR: 1.000e+00
Coefficient: −1.52e−01
Value: C
Clostridium.hathewayi
2.0
1.5
1.0
0.5
0.0
A (n=15)
B (n=16)
C (n=14)
surface

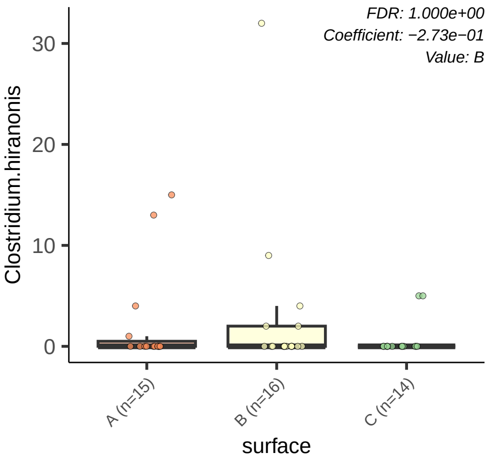

FDR: 1.000e+00
Coefficient: −2.73e−01
Value: B
Clostridium.hiranonis
30
20
10
0
A (n=15)
B (n=16)
C (n=14)
surface

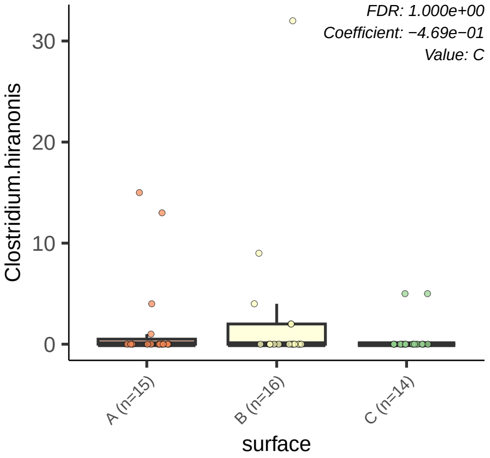
FDR: 1.000e+00
Coefficient: −4.69e−01
Value: C
Clostridium.hiranonis
0
10
20
30
A (n=15)
B (n=16)
C (n=14)
surface

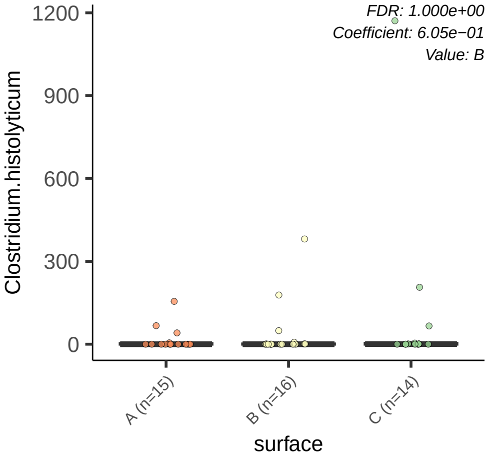
FDR: 1.000e+00
Coefficient: 6.05e−01
Value: B
1200
900
600
300
0
Clostridium.histolyticum
A (n=15)
B (n=16)
C (n=14)
surface

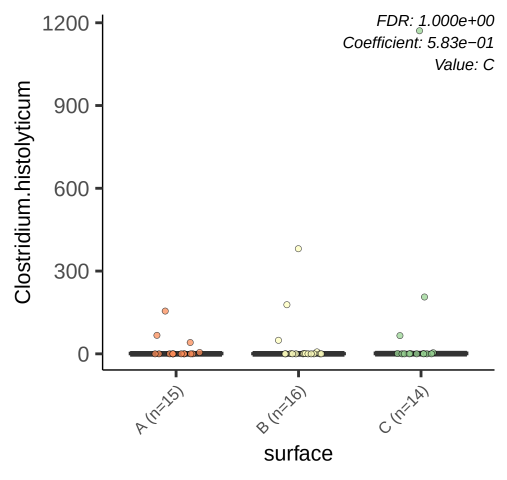
FDR: 1.000e+00
Coefficient: 5.83e−01
Value: C
1200
900
600
300
0
Clostridium.histolyticum
A (n=15)
B (n=16)
C (n=14)
surface

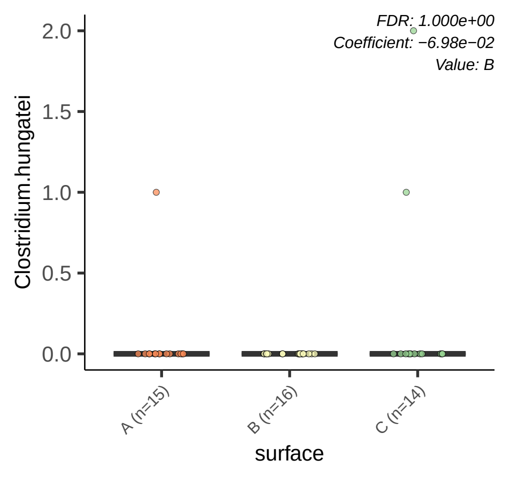

FDR: 1.000e+00
Coefficient: −6.98e−02
Value: B
Clostridium.hungatei
2.0
1.5
1.0
0.5
0.0
A (n=15)
B (n=16)
C (n=14)
surface

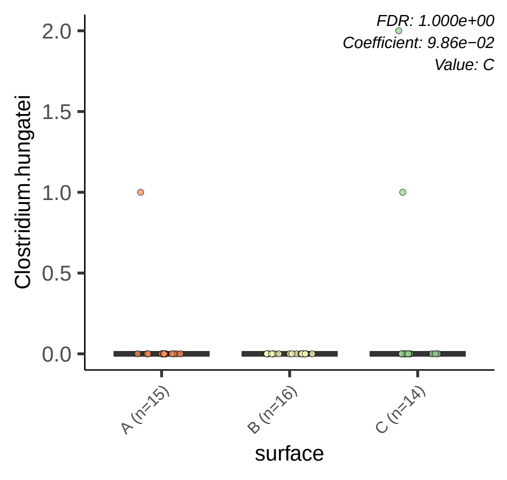

FDR: 1.000e+00
Coefficient: 9.86e−02
Value: C
Clostridium.hungatei
2.0
1.5
1.0
0.5
0.0
A (n=15)
B (n=16)
C (n=14)
surface

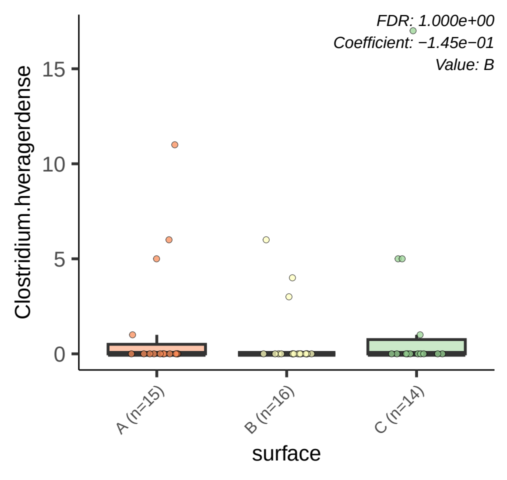
FDR: 1.000e+00
Coefficient: −1.45e−01
Value: B
Clostridium.hveragerdense
0
5
10
15
A (n=15)
B (n=16)
C (n=14)
surface

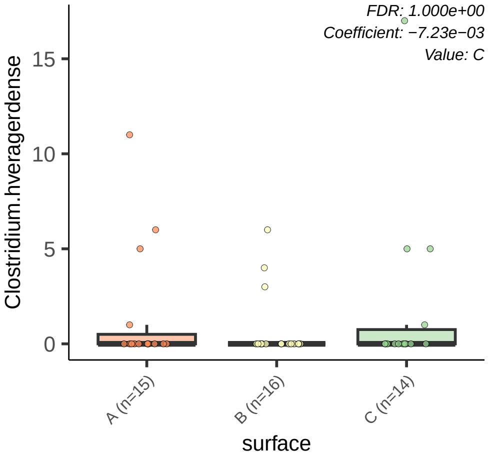
FDR: 1.000e+00
Coefficient: −7.23e−03
Value: C
Clostridium.hveragerdense
0
5
10
15
A (n=15)
B (n=16)
C (n=14)
surface

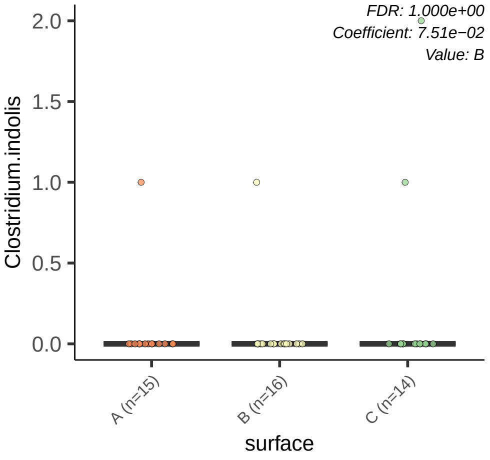
FDR: 1.000e+00
Coefficient: 7.51e−02
Value: B
Clostridium.indolis
2.0
1.5
1.0
0.5
0.0
A (n=15)
B (n=16)
C (n=14)
surface

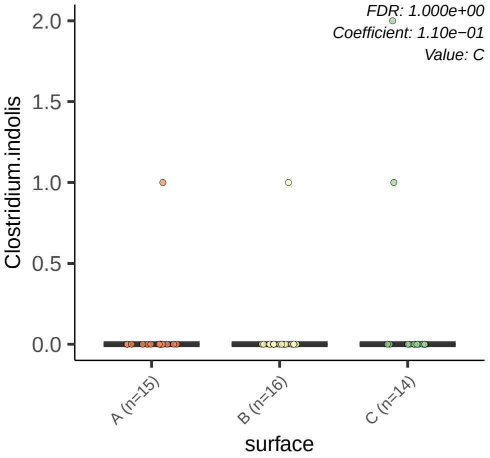
FDR: 1.000e+00
Coefficient: 1.10e−01
Value: C
Clostridium.indolis
2.0
1.5
1.0
0.5
0.0
A (n=15)
B (n=16)
C (n=14)
surface

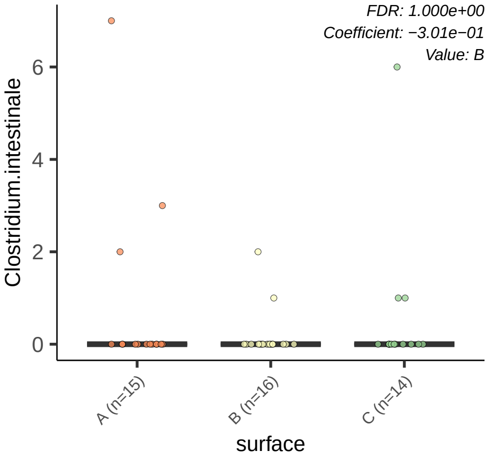
FDR: 1.000e+00
Coefficient: −3.01e−01
Value: B
Clostridium.intestinale
0
2
4
6
A (n=15)
B (n=16)
C (n=14)
surface

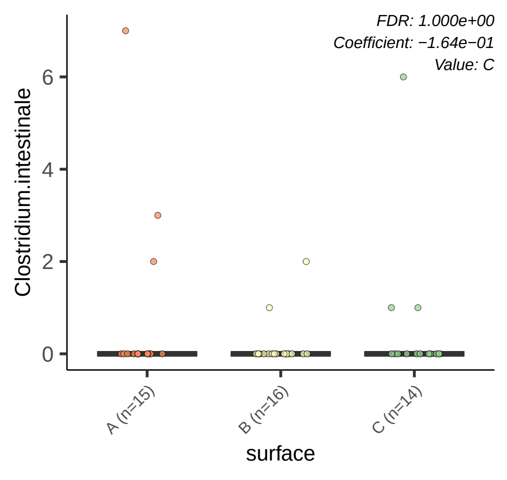
FDR: 1.000e+00
Coefficient: −1.64e−01
Value: C
Clostridium.intestinale
0
2
4
6
A (n=15)
B (n=16)
C (n=14)
surface

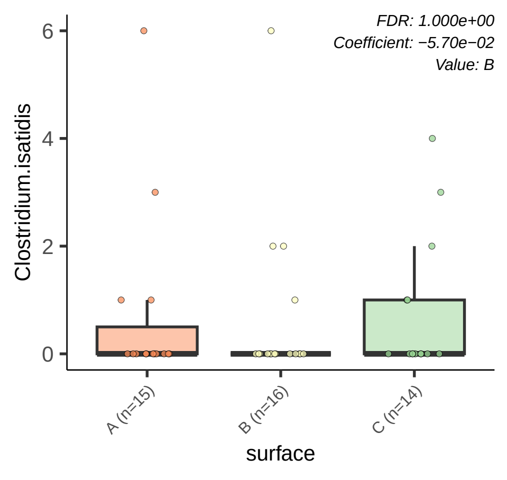
FDR: 1.000e+00
Coefficient: −5.70e−02
Value: B
Clostridium.isatidis
0
2
4
6
A (n=15)
B (n=16)
C (n=14)
surface

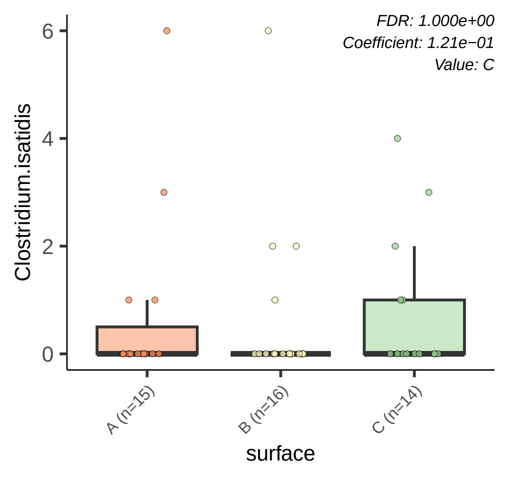
FDR: 1.000e+00
Coefficient: 1.21e−01
Value: C
Clostridium.isatidis
0
2
4
6
A (n=15)
B (n=16)
C (n=14)
surface

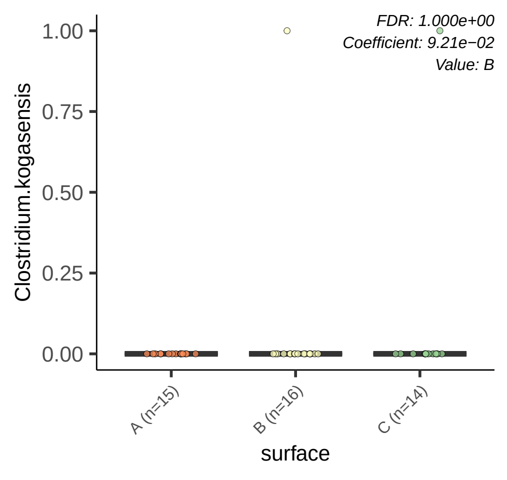
FDR: 1.000e+00
Coefficient: 9.21e−02
Value: B
Clostridium.kogasensis
1.00
0.75
0.50
0.25
0.00
A (n=15)
B (n=16)
C (n=14)
surface

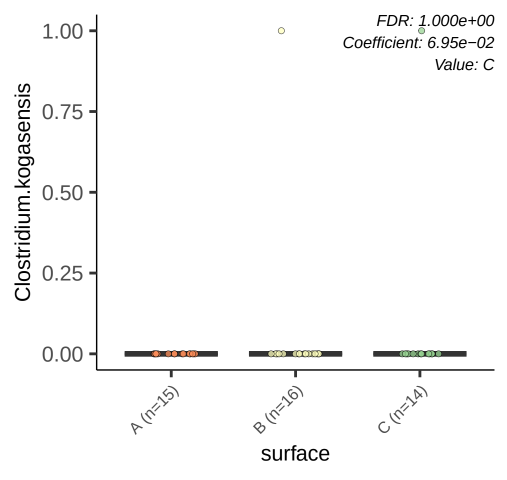
FDR: 1.000e+00
Coefficient: 6.95e−02
Value: C
Clostridium.kogasensis
1.00
0.75
0.50
0.25
0.00
A (n=15)
B (n=16)
C (n=14)
surface

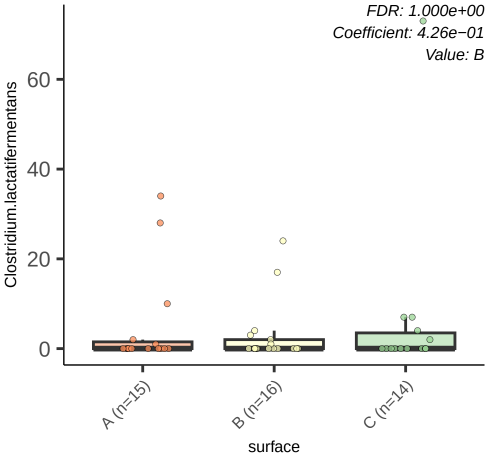

FDR: 1.000e+00
Coefficient: 4.26e−01
Value: B
Clostridium.lactatifermentans
0
20
40
60
A (n=15)
B (n=16)
C (n=14)
surface

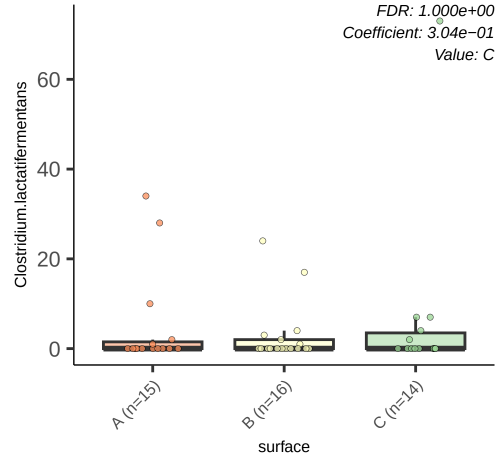

FDR: 1.000e+00
Coefficient: 3.04e−01
Value: C
Clostridium.lactatifermentans
60
40
20
0
A (n=15)
B (n=16)
C (n=14)
surface

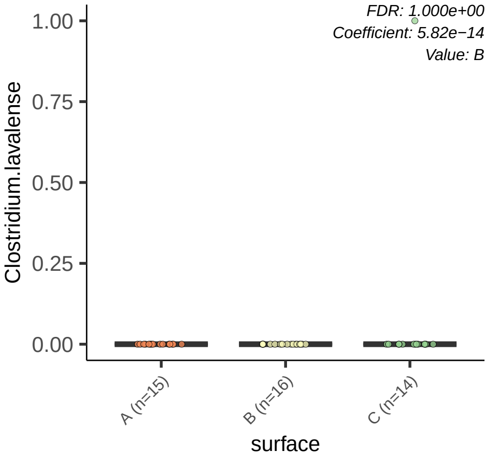
FDR: 1.000e+00
Coefficient: 5.82e−14
Value: B
Clostridium.lavalense
1.00
0.75
0.50
0.25
0.00
A (n=15)
B (n=16)
C (n=14)
surface

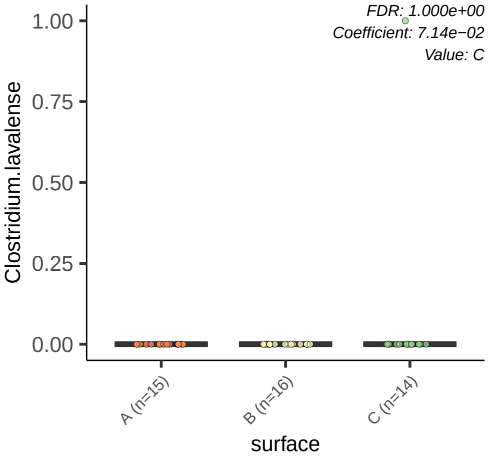

FDR: 1.000e+00
Coefficient: 7.14e−02
Value: C
Clostridium.lavalense
1.00
0.75
0.50
0.25
0.00
A (n=15)
B (n=16)
C (n=14)
surface

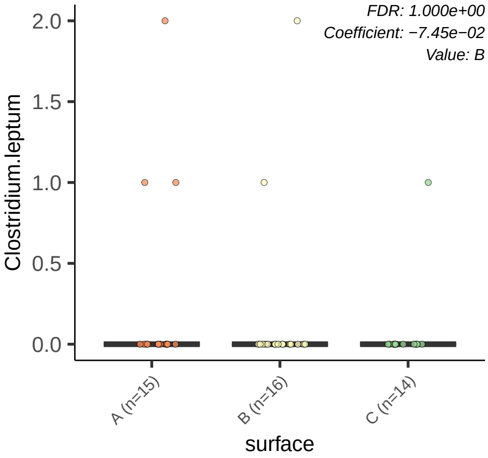

FDR: 1.000e+00
Coefficient: −7.45e−02
Value: B
Clostridium.leptum
2.0
1.5
1.0
0.5
0.0
A (n=15)
B (n=16)
C (n=14)
surface

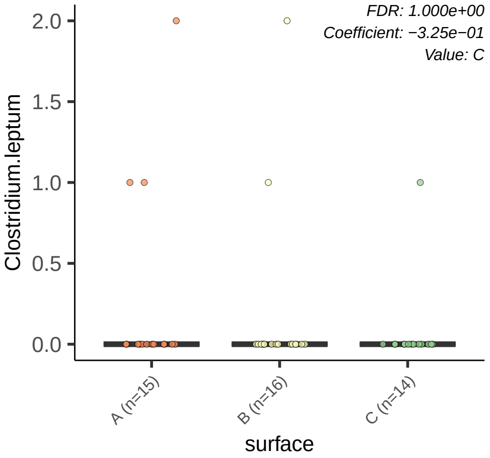

FDR: 1.000e+00
Coefficient: −3.25e−01
Value: C
Clostridium.leptum
2.0
1.5
1.0
0.5
0.0
A (n=15)
B (n=16)
C (n=14)
surface

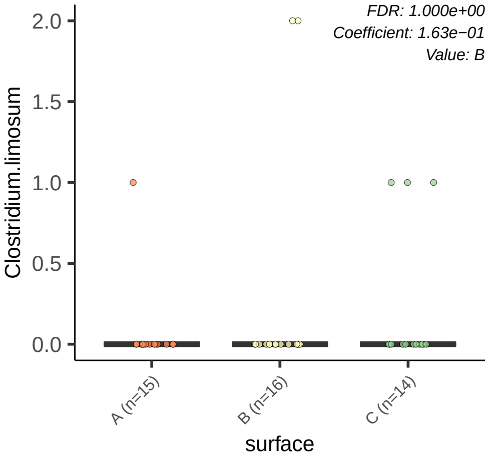
FDR: 1.000e+00
Coefficient: 1.63e−01
Value: B
Clostridium.limosum
2.0
1.5
1.0
0.5
0.0
A (n=15)
B (n=16)
C (n=14)
surface

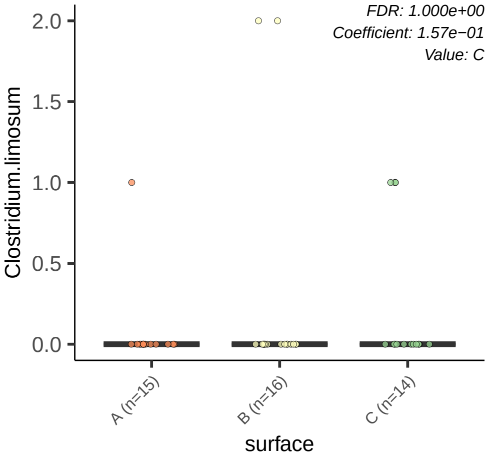

FDR: 1.000e+00
Coefficient: 1.57e−01
Value: C
Clostridium.limosum
2.0
1.5
1.0
0.5
0.0
A (n=15)
B (n=16)
C (n=14)
surface

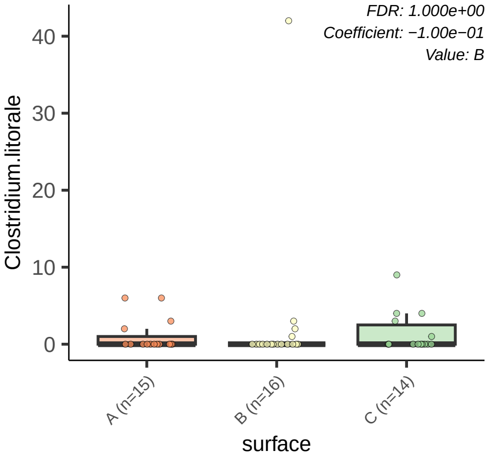

FDR: 1.000e+00
Coefficient: −1.00e−01
Value: B
Clostridium.litorale
40
30
20
10
0
A (n=15)
B (n=16)
C (n=14)
surface

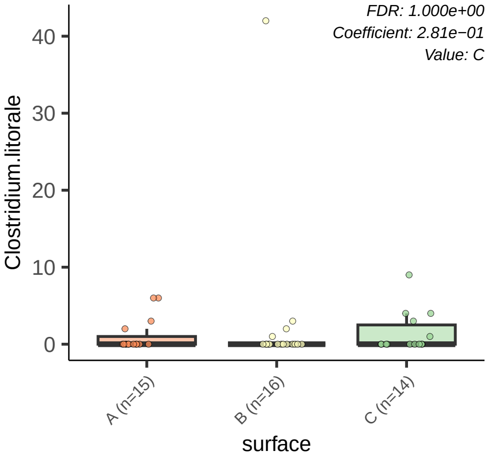

FDR: 1.000e+00
Coefficient: 2.81e−01
Value: C
Clostridium.litorale
40
30
20
10
0
A (n=15)
B (n=16)
C (n=14)
surface

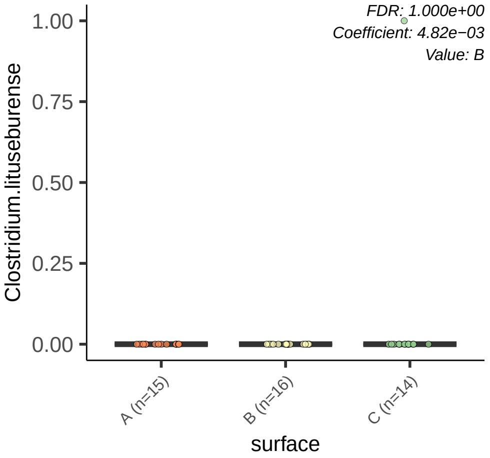
FDR: 1.000e+00
Coefficient: 4.82e−03
Value: B
Clostridium.lituseburense
1.00
0.75
0.50
0.25
0.00
A (n=15)
B (n=16)
C (n=14)
surface

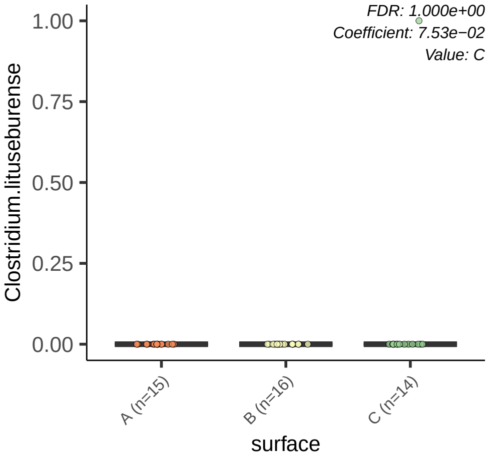

FDR: 1.000e+00
Coefficient: 7.53e−02
Value: C
Clostridium.litusebureense
1.00
0.75
0.50
0.25
0.00
A (n=15)
B (n=16)
C (n=14)
surface

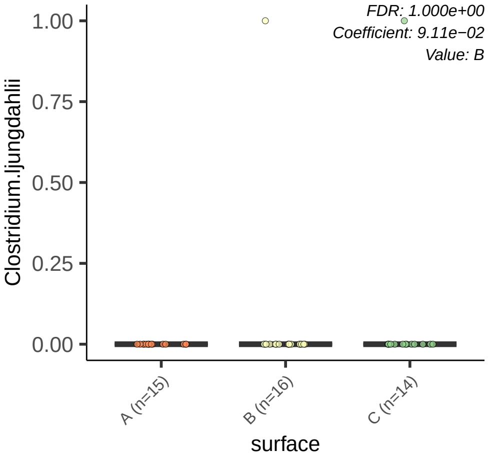

FDR: 1.000e+00
Coefficient: 9.11e−02
Value: B
Clostridium.ljungdahlii
1.00
0.75
0.50
0.25
0.00
A (n=15)
B (n=16)
C (n=14)
surface

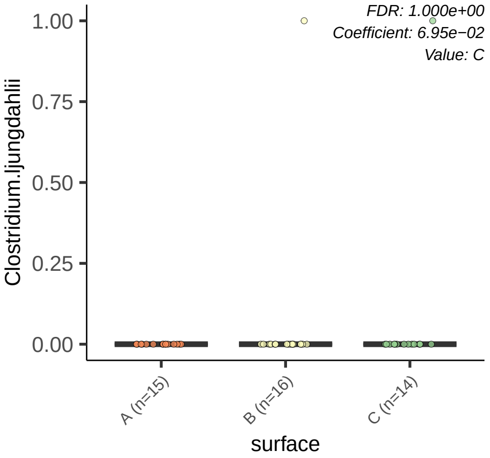

FDR: 1.000e+00
Coefficient: 6.95e−02
Value: C
Clostridium.ljungdahlii
1.00
0.75
0.50
0.25
0.00
A (n=15)
B (n=16)
C (n=14)
surface

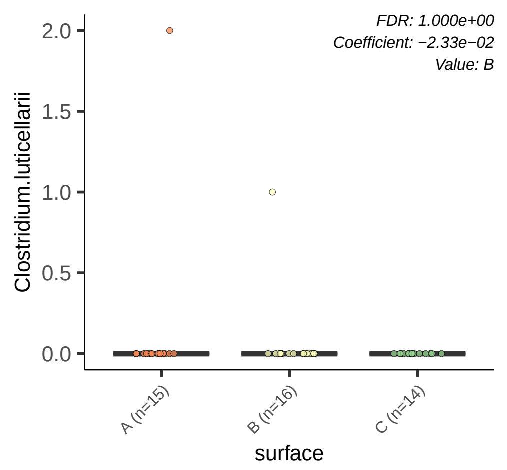
FDR: 1.000e+00
Coefficient: −2.33e−02
Value: B
Clostridium.luticellarii
2.0
1.5
1.0
0.5
0.0
A (n=15)
B (n=16)
C (n=14)
surface

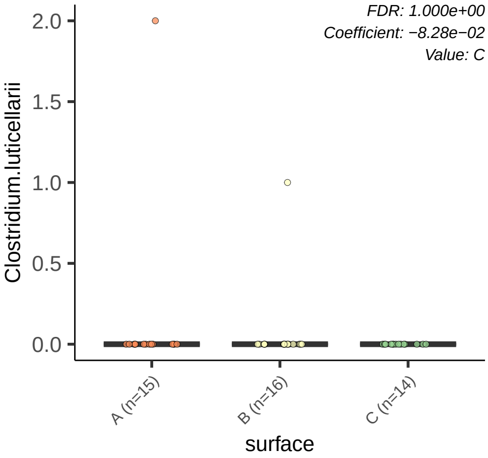

FDR: 1.000e+00
Coefficient: −8.28e−02
Value: C
Clostridium.luticellarii
2.0
1.5
1.0
0.5
0.0
A (n=15)
B (n=16)
C (n=14)
surface

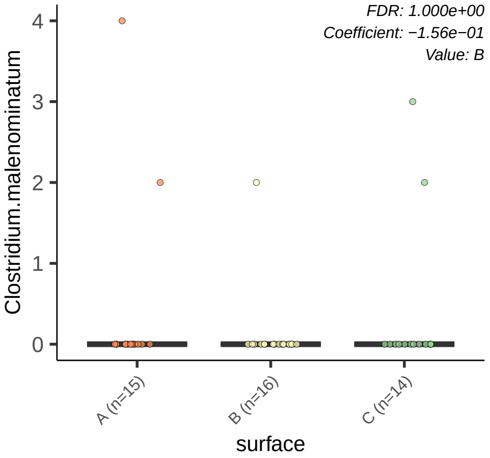
FDR: 1.000e+00
Coefficient: −1.56e−01
Value: B
Clostridium.malenominatum
0
1
2
3
4
A (n=15)
B (n=16)
C (n=14)
surface

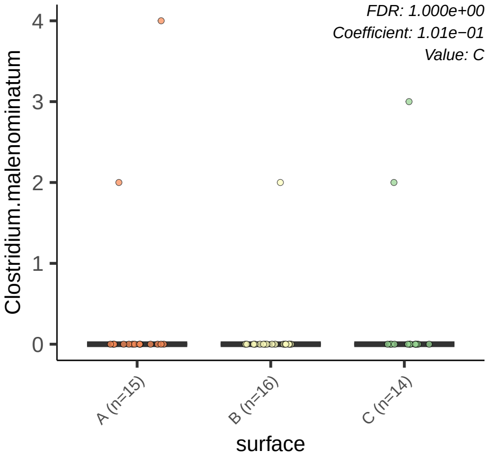
FDR: 1.000e+00
Coefficient: 1.01e−01
Value: C
Clostridium.malenominatum
4
3
2
1
0
A (n=15)
B (n=16)
C (n=14)
surface

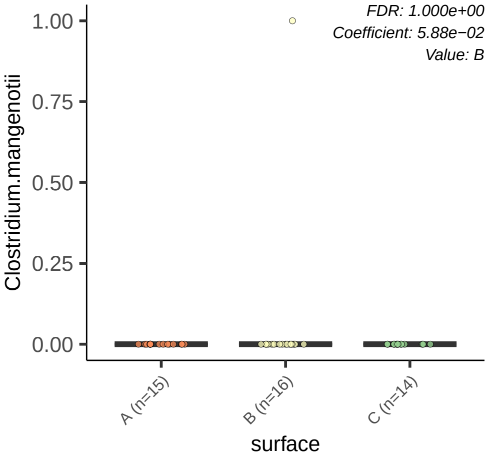

FDR: 1.000e+00
Coefficient: 5.88e−02
Value: B
Clostridium.mangenotii
1.00
0.75
0.50
0.25
0.00
A (n=15)
B (n=16)
C (n=14)
surface

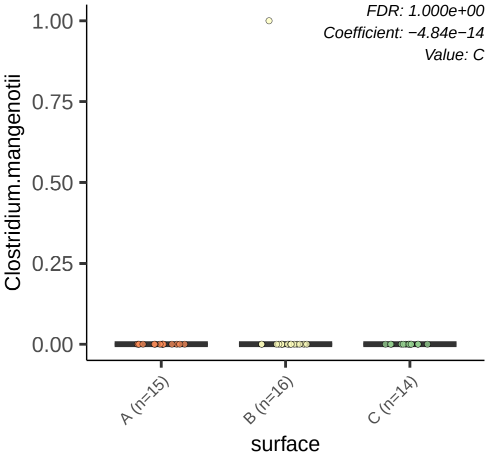

FDR: 1.000e+00
Coefficient: −4.84e−14
Value: C
Clostridium.mangenotii
1.00
0.75
0.50
0.25
0.00
A (n=15)
B (n=16)
C (n=14)
surface

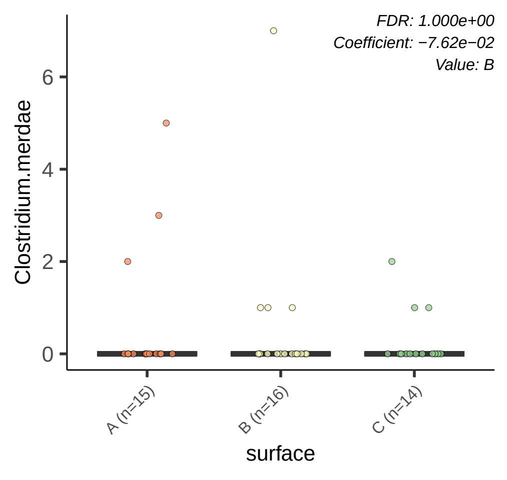

FDR: 1.000e+00
Coefficient: −7.62e−02
Value: B
Clostridium.merdae
0
2
4
6
A (n=15)
B (n=16)
C (n=14)
surface

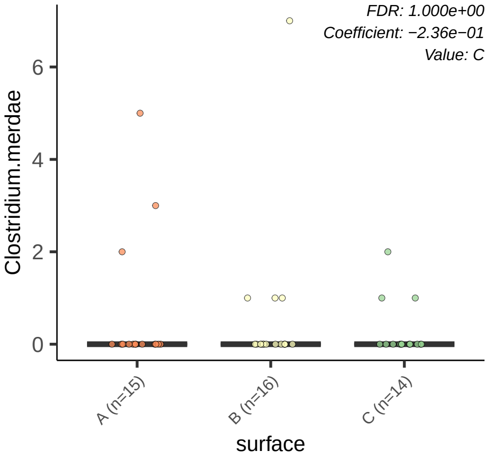
FDR: 1.000e+00
Coefficient: −2.36e−01
Value: C
Clostridium.merdae
0
2
4
6
A (n=15)
B (n=16)
C (n=14)
surface

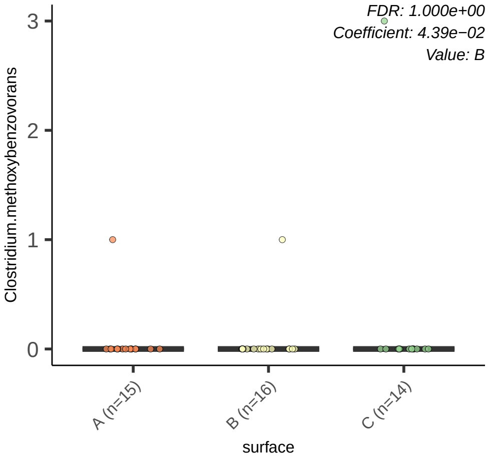

FDR: 1.000e+00
Coefficient: 4.39e−02
Value: B
Clostridium.methoxybenzovorans
0
1
2
3
A (n=15)
B (n=16)
C (n=14)
surface

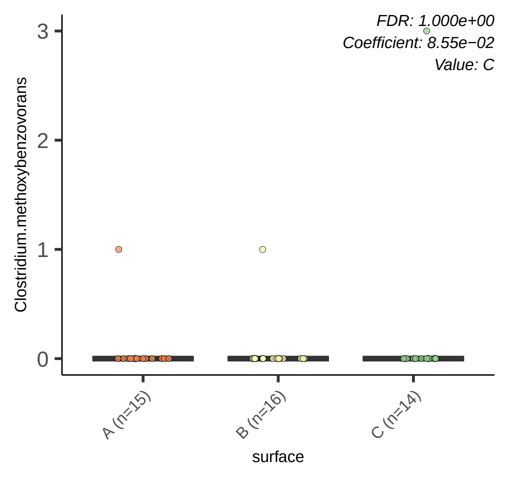
FDR: 1.000e+00
Coefficient: 8.55e−02
Value: C
Clostridium.methoxybenzovorans
3
2
1
0
A (n=15)
B (n=16)
C (n=14)
surface

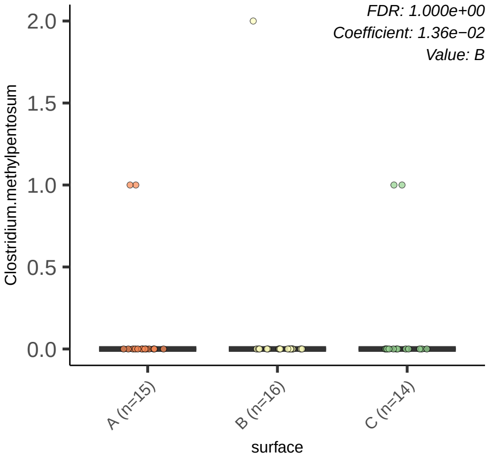
FDR: 1.000e+00
Coefficient: 1.36e−02
Value: B
Clostridium.methylpentosum
2.0
1.5
1.0
0.5
0.0
A (n=15)
B (n=16)
C (n=14)
surface

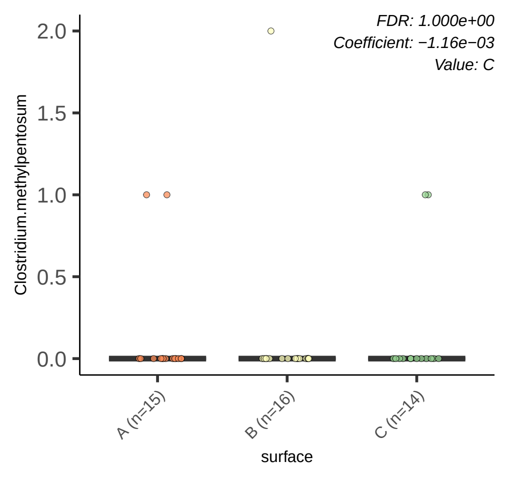

FDR: 1.000e+00
Coefficient: −1.16e−03
Value: C
2.0
1.5
1.0
0.5
0.0
Clostridium.methylpentosum
A (n=15)
B (n=16)
C (n=14)
surface

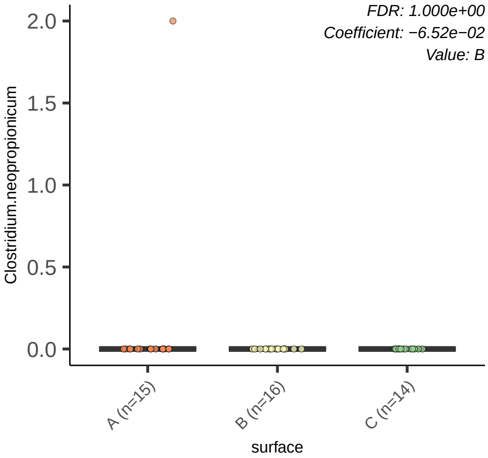

FDR: 1.000e+00
Coefficient: −6.52e−02
Value: B
Clostridium.neopropionicum
2.0
1.5
1.0
0.5
0.0
A (n=15)
B (n=16)
C (n=14)
surface

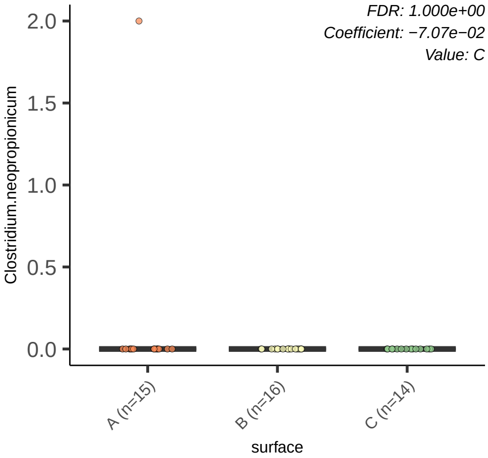
FDR: 1.000e+00
Coefficient: −7.07e−02
Value: C
2.0
1.5
1.0
0.5
0.0
Clostridium.neopropionicum
A (n=15)
B (n=16)
C (n=14)
surface

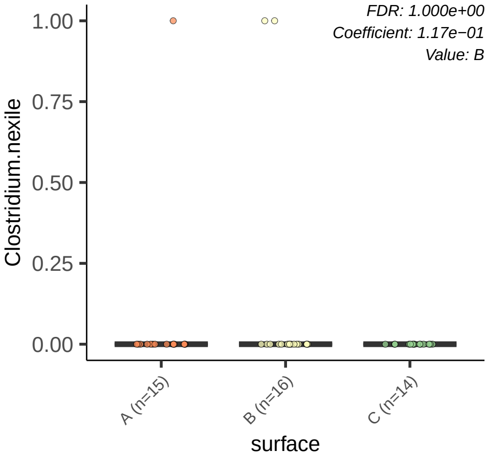

FDR: 1.000e+00
Coefficient: 1.17e−01
Value: B
Clostridium.nexile
1.00
0.75
0.50
0.25
0.00
A (n=15)
B (n=16)
C (n=14)
surface

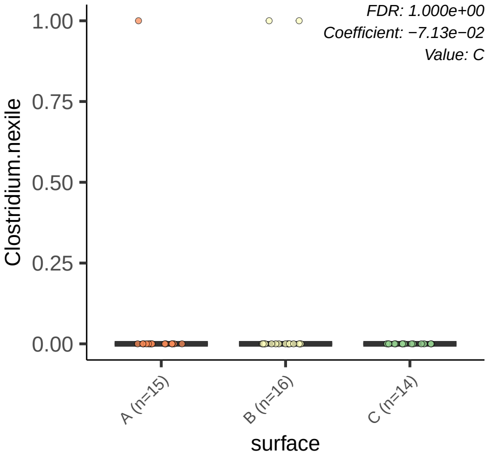
FDR: 1.000e+00
Coefficient: −7.13e−02
Value: C
Clostridium.nexile
1.00
0.75
0.50
0.25
0.00
A (n=15)
B (n=16)
C (n=14)
surface

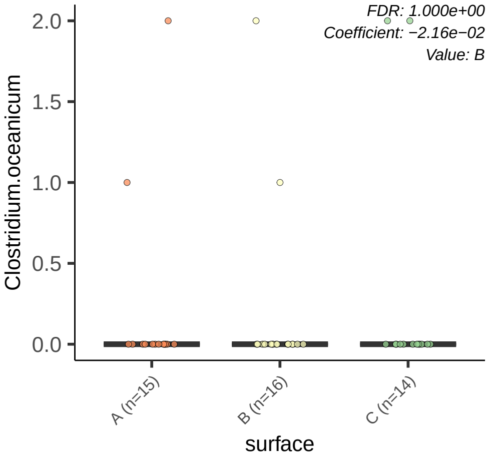

FDR: 1.000e+00
Coefficient: −2.16e−02
Value: B
Clostridium.oceanicum
2.0
1.5
1.0
0.5
0.0
A (n=15)
B (n=16)
C (n=14)
surface

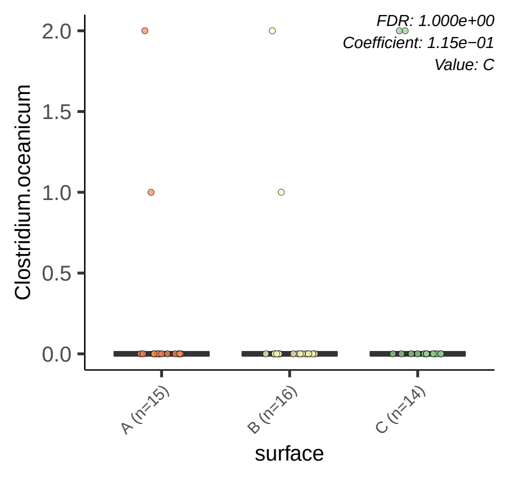
FDR: 1.000e+00
Coefficient: 1.15e−01
Value: C
Clostridium.oceanicum
2.0
1.5
1.0
0.5
0.0
A (n=15)
B (n=16)
C (n=14)
surface

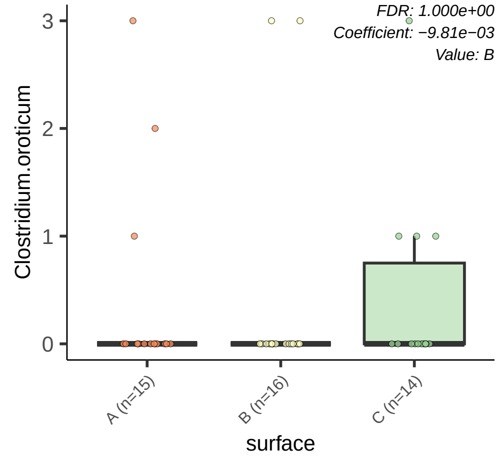
FDR: 1.000e+00
Coefficient: −9.81e−03
Value: B
Clostridium.oroticum
0
1
2
3
A (n=15)
B (n=16)
C (n=14)
surface

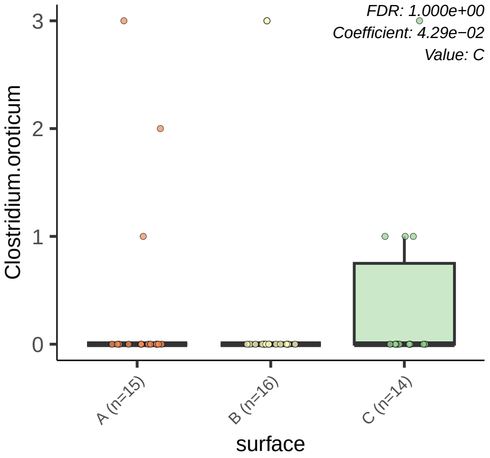
FDR: 1.000e+00
Coefficient: 4.29e−02
Value: C
Clostridium.oroticum
0
1
2
3
A (n=15)
B (n=16)
C (n=14)
surface

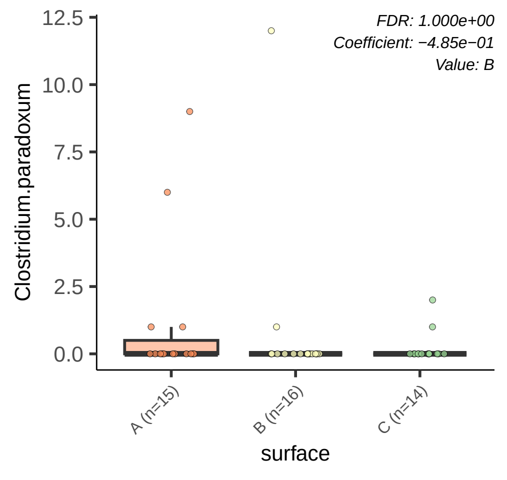

FDR: 1.000e+00
Coefficient: −4.85e−01
Value: B
Clostridium.paradoxum
12.5
10.0
7.5
5.0
2.5
0.0
A (n=15)
B (n=16)
C (n=14)
surface

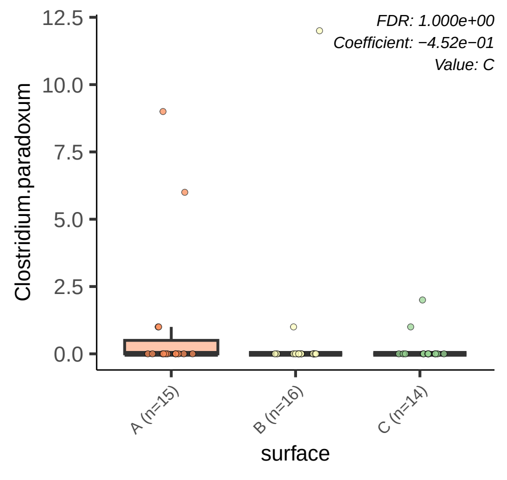

FDR: 1.000e+00
Coefficient: −4.52e−01
Value: C
12.5
10.0
7.5
5.0
2.5
0.0
Clostridium.paradoxum
A (n=15)
B (n=16)
C (n=14)
surface

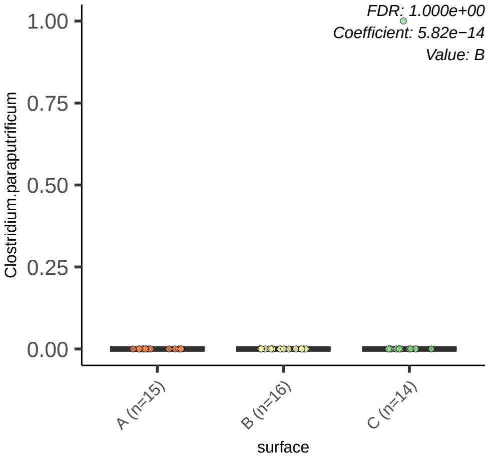
FDR: 1.000e+00
Coefficient: 5.82e−14
Value: B
1.00
0.75
0.50
0.25
0.00
Clostridium.paraputrificum
A (n=15)
B (n=16)
C (n=14)
surface

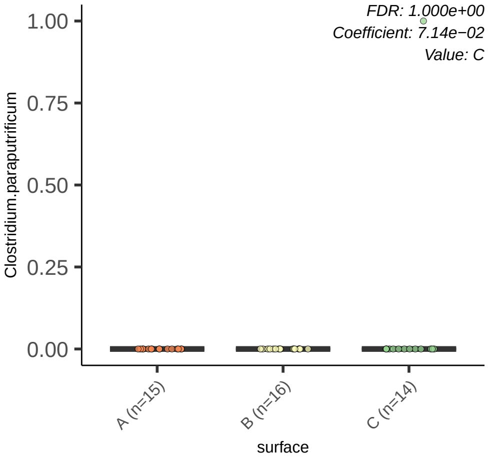
FDR: 1.000e+00
Coefficient: 7.14e−02
Value: C
Clostridium.paraputrificum
1.00
0.75
0.50
0.25
0.00
A (n=15)
B (n=16)
C (n=14)
surface

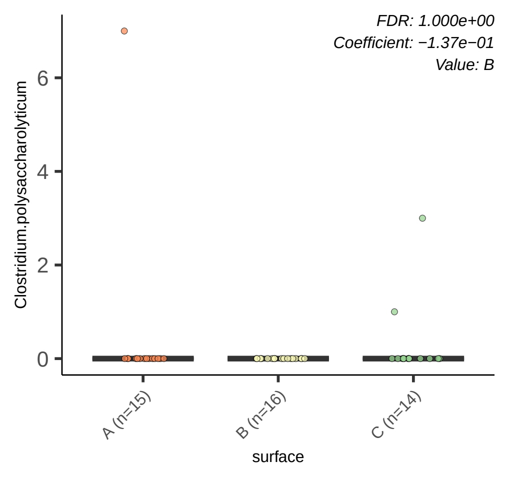
FDR: 1.000e+00
Coefficient: −1.37e−01
Value: B
Clostridium.polysaccharolyticum
6
4
2
0
A (n=15)
B (n=16)
C (n=14)
surface

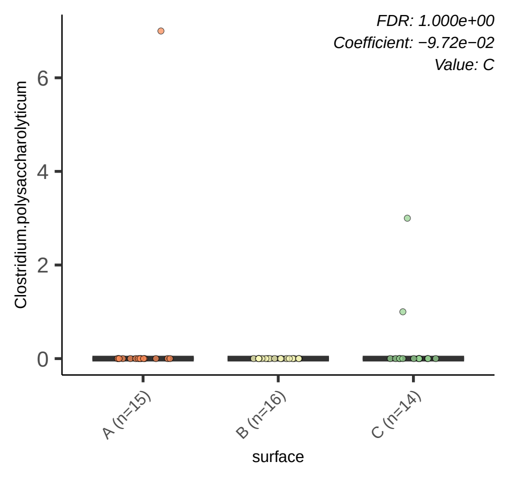
FDR: 1.000e+00
Coefficient: −9.72e−02
Value: C
Clostridium.polysaccharolyticum
0
2
4
6
A (n=15)
B (n=16)
C (n=14)
surface

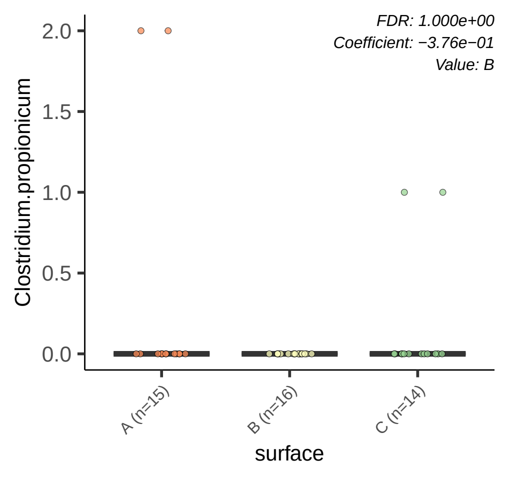
FDR: 1.000e+00
Coefficient: −3.76e−01
Value: B
Clostridium.propionicum
2.0
1.5
1.0
0.5
0.0
A (n=15)
B (n=16)
C (n=14)
surface

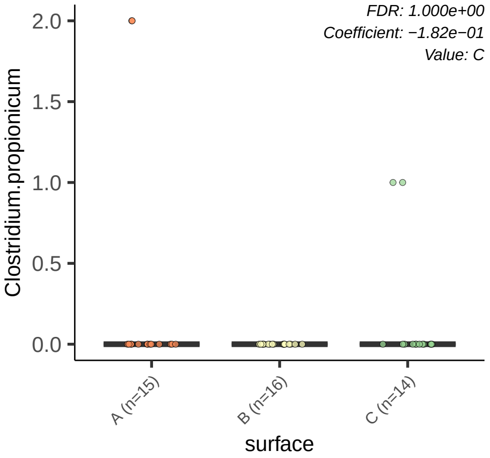

FDR: 1.000e+00
Coefficient: −1.82e−01
Value: C
Clostridium.propionicum
2.0
1.5
1.0
0.5
0.0
A (n=15)
B (n=16)
C (n=14)
surface

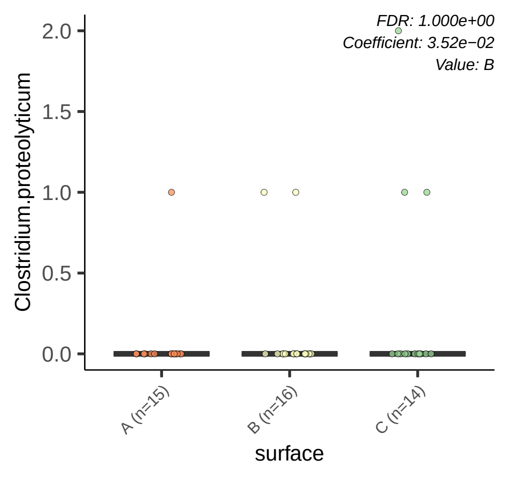
FDR: 1.000e+00
Coefficient: 3.52e−02
Value: B
Clostridium.proteolyticum
2.0
1.5
1.0
0.5
0.0
A (n=15)
B (n=16)
C (n=14)
surface

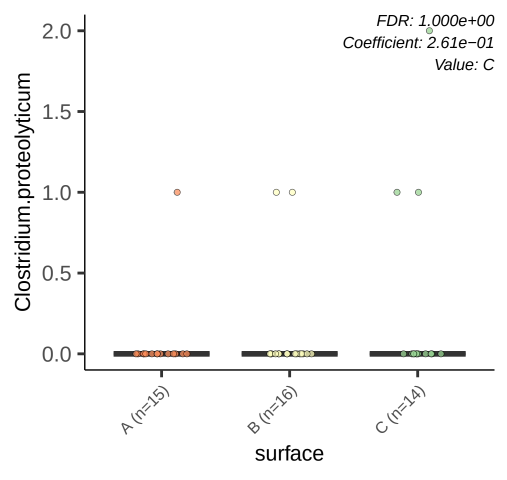
FDR: 1.000e+00
Coefficient: 2.61e−01
Value: C
Clostridium.proteolyticum
2.0
1.5
1.0
0.5
0.0
A (n=15)
B (n=16)
C (n=14)
surface

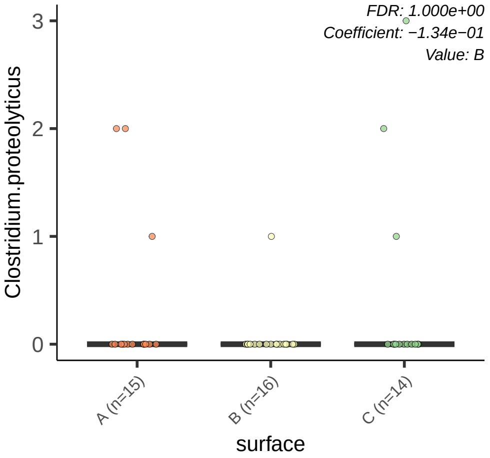
FDR: 1.000e+00
Coefficient: −1.34e−01
Value: B
Clostridium.proteolyticus
3
2
1
0
A (n=15)
B (n=16)
C (n=14)
surface

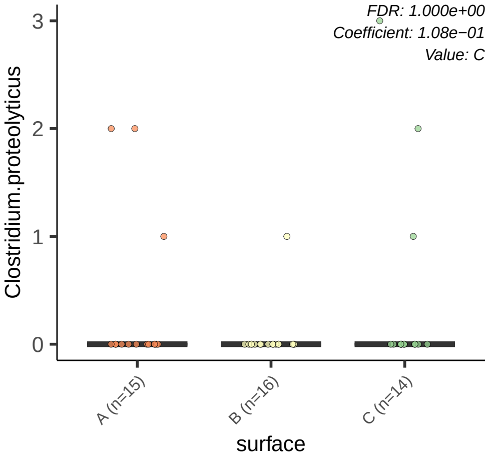

FDR: 1.000e+00
Coefficient: 1.08e−01
Value: C
3
2
1
0
Clostridium.proteolyticus
A (n=15)
B (n=16)
C (n=14)
surface

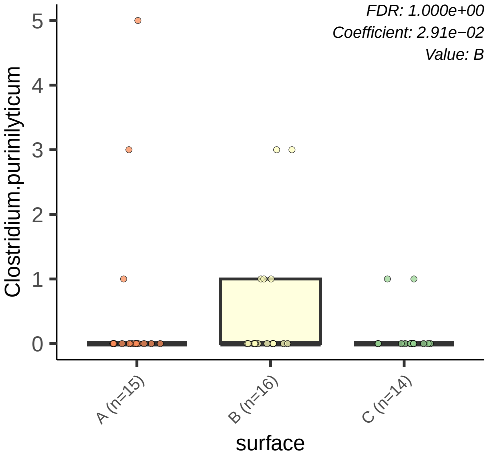

FDR: 1.000e+00
Coefficient: 2.91e−02
Value: B
Clostridium.purinilyticum
0
1
2
3
4
5
A (n=15)
B (n=16)
C (n=14)
surface

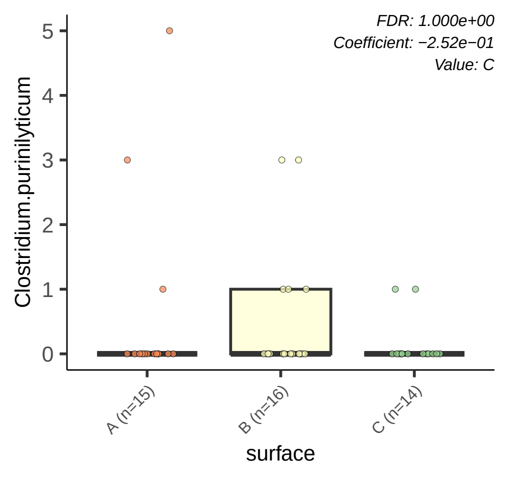
FDR: 1.000e+00
Coefficient: −2.52e−01
Value: C
Clostridium.purinilyticum
0
1
2
3
4
5
A (n=15)
B (n=16)
C (n=14)
surface

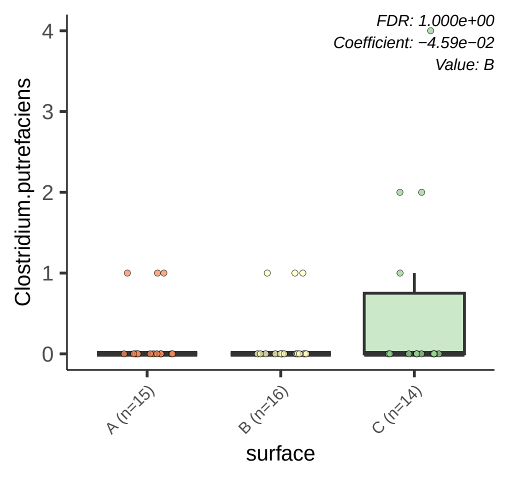
FDR: 1.000e+00
Coefficient: −4.59e−02
Value: B
Clostridium.putrefaciens
0
1
2
3
4
A (n=15)
B (n=16)
C (n=14)
surface

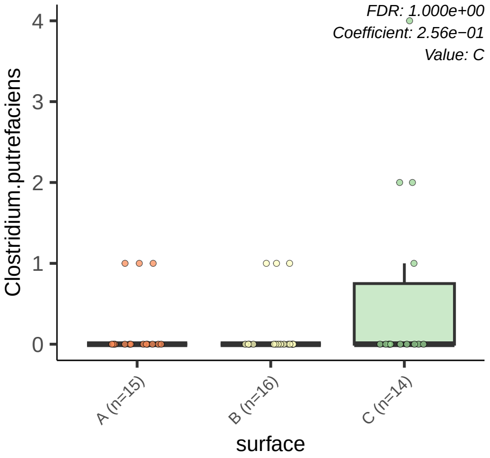

FDR: 1.000e+00
Coefficient: 2.56e−01
Value: C
Clostridium.putrefaciens
0
1
2
3
4
A (n=15)
B (n=16)
C (n=14)
surface

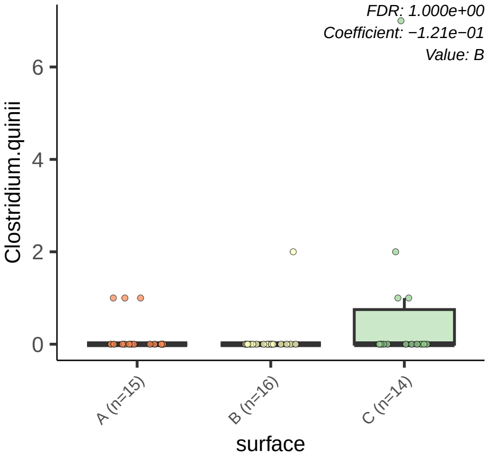

FDR: 1.000e+00
Coefficient: −1.21e−01
Value: B
Clostridium.quinii
0
2
4
6
A (n=15)
B (n=16)
C (n=14)
surface

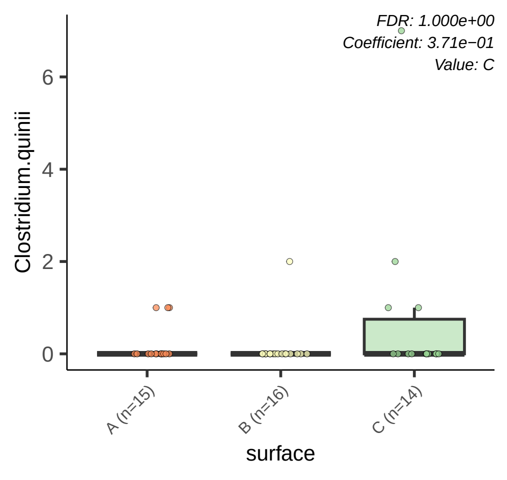
FDR: 1.000e+00
Coefficient: 3.71e−01
Value: C
Clostridium.quinii
0
2
4
6
A (n=15)
B (n=16)
C (n=14)
surface

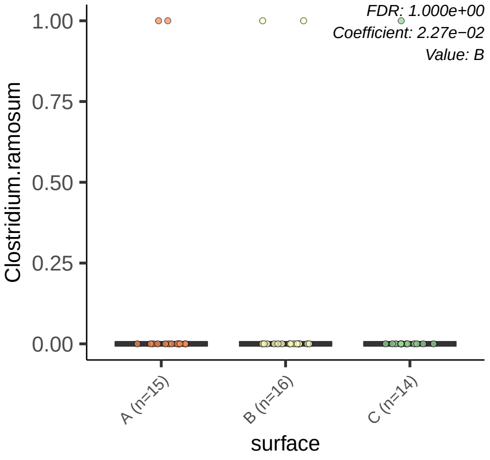

FDR: 1.000e+00
Coefficient: 2.27e−02
Value: B
Clostridium.ramosum
1.00
0.75
0.50
0.25
0.00
A (n=15)
B (n=16)
C (n=14)
surface

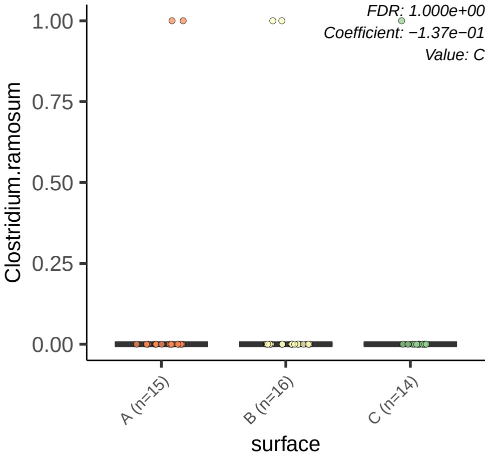
FDR: 1.000e+00
Coefficient: −1.37e−01
Value: C
Clostridium.ramosum
1.00
0.75
0.50
0.25
0.00
A (n=15)
B (n=16)
C (n=14)
surface

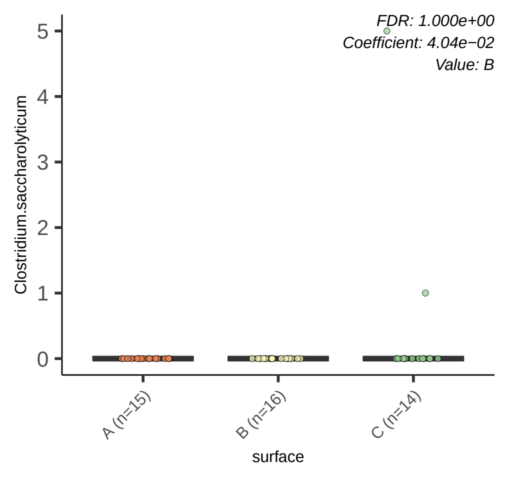
FDR: 1.000e+00
Coefficient: 4.04e−02
Value: B
Clostridium.saccharolyticum
0
1
2
3
4
5
A (n=15)
B (n=16)
C (n=14)
surface

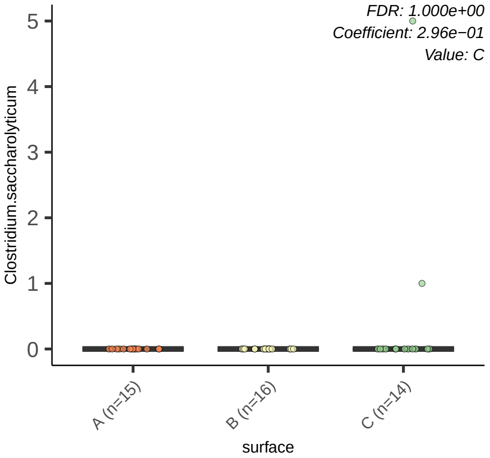

FDR: 1.000e+00
Coefficient: 2.96e−01
Value: C
Clostridium.saccharolyticum
0
1
2
3
4
5
A (n=15)
B (n=16)
C (n=14)
surface

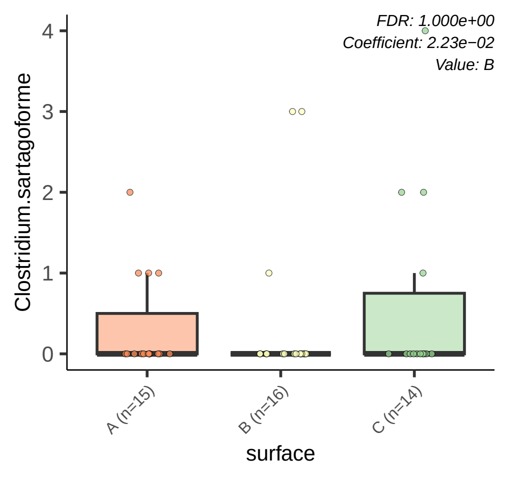
FDR: 1.000e+00
Coefficient: 2.23e−02
Value: B
Clostridium.sartagoforme
0
1
2
3
4
A (n=15)
B (n=16)
C (n=14)
surface

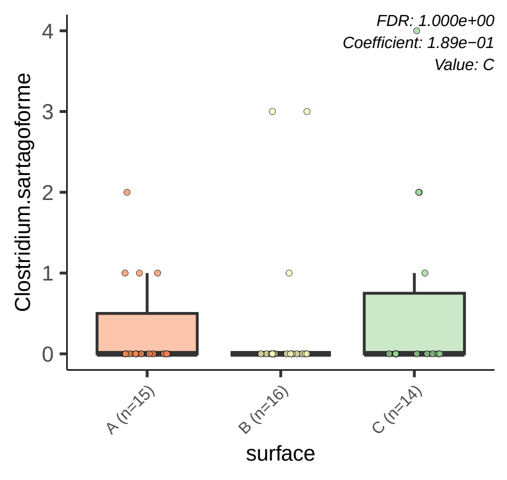
FDR: 1.000e+00
Coefficient: 1.89e−01
Value: C
Clostridium.sartagoforme
0
1
2
3
4
A (n=15)
B (n=16)
C (n=14)
surface

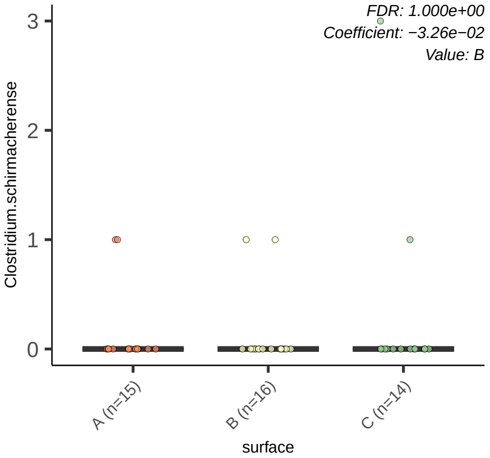
FDR: 1.000e+00
Coefficient: −3.26e−02
Value: B
Clostridium.schirmacherense
0
1
2
3
A (n=15)
B (n=16)
C (n=14)
surface

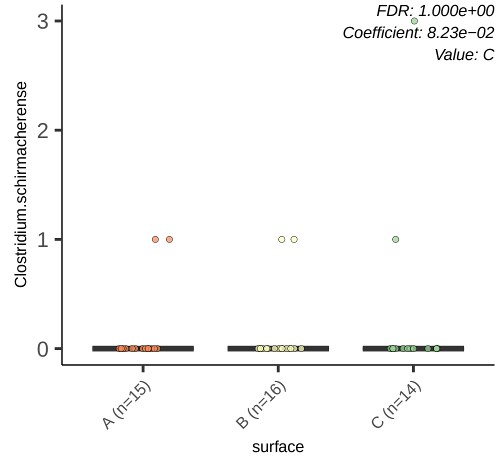
FDR: 1.000e+00
Coefficient: 8.23e−02
Value: C
Clostridium.schirmacherense
3
2
1
0
A (n=15)
B (n=16)
C (n=14)
surface

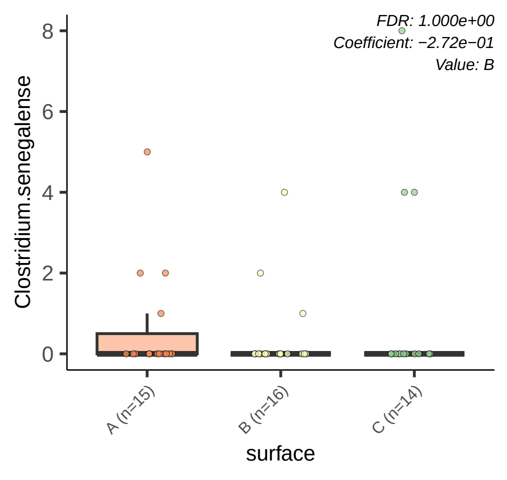
FDR: 1.000e+00
Coefficient: −2.72e−01
Value: B
Clostridium.senegalense
8
6
4
2
0
A (n=15)
B (n=16)
C (n=14)
surface

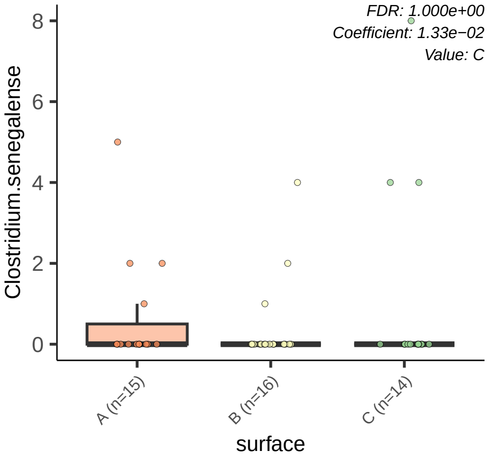

FDR: 1.000e+00
Coefficient: 1.33e−02
Value: C
Clostridium.senegalense
8
6
4
2
0
A (n=15)
B (n=16)
C (n=14)
surface

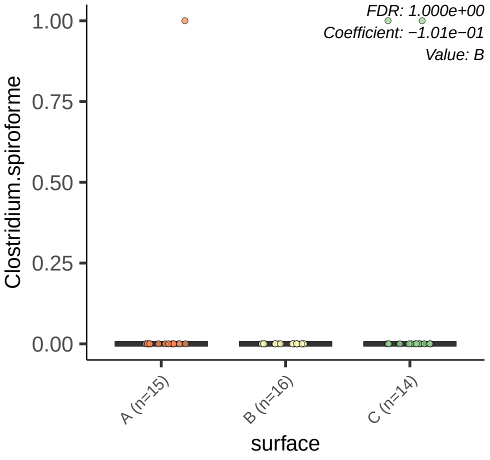

FDR: 1.000e+00
Coefficient: −1.01e−01
Value: B
Clostridium.spiroforme
1.00
0.75
0.50
0.25
0.00
A (n=15)
B (n=16)
C (n=14)
surface

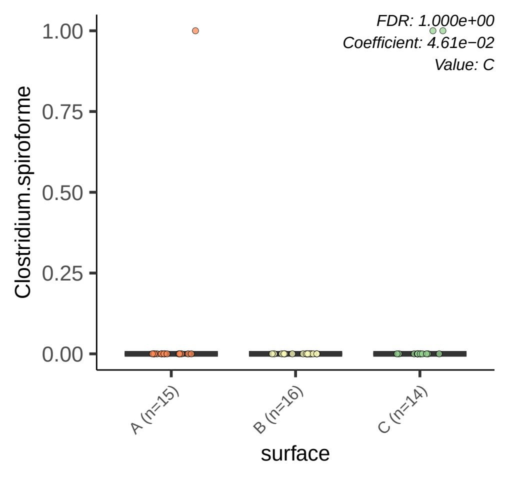
FDR: 1.000e+00
Coefficient: 4.61e−02
Value: C
Clostridium.spiroforme
1.00
0.75
0.50
0.25
0.00
A (n=15)
B (n=16)
C (n=14)
surface

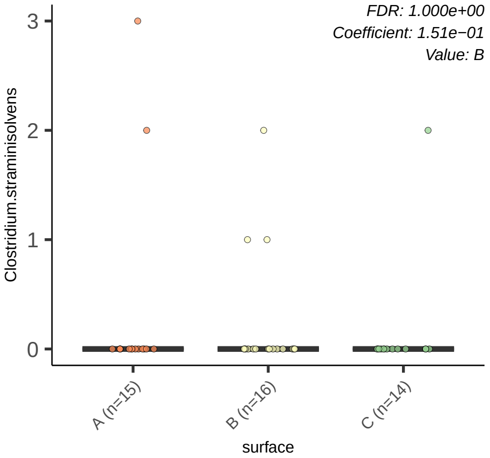

FDR: 1.000e+00
Coefficient: 1.51e−01
Value: B
Clostridium.straminisolvens
3
2
1
0
A (n=15)
B (n=16)
C (n=14)
surface

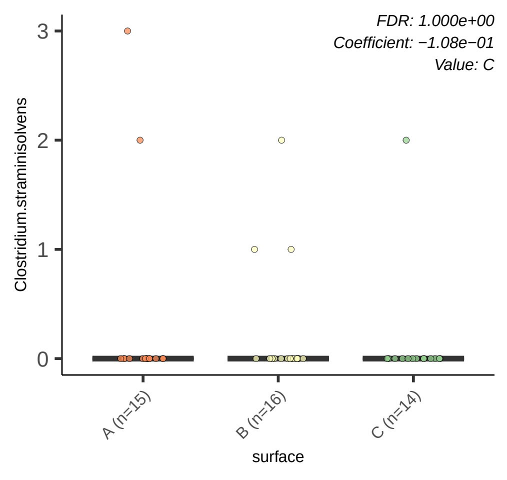

FDR: 1.000e+00
Coefficient: −1.08e−01
Value: C
Clostridium.straminisolvens
3
2
1
0
A (n=15)
B (n=16)
C (n=14)
surface

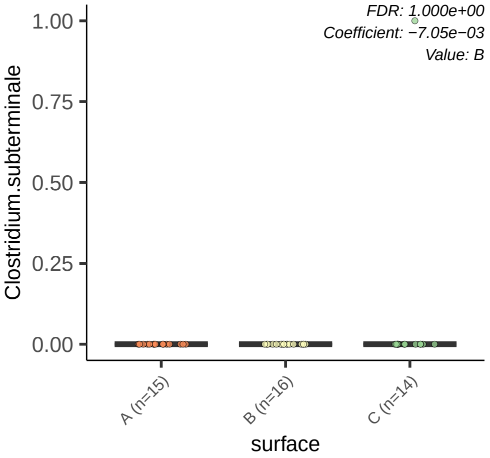

FDR: 1.000e+00
Coefficient: −7.05e−03
Value: B
Clostridium.subterminale
1.00
0.75
0.50
0.25
0.00
A (n=15)
B (n=16)
C (n=14)
surface

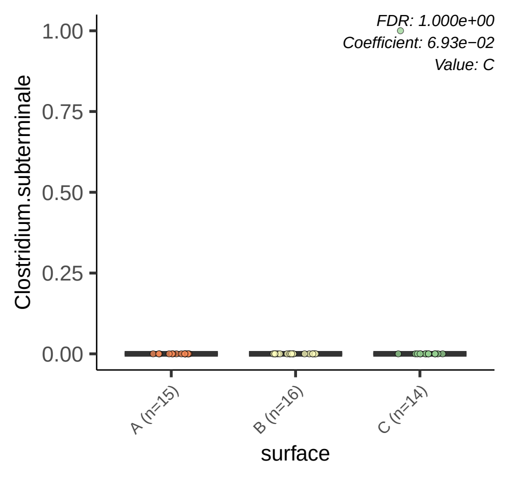
FDR: 1.000e+00
Coefficient: 6.93e−02
Value: C
Clostridium.subterminale
1.00
0.75
0.50
0.25
0.00
A (n=15)
B (n=16)
C (n=14)
surface

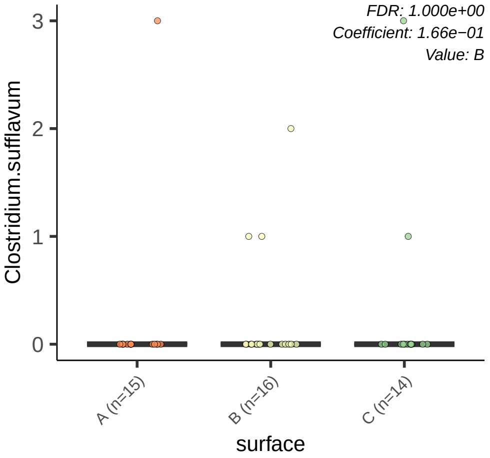
FDR: 1.000e+00
Coefficient: 1.66e−01
Value: B
Clostridium.sufflavum
3
2
1
0
A (n=15)
B (n=16)
C (n=14)
surface

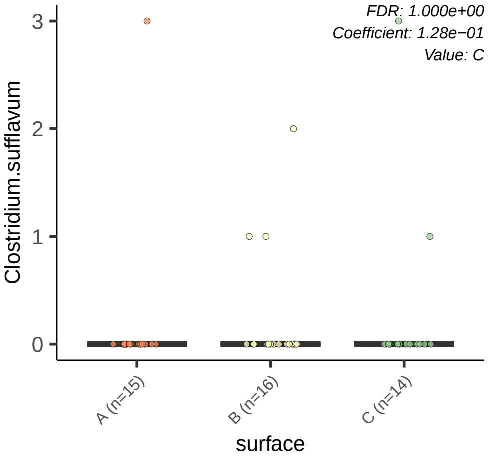
FDR: 1.000e+00
Coefficient: 1.28e−01
Value: C
Clostridium.sufflavum
3
2
1
0
A (n=15)
B (n=16)
C (n=14)
surface

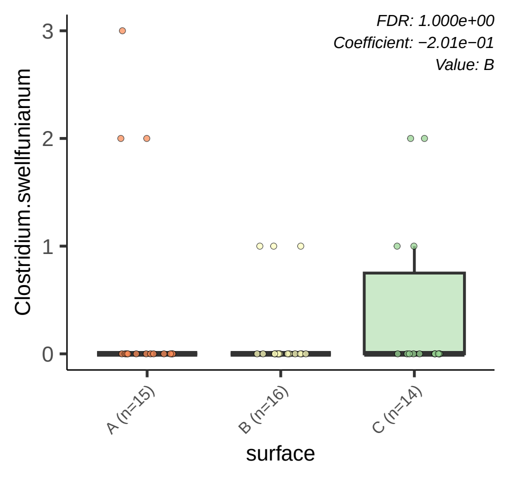

FDR: 1.000e+00
Coefficient: −2.01e−01
Value: B
Clostridium.swellfunianum
3
2
1
0
A (n=15)
B (n=16)
C (n=14)
surface

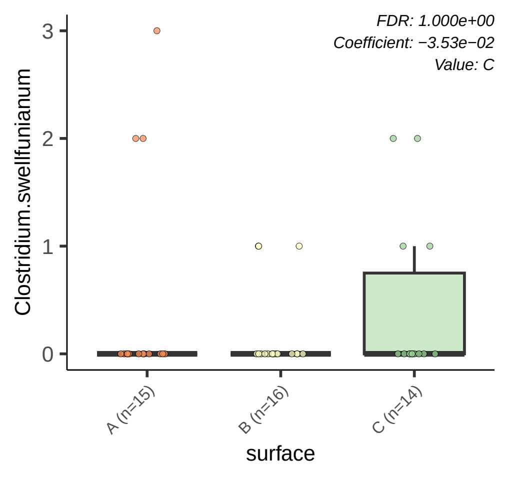

FDR: 1.000e+00
Coefficient: −3.53e−02
Value: C
Clostridium.swellfunianum
3
2
1
0
A (n=15)
B (n=16)
C (n=14)
surface

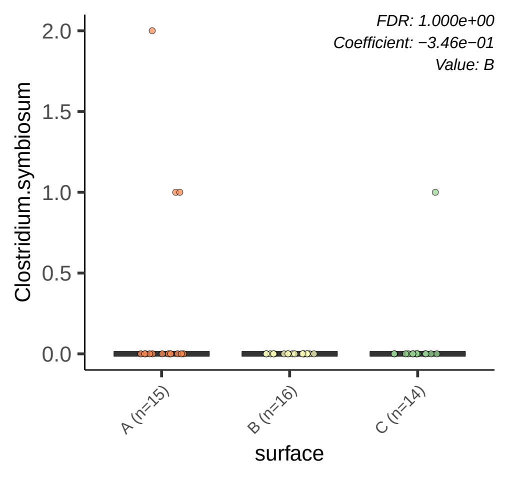

FDR: 1.000e+00
Coefficient: −3.46e−01
Value: B
Clostridium.symbiosum
2.0
1.5
1.0
0.5
0.0
A (n=15)
B (n=16)
C (n=14)
surface

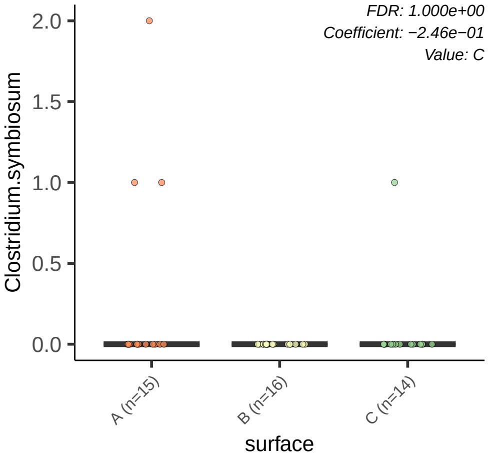
FDR: 1.000e+00
Coefficient: −2.46e−01
Value: C
Clostridium.symbiosum
2.0
1.5
1.0
0.5
0.0
A (n=15)
B (n=16)
C (n=14)
surface

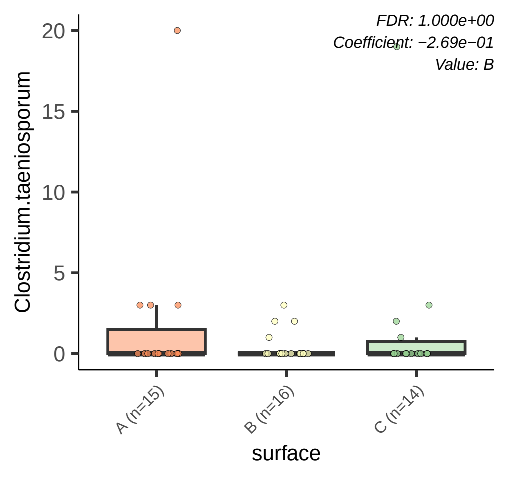
FDR: 1.000e+00
Coefficient: −2.69e−01
Value: B
Clostridium.taeniosporum
20
15
10
5
0
A (n=15)
B (n=16)
C (n=14)
surface

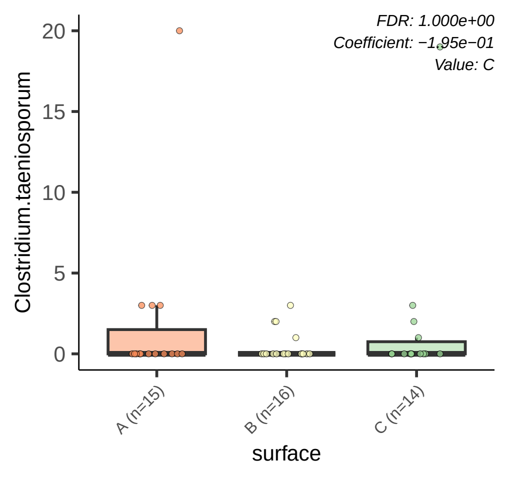

FDR: 1.000e+00
Coefficient: −1.95e−01
Value: C
Clostridium.taeniosporum
20
15
10
5
0
A (n=15)
B (n=16)
C (n=14)
surface

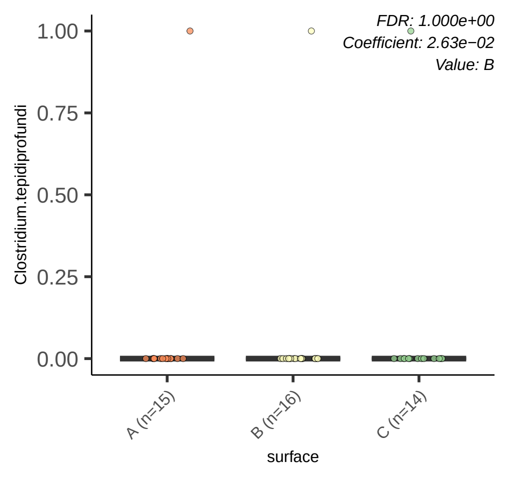
FDR: 1.000e+00
Coefficient: 2.63e−02
Value: B
Clostridium.tepidiprofundi
1.00
0.75
0.50
0.25
0.00
A (n=15)
B (n=16)
C (n=14)
surface

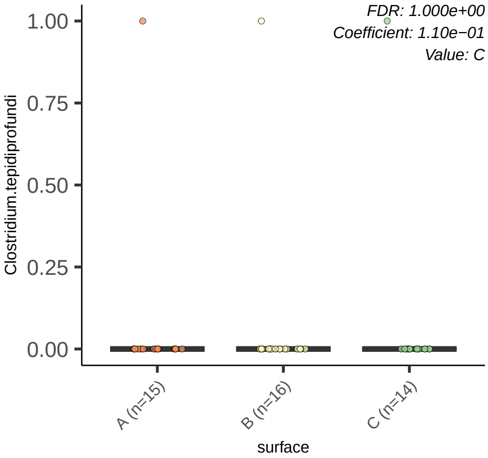

FDR: 1.000e+00
Coefficient: 1.10e−01
Value: C
Clostridium.tepidiprofundi
1.00
0.75
0.50
0.25
0.00
A (n=15)
B (n=16)
C (n=14)
surface

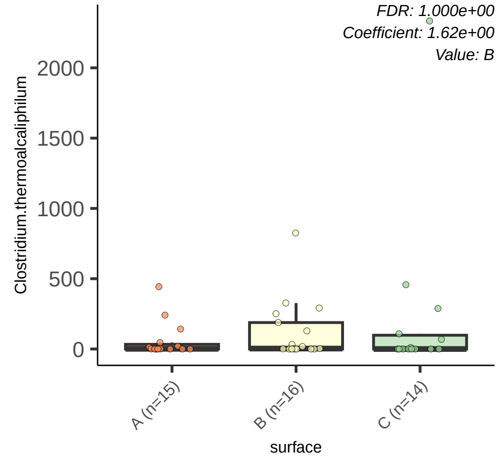
FDR: 1.000e+00
Coefficient: 1.62e+00
Value: B
Clostridium.thermoalcaliphilum
2000
1500
1000
500
0
A (n=15)
B (n=16)
C (n=14)
surface

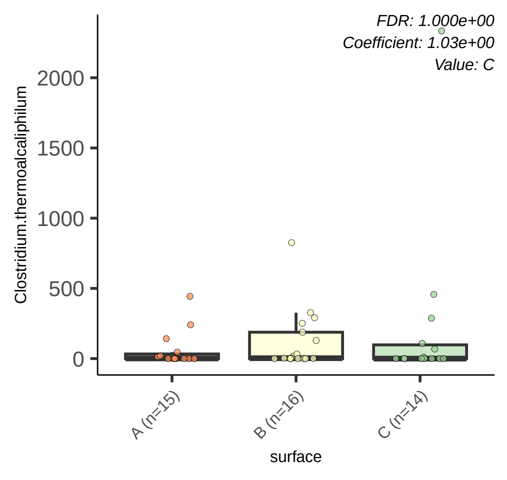
FDR: 1.000e+00
Coefficient: 1.03e+00
Value: C
Clostridium.thermoalcaliphilum
2000
1500
1000
500
0
A (n=15)
B (n=16)
C (n=14)
surface

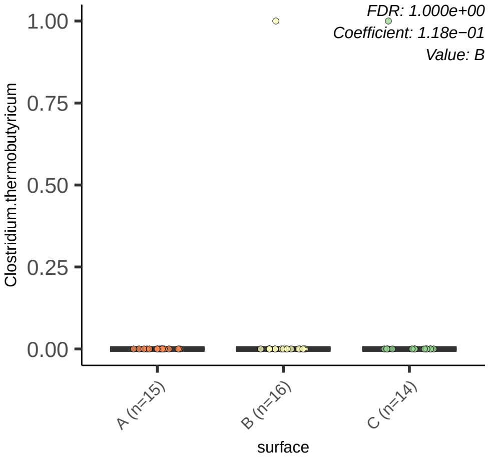
FDR: 1.000e+00
Coefficient: 1.18e−01
Value: B
1.00
0.75
0.50
0.25
0.00
Clostridium.thermobutyricum
A (n=15)
B (n=16)
C (n=14)
surface

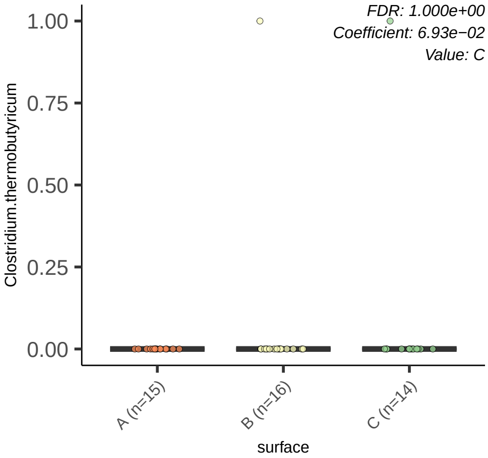

FDR: 1.000e+00
Coefficient: 6.93e−02
Value: C
1.00
0.75
0.50
0.25
0.00
Clostridium.thermobutyricum
A (n=15)
B (n=16)
C (n=14)
surface

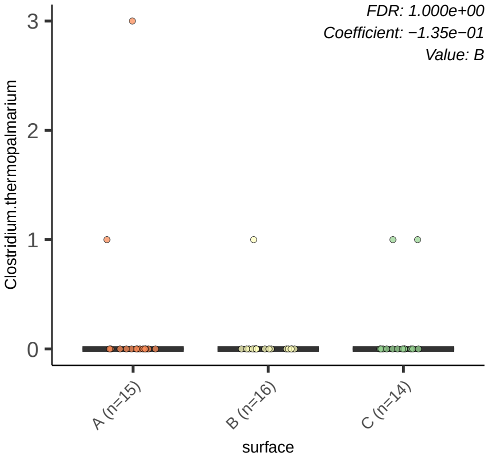

FDR: 1.000e+00
Coefficient: −1.35e−01
Value: B
Clostridium.thermopalmarium
0
1
2
3
A (n=15)
B (n=16)
C (n=14)
surface

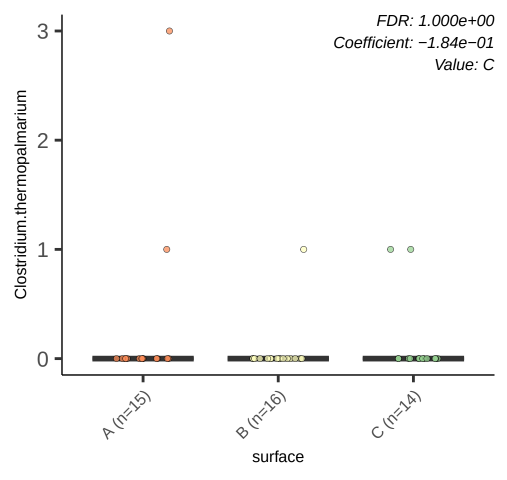

FDR: 1.000e+00
Coefficient: −1.84e−01
Value: C
Clostridium.thermopalmarium
0
1
2
3
A (n=15)
B (n=16)
C (n=14)
surface

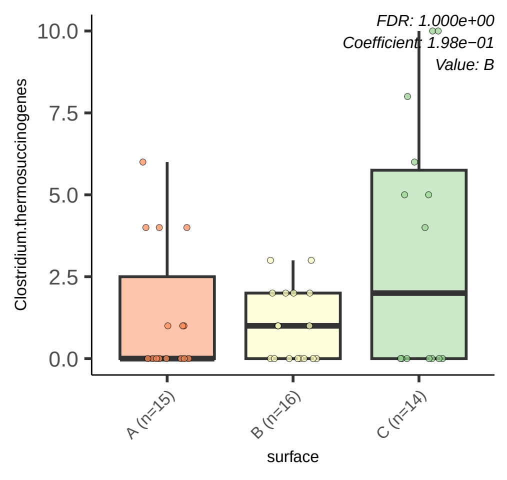

FDR: 1.000e+00
Coefficient: 1.98e−01
Value: B
10.0
7.5
5.0
2.5
0.0
Clostridium.thermosuccinogenes
A (n=15)
B (n=16)
C (n=14)
surface

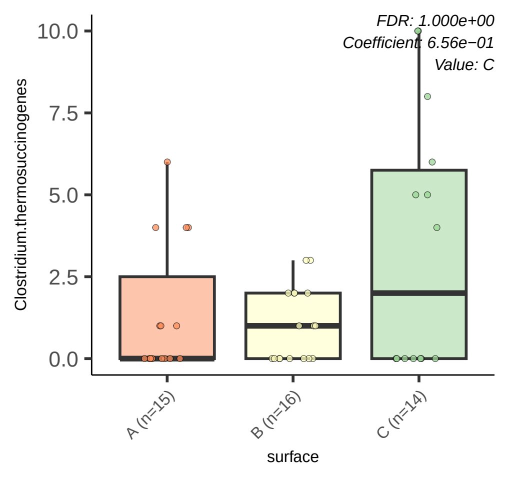

FDR: 1.000e+00
Coefficient: 6.56e−01
Value: C
10.0
7.5
5.0
2.5
0.0
Clostridium.thermosuccinogenes
A (n=15)
B (n=16)
C (n=14)
surface

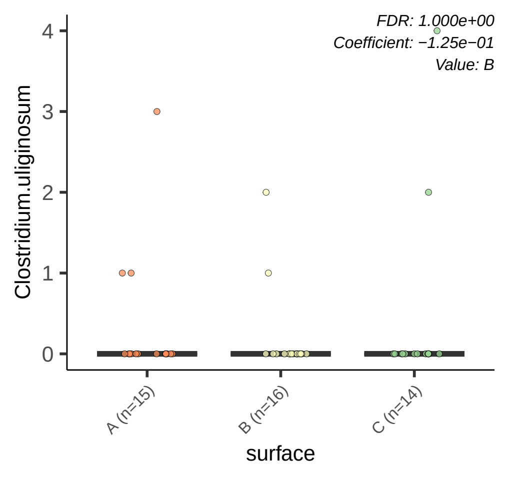

FDR: 1.000e+00
Coefficient: −1.25e−01
Value: B
Clostridium.uliginosum
0
1
2
3
4
A (n=15)
B (n=16)
C (n=14)
surface

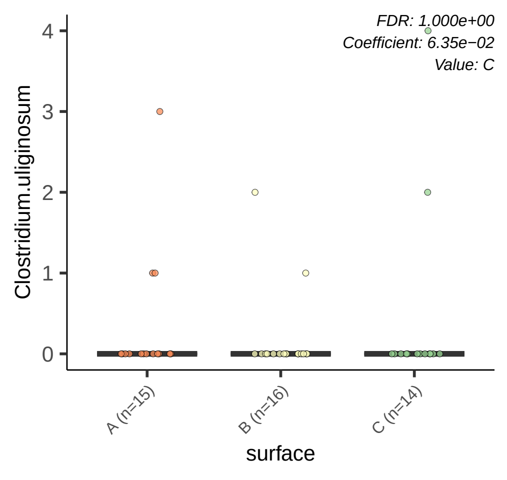
FDR: 1.000e+00
Coefficient: 6.35e−02
Value: C
Clostridium.uliginosum
4
3
2
1
0
A (n=15)
B (n=16)
C (n=14)
surface

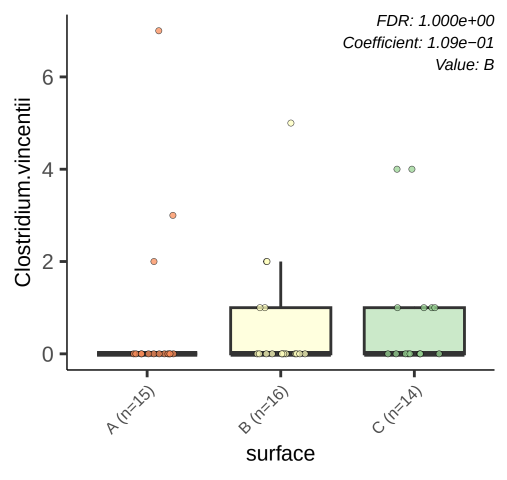

FDR: 1.000e+00
Coefficient: 1.09e−01
Value: B
Clostridium.vincentii
0
2
4
6
A (n=15)
B (n=16)
C (n=14)
surface

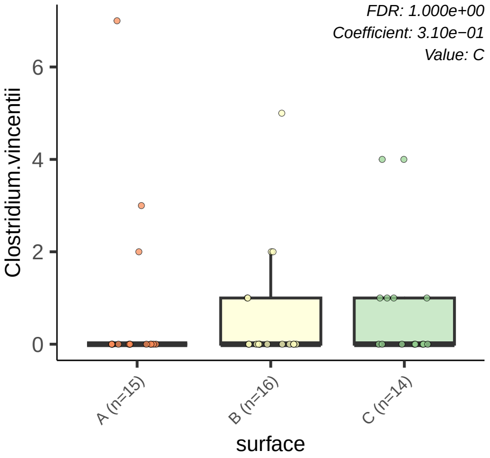
FDR: 1.000e+00
Coefficient: 3.10e−01
Value: C
Clostridium.vincentii
0
2
4
6
A (n=15)
B (n=16)
C (n=14)
surface

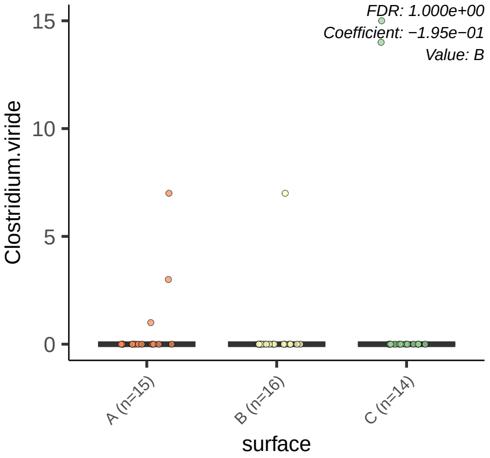
FDR: 1.000e+00
Coefficient: −1.95e−01
Value: B
Clostridium.viride
15
10
5
0
A (n=15)
B (n=16)
C (n=14)
surface

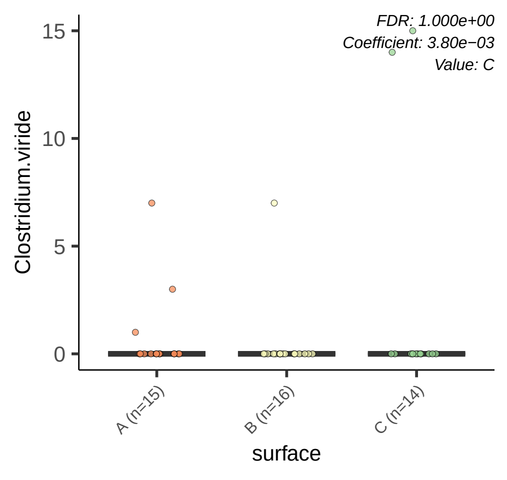
FDR: 1.000e+00
Coefficient: 3.80e−03
Value: C
Clostridium.viride
15
10
5
0
A (n=15)
B (n=16)
C (n=14)
surface

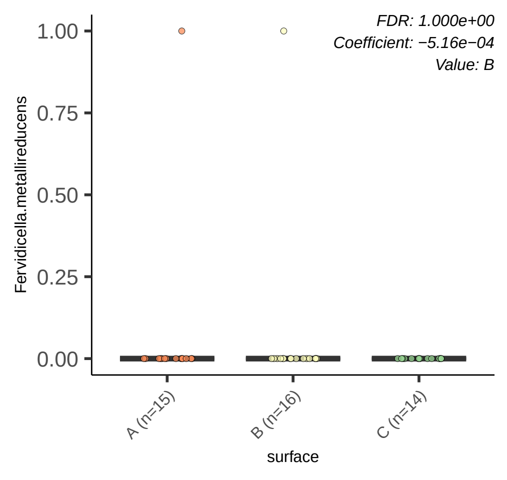
FDR: 1.000e+00
Coefficient: −5.16e−04
Value: B
Fervidicella.metallireducens
1.00
0.75
0.50
0.25
0.00
A (n=15)
B (n=16)
C (n=14)
surface

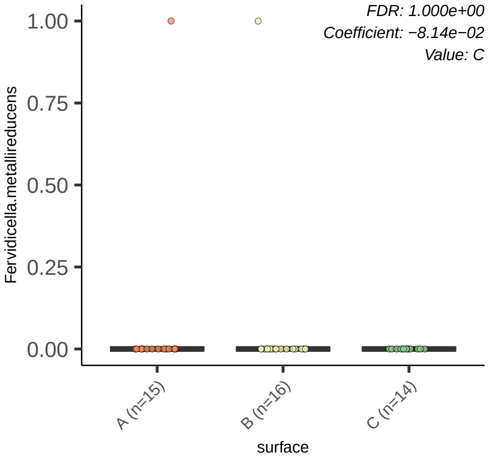

FDR: 1.000e+00
Coefficient: −8.14e−02
Value: C
Fervidicella.metallireducens
1.00
0.75
0.50
0.25
0.00
A (n=15)
B (n=16)
C (n=14)
surface

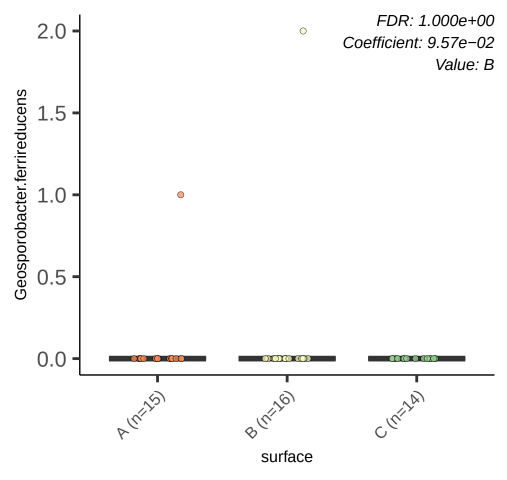
FDR: 1.000e+00
Coefficient: 9.57e−02
Value: B
Geosporobacter.ferrireducens
2.0
1.5
1.0
0.5
0.0
A (n=15)
B (n=16)
C (n=14)
surface

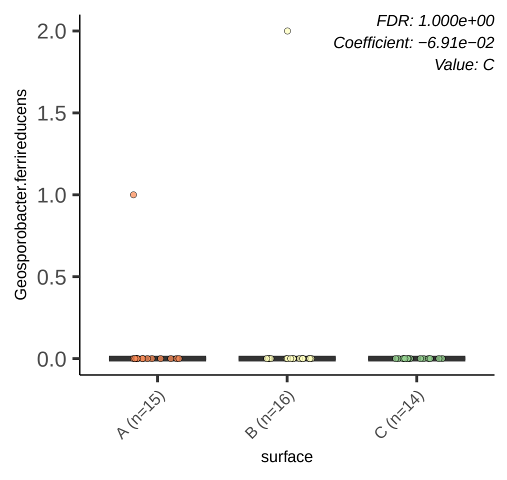

FDR: 1.000e+00
Coefficient: −6.91e−02
Value: C
Geosporobacter.ferrireducens
2.0
1.5
1.0
0.5
0.0
A (n=15)
B (n=16)
C (n=14)
surface

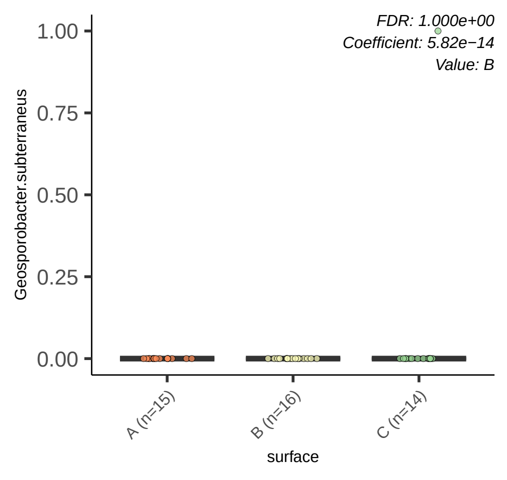

FDR: 1.000e+00
Coefficient: 5.82e−14
Value: B
Geosporobacter.subterraneus
1.00
0.75
0.50
0.25
0.00
A (n=15)
B (n=16)
C (n=14)
surface

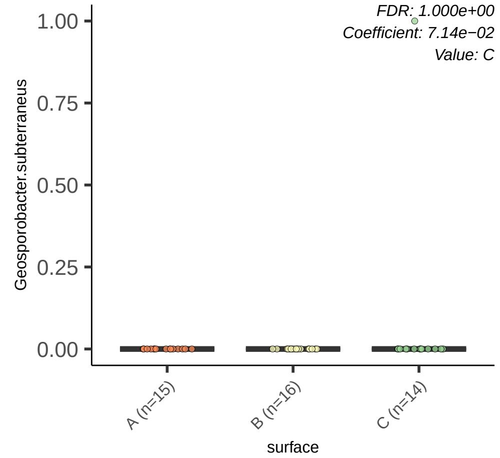
FDR: 1.000e+00
Coefficient: 7.14e−02
Value: C
1.00
0.75
0.50
0.25
0.00
Geosporobacter.subterraneus
A (n=15)
B (n=16)
C (n=14)
surface

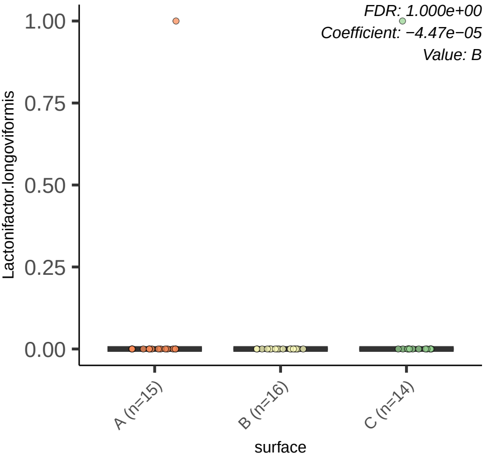
FDR: 1.000e+00
Coefficient: −4.47e−05
Value: B
Lactonifactor.longoviformis
1.00
0.75
0.50
0.25
0.00
A (n=15)
B (n=16)
C (n=14)
surface

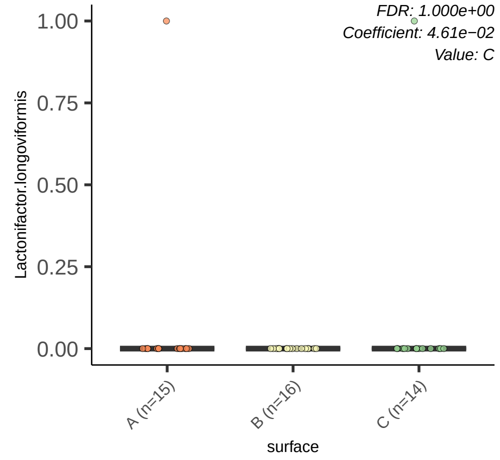

FDR: 1.000e+00
Coefficient: 4.61e−02
Value: C
1.00
0.75
0.50
0.25
0.00
Lactonifactor.longoviformis
A (n=15)
B (n=16)
C (n=14)
surface

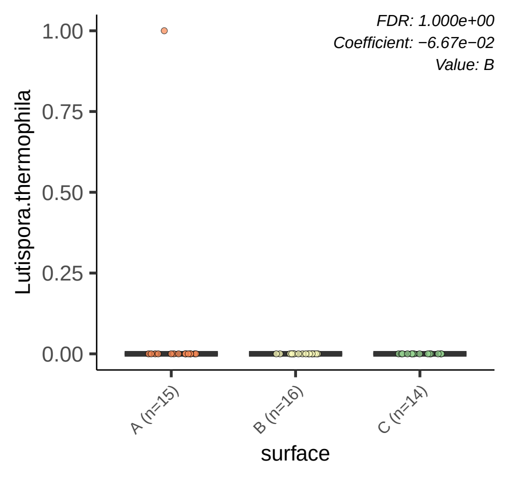

FDR: 1.000e+00
Coefficient: −6.67e−02
Value: B
Lutispora.thermophila
1.00
0.75
0.50
0.25
0.00
A (n=15)
B (n=16)
C (n=14)
surface

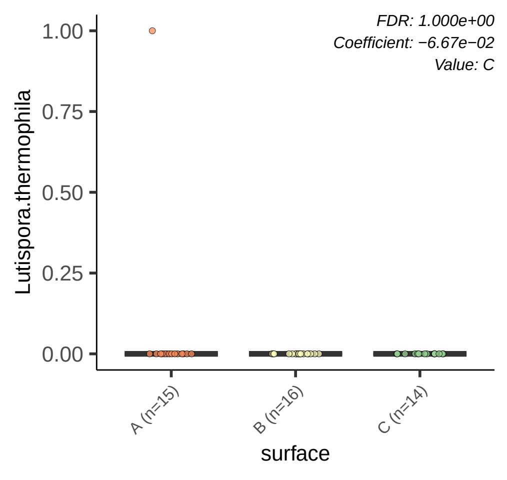
FDR: 1.000e+00
Coefficient: −6.67e−02
Value: C
Lutispora.thermophila
1.00
0.75
0.50
0.25
0.00
A (n=15)
B (n=16)
C (n=14)
surface

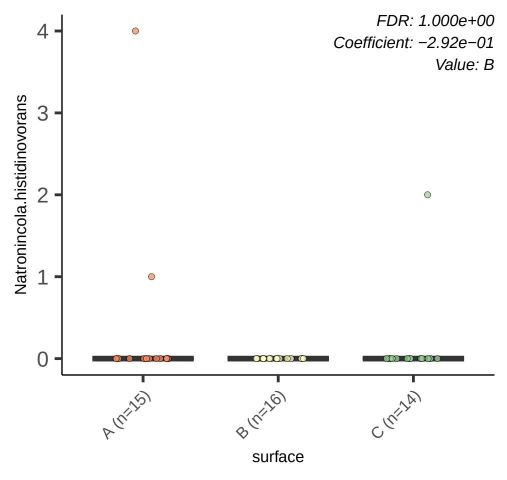
FDR: 1.000e+00
Coefficient: −2.92e−01
Value: B
Natronincola.histidinovorans
0
1
2
3
4
A (n=15)
B (n=16)
C (n=14)
surface

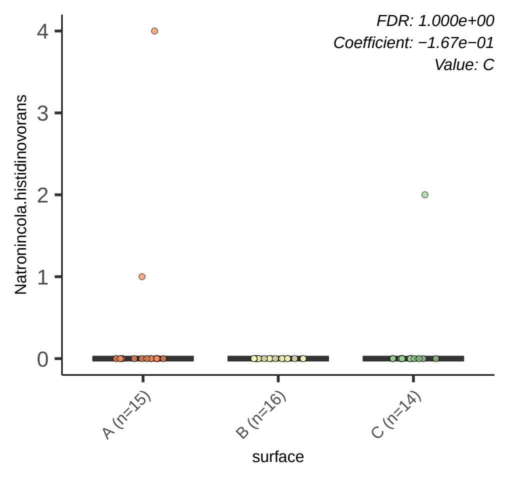
[truncated: 698,791 more chars]
